# Epigenetic regulation of transcription factor binding motifs promotes Th1 response in Chagas disease cardiomyopathy

**DOI:** 10.3389/fimmu.2022.958200

**Published:** 2022-08-22

**Authors:** Pauline Brochet, Barbara Maria Ianni, Laurie Laugier, Amanda Farage Frade, João Paulo Silva Nunes, Priscila Camillo Teixeira, Charles Mady, Ludmila Rodrigues Pinto Ferreira, Quentin Ferré, Ronaldo Honorato Barros Santos, Andreia Kuramoto, Sandrine Cabantous, Samuel Steffen, Antonio Noedir Stolf, Pablo Pomerantzeff, Alfredo Inacio Fiorelli, Edimar Alcides Bocchi, Cristina Wide Pissetti, Bruno Saba, Darlan da Silva Cândido, Fabrício C. Dias, Marcelo Ferraz Sampaio, Fabio Antônio Gaiotto, José Antonio Marin-Neto, Abílio Fragata, Ricardo Costa Fernandes Zaniratto, Sergio Siqueira, Giselle De Lima Peixoto, Vagner Oliveira-Carvalho Rigaud, Fernando Bacal, Paula Buck, Rafael Ribeiro Almeida, Hui Tzu Lin-Wang, André Schmidt, Martino Martinelli, Mario Hiroyuki Hirata, Eduardo Antonio Donadi, Alexandre Costa Pereira, Virmondes Rodrigues Junior, Denis Puthier, Jorge Kalil, Lionel Spinelli, Edecio Cunha-Neto, Christophe Chevillard

**Affiliations:** ^1^ Institut National de la Santé Et de la Recherche Médicale (INSERM), Unité Mixte de Recherche (UMR)_1090, Aix Marseille Université, TAGC Theories and Approaches of Genomic Complexity, Institut MarMaRa, Marseille, France; ^2^ Laboratory of Immunology, Heart Institute Instituto do Coração (InCor), University of São Paulo, School of Medicine, São Paulo, Brazil; ^3^ Aix Marseille Université, Génétique et Immunologie des Maladies Parasitaires, Inserm, UMR_906, Marseille, France; ^4^ Division of Clinical Immunology and Allergy, University of São Paulo, School of Medicine, São Paulo, Brazil; ^5^ Instituto Nacional de Ciência e Tecnologia, INCT, III- Institute for Investigation in Immunology, São Paulo, Brazil; ^6^ Myocardiopathies and Aortic Diseases Unit, Heart Institute Instituto do Coração (InCor), School of Medicine, University of São Paulo, São Paulo, Brazil; ^7^ RNA Systems Biology Laboratory (RSBL), Departamento de Morfologia, Instituto de Ciências Biológicas, Universidade Federal de Minas Gerais, Belo Horizonte, Minas Gerais, Brazil; ^8^ Division of Transplantation, Heart Institute Instituto do Coração (InCor), University of São Paulo, School of Medicine, São Paulo, Brazil; ^9^ Division of Surgery, Heart Institute Instituto do Coração (InCor), University of São Paulo, School of Medicine, São Paulo, Brazil; ^10^ Heart Institute (InCor), School of Medicine, University of São Paulo, São Paulo, São Paulo, Brazil; ^11^ Laboratory of Immunology, Universidade Federal Do Triângulo Mineiro (UFTM), Uberaba, Brazil; ^12^ Laboratório de Investigação Molecular em Cardiologia, Instituto de Cardiologia Dante Pazzanese (IDPC), São Paulo, Brazil; ^13^ School of Medicine of Ribeirão Preto Faculdade de Medicina de Ribeirão Preto (FMRP), University of São Paulo, Ribeirão Preto, Brazil; ^14^ Pacemaker Clinic, Heart Institute Instituto do Coração (InCor), School of Medicine, University of São Paulo, São Paulo, Brazil; ^15^ Heart Failure Unit, Heart Institute Instituto do Coração (InCor) School of Medicine, University of Sao Paulo, Sao Paulo, Brazil; ^16^ Department of Clinical and Toxicological Analyses, Faculty of Pharmaceutical Sciences, University of São Paulo (USP), São Paulo, Brazil; ^17^ Aix Marseille Université, CNRS, INSERM, Centre d'Immunologie de Marseille-Luminy, Marseille, France

**Keywords:** dilated cardiomyopathy, Chagas disease, epigenetic, methylation, Th1 response, transcription factors

## Abstract

Chagas disease, caused by the protozoan *Trypanosoma cruzi*, is an endemic parasitic disease of Latin America, affecting 7 million people. Although most patients are asymptomatic, 30% develop complications, including the often-fatal Chronic Chagasic Cardiomyopathy (CCC). Although previous studies have demonstrated some genetic deregulations associated with CCCs, the causes of their deregulations remain poorly described. Based on bulk RNA-seq and whole genome DNA methylation data, we investigated the genetic and epigenetic deregulations present in the moderate and severe stages of CCC. Analysis of heart tissue gene expression profile allowed us to identify 1407 differentially expressed transcripts (DEGs) specific from CCC patients. A tissue DNA methylation analysis done on the same tissue has permitted the identification of 92 regulatory Differentially Methylated Regions (DMR) localized in the promoter of DEGs. An in-depth study of the transcription factors binding sites (TFBS) in the DMRs corroborated the importance of TFBS’s DNA methylation for gene expression in CCC myocardium. TBX21, RUNX3 and EBF1 are the transcription factors whose binding motif appears to be affected by DNA methylation in the largest number of genes. By combining both transcriptomic and methylomic analysis on heart tissue, and methylomic analysis on blood, 4 biological processes affected by severe CCC have been identified, including immune response, ion transport, cardiac muscle processes and nervous system. An additional study on blood methylation of moderate CCC samples put forward the importance of ion transport and nervous system in the development of the disease.

## Introduction

Chagas disease is a neglected disease caused by the protozoan *Trypanosoma cruzi*. This parasite is endemic in 21 Latin America countries, where it affects around 7 million people through an insect vector, Reduviidae. With migratory flows, this disease can now be found in non-endemic countries (1). The clinical course of the disease comprises an acute phase, mostly asymptomatic, and a chronic phase, where 60% of the patients remain asymptomatic. However, 40% develop symptomatic disease, being 10% megaesophagus/megacolon, and 30% Chagas disease cardiomyopathy (CCC) with varying degrees of severity including refractory heart failure (1). This cardiomyopathy is the main cause of deaths from Chagas disease itself and is one of the most lethal cardiomyopathies (2). Some drugs are effective on *T. cruzi* only during the acute phase, but several side effects have been reported (3). The fact that the biological processes leading to CCC are not yet well understood has impaired the development of efficient therapeutic strategies.

The CCC myocardium displays a diffuse myocarditis with signs of inflammatory infiltrate and heart fiber damage, including significant fibrosis. The inflammatory infiltrate of CCC heart lesions is mainly composed of T cells displaying a Th1-like cytokine profile (4). This exacerbated Th1 response is characterized by a high expression of interferon-gamma (IFN-γ), tumor necrosis alpha (TNF-α) and TBX21 (T-bet) (5). Moreover, our group has previously demonstrated that CCC myocardium presents a unique gene expression profile, distinct from the other dilated cardiomyopathies (5, 6).

Many studies have highlighted the importance of DNA methylation in the regulation of gene expression in dilated cardiomyopathy (7), in particular by the methylation/demethylation of transcription factor binding site (TFBS) located in genes regulatory regions (8). Development of severe CCC is also dependent of epigenetic regulations such as DNA methylation (9), but also involving miRNAs (6, 10) and lncRNAs (11). To get a more complete picture of the epigenomic landscape of CCC myocardium, we performed gene expression analysis (RNA-seq) complemented with a methylation analysis (MethylationEPIC), covering 96% of gene loci, including lncRNA.

## Methods

### Ethical considerations

The protocol was approved by the institutional review boards of the University of São Paulo School of Medicine and INSERM (French National Institute of Health and Medical Research). Written informed consent was obtained from all patients. All experimental methods comply with the Helsinki Declaration.

### Patients and myocardial tissue collection

Human left ventricular free wall heart tissue samples were obtained from patients with end-stage heart failure CCC at the time of heart transplantation (n=8). CCC patients underwent a serological diagnosis of *T. cruzi* infection and standard electrocardiography and echocardiography (12). Biopsies from controls (n=6) were obtained from healthy hearts of organ donors having no suitable recipient, and biopsies for dilated cardiomyopathy (DCM) from end-stage patients (n=8) (**Supplementary Table 1**).

### RNA extraction and sequencing

Total RNAs were extracted from heart tissue samples as previously described (9). Ribosomal RNAs were depleted, and samples were prepared for sequencing according to the Illumina TruSeq RNA Preparation Kit and subjected to pairwise sequencing (2x150bp) with an Illumina HiSeq sequencer. This strategy allows to have information on only the most abundant non-coding RNAs. The RNA-seq data are available under the reference : (GEO accession: GSE191081).

### Quality control and alignment

Raw data quality was verified with FastQC (v0.11.5) and reads were filtered removing the adaptors and low-quality based using Trimmomatic (v0.39). Reads were aligned in paired-end mode on GRCh37 (hg19) human reference genome using STAR (v2.5.4b), and gene quantification was done with featureCounts (v2.0.0). All bioinformatic analyses are available at https://github.com/pbrochet/epiChagas.

### Differential expression analysis

Statistical analyses were performed using R (3.6.2). The *DESeq2* package (v1.26.0) was used for data normalization and differential gene expression analyses (13), using shrinkage function to correct log2 fold change (log2(FC)). Benjamini-Hochberg method was applied to obtain False Discovery Rate (FDR) for each analysis. Genes with an FDR ≤ 0.05 and an absolute log2(FC) greater than 1.5 were considered as differentially expressed (DEG).

### Functional enrichment

Gene Ontology Biological Process annotations enrichment was performed using ClueGO cytoscape plugin and KEGG pathway analysis was done with and GAGE (v2.36.0) (14) and pathview R package (v1.26.0). ncRNAs enrichment was realized with three databases: LncRNA2Target, LncTarD and LncRNADisease.

### Evaluation of cell types in heart tissue

RNAseq deconvolution was performed using ADAPTS R package. Using data coming from heart tissue microarray and PBMC single-cell RNA-seq datasets, signature matrices were generated with ADAPTS. A Wilcoxon test (FDR<0.05) was applied between healthy control and CCC.

### Tissue DNA methylation analysis

DNAs, extracted from whole tissue, were bisulfite converted and amplified with elongation of primers. Amplified DNAs were fragmented and hybridized in EPIC beads according to the protocol described by the manufacturer (Illumina, San Diego, California). Analysis of DNA methylation data was performed with the ChAMP package, using BMIQ for normalization and ComBat for batch-effect correction. Only genomic positions with an FDR<0.05 and a |Δβ|>0.2 were selected. A DMP (Differentially Methylated Position) is associated with a gene when the DMP is inside the gene body or in its promoter region (from gene TSS to 1.5kb upstream). The methylation data are available under the reference : (GEO accession: GSE191082).

### Transcription factor binding site characterization

An analysis of differentially methylated regions (DMR) was done with the ChAMP package, using the DMRCate method, with parameters lambda=400 and C=2. A DMR of interest was defined as a region containing at least 1 DMP located in TSS ([TSS - 1500bp; TSS]), 1st Exon or 5’UTR region of DEGs; and having an FDR ≤ 0.05. In order to identify transcription factor binding sites (TFBS) affected by a difference in methylation, the ReMap database was used (15). A total of 84 cell lines were selected (**Supplementary Table 2**), containing immune and heart-related cells, and including 151 transcription factors (TF). First, the transcription factors specifically associated with DMRs were identified with the OLOGRAM tool (16, 17) in a pairwise analysis, meaning we identify the individual TFs enriched with the DMRs. Only those with an FDR ≤ 0.05 were retained. In a second step, we studied the combinations of those selected TFs that were observed in the DMR by using the n-wise overlap option of OLOGRAM (option -more-bed-multiple-overlap, n ≤ 4). Finally, the known TFBS profiles of identified transcription factor were retrieved from JASPAR database. The location of each TFBS in DMR sequence was identified using FIMO tool.

### Blood DNA collection and DNA methylation analysis

Blood (5 to 15 ml of blood) from 96 CCC patients (48 moderate CCC (Left ventricular ejection fraction>40%) and 48 severe CCC (left ventricular ejection fraction<40%)) and 48 asymptomatic Chagas disease controls was also collected in EDTA tubes (**Supplementary Table 1**). Genomic DNA was isolated using standard salted methods and the methylation analysis was done using the same protocol as tissue DNAs.

## Results

### Headings disregulated genes associated to severe CCC

**Figure 1:**
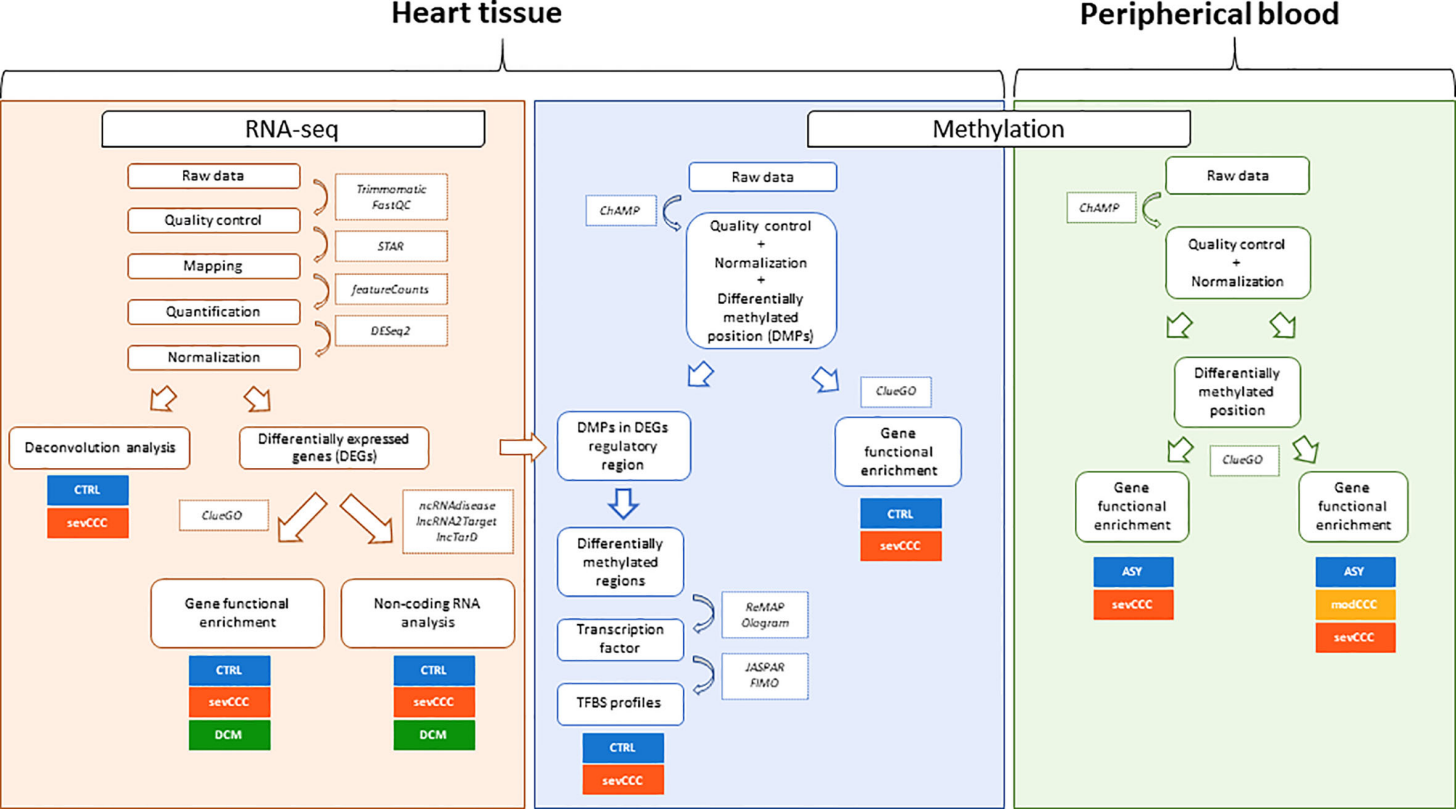
Workflow overview. Heart tissue RNAseq (orange) analysis was performed using classical steps: quality control, alignment, gene expression quantification and normalization. Then, three different analyses were done: deconvolution analysis, differentially expressed genes functional enrichment and non-coding RNAs characterization. Heart tissue (blue) and blood (green) methylation analysis followed the same first steps: quality control, normalization, batch effect correction, differential methylation position (DMP) test and DMPs associated genes functional enrichment. In tissue samples, a transcription factor binding site (TFBS) enrichment was carried out on differentially methylated regions (DMRs).

**Figure 2:**
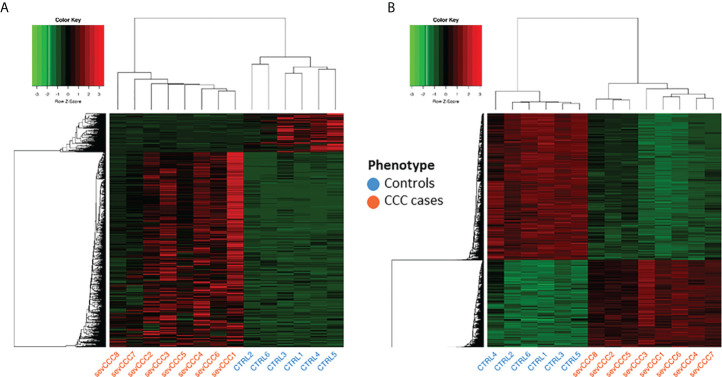
Analysis of samples clustering based on differentially expressed genes or differentially methylated CpG sites. Control samples identifiers are written in blue whereas case samples identifiers are written in red. **(A)** Hierarchical Clustering Analysis (HCA) performed on 6 control and 8 case samples, based on expression of 1409 differentially expressed genes. **(B)** Hierarchical Clustering Analysis (HCA) performed on the same samples as in A), based on methylation level of 16883 differentially methylated position.

Gene expression analysis was conducted on left ventricular free wall myocardial tissue from 8 severe CCC patients and 6 healthy organ donors (see workflow: **Figure 1**). For each sample, we got between 40 and 75 million sequencing reads. Sequences were aligned to the human reference genome GRCh37/hg19. The average mappable rate of the raw reads reached 90% (+/- 2%). No parasite RNA was detected. This is not surprising, since in chronic and end stage patients, the parasite ARN is no longer detected. Gene expression data were obtained from 43533 transcripts. A small fraction of these transcripts (1407/43533 (3.23%)) were considered as differentially expressed between control and CCC (**Supplementary Table 3**), most part being up-regulated (**Supplementary Figure 1A**). A specific enrichment occurs in protein coding and non-coding genes, as miRNAs (**Supplementary Table 4**). PCA and HCA (Hierarchical Clustering Analysis) analysis confirmed that CCC myocardial gene expression patterns were substantially different from controls (**Figure 2A** and **Supplementary Figure 2A**). The sex and the age of the patients have no impact on this clustering (**Supplementary Figures 2B**, **C**).

### Severe CCC is characterized by a strong inflammatory signature not present in DCM

To understand the pathogenic processes driving CCC, a comparative study was conducted between CCC patients and DCM patients. Among the 3188 genes are differentially expressed in DCM, mostly down-regulated (**Supplementary Figure 1B**), only 290 (9%) DEGs are in common with CCCs. (**Supplementary Table 5**). This further emphasizes that the mechanisms involved in CCC and DCM are not similar.

**Figure 3:**
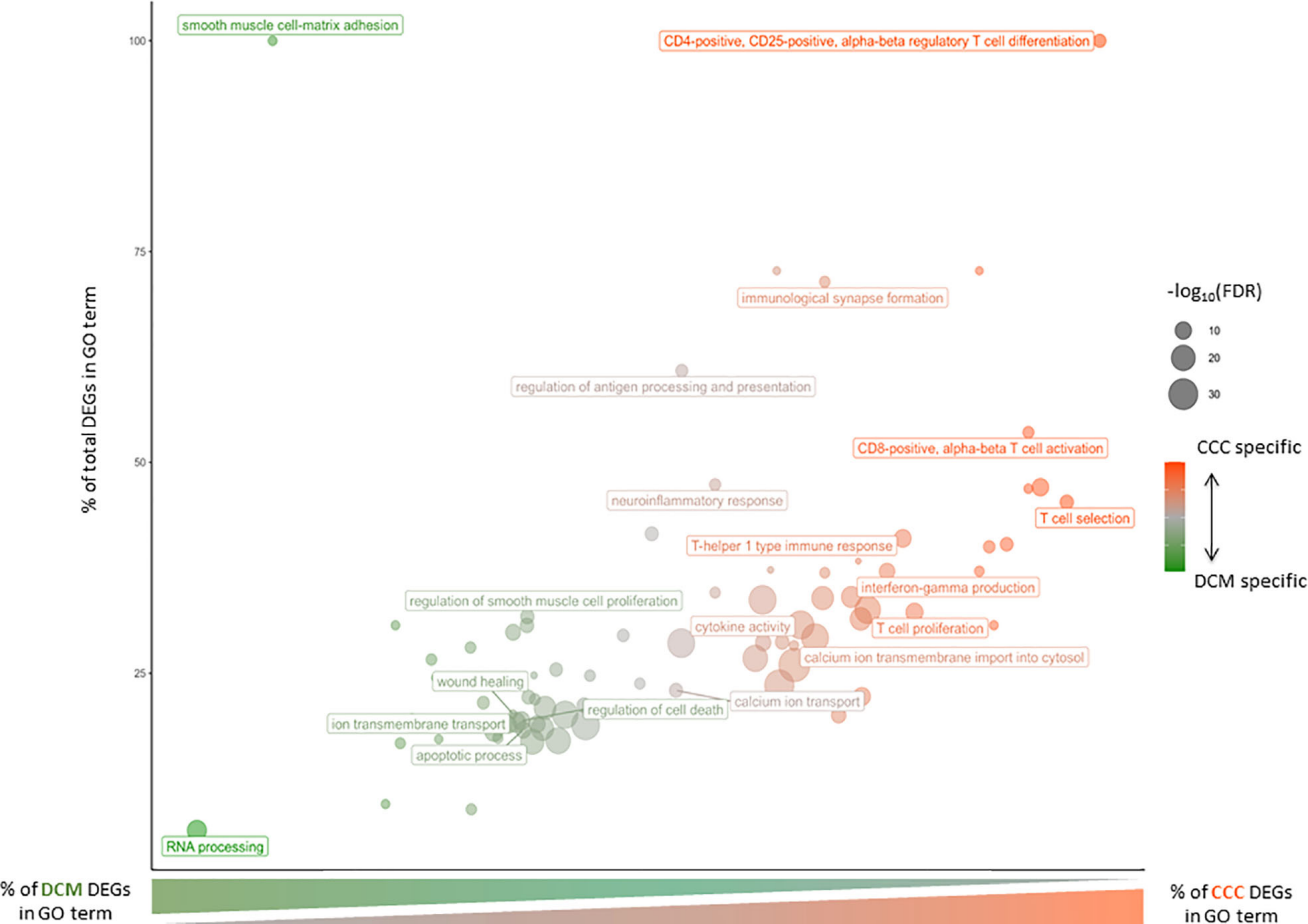
Gene Ontology Biological Process affected in severe CCC and/or DCM. Bubble chart of Gene Ontology Biological Process according to percent of severe CCC differentially expressed genes (DEG) and percent of total DEG (severe CCC + DCM) involved in each GO term. The size of each dot is associated to the enrichment of each GO term [-log_10_(FDR)] and its color to disease specificity (from green for DCM to red for severe CCC).

A functional analysis of the DEGs identified both in CCCs or DCMs was conducted (**Figure 3** and **Supplementary Table 6**). Some biological processes are shared by both diseases, like smooth muscle, ERK1/ERK2 cascade and ion transport. Interestingly, calcium ions are particularly affected in CCC, not in DCM. Regarding CCCs, DEGs are almost exclusively specific to the immune response (innate or adaptive). Most of enriched terms are related to T lymphocytes. More specifically, the T CD8+ and T CD4+ are associated to CCC, as well as Th1 response. Besides T cells, other immune cells seem to act in the pathogenic process of CCC, such as B cells, macrophages or NK cells. The regulation of interleukin production is also affected, included IL-1, IL-4, IL-6, IL-10 and IL-12. The chemokine CXCL9 was the most highly expressed among the cytokines (FC=78), followed by CCL19, IFN-gamma, CCL4, CXCL10, CCL17, CCL22, CXCL11, IL-26, LTA, IL-16 and IL-18. Together, these results provide an overview of the pathogenic process associated with CCCs, which is mostly related to the immune response. Furthermore, calcium ion transport seems to be particularly important in CCCs, compared to DCMs.

### Several non-coding RNA are specific to severe CCC

Non-coding RNAs are among the elements that can lead to genetic dysregulation. As gene expression analysis was performed by sequencing of total RNAs, we got information on non-coding RNAs (lincRNA, miRNA, snRNA, snoRNA, 3’overlapping ncRNA). Among the 3777 non-coding elements detected in our samples, 179, including 19 miRNAs and 145 lncRNAs; were differentially expressed. Those ncRNAs (**Supplementary Figure 3A**) were enough to classify samples according to their phenotype, demonstrating the importance of these non-coding elements in chronic Chagas cardiomyopathy. Only 6 had a known target (lncRNA-targeted gene: MIAT-miR-133a, RP11-276H19.1-GAS1, XIST-WNT1, MIR155HG-miR-155, LINC00707-ELAVL1 and KB-1732A1.1-E2F1). A similar analysis was performed on the ncRNAs associated to DCM (**Supplementary Table 7** and **Supplementary Figure 3B**). 143/179 ncRNAs are specific to CCC (**Supplementary Figure 3C**).

### DNA methylation directly affect the expression of some DEGs

Given the importance of methylation in the regulation of genetic response (18), we also performed several DNA methylation analyze in controls and CCC patients. Tissue sample DNA methylation analysis was conducted on the same samples as gene expression. Only 16883 CpGs (2.34%) were differentially methylated (FDR<0.05 and |Δ𝛃|>0.2) (**Supplementary Table 8**) and were associated to 5814 genes. PCA and HCA analysis confirmed the impact of those DMP on CCC (**Figure 2B** and **Supplementary Figure 2D**). The sex and the age of the patients seems to have no impact on this clustering (**Supplementary Figures 2E**, **F**).

Among the 16883 identified DMPs, 996 DMPs are associated to 390 DEGs. Interestingly, the upstream region of those DEGs (from TSS-1500 to TSS, 1st exon and 5’UTR) are significantly enriched in down-methylated DMPs (FDR ≤ 0.05). Moreover, these down-methylated DMPs are, in 86% of the cases, associated with over-expressed DEGs. We retained these regions called “regulatory region” for further analysis.

### Identification of transcription factors potentially involved in severe CCC

**Figure 4:**
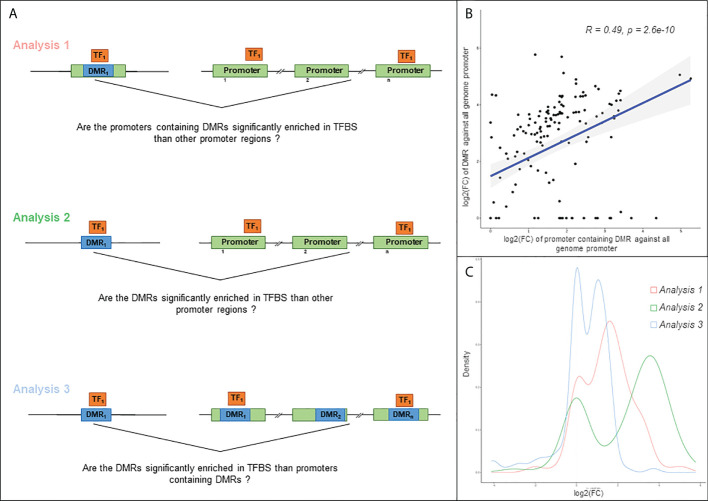
Analysis of the relation between TFBS (Transcription Factor Binding Site). **(A)** Schematic illustration of the three approaches used in this analysis. Differentially methylated region (DMR) is highlighted in blue, gene regulatory region in green, and transcription factor (TF) in orange. Analysis 1: TFBS enrichment in regulatory region containing at least one DMR, compared to all genome regulatory region. For each gene, a regulatory region is defined as the region from TSS-1500 to first exon. Analysis 2: TFBS enrichment in DMR compared to all genome regulatory region. Analysis 3: TFBS enrichment in DMR compared to regulatory region containing at least one DMR. **(B)** Scatter plot of the log2(FC) obtained with the analysis 1 and 2 and Spearman correlation of these values. The fold change is computed according to the observed S value compared to obtained S value, S corresponding to the number of overlapping bases between TFBS and query region. **(C)** Distribution of the log2(FC) obtained with the 3 approaches.

Based on the 409 DMPs associated with DEG regulatory regions, we were able to define 92 regulatory DMRs (**Supplementary Table 9**). These DMRs span on average 245bp and are in the promoter regions of 89 DEGs. Three analyses were performed with OLOGRAM to determine whether the enrichment in TF is significantly different of which would be expected by chance. Two analyses were performed: (A) with the promoters of the DEGs and (B) with the DMRs alone. For both analyses, enrichment was compared with all genomic promoters. (**Figure 4A**). The log2(FC)s of each TF in analysis (A) and (B) are significantly correlated (Spearman p-value ≤ 0.05), but the r2 is low (0.49) (**Figure 4B**) suggesting a different signal carried by the DMRs compared to all the promoters including the DMRs. Moreover, the log2(FC)s obtained in analysis B are significantly higher than the ones obtained in analysis A (Wilcoxon p-value=4.23E-07) (**Figure 4C**). These two analyses clearly show that there is a stronger enrichment of TFs in DMRs than in the promoter set. To confirm these results, a third analysis (C) was performed with the DMRs compared to the promoters of the DEGs containing these DMRs. The obtained distribution of the log2(FC) with this analysis showed two distinct peaks, one around 0 and another around 1.7 (**Figure 4C**), confirming that some TFs are specifically enriched in DMRs. A total of 30 TFs were found significantly associated to the DMRs in the analyses B and C. We also considered as a Cis-Regulatory Module (CRMs) the regions where at least 2 TFs bind to the genome according to ReMap. After data filtering and considering combinations of up to 4 TFs, we have identified 16 regions significantly associated with our DMRs, involving a total of 12 transcription factors (**Supplementary Table 10**). The top-regulators are BRD4, EED, BCLAF1, TBX21, RUNX3 and RUNX1.

### Immune, heart-relative or neurological processes affected by TFBS methylation

**Figure 5:**
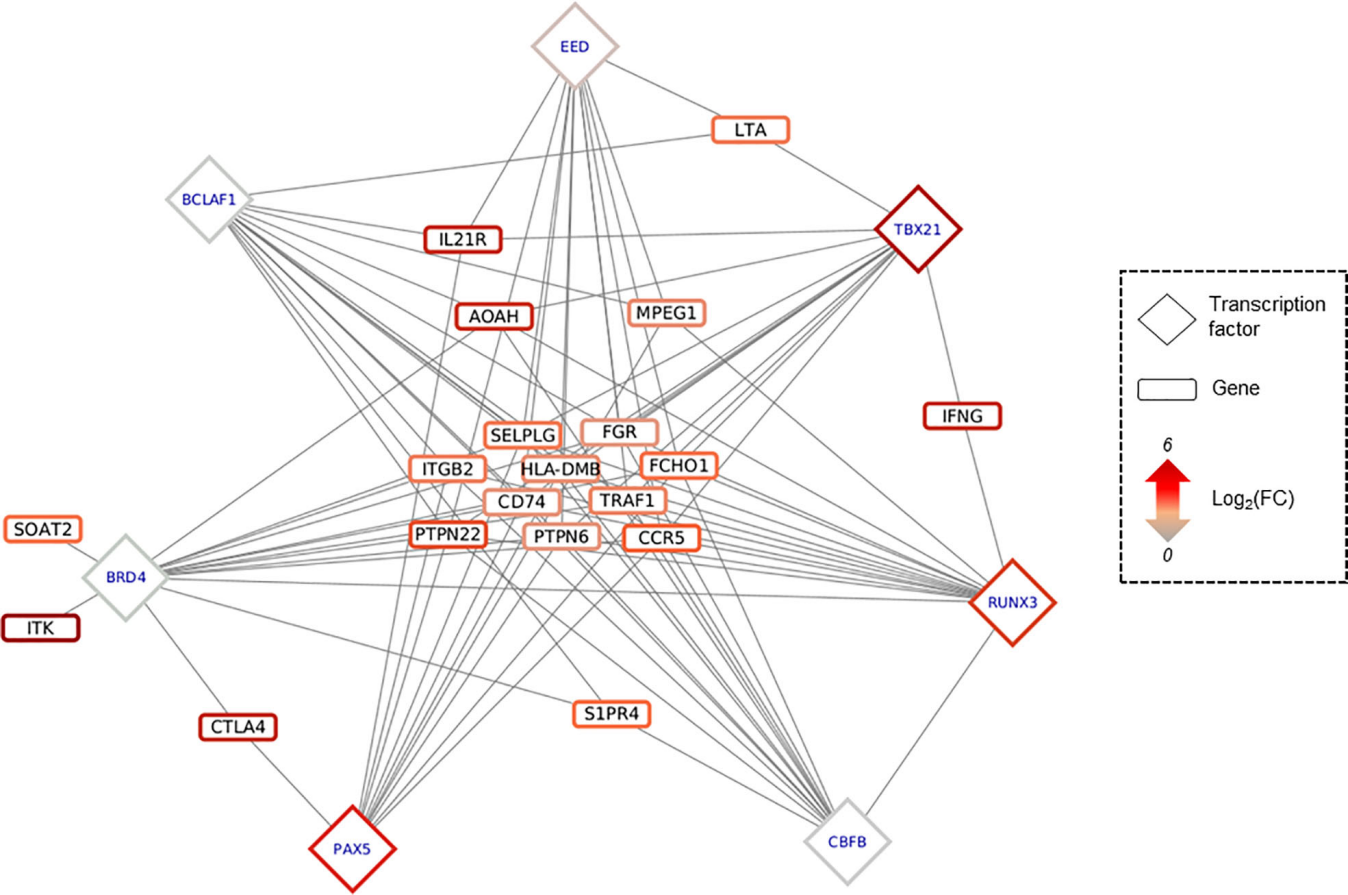
Predicted regulatory interaction in IFNy-Th1 pathway. Network composed by 19 genes involved in IFNy-Th1 pathway, and the top 7 TF predicted as targeting those 19 genes, according to *OLOGRAM* based on *ReMap* database. TF are written in blue in diamond, and genes in black in rectangle. Shapes borders are colored according to the fold change, from green to red.

The 30 TF previously identified are involved in several biological process, such as somatic recombination of immunoglobulin gene segments, regulation of cardiac muscle tissue growth or peripheral nervous system neuron development (**Supplementary Table 11**). Among the genes involved in the Th1/IFN-γ response, 19 genes are potentially targeted by 28 of the 30 TF previously identified, illustrating the important differential methylation of immune response-related genes in the pathogenic process associated with CCC. Seven TFs (BCLAF1, BRD4, CBFB, EED, PAX5, RUNX3 and TBX21) target at least 11/19 genes (**Figure 5**). A few of them are targeted by specific TFs (for example IFN-γ is targeted RUNX3+TBX21). Similar data were obtained for heart-relative or neurological process (**Supplementary Figure 4**).

### 5 TFBS identified in RUNX3 regulatory region

Of the 30 TFs of interest, 20 have at least one known motif in the Jaspar database, providing a total of 45 distinct motifs. After filtering predicted TFBS in our DMR overlapping at least one DMP, 423 TFBS have been identified, for all the 20 TFs (**Supplementary Table 12**). Interestingly, TBX21, RUNX3 and EBF1 are the TFs whose binding motif appears to be affected by DNA methylation in the largest number of genes. Because these TFs are involved in many complexes, regulation of their binding may affect the binding of all TFs in the complexes.

**Figure 6:**
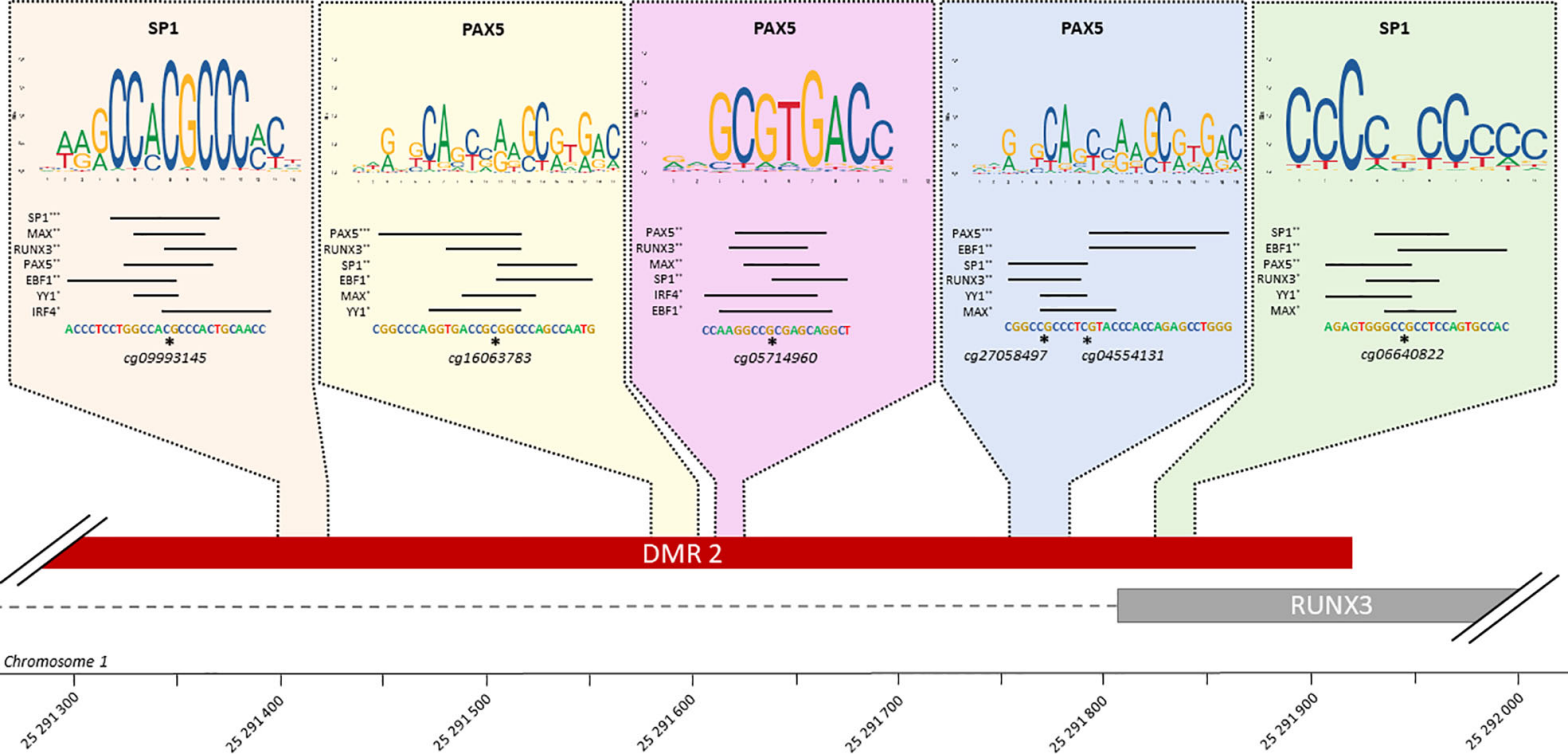
TFBS affected by methylation in RUNX3 regulatory region. Schematic representation of all TFBS found in RUNX3 regulatory region, using FIMO and Jaspar database. For each TFBS region, all the transcription factor predicted as affected by a differentiation of methylation in this region are rank by FIMO pvalue (***pvalue ≤ 0.001, **pvalue ≤ 0.01, *pvalue ≤ 0.05). The top-rank TF binding profile is shown, as well as the differentially methylated position in the TFBS.

Among the 25 genes with a low number of TFs binding in their promoter region (n ≤ 3), and thus being affected by specific TFs, 7 are targeted by TBX21 and 4 by RUNX3, showing again the importance of these TFs in CCC. Considering that RUNX3, a key regulator in CCCs, also has a DMR in its promoter region, further analysis was performed on this TF. On this 831 bp DMR, 5 TFBSs are present (**Figure 6**). This DMR is targeted by at least 6 of the following 7 TFs: RUNX3, PAX5, YY1, SP1, MAX, EBF1 and IRF4. SP1 and PAX5 seem to be the most affine with these sequences. PAX5 is always found associated to IRF4, suggesting an interaction between those 2 TFs.

### Immune cell type infiltration occurs in CCC heart tissue

Given the infiltration of immune cells in CCC myocardium, we characterized and quantified the proportions of these cell types in our samples. First of all, immune cell signatures (19) showed in general a higher proportion of immune cells in CCCs compared to controls (**Supplementary Figure 5A**), such as activated NKs, and more interestingly T CD8, T Cell memory and T follicular helper, with a reduced proportion of M2 macrophages. Secondly, a human heart tissue (left ventricle) cell signature (20) showed that the CCCs had fewer cardiomyocytes and smooth muscle than the controls (**Supplementary Figure 5B**). Moreover, CCC myocardium had a higher proportion of macrophages, in line with the immune cell infiltration in the cardiac tissues on CCC patients (**Supplementary Table 13**).

### Methylation sites in blood are associated to moderate or severe CCC

**Figure 7:**
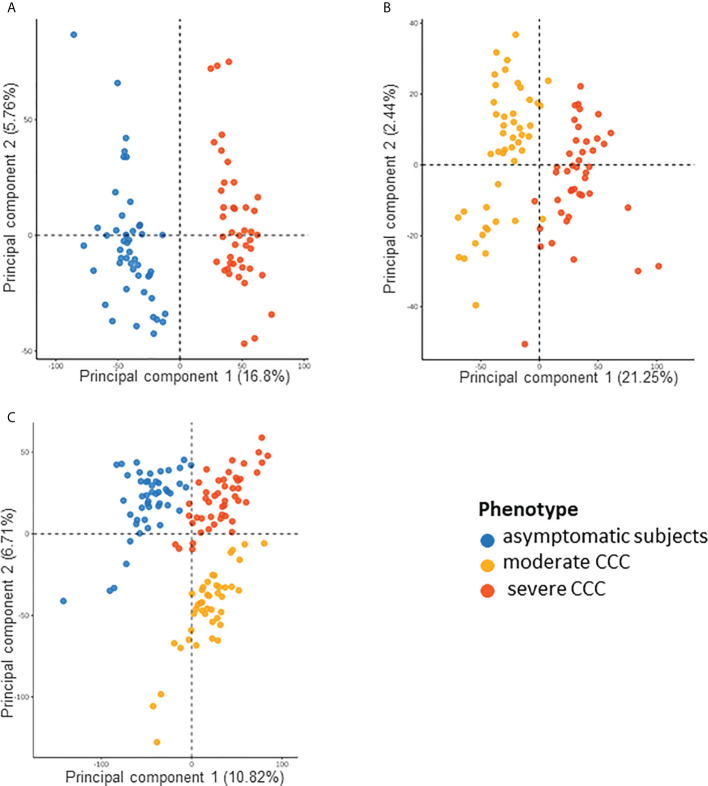
Analysis of samples distribution in the space of differentially methylated CpG sites for asymptomatic, moderate CCC and severe CCC samples. **(A)** Scatterplot of the two principal components of a PCA executed in the space of the 12624 CpG positions differentially methylated (DMP) between 48 asymptomatic blood samples and 90 CCC blood samples. **(B)** Scatterplot of the two principal components of a PCA executed in the space of the 6735 DMPs between 47 moderate CCC blood samples and 43 severe CCC blood samples. **(C)** Scatterplot of the two principal components of a PCA executed in the space of the 18889 CpG positions (union of the two previous sets) for the three groups of samples.

We also studied the DNA methylation in the blood of asymptomatic, moderate and severe CCC by hypothesizing that the blood data reflect the phenotype. We found 12624 DMPs between asymptomatic and severe CCC blood samples (FDR ≤ 0.05) (**Supplementary Table 14**). Despite the small variation in the level of DNA methylation detected in the blood (Δβ), the methylation of these 12624 DMPs was enough to separate controls from cases, either *via* PCA (**Figure 7A**) or HCA (**Supplementary Figure 6A**). Association was found in 6436 genes with at least one DMP, but only 139 genes are in common between the three analyses (RNA-seq in tissue and DNA methylation in tissue and blood).

6735 CpGs were found as DMPs between moderate and severe CCC (FDR<0.05) (**Supplementary Table 15**). They were enough to discriminate samples according to the stage of the disease on a PCA (**Figure 7B**) or HCA (**Supplementary Figure 6B**). Only 470 DMPs were also identified as DMP in controls vs severe CCC blood methylation analysis, and 1750 genes out of 3911 (44.75%) are shared by both blood methylation analysis (non-significant enrichment). Interestingly, looking at the DNA methylation level of all DMPs found in blood (merge of the two previous analysis, n=18889) allowed us to distinguish the three groups of individuals, according to their phenotype (**Figure 7C**), revealing a gradient of methylation from controls to severe CCC through moderate CCC.

### Nervous system related processes are strongly affected in moderate CCC

**Figure 8:**
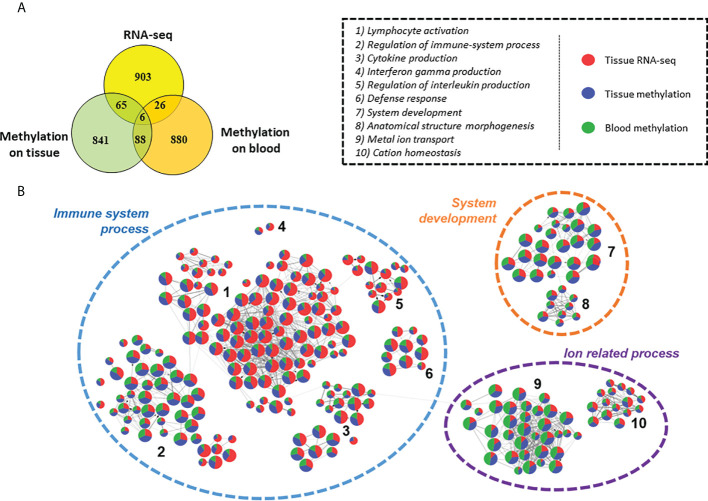
Comparison of differentially expressed genes, genes affected by methylation in tissue dataset and genes affected by methylation in blood dataset. **(A)** Venn diagram of top 1000 genes differentially expressed or methylated in previous tissue RNA-seq, tissue DNA methylation and blood DNA methylation analysis between control/asymptomatic and severe CCC samples. **(B)** Graph of the Gene ontology Biological Processes analysis of dysregulated element between control/asymptomatic and severe CCC. Nodes represents biological processes terms and are divided in 3 colors, according to the proportion of genes from RNA-seq (red), tissue methylation (blue) or blood methylation (green) analysis. Edges in the graph link GO terms having gene in common. 3 principal terms are highlighted in this synthesis. More precisely, several groups of gene ontology are enriched, involved in biological process related to: 1) lymphocyte activation; 2) Regulation of immune-system process; 3) Cytokine production; 4) Interferon gamma production; 5) Regulation of interleukin production; 6) Defense response; 7) System development; 8) Anatomical structure morphogenesis; 9) Metal ion transport; 10) Cation homeostasis.

The general pattern detected on heart tissues and on blood are different and seemed to be tissue/fluid specific (**Figure 8A**). However, we conducted a Gene ontology analysis of the top 1000 genes of each analysis (**Supplementary Table 16**). 3 major biological processes are affected in our analyses: immune system, system development and ion transport (**Figure 8B**). They are shared by all three analyses, showing that although few genes are found in common, they are involved in common biological functions. Therefore, it seemed reasonable to analyze the methylation differences between moderate and severe CCC to understand the development of the disease (**Supplementary Table 17**). Unlike to the results found between controls and severe CCC, here genes are mostly involved in neurogenesis, cardiovascular system development or actin filament organization. These genes are associated with the immune response, notably in adaptive immune response and also with ion-related processes.

## Discussion

This study focused on the study of epigenetic regulation in Chagas disease, and its impact on the development of the disease. Based on RNAseq and methylation data, 1409 differentially expressed genes (DEGs) were identified, among which 89 have differentially methylated regions (DMRs) in their regulatory region. 30 transcription factors were identified as potentially affected by these methylation differences, suggesting their involvement in genetic deregulation. Furthermore, similar biological processes are affected by differences in methylation in cardiac tissue or blood. Therefore, the pathogenic process in patients with moderate CCC could be studied.

In a previous analysis, combining microarray and methylation data, we showed that patients with CCC had significant differences in gene expression and DNA methylation compared to healthy controls (9). DEGs with DMPs were predominantly associated with the immune response, but also with several biological processes such as arrhythmia, muscle contraction, fibrosis and mitochondrial function. Here, we have set up a more advanced analysis, allowing us to have an in-depth analysis of genomic dysregulation. To do so, we were interested in both DNA methylation in order to identify transcription factors involved in the pathogenic process and in non-coding RNAs. Indeed, non-coding RNA, notably lncRNA and miRNA, are known to be involved in many of cardiovascular diseases (21, 22). The presented results extend the list of miRNAs associated to CCC, and not differentially expressed in DCM (10, 21, 23–27). Among the 19 miRNAs identified in this study, 3 have already been associated with severe CCC: miR-223, miR-208 and miR-151 (6). The involvement of the long non-coding RNA MIAT, previously associated to CCC (11), has also been confirmed. It acts as a sponge of miRNA-133 in breast cancer, dysregulated in CCC (10), and down-regulated in patients with heart failure (28).

Three distinct biological process were associated to CCC, either we look at gene dysregulation or methylation, in heart tissue or blood: immune response, ion transport and nervous system. Although ion transport and nervous system were also partially associated to DCM, immune response remains specific to CCC. Moreover, the genes affected in those pathways were not the same between these diseases, illustrating a specific response in CCC. For example, several genes associated to ion transport, and more precisely with potassium voltage-gated ion channel, like KCNA2, KCNA5, KCNAB2, KCNB2, KCNC2, KCNG3 and KCNN4, were found to be differentially expressed and/or methylated in CCC, and not in DCM. Of interest, while KCNB2, KCNC2 and KCNG3 are mainly expressed in neurons, KCNA5 is expressed in heart muscle and dendritic cells, while KCNA2, KCNAB2 are expressed in neurons and immune cells, indicating that potassium channels of neuronal, cardiomyocyte and inflammatory cells are modulated in CCC heart tissue. Dysregulation of potassium channel genes were reported in heart of 36 *T.cruzi* infected mice (21) and 25 severe CCC heart (9). Dampening of calcium and potassium ion channels in mouse heart tissue led to a reduced Ca2+ release and prolongation of action potential (29). TNF-α may amplify this dysfunction by inducing nitric oxide synthase (NOS2) and oxidant species, which promotes electrophysiological changes in rat ventricular myocyte (30). KCNN4 (KCa3.1), differentially expressed in CCC (not in DCM) and containing DMP in its promoter, acts as a regulator of membrane potential in T cells. After antigen recognition by the T cell repertoire, the Ca2+ enters in the cytosol and sequentially activate KCa3.1 and Kv1.3 (KCNA3), causing membrane depolarization (31). This calcium, present in cytosol, will activate the NFAT protein, which regulate genes involving in T cell activation (32). Moreover, high levels of Kv1.3 were found in multiple autoimmune diseases, such as multiple sclerosis or diabetes (33–35). In our data, these two potassium channels are up-regulated, and KCa3.1 seems to be targeted by RUNX3. The potential biomarker for heart disease Galectin-3 (Gal-3), upregulating the expression and activity of KCa3.1 channel in inflammatory cells and fibroblasts. Upregulated KCa3.1 facilitates inflammatory cell infiltration into the myocardium and fibroblast differentiation into activated form. Recently, it was shown that mice treated with TRAM-34, a KCa3.1 channel-specific inhibitor, either for 1- or 2-month period effectively reduced collagen deposition (36). Moreover, Gang et al. had shown that KCa3.1 inhibition by TRAM-34 therapy attenuated the increased inflammatory cell infiltration. Rezende de Castro et al. (37) studied gene expression profiling in acutely *T.cruzi* infected mice (15-day post-infection) and have found enriched pathways related to immunity and Th1 T cell immunity, cardiac conduction including potassium channels, protein synthesis and mitochondrial genes. Even though acute infection by *T.cruzi* (where parasites are very numerous in the myocardium) is not a perfect match for CCC, where parasites are scarce or absent from heart tissue, modulation of inflammatory cell and potassium channel gene expression profiles are similar in both situations.

On the other hand, our study underlined that several genes associated with moderate or severe CCC are involved in the nervous system. This enrichment is even stronger in moderate CCCs. Actually, the loss of neuron cells is a well-known phenomenon in digestive forms of Chagas disease (38, 39). In human hearts with dilated cardiomyopathy, the number of neuron cells is significantly reduced compared to controls. This neuronal depopulation is even more important in CCC (40), and during the acute phase in the mouse model (41). Similarly, a study showed that acute phase *T.cruzi* infected dogs displayed a rarefaction of the noradrenergic and acetylcholine nerve terminals accompanied by myocarditis, and the ventricles sympathetic denervation was present when the inflammatory process was moderate to intense (42). Moreover, in the rat model, moderate myocarditis lasting for two weeks caused complete denervation (43). A more recent study demonstrated that knockdown of acetylcholine in mice increased the Th1 response and clearance of *T.cruzi* parasitism in blood and tissue, but worsens the cardiac lesions and inflammatory infiltration (44). In the continuity of these observations, the increase in the amount of acetylcholine was associated with reduction of heart weight, inflammatory infiltration, and the fibrosis area, suggesting a reversing of cardiac hypertrophy (44). Moreover, macrophages, Th1, CD4 and B cells express adrenergic receptors (45–47). Their stimulation induces an increase of cAMP, which inhibits NF-kB activation, leading to the suppression of type 1 pro-inflammatory cytokines and promoting the production of type 2 anti-inflammatory cytokines, such as IL10. This cytokine inhibits the antigen presenting capacity of macrophages and dendritic cells, and thus the differentiation of CD4 T cells to Th1 (48). Finally, circulating antibodies binding acetylcholine and norepinephrine receptors have been found in Chagas patient serum, suggesting an autoimmune response (49), and correlated with the previous study, inducing the lack of neurotransmitters, and then the over production of Th1 cells. In our data, the genes related to acetylcholine production, or acetylcholine transporter are not differentially methylated or expressed in tissue samples according to fold change cut-off but present an FDR<0.05. In blood methylation samples, ACHE (acetylcholine production), and CHRNA3, CHRNA4 and CHRNA7 (acetylcholine receptors) genes have differentially methylated positions in the promoter or body. While CHRNA4 is mainly expressed in neurons, ACHE is found in neurons and cardiac/skeletal muscle, CHRNA7 is mainly expressed in neurons, cardiac/skeletal muscle and immune cells, and CHRNA3 is found in neurons, and thymus.

Both potassium voltage-gated ion channel and nervous system identified in the current and previous studies seem to be linked to immune response, and more precisely T-cell related process. Moreover, in our data, the immune response was strongly stimulated, especially the activation of T cells even in the absence of the parasite. This inflammatory infiltrate was already reported elsewhere (50) and is mainly composed of Th1 lymphocytes, macrophages, and NK-cells. In this work, we performed a deconvolution analysis on our heart tissue collection, and we confirmed the content of this infiltrate. Our group, among others, had already shown that the T-cell infiltrating heart tissue strongly produced IFN-γ and TNF-α (4). In parallel, lower quantities of IL-2, IL-4, IL-6 and IL-10 were detected in CCC heart tissue (51). Our results are consistent with current knowledge of the pathology, with high expression of genes involved in IFN-γ and cytokine production. According to Gene Ontology Biological Process enrichment, genes affected in CCC are also involved in interleukin production, including IL-2, IL-4 and IL-6. IFN-γ, which has been described simultaneously as a pathogen resistance and a disease tolerance gene is also acting as an upstream regulator (52). Indeed, IFN-γ stimulates the inflammatory response indirectly *via* the NF-κB pathway and activate the production of ROS and NOS, which, in an excessive quantity, have also deleterious effect on mitochondria and cardiomyocytes (5). Interestingly, IFN-γ, with IFN-β and TNF-α (53), induced expression of miR-155 (53). In our data, MIR155HG, coding for this miRNA, is up-regulated. miR-155 is also up-regulated in viral myocarditis; where it is expressed by infiltrating immune cells, and seems to be involved in TNF-α, IFN-γ and IL-6 production, as well as immune cell infiltration (54). The lack of this microRNA seems to decrease IFN-γ and TNF-α in the acute stage of Chagas cardiomyopathy in mice heart tissue (21). Moreover, we found a DMR in IFN-γ promoter region, targeted TBX21. According to Ologram, RUNX3 could also fix this DMR, but its TFBS wasn’t found in this DMR, as described in JASPAR database. Those two transcriptions factors are involved in Th1 differentiation, as well as GATA3 (55, 56). According to our analysis, the micro-RNA miR-142, associated to Th1 differentiation (57) in neuronal autoimmune disease, potentially targets RUNX3 and TBX21. In Chagas disease, TBX21 and IFN-γ expression are correlated with the left ventricular dilation, and the ratio between TBX21 and GATA3 expression is significantly higher in CCC than in non-inflammatory cardiomyopathy (58), which were also confirmed in our data in both control to CCC or DCM comparisons. Moreover, RUNX3 overexpression has been associated to the methylation of its promoter in CCC (9), as well as in our current analysis. In total, RUNX3 and TBX21 targets 29 genes, including 14 in common (AIM2, ARHGAP30, BATF, C16orf54, CCR5, CYTIP, DENND2D, DOK2, FGR, MIR142, PTPN22, TRAF1, TRAF3IP3 and WDFY4). Moreover, RUNX3 is targeted by 7 transcription factors: EBF1, IRF4, MAX, PAX5, RUNX3, SP1 and YY1.

We also found that XIST is one of the tops up-regulated lncRNA in Chagas disease. It is associated with the WNT1 gene, belonging to the Wnt family, but because of the wide sex dispersion in our dataset, we cannot conclude the direct involvement of this lncRNA in CCC. In our data, WNT1, as well as WNT10A and WNT10B are up-regulated in severe CCC. The Wnt pathway is involved in the differentiation of Th2 cells (59) and its inhibition in acute Chagas disease stage decrease the Th2 response (60) and increase inflammatory response, controlling the parasite (61). Studies have demonstrated that Wnt activation is related to pathological stages including inflammation, angiogenesis, and fibrosis and aberrant expression is associated with cardiovascular diseases (61, 62). Indeed, Analysis of the expression of Wnt proteins indicated that Wnt-2, Wnt-4, Wnt-10b, and Wnt-11 were significantly upregulated 5 days after myocardial infarction (63). Studies confirmed the interactions between the Wnt pathway and TGF-β signalling (64–66). TGF-β receptor overexpression has already been observed in acute phase of CCC (67). It has several role in CCC development, including parasitic invasion, inflammation, immune response, heart fibrosis and heart conduction (68). Our results had also shown the potential implication of NOTCH1 (downregulated and targeting DEGs with DMRs in their regulatory regions) in the genetic dysregulation in severe CCC. Activation of Notch signalling limits the range of cardiac damage by the improving of angiogenesis (69–71), cardiac regeneration and cardio protection (72), and reducing fibrosis (73), apoptosis (74), and oxidative stress (75).

Few studies have linked Notch signaling to immune response and parasitic infections. Tu et al. has shown that in *Trichuris muris* infection, a deficiency of Notch signaling in T cells led to a failure in initiating the Th2 response (76). It suggests that Notch signaling influences the Th2 profile and alters immune responses against parasites. Similarly, during Leishmania major infection, the Th1 response was induced in deficient mice, which presented high levels of IFN-ɣ that led to infection control (76). So, we can raise the hypothesis that a down expression of NOTCH1 in CCC patients promote the Th1 response associated to severe cardiomyopathy. To support this hypothesis, Guzmán-Rivera et al. had treated Chagas infected mice with simvastatin (77). Simvastatin activates the Notch 1 pathway in the hearts of *T.cruzi* infected mice and decreases the cellular infiltrate, inflammatory cytokines and prevents the increase in collagen deposition in cardiac tissue.

All the results obtained in our study converge towards a combined involvement of processes related to the immune response, ion transport, cardiac contraction and the nervous system. In particular, the nervous system appears to be strongly impacted between moderate and severe CCC, and the potassium-related process specific to CCC compared to DCM. Gene expression analysis alone has revealed some non-coding elements but has not provided so much new information about the disease. The inclusion of methylation put forward less obvious biological processes. Thus, most of the identified genes (differentially expressed and/or methylated) are generally involved in several of these processes, highlighting links between them. More precisely, all the previously described biological process seems to be linked to the immune response, and notably to the Th1 response, including IFN-γ and RUNX3. These results, combined with those obtained in previous analyses of CCC (9, 78), confirm the importance of DNA methylation in the development of CCC.

## Data availability statement

The datasets presented in this study can be found in online repositories. The names of the repository/repositories and accession number(s) can be found in the article/**Supplementary Material**.

## Ethics statement

The protocol was approved by the institutional review boards of the University of São Paulo School of Medicine and INSERM (French National Institute of Health and Medical Research). Written informed consent was obtained from all patients. All experimental methods comply with the Helsinki Declaration.

## Author contributions

All authors listed have made a substantial, direct, and intellectual contribution to the work and approved it for publication. Study design: PB, JK, LS, ECN, CC. Phenotype characterization: BMI, CM, RHBS, SS, ANS, PP, AIF, EAB, CWP, BS, FCD, MFS, FAG, JAMN, AF, SS, GDLP, FB, PB, HTLW, AS, MM, MHH, EAD, ACP, VRJ, Experimental analysis: PB, LL, AFF, JPSN, PCT, LRPF, AK, SC, DDSC, RCFZ, VOCR, RRA. Statistical analysis: PB, QF, DP, LS, ECN, CC. Manuscript preparation: PB, LS, ECN, CC.

## Funding

This work was supported by the Institut National de la Santé et de la Recherche Médicale (INSERM); the Aix-Marseille University (grant number: AMIDEX “International_2018” MITOMUTCHAGAS); the French Agency for Research (Agence Nationale de la Recherche-ANR (grant numbers: “Br-Fr-Chagas”, “landscardio”); the CNPq (Brazilian Council for Scientific and Technological Development); and the FAPESP (São Paulo State Research Funding Agency Brazil (grant numbers: 2013/50302-3, 2014/50890-5); the National Institutes of Health/USA (grant numbers: 2 P50 AI098461-02 and 2U19AI098461-06). This work was founded by the Inserm Cross-Cutting Project GOLD. This project has received funding from the Excellence Initiative of Aix-Marseille University - A*Midex a French “Investissements d’Avenir programme”- Institute MarMaRa AMX-19-IET-007. JPSN was a recipient of a MarMaRa fellowship. EC-N and JK are recipients of productivity awards by CNPq. The funders did not play any role in the study design, data collection and analysis, decision to publish, or preparation of the manuscript.

## Acknowledgments

Center de Calcul Intensif d’Aix-Marseille is acknowledged for granting access to its high performance computing resources. Authors thanks Delphine Potier, Pierre Milpied, Benoit Ballester for helpful discussions and comments.

## Conflict of interest

The authors declare that the research was conducted in the absence of any commercial or financial relationships that could be construed as a potential conflict of interest.

## Publisher’s note

All claims expressed in this article are solely those of the authors and do not necessarily represent those of their affiliated organizations, or those of the publisher, the editors and the reviewers. Any product that may be evaluated in this article, or claim that may be made by its manufacturer, is not guaranteed or endorsed by the publisher.

## Supplementary material

Supplementary Table 1 Biological samples included in this study.

Supplementary Table 2 Cell lines description.

Supplementary Table 3 List of unique differentially expressed genes identified by RNA-seq between 8 end stage CCC heart tissue samples and 6 heart tissue samples obtained from organ donors.

Supplementary Table 4 Statistics on coding sequences detected by RNA-seq and on differentially expressed genes.

Supplementary Table 5 List of unique differentially expressed genes identified by RNA-seq between 8 DCM heart tissue samples and 6 heart tissue samples obtained from organ donors.

Supplementary Table 6 Gene ontology analysis based on differentially expressed genes between control and severe CCC and/or between control and DCM.

Supplementary Table 7 List of differentially expressed ncRNAs between control and severe CCC, or between control and dilated cardiomyopathies.

Supplementary Table 8 List of the Differentially Methylated CpGs on heart tissues samples (controls versus CCC).

Supplementary Table 9 List of the regulatory DMRs associated to the 89 DEGs.

Supplementary Table 10 List of transcription factor and transcription factor complexes testing against our DMRs.

Supplementary Table 11 Gene ontology analysis of the TFs binding our DMRs.

Supplementary Table 12 For each DMR, list of predicted Transcription Factor Binding Site (TFBS) and their associated Transcription Factor (TF).

Supplementary Table 13 Cell type proportions in RNA-seq by comparison to cell type signatures.

Supplementary Table 14 List of the Differentially Methylated CpGs between blood asymptomatic samples and blood CCC samples.

Supplementary Table 15 List of the Differentially Methylated CpGs between blood moderate CCC samples and blood severe CCC samples.

Supplementary Table 16 Gene ontology analysis based on differentially expressed genes between control and severe CCC and/or genes affected by at least one differentially methylated CpGs between tissue control and severe CCC and/or genes affected by at least one differentially methylated CpGs between blood asymptomatic and severe CCC samples.

Supplementary Table 17 Gene ontology analysis based on genes affected by at least one differentially methylated CpGs between blood moderate CCC samples and blood severe CCC samples.

Supplementary Figure 1 Volcanopots of differential expression testing for CCC and DCM. Volcanoplot showing the difference of gene expression between **(A)** controls and CCCs or **(B)** controls and DCMs. On the x-axis is represented the log2(FC) and on the y-axis the -log10(adjusted pvalue). Overexpressed DEGs are in red, and underexpressed in green.

Supplementary Figure 2 Analysis of samples distribution in the space of differentially expressed genes or differentially methylated CpG sites between control and severe CCC. Age and sex impact on gene expression or on methylation. **(A)** Scatterplot of the two first principal component of a PCA of 6 Control and 8 Case samples executed in the space of the 1409 differentially expressed genes between the two conditions. **(B)** Scatterplot of the two first principal component of a PCA of 6 Control and 8 Case samples executed in the space of the 1409 differentially expressed genes between the two conditions colored by sex (blue: male, pink: female). **(C)** Scatterplot of the two first principal component of a PCA of 6 Control and 8 Case samples executed in the space of the 1409 differentially expressed genes between the two conditions colored by age (from grey to blue). **(D)** Scatterplot of the two first principal component of a PCA of 6 Control and 8 Case samples executed in the space of the 16883 differentially methylated CpG sites between the two conditions. **(E)** Scatterplot of the two first principal component of a PCA of 6 Control and 8 Case samples executed in the space of the 16883 differentially methylated CpG sites between the two conditions colored by sex (blue: male, pink: female). **(F)** Scatterplot of the two first principal component of a PCA of 6 Control and 8 Case samples executed in the space of the 16883 differentially methylated CpG sites between the two conditions colored by age (from grey to blue).

Supplementary Figure 3 ncRNA analysis in severe CCC and DCM. Control samples identifiers are written in blue, severe CCC samples identifiers in red and DCM samples in green. **(A)** Hierarchical Clustering Analysis (HCA) performed on 6 control and 8 severe CCC samples, based on expression of 179 differentially expressed ncRNAs. **(B)** Hierarchical Clustering Analysis (HCA) performed on 6 control and 8 DCM samples, based on expression of 327 differentially expressed ncRNAs. **(C)** Venn diagram of shared differentially expressed ncRNAs between severe CCC and DCM.

Supplementary Figure 4 Predicted regulatory interaction in cardiac muscular or nervous system process. Network composed by 6 transcription factors (TF) involved in cardiac muscular and/or nervous system process, and their 68 targeted genes, predicted by *OLOGRAM* according to *ReMap* database. TF are written in blue in diamond, and genes in black in rectangle. Shapes borders are colored according to the fold change, from green to red.

Supplementary Figure 5 Estimation of cell proportion in control and severe CCC heart tissue. Deconvolution of the RNA-seq bulk gene expressions of 6 control and 8 severe CCC samples to infer the proportion of cells in the samples. The * represent cell types whose proportion is significantly different (Wilcoxon test, FDR <0.05) between controls and cases. **(A)** Deconvolution using cell type signature with 22 immunological cell types (LM22 signature matrix). **(B)** Deconvolution using cell type signature with 5 left ventricle related cell types.

Supplementary Figure 6 Analysis of samples clustering based on differentially methylated CpG sites in blood samples. Asymptomatic samples identifiers are written in blue, moderate CCC in orange and severe CCC in red. **(A)** Hierarchical Clustering Analysis (HCA) performed on 48 asymptomatic and 90 severe CCC samples, based on expression of 12624 differentially methylated position. **(B)** Hierarchical Clustering Analysis (HCA) performed on 47 moderate CCC and 43 severe CCC samples, based on methylation level of 6735 differentially methylated position.
