# Supplementary material for: Epigenetic regulation of transcription factor binding motifs promotes Th1 response in Chagas disease cardiomyopathy
Source: Front Immunol. 2022 Aug 22;13:958200. doi: 10.3389/fimmu.2022.958200 (PMC9441916; doi:10.3389/fimmu.2022.958200)
Supplement: Supplementary Table 1 — Biological samples included in this study. [file DataSheet_1.zip › Supplementary Material/Supplementary Table 1.pdf]

Supplementary table 1. Biological samples included in the study.

ID	Origin	Phenotype	Age	Sex	EF
**CTRL1**	Heart tissue	CTRL	-	M	-
**CTRL2**	Heart tissue	CTRL	25	M	-
**CTRL3**	Heart tissue	CTRL	22	M	-
**CTRL4**	Heart tissue	CTRL	28	M	-
**CTRL5**	Heart tissue	CTRL	17	M	-
**CTRL6**	Heart tissue	CTRL	-	M	-
**DCM1**	Heart tissue	DCM	-	-	-
**DCM2**	Heart tissue	DCM	-	-	-
**DCM3**	Heart tissue	DCM	29	M	-
**DCM4**	Heart tissue	DCM	15	M	-
**DCM5**	Heart tissue	DCM	58	F	-
**DCM6**	Heart tissue	DCM	56	M	-
**DCM7**	Heart tissue	DCM	53	M	-
**DCM8**	Heart tissue	DCM	37	M	-
**sevCCC1**	Heart tissue	sevCCC	60	F	-
**sevCCC2**	Heart tissue	sevCCC	32	M	-
**sevCCC3**	Heart tissue	sevCCC	36	M	-
**sevCCC4**	Heart tissue	sevCCC	50	F	-
**sevCCC5**	Heart tissue	sevCCC	45	F	-
**sevCCC6**	Heart tissue	sevCCC	49	F	-
**sevCCC7**	Heart tissue	sevCCC	57	M	-
**sevCCC8**	Heart tissue	sevCCC	54	F	-
**SRR10822575**	Heart tissue	CTRL	-	-	-
**SRR10822576**	Heart tissue	CTRL	-	-	-
**SRR10822577**	Heart tissue	CTRL	-	-	-
**SRR10822578**	Heart tissue	CTRL	-	-	-
**SRR10822579**	Heart tissue	CTRL	-	-	-
**SRR10822580**	Heart tissue	CTRL	-	-	-
**SRR10822581**	Heart tissue	CTRL	-	-	-
**SRR10822582**	Heart tissue	CTRL	-	-	-
**SRR10822583**	Heart tissue	CTRL	-	-	-
**SRR10822584**	Heart tissue	CTRL	-	-	-
**SRR10822585**	Heart tissue	CTRL	-	-	-
**SRR10822586**	Heart tissue	CTRL	-	-	-
**SRR10822587**	Heart tissue	CTRL	-	-	-
**SRR10822588**	Heart tissue	CTRL	-	-	-
**SRR10822589**	Heart tissue	CTRL	-	-	-
**SRR10822590**	Heart tissue	CTRL	-	-	-
**SRR10822591**	Heart tissue	CTRL	-	-	-
**SRR10822592**	Heart tissue	CTRL	-	-	-
**SRR10822593**	Heart tissue	CTRL	-	-	-
**SRR10822594**	Heart tissue	CTRL	-	-	-
**SRR10822595**	Heart tissue	CTRL	-	-	-
**SRR10822596**	Heart tissue	CTRL	-	-	-

**SRR10822597**	Heart tissue	CTRL	-	-	-
**SRR10822598**	Heart tissue	CTRL	-	-	-
**SRR10822599**	Heart tissue	CTRL	-	-	-
**SRR10822600**	Heart tissue	CTRL	-	-	-
**SRR10822601**	Heart tissue	CTRL	-	-	-
**SRR10822602**	Heart tissue	CTRL	-	-	-
**SRR10822603**	Heart tissue	CTRL	-	-	-
**SRR10822604**	Heart tissue	CTRL	-	-	-
**CTRL8**	Peripherical blood	CTRL	-	F	0.67
**CTRL9**	Peripherical blood	CTRL	-	F	0.59
**CTRL10**	Peripherical blood	CTRL	-	F	0.67
**CTRL11**	Peripherical blood	CTRL	-	F	0.65
**CTRL12**	Peripherical blood	CTRL	-	M	0.67
**CTRL13**	Peripherical blood	CTRL	-	M	0.66
**CTRL14**	Peripherical blood	CTRL	-	M	0.7
**CTRL15**	Peripherical blood	CTRL	-	M	0.68
**CTRL16**	Peripherical blood	CTRL	-	F	0.65
**CTRL17**	Peripherical blood	CTRL	-	M	0.66
**CTRL18**	Peripherical blood	CTRL	-	F	0.65
**CTRL19**	Peripherical blood	CTRL	-	M	0.67
**CTRL20**	Peripherical blood	CTRL	-	F	0.62
**CTRL21**	Peripherical blood	CTRL	-	M	0.66
**CTRL22**	Peripherical blood	CTRL	-	M	0.72
**CTRL23**	Peripherical blood	CTRL	-	F	0.62
**CTRL24**	Peripherical blood	CTRL	-	F	0.63
**CTRL25**	Peripherical blood	CTRL	-	F	0.67
**CTRL26**	Peripherical blood	CTRL	-	M	0.62
**CTRL27**	Peripherical blood	CTRL	-	M	0.68
**CTRL28**	Peripherical blood	CTRL	-	M	0.61
**CTRL29**	Peripherical blood	CTRL	-	F	0.75
**CTRL30**	Peripherical blood	CTRL	-	F	0.65
**CTRL31**	Peripherical blood	CTRL	-	M	0.65
**CTRL32**	Peripherical blood	CTRL	-	F	0.68
**CTRL33**	Peripherical blood	CTRL	-	F	0.7
**CTRL34**	Peripherical blood	CTRL	-	F	0.72
**CTRL35**	Peripherical blood	CTRL	-	M	0.78
**CTRL36**	Peripherical blood	CTRL	-	F	0.64
**CTRL37**	Peripherical blood	CTRL	-	M	0.56
**CTRL38**	Peripherical blood	CTRL	-	F	0.74
**CTRL39**	Peripherical blood	CTRL	-	M	0.55
**CTRL40**	Peripherical blood	CTRL	-	M	0.68
**CTRL41**	Peripherical blood	CTRL	-	F	0.62
**CTRL42**	Peripherical blood	CTRL	-	F	0.67
**CTRL43**	Peripherical blood	CTRL	-	M	0.76
**CTRL44**	Peripherical blood	CTRL	-	M	0.69
**CTRL45**	Peripherical blood	CTRL	-	M	0.77
**CTRL46**	Peripherical blood	CTRL	-	F	0.69
**CTRL47**	Peripherical blood	CTRL	-	M	0.55
**CTRL48**	Peripherical blood	CTRL	-	F	0.81

**CTRL49**	Peripherical blood	CTRL	-	M	0.65
**CTRL50**	Peripherical blood	CTRL	-	M	0.68
**CTRL51**	Peripherical blood	CTRL	-	M	0.65
**CTRL52**	Peripherical blood	CTRL	-	F	0.77
**CTRL53**	Peripherical blood	CTRL	-	F	0.46
**CTRL54**	Peripherical blood	CTRL	-	M	0.63
**CTRL55**	Peripherical blood	CTRL	-	F	0.71
**modCCC1**	Peripherical blood	modCCC	-	F	0.68
**modCCC2**	Peripherical blood	modCCC	-	M	0.52
**modCCC3**	Peripherical blood	modCCC	-	F	0.68
**modCCC4**	Peripherical blood	modCCC	-	F	0.77
**modCCC5**	Peripherical blood	modCCC	-	F	0.67
**modCCC6**	Peripherical blood	modCCC	-	M	0.53
**modCCC7**	Peripherical blood	modCCC	-	F	0.62
**modCCC8**	Peripherical blood	modCCC	-	M	0.56
**modCCC9**	Peripherical blood	modCCC	-	F	0.68
**modCCC10**	Peripherical blood	modCCC	-	F	0.45
**modCCC11**	Peripherical blood	modCCC	-	M	0.63
**modCCC12**	Peripherical blood	modCCC	-	M	0.45
**modCCC13**	Peripherical blood	modCCC	-	F	0.67
**modCCC14**	Peripherical blood	modCCC	-	M	0.56
**modCCC15**	Peripherical blood	modCCC	-	F	0.58
**modCCC16**	Peripherical blood	modCCC	-	M	0.46
**modCCC17**	Peripherical blood	modCCC	-	M	0.3
**modCCC18**	Peripherical blood	modCCC	-	F	0.61
**modCCC19**	Peripherical blood	modCCC	-	F	0.57
**modCCC20**	Peripherical blood	modCCC	-	M	0.78
**modCCC21**	Peripherical blood	modCCC	-	M	0.69
**modCCC22**	Peripherical blood	modCCC	-	F	0.66
**modCCC23**	Peripherical blood	modCCC	-	F	0.47
**modCCC24**	Peripherical blood	modCCC	-	M	0.53
**modCCC25**	Peripherical blood	modCCC	-	M	0.58
**modCCC26**	Peripherical blood	modCCC	-	F	0.68
**modCCC27**	Peripherical blood	modCCC	-	M	0.52
**modCCC28**	Peripherical blood	modCCC	-	F	0.55
**modCCC29**	Peripherical blood	modCCC	-	M	0.7
**modCCC30**	Peripherical blood	modCCC	-	F	0.66
**modCCC31**	Peripherical blood	modCCC	-	F	0.49
**modCCC32**	Peripherical blood	modCCC	-	M	0.69
**modCCC33**	Peripherical blood	modCCC	-	F	0.65
**modCCC34**	Peripherical blood	modCCC	-	M	0.58
**modCCC35**	Peripherical blood	modCCC	-	M	0.5
**modCCC36**	Peripherical blood	modCCC	-	M	0.5
**modCCC37**	Peripherical blood	modCCC	-	F	0.66
**modCCC38**	Peripherical blood	modCCC	-	M	0.65
**modCCC39**	Peripherical blood	modCCC	-	F	0.56
**modCCC40**	Peripherical blood	modCCC	-	F	0.71
**modCCC41**	Peripherical blood	modCCC	-	F	0.48
**modCCC42**	Peripherical blood	modCCC	-	M	0.47

**modCCC43**	Peripherical blood	modCCC	-	M	0.7
**modCCC44**	Peripherical blood	modCCC	-	F	0.64
**modCCC45**	Peripherical blood	modCCC	-	F	0.67
**modCCC46**	Peripherical blood	modCCC	-	M	0.7
**modCCC47**	Peripherical blood	modCCC	-	F	0.64
**sevCCC11**	Peripherical blood	sevCCC	-	M	0.25
**sevCCC12**	Peripherical blood	sevCCC	-	M	0.3
**sevCCC13**	Peripherical blood	sevCCC	-	F	0.26
**sevCCC14**	Peripherical blood	sevCCC	-	M	0.25
**sevCCC15**	Peripherical blood	sevCCC	-	F	0.39
**sevCCC16**	Peripherical blood	sevCCC	-	M	0.26
**sevCCC17**	Peripherical blood	sevCCC	-	F	0.25
**sevCCC18**	Peripherical blood	sevCCC	-	M	0.28
**sevCCC19**	Peripherical blood	sevCCC	-	F	0.28
**sevCCC20**	Peripherical blood	sevCCC	-	F	0.25
**sevCCC21**	Peripherical blood	sevCCC	-	M	0.27
**sevCCC22**	Peripherical blood	sevCCC	-	M	0.2
**sevCCC23**	Peripherical blood	sevCCC	-	M	0.2
**sevCCC24**	Peripherical blood	sevCCC	-	M	0.32
**sevCCC25**	Peripherical blood	sevCCC	-	F	0.33
**sevCCC26**	Peripherical blood	sevCCC	-	F	0.3
**sevCCC27**	Peripherical blood	sevCCC	-	F	0.31
**sevCCC28**	Peripherical blood	sevCCC	-	M	0.19
**sevCCC29**	Peripherical blood	sevCCC	-	F	0.3
**sevCCC30**	Peripherical blood	sevCCC	-	F	0.22
**sevCCC31**	Peripherical blood	sevCCC	-	F	0.22
**sevCCC32**	Peripherical blood	sevCCC	-	F	0.3
**sevCCC33**	Peripherical blood	sevCCC	-	F	0.36
**sevCCC34**	Peripherical blood	sevCCC	-	F	0.23
**sevCCC35**	Peripherical blood	sevCCC	-	M	0.31
**sevCCC36**	Peripherical blood	sevCCC	-	M	0.34
**sevCCC37**	Peripherical blood	sevCCC	-	M	0.22
**sevCCC38**	Peripherical blood	sevCCC	-	M	0.29
**sevCCC39**	Peripherical blood	sevCCC	-	M	0.26
**sevCCC40**	Peripherical blood	sevCCC	-	M	0.26
**sevCCC41**	Peripherical blood	sevCCC	-	F	0.24
**sevCCC42**	Peripherical blood	sevCCC	-	M	0.18
**sevCCC43**	Peripherical blood	sevCCC	-	F	0.33
**sevCCC44**	Peripherical blood	sevCCC	-	F	0.27
**sevCCC45**	Peripherical blood	sevCCC	-	M	0.28
**sevCCC46**	Peripherical blood	sevCCC	-	M	0.23
**sevCCC47**	Peripherical blood	sevCCC	-	F	0.25
**sevCCC48**	Peripherical blood	sevCCC	-	F	0.14
**sevCCC49**	Peripherical blood	sevCCC	-	M	0.25
**sevCCC50**	Peripherical blood	sevCCC	-	M	0.25
**sevCCC51**	Peripherical blood	sevCCC	-	M	0.22
**sevCCC52**	Peripherical blood	sevCCC	-	F	0.34
**sevCCC53**	Peripherical blood	sevCCC	-	F	0.22

EF= Ejection fraction
