# Supplementary material for: Epigenetic regulation of transcription factor binding motifs promotes Th1 response in Chagas disease cardiomyopathy
Source: Front Immunol. 2022 Aug 22;13:958200. doi: 10.3389/fimmu.2022.958200 (PMC9441916; doi:10.3389/fimmu.2022.958200)
Supplement: Supplementary Table 1 — Biological samples included in this study. [file DataSheet_1.zip › Supplementary Material/Supplementary Table 10.pdf]

Supplementary table 10. List of transcription factor and transcription factor complexes testing against our DMRs

Transcription factor	S (summed of overlap bp)	log2(Fold change)	P-value	Corrected p-value	Number of transcription factor
ARID3A	347	0,6	2,0E-01	2,6E-01	1
ATF2	287	0,3	2,8E-01	3,3E-01	1
ATF3	80	1,3	1,1E-01	1,8E-01	1
BACH1	212	1,1	7,8E-02	1,5E-01	1
BACH2	290	1,4	9,1E-02	1,6E-01	1
BATF	788	0,7	9,9E-02	1,7E-01	1
BCL11A	823	0,9	5,1E-02	1,0E-01	1
BCL3	395	0,0	4,4E-01	4,6E-01	1
BCL6	1870	1,3	5,8E-03	2,0E-02	1
BCLAF1	2312	1,4	7,1E-04	3,4E-03	1
BHLHE40	1413	0,8	5,4E-02	1,1E-01	1
BRCA1	0	0,0	1,0E+00	1,0E+00	1
BRD2	191	1,2	1,3E-01	2,0E-01	1
BRD3	4	-4,0	1,7E-01	2,3E-01	1
BRD4	3090	1,3	7,9E-04	3,6E-03	1
CBFB	1597	0,9	1,4E-02	3,9E-02	1
CDK7	1269	1,6	1,1E-02	3,3E-02	1
CDK8	142	0,4	2,7E-01	3,1E-01	1
CDK9	60	-0,3	4,1E-01	4,5E-01	1
CEBPB	0	0,0	9,6E-02	1,7E-01	1
CEBPZ	0	0,0	1,5E-01	2,2E-01	1
CHD1	0	0,0	1,0E+00	1,0E+00	1
CHD2	4	-3,3	1,6E-01	2,3E-01	1
CREB1	486	0,7	1,6E-01	2,3E-01	1
CREM	672	0,8	7,4E-02	1,4E-01	1
CTCF	981	0,6	1,2E-01	1,9E-01	1
CUX1	58	0,9	1,5E-01	2,1E-01	1
E2F4	0	0,0	1,0E+00	1,0E+00	1
EBF1	2389	1,5	2,8E-04	1,4E-03	1
EED	2777	1,3	1,6E-05	8,3E-05	1
EGR1	309	1,3	4,6E-02	9,7E-02	1
ELF1	1256	1,2	1,2E-02	3,5E-02	1
ELK1	0	0,0	1,0E+00	1,0E+00	1

EP300	126	0,2	3,3E-01	3,8E-01	1
ERG	1599	1,6	8,5E-04	3,8E-03	1
ESRRA	105	1,0	1,3E-01	2,1E-01	1
ETS1	181	1,1	1,1E-01	1,8E-01	1
ETV6	821	1,2	2,5E-02	5,7E-02	1
EZH2	766	1,5	1,4E-02	3,9E-02	1
FANCL	0	0,0	1,0E+00	1,0E+00	1
FLI1	242	1,5	8,4E-02	1,6E-01	1
FOS	4	-2,7	4,5E-01	4,7E-01	1
FOXM1	31	-1,8	3,5E-01	3,9E-01	1
FOXP1	0	0,0	4,9E-01	4,9E-01	1
GABPA	1811	1,5	1,2E-03	5,1E-03	1
GATA1	0	0,0	1,0E+00	1,0E+00	1
GATA2	101	0,3	3,0E-01	3,4E-01	1
GATA3	1611	1,6	2,7E-03	1,1E-02	1
GTF2F1	914	1,4	9,7E-03	3,0E-02	1
HCFC1	577	1,6	1,4E-02	3,9E-02	1
HSF1	24	0,3	2,1E-01	2,7E-01	1
IKZF1	195	1,0	1,3E-01	2,1E-01	1
IRF3	4	-2,8	4,9E-01	4,9E-01	1
IRF4	1636	1,7	2,6E-03	1,1E-02	1
JUNB	359	0,8	1,4E-01	2,1E-01	1
JUND	4	-0,8	2,2E-01	2,8E-01	1
KDM6B	68	-1,4	2,6E-01	3,1E-01	1
KLF5	539	1,5	3,8E-02	8,3E-02	1
LMO2	554	1,8	2,4E-02	5,5E-02	1
LYL1	143	0,7	1,9E-01	2,5E-01	1
MAFF	0	0,0	1,0E+00	1,0E+00	1
MAFK	899	1,1	4,0E-02	8,7E-02	1
MAX	1277	1,2	1,7E-02	4,4E-02	1
MAZ	228	2,4	2,3E-02	5,5E-02	1
MED1	557	1,1	8,9E-02	1,6E-01	1
MEF2A	53	-0,9	4,7E-01	4,8E-01	1
MEF2B	0	0,0	3,0E-01	3,4E-01	1
MEF2C	0	0,0	7,5E-02	1,4E-01	1
MEIS1	31	-1,0	4,4E-01	4,6E-01	1

MTA3	268	0,0	4,2E-01	4,6E-01	1
MXI1	19	0,9	1,4E-01	2,1E-01	1
MYB	1910	1,5	2,7E-03	1,1E-02	1
MYC	1313	1,7	8,6E-03	2,8E-02	1
NCOR	0	0,0	2,5E-01	3,0E-01	1
NCOR2	0	0,0	2,6E-01	3,1E-01	1
NFATC1	54	-2,0	1,6E-01	2,2E-01	1
NFE2	0	0,0	1,0E+00	1,0E+00	1
NFIC	433	0,1	4,0E-01	4,4E-01	1
NFYA	4	-2,2	4,9E-01	4,9E-01	1
NFYB	293	0,8	1,7E-01	2,3E-01	1
NOTCH1	1016	1,2	1,5E-02	3,9E-02	1
NR2C2	0	0,0	1,0E+00	1,0E+00	1
NR3C1	131	-0,1	4,3E-01	4,6E-01	1
NRF1	4	-4,1	2,1E-01	2,7E-01	1
OCA2	540	0,8	1,2E-01	1,9E-01	1
PAX5	1277	1,4	5,6E-03	2,0E-02	1
PBX3	770	1,2	3,3E-02	7,5E-02	1
PML	351	-0,2	4,6E-01	4,7E-01	1
POU2F2	1637	0,9	2,2E-02	5,4E-02	1
PPARG	0	0,0	1,0E+00	1,0E+00	1
RAD21	35	-1,4	2,4E-01	2,9E-01	1
RARA	375	1,0	1,0E-01	1,8E-01	1
RB1	0	0,0	1,0E+00	1,0E+00	1
RBPJ	972	1,7	8,0E-03	2,7E-02	1
RCOR1	0	0,0	1,0E+00	1,0E+00	1
RELA	1241	0,9	4,6E-02	9,7E-02	1
REST	324	0,5	2,1E-01	2,7E-01	1
RFX5	0	0,0	1,0E+00	1,0E+00	1
RNF2	263	1,0	9,6E-02	1,7E-01	1
RUNX	0	0,0	1,0E+00	1,0E+00	1
RUNX1	2052	1,2	8,5E-03	2,8E-02	1
RUNX3	2092	1,1	4,7E-03	1,7E-02	1
RXR	291	0,9	1,5E-01	2,1E-01	1
RXRA	4	-4,1	1,0E-01	1,7E-01	1
SIN3A	0	0,0	1,0E+00	1,0E+00	1

SIX5	0	0,0	3,6E-01	4,0E-01	1
SMAD5	183	-0,7	2,8E-01	3,3E-01	1
SMC1A	283	0,8	1,6E-01	2,2E-01	1
SMC3	0	0,0	4,1E-01	4,4E-01	1
SOX11	0	0,0	1,0E+00	1,0E+00	1
SP1	986	1,4	1,1E-02	3,3E-02	1
SPI1	1457	1,5	3,7E-03	1,4E-02	1
SPIB	868	1,5	2,1E-02	5,4E-02	1
SREBF1	0	0,0	1,0E+00	1,0E+00	1
SREBF2	0	0,0	1,0E+00	1,0E+00	1
SRF	176	0,4	2,2E-01	2,8E-01	1
STAG1	0	0,0	5,5E-02	1,1E-01	1
STAT1	105	0,5	2,2E-01	2,8E-01	1
STAT3	749	1,0	4,7E-02	9,8E-02	1
STAT5A	47	-1,7	2,3E-01	2,8E-01	1
STAT5B	231	3,7	1,3E-02	3,6E-02	1
SUPT20H	0	0,0	1,0E+00	1,0E+00	1
SUZ12	0	0,0	1,0E+00	1,0E+00	1
TAF1	876	0,9	6,6E-02	1,3E-01	1
TAL1	798	0,9	5,5E-02	1,1E-01	1
TAL1_SCL	0	0,0	1,0E+00	1,0E+00	1
TARDBP	2314	1,0	2,2E-02	5,5E-02	1
TBL1XR1	0	0,0	1,0E+00	1,0E+00	1
TBP	246	0,2	3,1E-01	3,6E-01	1
TBX21	2271	1,2	3,5E-03	1,4E-02	1
TCF12	1286	0,9	2,3E-02	5,5E-02	1
TCF3	1333	1,2	1,1E-02	3,3E-02	1
TCF7	215	0,7	1,3E-01	2,1E-01	1
TP53	0	0,0	1,0E+00	1,0E+00	1
TRIM22	1347	1,1	3,7E-02	8,3E-02	1
UBTF	0	0,0	1,0E+00	1,0E+00	1
USF1	64	0,9	1,5E-01	2,1E-01	1
USF2	78	0,9	1,4E-01	2,1E-01	1
WRNIP1	0	0,0	1,0E+00	1,0E+00	1
YY1	1526	1,2	9,1E-03	2,9E-02	1
ZBED1	100	-0,7	4,3E-01	4,6E-01	1

ZBTB33	0	0,0	2,1E-01	2,7E-01	1
ZEB1	570	1,7	1,8E-02	4,6E-02	1
ZFP36	348	0,6	1,9E-01	2,5E-01	1
ZFX	0	0,0	1,0E+00	1,0E+00	1
ZNF143	0	0,0	4,6E-01	4,7E-01	1
ZNF207	770	0,6	1,4E-01	2,1E-01	1
ZNF274	0	0,0	1,0E+00	1,0E+00	1
ZNF335	0	0,0	1,0E+00	1,0E+00	1
ZNF384	131	0,8	1,7E-01	2,3E-01	1
ZZZ3	0	0,0	1,0E+00	1,0E+00	1
CBFB	2377	3,7	1,1E-06	1,1E-06	2
EED	3662	4,1	2,9E-07	2,9E-07	2
EBF1 + EED	2788	7,8	8,7E-08	8,8E-08	3
BCL6 + BRD4 + GATA3	1628	10,5	3,0E-10	3,1E-10	4
BCLAF1 + EBF1 + EED	2346	11,2	1,0E+00	1,0E+00	4
BRD4 + EED + ERG	1768	10,0	3,2E-12	3,2E-12	4
BRD4 + MYC + TBX21	1351	10,4	1,0E+00	1,0E+00	4
BCL6 + BRD4 + EBF1 + GATA3	1426	10,5	1,0E+00	1,0E+00	5
BCLAF1 + BRD4 + MYC + TBX21	1056	10,0	1,0E+00	1,0E+00	5
BCLAF1 + BRD4 + MYC + YY1	871	9,8	1,0E+00	1,0E+00	5
BCLAF1 + BRD4 + RBPJ + TBX21	1047	10,0	1,0E+00	1,0E+00	5
BCLAF1 + EBF1 + EED + TCF3	1678	10,7	1,0E+00	1,0E+00	5
BCLAF1 + EED + RUNX3 + TCF3	1744	10,8	1,0E+00	1,0E+00	5
BRD4 + EBF1 + EED + ERG	1574	10,6	1,0E+00	1,0E+00	5
BRD4 + EED + ERG + EZH2	521	9,0	1,0E+00	1,0E+00	5
BRD4 + MYC + RBPJ + TBX21	849	9,7	1,0E+00	1,0E+00	5
BCL6 + BRD4 + EBF1 + GATA3 + MYB	1333	10,4	1,0E+00	1,0E+00	6
BCL6 + BRD4 + GATA3 + NOTCH1 + PAX5	775	9,6	1,0E+00	1,0E+00	6
BCLAF1 + BRD4 + EBF1 + RBPJ + TBX21	956	9,9	1,0E+00	1,0E+00	6
BCLAF1 + BRD4 + EED + MYC + YY1	821	9,7	1,0E+00	1,0E+00	6
BCLAF1 + BRD4 + MYC + RBPJ + TBX21	607	9,2	1,0E+00	1,0E+00	6

BCLAF1 + BRD4 + MYC + TBX21 + YY1	740	9,5	1,0E+00	1,0E+00	6
BCLAF1 + EBF1 + EED + PAX5 + RUNX3	1649	10,7	1,0E+00	1,0E+00	6
BCLAF1 + EBF1 + EED + RUNX3 + TCF3	1497	10,5	1,0E+00	1,0E+00	6
BCLAF1 + EED + RUNX3 + TBX21 + TCF3	1541	10,6	1,0E+00	1,0E+00	6
BRD4 + EBF1 + EED + ERG + GTF2F1	1016	10,0	1,0E+00	1,0E+00	6
BRD4 + EED + GABPA + NOTCH1 + YY1	1117	10,1	1,0E+00	1,0E+00	6
EED + ELF1 + ERG + RUNX1 + SPI1	1165	10,2	1,0E+00	1,0E+00	6
EED + ELF1 + RUNX1 + RUNX3 + SPI1	1141	10,2	1,0E+00	1,0E+00	6
BCL6 + BRD4 + EBF1 + EZH2 + GATA3 + MYB	440	8,8	1,0E+00	1,0E+00	7
BCLAF1 + BRD4 + EED + MYC + TCF3 + YY1	651	9,3	1,0E+00	1,0E+00	7
BCLAF1 + CBFB + EED + RUNX3 + TBX21 + TCF3	1392	10,4	1,0E+00	1,0E+00	7
BCLAF1 + EBF1 + EED + PAX5 + RUNX3 + TCF3	1434	10,5	1,0E+00	1,0E+00	7
BRD4 + EBF1 + EED + ERG + GTF2F1 + HCFC1	655	9,4	1,0E+00	1,0E+00	7
BRD4 + EED + ELF1 + GABPA + NOTCH1 + YY1	908	9,8	1,0E+00	1,0E+00	7
BRD4 + EED + MYB + RUNX1 + TBX21 + YY1	1082	10,1	1,0E+00	1,0E+00	7
BRD4 + GATA3 + IRF4 + MYB + MYC + NOTCH1	742	9,5	1,0E+00	1,0E+00	7
EED + ELF1 + ERG + RUNX1 + RUNX3 + SPI1	1048	10,0	1,0E+00	1,0E+00	7
BCL6 + BRD4 + EBF1 + EZH2 + GATA3 + MYB + MYC	423	8,7	1,0E+00	1,0E+00	8

BCLAF1 + BRD4 + EBF1 + EED + IRF4 + MYB + TBX21	1267	10,3	1,0E+00	1,0E+00	8
BCLAF1 + BRD4 + EED + MYC + RUNX3 + TCF3 + YY1	643	9,3	1,0E+00	1,0E+00	8
BCLAF1 + BRD4 + EED + RUNX1 + RUNX3 + TBX21 + YY1	1275	10,3	1,0E+00	1,0E+00	8
BCLAF1 + CBFB + EED + ELF1 + RUNX3 + TBX21 + TCF3	938	9,9	1,0E+00	1,0E+00	8
BCLAF1 + EBF1 + EED + MAX + PAX5 + RUNX3 + TCF3	900	9,8	1,0E+00	1,0E+00	8
BRD4 + CDK7 + GATA3 + IRF4 + MYB + MYC + NOTCH1	736	9,5	1,0E+00	1,0E+00	8
BRD4 + EBF1 + EED + ELF1 + GTF2F1 + HCFC1 + SPI1	690	9,4	1,0E+00	1,0E+00	8
BRD4 + EBF1 + EED + ERG + GTF2F1 + HCFC1 + SPI1	597	9,2	1,0E+00	1,0E+00	8
BRD4 + EED + ELF1 + ERG + GABPA + NOTCH1 + YY1	818	9,7	1,0E+00	1,0E+00	8
BRD4 + EED + MYB + RUNX1 + RUNX3 + TBX21 + YY1	994	10,0	1,0E+00	1,0E+00	8
BCL6 + BRD4 + EBF1 + EZH2 + GATA3 + IRF4 + MYB + MYC	416	8,7	1,0E+00	1,0E+00	9
BCLAF1 + BRD4 + EBF1 + EED + ERG + IRF4 + MYB + TBX21	950	9,9	1,0E+00	1,0E+00	9
BCLAF1 + BRD4 + EBF1 + EED + RUNX1 + RUNX3 + TBX21 + YY1	1167	10,2	1,0E+00	1,0E+00	9
BCLAF1 + BRD4 + EED + MYB + MYC + RUNX3 + TCF3 + YY1	626	9,3	1,0E+00	1,0E+00	9
BCLAF1 + CBFB + EED + ELF1 + RUNX3 + TBX21 + TCF3 + YY1	824	9,7	1,0E+00	1,0E+00	9
BRD4 + CBFB + EBF1 + ERG + EZH2 + IRF4 + PAX5 + RUNX1	323	8,3	1,0E+00	1,0E+00	9
BRD4 + CDK7 + EED + MYB + RUNX1 + RUNX3 + TBX21 + YY1	894	9,8	1,0E+00	1,0E+00	9
BRD4 + CDK7 + GABPA + GATA3 + IRF4 + MYB + MYC + NOTCH1	734	9,5	1,0E+00	1,0E+00	9

BRD4 + EBF1 + EED + ELF1 + ERG + GTF2F1 + HCFC1 + SPI1	520	9,0	1,0E+00	1,0E+00	9
BRD4 + EBF1 + EED + ELF1 + EZH2 + GTF2F1 + HCFC1 + SPI1	278	8,1	1,0E+00	1,0E+00	9
BCL6 + BCLAF1 + BRD4 + EBF1 + EED + RUNX1 + RUNX3 + TBX21 + YY1	961	9,9	1,0E+00	1,0E+00	10
BCL6 + BRD4 + CDK7 + EBF1 + EZH2 + GATA3 + IRF4 + MYB + MYC	397	8,6	1,0E+00	1,0E+00	10
BCLAF1 + BRD4 + EBF1 + EED + ERG + IRF4 + MYB + TBX21 + YY1	882	9,8	1,0E+00	1,0E+00	10
BCLAF1 + CBFB + EBF1 + EED + IRF4 + PAX5 + RUNX3 + TBX21 + TCF3	1189	10,2	1,0E+00	1,0E+00	10
BRD4 + CBFB + EBF1 + ERG + EZH2 + IRF4 + PAX5 + RUNX1 + SPI1	271	8,1	1,0E+00	1,0E+00	10
BRD4 + CBFB + EBF1 + ERG + EZH2 + IRF4 + PAX5 + RUNX1 + TCF3	307	8,3	1,0E+00	1,0E+00	10
BRD4 + CDK7 + EBF1 + EED + MYB + RUNX1 + RUNX3 + TBX21 + YY1	893	9,8	1,0E+00	1,0E+00	10
BCL6 + BCLAF1 + BRD4 + CDK7 + EBF1 + EED + RUNX1 + RUNX3 + TBX21 + YY1	771	9,6	1,0E+00	1,0E+00	11
BCL6 + BRD4 + CDK7 + EBF1 + EZH2 + GATA3 + IRF4 + MYB + MYC + TBX21	354	8,5	1,0E+00	1,0E+00	11
BCLAF1 + BRD4 + CBFB + EBF1 + EED + IRF4 + PAX5 + RUNX3 + TBX21 + TCF3	1177	10,2	1,0E+00	1,0E+00	11
BCLAF1 + BRD4 + CDK7 + EBF1 + EED + ERG + IRF4 + MYB + TBX21 + YY1	780	9,6	1,0E+00	1,0E+00	11

BCLAF1 + BRD4 + CDK7 + EBF1 + EED + MYB + RUNX1 + RUNX3 + TBX21 + YY1	855	9,7	1,0E+00	1,0E+00	11
BCLAF1 + BRD4 + EBF1 + EED + ELF1 + EZH2 + GTF2F1 + HCFC1 + SPI1 + TCF3	258	8,0	1,0E+00	1,0E+00	11
BCLAF1 + CBFB + EBF1 + EED + GABPA + IRF4 + PAX5 + RUNX3 + TBX21 + TCF3	998	10,0	1,0E+00	1,0E+00	11
BRD4 + CBFB + EBF1 + ERG + EZH2 + IRF4 + MYB + PAX5 + RUNX1 + TCF3	250	8,0	1,0E+00	1,0E+00	11
BRD4 + CBFB + EBF1 + ERG + IRF4 + MYB + PAX5 + RUNX1 + RUNX3 + TCF3	874	9,8	1,0E+00	1,0E+00	11
BCL6 + BCLAF1 + BRD4 + CDK7 + EBF1 + EED + MYC + RUNX1 + RUNX3 + TBX21 + YY1	667	9,4	1,0E+00	1,0E+00	12
BCL6 + BCLAF1 + BRD4 + CDK7 + EED + GABPA + MYB + MYC + RUNX1 + RUNX3 + TBX21	722	9,5	1,0E+00	1,0E+00	12
BCL6 + BRD4 + CDK7 + EBF1 + EZH2 + GATA3 + IRF4 + MYB + MYC + RUNX1 + TBX21	345	8,4	1,0E+00	1,0E+00	12
BCLAF1 + BRD4 + CBFB + EBF1 + EED + IRF4 + PAX5 + RUNX3 + TBX21 + TCF3 + YY1	952	9,9	1,0E+00	1,0E+00	12
BCLAF1 + BRD4 + CBFB + EBF1 + ERG + IRF4 + MYB + PAX5 + RUNX1 + RUNX3 + TCF3	862	9,8	1,0E+00	1,0E+00	12
BCLAF1 + BRD4 + CDK7 + EBF1 + EED + ERG + HCFC1 + IRF4 + MYB + TBX21 + YY1	477	8,9	1,0E+00	1,0E+00	12
BCLAF1 + BRD4 + EBF1 + EED + ELF1 + EZH2 + GTF2F1 + HCFC1 + SP1 + SPI1 + TCF3	254	8,0	1,0E+00	1,0E+00	12

BRD4 + CBFB + EBF1 + ERG + EZH2 + IRF4 + MYB + PAX5 + RUNX1 + RUNX3 + TCF3	229	7,8	1,0E+00	1,0E+00	12
BCL6 + BCLAF1 + BRD4 + CBFB + CDK7 + EED + GABPA + MYB + MYC + RUNX1 + RUNX3 + TBX21	547	9,1	1,0E+00	1,0E+00	13
BCL6 + BCLAF1 + BRD4 + CDK7 + EBF1 + EED + GABPA + MYB + MYC + RUNX1 + RUNX3 + TBX21	697	9,4	1,0E+00	1,0E+00	13
BCL6 + BCLAF1 + BRD4 + CDK7 + EBF1 + EED + MYB + MYC + RUNX1 + RUNX3 + TBX21 + YY1	626	9,3	1,0E+00	1,0E+00	13
BCL6 + BRD4 + CDK7 + EBF1 + EZH2 + GATA3 + IRF4 + MYB + MYC + RBPJ + RUNX1 + TBX21	338	8,4	1,0E+00	1,0E+00	13
BCLAF1 + BRD4 + CBFB + EBF1 + EED + ERG + IRF4 + MYB + PAX5 + RUNX1 + RUNX3 + TCF3	854	9,7	1,0E+00	1,0E+00	13
BCLAF1 + BRD4 + EBF1 + EED + ELF1 + EZH2 + GATA3 + GTF2F1 + HCFC1 + SP1 + SPI1 + TCF3	253	8,0	1,0E+00	1,0E+00	13
BCL6 + BCLAF1 + BRD4 + CBFB + CDK7 + EBF1 + EED + GABPA + MYB + MYC + RUNX1 + RUNX3 + TBX21	543	9,1	1,0E+00	1,0E+00	14
BCL6 + BCLAF1 + BRD4 + CDK7 + EBF1 + EED + GABPA + MYB + MYC + RUNX1 + RUNX3 + TBX21 + YY1	606	9,2	1,0E+00	1,0E+00	14
BCL6 + BCLAF1 + BRD4 + EBF1 + GABPA + GTF2F1 + HCFC1 + IRF4 + MAX + MYB + SP1 + SPI1 + TBX21	548	9,1	1,0E+00	1,0E+00	14
BCL6 + BCLAF1 + BRD4 + EBF1 + GABPA + GTF2F1 + IRF4 + MAX + MYB + RBPJ + SP1 + SPI1 + TBX21	715	9,5	1,0E+00	1,0E+00	14

BCL6 + BRD4 + CBFB + EBF1 + EED + ERG + GABPA + GATA3 + IRF4 + MYB + RUNX1 + RUNX3 + YY1	755	9,6	1,0E+00	1,0E+00	14
BCL6 + BRD4 + CDK7 + EBF1 + EZH2 + GABPA + GATA3 + IRF4 + MYB + MYC + RBPJ + RUNX1 + TBX21	327	8,4	1,0E+00	1,0E+00	14
BCL6 + BRD4 + CDK7 + EED + ERG + EZH2 + GATA3 + MAX + MYB + NOTCH1 + RUNX1 + RUNX3 + SPI1	362	8,5	1,0E+00	1,0E+00	14
BCLAF1 + BRD4 + CBFB + CDK7 + EBF1 + EED + ERG + HCFC1 + IRF4 + MYB + TBX21 + YY1 + ZEB1	364	8,5	1,0E+00	1,0E+00	14
BCLAF1 + BRD4 + CBFB + EBF1 + EED + ERG + GATA3 + IRF4 + MYB + PAX5 + RUNX1 + RUNX3 + TCF3	819	9,7	1,0E+00	1,0E+00	14
BCLAF1 + BRD4 + CBFB + EED + ELF1 + GTF2F1 + HCFC1 + IRF4 + PAX5 + RUNX3 + TBX21 + TCF3 + YY1	567	9,1	1,0E+00	1,0E+00	14
BCLAF1 + BRD4 + CDK7 + EBF1 + EED + GATA3 + MYB + RUNX1 + RUNX3 + SP1 + SPI1 + TBX21 + YY1	587	9,2	1,0E+00	1,0E+00	14
BCLAF1 + BRD4 + EBF1 + EED + ELF1 + EZH2 + GATA3 + GTF2F1 + HCFC1 + PAX5 + SP1 + SPI1 + TCF3	250	8,0	1,0E+00	1,0E+00	14
BCL6 + BCLAF1 + BRD4 + CBFB + CDK7 + EBF1 + EED + GABPA + MYB + MYC + NOTCH1 + RUNX1 + RUNX3 + TBX21	529	9,0	1,0E+00	1,0E+00	15
BCL6 + BCLAF1 + BRD4 + CBFB + EBF1 + EED + ERG + GABPA + GATA3 + IRF4 + MYB + RUNX1 + RUNX3 + YY1	724	9,5	1,0E+00	1,0E+00	15

BCL6 + BCLAF1 + BRD4 + CBFB + EED + GABPA + IRF4 + MAX + MYB + RBPJ + RUNX3 + SP1 + SPI1 + TBX21	684	9,4	1,0E+00	1,0E+00	15
BCL6 + BCLAF1 + BRD4 + EBF1 + ERG + GTF2F1 + HCFC1 + IRF4 + MAX + MYB + SP1 + SPI1 + TBX21 + YY1	474	8,9	1,0E+00	1,0E+00	15
BCL6 + BCLAF1 + BRD4 + EBF1 + GABPA + GTF2F1 + HCFC1 + IRF4 + MAX + MYB + RBPJ + SP1 + SPI1 + TBX21	508	9,0	1,0E+00	1,0E+00	15
BCL6 + BCLAF1 + BRD4 + EBF1 + GABPA + GTF2F1 + HCFC1 + IRF4 + MAX + MYB + SP1 + SPI1 + TBX21 + YY1	470	8,9	1,0E+00	1,0E+00	15
BCL6 + BCLAF1 + BRD4 + EBF1 + GABPA + GTF2F1 + IRF4 + MAX + MYB + MYC + RBPJ + SP1 + SPI1 + TBX21	451	8,8	1,0E+00	1,0E+00	15
BCL6 + BRD4 + CDK7 + EBF1 + EZH2 + GABPA + GATA3 + IRF4 + MYB + MYC + RBPJ + RUNX1 + RUNX3 + TBX21	297	8,2	1,0E+00	1,0E+00	15
BCL6 + BRD4 + CDK7 + EED + ELF1 + ERG + EZH2 + GATA3 + MAX + MYB + NOTCH1 + RUNX1 + RUNX3 + SPI1	353	8,5	1,0E+00	1,0E+00	15
BCLAF1 + BRD4 + CBFB + CDK7 + EBF1 + EED + ERG + HCFC1 + IRF4 + MYB + NOTCH1 + TBX21 + YY1 + ZEB1	363	8,5	1,0E+00	1,0E+00	15
BCLAF1 + BRD4 + CBFB + EBF1 + EED + ERG + GABPA + GATA3 + IRF4 + MYB + PAX5 + RUNX1 + RUNX3 + TCF3	798	9,6	1,0E+00	1,0E+00	15

BCLAF1 + BRD4 + CBFB + EBF1 + EED + GABPA + GTF2F1 + IRF4 + MYB + NOTCH1 + PAX5 + RUNX3 + TBX21 + TCF3	658	9,4	1,0E+00	1,0E+00	15
BCLAF1 + BRD4 + CBFB + EED + ELF1 + GTF2F1 + HCFC1 + IRF4 + MYB + PAX5 + RUNX3 + TBX21 + TCF3 + YY1	463	8,9	1,0E+00	1,0E+00	15
BCLAF1 + BRD4 + CBFB + EED + ELF1 + GTF2F1 + HCFC1 + IRF4 + PAX5 + RUNX3 + TBX21 + TCF3 + YY1 + ZEB1	428	8,7	1,0E+00	1,0E+00	15
BCLAF1 + BRD4 + CDK7 + EBF1 + EED + GATA3 + MYB + PAX5 + RUNX1 + RUNX3 + SP1 + SPI1 + TBX21 + YY1	585	9,2	1,0E+00	1,0E+00	15
BCLAF1 + BRD4 + EBF1 + EED + ELF1 + EZH2 + GABPA + GATA3 + GTF2F1 + HCFC1 + PAX5 + SP1 + SPI1 + TCF3	248	8,0	1,0E+00	1,0E+00	15
BCL6 + BCLAF1 + BRD4 + CBFB + CDK7 + EBF1 + EED + GABPA + GATA3 + MYB + MYC + NOTCH1 + RUNX1 + RUNX3 + TBX21	499	9,0	1,0E+00	1,0E+00	16
BCL6 + BCLAF1 + BRD4 + CBFB + EBF1 + EED + ERG + GABPA + GATA3 + IRF4 + MYB + PAX5 + RUNX1 + RUNX3 + YY1	722	9,5	1,0E+00	1,0E+00	16
BCL6 + BCLAF1 + BRD4 + CBFB + EED + GABPA + IRF4 + MAX + MYB + RBPJ + RUNX3 + SP1 + SPI1 + TBX21 + TCF3	640	9,3	1,0E+00	1,0E+00	16
BCL6 + BCLAF1 + BRD4 + EBF1 + EED + GABPA + GTF2F1 + IRF4 + MAX + MYB + MYC + RBPJ + SP1 + SPI1 + TBX21	442	8,8	1,0E+00	1,0E+00	16

BCL6 + BCLAF1 + BRD4 + EBF1 + ERG + GABPA + GTF2F1 + HCFC1 + IRF4 + MAX + MYB + SP1 + SPI1 + TBX21 + YY1	465	8,9	1,0E+00	1,0E+00	16
BCL6 + BRD4 + CBFB + CDK7 + EED + ELF1 + ERG + EZH2 + GATA3 + MAX + MYB + NOTCH1 + RUNX1 + RUNX3 + SPI1	346	8,4	1,0E+00	1,0E+00	16
BCL6 + BRD4 + CDK7 + EBF1 + EED + ELF1 + GABPA + GATA3 + IRF4 + MYB + MYC + RUNX1 + RUNX3 + SPI1 + TCF3	548	9,1	1,0E+00	1,0E+00	16
BCL6 + BRD4 + CDK7 + EBF1 + EZH2 + GABPA + GATA3 + IRF4 + MAX + MYB + MYC + RBPJ + RUNX1 + RUNX3 + TBX21	295	8,2	1,0E+00	1,0E+00	16
BCLAF1 + BRD4 + CBFB + CDK7 + EBF1 + EED + ERG + HCFC1 + IRF4 + MYB + NOTCH1 + RUNX3 + TBX21 + YY1 + ZEB1	344	8,4	1,0E+00	1,0E+00	16
BCLAF1 + BRD4 + CBFB + EBF1 + EED + ELF1 + GABPA + GTF2F1 + IRF4 + MYB + NOTCH1 + PAX5 + RUNX3 + TBX21 + TCF3	478	8,9	1,0E+00	1,0E+00	16
BCLAF1 + BRD4 + CBFB + EBF1 + EED + ELF1 + GTF2F1 + HCFC1 + IRF4 + MYB + PAX5 + RUNX3 + TBX21 + TCF3 + YY1	429	8,7	1,0E+00	1,0E+00	16
BCLAF1 + BRD4 + CBFB + EBF1 + EED + ERG + GABPA + GATA3 + IRF4 + MYB + PAX5 + RUNX1 + RUNX3 + TCF3 + YY1	730	9,5	1,0E+00	1,0E+00	16
BCLAF1 + BRD4 + CBFB + EBF1 + EED + GABPA + GTF2F1 + IRF4 + MYB + NOTCH1 + PAX5 + RUNX3 + SPI1 + TBX21 + TCF3	577	9,2	1,0E+00	1,0E+00	16

BCLAF1 + BRD4 + CDK7 + EBF1 + EED + ELF1 + GATA3 + MYB + PAX5 + RUNX1 + RUNX3 + SP1 + SPI1 + TBX21 + YY1	552	9,1	1,0E+00	1,0E+00	16
BCLAF1 + BRD4 + EBF1 + EED + ELF1 + EZH2 + GABPA + GATA3 + GTF2F1 + HCFC1 + PAX5 + SP1 + SPI1 + TBX21 + TCF3	238	7,9	1,0E+00	1,0E+00	16
BCL6 + BCLAF1 + BRD4 + CBFB + CDK7 + EBF1 + EED + GABPA + GATA3 + MAX + MYB + MYC + NOTCH1 + RUNX1 + RUNX3 + TBX21	497	9,0	1,0E+00	1,0E+00	17
BCL6 + BCLAF1 + BRD4 + CBFB + EBF1 + EED + ERG + GABPA + GATA3 + IRF4 + MYB + PAX5 + RUNX1 + RUNX3 + TCF3 + YY1	687	9,4	1,0E+00	1,0E+00	17
BCL6 + BCLAF1 + BRD4 + CBFB + EED + GABPA + IRF4 + MAX + MYB + PAX5 + RBPJ + RUNX3 + SP1 + SPI1 + TBX21 + TCF3	622	9,3	1,0E+00	1,0E+00	17
BCL6 + BCLAF1 + BRD4 + EBF1 + EED + ELF1 + EZH2 + GABPA + GATA3 + GTF2F1 + HCFC1 + PAX5 + SP1 + SPI1 + TBX21 + TCF3	235	7,9	1,0E+00	1,0E+00	17
BCL6 + BCLAF1 + BRD4 + EBF1 + EED + GABPA + GTF2F1 + IRF4 + MAX + MYB + MYC + RBPJ + RUNX1 + SP1 + SPI1 + TBX21	416	8,7	1,0E+00	1,0E+00	17
BCL6 + BRD4 + CBFB + CDK7 + EED + ELF1 + ERG + EZH2 + GABPA + GATA3 + MAX + MYB + NOTCH1 + RUNX1 + RUNX3 + SPI1	341	8,4	1,0E+00	1,0E+00	17
BCL6 + BRD4 + CDK7 + EBF1 + EED + ELF1 + GABPA + GATA3 + IRF4 + MYB + MYC + NOTCH1 + RUNX1 + RUNX3 + SPI1 + TCF3	547	9,1	1,0E+00	1,0E+00	17

BCL6 + BRD4 + CDK7 + EBF1 + EZH2 + GABPA + GATA3 + IRF4 + MAX + MYB + MYC + RBPJ + RUNX1 + RUNX3 + TBX21 + ZEB1	274	8,1	1,0E+00	1,0E+00	17
BCLAF1 + BRD4 + CBFB + CDK7 + EBF1 + EED + ELF1 + GATA3 + MYB + PAX5 + RUNX1 + RUNX3 + SP1 + SPI1 + TBX21 + YY1	543	9,1	1,0E+00	1,0E+00	17
BCLAF1 + BRD4 + CBFB + CDK7 + EBF1 + EED + ERG + HCFC1 + IRF4 + MAX + MYB + NOTCH1 + RUNX3 + TBX21 + YY1 + ZEB1	341	8,4	1,0E+00	1,0E+00	17
BCLAF1 + BRD4 + CBFB + EBF1 + EED + ELF1 + GABPA + GTF2F1 + HCFC1 + IRF4 + MYB + NOTCH1 + PAX5 + RUNX3 + TBX21 + TCF3	308	8,3	1,0E+00	1,0E+00	17
BCLAF1 + BRD4 + CBFB + EBF1 + EED + ELF1 + GABPA + GTF2F1 + HCFC1 + IRF4 + MYB + PAX5 + RUNX3 + TBX21 + TCF3 + YY1	413	8,7	1,0E+00	1,0E+00	17
BCLAF1 + BRD4 + CBFB + EBF1 + EED + ELF1 + GTF2F1 + HCFC1 + IRF4 + PAX5 + RUNX3 + SP1 + TBX21 + TCF3 + YY1 + ZEB1	415	8,7	1,0E+00	1,0E+00	17
BCLAF1 + BRD4 + CBFB + EBF1 + EED + GABPA + GTF2F1 + IRF4 + MYB + NOTCH1 + PAX5 + RUNX3 + SP1 + SPI1 + TBX21 + TCF3	560	9,1	1,0E+00	1,0E+00	17
BCLAF1 + BRD4 + CBFB + EED + GABPA + GTF2F1 + HCFC1 + IRF4 + MYB + PAX5 + RUNX3 + SP1 + SPI1 + TBX21 + TCF3 + YY1	569	9,2	1,0E+00	1,0E+00	17

BCL6 + BCLAF1 + BRD4 + CBFB + EBF1 + EED + ELF1 + EZH2 + GABPA + GATA3 + GTF2F1 + HCFC1 + PAX5 + SP1 + SPI1 + TBX21 + TCF3	226	7,8	1,0E+00	1,0E+00	18
BCL6 + BCLAF1 + BRD4 + CBFB + EED + GABPA + IRF4 + MAX + MYB + PAX5 + RBPJ + RUNX3 + SP1 + SPI1 + TBX21 + TCF3 + ZEB1	547	9,1	1,0E+00	1,0E+00	18
BCL6 + BCLAF1 + BRD4 + EBF1 + EED + EZH2 + GABPA + GTF2F1 + IRF4 + MAX + MYB + MYC + RBPJ + RUNX1 + SP1 + SPI1 + TBX21	202	7,7	1,0E+00	1,0E+00	18
BCL6 + BRD4 + CBFB + CDK7 + EED + ELF1 + ERG + EZH2 + GABPA + GATA3 + MAX + MYB + MYC + NOTCH1 + RUNX1 + RUNX3 + SPI1	323	8,3	1,0E+00	1,0E+00	18
BCL6 + BRD4 + CDK7 + EBF1 + EED + ELF1 + GABPA + GATA3 + IRF4 + MYB + MYC + NOTCH1 + RUNX1 + RUNX3 + SPI1 + TBX21 + TCF3	475	8,9	1,0E+00	1,0E+00	18
BCL6 + BRD4 + CDK7 + EBF1 + EED + EZH2 + GABPA + GTF2F1 + IRF4 + MAX + MYB + MYC + RBPJ + RUNX1 + SP1 + SPI1 + TBX21	280	8,1	1,0E+00	1,0E+00	18
BCL6 + BRD4 + CDK7 + EBF1 + ERG + EZH2 + GABPA + GATA3 + IRF4 + MAX + MYB + MYC + RBPJ + RUNX1 + RUNX3 + TBX21 + ZEB1	273	8,1	1,0E+00	1,0E+00	18
BCL6 + CBFB + EBF1 + EED + ERG + EZH2 + GABPA + GATA3 + IRF4 + MYB + NOTCH1 + PAX5 + RUNX1 + RUNX3 + STAT5B + TBX21 + TCF3	89	6,5	1,0E+00	1,0E+00	18

BCLAF1 + BRD4 + CBFB + CDK7 + EBF1 + EED + ERG + HCFC1 + IRF4 + MAX + MYB + NOTCH1 + RUNX3 + SP1 + TBX21 + YY1 + ZEB1	338	8,4	1,0E+00	1,0E+00	18
BCLAF1 + BRD4 + CBFB + EBF1 + EED + ELF1 + GABPA + GTF2F1 + HCFC1 + IRF4 + MYB + NOTCH1 + PAX5 + RUNX3 + TBX21 + TCF3 + YY1	307	8,3	1,0E+00	1,0E+00	18
BCLAF1 + BRD4 + CBFB + EBF1 + EED + ELF1 + GTF2F1 + HCFC1 + IRF4 + PAX5 + RUNX3 + SP1 + SPI1 + TBX21 + TCF3 + YY1 + ZEB1	409	8,7	1,0E+00	1,0E+00	18
BCLAF1 + BRD4 + CBFB + EBF1 + EED + GABPA + GTF2F1 + HCFC1 + IRF4 + MYB + NOTCH1 + PAX5 + RUNX3 + SP1 + SPI1 + TBX21 + TCF3	404	8,7	1,0E+00	1,0E+00	18
BCLAF1 + BRD4 + CBFB + EBF1 + EED + GABPA + GTF2F1 + HCFC1 + IRF4 + MYB + PAX5 + RUNX3 + SP1 + SPI1 + TBX21 + TCF3 + YY1	562	9,1	1,0E+00	1,0E+00	18
BCL6 + BCLAF1 + BRD4 + CBFB + CDK7 + EBF1 + EED + ERG + GABPA + GATA3 + MAX + MYB + MYC + NOTCH1 + PAX5 + RUNX1 + RUNX3 + TBX21	451	8,8	1,0E+00	1,0E+00	19
BCL6 + BCLAF1 + BRD4 + CBFB + EBF1 + EED + ELF1 + EZH2 + GABPA + GATA3 + GTF2F1 + HCFC1 + PAX5 + SP1 + SPI1 + TBX21 + TCF3 + YY1	225	7,8	1,0E+00	1,0E+00	19

BCL6 + BCLAF1 + BRD4 + CBFB + EED + GABPA + GATA3 + IRF4 + MAX + MYB + PAX5 + RBPJ + RUNX3 + SP1 + SPI1 + TBX21 + TCF3 + ZEB1	499	9,0	1,0E+00	1,0E+00	19
BCL6 + BCLAF1 + BRD4 + CDK7 + EBF1 + EED + EZH2 + GABPA + GTF2F1 + IRF4 + MAX + MYB + MYC + RBPJ + RUNX1 + SP1 + SPI1 + TBX21	196	7,6	1,0E+00	1,0E+00	19
BCL6 + BRD4 + CBFB + CDK7 + EED + ELF1 + ERG + EZH2 + GABPA + GATA3 + MAX + MYB + MYC + NOTCH1 + PAX5 + RUNX1 + RUNX3 + SPI1	260	8,0	1,0E+00	1,0E+00	19
BCL6 + BRD4 + CDK7 + EBF1 + EED + ELF1 + GABPA + GATA3 + IRF4 + MYB + MYC + NOTCH1 + RUNX1 + RUNX3 + SPI1 + TBX21 + TCF3 + YY1	474	8,9	1,0E+00	1,0E+00	19
BCL6 + BRD4 + CDK7 + EBF1 + EED + EZH2 + GABPA + GTF2F1 + IRF4 + MAX + MYB + MYC + RBPJ + RUNX1 + SP1 + SPI1 + STAT5B + TBX21	118	6,9	1,0E+00	1,0E+00	19
BCL6 + BRD4 + CDK7 + EBF1 + ERG + EZH2 + GABPA + GATA3 + IRF4 + MAX + MYB + MYC + PAX5 + RBPJ + RUNX1 + RUNX3 + TBX21 + ZEB1	256	8,0	1,0E+00	1,0E+00	19
BCLAF1 + BRD4 + CBFB + CDK7 + EBF1 + EED + ELF1 + ERG + GABPA + GATA3 + MYB + PAX5 + RUNX1 + RUNX3 + SP1 + SPI1 + TBX21 + YY1	539	9,1	1,0E+00	1,0E+00	19

BCLAF1 + BRD4 + CBFB + CDK7 + EBF1 + EED + ERG + GTF2F1 + HCFC1 + IRF4 + MAX + MYB + NOTCH1 + RUNX3 + SP1 + TBX21 + YY1 + ZEB1	329	8,4	1,0E+00	1,0E+00	19
BCLAF1 + BRD4 + CBFB + EBF1 + EED + ELF1 + GTF2F1 + HCFC1 + IRF4 + MYC + PAX5 + RUNX3 + SP1 + SPI1 + TBX21 + TCF3 + YY1 + ZEB1	234	7,9	1,0E+00	1,0E+00	19
BCLAF1 + BRD4 + CBFB + EBF1 + EED + GABPA + GTF2F1 + HCFC1 + IRF4 + MYB + NOTCH1 + PAX5 + RUNX3 + SP1 + SPI1 + TBX21 + TCF3 + YY1	403	8,7	1,0E+00	1,0E+00	19
BCL6 + BCLAF1 + BRD4 + CBFB + CDK7 + EBF1 + EED + ELF1 + ERG + GABPA + GATA3 + MAX + MYB + MYC + NOTCH1 + PAX5 + RUNX1 + RUNX3 + TBX21	412	8,7	1,0E+00	1,0E+00	20
BCL6 + BCLAF1 + BRD4 + CBFB + EBF1 + EED + ELF1 + EZH2 + GABPA + GATA3 + GTF2F1 + HCFC1 + PAX5 + SP1 + SPI1 + TBX21 + TCF3 + YY1 + ZEB1	199	7,6	1,0E+00	1,0E+00	20
BCL6 + BRD4 + CDK7 + EBF1 + EED + ELF1 + GABPA + GATA3 + IRF4 + MAX + MYB + MYC + NOTCH1 + RUNX1 + RUNX3 + SPI1 + TBX21 + TCF3 + YY1	473	8,9	1,0E+00	1,0E+00	20
BCL6 + BRD4 + CDK7 + EBF1 + EED + ERG + EZH2 + GABPA + GATA3 + IRF4 + MAX + MYB + MYC + PAX5 + RBPJ + RUNX1 + RUNX3 + TBX21 + ZEB1	218	7,8	1,0E+00	1,0E+00	20

BCL6 + BRD4 + CDK7 + EBF1 + EED + EZH2 + GABPA + GTF2F1 + IRF4 + MAX + MYB + MYC + NOTCH1 + RBPJ + RUNX1 + SP1 + SPI1 + STAT5B + TBX21	103	6,7	1,0E+00	1,0E+00	20
BCLAF1 + BRD4 + CBFB + CDK7 + EBF1 + EED + ELF1 + ERG + GABPA + GATA3 + GTF2F1 + MYB + PAX5 + RUNX1 + RUNX3 + SP1 + SPI1 + TBX21 + YY1	523	9,0	1,0E+00	1,0E+00	20
BCLAF1 + BRD4 + CBFB + CDK7 + EBF1 + EED + ERG + GTF2F1 + HCFC1 + IRF4 + MAX + MYB + NOTCH1 + RUNX3 + SP1 + TBX21 + TCF3 + YY1 + ZEB1	310	8,3	1,0E+00	1,0E+00	20
BCLAF1 + BRD4 + CBFB + EBF1 + EED + ELF1 + GTF2F1 + HCFC1 + IRF4 + MYB + MYC + PAX5 + RUNX3 + SP1 + SPI1 + TBX21 + TCF3 + YY1 + ZEB1	230	7,8	1,0E+00	1,0E+00	20
BCL6 + BCLAF1 + BRD4 + CBFB + CDK7 + EBF1 + EED + ELF1 + ERG + GABPA + GATA3 + MAX + MYB + MYC + NOTCH1 + PAX5 + RUNX1 + RUNX3 + TBX21 + YY1	411	8,7	1,0E+00	1,0E+00	21
BCL6 + BCLAF1 + BRD4 + CBFB + EBF1 + EED + ELF1 + EZH2 + GABPA + GATA3 + GTF2F1 + HCFC1 + IRF4 + PAX5 + SP1 + SPI1 + TBX21 + TCF3 + YY1 + ZEB1	194	7,6	1,0E+00	1,0E+00	21
BCL6 + BCLAF1 + BRD4 + CBFB + EBF1 + EED + GABPA + GATA3 + GTF2F1 + IRF4 + MAX + MYB + PAX5 + RBPJ + RUNX1 + RUNX3 + SPI1 + TBX21 + TCF3 + ZEB1	514	9,0	1,0E+00	1,0E+00	21

BCL6 + BCLAF1 + BRD4 + CBFB + EED + GABPA + GATA3 + GTF2F1 + IRF4 + MAX + MYB + PAX5 + RBPJ + RUNX1 + RUNX3 + SP1 + SPI1 + TBX21 + TCF3 + ZEB1	479	8,9	1,0E+00	1,0E+00	21
BCL6 + BRD4 + CDK7 + EBF1 + EED + ELF1 + GABPA + GATA3 + IRF4 + MAX + MYB + MYC + NOTCH1 + RBPJ + RUNX1 + RUNX3 + SPI1 + TBX21 + TCF3 + YY1	472	8,9	1,0E+00	1,0E+00	21
BCL6 + BRD4 + CDK7 + EBF1 + EED + ERG + EZH2 + GABPA + GATA3 + IRF4 + MAX + MYB + MYC + NOTCH1 + PAX5 + RBPJ + RUNX1 + RUNX3 + TBX21 + ZEB1	206	7,7	1,0E+00	1,0E+00	21
BCL6 + BRD4 + CDK7 + EBF1 + EED + ERG + EZH2 + GABPA + GTF2F1 + IRF4 + MAX + MYB + MYC + NOTCH1 + RBPJ + RUNX1 + SP1 + SPI1 + STAT5B + TBX21	94	6,6	1,0E+00	1,0E+00	21
BCLAF1 + BRD4 + CBFB + CDK7 + EBF1 + EED + ELF1 + ERG + GABPA + GATA3 + GTF2F1 + MYB + NOTCH1 + PAX5 + RUNX1 + RUNX3 + SP1 + SPI1 + TBX21 + YY1	513	9,0	1,0E+00	1,0E+00	21
BCLAF1 + BRD4 + CBFB + CDK7 + EBF1 + EED + ERG + GTF2F1 + HCFC1 + IRF4 + MAX + MYB + NOTCH1 + PAX5 + RUNX3 + SP1 + TBX21 + TCF3 + YY1 + ZEB1	301	8,2	1,0E+00	1,0E+00	21
BCL6 + BCLAF1 + BRD4 + CBFB + CDK7 + EBF1 + EED + ELF1 + ERG + GABPA + GATA3 + IRF4 + MAX + MYB + MYC + NOTCH1 + PAX5 + RUNX1 + RUNX3 + TBX21 + YY1	404	8,7	1,0E+00	1,0E+00	22

BCL6 + BCLAF1 + BRD4 + CBFB + EBF1 + EED + ELF1 + ERG + EZH2 + GABPA + GATA3 + GTF2F1 + HCFC1 + IRF4 + PAX5 + SP1 + SPI1 + TBX21 + TCF3 + YY1 + ZEB1	189	7,6	1,0E+00	1,0E+00	22
BCL6 + BCLAF1 + BRD4 + CBFB + EBF1 + EED + ELF1 + GABPA + GATA3 + GTF2F1 + IRF4 + MAX + MYB + PAX5 + RBPJ + RUNX1 + RUNX3 + SPI1 + TBX21 + TCF3 + ZEB1	464	8,9	1,0E+00	1,0E+00	22
BCL6 + BCLAF1 + BRD4 + CBFB + EBF1 + EED + GABPA + GATA3 + GTF2F1 + IRF4 + MAX + MYB + PAX5 + RBPJ + RUNX1 + RUNX3 + SP1 + SPI1 + TBX21 + TCF3 + ZEB1	477	8,9	1,0E+00	1,0E+00	22
BCL6 + BCLAF1 + BRD4 + CDK7 + EBF1 + EED + ELF1 + GABPA + GATA3 + IRF4 + MAX + MYB + MYC + NOTCH1 + RBPJ + RUNX1 + RUNX3 + SPI1 + TBX21 + TCF3 + YY1	440	8,8	1,0E+00	1,0E+00	22
BCL6 + BCLAF1 + BRD4 + CDK7 + EBF1 + EED + ERG + EZH2 + GABPA + GATA3 + IRF4 + MAX + MYB + MYC + NOTCH1 + PAX5 + RBPJ + RUNX1 + RUNX3 + TBX21 + ZEB1	199	7,6	1,0E+00	1,0E+00	22
BCL6 + BCLAF1 + BRD4 + CDK7 + EBF1 + EED + ERG + GABPA + GATA3 + IRF4 + MAX + MYB + MYC + NOTCH1 + PAX5 + RBPJ + RUNX1 + RUNX3 + TBX21 + YY1 + ZEB1	407	8,7	1,0E+00	1,0E+00	22

BCL6 + BRD4 + CDK7 + EBF1 + EED + ERG + EZH2 + GABPA + GTF2F1 + IRF4 + MAX + MYB + MYC + NOTCH1 + RBPJ + RUNX1 + SP1 + SPI1 + STAT5B + TBX21 + YY1	83	6,4	1,0E+00	1,0E+00	22
BCLAF1 + BRD4 + CBFB + CDK7 + EBF1 + EED + ELF1 + ERG + GABPA + GATA3 + GTF2F1 + MYB + NOTCH1 + PAX5 + RUNX1 + RUNX3 + SP1 + SPI1 + TBX21 + TCF3 + YY1	466	8,9	1,0E+00	1,0E+00	22
BCLAF1 + BRD4 + CBFB + CDK7 + EBF1 + EED + ERG + GTF2F1 + HCFC1 + IRF4 + MAX + MYB + MYC + NOTCH1 + PAX5 + RUNX3 + SP1 + TBX21 + TCF3 + YY1 + ZEB1	294	8,2	1,0E+00	1,0E+00	22
BCL6 + BCLAF1 + BRD4 + CBFB + CDK7 + EBF1 + EED + ELF1 + ERG + EZH2 + GABPA + GATA3 + GTF2F1 + HCFC1 + IRF4 + PAX5 + SP1 + SPI1 + TBX21 + TCF3 + YY1 + ZEB1	184	7,5	1,0E+00	1,0E+00	23
BCL6 + BCLAF1 + BRD4 + CBFB + CDK7 + EBF1 + EED + ELF1 + ERG + GABPA + GATA3 + IRF4 + MAX + MYB + MYC + NOTCH1 + PAX5 + RUNX1 + RUNX3 + SP1 + TBX21 + YY1	394	8,6	1,0E+00	1,0E+00	23
BCL6 + BCLAF1 + BRD4 + CBFB + EBF1 + EED + ELF1 + ERG + GABPA + GATA3 + GTF2F1 + IRF4 + MAX + MYB + PAX5 + RBPJ + RUNX1 + RUNX3 + SPI1 + TBX21 + TCF3 + ZEB1	455	8,8	1,0E+00	1,0E+00	23

BCL6 + BCLAF1 + BRD4 + CDK7 + EBF1 + EED + ELF1 + ERG + GABPA + GATA3 + IRF4 + MAX + MYB + MYC + NOTCH1 + PAX5 + RBPJ + RUNX1 + RUNX3 + TBX21 + YY1 + ZEB1	365	8,5	1,0E+00	1,0E+00	23
BCL6 + BCLAF1 + BRD4 + CDK7 + EBF1 + EED + ELF1 + ERG + GABPA + GATA3 + IRF4 + MAX + MYB + MYC + NOTCH1 + RBPJ + RUNX1 + RUNX3 + SPI1 + TBX21 + TCF3 + YY1	431	8,8	1,0E+00	1,0E+00	23
BCL6 + BCLAF1 + BRD4 + CDK7 + EBF1 + EED + ERG + EZH2 + GABPA + GATA3 + IRF4 + MAX + MYB + MYC + NOTCH1 + PAX5 + RBPJ + RUNX1 + RUNX3 + TBX21 + YY1 + ZEB1	196	7,6	1,0E+00	1,0E+00	23
BCLAF1 + BRD4 + CBFB + CDK7 + EBF1 + EED + ELF1 + ERG + GABPA + GATA3 + GTF2F1 + MAX + MYB + NOTCH1 + PAX5 + RUNX1 + RUNX3 + SP1 + SPI1 + TBX21 + TCF3 + YY1	440	8,8	1,0E+00	1,0E+00	23
BCLAF1 + BRD4 + CBFB + CDK7 + EBF1 + EED + ERG + GABPA + GTF2F1 + HCFC1 + IRF4 + MAX + MYB + MYC + NOTCH1 + PAX5 + RUNX3 + SP1 + TBX21 + TCF3 + YY1 + ZEB1	292	8,2	1,0E+00	1,0E+00	23
BCL6 + BCLAF1 + BRD4 + CBFB + CDK7 + EBF1 + EED + ELF1 + ERG + EZH2 + GABPA + GATA3 + GTF2F1 + HCFC1 + IRF4 + PAX5 + RUNX1 + SP1 + SPI1 + TBX21 + TCF3 + YY1 + ZEB1	183	7,5	1,0E+00	1,0E+00	24

BCL6 + BCLAF1 + BRD4 + CBFB + CDK7 + EBF1 + EED + ELF1 + ERG + GABPA + GATA3 + HCFC1 + IRF4 + MAX + MYB + MYC + NOTCH1 + PAX5 + RUNX1 + RUNX3 + SP1 + TBX21 + YY1	296	8,2	1,0E+00	1,0E+00	24
BCL6 + BCLAF1 + BRD4 + CBFB + CDK7 + EBF1 + EED + ERG + GABPA + GTF2F1 + HCFC1 + IRF4 + MAX + MYB + MYC + NOTCH1 + PAX5 + RUNX3 + SP1 + TBX21 + TCF3 + YY1 + ZEB1	286	8,2	1,0E+00	1,0E+00	24
BCL6 + BCLAF1 + BRD4 + CBFB + EBF1 + EED + ELF1 + ERG + GABPA + GATA3 + GTF2F1 + IRF4 + MAX + MYB + PAX5 + RBPJ + RUNX1 + RUNX3 + SP1 + SPI1 + TBX21 + TCF3 + ZEB1	452	8,8	1,0E+00	1,0E+00	24
BCL6 + BCLAF1 + BRD4 + CBFB + EBF1 + EED + ELF1 + ERG + GABPA + GTF2F1 + HCFC1 + IRF4 + MAX + PAX5 + RBPJ + RUNX1 + RUNX3 + SP1 + SPI1 + TBX21 + TCF3 + YY1 + ZEB1	338	8,4	1,0E+00	1,0E+00	24
BCL6 + BCLAF1 + BRD4 + CDK7 + EBF1 + EED + ELF1 + ERG + GABPA + GATA3 + IRF4 + MAX + MYB + MYC + NOTCH1 + PAX5 + RBPJ + RUNX1 + RUNX3 + SPI1 + TBX21 + YY1 + ZEB1	362	8,5	1,0E+00	1,0E+00	24
BCL6 + BCLAF1 + BRD4 + CDK7 + EBF1 + EED + ELF1 + ERG + GABPA + GATA3 + IRF4 + MAX + MYB + MYC + NOTCH1 + RBPJ + RUNX1 + RUNX3 + SPI1 + TBX21 + TCF3 + YY1 + ZEB1	394	8,6	1,0E+00	1,0E+00	24

BCL6 + BCLAF1 + BRD4 + CDK7 + EBF1 + ELF1 + ERG + EZH2 + GABPA + GATA3 + IRF4 + MAX + MYB + MYC + NOTCH1 + PAX5 + RBPJ + RUNX1 + SP1 + SPI1 + TBX21 + TCF3 + YY1	196	7,6	1,0E+00	1,0E+00	24
BCL6 + BRD4 + CBFB + CDK7 + EBF1 + EED + ELF1 + ERG + EZH2 + GABPA + GTF2F1 + IRF4 + MAX + MYB + MYC + NOTCH1 + RBPJ + RUNX1 + SP1 + SPI1 + STAT5B + TBX21 + YY1	63	6,0	1,0E+00	1,0E+00	24
BCLAF1 + BRD4 + CBFB + CDK7 + EBF1 + EED + ELF1 + ERG + GABPA + GATA3 + GTF2F1 + IRF4 + MAX + MYB + NOTCH1 + PAX5 + RUNX1 + RUNX3 + SP1 + SPI1 + TBX21 + TCF3 + YY1	429	8,7	1,0E+00	1,0E+00	24
BCL6 + BCLAF1 + BRD4 + CBFB + CDK7 + EBF1 + EED + ELF1 + ERG + EZH2 + GABPA + GATA3 + GTF2F1 + HCFC1 + IRF4 + NOTCH1 + PAX5 + RUNX1 + SP1 + SPI1 + TBX21 + TCF3 + YY1 + ZEB1	168	7,4	1,0E+00	1,0E+00	25
BCL6 + BCLAF1 + BRD4 + CBFB + CDK7 + EBF1 + EED + ELF1 + ERG + GABPA + GATA3 + GTF2F1 + IRF4 + MAX + MYB + NOTCH1 + PAX5 + RUNX1 + RUNX3 + SP1 + SPI1 + TBX21 + TCF3 + YY1	428	8,7	1,0E+00	1,0E+00	25
BCL6 + BCLAF1 + BRD4 + CBFB + CDK7 + EBF1 + EED + ELF1 + ERG + GABPA + GATA3 + HCFC1 + IRF4 + MAX + MYB + MYC + NOTCH1 + PAX5 + RUNX1 + RUNX3 + SP1 + TBX21 + TCF3 + YY1	291	8,2	1,0E+00	1,0E+00	25

BCL6 + BCLAF1 + BRD4 + CBFB + CDK7 + EBF1 + EED + ERG + GABPA + GTF2F1 + HCFC1 + IRF4 + MAX + MYB + MYC + NOTCH1 + PAX5 + RUNX3 + SP1 + SPI1 + TBX21 + TCF3 + YY1 + ZEB1	284	8,1	1,0E+00	1,0E+00	25
BCL6 + BCLAF1 + BRD4 + CBFB + EBF1 + EED + ELF1 + ERG + GABPA + GATA3 + GTF2F1 + HCFC1 + IRF4 + MAX + MYB + PAX5 + RBPJ + RUNX1 + RUNX3 + SP1 + SPI1 + TBX21 + TCF3 + ZEB1	338	8,4	1,0E+00	1,0E+00	25
BCL6 + BCLAF1 + BRD4 + CBFB + EBF1 + EED + ELF1 + ERG + GABPA + GATA3 + GTF2F1 + HCFC1 + IRF4 + MAX + PAX5 + RBPJ + RUNX1 + RUNX3 + SP1 + SPI1 + TBX21 + TCF3 + YY1 + ZEB1	324	8,3	1,0E+00	1,0E+00	25
BCL6 + BCLAF1 + BRD4 + CDK7 + EBF1 + EED + ELF1 + ERG + GABPA + GATA3 + IRF4 + MAX + MYB + MYC + NOTCH1 + PAX5 + RBPJ + RUNX1 + RUNX3 + SPI1 + TBX21 + TCF3 + YY1 + ZEB1	354	8,5	1,0E+00	1,0E+00	25
BCL6 + BRD4 + CBFB + CDK7 + EBF1 + EED + ELF1 + ERG + EZH2 + GABPA + GATA3 + GTF2F1 + IRF4 + MAX + MYB + MYC + NOTCH1 + RBPJ + RUNX1 + SP1 + SPI1 + STAT5B + TBX21 + YY1	37	5,2	1,0E+00	1,0E+00	25
BCL6 + BCLAF1 + BRD4 + CBFB + CDK7 + EBF1 + EED + ELF1 + ERG + EZH2 + GABPA + GATA3 + GTF2F1 + HCFC1 + IRF4 + MYB + NOTCH1 + PAX5 + RUNX1 + SP1 + SPI1 + TBX21 + TCF3 + YY1 + ZEB1	154	7,3	1,0E+00	1,0E+00	26

BCL6 + BCLAF1 + BRD4 + CBFB + CDK7 + EBF1 + EED + ELF1 + ERG + EZH2 + GABPA + GATA3 + GTF2F1 + IRF4 + MAX + MYB + MYC + NOTCH1 + RBPJ + RUNX1 + SP1 + SPI1 + STAT5B + TBX21 + YY1	36	5,2	1,0E+00	1,0E+00	26
BCL6 + BCLAF1 + BRD4 + CBFB + CDK7 + EBF1 + EED + ELF1 + ERG + GABPA + GATA3 + GTF2F1 + HCFC1 + IRF4 + MAX + MYB + MYC + NOTCH1 + PAX5 + RUNX1 + RUNX3 + SP1 + TBX21 + TCF3 + YY1	249	8,0	1,0E+00	1,0E+00	26
BCL6 + BCLAF1 + BRD4 + CBFB + CDK7 + EBF1 + EED + ELF1 + ERG + GABPA + GATA3 + GTF2F1 + HCFC1 + IRF4 + MAX + MYB + NOTCH1 + PAX5 + RUNX1 + RUNX3 + SP1 + SPI1 + TBX21 + TCF3 + YY1	281	8,1	1,0E+00	1,0E+00	26
BCL6 + BCLAF1 + BRD4 + CBFB + CDK7 + EBF1 + EED + ERG + GABPA + GTF2F1 + HCFC1 + IRF4 + MAX + MYB + MYC + NOTCH1 + PAX5 + RBPJ + RUNX3 + SP1 + SPI1 + TBX21 + TCF3 + YY1 + ZEB1	281	8,1	1,0E+00	1,0E+00	26
BCL6 + BCLAF1 + BRD4 + CBFB + EBF1 + EED + ELF1 + ERG + GABPA + GATA3 + GTF2F1 + HCFC1 + IRF4 + MAX + MYB + PAX5 + RBPJ + RUNX1 + RUNX3 + SP1 + SPI1 + TBX21 + TCF3 + YY1 + ZEB1	315	8,3	1,0E+00	1,0E+00	26

BCL6 + BCLAF1 + BRD4 + CBFB + CDK7 + EBF1 + EED + ELF1 + ERG + EZH2 + GABPA + GATA3 + GTF2F1 + HCFC1 + IRF4 + MAX + MYB + NOTCH1 + PAX5 + RUNX1 + SP1 + SPI1 + TBX21 + TCF3 + YY1 + ZEB1	147	7,2	1,0E+00	1,0E+00	27
BCL6 + BCLAF1 + BRD4 + CBFB + CDK7 + EBF1 + EED + ELF1 + ERG + EZH2 + GABPA + GATA3 + GTF2F1 + IRF4 + MAX + MYB + MYC + NOTCH1 + RBPJ + RUNX1 + SP1 + SPI1 + STAT5B + TBX21 + TCF3 + YY1	31	5,0	1,0E+00	1,0E+00	27
BCL6 + BCLAF1 + BRD4 + CBFB + CDK7 + EBF1 + EED + ELF1 + ERG + GABPA + GATA3 + GTF2F1 + HCFC1 + IRF4 + MAX + MYB + MYC + NOTCH1 + PAX5 + RUNX1 + RUNX3 + SP1 + TBX21 + TCF3 + YY1 + ZEB1	218	7,8	1,0E+00	1,0E+00	27
BCL6 + BCLAF1 + BRD4 + CBFB + CDK7 + EBF1 + EED + ELF1 + ERG + GABPA + GATA3 + GTF2F1 + HCFC1 + IRF4 + MAX + MYB + NOTCH1 + PAX5 + RBPJ + RUNX1 + RUNX3 + SP1 + SPI1 + TBX21 + TCF3 + YY1	279	8,1	1,0E+00	1,0E+00	27
BCL6 + BCLAF1 + BRD4 + CBFB + CDK7 + EBF1 + EED + ERG + GABPA + GTF2F1 + HCFC1 + IRF4 + MAX + MYB + MYC + NOTCH1 + PAX5 + RBPJ + RUNX1 + RUNX3 + SP1 + SPI1 + TBX21 + TCF3 + YY1 + ZEB1	269	8,1	1,0E+00	1,0E+00	27

BCL6 + BCLAF1 + BRD4 + CBFB + CDK7 + EBF1 + EED + ELF1 + ERG + EZH2 + GABPA + GATA3 + GTF2F1 + IRF4 + MAX + MYB + MYC + NOTCH1 + PAX5 + RBPJ + RUNX1 + SP1 + SPI1 + STAT5B + TBX21 + TCF3 + YY1	27	4,8	1,0E+00	1,0E+00	28
BCL6 + BCLAF1 + BRD4 + CBFB + CDK7 + EBF1 + EED + ELF1 + ERG + GABPA + GATA3 + GTF2F1 + HCFC1 + IRF4 + MAX + MYB + MYC + NOTCH1 + PAX5 + RBPJ + RUNX1 + RUNX3 + SP1 + SPI1 + TBX21 + TCF3 + YY1	242	7,9	1,0E+00	1,0E+00	28
BCL6 + BCLAF1 + BRD4 + CBFB + CDK7 + EBF1 + EED + ELF1 + ERG + GABPA + GATA3 + GTF2F1 + IRF4 + MAX + MYB + MYC + NOTCH1 + PAX5 + RBPJ + RUNX1 + RUNX3 + SP1 + SPI1 + TBX21 + TCF3 + YY1 + ZEB1	293	8,2	1,0E+00	1,0E+00	28
BCL6 + BCLAF1 + BRD4 + CBFB + CDK7 + EBF1 + EED + ELF1 + ERG + GABPA + GATA3 + HCFC1 + IRF4 + MAX + MYB + MYC + NOTCH1 + PAX5 + RBPJ + RUNX1 + RUNX3 + SP1 + SPI1 + STAT5B + TCF3 + YY1 + ZEB1	58	5,9	1,0E+00	1,0E+00	28
BCL6 + BCLAF1 + BRD4 + CBFB + CDK7 + EBF1 + EED + ERG + GABPA + GATA3 + GTF2F1 + HCFC1 + IRF4 + MAX + MYB + MYC + NOTCH1 + PAX5 + RBPJ + RUNX1 + RUNX3 + SP1 + SPI1 + TBX21 + TCF3 + YY1 + ZEB1	241	7,9	1,0E+00	1,0E+00	28

BCL6 + BCLAF1 + BRD4 + CBFB + CDK7 + EBF1 + EED + ELF1 + ERG + EZH2 + GABPA + GATA3 + GTF2F1 + IRF4 + MAX + MYB + MYC + NOTCH1 + PAX5 + RBPJ + RUNX1 + RUNX3 + SP1 + SPI1 + STAT5B + TBX21 + TCF3 + YY1	26	4,7	1,0E+00	1,0E+00	29
BCL6 + BCLAF1 + BRD4 + CBFB + CDK7 + EBF1 + EED + ELF1 + ERG + EZH2 + GABPA + GATA3 + GTF2F1 + IRF4 + MAX + MYB + MYC + NOTCH1 + PAX5 + RBPJ + RUNX1 + RUNX3 + SP1 + SPI1 + TBX21 + TCF3 + YY1 + ZEB1	165	7,4	1,0E+00	1,0E+00	29
BCL6 + BCLAF1 + BRD4 + CBFB + CDK7 + EBF1 + EED + ELF1 + ERG + EZH2 + GABPA + GATA3 + HCFC1 + IRF4 + MAX + MYB + MYC + NOTCH1 + PAX5 + RBPJ + RUNX1 + RUNX3 + SP1 + SPI1 + STAT5B + TCF3 + YY1 + ZEB1	35	5,1	1,0E+00	1,0E+00	29
BCL6 + BCLAF1 + BRD4 + CBFB + CDK7 + EBF1 + EED + ELF1 + ERG + EZH2 + GABPA + GATA3 + IRF4 + MAX + MYB + MYC + NOTCH1 + PAX5 + RBPJ + RUNX1 + RUNX3 + SP1 + SPI1 + STAT5B + TBX21 + TCF3 + YY1 + ZEB1	30	4,9	1,0E+00	1,0E+00	29
BCL6 + BCLAF1 + BRD4 + CBFB + CDK7 + EBF1 + EED + ELF1 + ERG + GABPA + GATA3 + GTF2F1 + HCFC1 + IRF4 + MAX + MYB + MYC + NOTCH1 + PAX5 + RBPJ + RUNX1 + RUNX3 + SP1 + SPI1 + TBX21 + TCF3 + YY1 + ZEB1	217	7,8	1,0E+00	1,0E+00	29

BCL6 + BCLAF1 + BRD4 + CBFB + CDK7 + EBF1 + EED + ELF1 + ERG + EZH2 + GABPA + GATA3 + GTF2F1 + HCFC1 + IRF4 + MAX + MYB + MYC + NOTCH1 + PAX5 + RBPJ + RUNX1 + RUNX3 + SP1 + SPI1 + TBX21 + TCF3 + YY1 + ZEB1	138	7,1	1,0E+00	1,0E+00	30
BCL6 + BCLAF1 + BRD4 + CBFB + CDK7 + EBF1 + EED + ELF1 + ERG + EZH2 + GABPA + GATA3 + GTF2F1 + IRF4 + MAX + MYB + MYC + NOTCH1 + PAX5 + RBPJ + RUNX1 + RUNX3 + SP1 + SPI1 + STAT5B + TBX21 + TCF3 + YY1 + ZEB1	15	3,9	1,0E+00	1,0E+00	30
BCL6 + BCLAF1 + BRD4 + CBFB + CDK7 + EBF1 + EED + ELF1 + ERG + EZH2 + GABPA + GATA3 + HCFC1 + IRF4 + MAX + MYB + MYC + NOTCH1 + PAX5 + RBPJ + RUNX1 + RUNX3 + SP1 + SPI1 + STAT5B + TBX21 + TCF3 + YY1 + ZEB1	8	3,0	1,0E+00	1,0E+00	30
