# Supplementary material for: Epigenetic regulation of transcription factor binding motifs promotes Th1 response in Chagas disease cardiomyopathy
Source: Front Immunol. 2022 Aug 22;13:958200. doi: 10.3389/fimmu.2022.958200 (PMC9441916; doi:10.3389/fimmu.2022.958200)
Supplement: Supplementary Table 1 — Biological samples included in this study. [file DataSheet_1.zip › Supplementary Material/Supplementary Table 11.pdf]

Supplementary table 11. Gene ontology analysis of the TFs binding our DMRs

ID	Term	Corrected pvalue	Levels	Nb. TF	% Associated TF	Associated TF Found
GO:0006370	7-methylguanosine mRNA capping	1,45E-03	[7, 8, 9, 10]	2,00	5,88	[CDK7, GTF2F1]
GO:0034243	regulation of transcription elongation from RNA polymerase II promoter	4,58E-04	[7, 8, 9, 10, 11, 12]	3,00	9,09	[BRD4, EZH2, GTF2F1]
GO:0043011	myeloid dendritic cell differentiation	1,35E-04	[5, 6, 7, 8, 9, 10, 11]	3,00	14,29	[IRF4, RBPJ, SPI1]
GO:0070317	negative regulation of G0 to G1 transition	8,98E-04	[4, 5, 6, 7]	3,00	6,52	[EED, EZH2, MAX]
GO:0070734	histone H3-K27 methylation	1,43E-04	[7, 8, 9, 10, 11]	3,00	13,64	[EED, EZH2, GATA3]
GO:1901532	regulation of hematopoietic progenitor cell differentiation	6,73E-09	[4, 5, 6, 7, 8, 9]	7,00	7,29	[CBFB, GATA3, MYB, NOTCH1, RUNX1, SPI1, TCF3]
GO:0002320	lymphoid progenitor cell differentiation	1,58E-04	[5, 6, 7, 8, 9]	3,00	13,04	[GATA3, NOTCH1, SPI1]
GO:1903306	negative regulation of regulated secretory pathway	3,13E-03	[5, 6, 7, 8, 9]	2,00	8,00	[NOTCH1, SPI1]
GO:0003176	aortic valve development	7,06E-04	[5, 7, 8, 9]	3,00	7,50	[GATA3, NOTCH1, RBPJ]
GO:0045623	negative regulation of T-helper cell differentiation	2,41E-03	[4, 5, 6, 9, 10, 11, 12, 13, 14, 15, 16, 17]	2,00	11,11	[BCL6, TBX21]
GO:0010614	negative regulation of cardiac muscle hypertrophy	2,11E-03	[4, 5, 6, 7, 8]	2,00	6,90	[NOTCH1, YY1]
GO:0016447	somatic recombination of immunoglobulin gene segments	7,92E-05	[4, 5, 7, 8, 9, 10]	4,00	6,67	[BCL6, TBX21, TCF3, YY1]
GO:0033152	immunoglobulin V(D)J recombination	1,04E-03	[5, 6, 8, 9, 10, 11]	2,00	25,00	[TCF3, YY1]
GO:0043374	CD8-positive, alpha-beta T cell differentiation	5,39E-05	[7, 8, 9, 10, 11, 12, 13]	3,00	20,00	[CBFB, RUNX1, RUNX3]
GO:0045589	regulation of regulatory T cell differentiation	4,58E-04	[7, 8, 9, 10, 11, 12, 13]	3,00	9,09	[BCL6, CBFB, RUNX1]
GO:0030857	negative regulation of epithelial cell differentiation	8,98E-04	[4, 5, 6, 7]	3,00	6,52	[EZH2, NOTCH1, ZEB1]
GO:0045604	regulation of epidermal cell differentiation	1,02E-04	[4, 5, 6, 7]	4,00	6,15	[CBFB, EZH2, NOTCH1, RUNX1]
GO:1901532	regulation of hematopoietic progenitor cell differentiation	6,73E-09	[4, 5, 6, 7, 8, 9]	7,00	7,29	[CBFB, GATA3, MYB, NOTCH1, RUNX1, SPI1, TCF3]
GO:0046638	positive regulation of alpha-beta T cell differentiation	3,43E-05	[7, 8, 9, 10, 11, 12, 13, 14]	4,00	8,33	[CBFB, MYB, RUNX1, RUNX3]
GO:0045604	regulation of epidermal cell differentiation	1,02E-04	[4, 5, 6, 7]	4,00	6,15	[CBFB, EZH2, NOTCH1, RUNX1]
GO:1901532	regulation of hematopoietic progenitor cell differentiation	6,73E-09	[4, 5, 6, 7, 8, 9]	7,00	7,29	[CBFB, GATA3, MYB, NOTCH1, RUNX1, SPI1, TCF3]
GO:0002320	lymphoid progenitor cell differentiation	1,58E-04	[5, 6, 7, 8, 9]	3,00	13,04	[GATA3, NOTCH1, SPI1]
GO:1902036	regulation of hematopoietic stem cell differentiation	5,53E-06	[5, 6, 7, 8, 9, 10]	5,00	6,41	[CBFB, GATA3, MYB, RUNX1, TCF3]
GO:0045622	regulation of T-helper cell differentiation	1,52E-05	[4, 5, 9, 10, 11, 12, 13, 14, 15, 16]	4,00	10,26	[BCL6, IRF4, MYB, TBX21]
GO:0045623	negative regulation of T-helper cell differentiation	2,41E-03	[4, 5, 6, 9, 10, 11, 12, 13, 14, 15, 16, 17]	2,00	11,11	[BCL6, TBX21]
GO:0043371	negative regulation of CD4-positive, alpha-beta T cell differentiation	8,51E-09	[8, 9, 10, 11, 12, 13, 14, 15]	5,00	22,73	[BCL6, CBFB, RUNX1, RUNX3, TBX21]
GO:0045064	T-helper 2 cell differentiation	1,93E-03	[4, 10, 11, 12, 13, 14, 15, 16]	2,00	13,33	[BCL6, GATA3]
GO:0042093	T-helper cell differentiation	2,52E-06	[9, 10, 11, 12, 13, 14, 15]	5,00	7,58	[BCL6, GATA3, IRF4, MYB, TBX21]
GO:0072540	T-helper 17 cell lineage commitment	1,92E-03	[7, 8, 9, 10, 11, 12, 13, 14, 15, 16, 17]	2,00	14,29	[IRF4, TBX21]
GO:0032703	negative regulation of interleukin-2 production	2,74E-03	[4, 5, 6, 7, 8]	2,00	7,41	[GATA3, TBX21]
GO:0035745	T-helper 2 cell cytokine production	1,92E-03	[4, 6, 8, 9]	2,00	14,29	[GATA3, TBX21]
GO:1902106	negative regulation of leukocyte differentiation	6,03E-07	[4, 5, 6, 7, 8, 9, 10]	6,00	5,77	[BCL6, CBFB, MYC, RUNX1, RUNX3, TBX21]
GO:0016447	somatic recombination of immunoglobulin gene segments	7,92E-05	[4, 5, 7, 8, 9, 10]	4,00	6,67	[BCL6, TBX21, TCF3, YY1]
GO:0003160	endocardium morphogenesis	6,78E-04	[3, 4, 6, 7, 8]	2,00	33,33	[NOTCH1, RBPJ]
GO:0072132	mesenchyme morphogenesis	1,32E-03	[4, 5, 6, 7]	3,00	5,17	[MYC, NOTCH1, RBPJ]
GO:0010614	negative regulation of cardiac muscle hypertrophy	2,11E-03	[4, 5, 6, 7, 8]	2,00	6,90	[NOTCH1, YY1]
GO:0060251	regulation of glial cell proliferation	4,58E-04	[4, 5, 7, 8, 9]	3,00	9,09	[MYB, MYC, NOTCH1]
GO:0055021	regulation of cardiac muscle tissue growth	9,30E-05	[4, 5, 6, 7, 8, 9]	4,00	6,35	[NOTCH1, RBPJ, RUNX1, YY1]
GO:0061314	Notch signaling involved in heart development	1,45E-03	[5, 6, 7, 8]	2,00	18,18	[NOTCH1, RBPJ]
GO:0002320	lymphoid progenitor cell differentiation	1,58E-04	[5, 6, 7, 8, 9]	3,00	13,04	[GATA3, NOTCH1, SPI1]
GO:0003198	epithelial to mesenchymal transition involved in endocardial cushion formation	2,24E-03	[4, 5, 6, 7, 8, 9, 10]	2,00	10,53	[NOTCH1, RBPJ]
GO:1901533	negative regulation of hematopoietic progenitor cell differentiation	1,57E-03	[4, 5, 6, 7, 8, 9, 10]	2,00	16,67	[MYB, NOTCH1]
GO:1903306	negative regulation of regulated secretory pathway	3,13E-03	[5, 6, 7, 8, 9]	2,00	8,00	[NOTCH1, SPI1]
GO:0003176	aortic valve development	7,06E-04	[5, 7, 8, 9]	3,00	7,50	[GATA3, NOTCH1, RBPJ]
GO:0060842	arterial endothelial cell differentiation	6,78E-04	[5, 7, 8, 9]	2,00	33,33	[NOTCH1, RBPJ]
GO:0033152	immunoglobulin V(D)J recombination	1,04E-03	[5, 6, 8, 9, 10, 11]	2,00	25,00	[TCF3, YY1]
GO:0009912	auditory receptor cell fate commitment	5,32E-04	[5, 6, 7, 8, 9, 10, 11, 12]	2,00	40,00	[NOTCH1, RBPJ]
GO:0003256	regulation of transcription from RNA polymerase II promoter involved in myocardial precursor cell differentiation	8,92E-04	[6, 7, 8, 9, 10, 11, 12, 13]	2,00	28,57	[NOTCH1, RBPJ]
GO:0051569	regulation of histone H3-K4 methylation	2,11E-03	[7, 8, 9, 10, 11, 12]	2,00	6,90	[GATA3, MYB]
GO:0051570	regulation of histone H3-K9 methylation	2,74E-03	[7, 8, 9, 10, 11, 12]	2,00	7,41	[MYB, PAX5]
GO:0061419	positive regulation of transcription from RNA polymerase II promoter in response to hypoxia	6,78E-04	[5, 6, 7, 8, 9, 10, 11, 12, 13, 14]	2,00	33,33	[NOTCH1, RBPJ]
GO:0043367	CD4-positive, alpha-beta T cell differentiation	3,70E-11	[7, 8, 9, 10, 11, 12, 13]	8,00	9,41	[BCL6, CBFB, GATA3, IRF4, MYB, RUNX1, RUNX3, TBX21]
GO:0043374	CD8-positive, alpha-beta T cell differentiation	5,39E-05	[7, 8, 9, 10, 11, 12, 13]	3,00	20,00	[CBFB, RUNX1, RUNX3]
GO:0045589	regulation of regulatory T cell differentiation	4,58E-04	[7, 8, 9, 10, 11, 12, 13]	3,00	9,09	[BCL6, CBFB, RUNX1]
GO:0046638	positive regulation of alpha-beta T cell differentiation	3,43E-05	[7, 8, 9, 10, 11, 12, 13, 14]	4,00	8,33	[CBFB, MYB, RUNX1, RUNX3]
GO:0045622	regulation of T-helper cell differentiation	1,52E-05	[4, 5, 9, 10, 11, 12, 13, 14, 15, 16]	4,00	10,26	[BCL6, IRF4, MYB, TBX21]
GO:0043370	regulation of CD4-positive, alpha-beta T cell differentiation	5,34E-13	[8, 9, 10, 11, 12, 13, 14]	8,00	15,69	[BCL6, CBFB, GATA3, IRF4, MYB, RUNX1, RUNX3, TBX21]
GO:0045623	negative regulation of T-helper cell differentiation	2,41E-03	[4, 5, 6, 9, 10, 11, 12, 13, 14, 15, 16, 17]	2,00	11,11	[BCL6, TBX21]
GO:0043371	negative regulation of CD4-positive, alpha-beta T cell differentiation	8,51E-09	[8, 9, 10, 11, 12, 13, 14, 15]	5,00	22,73	[BCL6, CBFB, RUNX1, RUNX3, TBX21]
GO:0045064	T-helper 2 cell differentiation	1,93E-03	[4, 10, 11, 12, 13, 14, 15, 16]	2,00	13,33	[BCL6, GATA3]
GO:0042093	T-helper cell differentiation	2,52E-06	[9, 10, 11, 12, 13, 14, 15]	5,00	7,58	[BCL6, GATA3, IRF4, MYB, TBX21]
GO:0072540	T-helper 17 cell lineage commitment	1,92E-03	[7, 8, 9, 10, 11, 12, 13, 14, 15, 16, 17]	2,00	14,29	[IRF4, TBX21]
GO:0032663	regulation of interleukin-2 production	2,22E-06	[4, 5, 6, 7]	5,00	7,81	[GATA3, IRF4, RUNX1, STAT5B, TBX21]
GO:0045604	regulation of epidermal cell differentiation	1,02E-04	[4, 5, 6, 7]	4,00	6,15	[CBFB, EZH2, NOTCH1, RUNX1]
GO:0032703	negative regulation of interleukin-2 production	2,74E-03	[4, 5, 6, 7, 8]	2,00	7,41	[GATA3, TBX21]
GO:0032736	positive regulation of interleukin-13 production	1,57E-03	[4, 5, 6, 7, 8]	2,00	16,67	[GATA3, IRF4]
GO:0032743	positive regulation of interleukin-2 production	5,41E-04	[4, 5, 6, 7, 8]	3,00	8,33	[IRF4, RUNX1, STAT5B]
GO:0032753	positive regulation of interleukin-4 production	3,13E-03	[4, 5, 6, 7, 8]	2,00	8,00	[GATA3, IRF4]
GO:1901532	regulation of hematopoietic progenitor cell differentiation	6,73E-09	[4, 5, 6, 7, 8, 9]	7,00	7,29	[CBFB, GATA3, MYB, NOTCH1, RUNX1, SPI1, TCF3]
GO:0035745	T-helper 2 cell cytokine production	1,92E-03	[4, 6, 8, 9]	2,00	14,29	[GATA3, TBX21]
GO:1901533	negative regulation of hematopoietic progenitor cell differentiation	1,57E-03	[4, 5, 6, 7, 8, 9, 10]	2,00	16,67	[MYB, NOTCH1]
GO:1902106	negative regulation of leukocyte differentiation	6,03E-07	[4, 5, 6, 7, 8, 9, 10]	6,00	5,77	[BCL6, CBFB, MYC, RUNX1, RUNX3, TBX21]
GO:0048935	peripheral nervous system neuron development	1,57E-03	[5, 6, 7, 8, 9, 10]	2,00	16,67	[RUNX1, RUNX3]
GO:1902036	regulation of hematopoietic stem cell differentiation	5,53E-06	[5, 6, 7, 8, 9, 10]	5,00	6,41	[CBFB, GATA3, MYB, RUNX1, TCF3]
GO:0031060	regulation of histone methylation	1,41E-04	[6, 7, 8, 9, 10]	4,00	5,56	[BRD4, GATA3, MYB, PAX5]
GO:0050855	regulation of B cell receptor signaling pathway	4,07E-06	[5, 6, 7, 8, 9, 10, 11]	4,00	14,29	[CBFB, ELF1, PAX5, RUNX1]
GO:0061647	histone H3-K9 modification	1,16E-03	[7, 8, 9]	3,00	5,56	[GATA3, MYB, PAX5]

GO:0045058	T cell selection	1,19E-03	[2, 6, 7, 8, 9, 10, 11, 12]	3,00	5,66	[GATA3, IRF4, TBX21]
GO:0045580	regulation of T cell differentiation	1,41E-12	[6, 7, 8, 9, 10, 11, 12]	10,00	6,58	[BCL6, CBFB, GATA3, IRF4, MYB, RUNX1, RUNX3, STAT5B, TBX21, ZEB1]
GO:0045582	positive regulation of T cell differentiation	5,10E-09	[6, 7, 8, 9, 10, 11, 12, 13]	7,00	7,61	[BCL6, CBFB, GATA3, MYB, RUNX1, RUNX3, STAT5B]
GO:0051569	regulation of histone H3-K4 methylation	2,11E-03	[7, 8, 9, 10, 11, 12]	2,00	6,90	[GATA3, MYB]
GO:0051570	regulation of histone H3-K9 methylation	2,74E-03	[7, 8, 9, 10, 11, 12]	2,00	7,41	[MYB, PAX5]
