# Supplementary material for: Epigenetic regulation of transcription factor binding motifs promotes Th1 response in Chagas disease cardiomyopathy
Source: Front Immunol. 2022 Aug 22;13:958200. doi: 10.3389/fimmu.2022.958200 (PMC9441916; doi:10.3389/fimmu.2022.958200)
Supplement: Supplementary Table 1 — Biological samples included in this study. [file DataSheet_1.zip › Supplementary Material/Supplementary Table 12.pdf]

Supplementary table 12. For each DMR, list of predicted Transcription Factor Binding Site (TFBS) and their associated Transcription Factor (TF)

DMR id	Chromosome	Start	End	TF	Motif ID	p.value	TFBS predicted	Targeted Gene	DMP id
DMR2	chr1	25291462	25291473	PAX5	MA0014.3	0.00947	CAGAGTGGGCCG	RUNX3	cg06640822
DMR2	chr1	25291462	25291473	YY1	MA0095.2	0.027	CAGAGTGGGCCG	RUNX3	cg06640822
DMR2	chr1	25291468	25291477	RUNX3	MA0684.1	0.012	GGGCCGCCTC	RUNX3	cg06640822
DMR2	chr1	25291469	25291478	SP1	MA0079.2	0.00106	GGCCGCCTCC	RUNX3	cg06640822
DMR2	chr1	25291470	25291479	MAX	MA0058.3	0.033	GCCGCCTCCA	RUNX3	cg06640822
DMR2	chr1	25291470	25291479	SP1	MA0079.2	0.0371	GCCGCCTCCA	RUNX3	cg06640822
DMR2	chr1	25291472	25291486	EBF1	MA0154.4	0.00822	CGCCTCCAGTGCCAC	RUNX3	cg06640822
DMR2	chr1	25291522	25291540	PAX5	MA0014.2	0.000446	GTACCCACCAGAGCCTGGG	RUNX3	cg04554131
DMR2	chr1	25291527	25291540	EBF1	MA0154.3	0.0038	GTACCCACCAGAGC	RUNX3	cg04554131
DMR2	chr1	25291537	25291546	MAX	MA0058.1	0.0185	GCCCTCGTAC	RUNX3	cg27058497, cg04554131
DMR2	chr1	25291537	25291546	MAX	MA0058.2	0.0406	GCCCTCGTAC	RUNX3	cg27058497, cg04554131
DMR2	chr1	25291537	25291546	SP1	MA0079.2	0.0159	GCCCTCGTAC	RUNX3	cg27058497, cg04554131
DMR2	chr1	25291541	25291551	SP1	MA0079.3	0.0015	CGGCCGCCCTC	RUNX3	cg27058497
DMR2	chr1	25291541	25291546	YY1	MA0095.1	0.00953	GCCCTC	RUNX3	cg27058497
DMR2	chr1	25291542	25291551	RUNX3	MA0684.1	0.00516	CGGCCGCCCT	RUNX3	cg27058497
DMR2	chr1	25291542	25291551	SP1	MA0079.2	0.0497	CGGCCGCCCT	RUNX3	cg27058497
DMR2	chr1	25291544	25291558	EBF1	MA0154.4	0.00971	CCACCCCCGGCCGCC	RUNX3	cg27058497
DMR2	chr1	25291544	25291558	SP1	MA0079.4	0.0232	CCACCCCCGGCCGCC	RUNX3	cg27058497
DMR2	chr1	25291545	25291556	PAX5	MA0014.3	0.0155	ACCCCCGGCCGC	RUNX3	cg27058497
DMR2	chr1	25291687	25291696	SP1	MA0079.1	0.00765	CGAGCAGGCT	RUNX3	cg05714960
DMR2	chr1	25291689	25291703	EBF1	MA0154.4	0.0323	AAGGCCGCGAGCAGG	RUNX3	cg05714960
DMR2	chr1	25291690	25291701	PAX5	MA0014.3	0.00148	GGCCGCGAGCAG	RUNX3	cg05714960
DMR2	chr1	25291691	25291705	IRF4	MA1419.1	0.0186	CCAAGGCCGCGAGCA	RUNX3	cg05714960
DMR2	chr1	25291691	25291700	MAX	MA0058.3	0.00479	GCCGCGAGCA	RUNX3	cg05714960
DMR2	chr1	25291693	25291702	RUNX3	MA0684.1	0.0026	AGGCCGCGAG	RUNX3	cg05714960
DMR2	chr1	25291708	25291722	EBF1	MA0154.4	0.0162	GCGGCCCAGCCAATG	RUNX3	cg16063783
DMR2	chr1	25291708	25291722	SP1	MA0079.4	0.0141	GCGGCCCAGCCAATG	RUNX3	cg16063783
DMR2	chr1	25291710	25291720	SP1	MA0079.3	0.00411	GGCCCAGCCAA	RUNX3	cg16063783
DMR2	chr1	25291716	25291725	MAX	MA0058.3	0.0243	ACCGCGGCCC	RUNX3	cg16063783
DMR2	chr1	25291718	25291736	PAX5	MA0014.2	0.000835	CGGCCCAGGTGACCGCGGC	RUNX3	cg16063783
DMR2	chr1	25291718	25291727	RUNX3	MA0684.1	0.00128	TGACCGCGGC	RUNX3	cg16063783
DMR2	chr1	25291718	25291729	YY1	MA0095.2	0.0467	GGTGACCGCGGC	RUNX3	cg16063783
DMR2	chr1	25291892	25291906	IRF4	MA1419.1	0.0173	CGCCCACTGCAACCG	RUNX3	cg09993145
DMR2	chr1	25291897	25291906	MAX	MA0058.1	0.0422	CGCCCACTGC	RUNX3	cg09993145
DMR2	chr1	25291897	25291906	RUNX3	MA0684.1	0.00678	CGCCCACTGC	RUNX3	cg09993145
DMR2	chr1	25291899	25291913	SP1	MA0079.4	2.4e-08	CTGGCCACGCCCACT	RUNX3	cg09993145
DMR2	chr1	25291900	25291911	PAX5	MA0014.3	0.00714	GGCCACGCCCAC	RUNX3	cg09993145
DMR2	chr1	25291901	25291910	MAX	MA0058.3	0.00542	GCCACGCCCA	RUNX3	cg09993145
DMR2	chr1	25291901	25291910	SP1	MA0079.2	0.0085	GCCACGCCCA	RUNX3	cg09993145
DMR2	chr1	25291905	25291919	EBF1	MA0154.4	0.00924	ACCCTCCTGGCCACG	RUNX3	cg09993145
DMR2	chr1	25291905	25291910	YY1	MA0095.1	0.0142	GCCACG	RUNX3	cg09993145
DMR41	chr1	27668105	27668114	MYB	MA0100.3	0.0456	GCGAGCTGGC	SYTL1	cg01870058
DMR41	chr1	27668107	27668115	SP1	MA0079.5	0.00524	TGCGAGCTG	SYTL1	cg01870058
DMR41	chr1	27668108	27668117	RBPJ	MA1116.1	0.0232	GGTGCGAGCT	SYTL1	cg01870058
DMR41	chr1	27668108	27668117	SP1	MA0079.1	0.0305	GGTGCGAGCT	SYTL1	cg01870058
DMR41	chr1	27668110	27668124	EBF1	MA0154.4	0.00951	AGTGCCCGGTGCGAG	SYTL1	cg01870058
DMR41	chr1	27668110	27668119	SP1	MA0079.1	0.0348	CCGGTGCGAG	SYTL1	cg01870058
DMR41	chr1	27668110	27668119	TBX21	MA0690.1	0.00343	CCGGTGCGAG	SYTL1	cg01870058
DMR41	chr1	27668112	27668121	GABPA	MA0062.1	0.0392	GCCCGGTGCG	SYTL1	cg01870058
DMR41	chr1	27668112	27668121	SP1	MA0079.2	0.0136	GCCCGGTGCG	SYTL1	cg01870058
DMR41	chr1	27668423	27668433	EBF1	MA0154.2	0.0255	GCCCCCGCAGC	SYTL1	cg04608356

DMR41	chr1	27668424	27668433	MAX	MA0058.1	0.0358	GCCCCCGCAG	SYTL1	cg04608356
DMR41	chr1	27668424	27668433	SP1	MA0079.2	0.0275	GCCCCCGCAG	SYTL1	cg04608356
DMR51	chr1	27953015	27953026	RUNX3	MA0684.2	0.0189	GGAACCATCAGC	FGR	cg26707200
DMR51	chr1	27953021	27953032	YY1	MA0095.2	0.0119	TGTGACGGAACC	FGR	cg26707200
DMR51	chr1	27953023	27953031	SP1	MA0079.5	0.00621	GTGACGGAA	FGR	cg26707200
DMR51	chr1	27953025	27953034	TBX21	MA0690.1	0.0216	GATGTGACGG	FGR	cg26707200
DMR51	chr1	27953211	27953220	SP1	MA0079.2	0.0116	CCTTTGCCCC	FGR	cg25783189
DMR17	chr1	111743184	111743202	PAX5	MA0014.2	0.00143	CTGTGAAGCCAAGCCACGC	DENND2D	cg20317872, cg19269039
DMR17	chr1	111743186	111743200	IRF4	MA1419.1	0.0269	GTGAAGCCAAGCCAC	DENND2D	cg19269039
DMR17	chr1	111743194	111743203	RUNX3	MA0684.1	0.00484	AAGCCACGCG	DENND2D	cg20317872, cg19269039
DMR17	chr1	111743195	111743205	TBX21	MA0690.2	0.00685	AGCCACGCGCT	DENND2D	cg20317872, cg19269039
DMR17	chr1	111743196	111743206	TCF3	MA0522.3	0.000644	GCCACGCGCTG	DENND2D	cg20317872, cg19269039
DMR20	chr1	114414333	114414344	PAX5	MA0014.3	0.00802	AGGCGGCAGCAG	PTPN22	cg14385738
DMR20	chr1	114414334	114414343	ERG	MA0474.2	0.00424	GGCGGCAGCA	PTPN22	cg14385738
DMR20	chr1	114414334	114414343	GABPA	MA0062.1	0.00971	GGCGGCAGCA	PTPN22	cg14385738
DMR20	chr1	114414335	114414344	RUNX3	MA0684.1	0.0093	AGGCGGCAGC	PTPN22	cg14385738
DMR20	chr1	114414336	114414346	RUNX1	MA0002.1	0.0294	TCAGGCGGCAG	PTPN22	cg14385738
DMR20	chr1	114414336	114414345	TBX21	MA0690.1	0.0143	CAGGCGGCAG	PTPN22	cg14385738
DMR20	chr1	114414337	114414351	EBF1	MA0154.4	0.00394	TCTGCTCAGGCGGCA	PTPN22	cg14385738
DMR20	chr1	114414337	114414348	MYC	MA0147.3	0.0112	GCTCAGGCGGCA	PTPN22	cg14385738
DMR20	chr1	114414337	114414345	SP1	MA0079.5	0.00849	CAGGCGGCA	PTPN22	cg14385738
DMR20	chr1	114414338	114414347	MAX	MA0058.3	0.013	CTCAGGCGGC	PTPN22	cg14385738
DMR20	chr1	114414338	114414347	SP1	MA0079.2	0.0396	CTCAGGCGGC	PTPN22	cg14385738
DMR20	chr1	114414400	114414413	GABPA	MA0062.3	0.039	GTTACGTACTAATT	PTPN22	cg00041401
DMR20	chr1	114414401	114414416	BCL6	MA0463.2	0.0341	GTGGTTACGTACTAAT	PTPN22	cg00041401
DMR20	chr1	114414401	114414410	MYB	MA0100.3	0.0401	ACGTACTAAT	PTPN22	cg00041401
DMR20	chr1	114414402	114414416	SP1	MA0079.4	0.0108	GTGGTTACGTACTAA	PTPN22	cg00041401
DMR20	chr1	114414402	114414411	TBX21	MA0690.1	0.0251	TACGTACTAA	PTPN22	cg00041401
DMR20	chr1	114414403	114414414	MYC	MA0147.3	0.0363	GGTTACGTACTA	PTPN22	cg00041401
DMR20	chr1	114414405	114414414	MAX	MA0058.1	0.0156	GGTTACGTAC	PTPN22	cg00041401
DMR20	chr1	114414405	114414414	MAX	MA0058.2	0.032	GGTTACGTAC	PTPN22	cg00041401
DMR20	chr1	114414408	114414415	GATA3	MA0037.3	0.0257	TGGTTACG	PTPN22	cg00041401
DMR20	chr1	114414408	114414417	RBPJ	MA1116.1	0.00358	GGTGGTTACG	PTPN22	cg00041401
DMR3	chr1	159046762	159046776	IRF4	MA1419.1	0.0161	TAAAAAATGACCGCA	AIM2	cg00490406
DMR3	chr1	159046762	159046776	SP1	MA0079.4	0.0321	TAAAAAATGACCGCA	AIM2	cg00490406
DMR3	chr1	159046764	159046775	YY1	MA0095.2	0.000296	AAAAATGACCGC	AIM2	cg00490406
DMR3	chr1	159046769	159046783	EBF1	MA0154.4	0.00906	TGACCGCAGATGACT	AIM2	cg00490406
DMR3	chr1	159046769	159046778	RUNX3	MA0684.1	6.43e-05	TGACCGCAGA	AIM2	cg00490406
DMR3	chr1	159046769	159046777	SP1	MA0079.5	0.0161	TGACCGCAG	AIM2	cg00490406
DMR3	chr1	159046770	159046780	TBX21	MA0690.2	0.0316	GACCGCAGATG	AIM2	cg00490406
DMR3	chr1	159046928	159046941	EBF1	MA0154.3	0.000243	AGACCCAAGCGAAA	AIM2	cg17217296
DMR3	chr1	159046928	159046937	RUNX3	MA0684.1	0.0122	AGACCCAAGC	AIM2	cg17217296
DMR3	chr1	159046930	159046944	IRF4	MA1419.1	0.000131	ACCCAAGCGAAAGTA	AIM2	cg17217296
DMR3	chr1	159046931	159046940	EBF1	MA0154.1	0.0437	CCCAAGCGAA	AIM2	cg17217296
DMR3	chr1	159046931	159046940	SP1	MA0079.2	0.0144	CCCAAGCGAA	AIM2	cg17217296
DMR3	chr1	159046933	159046942	TBX21	MA0690.1	0.0213	CAAGCGAAAG	AIM2	cg17217296
DMR3	chr1	159046963	159046977	IRF4	MA1419.1	0.0113	GCAAAACCAACGACA	AIM2	cg10636246
DMR3	chr1	159046968	159046977	EBF1	MA0154.1	0.0202	ACCAACGACA	AIM2	cg10636246
DMR3	chr1	159046969	159046980	RUNX3	MA0684.2	0.00394	CCAACGACACCC	AIM2	cg10636246
DMR3	chr1	159046969	159046983	SP1	MA0079.4	0.00196	CCAACGACACCCTCA	AIM2	cg10636246
DMR3	chr1	159046971	159046981	TBX21	MA0690.2	0.00182	AACGACACCCT	AIM2	cg10636246
DMR3	chr1	159047027	159047041	SP1	MA0079.4	0.0204	TGGTTCACGTACTAT	AIM2	cg07195224
DMR3	chr1	159047029	159047042	EBF1	MA0154.3	0.0424	GTTCACGTACTATA	AIM2	cg07195224
DMR3	chr1	159047029	159047039	TBX21	MA0690.2	0.0149	GTTCACGTACT	AIM2	cg07195224

DMR3	chr1	159047158	159047168	SP1	MA0079.3	0.0366	GTTCCGGATTA	AIM2	cg17515347
DMR3	chr1	159047168	159047177	SP1	MA0079.2	0.0378	GCCATGACAG	AIM2	cg24145401
DMR3	chr1	159047172	159047177	YY1	MA0095.1	0.000627	GCCATG	AIM2	cg24145401
DMR50	chr1	160714371	160714382	PAX5	MA0014.3	0.0244	GTGTGTGGAAGC	SLAMF7	cg04009575
DMR16	chr1	161039680	161039695	BCL6	MA0463.2	0.0418	TCGCCCTCCTCGCCCC	ARHGAP30	cg00045607, cg03089651
DMR16	chr1	161039680	161039693	GABPA	MA0062.3	0.00558	TCGCCCTCCTCGCC	ARHGAP30	cg00045607
DMR16	chr1	161039681	161039690	RUNX3	MA0684.1	0.0237	CGCCCTCCTC	ARHGAP30	cg00045607
DMR16	chr1	161039681	161039691	SP1	MA0079.3	0.000145	CGCCCTCCTCG	ARHGAP30	cg00045607
DMR16	chr1	161039681	161039690	SP1	MA0079.2	0.00113	CGCCCTCCTC	ARHGAP30	cg00045607
DMR16	chr1	161039682	161039693	MYC	MA0147.3	0.0134	GCCCTCCTCGCC	ARHGAP30	cg00045607
DMR16	chr1	161039682	161039690	ZEB1	MA0103.2	0.0101	GCCCTCCTC	ARHGAP30	cg00045607
DMR16	chr1	161039683	161039693	EBF1	MA0154.2	0.0314	CCCTCCTCGCC	ARHGAP30	cg00045607
DMR16	chr1	161039683	161039692	MAX	MA0058.3	0.036	CCCTCCTCGC	ARHGAP30	cg00045607
DMR16	chr1	161039683	161039692	SP1	MA0079.2	0.01	CCCTCCTCGC	ARHGAP30	cg00045607
DMR16	chr1	161039683	161039692	TCF3	MA0522.2	0.0103	CCCTCCTCGC	ARHGAP30	cg00045607
DMR16	chr1	161039683	161039693	TCF3	MA0522.3	0.0172	CCCTCCTCGCC	ARHGAP30	cg00045607
DMR16	chr1	161039685	161039695	SP1	MA0079.3	0.0268	CTCCTCGCCCC	ARHGAP30	cg00045607, cg03089651
DMR16	chr1	161039685	161039695	TBX21	MA0690.2	0.00833	CTCCTCGCCCC	ARHGAP30	cg00045607, cg03089651
DMR16	chr1	161039686	161039695	MAX	MA0058.3	0.0276	TCCTCGCCCC	ARHGAP30	cg00045607, cg03089651
DMR16	chr1	161039686	161039695	SP1	MA0079.2	0.00226	TCCTCGCCCC	ARHGAP30	cg00045607, cg03089651
DMR16	chr1	161039690	161039708	PAX5	MA0014.2	0.0296	CGCCCCGCTCAGCTTTAGG	ARHGAP30	cg00045607, cg03089651
DMR16	chr1	161039691	161039700	RUNX3	MA0684.1	0.0146	GCCCCGCTCA	ARHGAP30	cg03089651
DMR16	chr1	161039691	161039700	SP1	MA0079.2	0.00604	GCCCCGCTCA	ARHGAP30	cg03089651
DMR16	chr1	161039692	161039701	SP1	MA0079.2	0.00031	CCCCGCTCAG	ARHGAP30	cg03089651
DMR16	chr1	161039692	161039701	TCF3	MA0522.2	0.0299	CCCCGCTCAG	ARHGAP30	cg03089651
DMR55	chr1	202128668	202128682	IRF4	MA1419.1	0.00811	GCCCCAGTGAAAACA	PTPN7	cg00903584
DMR55	chr1	202128670	202128682	ELF1	MA0473.1	0.0414	GCCCCAGTGAAAA	PTPN7	cg00903584
DMR55	chr1	202128671	202128682	MYC	MA0147.3	0.0148	GCCCCAGTGAAA	PTPN7	cg00903584
DMR55	chr1	202128673	202128682	EBF1	MA0154.1	0.00184	GCCCCAGTGA	PTPN7	cg00903584
DMR55	chr1	202128673	202128682	MAX	MA0058.1	0.00459	GCCCCAGTGA	PTPN7	cg00903584
DMR23	chr1	209929482	209929496	IRF4	MA1419.1	0.00661	TGCAAAAAGAAAGAC	TRAF3IP3	cg20694619
DMR23	chr1	209929482	209929501	SPI1	MA0080.5	0.00148	TGCAAAAAGAAAGACGGTAC	TRAF3IP3	cg20694619
DMR23	chr1	209929490	209929499	GABPA	MA0062.1	0.0364	GAAAGACGGT	TRAF3IP3	cg20694619
DMR23	chr1	209929491	209929501	RUNX1	MA0002.1	0.02	AAAGACGGTAC	TRAF3IP3	cg20694619
DMR23	chr1	209929491	209929502	YY1	MA0095.2	0.00138	AAAGACGGTACC	TRAF3IP3	cg20694619
DMR23	chr1	209929493	209929502	RUNX3	MA0684.1	0.02	AGACGGTACC	TRAF3IP3	cg20694619
DMR23	chr1	209929494	209929503	ERG	MA0474.2	0.0161	GACGGTACCA	TRAF3IP3	cg20694619
DMR23	chr1	209929495	209929504	TBX21	MA0690.1	0.0397	ACGGTACCAA	TRAF3IP3	cg20694619
DMR23	chr1	209929496	209929503	GATA3	MA0037.3	0.0397	CGGTACCA	TRAF3IP3	cg20694619
DMR23	chr1	209929533	209929552	SPI1	MA0080.5	0.0268	TATGGATTGAAAGCGTGTGC	TRAF3IP3	cg22436753
DMR23	chr1	209929538	209929547	GABPA	MA0062.1	0.0254	ATTGAAAGCG	TRAF3IP3	cg22436753
DMR23	chr1	209929539	209929553	IRF4	MA1419.1	0.00999	TTGAAAGCGTGTGCT	TRAF3IP3	cg22436753
DMR23	chr1	209929540	209929547	GATA3	MA0037.3	0.0241	TGAAAGCG	TRAF3IP3	cg22436753
DMR23	chr1	209929543	209929554	MYC	MA0147.3	0.0046	AAGCGTGTGCTT	TRAF3IP3	cg22436753
DMR23	chr1	209929544	209929553	MAX	MA0058.3	0.00241	AGCGTGTGCT	TRAF3IP3	cg22436753
DMR23	chr1	209929544	209929553	SP1	MA0079.1	0.0216	AGCGTGTGCT	TRAF3IP3	cg22436753
DMR23	chr1	209929544	209929553	TBX21	MA0690.1	0.0318	AGCGTGTGCT	TRAF3IP3	cg22436753
DMR23	chr1	209929545	209929555	RUNX1	MA0002.1	0.0152	GCGTGTGCTTT	TRAF3IP3	cg22436753
DMR23	chr1	209929614	209929622	SP1	MA0079.5	0.013	GGGAAAGCA	TRAF3IP3	cg01997629
DMR9	chr1	209941771	209941781	RUNX1	MA0002.1	0.0359	CGTTTTTGTAT	TRAF3IP3	cg03195100
DMR9	chr1	209941776	209941786	TBX21	MA0690.2	0.0419	GCTCTCGTTTT	TRAF3IP3	cg03195100
DMR9	chr1	209941805	209941814	MYB	MA0100.3	0.0128	AAGATCTGCG	TRAF3IP3	cg17990510
DMR9	chr1	209941808	209941818	RUNX1	MA0002.1	0.0209	ATCTGCGTCCT	TRAF3IP3	cg17990510
DMR9	chr1	209941811	209941824	EBF1	MA0154.3	0.0147	TGCGTCCTGGAATT	TRAF3IP3	cg17990510

DMR9	chr1	209941835	209941844	MYB	MA0100.3	0.0409	GATGACAGCG	TRAF3IP3	cg01000850
DMR9	chr1	209941835	209941845	TBX21	MA0690.2	0.0136	GATGACAGCGC	TRAF3IP3	cg01000850
DMR9	chr1	209941839	209941848	RUNX3	MA0684.1	0.0354	ACAGCGCAGC	TRAF3IP3	cg01000850, cg11705496
DMR8	chr10	49892944	49892954	TBX21	MA0690.2	0.0135	GGCCCCACATC	WDFY4	cg20504007
DMR8	chr10	49892946	49892955	RUNX3	MA0684.1	0.00559	CGGCCCCACA	WDFY4	cg20504007
DMR8	chr10	49892948	49892962	SP1	MA0079.4	0.000584	GAGGACCCGGCCCCA	WDFY4	cg20504007
DMR8	chr10	49892949	49892960	MYC	MA0147.3	0.0369	GGACCCGGCCCC	WDFY4	cg20504007
DMR8	chr10	49892949	49892960	PAX5	MA0014.3	0.0239	GGACCCGGCCCC	WDFY4	cg20504007
DMR8	chr10	49892949	49892960	YY1	MA0095.2	0.0355	GGACCCGGCCCC	WDFY4	cg20504007
DMR8	chr10	49892951	49892960	MAX	MA0058.1	0.0309	GGACCCGGCC	WDFY4	cg20504007
DMR8	chr10	49892951	49892969	PAX5	MA0014.2	0.00367	GGGGGAAGAGGACCCGGCC	WDFY4	cg20504007
DMR8	chr10	49892951	49892960	RUNX3	MA0684.1	0.0486	GGACCCGGCC	WDFY4	cg20504007
DMR8	chr10	49892951	49892961	TCF3	MA0522.3	0.0113	AGGACCCGGCC	WDFY4	cg20504007
DMR8	chr10	49892952	49892965	ELF1	MA0473.3	0.00569	GAAGAGGACCCGGC	WDFY4	cg20504007
DMR8	chr10	49892954	49892965	ELF1	MA0473.2	0.0249	GAAGAGGACCCG	WDFY4	cg20504007
DMR8	chr10	49892954	49892963	ERG	MA0474.2	0.00705	AGAGGACCCG	WDFY4	cg20504007
DMR8	chr10	49892954	49892965	PAX5	MA0014.3	0.034	GAAGAGGACCCG	WDFY4	cg20504007
DMR8	chr10	49892954	49892967	SPI1	MA0080.4	0.0028	GGGAAGAGGACCCG	WDFY4	cg20504007
DMR8	chr10	49892954	49892965	YY1	MA0095.2	0.0257	GAAGAGGACCCG	WDFY4	cg20504007
DMR8	chr10	49893324	49893343	SPI1	MA0080.5	0.0202	AGAGGTCGAGGAGTGCTGCT	WDFY4	cg12024826
DMR8	chr10	49893325	49893343	PAX5	MA0014.2	0.0275	AGAGGTCGAGGAGTGCTGC	WDFY4	cg12024826
DMR8	chr10	49893326	49893337	MYC	MA0147.3	0.0499	CGAGGAGTGCTG	WDFY4	cg12024826
DMR8	chr10	49893328	49893346	PAX5	MA0014.2	0.0209	GTCAGAGGTCGAGGAGTGC	WDFY4	cg12024826, cg04749316
DMR8	chr10	49893329	49893344	BCL6	MA0463.2	0.00131	CAGAGGTCGAGGAGTG	WDFY4	cg12024826
DMR8	chr10	49893329	49893348	SPI1	MA0080.5	0.0115	CCGTCAGAGGTCGAGGAGTG	WDFY4	cg12024826, cg04749316
DMR8	chr10	49893331	49893339	SP1	MA0079.5	0.00429	GTCGAGGAG	WDFY4	cg12024826
DMR8	chr10	49893332	49893345	ELF1	MA0473.3	0.0201	TCAGAGGTCGAGGA	WDFY4	cg12024826
DMR8	chr10	49893332	49893341	SP1	MA0079.1	0.0399	AGGTCGAGGA	WDFY4	cg12024826
DMR8	chr10	49893332	49893341	TBX21	MA0690.1	0.0293	AGGTCGAGGA	WDFY4	cg12024826
DMR8	chr10	49893334	49893343	ERG	MA0474.2	0.0352	AGAGGTCGAG	WDFY4	cg12024826
DMR8	chr10	49893338	49893351	ELF1	MA0473.3	0.0171	CTACCGTCAGAGGT	WDFY4	cg04749316
DMR8	chr10	49893338	49893347	MAX	MA0058.2	0.0342	CGTCAGAGGT	WDFY4	cg04749316
DMR8	chr10	49893340	49893349	ERG	MA0474.2	0.0123	ACCGTCAGAG	WDFY4	cg04749316
DMR8	chr10	49893342	49893351	RUNX3	MA0684.1	0.012	CTACCGTCAG	WDFY4	cg04749316
DMR8	chr10	49893343	49893357	SP1	MA0079.4	0.019	CTTGACCTACCGTCA	WDFY4	cg04749316
DMR8	chr10	49893344	49893349	YY1	MA0095.1	0.0119	ACCGTC	WDFY4	cg04749316
DMR8	chr10	49893346	49893356	TCF3	MA0522.3	0.00595	TTGACCTACCG	WDFY4	cg04749316
DMR8	chr10	49893449	49893463	IRF4	MA1419.1	0.029	AGGACAGTGAGGCTC	WDFY4	cg07345108
DMR8	chr10	49893450	49893463	ELF1	MA0473.3	0.0205	GGACAGTGAGGCTC	WDFY4	cg07345108
DMR8	chr10	49893456	49893465	ERG	MA0474.2	0.0414	TGAGGCTCGC	WDFY4	cg07345108
DMR8	chr10	49893458	49893469	MYC	MA0147.3	0.0275	AGGCTCGCCCTC	WDFY4	cg07345108
DMR8	chr10	49893458	49893469	PAX5	MA0014.3	0.0068	AGGCTCGCCCTC	WDFY4	cg07345108
DMR8	chr10	49893459	49893468	MAX	MA0058.3	0.027	GGCTCGCCCT	WDFY4	cg07345108
DMR8	chr10	49893459	49893469	SP1	MA0079.3	0.00126	GGCTCGCCCTC	WDFY4	cg07345108
DMR8	chr10	49893459	49893468	SP1	MA0079.2	0.0287	GGCTCGCCCT	WDFY4	cg07345108
DMR8	chr10	49893459	49893469	TCF3	MA0522.3	0.0432	GGCTCGCCCTC	WDFY4	cg07345108
DMR8	chr10	49893459	49893468	TCF3	MA0522.2	0.0477	GGCTCGCCCT	WDFY4	cg07345108
DMR8	chr10	49893460	49893470	SP1	MA0079.3	0.0128	GCTCGCCCTCC	WDFY4	cg07345108
DMR8	chr10	49893460	49893470	TBX21	MA0690.2	0.00911	GCTCGCCCTCC	WDFY4	cg07345108
DMR8	chr10	49893461	49893471	TCF3	MA0522.3	0.018	CTCGCCCTCCA	WDFY4	cg07345108
DMR8	chr10	49893462	49893477	BCL6	MA0463.2	0.00236	TCGCCCTCCAAGCTCC	WDFY4	cg07345108
DMR8	chr10	49893463	49893472	RUNX3	MA0684.1	0.00983	CGCCCTCCAA	WDFY4	cg07345108
DMR8	chr10	49893463	49893472	SP1	MA0079.2	0.00329	CGCCCTCCAA	WDFY4	cg07345108
DMR7	chr10	72362722	72362733	PAX5	MA0014.3	0.00216	GGGCGGGGCGCT	PRF1	cg12433559

DMR7	chr10	72362805	72362823	PAX5	MA0014.2	0.0146	CATCCCACACATGCGATGC	PRF1	cg02374486
DMR7	chr10	72362808	72362819	MYC	MA0147.3	0.000935	CCACACATGCGA	PRF1	cg02374486
DMR7	chr10	72362855	72362866	PAX5	MA0014.3	0.0251	GTGTAGGCCCAT	PRF1	cg15293582
DMR33	chr11	58981085	58981095	EBF1	MA0154.2	0.0188	GCATTCAAAGC	MPEG1	cg22750001
DMR5	chr11	60738985	60738998	GABPA	MA0062.3	0.0184	CACGCATCCATGCA	CD6	cg21939215
DMR5	chr11	60738987	60738996	MYB	MA0100.3	0.0219	CGCATCCATG	CD6	cg21939215
DMR5	chr11	60738989	60738998	ERG	MA0474.2	0.0467	CACGCATCCA	CD6	cg21939215
DMR5	chr11	60738989	60738998	MAX	MA0058.3	0.0221	CACGCATCCA	CD6	cg21939215
DMR5	chr11	60738991	60739000	MAX	MA0058.3	0.0479	CACACGCATC	CD6	cg21939215
DMR5	chr11	60738991	60739000	RUNX3	MA0684.1	0.02	CACACGCATC	CD6	cg21939215
DMR5	chr11	60738992	60739003	MYC	MA0147.3	0.00823	ATGCACACGCAT	CD6	cg21939215
DMR5	chr11	60738994	60739003	MAX	MA0058.2	0.00155	ATGCACACGC	CD6	cg21939215
DMR5	chr11	60738994	60739003	MAX	MA0058.1	0.0374	ATGCACACGC	CD6	cg21939215
DMR5	chr11	60738998	60739009	MYC	MA0147.3	0.00477	ACACGCATGCAC	CD6	cg27284288
DMR5	chr11	60738999	60739010	ELF1	MA0473.2	0.00709	CACACGCATGCA	CD6	cg27284288
DMR5	chr11	60738999	60739008	ERG	MA0474.2	0.0217	CACGCATGCA	CD6	cg27284288
DMR5	chr11	60738999	60739008	MAX	MA0058.3	0.00335	CACGCATGCA	CD6	cg27284288
DMR5	chr11	60739001	60739010	RUNX3	MA0684.1	0.0218	CACACGCATG	CD6	cg27284288
DMR5	chr11	60739004	60739013	MAX	MA0058.2	0.00169	AGACACACGC	CD6	cg27284288
DMR5	chr11	60739004	60739013	MAX	MA0058.1	0.00435	AGACACACGC	CD6	cg27284288
DMR5	chr11	60739005	60739024	SPI1	MA0080.5	0.0198	GTCACGGAGCCAGACACACG	CD6	cg27284288
DMR5	chr11	60739165	60739180	BCL6	MA0463.2	0.0125	ACGCATGCGATCAAAC	CD6	cg09902130, cg07380416
DMR5	chr11	60739166	60739173	GATA3	MA0037.3	0.00702	CGATCAAA	CD6	cg07380416
DMR5	chr11	60739166	60739173	GATA3	MA0037.2	0.0117	CGATCAAA	CD6	cg07380416
DMR5	chr11	60739168	60739178	RUNX1	MA0002.1	0.0185	GCATGCGATCA	CD6	cg09902130, cg07380416
DMR5	chr11	60739170	60739179	MAX	MA0058.3	0.00309	CGCATGCGAT	CD6	cg09902130, cg07380416
DMR5	chr11	60739171	60739182	MYC	MA0147.3	0.00711	ACACGCATGCGA	CD6	cg09902130, cg07380416
DMR5	chr11	60739172	60739183	ELF1	MA0473.2	0.00576	GACACGCATGCG	CD6	cg09902130, cg07380416, cg09153080
DMR5	chr11	60739172	60739181	ERG	MA0474.2	0.018	CACGCATGCG	CD6	cg09902130, cg07380416
DMR5	chr11	60739172	60739181	GABPA	MA0062.1	0.0137	CACGCATGCG	CD6	cg09902130, cg07380416
DMR5	chr11	60739172	60739181	MAX	MA0058.3	0.00327	CACGCATGCG	CD6	cg09902130, cg07380416
DMR5	chr11	60739173	60739182	MAX	MA0058.2	0.00367	ACACGCATGC	CD6	cg09902130
DMR5	chr11	60739173	60739182	MAX	MA0058.1	0.0064	ACACGCATGC	CD6	cg09902130
DMR5	chr11	60739174	60739183	RUNX3	MA0684.1	0.0369	GACACGCATG	CD6	cg09902130, cg09153080
DMR29	chr11	64107366	64107380	EBF1	MA0154.4	0.0117	ACTCACCACGCAAGG	CCDC88B	cg20975835
DMR38	chr11	118095538	118095547	MYB	MA0100.3	0.0247	TGCGATTGCA	AMICA1	cg03234777
DMR38	chr11	118095540	118095550	RUNX1	MA0002.1	0.00905	AGCTGCGATTG	AMICA1	cg03234777
DMR38	chr11	118095542	118095551	TBX21	MA0690.1	0.00116	AAGCTGCGAT	AMICA1	cg03234777
DMR61	chr12	7060378	7060386	SP1	MA0079.5	0.00598	GGGGCCCAC	PTPN6	cg26363363
DMR43	chr12	47610267	47610276	RBPJ	MA1116.1	0.0213	GGTGGCATCT	PCED1B-AS1	cg20515823
DMR43	chr12	47610268	47610282	EBF1	MA0154.4	0.00911	ATTTCCGGTGGCATC	PCED1B-AS1	cg20515823
DMR43	chr12	47610269	47610279	RUNX1	MA0002.1	0.0173	TCCGGTGGCAT	PCED1B-AS1	cg20515823
DMR43	chr12	47610269	47610278	TBX21	MA0690.1	0.0171	CCGGTGGCAT	PCED1B-AS1	cg20515823
DMR43	chr12	47610270	47610279	ERG	MA0474.2	0.0209	TCCGGTGGCA	PCED1B-AS1	cg20515823
DMR43	chr12	47610270	47610283	SPI1	MA0080.4	0.0212	CATTTCCGGTGGCA	PCED1B-AS1	cg20515823
DMR43	chr12	47610272	47610281	EBF1	MA0154.1	0.0153	TTTCCGGTGG	PCED1B-AS1	cg20515823
DMR43	chr12	47610272	47610285	GABPA	MA0062.3	0.000129	GACATTTCCGGTGG	PCED1B-AS1	cg20515823
DMR43	chr12	47610272	47610281	MAX	MA0058.1	0.0246	TTTCCGGTGG	PCED1B-AS1	cg20515823
DMR43	chr12	47610272	47610281	MAX	MA0058.2	0.0346	TTTCCGGTGG	PCED1B-AS1	cg20515823
DMR43	chr12	47610362	47610375	GABPA	MA0062.3	0.00739	GAGATCTCCCGTCT	PCED1B-AS1	cg00037681
DMR43	chr12	47610364	47610374	TBX21	MA0690.2	0.00746	GATCTCCCGTC	PCED1B-AS1	cg00037681
DMR43	chr12	47610366	47610380	EBF1	MA0154.4	0.0237	TCTCCCGTCTGGTCA	PCED1B-AS1	cg00037681
DMR43	chr12	47610366	47610375	MAX	MA0058.1	0.00778	TCTCCCGTCT	PCED1B-AS1	cg00037681
DMR43	chr12	47610366	47610375	MAX	MA0058.2	0.0442	TCTCCCGTCT	PCED1B-AS1	cg00037681

DMR43	chr12	47610367	47610382	BCL6	MA0463.2	0.0197	CTCCCGTCTGGTCACA	PCED1B-AS1	cg00037681
DMR43	chr12	47610369	47610378	ERG	MA0474.2	0.0326	CCCGTCTGGT	PCED1B-AS1	cg00037681
DMR43	chr12	47610369	47610378	MAX	MA0058.3	0.0168	CCCGTCTGGT	PCED1B-AS1	cg00037681
DMR43	chr12	47610370	47610380	RUNX1	MA0002.1	0.0253	CCGTCTGGTCA	PCED1B-AS1	cg00037681
DMR15	chr12	53496832	53496843	MYC	MA0147.3	0.0263	GCCCCAGTTCCG	SOAT2	cg04062576
DMR15	chr12	53496835	53496849	IRF4	MA1419.1	0.0469	CCAGTTCCGTGAGTA	SOAT2	cg04062576
DMR15	chr12	53496837	53496851	EBF1	MA0154.4	0.00554	AGTTCCGTGAGTAGC	SOAT2	cg04062576
DMR15	chr12	53496842	53496847	GATA3	MA0037.1	0.0473	CGTGAG	SOAT2	cg04062576
DMR15	chr12	53497128	53497139	MYC	MA0147.3	0.00648	GGGGATGTGGCG	SOAT2	cg17818435
DMR15	chr12	53497138	53497145	GATA3	MA0037.3	0.0159	CGATCCCT	SOAT2	cg17818435
DMR22	chr12	54891625	54891636	YY1	MA0095.2	0.00645	CTGAATGATCGC	NCKAP1L	cg21376733
DMR22	chr12	54891628	54891635	GATA3	MA0037.3	0.00915	CGATCATT	NCKAP1L	cg16509569
DMR22	chr12	54891628	54891642	IRF4	MA1419.1	0.0288	CTGACCGCGATCATT	NCKAP1L	cg16509569
DMR22	chr12	54891630	54891637	GATA3	MA0037.3	0.00836	TGATCGCG	NCKAP1L	cg21376733
DMR22	chr12	54891630	54891639	MAX	MA0058.3	0.0228	ACCGCGATCA	NCKAP1L	cg16509569
DMR22	chr12	54891632	54891641	TBX21	MA0690.1	0.0256	TGACCGCGAT	NCKAP1L	cg16509569
DMR22	chr12	54891633	54891646	GABPA	MA0062.3	0.0309	CCCCCTGACCGCGA	NCKAP1L	cg16509569
DMR22	chr12	54891634	54891648	EBF1	MA0154.4	0.011	CGCGGTCAGGGGGTT	NCKAP1L	cg21376733
DMR22	chr12	54891634	54891643	ERG	MA0474.2	0.0334	CGCGGTCAGG	NCKAP1L	cg21376733
DMR22	chr12	54891634	54891643	MYB	MA0100.3	0.0115	CCTGACCGCG	NCKAP1L	cg16509569
DMR26	chr12	68553817	68553826	TBX21	MA0690.1	0.02	CCAGGGCGAA	IFNG	cg05224770
DMR14	chr14	75988644	75988654	TBX21	MA0690.2	0.0157	TCCGACAGTTT	BATF	cg17747924
DMR14	chr14	75988646	75988655	RUNX3	MA0684.1	0.0223	TTCCGACAGT	BATF	cg17747924
DMR14	chr14	75988743	75988752	TBX21	MA0690.1	0.0124	TGGGCGGAAA	BATF	cg15645309
DMR89	chr15	38988745	38988755	EBF1	MA0154.2	0.0476	GGCCTATATTT	C15orf53	cg12609829
DMR18	chr16	27414410	27414419	SP1	MA0079.2	0.00188	CGCAGCCTCA	IL21R	cg00050618
DMR18	chr16	27414410	27414419	TCF3	MA0522.2	0.0139	CGCAGCCTCA	IL21R	cg00050618
DMR18	chr16	27414411	27414426	BCL6	MA0463.2	0.0198	TACCTCTCGCAGCCTC	IL21R	cg00050618
DMR18	chr16	27414412	27414422	TBX21	MA0690.2	0.00183	TCTCGCAGCCT	IL21R	cg00050618
DMR18	chr16	27414413	27414426	EBF1	MA0154.3	0.0313	TACCTCTCGCAGCC	IL21R	cg00050618
DMR18	chr16	27414526	27414536	TCF3	MA0522.3	0.0187	GACCCGTTCCC	IL21R	cg02787852
DMR80	chr16	29674165	29674184	SPI1	MA0080.5	0.0496	CTCTGAGGGCAGGAAATGGC	SPN	cg02914427
DMR80	chr16	29674171	29674184	ELF1	MA0473.3	8.14e-06	GGGCAGGAAATGGC	SPN	cg02914427
DMR80	chr16	29674175	29674184	MYB	MA0100.3	0.0471	AGGAAATGGC	SPN	cg02914427
DMR21	chr16	29757319	29757337	PAX5	MA0014.2	0.0116	TACGTCTGAGAGGCGGACC	C16orf54	cg23093496
DMR21	chr16	29757320	29757329	GABPA	MA0062.1	0.0135	AGAGGCGGAC	C16orf54	cg23093496
DMR21	chr16	29757322	29757336	EBF1	MA0154.4	0.0145	ACGTCTGAGAGGCGG	C16orf54	cg23093496
DMR21	chr16	29757322	29757329	GATA3	MA0037.2	0.0461	AGAGGCGG	C16orf54	cg23093496
DMR21	chr16	29757323	29757331	SP1	MA0079.5	0.0021	TGAGAGGCG	C16orf54	cg23093496
DMR21	chr16	29757357	29757376	SPI1	MA0080.5	0.00688	AGGCGGAAGGGAGGGCACCG	C16orf54	cg03750478
DMR21	chr16	29757368	29757382	SP1	MA0079.4	0.00151	AGGGCACCGCCTGTT	C16orf54	cg03750478
DMR21	chr16	29757369	29757380	PAX5	MA0014.3	0.0133	GGGCACCGCCTG	C16orf54	cg03750478
DMR21	chr16	29757370	29757383	GABPA	MA0062.3	0.000536	GGCACCGCCTGTTG	C16orf54	cg03750478
DMR21	chr16	29757370	29757380	TBX21	MA0690.2	0.0311	GGCACCGCCTG	C16orf54	cg03750478
DMR21	chr16	29757371	29757380	RUNX3	MA0684.1	0.00719	GCACCGCCTG	C16orf54	cg03750478
DMR21	chr16	29757373	29757382	ERG	MA0474.2	0.0207	ACCGCCTGTT	C16orf54	cg03750478
DMR21	chr16	29757373	29757382	GABPA	MA0062.1	0.0349	ACCGCCTGTT	C16orf54	cg03750478
DMR21	chr16	29757373	29757382	MYB	MA0100.3	0.00691	ACCGCCTGTT	C16orf54	cg03750478
DMR11	chr16	50715395	50715413	PAX5	MA0014.2	0.00221	GCGGCAGGGAAAGTGAGGC	SNX20	cg17619566
DMR11	chr16	50715401	50715415	EBF1	MA0154.4	0.00359	AGGCGGCAGGGAAAG	SNX20	cg17619566
DMR11	chr16	50715406	50715417	YY1	MA0095.2	0.0144	CAAGGCGGCAGG	SNX20	cg17619566
DMR11	chr16	50715407	50715416	TBX21	MA0690.1	0.00747	AAGGCGGCAG	SNX20	cg17619566
DMR11	chr16	50715515	50715529	EBF1	MA0154.4	0.0373	GGCCCCCATCATGCC	SNX20	cg27081230
DMR11	chr16	50715524	50715535	YY1	MA0095.2	0.00133	CATGACGGCCCC	SNX20	cg27081230

DMR11	chr16	50715525	50715535	TBX21	MA0690.2	0.016	CATGACGGCCC	SNX20	cg27081230
DMR11	chr16	50715527	50715538	PAX5	MA0014.3	0.000162	GGGCATGACGGC	SNX20	cg27081230
DMR11	chr16	50715527	50715538	YY1	MA0095.2	0.00295	GGGCATGACGGC	SNX20	cg27081230
DMR11	chr16	50715686	50715700	EBF1	MA0154.4	1.75e-05	AAGTCCCAGGGAAGC	SNX20	cg15393399
DMR37	chr17	56408997	56409012	BCL6	MA0463.2	0.0189	GCCGCTTCCAGCCACG	MIR142	cg26112797
DMR37	chr17	56409000	56409018	PAX5	MA0014.2	0.007	GCTTCCAGCCACGCTAGCC	MIR142	cg26112797
DMR37	chr17	56409003	56409013	TCF3	MA0522.3	0.00902	TCCAGCCACGC	MIR142	cg26112797
DMR37	chr17	56409005	56409014	RUNX3	MA0684.1	0.00573	CAGCCACGCT	MIR142	cg26112797
DMR37	chr17	56409006	56409015	MAX	MA0058.1	0.00933	AGCCACGCTA	MIR142	cg26112797
DMR37	chr17	56409006	56409015	MAX	MA0058.2	0.0471	AGCCACGCTA	MIR142	cg26112797
DMR37	chr17	56409006	56409015	MYB	MA0100.3	0.0289	AGCCACGCTA	MIR142	cg26112797
DMR37	chr17	56409006	56409017	MYC	MA0147.3	0.00641	AGCCACGCTAGC	MIR142	cg26112797
DMR37	chr17	56409006	56409016	TBX21	MA0690.2	0.0246	AGCCACGCTAG	MIR142	cg26112797
DMR37	chr17	56409007	56409018	ELF1	MA0473.2	0.04	GCCACGCTAGCC	MIR142	cg26112797
DMR37	chr17	56409007	56409016	MAX	MA0058.3	0.00712	GCCACGCTAG	MIR142	cg26112797
DMR37	chr17	56409007	56409016	TCF3	MA0522.2	0.0395	GCCACGCTAG	MIR142	cg26112797
DMR37	chr17	56409007	56409012	YY1	MA0095.1	0.0142	GCCACG	MIR142	cg26112797
DMR37	chr17	56409009	56409014	ZEB1	MA0103.1	0.0124	CACGCT	MIR142	cg26112797
DMR37	chr17	56409017	56409028	RUNX3	MA0684.2	0.00889	CCACCCTCCAGC	MIR142	cg10530767
DMR37	chr17	56409023	56409028	YY1	MA0095.1	0.0155	TCCAGC	MIR142	cg10530767
DMR45	chr19	3179533	3179545	ELF1	MA0473.1	0.00667	GCCTGCTGAAGAC	S1PR4	cg04490178
DMR45	chr19	3179540	3179551	ELF1	MA0473.2	0.0459	GAAGACGGTGCT	S1PR4	cg04490178
DMR45	chr19	3179540	3179551	YY1	MA0095.2	0.00168	GAAGACGGTGCT	S1PR4	cg04490178
DMR45	chr19	3179541	3179550	MAX	MA0058.1	0.0409	AAGACGGTGC	S1PR4	cg04490178
DMR25	chr2	10261900	10261911	MYC	MA0147.3	0.0124	GCCACCGCGCCC	RRM2	cg18623836
DMR27	chr2	158300481	158300490	RUNX3	MA0684.1	0.0283	AGTCCGCCAA	CYTIP	cg10559416
DMR27	chr2	158300481	158300490	TBX21	MA0690.1	0.0407	AGTCCGCCAA	CYTIP	cg10559416
DMR27	chr2	158300484	158300493	RUNX3	MA0684.1	0.0241	CCGCCAAATT	CYTIP	cg10559416
DMR27	chr2	158300485	158300494	MAX	MA0058.2	0.0273	CGCCAAATTG	CYTIP	cg10559416
DMR27	chr2	158300485	158300494	MAX	MA0058.1	0.0418	CGCCAAATTG	CYTIP	cg10559416
DMR27	chr2	158300485	158300496	MYC	MA0147.3	0.0243	CGCCAAATTGCC	CYTIP	cg10559416
DMR6	chr2	202125089	202125094	SPI1	MA0080.1	0.0467	CCGCAC	CASP8	cg14764819
DMR6	chr2	202125090	202125099	MYB	MA0100.3	0.0478	GGAGACCGCA	CASP8	cg14764819
DMR6	chr2	202125091	202125104	ELF1	MA0473.3	0.0055	GTCCAGGAGACCGC	CASP8	cg14764819
DMR6	chr2	202125162	202125177	BCL6	MA0463.2	0.0347	GCGCTTACCACTTGCC	CASP8	cg09421136
DMR6	chr2	202125165	202125178	GABPA	MA0062.3	0.0292	AGCGCTTACCACTT	CASP8	cg09421136
DMR6	chr2	202125165	202125175	TBX21	MA0690.2	0.0261	GCTTACCACTT	CASP8	cg09421136
DMR6	chr2	202125169	202125176	GATA3	MA0037.3	0.0298	CGCTTACC	CASP8	cg09421136
DMR6	chr2	202125171	202125181	RUNX1	MA0002.1	0.0176	AAGAGCGCTTA	CASP8	cg09421136
DMR6	chr2	202125173	202125192	SPI1	MA0080.5	0.00226	ACTAGAGGGGAAAGAGCGCT	CASP8	cg09421136
DMR6	chr2	202125173	202125184	YY1	MA0095.2	0.0437	GGAAAGAGCGCT	CASP8	cg09421136
DMR6	chr2	202125175	202125188	ELF1	MA0473.3	0.0104	GAGGGGAAAGAGCG	CASP8	cg09421136
DMR6	chr2	202125200	202125213	GABPA	MA0062.3	0.00911	CGAAACTCCTGAGT	CASP8	cg26842802
DMR6	chr2	202125204	202125213	MYB	MA0100.3	0.0437	CGAAACTCCT	CASP8	cg26842802
DMR6	chr2	202125206	202125220	IRF4	MA1419.1	0.00589	CCTGTTTCGAAACTC	CASP8	cg26842802
DMR6	chr2	202125207	202125222	BCL6	MA0463.2	0.0109	TTCCTGTTTCGAAACT	CASP8	cg26842802
DMR6	chr2	202125207	202125213	SPI1	MA0080.2	0.00715	CGAAACT	CASP8	cg26842802
DMR6	chr2	202125301	202125310	MAX	MA0058.3	0.0497	GGCCTGTGAC	CASP8	cg15174220
DMR6	chr2	202125301	202125310	TBX21	MA0690.1	0.00728	GGCCTGTGAC	CASP8	cg15174220
DMR35	chr2	225811633	225811647	IRF4	MA1419.1	0.0217	TTTAAACGGGAGCCC	DOCK10	cg16014076
DMR35	chr2	225811633	225811646	SPI1	MA0080.4	0.00378	TTAAACGGGAGCCC	DOCK10	cg16014076
DMR35	chr2	225811634	225811643	GABPA	MA0062.1	0.000948	AACGGGAGCC	DOCK10	cg16014076
DMR35	chr2	225811636	225811641	GATA3	MA0037.1	0.0444	CGGGAG	DOCK10	cg16014076
DMR35	chr2	225811636	225811641	SPI1	MA0080.1	0.0474	CGGGAG	DOCK10	cg16014076

DMR35	chr2	225811637	225811646	MYB	MA0100.3	0.0303	TTAAACGGGA	DOCK10	cg16014076
DMR42	chr20	56195556	56195574	PAX5	MA0014.2	0.01	GAATCTGGCCGGCCTGGCT	ZBP1	cg11460314
DMR42	chr20	56195563	56195574	PAX5	MA0014.3	0.0299	GAATCTGGCCGG	ZBP1	cg11460314
DMR42	chr20	56195563	56195574	YY1	MA0095.2	0.00808	GAATCTGGCCGG	ZBP1	cg11460314
DMR42	chr20	56195564	56195574	RUNX1	MA0002.1	0.0329	GAATCTGGCCG	ZBP1	cg11460314
DMR77	chr21	46334201	46334214	GABPA	MA0062.3	0.0458	CCTCAACCCTGCCC	ITGB2	cg01167274
DMR77	chr21	46334204	46334214	TCF3	MA0522.3	0.00553	CAACCCTGCCC	ITGB2	cg01167274
DMR77	chr21	46334205	46334214	RUNX3	MA0684.1	0.0411	AACCCTGCCC	ITGB2	cg01167274
DMR19	chr3	45984850	45984859	MYB	MA0100.3	0.0065	ACCAACCCGC	CXCR6	cg05979583
DMR24	chr3	46411436	46411447	RUNX3	MA0684.2	0.0303	GTGACCTTGGCT	CCR5	cg04131610
DMR24	chr3	46411440	46411451	PAX5	MA0014.3	0.000152	TTCCGTGACCTT	CCR5	cg04131610
DMR24	chr3	46411443	46411452	TBX21	MA0690.1	0.0336	CTTCCGTGAC	CCR5	cg04131610
DMR24	chr3	46411444	46411453	EBF1	MA0154.1	0.0166	GCTTCCGTGA	CCR5	cg04131610
DMR24	chr3	46411445	46411454	TCF3	MA0522.2	0.0174	GGCTTCCGTG	CCR5	cg04131610
DMR24	chr3	46411460	46411474	EBF1	MA0154.4	0.0335	GAGCCACAAGATGCC	CCR5	cg00803692
DMR24	chr3	46411465	46411474	RUNX3	MA0684.1	0.00186	GAGCCACAAG	CCR5	cg00803692
DMR85	chr4	100738002	100738011	TBX21	MA0690.1	0.0164	GCTCTGTGAG	DAPP1	cg10397389
DMR4	chr5	169407470	169407480	TBX21	MA0690.2	0.0139	CTCGGCAGCTA	FAM196B	cg00357551
DMR4	chr5	169407745	169407755	TBX21	MA0690.2	0.0402	AGTGACGGCGG	FAM196B	cg26972389
DMR1	chr6	31540021	31540031	TBX21	MA0690.2	0.02	ACTGCCGCTTC	LTA	cg17169196
DMR1	chr6	31540411	31540421	TBX21	MA0690.2	0.0227	CCCGACCCCCG	LTA	cg22318806
DMR1	chr6	31540452	31540461	TBX21	MA0690.1	0.0451	GCGGCGGAAG	LTA	cg26348243, cg17709873
DMR70	chr6	32909510	32909523	ELF1	MA0473.3	0.0229	GAAAGGCAGGTGAG	HLA-DMB	cg07623567
DMR70	chr6	32909510	32909523	SPI1	MA0080.4	0.0243	GAAAGGCAGGTGAG	HLA-DMB	cg07623567
DMR59	chr6	42391243	42391254	RUNX3	MA0684.2	0.0435	CTGGCTGCAAAC	TRERF1	cg03557441
DMR13	chr6	108145416	108145426	RUNX1	MA0002.1	0.00322	ATACTCGGTTT	SCML4	cg27353361
DMR13	chr6	108145532	108145547	BCL6	MA0463.2	0.0291	GGTAAATCGAGTTTTC	SCML4	cg27659622
DMR13	chr6	108145533	108145543	RUNX1	MA0002.1	0.0153	AATCGAGTTTT	SCML4	cg27659622
DMR13	chr6	108145535	108145544	MAX	MA0058.3	0.0332	AAATCGAGTT	SCML4	cg27659622
DMR13	chr6	108145570	108145585	BCL6	MA0463.2	0.00802	AAGCTGTCATCGTAAT	SCML4	cg05603896
DMR13	chr6	108145571	108145581	RUNX1	MA0002.1	0.0378	TGTCATCGTAA	SCML4	cg05603896
DMR86	chr7	36764063	36764082	SPI1	MA0080.5	0.0494	TGCACAGTGGCACAACTTCC	AOAH	cg25733272
DMR31	chr8	21771529	21771543	IRF4	MA1419.1	0.0192	TCCAAGCTGTACGCA	DOK2	cg08288130
DMR31	chr8	21771535	21771546	RUNX3	MA0684.2	0.0498	CTGTACGCAAGT	DOK2	cg08288130
DMR31	chr8	21771537	21771547	TBX21	MA0690.2	0.0218	GTACGCAAGTT	DOK2	cg08288130
DMR90	chr9	95726433	95726447	EBF1	MA0154.4	0.0442	GGGGGCAATGGCAAA	FGD3	cg22382309
DMR90	chr9	95726438	95726447	ERG	MA0474.2	0.0162	GGGGGCAATG	FGD3	cg22382309
DMR90	chr9	95726438	95726447	RBPJ	MA1116.1	0.0263	GGGGGCAATG	FGD3	cg22382309
DMR93	chr9	117692750	117692759	MAX	MA0058.3	0.0261	GCCTCTCGCC	TNFSF8	cg05185749
DMR12	chr9	123688735	123688745	TBX21	MA0690.2	0.0181	CTGGGCACATC	TRAF1	cg14064762
DMR12	chr9	123688738	123688749	RUNX3	MA0684.2	0.0377	GGCACATCGGAG	TRAF1	cg14064762
DMR12	chr9	123688740	123688751	YY1	MA0095.2	0.0333	CACATCGGAGGA	TRAF1	cg14064762
DMR12	chr9	123688741	123688760	SPI1	MA0080.5	0.00422	ACATCGGAGGAGGGGGCCAC	TRAF1	cg14064762
DMR12	chr9	123688881	123688890	RUNX3	MA0684.1	0.0387	AAAGCGCCTA	TRAF1	cg00894216
DMR12	chr9	123688881	123688891	TBX21	MA0690.2	0.0241	AAAAGCGCCTA	TRAF1	cg00894216
DMR12	chr9	123688882	123688901	SPI1	MA0080.5	0.00385	AGAGCAGAGCAAAAGCGCCT	TRAF1	cg00894216
DMR12	chr9	123689184	123689193	RUNX3	MA0684.1	0.0388	AGAACAAAGC	TRAF1	cg04517263
DMR12	chr9	123689187	123689193	SPI1	MA0080.2	0.0379	ACAAAGC	TRAF1	cg04517263
