# Supplementary material for: Epigenetic regulation of transcription factor binding motifs promotes Th1 response in Chagas disease cardiomyopathy
Source: Front Immunol. 2022 Aug 22;13:958200. doi: 10.3389/fimmu.2022.958200 (PMC9441916; doi:10.3389/fimmu.2022.958200)
Supplement: Supplementary Table 1 — Biological samples included in this study. [file DataSheet_1.zip › Supplementary Material/Supplementary Table 13.pdf]

Supplementary table 13. Cell type proportions in RNA-seq by comparison to cell type signatures.

Cell type	sevCCC1	sevCCC2	sevCCC3	sevCCC4	sevCCC5	sevCCC6	sevCCC7	sevCCC8	CTRL1	CTRL2	CTRL3	CTRL4	CTRL5	CTRL6	pvalue	Corrected pvalue
**Cardiomyocytes**	14,56	16,66	31,76	22,38	32,71	28,63	43,47	36,95	45,83	49,41	47,59	38,59	35,34	48,52	4,66E-03	1,17E-02
**Endothelial cells**	12,1	33,21	14,59	20,55	25,22	19,76	18,39	25,61	20,19	16,57	13,67	19,17	19,08	15,47	2,82E-01	2,82E-01
**Fibroblasts**	10,92	17,4	25,58	22,1	17,23	11,74	14,94	16,59	10,88	12,87	11,98	10,07	18,31	14,03	1,42E-01	1,77E-01
**Macrophages**	42,81	19,56	11,3	18,78	8,08	21,59	4,79	3,31	0	0	3,95	8,8	2,84	0	1,15E-02	1,91E-02
**Smooth muscle cells**	19,62	13,17	16,77	16,19	16,76	18,28	18,4	17,54	23,11	21,15	22,81	23,37	24,43	21,99	6,66E-04	3,33E-03
**Plasma cells**	0	0	0	0	0	1,64	0	2,52	0	0	0	0	0	0	2,44E-01	3,14E-01
**T cells CD8**	7,93	4,05	10,79	8,38	5,1	7,4	5,62	4,18	0	0	0	0	0	0	1,59E-03	1,07E-02
**T cells CD4 naive**	10,09	9,73	15,27	12,94	14,56	9,13	14,69	15,93	0	13,18	0	0	0	2,02	1,09E-02	2,45E-02
**T cells CD4 memory resting**	8,62	5,65	12,58	8,03	9,17	8,36	10,63	6,56	0	5,68	0	0	0	0	2,97E-03	1,07E-02
**T cells CD4 memory activated**	2,55	0	2,77	0	0	1,96	0	0	0	0	0	0	0	0	1,27E-01	1,76E-01
**T cells follicular helper**	5,5	3,1	9,78	4,14	3,48	3,49	3,48	0	0	0,87	0	0	0	0	7,13E-03	1,83E-02
**T cells regulatory Tregs**	3,38	0	4,8	0,14	0	1,55	0	0	0	0	0	0	0	0	6,29E-02	1,01E-01
**T cells gamma delta**	3,05	0	0	0	0	0	0	0	0	0	0	0	0	0	4,70E-01	5,52E-01
**NK cells resting**	5,44	2,55	4,07	3,17	1,2	3,07	1,53	0	0	0	0	0	0	0	4,71E-03	1,41E-02
**NK cells activated**	7,95	7,57	9,1	8,13	5,12	8,52	8,95	8,13	0	1,98	0	0	0	0	1,89E-03	1,07E-02
**Monocytes**	5,51	10,81	1,51	4,29	5,12	7,34	1,89	2,22	8,19	1,88	13,39	16,46	11,44	18,16	4,26E-02	7,67E-02
**Macrophages M0**	2,25	6,88	0	0,96	0,39	0	0	3,84	8,94	0	16,22	5,98	3,74	6,91	6,76E-02	1,01E-01
**Macrophages M1**	12,47	16,03	3,49	10,86	6,57	18,88	4,77	2,25	7,73	0	11,56	13,23	20,66	3,8	9,50E-01	9,50E-01
**Macrophages M2**	11,18	19,58	8,55	15,05	19,33	9,29	15,13	22,37	33,57	19,82	26,47	20,11	22,65	22,81	2,66E-03	1,07E-02
**Dendritic cells resting**	6,24	6,52	9,18	11,42	19,77	5,62	16,3	15,64	16,24	13,14	10,53	3	2,4	7,72	4,91E-01	5,52E-01
**Dendritic cells activated**	7,84	7,53	8	9,71	10,18	13,74	6,22	5,28	6,55	4,87	7,86	12,89	20,07	2,52	7,55E-01	7,99E-01
**Mast cells resting**	0	0	0	0	0	0	0	0	1,79	0	0	1,51	1,27	0	3,88E-02	7,67E-02
**others**	0	0	0,1	2,77	0	0	10,81	11,07	16,99	38,58	13,97	26,81	17,76	36,05	2,16E-03	1,07E-02
