# Supplementary material for: Epigenetic regulation of transcription factor binding motifs promotes Th1 response in Chagas disease cardiomyopathy
Source: Front Immunol. 2022 Aug 22;13:958200. doi: 10.3389/fimmu.2022.958200 (PMC9441916; doi:10.3389/fimmu.2022.958200)
Supplement: Supplementary Table 1 — Biological samples included in this study. [file DataSheet_1.zip › Supplementary Material/Supplementary Table 14.pdf]

Supplementary table 14. List of the Differentially Methylated CpGs between blood asymptomatic samples and blood CCC samples.

ID	Chromosome	Position	Gene	Feature	deltaBeta	pvalue	Corrected pvalue
cg07016094	7	11676721	THSD7A	Body	-0,067	2,46E-12	1,81E-06
cg06056382	5	50152744		IGR	0,032	3,09E-11	8,68E-06
cg26291818	8	61429582	RAB2A	5'UTR	0,021	3,54E-11	8,68E-06
cg01075810	21	33036345	SOD1	Body	-0,025	4,97E-11	9,16E-06
cg23817981	3	32993063	CCR4	TSS200	-0,016	1,63E-10	2,40E-05
cg08626876	5	140501540	PCDHB4	TSS200	-0,081	4,26E-10	5,23E-05
cg10824421	18	11368401		IGR	-0,048	5,33E-10	5,61E-05
cg16358698	1	34845080		IGR	-0,072	8,95E-10	7,51E-05
cg05595117	2	1860232	MYT1L	Body	-0,012	9,17E-10	7,51E-05
cg05385826	7	82646096	PCLO	Body	-0,021	1,13E-09	8,32E-05
cg23542758	7	130082359	CEP41	TSS1500	-0,014	1,70E-09	1,14E-04
cg21822325	9	139222119	GPSM1	5'UTR	0,03	2,43E-09	1,33E-04
cg25312873	14	69444960	ACTN1-AS1	TSS1500	-0,008	2,23E-09	1,33E-04
cg08041523	17	74947796		IGR	-0,025	2,54E-09	1,33E-04
cg07247419	20	21376484	NKX2-4	3'UTR	-0,028	2,74E-09	1,34E-04
cg24308082	11	102703383		IGR	-0,022	3,26E-09	1,50E-04
cg08531746	13	113512905	ATP11A	Body	-0,029	4,23E-09	1,83E-04
cg19209225	10	102756912	LZTS2	TSS200	0,021	4,85E-09	1,89E-04
cg16969227	13	44982026		IGR	-0,021	4,87E-09	1,89E-04
cg14449863	5	150053200	MYOZ3	Body	0,008	5,81E-09	2,02E-04
cg13746813	6	14911904		IGR	0,153	5,68E-09	2,02E-04
cg09235438	16	84294830		IGR	-0,014	6,02E-09	2,02E-04
cg17861653	5	131561363	P4HA2	5'UTR	-0,035	6,32E-09	2,02E-04
cg06087427	2	45572698		IGR	-0,039	7,06E-09	2,08E-04
cg23193153	6	34192178		IGR	-0,009	6,99E-09	2,08E-04
cg03052080	2	121342766		IGR	-0,022	7,77E-09	2,20E-04
cg23388844	11	90981521		IGR	-0,017	8,73E-09	2,38E-04
cg17253588	2	145866679		IGR	-0,015	1,02E-08	2,42E-04
cg09216620	3	178425722	KCNMB2	5'UTR	-0,041	1,02E-08	2,42E-04
cg19574017	15	63569834	APH1B	1stExon	0,025	9,96E-09	2,42E-04
cg09723881	17	33747829	SLFN12	Body	0,013	9,88E-09	2,42E-04
cg14400877	3	68681177		IGR	-0,079	1,12E-08	2,43E-04
cg24974158	8	129001476	PVT1	Body	0,011	1,12E-08	2,43E-04
cg06027282	16	30833292		IGR	-0,01	1,09E-08	2,43E-04
cg01991816	20	22293580		IGR	-0,016	1,29E-08	2,72E-04
cg13173797	4	37969628	TBC1D1	Body	0,068	1,37E-08	2,80E-04
cg05570946	6	112501487	LAMA4	Body	-0,021	1,62E-08	3,07E-04
cg12858274	8	133747448	TMEM71	Body	0,031	1,63E-08	3,07E-04
cg06401114	19	19627257	NDUFA13	Body	-0,007	1,56E-08	3,07E-04
cg21320412	18	30323279	KLHL14	Body	0,015	1,91E-08	3,52E-04
cg13075423	3	108943219		IGR	-0,023	2,22E-08	3,77E-04
cg04772776	5	50643767		IGR	0,044	2,12E-08	3,77E-04
cg00158401	10	127581141		IGR	-0,029	2,28E-08	3,77E-04
cg21083556	14	105193326	ADSSL1	Body	0,027	2,30E-08	3,77E-04
cg22511321	22	38013333	GGA1	Body	0,019	2,21E-08	3,77E-04
cg00650978	6	108684632	LACE1	Body	0,074	2,42E-08	3,87E-04
cg04080041	5	140811253	PCDHGA4	Body	-0,058	2,49E-08	3,91E-04
cg24293948	18	61670413		IGR	-0,049	2,62E-08	4,02E-04
cg22355554	4	125572022		IGR	0,013	2,84E-08	4,03E-04
cg17430199	5	76663105	PDE8B	Body	0,069	2,83E-08	4,03E-04
cg08269402	6	32549631	HLA-DRB1	Body	-0,219	2,76E-08	4,03E-04
cg19098906	22	19136238		IGR	-0,076	2,84E-08	4,03E-04
cg20368275	2	208549843		IGR	-0,009	3,68E-08	4,50E-04
cg16372928	4	5794286	EVC	Body	-0,013	3,93E-08	4,50E-04
cg25583503	4	69962676	UGT2B7	1stExon	-0,037	3,90E-08	4,50E-04
cg04239786	5	44316088	FGF10	Body	-0,013	3,90E-08	4,50E-04
cg01039154	5	167289371	TENM2	Body	-0,013	3,33E-08	4,50E-04
cg01048931	7	55639931	VOPP1	Body	-0,007	3,97E-08	4,50E-04
cg05567920	11	33183001	CSTF3	5'UTR	-0,008	3,95E-08	4,50E-04
cg11518240	12	2902790	FKBP4	TSS1500	-0,007	3,51E-08	4,50E-04
cg24342013	13	48891544	RB1	Body	-0,067	3,50E-08	4,50E-04
cg20344767	14	52326463	GNG2	TSS1500	-0,008	3,64E-08	4,50E-04
cg05587478	16	53980329	FTO	Body	0,058	3,51E-08	4,50E-04
cg00239838	19	35204549		IGR	-0,013	3,89E-08	4,50E-04
cg13122415	21	16596757		IGR	-0,025	3,66E-08	4,50E-04
cg26929161	20	50701266	ZFP64	Body	-0,027	4,16E-08	4,65E-04
cg00899354	5	174063572		IGR	-0,011	4,31E-08	4,74E-04
cg11233366	5	34915412	RAD1	Body	-0,006	4,46E-08	4,78E-04
cg00776960	15	65688118	IGDCC4	Body	-0,074	4,48E-08	4,78E-04
cg16740886	1	9593025		IGR	-0,017	4,82E-08	5,07E-04

cg10576878	1	46045910		IGR	-0,032	5,23E-08	5,35E-04
cg18641937	1	160924389	ITLN2	Body	0,017	5,30E-08	5,35E-04
cg12962191	5	140213924	PCDHA6	Body	-0,065	5,23E-08	5,35E-04
cg13008043	5	33531289	ADAMTS12	Body	-0,019	5,40E-08	5,38E-04
cg23657686	11	10565836		IGR	-0,028	5,71E-08	5,61E-04
cg05031931	7	214779	FAM20C	Body	-0,013	6,31E-08	6,12E-04
cg13098795	3	48369887	SPINK8	TSS200	0,028	6,50E-08	6,14E-04
cg08587989	22	44460345	PARVB	Body	0,029	6,43E-08	6,14E-04
cg04741746	6	41687340	TFEB	Body	0,014	6,67E-08	6,22E-04
cg07289727	4	187614428	FAT1	Body	-0,036	6,86E-08	6,26E-04
cg17477700	8	98142411	PGCP	Body	-0,013	6,89E-08	6,26E-04
cg03088705	10	111624928	XPNPEP1	Body	-0,031	7,07E-08	6,35E-04
cg07524997	5	140796892	PCDHGA4	Body	-0,087	7,72E-08	6,73E-04
cg23151649	7	151393469	PRKAG2	Body	0,078	7,67E-08	6,73E-04
cg00918794	15	64454941	PPIB	Body	-0,014	7,76E-08	6,73E-04
cg10576837	2	174891810		IGR	0,008	8,22E-08	6,88E-04
cg03583853	5	173142681	LINC01484	Body	0,025	8,22E-08	6,88E-04
cg05616242	12	54783416	ZNF385A	Body	0,018	8,31E-08	6,88E-04
cg16317295	14	50017009		IGR	-0,031	8,23E-08	6,88E-04
cg13467395	14	32215027	NUBPL	Body	-0,028	8,49E-08	6,95E-04
cg23999709	1	228888185		IGR	0,045	9,57E-08	7,19E-04
cg08528675	5	31737570		IGR	-0,012	9,52E-08	7,19E-04
cg00031042	6	158254767	SNX9	Body	-0,017	9,28E-08	7,19E-04
cg03734783	7	150745709	ACCN3	1stExon	-0,01	9,37E-08	7,19E-04
cg18428626	8	99956751	OSR2	5'UTR	-0,007	9,22E-08	7,19E-04
cg26599871	16	2749429	KCTD5	Body	0,012	8,94E-08	7,19E-04
cg03697708	19	13617549	CACNA1A	TSS1500	-0,021	9,21E-08	7,19E-04
cg17727639	22	44415701	PARVB	Body	0,014	9,42E-08	7,19E-04
cg09706365	9	19464573		IGR	0,027	9,74E-08	7,24E-04
cg09422531	17	21307337	KCNJ18	TSS1500	-0,022	9,90E-08	7,29E-04
cg05581802	12	106972569		IGR	-0,036	1,01E-07	7,39E-04
cg11113649	14	97054344		IGR	-0,008	1,04E-07	7,51E-04
cg25438876	5	85110900		IGR	-0,071	1,05E-07	7,55E-04
cg02462812	5	140501535	PCDHB4	TSS200	-0,081	1,08E-07	7,57E-04
cg21955686	17	6555093	C17orf100	5'UTR	-0,012	1,07E-07	7,57E-04
cg03543453	10	978919	LARP4B	TSS1500	0,01	1,10E-07	7,67E-04
cg03176698	4	86889760	ARHGAP24	Body	0,047	1,13E-07	7,81E-04
cg24591770	1	45082704	RNF220	Body	0,013	1,23E-07	7,83E-04
cg00341742	2	154335698	RPRM	TSS1500	-0,023	1,19E-07	7,83E-04
cg16339745	6	105285130	HACE1	Body	0,008	1,22E-07	7,83E-04
cg12578959	11	107328914	CWF19L2	TSS1500	-0,007	1,18E-07	7,83E-04
cg14481339	11	128737319	KCNJ1	TSS200	-0,012	1,19E-07	7,83E-04
cg23324048	14	60794590		IGR	-0,006	1,22E-07	7,83E-04
cg00866179	17	25710963		IGR	-0,026	1,16E-07	7,83E-04
cg12333112	17	38278849	MSL1	1stExon	-0,007	1,20E-07	7,83E-04
cg04996588	19	1107911	SBNO2	3'UTR	0,024	1,23E-07	7,83E-04
cg11022519	3	135967896	PCCB	TSS1500	-0,02	1,28E-07	7,91E-04
cg00562118	4	644805	PDE6B	Body	-0,069	1,29E-07	7,91E-04
cg13018725	6	33220041	VPS52	Body	-0,017	1,29E-07	7,91E-04
cg05482394	13	30882273	KATNAL1	TSS1500	0,033	1,27E-07	7,91E-04
cg15740385	17	80394974	HEXDC	Body	0,016	1,31E-07	7,95E-04
cg26129677	11	45714776		IGR	-0,019	1,34E-07	8,05E-04
cg02192390	19	47429282	ARHGAP35	Body	-0,008	1,34E-07	8,05E-04
cg22912997	12	130502283		IGR	-0,044	1,36E-07	8,06E-04
cg19260592	1	98872441		IGR	0,038	1,41E-07	8,30E-04
cg18755544	5	33339102		IGR	0,029	1,45E-07	8,43E-04
cg18607583	8	63387061	NKAIN3	Body	0,08	1,44E-07	8,43E-04
cg06773488	18	74726002	MBP	Body	0,052	1,47E-07	8,47E-04
cg10233866	6	94996919		IGR	-0,048	1,50E-07	8,55E-04
cg08370839	10	105678654	OBFC1	TSS1500	-0,023	1,51E-07	8,58E-04
cg17797229	13	110522297		IGR	-0,154	1,54E-07	8,68E-04
cg13369416	5	5881734		IGR	-0,075	1,57E-07	8,75E-04
cg03277828	1	6762102	DNAJC11	TSS200	-0,007	1,61E-07	8,75E-04
cg21501346	4	146296565		IGR	-0,015	1,63E-07	8,75E-04
cg08368538	8	101169276	SPAG1	TSS1500	0,045	1,58E-07	8,75E-04
cg03725192	16	1167740		IGR	0,05	1,60E-07	8,75E-04
cg07968906	17	40822942	PLEKHH3	Body	-0,09	1,62E-07	8,75E-04
cg07265976	4	2696715	FAM193A	Body	0,008	1,69E-07	8,87E-04
cg12602010	18	8963235		IGR	0,069	1,67E-07	8,87E-04
cg14816904	20	52844524		IGR	-0,025	1,68E-07	8,87E-04
cg17788475	3	186525220	RFC4	TSS1500	-0,01	1,73E-07	8,98E-04
cg11697123	5	83332828	EDIL3	Body	-0,02	1,73E-07	8,98E-04
cg18865445	13	110522265		IGR	-0,108	1,74E-07	8,98E-04

cg01497214	2	145277032	ZEB2-AS1	TSS200	-0,01	1,83E-07	9,23E-04
cg17739279	6	3849190	FAM50B	TSS1500	0,039	1,82E-07	9,23E-04
cg11906758	7	44789468	ZMIZ2	5'UTR	0,025	1,82E-07	9,23E-04
cg27371451	19	45060276	CEACAM22P	TSS200	0,056	1,84E-07	9,24E-04
cg08708382	8	121013706	DEPTOR	Body	-0,006	1,86E-07	9,26E-04
cg11256364	7	114562847	MDFIC	5'UTR	-0,007	1,91E-07	9,33E-04
cg03284642	13	30146493	SLC7A1	5'UTR	-0,008	1,91E-07	9,33E-04
cg18329931	19	17858255	FCHO1	TSS1500	-0,067	1,89E-07	9,33E-04
cg10830239	1	54043189	GLIS1	Body	0,018	2,01E-07	9,50E-04
cg19876963	7	18395942	HDAC9	Body	-0,013	1,98E-07	9,50E-04
cg20384759	12	116413613	MED13L	Body	-0,01	2,01E-07	9,50E-04
cg07728507	16	88349782		IGR	0,019	1,98E-07	9,50E-04
cg01043383	19	57630742	USP29	TSS1500	-0,01	2,01E-07	9,50E-04
cg09460553	13	110521956		IGR	-0,119	2,03E-07	9,55E-04
cg14464660	14	56313130		IGR	-0,031	2,12E-07	9,83E-04
cg09309286	17	32960898	TMEM132E	Body	-0,026	2,13E-07	9,83E-04
cg18449546	17	40168955	NKIRAS2	TSS1500	-0,004	2,13E-07	9,83E-04
cg10519903	1	6537604	PLEKHG5	Body	-0,026	2,18E-07	9,92E-04
cg10107322	10	134544617	INPP5A	Body	-0,015	2,18E-07	9,92E-04
cg03027535	2	49123683		IGR	-0,011	2,21E-07	9,92E-04
cg25424659	19	11450592	RAB3D	TSS1500	0,028	2,20E-07	9,92E-04
cg19342109	6	152958121	SYNE1	5'UTR	-0,013	2,30E-07	1,03E-03
cg21005108	7	74124574	LOC101926943	Body	-0,009	2,33E-07	1,03E-03
cg12808028	10	97522036	ENTPD1	Body	-0,021	2,35E-07	1,04E-03
cg25260352	5	137451732	NME5	Body	-0,03	2,38E-07	1,04E-03
cg01640590	20	40820367	PTPRT	Body	-0,07	2,38E-07	1,04E-03
cg14899711	15	49912605	FAM227B	5'UTR	-0,024	2,42E-07	1,05E-03
cg20383841	5	135732754		IGR	-0,02	2,48E-07	1,07E-03
cg15154540	1	167502324		IGR	-0,009	2,53E-07	1,08E-03
cg25430849	7	99699006	MCM7	1stExon	0,007	2,52E-07	1,08E-03
cg17806339	4	89544455	HERC3	Body	-0,025	2,60E-07	1,09E-03
cg05227343	10	89736642		IGR	-0,011	2,59E-07	1,09E-03
cg04203401	4	148978760	ARHGAP10	Body	0,021	2,65E-07	1,10E-03
cg05880758	13	68723704		IGR	-0,024	2,64E-07	1,10E-03
cg12589340	19	6004058	RFX2	Body	0,022	2,65E-07	1,10E-03
cg10725892	2	175460353	WIPF1	5'UTR	-0,01	2,70E-07	1,11E-03
cg18024493	2	18057814	KCNS3	TSS1500	-0,034	2,73E-07	1,11E-03
cg16400154	6	102068556	GRIK2	Body	-0,025	2,72E-07	1,11E-03
cg24289524	13	39199366		IGR	-0,044	2,82E-07	1,14E-03
cg16865618	4	27123753		IGR	-0,026	2,91E-07	1,15E-03
cg07852735	8	63951642	GGH	TSS200	-0,005	2,89E-07	1,15E-03
cg06697425	17	72915897	USH1G	Body	-0,071	2,91E-07	1,15E-03
cg20758651	19	33878901	PEPD	Body	0,011	2,90E-07	1,15E-03
cg01500266	5	176283546	UNC5A	Body	0,025	2,97E-07	1,17E-03
cg25700931	3	128039078	EEFSEC	Body	-0,008	3,03E-07	1,19E-03
cg18209470	7	116199599	CAV1	3'UTR	0,04	3,11E-07	1,19E-03
cg02291245	10	119780037	RAB11FIP2	Body	-0,023	3,10E-07	1,19E-03
cg10998122	11	64008466	FKBP2	TSS1500	0,011	3,08E-07	1,19E-03
cg04001333	14	76045348	FLVCR2	1stExon	-0,085	3,06E-07	1,19E-03
cg06498272	19	1192499		IGR	-0,029	3,06E-07	1,19E-03
cg01496759	14	44224266		IGR	-0,009	3,15E-07	1,19E-03
cg04909314	15	93467762	CHD2	ExonBnd	-0,008	3,13E-07	1,19E-03
cg14009778	19	29578525		IGR	-0,015	3,22E-07	1,21E-03
cg19535025	17	63491345		IGR	-0,062	3,26E-07	1,22E-03
cg15812596	2	190648578	PMS1	TSS1500	-0,005	3,32E-07	1,23E-03
cg00767842	18	56234780	ALPK2	Body	-0,009	3,36E-07	1,24E-03
cg12401651	1	150669681	GOLPH3L	TSS200	-0,013	3,40E-07	1,25E-03
cg07620533	5	140793470	PCDHGA4	Body	-0,063	3,41E-07	1,25E-03
cg13083943	11	3150633	OSBPL5	5'UTR	0,02	3,42E-07	1,25E-03
cg24636258	12	53068882	KRT1	3'UTR	-0,008	3,46E-07	1,25E-03
cg14058476	14	24835609	NFATC4	TSS1500	0,018	3,47E-07	1,25E-03
cg08255374	2	238848601		IGR	-0,021	3,50E-07	1,26E-03
cg03816625	16	12192430	SNX29	Body	0,014	3,56E-07	1,27E-03
cg01101663	5	140564161	PCDHB16	1stExon	-0,043	3,67E-07	1,31E-03
cg07045538	1	43914361	SZT2	Body	-0,007	3,84E-07	1,33E-03
cg04380228	2	47142555	MCFD2	5'UTR	-0,005	3,78E-07	1,33E-03
cg23839110	9	133973324	AIF1L	Body	0,027	3,84E-07	1,33E-03
cg05075416	15	99500703	IGF1R	3'UTR	0,036	3,83E-07	1,33E-03
cg14525131	17	4611128		IGR	0,026	3,79E-07	1,33E-03
cg09723388	17	50466815		IGR	0,006	3,95E-07	1,37E-03
cg14788660	1	155278430	FDPS	TSS200	-0,007	4,06E-07	1,40E-03
cg12297100	6	130341441	L3MBTL3	5'UTR	-0,009	4,13E-07	1,41E-03
cg04717621	15	102204782	TARSL2	Body	-0,007	4,15E-07	1,41E-03

cg17960980	2	224128063		IGR	-0,017	4,18E-07	1,42E-03
cg10554126	16	30851519	BCL7C	Body	0,013	4,19E-07	1,42E-03
cg09032748	1	32664949	CCDC28B	TSS1500	0,01	4,22E-07	1,42E-03
cg10884908	5	140614106	PCDHB18	Body	-0,03	4,26E-07	1,42E-03
cg05446629	11	134281742	B3GAT1	1stExon	-0,027	4,26E-07	1,42E-03
cg00749498	4	170144891	SH3RF1	Body	-0,005	4,30E-07	1,43E-03
cg05775451	1	228353712	C1orf69	1stExon	0,013	4,32E-07	1,43E-03
cg14762289	3	55305648		IGR	-0,071	4,34E-07	1,43E-03
cg27599391	11	108092406	ATM	TSS1500	-0,014	4,39E-07	1,44E-03
cg06417508	17	53352068	HLF	Body	-0,015	4,43E-07	1,44E-03
cg16880314	10	17726081	STAM	Body	0,032	4,48E-07	1,45E-03
cg14193409	16	67143991	C16orf70	5'UTR	-0,007	4,47E-07	1,45E-03
cg01513851	18	29407163		IGR	-0,021	4,53E-07	1,46E-03
cg24713063	20	35459146	SOGA1	Body	-0,019	4,58E-07	1,47E-03
cg25990982	18	11881080	GNAL	Body	-0,011	4,64E-07	1,48E-03
cg19172898	11	70693404	SHANK2	Body	-0,016	4,68E-07	1,49E-03
cg03363289	9	124990165	LHX6	Body	-0,213	4,77E-07	1,51E-03
cg27298425	15	79216880	CTSH	Body	0,015	4,80E-07	1,51E-03
cg27077106	16	57481227	CIAPIN1	5'UTR	-0,006	4,84E-07	1,52E-03
cg17609028	5	140479823	PCDHB3	TSS200	-0,039	4,89E-07	1,52E-03
cg01144969	10	5062467		IGR	-0,067	4,89E-07	1,52E-03
cg19021708	15	101085177	CERS3	TSS1500	-0,081	4,98E-07	1,54E-03
cg04076488	1	174669163	RABGAP1L	Body	0,051	5,09E-07	1,57E-03
cg26319038	2	218723627	TNS1	Body	-0,016	5,18E-07	1,57E-03
cg22127912	4	160656980		IGR	-0,05	5,17E-07	1,57E-03
cg08924212	7	152435333		IGR	-0,021	5,24E-07	1,57E-03
cg12235695	10	131969555	GLRX3	Body	-0,012	5,20E-07	1,57E-03
cg02443061	11	1691234		IGR	0,058	5,20E-07	1,57E-03
cg22583334	14	52183647	FRMD6	TSS1500	-0,025	5,22E-07	1,57E-03
cg24340285	19	1095545	POLR2E	TSS200	0,013	5,13E-07	1,57E-03
cg01709473	6	12010960		IGR	-0,017	5,27E-07	1,57E-03
cg07342012	6	81024395	BCKDHB	Body	-0,024	5,29E-07	1,57E-03
cg25714134	2	191873795	STAT1	Body	-0,009	5,35E-07	1,57E-03
cg23445604	3	46735801	ALS2CL	TSS1500	0,013	5,32E-07	1,57E-03
cg00385596	11	76088726	PRKRIR	Body	-0,012	5,36E-07	1,57E-03
cg13612365	3	53021495	SFMBT1	5'UTR	-0,024	5,50E-07	1,61E-03
cg21555240	5	89734950		IGR	0,022	5,53E-07	1,61E-03
cg24154336	11	64659044	MIR192	TSS1500	-0,009	5,56E-07	1,61E-03
cg16004357	14	89068370	ZC3H14	Body	-0,008	5,54E-07	1,61E-03
cg01273565	12	120128116	CIT	Body	0,01	5,61E-07	1,61E-03
cg10126903	16	29675214	SPN	Body	-0,006	5,62E-07	1,61E-03
cg06803853	16	2234031	CASKIN1	Body	0,016	5,68E-07	1,62E-03
cg22192489	5	134521633		IGR	-0,023	5,80E-07	1,65E-03
cg04703069	1	23126717	EPHB2	Body	0,021	5,86E-07	1,66E-03
cg04138346	6	32938946	BRD2	5'UTR	-0,012	5,92E-07	1,67E-03
cg18027903	7	15601624	TMEM195	1stExon	-0,026	5,94E-07	1,67E-03
cg08389628	1	6388849	ACOT7	Body	0,007	6,00E-07	1,67E-03
cg23636855	2	31919137		IGR	-0,032	6,03E-07	1,67E-03
cg16733545	9	133026410		IGR	-0,07	6,02E-07	1,67E-03
cg04528116	2	141755389	LRP1B	Body	0,015	6,18E-07	1,69E-03
cg09387617	7	78568601	MAGI2	Body	0,015	6,15E-07	1,69E-03
cg16903025	8	124549553	FBXO32	Body	0,036	6,13E-07	1,69E-03
cg10315978	15	45408736	DUOXA2	Body	-0,014	6,17E-07	1,69E-03
cg14656043	10	35419996	CREM	5'UTR	-0,025	6,35E-07	1,72E-03
cg07379617	11	15308187		IGR	-0,01	6,33E-07	1,72E-03
cg10350274	13	76112980	COMMD6	TSS1500	-0,013	6,33E-07	1,72E-03
cg13284946	15	89453670	MFGE8	Body	0,033	6,36E-07	1,72E-03
cg09698846	8	123964152	ZHX2	Body	-0,007	6,40E-07	1,72E-03
cg00678005	8	9597722	TNKS	Body	0,011	6,46E-07	1,73E-03
cg09387650	12	65713650	MSRB3	Body	-0,014	6,49E-07	1,73E-03
cg12518630	10	98740656	C10orf12	TSS1500	0,01	6,60E-07	1,75E-03
cg19595850	1	3712909	LRRC47	1stExon	0,012	6,66E-07	1,76E-03
cg05106892	15	99670731	SYNM	Body	0,038	6,76E-07	1,78E-03
cg22089374	21	47944402	DIP2A	Body	-0,023	6,80E-07	1,79E-03
cg26854140	3	11722255	VGLL4	Body	-0,012	6,85E-07	1,79E-03
cg17514558	5	140621375	PCDHB19P	Body	-0,073	6,87E-07	1,79E-03
cg19425237	1	150129151	PLEKHO1	5'UTR	0,009	6,93E-07	1,80E-03
cg22385827	2	211036478	C2orf67	TSS1500	-0,012	6,95E-07	1,80E-03
cg07158230	7	63391463		IGR	-0,071	7,09E-07	1,83E-03
cg05432453	5	57618609		IGR	0,027	7,18E-07	1,84E-03
cg07903036	10	57876642		IGR	-0,064	7,16E-07	1,84E-03
cg24007644	3	57095961	ARHGEF3	5'UTR	-0,009	7,28E-07	1,86E-03
cg14711869	4	2716524	FAM193A	Body	0,027	7,27E-07	1,86E-03

cg25065988	4	190882213	FRG1	Body	-0,009	7,63E-07	1,94E-03
cg09256126	11	64518158	PYGM	Body	0,015	7,69E-07	1,95E-03
cg18108507	3	51975897	RRP9	1stExon	-0,004	7,86E-07	1,98E-03
cg01058253	11	9748095	SWAP70	Body	0,073	7,85E-07	1,98E-03
cg19283778	6	96917485		IGR	-0,059	7,89E-07	1,98E-03
cg01487661	7	63643277		IGR	-0,073	8,08E-07	1,99E-03
cg23874145	7	101892216	CUX1	Body	0,007	8,15E-07	1,99E-03
cg00608965	12	91572191	DCN	1stExon	-0,05	8,11E-07	1,99E-03
cg08013260	12	112473249	NAA25	Body	0,015	8,12E-07	1,99E-03
cg27398640	15	77910606	LINGO1	Body	0,122	8,17E-07	1,99E-03
cg19606289	15	86000756	AKAP13	5'UTR	0,008	8,11E-07	1,99E-03
cg27028760	16	2042103	SYNGR3	Body	0,012	8,05E-07	1,99E-03
cg20473595	17	3848169	ATP2A3	Body	-0,042	8,02E-07	1,99E-03
cg08281371	17	76662056		IGR	0,006	8,23E-07	2,00E-03
cg03982541	14	105766837	PACS2	TSS1500	-0,004	8,32E-07	2,02E-03
cg09550558	1	20208553	OTUD3	TSS1500	-0,012	8,49E-07	2,05E-03
cg14832490	1	20957761		IGR	0,042	8,61E-07	2,05E-03
cg27630169	1	43772596	TIE1	Body	0,013	8,57E-07	2,05E-03
cg24010099	2	28124326	BRE	Body	-0,03	8,64E-07	2,05E-03
cg07043044	5	33835713	ADAMTS12	Body	-0,032	8,62E-07	2,05E-03
cg04872848	14	76045543	FLVCR2	1stExon	-0,053	8,61E-07	2,05E-03
cg08632711	17	1029277	ABR	Body	-0,013	8,84E-07	2,09E-03
cg21668964	3	182360510		IGR	-0,009	8,88E-07	2,10E-03
cg00435245	2	5812420		IGR	-0,037	8,99E-07	2,11E-03
cg07797337	4	86486686	ARHGAP24	5'UTR	-0,009	9,13E-07	2,11E-03
cg27393277	4	177673704	VEGFC	Body	0,036	9,15E-07	2,11E-03
cg06222419	5	140753491	PCDHGA6	TSS200	-0,035	9,17E-07	2,11E-03
cg05116545	9	124573788		IGR	0,027	9,13E-07	2,11E-03
cg10305989	11	73639428		IGR	0,017	9,12E-07	2,11E-03
cg01963061	14	65879906	FUT8	5'UTR	0,014	9,03E-07	2,11E-03
cg12704993	15	69325769	NOX5	Body	-0,014	9,14E-07	2,11E-03
cg21231909	17	49118881	SPAG9	Body	0,025	9,07E-07	2,11E-03
cg07001734	14	102829788	TECPR2	5'UTR	0,011	9,23E-07	2,11E-03
cg02114788	5	151088959		IGR	-0,016	9,31E-07	2,12E-03
cg19873731	4	146019757	ABCE1	5'UTR	-0,006	9,41E-07	2,13E-03
cg14313178	6	42983264	KLHDC3	5'UTR	0,02	9,39E-07	2,13E-03
cg08555556	6	163730796	PACRG	Body	0,036	9,47E-07	2,13E-03
cg14415214	11	2950403	PHLDA2	1stExon	-0,007	9,45E-07	2,13E-03
cg06067370	15	41099605	ZFYVE19	1stExon	0,013	9,42E-07	2,13E-03
cg23349798	6	148841330	SASH1	Body	0,014	9,66E-07	2,16E-03
cg08825190	19	18315256	RAB3A	TSS1500	-0,005	9,65E-07	2,16E-03
cg21904719	8	24956513		IGR	-0,028	9,73E-07	2,17E-03
cg12568245	6	157376879	ARID1B	Body	-0,029	9,76E-07	2,17E-03
cg16319129	6	91791565		IGR	-0,049	9,81E-07	2,17E-03
cg00489795	22	24041104	LOC91316	Body	0,011	9,86E-07	2,17E-03
cg08743899	6	15517343	JARID2	Body	-0,016	1,01E-06	2,20E-03
cg05725489	7	600658	PRKAR1B	Body	-0,019	1,01E-06	2,20E-03
cg21364882	4	48447800		IGR	-0,013	1,01E-06	2,21E-03
cg04756010	6	5778988		IGR	0,025	1,03E-06	2,25E-03
cg08477599	6	30654827	KIAA1949	5'UTR	-0,02	1,03E-06	2,25E-03
cg18426860	6	159592134	FNDC1	Body	0,055	1,04E-06	2,25E-03
cg07722722	1	45187551	C1orf228	Body	0,013	1,06E-06	2,27E-03
cg05954884	1	155279053	FDPS	5'UTR	-0,004	1,06E-06	2,27E-03
cg02644711	3	196713926		IGR	-0,026	1,05E-06	2,27E-03
cg25364517	5	98303844		IGR	-0,015	1,06E-06	2,27E-03
cg07154754	12	132514292	SNORA49	TSS1500	-0,012	1,06E-06	2,27E-03
cg09458618	7	83379803		IGR	-0,017	1,07E-06	2,27E-03
cg17935214	18	76643716		IGR	-0,018	1,08E-06	2,28E-03
cg09172043	10	101025916		IGR	-0,02	1,08E-06	2,29E-03
cg25697010	1	183773030	RGL1	TSS1500	0,023	1,09E-06	2,31E-03
cg02827386	1	97885839	DPYD	Body	-0,005	1,10E-06	2,32E-03
cg20176388	2	17724532	VSNL1	5'UTR	-0,067	1,11E-06	2,33E-03
cg17316760	1	198625883	PTPRC	Body	-0,011	1,13E-06	2,34E-03
cg04282082	9	124988720	LHX6	Body	-0,234	1,12E-06	2,34E-03
cg04178670	11	94973302		IGR	-0,008	1,12E-06	2,34E-03
cg00393814	17	34968449		IGR	0,013	1,12E-06	2,34E-03
cg26297808	7	1372924		IGR	-0,034	1,15E-06	2,38E-03
cg24367698	17	10453091	MYH2	TSS200	0,093	1,16E-06	2,39E-03
cg23251547	2	97577875	FAM178B	Body	0,017	1,17E-06	2,41E-03
cg04675616	1	245544178	KIF26B	Body	-0,023	1,18E-06	2,42E-03
cg05081498	4	190936289		IGR	-0,034	1,18E-06	2,42E-03
cg20719607	7	946806	ADAP1	Body	0,041	1,21E-06	2,46E-03
cg13581483	11	2019823	H19	TSS1500	0,017	1,21E-06	2,46E-03

cg08670210	11	10563297	RNF141	TSS1500	0,01	1,21E-06	2,46E-03
cg12946705	6	170727111		IGR	0,012	1,22E-06	2,48E-03
cg06942946	1	52604255		IGR	-0,013	1,23E-06	2,48E-03
cg19311448	2	11797017		IGR	-0,039	1,23E-06	2,48E-03
cg09225162	15	56757806	MNS1	TSS1500	-0,051	1,28E-06	2,58E-03
cg16552519	6	86159753	NT5E	5'UTR	0,007	1,29E-06	2,58E-03
cg16620364	19	536530	CDC34	Body	0,021	1,29E-06	2,58E-03
cg19280455	15	100385581		IGR	-0,033	1,30E-06	2,58E-03
cg04802018	2	12728102		IGR	-0,025	1,30E-06	2,58E-03
cg19035526	2	217107125		IGR	-0,042	1,32E-06	2,59E-03
cg00742851	3	3843342	LRRN1	5'UTR	-0,01	1,33E-06	2,59E-03
cg11500788	8	28355368	FZD3	5'UTR	-0,016	1,32E-06	2,59E-03
cg06928823	8	73533533	KCNB2	Body	-0,1	1,33E-06	2,59E-03
cg13893963	10	31323041		IGR	-0,015	1,31E-06	2,59E-03
cg15320811	12	50955961	DIP2B	Body	-0,007	1,31E-06	2,59E-03
cg12642414	15	63796736	USP3	TSS200	-0,004	1,33E-06	2,59E-03
cg17714423	22	26351163	MYO18B	ExonBnd	-0,02	1,33E-06	2,59E-03
cg01118078	20	33297179	TP53INP2	Body	-0,004	1,35E-06	2,61E-03
cg06571542	9	139620706	SNHG7	Body	0,02	1,37E-06	2,64E-03
cg07613095	14	51800249	LINC00640	Body	-0,012	1,37E-06	2,64E-03
cg18042079	17	30229074	UTP6	TSS1500	0,008	1,37E-06	2,64E-03
cg13918544	19	50921232	POLD1	3'UTR	0,02	1,37E-06	2,64E-03
cg18356337	2	145459205	TEX41	Body	-0,015	1,38E-06	2,64E-03
cg13651690	14	106320748		IGR	0,012	1,39E-06	2,65E-03
cg18246430	2	224266623		IGR	-0,018	1,39E-06	2,65E-03
cg07772435	22	40804545	SGSM3	Body	0,023	1,40E-06	2,65E-03
cg00173799	11	101001711	PGR	TSS1500	-0,05	1,41E-06	2,67E-03
cg26698749	7	98335450		IGR	-0,026	1,42E-06	2,68E-03
cg03840192	15	32965280	SCG5	Body	-0,008	1,42E-06	2,68E-03
cg14891543	2	231377628	SP100	Body	-0,027	1,43E-06	2,68E-03
cg14096615	12	16762843		IGR	-0,071	1,44E-06	2,68E-03
cg05933240	22	18051014	SLC25A18	5'UTR	0,035	1,43E-06	2,68E-03
cg05068548	3	160284382	KPNA4	TSS1500	0,083	1,44E-06	2,69E-03
cg02400799	6	116421257	NT5DC1	TSS1500	0,045	1,45E-06	2,69E-03
cg22751438	9	115333218	KIAA1958	5'UTR	-0,023	1,46E-06	2,70E-03
cg09400062	22	38151543	TRIOBP	Body	0,013	1,46E-06	2,70E-03
cg00202454	15	42371886	PLA2G4D	Body	-0,053	1,48E-06	2,74E-03
cg08770205	1	15380334	KAZN	Body	-0,014	1,50E-06	2,75E-03
cg25950704	8	17739686	FGL1	ExonBnd	0,008	1,50E-06	2,75E-03
cg02043424	10	43725386	RASGEF1A	TSS200	-0,011	1,49E-06	2,75E-03
cg02043697	12	132269737	SFRS8	Body	0,007	1,51E-06	2,76E-03
cg24042136	4	102109243	PPP3CA	Body	-0,04	1,54E-06	2,77E-03
cg02289322	5	176696395	NSD1	Body	-0,015	1,53E-06	2,77E-03
cg25383699	6	56111908	COL21A1	5'UTR	-0,031	1,52E-06	2,77E-03
cg16326034	6	72037706		IGR	-0,009	1,55E-06	2,77E-03
cg05102190	7	143078242	ZYX	TSS200	0,014	1,56E-06	2,77E-03
cg06945559	8	117564306		IGR	-0,009	1,54E-06	2,77E-03
cg08731760	11	15959742		IGR	-0,008	1,55E-06	2,77E-03
cg09548275	11	47282999	NR1H3	Body	0,048	1,56E-06	2,77E-03
cg07560506	19	54379618	MYADM	3'UTR	0,052	1,55E-06	2,77E-03
cg05720454	21	34442511	OLIG1	1stExon	-0,006	1,55E-06	2,77E-03
cg24731441	22	46481822	LOC400931	TSS200	0,016	1,55E-06	2,77E-03
cg19520087	5	140553020	PCDHB7	1stExon	-0,073	1,58E-06	2,80E-03
cg06327515	5	140603261	PCDHB14	1stExon	-0,052	1,58E-06	2,80E-03
cg06084835	1	65243433	RAVER2	Body	-0,023	1,59E-06	2,81E-03
cg09102079	1	53164386	COA7	TSS1500	0,053	1,63E-06	2,82E-03
cg11339837	1	90967211		IGR	0,042	1,64E-06	2,82E-03
cg20463292	1	161004671		IGR	-0,009	1,61E-06	2,82E-03
cg08312681	2	33644905		IGR	-0,007	1,64E-06	2,82E-03
cg05030593	3	108038761	HHLA2	5'UTR	-0,007	1,61E-06	2,82E-03
cg14321918	3	168526492	C3orf50	Body	0,012	1,64E-06	2,82E-03
cg19672694	5	40688524	PTGER4	Body	-0,005	1,62E-06	2,82E-03
cg11806367	5	71146767		IGR	0,058	1,64E-06	2,82E-03
cg07384522	6	31088208	CDSN	5'UTR	0,015	1,63E-06	2,82E-03
cg00142257	9	124990276	LHX6	Body	-0,108	1,63E-06	2,82E-03
cg22611089	11	134216258	GLB1L2	Body	0,023	1,62E-06	2,82E-03
cg00219833	14	52780896	PTGER2	TSS200	0,01	1,61E-06	2,82E-03
cg09174011	15	47241256		IGR	-0,016	1,60E-06	2,82E-03
cg14642620	3	142682883	PAQR9	TSS1500	-0,011	1,66E-06	2,83E-03
cg20427836	13	30466295		IGR	0,028	1,67E-06	2,84E-03
cg01940964	7	128471600	FLNC	Body	-0,022	1,67E-06	2,85E-03
cg03711717	2	18113753	KCNS3	3'UTR	-0,014	1,71E-06	2,90E-03
cg12948934	5	54686204	SKIV2L2	Body	-0,021	1,72E-06	2,91E-03

cg17053103	3	119061518	ARHGAP31	Body	-0,008	1,73E-06	2,92E-03
cg19299201	11	126872003	KIRREL3	TSS1500	-0,068	1,74E-06	2,93E-03
cg04685747	19	8458793	RAB11B	Body	0,017	1,77E-06	2,97E-03
cg16882206	8	115466108		IGR	-0,027	1,78E-06	2,98E-03
cg11698762	2	74648964	WDR54	5'UTR	-0,004	1,82E-06	2,99E-03
cg07862555	2	213159704	ERBB4	Body	-0,046	1,81E-06	2,99E-03
cg12192112	5	95469201		IGR	-0,015	1,82E-06	2,99E-03
cg04964098	5	137837276		IGR	-0,015	1,79E-06	2,99E-03
cg21357732	10	21774282		IGR	-0,007	1,80E-06	2,99E-03
cg04169306	10	103570611	MGEA5	Body	-0,023	1,82E-06	2,99E-03
cg06033186	16	89287356	ZNF778	5'UTR	0,008	1,81E-06	2,99E-03
cg02345961	18	34854574	BRUNOL4	Body	-0,056	1,80E-06	2,99E-03
cg02286329	1	152040891		IGR	-0,02	1,83E-06	3,00E-03
cg10338259	5	35430804		IGR	-0,012	1,83E-06	3,00E-03
cg22488426	7	12989338		IGR	-0,009	1,83E-06	3,00E-03
cg24937813	4	77636069	SHROOM3	Body	-0,013	1,85E-06	3,01E-03
cg26548077	7	134866975		IGR	-0,022	1,85E-06	3,01E-03
cg16497732	8	141405143	TRAPPC9	Body	-0,007	1,85E-06	3,01E-03
cg02315096	13	110522020		IGR	-0,128	1,85E-06	3,01E-03
cg14275835	6	126501843		IGR	-0,038	1,88E-06	3,04E-03
cg10060487	11	49071763		IGR	-0,074	1,88E-06	3,04E-03
cg20016411	11	113344987	DRD2	5'UTR	-0,047	1,89E-06	3,04E-03
cg08308725	4	1409543		IGR	-0,061	1,90E-06	3,06E-03
cg02145222	1	30322306		IGR	-0,031	1,91E-06	3,06E-03
cg12594341	2	50246340	NRXN1	Body	-0,019	1,93E-06	3,08E-03
cg09605494	10	43725411	RASGEF1A	5'UTR	-0,015	1,93E-06	3,08E-03
cg20040765	8	67358546	ADHFE1	Body	-0,006	1,94E-06	3,09E-03
cg26160310	10	69280873	CTNNA3	Body	-0,008	1,94E-06	3,09E-03
cg12397225	11	118758504	CXCR5	Body	0,026	1,96E-06	3,11E-03
cg21729825	2	112433296		IGR	-0,006	1,97E-06	3,11E-03
cg14334019	2	220955573		IGR	-0,01	1,97E-06	3,11E-03
cg12945621	4	1217147	CTBP1	Body	0,03	1,97E-06	3,11E-03
cg00837690	1	38293435	MTF1	Body	-0,014	1,98E-06	3,12E-03
cg02480604	19	19446463	MAU2	Body	0,006	1,99E-06	3,12E-03
cg03572287	3	50214881	SEMA3F	Body	0,019	2,00E-06	3,13E-03
cg24235862	21	27529710	APP	5'UTR	0,017	2,00E-06	3,13E-03
cg17958447	8	56873838	LYN	Body	-0,041	2,02E-06	3,15E-03
cg10591475	6	28109693	ZNF192	TSS200	-0,005	2,05E-06	3,17E-03
cg19110327	14	85589485		IGR	0,063	2,04E-06	3,17E-03
cg14047361	17	80015160	GPS1	3'UTR	0,017	2,05E-06	3,17E-03
cg20560396	19	49945779	SLC17A7	TSS1500	-0,066	2,05E-06	3,17E-03
cg08246800	11	73356353	PLEKHB1	TSS1500	0,039	2,08E-06	3,21E-03
cg19572947	1	182808556	DHX9	1stExon	-0,005	2,09E-06	3,22E-03
cg17659389	18	53087062	TCF4	Body	0,015	2,09E-06	3,22E-03
cg06882854	5	156083022	SGCD	Body	0,05	2,11E-06	3,23E-03
cg18558140	8	23104100	CHMP7	5'UTR	-0,005	2,11E-06	3,23E-03
cg13994785	14	31401769	STRN3	Body	0,035	2,12E-06	3,23E-03
cg11884089	7	111770158	DOCK4	Body	0,032	2,12E-06	3,24E-03
cg14228980	17	78122221	EIF4A3	TSS1500	0,009	2,13E-06	3,25E-03
cg25697702	3	40012603	MYRIP	5'UTR	-0,02	2,14E-06	3,25E-03
cg04427437	3	3081722	CNTN4	Body	-0,025	2,17E-06	3,27E-03
cg23766117	3	128731918	EFCC1	Body	0,02	2,17E-06	3,27E-03
cg12234226	6	90932450	BACH2	5'UTR	-0,024	2,18E-06	3,27E-03
cg26394930	6	94431180	TSG1	Body	-0,03	2,18E-06	3,27E-03
cg07870482	7	66440176		IGR	-0,022	2,19E-06	3,27E-03
cg15055109	7	127255737	PAX4	1stExon	-0,062	2,17E-06	3,27E-03
cg06952893	12	121188705		IGR	-0,036	2,18E-06	3,27E-03
cg07494888	18	76465958		IGR	-0,015	2,18E-06	3,27E-03
cg17667591	1	17719944	PADI6	Body	-0,128	2,24E-06	3,28E-03
cg03152916	2	198541731	RFTN2	TSS1500	0,011	2,23E-06	3,28E-03
cg12795310	3	6561264		IGR	-0,019	2,20E-06	3,28E-03
cg21173351	5	4135244		IGR	-0,052	2,22E-06	3,28E-03
cg13888441	6	37140088	PIM1	Body	-0,026	2,25E-06	3,28E-03
cg03524116	6	105942500		IGR	0,011	2,25E-06	3,28E-03
cg07759714	6	129662413	LAMA2	Body	0,029	2,25E-06	3,28E-03
cg01833771	6	143266788	HIVEP2	TSS1500	-0,007	2,23E-06	3,28E-03
cg24890816	9	74638077		IGR	0,01	2,21E-06	3,28E-03
cg18994606	10	111827966	ADD3	5'UTR	-0,011	2,20E-06	3,28E-03
cg18600604	12	112279610	MAPKAPK5-AS1	Body	-0,004	2,25E-06	3,28E-03
cg15825293	14	97432390		IGR	-0,011	2,25E-06	3,28E-03
cg18775118	18	11964049		IGR	-0,021	2,24E-06	3,28E-03
cg14025020	5	118510568	DMXL1	Body	0,04	2,26E-06	3,28E-03
cg04012027	6	27670844		IGR	0,031	2,28E-06	3,30E-03

cg19848538	12	112586276	TRAFD1	Body	0,018	2,28E-06	3,30E-03
cg12706086	18	26174021		IGR	-0,012	2,29E-06	3,30E-03
cg23016045	5	22853640	CDH12	1stExon	-0,009	2,31E-06	3,32E-03
cg06773966	6	76352329	SENP6	Body	-0,006	2,31E-06	3,33E-03
cg07177569	2	187454747	ITGAV	TSS200	0,007	2,32E-06	3,33E-03
cg17973977	14	91687281	C14orf159	Body	0,066	2,33E-06	3,34E-03
cg06418322	5	1765751		IGR	0,026	2,35E-06	3,36E-03
cg12577633	18	77623199	KCNG2	TSS1500	-0,067	2,37E-06	3,38E-03
cg03549563	20	61640931		IGR	-0,056	2,37E-06	3,38E-03
cg25367944	1	33710325		IGR	-0,014	2,40E-06	3,40E-03
cg21256257	5	141348243	RNF14	5'UTR	0,031	2,39E-06	3,40E-03
cg09514664	9	111727284	CTNNAL1	Body	0,031	2,40E-06	3,40E-03
cg13988606	1	113258121	PPM1J	TSS200	0,015	2,42E-06	3,42E-03
cg06280753	15	87097963	AGBL1	Body	-0,032	2,42E-06	3,42E-03
cg10422833	8	143614909	BAI1	Body	0,028	2,44E-06	3,43E-03
cg17469668	5	142261891	ARHGAP26	Body	0,006	2,44E-06	3,43E-03
cg07196044	6	20867280	CDKAL1	Body	0,016	2,46E-06	3,43E-03
cg08150181	7	68002814		IGR	-0,029	2,46E-06	3,43E-03
cg17466996	12	14437658		IGR	0,011	2,46E-06	3,43E-03
cg18412834	20	61885291	FLJ16779	TSS200	-0,007	2,46E-06	3,43E-03
cg06607089	21	19616551	CHODL	TSS1500	0,038	2,47E-06	3,43E-03
cg06562172	2	26927137	KCNK3	Body	0,011	2,50E-06	3,44E-03
cg03926968	3	142440284		IGR	-0,046	2,48E-06	3,44E-03
cg07935727	4	158496743	LOC340017	Body	-0,021	2,51E-06	3,44E-03
cg09268319	4	187520564	FAT1	Body	-0,019	2,50E-06	3,44E-03
cg23680535	6	36900905		IGR	-0,021	2,50E-06	3,44E-03
cg24877129	10	133606492		IGR	-0,019	2,49E-06	3,44E-03
cg01779270	21	44588834	CRYAA	TSS1500	0,022	2,49E-06	3,44E-03
cg02228073	1	170278944		IGR	-0,01	2,55E-06	3,47E-03
cg03164659	2	242901293	LINC01237	Body	0,068	2,53E-06	3,47E-03
cg14877820	3	16306698	OXNAD1	TSS200	0,004	2,54E-06	3,47E-03
cg03220091	14	22928550		IGR	0,064	2,54E-06	3,47E-03
cg09743193	22	33559963		IGR	-0,055	2,55E-06	3,47E-03
cg00563832	22	36908976	EIF3D	Body	0,009	2,55E-06	3,47E-03
cg03596877	6	30568668	PPP1R10	3'UTR	0,013	2,56E-06	3,48E-03
cg18619804	19	51611763	CTU1	TSS200	-0,004	2,57E-06	3,48E-03
cg03538833	3	101660066	LOC152225	Body	0,011	2,58E-06	3,48E-03
cg02421597	5	140753848	PCDHGA4	Body	-0,025	2,57E-06	3,48E-03
cg15869128	19	50016408	FCGRT	5'UTR	0,023	2,59E-06	3,49E-03
cg21737243	7	99012691	BUD31	Body	-0,022	2,61E-06	3,51E-03
cg20364368	6	30171483	TRIM26	5'UTR	-0,017	2,62E-06	3,52E-03
cg27402871	2	119916999	C1QL2	TSS1500	-0,063	2,65E-06	3,52E-03
cg05067162	3	115867558	LSAMP	Body	-0,008	2,64E-06	3,52E-03
cg11493553	7	156993697	UBE3C	Body	0,014	2,64E-06	3,52E-03
cg20449619	8	10581784	SOX7	3'UTR	-0,037	2,65E-06	3,52E-03
cg06783011	12	113575922		IGR	0,02	2,65E-06	3,52E-03
cg03620886	19	10420353	ZGLP1	TSS200	0,006	2,66E-06	3,52E-03
cg07920195	19	43530623	PSG11	5'UTR	-0,019	2,66E-06	3,52E-03
cg17924085	19	55996446	NAT14	TSS200	-0,005	2,65E-06	3,52E-03
cg12327691	10	101493322	COX15	TSS1500	-0,01	2,67E-06	3,52E-03
cg17537714	11	88104155		IGR	-0,019	2,67E-06	3,52E-03
cg03836831	16	969069	LMF1	Body	0,014	2,68E-06	3,52E-03
cg23815828	12	46885567		IGR	0,013	2,69E-06	3,54E-03
cg02978119	1	92960308		IGR	-0,006	2,72E-06	3,54E-03
cg12070783	6	27494859		IGR	-0,011	2,72E-06	3,54E-03
cg06251670	8	62561734	ASPH	Body	-0,018	2,72E-06	3,54E-03
cg02030350	14	102476349	DYNC1H1	Body	-0,006	2,71E-06	3,54E-03
cg14532444	15	59542638	MYO1E	Body	-0,011	2,72E-06	3,54E-03
cg26691434	16	29675160	SPN	Body	-0,01	2,72E-06	3,54E-03
cg19297703	16	79112473	WWOX	Body	0,008	2,74E-06	3,55E-03
cg11423281	1	226109692	PYCR2	Body	0,009	2,77E-06	3,57E-03
cg09707641	3	123597199	MYLK	5'UTR	-0,024	2,77E-06	3,57E-03
cg19103610	4	184585134	TRAPPC11	Body	-0,008	2,77E-06	3,57E-03
cg06291867	10	92617162	HTR7	1stExon	-0,037	2,78E-06	3,57E-03
cg02942766	2	42328937		IGR	-0,023	2,81E-06	3,59E-03
cg27488658	4	176592917	GPM6A	Body	-0,062	2,81E-06	3,59E-03
cg14023682	15	39877764	THBS1	ExonBnd	0,013	2,80E-06	3,59E-03
cg21661549	18	78005243	PARD6G	1stExon	-0,01	2,81E-06	3,59E-03
cg24080181	19	36036226	TMEM147	TSS1500	-0,004	2,82E-06	3,60E-03
cg00297432	1	6484784	ESPN	TSS200	0,021	2,83E-06	3,61E-03
cg25672398	1	22944795		IGR	-0,051	2,85E-06	3,62E-03
cg07107453	1	79114976	IFI44	TSS1500	0,057	2,85E-06	3,62E-03
cg10099397	1	182645784		IGR	0,021	2,87E-06	3,62E-03

cg11527921	2	166754028	TTC21B	Body	0,023	2,86E-06	3,62E-03
cg22350557	3	78115623		IGR	-0,022	2,86E-06	3,62E-03
cg22264386	4	47845952	LOC101927179	Body	-0,018	2,86E-06	3,62E-03
cg00550698	9	128980228		IGR	-0,028	2,89E-06	3,63E-03
cg02874691	3	169489835	MYNN	TSS1500	-0,006	2,90E-06	3,64E-03
cg02507726	10	60898087		IGR	-0,048	2,90E-06	3,64E-03
cg14869618	2	179500777	TTN	Body	-0,01	2,92E-06	3,66E-03
cg16138752	12	133128085	FBRSL1	Body	0,019	2,93E-06	3,66E-03
cg09094804	16	56485438	NUDT21	TSS200	0,025	2,92E-06	3,66E-03
cg15138902	17	66959525		IGR	-0,019	2,93E-06	3,66E-03
cg26960896	19	44270892	KCNN4	3'UTR	0,025	2,94E-06	3,66E-03
cg26393977	8	37612713	ERLIN2	3'UTR	0,009	2,96E-06	3,68E-03
cg10341625	15	64903123	ZNF609	Body	0,02	2,98E-06	3,68E-03
cg00623953	17	78451422	NPTX1	TSS1500	-0,031	2,98E-06	3,68E-03
cg22422911	19	47835195	C5AR2	TSS1500	-0,023	2,97E-06	3,68E-03
cg18968409	2	34633637		IGR	0,086	2,99E-06	3,69E-03
cg09584958	17	41456152	LINC00910	Body	-0,007	3,00E-06	3,70E-03
cg22941974	2	56410011	CCDC85A	TSS1500	0,03	3,02E-06	3,70E-03
cg02711800	7	158525801	ESYT2	3'UTR	-0,029	3,01E-06	3,70E-03
cg16678545	1	230492737	PGBD5	Body	0,005	3,06E-06	3,73E-03
cg18801303	2	107442467	ST6GAL2	Body	-0,006	3,06E-06	3,73E-03
cg26471191	2	134091049	MIR7853	Body	-0,03	3,06E-06	3,73E-03
cg10315303	3	176733109		IGR	-0,009	3,07E-06	3,73E-03
cg08295865	5	1790944		IGR	0,033	3,06E-06	3,73E-03
cg10549648	6	74189702	MTO1	Body	0,007	3,07E-06	3,73E-03
cg11316146	7	104624178		IGR	-0,011	3,06E-06	3,73E-03
cg02826729	2	170664958	SSB	Body	0,058	3,08E-06	3,74E-03
cg12361039	11	75486505	DGAT2	Body	0,01	3,10E-06	3,74E-03
cg27093379	12	78924937		IGR	-0,027	3,10E-06	3,74E-03
cg19800035	4	187621327	FAT1	Body	-0,016	3,11E-06	3,74E-03
cg18054674	5	140753542	PCDHGA4	Body	-0,063	3,12E-06	3,76E-03
cg23455102	12	103210837	LINC00485	Body	-0,021	3,13E-06	3,76E-03
cg05324803	22	43182795		IGR	-0,027	3,14E-06	3,77E-03
cg13709773	8	38206468	WHSC1L1	5'UTR	0,026	3,18E-06	3,80E-03
cg16143330	11	557454	LMNTD2	Body	0,012	3,18E-06	3,80E-03
cg07853663	17	70023371	LOC102723505	TSS1500	-0,048	3,18E-06	3,80E-03
cg06481887	10	85971900	PCDH21	Body	0,023	3,19E-06	3,80E-03
cg17808354	3	63953377	ATXN7	TSS200	-0,013	3,20E-06	3,80E-03
cg06325860	1	12592269		IGR	-0,007	3,22E-06	3,81E-03
cg04288544	1	93981941	FNBP1L	Body	-0,024	3,22E-06	3,81E-03
cg16773028	1	111148339	KCNA2	5'UTR	0,031	3,21E-06	3,81E-03
cg19907682	4	188231570	LOC339975	Body	-0,014	3,23E-06	3,81E-03
cg12562012	5	158305034	EBF1	Body	0,014	3,24E-06	3,81E-03
cg15611764	6	71383464	SMAP1	Body	-0,061	3,23E-06	3,81E-03
cg04622888	9	124990010	LHX6	TSS200	-0,221	3,24E-06	3,81E-03
cg07790680	19	18668394	C19orf50	TSS1500	0,01	3,24E-06	3,81E-03
cg03359095	20	61002866	C20orf151	TSS1500	0,063	3,25E-06	3,81E-03
cg25453614	2	174089409	ZAK	3'UTR	-0,01	3,25E-06	3,81E-03
cg01689592	16	30046769		IGR	-0,01	3,26E-06	3,81E-03
cg23984491	5	54900498		IGR	-0,013	3,27E-06	3,81E-03
cg04190037	13	113105962		IGR	0,036	3,27E-06	3,81E-03
cg20805999	4	119606266	METTL14	TSS1500	-0,015	3,29E-06	3,82E-03
cg20730770	5	140563241	PCDHB16	1stExon	-0,064	3,30E-06	3,82E-03
cg25344017	8	145509651	BOP1	Body	0,015	3,29E-06	3,82E-03
cg19806106	10	44069975	ZNF239	5'UTR	-0,006	3,29E-06	3,82E-03
cg03723001	12	117147681		IGR	-0,052	3,32E-06	3,83E-03
cg00455418	22	32340934	C22orf24	Body	0,012	3,31E-06	3,83E-03
cg14441787	8	40811496		IGR	0,008	3,33E-06	3,84E-03
cg12132029	19	11616515	ZNF653	1stExon	0,034	3,34E-06	3,84E-03
cg20839987	1	80841802		IGR	0,017	3,36E-06	3,85E-03
cg08293468	6	58777644		IGR	-0,02	3,35E-06	3,85E-03
cg08366144	12	40838289	MUC19	Body	-0,012	3,37E-06	3,85E-03
cg22499565	12	110152147	C12orf34	TSS200	-0,005	3,37E-06	3,85E-03
cg15694585	13	20703389		IGR	-0,06	3,37E-06	3,85E-03
cg17677402	11	61889979	INCENP	TSS1500	0,01	3,39E-06	3,87E-03
cg19628356	11	42261951	LOC100507205	Body	-0,014	3,41E-06	3,88E-03
cg13107794	22	19643042		IGR	-0,015	3,42E-06	3,89E-03
cg08383087	5	73247809		IGR	-0,015	3,43E-06	3,90E-03
cg03986574	11	64544080	SF1	Body	0,01	3,45E-06	3,90E-03
cg06067394	11	133789110	IGSF9B	Body	-0,1	3,46E-06	3,90E-03
cg06068118	12	133228397	POLE	Body	0,015	3,46E-06	3,90E-03
cg02788585	13	85386491		IGR	-0,018	3,45E-06	3,90E-03
cg01102193	1	235053806		IGR	-0,015	3,46E-06	3,90E-03

cg02087039	12	95867833	METAP2	1stExon	-0,007	3,48E-06	3,91E-03
cg02604720	7	151144508		IGR	0,012	3,51E-06	3,92E-03
cg24601370	11	12537746	PARVA	Body	0,011	3,50E-06	3,92E-03
cg01898166	12	72032063	ZFC3H1	Body	0,048	3,51E-06	3,92E-03
cg08796240	16	70733832	VAC14	Body	0,049	3,51E-06	3,92E-03
cg10038648	21	41634474	DSCAM	Body	-0,015	3,49E-06	3,92E-03
cg08483684	1	158913031	PYHIN1	Body	-0,02	3,55E-06	3,95E-03
cg21526011	5	150970510		IGR	-0,028	3,55E-06	3,95E-03
cg21337269	7	5166647	ZNF890P	Body	0,035	3,57E-06	3,95E-03
cg10371523	11	70395513	SHANK2	Body	-0,02	3,56E-06	3,95E-03
cg16250939	15	93482824	CHD2	ExonBnd	0,013	3,57E-06	3,95E-03
cg03040168	8	17828025	PCM1	Body	0,027	3,58E-06	3,95E-03
cg13618190	2	56210182	MIR217	Body	0,017	3,59E-06	3,96E-03
cg15108060	13	48974707	RB1	Body	0,021	3,58E-06	3,96E-03
cg18904161	6	137398100		IGR	-0,007	3,60E-06	3,97E-03
cg17619842	2	186073606		IGR	-0,051	3,61E-06	3,97E-03
cg22596895	1	93602507	MTF2	Body	0,008	3,66E-06	3,99E-03
cg19197669	1	223263197		IGR	0,008	3,70E-06	3,99E-03
cg08848003	3	98299487	CPOX	3'UTR	-0,006	3,73E-06	3,99E-03
cg05964940	5	83602601	EDIL3	Body	-0,015	3,73E-06	3,99E-03
cg03517570	5	150490911	ANXA6	Body	0,009	3,72E-06	3,99E-03
cg23104677	8	142366812	GPR20	3'UTR	0,023	3,68E-06	3,99E-03
cg24124620	8	142505085	FLJ43860	Body	-0,074	3,70E-06	3,99E-03
cg14211075	9	135818481	TSC1	5'UTR	0,04	3,72E-06	3,99E-03
cg02503633	10	134014536	DPYSL4	Body	0,017	3,70E-06	3,99E-03
cg13685129	11	334475		IGR	0,02	3,74E-06	3,99E-03
cg06807966	12	4274034		IGR	-0,004	3,70E-06	3,99E-03
cg07926066	13	90167263		IGR	-0,038	3,72E-06	3,99E-03
cg14617010	13	100092046		IGR	-0,011	3,66E-06	3,99E-03
cg10735475	13	112984976		IGR	-0,068	3,68E-06	3,99E-03
cg09272488	14	66425627		IGR	-0,028	3,64E-06	3,99E-03
cg06373083	15	28346972		IGR	0,023	3,64E-06	3,99E-03
cg01849531	16	8942712	PMM2	3'UTR	0,008	3,72E-06	3,99E-03
cg00627447	16	83233690	CDH13	5'UTR	-0,058	3,69E-06	3,99E-03
cg04665588	17	14243776	HS3ST3B1	Body	-0,013	3,67E-06	3,99E-03
cg05376962	1	110163523	AMPD2	5'UTR	-0,004	3,76E-06	4,00E-03
cg15122697	1	224401807	LOC101927143	TSS1500	0,019	3,76E-06	4,00E-03
cg06676621	4	110736369	GAR1	TSS1500	-0,004	3,77E-06	4,00E-03
cg01366622	5	173218550	LINC01485	TSS1500	-0,027	3,76E-06	4,00E-03
cg22898729	6	3742668	C6orf145	Body	0,011	3,77E-06	4,00E-03
cg14947466	11	2583705	KCNQ1	Body	0,022	3,77E-06	4,00E-03
cg02713586	18	72435348	ZNF407	Body	-0,006	3,79E-06	4,01E-03
cg02256560	10	38300033	ZNF33A	5'UTR	-0,004	3,81E-06	4,02E-03
cg07381885	14	62589035	LINC00643	Body	0,021	3,81E-06	4,02E-03
cg04829508	5	59675957	PDE4D	5'UTR	0,01	3,83E-06	4,03E-03
cg01459748	11	113817020	HTR3B	3'UTR	-0,036	3,83E-06	4,03E-03
cg04140807	15	99320300	IGF1R	Body	-0,098	3,84E-06	4,03E-03
cg09624807	16	57080116	NLRC5	Body	0,035	3,84E-06	4,03E-03
cg03164112	2	237174027	ASB18	TSS1500	-0,026	3,84E-06	4,03E-03
cg15685954	6	74175812	MTO1	Body	0,024	3,85E-06	4,03E-03
cg00302763	15	22442000		IGR	0,029	3,85E-06	4,03E-03
cg01950767	6	163775257		IGR	-0,03	3,87E-06	4,04E-03
cg16688582	7	28386631	CREB5	5'UTR	0,023	3,88E-06	4,04E-03
cg11806672	13	79176608	POU4F1	Body	-0,008	3,88E-06	4,04E-03
cg04948194	14	45840127		IGR	-0,032	3,91E-06	4,06E-03
cg06583873	2	236722183	AGAP1	Body	-0,023	3,92E-06	4,07E-03
cg26736929	20	57356450		IGR	-0,025	3,95E-06	4,09E-03
cg17987059	13	28752067	PAN3	Body	-0,007	3,97E-06	4,11E-03
cg07419314	22	20793602	SCARF2	TSS1500	0,026	3,98E-06	4,11E-03
cg16541075	2	242832796		IGR	-0,014	4,00E-06	4,11E-03
cg23644567	4	84519218	AGPAT9	ExonBnd	-0,007	4,01E-06	4,11E-03
cg07850533	9	4804779	RCL1	Body	-0,008	4,00E-06	4,11E-03
cg21678211	12	56863261	SPRYD4	Body	0,043	4,00E-06	4,11E-03
cg02964434	15	31617537		IGR	0,022	3,99E-06	4,11E-03
cg09641077	3	56835860	ARHGEF3	1stExon	-0,004	4,05E-06	4,14E-03
cg05095230	13	50367060	KPNA3	TSS200	-0,007	4,04E-06	4,14E-03
cg11160051	1	57045451	PPAP2B	TSS200	-0,029	4,08E-06	4,15E-03
cg02024097	5	67511519	PIK3R1	TSS200	0,007	4,08E-06	4,15E-03
cg09018491	9	16575200	BNC2	Body	-0,019	4,06E-06	4,15E-03
cg11891336	17	71152847		IGR	0,039	4,07E-06	4,15E-03
cg24085930	3	21832523		IGR	0,018	4,08E-06	4,15E-03
cg21608069	3	12602818	MKRN2	Body	0,017	4,14E-06	4,15E-03
cg01923873	4	154049200		IGR	-0,015	4,13E-06	4,15E-03

cg19641786	5	38310065	EGFLAM	Body	-0,01	4,12E-06	4,15E-03
cg24869241	6	80816217	BCKDHB	TSS200	-0,005	4,14E-06	4,15E-03
cg13721515	8	74335868	STAU2-AS1	Body	-0,01	4,13E-06	4,15E-03
cg26387297	13	47012328		IGR	-0,051	4,13E-06	4,15E-03
cg03776625	15	42840292	LRRC57	Body	-0,005	4,11E-06	4,15E-03
cg05989248	15	100530651	ADAMTS17	Body	0,041	4,10E-06	4,15E-03
cg17804302	16	50581891	NKD1	TSS1500	-0,004	4,13E-06	4,15E-03
cg26767443	19	41316244		IGR	0,011	4,09E-06	4,15E-03
cg01105427	4	8593507	CPZ	TSS1500	0,048	4,18E-06	4,18E-03
cg06070696	4	166662284	LINC01179	Body	-0,085	4,20E-06	4,18E-03
cg00742118	5	135359419		IGR	0,036	4,19E-06	4,18E-03
cg10790344	17	1693265	SMYD4	Body	0,014	4,20E-06	4,18E-03
cg12484037	17	68165632	KCNJ2	TSS200	-0,007	4,20E-06	4,18E-03
cg19659125	19	18794596	CRTC1	1stExon	0,017	4,18E-06	4,18E-03
cg06197252	2	191493581		IGR	-0,01	4,25E-06	4,22E-03
cg27237398	3	134572561	EPHB1	Body	-0,026	4,27E-06	4,23E-03
cg07589654	6	44432060		IGR	0,022	4,27E-06	4,23E-03
cg27319677	7	94139166	CASD1	TSS200	0,012	4,29E-06	4,24E-03
cg16508903	12	70574286		IGR	-0,011	4,29E-06	4,24E-03
cg03799736	5	93858506	KIAA0825	Body	0,035	4,31E-06	4,25E-03
cg26266966	15	72459944	GRAMD2	Body	-0,021	4,32E-06	4,25E-03
cg04510459	1	92048287		IGR	0,037	4,34E-06	4,26E-03
cg19743789	14	24900907	KHNYN	Body	0,017	4,34E-06	4,26E-03
cg12140786	15	73981070	CD276	5'UTR	-0,007	4,34E-06	4,26E-03
cg09335911	10	100027962	LOXL4	1stExon	-0,006	4,36E-06	4,27E-03
cg09083104	1	78607482		IGR	-0,013	4,38E-06	4,27E-03
cg06789677	10	108525625	SORCS1	Body	-0,022	4,37E-06	4,27E-03
cg15713086	20	54933689	C20orf108	TSS1500	-0,008	4,38E-06	4,27E-03
cg00044354	1	67389884	MIER1	TSS1500	0,075	4,41E-06	4,29E-03
cg18685681	15	71424228		IGR	-0,025	4,42E-06	4,29E-03
cg16528738	16	83968260		IGR	0,02	4,41E-06	4,29E-03
cg11370756	2	239988894	HDAC4	Body	0,011	4,45E-06	4,31E-03
cg14030285	3	9993744	PRRT3	5'UTR	-0,004	4,45E-06	4,31E-03
cg07398316	3	18168830	LOC339862	Body	0,026	4,45E-06	4,31E-03
cg19840589	1	89245410	PKN2	Body	0,024	4,47E-06	4,31E-03
cg25389187	4	153355136	FBXW7	5'UTR	-0,027	4,47E-06	4,31E-03
cg04035591	5	86564652	RASA1	1stExon	-0,004	4,49E-06	4,32E-03
cg26029416	15	32032548		IGR	-0,046	4,48E-06	4,32E-03
cg24441121	2	98262430	COX5B	TSS200	-0,016	4,50E-06	4,32E-03
cg11253871	8	695073	ERICH1-AS1	Body	0,01	4,51E-06	4,32E-03
cg17179794	9	129555970		IGR	0,009	4,51E-06	4,32E-03
cg06892006	12	131568441	GPR133	Body	-0,012	4,51E-06	4,32E-03
cg00339939	1	53815811		IGR	-0,015	4,53E-06	4,33E-03
cg01724479	7	156139261		IGR	-0,01	4,53E-06	4,33E-03
cg00793843	15	90777889	C15orf58	5'UTR	0,021	4,55E-06	4,34E-03
cg08455425	6	109658049		IGR	0,03	4,56E-06	4,34E-03
cg17184161	17	80873120	TBCD	Body	0,011	4,56E-06	4,34E-03
cg06699555	2	9173664		IGR	-0,024	4,61E-06	4,37E-03
cg11205502	2	37283136	HEATR5B	Body	0,024	4,65E-06	4,37E-03
cg05247193	3	101022426	IMPG2	Body	-0,057	4,67E-06	4,37E-03
cg03119233	4	7069300	GRPEL1	Body	0,008	4,60E-06	4,37E-03
cg03820148	6	37523234	MIR4462	TSS200	0,02	4,61E-06	4,37E-03
cg16497524	9	15421357	SNAPC3	TSS1500	-0,016	4,63E-06	4,37E-03
cg10575441	11	8751837	ST5	Body	-0,061	4,66E-06	4,37E-03
cg09592419	11	118521179	PHLDB1	Body	0,018	4,65E-06	4,37E-03
cg19269323	11	122752590	C11orf63	TSS1500	0,013	4,62E-06	4,37E-03
cg13412579	12	116398830	MED13L	3'UTR	0,034	4,62E-06	4,37E-03
cg18162480	14	57170889		IGR	-0,012	4,66E-06	4,37E-03
cg20901874	14	75745470	FOS	TSS200	-0,003	4,65E-06	4,37E-03
cg15921745	19	10777248	ILF3	5'UTR	0,005	4,63E-06	4,37E-03
cg10535632	13	28668167	FLT3	Body	-0,008	4,68E-06	4,37E-03
cg09710116	13	46708125	LCP1	Body	0,056	4,67E-06	4,37E-03
cg16114380	12	50606100	LIMA1	Body	-0,022	4,70E-06	4,38E-03
cg14204478	17	79213084	C17orf89	TSS200	-0,005	4,71E-06	4,39E-03
cg01959424	1	233840242		IGR	-0,019	4,72E-06	4,39E-03
cg04568519	7	158384490		IGR	-0,013	4,74E-06	4,40E-03
cg00247512	1	117115307		IGR	0,01	4,79E-06	4,43E-03
cg07022681	3	141929988	GK5	Body	-0,035	4,77E-06	4,43E-03
cg19293122	14	29077895		IGR	-0,039	4,79E-06	4,43E-03
cg09099653	22	20307061	DGCR6L	Body	0,014	4,78E-06	4,43E-03
cg25644624	6	105235535	HACE1	Body	0,018	4,81E-06	4,43E-03
cg27010208	11	93263413	SMCO4	5'UTR	-0,014	4,81E-06	4,43E-03
cg12893030	10	73769174	CHST3	3'UTR	0,041	4,82E-06	4,44E-03

cg04271960	4	23968911		IGR	-0,022	4,84E-06	4,45E-03
cg20921240	15	101604157	LRRK1	Body	0,009	4,85E-06	4,45E-03
cg17954229	3	189776543	P3H2	Body	-0,009	4,86E-06	4,45E-03
cg14873600	3	147072736		IGR	-0,013	4,87E-06	4,46E-03
cg03137972	1	173380251	LOC100506023	Body	0,035	4,89E-06	4,48E-03
cg26502715	2	187529796	ITGAV	Body	0,021	4,91E-06	4,48E-03
cg22755679	7	155920287		IGR	0,02	4,91E-06	4,48E-03
cg01411487	6	36369119	PXT1	Body	0,056	4,93E-06	4,48E-03
cg15959506	13	105113072		IGR	-0,01	4,94E-06	4,48E-03
cg05395323	19	21326526	ZNF431	Body	-0,02	4,94E-06	4,48E-03
cg00879218	19	38039666		IGR	-0,087	4,94E-06	4,48E-03
cg04772817	20	32316843		IGR	-0,005	4,94E-06	4,48E-03
cg17047253	10	100222966	HPSE2	Body	-0,011	4,96E-06	4,49E-03
cg03040581	1	8384718	SLC45A1	1stExon	-0,029	4,97E-06	4,49E-03
cg03707784	2	240965007	NDUFA10	TSS200	-0,005	4,97E-06	4,49E-03
cg17684765	2	3833816		IGR	-0,026	4,99E-06	4,50E-03
cg06539490	12	5152380	KCNA5	TSS1500	-0,051	4,99E-06	4,50E-03
cg17716961	12	120105711	PRKAB1	TSS200	-0,014	5,00E-06	4,50E-03
cg08875451	10	11322268	CUGBP2	Body	0,01	5,00E-06	4,50E-03
cg23994917	2	232791126	NPPC	TSS200	-0,005	5,02E-06	4,50E-03
cg11176737	12	5154148	KCNA5	1stExon	-0,053	5,01E-06	4,50E-03
cg04560810	5	138861847	TMEM173	5'UTR	-0,008	5,03E-06	4,51E-03
cg18702715	6	33871422		IGR	-0,008	5,04E-06	4,51E-03
cg23382806	4	74346243	AFM	TSS1500	-0,007	5,05E-06	4,51E-03
cg18131810	2	220348515	SPEG	Body	-0,042	5,07E-06	4,53E-03
cg26878995	1	168106731	GPR161	TSS1500	0,038	5,09E-06	4,53E-03
cg15379182	11	10591627	LYVE1	TSS1500	-0,007	5,09E-06	4,53E-03
cg09255521	11	125312257		IGR	0,05	5,10E-06	4,53E-03
cg22158547	1	223472245	SUSD4	Body	-0,04	5,15E-06	4,55E-03
cg21714507	2	238799378	RAMP1	Body	-0,036	5,19E-06	4,55E-03
cg16831002	5	140749358	PCDHGB3	TSS1500	-0,079	5,15E-06	4,55E-03
cg03217256	11	128838412	RICS	3'UTR	0,008	5,16E-06	4,55E-03
cg05309081	17	15990620	NCOR1	Body	0,035	5,18E-06	4,55E-03
cg05595885	17	73266870	MIF4GD	5'UTR	0,014	5,12E-06	4,55E-03
cg02678768	17	74002944	EVPL	3'UTR	0,074	5,18E-06	4,55E-03
cg26937943	18	8800248	KIAA0802	Body	-0,01	5,14E-06	4,55E-03
cg08520451	19	996720		IGR	-0,006	5,16E-06	4,55E-03
cg21681980	19	17221563	MYO9B	Body	0,033	5,16E-06	4,55E-03
cg10823956	19	54057671	ZNF331	TSS1500	0,024	5,13E-06	4,55E-03
cg01550271	22	31798207	DRG1	Body	-0,005	5,18E-06	4,55E-03
cg01079738	1	182922427	C1orf14	1stExon	-0,045	5,20E-06	4,56E-03
cg25379026	1	225117389	DNAH14	1stExon	-0,021	5,24E-06	4,56E-03
cg26217814	2	79252152	REG3G	TSS1500	-0,007	5,23E-06	4,56E-03
cg13933023	11	116166109		IGR	-0,008	5,23E-06	4,56E-03
cg09696486	16	7270014	A2BP1	Body	-0,02	5,24E-06	4,56E-03
cg20097520	18	8835912		IGR	0,078	5,22E-06	4,56E-03
cg04251571	10	35416385	CREM	5'UTR	-0,007	5,30E-06	4,59E-03
cg09751228	13	49049573	RB1	Body	-0,006	5,30E-06	4,59E-03
cg24010531	19	28400281		IGR	-0,074	5,29E-06	4,59E-03
cg27589058	20	45804311	EYA2	Body	0,067	5,29E-06	4,59E-03
cg19867917	2	3642629	COLEC11	TSS200	-0,089	5,32E-06	4,61E-03
cg08213479	9	116344184	RGS3	5'UTR	-0,04	5,33E-06	4,61E-03
cg24841879	9	127024965	NEK6	Body	0,021	5,33E-06	4,61E-03
cg19356943	2	69618507		IGR	-0,005	5,38E-06	4,61E-03
cg21979383	6	169260721		IGR	-0,008	5,40E-06	4,61E-03
cg06708390	8	141210798	TRAPPC9	Body	0,008	5,38E-06	4,61E-03
cg08750704	8	145216051	MROH1	5'UTR	-0,009	5,40E-06	4,61E-03
cg26789412	9	131247506	ODF2	Body	-0,014	5,37E-06	4,61E-03
cg07005513	11	61595956	FADS2	1stExon	-0,006	5,38E-06	4,61E-03
cg16489447	11	67211128	CORO1B	TSS200	-0,012	5,36E-06	4,61E-03
cg06672571	14	56331864		IGR	-0,01	5,40E-06	4,61E-03
cg12770187	16	46512538	ANKRD26P1	Body	0,049	5,36E-06	4,61E-03
cg25410121	19	18590625	ELL	Body	0,045	5,40E-06	4,61E-03
cg00714078	2	135809744	RAB3GAP1	TSS200	-0,007	5,42E-06	4,62E-03
cg06320503	4	125640345		IGR	0,009	5,43E-06	4,62E-03
cg10772244	12	6643804	GAPDH	TSS1500	0,011	5,44E-06	4,63E-03
cg12177141	1	208635571		IGR	0,008	5,46E-06	4,63E-03
cg25416479	6	84080564	ME1	Body	0,012	5,46E-06	4,63E-03
cg11036485	15	43924717	CATSPER2	Body	-0,09	5,47E-06	4,63E-03
cg14963000	14	87779342		IGR	-0,017	5,48E-06	4,64E-03
cg22561727	19	4719848	DPP9	Body	0,016	5,50E-06	4,65E-03
cg07409153	2	76839078		IGR	-0,041	5,54E-06	4,66E-03
cg26836573	5	43280399	MGC42105	Body	-0,02	5,53E-06	4,66E-03

cg25024993	5	140248610	PCDHA7	Body	-0,058	5,52E-06	4,66E-03
cg19820328	10	125332630		IGR	0,044	5,51E-06	4,66E-03
cg03417466	11	88910418	TYR	TSS1500	0,062	5,55E-06	4,66E-03
cg09842196	13	47012543		IGR	-0,059	5,55E-06	4,66E-03
cg03514666	19	47756853		IGR	-0,01	5,54E-06	4,66E-03
cg27191564	3	97722598	GABRR3	Body	0,076	5,56E-06	4,66E-03
cg13297560	15	99320054	IGF1R	Body	-0,081	5,59E-06	4,68E-03
cg09407712	7	139416287	HIPK2	Body	-0,009	5,61E-06	4,68E-03
cg13659419	9	140388523	PNPLA7	Body	0,014	5,61E-06	4,68E-03
cg12227505	17	78194145	SLC26A11	TSS200	0,015	5,61E-06	4,68E-03
cg25014228	18	55111605	ONECUT2	Body	-0,069	5,62E-06	4,68E-03
cg01957901	11	132116174	NTM	Body	-0,026	5,64E-06	4,70E-03
cg10230831	13	35487202		IGR	-0,059	5,65E-06	4,70E-03
cg23591140	10	57389301		IGR	-0,073	5,66E-06	4,70E-03
cg22946219	17	17714414	RAI1	3'UTR	0,011	5,67E-06	4,70E-03
cg27457535	22	44795116		IGR	0,034	5,68E-06	4,70E-03
cg06927960	1	89664552	GBP4	1stExon	-0,003	5,72E-06	4,72E-03
cg01745480	2	3683030	COLEC11	Body	-0,043	5,72E-06	4,72E-03
cg07362177	6	147981161		IGR	-0,008	5,72E-06	4,72E-03
cg06918569	15	70729653		IGR	-0,004	5,72E-06	4,72E-03
cg05338748	10	135276154	LOC619207	Body	-0,014	5,74E-06	4,73E-03
cg16051002	5	140579160	PCDHB11	TSS200	-0,026	5,76E-06	4,74E-03
cg17341313	14	31138796	SCFD1	Body	-0,012	5,78E-06	4,75E-03
cg22815056	20	4573300		IGR	0,016	5,78E-06	4,75E-03
cg25570494	20	61526495	DIDO1	Body	-0,006	5,83E-06	4,78E-03
cg25429830	2	67627842	ETAA1	Body	0,031	5,86E-06	4,79E-03
cg02571470	2	73294845	SFXN5	Body	-0,022	5,85E-06	4,79E-03
cg08410934	10	126108229	OAT	TSS1500	-0,007	5,86E-06	4,79E-03
cg27031099	8	126620534		IGR	0,061	5,88E-06	4,81E-03
cg22327690	17	57179774	TRIM37	Body	-0,008	5,91E-06	4,82E-03
cg08065063	2	11059462		IGR	0,016	5,93E-06	4,84E-03
cg16505702	8	132936219	EFR3A	Body	-0,047	5,94E-06	4,84E-03
cg15262352	7	5013468	RNF216L	TSS200	-0,012	5,96E-06	4,84E-03
cg01327395	17	73084978	SLC16A5	5'UTR	0,014	5,96E-06	4,84E-03
cg14858446	6	53783012	LRRC1	Body	-0,026	5,99E-06	4,85E-03
cg26328291	10	131200802		IGR	0,007	5,99E-06	4,85E-03
cg10195702	4	2758288	TNIP2	TSS1500	-0,012	6,00E-06	4,86E-03
cg26579892	11	63381077	PLA2G16	Body	0,016	6,01E-06	4,86E-03
cg27212650	3	25618791	RARB	Body	-0,046	6,04E-06	4,88E-03
cg16236688	10	9450208		IGR	-0,017	6,08E-06	4,89E-03
cg04426862	14	37131008	PAX9	5'UTR	0,007	6,08E-06	4,89E-03
cg20557069	21	47646604	LSS	Body	-0,006	6,08E-06	4,89E-03
cg27066288	1	162308401	NOS1AP	Body	0,051	6,10E-06	4,90E-03
cg13427149	1	217804379	GPATCH2	1stExon	-0,003	6,10E-06	4,90E-03
cg26514430	5	140249080	PCDHA6	Body	-0,06	6,09E-06	4,90E-03
cg06321588	11	3122878	OSBPL5	Body	0,013	6,12E-06	4,91E-03
cg24799218	7	87857148	SRI	TSS1500	0,034	6,14E-06	4,91E-03
cg08869031	15	90436841	AP3S2	Body	-0,005	6,14E-06	4,91E-03
cg16158296	21	45559077	C21orf33	Body	0,021	6,16E-06	4,92E-03
cg15875292	7	129691863	ZC3HC1	TSS1500	-0,005	6,21E-06	4,96E-03
cg00603356	7	107382211		IGR	0,018	6,22E-06	4,96E-03
cg25007781	14	105963610	C14orf80	Body	0,005	6,24E-06	4,96E-03
cg18498739	21	30360186	LTN1	Body	-0,009	6,23E-06	4,96E-03
cg16513416	3	139655283	CLSTN2	Body	-0,066	6,25E-06	4,97E-03
cg10245910	13	108519547	FAM155A	TSS200	0,02	6,28E-06	4,98E-03
cg18876007	1	75606694	LHX8	Body	-0,069	6,32E-06	5,00E-03
cg18856995	1	183667383	RGL1	5'UTR	-0,009	6,31E-06	5,00E-03
cg25902823	2	158957800	UPP2	TSS1500	0,047	6,31E-06	5,00E-03
cg05588228	5	145132641		IGR	0,007	6,32E-06	5,00E-03
cg11827190	1	180475267		IGR	0,04	6,36E-06	5,01E-03
cg02735058	6	16181250		IGR	-0,05	6,36E-06	5,01E-03
cg15984406	8	703870		IGR	-0,093	6,37E-06	5,01E-03
cg17485711	11	69240275		IGR	-0,006	6,35E-06	5,01E-03
cg16923865	14	37999024	MIPOL1	Body	-0,007	6,37E-06	5,01E-03
cg23889086	19	47731562	BBC3	Body	0,011	6,38E-06	5,01E-03
cg24430147	1	115885042		IGR	0,006	6,46E-06	5,03E-03
cg17736879	3	120626107	STXBP5L	TSS1500	-0,033	6,46E-06	5,03E-03
cg05845376	5	140683632	SLC25A2	TSS200	-0,091	6,43E-06	5,03E-03
cg25287375	9	77703619	C9orf95	TSS1500	-0,005	6,44E-06	5,03E-03
cg22466590	9	134403954	UCK1	Body	-0,016	6,48E-06	5,03E-03
cg05698098	11	61595494	FADS2	TSS1500	0,026	6,43E-06	5,03E-03
cg04813677	13	21498178		IGR	0,047	6,47E-06	5,03E-03
cg11178686	13	111599247		IGR	-0,006	6,48E-06	5,03E-03

cg02282237	14	61788862	PRKCH	1stExon	-0,006	6,47E-06	5,03E-03
cg05103581	15	99499605	IGF1R	Body	0,058	6,48E-06	5,03E-03
cg09812689	16	1727431	CRAMP1L	3'UTR	-0,012	6,44E-06	5,03E-03
cg09758869	16	12010052	GSPT1	TSS1500	0,004	6,43E-06	5,03E-03
cg16646428	13	65866657		IGR	0,015	6,50E-06	5,03E-03
cg02610431	14	79668270	NRXN3	Body	-0,044	6,50E-06	5,03E-03
cg19583880	1	27143747		IGR	0,038	6,55E-06	5,04E-03
cg26688155	5	64778185	ADAMTS6	TSS1500	-0,005	6,55E-06	5,04E-03
cg18326610	7	148680126		IGR	-0,005	6,55E-06	5,04E-03
cg20903136	8	62575678	ASPH	3'UTR	0,023	6,54E-06	5,04E-03
cg16000824	11	1143665		IGR	-0,019	6,55E-06	5,04E-03
cg10487387	11	67298456		IGR	-0,018	6,55E-06	5,04E-03
cg13044223	1	45240849	SNORD55	TSS1500	-0,006	6,57E-06	5,04E-03
cg23596233	11	134281663	B3GAT1	5'UTR	-0,011	6,57E-06	5,04E-03
cg14633517	1	226951710		IGR	0,03	6,60E-06	5,05E-03
cg18335505	2	131514588	FAM123C	5'UTR	-0,049	6,60E-06	5,05E-03
cg16359312	6	107833469	SOBP	Body	0,016	6,59E-06	5,05E-03
cg26615224	19	1621124	TCF3	Body	0,017	6,59E-06	5,05E-03
cg17872886	2	3642732	COLEC11	1stExon	-0,074	6,64E-06	5,07E-03
cg05074873	12	27430870	STK38L	5'UTR	0,032	6,65E-06	5,07E-03
cg06499214	6	49712910	CRISP3	TSS1500	0,035	6,68E-06	5,07E-03
cg27338512	6	157970968	ZDHHC14	Body	0,008	6,66E-06	5,07E-03
cg23234556	7	4722035	FOXK1	1stExon	0,017	6,68E-06	5,07E-03
cg21199038	9	101644362		IGR	0,013	6,67E-06	5,07E-03
cg13573358	5	3853734		IGR	-0,021	6,69E-06	5,08E-03
cg01862688	5	140480872	PCDHB3	1stExon	-0,047	6,71E-06	5,08E-03
cg04330057	6	16306377	ATXN1	3'UTR	0,015	6,71E-06	5,08E-03
cg09639369	14	92914941	SLC24A4	Body	-0,011	6,71E-06	5,08E-03
cg06691743	19	56288887		IGR	-0,041	6,73E-06	5,08E-03
cg07106169	2	95740362		IGR	-0,038	6,74E-06	5,09E-03
cg00165078	1	94799132		IGR	-0,014	6,76E-06	5,09E-03
cg04181327	2	91776322		IGR	-0,039	6,77E-06	5,10E-03
cg15026780	16	4784446	C16orf71	1stExon	-0,007	6,77E-06	5,10E-03
cg05686197	2	141787134	LRP1B	Body	-0,011	6,79E-06	5,10E-03
cg25950936	8	26279953		IGR	-0,012	6,79E-06	5,10E-03
cg23190649	18	77545050		IGR	-0,056	6,79E-06	5,10E-03
cg19834701	2	241158076		IGR	-0,014	6,80E-06	5,10E-03
cg24394909	5	91377816		IGR	0,041	6,82E-06	5,10E-03
cg14309385	20	57427010	GNAS	TSS1500	0,013	6,82E-06	5,10E-03
cg18512769	22	50825141	PPP6R2	5'UTR	-0,04	6,83E-06	5,10E-03
cg15430995	3	139008912		IGR	0,046	6,85E-06	5,10E-03
cg02773770	3	184348487		IGR	-0,013	6,84E-06	5,10E-03
cg09371693	12	57855541	GLI1	5'UTR	0,009	6,84E-06	5,10E-03
cg23467944	19	35829110	CD22	Body	0,016	6,86E-06	5,10E-03
cg01003595	2	86426930	MRPL35	Body	-0,017	6,87E-06	5,11E-03
cg23727336	6	57178595	PRIM2	TSS1500	-0,02	6,92E-06	5,14E-03
cg05881745	4	1409557		IGR	-0,049	6,94E-06	5,14E-03
cg05740180	10	86189566	CCSER2	Body	0,049	6,94E-06	5,14E-03
cg06477663	13	46757415	LCP1	TSS1500	0,067	6,95E-06	5,14E-03
cg26841114	16	88871987	CDT1	Body	0,016	6,97E-06	5,15E-03
cg12301301	1	68564241	GPR177	3'UTR	0,011	7,00E-06	5,17E-03
cg01239717	6	107077302	RTN4IP1	5'UTR	0,035	7,01E-06	5,17E-03
cg21344322	14	35294119	BAZ1A	Body	0,012	7,01E-06	5,17E-03
cg02983885	19	42772892		IGR	-0,004	7,01E-06	5,17E-03
cg03351248	15	100880064	ADAMTS17	Body	-0,039	7,03E-06	5,18E-03
cg01195404	2	467135		IGR	-0,024	7,06E-06	5,19E-03
cg10860760	7	71260527	CALN1	Body	0,016	7,08E-06	5,20E-03
cg19711816	15	77545023	PEAK1	5'UTR	0,013	7,09E-06	5,20E-03
cg21110966	22	23263903		IGR	0,016	7,10E-06	5,21E-03
cg06644655	13	112333521		IGR	0,01	7,13E-06	5,22E-03
cg07380384	2	10360574		IGR	-0,02	7,15E-06	5,23E-03
cg08465351	14	77808230	TMED8	Body	-0,007	7,15E-06	5,23E-03
cg12251659	12	122018385	KDM2B	TSS200	0,005	7,23E-06	5,28E-03
cg16594986	3	187661742		IGR	0,007	7,24E-06	5,28E-03
cg19214977	17	2777960	RAP1GAP2	Body	0,029	7,28E-06	5,31E-03
cg09670566	10	28507577	MPP7	Body	-0,006	7,33E-06	5,33E-03
cg06292902	14	68212234		IGR	-0,007	7,34E-06	5,34E-03
cg18131141	1	36043002	TFAP2E	Body	0,008	7,38E-06	5,35E-03
cg23017722	8	107783672	ABRA	TSS1500	0,037	7,38E-06	5,35E-03
cg26066277	17	79792777	DYSFIP1	1stExon	0,015	7,36E-06	5,35E-03
cg19168526	4	53801801	SCFD2	Body	-0,018	7,40E-06	5,36E-03
cg15277378	7	5272208	WIPI2	3'UTR	0,012	7,42E-06	5,37E-03
cg15926099	6	31803111	C6orf48	5'UTR	-0,004	7,48E-06	5,38E-03

cg00528492	6	32408863	HLA-DRA	Body	0,044	7,47E-06	5,38E-03
cg01924868	8	26305569		IGR	-0,039	7,52E-06	5,38E-03
cg13219104	8	70534097	SULF1	Body	-0,02	7,50E-06	5,38E-03
cg12773368	14	35591473	PPP2R3C	1stExon	0,008	7,53E-06	5,38E-03
cg05157120	14	69727352	GALNTL1	Body	-0,006	7,46E-06	5,38E-03
cg05488279	17	1819770		IGR	-0,024	7,53E-06	5,38E-03
cg18732892	17	57037342	PPM1E	Body	0,028	7,51E-06	5,38E-03
cg23850904	19	40730628	CNTD2	Body	-0,055	7,51E-06	5,38E-03
cg25355465	20	36576591		IGR	-0,025	7,45E-06	5,38E-03
cg03015225	21	38581371	DSCR9	Body	0,024	7,52E-06	5,38E-03
cg00071692	22	32006800	SFI1	Body	0,02	7,48E-06	5,38E-03
cg13760881	9	72812874	MAMDC2	Body	0,034	7,54E-06	5,38E-03
cg08224779	20	45629954	EYA2	Body	-0,038	7,55E-06	5,39E-03
cg22985373	6	117859232	DCBLD1	Body	0,035	7,57E-06	5,40E-03
cg15076368	3	39224943	XIRP1	3'UTR	-0,012	7,62E-06	5,43E-03
cg19454651	12	121772206	ANAPC5	Body	-0,006	7,63E-06	5,43E-03
cg09341892	1	203379814		IGR	0,059	7,65E-06	5,44E-03
cg05373657	7	130126637	MEST	5'UTR	0,011	7,67E-06	5,44E-03
cg13783641	17	73898112	MRPL38	Body	-0,01	7,66E-06	5,44E-03
cg18649995	15	27012710	GABRB3	Body	-0,01	7,69E-06	5,46E-03
cg22674497	17	72916000	USH1G	Body	-0,083	7,71E-06	5,46E-03
cg25614364	4	129747274	PHF17	5'UTR	0,025	7,72E-06	5,46E-03
cg22867714	12	6169044	VWF	Body	0,016	7,73E-06	5,47E-03
cg02776313	22	50965782	TYMP	Body	0,048	7,76E-06	5,48E-03
cg14438445	2	39221176	SOS1	Body	-0,023	7,79E-06	5,49E-03
cg27022615	11	70049105	FADD	TSS200	-0,004	7,80E-06	5,49E-03
cg15368722	18	55470404		IGR	-0,005	7,79E-06	5,49E-03
cg03773731	19	15310387	NOTCH3	Body	0,014	7,79E-06	5,49E-03
cg21856603	17	3713823		IGR	0,019	7,81E-06	5,49E-03
cg11569930	1	155113381	DPM3	TSS1500	-0,094	7,85E-06	5,51E-03
cg05414964	7	33830854		IGR	0,006	7,96E-06	5,59E-03
cg05574313	4	155338166	DCHS2	Body	-0,02	7,97E-06	5,59E-03
cg24407907	8	138116159		IGR	0,025	8,03E-06	5,59E-03
cg26053066	9	96080065	WNK2	Body	-0,045	8,04E-06	5,59E-03
cg02686331	13	110296524		IGR	-0,008	8,00E-06	5,59E-03
cg10190509	17	34308137	CCL16	Body	0,019	8,03E-06	5,59E-03
cg03745114	17	46017593	PNPO	TSS1500	0,022	8,02E-06	5,59E-03
cg11922371	17	48226442	PPP1R9B	Body	-0,005	8,00E-06	5,59E-03
cg24916020	19	33096688	ANKRD27	Body	0,009	8,03E-06	5,59E-03
cg22441543	22	50355555	PIM3	Body	-0,006	8,00E-06	5,59E-03
cg05075887	2	3650748	COLEC11	5'UTR	-0,022	8,07E-06	5,61E-03
cg09099177	15	68499367	CLN6	3'UTR	0,03	8,07E-06	5,61E-03
cg21288091	8	105524569	LRP12	Body	0,01	8,10E-06	5,62E-03
cg07874755	8	703701		IGR	-0,07	8,14E-06	5,64E-03
cg03953506	12	17732825		IGR	-0,027	8,15E-06	5,64E-03
cg26978691	10	105128013	TAF5	1stExon	-0,005	8,19E-06	5,66E-03
cg07420961	1	6052177	NPHP4	5'UTR	-0,004	8,21E-06	5,67E-03
cg20230572	13	31308809	ALOX5AP	TSS1500	0,031	8,21E-06	5,67E-03
cg17845200	13	113819380	PROZ	Body	0,013	8,24E-06	5,67E-03
cg15459633	14	77740082		IGR	-0,02	8,22E-06	5,67E-03
cg00545513	16	88929283		IGR	-0,009	8,24E-06	5,67E-03
cg13375624	15	96829126		IGR	0,042	8,25E-06	5,68E-03
cg27295621	2	44880258	CAMKMT	Body	-0,009	8,28E-06	5,69E-03
cg10475970	5	140772512	PCDHGA4	Body	-0,032	8,30E-06	5,69E-03
cg04747226	11	105481319	GRIA4	5'UTR	-0,011	8,30E-06	5,69E-03
cg08248319	16	47766189		IGR	-0,016	8,30E-06	5,69E-03
cg12745003	2	23659593	KLHL29	5'UTR	-0,056	8,33E-06	5,71E-03
cg23213872	1	242613203	PLD5	5'UTR	-0,029	8,35E-06	5,71E-03
cg25383479	6	108491702	NR2E1	Body	-0,025	8,37E-06	5,71E-03
cg19113906	7	143582609	TCAF1	TSS200	-0,035	8,38E-06	5,71E-03
cg06461981	11	6890151	OR10A2	TSS1500	0,042	8,37E-06	5,71E-03
cg01990304	19	46088010	OPA3	1stExon	-0,003	8,39E-06	5,72E-03
cg27000659	6	31617530	BAT3	Body	0,013	8,42E-06	5,73E-03
cg11159417	5	148822491		IGR	0,01	8,48E-06	5,76E-03
cg20562292	17	583825	VPS53	Body	0,036	8,48E-06	5,76E-03
cg12610917	19	46387992	IRF2BP1	1stExon	-0,036	8,49E-06	5,76E-03
cg11443986	17	7487198	MPDU1	1stExon	-0,004	8,51E-06	5,77E-03
cg08094128	21	30565706	NCRNA00189	TSS200	-0,032	8,58E-06	5,81E-03
cg16766632	12	57530100	LRP1	Body	0,019	8,62E-06	5,82E-03
cg14209540	12	98937392	TMPO	Body	0,03	8,61E-06	5,82E-03
cg02005039	16	87206291		IGR	-0,012	8,62E-06	5,82E-03
cg15383979	17	78362278	RNF213	Body	0,008	8,63E-06	5,83E-03
cg14976684	1	101492856	DPH5	TSS1500	-0,005	8,76E-06	5,83E-03

cg13515254	1	178089126	RASAL2	Body	0,048	8,71E-06	5,83E-03
cg26524446	3	45408913		IGR	-0,012	8,70E-06	5,83E-03
cg21611868	3	135969116	PCCB	TSS200	0,004	8,76E-06	5,83E-03
cg02114451	4	35543696		IGR	-0,012	8,76E-06	5,83E-03
cg11404039	4	155471778	PLRG1	TSS1500	-0,006	8,74E-06	5,83E-03
cg07164639	6	110736958	DDO	TSS1500	0,076	8,69E-06	5,83E-03
cg24962544	6	157467221	ARID1B	Body	0,01	8,76E-06	5,83E-03
cg00675001	7	12713893		IGR	0,028	8,76E-06	5,83E-03
cg12816747	9	134884962	MED27	Body	-0,02	8,70E-06	5,83E-03
cg18059464	10	134915277	GPR123	Body	0,036	8,71E-06	5,83E-03
cg24963613	11	113149755		IGR	-0,01	8,71E-06	5,83E-03
cg04229722	13	107190457		IGR	0,014	8,66E-06	5,83E-03
cg05147509	16	2339536	ABCA3	Body	0,017	8,70E-06	5,83E-03
cg05173383	16	69422211		IGR	-0,007	8,76E-06	5,83E-03
cg25715073	22	50452430	IL17REL	TSS1500	0,025	8,71E-06	5,83E-03
cg26540127	2	114647103	ACTR3	TSS1500	-0,004	8,78E-06	5,83E-03
cg21733911	3	180397238	CCDC39	5'UTR	-0,004	8,78E-06	5,83E-03
cg16777216	2	23602341		IGR	0,022	8,81E-06	5,84E-03
cg25822388	5	139174207	PSD2	TSS1500	-0,048	8,82E-06	5,84E-03
cg10418263	6	35265544	DEF6	TSS200	-0,004	8,80E-06	5,84E-03
cg01442943	6	41215914	TREML2P	TSS1500	0,015	8,81E-06	5,84E-03
cg12849978	11	93734156		IGR	-0,031	8,83E-06	5,84E-03
cg25081232	5	132001119		IGR	0,012	8,85E-06	5,84E-03
cg25273520	15	59713427		IGR	-0,045	8,87E-06	5,86E-03
cg04481478	2	179567366	TTN	Body	0,012	8,90E-06	5,86E-03
cg13940534	5	140909712	DIAPH1	Body	0,033	8,90E-06	5,86E-03
cg04863300	1	11010604	C1orf127	Body	0,016	8,92E-06	5,87E-03
cg21752469	8	57594909		IGR	-0,014	8,93E-06	5,87E-03
cg26205979	11	46443014	AMBRA1	Body	-0,007	8,91E-06	5,87E-03
cg01031319	14	75388007	RPS6KL1	Body	0,01	8,93E-06	5,87E-03
cg07017261	19	55770040	PPP6R1	TSS200	-0,005	8,95E-06	5,87E-03
cg05106058	4	172735924	GALNTL6	Body	-0,035	8,98E-06	5,88E-03
cg04227758	6	86186351	NT5E	Body	-0,017	8,98E-06	5,88E-03
cg19434167	12	116398767	MED13L	3'UTR	0,028	9,00E-06	5,88E-03
cg16831461	14	50470026	C14orf182	Body	-0,004	9,00E-06	5,88E-03
cg04241305	5	137689053	KDM3B	Body	0,005	9,01E-06	5,89E-03
cg14234302	19	50391354	TBC1D17	Body	-0,049	9,03E-06	5,89E-03
cg05912220	3	113546976		IGR	-0,019	9,05E-06	5,90E-03
cg04509429	5	138441649	SIL1	Body	-0,011	9,07E-06	5,91E-03
cg07829799	22	45034455		IGR	-0,024	9,08E-06	5,91E-03
cg07848657	4	59843950		IGR	-0,017	9,09E-06	5,91E-03
cg16047823	10	80538951		IGR	0,019	9,10E-06	5,91E-03
cg27143209	15	78354460	TBC1D2B	Body	-0,006	9,10E-06	5,91E-03
cg25941598	15	38839973	RASGRP1	Body	-0,017	9,12E-06	5,91E-03
cg27014546	14	96743154		IGR	-0,022	9,13E-06	5,92E-03
cg05237374	14	66980127	GPHN	Body	-0,037	9,23E-06	5,98E-03
cg26903744	4	102710414	BANK1	TSS1500	0,015	9,28E-06	6,00E-03
cg13336521	16	2043558	SYNGR3	3'UTR	0,018	9,28E-06	6,00E-03
cg16490062	16	12211322	SNX29	Body	0,026	9,31E-06	6,01E-03
cg27402766	3	100468242	ABI3BP	3'UTR	0,017	9,38E-06	6,05E-03
cg09896867	12	113734442	TPCN1	3'UTR	-0,012	9,38E-06	6,05E-03
cg07899263	13	99293508		IGR	-0,023	9,39E-06	6,05E-03
cg25690118	1	151497318	CGN	Body	0,017	9,43E-06	6,05E-03
cg07102435	11	102096771	YAP1	Body	0,013	9,42E-06	6,05E-03
cg01507342	17	65387096	PITPNC1	Body	-0,064	9,42E-06	6,05E-03
cg00530268	3	126921916		IGR	-0,069	9,46E-06	6,06E-03
cg11677852	5	1108876	SLC12A7	Body	0,047	9,45E-06	6,06E-03
cg22497960	9	66859433		IGR	-0,022	9,45E-06	6,06E-03
cg10254317	17	80254303		IGR	0,006	9,44E-06	6,06E-03
cg06462174	17	30813688	CDK5R1	TSS1500	0,018	9,47E-06	6,06E-03
cg03458695	10	78163703	C10orf11	Body	-0,031	9,52E-06	6,08E-03
cg05237266	3	159733828	IL12A-AS1	Body	-0,005	9,54E-06	6,09E-03
cg05968467	11	128482094		IGR	-0,007	9,55E-06	6,09E-03
cg13269032	20	47274909	PREX1	Body	0,025	9,57E-06	6,10E-03
cg22249932	17	4545721	ALOX15	TSS1500	-0,061	9,58E-06	6,10E-03
cg01291513	3	12571276	TSEN2	ExonBnd	-0,028	9,63E-06	6,11E-03
cg16284674	10	135123337	ZNF511	Body	0,02	9,62E-06	6,11E-03
cg03456468	17	8077382	TMEM107	3'UTR	0,032	9,62E-06	6,11E-03
cg21920221	5	171612889	STK10	Body	-0,01	9,68E-06	6,14E-03
cg13062095	14	72307273		IGR	-0,017	9,68E-06	6,14E-03
cg12528981	5	170919715		IGR	0,005	9,73E-06	6,14E-03
cg12693101	9	96352183	PHF2	Body	-0,078	9,71E-06	6,14E-03
cg24117442	12	93963504	SOCS2	TSS200	-0,013	9,73E-06	6,14E-03

cg20383501	20	43805939		IGR	0,013	9,72E-06	6,14E-03
cg25846372	21	38608601	DSCR3	Body	0,023	9,72E-06	6,14E-03
cg00339300	1	61508924		IGR	-0,007	9,81E-06	6,14E-03
cg08425449	1	204042326	SOX13	1stExon	-0,006	9,78E-06	6,14E-03
cg09836344	4	1243392	C4orf42	TSS1500	0,033	9,79E-06	6,14E-03
cg21203097	9	38336757		IGR	-0,008	9,77E-06	6,14E-03
cg10605064	11	8290815		IGR	-0,065	9,77E-06	6,14E-03
cg01525669	13	107211833	ARGLU1	Body	0,008	9,78E-06	6,14E-03
cg16080697	17	35732698	C17orf78	TSS1500	0,013	9,79E-06	6,14E-03
cg20758756	18	9321560		IGR	0,141	9,79E-06	6,14E-03
cg22788747	20	35480780	SOGA1	Body	0,011	9,80E-06	6,14E-03
cg01522478	1	234972737		IGR	0,016	9,83E-06	6,15E-03
cg24796507	4	170190311	SH3RF1	Body	-0,008	9,86E-06	6,16E-03
cg05971912	6	150185499	LRP11	TSS200	-0,004	9,85E-06	6,16E-03
cg03563298	2	242990263		IGR	-0,015	9,87E-06	6,16E-03
cg24798589	6	20445170	E2F3	Body	-0,027	9,88E-06	6,16E-03
cg02246180	14	21945391	TOX4	1stExon	-0,005	9,90E-06	6,17E-03
cg06493559	17	13638964		IGR	-0,019	9,93E-06	6,18E-03
cg13310598	3	87119326		IGR	-0,023	9,96E-06	6,19E-03
cg20099552	3	88068427		IGR	-0,032	9,97E-06	6,19E-03
cg18840151	7	6098981	EIF2AK1	TSS200	-0,006	9,97E-06	6,19E-03
cg04617549	19	51569622	KLK13	TSS1500	-0,036	9,96E-06	6,19E-03
cg15674627	11	1509252	MOB2	TSS1500	0,015	9,99E-06	6,19E-03
cg06880930	16	57147300	CPNE2	Body	0,008	1,00E-05	6,19E-03
cg27052498	17	72413843		IGR	0,022	1,00E-05	6,20E-03
cg19915618	1	18902229		IGR	0,075	1,00E-05	6,21E-03
cg04503600	5	140798188	PCDHGA4	Body	-0,06	1,01E-05	6,22E-03
cg04471822	2	71249764	OR7E91P	TSS1500	-0,017	1,01E-05	6,25E-03
cg26469586	11	2019626	H19	TSS1500	0,022	1,01E-05	6,25E-03
cg07965110	1	24863166		IGR	-0,013	1,02E-05	6,27E-03
cg22026853	6	99282887	POU3F2	1stExon	-0,011	1,02E-05	6,27E-03
cg14999911	7	130353086	COPG2	Body	-0,008	1,02E-05	6,27E-03
cg02477523	3	180456508		IGR	-0,019	1,02E-05	6,27E-03
cg18011946	5	65440816	SFRS12	5'UTR	-0,004	1,03E-05	6,27E-03
cg01113488	6	119027122		IGR	-0,046	1,03E-05	6,27E-03
cg10811195	7	36433157	ANLN	Body	0,034	1,02E-05	6,27E-03
cg02397167	11	12274599	MICAL2	Body	-0,007	1,02E-05	6,27E-03
cg20372511	11	73915489	PPME1	ExonBnd	-0,008	1,02E-05	6,27E-03
cg13728449	11	129014781	RICS	Body	0,019	1,02E-05	6,27E-03
cg06675348	16	11276415		IGR	0,015	1,03E-05	6,27E-03
cg18879950	6	141473527		IGR	-0,03	1,03E-05	6,28E-03
cg24459800	2	234221241	SAG	Body	-0,027	1,04E-05	6,29E-03
cg22980079	4	2627118	FAM193A	TSS200	-0,006	1,04E-05	6,29E-03
cg03415545	4	100274039	ADH1C	TSS200	0,025	1,03E-05	6,29E-03
cg04017512	10	35195138		IGR	0,074	1,04E-05	6,29E-03
cg25406773	16	67678852	RLTPR	TSS200	0,005	1,03E-05	6,29E-03
cg19400076	16	75681741	KARS	TSS200	-0,003	1,04E-05	6,29E-03
cg25393494	17	17109936	PLD6	TSS1500	-0,093	1,04E-05	6,29E-03
cg24242002	18	21594044	TTC39C	5'UTR	-0,005	1,04E-05	6,29E-03
cg01839850	22	38240395	ANKRD54	TSS200	-0,005	1,04E-05	6,29E-03
cg15821589	1	78444904	FUBP1	TSS200	0,093	1,04E-05	6,30E-03
cg06821212	3	156807121	LINC00881	TSS1500	-0,003	1,04E-05	6,30E-03
cg22537859	10	101948986	CHUK	3'UTR	0,042	1,04E-05	6,30E-03
cg15174075	11	36616318	C11orf74	TSS200	-0,005	1,05E-05	6,30E-03
cg10265503	16	1494476	CCDC154	1stExon	0,028	1,04E-05	6,30E-03
cg19453059	20	29955259	DEFB118	TSS1500	0,034	1,04E-05	6,30E-03
cg27529037	20	44575021	PCIF1	Body	0,048	1,05E-05	6,30E-03
cg13670883	3	70047594	LINC01212	TSS1500	0,042	1,05E-05	6,31E-03
cg05291677	5	1744919		IGR	-0,038	1,05E-05	6,31E-03
cg18537721	2	191791548	GLS	Body	0,024	1,05E-05	6,32E-03
cg14157768	2	239414104		IGR	0,051	1,05E-05	6,32E-03
cg15468403	4	95526836	PDLIM5	Body	-0,007	1,05E-05	6,32E-03
cg24241128	5	180071734	FLT4	Body	0,014	1,05E-05	6,32E-03
cg00180806	13	114324947	GRK1	Body	-0,048	1,05E-05	6,32E-03
cg26516954	17	81009758	B3GNTL1	TSS200	-0,004	1,06E-05	6,32E-03
cg16442638	2	120989893		IGR	-0,006	1,06E-05	6,33E-03
cg25133685	6	29013336	OR2W1	TSS1500	0,091	1,06E-05	6,33E-03
cg13320538	8	2419822		IGR	-0,03	1,06E-05	6,33E-03
cg18555295	10	17211572	TRDMT1	Body	0,032	1,06E-05	6,35E-03
cg10660635	11	1780294	CTSD	Body	0,034	1,07E-05	6,37E-03
cg01214260	1	163307616	NUF2	Body	0,014	1,07E-05	6,40E-03
cg22333214	1	98511789	MIR137	TSS200	-0,014	1,08E-05	6,41E-03
cg07527547	10	127026279		IGR	-0,014	1,08E-05	6,42E-03

cg04596558	16	1498758	CLCN7	ExonBnd	0,025	1,08E-05	6,42E-03
cg07315623	1	28735095	PHACTR4	Body	0,008	1,10E-05	6,43E-03
cg04392073	1	60311151	HOOK1	Body	-0,01	1,09E-05	6,43E-03
cg12387700	1	85514134	MCOLN3	1stExon	-0,005	1,09E-05	6,43E-03
cg17708747	2	43308536		IGR	-0,009	1,10E-05	6,43E-03
cg10641613	4	3387994	RGS12	Body	-0,039	1,09E-05	6,43E-03
cg15536489	7	1120860	C7orf50	Body	0,012	1,09E-05	6,43E-03
cg26901873	8	28811004	HMBOX1	5'UTR	-0,038	1,09E-05	6,43E-03
cg21770758	10	112440394	RBM20	Body	0,042	1,10E-05	6,43E-03
cg08406082	12	5378296		IGR	-0,009	1,08E-05	6,43E-03
cg16618389	12	46865385		IGR	-0,007	1,10E-05	6,43E-03
cg05271910	13	100037488	UBAC2	Body	-0,009	1,09E-05	6,43E-03
cg25018731	14	73181273	DPF3	Body	0,026	1,10E-05	6,43E-03
cg20497936	16	88770188	RNF166	TSS200	-0,004	1,09E-05	6,43E-03
cg13333761	16	89782916	VPS9D1	Body	-0,008	1,09E-05	6,43E-03
cg22369607	18	13821885		IGR	-0,01	1,09E-05	6,43E-03
cg00655184	18	19997912	CTAGE1	TSS200	-0,053	1,10E-05	6,43E-03
cg25706478	22	45636726	KIAA0930	TSS200	0,026	1,09E-05	6,43E-03
cg05468064	22	46423449		IGR	0,05	1,09E-05	6,43E-03
cg04552378	1	214782555	CENPF	5'UTR	-0,005	1,11E-05	6,47E-03
cg00835279	2	3642710	COLEC11	1stExon	-0,092	1,10E-05	6,47E-03
cg03268613	3	88188039	ZNF654	TSS1500	0,018	1,11E-05	6,48E-03
cg25416270	6	17505703	CAP2	Body	-0,016	1,11E-05	6,48E-03
cg16291917	6	31590576	SNORA38	TSS1500	0,014	1,11E-05	6,48E-03
cg17414718	11	1444836	BRSK2	Body	-0,022	1,11E-05	6,48E-03
cg18476795	18	56455224		IGR	0,008	1,11E-05	6,48E-03
cg17010825	2	76301649		IGR	0,012	1,12E-05	6,49E-03
cg06212122	10	14136368	FRMD4A	Body	-0,045	1,12E-05	6,49E-03
cg09717029	15	93383415		IGR	0,029	1,12E-05	6,49E-03
cg03445220	1	244747323	C1orf101	Body	-0,023	1,12E-05	6,50E-03
cg08897637	8	143530646		IGR	-0,015	1,12E-05	6,50E-03
cg06765630	20	44847566	CDH22	Body	0,01	1,12E-05	6,50E-03
cg15364450	3	66119154	SLC25A26	TSS200	-0,023	1,13E-05	6,51E-03
cg24020806	10	7448857	SFMBT2	5'UTR	-0,008	1,13E-05	6,51E-03
cg06349752	19	16432781		IGR	0,011	1,13E-05	6,51E-03
cg27403957	19	40786376	AKT2	5'UTR	-0,033	1,12E-05	6,51E-03
cg24454553	12	105380171	C12orf45	1stExon	-0,006	1,13E-05	6,53E-03
cg24119077	1	40349270	TRIT1	TSS200	-0,005	1,13E-05	6,53E-03
cg04770944	12	69069079	LOC100507250	Body	-0,012	1,13E-05	6,53E-03
cg08255233	6	87607178		IGR	0,023	1,13E-05	6,54E-03
cg04136787	9	32574247	NDUFB6	TSS1500	0,015	1,13E-05	6,54E-03
cg23356876	1	178403869	RASAL2	Body	0,024	1,14E-05	6,54E-03
cg05755354	10	14372596	FRMD4A	1stExon	0,017	1,14E-05	6,54E-03
cg19823712	14	66783492		IGR	-0,018	1,14E-05	6,54E-03
cg07035442	17	81028481		IGR	0,029	1,14E-05	6,54E-03
cg02840044	19	30299177		IGR	-0,011	1,14E-05	6,54E-03
cg08100159	2	9560753	ITGB1BP1	5'UTR	0,012	1,14E-05	6,55E-03
cg10788417	7	100198382	PCOLCE	TSS1500	0,016	1,14E-05	6,55E-03
cg08370757	16	84230590	ADAD2	3'UTR	-0,039	1,15E-05	6,55E-03
cg06500281	17	41093246		IGR	0,02	1,15E-05	6,55E-03
cg22391913	18	24980040		IGR	-0,067	1,14E-05	6,55E-03
cg08499756	16	1525669	CLCN7	TSS1500	-0,033	1,15E-05	6,56E-03
cg23445482	11	112040566	TEX12	Body	0,02	1,15E-05	6,57E-03
cg11404156	6	46658344	TDRD6	1stExon	-0,007	1,15E-05	6,58E-03
cg22109433	18	13472332	LDLRAD4	Body	0,041	1,16E-05	6,59E-03
cg08381480	2	224879566	SERPINE2	Body	-0,007	1,16E-05	6,60E-03
cg18026479	2	174222196	CDCA7	Body	0,024	1,17E-05	6,61E-03
cg07451446	8	107012924	ZFPM2-AS1	Body	0,023	1,16E-05	6,61E-03
cg10787824	8	131289278	ASAP1	Body	0,015	1,16E-05	6,61E-03
cg01110668	10	71218586	TSPAN15	Body	0,009	1,17E-05	6,61E-03
cg18305652	10	134549665	INPP5A	Body	-0,046	1,17E-05	6,61E-03
cg09544081	8	133109689	HHLA1	Body	-0,016	1,17E-05	6,61E-03
cg05290740	17	32292107	ASIC2	Body	0,021	1,17E-05	6,62E-03
cg01394989	11	114255393		IGR	-0,018	1,17E-05	6,63E-03
cg24791575	3	149902148		IGR	-0,008	1,17E-05	6,63E-03
cg21508457	6	30311316	TRIM39	3'UTR	-0,01	1,18E-05	6,65E-03
cg23082339	5	140810051	PCDHGA4	Body	-0,016	1,18E-05	6,66E-03
cg25977898	8	63022662		IGR	-0,018	1,18E-05	6,66E-03
cg25512683	17	41003399	AOC3	1stExon	0,015	1,18E-05	6,66E-03
cg14243010	9	127117328	LOC100129034	Body	0,029	1,19E-05	6,71E-03
cg08549781	2	224681209	AP1S3	Body	0,06	1,20E-05	6,73E-03
cg13762474	15	42371808	PLA2G4D	Body	-0,061	1,20E-05	6,73E-03
cg00917156	16	57831841	KIFC3	Body	-0,039	1,20E-05	6,73E-03

cg04754260	16	88455670		IGR	0,033	1,20E-05	6,73E-03
cg01015354	17	21823975	FAM27L	TSS1500	-0,049	1,20E-05	6,73E-03
cg19738962	9	132022957		IGR	-0,014	1,20E-05	6,74E-03
cg27379787	2	239907658		IGR	-0,065	1,21E-05	6,74E-03
cg13169766	3	134034554		IGR	-0,005	1,21E-05	6,74E-03
cg21073160	6	44275059	AARS2	Body	0,034	1,21E-05	6,74E-03
cg13931396	7	65615910	CRCP	Body	0,017	1,21E-05	6,74E-03
cg10765507	8	674899	ERICH1	Body	-0,027	1,21E-05	6,74E-03
cg05452419	12	56863023	SPRYD4	Body	0,048	1,21E-05	6,74E-03
cg25543578	13	62516040		IGR	-0,009	1,21E-05	6,74E-03
cg15628498	15	53082902	ONECUT1	TSS1500	-0,005	1,21E-05	6,74E-03
cg17536310	17	25925533	KSR1	Body	0,013	1,21E-05	6,74E-03
cg09003917	19	1008388	GRIN3B	Body	0,024	1,21E-05	6,74E-03
cg18011291	19	17282314	MYO9B	Body	0,008	1,21E-05	6,74E-03
cg15542994	19	52408371	ZNF649	TSS200	-0,01	1,21E-05	6,74E-03
cg07262555	20	25850253		IGR	-0,028	1,21E-05	6,74E-03
cg26296524	1	15124175	KIAA1026	Body	-0,022	1,22E-05	6,76E-03
cg18408873	12	122240842	LINC01089	Body	-0,008	1,22E-05	6,76E-03
cg20395153	10	28031385	MKX	Body	-0,006	1,22E-05	6,76E-03
cg17613592	3	5285234		IGR	-0,01	1,22E-05	6,77E-03
cg02720600	6	76425457	SENP6	3'UTR	0,016	1,22E-05	6,77E-03
cg19548912	6	138299068		IGR	-0,092	1,23E-05	6,77E-03
cg00099091	16	776809	HAGHL	TSS1500	-0,007	1,22E-05	6,77E-03
cg13017801	1	214499900	SMYD2	Body	0,047	1,23E-05	6,77E-03
cg23626895	11	101800354	CEP126	Body	-0,008	1,23E-05	6,77E-03
cg15131789	1	18060276		IGR	0,049	1,23E-05	6,78E-03
cg08724891	2	219826145	CDK5R2	3'UTR	0,066	1,23E-05	6,78E-03
cg14960882	20	31185166	LOC149950	Body	0,007	1,23E-05	6,79E-03
cg13992976	8	125676472	MTSS1	Body	0,011	1,24E-05	6,80E-03
cg02611672	14	89026119		IGR	-0,029	1,24E-05	6,80E-03
cg03662860	2	183887702	NCKAP1	Body	0,033	1,24E-05	6,82E-03
cg14543959	3	113557658	GRAMD1C	TSS200	0,01	1,24E-05	6,82E-03
cg19303716	2	1891338	MYT1L	Body	-0,011	1,24E-05	6,82E-03
cg22776675	4	7436259	PSAPL1	1stExon	-0,022	1,25E-05	6,85E-03
cg00351443	10	105252720	NEURL	TSS1500	0,03	1,26E-05	6,87E-03
cg20038355	11	95431082		IGR	0,036	1,26E-05	6,88E-03
cg04402122	16	1544648	TELO2	Body	0,013	1,26E-05	6,88E-03
cg13437554	19	2272716	OAZ1	Body	0,034	1,26E-05	6,88E-03
cg05025391	6	16583476	ATXN1	5'UTR	0,006	1,26E-05	6,89E-03
cg21115527	7	75931838	HSPB1	TSS200	-0,006	1,26E-05	6,89E-03
cg09574498	17	48944758		IGR	-0,007	1,26E-05	6,89E-03
cg22302420	19	45664198	NKPD1	TSS1500	0,049	1,26E-05	6,89E-03
cg09293652	14	20794803	SNORD126	TSS200	0,039	1,27E-05	6,89E-03
cg27226618	2	265872	ACP1	Body	-0,027	1,28E-05	6,91E-03
cg21265776	3	142519228	TRPC1	Body	-0,014	1,27E-05	6,91E-03
cg14245109	3	182082522		IGR	-0,015	1,28E-05	6,91E-03
cg16145303	4	98040667		IGR	0,035	1,27E-05	6,91E-03
cg09859756	5	149384153	HMGXB3	5'UTR	0,008	1,27E-05	6,91E-03
cg23446033	10	22880692	PIP4K2A	ExonBnd	0,026	1,27E-05	6,91E-03
cg27184649	11	70935963	SHANK2	TSS200	-0,009	1,28E-05	6,91E-03
cg20343312	5	60917264		IGR	-0,012	1,28E-05	6,92E-03
cg11911736	18	76764262		IGR	-0,027	1,28E-05	6,92E-03
cg23718418	6	33377739	PHF1	TSS1500	-0,009	1,29E-05	6,93E-03
cg07914267	9	35670549		IGR	-0,007	1,29E-05	6,93E-03
cg16487481	10	126796015	CTBP2	5'UTR	0,012	1,28E-05	6,93E-03
cg05184938	17	75369939	sept-09	5'UTR	-0,005	1,28E-05	6,93E-03
cg08876826	19	35634102	FXYD7	TSS200	-0,004	1,29E-05	6,93E-03
cg04903883	14	106321551		IGR	0,019	1,29E-05	6,94E-03
cg27159031	1	244192443	LOC339529	Body	0,019	1,29E-05	6,94E-03
cg19175993	1	51821069	EPS15	3'UTR	0,031	1,29E-05	6,94E-03
cg19097648	5	115177461	ATG12	1stExon	0,011	1,29E-05	6,94E-03
cg09273779	9	35605990	TESK1	Body	-0,005	1,30E-05	6,94E-03
cg14331899	11	61125360	CYBASC3	TSS1500	0,018	1,30E-05	6,94E-03
cg03292213	17	17109640	PLD6	1stExon	-0,057	1,29E-05	6,94E-03
cg24452392	2	204732461	CTLA4	TSS200	-0,01	1,30E-05	6,94E-03
cg26259546	3	57113458	ARHGEF3	TSS200	-0,005	1,30E-05	6,94E-03
cg23638070	8	6569043	AGPAT5	Body	0,016	1,30E-05	6,94E-03
cg25304134	1	215293949	KCNK2	Body	0,041	1,30E-05	6,94E-03
cg14101038	9	95991922	WNK2	Body	0,009	1,31E-05	6,97E-03
cg26991064	18	22932086	ZNF521	5'UTR	-0,003	1,31E-05	6,98E-03
cg05659314	1	153590020	S100A14	TSS1500	0,015	1,31E-05	6,99E-03
cg10264935	3	184261694		IGR	0,038	1,32E-05	7,02E-03
cg16100915	1	40157375	HPCAL4	TSS1500	-0,005	1,32E-05	7,03E-03

cg17194100	15	96949312		IGR	-0,017	1,32E-05	7,03E-03
cg13773689	17	5372292	DHX33	TSS200	0,005	1,32E-05	7,03E-03
cg07221224	17	25757627	TBC1D3P5	Body	-0,022	1,33E-05	7,05E-03
cg06696623	17	20956123		IGR	0,011	1,34E-05	7,10E-03
cg15393490	1	207996459		IGR	0,038	1,34E-05	7,11E-03
cg16138161	6	27635891		IGR	-0,017	1,34E-05	7,12E-03
cg16364833	2	70121066	SNRNP27	TSS200	0,008	1,35E-05	7,13E-03
cg08442737	2	171842013		IGR	-0,006	1,36E-05	7,13E-03
cg06391473	2	238875301	UBE2F	TSS1500	0,013	1,35E-05	7,13E-03
cg14460963	3	99500641	COL8A1	5'UTR	-0,008	1,36E-05	7,13E-03
cg10599156	3	141145231	ZBTB38	5'UTR	0,126	1,35E-05	7,13E-03
cg24347838	3	180398017	CCDC39	TSS1500	-0,012	1,36E-05	7,13E-03
cg16777106	4	158281194	GRIA2	Body	-0,025	1,36E-05	7,13E-03
cg21754609	11	125769975	HYLS1	Body	-0,007	1,35E-05	7,13E-03
cg14175970	14	59888642		IGR	0,008	1,35E-05	7,13E-03
cg26558263	16	89961472	TCF25	Body	0,01	1,36E-05	7,13E-03
cg27423411	19	6424990	KHSRP	TSS200	-0,004	1,35E-05	7,13E-03
cg23070297	19	49844652	TEAD2	Body	0,019	1,36E-05	7,13E-03
cg04864577	21	17657869	MIR99AHG	Body	-0,009	1,35E-05	7,13E-03
cg06118507	21	41948887	DSCAM	Body	-0,012	1,36E-05	7,13E-03
cg10307507	15	64647710	CSNK1G1	5'UTR	0,006	1,36E-05	7,13E-03
cg25472174	11	55904782	OR8J3	1stExon	-0,023	1,36E-05	7,14E-03
cg16119246	1	210502114	HHAT	TSS1500	0,012	1,37E-05	7,15E-03
cg27121870	11	134257030	B3GAT1	Body	-0,006	1,37E-05	7,15E-03
cg07180355	1	16264490	SPEN	Body	0,016	1,37E-05	7,15E-03
cg11277143	2	10334372	C2orf48	Body	0,013	1,37E-05	7,15E-03
cg05058115	16	15744009	NDE1	TSS200	-0,003	1,37E-05	7,15E-03
cg06615667	9	132816229	GPR107	1stExon	-0,005	1,37E-05	7,15E-03
cg01612545	8	114419963	CSMD3	Body	-0,031	1,37E-05	7,16E-03
cg06381133	6	41747367	FRS3	5'UTR	-0,006	1,38E-05	7,16E-03
cg00282787	10	103985036	ELOVL3	TSS1500	0,018	1,38E-05	7,16E-03
cg24130284	12	3019650	TULP3	Body	0,027	1,38E-05	7,16E-03
cg27185997	15	92867665		IGR	-0,008	1,38E-05	7,16E-03
cg07302599	15	99487021	IGF1R	Body	0,04	1,38E-05	7,16E-03
cg11963753	2	178788851	PDE11A	TSS1500	0,044	1,38E-05	7,17E-03
cg19446340	4	101208932		IGR	0,01	1,38E-05	7,17E-03
cg26650480	20	4796176	RASSF2	5'UTR	0,028	1,38E-05	7,17E-03
cg14698025	10	1245418	ADARB2	Body	-0,05	1,39E-05	7,17E-03
cg08507436	11	77409695	RSF1	Body	0,028	1,39E-05	7,17E-03
cg22353329	17	77814357	CBX4	TSS1500	-0,019	1,39E-05	7,17E-03
cg24619135	7	99510609	TRIM4	Body	0,037	1,39E-05	7,19E-03
cg15251374	8	41910035	MYST3	TSS1500	-0,025	1,39E-05	7,19E-03
cg06379095	19	7735239	RETN	3'UTR	-0,007	1,39E-05	7,19E-03
cg00650877	20	39974102	LPIN3	5'UTR	0,022	1,39E-05	7,19E-03
cg26972207	11	27352501		IGR	-0,008	1,40E-05	7,21E-03
cg07816145	7	116555414	CAPZA2	Body	0,015	1,40E-05	7,22E-03
cg00210939	17	79546777	NPLOC4	Body	0,027	1,40E-05	7,22E-03
cg19751789	19	11199944	LDLR	TSS200	-0,01	1,41E-05	7,23E-03
cg22688348	2	182514316	CERKL	Body	-0,016	1,41E-05	7,24E-03
cg08468772	5	139174423	PSD2	TSS1500	0,017	1,41E-05	7,26E-03
cg12853613	8	6575798	AGPAT5	Body	-0,045	1,42E-05	7,30E-03
cg05055844	11	126275997	ST3GAL4	5'UTR	0,032	1,42E-05	7,30E-03
cg07724140	12	128642958		IGR	-0,065	1,43E-05	7,31E-03
cg01116906	13	98923476	FARP1	Body	-0,013	1,43E-05	7,32E-03
cg14998312	5	53317792	ARL15	Body	-0,011	1,44E-05	7,36E-03
cg27002185	11	2907067	CDKN1C	TSS200	0,006	1,44E-05	7,36E-03
cg00895836	13	25643450		IGR	-0,017	1,44E-05	7,36E-03
cg13276570	5	10567643	ANKRD33B	Body	0,021	1,44E-05	7,36E-03
cg26719024	10	105156444	PDCD11	1stExon	-0,005	1,44E-05	7,36E-03
cg24900225	8	116339484		IGR	-0,033	1,45E-05	7,38E-03
cg20245116	1	55522013	PCSK9	Body	-0,114	1,45E-05	7,40E-03
cg09629119	3	71547974	FOXP1	5'UTR	-0,02	1,46E-05	7,41E-03
cg11742202	6	74364667	SLC17A5	TSS1500	0,042	1,46E-05	7,41E-03
cg12402966	1	10006802	NMNAT1	5'UTR	-0,013	1,46E-05	7,42E-03
cg03267668	10	104829106	CNNM2	Body	0,025	1,47E-05	7,45E-03
cg08080287	2	175419681		IGR	0,029	1,47E-05	7,45E-03
cg16698116	1	149981734	OTUD7B	5'UTR	-0,009	1,47E-05	7,45E-03
cg09198138	6	158483047	SYNJ2	Body	-0,019	1,47E-05	7,46E-03
cg13968390	2	108904812	SULT1C2	TSS1500	-0,132	1,47E-05	7,47E-03
cg23233843	4	41878739		IGR	-0,046	1,47E-05	7,47E-03
cg22389545	2	234179183	ATG16L1	Body	-0,025	1,48E-05	7,48E-03
cg09778963	12	27032649		IGR	0,008	1,48E-05	7,50E-03
cg00799033	20	56667555		IGR	-0,008	1,48E-05	7,50E-03

cg05950923	10	93709228	BTAF1	Body	0,006	1,49E-05	7,52E-03
cg05497240	5	154199788	C5orf4	3'UTR	0,009	1,50E-05	7,55E-03
cg10883303	7	27239024	HOXA13	1stExon	-0,022	1,50E-05	7,56E-03
cg26712548	19	58176079		IGR	0,008	1,50E-05	7,56E-03
cg00022235	16	11764847	SNN	5'UTR	0,013	1,51E-05	7,62E-03
cg12410921	1	39249664		IGR	-0,054	1,52E-05	7,63E-03
cg05893865	2	157740929		IGR	-0,021	1,52E-05	7,63E-03
cg11064833	7	134361658	BPGM	Body	0,023	1,52E-05	7,63E-03
cg18139178	10	134997342	KNDC1	Body	0,016	1,52E-05	7,63E-03
cg03545677	2	47403996	CALM2	TSS1500	-0,004	1,52E-05	7,64E-03
cg08950930	16	1198408		IGR	-0,013	1,52E-05	7,64E-03
cg24349804	17	79400831	BAHCC1	Body	0,012	1,53E-05	7,64E-03
cg23404573	19	7991114	CTXN1	TSS200	0,01	1,52E-05	7,64E-03
cg25315503	21	45247038		IGR	0,025	1,52E-05	7,64E-03
cg03441844	1	161368947		IGR	0,096	1,53E-05	7,65E-03
cg08386913	8	29941090	SARAF	TSS1500	-0,029	1,53E-05	7,65E-03
cg01558777	10	85933798	C10orf99	Body	-0,01	1,53E-05	7,65E-03
cg26278080	11	67065285	ANKRD13D	Body	-0,016	1,53E-05	7,65E-03
cg09564493	16	67881212	CENPT	5'UTR	-0,005	1,53E-05	7,65E-03
cg25404433	16	79709458		IGR	-0,033	1,53E-05	7,65E-03
cg23996000	6	157721067	C6orf35	Body	-0,005	1,54E-05	7,67E-03
cg11647481	7	4781704	FOXK1	Body	-0,011	1,54E-05	7,68E-03
cg08441314	11	121446689	SORL1	Body	0,028	1,55E-05	7,70E-03
cg10968102	2	53037743		IGR	-0,015	1,55E-05	7,70E-03
cg05432848	8	75262469	GDAP1	TSS200	-0,016	1,55E-05	7,72E-03
cg19996105	1	190723988	LOC440704	Body	-0,038	1,56E-05	7,72E-03
cg05130355	7	41982337		IGR	-0,061	1,56E-05	7,72E-03
cg26464626	19	35323752		IGR	-0,016	1,56E-05	7,72E-03
cg22009923	11	832065	CD151	TSS1500	0,031	1,56E-05	7,73E-03
cg08742578	2	138721303	HNMT	TSS1500	-0,009	1,56E-05	7,73E-03
cg00652726	3	68922188	FAM19A4	Body	0,009	1,56E-05	7,73E-03
cg02349373	19	38281559		IGR	-0,078	1,56E-05	7,73E-03
cg24128226	2	55329326		IGR	0,046	1,56E-05	7,73E-03
cg00339913	8	22085227	PHYHIP	Body	-0,052	1,57E-05	7,73E-03
cg05305140	8	57233822	SDR16C5	TSS1500	-0,032	1,57E-05	7,73E-03
cg20500869	17	78422484		IGR	0,019	1,57E-05	7,73E-03
cg19203457	6	114181798	MARCKS	3'UTR	0,062	1,57E-05	7,74E-03
cg20955757	6	122931037	PKIB	TSS1500	-0,015	1,58E-05	7,74E-03
cg17420110	7	3995993	SDK1	Body	-0,055	1,58E-05	7,74E-03
cg14417372	11	119455230		IGR	0,006	1,57E-05	7,74E-03
cg20412736	12	89745410	DUSP6	Body	-0,004	1,57E-05	7,74E-03
cg12825577	16	22825740	HS3ST2	TSS200	-0,014	1,58E-05	7,74E-03
cg07509872	17	26574551	PPY2	Body	-0,015	1,58E-05	7,74E-03
cg04585822	17	72916509	USH1G	Body	-0,08	1,57E-05	7,74E-03
cg00635343	18	5193969	LOC642597	Body	-0,044	1,57E-05	7,74E-03
cg25531473	3	52810463	ITIH1	TSS1500	-0,022	1,58E-05	7,74E-03
cg13908076	14	43247814		IGR	0,043	1,58E-05	7,76E-03
cg26008643	2	25380006	EFR3B	3'UTR	0,031	1,59E-05	7,76E-03
cg09274402	7	77166647	PTPN12	TSS200	-0,013	1,59E-05	7,78E-03
cg08830379	12	81110208	MYF5	TSS1500	-0,072	1,59E-05	7,78E-03
cg19769036	13	31685197		IGR	0,015	1,60E-05	7,78E-03
cg24126567	17	74525046	CYGB	Body	0,016	1,59E-05	7,78E-03
cg22645983	18	64598632		IGR	-0,012	1,59E-05	7,78E-03
cg12005407	3	183847005		IGR	0,036	1,60E-05	7,79E-03
cg14332346	11	18548210	TSG101	Body	-0,018	1,60E-05	7,79E-03
cg24134285	7	783171	DNAAF5	Body	-0,008	1,60E-05	7,80E-03
cg27589088	16	80966033		IGR	-0,009	1,60E-05	7,80E-03
cg16056486	2	231998018	PSMD1	Body	0,01	1,61E-05	7,80E-03
cg22635088	5	2096922		IGR	-0,046	1,61E-05	7,81E-03
cg03754926	16	17195865		IGR	-0,011	1,61E-05	7,81E-03
cg18759429	5	112139326	APC	Body	0,01	1,61E-05	7,83E-03
cg25678095	8	41833374	KAT6A	Body	-0,011	1,61E-05	7,83E-03
cg22337904	8	64374723		IGR	0,006	1,62E-05	7,83E-03
cg05957546	11	7819874	OR5P2	TSS1500	-0,038	1,62E-05	7,83E-03
cg22064129	18	43194482	SLC14A2	TSS1500	-0,017	1,62E-05	7,83E-03
cg21677747	15	91180932	CRTC3	Body	0,008	1,62E-05	7,85E-03
cg19942138	4	114192607	ANK2	Body	-0,006	1,63E-05	7,86E-03
cg10778636	1	20878361	FAM43B	TSS1500	0,017	1,64E-05	7,88E-03
cg24339193	1	205399803		IGR	-0,006	1,64E-05	7,88E-03
cg12251803	2	238639781	LRRFIP1	Body	0,028	1,64E-05	7,88E-03
cg04373034	3	8546989	LMCD1	Body	-0,009	1,64E-05	7,88E-03
cg18075011	3	52537803	STAB1	Body	0,006	1,64E-05	7,88E-03
cg08984202	6	30421649		IGR	-0,006	1,64E-05	7,88E-03

cg18338863	6	114178495	MARCKS	TSS200	-0,006	1,63E-05	7,88E-03
cg07835052	7	117499847	CTTNBP2	Body	-0,017	1,64E-05	7,88E-03
cg12487162	11	12845864	TEAD1	Body	-0,063	1,64E-05	7,88E-03
cg09924998	12	122751933	VPS33A	TSS1500	0,034	1,64E-05	7,88E-03
cg12942690	12	123957029	RILPL1	3'UTR	0,008	1,64E-05	7,88E-03
cg23921860	17	4608406	PELP1	TSS1500	-0,022	1,64E-05	7,88E-03
cg00201978	22	49178443		IGR	-0,006	1,64E-05	7,88E-03
cg08228917	13	40177062	LHFP	5'UTR	-0,014	1,65E-05	7,89E-03
cg26321462	5	10540223		IGR	0,007	1,65E-05	7,89E-03
cg04343509	11	68135744	LRP5	Body	-0,014	1,65E-05	7,90E-03
cg09145990	20	46438193		IGR	0,022	1,65E-05	7,91E-03
cg15963109	17	5371657	DHX33	Body	0,011	1,66E-05	7,91E-03
cg20031401	11	86748885	TMEM135	1stExon	0,005	1,66E-05	7,92E-03
cg20509703	6	5488082	FARS2	Body	0,012	1,66E-05	7,93E-03
cg14707834	11	415457	SIGIRR	5'UTR	-0,01	1,66E-05	7,93E-03
cg01214900	4	66784395		IGR	-0,011	1,67E-05	7,93E-03
cg04863947	6	48416969		IGR	0,043	1,67E-05	7,93E-03
cg20226154	6	150346787	RAET1L	TSS200	-0,021	1,67E-05	7,94E-03
cg24633745	1	246930955	SCCPDH	3'UTR	0,008	1,67E-05	7,94E-03
cg03744743	2	96951129	SNRNP200	Body	-0,007	1,67E-05	7,94E-03
cg12990411	4	22119459		IGR	0,028	1,67E-05	7,94E-03
cg15547850	14	22457548		IGR	-0,004	1,67E-05	7,94E-03
cg01016533	16	31123892	BCKDK	3'UTR	0,013	1,67E-05	7,94E-03
cg08917208	2	24149416	ATAD2B	Body	-0,004	1,68E-05	7,95E-03
cg17671982	10	31643505	ZEB1	Body	0,047	1,68E-05	7,95E-03
cg12342694	6	137224034	PEX7	Body	0,032	1,68E-05	7,95E-03
cg23774975	11	3848848	RHOG	Body	0,032	1,68E-05	7,95E-03
cg04588708	16	811078	MSLN	TSS200	0,022	1,68E-05	7,95E-03
cg04919223	16	21973340	UQCRC2	Body	-0,017	1,68E-05	7,95E-03
cg06217494	4	156680769	GUCY1B3	Body	-0,006	1,69E-05	7,95E-03
cg04867226	7	5145248		IGR	-0,029	1,69E-05	7,95E-03
cg18371293	20	3997524	RNF24	TSS1500	0,039	1,68E-05	7,95E-03
cg12369537	12	112324408	MAPKAPK5	Body	-0,005	1,69E-05	7,96E-03
cg04550827	5	76369467		IGR	0,034	1,69E-05	7,97E-03
cg14735166	3	31949558	OSBPL10	Body	0,014	1,70E-05	7,98E-03
cg27425268	4	40469636	RBM47	5'UTR	0,005	1,70E-05	7,99E-03
cg26847878	3	48754813	IP6K2	TSS200	-0,007	1,70E-05	7,99E-03
cg16417606	2	72437509	EXOC6B	Body	-0,007	1,70E-05	8,00E-03
cg17487741	13	50107026	RCBTB1	3'UTR	0,032	1,70E-05	8,00E-03
cg11721273	1	176126003	RFWD2	Body	0,029	1,71E-05	8,01E-03
cg23185751	7	30720163	CRHR2	Body	0,012	1,71E-05	8,01E-03
cg20090301	12	77756101		IGR	-0,059	1,71E-05	8,01E-03
cg06030459	12	100986206	GAS2L3	TSS1500	0,023	1,71E-05	8,01E-03
cg09166898	15	41167165	RHOV	TSS1500	0,023	1,71E-05	8,01E-03
cg14599326	18	63648951		IGR	0,033	1,71E-05	8,01E-03
cg13425515	18	2571394	NDC80	TSS200	-0,006	1,72E-05	8,02E-03
cg24929648	1	51768832	TTC39A	Body	0,012	1,73E-05	8,02E-03
cg07378590	3	48541910	SHISA5	TSS1500	-0,006	1,73E-05	8,02E-03
cg07766803	5	2746667	IRX2	3'UTR	-0,079	1,72E-05	8,02E-03
cg22801819	7	52736648		IGR	-0,023	1,73E-05	8,02E-03
cg13544184	9	71395167	PIP5K1B	5'UTR	-0,014	1,72E-05	8,02E-03
cg02264416	11	104215127		IGR	0,056	1,73E-05	8,02E-03
cg15405572	12	78224552	NAV3	TSS1500	-0,039	1,72E-05	8,02E-03
cg12957265	15	57026014	ZNF280D	TSS1500	-0,053	1,72E-05	8,02E-03
cg25806159	19	2236785	PLEKHJ1	TSS1500	-0,006	1,73E-05	8,02E-03
cg25201673	19	44331124	ZNF283	TSS1500	-0,036	1,72E-05	8,02E-03
cg11434050	3	32185816	GPD1L	Body	-0,009	1,73E-05	8,04E-03
cg01674800	5	106603996		IGR	0,022	1,73E-05	8,04E-03
cg11907972	22	18504177	MICAL3	5'UTR	0,01	1,73E-05	8,04E-03
cg23972829	4	119507067		IGR	-0,067	1,74E-05	8,04E-03
cg01663407	2	64754308	AFTPH	5'UTR	0,018	1,74E-05	8,05E-03
cg05950192	8	1207710		IGR	-0,023	1,74E-05	8,06E-03
cg22579317	18	650558	TYMSOS	3'UTR	-0,007	1,74E-05	8,06E-03
cg23538749	2	231615143	CAB39	5'UTR	-0,023	1,75E-05	8,06E-03
cg11206788	11	133658715		IGR	-0,011	1,74E-05	8,06E-03
cg10064339	11	73693792	UCP2	1stExon	-0,005	1,75E-05	8,07E-03
cg04383836	17	9559049	USP43	Body	-0,029	1,75E-05	8,07E-03
cg27318106	5	108587200		IGR	-0,01	1,75E-05	8,08E-03
cg04902302	2	175594739		IGR	-0,022	1,76E-05	8,11E-03
cg18051433	4	137105305		IGR	-0,017	1,76E-05	8,11E-03
cg19336573	5	82696331		IGR	-0,013	1,76E-05	8,11E-03
cg14872693	11	65190203	NEAT1	TSS200	0,005	1,76E-05	8,11E-03
cg15710971	17	25621084	WSB1	TSS200	0,007	1,76E-05	8,11E-03

cg13401720	13	30790507	KATNAL1	Body	-0,008	1,77E-05	8,11E-03
cg11381211	1	146716901	CHD1L	5'UTR	0,014	1,77E-05	8,12E-03
cg24763568	1	218518565	TGFB2-AS1	Body	0,03	1,77E-05	8,13E-03
cg05106659	4	152682899	PET112L	TSS1500	-0,009	1,77E-05	8,13E-03
cg21144063	7	64035529		IGR	-0,112	1,77E-05	8,13E-03
cg24699699	1	93258316	EVI5	TSS1500	-0,018	1,78E-05	8,13E-03
cg25069618	15	81195797	CEMIP	Body	0,014	1,78E-05	8,14E-03
cg07756691	11	75239134		IGR	-0,026	1,78E-05	8,15E-03
cg06533086	5	39139105	FYB	Body	0,06	1,78E-05	8,15E-03
cg04322064	20	20213959	CFAP61	Body	0,047	1,78E-05	8,15E-03
cg02155558	15	43621948	ADAL	TSS1500	0,063	1,79E-05	8,16E-03
cg19695900	1	31671279	NKAIN1	Body	0,025	1,79E-05	8,16E-03
cg16534103	4	166300252	CPE	5'UTR	-0,007	1,80E-05	8,19E-03
cg17370714	2	53995389	CHAC2	Body	-0,004	1,81E-05	8,21E-03
cg15518542	10	1444784	ADARB2	Body	0,006	1,81E-05	8,21E-03
cg10572659	11	65565292		IGR	-0,02	1,81E-05	8,21E-03
cg19894076	12	101187957	ANO4	TSS1500	-0,034	1,81E-05	8,21E-03
cg13562917	15	90776873	CIB1	Body	-0,005	1,81E-05	8,21E-03
cg10822545	19	13985513	MIR181D	TSS200	0,014	1,80E-05	8,21E-03
cg10502892	8	144499683	MAFA-AS1	TSS200	0,023	1,81E-05	8,22E-03
cg07446376	1	3263569	PRDM16	Body	0,013	1,82E-05	8,22E-03
cg10292915	11	6790768	OR2AG2	TSS1500	-0,009	1,81E-05	8,22E-03
cg03479403	17	18149067	FLII	ExonBnd	0,015	1,81E-05	8,22E-03
cg01980174	18	29027729	DSG3	TSS200	-0,034	1,82E-05	8,22E-03
cg25813942	18	2843518		IGR	0,046	1,82E-05	8,25E-03
cg13960778	2	124440686		IGR	-0,018	1,83E-05	8,25E-03
cg02965180	7	94285784	SGCE	TSS1500	0,012	1,83E-05	8,25E-03
cg13116789	12	111883369	SH2B3	Body	0,014	1,83E-05	8,25E-03
cg27168444	18	3126670	MYOM1	Body	0,022	1,83E-05	8,25E-03
cg11858843	22	30181185		IGR	0,006	1,82E-05	8,25E-03
cg24915468	3	98215011		IGR	0,029	1,83E-05	8,25E-03
cg05926110	15	94443587	LINC01580	TSS1500	-0,01	1,84E-05	8,26E-03
cg06593910	16	88709927	CYBA	Body	0,01	1,83E-05	8,26E-03
cg17976205	20	61002857	C20orf151	TSS1500	0,044	1,83E-05	8,26E-03
cg15124400	9	124989839	LHX6	1stExon	-0,213	1,85E-05	8,33E-03
cg09553058	12	92539873	BTG1	TSS1500	-0,006	1,86E-05	8,34E-03
cg02953927	15	44038812	PDIA3	1stExon	0,031	1,86E-05	8,34E-03
cg03955365	3	171490386	PLD1	5'UTR	-0,017	1,87E-05	8,34E-03
cg10823173	5	2633471		IGR	-0,017	1,86E-05	8,34E-03
cg01950524	5	38135043		IGR	-0,011	1,86E-05	8,34E-03
cg05170275	6	106534146	PRDM1	TSS200	-0,007	1,86E-05	8,34E-03
cg18652371	12	123718111	MPHOSPH9	TSS1500	-0,004	1,87E-05	8,34E-03
cg06438489	15	86244167	AKAP13	Body	0,023	1,86E-05	8,34E-03
cg03325767	17	80279477	SECTM1	3'UTR	0,01	1,87E-05	8,34E-03
cg03982507	18	44496911	PIAS2	Body	-0,003	1,87E-05	8,34E-03
cg07688434	19	30448200	URI1	Body	0,019	1,87E-05	8,34E-03
cg13584095	20	44827392	CDH22	Body	-0,023	1,87E-05	8,34E-03
cg26163253	2	235130234		IGR	-0,006	1,87E-05	8,34E-03
cg16243197	1	182807962	DHX9	TSS1500	0,027	1,87E-05	8,35E-03
cg26770470	2	165352228	GRB14	Body	0,013	1,88E-05	8,35E-03
cg13878093	6	134274457	TBPL1	1stExon	-0,005	1,88E-05	8,35E-03
cg04960492	7	7111213		IGR	-0,02	1,88E-05	8,35E-03
cg08411443	11	43958160		IGR	0,017	1,88E-05	8,35E-03
cg16136385	11	46848389	CKAP5	5'UTR	0,016	1,88E-05	8,35E-03
cg04223548	13	22247214	FGF9	Body	-0,049	1,88E-05	8,35E-03
cg13489987	14	67292488	GPHN	Body	-0,03	1,88E-05	8,35E-03
cg09661437	15	52960246	FAM214A	Body	-0,018	1,88E-05	8,35E-03
cg10109421	16	52641824		IGR	-0,025	1,88E-05	8,35E-03
cg21900035	3	197602481		IGR	0,013	1,89E-05	8,36E-03
cg12903911	7	155170812		IGR	-0,02	1,89E-05	8,36E-03
cg11763723	15	75971951	CSPG4	Body	0,007	1,89E-05	8,36E-03
cg05069228	3	61793623	PTPRG	Body	-0,021	1,89E-05	8,37E-03
cg24180480	17	79852289	ANAPC11	5'UTR	0,016	1,89E-05	8,37E-03
cg16331482	2	240428335		IGR	-0,008	1,90E-05	8,41E-03
cg24586758	5	140480200	PCDHB3	TSS200	-0,051	1,91E-05	8,41E-03
cg09053087	2	239748358		IGR	-0,009	1,91E-05	8,41E-03
cg13107884	1	227625934		IGR	0,023	1,91E-05	8,42E-03
cg10042938	2	198158674	ANKRD44-IT1	Body	-0,015	1,91E-05	8,42E-03
cg18172411	10	120485687	CACUL1	Body	-0,006	1,91E-05	8,42E-03
cg13745645	15	29862189	FAM189A1	Body	-0,007	1,91E-05	8,42E-03
cg21132229	17	40757728	FAM134C	Body	-0,01	1,91E-05	8,42E-03
cg15288816	2	110418247		IGR	-0,017	1,92E-05	8,43E-03
cg27311970	1	54195830	GLIS1	5'UTR	0,005	1,92E-05	8,43E-03

cg00909668	8	141582617	AGO2	Body	-0,037	1,92E-05	8,43E-03
cg23984228	11	12562564		IGR	-0,018	1,92E-05	8,43E-03
cg11214507	1	210419938		IGR	-0,008	1,93E-05	8,44E-03
cg17678877	2	105654366	MRPS9	TSS200	-0,008	1,93E-05	8,44E-03
cg08677932	3	167967462	C3orf50	Body	-0,007	1,93E-05	8,44E-03
cg01967637	5	142784019	NR3C1	5'UTR	0,014	1,94E-05	8,44E-03
cg13847825	5	176513311	FGFR4	TSS1500	0,024	1,94E-05	8,44E-03
cg03328393	7	1610694	PSMG3	TSS1500	0,04	1,93E-05	8,44E-03
cg21926039	7	134331413	BPGM	TSS200	-0,006	1,92E-05	8,44E-03
cg12580801	8	67379873	ADHFE1	Body	-0,006	1,93E-05	8,44E-03
cg03463446	10	127990580	ADAM12	Body	-0,013	1,94E-05	8,44E-03
cg26056477	12	76963168		IGR	0,029	1,93E-05	8,44E-03
cg26740481	15	23931011	NDN	3'UTR	-0,023	1,93E-05	8,44E-03
cg16578496	19	4311154	FSD1	Body	0,01	1,93E-05	8,44E-03
cg19340420	19	50037079	RCN3	Body	-0,008	1,94E-05	8,44E-03
cg11384188	6	166797300	BRP44L	TSS1500	-0,003	1,94E-05	8,46E-03
cg05932980	13	103495884	BIVM-ERCC5	Body	-0,019	1,95E-05	8,47E-03
cg19948167	13	55819841		IGR	0,039	1,95E-05	8,50E-03
cg20073050	4	106395643	PPA2	TSS1500	0,038	1,96E-05	8,52E-03
cg15961904	8	1379994		IGR	-0,012	1,96E-05	8,52E-03
cg25021969	6	81175499		IGR	-0,032	1,97E-05	8,54E-03
cg15735078	2	226445764	KIAA1486	Body	-0,01	1,98E-05	8,58E-03
cg15909951	2	242743702		IGR	-0,006	1,98E-05	8,58E-03
cg03660010	4	48342263	SLAIN2	TSS1500	-0,01	1,98E-05	8,58E-03
cg17187705	13	35735300	NBEA	Body	-0,018	1,99E-05	8,60E-03
cg02342892	6	29431208	OR2H1	3'UTR	-0,01	2,00E-05	8,64E-03
cg01646718	8	145580727	FBXL6	Body	0,008	2,00E-05	8,64E-03
cg22065884	11	63685188	RCOR2	TSS1500	-0,007	2,00E-05	8,64E-03
cg06745695	11	68519060	MTL5	TSS200	-0,009	2,00E-05	8,64E-03
cg02386293	11	69523781		IGR	0,037	2,00E-05	8,64E-03
cg26758338	19	37382272	ZNF829	3'UTR	0,012	2,00E-05	8,64E-03
cg14238757	20	46705449		IGR	0,014	2,00E-05	8,64E-03
cg13315923	3	171921695	FNDC3B	Body	0,017	2,01E-05	8,68E-03
cg06663310	5	176153056		IGR	0,045	2,01E-05	8,68E-03
cg02612782	3	33454240	UBP1	Body	-0,005	2,02E-05	8,69E-03
cg05047569	22	41716384	ZC3H7B	5'UTR	0,017	2,02E-05	8,69E-03
cg06063517	11	575810	LOC143666	Body	-0,004	2,02E-05	8,70E-03
cg16629749	13	112631042		IGR	-0,03	2,02E-05	8,70E-03
cg26570254	7	76983221	GSAP	Body	0,042	2,03E-05	8,72E-03
cg20641865	10	113744016		IGR	-0,056	2,03E-05	8,72E-03
cg19683073	5	79553267	SERINC5	TSS1500	0,037	2,03E-05	8,72E-03
cg21861192	6	34214912	C6orf1	Body	0,01	2,04E-05	8,72E-03
cg13490403	9	124982413	LHX6	Body	0,062	2,04E-05	8,72E-03
cg07088388	11	109816936		IGR	-0,017	2,04E-05	8,73E-03
cg10095517	7	6916089		IGR	-0,05	2,04E-05	8,73E-03
cg26510945	19	39305100	LGALS4	TSS1500	-0,011	2,04E-05	8,73E-03
cg04519671	13	61228741		IGR	-0,06	2,04E-05	8,74E-03
cg10494844	9	131181156	CERCAM	TSS1500	-0,024	2,05E-05	8,74E-03
cg04775668	2	127535185		IGR	-0,003	2,05E-05	8,76E-03
cg00963786	16	69830352	WWP2	Body	-0,009	2,05E-05	8,76E-03
cg16612423	5	6491884	UBE2QL1	3'UTR	0,005	2,05E-05	8,77E-03
cg23197837	17	79246549	SLC38A10	Body	0,078	2,06E-05	8,80E-03
cg11416379	2	61200075	PUS10	Body	0,008	2,07E-05	8,81E-03
cg21123833	5	138180774	CTNNA1	Body	-0,008	2,07E-05	8,82E-03
cg07805777	11	15962932		IGR	-0,047	2,08E-05	8,84E-03
cg04117480	1	120439522	ADAM30	TSS1500	-0,009	2,08E-05	8,85E-03
cg24134141	1	156781800	SH2D2A	Body	0,023	2,08E-05	8,86E-03
cg24125134	2	241930655		IGR	0,018	2,08E-05	8,86E-03
cg10272857	1	217695788	GPATCH2	Body	0,026	2,09E-05	8,86E-03
cg05859039	4	103746835	UBE2D3	Body	-0,023	2,09E-05	8,86E-03
cg02553537	5	140682911	SLC25A2	1stExon	-0,019	2,09E-05	8,87E-03
cg27191184	16	580239	SOLH	5'UTR	0,029	2,09E-05	8,87E-03
cg11876191	15	99321381	IGF1R	Body	-0,011	2,09E-05	8,87E-03
cg20220034	6	94157674		IGR	0,048	2,10E-05	8,89E-03
cg27052575	3	105553809	CBLB	Body	0,008	2,10E-05	8,89E-03
cg00843326	5	63800306		IGR	-0,032	2,10E-05	8,89E-03
cg10171609	10	5405573	UCN3	TSS1500	-0,009	2,10E-05	8,89E-03
cg13148429	18	46688124	DYM	Body	-0,017	2,12E-05	8,93E-03
cg06757585	5	140753531	PCDHGA4	Body	-0,054	2,12E-05	8,94E-03
cg22785136	6	117609650	ROS1	3'UTR	0,02	2,12E-05	8,95E-03
cg08497311	16	88806610	FAM38A	Body	0,015	2,12E-05	8,95E-03
cg26390512	6	6714166		IGR	-0,024	2,12E-05	8,95E-03
cg07372023	5	148550001	ABLIM3	5'UTR	-0,012	2,13E-05	8,95E-03

cg20548043	8	104426639	DCAF13	TSS1500	-0,01	2,13E-05	8,96E-03
cg21718826	16	4742732	NUDT16L1	TSS1500	0,024	2,13E-05	8,96E-03
cg25689167	2	8500033		IGR	0,008	2,13E-05	8,97E-03
cg27463004	22	39716675	RPL3	TSS1500	-0,021	2,13E-05	8,97E-03
cg15613067	2	242053881	PASK	Body	-0,007	2,14E-05	8,99E-03
cg01774878	12	122063005	ORAI1	TSS1500	0,033	2,14E-05	8,99E-03
cg19343233	5	54397868	GZMA	TSS1500	-0,005	2,15E-05	9,01E-03
cg14815086	15	68492786	CALML4	Body	0,008	2,15E-05	9,01E-03
cg10326891	4	88343821	NUDT9	1stExon	0,015	2,15E-05	9,01E-03
cg14777341	17	78992323		IGR	0,008	2,16E-05	9,03E-03
cg18219904	18	77018833	ATP9B	Body	0,015	2,16E-05	9,04E-03
cg23286079	5	56512693	GPBP1	Body	0,025	2,17E-05	9,04E-03
cg14468658	5	140723509	PCDHGA2	Body	-0,046	2,17E-05	9,04E-03
cg20715606	6	56679248	DST	Body	0,039	2,16E-05	9,04E-03
cg17849626	6	88049878	SMIM8	Body	0,015	2,17E-05	9,04E-03
cg26928720	8	26375300	DPYSL2	Body	-0,022	2,16E-05	9,04E-03
cg21585845	12	72329516		IGR	-0,041	2,17E-05	9,04E-03
cg25275308	15	94443609	LINC01580	TSS1500	-0,018	2,17E-05	9,04E-03
cg03493083	17	10762190		IGR	-0,008	2,17E-05	9,04E-03
cg04722168	1	26324647	PAFAH2	5'UTR	-0,003	2,17E-05	9,05E-03
cg16277169	12	115112189	TBX3	Body	-0,055	2,18E-05	9,06E-03
cg21105875	1	202778869	KDM5B	TSS1500	0,055	2,19E-05	9,10E-03
cg18674306	12	6483152	LTBR	TSS1500	0,019	2,19E-05	9,10E-03
cg05820435	1	19701756	CAPZB	Body	0,014	2,21E-05	9,12E-03
cg08544271	2	41944895		IGR	0,036	2,21E-05	9,12E-03
cg06813824	2	96511628		IGR	-0,04	2,21E-05	9,12E-03
cg25838818	2	108905173	SULT1C2	5'UTR	-0,107	2,21E-05	9,12E-03
cg04914600	3	43248410		IGR	0,006	2,20E-05	9,12E-03
cg12356120	3	135870113	MSL2	Body	-0,004	2,21E-05	9,12E-03
cg06211378	3	150140268	TSC22D2	Body	0,018	2,21E-05	9,12E-03
cg03060468	5	140683196	SLC25A2	1stExon	-0,056	2,20E-05	9,12E-03
cg04026699	6	28834513		IGR	0,019	2,21E-05	9,12E-03
cg06680906	15	69325560	MIR548H4	Body	-0,072	2,20E-05	9,12E-03
cg01928867	17	807384	NXN	Body	-0,052	2,21E-05	9,12E-03
cg18493449	17	74467972	RHBDF2	Body	0,009	2,20E-05	9,12E-03
cg05321495	19	34167989	CHST8	5'UTR	0,063	2,20E-05	9,12E-03
cg18356679	6	126068737		IGR	-0,023	2,21E-05	9,12E-03
cg02074878	12	10961294		IGR	-0,009	2,21E-05	9,12E-03
cg02477974	14	20389177	OR4K5	1stExon	-0,014	2,21E-05	9,12E-03
cg21523680	12	118451332		IGR	-0,008	2,22E-05	9,13E-03
cg03815900	17	7846788	CNTROB	Body	-0,006	2,22E-05	9,13E-03
cg18350885	4	158623024		IGR	-0,044	2,22E-05	9,13E-03
cg22841336	12	57040011	ATP5B	TSS200	-0,005	2,22E-05	9,14E-03
cg04660829	4	1505559		IGR	0,011	2,23E-05	9,17E-03
cg13537510	6	29623721	MOG	TSS1500	0,011	2,23E-05	9,17E-03
cg25756044	13	111340191	CARS2	Body	-0,016	2,23E-05	9,17E-03
cg24446178	12	100750702	SLC17A8	TSS200	0,039	2,24E-05	9,17E-03
cg09046813	22	37172713	RABL4	TSS1500	0,01	2,24E-05	9,17E-03
cg17872753	1	159044095	AIM2	5'UTR	0,045	2,25E-05	9,20E-03
cg06118478	12	48389984	COL2A1	Body	-0,013	2,25E-05	9,21E-03
cg07373946	16	7563521	RBFOX1	Body	-0,029	2,25E-05	9,21E-03
cg10992704	1	162795932		IGR	-0,02	2,25E-05	9,21E-03
cg03767653	8	144983293		IGR	-0,006	2,26E-05	9,23E-03
cg22069341	14	101467596		IGR	-0,01	2,26E-05	9,23E-03
cg04895936	10	98832719	SLIT1	Body	0,009	2,26E-05	9,23E-03
cg06562029	10	94000570	CPEB3	5'UTR	-0,005	2,27E-05	9,25E-03
cg06157539	18	32621265	MAPRE2	TSS200	-0,005	2,27E-05	9,25E-03
cg25554042	3	50230878	GNAT1	Body	-0,058	2,27E-05	9,26E-03
cg02557269	7	54826467	SEC61G	5'UTR	-0,012	2,28E-05	9,26E-03
cg16445596	16	90075204	DBNDD1	Body	0,017	2,28E-05	9,26E-03
cg04494602	17	62982045		IGR	-0,004	2,27E-05	9,26E-03
cg04783231	19	57050834	ZFP28	Body	0,034	2,28E-05	9,26E-03
cg09870910	1	1222942	SCNN1D	Body	0,015	2,28E-05	9,27E-03
cg04047016	18	43306852	SLC14A1	5'UTR	0,033	2,28E-05	9,27E-03
cg07238832	11	27681475	BDNF	Body	-0,012	2,28E-05	9,27E-03
cg05307228	3	40085665	MYRIP	Body	-0,008	2,29E-05	9,29E-03
cg16123062	4	170150924	SH3RF1	Body	-0,024	2,29E-05	9,29E-03
cg02590614	11	130343077	ADAMTS15	Body	-0,02	2,29E-05	9,29E-03
cg21386414	1	26421349		IGR	0,059	2,30E-05	9,32E-03
cg14863944	13	22740131		IGR	-0,011	2,30E-05	9,33E-03
cg11096857	14	24563257	PCK2	TSS1500	-0,005	2,31E-05	9,33E-03
cg24866110	22	21216897	SNAP29	Body	0,015	2,30E-05	9,33E-03
cg11912306	19	48248602	GLTSCR2	TSS200	0,006	2,31E-05	9,34E-03

cg18796287	5	138730886	LOC389333	TSS200	-0,004	2,31E-05	9,35E-03
cg25978374	8	144658337	NAPRT	Body	0,012	2,31E-05	9,35E-03
cg09064570	4	151462241	LRBA	Body	0,02	2,32E-05	9,37E-03
cg02390588	12	53722253	SP7	Body	-0,017	2,32E-05	9,37E-03
cg04811540	7	1351903		IGR	-0,028	2,32E-05	9,37E-03
cg10306866	5	107716890	FBXL17	1stExon	-0,005	2,33E-05	9,37E-03
cg20001666	1	20990821	KIF17	3'UTR	-0,022	2,33E-05	9,38E-03
cg11465897	5	147043855	JAKMIP2	Body	0,012	2,33E-05	9,38E-03
cg01739845	15	77989284		IGR	-0,036	2,33E-05	9,39E-03
cg05161243	19	47257181	FKRP	5'UTR	-0,039	2,33E-05	9,39E-03
cg08253842	1	1316981		IGR	-0,009	2,34E-05	9,40E-03
cg14645995	11	8703877	RPL27A	TSS200	-0,007	2,34E-05	9,40E-03
cg16003659	11	43575929		IGR	-0,055	2,34E-05	9,40E-03
cg12177551	11	65190180	NEAT1	TSS200	0,011	2,34E-05	9,40E-03
cg01707469	15	32935693	SCG5	5'UTR	-0,008	2,34E-05	9,40E-03
cg07893836	2	104486874		IGR	-0,02	2,35E-05	9,40E-03
cg20433989	13	49683801	FNDC3A	Body	0,027	2,35E-05	9,40E-03
cg00864916	1	180886483	KIAA1614	Body	-0,1	2,35E-05	9,41E-03
cg05641932	9	124984031	LHX6	TSS200	0,041	2,35E-05	9,42E-03
cg13166025	1	18506652	IGSF21	Body	0,011	2,36E-05	9,42E-03
cg04167299	10	52611821	A1CF	Body	-0,015	2,36E-05	9,42E-03
cg14844624	18	44100475	LOXHD1	TSS1500	-0,015	2,36E-05	9,42E-03
cg21436055	1	227746111		IGR	-0,048	2,37E-05	9,44E-03
cg08405984	7	70111413	AUTS2	Body	0,007	2,37E-05	9,44E-03
cg20001215	9	5833864	ERMP1	TSS1500	-0,008	2,36E-05	9,44E-03
cg06553975	12	133085262	FBRSL1	Body	0,009	2,37E-05	9,44E-03
cg24153199	14	50698328	SOS2	TSS1500	-0,005	2,37E-05	9,44E-03
cg25118545	21	29055722	MIR5009	Body	-0,016	2,37E-05	9,44E-03
cg06427759	5	115419633	COMMD10	TSS1500	0,015	2,38E-05	9,45E-03
cg17095762	16	84050535	SLC38A8	Body	-0,037	2,38E-05	9,45E-03
cg16695714	6	11763786	C6orf105	Body	-0,011	2,38E-05	9,48E-03
cg20324199	9	96080326	WNK2	Body	-0,062	2,39E-05	9,48E-03
cg15205546	3	21316168		IGR	-0,017	2,39E-05	9,49E-03
cg18518722	22	20786717	SCARF2	Body	0,018	2,39E-05	9,49E-03
cg12278684	19	43100413	CEACAM8	TSS1500	0,009	2,39E-05	9,49E-03
cg01914991	3	132136325	DNAJC13	TSS1500	-0,003	2,40E-05	9,50E-03
cg08562215	2	241725902	KIF1A	Body	0,007	2,41E-05	9,53E-03
cg17995967	3	129275655	PLXND1	Body	0,006	2,41E-05	9,53E-03
cg07901857	4	35464028		IGR	-0,104	2,41E-05	9,53E-03
cg22701361	10	116471259		IGR	-0,008	2,41E-05	9,53E-03
cg19483696	22	37935280		IGR	0,01	2,41E-05	9,53E-03
cg08547200	6	144284774	PLAGL1	5'UTR	-0,006	2,41E-05	9,53E-03
cg26848957	17	70507711		IGR	-0,023	2,41E-05	9,53E-03
cg14438363	9	114391251		IGR	-0,01	2,42E-05	9,54E-03
cg01936977	16	1664571	CRAMP1L	TSS200	-0,005	2,42E-05	9,54E-03
cg04285727	1	3350440	PRDM16	3'UTR	-0,013	2,42E-05	9,54E-03
cg14684554	10	99394140	MORN4	TSS1500	-0,014	2,42E-05	9,54E-03
cg00643076	18	77948950	PARD6G	Body	0,014	2,43E-05	9,57E-03
cg21694330	3	96013039		IGR	-0,015	2,43E-05	9,57E-03
cg04066178	3	141524333	GRK7	Body	0,024	2,44E-05	9,57E-03
cg27166718	6	118880109	PLN	Body	-0,017	2,44E-05	9,57E-03
cg26031047	8	127570723	FAM84B	TSS1500	-0,005	2,43E-05	9,57E-03
cg26891088	19	36757635		IGR	-0,049	2,44E-05	9,57E-03
cg24070213	2	121070622		IGR	0,023	2,44E-05	9,58E-03
cg17075102	5	176504763	ZNF346	Body	-0,005	2,44E-05	9,60E-03
cg01858828	6	14921628		IGR	-0,04	2,45E-05	9,61E-03
cg05128386	16	47409367	ITFG1	Body	0,048	2,45E-05	9,62E-03
cg08339887	20	419393	TBC1D20	Body	-0,004	2,46E-05	9,64E-03
cg27284288	11	60739005	CD6	TSS200	-0,005	2,46E-05	9,65E-03
cg08261620	11	112484541		IGR	-0,017	2,46E-05	9,65E-03
cg07358136	13	113007810		IGR	0,01	2,47E-05	9,66E-03
cg17778556	2	224773702	WDFY1	Body	0,056	2,47E-05	9,67E-03
cg18135341	6	137341992	IL20RA	Body	0,026	2,48E-05	9,67E-03
cg16736827	7	129142266	SMKR1	TSS200	0,009	2,48E-05	9,67E-03
cg19287297	8	127652045		IGR	-0,006	2,48E-05	9,67E-03
cg18388840	11	100490454		IGR	0,042	2,48E-05	9,67E-03
cg13081526	6	32449961		IGR	-0,172	2,48E-05	9,68E-03
cg23034245	12	102134031	SYCP3	TSS1500	0,023	2,48E-05	9,68E-03
cg09674093	2	23646462	KLHL29	5'UTR	0,019	2,49E-05	9,69E-03
cg07551022	1	205760086	SLC41A1	3'UTR	-0,006	2,49E-05	9,70E-03
cg26950898	6	32164380	GPSM3	TSS1500	-0,006	2,49E-05	9,70E-03
cg03394683	15	77927954	LINGO1	5'UTR	0,015	2,49E-05	9,70E-03
cg17792144	1	220701757	MARK1	5'UTR	-0,005	2,50E-05	9,70E-03

cg07808302	2	208714745	PLEKHM3	Body	-0,022	2,51E-05	9,70E-03
cg12515314	3	169379266	MECOM	5'UTR	-0,004	2,51E-05	9,70E-03
cg26210267	4	668877	ATP5I	TSS1500	0,021	2,50E-05	9,70E-03
cg00179663	4	92189705	FAM190A	Body	0,02	2,50E-05	9,70E-03
cg02232340	6	85377714		IGR	-0,03	2,50E-05	9,70E-03
cg11689994	10	63887754		IGR	-0,026	2,50E-05	9,70E-03
cg11076280	17	64733766	PRKCA	Body	-0,069	2,50E-05	9,70E-03
cg11468012	19	1065809	HMHA1	TSS1500	0,008	2,50E-05	9,70E-03
cg04332442	22	46770063	CELSR1	Body	-0,008	2,50E-05	9,70E-03
cg14885187	6	47445511	CD2AP	TSS200	0,007	2,51E-05	9,71E-03
cg15794873	10	127059374		IGR	-0,052	2,51E-05	9,71E-03
cg18190746	15	72337075	MYO9A	Body	-0,005	2,51E-05	9,71E-03
cg09180903	4	170506612	NEK1	Body	0,013	2,52E-05	9,74E-03
cg13963658	22	50697503	MAPK12	Body	0,019	2,52E-05	9,74E-03
cg12911915	11	1588478	DUSP8	5'UTR	0,016	2,53E-05	9,75E-03
cg22502704	11	46137523	PHF21A	5'UTR	-0,018	2,53E-05	9,75E-03
cg25698089	15	65321903	MTFMT	1stExon	-0,006	2,53E-05	9,76E-03
cg05199331	17	2740851	RAP1GAP2	Body	0,026	2,54E-05	9,76E-03
cg25141766	17	79826316	ARHGDIA	3'UTR	0,018	2,54E-05	9,76E-03
cg13499300	19	54369556	MYADM	TSS200	-0,01	2,54E-05	9,76E-03
cg12284761	2	103216004		IGR	-0,01	2,54E-05	9,78E-03
cg22409558	7	79082802	MAGI2-AS3	TSS1500	-0,015	2,54E-05	9,78E-03
cg22507989	22	21356069	FLJ39582	TSS200	0,004	2,55E-05	9,79E-03
cg06931515	1	67012301	SGIP1	Body	-0,012	2,55E-05	9,79E-03
cg27358947	10	97619195	ENTPD1	Body	0,029	2,55E-05	9,80E-03
cg27444414	1	184122874		IGR	0,011	2,56E-05	9,80E-03
cg03689130	3	8613360	LINC00312	TSS200	0,008	2,56E-05	9,80E-03
cg14229264	3	24130128		IGR	-0,052	2,56E-05	9,80E-03
cg16508957	7	29415903	CHN2	Body	0,017	2,56E-05	9,80E-03
cg23795953	11	119600400	PVRL1	TSS1500	-0,005	2,56E-05	9,80E-03
cg14398214	17	56493260	RNF43	5'UTR	0,009	2,56E-05	9,80E-03
cg18092363	18	77202678	NFATC1	Body	0,01	2,56E-05	9,80E-03
cg00050402	7	55073022		IGR	-0,095	2,57E-05	9,80E-03
cg08169311	11	69706622		IGR	0,049	2,57E-05	9,80E-03
cg14900773	17	80935431	B3GNTL1	Body	0,018	2,57E-05	9,80E-03
cg11754420	13	112979874		IGR	-0,062	2,57E-05	9,81E-03
cg17412901	1	3332705	PRDM16	Body	0,02	2,58E-05	9,85E-03
cg06726155	2	139914003		IGR	-0,014	2,58E-05	9,85E-03
cg26539818	17	17109691	PLD6	TSS200	-0,056	2,60E-05	9,90E-03
cg16330066	4	8399372	ACOX3	Body	0,009	2,60E-05	9,91E-03
cg09861034	2	204608894		IGR	-0,008	2,61E-05	9,92E-03
cg21778229	4	125860482		IGR	0,017	2,61E-05	9,92E-03
cg02973156	12	127544951		IGR	-0,024	2,61E-05	9,92E-03
cg23769193	14	72914835	RGS6	Body	-0,009	2,61E-05	9,92E-03
cg04679515	5	62113067		IGR	-0,037	2,62E-05	9,94E-03
cg08665844	4	113485177	C4orf21	Body	0,05	2,62E-05	9,95E-03
cg17836499	8	29953066	LEPROTL1	Body	-0,004	2,62E-05	9,95E-03
cg12580847	5	39425450	DAB2	TSS200	-0,006	2,63E-05	9,95E-03
cg14514704	6	170713208	FAM120B	Body	-0,024	2,63E-05	9,95E-03
cg11545635	1	85610457		IGR	-0,018	2,63E-05	9,96E-03
cg11568498	11	63719508	NAA40	Body	-0,007	2,64E-05	9,97E-03
cg13281312	12	77719842		IGR	-0,111	2,63E-05	9,97E-03
cg23171477	2	135922344	RAB3GAP1	Body	-0,008	2,64E-05	9,98E-03
cg03135515	5	172483116	C5orf41	TSS1500	-0,003	2,64E-05	9,98E-03
cg04720886	7	148035641	CNTNAP2	Body	-0,043	2,65E-05	9,98E-03
cg25579620	13	88840548	LOC105370306	Body	0,023	2,64E-05	9,98E-03
cg07725204	14	40297066		IGR	-0,028	2,64E-05	9,98E-03
cg04187820	18	72732056	ZNF407	Body	-0,006	2,64E-05	9,98E-03
cg12832418	19	55625129	PPP1R12C	Body	0,021	2,65E-05	9,98E-03
cg02948259	22	24988020	C22orf36	Body	0,019	2,65E-05	9,98E-03
cg15819846	19	17372056	USHBP1	Body	0,015	2,65E-05	9,98E-03
cg03717277	3	12525802	TSEN2	TSS1500	-0,005	2,66E-05	9,98E-03
cg11411884	4	1016333	FGFRL1	Body	0,008	2,66E-05	9,98E-03
cg08981228	6	101840370		IGR	-0,014	2,66E-05	9,98E-03
cg26951958	8	9912981	MSRA	Body	-0,003	2,66E-05	9,98E-03
cg15733810	14	67819734	ATP6V1D	Body	0,041	2,66E-05	9,98E-03
cg13784796	15	37190998	MEIS2	Body	-0,037	2,65E-05	9,98E-03
cg02233076	22	46283680		IGR	0,02	2,65E-05	9,98E-03
cg23677882	7	157281167		IGR	-0,017	2,66E-05	9,98E-03
cg22181201	11	76571587	ACER3	TSS1500	-0,042	2,66E-05	9,98E-03
cg04890237	1	2537585	MMEL1	Body	-0,017	2,67E-05	9,99E-03
cg11948800	20	62186566	C20orf195	5'UTR	0,013	2,67E-05	9,99E-03
cg26296878	16	89284452	ZNF778	5'UTR	-0,006	2,67E-05	1,00E-02

cg12384861	2	201875008	FAM126B	Body	0,015	2,68E-05	1,00E-02
cg07604117	6	152702365	SYNE1	Body	-0,011	2,68E-05	1,00E-02
cg15784997	19	3196307	NCLN	Body	-0,008	2,68E-05	1,00E-02
cg14638957	3	196218787	RNF168	Body	-0,007	2,68E-05	1,00E-02
cg04354433	6	32256343		IGR	-0,015	2,68E-05	1,00E-02
cg24839145	8	5028854		IGR	-0,01	2,68E-05	1,00E-02
cg00817900	10	22519527		IGR	-0,009	2,68E-05	1,00E-02
cg08564027	20	61660810		IGR	-0,135	2,68E-05	1,00E-02
cg19087530	8	69607247	C8orf34	Body	0,033	2,69E-05	1,00E-02
cg07059889	4	168230933		IGR	0,053	2,69E-05	1,00E-02
cg07735163	13	30975916		IGR	-0,006	2,69E-05	1,00E-02
cg17269277	4	1796142	FGFR3	Body	-0,008	2,70E-05	1,00E-02
cg16904399	10	22902615	PIP4K2A	Body	0,01	2,70E-05	1,00E-02
cg13142917	11	7749398		IGR	0,046	2,70E-05	1,00E-02
cg24035682	12	121958802	KDM2B	Body	0,009	2,70E-05	1,00E-02
cg18623022	6	113468914		IGR	-0,011	2,70E-05	1,00E-02
cg25578609	17	5974066	WSCD1	5'UTR	-0,006	2,71E-05	1,01E-02
cg09944427	4	157496734		IGR	-0,01	2,72E-05	1,01E-02
cg15279475	2	152688701		IGR	0,023	2,73E-05	1,01E-02
cg12998385	4	88444606	SPARCL1	5'UTR	0,025	2,73E-05	1,01E-02
cg18405915	4	96345480	UNC5C	Body	-0,009	2,73E-05	1,01E-02
cg20861881	11	94473439		IGR	-0,006	2,73E-05	1,01E-02
cg24084871	12	52428202	NR4A1	5'UTR	0,011	2,73E-05	1,01E-02
cg27574786	1	89990235	LRRC8B	TSS200	-0,004	2,74E-05	1,01E-02
cg09161320	13	98330268		IGR	-0,009	2,74E-05	1,01E-02
cg05735955	19	11087842	SMARCA4	5'UTR	0,014	2,75E-05	1,01E-02
cg27239147	3	50388469	TUSC4	5'UTR	-0,004	2,76E-05	1,02E-02
cg02891314	5	179741120	GFPT2	Body	-0,209	2,76E-05	1,02E-02
cg17515844	6	158244686	SNX9	Body	-0,004	2,76E-05	1,02E-02
cg12682916	5	140420926		IGR	-0,007	2,77E-05	1,02E-02
cg05615443	13	47012103		IGR	-0,031	2,77E-05	1,02E-02
cg11132443	3	130569560	ATP2C1	5'UTR	-0,005	2,78E-05	1,02E-02
cg05093728	2	129069094	HS6ST1	Body	-0,008	2,78E-05	1,02E-02
cg22827465	19	3094947	GNA11	Body	0,013	2,78E-05	1,02E-02
cg22116842	8	60671550		IGR	0,025	2,78E-05	1,02E-02
cg15153770	6	33235079	VPS52	Body	0,004	2,78E-05	1,02E-02
cg09466397	16	4749247	ANKS3	Body	0,019	2,79E-05	1,02E-02
cg17015290	20	36850842	KIAA1755	ExonBnd	0,027	2,79E-05	1,02E-02
cg23511816	2	225137328		IGR	-0,022	2,79E-05	1,02E-02
cg00261832	6	132270033	CTGF	3'UTR	-0,012	2,79E-05	1,02E-02
cg27095467	16	27643239	KIAA0556	Body	-0,009	2,79E-05	1,02E-02
cg26332213	22	26959102	TPST2	5'UTR	0,02	2,79E-05	1,02E-02
cg13750474	13	28364188		IGR	-0,006	2,80E-05	1,02E-02
cg00530593	18	43684146	ATP5A1	5'UTR	-0,007	2,80E-05	1,03E-02
cg05934012	1	1533852		IGR	0,006	2,81E-05	1,03E-02
cg20110257	6	30657219	NRM	Body	-0,006	2,81E-05	1,03E-02
cg12668049	1	190323079	BRINP3	Body	-0,019	2,81E-05	1,03E-02
cg06380123	12	51717978	BIN2	TSS200	-0,005	2,82E-05	1,03E-02
cg19475020	12	119119725		IGR	-0,043	2,82E-05	1,03E-02
cg15908975	7	126698829	MIR592	TSS1500	0,078	2,82E-05	1,03E-02
cg14822216	9	128188454		IGR	-0,01	2,83E-05	1,03E-02
cg16159886	19	35214276		IGR	-0,007	2,83E-05	1,03E-02
cg21071763	2	152370194	NEB	ExonBnd	-0,008	2,83E-05	1,03E-02
cg08202720	2	239196219	PER2	5'UTR	0,016	2,83E-05	1,03E-02
cg07483432	12	72079704	TMEM19	TSS200	-0,004	2,83E-05	1,03E-02
cg10251048	10	99447092	AVPI1	TSS200	-0,004	2,83E-05	1,03E-02
cg27091836	6	11708927		IGR	-0,009	2,84E-05	1,03E-02
cg18260869	4	187619486	FAT1	Body	-0,007	2,84E-05	1,03E-02
cg18313661	7	63560590		IGR	-0,061	2,84E-05	1,03E-02
cg03589768	15	65577961	PARP16	Body	0,012	2,84E-05	1,03E-02
cg02723107	4	55987799	KDR	Body	0,012	2,85E-05	1,03E-02
cg00857222	3	41724788	ULK4	Body	-0,006	2,85E-05	1,03E-02
cg00714547	11	70948123		IGR	-0,036	2,85E-05	1,03E-02
cg01743095	16	14725172	PARN	TSS1500	-0,004	2,85E-05	1,03E-02
cg24639732	1	46217587	IPP	TSS1500	-0,006	2,86E-05	1,03E-02
cg18642459	12	4560908		IGR	0,04	2,85E-05	1,03E-02
cg23260111	10	46158095	ANUBL1	Body	0,039	2,86E-05	1,04E-02
cg22281754	16	72046030	DHODH	Body	-0,009	2,86E-05	1,04E-02
cg07090057	3	108837484	MORC1	TSS1500	-0,007	2,86E-05	1,04E-02
cg10234282	1	207975342	MIR29C	TSS200	0,01	2,87E-05	1,04E-02
cg04011754	16	27500185	GTF3C1	Body	-0,008	2,88E-05	1,04E-02
cg05872643	12	32112763	C12orf35	5'UTR	-0,004	2,88E-05	1,04E-02
cg01946548	3	149469835	COMMD2	Body	-0,006	2,88E-05	1,04E-02

cg14197268	6	154475681	IPCEF1	3'UTR	-0,022	2,88E-05	1,04E-02
cg07598034	7	134001087	SLC35B4	Body	-0,004	2,89E-05	1,04E-02
cg17286932	12	114361659	RBM19	Body	0,007	2,89E-05	1,04E-02
cg25692290	3	47844315	DHX30	TSS200	0,004	2,89E-05	1,04E-02
cg05830870	18	44677236	HDHD2	TSS1500	-0,002	2,90E-05	1,04E-02
cg26369098	6	111804425	REV3L	TSS200	-0,006	2,91E-05	1,04E-02
cg27393597	7	126105476	GRM8	Body	0,053	2,91E-05	1,04E-02
cg15451666	7	150414187	GIMAP1	5'UTR	-0,007	2,91E-05	1,04E-02
cg17355400	12	46844645		IGR	0,043	2,91E-05	1,04E-02
cg17444638	15	45247942	C15orf43	TSS1500	0,022	2,90E-05	1,04E-02
cg03814474	15	79405172		IGR	-0,007	2,91E-05	1,04E-02
cg12550705	2	196313052		IGR	0,047	2,91E-05	1,05E-02
cg20887363	4	184908582	STOX2	Body	-0,033	2,91E-05	1,05E-02
cg09664824	19	11517953	RGL3	Body	-0,023	2,92E-05	1,05E-02
cg16974516	2	237640723		IGR	-0,026	2,92E-05	1,05E-02
cg01114915	15	57595397	LINC00926	Body	0,046	2,92E-05	1,05E-02
cg21781703	1	116040184		IGR	-0,006	2,92E-05	1,05E-02
cg26867765	3	128448775	RAB7A	5'UTR	0,035	2,93E-05	1,05E-02
cg00337099	2	25463776	DNMT3A	Body	0,011	2,93E-05	1,05E-02
cg20344326	6	137736599		IGR	0,007	2,94E-05	1,05E-02
cg05624869	11	57268880	SLC43A1	Body	-0,01	2,94E-05	1,05E-02
cg20378687	3	160281681	KPNA4	Body	0,045	2,94E-05	1,05E-02
cg08345776	10	71993218	PPA1	TSS200	0,003	2,94E-05	1,05E-02
cg02646072	1	28267426	SMPDL3B	5'UTR	-0,007	2,95E-05	1,05E-02
cg20193104	6	31588802	BAT2	5'UTR	0,007	2,95E-05	1,05E-02
cg17598552	6	31094068	PSORS1C1	5'UTR	0,029	2,96E-05	1,05E-02
cg04350064	13	51722426	LINC00371	Body	-0,057	2,96E-05	1,06E-02
cg07967454	2	33003024	TTC27	Body	-0,005	2,96E-05	1,06E-02
cg22857746	7	38433588	AMPH	ExonBnd	0,009	2,97E-05	1,06E-02
cg21194128	20	52740548		IGR	-0,006	2,97E-05	1,06E-02
cg17017272	15	91447374	MAN2A2	TSS200	0,024	2,98E-05	1,06E-02
cg17549292	2	85578312	RETSAT	Body	-0,007	2,99E-05	1,06E-02
cg04755386	2	109622481		IGR	0,029	2,99E-05	1,06E-02
cg17813930	5	478174	LOC100288152	Body	0,032	2,99E-05	1,06E-02
cg04749840	6	108095067	SCML4	5'UTR	-0,011	2,99E-05	1,06E-02
cg05898179	15	65954293	DENND4A	Body	0,023	2,99E-05	1,06E-02
cg12666423	2	843922	LINC01115	Body	0,031	2,99E-05	1,06E-02
cg07749074	3	186383386	HRG	TSS1500	-0,053	3,00E-05	1,06E-02
cg23720528	10	92501798	HTR7	3'UTR	-0,008	3,00E-05	1,06E-02
cg16334425	11	67210033	CORO1B	Body	0,014	3,00E-05	1,06E-02
cg08457029	16	75313404		IGR	-0,095	3,00E-05	1,06E-02
cg13166107	1	113939376	MAGI3	Body	-0,006	3,02E-05	1,06E-02
cg16391801	3	135794793	PPP2R3A	Body	0,098	3,01E-05	1,06E-02
cg19599776	7	156433333	C7orf13	Body	-0,007	3,01E-05	1,06E-02
cg25327401	8	13462871		IGR	-0,019	3,02E-05	1,06E-02
cg08521570	9	71628408	PRKACG	1stExon	-0,008	3,01E-05	1,06E-02
cg06654431	16	1946951		IGR	-0,018	3,01E-05	1,06E-02
cg06366151	17	41150311	RPL27	TSS200	-0,005	3,02E-05	1,06E-02
cg04727725	2	37461736	NDUFAF7	Body	-0,008	3,02E-05	1,06E-02
cg24544889	1	177537910		IGR	-0,028	3,02E-05	1,07E-02
cg06206419	18	72176176	CNDP2	ExonBnd	-0,013	3,03E-05	1,07E-02
cg20413433	2	101805157		IGR	-0,006	3,03E-05	1,07E-02
cg01398428	11	68178928	LRP5	Body	-0,007	3,03E-05	1,07E-02
cg23327896	11	73669290	DNAJB13	Body	-0,03	3,03E-05	1,07E-02
cg05095863	20	45465311		IGR	0,045	3,04E-05	1,07E-02
cg01748832	1	1911471	KIAA1751	Body	-0,006	3,05E-05	1,07E-02
cg04553231	1	244102909	LOC339529	Body	0,016	3,05E-05	1,07E-02
cg04993628	2	232428244		IGR	-0,046	3,05E-05	1,07E-02
cg23664783	4	47846514	LOC101927179	Body	-0,005	3,05E-05	1,07E-02
cg12050504	9	101568592	GALNT12	TSS1500	0,007	3,05E-05	1,07E-02
cg13639866	14	101430211	SNORD114-8	TSS1500	-0,007	3,05E-05	1,07E-02
cg13255083	2	210444162	MAP2	TSS1500	0,049	3,06E-05	1,07E-02
cg22772380	7	6747037	ZNF12	TSS1500	-0,131	3,06E-05	1,07E-02
cg22667205	17	59079952	BCAS3	Body	-0,02	3,06E-05	1,07E-02
cg04741316	13	27404195		IGR	-0,013	3,06E-05	1,07E-02
cg21605452	3	176915282	TBL1XR1	TSS1500	-0,004	3,07E-05	1,07E-02
cg00531786	3	111638600	PHLDB2	Body	0,015	3,08E-05	1,08E-02
cg08142860	7	155089394	INSIG1	TSS200	-0,006	3,08E-05	1,08E-02
cg02104130	11	45937020	PEX16	Body	0,01	3,09E-05	1,08E-02
cg07241917	1	221069699		IGR	-0,013	3,10E-05	1,08E-02
cg18586470	15	100882540	ADAMTS17	TSS1500	-0,01	3,10E-05	1,08E-02
cg02233864	3	61515863		IGR	-0,028	3,11E-05	1,08E-02
cg13370193	4	158986696		IGR	0,009	3,11E-05	1,08E-02

cg25707676	7	96643539	DLX6AS	TSS200	-0,018	3,11E-05	1,08E-02
cg24480030	7	98030860	BAIAP2L1	TSS1500	-0,014	3,11E-05	1,08E-02
cg18386186	7	108456819		IGR	-0,007	3,11E-05	1,08E-02
cg21826837	16	87347499	C16orf95	Body	0,008	3,11E-05	1,08E-02
cg06580879	17	4443121	SPNS2	3'UTR	0,016	3,11E-05	1,08E-02
cg09870606	16	74641419	GLG1	TSS1500	-0,008	3,12E-05	1,08E-02
cg14976468	6	29056092	OR2B3	TSS1500	0,053	3,13E-05	1,09E-02
cg08675498	2	71295586	NAGK	5'UTR	-0,004	3,13E-05	1,09E-02
cg00914990	3	132360928	ACAD11	Body	0,017	3,13E-05	1,09E-02
cg06050191	4	183696126	TENM3	Body	-0,022	3,13E-05	1,09E-02
cg14030158	6	25419323	LRRC16A	Body	-0,011	3,14E-05	1,09E-02
cg00475761	3	70301132		IGR	-0,041	3,14E-05	1,09E-02
cg11240327	6	90348503	LYRM2	TSS1500	0,01	3,15E-05	1,09E-02
cg05210227	8	37824187	ADRB3	TSS200	-0,043	3,15E-05	1,09E-02
cg21126680	12	118589026	TAOK3	Body	-0,023	3,15E-05	1,09E-02
cg08438366	20	37230612	C20orf95	1stExon	-0,031	3,15E-05	1,09E-02
cg26051165	7	120742600	C7orf58	Body	-0,031	3,16E-05	1,09E-02
cg13303177	11	9405274	IPO7	TSS1500	0,011	3,16E-05	1,09E-02
cg23889013	19	48710006		IGR	-0,021	3,17E-05	1,10E-02
cg01569592	3	185842634		IGR	-0,057	3,18E-05	1,10E-02
cg01849987	8	73449509	KCNB2	TSS200	-0,007	3,18E-05	1,10E-02
cg25605773	4	140443230	SETD7	Body	-0,022	3,18E-05	1,10E-02
cg05759424	2	42721296	KCNG3	TSS200	0,009	3,18E-05	1,10E-02
cg13393601	2	69534855		IGR	-0,011	3,18E-05	1,10E-02
cg02748047	6	32373022	BTNL2	Body	0,039	3,19E-05	1,10E-02
cg05506088	9	118000103	DEC1	5'UTR	-0,018	3,19E-05	1,10E-02
cg00850443	3	195793870	TFRC	Body	0,032	3,20E-05	1,10E-02
cg17118125	3	127509531	MGLL	Body	0,008	3,20E-05	1,10E-02
cg21219996	9	77113070	RORB	Body	-0,01	3,20E-05	1,10E-02
cg07387570	22	46663686	TTC38	TSS200	-0,004	3,20E-05	1,10E-02
cg04870227	3	6003102		IGR	-0,011	3,21E-05	1,10E-02
cg04357242	9	706836	KANK1	1stExon	-0,006	3,21E-05	1,10E-02
cg17559110	11	984447	AP2A2	Body	-0,007	3,21E-05	1,10E-02
cg17333424	17	40306910	RAB5C	5'UTR	-0,006	3,21E-05	1,10E-02
cg23282442	8	11225832	C8orf12	TSS200	-0,013	3,22E-05	1,10E-02
cg18616525	10	2215471		IGR	-0,025	3,22E-05	1,10E-02
cg01548889	16	17173095		IGR	-0,052	3,22E-05	1,10E-02
cg11108432	8	10697698	PINX1	TSS1500	-0,003	3,23E-05	1,10E-02
cg00865865	15	70995876	UACA	TSS1500	-0,015	3,23E-05	1,10E-02
cg09754122	13	100741804	PCCA	Body	-0,005	3,23E-05	1,10E-02
cg04010248	2	242814898	C2orf85	Body	0,028	3,23E-05	1,10E-02
cg16404074	15	78934580	CHRNB4	TSS1500	-0,011	3,23E-05	1,10E-02
cg12135269	2	3642586	COLEC11	1stExon	-0,077	3,24E-05	1,11E-02
cg07472159	6	116447667	COL10A1	TSS1500	0,01	3,24E-05	1,11E-02
cg10336154	9	36030377		IGR	-0,027	3,24E-05	1,11E-02
cg25733708	19	54393220	PRKCG	Body	-0,008	3,24E-05	1,11E-02
cg13057359	3	196190691		IGR	-0,005	3,25E-05	1,11E-02
cg24461183	9	85107437		IGR	-0,011	3,25E-05	1,11E-02
cg09123298	11	32008659		IGR	-0,008	3,25E-05	1,11E-02
cg22665594	11	32093759		IGR	0,025	3,25E-05	1,11E-02
cg01388649	17	46810768		IGR	-0,015	3,25E-05	1,11E-02
cg11320690	20	17550474	DSTN	TSS200	0,005	3,25E-05	1,11E-02
cg12913956	4	108383	ZNF718	Body	-0,013	3,26E-05	1,11E-02
cg24133647	3	40208897	MYRIP	Body	-0,007	3,26E-05	1,11E-02
cg13277159	3	142442730	TRPC1	TSS1500	-0,002	3,27E-05	1,11E-02
cg14003984	4	38806733	TLR1	TSS1500	0,054	3,26E-05	1,11E-02
cg24060908	4	48991973	CWH43	Body	-0,02	3,27E-05	1,11E-02
cg05044303	12	52476017	OR7E47P	Body	0,025	3,27E-05	1,11E-02
cg18561199	14	95027379	SERPINA4	TSS1500	-0,229	3,26E-05	1,11E-02
cg01349088	20	30012193	DEFB122	Body	-0,061	3,27E-05	1,11E-02
cg09039201	22	40948360	MKL1	ExonBnd	-0,007	3,26E-05	1,11E-02
cg05388468	1	193074664	GLRX2	1stExon	-0,004	3,27E-05	1,11E-02
cg06446456	6	91865691		IGR	0,014	3,27E-05	1,11E-02
cg14692457	7	116554		IGR	0,007	3,28E-05	1,11E-02
cg12439789	10	28820707	WAC	TSS1500	0,036	3,28E-05	1,11E-02
cg22457860	12	71149597	PTPRR	Body	0,016	3,28E-05	1,11E-02
cg20085801	14	65481081	FNTB	Body	-0,007	3,28E-05	1,11E-02
cg06765321	2	70057479	GMCL1	Body	-0,004	3,29E-05	1,11E-02
cg15212455	7	39170539	POU6F2	Body	-0,126	3,29E-05	1,11E-02
cg25508228	3	142166203	XRN1	Body	-0,005	3,29E-05	1,11E-02
cg18921541	13	27975612		IGR	0,006	3,30E-05	1,11E-02
cg19953728	2	206981173	LOC100329109	Body	-0,013	3,30E-05	1,11E-02
cg07399685	5	24178148	C5orf17	Body	-0,041	3,30E-05	1,11E-02

cg12359279	21	42797953	MX1	TSS200	-0,005	3,31E-05	1,11E-02
cg25247453	3	167449639	PDCD10	5'UTR	0,008	3,32E-05	1,12E-02
cg11789820	14	58059860	SLC35F4	Body	-0,045	3,32E-05	1,12E-02
cg20646757	16	2039105	SYNGR3	TSS1500	-0,023	3,32E-05	1,12E-02
cg14797032	2	155564519	KCNJ3	Body	0,055	3,34E-05	1,12E-02
cg13628807	2	161128446	LOC100505984	TSS200	0,011	3,33E-05	1,12E-02
cg19651585	3	137716784	CLDN18	TSS1500	-0,013	3,33E-05	1,12E-02
cg11154476	9	12922803		IGR	-0,03	3,33E-05	1,12E-02
cg22571217	9	90112519	DAPK1	TSS1500	-0,004	3,33E-05	1,12E-02
cg13555047	12	115610683		IGR	-0,009	3,34E-05	1,12E-02
cg27413820	17	17701697	RAI1	Body	-0,008	3,34E-05	1,12E-02
cg25905674	17	43047856		IGR	-0,005	3,33E-05	1,12E-02
cg18149657	19	35773231	HAMP	TSS200	-0,01	3,33E-05	1,12E-02
cg26551569	2	230873081	FBXO36	Body	-0,007	3,34E-05	1,12E-02
cg27127146	3	146963668		IGR	0,013	3,35E-05	1,12E-02
cg10204951	7	910479	UNC84A	Body	0,005	3,35E-05	1,12E-02
cg15998961	12	133360718	GOLGA3	Body	0,009	3,35E-05	1,12E-02
cg11368182	19	436541	SHC2	Body	0,03	3,34E-05	1,12E-02
cg02050915	19	18209584	IL12RB1	1stExon	0,022	3,34E-05	1,12E-02
cg26982651	11	28373411		IGR	-0,021	3,37E-05	1,13E-02
cg08138366	19	2217980	DOT1L	Body	0,008	3,38E-05	1,13E-02
cg08572336	19	51165404	SHANK1	Body	-0,049	3,38E-05	1,13E-02
cg26003818	1	231474059	C1orf124	5'UTR	-0,004	3,39E-05	1,13E-02
cg09943560	14	91581064	C14orf159	5'UTR	-0,005	3,40E-05	1,13E-02
cg11440987	3	72621022		IGR	-0,014	3,41E-05	1,13E-02
cg15246686	9	72715990	MAMDC2	Body	-0,021	3,41E-05	1,13E-02
cg20123916	16	81812711	PLCG2	TSS200	-0,007	3,40E-05	1,13E-02
cg07553098	2	182437764	CERKL	Body	0,031	3,41E-05	1,13E-02
cg00445757	1	44226088	ST3GAL3	Body	0,007	3,41E-05	1,13E-02
cg25828334	19	18545568	ISYNA1	3'UTR	0,027	3,41E-05	1,13E-02
cg09256413	1	72566690	NEGR1	Body	0,032	3,42E-05	1,13E-02
cg07231615	5	52647228		IGR	-0,017	3,42E-05	1,13E-02
cg09213184	5	72145065	TNPO1	Body	-0,004	3,42E-05	1,13E-02
cg02726585	9	124984157	LHX6	TSS200	0,016	3,42E-05	1,13E-02
cg09862165	18	45245512		IGR	0,018	3,42E-05	1,13E-02
cg00347584	11	47283025	NR1H3	Body	0,039	3,42E-05	1,13E-02
cg26624732	5	70746330		IGR	-0,065	3,43E-05	1,14E-02
cg13775991	15	66794446	SNORD18C	TSS1500	0,018	3,43E-05	1,14E-02
cg08383709	17	61627648	DCAF7	TSS200	0,006	3,43E-05	1,14E-02
cg25470324	2	43546568	THADA	Body	0,007	3,44E-05	1,14E-02
cg26643813	12	119813750	CCDC60	Body	0,041	3,45E-05	1,14E-02
cg10367468	20	48786036		IGR	0,005	3,45E-05	1,14E-02
cg11186432	6	167821229		IGR	-0,044	3,45E-05	1,14E-02
cg23711273	22	42074566	SNU13	Body	0,015	3,45E-05	1,14E-02
cg08123705	2	36873276		IGR	0,036	3,45E-05	1,14E-02
cg11948054	17	75149428	SEC14L1	Body	-0,019	3,46E-05	1,14E-02
cg07747306	14	60975174	SIX6	TSS1500	-0,019	3,46E-05	1,14E-02
cg27361397	2	97426627	CNNM4	TSS200	-0,003	3,46E-05	1,14E-02
cg18724778	1	2452358	PANK4	ExonBnd	-0,004	3,47E-05	1,14E-02
cg26218269	4	151503227	LRBA	Body	0,008	3,47E-05	1,14E-02
cg03754403	6	40567579		IGR	-0,032	3,46E-05	1,14E-02
cg24004370	11	78177439	NARS2	Body	-0,011	3,47E-05	1,14E-02
cg09129925	17	17035323	MPRIP	Body	0,01	3,47E-05	1,14E-02
cg10124255	7	124286338		IGR	-0,018	3,48E-05	1,14E-02
cg06889975	8	22558722		IGR	0,023	3,48E-05	1,14E-02
cg14280283	9	139903861	ABCA2	Body	-0,004	3,48E-05	1,14E-02
cg07710907	8	6688074	XKR5	Body	-0,015	3,49E-05	1,14E-02
cg27355597	1	55051518	ACOT11	ExonBnd	0,018	3,49E-05	1,14E-02
cg24515946	6	168838659		IGR	-0,023	3,49E-05	1,14E-02
cg00323535	9	139305148	PMPCA	1stExon	0,016	3,49E-05	1,14E-02
cg15809520	5	102465626	PPIP5K2	Body	0,007	3,50E-05	1,15E-02
cg09887415	2	629016		IGR	-0,063	3,51E-05	1,15E-02
cg24867502	4	3486234	DOK7	TSS200	0,043	3,51E-05	1,15E-02
cg24271393	5	78469793		IGR	-0,009	3,51E-05	1,15E-02
cg20165252	6	150919919	PLEKHG1	TSS1500	0,035	3,51E-05	1,15E-02
cg19970430	7	1637167		IGR	-0,012	3,50E-05	1,15E-02
cg03220365	11	61174036	CPSF7	3'UTR	0,027	3,50E-05	1,15E-02
cg20003635	12	75707098	CAPS2	Body	-0,012	3,50E-05	1,15E-02
cg26526113	14	35180593	CFL2	3'UTR	0,008	3,51E-05	1,15E-02
cg15463692	3	185245491	LIPH	Body	-0,005	3,51E-05	1,15E-02
cg08699327	11	111597521	SIK2	3'UTR	-0,008	3,52E-05	1,15E-02
cg00786305	2	176977220		IGR	-0,023	3,52E-05	1,15E-02
cg23897367	17	7796962	CHD3	Body	0,02	3,52E-05	1,15E-02

cg20816204	3	122312333	PARP15	Body	0,037	3,52E-05	1,15E-02
cg10166392	6	42747983		IGR	0,028	3,53E-05	1,15E-02
cg03314079	7	100025948	MEPCE	TSS1500	-0,006	3,53E-05	1,15E-02
cg05649724	14	102415204		IGR	0,034	3,53E-05	1,15E-02
cg08778287	15	99499615	IGF1R	Body	0,061	3,53E-05	1,15E-02
cg23666072	2	239631410		IGR	-0,018	3,54E-05	1,15E-02
cg18046698	22	42196727	CCDC134	5'UTR	-0,004	3,54E-05	1,15E-02
cg03260858	3	49140771	QARS	ExonBnd	0,009	3,54E-05	1,15E-02
cg16393107	1	175985758	RFWD2	Body	0,024	3,56E-05	1,15E-02
cg27251916	8	125954285	LINC00964	Body	0,047	3,56E-05	1,15E-02
cg13526784	10	21624218		IGR	-0,006	3,56E-05	1,15E-02
cg16075775	14	55241716	SAMD4A	Body	0,004	3,56E-05	1,15E-02
cg26219497	15	86244192	AKAP13	Body	0,042	3,57E-05	1,16E-02
cg24736274	6	33868438		IGR	0,032	3,57E-05	1,16E-02
cg16327885	12	69741669	LYZ	TSS1500	0,041	3,57E-05	1,16E-02
cg05357108	19	10505842	CDC37	Body	-0,011	3,57E-05	1,16E-02
cg03876904	7	158826341	VIPR2	Body	0,012	3,58E-05	1,16E-02
cg12360113	9	116102758	WDR31	TSS200	-0,003	3,58E-05	1,16E-02
cg09722397	17	72855943	GRIN2C	5'UTR	-0,004	3,58E-05	1,16E-02
cg10035922	1	36916039	OSCP1	1stExon	-0,005	3,58E-05	1,16E-02
cg17520051	2	216248296	FN1	Body	-0,013	3,59E-05	1,16E-02
cg13344754	3	72353595		IGR	0,008	3,59E-05	1,16E-02
cg24887262	11	120040134		IGR	-0,006	3,60E-05	1,16E-02
cg15924582	14	102356845	PPP2R5C	Body	-0,005	3,60E-05	1,16E-02
cg03083227	16	16235187	ABCC1	3'UTR	0,021	3,60E-05	1,16E-02
cg12004575	5	150003580	SYNPO	Body	0,026	3,60E-05	1,16E-02
cg23124919	5	134015344	SEC24A	Body	0,025	3,61E-05	1,16E-02
cg08841897	3	18889671		IGR	0,009	3,61E-05	1,16E-02
cg08151857	17	46691820	HOXB8	1stExon	-0,005	3,61E-05	1,16E-02
cg17565365	10	82343645	SH2D4B	Body	0,04	3,62E-05	1,16E-02
cg24126630	8	1110039		IGR	-0,051	3,62E-05	1,16E-02
cg14051367	2	241696955	KIF1A	Body	0,015	3,63E-05	1,17E-02
cg23191253	11	116041814		IGR	0,025	3,63E-05	1,17E-02
cg22942908	1	110578259	FAM40A	Body	-0,015	3,66E-05	1,17E-02
cg02944048	1	205113116	DSTYK	3'UTR	-0,007	3,66E-05	1,17E-02
cg20795999	3	184074817	CLCN2	Body	0,019	3,64E-05	1,17E-02
cg25935176	3	187388389	SST	TSS200	0,038	3,64E-05	1,17E-02
cg07521988	7	90393740	CDK14	Body	-0,013	3,66E-05	1,17E-02
cg10710988	10	21108410	NEBL	Body	-0,01	3,65E-05	1,17E-02
cg24780185	10	74653173	OIT3	TSS200	-0,054	3,65E-05	1,17E-02
cg13595605	10	119478873		IGR	-0,05	3,65E-05	1,17E-02
cg05178711	14	74428069	COQ6	Body	-0,007	3,65E-05	1,17E-02
cg08582629	18	21719467	CABYR	5'UTR	-0,03	3,65E-05	1,17E-02
cg10003060	20	19569380	SLC24A3	Body	-0,014	3,65E-05	1,17E-02
cg02412616	22	46422752		IGR	0,033	3,66E-05	1,17E-02
cg27140441	4	184328596		IGR	-0,006	3,66E-05	1,17E-02
cg10609524	5	100115478		IGR	-0,01	3,66E-05	1,17E-02
cg25726513	4	1340596	KIAA1530	TSS1500	0,016	3,67E-05	1,17E-02
cg17693122	5	83492287	EDIL3	Body	0,026	3,67E-05	1,17E-02
cg12190980	9	131790493	SH3GLB2	1stExon	-0,004	3,67E-05	1,17E-02
cg22447778	3	88106558	CGGBP1	5'UTR	0,033	3,67E-05	1,17E-02
cg03511261	7	39961257		IGR	-0,008	3,68E-05	1,17E-02
cg03602297	2	30142128	ALK	Body	-0,055	3,68E-05	1,17E-02
cg03783628	6	7939976	BLOC1S5-TXNDC5	Body	0,024	3,68E-05	1,17E-02
cg21141264	1	25188911		IGR	0,056	3,68E-05	1,17E-02
cg09469033	2	32391091	SLC30A6	Body	0,011	3,69E-05	1,17E-02
cg09439238	6	32096206	ATF6B	TSS200	-0,005	3,69E-05	1,17E-02
cg01139808	6	170549344		IGR	0,021	3,69E-05	1,17E-02
cg13786567	9	137258920	RXRA	Body	-0,051	3,69E-05	1,17E-02
cg11057513	12	116835153		IGR	-0,015	3,69E-05	1,17E-02
cg06146665	17	76799964	USP36	Body	0,054	3,69E-05	1,17E-02
cg27416412	4	2435825		IGR	-0,012	3,70E-05	1,17E-02
cg03566822	10	64578476	EGR2	5'UTR	-0,009	3,70E-05	1,17E-02
cg27091100	19	36128359	RBM42	Body	0,013	3,70E-05	1,17E-02
cg23783653	6	27059850		IGR	0,011	3,70E-05	1,17E-02
cg05673892	3	38033934	VILL	TSS1500	0,014	3,72E-05	1,18E-02
cg04972244	16	57117433	NLRC5	3'UTR	-0,021	3,72E-05	1,18E-02
cg06937068	5	140615329	PCDHB18	Body	-0,086	3,73E-05	1,18E-02
cg21469772	9	124989294	LHX6	Body	-0,141	3,73E-05	1,18E-02
cg18643460	19	2740371	SLC39A3	TSS1500	-0,006	3,73E-05	1,18E-02
cg05865670	22	21355860	THAP7	Body	-0,004	3,73E-05	1,18E-02
cg26717675	12	121007295	RNF10	Body	-0,006	3,74E-05	1,18E-02
cg27133504	7	156917432		IGR	0,012	3,75E-05	1,18E-02

cg02315187	21	46317913	ITGB2	Body	-0,049	3,75E-05	1,18E-02
cg03453035	6	4056856	PRPF4B	Body	-0,006	3,75E-05	1,18E-02
cg25847024	1	111991376	WDR77	Body	0,009	3,76E-05	1,18E-02
cg01829945	2	192540664		IGR	-0,004	3,76E-05	1,18E-02
cg03805782	3	124699654	HEG1	Body	-0,015	3,76E-05	1,18E-02
cg08182198	22	43044351	CYB5R3	TSS1500	0,025	3,76E-05	1,18E-02
cg06672120	16	85932383	IRF8	TSS1500	-0,005	3,76E-05	1,19E-02
cg13266018	7	5637739	FSCN1	Body	0,022	3,77E-05	1,19E-02
cg03656459	7	123635440		IGR	-0,028	3,77E-05	1,19E-02
cg10692863	13	97896008	MBNL2	5'UTR	-0,024	3,77E-05	1,19E-02
cg03789871	15	74159515		IGR	0,026	3,77E-05	1,19E-02
cg14662967	2	193039325	TMEFF2	Body	0,011	3,78E-05	1,19E-02
cg19166043	11	76003666		IGR	0,013	3,78E-05	1,19E-02
cg12447589	1	17765625	RCC2	5'UTR	-0,006	3,80E-05	1,19E-02
cg11125107	3	124998037	ZNF148	Body	0,031	3,80E-05	1,19E-02
cg20670020	10	134250521		IGR	0,047	3,80E-05	1,19E-02
cg10996014	3	49449848	RHOA	TSS1500	-0,005	3,81E-05	1,19E-02
cg14022183	4	187729190		IGR	0,012	3,81E-05	1,19E-02
cg19270858	12	53342770	KRT18	TSS200	0,013	3,81E-05	1,19E-02
cg18373580	6	14783197		IGR	0,07	3,81E-05	1,19E-02
cg06459669	10	111683143	XPNPEP1	Body	-0,013	3,82E-05	1,19E-02
cg02442619	12	91572142	DCN	1stExon	-0,04	3,82E-05	1,19E-02
cg21385822	15	75661000	MAN2C1	TSS200	0,005	3,82E-05	1,19E-02
cg08266462	1	59228879		IGR	0,018	3,82E-05	1,19E-02
cg07253870	12	7013813	LRRC23	TSS200	-0,005	3,83E-05	1,20E-02
cg03986322	1	9789699	CLSTN1	3'UTR	0,021	3,83E-05	1,20E-02
cg03773594	16	27383830		IGR	0,015	3,83E-05	1,20E-02
cg20392110	17	79142629	AATK-AS1	Body	0,01	3,83E-05	1,20E-02
cg11170796	19	1650224	TCF3	Body	0,012	3,83E-05	1,20E-02
cg07393508	8	49173116		IGR	-0,007	3,84E-05	1,20E-02
cg22529504	20	59743569		IGR	-0,046	3,84E-05	1,20E-02
cg01062913	1	149169973		IGR	-0,025	3,85E-05	1,20E-02
cg01485378	3	17780992	TBC1D5	1stExon	0,045	3,85E-05	1,20E-02
cg02808430	3	27498448	SLC4A7	TSS1500	0,036	3,86E-05	1,20E-02
cg06955420	12	125012405	NCOR2	5'UTR	-0,01	3,85E-05	1,20E-02
cg05847200	14	57355176	OTX2-AS1	Body	-0,05	3,85E-05	1,20E-02
cg03020572	14	98087004		IGR	-0,008	3,86E-05	1,20E-02
cg19091719	5	149997515	SYNPO	5'UTR	0,012	3,86E-05	1,20E-02
cg21173721	9	130743698	FAM102A	TSS1500	-0,006	3,86E-05	1,20E-02
cg17741539	1	3394478	ARHGEF16	Body	0,03	3,87E-05	1,20E-02
cg18002751	6	71136714	FAM135A	5'UTR	-0,022	3,87E-05	1,20E-02
cg17070935	8	51364244	SNTG1	Body	-0,02	3,87E-05	1,20E-02
cg20091114	19	17213063	MYO9B	Body	0,008	3,87E-05	1,20E-02
cg13881108	7	55073432		IGR	-0,093	3,88E-05	1,20E-02
cg03504160	4	6659194		IGR	-0,02	3,88E-05	1,20E-02
cg13766589	9	139331009	INPP5E	Body	0,01	3,88E-05	1,20E-02
cg03065803	10	73576273	PSAP	3'UTR	-0,011	3,89E-05	1,20E-02
cg22274745	2	182451537	CERKL	Body	-0,012	3,90E-05	1,21E-02
cg14076977	15	40600284	PLCB2	TSS200	-0,004	3,90E-05	1,21E-02
cg04413130	4	153857909		IGR	-0,006	3,90E-05	1,21E-02
cg17663907	2	16081485	MYCNOS	Body	-0,006	3,90E-05	1,21E-02
cg01690878	3	98250181	GPR15	TSS1500	0,048	3,91E-05	1,21E-02
cg26040745	8	55369129	SOX17	TSS1500	-0,045	3,91E-05	1,21E-02
cg25472554	3	196051315	TM4SF19	ExonBnd	-0,01	3,93E-05	1,21E-02
cg08453205	5	179588440	RASGEF1C	5'UTR	-0,061	3,93E-05	1,21E-02
cg10648543	11	122673964	UBASH3B	Body	0,007	3,93E-05	1,21E-02
cg03943674	18	77643329	KCNG2	Body	-0,057	3,93E-05	1,21E-02
cg13610108	22	39928902	RPS19BP1	TSS200	-0,004	3,95E-05	1,22E-02
cg06596711	19	19148759	ARMC6	5'UTR	-0,021	3,95E-05	1,22E-02
cg06199212	12	50298517	FAIM2	TSS1500	0,01	3,96E-05	1,22E-02
cg05142929	18	59997009	TNFRSF11A	Body	-0,034	3,96E-05	1,22E-02
cg05896849	17	29188456	ATAD5	Body	-0,009	3,97E-05	1,22E-02
cg16090291	2	233361742		IGR	0,01	3,98E-05	1,22E-02
cg09107155	13	41556139	ELF1	1stExon	-0,022	3,98E-05	1,22E-02
cg01709619	3	129346694		IGR	0,012	3,98E-05	1,22E-02
cg26073987	4	6783056	KIAA0232	TSS1500	-0,004	3,98E-05	1,22E-02
cg21964798	5	1503259	LPCAT1	Body	0,019	3,99E-05	1,22E-02
cg22896996	12	119644232		IGR	-0,044	3,99E-05	1,22E-02
cg18688293	6	142409417	NMBR	1stExon	-0,032	3,99E-05	1,22E-02
cg06198312	1	27895943	AHDC1	5'UTR	0,018	4,00E-05	1,23E-02
cg09037197	3	148760012	HLTF	Body	0,024	4,01E-05	1,23E-02
cg01250954	3	194909020	XXYLT1	Body	-0,022	4,01E-05	1,23E-02
cg11101639	4	94754985		IGR	-0,042	4,00E-05	1,23E-02

cg04548688	4	138727652		IGR	0,029	4,00E-05	1,23E-02
cg02874031	12	106988340	RFX4	Body	-0,019	4,01E-05	1,23E-02
cg06427621	16	55906624	CES5A	Body	-0,009	4,01E-05	1,23E-02
cg12493107	20	23344079	GZF1	TSS1500	0,017	4,01E-05	1,23E-02
cg23457506	2	30687453	LCLAT1	5'UTR	0,011	4,01E-05	1,23E-02
cg13489722	1	46738006	RAD54L	Body	-0,016	4,03E-05	1,23E-02
cg05479750	3	151869793		IGR	-0,005	4,04E-05	1,23E-02
cg00047845	3	147877708		IGR	-0,022	4,04E-05	1,23E-02
cg08510234	17	738869	NXN	Body	-0,025	4,04E-05	1,23E-02
cg02994654	3	131758853	CPNE4	TSS1500	-0,048	4,04E-05	1,23E-02
cg11394947	1	19074199	PAX7	3'UTR	-0,012	4,05E-05	1,23E-02
cg23984906	1	54770346	SSBP3	Body	0,021	4,05E-05	1,23E-02
cg16254375	4	93105176		IGR	0,009	4,05E-05	1,23E-02
cg07148032	6	32935354	BRD2	TSS1500	-0,005	4,05E-05	1,23E-02
cg17652600	7	129436516		IGR	0,016	4,05E-05	1,23E-02
cg15417287	12	31926899		IGR	0,016	4,05E-05	1,23E-02
cg07423765	12	121147730	UNC119B	TSS200	0,021	4,06E-05	1,23E-02
cg23341488	11	10436501	CAND1.11	Body	-0,005	4,07E-05	1,24E-02
cg13216080	8	37968048	ASH2L	Body	-0,008	4,07E-05	1,24E-02
cg09072052	8	144070960	LOC100133669	Body	0,008	4,07E-05	1,24E-02
cg16176378	8	34843233		IGR	-0,042	4,07E-05	1,24E-02
cg09303201	16	85674253	KIAA0182	5'UTR	0,011	4,08E-05	1,24E-02
cg17942096	19	19301711	LOC729991	Body	-0,017	4,07E-05	1,24E-02
cg10326213	2	220713767		IGR	-0,006	4,08E-05	1,24E-02
cg17617535	11	117391948	DSCAML1	Body	0,007	4,08E-05	1,24E-02
cg02931398	1	156637105		IGR	0,009	4,09E-05	1,24E-02
cg25438652	7	106804854		IGR	0,014	4,09E-05	1,24E-02
cg15057446	11	74951923		IGR	-0,008	4,09E-05	1,24E-02
cg03511628	18	77280586	NFATC1	Body	0,068	4,09E-05	1,24E-02
cg00030420	5	140683772	SLC25A2	TSS200	-0,045	4,10E-05	1,24E-02
cg25654577	15	60197332		IGR	-0,035	4,10E-05	1,24E-02
cg17624892	6	26537952	HMGN4	TSS1500	-0,004	4,11E-05	1,24E-02
cg27531276	8	145830821	KIAA1688	Body	-0,01	4,11E-05	1,24E-02
cg10883123	13	113436651	ATP11A	Body	-0,009	4,11E-05	1,24E-02
cg11649068	17	34136647	TAF15	Body	0,017	4,11E-05	1,24E-02
cg03548463	1	1603847	LOC728661	Body	0,007	4,12E-05	1,24E-02
cg07636088	13	31734946	HSPH1	Body	-0,018	4,13E-05	1,25E-02
cg12118843	6	28641917		IGR	-0,02	4,13E-05	1,25E-02
cg01067010	11	132115847	NTM	Body	-0,032	4,14E-05	1,25E-02
cg08481125	20	17914638		IGR	0,009	4,14E-05	1,25E-02
cg18539115	7	143078024	ZYX	TSS1500	0,006	4,14E-05	1,25E-02
cg01880829	10	103867449	LDB1	3'UTR	0,022	4,14E-05	1,25E-02
cg09098905	16	3074344	HCFC1R1	TSS200	-0,005	4,15E-05	1,25E-02
cg02489630	13	78620363	LINC00446	Body	0,044	4,15E-05	1,25E-02
cg26228696	1	150849189	ARNT	TSS200	-0,005	4,17E-05	1,25E-02
cg05248542	4	110484104	CCDC109B	Body	-0,011	4,16E-05	1,25E-02
cg08764163	5	63258768	HTR1A	TSS1500	-0,022	4,17E-05	1,25E-02
cg22653915	8	52722023	PXDNL	TSS200	-0,017	4,17E-05	1,25E-02
cg12504804	17	30813443	CDK5R1	TSS1500	-0,008	4,17E-05	1,25E-02
cg23801435	5	67692436		IGR	-0,007	4,18E-05	1,25E-02
cg09232103	3	94656226	LOC255025	TSS1500	-0,011	4,18E-05	1,25E-02
cg23436042	5	175322631		IGR	0,028	4,18E-05	1,25E-02
cg13704504	11	2403018	CD81	5'UTR	0,009	4,19E-05	1,25E-02
cg14741143	3	156273315	SSR3	TSS1500	-0,073	4,19E-05	1,25E-02
cg08331832	14	85273270		IGR	-0,01	4,19E-05	1,25E-02
cg26217356	8	142076228		IGR	-0,011	4,19E-05	1,25E-02
cg13710846	3	63912454	ATXN7	Body	0,007	4,20E-05	1,25E-02
cg09246253	7	5643021	FSCN1	Body	0,007	4,20E-05	1,25E-02
cg26771998	10	75490334	BMS1P4	TSS200	-0,017	4,20E-05	1,25E-02
cg09755438	15	73531857	NEO1	Body	-0,007	4,20E-05	1,25E-02
cg24824178	5	141263222		IGR	-0,014	4,21E-05	1,26E-02
cg11343941	2	39235194	SOS1	Body	-0,007	4,23E-05	1,26E-02
cg25099021	2	216249662	FN1	Body	0,017	4,22E-05	1,26E-02
cg05767754	3	112709667	GTPBP8	TSS200	-0,006	4,23E-05	1,26E-02
cg19778582	5	7900364	MTRR	3'UTR	0,01	4,22E-05	1,26E-02
cg12590791	6	89931408	GABRR1	5'UTR	-0,025	4,23E-05	1,26E-02
cg11883881	7	101468117	CUX1	Body	-0,007	4,21E-05	1,26E-02
cg23696886	8	22437193	PDLIM2	TSS1500	-0,005	4,21E-05	1,26E-02
cg26159428	9	110926162		IGR	0,016	4,22E-05	1,26E-02
cg14331281	10	51491984		IGR	-0,023	4,22E-05	1,26E-02
cg16841674	10	133317015		IGR	-0,015	4,22E-05	1,26E-02
cg10829210	17	15685104		IGR	-0,018	4,23E-05	1,26E-02
cg11523191	20	62187126	C20orf195	Body	0,01	4,23E-05	1,26E-02

cg15471815	1	228645486	HIST3H2BB	TSS1500	-0,004	4,23E-05	1,26E-02
cg10826006	12	115775938		IGR	-0,032	4,24E-05	1,26E-02
cg13155114	2	3523533	ADI1	TSS200	-0,006	4,24E-05	1,26E-02
cg04340928	16	55067613		IGR	-0,006	4,24E-05	1,26E-02
cg02560447	14	60794991		IGR	-0,037	4,25E-05	1,26E-02
cg05675785	7	128398229	CALU	Body	-0,009	4,26E-05	1,26E-02
cg23739762	8	58487982		IGR	-0,045	4,26E-05	1,26E-02
cg23825092	10	115938972	TDRD1	TSS200	-0,015	4,26E-05	1,26E-02
cg27340529	11	94245309	C11orf97	TSS1500	-0,042	4,26E-05	1,26E-02
cg09104263	13	101232684	GGACT	5'UTR	-0,015	4,26E-05	1,26E-02
cg10359333	19	17988635	SLC5A5	Body	-0,004	4,26E-05	1,26E-02
cg25117123	2	233471018	EFHD1	Body	0,009	4,27E-05	1,26E-02
cg19032662	8	94242042		IGR	0,014	4,27E-05	1,26E-02
cg09747162	1	35227074	GJB4	Body	-0,017	4,28E-05	1,26E-02
cg03681562	6	151222659	MTHFD1L	Body	-0,008	4,28E-05	1,26E-02
cg11791531	12	78573360	NAV3	Body	-0,038	4,28E-05	1,26E-02
cg20711274	15	97440396		IGR	-0,009	4,28E-05	1,26E-02
cg09318521	1	224301995	FBXO28	1stExon	-0,004	4,30E-05	1,27E-02
cg04947801	4	46394634		IGR	-0,047	4,29E-05	1,27E-02
cg08773007	6	152599208	SYNE1	ExonBnd	-0,012	4,30E-05	1,27E-02
cg03791074	7	1117852	C7orf50	Body	0,01	4,29E-05	1,27E-02
cg07074976	8	37924550		IGR	-0,03	4,30E-05	1,27E-02
cg16785690	9	135037323	NTNG2	TSS200	-0,007	4,30E-05	1,27E-02
cg08511757	16	89342065	ANKRD11	Body	0,011	4,30E-05	1,27E-02
cg03321231	17	157219	RPH3AL	Body	-0,018	4,30E-05	1,27E-02
cg02484511	17	33914243	AP2B1	TSS200	-0,005	4,30E-05	1,27E-02
cg18861421	19	11450590	RAB3D	TSS1500	0,017	4,30E-05	1,27E-02
cg01702474	16	2790808	SRRM2-AS1	Body	0,02	4,31E-05	1,27E-02
cg16209303	14	103405847	CDC42BPB	Body	-0,009	4,31E-05	1,27E-02
cg08853056	5	84716347		IGR	-0,012	4,33E-05	1,27E-02
cg08755283	5	118324104	DTWD2	1stExon	0,016	4,33E-05	1,27E-02
cg21671867	6	38449346	BTBD9	Body	-0,008	4,32E-05	1,27E-02
cg23979472	8	22421913	SORBS3	TSS1500	0,005	4,33E-05	1,27E-02
cg25674586	14	26673749		IGR	-0,035	4,33E-05	1,27E-02
cg21150347	15	50783575	USP8	Body	0,048	4,33E-05	1,27E-02
cg13294846	5	2112351		IGR	-0,01	4,34E-05	1,27E-02
cg13929598	7	29845596	WIPF3	TSS1500	0,02	4,34E-05	1,27E-02
cg20897479	11	12292577		IGR	0,032	4,34E-05	1,27E-02
cg26827394	16	1362626	UBE2I	5'UTR	0,008	4,35E-05	1,27E-02
cg24839693	17	43212892	ACBD4	TSS200	-0,003	4,34E-05	1,27E-02
cg23422865	17	79410408	BAHCC1	Body	0,017	4,35E-05	1,27E-02
cg09100996	1	231368950	C1orf131	Body	0,012	4,35E-05	1,27E-02
cg02068690	2	25600451	DTNB	3'UTR	-0,021	4,35E-05	1,27E-02
cg07253079	2	120995104		IGR	0,015	4,36E-05	1,27E-02
cg05124077	4	41983325	DCAF4L1	TSS1500	-0,012	4,36E-05	1,27E-02
cg07973591	15	78911824	CHRNA3	Body	0,03	4,35E-05	1,27E-02
cg24240077	11	77574320	C11orf67	Body	-0,016	4,37E-05	1,27E-02
cg23074647	12	28935367		IGR	-0,035	4,37E-05	1,27E-02
cg14847917	12	78635247		IGR	-0,04	4,37E-05	1,27E-02
cg14313213	1	242854361		IGR	-0,024	4,38E-05	1,28E-02
cg19717060	17	70553078		IGR	-0,051	4,38E-05	1,28E-02
cg08638876	15	63569511	APH1B	TSS1500	-0,004	4,38E-05	1,28E-02
cg14644761	5	141082361		IGR	-0,005	4,39E-05	1,28E-02
cg27117695	4	68415072		IGR	-0,018	4,39E-05	1,28E-02
cg15221642	5	140598706		IGR	-0,064	4,39E-05	1,28E-02
cg16621189	19	50006799		IGR	0,025	4,39E-05	1,28E-02
cg11653179	1	65209572	RAVER2	TSS1500	0,021	4,39E-05	1,28E-02
cg01051327	1	26946744		IGR	-0,003	4,40E-05	1,28E-02
cg04212884	16	67876131	CENPT	5'UTR	-0,005	4,40E-05	1,28E-02
cg21230793	8	93920498	C8orf83	Body	-0,033	4,41E-05	1,28E-02
cg18656127	1	22883519		IGR	0,019	4,41E-05	1,28E-02
cg19399805	12	22643079	C2CD5	Body	0,041	4,41E-05	1,28E-02
cg17987649	6	29526534	UBD	Body	-0,019	4,42E-05	1,28E-02
cg26777826	11	10287775	SBF2	Body	-0,004	4,42E-05	1,28E-02
cg08517686	5	158634925	RNF145	5'UTR	-0,02	4,42E-05	1,28E-02
cg18745752	10	114822234	TCF7L2	Body	0,014	4,42E-05	1,28E-02
cg17267880	20	31126718	NOL4L	Body	-0,009	4,43E-05	1,28E-02
cg05225001	2	20102464	TTC32	TSS1500	-0,021	4,44E-05	1,28E-02
cg21074101	13	114908155		IGR	-0,013	4,44E-05	1,28E-02
cg21879513	20	61927203	COL20A1	Body	-0,032	4,44E-05	1,28E-02
cg13367219	12	28764033		IGR	-0,069	4,45E-05	1,29E-02
cg14542064	18	42400400	SETBP1	Body	0,015	4,46E-05	1,29E-02
cg17511936	6	155566057	TIAM2	Body	0,054	4,46E-05	1,29E-02

cg08282924	20	47423229	PREX1	Body	-0,008	4,47E-05	1,29E-02
cg10900048	4	73935377	COX18	5'UTR	-0,003	4,47E-05	1,29E-02
cg19088553	6	101901884	GRIK2	Body	-0,042	4,47E-05	1,29E-02
cg25862072	6	164520845		IGR	0,006	4,47E-05	1,29E-02
cg16538471	7	20153546		IGR	0,034	4,49E-05	1,29E-02
cg15071899	7	156228452		IGR	-0,05	4,49E-05	1,29E-02
cg12987790	9	90112590	DAPK1	5'UTR	-0,01	4,49E-05	1,29E-02
cg14869388	18	34892350	CELF4	Body	-0,014	4,49E-05	1,29E-02
cg03426349	3	155007942		IGR	0,036	4,50E-05	1,29E-02
cg00859129	1	109422184	GPSM2	5'UTR	0,04	4,50E-05	1,29E-02
cg02644494	19	6412686		IGR	-0,038	4,51E-05	1,30E-02
cg12304520	5	140810123	PCDHGA4	Body	-0,05	4,51E-05	1,30E-02
cg00570469	19	36602113		IGR	0,014	4,52E-05	1,30E-02
cg05690388	2	224392870		IGR	0,007	4,52E-05	1,30E-02
cg16816603	22	45403047	PHF21B	Body	-0,017	4,52E-05	1,30E-02
cg21082033	5	177591349		IGR	-0,004	4,53E-05	1,30E-02
cg22205640	11	13072895		IGR	-0,018	4,54E-05	1,30E-02
cg08712722	4	153601180	TMEM154	1stExon	-0,01	4,55E-05	1,30E-02
cg02331407	8	54856825	RGS20	Body	0,008	4,54E-05	1,30E-02
cg16533146	12	27901072	MRPS35	Body	0,056	4,54E-05	1,30E-02
cg14526028	8	139840496	COL22A1	Body	-0,007	4,56E-05	1,30E-02
cg16025584	9	139887217	C9orf142	Body	-0,005	4,55E-05	1,30E-02
cg05492752	5	81266947	ATG10	TSS1500	-0,016	4,57E-05	1,31E-02
cg23842996	6	70956109	COL9A1	Body	-0,01	4,57E-05	1,31E-02
cg09896340	8	1440147		IGR	-0,015	4,57E-05	1,31E-02
cg06903472	9	130930030	CIZ1	Body	0,013	4,57E-05	1,31E-02
cg17087356	3	59749626	FHIT	Body	0,01	4,58E-05	1,31E-02
cg11274119	4	170948608	MFAP3L	TSS1500	-0,004	4,58E-05	1,31E-02
cg11783318	6	133947056	TARID	Body	-0,006	4,58E-05	1,31E-02
cg23181443	11	102740504	MMP12	Body	0,035	4,58E-05	1,31E-02
cg09437538	12	119509583	SRRM4	Body	-0,035	4,58E-05	1,31E-02
cg18053948	12	132936807		IGR	0,009	4,58E-05	1,31E-02
cg10026431	5	81282123	ATG10	5'UTR	-0,009	4,59E-05	1,31E-02
cg21573359	1	220132728		IGR	0,01	4,60E-05	1,31E-02
cg06150007	3	114998690		IGR	-0,015	4,60E-05	1,31E-02
cg13771271	4	65180301	TECRL	Body	-0,053	4,60E-05	1,31E-02
cg17280782	5	149012152	ARHGEF37	3'UTR	0,025	4,60E-05	1,31E-02
cg19722949	5	156362978	TIMD4	Body	0,009	4,60E-05	1,31E-02
cg21421172	6	168045888		IGR	-0,056	4,60E-05	1,31E-02
cg06053850	11	76510871		IGR	-0,023	4,60E-05	1,31E-02
cg18920097	15	35045695	GJD2	Body	-0,028	4,60E-05	1,31E-02
cg12759986	5	156948867	ADAM19	Body	-0,017	4,61E-05	1,31E-02
cg13053332	9	94587071	ROR2	Body	0,012	4,61E-05	1,31E-02
cg02859866	10	13760165	FRMD4A	Body	-0,01	4,62E-05	1,31E-02
cg05030328	10	45849898		IGR	0,023	4,61E-05	1,31E-02
cg21039438	11	528271		IGR	-0,013	4,62E-05	1,31E-02
cg25689961	11	3148731	OSBPL5	Body	0,027	4,61E-05	1,31E-02
cg03355159	14	93137424	RIN3	Body	0,019	4,62E-05	1,31E-02
cg10266524	19	37711193		IGR	0,033	4,62E-05	1,31E-02
cg08508864	2	137371349		IGR	-0,02	4,63E-05	1,31E-02
cg19277389	4	7802545	AFAP1	Body	0,018	4,63E-05	1,31E-02
cg26856527	7	4247868	SDK1	Body	-0,01	4,63E-05	1,31E-02
cg13714378	11	34702946		IGR	-0,009	4,65E-05	1,31E-02
cg07118866	11	36531924	TRAF6	TSS200	-0,006	4,65E-05	1,31E-02
cg00316828	16	87985026	BANP	TSS200	0,003	4,65E-05	1,31E-02
cg05037505	9	124989550	LHX6	Body	-0,129	4,66E-05	1,32E-02
cg17452291	7	86781755	DMTF1	TSS200	0,014	4,67E-05	1,32E-02
cg24169009	1	232033322	DISC1	Body	-0,005	4,67E-05	1,32E-02
cg15697036	4	1808923	FGFR3	Body	0,017	4,67E-05	1,32E-02
cg05369347	13	112057814		IGR	0,007	4,68E-05	1,32E-02
cg02817601	16	89144489		IGR	-0,008	4,68E-05	1,32E-02
cg19902483	4	148503322		IGR	-0,022	4,68E-05	1,32E-02
cg03654045	14	78617230		IGR	0,025	4,68E-05	1,32E-02
cg14611555	9	90689720		IGR	-0,019	4,69E-05	1,32E-02
cg09666561	16	30406910	ZNF48	TSS200	0,01	4,69E-05	1,32E-02
cg21010407	1	46955679		IGR	-0,015	4,69E-05	1,32E-02
cg07896619	9	98269081	PTCH1	Body	0,005	4,69E-05	1,32E-02
cg08522085	7	44102657	PGAM2	Body	0,022	4,70E-05	1,32E-02
cg01406080	20	42885970	GDAP1L1	ExonBnd	0,01	4,70E-05	1,32E-02
cg14331858	17	26694306	VTN	3'UTR	0,031	4,70E-05	1,32E-02
cg22062191	12	2087331	DCP1B	Body	0,011	4,71E-05	1,32E-02
cg10084324	3	183353251	KLHL24	TSS200	-0,008	4,71E-05	1,32E-02
cg14557714	9	90341385	CTSL1	5'UTR	-0,016	4,72E-05	1,32E-02

cg12419348	17	54991398	TRIM25	5'UTR	-0,004	4,72E-05	1,32E-02
cg23017261	5	140431882	PCDHB1	1stExon	-0,013	4,73E-05	1,33E-02
cg14749705	9	137365759		IGR	-0,008	4,74E-05	1,33E-02
cg15903395	2	88824153	C2orf51	TSS200	-0,019	4,74E-05	1,33E-02
cg03929563	17	74513794		IGR	0,009	4,74E-05	1,33E-02
cg19692240	13	21748982	MRPL57	TSS1500	0,074	4,74E-05	1,33E-02
cg14177401	9	124074692	GSN	Body	-0,011	4,76E-05	1,33E-02
cg20007622	11	14340592	RRAS2	Body	0,014	4,76E-05	1,33E-02
cg08401981	11	34379677	ABTB2	TSS200	-0,005	4,76E-05	1,33E-02
cg21684588	1	67600265		IGR	-0,033	4,77E-05	1,33E-02
cg10828159	1	152020549		IGR	-0,004	4,77E-05	1,33E-02
cg06900404	20	54919037		IGR	-0,024	4,77E-05	1,33E-02
cg01375154	16	69139327	HAS3	TSS1500	-0,013	4,77E-05	1,33E-02
cg17392047	11	65247138		IGR	0,032	4,78E-05	1,33E-02
cg01894780	17	74466043	AANAT	Body	0,013	4,78E-05	1,33E-02
cg23466118	20	49507816	ADNP	3'UTR	0,007	4,79E-05	1,34E-02
cg26501714	22	50970562	ODF3B	5'UTR	-0,005	4,79E-05	1,34E-02
cg06912282	1	1563001	MIB2	Body	0,022	4,80E-05	1,34E-02
cg27564939	17	2207243	SMG6	TSS1500	-0,005	4,80E-05	1,34E-02
cg13453139	17	8815262	PIK3R5	5'UTR	0,007	4,80E-05	1,34E-02
cg23919678	6	155476467	TIAM2	Body	-0,019	4,81E-05	1,34E-02
cg27349460	18	76000984		IGR	-0,045	4,81E-05	1,34E-02
cg18423935	3	120626667	STXBP5L	TSS1500	-0,044	4,81E-05	1,34E-02
cg00816544	8	146032931	ZNF517	Body	-0,006	4,82E-05	1,34E-02
cg08624093	1	2733491		IGR	0,009	4,83E-05	1,34E-02
cg20152755	9	124984098	LHX6	TSS200	0,036	4,84E-05	1,34E-02
cg07285048	11	13947573		IGR	-0,006	4,84E-05	1,34E-02
cg05119200	21	36478670		IGR	-0,071	4,84E-05	1,34E-02
cg06826046	17	3686339	ITGAE	Body	-0,004	4,85E-05	1,35E-02
cg22617643	8	74206753	RPL7	TSS1500	0,016	4,85E-05	1,35E-02
cg15565809	2	182290868		IGR	-0,012	4,87E-05	1,35E-02
cg07460717	13	112806931		IGR	-0,019	4,87E-05	1,35E-02
cg15287095	2	23584501		IGR	-0,017	4,89E-05	1,35E-02
cg15414940	2	44619580	CAMKMT	Body	-0,051	4,89E-05	1,35E-02
cg08963067	3	115502443		IGR	-0,05	4,88E-05	1,35E-02
cg21875655	6	10660286		IGR	-0,061	4,88E-05	1,35E-02
cg22312220	10	105391771	SH3PXD2A	Body	-0,018	4,88E-05	1,35E-02
cg10436540	17	4047319	ZZEF1	TSS1500	-0,005	4,88E-05	1,35E-02
cg11954313	17	28921927	LRRC37B2	Body	-0,02	4,88E-05	1,35E-02
cg17656881	17	79202227	ENTHD2	3'UTR	0,041	4,88E-05	1,35E-02
cg02653117	6	32938824	BRD2	5'UTR	0,016	4,90E-05	1,35E-02
cg05877222	6	156952186		IGR	0,006	4,90E-05	1,35E-02
cg03715259	7	50935274		IGR	-0,005	4,89E-05	1,35E-02
cg25643132	10	101956546	CHUK	Body	0,005	4,90E-05	1,35E-02
cg10437577	15	75917396	SNUPN	5'UTR	0,014	4,90E-05	1,35E-02
cg21410568	17	1084451	ABR	TSS1500	0,025	4,90E-05	1,35E-02
cg25164226	22	47158357	TBC1D22A	TSS200	-0,006	4,90E-05	1,35E-02
cg01805469	14	74357777	ZNF410	5'UTR	-0,007	4,91E-05	1,35E-02
cg09891198	2	109855261	SH3RF3	Body	0,005	4,92E-05	1,35E-02
cg26774043	11	1647150	HCCA2	Body	0,017	4,92E-05	1,35E-02
cg19567758	17	80136000	CCDC57	Body	0,007	4,92E-05	1,35E-02
cg26134159	17	14201024		IGR	-0,009	4,92E-05	1,35E-02
cg16739092	20	17519424	BFSP1	Body	0,034	4,92E-05	1,35E-02
cg17550721	5	176375408	UIMC1	Body	-0,008	4,93E-05	1,36E-02
cg02627966	7	54899537		IGR	0,05	4,93E-05	1,36E-02
cg03034277	11	47885912		IGR	0,007	4,93E-05	1,36E-02
cg20818806	13	110885926	COL4A1	Body	-0,008	4,94E-05	1,36E-02
cg07137170	22	32343451	YWHAH	Body	-0,007	4,94E-05	1,36E-02
cg16000504	3	21706663	ZNF385D	Body	0,009	4,94E-05	1,36E-02
cg00442390	6	56584334	RNU6-71P	Body	-0,055	4,94E-05	1,36E-02
cg00786406	1	114474389	HIPK1	5'UTR	-0,012	4,95E-05	1,36E-02
cg14150727	1	202182417	LGR6	TSS1500	0,03	4,96E-05	1,36E-02
cg14382976	7	117067624	ASZ1	TSS200	-0,045	4,96E-05	1,36E-02
cg22721998	13	110522149		IGR	-0,065	4,95E-05	1,36E-02
cg11261667	19	15132411	CCDC105	Body	-0,03	4,95E-05	1,36E-02
cg01667687	2	175804030	CHN1	Body	0,016	4,97E-05	1,36E-02
cg00352576	9	135454197		IGR	-0,048	4,96E-05	1,36E-02
cg08307350	11	74445385		IGR	-0,033	4,97E-05	1,36E-02
cg10846857	15	77907603	LINGO1	Body	-0,023	4,97E-05	1,36E-02
cg14600786	19	12902996	JUNB	1stExon	-0,003	4,96E-05	1,36E-02
cg01596778	3	168619570	LOC100507661	TSS200	-0,007	4,97E-05	1,36E-02
cg00436051	22	29663508	EWSR1	TSS1500	-0,003	4,97E-05	1,36E-02
cg20685276	6	52109654	IL17F	TSS1500	-0,006	4,98E-05	1,36E-02

cg15346802	1	56998626	PPAP2B	Body	-0,034	4,99E-05	1,36E-02
cg03718662	1	178244359	RASAL2	Body	0,037	4,99E-05	1,36E-02
cg01256539	5	119802022	PRR16	5'UTR	0,067	4,99E-05	1,36E-02
cg21024089	10	91042116		IGR	0,008	5,00E-05	1,36E-02
cg18176307	12	387235		IGR	-0,005	4,99E-05	1,36E-02
cg04510639	17	74722764	JMJD6	1stExon	0,015	4,99E-05	1,36E-02
cg26145166	20	35990022	SRC	5'UTR	0,018	5,00E-05	1,36E-02
cg18741439	8	19310134	CSGALNACT1	Body	0,027	5,00E-05	1,36E-02
cg20417180	1	22435805		IGR	-0,006	5,01E-05	1,36E-02
cg06888261	2	175130314		IGR	-0,018	5,01E-05	1,36E-02
cg09034601	6	28175888		IGR	-0,009	5,01E-05	1,36E-02
cg18763875	11	67291278	CABP2	TSS1500	0,02	5,01E-05	1,36E-02
cg12618499	19	23619280		IGR	-0,042	5,01E-05	1,36E-02
cg18630243	22	24059634	LOC91316	TSS200	0,015	5,01E-05	1,36E-02
cg10104676	5	150444853	TNIP1	TSS200	-0,005	5,02E-05	1,36E-02
cg27302421	7	154812894		IGR	-0,063	5,02E-05	1,36E-02
cg20546518	14	105195480	ADSSL1	TSS1500	0,017	5,02E-05	1,36E-02
cg08830672	16	67516286	ATP6V0D1	TSS1500	0,036	5,02E-05	1,36E-02
cg12652108	18	53072177	TCF4	TSS1500	0,01	5,02E-05	1,36E-02
cg06080068	1	10567799	PEX14	Body	-0,067	5,03E-05	1,36E-02
cg11308227	17	79202435	ENTHD2	3'UTR	0,05	5,03E-05	1,36E-02
cg00048743	5	140782367	PCDHGA4	Body	-0,049	5,03E-05	1,36E-02
cg04558601	7	21502552	SP4	Body	0,024	5,03E-05	1,36E-02
cg16854826	10	80889523	ZMIZ1	5'UTR	0,017	5,03E-05	1,36E-02
cg12772509	2	3681758	COLEC11	Body	-0,021	5,04E-05	1,36E-02
cg13368274	2	172136124		IGR	-0,011	5,05E-05	1,36E-02
cg11256055	6	52016326	LINCMD1	TSS1500	-0,007	5,05E-05	1,36E-02
cg17177816	10	96304193	HELLS	TSS1500	-0,006	5,05E-05	1,36E-02
cg09556292	14	103058815	RCOR1	TSS1500	-0,006	5,05E-05	1,36E-02
cg27394057	19	54135129	DPRX	TSS200	-0,008	5,05E-05	1,36E-02
cg13232176	22	25503378	KIAA1671	Body	0,027	5,05E-05	1,36E-02
cg23415057	11	410032	SIGIRR	5'UTR	0,025	5,06E-05	1,36E-02
cg19172447	12	132551483	EP400	Body	0,012	5,07E-05	1,37E-02
cg21009824	1	38477009	UTP11L	TSS1500	0,005	5,07E-05	1,37E-02
cg13468249	17	7154818	DULLARD	5'UTR	0,029	5,07E-05	1,37E-02
cg14628708	7	106865205	COG5	Body	-0,009	5,08E-05	1,37E-02
cg13083004	1	23809994	ASAP3	Body	0,015	5,08E-05	1,37E-02
cg09346503	2	11968388		IGR	-0,019	5,09E-05	1,37E-02
cg02230408	1	2722326		IGR	0,03	5,10E-05	1,37E-02
cg22390041	1	19226319	ALDH4A1	Body	0,006	5,10E-05	1,37E-02
cg08534653	2	1747700	PXDN	Body	-0,027	5,10E-05	1,37E-02
cg13136648	4	38990279	TMEM156	Body	-0,072	5,10E-05	1,37E-02
cg17300125	9	95202490	CENPP	Body	-0,011	5,10E-05	1,37E-02
cg10110432	11	1311352	TOLLIP	Body	-0,01	5,09E-05	1,37E-02
cg09473364	13	112927190		IGR	0,007	5,09E-05	1,37E-02
cg19803550	17	1637391	WDR81	Body	0,005	5,09E-05	1,37E-02
cg06633615	10	131592944		IGR	0,011	5,11E-05	1,37E-02
cg27083740	16	6071073	A2BP1	5'UTR	-0,043	5,11E-05	1,37E-02
cg23593208	19	22778462	GOLGA2P9	TSS1500	-0,074	5,11E-05	1,37E-02
cg14989164	1	180205157	LHX4	Body	0,01	5,13E-05	1,37E-02
cg15733705	2	72017902		IGR	0,024	5,12E-05	1,37E-02
cg25368586	13	111561018	ANKRD10	Body	0,026	5,12E-05	1,37E-02
cg22304190	17	12906955	ELAC2	Body	0,017	5,13E-05	1,37E-02
cg11862246	15	78730200	IREB2	TSS1500	-0,006	5,13E-05	1,37E-02
cg27105304	7	157484751	PTPRN2	Body	-0,006	5,14E-05	1,37E-02
cg07909223	11	82747090	RAB30	TSS1500	0,007	5,14E-05	1,37E-02
cg05766965	15	67547620	AAGAB	TSS1500	-0,007	5,14E-05	1,37E-02
cg09358088	18	55165284		IGR	-0,022	5,14E-05	1,37E-02
cg11599078	12	91194639		IGR	0,017	5,15E-05	1,38E-02
cg01166310	2	8732965		IGR	-0,014	5,16E-05	1,38E-02
cg05287481	11	17249967		IGR	0,016	5,16E-05	1,38E-02
cg08962185	8	143336052	TSNARE1	Body	-0,05	5,16E-05	1,38E-02
cg17790400	4	25314231	ZCCHC4	TSS200	-0,005	5,18E-05	1,38E-02
cg19014792	7	3019159	CARD11	5'UTR	0,047	5,17E-05	1,38E-02
cg22343429	14	67879031	PLEK2	TSS1500	-0,03	5,17E-05	1,38E-02
cg22675767	1	1234367	ACAP3	Body	0,025	5,18E-05	1,38E-02
cg10703763	3	12815501		IGR	-0,009	5,18E-05	1,38E-02
cg05354099	19	54465346	CACNG8	TSS1500	-0,021	5,19E-05	1,38E-02
cg02006966	21	46827403	COL18A1-AS2	Body	-0,007	5,19E-05	1,38E-02
cg07787995	17	16492746		IGR	-0,004	5,19E-05	1,38E-02
cg10075774	5	127626430	FBN2	ExonBnd	-0,019	5,20E-05	1,38E-02
cg22101921	3	9851381	TTLL3	TSS1500	0,006	5,20E-05	1,38E-02
cg04098297	13	114312482	ATP4B	1stExon	0,016	5,20E-05	1,38E-02

cg07146773	15	80466042	FAH	Body	0,013	5,20E-05	1,38E-02
cg20725245	17	48297235		IGR	-0,011	5,21E-05	1,38E-02
cg15579376	3	138154754	ESYT3	Body	0,01	5,22E-05	1,39E-02
cg10453948	2	208482295	METTL21A	Body	0,004	5,22E-05	1,39E-02
cg25281562	12	121454272	C12orf43	1stExon	-0,004	5,22E-05	1,39E-02
cg01793387	8	22461396	KIAA1967	TSS1500	-0,006	5,23E-05	1,39E-02
cg14965300	10	14951152	DCLRE1C	Body	-0,006	5,23E-05	1,39E-02
cg05919661	12	49504308	LMBR1L	1stExon	-0,004	5,23E-05	1,39E-02
cg14458068	5	140772256	PCDHGA4	Body	-0,053	5,24E-05	1,39E-02
cg08029832	11	65732239	SART1	Body	0,006	5,24E-05	1,39E-02
cg11575892	12	57908774	MARS	Body	0,02	5,24E-05	1,39E-02
cg13064751	8	110357011	ENY2	Body	-0,017	5,25E-05	1,39E-02
cg24091761	1	94057755	BCAR3	Body	0,082	5,26E-05	1,39E-02
cg17965230	6	106534206	PRDM1	5'UTR	-0,004	5,26E-05	1,39E-02
cg12735653	8	22487315	BIN3	Body	0,008	5,26E-05	1,39E-02
cg10550387	10	61846916	ANK3	Body	0,035	5,26E-05	1,39E-02
cg26765337	18	21396888	LAMA3	Body	0,016	5,26E-05	1,39E-02
cg02079700	22	43166347		IGR	0,024	5,27E-05	1,39E-02
cg17050984	5	14397222	TRIO	Body	-0,006	5,27E-05	1,39E-02
cg03646542	5	172076155	NEURL1B	Body	0,038	5,27E-05	1,39E-02
cg09399498	7	86782128	DMTF1	5'UTR	0,007	5,27E-05	1,39E-02
cg21089806	8	32463093	NRG1	ExonBnd	0,015	5,27E-05	1,39E-02
cg23649326	5	80604119	ZCCHC9	Body	0,039	5,28E-05	1,39E-02
cg26125550	7	141695623	MGAM	TSS200	-0,009	5,28E-05	1,39E-02
cg08559772	13	28518317	ATP5EP2	TSS1500	-0,023	5,28E-05	1,39E-02
cg22235926	5	56131369	MAP3K1	Body	0,038	5,29E-05	1,39E-02
cg12587213	1	206589868	SRGAP2	Body	-0,007	5,29E-05	1,39E-02
cg25875824	1	29063431	YTHDF2	5'UTR	-0,006	5,31E-05	1,39E-02
cg11343260	2	61225096	PUS10	Body	0,013	5,31E-05	1,39E-02
cg05570059	2	177345796		IGR	0,01	5,30E-05	1,39E-02
cg21819722	4	42429072	ATP8A1	Body	0,013	5,31E-05	1,39E-02
cg10703676	9	117140024	AKNA	Body	-0,014	5,30E-05	1,39E-02
cg12895546	11	68202244	LRP5	Body	-0,012	5,31E-05	1,39E-02
cg14782991	12	6833168	COPS7A	1stExon	-0,005	5,30E-05	1,39E-02
cg27307942	12	120378819		IGR	-0,04	5,31E-05	1,39E-02
cg04438332	15	63889271	FBXL22	TSS1500	0,039	5,31E-05	1,39E-02
cg10572858	15	72742016		IGR	0,019	5,31E-05	1,39E-02
cg15698598	16	56896608		IGR	0,055	5,29E-05	1,39E-02
cg16119522	20	37433745	PPP1R16B	TSS1500	-0,004	5,31E-05	1,39E-02
cg19245011	1	92414221	BRDT	TSS1500	-0,04	5,32E-05	1,39E-02
cg23243867	1	77334045	ST6GALNAC5	Body	-0,006	5,33E-05	1,39E-02
cg19995891	2	191042691	C2orf88	5'UTR	-0,041	5,33E-05	1,39E-02
cg16230878	6	79577175	IRAK1BP1	TSS200	-0,004	5,33E-05	1,39E-02
cg20101398	6	114384045	HS3ST5	TSS200	-0,01	5,33E-05	1,39E-02
cg09896999	7	6746977	ZNF12	TSS1500	-0,03	5,34E-05	1,39E-02
cg09461240	11	77996112	GAB2	5'UTR	-0,022	5,33E-05	1,39E-02
cg13418562	12	24932305		IGR	0,008	5,32E-05	1,39E-02
cg23825570	12	42633508	YAF2	TSS1500	0,006	5,33E-05	1,39E-02
cg12873036	13	46347470		IGR	-0,016	5,34E-05	1,39E-02
cg00145958	14	45723370	MIS18BP1	TSS1500	0,051	5,34E-05	1,39E-02
cg27578637	16	30775658	RNF40	Body	-0,004	5,32E-05	1,39E-02
cg24330765	5	151151190	G3BP1	TSS1500	-0,004	5,34E-05	1,39E-02
cg08253748	10	54531709	MBL2	TSS1500	-0,039	5,34E-05	1,39E-02
cg13867541	6	53552049		IGR	-0,006	5,34E-05	1,39E-02
cg00800759	16	21291944	CRYM	5'UTR	-0,026	5,36E-05	1,40E-02
cg00521598	1	39339413	MYCBP	TSS1500	-0,007	5,36E-05	1,40E-02
cg17410930	12	83021797		IGR	-0,048	5,36E-05	1,40E-02
cg23744755	10	106096667	ITPRIP	5'UTR	-0,025	5,36E-05	1,40E-02
cg25329296	4	113486062	C4orf21	Body	0,014	5,38E-05	1,40E-02
cg15466493	5	178618516	ADAMTS2	Body	0,011	5,39E-05	1,40E-02
cg24710048	6	170532661		IGR	0,102	5,38E-05	1,40E-02
cg13350855	11	6643752	DCHS1	Body	0,018	5,39E-05	1,40E-02
cg26132512	11	117104857	RNF214	5'UTR	0,014	5,39E-05	1,40E-02
cg04162059	12	64784177	C12orf56	1stExon	-0,009	5,38E-05	1,40E-02
cg11314826	12	114270851	RBM19	Body	-0,014	5,39E-05	1,40E-02
cg01427852	12	116886173		IGR	-0,007	5,39E-05	1,40E-02
cg04362655	14	96001355	GLRX5	1stExon	-0,005	5,38E-05	1,40E-02
cg04894259	21	43225016	PRDM15	Body	0,013	5,39E-05	1,40E-02
cg04907724	14	70170645	SUSD6	Body	0,018	5,39E-05	1,40E-02
cg03774957	5	141227950		IGR	0,027	5,39E-05	1,40E-02
cg14592399	4	41881933		IGR	-0,036	5,41E-05	1,40E-02
cg18059449	4	106629662	INTS12	5'UTR	-0,008	5,41E-05	1,40E-02
cg01126080	4	186847298	SORBS2	5'UTR	-0,013	5,41E-05	1,40E-02

cg15642200	5	146833470	DPYSL3	TSS1500	-0,004	5,41E-05	1,40E-02
cg06041324	9	101704337		IGR	-0,009	5,41E-05	1,40E-02
cg15233530	11	67889369	CHKA	TSS1500	-0,006	5,41E-05	1,40E-02
cg10209481	16	4226328		IGR	-0,017	5,40E-05	1,40E-02
cg16068401	18	54317872	WDR7	TSS1500	-0,012	5,42E-05	1,40E-02
cg04607679	6	32935803	BRD2	TSS1500	-0,004	5,43E-05	1,40E-02
cg01867925	11	705120	EPS8L2	TSS1500	0,023	5,43E-05	1,40E-02
cg03919650	16	3306855	MEFV	TSS1500	0,028	5,43E-05	1,40E-02
cg18619300	2	7009129		IGR	-0,005	5,44E-05	1,41E-02
cg23599385	2	106637299		IGR	0,017	5,44E-05	1,41E-02
cg22200270	10	34662183	PARD3	Body	0,031	5,45E-05	1,41E-02
cg26524347	12	121738449		IGR	0,008	5,45E-05	1,41E-02
cg15213988	17	4997134	ZFP3	3'UTR	-0,01	5,46E-05	1,41E-02
cg09241841	11	111459818		IGR	-0,013	5,46E-05	1,41E-02
cg08808654	10	105841289	COL17A1	5'UTR	-0,02	5,47E-05	1,41E-02
cg05213745	16	22308244	POLR3E	TSS1500	-0,022	5,48E-05	1,41E-02
cg06812722	8	132637826		IGR	-0,052	5,49E-05	1,41E-02
cg07085632	11	125974404		IGR	0,049	5,48E-05	1,41E-02
cg24947106	5	525233	SLC9A3	TSS1500	-0,055	5,49E-05	1,41E-02
cg13249701	12	125302755	SCARB1	Body	-0,007	5,49E-05	1,41E-02
cg27302994	1	30240265		IGR	-0,062	5,50E-05	1,41E-02
cg14030525	7	119960959	KCND2	Body	-0,06	5,50E-05	1,41E-02
cg12943115	14	70345911	SMOC1	TSS1500	-0,024	5,50E-05	1,41E-02
cg05541779	15	45943467	SQRDL	5'UTR	-0,013	5,50E-05	1,41E-02
cg21848981	4	190597409		IGR	-0,039	5,51E-05	1,41E-02
cg14660998	10	127227778		IGR	-0,056	5,51E-05	1,41E-02
cg04423621	16	83390001	CDH13	Body	-0,011	5,51E-05	1,41E-02
cg10240853	7	100210548	MOSPD3	Body	-0,021	5,51E-05	1,41E-02
cg02409234	12	114246083		IGR	-0,017	5,52E-05	1,42E-02
cg01621268	7	75272341	HIP1	Body	0,016	5,52E-05	1,42E-02
cg21518023	12	81845232	PPFIA2	5'UTR	-0,016	5,53E-05	1,42E-02
cg25345603	1	6450174	ACOT7	Body	0,015	5,54E-05	1,42E-02
cg27324066	3	151839230		IGR	0,053	5,54E-05	1,42E-02
cg17391518	19	59028285	ZBTB45	Body	-0,019	5,54E-05	1,42E-02
cg16502517	3	46718727	ALS2CL	5'UTR	0,016	5,54E-05	1,42E-02
cg09137243	15	69580837		IGR	0,018	5,54E-05	1,42E-02
cg08213963	15	90439967	ARPIN	3'UTR	0,023	5,55E-05	1,42E-02
cg27079341	5	154033185		IGR	0,013	5,55E-05	1,42E-02
cg03625665	15	99602038		IGR	-0,012	5,55E-05	1,42E-02
cg20553766	20	54824583	MC3R	1stExon	0,081	5,56E-05	1,42E-02
cg22282744	9	125591114	PDCL	TSS200	-0,006	5,56E-05	1,42E-02
cg08021244	12	123001969	RSRC2	ExonBnd	0,012	5,57E-05	1,42E-02
cg04464062	16	88540349	ZFPM1	Body	0,033	5,57E-05	1,42E-02
cg25667751	1	10567686	PEX14	Body	-0,057	5,58E-05	1,42E-02
cg19284186	3	12586998	MKRN2OS	TSS200	0,008	5,58E-05	1,42E-02
cg23206822	1	19182586	TAS1R2	Body	0,047	5,60E-05	1,42E-02
cg08696989	2	8020930		IGR	0,02	5,59E-05	1,42E-02
cg17149980	2	97426598	CNNM4	TSS200	0,015	5,60E-05	1,42E-02
cg13358362	10	105072446	PCGF6	Body	-0,016	5,60E-05	1,42E-02
cg16594214	11	70035873		IGR	-0,015	5,59E-05	1,42E-02
cg19631576	19	15132007	CCDC105	Body	-0,022	5,59E-05	1,42E-02
cg21937462	21	45561490	C21orf33	Body	0,029	5,59E-05	1,42E-02
cg04065236	1	1023150	C1orf159	Body	-0,011	5,60E-05	1,42E-02
cg03506106	2	20153664	WDR35	Body	0,014	5,61E-05	1,42E-02
cg01288070	2	35940133		IGR	0,038	5,61E-05	1,42E-02
cg22299960	5	147202497		IGR	-0,007	5,61E-05	1,42E-02
cg12768018	10	127213259		IGR	-0,015	5,61E-05	1,42E-02
cg06420305	16	78133211	WWOX	TSS1500	-0,004	5,61E-05	1,42E-02
cg08585379	5	17058214		IGR	0,007	5,62E-05	1,43E-02
cg12543807	11	85355143		IGR	-0,018	5,62E-05	1,43E-02
cg24237600	1	155178849	THBS3	TSS1500	0,014	5,62E-05	1,43E-02
cg25647415	3	50304250	SEMA3B	TSS1500	0,015	5,63E-05	1,43E-02
cg10687350	19	32718629		IGR	-0,015	5,64E-05	1,43E-02
cg11621243	20	20200773	CFAP61	Body	-0,023	5,64E-05	1,43E-02
cg00389463	19	10397620	ICAM4	TSS200	0,022	5,64E-05	1,43E-02
cg09530861	6	107776494	PDSS2	Body	0,026	5,65E-05	1,43E-02
cg10968396	12	1352926	ERC1	Body	0,015	5,66E-05	1,43E-02
cg02428538	16	24856791	SLC5A11	TSS1500	-0,013	5,66E-05	1,43E-02
cg26822551	1	179544476	NPHS2	Body	-0,071	5,67E-05	1,43E-02
cg06470855	13	112997365		IGR	-0,082	5,67E-05	1,43E-02
cg07998323	2	61296459	KIAA1841	5'UTR	-0,006	5,69E-05	1,44E-02
cg16917713	4	22340824	LOC100505912	Body	0,007	5,69E-05	1,44E-02
cg16569972	5	92004163		IGR	-0,013	5,69E-05	1,44E-02

cg10738714	15	91033215	IQGAP1	Body	-0,007	5,69E-05	1,44E-02
cg23535449	17	79107259	MIR1250	TSS200	0,008	5,69E-05	1,44E-02
cg09037962	22	20776817		IGR	-0,04	5,69E-05	1,44E-02
cg11195689	15	93127878		IGR	-0,011	5,70E-05	1,44E-02
cg03915569	6	18571016	MIR548A1	TSS1500	0,012	5,71E-05	1,44E-02
cg02456219	6	41754918	PRICKLE4	3'UTR	-0,005	5,70E-05	1,44E-02
cg27086879	4	172801611	GALNTL6	Body	-0,027	5,71E-05	1,44E-02
cg06151968	19	36612911	TBCB	Body	0,032	5,71E-05	1,44E-02
cg14365126	9	139545538		IGR	0,046	5,72E-05	1,44E-02
cg04596640	3	57533508		IGR	0,012	5,74E-05	1,44E-02
cg02909206	3	194574434		IGR	0,07	5,73E-05	1,44E-02
cg02010004	5	1159392		IGR	-0,032	5,73E-05	1,44E-02
cg15676735	11	62521955	ZBTB3	TSS1500	-0,004	5,74E-05	1,44E-02
cg14180581	11	82746478	RAB30	TSS1500	0,012	5,73E-05	1,44E-02
cg05679709	11	128044574		IGR	0,009	5,74E-05	1,44E-02
cg09764773	12	120486023	CCDC64	Body	-0,023	5,74E-05	1,44E-02
cg25897632	16	77224911	MON1B	5'UTR	-0,003	5,73E-05	1,44E-02
cg27658048	7	4201594	SDK1	Body	-0,017	5,75E-05	1,44E-02
cg25824052	3	12852040	CAND2	Body	0,009	5,75E-05	1,44E-02
cg05051876	6	132676184	MOXD1	Body	0,014	5,75E-05	1,44E-02
cg05724271	7	8151066		IGR	-0,023	5,75E-05	1,44E-02
cg04746945	2	54481922	TSPYL6	1stExon	-0,006	5,76E-05	1,44E-02
cg21178653	12	67876529		IGR	-0,03	5,77E-05	1,45E-02
cg27346444	5	72156443	TNPO1	Body	-0,007	5,77E-05	1,45E-02
cg25728350	7	123345457	WASL	Body	-0,008	5,78E-05	1,45E-02
cg02547883	9	121098974		IGR	-0,043	5,78E-05	1,45E-02
cg07320684	14	39486072		IGR	0,044	5,79E-05	1,45E-02
cg24457614	2	51171084	NRXN1	Body	0,03	5,80E-05	1,45E-02
cg05890386	7	73256582	WBSCR27	5'UTR	0,006	5,80E-05	1,45E-02
cg03506216	8	11550001		IGR	-0,014	5,80E-05	1,45E-02
cg23071408	14	74537090	ALDH6A1	Body	-0,025	5,80E-05	1,45E-02
cg16431961	14	91806143	CCDC88C	Body	-0,015	5,80E-05	1,45E-02
cg06549871	16	89939812	TCF25	TSS200	-0,006	5,80E-05	1,45E-02
cg24605370	13	99667976	DOCK9	Body	-0,009	5,81E-05	1,45E-02
cg04934867	3	2933549	CNTN4	Body	-0,013	5,82E-05	1,45E-02
cg25739943	7	11291686		IGR	-0,01	5,82E-05	1,45E-02
cg06755373	16	3225493		IGR	0,014	5,82E-05	1,45E-02
cg04598611	1	171640223		IGR	-0,008	5,83E-05	1,45E-02
cg08978835	10	1402076	ADARB2	Body	0,006	5,83E-05	1,45E-02
cg11753157	14	99734018	BCL11B	Body	-0,068	5,83E-05	1,45E-02
cg27048067	8	674560	ERICH1	Body	-0,113	5,84E-05	1,45E-02
cg12354960	1	156816460	NTRK1	Body	0,008	5,85E-05	1,45E-02
cg09876574	9	126561628	DENND1A	Body	-0,017	5,85E-05	1,45E-02
cg09570913	17	15344763	CDRT4	5'UTR	-0,006	5,85E-05	1,45E-02
cg10592619	19	5104336	KDM4B	Body	-0,008	5,85E-05	1,45E-02
cg06723553	22	24181428	DERL3	TSS1500	-0,004	5,84E-05	1,45E-02
cg06342490	2	42795193	MTA3	TSS1500	0,017	5,85E-05	1,45E-02
cg19411734	1	13877579		IGR	0,031	5,88E-05	1,46E-02
cg02452944	5	140810109	PCDHGA4	Body	-0,049	5,87E-05	1,46E-02
cg26944151	9	139607602	FAM69B	Body	0,016	5,87E-05	1,46E-02
cg06159404	10	43846376		IGR	-0,078	5,86E-05	1,46E-02
cg10839265	12	11639711		IGR	-0,005	5,88E-05	1,46E-02
cg11777437	12	32549333		IGR	0,039	5,87E-05	1,46E-02
cg00426486	16	86605100		IGR	0,023	5,87E-05	1,46E-02
cg05080578	18	6729112		IGR	0,01	5,86E-05	1,46E-02
cg03320758	18	57678429		IGR	0,014	5,86E-05	1,46E-02
cg05338009	19	54041398	ZNF331	1stExon	0,053	5,88E-05	1,46E-02
cg08620074	6	38698525	DNAH8	Body	-0,007	5,88E-05	1,46E-02
cg18036235	19	2721669	DIRAS1	TSS1500	0,011	5,89E-05	1,46E-02
cg18406000	6	114650610		IGR	-0,072	5,90E-05	1,46E-02
cg16025792	12	121948003	KDM2B	Body	-0,009	5,90E-05	1,46E-02
cg13444307	3	49593425	BSN	Body	0,011	5,90E-05	1,46E-02
cg01829017	5	38490321	LIFR	Body	0,027	5,91E-05	1,46E-02
cg00448814	6	28227482	NKAPL	1stExon	-0,013	5,90E-05	1,46E-02
cg08722103	12	133197668	P2RX2	Body	-0,038	5,91E-05	1,46E-02
cg16185223	14	64688013	SYNE2	Body	0,043	5,91E-05	1,46E-02
cg27105949	20	11897536	BTBD3	TSS1500	0,014	5,91E-05	1,46E-02
cg26345444	12	16430861	SLC15A5	TSS1500	0,008	5,91E-05	1,46E-02
cg13185308	11	17498819	ABCC8	TSS1500	-0,058	5,92E-05	1,46E-02
cg21846973	20	24953547	APMAP	Body	0,008	5,92E-05	1,46E-02
cg24003560	1	220863347	C1orf115	TSS1500	0,053	5,95E-05	1,46E-02
cg23576092	2	28543298	BRE	3'UTR	0,006	5,94E-05	1,46E-02
cg11857517	5	9167289	SEMA5A	Body	0,043	5,94E-05	1,46E-02

cg26694538	13	49458503		IGR	-0,017	5,94E-05	1,46E-02
cg09635053	16	1031944	SOX8	1stExon	-0,019	5,95E-05	1,46E-02
cg22624572	18	13886087	MC2R	5'UTR	-0,031	5,94E-05	1,46E-02
cg08729407	17	28431896	EFCAB5	Body	-0,005	5,95E-05	1,46E-02
cg22248648	11	17626394	OTOG	Body	-0,06	5,96E-05	1,47E-02
cg12188482	17	4046863	ZZEF1	TSS1500	-0,003	5,97E-05	1,47E-02
cg21616405	12	109246512	SSH1	Body	0,008	5,97E-05	1,47E-02
cg23998987	16	2021791	TBL3	TSS1500	-0,003	5,97E-05	1,47E-02
cg13436451	6	168378673	HGC6.3	TSS1500	0,006	5,98E-05	1,47E-02
cg03314338	7	130738644	LINC-PINT	Body	-0,006	5,98E-05	1,47E-02
cg16811695	10	115999009	VWA2	TSS200	-0,01	5,98E-05	1,47E-02
cg00267746	20	57463984	GNAS	3'UTR	0,022	5,99E-05	1,47E-02
cg17347633	8	126379036	NSMCE2	Body	-0,005	5,99E-05	1,47E-02
cg14076329	1	244882957		IGR	-0,02	5,99E-05	1,47E-02
cg23365999	1	198460992		IGR	0,047	6,01E-05	1,47E-02
cg10868218	3	113187660	SPICE1	Body	0,011	6,01E-05	1,47E-02
cg16423770	9	115036366	PTBP3	Body	0,018	6,00E-05	1,47E-02
cg13862711	9	124989915	LHX6	Body	-0,209	6,01E-05	1,47E-02
cg17579242	10	43394705		IGR	0,005	6,00E-05	1,47E-02
cg08251636	12	46653890	SLC38A1	5'UTR	0,036	6,01E-05	1,47E-02
cg00007540	14	103609265		IGR	0,025	6,01E-05	1,47E-02
cg26914616	6	127980151		IGR	-0,01	6,02E-05	1,47E-02
cg04286158	3	140732678		IGR	0,037	6,02E-05	1,47E-02
cg23046990	3	177340437	LINC00578	Body	0,006	6,03E-05	1,47E-02
cg14622996	6	32109801		IGR	0,025	6,03E-05	1,47E-02
cg01606197	6	33679137	C6orf125	Body	-0,004	6,03E-05	1,47E-02
cg00955432	9	133491847	FUBP3	Body	-0,007	6,03E-05	1,47E-02
cg04511352	4	12958421		IGR	-0,042	6,04E-05	1,47E-02
cg23895495	7	47539837	TNS3	5'UTR	0,042	6,03E-05	1,47E-02
cg02235123	10	11944159		IGR	-0,019	6,04E-05	1,47E-02
cg26869177	13	21994997	ZDHHC20	Body	0,033	6,04E-05	1,47E-02
cg00107631	16	86741267		IGR	-0,016	6,04E-05	1,47E-02
cg00359683	22	26423016	MYO18B	Body	-0,021	6,04E-05	1,47E-02
cg12197062	3	114402662	ZBTB20	Body	-0,01	6,06E-05	1,47E-02
cg13196143	9	35748228	GBA2	Body	0,033	6,05E-05	1,47E-02
cg20667128	18	6018560	L3MBTL4	Body	-0,024	6,06E-05	1,47E-02
cg13384453	1	174968388	CACYBP	TSS1500	-0,004	6,07E-05	1,47E-02
cg02049753	9	94186939	NFIL3	TSS1500	-0,004	6,07E-05	1,47E-02
cg04456664	14	29692072		IGR	-0,035	6,07E-05	1,47E-02
cg14524176	16	27780757	KIAA0556	Body	0,008	6,07E-05	1,47E-02
cg01293850	16	30086387	PPP4C	TSS1500	0,011	6,06E-05	1,47E-02
cg22676000	19	42259081	CEACAM6	TSS1500	0,022	6,06E-05	1,47E-02
cg16707423	1	227167020	CABC1	Body	0,006	6,08E-05	1,48E-02
cg25907642	5	78260853	ARSB	Body	0,021	6,08E-05	1,48E-02
cg01551441	5	158477707	EBF1	Body	0,022	6,08E-05	1,48E-02
cg19720179	6	155451057	TIAM2	Body	-0,015	6,08E-05	1,48E-02
cg13000555	12	104460455	HCFC2	Body	-0,018	6,08E-05	1,48E-02
cg06985153	15	90438687	AP3S2	TSS1500	-0,007	6,09E-05	1,48E-02
cg27419138	6	117016617	KPNA5	Body	0,026	6,09E-05	1,48E-02
cg12397205	11	1244002	MUC5B	TSS1500	0,027	6,09E-05	1,48E-02
cg16688269	12	83496609	TMTC2	Body	0,013	6,10E-05	1,48E-02
cg10928426	7	127997529	PRRT4	Body	0,014	6,10E-05	1,48E-02
cg02616786	10	135279147	LOC619207	Body	-0,065	6,11E-05	1,48E-02
cg04636811	11	134048956	NCAPD3	Body	0,009	6,12E-05	1,48E-02
cg08632088	20	47864756	ZNFX1	Body	0,016	6,12E-05	1,48E-02
cg03546873	16	10851555	NUBP1	Body	0,007	6,13E-05	1,48E-02
cg26050798	4	71875339	DCK	Body	0,036	6,14E-05	1,48E-02
cg22874772	19	865897		IGR	-0,012	6,14E-05	1,48E-02
cg25229964	1	26503623	CNKSR1	TSS1500	0,009	6,15E-05	1,48E-02
cg22128197	1	154529357	UBE2Q1	Body	0,031	6,14E-05	1,48E-02
cg27663464	4	1005900	FGFRL1	TSS1500	0,005	6,14E-05	1,48E-02
cg04939222	5	153899878		IGR	0,006	6,15E-05	1,48E-02
cg01139966	1	150669796	GOLPH3L	TSS200	-0,004	6,16E-05	1,48E-02
cg06905766	1	232306788		IGR	-0,017	6,16E-05	1,48E-02
cg23218382	2	48469429		IGR	-0,019	6,16E-05	1,48E-02
cg06070951	3	48506432	TREX1	TSS1500	0,009	6,16E-05	1,48E-02
cg14391684	6	116967245	ZUFSP	Body	0,046	6,15E-05	1,48E-02
cg07500713	6	168896207	SMOC2	Body	-0,013	6,16E-05	1,48E-02
cg13832457	7	590072	PRKAR1B	Body	0,017	6,15E-05	1,48E-02
cg14774086	22	24181270	DERL3	TSS200	-0,004	6,15E-05	1,48E-02
cg02683509	6	156950855		IGR	-0,01	6,17E-05	1,48E-02
cg21600154	11	125972724		IGR	0,037	6,17E-05	1,48E-02
cg22009136	14	94755491	SERPINA10	Body	0,057	6,17E-05	1,48E-02

cg00393533	22	26801377		IGR	0,05	6,17E-05	1,48E-02
cg13972353	9	139354348	SEC16A	Body	-0,008	6,18E-05	1,48E-02
cg18645297	7	130419594	KLF14	TSS1500	-0,01	6,18E-05	1,49E-02
cg02873048	2	37559202		IGR	-0,006	6,19E-05	1,49E-02
cg20785915	10	79161677	KCNMA1	Body	0,015	6,19E-05	1,49E-02
cg26888807	3	79634890	ROBO1	Body	0,044	6,19E-05	1,49E-02
cg07504581	5	1196279		IGR	-0,031	6,19E-05	1,49E-02
cg23221681	1	89358385	GTF2B	TSS1500	-0,024	6,21E-05	1,49E-02
cg06716807	2	27632449	PPM1G	TSS200	0,007	6,20E-05	1,49E-02
cg16758272	3	42258157	TRAK1	Body	-0,028	6,20E-05	1,49E-02
cg13389209	9	137689064	COL5A1	Body	0,014	6,20E-05	1,49E-02
cg27143842	12	671027	B4GALNT3	3'UTR	0,014	6,21E-05	1,49E-02
cg21058710	12	125349752	SCARB1	TSS1500	0,014	6,21E-05	1,49E-02
cg08347042	19	45579378	ZNF296	1stExon	-0,004	6,21E-05	1,49E-02
cg06710735	3	48264567	CAMP	TSS1500	0,041	6,22E-05	1,49E-02
cg10548410	5	27369586		IGR	-0,035	6,22E-05	1,49E-02
cg13832372	9	124983482	LHX6	Body	0,036	6,22E-05	1,49E-02
cg14449789	14	104408191	RD3L	5'UTR	0,031	6,22E-05	1,49E-02
cg08682936	15	98417417	LINC00923	Body	-0,008	6,22E-05	1,49E-02
cg11216176	1	230493133	PGBD5	Body	-0,021	6,24E-05	1,49E-02
cg15387777	2	201809187	ORC2	Body	-0,009	6,23E-05	1,49E-02
cg21910367	4	6557423	PPP2R2C	TSS200	-0,017	6,24E-05	1,49E-02
cg22324709	5	31825953	PDZD2	Body	-0,007	6,24E-05	1,49E-02
cg03141756	6	31651676		IGR	0,019	6,25E-05	1,49E-02
cg09548390	8	51555740	SNTG1	Body	-0,041	6,24E-05	1,49E-02
cg08284758	15	29552456	FAM189A1	Body	-0,021	6,24E-05	1,49E-02
cg05522885	19	4471596	HDGF2	TSS1500	-0,011	6,25E-05	1,49E-02
cg22415594	13	46626866	ZC3H13	5'UTR	0,008	6,25E-05	1,49E-02
cg08827427	19	412912		IGR	-0,032	6,26E-05	1,49E-02
cg11867599	4	128887130	MFSD8	5'UTR	0,014	6,26E-05	1,49E-02
cg20724184	13	22178880	EFHA1	TSS1500	-0,013	6,26E-05	1,49E-02
cg19690494	13	74182598		IGR	-0,008	6,27E-05	1,49E-02
cg04523603	1	3622085	TP73	Body	-0,012	6,27E-05	1,49E-02
cg16117910	2	109745648	SH3RF3	TSS1500	-0,005	6,28E-05	1,49E-02
cg25660890	16	2069394	NPW	TSS200	0,009	6,29E-05	1,49E-02
cg13875133	9	118917650	PAPPA	Body	-0,011	6,29E-05	1,49E-02
cg00189366	10	43846705		IGR	-0,049	6,29E-05	1,49E-02
cg15400997	12	94580329	PLXNC1	Body	0,014	6,29E-05	1,49E-02
cg20302133	1	111217194	KCNA3	1stExon	0,008	6,30E-05	1,49E-02
cg22247748	2	134326204	NCKAP5	TSS200	-0,023	6,30E-05	1,49E-02
cg23244790	5	140811102	PCDHGA4	Body	-0,021	6,30E-05	1,49E-02
cg04509542	11	29358540		IGR	-0,036	6,30E-05	1,49E-02
cg16541929	15	40214068	GPR176	TSS1500	0,007	6,31E-05	1,49E-02
cg05138892	16	86327786		IGR	-0,044	6,30E-05	1,49E-02
cg17236853	1	156792882	NTRK1	Body	0,016	6,31E-05	1,50E-02
cg13628006	10	34446040	PARD3	Body	0,019	6,31E-05	1,50E-02
cg17563271	17	77632749		IGR	-0,06	6,32E-05	1,50E-02
cg07309361	3	52001477	PCBP4	TSS200	-0,006	6,33E-05	1,50E-02
cg22195209	6	108372344	OSTM1	Body	0,045	6,32E-05	1,50E-02
cg22644163	7	100421510	EPHB4	Body	0,008	6,33E-05	1,50E-02
cg14524337	9	100459217	XPA	Body	-0,005	6,33E-05	1,50E-02
cg04665351	12	3000000	TULP3	TSS200	0,009	6,33E-05	1,50E-02
cg06051392	4	776055	LOC100129917	TSS1500	-0,003	6,35E-05	1,50E-02
cg01028287	7	44275556	CAMK2B	Body	0,016	6,34E-05	1,50E-02
cg06866686	14	95236879	GSC	TSS1500	-0,018	6,34E-05	1,50E-02
cg21155461	19	58740253	ZNF544	5'UTR	-0,008	6,34E-05	1,50E-02
cg05628436	22	39186607	DNAL4	5'UTR	-0,005	6,35E-05	1,50E-02
cg08862774	1	22110598	USP48	TSS1500	-0,02	6,36E-05	1,50E-02
cg02907737	22	46519334		IGR	0,025	6,36E-05	1,50E-02
cg11390982	7	1462773		IGR	-0,021	6,37E-05	1,50E-02
cg01048764	1	201838945	IPO9	Body	-0,007	6,37E-05	1,50E-02
cg21975462	11	95032879		IGR	0,017	6,37E-05	1,50E-02
cg13537146	3	12926674		IGR	0,018	6,38E-05	1,50E-02
cg21741806	13	89719325		IGR	-0,02	6,38E-05	1,50E-02
cg10415968	3	175926854		IGR	-0,014	6,39E-05	1,50E-02
cg13499966	2	10220719	CYS1	TSS200	0,009	6,40E-05	1,50E-02
cg08904194	6	159295138		IGR	0,036	6,40E-05	1,50E-02
cg26102806	7	43183715	HECW1	5'UTR	-0,018	6,40E-05	1,50E-02
cg14916389	7	135371462	SLC13A4	Body	0,007	6,40E-05	1,50E-02
cg03228945	9	130287882	FAM129B	Body	-0,014	6,40E-05	1,50E-02
cg05775658	10	49482723	FRMPD2	5'UTR	-0,007	6,39E-05	1,50E-02
cg04596539	20	55963005		IGR	0,008	6,40E-05	1,50E-02
cg23060193	1	235490959	ARID4B	5'UTR	0,007	6,42E-05	1,51E-02

cg00610000	2	58272914	VRK2	TSS1500	0,015	6,42E-05	1,51E-02
cg24335984	3	50361554	HYAL2	TSS1500	0,015	6,43E-05	1,51E-02
cg15155239	6	132273155	CTGF	TSS1500	-0,012	6,42E-05	1,51E-02
cg05953394	7	151573773	PRKAG2	5'UTR	0,007	6,42E-05	1,51E-02
cg20424239	11	129855994	PRDM10	5'UTR	0,017	6,41E-05	1,51E-02
cg19188370	12	125103181		IGR	-0,014	6,42E-05	1,51E-02
cg01856053	13	42728142	DGKH	5'UTR	-0,005	6,42E-05	1,51E-02
cg17387838	19	54515811	CACNG6	3'UTR	-0,031	6,42E-05	1,51E-02
cg07072257	6	13625427	RANBP9	Body	-0,026	6,43E-05	1,51E-02
cg06203959	22	39760924	SYNGR1	Body	-0,018	6,43E-05	1,51E-02
cg06507232	3	187838411		IGR	0,005	6,44E-05	1,51E-02
cg05721515	10	8095288	FLJ45983	Body	-0,021	6,44E-05	1,51E-02
cg26894435	2	203242089	BMPR2	5'UTR	-0,004	6,45E-05	1,51E-02
cg07353006	8	10405104		IGR	0,016	6,44E-05	1,51E-02
cg23679432	12	32836024	DNM1L	Body	0,009	6,44E-05	1,51E-02
cg27619796	16	86756180		IGR	-0,027	6,45E-05	1,51E-02
cg08575141	20	54923208		IGR	0,056	6,46E-05	1,51E-02
cg24941435	6	159535051		IGR	-0,024	6,46E-05	1,51E-02
cg15571162	12	32368230	BICD1	Body	0,009	6,46E-05	1,51E-02
cg03095607	2	1846569	MYT1L	Body	-0,022	6,47E-05	1,51E-02
cg18686498	13	102069257	NALCN	TSS1500	-0,016	6,48E-05	1,51E-02
cg02220284	3	71013485	FOXP1	Body	0,035	6,49E-05	1,51E-02
cg03095527	19	1230768	C19orf26	3'UTR	0,013	6,49E-05	1,51E-02
cg16956232	7	127292550	SND1	Body	-0,005	6,51E-05	1,52E-02
cg09806151	2	23358544		IGR	0,04	6,53E-05	1,52E-02
cg00917642	4	1208960	CTBP1	Body	0,012	6,53E-05	1,52E-02
cg13023584	5	140821425	PCDHGA4	Body	-0,04	6,53E-05	1,52E-02
cg05500783	6	32410873	HLA-DRA	Body	0,031	6,53E-05	1,52E-02
cg05804440	7	64709189		IGR	0,022	6,52E-05	1,52E-02
cg01886151	10	59804100		IGR	-0,027	6,53E-05	1,52E-02
cg04980297	12	68717066	MDM1	Body	0,031	6,52E-05	1,52E-02
cg09000356	17	75084634	SCARNA16	TSS1500	-0,004	6,52E-05	1,52E-02
cg15988320	5	140868422	PCDHGA4	Body	-0,048	6,53E-05	1,52E-02
cg21348586	8	141461100	TRAPPC9	Body	0,006	6,54E-05	1,52E-02
cg27021587	1	1758687	GNB1	5'UTR	0,015	6,55E-05	1,52E-02
cg18301048	6	30685243	MDC1	1stExon	-0,003	6,54E-05	1,52E-02
cg16829236	7	94123583		IGR	-0,008	6,54E-05	1,52E-02
cg06742341	11	75380355	MAP6	TSS1500	-0,039	6,55E-05	1,52E-02
cg01482958	1	85357136	LPAR3	5'UTR	-0,008	6,56E-05	1,52E-02
cg05006142	6	170536124		IGR	0,06	6,56E-05	1,52E-02
cg16252312	8	72523563		IGR	-0,008	6,56E-05	1,52E-02
cg01797704	16	81577049	CMIP	Body	0,016	6,55E-05	1,52E-02
cg04878851	17	76778451	CYTH1	TSS200	0,012	6,55E-05	1,52E-02
cg23248424	5	179741104	GFPT2	Body	-0,193	6,57E-05	1,52E-02
cg27611994	7	995984	ADAP1	TSS1500	0,011	6,57E-05	1,52E-02
cg04503160	19	50431843	ATF5	TSS1500	0,028	6,58E-05	1,52E-02
cg05068322	6	105405275	LIN28B	Body	-0,015	6,59E-05	1,52E-02
cg02087005	7	156837151		IGR	-0,035	6,59E-05	1,52E-02
cg11836585	15	59225166	SLTM	Body	-0,007	6,59E-05	1,52E-02
cg11517017	22	26156974	MYO18B	5'UTR	0,017	6,59E-05	1,52E-02
cg17668274	5	31162289		IGR	-0,034	6,60E-05	1,52E-02
cg23138980	5	72728265		IGR	0,02	6,61E-05	1,53E-02
cg09075525	13	25940208		IGR	-0,007	6,61E-05	1,53E-02
cg02648999	2	105946563	TGFBRAP1	TSS1500	-0,006	6,62E-05	1,53E-02
cg15061569	19	53832670		IGR	-0,074	6,62E-05	1,53E-02
cg06858239	3	195012474	ACAP2	Body	0,036	6,64E-05	1,53E-02
cg25283850	8	29713495		IGR	-0,009	6,63E-05	1,53E-02
cg01962498	8	30299680	RBPMS	Body	-0,006	6,64E-05	1,53E-02
cg04332672	9	139690028	CCDC183	TSS1500	0,016	6,63E-05	1,53E-02
cg16066696	14	77843743	C14orf174	TSS200	0,006	6,64E-05	1,53E-02
cg03549163	16	68397790	SMPD3	Body	-0,028	6,64E-05	1,53E-02
cg17382459	19	56348216	NLRP4	1stExon	-0,039	6,64E-05	1,53E-02
cg25826386	22	31285774	OSBP2	Body	0,013	6,63E-05	1,53E-02
cg05368762	12	50135785	TMBIM6	5'UTR	0,005	6,65E-05	1,53E-02
cg09364660	1	39338525	MYCBP	Body	-0,003	6,65E-05	1,53E-02
cg07802710	5	140810260	PCDHGA4	Body	-0,029	6,66E-05	1,53E-02
cg08613370	12	6270824		IGR	0,024	6,66E-05	1,53E-02
cg00000109	3	171916037	FNDC3B	Body	0,011	6,68E-05	1,54E-02
cg19698675	5	150467411	TNIP1	TSS1500	-0,008	6,68E-05	1,54E-02
cg27216355	16	71599010		IGR	-0,005	6,70E-05	1,54E-02
cg02366519	4	8145692	ABLIM2	Body	0,035	6,71E-05	1,54E-02
cg21312906	16	24857529	SLC5A11	5'UTR	0,017	6,71E-05	1,54E-02
cg15077085	18	78004728	PARD6G	Body	-0,005	6,71E-05	1,54E-02

cg13177860	6	79881191		IGR	0,015	6,71E-05	1,54E-02
cg06800849	16	89180587	ACSF3	Body	0,03	6,72E-05	1,54E-02
cg20546331	19	36450358		IGR	-0,013	6,72E-05	1,54E-02
cg12550074	7	51698710		IGR	-0,007	6,72E-05	1,54E-02
cg11859203	20	31316280	COMMD7	Body	-0,028	6,72E-05	1,54E-02
cg02901159	11	77348811	CLNS1A	1stExon	-0,002	6,72E-05	1,54E-02
cg01351925	5	141276282	LOC729080	TSS200	0,006	6,73E-05	1,54E-02
cg16874804	2	105915145	TGFBRAP1	ExonBnd	-0,005	6,73E-05	1,54E-02
cg01905102	1	244815677	PPPDE1	TSS1500	0,034	6,73E-05	1,54E-02
cg15653044	17	4545718	ALOX15	TSS1500	-0,055	6,74E-05	1,54E-02
cg07231479	5	140794359	PCDHGA4	Body	-0,038	6,74E-05	1,54E-02
cg11601955	8	141931458	PTK2	Body	-0,009	6,75E-05	1,54E-02
cg15345107	7	55198494	EGFR	Body	-0,036	6,75E-05	1,54E-02
cg22180100	1	151566791		IGR	-0,003	6,75E-05	1,54E-02
cg01904151	3	156446700		IGR	-0,005	6,76E-05	1,54E-02
cg16393844	3	191750099		IGR	-0,042	6,76E-05	1,54E-02
cg24228133	5	102343253	PAM	Body	0,007	6,76E-05	1,54E-02
cg20369513	6	31597496	BAT2	Body	0,008	6,77E-05	1,54E-02
cg04520078	8	43101622		IGR	-0,074	6,77E-05	1,54E-02
cg02424858	11	78295329		IGR	0,023	6,77E-05	1,54E-02
cg08342675	2	201676391	BZW1	TSS1500	-0,006	6,77E-05	1,54E-02
cg10172476	20	30844736		IGR	-0,017	6,77E-05	1,54E-02
cg00482898	2	70521446	SNRPG	TSS1500	-0,018	6,78E-05	1,55E-02
cg07078644	1	109656737	KIAA1324	5'UTR	-0,003	6,78E-05	1,55E-02
cg23344409	19	16997461		IGR	0,015	6,79E-05	1,55E-02
cg04012600	1	109850126	MYBPHL	TSS1500	0,034	6,79E-05	1,55E-02
cg11267359	6	1986540	GMDS	Body	-0,006	6,79E-05	1,55E-02
cg26407316	14	50801745	CDKL1	Body	0,026	6,79E-05	1,55E-02
cg16652328	3	33138982	GLB1	TSS1500	-0,004	6,80E-05	1,55E-02
cg19778785	14	53145161	ERO1A	ExonBnd	0,011	6,80E-05	1,55E-02
cg23762900	15	63888599	FBXL22	TSS1500	0,013	6,81E-05	1,55E-02
cg11036715	15	71185960	THAP10	TSS1500	0,038	6,81E-05	1,55E-02
cg21180345	17	58823065	BCAS3	Body	0,023	6,81E-05	1,55E-02
cg21970632	18	76272071		IGR	0,017	6,81E-05	1,55E-02
cg01959980	2	73616832	ALMS1	Body	0,02	6,82E-05	1,55E-02
cg00705922	19	48677585		IGR	-0,01	6,81E-05	1,55E-02
cg15477738	2	174824642	SP3	Body	-0,012	6,83E-05	1,55E-02
cg06520285	10	35345625	CUL2	Body	-0,007	6,83E-05	1,55E-02
cg13048425	5	163223354		IGR	-0,025	6,83E-05	1,55E-02
cg15094117	5	147184031		IGR	0,021	6,84E-05	1,55E-02
cg27506098	10	32649455	EPC1	Body	-0,005	6,84E-05	1,55E-02
cg05120716	16	23881993	PRKCB	Body	0,009	6,84E-05	1,55E-02
cg08941873	5	118308850	DTWD2	Body	-0,024	6,85E-05	1,55E-02
cg12977346	8	8621874		IGR	-0,017	6,85E-05	1,55E-02
cg21762727	17	7163136	C17orf81	3'UTR	-0,009	6,86E-05	1,55E-02
cg10037204	2	240655816		IGR	-0,007	6,86E-05	1,55E-02
cg18006179	4	68622740	GNRHR	TSS1500	0,016	6,86E-05	1,55E-02
cg20244386	8	37804398		IGR	0,007	6,86E-05	1,55E-02
cg02267483	5	140810404	PCDHGA4	Body	-0,044	6,87E-05	1,55E-02
cg17472152	6	143486011	AIG1	Body	-0,035	6,88E-05	1,55E-02
cg18456139	3	195001478	ACAP2	Body	0,025	6,89E-05	1,56E-02
cg10218605	7	158379132	PTPRN2	Body	-0,051	6,89E-05	1,56E-02
cg25317548	2	114481691	SLC35F5	Body	-0,041	6,90E-05	1,56E-02
cg05587359	8	83219871		IGR	0,014	6,90E-05	1,56E-02
cg23743252	5	141348279	RNF14	5'UTR	0,036	6,91E-05	1,56E-02
cg13264672	7	39993440	CDK13	Body	0,091	6,91E-05	1,56E-02
cg16875813	1	179057836	TOR3A	Body	0,017	6,91E-05	1,56E-02
cg12869958	11	67017687	KDM2A	Body	-0,009	6,92E-05	1,56E-02
cg03772739	7	28930250		IGR	0,063	6,93E-05	1,56E-02
cg16703673	16	50241762	PAPD5	Body	-0,03	6,93E-05	1,56E-02
cg20254011	1	221717084		IGR	-0,041	6,95E-05	1,57E-02
cg08906298	7	53968788		IGR	-0,009	6,95E-05	1,57E-02
cg19384852	9	6758071	KDM4C	1stExon	-0,004	6,95E-05	1,57E-02
cg08330117	8	90770456	RIPK2	1stExon	-0,004	6,96E-05	1,57E-02
cg10909749	20	16333615	KIF16B	Body	-0,01	6,96E-05	1,57E-02
cg23465999	2	22333202		IGR	-0,016	6,97E-05	1,57E-02
cg24417988	10	102955697		IGR	0,019	6,97E-05	1,57E-02
cg26883095	16	80858912		IGR	-0,028	6,97E-05	1,57E-02
cg18701598	10	91011892	LIPA	TSS1500	-0,005	6,98E-05	1,57E-02
cg03180404	11	126907483		IGR	-0,007	6,98E-05	1,57E-02
cg24027844	1	102375748	OLFM3	Body	-0,008	6,99E-05	1,57E-02
cg16316407	1	232121887	DISC1	Body	-0,007	6,98E-05	1,57E-02
cg16438246	8	145532913	HSF1	Body	0,009	6,99E-05	1,57E-02

cg23670372	1	10732290	CASZ1	Body	0,014	7,00E-05	1,57E-02
cg14432474	1	212606122	NENF	TSS200	-0,01	7,00E-05	1,57E-02
cg12446286	4	26050316		IGR	-0,005	7,00E-05	1,57E-02
cg08473883	17	42374698		IGR	-0,009	7,01E-05	1,57E-02
cg10655534	3	152101463	MBNL1	Body	0,008	7,01E-05	1,57E-02
cg07881458	14	106692325		IGR	-0,024	7,02E-05	1,57E-02
cg16301256	10	73045356	UNC5B	Body	0,022	7,02E-05	1,57E-02
cg14477114	3	65914296	MAGI1	Body	-0,008	7,03E-05	1,57E-02
cg03925904	4	187192739	F11	Body	0,009	7,03E-05	1,57E-02
cg20724494	1	35736459	ZMYM4	Body	0,015	7,04E-05	1,57E-02
cg09330242	9	125281532	OR1J4	1stExon	-0,034	7,03E-05	1,57E-02
cg13866149	9	127036966	NEK6	5'UTR	0,01	7,04E-05	1,57E-02
cg23454427	6	45966169	CLIC5	Body	-0,006	7,04E-05	1,57E-02
cg14871096	22	22120295	MAPK1	3'UTR	-0,008	7,04E-05	1,57E-02
cg24759279	10	7128104		IGR	-0,015	7,05E-05	1,57E-02
cg25135457	17	40715244	COASY	1stExon	0,024	7,05E-05	1,57E-02
cg06906598	17	80546637	FOXK2	Body	0,009	7,05E-05	1,57E-02
cg06084526	2	47445633	LOC101927043	Body	0,033	7,06E-05	1,57E-02
cg06398390	7	50815700	GRB10	5'UTR	-0,021	7,06E-05	1,57E-02
cg11996870	7	91795403	LOC401387	TSS1500	-0,007	7,06E-05	1,57E-02
cg05816827	11	78960662	TENM4	5'UTR	-0,015	7,06E-05	1,57E-02
cg02581146	19	9846701		IGR	-0,011	7,06E-05	1,57E-02
cg27301228	1	97188081	PTBP2	TSS200	-0,013	7,07E-05	1,58E-02
cg04750910	10	102416409		IGR	-0,006	7,07E-05	1,58E-02
cg26005165	20	62339510	ZGPAT	5'UTR	-0,003	7,08E-05	1,58E-02
cg10985150	6	150989765	PLEKHG1	5'UTR	0,021	7,08E-05	1,58E-02
cg08496872	1	10290272	KIF1B	5'UTR	-0,008	7,09E-05	1,58E-02
cg06668300	2	95691755	MAL	Body	-0,007	7,10E-05	1,58E-02
cg11211103	5	123155696		IGR	-0,009	7,09E-05	1,58E-02
cg00157109	7	151569191	PRKAG2	Body	0,034	7,09E-05	1,58E-02
cg01575096	2	238969261	SCLY	TSS1500	-0,004	7,10E-05	1,58E-02
cg09771226	17	45786313	TBKBP1	Body	-0,006	7,11E-05	1,58E-02
cg24578428	4	8621226	CPZ	Body	0,013	7,12E-05	1,58E-02
cg21272827	4	169048496	ANXA10	Body	0,028	7,12E-05	1,58E-02
cg10403906	6	31697280	DDAH2	5'UTR	-0,005	7,12E-05	1,58E-02
cg11635557	20	49493097	BCAS4	Body	0,01	7,12E-05	1,58E-02
cg12610744	12	53208762	KRT4	TSS1500	-0,012	7,13E-05	1,58E-02
cg25735648	15	35998009		IGR	-0,039	7,13E-05	1,58E-02
cg07088913	4	37635937	RELL1	Body	0,017	7,14E-05	1,58E-02
cg02600653	3	19431990	KCNH8	ExonBnd	0,014	7,14E-05	1,58E-02
cg22826038	10	121169671	GRK5	Body	-0,013	7,15E-05	1,59E-02
cg04272632	1	34629400	CSMD2	Body	-0,01	7,16E-05	1,59E-02
cg07633367	2	48676137	PPP1R21	Body	-0,008	7,16E-05	1,59E-02
cg24852777	12	111488026	CUX2	Body	0,022	7,16E-05	1,59E-02
cg08896420	15	89453807	MFGE8	Body	0,034	7,16E-05	1,59E-02
cg25333586	4	122251075	QRFPR	Body	-0,015	7,17E-05	1,59E-02
cg21513009	2	72079294		IGR	-0,056	7,17E-05	1,59E-02
cg07175985	13	24007965	SACS	TSS200	-0,02	7,17E-05	1,59E-02
cg01221593	2	24492531	ITSN2	Body	-0,004	7,22E-05	1,59E-02
cg00712943	2	31806781	SRD5A2	TSS1500	-0,073	7,21E-05	1,59E-02
cg20337959	2	71014835	FIGLA	Body	0,024	7,20E-05	1,59E-02
cg17796534	2	216410812		IGR	0,031	7,23E-05	1,59E-02
cg23146699	3	66020673	MAGI1	Body	0,042	7,18E-05	1,59E-02
cg04258358	4	728867	PCGF3	Body	0,008	7,22E-05	1,59E-02
cg15717917	5	117661944		IGR	-0,026	7,18E-05	1,59E-02
cg20825168	5	132050793	KIF3A	Body	-0,024	7,19E-05	1,59E-02
cg12043062	5	140729723	PCDHGB1	TSS200	-0,04	7,22E-05	1,59E-02
cg23305428	7	152009390	KMT2C	Body	0,015	7,20E-05	1,59E-02
cg09933929	7	154677023	DPP6	Body	-0,025	7,23E-05	1,59E-02
cg06948120	8	17743177	FGL1	5'UTR	0,009	7,19E-05	1,59E-02
cg25283565	8	54756094	ATP6V1H	TSS1500	-0,003	7,22E-05	1,59E-02
cg05226457	9	115095963	PTBP3	TSS1500	-0,004	7,19E-05	1,59E-02
cg09279228	10	1034461	GTPBP4	1stExon	-0,004	7,19E-05	1,59E-02
cg14002159	10	134992030	KNDC1	Body	0,012	7,20E-05	1,59E-02
cg08448080	11	3892376	STIM1	Body	-0,026	7,22E-05	1,59E-02
cg24255209	11	72863808		IGR	0,016	7,21E-05	1,59E-02
cg19696970	11	91944842		IGR	-0,011	7,21E-05	1,59E-02
cg25502233	12	132851931	LOC100130238	TSS200	0,038	7,18E-05	1,59E-02
cg16676734	14	93582668	ITPK1	TSS1500	-0,006	7,23E-05	1,59E-02
cg02713760	15	83953998	BNC1	TSS1500	-0,021	7,22E-05	1,59E-02
cg07511128	17	59408248	BCAS3	Body	0,024	7,21E-05	1,59E-02
cg15824864	17	77812175	CBX4	Body	-0,005	7,22E-05	1,59E-02
cg25213718	21	25333353		IGR	0,031	7,20E-05	1,59E-02

cg10209441	22	45065052	PRR5	5'UTR	-0,004	7,22E-05	1,59E-02
cg01439264	5	140792540	PCDHGA10	TSS1500	-0,018	7,23E-05	1,59E-02
cg22944932	14	93260360	GOLGA5	TSS1500	0,004	7,24E-05	1,59E-02
cg01637548	20	62574518	UCKL1	Body	0,018	7,24E-05	1,59E-02
cg01753468	1	56598336		IGR	-0,007	7,24E-05	1,59E-02
cg26821344	2	43805673	THADA	Body	0,018	7,25E-05	1,59E-02
cg09395732	3	10857456	SLC6A11	TSS1500	-0,03	7,26E-05	1,59E-02
cg03344068	3	125154426		IGR	0,011	7,26E-05	1,59E-02
cg04097219	5	142629749		IGR	0,007	7,26E-05	1,59E-02
cg20830615	9	136753686	VAV2	Body	0,016	7,26E-05	1,59E-02
cg19016062	10	5904561	ANKRD16	3'UTR	0,017	7,26E-05	1,59E-02
cg16698903	10	74167485	MICU1	Body	0,015	7,25E-05	1,59E-02
cg05299140	17	47972401		IGR	0,028	7,25E-05	1,59E-02
cg08744476	20	32226532	CBFA2T2	Body	0,004	7,25E-05	1,59E-02
cg06369493	22	22399223		IGR	-0,008	7,26E-05	1,59E-02
cg16065784	8	144870949		IGR	0,011	7,27E-05	1,59E-02
cg07043797	2	101642961	TBC1D8	Body	-0,064	7,28E-05	1,59E-02
cg03823722	10	3214957	PITRM1	1stExon	-0,004	7,27E-05	1,59E-02
cg26236741	10	130610237		IGR	-0,024	7,27E-05	1,59E-02
cg02931353	20	17294918	PCSK2	Body	-0,014	7,27E-05	1,59E-02
cg19478951	10	133879084		IGR	0,04	7,28E-05	1,59E-02
cg14298242	8	2018128	MYOM2	Body	-0,017	7,29E-05	1,59E-02
cg05779818	2	20514510	PUM2	Body	0,012	7,29E-05	1,59E-02
cg11091731	11	9701847	SWAP70	Body	0,017	7,29E-05	1,59E-02
cg01480638	18	13810979		IGR	-0,044	7,29E-05	1,59E-02
cg09624514	7	44103435	PGAM2	Body	0,018	7,31E-05	1,59E-02
cg14638453	11	63804481	MACROD1	Body	0,016	7,31E-05	1,59E-02
cg22044630	12	133127993	FBRSL1	Body	0,01	7,31E-05	1,59E-02
cg16307144	19	38704933	DPF1	Body	-0,047	7,31E-05	1,59E-02
cg26199434	15	32969882	SCG5	Body	-0,006	7,32E-05	1,59E-02
cg12868544	6	31687926	LY6G6C	Body	-0,009	7,32E-05	1,59E-02
cg07831198	19	39148414	ACTN4	Body	0,025	7,33E-05	1,59E-02
cg16514115	9	74600633		IGR	-0,005	7,33E-05	1,59E-02
cg09610614	11	1104473		IGR	-0,008	7,34E-05	1,59E-02
cg04698521	2	240197864	HDAC4	Body	0,022	7,35E-05	1,60E-02
cg10154826	6	17600994	FAM8A1	1stExon	0,086	7,35E-05	1,60E-02
cg03157150	16	4738680	MGRN1	3'UTR	0,009	7,35E-05	1,60E-02
cg05724777	6	32634362	HLA-DQB1	1stExon	0,032	7,36E-05	1,60E-02
cg13510360	9	139795609	TRAF2	Body	0,06	7,36E-05	1,60E-02
cg17339617	5	146608294		IGR	-0,046	7,37E-05	1,60E-02
cg06805323	6	31634821	CSNK2B	Body	0,022	7,38E-05	1,60E-02
cg04577276	13	99740157	DOCK9	TSS1500	-0,004	7,38E-05	1,60E-02
cg04900856	19	6534763	TNFSF9	Body	0,01	7,38E-05	1,60E-02
cg03602029	6	137814078	OLIG3	1stExon	-0,041	7,39E-05	1,60E-02
cg11170552	11	69307431		IGR	-0,013	7,39E-05	1,60E-02
cg14892570	19	15559628	MIR1470	TSS1500	0,022	7,39E-05	1,60E-02
cg13349742	2	235080074		IGR	0,012	7,40E-05	1,60E-02
cg24847541	13	92051154	GPC5	5'UTR	-0,004	7,40E-05	1,60E-02
cg10775230	3	46742491	TMIE	TSS1500	0,018	7,40E-05	1,60E-02
cg11811828	6	31148666		IGR	-0,1	7,41E-05	1,60E-02
cg26803100	14	35344842	BAZ1A	5'UTR	-0,019	7,41E-05	1,60E-02
cg27586071	2	230453157	DNER	Body	-0,017	7,41E-05	1,60E-02
cg05618934	4	1407592		IGR	-0,051	7,43E-05	1,61E-02
cg27243847	5	131730593	SLC22A5	3'UTR	0,027	7,43E-05	1,61E-02
cg21139713	1	27675587	SYTL1	Body	0,029	7,44E-05	1,61E-02
cg00680998	9	119185008		IGR	-0,034	7,44E-05	1,61E-02
cg16116203	12	6876922	PTMS	Body	0,041	7,44E-05	1,61E-02
cg10327484	12	103720299	C12orf42	Body	-0,018	7,45E-05	1,61E-02
cg23648347	15	91447241	MAN2A2	TSS200	0,03	7,45E-05	1,61E-02
cg08025214	8	101704984		IGR	0,035	7,46E-05	1,61E-02
cg14848963	14	94443691	ASB2	TSS1500	0,014	7,46E-05	1,61E-02
cg02910054	16	12241554	SNX29	Body	0,073	7,46E-05	1,61E-02
cg08932533	3	107812758		IGR	-0,016	7,47E-05	1,61E-02
cg14056864	3	146187210	PLSCR2	TSS200	-0,044	7,47E-05	1,61E-02
cg24686379	19	52511775	ZNF615	TSS1500	0,013	7,47E-05	1,61E-02
cg04862038	22	19442763	UFD1L	Body	0,007	7,48E-05	1,61E-02
cg03529595	16	7056916	A2BP1	5'UTR	-0,012	7,48E-05	1,61E-02
cg09025002	17	7588507	WRAP53	TSS1500	0,029	7,48E-05	1,61E-02
cg09536555	14	94184859	PRIMA1	3'UTR	-0,023	7,49E-05	1,61E-02
cg05722872	3	19989128	RAB5A	5'UTR	-0,004	7,49E-05	1,61E-02
cg13296371	4	4250746	TMEM128	TSS1500	-0,04	7,51E-05	1,62E-02
cg06847021	16	89165406	ACSF3	Body	0,02	7,52E-05	1,62E-02
cg25811867	2	3488468		IGR	0,009	7,53E-05	1,62E-02

cg00885461	6	37465801	C6orf129	Body	0,009	7,52E-05	1,62E-02
cg00689133	6	153316553	MTRF1L	Body	0,042	7,53E-05	1,62E-02
cg13465292	7	9766677		IGR	0,007	7,53E-05	1,62E-02
cg13905089	18	46247183	CTIF	Body	0,014	7,55E-05	1,62E-02
cg24662205	1	95155994	LINC01057	Body	0,017	7,55E-05	1,62E-02
cg18733215	2	148390025		IGR	0,024	7,55E-05	1,62E-02
cg17304336	11	65343565	EHBP1L1	5'UTR	-0,003	7,55E-05	1,62E-02
cg24435866	1	154976595	ZBTB7B	5'UTR	0,007	7,56E-05	1,62E-02
cg08861957	5	130596889		IGR	0,048	7,56E-05	1,62E-02
cg19847038	3	93747528	ARL13B	Body	0,027	7,56E-05	1,62E-02
cg12071474	18	71900359		IGR	0,016	7,57E-05	1,62E-02
cg25717113	8	1993088	MYOM2	TSS200	-0,004	7,58E-05	1,62E-02
cg17390484	1	32667225	CCDC28B	5'UTR	-0,005	7,59E-05	1,62E-02
cg07644184	3	71265048	FOXP1	5'UTR	-0,008	7,59E-05	1,62E-02
cg00845775	14	104172974	XRCC3	Body	-0,011	7,59E-05	1,62E-02
cg11769850	8	914386		IGR	-0,006	7,60E-05	1,62E-02
cg21558977	13	62239257		IGR	-0,021	7,60E-05	1,62E-02
cg17123029	17	43025585	KIF18B	TSS1500	-0,02	7,60E-05	1,62E-02
cg20423693	4	176755401	GPM6A	5'UTR	-0,028	7,61E-05	1,63E-02
cg20145897	4	188231076	LOC339975	Body	-0,02	7,61E-05	1,63E-02
cg23756169	18	42896264	SLC14A2	5'UTR	-0,024	7,62E-05	1,63E-02
cg07531072	19	15560283	WIZ	5'UTR	-0,008	7,62E-05	1,63E-02
cg13633669	14	68086500	ARG2	TSS200	-0,005	7,63E-05	1,63E-02
cg03445151	2	23516881		IGR	0,05	7,64E-05	1,63E-02
cg22220728	6	84907643	CEP162	Body	0,014	7,64E-05	1,63E-02
cg00819029	7	92777530	SAMD9L	1stExon	-0,017	7,64E-05	1,63E-02
cg06884495	14	104394621	TDRD9	TSS200	-0,057	7,64E-05	1,63E-02
cg05109659	7	73794323	CLIP2	Body	0,006	7,65E-05	1,63E-02
cg27645129	2	192111659	MYO1B	5'UTR	-0,042	7,65E-05	1,63E-02
cg26391277	17	48071845	DLX3	Body	0,013	7,65E-05	1,63E-02
cg13133492	4	7985912	ABLIM2	Body	-0,021	7,68E-05	1,63E-02
cg15838042	5	176022759	PCDH24	3'UTR	0,013	7,67E-05	1,63E-02
cg18850127	7	39170497	POU6F2	Body	-0,106	7,67E-05	1,63E-02
cg04048557	8	11325201	FAM167A	TSS1500	-0,003	7,68E-05	1,63E-02
cg19129839	8	144854795		IGR	-0,041	7,67E-05	1,63E-02
cg25881985	10	131367501	MGMT	Body	-0,023	7,66E-05	1,63E-02
cg15948326	11	128775691	KCNJ5	5'UTR	-0,003	7,67E-05	1,63E-02
cg00812236	16	2210434	TRAF7	5'UTR	0,052	7,68E-05	1,63E-02
cg18955753	17	38256932	NR1D1	1stExon	-0,006	7,67E-05	1,63E-02
cg15233183	19	16187102	TPM4	Body	-0,003	7,68E-05	1,63E-02
cg03120801	1	198712602	PTPRC	Body	0,019	7,69E-05	1,63E-02
cg18666853	18	25616621	CDH2	TSS200	0,008	7,69E-05	1,63E-02
cg27341846	6	54110008	MLIP	Body	-0,01	7,70E-05	1,63E-02
cg22398392	7	30810439	FAM188B	TSS1500	0,009	7,70E-05	1,63E-02
cg10289843	9	90340776	CTSL	TSS200	-0,01	7,71E-05	1,63E-02
cg10314439	2	242020487	SNED1	Body	0,019	7,71E-05	1,64E-02
cg14256726	9	139342721	SEC16A	Body	-0,009	7,71E-05	1,64E-02
cg03666300	16	88448381		IGR	0,008	7,72E-05	1,64E-02
cg22549881	2	31806898	SRD5A2	TSS1500	-0,06	7,73E-05	1,64E-02
cg10846321	2	217443122		IGR	0,009	7,73E-05	1,64E-02
cg20365193	11	68923088	LOC338694	Body	0,01	7,73E-05	1,64E-02
cg15965212	20	55102313	GCNT7	TSS1500	-0,012	7,73E-05	1,64E-02
cg09310311	22	42999134	POLDIP3	Body	0,008	7,72E-05	1,64E-02
cg02607340	7	100494521	ACHE	TSS1500	-0,012	7,73E-05	1,64E-02
cg06933862	16	2341628	ABCA3	Body	0,01	7,76E-05	1,64E-02
cg27578869	1	17252657	CROCC	Body	-0,013	7,81E-05	1,64E-02
cg05350384	1	94485118	ABCA4	ExonBnd	0,034	7,79E-05	1,64E-02
cg26033349	2	187713554	ZSWIM2	Body	0,021	7,80E-05	1,64E-02
cg19526237	3	170410487		IGR	-0,006	7,80E-05	1,64E-02
cg16081651	3	177393409	LINC00578	Body	-0,006	7,81E-05	1,64E-02
cg16961923	4	153146998		IGR	-0,004	7,78E-05	1,64E-02
cg24899451	6	31852522	EHMT2	Body	-0,004	7,81E-05	1,64E-02
cg22397796	6	170462619		IGR	0,024	7,78E-05	1,64E-02
cg14379735	7	3458538	SDK1	Body	-0,019	7,80E-05	1,64E-02
cg15256491	7	4729610	FOXK1	Body	0,023	7,82E-05	1,64E-02
cg26959945	7	20233217	MACC1	5'UTR	0,033	7,81E-05	1,64E-02
cg00269140	7	86389542	GRM3	5'UTR	-0,011	7,80E-05	1,64E-02
cg24576174	11	65828083	SF3B2	Body	-0,006	7,79E-05	1,64E-02
cg21640587	11	117668038	DSCAML1	TSS200	-0,005	7,79E-05	1,64E-02
cg10800369	12	16761930	LMO3	TSS1500	-0,064	7,80E-05	1,64E-02
cg09614264	12	32186697		IGR	0,047	7,78E-05	1,64E-02
cg27266129	15	91382476		IGR	0,025	7,79E-05	1,64E-02
cg02346901	17	7480149	EIF4A1	Body	0,018	7,79E-05	1,64E-02

cg25677314	17	21192412	MAP2K3	Body	0,015	7,81E-05	1,64E-02
cg17440336	18	48679732		IGR	0,019	7,82E-05	1,64E-02
cg02964213	1	211434045	RCOR3	Body	-0,003	7,83E-05	1,65E-02
cg00572586	17	17109740	PLD6	TSS200	-0,054	7,83E-05	1,65E-02
cg11334300	13	114152914	TMCO3	Body	0,048	7,84E-05	1,65E-02
cg15787377	3	52810468	ITIH1	TSS1500	-0,017	7,85E-05	1,65E-02
cg02843235	5	122283728	SNX24	Body	-0,026	7,84E-05	1,65E-02
cg21041620	12	49076286	KANSL2	TSS1500	0,004	7,84E-05	1,65E-02
cg23240895	22	45071884	PRR5	5'UTR	0,019	7,85E-05	1,65E-02
cg23463099	3	140229290	CLSTN2	Body	0,028	7,85E-05	1,65E-02
cg24286200	7	139481268	TBXAS1	5'UTR	-0,007	7,86E-05	1,65E-02
cg24662195	14	23263927	SLC7A7	Body	0,024	7,86E-05	1,65E-02
cg20788426	2	219194421	PNKD	Body	0,034	7,87E-05	1,65E-02
cg00035347	10	104953608	NT5C2	TSS1500	0,047	7,87E-05	1,65E-02
cg06401643	11	66701529	PC	5'UTR	-0,018	7,87E-05	1,65E-02
cg23469963	2	9453539	ASAP2	Body	-0,014	7,87E-05	1,65E-02
cg02868468	14	105045347	C14orf180	TSS1500	0,02	7,87E-05	1,65E-02
cg07549373	6	33232598	VPS52	Body	-0,018	7,88E-05	1,65E-02
cg18187680	10	8095825	FLJ45983	TSS1500	-0,004	7,88E-05	1,65E-02
cg01095482	1	43765796	TIE1	TSS1500	0,017	7,89E-05	1,65E-02
cg03774868	2	115442503	DPP10	Body	0,026	7,90E-05	1,65E-02
cg13607248	3	183874556	DVL3	Body	0,029	7,90E-05	1,65E-02
cg03790959	7	155867630		IGR	-0,023	7,91E-05	1,65E-02
cg08599688	9	35605128	TESK1	TSS200	-0,005	7,91E-05	1,65E-02
cg06775644	17	54991465	TRIM25	TSS200	-0,007	7,92E-05	1,65E-02
cg03970039	1	6550645	PLEKHG5	5'UTR	-0,005	7,93E-05	1,65E-02
cg24660828	1	235668414	B3GALNT2	TSS1500	-0,003	7,93E-05	1,65E-02
cg16292778	4	106370657	PPA2	Body	0,015	7,93E-05	1,65E-02
cg10012711	6	28553927	SCAND3	Body	0,013	7,93E-05	1,65E-02
cg10403266	15	101987007	PCSK6	Body	0,016	7,92E-05	1,65E-02
cg24492876	3	189703833	P3H2	Body	0,011	7,94E-05	1,66E-02
cg13453731	2	182049434	LOC101927156	Body	0,01	7,96E-05	1,66E-02
cg09723833	6	105401186		IGR	-0,014	7,95E-05	1,66E-02
cg06837731	10	101945906	ERLIN1	TSS200	-0,005	7,96E-05	1,66E-02
cg07548925	17	38474425	RARA	5'UTR	0,005	7,96E-05	1,66E-02
cg00854273	17	75959756		IGR	-0,012	7,96E-05	1,66E-02
cg06123179	13	81447974		IGR	0,011	7,96E-05	1,66E-02
cg08014100	15	40108340	GPR176	Body	-0,009	7,97E-05	1,66E-02
cg11184682	3	50365769	TUSC2	TSS200	-0,005	7,98E-05	1,66E-02
cg00344411	3	64547108	ADAMTS9	Body	-0,006	7,99E-05	1,66E-02
cg15791948	10	104484669	SFXN2	5'UTR	0,038	7,99E-05	1,66E-02
cg27303948	5	154150986	LARP1	Body	0,021	8,00E-05	1,66E-02
cg08341178	3	44596754	ZKSCAN7	1stExon	-0,004	8,01E-05	1,67E-02
cg27483767	11	1098080	MUC2	Body	-0,034	8,02E-05	1,67E-02
cg13912318	17	76506686	DNAH17	Body	-0,02	8,02E-05	1,67E-02
cg11908659	2	62736315		IGR	0,008	8,05E-05	1,67E-02
cg20900097	3	50230885	GNAT1	Body	-0,052	8,05E-05	1,67E-02
cg24754507	6	28481862	GPX6	Body	-0,009	8,04E-05	1,67E-02
cg23191292	6	157099924	ARID1B	1stExon	-0,007	8,04E-05	1,67E-02
cg09983640	12	26435700		IGR	0,017	8,04E-05	1,67E-02
cg20800039	15	58891233	ADAM10	Body	0,01	8,05E-05	1,67E-02
cg15561223	16	53576384		IGR	-0,05	8,04E-05	1,67E-02
cg05959377	22	50963918	SCO2	5'UTR	-0,004	8,04E-05	1,67E-02
cg22851415	1	112296918	C1orf183	Body	-0,006	8,05E-05	1,67E-02
cg09589202	8	101031817	RGS22	Body	0,008	8,05E-05	1,67E-02
cg12835689	1	94057740	BCAR3	Body	0,071	8,06E-05	1,67E-02
cg13627968	3	129315176	PLXND1	Body	0,022	8,07E-05	1,67E-02
cg23615201	6	41467243		IGR	0,02	8,07E-05	1,67E-02
cg24266872	10	27437414	YME1L1	Body	-0,017	8,07E-05	1,67E-02
cg20941237	14	91686100	C14orf159	Body	0,014	8,07E-05	1,67E-02
cg07084709	15	57883511	GCOM1	TSS1500	0,007	8,06E-05	1,67E-02
cg23280506	17	14201938		IGR	0,068	8,07E-05	1,67E-02
cg22231909	4	86582384	ARHGAP24	Body	0,026	8,08E-05	1,67E-02
cg19500979	11	67287273	CABP2	Body	-0,044	8,08E-05	1,67E-02
cg04571321	19	38924347	RYR1	1stExon	0,007	8,08E-05	1,67E-02
cg02480016	10	79537097		IGR	-0,016	8,09E-05	1,67E-02
cg09248019	21	42732441		IGR	-0,007	8,10E-05	1,67E-02
cg20739360	7	91833683	KRIT1	Body	-0,017	8,10E-05	1,67E-02
cg12017577	1	53387351	ECHDC2	1stExon	-0,005	8,11E-05	1,67E-02
cg07331152	5	7983756		IGR	-0,008	8,11E-05	1,67E-02
cg12529637	7	143083425	ZYX	Body	-0,009	8,12E-05	1,67E-02
cg10282571	5	9927323		IGR	-0,028	8,12E-05	1,67E-02
cg18709881	18	72837627		IGR	-0,061	8,12E-05	1,67E-02

cg12833662	1	42197894	HIVEP3	5'UTR	-0,019	8,14E-05	1,67E-02
cg15122936	4	163407197		IGR	-0,008	8,14E-05	1,67E-02
cg22153080	8	49180382		IGR	-0,009	8,14E-05	1,67E-02
cg18898282	20	39706949	TOP1	Body	-0,034	8,14E-05	1,67E-02
cg20274430	22	41075992	MCHR1	Body	0,029	8,13E-05	1,67E-02
cg22768487	22	46770253	CELSR1	Body	-0,004	8,14E-05	1,67E-02
cg02700360	3	11874932	C3orf31	Body	0,005	8,15E-05	1,68E-02
cg20560906	3	47459876	SCAP	Body	0,005	8,15E-05	1,68E-02
cg14223053	3	107242624	BBX	5'UTR	-0,005	8,16E-05	1,68E-02
cg02271380	19	38040258	ZNF571-AS1	Body	-0,006	8,16E-05	1,68E-02
cg14680655	21	43501580	UMODL1	Body	0,04	8,15E-05	1,68E-02
cg01697719	22	19754125	TBX1	Body	-0,005	8,16E-05	1,68E-02
cg25342861	17	45177598		IGR	0,011	8,16E-05	1,68E-02
cg22013195	2	4601053		IGR	-0,036	8,17E-05	1,68E-02
cg12349571	15	70364359	TLE3	Body	0,014	8,19E-05	1,68E-02
cg22705589	16	73865191		IGR	0,034	8,19E-05	1,68E-02
cg24231834	1	171182100		IGR	-0,019	8,21E-05	1,68E-02
cg18649310	21	36245862	RUNX1	Body	0,024	8,22E-05	1,69E-02
cg25753473	6	170532835		IGR	0,074	8,23E-05	1,69E-02
cg07683804	2	29093162	TRMT61B	5'UTR	0,006	8,23E-05	1,69E-02
cg11533888	3	185303603	SENP2	TSS1500	-0,028	8,23E-05	1,69E-02
cg09148738	1	153950187	JTB	1stExon	0,008	8,24E-05	1,69E-02
cg02219519	17	81036100	METRNL	TSS1500	0,014	8,24E-05	1,69E-02
cg10413833	6	150068040	NUP43	TSS1500	0,029	8,25E-05	1,69E-02
cg08765940	11	133789708	IGSF9B	Body	-0,06	8,25E-05	1,69E-02
cg10345716	18	19931103		IGR	-0,052	8,25E-05	1,69E-02
cg23063807	2	506174		IGR	-0,043	8,27E-05	1,69E-02
cg11928668	2	206754192		IGR	0,022	8,27E-05	1,69E-02
cg10880709	4	1283506	MAEA	TSS200	0,011	8,27E-05	1,69E-02
cg03794323	5	177026973	B4GALT7	TSS200	-0,009	8,27E-05	1,69E-02
cg18600110	20	33567304	MYH7B	ExonBnd	-0,014	8,27E-05	1,69E-02
cg11613015	5	112073406	APC	TSS200	-0,009	8,28E-05	1,69E-02
cg10652508	19	2809933	THOP1	Body	0,021	8,28E-05	1,69E-02
cg07929996	4	7761820	AFAP1	3'UTR	-0,023	8,28E-05	1,69E-02
cg01222408	15	63570099	APH1B	Body	-0,006	8,28E-05	1,69E-02
cg24480453	6	166232637		IGR	-0,051	8,29E-05	1,69E-02
cg05342853	11	101929189	C11orf70	Body	-0,041	8,29E-05	1,69E-02
cg11460029	17	7608747	EFNB3	5'UTR	-0,005	8,29E-05	1,69E-02
cg09185231	10	81056385	ZMIZ1	Body	0,007	8,30E-05	1,69E-02
cg13143213	11	75040656	ARRB1	Body	0,026	8,29E-05	1,69E-02
cg07271818	12	57693843	R3HDM2	Body	-0,005	8,30E-05	1,69E-02
cg04208787	11	118966294	H2AFX	TSS200	-0,006	8,30E-05	1,69E-02
cg24244235	20	37702438		IGR	-0,017	8,31E-05	1,69E-02
cg20232307	1	152539753	LCE3E	TSS1500	0,076	8,32E-05	1,69E-02
cg05361709	15	75494287	C15orf39	1stExon	-0,004	8,32E-05	1,69E-02
cg11081694	1	91871709	HFM1	TSS1500	0,008	8,34E-05	1,69E-02
cg19961339	3	128841447	RAB43	TSS1500	0,011	8,33E-05	1,69E-02
cg21435470	5	131810285	C5orf56	Body	0,01	8,33E-05	1,69E-02
cg22652190	6	44095455	TMEM63B	5'UTR	-0,011	8,34E-05	1,69E-02
cg08419879	6	151152916	PLEKHG1	Body	-0,006	8,34E-05	1,69E-02
cg19872681	14	23284518	SLC7A7	5'UTR	0,019	8,34E-05	1,69E-02
cg04589783	17	51353136		IGR	-0,01	8,33E-05	1,69E-02
cg13811232	19	40866273	PLD3	5'UTR	-0,009	8,33E-05	1,69E-02
cg11610763	17	64561356	PRKCA	Body	0,033	8,34E-05	1,69E-02
cg04797575	4	176709392	GPM6A	TSS1500	-0,027	8,36E-05	1,70E-02
cg03103178	8	37824080	ADRB3	1stExon	-0,029	8,36E-05	1,70E-02
cg08416661	11	65647279	CTSW	TSS200	-0,006	8,36E-05	1,70E-02
cg08384574	16	8955551	CARHSP1	5'UTR	0,03	8,36E-05	1,70E-02
cg27175380	1	35446563		IGR	0,031	8,37E-05	1,70E-02
cg03039218	17	43025639	KIF18B	TSS1500	-0,05	8,37E-05	1,70E-02
cg26009832	1	169081894	ATP1B1	Body	0,036	8,38E-05	1,70E-02
cg13065228	8	145701192	FOXH1	1stExon	0,058	8,39E-05	1,70E-02
cg20100381	16	66864408	NAE1	5'UTR	-0,008	8,40E-05	1,70E-02
cg24260662	2	133281441	GPR39	Body	-0,032	8,40E-05	1,70E-02
cg17566917	6	33842689		IGR	-0,044	8,40E-05	1,70E-02
cg24676534	5	132201913	GDF9	TSS1500	0,01	8,40E-05	1,70E-02
cg07607553	12	29944570		IGR	-0,015	8,42E-05	1,70E-02
cg14733637	5	1342200	CLPTM1L	Body	0,018	8,42E-05	1,70E-02
cg00081919	2	43187471		IGR	0,019	8,43E-05	1,70E-02
cg21043808	17	32683249	CCL13	TSS1500	0,013	8,43E-05	1,70E-02
cg09451738	2	242852978		IGR	-0,024	8,45E-05	1,71E-02
cg23524294	6	26158342	HIST1H2BD	TSS200	-0,005	8,45E-05	1,71E-02
cg14444103	4	147367017	SLC10A7	Body	-0,008	8,45E-05	1,71E-02

cg01742249	4	6568120		IGR	0,043	8,47E-05	1,71E-02
cg14266527	4	151501298	LRBA	Body	-0,007	8,47E-05	1,71E-02
cg04960065	4	185189300		IGR	-0,003	8,46E-05	1,71E-02
cg21574556	5	108350932	FER	Body	0,018	8,46E-05	1,71E-02
cg05639234	5	132184036		IGR	0,009	8,47E-05	1,71E-02
cg16102240	10	60898030		IGR	-0,038	8,48E-05	1,71E-02
cg18621433	10	91222148	SLC16A12	ExonBnd	-0,018	8,48E-05	1,71E-02
cg27601855	11	47197938	ARFGAP2	Body	0,004	8,46E-05	1,71E-02
cg09887687	12	10018275	CLEC2B	5'UTR	-0,008	8,46E-05	1,71E-02
cg09600820	14	61070249		IGR	-0,022	8,46E-05	1,71E-02
cg00132716	16	65735154		IGR	-0,015	8,48E-05	1,71E-02
cg12311711	19	3482660		IGR	-0,008	8,47E-05	1,71E-02
cg19354148	20	2732612	EBF4	Body	-0,007	8,48E-05	1,71E-02
cg21973907	7	155142741		IGR	-0,023	8,49E-05	1,71E-02
cg15708424	2	96068732	FAHD2A	5'UTR	-0,006	8,50E-05	1,71E-02
cg27140006	6	109812585		IGR	-0,042	8,49E-05	1,71E-02
cg15952389	16	70381054	LOC100506083	TSS1500	-0,003	8,49E-05	1,71E-02
cg27255237	17	60557268	TLK2	5'UTR	-0,004	8,50E-05	1,71E-02
cg08828594	15	99558786		IGR	-0,007	8,50E-05	1,71E-02
cg22381317	12	52380850	ACVR1B	Body	-0,011	8,52E-05	1,71E-02
cg23337046	9	21032451	HACD4	TSS1500	0,01	8,53E-05	1,71E-02
cg12112264	20	39591787		IGR	-0,005	8,53E-05	1,71E-02
cg05712903	5	178977398	RUFY1	TSS200	-0,006	8,54E-05	1,71E-02
cg23819092	1	156047143	MEX3A	Body	-0,034	8,54E-05	1,71E-02
cg07695556	5	7653389	ADCY2	Body	-0,029	8,55E-05	1,71E-02
cg08082788	14	101618406		IGR	-0,048	8,55E-05	1,71E-02
cg00274051	21	29600801	LOC101927973	Body	0,022	8,55E-05	1,71E-02
cg15767719	18	45276176		IGR	-0,016	8,56E-05	1,71E-02
cg16402161	4	185677585	ACSL1	3'UTR	-0,009	8,56E-05	1,72E-02
cg22068494	2	242449026	STK25	TSS1500	0,034	8,57E-05	1,72E-02
cg14094614	5	171490780	STK10	Body	0,008	8,57E-05	1,72E-02
cg17171826	15	62184442	VPS13C	Body	0,007	8,57E-05	1,72E-02
cg14747115	19	3051074		IGR	0,017	8,56E-05	1,72E-02
cg15102595	3	58128652	FLNB	Body	0,013	8,57E-05	1,72E-02
cg03040522	1	154599652	ADAR	5'UTR	-0,014	8,59E-05	1,72E-02
cg24233332	6	31673800	LY6G6F	TSS1500	0,009	8,58E-05	1,72E-02
cg00226831	15	42371511	PLA2G4D	Body	-0,036	8,59E-05	1,72E-02
cg16777493	16	84483628	ATP2C2	Body	-0,01	8,59E-05	1,72E-02
cg04013970	17	43158427	NMT1	Body	-0,013	8,58E-05	1,72E-02
cg19841183	21	15953494	SAMSN1-AS1	TSS1500	-0,007	8,58E-05	1,72E-02
cg16522602	5	79964079	MSH3	Body	0,016	8,59E-05	1,72E-02
cg14579239	5	140220822	PCDHA6	Body	-0,053	8,59E-05	1,72E-02
cg27105767	21	45673047	DNMT3L	Body	0,018	8,61E-05	1,72E-02
cg10474881	6	114181686	MARCKS	Body	0,069	8,62E-05	1,72E-02
cg10133248	10	35893437	GJD4	TSS1500	-0,008	8,62E-05	1,72E-02
cg14395744	18	72837693		IGR	-0,082	8,63E-05	1,72E-02
cg08200159	1	37216615		IGR	-0,013	8,65E-05	1,72E-02
cg23870876	9	117484049		IGR	-0,033	8,65E-05	1,72E-02
cg00705613	12	132661528		IGR	-0,019	8,65E-05	1,72E-02
cg01693246	15	31512162		IGR	-0,013	8,65E-05	1,72E-02
cg07586725	1	95007356	F3	1stExon	0,012	8,66E-05	1,72E-02
cg10562586	2	210444154	MAP2	TSS1500	0,058	8,67E-05	1,72E-02
cg00666696	7	157319754		IGR	-0,018	8,66E-05	1,72E-02
cg00167717	8	144676961	EEF1D	5'UTR	0,017	8,66E-05	1,72E-02
cg27235072	3	20049601	C3orf48	Body	-0,007	8,68E-05	1,73E-02
cg11152884	5	170850020	FGF18	Body	-0,008	8,68E-05	1,73E-02
cg06344576	2	220316415	SPEG	Body	0,011	8,70E-05	1,73E-02
cg05954640	4	102711629	BANK1	TSS200	-0,012	8,70E-05	1,73E-02
cg07472579	6	10660390		IGR	-0,057	8,70E-05	1,73E-02
cg14500563	9	140735058		IGR	-0,011	8,70E-05	1,73E-02
cg15684811	1	59250924	JUN	TSS1500	-0,007	8,71E-05	1,73E-02
cg04333485	3	113871401	DRD3	Body	-0,019	8,71E-05	1,73E-02
cg11147251	8	132870146		IGR	-0,006	8,71E-05	1,73E-02
cg15194925	22	30987935	PES1	TSS200	-0,004	8,70E-05	1,73E-02
cg26944562	10	38070165		IGR	-0,015	8,72E-05	1,73E-02
cg20697926	18	77377589		IGR	-0,104	8,72E-05	1,73E-02
cg13155106	2	75938138	C2orf3	TSS200	0,006	8,72E-05	1,73E-02
cg23162310	5	172199255	DUSP1	TSS1500	-0,003	8,73E-05	1,73E-02
cg20387347	7	98554099	TRRAP	Body	-0,006	8,73E-05	1,73E-02
cg19221903	7	124996163	LOC101928283	Body	0,054	8,73E-05	1,73E-02
cg26739084	10	126036630		IGR	-0,011	8,73E-05	1,73E-02
cg17492891	22	41843204	TOB2	TSS200	-0,003	8,72E-05	1,73E-02
cg03721641	22	50451245	IL17REL	TSS200	0,02	8,73E-05	1,73E-02

cg09852697	1	3616642	TP73	Body	0,041	8,75E-05	1,73E-02
cg02180182	4	180546057		IGR	-0,026	8,74E-05	1,73E-02
cg04795246	9	135227209	SETX	5'UTR	-0,013	8,75E-05	1,73E-02
cg13627406	10	103901363	PPRC1	Body	0,014	8,74E-05	1,73E-02
cg08157758	14	20614511		IGR	-0,039	8,75E-05	1,73E-02
cg25744853	16	20794024	ACSM3	Body	-0,006	8,75E-05	1,73E-02
cg12531485	8	48293636	SPIDR	Body	-0,014	8,76E-05	1,73E-02
cg06395988	2	242041550	MTERFD2	Body	-0,006	8,77E-05	1,73E-02
cg19152887	3	187460723	BCL6	5'UTR	-0,005	8,77E-05	1,73E-02
cg10129944	6	12718408	PHACTR1	5'UTR	-0,043	8,78E-05	1,73E-02
cg02413149	9	128287210	MAPKAP1	Body	-0,012	8,78E-05	1,73E-02
cg17108752	3	105070689		IGR	-0,027	8,78E-05	1,73E-02
cg11236289	17	56411283	BZRAP1-AS1	Body	-0,004	8,79E-05	1,74E-02
cg26521402	7	89842741	STEAP2	5'UTR	0,021	8,80E-05	1,74E-02
cg16339097	3	82293457		IGR	-0,016	8,80E-05	1,74E-02
cg13466265	3	191053398	CCDC50	Body	0,021	8,80E-05	1,74E-02
cg08389497	10	73820491	SPOCK2	3'UTR	0,008	8,80E-05	1,74E-02
cg23371476	2	105877824		IGR	0,042	8,82E-05	1,74E-02
cg03884014	7	1611147	PSMG3	TSS1500	0,021	8,82E-05	1,74E-02
cg21123913	4	89513664	HERC3	5'UTR	0,01	8,83E-05	1,74E-02
cg26901169	13	51483776	RNASEH2B	TSS200	-0,003	8,83E-05	1,74E-02
cg07741809	20	59966734	CDH4	Body	0,061	8,83E-05	1,74E-02
cg25920676	1	9990240	LZIC	3'UTR	0,005	8,84E-05	1,74E-02
cg17783135	3	194876491	C3orf21	Body	0,019	8,85E-05	1,74E-02
cg25134515	4	184020452	WWC2	TSS200	-0,007	8,84E-05	1,74E-02
cg10747656	6	159050738	TMEM181	Body	-0,005	8,85E-05	1,74E-02
cg19730972	14	102176671		IGR	0,033	8,85E-05	1,74E-02
cg26872588	1	230992820	C1orf198	Body	-0,021	8,85E-05	1,74E-02
cg19002058	4	6347514	PPP2R2C	Body	0,013	8,86E-05	1,74E-02
cg22951411	19	57375307		IGR	-0,038	8,86E-05	1,74E-02
cg16142698	3	132136350	DNAJC13	TSS1500	-0,005	8,87E-05	1,74E-02
cg00915244	12	133378492	GOLGA3	Body	0,007	8,87E-05	1,74E-02
cg08612082	16	86334503		IGR	-0,006	8,87E-05	1,74E-02
cg02028332	19	19335919	NCAN	Body	-0,007	8,87E-05	1,74E-02
cg02153584	22	29168773	CCDC117	1stExon	-0,005	8,87E-05	1,74E-02
cg08242679	2	240761684		IGR	-0,008	8,88E-05	1,74E-02
cg24454932	6	170532633		IGR	0,091	8,88E-05	1,74E-02
cg04685110	5	92409635		IGR	0,011	8,89E-05	1,74E-02
cg06746220	3	195634940	TNK2	5'UTR	-0,006	8,90E-05	1,74E-02
cg01620486	8	47015640		IGR	-0,021	8,90E-05	1,74E-02
cg12732899	9	112887765	AKAP2	TSS200	0,024	8,90E-05	1,74E-02
cg24053992	1	119554564		IGR	-0,022	8,93E-05	1,74E-02
cg10311562	2	25070805	ADCY3	Body	-0,008	8,92E-05	1,74E-02
cg17871966	4	106629496	INTS12	5'UTR	-0,004	8,93E-05	1,74E-02
cg03789088	6	3287259	SLC22A23	Body	0,006	8,91E-05	1,74E-02
cg19608620	7	150362308		IGR	-0,02	8,91E-05	1,74E-02
cg00315955	8	124072387		IGR	-0,006	8,92E-05	1,74E-02
cg12855310	10	134215035	PWWP2B	Body	0,022	8,92E-05	1,74E-02
cg07675998	11	64268315		IGR	0,036	8,92E-05	1,74E-02
cg12429629	15	93152363		IGR	-0,022	8,91E-05	1,74E-02
cg11392858	17	17109651	PLD6	TSS200	-0,045	8,92E-05	1,74E-02
cg24190091	1	20835240	MUL1	TSS1500	-0,009	8,93E-05	1,74E-02
cg03044510	21	27107978	ATP5J	TSS200	0,006	8,94E-05	1,74E-02
cg18944014	2	239799440	TWIST2	3'UTR	-0,018	8,94E-05	1,75E-02
cg23141851	3	14615941		IGR	-0,069	8,94E-05	1,75E-02
cg02294690	4	17581984	LAP3	Body	-0,096	8,95E-05	1,75E-02
cg09821763	10	82218906	TSPAN14	5'UTR	-0,003	8,95E-05	1,75E-02
cg10813070	16	2228344	CASKIN1	3'UTR	-0,044	8,95E-05	1,75E-02
cg02022808	5	140744914	PCDHGA4	Body	-0,039	8,96E-05	1,75E-02
cg24031277	7	33102336	NT5C3	1stExon	0,009	8,96E-05	1,75E-02
cg16661271	1	6561199	PLEKHG5	Body	-0,012	8,97E-05	1,75E-02
cg07827432	2	208576357	CCNYL1	1stExon	-0,01	8,97E-05	1,75E-02
cg05862027	16	46836742	C16orf87	3'UTR	0,005	8,97E-05	1,75E-02
cg00485707	15	89198782	ISG20	3'UTR	-0,006	8,97E-05	1,75E-02
cg23543912	18	74667616	ZNF236	Body	-0,006	8,97E-05	1,75E-02
cg10404265	2	44034401	DYNC2LI1	Body	0,008	8,98E-05	1,75E-02
cg17239140	15	41836578	RPAP1	TSS200	0,01	8,98E-05	1,75E-02
cg07635854	19	11374482	DOCK6	TSS1500	0,005	8,98E-05	1,75E-02
cg00042969	19	40882392	PLD3	Body	0,057	8,98E-05	1,75E-02
cg17930194	9	124982834	LHX6	Body	0,064	8,99E-05	1,75E-02
cg26399996	11	133488488		IGR	-0,024	8,99E-05	1,75E-02
cg08691567	12	53183930	KRT3	Body	-0,072	8,99E-05	1,75E-02
cg14295437	6	154651280	IPCEF1	5'UTR	0,021	9,00E-05	1,75E-02

cg15047610	19	47750787		IGR	-0,016	9,00E-05	1,75E-02
cg24024660	7	99195788		IGR	-0,054	9,00E-05	1,75E-02
cg16323293	6	163612985	PACRG	Body	0,011	9,01E-05	1,75E-02
cg21265853	22	32779470	LOC339666	Body	-0,009	9,01E-05	1,75E-02
cg04468640	15	44220143	FRMD5	Body	-0,02	9,02E-05	1,75E-02
cg16209042	6	42016765	CCND3	TSS200	-0,002	9,03E-05	1,75E-02
cg08835956	7	39171034	POU6F2	Body	-0,073	9,03E-05	1,75E-02
cg11694223	8	27473467	CLU	TSS1500	0,008	9,03E-05	1,75E-02
cg12650715	14	96371984	TUNAR	Body	-0,021	9,03E-05	1,75E-02
cg09884223	15	93427461	LINC01578	Body	-0,011	9,03E-05	1,75E-02
cg27392413	11	12291934		IGR	0,041	9,03E-05	1,75E-02
cg12742468	6	128841561	PTPRK	1stExon	0,012	9,05E-05	1,75E-02
cg04428805	17	65239522	HELZ	5'UTR	-0,011	9,05E-05	1,75E-02
cg08172839	22	40141721	ENTHD1	Body	0,012	9,05E-05	1,75E-02
cg17882795	1	25071642	CLIC4	TSS200	0,006	9,06E-05	1,75E-02
cg01396065	3	193789440		IGR	0,04	9,06E-05	1,75E-02
cg20713929	10	103052532		IGR	-0,005	9,06E-05	1,75E-02
cg16974433	11	35198159	CD44	Body	-0,006	9,06E-05	1,75E-02
cg20770803	17	40950659	CNTD1	TSS200	-0,002	9,07E-05	1,75E-02
cg12167263	19	38657419	SIPA1L3	Body	0,007	9,07E-05	1,75E-02
cg10398116	19	48958792	KCNJ14	TSS200	-0,01	9,07E-05	1,75E-02
cg23648015	19	49519116	RUVBL2	3'UTR	-0,024	9,09E-05	1,75E-02
cg05946513	1	15467616	C1orf126	Body	-0,017	9,09E-05	1,76E-02
cg02016771	4	89069031	ABCG2	5'UTR	-0,011	9,10E-05	1,76E-02
cg22564448	8	100559503	VPS13B	Body	-0,036	9,10E-05	1,76E-02
cg19570545	1	201252472	PKP1	TSS200	-0,004	9,11E-05	1,76E-02
cg26479436	12	51983478		IGR	-0,02	9,11E-05	1,76E-02
cg08209664	8	134564512	ST3GAL1	5'UTR	0,033	9,11E-05	1,76E-02
cg22021160	12	76478621	NAP1L1	5'UTR	0,005	9,11E-05	1,76E-02
cg11703701	16	228424		IGR	0,044	9,11E-05	1,76E-02
cg06625816	15	48484302	CTXN2	5'UTR	-0,031	9,12E-05	1,76E-02
cg04798810	1	202245619	LGR6	Body	0,006	9,13E-05	1,76E-02
cg14331404	2	97482184	CNNM3	1stExon	0,005	9,13E-05	1,76E-02
cg26048140	20	46365812	SULF2	Body	-0,015	9,13E-05	1,76E-02
cg21686386	1	222614476		IGR	-0,014	9,14E-05	1,76E-02
cg22547485	4	140004218	ELF2	Body	-0,004	9,14E-05	1,76E-02
cg14989243	6	76203530	FILIP1	TSS200	-0,066	9,14E-05	1,76E-02
cg03704960	3	107886156	IFT57	Body	0,007	9,16E-05	1,76E-02
cg24851586	6	9897068		IGR	-0,014	9,16E-05	1,76E-02
cg00538212	7	158751591		IGR	-0,085	9,15E-05	1,76E-02
cg20936291	9	130679328	ST6GALNAC4	TSS200	0,014	9,16E-05	1,76E-02
cg20958804	11	130721851		IGR	-0,033	9,17E-05	1,76E-02
cg25822402	13	39260877	FREM2	TSS1500	-0,007	9,16E-05	1,76E-02
cg26897283	16	67427183	TPPP3	5'UTR	-0,024	9,15E-05	1,76E-02
cg08502240	19	984437	WDR18	1stExon	-0,004	9,15E-05	1,76E-02
cg22598607	21	45374311	AGPAT3	5'UTR	-0,007	9,15E-05	1,76E-02
cg01721954	21	46954883	SLC19A1	TSS1500	0,027	9,16E-05	1,76E-02
cg15174246	22	30663901	OSM	TSS1500	-0,004	9,15E-05	1,76E-02
cg20010635	1	7811409	CAMTA1	Body	0,01	9,21E-05	1,76E-02
cg12339351	1	17338383	ATP13A2	1stExon	-0,004	9,18E-05	1,76E-02
cg04768501	3	9956823	IL17RE	Body	0,026	9,17E-05	1,76E-02
cg15233126	3	40566533	ZNF621	5'UTR	-0,003	9,21E-05	1,76E-02
cg27084712	3	42977845		IGR	-0,066	9,19E-05	1,76E-02
cg08133229	3	196626891	SENP5	Body	-0,01	9,20E-05	1,76E-02
cg16361921	5	106599492		IGR	-0,233	9,19E-05	1,76E-02
cg22191665	5	127761982	FBN2	Body	-0,008	9,21E-05	1,76E-02
cg00400340	5	135311642		IGR	0,023	9,20E-05	1,76E-02
cg24377133	8	144170375		IGR	0,053	9,17E-05	1,76E-02
cg25449938	10	102762401	LZTS2	Body	0,013	9,17E-05	1,76E-02
cg01714160	11	617467	IRF7	TSS1500	-0,003	9,21E-05	1,76E-02
cg13587325	12	6977202	TPI1	Body	0,008	9,21E-05	1,76E-02
cg11906768	14	89674643	FOXN3	Body	0,007	9,21E-05	1,76E-02
cg15268101	15	90704119		IGR	0,011	9,21E-05	1,76E-02
cg06082141	17	10604565	C17orf48	5'UTR	0,03	9,20E-05	1,76E-02
cg13220123	18	76160277		IGR	-0,017	9,18E-05	1,76E-02
cg14737370	19	9271995	ZNF317	Body	-0,023	9,20E-05	1,76E-02
cg04277172	20	44410366	WFDC3	Body	-0,018	9,21E-05	1,76E-02
cg12876261	7	56119223	PSPH	5'UTR	0,008	9,22E-05	1,76E-02
cg03835140	19	45662154	NKPD1	Body	-0,039	9,22E-05	1,76E-02
cg01028360	8	12869510	C8orf79	Body	0,005	9,22E-05	1,76E-02
cg07044147	1	77636502	PIGK	Body	0,024	9,23E-05	1,76E-02
cg08078265	2	173470725	PDK1	Body	-0,005	9,24E-05	1,76E-02
cg21066554	2	240687886	LOC150935	Body	0,008	9,24E-05	1,76E-02

cg25738786	6	170532786		IGR	0,1	9,24E-05	1,76E-02
cg25161161	17	78190898	SGSH	Body	0,006	9,23E-05	1,76E-02
cg12880874	2	2942535	LINC01250	Body	0,143	9,26E-05	1,76E-02
cg12601456	18	24032748		IGR	-0,008	9,26E-05	1,76E-02
cg23843797	16	2479325	CCNF	TSS200	0,019	9,26E-05	1,76E-02
cg03330302	2	26468406	HADHA	TSS1500	-0,003	9,27E-05	1,76E-02
cg00864550	17	78055278	CCDC40	Body	0,006	9,28E-05	1,77E-02
cg11555708	11	108277527	C11orf65	Body	0,037	9,28E-05	1,77E-02
cg05355757	12	16762831		IGR	-0,052	9,29E-05	1,77E-02
cg17750252	2	136567154	LCT	Body	-0,006	9,30E-05	1,77E-02
cg05207635	3	184055142	FAM131A	TSS200	0,016	9,30E-05	1,77E-02
cg13126652	8	105368434	TM7SF4	3'UTR	-0,011	9,31E-05	1,77E-02
cg00438616	15	76304843	NRG4	TSS200	-0,007	9,31E-05	1,77E-02
cg00374492	6	42015867	CCND3	5'UTR	-0,005	9,31E-05	1,77E-02
cg12871652	12	78581770	NAV3	Body	-0,067	9,32E-05	1,77E-02
cg09648459	11	130479188		IGR	-0,01	9,32E-05	1,77E-02
cg23044176	3	55002570	LRTM1	TSS1500	-0,006	9,34E-05	1,77E-02
cg23264877	7	37366753	ELMO1	Body	0,03	9,33E-05	1,77E-02
cg04326198	14	36994456		IGR	-0,021	9,34E-05	1,77E-02
cg07236061	5	73720202	LINC01331	Body	0,006	9,34E-05	1,77E-02
cg02756939	1	211556576	C1orf97	Body	0,039	9,37E-05	1,77E-02
cg06530497	4	10057605		IGR	-0,007	9,36E-05	1,77E-02
cg08820104	5	54183619		IGR	0,019	9,36E-05	1,77E-02
cg05431171	6	35461405	TEAD3	5'UTR	0,023	9,36E-05	1,77E-02
cg14031178	6	69344423	BAI3	TSS1500	-0,011	9,35E-05	1,77E-02
cg07520919	6	160721435		IGR	-0,044	9,37E-05	1,77E-02
cg02468649	7	45145961	TBRG4	Body	-0,01	9,36E-05	1,77E-02
cg11495285	10	90636202		IGR	0,009	9,37E-05	1,77E-02
cg12094965	12	109749643		IGR	0,011	9,37E-05	1,77E-02
cg21115151	15	56788981		IGR	-0,015	9,35E-05	1,77E-02
cg25192916	18	51751750	MBD2	TSS1500	0,014	9,35E-05	1,77E-02
cg27041980	20	25291797	ABHD12	Body	-0,023	9,37E-05	1,77E-02
cg26449717	16	84494287	ATP2C2	Body	-0,008	9,38E-05	1,77E-02
cg12819931	10	30663455	LOC729668	TSS200	-0,022	9,39E-05	1,77E-02
cg11082847	7	4056929	SDK1	Body	-0,008	9,40E-05	1,77E-02
cg13025422	7	55433036	LANCL2	TSS200	-0,005	9,40E-05	1,77E-02
cg12874971	5	157502673		IGR	-0,052	9,41E-05	1,78E-02
cg21406046	10	120451110	CACUL1	Body	0,018	9,41E-05	1,78E-02
cg16203381	18	55108965	ONECUT2	Body	-0,016	9,42E-05	1,78E-02
cg11752783	1	115060374		IGR	-0,004	9,43E-05	1,78E-02
cg11832804	5	1279449	TERT	Body	0,005	9,43E-05	1,78E-02
cg06040838	6	157469646	ARID1B	Body	0,025	9,43E-05	1,78E-02
cg22803095	5	12575112	LINC01194	Body	0,013	9,44E-05	1,78E-02
cg06422145	11	61579991	FADS1	Body	0,018	9,44E-05	1,78E-02
cg15288451	18	45251516		IGR	0,02	9,44E-05	1,78E-02
cg12965023	5	139170217		IGR	0,018	9,45E-05	1,78E-02
cg26803670	12	123414556	ABCB9	Body	0,008	9,45E-05	1,78E-02
cg25273619	13	114123037	DCUN1D2	Body	0,003	9,46E-05	1,78E-02
cg18683453	19	49626988	PPFIA3	5'UTR	-0,008	9,46E-05	1,78E-02
cg20513643	12	49393348	DDN	TSS1500	-0,007	9,46E-05	1,78E-02
cg08944929	16	67979129	SLC12A4	Body	0,015	9,46E-05	1,78E-02
cg04593941	1	198983857	LINC01221	TSS1500	-0,007	9,46E-05	1,78E-02
cg20617049	1	11544453	PTCHD2	5'UTR	0,025	9,47E-05	1,78E-02
cg25824462	7	116271183		IGR	0,028	9,49E-05	1,78E-02
cg23700044	10	98945809	SLIT1	TSS200	-0,007	9,49E-05	1,78E-02
cg02264057	7	40516569	SUGCT	Body	0,03	9,50E-05	1,78E-02
cg27445072	16	82921787	CDH13	Body	-0,009	9,50E-05	1,78E-02
cg05432303	16	362789	AXIN1	Body	-0,017	9,50E-05	1,78E-02
cg15931233	5	53455496	ARL15	Body	-0,006	9,50E-05	1,78E-02
cg18923105	14	64675705	SYNE2	Body	0,008	9,52E-05	1,79E-02
cg05842524	1	78415328	FUBP1	Body	0,049	9,56E-05	1,79E-02
cg13204762	2	135205348	MGAT5	Body	-0,007	9,54E-05	1,79E-02
cg08172649	4	68831201		IGR	-0,01	9,55E-05	1,79E-02
cg00669594	6	33156845	COL11A2	Body	0,019	9,54E-05	1,79E-02
cg15795748	9	134488511	RAPGEF1	Body	0,016	9,55E-05	1,79E-02
cg09754422	10	93162972	HECTD2-AS1	Body	0,017	9,53E-05	1,79E-02
cg10113589	10	98118853	OPALIN	1stExon	-0,046	9,55E-05	1,79E-02
cg06021522	11	17898125	SERGEF	Body	0,008	9,53E-05	1,79E-02
cg15256208	11	60690017	TMEM109	3'UTR	0,016	9,55E-05	1,79E-02
cg20177315	11	69634648	FGF3	TSS1500	-0,019	9,55E-05	1,79E-02
cg02528196	13	99383653	SLC15A1	Body	0,005	9,56E-05	1,79E-02
cg05592086	16	10336772		IGR	0,022	9,54E-05	1,79E-02
cg03184776	17	41522069	MIR2117	TSS200	0,032	9,53E-05	1,79E-02

cg07778837	19	55996461	NAT14	TSS200	0,008	9,56E-05	1,79E-02
cg25709972	19	39882091	MED29	1stExon	-0,005	9,56E-05	1,79E-02
cg20372956	6	160148392	WTAP	5'UTR	-0,012	9,58E-05	1,79E-02
cg15721142	12	133354410	GOLGA3	Body	0,013	9,58E-05	1,79E-02
cg14517721	14	99741343		IGR	-0,005	9,58E-05	1,79E-02
cg12428622	1	43241260	C1orf50	3'UTR	-0,006	9,59E-05	1,79E-02
cg05126264	9	21077879	IFNB1	1stExon	-0,009	9,59E-05	1,79E-02
cg14761454	11	108092087	ATM	TSS1500	-0,008	9,59E-05	1,79E-02
cg11737403	19	57791287	ZNF460	TSS1500	-0,005	9,60E-05	1,79E-02
cg13479842	3	181488778		IGR	-0,012	9,61E-05	1,79E-02
cg00003181	6	146157367		IGR	-0,015	9,61E-05	1,79E-02
cg22511564	16	46809514		IGR	-0,017	9,61E-05	1,79E-02
cg24559983	21	38592988		IGR	-0,004	9,61E-05	1,79E-02
cg16542956	12	46766741	SLC38A2	TSS200	-0,007	9,62E-05	1,79E-02
cg09640960	20	60794676	HRH3	Body	-0,006	9,62E-05	1,79E-02
cg26240235	1	197607830	DENND1B	Body	0,015	9,63E-05	1,79E-02
cg18890544	1	242220538		IGR	-0,035	9,63E-05	1,79E-02
cg07323447	16	67022719	CES8	1stExon	0,013	9,63E-05	1,79E-02
cg22534690	9	1727455		IGR	-0,057	9,64E-05	1,79E-02
cg05238074	4	43135067		IGR	0,026	9,65E-05	1,80E-02
cg08608031	13	37398113	RFXAP	Body	0,017	9,65E-05	1,80E-02
cg03237356	4	178230943	NEIL3	TSS200	0,004	9,66E-05	1,80E-02
cg08935238	5	140798161	PCDHGA4	Body	-0,053	9,68E-05	1,80E-02
cg06935967	6	147605330	STXBP5	Body	-0,005	9,69E-05	1,80E-02
cg00995147	1	221135452		IGR	-0,018	9,70E-05	1,80E-02
cg05398047	2	165953894	SCN3A	Body	-0,027	9,71E-05	1,80E-02
cg17934277	2	216982485	XRCC5	Body	0,018	9,72E-05	1,80E-02
cg00311760	3	9877940	ARPC4-TTLL3	3'UTR	-0,018	9,71E-05	1,80E-02
cg11779646	12	97824438		IGR	-0,011	9,71E-05	1,80E-02
cg08683697	17	72152565		IGR	0,042	9,72E-05	1,80E-02
cg17351116	20	25371719	ABHD12	TSS1500	-0,005	9,71E-05	1,80E-02
cg27665767	5	140764485	PCDHGA4	Body	-0,047	9,72E-05	1,80E-02
cg11796194	7	148267646		IGR	0,021	9,74E-05	1,81E-02
cg19957094	21	33280633	HUNK	Body	-0,036	9,74E-05	1,81E-02
cg18938534	7	42214361	GLI3	Body	-0,012	9,75E-05	1,81E-02
cg03668686	1	214139722	PROX1-AS1	Body	-0,01	9,76E-05	1,81E-02
cg12848295	8	655910	ERICH1	Body	-0,007	9,78E-05	1,81E-02
cg07849735	1	203097253	ADORA1	5'UTR	-0,007	9,79E-05	1,81E-02
cg23598089	1	203652079	ATP2B4	5'UTR	0,027	9,78E-05	1,81E-02
cg25407831	6	36749984	CPNE5	Body	0,012	9,79E-05	1,81E-02
cg11688511	14	96949173	AK7	Body	-0,021	9,79E-05	1,81E-02
cg21204024	1	150669581	GOLPH3L	5'UTR	-0,02	9,81E-05	1,81E-02
cg04348561	1	167738686	MPZL1	Body	0,007	9,81E-05	1,81E-02
cg10894222	3	149543247	RNF13	5'UTR	-0,005	9,81E-05	1,81E-02
cg22458640	5	79703067	ZFYVE16	TSS1500	0,016	9,81E-05	1,81E-02
cg15670475	19	18905020		IGR	0,034	9,81E-05	1,81E-02
cg26374122	2	167998704	XIRP2-AS1	TSS1500	-0,017	9,82E-05	1,81E-02
cg06517189	1	18103667	ACTL8	5'UTR	-0,012	9,83E-05	1,82E-02
cg03107336	11	6325202		IGR	-0,01	9,83E-05	1,82E-02
cg02486415	12	52263387		IGR	0,031	9,83E-05	1,82E-02
cg01383203	16	1220524	CACNA1H	Body	0,03	9,84E-05	1,82E-02
cg10035224	1	9967106	CTNNBIP1	5'UTR	0,006	9,85E-05	1,82E-02
cg18749800	1	214501057	SMYD2	ExonBnd	0,047	9,88E-05	1,82E-02
cg13356253	2	54087189	LOC100302652	TSS200	-0,026	9,87E-05	1,82E-02
cg19114721	2	103126336	SLC9A4	Body	0,036	9,87E-05	1,82E-02
cg23397572	5	110853455	STARD4-AS1	Body	0,01	9,85E-05	1,82E-02
cg18725291	5	133885985	JADE2	Body	0,019	9,87E-05	1,82E-02
cg25337148	5	134834579		IGR	-0,009	9,86E-05	1,82E-02
cg24494723	5	149160911	PPARGC1B	Body	0,02	9,88E-05	1,82E-02
cg04918082	7	100547176		IGR	-0,019	9,87E-05	1,82E-02
cg10300895	8	10283858	MSRA	Body	-0,015	9,88E-05	1,82E-02
cg00793342	11	67412293	ACY3	Body	0,02	9,87E-05	1,82E-02
cg02507181	12	10251536	CLEC1A	5'UTR	-0,005	9,87E-05	1,82E-02
cg14555233	13	50637481	DLEU2	Body	0,017	9,88E-05	1,82E-02
cg16720508	13	51836152	FAM124A	Body	0,023	9,85E-05	1,82E-02
cg17037640	13	95671490		IGR	0,016	9,85E-05	1,82E-02
cg18279358	17	4383006	SPNS3	Body	0,019	9,86E-05	1,82E-02
cg01123729	17	40169510	DNAJC7	TSS1500	-0,004	9,88E-05	1,82E-02
cg07797231	2	232791732	NPPC	TSS1500	0,005	9,89E-05	1,82E-02
cg11128254	17	428311	VPS53	Body	0,043	9,90E-05	1,82E-02
cg14878716	2	8026595		IGR	-0,004	9,92E-05	1,82E-02
cg05801578	13	111267397	CARKD	TSS1500	-0,004	9,92E-05	1,82E-02
cg10792161	17	8553054		IGR	-0,02	9,92E-05	1,82E-02

cg24011488	14	68324675	RAD51B	Body	-0,005	9,93E-05	1,82E-02
cg10410832	1	27808895	WASF2	5'UTR	0,01	9,94E-05	1,82E-02
cg23741360	2	9511764	ASAP2	Body	0,028	9,95E-05	1,82E-02
cg24019521	5	137801041	EGR1	TSS200	0,004	9,94E-05	1,82E-02
cg10786916	14	103442041	CDC42BPB	Body	-0,006	9,95E-05	1,83E-02
cg14416903	21	45409196		IGR	0,016	9,96E-05	1,83E-02
cg04981439	5	38808931		IGR	-0,042	9,98E-05	1,83E-02
cg04617914	6	41512853	FOXP4	TSS1500	0,023	9,98E-05	1,83E-02
cg21745091	11	118992183	HINFP	TSS200	0,006	9,98E-05	1,83E-02
cg17512133	4	77973813	CCNI	Body	0,021	9,99E-05	1,83E-02
cg02144258	2	220391374	ACCN4	Body	0,037	9,99E-05	1,83E-02
cg11786966	1	64508232	ROR1	Body	-0,007	1,00E-04	1,83E-02
cg14237989	1	197329322	CRB1	Body	-0,008	1,00E-04	1,83E-02
cg19402238	2	26407200	FAM59B	Body	0,006	1,00E-04	1,83E-02
cg14879337	3	113046688	CFAP44	Body	0,011	1,00E-04	1,83E-02
cg24030680	8	24749345		IGR	0,054	1,00E-04	1,83E-02
cg00015639	14	24554539	NRL	TSS1500	0,009	1,00E-04	1,83E-02
cg01963748	18	75104820		IGR	-0,007	1,00E-04	1,83E-02
cg09120986	19	47249219	FKRP	TSS200	-0,008	1,00E-04	1,83E-02
cg03664992	1	39957393	BMP8A	1stExon	-0,014	1,00E-04	1,83E-02
cg18882653	1	195683538		IGR	-0,013	1,00E-04	1,83E-02
cg02949481	16	131562	MPG	Body	0,01	1,00E-04	1,83E-02
cg09124558	1	162726620	DDR2	Body	0,006	1,00E-04	1,83E-02
cg06287520	17	77487593	RBFOX3	5'UTR	-0,016	1,00E-04	1,83E-02
cg03713668	15	41316752	INO80	Body	0,016	1,00E-04	1,83E-02
cg08148052	11	63953512	STIP1	TSS200	-0,003	1,01E-04	1,83E-02
cg25725261	4	66746626		IGR	-0,024	1,01E-04	1,83E-02
cg24391982	6	169629902	THBS2	Body	0,055	1,01E-04	1,84E-02
cg05531437	13	100379580	CLYBL-AS1	Body	0,014	1,01E-04	1,84E-02
cg05956608	1	207999258		IGR	-0,003	1,01E-04	1,84E-02
cg02412505	2	232467940		IGR	-0,019	1,01E-04	1,84E-02
cg26838532	4	128983634	LARP1B	5'UTR	-0,008	1,01E-04	1,84E-02
cg13526518	17	72349697	KIF19	Body	0,023	1,01E-04	1,84E-02
cg01219087	20	4572227		IGR	-0,019	1,01E-04	1,84E-02
cg13648937	12	25204249	LRMP	TSS1500	-0,014	1,01E-04	1,84E-02
cg00733838	4	7473297	SORCS2	Body	-0,008	1,01E-04	1,84E-02
cg03038853	10	285218	ZMYND11	Body	-0,01	1,01E-04	1,84E-02
cg18787229	14	25519597	STXBP6	TSS1500	-0,008	1,01E-04	1,84E-02
cg14922328	2	197080641	HECW2	Body	-0,007	1,01E-04	1,84E-02
cg25932019	9	5430931	PLGRKT	Body	-0,013	1,01E-04	1,84E-02
cg23839081	11	14709750	PDE3B	Body	0,038	1,01E-04	1,84E-02
cg21291641	17	31204424	MYO1D	TSS1500	-0,003	1,01E-04	1,84E-02
cg20440596	5	98207622	CHD1	Body	0,013	1,01E-04	1,84E-02
cg22044207	3	150140824	TSC22D2	ExonBnd	0,019	1,01E-04	1,84E-02
cg07048649	17	72890079	FADS6	TSS200	0,024	1,01E-04	1,84E-02
cg05527368	17	71334936	SDK2	Body	-0,01	1,01E-04	1,84E-02
cg00148063	8	4853360	CSMD1	TSS1500	-0,07	1,02E-04	1,84E-02
cg14890726	3	113233993	CCDC52	5'UTR	-0,004	1,02E-04	1,85E-02
cg01970804	12	121544875		IGR	0,014	1,02E-04	1,85E-02
cg08000487	1	185108577	TRMT1L	Body	0,021	1,02E-04	1,85E-02
cg23798983	1	181290535		IGR	-0,006	1,02E-04	1,85E-02
cg19461687	1	207143611	FCAMR	1stExon	-0,007	1,02E-04	1,85E-02
cg21157923	9	139637747	LCN10	TSS1500	0,018	1,02E-04	1,85E-02
cg21553656	3	49569379	DAG1	Body	-0,015	1,02E-04	1,85E-02
cg03719380	3	49758394	AMIGO3	TSS1500	0,016	1,02E-04	1,85E-02
cg23090258	18	65839618		IGR	-0,013	1,02E-04	1,85E-02
cg01786992	20	44715748	NCOA5	5'UTR	0,031	1,02E-04	1,85E-02
cg18945794	4	3319669	RGS12	Body	0,004	1,02E-04	1,85E-02
cg15163987	4	186349453	C4orf47	TSS1500	0,018	1,02E-04	1,85E-02
cg13203499	11	80560404		IGR	-0,028	1,03E-04	1,85E-02
cg11301354	5	133860277		IGR	-0,029	1,03E-04	1,86E-02
cg26338202	6	163768326		IGR	-0,022	1,03E-04	1,86E-02
cg25000693	7	12646744	SCIN	Body	-0,008	1,03E-04	1,86E-02
cg20516332	5	88133256	MEF2C	5'UTR	0,015	1,03E-04	1,86E-02
cg11205079	1	16046180	PLEKHM2	Body	0,008	1,03E-04	1,86E-02
cg13609694	9	131058235		IGR	0,006	1,03E-04	1,86E-02
cg12847548	11	74179208	KCNE3	TSS1500	0,017	1,03E-04	1,86E-02
cg25284446	11	125208592	PKNOX2	5'UTR	-0,036	1,03E-04	1,86E-02
cg05286196	3	12994919	IQSEC1	Body	-0,006	1,03E-04	1,86E-02
cg02613937	19	45395297	TOMM40	Body	0,046	1,03E-04	1,86E-02
cg00106302	19	51846803	VSIG10L	TSS1500	0,022	1,03E-04	1,86E-02
cg07234962	9	120038097	ASTN2	Body	-0,015	1,03E-04	1,86E-02
cg09123257	5	180670541	GNB2L1	Body	-0,002	1,03E-04	1,86E-02

cg19181996	3	167338427	WDR49	5'UTR	0,013	1,03E-04	1,86E-02
cg08622675	7	6524998	KDELR2	TSS1500	0,007	1,03E-04	1,86E-02
cg02734782	11	118272031	ATP5L	TSS200	0,004	1,03E-04	1,86E-02
cg14794915	3	57933977		IGR	0,009	1,03E-04	1,86E-02
cg02098293	7	106709269	PRKAR2B	Body	0,054	1,03E-04	1,86E-02
cg12228061	12	116382677		IGR	-0,009	1,03E-04	1,86E-02
cg11454719	2	223155841	PAX3	Body	-0,006	1,04E-04	1,86E-02
cg17151420	2	27580080	GTF3C2	TSS200	-0,003	1,04E-04	1,87E-02
cg07221454	11	60552841	MS4A10	5'UTR	-0,009	1,04E-04	1,87E-02
cg03217915	21	44840965	SIK1	Body	0,003	1,04E-04	1,87E-02
cg22532853	22	23197527		IGR	-0,029	1,04E-04	1,87E-02
cg03294098	1	12677753	DHRS3	TSS200	-0,012	1,04E-04	1,87E-02
cg20319698	2	238644099	LRRFIP1	Body	0,047	1,04E-04	1,87E-02
cg05713044	5	16671496	MYO10	Body	-0,008	1,04E-04	1,87E-02
cg23685155	12	54396440	HOXC9	Body	-0,043	1,04E-04	1,87E-02
cg20773127	4	111397155	ENPEP	TSS200	-0,051	1,04E-04	1,87E-02
cg02874533	7	65882633		IGR	0,014	1,04E-04	1,87E-02
cg17848335	19	2675185	GNG7	5'UTR	0,05	1,04E-04	1,87E-02
cg05823563	9	104249936	C9orf125	TSS1500	-0,049	1,04E-04	1,87E-02
cg24772001	7	148287941	C7orf33	1stExon	-0,005	1,04E-04	1,87E-02
cg09868780	5	115299071	LVRN	Body	0,025	1,04E-04	1,87E-02
cg24281189	9	95021153	IARS	ExonBnd	-0,007	1,04E-04	1,87E-02
cg27517238	10	132284984		IGR	-0,02	1,04E-04	1,87E-02
cg00619519	1	10093261	UBE4B	5'UTR	-0,01	1,04E-04	1,87E-02
cg22031522	19	40420037	FCGBP	Body	0,021	1,04E-04	1,87E-02
cg01421119	1	211555733	C1orf97	TSS1500	0,046	1,05E-04	1,87E-02
cg22019569	1	246599230	SMYD3	Body	0,022	1,05E-04	1,87E-02
cg25213418	17	27773707	TAOK1	5'UTR	-0,028	1,05E-04	1,87E-02
cg03643143	1	93399212	FAM69A	Body	0,009	1,05E-04	1,87E-02
cg05088331	1	170045171	KIFAP3	TSS1500	0,02	1,05E-04	1,87E-02
cg25761326	10	11206792	CUGBP2	TSS200	-0,004	1,05E-04	1,87E-02
cg01878940	16	54159705		IGR	0,03	1,05E-04	1,87E-02
cg14454577	1	117044461		IGR	-0,008	1,05E-04	1,88E-02
cg24360978	3	77140685	ROBO2	5'UTR	0,013	1,05E-04	1,88E-02
cg19764061	15	59069664	FAM63B	Body	0,01	1,05E-04	1,88E-02
cg26681081	19	36606109	POLR2I	5'UTR	-0,003	1,05E-04	1,88E-02
cg15088491	5	35195437	PRLR	5'UTR	-0,008	1,05E-04	1,88E-02
cg13173536	2	3403300	TTC15	Body	-0,009	1,05E-04	1,88E-02
cg09858176	1	16345293	HSPB7	TSS200	0,009	1,05E-04	1,88E-02
cg24939980	19	40476778	PSMC4	TSS200	-0,005	1,05E-04	1,88E-02
cg17545652	10	6261011	PFKFB3	Body	0,018	1,05E-04	1,88E-02
cg01137401	3	147107349	ZIC4	Body	-0,036	1,05E-04	1,88E-02
cg03031383	4	69312104	TMPRSS11E	TSS1500	0,012	1,05E-04	1,88E-02
cg25724604	10	82218377	TSPAN14	5'UTR	-0,012	1,05E-04	1,88E-02
cg17794320	15	90190822	KIF7	Body	-0,046	1,06E-04	1,88E-02
cg23149687	5	119801643	PRR16	5'UTR	0,048	1,06E-04	1,89E-02
cg13689681	9	99382204	CDC14B	TSS200	-0,009	1,06E-04	1,89E-02
cg09375809	9	134160579		IGR	0,016	1,06E-04	1,89E-02
cg07137888	13	92711204	GPC5	Body	0,008	1,06E-04	1,89E-02
cg22792063	14	23067579	ABHD4	Body	-0,005	1,06E-04	1,89E-02
cg14874703	14	45366363	C14orf28	TSS200	-0,003	1,06E-04	1,89E-02
cg05249443	20	46172182	NCOA3	5'UTR	0,032	1,06E-04	1,89E-02
cg03784329	1	11175571	MTOR	Body	0,007	1,06E-04	1,89E-02
cg02113184	3	186285622	TBCCD1	5'UTR	-0,006	1,06E-04	1,89E-02
cg01156370	6	33138482	COL11A2	Body	0,007	1,06E-04	1,89E-02
cg23416895	7	985505	ADAP1	TSS200	0,014	1,06E-04	1,89E-02
cg04495336	8	2419119		IGR	-0,035	1,06E-04	1,89E-02
cg04067517	2	76017724		IGR	-0,016	1,06E-04	1,89E-02
cg24594305	17	70054286	LOC102723517	TSS1500	-0,037	1,06E-04	1,89E-02
cg17582444	3	179110334	MFN1	3'UTR	0,012	1,06E-04	1,89E-02
cg07417683	1	159440267		IGR	-0,063	1,06E-04	1,89E-02
cg25695000	9	140009393	DPP7	TSS200	0,014	1,06E-04	1,89E-02
cg25165657	1	110992885	PROK1	TSS1500	0,013	1,06E-04	1,89E-02
cg13858685	12	3085618	TEAD4	5'UTR	0,015	1,07E-04	1,89E-02
cg01264332	12	4405469	CCND2	Body	-0,01	1,07E-04	1,89E-02
cg08179621	18	21119789	NPC1	ExonBnd	-0,01	1,07E-04	1,89E-02
cg21419475	12	124118052	EIF2B1	Body	-0,004	1,07E-04	1,89E-02
cg23476801	4	140037891	ELF2	Body	0,01	1,07E-04	1,89E-02
cg06475052	1	161658798		IGR	0,011	1,07E-04	1,89E-02
cg09885728	5	137413510		IGR	-0,007	1,07E-04	1,89E-02
cg08957544	9	128191269		IGR	0,018	1,07E-04	1,89E-02
cg22391888	5	140749929	PCDHGB3	5'UTR	-0,033	1,07E-04	1,90E-02
cg07898446	6	3849294	FAM50B	TSS1500	0,019	1,07E-04	1,90E-02

cg10141830	11	46192994	LOC101928894	TSS1500	0,014	1,07E-04	1,90E-02
cg24662880	22	49351763		IGR	-0,03	1,07E-04	1,90E-02
cg09060129	3	3868974	LRRN1	5'UTR	-0,012	1,07E-04	1,90E-02
cg09949845	2	9562978	ITGB1BP1	5'UTR	-0,004	1,07E-04	1,90E-02
cg11744351	13	30424153	UBL3	1stExon	-0,006	1,07E-04	1,90E-02
cg00407017	21	17657235	MIR99AHG	Body	0,057	1,07E-04	1,90E-02
cg04607281	11	85439999	SYTL2	Body	0,027	1,07E-04	1,90E-02
cg23633413	12	133425336	CHFR	Body	-0,007	1,07E-04	1,90E-02
cg06682556	13	65533139		IGR	-0,007	1,07E-04	1,90E-02
cg09760403	16	17090863		IGR	-0,014	1,08E-04	1,90E-02
cg14254597	10	98471304	PIK3AP1	Body	0,027	1,08E-04	1,90E-02
cg02258614	19	38975245	RYR1	Body	0,014	1,08E-04	1,90E-02
cg07836226	1	8687954	RERE	Body	0,027	1,09E-04	1,90E-02
cg10134268	1	25382777		IGR	-0,006	1,08E-04	1,90E-02
cg23558456	1	45262537		IGR	0,044	1,08E-04	1,90E-02
cg10092516	1	240303037	FMN2	Body	0,041	1,08E-04	1,90E-02
cg03224901	2	85086620	C2orf89	Body	-0,005	1,08E-04	1,90E-02
cg10572794	3	58223527	ABHD6	1stExon	-0,006	1,08E-04	1,90E-02
cg19388273	3	150064304		IGR	0,019	1,08E-04	1,90E-02
cg00408646	3	193130580	ATP13A4	Body	-0,033	1,09E-04	1,90E-02
cg17342807	4	379766		IGR	-0,025	1,08E-04	1,90E-02
cg22886896	5	151141323		IGR	0,032	1,09E-04	1,90E-02
cg20346876	6	125257916	RNF217-AS1	Body	0,017	1,09E-04	1,90E-02
cg10614773	8	144668349	EEF1D	Body	0,016	1,08E-04	1,90E-02
cg21746459	9	104160900	MRPL50	5'UTR	-0,004	1,09E-04	1,90E-02
cg07240236	9	140205100	EXD3	Body	0,023	1,08E-04	1,90E-02
cg18387270	10	89625287	PTEN	5'UTR	-0,021	1,09E-04	1,90E-02
cg21107950	10	128469772		IGR	-0,008	1,08E-04	1,90E-02
cg15226102	11	69469719		IGR	-0,032	1,09E-04	1,90E-02
cg15236696	11	95883435	MAML2	Body	-0,037	1,08E-04	1,90E-02
cg13972906	11	129719560	TMEM45B	5'UTR	-0,015	1,08E-04	1,90E-02
cg22721644	12	47427216		IGR	0,023	1,08E-04	1,90E-02
cg05628616	12	80794579		IGR	-0,051	1,08E-04	1,90E-02
cg15839831	14	31401915	STRN3	Body	0,024	1,08E-04	1,90E-02
cg24108579	14	32475172		IGR	-0,007	1,08E-04	1,90E-02
cg25095171	14	93577304	ITPK1	Body	0,016	1,08E-04	1,90E-02
cg02760164	15	42371967	PLA2G4D	Body	-0,04	1,08E-04	1,90E-02
cg18991015	15	70532902		IGR	0,02	1,08E-04	1,90E-02
cg09180926	15	91537654	PRC1	5'UTR	-0,003	1,08E-04	1,90E-02
cg06890619	16	29888152	SEZ6L2	Body	-0,009	1,08E-04	1,90E-02
cg02484335	16	84623830	COTL1	Body	0,012	1,09E-04	1,90E-02
cg07299526	16	89702762	DPEP1	Body	0,008	1,08E-04	1,90E-02
cg04591032	17	46827458		IGR	0,025	1,08E-04	1,90E-02
cg24084719	19	39171905	ACTN4	Body	-0,004	1,08E-04	1,90E-02
cg01243992	20	31378276	DNMT3B	Body	-0,005	1,09E-04	1,90E-02
cg09360075	20	39987465	LPIN3	Body	0,013	1,08E-04	1,90E-02
cg07626553	22	44494494	PARVB	Body	0,021	1,08E-04	1,90E-02
cg21963179	10	90671015	STAMBPL1	Body	0,016	1,09E-04	1,90E-02
cg04493169	7	27912112	JAZF1	Body	-0,008	1,09E-04	1,90E-02
cg12594911	1	144931379	PDE4DIP	1stExon	0,015	1,09E-04	1,90E-02
cg12656628	1	46016485	AKR1A1	TSS200	-0,003	1,09E-04	1,90E-02
cg17683948	3	45429800	LARS2	TSS1500	0,008	1,09E-04	1,90E-02
cg19282251	5	79866542	ANKRD34B	TSS1500	-0,048	1,09E-04	1,90E-02
cg04272613	14	59721173	DAAM1	5'UTR	0,008	1,09E-04	1,90E-02
cg14924940	4	56716151		IGR	-0,034	1,09E-04	1,91E-02
cg15276815	10	133918387	JAKMIP3	1stExon	-0,021	1,09E-04	1,91E-02
cg14308029	11	69840204		IGR	0,021	1,09E-04	1,91E-02
cg16822754	15	42130883	PLA2G4B	TSS200	0,018	1,09E-04	1,91E-02
cg22995077	10	27796313	RAB18	Body	0,009	1,09E-04	1,91E-02
cg10304358	3	15475904	EAF1	Body	-0,007	1,10E-04	1,91E-02
cg23216724	6	167571584	GPR31	TSS1500	0,062	1,09E-04	1,91E-02
cg00296005	6	169423509		IGR	-0,011	1,10E-04	1,91E-02
cg17136839	7	139040622		IGR	0,009	1,09E-04	1,91E-02
cg04084476	11	1527081		IGR	0,01	1,09E-04	1,91E-02
cg21786381	11	75234078	GDPD5	5'UTR	0,021	1,09E-04	1,91E-02
cg04172826	12	129675569	TMEM132D	Body	0,079	1,10E-04	1,91E-02
cg00175441	17	76100646	TNRC6C	Body	0,007	1,09E-04	1,91E-02
cg06189089	5	149325756	PDE6A	TSS1500	-0,01	1,10E-04	1,91E-02
cg14768955	16	88320874		IGR	-0,039	1,10E-04	1,91E-02
cg20121944	11	74996242	ARRB1	Body	-0,006	1,10E-04	1,91E-02
cg00583022	1	163309861	NUF2	Body	0,037	1,10E-04	1,91E-02
cg26859158	2	202127542	CASP8	Body	-0,011	1,10E-04	1,91E-02
cg06752928	3	151693727		IGR	-0,036	1,10E-04	1,91E-02

cg10953002	4	74241932		IGR	0,017	1,10E-04	1,91E-02
cg14422446	4	184858353	STOX2	Body	-0,007	1,10E-04	1,91E-02
cg05353134	16	24819931	TNRC6A	Body	0,011	1,10E-04	1,91E-02
cg11564670	18	13915006	MC2R	5'UTR	-0,036	1,10E-04	1,91E-02
cg24926355	21	40018791	ERG	5'UTR	-0,016	1,10E-04	1,91E-02
cg20497026	12	58170306	FAM119B	Body	0,014	1,10E-04	1,91E-02
cg11028188	7	24939923	OSBPL3	5'UTR	0,01	1,10E-04	1,91E-02
cg16132315	9	107662475	ABCA1	Body	-0,032	1,10E-04	1,91E-02
cg16185211	19	8099158		IGR	-0,008	1,10E-04	1,91E-02
cg04364695	8	40618425	ZMAT4	Body	-0,107	1,10E-04	1,91E-02
cg13766059	2	47243551	TTC7A	Body	0,025	1,10E-04	1,92E-02
cg11940973	5	145939975		IGR	0,024	1,10E-04	1,92E-02
cg15168615	12	113599683	DDX54	Body	0,015	1,10E-04	1,92E-02
cg01140102	5	176766085	LMAN2	Body	0,015	1,10E-04	1,92E-02
cg16252308	7	134862183		IGR	0,02	1,10E-04	1,92E-02
cg26229249	16	11608558		IGR	-0,007	1,11E-04	1,92E-02
cg16548699	7	98007687	BAIAP2L1	Body	-0,017	1,11E-04	1,92E-02
cg03879918	10	131686425	EBF3	Body	0,011	1,11E-04	1,92E-02
cg15573413	3	40806770		IGR	-0,011	1,11E-04	1,92E-02
cg16364717	11	86024087	C11orf73	Body	0,023	1,11E-04	1,92E-02
cg08105328	12	58266446		IGR	-0,005	1,11E-04	1,92E-02
cg15617706	12	129788159	TMEM132D	Body	-0,01	1,11E-04	1,92E-02
cg13834500	14	24665445	TM9SF1	TSS1500	-0,004	1,11E-04	1,92E-02
cg17396676	19	16583990	EPS15L1	TSS1500	0,009	1,11E-04	1,92E-02
cg02206157	14	97500175		IGR	-0,04	1,11E-04	1,92E-02
cg22684968	7	121784356	AASS	TSS200	-0,006	1,11E-04	1,92E-02
cg25499769	12	128245397		IGR	0,018	1,11E-04	1,92E-02
cg26554385	1	154977246	ZBTB7B	5'UTR	0,018	1,11E-04	1,92E-02
cg08709884	11	64573220	MEN1	Body	0,006	1,11E-04	1,92E-02
cg23762110	10	129478323		IGR	-0,017	1,11E-04	1,92E-02
cg06642389	4	38384197		IGR	0,005	1,11E-04	1,92E-02
cg02700271	4	128765248		IGR	-0,008	1,11E-04	1,92E-02
cg11028622	3	94093345		IGR	0,022	1,11E-04	1,93E-02
cg01828067	5	140628308		IGR	-0,075	1,11E-04	1,93E-02
cg22944476	17	8021815	ALOXE3	5'UTR	-0,003	1,12E-04	1,93E-02
cg01224715	5	140811520	PCDHGA4	Body	-0,061	1,12E-04	1,93E-02
cg00866976	16	56224782	GNAO1	TSS1500	-0,007	1,12E-04	1,93E-02
cg13125537	17	7037323		IGR	-0,011	1,12E-04	1,93E-02
cg14093894	5	59967842	DEPDC1B	Body	-0,006	1,12E-04	1,93E-02
cg19980912	5	133304180	C5orf15	1stExon	0,011	1,12E-04	1,93E-02
cg18213268	8	8473069		IGR	0,023	1,12E-04	1,93E-02
cg14138312	9	6869699	KDM4C	Body	-0,008	1,12E-04	1,93E-02
cg25519033	11	695585	TMEM80	TSS200	-0,004	1,12E-04	1,93E-02
cg23777302	11	69706954		IGR	0,045	1,12E-04	1,93E-02
cg21610385	11	102461989	MMP20	Body	0,008	1,12E-04	1,93E-02
cg06740710	15	68346336	PIAS1	TSS1500	-0,008	1,12E-04	1,93E-02
cg06778713	15	98937852		IGR	-0,012	1,12E-04	1,93E-02
cg08060876	19	23469437		IGR	-0,026	1,12E-04	1,93E-02
cg25115034	5	21391623		IGR	-0,051	1,12E-04	1,93E-02
cg21858649	5	52636046		IGR	0,045	1,12E-04	1,93E-02
cg01375417	1	226549728	PARP1	Body	0,006	1,12E-04	1,93E-02
cg22909901	3	182981707	MCF2L2	Body	0,027	1,12E-04	1,93E-02
cg14378824	12	47747652		IGR	0,008	1,12E-04	1,93E-02
cg04003839	8	1805190	ARHGEF10	Body	-0,004	1,13E-04	1,93E-02
cg18993778	7	139313985	HIPK2	Body	-0,067	1,13E-04	1,94E-02
cg01748244	1	2179352	SKI	Body	0,009	1,13E-04	1,94E-02
cg06552037	2	220435838	INHA	TSS1500	-0,006	1,13E-04	1,94E-02
cg10533098	9	102862637	INVS	5'UTR	0,028	1,13E-04	1,94E-02
cg03756778	15	92407388	SLCO3A1	Body	0,006	1,13E-04	1,94E-02
cg19388050	11	2562647	KCNQ1	Body	-0,009	1,13E-04	1,94E-02
cg16696400	16	7364322	A2BP1	Body	-0,025	1,13E-04	1,94E-02
cg12305855	4	160216262	RAPGEF2	Body	0,015	1,13E-04	1,94E-02
cg10289901	7	123198384	NDUFA5	TSS1500	-0,056	1,13E-04	1,94E-02
cg25240707	2	26265079	RAB10	Body	-0,005	1,13E-04	1,94E-02
cg13997788	2	46593223	EPAS1	Body	0,013	1,13E-04	1,94E-02
cg19234858	2	74756372	HTRA2	TSS200	-0,002	1,13E-04	1,94E-02
cg19547217	2	231457660		IGR	-0,026	1,13E-04	1,94E-02
cg23319258	3	98243150	CLDND1	TSS1500	-0,014	1,13E-04	1,94E-02
cg00602992	7	107541870	DLD	5'UTR	0,008	1,13E-04	1,94E-02
cg27196925	12	9412289		IGR	0,021	1,13E-04	1,94E-02
cg25270205	12	109208726	SSH1	Body	0,011	1,13E-04	1,94E-02
cg09046427	12	128754675	TMEM132C	Body	-0,052	1,13E-04	1,94E-02
cg02765864	14	101875672	LINC00524	TSS1500	-0,02	1,13E-04	1,94E-02

cg22312354	19	56158714	CCDC106	TSS1500	0,017	1,13E-04	1,94E-02
cg09167115	8	37659174	ADGRA2	Body	0,019	1,13E-04	1,94E-02
cg04101729	1	41203400	NFYC	TSS1500	-0,006	1,14E-04	1,94E-02
cg19436804	8	8560866	CLDN23	1stExon	-0,009	1,14E-04	1,94E-02
cg07602984	1	36790927	FAM176B	TSS1500	0,011	1,14E-04	1,94E-02
cg24879592	15	47014072		IGR	0,014	1,14E-04	1,95E-02
cg19166896	15	92410356	SLCO3A1	Body	0,004	1,14E-04	1,95E-02
cg05164350	14	75537863	FAM164C	Body	-0,005	1,14E-04	1,95E-02
cg26982271	19	36365679	APLP1	Body	-0,004	1,14E-04	1,95E-02
cg16902425	6	29526459	UBD	Body	-0,023	1,14E-04	1,95E-02
cg08732684	6	32095128	ATF6B	Body	0,015	1,14E-04	1,95E-02
cg00018682	8	11359569	BLK	5'UTR	0,016	1,14E-04	1,95E-02
cg14559482	14	73364767		IGR	-0,02	1,14E-04	1,95E-02
cg22607707	12	88221160		IGR	-0,024	1,14E-04	1,95E-02
cg15533935	12	93774767	NUDT4P2	Body	-0,029	1,15E-04	1,95E-02
cg27158340	14	105603389		IGR	-0,029	1,15E-04	1,95E-02
cg02501094	13	103556783		IGR	-0,007	1,15E-04	1,95E-02
cg12728588	1	36025489	NCDN	Body	0,02	1,15E-04	1,95E-02
cg18164784	5	140762229	PCDHGA4	Body	-0,042	1,15E-04	1,95E-02
cg08021716	2	37434054	CEBPZ	Body	-0,017	1,15E-04	1,95E-02
cg26662493	6	6462060	LY86-AS1	Body	-0,021	1,15E-04	1,95E-02
cg12146763	17	14483597		IGR	-0,012	1,15E-04	1,96E-02
cg04847386	17	79445297		IGR	0,011	1,15E-04	1,96E-02
cg12605148	15	28356800	HERC2	3'UTR	-0,007	1,15E-04	1,96E-02
cg01380607	19	996373		IGR	-0,038	1,15E-04	1,96E-02
cg18209212	12	130646256	FZD10	TSS1500	-0,01	1,15E-04	1,96E-02
cg09317554	4	151505084	LRBA	Body	-0,008	1,15E-04	1,96E-02
cg06975395	11	34204323	ABTB2	Body	0,01	1,15E-04	1,96E-02
cg16984619	11	6633451	TAF10	TSS200	0,006	1,15E-04	1,96E-02
cg24065398	2	107442387	ST6GAL2	Body	0,01	1,15E-04	1,96E-02
cg26221131	14	69445993	ACTN1-AS1	TSS1500	-0,005	1,15E-04	1,96E-02
cg13064555	2	241451445	ANKMY1	Body	-0,013	1,15E-04	1,96E-02
cg27236896	7	80128051	GNAT3	Body	0,017	1,15E-04	1,96E-02
cg24355665	7	86549240	KIAA1324L	Body	-0,015	1,15E-04	1,96E-02
cg25985724	6	26104396	HIST1H4C	1stExon	-0,005	1,16E-04	1,96E-02
cg24766816	6	30568535	PPP1R10	3'UTR	0,007	1,16E-04	1,96E-02
cg01287209	2	187561243	FAM171B	Body	-0,011	1,16E-04	1,96E-02
cg20093661	9	140437771	PNPLA7	Body	0,006	1,16E-04	1,96E-02
cg13742390	5	55289675	FLJ31104	TSS1500	-0,038	1,16E-04	1,96E-02
cg18163106	7	17414387	KCCAT333	TSS200	-0,007	1,16E-04	1,96E-02
cg15080866	1	2705124		IGR	0,035	1,16E-04	1,97E-02
cg04276723	7	38670597	AMPH	Body	-0,006	1,16E-04	1,97E-02
cg16689481	1	152079713	TCHH	3'UTR	-0,069	1,16E-04	1,97E-02
cg01991514	14	102391727	PPP2R5C	3'UTR	-0,008	1,16E-04	1,97E-02
cg09443855	22	41602903	L3MBTL2	Body	0,011	1,16E-04	1,97E-02
cg03386903	17	7832479	KCNAB3	Body	0,005	1,16E-04	1,97E-02
cg22962811	4	181868775		IGR	-0,017	1,16E-04	1,97E-02
cg17431659	3	185697805	LOC344887	Body	0,043	1,16E-04	1,97E-02
cg08084454	3	176915592	TBL1XR1	TSS1500	-0,006	1,16E-04	1,97E-02
cg15204975	5	133883856	JADE2	Body	0,04	1,16E-04	1,97E-02
cg13166213	8	11560563	GATA4	TSS1500	-0,016	1,16E-04	1,97E-02
cg12074247	13	34675978		IGR	-0,027	1,16E-04	1,97E-02
cg24083695	19	3030090	TLE2	TSS1500	-0,009	1,16E-04	1,97E-02
cg24995941	20	35936259	MANBAL	Body	0,016	1,16E-04	1,97E-02
cg08532922	10	81939751	ANXA11	5'UTR	-0,008	1,17E-04	1,97E-02
cg14561362	4	146461062	SMAD1	Body	0,04	1,17E-04	1,97E-02
cg23661344	17	17109678	PLD6	TSS200	-0,033	1,17E-04	1,97E-02
cg10419511	20	31170984		IGR	0,009	1,17E-04	1,97E-02
cg07780517	13	30077489	MTUS2	3'UTR	-0,052	1,17E-04	1,97E-02
cg15676837	1	202137199	PTPRV	Body	-0,007	1,17E-04	1,97E-02
cg21199659	9	133366752	ASS1	Body	-0,016	1,17E-04	1,97E-02
cg17491697	1	6305588	HES3	3'UTR	-0,005	1,17E-04	1,97E-02
cg21806985	5	112824646	MCC	TSS200	-0,073	1,17E-04	1,97E-02
cg21149863	7	13764990		IGR	-0,012	1,17E-04	1,97E-02
cg18019042	10	17428821	ST8SIA6-AS1	TSS200	0,034	1,17E-04	1,97E-02
cg22715398	15	52968154	KIAA1370	Body	0,035	1,17E-04	1,97E-02
cg03605666	19	3383594	NFIC	Body	0,013	1,17E-04	1,97E-02
cg06894549	1	155890816	SNORA42	TSS1500	-0,007	1,17E-04	1,98E-02
cg00120810	3	11195317	HRH1	5'UTR	0,016	1,17E-04	1,98E-02
cg21756587	5	2867363		IGR	-0,015	1,17E-04	1,98E-02
cg11840968	5	176167283		IGR	0,016	1,17E-04	1,98E-02
cg10754705	8	22085381	PHYHIP	Body	-0,031	1,17E-04	1,98E-02
cg18215027	10	106824070	SORCS3	Body	-0,039	1,17E-04	1,98E-02

cg18535611	13	28505434		IGR	-0,067	1,17E-04	1,98E-02
cg02711212	15	69854024	LOC145837	TSS200	-0,006	1,17E-04	1,98E-02
cg03171659	16	89120700		IGR	-0,007	1,17E-04	1,98E-02
cg02626476	17	80595110	WDR45B	Body	0,02	1,17E-04	1,98E-02
cg16004875	1	18902086		IGR	0,092	1,18E-04	1,98E-02
cg12810325	7	131630318		IGR	-0,004	1,18E-04	1,98E-02
cg24548191	13	54725166		IGR	-0,017	1,18E-04	1,98E-02
cg13348877	18	78005237	PARD6G	1stExon	-0,007	1,18E-04	1,98E-02
cg17080180	14	77788400	GSTZ1	Body	-0,003	1,18E-04	1,98E-02
cg16439795	12	48185867	HDAC7	Body	-0,005	1,18E-04	1,98E-02
cg11308572	4	2926298	ADD1	Body	0,006	1,18E-04	1,98E-02
cg09053419	11	7695679	CYB5R2	TSS1500	-0,042	1,18E-04	1,98E-02
cg02206526	3	123134800	ADCY5	Body	0,005	1,18E-04	1,98E-02
cg00560645	9	77449298	TRPM6	Body	-0,008	1,18E-04	1,98E-02
cg03642635	11	133788233	IGSF9B	3'UTR	-0,064	1,18E-04	1,98E-02
cg19241322	12	48746089	ZNF641	TSS1500	0,007	1,18E-04	1,98E-02
cg13986498	13	24917418		IGR	-0,022	1,18E-04	1,98E-02
cg02476339	22	50009156		IGR	0,004	1,18E-04	1,98E-02
cg26778584	3	149691960		IGR	0,007	1,18E-04	1,98E-02
cg13301956	11	6240838	FAM160A2	Body	-0,035	1,18E-04	1,98E-02
cg10657965	7	101888125	CUX1	Body	0,019	1,18E-04	1,98E-02
cg21034274	11	2404891	CD81	5'UTR	0,016	1,18E-04	1,98E-02
cg05036191	2	1052729	SNTG2	Body	-0,014	1,18E-04	1,98E-02
cg25066643	12	120729786		IGR	-0,007	1,18E-04	1,98E-02
cg06800545	1	219503241		IGR	0,04	1,19E-04	1,99E-02
cg14317075	3	46653653	LOC100132146	TSS1500	-0,018	1,19E-04	1,99E-02
cg16355211	3	194372297	LSG1	Body	-0,007	1,19E-04	1,99E-02
cg25722073	1	172351357	DNM3	Body	-0,013	1,19E-04	1,99E-02
cg04925220	6	107807442		IGR	-0,02	1,19E-04	1,99E-02
cg06571218	20	50368955	ATP9A	Body	0,017	1,19E-04	1,99E-02
cg21230392	4	16077697	PROM1	1stExon	0,004	1,19E-04	1,99E-02
cg24746838	7	64452895	ZNF117	TSS1500	-0,011	1,19E-04	1,99E-02
cg18197540	8	133814746	PHF20L1	Body	0,009	1,19E-04	1,99E-02
cg21129641	2	24232750	MFSD2B	TSS1500	0,01	1,19E-04	1,99E-02
cg01087419	12	14959261	C12orf69	Body	0,014	1,19E-04	1,99E-02
cg26402799	12	43895441	ADAMTS20	Body	-0,009	1,19E-04	1,99E-02
cg21171549	6	36830428	PPIL1	Body	-0,01	1,19E-04	1,99E-02
cg04694155	12	95397486	NDUFA12	5'UTR	-0,005	1,19E-04	1,99E-02
cg14539210	17	27331785	SEZ6	Body	0,032	1,19E-04	1,99E-02
cg01589157	12	133526768	ZNF605	5'UTR	-0,007	1,19E-04	1,99E-02
cg21889703	6	136607649	BCLAF1	5'UTR	0,042	1,20E-04	1,99E-02
cg11917783	12	56863122	SPRYD4	Body	0,036	1,20E-04	1,99E-02
cg06690525	2	170430736	FASTKD1	TSS1500	0,023	1,20E-04	1,99E-02
cg01913165	10	51042324	PARG	Body	0,025	1,20E-04	1,99E-02
cg11294276	15	59608231	MYO1E	Body	-0,024	1,20E-04	1,99E-02
cg23142990	17	48879522		IGR	-0,019	1,20E-04	1,99E-02
cg13590687	18	42921844	SLC14A2	5'UTR	-0,018	1,20E-04	1,99E-02
cg03456771	7	56242072		IGR	-0,064	1,20E-04	1,99E-02
cg02987814	20	55675459		IGR	-0,024	1,20E-04	1,99E-02
cg04293820	2	61483481	USP34	Body	-0,008	1,20E-04	1,99E-02
cg07260333	16	89986946	MC1R	1stExon	0,021	1,20E-04	1,99E-02
cg07926074	7	112406584	TMEM168	3'UTR	0,014	1,20E-04	2,00E-02
cg18521771	9	117373239	C9orf91	TSS1500	0,02	1,20E-04	2,00E-02
cg15138339	17	40715222	COASY	1stExon	0,028	1,20E-04	2,00E-02
cg11593729	3	150690251	CLRN1	1stExon	-0,012	1,20E-04	2,00E-02
cg22141702	4	78079462	CCNG2	5'UTR	-0,004	1,20E-04	2,00E-02
cg20754980	1	227154520	ADCK3	Body	-0,006	1,20E-04	2,00E-02
cg01975338	14	97326473	VRK1	Body	0,012	1,20E-04	2,00E-02
cg22524722	16	2933076	FLYWCH2	TSS200	0,006	1,20E-04	2,00E-02
cg24361121	17	77966786	TBC1D16	Body	0,008	1,20E-04	2,00E-02
cg00086429	2	206566025	NRP2	Body	-0,017	1,20E-04	2,00E-02
cg15481845	10	112396781		IGR	0,019	1,20E-04	2,00E-02
cg08246316	16	30751261	SRCAP	3'UTR	0,024	1,20E-04	2,00E-02
cg15441164	1	33591993		IGR	-0,006	1,21E-04	2,00E-02
cg08826460	19	11201433	LDLR	Body	-0,006	1,21E-04	2,00E-02
cg27279713	11	109994010	ZC3H12C	Body	0,026	1,21E-04	2,00E-02
cg19508823	2	116315952	DPP10	Body	-0,008	1,21E-04	2,00E-02
cg00582714	3	129373072	TMCC1	Body	0,008	1,21E-04	2,00E-02
cg02343064	4	907595	GAK	Body	-0,007	1,21E-04	2,00E-02
cg18313899	6	118228498	SLC35F1	TSS200	-0,011	1,21E-04	2,00E-02
cg13193606	4	2408478	ZFYVE28	Body	0,013	1,21E-04	2,00E-02
cg02392258	11	117198878	CEP164	5'UTR	-0,003	1,21E-04	2,00E-02
cg20980615	17	6939347	SLC16A13	TSS200	-0,004	1,21E-04	2,00E-02

cg19782446	17	27921665	ANKRD13B	Body	0,014	1,21E-04	2,00E-02
cg21102950	3	155524173	C3orf33	TSS200	-0,004	1,21E-04	2,00E-02
cg04864659	17	39471952	KRTAP17-1	TSS200	-0,011	1,21E-04	2,00E-02
cg09207310	12	46930514	LOC100288798	Body	0,009	1,21E-04	2,00E-02
cg13045813	2	196933518	DNAH7	5'UTR	0,007	1,21E-04	2,00E-02
cg10986849	2	206750583		IGR	-0,032	1,21E-04	2,00E-02
cg10453019	3	16216215	GALNTL2	5'UTR	-0,042	1,21E-04	2,00E-02
cg05947699	5	173345670	CPEB4	Body	-0,007	1,21E-04	2,00E-02
cg26694831	5	178763419	ADAMTS2	Body	-0,044	1,21E-04	2,00E-02
cg06696596	6	122391246		IGR	-0,087	1,21E-04	2,00E-02
cg25263189	7	4850105	RADIL	Body	0,02	1,21E-04	2,00E-02
cg02184125	7	105192952	RINT1	Body	0,006	1,21E-04	2,00E-02
cg12983042	8	62823894		IGR	-0,007	1,21E-04	2,00E-02
cg25355416	9	72926759	SMC5	Body	0,023	1,21E-04	2,00E-02
cg14081634	12	20661977	PDE3A	Body	0,011	1,21E-04	2,00E-02
cg22302446	4	125243557		IGR	-0,014	1,22E-04	2,00E-02
cg06417987	11	35265310		IGR	-0,005	1,22E-04	2,00E-02
cg04561389	13	103052468	FGF14	Body	-0,006	1,22E-04	2,00E-02
cg27195304	19	39980647	TIMM50	3'UTR	0,011	1,22E-04	2,00E-02
cg11732360	21	40721090	HMGN1	TSS200	0,008	1,22E-04	2,00E-02
cg12742294	22	19868885	TXNRD2	Body	-0,008	1,22E-04	2,00E-02
cg14067873	12	81110706	MYF5	TSS200	-0,031	1,22E-04	2,00E-02
cg15282732	6	150346863	RAET1L	TSS200	-0,017	1,22E-04	2,01E-02
cg25349828	10	21809392	SKIDA1	5'UTR	-0,029	1,22E-04	2,01E-02
cg22081930	8	115309470		IGR	0,037	1,22E-04	2,01E-02
cg05966255	17	38758057		IGR	-0,035	1,22E-04	2,01E-02
cg02490582	19	38964685	RYR1	Body	-0,018	1,22E-04	2,01E-02
cg19168631	14	59655243	DAAM1	TSS200	-0,003	1,22E-04	2,01E-02
cg08611946	9	4835799	RCL1	Body	0,016	1,22E-04	2,01E-02
cg13930557	9	139905509	ABCA2	Body	0,024	1,22E-04	2,01E-02
cg24270182	8	9046418		IGR	-0,008	1,22E-04	2,01E-02
cg02230791	1	65625801	AK4	Body	0,006	1,22E-04	2,01E-02
cg14220277	21	38338958	HLCS	5'UTR	0,01	1,22E-04	2,01E-02
cg07744561	5	6022991		IGR	-0,007	1,23E-04	2,01E-02
cg25905348	1	40733757	ZMPSTE24	Body	0,015	1,23E-04	2,01E-02
cg21733531	7	63560711		IGR	-0,049	1,23E-04	2,02E-02
cg08859206	1	53392774	SCP2	TSS200	-0,017	1,23E-04	2,02E-02
cg18545120	19	47242589	STRN4	Body	-0,015	1,23E-04	2,02E-02
cg06716048	1	158534444	OR6P1	TSS1500	0,041	1,23E-04	2,02E-02
cg05665093	2	192547246	OBFC2A	Body	-0,013	1,23E-04	2,02E-02
cg23123838	14	105886072	MTA1	TSS200	0,013	1,23E-04	2,02E-02
cg20551922	17	4391282	SPNS3	3'UTR	0,034	1,23E-04	2,02E-02
cg10653997	17	7583279	TP53	5'UTR	0,006	1,23E-04	2,02E-02
cg24372629	19	19228094		IGR	-0,008	1,23E-04	2,02E-02
cg25699964	3	46364062		IGR	-0,019	1,23E-04	2,02E-02
cg05206840	5	38695848		IGR	-0,019	1,23E-04	2,02E-02
cg14104626	5	156988400	ADAM19	Body	0,009	1,23E-04	2,02E-02
cg12744916	11	5864605	OR52E6	TSS1500	0,054	1,23E-04	2,02E-02
cg07055694	16	65160241		IGR	-0,034	1,23E-04	2,02E-02
cg18355337	19	55549722	GP6	TSS200	0,071	1,23E-04	2,02E-02
cg16026189	1	983039	AGRN	Body	0,007	1,23E-04	2,02E-02
cg04141270	11	33217686		IGR	-0,008	1,23E-04	2,02E-02
cg09427016	16	89167018	ACSF3	Body	0,044	1,23E-04	2,02E-02
cg12822891	14	103464954	CDC42BPB	Body	-0,016	1,23E-04	2,02E-02
cg13190694	12	131343760		IGR	-0,005	1,23E-04	2,02E-02
cg03682112	18	77623598	KCNG2	TSS200	-0,059	1,24E-04	2,02E-02
cg12917056	1	186416576	LOC102724919	Body	0,028	1,24E-04	2,02E-02
cg11474358	20	61517784	DIDO1	Body	0,02	1,24E-04	2,02E-02
cg02531969	11	128666538	FLI1	Body	0,026	1,24E-04	2,02E-02
cg10442572	16	4675384	MGRN1	Body	-0,005	1,24E-04	2,02E-02
cg19780430	5	97685493		IGR	-0,039	1,24E-04	2,02E-02
cg13362694	5	171646800	UBTD2	Body	0,004	1,24E-04	2,03E-02
cg21657010	9	98080123	FANCC	TSS1500	-0,007	1,24E-04	2,03E-02
cg05587756	12	10874734	CSDA	Body	-0,005	1,24E-04	2,03E-02
cg00979695	7	138363322	SVOPL	Body	0,013	1,24E-04	2,03E-02
cg05164937	10	135498991		IGR	-0,063	1,24E-04	2,03E-02
cg00557709	1	82268801	LPHN2	5'UTR	-0,008	1,24E-04	2,03E-02
cg17361449	20	43104463	TTPAL	TSS200	0,013	1,24E-04	2,03E-02
cg08553136	5	32090873	PDZD2	Body	0,006	1,24E-04	2,03E-02
cg02568347	13	34071080	STARD13	Body	0,048	1,24E-04	2,03E-02
cg26836793	7	32982953	RP9P	TSS200	0,007	1,24E-04	2,03E-02
cg06651313	8	144695921	TSTA3	Body	0,006	1,25E-04	2,03E-02
cg06351643	11	69624649		IGR	0,006	1,25E-04	2,03E-02

cg13964104	1	66820623	PDE4B	Body	0,025	1,25E-04	2,03E-02
cg05855588	13	33590273	KL	TSS1500	-0,006	1,25E-04	2,03E-02
cg26673496	8	29128725		IGR	0,017	1,25E-04	2,03E-02
cg23163993	19	10829339	DNM2	Body	-0,004	1,25E-04	2,03E-02
cg06771188	6	27521477		IGR	-0,017	1,25E-04	2,03E-02
cg19400295	2	174214359		IGR	0,006	1,25E-04	2,03E-02
cg15380539	15	77861116		IGR	0,013	1,25E-04	2,03E-02
cg18047082	2	167232696	SCN9A	TSS200	-0,009	1,25E-04	2,03E-02
cg04458627	17	46115738	MIR152	TSS1500	0,026	1,25E-04	2,03E-02
cg08188330	19	50666484	C19orf41	1stExon	-0,023	1,25E-04	2,03E-02
cg17345283	12	132280296	SFSWAP	Body	0,054	1,25E-04	2,03E-02
cg09984469	2	72372162	CYP26B1	Body	0,01	1,25E-04	2,03E-02
cg26037505	5	138862156	TMEM173	5'UTR	-0,006	1,25E-04	2,04E-02
cg08436959	6	138617156	KIAA1244	Body	0,007	1,25E-04	2,04E-02
cg01508568	12	92880499		IGR	0,01	1,26E-04	2,04E-02
cg21850832	7	130081170	CEP41	TSS200	0,016	1,26E-04	2,04E-02
cg02300050	11	110056341	RDX	3'UTR	0,026	1,26E-04	2,04E-02
cg21031128	8	21771278	DOK2	TSS200	-0,005	1,26E-04	2,04E-02
cg11888265	9	100277011	TMOD1	5'UTR	0,02	1,26E-04	2,04E-02
cg21380590	14	23624778	SLC7A8	TSS1500	0,021	1,26E-04	2,04E-02
cg15986194	14	57961453	C14orf105	TSS1500	-0,013	1,26E-04	2,04E-02
cg08955448	18	6728895		IGR	0,049	1,26E-04	2,04E-02
cg22688351	21	41253706	PCP4	Body	-0,05	1,26E-04	2,04E-02
cg07695269	4	7394902	SORCS2	Body	0,03	1,26E-04	2,04E-02
cg25359478	10	78989901	KCNMA1	Body	-0,022	1,26E-04	2,04E-02
cg08348387	2	242158123	ANO7	Body	0,012	1,26E-04	2,04E-02
cg23619399	13	79169753		IGR	-0,017	1,26E-04	2,04E-02
cg02350428	22	49763410		IGR	-0,048	1,26E-04	2,04E-02
cg23699989	6	170120696	PHF10	Body	-0,017	1,26E-04	2,04E-02
cg00728346	7	95002891	PON3	Body	0,03	1,26E-04	2,04E-02
cg21383720	9	96214543	FAM120AOS	1stExon	-0,004	1,26E-04	2,04E-02
cg07427211	16	696003	FAM195A	Body	0,03	1,26E-04	2,04E-02
cg04350092	19	11290464	KANK2	Body	0,021	1,26E-04	2,04E-02
cg12245717	20	22555206	C20orf56	Body	-0,053	1,26E-04	2,04E-02
cg07376775	2	100649891	AFF3	Body	0,036	1,26E-04	2,04E-02
cg11214141	6	52906363	ICK	5'UTR	-0,005	1,26E-04	2,04E-02
cg20216802	3	154797958	MME	TSS200	-0,007	1,27E-04	2,04E-02
cg18183961	4	40519094	RBM47	TSS1500	0,033	1,26E-04	2,04E-02
cg20800450	6	14790281		IGR	0,027	1,26E-04	2,04E-02
cg00830420	12	5758014	ANO2	Body	-0,037	1,26E-04	2,04E-02
cg14400871	16	75018461	WDR59	Body	-0,004	1,27E-04	2,04E-02
cg10189885	2	198176433	ANKRD44	TSS1500	-0,034	1,27E-04	2,04E-02
cg03666309	19	39234827	CAPN12	5'UTR	0,032	1,27E-04	2,04E-02
cg00568792	11	125462554	STT3A	TSS200	-0,004	1,27E-04	2,04E-02
cg07263509	2	63665072	C2orf86	5'UTR	0,017	1,27E-04	2,04E-02
cg20281573	5	114516427	TRIM36	TSS200	-0,045	1,27E-04	2,04E-02
cg20715090	4	160188010	RAPGEF2	TSS1500	0,03	1,27E-04	2,04E-02
cg25135812	7	103076726	SLC26A5	5'UTR	0,005	1,27E-04	2,04E-02
cg13376046	12	13197196	KIAA1467	TSS200	-0,004	1,27E-04	2,04E-02
cg16585554	17	65386854	PITPNC1	Body	-0,147	1,27E-04	2,04E-02
cg01287037	15	65594648		IGR	-0,007	1,27E-04	2,05E-02
cg23855428	12	119326522		IGR	-0,014	1,27E-04	2,05E-02
cg04841371	1	152958102	SPRR1A	3'UTR	-0,014	1,27E-04	2,05E-02
cg24408469	22	46770440	CELSR1	Body	-0,011	1,27E-04	2,05E-02
cg22448433	13	76123478	UCHL3	TSS1500	-0,003	1,27E-04	2,05E-02
cg25229252	3	50217543	SEMA3F	Body	-0,02	1,27E-04	2,05E-02
cg00979348	4	84256345	HPSE	TSS1500	0,004	1,27E-04	2,05E-02
cg10374248	10	99083645		IGR	-0,005	1,27E-04	2,05E-02
cg21071117	11	124747075	ROBO3	Body	-0,02	1,27E-04	2,05E-02
cg24849648	7	120964019	WNT16	TSS1500	-0,036	1,27E-04	2,05E-02
cg01791597	8	77911072	PEX2	5'UTR	0,028	1,28E-04	2,05E-02
cg00312125	4	84030111	PLAC8	5'UTR	0,045	1,28E-04	2,05E-02
cg13501117	1	3566577	WDR8	1stExon	-0,005	1,28E-04	2,05E-02
cg03208218	5	79551238	SERINC5	Body	0,004	1,28E-04	2,05E-02
cg02796638	7	155142689		IGR	-0,041	1,28E-04	2,05E-02
cg15201549	16	14723996	PARN	5'UTR	-0,006	1,28E-04	2,05E-02
cg07017875	5	140789247	PCDHGA4	Body	-0,034	1,28E-04	2,05E-02
cg08364654	3	42978180		IGR	-0,074	1,28E-04	2,05E-02
cg11873026	3	129326664	PLXND1	TSS1500	0,025	1,28E-04	2,05E-02
cg14817370	6	160390042	IGF2R	TSS200	-0,004	1,28E-04	2,05E-02
cg03492808	22	18915145	PRODH	Body	0,018	1,28E-04	2,05E-02
cg08904369	6	167704188	UNC93A	TSS1500	-0,021	1,28E-04	2,05E-02
cg17364238	10	96255424	TBC1D12	Body	-0,005	1,28E-04	2,05E-02

cg12111104	13	33860756	STARD13	TSS1500	-0,007	1,28E-04	2,05E-02
cg21446605	15	97699995		IGR	-0,008	1,28E-04	2,05E-02
cg26119330	2	64677720		IGR	-0,007	1,28E-04	2,06E-02
cg25111777	1	208337399	PLXNA2	Body	0,015	1,28E-04	2,06E-02
cg07660894	17	28294675	EFCAB5	Body	-0,005	1,29E-04	2,06E-02
cg07068976	4	106630329	GSTCD	5'UTR	-0,005	1,29E-04	2,06E-02
cg01182271	1	54766972	SSBP3	Body	0,005	1,29E-04	2,06E-02
cg27596010	21	44892625	LINC00313	Body	-0,008	1,29E-04	2,06E-02
cg23715056	10	97982467	BLNK	Body	0,024	1,29E-04	2,06E-02
cg02856462	13	46283374	SPERT	Body	-0,011	1,29E-04	2,06E-02
cg20999084	17	8124252	C17orf44	Body	-0,002	1,29E-04	2,06E-02
cg05063412	12	114917737		IGR	-0,006	1,29E-04	2,06E-02
cg12794758	1	203097234	ADORA1	5'UTR	-0,029	1,29E-04	2,06E-02
cg01654770	15	80543586		IGR	-0,044	1,29E-04	2,06E-02
cg18802288	4	649308	PDE6B	Body	0,029	1,29E-04	2,06E-02
cg20740769	1	101701819	S1PR1	TSS1500	0,005	1,29E-04	2,06E-02
cg03913299	2	191969969	STAT4	Body	0,015	1,29E-04	2,06E-02
cg14170787	9	112889386	AKAP2	Body	0,037	1,29E-04	2,06E-02
cg09675853	2	239306169	TRAF3IP1	Body	0,006	1,29E-04	2,06E-02
cg07143110	2	241530344	CAPN10	Body	-0,006	1,29E-04	2,06E-02
cg21488156	13	111288822	CARKD	Body	-0,005	1,29E-04	2,06E-02
cg12215975	1	53107021	FAM159A	Body	0,02	1,30E-04	2,07E-02
cg08567662	2	176867156	KIAA1715	TSS200	-0,003	1,30E-04	2,07E-02
cg25310430	7	158649243	WDR60	TSS200	-0,005	1,30E-04	2,07E-02
cg00456774	19	40857787	PLD3	5'UTR	-0,011	1,30E-04	2,07E-02
cg13250388	22	39641078	PDGFB	TSS200	-0,005	1,30E-04	2,07E-02
cg06054726	10	73956853	ASCC1	Body	0,01	1,30E-04	2,07E-02
cg00741016	11	1249607	MUC5B	Body	0,036	1,30E-04	2,07E-02
cg22712641	22	20094086	DGCR8	Body	0,012	1,30E-04	2,07E-02
cg12166292	2	192542908	NABP1	1stExon	-0,003	1,30E-04	2,07E-02
cg07766779	6	149887504	C6orf72	TSS200	0,012	1,30E-04	2,07E-02
cg04254198	17	77679849		IGR	0,008	1,30E-04	2,07E-02
cg14667685	1	39249604		IGR	-0,083	1,30E-04	2,07E-02
cg15625634	5	82393590	XRCC4	5'UTR	0,032	1,30E-04	2,07E-02
cg05038241	10	121090736	GRK5	Body	0,008	1,30E-04	2,07E-02
cg20432810	13	49008488	LPAR6	5'UTR	0,043	1,30E-04	2,07E-02
cg16787365	14	77499911		IGR	-0,005	1,30E-04	2,07E-02
cg23460707	10	133558971		IGR	0,057	1,30E-04	2,07E-02
cg06801163	17	4686500	TM4SF5	3'UTR	-0,039	1,30E-04	2,07E-02
cg11337388	1	44446092	B4GALT2	5'UTR	0,021	1,30E-04	2,07E-02
cg15019661	2	10612098		IGR	-0,02	1,30E-04	2,07E-02
cg20707991	7	157333949	PTPRN2	Body	0,026	1,30E-04	2,07E-02
cg07279094	19	34293132	KCTD15	Body	-0,006	1,30E-04	2,07E-02
cg14371343	7	122097744	CADPS2	Body	0,021	1,31E-04	2,07E-02
cg15629162	18	12304383		IGR	-0,009	1,31E-04	2,07E-02
cg17382158	3	73629535	PDZRN3	Body	-0,021	1,31E-04	2,07E-02
cg09429345	3	138327826	FAIM	5'UTR	0,008	1,31E-04	2,07E-02
cg24043607	3	155012093	LOC100507537	TSS1500	0,021	1,31E-04	2,07E-02
cg04241582	5	162930306	MAT2B	1stExon	-0,004	1,31E-04	2,07E-02
cg07216054	7	100823966	NAT16	TSS1500	-0,047	1,31E-04	2,07E-02
cg25890048	11	55703443	OR5I1	1stExon	-0,011	1,31E-04	2,07E-02
cg13663385	16	34429877		IGR	-0,041	1,31E-04	2,07E-02
cg15655500	18	12407812	SLMO1	TSS200	-0,005	1,31E-04	2,07E-02
cg07688933	19	47551971	TMEM160	TSS200	-0,005	1,31E-04	2,07E-02
cg16712060	1	203842506		IGR	0,011	1,31E-04	2,07E-02
cg25499748	9	139886815	C9orf142	TSS200	-0,004	1,31E-04	2,07E-02
cg15262952	16	30048001		IGR	0,039	1,31E-04	2,07E-02
cg05145585	1	78021977	AK5	Body	-0,01	1,31E-04	2,08E-02
cg17868674	1	29546373	MECR	Body	0,012	1,31E-04	2,08E-02
cg02212456	3	154688152		IGR	-0,008	1,31E-04	2,08E-02
cg19770749	11	98886917		IGR	-0,015	1,31E-04	2,08E-02
cg02645985	15	63773096		IGR	0,01	1,31E-04	2,08E-02
cg16130075	8	93276042		IGR	-0,009	1,32E-04	2,08E-02
cg10529778	12	29898829	TMTC1	Body	0,008	1,32E-04	2,08E-02
cg00200298	6	89790339	PNRC1	TSS200	-0,007	1,32E-04	2,08E-02
cg21226225	12	104680742	TXNRD1	1stExon	-0,004	1,32E-04	2,08E-02
cg20888435	2	27662669	NRBP1	Body	-0,004	1,32E-04	2,08E-02
cg06113913	4	84060418		IGR	-0,035	1,32E-04	2,08E-02
cg13406339	8	811003		IGR	-0,015	1,32E-04	2,08E-02
cg12836175	9	120171722	ASTN2	Body	-0,042	1,32E-04	2,08E-02
cg25157001	12	58241890	CTDSP2	TSS1500	0,016	1,32E-04	2,08E-02
cg08830485	11	17410707	KCNJ11	5'UTR	0,007	1,32E-04	2,08E-02
cg26891135	1	89521065	GBP1	Body	-0,005	1,32E-04	2,08E-02

cg09107603	4	39560775	C4orf34	Body	-0,013	1,32E-04	2,08E-02
cg01382900	6	13615583	NOL7	5'UTR	-0,003	1,32E-04	2,08E-02
cg11003660	9	21027780	HACD4	Body	0,005	1,32E-04	2,08E-02
cg15808814	16	24925253		IGR	-0,007	1,32E-04	2,08E-02
cg18813777	1	154297271	ATP8B2	TSS1500	-0,003	1,32E-04	2,08E-02
cg11471469	6	31940522	STK19	Body	-0,003	1,32E-04	2,08E-02
cg12052846	7	9697175		IGR	0,014	1,32E-04	2,08E-02
cg17233620	12	54698071		IGR	-0,004	1,32E-04	2,09E-02
cg21811527	6	29465107		IGR	-0,016	1,32E-04	2,09E-02
cg22084182	7	27973667	JAZF1	Body	0,027	1,33E-04	2,09E-02
cg07495530	21	42804101	MX1	1stExon	0,018	1,32E-04	2,09E-02
cg03730490	19	33998371	PEPD	Body	0,032	1,33E-04	2,09E-02
cg00612299	19	14584761	PTGER1	Body	-0,024	1,33E-04	2,09E-02
cg11321691	8	145631770	CPSF1	Body	-0,011	1,33E-04	2,09E-02
cg05839875	5	39203190	FYB	5'UTR	-0,014	1,33E-04	2,09E-02
cg09743500	2	238644037	LRRFIP1	Body	0,025	1,33E-04	2,09E-02
cg00219947	14	39420648		IGR	0,031	1,33E-04	2,09E-02
cg08651894	1	154832235	KCNN3	Body	0,016	1,33E-04	2,09E-02
cg16558333	7	30347696	ZNRF2	Body	0,009	1,33E-04	2,09E-02
cg13127542	20	45324833		IGR	-0,061	1,33E-04	2,09E-02
cg26664844	2	169925400	DHRS9	5'UTR	-0,016	1,33E-04	2,09E-02
cg16508998	2	147890920		IGR	-0,012	1,33E-04	2,09E-02
cg03099790	16	56456455	AMFR	Body	0,014	1,33E-04	2,09E-02
cg12103265	19	34114048	CHST8	5'UTR	0,012	1,33E-04	2,09E-02
cg18737506	11	65413176	SIPA1	Body	0,013	1,33E-04	2,09E-02
cg25674438	15	52194280	TMOD3	Body	0,015	1,33E-04	2,09E-02
cg25415605	16	19181035	SYT17	Body	-0,035	1,33E-04	2,09E-02
cg00489486	7	130598638	LOC100506860	Body	-0,009	1,33E-04	2,09E-02
cg07945148	22	24666632	CYTSA	TSS200	-0,004	1,34E-04	2,10E-02
cg04341065	3	8889974		IGR	-0,006	1,34E-04	2,10E-02
cg22154024	12	103351180	ASCL1	TSS1500	-0,007	1,34E-04	2,10E-02
cg03295187	19	7587289	MCOLN1	TSS1500	-0,003	1,34E-04	2,10E-02
cg16241107	22	50325639		IGR	0,019	1,34E-04	2,10E-02
cg05563292	5	9474146	SEMA5A	5'UTR	-0,014	1,34E-04	2,10E-02
cg02387403	10	130511408		IGR	-0,014	1,34E-04	2,10E-02
cg23521980	3	107779873	CD47	Body	0,007	1,34E-04	2,10E-02
cg07180616	1	8823146	RERE	5'UTR	0,048	1,34E-04	2,10E-02
cg23435387	13	25416561	RNF17	Body	-0,017	1,34E-04	2,10E-02
cg12337669	4	7881857	AFAP1	5'UTR	-0,034	1,34E-04	2,10E-02
cg14236900	5	66600854		IGR	0,038	1,34E-04	2,10E-02
cg02009601	8	93979580	C8orf83	TSS1500	0,016	1,34E-04	2,10E-02
cg03700492	17	73874623	TRIM47	1stExon	-0,007	1,35E-04	2,10E-02
cg01292265	20	2633870	NOP56	Body	-0,004	1,35E-04	2,10E-02
cg06892164	6	123033822	PKIB	Body	-0,028	1,35E-04	2,10E-02
cg17918944	7	44229914	GCK	TSS1500	0,013	1,35E-04	2,10E-02
cg04666031	10	125232798		IGR	-0,018	1,35E-04	2,10E-02
cg24875593	21	45153009	PDXK	Body	-0,119	1,35E-04	2,10E-02
cg18750782	10	102374055		IGR	-0,031	1,35E-04	2,11E-02
cg26106417	4	3425381	RGS12	Body	-0,01	1,35E-04	2,11E-02
cg22887730	11	44914588	TSPAN18	5'UTR	0,006	1,35E-04	2,11E-02
cg22535216	15	41837601	RPAP1	TSS1500	0,02	1,35E-04	2,11E-02
cg22671691	10	24753693	KIAA1217	Body	0,013	1,35E-04	2,11E-02
cg14685990	17	37910900		IGR	-0,004	1,35E-04	2,11E-02
cg05318600	7	6695121		IGR	0,007	1,35E-04	2,11E-02
cg03013172	19	5688456	HSD11B1L	3'UTR	0,007	1,35E-04	2,11E-02
cg12129639	5	153381939	FAM114A2	ExonBnd	-0,005	1,35E-04	2,11E-02
cg08237971	1	27290846		IGR	0,024	1,35E-04	2,11E-02
cg14766621	2	1891057	MYT1L	Body	0,015	1,35E-04	2,11E-02
cg23417521	20	18679476	LOC101929526	TSS200	-0,01	1,35E-04	2,11E-02
cg12412323	20	62182604		IGR	0,016	1,35E-04	2,11E-02
cg22249705	6	43617977	RSPH9	Body	0,024	1,36E-04	2,11E-02
cg09305898	1	231762372	DISC1	TSS200	-0,011	1,36E-04	2,11E-02
cg10243958	7	112423832	TMEM168	Body	0,024	1,36E-04	2,11E-02
cg27277183	12	121688441	CAMKK2	Body	0,012	1,36E-04	2,11E-02
cg05916707	14	37058256		IGR	-0,01	1,36E-04	2,11E-02
cg07772147	16	19486178	TMC5	Body	-0,034	1,36E-04	2,11E-02
cg05779815	10	71912030	SAR1A	3'UTR	-0,01	1,36E-04	2,11E-02
cg11008690	8	54934459	TCEA1	Body	0,005	1,36E-04	2,11E-02
cg11671925	11	67806426	TCIRG1	TSS200	-0,003	1,36E-04	2,11E-02
cg02606127	2	238621012	LRRFIP1	Body	-0,011	1,36E-04	2,12E-02
cg04528333	4	6123295	JAKMIP1	5'UTR	0,008	1,36E-04	2,12E-02
cg19559984	7	28848933	CREB5	Body	0,011	1,36E-04	2,12E-02
cg19816778	9	77229115	RORB	Body	0,046	1,36E-04	2,12E-02

cg26954543	10	121307123		IGR	-0,017	1,36E-04	2,12E-02
cg23424146	12	25056071	BCAT1	TSS1500	-0,005	1,36E-04	2,12E-02
cg00729815	12	90014966	ATP2B1	Body	0,017	1,36E-04	2,12E-02
cg17474241	14	103058813	RCOR1	TSS200	-0,007	1,36E-04	2,12E-02
cg25561291	19	4396942	SH3GL1	Body	-0,011	1,36E-04	2,12E-02
cg08054383	19	56717414		IGR	0,028	1,36E-04	2,12E-02
cg19494181	22	38656889	TMEM184B	5'UTR	0,03	1,36E-04	2,12E-02
cg07310116	5	179863147		IGR	-0,018	1,36E-04	2,12E-02
cg25073686	11	38615287		IGR	-0,018	1,36E-04	2,12E-02
cg16359174	19	49222967	MAMSTR	5'UTR	-0,023	1,36E-04	2,12E-02
cg22350171	1	228675139	RNF187	5'UTR	0,012	1,37E-04	2,12E-02
cg03915372	5	140557249	PCDHB8	TSS200	-0,077	1,37E-04	2,12E-02
cg07158393	7	85104015		IGR	0,027	1,37E-04	2,12E-02
cg01348977	9	114679784	UGCG	Body	0,017	1,37E-04	2,12E-02
cg03811519	9	134001305	NUP214	Body	-0,003	1,37E-04	2,12E-02
cg14186349	15	96964022		IGR	-0,057	1,37E-04	2,12E-02
cg20554643	19	36557314	WDR62	ExonBnd	0,014	1,37E-04	2,12E-02
cg18023817	1	95730208		IGR	-0,014	1,37E-04	2,12E-02
cg03931444	4	76439695	RCHY1	TSS200	-0,004	1,37E-04	2,12E-02
cg02786309	7	136089594		IGR	-0,014	1,37E-04	2,12E-02
cg00602655	10	35979870		IGR	-0,012	1,37E-04	2,12E-02
cg10193817	11	115375226	CADM1	5'UTR	-0,014	1,37E-04	2,12E-02
cg16758809	12	43151825		IGR	-0,014	1,37E-04	2,12E-02
cg00742738	14	104638843	KIF26A	Body	0,007	1,37E-04	2,12E-02
cg07553663	16	34741823	LOC100130700	TSS1500	0,046	1,37E-04	2,12E-02
cg17456644	17	25879003	KSR1	5'UTR	0,014	1,37E-04	2,12E-02
cg03070741	19	2650727	GNG7	5'UTR	-0,008	1,37E-04	2,12E-02
cg01803101	19	39589954	PAPL	Body	-0,03	1,37E-04	2,12E-02
cg11391637	1	39249315		IGR	-0,038	1,37E-04	2,12E-02
cg00021264	2	85779671	GGCX	ExonBnd	0,018	1,37E-04	2,12E-02
cg15559578	1	222885920	AIDA	TSS200	-0,005	1,37E-04	2,12E-02
cg16974878	1	231622987		IGR	-0,045	1,37E-04	2,12E-02
cg14343266	9	139379656	C9orf163	3'UTR	0,004	1,37E-04	2,12E-02
cg10064871	11	6341908	PRKCDBP	TSS200	0,013	1,38E-04	2,12E-02
cg06060054	16	19097732		IGR	-0,025	1,38E-04	2,12E-02
cg01409643	15	78913615	CHRNA3	1stExon	-0,007	1,38E-04	2,12E-02
cg12117658	17	56406294	BZRAP1	TSS200	-0,003	1,38E-04	2,12E-02
cg07967246	1	197305177	CRB1	Body	0,047	1,38E-04	2,13E-02
cg09032914	16	73913076		IGR	-0,017	1,38E-04	2,13E-02
cg00850971	6	7052187		IGR	-0,005	1,38E-04	2,13E-02
cg26234980	17	74534419	CYGB	TSS1500	0,008	1,38E-04	2,13E-02
cg05289496	21	44444715	PKNOX1	Body	-0,003	1,38E-04	2,13E-02
cg24672565	3	14382884		IGR	0,057	1,38E-04	2,13E-02
cg16681559	20	62788807		IGR	0,017	1,38E-04	2,13E-02
cg02272395	5	140419666		IGR	-0,052	1,39E-04	2,13E-02
cg17216492	11	45743936	LOC100507384	Body	-0,052	1,39E-04	2,13E-02
cg06172077	3	195639973		IGR	0,028	1,39E-04	2,13E-02
cg05937787	16	72594726	LINC01572	Body	0,026	1,39E-04	2,13E-02
cg12656227	7	157334162	PTPRN2	Body	-0,007	1,39E-04	2,13E-02
cg23544294	1	44798990	ERI3	5'UTR	-0,005	1,39E-04	2,13E-02
cg06179260	3	49384492		IGR	-0,023	1,39E-04	2,13E-02
cg07546427	5	66174128	MAST4	Body	-0,007	1,39E-04	2,13E-02
cg18085549	5	141901577		IGR	0,008	1,39E-04	2,13E-02
cg20884984	11	119292770	THY1	5'UTR	0,016	1,39E-04	2,13E-02
cg13344757	17	26904381	ALDOC	TSS1500	0,021	1,39E-04	2,13E-02
cg09961085	18	46765076	DYM	Body	-0,011	1,39E-04	2,13E-02
cg23783695	6	100062259	PRDM13	Body	-0,01	1,39E-04	2,13E-02
cg09903188	1	223356832		IGR	-0,027	1,39E-04	2,14E-02
cg02847116	15	27229446	GABRG3	Body	0,015	1,39E-04	2,14E-02
cg01493016	4	25657403	SLC34A2	TSS200	-0,013	1,40E-04	2,14E-02
cg04910677	11	129245689	BARX2	TSS200	-0,008	1,40E-04	2,14E-02
cg08659629	12	461643	KDM5A	Body	0,005	1,40E-04	2,14E-02
cg12078527	8	21721004		IGR	0,041	1,40E-04	2,14E-02
cg16389474	20	60878197	ADRM1	5'UTR	0,006	1,40E-04	2,15E-02
cg12552337	2	235988130		IGR	0,028	1,40E-04	2,15E-02
cg04057956	12	6339200	CD9	Body	0,05	1,40E-04	2,15E-02
cg06779709	15	79197402		IGR	0,012	1,40E-04	2,15E-02
cg13649960	1	69189862		IGR	-0,009	1,40E-04	2,15E-02
cg12024649	15	50875286	TRPM7	ExonBnd	0,023	1,40E-04	2,15E-02
cg22483919	5	5524013		IGR	-0,012	1,40E-04	2,15E-02
cg17335701	5	96142712	ERAP1	5'UTR	-0,005	1,40E-04	2,15E-02
cg01103717	22	32145601	C22orf30	5'UTR	-0,006	1,41E-04	2,15E-02
cg02207673	1	11137617	EXOSC10	Body	0,006	1,41E-04	2,15E-02

cg06621900	2	240239815	HDAC4	Body	0,009	1,41E-04	2,15E-02
cg21831463	7	132372840		IGR	-0,06	1,41E-04	2,15E-02
cg13191008	11	4629912	TRIM68	TSS1500	-0,042	1,41E-04	2,15E-02
cg09556823	19	44675785	ZNF226	Body	0,005	1,41E-04	2,15E-02
cg20841073	5	173315978	CPEB4	5'UTR	-0,006	1,41E-04	2,16E-02
cg26365399	6	127587909	RNF146	TSS200	0,007	1,41E-04	2,16E-02
cg14403574	22	44136822	EFCAB6	Body	-0,042	1,41E-04	2,16E-02
cg08333904	17	21729463		IGR	0,041	1,41E-04	2,16E-02
cg20370505	13	111358522	CARS2	TSS200	0,013	1,41E-04	2,16E-02
cg12375722	5	140729719	PCDHGA2	Body	-0,049	1,41E-04	2,16E-02
cg13068285	8	80522759	STMN2	TSS1500	-0,047	1,41E-04	2,16E-02
cg02985343	12	19335592	PLEKHA5	Body	0,018	1,41E-04	2,16E-02
cg15943396	5	159436103	TTC1	TSS200	-0,004	1,42E-04	2,16E-02
cg09826002	1	16174366	SPEN	1stExon	0,004	1,42E-04	2,16E-02
cg24532400	5	64064502	SDCCAG10	TSS1500	0,004	1,42E-04	2,16E-02
cg02483462	9	140939579	CACNA1B	Body	0,02	1,42E-04	2,16E-02
cg15330584	11	69455512	CCND1	TSS1500	0,01	1,42E-04	2,16E-02
cg03312661	12	16997961		IGR	-0,045	1,42E-04	2,16E-02
cg03773631	14	29580937		IGR	-0,036	1,42E-04	2,16E-02
cg04418434	6	7110773	RREB1	5'UTR	-0,027	1,42E-04	2,16E-02
cg00900302	2	149944465	LYPD6B	5'UTR	0,023	1,42E-04	2,17E-02
cg17588578	5	140723583	PCDHGA2	Body	-0,06	1,42E-04	2,17E-02
cg04526020	11	64948989	CAPN1	TSS1500	0,005	1,42E-04	2,17E-02
cg05364651	16	1267876	CACNA1H	Body	0,015	1,42E-04	2,17E-02
cg09967523	18	18943072	GREB1L	5'UTR	-0,036	1,42E-04	2,17E-02
cg16553796	18	78005475	PARD6G	TSS200	0,023	1,42E-04	2,17E-02
cg16478012	16	87425779	FBXO31	TSS200	-0,004	1,42E-04	2,17E-02
cg21549927	8	110987617	KCNV1	TSS1500	-0,025	1,42E-04	2,17E-02
cg17283327	10	129239859	DOCK1	Body	0,012	1,42E-04	2,17E-02
cg03504865	4	107958152	DKK2	TSS1500	-0,018	1,43E-04	2,17E-02
cg27266027	21	40555129	PSMG1	Body	-0,003	1,43E-04	2,17E-02
cg15619643	1	45270421	PLK3	Body	0,007	1,43E-04	2,17E-02
cg10785170	14	101451162	SNORD114-25	TSS1500	-0,011	1,43E-04	2,17E-02
cg23918953	12	68915528		IGR	0,037	1,43E-04	2,17E-02
cg03098712	11	68860771		IGR	-0,006	1,43E-04	2,17E-02
cg08682607	14	97208170		IGR	-0,039	1,43E-04	2,17E-02
cg01527551	22	44703653	KIAA1644	5'UTR	0,019	1,43E-04	2,17E-02
cg09332230	10	105192586	PDCD11	Body	-0,005	1,43E-04	2,17E-02
cg12219496	4	85604949	WDFY3	TSS1500	-0,01	1,43E-04	2,17E-02
cg05827943	7	26241126	HNRNPA2B1	TSS1500	-0,006	1,43E-04	2,17E-02
cg12130067	8	37740548	RAB11FIP1	Body	0,027	1,43E-04	2,17E-02
cg20220121	10	102477660		IGR	-0,023	1,43E-04	2,17E-02
cg00676360	6	49917175	DEFB133	TSS200	-0,014	1,43E-04	2,18E-02
cg18174683	2	68675870		IGR	-0,01	1,43E-04	2,18E-02
cg20645472	7	13899782		IGR	0,02	1,44E-04	2,18E-02
cg17184704	10	11727286		IGR	0,003	1,44E-04	2,18E-02
cg04704631	10	75541698	CHCHD1	TSS200	-0,004	1,44E-04	2,18E-02
cg16820724	11	73586905	COA4	5'UTR	0,027	1,44E-04	2,18E-02
cg26996703	12	125256752		IGR	-0,026	1,44E-04	2,18E-02
cg17368581	5	72512191		IGR	-0,006	1,44E-04	2,18E-02
cg03582135	6	42096968	C6orf132	Body	0,012	1,44E-04	2,18E-02
cg26036375	2	122660039		IGR	-0,033	1,44E-04	2,18E-02
cg09719331	20	44486197	ZSWIM3	TSS200	-0,005	1,44E-04	2,18E-02
cg19526450	5	142254301	ARHGAP26	Body	0,034	1,44E-04	2,18E-02
cg02768742	5	145562530	LARS	TSS1500	0,003	1,44E-04	2,18E-02
cg13270055	22	36960499	CACNG2	Body	-0,074	1,44E-04	2,18E-02
cg22054580	8	144811385	FAM83H	Body	0,017	1,44E-04	2,18E-02
cg18188134	16	71757035	PHLPP2	5'UTR	-0,007	1,44E-04	2,18E-02
cg15002641	1	204092001	SOX13	Body	0,011	1,44E-04	2,18E-02
cg15514393	10	88403555		IGR	-0,019	1,44E-04	2,18E-02
cg13185005	11	268950		IGR	-0,004	1,44E-04	2,18E-02
cg25744649	1	145530939	ITGA10	Body	-0,01	1,45E-04	2,18E-02
cg05778559	3	16646375	DAZL	Body	-0,008	1,45E-04	2,18E-02
cg04159246	11	131531178	NTM	Body	0,029	1,45E-04	2,18E-02
cg25903453	15	55972199	PRTG	Body	0,012	1,45E-04	2,18E-02
cg01890352	18	77500656	CTDP1	Body	0,026	1,45E-04	2,18E-02
cg04650417	19	15792035	CYP4F12	Body	-0,047	1,45E-04	2,18E-02
cg14473312	8	145160216	MAF1	5'UTR	0,03	1,45E-04	2,18E-02
cg23335946	1	185125966	C1orf25	1stExon	-0,005	1,45E-04	2,18E-02
cg09915377	7	97821499	LMTK2	Body	0,005	1,45E-04	2,19E-02
cg03327325	10	43846574		IGR	-0,086	1,45E-04	2,19E-02
cg12098645	12	69326716	CPM	5'UTR	0,011	1,45E-04	2,19E-02
cg06592080	16	3543548	C16orf90	3'UTR	-0,007	1,45E-04	2,19E-02

cg05439318	16	69458329	CYB5B	TSS200	-0,003	1,45E-04	2,19E-02
cg24633027	5	140754314	PCDHGA4	Body	-0,047	1,45E-04	2,19E-02
cg15644231	11	6263479	CNGA4	Body	-0,016	1,45E-04	2,19E-02
cg16681268	7	117067616	ASZ1	TSS200	-0,028	1,45E-04	2,19E-02
cg03557441	6	42391254	TRERF1	5'UTR	-0,031	1,45E-04	2,19E-02
cg18746719	11	100092054	CNTN5	Body	-0,02	1,46E-04	2,19E-02
cg18844090	12	115832476		IGR	0,034	1,46E-04	2,19E-02
cg10574499	16	67918754	NRN1L	TSS200	-0,011	1,46E-04	2,19E-02
cg07185247	1	248097461		IGR	-0,012	1,46E-04	2,19E-02
cg09120054	16	1581306	TMEM204	5'UTR	0,012	1,46E-04	2,19E-02
cg20238243	1	220998876		IGR	0,02	1,46E-04	2,20E-02
cg00146698	4	113431261		IGR	-0,005	1,46E-04	2,20E-02
cg03399137	14	50175659	KLHDC1	Body	0,038	1,46E-04	2,20E-02
cg14999189	17	2908369	RAP1GAP2	Body	0,025	1,46E-04	2,20E-02
cg04689082	3	194406572	FAM43A	TSS200	-0,004	1,46E-04	2,20E-02
cg08979696	7	36407077	KIAA0895	TSS200	-0,004	1,46E-04	2,20E-02
cg27207920	13	50380012		IGR	0,018	1,46E-04	2,20E-02
cg02045894	8	74361398	STAU2	Body	0,005	1,46E-04	2,20E-02
cg02256855	6	25084106	CMAHP	Body	-0,006	1,47E-04	2,20E-02
cg15594528	11	112096869	PTS	TSS1500	-0,005	1,47E-04	2,20E-02
cg09993711	14	64746129	ESR2	Body	0,035	1,47E-04	2,20E-02
cg18782716	15	41709119	RTF1	TSS200	-0,005	1,47E-04	2,20E-02
cg13462158	1	55522104	PCSK9	Body	-0,092	1,47E-04	2,20E-02
cg18079538	14	36947368	SFTA3	Body	-0,008	1,47E-04	2,20E-02
cg11072700	22	18306908	MICAL3	Body	0,011	1,47E-04	2,20E-02
cg11459997	9	5655592	RIC1	Body	-0,013	1,47E-04	2,20E-02
cg19834098	13	25873837		IGR	0,011	1,47E-04	2,20E-02
cg06405299	9	127624623	RPL35	TSS1500	-0,003	1,47E-04	2,20E-02
cg17351327	10	105459568	SH3PXD2A	Body	-0,008	1,47E-04	2,20E-02
cg15656560	19	16222031	RAB8A	TSS1500	0,014	1,47E-04	2,20E-02
cg17895286	1	78279007	FAM73A	Body	0,007	1,47E-04	2,21E-02
cg27229596	2	112433462		IGR	-0,023	1,48E-04	2,21E-02
cg17744722	6	3258989	PSMG4	TSS200	-0,005	1,48E-04	2,21E-02
cg16752400	8	66131741		IGR	-0,03	1,47E-04	2,21E-02
cg05919650	14	75719068		IGR	0,014	1,47E-04	2,21E-02
cg09766769	16	4852830	ROGDI	TSS200	-0,004	1,47E-04	2,21E-02
cg23975975	18	76192780		IGR	-0,013	1,48E-04	2,21E-02
cg12024887	19	6737490	GPR108	1stExon	-0,006	1,48E-04	2,21E-02
cg11400613	19	10491835	TYK2	TSS1500	0,009	1,47E-04	2,21E-02
cg20290017	9	113831239		IGR	-0,014	1,48E-04	2,21E-02
cg25099389	5	71706309		IGR	-0,012	1,48E-04	2,21E-02
cg19836803	1	11175735	MTOR	Body	-0,011	1,48E-04	2,21E-02
cg07653189	6	1703190	GMDS	Body	0,013	1,48E-04	2,21E-02
cg06086073	6	28549832	SCAND3	Body	0,01	1,48E-04	2,21E-02
cg20619563	6	84621458	CYB5R4	Body	0,023	1,48E-04	2,21E-02
cg06567227	6	100909121	SIM1	Body	-0,008	1,48E-04	2,21E-02
cg15236240	10	79781362	POLR3A	Body	-0,007	1,48E-04	2,21E-02
cg19042848	15	83948352	BNC1	Body	-0,009	1,48E-04	2,21E-02
cg22271697	19	1042536	ABCA7	Body	0,013	1,48E-04	2,21E-02
cg04366994	10	134361203	INPP5A	Body	0,022	1,48E-04	2,21E-02
cg16304097	6	90276732	ANKRD6	1stExon	-0,005	1,48E-04	2,21E-02
cg00095526	1	201081686	CACNA1S	1stExon	0,036	1,48E-04	2,21E-02
cg09189780	3	141205007	RASA2	TSS1500	-0,007	1,48E-04	2,21E-02
cg17727632	3	149589738	RNF13	Body	0,043	1,48E-04	2,21E-02
cg07251136	5	95158433	GLRX	1stExon	-0,003	1,48E-04	2,21E-02
cg00200170	7	8259071	ICA1	Body	0,026	1,48E-04	2,21E-02
cg08219003	16	19165392		IGR	0,03	1,48E-04	2,21E-02
cg03349779	17	11204994	SHISA6	Body	-0,039	1,49E-04	2,21E-02
cg03730946	19	6475456	DENND1C	Body	0,013	1,49E-04	2,21E-02
cg26278693	22	38867491	KDELR3	Body	0,006	1,49E-04	2,21E-02
cg25654517	6	29627131	MOG	Body	-0,012	1,49E-04	2,21E-02
cg20638426	19	19843991	ZNF14	TSS200	-0,005	1,49E-04	2,21E-02
cg01113896	10	34836433	PARD3	Body	-0,018	1,49E-04	2,21E-02
cg00413066	15	52405464	BCL2L10	TSS1500	0,008	1,49E-04	2,21E-02
cg17256679	17	32647709	CCL8	Body	-0,01	1,49E-04	2,22E-02
cg18528621	17	60885154	MARCH10	TSS1500	-0,004	1,49E-04	2,22E-02
cg14508973	9	96209198	FAM120AOS	3'UTR	-0,006	1,49E-04	2,22E-02
cg23112326	10	8087098		IGR	-0,005	1,49E-04	2,22E-02
cg03770575	12	24414339	SOX5	5'UTR	-0,014	1,49E-04	2,22E-02
cg00380642	2	43382918		IGR	-0,006	1,49E-04	2,22E-02
cg16743005	16	50403128	BRD7	TSS1500	-0,004	1,49E-04	2,22E-02
cg23656322	1	153533922	S100A2	Body	0,073	1,49E-04	2,22E-02
cg22712383	1	230926883	CAPN9	Body	-0,037	1,50E-04	2,22E-02

cg03985801	1	202182603	LGR6	TSS1500	0,026	1,50E-04	2,22E-02
cg16347927	2	191354323	MFSD6	Body	-0,021	1,50E-04	2,22E-02
cg18319967	5	173416427	C5orf47	1stExon	-0,008	1,50E-04	2,22E-02
cg03867877	8	29128303		IGR	0,019	1,50E-04	2,22E-02
cg26820840	19	36336980	NPHS1	Body	-0,004	1,50E-04	2,22E-02
cg23221052	5	179740743	GFPT2	Body	-0,137	1,50E-04	2,22E-02
cg04799410	3	187717711		IGR	-0,046	1,50E-04	2,22E-02
cg11479503	9	124989052	LHX6	Body	-0,093	1,50E-04	2,22E-02
cg00814985	13	114801132	RASA3	Body	0,022	1,50E-04	2,22E-02
cg06568490	3	13525965	HDAC11	Body	-0,03	1,50E-04	2,22E-02
cg08998166	9	85025698		IGR	-0,013	1,50E-04	2,22E-02
cg10483960	16	76311511	CNTNAP4	5'UTR	-0,045	1,50E-04	2,22E-02
cg19843429	7	25019751	OSBPL3	5'UTR	-0,005	1,50E-04	2,22E-02
cg17780098	1	201476311	CSRP1	5'UTR	-0,004	1,50E-04	2,22E-02
cg15466144	2	210866282	RPE	TSS1500	-0,008	1,50E-04	2,22E-02
cg03643302	22	21343156	LZTR1	ExonBnd	-0,007	1,50E-04	2,22E-02
cg02708940	2	742423		IGR	0,012	1,50E-04	2,23E-02
cg26942047	1	245185304	EFCAB2	Body	-0,006	1,51E-04	2,23E-02
cg25704749	2	241526252	CAPN10	1stExon	0,016	1,51E-04	2,23E-02
cg22928365	3	42099175		IGR	0,022	1,51E-04	2,23E-02
cg24317857	7	38711703		IGR	0,037	1,51E-04	2,23E-02
cg05269024	8	38085392		IGR	0,005	1,51E-04	2,23E-02
cg18144936	17	53345169	HLF	Body	-0,024	1,51E-04	2,23E-02
cg05718270	8	73920493	TERF1	TSS1500	0,035	1,51E-04	2,23E-02
cg16357921	9	90589634	CDK20	1stExon	-0,01	1,51E-04	2,23E-02
cg13123998	16	12391473	SNX29	Body	-0,018	1,51E-04	2,23E-02
cg26008464	6	134373624	SLC2A12	1stExon	-0,003	1,51E-04	2,23E-02
cg11807261	4	13991309		IGR	-0,016	1,51E-04	2,23E-02
cg07370650	5	5315111	ADAMTS16	Body	-0,018	1,51E-04	2,23E-02
cg22244725	5	136352768	SPOCK1	Body	-0,026	1,51E-04	2,23E-02
cg00720829	6	30131219	TRIM15	5'UTR	-0,029	1,51E-04	2,23E-02
cg02872426	6	110736772	DDO	TSS200	0,086	1,51E-04	2,23E-02
cg14634336	6	111804804	REV3L	TSS1500	-0,007	1,51E-04	2,23E-02
cg05237489	10	108623805	SORCS1	Body	-0,008	1,51E-04	2,23E-02
cg10297023	11	115631855	LINC00900	TSS1500	0,015	1,51E-04	2,23E-02
cg15596810	13	41554217	ELF1	Body	-0,009	1,51E-04	2,23E-02
cg04862249	17	4982244	ZFP3	5'UTR	-0,003	1,51E-04	2,23E-02
cg11450671	19	595812	HCN2	Body	0,032	1,51E-04	2,23E-02
cg00420275	6	14912895		IGR	0,03	1,51E-04	2,23E-02
cg17974674	2	241451567	ANKMY1	Body	-0,007	1,52E-04	2,23E-02
cg02631879	5	5423457	KIAA0947	Body	-0,005	1,52E-04	2,23E-02
cg08642198	8	2419444		IGR	-0,052	1,52E-04	2,23E-02
cg09102623	9	37079866	EBLN3	TSS200	-0,004	1,52E-04	2,23E-02
cg01017127	10	29408566		IGR	-0,007	1,52E-04	2,23E-02
cg27353381	11	1221141		IGR	-0,04	1,52E-04	2,23E-02
cg16878676	11	14309451	RRAS2	Body	0,021	1,52E-04	2,23E-02
cg25942539	13	89539539		IGR	-0,006	1,52E-04	2,23E-02
cg18984211	2	73498074	FBXO41	TSS200	0,019	1,52E-04	2,24E-02
cg24856518	2	120517676	PTPN4	5'UTR	-0,006	1,52E-04	2,24E-02
cg00669531	11	34367871	ABTB2	Body	-0,007	1,52E-04	2,24E-02
cg09408383	15	49726332	FGF7	Body	-0,006	1,52E-04	2,24E-02
cg23604606	2	21638254		IGR	-0,032	1,52E-04	2,24E-02
cg18222517	11	107067568		IGR	-0,023	1,52E-04	2,24E-02
cg20116420	13	78272830	MIR3665	TSS1500	-0,004	1,53E-04	2,24E-02
cg26084442	2	164202588		IGR	-0,021	1,53E-04	2,24E-02
cg16930880	2	177373410		IGR	-0,023	1,53E-04	2,24E-02
cg27504193	9	104757854		IGR	-0,023	1,53E-04	2,24E-02
cg25704110	12	4967040		IGR	-0,004	1,53E-04	2,24E-02
cg20222925	7	107645075	LAMB1	TSS1500	-0,032	1,53E-04	2,25E-02
cg15552016	22	50717404	PLXNB2	Body	0,015	1,53E-04	2,25E-02
cg13879247	6	145984613	EPM2A	Body	-0,008	1,53E-04	2,25E-02
cg18036270	11	125384310		IGR	-0,006	1,53E-04	2,25E-02
cg19123622	1	9923630	CTNNBIP1	Body	0,008	1,53E-04	2,25E-02
cg02138348	7	66255554	RABGEF1	Body	-0,021	1,53E-04	2,25E-02
cg11174851	11	69634592	FGF3	TSS1500	-0,01	1,53E-04	2,25E-02
cg20956174	1	65729786	DNAJC6	TSS1500	0,021	1,53E-04	2,25E-02
cg20895553	6	149882115		IGR	-0,015	1,53E-04	2,25E-02
cg01334824	15	27787650		IGR	-0,011	1,53E-04	2,25E-02
cg19984742	20	54824490	MC3R	1stExon	0,045	1,53E-04	2,25E-02
cg11312554	6	31832650	SLC44A4	Body	-0,006	1,54E-04	2,25E-02
cg07279955	6	99275764		IGR	-0,018	1,54E-04	2,25E-02
cg20642712	7	96745340	ACN9	TSS1500	-0,004	1,54E-04	2,25E-02
cg05938683	1	85156187	SSX2IP	TSS200	0,016	1,54E-04	2,25E-02

cg04186868	12	57611144	NXPH4	Body	-0,007	1,54E-04	2,26E-02
cg00043080	19	40853155	PLD3	TSS1500	0,007	1,54E-04	2,26E-02
cg04315980	5	79776985		IGR	-0,007	1,54E-04	2,26E-02
cg22924015	10	10687311		IGR	-0,006	1,54E-04	2,26E-02
cg02925222	11	280445	NLRP6	Body	-0,019	1,54E-04	2,26E-02
cg14263553	8	8120624		IGR	-0,006	1,54E-04	2,26E-02
cg00753252	8	1942847	KBTBD11	5'UTR	-0,007	1,55E-04	2,26E-02
cg24380121	1	220837951		IGR	0,019	1,55E-04	2,26E-02
cg14755054	2	201940010	NDUFB3	5'UTR	0,014	1,55E-04	2,26E-02
cg08760541	8	674123	ERICH1	Body	-0,051	1,55E-04	2,26E-02
cg02701112	10	29717546	SVIL-AS1	Body	-0,013	1,55E-04	2,26E-02
cg15236504	10	80540483		IGR	0,013	1,55E-04	2,26E-02
cg20037328	11	78003389	GAB2	5'UTR	-0,01	1,55E-04	2,26E-02
cg23645410	11	93868198	PANX1	Body	0,016	1,55E-04	2,26E-02
cg09119416	12	108142333	PRDM4	Body	0,006	1,55E-04	2,26E-02
cg20901959	12	130502552		IGR	-0,024	1,55E-04	2,26E-02
cg13509849	14	90527972	KCNK13	TSS200	-0,009	1,55E-04	2,26E-02
cg07661156	14	92072013	CATSPERB	Body	0,008	1,55E-04	2,26E-02
cg06144743	16	19721014	C16orf88	Body	0,009	1,55E-04	2,26E-02
cg21388793	19	2427982	TIMM13	TSS200	-0,004	1,55E-04	2,26E-02
cg17000427	4	981041	IDUA	Body	-0,004	1,55E-04	2,26E-02
cg15728601	4	8510291		IGR	0,016	1,55E-04	2,26E-02
cg27305895	9	86594214	HNRNPK	5'UTR	-0,005	1,55E-04	2,26E-02
cg19984833	16	25228102	AQP8	TSS200	-0,012	1,56E-04	2,26E-02
cg14200846	18	76744969	SALL3	Body	-0,038	1,55E-04	2,26E-02
cg05115902	17	13923193		IGR	0,033	1,56E-04	2,26E-02
cg24277994	19	5524603		IGR	-0,068	1,56E-04	2,26E-02
cg21936545	14	86502432	LOC101928767	Body	-0,048	1,56E-04	2,27E-02
cg09984146	7	64336329		IGR	-0,024	1,56E-04	2,27E-02
cg25281623	9	107509848	NIPSNAP3A	TSS200	-0,006	1,56E-04	2,27E-02
cg04661001	19	19217217	SLC25A42	Body	0,036	1,56E-04	2,27E-02
cg08497332	3	88040260	HTR1F	Body	-0,01	1,56E-04	2,27E-02
cg20163980	22	17647332	CECR5	TSS1500	0,019	1,56E-04	2,27E-02
cg01335363	13	46810229	LRRC63	Body	0,041	1,56E-04	2,27E-02
cg12781686	11	131776986	NTM	Body	-0,028	1,56E-04	2,27E-02
cg21558005	16	11034124	DEXI	3'UTR	-0,021	1,56E-04	2,27E-02
cg05106053	3	121647021	SLC15A2	Body	-0,005	1,56E-04	2,27E-02
cg21286622	20	61201650		IGR	0,02	1,56E-04	2,27E-02
cg23484980	22	23100742		IGR	0,036	1,56E-04	2,27E-02
cg13776199	4	183455525	TENM3	Body	-0,018	1,57E-04	2,27E-02
cg16792464	1	22250284	HSPG2	Body	0,026	1,57E-04	2,27E-02
cg13357229	4	154711171	SFRP2	TSS1500	-0,03	1,57E-04	2,27E-02
cg06983502	4	99750177		IGR	-0,009	1,57E-04	2,27E-02
cg23859630	6	32160455	GPSM3	5'UTR	-0,006	1,57E-04	2,27E-02
cg10730102	8	142915446		IGR	-0,041	1,57E-04	2,27E-02
cg16447925	12	111156525		IGR	0,02	1,57E-04	2,27E-02
cg09727611	9	112887685	AKAP2	TSS200	0,036	1,57E-04	2,28E-02
cg03129643	1	37940147	ZC3H12A	1stExon	-0,004	1,57E-04	2,28E-02
cg02459163	8	101609045	SNX31	Body	0,025	1,57E-04	2,28E-02
cg21711333	16	1425548	UNKL	5'UTR	-0,05	1,57E-04	2,28E-02
cg17711567	19	44325008	LYPD5	TSS1500	-0,046	1,57E-04	2,28E-02
cg02097318	10	99268011	UBTD1	Body	-0,016	1,57E-04	2,28E-02
cg05420994	6	32807296	TAP2	TSS1500	0,016	1,57E-04	2,28E-02
cg01947983	13	23985667	SACS	5'UTR	0,007	1,57E-04	2,28E-02
cg13139631	17	78454044		IGR	-0,025	1,57E-04	2,28E-02
cg21028608	2	32464513	NLRC4	Body	-0,007	1,58E-04	2,28E-02
cg17840363	4	71780347	MOBKL1A	Body	0,01	1,58E-04	2,28E-02
cg01930404	15	93649702		IGR	-0,01	1,58E-04	2,28E-02
cg01889129	10	119198887		IGR	0,068	1,58E-04	2,28E-02
cg15972264	6	11279680	NEDD9	Body	0,005	1,58E-04	2,28E-02
cg21534994	5	1881655	IRX4	Body	-0,024	1,58E-04	2,28E-02
cg26130645	7	116502393	CAPZA2	TSS200	0,003	1,58E-04	2,28E-02
cg14381948	7	154684893	DPP6	3'UTR	-0,054	1,58E-04	2,28E-02
cg08211061	10	74115895	DNAJB12	TSS1500	0,041	1,58E-04	2,28E-02
cg27046148	4	166131112	KLHL2	TSS200	-0,015	1,58E-04	2,29E-02
cg15429990	7	19129870		IGR	-0,017	1,58E-04	2,29E-02
cg02319883	12	102259350		IGR	-0,015	1,58E-04	2,29E-02
cg24806383	8	617007	ERICH1	Body	-0,006	1,58E-04	2,29E-02
cg16037569	1	9710867	PIK3CD	TSS1500	0,007	1,59E-04	2,29E-02
cg17510922	2	182386517	ITGA4	Body	0,029	1,59E-04	2,29E-02
cg10998119	9	88357125	AGTPBP1	TSS200	-0,003	1,59E-04	2,29E-02
cg15557084	5	115853116	SEMA6A	5'UTR	0,029	1,59E-04	2,29E-02
cg01925498	1	148901580		IGR	-0,031	1,59E-04	2,29E-02

cg27628952	11	67229131	CABP4	3'UTR	0,016	1,59E-04	2,29E-02
cg15889260	11	121891076		IGR	0,008	1,59E-04	2,29E-02
cg17832732	15	95976254	LINC00924	TSS200	0,029	1,59E-04	2,29E-02
cg24622747	16	72683456	LINC01572	Body	-0,011	1,59E-04	2,29E-02
cg07315010	1	242161780	MAP1LC3C	Body	0,024	1,59E-04	2,29E-02
cg05076755	11	76381148	LRRC32	5'UTR	0,022	1,59E-04	2,29E-02
cg04123472	12	118982122		IGR	-0,008	1,59E-04	2,29E-02
cg23301322	15	81241280	CEMIP	3'UTR	0,012	1,59E-04	2,29E-02
cg17581065	17	8642478	CCDC42	Body	0,012	1,59E-04	2,29E-02
cg09111198	19	8384146	NDUFA7	Body	0,006	1,59E-04	2,29E-02
cg27178512	14	74877446	SYNDIG1L	5'UTR	-0,014	1,59E-04	2,29E-02
cg00899463	4	108784393	SGMS2	5'UTR	-0,008	1,59E-04	2,29E-02
cg16360668	1	19265059	IFFO2	Body	0,009	1,60E-04	2,29E-02
cg22077046	3	10452678	ATP2B2	Body	-0,017	1,60E-04	2,29E-02
cg20272918	3	47555463	C3orf75	TSS1500	0,005	1,60E-04	2,29E-02
cg05687848	4	170487879	NEK1	Body	0,013	1,60E-04	2,29E-02
cg04074363	5	112293410		IGR	0,019	1,59E-04	2,29E-02
cg09368129	5	140071347	HARS2	Body	-0,004	1,60E-04	2,29E-02
cg17781494	7	101183115	COL26A1	Body	0,022	1,59E-04	2,29E-02
cg06815817	7	148936572	ZNF212	TSS1500	-0,003	1,60E-04	2,29E-02
cg09912768	15	66585646	DIS3L	1stExon	-0,004	1,60E-04	2,29E-02
cg08898116	18	30091445	WBP11P1	TSS200	-0,009	1,60E-04	2,29E-02
cg21983579	19	36630882	CAPNS1	TSS200	-0,003	1,60E-04	2,29E-02
cg11928630	19	39649618	PAK4	5'UTR	0,036	1,59E-04	2,29E-02
cg25737323	2	29338100	CLIP4	TSS1500	-0,004	1,60E-04	2,29E-02
cg12950438	11	61111006	TKFC	Body	0,008	1,60E-04	2,29E-02
cg03531512	22	26908677	TFIP11	TSS1500	-0,004	1,60E-04	2,29E-02
cg27321658	19	48402181		IGR	-0,051	1,60E-04	2,29E-02
cg10542127	1	2378521		IGR	0,038	1,60E-04	2,29E-02
cg05214771	1	220328606	RAB3GAP2	Body	-0,005	1,60E-04	2,29E-02
cg00057376	2	44232247		IGR	-0,041	1,60E-04	2,29E-02
cg17205466	4	77133692	FAM47E	TSS1500	-0,016	1,60E-04	2,29E-02
cg22961212	5	66564534		IGR	-0,013	1,60E-04	2,29E-02
cg01368780	6	169937196	WDR27	Body	-0,006	1,60E-04	2,29E-02
cg20424204	9	135377132	C9orf171	Body	0,022	1,60E-04	2,29E-02
cg07968094	14	64901479	MIR548AZ	Body	-0,009	1,60E-04	2,29E-02
cg09773058	17	21364477		IGR	-0,013	1,60E-04	2,29E-02
cg18315160	19	815275	LPPR3	Body	0,036	1,60E-04	2,29E-02
cg21669966	6	42224838	TRERF1	Body	-0,005	1,60E-04	2,29E-02
cg20650029	8	133204098	KCNQ3	Body	-0,026	1,60E-04	2,29E-02
cg17155697	20	30440639		IGR	-0,004	1,60E-04	2,29E-02
cg03212797	5	150827313	SLC36A1	1stExon	-0,007	1,60E-04	2,30E-02
cg02064275	17	39465713		IGR	0,025	1,60E-04	2,30E-02
cg25714432	5	2887968		IGR	-0,039	1,61E-04	2,30E-02
cg03265267	2	192015389	STAT4	5'UTR	-0,004	1,61E-04	2,30E-02
cg18099260	6	41687384	TFEB	Body	0,018	1,61E-04	2,30E-02
cg09465168	15	45997356		IGR	0,01	1,61E-04	2,30E-02
cg20243838	1	2398837		IGR	-0,005	1,61E-04	2,30E-02
cg13632142	1	163430188		IGR	-0,022	1,61E-04	2,30E-02
cg21610805	1	198635026	PTPRC	Body	-0,012	1,61E-04	2,30E-02
cg09968329	5	150667818	SLC36A3	Body	-0,018	1,61E-04	2,30E-02
cg07505373	7	4748942	FOXK1	Body	0,006	1,61E-04	2,30E-02
cg04820270	11	64319262		IGR	0,02	1,61E-04	2,30E-02
cg08322034	6	80657412	ELOVL4	TSS200	-0,011	1,61E-04	2,30E-02
cg00334349	7	127928611		IGR	-0,019	1,61E-04	2,30E-02
cg12756411	2	65273162		IGR	0,003	1,61E-04	2,30E-02
cg25590724	14	68799979	RAD51B	Body	0,017	1,61E-04	2,30E-02
cg11537811	2	96944360	SNRNP200	Body	0,009	1,61E-04	2,30E-02
cg24266851	3	19214779	KCNH8	Body	0,01	1,61E-04	2,30E-02
cg09265417	7	94294798	PEG10	Body	0,008	1,61E-04	2,30E-02
cg16031718	7	64333540		IGR	0,011	1,61E-04	2,30E-02
cg02540593	11	103908576	PDGFD	Body	0,009	1,62E-04	2,30E-02
cg03469793	1	180199269	LHX4	TSS200	0,028	1,62E-04	2,30E-02
cg13852407	10	7153900		IGR	-0,006	1,62E-04	2,30E-02
cg12798259	11	31823282	PAX6	Body	-0,032	1,62E-04	2,30E-02
cg09649356	20	46822547		IGR	0,014	1,62E-04	2,30E-02
cg23113826	21	15580581	LIPI	TSS1500	-0,014	1,62E-04	2,30E-02
cg25178565	10	22990128	PIP4K2A	Body	0,018	1,62E-04	2,30E-02
cg10840064	1	42800299	FOXJ3	5'UTR	-0,017	1,62E-04	2,30E-02
cg03045720	2	162413950		IGR	-0,023	1,62E-04	2,30E-02
cg07976263	16	81773196		IGR	0,05	1,62E-04	2,30E-02
cg07010930	22	31485543	SMTN	Body	0,02	1,62E-04	2,30E-02
cg08885800	1	201084119		IGR	-0,024	1,62E-04	2,31E-02

cg27353352	4	146054604		IGR	-0,046	1,62E-04	2,31E-02
cg08692130	10	18948698	ARL5B	Body	0,008	1,62E-04	2,31E-02
cg07888661	10	73759402	CHST3	5'UTR	0,031	1,62E-04	2,31E-02
cg15489005	1	39044328		IGR	0,014	1,62E-04	2,31E-02
cg10079538	2	5217722		IGR	-0,025	1,62E-04	2,31E-02
cg00973737	4	100128186	ADH6	Body	0,013	1,62E-04	2,31E-02
cg10450733	6	50815047	TFAP2B	3'UTR	-0,022	1,62E-04	2,31E-02
cg24325790	17	34345392	CCL23	TSS1500	-0,015	1,62E-04	2,31E-02
cg24702253	11	3240068	MRGPRG	TSS200	0,022	1,63E-04	2,31E-02
cg03557226	17	45693418	NPEPPS	Body	-0,02	1,63E-04	2,31E-02
cg00147079	16	48132552	ABCC12	Body	-0,015	1,63E-04	2,31E-02
cg11367627	16	74653911		IGR	0,005	1,63E-04	2,31E-02
cg04201752	3	50360397	HYAL2	TSS200	0,034	1,63E-04	2,31E-02
cg19731340	5	141704709	SPRY4	TSS200	-0,004	1,63E-04	2,31E-02
cg06718928	6	166490246		IGR	0,033	1,63E-04	2,31E-02
cg14527276	9	118948957	PAPPA	Body	-0,014	1,63E-04	2,31E-02
cg17950697	10	10099747	LOC101928298	TSS1500	0,022	1,63E-04	2,31E-02
cg06677367	11	75977514		IGR	-0,02	1,63E-04	2,31E-02
cg24983628	14	103670161		IGR	-0,035	1,63E-04	2,31E-02
cg01416657	1	205392241	LEMD1	TSS1500	-0,059	1,63E-04	2,31E-02
cg26426774	8	33356290	C8orf41	3'UTR	-0,023	1,63E-04	2,31E-02
cg16972043	16	46932066	GPT2	Body	-0,03	1,63E-04	2,31E-02
cg08749419	4	6130359	JAKMIP1	5'UTR	-0,017	1,64E-04	2,32E-02
cg27510474	1	60039802	FGGY	Body	-0,013	1,64E-04	2,32E-02
cg12594107	1	85121730	SSX2IP	Body	0,008	1,64E-04	2,32E-02
cg07211044	8	60032983	TOX	TSS1500	0,051	1,64E-04	2,32E-02
cg14291256	10	127461065	MMP21	Body	-0,01	1,64E-04	2,32E-02
cg18226049	11	2424791	TSSC4	Body	0,008	1,64E-04	2,32E-02
cg10827163	1	233539840		IGR	-0,021	1,64E-04	2,32E-02
cg23200746	18	2897548	EMILIN2	Body	-0,006	1,64E-04	2,32E-02
cg16873271	19	8390059	KANK3	Body	0,022	1,64E-04	2,32E-02
cg03712843	13	39565534	STOML3	TSS1500	-0,007	1,64E-04	2,32E-02
cg27335300	1	35350950	DLGAP3	Body	-0,026	1,65E-04	2,33E-02
cg25600973	5	168006775	PANK3	TSS200	0,007	1,65E-04	2,33E-02
cg26071963	14	88945568	PTPN21	Body	-0,01	1,65E-04	2,33E-02
cg00171166	20	35444275	C20orf117	Body	0,005	1,65E-04	2,33E-02
cg21511036	2	227663538	IRS1	TSS200	-0,007	1,65E-04	2,33E-02
cg14584254	3	191750463		IGR	-0,022	1,65E-04	2,33E-02
cg25103197	5	90672527	ARRDC3	Body	0,021	1,65E-04	2,33E-02
cg24878005	6	150160468	LRP11	Body	0,013	1,65E-04	2,33E-02
cg20663241	8	28197671	PNOC	3'UTR	0,066	1,65E-04	2,33E-02
cg10318906	9	96717995	BARX1	TSS1500	-0,005	1,65E-04	2,33E-02
cg01803766	11	131561541	NTM	Body	-0,05	1,65E-04	2,33E-02
cg20194805	15	86499438		IGR	-0,018	1,65E-04	2,33E-02
cg21899596	4	57522493	HOPX	1stExon	-0,008	1,65E-04	2,33E-02
cg00424923	19	48297800		IGR	-0,014	1,65E-04	2,33E-02
cg07150925	3	49591642	BSN	TSS1500	-0,006	1,65E-04	2,33E-02
cg02569554	3	197280980	BDH1	5'UTR	0,014	1,65E-04	2,33E-02
cg02788195	10	60966072	PHYHIPL	Body	-0,026	1,65E-04	2,33E-02
cg19951283	13	99743631		IGR	0,016	1,65E-04	2,33E-02
cg18231690	15	85294586	ZNF592	5'UTR	0,024	1,65E-04	2,33E-02
cg03959925	20	56377456		IGR	0,021	1,65E-04	2,33E-02
cg21248296	2	104487246		IGR	0,037	1,66E-04	2,33E-02
cg10968649	1	44115804	KDM4A	1stExon	-0,003	1,66E-04	2,33E-02
cg21672780	2	42737598	MTA3	5'UTR	-0,006	1,66E-04	2,33E-02
cg01491766	3	143060927	SLC9A9-AS1	TSS200	-0,016	1,66E-04	2,33E-02
cg15823482	5	110846605	STARD4	5'UTR	-0,017	1,66E-04	2,33E-02
cg12171484	8	8679835	MFHAS1	Body	0,019	1,66E-04	2,33E-02
cg22871542	8	10331660	LINCR-0001	TSS1500	-0,015	1,66E-04	2,33E-02
cg21896766	8	114444395	CSMD3	Body	-0,03	1,66E-04	2,33E-02
cg17646666	11	76704109	ACER3	Body	-0,004	1,66E-04	2,33E-02
cg17140262	12	95618791	VEZT	Body	0,01	1,66E-04	2,33E-02
cg04885331	19	39056594	RYR1	Body	-0,022	1,66E-04	2,33E-02
cg17671559	20	33531811	GSS	Body	0,019	1,66E-04	2,33E-02
cg06071083	16	68002365	SLC12A4	1stExon	-0,003	1,66E-04	2,33E-02
cg22451887	20	60969143	CABLES2	Body	0,011	1,66E-04	2,33E-02
cg15478390	7	82792345	PCLO	TSS200	-0,01	1,66E-04	2,33E-02
cg15238246	10	6191207	PFKFB3	Body	0,012	1,66E-04	2,33E-02
cg19282408	15	81624074	TMC3-AS1	Body	-0,016	1,66E-04	2,33E-02
cg05547993	16	70835035	VAC14	1stExon	-0,003	1,66E-04	2,33E-02
cg20736821	1	44393218	ST3GAL3	3'UTR	0,006	1,66E-04	2,33E-02
cg07144560	5	148865756		IGR	0,024	1,66E-04	2,33E-02
cg07026882	13	113417167	ATP11A	Body	-0,014	1,66E-04	2,33E-02

cg06758681	16	89723958	SPATA33	TSS1500	0,005	1,66E-04	2,33E-02
cg23373558	1	224437996	NVL	Body	0,006	1,67E-04	2,33E-02
cg25799109	3	57102900	ARHGEF3	5'UTR	0,043	1,67E-04	2,33E-02
cg25013138	1	77768670	AK5	Body	-0,005	1,67E-04	2,33E-02
cg09992204	15	99500303	IGF1R	Body	0,012	1,67E-04	2,33E-02
cg23475625	7	155259680		IGR	-0,007	1,67E-04	2,34E-02
cg20341504	2	54900998		IGR	0,025	1,67E-04	2,34E-02
cg09913932	7	1771884	ELFN1	5'UTR	0,021	1,67E-04	2,34E-02
cg10770533	1	2997542	PRDM16	Body	-0,011	1,67E-04	2,34E-02
cg22199615	2	167233070	SCN9A	TSS1500	-0,01	1,67E-04	2,34E-02
cg03927812	11	130271903		IGR	0,028	1,67E-04	2,34E-02
cg09307521	1	184791362	FAM129A	Body	0,006	1,67E-04	2,34E-02
cg11251048	9	32292856		IGR	0,012	1,67E-04	2,34E-02
cg02618689	2	104764029		IGR	0,024	1,68E-04	2,34E-02
cg05335107	2	109400084	RANBP2	Body	0,013	1,68E-04	2,34E-02
cg06544512	4	38159082		IGR	-0,017	1,68E-04	2,34E-02
cg22274904	5	176111506		IGR	-0,021	1,68E-04	2,34E-02
cg04413724	6	31789540		IGR	0,006	1,68E-04	2,34E-02
cg09292244	8	8182618	PRAGMIN	Body	0,01	1,68E-04	2,34E-02
cg27607372	11	45168563	PRDM11	Body	0,008	1,68E-04	2,34E-02
cg20783780	11	112191546	LOC283140	Body	0,032	1,68E-04	2,34E-02
cg00049528	11	122013067	MIR100HG	Body	-0,007	1,68E-04	2,34E-02
cg15056189	12	49176428	ADCY6	1stExon	0,013	1,68E-04	2,34E-02
cg12050728	17	2265392	SGSM2	Body	-0,007	1,68E-04	2,34E-02
cg25750507	17	46684004		IGR	0,017	1,67E-04	2,34E-02
cg00346503	19	53377398		IGR	-0,028	1,68E-04	2,34E-02
cg14112372	1	172314302	DNM3	Body	0,036	1,68E-04	2,34E-02
cg05847835	2	139541515		IGR	-0,021	1,68E-04	2,34E-02
cg21574204	6	28048958	ZNF165	5'UTR	-0,004	1,68E-04	2,34E-02
cg01118541	8	144483004		IGR	0,032	1,68E-04	2,34E-02
cg15785720	9	139964984	C9orf140	5'UTR	-0,004	1,68E-04	2,34E-02
cg13217791	10	6196522	PFKFB3	Body	0,009	1,68E-04	2,34E-02
cg01452777	16	8715930	C16orf68	5'UTR	-0,002	1,68E-04	2,34E-02
cg10980921	8	103705294		IGR	0,017	1,68E-04	2,34E-02
cg26394221	2	187455488	ITGAV	Body	-0,008	1,68E-04	2,34E-02
cg03943568	16	65105747	CDH11	5'UTR	-0,012	1,68E-04	2,34E-02
cg05463042	2	77974053	LOC101927967	Body	-0,038	1,69E-04	2,35E-02
cg06297614	4	108664968		IGR	-0,016	1,69E-04	2,35E-02
cg25043279	7	148287142	C7orf33	TSS1500	0,007	1,68E-04	2,35E-02
cg08622677	12	3601306	PRMT8	Body	-0,018	1,69E-04	2,35E-02
cg25941520	14	68035891	PLEKHH1	Body	0,006	1,69E-04	2,35E-02
cg05525297	16	1533807		IGR	-0,007	1,69E-04	2,35E-02
cg25016143	18	64736132		IGR	0,046	1,69E-04	2,35E-02
cg08419827	19	54384913	PRKCG	TSS1500	0,009	1,68E-04	2,35E-02
cg01095667	20	11830555	LINC00687	Body	-0,013	1,69E-04	2,35E-02
cg24603576	2	102607752	IL1R2	TSS1500	0,008	1,69E-04	2,35E-02
cg22526705	1	2335890	RER1	3'UTR	-0,007	1,69E-04	2,35E-02
cg00045114	1	172674159		IGR	-0,038	1,69E-04	2,35E-02
cg21669109	20	1324311	FKBP1A-SDCBP2	Body	-0,007	1,69E-04	2,35E-02
cg19652483	6	76191813	FILIP1	5'UTR	-0,04	1,69E-04	2,35E-02
cg02230085	17	900491	TIMM22	1stExon	-0,003	1,69E-04	2,35E-02
cg06878786	18	21693037	TTC39C	Body	0,018	1,69E-04	2,35E-02
cg02217895	3	140179968	CLSTN2	Body	0,02	1,69E-04	2,35E-02
cg22589073	6	140406867	LOC100507477	Body	0,036	1,69E-04	2,35E-02
cg20784733	7	66205729	RABGEF1	1stExon	0,008	1,69E-04	2,35E-02
cg20298425	8	95892357	INTS8	Body	0,011	1,69E-04	2,35E-02
cg20198948	17	42297002	UBTF	TSS200	0,007	1,69E-04	2,35E-02
cg13357341	1	150849964	ARNT	TSS1500	0,037	1,69E-04	2,35E-02
cg01364833	3	121380236	HCLS1	TSS1500	-0,005	1,69E-04	2,35E-02
cg21103075	21	36437366		IGR	-0,007	1,70E-04	2,35E-02
cg02379052	14	105486781	CDCA4	5'UTR	-0,003	1,70E-04	2,35E-02
cg08166982	1	115300440	CSDE1	5'UTR	-0,003	1,70E-04	2,35E-02
cg11239445	7	134625906	CALD1	Body	0,017	1,70E-04	2,35E-02
cg03293513	16	1821253	NME3	Body	0,006	1,70E-04	2,35E-02
cg24217144	6	65939848	EYS	Body	-0,011	1,70E-04	2,35E-02
cg01272967	10	386708	DIP2C	Body	-0,017	1,70E-04	2,35E-02
cg01786248	12	109740038	FOXN4	Body	-0,02	1,70E-04	2,35E-02
cg10892497	8	134244175		IGR	0,009	1,70E-04	2,35E-02
cg00002028	1	20960010	PINK1	1stExon	0,008	1,70E-04	2,35E-02
cg05785348	1	211555829	C1orf97	TSS1500	0,011	1,71E-04	2,35E-02
cg19354838	2	98286165	LINC01125	TSS200	0,015	1,71E-04	2,35E-02
cg00326836	3	64248100		IGR	-0,015	1,70E-04	2,35E-02
cg02797353	3	119531385	NR1I2	Body	-0,011	1,71E-04	2,35E-02

cg16975599	3	158962761	IQCJ	Body	0,039	1,70E-04	2,35E-02
cg27523654	3	197044577		IGR	-0,006	1,71E-04	2,35E-02
cg07073803	5	110796953	CAMK4	Body	-0,046	1,71E-04	2,35E-02
cg09171621	6	27569367		IGR	-0,049	1,71E-04	2,35E-02
cg10994819	6	99399304		IGR	-0,024	1,71E-04	2,35E-02
cg16785000	6	100684047		IGR	0,019	1,71E-04	2,35E-02
cg05813781	7	4308209	SDK1	Body	-0,018	1,70E-04	2,35E-02
cg19449969	12	42816769	PPHLN1	Body	0,039	1,70E-04	2,35E-02
cg05934015	12	129280265	SLC15A4	Body	0,029	1,70E-04	2,35E-02
cg09163989	13	111045415	COL4A2	Body	-0,044	1,71E-04	2,35E-02
cg06806214	16	87392029	FBXO31	Body	-0,005	1,70E-04	2,35E-02
cg18736736	17	19650063	ALDH3A1	5'UTR	0,006	1,71E-04	2,35E-02
cg20702887	17	72246008	TTYH2	5'UTR	-0,031	1,71E-04	2,35E-02
cg00715343	17	76171845	TK1	Body	0,004	1,71E-04	2,35E-02
cg14176339	17	79244542	SLC38A10	Body	0,041	1,70E-04	2,35E-02
cg14725803	22	19035997	DGCR2	Body	-0,01	1,71E-04	2,35E-02
cg03468353	16	76296523		IGR	0,009	1,71E-04	2,36E-02
cg08147389	14	73756251	NUMB	Body	-0,048	1,71E-04	2,36E-02
cg17361314	1	45480186	UROD	Body	0,023	1,71E-04	2,36E-02
cg09172991	2	69794389	AAK1	Body	0,022	1,71E-04	2,36E-02
cg15536804	12	26647178	ITPR2	Body	0,017	1,71E-04	2,36E-02
cg10596237	4	113436566	NEUROG2	Body	0,011	1,71E-04	2,36E-02
cg12917258	10	99258018	UBTD1	TSS1500	-0,006	1,71E-04	2,36E-02
cg02557933	2	220351040	SPEG	Body	0,032	1,71E-04	2,36E-02
cg04559779	8	65291730	MIR124-2	Body	-0,026	1,71E-04	2,36E-02
cg19195949	2	73383912		IGR	-0,003	1,72E-04	2,36E-02
cg27166577	6	80656754	ELOVL4	Body	-0,006	1,72E-04	2,36E-02
cg09647790	2	48533876		IGR	0,009	1,72E-04	2,36E-02
cg02741393	2	241501875	ANKMY1	TSS1500	-0,017	1,72E-04	2,36E-02
cg07244268	3	164924675		IGR	-0,005	1,72E-04	2,36E-02
cg11093762	7	930723	C7orf20	Body	-0,007	1,72E-04	2,36E-02
cg17151604	10	75197928	PPP3CB	3'UTR	-0,006	1,72E-04	2,36E-02
cg09806556	15	77588060	PEAK1	5'UTR	0,008	1,72E-04	2,36E-02
cg26392328	17	53827880	PCTP	TSS1500	-0,008	1,72E-04	2,36E-02
cg17744261	17	71252029	CPSF4L	Body	0,023	1,72E-04	2,36E-02
cg11270815	19	9546371	ZNF266	TSS200	-0,003	1,72E-04	2,36E-02
cg16868591	19	12803493	FBXW9	Body	-0,024	1,72E-04	2,36E-02
cg20820128	2	20595658		IGR	-0,01	1,72E-04	2,36E-02
cg18753020	1	236288284		IGR	0,009	1,72E-04	2,36E-02
cg12843387	4	41144651	APBB2	5'UTR	-0,008	1,72E-04	2,36E-02
cg06894599	16	34984393	FLJ26245	Body	-0,036	1,72E-04	2,36E-02
cg11529567	2	230807409	FBXO36	Body	-0,009	1,72E-04	2,36E-02
cg11333117	1	212457740	PPP2R5A	TSS1500	-0,044	1,72E-04	2,36E-02
cg22519233	4	1806633	FGFR3	Body	0,005	1,72E-04	2,36E-02
cg20751430	6	112081257	FYN	TSS1500	-0,017	1,72E-04	2,36E-02
cg03265014	5	7897340	MTRR	ExonBnd	-0,048	1,72E-04	2,37E-02
cg00313401	15	74219948	LOXL1	1stExon	-0,005	1,73E-04	2,37E-02
cg21496511	17	56232384	OR4D1	TSS200	0,008	1,73E-04	2,37E-02
cg16049584	10	93735638	BTAF1	Body	-0,004	1,73E-04	2,37E-02
cg20398378	2	190414043		IGR	-0,03	1,73E-04	2,37E-02
cg02297747	17	74519321		IGR	0,024	1,73E-04	2,37E-02
cg07179872	5	140802135	PCDHGA4	Body	-0,052	1,73E-04	2,37E-02
cg08661112	10	91405677	PANK1	TSS1500	-0,018	1,73E-04	2,37E-02
cg23674882	2	74780272	LOXL3	5'UTR	0,025	1,73E-04	2,37E-02
cg00937568	21	34602128	IFNAR2	TSS200	0,01	1,73E-04	2,37E-02
cg04465974	1	172501480	SUCO	TSS1500	-0,007	1,74E-04	2,37E-02
cg06689372	3	58645085	FAM3D	5'UTR	0,019	1,74E-04	2,37E-02
cg13136655	6	149806131	ZC3H12D	5'UTR	-0,026	1,73E-04	2,37E-02
cg09184315	8	127748477		IGR	0,036	1,74E-04	2,37E-02
cg01871631	8	129807058		IGR	-0,015	1,74E-04	2,37E-02
cg01302240	9	116263669	RGS3	TSS200	-0,021	1,74E-04	2,37E-02
cg05487290	11	2029538		IGR	0,015	1,74E-04	2,37E-02
cg15172729	11	67056446	ANKRD13D	TSS1500	-0,004	1,74E-04	2,37E-02
cg13234696	12	89355145		IGR	-0,031	1,74E-04	2,37E-02
cg24834886	13	50021605	SETDB2	5'UTR	0,048	1,74E-04	2,37E-02
cg05390137	14	35231142	BAZ1A	Body	-0,021	1,74E-04	2,37E-02
cg09513224	16	784431	NARFL	Body	0,006	1,73E-04	2,37E-02
cg02352398	16	19869374	IQCK	3'UTR	-0,036	1,74E-04	2,37E-02
cg12107369	16	81295075	BCO1	Body	0,016	1,74E-04	2,37E-02
cg17298666	18	57364491	CCBE1	1stExon	-0,009	1,74E-04	2,37E-02
cg09337248	20	36572562	VSTM2L	Body	-0,011	1,74E-04	2,37E-02
cg10921149	21	34971033	CRYZL1	Body	0,014	1,74E-04	2,37E-02
cg04930288	22	25393922		IGR	-0,007	1,74E-04	2,37E-02

cg04941033	4	41983923	DCAF4L1	1stExon	0,012	1,74E-04	2,37E-02
cg01599781	9	136392594		IGR	0,023	1,74E-04	2,37E-02
cg19896639	1	9599276	SLC25A33	TSS1500	-0,004	1,74E-04	2,37E-02
cg13706730	4	3326208	RGS12	Body	-0,014	1,74E-04	2,37E-02
cg17156809	19	30989605	ZNF536	Body	-0,018	1,74E-04	2,37E-02
cg06534553	15	72608241	BRUNOL6	Body	0,023	1,74E-04	2,38E-02
cg01941814	6	32130562	PPT2	Body	0,009	1,74E-04	2,38E-02
cg13641144	16	58175324		IGR	0,01	1,75E-04	2,38E-02
cg08714767	4	25770960	SEL1L3	Body	-0,016	1,75E-04	2,38E-02
cg02148796	2	29548577	ALK	Body	0,096	1,75E-04	2,38E-02
cg11098970	2	196535378	SLC39A10	5'UTR	0,021	1,75E-04	2,38E-02
cg17694165	11	58335521	LPXN	Body	-0,014	1,75E-04	2,38E-02
cg22479481	17	16120428	PIGL	TSS200	-0,006	1,75E-04	2,38E-02
cg17367688	20	51422235		IGR	-0,032	1,75E-04	2,38E-02
cg16222850	15	64989523	OAZ2	Body	-0,029	1,75E-04	2,38E-02
cg22044018	20	60877802	ADRM1	TSS200	-0,005	1,75E-04	2,38E-02
cg10586672	3	14514657	SLC6A6	Body	-0,007	1,75E-04	2,38E-02
cg11458650	4	152024299	RPS3A	Body	0,008	1,75E-04	2,38E-02
cg22219494	1	25201805		IGR	0,022	1,75E-04	2,38E-02
cg04407137	20	61713234		IGR	-0,012	1,75E-04	2,39E-02
cg10567034	11	70049203	FADD	TSS200	-0,003	1,76E-04	2,39E-02
cg04629867	16	1495835	CCDC154	TSS1500	0,02	1,76E-04	2,39E-02
cg22251211	5	138265577	CTNNA1	Body	-0,003	1,76E-04	2,39E-02
cg15884789	6	28047400	ZNF165	TSS1500	0,025	1,76E-04	2,39E-02
cg12870041	5	95883446	LOC101929710	Body	-0,006	1,76E-04	2,39E-02
cg26117186	13	88464367		IGR	0,016	1,76E-04	2,39E-02
cg04535320	2	45172172	SIX3	3'UTR	-0,021	1,76E-04	2,39E-02
cg06586882	6	38831548	DNAH8	Body	0,024	1,76E-04	2,39E-02
cg01969748	1	109420034	GPSM2	5'UTR	-0,005	1,76E-04	2,39E-02
cg01473249	6	166219132		IGR	-0,055	1,76E-04	2,39E-02
cg23028307	1	235117202		IGR	0,032	1,77E-04	2,39E-02
cg00510849	9	22446834	DMRTA1	TSS200	-0,006	1,77E-04	2,39E-02
cg25597878	22	40099982		IGR	0,028	1,77E-04	2,40E-02
cg03693068	7	18810870	HDAC9	Body	0,012	1,77E-04	2,40E-02
cg18362486	11	111649972		IGR	-0,012	1,77E-04	2,40E-02
cg16248765	13	45578845	GPALPP1	Body	0,021	1,77E-04	2,40E-02
cg12697266	20	7723304		IGR	-0,021	1,77E-04	2,40E-02
cg23462257	4	103421943	NFKB1	TSS1500	0,023	1,77E-04	2,40E-02
cg02816067	11	119539167	PVRL1	Body	0,019	1,77E-04	2,40E-02
cg17651653	14	30066889	MIR548AI	Body	-0,02	1,77E-04	2,40E-02
cg25887236	18	74844781	MBP	TSS200	0,013	1,77E-04	2,40E-02
cg09172766	16	89788316	C16orf7	TSS1500	-0,003	1,77E-04	2,40E-02
cg06747982	4	100864777	DNAJB14	Body	-0,016	1,77E-04	2,40E-02
cg10517798	2	189715453		IGR	-0,014	1,77E-04	2,40E-02
cg01662083	16	79437712		IGR	-0,013	1,77E-04	2,40E-02
cg08317252	19	1223163	STK11	Body	0,02	1,77E-04	2,40E-02
cg23327200	17	72246037	TTYH2	5'UTR	-0,028	1,77E-04	2,40E-02
cg13869872	2	197457420	HECW2	TSS200	-0,008	1,77E-04	2,40E-02
cg02331450	3	33155354	CRTAP	TSS200	-0,004	1,78E-04	2,40E-02
cg23996767	5	64920638	TRIM23	TSS1500	-0,007	1,78E-04	2,40E-02
cg27277859	19	51165632	SHANK1	Body	-0,049	1,78E-04	2,40E-02
cg13540417	16	11126178	CLEC16A	Body	0,007	1,78E-04	2,40E-02
cg26975834	16	12719124		IGR	-0,038	1,78E-04	2,40E-02
cg05536782	2	3676611	COLEC11	Body	-0,08	1,78E-04	2,40E-02
cg10936841	5	175295613	CPLX2	5'UTR	-0,05	1,78E-04	2,40E-02
cg11715862	6	88102731		IGR	-0,006	1,78E-04	2,40E-02
cg04744409	6	105388191		IGR	-0,038	1,78E-04	2,40E-02
cg26620728	10	83808891	NRG3	Body	0,015	1,78E-04	2,40E-02
cg19610561	13	58132491		IGR	-0,021	1,78E-04	2,40E-02
cg09302448	16	67562672	FAM65A	TSS200	0,007	1,78E-04	2,40E-02
cg09542006	16	26241483		IGR	-0,021	1,78E-04	2,40E-02
cg19707450	7	129647455		IGR	-0,007	1,78E-04	2,40E-02
cg21521438	3	32417973		IGR	-0,013	1,78E-04	2,40E-02
cg10436759	1	204226557	PLEKHA6	Body	0,007	1,78E-04	2,41E-02
cg01057126	12	122112751		IGR	0,02	1,78E-04	2,41E-02
cg05772319	16	323063	RGS11	Body	0,014	1,78E-04	2,41E-02
cg07882010	12	114126511		IGR	-0,019	1,79E-04	2,41E-02
cg23804099	1	79506422		IGR	-0,015	1,79E-04	2,41E-02
cg23943415	8	16369650		IGR	0,027	1,79E-04	2,41E-02
cg05490132	15	43661835	TUBGCP4	TSS1500	0,01	1,79E-04	2,41E-02
cg00693172	17	8053426	PER1	Body	0,014	1,79E-04	2,41E-02
cg27574547	20	58280745	PHACTR3	5'UTR	-0,063	1,79E-04	2,41E-02
cg07304894	9	133280772	HMCN2	Body	-0,007	1,79E-04	2,41E-02

cg26315263	15	59665250	MYO1E	TSS200	0,008	1,79E-04	2,41E-02
cg08197069	2	70343944		IGR	0,018	1,79E-04	2,41E-02
cg04621962	14	39937815		IGR	-0,019	1,79E-04	2,41E-02
cg12450006	5	141913920		IGR	0,007	1,79E-04	2,41E-02
cg02263705	9	4683957	CDC37L1	Body	0,005	1,79E-04	2,41E-02
cg03745912	10	120924721	SFXN4	Body	-0,003	1,79E-04	2,41E-02
cg27081704	11	118574745		IGR	0,019	1,79E-04	2,41E-02
cg10516516	3	54231340	CACNA2D3	Body	0,034	1,80E-04	2,41E-02
cg18284616	4	103477551	NFKB1	Body	-0,007	1,80E-04	2,41E-02
cg04910295	6	4181334		IGR	-0,007	1,79E-04	2,41E-02
cg27652887	10	12083919	UPF2	5'UTR	-0,003	1,80E-04	2,41E-02
cg25598488	12	34258505		IGR	-0,017	1,80E-04	2,41E-02
cg25083402	14	104392395		IGR	0,017	1,80E-04	2,41E-02
cg00787008	17	56198423		IGR	0,039	1,79E-04	2,41E-02
cg18693889	1	104095954	RNPC3	3'UTR	0,035	1,80E-04	2,41E-02
cg23329739	2	149956027	LYPD6B	5'UTR	-0,011	1,80E-04	2,41E-02
cg20669834	3	123339417	MYLK	1stExon	-0,005	1,80E-04	2,41E-02
cg24601517	6	5004202	RPP40	1stExon	-0,005	1,80E-04	2,41E-02
cg20092399	7	51192342	COBL	Body	-0,063	1,80E-04	2,41E-02
cg09722396	11	15213350	INSC	Body	-0,005	1,80E-04	2,41E-02
cg27172877	11	124806777	HEPACAM	TSS1500	-0,025	1,80E-04	2,41E-02
cg27047829	17	60872206	MARCH10	Body	-0,009	1,80E-04	2,41E-02
cg03776082	2	136884116		IGR	0,027	1,80E-04	2,41E-02
cg15358052	14	69865456	SLC39A9	5'UTR	-0,005	1,80E-04	2,42E-02
cg09410380	19	641856	FGF22	Body	0,008	1,80E-04	2,42E-02
cg21127189	4	39368001	RFC1	TSS200	-0,005	1,80E-04	2,42E-02
cg16579269	16	62397449		IGR	-0,024	1,80E-04	2,42E-02
cg06033579	1	12662841	DHRS3	Body	0,006	1,81E-04	2,42E-02
cg03612435	22	19160780		IGR	0,015	1,81E-04	2,42E-02
cg22760710	3	185083290	MAP3K13	5'UTR	0,004	1,81E-04	2,42E-02
cg12053709	9	138455431	PAEP	Body	-0,008	1,81E-04	2,42E-02
cg08158024	14	24626558	RNF31	Body	-0,006	1,81E-04	2,42E-02
cg06478457	7	29604764	PRR15	5'UTR	-0,006	1,81E-04	2,42E-02
cg14250833	6	10882240	GCM2	TSS200	-0,018	1,81E-04	2,42E-02
cg00935887	2	242844000		IGR	-0,098	1,81E-04	2,42E-02
cg03969079	10	63759909	ARID5B	Body	0,018	1,81E-04	2,42E-02
cg13784878	1	28699088	PHACTR4	5'UTR	0,015	1,82E-04	2,43E-02
cg05780293	5	153767134	GALNT10	Body	-0,014	1,82E-04	2,43E-02
cg15445725	3	194469106		IGR	-0,017	1,82E-04	2,43E-02
cg25104285	7	29287715	CHN2	Body	0,013	1,82E-04	2,43E-02
cg15283212	18	21014021	C18orf45	Body	0,015	1,82E-04	2,43E-02
cg02508210	2	44314563		IGR	-0,004	1,82E-04	2,43E-02
cg08892382	12	6635112	NCAPD2	Body	-0,005	1,82E-04	2,43E-02
cg07365439	13	19581746	LINC00442	TSS1500	-0,067	1,82E-04	2,43E-02
cg19311443	3	113382256	KIAA2018	Body	0,008	1,82E-04	2,43E-02
cg06268161	5	140595879	PCDHB13	1stExon	-0,035	1,82E-04	2,43E-02
cg06986837	12	78231974	NAV3	Body	-0,051	1,82E-04	2,43E-02
cg24684166	12	80836727	PTPRQ	TSS1500	0,033	1,82E-04	2,43E-02
cg18578970	14	79370439	NRXN3	Body	-0,009	1,82E-04	2,43E-02
cg05063616	17	42147940	G6PC3	TSS200	-0,006	1,82E-04	2,43E-02
cg22746808	22	32364803		IGR	-0,013	1,82E-04	2,43E-02
cg11333664	1	74215505		IGR	0,012	1,82E-04	2,43E-02
cg07660890	2	37881124	CDC42EP3	5'UTR	0,05	1,82E-04	2,43E-02
cg02048657	5	1293231	TERT	Body	0,016	1,82E-04	2,43E-02
cg18131041	14	61814530	PRKCH	Body	-0,009	1,82E-04	2,43E-02
cg06217323	3	75445502		IGR	-0,052	1,82E-04	2,43E-02
cg13958614	5	72744196	FOXD1	5'UTR	-0,009	1,82E-04	2,43E-02
cg11192541	6	30434109		IGR	-0,021	1,82E-04	2,43E-02
cg08192767	7	20614517		IGR	0,005	1,83E-04	2,43E-02
cg08264579	5	89703833	CETN3	Body	0,017	1,83E-04	2,43E-02
cg03115835	1	26758846	DHDDS	1stExon	-0,006	1,83E-04	2,43E-02
cg11807529	1	17958884	ARHGEF10L	Body	0,006	1,83E-04	2,43E-02
cg03883572	5	66461884	MAST4	Body	-0,043	1,83E-04	2,43E-02
cg19106489	5	72649639		IGR	-0,046	1,83E-04	2,43E-02
cg16136774	5	91976489		IGR	0,014	1,83E-04	2,43E-02
cg19063654	5	156516798	HAVCR2	Body	-0,011	1,83E-04	2,43E-02
cg25137674	7	38450808	AMPH	Body	-0,006	1,83E-04	2,43E-02
cg25708403	8	9597811	MIR597	TSS1500	0,017	1,83E-04	2,43E-02
cg00387552	10	7555607		IGR	0,006	1,83E-04	2,43E-02
cg07141452	20	3775639	CDC25B	TSS1500	-0,015	1,83E-04	2,43E-02
cg10526704	9	138593952	KCNT1	TSS200	0,005	1,83E-04	2,43E-02
cg07013177	12	58166795	METTL1	TSS1500	0,004	1,83E-04	2,43E-02
cg25573095	18	74889109		IGR	0,016	1,83E-04	2,43E-02

cg19940332	6	44281100	AARS2	TSS200	-0,005	1,83E-04	2,43E-02
cg04004930	7	111496215	DOCK4	Body	-0,027	1,83E-04	2,43E-02
cg15303490	9	80850951	CEP78	TSS200	-0,004	1,83E-04	2,44E-02
cg16542960	6	702344		IGR	0,028	1,84E-04	2,44E-02
cg14859667	10	58121125	ZWINT	TSS200	-0,004	1,84E-04	2,44E-02
cg09430095	12	4646531	C12orf4	5'UTR	-0,01	1,84E-04	2,44E-02
cg01902584	4	39399553	MIR1273H	Body	-0,008	1,84E-04	2,44E-02
cg05136264	9	124989408	LHX6	Body	-0,114	1,84E-04	2,44E-02
cg21649569	21	40752157	WRB	TSS200	0,011	1,84E-04	2,44E-02
cg09468386	2	103418024	TMEM182	Body	-0,023	1,84E-04	2,44E-02
cg11189353	2	122543547		IGR	-0,008	1,84E-04	2,44E-02
cg02076818	4	32351541		IGR	0,045	1,84E-04	2,44E-02
cg19491768	10	123441249		IGR	0,049	1,84E-04	2,44E-02
cg09912203	16	74904865		IGR	-0,01	1,84E-04	2,44E-02
cg17633774	18	46194291	CTIF	Body	0,009	1,84E-04	2,44E-02
cg11607339	18	72461323	ZNF407	Body	-0,04	1,84E-04	2,44E-02
cg04862324	22	50900009	SBF1	Body	0,007	1,84E-04	2,44E-02
cg05726854	7	4063608	SDK1	Body	-0,024	1,84E-04	2,44E-02
cg11432443	1	6548491	PLEKHG5	5'UTR	0,015	1,84E-04	2,44E-02
cg23080538	1	6537569	PLEKHG5	Body	-0,015	1,84E-04	2,44E-02
cg04352092	1	56410764		IGR	-0,009	1,84E-04	2,44E-02
cg19584027	12	94529752		IGR	0,005	1,84E-04	2,44E-02
cg23911465	14	75469330	EIF2B2	TSS1500	-0,004	1,84E-04	2,44E-02
cg04653226	17	9725928		IGR	-0,021	1,84E-04	2,44E-02
cg05091734	21	46706579	POFUT2	Body	0,008	1,84E-04	2,44E-02
cg27269148	13	113735331	MCF2L	Body	0,009	1,85E-04	2,44E-02
cg25011388	14	20920574	OSGEP	Body	-0,006	1,85E-04	2,44E-02
cg07029404	6	53358356		IGR	-0,042	1,85E-04	2,44E-02
cg25322626	7	69227635	AUTS2	Body	0,022	1,85E-04	2,44E-02
cg19795556	10	60494185	BICC1	Body	0,014	1,85E-04	2,44E-02
cg23219905	10	122756385	MIR5694	Body	-0,041	1,85E-04	2,44E-02
cg02225490	11	44932583	TSPAN18	Body	0,008	1,85E-04	2,44E-02
cg12383453	11	134254877	B3GAT1	Body	-0,016	1,85E-04	2,44E-02
cg05679836	12	57472765	TMEM194A	TSS200	-0,005	1,85E-04	2,44E-02
cg20898283	14	75920198	JDP2	Body	0,034	1,85E-04	2,44E-02
cg13486556	15	52044539	LYSMD2	TSS1500	0,026	1,85E-04	2,45E-02
cg05189754	17	20173189	SPECC1	Body	-0,009	1,85E-04	2,45E-02
cg17913405	17	25290204		IGR	-0,075	1,85E-04	2,45E-02
cg27126505	13	51375420	DLEU7	Body	0,007	1,85E-04	2,45E-02
cg06047040	3	27753347		IGR	-0,01	1,85E-04	2,45E-02
cg06511482	1	882546	NOC2L	Body	0,013	1,85E-04	2,45E-02
cg25212131	5	140683670	SLC25A2	TSS200	-0,052	1,86E-04	2,45E-02
cg15143370	9	132625508	USP20	Body	-0,009	1,86E-04	2,45E-02
cg25223095	3	16927003	PLCL2	Body	0,004	1,86E-04	2,45E-02
cg08286554	10	70940093	SUPV3L1	1stExon	-0,005	1,86E-04	2,45E-02
cg13939640	6	125303753	RNF217	TSS1500	0,059	1,86E-04	2,45E-02
cg00724572	5	140071908	HARS2	5'UTR	0,025	1,86E-04	2,45E-02
cg16150605	12	57037148	ATP5B	Body	0,008	1,86E-04	2,45E-02
cg16709457	11	27745361		IGR	-0,041	1,86E-04	2,45E-02
cg13383536	11	44972583	TP53I11	5'UTR	0,007	1,86E-04	2,45E-02
cg15423862	3	155838109	KCNAB1	TSS1500	0,007	1,86E-04	2,45E-02
cg21540266	4	190936644		IGR	0,013	1,86E-04	2,45E-02
cg25748726	3	24023098		IGR	-0,015	1,87E-04	2,46E-02
cg15197582	18	59997096	TNFRSF11A	Body	-0,029	1,87E-04	2,46E-02
cg26060655	4	181681422		IGR	-0,019	1,87E-04	2,46E-02
cg21223625	14	59332349		IGR	0,014	1,87E-04	2,46E-02
cg16058403	8	28911626		IGR	0,013	1,87E-04	2,46E-02
cg22188408	9	130098419	GARNL3	ExonBnd	-0,005	1,87E-04	2,46E-02
cg12500602	15	92525909	SLCO3A1	Body	0,016	1,87E-04	2,46E-02
cg08266177	14	94432162		IGR	-0,006	1,87E-04	2,46E-02
cg02344334	3	122974116	SEC22A	Body	-0,006	1,88E-04	2,46E-02
cg20677627	16	5246141		IGR	-0,017	1,88E-04	2,46E-02
cg01714383	16	87703576	JPH3	Body	0,016	1,88E-04	2,46E-02
cg15086925	20	45906236	ZMYND8	Body	0,007	1,88E-04	2,46E-02
cg18244483	4	107423468		IGR	-0,029	1,88E-04	2,47E-02
cg05066973	16	89661800	CPNE7	Body	0,017	1,88E-04	2,47E-02
cg02035300	1	204874132	NFASC	5'UTR	-0,008	1,88E-04	2,47E-02
cg27418906	20	34441971	PHF20	Body	-0,011	1,88E-04	2,47E-02
cg08503808	8	141348605	TRAPPC9	Body	0,028	1,88E-04	2,47E-02
cg11395431	4	187070481	FAM149A	5'UTR	0,041	1,88E-04	2,47E-02
cg14115785	5	75737331	IQGAP2	Body	-0,003	1,88E-04	2,47E-02
cg15333689	5	159846543	SLU7	TSS1500	-0,005	1,88E-04	2,47E-02
cg26717016	6	33424210	ZBTB9	Body	-0,008	1,88E-04	2,47E-02

cg07632068	15	40179641	GPR176	Body	0,02	1,88E-04	2,47E-02
cg04251370	19	48846867	TMEM143	Body	0,006	1,88E-04	2,47E-02
cg17147211	1	119544070		IGR	-0,006	1,88E-04	2,47E-02
cg14702390	18	9917748	VAPA	Body	-0,009	1,88E-04	2,47E-02
cg08795904	22	50695594	MAPK12	Body	0,013	1,89E-04	2,47E-02
cg01420022	4	40104428	N4BP2	Body	0,006	1,89E-04	2,47E-02
cg12768613	9	139659883	LCN15	TSS1500	0,008	1,89E-04	2,47E-02
cg07093456	10	118380177	PNLIPRP2	TSS1500	-0,014	1,89E-04	2,47E-02
cg10029377	5	140792514	PCDHGA10	TSS1500	-0,043	1,89E-04	2,47E-02
cg19359929	1	3541763	TPRG1L	1stExon	-0,003	1,89E-04	2,47E-02
cg25652610	19	4648335	TNFAIP8L1	5'UTR	0,024	1,89E-04	2,47E-02
cg22360704	2	209000030	LOC100507443	Body	0,028	1,89E-04	2,47E-02
cg01511655	3	58182789	DNASE1L3	Body	0,006	1,89E-04	2,47E-02
cg13920435	9	90589640	CDK20	1stExon	-0,013	1,89E-04	2,47E-02
cg11674432	10	43572424	RET	TSS200	0,01	1,89E-04	2,47E-02
cg01143197	11	56000767	OR5T2	TSS200	-0,043	1,89E-04	2,47E-02
cg17439237	15	59377590	RNF111	Body	0,02	1,89E-04	2,47E-02
cg09528372	19	41257673	SNRPA	Body	-0,003	1,89E-04	2,47E-02
cg24901447	12	117036895		IGR	-0,016	1,89E-04	2,47E-02
cg18114463	1	1476551	C1orf70	TSS1500	0,015	1,89E-04	2,47E-02
cg23682200	12	130677376		IGR	-0,015	1,89E-04	2,47E-02
cg25893869	1	70687106	SFRS11	5'UTR	-0,007	1,90E-04	2,48E-02
cg23847967	1	78421529	FUBP1	Body	0,028	1,90E-04	2,48E-02
cg07943152	15	75391193		IGR	0,026	1,90E-04	2,48E-02
cg20959920	17	75238948		IGR	0,016	1,90E-04	2,48E-02
cg15433588	4	100763828	DAPP1	Body	-0,006	1,90E-04	2,48E-02
cg11842199	3	86317417		IGR	0,024	1,90E-04	2,48E-02
cg09645743	7	117067136	ASZ1	Body	0,035	1,90E-04	2,48E-02
cg25792271	2	74779889	LOXL3	5'UTR	0,021	1,90E-04	2,48E-02
cg24119990	3	187386810	SST	3'UTR	-0,026	1,90E-04	2,48E-02
cg09277744	12	4386203	CCND2	Body	-0,004	1,90E-04	2,48E-02
cg02129552	16	75000746	WDR59	Body	0,006	1,90E-04	2,48E-02
cg24382918	3	141634157	ATP1B3	Body	-0,004	1,90E-04	2,48E-02
cg12609030	6	129083238		IGR	-0,009	1,90E-04	2,48E-02
cg13908518	1	104096517	RNPC3	3'UTR	0,005	1,91E-04	2,48E-02
cg19119231	2	38155379	RMDN2	5'UTR	0,018	1,91E-04	2,48E-02
cg18514279	10	108369835	SORCS1	Body	-0,036	1,91E-04	2,48E-02
cg06533679	11	47374222	MYBPC3	5'UTR	0,019	1,91E-04	2,48E-02
cg20883850	16	87272191		IGR	-0,004	1,91E-04	2,48E-02
cg11082512	17	48322441		IGR	0,007	1,91E-04	2,48E-02
cg19293581	6	78645817		IGR	-0,018	1,91E-04	2,48E-02
cg07356135	8	49965470	C8orf22	TSS1500	0,053	1,91E-04	2,48E-02
cg13898318	13	25754718		IGR	-0,041	1,91E-04	2,48E-02
cg17938265	17	17696257	RAI1	ExonBnd	-0,006	1,91E-04	2,48E-02
cg21533476	18	48910804		IGR	-0,009	1,91E-04	2,48E-02
cg09994057	10	14598259	FAM107B	5'UTR	0,029	1,91E-04	2,49E-02
cg03440529	2	65279215		IGR	0,021	1,92E-04	2,49E-02
cg04124966	3	133880809	RYK	Body	-0,004	1,91E-04	2,49E-02
cg14023672	4	62294149		IGR	0,012	1,91E-04	2,49E-02
cg08363278	6	28829746		IGR	0,004	1,91E-04	2,49E-02
cg19758859	6	148869489	SASH1	Body	-0,009	1,91E-04	2,49E-02
cg24311564	7	154542313	DPP6	Body	-0,009	1,92E-04	2,49E-02
cg20776317	9	139292793	SNAPC4	1stExon	0,012	1,91E-04	2,49E-02
cg26872475	11	119039430	NLRX1	TSS200	-0,004	1,91E-04	2,49E-02
cg24172269	11	133826329	IGSF9B	Body	-0,004	1,92E-04	2,49E-02
cg26225496	14	67879166	PLEK2	TSS1500	-0,032	1,92E-04	2,49E-02
cg11684551	15	43689395	TUBGCP4	ExonBnd	-0,005	1,91E-04	2,49E-02
cg07610074	19	10160712	C3P1	Body	0,049	1,91E-04	2,49E-02
cg06296773	3	175338547	NAALADL2	Body	-0,011	1,92E-04	2,49E-02
cg14117166	9	140064014	LRRC26	1stExon	-0,005	1,92E-04	2,49E-02
cg02043077	2	118845769	INSIG2	TSS1500	-0,005	1,92E-04	2,49E-02
cg06230615	8	113900516	CSMD3	Body	-0,06	1,92E-04	2,49E-02
cg05011589	20	35028834	DLGAP4	5'UTR	-0,032	1,92E-04	2,49E-02
cg25226555	5	98714340		IGR	0,056	1,92E-04	2,49E-02
cg08462478	8	87135788	ATP6V0D2	Body	0,012	1,92E-04	2,49E-02
cg06900676	2	61293330	KIAA1841	5'UTR	-0,003	1,92E-04	2,49E-02
cg21129965	2	238499456	RAB17	5'UTR	-0,014	1,92E-04	2,49E-02
cg11677533	1	70828047	HHLA3	Body	0,021	1,92E-04	2,49E-02
cg01896085	2	9279786		IGR	0,02	1,92E-04	2,49E-02
cg02269094	5	55061610	DDX4	TSS1500	0,011	1,92E-04	2,49E-02
cg05935800	1	226023590	EPHX1	Body	0,023	1,93E-04	2,49E-02
cg11027568	4	8456748	TRMT44	Body	-0,008	1,93E-04	2,49E-02
cg02609724	5	139506447	C5orf53	1stExon	-0,005	1,93E-04	2,49E-02

cg10314295	10	75936245	ADK	TSS200	-0,003	1,92E-04	2,49E-02
cg23483798	18	32958206	ZNF396	TSS1500	0,009	1,93E-04	2,49E-02
cg26144246	20	42100181		IGR	-0,006	1,93E-04	2,49E-02
cg08638044	15	27819924		IGR	-0,028	1,93E-04	2,49E-02
cg12429471	7	114872705	LINC01392	TSS1500	0,016	1,93E-04	2,49E-02
cg19309079	2	133175311	GPR39	1stExon	-0,017	1,93E-04	2,49E-02
cg05866214	2	240600475		IGR	-0,005	1,93E-04	2,49E-02
cg16991944	3	14330859		IGR	-0,012	1,93E-04	2,49E-02
cg13333304	3	170136200	CLDN11	TSS1500	-0,03	1,93E-04	2,49E-02
cg21933652	5	136141612		IGR	-0,029	1,93E-04	2,49E-02
cg09744448	5	156998546	ADAM19	Body	-0,009	1,93E-04	2,49E-02
cg09919882	11	129764541	NFRKB	5'UTR	0,083	1,93E-04	2,49E-02
cg03710176	15	62352459	VPS13C	Body	-0,002	1,93E-04	2,49E-02
cg21075678	7	137557164		IGR	-0,067	1,93E-04	2,49E-02
cg09532331	9	15422570	SNAPC3	TSS1500	-0,004	1,93E-04	2,49E-02
cg16778148	11	2817922	KCNQ1	Body	-0,009	1,93E-04	2,49E-02
cg17783031	3	48673137	SLC26A6	TSS1500	-0,004	1,93E-04	2,49E-02
cg15599642	3	98515469	DCBLD2	3'UTR	-0,005	1,93E-04	2,49E-02
cg22228457	3	176095343		IGR	0,01	1,93E-04	2,49E-02
cg23524474	5	74212788		IGR	-0,011	1,93E-04	2,49E-02
cg00168502	5	178310723	ZNF354B	Body	0,008	1,93E-04	2,49E-02
cg26303870	14	69443683	ACTN1	Body	0,021	1,93E-04	2,49E-02
cg02926035	15	57618604		IGR	-0,007	1,93E-04	2,49E-02
cg09596025	16	84090260	MBTPS1	Body	0,008	1,93E-04	2,49E-02
cg15571423	3	130158846	COL29A1	Body	-0,008	1,94E-04	2,49E-02
cg00518122	1	53678968	CPT2	Body	0,008	1,94E-04	2,50E-02
cg00901946	2	97372840	LMAN2L	Body	0,004	1,94E-04	2,50E-02
cg04585364	2	171628203		IGR	-0,021	1,94E-04	2,50E-02
cg06025456	6	32120863	PPT2	TSS1500	0,029	1,94E-04	2,50E-02
cg10432632	6	160182818	ACAT2	TSS200	-0,003	1,94E-04	2,50E-02
cg19587003	9	35062116	VCP	ExonBnd	-0,007	1,94E-04	2,50E-02
cg14711570	18	52591974	CCDC68	Body	0,027	1,94E-04	2,50E-02
cg10244649	1	17775872		IGR	-0,006	1,94E-04	2,50E-02
cg00802302	11	45376880	LOC101928812	TSS200	-0,009	1,94E-04	2,50E-02
cg23400639	18	2540792	METTL4	Body	0,01	1,94E-04	2,50E-02
cg19254328	3	126113327	CCDC37	TSS1500	0,026	1,94E-04	2,50E-02
cg16021364	10	51042276	PARG	Body	0,021	1,94E-04	2,50E-02
cg04472859	6	119789081	LOC285762	Body	0,042	1,94E-04	2,50E-02
cg08471699	2	63816291	WDPCP	TSS1500	-0,004	1,94E-04	2,50E-02
cg08167132	1	82063629		IGR	-0,007	1,94E-04	2,50E-02
cg21295827	1	178708719	RALGPS2	5'UTR	-0,009	1,95E-04	2,50E-02
cg12337143	2	69879666		IGR	-0,016	1,95E-04	2,50E-02
cg16739160	3	57286158	APPL1	Body	0,015	1,95E-04	2,50E-02
cg27666108	3	88054104		IGR	0,041	1,95E-04	2,50E-02
cg22967122	5	156569777	MED7	1stExon	-0,005	1,95E-04	2,50E-02
cg09588476	11	129054197	ARHGAP32	Body	0,042	1,95E-04	2,50E-02
cg09783353	20	48040899	KCNB1	Body	-0,008	1,95E-04	2,50E-02
cg13067649	3	130300740	COL6A6	Body	-0,021	1,95E-04	2,50E-02
cg04076387	16	597434	SOLH	Body	0,049	1,95E-04	2,50E-02
cg14672407	16	68163196	NFATC3	Body	-0,029	1,95E-04	2,50E-02
cg00750166	17	71228632	C17orf80	TSS200	0,005	1,95E-04	2,50E-02
cg12366301	13	21562729	LATS2	Body	-0,006	1,95E-04	2,51E-02
cg09779924	19	2755476	SGTA	3'UTR	-0,006	1,95E-04	2,51E-02
cg09918428	7	128562		IGR	0,008	1,96E-04	2,51E-02
cg18162549	4	136836142	LINC00613	TSS1500	0,055	1,96E-04	2,51E-02
cg24598187	5	96253798	ERAP2	3'UTR	-0,005	1,96E-04	2,51E-02
cg14566904	11	105386196		IGR	-0,008	1,96E-04	2,51E-02
cg22019242	14	73702950	PAPLN	TSS1500	0,029	1,96E-04	2,51E-02
cg00155622	15	41710064	RTF1	Body	-0,006	1,96E-04	2,51E-02
cg14247763	2	74600428	DCTN1	Body	0,014	1,96E-04	2,51E-02
cg02463137	10	125778039	CHST15	Body	-0,015	1,96E-04	2,51E-02
cg08307369	7	128379382	CALU	1stExon	0,007	1,96E-04	2,51E-02
cg12085473	8	20786621		IGR	0,005	1,96E-04	2,51E-02
cg23915527	1	161368787		IGR	0,071	1,96E-04	2,51E-02
cg07990805	16	57392173	CCL22	TSS1500	0,025	1,96E-04	2,51E-02
cg21933626	3	123026636	ADCY5	Body	-0,04	1,96E-04	2,51E-02
cg13880897	6	18336302		IGR	0,016	1,96E-04	2,51E-02
cg21897367	7	142759657	OR6W1P	Body	-0,013	1,96E-04	2,51E-02
cg22250642	22	38349509	C22orf23	5'UTR	-0,004	1,96E-04	2,51E-02
cg15751204	3	138568802		IGR	0,016	1,96E-04	2,51E-02
cg26430287	15	67439567	SMAD3	Body	-0,029	1,96E-04	2,51E-02
cg17040068	7	102715209	ARMC10	TSS200	-0,005	1,97E-04	2,51E-02
cg25374115	22	24564525	CABIN1	Body	0,008	1,97E-04	2,51E-02

cg27071830	10	125934535		IGR	0,018	1,97E-04	2,51E-02
cg07112693	10	73780924		IGR	0,028	1,97E-04	2,51E-02
cg06492144	2	31806352	SRD5A2	TSS1500	-0,048	1,97E-04	2,51E-02
cg20609411	5	7869068	FASTKD3	5'UTR	-0,003	1,97E-04	2,51E-02
cg12797413	17	38217603	THRA	TSS1500	0,015	1,97E-04	2,51E-02
cg06070002	1	45986120	PRDX1	5'UTR	0,045	1,97E-04	2,52E-02
cg07554041	2	121992375	TFCP2L1	Body	-0,016	1,97E-04	2,52E-02
cg17322784	3	176757859	TBL1XR1	Body	-0,018	1,97E-04	2,52E-02
cg16222641	4	150687060		IGR	-0,016	1,97E-04	2,52E-02
cg02708626	6	42055332		IGR	0,006	1,97E-04	2,52E-02
cg06951157	9	130679334	ST6GALNAC4	TSS200	0,014	1,97E-04	2,52E-02
cg27110849	11	86519306	PRSS23	Body	-0,006	1,97E-04	2,52E-02
cg02633398	16	88974944	CBFA2T3	5'UTR	0,023	1,97E-04	2,52E-02
cg20584874	17	39974945	FKBP10	Body	0,009	1,97E-04	2,52E-02
cg04870162	18	72837685		IGR	-0,057	1,97E-04	2,52E-02
cg06624291	20	21494689	NKX2-2	TSS200	-0,013	1,98E-04	2,52E-02
cg10918016	12	124809934	NCOR2	3'UTR	-0,005	1,98E-04	2,52E-02
cg07768118	2	8280378	LINC00299	Body	0,023	1,98E-04	2,52E-02
cg06205922	1	1473141	C1orf70	Body	-0,006	1,98E-04	2,52E-02
cg06440056	5	102878639	LOC102467212	TSS1500	0,024	1,98E-04	2,52E-02
cg03789934	19	56342665	NLRP11	5'UTR	-0,045	1,98E-04	2,52E-02
cg10838844	21	44480699	CBS	Body	0,009	1,98E-04	2,52E-02
cg11022791	12	68051679	DYRK2	Body	-0,008	1,98E-04	2,52E-02
cg00179082	7	73698370		IGR	0,032	1,98E-04	2,53E-02
cg05958346	15	91422794	FURIN	Body	-0,006	1,98E-04	2,53E-02
cg24703400	2	21880071		IGR	-0,014	1,98E-04	2,53E-02
cg09155774	9	96723033		IGR	-0,005	1,99E-04	2,53E-02
cg15652989	16	69222106	SNTB2	Body	-0,005	1,99E-04	2,53E-02
cg21219851	17	78898189	RPTOR	Body	-0,005	1,99E-04	2,53E-02
cg22842444	11	98815846		IGR	-0,029	1,99E-04	2,53E-02
cg13734707	2	10540304	HPCAL1	5'UTR	-0,021	1,99E-04	2,53E-02
cg25557534	3	137237384		IGR	-0,01	2,00E-04	2,53E-02
cg01942790	4	1211419	CTBP1	Body	0,012	1,99E-04	2,53E-02
cg11390255	4	13785203	LINC01182	Body	-0,008	2,00E-04	2,53E-02
cg15612517	5	80032595	MSH3	Body	-0,014	1,99E-04	2,53E-02
cg23450373	5	140254752	PCDHA12	TSS200	-0,05	1,99E-04	2,53E-02
cg07829731	7	34338514		IGR	-0,034	1,99E-04	2,53E-02
cg04752891	7	130969341	MKLN1	Body	0,009	1,99E-04	2,53E-02
cg00698575	9	140388604	PNPLA7	Body	0,039	1,99E-04	2,53E-02
cg13448197	10	43846281		IGR	-0,073	2,00E-04	2,53E-02
cg12851635	11	2826951	KCNQ1	Body	-0,006	1,99E-04	2,53E-02
cg23596308	13	111747937		IGR	-0,029	1,99E-04	2,53E-02
cg10431604	14	24809315	RIPK3	TSS200	-0,004	1,99E-04	2,53E-02
cg08618981	16	15744397	NDE1	5'UTR	-0,005	1,99E-04	2,53E-02
cg12278277	20	34773198	EPB41L1	Body	-0,005	1,99E-04	2,53E-02
cg18579806	21	43758049		IGR	0,014	1,99E-04	2,53E-02
cg18132256	22	24190340		IGR	0,011	1,99E-04	2,53E-02
cg14404318	17	74538788	PRCD	3'UTR	0,013	2,00E-04	2,53E-02
cg06927370	8	127569938	FAM84B	5'UTR	-0,003	2,00E-04	2,53E-02
cg02116437	2	219126018	GPBAR1	5'UTR	0,027	2,00E-04	2,53E-02
cg18031986	3	98001070	OR5H2	TSS1500	-0,024	2,00E-04	2,53E-02
cg14097382	5	140868651	PCDHGC5	TSS200	-0,029	2,00E-04	2,53E-02
cg13611783	6	71377394	SMAP1	TSS200	-0,005	2,00E-04	2,53E-02
cg06134936	10	69834491	HERC4	5'UTR	-0,004	2,00E-04	2,53E-02
cg23318736	14	101779813		IGR	-0,046	2,00E-04	2,53E-02
cg04755227	16	88449254		IGR	0,025	2,00E-04	2,53E-02
cg19455377	20	49942771		IGR	0,019	2,00E-04	2,53E-02
cg09772382	20	57463775	GNAS	3'UTR	0,01	2,00E-04	2,53E-02
cg21665744	7	39171113	POU6F2	Body	-0,076	2,00E-04	2,54E-02
cg06899054	12	90288912		IGR	0,037	2,00E-04	2,54E-02
cg13611742	7	115778454		IGR	-0,013	2,01E-04	2,54E-02
cg03242547	12	111127004	HVCN1	5'UTR	0,006	2,01E-04	2,54E-02
cg20659485	5	132204398	UQCRQ	3'UTR	-0,008	2,01E-04	2,54E-02
cg08234953	16	4817088	ZNF500	5'UTR	0,003	2,01E-04	2,54E-02
cg00005631	6	134957462		IGR	-0,004	2,01E-04	2,54E-02
cg22239605	11	237153	PSMD13	Body	0,007	2,01E-04	2,54E-02
cg04191934	12	48645009		IGR	-0,046	2,01E-04	2,54E-02
cg09574009	13	55211764		IGR	-0,027	2,01E-04	2,54E-02
cg25342674	1	31881983	SERINC2	TSS1500	0,038	2,01E-04	2,54E-02
cg19744691	19	12777225	MORG1	TSS1500	0,021	2,01E-04	2,54E-02
cg05810074	20	58178556	PHACTR3	TSS1500	-0,042	2,01E-04	2,54E-02
cg12256771	9	123691114	TRAF1	5'UTR	-0,008	2,01E-04	2,54E-02
cg11279102	1	110714360	SLC6A17	Body	-0,031	2,02E-04	2,54E-02

cg06282247	2	151281841		IGR	-0,043	2,02E-04	2,54E-02
cg04802959	2	219118769	ARPC2	3'UTR	-0,011	2,02E-04	2,54E-02
cg04498580	4	140478204	SETD7	TSS1500	-0,003	2,02E-04	2,54E-02
cg02104893	6	123109391	SMPDL3A	TSS1500	-0,051	2,02E-04	2,54E-02
cg08163128	7	45744196	ADCY1	ExonBnd	-0,006	2,02E-04	2,54E-02
cg27468419	8	57309109	SDR16C6	TSS1500	-0,021	2,02E-04	2,54E-02
cg14085430	11	114224871		IGR	0,013	2,01E-04	2,54E-02
cg07180351	14	22590742		IGR	-0,009	2,01E-04	2,54E-02
cg13051728	14	68086375	ARG2	TSS1500	-0,003	2,02E-04	2,54E-02
cg12350388	15	57972079	GCOM1	Body	-0,011	2,02E-04	2,54E-02
cg00963305	16	58059520	MMP15	5'UTR	-0,004	2,01E-04	2,54E-02
cg01707233	16	58079272	MMP15	Body	0,011	2,02E-04	2,54E-02
cg08703522	17	44898785		IGR	0,036	2,02E-04	2,54E-02
cg02474116	19	55625184	PPP1R12C	Body	0,011	2,02E-04	2,54E-02
cg01963375	4	70998697	CSN1S2BP	TSS1500	0,036	2,02E-04	2,54E-02
cg13767819	5	64254427	CWC27	Body	0,023	2,02E-04	2,54E-02
cg11662745	5	146020768	PPP2R2B	Body	0,018	2,02E-04	2,54E-02
cg03098614	12	131865659		IGR	-0,042	2,02E-04	2,54E-02
cg06983322	16	728084	RHBDL1	3'UTR	0,014	2,02E-04	2,54E-02
cg24045385	17	13972156	COX10	TSS1500	0,021	2,02E-04	2,54E-02
cg18221304	6	31741184	C6orf27	Body	0,006	2,02E-04	2,54E-02
cg26029682	1	117452580	PTGFRN	TSS200	-0,01	2,03E-04	2,55E-02
cg13613180	11	74560694	XRRA1	Body	0,019	2,03E-04	2,55E-02
cg03427171	1	179053297	TOR3A	Body	0,007	2,03E-04	2,55E-02
cg02046218	2	69747402	SNORA36C	TSS200	-0,004	2,03E-04	2,55E-02
cg22687547	2	168401402		IGR	0,004	2,03E-04	2,55E-02
cg17515245	3	149260247	WWTR1	Body	-0,006	2,03E-04	2,55E-02
cg03868206	3	160284293	KPNA4	TSS1500	0,062	2,03E-04	2,55E-02
cg06005688	4	41872254		IGR	-0,032	2,03E-04	2,55E-02
cg06874945	5	26737262		IGR	-0,023	2,03E-04	2,55E-02
cg08077736	6	33131831	COL11A2	Body	0,017	2,03E-04	2,55E-02
cg17944372	6	76086013	FILIP1	Body	-0,011	2,03E-04	2,55E-02
cg12228863	13	95069222		IGR	-0,044	2,03E-04	2,55E-02
cg18050634	17	79420145	BAHCC1	Body	0,033	2,03E-04	2,55E-02
cg26932594	20	25966424		IGR	-0,043	2,03E-04	2,55E-02
cg19302347	2	236918009	AGAP1	Body	-0,015	2,03E-04	2,55E-02
cg20758814	5	148389337	SH3TC2	Body	-0,006	2,03E-04	2,55E-02
cg13799581	7	117835958	NAA38	3'UTR	-0,05	2,03E-04	2,55E-02
cg01943632	11	118764337	CXCR5	5'UTR	0,015	2,03E-04	2,55E-02
cg16552066	17	65430307	PITPNC1	Body	-0,013	2,03E-04	2,55E-02
cg22794549	21	38339120	HLCS	TSS200	0,018	2,03E-04	2,55E-02
cg21199638	15	74689453		IGR	0,01	2,03E-04	2,55E-02
cg01979389	9	136124866		IGR	-0,017	2,03E-04	2,55E-02
cg17831882	11	41736031	LINC01499	TSS200	-0,021	2,04E-04	2,55E-02
cg09426825	13	52305991	WDFY2	Body	-0,005	2,04E-04	2,55E-02
cg21686532	10	78089701	C10orf11	Body	0,011	2,04E-04	2,55E-02
cg03186986	2	69869962	AAK1	Body	-0,006	2,04E-04	2,55E-02
cg26837161	11	17315992	NUCB2	5'UTR	-0,025	2,04E-04	2,55E-02
cg07794413	5	175829710	CLTB	Body	0,018	2,04E-04	2,55E-02
cg19061957	16	72042348	DHODH	TSS1500	-0,007	2,04E-04	2,55E-02
cg26859261	2	17211580		IGR	-0,033	2,04E-04	2,55E-02
cg13863103	9	98902324		IGR	0,013	2,04E-04	2,55E-02
cg16555533	5	140243006	PCDHA6	Body	-0,053	2,04E-04	2,55E-02
cg16471660	21	30630207	LINC00189	Body	0,006	2,04E-04	2,55E-02
cg12631766	1	184025029	TSEN15	Body	0,019	2,04E-04	2,55E-02
cg02879515	7	92147135	PEX1	Body	0,024	2,04E-04	2,55E-02
cg03027459	5	148408510	SH3TC2	Body	-0,006	2,04E-04	2,55E-02
cg17039369	21	46341006	ITGB2	TSS200	-0,004	2,04E-04	2,55E-02
cg03184001	5	53386708	ARL15	Body	-0,041	2,04E-04	2,55E-02
cg15510325	7	150669058	KCNH2	Body	0,005	2,05E-04	2,55E-02
cg01630519	17	43992416	MAPT	5'UTR	0,022	2,05E-04	2,55E-02
cg21252552	9	139144428		IGR	-0,009	2,05E-04	2,56E-02
cg16696306	1	32691278	EIF3I	Body	-0,007	2,05E-04	2,56E-02
cg16815651	10	1466996	ADARB2	Body	0,03	2,05E-04	2,56E-02
cg02775854	3	111396833	PLCXD2-AS1	TSS1500	-0,007	2,05E-04	2,56E-02
cg12229899	2	113089657	ZC3H6	Body	0,011	2,05E-04	2,56E-02
cg26619047	5	139028068	CXXC5	TSS1500	-0,004	2,05E-04	2,56E-02
cg24880997	6	30079662	TRIM31	Body	0,024	2,05E-04	2,56E-02
cg10583931	5	75379800	SV2C	5'UTR	-0,009	2,05E-04	2,56E-02
cg13813770	9	139001804		IGR	-0,003	2,05E-04	2,56E-02
cg08935663	5	140309344	PCDHAC1	1stExon	0,01	2,05E-04	2,56E-02
cg14543953	11	102669090	MMP1	TSS200	-0,012	2,05E-04	2,56E-02
cg27168493	1	67636724	IL23R	Body	-0,007	2,06E-04	2,56E-02

cg21290042	1	224804053	CNIH3	TSS200	-0,014	2,06E-04	2,56E-02
cg07472196	3	99801503	C3orf26	Body	0,011	2,06E-04	2,56E-02
cg16051275	6	4603564		IGR	0,045	2,06E-04	2,56E-02
cg26091290	6	19182028		IGR	-0,008	2,06E-04	2,56E-02
cg02229292	6	134583282	SGK1	ExonBnd	-0,016	2,06E-04	2,56E-02
cg22664307	6	144471564	STX11	TSS200	-0,004	2,06E-04	2,56E-02
cg22820726	11	1401914		IGR	-0,033	2,06E-04	2,56E-02
cg09917056	12	69084425	NUP107	ExonBnd	0,018	2,06E-04	2,56E-02
cg09750057	16	82206435		IGR	-0,016	2,06E-04	2,56E-02
cg19747604	19	46915570	CCDC8	1stExon	0,037	2,06E-04	2,56E-02
cg04100007	1	2075739	PRKCZ	5'UTR	0,007	2,06E-04	2,56E-02
cg08359167	11	2721351	KCNQ1OT1	TSS200	0,013	2,06E-04	2,56E-02
cg17730424	3	107869372		IGR	0,007	2,06E-04	2,56E-02
cg00791249	19	10426722	FDX1L	TSS200	-0,004	2,06E-04	2,56E-02
cg25940557	1	158575177	OR10Z1	TSS1500	-0,017	2,06E-04	2,57E-02
cg12170514	2	37663557		IGR	0,011	2,06E-04	2,57E-02
cg03809403	4	100571622		IGR	0,008	2,06E-04	2,57E-02
cg17926234	2	61404587	AHSA2	1stExon	0,007	2,07E-04	2,57E-02
cg22115076	5	50673588		IGR	0,032	2,07E-04	2,57E-02
cg24336646	6	132834059	STX7	5'UTR	-0,004	2,07E-04	2,57E-02
cg01404850	15	43533460	TGM5	Body	0,019	2,07E-04	2,57E-02
cg22761607	19	4494084	HDGF2	Body	-0,004	2,07E-04	2,57E-02
cg15116481	19	51330265	KLK15	Body	-0,052	2,07E-04	2,57E-02
cg15156367	1	92495004	EPHX4	TSS1500	-0,018	2,07E-04	2,57E-02
cg04735635	2	179032767		IGR	0,012	2,07E-04	2,57E-02
cg21393986	3	196159244	UBXN7	1stExon	-0,005	2,07E-04	2,57E-02
cg10702113	3	196664466	NCBP2	Body	0,004	2,07E-04	2,57E-02
cg09456340	5	5288296	ADAMTS16	Body	-0,01	2,07E-04	2,57E-02
cg01798062	5	125379942		IGR	-0,043	2,07E-04	2,57E-02
cg09708488	8	21882013	NPM2	TSS1500	-0,008	2,07E-04	2,57E-02
cg09461494	8	100262768	VPS13B	Body	0,045	2,07E-04	2,57E-02
cg25038330	10	463561	DIP2C	Body	-0,03	2,07E-04	2,57E-02
cg21404224	15	23086897	NIPA1	TSS200	-0,003	2,07E-04	2,57E-02
cg05620814	16	74640948	GLG1	Body	-0,004	2,07E-04	2,57E-02
cg19497548	1	26249349		IGR	-0,007	2,08E-04	2,57E-02
cg18415320	19	34576879		IGR	-0,025	2,08E-04	2,57E-02
cg14793596	1	1290092	MXRA8	Body	-0,036	2,08E-04	2,57E-02
cg04421631	3	72495876	RYBP	TSS200	-0,004	2,08E-04	2,57E-02
cg24175137	3	192239164	FGF12	Body	0,035	2,08E-04	2,57E-02
cg11613229	5	119800742	PRR16	5'UTR	0,011	2,08E-04	2,57E-02
cg22146644	6	155184017		IGR	0,02	2,08E-04	2,57E-02
cg17604071	12	114195388	LINC01234	Body	-0,041	2,08E-04	2,57E-02
cg20212077	13	68194226		IGR	-0,029	2,08E-04	2,57E-02
cg04738447	16	708575	WDR90	Body	-0,008	2,08E-04	2,57E-02
cg15035117	16	27410868		IGR	0,034	2,08E-04	2,57E-02
cg01295034	16	48576092	N4BP1	3'UTR	0,035	2,08E-04	2,57E-02
cg05746370	16	87746161	KLHDC4	Body	0,01	2,08E-04	2,57E-02
cg14976348	1	217448814		IGR	-0,03	2,08E-04	2,57E-02
cg18257840	15	70820860		IGR	-0,01	2,08E-04	2,58E-02
cg22876643	1	68962318	DEPDC1	Body	0,009	2,08E-04	2,58E-02
cg03793846	5	140894047		IGR	0,036	2,08E-04	2,58E-02
cg02173128	1	6202177	CHD5	Body	0,007	2,09E-04	2,58E-02
cg17719126	6	33383819	PHF1	Body	0,014	2,09E-04	2,58E-02
cg09668400	13	26625325	SHISA2	TSS200	-0,013	2,09E-04	2,58E-02
cg03049399	11	82429840		IGR	0,006	2,09E-04	2,58E-02
cg10152198	1	19711006	CAPZB	Body	0,032	2,09E-04	2,58E-02
cg02005157	11	34663205	EHF	5'UTR	0,011	2,09E-04	2,58E-02
cg10973879	13	79944111	RBM26	Body	-0,009	2,09E-04	2,58E-02
cg01388889	13	112508206		IGR	-0,04	2,09E-04	2,58E-02
cg17695831	19	55038337		IGR	0,013	2,09E-04	2,58E-02
cg05799343	1	33635646	TRIM62	Body	0,013	2,09E-04	2,58E-02
cg13946500	19	10217312	PPAN-P2RY11	Body	-0,004	2,09E-04	2,58E-02
cg24088764	4	126641308		IGR	0,017	2,09E-04	2,58E-02
cg21972470	3	108308413	KIAA1524	1stExon	-0,005	2,10E-04	2,58E-02
cg05422630	22	34119540	LARGE-AS1	TSS1500	-0,024	2,10E-04	2,59E-02
cg08065241	3	56502265	ERC2	5'UTR	-0,008	2,10E-04	2,59E-02
cg03120361	11	16946921	PLEKHA7	Body	0,008	2,10E-04	2,59E-02
cg09163745	5	131429287		IGR	0,011	2,10E-04	2,59E-02
cg11513483	13	90378588		IGR	-0,042	2,10E-04	2,59E-02
cg09006915	9	126960331		IGR	0,036	2,10E-04	2,59E-02
cg01980562	19	1174207	SBNO2	1stExon	0,004	2,10E-04	2,59E-02
cg00259367	1	21057579	SH2D5	5'UTR	0,017	2,10E-04	2,59E-02
cg23953309	6	112193859	FYN	5'UTR	-0,029	2,11E-04	2,59E-02

cg21467904	8	118532775	MED30	TSS200	-0,004	2,11E-04	2,60E-02
cg18666327	17	53279016		IGR	-0,021	2,11E-04	2,60E-02
cg16381776	12	4680531		IGR	0,015	2,11E-04	2,60E-02
cg19539972	4	7069911	GRPEL1	TSS200	-0,003	2,11E-04	2,60E-02
cg10462187	10	124067402	BTBD16	Body	0,04	2,11E-04	2,60E-02
cg12451530	2	54087209	LOC100302652	TSS200	-0,034	2,11E-04	2,60E-02
cg16947612	2	56103666	EFEMP1	Body	0,03	2,11E-04	2,60E-02
cg15453599	14	50155052	POLE2	TSS200	-0,005	2,11E-04	2,60E-02
cg03436453	1	27816827	WASF2	TSS200	-0,007	2,11E-04	2,60E-02
cg09680131	13	111100754	COL4A2	Body	-0,006	2,11E-04	2,60E-02
cg14428139	19	21439793		IGR	-0,021	2,12E-04	2,60E-02
cg22820238	20	11826062	LINC00687	Body	-0,019	2,12E-04	2,60E-02
cg16784006	2	21874939		IGR	-0,017	2,12E-04	2,60E-02
cg09393551	14	31764703	HEATR5A	Body	-0,02	2,12E-04	2,60E-02
cg27353579	17	49337712	MBTD1	TSS1500	-0,003	2,12E-04	2,60E-02
cg20486563	17	79256081	SLC38A10	Body	0,02	2,12E-04	2,60E-02
cg15226160	19	11727694	ZNF627	Body	-0,009	2,12E-04	2,60E-02
cg21591174	4	12867006		IGR	0,051	2,12E-04	2,60E-02
cg12768483	17	1268969	YWHAE	Body	-0,011	2,12E-04	2,60E-02
cg08951834	17	38656877	TNS4	5'UTR	0,009	2,12E-04	2,60E-02
cg14804102	16	89339261	ANKRD11	Body	-0,017	2,12E-04	2,60E-02
cg20191360	1	27930944	AHDC1	TSS1500	-0,006	2,12E-04	2,60E-02
cg21907625	22	37098833	CACNG2	TSS200	-0,061	2,12E-04	2,60E-02
cg13915156	2	39958154		IGR	-0,009	2,12E-04	2,60E-02
cg10268784	1	45113588	RNF220	Body	0,016	2,12E-04	2,60E-02
cg14651910	7	105163122	PUS7	TSS1500	0,051	2,12E-04	2,60E-02
cg14667123	8	35235594	UNC5D	Body	-0,051	2,13E-04	2,60E-02
cg12956209	11	32851240	PRRG4	TSS1500	-0,002	2,12E-04	2,60E-02
cg02530375	11	69706831		IGR	0,04	2,13E-04	2,60E-02
cg15736240	12	126727103		IGR	-0,012	2,13E-04	2,60E-02
cg16243388	12	118812981	SUDS3	TSS1500	0,012	2,13E-04	2,60E-02
cg21100518	6	29595002	GABBR1	Body	0,048	2,13E-04	2,61E-02
cg12157788	7	4389134		IGR	-0,026	2,13E-04	2,61E-02
cg19969431	14	74321094	PTGR2	5'UTR	0,009	2,13E-04	2,61E-02
cg04862264	3	184086153	POLR2H	3'UTR	-0,004	2,13E-04	2,61E-02
cg11314617	8	72757456	MSC	TSS1500	-0,038	2,13E-04	2,61E-02
cg23081079	3	33138872	GLB1	TSS200	-0,004	2,13E-04	2,61E-02
cg00414166	11	67777664	ALDH3B1	TSS200	-0,011	2,13E-04	2,61E-02
cg08397665	5	140944122	LOC100505658	Body	-0,034	2,13E-04	2,61E-02
cg20630633	15	91100491	CRTC3	Body	-0,033	2,13E-04	2,61E-02
cg27334938	18	77167042	NFATC1	Body	-0,008	2,13E-04	2,61E-02
cg00007932	13	47171977	LRCH1	Body	0,032	2,13E-04	2,61E-02
cg21433330	11	31390770	DNAJC24	TSS1500	-0,052	2,14E-04	2,61E-02
cg16554933	16	4401344	Magmas	TSS200	-0,004	2,14E-04	2,61E-02
cg07989514	14	77495178	C14orf4	TSS200	-0,004	2,14E-04	2,61E-02
cg08236017	17	7452258	TNFSF12	TSS200	-0,003	2,14E-04	2,61E-02
cg15197068	3	10068042	FANCD2	TSS200	-0,005	2,14E-04	2,61E-02
cg16962392	19	50755467	MYH14	Body	0,014	2,14E-04	2,61E-02
cg14426510	17	48796535	LUC7L3	TSS1500	0,014	2,14E-04	2,61E-02
cg24205332	11	130298700	ADAMTS8	TSS200	0,011	2,14E-04	2,61E-02
cg06296640	2	47270917	TTC7A	Body	0,025	2,14E-04	2,61E-02
cg10673243	3	111602669	PHLDB2	5'UTR	0,008	2,14E-04	2,62E-02
cg06555319	5	1408879	SLC6A3	Body	-0,026	2,14E-04	2,62E-02
cg13984181	1	92495720	EPHX4	1stExon	-0,005	2,15E-04	2,62E-02
cg08975641	2	189959552	COL5A2	Body	-0,061	2,15E-04	2,62E-02
cg07583420	11	2158555	INS-IGF2	Body	-0,006	2,15E-04	2,62E-02
cg05231763	19	4311907	FSD1	Body	0,006	2,15E-04	2,62E-02
cg20348680	7	16720938	BZW2	ExonBnd	-0,009	2,15E-04	2,62E-02
cg07670676	10	100171689	PYROXD2	Body	-0,008	2,15E-04	2,62E-02
cg08942716	17	7835080	TRAPPC1	Body	-0,003	2,15E-04	2,62E-02
cg07454239	20	3219465	SLC4A11	TSS1500	-0,003	2,15E-04	2,62E-02
cg06300141	3	32612708	DYNC1LI1	TSS1500	-0,014	2,15E-04	2,62E-02
cg06072914	3	45730538	SACM1L	TSS1500	0,02	2,15E-04	2,62E-02
cg14948436	6	39290159	KCNK16	1stExon	0,013	2,15E-04	2,62E-02
cg09340485	9	112670245	PALM2	Body	0,013	2,15E-04	2,62E-02
cg24152351	9	124982326	LHX6	5'UTR	0,047	2,15E-04	2,62E-02
cg22017900	11	28855503		IGR	-0,048	2,15E-04	2,62E-02
cg21707740	14	61501895	SLC38A6	Body	0,023	2,15E-04	2,62E-02
cg12346637	15	93564113	CHD2	Body	0,008	2,15E-04	2,62E-02
cg26573952	6	17987707	KIF13A	1stExon	-0,006	2,16E-04	2,62E-02
cg12557447	6	143091154	HIVEP2	Body	-0,008	2,16E-04	2,62E-02
cg14908986	11	70120720	PPFIA1	Body	-0,016	2,16E-04	2,62E-02
cg18642567	14	21755798	RPGRIP1	TSS1500	-0,022	2,16E-04	2,62E-02

cg13050144	19	55427040		IGR	0,023	2,16E-04	2,62E-02
cg08796640	8	119123054	EXT1	1stExon	0,008	2,16E-04	2,62E-02
cg11200414	1	213405366	RPS6KC1	Body	-0,011	2,16E-04	2,62E-02
cg21376738	3	10289910	TATDN2	TSS1500	-0,004	2,16E-04	2,62E-02
cg23797995	11	61131994	TMEM138	Body	0,008	2,16E-04	2,62E-02
cg15331260	12	110461411	ANKRD13A	Body	-0,021	2,16E-04	2,62E-02
cg13114145	22	17847774		IGR	-0,016	2,16E-04	2,62E-02
cg01081634	11	65490737		IGR	0,019	2,16E-04	2,62E-02
cg17844553	12	11322611	PRR4	5'UTR	-0,009	2,16E-04	2,62E-02
cg13995022	17	21075124	DHRS7B	Body	0,026	2,16E-04	2,62E-02
cg22367962	12	132195279	SFRS8	TSS1500	-0,003	2,16E-04	2,62E-02
cg05051369	1	229404111		IGR	-0,006	2,16E-04	2,62E-02
cg01965247	15	40702776	IVD	Body	-0,005	2,16E-04	2,62E-02
cg19309752	1	117695016	VTCN1	Body	0,014	2,16E-04	2,63E-02
cg04829878	9	128464166	MAPKAP1	5'UTR	-0,006	2,16E-04	2,63E-02
cg00148035	12	111543137	CUX2	Body	0,029	2,17E-04	2,63E-02
cg03860494	5	123143225		IGR	0,011	2,17E-04	2,63E-02
cg15706657	6	167571324	GPR31	TSS200	0,035	2,17E-04	2,63E-02
cg25898710	12	113456875		IGR	0,007	2,17E-04	2,63E-02
cg18175468	13	78137493	SCEL	Body	-0,047	2,17E-04	2,63E-02
cg17004294	3	183220738	KLHL6	Body	-0,016	2,17E-04	2,63E-02
cg27199295	1	241162312	RGS7	Body	-0,006	2,17E-04	2,63E-02
cg20460852	8	23082822	TNFRSF10A	TSS200	0,017	2,17E-04	2,63E-02
cg20595215	3	8543014	LMCD1	TSS1500	0,012	2,17E-04	2,63E-02
cg03889013	10	134143489		IGR	-0,009	2,17E-04	2,63E-02
cg11634827	9	95858267	C9orf89	TSS200	0,032	2,17E-04	2,63E-02
cg22877411	3	48412890	FBXW12	TSS1500	0,01	2,17E-04	2,63E-02
cg09832661	7	151145737		IGR	-0,007	2,17E-04	2,63E-02
cg09123813	22	31836032	EIF4ENIF1	Body	0,007	2,17E-04	2,63E-02
cg12585516	4	83123547		IGR	-0,016	2,18E-04	2,63E-02
cg00643253	16	28844428	ATXN2L	Body	-0,015	2,18E-04	2,63E-02
cg08546829	3	186819195		IGR	-0,005	2,18E-04	2,63E-02
cg06819431	1	153605367	C1orf77	TSS1500	0,006	2,18E-04	2,64E-02
cg13809915	2	158789111		IGR	-0,005	2,18E-04	2,64E-02
cg00911289	3	128840257	RAB43	1stExon	-0,004	2,18E-04	2,64E-02
cg00539817	5	179831375		IGR	-0,02	2,18E-04	2,64E-02
cg15415953	6	2636435	C6orf195	TSS1500	-0,023	2,18E-04	2,64E-02
cg12103547	6	169050956	SMOC2	Body	-0,016	2,18E-04	2,64E-02
cg16340422	17	17110120	PLD6	TSS1500	-0,074	2,18E-04	2,64E-02
cg27581965	20	50179190	NFATC2	TSS200	-0,004	2,18E-04	2,64E-02
cg11406373	5	156751406	CYFIP2	Body	-0,004	2,18E-04	2,64E-02
cg20982209	3	17807615		IGR	0,011	2,18E-04	2,64E-02
cg13922121	11	19575262	NAV2	Body	0,032	2,18E-04	2,64E-02
cg07272042	16	85860163		IGR	0,018	2,18E-04	2,64E-02
cg12825303	6	3153338	BPHL	3'UTR	0,019	2,18E-04	2,64E-02
cg02459107	1	67143332	SGIP1	Body	-0,018	2,19E-04	2,64E-02
cg14635629	9	140768247		IGR	-0,033	2,19E-04	2,64E-02
cg20407067	3	46907844		IGR	0,017	2,19E-04	2,64E-02
cg15081667	5	132058126	KIF3A	Body	0,009	2,19E-04	2,64E-02
cg20975297	12	77719732		IGR	-0,029	2,19E-04	2,64E-02
cg02269431	16	67738256	GFOD2	Body	-0,007	2,19E-04	2,64E-02
cg16923536	17	29926485		IGR	-0,016	2,19E-04	2,64E-02
cg07777362	12	88785541		IGR	-0,017	2,19E-04	2,64E-02
cg01231025	7	44269984	CAMK2B	Body	0,008	2,19E-04	2,64E-02
cg01553675	14	76299110	TTLL5	Body	-0,009	2,19E-04	2,64E-02
cg06412358	21	34392373		IGR	-0,006	2,19E-04	2,64E-02
cg17067993	17	38721675	CCR7	5'UTR	-0,003	2,19E-04	2,64E-02
cg10489284	5	1088244	SLC12A7	Body	0,006	2,19E-04	2,64E-02
cg27354635	5	75770361	IQGAP2	Body	0,023	2,20E-04	2,64E-02
cg24636944	5	92615296		IGR	-0,03	2,20E-04	2,64E-02
cg20464732	8	111073225		IGR	-0,013	2,19E-04	2,64E-02
cg15017865	17	39933462	JUP	5'UTR	0,025	2,19E-04	2,64E-02
cg00339695	16	24857497	SLC5A11	TSS200	0,039	2,20E-04	2,64E-02
cg01141656	3	126840322		IGR	-0,03	2,20E-04	2,65E-02
cg27343208	4	2363117	ZFYVE28	Body	-0,007	2,20E-04	2,65E-02
cg07109603	12	24049014	SOX5	Body	-0,084	2,20E-04	2,65E-02
cg24858738	6	13711938	RANBP9	TSS200	0,012	2,20E-04	2,65E-02
cg19288356	10	29950329	SVIL	5'UTR	0,009	2,20E-04	2,65E-02
cg05128566	7	95401838	DYNC1I1	1stExon	0,012	2,20E-04	2,65E-02
cg19819774	14	52437206		IGR	0,007	2,20E-04	2,65E-02
cg10473367	16	1309550		IGR	-0,037	2,20E-04	2,65E-02
cg27059302	17	61227755	TANC2	Body	0,015	2,20E-04	2,65E-02
cg07434260	19	10698036	AP1M2	TSS200	-0,028	2,20E-04	2,65E-02

cg21537729	4	15682922	LOC285550	TSS1500	-0,004	2,20E-04	2,65E-02
cg21882990	6	111902385	TRAF3IP2-AS1	Body	0,008	2,20E-04	2,65E-02
cg01253160	11	124747172	ROBO3	Body	-0,034	2,20E-04	2,65E-02
cg03575602	10	12390599	CAMK1D	TSS1500	0,024	2,21E-04	2,65E-02
cg25312694	7	17500466	KCCAT333	Body	-0,017	2,21E-04	2,65E-02
cg18750738	5	72595322		IGR	-0,006	2,21E-04	2,65E-02
cg01704862	7	150780657	TMUB1	TSS200	0,006	2,21E-04	2,65E-02
cg03844063	20	43992051	SYS1	5'UTR	-0,005	2,21E-04	2,65E-02
cg05338505	7	5609674		IGR	-0,003	2,21E-04	2,65E-02
cg24840042	1	77550315		IGR	-0,005	2,21E-04	2,65E-02
cg13944838	5	179740914	GFPT2	Body	-0,123	2,21E-04	2,65E-02
cg20656604	6	41800452	USP49	5'UTR	0,011	2,21E-04	2,65E-02
cg14326472	9	126164083	DENND1A	3'UTR	0,013	2,21E-04	2,65E-02
cg00045910	10	23466070		IGR	-0,063	2,21E-04	2,65E-02
cg22301418	13	113514151	ATP11A	Body	0,017	2,21E-04	2,65E-02
cg08214029	17	34391478	CCL18	TSS200	0,022	2,21E-04	2,65E-02
cg14102530	20	52680924	BCAS1	5'UTR	-0,113	2,21E-04	2,65E-02
cg07919145	22	39780408	SYNGR1	3'UTR	0,03	2,21E-04	2,65E-02
cg20757133	3	21370390		IGR	-0,025	2,21E-04	2,65E-02
cg00554352	15	90200115	KIF7	TSS1500	0,009	2,21E-04	2,65E-02
cg20105042	16	71470299		IGR	-0,031	2,22E-04	2,65E-02
cg13084560	1	8418823	RERE	Body	0,004	2,22E-04	2,65E-02
cg25500028	3	187336889		IGR	-0,04	2,22E-04	2,65E-02
cg23195199	4	89723908	FAM13A	Body	-0,011	2,22E-04	2,65E-02
cg07148341	6	43714275		IGR	0,016	2,22E-04	2,65E-02
cg02103895	6	127665881	ECHDC1	TSS1500	0,006	2,22E-04	2,65E-02
cg17092391	7	73515902	LIMK1	Body	0,021	2,22E-04	2,65E-02
cg01250407	7	116964802	WNT2	TSS1500	-0,009	2,22E-04	2,65E-02
cg09332295	8	11201258	TDH	Body	0,013	2,22E-04	2,65E-02
cg20299200	9	15738284	CCDC171	Body	0,026	2,22E-04	2,65E-02
cg21791725	12	120222364	CIT	Body	-0,003	2,22E-04	2,65E-02
cg24167790	14	66106053	FUT8	Body	0,011	2,22E-04	2,65E-02
cg12406047	16	59912592	LOC101927580	Body	-0,013	2,22E-04	2,65E-02
cg12318408	17	2699802	RAP1GAP2	1stExon	-0,003	2,22E-04	2,65E-02
cg14121185	17	64488849	PRKCA	Body	0,049	2,22E-04	2,65E-02
cg25622125	15	28365984	HERC2	Body	-0,005	2,22E-04	2,65E-02
cg04578489	2	137913401	THSD7B	Body	-0,025	2,22E-04	2,65E-02
cg23203817	17	76433758	DNAH17	Body	0,009	2,22E-04	2,65E-02
cg12306444	21	29093202	NCRNA00113	TSS1500	-0,01	2,22E-04	2,65E-02
cg13641532	1	29502946	SRSF4	Body	-0,006	2,23E-04	2,65E-02
cg25404394	1	109102539	FAM102B	TSS1500	-0,005	2,23E-04	2,65E-02
cg00951717	1	210651095	HHAT	Body	0,014	2,23E-04	2,65E-02
cg19631792	8	74222249	RDH10	Body	-0,022	2,22E-04	2,65E-02
cg03638886	12	68381781	IFNG-AS1	TSS1500	0,018	2,23E-04	2,65E-02
cg18492839	13	20138761		IGR	0,011	2,22E-04	2,65E-02
cg18519993	15	65046138	RBPMS2	Body	0,02	2,22E-04	2,65E-02
cg22684787	16	766403	METRN	Body	0,034	2,22E-04	2,65E-02
cg22176903	16	57843979	LOC388282	TSS1500	0,031	2,22E-04	2,65E-02
cg08952590	19	53466322	ZNF816A	TSS1500	-0,003	2,23E-04	2,65E-02
cg07376282	7	124404324	GPR37	1stExon	-0,033	2,23E-04	2,66E-02
cg23802307	13	112575212		IGR	-0,04	2,23E-04	2,66E-02
cg04778907	9	125314394	OR1N2	TSS1500	-0,042	2,23E-04	2,66E-02
cg23564309	21	45582448		IGR	-0,018	2,23E-04	2,66E-02
cg21715655	1	223816422	CAPN8	Body	-0,01	2,23E-04	2,66E-02
cg01132589	2	210444475	MAP2	1stExon	0,034	2,23E-04	2,66E-02
cg20111187	4	3882339		IGR	-0,01	2,23E-04	2,66E-02
cg21740359	6	30434324		IGR	-0,018	2,23E-04	2,66E-02
cg22681721	6	35477358	TULP1	Body	0,022	2,23E-04	2,66E-02
cg12463893	9	139259372	DNLZ	TSS1500	0,02	2,23E-04	2,66E-02
cg25958158	10	105041668	INA	Body	-0,008	2,23E-04	2,66E-02
cg05469396	15	91419421	FURIN	Body	-0,008	2,23E-04	2,66E-02
cg10180052	16	2018558	RNF151	Body	-0,056	2,23E-04	2,66E-02
cg27017875	16	12153035	SNX29	Body	0,007	2,23E-04	2,66E-02
cg22212727	17	67589937	LINC01483	TSS200	0,011	2,23E-04	2,66E-02
cg03632204	19	17212818	MYO9B	Body	0,01	2,23E-04	2,66E-02
cg22327543	22	37536156	IL2RB	Body	0,019	2,23E-04	2,66E-02
cg04059695	4	139787977		IGR	0,008	2,24E-04	2,66E-02
cg25358913	7	73511246	LIMK1	Body	0,006	2,24E-04	2,66E-02
cg21486779	15	34530741	SLC12A6	Body	-0,007	2,24E-04	2,66E-02
cg15482003	16	2039745	SYNGR3	TSS200	-0,018	2,24E-04	2,66E-02
cg02377915	22	22307573	PPM1F	TSS1500	0,015	2,24E-04	2,66E-02
cg12227008	22	35947609	RASD2	Body	-0,008	2,24E-04	2,66E-02
cg11661318	8	11303067	FAM167A	5'UTR	0,012	2,24E-04	2,66E-02

cg21609975	5	95409135		IGR	-0,007	2,24E-04	2,66E-02
cg25717214	15	74521182		IGR	0,039	2,24E-04	2,66E-02
cg24237381	15	67296121		IGR	-0,008	2,24E-04	2,66E-02
cg04900093	1	37791904		IGR	0,053	2,24E-04	2,66E-02
cg21895377	5	148293288		IGR	-0,008	2,24E-04	2,66E-02
cg11723270	2	154450473		IGR	0,011	2,24E-04	2,66E-02
cg27530704	1	27278570	C1orf172	Body	0,015	2,24E-04	2,66E-02
cg08574044	5	142361829	ARHGAP26	Body	0,034	2,24E-04	2,66E-02
cg13362574	15	65596329		IGR	-0,007	2,24E-04	2,66E-02
cg23026896	15	36723660		IGR	-0,019	2,24E-04	2,66E-02
cg25213550	7	151692197	GALNTL5	Body	0,013	2,24E-04	2,66E-02
cg23134075	14	61517513	SLC38A6	Body	-0,007	2,25E-04	2,66E-02
cg05412045	2	3337680	TSSC1	Body	0,007	2,25E-04	2,66E-02
cg09351035	11	748060	TALDO1	Body	-0,004	2,25E-04	2,66E-02
cg20067801	17	15401641		IGR	0,026	2,25E-04	2,66E-02
cg25101939	3	183860430	EIF2B5	Body	-0,007	2,25E-04	2,66E-02
cg12677723	19	15559494	MIR1470	TSS1500	0,016	2,25E-04	2,66E-02
cg18174779	1	57822544	DAB1	5'UTR	-0,02	2,25E-04	2,67E-02
cg01934258	7	36546644		IGR	-0,033	2,25E-04	2,67E-02
cg16666160	9	127269631	NR5A1	1stExon	-0,007	2,25E-04	2,67E-02
cg09849246	14	96241403		IGR	-0,007	2,25E-04	2,67E-02
cg27147164	14	103305463	TRAF3	5'UTR	0,005	2,25E-04	2,67E-02
cg08340583	4	178302630		IGR	-0,017	2,25E-04	2,67E-02
cg11622008	3	140769610	SPSB4	TSS1500	-0,01	2,26E-04	2,67E-02
cg09532503	4	119711185	SEC24D	Body	0,015	2,26E-04	2,67E-02
cg27219662	6	28891728	TRIM27	5'UTR	0,009	2,26E-04	2,67E-02
cg22659772	8	49221193		IGR	0,03	2,26E-04	2,67E-02
cg03872905	17	61539517		IGR	0,006	2,26E-04	2,67E-02
cg01234517	7	150021553	ACTR3C	TSS1500	0,015	2,26E-04	2,67E-02
cg06208615	12	58115115	OS9	3'UTR	0,033	2,26E-04	2,67E-02
cg04191437	1	36772694	SH3D21	TSS200	-0,005	2,26E-04	2,67E-02
cg14800014	5	125800764	GRAMD3	Body	-0,01	2,26E-04	2,67E-02
cg05557618	9	136326006	CACFD1	Body	0,034	2,26E-04	2,67E-02
cg06454410	19	10420374	ZGLP1	TSS200	0,005	2,26E-04	2,67E-02
cg14294444	9	128003255	HSPA5	Body	-0,003	2,26E-04	2,67E-02
cg24906129	6	169364525		IGR	0,016	2,27E-04	2,68E-02
cg17094014	6	41605343	MDFI	TSS1500	0,016	2,27E-04	2,68E-02
cg27596843	11	27218405	BBOX1-AS1	Body	-0,047	2,27E-04	2,68E-02
cg06251561	17	438379	VPS53	Body	0,024	2,27E-04	2,68E-02
cg13229608	7	95937185	SLC25A13	Body	-0,009	2,27E-04	2,68E-02
cg03082830	7	1165370	C7orf50	Body	-0,018	2,27E-04	2,68E-02
cg20014207	2	241234933		IGR	-0,005	2,28E-04	2,68E-02
cg08074480	1	160222204	DCAF8	Body	-0,035	2,28E-04	2,68E-02
cg09657260	9	21690064		IGR	0,06	2,28E-04	2,68E-02
cg13301731	12	128796190	TMEM132C	Body	-0,025	2,28E-04	2,68E-02
cg12367415	6	33868187		IGR	0,011	2,28E-04	2,68E-02
cg20947088	9	138811046		IGR	-0,019	2,28E-04	2,69E-02
cg08470393	14	74569727	LIN52	Body	0,009	2,28E-04	2,69E-02
cg07031551	11	72463331	ARAP1	5'UTR	-0,004	2,28E-04	2,69E-02
cg04310802	5	2005957		IGR	-0,019	2,28E-04	2,69E-02
cg16652817	7	54956598		IGR	-0,063	2,28E-04	2,69E-02
cg01181485	4	153454027	FBXW7	5'UTR	0,018	2,28E-04	2,69E-02
cg07898084	13	53029385	CKAP2	TSS200	-0,003	2,28E-04	2,69E-02
cg21528528	1	20703949	LINC01141	Body	0,012	2,28E-04	2,69E-02
cg05924987	4	1407796		IGR	-0,027	2,28E-04	2,69E-02
cg01403103	16	112852	RHBDF1	Body	0,009	2,29E-04	2,69E-02
cg20111340	19	39935944	SUPT5H	TSS1500	0,006	2,29E-04	2,69E-02
cg01143774	7	31483139		IGR	-0,005	2,29E-04	2,69E-02
cg22901226	19	40597369	ZNF780A	TSS1500	-0,01	2,29E-04	2,69E-02
cg19049344	2	166095825	SCN2A	TSS200	-0,004	2,29E-04	2,69E-02
cg25891355	13	58205111	PCDH17	TSS1500	-0,015	2,29E-04	2,69E-02
cg07876882	5	132151047	ANKRD43	3'UTR	-0,006	2,29E-04	2,70E-02
cg01931502	1	161284176	SDHC	5'UTR	-0,005	2,29E-04	2,70E-02
cg00171704	14	67042580	GPHN	Body	-0,015	2,29E-04	2,70E-02
cg04282723	11	975233	AP2A2	Body	-0,018	2,30E-04	2,70E-02
cg17058616	17	73102145	SLC16A5	3'UTR	0,017	2,30E-04	2,70E-02
cg03559813	4	189923233		IGR	-0,021	2,30E-04	2,70E-02
cg04538551	15	25344249	SNORD116-26	TSS1500	-0,062	2,30E-04	2,70E-02
cg08809956	1	229256312		IGR	0,006	2,30E-04	2,70E-02
cg07406797	2	10818165	NOL10	Body	0,01	2,30E-04	2,70E-02
cg17933893	2	179401935	TTN-AS1	Body	0,025	2,30E-04	2,70E-02
cg13483431	3	124949647	ZNF148	3'UTR	0,011	2,30E-04	2,70E-02
cg25687071	3	136751404		IGR	-0,005	2,30E-04	2,70E-02

cg23843620	6	33238264	VPS52	Body	0,006	2,30E-04	2,70E-02
cg17995050	7	55073248		IGR	-0,059	2,30E-04	2,70E-02
cg16683015	8	143171268		IGR	-0,017	2,30E-04	2,70E-02
cg25548966	11	68351732	PPP6R3	Body	0,024	2,30E-04	2,70E-02
cg16986720	11	84634735	DLG2	TSS1500	-0,008	2,30E-04	2,70E-02
cg09120724	17	56494602	RNF43	5'UTR	0,046	2,30E-04	2,70E-02
cg04967200	10	134115376	STK32C	Body	0,02	2,30E-04	2,70E-02
cg03353971	17	31254616	TMEM98	TSS1500	0,028	2,30E-04	2,70E-02
cg20114462	20	11830300	LINC00687	Body	-0,006	2,31E-04	2,70E-02
cg11321030	10	103326711		IGR	0,043	2,31E-04	2,70E-02
cg07621727	14	57496603		IGR	-0,048	2,31E-04	2,70E-02
cg16992594	1	1970955		IGR	0,004	2,31E-04	2,71E-02
cg03672752	2	219128071	GPBAR1	Body	0,005	2,31E-04	2,71E-02
cg14220146	9	96068079	WNK2	Body	-0,01	2,31E-04	2,71E-02
cg23151504	10	102671543	SLF2	TSS1500	0,009	2,31E-04	2,71E-02
cg09307689	11	557469	C11orf35	Body	0,022	2,31E-04	2,71E-02
cg22677180	15	57537929	TCF12	Body	-0,008	2,31E-04	2,71E-02
cg16865475	19	39051817	RYR1	Body	0,006	2,31E-04	2,71E-02
cg07927484	5	153826296	SAP30L	1stExon	-0,003	2,31E-04	2,71E-02
cg22490454	2	468934		IGR	-0,044	2,32E-04	2,71E-02
cg01266688	2	98274472	ACTR1B	Body	-0,006	2,32E-04	2,71E-02
cg15110481	2	134277486	NCKAP5	5'UTR	-0,061	2,32E-04	2,71E-02
cg21447540	4	120400587	LOC645513	Body	-0,004	2,32E-04	2,71E-02
cg06617414	6	14211210		IGR	-0,004	2,32E-04	2,71E-02
cg20852490	7	96634824	DLX6	TSS1500	-0,01	2,32E-04	2,71E-02
cg01514916	10	63865279		IGR	0,022	2,32E-04	2,71E-02
cg11264564	11	59387768		IGR	-0,013	2,32E-04	2,71E-02
cg12472597	11	67141742	LOC100130987	Body	0,012	2,32E-04	2,71E-02
cg08933115	11	69487063	ORAOV1	Body	-0,006	2,32E-04	2,71E-02
cg06719428	16	24943040	ARHGAP17	Body	-0,013	2,32E-04	2,71E-02
cg15981875	18	59997120	TNFRSF11A	Body	-0,03	2,32E-04	2,71E-02
cg11751810	17	88286	RPH3AL	Body	0,013	2,32E-04	2,71E-02
cg10984680	4	118009982		IGR	-0,012	2,32E-04	2,71E-02
cg05074194	10	11990936	UPF2	Body	0,019	2,32E-04	2,71E-02
cg15201218	12	99548301	ANKS1B	1stExon	-0,034	2,32E-04	2,71E-02
cg20201876	21	43134276	LINC00479	Body	0,039	2,32E-04	2,71E-02
cg16201970	2	191448492		IGR	0,009	2,32E-04	2,71E-02
cg06272329	1	6104498	KCNAB2	TSS1500	0,018	2,33E-04	2,71E-02
cg19771599	1	11070084		IGR	0,005	2,33E-04	2,71E-02
cg20506421	1	175009994		IGR	-0,014	2,33E-04	2,71E-02
cg22218444	1	177867292		IGR	-0,042	2,33E-04	2,71E-02
cg18603406	2	85574124	RETSAT	Body	0,007	2,33E-04	2,71E-02
cg07612923	3	117604196		IGR	-0,008	2,33E-04	2,71E-02
cg00569482	3	117835810		IGR	-0,015	2,33E-04	2,71E-02
cg23499378	4	84883534		IGR	-0,007	2,33E-04	2,71E-02
cg11469319	5	6667352	SRD5A1	Body	-0,007	2,33E-04	2,71E-02
cg25429578	5	176938407	DOK3	TSS1500	0,023	2,33E-04	2,71E-02
cg12229404	7	130760649	FLJ43663	Body	-0,014	2,33E-04	2,71E-02
cg25520302	9	129038685	LOC101929116	TSS1500	0,006	2,33E-04	2,71E-02
cg11930279	12	12022652	ETV6	Body	0,004	2,33E-04	2,71E-02
cg14648877	12	124434035	CCDC92	5'UTR	-0,014	2,33E-04	2,71E-02
cg00170188	17	42441553	FAM171A2	TSS1500	-0,006	2,33E-04	2,71E-02
cg24938632	17	46830125		IGR	0,012	2,33E-04	2,71E-02
cg04313968	19	1821895	REXO1	Body	0,009	2,33E-04	2,71E-02
cg07068709	1	1772952	GNB1	5'UTR	0,02	2,33E-04	2,71E-02
cg24702651	1	172582785		IGR	0,007	2,33E-04	2,71E-02
cg26758810	6	148884544		IGR	-0,016	2,33E-04	2,71E-02
cg13115376	7	63391680		IGR	-0,043	2,33E-04	2,71E-02
cg11833660	12	109181397	SSH1	3'UTR	0,008	2,33E-04	2,71E-02
cg02027651	14	93897981	UNC79	5'UTR	-0,041	2,33E-04	2,71E-02
cg04230582	16	2813667	SRRM2	Body	0,01	2,33E-04	2,71E-02
cg27070061	18	49837827		IGR	-0,029	2,33E-04	2,71E-02
cg27248643	3	16646435	DAZL	Body	-0,008	2,34E-04	2,71E-02
cg18677871	5	1103325	SLC12A7	Body	0,025	2,34E-04	2,71E-02
cg15097530	1	204486394	MDM4	5'UTR	0,023	2,34E-04	2,71E-02
cg06691214	2	208394839	CREB1	5'UTR	-0,006	2,34E-04	2,71E-02
cg26181818	2	233323935	ALPI	3'UTR	-0,069	2,34E-04	2,71E-02
cg17407969	10	134992545	KNDC1	Body	-0,019	2,34E-04	2,71E-02
cg11625476	17	4795410	MINK1	Body	-0,011	2,34E-04	2,71E-02
cg15968021	18	56363161	MALT1	Body	-0,016	2,34E-04	2,71E-02
cg20019658	18	66382486	CCDC102B	TSS200	0,006	2,34E-04	2,71E-02
cg03711854	19	45663488	NKPD1	TSS200	0,015	2,34E-04	2,71E-02
cg13374297	20	52138135		IGR	-0,007	2,34E-04	2,71E-02

cg13828068	9	86615267	RMI1	5'UTR	0,01	2,34E-04	2,71E-02
cg23721140	2	95825033	ZNF514	5'UTR	-0,003	2,34E-04	2,72E-02
cg08091666	17	79961769	ASPSCR1	Body	0,02	2,34E-04	2,72E-02
cg01536355	4	10624057	CLNK	Body	-0,008	2,34E-04	2,72E-02
cg09994595	1	161089339	NIT1	Body	0,015	2,34E-04	2,72E-02
cg22782446	6	30685087	MDC1	1stExon	-0,003	2,34E-04	2,72E-02
cg10071690	10	666063	DIP2C	Body	0,036	2,34E-04	2,72E-02
cg16499415	14	61669675		IGR	-0,013	2,34E-04	2,72E-02
cg14183184	14	99740091		IGR	-0,005	2,34E-04	2,72E-02
cg01433152	16	75032171	ZNRF1	TSS1500	0,03	2,35E-04	2,72E-02
cg08955461	2	38006837		IGR	0,025	2,35E-04	2,72E-02
cg12561408	6	153450334	RGS17	5'UTR	-0,02	2,35E-04	2,72E-02
cg25898281	7	88964363	ZNF804B	Body	-0,051	2,35E-04	2,72E-02
cg13436449	14	59600377		IGR	0,005	2,35E-04	2,72E-02
cg10687823	19	36103576	HAUS5	TSS200	-0,004	2,35E-04	2,72E-02
cg26120617	12	6603256	NCAPD2	TSS200	-0,003	2,35E-04	2,72E-02
cg01152936	22	19157086		IGR	0,006	2,35E-04	2,72E-02
cg16501436	6	32821039	PSMB9	TSS1500	-0,007	2,35E-04	2,72E-02
cg08743812	5	71014736	CARTPT	TSS1500	-0,041	2,35E-04	2,72E-02
cg16952407	1	881269	NOC2L	Body	0,009	2,35E-04	2,72E-02
cg10193018	4	22755948	GBA3	Body	0,01	2,36E-04	2,72E-02
cg00023196	7	158107180	PTPRN2	Body	-0,009	2,36E-04	2,72E-02
cg26121639	10	131639315	EBF3	Body	-0,026	2,36E-04	2,72E-02
cg14685131	17	26880453	UNC119	TSS1500	-0,007	2,36E-04	2,73E-02
cg10888830	7	137556243		IGR	-0,057	2,36E-04	2,73E-02
cg23759189	11	131410868	NTM	Body	-0,034	2,36E-04	2,73E-02
cg17931529	19	2080023	MOBKL2A	5'UTR	0,015	2,36E-04	2,73E-02
cg03078062	6	57375853	PRIM2	Body	0,015	2,36E-04	2,73E-02
cg05670935	4	39368862	RFC1	TSS1500	-0,004	2,37E-04	2,73E-02
cg26777345	4	99877093		IGR	0,025	2,37E-04	2,73E-02
cg19119609	20	57331394		IGR	-0,013	2,37E-04	2,73E-02
cg02045315	8	408443	FBXO25	Body	-0,005	2,37E-04	2,73E-02
cg26137290	2	1776737		IGR	-0,009	2,37E-04	2,73E-02
cg14229436	2	3652023	COLEC11	Body	-0,007	2,37E-04	2,73E-02
cg18382401	5	91623634		IGR	0,027	2,37E-04	2,73E-02
cg15132611	6	63921458		IGR	0,021	2,37E-04	2,73E-02
cg10332972	11	66278120	BBS1	5'UTR	-0,005	2,37E-04	2,73E-02
cg10966500	12	110338339	TCHP	5'UTR	-0,004	2,37E-04	2,73E-02
cg07455713	17	43487978	ARHGAP27	Body	-0,004	2,37E-04	2,73E-02
cg09244349	17	44290388	KANSL1	5'UTR	-0,011	2,37E-04	2,73E-02
cg15410236	19	948452	ARID3A	Body	0,014	2,37E-04	2,73E-02
cg01804363	19	1568552	MEX3D	TSS1500	-0,004	2,37E-04	2,73E-02
cg24299136	19	2511707	GNG7	3'UTR	0,009	2,37E-04	2,73E-02
cg18595911	21	40722699		IGR	-0,003	2,37E-04	2,73E-02
cg14120667	9	138640726	KCNT1	Body	-0,022	2,37E-04	2,73E-02
cg07119871	14	94253356	PRIMA1	Body	-0,027	2,37E-04	2,73E-02
cg04969657	1	112031526	TMIGD3	Body	0,005	2,37E-04	2,73E-02
cg24518794	4	38869206	MIR574	TSS1500	0,012	2,37E-04	2,73E-02
cg25577497	12	130937299	RIMBP2	Body	0,029	2,37E-04	2,73E-02
cg20782816	3	9808060	OGG1	3'UTR	0,009	2,37E-04	2,73E-02
cg02326566	5	140220686	PCDHA6	Body	-0,065	2,37E-04	2,73E-02
cg23620639	10	71561211	COL13A1	TSS1500	-0,004	2,37E-04	2,73E-02
cg12072409	3	122712131	SEMA5B	TSS200	0,028	2,38E-04	2,74E-02
cg14791221	5	14595490	FAM105A	Body	-0,003	2,38E-04	2,74E-02
cg04683496	10	443561	DIP2C	Body	0,011	2,38E-04	2,74E-02
cg21250298	1	97163096		IGR	-0,025	2,38E-04	2,74E-02
cg15496268	14	34600893		IGR	-0,03	2,38E-04	2,74E-02
cg11340363	19	17985049	SLC5A5	Body	0,007	2,38E-04	2,74E-02
cg02305203	4	57548273	HOPX	TSS1500	0,012	2,38E-04	2,74E-02
cg10985914	1	212769641	ATF3	5'UTR	-0,025	2,38E-04	2,74E-02
cg07207080	4	158559183		IGR	0,02	2,38E-04	2,74E-02
cg06936320	20	43272141	ADA	Body	0,021	2,38E-04	2,74E-02
cg17828988	10	81951504	ANXA11	5'UTR	0,061	2,39E-04	2,74E-02
cg27391744	12	50357142	AQP5	Body	0,011	2,39E-04	2,74E-02
cg08671280	16	1934468	LINC00254	TSS1500	-0,027	2,39E-04	2,74E-02
cg15808558	2	222437611	EPHA4	TSS1500	-0,004	2,39E-04	2,74E-02
cg00887018	1	201327830		IGR	0,02	2,39E-04	2,74E-02
cg27094813	1	212624500		IGR	0,029	2,39E-04	2,74E-02
cg09047680	8	130832998		IGR	0,012	2,39E-04	2,74E-02
cg02380775	3	9475571	SETD5	5'UTR	-0,006	2,39E-04	2,74E-02
cg00926226	8	33462191		IGR	-0,043	2,39E-04	2,74E-02
cg00584022	12	25403167	KRAS	5'UTR	-0,006	2,39E-04	2,74E-02
cg24070867	16	1494523	CCDC154	TSS200	0,011	2,39E-04	2,74E-02

cg02498477	18	44471411	PIAS2	Body	0,042	2,39E-04	2,74E-02
cg03009437	22	31672836	LIMK2	Body	-0,018	2,39E-04	2,74E-02
cg16243886	22	37892903	CARD10	Body	0,013	2,39E-04	2,74E-02
cg23665710	8	28476677		IGR	0,053	2,40E-04	2,75E-02
cg16506798	20	23072496		IGR	0,018	2,40E-04	2,75E-02
cg19828789	5	71079151		IGR	0,017	2,40E-04	2,75E-02
cg10947350	1	31125561		IGR	-0,011	2,40E-04	2,75E-02
cg12985235	19	4345965	MPND	Body	0,006	2,40E-04	2,75E-02
cg04797843	17	1171669		IGR	-0,003	2,40E-04	2,75E-02
cg08249934	6	52878460	ICK	Body	-0,006	2,40E-04	2,75E-02
cg19360316	17	21029189	DHRS7B	TSS1500	-0,007	2,40E-04	2,75E-02
cg03563403	9	35079882	FANCG	5'UTR	-0,003	2,40E-04	2,75E-02
cg04693437	10	8446659		IGR	-0,01	2,40E-04	2,75E-02
cg04876431	15	96394274		IGR	-0,013	2,40E-04	2,75E-02
cg18407292	1	3840092		IGR	-0,01	2,41E-04	2,75E-02
cg13672525	1	204485087	MDM4	TSS1500	0,031	2,41E-04	2,75E-02
cg02763234	3	49764811	IP6K1	Body	0,01	2,41E-04	2,75E-02
cg02200481	3	50421338	CACNA2D2	Body	0,018	2,41E-04	2,75E-02
cg11088968	5	176050495	SNCB	Body	0,015	2,41E-04	2,75E-02
cg16655765	6	110680256	C6orf186	TSS1500	0,08	2,41E-04	2,75E-02
cg22902532	11	74440516	CHRDL2	Body	-0,029	2,41E-04	2,75E-02
cg20093339	11	127896734		IGR	-0,012	2,41E-04	2,75E-02
cg10171230	15	79856365		IGR	-0,027	2,41E-04	2,75E-02
cg26834436	16	57383592		IGR	-0,013	2,41E-04	2,75E-02
cg20059407	15	79234318	CTSH	Body	0,017	2,41E-04	2,75E-02
cg06167953	11	86170558	ME3	Body	0,012	2,41E-04	2,75E-02
cg00450475	10	75118604	TTC18	1stExon	0,01	2,41E-04	2,75E-02
cg20012172	22	38902550	DDX17	TSS1500	-0,005	2,41E-04	2,76E-02
cg02727741	2	136373129	R3HDM1	Body	0,011	2,41E-04	2,76E-02
cg02929350	11	5154140	OR52A5	TSS1500	0,042	2,41E-04	2,76E-02
cg26529856	13	113740490	MCF2L	Body	0,02	2,42E-04	2,76E-02
cg15919258	14	90849921		IGR	-0,004	2,42E-04	2,76E-02
cg04281401	11	63687862		IGR	0,016	2,42E-04	2,76E-02
cg19639925	20	45217449	SLC13A3	Body	-0,022	2,42E-04	2,76E-02
cg13748640	9	102858493	ERP44	Body	-0,005	2,42E-04	2,76E-02
cg24444091	21	27462541	APP	Body	-0,005	2,43E-04	2,77E-02
cg24844449	22	29706704	GAS2L1	Body	0,017	2,43E-04	2,77E-02
cg26895706	9	95420444	IPPK	Body	-0,009	2,43E-04	2,77E-02
cg09527680	8	49747745		IGR	-0,014	2,43E-04	2,77E-02
cg13895085	16	49707702	ZNF423	Body	-0,009	2,43E-04	2,77E-02
cg24462596	11	94706862	KDM4D	1stExon	0,011	2,43E-04	2,77E-02
cg00213822	6	10210316		IGR	0,03	2,43E-04	2,77E-02
cg00702381	6	88117996	C6orf165	5'UTR	-0,004	2,43E-04	2,77E-02
cg10934670	3	35719557	ARPP21	5'UTR	0,008	2,43E-04	2,77E-02
cg04729070	8	67837953	SNHG6	TSS200	-0,006	2,43E-04	2,77E-02
cg07084004	14	57506426		IGR	-0,024	2,44E-04	2,77E-02
cg04990202	1	153581750	S100A16	5'UTR	0,014	2,44E-04	2,77E-02
cg13833439	7	45963024		IGR	-0,011	2,44E-04	2,77E-02
cg04181401	2	220067795		IGR	-0,005	2,44E-04	2,77E-02
cg25061610	6	32976049	HLA-DOA	Body	0,018	2,44E-04	2,77E-02
cg03771070	6	131552568	AKAP7	Body	0,026	2,44E-04	2,77E-02
cg23611299	7	133285818	EXOC4	Body	0,034	2,44E-04	2,77E-02
cg09655100	10	114709926	TCF7L2	TSS200	0,006	2,44E-04	2,77E-02
cg05378452	17	68037743		IGR	-0,005	2,44E-04	2,77E-02
cg10567718	2	85878529		IGR	-0,011	2,44E-04	2,78E-02
cg23172215	3	4393231		IGR	-0,031	2,44E-04	2,78E-02
cg20459126	3	142443247	TRPC1	TSS200	-0,003	2,44E-04	2,78E-02
cg14624145	17	37024169		IGR	0,066	2,44E-04	2,78E-02
cg26865853	10	105654499	OBFC1	Body	-0,052	2,44E-04	2,78E-02
cg04408925	21	16032484		IGR	0,055	2,44E-04	2,78E-02
cg21185686	5	140810433	PCDHGA4	Body	-0,03	2,44E-04	2,78E-02
cg14982653	15	99524416	PGPEP1L	Body	0,027	2,44E-04	2,78E-02
cg09204942	2	26368950		IGR	0,017	2,45E-04	2,78E-02
cg01358522	1	46921287		IGR	0,024	2,45E-04	2,78E-02
cg15216801	11	61647647	FADS3	Body	0,023	2,45E-04	2,78E-02
cg22594126	14	81235209	CEP128	Body	0,032	2,45E-04	2,78E-02
cg13399399	16	89961344	TCF25	Body	-0,005	2,45E-04	2,78E-02
cg18994438	17	43318045	FMNL1	Body	0,013	2,45E-04	2,78E-02
cg04172049	17	47931091		IGR	0,016	2,45E-04	2,78E-02
cg13173564	19	4343352	MPND	TSS200	-0,007	2,45E-04	2,78E-02
cg07143525	6	44205913		IGR	-0,004	2,45E-04	2,78E-02
cg16990628	1	89739300	GBP5	TSS1500	-0,009	2,45E-04	2,78E-02
cg24278165	8	23083551	TNFRSF10A	TSS1500	0,027	2,45E-04	2,78E-02

cg01848374	22	47202104	TBC1D22A	Body	-0,006	2,45E-04	2,78E-02
cg09223687	4	1027885		IGR	0,025	2,45E-04	2,78E-02
cg02452495	9	126830788		IGR	0,025	2,45E-04	2,78E-02
cg12873454	1	14924611	KIAA1026	TSS1500	-0,025	2,46E-04	2,79E-02
cg08780166	2	40470864	SLC8A1	Body	0,015	2,46E-04	2,79E-02
cg18922793	4	127876757		IGR	-0,027	2,46E-04	2,79E-02
cg02161292	5	140821687	PCDHGA4	Body	-0,051	2,46E-04	2,79E-02
cg17824679	6	29113955		IGR	-0,008	2,46E-04	2,79E-02
cg07374509	6	30419070		IGR	-0,016	2,46E-04	2,79E-02
cg22774829	7	127033083	ZNF800	TSS1500	-0,005	2,46E-04	2,79E-02
cg23567315	12	7924652	NANOGNB	Body	-0,022	2,46E-04	2,79E-02
cg11136205	12	133351421	GOLGA3	Body	0,009	2,46E-04	2,79E-02
cg08025954	22	24823455	ADORA2A	TSS200	-0,004	2,46E-04	2,79E-02
cg14864167	8	66751182	PDE7A	Body	-0,059	2,46E-04	2,79E-02
cg12573766	11	75110826	SNORD15A	TSS1500	-0,003	2,46E-04	2,79E-02
cg24695011	3	125781947	SLC41A3	Body	0,02	2,46E-04	2,79E-02
cg10499102	6	564040	EXOC2	Body	0,009	2,46E-04	2,79E-02
cg25191231	17	30677038	ZNF207	TSS200	0,006	2,46E-04	2,79E-02
cg09233377	13	44714325	SMIM2-AS1	Body	-0,006	2,46E-04	2,79E-02
cg07306531	16	3355411	ZNF75A	TSS200	-0,004	2,47E-04	2,79E-02
cg14190264	17	635361	FAM57A	TSS1500	0,007	2,47E-04	2,79E-02
cg05119641	2	183387476	PDE1A	5'UTR	-0,015	2,47E-04	2,79E-02
cg08353915	9	33511604	SUGT1P1	TSS1500	-0,015	2,47E-04	2,79E-02
cg20880193	19	6071224	RFX2	5'UTR	0,028	2,47E-04	2,79E-02
cg24541835	1	12651540	DHRS3	Body	0,009	2,47E-04	2,79E-02
cg20313362	1	43158569	YBX1	Body	0,015	2,47E-04	2,79E-02
cg10694630	12	809896		IGR	-0,007	2,47E-04	2,79E-02
cg01496820	12	5674808	ANO2	Body	-0,008	2,47E-04	2,79E-02
cg02729758	9	89695922		IGR	-0,045	2,47E-04	2,79E-02
cg25143099	21	46979544		IGR	0,013	2,47E-04	2,79E-02
cg18050733	6	32904889	HLA-DMB	Body	0,008	2,47E-04	2,79E-02
cg07153421	5	75622396	SV2C	3'UTR	0,015	2,48E-04	2,79E-02
cg24614660	5	157601396		IGR	-0,013	2,47E-04	2,79E-02
cg20321706	12	132845874	GALNT9	Body	0,008	2,48E-04	2,79E-02
cg11919810	13	39586956	PROSER1	Body	0,014	2,48E-04	2,79E-02
cg11765362	2	31806551	SRD5A2	TSS1500	-0,065	2,48E-04	2,79E-02
cg07919162	19	19431259	SF4	Body	-0,006	2,48E-04	2,79E-02
cg15315630	1	35497506	ZMYM6	5'UTR	-0,004	2,48E-04	2,79E-02
cg09979763	1	245499904	KIF26B	Body	-0,014	2,48E-04	2,79E-02
cg10239186	6	166756509	SFT2D1	TSS1500	-0,006	2,48E-04	2,79E-02
cg04544533	12	124811985	NCOR2	Body	0,007	2,48E-04	2,79E-02
cg00825130	13	60664311	DIAPH3	Body	-0,003	2,48E-04	2,79E-02
cg00481742	14	92302542	TC2N	1stExon	-0,004	2,48E-04	2,79E-02
cg01927645	15	75423359		IGR	-0,017	2,48E-04	2,79E-02
cg23689985	17	36112892		IGR	-0,012	2,48E-04	2,79E-02
cg20506400	18	7723056	PTPRM	Body	0,033	2,48E-04	2,79E-02
cg21144941	22	17680575	CECR1	TSS200	-0,008	2,48E-04	2,79E-02
cg01243653	11	77158483	PAK1	5'UTR	-0,038	2,48E-04	2,79E-02
cg04931573	14	104335108		IGR	0,016	2,48E-04	2,79E-02
cg27009392	16	69385547	TMED6	1stExon	-0,006	2,48E-04	2,79E-02
cg03731616	3	123603618	MYLK	TSS1500	-0,021	2,48E-04	2,80E-02
cg14576128	12	1100379	ERC1	TSS200	0,007	2,48E-04	2,80E-02
cg13525592	4	647542	PDE6B	5'UTR	0,019	2,49E-04	2,80E-02
cg08255572	4	127662005		IGR	-0,006	2,49E-04	2,80E-02
cg14587044	14	52066678	FRMD6-AS2	TSS200	-0,012	2,49E-04	2,80E-02
cg23993521	19	54156392		IGR	-0,014	2,49E-04	2,80E-02
cg15493789	22	25565455	KIAA1671	Body	-0,014	2,49E-04	2,80E-02
cg05723989	1	210407677	C1orf133	TSS1500	-0,023	2,49E-04	2,80E-02
cg12197666	13	40511553		IGR	-0,017	2,49E-04	2,80E-02
cg11949866	1	10754891	CASZ1	5'UTR	0,01	2,49E-04	2,80E-02
cg23720516	10	118082855	CCDC172	TSS1500	0,018	2,49E-04	2,80E-02
cg14743812	13	113006248		IGR	0,04	2,49E-04	2,80E-02
cg05922057	17	4047571	ZZEF1	TSS1500	-0,006	2,49E-04	2,80E-02
cg24902174	2	242707603	D2HGDH	3'UTR	0,023	2,49E-04	2,80E-02
cg18920289	20	447168		IGR	0,024	2,49E-04	2,80E-02
cg06341103	1	94713310		IGR	0,009	2,49E-04	2,80E-02
cg15623062	6	31747133	VARS	Body	0,02	2,49E-04	2,80E-02
cg01821113	2	218277531	DIRC3	Body	-0,008	2,49E-04	2,80E-02
cg04403629	3	114867274	ZBTB20	TSS1500	-0,009	2,50E-04	2,80E-02
cg18089670	18	660756	TYMS	Body	-0,006	2,50E-04	2,80E-02
cg15816911	14	71606274		IGR	-0,024	2,50E-04	2,80E-02
cg12055459	2	97652489	FAM178B	TSS200	-0,004	2,50E-04	2,80E-02
cg04148467	2	207022459	NDUFS1	5'UTR	-0,005	2,50E-04	2,80E-02

cg23687258	6	106808749		IGR	0,011	2,50E-04	2,80E-02
cg02351655	16	4442918	CORO7	Body	0,008	2,50E-04	2,80E-02
cg18308351	17	19552182	ALDH3A2	5'UTR	-0,004	2,50E-04	2,80E-02
cg07584805	17	56738915	TEX14	5'UTR	0,008	2,50E-04	2,80E-02
cg05632277	3	136149487	STAG1	Body	0,011	2,50E-04	2,80E-02
cg22741997	17	6900356	ALOX12	Body	-0,007	2,50E-04	2,80E-02
cg13680227	3	166796103		IGR	-0,016	2,50E-04	2,81E-02
cg00928088	5	40268273		IGR	-0,013	2,50E-04	2,81E-02
cg13354811	7	96045954		IGR	-0,02	2,50E-04	2,81E-02
cg02863842	19	45349685	PVRL2	5'UTR	-0,008	2,51E-04	2,81E-02
cg10039268	1	198189716	NEK7	5'UTR	-0,012	2,51E-04	2,81E-02
cg00641828	8	141715451	PTK2	Body	-0,008	2,51E-04	2,81E-02
cg16862595	15	54210416		IGR	-0,015	2,51E-04	2,81E-02
cg15292009	14	50785556	ATP5S	Body	-0,051	2,51E-04	2,81E-02
cg14794445	15	29396429	APBA2	Body	0,021	2,51E-04	2,81E-02
cg03752439	1	38087878	RSPO1	Body	0,053	2,51E-04	2,81E-02
cg12233379	1	151812524	LOC100132111	Body	-0,01	2,51E-04	2,81E-02
cg11960189	2	60580258		IGR	0,011	2,51E-04	2,81E-02
cg10672884	2	171785479	GORASP2	TSS1500	-0,005	2,51E-04	2,81E-02
cg08347626	5	1850140		IGR	-0,062	2,51E-04	2,81E-02
cg16846489	9	19049460	SAXO1	TSS200	-0,004	2,51E-04	2,81E-02
cg25875280	16	67768836	RANBP10	Body	0,014	2,52E-04	2,81E-02
cg15468423	19	2700763	GNG7	5'UTR	0,013	2,52E-04	2,81E-02
cg05927579	20	43378669	KCNK15	Body	-0,067	2,51E-04	2,81E-02
cg20780708	1	53662814	CPT2	Body	-0,003	2,52E-04	2,81E-02
cg04106862	16	22309278	POLR3E	5'UTR	0,005	2,52E-04	2,81E-02
cg20196453	16	72142455	DHX38	Body	-0,008	2,52E-04	2,81E-02
cg01166399	6	119346816	FAM184A	5'UTR	0,036	2,52E-04	2,81E-02
cg04303335	14	82000858	SEL1L	TSS1500	-0,007	2,52E-04	2,81E-02
cg14479268	4	154498113	KIAA0922	Body	0,007	2,52E-04	2,81E-02
cg14508567	7	131734246		IGR	0,043	2,52E-04	2,81E-02
cg03034820	20	42649372	TOX2	Body	0,021	2,52E-04	2,81E-02
cg24861261	18	11981455	IMPA2	5'UTR	0,016	2,52E-04	2,82E-02
cg20406003	13	103242942		IGR	0,017	2,52E-04	2,82E-02
cg15252509	18	56337165	MALT1	TSS1500	-0,033	2,53E-04	2,82E-02
cg17669433	1	93594340	MTF2	Body	-0,006	2,53E-04	2,82E-02
cg12342178	1	238282400		IGR	-0,026	2,53E-04	2,82E-02
cg13165620	12	110923906	FAM216A	Body	-0,005	2,53E-04	2,82E-02
cg18318655	13	31713793	HSPH1	Body	-0,006	2,53E-04	2,82E-02
cg00376910	15	89765061	RLBP1	TSS200	-0,026	2,53E-04	2,82E-02
cg21875234	16	12009405	GSPT1	1stExon	0,003	2,53E-04	2,82E-02
cg07987191	19	46191614	SNRPD2	Body	-0,005	2,53E-04	2,82E-02
cg23680065	10	99773078	CRTAC1	Body	-0,036	2,53E-04	2,82E-02
cg23953396	17	72427941	GPRC5C	1stExon	-0,007	2,53E-04	2,82E-02
cg12901052	12	131040681		IGR	-0,07	2,53E-04	2,82E-02
cg27612914	17	40738101	FAM134C	Body	-0,007	2,53E-04	2,82E-02
cg23453420	18	19180773	ESCO1	TSS200	-0,004	2,53E-04	2,82E-02
cg06156640	2	3704363	ALLC	TSS1500	0,03	2,53E-04	2,82E-02
cg15564865	6	4021423	PRPF4B	TSS200	-0,004	2,54E-04	2,82E-02
cg02393606	10	135033364	KNDC1	Body	0,032	2,54E-04	2,82E-02
cg17219038	11	71791565	LRTOMT	5'UTR	-0,004	2,53E-04	2,82E-02
cg13664146	17	71228606	C17orf80	TSS200	-0,003	2,53E-04	2,82E-02
cg11362062	2	220036240	SLC23A3	TSS1500	-0,016	2,54E-04	2,82E-02
cg10459751	5	123780725		IGR	0,039	2,54E-04	2,82E-02
cg00669776	6	12047856	HIVEP1	Body	0,02	2,54E-04	2,82E-02
cg11290183	7	152456743	ACTR3B	TSS200	-0,002	2,54E-04	2,82E-02
cg08283408	3	49949060	MON1A	Body	-0,006	2,54E-04	2,83E-02
cg10929784	3	79773602	ROBO1	5'UTR	-0,044	2,54E-04	2,83E-02
cg07359208	11	7320836	SYT9	Body	-0,009	2,54E-04	2,83E-02
cg07758275	17	5327986	RPAIN	Body	-0,007	2,54E-04	2,83E-02
cg06203059	4	48108327	TXK	Body	0,011	2,54E-04	2,83E-02
cg22047262	12	50189301	NCKAP5L	Body	0,006	2,54E-04	2,83E-02
cg08111158	1	227746191		IGR	-0,051	2,55E-04	2,83E-02
cg20876987	6	28553998	SCAND3	Body	-0,009	2,55E-04	2,83E-02
cg27627876	16	88102820	BANP	Body	0,009	2,55E-04	2,83E-02
cg14443640	18	3009125	LPIN2	5'UTR	0,031	2,55E-04	2,83E-02
cg13754355	9	139635449	LCN10	Body	0,016	2,55E-04	2,83E-02
cg07895499	3	156409317	TIPARP	Body	-0,014	2,55E-04	2,83E-02
cg21564242	3	179754761	PEX5L	TSS1500	-0,007	2,55E-04	2,83E-02
cg05924733	4	160277162	RAPGEF2	Body	-0,004	2,55E-04	2,83E-02
cg04186465	6	12125962	HIVEP1	Body	-0,005	2,55E-04	2,83E-02
cg23424194	12	112150215	ACAD10	Body	0,017	2,55E-04	2,83E-02
cg01168201	17	1958412	HIC1	TSS1500	0,004	2,56E-04	2,84E-02

cg10734044	3	16245632	GALNTL2	Body	-0,009	2,56E-04	2,84E-02
cg18764814	5	1974131		IGR	-0,038	2,56E-04	2,84E-02
cg21678834	17	79787035	FAM195B	5'UTR	0,019	2,56E-04	2,84E-02
cg23117180	1	157973398	KIRREL	Body	-0,026	2,56E-04	2,84E-02
cg04525851	10	43128215	ZNF33B	Body	0,027	2,56E-04	2,84E-02
cg04666830	1	156786617	SH2D2A	TSS1500	-0,003	2,56E-04	2,84E-02
cg15483386	11	72524600	ATG16L2	TSS1500	-0,003	2,56E-04	2,84E-02
cg08105469	13	94996756	GPC6	Body	0,006	2,56E-04	2,84E-02
cg18998363	16	31493372	SLC5A2	TSS1500	0,006	2,56E-04	2,84E-02
cg02117228	3	189721131	P3H2	Body	-0,022	2,56E-04	2,84E-02
cg24129475	4	141280375	SCOC-AS1	Body	-0,019	2,56E-04	2,84E-02
cg00260680	21	38641729		IGR	-0,005	2,57E-04	2,84E-02
cg25639095	17	77078175	ENGASE	Body	0,01	2,57E-04	2,84E-02
cg22902223	5	168240040	SLIT3	Body	-0,008	2,57E-04	2,85E-02
cg07588996	6	18250021	DEK	Body	-0,006	2,57E-04	2,85E-02
cg27376339	15	25841862		IGR	0,006	2,57E-04	2,85E-02
cg11787855	19	50180430	PRMT1	TSS200	-0,006	2,57E-04	2,85E-02
cg15144068	14	106321751		IGR	0,025	2,57E-04	2,85E-02
cg23051299	2	75938289	C2orf3	TSS200	0,033	2,57E-04	2,85E-02
cg10376468	1	78245116	FAM73A	TSS200	-0,011	2,58E-04	2,85E-02
cg23868571	6	31337262		IGR	0,023	2,58E-04	2,85E-02
cg25648091	14	71108484	LOC101928075	TSS1500	-0,004	2,58E-04	2,85E-02
cg20884524	6	76108836	FILIP1	Body	-0,058	2,58E-04	2,85E-02
cg00108980	1	59118461		IGR	-0,015	2,58E-04	2,85E-02
cg05563450	7	138603324	KIAA1549	Body	-0,017	2,58E-04	2,85E-02
cg04282558	7	149124500		IGR	0,006	2,58E-04	2,85E-02
cg01526745	17	32448038	ASIC2	Body	-0,023	2,58E-04	2,85E-02
cg00092235	17	70154920		IGR	-0,06	2,58E-04	2,85E-02
cg27662875	19	50165232	IRF3	Body	0,01	2,58E-04	2,85E-02
cg19892963	22	39111285	GTPBP1	Body	0,026	2,58E-04	2,85E-02
cg13203239	1	31192158	MATN1	Body	-0,004	2,58E-04	2,85E-02
cg21436120	1	164536135	PBX1	Body	0,014	2,58E-04	2,85E-02
cg08162294	1	201123766	TMEM9	TSS200	0,004	2,58E-04	2,85E-02
cg14517616	5	68258521		IGR	-0,006	2,58E-04	2,85E-02
cg00798993	11	123940777		IGR	-0,028	2,58E-04	2,85E-02
cg24575235	14	73149487	DPF3	Body	-0,013	2,58E-04	2,85E-02
cg18065204	18	77497004	CTDP1	Body	-0,006	2,58E-04	2,85E-02
cg06930757	19	51216389	SHANK1	Body	-0,027	2,58E-04	2,85E-02
cg21990021	20	35155565	DLGAP4	3'UTR	-0,008	2,58E-04	2,85E-02
cg16357147	1	200849323		IGR	-0,006	2,59E-04	2,85E-02
cg08108518	9	35651544	SIT1	TSS1500	-0,007	2,59E-04	2,85E-02
cg13781350	17	19582026	SLC47A2	Body	-0,007	2,59E-04	2,85E-02
cg00655231	19	3400148	NFIC	Body	-0,016	2,59E-04	2,85E-02
cg23937078	1	31246279		IGR	0,016	2,59E-04	2,85E-02
cg07622342	14	35873382	NFKBIA	Body	-0,004	2,59E-04	2,85E-02
cg18191092	8	64331042		IGR	-0,029	2,59E-04	2,85E-02
cg02777633	3	64009154	PSMD6	TSS200	0,005	2,59E-04	2,85E-02
cg14215082	8	9055850		IGR	-0,008	2,59E-04	2,85E-02
cg08251925	7	30032891		IGR	0,014	2,59E-04	2,86E-02
cg23301142	19	50354157	PTOV1	TSS1500	0,005	2,59E-04	2,86E-02
cg08220686	6	166742990	SFT2D1	ExonBnd	0,013	2,60E-04	2,86E-02
cg21928385	2	7948150		IGR	0,01	2,60E-04	2,86E-02
cg20193781	2	128589932		IGR	0,009	2,60E-04	2,86E-02
cg06609282	3	86283974		IGR	-0,013	2,60E-04	2,86E-02
cg05685587	6	1312577	FOXQ1	TSS200	-0,013	2,60E-04	2,86E-02
cg14052997	10	89575546	ATAD1	5'UTR	-0,02	2,60E-04	2,86E-02
cg22736271	11	120113924	POU2F3	Body	0,027	2,60E-04	2,86E-02
cg24308698	13	75877949	TBC1D4	Body	-0,005	2,60E-04	2,86E-02
cg20100542	16	27501818	GTF3C1	Body	-0,008	2,60E-04	2,86E-02
cg24557281	4	4547745		IGR	-0,006	2,60E-04	2,86E-02
cg23512763	19	3942969	MIR1268A	Body	-0,016	2,60E-04	2,86E-02
cg03718416	12	15917626	EPS8	5'UTR	0,021	2,60E-04	2,86E-02
cg19623875	1	33647561	TRIM62	5'UTR	0,005	2,60E-04	2,86E-02
cg24084358	20	42984276	HNF4A	TSS200	-0,01	2,60E-04	2,86E-02
cg24268822	3	49059985	NDUFAF3	Body	-0,004	2,61E-04	2,86E-02
cg12935960	1	94059570	BCAR3	Body	0,006	2,61E-04	2,86E-02
cg01276169	12	57569898	LRP1	Body	-0,02	2,61E-04	2,86E-02
cg00869550	16	58046537	USB1	3'UTR	0,019	2,61E-04	2,86E-02
cg15050473	5	31796646		IGR	-0,005	2,61E-04	2,86E-02
cg11105375	14	55595969	LGALS3	1stExon	0,004	2,61E-04	2,86E-02
cg17792616	15	93580327		IGR	0,06	2,61E-04	2,86E-02
cg22586102	1	26668540	AIM1L	Body	0,01	2,61E-04	2,87E-02
cg21264372	1	205561090	MFSD4	Body	-0,003	2,61E-04	2,87E-02

cg04083496	4	140148740		IGR	0,01	2,61E-04	2,87E-02
cg02586423	5	154392124	KIF4B	TSS1500	0,043	2,61E-04	2,87E-02
cg14707961	6	129691120	LAMA2	ExonBnd	0,012	2,61E-04	2,87E-02
cg10918676	8	144906810	PUF60	5'UTR	0,015	2,61E-04	2,87E-02
cg01300175	12	85842498		IGR	-0,024	2,61E-04	2,87E-02
cg16621633	16	19296418	LOC728276	TSS1500	0,039	2,61E-04	2,87E-02
cg07031797	22	50483123	TTLL8	Body	-0,023	2,61E-04	2,87E-02
cg27118612	1	202788667	MGAT4EP	TSS1500	0,01	2,62E-04	2,87E-02
cg13863078	4	177244109	SPCS3	Body	0,035	2,62E-04	2,87E-02
cg21627409	5	140810106	PCDHGA4	Body	-0,031	2,62E-04	2,87E-02
cg26123258	7	149470305	ZNF467	TSS200	-0,004	2,62E-04	2,87E-02
cg01266362	9	98265802	PTCH1	Body	-0,008	2,62E-04	2,87E-02
cg24983858	10	80887603	ZMIZ1	5'UTR	0,028	2,62E-04	2,87E-02
cg06610988	18	42262499	SETBP1	5'UTR	0,041	2,62E-04	2,87E-02
cg17431382	1	78024449	AK5	3'UTR	-0,009	2,62E-04	2,87E-02
cg01755009	1	247978690	OR14A16	1stExon	-0,007	2,62E-04	2,87E-02
cg01153972	13	20721044	GJA3	5'UTR	-0,01	2,62E-04	2,87E-02
cg23014794	14	77426840		IGR	0,011	2,62E-04	2,87E-02
cg14830915	20	30869579	KIF3B	5'UTR	0,012	2,62E-04	2,87E-02
cg25839745	4	101969341	PPP3CA	Body	-0,006	2,62E-04	2,87E-02
cg10520319	1	32666035	CCDC28B	TSS200	-0,004	2,63E-04	2,87E-02
cg18516268	17	37010922	RPL23	TSS1500	0,007	2,63E-04	2,87E-02
cg19741107	4	140872103	MAML3	Body	-0,015	2,63E-04	2,87E-02
cg25581090	14	65009707	HSPA2	3'UTR	0,041	2,63E-04	2,88E-02
cg05869611	7	80096608	GNAT3	Body	0,018	2,63E-04	2,88E-02
cg17354476	10	29698255	LOC387647	TSS1500	0,044	2,63E-04	2,88E-02
cg10158839	14	38191362	TTC6	Body	-0,03	2,63E-04	2,88E-02
cg08899266	19	7910060	EVI5L	TSS1500	0,017	2,63E-04	2,88E-02
cg11037787	1	20307002	PLA2G2A	TSS1500	-0,007	2,63E-04	2,88E-02
cg01052276	2	97200489		IGR	0,023	2,63E-04	2,88E-02
cg00184005	5	151328931		IGR	-0,028	2,63E-04	2,88E-02
cg09015232	20	44657115	SLC12A5	TSS1500	-0,067	2,64E-04	2,88E-02
cg17537241	18	13590450	LDLRAD4	Body	-0,006	2,64E-04	2,88E-02
cg18477362	3	166452764		IGR	-0,01	2,64E-04	2,88E-02
cg16525461	8	38645456	TACC1	5'UTR	-0,005	2,64E-04	2,88E-02
cg24156854	19	7983776	SNAPC2	TSS1500	-0,043	2,64E-04	2,88E-02
cg09086615	4	127966639		IGR	-0,033	2,64E-04	2,88E-02
cg10653296	13	100318130	CLYBL	Body	0,013	2,64E-04	2,88E-02
cg14849526	14	100842590	WDR25	TSS200	0,004	2,64E-04	2,88E-02
cg06459327	15	34635538	NOP10	TSS200	-0,005	2,64E-04	2,88E-02
cg16017471	17	30579985		IGR	0,008	2,64E-04	2,88E-02
cg14453935	18	77398086		IGR	-0,051	2,64E-04	2,88E-02
cg11665583	22	26893201	TFIP11	Body	-0,015	2,64E-04	2,88E-02
cg07147748	13	43936537	ENOX1	Body	0,009	2,65E-04	2,88E-02
cg24262066	21	28217060	ADAMTS1	1stExon	-0,011	2,65E-04	2,88E-02
cg09002469	12	123459295	OGFOD2	TSS200	-0,004	2,65E-04	2,88E-02
cg02689107	5	112157485	APC	Body	0,012	2,65E-04	2,89E-02
cg13131859	4	4323012	ZNF509	Body	-0,006	2,65E-04	2,89E-02
cg21508673	13	113818634	PROZ	Body	0,051	2,65E-04	2,89E-02
cg19791630	19	6772811	VAV1	5'UTR	-0,005	2,65E-04	2,89E-02
cg06021832	1	41329406	CITED4	TSS1500	0,019	2,65E-04	2,89E-02
cg19344838	4	185863872		IGR	-0,009	2,65E-04	2,89E-02
cg18506679	9	127563860	OLFML2A	Body	-0,007	2,65E-04	2,89E-02
cg14205519	9	139925750	C9orf139	5'UTR	0,016	2,65E-04	2,89E-02
cg19678730	12	9280567		IGR	-0,008	2,65E-04	2,89E-02
cg03272982	16	66508061	BEAN1-AS1	Body	0,023	2,65E-04	2,89E-02
cg21711958	9	12735046	LURAP1L-AS1	Body	-0,021	2,65E-04	2,89E-02
cg15070709	1	11579475	PTCHD2	Body	0,018	2,66E-04	2,89E-02
cg10290291	4	120415417	LOC645513	Body	0,012	2,66E-04	2,89E-02
cg03054605	1	44232845	ST3GAL3	Body	-0,024	2,66E-04	2,89E-02
cg17059414	1	113010011	WNT2B	5'UTR	-0,02	2,66E-04	2,89E-02
cg00275503	6	133908606		IGR	-0,024	2,66E-04	2,89E-02
cg08396782	6	165240591		IGR	-0,027	2,66E-04	2,89E-02
cg19374961	8	60019164	TOX	Body	-0,02	2,66E-04	2,89E-02
cg20444525	11	43919933	LOC729799	Body	-0,004	2,66E-04	2,89E-02
cg22627158	11	65414798	SIPA1	Body	0,012	2,66E-04	2,89E-02
cg26362272	1	22108717	USP48	Body	-0,011	2,67E-04	2,89E-02
cg02210006	2	98861881	VWA3B	Body	0,043	2,66E-04	2,89E-02
cg16363684	4	184264223		IGR	-0,053	2,66E-04	2,89E-02
cg27543327	5	148601084	ABLIM3	Body	0,019	2,67E-04	2,89E-02
cg05883747	8	140839863	TRAPPC9	Body	0,005	2,66E-04	2,89E-02
cg08290614	11	111398249	C11orf88	Body	0,01	2,66E-04	2,89E-02
cg09379965	12	120541737	RAB35	Body	-0,009	2,67E-04	2,89E-02

cg05355674	20	40328860		IGR	-0,008	2,67E-04	2,89E-02
cg02925707	18	32621224	MAPRE2	TSS200	-0,004	2,67E-04	2,89E-02
cg17373374	4	110090515	COL25A1	Body	0,01	2,67E-04	2,90E-02
cg05660893	6	147704068	STXBP5	Body	0,005	2,67E-04	2,90E-02
cg06132598	9	138899436	NACC2	3'UTR	-0,067	2,67E-04	2,90E-02
cg25752087	17	12523042		IGR	-0,042	2,67E-04	2,90E-02
cg06632557	11	61313548	SYT7	Body	0,007	2,67E-04	2,90E-02
cg22562234	2	45667501	SRBD1	Body	0,009	2,68E-04	2,90E-02
cg01084189	2	134865337		IGR	-0,006	2,67E-04	2,90E-02
cg10306925	3	153161634		IGR	0,033	2,68E-04	2,90E-02
cg15612951	12	121544388		IGR	0,005	2,67E-04	2,90E-02
cg23998114	19	33798340		IGR	-0,025	2,68E-04	2,90E-02
cg20176989	19	55362650	KIR3DL2	Body	0,064	2,68E-04	2,90E-02
cg17219660	1	202091880	GPR37L1	TSS200	-0,008	2,68E-04	2,90E-02
cg04581293	3	156889197		IGR	-0,012	2,68E-04	2,90E-02
cg13296847	5	118352190		IGR	0,029	2,68E-04	2,90E-02
cg07675782	15	80484505		IGR	-0,006	2,68E-04	2,90E-02
cg07481784	17	43138517	DCAKD	TSS200	-0,007	2,68E-04	2,90E-02
cg08344281	19	6042039	RFX2	Body	0,013	2,68E-04	2,90E-02
cg17926016	8	145739374	RECQL4	Body	0,019	2,68E-04	2,90E-02
cg00438486	17	30620277	RHBDL3	Body	0,008	2,68E-04	2,90E-02
cg23885512	19	45515293	RELB	Body	0,006	2,68E-04	2,90E-02
cg06475339	2	149351829		IGR	-0,008	2,68E-04	2,90E-02
cg05438638	2	27428908	SLC5A6	ExonBnd	-0,007	2,68E-04	2,90E-02
cg03339123	2	176866845	KIAA1715	5'UTR	-0,003	2,68E-04	2,90E-02
cg11754900	4	101758860		IGR	-0,006	2,68E-04	2,90E-02
cg24711774	7	63643958		IGR	-0,059	2,69E-04	2,90E-02
cg17861206	10	103826705	HPS6	1stExon	0,019	2,69E-04	2,90E-02
cg02777535	7	152161078	LOC100128822	TSS200	0,011	2,69E-04	2,90E-02
cg27337539	11	76900304	MYO7A	Body	0,012	2,69E-04	2,90E-02
cg15210447	18	33615795	RPRD1A	Body	-0,019	2,69E-04	2,90E-02
cg08638886	6	28704807		IGR	-0,079	2,69E-04	2,91E-02
cg16956153	3	107943906		IGR	0,027	2,69E-04	2,91E-02
cg00147339	6	7590875	SNRNP48	Body	-0,005	2,69E-04	2,91E-02
cg09538287	10	69456036	CTNNA3	TSS200	0,032	2,70E-04	2,91E-02
cg08384432	4	7349755	SORCS2	Body	-0,017	2,70E-04	2,91E-02
cg14288287	10	14633741	FAM107B	5'UTR	0,019	2,70E-04	2,91E-02
cg02615599	7	150725389	ABCB8	TSS200	-0,014	2,70E-04	2,91E-02
cg03598731	8	130996123		IGR	-0,01	2,70E-04	2,91E-02
cg16113254	14	75777372		IGR	0,022	2,70E-04	2,91E-02
cg09097152	6	25411727	LRRC16A	Body	0,01	2,70E-04	2,91E-02
cg02110776	17	983522	ABR	Body	0,006	2,70E-04	2,91E-02
cg02872767	19	1525453	PLK5P	Body	-0,178	2,70E-04	2,91E-02
cg09409957	2	234683067	MROH2A	TSS1500	-0,035	2,70E-04	2,91E-02
cg07048256	14	24733374	TGM1	TSS1500	0,022	2,70E-04	2,91E-02
cg21960110	16	202482	HBZ	TSS1500	0,031	2,70E-04	2,91E-02
cg16517195	16	66429972	CDH5	ExonBnd	-0,005	2,70E-04	2,91E-02
cg09479650	16	85578516		IGR	0,033	2,70E-04	2,91E-02
cg10296867	17	10048989	GAS7	Body	0,016	2,70E-04	2,91E-02
cg03567001	20	32668840	RALY	3'UTR	0,029	2,70E-04	2,91E-02
cg07089687	5	71621738	PTCD2	5'UTR	-0,013	2,71E-04	2,92E-02
cg17588181	8	137808438		IGR	0,03	2,71E-04	2,92E-02
cg16313230	11	66358016	CCDC87	1stExon	-0,01	2,71E-04	2,92E-02
cg22536016	3	38387764	XYLB	TSS1500	0,012	2,71E-04	2,92E-02
cg13293246	2	127818101	BIN1	Body	0,01	2,72E-04	2,92E-02
cg14036627	6	31148657		IGR	-0,085	2,72E-04	2,92E-02
cg20575909	16	31468456	ARMC5	TSS1500	0,006	2,72E-04	2,92E-02
cg14898243	10	70863693	SRGN	Body	0,043	2,72E-04	2,93E-02
cg14694901	10	75255186	PPP3CB	Body	-0,004	2,72E-04	2,93E-02
cg01543974	16	9160649		IGR	-0,012	2,72E-04	2,93E-02
cg15888693	17	4074172	ANKFY1	Body	-0,014	2,72E-04	2,93E-02
cg09247193	17	47075095	IGF2BP1	5'UTR	-0,006	2,72E-04	2,93E-02
cg26465666	2	183386928	PDE1A	Body	0,044	2,72E-04	2,93E-02
cg23901857	2	176993221	HOXD8	TSS1500	0,011	2,72E-04	2,93E-02
cg17050724	17	79008885	FLJ90757	TSS1500	0,014	2,72E-04	2,93E-02
cg01792902	3	178864926	PIK3CA	TSS1500	-0,006	2,72E-04	2,93E-02
cg05914069	10	101541451	ABCC2	TSS1500	-0,018	2,72E-04	2,93E-02
cg04763468	18	77393042		IGR	-0,009	2,72E-04	2,93E-02
cg12474981	19	48751505	CARD8	5'UTR	0,054	2,72E-04	2,93E-02
cg09132335	1	79020146		IGR	-0,027	2,73E-04	2,93E-02
cg06664037	2	231089039	SP110	5'UTR	0,012	2,73E-04	2,93E-02
cg10105792	8	6676807	XKR5	Body	-0,009	2,73E-04	2,93E-02
cg01014615	2	177027621	HOXD3	TSS1500	-0,025	2,73E-04	2,93E-02

cg18838675	6	34173886		IGR	0,009	2,73E-04	2,93E-02
cg25721516	17	42276931	ATXN7L3	TSS1500	0,006	2,73E-04	2,93E-02
cg13989964	20	10595286	SLX4IP	Body	-0,006	2,73E-04	2,93E-02
cg15388355	3	194432314	LOC100507391	Body	0,009	2,73E-04	2,93E-02
cg03412967	7	143825736	OR2A14	TSS1500	-0,043	2,73E-04	2,93E-02
cg07887752	11	67029601		IGR	0,017	2,73E-04	2,93E-02
cg18507129	3	142839903	CHST2	Body	-0,004	2,73E-04	2,93E-02
cg06657841	8	13707373		IGR	-0,011	2,73E-04	2,93E-02
cg14945214	12	120548863	RAB35	Body	0,035	2,73E-04	2,93E-02
cg08425603	17	72589089	CD300LD	TSS1500	0,023	2,73E-04	2,93E-02
cg16989340	1	1084147		IGR	0,02	2,73E-04	2,93E-02
cg19642039	3	154052879		IGR	-0,018	2,74E-04	2,93E-02
cg11552025	6	3383656	SLC22A23	Body	-0,011	2,73E-04	2,93E-02
cg00646615	7	142362255		IGR	0,044	2,74E-04	2,93E-02
cg23962436	11	60634092	ZP1	TSS1500	-0,009	2,73E-04	2,93E-02
cg16453673	12	105630771	APPL2	TSS1500	0,011	2,74E-04	2,93E-02
cg13910689	17	66968123		IGR	0,021	2,73E-04	2,93E-02
cg10873538	20	25292112	ABHD12	Body	-0,015	2,74E-04	2,93E-02
cg27187252	22	40754306	ADSL	Body	0,005	2,73E-04	2,93E-02
cg02755935	19	44119993	ZNF428	5'UTR	-0,016	2,74E-04	2,93E-02
cg02254389	1	238083979	LOC100130331	Body	-0,013	2,74E-04	2,93E-02
cg18197963	8	29195838	DUSP4	Body	0,01	2,74E-04	2,93E-02
cg23906264	8	42705540	RNF170	3'UTR	-0,008	2,74E-04	2,93E-02
cg18243346	5	25298993		IGR	-0,018	2,74E-04	2,94E-02
cg03709892	1	19739396	CAPZB	Body	0,006	2,75E-04	2,94E-02
cg02172531	1	78634501		IGR	-0,018	2,75E-04	2,94E-02
cg18278714	2	127752644		IGR	-0,007	2,75E-04	2,94E-02
cg14662379	2	241759279	KIF1A	5'UTR	-0,006	2,74E-04	2,94E-02
cg01342226	4	141034510	MAML3	Body	0,023	2,75E-04	2,94E-02
cg13530485	10	28922671		IGR	-0,01	2,75E-04	2,94E-02
cg02450004	10	102891568	TLX1NB	TSS1500	-0,007	2,74E-04	2,94E-02
cg02106188	12	26205058	RASSF8	TSS1500	-0,005	2,75E-04	2,94E-02
cg23028436	12	27396983	STK38L	TSS200	0,01	2,75E-04	2,94E-02
cg05241431	16	49895551		IGR	0,013	2,75E-04	2,94E-02
cg09291387	17	64636403	PRKCA	Body	-0,009	2,75E-04	2,94E-02
cg15127832	4	184930913	STOX2	Body	-0,007	2,75E-04	2,94E-02
cg22624278	12	131237708		IGR	0,014	2,75E-04	2,94E-02
cg15063715	1	10857436	CASZ1	TSS1500	0,007	2,75E-04	2,94E-02
cg14801768	10	28622505		IGR	-0,012	2,75E-04	2,94E-02
cg16490015	14	104742009		IGR	-0,035	2,75E-04	2,94E-02
cg03369196	3	9771984	BRPF1	TSS1500	0,033	2,75E-04	2,94E-02
cg13118601	11	94597879	AMOTL1	ExonBnd	0,006	2,75E-04	2,94E-02
cg20284025	1	119683198	WARS2	1stExon	-0,004	2,76E-04	2,94E-02
cg02424007	6	110967726	CDK19	Body	-0,008	2,76E-04	2,94E-02
cg27409514	19	1169138	SBNO2	5'UTR	0,017	2,76E-04	2,94E-02
cg15622014	2	44392717		IGR	-0,005	2,76E-04	2,94E-02
cg09145053	16	84693437	KLHL36	Body	-0,011	2,76E-04	2,94E-02
cg10743564	9	33001898	APTX	TSS1500	-0,002	2,76E-04	2,94E-02
cg07975705	5	114961891	TMED7-TICAM2	TSS200	-0,003	2,76E-04	2,94E-02
cg18384402	6	33140761	COL11A2	Body	0,026	2,76E-04	2,94E-02
cg09900758	11	3236849		IGR	-0,014	2,76E-04	2,94E-02
cg00939226	11	133938801	JAM3	TSS200	-0,005	2,76E-04	2,94E-02
cg08759899	5	101831271	SLCO6A1	Body	-0,006	2,76E-04	2,94E-02
cg22506490	1	177002228	ASTN1	Body	0,015	2,76E-04	2,94E-02
cg08898859	9	127876525	SCAI	Body	0,012	2,76E-04	2,94E-02
cg16824858	14	52309194		IGR	-0,02	2,76E-04	2,94E-02
cg03251675	20	18124569	CSRP2BP	Body	0,008	2,76E-04	2,94E-02
cg27139943	2	219502866	ZNF142	3'UTR	0,008	2,76E-04	2,94E-02
cg14886963	5	78002642		IGR	0,008	2,76E-04	2,94E-02
cg06541043	3	127676589	KBTBD12	Body	-0,006	2,77E-04	2,94E-02
cg16250145	1	53906825	FLJ40434	TSS1500	0,014	2,77E-04	2,94E-02
cg25807280	7	54624609	VSTM2A-OT1	TSS200	-0,053	2,77E-04	2,94E-02
cg16775985	7	101435832		IGR	0,014	2,77E-04	2,95E-02
cg20801532	2	241186121		IGR	-0,025	2,77E-04	2,95E-02
cg20293685	10	35363893	CUL2	5'UTR	0,012	2,77E-04	2,95E-02
cg00909073	12	48302254		IGR	-0,011	2,77E-04	2,95E-02
cg07675682	17	78195515	SLC26A11	Body	0,012	2,77E-04	2,95E-02
cg06456376	19	7856679		IGR	0,008	2,77E-04	2,95E-02
cg01364826	1	205392200	LEMD1	TSS1500	-0,039	2,77E-04	2,95E-02
cg04035392	3	47866155	DHX30	Body	0,022	2,77E-04	2,95E-02
cg02185727	16	16043857	ABCC1	Body	-0,003	2,77E-04	2,95E-02
cg18835048	5	10249761	FAM173B	Body	-0,003	2,78E-04	2,95E-02
cg15686393	6	33760988		IGR	0,052	2,78E-04	2,95E-02

cg11131703	14	100847897	WDR25	Body	-0,018	2,78E-04	2,95E-02
cg11653071	20	17949732	SNX5	TSS1500	0,003	2,78E-04	2,95E-02
cg22506343	3	127173672		IGR	-0,013	2,78E-04	2,95E-02
cg07960232	2	142923646		IGR	0,039	2,78E-04	2,95E-02
cg05596911	5	118502651	DMXL1	Body	0,034	2,78E-04	2,95E-02
cg06525498	6	108391237	OSTM1	Body	-0,006	2,78E-04	2,95E-02
cg11603096	22	36925444	EIF3D	TSS200	0,005	2,78E-04	2,95E-02
cg11087774	10	134730605		IGR	-0,017	2,78E-04	2,95E-02
cg27367618	4	110900218	EGF	Body	-0,009	2,78E-04	2,95E-02
cg21609024	1	53795111	LRP8	TSS1500	0,013	2,79E-04	2,96E-02
cg18806266	6	41507480	FOXP4-AS1	Body	0,006	2,79E-04	2,96E-02
cg03773192	10	120965975	GRK5	TSS1500	-0,032	2,79E-04	2,96E-02
cg03838327	9	124991656	LHX6	TSS1500	-0,03	2,79E-04	2,96E-02
cg03622996	19	6422198	KHSRP	Body	0,016	2,79E-04	2,96E-02
cg00094006	1	47976007		IGR	0,004	2,79E-04	2,96E-02
cg14927712	2	140443019		IGR	-0,036	2,79E-04	2,96E-02
cg19698399	15	55700718	CCPG1	TSS200	-0,003	2,79E-04	2,96E-02
cg03226245	19	36486125	SDHAF1	1stExon	-0,004	2,79E-04	2,96E-02
cg27440659	16	57661882	ADGRG1	TSS1500	0,013	2,79E-04	2,96E-02
cg26190476	1	113261723	FAM19A3	TSS1500	0,016	2,79E-04	2,96E-02
cg12435056	3	36918206	TRANK1	Body	-0,019	2,79E-04	2,96E-02
cg11805138	6	31148332		IGR	-0,071	2,79E-04	2,96E-02
cg00083042	10	30404617		IGR	-0,008	2,79E-04	2,96E-02
cg05743112	17	73850681	WBP2	Body	0,029	2,79E-04	2,96E-02
cg27659095	3	18786206		IGR	-0,025	2,79E-04	2,96E-02
cg19649564	11	124932892	SLC37A2	TSS200	0,008	2,80E-04	2,96E-02
cg25140345	1	2440291	PANK4	Body	-0,004	2,80E-04	2,96E-02
cg21338523	2	144105257	ARHGAP15	Body	0,007	2,80E-04	2,96E-02
cg05670323	2	111951702		IGR	-0,005	2,80E-04	2,96E-02
cg24707825	5	49994003	PARP8	Body	0,012	2,80E-04	2,96E-02
cg01382141	6	12151290	HIVEP1	Body	0,021	2,80E-04	2,96E-02
cg13930665	9	10371306	PTPRD	5'UTR	-0,015	2,80E-04	2,96E-02
cg06766960	11	133703094		IGR	-0,057	2,80E-04	2,96E-02
cg26719670	13	88080867		IGR	-0,031	2,80E-04	2,96E-02
cg18318818	17	48640257	CACNA1G	Body	0,03	2,80E-04	2,96E-02
cg13374658	17	77644717		IGR	0,029	2,80E-04	2,96E-02
cg06052200	18	49870300	DCC	Body	-0,07	2,80E-04	2,96E-02
cg18882399	19	42758880	ERF	Body	-0,005	2,80E-04	2,96E-02
cg10149296	2	20795912		IGR	0,031	2,80E-04	2,96E-02
cg18534458	2	42998	FAM110C	Body	-0,028	2,81E-04	2,96E-02
cg20641391	7	54624237	VSTM2A	Body	-0,02	2,81E-04	2,96E-02
cg02625745	2	37458728	CEBPZ	5'UTR	0,006	2,81E-04	2,97E-02
cg04918684	2	39251573	SOS1	Body	0,018	2,81E-04	2,97E-02
cg02606403	2	63276268	LOC100132215	TSS1500	-0,015	2,81E-04	2,97E-02
cg15989168	4	114221696	ANK2	Body	-0,019	2,81E-04	2,97E-02
cg26537431	6	16327119	ATXN1	Body	0,007	2,81E-04	2,97E-02
cg12037550	10	1705054	ADARB2	Body	0,036	2,81E-04	2,97E-02
cg08624200	12	3874722		IGR	-0,01	2,81E-04	2,97E-02
cg07135335	13	101420872	NALCN-AS1	Body	-0,007	2,81E-04	2,97E-02
cg14368592	20	61981657	CHRNA4	Body	0,003	2,81E-04	2,97E-02
cg01118730	10	133767507	PPP2R2D	Body	-0,009	2,81E-04	2,97E-02
cg15298833	12	94541465	PLXNC1	TSS1500	0,011	2,81E-04	2,97E-02
cg22035501	5	112073398	APC	TSS200	0,006	2,81E-04	2,97E-02
cg12523272	12	32137361	KIAA1551	Body	-0,005	2,82E-04	2,97E-02
cg20877181	12	110941087	RAD9B	Body	-0,012	2,82E-04	2,97E-02
cg10140728	15	63569646	APH1B	TSS200	0,012	2,82E-04	2,97E-02
cg17031543	4	52918471	SPATA18	Body	-0,033	2,82E-04	2,97E-02
cg20017891	7	1423720		IGR	0,012	2,82E-04	2,97E-02
cg22524174	19	31820763	TSHZ3	Body	0,006	2,82E-04	2,97E-02
cg17833939	3	100364805	ADGRG7	ExonBnd	0,007	2,82E-04	2,97E-02
cg19321263	6	30160045	TRIM26	Body	0,007	2,82E-04	2,97E-02
cg27415928	8	48109917		IGR	0,017	2,82E-04	2,97E-02
cg11139684	14	35515356	FAM177A1	TSS1500	0,003	2,82E-04	2,97E-02
cg14806867	13	96329355	DNAJC3	TSS200	0,014	2,82E-04	2,97E-02
cg26724375	10	118976795		IGR	-0,005	2,82E-04	2,97E-02
cg09088406	15	27819306		IGR	-0,045	2,83E-04	2,97E-02
cg16054280	3	122517974	DIRC2	Body	0,008	2,83E-04	2,98E-02
cg07727297	3	148027287		IGR	-0,041	2,83E-04	2,98E-02
cg01729125	4	78996164	FRAS1	Body	-0,01	2,83E-04	2,98E-02
cg08814749	5	19478817	CDH18	3'UTR	-0,024	2,83E-04	2,98E-02
cg23977883	5	138749387	DNAJC18	3'UTR	-0,007	2,83E-04	2,98E-02
cg18756426	6	163270478	PACRG	Body	-0,04	2,83E-04	2,98E-02
cg21577169	8	93547498		IGR	-0,016	2,83E-04	2,98E-02

cg14080075	12	82152625	PPFIA2	5'UTR	-0,007	2,83E-04	2,98E-02
cg03207593	13	112695111		IGR	-0,032	2,83E-04	2,98E-02
cg02976694	14	93416999	ITPK1	Body	-0,014	2,83E-04	2,98E-02
cg06358270	20	4796462	RASSF2	TSS1500	0,029	2,83E-04	2,98E-02
cg04314130	2	2692944		IGR	-0,006	2,83E-04	2,98E-02
cg22098076	4	83906009	LIN54	ExonBnd	0,02	2,83E-04	2,98E-02
cg26137432	11	112021514	IL18	Body	-0,005	2,83E-04	2,98E-02
cg08321129	13	78493878	EDNRB	TSS1500	-0,011	2,83E-04	2,98E-02
cg06332127	3	158763606		IGR	-0,017	2,84E-04	2,98E-02
cg15148513	8	142185389	DENND3	Body	0,008	2,84E-04	2,98E-02
cg07206293	1	11768057	C1orf187	Body	0,035	2,84E-04	2,98E-02
cg15111398	1	42504277		IGR	-0,005	2,84E-04	2,98E-02
cg00151525	1	52608409	ZFYVE9	5'UTR	-0,003	2,84E-04	2,98E-02
cg01190931	4	6353157	PPP2R2C	Body	0,006	2,84E-04	2,98E-02
cg22965752	6	29425885	OR2H1	TSS1500	-0,026	2,84E-04	2,98E-02
cg12281249	7	90770562	CDK14	3'UTR	0,031	2,84E-04	2,98E-02
cg26964636	10	24639300	KIAA1217	Body	-0,049	2,84E-04	2,98E-02
cg23509194	18	70803217		IGR	-0,048	2,84E-04	2,98E-02
cg26154812	3	52321085	GLYCTK	TSS1500	0,012	2,84E-04	2,98E-02
cg23867624	6	1610197	FOXC1	TSS1500	-0,004	2,84E-04	2,98E-02
cg03630015	2	219747459	WNT10A	Body	0,031	2,84E-04	2,98E-02
cg14899088	3	184020451	PSMD2	ExonBnd	0,007	2,84E-04	2,98E-02
cg26065929	15	29283544	APBA2	5'UTR	0,013	2,84E-04	2,98E-02
cg17691263	10	38637466		IGR	-0,034	2,84E-04	2,98E-02
cg18802268	12	18735475	PIK3C2G	Body	-0,016	2,84E-04	2,98E-02
cg11371681	1	26606896	SH3BGRL3	Body	-0,005	2,85E-04	2,98E-02
cg27295033	1	27114831	PIGV	5'UTR	-0,005	2,85E-04	2,98E-02
cg00887518	7	50411455	IKZF1	Body	-0,025	2,85E-04	2,98E-02
cg18494812	20	43561748	PABPC1L	Body	0,012	2,85E-04	2,98E-02
cg06094745	2	240118084	HDAC4	Body	0,024	2,85E-04	2,98E-02
cg10606776	5	55610640		IGR	-0,062	2,85E-04	2,98E-02
cg17295447	10	129922685	MKI67	Body	-0,008	2,85E-04	2,98E-02
cg17834443	8	19674713	INTS10	TSS1500	-0,003	2,85E-04	2,98E-02
cg25012675	9	73477878	TRPM3	5'UTR	-0,015	2,85E-04	2,98E-02
cg00547614	6	7107703	RREB1	TSS1500	-0,003	2,85E-04	2,98E-02
cg22023169	15	63606774		IGR	-0,026	2,85E-04	2,98E-02
cg02457826	20	30310733	BCL2L1	TSS200	0,009	2,85E-04	2,98E-02
cg01729887	3	158978383	IQCJ	Body	0,041	2,86E-04	2,99E-02
cg02081364	1	247267551	ZNF669	5'UTR	-0,003	2,86E-04	2,99E-02
cg16238336	3	65465873	MAGI1	Body	-0,068	2,86E-04	2,99E-02
cg11263624	6	35227228	ZNF76	TSS1500	-0,005	2,86E-04	2,99E-02
cg23406971	19	1474586	C19orf25	3'UTR	0,018	2,86E-04	2,99E-02
cg04940655	13	98829550	FARP1	5'UTR	0,01	2,86E-04	2,99E-02
cg15739658	13	97619052	LINC00359	Body	-0,018	2,86E-04	2,99E-02
cg16747903	1	18010399	ARHGEF10L	Body	-0,004	2,86E-04	2,99E-02
cg17673486	2	217790633		IGR	-0,02	2,86E-04	2,99E-02
cg10724261	5	146777290	DPYSL3	Body	-0,006	2,86E-04	2,99E-02
cg07567823	10	95171226	MYOF	Body	0,016	2,87E-04	3,00E-02
cg14976992	2	172335867	DCAF17	Body	0,029	2,87E-04	3,00E-02
cg27579181	5	4970693		IGR	-0,012	2,87E-04	3,00E-02
cg06172191	12	21728772	GYS2	Body	-0,007	2,87E-04	3,00E-02
cg14006031	12	104747135		IGR	-0,011	2,87E-04	3,00E-02
cg20497246	19	49844896	TEAD2	Body	0,007	2,87E-04	3,00E-02
cg00622819	11	417917	SIGIRR	TSS1500	0,024	2,87E-04	3,00E-02
cg08527721	9	3888146	GLIS3	Body	0,046	2,88E-04	3,00E-02
cg21227771	13	22392996		IGR	-0,006	2,88E-04	3,00E-02
cg21348526	15	38647056	SPRED1	3'UTR	0,022	2,88E-04	3,00E-02
cg11917991	19	18345112	PDE4C	5'UTR	-0,051	2,88E-04	3,00E-02
cg09365002	6	33288329	DAXX	Body	0,011	2,88E-04	3,00E-02
cg26460647	4	139772746		IGR	-0,038	2,88E-04	3,00E-02
cg06513244	8	95954357	TP53INP1	5'UTR	-0,022	2,88E-04	3,00E-02
cg19935055	11	66935121	KDM2A	Body	0,009	2,88E-04	3,00E-02
cg05612094	14	45722890	MIS18BP1	TSS1500	0,051	2,88E-04	3,01E-02
cg00459440	6	66496955	MCART3P	TSS1500	-0,008	2,88E-04	3,01E-02
cg17067577	4	113993641	ANK2	Body	0,011	2,88E-04	3,01E-02
cg17360849	1	201688419	MIR5191	TSS1500	0,08	2,89E-04	3,01E-02
cg01577511	3	10987555		IGR	-0,002	2,89E-04	3,01E-02
cg10573303	19	13690312		IGR	-0,054	2,89E-04	3,01E-02
cg14733720	19	44325004	LYPD5	TSS200	-0,034	2,89E-04	3,01E-02
cg14571620	12	123717888	C12orf65	1stExon	-0,003	2,89E-04	3,01E-02
cg01425746	11	45671369	CHST1	Body	-0,069	2,89E-04	3,01E-02
cg00840807	11	394096	PKP3	TSS200	0,014	2,89E-04	3,01E-02
cg17015469	14	103921092	MARK3	Body	0,004	2,89E-04	3,01E-02

cg03299065	13	19919394	LOC100101938	TSS1500	-0,047	2,89E-04	3,01E-02
cg25594486	19	51165441	SHANK1	Body	-0,039	2,89E-04	3,01E-02
cg23789846	21	30375227		IGR	-0,005	2,89E-04	3,01E-02
cg06765111	7	44925077	PURB	TSS200	0,016	2,90E-04	3,01E-02
cg09830206	11	65987958	PACS1	Body	0,016	2,90E-04	3,01E-02
cg09331184	12	53784584	SP1	Body	0,03	2,90E-04	3,01E-02
cg24527382	2	49631579		IGR	-0,043	2,90E-04	3,02E-02
cg04141610	7	558976	PDGFA	5'UTR	0,005	2,90E-04	3,02E-02
cg06048154	16	1311632		IGR	-0,041	2,91E-04	3,02E-02
cg07031748	17	17184658	COPS3	TSS200	-0,003	2,91E-04	3,02E-02
cg01654463	22	24504055	CABIN1	Body	0,011	2,91E-04	3,02E-02
cg01670098	1	39748831	MACF1	Body	-0,011	2,91E-04	3,02E-02
cg08181572	5	38854383	OSMR	5'UTR	-0,029	2,91E-04	3,02E-02
cg05013942	17	36003336	DDX52	5'UTR	0,01	2,91E-04	3,02E-02
cg03790394	3	10465377	ATP2B2	Body	0,05	2,91E-04	3,02E-02
cg18001327	13	71849589		IGR	0,032	2,91E-04	3,02E-02
cg23403388	2	38796085	HNRNPLL	Body	0,03	2,91E-04	3,02E-02
cg12986236	6	37784591		IGR	0,008	2,91E-04	3,02E-02
cg12733656	7	6388695	C7orf70	TSS200	-0,004	2,91E-04	3,02E-02
cg07156394	7	55623053	VOPP1	Body	0,011	2,91E-04	3,02E-02
cg13335691	7	56242142		IGR	-0,064	2,91E-04	3,02E-02
cg03063274	14	69002262	RAD51B	Body	-0,006	2,91E-04	3,02E-02
cg09989938	16	28944403	CD19	Body	0,015	2,91E-04	3,02E-02
cg14813588	19	12595728	ZNF709	TSS200	-0,002	2,91E-04	3,02E-02
cg02507175	21	44394436	PKNOX1	TSS1500	-0,004	2,91E-04	3,02E-02
cg00885365	2	215702120		IGR	0,029	2,91E-04	3,02E-02
cg24782486	12	46384666	SFRS2IP	TSS1500	0,006	2,92E-04	3,02E-02
cg24744172	2	236402993	AGAP1	1stExon	-0,01	2,92E-04	3,02E-02
cg10391424	22	49417369		IGR	0,025	2,92E-04	3,02E-02
cg11374733	15	66794527	RPL4	Body	0,009	2,92E-04	3,02E-02
cg18798372	8	124557975		IGR	-0,007	2,92E-04	3,02E-02
cg12145739	14	24912198	SDR39U1	TSS200	-0,02	2,92E-04	3,02E-02
cg21894287	18	72837531		IGR	-0,073	2,92E-04	3,02E-02
cg09726509	11	57286633		IGR	-0,005	2,92E-04	3,02E-02
cg04235361	11	120727936	GRIK4	Body	-0,013	2,92E-04	3,02E-02
cg15759147	14	104604243	KIF26A	TSS1500	-0,006	2,92E-04	3,02E-02
cg21213617	9	124989241	LHX6	Body	-0,056	2,92E-04	3,02E-02
cg14022995	11	2397350	CD81	TSS1500	0,007	2,92E-04	3,02E-02
cg17064981	11	126936873		IGR	0,061	2,92E-04	3,02E-02
cg01938476	12	7076853	SCARNA12	TSS200	0,012	2,92E-04	3,02E-02
cg19153003	12	121698481	CAMKK2	Body	-0,008	2,92E-04	3,02E-02
cg07573285	6	1250977		IGR	-0,064	2,92E-04	3,03E-02
cg04628802	16	71420541	CALB2	Body	-0,044	2,92E-04	3,03E-02
cg19878627	2	54087184	LOC100302652	TSS200	-0,033	2,93E-04	3,03E-02
cg17832639	6	32078133	TNXB	TSS1500	0,018	2,93E-04	3,03E-02
cg15202115	8	79305876		IGR	-0,009	2,93E-04	3,03E-02
cg06817690	3	9882439	RPUSD3	Body	0,015	2,93E-04	3,03E-02
cg18686217	3	49546906	DAG1	5'UTR	0,006	2,93E-04	3,03E-02
cg15332128	7	17514262	LOC101927630	Body	-0,007	2,93E-04	3,03E-02
cg11343271	11	69041495		IGR	0,022	2,93E-04	3,03E-02
cg22110428	19	51980908	CEACAM18	TSS1500	0,091	2,93E-04	3,03E-02
cg09470268	1	18617697	IGSF21	Body	-0,032	2,93E-04	3,03E-02
cg06223422	2	158913237	UPP2	Body	-0,008	2,93E-04	3,03E-02
cg25498973	5	179867108		IGR	0,044	2,93E-04	3,03E-02
cg12648671	18	77377538		IGR	-0,085	2,93E-04	3,03E-02
cg20406979	6	167373233		IGR	0,038	2,93E-04	3,03E-02
cg16300565	5	32388665	ZFR	Body	-0,032	2,93E-04	3,03E-02
cg05196487	8	28929078	KIF13B	Body	-0,017	2,93E-04	3,03E-02
cg17735716	19	7692222	XAB2	Body	-0,007	2,93E-04	3,03E-02
cg05596350	7	1802343		IGR	-0,026	2,94E-04	3,03E-02
cg27388567	1	221997465		IGR	-0,025	2,94E-04	3,03E-02
cg18608609	2	47706460	MSH2	Body	-0,012	2,94E-04	3,03E-02
cg20263236	3	61993451	PTPRG	Body	-0,02	2,94E-04	3,03E-02
cg24308312	4	164910272	MARCH1	5'UTR	-0,061	2,94E-04	3,03E-02
cg07985227	10	2936434		IGR	-0,006	2,94E-04	3,03E-02
cg19618279	17	40715228	COASY	1stExon	0,02	2,94E-04	3,03E-02
cg17854544	5	141344968	RNF14	TSS1500	0,007	2,94E-04	3,03E-02
cg13429194	12	96661032	ELK3	3'UTR	0,02	2,94E-04	3,03E-02
cg12167239	22	19166808	SLC25A1	TSS1500	-0,003	2,94E-04	3,03E-02
cg20632978	17	79167828	AZI1	Body	0,004	2,94E-04	3,03E-02
cg01756063	2	18742450	RDH14	TSS1500	-0,021	2,94E-04	3,03E-02
cg25890827	6	130704571	TMEM200A	5'UTR	0,011	2,94E-04	3,03E-02
cg12970008	11	3186095	OSBPL5	5'UTR	-0,006	2,94E-04	3,03E-02

cg24517863	1	233601965		IGR	-0,012	2,95E-04	3,03E-02
cg25687021	3	193753082		IGR	0,018	2,94E-04	3,03E-02
cg01493038	7	107818639	NRCAM	Body	-0,012	2,95E-04	3,03E-02
cg11658975	6	170700107	FAM120B	Body	0,009	2,95E-04	3,03E-02
cg10827226	1	20977640	PINK1	3'UTR	0,006	2,95E-04	3,03E-02
cg10284115	3	15841404	ANKRD28	Body	0,022	2,95E-04	3,03E-02
cg09831413	4	157670994		IGR	0,005	2,95E-04	3,04E-02
cg13963807	11	67165707	PPP1CA	3'UTR	0,013	2,95E-04	3,04E-02
cg25241573	19	28237512		IGR	-0,042	2,95E-04	3,04E-02
cg14044930	14	35509692		IGR	-0,011	2,95E-04	3,04E-02
cg17758563	6	16323116	ATXN1	Body	-0,041	2,95E-04	3,04E-02
cg16399182	6	99842025	COQ3	1stExon	-0,008	2,95E-04	3,04E-02
cg14275207	15	74495401	STRA6	5'UTR	-0,032	2,95E-04	3,04E-02
cg17348722	17	7588137	WRAP53	TSS1500	0,041	2,96E-04	3,04E-02
cg24366429	17	77207424	HRNBP3	5'UTR	0,032	2,96E-04	3,04E-02
cg07470708	19	49564932	NTF4	Body	-0,027	2,95E-04	3,04E-02
cg14416269	4	6271139	WFS1	TSS1500	-0,034	2,96E-04	3,04E-02
cg12417603	12	130502586		IGR	-0,058	2,96E-04	3,04E-02
cg16648579	22	23041412		IGR	-0,018	2,96E-04	3,04E-02
cg21908635	12	105142673	CHST11	Body	0,019	2,96E-04	3,04E-02
cg04144183	21	33709438	URB1	Body	0,012	2,96E-04	3,04E-02
cg02113385	18	77203443	NFATC1	Body	0,017	2,96E-04	3,04E-02
cg05766539	3	55572619	ERC2	3'UTR	-0,024	2,96E-04	3,04E-02
cg21909090	9	79521848	PRUNE2	TSS1500	0,036	2,96E-04	3,04E-02
cg15423587	5	115169714	ATG12	Body	-0,006	2,96E-04	3,04E-02
cg27452068	19	45895808	PPP1R13L	Body	0,005	2,96E-04	3,04E-02
cg13723853	14	69322248		IGR	-0,015	2,96E-04	3,04E-02
cg15651727	1	18703121	IGSF21	Body	-0,042	2,97E-04	3,04E-02
cg22470838	3	165501377	BCHE	Body	-0,042	2,97E-04	3,04E-02
cg17397671	7	80549872	SEMA3C	TSS1500	-0,01	2,97E-04	3,04E-02
cg07997221	11	63917707	MACROD1	Body	-0,027	2,97E-04	3,04E-02
cg25103726	12	130892289	RIMBP2	Body	0,004	2,97E-04	3,04E-02
cg13468874	18	70987115		IGR	0,009	2,97E-04	3,04E-02
cg23118401	20	36032645	SRC	3'UTR	0,004	2,97E-04	3,04E-02
cg24204949	3	64085259	PRICKLE2-AS1	Body	-0,01	2,97E-04	3,04E-02
cg08028091	10	44144199	ZNF32	5'UTR	-0,004	2,97E-04	3,04E-02
cg03768897	10	127588272	FANK1	Body	0,038	2,97E-04	3,04E-02
cg20302660	11	5894127		IGR	0,053	2,97E-04	3,04E-02
cg01694696	12	56321112	WIBG	TSS1500	-0,003	2,97E-04	3,04E-02
cg03807590	12	105550162	KIAA1033	Body	0,011	2,97E-04	3,04E-02
cg22820425	16	57831837	KIFC3	1stExon	-0,026	2,97E-04	3,04E-02
cg17035844	1	171640211		IGR	-0,015	2,98E-04	3,04E-02
cg20322766	2	7999224		IGR	0,012	2,98E-04	3,04E-02
cg04261937	4	185450622		IGR	-0,004	2,98E-04	3,04E-02
cg00554754	5	81715144		IGR	-0,026	2,97E-04	3,04E-02
cg00127047	5	142150919	ARHGAP26	Body	-0,004	2,98E-04	3,04E-02
cg07635744	6	13365049	GFOD1	Body	-0,009	2,97E-04	3,04E-02
cg16991860	6	73358990	KCNQ5-IT1	Body	0,032	2,98E-04	3,04E-02
cg10673833	7	45018849	MYO1G	TSS200	0,006	2,97E-04	3,04E-02
cg17886471	8	10339410		IGR	0,005	2,98E-04	3,04E-02
cg02636041	10	43698008	RASGEF1A	Body	-0,005	2,98E-04	3,04E-02
cg05308498	10	62174599	ANK3	Body	-0,009	2,98E-04	3,04E-02
cg05671385	11	104915759	CASP1	Body	-0,005	2,97E-04	3,04E-02
cg20157841	12	6333176	CD9	Body	0,012	2,98E-04	3,04E-02
cg04010286	17	30697051	ZNF207	3'UTR	0,008	2,98E-04	3,04E-02
cg22290966	18	52495613	RAB27B	TSS200	-0,004	2,97E-04	3,04E-02
cg03457587	19	54694791	MBOAT7	TSS1500	-0,005	2,98E-04	3,04E-02
cg14758556	20	62440591		IGR	-0,02	2,98E-04	3,04E-02
cg00046018	1	202318086	PPP1R12B	1stExon	-0,005	2,98E-04	3,05E-02
cg00400028	3	140949916	PXYLP1	TSS1500	0,022	2,98E-04	3,05E-02
cg15410528	19	35617470	LGI4	Body	-0,007	2,98E-04	3,05E-02
cg08757742	5	80256371	RASGRF2	TSS200	-0,014	2,98E-04	3,05E-02
cg07743359	8	72248546	EYA1	5'UTR	-0,03	2,98E-04	3,05E-02
cg12554051	12	665863	B4GALNT3	Body	-0,003	2,98E-04	3,05E-02
cg14220272	21	38445791	PIGP	TSS1500	-0,006	2,98E-04	3,05E-02
cg23023178	19	15236599	ILVBL	TSS200	-0,003	2,98E-04	3,05E-02
cg00145904	1	10697703	CASZ1	3'UTR	0,018	2,99E-04	3,05E-02
cg25314486	1	21830411		IGR	0,009	2,99E-04	3,05E-02
cg26606373	1	54618779	CDCP2	TSS200	0,018	2,99E-04	3,05E-02
cg19161000	1	177151620	FAM5B	5'UTR	0,019	2,99E-04	3,05E-02
cg15850484	2	42104651	LOC388942	TSS200	-0,017	2,99E-04	3,05E-02
cg10466962	2	109526581	EDAR	Body	-0,006	2,99E-04	3,05E-02
cg00367488	2	120020686	STEAP3	Body	0,006	3,00E-04	3,05E-02

cg21913580	4	6772859		IGR	0,015	2,99E-04	3,05E-02
cg23506964	4	56413211	CLOCK	TSS1500	-0,003	2,99E-04	3,05E-02
cg09351288	5	180625118	TRIM7	Body	0,028	2,99E-04	3,05E-02
cg21113768	6	144386848	PLAGL1	TSS1500	0,024	2,99E-04	3,05E-02
cg09432202	6	163768551		IGR	0,009	2,99E-04	3,05E-02
cg01650433	7	143815533		IGR	-0,025	2,99E-04	3,05E-02
cg15550363	10	72318226	PALD1	Body	-0,005	2,99E-04	3,05E-02
cg25345625	10	131576889		IGR	-0,007	2,99E-04	3,05E-02
cg12999267	12	94376970		IGR	-0,044	2,99E-04	3,05E-02
cg02269049	12	104680606	TXNRD1	Body	-0,004	2,99E-04	3,05E-02
cg01705312	12	123437313	ABCB9	Body	0,014	2,99E-04	3,05E-02
cg27537570	12	133179978		IGR	-0,053	2,99E-04	3,05E-02
cg07819175	21	30359239	LTN1	ExonBnd	0,008	2,99E-04	3,05E-02
cg16497828	22	39053541	FAM227A	TSS1500	-0,04	3,00E-04	3,05E-02
cg22884899	17	6939299	SLC16A13	TSS200	-0,004	3,00E-04	3,05E-02
cg16111737	12	117408163	FBXW8	Body	0,006	3,00E-04	3,05E-02
cg02627824	11	66756107		IGR	0,016	3,00E-04	3,05E-02
cg22893486	1	31044446		IGR	-0,041	3,00E-04	3,05E-02
cg20768445	18	76601755		IGR	-0,02	3,00E-04	3,05E-02
cg16233489	5	37379269	WDR70	TSS200	-0,008	3,00E-04	3,06E-02
cg04610601	5	168174897	SLIT3	Body	-0,006	3,00E-04	3,06E-02
cg17071957	9	124029926	GSN	TSS1500	0,021	3,01E-04	3,06E-02
cg19597529	3	122399230	PARP14	TSS1500	-0,015	3,01E-04	3,06E-02
cg09495953	4	7688794	SORCS2	Body	0,027	3,01E-04	3,06E-02
cg15862514	1	9034468	CA6	Body	-0,013	3,01E-04	3,06E-02
cg10124440	12	6960295	USP5	TSS1500	0,022	3,01E-04	3,06E-02
cg22425341	10	99582305		IGR	0,007	3,01E-04	3,06E-02
cg07636194	6	27107282	HIST1H2BK	3'UTR	-0,007	3,01E-04	3,06E-02
cg12011711	17	65496598	PITPNC1	Body	0,035	3,01E-04	3,06E-02
cg08017249	1	2289112	MORN1	Body	-0,02	3,02E-04	3,06E-02
cg24067958	6	148761328	SASH1	ExonBnd	-0,007	3,02E-04	3,06E-02
cg08081859	8	97400276	LOC102724804	TSS1500	-0,006	3,02E-04	3,06E-02
cg07319998	12	694784	NINJ2	Body	0,009	3,02E-04	3,06E-02
cg20524889	13	73649835	KLF5	Body	-0,031	3,02E-04	3,06E-02
cg10388716	5	169187711	DOCK2	Body	-0,004	3,02E-04	3,06E-02
cg24687051	6	73332073	KCNQ5	1stExon	-0,007	3,02E-04	3,06E-02
cg08098650	14	104409977	RD3L	TSS1500	0,007	3,02E-04	3,06E-02
cg04345744	1	196507743	KCNT2	Body	0,086	3,02E-04	3,06E-02
cg25770249	12	56719867	PAN2	Body	-0,009	3,02E-04	3,06E-02
cg23942665	5	151250531	GLRA1	Body	-0,015	3,03E-04	3,07E-02
cg12900185	6	152731028	SYNE1	Body	0,005	3,03E-04	3,07E-02
cg17179911	8	674434	ERICH1	Body	-0,095	3,03E-04	3,07E-02
cg16485474	8	2549080		IGR	-0,019	3,02E-04	3,07E-02
cg18230995	11	12109751		IGR	-0,038	3,03E-04	3,07E-02
cg09480307	16	77756392	NUDT7	TSS200	-0,005	3,03E-04	3,07E-02
cg07978456	16	86817342		IGR	-0,009	3,03E-04	3,07E-02
cg01339697	17	27601409	NUFIP2	Body	-0,015	3,03E-04	3,07E-02
cg19751245	17	27919632	ANKRD13B	TSS1500	0,034	3,02E-04	3,07E-02
cg05845689	11	67499849		IGR	-0,045	3,03E-04	3,07E-02
cg18852567	1	35351627	DLGAP3	Body	-0,019	3,03E-04	3,07E-02
cg11772355	2	216363412		IGR	-0,01	3,03E-04	3,07E-02
cg25969272	4	78034410		IGR	-0,009	3,03E-04	3,07E-02
cg02698620	7	45026579	C7orf40	TSS1500	-0,006	3,03E-04	3,07E-02
cg25947998	8	42948467	SGK196	TSS200	-0,003	3,03E-04	3,07E-02
cg12640713	9	139971854	UAP1L1	TSS200	0,008	3,03E-04	3,07E-02
cg08544355	13	113893067	CUL4A	Body	0,025	3,03E-04	3,07E-02
cg03612350	16	2500696	CCNF	Body	-0,005	3,03E-04	3,07E-02
cg24638851	18	6406993	L3MBTL4	5'UTR	-0,007	3,03E-04	3,07E-02
cg12605354	8	93895711		IGR	0,022	3,03E-04	3,07E-02
cg16289029	12	31473577	FAM60A	5'UTR	0,023	3,03E-04	3,07E-02
cg09598285	20	4741866		IGR	-0,035	3,03E-04	3,07E-02
cg26754187	3	82207271		IGR	0,011	3,04E-04	3,07E-02
cg27629807	5	109812946	TMEM232	Body	0,023	3,04E-04	3,07E-02
cg23303108	8	23083578	TNFRSF10A	TSS1500	0,02	3,04E-04	3,07E-02
cg23413475	10	95750037		IGR	-0,012	3,04E-04	3,07E-02
cg14546261	12	7052696	C12orf57	TSS1500	-0,004	3,04E-04	3,07E-02
cg27432036	17	7382829	ZBTB4	1stExon	0,014	3,04E-04	3,07E-02
cg25182784	15	51413285		IGR	-0,007	3,04E-04	3,07E-02
cg00370820	22	39101671	GTPBP1	TSS200	0,009	3,04E-04	3,07E-02
cg12935209	10	108924553	SORCS1	TSS200	0,025	3,04E-04	3,07E-02
cg23478124	11	85776966	PICALM	Body	0,03	3,04E-04	3,07E-02
cg01937601	16	89130931		IGR	0,009	3,04E-04	3,07E-02
cg12678351	9	129289500		IGR	0,005	3,04E-04	3,07E-02

cg11915620	1	25380219		IGR	0,007	3,04E-04	3,07E-02
cg10493506	2	230340591	DNER	Body	-0,045	3,04E-04	3,07E-02
cg22912044	15	57948188	GCOM1	Body	0,019	3,04E-04	3,07E-02
cg04330730	1	48472619		IGR	-0,016	3,05E-04	3,07E-02
cg06742800	2	27255708	TMEM214	TSS200	-0,009	3,05E-04	3,07E-02
cg19936051	10	110063192		IGR	0,008	3,05E-04	3,07E-02
cg05935360	19	11639865	ECSIT	5'UTR	-0,003	3,05E-04	3,07E-02
cg05467845	12	25249040	LRMP	Body	0,027	3,05E-04	3,07E-02
cg11665185	17	7499868	FXR2	Body	-0,006	3,05E-04	3,07E-02
cg22284448	2	27008234	CENPA	TSS1500	0,051	3,05E-04	3,08E-02
cg12337559	4	3449904	HGFAC	Body	0,003	3,05E-04	3,08E-02
cg00723553	6	20218373		IGR	-0,005	3,05E-04	3,08E-02
cg02324703	10	26954087		IGR	-0,007	3,05E-04	3,08E-02
cg14596352	17	78777181	RPTOR	Body	-0,013	3,05E-04	3,08E-02
cg19450531	3	47422572	PTPN23	1stExon	0,003	3,05E-04	3,08E-02
cg26459588	11	11838030		IGR	-0,02	3,06E-04	3,08E-02
cg06679777	13	30881210	KATNAL1	TSS200	0,008	3,06E-04	3,08E-02
cg25548316	11	2922812	SLC22A18AS	5'UTR	-0,014	3,06E-04	3,08E-02
cg24769369	5	140502262	PCDHB4	1stExon	-0,009	3,06E-04	3,08E-02
cg00575879	9	34478186	DNAI1	Body	-0,028	3,06E-04	3,08E-02
cg10274141	11	76092112	LOC100506127	TSS1500	-0,005	3,06E-04	3,08E-02
cg24818895	14	72043073	SIPA1L1	5'UTR	-0,005	3,06E-04	3,08E-02
cg27134767	16	89713339	CHMP1A	Body	0,021	3,06E-04	3,08E-02
cg06406536	18	76766588		IGR	-0,015	3,06E-04	3,08E-02
cg08977883	9	104145989	BAAT	TSS200	-0,019	3,06E-04	3,08E-02
cg01835489	12	53299310	KRT8	TSS1500	-0,005	3,06E-04	3,08E-02
cg19655070	17	43237981	HEXIM2	TSS1500	-0,005	3,06E-04	3,08E-02
cg01529419	19	14682919	NDUFB7	TSS200	-0,003	3,06E-04	3,08E-02
cg14610884	6	116575311	TSPYL4	TSS200	-0,004	3,06E-04	3,08E-02
cg04376055	8	27214274	PTK2B	5'UTR	-0,016	3,06E-04	3,08E-02
cg12731325	17	7671330	DNAH2	Body	-0,015	3,06E-04	3,08E-02
cg19624873	1	201857955	SHISA4	5'UTR	-0,007	3,07E-04	3,08E-02
cg03953513	7	96287204		IGR	-0,014	3,07E-04	3,08E-02
cg13153540	7	47128394		IGR	0,017	3,07E-04	3,08E-02
cg15188908	5	1840272		IGR	0,005	3,07E-04	3,08E-02
cg14466442	10	126242636	LHPP	Body	-0,02	3,07E-04	3,08E-02
cg13078421	19	2624622	GNG7	5'UTR	-0,027	3,07E-04	3,08E-02
cg22604833	6	103429659		IGR	-0,055	3,07E-04	3,08E-02
cg24235613	6	31765083	VARS	TSS1500	0,023	3,07E-04	3,08E-02
cg00728090	16	87552701		IGR	0,037	3,07E-04	3,08E-02
cg18519323	19	39728296		IGR	0,024	3,07E-04	3,09E-02
cg10528346	5	130274876		IGR	0,013	3,08E-04	3,09E-02
cg19946085	15	51559439	CYP19A1	5'UTR	-0,009	3,08E-04	3,09E-02
cg04215385	21	37445335	CBR1	3'UTR	0,005	3,08E-04	3,09E-02
cg01861131	16	4845708	LOC440335	Body	0,022	3,08E-04	3,09E-02
cg13545645	1	100732082	RTCA-AS1	TSS1500	-0,004	3,09E-04	3,09E-02
cg17881406	1	156442152	MEF2D	Body	0,013	3,09E-04	3,09E-02
cg05237432	1	230804229	COG2	Body	-0,005	3,09E-04	3,09E-02
cg00580613	2	38657763		IGR	-0,032	3,08E-04	3,09E-02
cg09826605	2	177843826		IGR	0,026	3,09E-04	3,09E-02
cg07204522	3	63964946	ATXN7	Body	-0,01	3,08E-04	3,09E-02
cg24725647	3	127544607		IGR	-0,01	3,09E-04	3,09E-02
cg22539471	3	190049044		IGR	-0,007	3,09E-04	3,09E-02
cg09670276	3	195997776	PCYT1A	5'UTR	-0,008	3,09E-04	3,09E-02
cg03365190	4	1987655	WHSC2	Body	0,008	3,09E-04	3,09E-02
cg09986977	8	71520066	LACTB2-AS1	TSS1500	0,003	3,08E-04	3,09E-02
cg10630212	10	13236960	MCM10	Body	0,006	3,09E-04	3,09E-02
cg09793376	12	130627354		IGR	-0,024	3,08E-04	3,09E-02
cg19911719	17	7689569	DNAH2	Body	-0,011	3,09E-04	3,09E-02
cg21737734	18	44236847	LOXHD1	Body	-0,011	3,09E-04	3,09E-02
cg22078572	18	61033618	KDSR	Body	-0,003	3,09E-04	3,09E-02
cg05480894	7	143582605	TCAF1	TSS200	-0,03	3,09E-04	3,09E-02
cg01193320	16	53926049	FTO	Body	-0,021	3,09E-04	3,09E-02
cg16577665	1	119702694		IGR	-0,009	3,09E-04	3,09E-02
cg14516946	5	65056260	NLN	Body	-0,026	3,10E-04	3,09E-02
cg06823965	7	101632645	CUX1	Body	-0,003	3,09E-04	3,09E-02
cg24494316	7	113726705		IGR	-0,006	3,10E-04	3,09E-02
cg07674511	8	49219836		IGR	-0,039	3,10E-04	3,09E-02
cg13706769	9	140400693	PNPLA7	Body	0,006	3,10E-04	3,09E-02
cg26690331	12	105465008	ALDH1L2	Body	-0,035	3,09E-04	3,09E-02
cg19831775	12	108054679		IGR	-0,02	3,09E-04	3,09E-02
cg04605759	12	112550483		IGR	-0,005	3,10E-04	3,09E-02
cg08968830	12	115255983		IGR	-0,02	3,09E-04	3,09E-02

cg08123903	13	114792210	RASA3	Body	-0,005	3,09E-04	3,09E-02
cg12712374	14	23398828	PRMT5	TSS200	-0,002	3,09E-04	3,09E-02
cg11011736	17	33934776	AP2B1	Body	0,013	3,09E-04	3,09E-02
cg02224329	2	185740590	ZNF804A	Body	-0,013	3,10E-04	3,09E-02
cg22646782	19	5152272	KDM4B	3'UTR	-0,011	3,10E-04	3,09E-02
cg25586816	13	88841040	LOC105370306	Body	-0,005	3,10E-04	3,09E-02
cg15461431	2	43836757		IGR	0,008	3,10E-04	3,10E-02
cg06805387	7	88339486		IGR	-0,015	3,10E-04	3,10E-02
cg06492412	20	634850	SRXN1	TSS1500	0,031	3,10E-04	3,10E-02
cg23431697	17	16789912		IGR	-0,006	3,10E-04	3,10E-02
cg06812361	2	66591811		IGR	-0,032	3,11E-04	3,10E-02
cg16579969	6	70992823	COL9A1	1stExon	-0,005	3,10E-04	3,10E-02
cg19359842	6	158316250	SNX9	Body	-0,016	3,11E-04	3,10E-02
cg04342458	10	104781910	CNNM2	Body	0,021	3,11E-04	3,10E-02
cg18801045	5	10751081	DAP	Body	0,008	3,11E-04	3,10E-02
cg12673638	9	134102723	NUP214	Body	0,025	3,11E-04	3,10E-02
cg12382333	17	47115593	IGF2BP1	Body	-0,007	3,11E-04	3,10E-02
cg01983185	19	8428990	ANGPTL4	TSS200	-0,01	3,11E-04	3,10E-02
cg27156267	12	133758001	ZNF268	1stExon	-0,005	3,11E-04	3,10E-02
cg00956759	17	30023399		IGR	-0,006	3,11E-04	3,10E-02
cg25332502	18	74832372	MBP	5'UTR	0,022	3,11E-04	3,10E-02
cg18333018	12	106472544	NUAK1	Body	-0,014	3,11E-04	3,10E-02
cg01245102	20	62236331	GMEB2	Body	-0,007	3,11E-04	3,10E-02
cg07318627	16	88820993	FAM38A	Body	0,023	3,11E-04	3,10E-02
cg15533961	2	74731376	LBX2-AS1	Body	-0,006	3,12E-04	3,10E-02
cg15973470	3	189706355	P3H2	Body	0,046	3,12E-04	3,10E-02
cg26124717	5	90675823	LOC100129716	TSS1500	-0,003	3,12E-04	3,10E-02
cg17213919	6	69040829	LOC101928280	TSS1500	0,042	3,12E-04	3,10E-02
cg07061255	7	154991498		IGR	-0,011	3,12E-04	3,10E-02
cg13452652	9	124070037	GSN	Body	-0,006	3,12E-04	3,10E-02
cg03601752	19	2621851	GNG7	5'UTR	0,01	3,12E-04	3,10E-02
cg07728212	22	32022290	PISD	5'UTR	0,021	3,12E-04	3,10E-02
cg27505684	1	174968509	CACYBP	TSS1500	-0,004	3,12E-04	3,10E-02
cg03787603	7	101768610	CUX1	Body	0,006	3,12E-04	3,10E-02
cg09414280	12	120130253	CIT	Body	0,005	3,12E-04	3,10E-02
cg19678940	7	66255551	RABGEF1	Body	-0,013	3,12E-04	3,10E-02
cg22282477	15	92952569	ST8SIA2	Body	0,011	3,12E-04	3,10E-02
cg07180674	11	102959223	DCUN1D5	Body	0,008	3,12E-04	3,11E-02
cg25115537	8	123801352	ZHX2	5'UTR	0,02	3,13E-04	3,11E-02
cg26621248	11	831888	CD151	TSS1500	0,034	3,12E-04	3,11E-02
cg04543124	11	134897577		IGR	-0,057	3,12E-04	3,11E-02
cg04744810	12	40618786	LRRK2	TSS200	-0,004	3,13E-04	3,11E-02
cg07379480	19	46149243	EML2	TSS1500	-0,003	3,13E-04	3,11E-02
cg01128422	12	129594574	LOC283352	Body	0,05	3,13E-04	3,11E-02
cg20884502	12	112546705	NAA25	TSS200	-0,004	3,13E-04	3,11E-02
cg02719427	11	2151725	INS-IGF2	Body	0,005	3,13E-04	3,11E-02
cg27445281	6	6956307		IGR	-0,011	3,13E-04	3,11E-02
cg19914124	1	3073626	PRDM16	Body	-0,012	3,13E-04	3,11E-02
cg21212034	3	194390306	LSG1	Body	0,008	3,13E-04	3,11E-02
cg05293407	6	28891967	TRIM27	TSS200	-0,008	3,13E-04	3,11E-02
cg14974807	7	79739813		IGR	0,031	3,13E-04	3,11E-02
cg11180638	11	17948881	SERGEF	Body	0,012	3,14E-04	3,11E-02
cg17208425	11	122756965	C11orf63	Body	-0,005	3,14E-04	3,11E-02
cg18567954	12	113496168	DTX1	1stExon	0,019	3,13E-04	3,11E-02
cg05086975	17	48945232		IGR	-0,004	3,13E-04	3,11E-02
cg16106133	19	39233699	CAPN12	Body	0,008	3,13E-04	3,11E-02
cg05890956	20	31668448	C20orf186	TSS1500	-0,048	3,13E-04	3,11E-02
cg25406699	1	54744131	SSBP3	Body	0,014	3,14E-04	3,11E-02
cg02820104	8	123938870	ZHX2	5'UTR	-0,007	3,14E-04	3,11E-02
cg10186039	15	66543546	MEGF11	5'UTR	0,008	3,14E-04	3,11E-02
cg11262850	5	154071158		IGR	0,04	3,14E-04	3,11E-02
cg16896205	1	111413643	CD53	TSS200	0,015	3,14E-04	3,11E-02
cg25681122	3	194832600	C3orf21	Body	0,011	3,14E-04	3,11E-02
cg07976297	8	78310675		IGR	-0,024	3,14E-04	3,11E-02
cg19782407	10	105257095	NEURL	Body	-0,015	3,14E-04	3,11E-02
cg07592963	11	2890649	KCNQ1DN	TSS1500	-0,021	3,14E-04	3,11E-02
cg11779145	12	119327268		IGR	-0,045	3,14E-04	3,11E-02
cg09219421	19	13779879		IGR	-0,02	3,14E-04	3,11E-02
cg09308536	10	134016855	DPYSL4	Body	0,019	3,14E-04	3,11E-02
cg14923547	7	155842198		IGR	-0,022	3,15E-04	3,11E-02
cg21935292	12	65152931	GNS	1stExon	-0,004	3,15E-04	3,12E-02
cg18832655	10	11805122	ECHDC3	Body	0,02	3,15E-04	3,12E-02
cg22772438	22	44556035	PARVB	Body	0,027	3,15E-04	3,12E-02

cg22273744	4	95972614	BMPR1B	TSS1500	-0,008	3,15E-04	3,12E-02
cg27066321	1	10534234	PEX14	TSS1500	0,011	3,15E-04	3,12E-02
cg07106095	3	122297436	PARP15	Body	0,024	3,15E-04	3,12E-02
cg27264126	10	4379534		IGR	0,007	3,15E-04	3,12E-02
cg07909359	16	67208405	NOL3	Body	0,006	3,15E-04	3,12E-02
cg25631551	18	6682636		IGR	-0,012	3,15E-04	3,12E-02
cg05081346	2	227828214	RHBDD1	Body	-0,013	3,16E-04	3,12E-02
cg14671069	6	152793813	SYNE1	Body	0,031	3,16E-04	3,12E-02
cg03340215	15	83315615	CPEB1	Body	-0,049	3,16E-04	3,12E-02
cg07872108	4	33882186		IGR	-0,008	3,16E-04	3,12E-02
cg22607981	2	175553284		IGR	0,025	3,16E-04	3,12E-02
cg24140528	17	30158473		IGR	-0,028	3,16E-04	3,12E-02
cg26825569	6	46704077	PLA2G7	TSS1500	-0,017	3,16E-04	3,12E-02
cg23501196	3	12358936	PPARG	5'UTR	0,021	3,16E-04	3,12E-02
cg20170405	10	27099671	ABI1	Body	0,006	3,16E-04	3,12E-02
cg16975973	12	77459865	E2F7	TSS1500	-0,006	3,16E-04	3,12E-02
cg14038594	1	159793925		IGR	0,006	3,16E-04	3,12E-02
cg00087355	2	239216996		IGR	0,021	3,17E-04	3,13E-02
cg24250435	3	182583252	ATP11B	ExonBnd	0,011	3,17E-04	3,13E-02
cg00141479	10	23003092	PIP4K2A	Body	-0,004	3,17E-04	3,13E-02
cg05370094	16	88946905	CBFA2T3	Body	0,013	3,17E-04	3,13E-02
cg25655069	2	73114919	SPR	Body	-0,016	3,17E-04	3,13E-02
cg02625502	6	4491606		IGR	-0,013	3,17E-04	3,13E-02
cg07965823	14	77964712	ISM2	Body	-0,012	3,17E-04	3,13E-02
cg07066794	14	89014677	PTPN21	Body	0,015	3,17E-04	3,13E-02
cg26477844	19	55691742	SYT5	TSS200	-0,003	3,17E-04	3,13E-02
cg00972922	3	168866785	MECOM	TSS1500	-0,02	3,17E-04	3,13E-02
cg26619106	2	137119539		IGR	-0,006	3,17E-04	3,13E-02
cg09061047	2	53733249		IGR	0,008	3,17E-04	3,13E-02
cg06372632	10	1768665	ADARB2	Body	-0,019	3,17E-04	3,13E-02
cg06494284	9	136738660	VAV2	Body	0,007	3,18E-04	3,13E-02
cg21407701	3	153118398		IGR	0,015	3,18E-04	3,13E-02
cg08102602	12	6875642	PTMS	1stExon	0,01	3,18E-04	3,13E-02
cg14433213	9	114706631		IGR	0,012	3,18E-04	3,13E-02
cg06390664	5	55196307	IL31RA	Body	-0,012	3,18E-04	3,13E-02
cg11782516	8	56669017	TMEM68	Body	0,033	3,18E-04	3,13E-02
cg24203376	10	115938868	TDRD1	TSS200	-0,016	3,18E-04	3,14E-02
cg14417676	2	202316517	TRAK2	TSS200	0,025	3,18E-04	3,14E-02
cg06680465	4	3113829	HTT	Body	0,007	3,18E-04	3,14E-02
cg18298050	22	26829938	ASPHD2	Body	-0,02	3,19E-04	3,14E-02
cg05972298	6	4348932		IGR	0,049	3,19E-04	3,14E-02
cg15436096	14	59931922	GPR135	1stExon	-0,005	3,19E-04	3,14E-02
cg01083135	16	81122724	GCSH	Body	-0,007	3,19E-04	3,14E-02
cg08806779	16	86965605		IGR	-0,007	3,19E-04	3,14E-02
cg20061882	2	25046245	ADCY3	Body	-0,007	3,19E-04	3,14E-02
cg16967003	7	1293599		IGR	-0,059	3,19E-04	3,14E-02
cg22986222	6	32095276	ATF6B	Body	0,024	3,19E-04	3,14E-02
cg22343980	1	168148117	TIPRL	TSS200	0,005	3,20E-04	3,14E-02
cg18499883	8	37395908		IGR	-0,009	3,19E-04	3,14E-02
cg22844018	14	107200026		IGR	-0,016	3,20E-04	3,14E-02
cg18023251	20	56281839	PMEPA1	5'UTR	0,014	3,20E-04	3,14E-02
cg06994786	8	105162043	RIMS2	Body	0,026	3,20E-04	3,14E-02
cg11760016	8	110347041	NUDCD1	TSS1500	-0,008	3,20E-04	3,14E-02
cg12900790	1	114375675	PTPN22	3'UTR	0,044	3,20E-04	3,14E-02
cg23974473	5	140625794	PCDHB15	1stExon	-0,06	3,20E-04	3,14E-02
cg18792584	1	179851234	TOR1AIP1	TSS200	-0,002	3,20E-04	3,14E-02
cg14054781	1	1663781	SLC35E2	Body	0,004	3,20E-04	3,14E-02
cg00769845	1	35435704		IGR	-0,005	3,20E-04	3,15E-02
cg15487791	4	26585040	TBC1D19	TSS1500	-0,05	3,20E-04	3,15E-02
cg06723284	15	41063963	C15orf62	1stExon	0,005	3,20E-04	3,15E-02
cg05979549	1	54629222		IGR	-0,017	3,21E-04	3,15E-02
cg11647681	5	140810161	PCDHGA4	Body	-0,047	3,21E-04	3,15E-02
cg00773446	7	23164507	KLHL7	Body	0,027	3,21E-04	3,15E-02
cg21130998	7	75677243	STYXL1	1stExon	-0,003	3,21E-04	3,15E-02
cg15448098	7	100083084	NYAP1	Body	0,016	3,21E-04	3,15E-02
cg17622297	9	33983558	UBAP2	5'UTR	0,011	3,21E-04	3,15E-02
cg14364186	9	135619500	C9orf98	Body	0,039	3,21E-04	3,15E-02
cg07342618	10	3300407		IGR	-0,004	3,21E-04	3,15E-02
cg10383724	11	12228521	MICAL2	Body	0,013	3,21E-04	3,15E-02
cg09214535	13	30092023	SLC7A1	Body	0,007	3,21E-04	3,15E-02
cg13446496	14	70051570		IGR	0,04	3,21E-04	3,15E-02
cg20600338	14	71786811		IGR	-0,003	3,21E-04	3,15E-02
cg17287585	15	65627108	IGDCC3	Body	0,014	3,21E-04	3,15E-02

cg00483282	15	99702301	TTC23	Body	0,005	3,21E-04	3,15E-02
cg27160920	16	10755037	TEKT5	Body	-0,02	3,21E-04	3,15E-02
cg07449655	18	13073110	CEP192	Body	0,01	3,21E-04	3,15E-02
cg12475921	1	174787829	RABGAP1L	Body	-0,009	3,21E-04	3,15E-02
cg16808912	3	99603495	MIR548G	Body	-0,048	3,21E-04	3,15E-02
cg26547506	12	56863038	SPRYD4	Body	0,034	3,22E-04	3,15E-02
cg26656684	8	59465595	SDCBP	TSS200	0,006	3,22E-04	3,15E-02
cg04856586	11	26309744		IGR	-0,017	3,22E-04	3,15E-02
cg08768873	16	12213533	SNX29	Body	-0,021	3,22E-04	3,15E-02
cg10491368	5	172570703	BNIP1	TSS1500	-0,01	3,22E-04	3,15E-02
cg04391722	8	81397601	ZBTB10	TSS1500	-0,011	3,22E-04	3,15E-02
cg22397625	11	119044725	NLRX1	Body	0,008	3,22E-04	3,15E-02
cg14850601	18	44143050	LOXHD1	Body	0,014	3,22E-04	3,15E-02
cg17649532	22	50880360	PPP6R2	Body	0,007	3,22E-04	3,15E-02
cg27125439	10	29311888		IGR	-0,01	3,22E-04	3,15E-02
cg18143333	15	89225506		IGR	-0,017	3,22E-04	3,15E-02
cg14573810	15	66270794	MEGF11	Body	0,027	3,22E-04	3,15E-02
cg15006883	2	153520430	PRPF40A	Body	0,015	3,22E-04	3,15E-02
cg24148044	16	85061578	KIAA0513	5'UTR	0,006	3,23E-04	3,15E-02
cg11776773	17	61767711	MAP3K3	Body	-0,003	3,23E-04	3,15E-02
cg11001092	3	93686084	PROS1	Body	0,007	3,23E-04	3,16E-02
cg25645199	5	171533681	STK10	Body	0,003	3,23E-04	3,16E-02
cg06613817	1	119910670	HAO2	TSS1500	0,006	3,23E-04	3,16E-02
cg15972506	3	11079298	SLC6A1	3'UTR	0,022	3,23E-04	3,16E-02
cg03533390	6	154756615	CNKSR3	Body	-0,006	3,23E-04	3,16E-02
cg11488101	10	43313023	BMS1	Body	-0,019	3,24E-04	3,16E-02
cg09169514	2	233741082	C2orf82	3'UTR	-0,004	3,24E-04	3,16E-02
cg14452314	12	70341552	MYRFL	Body	-0,022	3,24E-04	3,16E-02
cg03875571	3	48718585	NCKIPSD	Body	0,012	3,24E-04	3,17E-02
cg23228491	7	56020011	MRPS17	5'UTR	-0,005	3,24E-04	3,17E-02
cg01408558	19	10563212	PDE4A	Body	0,016	3,24E-04	3,17E-02
cg06303867	4	118055551		IGR	-0,021	3,24E-04	3,17E-02
cg19226872	2	19558542	OSR1	TSS200	-0,005	3,25E-04	3,17E-02
cg27448124	14	37669932	MIPOL1	5'UTR	0,037	3,25E-04	3,17E-02
cg19254240	14	74206265	ELMSAN1	Body	0,007	3,25E-04	3,17E-02
cg07572773	17	39472015	KRTAP17-1	TSS200	-0,017	3,25E-04	3,17E-02
cg02722429	18	21017489	C18orf45	Body	-0,007	3,25E-04	3,17E-02
cg03030685	20	2853339	PTPRA	TSS1500	-0,003	3,25E-04	3,17E-02
cg21042902	5	176873577	PRR7	TSS1500	-0,006	3,25E-04	3,17E-02
cg13229363	7	157961051	PTPRN2	Body	-0,026	3,25E-04	3,17E-02
cg19433860	21	45822237	TRPM2	Body	0,016	3,25E-04	3,17E-02
cg06764674	9	7935778		IGR	-0,006	3,25E-04	3,17E-02
cg10471832	2	238707401	RBM44	1stExon	-0,021	3,25E-04	3,17E-02
cg08818893	19	50476388	SIGLEC16	Body	0,015	3,25E-04	3,17E-02
cg02628631	2	8718510	LOC101929567	Body	-0,003	3,25E-04	3,17E-02
cg11525210	5	149445234	CSF1R	Body	0,011	3,25E-04	3,17E-02
cg07184401	10	3307763		IGR	0,024	3,26E-04	3,17E-02
cg13539803	13	110424782	IRS2	Body	-0,011	3,26E-04	3,17E-02
cg09906193	4	38664952	FLJ13197	Body	-0,003	3,26E-04	3,17E-02
cg26708920	10	13826317	FRMD4A	Body	0,134	3,26E-04	3,17E-02
cg26221766	4	42305072		IGR	-0,007	3,26E-04	3,17E-02
cg15698851	8	29940391	TMEM66	1stExon	0,01	3,26E-04	3,17E-02
cg23391574	11	66232362	PELI3	TSS1500	0,007	3,26E-04	3,17E-02
cg13333625	17	6070023		IGR	0,036	3,26E-04	3,17E-02
cg00565242	3	167582594		IGR	0,039	3,26E-04	3,17E-02
cg08407503	12	30842614	IPO8	Body	-0,008	3,26E-04	3,17E-02
cg21827277	12	67913815	LOC100507175	TSS200	-0,007	3,26E-04	3,17E-02
cg23677352	4	149715675		IGR	-0,023	3,26E-04	3,17E-02
cg03894037	13	102391911	FGF14	Body	-0,026	3,26E-04	3,17E-02
cg00115313	1	193029121	TROVE2	5'UTR	-0,009	3,26E-04	3,17E-02
cg08976908	15	75116357	LMAN1L	Body	0,017	3,27E-04	3,18E-02
cg00854995	5	133512930	SKP1	TSS1500	-0,003	3,27E-04	3,18E-02
cg14749123	19	8667984	ADAMTS10	Body	-0,006	3,27E-04	3,18E-02
cg27075055	3	179131551	GNB4	ExonBnd	0,044	3,27E-04	3,18E-02
cg00969565	17	79946805	ASPSCR1	Body	-0,005	3,27E-04	3,18E-02
cg14231959	8	1897035	ARHGEF10	Body	0,036	3,27E-04	3,18E-02
cg13331591	10	112466738	RBM20	Body	0,029	3,27E-04	3,18E-02
cg16802932	17	6653041		IGR	0,028	3,27E-04	3,18E-02
cg16011852	1	150531225	ADAMTSL4	Body	0,008	3,28E-04	3,18E-02
cg16259229	5	140420475		IGR	-0,046	3,28E-04	3,18E-02
cg17619965	6	35216799	SCUBE3	3'UTR	-0,026	3,28E-04	3,18E-02
cg10905699	11	77531602	AAMDC	TSS1500	-0,007	3,28E-04	3,18E-02
cg05180206	16	74249500		IGR	-0,01	3,28E-04	3,18E-02

cg05560575	12	116919971		IGR	-0,027	3,28E-04	3,18E-02
cg03813487	6	33025746		IGR	0,048	3,28E-04	3,18E-02
cg11357787	15	63354797	TPM1	Body	-0,003	3,28E-04	3,18E-02
cg03499659	1	74100712		IGR	-0,023	3,28E-04	3,18E-02
cg24077260	4	183068417		IGR	0,039	3,28E-04	3,18E-02
cg09338797	6	90603585	GJA10	TSS1500	-0,007	3,29E-04	3,19E-02
cg13680864	10	17686119	STAM	TSS200	0,007	3,29E-04	3,19E-02
cg02352325	12	68644392	IL22	Body	-0,033	3,29E-04	3,19E-02
cg24680415	17	70358572		IGR	-0,012	3,29E-04	3,19E-02
cg25749306	19	46997775	PNMAL2	1stExon	-0,017	3,28E-04	3,19E-02
cg17916267	10	99513736	ZFYVE27	Body	-0,006	3,29E-04	3,19E-02
cg24678438	17	79484403		IGR	0,027	3,29E-04	3,19E-02
cg08325413	7	114717840	LINC01393	TSS1500	0,046	3,29E-04	3,19E-02
cg17436460	10	17028480	CUBN	Body	-0,007	3,29E-04	3,19E-02
cg16705777	19	18758056	KLHL26	Body	0,007	3,29E-04	3,19E-02
cg22489099	2	200322556	SATB2	5'UTR	0,008	3,29E-04	3,19E-02
cg15574890	15	60386144		IGR	-0,04	3,29E-04	3,19E-02
cg27537600	8	101170391	SPAG1	5'UTR	-0,004	3,30E-04	3,19E-02
cg19307389	1	1028583	C1orf159	5'UTR	0,02	3,30E-04	3,19E-02
cg07317529	4	23883895	PPARGC1A	Body	-0,005	3,30E-04	3,19E-02
cg11371551	6	56380508	RNU6-71P	Body	-0,049	3,30E-04	3,19E-02
cg12669354	11	831584	CD151	TSS1500	0,018	3,30E-04	3,19E-02
cg05912053	15	60919520	RORA	Body	0,014	3,30E-04	3,19E-02
cg23825213	18	77623475	KCNG2	TSS200	-0,071	3,30E-04	3,19E-02
cg04383400	11	1945162	TNNT3	Body	-0,011	3,30E-04	3,19E-02
cg12035374	13	22621217		IGR	-0,012	3,30E-04	3,19E-02
cg23221414	16	8992664	USP7	Body	-0,005	3,30E-04	3,19E-02
cg07451261	10	102415086		IGR	-0,022	3,31E-04	3,20E-02
cg13189979	12	80987359	PTPRQ	Body	-0,012	3,31E-04	3,20E-02
cg04904276	16	11835562	TXNDC11	Body	0,043	3,30E-04	3,20E-02
cg26194477	14	29234890	FOXG1	TSS1500	-0,026	3,31E-04	3,20E-02
cg08613597	16	56224627	GNAO1	TSS1500	-0,013	3,31E-04	3,20E-02
cg21217221	14	65881787	FUT8	5'UTR	0,029	3,31E-04	3,20E-02
cg24474958	6	53975391	MLIP	TSS1500	0,022	3,31E-04	3,20E-02
cg21335509	7	123389943	WASL	TSS1500	-0,047	3,31E-04	3,20E-02
cg07160793	2	382090		IGR	-0,043	3,31E-04	3,20E-02
cg02620388	7	100271115	GNB2	TSS1500	-0,004	3,31E-04	3,20E-02
cg00617876	13	42848057	AKAP11	5'UTR	0,008	3,31E-04	3,20E-02
cg07276356	22	20007899	TANGO2	TSS1500	0,007	3,31E-04	3,20E-02
cg02891633	3	119343091	PLA1A	Body	0,009	3,31E-04	3,20E-02
cg10657739	2	170550809	C2orf77	1stExon	-0,003	3,31E-04	3,20E-02
cg05306831	11	56467759	OR9G1	TSS200	-0,017	3,32E-04	3,20E-02
cg10070442	21	40822525	SH3BGR	TSS1500	-0,013	3,32E-04	3,20E-02
cg26628410	10	61563135	CCDC6	Body	0,004	3,32E-04	3,20E-02
cg23855392	15	80189782	MTHFS	TSS1500	0,006	3,32E-04	3,20E-02
cg06468780	21	31798236	KRTAP13-3	TSS200	-0,014	3,32E-04	3,20E-02
cg11399053	11	130781648	SNX19	Body	-0,02	3,32E-04	3,20E-02
cg09970565	1	38354103	INPP5B	Body	-0,006	3,32E-04	3,21E-02
cg17895870	19	16254474	HSH2D	5'UTR	0,007	3,33E-04	3,21E-02
cg16444942	12	129309117	SLC15A4	TSS1500	0,025	3,33E-04	3,21E-02
cg14036069	9	127047671	NEK6	5'UTR	-0,015	3,33E-04	3,21E-02
cg04349177	11	62172512		IGR	0,019	3,33E-04	3,21E-02
cg02759713	10	71868555	H2AFY2	Body	0,005	3,33E-04	3,21E-02
cg02310882	16	73093296	ZFHX3	TSS1500	-0,012	3,33E-04	3,21E-02
cg18318518	20	60871831		IGR	0,014	3,33E-04	3,21E-02
cg17248752	19	39838214	SAMD4B	5'UTR	-0,022	3,33E-04	3,21E-02
cg13008977	5	168271613	SLIT3	Body	-0,007	3,34E-04	3,21E-02
cg07001877	1	211504385	TRAF5	5'UTR	0,007	3,34E-04	3,21E-02
cg05048372	18	60382543	PHLPP1	TSS200	-0,003	3,34E-04	3,22E-02
cg04630412	4	24009753		IGR	-0,042	3,34E-04	3,22E-02
cg01743873	11	833708	CD151	5'UTR	0,036	3,34E-04	3,22E-02
cg07394446	15	100881458	ADAMTS17	Body	-0,012	3,34E-04	3,22E-02
cg13293524	17	46651822	HOXB3	TSS200	0,014	3,34E-04	3,22E-02
cg09247219	1	185120505	TRMT1L	Body	0,033	3,34E-04	3,22E-02
cg20745987	4	155702760	RBM46	1stExon	-0,065	3,34E-04	3,22E-02
cg24817154	6	27858279	HIST1H3J	1stExon	-0,009	3,34E-04	3,22E-02
cg10304506	7	127014560	ZNF800	Body	0,012	3,35E-04	3,22E-02
cg04890646	3	150480280	SIAH2	1stExon	-0,003	3,35E-04	3,22E-02
cg27143142	12	79514170	SYT1	5'UTR	-0,012	3,35E-04	3,22E-02
cg10240348	19	2182961	DOT1L	Body	0,018	3,35E-04	3,22E-02
cg03708790	22	43084979		IGR	-0,008	3,35E-04	3,22E-02
cg22805796	17	79829393	ARHGDIA	TSS200	-0,004	3,35E-04	3,22E-02
cg26220061	1	70819839	HHLA3	TSS1500	-0,006	3,35E-04	3,22E-02

cg01215495	1	144931367	PDE4DIP	1stExon	0,022	3,35E-04	3,22E-02
cg19616851	7	63363811		IGR	0,063	3,35E-04	3,22E-02
cg23473632	6	160417673	IGF2R	Body	-0,012	3,35E-04	3,22E-02
cg25914621	12	49583559	TUBA1A	TSS1500	0,007	3,35E-04	3,22E-02
cg23601397	2	227665895		IGR	-0,005	3,36E-04	3,22E-02
cg01079779	3	57176708	IL17RD	Body	0,028	3,36E-04	3,22E-02
cg07038689	14	81690541		IGR	0,016	3,36E-04	3,22E-02
cg27118298	14	103272938	TRAF3	5'UTR	0,019	3,36E-04	3,22E-02
cg05368134	1	24993975	SRRM1	Body	0,013	3,36E-04	3,23E-02
cg02248355	10	73078591	SLC29A3	TSS1500	-0,005	3,36E-04	3,23E-02
cg14695540	19	9904086		IGR	-0,033	3,36E-04	3,23E-02
cg15706223	1	1141934	TNFRSF18	1stExon	-0,005	3,36E-04	3,23E-02
cg18815589	13	66981290	PCDH9	Body	-0,027	3,36E-04	3,23E-02
cg18708075	19	52074312	ZNF175	TSS1500	-0,006	3,36E-04	3,23E-02
cg24668155	15	76629156	ISL2	5'UTR	0,014	3,36E-04	3,23E-02
cg13460311	18	72837782		IGR	-0,044	3,36E-04	3,23E-02
cg14561934	10	15412027	FAM171A1	Body	0,036	3,36E-04	3,23E-02
cg02957305	14	51452451	TRIM9	Body	-0,007	3,36E-04	3,23E-02
cg03108238	17	7340375	TMEM102	Body	-0,006	3,37E-04	3,23E-02
cg02015800	22	20937702	MED15	Body	-0,006	3,37E-04	3,23E-02
cg26940100	1	228395760	OBSCN	TSS200	0,022	3,37E-04	3,23E-02
cg08435945	2	169324661	LASS6	Body	-0,015	3,37E-04	3,23E-02
cg16659538	3	108208917	MYH15	Body	-0,037	3,37E-04	3,23E-02
cg21484573	6	163730853	PACRG	Body	0,038	3,37E-04	3,23E-02
cg10825645	10	43846235		IGR	-0,055	3,37E-04	3,23E-02
cg20446948	11	15963167		IGR	-0,04	3,37E-04	3,23E-02
cg01729592	16	54035302	FTO	Body	0,01	3,37E-04	3,23E-02
cg11952344	17	80398854	HEXDC	Body	0,028	3,37E-04	3,23E-02
cg19459212	17	7998770		IGR	0,015	3,37E-04	3,23E-02
cg02157334	19	46073383	OPA3	Body	0,024	3,37E-04	3,23E-02
cg16093863	3	144393272		IGR	-0,03	3,37E-04	3,23E-02
cg13031545	6	13284862	LOC100130357	Body	-0,008	3,38E-04	3,23E-02
cg03094728	7	91687463	AKAP9	Body	-0,031	3,38E-04	3,23E-02
cg13361585	1	246959476		IGR	0,009	3,38E-04	3,23E-02
cg15523543	2	192503429		IGR	-0,019	3,38E-04	3,24E-02
cg15776615	3	43538738	ANO10	Body	0,009	3,38E-04	3,24E-02
cg00320625	6	30584596	MRPS18B	TSS1500	-0,008	3,38E-04	3,24E-02
cg14114043	6	170531435		IGR	0,048	3,38E-04	3,24E-02
cg07410266	7	103848657	ORC5	TSS200	-0,006	3,38E-04	3,24E-02
cg24135583	7	157353191	PTPRN2	Body	0,018	3,38E-04	3,24E-02
cg13934792	9	139694739	KIAA1984	Body	-0,007	3,38E-04	3,24E-02
cg22488904	1	6661399	KLHL21	Body	-0,004	3,38E-04	3,24E-02
cg22062555	19	45664251	NKPD1	TSS1500	0,045	3,38E-04	3,24E-02
cg11894898	5	31007981		IGR	0,014	3,39E-04	3,24E-02
cg00745835	10	80778842	LOC283050	Body	-0,006	3,39E-04	3,24E-02
cg06923616	18	66381343	CCDC102B	TSS1500	0,041	3,39E-04	3,24E-02
cg27559724	3	186524679	RFC4	TSS200	0,005	3,39E-04	3,24E-02
cg16773043	7	1963149	MAD1L1	Body	-0,004	3,39E-04	3,24E-02
cg13185239	2	111526591	ACOXL	Body	0,014	3,39E-04	3,24E-02
cg10407646	13	27957896		IGR	0,014	3,39E-04	3,24E-02
cg01819910	1	201687738	NAV1	Body	-0,005	3,39E-04	3,24E-02
cg12762583	10	35463740	CREM	5'UTR	-0,011	3,39E-04	3,24E-02
cg09927529	12	10365082	GABARAPL1	TSS1500	-0,004	3,39E-04	3,24E-02
cg17450434	20	43350973	WISP2	Body	0,005	3,39E-04	3,24E-02
cg18039340	12	7261952	C1RL	TSS200	-0,004	3,39E-04	3,24E-02
cg22526139	15	90030153	RHCG	Body	-0,052	3,40E-04	3,24E-02
cg12142438	3	185427184	IGF2BP2	Body	0,018	3,40E-04	3,24E-02
cg14103772	6	131204270	EPB41L2	Body	-0,009	3,40E-04	3,24E-02
cg13104618	10	18951094	ARL5B	Body	0,005	3,40E-04	3,24E-02
cg06007291	11	7534103	PPFIBP2	TSS1500	0,034	3,40E-04	3,24E-02
cg23239812	11	44626949	CD82	Body	0,008	3,40E-04	3,24E-02
cg26859986	12	95274215		IGR	0,046	3,40E-04	3,24E-02
cg05929117	1	1983033	PRKCZ	Body	0,04	3,40E-04	3,24E-02
cg01380747	3	127973356	EEFSEC	Body	0,008	3,40E-04	3,24E-02
cg21459419	3	138680674		IGR	0,016	3,40E-04	3,24E-02
cg01105385	5	67584222	PIK3R1	Body	-0,004	3,40E-04	3,24E-02
cg18776945	5	147024842	JAKMIP2	Body	0,007	3,40E-04	3,24E-02
cg01177488	12	7282511	CLSTN3	TSS1500	-0,003	3,40E-04	3,24E-02
cg22861340	1	29101849		IGR	0,014	3,41E-04	3,24E-02
cg00376141	13	110450377		IGR	0,028	3,41E-04	3,24E-02
cg24843790	21	44727494		IGR	0,028	3,41E-04	3,24E-02
cg00041084	5	64398983		IGR	-0,004	3,41E-04	3,25E-02
cg04242525	8	144236576		IGR	-0,01	3,41E-04	3,25E-02

cg11374145	12	108934086	SART3	Body	0,027	3,41E-04	3,25E-02
cg21391551	16	66960351	RRAD	TSS1500	0,013	3,41E-04	3,25E-02
cg24785473	3	35681883		IGR	-0,042	3,41E-04	3,25E-02
cg01449756	4	128125758		IGR	-0,012	3,41E-04	3,25E-02
cg18943676	15	33840807	RYR3	Body	0,006	3,41E-04	3,25E-02
cg13932046	10	78343530		IGR	0,035	3,42E-04	3,25E-02
cg21253881	15	68867830		IGR	-0,016	3,42E-04	3,25E-02
cg22811350	8	655845	ERICH1	Body	-0,007	3,42E-04	3,25E-02
cg08747583	19	55144503	LILRB1	Body	0,012	3,42E-04	3,25E-02
cg26262900	3	46768322		IGR	-0,018	3,42E-04	3,25E-02
cg09987982	3	48343027	NME6	5'UTR	-0,005	3,42E-04	3,25E-02
cg17385077	6	1439033		IGR	-0,042	3,42E-04	3,25E-02
cg13247871	7	88632493	ZNF804B	Body	-0,006	3,42E-04	3,25E-02
cg20353489	12	34758307		IGR	0,064	3,42E-04	3,25E-02
cg00011350	12	49444296	MLL2	Body	0,007	3,42E-04	3,25E-02
cg26184409	9	134614177	RAPGEF1	TSS1500	0,028	3,42E-04	3,25E-02
cg10008328	16	52655368		IGR	-0,041	3,42E-04	3,25E-02
cg11214251	5	57100485		IGR	-0,013	3,43E-04	3,25E-02
cg14920716	19	36726985	ZNF146	5'UTR	-0,006	3,43E-04	3,25E-02
cg11196034	15	76053406		IGR	-0,008	3,43E-04	3,26E-02
cg08823065	17	26345670		IGR	-0,007	3,43E-04	3,26E-02
cg04072648	15	25200145	SNURF	5'UTR	0,016	3,43E-04	3,26E-02
cg04780697	17	16120448	PIGL	TSS200	-0,003	3,43E-04	3,26E-02
cg14479751	19	48867168	SYNGR4	TSS1500	-0,01	3,43E-04	3,26E-02
cg01468272	4	188534677	LOC100506272	Body	-0,012	3,43E-04	3,26E-02
cg11094112	8	111777660		IGR	0,02	3,43E-04	3,26E-02
cg05492845	11	64002298	VEGFB	5'UTR	-0,004	3,43E-04	3,26E-02
cg10215604	19	15375418	BRD4	Body	0,007	3,43E-04	3,26E-02
cg12305870	20	398182	RBCK1	Body	0,008	3,43E-04	3,26E-02
cg08686931	4	139360705		IGR	-0,016	3,44E-04	3,26E-02
cg20096490	13	110873982	COL4A1	Body	0,015	3,44E-04	3,26E-02
cg05673146	5	133860019	JADE2	TSS1500	0,006	3,44E-04	3,26E-02
cg04800909	7	24784732	DFNA5	Body	-0,009	3,44E-04	3,26E-02
cg23094251	7	136088596		IGR	-0,011	3,44E-04	3,26E-02
cg00615852	6	111137376	CDK19	TSS1500	-0,003	3,44E-04	3,26E-02
cg03444737	2	72074179		IGR	0,046	3,44E-04	3,26E-02
cg15337578	12	6655355	IFFO1	Body	0,014	3,44E-04	3,26E-02
cg07434500	21	30671675	BACH1	TSS200	0,007	3,44E-04	3,26E-02
cg00504690	1	64240202	ROR1	Body	-0,006	3,44E-04	3,26E-02
cg18384097	1	202129566	PTPN7	TSS1500	-0,009	3,44E-04	3,26E-02
cg08732879	17	41180520	RND2	Body	0,014	3,44E-04	3,26E-02
cg21597180	11	134940129		IGR	0,026	3,44E-04	3,26E-02
cg18201392	1	185023741	RNF2	5'UTR	-0,004	3,44E-04	3,26E-02
cg02335971	8	121457381	MTBP	TSS1500	0,007	3,45E-04	3,26E-02
cg04251669	5	1252302		IGR	0,023	3,45E-04	3,26E-02
cg25817867	9	136205820		IGR	-0,007	3,45E-04	3,26E-02
cg23549693	11	65746292	SART1	Body	-0,014	3,45E-04	3,27E-02
cg00530905	1	9582655		IGR	0,027	3,45E-04	3,27E-02
cg25863732	14	96136582	TCL6	Body	-0,044	3,45E-04	3,27E-02
cg06842886	3	186572382	ADIPOQ	Body	-0,006	3,45E-04	3,27E-02
cg06956784	1	18060505		IGR	0,028	3,46E-04	3,27E-02
cg23198568	10	33623905	NRP1	TSS200	-0,005	3,46E-04	3,27E-02
cg09951445	12	6419280	PLEKHG6	TSS1500	0,008	3,46E-04	3,27E-02
cg09996789	16	1310584		IGR	-0,023	3,46E-04	3,27E-02
cg17362819	3	45689110	LIMD1	Body	0,035	3,46E-04	3,27E-02
cg02444961	7	94284539	SGCE	Body	-0,009	3,46E-04	3,27E-02
cg06053417	16	591589	SOLH	5'UTR	0,005	3,46E-04	3,27E-02
cg12111166	16	88751442	SNAI3	Body	0,021	3,46E-04	3,27E-02
cg21017352	1	232220804		IGR	-0,032	3,46E-04	3,27E-02
cg14717796	8	27348650	EPHX2	5'UTR	-0,008	3,46E-04	3,27E-02
cg26371471	8	56662092	TMEM68	Body	-0,011	3,46E-04	3,27E-02
cg27546017	9	120480847		IGR	0,033	3,46E-04	3,27E-02
cg27032142	15	85201545	NMB	1stExon	-0,003	3,46E-04	3,27E-02
cg02834465	20	37820003		IGR	-0,014	3,46E-04	3,27E-02
cg16837662	21	46712073	LOC642852	Body	0,027	3,46E-04	3,27E-02
cg22362386	21	35284672	ATP5O	Body	0,021	3,46E-04	3,27E-02
cg06971517	9	112012724	EPB41L4B	Body	-0,006	3,47E-04	3,27E-02
cg06086465	9	137543933	COL5A1	Body	-0,025	3,47E-04	3,27E-02
cg21333964	11	118955461	HMBS	TSS200	-0,006	3,47E-04	3,27E-02
cg02510428	22	21318763	AIFM3	TSS1500	-0,036	3,47E-04	3,27E-02
cg12297077	6	43277256	CRIP3	TSS1500	-0,02	3,47E-04	3,27E-02
cg07140660	5	171433509	FBXW11	1stExon	-0,005	3,47E-04	3,27E-02
cg26623696	11	90797970		IGR	0,028	3,47E-04	3,27E-02

cg11144644	4	110409433	SEC24B	Body	0,019	3,47E-04	3,28E-02
cg06321045	6	31088343	CDSN	TSS200	0,04	3,47E-04	3,28E-02
cg27142354	17	53713264		IGR	-0,006	3,47E-04	3,28E-02
cg18430156	2	187558795	FAM171B	5'UTR	-0,006	3,48E-04	3,28E-02
cg02852182	6	14075688		IGR	-0,01	3,48E-04	3,28E-02
cg27274463	12	110338238	TCHP	5'UTR	-0,006	3,48E-04	3,28E-02
cg03437924	16	25123326	LCMT1	Body	-0,011	3,48E-04	3,28E-02
cg01893041	5	67583972	PIK3R1	Body	-0,006	3,48E-04	3,28E-02
cg04038932	9	135286214	C9orf171	Body	0,022	3,48E-04	3,28E-02
cg02858510	14	51818419		IGR	-0,014	3,48E-04	3,28E-02
cg26394071	20	18964864		IGR	-0,016	3,48E-04	3,28E-02
cg26937148	5	92919647	NR2F1	1stExon	-0,01	3,48E-04	3,28E-02
cg06218228	2	238875540	UBE2F	TSS200	0,005	3,49E-04	3,28E-02
cg24183575	3	49497929		IGR	0,011	3,48E-04	3,28E-02
cg04288388	4	114199108	ANK2	ExonBnd	-0,011	3,48E-04	3,28E-02
cg05357958	12	15838065	EPS8	5'UTR	-0,025	3,49E-04	3,28E-02
cg21317687	12	124118107	GTF2H3	TSS200	-0,004	3,48E-04	3,28E-02
cg14669515	1	9386821	SPSB1	5'UTR	0,013	3,49E-04	3,28E-02
cg20276503	1	111992326	WDR77	TSS1500	-0,009	3,49E-04	3,28E-02
cg06725695	5	109155778	MAN2A1	Body	0,009	3,49E-04	3,28E-02
cg10447080	6	76203225	FILIP1	5'UTR	-0,051	3,49E-04	3,28E-02
cg24423424	22	36718563	MYH9	Body	-0,01	3,49E-04	3,28E-02
cg10216790	2	46553115	EPAS1	Body	0,007	3,49E-04	3,28E-02
cg25649765	2	11672761	GREB1	TSS1500	0,039	3,49E-04	3,28E-02
cg14995507	15	41136797	SPINT1	1stExon	0,007	3,49E-04	3,28E-02
cg02206983	18	7857178	PTPRM	Body	-0,009	3,49E-04	3,28E-02
cg02167945	1	26028827	MAN1C1	Body	0,015	3,49E-04	3,29E-02
cg24751551	21	39627840	KCNJ15	TSS1500	0,037	3,50E-04	3,29E-02
cg21926390	22	51019849	CHKB	ExonBnd	0,007	3,50E-04	3,29E-02
cg16774853	17	74169640	RNF157	Body	-0,005	3,50E-04	3,29E-02
cg11649182	1	1539923		IGR	-0,017	3,50E-04	3,29E-02
cg10878768	1	33430605	RNF19B	TSS1500	-0,003	3,50E-04	3,29E-02
cg06318676	1	92077357		IGR	0,013	3,50E-04	3,29E-02
cg10130187	4	16766526	LDB2	Body	-0,007	3,50E-04	3,29E-02
cg00537185	10	75258323	PPP3CB-AS1	Body	-0,006	3,50E-04	3,29E-02
cg20272558	12	56914153	RBMS2	TSS1500	-0,013	3,50E-04	3,29E-02
cg09259018	21	37954631		IGR	-0,061	3,50E-04	3,29E-02
cg02972064	15	57544255	TCF12	Body	0,014	3,50E-04	3,29E-02
cg25610294	14	24777910	LTB4R2	TSS1500	0,017	3,51E-04	3,29E-02
cg13588826	21	47533197	COL6A2	Body	0,008	3,51E-04	3,29E-02
cg03700624	5	2629454		IGR	-0,033	3,51E-04	3,29E-02
cg06113917	4	66809014		IGR	0,069	3,51E-04	3,29E-02
cg16266268	19	54197067	MIR519B	TSS1500	0,008	3,51E-04	3,29E-02
cg20180061	3	181445385	SOX2OT	Body	-0,013	3,51E-04	3,29E-02
cg12027399	4	71049542		IGR	-0,008	3,51E-04	3,29E-02
cg20698105	4	178501543		IGR	0,028	3,51E-04	3,29E-02
cg04670132	5	95203005		IGR	-0,01	3,51E-04	3,29E-02
cg05108054	7	50134416	C7orf72	TSS1500	0,017	3,51E-04	3,29E-02
cg20405893	10	50822158	CHAT	1stExon	-0,012	3,51E-04	3,29E-02
cg19080964	16	924036	LMF1	Body	-0,01	3,51E-04	3,29E-02
cg13750905	16	57832000	KIFC3	TSS200	-0,044	3,51E-04	3,29E-02
cg02843535	17	18759628		IGR	-0,003	3,51E-04	3,29E-02
cg00714068	18	3513534	DLGAP1	Body	0,006	3,51E-04	3,29E-02
cg15701281	7	1462569		IGR	-0,023	3,52E-04	3,30E-02
cg23922228	10	4110650	LOC101927964	Body	0,017	3,52E-04	3,30E-02
cg06026375	12	102591323	PMCH	1stExon	0,016	3,52E-04	3,30E-02
cg21486646	12	129417379	GLT1D1	Body	-0,034	3,52E-04	3,30E-02
cg15721541	15	102037736		IGR	-0,016	3,52E-04	3,30E-02
cg17398836	17	75314830	sept-09	TSS1500	0,02	3,52E-04	3,30E-02
cg12839275	19	58951698	ZNF132	TSS200	-0,002	3,52E-04	3,30E-02
cg26726589	20	306364	SOX12	5'UTR	0,016	3,52E-04	3,30E-02
cg25433682	16	30407049	ZNF48	TSS200	-0,008	3,52E-04	3,30E-02
cg11819469	5	68715702	MARVELD2	Body	-0,004	3,52E-04	3,30E-02
cg22691824	7	1364975		IGR	0,028	3,52E-04	3,30E-02
cg00530592	8	37639753		IGR	-0,023	3,52E-04	3,30E-02
cg02590750	6	30973562	MUC22	TSS200	0,006	3,53E-04	3,30E-02
cg11476726	9	136605158	SARDH	TSS200	-0,013	3,53E-04	3,30E-02
cg20000562	14	36978633	SFTA3	Body	-0,013	3,53E-04	3,30E-02
cg26338113	2	44595761	CAMKMT	Body	0,006	3,53E-04	3,30E-02
cg03287740	8	66546718	ARMC1	TSS1500	-0,007	3,53E-04	3,30E-02
cg21315209	5	11034429	CTNND2	Body	0,02	3,53E-04	3,30E-02
cg17077639	6	31055492		IGR	-0,005	3,53E-04	3,30E-02
cg26520012	10	42672589		IGR	-0,036	3,53E-04	3,30E-02

cg21859597	1	115323395	SIKE1	TSS200	-0,003	3,54E-04	3,30E-02
cg01154254	2	71205159	ANKRD53	TSS1500	0,019	3,54E-04	3,30E-02
cg07032358	4	68589747		IGR	-0,008	3,54E-04	3,30E-02
cg22412072	6	30655217	KIAA1949	5'UTR	0,011	3,53E-04	3,30E-02
cg06433816	9	140311437	EXD3	5'UTR	-0,066	3,54E-04	3,30E-02
cg03851398	10	135171564	C10orf125	TSS200	0,011	3,54E-04	3,30E-02
cg17282830	12	88427966	C12orf29	TSS1500	-0,019	3,53E-04	3,30E-02
cg18537660	13	112568392		IGR	-0,015	3,53E-04	3,30E-02
cg06489804	19	12807371	FBXW9	1stExon	-0,004	3,53E-04	3,30E-02
cg25491456	19	18319564	PDE4C	3'UTR	-0,039	3,54E-04	3,30E-02
cg22122680	1	220219742	EPRS	1stExon	-0,005	3,54E-04	3,30E-02
cg19267144	17	40956947	CNTD1	Body	-0,015	3,54E-04	3,30E-02
cg24878478	19	11319376	DOCK6	Body	0,016	3,54E-04	3,30E-02
cg19343034	19	49223253	MAMSTR	TSS1500	-0,017	3,54E-04	3,30E-02
cg19462286	1	160256163	PEX19	TSS1500	-0,039	3,54E-04	3,30E-02
cg20805735	1	172855455		IGR	-0,006	3,54E-04	3,30E-02
cg20595490	2	149215642	MBD5	TSS1500	-0,006	3,54E-04	3,30E-02
cg08315524	5	57542458		IGR	0,026	3,54E-04	3,30E-02
cg01178106	9	131475831	PKN3	Body	0,014	3,54E-04	3,30E-02
cg24346342	15	52463713	GNB5	Body	-0,01	3,54E-04	3,30E-02
cg03121467	5	111539903	EPB41L4A	Body	0,014	3,54E-04	3,30E-02
cg05411749	7	37772436		IGR	0,026	3,54E-04	3,30E-02
cg02969418	8	120780847	TAF2	Body	-0,015	3,54E-04	3,30E-02
cg17703658	12	57914352	DDIT3	TSS200	-0,004	3,54E-04	3,30E-02
cg23781122	12	91605181		IGR	0,014	3,54E-04	3,30E-02
cg03079917	15	84422632	ADAMTSL3	Body	-0,033	3,54E-04	3,30E-02
cg15934752	15	91497429	RCCD1	TSS1500	0,013	3,54E-04	3,30E-02
cg02804655	2	134601854		IGR	-0,037	3,55E-04	3,30E-02
cg19701416	10	28592092		IGR	-0,004	3,55E-04	3,30E-02
cg02212638	8	33458791	DUSP26	TSS1500	0,007	3,55E-04	3,31E-02
cg00972313	10	1531243	ADARB2	Body	0,031	3,55E-04	3,31E-02
cg19852827	2	176934369		IGR	-0,041	3,55E-04	3,31E-02
cg20133629	18	14979467		IGR	-0,065	3,55E-04	3,31E-02
cg08872632	2	242218975	HDLBP	5'UTR	-0,014	3,55E-04	3,31E-02
cg18285105	9	10633641		IGR	0,037	3,55E-04	3,31E-02
cg19987863	9	134042691	NUP214	Body	0,012	3,55E-04	3,31E-02
cg23449931	16	31409292	ITGAD	Body	0,009	3,56E-04	3,31E-02
cg05514401	13	110436111	IRS2	1stExon	-0,015	3,56E-04	3,31E-02
cg13265331	1	15671522	FHAD1	Body	0,018	3,56E-04	3,31E-02
cg10815657	19	289902	PPAP2C	Body	0,095	3,56E-04	3,31E-02
cg06720117	11	117571329	DSCAML1	Body	0,011	3,56E-04	3,31E-02
cg00746623	19	51105087		IGR	-0,04	3,56E-04	3,31E-02
cg10130636	10	92179206	LOC101926942	Body	0,007	3,56E-04	3,31E-02
cg10294363	12	6480192	SCNN1A	Body	0,019	3,56E-04	3,31E-02
cg17660078	3	149688926	PFN2	TSS200	-0,004	3,57E-04	3,31E-02
cg14929208	3	156273297	SSR3	TSS1500	-0,055	3,57E-04	3,31E-02
cg19517774	17	1085023	ABR	5'UTR	0,014	3,57E-04	3,31E-02
cg26863345	5	36264490	RANBP3L	Body	-0,009	3,57E-04	3,32E-02
cg05806645	8	9009352	PPP1R3B	TSS1500	-0,045	3,57E-04	3,32E-02
cg06459414	16	9012968	USP7	Body	-0,006	3,57E-04	3,32E-02
cg09768571	1	212551106	TMEM206	Body	-0,005	3,57E-04	3,32E-02
cg01628067	1	2222419	SKI	Body	-0,002	3,57E-04	3,32E-02
cg25510569	3	171506308	PLD1	5'UTR	0,01	3,57E-04	3,32E-02
cg08376647	4	169716197	PALLD	Body	-0,011	3,57E-04	3,32E-02
cg11411943	7	91721219	AKAP9	Body	0,038	3,57E-04	3,32E-02
cg10214968	16	34252958		IGR	0,035	3,57E-04	3,32E-02
cg13522279	2	144635077		IGR	0,027	3,58E-04	3,32E-02
cg01289861	3	10182921	VHL	TSS1500	0,005	3,58E-04	3,32E-02
cg12366895	13	112613543		IGR	-0,025	3,58E-04	3,32E-02
cg25117062	2	10442836	HPCAL1	TSS1500	0,008	3,58E-04	3,32E-02
cg09152824	3	18929965		IGR	-0,026	3,58E-04	3,32E-02
cg21554704	2	225282008		IGR	-0,007	3,58E-04	3,32E-02
cg15764052	5	115962584		IGR	0,038	3,58E-04	3,32E-02
cg17368726	7	108169094		IGR	0,023	3,58E-04	3,32E-02
cg06269299	2	169756942	G6PC2	TSS1500	-0,008	3,59E-04	3,32E-02
cg16311192	7	91875427	KRIT1	TSS200	0,011	3,59E-04	3,32E-02
cg01303655	17	76102354	TNRC6C	3'UTR	0,014	3,59E-04	3,32E-02
cg13513288	9	4299830	GLIS3	5'UTR	-0,005	3,59E-04	3,32E-02
cg04555139	1	198603309		IGR	-0,051	3,59E-04	3,32E-02
cg04170065	16	72153204	PMFBP1	3'UTR	0,021	3,59E-04	3,32E-02
cg14846324	15	72585803	BRUNOL6	Body	-0,004	3,59E-04	3,33E-02
cg04368994	7	100732819	TRIM56	Body	0,008	3,59E-04	3,33E-02
cg24167624	1	161993686	OLFML2B	1stExon	0,026	3,59E-04	3,33E-02

cg07966140	13	33860036	STARD13	TSS200	-0,012	3,59E-04	3,33E-02
cg19142133	17	73827687	UNC13D	Body	0,008	3,59E-04	3,33E-02
cg07121807	1	18152750	ACTL8	Body	-0,023	3,60E-04	3,33E-02
cg08901704	4	8023314	ABLIM2	Body	-0,007	3,60E-04	3,33E-02
cg17606003	10	102989376	FLJ41350	Body	-0,008	3,59E-04	3,33E-02
cg22380508	11	63381144	PLA2G16	Body	0,018	3,59E-04	3,33E-02
cg24346328	14	59238398		IGR	0,007	3,60E-04	3,33E-02
cg06907898	20	20950047		IGR	-0,007	3,60E-04	3,33E-02
cg19815317	22	17611205	LOC100996342	Body	-0,015	3,60E-04	3,33E-02
cg11048961	22	37499853	TMPRSS6	TSS200	0,016	3,60E-04	3,33E-02
cg00009167	1	109656433	KIAA1324	TSS200	-0,005	3,60E-04	3,33E-02
cg11328022	20	34207568	SPAG4	Body	-0,003	3,60E-04	3,33E-02
cg15380178	22	18876079		IGR	-0,027	3,60E-04	3,33E-02
cg15209246	1	4133569		IGR	-0,039	3,60E-04	3,33E-02
cg08700932	2	8011432		IGR	0,034	3,60E-04	3,33E-02
cg12930727	3	50341293	HYAL1	TSS1500	0,004	3,60E-04	3,33E-02
cg17974398	6	31239324	HLA-C	Body	0,013	3,60E-04	3,33E-02
cg02025177	6	168593649		IGR	-0,005	3,60E-04	3,33E-02
cg17560129	8	97340620	PTDSS1	Body	0,011	3,60E-04	3,33E-02
cg07396272	12	21014317	SLCO1B3	Body	0,02	3,60E-04	3,33E-02
cg27647454	12	24868482		IGR	-0,007	3,60E-04	3,33E-02
cg03061758	3	14674355		IGR	0,027	3,60E-04	3,33E-02
cg04482948	2	228190415		IGR	-0,002	3,61E-04	3,33E-02
cg27065186	16	22076566	C16orf52	Body	-0,005	3,61E-04	3,33E-02
cg22850007	1	46512449	PIK3R3	Body	-0,005	3,61E-04	3,33E-02
cg05171729	6	168627236		IGR	-0,014	3,61E-04	3,33E-02
cg23457546	9	139415972	NOTCH1	Body	0,02	3,61E-04	3,33E-02
cg04876500	10	131367623	MGMT	Body	-0,015	3,61E-04	3,33E-02
cg15175446	11	62420778	INTS5	TSS200	-0,007	3,61E-04	3,33E-02
cg02196734	15	75018032	CYP1A1	TSS200	-0,005	3,61E-04	3,33E-02
cg04787571	3	31744108	OSBPL10	Body	-0,02	3,62E-04	3,34E-02
cg27222785	1	1008091		IGR	0,013	3,62E-04	3,34E-02
cg16842187	2	26951473	KCNK3	3'UTR	-0,071	3,62E-04	3,34E-02
cg19037350	5	140734648	PCDHGA4	TSS200	-0,028	3,62E-04	3,34E-02
cg03162913	12	58401753		IGR	-0,011	3,62E-04	3,34E-02
cg16490230	10	123747524	TACC2	TSS1500	-0,019	3,62E-04	3,34E-02
cg03543332	17	47707495	SPOP	5'UTR	0,018	3,62E-04	3,34E-02
cg14977267	12	121426656	HNF1A	Body	-0,005	3,62E-04	3,34E-02
cg23063910	1	8268664	LOC102724539	TSS200	-0,01	3,63E-04	3,34E-02
cg25107000	6	31275643		IGR	-0,076	3,63E-04	3,34E-02
cg03186339	6	108439752		IGR	0,011	3,63E-04	3,34E-02
cg04454857	1	114471572	HIPK1-AS1	Body	0,005	3,63E-04	3,34E-02
cg13059337	16	2817391	SRRM2	Body	-0,005	3,63E-04	3,34E-02
cg27292866	2	14525090	LINC00276	Body	-0,01	3,63E-04	3,34E-02
cg19074493	2	88367018	SMYD1	TSS1500	-0,006	3,63E-04	3,34E-02
cg21887095	2	139899031		IGR	-0,017	3,63E-04	3,34E-02
cg21304813	2	242002695	SNED1	Body	0,046	3,64E-04	3,34E-02
cg21729377	3	158361586	GFM1	TSS1500	-0,008	3,63E-04	3,34E-02
cg15032849	6	150757028		IGR	-0,014	3,63E-04	3,34E-02
cg16944203	10	24931755	ARHGAP21	Body	0,004	3,63E-04	3,34E-02
cg20919799	11	2906667	CDKN1C	Body	-0,005	3,64E-04	3,34E-02
cg05685435	12	57851749	INHBE	3'UTR	0,023	3,63E-04	3,34E-02
cg00994907	12	114273586	RBM19	Body	-0,013	3,63E-04	3,34E-02
cg22229206	13	113216305	TUBGCP3	Body	0,009	3,63E-04	3,34E-02
cg18585512	14	69379000	ACTN1	Body	-0,013	3,63E-04	3,34E-02
cg21737020	17	6473106		IGR	0,024	3,63E-04	3,34E-02
cg04705499	19	46796459		IGR	-0,019	3,63E-04	3,34E-02
cg09012594	10	21464258	NEBL	TSS1500	0,036	3,64E-04	3,34E-02
cg05994458	11	13484274	BTBD10	5'UTR	-0,004	3,64E-04	3,34E-02
cg04966682	15	27772702	GABRG3	Body	-0,048	3,64E-04	3,34E-02
cg01712452	20	56736072	C20orf85	3'UTR	0,012	3,64E-04	3,34E-02
cg15455643	13	111040242	COL4A2	Body	-0,06	3,64E-04	3,34E-02
cg00398872	9	139615154	FAM69B	Body	0,017	3,64E-04	3,34E-02
cg20620307	1	84011474		IGR	0,061	3,64E-04	3,35E-02
cg12766402	12	55614536	OR10A7	TSS1500	0,022	3,64E-04	3,35E-02
cg07432969	2	241569557	GPR35	1stExon	0,009	3,65E-04	3,35E-02
cg20302533	7	39170763	POU6F2	Body	-0,074	3,64E-04	3,35E-02
cg05925952	11	62564125	NXF1	3'UTR	-0,006	3,65E-04	3,35E-02
cg01488862	12	108012646	BTBD11	Body	-0,01	3,65E-04	3,35E-02
cg04657735	11	17078312		IGR	0,01	3,65E-04	3,35E-02
cg01972495	10	121152892	GRK5	Body	0,025	3,65E-04	3,35E-02
cg25610945	1	236255094		IGR	0,09	3,65E-04	3,35E-02
cg24484268	7	127984063	RBM28	TSS200	0,007	3,66E-04	3,35E-02

cg22697386	13	86373320	SLITRK6	1stExon	-0,034	3,65E-04	3,35E-02
cg17410668	14	75958146		IGR	0,007	3,65E-04	3,35E-02
cg24571830	6	108977456	FOXO3	Body	0,027	3,66E-04	3,36E-02
cg21047473	16	87250042	LOC101928708	Body	-0,034	3,66E-04	3,36E-02
cg13729147	10	118591438		IGR	-0,005	3,66E-04	3,36E-02
cg05614408	5	94901279	ARSK	Body	-0,004	3,66E-04	3,36E-02
cg14387256	2	224812501		IGR	-0,021	3,66E-04	3,36E-02
cg01576360	7	23431686	IGF2BP3	Body	0,006	3,66E-04	3,36E-02
cg07992377	11	3173953	OSBPL5	5'UTR	0,015	3,66E-04	3,36E-02
cg13910625	10	111936014		IGR	0,006	3,66E-04	3,36E-02
cg15099410	1	28585832	SESN2	TSS200	-0,003	3,66E-04	3,36E-02
cg03797505	4	148742150	ARHGAP10	Body	-0,006	3,66E-04	3,36E-02
cg27606022	6	53179101	ELOVL5	5'UTR	0,022	3,67E-04	3,36E-02
cg01363324	9	124979353	LHX6	Body	-0,019	3,66E-04	3,36E-02
cg27111836	22	34053857	LARGE	Body	-0,027	3,66E-04	3,36E-02
cg13378083	1	23142734	EPHB2	Body	0,023	3,67E-04	3,36E-02
cg18462784	7	127218505		IGR	-0,026	3,67E-04	3,36E-02
cg01802827	12	34474362		IGR	-0,023	3,67E-04	3,36E-02
cg10407598	17	73629365	RECQL5	Body	0,02	3,67E-04	3,36E-02
cg05811362	21	46548047	ADARB1	5'UTR	-0,005	3,67E-04	3,36E-02
cg23220148	5	96227843	ERAP2	Body	0,012	3,67E-04	3,36E-02
cg25021709	12	119818931	CCDC60	Body	0,006	3,67E-04	3,36E-02
cg13477541	5	33072822		IGR	-0,004	3,67E-04	3,36E-02
cg14980183	7	121187704		IGR	-0,014	3,67E-04	3,36E-02
cg15301752	19	43910076	TEX101	5'UTR	0,006	3,67E-04	3,36E-02
cg05298088	22	50469442	TTLL8	Body	-0,036	3,67E-04	3,36E-02
cg17320866	2	46716084		IGR	-0,009	3,67E-04	3,36E-02
cg20573828	2	206301002	PARD3B	Body	-0,008	3,68E-04	3,36E-02
cg11111991	6	148466250		IGR	-0,009	3,68E-04	3,36E-02
cg18799748	11	7109806	RBMXL2	TSS1500	0,021	3,68E-04	3,36E-02
cg24273318	14	35409083	C14orf19	TSS200	0,005	3,68E-04	3,36E-02
cg26431811	12	49745236	DNAJC22	Body	0,023	3,68E-04	3,36E-02
cg25296891	3	185071842	MAP3K13	5'UTR	-0,004	3,68E-04	3,36E-02
cg16722302	7	5461524	TNRC18	5'UTR	-0,003	3,68E-04	3,36E-02
cg01011550	20	22411498		IGR	-0,042	3,68E-04	3,36E-02
cg14991792	11	26493999	ANO3	Body	-0,018	3,68E-04	3,36E-02
cg03870746	7	1068244	C7orf50	Body	-0,006	3,68E-04	3,36E-02
cg05529201	16	87368205	FBXO31	Body	0,014	3,68E-04	3,36E-02
cg16050974	1	10057700	RBP7	Body	-0,013	3,69E-04	3,37E-02
cg27137363	2	161141507	RBMS1	ExonBnd	-0,007	3,69E-04	3,37E-02
cg05287399	6	113410438		IGR	0,006	3,69E-04	3,37E-02
cg03260104	6	40346509	TDRG1	Body	-0,008	3,69E-04	3,37E-02
cg17338133	3	183888141	DVL3	Body	-0,016	3,69E-04	3,37E-02
cg22870642	11	8076898	TUB	Body	-0,051	3,69E-04	3,37E-02
cg09132404	11	11960273	USP47	Body	-0,006	3,69E-04	3,37E-02
cg08602190	10	128076260	ADAM12	Body	-0,01	3,69E-04	3,37E-02
cg13044077	11	75380327	MAP6	TSS1500	-0,027	3,69E-04	3,37E-02
cg15703377	17	71282593	CDC42EP4	Body	-0,008	3,69E-04	3,37E-02
cg07679230	19	3586905	GIPC3	Body	0,011	3,70E-04	3,37E-02
cg01306662	4	146022320	ABCE1	5'UTR	-0,005	3,70E-04	3,37E-02
cg26690993	7	35406292	LOC401324	Body	-0,016	3,70E-04	3,37E-02
cg19398349	7	69642889	AUTS2	Body	-0,014	3,70E-04	3,37E-02
cg00761755	17	1531579	SLC43A2	5'UTR	-0,003	3,70E-04	3,37E-02
cg25662173	1	204463867		IGR	-0,004	3,70E-04	3,37E-02
cg08260286	19	55987563	ZNF628	TSS200	-0,004	3,70E-04	3,37E-02
cg10503655	20	45443689		IGR	-0,027	3,70E-04	3,37E-02
cg24080529	22	33197034	SYN3	Body	0,025	3,70E-04	3,37E-02
cg04461219	15	96940091		IGR	-0,034	3,71E-04	3,38E-02
cg14576319	3	171175713	TNIK	Body	-0,022	3,71E-04	3,38E-02
cg15509177	13	19919208	LOC100101938	TSS200	-0,088	3,71E-04	3,38E-02
cg04018214	12	91572199	DCN	1stExon	-0,028	3,71E-04	3,38E-02
cg22728629	4	1562625		IGR	-0,008	3,71E-04	3,38E-02
cg24930541	5	118664652	TNFAIP8	5'UTR	-0,035	3,71E-04	3,38E-02
cg21646520	7	139614808	TBXAS1	Body	-0,006	3,71E-04	3,38E-02
cg24109093	15	92400930	SLCO3A1	Body	-0,029	3,71E-04	3,38E-02
cg00653312	16	87863785	SLC7A5	3'UTR	0,01	3,71E-04	3,38E-02
cg25996584	6	112940409		IGR	-0,01	3,71E-04	3,38E-02
cg04664127	8	95731971	DPY19L4	TSS200	-0,003	3,71E-04	3,38E-02
cg15464727	14	75350039	DLST	Body	0,012	3,71E-04	3,38E-02
cg04461705	2	24346867	LOC375190	5'UTR	-0,003	3,71E-04	3,38E-02
cg09352738	8	84358274		IGR	-0,007	3,71E-04	3,38E-02
cg25756166	22	42372528	sept-03	TSS1500	-0,004	3,71E-04	3,38E-02
cg12318392	2	226265684	NYAP2	1stExon	-0,035	3,72E-04	3,38E-02

cg19278414	3	50570733		IGR	0,005	3,72E-04	3,38E-02
cg16359799	3	138739900	PRR23B	TSS200	-0,048	3,72E-04	3,38E-02
cg27554556	3	148804650	HLTF	TSS1500	-0,023	3,72E-04	3,38E-02
cg23993345	5	271700	PDCD6	TSS200	-0,006	3,72E-04	3,38E-02
cg03921542	9	95480578	BICD2	Body	0,011	3,72E-04	3,38E-02
cg26831968	10	104005242	GBF1	TSS200	-0,003	3,71E-04	3,38E-02
cg09137453	12	7282081	CLSTN3	TSS1500	0,02	3,72E-04	3,38E-02
cg13766329	22	45596980	C22orf9	Body	-0,004	3,72E-04	3,38E-02
cg26675212	2	23780305	KLHL29	5'UTR	0,019	3,72E-04	3,38E-02
cg06342317	2	121105259	INHBB	Body	-0,009	3,72E-04	3,38E-02
cg12405190	3	104283272		IGR	0,03	3,72E-04	3,38E-02
cg09225442	16	30662040	PRR14	TSS200	0,006	3,72E-04	3,38E-02
cg11449409	16	46772242	MYLK3	Body	-0,012	3,72E-04	3,38E-02
cg05969743	2	236890318	AGAP1	Body	-0,044	3,72E-04	3,38E-02
cg04682600	14	31874845	HEATR5A	5'UTR	0,038	3,72E-04	3,38E-02
cg00238391	7	150977475		IGR	-0,019	3,72E-04	3,38E-02
cg12227166	8	2366868		IGR	-0,072	3,72E-04	3,38E-02
cg09744867	15	49535482	GALK2	Body	0,006	3,72E-04	3,38E-02
cg04168762	4	99722985		IGR	-0,012	3,72E-04	3,38E-02
cg05675373	1	110754257	KCNC4	1stExon	-0,022	3,73E-04	3,38E-02
cg18110883	3	9009066		IGR	-0,021	3,73E-04	3,38E-02
cg22015888	5	132459199		IGR	0,014	3,73E-04	3,38E-02
cg10012599	11	46744754	F2	Body	0,007	3,73E-04	3,38E-02
cg20313642	12	74688000		IGR	0,008	3,73E-04	3,38E-02
cg20495009	12	105323744	SLC41A2	TSS1500	0,02	3,73E-04	3,38E-02
cg27443491	11	67912720		IGR	0,028	3,73E-04	3,38E-02
cg03870432	3	139393257	NMNAT3	5'UTR	-0,005	3,73E-04	3,38E-02
cg07934031	5	179105728	CBY3	Body	-0,003	3,73E-04	3,38E-02
cg26157028	15	85144168	ZSCAN2	TSS200	-0,005	3,73E-04	3,38E-02
cg15142890	5	1212545	SLC6A19	Body	0,006	3,73E-04	3,38E-02
cg11682702	1	235416786	ARID4B	Body	-0,022	3,74E-04	3,38E-02
cg23841631	2	45438090	LINC01121	Body	0,021	3,74E-04	3,38E-02
cg13690169	9	129096404	MVB12B	Body	-0,004	3,74E-04	3,38E-02
cg00548712	9	136891075	LINC00094	Body	-0,003	3,74E-04	3,38E-02
cg04217534	11	82354913		IGR	-0,014	3,74E-04	3,38E-02
cg03208934	12	76865269	OSBPL8	Body	-0,007	3,74E-04	3,38E-02
cg00480497	14	69050409	RAD51B	Body	0,007	3,74E-04	3,38E-02
cg22412481	15	69709830	KIF23	Body	0,013	3,74E-04	3,38E-02
cg02114927	17	60256189		IGR	-0,009	3,74E-04	3,38E-02
cg14129266	18	2576770	NDC80	Body	0,016	3,74E-04	3,38E-02
cg13643356	19	2202660	DOT1L	Body	0,018	3,74E-04	3,38E-02
cg23878490	14	62265250		IGR	0,018	3,74E-04	3,39E-02
cg20393502	16	31044354	STX4	TSS1500	-0,005	3,74E-04	3,39E-02
cg08227290	14	75908005	JDP2	Body	0,025	3,74E-04	3,39E-02
cg16495696	15	90792869	TTLL13	5'UTR	0,016	3,74E-04	3,39E-02
cg17539492	4	122721218	EXOSC9	TSS1500	-0,008	3,75E-04	3,39E-02
cg21226087	12	91574008	DCN	5'UTR	0,027	3,75E-04	3,39E-02
cg22404044	10	96083764	PLCE1	Body	-0,005	3,75E-04	3,39E-02
cg22814042	19	7688694	XAB2	Body	0,003	3,75E-04	3,39E-02
cg27296459	3	141464595	RNF7	3'UTR	0,008	3,75E-04	3,39E-02
cg01998047	13	30982651		IGR	-0,002	3,75E-04	3,39E-02
cg12926300	5	143253949		IGR	0,032	3,75E-04	3,39E-02
cg11322655	8	1713005	CLN8	5'UTR	0,047	3,75E-04	3,39E-02
cg18986078	14	21510323	RNASE7	TSS200	0,017	3,75E-04	3,39E-02
cg25953715	10	68375590	CTNNA3	Body	-0,01	3,75E-04	3,39E-02
cg03826976	11	7695433	CYB5R2	TSS1500	-0,027	3,76E-04	3,39E-02
cg07585765	11	74022326	LOC101928580	TSS200	-0,043	3,76E-04	3,39E-02
cg16549108	16	21226511	ZP2	TSS1500	0,027	3,76E-04	3,39E-02
cg06403888	4	170304407		IGR	-0,016	3,76E-04	3,39E-02
cg25479151	7	3285595		IGR	0,006	3,76E-04	3,39E-02
cg09153253	12	108007355	BTBD11	Body	-0,007	3,76E-04	3,39E-02
cg11078198	14	21572134	ZNF219	5'UTR	-0,005	3,76E-04	3,39E-02
cg09564133	5	176738847	MXD3	1stExon	-0,006	3,76E-04	3,39E-02
cg02124890	15	35472724		IGR	-0,009	3,76E-04	3,39E-02
cg01192190	9	134305483	PRRC2B	5'UTR	0,006	3,76E-04	3,39E-02
cg12332795	1	158800873	MNDA	TSS1500	0,035	3,76E-04	3,40E-02
cg04735168	3	112727794	C3orf17	Body	-0,004	3,77E-04	3,40E-02
cg26196336	6	130702783	TMEM200A	5'UTR	0,017	3,76E-04	3,40E-02
cg02498602	1	26633876	UBXN11	TSS1500	0,02	3,77E-04	3,40E-02
cg03700287	5	140621697	PCDHB19P	Body	-0,032	3,77E-04	3,40E-02
cg18401108	2	238116756		IGR	0,016	3,77E-04	3,40E-02
cg11457640	6	40567376		IGR	-0,046	3,77E-04	3,40E-02
cg18271913	1	205561852	MFSD4	Body	0,015	3,77E-04	3,40E-02

cg21685789	5	161561256	GABRG2	Body	-0,009	3,77E-04	3,40E-02
cg21687591	3	169492002	MYNN	5'UTR	0,036	3,77E-04	3,40E-02
cg13892088	7	154863323	HTR5A	1stExon	-0,054	3,77E-04	3,40E-02
cg00576433	2	232257914		IGR	0,022	3,78E-04	3,40E-02
cg12093136	4	76439140	RCHY1	Body	-0,007	3,78E-04	3,40E-02
cg07660570	17	572542	VPS53	Body	-0,005	3,78E-04	3,40E-02
cg07748774	1	236287045		IGR	0,019	3,78E-04	3,40E-02
cg24883601	2	240112434	HDAC4	Body	-0,004	3,78E-04	3,40E-02
cg03684977	17	37893803	GRB7	TSS1500	0,012	3,78E-04	3,40E-02
cg10643271	12	129309330	SLC15A4	TSS1500	0,055	3,78E-04	3,40E-02
cg21622536	13	112997643		IGR	-0,032	3,78E-04	3,40E-02
cg14718741	4	189140097		IGR	-0,006	3,78E-04	3,41E-02
cg07328400	10	70163809	RUFY2	Body	0,007	3,78E-04	3,41E-02
cg17163841	19	18332997	PDE4C	Body	0,019	3,78E-04	3,41E-02
cg01372694	7	65878352		IGR	-0,021	3,79E-04	3,41E-02
cg01778908	16	70759095	VAC14	Body	-0,01	3,79E-04	3,41E-02
cg10473171	5	106951240	EFNA5	Body	-0,012	3,79E-04	3,41E-02
cg22868518	11	507468	RNH1	TSS1500	-0,043	3,79E-04	3,41E-02
cg15447512	1	110163209	AMPD2	Body	0,029	3,79E-04	3,41E-02
cg01621716	2	11636147		IGR	0,035	3,79E-04	3,41E-02
cg12387646	12	49258726	RND1	Body	-0,035	3,79E-04	3,41E-02
cg17358883	4	111559134	PITX2	TSS1500	-0,025	3,79E-04	3,41E-02
cg06934654	16	89180742	ACSF3	Body	0,021	3,79E-04	3,41E-02
cg26969102	19	56954002	ZNF667	Body	0,029	3,79E-04	3,41E-02
cg03536202	13	30120364	SLC7A1	5'UTR	0,005	3,79E-04	3,41E-02
cg07492055	18	59757373	PIGN	Body	0,035	3,79E-04	3,41E-02
cg18307996	22	31581270	RNF185	5'UTR	-0,007	3,79E-04	3,41E-02
cg26951680	15	75526720		IGR	0,021	3,80E-04	3,41E-02
cg17890039	7	14852822	DGKB	Body	-0,012	3,80E-04	3,41E-02
cg04926712	9	37577655	FBXO10	TSS1500	0,005	3,80E-04	3,41E-02
cg09667467	9	125030469	MRRF	5'UTR	0,014	3,80E-04	3,41E-02
cg07115206	16	6425686	A2BP1	5'UTR	-0,062	3,80E-04	3,41E-02
cg17839113	3	133749257	SLCO2A1	TSS1500	-0,018	3,80E-04	3,41E-02
cg23970377	3	52336681		IGR	0,016	3,80E-04	3,41E-02
cg13052453	14	105885138	MTA1	TSS1500	0,01	3,80E-04	3,41E-02
cg20094201	20	44520771	CTSA	Body	0,029	3,80E-04	3,41E-02
cg17375267	19	46915776	CCDC8	1stExon	0,051	3,80E-04	3,41E-02
cg05070709	19	51487463	KLK7	TSS1500	-0,042	3,81E-04	3,41E-02
cg06258196	7	101852361	CUX1	Body	0,005	3,81E-04	3,41E-02
cg03484402	10	21186180	NEBL	1stExon	0,024	3,81E-04	3,41E-02
cg11164293	16	56817380	NUP93	TSS200	0,038	3,81E-04	3,41E-02
cg12168389	1	31881988	SERINC2	TSS1500	0,031	3,81E-04	3,41E-02
cg24450312	1	206681158	RASSF5	1stExon	0,008	3,81E-04	3,42E-02
cg08670647	2	144358314	ARHGAP15	Body	0,012	3,81E-04	3,42E-02
cg23060586	3	188822100		IGR	-0,008	3,81E-04	3,42E-02
cg21010350	7	150445533	LOC100128542	TSS1500	-0,023	3,81E-04	3,42E-02
cg03320594	11	12237374	MICAL2	Body	0,005	3,81E-04	3,42E-02
cg09480227	13	50600044	DLEU2	Body	0,015	3,81E-04	3,42E-02
cg01767732	14	72399591	RGS6	TSS200	-0,017	3,81E-04	3,42E-02
cg07181627	16	22705329		IGR	0,015	3,81E-04	3,42E-02
cg04756515	18	72837700		IGR	-0,071	3,81E-04	3,42E-02
cg14842771	7	142558879	EPHB6	5'UTR	-0,005	3,81E-04	3,42E-02
cg17032533	5	175964269	RNF44	1stExon	0,008	3,82E-04	3,42E-02
cg15966198	7	73668926	RFC2	TSS200	0,007	3,82E-04	3,42E-02
cg15787744	6	44234175	NFKBIE	TSS1500	0,027	3,82E-04	3,42E-02
cg13487185	1	29213677	EPB41	1stExon	0,012	3,82E-04	3,42E-02
cg23918287	4	157894506		IGR	-0,009	3,82E-04	3,42E-02
cg01485627	14	86089263	FLRT2	Body	-0,004	3,82E-04	3,42E-02
cg14920920	16	90094183	GAS8	5'UTR	-0,007	3,82E-04	3,42E-02
cg00595212	18	11755774	GNAL	Body	0,02	3,82E-04	3,42E-02
cg00794648	1	222800850	MIA3	Body	-0,008	3,82E-04	3,42E-02
cg27560091	13	20357708	PSPC1	TSS1500	0,015	3,82E-04	3,42E-02
cg05322344	10	118765422	SHTN1	TSS1500	-0,029	3,82E-04	3,42E-02
cg09855906	9	136930274	BRD3	5'UTR	0,01	3,82E-04	3,42E-02
cg14329350	11	62573880	NXF1	TSS1500	0,023	3,82E-04	3,42E-02
cg00402812	1	109633307	TMEM167B	TSS200	-0,004	3,82E-04	3,42E-02
cg17503823	11	49917069		IGR	-0,023	3,82E-04	3,42E-02
cg16917406	15	85114159	UBE2QP1	TSS200	-0,004	3,82E-04	3,42E-02
cg13999688	14	76837510	ESRRB	TSS200	-0,008	3,83E-04	3,42E-02
cg23758089	3	16424142	RFTN1	Body	-0,005	3,83E-04	3,42E-02
cg15856832	3	49127364	QRICH1	5'UTR	0,016	3,83E-04	3,42E-02
cg22782074	16	49814608	ZNF423	Body	-0,012	3,83E-04	3,42E-02
cg02151609	17	1957529	HIC1	TSS1500	-0,006	3,83E-04	3,42E-02

cg09927065	4	176976659		IGR	-0,017	3,83E-04	3,42E-02
cg18786718	16	2868001	PRSS21	Body	-0,035	3,83E-04	3,42E-02
cg23330543	6	46655690	TDRD6	5'UTR	-0,009	3,83E-04	3,42E-02
cg11018432	12	124832427	NCOR2	Body	0,004	3,84E-04	3,42E-02
cg18778658	6	31854450	EHMT2	Body	0,007	3,84E-04	3,43E-02
cg09522374	1	45025145	RNF220	Body	0,011	3,84E-04	3,43E-02
cg13874317	3	17686961	TBC1D5	5'UTR	-0,026	3,84E-04	3,43E-02
cg13026328	16	31496045	SLC5A2	Body	0,028	3,84E-04	3,43E-02
cg02764515	19	39400205	CCER2	Body	0,021	3,84E-04	3,43E-02
cg11324184	4	139964223	NOCT	ExonBnd	0,025	3,84E-04	3,43E-02
cg02128191	10	669285	DIP2C	Body	-0,009	3,84E-04	3,43E-02
cg13025566	3	183905972	ABCF3	Body	0,006	3,84E-04	3,43E-02
cg07737814	13	20438241	ZMYM5	TSS1500	-0,004	3,85E-04	3,43E-02
cg18229848	13	28491326		IGR	-0,011	3,85E-04	3,43E-02
cg11214757	5	176024225	GPRIN1	Body	-0,027	3,85E-04	3,43E-02
cg05555502	9	96943707	MIRLET7DHG	Body	0,015	3,85E-04	3,43E-02
cg19678266	1	203317248	FMOD	Body	-0,016	3,85E-04	3,43E-02
cg25862117	6	131504781	AKAP7	Body	-0,018	3,85E-04	3,44E-02
cg01295392	12	108169569	ASCL4	1stExon	-0,021	3,86E-04	3,44E-02
cg26979179	2	217366073	RPL37A	Body	-0,008	3,86E-04	3,44E-02
cg13520475	10	113905087		IGR	-0,008	3,86E-04	3,44E-02
cg26201819	18	42835380	SLC14A2	5'UTR	-0,013	3,86E-04	3,44E-02
cg26697158	15	48442242	MYEF2	Body	-0,006	3,86E-04	3,44E-02
cg25665331	3	31706119	OSBPL10	Body	-0,006	3,86E-04	3,44E-02
cg06179039	16	67263524	FHOD1	3'UTR	0,009	3,86E-04	3,44E-02
cg06560836	17	29697553	NF1	Body	0,011	3,86E-04	3,44E-02
cg22284614	17	42342552	SLC4A1	5'UTR	-0,01	3,86E-04	3,44E-02
cg02781074	13	101241206	GGACT	TSS200	-0,068	3,86E-04	3,44E-02
cg24699005	19	1192342		IGR	-0,062	3,87E-04	3,44E-02
cg26305160	6	69471490	ADGRB3	Body	0,029	3,87E-04	3,44E-02
cg27459530	16	87744906	KLHDC4	Body	0,004	3,87E-04	3,44E-02
cg11816345	17	61700755	MAP3K3	Body	-0,01	3,87E-04	3,44E-02
cg07676300	19	11285258	KANK2	Body	0,004	3,87E-04	3,44E-02
cg22608202	6	138777028	NHSL1	Body	-0,027	3,87E-04	3,44E-02
cg08381274	8	97169621	GDF6	Body	-0,037	3,87E-04	3,44E-02
cg22770294	4	3308933		IGR	0,004	3,87E-04	3,44E-02
cg19064846	7	157273391		IGR	0,029	3,87E-04	3,44E-02
cg11912179	11	134026542	NCAPD3	Body	-0,019	3,87E-04	3,44E-02
cg07420799	21	45749993	C21orf2	3'UTR	0,003	3,87E-04	3,44E-02
cg01444309	1	80083521		IGR	-0,024	3,87E-04	3,44E-02
cg09763347	8	133574010	HPYR1	TSS1500	-0,008	3,87E-04	3,44E-02
cg17828999	14	89878824	FOXN3	ExonBnd	-0,013	3,87E-04	3,44E-02
cg20956520	12	88242651		IGR	-0,019	3,87E-04	3,44E-02
cg04176448	5	149505943	PDGFRB	Body	0,014	3,88E-04	3,44E-02
cg20046143	7	132344174	FLJ40288	Body	-0,007	3,88E-04	3,44E-02
cg25217966	11	30040572		IGR	0,01	3,88E-04	3,44E-02
cg20181513	12	99548893	ANKS1B	TSS200	-0,033	3,88E-04	3,45E-02
cg00075967	15	74495354	STRA6	5'UTR	-0,03	3,88E-04	3,45E-02
cg19805933	2	38663108		IGR	0,01	3,88E-04	3,45E-02
cg20499714	12	78140169		IGR	0,037	3,88E-04	3,45E-02
cg27509005	15	27606952	GABRG3	Body	-0,018	3,88E-04	3,45E-02
cg00059165	6	36926828	PI16	Body	0,01	3,88E-04	3,45E-02
cg05948029	7	50534420	DDC	Body	0,009	3,88E-04	3,45E-02
cg10394859	7	124673583	POT1-AS1	Body	-0,014	3,88E-04	3,45E-02
cg22692196	16	828854		IGR	0,019	3,88E-04	3,45E-02
cg12283145	12	123277570	CCDC62	Body	-0,008	3,88E-04	3,45E-02
cg24532901	18	77782597	TXNL4A	5'UTR	0,05	3,89E-04	3,45E-02
cg01913259	15	27189193	GABRA5	Body	-0,053	3,89E-04	3,45E-02
cg13673960	4	1742301	TACC3	Body	0,044	3,89E-04	3,45E-02
cg03757387	4	109770908	COL25A1	Body	-0,016	3,89E-04	3,45E-02
cg15035364	7	102937814	PMPCB	TSS200	0,007	3,89E-04	3,45E-02
cg20866193	13	93118732	GPC5	Body	0,017	3,89E-04	3,45E-02
cg23708211	18	48513899	ELAC1	3'UTR	-0,012	3,89E-04	3,45E-02
cg24526470	22	46866398	CELSR1	Body	0,019	3,89E-04	3,45E-02
cg21108029	7	4191467	SDK1	Body	-0,022	3,90E-04	3,45E-02
cg14204430	4	116035217	NDST4	TSS200	0,02	3,90E-04	3,45E-02
cg20968137	6	117774748		IGR	0,01	3,90E-04	3,45E-02
cg03276795	1	109167248	FAM102B	ExonBnd	-0,01	3,90E-04	3,45E-02
cg23718283	17	71308304	CDC42EP4	TSS200	-0,003	3,90E-04	3,45E-02
cg21919420	1	222794514	MIA3	Body	-0,009	3,90E-04	3,45E-02
cg19746887	3	71190489	FOXP1	Body	-0,007	3,90E-04	3,46E-02
cg12938436	5	106608874		IGR	0,036	3,90E-04	3,46E-02
cg18821316	1	153931119	CRTC2	TSS200	0,011	3,90E-04	3,46E-02

cg14783993	12	87106229	MGAT4C	5'UTR	-0,042	3,90E-04	3,46E-02
cg04087167	8	141537188		IGR	-0,01	3,91E-04	3,46E-02
cg24873171	15	43501932	EPB42	Body	0,011	3,90E-04	3,46E-02
cg09530217	15	101728353	CHSY1	Body	0,049	3,91E-04	3,46E-02
cg21641231	1	100315925	AGL	TSS1500	-0,004	3,91E-04	3,46E-02
cg03257739	7	43918221	URGCP	Body	0,009	3,91E-04	3,46E-02
cg00278329	16	31440531	COX6A2	TSS1500	0,008	3,91E-04	3,46E-02
cg21683284	1	65774857	DNAJC6	Body	-0,026	3,91E-04	3,46E-02
cg11579630	4	78082718	CCNG2	Body	0,029	3,91E-04	3,46E-02
cg20850604	9	123342833	CDK5RAP2	TSS1500	-0,013	3,91E-04	3,46E-02
cg15851734	16	80753231	CDYL2	Body	0,006	3,91E-04	3,46E-02
cg02060919	4	32932085		IGR	-0,023	3,92E-04	3,46E-02
cg11696073	5	5420754		IGR	-0,009	3,92E-04	3,46E-02
cg04648334	6	27236866		IGR	-0,048	3,91E-04	3,46E-02
cg06035134	8	128311181	CASC21	Body	-0,016	3,92E-04	3,46E-02
cg15932817	11	15122340		IGR	-0,056	3,92E-04	3,46E-02
cg01162550	12	11853747	ETV6	Body	-0,01	3,92E-04	3,46E-02
cg20524211	13	111366070	ING1	Body	-0,005	3,91E-04	3,46E-02
cg01874084	12	56320851	WIBG	Body	-0,004	3,92E-04	3,46E-02
cg05565809	6	31740787	C6orf27	Body	0,014	3,92E-04	3,46E-02
cg19564893	4	81048483		IGR	-0,044	3,92E-04	3,46E-02
cg22553158	11	83985070	DLG2	TSS1500	0,026	3,92E-04	3,46E-02
cg07747324	14	62009578	PRKCH	Body	-0,004	3,92E-04	3,46E-02
cg16898289	2	182818960	PPP1R1C	TSS200	-0,008	3,93E-04	3,46E-02
cg20928427	3	110363228		IGR	-0,006	3,93E-04	3,46E-02
cg15735239	3	162901941	LINC01192	Body	0,017	3,93E-04	3,46E-02
cg20676578	4	83719449	SCD5	Body	-0,004	3,93E-04	3,46E-02
cg11477892	4	106580295		IGR	-0,015	3,93E-04	3,46E-02
cg11848592	6	32300765	C6orf10	Body	0,008	3,93E-04	3,46E-02
cg07627726	7	93495		IGR	-0,008	3,93E-04	3,46E-02
cg03265969	7	54617631	VSTM2A	Body	-0,019	3,93E-04	3,46E-02
cg02561081	7	95116255	ASB4	Body	0,022	3,92E-04	3,46E-02
cg23619829	8	66556444	MTFR1	TSS1500	-0,007	3,92E-04	3,46E-02
cg16468389	17	46831742		IGR	-0,035	3,93E-04	3,46E-02
cg08351914	21	21354552		IGR	0,034	3,93E-04	3,46E-02
cg12709329	13	49465897		IGR	-0,055	3,93E-04	3,46E-02
cg04024209	3	43415428	ANO10	Body	0,011	3,93E-04	3,46E-02
cg16574871	8	97165770	GDF6	Body	-0,016	3,93E-04	3,46E-02
cg00550493	6	64928665	EYS	Body	-0,008	3,93E-04	3,46E-02
cg26945867	16	820155	MSLNL	Body	0,016	3,93E-04	3,46E-02
cg10368507	17	40346798	GHDC	TSS1500	-0,019	3,93E-04	3,46E-02
cg21831836	11	107819630	RAB39	Body	-0,006	3,93E-04	3,46E-02
cg27132071	14	33030722	AKAP6	Body	-0,008	3,93E-04	3,46E-02
cg05016975	17	62461992	C17orf60	Body	0,01	3,93E-04	3,47E-02
cg21779088	1	1000856		IGR	0,016	3,94E-04	3,47E-02
cg08845001	1	229363470		IGR	-0,004	3,94E-04	3,47E-02
cg27226107	6	15491805	JARID2	Body	-0,011	3,94E-04	3,47E-02
cg05738687	17	290137	FAM101B	3'UTR	0,014	3,94E-04	3,47E-02
cg08355428	5	120032976		IGR	0,014	3,94E-04	3,47E-02
cg07851632	5	140682503	SLC25A2	1stExon	-0,017	3,94E-04	3,47E-02
cg03487407	14	105904741	MTA1	Body	0,007	3,94E-04	3,47E-02
cg14575950	17	45382681	ITGB3	Body	-0,033	3,94E-04	3,47E-02
cg02484352	5	131599890	PDLIM4	Body	0,022	3,95E-04	3,47E-02
cg04936970	1	40974132	DEM1	TSS1500	-0,006	3,95E-04	3,47E-02
cg12746356	10	124220874	HTRA1	TSS200	-0,012	3,95E-04	3,47E-02
cg09334399	1	231750709	TSNAX-DISC1	Body	-0,032	3,95E-04	3,48E-02
cg06110728	12	45270886	NELL2	Body	-0,011	3,95E-04	3,48E-02
cg21763598	7	92219715	FAM133B	TSS200	-0,003	3,95E-04	3,48E-02
cg08515328	8	48960728	UBE2V2	Body	0,019	3,95E-04	3,48E-02
cg27178849	2	8944298	KIDINS220	Body	0,029	3,95E-04	3,48E-02
cg07702696	12	133335488	ANKLE2	Body	-0,019	3,95E-04	3,48E-02
cg07232688	1	100644688	LRRC39	TSS1500	0,012	3,96E-04	3,48E-02
cg11638553	7	137605005	CREB3L2	Body	-0,004	3,96E-04	3,48E-02
cg25794858	14	93760500	BTBD7	Body	0,01	3,96E-04	3,48E-02
cg05109049	17	29641333	NF1	Body	-0,008	3,96E-04	3,48E-02
cg23220105	2	219990589	NHEJ1	Body	0,027	3,96E-04	3,48E-02
cg20030243	5	76011643	F2R	TSS1500	-0,004	3,96E-04	3,48E-02
cg01935400	17	80393124	HEXDC	Body	0,009	3,96E-04	3,48E-02
cg08493324	3	11208187	HRH1	5'UTR	0,007	3,97E-04	3,48E-02
cg09057473	4	84177172		IGR	0,038	3,97E-04	3,48E-02
cg21548029	5	140515675	PCDHB5	1stExon	-0,065	3,97E-04	3,48E-02
cg26714514	8	81889651	PAG1	Body	-0,017	3,97E-04	3,48E-02
cg23820285	10	23003250	PIP4K2A	1stExon	-0,003	3,97E-04	3,48E-02

cg15854606	19	18654548	FKBP8	TSS200	0,005	3,97E-04	3,48E-02
cg20789674	9	2621597	VLDLR	TSS200	-0,005	3,97E-04	3,48E-02
cg20451748	12	27159886	TM7SF3	Body	0,011	3,97E-04	3,48E-02
cg14298866	4	115184444		IGR	0,021	3,97E-04	3,48E-02
cg06643115	10	16517689	PTER	5'UTR	-0,005	3,97E-04	3,48E-02
cg08751623	7	8849132		IGR	0,043	3,97E-04	3,48E-02
cg16626387	9	98268788	PTCH1	5'UTR	-0,004	3,97E-04	3,48E-02
cg04039434	17	11838982	DNAH9	Body	0,019	3,97E-04	3,48E-02
cg00890632	10	6257204	PFKFB3	Body	0,012	3,97E-04	3,49E-02
cg11153057	1	147380334	GJA8	Body	-0,014	3,98E-04	3,49E-02
cg16223190	17	56380051	BZRAP1	3'UTR	0,01	3,98E-04	3,49E-02
cg15796595	1	55069723	ACOT11	Body	0,022	3,98E-04	3,49E-02
cg18493709	16	700657	WDR90	Body	0,007	3,98E-04	3,49E-02
cg24166763	18	54624442	WDR7	Body	0,006	3,98E-04	3,49E-02
cg16401668	1	48296905		IGR	0,008	3,98E-04	3,49E-02
cg27405362	21	43230942	PRDM15	Body	-0,007	3,98E-04	3,49E-02
cg21452088	11	16360202	SOX6	Body	0,029	3,98E-04	3,49E-02
cg00464210	3	136194317	STAG1	Body	-0,005	3,98E-04	3,49E-02
cg06726287	7	81681115	CACNA2D1	Body	-0,011	3,98E-04	3,49E-02
cg23482582	8	146079270	COMMD5	TSS1500	-0,02	3,98E-04	3,49E-02
cg01068691	16	31420927	ITGAD	Body	0,014	3,98E-04	3,49E-02
cg03691418	17	80962847	B3GNTL1	Body	-0,012	3,98E-04	3,49E-02
cg03434609	21	43711038	ABCG1	Body	0,03	3,98E-04	3,49E-02
cg08893641	6	100016580	CCNC	5'UTR	0,01	3,98E-04	3,49E-02
cg09520537	12	30916436		IGR	-0,01	3,98E-04	3,49E-02
cg05063577	2	40825501		IGR	-0,026	3,99E-04	3,49E-02
cg22954484	3	44804219	KIAA1143	TSS1500	-0,033	3,99E-04	3,49E-02
cg25800707	11	118401956	LOC101929089	TSS1500	0,004	3,99E-04	3,49E-02
cg09177080	17	73840822	UNC13D	TSS200	-0,004	3,99E-04	3,49E-02
cg16831361	21	39668954	KCNJ15	5'UTR	0,025	3,99E-04	3,49E-02
cg21827239	8	21059807		IGR	-0,008	3,99E-04	3,49E-02
cg21611112	11	125253048	PKNOX2	Body	0,009	3,99E-04	3,49E-02
cg07750969	9	131486582	ZDHHC12	TSS200	-0,004	3,99E-04	3,49E-02
cg10236261	12	110573183	IFT81	Body	-0,026	3,99E-04	3,49E-02
cg11882101	1	26387609	TRIM63	Body	0,044	3,99E-04	3,49E-02
cg07207982	6	34984930	ANKS1A	Body	0,044	3,99E-04	3,49E-02
cg11209106	13	35325620		IGR	-0,006	3,99E-04	3,49E-02
cg04547433	16	19052983	TMC7	Body	0,013	3,99E-04	3,49E-02
cg18362495	21	47401153	COL6A1	TSS1500	-0,006	3,99E-04	3,49E-02
cg05602567	22	32871203	FBXO7	TSS200	-0,003	3,99E-04	3,49E-02
cg04939510	22	42833864		IGR	-0,003	3,99E-04	3,49E-02
cg16231954	3	77089765	ROBO2	5'UTR	-0,037	3,99E-04	3,49E-02
cg03356335	17	71848081		IGR	-0,006	4,00E-04	3,49E-02
cg00683329	18	77545136		IGR	-0,036	4,00E-04	3,49E-02
cg08356572	6	28891055	TRIM27	1stExon	-0,006	4,00E-04	3,49E-02
cg06813578	15	51386254	TNFAIP8L3	Body	-0,007	4,00E-04	3,49E-02
cg17123508	3	71804002	EIF4E3	TSS1500	0,041	4,00E-04	3,49E-02
cg27267304	3	141866549	TFDP2	5'UTR	-0,019	4,00E-04	3,49E-02
cg02291164	14	59296302		IGR	-0,086	4,00E-04	3,49E-02
cg15156844	18	3880020	DLGAP1	1stExon	0,004	4,00E-04	3,49E-02
cg02262221	4	91047852	FAM190A	TSS1500	0,038	4,00E-04	3,49E-02
cg07696485	3	36986642	TRANK1	TSS200	0,008	4,00E-04	3,49E-02
cg09347991	22	42294630	SREBF2	Body	0,011	4,00E-04	3,49E-02
cg09561351	12	42876655	PRICKLE1	5'UTR	-0,004	4,00E-04	3,49E-02
cg13687837	2	132404694		IGR	-0,019	4,00E-04	3,49E-02
cg15640580	10	115076373		IGR	-0,008	4,00E-04	3,49E-02
cg17619638	15	22958213	CYFIP1	Body	0,004	4,00E-04	3,49E-02
cg24206614	1	205326391	KLHDC8A	TSS200	-0,007	4,01E-04	3,49E-02
cg15414924	14	36291501		IGR	0,009	4,01E-04	3,49E-02
cg19423907	4	39699804	UBE2K	5'UTR	0,007	4,01E-04	3,49E-02
cg02151068	9	115113524	MIR3134	TSS1500	-0,01	4,01E-04	3,49E-02
cg10304587	10	114582480	LOC103344931	TSS1500	-0,008	4,01E-04	3,49E-02
cg20239740	19	55690495	SYT5	5'UTR	-0,004	4,01E-04	3,49E-02
cg04545636	10	11208973	CUGBP2	Body	-0,006	4,01E-04	3,49E-02
cg22564748	6	112029200	FYN	Body	-0,006	4,01E-04	3,49E-02
cg08414984	6	72927445	RIMS1	5'UTR	0,044	4,01E-04	3,49E-02
cg00589305	11	19367621		IGR	-0,065	4,01E-04	3,49E-02
cg25684933	11	65680635		IGR	0,015	4,01E-04	3,50E-02
cg09789239	13	46614084	ZC3H13	Body	0,007	4,02E-04	3,50E-02
cg02891048	1	8044991	PARK7	Body	0,008	4,02E-04	3,50E-02
cg16382392	4	156294474	MAP9	Body	0,028	4,02E-04	3,50E-02
cg15104699	10	127063738		IGR	-0,008	4,02E-04	3,50E-02
cg01620570	17	15165908	PMP22	TSS200	-0,003	4,02E-04	3,50E-02

cg19766369	13	83408291		IGR	0,015	4,02E-04	3,50E-02
cg11470307	19	50728854	MYH14	ExonBnd	-0,03	4,02E-04	3,50E-02
cg23028858	22	39639060	PDGFB	Body	-0,01	4,02E-04	3,50E-02
cg13970093	9	95873501	C9orf89	Body	0,017	4,02E-04	3,50E-02
cg21032929	2	187561321	FAM171B	Body	0,009	4,03E-04	3,50E-02
cg16943234	1	145515802	GNRHR2	Body	0,005	4,03E-04	3,50E-02
cg17617784	14	75276278	YLPM1	Body	-0,005	4,03E-04	3,50E-02
cg08111090	2	147892016		IGR	-0,01	4,03E-04	3,50E-02
cg18417966	4	170580702	CLCN3	TSS1500	0,045	4,03E-04	3,50E-02
cg17675088	5	93013224	FAM172A	Body	-0,014	4,03E-04	3,50E-02
cg16788890	13	50867394	DLEU1	Body	-0,005	4,03E-04	3,50E-02
cg17923984	15	67213027		IGR	-0,006	4,03E-04	3,50E-02
cg00970984	16	57122796		IGR	-0,004	4,03E-04	3,50E-02
cg00921138	16	66503519	BEAN1	ExonBnd	0,011	4,03E-04	3,50E-02
cg06557406	19	5497463		IGR	-0,025	4,03E-04	3,50E-02
cg04766043	13	114770710	RASA3	Body	0,008	4,03E-04	3,50E-02
cg24383300	6	246530		IGR	-0,005	4,04E-04	3,51E-02
cg19763809	2	98703475	VWA3B	TSS200	0,01	4,04E-04	3,51E-02
cg15509013	5	16489266	FAM134B	Body	-0,011	4,04E-04	3,51E-02
cg15561056	22	39931051		IGR	0,008	4,04E-04	3,51E-02
cg02432628	21	42853308	TMPRSS2	Body	-0,008	4,04E-04	3,51E-02
cg00646546	1	2853362		IGR	-0,01	4,04E-04	3,51E-02
cg27598956	8	9758788	LOC157627	Body	0,031	4,04E-04	3,51E-02
cg06118587	11	10655869	MRVI1	5'UTR	-0,016	4,04E-04	3,51E-02
cg00000714	19	54695678	TSEN34	Body	0,016	4,04E-04	3,51E-02
cg06452451	5	74062824	NSA2	TSS1500	-0,003	4,04E-04	3,51E-02
cg06839650	6	112971587		IGR	-0,018	4,04E-04	3,51E-02
cg26615126	2	3642867	COLEC11	5'UTR	-0,036	4,05E-04	3,51E-02
cg08342886	6	33240066	VPS52	TSS1500	-0,005	4,05E-04	3,51E-02
cg15044322	11	67291020	CABP2	TSS200	0,004	4,05E-04	3,51E-02
cg14778560	1	1693368	NADK	Body	-0,01	4,05E-04	3,51E-02
cg01013002	6	35994101	MAPK14	TSS1500	-0,007	4,05E-04	3,51E-02
cg22053068	7	156469314	RNF32	3'UTR	0,006	4,05E-04	3,51E-02
cg10042335	11	21000389	NELL1	Body	-0,033	4,06E-04	3,51E-02
cg04630461	11	56948658	LRRC55	TSS1500	-0,04	4,05E-04	3,51E-02
cg08865886	14	76127443	C14orf1	5'UTR	-0,003	4,06E-04	3,51E-02
cg18224804	15	44487667	FRMD5	TSS1500	-0,003	4,05E-04	3,51E-02
cg21622750	16	20340337	GP2	TSS1500	0,007	4,05E-04	3,51E-02
cg20550890	17	19674148	ULK2	3'UTR	-0,006	4,05E-04	3,51E-02
cg21481658	17	33396459	RFFL	Body	0,009	4,05E-04	3,51E-02
cg20210637	20	1246100	SNPH	TSS1500	-0,003	4,05E-04	3,51E-02
cg01436363	3	155500475	C3orf33	Body	0,023	4,06E-04	3,51E-02
cg10271547	4	7430936	SORCS2	Body	0,012	4,06E-04	3,51E-02
cg07250198	6	33858087	LINC01016	Body	-0,007	4,06E-04	3,51E-02
cg05027444	10	43050881		IGR	0,006	4,06E-04	3,51E-02
cg06897650	15	80178743	MTHFS	Body	-0,009	4,06E-04	3,51E-02
cg11664861	2	54197786	PSME4	1stExon	0,004	4,06E-04	3,51E-02
cg19680908	5	119992045	PRR16	Body	-0,046	4,06E-04	3,51E-02
cg03202009	14	60031299	CCDC175	Body	-0,065	4,06E-04	3,51E-02
cg11227853	21	36218936	RUNX1	Body	-0,013	4,06E-04	3,52E-02
cg01514538	1	2980380	FLJ42875	Body	0,003	4,07E-04	3,52E-02
cg18066158	6	45155906	SUPT3H	Body	0,009	4,07E-04	3,52E-02
cg20018401	7	134456101		IGR	-0,032	4,07E-04	3,52E-02
cg09895547	13	106635186		IGR	-0,009	4,07E-04	3,52E-02
cg12961236	14	45369477	C14orf28	5'UTR	0,011	4,07E-04	3,52E-02
cg07376696	19	51467708	KLK6	Body	0,013	4,07E-04	3,52E-02
cg17884169	20	31446444	EFCAB8	TSS1500	0,006	4,07E-04	3,52E-02
cg06126588	22	44744913		IGR	0,019	4,07E-04	3,52E-02
cg17988641	18	71829872		IGR	-0,009	4,07E-04	3,52E-02
cg16276856	7	54614796	VSTM2A	Body	-0,008	4,07E-04	3,52E-02
cg19773296	19	37806526		IGR	-0,013	4,07E-04	3,52E-02
cg18135436	15	65946740	SLC24A1	3'UTR	-0,004	4,08E-04	3,52E-02
cg24524403	17	79048599	BAIAP2	Body	-0,015	4,07E-04	3,52E-02
cg03695597	12	70174183	RAB3IP	Body	-0,015	4,08E-04	3,52E-02
cg24082339	5	167372659	TENM2	Body	0,006	4,08E-04	3,52E-02
cg20957428	11	116700436	APOC3	TSS200	0,021	4,08E-04	3,52E-02
cg24852972	3	59352714		IGR	-0,01	4,08E-04	3,52E-02
cg05153782	2	186665621	FSIP2	Body	-0,009	4,08E-04	3,52E-02
cg10070101	17	78963290		IGR	-0,042	4,08E-04	3,52E-02
cg27592761	18	43244965	SLC14A2	Body	0,013	4,08E-04	3,52E-02
cg04973273	4	153274424	FBXW7	TSS1500	0,016	4,08E-04	3,52E-02
cg21507987	11	10527506	AMPD3	3'UTR	0,035	4,08E-04	3,52E-02
cg20084602	11	304489		IGR	-0,049	4,09E-04	3,53E-02

cg14729930	17	38345453	RAPGEFL1	Body	0,016	4,09E-04	3,53E-02
cg23009468	5	38061074		IGR	-0,009	4,09E-04	3,53E-02
cg26475124	1	53608399	SLC1A7	TSS200	-0,013	4,09E-04	3,53E-02
cg16518104	5	123873274		IGR	-0,006	4,09E-04	3,53E-02
cg16111073	12	124344237	DNAH10	Body	-0,006	4,09E-04	3,53E-02
cg19108485	17	75319068	sept-09	Body	0,022	4,09E-04	3,53E-02
cg03990551	4	122791477	BBS7	5'UTR	-0,003	4,10E-04	3,53E-02
cg01750172	10	12392385	CAMK1D	Body	-0,003	4,10E-04	3,53E-02
cg26629982	5	100227566	ST8SIA4	Body	0,027	4,10E-04	3,53E-02
cg18319651	11	114318549	REXO2	ExonBnd	-0,007	4,10E-04	3,53E-02
cg18986218	14	91225366	TTC7B	Body	0,014	4,10E-04	3,53E-02
cg11267431	22	38073001	LGALS1	Body	-0,005	4,10E-04	3,53E-02
cg04188348	1	175163165	KIAA0040	TSS1500	0,016	4,10E-04	3,53E-02
cg18573244	12	10956452	TAS2R7	TSS1500	0,023	4,10E-04	3,53E-02
cg00590036	6	158957433	TMEM181	TSS200	-0,01	4,10E-04	3,53E-02
cg01223499	14	93107823	RIN3	Body	0,019	4,10E-04	3,53E-02
cg15061245	4	73921372	COX18	3'UTR	-0,018	4,10E-04	3,53E-02
cg25386639	14	37026514		IGR	-0,045	4,10E-04	3,53E-02
cg15090644	12	94954628	MIR5700	TSS1500	0,039	4,10E-04	3,53E-02
cg19853210	19	12098259		IGR	-0,006	4,11E-04	3,53E-02
cg20620508	5	159613044	FABP6	TSS1500	0,028	4,11E-04	3,53E-02
cg22590868	3	10333043	GHRLOS	Body	0,017	4,11E-04	3,54E-02
cg04493259	2	42968	FAM110C	Body	-0,023	4,11E-04	3,54E-02
cg14498972	3	155523948	C3orf33	5'UTR	0,003	4,11E-04	3,54E-02
cg13833040	12	110784179	ATP2A2	Body	-0,01	4,11E-04	3,54E-02
cg05968393	15	31672998	KLF13	Body	-0,004	4,11E-04	3,54E-02
cg05935476	6	157400180	ARID1B	Body	0,02	4,11E-04	3,54E-02
cg01260933	22	46750582	TRMU	Body	0,005	4,11E-04	3,54E-02
cg08366132	1	27191899		IGR	-0,003	4,11E-04	3,54E-02
cg20945581	7	30801378	INMT-FAM188B	Body	-0,006	4,11E-04	3,54E-02
cg09854852	6	27289918		IGR	-0,018	4,12E-04	3,54E-02
cg01365997	9	86955743	SLC28A3	TSS200	0,024	4,12E-04	3,54E-02
cg04398909	6	70985871	COL9A1	Body	-0,018	4,12E-04	3,54E-02
cg20653128	17	40936820	WNK4	Body	-0,045	4,12E-04	3,54E-02
cg01393184	2	9696108	ADAM17	TSS200	-0,003	4,12E-04	3,54E-02
cg18058524	2	228498185	C2orf83	TSS200	0,01	4,12E-04	3,54E-02
cg23804764	4	150038122		IGR	-0,009	4,12E-04	3,54E-02
cg24736380	10	3144101	PFKP	Body	0,009	4,12E-04	3,54E-02
cg12266841	11	45687263	CHST1	TSS200	-0,003	4,12E-04	3,54E-02
cg12982131	17	48172957	PDK2	5'UTR	0,021	4,12E-04	3,54E-02
cg11656509	19	11172073	SMARCA4	Body	-0,005	4,12E-04	3,54E-02
cg22502856	1	209825678	LAMB3	TSS1500	0,009	4,13E-04	3,54E-02
cg05215130	2	186525540		IGR	0,033	4,13E-04	3,54E-02
cg24855295	4	146540341	MMAA	TSS200	0,004	4,13E-04	3,54E-02
cg02357153	12	55073935		IGR	-0,013	4,13E-04	3,54E-02
cg09907628	16	89498634	ANKRD11	5'UTR	0,022	4,13E-04	3,54E-02
cg26550851	1	3701203	LRRC47	Body	-0,009	4,13E-04	3,54E-02
cg16834496	10	115438884	CASP7	TSS1500	-0,004	4,13E-04	3,54E-02
cg00204532	3	4533398		IGR	0,03	4,13E-04	3,55E-02
cg11951604	18	33709151	SLC39A6	1stExon	0,006	4,14E-04	3,55E-02
cg03044471	11	1321009	TOLLIP	Body	0,016	4,14E-04	3,55E-02
cg08707112	10	8095764	FLJ45983	TSS1500	-0,006	4,14E-04	3,55E-02
cg10047019	8	21822950	XPO7	Body	-0,008	4,14E-04	3,55E-02
cg17172946	10	112440727	RBM20	Body	-0,061	4,14E-04	3,55E-02
cg03803873	1	28562511	ATPIF1	TSS200	0,003	4,14E-04	3,55E-02
cg05189127	10	127059333		IGR	-0,056	4,14E-04	3,55E-02
cg16566825	2	182445854	CERKL	Body	-0,014	4,14E-04	3,55E-02
cg18091046	13	30077344	MTUS2	3'UTR	-0,057	4,14E-04	3,55E-02
cg11034191	4	143767672	INPP4B	TSS200	-0,005	4,14E-04	3,55E-02
cg00711959	2	13962486		IGR	-0,026	4,15E-04	3,55E-02
cg05350601	6	125004781	NKAIN2	Body	-0,023	4,15E-04	3,55E-02
cg15948871	6	31139620	POU5F1	TSS1500	0,014	4,15E-04	3,55E-02
cg06019792	9	71815029	TJP2	5'UTR	-0,023	4,15E-04	3,55E-02
cg09118485	16	4460917	CORO7-PAM16	Body	0,024	4,15E-04	3,55E-02
cg20088138	17	78010268	CCDC40	TSS200	-0,005	4,15E-04	3,55E-02
cg12607945	2	169347230	CERS6	Body	-0,003	4,15E-04	3,55E-02
cg23448811	1	3311656	PRDM16	Body	-0,011	4,15E-04	3,55E-02
cg17938601	18	13480993	LDLRAD4	Body	-0,006	4,15E-04	3,55E-02
cg24601355	18	72799885		IGR	-0,004	4,15E-04	3,55E-02
cg12220753	1	53393030	SCP2	5'UTR	-0,003	4,15E-04	3,56E-02
cg00592871	8	118738517		IGR	-0,016	4,16E-04	3,56E-02
cg23605373	3	137717380	CLDN18	TSS1500	-0,015	4,16E-04	3,56E-02
cg02141631	3	150331040	SELT	Body	0,008	4,16E-04	3,56E-02

cg24827258	5	1800961	NDUFS6	TSS1500	-0,011	4,16E-04	3,56E-02
cg17943107	5	126250722	MARCH3	Body	-0,016	4,16E-04	3,56E-02
cg01397325	11	20386022	HTATIP2	Body	0,042	4,16E-04	3,56E-02
cg02208793	11	84634615	DLG2	TSS200	-0,045	4,16E-04	3,56E-02
cg06651119	16	83640436	CDH13	Body	-0,027	4,16E-04	3,56E-02
cg00146805	7	52339555		IGR	-0,01	4,16E-04	3,56E-02
cg25643118	6	80657428	ELOVL4	TSS200	-0,012	4,16E-04	3,56E-02
cg22207479	18	43195041	SLC14A2	5'UTR	-0,013	4,16E-04	3,56E-02
cg11557886	5	44811891	MRPS30	Body	0,019	4,16E-04	3,56E-02
cg02107816	1	7612135	CAMTA1	Body	-0,007	4,16E-04	3,56E-02
cg06936746	11	105879122	KIAA1826	3'UTR	-0,005	4,17E-04	3,56E-02
cg06520508	1	1261347	GLTPD1	5'UTR	0,022	4,17E-04	3,56E-02
cg00948114	6	157745216	C6orf35	5'UTR	-0,008	4,17E-04	3,56E-02
cg27336178	9	139971800	UAP1L1	TSS200	-0,004	4,17E-04	3,56E-02
cg05182503	14	24721651	TGM1	Body	0,012	4,17E-04	3,56E-02
cg13790268	14	100125548	HHIPL1	Body	-0,036	4,17E-04	3,56E-02
cg05254609	14	102687845	WDR20	Body	0,031	4,17E-04	3,56E-02
cg02136209	16	12746288		IGR	-0,051	4,17E-04	3,56E-02
cg26521813	17	38638625	TNS4	Body	0,02	4,17E-04	3,56E-02
cg20189808	2	242756029	NEU4	Body	-0,018	4,17E-04	3,56E-02
cg00722180	14	73525139	RBM25	TSS200	0,003	4,17E-04	3,56E-02
cg26989950	18	64181521	CDH19	Body	0,029	4,17E-04	3,56E-02
cg11591992	1	24621076		IGR	0,03	4,18E-04	3,56E-02
cg21604970	1	77505574	ST6GALNAC5	Body	-0,005	4,18E-04	3,56E-02
cg09358751	14	102785836	ZNF839	TSS1500	-0,003	4,18E-04	3,56E-02
cg17424654	6	89673513	RNGTT	TSS200	-0,005	4,18E-04	3,56E-02
cg15171398	6	132826295	STX7	5'UTR	-0,004	4,18E-04	3,56E-02
cg18502477	11	57946199	OR9Q1	5'UTR	-0,021	4,18E-04	3,56E-02
cg03554749	4	85405132		IGR	-0,024	4,18E-04	3,56E-02
cg14095720	1	201195051	IGFN1	Body	0,003	4,18E-04	3,56E-02
cg14924165	4	176575489	GPM6A	Body	0,037	4,18E-04	3,56E-02
cg14308254	8	124219699	FAM83A	Body	-0,018	4,18E-04	3,56E-02
cg07455238	12	72480987		IGR	-0,013	4,18E-04	3,56E-02
cg24721673	7	111651276	DOCK4	Body	0,03	4,18E-04	3,57E-02
cg01498231	10	73847259	SPOCK2	Body	-0,004	4,18E-04	3,57E-02
cg27252086	15	43621643	LCMT2	1stExon	0,048	4,19E-04	3,57E-02
cg16380846	9	30773390		IGR	-0,018	4,19E-04	3,57E-02
cg13437522	16	66518730	BEAN1	Body	-0,006	4,19E-04	3,57E-02
cg03818793	18	24129558	KCTD1	TSS1500	0,018	4,19E-04	3,57E-02
cg06578098	4	32750186		IGR	-0,006	4,19E-04	3,57E-02
cg07408607	12	24199071	SOX5	5'UTR	-0,011	4,19E-04	3,57E-02
cg11049042	19	47334821	SNAR-E	TSS1500	-0,006	4,19E-04	3,57E-02
cg22240695	5	140729700	PCDHGB1	TSS200	-0,036	4,19E-04	3,57E-02
cg02669193	6	167173257	RPS6KA2	Body	-0,034	4,19E-04	3,57E-02
cg26167518	8	25324694	CDCA2	Body	0,042	4,20E-04	3,57E-02
cg15781176	8	120777204	TAF2	Body	0,043	4,20E-04	3,57E-02
cg23359810	10	51572113	NCOA4	5'UTR	-0,012	4,20E-04	3,57E-02
cg03874344	11	4210607	LOC100506082	Body	-0,01	4,20E-04	3,57E-02
cg19005438	15	60295158	FOXB1	TSS1500	-0,03	4,19E-04	3,57E-02
cg04771023	16	75617114		IGR	0,014	4,20E-04	3,57E-02
cg02713753	20	47438518	PREX1	Body	0,014	4,20E-04	3,57E-02
cg12110216	11	119455041		IGR	-0,004	4,20E-04	3,57E-02
cg00613198	14	62010028	PRKCH	Body	-0,014	4,20E-04	3,57E-02
cg08621656	8	10671003	PINX1	Body	-0,013	4,20E-04	3,57E-02
cg05435286	3	27411063	NEK10	TSS200	-0,003	4,20E-04	3,57E-02
cg05567191	5	157911719		IGR	-0,023	4,20E-04	3,57E-02
cg20181492	2	172082104	TLK1	5'UTR	0,012	4,21E-04	3,57E-02
cg09061045	5	140071322	HARS	TSS1500	-0,003	4,21E-04	3,57E-02
cg03306780	8	20798259		IGR	-0,006	4,21E-04	3,57E-02
cg10458392	19	3600539	TBXA2R	Body	-0,016	4,21E-04	3,57E-02
cg21140986	12	55888111		IGR	0,023	4,21E-04	3,57E-02
cg02557110	5	1089654	SLC12A7	Body	-0,01	4,21E-04	3,57E-02
cg22473235	5	59154744	PDE4D	Body	0,013	4,21E-04	3,57E-02
cg02939242	21	39699129	LINC01423	Body	0,007	4,21E-04	3,57E-02
cg16400897	2	31208719	GALNT14	Body	-0,017	4,21E-04	3,57E-02
cg12758847	11	10423210	CAND1.11	Body	0,013	4,21E-04	3,57E-02
cg02481778	13	77460607	KCTD12	TSS200	0,005	4,21E-04	3,57E-02
cg17237460	9	79793327	VPS13A	Body	-0,006	4,21E-04	3,58E-02
cg12160448	8	25904511		IGR	-0,007	4,21E-04	3,58E-02
cg19214252	3	169352742	MECOM	5'UTR	-0,019	4,22E-04	3,58E-02
cg16063308	10	11576398	USP6NL	Body	0,017	4,22E-04	3,58E-02
cg09529287	7	88294333		IGR	-0,013	4,22E-04	3,58E-02
cg24980904	18	3248854	MYL12A	5'UTR	-0,005	4,22E-04	3,58E-02

cg00259945	19	38809881	KCNK6	TSS1500	0,02	4,22E-04	3,58E-02
cg23092927	4	133293183		IGR	-0,011	4,22E-04	3,58E-02
cg26795011	8	145027226	PLEC1	Body	-0,003	4,22E-04	3,58E-02
cg10941635	12	308722	SLC6A12	Body	-0,041	4,23E-04	3,58E-02
cg17751438	2	145275197	ZEB2	Body	-0,005	4,23E-04	3,58E-02
cg11729235	8	59634418		IGR	-0,008	4,23E-04	3,58E-02
cg14384748	12	132859951	GALNT9	Body	0,069	4,23E-04	3,58E-02
cg14988214	2	8977332	KIDINS220	5'UTR	-0,002	4,23E-04	3,59E-02
cg19794706	14	75643232	TMED10	1stExon	0,008	4,23E-04	3,59E-02
cg02368953	2	75141774	LINC01291	Body	0,009	4,23E-04	3,59E-02
cg17376288	3	190993197	UTS2D	Body	0,01	4,23E-04	3,59E-02
cg14341520	9	2717338	KCNV2	TSS200	-0,007	4,24E-04	3,59E-02
cg03693861	2	33701285	RASGRP3	5'UTR	-0,005	4,24E-04	3,59E-02
cg06825957	3	49591628	BSN	TSS1500	-0,007	4,24E-04	3,59E-02
cg19464155	4	8437763	ACOX3	5'UTR	-0,02	4,24E-04	3,59E-02
cg08163193	9	34692051	CCL19	TSS1500	0,032	4,24E-04	3,59E-02
cg06947488	1	154524643	UBE2Q1	Body	-0,004	4,24E-04	3,59E-02
cg05677724	12	131125565		IGR	-0,013	4,24E-04	3,59E-02
cg05830569	1	37332231	GRIK3	Body	-0,012	4,24E-04	3,59E-02
cg22883525	17	78069719	CCDC40	Body	0,021	4,24E-04	3,59E-02
cg23544472	11	131707666	NTM	Body	-0,007	4,25E-04	3,59E-02
cg05807826	11	96117376	CCDC82	Body	-0,006	4,25E-04	3,59E-02
cg00676984	7	2352141	SNX8	Body	0,018	4,25E-04	3,60E-02
cg27054424	7	105094993		IGR	0,016	4,25E-04	3,60E-02
cg04257469	14	57359877	OTX2-AS1	Body	-0,047	4,25E-04	3,60E-02
cg11795947	10	63781425	ARID5B	Body	0,029	4,25E-04	3,60E-02
cg25363550	14	69102176		IGR	-0,024	4,25E-04	3,60E-02
cg02743569	5	2304028		IGR	-0,044	4,25E-04	3,60E-02
cg14402574	9	108421903		IGR	-0,005	4,25E-04	3,60E-02
cg05263883	19	55173353	LILRB4	TSS1500	0,009	4,25E-04	3,60E-02
cg14443472	9	129266352	FAM125B	3'UTR	0,009	4,25E-04	3,60E-02
cg03962316	12	82892515		IGR	0,03	4,25E-04	3,60E-02
cg25822376	7	119945494	KCND2	Body	-0,024	4,26E-04	3,60E-02
cg23262361	9	97137570	HIATL1	Body	-0,003	4,26E-04	3,60E-02
cg02190636	19	541711	CDC34	3'UTR	0,013	4,26E-04	3,60E-02
cg00526711	15	91477898	UNC45A	5'UTR	-0,022	4,26E-04	3,60E-02
cg22933154	14	65381118	CHURC1	TSS200	0,003	4,26E-04	3,60E-02
cg24165921	12	79941506		IGR	-0,027	4,26E-04	3,60E-02
cg03168497	17	48586147	MYCBPAP	Body	0,065	4,26E-04	3,60E-02
cg23418180	11	128729960	KCNJ1	5'UTR	0,026	4,26E-04	3,60E-02
cg04630574	16	67979792	SLC12A4	Body	0,006	4,26E-04	3,60E-02
cg12551813	1	200781121	CAMSAP1L1	Body	0,032	4,26E-04	3,60E-02
cg04978426	16	70571654	SNORD111	TSS1500	-0,014	4,26E-04	3,60E-02
cg24030138	18	43419102	SIGLEC15	Body	-0,046	4,26E-04	3,60E-02
cg16220931	1	170253981	LINC01142	TSS1500	-0,005	4,26E-04	3,60E-02
cg13397387	12	72032132	ZFC3H1	Body	0,026	4,26E-04	3,60E-02
cg10964761	4	169815479	PALLD	Body	-0,006	4,27E-04	3,60E-02
cg06252875	7	158828750	VIPR2	Body	0,008	4,27E-04	3,60E-02
cg11743591	10	52053390		IGR	-0,007	4,27E-04	3,60E-02
cg18762249	17	77283027	RBFOX3	5'UTR	0,052	4,27E-04	3,60E-02
cg13406113	3	143153022	SLC9A9	Body	-0,006	4,27E-04	3,60E-02
cg26860198	9	88559365	NAA35	Body	-0,005	4,27E-04	3,60E-02
cg03310027	15	43941072	CATSPER2	TSS200	-0,03	4,27E-04	3,60E-02
cg00064080	10	122598254		IGR	-0,012	4,27E-04	3,60E-02
cg23055396	11	62538850	TAF6L	TSS200	-0,006	4,27E-04	3,60E-02
cg26882009	5	140346106	PCDHAC2	1stExon	0,009	4,27E-04	3,60E-02
cg04661382	11	109294584	C11orf87	Body	-0,036	4,27E-04	3,60E-02
cg19083265	17	75300284	sept-09	5'UTR	-0,008	4,27E-04	3,60E-02
cg08469616	9	86365438	GKAP1	Body	-0,005	4,28E-04	3,60E-02
cg24138528	6	141782625		IGR	0,015	4,28E-04	3,60E-02
cg08218445	3	192543630	MB21D2	Body	-0,024	4,28E-04	3,60E-02
cg14587062	6	157800824		IGR	0,008	4,28E-04	3,60E-02
cg17056048	2	144271431	ARHGAP15	Body	-0,005	4,28E-04	3,60E-02
cg23202262	12	131198244		IGR	0,044	4,28E-04	3,60E-02
cg26985447	14	105343315	KIAA0284	5'UTR	0,013	4,28E-04	3,60E-02
cg18176599	17	47014454	SNF8	Body	-0,011	4,28E-04	3,61E-02
cg18319689	1	46016267	AKR1A1	TSS1500	-0,003	4,28E-04	3,61E-02
cg27070458	9	130547825	MIR3960	TSS1500	0,009	4,29E-04	3,61E-02
cg21363699	11	120894693	TBCEL	TSS200	-0,004	4,28E-04	3,61E-02
cg16245035	1	10684392	PEX14	Body	0,003	4,29E-04	3,61E-02
cg23898184	1	192921219		IGR	0,058	4,29E-04	3,61E-02
cg14775958	6	170754745		IGR	0,017	4,29E-04	3,61E-02
cg22056218	11	63973536	FERMT3	TSS1500	0,028	4,29E-04	3,61E-02

cg13507983	11	5128882		IGR	-0,045	4,29E-04	3,61E-02
cg10602537	5	172260815	ERGIC1	TSS1500	-0,065	4,29E-04	3,61E-02
cg01955377	2	188392251	TFPI	5'UTR	0,019	4,29E-04	3,61E-02
cg26777800	4	39530705	UGDH	TSS1500	-0,011	4,29E-04	3,61E-02
cg03132592	9	134955718	MED27	TSS1500	-0,019	4,29E-04	3,61E-02
cg00415077	16	1408126	GNPTG	Body	0,018	4,29E-04	3,61E-02
cg13246339	18	11990145	IMPA2	Body	-0,039	4,29E-04	3,61E-02
cg15616998	1	40435396	MFSD2A	3'UTR	0,017	4,29E-04	3,61E-02
cg11830061	9	5185415	INSL6	1stExon	-0,019	4,30E-04	3,61E-02
cg25892778	9	92448452		IGR	-0,013	4,30E-04	3,61E-02
cg13556057	11	62267946	AHNAK	Body	0,015	4,30E-04	3,61E-02
cg00701514	16	1183462		IGR	0,008	4,30E-04	3,61E-02
cg17929959	18	19873627		IGR	-0,048	4,30E-04	3,61E-02
cg19132827	8	11126310		IGR	-0,027	4,30E-04	3,61E-02
cg08106164	2	140286780		IGR	-0,044	4,30E-04	3,61E-02
cg12462097	9	119025685	PAPPA	Body	-0,01	4,30E-04	3,61E-02
cg25589139	18	44042039		IGR	0,012	4,30E-04	3,61E-02
cg20249550	19	13001990	GCDH	1stExon	-0,003	4,30E-04	3,61E-02
cg18126938	1	241376368	RGS7	Body	-0,029	4,31E-04	3,61E-02
cg12167197	2	203798125	CARF	5'UTR	-0,006	4,30E-04	3,61E-02
cg12816562	2	152634098		IGR	-0,004	4,31E-04	3,61E-02
cg22904096	2	211180386	MYL1	TSS1500	-0,027	4,31E-04	3,61E-02
cg13164087	6	87832666		IGR	-0,07	4,31E-04	3,61E-02
cg08167158	11	44546253		IGR	-0,008	4,31E-04	3,61E-02
cg03215500	14	81371212	CEP128	Body	-0,007	4,31E-04	3,61E-02
cg06685910	18	44710932		IGR	0,023	4,31E-04	3,61E-02
cg18588441	2	175816203	CHN1	Body	0,014	4,31E-04	3,61E-02
cg04695939	3	43573746	ANO10	Body	-0,004	4,31E-04	3,61E-02
cg09837037	5	1201602	SLC6A19	TSS200	0,012	4,31E-04	3,61E-02
cg03136712	5	140053068	DND1	Body	-0,004	4,31E-04	3,61E-02
cg07158312	8	95565682	KIAA1429	1stExon	0,006	4,31E-04	3,61E-02
cg00512287	11	118512372	PHLDB1	Body	0,02	4,31E-04	3,61E-02
cg20006624	19	53789950		IGR	-0,051	4,31E-04	3,62E-02
cg01400516	16	47175936	NETO2	Body	-0,004	4,32E-04	3,62E-02
cg04950965	1	68202520	GNG12	5'UTR	0,03	4,32E-04	3,62E-02
cg15850954	10	26505245	GAD2	5'UTR	-0,005	4,32E-04	3,62E-02
cg04437232	20	36068557		IGR	0,011	4,32E-04	3,62E-02
cg24388969	15	57261083	TCF12	Body	-0,009	4,32E-04	3,62E-02
cg22232554	2	8731190		IGR	0,008	4,32E-04	3,62E-02
cg16286251	1	167051948	GPA33	Body	0,008	4,32E-04	3,62E-02
cg09462438	2	144141676	ARHGAP15	Body	0,011	4,32E-04	3,62E-02
cg06598877	1	101179462		IGR	-0,015	4,32E-04	3,62E-02
cg02875558	17	37309177	PLXDC1	TSS1500	-0,002	4,32E-04	3,62E-02
cg07533488	12	117287121	RNFT2	Body	-0,03	4,32E-04	3,62E-02
cg27590105	6	150346816	RAET1L	TSS200	-0,011	4,32E-04	3,62E-02
cg09637470	11	109963583	ZC3H12C	TSS1500	0,005	4,32E-04	3,62E-02
cg26916927	6	38671019	GLO1	TSS200	-0,004	4,33E-04	3,62E-02
cg13690588	1	28415118	EYA3	1stExon	0,018	4,33E-04	3,62E-02
cg19430878	5	152780823		IGR	-0,017	4,33E-04	3,62E-02
cg24959475	7	48144336	UPP1	Body	0,006	4,33E-04	3,62E-02
cg10457472	13	97874507	MBNL2	TSS200	-0,008	4,33E-04	3,62E-02
cg10935889	3	123603624	MYLK	TSS1500	-0,037	4,33E-04	3,62E-02
cg25849268	13	50511071	C13orf1	TSS1500	-0,004	4,33E-04	3,62E-02
cg15070507	18	45663129		IGR	-0,007	4,33E-04	3,62E-02
cg12098015	19	38270616	ZNF573	TSS1500	-0,006	4,33E-04	3,62E-02
cg22127075	1	32135179	COL16A1	Body	0,018	4,34E-04	3,62E-02
cg13520532	10	135273122	LOC619207	Body	-0,036	4,34E-04	3,62E-02
cg02617837	12	107349530	C12orf23	TSS200	0,005	4,34E-04	3,62E-02
cg20664285	16	16181129	ABCC1	Body	0,05	4,34E-04	3,62E-02
cg17009399	19	47852367	DHX34	TSS200	-0,003	4,34E-04	3,62E-02
cg24562819	20	23029806	THBD	1stExon	-0,007	4,34E-04	3,62E-02
cg23558208	21	44589537	CRYAA	Body	0,024	4,34E-04	3,62E-02
cg19684296	15	67008514	SMAD6	Body	0,073	4,34E-04	3,62E-02
cg00881032	2	169996363	LRP2	Body	0,023	4,34E-04	3,62E-02
cg13980458	6	39174673	KCNK5	Body	0,019	4,34E-04	3,62E-02
cg18075315	12	106988393	RFX4	Body	-0,035	4,34E-04	3,62E-02
cg00154335	22	37554221		IGR	0,007	4,34E-04	3,62E-02
cg06098138	3	53912235	ACTR8	Body	0,021	4,34E-04	3,62E-02
cg07168079	2	232650383	COPS7B	TSS1500	0,009	4,34E-04	3,62E-02
cg15687530	5	1725135		IGR	-0,042	4,34E-04	3,62E-02
cg20331241	6	32052409	TNXB	Body	-0,006	4,34E-04	3,62E-02
cg25840057	13	113730354	MCF2L	Body	0,006	4,34E-04	3,62E-02
cg22736379	14	67831489	EIF2S1	ExonBnd	0,023	4,34E-04	3,62E-02

cg01561194	10	1595633	ADARB2	Body	-0,032	4,35E-04	3,63E-02
cg25357666	15	100517183	ADAMTS17	Body	-0,023	4,35E-04	3,63E-02
cg07938281	16	326083	RGS11	TSS200	-0,025	4,35E-04	3,63E-02
cg00346205	1	214169844	PROX1	5'UTR	-0,017	4,35E-04	3,63E-02
cg11034619	10	49482767	FRMPD2	5'UTR	0,017	4,35E-04	3,63E-02
cg19502936	17	1553591	RILP	TSS200	0,008	4,35E-04	3,63E-02
cg09058991	16	88698872		IGR	0,019	4,35E-04	3,63E-02
cg03784375	7	157570978	PTPRN2	Body	-0,007	4,36E-04	3,63E-02
cg00900202	12	49391977	DDN	Body	-0,005	4,36E-04	3,63E-02
cg01641509	2	136844587		IGR	-0,006	4,36E-04	3,63E-02
cg25620102	2	241244553		IGR	0,034	4,36E-04	3,63E-02
cg08240881	13	112333627		IGR	0,007	4,36E-04	3,63E-02
cg07169712	6	29571419	GABBR1	Body	-0,005	4,36E-04	3,63E-02
cg12488810	6	75884860	COL12A1	Body	-0,026	4,36E-04	3,63E-02
cg15029735	10	79681906	DLG5	Body	-0,01	4,36E-04	3,63E-02
cg01511742	3	71112437	FOXP1	Body	-0,007	4,36E-04	3,63E-02
cg16288814	2	216898713		IGR	-0,017	4,36E-04	3,63E-02
cg22636914	7	136849321	LOC349160	TSS1500	-0,018	4,37E-04	3,63E-02
cg27113868	11	10921952		IGR	-0,033	4,37E-04	3,63E-02
cg08321576	12	10022229	CLEC2B	1stExon	-0,004	4,37E-04	3,63E-02
cg08423052	14	52780921	PTGER2	TSS200	-0,004	4,37E-04	3,63E-02
cg16151541	2	223182775		IGR	-0,013	4,37E-04	3,63E-02
cg27465994	8	134470913	ST3GAL1	3'UTR	-0,017	4,37E-04	3,63E-02
cg25138076	6	149766127		IGR	-0,005	4,37E-04	3,64E-02
cg24874389	17	79609095	TSPAN10	TSS1500	-0,014	4,37E-04	3,64E-02
cg19427600	12	94683151	PLXNC1	Body	0,005	4,37E-04	3,64E-02
cg01885814	21	46910559	COL18A1	Body	0,011	4,37E-04	3,64E-02
cg23276673	2	136577322	LCT	Body	0,011	4,38E-04	3,64E-02
cg17245223	10	131647483	EBF3	Body	-0,013	4,38E-04	3,64E-02
cg13383608	19	47157260	DACT3	Body	0,023	4,38E-04	3,64E-02
cg19893409	18	61009665	KDSR	Body	-0,042	4,38E-04	3,64E-02
cg06830450	12	58121004	LOC100130776	Body	-0,006	4,38E-04	3,64E-02
cg19636411	12	95909538	METAP2	3'UTR	0,01	4,38E-04	3,64E-02
cg15767676	1	2115675	PRKCZ	Body	0,019	4,39E-04	3,64E-02
cg01007483	2	241385321	GPC1	Body	-0,013	4,39E-04	3,64E-02
cg11784053	3	107517460	BBX	Body	0,008	4,39E-04	3,64E-02
cg14305798	3	145236908		IGR	-0,024	4,39E-04	3,64E-02
cg27611274	7	157512892	PTPRN2	Body	-0,005	4,39E-04	3,64E-02
cg07086762	8	87007480		IGR	0,039	4,39E-04	3,64E-02
cg02932978	9	126978469		IGR	0,016	4,39E-04	3,64E-02
cg24511782	12	110841169	ANAPC7	Body	-0,003	4,39E-04	3,64E-02
cg03867790	12	131887519		IGR	-0,026	4,39E-04	3,64E-02
cg24129115	14	23352488	REM2	1stExon	0,057	4,39E-04	3,64E-02
cg21663667	2	242169936	HDLBP	Body	0,01	4,39E-04	3,64E-02
cg27401361	3	47338836	KLHL18	Body	0,02	4,39E-04	3,64E-02
cg14938148	3	179373956	USP13	Body	-0,007	4,39E-04	3,64E-02
cg25199667	5	151082206		IGR	-0,016	4,39E-04	3,64E-02
cg00604199	8	26481284	DPYSL2	Body	-0,008	4,39E-04	3,64E-02
cg08602378	10	79829044		IGR	-0,007	4,39E-04	3,64E-02
cg26480228	12	48921775	OR8S1	Body	-0,017	4,39E-04	3,64E-02
cg07203320	3	139108984	COPB2	TSS1500	0,009	4,40E-04	3,65E-02
cg11271406	15	43330997	UBR1	Body	0,028	4,40E-04	3,65E-02
cg23243821	1	33133661	RBBP4	Body	0,006	4,40E-04	3,65E-02
cg23954953	1	97187154	PTBP2	TSS200	0,01	4,40E-04	3,65E-02
cg16901991	2	24272451	FKBP1B	TSS200	-0,003	4,40E-04	3,65E-02
cg19251280	8	98657731	MTDH	Body	-0,004	4,40E-04	3,65E-02
cg05184381	18	60445513	PHLPP1	Body	-0,004	4,40E-04	3,65E-02
cg02011981	11	70256681	CTTN	Body	0,007	4,41E-04	3,65E-02
cg05052384	1	155920254	ARHGEF2	Body	-0,005	4,41E-04	3,65E-02
cg16325777	10	43250569		IGR	-0,028	4,41E-04	3,65E-02
cg18527673	17	19469328	SLC47A1	Body	-0,007	4,41E-04	3,65E-02
cg09404905	4	88342280	NUDT9	TSS1500	-0,006	4,41E-04	3,65E-02
cg07697348	8	125384179	TMEM65	1stExon	-0,004	4,41E-04	3,65E-02
cg21782529	5	2241205		IGR	-0,015	4,41E-04	3,65E-02
cg27603338	5	5886845		IGR	-0,031	4,41E-04	3,65E-02
cg26216141	6	24476586	GPLD1	Body	-0,01	4,41E-04	3,65E-02
cg17892588	8	144162379		IGR	0,009	4,41E-04	3,65E-02
cg09324662	14	23374466	RBM23	Body	-0,004	4,41E-04	3,65E-02
cg25440534	5	108137789	FER	5'UTR	-0,017	4,41E-04	3,65E-02
cg12982932	6	1016638	LOC285768	Body	0,013	4,41E-04	3,65E-02
cg11809464	6	100054515	PRDM13	TSS200	-0,039	4,42E-04	3,65E-02
cg18627650	12	8202474	FOXJ2	Body	-0,006	4,42E-04	3,65E-02
cg16103990	10	26500721	MYO3A	Body	-0,016	4,42E-04	3,66E-02

cg08087034	16	90034285	DEF8	3'UTR	0,007	4,42E-04	3,66E-02
cg03682265	6	134911686		IGR	-0,019	4,42E-04	3,66E-02
cg06094924	6	73458099	KCNQ5	Body	0,018	4,42E-04	3,66E-02
cg27005118	17	13972210	COX10	TSS1500	-0,059	4,43E-04	3,66E-02
cg22935319	17	38545021	TOP2A	3'UTR	0,026	4,43E-04	3,66E-02
cg03624321	3	69058454	EOGT	Body	-0,023	4,43E-04	3,66E-02
cg07788140	12	113921202		IGR	-0,046	4,43E-04	3,66E-02
cg08119256	13	113483183	ATP11A	Body	0,004	4,43E-04	3,66E-02
cg12010135	7	45475682		IGR	0,036	4,43E-04	3,66E-02
cg03753299	18	11134436	PIEZO2	Body	-0,007	4,43E-04	3,66E-02
cg09244338	1	18622294	IGSF21	Body	-0,039	4,44E-04	3,67E-02
cg21419945	4	7612833	SORCS2	Body	0,015	4,44E-04	3,67E-02
cg11640106	4	93192395	LOC101929194	Body	-0,013	4,44E-04	3,67E-02
cg24504446	7	63765270		IGR	0,017	4,44E-04	3,67E-02
cg10717425	12	121296406	SPPL3	Body	0,016	4,44E-04	3,67E-02
cg24916284	2	105372789		IGR	-0,009	4,44E-04	3,67E-02
cg00590481	4	174913266		IGR	0,013	4,44E-04	3,67E-02
cg23329272	5	180085924		IGR	0,077	4,44E-04	3,67E-02
cg15706776	13	67374653	PCDH9	Body	-0,009	4,44E-04	3,67E-02
cg20118557	19	41168801		IGR	-0,003	4,44E-04	3,67E-02
cg13611785	5	12936481		IGR	-0,006	4,44E-04	3,67E-02
cg17970936	11	130356135		IGR	-0,005	4,45E-04	3,67E-02
cg26286854	5	92769578	NR2F1-AS1	Body	-0,012	4,45E-04	3,67E-02
cg18416374	1	8384261	SLC45A1	Body	-0,007	4,45E-04	3,67E-02
cg23256476	11	69037846		IGR	0,011	4,45E-04	3,67E-02
cg20667913	17	564900	VPS53	Body	0,007	4,45E-04	3,67E-02
cg08805081	1	205389582	LEMD1	ExonBnd	0,011	4,46E-04	3,67E-02
cg08132931	2	70994962	ADD2	5'UTR	-0,004	4,46E-04	3,67E-02
cg20809690	4	2757764	TNIP2	1stExon	0,019	4,46E-04	3,67E-02
cg19442021	5	119432237		IGR	0,087	4,45E-04	3,67E-02
cg18902644	6	2460774		IGR	-0,008	4,45E-04	3,67E-02
cg05642338	7	90357638	CDK14	Body	0,005	4,46E-04	3,67E-02
cg15990743	8	22743866	LOC101929237	Body	0,011	4,45E-04	3,67E-02
cg06216926	14	92572917	ATXN3	5'UTR	-0,004	4,45E-04	3,67E-02
cg05782457	16	10983791	CIITA	Body	0,02	4,45E-04	3,67E-02
cg16262623	17	42297029	UBTF	TSS200	-0,004	4,45E-04	3,67E-02
cg08664615	17	46132018	NFE2L1	Body	-0,008	4,45E-04	3,67E-02
cg14823865	17	48475505	LRRC59	TSS1500	0,045	4,45E-04	3,67E-02
cg22010774	19	38956915	RYR1	Body	0,007	4,46E-04	3,67E-02
cg14773971	22	31924660	SFI1	ExonBnd	-0,004	4,45E-04	3,67E-02
cg04527369	16	34256428		IGR	0,048	4,46E-04	3,67E-02
cg15591629	1	60018296	FGGY	5'UTR	0,018	4,46E-04	3,67E-02
cg13801687	2	27426245	SLC5A6	Body	0,022	4,46E-04	3,67E-02
cg05316290	9	72036933		IGR	-0,026	4,46E-04	3,67E-02
cg22114560	14	90101398		IGR	0,006	4,46E-04	3,67E-02
cg22251679	5	16594309	FAM134B	Body	-0,008	4,46E-04	3,67E-02
cg16654441	8	124795479	FAM91A1	Body	0,016	4,46E-04	3,67E-02
cg19780563	10	94594494	EXOC6	5'UTR	0,005	4,46E-04	3,67E-02
cg22375082	22	32454420	SLC5A1	TSS1500	0,021	4,46E-04	3,67E-02
cg27392286	3	87424754		IGR	-0,012	4,47E-04	3,67E-02
cg13681940	6	134159412	MGC34034	Body	-0,011	4,47E-04	3,67E-02
cg02973823	17	73552728	LLGL2	Body	-0,004	4,47E-04	3,67E-02
cg18891387	19	5903977	NDUFA11	1stExon	-0,004	4,47E-04	3,67E-02
cg02943129	12	54174761		IGR	-0,029	4,47E-04	3,68E-02
cg10633210	19	1316695		IGR	0,027	4,47E-04	3,68E-02
cg19593344	7	157680809	PTPRN2	Body	0,018	4,47E-04	3,68E-02
cg27528222	1	2138953	C1orf86	5'UTR	0,01	4,48E-04	3,68E-02
cg20246229	5	178419495	GRM6	Body	-0,013	4,48E-04	3,68E-02
cg07028390	2	219433579	RQCD1	TSS200	-0,005	4,48E-04	3,68E-02
cg07824663	19	1163804	SBNO2	5'UTR	0,012	4,48E-04	3,68E-02
cg16044810	7	151137746	CRYGN	TSS1500	0,026	4,48E-04	3,68E-02
cg02174092	10	43846539		IGR	-0,083	4,48E-04	3,68E-02
cg06842116	22	17182794		IGR	0,008	4,48E-04	3,68E-02
cg04385598	6	33424048	ZBTB9	Body	0,006	4,48E-04	3,68E-02
cg04787525	10	663015	DIP2C	Body	-0,031	4,48E-04	3,68E-02
cg07447260	10	103600544	KCNIP2	Body	-0,003	4,48E-04	3,68E-02
cg11444704	5	159738916	CCNJL	Body	-0,016	4,49E-04	3,69E-02
cg26194484	6	158283187	SNX9	Body	-0,01	4,49E-04	3,69E-02
cg03504078	5	140480218	PCDHB3	TSS200	-0,066	4,49E-04	3,69E-02
cg16358257	10	112836609	ADRA2A	TSS200	-0,003	4,49E-04	3,69E-02
cg26833395	20	30102102	HM13	TSS200	-0,003	4,49E-04	3,69E-02
cg12448285	1	245534821	KIF26B	Body	-0,006	4,49E-04	3,69E-02
cg26856676	10	102791011	PDZD7	TSS200	-0,005	4,49E-04	3,69E-02

cg09891316	9	18208448		IGR	-0,042	4,49E-04	3,69E-02
cg25368100	11	73668348	DNAJB13	Body	-0,055	4,49E-04	3,69E-02
cg24106215	1	202927166	ADIPOR1	5'UTR	-0,003	4,49E-04	3,69E-02
cg04713358	3	14167416	CHCHD4	TSS1500	-0,013	4,50E-04	3,69E-02
cg27531206	1	165820531	UCK2	Body	-0,006	4,50E-04	3,69E-02
cg15836705	10	88476505	LDB3	Body	0,008	4,50E-04	3,69E-02
cg08015278	3	73976278		IGR	-0,046	4,50E-04	3,69E-02
cg01050997	1	32446793		IGR	-0,013	4,51E-04	3,69E-02
cg14181387	1	36022775	NCDN	TSS1500	0,009	4,50E-04	3,69E-02
cg17112200	1	240725615	GREM2	5'UTR	-0,038	4,50E-04	3,69E-02
cg04967250	2	180872164	CWC22	TSS1500	-0,004	4,50E-04	3,69E-02
cg23030903	3	9908764	CIDEC	3'UTR	0,014	4,50E-04	3,69E-02
cg23295955	4	26492123	CCKAR	TSS200	-0,014	4,51E-04	3,69E-02
cg13641956	4	84558291		IGR	-0,013	4,51E-04	3,69E-02
cg10977845	12	110767130	ATP2A2	Body	-0,01	4,50E-04	3,69E-02
cg22775642	12	125332183	SCARB1	Body	-0,008	4,50E-04	3,69E-02
cg09858237	17	9479929	WDR16	TSS200	-0,005	4,50E-04	3,69E-02
cg22855255	17	50237267	CA10	TSS1500	-0,038	4,50E-04	3,69E-02
cg03126817	19	14901631		IGR	-0,027	4,51E-04	3,69E-02
cg15367253	19	49534973	SNAR-G2	Body	0,064	4,50E-04	3,69E-02
cg05931192	20	2134237		IGR	-0,012	4,50E-04	3,69E-02
cg00202561	7	157053587	UBE3C	Body	0,006	4,51E-04	3,69E-02
cg15228932	2	54754957	RPL23AP32	TSS1500	0,005	4,51E-04	3,69E-02
cg09467976	7	31167122		IGR	-0,022	4,51E-04	3,69E-02
cg04916200	12	107168633	RIC8B	Body	-0,004	4,51E-04	3,69E-02
cg02724377	14	104237510	PPP1R13B	Body	-0,01	4,51E-04	3,69E-02
cg01619796	21	38790095	DYRK1A	5'UTR	-0,029	4,51E-04	3,69E-02
cg01968657	1	237207256	RYR2	Body	-0,026	4,51E-04	3,69E-02
cg00121884	20	3799732	C20orf29	TSS1500	0,01	4,51E-04	3,69E-02
cg03761141	12	72078615	TMEM19	TSS1500	0,013	4,52E-04	3,69E-02
cg02867305	12	117539595		IGR	0,015	4,52E-04	3,70E-02
cg12729307	17	27893731	ABHD15	1stExon	-0,006	4,52E-04	3,70E-02
cg00050629	22	19132453	DGCR14	TSS1500	-0,004	4,52E-04	3,70E-02
cg05157376	1	92781750	RPAP2	Body	-0,043	4,52E-04	3,70E-02
cg10638044	1	110170914	AMPD2	Body	0,007	4,52E-04	3,70E-02
cg25642673	4	185353115	IRF2	5'UTR	0,012	4,52E-04	3,70E-02
cg05756446	1	65209545	RAVER2	TSS1500	0,034	4,52E-04	3,70E-02
cg08293102	14	51387478	PYGL	Body	0,016	4,52E-04	3,70E-02
cg16430428	2	3642847	COLEC11	5'UTR	-0,051	4,53E-04	3,70E-02
cg18021506	2	219271208		IGR	0,007	4,53E-04	3,70E-02
cg14128315	4	1227091	CTBP1	Body	0,02	4,53E-04	3,70E-02
cg13508208	7	111030821	IMMP2L	Body	0,016	4,53E-04	3,70E-02
cg07862582	8	124581950		IGR	-0,009	4,53E-04	3,70E-02
cg09542991	11	46260795		IGR	0,006	4,53E-04	3,70E-02
cg26320772	22	21328392	AIFM3	Body	0,015	4,53E-04	3,70E-02
cg19035215	5	2186659		IGR	-0,035	4,53E-04	3,70E-02
cg20773929	3	15100395	MRPS25	Body	-0,004	4,53E-04	3,70E-02
cg04966354	5	23631976		IGR	-0,015	4,53E-04	3,70E-02
cg24652994	7	122011261	CADPS2	Body	-0,012	4,53E-04	3,70E-02
cg01538397	9	32784265	TMEM215	Body	-0,048	4,53E-04	3,70E-02
cg10137240	21	26758274	LINC00158	Body	-0,023	4,53E-04	3,70E-02
cg12124527	1	61818009	NFIA	Body	0,011	4,54E-04	3,70E-02
cg07794170	1	233492333	KIAA1804	Body	0,05	4,54E-04	3,70E-02
cg25020728	12	123011538	RSRC2	1stExon	-0,004	4,54E-04	3,70E-02
cg07728870	2	199922848		IGR	-0,013	4,54E-04	3,70E-02
cg27409499	6	31628339	C6orf47	5'UTR	-0,005	4,54E-04	3,70E-02
cg22725359	10	36891396		IGR	-0,02	4,54E-04	3,70E-02
cg14207586	12	122916266		IGR	-0,025	4,54E-04	3,70E-02
cg00840694	8	1994574	MYOM2	5'UTR	0,036	4,54E-04	3,70E-02
cg01977486	11	2019736	H19	TSS1500	0,02	4,54E-04	3,70E-02
cg24089250	1	86116497	ZNHIT6	3'UTR	0,011	4,54E-04	3,70E-02
cg25855217	2	3251064	TSSC1	Body	-0,008	4,54E-04	3,70E-02
cg01228206	1	10762064	CASZ1	5'UTR	0,006	4,54E-04	3,70E-02
cg00293519	2	216275155	FN1	Body	-0,009	4,55E-04	3,70E-02
cg24723255	12	118591326	TAOK3	Body	0,006	4,55E-04	3,70E-02
cg17824615	12	132413775	PUS1	5'UTR	0,007	4,55E-04	3,70E-02
cg11047369	14	59797418	DAAM1	ExonBnd	0,025	4,55E-04	3,70E-02
cg06618418	17	22012690		IGR	0,009	4,55E-04	3,70E-02
cg27614742	17	53514682		IGR	0,009	4,55E-04	3,70E-02
cg14259594	22	18958660	DGCR5	Body	-0,013	4,55E-04	3,70E-02
cg15062470	2	27718710	FNDC4	TSS1500	-0,037	4,55E-04	3,70E-02
cg07101432	8	140968284	TRAPPC9	Body	-0,019	4,55E-04	3,70E-02
cg03688938	13	24143335	TNFRSF19	TSS1500	0,033	4,55E-04	3,70E-02

cg25878151	15	77308126	PSTPIP1	Body	0,035	4,55E-04	3,70E-02
cg11788465	5	134370313	PITX1	TSS1500	0,005	4,55E-04	3,70E-02
cg20441130	7	834565		IGR	-0,01	4,55E-04	3,70E-02
cg23793965	8	91841347	NECAB1	Body	0,044	4,55E-04	3,70E-02
cg11293312	19	49252020	FUT1	3'UTR	0,021	4,55E-04	3,70E-02
cg09540952	6	28616640		IGR	-0,004	4,56E-04	3,71E-02
cg01758539	8	56981229	RPS20	3'UTR	0,021	4,56E-04	3,71E-02
cg21650737	8	674525	ERICH1	Body	-0,105	4,56E-04	3,71E-02
cg26388153	5	115962086		IGR	0,033	4,56E-04	3,71E-02
cg19695507	10	13526193	BEND7	Body	0,016	4,56E-04	3,71E-02
cg17541483	11	521046		IGR	0,014	4,56E-04	3,71E-02
cg10318458	1	47407158	CYP4A11	TSS200	-0,034	4,56E-04	3,71E-02
cg23415916	5	14178469	TRIO	Body	0,007	4,56E-04	3,71E-02
cg03821671	8	71582674	XKR9	5'UTR	0,016	4,56E-04	3,71E-02
cg14033108	14	81735878	STON2	3'UTR	-0,015	4,57E-04	3,71E-02
cg10010183	1	29651806	PTPRU	Body	0,012	4,57E-04	3,71E-02
cg08394070	10	96087958	PLCE1	3'UTR	0,006	4,57E-04	3,71E-02
cg12625296	7	100861373	ZNHIT1	5'UTR	-0,005	4,57E-04	3,71E-02
cg22169680	1	997269		IGR	0,021	4,57E-04	3,71E-02
cg15797223	14	104551994	ASPG	TSS200	0,019	4,57E-04	3,71E-02
cg05979484	7	55432630	LANCL2	TSS1500	0,027	4,58E-04	3,72E-02
cg06701210	7	115779118		IGR	0,018	4,57E-04	3,72E-02
cg03411494	10	96336485	HELLS	ExonBnd	0,015	4,58E-04	3,72E-02
cg11163963	4	189101683		IGR	-0,027	4,58E-04	3,72E-02
cg02341271	18	21960369	OSBPL1A	5'UTR	0,025	4,58E-04	3,72E-02
cg21721107	10	11623423	USP6NL	Body	-0,034	4,59E-04	3,72E-02
cg25816696	2	239427974		IGR	-0,013	4,59E-04	3,72E-02
cg11889076	4	64987721		IGR	-0,011	4,59E-04	3,72E-02
cg05497435	9	124945923	MORN5	Body	-0,016	4,59E-04	3,72E-02
cg20269993	1	227512957		IGR	-0,004	4,59E-04	3,73E-02
cg15362398	2	203242106	BMPR2	5'UTR	-0,003	4,59E-04	3,73E-02
cg11878146	7	84161222		IGR	0,028	4,59E-04	3,73E-02
cg13857707	12	110887879	ARPC3	Body	-0,007	4,59E-04	3,73E-02
cg11612700	13	112985677	LINC01044	TSS200	-0,062	4,59E-04	3,73E-02
cg15619618	14	21878144	CHD8	ExonBnd	0,009	4,59E-04	3,73E-02
cg04154354	14	38069659	TTC6	5'UTR	-0,012	4,59E-04	3,73E-02
cg17022038	19	18260479	MAST3	Body	0,033	4,59E-04	3,73E-02
cg07261190	2	89173636		IGR	-0,034	4,60E-04	3,73E-02
cg16501033	5	155872537	SGCD	Body	0,03	4,60E-04	3,73E-02
cg02376952	16	89569221		IGR	0,006	4,60E-04	3,73E-02
cg10576139	19	3435534	NFIC	Body	0,026	4,60E-04	3,73E-02
cg00246303	10	61195313		IGR	0,025	4,60E-04	3,73E-02
cg23758205	20	55279846		IGR	0,013	4,60E-04	3,73E-02
cg08033244	11	1781505	CTSD	Body	0,022	4,60E-04	3,73E-02
cg26787379	14	51133335	SAV1	Body	0,039	4,60E-04	3,73E-02
cg22898160	16	58496589	NDRG4	TSS1500	-0,054	4,60E-04	3,73E-02
cg17772680	12	133653416		IGR	0,046	4,61E-04	3,73E-02
cg10606856	15	27113759	GABRA5	5'UTR	-0,031	4,61E-04	3,73E-02
cg22295169	11	61615526	FADS2	Body	0,015	4,61E-04	3,73E-02
cg21278129	19	7797355	CLEC4G	TSS1500	0,031	4,61E-04	3,73E-02
cg20515136	3	159707145	IL12A	Body	0,027	4,61E-04	3,73E-02
cg10623560	1	22843655	ZBTB40	Body	-0,005	4,61E-04	3,73E-02
cg15488570	11	888316	CHID1	Body	-0,006	4,61E-04	3,73E-02
cg03159156	11	64026276	PLCB3	Body	0,014	4,61E-04	3,73E-02
cg06051311	6	30131001	TRIM15	5'UTR	-0,02	4,61E-04	3,73E-02
cg05452385	3	172030073	FNDC3B	Body	0,014	4,62E-04	3,73E-02
cg15925090	4	4874042		IGR	-0,009	4,62E-04	3,73E-02
cg19899836	4	184668369		IGR	0,005	4,62E-04	3,73E-02
cg00962793	14	104852641		IGR	0,017	4,62E-04	3,73E-02
cg25214376	10	1781920		IGR	-0,044	4,62E-04	3,73E-02
cg14043335	19	18607546	ELL	Body	0,03	4,62E-04	3,74E-02
cg23706108	8	74475489	STAU2	Body	-0,011	4,62E-04	3,74E-02
cg21356834	14	32400097		IGR	-0,019	4,62E-04	3,74E-02
cg12686605	15	102212626	TARSL2	Body	-0,02	4,62E-04	3,74E-02
cg19942640	5	33440671	TARS	TSS1500	0,007	4,62E-04	3,74E-02
cg25910261	7	157405965	PTPRN2	Body	-0,067	4,62E-04	3,74E-02
cg26394233	7	157444480	PTPRN2	Body	-0,022	4,62E-04	3,74E-02
cg21636610	5	55655747		IGR	-0,01	4,63E-04	3,74E-02
cg13090345	6	80318205		IGR	-0,024	4,63E-04	3,74E-02
cg06103338	20	61667933	LINC00029	Body	-0,067	4,63E-04	3,74E-02
cg20020490	7	5822218	RNF216	TSS1500	-0,015	4,63E-04	3,74E-02
cg06832950	14	51025891	ATL1	TSS1500	0,006	4,63E-04	3,74E-02
cg19176897	1	236686473	LGALS8	TSS1500	0,064	4,63E-04	3,74E-02

cg14071599	3	115770708	LSAMP	Body	0,008	4,63E-04	3,74E-02
cg12869195	4	166546664		IGR	0,013	4,63E-04	3,74E-02
cg06526721	5	119799384	PRR16	TSS1500	0,062	4,63E-04	3,74E-02
cg17560165	6	151457473		IGR	-0,022	4,63E-04	3,74E-02
cg14216201	7	95883422	SLC25A13	Body	-0,005	4,63E-04	3,74E-02
cg15642285	11	45906138	MAPK8IP1	TSS1500	-0,025	4,63E-04	3,74E-02
cg09584188	16	58364996		IGR	0,043	4,63E-04	3,74E-02
cg13713152	18	32665752	MAPRE2	Body	0,035	4,63E-04	3,74E-02
cg10864987	16	18813117	ARL6IP1	TSS1500	-0,004	4,64E-04	3,74E-02
cg06073499	1	34368645	CSMD2	Body	-0,003	4,64E-04	3,74E-02
cg17872570	3	71493242	FOXP1	5'UTR	0,004	4,64E-04	3,74E-02
cg15104484	22	18560503	PEX26	TSS200	-0,002	4,64E-04	3,74E-02
cg14224587	2	74736118	PCGF1	TSS1500	0,008	4,65E-04	3,75E-02
cg05527034	4	155250680	DCHS2	Body	-0,005	4,65E-04	3,75E-02
cg22697239	11	44626708	CD82	Body	0,022	4,65E-04	3,75E-02
cg26573334	12	113669935	TPCN1	Body	0,008	4,65E-04	3,75E-02
cg20075807	17	17324178		IGR	0,025	4,65E-04	3,75E-02
cg20994912	20	59702512		IGR	-0,009	4,65E-04	3,75E-02
cg09851883	8	101162726	POLR2K	TSS200	-0,005	4,65E-04	3,75E-02
cg01992464	16	89913768	SPIRE2	Body	-0,008	4,66E-04	3,75E-02
cg06598942	4	139860154		IGR	0,032	4,66E-04	3,75E-02
cg06922858	2	223448132	FARSB	Body	0,01	4,66E-04	3,75E-02
cg07883537	17	47296912	ABI3	Body	-0,036	4,66E-04	3,75E-02
cg04471171	1	34577639	CSMD2	Body	0,046	4,66E-04	3,76E-02
cg04376815	1	35121781		IGR	0,019	4,66E-04	3,76E-02
cg15870857	14	104742172		IGR	-0,038	4,66E-04	3,76E-02
cg20467418	4	3297777		IGR	-0,016	4,66E-04	3,76E-02
cg00480686	6	42911980		IGR	0,007	4,67E-04	3,76E-02
cg07249342	2	73340897	RAB11FIP5	TSS1500	-0,026	4,67E-04	3,76E-02
cg25088790	5	132723448	FSTL4	Body	-0,018	4,67E-04	3,76E-02
cg13417450	17	44871378	WNT3	Body	0,009	4,67E-04	3,76E-02
cg02910344	5	180215930		IGR	-0,003	4,67E-04	3,76E-02
cg26393850	17	74651114		IGR	-0,016	4,67E-04	3,76E-02
cg14017034	4	170159425	SH3RF1	Body	0,006	4,67E-04	3,76E-02
cg27277314	3	188991266	TPRG1	Body	0,014	4,67E-04	3,76E-02
cg02769252	17	25932535	KSR1	Body	-0,005	4,68E-04	3,76E-02
cg06150302	12	121681590	CAMKK2	Body	0,011	4,68E-04	3,76E-02
cg18094967	8	124226735		IGR	0,008	4,68E-04	3,76E-02
cg15311651	6	31561732	NCR3	TSS1500	-0,008	4,68E-04	3,76E-02
cg04903691	11	118763984	CXCR5	Body	0,015	4,68E-04	3,76E-02
cg08204194	1	42211293	HIVEP3	5'UTR	0,014	4,68E-04	3,76E-02
cg26758722	4	57859050	POLR2B	Body	-0,004	4,68E-04	3,76E-02
cg05093781	6	7641451		IGR	-0,019	4,68E-04	3,76E-02
cg05427639	7	33080496	NT5C3	1stExon	-0,009	4,68E-04	3,76E-02
cg03178071	19	44507379	ZNF230	5'UTR	-0,005	4,68E-04	3,76E-02
cg22127213	20	52374247		IGR	-0,007	4,68E-04	3,76E-02
cg26657739	6	76375351	SENP6	Body	0,016	4,69E-04	3,77E-02
cg20860996	7	143582536	TCAF1	TSS200	-0,017	4,69E-04	3,77E-02
cg03801213	20	31318607	COMMD7	Body	-0,007	4,69E-04	3,77E-02
cg09588284	11	125551643	ACRV1	TSS1500	-0,015	4,69E-04	3,77E-02
cg10961551	1	84764612	SAMD13	Body	0,004	4,69E-04	3,77E-02
cg22939456	2	157450029		IGR	-0,008	4,70E-04	3,77E-02
cg08288226	5	56246733	MIER3	Body	-0,003	4,69E-04	3,77E-02
cg11017765	5	104075794		IGR	-0,03	4,69E-04	3,77E-02
cg22411618	6	47406972		IGR	0,032	4,69E-04	3,77E-02
cg15309066	7	25164988	CYCS	TSS200	-0,003	4,70E-04	3,77E-02
cg16233074	17	42445042		IGR	0,01	4,69E-04	3,77E-02
cg20667124	19	3187880	NCLN	Body	0,007	4,69E-04	3,77E-02
cg21536992	19	49622564	C19orf73	TSS200	0,007	4,70E-04	3,77E-02
cg07522981	19	51071307	LRRC4B	TSS200	-0,005	4,69E-04	3,77E-02
cg26558408	9	124748751	TTLL11	Body	0,015	4,70E-04	3,77E-02
cg05501509	17	75789529		IGR	-0,034	4,70E-04	3,77E-02
cg05303999	20	47988955	KCNB1	3'UTR	-0,009	4,70E-04	3,77E-02
cg22690321	11	125200855	PKNOX2	5'UTR	-0,008	4,70E-04	3,77E-02
cg18894782	22	31218485	OSBP2	TSS200	-0,005	4,70E-04	3,77E-02
cg17558169	11	67057871	ANKRD13D	Body	0,02	4,70E-04	3,77E-02
cg23457837	5	74532835	ANKRD31	TSS200	-0,004	4,70E-04	3,77E-02
cg14294135	20	14929390	MACROD2	Body	-0,036	4,70E-04	3,77E-02
cg00160583	1	32167512	COL16A1	Body	0,013	4,71E-04	3,77E-02
cg07192882	1	154794919	KCNN3	Body	0,01	4,71E-04	3,77E-02
cg25905016	2	24267482	C2orf44	5'UTR	0,011	4,71E-04	3,77E-02
cg22974681	6	108444105		IGR	-0,037	4,71E-04	3,77E-02
cg09271729	13	86159111		IGR	0,019	4,71E-04	3,77E-02

cg03641134	7	67837		IGR	-0,021	4,71E-04	3,77E-02
cg13430898	9	137458253		IGR	0,01	4,71E-04	3,77E-02
cg15910323	12	129308685	SLC15A4	TSS200	-0,004	4,71E-04	3,77E-02
cg26547516	12	46837215	LOC100288798	Body	-0,01	4,71E-04	3,77E-02
cg17155850	1	26327785		IGR	-0,009	4,71E-04	3,77E-02
cg18483611	5	170239551	GABRP	3'UTR	0,005	4,71E-04	3,77E-02
cg01572267	9	23692860	ELAVL2	Body	-0,011	4,71E-04	3,77E-02
cg23948730	17	2496664	PAFAH1B1	TSS1500	-0,006	4,71E-04	3,77E-02
cg14500211	20	20391461	RALGAPA2	Body	0,034	4,71E-04	3,77E-02
cg12175713	4	6973386	TBC1D14	Body	-0,014	4,71E-04	3,77E-02
cg24089715	2	25017714	PTRHD1	TSS1500	0,03	4,71E-04	3,77E-02
cg16557097	6	33423398	ZBTB9	Body	-0,007	4,72E-04	3,77E-02
cg21854649	8	124279889	ZHX1-C8orf76	5'UTR	0,017	4,72E-04	3,77E-02
cg20235051	17	59489852	C17orf82	1stExon	-0,003	4,72E-04	3,77E-02
cg03201096	7	29518198	CHN2	TSS1500	0,012	4,72E-04	3,77E-02
cg06503558	20	7703395		IGR	0,01	4,72E-04	3,78E-02
cg09899557	4	23882563	PPARGC1A	Body	0,061	4,72E-04	3,78E-02
cg26660472	20	60805885		IGR	-0,024	4,72E-04	3,78E-02
cg08955941	5	140420502		IGR	-0,036	4,73E-04	3,78E-02
cg07098680	1	9372708	SPSB1	5'UTR	0,019	4,73E-04	3,78E-02
cg17937726	3	163722051		IGR	-0,043	4,73E-04	3,78E-02
cg03227259	8	140914351	TRAPPC9	Body	-0,013	4,73E-04	3,78E-02
cg18612482	10	97454618	TCTN3	TSS1500	0,018	4,73E-04	3,78E-02
cg02010682	8	99954432		IGR	-0,008	4,73E-04	3,78E-02
cg25023379	14	70138712	KIAA0247	Body	0,009	4,73E-04	3,78E-02
cg22528339	13	49011342	LPAR6	5'UTR	0,01	4,73E-04	3,78E-02
cg09789536	1	896226	KLHL17	Body	0,013	4,74E-04	3,78E-02
cg19484183	10	1102611	WDR37	TSS200	-0,005	4,74E-04	3,78E-02
cg08000733	14	54831673		IGR	0,031	4,74E-04	3,78E-02
cg14287071	4	143394762	INPP4B	5'UTR	-0,016	4,74E-04	3,78E-02
cg25803859	16	693227	FAM195A	Body	0,02	4,74E-04	3,78E-02
cg00324018	19	56048593	SBK2	TSS200	-0,025	4,74E-04	3,78E-02
cg05938285	3	196695938	PIGZ	TSS1500	-0,005	4,74E-04	3,78E-02
cg23163573	2	108905468	SULT1C2	5'UTR	-0,064	4,74E-04	3,79E-02
cg21170085	9	137645730	COL5A1	Body	0,014	4,74E-04	3,79E-02
cg00554773	4	154047992		IGR	-0,007	4,75E-04	3,79E-02
cg01089425	5	10655518	ANKRD33B	3'UTR	0,019	4,75E-04	3,79E-02
cg04997435	5	153588790	GALNT10	Body	-0,015	4,75E-04	3,79E-02
cg08964756	8	132924803	EFR3A	Body	0,043	4,75E-04	3,79E-02
cg16698571	9	25680226	TUSC1	TSS1500	0,012	4,75E-04	3,79E-02
cg08384843	14	57671663	EXOC5	3'UTR	0,016	4,75E-04	3,79E-02
cg09171530	21	35987539	RCAN1	TSS1500	0,005	4,75E-04	3,79E-02
cg08225137	3	54915807	CACNA2D3	Body	0,013	4,75E-04	3,79E-02
cg04880558	6	31707613	MSH5	TSS200	-0,003	4,75E-04	3,79E-02
cg01048576	1	202958016	LOC100506747	Body	-0,02	4,75E-04	3,79E-02
cg07815878	4	184872086	STOX2	Body	0,008	4,75E-04	3,79E-02
cg13917245	10	71645393	COL13A1	Body	-0,006	4,75E-04	3,79E-02
cg22327778	11	1860218	TNNI2	TSS1500	-0,009	4,75E-04	3,79E-02
cg03164991	12	18891971	CAPZA3	1stExon	-0,008	4,75E-04	3,79E-02
cg09245872	16	30016874	INO80E	3'UTR	0,022	4,75E-04	3,79E-02
cg10393578	20	31880141	BPIFB1	Body	0,04	4,75E-04	3,79E-02
cg09330551	19	51334432	KLK15	Body	0,028	4,75E-04	3,79E-02
cg11771408	5	132024049		IGR	-0,03	4,76E-04	3,79E-02
cg04243581	7	107301842	SLC26A4	5'UTR	-0,004	4,76E-04	3,79E-02
cg03462376	10	79609388	DLG5	Body	0,012	4,76E-04	3,79E-02
cg12285834	3	72345017		IGR	-0,01	4,76E-04	3,79E-02
cg00561674	11	92615215	FAT3	Body	-0,015	4,76E-04	3,79E-02
cg16356068	15	29233465	APBA2	5'UTR	0,008	4,76E-04	3,79E-02
cg09640447	12	47549530		IGR	-0,006	4,76E-04	3,79E-02
cg08903121	21	42002736	DSCAM-IT1	TSS200	-0,007	4,77E-04	3,79E-02
cg21148892	2	71047649	CLEC4F	1stExon	0,015	4,77E-04	3,79E-02
cg10019083	4	7985149	ABLIM2	Body	-0,018	4,77E-04	3,79E-02
cg04586987	17	79539111	NPLOC4	ExonBnd	0,012	4,77E-04	3,79E-02
cg08020330	18	57084067		IGR	0,007	4,77E-04	3,79E-02
cg22469435	6	88412272	NCRNA00120	TSS1500	-0,005	4,77E-04	3,79E-02
cg26963253	10	5741251	FAM208B	5'UTR	0,006	4,77E-04	3,80E-02
cg07405362	20	60967344	CABLES2	Body	0,017	4,77E-04	3,80E-02
cg23342296	3	54317727	CACNA2D3	Body	0,012	4,78E-04	3,80E-02
cg23516560	3	54934591	CACNA2D3	Body	-0,021	4,78E-04	3,80E-02
cg24275379	4	8233126	SH3TC1	Body	0,005	4,78E-04	3,80E-02
cg08282428	4	155702411	RBM46	TSS200	-0,056	4,78E-04	3,80E-02
cg23500924	5	140465235		IGR	-0,068	4,78E-04	3,80E-02
cg26780915	7	105519144		IGR	0,012	4,78E-04	3,80E-02

cg04376453	10	124891159		IGR	0,006	4,78E-04	3,80E-02
cg08279220	12	77720134		IGR	-0,092	4,78E-04	3,80E-02
cg08355895	14	93557609	ITPK1	Body	-0,013	4,78E-04	3,80E-02
cg20161988	15	65207617	ANKDD1A	Body	0,019	4,78E-04	3,80E-02
cg00454305	16	1429905	UNKL	TSS1500	-0,044	4,77E-04	3,80E-02
cg10227358	17	4545146	ALOX15	TSS200	-0,017	4,78E-04	3,80E-02
cg18454602	19	46889889	PPP5C	Body	0,007	4,78E-04	3,80E-02
cg09735146	16	85845936		IGR	-0,045	4,78E-04	3,80E-02
cg00255921	9	91933654	SECISBP2	5'UTR	-0,004	4,78E-04	3,80E-02
cg10507281	14	55230528	SAMD4A	Body	-0,007	4,78E-04	3,80E-02
cg21113846	7	837536		IGR	-0,005	4,78E-04	3,80E-02
cg21608605	6	152128258	ESR1	TSS1500	-0,008	4,78E-04	3,80E-02
cg05680417	9	123770503	C5	Body	-0,008	4,79E-04	3,80E-02
cg05107650	3	141160397	ZBTB38	5'UTR	0,116	4,79E-04	3,80E-02
cg19961545	2	54087066	GPR75	5'UTR	-0,032	4,79E-04	3,80E-02
cg08168258	14	62588083	LINC00643	Body	-0,011	4,79E-04	3,80E-02
cg21315783	2	53725202		IGR	-0,008	4,79E-04	3,80E-02
cg08726446	12	108237937		IGR	-0,007	4,79E-04	3,80E-02
cg02656743	1	93804673	LOC100131564	Body	-0,008	4,80E-04	3,80E-02
cg09786053	2	84525942		IGR	-0,03	4,80E-04	3,81E-02
cg04974097	5	169331330	DOCK2	Body	0,028	4,80E-04	3,81E-02
cg07355049	2	191067602	C2orf88	3'UTR	-0,006	4,80E-04	3,81E-02
cg17550357	5	148998278	ARHGEF37	Body	0,007	4,80E-04	3,81E-02
cg10546814	11	102347727		IGR	-0,006	4,80E-04	3,81E-02
cg11122301	12	122255844	SETD1B	Body	-0,007	4,80E-04	3,81E-02
cg21097105	17	19265900	B9D1	1stExon	0,006	4,80E-04	3,81E-02
cg13610835	19	39904138	PLEKHG2	5'UTR	0,008	4,80E-04	3,81E-02
cg03770290	19	34175247	CHST8	5'UTR	-0,013	4,80E-04	3,81E-02
cg15736978	20	2673202	EBF4	TSS1500	0,011	4,80E-04	3,81E-02
cg00886909	4	110724358	CFI	TSS1500	-0,007	4,81E-04	3,81E-02
cg05133691	2	58434481	FANCL	Body	-0,011	4,81E-04	3,81E-02
cg06524509	19	22712026	LOC101929124	TSS1500	-0,029	4,81E-04	3,81E-02
cg08442922	17	42015422		IGR	-0,023	4,81E-04	3,81E-02
cg00249614	1	59593751		IGR	-0,007	4,81E-04	3,81E-02
cg09304854	7	2250996	MAD1L1	Body	0,007	4,81E-04	3,81E-02
cg27218829	11	126138729	FOXRED1	TSS1500	0,004	4,81E-04	3,81E-02
cg02483911	17	3732624	C17orf85	5'UTR	-0,008	4,82E-04	3,81E-02
cg02338963	1	53707922		IGR	0,031	4,82E-04	3,82E-02
cg16965697	11	118890108	RPS25	TSS1500	0,003	4,82E-04	3,82E-02
cg12542148	12	65626516	LEMD3	Body	-0,014	4,82E-04	3,82E-02
cg04597389	17	79935740	ASPSCR1	Body	0,004	4,82E-04	3,82E-02
cg26797679	8	52580320	PXDNL	Body	-0,023	4,82E-04	3,82E-02
cg09621648	9	133661035	ABL1	Body	-0,011	4,82E-04	3,82E-02
cg15806989	8	60214969		IGR	0,028	4,83E-04	3,82E-02
cg17890828	19	36103534	HAUS5	TSS200	-0,003	4,83E-04	3,82E-02
cg03229538	1	173065092		IGR	-0,005	4,83E-04	3,82E-02
cg23123262	2	98280693	ACTR1B	TSS200	-0,003	4,83E-04	3,82E-02
cg17698117	12	51751381	GALNT6	Body	0,038	4,83E-04	3,82E-02
cg14201291	1	208274624	PLXNA2	Body	0,016	4,83E-04	3,82E-02
cg03728046	7	6566428	GRID2IP	Body	0,005	4,83E-04	3,82E-02
cg23702228	7	95130195	ASB4	Body	-0,019	4,84E-04	3,82E-02
cg17879823	9	131291639	GLE1	Body	0,02	4,84E-04	3,82E-02
cg00774724	14	76647716	GPATCH2L	Body	-0,016	4,84E-04	3,82E-02
cg09240286	5	140718526	PCDHGA2	5'UTR	-0,034	4,84E-04	3,82E-02
cg02446672	4	39408371	KLB	TSS200	0,019	4,84E-04	3,82E-02
cg03930369	13	111090821	COL4A2	Body	0,005	4,84E-04	3,82E-02
cg15602036	9	132346274		IGR	-0,004	4,84E-04	3,82E-02
cg06813759	22	31573617	RNF185	5'UTR	-0,004	4,84E-04	3,82E-02
cg13412498	9	36037340	RECK	Body	-0,005	4,84E-04	3,82E-02
cg14701504	17	59560480	TBX4	Body	-0,008	4,84E-04	3,82E-02
cg13441599	7	102715243	ARMC10	TSS200	0,005	4,84E-04	3,82E-02
cg00043284	17	77955342	TBC1D16	Body	-0,005	4,84E-04	3,82E-02
cg20964856	19	41767669	HNRNPUL1	TSS1500	0,026	4,84E-04	3,82E-02
cg05073702	20	3997189	RNF24	TSS1500	0,02	4,84E-04	3,82E-02
cg10415729	4	17175083	LOC101929123	Body	-0,038	4,84E-04	3,82E-02
cg04684864	17	74667833		IGR	-0,004	4,85E-04	3,82E-02
cg22860181	1	182922502	C1orf14	1stExon	-0,017	4,85E-04	3,82E-02
cg23484948	13	33113010	N4BP2L2	TSS200	0,007	4,85E-04	3,82E-02
cg05119290	17	4439394	SPNS2	Body	0,01	4,85E-04	3,83E-02
cg19084362	1	156811518	NTRK1	Body	0,022	4,85E-04	3,83E-02
cg00894093	5	65018509	SGTB	TSS1500	-0,004	4,85E-04	3,83E-02
cg09679574	6	53139986	ELOVL5	Body	-0,006	4,85E-04	3,83E-02
cg06446261	14	63767733		IGR	0,016	4,85E-04	3,83E-02

cg05120280	18	3742168	DLGAP1	Body	0,005	4,85E-04	3,83E-02
cg08154591	7	158736033	WDR60	Body	0,006	4,86E-04	3,83E-02
cg22898785	9	140561736	EHMT1	Body	-0,019	4,86E-04	3,83E-02
cg17413846	16	25227013	AQP8	TSS1500	0,038	4,85E-04	3,83E-02
cg13938107	11	63812843	MACROD1	Body	-0,022	4,86E-04	3,83E-02
cg14166832	2	3321998	TSSC1	Body	0,007	4,86E-04	3,83E-02
cg05110787	11	61447839	DAGLA	TSS200	0,007	4,86E-04	3,83E-02
cg10517121	12	42719875	PPHLN1	TSS200	-0,004	4,86E-04	3,83E-02
cg06949789	3	23958708	NKIRAS1	TSS200	-0,002	4,86E-04	3,83E-02
cg05822031	16	1980154		IGR	-0,006	4,86E-04	3,83E-02
cg08112064	1	110927930	SLC16A4	Body	0,037	4,86E-04	3,83E-02
cg05370693	9	706788	KANK1	TSS200	-0,005	4,86E-04	3,83E-02
cg01428437	1	1852164	TMEM52	TSS1500	0,017	4,87E-04	3,83E-02
cg16075945	2	153781560		IGR	-0,018	4,87E-04	3,83E-02
cg26863180	5	58913251	PDE4D	Body	-0,004	4,87E-04	3,83E-02
cg22497014	7	894009	UNC84A	Body	0,005	4,87E-04	3,83E-02
cg09640577	7	139865794	KDM7A	Body	0,005	4,87E-04	3,83E-02
cg08499158	17	42289980	UBTF	Body	-0,011	4,87E-04	3,83E-02
cg20052268	19	1752160		IGR	-0,003	4,87E-04	3,83E-02
cg08951855	20	59049900		IGR	0,037	4,87E-04	3,83E-02
cg12938818	7	120602109	ING3	Body	0,01	4,87E-04	3,83E-02
cg17573586	3	125990056		IGR	0,016	4,87E-04	3,83E-02
cg06812793	4	92516304	FAM190A	Body	-0,009	4,87E-04	3,83E-02
cg15184187	1	16722920	SZRD1	3'UTR	-0,007	4,88E-04	3,84E-02
cg26192943	1	204217974	PLEKHA6	Body	-0,012	4,88E-04	3,84E-02
cg22860762	1	223088637	DISP1	5'UTR	-0,005	4,88E-04	3,84E-02
cg12539350	2	2590640		IGR	-0,008	4,88E-04	3,84E-02
cg25673474	4	75565779		IGR	0,008	4,88E-04	3,84E-02
cg10935175	5	140614727	PCDHB18	Body	-0,06	4,88E-04	3,84E-02
cg18432149	7	140715197	MRPS33	TSS1500	-0,042	4,88E-04	3,84E-02
cg15884551	8	9790757		IGR	-0,023	4,88E-04	3,84E-02
cg24129947	15	43213015	TTBK2	TSS200	-0,004	4,88E-04	3,84E-02
cg06536378	19	53807723		IGR	-0,05	4,88E-04	3,84E-02
cg01121603	2	242005101	SNED1	Body	-0,029	4,88E-04	3,84E-02
cg11616100	4	54654511		IGR	-0,009	4,88E-04	3,84E-02
cg00584549	6	29796439	HLA-G	Body	-0,036	4,88E-04	3,84E-02
cg01202950	15	74943647	EDC3	Body	-0,011	4,88E-04	3,84E-02
cg15037963	4	40379638		IGR	-0,009	4,89E-04	3,84E-02
cg14272175	3	58318960	PXK	Body	-0,004	4,89E-04	3,84E-02
cg14039865	20	30326367	TPX2	TSS1500	-0,041	4,89E-04	3,84E-02
cg24485038	1	9657892	TMEM201	Body	0,006	4,89E-04	3,84E-02
cg12525514	1	1343260	MRPL20	TSS1500	0,006	4,89E-04	3,84E-02
cg03466415	2	167168852	SCN9A	5'UTR	-0,013	4,89E-04	3,84E-02
cg18721683	3	195740172		IGR	-0,006	4,89E-04	3,84E-02
cg04663194	11	66315239	ACTN3	Body	0,007	4,89E-04	3,84E-02
cg23181059	11	69486959	ORAOV1	Body	-0,011	4,89E-04	3,84E-02
cg12339921	14	59823687	DAAM1	Body	0,025	4,89E-04	3,84E-02
cg03767807	16	1764432	MAPK8IP3	Body	0,011	4,89E-04	3,84E-02
cg26608663	16	4007772		IGR	-0,033	4,89E-04	3,84E-02
cg17846621	17	78113000	EIF4A3	Body	-0,006	4,89E-04	3,84E-02
cg03873659	3	123304056	PTPLB	TSS200	-0,003	4,89E-04	3,84E-02
cg04655303	22	50699863	MAPK12	1stExon	0,005	4,89E-04	3,84E-02
cg00907715	1	22233389	HSPG2	Body	0,031	4,90E-04	3,84E-02
cg05624786	2	226534123		IGR	-0,012	4,90E-04	3,84E-02
cg03578464	3	8928666	RAD18	Body	0,007	4,90E-04	3,84E-02
cg13012862	3	148599303	CPA3	ExonBnd	-0,006	4,90E-04	3,84E-02
cg10825847	11	104894216	CASP5	TSS1500	0,035	4,90E-04	3,84E-02
cg17159125	7	65540622	ASL	TSS1500	0,012	4,90E-04	3,84E-02
cg18184219	1	243388524	CEP170	Body	0,014	4,90E-04	3,84E-02
cg15921099	2	121010219	RALB	TSS200	0,005	4,90E-04	3,84E-02
cg15125963	3	176868150	TBL1XR1	5'UTR	0,024	4,91E-04	3,84E-02
cg04756039	4	48323292		IGR	-0,018	4,90E-04	3,84E-02
cg09514817	7	38947032	VPS41	Body	0,023	4,90E-04	3,84E-02
cg18017575	7	75213863	HIP1	Body	0,035	4,90E-04	3,84E-02
cg27440965	20	43954866	SDC4	3'UTR	0,024	4,91E-04	3,84E-02
cg13334819	7	99746414	C7orf59	TSS200	-0,003	4,91E-04	3,84E-02
cg20388901	8	130091589		IGR	-0,013	4,91E-04	3,84E-02
cg15564131	3	18476925	SATB1	5'UTR	-0,027	4,91E-04	3,84E-02
cg23697780	8	65712412	CYP7B1	TSS1500	-0,014	4,91E-04	3,84E-02
cg14261395	9	135134239		IGR	-0,036	4,91E-04	3,84E-02
cg12432006	17	71192905	COG1	Body	-0,021	4,91E-04	3,84E-02
cg01181758	16	56485501	OGFOD1	1stExon	-0,006	4,91E-04	3,84E-02
cg01573766	1	235580609	TBCE	Body	-0,005	4,91E-04	3,85E-02

cg21755381	6	32938280	BRD2	TSS1500	-0,009	4,91E-04	3,85E-02
cg07169826	7	142334043		IGR	0,029	4,92E-04	3,85E-02
cg23234498	1	50569329	ELAVL4	TSS1500	0,02	4,92E-04	3,85E-02
cg04277903	22	19107699	DGCR2	Body	0,023	4,92E-04	3,85E-02
cg11362076	2	190648200	PMS1	TSS1500	-0,01	4,92E-04	3,85E-02
cg17592426	8	126744098		IGR	0,006	4,92E-04	3,85E-02
cg05661667	9	37239414	ZCCHC7	Body	0,029	4,92E-04	3,85E-02
cg04568339	1	101491652	DPH5	TSS1500	-0,003	4,93E-04	3,85E-02
cg15921319	2	9562408	ITGB1BP1	5'UTR	0,029	4,93E-04	3,85E-02
cg03464429	5	139234348	NRG2	Body	0,014	4,93E-04	3,85E-02
cg22347605	7	114060160	FOXP2	5'UTR	-0,013	4,93E-04	3,85E-02
cg14906455	17	2628088		IGR	-0,005	4,93E-04	3,85E-02
cg12337689	15	91454269	MAN2A2	Body	0,006	4,93E-04	3,85E-02
cg08910914	9	74526678	ABHD17B	TSS1500	-0,005	4,93E-04	3,85E-02
cg16943920	17	3631446	ITGAE	ExonBnd	0,004	4,93E-04	3,85E-02
cg18652346	6	28733673		IGR	-0,037	4,93E-04	3,85E-02
cg03476875	8	98182787	LOC101927066	Body	-0,007	4,93E-04	3,85E-02
cg21219903	1	43920090	HYI	TSS200	-0,039	4,93E-04	3,85E-02
cg12758444	5	104729451		IGR	0,025	4,93E-04	3,85E-02
cg00631185	14	23504548	PSMB5	TSS200	-0,003	4,93E-04	3,85E-02
cg22664064	2	152146702	NMI	TSS1500	-0,007	4,94E-04	3,85E-02
cg00994876	2	242663243	ING5	Body	0,006	4,94E-04	3,85E-02
cg13046221	12	131055901		IGR	-0,118	4,94E-04	3,85E-02
cg11113363	1	3184547	PRDM16	Body	0,005	4,95E-04	3,86E-02
cg18926317	1	24980268	SRRM1	Body	-0,007	4,94E-04	3,86E-02
cg07921235	2	3642098	COLEC11	TSS1500	-0,01	4,95E-04	3,86E-02
cg06732439	2	239140318	LOC151174	TSS200	-0,006	4,94E-04	3,86E-02
cg03855994	3	50230988	GNAT1	Body	-0,055	4,94E-04	3,86E-02
cg20575176	9	89598792	LOC100506834	Body	-0,015	4,94E-04	3,86E-02
cg02894760	9	114359776	PTGR1	5'UTR	0,007	4,94E-04	3,86E-02
cg13903583	10	78629865	KCNMA1	3'UTR	0,038	4,94E-04	3,86E-02
cg08781069	10	100371255	HPSE2	Body	-0,012	4,94E-04	3,86E-02
cg02738977	18	8073218	PTPRM	Body	0,014	4,94E-04	3,86E-02
cg15900011	19	36235235	U2AF1L4	Body	0,023	4,94E-04	3,86E-02
cg22190774	12	112849407		IGR	-0,011	4,95E-04	3,86E-02
cg11533740	5	55548290		IGR	0,008	4,95E-04	3,86E-02
cg12514963	7	2580905	C7orf27	Body	0,007	4,95E-04	3,86E-02
cg04707327	14	50159532	KLHDC1	TSS1500	0,021	4,95E-04	3,86E-02
cg23601586	17	73517413	TSEN54	Body	-0,01	4,95E-04	3,86E-02
cg05315920	19	18589158	ELL	Body	0,027	4,95E-04	3,86E-02
cg02535223	16	77952576	VAT1L	Body	-0,02	4,96E-04	3,86E-02
cg21373806	17	79419834	BAHCC1	Body	0,02	4,96E-04	3,86E-02
cg11322509	10	73157773	CDH23	5'UTR	-0,008	4,96E-04	3,86E-02
cg08719551	6	30876159	GTF2H4	1stExon	-0,005	4,96E-04	3,86E-02
cg22344819	14	75000642	LTBP2	Body	0,013	4,96E-04	3,86E-02
cg21695704	6	9141452		IGR	-0,022	4,96E-04	3,86E-02
cg22090404	13	51489972	RNASEH2B	Body	0,044	4,96E-04	3,86E-02
cg07275648	3	148804790	HLTF	TSS1500	-0,006	4,96E-04	3,86E-02
cg16740311	20	61991088	CHRNA4	5'UTR	0,036	4,96E-04	3,86E-02
cg27481747	1	111010056		IGR	-0,009	4,97E-04	3,87E-02
cg13860293	8	144355357	GLI4	Body	0,014	4,97E-04	3,87E-02
cg07707598	22	43274627	PACSIN2	Body	0,005	4,97E-04	3,87E-02
cg24982582	8	67977447	CSPP1	Body	-0,004	4,97E-04	3,87E-02
cg22046166	7	107771104	LAMB4	TSS1500	-0,005	4,97E-04	3,87E-02
cg04838147	10	26505069	GAD2	TSS200	-0,005	4,97E-04	3,87E-02
cg23826681	1	169074810	ATP1B1	TSS1500	-0,005	4,98E-04	3,87E-02
cg20145176	11	77436649	RSF1	Body	0,032	4,98E-04	3,87E-02
cg21697378	21	43947935	SLC37A1	Body	-0,006	4,98E-04	3,87E-02
cg26633127	10	76847300		IGR	-0,01	4,98E-04	3,87E-02
cg01872324	19	17684869	GLT25D1	Body	0,013	4,98E-04	3,87E-02
cg14153069	9	131683257	PHYHD1	1stExon	0,016	4,98E-04	3,87E-02
cg05668615	5	68413095	SLC30A5	Body	-0,007	4,98E-04	3,87E-02
cg17982866	13	113637695	MCF2L	Body	0,011	4,98E-04	3,87E-02
cg21820759	17	63039150	GNA13	Body	0,008	4,98E-04	3,87E-02
cg03339674	1	16058504	PLEKHM2	Body	0,004	4,99E-04	3,88E-02
cg08358816	6	166578088	T	ExonBnd	-0,039	4,99E-04	3,88E-02
cg07614271	11	75023322	ARRB1	Body	0,015	4,99E-04	3,88E-02
cg08673947	13	49156436	LINC00462	TSS1500	0,005	4,99E-04	3,88E-02
cg24166520	17	16342669	NCRNA00188	Body	-0,003	4,99E-04	3,88E-02
cg22262704	20	23861280	CST5	TSS1500	-0,039	4,99E-04	3,88E-02
cg05541916	14	101299147	MEG3	Body	0,005	4,99E-04	3,88E-02
cg15555727	8	144692268	PYCRL	TSS1500	-0,003	4,99E-04	3,88E-02
cg03732020	11	47282968	NR1H3	Body	0,026	4,99E-04	3,88E-02

cg09496391	8	701366		IGR	-0,019	4,99E-04	3,88E-02
cg13135056	9	124413801	DAB2IP	Body	-0,007	5,00E-04	3,88E-02
cg15378449	12	46765285	SLC38A2	5'UTR	-0,003	4,99E-04	3,88E-02
cg01647734	11	100722194	ARHGAP42	Body	-0,025	5,00E-04	3,88E-02
cg01743538	3	47324986	KIF9	TSS1500	-0,005	5,00E-04	3,88E-02
cg02718120	4	31172146	LOC102723778	TSS1500	-0,029	5,00E-04	3,88E-02
cg23142529	11	120435978		IGR	0,012	5,00E-04	3,88E-02
cg21041608	17	1531717	SLC43A2	5'UTR	0,007	5,00E-04	3,88E-02
cg14216029	19	42901307		IGR	-0,004	5,00E-04	3,88E-02
cg16417118	1	2162931	SKI	Body	-0,035	5,00E-04	3,88E-02
cg16351077	14	93160501		IGR	-0,022	5,00E-04	3,88E-02
cg07532839	16	30194776	CORO1A	TSS200	0,005	5,01E-04	3,88E-02
cg08124030	3	149095283	TM4SF1	1stExon	0,112	5,01E-04	3,88E-02
cg03025176	4	7086303		IGR	-0,039	5,01E-04	3,88E-02
cg18580385	10	104364518	SUFU	Body	0,018	5,01E-04	3,88E-02
cg19161859	1	84327426		IGR	0,014	5,01E-04	3,88E-02
cg16509443	1	165513253	LOC400794	Body	0,008	5,01E-04	3,88E-02
cg17899373	10	118032759	GFRA1	5'UTR	-0,007	5,01E-04	3,88E-02
cg11396329	14	21573039	ZNF219	TSS200	0,024	5,01E-04	3,88E-02
cg26179667	3	128905252		IGR	-0,01	5,01E-04	3,89E-02
cg24680280	6	124129877	NKAIN2	5'UTR	-0,037	5,02E-04	3,89E-02
cg07102406	16	2294639	DCI	Body	0,008	5,02E-04	3,89E-02
cg18898440	17	5974064	WSCD1	5'UTR	-0,006	5,02E-04	3,89E-02
cg20489453	7	128845217	SMO	Body	0,006	5,02E-04	3,89E-02
cg00141611	5	172264364	ERGIC1	Body	-0,015	5,02E-04	3,89E-02
cg02637432	11	129288812	BARX2	Body	-0,014	5,02E-04	3,89E-02
cg25322086	21	47575547	FTCD	TSS200	0,012	5,02E-04	3,89E-02
cg14225039	7	123916407		IGR	-0,049	5,03E-04	3,89E-02
cg13383335	11	35704643	TRIM44	Body	-0,024	5,03E-04	3,89E-02
cg19644850	11	62571803	NXF1	Body	0,019	5,02E-04	3,89E-02
cg01663018	15	53097777		IGR	-0,005	5,03E-04	3,89E-02
cg00667265	15	93884571		IGR	-0,007	5,03E-04	3,89E-02
cg06946880	2	71163123	ATP6V1B1	1stExon	0,017	5,03E-04	3,89E-02
cg11472297	22	35792396		IGR	-0,009	5,03E-04	3,89E-02
cg09726469	1	150552014	MCL1	1stExon	-0,005	5,03E-04	3,89E-02
cg09004241	2	232259431	B3GNT7	TSS1500	0,027	5,03E-04	3,89E-02
cg21129460	6	25450346	LRRC16A	Body	-0,005	5,03E-04	3,89E-02
cg05287529	10	101380128	SLC25A28	1stExon	0,015	5,03E-04	3,89E-02
cg20541753	11	34938014	PDHX	5'UTR	-0,004	5,03E-04	3,89E-02
cg20853889	15	93580227		IGR	0,041	5,03E-04	3,89E-02
cg17563937	1	243644674	SDCCAG8	Body	-0,003	5,03E-04	3,89E-02
cg07931190	19	55858037	SUV420H2	Body	0,003	5,04E-04	3,89E-02
cg25134306	5	138945277	UBE2D2	5'UTR	-0,018	5,04E-04	3,89E-02
cg05037024	5	155848920	SGCD	Body	-0,023	5,04E-04	3,89E-02
cg24324190	4	147631269	TTC29	Body	0,045	5,04E-04	3,89E-02
cg04838050	12	53172395	KRT76	TSS1500	0,005	5,04E-04	3,90E-02
cg13411923	12	122396677	WDR66	Body	0,004	5,04E-04	3,90E-02
cg24244927	18	57505054		IGR	-0,005	5,04E-04	3,90E-02
cg18852990	6	170065310	WDR27	Body	-0,007	5,04E-04	3,90E-02
cg20218520	16	25123425	LCMT1	Body	-0,007	5,04E-04	3,90E-02
cg07422398	19	38722025		IGR	0,03	5,04E-04	3,90E-02
cg15281331	10	133879497		IGR	0,046	5,05E-04	3,90E-02
cg22114309	1	895869	KLHL17	TSS200	0,015	5,05E-04	3,90E-02
cg04248373	10	97145431	SORBS1	Body	0,021	5,05E-04	3,90E-02
cg06021884	15	89660742	ABHD2	Body	-0,021	5,05E-04	3,90E-02
cg02530794	16	70759288	VAC14	Body	0,026	5,05E-04	3,90E-02
cg14789963	17	75048699		IGR	0,016	5,05E-04	3,90E-02
cg25974360	20	54987255	CASS4	TSS200	-0,014	5,05E-04	3,90E-02
cg16532223	1	6542083	PLEKHG5	5'UTR	0,007	5,05E-04	3,90E-02
cg09609376	7	4788854	FOXK1	Body	-0,006	5,06E-04	3,90E-02
cg09038916	16	1580043	TMEM204	5'UTR	0,01	5,06E-04	3,90E-02
cg13273136	2	113931565	PSD4	1stExon	-0,004	5,06E-04	3,90E-02
cg23132748	12	23738791	SOX5	TSS1500	-0,018	5,06E-04	3,90E-02
cg24345349	20	24944010	C20orf3	3'UTR	-0,007	5,06E-04	3,90E-02
cg09010340	11	34606863		IGR	0,033	5,06E-04	3,90E-02
cg16267624	7	103798039	ORC5	Body	0,017	5,06E-04	3,90E-02
cg11001059	17	74380860	SPHK1	5'UTR	-0,006	5,06E-04	3,91E-02
cg08035323	2	9843525		IGR	-0,049	5,07E-04	3,91E-02
cg14030586	9	136658254	VAV2	Body	0,007	5,07E-04	3,91E-02
cg26767001	12	966836	WNK1	Body	-0,008	5,07E-04	3,91E-02
cg13797950	12	56615544	RNF41	5'UTR	-0,017	5,07E-04	3,91E-02
cg02528150	12	62940760	MON2	Body	0,012	5,07E-04	3,91E-02
cg01934790	16	756400	FBXL16	TSS1500	-0,012	5,07E-04	3,91E-02

cg25602603	19	22320744		IGR	-0,057	5,07E-04	3,91E-02
cg13519653	22	43929507	EFCAB6-AS1	Body	0,007	5,07E-04	3,91E-02
cg23361957	3	139066304	MRPS22	Body	-0,007	5,07E-04	3,91E-02
cg16408267	4	74486668	RASSF6	TSS1500	-0,034	5,08E-04	3,91E-02
cg23203884	1	114355244	RSBN1	TSS200	-0,005	5,08E-04	3,91E-02
cg09640764	1	183106444	LAMC1	Body	0,012	5,08E-04	3,91E-02
cg01350032	1	245961269	SMYD3	Body	0,007	5,08E-04	3,91E-02
cg08988364	3	143566850	SLC9A9	Body	-0,004	5,08E-04	3,91E-02
cg21135307	7	78901619	MAGI2	Body	-0,031	5,08E-04	3,91E-02
cg14862378	10	81965171	ANXA11	1stExon	0,003	5,08E-04	3,91E-02
cg22245592	16	70488341	FUK	TSS200	0,005	5,08E-04	3,91E-02
cg22862047	20	57288721	STX16-NPEPL1	Body	0,011	5,08E-04	3,91E-02
cg22307649	11	118889171	TRAPPC4	TSS200	-0,004	5,08E-04	3,91E-02
cg04739936	8	103447304		IGR	-0,007	5,08E-04	3,91E-02
cg08470875	2	26401718	FAM59B	Body	-0,024	5,08E-04	3,91E-02
cg17319788	7	55121401	EGFR	Body	-0,014	5,09E-04	3,91E-02
cg11132209	20	23141943		IGR	0,016	5,09E-04	3,91E-02
cg09737668	1	159923525	SLAMF9	Body	0,007	5,09E-04	3,92E-02
cg16998336	10	63589943		IGR	0,006	5,09E-04	3,92E-02
cg07303187	3	126194934	ZXDC	TSS200	0,014	5,09E-04	3,92E-02
cg10883411	18	8701877	GACAT2	Body	0,029	5,10E-04	3,92E-02
cg02346135	19	648389	RNF126	Body	0,014	5,10E-04	3,92E-02
cg12832482	9	134509057	RAPGEF1	Body	0,012	5,10E-04	3,92E-02
cg08843804	13	22102098	MICU2	Body	0,032	5,10E-04	3,92E-02
cg15967501	19	49143404	SEC1	Body	-0,044	5,10E-04	3,92E-02
cg07488600	5	174906281	SFXN1	5'UTR	-0,005	5,10E-04	3,92E-02
cg00510094	13	113729377	MCF2L	Body	0,01	5,10E-04	3,92E-02
cg23139592	1	91407092	ZNF644	Body	0,009	5,10E-04	3,92E-02
cg10656016	3	111448206	PLCXD2	Body	-0,012	5,10E-04	3,92E-02
cg22741544	5	118374225		IGR	0,006	5,10E-04	3,92E-02
cg21859425	21	18884184	CXADR	TSS1500	-0,037	5,10E-04	3,92E-02
cg16441388	7	92849458	HEPACAM2	TSS1500	-0,015	5,11E-04	3,92E-02
cg23607077	7	2165812	MAD1L1	Body	0,015	5,11E-04	3,92E-02
cg21367838	20	47805154	STAU1	TSS1500	-0,009	5,11E-04	3,92E-02
cg10030658	6	56298004		IGR	-0,051	5,11E-04	3,92E-02
cg06550100	7	72852691		IGR	0,021	5,11E-04	3,92E-02
cg02875965	16	80773937	CDYL2	Body	-0,014	5,11E-04	3,92E-02
cg16747052	7	157842188	PTPRN2	Body	0,02	5,11E-04	3,92E-02
cg00155429	1	154155697	TPM3	1stExon	0,003	5,11E-04	3,92E-02
cg15256492	12	115125475		IGR	-0,043	5,11E-04	3,92E-02
cg19496414	8	59251861		IGR	0,028	5,12E-04	3,93E-02
cg17986701	20	44574422	PCIF1	Body	0,034	5,12E-04	3,93E-02
cg17018151	1	169779161	C1orf112	Body	-0,006	5,12E-04	3,93E-02
cg10724771	4	83720102	SCD5	TSS200	-0,005	5,12E-04	3,93E-02
cg20895609	5	43289455	HMGCS1	3'UTR	0,026	5,12E-04	3,93E-02
cg10047905	8	133580350		IGR	-0,014	5,12E-04	3,93E-02
cg18562525	17	7232547	NEURL4	1stExon	0,034	5,12E-04	3,93E-02
cg22949004	19	50412996	NUP62	Body	0,016	5,12E-04	3,93E-02
cg02321197	2	158755934		IGR	-0,032	5,12E-04	3,93E-02
cg05498103	6	166689361		IGR	-0,005	5,12E-04	3,93E-02
cg05384514	10	92758152		IGR	-0,007	5,12E-04	3,93E-02
cg03400849	11	31149006		IGR	-0,04	5,12E-04	3,93E-02
cg01585348	2	102862214		IGR	-0,017	5,12E-04	3,93E-02
cg19793131	11	96209225	JRKL-AS1	Body	-0,017	5,13E-04	3,93E-02
cg18333152	14	24025304	THTPA	1stExon	-0,011	5,13E-04	3,93E-02
cg17711068	2	138721306	HNMT	TSS1500	-0,013	5,13E-04	3,93E-02
cg16795827	6	162764426	PARK2	Body	-0,044	5,13E-04	3,93E-02
cg01482087	7	100490570	ACHE	Body	0,014	5,13E-04	3,93E-02
cg02609817	12	104179281	NT5DC3	Body	-0,01	5,13E-04	3,93E-02
cg08578505	19	45582924	GEMIN7	5'UTR	-0,004	5,13E-04	3,93E-02
cg12627537	22	43485414	TTLL1	Body	0,009	5,13E-04	3,93E-02
cg25622749	1	224517955	NVL	TSS200	0,01	5,13E-04	3,93E-02
cg16787262	12	13124381		IGR	0,007	5,14E-04	3,93E-02
cg13345220	5	68715517	MARVELD2	Body	-0,005	5,14E-04	3,93E-02
cg01851990	1	27177295	ZDHHC18	Body	-0,005	5,14E-04	3,93E-02
cg08135368	13	113614263		IGR	-0,013	5,14E-04	3,93E-02
cg07781401	17	19773621		IGR	-0,011	5,14E-04	3,93E-02
cg03901475	3	191086850	CCDC50	Body	0,008	5,14E-04	3,93E-02
cg26315509	9	139795523	TRAF2	Body	0,057	5,14E-04	3,93E-02
cg16478734	12	128602715	LOC101927694	TSS200	-0,017	5,14E-04	3,93E-02
cg17886546	20	43945735	RBPJL	3'UTR	-0,03	5,14E-04	3,93E-02
cg10496560	6	30640525	DHX16	1stExon	0,003	5,14E-04	3,93E-02
cg06516981	6	19852162		IGR	-0,005	5,14E-04	3,93E-02

cg04817148	3	45884404	LZTFL1	TSS1500	-0,006	5,15E-04	3,93E-02
cg14812146	4	140957709	MAML3	Body	-0,011	5,15E-04	3,93E-02
cg06518177	3	48697561	CELSR3	1stExon	0,01	5,15E-04	3,93E-02
cg13160528	6	166140118		IGR	-0,014	5,15E-04	3,94E-02
cg25311764	15	59907427	GCNT3	5'UTR	0,027	5,15E-04	3,94E-02
cg19906777	1	2023482	PRKCZ	Body	-0,01	5,15E-04	3,94E-02
cg25744127	11	109292505	C11orf87	TSS1500	-0,048	5,15E-04	3,94E-02
cg20295000	16	48278285	LONP2	5'UTR	-0,003	5,16E-04	3,94E-02
cg07643370	18	21091857	C18orf8	Body	0,017	5,16E-04	3,94E-02
cg01745867	6	30710816	FLOT1	TSS1500	-0,005	5,16E-04	3,94E-02
cg17227395	7	18678293	HDAC9	Body	-0,009	5,16E-04	3,94E-02
cg02142898	7	19787793	TMEM196	Body	0,021	5,16E-04	3,94E-02
cg00447574	8	1041727		IGR	-0,014	5,16E-04	3,94E-02
cg18347656	14	57359959	OTX2-AS1	Body	-0,061	5,16E-04	3,94E-02
cg12012500	15	99499719	IGF1R	Body	0,041	5,16E-04	3,94E-02
cg04185884	16	85666910	KIAA0182	5'UTR	-0,007	5,16E-04	3,94E-02
cg10019429	19	32836659	ZNF507	1stExon	-0,004	5,16E-04	3,94E-02
cg02370467	2	59470247	LOC101927285	Body	0,008	5,16E-04	3,94E-02
cg05730027	6	159654415	FNDC1	Body	-0,045	5,16E-04	3,94E-02
cg00700373	2	237970256		IGR	0,016	5,17E-04	3,94E-02
cg08605930	11	74017513	P4HA3	Body	-0,019	5,17E-04	3,94E-02
cg19234880	9	15476439	PSIP1	Body	0,009	5,17E-04	3,94E-02
cg08934319	3	79174694	ROBO1	Body	-0,057	5,17E-04	3,94E-02
cg02997881	3	159411756	IQCJ-SCHIP1	Body	-0,025	5,17E-04	3,94E-02
cg23470862	3	171618536	TMEM212-AS1	TSS200	0,028	5,17E-04	3,94E-02
cg20339715	8	27757965	SCARA5	Body	-0,034	5,17E-04	3,94E-02
cg26265174	16	67552279	LOC100505942	Body	0,019	5,17E-04	3,94E-02
cg24962545	3	50606606	HEMK1	TSS1500	-0,005	5,17E-04	3,94E-02
cg18024160	5	174054795		IGR	-0,007	5,17E-04	3,94E-02
cg22454660	1	68696988	GPR177	Body	-0,009	5,17E-04	3,94E-02
cg18279842	1	200879897	C1orf106	Body	-0,005	5,17E-04	3,94E-02
cg22189548	20	31341789		IGR	0,014	5,17E-04	3,94E-02
cg06133908	20	36156936	BLCAP	TSS1500	0,036	5,17E-04	3,94E-02
cg05809734	21	45079336	HSF2BP	1stExon	0,004	5,18E-04	3,94E-02
cg26613956	21	39528492	DSCR8	Body	0,008	5,18E-04	3,94E-02
cg25163906	11	128784508	KCNJ5	Body	0,01	5,18E-04	3,94E-02
cg14055374	16	79634410	MAF	5'UTR	-0,002	5,18E-04	3,94E-02
cg03158400	21	42688886	FAM3B	Body	0,008	5,18E-04	3,94E-02
cg16633852	22	37794256	ELFN2	5'UTR	0,013	5,18E-04	3,94E-02
cg26786037	12	109825623	MYO1H	TSS1500	-0,021	5,18E-04	3,94E-02
cg20206277	21	43098901	NCRNA00111	TSS1500	0,034	5,18E-04	3,94E-02
cg12753558	1	247464072	ZNF496	Body	0,003	5,18E-04	3,94E-02
cg05709063	5	64859142	CENPK	TSS200	0,007	5,19E-04	3,94E-02
cg01775523	8	143460155	TSNARE1	5'UTR	0,008	5,18E-04	3,94E-02
cg02623259	9	92704690		IGR	0,007	5,18E-04	3,94E-02
cg19780571	10	101828642	CPN1	Body	-0,019	5,18E-04	3,94E-02
cg10314270	11	76672062	ACER3	5'UTR	-0,015	5,18E-04	3,94E-02
cg24912701	15	58270510	ALDH1A2	Body	-0,016	5,18E-04	3,94E-02
cg02984151	15	58879456		IGR	0,026	5,18E-04	3,94E-02
cg24978087	16	841107	CHTF18	Body	0,006	5,19E-04	3,94E-02
cg03565323	17	16472866	ZNF287	TSS1500	-0,018	5,18E-04	3,94E-02
cg15905579	13	27335084	GPR12	TSS200	0,011	5,19E-04	3,94E-02
cg07159124	3	62234072	PTPRG	Body	-0,012	5,19E-04	3,95E-02
cg20270599	3	189838996	LEPREL1	5'UTR	0,023	5,19E-04	3,95E-02
cg00355281	7	24323767	NPY	TSS200	-0,008	5,19E-04	3,95E-02
cg05776274	7	30266237		IGR	0,036	5,19E-04	3,95E-02
cg04528900	7	156761231	NOM1	Body	-0,009	5,19E-04	3,95E-02
cg05493509	10	134041783	STK32C	Body	0,009	5,19E-04	3,95E-02
cg19354045	12	111051792	TCTN1	TSS200	0,004	5,19E-04	3,95E-02
cg11372563	19	27737627		IGR	-0,013	5,19E-04	3,95E-02
cg18773392	11	44960033	TP53I11	5'UTR	0,016	5,19E-04	3,95E-02
cg08060515	15	49448048	GALK2	5'UTR	0,003	5,19E-04	3,95E-02
cg00267422	19	42772781	CIC	5'UTR	-0,003	5,20E-04	3,95E-02
cg10892375	14	21944853	RAB2B	Body	-0,009	5,20E-04	3,95E-02
cg06435847	17	3562636	CTNS	Body	0,014	5,20E-04	3,95E-02
cg08328927	11	443853		IGR	0,009	5,20E-04	3,95E-02
cg07718813	1	6640976	ZBTB48	Body	0,023	5,20E-04	3,95E-02
cg09316406	8	134414768		IGR	0,017	5,20E-04	3,95E-02
cg19021428	10	84983301		IGR	-0,046	5,20E-04	3,95E-02
cg00038436	16	4323541	TFAP4	TSS1500	-0,004	5,20E-04	3,95E-02
cg27560818	16	87886933	SLC7A5	Body	-0,005	5,20E-04	3,95E-02
cg18919017	5	146796877	DPYSL3	Body	0,019	5,20E-04	3,95E-02
cg20364471	6	39607481	KIF6	Body	0,028	5,21E-04	3,95E-02

cg06512815	3	58370946	PXK	Body	-0,005	5,21E-04	3,95E-02
cg18422058	14	78951762	NRXN3	5'UTR	-0,067	5,21E-04	3,95E-02
cg26851496	15	81293049	MESDC1	TSS1500	0,007	5,21E-04	3,95E-02
cg02576395	1	27884179	AHDC1	5'UTR	0,019	5,21E-04	3,95E-02
cg18802244	5	91378333		IGR	-0,006	5,21E-04	3,95E-02
cg26661319	11	99941071	CNTN5	Body	0,018	5,21E-04	3,95E-02
cg12058092	12	120668276	PXN	Body	0,023	5,21E-04	3,95E-02
cg22902669	17	27192345		IGR	-0,035	5,22E-04	3,96E-02
cg12981595	17	39254427	KRTAP4-8	TSS200	-0,032	5,22E-04	3,96E-02
cg08157418	1	3059826	PRDM16	Body	-0,007	5,22E-04	3,96E-02
cg17788125	6	36953807	MTCH1	1stExon	0,003	5,22E-04	3,96E-02
cg23679332	8	38129652		IGR	-0,006	5,22E-04	3,96E-02
cg13814349	9	89627678	LOC440173	Body	-0,007	5,22E-04	3,96E-02
cg09994891	10	2173024		IGR	-0,048	5,22E-04	3,96E-02
cg17904801	2	3642263	COLEC11	TSS200	-0,009	5,22E-04	3,96E-02
cg23415789	17	59445792	BCAS3	Body	-0,014	5,22E-04	3,96E-02
cg15012864	1	226891415	ITPKB	Body	-0,006	5,23E-04	3,96E-02
cg27227671	5	104109665		IGR	0,021	5,23E-04	3,96E-02
cg05681996	8	140376014		IGR	-0,016	5,23E-04	3,96E-02
cg02619087	12	103838906	C12orf42	Body	-0,011	5,23E-04	3,96E-02
cg21785920	20	36983626	LBP	Body	-0,009	5,23E-04	3,96E-02
cg13493295	5	67067709		IGR	-0,018	5,23E-04	3,96E-02
cg27283457	18	111676	ROCK1P1	Body	-0,062	5,23E-04	3,96E-02
cg12970730	3	64208023	PRICKLE2	5'UTR	-0,012	5,23E-04	3,96E-02
cg20076181	3	130745893	NEK11	5'UTR	-0,004	5,23E-04	3,96E-02
cg06335220	4	100132683	LOC100507053	Body	0,014	5,23E-04	3,96E-02
cg02680050	12	133179887		IGR	-0,06	5,23E-04	3,96E-02
cg06998361	16	58144503		IGR	0,021	5,23E-04	3,96E-02
cg16276909	16	68712097	CDH3	Body	-0,007	5,23E-04	3,96E-02
cg16685041	3	12926742		IGR	0,01	5,23E-04	3,96E-02
cg10858687	11	128777352	C11orf45	TSS1500	0,013	5,23E-04	3,96E-02
cg21238238	4	7152894		IGR	-0,033	5,23E-04	3,96E-02
cg01642461	14	65289728	SPTB	1stExon	0,009	5,24E-04	3,96E-02
cg20001039	21	43442215	ZNF295-AS1	Body	-0,01	5,24E-04	3,96E-02
cg14625975	9	139909019	ABCA2	Body	0,018	5,24E-04	3,96E-02
cg24170720	15	65596249		IGR	0,003	5,24E-04	3,96E-02
cg04560062	1	180889023	KIAA1614	Body	0,005	5,24E-04	3,96E-02
cg19521420	15	67358109	SMAD3	TSS200	0,004	5,24E-04	3,96E-02
cg21064315	1	43917647	SZT2	3'UTR	0,013	5,25E-04	3,97E-02
cg09896762	1	95100713		IGR	0,016	5,25E-04	3,97E-02
cg27253757	6	31852939	EHMT2	Body	-0,005	5,25E-04	3,97E-02
cg03130056	20	44521483	CTSA	Body	0,027	5,25E-04	3,97E-02
cg13869942	1	151878061	THEM4	Body	-0,03	5,25E-04	3,97E-02
cg13973086	2	469105		IGR	-0,056	5,25E-04	3,97E-02
cg24793584	17	80557532	FOXK2	Body	0,005	5,25E-04	3,97E-02
cg22143115	6	122792161	PKIB	TSS1500	-0,027	5,26E-04	3,97E-02
cg10680793	1	23913713		IGR	-0,025	5,26E-04	3,97E-02
cg20536431	1	46329930	MAST2	Body	0,014	5,26E-04	3,97E-02
cg23067782	4	170913027	MFAP3L	Body	-0,007	5,26E-04	3,97E-02
cg01979620	7	6295168	CYTH3	Body	-0,007	5,26E-04	3,97E-02
cg16941413	11	58394604		IGR	-0,025	5,26E-04	3,97E-02
cg16749120	15	69881030		IGR	0,009	5,26E-04	3,97E-02
cg07095737	16	14458471		IGR	0,006	5,26E-04	3,97E-02
cg10500667	16	64187788		IGR	-0,012	5,26E-04	3,97E-02
cg04486388	16	85972959		IGR	0,007	5,26E-04	3,97E-02
cg07673383	21	37703224	MORC3	Body	0,017	5,26E-04	3,97E-02
cg25306991	22	51001617	C22orf41	TSS1500	0,031	5,26E-04	3,97E-02
cg04074557	1	245134710	EFCAB2	Body	-0,005	5,27E-04	3,97E-02
cg06728232	3	172858390	SPATA16	5'UTR	-0,034	5,27E-04	3,97E-02
cg12439157	1	170628856		IGR	0,032	5,27E-04	3,97E-02
cg21499787	16	21133180	DNAH3	Body	-0,027	5,27E-04	3,97E-02
cg01824401	5	137774650	REEP2	TSS200	-0,006	5,27E-04	3,97E-02
cg17188688	22	22333247	TOP3B	5'UTR	0,023	5,27E-04	3,98E-02
cg14734994	2	88468372	THNSL2	TSS1500	-0,007	5,27E-04	3,98E-02
cg01204634	5	1395156	SLC6A3	Body	-0,016	5,27E-04	3,98E-02
cg14220329	1	45050807	RNF220	Body	0,014	5,27E-04	3,98E-02
cg04659628	12	23738855	SOX5	TSS1500	-0,025	5,28E-04	3,98E-02
cg03780079	19	52097905		IGR	-0,004	5,28E-04	3,98E-02
cg00424166	6	150045504	NUP43	3'UTR	0,016	5,28E-04	3,98E-02
cg12722429	14	25149059		IGR	-0,021	5,28E-04	3,98E-02
cg13780562	21	46330607	ITGB2	Body	0,007	5,28E-04	3,98E-02
cg03630148	5	1933941		IGR	-0,053	5,28E-04	3,98E-02
cg16619614	12	44200691	TWF1	TSS1500	-0,01	5,28E-04	3,98E-02

cg05123333	16	28070454	GSG1L	Body	-0,023	5,28E-04	3,98E-02
cg26775538	19	815090	LPPR3	Body	0,032	5,28E-04	3,98E-02
cg22141389	6	87646922	HTR1E	TSS200	-0,01	5,29E-04	3,98E-02
cg25933456	6	166190100		IGR	-0,042	5,29E-04	3,98E-02
cg12171846	12	56981517	RBMS2	Body	-0,006	5,28E-04	3,98E-02
cg22124697	12	93708430	LOC643339	Body	-0,01	5,28E-04	3,98E-02
cg20254998	6	136976783	MAP3K5	Body	-0,034	5,29E-04	3,98E-02
cg16157468	17	64792460	PRKCA	Body	-0,006	5,29E-04	3,98E-02
cg12241979	3	28182184		IGR	0,006	5,29E-04	3,98E-02
cg00154322	4	146829190	ZNF827	Body	-0,031	5,29E-04	3,98E-02
cg01128511	4	40440442	RBM47	Body	-0,009	5,29E-04	3,98E-02
cg15778145	1	24974607	SRRM1	Body	0,014	5,30E-04	3,98E-02
cg27299484	4	42418956	ATP8A1	Body	0,005	5,29E-04	3,98E-02
cg11524415	7	30059859	FKBP14	Body	0,009	5,29E-04	3,98E-02
cg26535366	11	58274247		IGR	-0,02	5,30E-04	3,98E-02
cg21806498	15	66149811		IGR	-0,01	5,29E-04	3,98E-02
cg05716290	19	3977807	EEF2	ExonBnd	0,013	5,29E-04	3,98E-02
cg21009572	7	92464647	CDK6	5'UTR	-0,006	5,30E-04	3,98E-02
cg03033688	16	22824489	HS3ST2	TSS1500	0,041	5,30E-04	3,98E-02
cg18381490	1	29751343		IGR	0,048	5,30E-04	3,98E-02
cg21082272	4	1202966	LOC100130872	TSS1500	0,003	5,30E-04	3,98E-02
cg16585715	17	55601342	LOC101927557	TSS1500	0,015	5,30E-04	3,98E-02
cg26706187	11	115024626		IGR	-0,025	5,30E-04	3,99E-02
cg01935813	2	62238115	COMMD1	Body	0,024	5,30E-04	3,99E-02
cg04843660	1	228468399	OBSCN	Body	0,018	5,31E-04	3,99E-02
cg14101687	2	112917234	FBLN7	Body	0,008	5,31E-04	3,99E-02
cg14824740	1	70006172		IGR	0,017	5,31E-04	3,99E-02
cg10020445	3	49830420	CDHR4	Body	0,009	5,31E-04	3,99E-02
cg27640133	6	10190462		IGR	-0,044	5,31E-04	3,99E-02
cg24870850	8	93979504	TRIQK	TSS1500	0,014	5,31E-04	3,99E-02
cg14640354	13	32605599	FRY	5'UTR	-0,003	5,31E-04	3,99E-02
cg14531522	16	3451969	ZNF174	5'UTR	0,032	5,31E-04	3,99E-02
cg26642585	3	183961869	ALG3	Body	0,022	5,31E-04	3,99E-02
cg16669842	10	115523468	PLEKHS1	5'UTR	0,025	5,31E-04	3,99E-02
cg10910644	2	33298134	LTBP1	Body	-0,009	5,31E-04	3,99E-02
cg05316615	2	64113477	UGP2	ExonBnd	0,007	5,32E-04	3,99E-02
cg23088318	15	25093985	SNRPN	5'UTR	0,086	5,32E-04	3,99E-02
cg15185106	6	111195554	AMD1	TSS1500	-0,003	5,32E-04	3,99E-02
cg18145070	8	53106876	ST18	Body	-0,083	5,32E-04	3,99E-02
cg17916021	15	25200133	SNRPN	TSS200	0,012	5,32E-04	3,99E-02
cg00132135	12	107155510	RFX4	3'UTR	-0,005	5,32E-04	3,99E-02
cg23144668	10	116853333	ATRNL1	5'UTR	-0,006	5,33E-04	3,99E-02
cg11697588	11	14995231	CALCA	TSS1500	0,006	5,33E-04	3,99E-02
cg14028877	22	19937281	COMT	TSS1500	0,017	5,32E-04	3,99E-02
cg07497511	17	59457396	BCAS3	Body	-0,026	5,33E-04	3,99E-02
cg04199303	7	156332650	C7orf4	TSS1500	-0,053	5,33E-04	3,99E-02
cg00230120	12	1609346	LOC100292680	TSS1500	0,004	5,33E-04	3,99E-02
cg23922708	18	78005180	PARD6G	1stExon	-0,004	5,33E-04	4,00E-02
cg01099150	2	8746519		IGR	-0,014	5,34E-04	4,00E-02
cg17587427	5	159904740		IGR	-0,03	5,34E-04	4,00E-02
cg00588393	6	46326932		IGR	0,006	5,34E-04	4,00E-02
cg26834192	6	161561031	AGPAT4	Body	0,017	5,34E-04	4,00E-02
cg04300377	13	63056558		IGR	-0,012	5,34E-04	4,00E-02
cg00001520	14	37666489	MIPOL1	TSS1500	0,024	5,34E-04	4,00E-02
cg08576390	16	20878583	DCUN1D3	5'UTR	-0,006	5,33E-04	4,00E-02
cg13976853	19	2945039	ZNF77	TSS200	-0,005	5,34E-04	4,00E-02
cg22103164	4	155702409	RBM46	TSS200	-0,06	5,34E-04	4,00E-02
cg07515350	15	69745013	RPLP1	TSS200	-0,003	5,34E-04	4,00E-02
cg03461704	1	205818484	PM20D1	Body	0,032	5,34E-04	4,00E-02
cg02798621	6	82462430	FAM46A	TSS200	-0,005	5,34E-04	4,00E-02
cg19021328	10	45869294	ALOX5	TSS1500	0,008	5,34E-04	4,00E-02
cg15401892	12	68790405		IGR	-0,005	5,34E-04	4,00E-02
cg14199148	13	52158669	WDFY2	1stExon	-0,004	5,34E-04	4,00E-02
cg09313046	16	74331618	PSMD7	Body	-0,044	5,34E-04	4,00E-02
cg01205058	6	167508191		IGR	0,021	5,34E-04	4,00E-02
cg24494617	8	22735388	PEBP4	Body	-0,029	5,34E-04	4,00E-02
cg00712390	17	79373624	BAHCC1	1stExon	-0,011	5,35E-04	4,00E-02
cg21850903	1	33820100	PHC2	Body	0,009	5,35E-04	4,00E-02
cg12070221	10	62407154	ANK3	Body	-0,009	5,35E-04	4,00E-02
cg15704807	12	96230261		IGR	0,014	5,35E-04	4,00E-02
cg24076669	6	159342791		IGR	-0,011	5,35E-04	4,00E-02
cg22432269	15	22892697	CYFIP1	5'UTR	-0,003	5,36E-04	4,00E-02
cg22129639	15	43785364	TP53BP1	TSS200	-0,005	5,36E-04	4,00E-02

cg27334900	16	56677335	MT1DP	TSS1500	0,006	5,36E-04	4,00E-02
cg22150971	5	149402683	HMGXB3	Body	-0,004	5,36E-04	4,00E-02
cg05441198	13	61237593		IGR	-0,016	5,36E-04	4,00E-02
cg12846407	7	7758156	RPA3	1stExon	0,024	5,36E-04	4,01E-02
cg01328119	1	228783545	DUSP5P	Body	-0,003	5,36E-04	4,01E-02
cg05559067	2	231570641		IGR	-0,004	5,36E-04	4,01E-02
cg03132185	3	134198480	ANAPC13	Body	0,026	5,36E-04	4,01E-02
cg01135863	4	17788258		IGR	-0,023	5,36E-04	4,01E-02
cg13453634	6	82643472		IGR	-0,011	5,36E-04	4,01E-02
cg11838542	7	2128505	MAD1L1	Body	0,015	5,36E-04	4,01E-02
cg13426096	8	37887990	EIF4EBP1	TSS200	0,007	5,36E-04	4,01E-02
cg00430138	11	11688731		IGR	-0,015	5,36E-04	4,01E-02
cg25632983	12	132568608	EP400NL	TSS1500	-0,004	5,36E-04	4,01E-02
cg27345024	16	3728415	TRAP1	Body	-0,014	5,36E-04	4,01E-02
cg15729697	21	17911454	MIRLET7C	TSS1500	0,004	5,36E-04	4,01E-02
cg03576411	14	99172002		IGR	-0,04	5,37E-04	4,01E-02
cg10989806	2	131832659	FAM168B	Body	0,009	5,37E-04	4,01E-02
cg01142693	8	144696044	TSTA3	Body	-0,024	5,37E-04	4,01E-02
cg16756442	12	49346866	ARF3	5'UTR	-0,007	5,37E-04	4,01E-02
cg09633764	16	52289925		IGR	-0,005	5,37E-04	4,01E-02
cg25367758	19	588447	HCN2	TSS1500	-0,014	5,37E-04	4,01E-02
cg24309609	14	66607416		IGR	0,021	5,37E-04	4,01E-02
cg27336554	5	126986758	CTXN3	5'UTR	0,013	5,37E-04	4,01E-02
cg19366463	9	94959115		IGR	-0,019	5,38E-04	4,01E-02
cg10988169	8	54632888	ATP6V1H	Body	0,031	5,38E-04	4,01E-02
cg14609556	7	102669158	FBXL13	Body	0,033	5,38E-04	4,01E-02
cg06228183	8	128095546	PRNCR1	Body	-0,006	5,38E-04	4,01E-02
cg06525016	5	133473501	TCF7	Body	0,01	5,38E-04	4,01E-02
cg19968202	11	121365727	SORL1	Body	-0,032	5,38E-04	4,01E-02
cg16417831	21	33958411	TCP10L	TSS1500	0,026	5,38E-04	4,01E-02
cg17389237	1	26882504	RPS6KA1	Body	0,015	5,38E-04	4,01E-02
cg13306401	3	30054583		IGR	-0,006	5,38E-04	4,01E-02
cg12344249	5	16478970	FAM134B	Body	-0,016	5,38E-04	4,01E-02
cg18844163	5	142362123	ARHGAP26	Body	0,024	5,38E-04	4,01E-02
cg07085403	6	90531629		IGR	-0,006	5,39E-04	4,01E-02
cg18238340	14	105899368	MTA1	Body	0,014	5,38E-04	4,01E-02
cg09062397	20	62289856	RTEL1	5'UTR	-0,006	5,39E-04	4,01E-02
cg19850333	3	46448579	CCRL2	TSS1500	-0,007	5,39E-04	4,01E-02
cg23596620	12	113229465	RPH3A	TSS200	-0,01	5,39E-04	4,02E-02
cg22233660	22	44258651	SULT4A1	TSS1500	-0,024	5,39E-04	4,02E-02
cg12830684	1	10613540	PEX14	Body	-0,042	5,40E-04	4,02E-02
cg27124276	8	10005474	MSRA	Body	-0,012	5,40E-04	4,02E-02
cg01716380	13	41885273	NAA16	TSS200	-0,004	5,40E-04	4,02E-02
cg06447474	16	6067747	A2BP1	TSS1500	-0,017	5,40E-04	4,02E-02
cg23529259	16	11704571		IGR	-0,004	5,40E-04	4,02E-02
cg21045485	6	131252985	EPB41L2	Body	0,037	5,40E-04	4,02E-02
cg26361533	12	2445561	CACNA1C	Body	0,021	5,40E-04	4,02E-02
cg14251771	14	90701431		IGR	0,011	5,40E-04	4,02E-02
cg22463553	5	140788504	PCDHGA4	Body	-0,05	5,41E-04	4,02E-02
cg24029336	8	101911063		IGR	-0,006	5,41E-04	4,02E-02
cg16655778	12	132981736		IGR	0,025	5,41E-04	4,02E-02
cg07349222	16	54096679	FTO	Body	0,02	5,41E-04	4,02E-02
cg00016968	1	113250448	RHOC	TSS1500	0,02	5,41E-04	4,02E-02
cg27398423	2	241815057	AGXT	Body	0,013	5,41E-04	4,02E-02
cg07729811	5	156712286	CYFIP2	5'UTR	0,006	5,41E-04	4,02E-02
cg04673701	13	52188823	WDFY2	Body	0,04	5,41E-04	4,02E-02
cg24820672	13	112984728		IGR	-0,088	5,41E-04	4,02E-02
cg09186143	22	28387397	TTC28-AS1	Body	-0,009	5,41E-04	4,02E-02
cg23384406	15	83360963	AP3B2	Body	-0,007	5,41E-04	4,02E-02
cg23580358	19	14180843		IGR	-0,014	5,41E-04	4,02E-02
cg24448113	5	140475611	PCDHB2	1stExon	-0,063	5,42E-04	4,02E-02
cg21155973	12	72916569	TRHDE	Body	-0,032	5,42E-04	4,03E-02
cg10764236	18	286625		IGR	-0,042	5,42E-04	4,03E-02
cg22821709	10	34229048		IGR	0,016	5,43E-04	4,03E-02
cg03299095	15	25434533	SNORD115-11	TSS200	0,006	5,42E-04	4,03E-02
cg04987465	17	45056797	RPRML	TSS200	0,003	5,43E-04	4,03E-02
cg17727469	2	242293577		IGR	0,011	5,43E-04	4,03E-02
cg06290506	4	76977236	ART3	5'UTR	0,024	5,43E-04	4,03E-02
cg21266908	6	46460471	RCAN2	TSS1500	-0,05	5,43E-04	4,03E-02
cg24594452	11	47776214	FNBP4	Body	-0,006	5,43E-04	4,03E-02
cg05254946	19	39052112	RYR1	Body	0,007	5,43E-04	4,03E-02
cg06615699	13	78832424		IGR	-0,045	5,43E-04	4,03E-02
cg01541645	14	61104329		IGR	-0,011	5,43E-04	4,03E-02

cg12798649	14	64749776	ESR2	5'UTR	-0,008	5,44E-04	4,03E-02
cg02979335	4	55313518		IGR	-0,007	5,44E-04	4,03E-02
cg23097488	16	67062968	CBFB	TSS200	0,004	5,44E-04	4,03E-02
cg05935156	3	49904706	CAMKV	5'UTR	0,017	5,44E-04	4,03E-02
cg21305315	4	3527935	LRPAP1	Body	0,014	5,44E-04	4,03E-02
cg27212978	4	38869221	FAM114A1	TSS1500	0,01	5,44E-04	4,03E-02
cg04026169	4	85422265		IGR	0,03	5,44E-04	4,03E-02
cg04105876	17	77195907	HRNBP3	5'UTR	0,011	5,44E-04	4,03E-02
cg02032593	3	127242401	LINC01471	Body	-0,017	5,44E-04	4,03E-02
cg15350314	3	184475101	LOC101928992	Body	0,056	5,44E-04	4,03E-02
cg21982119	7	140373931	ADCK2	1stExon	0,041	5,44E-04	4,03E-02
cg16019436	13	113825824	PROZ	Body	-0,012	5,44E-04	4,03E-02
cg18400889	2	234367790	DGKD	Body	-0,005	5,45E-04	4,04E-02
cg15798823	7	22866267		IGR	-0,034	5,45E-04	4,04E-02
cg13599685	7	28133964	JAZF1	Body	-0,008	5,45E-04	4,04E-02
cg09348431	8	144898112	SCRIB	TSS1500	0,008	5,45E-04	4,04E-02
cg06526832	9	126349160	DENND1A	Body	-0,008	5,44E-04	4,04E-02
cg16557250	10	35842204	CCNY	Body	-0,005	5,45E-04	4,04E-02
cg00806552	20	18478084	RBBP9	TSS200	-0,007	5,45E-04	4,04E-02
cg27506401	4	101856463		IGR	0,016	5,45E-04	4,04E-02
cg11994052	11	112916105	NCAM1	Body	-0,026	5,45E-04	4,04E-02
cg19089314	22	50919938	ADM2	TSS200	0,009	5,45E-04	4,04E-02
cg18402590	21	37631948	DOPEY2	Body	0,006	5,46E-04	4,04E-02
cg22847228	5	140792700	PCDHGA4	Body	-0,011	5,46E-04	4,04E-02
cg21822335	8	102405915		IGR	-0,007	5,46E-04	4,04E-02
cg13396967	8	134115055	SLA	1stExon	-0,006	5,46E-04	4,04E-02
cg20255711	19	36236366	PSENEN	TSS200	-0,003	5,46E-04	4,04E-02
cg01632597	7	115840881		IGR	0,014	5,46E-04	4,04E-02
cg10215763	7	142636470	C7orf34	TSS200	-0,007	5,46E-04	4,04E-02
cg01959995	13	110790566		IGR	-0,003	5,46E-04	4,04E-02
cg08896772	15	42101579	MAPKBP1	Body	0,028	5,46E-04	4,04E-02
cg20259501	20	48939655		IGR	0,009	5,46E-04	4,04E-02
cg11996741	22	30769389	CCDC157	Body	0,021	5,46E-04	4,04E-02
cg05726254	2	47305017		IGR	0,007	5,47E-04	4,04E-02
cg19510206	12	133101587	FBRSL1	Body	0,01	5,47E-04	4,04E-02
cg09504482	13	41238655	FOXO1	Body	-0,005	5,46E-04	4,04E-02
cg00090091	13	107036741		IGR	0,005	5,46E-04	4,04E-02
cg27250981	4	91058700	CCSER1	5'UTR	-0,011	5,47E-04	4,04E-02
cg01231611	1	120354235	REG4	TSS200	0,009	5,47E-04	4,04E-02
cg27403612	6	155231129		IGR	0,008	5,47E-04	4,04E-02
cg20642756	10	45497470	ZNF22	5'UTR	0,025	5,47E-04	4,04E-02
cg19393677	15	67841407	MAP2K5	Body	-0,032	5,47E-04	4,05E-02
cg16310262	15	65903328	C15orf44	1stExon	-0,004	5,48E-04	4,05E-02
cg11959156	20	62865391	MYT1	Body	-0,009	5,48E-04	4,05E-02
cg07376301	22	42045469	XRCC6	Body	-0,005	5,48E-04	4,05E-02
cg17388021	1	155942085	ARHGEF2	Body	0,018	5,48E-04	4,05E-02
cg14286144	2	53285758		IGR	-0,007	5,48E-04	4,05E-02
cg04204975	11	61891341	INCENP	TSS200	-0,003	5,48E-04	4,05E-02
cg02320084	16	85769350	C16orf74	5'UTR	-0,025	5,48E-04	4,05E-02
cg24886257	19	52452447		IGR	-0,035	5,48E-04	4,05E-02
cg09943309	2	103380653	TMEM182	Body	-0,006	5,49E-04	4,05E-02
cg27494897	2	151061125		IGR	-0,03	5,49E-04	4,05E-02
cg11126068	11	67195806	RPS6KB2	TSS200	-0,004	5,49E-04	4,05E-02
cg18831169	13	73670290		IGR	-0,013	5,50E-04	4,06E-02
cg24511375	15	75940328	SNX33	TSS1500	0,004	5,50E-04	4,06E-02
cg17089214	22	22089827	YPEL1	5'UTR	-0,004	5,49E-04	4,06E-02
cg25430507	2	139539181	NXPH2	TSS1500	0,03	5,50E-04	4,06E-02
cg07123870	5	153745126	GALNT10	Body	-0,012	5,50E-04	4,06E-02
cg04830814	5	32174484	GOLPH3	TSS200	0,005	5,51E-04	4,06E-02
cg27353957	16	78055642	CLEC3A	TSS1500	0,012	5,51E-04	4,06E-02
cg12021257	17	17650880	RAI1	5'UTR	0,015	5,51E-04	4,06E-02
cg22937891	5	114506309	TRIM36	Body	-0,004	5,51E-04	4,06E-02
cg17244673	10	17166078	CUBN	Body	-0,006	5,51E-04	4,06E-02
cg13215970	4	74864596	CXCL5	TSS200	0,015	5,51E-04	4,07E-02
cg11996652	11	70882283	SHANK2	5'UTR	-0,01	5,51E-04	4,07E-02
cg09055202	14	61944806	PRKCH	Body	0,014	5,51E-04	4,07E-02
cg01220134	16	960424	LMF1	Body	-0,015	5,51E-04	4,07E-02
cg07178441	2	187221600		IGR	0,009	5,52E-04	4,07E-02
cg12701846	21	32887062	TIAM1	5'UTR	-0,035	5,52E-04	4,07E-02
cg14927126	11	116860925	SIK3	Body	-0,021	5,52E-04	4,07E-02
cg21048949	11	117187053	BACE1	TSS200	-0,003	5,52E-04	4,07E-02
cg24483930	5	125803871	GRAMD3	Body	0,015	5,52E-04	4,07E-02
cg18649737	14	102680518	WDR20	Body	-0,013	5,52E-04	4,07E-02

cg07243038	19	44952801	ZNF229	TSS200	0,012	5,52E-04	4,07E-02
cg15622820	20	60605320	TAF4	Body	-0,007	5,52E-04	4,07E-02
cg00162421	1	10508962	APITD1	Body	-0,005	5,53E-04	4,07E-02
cg23495059	1	39324743	RRAGC	Body	0,002	5,53E-04	4,07E-02
cg05040656	1	68200130	GNG12	5'UTR	0,031	5,53E-04	4,07E-02
cg22985204	9	111876664	TMEM245	Body	0,02	5,53E-04	4,07E-02
cg13073052	1	9404377	SPSB1	5'UTR	-0,011	5,53E-04	4,07E-02
cg14832908	1	6479424	HES2	Body	-0,021	5,54E-04	4,07E-02
cg13863764	1	11561908	PTCHD2	Body	-0,046	5,53E-04	4,07E-02
cg04704294	1	66257822	PDE4B	TSS1500	0,037	5,54E-04	4,07E-02
cg21490808	1	164442378		IGR	0,033	5,54E-04	4,07E-02
cg24628446	2	146090028		IGR	-0,008	5,54E-04	4,07E-02
cg06199336	2	200329942	SATB2	TSS200	-0,012	5,54E-04	4,07E-02
cg06061536	3	41405770	ULK4	Body	0,008	5,54E-04	4,07E-02
cg06344162	3	58395271	PXK	ExonBnd	0,016	5,54E-04	4,07E-02
cg02910031	3	149672627	RNF13	Body	-0,01	5,54E-04	4,07E-02
cg20887711	4	1340912	KIAA1530	TSS200	-0,004	5,53E-04	4,07E-02
cg02354107	4	77135186	SCARB2	TSS200	-0,006	5,54E-04	4,07E-02
cg00770663	4	130017173	C4orf33	TSS200	-0,01	5,53E-04	4,07E-02
cg07774410	5	1323142	CLPTM1L	Body	0,005	5,54E-04	4,07E-02
cg11966651	6	15248167	JARID2	TSS1500	-0,002	5,53E-04	4,07E-02
cg04761653	6	82463237	FAM46A	TSS1500	0,009	5,53E-04	4,07E-02
cg03401309	7	150749938		IGR	0,017	5,54E-04	4,07E-02
cg11960212	8	95133021		IGR	-0,013	5,53E-04	4,07E-02
cg22280333	11	193016	SCGB1C1	TSS200	0,015	5,54E-04	4,07E-02
cg22218464	15	83329551	CPEB1-AS1	Body	0,018	5,54E-04	4,07E-02
cg09183146	16	1429863	UNKL	TSS200	-0,037	5,53E-04	4,07E-02
cg06485706	16	28996270	LAT	Body	-0,004	5,54E-04	4,07E-02
cg24087796	17	46871873	TTLL6	Body	0,01	5,53E-04	4,07E-02
cg21005054	17	78812115	RPTOR	Body	-0,006	5,53E-04	4,07E-02
cg17425144	1	10567563	PEX14	Body	-0,039	5,55E-04	4,07E-02
cg09319612	3	125038862	ZNF148	5'UTR	-0,004	5,55E-04	4,07E-02
cg01498530	3	177159709	LINC00578	Body	0,01	5,55E-04	4,07E-02
cg13177026	4	96115338	UNC5C	Body	-0,013	5,55E-04	4,07E-02
cg13234647	5	6437454		IGR	-0,034	5,55E-04	4,07E-02
cg04519403	5	79298951		IGR	-0,023	5,55E-04	4,07E-02
cg07914959	6	91006321	BACH2	1stExon	-0,004	5,54E-04	4,07E-02
cg13603955	7	22590003		IGR	-0,034	5,55E-04	4,07E-02
cg13810766	7	151542452	PRKAG2	Body	-0,016	5,55E-04	4,07E-02
cg16279003	8	126010448	SQLE	TSS1500	-0,003	5,55E-04	4,07E-02
cg20090108	11	88242488	GRM5	Body	-0,018	5,55E-04	4,07E-02
cg12726989	14	58831884	ARID4A	Body	-0,004	5,55E-04	4,07E-02
cg02319068	14	96728517	BDKRB1	5'UTR	-0,017	5,55E-04	4,07E-02
cg01006474	16	86123325		IGR	-0,012	5,54E-04	4,07E-02
cg14224882	17	77824019		IGR	0,012	5,55E-04	4,07E-02
cg21420044	18	74497570		IGR	0,004	5,55E-04	4,07E-02
cg16546864	21	46392580	C21orf70	Body	0,007	5,55E-04	4,07E-02
cg23830052	21	46481044		IGR	-0,025	5,55E-04	4,07E-02
cg10788355	13	41558816	ELF1	5'UTR	-0,014	5,55E-04	4,07E-02
cg05218653	16	2334964	ABCA3	Body	0,003	5,56E-04	4,07E-02
cg23651088	17	68071012	KCNJ16	TSS1500	-0,008	5,56E-04	4,08E-02
cg21931947	6	25193934		IGR	0,025	5,56E-04	4,08E-02
cg09819651	10	5454355	NET1	TSS200	-0,005	5,56E-04	4,08E-02
cg26314621	17	26940351	SGK494	Body	-0,021	5,56E-04	4,08E-02
cg05704183	2	145764890		IGR	0,02	5,56E-04	4,08E-02
cg06292396	10	72259703	PALD1	5'UTR	0,014	5,56E-04	4,08E-02
cg06297571	12	132270829	SFRS8	Body	0,009	5,57E-04	4,08E-02
cg17399352	17	74379779	SPHK1	TSS1500	-0,003	5,57E-04	4,08E-02
cg26182202	2	237101915		IGR	-0,049	5,57E-04	4,08E-02
cg13682152	19	16996186		IGR	0,026	5,57E-04	4,08E-02
cg10355466	8	1731776	CLN8	3'UTR	-0,007	5,57E-04	4,08E-02
cg13722794	8	17822079	PCM1	ExonBnd	0,015	5,57E-04	4,08E-02
cg11717817	10	44786986		IGR	0,009	5,58E-04	4,08E-02
cg14034135	2	55808743	PPP4R3B	Body	0,041	5,58E-04	4,08E-02
cg17605100	3	12782862	TMEM40	Body	0,023	5,58E-04	4,08E-02
cg14241509	4	6196512	JAKMIP1	5'UTR	0,039	5,58E-04	4,08E-02
cg15781525	6	564561	EXOC2	Body	0,008	5,58E-04	4,08E-02
cg25196881	15	39780412		IGR	-0,004	5,58E-04	4,08E-02
cg03751681	15	58306537	ALDH1A2	TSS1500	-0,039	5,58E-04	4,08E-02
cg10056718	16	12001429	GSPT1	Body	0,005	5,58E-04	4,08E-02
cg13710361	19	45555397	CLASRP	Body	0,007	5,58E-04	4,08E-02
cg01899676	12	106988413	RFX4	Body	-0,044	5,59E-04	4,09E-02
cg22531801	1	235806070	GNG4	5'UTR	-0,052	5,59E-04	4,09E-02

cg21436413	4	25861586	SEL1L3	Body	-0,004	5,59E-04	4,09E-02
cg05422369	18	32924550	ZNF24	TSS200	-0,009	5,59E-04	4,09E-02
cg22808989	20	39766165	PLCG1	5'UTR	0,009	5,59E-04	4,09E-02
cg09552183	12	133614153	ZNF84	5'UTR	-0,005	5,59E-04	4,09E-02
cg04656222	17	7690529	DNAH2	Body	-0,005	5,59E-04	4,09E-02
cg25028180	7	96017850		IGR	-0,05	5,59E-04	4,09E-02
cg13346671	12	101062631		IGR	-0,016	5,59E-04	4,09E-02
cg07415388	17	17717276	MIR33B	TSS200	-0,014	5,59E-04	4,09E-02
cg02957962	19	542548	GZMM	TSS1500	0,025	5,59E-04	4,09E-02
cg02868743	1	218667927	MIR548F3	Body	-0,023	5,60E-04	4,09E-02
cg16905306	3	52574451	LOC440957	Body	0,007	5,60E-04	4,09E-02
cg20840783	6	138655440	KIAA1244	Body	-0,02	5,60E-04	4,09E-02
cg17861473	12	28970616		IGR	-0,021	5,60E-04	4,09E-02
cg18580117	15	42787236	SNAP23	TSS1500	0,027	5,60E-04	4,09E-02
cg10285464	1	30481962		IGR	0,057	5,60E-04	4,09E-02
cg05981379	10	133030722	TCERG1L	Body	-0,012	5,60E-04	4,09E-02
cg12696047	16	10187350	GRIN2A	Body	-0,011	5,60E-04	4,09E-02
cg26796873	11	85359560	TMEM126A	5'UTR	-0,071	5,60E-04	4,09E-02
cg11115318	12	102087240		IGR	0,01	5,60E-04	4,09E-02
cg10642948	19	58929155	ZNF584	3'UTR	-0,005	5,61E-04	4,09E-02
cg23352030	20	62198469	PRIC285	Body	0,046	5,61E-04	4,09E-02
cg18811621	2	208207188		IGR	-0,007	5,61E-04	4,09E-02
cg26350446	7	111202682	IMMP2L	TSS1500	-0,003	5,61E-04	4,09E-02
cg10821841	13	112200892		IGR	-0,014	5,61E-04	4,09E-02
cg11810498	9	130477698	PTRH1	Body	-0,004	5,61E-04	4,10E-02
cg11381588	2	64202799	VPS54	Body	-0,016	5,62E-04	4,10E-02
cg21795549	5	138486473	SIL1	5'UTR	0,014	5,61E-04	4,10E-02
cg20833872	16	1525644	CLCN7	TSS1500	-0,046	5,62E-04	4,10E-02
cg02927961	19	11271097		IGR	0,009	5,62E-04	4,10E-02
cg13632994	1	94080251	BCAR3	TSS1500	0,005	5,62E-04	4,10E-02
cg08906307	1	221065434		IGR	-0,026	5,62E-04	4,10E-02
cg27199022	2	120234000	SCTR	Body	-0,004	5,62E-04	4,10E-02
cg08791400	6	9199875		IGR	-0,031	5,62E-04	4,10E-02
cg13641082	7	2319604	SNX8	Body	-0,025	5,62E-04	4,10E-02
cg26557744	3	116515339		IGR	-0,02	5,62E-04	4,10E-02
cg08593216	1	153609573	CHTOP	Body	-0,004	5,63E-04	4,10E-02
cg10532647	2	119381315		IGR	-0,019	5,63E-04	4,10E-02
cg24252262	5	23014515		IGR	-0,017	5,63E-04	4,10E-02
cg25665749	5	134442830	C5orf66	5'UTR	0,022	5,63E-04	4,10E-02
cg12368241	6	64355267	PHF3	TSS1500	0,013	5,63E-04	4,10E-02
cg13445036	8	67974862	COPS5	TSS1500	-0,008	5,63E-04	4,10E-02
cg13390724	9	73864728		IGR	-0,005	5,63E-04	4,10E-02
cg03258760	13	24152560	TNFRSF19	TSS1500	-0,009	5,63E-04	4,10E-02
cg25480971	20	36022108	SRC	Body	0,009	5,63E-04	4,10E-02
cg06626338	20	56678844		IGR	-0,022	5,63E-04	4,10E-02
cg02682981	22	39151639	SUN2	5'UTR	0,004	5,63E-04	4,10E-02
cg13623887	7	86849946	C7orf23	TSS1500	-0,004	5,63E-04	4,10E-02
cg25123814	11	46494341	AMBRA1	Body	-0,005	5,64E-04	4,10E-02
cg16596957	16	87575150		IGR	0,024	5,64E-04	4,10E-02
cg18348952	3	53891842	IL17RB	Body	-0,005	5,64E-04	4,10E-02
cg02956254	10	126606550		IGR	-0,003	5,64E-04	4,10E-02
cg09011256	20	37074631	SNHG11	TSS1500	-0,004	5,64E-04	4,10E-02
cg17752270	5	169706238	LCP2	Body	-0,003	5,64E-04	4,11E-02
cg13801575	3	49752362	RNF123	Body	0,008	5,64E-04	4,11E-02
cg12871059	5	1498999	LPCAT1	Body	0,018	5,64E-04	4,11E-02
cg21799053	8	22856801	RHOBTB2	Body	-0,006	5,64E-04	4,11E-02
cg07830813	15	45630898		IGR	-0,048	5,64E-04	4,11E-02
cg22128527	1	39510999		IGR	-0,072	5,65E-04	4,11E-02
cg05462233	10	78191046	C10orf11	Body	-0,006	5,65E-04	4,11E-02
cg04975876	10	112726882	SHOC2	Body	0,034	5,65E-04	4,11E-02
cg18374299	11	14996911		IGR	-0,008	5,65E-04	4,11E-02
cg12917790	14	93604305		IGR	0,012	5,65E-04	4,11E-02
cg03305830	11	17192241	PIK3C2A	TSS1500	0,027	5,65E-04	4,11E-02
cg12703042	11	111259829		IGR	0,029	5,65E-04	4,11E-02
cg10161470	16	2384061	ABCA3	5'UTR	0,015	5,65E-04	4,11E-02
cg20192478	4	42334339		IGR	-0,012	5,65E-04	4,11E-02
cg09772831	19	12175160	ZNF844	TSS1500	0,032	5,65E-04	4,11E-02
cg04122371	14	35876447		IGR	-0,012	5,65E-04	4,11E-02
cg20346534	6	46839671	ADGRF5	Body	-0,01	5,65E-04	4,11E-02
cg03659048	7	151107958	WDR86	TSS1500	0,053	5,65E-04	4,11E-02
cg11870173	19	53891732		IGR	-0,016	5,66E-04	4,11E-02
cg01705612	3	155455825		IGR	0,012	5,66E-04	4,11E-02
cg11522767	6	157342961	ARID1B	Body	-0,007	5,66E-04	4,11E-02

cg24932305	2	218875016		IGR	0,007	5,66E-04	4,11E-02
cg02201969	4	6955849	TBC1D14	Body	-0,008	5,67E-04	4,11E-02
cg22196977	2	128263013	IWS1	Body	-0,018	5,67E-04	4,11E-02
cg23737239	4	95174216	SMARCAD1	TSS1500	0,005	5,67E-04	4,11E-02
cg25493948	15	33154262	FMN1	Body	-0,036	5,67E-04	4,11E-02
cg09741930	19	13115241	NFIX	Body	0,016	5,67E-04	4,11E-02
cg26671334	21	45913090		IGR	0,007	5,67E-04	4,11E-02
cg09790035	5	31844940	PDZD2	Body	0,013	5,67E-04	4,12E-02
cg00981975	7	150974613	SMARCD3	TSS1500	-0,021	5,67E-04	4,12E-02
cg24571755	12	105086144	CHST11	Body	0,025	5,67E-04	4,12E-02
cg24364908	17	34897161		IGR	0,006	5,67E-04	4,12E-02
cg21106603	6	140758082		IGR	-0,008	5,67E-04	4,12E-02
cg11502745	11	18742694	IGSF22	Body	-0,005	5,67E-04	4,12E-02
cg10794086	6	30455964	HLA-E	TSS1500	-0,01	5,68E-04	4,12E-02
cg06822360	8	2242304		IGR	-0,009	5,68E-04	4,12E-02
cg22728616	2	235680816		IGR	-0,01	5,69E-04	4,12E-02
cg24356977	3	48339943	NME6	Body	-0,005	5,69E-04	4,12E-02
cg08097657	3	50313996	SEMA3B	Body	-0,031	5,68E-04	4,12E-02
cg19671561	3	195938283	ZDHHC19	1stExon	0,015	5,68E-04	4,12E-02
cg25069361	5	137779871	REEP2	Body	0,019	5,68E-04	4,12E-02
cg10706611	6	147526094	STXBP5-AS1	TSS1500	-0,014	5,68E-04	4,12E-02
cg26019412	8	56657461	TMEM68	Body	0,051	5,68E-04	4,12E-02
cg23206523	9	131607977	CCBL1	5'UTR	0,012	5,69E-04	4,12E-02
cg21242647	10	99625095	CRTAC1	3'UTR	-0,006	5,68E-04	4,12E-02
cg07323979	16	12618485	SNX29	Body	-0,007	5,69E-04	4,12E-02
cg01861389	16	57481027	COQ9	TSS1500	0,008	5,68E-04	4,12E-02
cg12534176	17	58574569	APPBP2	Body	-0,007	5,69E-04	4,12E-02
cg15999544	22	22116329	MAPK1	3'UTR	-0,005	5,68E-04	4,12E-02
cg03062033	22	24562887	CABIN1	Body	0,008	5,68E-04	4,12E-02
cg11363745	14	39954022		IGR	-0,009	5,69E-04	4,12E-02
cg21404124	2	167960090	XIRP2	Body	-0,021	5,69E-04	4,12E-02
cg14948825	4	178138573		IGR	-0,008	5,69E-04	4,12E-02
cg01838658	6	53036881		IGR	-0,004	5,69E-04	4,12E-02
cg08767171	13	42491015	VWA8	Body	-0,008	5,69E-04	4,12E-02
cg10798655	19	2217953	DOT1L	Body	0,011	5,70E-04	4,12E-02
cg01101647	4	66535732	EPHA5	TSS200	-0,02	5,70E-04	4,12E-02
cg10253639	17	13266346		IGR	-0,008	5,70E-04	4,12E-02
cg00784051	16	69352641	VPS4A	Body	0,003	5,70E-04	4,12E-02
cg05413022	11	4081545	STIM1	Body	-0,047	5,70E-04	4,12E-02
cg21594278	5	72815513		IGR	0,047	5,70E-04	4,13E-02
cg09796089	14	104668921		IGR	-0,05	5,70E-04	4,13E-02
cg18630511	3	155462425		IGR	-0,005	5,70E-04	4,13E-02
cg05197360	7	977580	ADAP1	Body	0,008	5,70E-04	4,13E-02
cg03885646	3	156273458	SSR3	TSS1500	-0,053	5,71E-04	4,13E-02
cg08598287	10	43836083		IGR	-0,023	5,71E-04	4,13E-02
cg13214128	14	61607559		IGR	0,034	5,71E-04	4,13E-02
cg23854667	15	32321833	CHRNA7	TSS1500	-0,007	5,71E-04	4,13E-02
cg23631062	1	102462806	OLFM3	TSS200	-0,027	5,71E-04	4,13E-02
cg16850067	9	117111004	AKNA	Body	0,015	5,71E-04	4,13E-02
cg19979370	11	128772355	KCNJ5	5'UTR	0,017	5,71E-04	4,13E-02
cg13146294	12	55079205		IGR	-0,021	5,71E-04	4,13E-02
cg14519372	21	29625908		IGR	0,02	5,72E-04	4,13E-02
cg03143959	16	30670728		IGR	-0,003	5,72E-04	4,13E-02
cg27524103	3	187957576	LPP	5'UTR	0,004	5,72E-04	4,13E-02
cg11576645	2	113642520		IGR	0,025	5,72E-04	4,13E-02
cg07222421	1	155718648	MSTO2P	Body	-0,02	5,72E-04	4,13E-02
cg20140452	4	149363715	NR3C2	TSS200	-0,006	5,72E-04	4,13E-02
cg00932677	4	187776068		IGR	-0,008	5,72E-04	4,13E-02
cg12661262	6	21691612	FLJ22536	Body	0,013	5,72E-04	4,13E-02
cg25457000	10	4813681		IGR	0,029	5,72E-04	4,13E-02
cg13435189	11	107324751	CWF19L2	Body	-0,005	5,72E-04	4,13E-02
cg24106124	2	232645314	PDE6D	Body	-0,003	5,73E-04	4,13E-02
cg23039660	4	1005884	FGFRL1	5'UTR	0,009	5,72E-04	4,13E-02
cg16412772	8	65500144		IGR	-0,029	5,72E-04	4,13E-02
cg24542613	12	123520574	PITPNM2	5'UTR	0,008	5,72E-04	4,13E-02
cg08253494	19	33696285	LRP3	Body	0,006	5,72E-04	4,13E-02
cg18987431	2	104000490		IGR	-0,016	5,73E-04	4,13E-02
cg21946672	6	107830934	SOBP	Body	-0,004	5,73E-04	4,13E-02
cg25947758	2	223581062		IGR	-0,005	5,73E-04	4,13E-02
cg26740639	3	10805956	LOC285370	TSS200	0,02	5,73E-04	4,13E-02
cg03022368	16	83986702	OSGIN1	TSS200	-0,003	5,73E-04	4,13E-02
cg03367519	5	58334910	PDE4D	Body	-0,007	5,73E-04	4,13E-02
cg14061069	19	46274453	DMPK	Body	0,076	5,73E-04	4,13E-02

cg01943216	4	181055419		IGR	-0,014	5,74E-04	4,13E-02
cg12499294	6	26373094	BTN3A2	Body	-0,006	5,74E-04	4,13E-02
cg11655629	7	113724864		IGR	-0,01	5,74E-04	4,13E-02
cg15092703	10	72678058		IGR	-0,043	5,74E-04	4,13E-02
cg15830441	13	21348144	N6AMT2	TSS200	-0,003	5,74E-04	4,13E-02
cg21803813	19	45579818	ZNF296	TSS200	0,007	5,74E-04	4,13E-02
cg08109624	1	242220925		IGR	-0,03	5,74E-04	4,14E-02
cg01571383	5	3740754		IGR	-0,006	5,74E-04	4,14E-02
cg26829379	6	32812012	PSMB8	Body	-0,002	5,74E-04	4,14E-02
cg09206496	6	38945875	DNAH8	Body	-0,009	5,74E-04	4,14E-02
cg14470629	12	97041389	CFAP54	Body	0,028	5,74E-04	4,14E-02
cg00127037	6	19326520		IGR	-0,016	5,74E-04	4,14E-02
cg13802344	1	41433256		IGR	-0,017	5,75E-04	4,14E-02
cg17203220	16	67038724	CES4A	Body	0,01	5,75E-04	4,14E-02
cg19267925	21	44516208	U2AF1	5'UTR	0,01	5,75E-04	4,14E-02
cg06233904	16	31044135	STX4	TSS1500	-0,003	5,75E-04	4,14E-02
cg07639783	7	1610747	PSMG3	TSS1500	0,042	5,75E-04	4,14E-02
cg02888748	11	27381665	CCDC34	Body	0,011	5,75E-04	4,14E-02
cg15052383	2	172864476	METAP1D	TSS1500	-0,005	5,75E-04	4,14E-02
cg02888092	2	238865252		IGR	0,036	5,75E-04	4,14E-02
cg02043758	17	28504682	NSRP1	Body	0,009	5,75E-04	4,14E-02
cg04154802	5	60595215		IGR	-0,009	5,76E-04	4,14E-02
cg06386236	1	21354302	EIF4G3	5'UTR	-0,008	5,76E-04	4,14E-02
cg19672261	1	151681524	CELF3	Body	-0,009	5,76E-04	4,14E-02
cg12972643	3	197024709	DLG1	5'UTR	-0,004	5,76E-04	4,14E-02
cg13564064	9	126154367	DENND1A	Body	-0,01	5,76E-04	4,14E-02
cg21919011	10	69827094	HERC4	5'UTR	0,01	5,76E-04	4,14E-02
cg24924875	12	59988744	SLC16A7	TSS1500	0,032	5,76E-04	4,14E-02
cg19384697	7	76139922	UPK3B	5'UTR	-0,036	5,76E-04	4,14E-02
cg23600500	21	43347260	C2CD2	TSS1500	0,027	5,77E-04	4,15E-02
cg01217409	14	71260575	MAP3K9	Body	-0,004	5,77E-04	4,15E-02
cg04000902	21	36211765	RUNX1	Body	-0,004	5,77E-04	4,15E-02
cg14378564	1	28417750		IGR	-0,079	5,77E-04	4,15E-02
cg15264642	10	23647478		IGR	-0,02	5,77E-04	4,15E-02
cg04600984	1	3589862	TP73	5'UTR	0,019	5,77E-04	4,15E-02
cg16707227	2	19157847		IGR	0,006	5,77E-04	4,15E-02
cg05958206	2	236784116	AGAP1	Body	-0,029	5,78E-04	4,15E-02
cg17936062	3	50290579	GNAI2	Body	0,011	5,78E-04	4,15E-02
cg20785661	6	13289660	LOC100130357	Body	-0,005	5,78E-04	4,15E-02
cg11212920	9	133814433	FIBCD1	5'UTR	-0,005	5,78E-04	4,15E-02
cg25946869	12	113796432	PLBD2	1stExon	0,01	5,77E-04	4,15E-02
cg12317470	15	67143691		IGR	0,006	5,77E-04	4,15E-02
cg12439172	16	76742691		IGR	-0,02	5,78E-04	4,15E-02
cg15713809	17	78322484	RNF213	Body	-0,005	5,77E-04	4,15E-02
cg13592982	18	48876658		IGR	0,037	5,77E-04	4,15E-02
cg23640193	18	11750277	GNAL	TSS1500	-0,019	5,78E-04	4,15E-02
cg16618252	9	33232834		IGR	0,019	5,78E-04	4,15E-02
cg05067156	16	88591451	ZFPM1	Body	0,016	5,78E-04	4,15E-02
cg09108969	15	102094562		IGR	-0,019	5,78E-04	4,15E-02
cg15095654	9	75242161	TMC1	5'UTR	-0,007	5,78E-04	4,15E-02
cg17357149	16	30761560	PHKG2	Body	0,012	5,78E-04	4,15E-02
cg10574172	1	163187713	RGS5	5'UTR	0,006	5,79E-04	4,15E-02
cg07262001	22	42734088		IGR	0,007	5,79E-04	4,15E-02
cg22213622	12	22090730	ABCC9	TSS1500	0,01	5,79E-04	4,15E-02
cg06891458	15	41951943	MGA	TSS1500	-0,03	5,80E-04	4,16E-02
cg11203538	5	16794797	MYO10	Body	0,004	5,80E-04	4,16E-02
cg16402452	12	58129855	AGAP2	Body	0,008	5,80E-04	4,16E-02
cg22591002	2	97530695	SEMA4C	Body	0,011	5,80E-04	4,16E-02
cg23249717	6	167507105		IGR	0,011	5,80E-04	4,16E-02
cg27313690	10	21634014		IGR	-0,011	5,81E-04	4,16E-02
cg17269348	6	11486909		IGR	-0,019	5,81E-04	4,16E-02
cg02828514	13	45993412	SLC25A30	TSS1500	0,036	5,81E-04	4,16E-02
cg04019914	20	57463357	GNAS	3'UTR	-0,035	5,81E-04	4,16E-02
cg04793497	2	20550749	PUM2	TSS200	0,015	5,81E-04	4,16E-02
cg15244633	19	50846611	NAPSB	Body	0,015	5,81E-04	4,16E-02
cg16914149	3	189873585		IGR	0,052	5,82E-04	4,17E-02
cg20988331	11	60955214		IGR	-0,005	5,81E-04	4,17E-02
cg03926983	16	1376555		IGR	0,009	5,82E-04	4,17E-02
cg12206632	19	9004188	MUC16	Body	-0,03	5,82E-04	4,17E-02
cg07126559	13	23754937	SGCG	TSS200	0,007	5,82E-04	4,17E-02
cg03832688	5	124228107		IGR	-0,011	5,82E-04	4,17E-02
cg02106043	1	161575319	HSPA7	TSS1500	-0,01	5,82E-04	4,17E-02
cg13736685	2	9619835	IAH1	Body	0,018	5,82E-04	4,17E-02

cg26076960	13	48669388	MED4	TSS200	-0,006	5,82E-04	4,17E-02
cg09825670	1	1611999	CDK11B	Body	-0,005	5,82E-04	4,17E-02
cg16112248	7	54164018		IGR	-0,015	5,82E-04	4,17E-02
cg19674032	13	111682796		IGR	0,024	5,83E-04	4,17E-02
cg21947103	16	4466648	CORO7	1stExon	0,003	5,83E-04	4,17E-02
cg22241638	2	44364503		IGR	-0,005	5,83E-04	4,17E-02
cg17936862	2	61245788	PUS10	TSS1500	-0,018	5,83E-04	4,17E-02
cg25830660	3	197026096	DLG1	TSS1500	-0,012	5,83E-04	4,17E-02
cg00612202	7	926974	C7orf20	Body	0,011	5,83E-04	4,17E-02
cg25862752	7	81392774	HGF	Body	0,042	5,83E-04	4,17E-02
cg10316510	7	158951344		IGR	-0,014	5,83E-04	4,17E-02
cg13571460	9	124989337	LHX6	Body	-0,118	5,83E-04	4,17E-02
cg20552747	17	50237576	CA10	TSS1500	-0,029	5,83E-04	4,17E-02
cg23687169	20	31564594		IGR	-0,019	5,83E-04	4,17E-02
cg06615843	7	12535447		IGR	0,039	5,83E-04	4,17E-02
cg02184047	1	39733224	MACF1	Body	-0,02	5,84E-04	4,17E-02
cg16738194	4	1076636	RNF212	Body	0,023	5,84E-04	4,17E-02
cg22976507	10	15927427		IGR	0,005	5,83E-04	4,17E-02
cg19815720	3	183770650	HTR3C	TSS200	0,031	5,84E-04	4,17E-02
cg18273566	1	23695031	C1orf213	TSS1500	-0,002	5,84E-04	4,17E-02
cg14990071	3	62975961		IGR	0,007	5,84E-04	4,17E-02
cg00950552	7	140347117		IGR	-0,03	5,84E-04	4,17E-02
cg08685581	10	54359650		IGR	-0,007	5,84E-04	4,17E-02
cg22533244	2	135548324		IGR	0,007	5,84E-04	4,17E-02
cg22005405	4	164676460	MARCH1	Body	0,011	5,84E-04	4,17E-02
cg05485379	3	197121410		IGR	0,005	5,85E-04	4,17E-02
cg20734030	12	95111263		IGR	0,051	5,85E-04	4,18E-02
cg05389024	2	170366131	KBTBD10	TSS200	-0,017	5,85E-04	4,18E-02
cg03862488	5	167723042	WWC1	Body	0,009	5,85E-04	4,18E-02
cg15522298	9	33168523	B4GALT1	TSS1500	0,03	5,85E-04	4,18E-02
cg00918917	17	61819349	STRADA	TSS200	0,007	5,85E-04	4,18E-02
cg16208203	20	46670972		IGR	-0,009	5,85E-04	4,18E-02
cg11598561	3	128181322	DNAJB8	3'UTR	0,009	5,85E-04	4,18E-02
cg05475027	16	78274575	WWOX	Body	-0,005	5,85E-04	4,18E-02
cg10567638	17	78183746	SGSH	3'UTR	0,006	5,85E-04	4,18E-02
cg13890442	1	4672897		IGR	-0,008	5,86E-04	4,18E-02
cg04351049	1	165325673	LMX1A	TSS1500	-0,004	5,86E-04	4,18E-02
cg18556129	19	2257204	JSRP1	TSS1500	-0,013	5,86E-04	4,18E-02
cg19132680	12	95449869	NR2C1	Body	0,007	5,86E-04	4,18E-02
cg06323216	6	78180963		IGR	-0,009	5,86E-04	4,18E-02
cg06677660	19	49140777	SEC1	TSS1500	-0,004	5,86E-04	4,18E-02
cg03765047	2	9620903	IAH1	Body	-0,007	5,86E-04	4,18E-02
cg08595011	3	43147479	C3orf39	5'UTR	-0,006	5,86E-04	4,18E-02
cg14304922	15	93182492	FAM174B	Body	-0,009	5,86E-04	4,18E-02
cg17465881	1	7844076	PER3	TSS1500	-0,005	5,86E-04	4,18E-02
cg10178647	22	39168827		IGR	-0,007	5,86E-04	4,18E-02
cg12902803	12	78486615	NAV3	Body	0,013	5,87E-04	4,18E-02
cg22315657	18	56189362	ALPK2	Body	-0,019	5,87E-04	4,18E-02
cg18041406	19	1432955	DAZAP1	Body	0,007	5,86E-04	4,18E-02
cg07608848	2	1647185	PXDN	Body	0,005	5,87E-04	4,18E-02
cg19638060	10	71048428	HK1	ExonBnd	0,016	5,87E-04	4,18E-02
cg17834219	12	34480142		IGR	-0,03	5,87E-04	4,18E-02
cg04859263	19	12865764	BEST2	Body	-0,026	5,87E-04	4,18E-02
cg01131004	20	38884693		IGR	-0,019	5,87E-04	4,18E-02
cg02133557	20	61560434	DIDO1	5'UTR	0,005	5,87E-04	4,18E-02
cg21089646	5	179373290		IGR	0,004	5,87E-04	4,18E-02
cg17289863	6	44213050	HSP90AB1	TSS1500	0,011	5,87E-04	4,18E-02
cg22587293	2	637620		IGR	-0,011	5,88E-04	4,18E-02
cg12455681	12	15059241		IGR	-0,005	5,88E-04	4,18E-02
cg00963378	13	20716638	GJA3	Body	-0,065	5,88E-04	4,18E-02
cg05673940	15	61334658	RORA	Body	-0,006	5,88E-04	4,18E-02
cg07037509	1	112285979	C1orf183	Body	0,015	5,88E-04	4,18E-02
cg15461842	2	170748176	UBR3	Body	-0,009	5,88E-04	4,18E-02
cg22503466	6	46655976	TDRD6	1stExon	-0,02	5,88E-04	4,18E-02
cg13002986	8	64377131	LOC102724612	TSS1500	-0,013	5,88E-04	4,18E-02
cg05402948	10	98681487	LCOR	5'UTR	-0,006	5,88E-04	4,18E-02
cg01922891	16	4714647	MGRN1	Body	0,028	5,88E-04	4,18E-02
cg12100006	11	35964190	LDLRAD3	TSS1500	-0,012	5,88E-04	4,18E-02
cg13764763	5	142785501	NR3C1	TSS1500	-0,029	5,88E-04	4,18E-02
cg09699430	6	37441431	CMTR1	Body	0,012	5,89E-04	4,18E-02
cg08448170	4	183769251		IGR	-0,051	5,89E-04	4,19E-02
cg07977661	16	81567375	CMIP	Body	-0,007	5,89E-04	4,19E-02
cg20997239	3	126960539		IGR	-0,011	5,89E-04	4,19E-02

cg07390791	2	239464827		IGR	0,012	5,89E-04	4,19E-02
cg19802477	19	54850444	LILRA4	TSS200	0,038	5,89E-04	4,19E-02
cg06359086	6	10723648	TMEM14C	5'UTR	0,013	5,90E-04	4,19E-02
cg19076479	6	130342336	L3MBTL3	5'UTR	-0,006	5,90E-04	4,19E-02
cg18844118	7	26191489	NFE2L3	TSS1500	-0,006	5,90E-04	4,19E-02
cg00151565	1	173961904	RC3H1	1stExon	-0,006	5,90E-04	4,19E-02
cg09587069	20	31407260	MAPRE1	TSS1500	-0,035	5,90E-04	4,19E-02
cg03988107	22	21802598	HIC2	3'UTR	0,024	5,91E-04	4,19E-02
cg08418332	9	34691001	CCL19	Body	0,021	5,91E-04	4,20E-02
cg12269111	2	168554345		IGR	-0,007	5,91E-04	4,20E-02
cg27357918	4	645256	PDE6B	Body	-0,058	5,91E-04	4,20E-02
cg06927337	7	2557028	LFNG	Body	0,017	5,91E-04	4,20E-02
cg11948874	20	36147327	BLCAP	Body	0,027	5,91E-04	4,20E-02
cg20008140	20	57463455	GNAS	3'UTR	-0,04	5,91E-04	4,20E-02
cg19215386	18	72921446	TSHZ1	TSS1500	0,008	5,91E-04	4,20E-02
cg08134504	2	16245936		IGR	0,009	5,91E-04	4,20E-02
cg17809267	7	16119982		IGR	-0,005	5,92E-04	4,20E-02
cg10424229	10	131665015	EBF3	Body	-0,008	5,92E-04	4,20E-02
cg17957186	13	79177895	POU4F1	TSS1500	-0,005	5,92E-04	4,20E-02
cg21965980	19	35614992	FXYD3	3'UTR	0,013	5,92E-04	4,20E-02
cg02917359	4	185624989	CENPU	Body	-0,008	5,92E-04	4,20E-02
cg16786717	15	88994114		IGR	-0,046	5,92E-04	4,20E-02
cg10960709	7	140117543	RAB19	Body	-0,029	5,92E-04	4,20E-02
cg03422806	20	55292557		IGR	-0,036	5,93E-04	4,20E-02
cg08925512	1	156075138	LMNA	5'UTR	0,021	5,93E-04	4,20E-02
cg11513563	5	141046296	ARAP3	Body	-0,008	5,93E-04	4,20E-02
cg16328079	3	65706740	MAGI1	Body	-0,011	5,93E-04	4,21E-02
cg20982112	3	110911278		IGR	-0,004	5,94E-04	4,21E-02
cg11099463	3	63739116		IGR	-0,025	5,94E-04	4,21E-02
cg15105899	4	129194226	PGRMC2	Body	0,012	5,94E-04	4,21E-02
cg18346182	10	734399	DIP2C	Body	-0,021	5,94E-04	4,21E-02
cg09551147	10	106399957	SORCS3	TSS1500	-0,003	5,95E-04	4,21E-02
cg14669919	11	65340482	FAM89B	Body	0,004	5,95E-04	4,22E-02
cg06414561	3	15466704	METTL6	Body	-0,006	5,95E-04	4,22E-02
cg06211164	3	50359065	HYAL2	TSS200	-0,004	5,95E-04	4,22E-02
cg03546235	10	102590290		IGR	-0,013	5,95E-04	4,22E-02
cg17576140	11	11172009		IGR	-0,004	5,95E-04	4,22E-02
cg12651154	12	26820570	ITPR2	Body	0,017	5,95E-04	4,22E-02
cg08310597	3	18911824		IGR	-0,022	5,95E-04	4,22E-02
cg04040779	11	47844773	NUP160	Body	0,012	5,96E-04	4,22E-02
cg09799538	11	68093246	LRP5	5'UTR	0,008	5,96E-04	4,22E-02
cg24805654	1	236319812	GPR137B	Body	0,023	5,96E-04	4,22E-02
cg10244503	1	87691781		IGR	0,03	5,96E-04	4,22E-02
cg03040736	12	109097262	CORO1C	TSS1500	-0,007	5,96E-04	4,22E-02
cg08469326	16	833382		IGR	0,014	5,96E-04	4,22E-02
cg00483459	3	46735782	ALS2CL	TSS1500	0,017	5,96E-04	4,22E-02
cg26723048	3	36910475	TRANK1	Body	-0,025	5,97E-04	4,22E-02
cg09025432	7	153083911		IGR	-0,041	5,97E-04	4,22E-02
cg02090014	7	156802340	MNX1	TSS1500	-0,01	5,97E-04	4,22E-02
cg27540038	15	71385496		IGR	-0,01	5,97E-04	4,22E-02
cg24931017	20	59600990		IGR	-0,006	5,97E-04	4,22E-02
cg18852857	22	26118876	ADRBK2	3'UTR	-0,01	5,97E-04	4,22E-02
cg07368518	1	205464823		IGR	0,02	5,97E-04	4,22E-02
cg12031103	21	19289397	CHODL	TSS1500	-0,014	5,97E-04	4,22E-02
cg27364560	9	12698601	TYRP1	Body	-0,016	5,98E-04	4,23E-02
cg03924196	14	64386420	SYNE2	Body	0,032	5,98E-04	4,23E-02
cg10437704	9	130873030		IGR	0,017	5,98E-04	4,23E-02
cg18576013	21	45403468	AGPAT3	3'UTR	0,007	5,98E-04	4,23E-02
cg06713633	3	49050825	WDR6	Body	0,009	5,98E-04	4,23E-02
cg27506547	7	73635328	LAT2	Body	-0,024	5,98E-04	4,23E-02
cg16374347	7	4815408	KIAA0415	Body	-0,008	5,98E-04	4,23E-02
cg12851039	10	11684318		IGR	-0,008	5,98E-04	4,23E-02
cg12084011	7	107220368	BCAP29	TSS200	-0,006	5,98E-04	4,23E-02
cg01481910	4	186888001		IGR	-0,006	5,99E-04	4,23E-02
cg01204058	16	28109836	XPO6	3'UTR	0,012	5,99E-04	4,23E-02
cg14842816	10	3192898	PITRM1	Body	0,01	5,99E-04	4,23E-02
cg25922935	3	156747491	LEKR1	Body	0,036	5,99E-04	4,23E-02
cg06252669	5	146464484		IGR	-0,013	5,99E-04	4,23E-02
cg00123238	10	68863995	CTNNA3	Body	0,01	5,99E-04	4,23E-02
cg04176254	16	67517429	AGRP	TSS200	0,013	5,99E-04	4,23E-02
cg14012686	2	74785750	C2orf65	3'UTR	0,027	6,00E-04	4,23E-02
cg26021742	2	131862796	PLEKHB2	1stExon	0,006	6,00E-04	4,23E-02
cg02438950	3	176816524	TBL1XR1	5'UTR	0,029	6,00E-04	4,23E-02

cg05295696	4	8345406		IGR	-0,011	6,00E-04	4,23E-02
cg21969116	6	99801200		IGR	-0,048	6,00E-04	4,23E-02
cg07649832	12	18082030		IGR	-0,033	6,00E-04	4,23E-02
cg25460059	11	9595544	WEE1	1stExon	-0,005	6,00E-04	4,23E-02
cg18554439	16	6464930	RBFOX1	5'UTR	-0,054	6,00E-04	4,24E-02
cg23374892	19	51165845	SHANK1	Body	-0,057	6,00E-04	4,24E-02
cg18898492	7	51091507	COBL	Body	-0,014	6,01E-04	4,24E-02
cg23454204	11	75203194	GDPD5	5'UTR	0,015	6,01E-04	4,24E-02
cg04130356	16	810643	MSLN	TSS1500	0,015	6,00E-04	4,24E-02
cg02242894	16	31494799	SLC5A2	Body	-0,007	6,01E-04	4,24E-02
cg07942932	19	1240652	ATP5D	TSS1500	-0,004	6,01E-04	4,24E-02
cg16355534	1	215041899		IGR	-0,006	6,01E-04	4,24E-02
cg00703445	17	53500432	MMD	TSS1500	-0,005	6,01E-04	4,24E-02
cg12765340	11	62522365	ZBTB3	TSS1500	0,023	6,01E-04	4,24E-02
cg23045276	11	69008732		IGR	-0,005	6,01E-04	4,24E-02
cg01931222	12	54623795		IGR	-0,005	6,01E-04	4,24E-02
cg07038357	16	26625928		IGR	-0,009	6,01E-04	4,24E-02
cg21075261	7	155633421		IGR	-0,009	6,01E-04	4,24E-02
cg07794804	6	68948686		IGR	0,009	6,02E-04	4,24E-02
cg12860374	10	105992228	C10orf79	TSS200	-0,004	6,02E-04	4,24E-02
cg13534901	7	17339481	AHR	Body	-0,006	6,02E-04	4,24E-02
cg10718608	17	19438221	SLC47A1	Body	-0,021	6,02E-04	4,24E-02
cg18302291	5	139007130	UBE2D2	3'UTR	0,005	6,02E-04	4,24E-02
cg23511118	3	67840535		IGR	0,016	6,02E-04	4,24E-02
cg01690274	12	33097414		IGR	0,037	6,02E-04	4,24E-02
cg05365000	21	44735596		IGR	0,022	6,02E-04	4,24E-02
cg21329920	9	2119875	SMARCA2	Body	-0,004	6,03E-04	4,24E-02
cg23642737	12	7938159		IGR	0,019	6,03E-04	4,24E-02
cg20770656	16	15626885	C16orf45	Body	-0,008	6,03E-04	4,24E-02
cg08156924	1	182558875	RNASEL	TSS1500	-0,007	6,03E-04	4,24E-02
cg09187217	4	17586185	LAP3	Body	0,005	6,03E-04	4,24E-02
cg25980539	5	123090970		IGR	-0,017	6,03E-04	4,24E-02
cg02286547	11	75920278		IGR	-0,01	6,03E-04	4,24E-02
cg02805814	19	53088482		IGR	-0,008	6,03E-04	4,24E-02
cg06916591	2	73208472	SFXN5	Body	-0,014	6,04E-04	4,24E-02
cg01207311	2	85594391	ELMOD3	Body	-0,015	6,03E-04	4,24E-02
cg03988660	3	57316479	ASB14	Body	-0,021	6,03E-04	4,24E-02
cg18566197	3	143393375	SLC9A9	Body	-0,005	6,03E-04	4,24E-02
cg14255617	6	32729118	HLA-DQB2	Body	-0,044	6,04E-04	4,24E-02
cg14956327	6	110737053	DDO	TSS1500	0,046	6,04E-04	4,24E-02
cg11260715	7	22602834		IGR	-0,004	6,04E-04	4,24E-02
cg26073542	11	3174128	OSBPL5	5'UTR	0,02	6,03E-04	4,24E-02
cg24354612	19	10384847	ICAM1	Body	0,014	6,03E-04	4,24E-02
cg24198896	6	31770844	LSM2	Body	0,015	6,04E-04	4,24E-02
cg24043192	5	43515527	C5orf34	TSS1500	-0,007	6,04E-04	4,24E-02
cg20052429	8	98962902	MATN2	Body	-0,009	6,04E-04	4,24E-02
cg06487775	1	247595137	NLRP3	Body	0,011	6,04E-04	4,25E-02
cg09997497	2	183902928	NCKAP1	5'UTR	-0,003	6,04E-04	4,25E-02
cg09515372	5	146101357	PPP2R2B	Body	0,015	6,04E-04	4,25E-02
cg17369510	8	53321188	ST18	5'UTR	-0,009	6,05E-04	4,25E-02
cg13743091	9	129948099	RALGPS1	Body	-0,026	6,04E-04	4,25E-02
cg00701940	12	86422099	MGAT4C	5'UTR	-0,04	6,04E-04	4,25E-02
cg06327920	12	93323225	EEA1	TSS200	-0,005	6,04E-04	4,25E-02
cg25147835	19	8510289	HNRNPM	Body	-0,004	6,05E-04	4,25E-02
cg19149819	1	84893084		IGR	-0,035	6,05E-04	4,25E-02
cg14898260	8	61194099	CA8	TSS200	0,006	6,05E-04	4,25E-02
cg08965559	12	106715303	TCP11L2	Body	-0,003	6,05E-04	4,25E-02
cg01173291	15	85144040	ZSCAN2	TSS1500	-0,003	6,05E-04	4,25E-02
cg07392829	21	31964616	KRTAP6-3	TSS200	-0,016	6,05E-04	4,25E-02
cg03522035	11	76370510	LRRC32	3'UTR	0,014	6,05E-04	4,25E-02
cg02507677	8	73160775		IGR	0,013	6,05E-04	4,25E-02
cg13404577	2	122482164	NIFK-AS1	Body	-0,005	6,06E-04	4,25E-02
cg25607592	3	126649987	CHCHD6	Body	0,009	6,05E-04	4,25E-02
cg13727338	7	127022811	ZNF800	Body	-0,009	6,05E-04	4,25E-02
cg03328009	10	119102596	PDZD8	Body	0,015	6,06E-04	4,25E-02
cg00211087	17	73805065	UNK	Body	0,013	6,06E-04	4,25E-02
cg26113488	1	39249433		IGR	-0,078	6,06E-04	4,25E-02
cg00860726	2	129508082		IGR	-0,037	6,06E-04	4,25E-02
cg10817929	2	125673604		IGR	-0,029	6,06E-04	4,25E-02
cg05374756	20	37586943		IGR	0,007	6,06E-04	4,25E-02
cg13597025	6	166088232		IGR	-0,041	6,06E-04	4,25E-02
cg22667509	11	92787048		IGR	-0,03	6,06E-04	4,25E-02
cg23088846	11	18113918	SAAL1	Body	-0,007	6,07E-04	4,25E-02

cg12726014	13	57484467		IGR	-0,008	6,07E-04	4,25E-02
cg14766675	1	110198695	GSTM4	TSS200	0,005	6,07E-04	4,25E-02
cg01102158	2	3381484	TSSC1	1stExon	-0,004	6,07E-04	4,25E-02
cg16500704	2	169816472	ABCB11	Body	-0,008	6,07E-04	4,25E-02
cg03940169	3	58121953	FLNB	Body	-0,006	6,07E-04	4,25E-02
cg15796536	5	140873082	PCDHGA8	Body	-0,014	6,07E-04	4,25E-02
cg00151370	6	16323285	ATXN1	Body	-0,036	6,07E-04	4,25E-02
cg08578703	6	25042495		IGR	0,008	6,07E-04	4,25E-02
cg07432550	7	46725064		IGR	0,031	6,07E-04	4,25E-02
cg06373342	17	67808872	LINC01483	Body	-0,02	6,07E-04	4,25E-02
cg01013155	1	11006392		IGR	0,017	6,08E-04	4,25E-02
cg11894100	4	71705650	GRSF1	TSS200	-0,005	6,08E-04	4,25E-02
cg03062245	20	43858493		IGR	-0,033	6,07E-04	4,25E-02
cg05958166	5	156569898	MED7	TSS200	-0,003	6,08E-04	4,25E-02
cg12453378	22	50455857	TTLL8	Body	0,021	6,08E-04	4,25E-02
cg12711974	3	160473029	PPM1L	TSS1500	-0,002	6,08E-04	4,25E-02
cg08728384	13	66467508		IGR	-0,018	6,08E-04	4,25E-02
cg19816994	1	1203436	UBE2J2	5'UTR	0,005	6,08E-04	4,25E-02
cg12467098	9	119356798	ASTN2	Body	-0,022	6,08E-04	4,25E-02
cg25647784	17	40934907	WNK4	Body	-0,048	6,08E-04	4,25E-02
cg09578275	18	6511511	LINC01387	Body	-0,017	6,08E-04	4,25E-02
cg14276006	19	1289881	EFNA2	Body	0,024	6,08E-04	4,25E-02
cg06087537	9	36401074	RNF38	TSS200	-0,006	6,08E-04	4,26E-02
cg27589663	20	61733633	HAR1B	Body	-0,004	6,08E-04	4,26E-02
cg05719566	14	43324599		IGR	-0,011	6,09E-04	4,26E-02
cg22021882	8	52756855	PCMTD1	Body	0,045	6,09E-04	4,26E-02
cg00874817	16	46824055		IGR	-0,034	6,09E-04	4,26E-02
cg03473046	22	26908215	TFIP11	5'UTR	-0,005	6,09E-04	4,26E-02
cg17836129	11	62495056	TTC9C	TSS1500	-0,003	6,09E-04	4,26E-02
cg20061812	2	103149495	SLC9A4	3'UTR	0,009	6,09E-04	4,26E-02
cg13854688	3	43821635		IGR	0,027	6,09E-04	4,26E-02
cg18102575	5	173472717	HMP19	TSS200	0,006	6,09E-04	4,26E-02
cg05236658	9	15374688		IGR	-0,023	6,09E-04	4,26E-02
cg17086421	10	3214354	PITRM1	Body	-0,017	6,09E-04	4,26E-02
cg00212811	16	65159613		IGR	-0,051	6,10E-04	4,26E-02
cg18503728	17	13364556		IGR	-0,015	6,10E-04	4,26E-02
cg23526147	19	13134813	NFIX	Body	-0,015	6,09E-04	4,26E-02
cg24629649	4	581655		IGR	-0,033	6,10E-04	4,26E-02
cg07688972	11	14441233		IGR	-0,008	6,10E-04	4,26E-02
cg26546229	16	89385611	ANKRD11	5'UTR	-0,007	6,10E-04	4,26E-02
cg19358594	1	10532567	DFFA	5'UTR	-0,003	6,10E-04	4,26E-02
cg14347781	1	17786764		IGR	-0,015	6,10E-04	4,26E-02
cg17249210	10	103113752	BTRC	TSS200	-0,005	6,10E-04	4,26E-02
cg16208206	20	31446363	EFCAB8	TSS1500	-0,014	6,10E-04	4,26E-02
cg14467840	1	153600972	S100A13	TSS1500	0,015	6,11E-04	4,26E-02
cg00971582	9	86643895		IGR	0,017	6,11E-04	4,27E-02
cg26737030	10	101654666	DNMBP	Body	-0,007	6,11E-04	4,27E-02
cg15547661	22	38840933	KCNJ4	TSS1500	0,015	6,11E-04	4,27E-02
cg13434757	6	160541976	SLC22A1	TSS1500	0,01	6,12E-04	4,27E-02
cg26111002	12	27395963	STK38L	TSS1500	0,006	6,12E-04	4,27E-02
cg21667943	12	56210943	ORMDL2	TSS1500	-0,006	6,11E-04	4,27E-02
cg23325796	12	115442768		IGR	0,031	6,12E-04	4,27E-02
cg08243619	1	11561662	PTCHD2	Body	-0,05	6,12E-04	4,27E-02
cg12396106	11	70232216		IGR	0,034	6,12E-04	4,27E-02
cg02366002	6	109416520	SESN1	TSS1500	-0,004	6,12E-04	4,27E-02
cg05521603	5	125951315	PHAX	Body	0,006	6,12E-04	4,27E-02
cg20174256	4	2845801	ADD1	TSS200	0,011	6,12E-04	4,27E-02
cg17356750	7	114329849	FOXP2	Body	-0,028	6,12E-04	4,27E-02
cg27346969	11	118799854		IGR	-0,002	6,12E-04	4,27E-02
cg07775917	20	40820131	PTPRT	Body	-0,043	6,12E-04	4,27E-02
cg09373171	1	240501227	FMN2	Body	0,057	6,12E-04	4,27E-02
cg05074554	5	50262995		IGR	-0,012	6,13E-04	4,27E-02
cg23999900	5	137501677	BRD8	Body	-0,005	6,13E-04	4,27E-02
cg00118597	12	15502027	PTPRO	Body	0,031	6,13E-04	4,27E-02
cg23940014	15	58985459	HSP90AB4P	TSS200	0,007	6,13E-04	4,27E-02
cg04108328	8	27634490	ESCO2	Body	0,014	6,13E-04	4,27E-02
cg17039936	5	89769860	MBLAC2	1stExon	-0,003	6,14E-04	4,27E-02
cg26569309	5	140563400	PCDHB16	1stExon	-0,066	6,14E-04	4,27E-02
cg00538298	11	1084334	MUC2	Body	0,013	6,14E-04	4,27E-02
cg22814353	11	6704824	MRPL17	TSS200	-0,003	6,14E-04	4,27E-02
cg26540367	16	215864	HBM	TSS200	-0,008	6,13E-04	4,27E-02
cg00548989	20	3663290	ADAM33	TSS1500	-0,032	6,14E-04	4,27E-02
cg04141948	7	1023156	CYP2W1	Body	-0,022	6,14E-04	4,27E-02

cg14619168	16	20339035	GP2	TSS200	0,005	6,14E-04	4,27E-02
cg14428055	1	161111946		IGR	0,024	6,14E-04	4,27E-02
cg07843065	1	150265600	MRPS21	TSS1500	-0,009	6,15E-04	4,27E-02
cg03603234	2	20792663	HS1BP3-IT1	TSS1500	-0,01	6,15E-04	4,27E-02
cg17891495	2	220408737	TMEM198	TSS200	-0,004	6,15E-04	4,27E-02
cg21222826	3	69062762	C3orf64	1stExon	0,009	6,15E-04	4,27E-02
cg20721270	3	181016211	SOX2-OT	Body	-0,031	6,15E-04	4,27E-02
cg25756469	4	3387865	RGS12	Body	-0,028	6,15E-04	4,27E-02
cg20290061	6	25010034	FAM65B	Body	-0,034	6,15E-04	4,27E-02
cg20909399	6	108914184	FOXO3	Body	-0,023	6,15E-04	4,27E-02
cg11619434	8	106466767	ZFPM2	Body	0,011	6,15E-04	4,27E-02
cg08723176	12	120703615	PXN	TSS200	-0,004	6,14E-04	4,27E-02
cg14559537	13	77430364		IGR	0,029	6,15E-04	4,27E-02
cg10536303	14	24583056	DCAF11	TSS1500	0,004	6,15E-04	4,27E-02
cg18019500	14	81503966	TSHR	Body	-0,019	6,15E-04	4,27E-02
cg11197281	15	90324455		IGR	0,009	6,15E-04	4,27E-02
cg15489932	16	73266628		IGR	-0,01	6,15E-04	4,27E-02
cg16200799	17	60717850	MRC2	Body	0,018	6,15E-04	4,27E-02
cg15095482	17	61190825	TANC2	Body	0,033	6,14E-04	4,27E-02
cg14157461	18	3283177		IGR	-0,007	6,15E-04	4,27E-02
cg13477582	6	32806909	TAP2	TSS1500	0,013	6,15E-04	4,28E-02
cg14359478	19	15529690	AKAP8L	Body	-0,002	6,16E-04	4,28E-02
cg16022942	22	23536136	BCR	Body	-0,011	6,16E-04	4,28E-02
cg24848259	1	223827339	CAPN8	Body	-0,007	6,16E-04	4,28E-02
cg15418419	6	32916606	HLA-DMA	3'UTR	0,01	6,16E-04	4,28E-02
cg07270857	16	63093889		IGR	0,021	6,16E-04	4,28E-02
cg01759679	5	180619664	LOC102577426	TSS1500	-0,005	6,16E-04	4,28E-02
cg20118422	15	64455210	PPIB	5'UTR	0,011	6,16E-04	4,28E-02
cg11619390	1	32931179	ZBTB8B	5'UTR	0,028	6,16E-04	4,28E-02
cg20100147	6	108436275		IGR	-0,003	6,16E-04	4,28E-02
cg05831784	20	7917317	HAO1	Body	-0,006	6,16E-04	4,28E-02
cg01853986	6	161350484		IGR	-0,024	6,17E-04	4,28E-02
cg14151317	9	139916869	ABCA2	Body	0,005	6,17E-04	4,28E-02
cg16605553	11	62649157	SLC3A2	TSS200	-0,003	6,17E-04	4,28E-02
cg07523043	6	30175299	TRIM26	5'UTR	0,019	6,17E-04	4,28E-02
cg17159699	8	53081140	ST18	Body	0,029	6,18E-04	4,28E-02
cg07374224	21	43809848	TMPRSS3	Body	-0,034	6,18E-04	4,28E-02
cg09999998	22	26743566	SEZ6L	Body	-0,01	6,18E-04	4,28E-02
cg22062131	7	100962659	RABL5	5'UTR	-0,022	6,18E-04	4,28E-02
cg12668824	3	172063592	FNDC3B	Body	0,012	6,18E-04	4,28E-02
cg27469081	6	170368455		IGR	0,009	6,18E-04	4,28E-02
cg02760672	20	49954263		IGR	0,007	6,18E-04	4,28E-02
cg01944810	2	240879397		IGR	-0,011	6,18E-04	4,28E-02
cg10101634	3	32432642	CMTM7	TSS1500	0,036	6,18E-04	4,28E-02
cg27384769	19	14142958	IL27RA	Body	0,006	6,19E-04	4,29E-02
cg11361127	2	46563062	EPAS1	Body	-0,005	6,19E-04	4,29E-02
cg13999304	3	129607809	TMCC1	5'UTR	-0,026	6,19E-04	4,29E-02
cg09500043	11	61686096	RAB3IL1	Body	0,015	6,19E-04	4,29E-02
cg27665769	4	2757761	TNIP2	1stExon	0,015	6,19E-04	4,29E-02
cg20405811	13	41553626	ELF1	Body	-0,026	6,19E-04	4,29E-02
cg06240412	20	52774697	CYP24A1	ExonBnd	0,006	6,19E-04	4,29E-02
cg18885365	1	40420800	MFSD2A	5'UTR	0,014	6,19E-04	4,29E-02
cg02792460	2	69322038	ANTXR1	Body	-0,006	6,20E-04	4,29E-02
cg16434951	5	34914928	BRIX1	TSS1500	-0,004	6,20E-04	4,29E-02
cg02672378	6	41506029		IGR	0,015	6,20E-04	4,29E-02
cg00117436	6	105560688	BVES	Body	0,011	6,19E-04	4,29E-02
cg02775377	15	61476554	RORA	Body	0,014	6,20E-04	4,29E-02
cg09645572	17	64297761	PRKCA	TSS1500	0,033	6,20E-04	4,29E-02
cg09401683	5	65466841	SREK1	Body	-0,008	6,20E-04	4,29E-02
cg00561831	3	107711933		IGR	-0,007	6,20E-04	4,29E-02
cg25806249	13	24297754		IGR	-0,037	6,20E-04	4,29E-02
cg07269843	1	1200858	UBE2J2	5'UTR	-0,005	6,21E-04	4,29E-02
cg26561438	2	213793434		IGR	0,037	6,21E-04	4,29E-02
cg27179353	4	156364125		IGR	-0,017	6,20E-04	4,29E-02
cg02717486	5	133512833	SKP1	TSS200	0,018	6,21E-04	4,29E-02
cg11488168	5	140226860	PCDHA9	TSS1500	-0,059	6,21E-04	4,29E-02
cg22655190	9	5833487	ERMP1	TSS1500	-0,032	6,21E-04	4,29E-02
cg00998075	11	2419748		IGR	0,021	6,21E-04	4,29E-02
cg25320816	13	114099871	ADPRHL1	5'UTR	0,024	6,21E-04	4,29E-02
cg09272864	14	31553783	AP4S1	3'UTR	0,02	6,20E-04	4,29E-02
cg18878432	17	46683409	LOC404266	Body	0,012	6,21E-04	4,29E-02
cg10731362	12	49801790	SPATS2	5'UTR	-0,004	6,21E-04	4,29E-02
cg07448742	6	30657984	NRM	Body	0,026	6,21E-04	4,29E-02

cg22451356	1	6159517	KCNAB2	3'UTR	0,015	6,21E-04	4,29E-02
cg27370997	2	61921752		IGR	-0,004	6,21E-04	4,29E-02
cg24877298	6	126362546		IGR	0,012	6,21E-04	4,29E-02
cg01851874	8	93074929	RUNX1T1	Body	-0,007	6,21E-04	4,29E-02
cg20023884	1	94429423		IGR	-0,019	6,22E-04	4,29E-02
cg09957135	11	123526101	SCN3B	TSS1500	0,035	6,22E-04	4,29E-02
cg16258018	11	134025732	NCAPD3	Body	0,007	6,22E-04	4,29E-02
cg02796873	19	10056105		IGR	0,012	6,22E-04	4,29E-02
cg17009842	5	153854401		IGR	-0,016	6,22E-04	4,30E-02
cg05984456	7	102516271	FBXL13	Body	0,019	6,22E-04	4,30E-02
cg14606256	12	54132568		IGR	-0,009	6,22E-04	4,30E-02
cg21658946	16	69364455	COG8	3'UTR	-0,003	6,22E-04	4,30E-02
cg22325823	17	72667062	RAB37	TSS200	0,05	6,22E-04	4,30E-02
cg05183366	20	25025765	ACSS1	Body	0,012	6,22E-04	4,30E-02
cg10761642	1	157389551		IGR	-0,015	6,22E-04	4,30E-02
cg23252622	7	143127640	EPHA1-AS1	Body	-0,022	6,23E-04	4,30E-02
cg25688990	13	105635494		IGR	-0,007	6,23E-04	4,30E-02
cg09319202	1	111506715	C1orf103	TSS200	-0,005	6,23E-04	4,30E-02
cg03842687	3	5050278		IGR	0,01	6,23E-04	4,30E-02
cg25805368	7	1802235		IGR	-0,007	6,23E-04	4,30E-02
cg12148993	11	114269290	C11orf71	Body	0,006	6,23E-04	4,30E-02
cg15433343	15	66085093	DENND4A	TSS1500	-0,003	6,23E-04	4,30E-02
cg01016497	7	102725810	ARMC10	Body	0,006	6,24E-04	4,30E-02
cg18002480	8	672007	ERICH1	Body	-0,072	6,24E-04	4,30E-02
cg03200095	3	53213700	PRKCD	Body	0,004	6,24E-04	4,30E-02
cg01367140	1	54618821	CDCP2	TSS200	0,019	6,24E-04	4,30E-02
cg00901543	2	2667584		IGR	-0,017	6,24E-04	4,30E-02
cg08826839	2	163100125	FAP	TSS200	0,012	6,24E-04	4,30E-02
cg11887555	4	6955477	TBC1D14	Body	0,012	6,24E-04	4,30E-02
cg22777467	5	125826160	GRAMD3	Body	0,01	6,24E-04	4,30E-02
cg06790566	20	44922245	CDH22	5'UTR	-0,056	6,24E-04	4,30E-02
cg26464892	20	55966440	RBM38	TSS200	-0,01	6,24E-04	4,30E-02
cg08063838	21	43442235	ZNF295-AS1	Body	-0,013	6,24E-04	4,30E-02
cg25989400	1	18386994		IGR	-0,014	6,24E-04	4,30E-02
cg16834011	22	19931790	COMT	5'UTR	0,035	6,24E-04	4,30E-02
cg10218279	1	46278418	MAST2	Body	-0,004	6,25E-04	4,30E-02
cg19907625	12	10876053	CSDA	TSS200	-0,003	6,25E-04	4,30E-02
cg08111960	11	59822765	MS4A3	TSS1500	0,022	6,25E-04	4,30E-02
cg18034049	15	63613668		IGR	-0,011	6,25E-04	4,31E-02
cg04681219	19	36135762	ETV2	3'UTR	-0,007	6,25E-04	4,31E-02
cg03915164	2	238678231	LRRFIP1	Body	-0,005	6,25E-04	4,31E-02
cg07730173	17	79483318		IGR	0,022	6,25E-04	4,31E-02
cg13835979	3	127608818		IGR	-0,033	6,26E-04	4,31E-02
cg06642945	6	163570860	PACRG	Body	-0,015	6,26E-04	4,31E-02
cg10388873	2	68416085	PPP3R1	Body	0,009	6,26E-04	4,31E-02
cg26424318	2	46380726	PRKCE	Body	0,018	6,26E-04	4,31E-02
cg13684955	14	89647182	FOXN3	Body	-0,013	6,26E-04	4,31E-02
cg19873382	6	127439539	RSPO3	TSS1500	-0,007	6,26E-04	4,31E-02
cg14727131	1	118725249	SPAG17	Body	0,012	6,26E-04	4,31E-02
cg16965605	12	66312027	HMGA2	Body	-0,012	6,26E-04	4,31E-02
cg15080870	19	47770746	CCDC9	Body	-0,004	6,26E-04	4,31E-02
cg06282219	1	36508853	AGO3	Body	0,003	6,27E-04	4,31E-02
cg13976375	1	39281613		IGR	0,022	6,27E-04	4,31E-02
cg00808909	6	139114316	CCDC28A	3'UTR	0,01	6,27E-04	4,31E-02
cg11672144	8	48566057	SPIDR	Body	0,004	6,27E-04	4,31E-02
cg10122855	11	14795513	PDE3B	Body	0,029	6,27E-04	4,31E-02
cg02052956	20	62455321		IGR	0,038	6,27E-04	4,31E-02
cg00149397	22	26986241	TPST2	TSS200	0,003	6,27E-04	4,31E-02
cg26394196	10	1453818	ADARB2	Body	-0,023	6,27E-04	4,31E-02
cg02194715	16	492818	RAB11FIP3	Body	0,028	6,27E-04	4,31E-02
cg05367052	17	76518346	DNAH17	Body	-0,006	6,28E-04	4,31E-02
cg01229610	1	161047662	PVRL4	Body	0,015	6,28E-04	4,32E-02
cg04605291	2	27797070		IGR	-0,02	6,28E-04	4,32E-02
cg27178948	12	48515787	PFKM	TSS1500	-0,005	6,28E-04	4,32E-02
cg11323186	17	45266801	CDC27	TSS200	0,007	6,28E-04	4,32E-02
cg25024210	18	2912429	EMILIN2	Body	-0,007	6,28E-04	4,32E-02
cg02914667	11	63894453	MACROD1	Body	-0,011	6,28E-04	4,32E-02
cg02484776	15	42567603	GANC	Body	0,044	6,28E-04	4,32E-02
cg17210933	5	3428696		IGR	-0,05	6,29E-04	4,32E-02
cg05453679	6	97247912	GPR63	5'UTR	0,028	6,29E-04	4,32E-02
cg18521925	6	110798323	SLC22A16	TSS1500	0,016	6,29E-04	4,32E-02
cg02842701	1	62373926	INADL	Body	0,011	6,29E-04	4,32E-02
cg01811272	2	47276456	TTC7A	Body	0,025	6,29E-04	4,32E-02

cg07776569	1	211843210	NEK2	Body	0,006	6,30E-04	4,32E-02
cg24064079	2	120301053	CFAP221	TSS1500	-0,006	6,30E-04	4,32E-02
cg20919596	7	2727028	AMZ1	TSS1500	0,008	6,29E-04	4,32E-02
cg19275220	7	108168721		IGR	-0,005	6,29E-04	4,32E-02
cg00213663	8	42428333		IGR	0,014	6,30E-04	4,32E-02
cg07611812	8	53627203	RB1CC1	TSS200	-0,004	6,30E-04	4,32E-02
cg04633409	12	44195947	TWF1	Body	0,008	6,29E-04	4,32E-02
cg22619774	12	111169761	PPP1CC	Body	0,012	6,30E-04	4,32E-02
cg10266418	16	66636477	CMTM3	TSS1500	0,015	6,29E-04	4,32E-02
cg09435109	19	53970585	ZNF813	TSS1500	0,01	6,29E-04	4,32E-02
cg24203465	20	57425986	GNASAS	TSS200	0,016	6,30E-04	4,32E-02
cg05436055	20	58203488	LOC100506384	TSS200	-0,024	6,30E-04	4,32E-02
cg12089712	6	31047598		IGR	-0,005	6,30E-04	4,32E-02
cg14328241	6	53976336	MLIP	1stExon	-0,017	6,30E-04	4,32E-02
cg01150458	21	34070599	SYNJ1	Body	-0,006	6,30E-04	4,32E-02
cg24106661	2	61886946		IGR	0,007	6,31E-04	4,32E-02
cg00229359	2	70556142		IGR	-0,006	6,31E-04	4,32E-02
cg15704204	5	167689728	TENM2	Body	-0,01	6,31E-04	4,32E-02
cg14771144	12	48491955	SENP1	Body	-0,007	6,31E-04	4,32E-02
cg11679762	15	92074942		IGR	-0,005	6,30E-04	4,32E-02
cg02693870	16	55689324	SLC6A2	TSS1500	-0,045	6,31E-04	4,32E-02
cg01150124	20	46095646		IGR	-0,013	6,30E-04	4,32E-02
cg19368625	2	1452665	TPO	Body	-0,031	6,31E-04	4,32E-02
cg01779765	14	62583795	FLJ43390	TSS1500	0,018	6,31E-04	4,32E-02
cg03764976	19	15295235	NOTCH3	Body	0,017	6,31E-04	4,32E-02
cg12922647	15	31396316	TRPM1	Body	-0,02	6,31E-04	4,32E-02
cg13744917	1	2986538	PRDM16	Body	-0,003	6,31E-04	4,32E-02
cg23242429	17	35879310	SYNRG	Body	-0,006	6,32E-04	4,33E-02
cg05554991	1	67772320	IL12RB2	TSS1500	0,019	6,32E-04	4,33E-02
cg26355552	3	9183074	SRGAP3	Body	-0,031	6,32E-04	4,33E-02
cg05574795	2	242065411	PASK	Body	0,006	6,32E-04	4,33E-02
cg12715266	12	60812459		IGR	0,007	6,32E-04	4,33E-02
cg05383838	20	46256408	NCOA3	Body	0,007	6,32E-04	4,33E-02
cg03354925	1	72035775	NEGR1	Body	0,015	6,32E-04	4,33E-02
cg22361050	10	48354460	ZNF488	TSS1500	-0,007	6,32E-04	4,33E-02
cg06930464	1	171510692	BAT2L2	Body	-0,007	6,33E-04	4,33E-02
cg17000939	12	75688237	CAPS2	Body	-0,025	6,32E-04	4,33E-02
cg01511823	12	25348048	CASC1	1stExon	-0,002	6,33E-04	4,33E-02
cg05711131	19	36231948	TMEM149	Body	-0,003	6,33E-04	4,33E-02
cg16761327	4	166893140	TLL1	Body	0,026	6,33E-04	4,33E-02
cg07971007	17	79127372	AATK	Body	0,022	6,33E-04	4,33E-02
cg12212453	14	38058639		IGR	-0,021	6,33E-04	4,33E-02
cg19176072	10	135083742	ADAM8	Body	0,007	6,33E-04	4,33E-02
cg09627423	4	54579899	LOC100506444	Body	-0,009	6,34E-04	4,34E-02
cg00123503	6	80604867		IGR	0,018	6,34E-04	4,34E-02
cg14343457	6	149716235	MAP3K7IP2	Body	-0,009	6,34E-04	4,34E-02
cg05273205	8	25907918		IGR	-0,022	6,34E-04	4,34E-02
cg13813191	12	113863892	SDSL	5'UTR	0,034	6,34E-04	4,34E-02
cg05628259	5	127716558	FBN2	Body	0,046	6,35E-04	4,34E-02
cg18683500	13	98233551		IGR	-0,013	6,35E-04	4,34E-02
cg14767402	13	103426324	C13orf27	TSS200	-0,006	6,35E-04	4,34E-02
cg00645628	1	101213654		IGR	0,006	6,35E-04	4,34E-02
cg26414014	6	91387292		IGR	-0,007	6,35E-04	4,34E-02
cg02011446	19	13213716	LYL1	TSS200	0,006	6,35E-04	4,34E-02
cg21618635	4	1984950	WHSC2	3'UTR	-0,012	6,36E-04	4,34E-02
cg26774557	12	55979866		IGR	-0,008	6,36E-04	4,34E-02
cg13392874	2	215493830		IGR	-0,012	6,36E-04	4,34E-02
cg13016773	12	72081013	TMEM19	Body	0,014	6,36E-04	4,34E-02
cg08201238	16	560855	RAB11FIP3	Body	-0,016	6,36E-04	4,34E-02
cg25304816	1	36627734	MAP7D1	Body	-0,003	6,36E-04	4,34E-02
cg01819982	4	81104878		IGR	0,008	6,36E-04	4,34E-02
cg07835594	14	22475377		IGR	-0,016	6,36E-04	4,34E-02
cg10811429	15	80189771	MTHFS	TSS1500	0,031	6,36E-04	4,34E-02
cg01479473	16	1524174	CLCN7	Body	-0,051	6,37E-04	4,34E-02
cg12188614	7	54697527		IGR	-0,035	6,37E-04	4,35E-02
cg19402229	2	230451698	DNER	Body	-0,026	6,37E-04	4,35E-02
cg05514656	5	35778476	SPEF2	Body	-0,008	6,37E-04	4,35E-02
cg17704343	17	43025655	KIF18B	TSS1500	-0,045	6,37E-04	4,35E-02
cg18235026	7	129419139		IGR	-0,006	6,38E-04	4,35E-02
cg19758563	10	134980426	KNDC1	Body	-0,005	6,38E-04	4,35E-02
cg22650197	11	44227997	EXT2	Body	0,02	6,38E-04	4,35E-02
cg15537567	4	23017280		IGR	0,018	6,38E-04	4,35E-02
cg09550136	8	117491607		IGR	0,012	6,38E-04	4,35E-02

cg09328012	22	46481677	LOC400931	TSS200	0,012	6,38E-04	4,35E-02
cg10493479	1	247835667	OR13G1	1stExon	-0,009	6,38E-04	4,35E-02
cg13944939	9	130679246	ST6GALNAC4	5'UTR	0,006	6,38E-04	4,35E-02
cg19814100	11	111322009		IGR	0,009	6,38E-04	4,35E-02
cg06388946	2	46525020	EPAS1	5'UTR	-0,006	6,39E-04	4,35E-02
cg04991718	3	51849844		IGR	0,036	6,39E-04	4,35E-02
cg04890856	4	33724724		IGR	-0,018	6,39E-04	4,35E-02
cg10254698	14	93669495	C14orf142	3'UTR	0,036	6,39E-04	4,35E-02
cg03097336	16	84178939	LRRC50	5'UTR	0,004	6,39E-04	4,35E-02
cg13676099	20	19281324	SLC24A3	Body	0,031	6,39E-04	4,35E-02
cg24308915	9	140017977		IGR	0,008	6,39E-04	4,35E-02
cg20277616	17	29701389	NF1	3'UTR	0,026	6,39E-04	4,35E-02
cg11731114	10	8096064	FLJ45983	TSS1500	-0,006	6,39E-04	4,35E-02
cg00318416	12	50470384	ASIC1	Body	0,013	6,39E-04	4,35E-02
cg08381620	13	24062873		IGR	-0,058	6,39E-04	4,35E-02
cg23792962	20	7589134		IGR	-0,046	6,39E-04	4,35E-02
cg16998655	21	42621363	BACE2	Body	0,009	6,39E-04	4,35E-02
cg17883677	1	32281856	SPOCD1	TSS1500	-0,011	6,39E-04	4,35E-02
cg06505010	5	1923653		IGR	-0,026	6,40E-04	4,35E-02
cg26070824	15	49715415	FGF7	1stExon	-0,059	6,40E-04	4,35E-02
cg06952578	7	150745926	ASIC3	5'UTR	-0,017	6,40E-04	4,36E-02
cg14515211	4	174292111	SAP30	5'UTR	-0,004	6,40E-04	4,36E-02
cg18185081	22	37172374	IFT27	TSS200	-0,003	6,40E-04	4,36E-02
cg24730328	10	99910477	R3HCC1L	5'UTR	-0,01	6,40E-04	4,36E-02
cg18313396	8	18471431	PSD3	Body	-0,01	6,40E-04	4,36E-02
cg17168830	11	3400651	ZNF195	TSS1500	-0,008	6,41E-04	4,36E-02
cg10832581	3	102474847		IGR	0,023	6,41E-04	4,36E-02
cg11160408	3	123750917		IGR	-0,007	6,41E-04	4,36E-02
cg09249657	4	40518806	RBM47	TSS1500	0,012	6,41E-04	4,36E-02
cg22846391	5	176310124	HK3	Body	0,009	6,41E-04	4,36E-02
cg20107824	10	78868270	KCNMA1	Body	-0,03	6,41E-04	4,36E-02
cg18217874	7	133531244	EXOC4	Body	-0,037	6,41E-04	4,36E-02
cg12980419	6	139564250	TXLNB	Body	0,005	6,41E-04	4,36E-02
cg12080364	16	69354837	VPS4A	Body	-0,006	6,41E-04	4,36E-02
cg05244369	10	105308523	NEURL1	Body	0,012	6,41E-04	4,36E-02
cg20513178	1	43995300	PTPRF	TSS1500	-0,007	6,42E-04	4,36E-02
cg12415902	1	5931813	NPHP4	Body	0,005	6,42E-04	4,36E-02
cg26518884	4	166322679	CPE	Body	-0,008	6,42E-04	4,36E-02
cg02410867	19	16198843	TPM4	Body	0,006	6,42E-04	4,36E-02
cg19221136	1	110950697	LAMTOR5	TSS200	-0,003	6,42E-04	4,36E-02
cg18742550	2	164030043		IGR	-0,007	6,42E-04	4,36E-02
cg10750859	4	152622689	GATB	ExonBnd	-0,006	6,42E-04	4,36E-02
cg18956271	5	168844191		IGR	0,023	6,42E-04	4,36E-02
cg21988633	19	23383482		IGR	0,01	6,42E-04	4,36E-02
cg13895316	20	335543		IGR	0,015	6,42E-04	4,36E-02
cg10767420	2	2581557		IGR	0,011	6,42E-04	4,36E-02
cg11718317	11	126873689		IGR	-0,008	6,42E-04	4,36E-02
cg01885906	5	179306815	TBC1D9B	Body	0,008	6,43E-04	4,36E-02
cg01707905	19	51288648		IGR	-0,016	6,43E-04	4,36E-02
cg11729019	12	7188330	C1R	Body	0,006	6,43E-04	4,36E-02
cg26957633	5	180527073		IGR	0,048	6,43E-04	4,37E-02
cg27213238	6	10530035	GCNT2	Body	0,069	6,43E-04	4,37E-02
cg18853775	19	15218139	SYDE1	TSS200	-0,007	6,43E-04	4,37E-02
cg23979832	17	79031550	BAIAP2	Body	-0,019	6,43E-04	4,37E-02
cg05983557	2	136912140		IGR	0,021	6,43E-04	4,37E-02
cg16850288	3	42108299		IGR	0,022	6,43E-04	4,37E-02
cg10291205	13	46856182		IGR	0,045	6,43E-04	4,37E-02
cg04822876	1	110019431	SYPL2	Body	-0,031	6,44E-04	4,37E-02
cg05702851	4	110481083	CCDC109B	TSS1500	0,003	6,44E-04	4,37E-02
cg23672472	7	95951587	SLC25A13	TSS200	0,006	6,44E-04	4,37E-02
cg26777712	1	89151226	PKN2-AS1	TSS1500	-0,005	6,44E-04	4,37E-02
cg19423197	3	113465120	ATP6V1A	TSS1500	-0,004	6,44E-04	4,37E-02
cg27406845	17	56592101	MTMR4	Body	-0,003	6,44E-04	4,37E-02
cg01726295	7	135938950		IGR	0,05	6,44E-04	4,37E-02
cg19315653	1	203096230	ADORA1	TSS1500	-0,065	6,44E-04	4,37E-02
cg17693183	3	112951443	BOC	5'UTR	0,034	6,44E-04	4,37E-02
cg24796651	4	76912348	SDAD1	TSS1500	0,056	6,44E-04	4,37E-02
cg12545295	12	48551391	ASB8	TSS200	-0,004	6,44E-04	4,37E-02
cg17652718	21	28733016	MIR5009	Body	-0,011	6,44E-04	4,37E-02
cg16858957	11	117072915	TAGLN	5'UTR	0,017	6,44E-04	4,37E-02
cg04097078	22	20784542	SCARF2	Body	0,045	6,44E-04	4,37E-02
cg19760563	18	41168321		IGR	-0,007	6,45E-04	4,37E-02
cg22370694	1	228395660	OBSCN	TSS200	0,029	6,45E-04	4,37E-02

cg17019053	2	74875263	C2orf65	TSS200	0,036	6,45E-04	4,37E-02
cg07245477	6	23338407		IGR	-0,029	6,45E-04	4,37E-02
cg12203251	8	19629562		IGR	-0,019	6,45E-04	4,37E-02
cg18493244	9	134298906		IGR	0,011	6,45E-04	4,37E-02
cg08359855	12	24510637	SOX5	5'UTR	-0,011	6,45E-04	4,37E-02
cg10728060	13	53029403	CKAP2	TSS200	-0,003	6,45E-04	4,37E-02
cg05351887	16	3988869		IGR	-0,07	6,45E-04	4,37E-02
cg13854874	21	37757525	CHAF1B	TSS200	-0,037	6,45E-04	4,37E-02
cg04035031	22	46067830	ATXN10	1stExon	-0,003	6,45E-04	4,37E-02
cg25255232	5	95188508	LINC01554	Body	0,019	6,45E-04	4,37E-02
cg02056891	17	1975419	SMG6	Body	0,024	6,45E-04	4,37E-02
cg02107251	17	4853353	PFN1	TSS1500	-0,005	6,45E-04	4,37E-02
cg06082822	6	18220421	KDM1B	Body	-0,006	6,46E-04	4,37E-02
cg18172281	8	21627806	GFRA2	Body	-0,008	6,46E-04	4,37E-02
cg03524264	1	115839827	NGF	5'UTR	-0,035	6,46E-04	4,37E-02
cg11036041	4	41365307	LIMCH1	Body	0,075	6,46E-04	4,37E-02
cg05884756	17	73127913	NT5C	TSS200	0,003	6,46E-04	4,37E-02
cg24734575	19	33360626	SLC7A9	1stExon	-0,005	6,46E-04	4,37E-02
cg01135165	3	108191083	MYH15	Body	-0,006	6,46E-04	4,37E-02
cg19653589	19	2614177	GNG7	5'UTR	-0,021	6,46E-04	4,37E-02
cg25826226	15	41953061	MGA	5'UTR	-0,006	6,46E-04	4,37E-02
cg26451835	6	117996421	NUS1	TSS200	-0,004	6,47E-04	4,37E-02
cg20776947	12	133197827	P2RX2	Body	0,027	6,46E-04	4,37E-02
cg21954459	17	19319617	RNF112	3'UTR	0,006	6,46E-04	4,37E-02
cg18944966	19	6023511	RFX2	Body	0,026	6,46E-04	4,37E-02
cg06962884	16	90042262	AFG3L1	Body	-0,004	6,47E-04	4,37E-02
cg20972555	1	113160916	CAPZA1	TSS1500	-0,004	6,47E-04	4,37E-02
cg09684637	10	84983186		IGR	-0,02	6,47E-04	4,37E-02
cg04338250	19	46543685	IGFL4	Body	0,015	6,47E-04	4,37E-02
cg25808926	13	21714709	SAP18	1stExon	0,005	6,47E-04	4,37E-02
cg11588317	11	95837120	MAML2	Body	0,01	6,47E-04	4,37E-02
cg06437206	8	144887347	SCRIB	Body	0,008	6,48E-04	4,38E-02
cg26286077	11	18825048		IGR	-0,031	6,48E-04	4,38E-02
cg19396253	7	77584545	PHTF2	3'UTR	0,01	6,48E-04	4,38E-02
cg02547521	7	44147661	AEBP1	Body	0,008	6,48E-04	4,38E-02
cg07720645	6	90596316		IGR	-0,004	6,48E-04	4,38E-02
cg27048142	19	46544240	IGFL4	1stExon	-0,022	6,49E-04	4,38E-02
cg18826718	3	38780151	SCN10A	Body	-0,007	6,49E-04	4,38E-02
cg17471471	6	135308927	HBS1L	ExonBnd	-0,006	6,49E-04	4,38E-02
cg10031532	6	28555021	SCAND3	1stExon	-0,004	6,49E-04	4,38E-02
cg25767701	6	68643388		IGR	0,04	6,49E-04	4,38E-02
cg16425829	12	119632544	HSPB8	3'UTR	-0,025	6,49E-04	4,38E-02
cg03325346	11	985936	AP2A2	Body	-0,006	6,49E-04	4,38E-02
cg04016985	7	135123085	CNOT4	5'UTR	0,01	6,49E-04	4,38E-02
cg11530914	16	67281528	FHOD1	TSS200	-0,005	6,49E-04	4,38E-02
cg11140785	19	57703301	ZNF264	1stExon	0,005	6,49E-04	4,38E-02
cg26210511	1	47184937	EFCAB14	TSS1500	-0,013	6,50E-04	4,38E-02
cg02974808	6	104973735		IGR	-0,008	6,50E-04	4,38E-02
cg19875370	7	25312085		IGR	0,043	6,50E-04	4,38E-02
cg16217427	11	12069574		IGR	0,031	6,50E-04	4,38E-02
cg11004222	19	50957350	MYBPC2	Body	-0,004	6,50E-04	4,38E-02
cg09362608	4	6659805		IGR	-0,056	6,50E-04	4,38E-02
cg16502380	19	49254621	FUT1	5'UTR	0,032	6,50E-04	4,38E-02
cg01800614	6	29719096	IFITM4P	TSS200	0,004	6,50E-04	4,39E-02
cg00908192	1	201283916	PKP1	Body	0,009	6,51E-04	4,39E-02
cg04353483	4	26493496	CCKAR	TSS1500	-0,005	6,51E-04	4,39E-02
cg04618263	4	81017725		IGR	-0,007	6,51E-04	4,39E-02
cg12526549	13	80506626		IGR	-0,015	6,51E-04	4,39E-02
cg14819504	2	160761413	LY75	TSS200	-0,004	6,51E-04	4,39E-02
cg18863105	3	183149726		IGR	0,006	6,51E-04	4,39E-02
cg04875619	4	177926577		IGR	0,026	6,51E-04	4,39E-02
cg11497952	4	185940969	HELT	Body	-0,008	6,51E-04	4,39E-02
cg03168711	17	8827675	PIK3R5	5'UTR	0,021	6,51E-04	4,39E-02
cg21447167	22	46364407	WNT7B	Body	0,016	6,51E-04	4,39E-02
cg08607780	1	53556701	SLC1A7	Body	0,045	6,52E-04	4,39E-02
cg13638257	6	33280436	TAPBP	Body	-0,026	6,52E-04	4,39E-02
cg14128332	8	134706976		IGR	0,012	6,52E-04	4,39E-02
cg06741005	8	145831211	KIAA1688	5'UTR	-0,01	6,52E-04	4,39E-02
cg07702770	10	21260833	NEBL	Body	0,031	6,52E-04	4,39E-02
cg24756403	10	115860310		IGR	-0,039	6,52E-04	4,39E-02
cg06131833	10	118378224		IGR	-0,035	6,52E-04	4,39E-02
cg12254216	15	41102135	ZFYVE19	Body	0,005	6,52E-04	4,39E-02
cg05719831	16	11165205	CLEC16A	Body	-0,004	6,52E-04	4,39E-02

cg09349298	3	88558623		IGR	-0,013	6,52E-04	4,39E-02
cg24443692	8	63015631		IGR	-0,016	6,52E-04	4,39E-02
cg06741336	14	101696494		IGR	0,024	6,52E-04	4,39E-02
cg06947941	1	101897709		IGR	-0,008	6,53E-04	4,39E-02
cg02828078	22	40599692	TNRC6B	Body	-0,009	6,53E-04	4,39E-02
cg04126391	9	92120836		IGR	-0,006	6,53E-04	4,39E-02
cg14733672	22	35777245	HMOX1	Body	-0,003	6,53E-04	4,39E-02
cg24893930	2	139361554		IGR	-0,007	6,53E-04	4,39E-02
cg10981747	10	115438129	CASP7	TSS1500	-0,004	6,53E-04	4,39E-02
cg02663540	17	47472466		IGR	-0,004	6,53E-04	4,39E-02
cg17789138	19	49936880	SLC17A7	Body	-0,018	6,53E-04	4,39E-02
cg15039797	7	139320346	HIPK2	Body	-0,034	6,53E-04	4,39E-02
cg05273780	18	56603531	ZNF532	Body	0,028	6,54E-04	4,39E-02
cg13186228	20	57225195	STX16	TSS1500	0,004	6,54E-04	4,39E-02
cg04442407	12	69633209	CPSF6	TSS200	-0,006	6,54E-04	4,39E-02
cg13584000	20	10448859	SLX4IP	Body	-0,006	6,54E-04	4,39E-02
cg05649864	15	61601669		IGR	-0,052	6,54E-04	4,39E-02
cg24575083	11	7695485	CYB5R2	TSS1500	-0,027	6,54E-04	4,39E-02
cg17558214	7	11101436	PHF14	Body	0,021	6,54E-04	4,40E-02
cg00218090	14	100485810		IGR	-0,029	6,54E-04	4,40E-02
cg23510449	16	83715995	CDH13	Body	-0,04	6,55E-04	4,40E-02
cg04623987	19	32816062		IGR	0,006	6,55E-04	4,40E-02
cg15149938	22	50018392	C22orf34	Body	-0,007	6,55E-04	4,40E-02
cg03084949	11	67232511	TMEM134	Body	-0,003	6,55E-04	4,40E-02
cg24367228	5	131915055	RAD50	Body	-0,007	6,56E-04	4,40E-02
cg03347015	11	62490152	HNRNPUL2	Body	0,005	6,56E-04	4,40E-02
cg15554554	11	124199796		IGR	-0,026	6,55E-04	4,40E-02
cg18748332	12	54020076	ATF7	5'UTR	-0,003	6,56E-04	4,40E-02
cg00315781	12	110213263		IGR	-0,002	6,55E-04	4,40E-02
cg04868518	16	11771778	SNN	3'UTR	-0,011	6,56E-04	4,40E-02
cg19422300	20	61989375	CHRNA4	Body	0,017	6,56E-04	4,40E-02
cg03813230	2	121730358	GLI2	Body	0,005	6,56E-04	4,40E-02
cg25453556	11	58911101	FAM111A	5'UTR	0,03	6,56E-04	4,40E-02
cg00001269	20	48959004		IGR	0,015	6,56E-04	4,40E-02
cg21702441	5	153537098		IGR	-0,045	6,56E-04	4,40E-02
cg21016004	12	16759591	LMO3	TSS200	-0,005	6,56E-04	4,40E-02
cg22553717	12	31249812	DDX11	Body	0,014	6,57E-04	4,40E-02
cg13150341	19	44953124	ZNF229	TSS1500	0,043	6,57E-04	4,40E-02
cg01647837	1	23343075	C1orf234	TSS1500	0,004	6,57E-04	4,40E-02
cg00014118	1	1935561	KIAA1751	TSS1500	-0,031	6,57E-04	4,40E-02
cg13254203	1	35585672		IGR	-0,019	6,57E-04	4,40E-02
cg22988305	3	52240549	ALAS1	Body	-0,005	6,57E-04	4,40E-02
cg14978135	11	117237349	CEP164	Body	0,007	6,57E-04	4,40E-02
cg10069353	12	56510054	RPL41	TSS1500	-0,01	6,57E-04	4,40E-02
cg13972460	20	1165703	C20orf46	TSS1500	0,018	6,57E-04	4,40E-02
cg13909328	20	42611749	TOX2	Body	0,009	6,57E-04	4,40E-02
cg11958173	1	237729946	RYR2	Body	-0,005	6,57E-04	4,41E-02
cg07478785	2	796359		IGR	0,014	6,58E-04	4,41E-02
cg14176755	2	176803677	KIAA1715	Body	0,016	6,58E-04	4,41E-02
cg16662408	6	32053637	TNXB	Body	0,02	6,58E-04	4,41E-02
cg10899768	8	102506635	GRHL2	Body	0,025	6,58E-04	4,41E-02
cg03201492	16	1096348		IGR	0,022	6,58E-04	4,41E-02
cg25774900	17	39968875	P3H4	TSS1500	0,005	6,58E-04	4,41E-02
cg10268161	19	37690673	ZNF585B	Body	0,008	6,58E-04	4,41E-02
cg15550297	5	122885981	CSNK1G3	5'UTR	0,006	6,58E-04	4,41E-02
cg03052071	10	134765099		IGR	-0,034	6,58E-04	4,41E-02
cg21240272	9	91174472	NXNL2	Body	-0,009	6,58E-04	4,41E-02
cg06745865	5	172710181		IGR	0,017	6,58E-04	4,41E-02
cg01232145	3	71027078	FOXP1	Body	0,009	6,58E-04	4,41E-02
cg22680591	11	2922051	SLC22A18AS	5'UTR	-0,006	6,58E-04	4,41E-02
cg08412699	7	20449718	ITGB8	3'UTR	0,011	6,59E-04	4,41E-02
cg24783785	17	619036	VPS53	TSS1500	-0,044	6,59E-04	4,41E-02
cg26132356	11	62284877	AHNAK	Body	0,016	6,59E-04	4,41E-02
cg22266511	16	30079978	ALDOA	ExonBnd	0,02	6,59E-04	4,41E-02
cg27042271	17	44269794	KANSL1	1stExon	-0,003	6,59E-04	4,41E-02
cg00604945	19	9214061	OR7G2	TSS200	0,023	6,59E-04	4,41E-02
cg01027365	2	191184571	HIBCH	TSS200	-0,007	6,60E-04	4,41E-02
cg18268492	10	99185986	PGAM1	TSS200	0,01	6,60E-04	4,41E-02
cg24892871	19	10611043	KEAP1	5'UTR	0,012	6,60E-04	4,41E-02
cg01337997	1	212694273		IGR	-0,006	6,60E-04	4,41E-02
cg18351858	3	184053569	FAM131A	TSS200	-0,013	6,60E-04	4,41E-02
cg07793369	9	139377940	SEC16A	5'UTR	0,017	6,60E-04	4,41E-02
cg14244483	10	772088		IGR	-0,035	6,60E-04	4,41E-02

cg21429102	15	58429769	AQP9	TSS1500	0,005	6,60E-04	4,41E-02
cg13507893	22	32650997	SLC5A4	Body	0,007	6,60E-04	4,41E-02
cg16128480	12	91201770		IGR	0,04	6,60E-04	4,41E-02
cg02773588	3	48698519	CELSR3	1stExon	-0,038	6,61E-04	4,41E-02
cg13514578	12	50016646	PRPF40B	TSS1500	-0,002	6,61E-04	4,41E-02
cg22373225	11	45234334	PRDM11	Body	-0,005	6,61E-04	4,41E-02
cg03063746	5	32353017		IGR	-0,005	6,61E-04	4,41E-02
cg24721350	7	75511202	RHBDD2	Body	0,004	6,61E-04	4,41E-02
cg11336590	1	166958322	MAEL	TSS200	-0,009	6,61E-04	4,41E-02
cg10042864	5	50045240	PARP8	Body	0,038	6,61E-04	4,41E-02
cg15081786	12	7172505	C1S	Body	-0,015	6,61E-04	4,42E-02
cg11770888	6	105088848		IGR	-0,022	6,61E-04	4,42E-02
cg24825276	12	122766629	CLIP1	Body	-0,011	6,61E-04	4,42E-02
cg13929970	4	15939862	FGFBP1	5'UTR	-0,006	6,61E-04	4,42E-02
cg00402524	16	89710345		IGR	0,018	6,62E-04	4,42E-02
cg05932042	17	40822653	PLEKHH3	Body	-0,045	6,62E-04	4,42E-02
cg07415610	8	77343706	LINC01111	Body	-0,008	6,62E-04	4,42E-02
cg08744435	8	135703703	ZFAT	5'UTR	-0,004	6,62E-04	4,42E-02
cg00086113	15	75760823	PTPN9	3'UTR	-0,006	6,62E-04	4,42E-02
cg08714996	5	140729928	PCDHGB1	1stExon	-0,032	6,62E-04	4,42E-02
cg12771352	11	946926	AP2A2	Body	-0,004	6,62E-04	4,42E-02
cg08162562	16	14280449	MKL2	Body	-0,016	6,62E-04	4,42E-02
cg07758936	3	27525996	SLC4A7	TSS200	0,005	6,63E-04	4,42E-02
cg15842894	3	194500070	LOC100507391	Body	0,028	6,63E-04	4,42E-02
cg19306866	21	31970997		IGR	-0,02	6,63E-04	4,42E-02
cg14207954	2	96987397		IGR	-0,003	6,63E-04	4,42E-02
cg23059689	13	111288257	CARKD	Body	-0,008	6,63E-04	4,42E-02
cg08732693	4	77343547		IGR	0,029	6,63E-04	4,42E-02
cg14841103	16	2284586	E4F1	ExonBnd	0,015	6,63E-04	4,42E-02
cg00168439	3	55926920	ERC2	Body	0,021	6,63E-04	4,42E-02
cg20100445	10	134756707	C10orf93	TSS1500	-0,042	6,63E-04	4,42E-02
cg25760229	16	1203595	CACNA1H	5'UTR	-0,004	6,63E-04	4,42E-02
cg11568518	7	51004273		IGR	-0,007	6,64E-04	4,42E-02
cg07851575	13	41387748	TPTE2P5	Body	-0,014	6,64E-04	4,42E-02
cg07565099	2	32502226	YIPF4	TSS1500	-0,005	6,64E-04	4,42E-02
cg17199223	1	178063098	RASAL2	5'UTR	0,009	6,64E-04	4,42E-02
cg04990962	14	75296265	YLPM1	Body	-0,006	6,64E-04	4,42E-02
cg07958984	1	39249567		IGR	-0,063	6,64E-04	4,42E-02
cg13049917	6	25489821	LRRC16A	Body	0,014	6,65E-04	4,43E-02
cg08820315	15	79394430		IGR	-0,043	6,65E-04	4,43E-02
cg00156743	2	95722243		IGR	-0,012	6,65E-04	4,43E-02
cg11693410	12	49378904		IGR	0,013	6,65E-04	4,43E-02
cg17026052	6	35464571	TEAD3	5'UTR	-0,005	6,66E-04	4,43E-02
cg22121570	15	37176940	LOC145845	Body	0,036	6,66E-04	4,43E-02
cg15796392	20	31062480	NOL4L	Body	0,026	6,66E-04	4,43E-02
cg09988995	14	73700839		IGR	-0,027	6,66E-04	4,43E-02
cg13541540	19	56124269	ZNF865	TSS1500	-0,007	6,66E-04	4,43E-02
cg13554708	15	75871745	PTPN9	TSS200	-0,003	6,66E-04	4,43E-02
cg14998917	12	22777902	ETNK1	TSS200	-0,005	6,66E-04	4,43E-02
cg19900314	12	27728757	PPFIBP1	5'UTR	-0,023	6,66E-04	4,43E-02
cg21104040	13	112856976		IGR	-0,01	6,66E-04	4,43E-02
cg14472708	3	101545039	NXPE3	3'UTR	0,039	6,66E-04	4,43E-02
cg08316204	20	35973919	SRC	TSS1500	0,027	6,66E-04	4,43E-02
cg23567583	6	29932743		IGR	-0,004	6,67E-04	4,43E-02
cg23817866	1	218512841		IGR	-0,022	6,67E-04	4,43E-02
cg01591405	17	18024867	MYO15A	Body	-0,011	6,67E-04	4,43E-02
cg23882790	19	21203004	ZNF430	TSS1500	0,065	6,67E-04	4,44E-02
cg15966253	3	23847480	UBE2E1	5'UTR	-0,003	6,68E-04	4,44E-02
cg17696468	11	31742528	ELP4	Body	0,006	6,68E-04	4,44E-02
cg26232187	15	88799300	NTRK3	Body	-0,004	6,68E-04	4,44E-02
cg04984644	16	3159459		IGR	0,011	6,68E-04	4,44E-02
cg26830473	21	47744718	C21orf58	TSS1500	0,01	6,68E-04	4,44E-02
cg18802758	2	202646102	ALS2	TSS1500	-0,002	6,68E-04	4,44E-02
cg21772161	2	166484055	CSRNP3	Body	-0,006	6,69E-04	4,44E-02
cg25258002	11	57245109		IGR	0,013	6,69E-04	4,44E-02
cg23989584	11	120038521		IGR	-0,011	6,69E-04	4,44E-02
cg26279366	19	40948589	SERTAD3	5'UTR	0,013	6,69E-04	4,44E-02
cg10592563	2	178974175	PDE11A	TSS1500	0,011	6,69E-04	4,44E-02
cg10955522	7	5274975		IGR	-0,024	6,69E-04	4,44E-02
cg21265249	22	45573528	NUP50	Body	-0,006	6,69E-04	4,44E-02
cg08761909	10	95796595	PLCE1	Body	-0,042	6,69E-04	4,44E-02
cg24738274	16	67022428	CES4A	TSS200	0,011	6,69E-04	4,44E-02
cg03478070	20	3689084	SIGLEC1	TSS1500	-0,016	6,69E-04	4,44E-02

cg07667906	14	106329752		IGR	0,047	6,69E-04	4,45E-02
cg01163994	8	57363966		IGR	0,016	6,70E-04	4,45E-02
cg14608778	15	39933963	FSIP1	Body	0,026	6,70E-04	4,45E-02
cg18419125	5	162866149	CCNG1	5'UTR	0,044	6,70E-04	4,45E-02
cg24074448	19	18768688	KLHL26	Body	0,015	6,70E-04	4,45E-02
cg04541617	8	126103739	KIAA0196	5'UTR	-0,002	6,70E-04	4,45E-02
cg04249665	11	8960702	ASCL3	5'UTR	-0,021	6,70E-04	4,45E-02
cg08490387	14	77392402		IGR	0,024	6,70E-04	4,45E-02
cg13033164	15	89079634	DET1	Body	-0,006	6,70E-04	4,45E-02
cg06622857	9	24545490	IZUMO3	1stExon	-0,023	6,70E-04	4,45E-02
cg25781385	6	5507831	FARS2	Body	-0,017	6,71E-04	4,45E-02
cg10328047	8	145024774	PLEC1	Body	0,012	6,70E-04	4,45E-02
cg02872545	6	30923697		IGR	-0,003	6,71E-04	4,45E-02
cg21053135	7	150499737	TMEM176B	TSS1500	-0,007	6,71E-04	4,45E-02
cg26337602	17	792516	NXN	Body	-0,039	6,71E-04	4,45E-02
cg11005982	4	27140053		IGR	-0,022	6,71E-04	4,45E-02
cg04265672	5	179402240	RNF130	Body	0,01	6,71E-04	4,45E-02
cg13817518	9	2424726		IGR	-0,012	6,71E-04	4,45E-02
cg01339103	1	64758157		IGR	0,04	6,72E-04	4,45E-02
cg25859441	4	21953738		IGR	-0,007	6,72E-04	4,45E-02
cg17414511	2	226557276		IGR	0,037	6,72E-04	4,45E-02
cg27180890	22	48820213		IGR	-0,02	6,72E-04	4,45E-02
cg12647821	2	178129899	NFE2L2	TSS1500	-0,004	6,72E-04	4,45E-02
cg12930100	3	181428697	SOX2OT	Body	-0,005	6,72E-04	4,45E-02
cg20774191	4	165851859		IGR	-0,007	6,72E-04	4,45E-02
cg22302675	7	157742562	PTPRN2	Body	-0,042	6,72E-04	4,45E-02
cg14977875	5	2008364		IGR	-0,038	6,72E-04	4,45E-02
cg24899508	1	11938116		IGR	0,023	6,73E-04	4,45E-02
cg05723902	14	104577028	ASPG	Body	0,023	6,72E-04	4,45E-02
cg24979576	1	179744527	FAM163A	5'UTR	-0,018	6,73E-04	4,46E-02
cg06331115	5	162383753		IGR	-0,026	6,73E-04	4,46E-02
cg26286043	13	49799710		IGR	0,057	6,73E-04	4,46E-02
cg18582689	6	55192338	GFRAL	5'UTR	0,034	6,73E-04	4,46E-02
cg02620645	9	28439802	LINGO2	5'UTR	-0,024	6,73E-04	4,46E-02
cg17232592	1	89829463	GBP6	1stExon	-0,029	6,74E-04	4,46E-02
cg05082203	2	164012634		IGR	0,031	6,74E-04	4,46E-02
cg01214711	10	134527428	INPP5A	Body	-0,011	6,74E-04	4,46E-02
cg02330339	15	40558247	PAK6	Body	0,005	6,74E-04	4,46E-02
cg10354614	16	81773158		IGR	0,029	6,73E-04	4,46E-02
cg10542482	18	74784021	MBP	Body	-0,031	6,74E-04	4,46E-02
cg18945842	22	42306589		IGR	-0,006	6,74E-04	4,46E-02
cg19677630	3	10527231	ATP2B2	5'UTR	0,032	6,74E-04	4,46E-02
cg01113040	17	42440939	FAM171A2	Body	0,005	6,74E-04	4,46E-02
cg03630596	9	139925856	C9orf139	5'UTR	0,023	6,74E-04	4,46E-02
cg01377128	1	20513402	UBXN10-AS1	TSS1500	0,029	6,76E-04	4,46E-02
cg16395432	1	26880807	MIR1976	TSS1500	0,037	6,75E-04	4,46E-02
cg22699447	1	107359588		IGR	0,034	6,76E-04	4,46E-02
cg01301044	1	110950379	LAMTOR5	1stExon	0,004	6,76E-04	4,46E-02
cg15538837	1	226250361	H3F3A	TSS200	-0,004	6,76E-04	4,46E-02
cg04809596	2	40078792		IGR	-0,007	6,75E-04	4,46E-02
cg25068958	3	33931123		IGR	0,017	6,76E-04	4,46E-02
cg18144654	3	127995346	EEFSEC	Body	-0,015	6,75E-04	4,46E-02
cg04102784	5	81152212		IGR	0,009	6,75E-04	4,46E-02
cg14446066	7	78007112	MAGI2	Body	-0,007	6,76E-04	4,46E-02
cg12599168	7	142554539	EPHB6	5'UTR	-0,025	6,76E-04	4,46E-02
cg02846517	8	53106786	ST18	Body	-0,072	6,75E-04	4,46E-02
cg19459876	11	2292477	ASCL2	TSS1500	-0,006	6,75E-04	4,46E-02
cg05226335	11	70253499	CTTN	Body	0,073	6,75E-04	4,46E-02
cg07617482	13	36245096	MIR548F5	Body	-0,011	6,75E-04	4,46E-02
cg11435848	16	21315232	CRYM-AS1	Body	-0,007	6,76E-04	4,46E-02
cg04393637	17	75790091		IGR	-0,011	6,76E-04	4,46E-02
cg11558566	19	7413805		IGR	-0,002	6,75E-04	4,46E-02
cg23323816	20	57181782	APCDD1L-AS1	Body	-0,009	6,76E-04	4,46E-02
cg02571448	21	47294762	PCBP3	Body	-0,028	6,75E-04	4,46E-02
cg03389890	22	16868045		IGR	-0,051	6,76E-04	4,46E-02
cg06574501	19	512497	TPGS1	Body	-0,073	6,76E-04	4,46E-02
cg14318680	9	95219836	CENPP	Body	0,012	6,77E-04	4,47E-02
cg05312712	22	24563123	CABIN1	Body	0,017	6,77E-04	4,47E-02
cg05803265	1	54352207	YIPF1	Body	0,012	6,77E-04	4,47E-02
cg08598454	12	25056127	BCAT1	TSS1500	-0,003	6,77E-04	4,47E-02
cg16332332	1	11592420	PTCHD2	Body	0,024	6,77E-04	4,47E-02
cg07396407	1	178514897	C1orf220	Body	0,003	6,77E-04	4,47E-02
cg12561746	1	219607263		IGR	0,043	6,77E-04	4,47E-02

cg16006934	12	120907680	DYNLL1	1stExon	0,007	6,77E-04	4,47E-02
cg15437457	12	1100385	ERC1	TSS200	0,004	6,77E-04	4,47E-02
cg20821187	1	200452917		IGR	-0,042	6,78E-04	4,47E-02
cg06741198	6	150039666	LATS1	TSS1500	-0,004	6,78E-04	4,47E-02
cg07898656	11	70173356	PPFIA1	Body	0,01	6,78E-04	4,47E-02
cg26884787	16	88543447	ZFPM1	Body	-0,015	6,78E-04	4,47E-02
cg02422689	19	45873808	ERCC2	5'UTR	-0,005	6,78E-04	4,47E-02
cg02539911	17	58677246	PPM1D	TSS1500	-0,005	6,78E-04	4,47E-02
cg01922269	4	111219469		IGR	0,024	6,79E-04	4,47E-02
cg10547505	5	149590096	SLC6A7	3'UTR	0,008	6,78E-04	4,47E-02
cg13468318	9	133874121		IGR	-0,026	6,79E-04	4,47E-02
cg15387099	10	71624209	COL13A1	Body	0,009	6,78E-04	4,47E-02
cg06930095	15	85259484	SEC11A	1stExon	-0,004	6,79E-04	4,47E-02
cg25013053	17	33475031	UNC45B	5'UTR	0,007	6,79E-04	4,47E-02
cg23233468	17	37027923	LASP1	5'UTR	-0,003	6,79E-04	4,47E-02
cg00510948	19	2730590		IGR	0,013	6,79E-04	4,47E-02
cg24494876	19	47921401	MEIS3	Body	-0,014	6,79E-04	4,47E-02
cg13042802	5	22889930		IGR	0,039	6,79E-04	4,47E-02
cg01519900	4	155265901	DCHS2	Body	0,007	6,79E-04	4,47E-02
cg20797316	5	81670873		IGR	-0,011	6,79E-04	4,47E-02
cg12305431	7	27157855	HOXA3	5'UTR	0,006	6,79E-04	4,47E-02
cg16509392	11	110562137	ARHGAP20	5'UTR	-0,007	6,79E-04	4,47E-02
cg07535903	3	97594844	CRYBG3	TSS1500	-0,007	6,79E-04	4,47E-02
cg02150126	7	20443181	ITGB8	Body	0,011	6,79E-04	4,47E-02
cg21094614	7	16691852	BZW2	5'UTR	-0,013	6,79E-04	4,47E-02
cg08967516	19	11851060	ZNF823	TSS1500	0,009	6,80E-04	4,47E-02
cg27552081	17	25621774	WSB1	Body	-0,005	6,80E-04	4,48E-02
cg07792529	1	200143149	NR5A2	Body	0,014	6,80E-04	4,48E-02
cg06348206	2	198062669	ANKRD44	Body	0,021	6,80E-04	4,48E-02
cg16263705	7	143805919	OR2A2	TSS1500	0,018	6,80E-04	4,48E-02
cg08363684	6	761547		IGR	-0,02	6,80E-04	4,48E-02
cg03104878	1	67240174	TCTEX1D1	Body	0,022	6,80E-04	4,48E-02
cg14275894	3	69436791	FRMD4B	TSS1500	-0,005	6,81E-04	4,48E-02
cg13134407	3	176535538	LINC01209	TSS1500	-0,014	6,81E-04	4,48E-02
cg06061240	1	51800535	TTC39A	Body	0,011	6,81E-04	4,48E-02
cg22510961	2	169668175	NOSTRIN	ExonBnd	0,039	6,81E-04	4,48E-02
cg11954557	6	32809266	PSMB8	Body	0,023	6,81E-04	4,48E-02
cg11044316	9	91768792	SHC3	Body	-0,027	6,81E-04	4,48E-02
cg14011554	9	128657810	PBX3	Body	0,009	6,81E-04	4,48E-02
cg09252596	10	105811592	COL17A1	Body	0,021	6,81E-04	4,48E-02
cg16746770	12	15580907	PTPRO	Body	0,026	6,81E-04	4,48E-02
cg04770392	12	49226625	DDX23	Body	-0,02	6,81E-04	4,48E-02
cg24388941	12	77718302		IGR	-0,016	6,81E-04	4,48E-02
cg11736942	14	105054436	C14orf180	Body	0,015	6,81E-04	4,48E-02
cg24136928	20	21140654	KIZ	Body	0,006	6,81E-04	4,48E-02
cg06856282	20	51217902		IGR	0,012	6,81E-04	4,48E-02
cg22477023	2	213038668	ERBB4	Body	-0,008	6,82E-04	4,48E-02
cg03105520	3	114070056	ZBTB20-AS1	TSS1500	-0,005	6,82E-04	4,48E-02
cg02106828	1	236959224	MTR	Body	0,005	6,82E-04	4,48E-02
cg05131207	15	100223274	MEF2A	Body	-0,037	6,82E-04	4,48E-02
cg05577736	18	8766439	MTCL1	Body	-0,012	6,82E-04	4,48E-02
cg09856472	15	39803760		IGR	-0,023	6,82E-04	4,48E-02
cg19718471	7	73735689	CLIP2	Body	0,008	6,82E-04	4,48E-02
cg08794582	5	1524227	LPCAT1	TSS200	-0,003	6,83E-04	4,48E-02
cg25962947	11	102323538	TMEM123	1stExon	0,013	6,83E-04	4,48E-02
cg19192981	15	57664704		IGR	-0,144	6,83E-04	4,48E-02
cg20256375	13	112730681		IGR	-0,078	6,83E-04	4,48E-02
cg20216887	17	49323893	MBTD1	5'UTR	0,017	6,83E-04	4,48E-02
cg21041476	22	34415292		IGR	-0,025	6,83E-04	4,48E-02
cg20704450	1	228658371		IGR	-0,065	6,84E-04	4,48E-02
cg27258823	5	151152059	G3BP1	5'UTR	-0,002	6,84E-04	4,48E-02
cg22797285	10	113786712		IGR	-0,029	6,84E-04	4,48E-02
cg09492169	17	43482735	ARHGAP27	Body	-0,012	6,83E-04	4,48E-02
cg01105969	2	106721344	UXS1	Body	0,016	6,84E-04	4,48E-02
cg00088445	2	74361349		IGR	0,007	6,84E-04	4,49E-02
cg18458331	2	239321216		IGR	-0,013	6,84E-04	4,49E-02
cg17591926	12	59136604	LOC101927653	Body	-0,022	6,84E-04	4,49E-02
cg13331246	1	6521856	TNFRSF25	Body	-0,009	6,84E-04	4,49E-02
cg11164846	6	34005998	GRM4	Body	0,006	6,84E-04	4,49E-02
cg06403510	8	120868436	DSCC1	TSS1500	-0,004	6,85E-04	4,49E-02
cg24838864	19	11598009	ZNF653	Body	0,011	6,84E-04	4,49E-02
cg03343681	1	2731578		IGR	0,014	6,85E-04	4,49E-02
cg15003812	5	34899275	TTC23L	3'UTR	-0,008	6,85E-04	4,49E-02

cg12831116	6	139094969	CCDC28A	1stExon	-0,004	6,85E-04	4,49E-02
cg22685901	6	148668023	SASH1	Body	-0,026	6,85E-04	4,49E-02
cg25473181	2	11300636	PQLC3	Body	-0,005	6,85E-04	4,49E-02
cg01889554	2	136362398	R3HDM1	5'UTR	0,037	6,86E-04	4,49E-02
cg02592999	8	93890266		IGR	0,02	6,85E-04	4,49E-02
cg11549894	8	111907068		IGR	-0,016	6,85E-04	4,49E-02
cg06417962	11	507970	RNH1	TSS1500	-0,032	6,85E-04	4,49E-02
cg19783150	12	22491533		IGR	0,008	6,86E-04	4,49E-02
cg03523485	12	132852593	GALNT9	Body	-0,036	6,85E-04	4,49E-02
cg20446176	17	6922932	MIR497HG	Body	0,019	6,85E-04	4,49E-02
cg04348305	20	25230959	PYGB	Body	-0,009	6,86E-04	4,49E-02
cg15061287	7	99047880	CPSF4	ExonBnd	0,013	6,86E-04	4,49E-02
cg15553268	17	19146105	EPN2	5'UTR	0,015	6,86E-04	4,49E-02
cg10365572	5	141245930	PCDH1	TSS200	0,013	6,86E-04	4,49E-02
cg14826721	9	33023719	DNAJA1	TSS1500	-0,004	6,86E-04	4,49E-02
cg26051775	1	223945170	CAPN2	Body	-0,008	6,86E-04	4,49E-02
cg10459430	1	93685227	CCDC18	Body	-0,05	6,87E-04	4,49E-02
cg13800802	9	98619666	C9orf130	Body	0,025	6,87E-04	4,49E-02
cg03299208	15	80452147	FAH	Body	0,023	6,87E-04	4,49E-02
cg10036075	16	85345476		IGR	0,019	6,87E-04	4,49E-02
cg07356861	16	89184285	ACSF3	Body	0,006	6,87E-04	4,49E-02
cg14153771	22	46753015	TRMU	3'UTR	-0,012	6,87E-04	4,49E-02
cg14585694	11	120081506	OAF	TSS1500	-0,004	6,87E-04	4,49E-02
cg26782884	2	148804219	MBD5	5'UTR	0,006	6,87E-04	4,49E-02
cg03253831	4	119199842	SNHG8	TSS200	0,008	6,87E-04	4,50E-02
cg01067385	7	121634758	PTPRZ1	Body	-0,009	6,88E-04	4,50E-02
cg04244624	1	85991560	DDAH1	5'UTR	-0,006	6,88E-04	4,50E-02
cg10575128	3	179881046		IGR	-0,015	6,88E-04	4,50E-02
cg09080361	4	163969292		IGR	-0,009	6,88E-04	4,50E-02
cg06144274	10	15174993	NMT2	Body	-0,006	6,88E-04	4,50E-02
cg23714773	7	808081	HEATR2	Body	-0,009	6,88E-04	4,50E-02
cg09663430	22	18251942	BID	5'UTR	0,027	6,88E-04	4,50E-02
cg01272565	1	233497906	KIAA1804	Body	0,01	6,88E-04	4,50E-02
cg20759626	13	74250870		IGR	0,008	6,88E-04	4,50E-02
cg15927863	15	61473418	RORA	Body	-0,008	6,89E-04	4,50E-02
cg18765724	17	46227039	SKAP1	Body	-0,01	6,89E-04	4,50E-02
cg26025657	11	46655595	ATG13	5'UTR	0,011	6,89E-04	4,50E-02
cg20461738	2	134877431		IGR	0,006	6,89E-04	4,50E-02
cg23130368	3	57479362	DNAH12	Body	0,046	6,89E-04	4,50E-02
cg23524184	17	48129754		IGR	0,004	6,89E-04	4,50E-02
cg26427915	10	33224866	ITGB1	TSS1500	0,028	6,90E-04	4,50E-02
cg24172509	5	140778424	PCDHGA4	Body	-0,049	6,90E-04	4,51E-02
cg17483598	12	10538268	LOC101928100	Body	-0,025	6,90E-04	4,51E-02
cg16044222	15	48280471		IGR	0,007	6,90E-04	4,51E-02
cg08977842	17	81008497	B3GNTL1	Body	0,012	6,90E-04	4,51E-02
cg20039979	19	52960806	ZNF578	5'UTR	-0,027	6,90E-04	4,51E-02
cg02799404	1	25501453		IGR	0,014	6,91E-04	4,51E-02
cg22339151	13	24721982	SPATA13	Body	-0,015	6,91E-04	4,51E-02
cg10512498	4	176986333	WDR17	TSS1500	0,014	6,91E-04	4,51E-02
cg11226904	2	214671150	SPAG16	Body	0,04	6,91E-04	4,51E-02
cg04845185	20	61696220		IGR	-0,012	6,91E-04	4,51E-02
cg16102251	1	181639003	CACNA1E	Body	-0,024	6,92E-04	4,51E-02
cg06183267	2	100759134	AFF3	TSS200	-0,015	6,92E-04	4,51E-02
cg09649266	2	88583529		IGR	-0,041	6,92E-04	4,51E-02
cg12216448	22	46759962	CELSR1	Body	0,006	6,92E-04	4,51E-02
cg09998650	2	227292478		IGR	-0,019	6,92E-04	4,51E-02
cg26902746	1	180941643		IGR	-0,007	6,92E-04	4,51E-02
cg00665816	9	72131278	APBA1	Body	-0,007	6,92E-04	4,51E-02
cg15838464	17	38149252	PSMD3	Body	0,021	6,92E-04	4,51E-02
cg00630115	19	45560598	CLASRP	Body	0,016	6,92E-04	4,51E-02
cg07493157	4	40609287	RBM47	5'UTR	-0,01	6,93E-04	4,51E-02
cg04208996	8	144488011		IGR	0,004	6,93E-04	4,51E-02
cg27146731	11	30314381		IGR	0,007	6,93E-04	4,51E-02
cg26066917	11	117072446	LOC100652768	Body	0,026	6,93E-04	4,51E-02
cg25315648	5	159378478	ADRA1B	Body	-0,041	6,93E-04	4,52E-02
cg18534115	12	52295400		IGR	-0,021	6,93E-04	4,52E-02
cg26545489	3	130746981	NEK11	5'UTR	-0,006	6,93E-04	4,52E-02
cg12787185	21	28821354	MIR5009	Body	-0,023	6,93E-04	4,52E-02
cg02647406	4	20704940	PACRGL	5'UTR	-0,01	6,93E-04	4,52E-02
cg15006101	5	140627399	PCDHB15	1stExon	-0,04	6,93E-04	4,52E-02
cg00583513	16	80637780	CDYL2	3'UTR	0,006	6,94E-04	4,52E-02
cg13408214	17	40823572	PLEKHH3	Body	-0,047	6,94E-04	4,52E-02
cg17172090	6	2926650		IGR	-0,017	6,94E-04	4,52E-02

cg25924421	7	23695323		IGR	0,01	6,94E-04	4,52E-02
cg12489205	9	125156765	PTGS1	3'UTR	-0,01	6,94E-04	4,52E-02
cg21221497	4	154049212		IGR	-0,01	6,94E-04	4,52E-02
cg23626670	4	185767962	LOC731424	Body	0,012	6,94E-04	4,52E-02
cg16552437	5	139554476	C5orf32	TSS200	-0,009	6,94E-04	4,52E-02
cg00396677	11	33278679	HIPK3	TSS1500	-0,003	6,94E-04	4,52E-02
cg05595517	17	12763025	ARHGAP44	Body	-0,041	6,94E-04	4,52E-02
cg00582080	10	5810308	GDI2	Body	-0,009	6,95E-04	4,52E-02
cg18752327	3	169404841		IGR	-0,012	6,95E-04	4,52E-02
cg16851467	4	4427496	STX18	Body	-0,018	6,95E-04	4,52E-02
cg02216202	6	35477666	TULP1	Body	0,019	6,95E-04	4,52E-02
cg15466977	7	6728225	ZNF12	3'UTR	-0,005	6,95E-04	4,52E-02
cg17479357	7	43769596	COA1	TSS1500	-0,01	6,95E-04	4,52E-02
cg02568083	8	71516655	TRAM1	Body	0,022	6,95E-04	4,52E-02
cg13022129	9	131683862	PHYHD1	1stExon	0,011	6,95E-04	4,52E-02
cg02302348	10	3542628		IGR	-0,01	6,95E-04	4,52E-02
cg13753386	13	41040865	LINC00598	Body	-0,009	6,95E-04	4,52E-02
cg26862469	14	22217315		IGR	-0,006	6,95E-04	4,52E-02
cg01718322	14	96342365		IGR	-0,026	6,95E-04	4,52E-02
cg12246608	17	86461	RPH3AL	Body	-0,007	6,95E-04	4,52E-02
cg05794042	18	45687771		IGR	0,01	6,95E-04	4,52E-02
cg10996527	8	141994633	PTK2	5'UTR	0,009	6,96E-04	4,52E-02
cg23914328	15	29393367	APBA2	Body	0,015	6,96E-04	4,52E-02
cg09116560	11	18814421	PTPN5	TSS1500	-0,02	6,96E-04	4,52E-02
cg05783554	2	232466435		IGR	0,01	6,96E-04	4,52E-02
cg00306378	2	239925720		IGR	-0,006	6,97E-04	4,53E-02
cg04687337	4	184431586	ING2	Body	-0,005	6,97E-04	4,53E-02
cg11825501	7	4168039	SDK1	Body	-0,012	6,97E-04	4,53E-02
cg11715064	8	128095984	PRNCR1	Body	-0,006	6,97E-04	4,53E-02
cg16159605	10	17109039	CUBN	Body	-0,006	6,97E-04	4,53E-02
cg26160008	12	6451035	TNFRSF1A	5'UTR	-0,004	6,97E-04	4,53E-02
cg12393861	13	102851292	FGF14	Body	-0,034	6,97E-04	4,53E-02
cg22687511	13	112883974		IGR	-0,019	6,97E-04	4,53E-02
cg12475170	18	33075258	INO80C	Body	-0,012	6,97E-04	4,53E-02
cg12111164	5	132868977	FSTL4	Body	-0,013	6,97E-04	4,53E-02
cg07790813	18	46225994	CTIF	Body	-0,013	6,97E-04	4,53E-02
cg24580782	1	27240365	NR0B2	1stExon	-0,011	6,97E-04	4,53E-02
cg26004639	4	8389845	ACOX3	Body	-0,007	6,98E-04	4,53E-02
cg19562210	6	43274578	CRIP3	Body	0,014	6,98E-04	4,53E-02
cg12738981	1	155828357	SYT11	TSS1500	0,015	6,98E-04	4,53E-02
cg25374629	9	102131421	NAMA	Body	-0,005	6,98E-04	4,53E-02
cg08567104	13	111359719	CARS2	TSS1500	0,008	6,98E-04	4,53E-02
cg24083367	20	48428997	SLC9A8	TSS1500	-0,023	6,98E-04	4,53E-02
cg10009757	17	77207289	HRNBP3	5'UTR	0,02	6,98E-04	4,53E-02
cg15247955	8	104497747		IGR	0,025	6,99E-04	4,53E-02
cg05897505	20	5752329	C20orf196	5'UTR	0,014	6,99E-04	4,53E-02
cg06880836	4	54441786	LNX1	5'UTR	0,012	6,99E-04	4,53E-02
cg12179044	12	120632364	GCN1L1	Body	-0,002	6,99E-04	4,54E-02
cg04984128	3	110062428		IGR	-0,007	7,00E-04	4,54E-02
cg14421717	5	54603624	DHX29	TSS200	-0,002	7,00E-04	4,54E-02
cg20574687	8	116111705		IGR	-0,021	7,00E-04	4,54E-02
cg09814233	9	33447628	AQP3	1stExon	-0,003	7,00E-04	4,54E-02
cg00846799	11	65316546	LTBP3	Body	-0,008	7,00E-04	4,54E-02
cg25535982	11	74030400		IGR	-0,019	7,00E-04	4,54E-02
cg21448167	12	3333872	TSPAN9	Body	0,004	7,00E-04	4,54E-02
cg14058711	12	106751671	POLR3B	TSS200	-0,003	7,00E-04	4,54E-02
cg03051310	12	108954266	SART3	Body	-0,003	7,00E-04	4,54E-02
cg21935521	22	20869479	MED15	5'UTR	0,03	7,00E-04	4,54E-02
cg12104689	17	58600131	APPBP2	Body	-0,006	7,01E-04	4,54E-02
cg02738205	16	82741062	CDH13	Body	-0,023	7,01E-04	4,54E-02
cg18849036	10	79182708	KCNMA1	Body	-0,041	7,01E-04	4,54E-02
cg24831125	7	77687711	MAGI2	Body	-0,046	7,01E-04	4,54E-02
cg26636103	11	83502111	DLG2	Body	-0,007	7,01E-04	4,54E-02
cg23182573	2	9983397	TAF1B	TSS200	-0,007	7,01E-04	4,54E-02
cg14473123	8	48921407	UBE2V2	Body	-0,004	7,01E-04	4,54E-02
cg25590628	11	67291280	CABP2	TSS1500	0,008	7,01E-04	4,54E-02
cg24927883	15	89973967		IGR	-0,004	7,01E-04	4,54E-02
cg00716848	2	3633875		IGR	-0,006	7,02E-04	4,54E-02
cg12554481	6	56347485	DST	ExonBnd	-0,007	7,01E-04	4,54E-02
cg25937919	1	210614432	HHAT	Body	0,012	7,02E-04	4,54E-02
cg18394684	3	46224928		IGR	0,013	7,02E-04	4,54E-02
cg07163456	3	192636424	C3orf59	TSS1500	-0,053	7,02E-04	4,54E-02
cg10647661	5	142252696	ARHGAP26	Body	0,035	7,02E-04	4,54E-02

cg27422345	7	1715242		IGR	0,024	7,02E-04	4,54E-02
cg11737750	7	71730541	CALN1	Body	-0,027	7,02E-04	4,54E-02
cg09074468	9	90589806	CDK20	TSS200	-0,007	7,02E-04	4,54E-02
cg13573626	14	105858487	PACS2	Body	0,015	7,02E-04	4,54E-02
cg04903916	6	33638413	ITPR3	Body	0,008	7,03E-04	4,54E-02
cg19593949	1	205312444	KLHDC8A	Body	-0,02	7,03E-04	4,55E-02
cg09918756	2	122283716	CLASP1	Body	-0,005	7,03E-04	4,55E-02
cg02307664	2	134586281		IGR	-0,013	7,03E-04	4,55E-02
cg03545101	4	109894258	COL25A1	Body	-0,022	7,03E-04	4,55E-02
cg17386433	1	28052708	FAM76A	Body	0,003	7,03E-04	4,55E-02
cg08911852	16	7917211		IGR	-0,039	7,03E-04	4,55E-02
cg18497492	16	68015076	DPEP3	TSS1500	-0,007	7,03E-04	4,55E-02
cg26402527	3	10526551	ATP2B2	5'UTR	0,029	7,04E-04	4,55E-02
cg22994555	10	73111419	SLC29A3	Body	-0,065	7,04E-04	4,55E-02
cg03897905	2	137814066	THSD7B	Body	-0,02	7,04E-04	4,55E-02
cg02968405	5	168690633	SLIT3	Body	-0,015	7,04E-04	4,55E-02
cg26203738	2	216673958	LINC00607	Body	0,018	7,04E-04	4,55E-02
cg07603382	7	93521153	TFPI2	TSS1500	0,05	7,04E-04	4,55E-02
cg02481848	8	91942157	NECAB1	Body	-0,013	7,04E-04	4,55E-02
cg11648212	2	55091021	EML6	ExonBnd	0,011	7,05E-04	4,55E-02
cg27323568	3	49451217	TCTA	Body	0,014	7,05E-04	4,55E-02
cg21071793	3	11421381	ATG7	Body	-0,024	7,05E-04	4,55E-02
cg05652141	3	127058958		IGR	0,041	7,05E-04	4,55E-02
cg24153132	11	75110278	RPS3	TSS1500	-0,007	7,05E-04	4,55E-02
cg06444189	12	6953740	GNB3	Body	-0,017	7,05E-04	4,55E-02
cg01107240	16	74054182		IGR	0,038	7,05E-04	4,55E-02
cg04231306	18	25358921		IGR	-0,036	7,05E-04	4,55E-02
cg00981651	20	44574847	PCIF1	Body	0,021	7,06E-04	4,55E-02
cg07106432	2	27593462	SNX17	1stExon	-0,004	7,06E-04	4,55E-02
cg00647899	5	62983112		IGR	-0,008	7,06E-04	4,55E-02
cg04006554	6	46138530	ENPP5	5'UTR	-0,003	7,06E-04	4,55E-02
cg10824926	11	58651984		IGR	0,016	7,06E-04	4,55E-02
cg07045199	13	24463743	PCOTH	5'UTR	-0,007	7,06E-04	4,55E-02
cg19846825	17	4465386	GGT6	TSS1500	0,024	7,06E-04	4,55E-02
cg19772783	18	32211127	DTNA	5'UTR	0,011	7,06E-04	4,55E-02
cg01076704	20	31127744		IGR	-0,004	7,06E-04	4,55E-02
cg04720797	5	140480223	PCDHB3	TSS200	-0,066	7,06E-04	4,55E-02
cg08984686	6	33267143	RGL2	5'UTR	-0,004	7,06E-04	4,55E-02
cg00760847	18	3262519	MYL12B	5'UTR	-0,005	7,06E-04	4,55E-02
cg07833865	1	46657852	POMGNT1	Body	-0,005	7,07E-04	4,56E-02
cg07134367	10	91404689	PANK1	TSS1500	-0,004	7,07E-04	4,56E-02
cg11455895	1	94074463	BCAR3	Body	0,01	7,07E-04	4,56E-02
cg16688437	2	208632990	FZD5	Body	0,021	7,07E-04	4,56E-02
cg02207312	11	60674164	PRPF19	TSS200	0,004	7,08E-04	4,56E-02
cg02532113	6	82479357		IGR	-0,016	7,08E-04	4,56E-02
cg02387346	16	31503940	C16orf58	Body	-0,005	7,08E-04	4,56E-02
cg14022857	7	11580000	THSD7A	Body	-0,014	7,08E-04	4,56E-02
cg21626923	10	15493985		IGR	-0,048	7,08E-04	4,56E-02
cg23825522	3	45646427	LIMD1	Body	-0,005	7,08E-04	4,56E-02
cg17929169	6	112195522	FYN	TSS1500	0,008	7,08E-04	4,56E-02
cg06800578	1	44683416	DMAP1	Body	-0,015	7,09E-04	4,57E-02
cg20451868	1	64240845	ROR1	Body	-0,008	7,09E-04	4,57E-02
cg00765967	3	98104239		IGR	-0,021	7,09E-04	4,57E-02
cg01488865	20	61569319	C20orf11	TSS200	0,003	7,09E-04	4,57E-02
cg11110570	22	45405067	PHF21B	TSS200	-0,004	7,09E-04	4,57E-02
cg01423147	1	89458345	RBMXL1	1stExon	-0,003	7,09E-04	4,57E-02
cg26151662	11	86884341	TMEM135	Body	-0,006	7,09E-04	4,57E-02
cg14231861	3	145613781		IGR	0,01	7,10E-04	4,57E-02
cg00624150	5	134240771	PCBD2	TSS200	-0,004	7,10E-04	4,57E-02
cg26764953	6	53591212		IGR	0,076	7,09E-04	4,57E-02
cg23297909	9	36471011		IGR	-0,002	7,09E-04	4,57E-02
cg16570051	7	44145692	AEBP1	Body	0,022	7,10E-04	4,57E-02
cg07268200	1	231337208	TRIM67	Body	-0,007	7,10E-04	4,57E-02
cg04049225	16	85672175	KIAA0182	5'UTR	0,019	7,10E-04	4,57E-02
cg01831872	2	36682726	CRIM1	Body	0,011	7,10E-04	4,57E-02
cg21918062	14	79177505	NRXN3	Body	-0,024	7,10E-04	4,57E-02
cg25986116	1	213571266		IGR	-0,033	7,11E-04	4,57E-02
cg11186468	19	36110654	HAUS5	ExonBnd	-0,022	7,11E-04	4,57E-02
cg26086271	15	41913343		IGR	0,006	7,11E-04	4,57E-02
cg15061260	6	93498994		IGR	0,011	7,11E-04	4,57E-02
cg13738384	20	45216795	SLC13A3	ExonBnd	0,012	7,11E-04	4,57E-02
cg17645978	5	86042255		IGR	0,015	7,11E-04	4,57E-02
cg23461604	12	49239381	DDX23	Body	-0,004	7,11E-04	4,57E-02

cg07459363	5	11271631	CTNND2	Body	-0,015	7,11E-04	4,57E-02
cg22572902	4	95972790	BMPR1B	TSS200	-0,011	7,12E-04	4,58E-02
cg06934092	7	93205284	CALCR	TSS1500	-0,023	7,12E-04	4,58E-02
cg07339200	10	29999893	SVIL	5'UTR	0,005	7,12E-04	4,58E-02
cg26036199	1	153940256	SLC39A1	TSS200	-0,006	7,13E-04	4,58E-02
cg24120669	5	140529819	PCDHB6	TSS200	-0,064	7,12E-04	4,58E-02
cg10233076	6	153184671		IGR	0,009	7,13E-04	4,58E-02
cg15895294	7	38756104		IGR	-0,006	7,12E-04	4,58E-02
cg18609971	7	46426616		IGR	-0,035	7,12E-04	4,58E-02
cg02985515	7	51208083	COBL	Body	0,016	7,12E-04	4,58E-02
cg10029396	7	137718047		IGR	-0,006	7,12E-04	4,58E-02
cg23541232	11	90222171	DISC1FP1	Body	-0,026	7,13E-04	4,58E-02
cg03985140	12	7183010		IGR	0,019	7,12E-04	4,58E-02
cg24744710	12	75524725	KCNC2	Body	0,02	7,13E-04	4,58E-02
cg12642431	15	70994672	UACA	Body	0,006	7,13E-04	4,58E-02
cg23226397	17	1657480	SERPINF2	Body	0,004	7,12E-04	4,58E-02
cg04745442	9	100365943	TSTD2	Body	-0,009	7,13E-04	4,58E-02
cg12592359	20	30944839	ASXL1	TSS1500	0,047	7,13E-04	4,58E-02
cg18958244	2	216340596		IGR	0,013	7,13E-04	4,58E-02
cg02918483	4	84406202	FAM175A	1stExon	0,006	7,13E-04	4,58E-02
cg06246312	4	86965186	MAPK10	Body	-0,005	7,13E-04	4,58E-02
cg08658781	10	131570172		IGR	-0,007	7,13E-04	4,58E-02
cg03087937	11	26593556	MUC15	5'UTR	-0,008	7,14E-04	4,58E-02
cg06975311	11	65407399	SIPA1	TSS200	0,011	7,14E-04	4,58E-02
cg13672871	13	27317381		IGR	-0,05	7,14E-04	4,58E-02
cg16008164	18	218233	THOC1	Body	-0,005	7,13E-04	4,58E-02
cg08467643	3	183890761	DVL3	3'UTR	0,005	7,14E-04	4,58E-02
cg19067800	5	4059295		IGR	-0,042	7,14E-04	4,58E-02
cg23155625	8	141001217	TRAPPC9	Body	0,008	7,14E-04	4,58E-02
cg15450991	12	2879628	LOC283440	Body	0,006	7,14E-04	4,58E-02
cg08327349	22	42483140	NDUFA6	Body	-0,006	7,14E-04	4,58E-02
cg11413763	1	9365494	SPSB1	5'UTR	0,013	7,14E-04	4,58E-02
cg14049858	7	22772224		IGR	0,034	7,14E-04	4,58E-02
cg07369363	12	32112103	C12orf35	TSS1500	-0,002	7,14E-04	4,58E-02
cg02341182	3	33480109	UBP1	Body	0,015	7,15E-04	4,58E-02
cg00615473	21	32930423	TIAM1	5'UTR	-0,008	7,15E-04	4,58E-02
cg27120683	6	26022273	HIST1H4A	3'UTR	-0,004	7,15E-04	4,58E-02
cg27055486	8	21991648		IGR	0,014	7,15E-04	4,58E-02
cg00047753	17	8244936	ODF4	Body	-0,009	7,15E-04	4,58E-02
cg22381128	11	69275403		IGR	-0,012	7,15E-04	4,58E-02
cg05035892	6	25992641		IGR	-0,007	7,15E-04	4,58E-02
cg00222175	22	34049875	LARGE	Body	-0,039	7,15E-04	4,58E-02
cg03821253	19	12911205	PRDX2	Body	0,009	7,16E-04	4,58E-02
cg20183778	7	102105344	ALKBH4	TSS200	0,003	7,16E-04	4,58E-02
cg18655522	14	69424756	ACTN1	Body	0,018	7,16E-04	4,58E-02
cg07231911	1	94141047	BCAR3	5'UTR	0,014	7,16E-04	4,59E-02
cg25774801	1	230516711	PGBD5	Body	-0,006	7,16E-04	4,59E-02
cg01015315	2	39222043	SOS1	Body	0,029	7,16E-04	4,59E-02
cg13501263	15	91498614	RCCD1	5'UTR	-0,003	7,16E-04	4,59E-02
cg24851318	19	38160644	ZNF781	Body	0,008	7,16E-04	4,59E-02
cg24899209	11	58346540	LPXN	TSS1500	-0,005	7,17E-04	4,59E-02
cg10175712	2	210840859	UNC80	Body	-0,011	7,17E-04	4,59E-02
cg26633695	5	139838295	ANKHD1	Body	-0,005	7,17E-04	4,59E-02
cg15843804	2	24788694		IGR	-0,006	7,17E-04	4,59E-02
cg07147383	17	79497869	FSCN2	Body	0,027	7,17E-04	4,59E-02
cg27585939	11	108983607		IGR	0,032	7,18E-04	4,59E-02
cg14236043	10	45567048		IGR	-0,015	7,18E-04	4,59E-02
cg27256468	1	201252824	PKP1	5'UTR	-0,029	7,18E-04	4,59E-02
cg24316913	2	84880828	DNAH6	Body	0,005	7,18E-04	4,59E-02
cg04516858	3	164404063		IGR	-0,013	7,18E-04	4,59E-02
cg15982595	13	113301180	C13orf35	TSS200	0,007	7,18E-04	4,59E-02
cg02191238	1	41296126	KCNQ4	Body	0,008	7,18E-04	4,59E-02
cg10933371	2	227355039		IGR	-0,026	7,18E-04	4,59E-02
cg13803245	12	4061025		IGR	-0,019	7,18E-04	4,59E-02
cg02604346	11	119932041		IGR	-0,033	7,18E-04	4,59E-02
cg13408421	3	122469641	HSPBAP1	Body	0,009	7,18E-04	4,59E-02
cg14997048	20	43991673	SYS1	TSS200	-0,004	7,18E-04	4,59E-02
cg08762919	16	85328980		IGR	-0,006	7,19E-04	4,59E-02
cg12042508	22	35845202		IGR	0,005	7,19E-04	4,59E-02
cg21201294	7	47611926		IGR	0,012	7,19E-04	4,59E-02
cg09173152	17	78359105	RNF213	Body	-0,005	7,19E-04	4,59E-02
cg27098574	16	75285489	BCAR1	Body	0,017	7,19E-04	4,59E-02
cg23531127	21	40289264	LOC400867	Body	-0,011	7,19E-04	4,59E-02

cg00230282	2	219331047	USP37	Body	0,027	7,19E-04	4,59E-02
cg14023926	5	140739532	PCDHGB2	TSS200	-0,013	7,20E-04	4,59E-02
cg22756386	5	176225714		IGR	-0,04	7,19E-04	4,59E-02
cg01951240	10	135122313	ZNF511	TSS200	0,004	7,19E-04	4,59E-02
cg22245980	12	132976722		IGR	0,033	7,19E-04	4,59E-02
cg22474314	15	96870959	NR2F2-AS1	TSS1500	-0,004	7,19E-04	4,59E-02
cg26052783	17	31529254	ASIC2	Body	-0,042	7,19E-04	4,59E-02
cg08019151	3	38836991	SCN10A	TSS1500	0,011	7,20E-04	4,59E-02
cg17286076	8	38785410	PLEKHA2	Body	0,018	7,20E-04	4,59E-02
cg17275287	2	120975021		IGR	0,005	7,20E-04	4,59E-02
cg14383053	2	230451648	DNER	Body	-0,04	7,20E-04	4,59E-02
cg24063411	6	135449953		IGR	-0,036	7,20E-04	4,59E-02
cg24933115	10	75492863		IGR	0,003	7,20E-04	4,59E-02
cg09111484	12	80749651		IGR	-0,02	7,20E-04	4,59E-02
cg25537060	12	99486183	LOC101928937	TSS1500	0,009	7,20E-04	4,59E-02
cg01650266	15	25626275	UBE3A	Body	-0,005	7,20E-04	4,59E-02
cg01555151	14	80679979	DIO2	TSS1500	-0,018	7,20E-04	4,60E-02
cg01145910	1	5729401		IGR	-0,03	7,21E-04	4,60E-02
cg16016583	7	158248795	PTPRN2	Body	0,018	7,21E-04	4,60E-02
cg09270257	17	48572299		IGR	0,025	7,21E-04	4,60E-02
cg08289189	22	19937277	COMT	5'UTR	0,013	7,21E-04	4,60E-02
cg25994838	5	180615482		IGR	-0,01	7,21E-04	4,60E-02
cg02312170	10	122610835	WDR11	5'UTR	-0,003	7,21E-04	4,60E-02
cg05120641	15	41245706	CHAC1	1stExon	-0,003	7,21E-04	4,60E-02
cg24776480	1	2066446	PRKCZ	5'UTR	-0,011	7,21E-04	4,60E-02
cg04785972	3	63263828	SYNPR	TSS200	-0,025	7,21E-04	4,60E-02
cg27454136	4	5889876	CRMP1	Body	-0,01	7,21E-04	4,60E-02
cg01006609	17	3377234	ASPA	TSS200	0,023	7,21E-04	4,60E-02
cg11954813	2	55329546		IGR	0,009	7,22E-04	4,60E-02
cg20692810	2	160472418	BAZ2B	5'UTR	-0,002	7,22E-04	4,60E-02
cg04809899	2	223824915		IGR	0,024	7,22E-04	4,60E-02
cg01363734	12	117482953	TESC	Body	0,056	7,22E-04	4,60E-02
cg23590302	1	171753185	METTL13	Body	0,007	7,22E-04	4,60E-02
cg23387597	10	106093778	ITPRIP	TSS200	-0,011	7,22E-04	4,60E-02
cg20813493	11	118436660	C11orf60	5'UTR	0,007	7,22E-04	4,60E-02
cg21450215	4	147577630		IGR	-0,036	7,22E-04	4,60E-02
cg08391020	5	74344779		IGR	-0,022	7,23E-04	4,60E-02
cg14940260	6	16217882		IGR	0,057	7,23E-04	4,60E-02
cg20105652	7	121512395	PTPRZ1	TSS1500	0,025	7,22E-04	4,60E-02
cg09703323	11	65197876		IGR	-0,008	7,23E-04	4,60E-02
cg17655970	13	112985463		IGR	-0,047	7,23E-04	4,60E-02
cg03219694	19	9416667	ZNF699	TSS1500	-0,008	7,23E-04	4,60E-02
cg02869559	21	36259067	RUNX1	Body	0,054	7,23E-04	4,60E-02
cg17881312	1	17417545	PADI2	Body	-0,016	7,23E-04	4,60E-02
cg16627764	2	545655		IGR	0,029	7,23E-04	4,60E-02
cg17890705	5	54878170		IGR	-0,026	7,23E-04	4,60E-02
cg05803761	6	30737831		IGR	0,019	7,24E-04	4,60E-02
cg27262003	6	155011318		IGR	0,01	7,23E-04	4,60E-02
cg16867817	7	82057703	CACNA2D1	Body	-0,011	7,23E-04	4,60E-02
cg05188967	7	122226613	CADPS2	Body	-0,038	7,23E-04	4,60E-02
cg25260564	10	133985960	JAKMIP3	3'UTR	0,015	7,23E-04	4,60E-02
cg06184251	11	109514984		IGR	-0,052	7,23E-04	4,60E-02
cg12524899	19	31117745		IGR	-0,022	7,23E-04	4,60E-02
cg06736134	5	147040725	JAKMIP2	Body	-0,012	7,24E-04	4,60E-02
cg16015000	6	167623149		IGR	-0,016	7,24E-04	4,60E-02
cg27353293	14	77925696	AHSA1	Body	-0,012	7,24E-04	4,60E-02
cg05720182	1	155239307	CLK2	Body	-0,009	7,24E-04	4,60E-02
cg06852942	5	476497	SLC9A3	Body	0,018	7,24E-04	4,60E-02
cg12968684	5	158246281	EBF1	Body	-0,029	7,24E-04	4,60E-02
cg03953460	14	32662093		IGR	-0,01	7,24E-04	4,60E-02
cg09750478	22	23025767		IGR	-0,005	7,24E-04	4,60E-02
cg21159068	1	231295910		IGR	0,023	7,25E-04	4,61E-02
cg06064716	2	201164258		IGR	0,017	7,25E-04	4,61E-02
cg08795149	7	49167921		IGR	-0,052	7,25E-04	4,61E-02
cg10117057	11	120369945		IGR	-0,01	7,25E-04	4,61E-02
cg10677017	11	132184930	NTM	Body	-0,017	7,25E-04	4,61E-02
cg23772874	13	91828095		IGR	-0,016	7,25E-04	4,61E-02
cg05446615	16	88547861	ZFPM1	Body	0,012	7,25E-04	4,61E-02
cg07374243	7	127233525	FSCN3	TSS200	0,01	7,25E-04	4,61E-02
cg20566657	4	10021355	SLC2A9	Body	0,023	7,25E-04	4,61E-02
cg16603509	20	34213862		IGR	-0,006	7,26E-04	4,61E-02
cg13175532	10	127584967	FANK1	TSS200	0,014	7,26E-04	4,61E-02
cg07297954	11	72526097	ATG16L2	Body	-0,003	7,26E-04	4,61E-02

cg03061518	12	123738291	C12orf65	Body	0,006	7,26E-04	4,61E-02
cg07667885	13	103285657	TPP2	Body	0,011	7,26E-04	4,61E-02
cg05363451	13	110526236		IGR	-0,006	7,26E-04	4,61E-02
cg22237937	1	26663502	AIM1L	Body	0,007	7,27E-04	4,61E-02
cg13557373	1	203567067		IGR	0,01	7,27E-04	4,61E-02
cg01131395	13	47207812	LRCH1	Body	-0,006	7,27E-04	4,61E-02
cg21245483	15	74890681		IGR	-0,004	7,27E-04	4,61E-02
cg16366686	17	75315837	sept-09	5'UTR	0,039	7,27E-04	4,61E-02
cg02094789	20	62814303	MYT1	5'UTR	-0,01	7,27E-04	4,61E-02
cg10908738	5	10494233	LOC101929412	Body	0,028	7,27E-04	4,61E-02
cg05345586	15	69110457	ANP32A	Body	-0,003	7,27E-04	4,61E-02
cg24918705	5	140501321	PCDHB4	TSS1500	-0,011	7,27E-04	4,61E-02
cg06357371	1	40136763	NT5C1A	Body	0,049	7,27E-04	4,61E-02
cg27094426	6	122906995	PKIB	5'UTR	0,013	7,27E-04	4,61E-02
cg02120577	10	116061887	AFAP1L2	Body	0,011	7,28E-04	4,61E-02
cg00431655	17	72445121		IGR	0,013	7,27E-04	4,61E-02
cg13704678	3	10362667	SEC13	1stExon	-0,004	7,28E-04	4,61E-02
cg15658344	12	50720139		IGR	0,014	7,28E-04	4,61E-02
cg25105555	17	80540647	FOXK2	Body	0,005	7,28E-04	4,61E-02
cg02456552	19	10426933	FDX1L	TSS1500	0,016	7,28E-04	4,61E-02
cg18638914	4	779568	CPLX1	3'UTR	0,008	7,29E-04	4,62E-02
cg11290092	6	44213713	HSP90AB1	TSS1500	0,021	7,29E-04	4,62E-02
cg16136400	6	119032005	CEP85L	TSS1500	-0,018	7,29E-04	4,62E-02
cg25864762	7	1850388		IGR	0,007	7,29E-04	4,62E-02
cg16059473	9	29831191		IGR	-0,015	7,29E-04	4,62E-02
cg03740864	10	120660492		IGR	-0,047	7,29E-04	4,62E-02
cg03493547	11	47201858	PACSIN3	Body	-0,009	7,29E-04	4,62E-02
cg26595177	11	67187315	CARNS1	ExonBnd	0,013	7,29E-04	4,62E-02
cg05973881	16	57653180	ADGRG1	TSS1500	0,019	7,29E-04	4,62E-02
cg24315815	3	145969579	PLSCR4	TSS1500	-0,009	7,29E-04	4,62E-02
cg12294026	5	14452155	TRIO	Body	-0,009	7,29E-04	4,62E-02
cg05329280	20	5192635		IGR	0,029	7,29E-04	4,62E-02
cg00971332	11	117800571	TMPRSS13	TSS1500	0,007	7,30E-04	4,62E-02
cg14617054	19	289762	PPAP2C	Body	0,02	7,30E-04	4,62E-02
cg14963812	3	15251896	CAPN7	Body	0,021	7,30E-04	4,62E-02
cg10487318	8	67974566	COPS5	TSS200	-0,004	7,30E-04	4,62E-02
cg05086789	10	29701805	LOC387647	Body	0,028	7,30E-04	4,62E-02
cg16990945	18	74832261	MBP	5'UTR	0,012	7,30E-04	4,62E-02
cg00897921	22	47355327	TBC1D22A	Body	0,003	7,30E-04	4,62E-02
cg03549240	8	42701169		IGR	-0,008	7,30E-04	4,62E-02
cg00712582	1	37551235		IGR	-0,042	7,31E-04	4,62E-02
cg17157462	13	37268404	C13orf36	5'UTR	-0,015	7,31E-04	4,62E-02
cg26118737	1	224518226	NVL	TSS1500	-0,002	7,31E-04	4,62E-02
cg06394478	7	44530239	NUDCD3	1stExon	-0,004	7,31E-04	4,62E-02
cg05768747	11	15574255		IGR	-0,007	7,31E-04	4,62E-02
cg03012280	15	41098255	ZFYVE19	TSS1500	-0,084	7,31E-04	4,62E-02
cg19482754	12	110224547	TRPV4	Body	0,014	7,31E-04	4,62E-02
cg03785827	9	16423719	BNC2	Body	-0,006	7,31E-04	4,62E-02
cg19705840	8	121065704		IGR	0,025	7,31E-04	4,62E-02
cg09964575	4	1695311	SLBP	3'UTR	-0,006	7,31E-04	4,62E-02
cg20051224	16	72115941		IGR	0,005	7,31E-04	4,62E-02
cg19726894	5	172367362	ERGIC1	Body	0,007	7,32E-04	4,62E-02
cg01684805	3	39508348	MOBP	TSS1500	-0,008	7,32E-04	4,62E-02
cg24554572	11	68442027		IGR	-0,006	7,32E-04	4,62E-02
cg14669500	11	46868024	CKAP5	TSS200	-0,003	7,32E-04	4,63E-02
cg18723873	15	75018222	CYP1A1	TSS1500	-0,004	7,32E-04	4,63E-02
cg07792822	1	157961914	KIRREL	TSS1500	-0,01	7,33E-04	4,63E-02
cg08687088	10	128993051	DOCK1	Body	-0,034	7,33E-04	4,63E-02
cg00997172	20	32399000	CHMP4B	TSS200	-0,004	7,32E-04	4,63E-02
cg12762029	11	119191624		IGR	-0,005	7,33E-04	4,63E-02
cg09533868	7	807608	HEATR2	Body	-0,017	7,33E-04	4,63E-02
cg15415255	8	125979668		IGR	0,009	7,33E-04	4,63E-02
cg19722781	19	53662958	ZNF347	TSS1500	-0,022	7,33E-04	4,63E-02
cg02693744	13	111165457		IGR	-0,005	7,33E-04	4,63E-02
cg00356134	14	64520322	SYNE2	Body	-0,007	7,34E-04	4,63E-02
cg20517396	22	36004529	MB	Body	0,009	7,34E-04	4,63E-02
cg22305209	22	37256352	NCF4	TSS1500	0,026	7,34E-04	4,63E-02
cg11754897	13	28194657	POLR1D	TSS1500	-0,003	7,34E-04	4,63E-02
cg11129066	6	8048354	EEF1E1-BLOC1S5	Body	0,005	7,34E-04	4,63E-02
cg06050631	6	140098850		IGR	-0,012	7,34E-04	4,63E-02
cg19637903	9	71413583	PIP5K1B	5'UTR	-0,013	7,35E-04	4,63E-02
cg20315577	10	74075151		IGR	0,02	7,35E-04	4,63E-02
cg04142692	11	67379534	NDUFV1	Body	0,009	7,34E-04	4,63E-02

cg02028568	22	25170368	PIWIL3	1stExon	-0,043	7,35E-04	4,63E-02
cg02017815	7	173621		IGR	-0,014	7,35E-04	4,63E-02
cg12120741	13	78492306	EDNRB	1stExon	-0,031	7,35E-04	4,63E-02
cg06919553	16	84151088	MBTPS1	TSS1500	-0,01	7,35E-04	4,63E-02
cg12934281	5	180565439		IGR	-0,059	7,35E-04	4,64E-02
cg11503425	2	101925483	RNF149	TSS1500	0,003	7,35E-04	4,64E-02
cg21976650	5	98172189		IGR	-0,012	7,35E-04	4,64E-02
cg04396550	8	133493434	KCNQ3	TSS1500	-0,003	7,35E-04	4,64E-02
cg19931583	3	141711382	TFDP2	5'UTR	-0,019	7,35E-04	4,64E-02
cg18444028	3	169782040	GPR160	5'UTR	0,01	7,35E-04	4,64E-02
cg05532157	5	40791428	PRKAA1	Body	0,064	7,36E-04	4,64E-02
cg22091421	6	150359130		IGR	-0,01	7,36E-04	4,64E-02
cg04507029	14	77539443	LOC102724190	Body	-0,01	7,36E-04	4,64E-02
cg05612325	17	14110411	COX10	Body	-0,004	7,36E-04	4,64E-02
cg25790298	3	172031063	FNDC3B	Body	0,023	7,36E-04	4,64E-02
cg14028447	1	95401503		IGR	-0,013	7,36E-04	4,64E-02
cg01557772	3	21258764		IGR	-0,031	7,36E-04	4,64E-02
cg10963167	15	40049514	FSIP1	Body	0,019	7,36E-04	4,64E-02
cg01215339	22	50684054	HDAC10	Body	-0,011	7,36E-04	4,64E-02
cg01516446	1	201859688	SHISA4	Body	-0,017	7,37E-04	4,64E-02
cg01725704	22	47159108	TBC1D22A	TSS1500	-0,006	7,37E-04	4,64E-02
cg03301460	20	45618719	EYA2	Body	-0,055	7,37E-04	4,64E-02
cg23680832	7	75428501		IGR	0,007	7,37E-04	4,64E-02
cg05114970	3	100144035	LNP1	5'UTR	-0,006	7,37E-04	4,64E-02
cg07022494	14	77942236	ISM2	Body	0,007	7,37E-04	4,64E-02
cg14712313	2	206949263	INO80D	5'UTR	-0,009	7,38E-04	4,64E-02
cg23401461	2	47058307	LOC100134259	Body	-0,012	7,38E-04	4,64E-02
cg12389643	2	17953921	GEN1	Body	0,01	7,38E-04	4,64E-02
cg17326647	2	208347711		IGR	0,015	7,38E-04	4,64E-02
cg18512156	5	172661970	NKX2-5	1stExon	-0,006	7,38E-04	4,65E-02
cg09957624	17	63317229		IGR	0,037	7,38E-04	4,65E-02
cg05299185	3	187872455	LPP	5'UTR	0,004	7,39E-04	4,65E-02
cg01502530	5	106406765		IGR	-0,015	7,39E-04	4,65E-02
cg10531975	5	147559173		IGR	-0,008	7,39E-04	4,65E-02
cg14841847	6	108616810	LACE1	Body	0,018	7,39E-04	4,65E-02
cg05675621	7	157630250	PTPRN2	Body	0,019	7,39E-04	4,65E-02
cg12456539	10	118246635		IGR	-0,008	7,39E-04	4,65E-02
cg15309028	11	10772429	CTR9	TSS1500	0,005	7,39E-04	4,65E-02
cg12625670	11	63278360	LGALS12	Body	0,005	7,39E-04	4,65E-02
cg20872252	11	134287326		IGR	-0,025	7,39E-04	4,65E-02
cg09662304	13	112754881		IGR	-0,026	7,39E-04	4,65E-02
cg10022349	14	75041541	LTBP2	Body	0,031	7,39E-04	4,65E-02
cg04555717	15	27606550	GABRG3	Body	-0,01	7,39E-04	4,65E-02
cg22923630	17	20880927	LOC339260	Body	-0,006	7,39E-04	4,65E-02
cg23402926	17	73083781	SLC16A5	TSS1500	-0,008	7,39E-04	4,65E-02
cg14911690	19	19729395	PBX4	1stExon	-0,012	7,39E-04	4,65E-02
cg06658673	20	60035550	CDH4	Body	-0,042	7,39E-04	4,65E-02
cg05656213	2	26876587		IGR	0,01	7,40E-04	4,65E-02
cg04907070	8	63057336		IGR	-0,02	7,40E-04	4,65E-02
cg23175907	8	96034539		IGR	0,031	7,40E-04	4,65E-02
cg08207365	1	94609734		IGR	-0,018	7,40E-04	4,65E-02
cg03672119	2	236918394	AGAP1	Body	-0,006	7,40E-04	4,65E-02
cg12529140	17	26369940	NLK	1stExon	0,004	7,40E-04	4,65E-02
cg17387737	4	103932949	SLC9B1	5'UTR	-0,013	7,41E-04	4,65E-02
cg06397347	11	57829011	OR9Q1	5'UTR	-0,025	7,41E-04	4,65E-02
cg25780632	20	52687924	BCAS1	TSS1500	0,01	7,41E-04	4,65E-02
cg25963980	11	35442225	SLC1A2	TSS1500	-0,024	7,41E-04	4,65E-02
cg25496680	19	16604089	CALR3	Body	-0,01	7,41E-04	4,65E-02
cg00184240	12	122666660	LRRC43	5'UTR	0,011	7,41E-04	4,65E-02
cg18564888	15	73734744	C15orf60	TSS1500	-0,006	7,41E-04	4,65E-02
cg09232727	22	29140725	HSCB	Body	0,028	7,41E-04	4,65E-02
cg14981137	5	50678697	ISL1	TSS1500	-0,007	7,42E-04	4,65E-02
cg18439474	4	62804350	ADGRL3	Body	-0,029	7,42E-04	4,65E-02
cg05646433	12	104945170	CHST11	Body	0,024	7,42E-04	4,65E-02
cg20713035	5	14410284	TRIO	Body	-0,004	7,42E-04	4,66E-02
cg09487998	5	14482334	TRIO	Body	-0,02	7,42E-04	4,66E-02
cg26135528	6	30524499	PRR3	5'UTR	-0,007	7,42E-04	4,66E-02
cg04935598	14	71631054		IGR	-0,004	7,42E-04	4,66E-02
cg13605338	12	81675069	LOC102724663	Body	0,029	7,42E-04	4,66E-02
cg23230276	9	134752305	MED27	Body	0,007	7,43E-04	4,66E-02
cg19353660	1	226570717	PARP1	ExonBnd	-0,006	7,43E-04	4,66E-02
cg11401986	9	91604088		IGR	-0,012	7,43E-04	4,66E-02
cg09456992	17	77164776	RBFOX3	5'UTR	0,008	7,43E-04	4,66E-02

cg06570125	5	179723634		IGR	0,015	7,43E-04	4,66E-02
cg05350839	1	204926713	NFASC	Body	-0,005	7,44E-04	4,66E-02
cg08652985	17	73150004	HN1	5'UTR	-0,003	7,43E-04	4,66E-02
cg26993348	1	156308302	TSACC	5'UTR	-0,003	7,44E-04	4,66E-02
cg17963289	8	30762212		IGR	0,042	7,44E-04	4,66E-02
cg26634243	2	236701783	AGAP1	Body	0,021	7,44E-04	4,66E-02
cg18983979	14	91779766	CCDC88C	Body	-0,004	7,44E-04	4,66E-02
cg20002209	16	3004363		IGR	0,016	7,44E-04	4,66E-02
cg18362112	5	50669876	LOC642366	Body	-0,044	7,44E-04	4,66E-02
cg08949668	6	33167969	SLC39A7	TSS1500	-0,004	7,44E-04	4,66E-02
cg25388882	1	85098598	C1orf180	Body	0,024	7,44E-04	4,66E-02
cg07170641	11	59950068	MS4A6A	5'UTR	-0,02	7,44E-04	4,66E-02
cg08181258	14	67710831	MPP5	5'UTR	0,007	7,44E-04	4,66E-02
cg17763197	19	44720946	ZNF227	5'UTR	0,008	7,44E-04	4,66E-02
cg03108928	1	44418720	IPO13	Body	0,012	7,45E-04	4,66E-02
cg05948372	18	19283268	ABHD3	Body	-0,005	7,45E-04	4,66E-02
cg09687907	12	57915636	MBD6	TSS1500	0,02	7,45E-04	4,66E-02
cg18769770	20	30424434		IGR	0,026	7,45E-04	4,66E-02
cg16405169	3	170078979	SKIL	1stExon	0,009	7,45E-04	4,66E-02
cg03443357	5	14168374	TRIO	Body	0,027	7,45E-04	4,66E-02
cg24735489	6	31088352	CDSN	TSS200	0,043	7,45E-04	4,66E-02
cg07051257	19	39897430	ZFP36	TSS200	0,007	7,45E-04	4,66E-02
cg09100988	11	66725961	PC	TSS200	-0,013	7,46E-04	4,67E-02
cg17478615	1	185285364	IVNS1ABP	5'UTR	0,005	7,46E-04	4,67E-02
cg12534150	5	112073613	APC	1stExon	0,005	7,46E-04	4,67E-02
cg08098550	5	131391828		IGR	0,034	7,46E-04	4,67E-02
cg11006028	11	124807188	HEPACAM	TSS1500	-0,008	7,46E-04	4,67E-02
cg04264018	6	27100671	HIST1H2BJ	TSS200	-0,002	7,47E-04	4,67E-02
cg01492649	11	86749237	TMEM135	Body	-0,005	7,46E-04	4,67E-02
cg17002138	1	54303960	TMEM48	5'UTR	-0,003	7,47E-04	4,67E-02
cg07418563	13	69459858	LINC00550	TSS1500	-0,011	7,47E-04	4,67E-02
cg22087540	8	87006408		IGR	-0,006	7,47E-04	4,67E-02
cg01003494	2	60618524		IGR	-0,018	7,47E-04	4,67E-02
cg17140940	5	20833387		IGR	-0,02	7,47E-04	4,67E-02
cg21001641	12	120729728		IGR	-0,002	7,47E-04	4,67E-02
cg04902669	17	6329050	AIPL1	Body	-0,011	7,47E-04	4,67E-02
cg17082721	19	38139016	ZFP30	5'UTR	-0,004	7,47E-04	4,67E-02
cg02934563	14	93647474		IGR	0,004	7,47E-04	4,67E-02
cg21661392	19	19735040	LPAR2	3'UTR	0,004	7,47E-04	4,67E-02
cg17019513	7	132824219		IGR	-0,04	7,48E-04	4,67E-02
cg02095498	8	142368264	GPR20	5'UTR	0,009	7,48E-04	4,67E-02
cg18438823	11	4676365	OR51E1	3'UTR	-0,016	7,48E-04	4,67E-02
cg26285694	3	167099430	ZBBX	TSS1500	-0,005	7,48E-04	4,67E-02
cg10941445	2	109934856	SH3RF3	Body	-0,009	7,49E-04	4,68E-02
cg13966557	4	184908884	STOX2	Body	-0,048	7,49E-04	4,68E-02
cg06436631	14	51835289		IGR	0,018	7,49E-04	4,68E-02
cg15290779	7	111202666	IMMP2L	TSS1500	-0,004	7,49E-04	4,68E-02
cg13214295	17	75789279		IGR	-0,046	7,49E-04	4,68E-02
cg09390391	3	50474115	CACNA2D2	Body	0,009	7,49E-04	4,68E-02
cg11299371	6	160224288	PNLDC1	Body	-0,006	7,49E-04	4,68E-02
cg03874438	13	38322203	TRPC4	Body	-0,01	7,49E-04	4,68E-02
cg10346121	10	104179484	FBXL15	TSS200	-0,005	7,50E-04	4,68E-02
cg18196797	6	152865908	SYNE1	Body	0,008	7,50E-04	4,68E-02
cg07521475	9	135994613	RALGDS	Body	0,017	7,50E-04	4,68E-02
cg23031135	11	68779553	MRGPRF	5'UTR	0,007	7,50E-04	4,68E-02
cg20147645	13	28023563	MTIF3	5'UTR	0,035	7,50E-04	4,68E-02
cg16392310	19	7913967	EVI5L	Body	-0,014	7,50E-04	4,68E-02
cg03127558	19	49223278	MAMSTR	TSS1500	-0,013	7,50E-04	4,68E-02
cg23855752	12	10322040	OLR1	Body	-0,018	7,50E-04	4,68E-02
cg07809953	16	61348146		IGR	-0,02	7,50E-04	4,68E-02
cg08937323	11	118245731	UBE4A	Body	0,006	7,51E-04	4,68E-02
cg25958733	15	33421010		IGR	-0,011	7,51E-04	4,68E-02
cg19774820	2	209100943		IGR	0,042	7,51E-04	4,68E-02
cg13835205	12	6961384	CDCA3	TSS200	0,005	7,51E-04	4,68E-02
cg11508942	16	3108076	MMP25	Body	0,018	7,51E-04	4,68E-02
cg04307216	16	79897930		IGR	-0,008	7,51E-04	4,68E-02
cg23725958	20	2074024		IGR	-0,026	7,51E-04	4,68E-02
cg16722154	10	120481145	CACUL1	Body	0,008	7,51E-04	4,68E-02
cg17931227	6	31148370		IGR	-0,046	7,51E-04	4,68E-02
cg04752023	1	45108655	RNF220	Body	0,013	7,51E-04	4,68E-02
cg27042842	12	32630868		IGR	0,03	7,52E-04	4,68E-02
cg06769116	1	244217538	ZNF238	Body	-0,014	7,52E-04	4,69E-02
cg07434438	16	72961899	ZFHX3	Body	0,031	7,52E-04	4,69E-02

cg03139543	1	246935454		IGR	0,011	7,53E-04	4,69E-02
cg26560981	4	87039369	MAPK10	Body	0,008	7,53E-04	4,69E-02
cg07215575	6	128348209	LOC101928140	TSS1500	0,008	7,52E-04	4,69E-02
cg27229251	7	2680532	TTYH3	Body	0,007	7,52E-04	4,69E-02
cg03906047	7	47989412	PKD1L1	TSS1500	-0,014	7,52E-04	4,69E-02
cg05242172	14	67462583	GPHN	Body	0,013	7,53E-04	4,69E-02
cg01419469	17	8092488	C17orf59	1stExon	0,026	7,52E-04	4,69E-02
cg16927606	19	36233324	TMEM149	1stExon	-0,004	7,52E-04	4,69E-02
cg02386644	19	44616907	ZNF225	TSS1500	-0,008	7,53E-04	4,69E-02
cg16227703	1	38478221	UTP11L	TSS200	-0,003	7,53E-04	4,69E-02
cg20921479	2	59303635		IGR	0,018	7,53E-04	4,69E-02
cg21111416	3	148804720	HLTF	TSS1500	-0,019	7,53E-04	4,69E-02
cg08959725	10	91621365		IGR	-0,021	7,53E-04	4,69E-02
cg23933698	11	12926510	TEAD1	Body	-0,027	7,53E-04	4,69E-02
cg00934864	19	46916145	CCDC8	1stExon	0,057	7,53E-04	4,69E-02
cg05696603	7	84644475	SEMA3D	Body	-0,005	7,54E-04	4,69E-02
cg12873784	16	73130218		IGR	0,023	7,54E-04	4,69E-02
cg24336358	12	3313713	TSPAN9	Body	0,01	7,54E-04	4,69E-02
cg20836822	13	43344011		IGR	-0,008	7,54E-04	4,69E-02
cg10437787	17	7904871	GUCY2D	TSS1500	0,023	7,55E-04	4,70E-02
cg21940708	19	50191683	PRMT1	3'UTR	0,009	7,55E-04	4,70E-02
cg09853898	19	591536	HCN2	Body	-0,013	7,55E-04	4,70E-02
cg17953300	11	65418265	SIPA1	3'UTR	0,013	7,56E-04	4,70E-02
cg09329151	12	42632601	YAF2	TSS1500	-0,018	7,56E-04	4,70E-02
cg22134476	11	67874294	CHKA	Body	-0,005	7,56E-04	4,70E-02
cg03704006	16	88994295	CBFA2T3	5'UTR	-0,004	7,56E-04	4,70E-02
cg01108112	9	139254189		IGR	0,018	7,56E-04	4,70E-02
cg16312647	15	64455677	PPIB	TSS1500	-0,003	7,57E-04	4,70E-02
cg18439358	17	41910968	MPP3	TSS1500	-0,004	7,57E-04	4,70E-02
cg19950556	21	46439470		IGR	0,014	7,57E-04	4,71E-02
cg24218609	8	5366654		IGR	-0,013	7,57E-04	4,71E-02
cg20612875	17	1094308		IGR	0,015	7,57E-04	4,71E-02
cg01525195	7	46820511		IGR	-0,023	7,58E-04	4,71E-02
cg21905390	8	14176154	SGCZ	Body	0,038	7,58E-04	4,71E-02
cg23543885	8	123168675		IGR	-0,018	7,58E-04	4,71E-02
cg21699184	19	14630506	DNAJB1	TSS1500	-0,011	7,58E-04	4,71E-02
cg02315619	3	62031063	PTPRG	Body	0,009	7,58E-04	4,71E-02
cg07521193	15	27819422		IGR	-0,062	7,58E-04	4,71E-02
cg08631398	2	220097524	ANKZF1	5'UTR	0,026	7,58E-04	4,71E-02
cg04874326	6	2634942	C6orf195	1stExon	-0,004	7,59E-04	4,71E-02
cg00141366	10	73074590		IGR	0,031	7,59E-04	4,71E-02
cg12761885	15	39136567		IGR	-0,012	7,59E-04	4,71E-02
cg09925467	10	114043123	TECTB	TSS1500	-0,028	7,59E-04	4,71E-02
cg01938856	19	1591832	MBD3	Body	-0,012	7,59E-04	4,71E-02
cg23599125	2	47557159	LOC101927043	Body	-0,009	7,60E-04	4,71E-02
cg13516513	16	68670061		IGR	0,016	7,60E-04	4,72E-02
cg03217906	6	127838893	SOGA3	5'UTR	-0,015	7,60E-04	4,72E-02
cg23389023	13	113784263	F10	Body	-0,035	7,60E-04	4,72E-02
cg02288964	1	967561	AGRN	Body	0,028	7,60E-04	4,72E-02
cg21522295	19	17983747	SLC5A5	Body	-0,006	7,61E-04	4,72E-02
cg13614606	11	864739	TSPAN4	Body	0,008	7,61E-04	4,72E-02
cg26732155	15	37236977	MEIS2	Body	-0,007	7,61E-04	4,72E-02
cg19070873	14	96505448	C14orf132	TSS1500	-0,008	7,62E-04	4,72E-02
cg23562858	17	26845789		IGR	0,02	7,61E-04	4,72E-02
cg03238162	18	29027701	DSG3	TSS200	-0,015	7,62E-04	4,72E-02
cg06625721	12	33205814		IGR	-0,014	7,62E-04	4,72E-02
cg03183451	2	46220673	PRKCE	Body	-0,006	7,62E-04	4,72E-02
cg12211471	3	127681342	KBTBD12	Body	0,035	7,62E-04	4,72E-02
cg03583975	11	85219848	DLG2	Body	0,024	7,62E-04	4,72E-02
cg10195929	12	58019223	SLC26A10	Body	-0,014	7,62E-04	4,72E-02
cg21755457	12	54020320	ATF7	TSS200	-0,003	7,62E-04	4,72E-02
cg05667401	16	31073266	ZNF668	Body	-0,003	7,62E-04	4,72E-02
cg18158709	2	235598570		IGR	-0,016	7,63E-04	4,73E-02
cg19539278	12	18414015	PIK3C2G	TSS1500	0,039	7,63E-04	4,73E-02
cg20352410	11	8710644	RPL27A	3'UTR	-0,005	7,63E-04	4,73E-02
cg08585380	6	86388840	SNORD50B	TSS1500	-0,003	7,64E-04	4,73E-02
cg17474216	12	88427963	C12orf29	TSS1500	-0,021	7,64E-04	4,73E-02
cg06582411	7	100229865	TFR2	Body	0,019	7,64E-04	4,73E-02
cg14420767	5	135380490	TGFBI	Body	-0,008	7,64E-04	4,73E-02
cg02470108	16	58577764	CNOT1	Body	0,007	7,64E-04	4,73E-02
cg08000336	1	36621206	MAP7D1	TSS1500	-0,003	7,64E-04	4,73E-02
cg16306253	1	48912625	SPATA6	Body	-0,019	7,65E-04	4,73E-02
cg27224018	3	177234173	LINC00578	Body	-0,007	7,65E-04	4,73E-02

cg26129303	8	142095375		IGR	0,007	7,65E-04	4,73E-02
cg10608255	10	6042325		IGR	0,006	7,65E-04	4,73E-02
cg14253018	16	29722049		IGR	0,028	7,65E-04	4,73E-02
cg19409920	2	240877826		IGR	0,039	7,65E-04	4,74E-02
cg02719956	11	306324		IGR	-0,01	7,65E-04	4,74E-02
cg01546397	16	451643	DECR2	TSS1500	0,004	7,65E-04	4,74E-02
cg24323597	3	123813504	KALRN	TSS200	-0,013	7,66E-04	4,74E-02
cg03538020	5	156363442	TIMD4	Body	0,012	7,66E-04	4,74E-02
cg09264584	6	128686735	PTPRK	Body	0,019	7,66E-04	4,74E-02
cg09532836	17	34180621		IGR	0,005	7,66E-04	4,74E-02
cg00657440	10	124091406	BTBD16	Body	-0,018	7,66E-04	4,74E-02
cg13604777	19	10047205	OLFM2	TSS200	0,007	7,66E-04	4,74E-02
cg03891268	3	195920019		IGR	0,018	7,66E-04	4,74E-02
cg15024936	2	101925327	RNF149	TSS200	-0,005	7,67E-04	4,74E-02
cg21250206	2	109832952	SH3RF3	Body	0,029	7,67E-04	4,74E-02
cg00294196	15	93572609		IGR	0,026	7,67E-04	4,74E-02
cg19419789	16	2255584	MLST8	5'UTR	0,007	7,67E-04	4,74E-02
cg15785580	16	31119650	BCKDK	TSS200	-0,003	7,67E-04	4,74E-02
cg16652241	16	58059321	MMP15	5'UTR	-0,006	7,67E-04	4,74E-02
cg03355204	19	1008063	GRIN3B	Body	0,009	7,67E-04	4,74E-02
cg11820497	19	10654946	ATG4D	1stExon	-0,003	7,67E-04	4,74E-02
cg11983420	20	25726268		IGR	-0,01	7,67E-04	4,74E-02
cg03313666	22	38142162	TRIOBP	TSS200	0,006	7,67E-04	4,74E-02
cg08964643	7	63560420		IGR	-0,06	7,67E-04	4,74E-02
cg23127689	12	130681639		IGR	-0,028	7,67E-04	4,74E-02
cg23806457	21	46626969	ADARB1	Body	-0,004	7,67E-04	4,74E-02
cg22250332	4	147020563		IGR	0,014	7,68E-04	4,74E-02
cg20240790	16	27734275	KIAA0556	Body	-0,007	7,68E-04	4,74E-02
cg19232414	6	34048228	GRM4	Body	-0,019	7,68E-04	4,75E-02
cg11687286	15	100331564	DNM1P46	Body	-0,007	7,68E-04	4,75E-02
cg12542281	13	33113069	N4BP2L2	TSS200	-0,003	7,68E-04	4,75E-02
cg19705159	7	102109446	LRWD1	Body	0,008	7,68E-04	4,75E-02
cg19956872	3	147088456		IGR	-0,019	7,69E-04	4,75E-02
cg14434028	2	70452453	TIA1	Body	0,034	7,69E-04	4,75E-02
cg12184344	4	95115189	LOC101929210	Body	-0,01	7,69E-04	4,75E-02
cg14654935	7	39644725	YAE1D1	Body	0,009	7,69E-04	4,75E-02
cg19267057	10	4447810	LINC00703	TSS1500	-0,015	7,69E-04	4,75E-02
cg10363005	11	126867352	KIRREL3	Body	-0,046	7,69E-04	4,75E-02
cg26988241	14	50087290	RPL36AL	1stExon	-0,004	7,69E-04	4,75E-02
cg12724546	14	69484295		IGR	0,011	7,69E-04	4,75E-02
cg23746045	14	95354735		IGR	0,013	7,69E-04	4,75E-02
cg27341832	15	84277424	SH3GL3	Body	-0,006	7,69E-04	4,75E-02
cg03536664	3	48636487	UQCRC1	3'UTR	0,014	7,69E-04	4,75E-02
cg11881990	11	832067	CD151	TSS1500	0,02	7,69E-04	4,75E-02
cg22183373	4	24806777		IGR	-0,005	7,70E-04	4,75E-02
cg19642292	8	11660393	FDFT1	TSS1500	-0,004	7,70E-04	4,75E-02
cg07915896	15	65129816		IGR	-0,008	7,70E-04	4,75E-02
cg13721464	1	112257233	RAP1A	3'UTR	0,019	7,71E-04	4,75E-02
cg19351336	3	176762502	TBL1XR1	Body	0,016	7,71E-04	4,75E-02
cg22863456	5	51430203		IGR	-0,015	7,71E-04	4,75E-02
cg13208732	8	57124347	CHCHD7	1stExon	-0,003	7,71E-04	4,75E-02
cg15903421	9	117267460	DFNB31	5'UTR	-0,019	7,71E-04	4,75E-02
cg16109953	14	24701739	GMPR2	1stExon	-0,003	7,71E-04	4,75E-02
cg13131686	19	8302808	CERS4	5'UTR	0,012	7,71E-04	4,75E-02
cg16715953	19	34396333		IGR	0,017	7,71E-04	4,75E-02
cg22507402	4	26321356	RBPJ	5'UTR	0,003	7,71E-04	4,75E-02
cg03671802	18	56941473	RAX	TSS1500	-0,023	7,71E-04	4,75E-02
cg11177680	12	1192038	ERC1	Body	0,012	7,71E-04	4,75E-02
cg15421236	8	125384727	TMEM65	5'UTR	-0,008	7,72E-04	4,75E-02
cg14282612	10	102046509	BLOC1S2	TSS1500	-0,004	7,71E-04	4,75E-02
cg18884388	7	190334		IGR	0,009	7,72E-04	4,75E-02
cg05546064	6	30685074	MDC1	1stExon	-0,006	7,72E-04	4,75E-02
cg16359235	6	116697339	DSE	5'UTR	-0,009	7,72E-04	4,76E-02
cg26680763	19	39739565	IFNL4	TSS200	-0,014	7,72E-04	4,76E-02
cg22553313	1	234746318	IRF2BP2	TSS1500	-0,003	7,72E-04	4,76E-02
cg00885474	12	56524830	ESYT1	ExonBnd	-0,004	7,73E-04	4,76E-02
cg23166357	14	64010079	PPP2R5E	TSS200	-0,002	7,73E-04	4,76E-02
cg12837729	16	11325312		IGR	-0,018	7,73E-04	4,76E-02
cg26943759	16	87100606		IGR	0,031	7,72E-04	4,76E-02
cg01451880	3	45801044	SLC6A20	Body	0,011	7,73E-04	4,76E-02
cg09153398	2	7150625	RNF144A	Body	0,049	7,73E-04	4,76E-02
cg16844164	21	36953232	LOC100506403	TSS200	-0,006	7,73E-04	4,76E-02
cg10504588	2	61104882		IGR	-0,009	7,73E-04	4,76E-02

cg19706169	22	37265071	NCF4	Body	-0,011	7,73E-04	4,76E-02
cg27048363	5	6348254		IGR	-0,011	7,74E-04	4,76E-02
cg18839637	5	140723684	PCDHGA2	Body	-0,037	7,74E-04	4,76E-02
cg24907011	8	24855940		IGR	0,038	7,74E-04	4,76E-02
cg03887279	20	62148921		IGR	0,008	7,74E-04	4,76E-02
cg17075352	4	71533675	IGJ	TSS1500	0,035	7,74E-04	4,76E-02
cg05254221	10	61050062	FAM13C	Body	-0,048	7,75E-04	4,76E-02
cg13311154	1	7926252		IGR	-0,014	7,75E-04	4,76E-02
cg18909235	17	72948506	C17orf28	Body	-0,048	7,75E-04	4,77E-02
cg13765660	3	48281517	ZNF589	TSS1500	0,023	7,75E-04	4,77E-02
cg02063961	5	3346841		IGR	-0,011	7,75E-04	4,77E-02
cg21103758	8	41568929	ANK1	Body	-0,005	7,75E-04	4,77E-02
cg05598742	10	82173622	FAM213A	5'UTR	0,019	7,75E-04	4,77E-02
cg15369377	16	2767942	PRSS27	Body	0,029	7,76E-04	4,77E-02
cg08662890	18	71752067	FBXO15	Body	-0,008	7,76E-04	4,77E-02
cg14532872	19	52150681	SIGLEC14	TSS1500	-0,009	7,76E-04	4,77E-02
cg22748452	20	634604	SRXN1	TSS1500	0,041	7,76E-04	4,77E-02
cg07805952	17	38245719	THRA	Body	0,016	7,76E-04	4,77E-02
cg10949330	1	53906948	FLJ40434	TSS1500	-0,006	7,76E-04	4,77E-02
cg05468349	1	238649828	LINC01139	TSS1500	0,027	7,76E-04	4,77E-02
cg06039573	2	64590619		IGR	-0,005	7,76E-04	4,77E-02
cg18083402	8	100675936	VPS13B	Body	-0,032	7,76E-04	4,77E-02
cg21513686	4	52884431	LRRC66	TSS1500	0,023	7,76E-04	4,77E-02
cg11856021	2	71780175	DYSF	Body	0,067	7,77E-04	4,77E-02
cg15422226	1	156486717		IGR	-0,016	7,77E-04	4,77E-02
cg02536588	2	26568601	EPT1	TSS1500	-0,003	7,77E-04	4,77E-02
cg00563045	6	30878494	GTF2H4	Body	-0,009	7,77E-04	4,77E-02
cg20347101	7	66147509		IGR	-0,003	7,77E-04	4,77E-02
cg19458078	1	153232069	LOR	TSS200	-0,011	7,77E-04	4,77E-02
cg04689734	9	96919025		IGR	0,013	7,78E-04	4,77E-02
cg09362332	14	31349663	COCH	ExonBnd	-0,006	7,78E-04	4,77E-02
cg24451737	17	80616762	RAB40B	Body	0,017	7,78E-04	4,77E-02
cg18557261	12	1612672	LOC100292680	Body	0,005	7,78E-04	4,77E-02
cg15921461	7	158373792	PTPRN2	Body	0,012	7,78E-04	4,78E-02
cg11956108	1	1895061	KIAA1751	Body	0,013	7,78E-04	4,78E-02
cg04107533	1	201084506	ASCL5	TSS200	-0,023	7,79E-04	4,78E-02
cg25795625	2	209120001	IDH1	TSS200	-0,004	7,79E-04	4,78E-02
cg20447920	6	131555431	AKAP7	Body	-0,02	7,79E-04	4,78E-02
cg08112137	6	170040005	WDR27	Body	-0,018	7,79E-04	4,78E-02
cg03486847	7	6567103	GRID2IP	Body	0,021	7,79E-04	4,78E-02
cg21466789	8	29555808		IGR	0,009	7,79E-04	4,78E-02
cg21057254	9	4295758	GLIS3	5'UTR	0,014	7,79E-04	4,78E-02
cg14264571	9	35754691	MSMP	TSS1500	-0,011	7,80E-04	4,78E-02
cg08521987	10	119000927	SLC18A2	5'UTR	-0,009	7,80E-04	4,78E-02
cg25558737	11	61075288	DDB1	Body	-0,016	7,79E-04	4,78E-02
cg02152620	14	44008450		IGR	-0,03	7,79E-04	4,78E-02
cg18166564	15	25492082	SNORD115-10	TSS1500	0,024	7,80E-04	4,78E-02
cg17196903	16	66637857	CMTM3	TSS1500	-0,004	7,79E-04	4,78E-02
cg14035166	16	66946430	CDH16	Body	0,016	7,80E-04	4,78E-02
cg17980432	17	28591078	BLMH	Body	0,009	7,79E-04	4,78E-02
cg07938316	18	47810808	CXXC1	Body	-0,006	7,79E-04	4,78E-02
cg05137810	19	42513374	GRIK5	Body	-0,026	7,79E-04	4,78E-02
cg02363600	19	42954668	LIPE-AS1	Body	-0,004	7,79E-04	4,78E-02
cg21765224	20	34359771	PHF20	TSS200	-0,004	7,79E-04	4,78E-02
cg07550275	21	29601135	LOC101927973	TSS200	0,016	7,80E-04	4,78E-02
cg15411403	5	92922043	NR2F1	Body	-0,034	7,80E-04	4,78E-02
cg09404334	6	86304180	SNX14	TSS1500	-0,005	7,80E-04	4,78E-02
cg25105919	18	21083459	C18orf8	TSS200	0,005	7,80E-04	4,78E-02
cg02016764	4	38805732	TLR1	5'UTR	0,025	7,80E-04	4,78E-02
cg04799232	4	103475833	NFKB1	Body	-0,006	7,80E-04	4,78E-02
cg07595346	1	24232748	CNR2	5'UTR	0,02	7,81E-04	4,78E-02
cg14839257	6	167275999	RPS6KA2	TSS1500	-0,004	7,81E-04	4,78E-02
cg21638357	7	100285711	GIGYF1	Body	0,011	7,81E-04	4,78E-02
cg00925766	14	69676183	EXD2	TSS1500	-0,009	7,81E-04	4,78E-02
cg26849077	15	65914281	SLC24A1	5'UTR	-0,012	7,81E-04	4,78E-02
cg04307587	19	44104283	ZNF576	3'UTR	-0,008	7,81E-04	4,78E-02
cg19449111	2	55585089	CCDC88A	Body	-0,006	7,81E-04	4,78E-02
cg22179564	5	74199422		IGR	-0,049	7,81E-04	4,78E-02
cg05740473	1	84988095	SPATA1	Body	0,046	7,81E-04	4,78E-02
cg02889971	5	102589620		IGR	-0,006	7,81E-04	4,78E-02
cg05070268	16	1414687	UNKL	3'UTR	0,014	7,81E-04	4,78E-02
cg25355010	2	87018929	CD8A	TSS200	-0,036	7,81E-04	4,78E-02
cg01109707	11	47166816	C11orf49	Body	-0,016	7,82E-04	4,78E-02

cg12776836	9	1232682		IGR	0,028	7,82E-04	4,78E-02
cg02300840	9	23829435		IGR	-0,069	7,82E-04	4,78E-02
cg24064019	20	57385278		IGR	0,029	7,82E-04	4,78E-02
cg03752471	8	145701266	FOXH1	1stExon	0,036	7,82E-04	4,78E-02
cg11724516	9	115653222	SLC46A2	TSS200	-0,005	7,82E-04	4,78E-02
cg17842189	22	47425596	TBC1D22A	Body	-0,01	7,82E-04	4,78E-02
cg18663033	2	241034966		IGR	-0,045	7,82E-04	4,78E-02
cg08047123	3	154475326		IGR	-0,017	7,82E-04	4,78E-02
cg11283869	20	42874963	GDAP1L1	TSS1500	0,027	7,82E-04	4,78E-02
cg16305609	1	117837480	LINC01525	TSS1500	-0,01	7,83E-04	4,78E-02
cg27528695	1	109643479		IGR	0,016	7,83E-04	4,78E-02
cg00978427	5	140736762	PCDHGA2	Body	-0,038	7,83E-04	4,78E-02
cg01504108	17	3802384	P2RX1	Body	-0,009	7,83E-04	4,78E-02
cg15989091	2	74780172	LOXL3	5'UTR	0,02	7,83E-04	4,78E-02
cg27079740	4	176733877	GPM6A	1stExon	-0,045	7,83E-04	4,78E-02
cg22317989	11	1940912	TNNT3	1stExon	0,021	7,83E-04	4,78E-02
cg26839356	15	99603018		IGR	-0,029	7,83E-04	4,78E-02
cg04733382	2	62424219	B3GNT2	5'UTR	-0,003	7,83E-04	4,79E-02
cg07327144	17	48450912	EME1	5'UTR	0,028	7,84E-04	4,79E-02
cg01833485	1	216860692	ESRRG	5'UTR	-0,05	7,84E-04	4,79E-02
cg16355771	3	13242707		IGR	0,018	7,84E-04	4,79E-02
cg21001906	1	45923414	TESK2	Body	0,024	7,84E-04	4,79E-02
cg20530170	2	64805438	AFTPH	Body	0,034	7,84E-04	4,79E-02
cg15720975	2	242625759	DTYMK	Body	-0,007	7,84E-04	4,79E-02
cg18870532	8	142139341	DENND3	5'UTR	-0,003	7,84E-04	4,79E-02
cg14037037	14	69524477	DCAF5	Body	0,004	7,84E-04	4,79E-02
cg16538178	16	85647585	KIAA0182	5'UTR	0,007	7,84E-04	4,79E-02
cg01229464	2	29320782	CLIP4	1stExon	-0,016	7,85E-04	4,79E-02
cg14718370	12	56818609	TIMELESS	Body	-0,006	7,85E-04	4,79E-02
cg04637204	12	106532491	NUAK1	5'UTR	-0,007	7,85E-04	4,79E-02
cg00408605	8	141466079	TRAPPC9	5'UTR	0,013	7,85E-04	4,79E-02
cg01088089	12	127245854	LINC00944	Body	0,014	7,85E-04	4,79E-02
cg25787588	14	50784952	ATP5S	Body	-0,09	7,86E-04	4,79E-02
cg08111770	16	14539320	PARN	Body	0,007	7,86E-04	4,79E-02
cg03991250	1	17326988	ATP13A2	Body	0,006	7,86E-04	4,79E-02
cg16837310	4	185205551		IGR	0,006	7,86E-04	4,79E-02
cg08293824	3	172313318		IGR	-0,034	7,86E-04	4,79E-02
cg09912806	4	88343654	NUDT9	TSS200	-0,003	7,86E-04	4,79E-02
cg06063962	1	15223110	KAZN	Body	0,013	7,86E-04	4,79E-02
cg06876479	1	222791170	MIA3	TSS1500	-0,003	7,86E-04	4,79E-02
cg19996027	5	106717374	EFNA5	Body	0,028	7,87E-04	4,80E-02
cg12502286	8	1899127	ARHGEF10	Body	0,007	7,87E-04	4,80E-02
cg03863046	10	32116054	ARHGAP12	Body	0,007	7,87E-04	4,80E-02
cg14224569	22	39567277		IGR	-0,002	7,87E-04	4,80E-02
cg16079364	10	103532798	FGF8	Body	0,023	7,87E-04	4,80E-02
cg21153648	16	30394785	sept-01	TSS1500	-0,017	7,87E-04	4,80E-02
cg17809974	21	43720804		IGR	0,011	7,87E-04	4,80E-02
cg19335130	5	146256776	PPP2R2B	Body	-0,029	7,87E-04	4,80E-02
cg20761400	12	4119359		IGR	0,008	7,88E-04	4,80E-02
cg20391058	2	2295451	MYT1L	5'UTR	-0,016	7,88E-04	4,80E-02
cg23995931	12	53973619	ATF7	Body	0,004	7,88E-04	4,80E-02
cg07588355	1	112782447		IGR	0,032	7,88E-04	4,80E-02
cg10774974	3	130649266	ATP2C1	ExonBnd	0,031	7,88E-04	4,80E-02
cg12095968	1	151739679	OAZ3	Body	-0,017	7,89E-04	4,80E-02
cg17264818	2	95612985		IGR	0,011	7,89E-04	4,80E-02
cg00996758	3	137486536		IGR	-0,011	7,89E-04	4,80E-02
cg26369576	7	31276097		IGR	-0,008	7,89E-04	4,80E-02
cg13616215	7	133619041	EXOC4	Body	0,013	7,89E-04	4,80E-02
cg04290666	10	102242134	WNT8B	Body	-0,005	7,88E-04	4,80E-02
cg05297002	12	11788574		IGR	-0,004	7,88E-04	4,80E-02
cg20245503	12	58026705	B4GALNT1	5'UTR	-0,004	7,88E-04	4,80E-02
cg19272195	14	38028789		IGR	0,007	7,88E-04	4,80E-02
cg13402936	15	50471108		IGR	0,02	7,88E-04	4,80E-02
cg02535796	22	42755569		IGR	0,012	7,88E-04	4,80E-02
cg10056799	5	68411157	SLC30A5	Body	0,035	7,89E-04	4,80E-02
cg08038409	20	825155	FAM110A	TSS200	-0,003	7,89E-04	4,80E-02
cg12214830	2	47745462		IGR	0,009	7,90E-04	4,80E-02
cg11542063	2	178129680	NFE2L2	5'UTR	-0,005	7,90E-04	4,80E-02
cg03363248	3	27754391		IGR	-0,005	7,90E-04	4,80E-02
cg15450966	4	3486039	DOK7	Body	0,034	7,90E-04	4,80E-02
cg23179168	8	127570165	FAM84B	5'UTR	-0,003	7,90E-04	4,80E-02
cg20998041	12	45949663		IGR	-0,009	7,90E-04	4,80E-02
cg01625621	15	34260433	AVEN	Body	-0,015	7,90E-04	4,80E-02

cg17147728	15	55880224	PYGO1	Body	-0,003	7,90E-04	4,80E-02
cg04636748	15	75117708	CPLX3	TSS1500	-0,017	7,90E-04	4,80E-02
cg26925226	15	74751138	UBL7	Body	-0,003	7,90E-04	4,80E-02
cg18872441	5	620334	CEP72	Body	-0,004	7,90E-04	4,80E-02
cg04562396	14	55518417	MAPK1IP1L	1stExon	0,007	7,90E-04	4,80E-02
cg16622517	4	63075650		IGR	-0,034	7,91E-04	4,81E-02
cg24584439	10	60087111		IGR	-0,026	7,91E-04	4,81E-02
cg16521028	12	75874414	GLIPR1	TSS200	-0,006	7,91E-04	4,81E-02
cg19474267	17	64306194	PRKCA	Body	-0,007	7,91E-04	4,81E-02
cg03217434	19	2183142	DOT1L	Body	0,027	7,91E-04	4,81E-02
cg14934826	6	91210357		IGR	-0,016	7,91E-04	4,81E-02
cg03562592	8	42294806	SLC20A2	Body	-0,009	7,91E-04	4,81E-02
cg13667314	11	75546440	UVRAG	Body	0,009	7,91E-04	4,81E-02
cg00565290	1	232848116		IGR	-0,006	7,92E-04	4,81E-02
cg06107942	2	74372634	BOLA3	Body	0,008	7,92E-04	4,81E-02
cg15448481	9	103350508	MURC	3'UTR	0,022	7,92E-04	4,81E-02
cg02242919	16	2264594	PGP	1stExon	-0,003	7,92E-04	4,81E-02
cg00912757	13	46597943	ZC3H13	Body	-0,003	7,92E-04	4,81E-02
cg00510991	19	19656163	CILP2	Body	0,011	7,92E-04	4,81E-02
cg05797890	13	60763264		IGR	0,025	7,92E-04	4,81E-02
cg22449980	4	157701149	PDGFC	Body	0,009	7,92E-04	4,81E-02
cg04363633	6	46655453	TDRD6	TSS200	-0,006	7,93E-04	4,81E-02
cg20065005	1	16712408	C1orf144	Body	-0,019	7,93E-04	4,81E-02
cg23374955	2	45902742	PRKCE	Body	0,024	7,93E-04	4,81E-02
cg05686542	5	102895791	NUDT12	Body	0,007	7,93E-04	4,81E-02
cg11127606	6	27866735		IGR	0,007	7,93E-04	4,81E-02
cg02965892	7	2402223	EIF3B	Body	0,005	7,93E-04	4,81E-02
cg01433189	15	45544230	SLC28A2	TSS200	-0,005	7,93E-04	4,81E-02
cg02248833	19	50425434	IL4I1	Body	0,019	7,93E-04	4,81E-02
cg17098952	20	866396	ANGPT4	Body	0,018	7,93E-04	4,81E-02
cg18158172	5	177819485	COL23A1	Body	-0,029	7,94E-04	4,81E-02
cg13904308	11	58904869		IGR	0,008	7,94E-04	4,81E-02
cg23158363	16	4500957	DNAJA3	Body	-0,009	7,94E-04	4,81E-02
cg10537220	10	64133795	ZNF365	TSS200	0,011	7,94E-04	4,81E-02
cg22827685	12	117768981	NOS1	5'UTR	-0,015	7,94E-04	4,81E-02
cg06396496	3	53289970	TKT	1stExon	-0,004	7,94E-04	4,81E-02
cg23114594	10	13544648	BEND7	5'UTR	0,012	7,94E-04	4,81E-02
cg10384558	10	16851329	RSU1	5'UTR	0,021	7,94E-04	4,81E-02
cg01661284	14	77842107	TMED8	Body	0,026	7,94E-04	4,81E-02
cg12051081	1	117265030		IGR	-0,006	7,95E-04	4,81E-02
cg16989180	1	145097220	SEC22B	Body	-0,019	7,95E-04	4,81E-02
cg01242123	4	944277	TMEM175	Body	0,013	7,95E-04	4,81E-02
cg08552446	10	52898733	PRKG1	Body	-0,007	7,95E-04	4,81E-02
cg01548456	22	50352384		IGR	0,01	7,95E-04	4,81E-02
cg18145921	18	12059782		IGR	-0,008	7,95E-04	4,81E-02
cg23756763	2	24944936	NCOA1	Body	0,014	7,95E-04	4,82E-02
cg01165683	3	24430957	THRB	5'UTR	0,012	7,95E-04	4,82E-02
cg01312999	5	131730715	SLC22A5	3'UTR	0,012	7,95E-04	4,82E-02
cg11749367	11	44359222		IGR	-0,033	7,95E-04	4,82E-02
cg04946387	11	134253884	B3GAT1	Body	-0,032	7,95E-04	4,82E-02
cg01438757	13	25718667		IGR	0,036	7,95E-04	4,82E-02
cg15725771	19	47798258		IGR	0,008	7,95E-04	4,82E-02
cg06917451	1	177138584		IGR	-0,053	7,96E-04	4,82E-02
cg07772309	9	140353475	NELF	Body	-0,003	7,96E-04	4,82E-02
cg00409844	5	68798649	OCLN	5'UTR	0,018	7,96E-04	4,82E-02
cg23609185	13	23363031		IGR	0,012	7,96E-04	4,82E-02
cg21737976	20	1294361	SDCBP2	TSS200	0,007	7,96E-04	4,82E-02
cg03901462	11	62572874	NXF1	1stExon	0,01	7,97E-04	4,82E-02
cg18007049	17	31617566	ACCN1	Body	-0,038	7,97E-04	4,82E-02
cg00393263	1	228336474	GJC2	TSS1500	0,007	7,97E-04	4,82E-02
cg07641653	16	67063654	CBFB	Body	0,006	7,97E-04	4,82E-02
cg03032551	2	27632711	PPM1G	TSS200	0,004	7,97E-04	4,82E-02
cg09833737	2	105859057	GPR45	1stExon	-0,011	7,97E-04	4,82E-02
cg24884166	18	72695518	ZNF407	Body	0,079	7,97E-04	4,82E-02
cg06522562	2	203500023	FAM117B	1stExon	-0,003	7,98E-04	4,82E-02
cg08028669	1	21439368	EIF4G3	TSS1500	0,009	7,98E-04	4,82E-02
cg03568039	20	52561293	BCAS1	3'UTR	-0,012	7,98E-04	4,82E-02
cg04649336	10	72439480	ADAMTS14	Body	0,013	7,98E-04	4,82E-02
cg27100085	11	62528196	POLR2G	TSS1500	0,014	7,98E-04	4,82E-02
cg09865786	12	105601850	APPL2	Body	-0,008	7,98E-04	4,83E-02
cg00599850	16	88683223	ZC3H18	Body	-0,005	7,98E-04	4,83E-02
cg19725044	2	128786239	SAP130	TSS1500	-0,004	7,99E-04	4,83E-02
cg27436049	13	105490607		IGR	0,043	7,99E-04	4,83E-02

cg06772717	8	72466764		IGR	0,022	7,99E-04	4,83E-02
cg08511716	5	94621030	MCTP1	TSS1500	-0,007	7,99E-04	4,83E-02
cg15961105	12	55370186	KIAA0748	5'UTR	-0,006	8,00E-04	4,83E-02
cg24300168	16	87800494	KLHDC4	TSS1500	-0,029	8,00E-04	4,83E-02
cg20737210	19	1918832	SCAMP4	Body	0,004	8,00E-04	4,83E-02
cg19197515	4	122686430	PP12613	Body	-0,003	8,00E-04	4,83E-02
cg09661926	6	35911587	SLC26A8	3'UTR	0,02	8,00E-04	4,83E-02
cg02856379	6	119595549	MAN1A1	Body	0,033	8,00E-04	4,83E-02
cg08575136	10	47148432	LINC00842	Body	-0,009	8,00E-04	4,83E-02
cg07182357	11	63275003	LGALS12	TSS1500	0,021	8,00E-04	4,83E-02
cg09302213	17	67515558	MAP2K6	ExonBnd	-0,005	8,00E-04	4,83E-02
cg05080815	19	35379312		IGR	-0,029	8,01E-04	4,83E-02
cg03127182	1	26421108		IGR	0,037	8,01E-04	4,83E-02
cg10174867	1	225117431	DNAH14	1stExon	-0,017	8,01E-04	4,83E-02
cg16090375	4	170913579	MFAP3L	Body	-0,006	8,01E-04	4,83E-02
cg23054426	7	19185267	FERD3L	TSS1500	0,011	8,01E-04	4,83E-02
cg08465795	13	27526037		IGR	-0,005	8,01E-04	4,83E-02
cg20541961	2	29033199	SPDYA	TSS1500	-0,004	8,01E-04	4,83E-02
cg15221192	5	60821600	ZSWIM6	Body	0,005	8,01E-04	4,83E-02
cg05861705	6	82417175		IGR	0,007	8,02E-04	4,83E-02
cg12730381	7	75988212	YWHAG	1stExon	-0,005	8,01E-04	4,83E-02
cg07865065	7	100146527	AGFG2	ExonBnd	-0,026	8,01E-04	4,83E-02
cg04578612	8	11365915	BLK	5'UTR	0,014	8,01E-04	4,83E-02
cg14335121	20	44508832	ZSWIM1	TSS1500	-0,017	8,02E-04	4,83E-02
cg07923758	3	32941766		IGR	-0,003	8,02E-04	4,83E-02
cg10473059	7	18003093		IGR	0,02	8,02E-04	4,84E-02
cg16088485	8	100353039	VPS13B	Body	-0,018	8,02E-04	4,84E-02
cg16090056	16	58586898	CNOT1	Body	-0,005	8,02E-04	4,84E-02
cg01435344	10	99610084	GOLGA7B	Body	-0,013	8,02E-04	4,84E-02
cg22821947	11	133837401		IGR	-0,018	8,02E-04	4,84E-02
cg09618385	16	87864826	SLC7A5	3'UTR	0,016	8,03E-04	4,84E-02
cg14520588	19	3963403	DAPK3	Body	0,007	8,03E-04	4,84E-02
cg17823343	20	14883307	MACROD2-AS1	Body	-0,034	8,02E-04	4,84E-02
cg00377591	11	36527638	TRAF6	5'UTR	0,022	8,03E-04	4,84E-02
cg00659527	3	111390171		IGR	-0,005	8,03E-04	4,84E-02
cg21338642	1	23695349	ZNF436-AS1	TSS1500	-0,003	8,04E-04	4,84E-02
cg04732548	7	63601086		IGR	0,056	8,04E-04	4,84E-02
cg19507638	5	93509721	C5orf36	Body	-0,007	8,04E-04	4,84E-02
cg05507902	5	14609411	FAM105A	Body	0,013	8,04E-04	4,84E-02
cg03095319	6	122280693		IGR	-0,023	8,04E-04	4,84E-02
cg12179661	9	140333805	ENTPD8	5'UTR	0,028	8,05E-04	4,85E-02
cg14285128	3	120218231		IGR	0,027	8,05E-04	4,85E-02
cg25851417	12	91395391	EPYC	Body	0,01	8,05E-04	4,85E-02
cg15734839	5	38469117		IGR	-0,025	8,06E-04	4,85E-02
cg17341703	6	88182369	SLC35A1	TSS1500	-0,004	8,06E-04	4,85E-02
cg07629776	10	13972210	FRMD4A	Body	-0,11	8,06E-04	4,85E-02
cg10206882	14	25411743	STXBP6	Body	-0,015	8,06E-04	4,85E-02
cg03062865	16	89720506	CHMP1A	Body	0,016	8,06E-04	4,85E-02
cg19923798	17	26698570	SARM1	TSS1500	0,004	8,06E-04	4,85E-02
cg07124322	18	30105947		IGR	0,009	8,06E-04	4,85E-02
cg24360457	21	29891060		IGR	-0,016	8,06E-04	4,85E-02
cg10861541	2	210444201	MAP2	TSS1500	0,05	8,06E-04	4,85E-02
cg13071563	2	240520538		IGR	0,008	8,06E-04	4,85E-02
cg25011019	3	77088311	ROBO2	TSS1500	-0,008	8,06E-04	4,85E-02
cg00441269	4	120489516	PDE5A	Body	-0,02	8,07E-04	4,85E-02
cg27079346	9	99881718		IGR	-0,017	8,06E-04	4,85E-02
cg12991791	11	3863056	RHOG	TSS1500	-0,003	8,06E-04	4,85E-02
cg01075132	17	59805883	BRIP1	Body	0,031	8,07E-04	4,85E-02
cg20159112	7	75911119	SRRM3	Body	-0,012	8,07E-04	4,85E-02
cg12705997	12	123594792	PITPNM2	5'UTR	0,004	8,07E-04	4,85E-02
cg16215259	6	4954159	CDYL	Body	-0,008	8,07E-04	4,85E-02
cg21862372	12	24104007	SOX5	5'UTR	-0,02	8,07E-04	4,85E-02
cg12831869	12	88547758	TMTC3	Body	0,023	8,07E-04	4,85E-02
cg24376802	11	2485050	KCNQ1	Body	0,009	8,07E-04	4,85E-02
cg23830082	3	43196848		IGR	-0,029	8,07E-04	4,85E-02
cg17121002	9	138130787		IGR	-0,004	8,08E-04	4,86E-02
cg22861279	14	105953244	CRIP1	TSS200	0,007	8,08E-04	4,86E-02
cg25205321	13	111096339	COL4A2	Body	0,04	8,08E-04	4,86E-02
cg20566420	12	122327043	PSMD9	Body	-0,006	8,08E-04	4,86E-02
cg02943050	3	170477868		IGR	-0,008	8,08E-04	4,86E-02
cg21297695	7	151456710	PRKAG2	Body	-0,021	8,08E-04	4,86E-02
cg26357344	12	53300293	KRT8	TSS1500	0,021	8,08E-04	4,86E-02
cg23735592	17	62701073		IGR	-0,008	8,08E-04	4,86E-02

cg04215179	7	23719682	C7orf46	TSS200	0,013	8,08E-04	4,86E-02
cg10053134	1	16203102	MIR5096	Body	-0,007	8,09E-04	4,86E-02
cg14938806	12	103965445		IGR	-0,02	8,09E-04	4,86E-02
cg06518763	17	7758421	TMEM88	1stExon	0,01	8,09E-04	4,86E-02
cg11091250	6	32056600	TNXB	Body	0,016	8,09E-04	4,86E-02
cg09657667	7	28530206	CREB5	5'UTR	-0,011	8,09E-04	4,86E-02
cg17705334	10	81002615	ZMIZ1	Body	-0,006	8,09E-04	4,86E-02
cg25091757	8	82634625	ZFAND1	TSS1500	-0,004	8,09E-04	4,86E-02
cg20148850	6	10422227		IGR	0,021	8,10E-04	4,86E-02
cg06480224	2	203013739		IGR	-0,009	8,10E-04	4,86E-02
cg04677581	7	158625220		IGR	-0,017	8,10E-04	4,86E-02
cg23033619	20	21927768		IGR	-0,01	8,10E-04	4,86E-02
cg09355428	1	212641399		IGR	0,013	8,10E-04	4,86E-02
cg11299207	6	31865794	EHMT2	TSS1500	-0,003	8,10E-04	4,86E-02
cg07019442	15	40452378	BUB1B	TSS1500	0,005	8,10E-04	4,86E-02
cg16287930	1	173324151	LOC100506023	Body	-0,013	8,11E-04	4,86E-02
cg02374837	16	8891602	PMM2	TSS200	-0,005	8,11E-04	4,86E-02
cg22176014	8	120725022		IGR	0,012	8,11E-04	4,86E-02
cg14871476	3	72191365		IGR	-0,033	8,11E-04	4,87E-02
cg24524702	15	100109440	MEF2A	5'UTR	0,004	8,11E-04	4,87E-02
cg00930833	8	41168264	SFRP1	TSS1500	-0,016	8,12E-04	4,87E-02
cg05131526	15	91447195	MAN2A2	TSS1500	0,033	8,12E-04	4,87E-02
cg13717768	16	10713540		IGR	-0,065	8,12E-04	4,87E-02
cg02485389	2	85839055	C2orf68	1stExon	-0,004	8,12E-04	4,87E-02
cg18633060	3	101404648	RPL24	ExonBnd	0,042	8,12E-04	4,87E-02
cg00583828	22	24911433	UPB1	Body	-0,01	8,12E-04	4,87E-02
cg22375283	2	114648348	ACTR3	1stExon	-0,002	8,13E-04	4,87E-02
cg25807199	3	193345871	OPA1-AS1	TSS1500	0,008	8,13E-04	4,87E-02
cg06735892	3	196725337		IGR	0,005	8,13E-04	4,87E-02
cg06015329	8	86131704	C8orf59	Body	-0,007	8,13E-04	4,87E-02
cg00400964	22	42325444		IGR	0,013	8,14E-04	4,88E-02
cg23156226	8	103132625	NCALD	5'UTR	0,009	8,14E-04	4,88E-02
cg20652681	1	53097272		IGR	-0,006	8,14E-04	4,88E-02
cg12429431	2	239305997	TRAF3IP1	Body	0,005	8,14E-04	4,88E-02
cg01020556	5	73248291		IGR	0,009	8,14E-04	4,88E-02
cg08835299	6	168965457	SMOC2	Body	-0,013	8,14E-04	4,88E-02
cg25492350	7	5280248		IGR	-0,004	8,14E-04	4,88E-02
cg05975755	8	108633393		IGR	0,01	8,14E-04	4,88E-02
cg26388423	14	72398740	RGS6	TSS1500	-0,005	8,14E-04	4,88E-02
cg10935882	18	53176051	TCF4	Body	-0,012	8,14E-04	4,88E-02
cg16030541	21	20716658		IGR	0,03	8,14E-04	4,88E-02
cg26083458	6	33280424	TAPBP	Body	-0,047	8,14E-04	4,88E-02
cg20798017	11	112214137	LOC283140	Body	0,009	8,15E-04	4,88E-02
cg10788301	17	4401722	SPNS2	TSS1500	0,025	8,15E-04	4,88E-02
cg04180299	16	67678801	RLTPR	TSS1500	0,004	8,15E-04	4,88E-02
cg22923415	10	30570487		IGR	-0,03	8,15E-04	4,88E-02
cg11759454	20	34959563	DLGAP4	5'UTR	-0,031	8,15E-04	4,88E-02
cg20676561	4	24981863	CCDC149	TSS200	-0,003	8,15E-04	4,88E-02
cg00506513	8	39542886	ADAM18	Body	-0,005	8,15E-04	4,88E-02
cg16100749	17	78972049	CHMP6	Body	0,007	8,15E-04	4,88E-02
cg04831900	1	94348103		IGR	-0,028	8,16E-04	4,88E-02
cg17829673	7	21985366	CDCA7L	Body	-0,004	8,16E-04	4,88E-02
cg06427906	16	80934298		IGR	-0,03	8,16E-04	4,88E-02
cg16693872	5	176882643	PRR7	Body	-0,003	8,16E-04	4,88E-02
cg00310201	2	98351994	ZAP70	Body	0,008	8,16E-04	4,88E-02
cg10709267	14	76841886	ESRRB	5'UTR	-0,04	8,16E-04	4,88E-02
cg14057760	12	12982506	DDX47	3'UTR	0,025	8,17E-04	4,89E-02
cg02095722	19	47421928	ARHGAP35	TSS200	-0,013	8,17E-04	4,89E-02
cg11372447	6	137676143		IGR	0,01	8,17E-04	4,89E-02
cg27198497	6	33870701		IGR	0,025	8,18E-04	4,89E-02
cg15515224	1	154063657	NUP210L	Body	0,013	8,18E-04	4,89E-02
cg10848065	4	3814739		IGR	0,014	8,18E-04	4,89E-02
cg10372829	13	20751257		IGR	-0,045	8,18E-04	4,89E-02
cg16709541	19	663602	RNF126	TSS1500	0,009	8,18E-04	4,89E-02
cg18729787	6	33246307	B3GALT4	1stExon	0,015	8,18E-04	4,89E-02
cg23997156	21	44144392	PDE9A	5'UTR	0,01	8,18E-04	4,89E-02
cg03517341	22	43873298	MPPED1	Body	0,014	8,19E-04	4,89E-02
cg27474707	5	140260937	PCDHA13	TSS1500	-0,041	8,19E-04	4,89E-02
cg08166767	1	152671348	LCE2A	5'UTR	-0,043	8,19E-04	4,89E-02
cg08350814	2	191045309	C2orf88	TSS1500	-0,006	8,19E-04	4,89E-02
cg08389300	10	120671241		IGR	0,01	8,19E-04	4,89E-02
cg25150773	11	75308706	MAP6	Body	0,023	8,19E-04	4,89E-02
cg25232967	15	86124656	AKAP13	Body	0,026	8,19E-04	4,89E-02

cg26434090	11	117665395	DSCAML1	Body	-0,047	8,20E-04	4,90E-02
cg17867243	15	42371653	PLA2G4D	Body	-0,063	8,20E-04	4,90E-02
cg26665055	1	201924479	TIMM17A	TSS200	-0,003	8,20E-04	4,90E-02
cg02165556	4	121684361	PRDM5	Body	0,01	8,20E-04	4,90E-02
cg21142942	6	36193115	BRPF3	Body	-0,007	8,20E-04	4,90E-02
cg25462229	6	163731242	PACRG	Body	-0,01	8,20E-04	4,90E-02
cg25797568	21	27106535	ATP5J	5'UTR	-0,005	8,20E-04	4,90E-02
cg01634142	12	28471791	CCDC91	Body	0,039	8,21E-04	4,90E-02
cg22869011	2	179152543	OSBPL6	5'UTR	0,042	8,21E-04	4,90E-02
cg20857372	6	15524714	DTNBP1	Body	-0,004	8,21E-04	4,90E-02
cg15627410	19	40972132	SPTBN4	TSS1500	0,016	8,21E-04	4,90E-02
cg11395402	14	32362889		IGR	-0,015	8,21E-04	4,90E-02
cg24141233	1	212781895	ATF3	TSS200	0,009	8,21E-04	4,90E-02
cg03084476	1	230719650		IGR	-0,018	8,21E-04	4,90E-02
cg20122488	17	17396436	MED9	3'UTR	0,022	8,21E-04	4,90E-02
cg05536998	18	46500646		IGR	0,011	8,21E-04	4,90E-02
cg27495336	19	11708223	ZNF627	TSS200	-0,004	8,21E-04	4,90E-02
cg01205372	1	109778208	SARS	Body	0,011	8,22E-04	4,90E-02
cg00539654	4	110910455	EGF	Body	-0,008	8,22E-04	4,90E-02
cg01904977	12	103816964	C12orf42	Body	-0,051	8,22E-04	4,90E-02
cg26273310	21	38104779	SIM2	Body	-0,012	8,22E-04	4,90E-02
cg09848297	2	44995262	CAMKMT	Body	-0,032	8,22E-04	4,90E-02
cg14453945	1	161327008	SDHC	Body	0,006	8,22E-04	4,90E-02
cg00480298	17	44068857	MAPT	Body	-0,022	8,22E-04	4,90E-02
cg16716883	10	74079772		IGR	-0,004	8,22E-04	4,90E-02
cg23055496	3	6906371	GRM7	Body	-0,034	8,23E-04	4,90E-02
cg17566161	7	44794762	ZMIZ2	TSS1500	0,015	8,23E-04	4,90E-02
cg26263340	2	73511667		IGR	0,005	8,23E-04	4,90E-02
cg16311881	15	96389537		IGR	0,016	8,23E-04	4,90E-02
cg13965328	20	2539876	TMC2	Body	-0,029	8,23E-04	4,90E-02
cg16860556	9	133932814	LAMC3	Body	0,01	8,23E-04	4,90E-02
cg21535761	2	201305436	SPATS2L	Body	0,021	8,24E-04	4,90E-02
cg03629429	5	230956	SDHA	Body	-0,004	8,23E-04	4,90E-02
cg02226953	7	150525449		IGR	-0,018	8,24E-04	4,90E-02
cg10754697	17	1504809	SLC43A2	Body	-0,011	8,23E-04	4,90E-02
cg15788533	2	106720330	UXS1	Body	-0,01	8,24E-04	4,91E-02
cg21301831	7	6647127	C7orf26	Body	-0,008	8,24E-04	4,91E-02
cg24509584	11	29983897		IGR	-0,022	8,24E-04	4,91E-02
cg11637017	6	116566328	NT5DC1	3'UTR	0,035	8,24E-04	4,91E-02
cg18669139	7	50346768	IKZF1	5'UTR	-0,008	8,24E-04	4,91E-02
cg02586689	11	22883151	CCDC179	TSS1500	-0,005	8,25E-04	4,91E-02
cg25999309	15	43940386	CATSPER2	5'UTR	-0,017	8,25E-04	4,91E-02
cg13040921	19	33863415	CEBPG	TSS1500	0,012	8,25E-04	4,91E-02
cg22272803	7	155989369		IGR	0,023	8,25E-04	4,91E-02
cg24733530	8	24799771		IGR	-0,005	8,25E-04	4,91E-02
cg22370022	19	33937297	PEPD	Body	-0,004	8,25E-04	4,91E-02
cg16992985	1	93270270		IGR	0,014	8,25E-04	4,91E-02
cg01827910	2	31441588	CAPN14	TSS1500	-0,005	8,25E-04	4,91E-02
cg02135170	2	110364520	sept-10	Body	-0,035	8,25E-04	4,91E-02
cg12598905	8	125672004	MTSS1	Body	0,003	8,26E-04	4,91E-02
cg18214513	2	10700146		IGR	-0,005	8,26E-04	4,91E-02
cg21707175	4	22692551		IGR	-0,011	8,26E-04	4,91E-02
cg02202377	10	20380851	PLXDC2	Body	0,042	8,26E-04	4,91E-02
cg06454894	2	88415702		IGR	-0,015	8,27E-04	4,91E-02
cg00698535	16	81812719	PLCG2	TSS1500	-0,003	8,27E-04	4,91E-02
cg14400946	2	25805233	DTNB	Body	0,019	8,27E-04	4,92E-02
cg13635072	6	72861782	RIMS1	Body	-0,025	8,27E-04	4,92E-02
cg02261480	12	4529962		IGR	-0,012	8,27E-04	4,92E-02
cg23012731	1	33438978		IGR	-0,01	8,28E-04	4,92E-02
cg15265313	1	61548170	NFIA	5'UTR	-0,003	8,27E-04	4,92E-02
cg20784768	1	234041456	SLC35F3	Body	-0,042	8,28E-04	4,92E-02
cg25878744	1	244303505		IGR	-0,005	8,28E-04	4,92E-02
cg12780678	2	1746846	PXDN	Body	-0,005	8,27E-04	4,92E-02
cg18947710	3	16216045	GALNT15	TSS200	-0,039	8,28E-04	4,92E-02
cg03084350	3	38065265	PLCD1	Body	0,018	8,28E-04	4,92E-02
cg07493874	5	1342172	CLPTM1L	Body	0,027	8,28E-04	4,92E-02
cg17557357	5	140729608	PCDHGA2	Body	-0,014	8,28E-04	4,92E-02
cg05896606	5	174891608		IGR	-0,006	8,28E-04	4,92E-02
cg23146346	6	17282859	RBM24	TSS200	0,033	8,28E-04	4,92E-02
cg12093799	7	35708408	HERPUD2	Body	0,021	8,28E-04	4,92E-02
cg08606227	7	128095950	C7orf68	1stExon	-0,002	8,27E-04	4,92E-02
cg19029747	13	67805458	PCDH9	TSS1500	0,004	8,27E-04	4,92E-02
cg15430743	18	31676948	NOL4	Body	0,024	8,28E-04	4,92E-02

cg03660901	18	76473146		IGR	-0,066	8,28E-04	4,92E-02
cg09676033	20	21120677	KIZ	Body	-0,007	8,28E-04	4,92E-02
cg26930889	14	39519326	SEC23A	Body	-0,007	8,29E-04	4,92E-02
cg25142582	2	27435069	SLC5A6	5'UTR	-0,005	8,29E-04	4,92E-02
cg16352333	8	28967565	KIF13B	Body	0,014	8,29E-04	4,92E-02
cg14031033	12	48119770	P11	TSS1500	0,007	8,29E-04	4,92E-02
cg22307663	12	62916317	MON2	Body	-0,02	8,29E-04	4,92E-02
cg07338200	2	8360098	LINC00299	Body	0,013	8,30E-04	4,92E-02
cg21685048	2	17771856	VSNL1	5'UTR	-0,032	8,30E-04	4,92E-02
cg14257839	17	9924479	GAS7	5'UTR	0,026	8,30E-04	4,92E-02
cg25664220	3	72788482		IGR	0,044	8,30E-04	4,92E-02
cg24728949	5	106820169	EFNA5	Body	0,019	8,30E-04	4,92E-02
cg17605713	12	30571008		IGR	-0,006	8,30E-04	4,92E-02
cg17879376	1	32212024	BAI2	Body	0,02	8,30E-04	4,92E-02
cg00040426	9	138413253	LCN1	TSS200	0,026	8,30E-04	4,92E-02
cg13039393	6	151639747	AKAP12	Body	-0,015	8,31E-04	4,92E-02
cg11983727	11	128801648		IGR	-0,009	8,31E-04	4,93E-02
cg26055840	14	100118833	HHIPL1	Body	0,014	8,31E-04	4,93E-02
cg02182794	4	13369795	RAB28	3'UTR	0,007	8,31E-04	4,93E-02
cg01811325	10	131757672	EBF3	Body	-0,018	8,31E-04	4,93E-02
cg03751149	7	135443227		IGR	-0,033	8,31E-04	4,93E-02
cg04462898	4	120328372	LINC01061	Body	0,02	8,31E-04	4,93E-02
cg00217442	8	23022694	TNFRSF10D	TSS1500	-0,019	8,31E-04	4,93E-02
cg13615839	1	218094953		IGR	0,048	8,32E-04	4,93E-02
cg13491563	3	183903302	ABCF3	TSS1500	-0,019	8,32E-04	4,93E-02
cg00016223	6	36515007	STK38	1stExon	-0,003	8,32E-04	4,93E-02
cg01986070	6	89600688	RNGTT	Body	0,011	8,32E-04	4,93E-02
cg12055053	17	19659407		IGR	0,016	8,32E-04	4,93E-02
cg05477778	19	16480028	EPS15L1	Body	-0,037	8,32E-04	4,93E-02
cg08805210	10	8441710		IGR	0,011	8,32E-04	4,93E-02
cg15960924	3	32151282	GPD1L	Body	-0,007	8,32E-04	4,93E-02
cg26525861	11	68205594	LRP5	Body	0,006	8,32E-04	4,93E-02
cg08697406	5	143519592		IGR	-0,015	8,33E-04	4,93E-02
cg04766314	13	100949400	PCCA	Body	0,011	8,33E-04	4,93E-02
cg15012939	7	158278869	PTPRN2	Body	-0,023	8,33E-04	4,93E-02
cg17690202	8	145065187	GRINA	5'UTR	0,01	8,33E-04	4,93E-02
cg05707458	1	205425241		IGR	0,008	8,33E-04	4,93E-02
cg20800221	12	113443790	OAS2	Body	0,012	8,33E-04	4,93E-02
cg22593095	5	135363107	TGFBI	TSS1500	0,013	8,33E-04	4,93E-02
cg09258067	6	100529812		IGR	-0,011	8,33E-04	4,93E-02
cg20431676	16	88815953	FAM38A	Body	-0,014	8,34E-04	4,93E-02
cg10259462	17	72336179	KIF19	Body	-0,021	8,34E-04	4,93E-02
cg18456138	3	4588688	ITPR1	Body	0,033	8,34E-04	4,93E-02
cg01798097	19	44644849	ZNF234	TSS1500	0,045	8,34E-04	4,93E-02
cg15158924	4	21710948	KCNIP4	5'UTR	0,014	8,34E-04	4,93E-02
cg18683143	3	184429764	MAGEF1	1stExon	0,012	8,34E-04	4,93E-02
cg11052202	6	99290546		IGR	-0,02	8,34E-04	4,93E-02
cg09488838	8	17581251	MTUS1	TSS1500	0,042	8,34E-04	4,93E-02
cg08496775	8	80523226	STMN2	TSS200	-0,045	8,34E-04	4,93E-02
cg02273875	9	130679474	ST6GALNAC4	TSS200	0,005	8,34E-04	4,93E-02
cg15995714	10	134222445	PWWP2B	3'UTR	0,022	8,34E-04	4,93E-02
cg07143919	6	43351643		IGR	-0,002	8,34E-04	4,93E-02
cg17523253	17	56072358		IGR	0,008	8,34E-04	4,93E-02
cg09330643	10	72142276	LRRC20	5'UTR	-0,005	8,35E-04	4,93E-02
cg05214947	10	129816761	PTPRE	5'UTR	-0,011	8,35E-04	4,93E-02
cg20829454	12	76630497		IGR	-0,009	8,35E-04	4,93E-02
cg19716981	8	86157450	CA13	TSS1500	-0,003	8,35E-04	4,94E-02
cg19845715	13	20703121		IGR	-0,006	8,35E-04	4,94E-02
cg11170479	14	93517493	ITPK1	Body	-0,004	8,35E-04	4,94E-02
cg24435407	1	11909677		IGR	0,006	8,35E-04	4,94E-02
cg16138024	12	50068554	FMNL3	Body	-0,024	8,36E-04	4,94E-02
cg06391982	3	13936753		IGR	-0,02	8,36E-04	4,94E-02
cg16364802	2	33069143	LINC00486	Body	0,008	8,37E-04	4,94E-02
cg20268018	2	210867683	RPE	5'UTR	0,004	8,37E-04	4,94E-02
cg21305371	4	159927510	C4orf45	Body	-0,013	8,36E-04	4,94E-02
cg03815571	6	170599840	DLL1	TSS200	-0,005	8,36E-04	4,94E-02
cg15195837	3	65675612	MAGI1	Body	0,007	8,37E-04	4,94E-02
cg22447539	7	5633086	FSCN1	1stExon	-0,008	8,37E-04	4,94E-02
cg25549811	8	73373122		IGR	0,022	8,37E-04	4,94E-02
cg22158388	4	151879133	LRBA	Body	-0,019	8,37E-04	4,94E-02
cg04223956	1	198201614	NEK7	5'UTR	-0,007	8,38E-04	4,94E-02
cg20039263	4	157218394		IGR	-0,017	8,38E-04	4,94E-02
cg15133477	9	34839982	FAM205BP	TSS1500	0,025	8,38E-04	4,94E-02

cg14343178	9	127614469	WDR38	TSS1500	0,01	8,38E-04	4,94E-02
cg13441753	9	139127333	QSOX2	Body	-0,005	8,37E-04	4,94E-02
cg05344692	16	68544404		IGR	0,014	8,38E-04	4,94E-02
cg19277521	21	34018085	SYNJ1	ExonBnd	-0,007	8,38E-04	4,94E-02
cg09017718	1	10758995	CASZ1	5'UTR	0,007	8,38E-04	4,94E-02
cg25909730	6	88930271		IGR	-0,039	8,38E-04	4,95E-02
cg27451089	18	63605791		IGR	-0,024	8,38E-04	4,95E-02
cg16114709	2	23721660	KLHL29	5'UTR	0,018	8,39E-04	4,95E-02
cg11923054	4	4250051	TMEM128	TSS200	-0,003	8,39E-04	4,95E-02
cg17763553	8	18953483		IGR	0,039	8,39E-04	4,95E-02
cg04308589	10	79110881	KCNMA1	Body	0,025	8,39E-04	4,95E-02
cg18829134	3	172115678	FNDC3B	3'UTR	0,034	8,39E-04	4,95E-02
cg25459301	8	10941183	XKR6	Body	0,008	8,39E-04	4,95E-02
cg17163179	1	70497127	LRRC7	Body	0,035	8,40E-04	4,95E-02
cg07642569	3	151910095		IGR	-0,023	8,40E-04	4,95E-02
cg10977869	5	124470315	LOC101927421	Body	-0,009	8,40E-04	4,95E-02
cg07663788	12	109250445	SSH1	Body	-0,024	8,40E-04	4,95E-02
cg22725947	13	52158432	WDFY2	TSS200	-0,004	8,40E-04	4,95E-02
cg05176996	9	98637794	C9orf130	Body	-0,003	8,40E-04	4,95E-02
cg03651715	6	151645054	AKAP12	Body	0,034	8,41E-04	4,95E-02
cg08608240	16	1080808		IGR	-0,008	8,41E-04	4,95E-02
cg02453603	2	128615626	POLR2D	1stExon	-0,003	8,41E-04	4,95E-02
cg27438767	15	81589187	IL16	TSS200	-0,002	8,41E-04	4,96E-02
cg12530140	1	73183527		IGR	0,033	8,41E-04	4,96E-02
cg27226525	8	145981945	ZNF251	TSS1500	0,006	8,41E-04	4,96E-02
cg06352218	12	8996607	A2ML1	TSS1500	-0,005	8,41E-04	4,96E-02
cg08234376	10	70100076	HNRNPH3	Body	-0,004	8,41E-04	4,96E-02
cg04192867	10	93647053		IGR	0,006	8,42E-04	4,96E-02
cg22881573	3	49638532	BSN	Body	-0,006	8,42E-04	4,96E-02
cg25052619	5	53843486		IGR	0,019	8,42E-04	4,96E-02
cg12007236	14	50999402	ATL1	TSS1500	-0,003	8,42E-04	4,96E-02
cg12334758	1	212391504	LOC101929541	Body	0,01	8,42E-04	4,96E-02
cg06501267	14	95577676	DICER1	Body	0,006	8,42E-04	4,96E-02
cg15697225	12	133186923		IGR	-0,046	8,42E-04	4,96E-02
cg04204557	21	46065774	KRTAP10-11	TSS1500	-0,027	8,42E-04	4,96E-02
cg19193574	6	155226498		IGR	-0,007	8,42E-04	4,96E-02
cg01982788	12	56556339	SMARCC2	3'UTR	0,014	8,42E-04	4,96E-02
cg09222890	12	6494180	LTBR	Body	0,027	8,43E-04	4,96E-02
cg03339618	16	78455152	WWOX	Body	0,01	8,43E-04	4,96E-02
cg14801238	6	31275664		IGR	-0,046	8,43E-04	4,96E-02
cg01486694	1	204121458	ETNK2	TSS200	-0,006	8,44E-04	4,96E-02
cg10121586	2	175436504	WIPF1	Body	0,008	8,44E-04	4,96E-02
cg18342425	13	25337983	RNF17	TSS1500	0,022	8,44E-04	4,96E-02
cg21777415	3	9446652	SETD5	5'UTR	-0,023	8,44E-04	4,96E-02
cg18765127	6	30128254	TRIM10	1stExon	0,017	8,44E-04	4,96E-02
cg00462443	7	142424877		IGR	0,039	8,44E-04	4,96E-02
cg07794665	10	30224680		IGR	-0,009	8,44E-04	4,96E-02
cg08161922	12	2163608	CACNA1C	Body	-0,019	8,44E-04	4,96E-02
cg19784903	17	45786737	TBKBP1	Body	-0,004	8,44E-04	4,96E-02
cg07896268	17	72428187	GPRC5C	1stExon	-0,007	8,44E-04	4,96E-02
cg06749753	20	48403009		IGR	0,044	8,45E-04	4,96E-02
cg26157361	3	105434386	CBLB	Body	0,032	8,45E-04	4,97E-02
cg17876308	5	162864496	CCNG1	TSS200	-0,004	8,45E-04	4,97E-02
cg19457556	10	62494027	ANK3	TSS1500	-0,008	8,45E-04	4,97E-02
cg08649179	10	99570368		IGR	-0,022	8,45E-04	4,97E-02
cg18133074	11	1473084	BRSK2	Body	0,011	8,45E-04	4,97E-02
cg21300659	15	59994115		IGR	-0,006	8,45E-04	4,97E-02
cg16722931	17	75312494	sept-09	5'UTR	0,015	8,45E-04	4,97E-02
cg05196982	21	20162412		IGR	0,025	8,45E-04	4,97E-02
cg14057241	9	132402804	ASB6	Body	-0,024	8,46E-04	4,97E-02
cg03348466	15	91104770	CRTC3	Body	-0,018	8,46E-04	4,97E-02
cg15273270	4	36324571	DTHD1	Body	-0,034	8,46E-04	4,97E-02
cg17524498	12	80447515		IGR	0,011	8,46E-04	4,97E-02
cg12575672	10	13968042	FRMD4A	Body	-0,011	8,46E-04	4,97E-02
cg11059617	14	61191084	SIX4	TSS1500	-0,009	8,46E-04	4,97E-02
cg06796120	17	80772942	TBCD	Body	-0,005	8,46E-04	4,97E-02
cg06328061	8	116681622	TRPS1	TSS1500	-0,002	8,47E-04	4,97E-02
cg03750061	10	119131167	PDZD8	Body	0,035	8,47E-04	4,97E-02
cg26426437	12	121686502	CAMKK2	Body	0,014	8,47E-04	4,97E-02
cg24409442	17	10521903	MYHAS	Body	0,019	8,47E-04	4,97E-02
cg22757270	16	9891418	GRIN2A	Body	0,044	8,47E-04	4,97E-02
cg15433411	17	40086473	TTC25	TSS1500	-0,003	8,47E-04	4,97E-02
cg06111254	1	66378294	PDE4B	TSS1500	0,013	8,47E-04	4,97E-02

cg01682157	6	1516643		IGR	0,01	8,47E-04	4,97E-02
cg13719712	11	43959984		IGR	-0,024	8,47E-04	4,97E-02
cg24531077	12	113440283	OAS2	Body	0,005	8,48E-04	4,97E-02
cg21827969	4	56212299	SRD5A3	TSS200	-0,005	8,48E-04	4,97E-02
cg20027578	6	168476697	FRMD1	TSS200	-0,011	8,48E-04	4,97E-02
cg10509076	7	104503457	LHFPL3	Body	-0,005	8,48E-04	4,97E-02
cg11334510	10	17104215	CUBN	Body	0,01	8,48E-04	4,97E-02
cg01067446	12	122190026	TMEM120B	ExonBnd	-0,005	8,48E-04	4,97E-02
cg03125605	16	67236337	MIR328	TSS200	0,012	8,48E-04	4,97E-02
cg15314164	1	100459183	SLC35A3	Body	0,018	8,49E-04	4,98E-02
cg16865492	19	34895283	PDCD2L	TSS200	-0,007	8,49E-04	4,98E-02
cg11560672	6	92526239		IGR	-0,017	8,49E-04	4,98E-02
cg21919766	11	119051923	NLRX1	Body	0,028	8,49E-04	4,98E-02
cg11682959	7	84505961		IGR	-0,013	8,49E-04	4,98E-02
cg11863217	7	157281332		IGR	-0,008	8,49E-04	4,98E-02
cg25245133	17	46802064	C17orf93	Body	-0,004	8,50E-04	4,98E-02
cg07096051	11	45703871		IGR	-0,009	8,50E-04	4,98E-02
cg05237369	1	219617291		IGR	-0,02	8,50E-04	4,98E-02
cg00582970	17	60947793		IGR	-0,041	8,50E-04	4,98E-02
cg14792081	1	65468460		IGR	0,003	8,50E-04	4,98E-02
cg02466008	5	14508414	TRIO	Body	0,007	8,50E-04	4,98E-02
cg19856013	6	51342462		IGR	-0,008	8,50E-04	4,98E-02
cg16807089	7	143582499	FAM115A	5'UTR	-0,014	8,50E-04	4,98E-02
cg06981391	10	14050572	FRMD4A	Body	0,026	8,50E-04	4,98E-02
cg13666041	14	69442697	ACTN1	Body	0,023	8,50E-04	4,98E-02
cg14249036	18	57477963		IGR	-0,005	8,50E-04	4,98E-02
cg16701228	2	216002243	ABCA12	Body	0,04	8,50E-04	4,98E-02
cg24399432	1	24796905	NIPAL3	3'UTR	-0,004	8,51E-04	4,98E-02
cg04251802	1	248159286	OR2L13	5'UTR	-0,013	8,51E-04	4,98E-02
cg14358282	2	219866628	MIR375	TSS200	0,012	8,51E-04	4,98E-02
cg03386956	8	25187433	DOCK5	Body	0,006	8,51E-04	4,98E-02
cg13088102	20	10200189	SNAP25	5'UTR	-0,005	8,51E-04	4,98E-02
cg06085476	4	56413854	CLOCK	TSS1500	-0,011	8,51E-04	4,98E-02
cg02092328	15	26261124	LOC100128714	Body	0,042	8,51E-04	4,98E-02
cg01272296	18	21476793	LAMA3	Body	-0,007	8,52E-04	4,98E-02
cg25503542	3	10240318	IRAK2	Body	0,018	8,52E-04	4,98E-02
cg17311528	12	125092428		IGR	0,01	8,52E-04	4,98E-02
cg01413780	2	203749516	WDR12	Body	-0,005	8,52E-04	4,99E-02
cg24726946	10	52611825	A1CF	Body	-0,006	8,52E-04	4,99E-02
cg11409060	12	14518540	ATF7IP	TSS200	-0,006	8,52E-04	4,99E-02
cg15083678	12	2724200	CACNA1C	Body	-0,016	8,52E-04	4,99E-02
cg20236625	16	57653092	ADGRG1	TSS1500	0,014	8,52E-04	4,99E-02
cg21622764	1	145589449	NUDT17	TSS200	0,004	8,53E-04	4,99E-02
cg19550574	2	217919783		IGR	0,027	8,53E-04	4,99E-02
cg04411052	14	72219061		IGR	0,004	8,53E-04	4,99E-02
cg09147880	16	56716367	MT1X	TSS200	0,006	8,53E-04	4,99E-02
cg01658333	10	97364228		IGR	0,012	8,54E-04	4,99E-02
cg24727383	13	102140931	ITGBL1	TSS1500	-0,006	8,54E-04	4,99E-02
cg13999554	14	74188507	ELMSAN1	Body	0,011	8,54E-04	4,99E-02
cg08281866	8	32539517	NRG1	Body	-0,032	8,54E-04	4,99E-02
cg12381317	12	48500521	PFKM	5'UTR	-0,064	8,54E-04	4,99E-02
cg20562176	19	8008963	TIMM44	TSS1500	-0,003	8,54E-04	4,99E-02
cg09247362	11	123994685	VWA5A	Body	0,011	8,54E-04	4,99E-02
cg18260919	3	135869103	MSL2	3'UTR	0,024	8,55E-04	4,99E-02
cg24820916	14	52313772		IGR	-0,003	8,55E-04	4,99E-02
cg06879567	18	3594243	DLGAP1	Body	-0,004	8,55E-04	4,99E-02
cg04687439	19	996220		IGR	-0,036	8,55E-04	4,99E-02
cg02280503	20	62375973	ZBTB46	3'UTR	0,027	8,55E-04	4,99E-02
cg12064083	22	43197590	ARFGAP3	Body	0,006	8,55E-04	4,99E-02
cg06382831	1	210008499	DIEXF	ExonBnd	-0,018	8,55E-04	4,99E-02
cg01691063	2	210555274	MAP2	Body	0,033	8,55E-04	4,99E-02
cg01763902	6	112680698		IGR	0,011	8,55E-04	4,99E-02
cg02091951	20	33410342	NCOA6	5'UTR	-0,006	8,55E-04	4,99E-02
cg10911865	3	44903235	MIR564	TSS200	-0,005	8,56E-04	5,00E-02
cg23378491	6	33754947	LEMD2	TSS1500	0,01	8,56E-04	5,00E-02
cg07792815	16	19474834	TMC5	Body	-0,014	8,56E-04	5,00E-02
cg14519997	9	14321079		IGR	-0,049	8,56E-04	5,00E-02
cg17782950	20	44045785	PIGT	Body	0,035	8,56E-04	5,00E-02
cg13155079	11	44286499	ALX4	Body	0,013	8,56E-04	5,00E-02
cg14563732	8	143474839	TSNARE1	5'UTR	-0,014	8,57E-04	5,00E-02
cg19272754	11	66631469	PC	Body	0,022	8,57E-04	5,00E-02
