# Supplementary material for: Epigenetic regulation of transcription factor binding motifs promotes Th1 response in Chagas disease cardiomyopathy
Source: Front Immunol. 2022 Aug 22;13:958200. doi: 10.3389/fimmu.2022.958200 (PMC9441916; doi:10.3389/fimmu.2022.958200)
Supplement: Supplementary Table 1 — Biological samples included in this study. [file DataSheet_1.zip › Supplementary Material/Supplementary Table 15.pdf]

Supplementary table 15. List of the Differentially Methylated CpGs between blood moderate CCC samples and blood severe CCC sa

ID	Chromosome	Position	Gene	Feature	deltaBeta	pvalue	Corrected pvalue
cg19209225	10	102756912	LZTS2	TSS200	0,027	8,42E-14	6,21E-08
cg20141578	12	12225262	BCL2L14	5'UTR	0,137	1,48E-11	5,44E-06
cg18015640	1	93310900	FAM69A	Body	0,012	8,46E-11	2,08E-05
cg15140996	15	95038079		IGR	0,013	3,01E-10	5,54E-05
cg24045875	20	61425320		IGR	0,013	4,06E-09	5,98E-04
cg10821332	3	38164739	ACAA1	Body	-0,009	6,04E-09	7,42E-04
cg07361047	6	167533317	CCR6	5'UTR	0,061	7,46E-09	7,85E-04
cg25880573	1	24073129	TCEB3	Body	0,033	9,54E-09	8,78E-04
cg14801238	6	31275664		IGR	-0,074	1,64E-08	1,12E-03
cg17835356	8	41387960	GINS4	Body	0,045	1,56E-08	1,12E-03
cg13471932	9	135992243	RALGDS	Body	-0,022	1,90E-08	1,12E-03
cg21150100	10	103132349	BTRC	Body	0,015	1,89E-08	1,12E-03
cg23731908	15	48687756		IGR	-0,009	1,97E-08	1,12E-03
cg26916851	4	141117172		IGR	0,056	2,62E-08	1,21E-03
cg15636086	6	35057214	ANKS1A	3'UTR	-0,015	2,75E-08	1,21E-03
cg09144424	10	97050675	PDLIM1	1stExon	0,006	2,95E-08	1,21E-03
cg18797859	12	96253319	SNRPF	Body	-0,017	2,86E-08	1,21E-03
cg23195373	19	45954891		IGR	0,039	2,69E-08	1,21E-03
cg25998667	3	156841889	LINC00880	TSS1500	-0,028	4,03E-08	1,25E-03
cg12140212	4	188477987	)C100506272	Body	0,06	3,57E-08	1,25E-03
cg00581130	5	60240568	ERCC8	Body	0,014	3,73E-08	1,25E-03
cg22792014	10	134267908		IGR	-0,018	3,24E-08	1,25E-03
cg21971804	11	61829023		IGR	0,014	4,09E-08	1,25E-03
cg12251659	12	122018385	KDM2B	TSS200	0,006	4,01E-08	1,25E-03
cg07864883	3	42743077	HHATL	5'UTR	0,023	4,91E-08	1,45E-03
cg08486042	1	1200662	UBE2J2	5'UTR	0,008	5,65E-08	1,60E-03
cg25773490	3	5020685	BHLHE40	TSS1500	0,003	6,22E-08	1,64E-03
cg22432580	14	54955579	GMFB	Body	-0,006	6,08E-08	1,64E-03
cg25516604	3	156871707	CCNL1	Body	-0,037	7,19E-08	1,71E-03
cg20031843	8	17656574	MTUS1	5'UTR	0,024	7,20E-08	1,71E-03
cg10369169	17	7312041	NLGN2	Body	-0,035	6,87E-08	1,71E-03
cg08322923	5	172884008		IGR	0,046	7,49E-08	1,72E-03
cg14794786	7	150815320	AGAP3	Body	0,007	7,76E-08	1,73E-03
cg16594214	11	70035873		IGR	-0,019	8,07E-08	1,75E-03
cg07849438	12	19593035	AEBP2	1stExon	0,013	9,34E-08	1,97E-03
cg07870479	16	1813987	MAPK8IP3	Body	-0,012	1,00E-07	2,05E-03
cg20198768	6	29635579	MOG	3'UTR	0,093	1,05E-07	2,10E-03
cg25905016	2	24267482	C2orf44	5'UTR	0,019	1,14E-07	2,14E-03
cg11499025	5	140739655	PCDHGA4	Body	-0,05	1,11E-07	2,14E-03
cg13263947	2	191443609		IGR	0,191	1,47E-07	2,35E-03
cg05588228	5	145132641		IGR	0,007	1,47E-07	2,35E-03
cg21093789	7	17070291		IGR	-0,023	1,34E-07	2,35E-03
cg01449168	7	36339405	EEPD1	3'UTR	0,008	1,40E-07	2,35E-03
cg12509268	12	124911444	NCOR2	Body	-0,023	1,43E-07	2,35E-03
cg22795788	13	26669386		IGR	0,087	1,39E-07	2,35E-03
cg10398116	19	48958792	KCNJ14	TSS200	-0,014	1,41E-07	2,35E-03
cg23328066	1	92951681	GFI1	5'UTR	0,035	1,56E-07	2,45E-03
cg11195689	15	93127878		IGR	-0,014	1,60E-07	2,46E-03
cg00695694	2	171638962	ERICH2	TSS1500	-0,012	1,95E-07	2,67E-03
cg05725489	7	600658	PRKAR1B	Body	-0,02	1,87E-07	2,67E-03
cg23477281	10	89263190	MIR4678	TSS1500	-0,008	1,80E-07	2,67E-03
cg20434012	13	99096515	FARP1	Body	-0,011	1,90E-07	2,67E-03
cg19728741	14	105953457	CRIP1	5'UTR	0,011	1,84E-07	2,67E-03
cg15268101	15	90704119		IGR	0,014	1,96E-07	2,67E-03
cg15777212	7	28278486	JAZF1-AS1	Body	-0,02	2,03E-07	2,67E-03
cg19683251	17	60762219	MRC2	Body	-0,009	2,02E-07	2,67E-03
cg16622899	7	1577003	MAFK	5'UTR	-0,006	2,08E-07	2,69E-03
cg05980603	2	33556879	LTBP1	Body	-0,014	2,25E-07	2,86E-03

cg05321495	19	34167989	CHST8	5'UTR	0,072	2,32E-07	2,90E-03
cg13890859	6	30595618	ATAT1	ExonBnd	-0,018	2,45E-07	2,97E-03
cg26048140	20	46365812	SULF2	Body	-0,018	2,50E-07	2,97E-03
cg07395574	22	24034867	RGL4	ExonBnd	-0,02	2,46E-07	2,97E-03
cg19302283	4	75720398	BTC	TSS1500	-0,025	2,75E-07	3,03E-03
cg12528649	11	46407116	CHRM4	1stExon	-0,009	2,73E-07	3,03E-03
cg19511862	12	125478458	BRI3BP	1stExon	0,005	2,65E-07	3,03E-03
cg02383368	19	7684468	XAB2	3'UTR	-0,012	2,74E-07	3,03E-03
cg13313314	22	45566793	NUP50	5'UTR	-0,032	2,76E-07	3,03E-03
cg27013063	5	122848414	CSNK1G3	5'UTR	0,011	3,05E-07	3,28E-03
cg07876131	16	47921600		IGR	-0,065	3,11E-07	3,28E-03
cg07261621	19	1065860	HMHA1	TSS1500	-0,015	3,08E-07	3,28E-03
cg15466587	1	174992776	MRPS14	TSS1500	0,006	3,19E-07	3,30E-03
cg16242336	4	47846504	)C101927179	Body	-0,007	3,40E-07	3,43E-03
cg16113772	6	2972824	SERPINB6	TSS1500	-0,04	3,40E-07	3,43E-03
cg22664798	10	134750215	C10orf93	Body	-0,022	3,45E-07	3,44E-03
cg19036016	7	151077005		IGR	-0,008	3,59E-07	3,48E-03
cg21278129	19	7797355	CLEC4G	TSS1500	0,05	3,56E-07	3,48E-03
cg17910274	13	102068234	NALCN	5'UTR	-0,049	3,85E-07	3,68E-03
cg07703378	1	153505275		IGR	0,054	4,07E-07	3,69E-03
cg16814895	3	184529876	VPS8	TSS200	0,007	4,51E-07	3,69E-03
cg27556598	5	118836944	HSD17B4	Body	-0,012	4,41E-07	3,69E-03
cg12766777	6	42112884		IGR	0,031	4,37E-07	3,69E-03
cg25995854	6	169690271		IGR	0,044	4,56E-07	3,69E-03
cg15646967	7	43795266		IGR	-0,015	4,58E-07	3,69E-03
cg22675550	7	78841107	MAGI2	Body	-0,013	4,22E-07	3,69E-03
cg23910634	7	116166337	CAV1	5'UTR	-0,008	4,44E-07	3,69E-03
cg03083414	7	143602071		IGR	0,014	4,46E-07	3,69E-03
cg02660271	8	18815426	PSD3	Body	0,049	4,48E-07	3,69E-03
cg07088328	8	38089161	DDHD2	1stExon	0,006	4,60E-07	3,69E-03
cg16066696	14	77843743	C14orf174	TSS200	0,008	4,39E-07	3,69E-03
cg23114881	14	88851550	SPATA7	TSS1500	-0,01	4,14E-07	3,69E-03
cg18605086	21	30243913		IGR	-0,008	4,48E-07	3,69E-03
cg01933487	22	39795565	MAP3K7IP1	TSS200	0,006	4,32E-07	3,69E-03
cg09184819	10	113113338		IGR	-0,026	4,67E-07	3,70E-03
cg02724667	1	161044980	PVRL4	Body	-0,019	5,08E-07	3,75E-03
cg26900875	4	3443608	HGFAC	TSS200	-0,014	4,85E-07	3,75E-03
cg22387286	4	185322916	IRF2	Body	0,015	4,79E-07	3,75E-03
cg16182517	7	83906997		IGR	0,029	5,09E-07	3,75E-03
cg07105285	11	92702663	MTNR1B	TSS200	-0,045	5,10E-07	3,75E-03
cg06875181	17	76649257		IGR	0,16	4,92E-07	3,75E-03
cg17845732	19	36130321		IGR	0,046	4,96E-07	3,75E-03
cg25407119	9	138116761		IGR	-0,034	5,24E-07	3,82E-03
cg09005886	12	15517124	PTPRO	Body	0,04	5,34E-07	3,82E-03
cg18496289	19	38909309	RASGRP4	Body	-0,018	5,32E-07	3,82E-03
cg22499994	10	17071728	CUBN	Body	0,007	5,64E-07	3,89E-03
cg14555810	12	88444663	CEP290	Body	-0,035	5,59E-07	3,89E-03
cg09319797	16	12061715	TNFRSF17	3'UTR	0,016	5,62E-07	3,89E-03
cg08487581	16	89100852		IGR	-0,008	5,62E-07	3,89E-03
cg15623866	1	156386933	C1orf61	5'UTR	0,044	5,82E-07	3,93E-03
cg25589352	8	110988168	KCNV1	TSS1500	0,011	5,84E-07	3,93E-03
cg13822122	21	47849174	PCNT	Body	0,005	5,87E-07	3,93E-03
cg01279902	14	104171040	XRCC3	Body	0,014	6,06E-07	4,02E-03
cg25649765	2	11672761	GREB1	TSS1500	0,058	6,49E-07	4,12E-03
cg08365618	3	25831282	OXSM	TSS1500	-0,014	6,54E-07	4,12E-03
cg09433113	7	28701898	CREB5	Body	0,069	6,47E-07	4,12E-03
cg08672675	12	72331271	TPH2	TSS1500	0,069	6,44E-07	4,12E-03
cg09489686	16	1444138		IGR	-0,014	6,37E-07	4,12E-03
cg22477428	16	2837938	PRSS33	TSS1500	0,035	6,27E-07	4,12E-03
cg26586164	2	25895850	DTNB	5'UTR	0,006	6,90E-07	4,20E-03
cg19419106	5	10502967	)C101929412	TSS200	-0,016	6,90E-07	4,20E-03

cg22854219	5	134388915	C5orf66	5'UTR	0,023	6,84E-07	4,20E-03
cg10679756	7	151191536	RHEB	Body	-0,033	6,77E-07	4,20E-03
cg17842936	1	153250273		IGR	-0,037	7,18E-07	4,29E-03
cg26776018	8	70575849		IGR	0,056	7,13E-07	4,29E-03
cg18832388	12	73524285		IGR	-0,033	7,28E-07	4,29E-03
cg06903478	17	76183632	TK1	TSS1500	-0,028	7,26E-07	4,29E-03
cg15550100	2	242606289	ATG4B	Body	0,008	7,66E-07	4,48E-03
cg24431318	2	47403193	CALM2	Body	0,008	7,93E-07	4,50E-03
cg26938522	6	158497675	SYNJ2	ExonBnd	0,009	7,93E-07	4,50E-03
cg13211181	12	25801455	IFLTD1	1stExon	0,042	7,95E-07	4,50E-03
cg22721434	14	66136108	FUT8	Body	-0,006	8,00E-07	4,50E-03
cg21058182	15	90401931	AP3S2	Body	0,017	7,89E-07	4,50E-03
cg10037204	2	240655816		IGR	-0,008	8,11E-07	4,52E-03
cg07733576	3	187462173	BCL6	5'UTR	0,012	8,52E-07	4,65E-03
cg11497952	4	185940969	HELT	Body	-0,015	8,55E-07	4,65E-03
cg04920704	11	1392099		IGR	-0,024	8,64E-07	4,65E-03
cg03908243	14	62103344	FLJ22447	Body	-0,015	8,66E-07	4,65E-03
cg01154336	14	101539195		IGR	-0,024	8,70E-07	4,65E-03
cg14710017	15	93799886		IGR	-0,009	8,42E-07	4,65E-03
cg19680363	2	83509701		IGR	-0,011	8,95E-07	4,74E-03
cg01678309	15	68930255	CORO2B	Body	0,056	9,03E-07	4,75E-03
cg25107000	6	31275643		IGR	-0,095	9,11E-07	4,75E-03
cg15698598	16	56896608		IGR	0,065	9,16E-07	4,75E-03
cg08851723	11	77728180	KCTD14	Body	-0,011	9,34E-07	4,77E-03
cg06527889	16	50784048	CYLD	Body	0,018	9,33E-07	4,77E-03
cg09455154	17	9532142	CFAP52	ExonBnd	0,022	9,38E-07	4,77E-03
cg05064121	3	195636243	TNK2	TSS1500	0,011	9,74E-07	4,78E-03
cg11507821	7	29844982	WIPF3	TSS1500	-0,019	9,91E-07	4,78E-03
cg02627966	7	54899537		IGR	0,059	9,57E-07	4,78E-03
cg09047553	11	19138639	ZDHHC13	TSS200	0,012	9,76E-07	4,78E-03
cg09010323	11	75379568	MAP6	TSS200	-0,017	9,92E-07	4,78E-03
cg15156382	12	4438071	TIGAR	Body	0,021	9,81E-07	4,78E-03
cg02157052	13	101326091	TMTC4	5'UTR	0,014	9,64E-07	4,78E-03
cg17593893	17	73867015		IGR	-0,021	9,81E-07	4,78E-03
cg21219191	9	74280273		IGR	-0,012	1,00E-06	4,80E-03
cg04793096	8	23077202	TNFRSF10A	Body	0,016	1,05E-06	4,95E-03
cg07607625	16	3422627	MTRNR2L4	TSS1500	0,011	1,04E-06	4,95E-03
cg08114317	1	1935535	KIAA1751	TSS1500	-0,038	1,08E-06	4,98E-03
cg19114721	2	103126336	SLC9A4	Body	0,047	1,07E-06	4,98E-03
cg16382960	3	33602534	CLASP2	Body	0,006	1,08E-06	4,98E-03
cg19559506	10	16587199		IGR	0,009	1,07E-06	4,98E-03
cg03568562	8	72872918	MSC-AS1	Body	0,011	1,09E-06	4,99E-03
cg11390381	1	151800946	RORC	Body	0,052	1,14E-06	5,08E-03
cg13724820	2	44104732	ABCG8	Body	-0,027	1,12E-06	5,08E-03
cg15862020	2	198326124	COQ10B	Body	0,009	1,13E-06	5,08E-03
cg13575638	3	9440081	SETD5	5'UTR	0,009	1,14E-06	5,08E-03
cg25279509	5	72884112		IGR	0,049	1,15E-06	5,08E-03
cg03067182	7	1515983	INTS1	Body	-0,014	1,15E-06	5,08E-03
cg22992837	11	113643339	ZW10	Body	0,025	1,17E-06	5,12E-03
cg01807127	20	22800500		IGR	0,01	1,19E-06	5,17E-03
cg11321030	10	103326711		IGR	0,049	1,20E-06	5,21E-03
cg25662676	1	28461218		IGR	-0,008	1,22E-06	5,22E-03
cg12098401	2	220247547	DNPEP	Body	-0,014	1,21E-06	5,22E-03
cg01502930	3	31993928	OSBPL10	Body	0,059	1,25E-06	5,22E-03
cg06745865	5	172710181		IGR	0,026	1,26E-06	5,22E-03
cg15536489	7	1120860	C7orf50	Body	0,011	1,25E-06	5,22E-03
cg04355791	11	43602436	MIR129-2	TSS1500	0,017	1,24E-06	5,22E-03
cg10617091	12	46273255	ARID2	Body	-0,049	1,26E-06	5,22E-03
cg04320316	17	41712200		IGR	-0,014	1,26E-06	5,22E-03
cg10265982	1	6837223		IGR	0,019	1,27E-06	5,23E-03
cg02931642	3	184320734		IGR	0,104	1,28E-06	5,23E-03

cg07016939	1	16069855	TMEM82	Body	-0,009	1,29E-06	5,26E-03
cg20719607	7	946806	ADAP1	Body	0,043	1,31E-06	5,30E-03
cg00613753	10	6622433	PRKCQ	TSS200	0,007	1,33E-06	5,34E-03
cg17280525	22	18310515	MICAL3	Body	-0,016	1,36E-06	5,46E-03
cg16831361	21	39668954	KCNJ15	5'UTR	0,034	1,37E-06	5,46E-03
cg09504313	2	47401358	CALM2	TSS200	0,048	1,40E-06	5,52E-03
cg00597666	6	105335437		IGR	0,056	1,40E-06	5,52E-03
cg25302257	10	115345867	HABP2	Body	0,034	1,42E-06	5,56E-03
cg18712892	9	34521333	ENHO	3'UTR	-0,013	1,44E-06	5,60E-03
cg17781983	7	13506380		IGR	-0,025	1,47E-06	5,71E-03
cg23715749	1	37413867	GRIK3	Body	0,051	1,49E-06	5,72E-03
cg26687031	1	38269629	YRDC	Body	0,005	1,50E-06	5,72E-03
cg23558456	1	45262537		IGR	0,065	1,49E-06	5,72E-03
cg22194807	18	32957683	ZNF396	TSS1500	-0,01	1,52E-06	5,76E-03
cg21285198	1	1360970	TMEM88B	TSS1500	0,036	1,65E-06	5,86E-03
cg07354895	1	53580047	SLC1A7	Body	-0,013	1,63E-06	5,86E-03
cg25610945	1	236255094		IGR	0,126	1,63E-06	5,86E-03
cg04779211	2	11484111	ROCK2	Body	0,006	1,56E-06	5,86E-03
cg12756490	3	49094418	QRICH1	Body	-0,011	1,66E-06	5,86E-03
cg03538833	3	101660066	LOC152225	Body	0,011	1,58E-06	5,86E-03
cg02898423	4	189081246		IGR	0,008	1,67E-06	5,86E-03
cg04130455	4	189580331		IGR	-0,033	1,78E-06	5,86E-03
cg04225172	5	31767552		IGR	0,039	1,76E-06	5,86E-03
cg17792994	5	121478708	ZNF474	5'UTR	-0,009	1,60E-06	5,86E-03
cg07110487	6	27515380		IGR	-0,076	1,57E-06	5,86E-03
cg01504489	7	1131626	GPER	Body	-0,017	1,78E-06	5,86E-03
cg05336893	7	117745095		IGR	0,031	1,71E-06	5,86E-03
cg14202601	7	127983956	RBM28	1stExon	0,009	1,73E-06	5,86E-03
cg16172099	8	103750821		IGR	0,042	1,70E-06	5,86E-03
cg18059464	10	134915277	GPR123	Body	0,043	1,72E-06	5,86E-03
cg09297375	11	1396193		IGR	-0,017	1,67E-06	5,86E-03
cg10911997	11	34653201	EHF	TSS1500	-0,014	1,65E-06	5,86E-03
cg00535257	12	26527310	ITPR2	Body	0,012	1,76E-06	5,86E-03
cg25462669	13	74192469		IGR	0,044	1,60E-06	5,86E-03
cg20255590	14	89022324	PTPN21	TSS1500	-0,024	1,71E-06	5,86E-03
cg15530474	14	99696161	BCL11B	Body	0,019	1,72E-06	5,86E-03
cg10157936	14	105220767	SIVA1	Body	-0,023	1,75E-06	5,86E-03
cg08496964	16	8729419	C16orf68	Body	-0,011	1,64E-06	5,86E-03
cg07272042	16	85860163		IGR	0,025	1,75E-06	5,86E-03
cg03346892	16	89160896	ACSF3	5'UTR	0,008	1,65E-06	5,86E-03
cg20356664	18	3262530	MYL12B	5'UTR	0,007	1,72E-06	5,86E-03
cg20026651	20	3776748	CDC25B	1stExon	0,005	1,63E-06	5,86E-03
cg06328217	21	40114384	LINC00114	Body	-0,02	1,75E-06	5,86E-03
cg09135500	21	44202452		IGR	-0,014	1,78E-06	5,86E-03
cg22856539	1	25559038	SYF2	TSS200	-0,022	1,82E-06	5,89E-03
cg00472519	2	88955010		IGR	-0,016	1,81E-06	5,89E-03
cg12885549	2	179279527	MIR548N	Body	0,007	1,82E-06	5,89E-03
cg11184021	19	16490552	EPS15L1	Body	-0,017	1,82E-06	5,89E-03
cg21616089	17	47089952	IGF2BP1	Body	0,032	1,89E-06	6,06E-03
cg02984790	21	48090128		IGR	0,027	1,89E-06	6,06E-03
cg20777630	10	71396029		IGR	-0,017	1,91E-06	6,07E-03
cg10658853	11	2233512		IGR	-0,023	1,91E-06	6,07E-03
cg01173563	15	73027500	BBS4	Body	-0,01	1,93E-06	6,10E-03
cg08373303	6	76066476	FILIP1	Body	-0,017	1,98E-06	6,24E-03
cg11600991	7	966196	ADAP1	Body	-0,017	1,99E-06	6,24E-03
cg25776467	3	124647658	MUC13	Body	0,013	2,00E-06	6,26E-03
cg15235185	1	193028809	TROVE2	TSS200	0,005	2,02E-06	6,27E-03
cg26817972	1	165735689	TMCO1	Body	0,012	2,03E-06	6,28E-03
cg07850533	9	4804779	RCL1	Body	-0,008	2,04E-06	6,29E-03
cg24253771	9	86237816	IDNK	TSS200	0,006	2,08E-06	6,39E-03
cg02107558	1	2000679	PRKCZ	Body	-0,019	2,29E-06	6,41E-03

cg13302864	3	49143828	QARS	TSS1500	-0,017	2,20E-06	6,41E-03
cg19606569	4	39029049	TMEM156	Body	-0,012	2,26E-06	6,41E-03
cg18155353	5	172614178		IGR	0,031	2,26E-06	6,41E-03
cg09871008	6	32798179	TAP2	Body	-0,008	2,25E-06	6,41E-03
cg25407831	6	36749984	CPNE5	Body	0,014	2,14E-06	6,41E-03
cg15729404	6	133561614	EYA4	TSS1500	-0,012	2,32E-06	6,41E-03
cg27614489	6	170593078	DLL1	Body	-0,009	2,18E-06	6,41E-03
cg03311036	7	142985443	CASP2	1stExon	0,005	2,20E-06	6,41E-03
cg05288172	8	103751006		IGR	0,049	2,20E-06	6,41E-03
cg07790896	9	92001668	SEMA4D	Body	0,01	2,31E-06	6,41E-03
cg06733794	10	123922970	TACC2	Body	0,011	2,23E-06	6,41E-03
cg12929346	10	126215173	LHPP	Body	-0,018	2,27E-06	6,41E-03
cg15075597	11	44979326		IGR	0,018	2,32E-06	6,41E-03
cg09966895	11	78738326	ODZ4	Body	-0,006	2,31E-06	6,41E-03
cg15848364	11	131734198	NTM	Body	-0,018	2,20E-06	6,41E-03
cg06107469	12	56862350	SPRYD4	1stExon	0,004	2,27E-06	6,41E-03
cg25306170	13	37680502	CSNK1A1L	TSS1500	-0,005	2,30E-06	6,41E-03
cg14936543	15	87096486	AGBL1	Body	0,028	2,29E-06	6,41E-03
cg08957564	16	12071814	RUNDC2A	Body	0,013	2,17E-06	6,41E-03
cg17922749	16	27985013	GSG1L	Body	0,035	2,16E-06	6,41E-03
cg14702840	16	84074531	SLC38A8	Body	0,056	2,10E-06	6,41E-03
cg22806907	17	40826212	PLEKHH3	Body	0,008	2,14E-06	6,41E-03
cg23654043	18	29841319		IGR	-0,009	2,11E-06	6,41E-03
cg16586648	19	39659203	PAK4	5'UTR	-0,016	2,26E-06	6,41E-03
cg01026094	20	55310327		IGR	0,047	2,28E-06	6,41E-03
cg17809471	22	37882605	MFNG	TSS200	-0,005	2,14E-06	6,41E-03
cg21867846	1	235247523		IGR	0,037	2,37E-06	6,44E-03
cg06961054	5	56204405	SETD9	TSS1500	-0,111	2,37E-06	6,44E-03
cg26601533	14	93260649	GOLGA5	1stExon	0,006	2,37E-06	6,44E-03
cg06345462	16	13263104	SHISA9	Body	0,012	2,36E-06	6,44E-03
cg21300187	17	38073560	GSDMB	Body	-0,008	2,38E-06	6,44E-03
cg11253514	1	228604240	TRIM17	1stExon	-0,007	2,43E-06	6,50E-03
cg14544448	6	150657685		IGR	0,055	2,41E-06	6,50E-03
cg12887863	11	13325247	ARNTL	5'UTR	0,012	2,42E-06	6,50E-03
cg04496580	1	46639801	TSPAN1	TSS1500	-0,011	2,56E-06	6,55E-03
cg11936040	1	75598867	LHX8	Body	-0,031	2,48E-06	6,55E-03
cg14523881	1	213164074	VASH2	3'UTR	-0,007	2,58E-06	6,55E-03
cg12324353	5	1269197	TERT	Body	0,041	2,58E-06	6,55E-03
cg11905821	6	31770932	LSM2	Body	0,008	2,57E-06	6,55E-03
cg22176954	7	22589311		IGR	-0,035	2,55E-06	6,55E-03
cg03266488	9	25679914	TUSC1	TSS1500	0,017	2,50E-06	6,55E-03
cg07447910	9	71820036	TJP2	TSS200	-0,014	2,53E-06	6,55E-03
cg00458212	9	100680821	C9orf156	Body	0,055	2,54E-06	6,55E-03
cg19648686	10	5044992	AKR1C2	Body	-0,02	2,60E-06	6,55E-03
cg08577187	10	112402638		IGR	-0,053	2,45E-06	6,55E-03
cg24576174	11	65828083	SF3B2	Body	-0,007	2,59E-06	6,55E-03
cg16404879	11	70165172	PPFIA1	Body	-0,018	2,56E-06	6,55E-03
cg08014100	15	40108340	GPR176	Body	-0,011	2,51E-06	6,55E-03
cg10081138	17	7604803	WRAP53	Body	0,005	2,55E-06	6,55E-03
cg06394209	17	54738946		IGR	-0,016	2,52E-06	6,55E-03
cg06255227	20	62738125	NPBWR2	1stExon	0,023	2,52E-06	6,55E-03
cg15685783	19	10793259	ILF3	Body	0,009	2,61E-06	6,56E-03
cg18508935	2	20307733		IGR	0,009	2,65E-06	6,57E-03
cg18455678	6	36981809	FGD2	Body	0,007	2,64E-06	6,57E-03
cg16950895	11	77898518	USP35	TSS1500	0,045	2,65E-06	6,57E-03
cg04313756	14	96180842	TCL1A	TSS1500	-0,05	2,65E-06	6,57E-03
cg10985026	1	16272042	ZBTB17	Body	-0,012	2,68E-06	6,64E-03
cg25309546	6	38693390	DNAH8	Body	0,031	2,74E-06	6,76E-03
cg00221462	7	42293921		IGR	0,008	2,80E-06	6,80E-03
cg05675338	11	100609146	ARHGAP42	Body	-0,019	2,80E-06	6,80E-03
cg06528591	12	131978899		IGR	-0,021	2,80E-06	6,80E-03

cg04705318	14	100138306	HHIPL1	Body	0,009	2,77E-06	6,80E-03
cg00531592	13	42557456		IGR	0,011	2,83E-06	6,86E-03
cg16974966	18	76984589	ATP9B	Body	0,017	2,88E-06	6,95E-03
cg01433381	1	39592860	MACF1	Body	0,007	2,98E-06	7,05E-03
cg12439157	1	170628856		IGR	0,043	2,99E-06	7,05E-03
cg08866665	6	169689892		IGR	0,035	2,96E-06	7,05E-03
cg25360211	12	9361610	PZP	TSS1500	0,05	2,99E-06	7,05E-03
cg19330592	14	91580380	C14orf159	1stExon	0,012	2,93E-06	7,05E-03
cg02184697	16	3013025	KREMEN2	TSS1500	0,039	2,99E-06	7,05E-03
cg02910054	16	12241554	SNX29	Body	0,085	2,96E-06	7,05E-03
cg03359095	20	61002866	C20orf151	TSS1500	0,064	2,99E-06	7,05E-03
cg05770091	7	100875312		IGR	-0,016	3,01E-06	7,06E-03
cg16335926	5	142784462	NR3C1	TSS1500	-0,008	3,08E-06	7,19E-03
cg08743899	6	15517343	JARID2	Body	-0,015	3,08E-06	7,19E-03
cg03088705	10	111624928	XPNPEP1	Body	-0,026	3,13E-06	7,28E-03
cg20613872	17	20205653	SPECC1	Body	-0,035	3,16E-06	7,32E-03
cg03816625	16	12192430	SNX29	Body	0,012	3,18E-06	7,33E-03
cg19858173	2	128099688	MAP3K2	Body	-0,01	3,19E-06	7,34E-03
cg24035931	8	70949392		IGR	0,036	3,21E-06	7,36E-03
cg09283925	3	167457335	SERPINI1	5'UTR	-0,01	3,23E-06	7,37E-03
cg10998291	3	194876449	C3orf21	Body	0,039	3,22E-06	7,37E-03
cg02101203	10	14050521	FRMD4A	Body	0,026	3,24E-06	7,37E-03
cg06679562	3	14220594	XPC	TSS1500	-0,02	3,27E-06	7,41E-03
cg20984590	12	49048478	SNORA34	TSS200	0,007	3,28E-06	7,41E-03
cg14202937	1	110170797	AMPD2	Body	-0,017	3,33E-06	7,46E-03
cg19029182	1	217958615	SPATA17-AS1	TSS200	0,017	3,38E-06	7,46E-03
cg18243346	5	25298993		IGR	-0,024	3,39E-06	7,46E-03
cg07635744	6	13365049	GFOD1	Body	-0,012	3,40E-06	7,46E-03
cg19491768	10	123441249		IGR	0,061	3,37E-06	7,46E-03
cg20495009	12	105323744	SLC41A2	TSS1500	0,03	3,37E-06	7,46E-03
cg26346721	14	52159181	FRMD6	Body	-0,03	3,36E-06	7,46E-03
cg04435828	15	65667529	IGDCC3	Body	0,019	3,34E-06	7,46E-03
cg02791851	19	8401884	KANK3	Body	0,03	3,33E-06	7,46E-03
cg02356872	19	49617495	LIN7B	TSS200	0,019	3,39E-06	7,46E-03
cg25949191	1	168051566	GPR161	3'UTR	-0,004	3,50E-06	7,47E-03
cg04279411	1	230645569		IGR	0,073	3,43E-06	7,47E-03
cg17104149	4	26492421	CCKAR	TSS1500	0,038	3,45E-06	7,47E-03
cg06883126	5	140457585		IGR	-0,059	3,52E-06	7,47E-03
cg26343358	6	31275666		IGR	-0,086	3,47E-06	7,47E-03
cg12047354	6	49490175	GLYATL3	Body	0,054	3,44E-06	7,47E-03
cg07673285	10	95985675	PLCE1	Body	-0,014	3,52E-06	7,47E-03
cg06489500	11	1436059	BRSK2	Body	-0,006	3,46E-06	7,47E-03
cg15294454	11	2223951		IGR	-0,022	3,48E-06	7,47E-03
cg17310977	13	38829325		IGR	-0,018	3,52E-06	7,47E-03
cg20239485	15	99215867	IGF1R	Body	0,013	3,47E-06	7,47E-03
cg11645313	9	136102164		IGR	-0,009	3,54E-06	7,47E-03
cg12762583	10	35463740	CREM	5'UTR	-0,014	3,54E-06	7,47E-03
cg15262352	7	5013468	RNF216L	TSS200	-0,013	3,58E-06	7,49E-03
cg05233622	11	75379501	MAP6	TSS200	0,006	3,57E-06	7,49E-03
cg05128498	21	47532820	COL6A2	Body	-0,021	3,57E-06	7,49E-03
cg02225054	16	12210834	SNX29	Body	0,006	3,63E-06	7,57E-03
cg14753432	11	44324833	ALX4	Body	0,034	3,64E-06	7,57E-03
cg01729887	3	158978383	IQCJ	Body	0,054	3,68E-06	7,61E-03
cg07545161	12	64216622	TMEM5-AS1	TSS1500	-0,008	3,67E-06	7,61E-03
cg15125476	8	28894556	HMBOX1	Body	-0,026	3,77E-06	7,78E-03
cg07726222	16	2125875	TSC2	Body	-0,012	3,78E-06	7,78E-03
cg16844818	2	242306625	FARP2	5'UTR	-0,018	3,80E-06	7,80E-03
cg02067350	2	106509528	NCK2	Body	-0,007	3,86E-06	7,90E-03
cg25595319	19	16143244	FLJ25328	TSS1500	-0,043	3,87E-06	7,90E-03
cg14763906	4	186391968	CCDC110	Body	0,006	3,89E-06	7,91E-03
cg16331972	17	8791471	PIK3R5	Body	0,006	3,90E-06	7,91E-03

cg10236302	15	67406806	SMAD3	Body	-0,009	3,95E-06	8,00E-03
cg14355428	7	91762876	CYP51A1	5'UTR	0,013	3,97E-06	8,01E-03
cg10570941	6	26273029	HIST1H3G	TSS1500	-0,018	4,02E-06	8,10E-03
cg19663928	8	71693264		IGR	-0,014	4,05E-06	8,10E-03
cg06848244	9	94249886		IGR	0,012	4,04E-06	8,10E-03
cg09118740	2	136743225	DARS	1stExon	0,005	4,07E-06	8,12E-03
cg05268564	2	209131274	PIKFYVE	5'UTR	-0,006	4,08E-06	8,12E-03
cg01093212	22	39715156	SNORD43	TSS200	0,005	4,13E-06	8,20E-03
cg02085186	16	23795768		IGR	-0,008	4,14E-06	8,21E-03
cg22734934	20	43552448	PABPC1L	Body	-0,018	4,20E-06	8,30E-03
cg06027600	1	28208814	THEMIS2	Body	-0,021	4,23E-06	8,31E-03
cg00568763	4	186281460	SNX25	Body	-0,01	4,23E-06	8,31E-03
cg09425471	2	40430224	SLC8A1-AS1	Body	0,03	4,28E-06	8,38E-03
cg20301074	14	65746603		IGR	0,008	4,32E-06	8,45E-03
cg21041694	3	9623509		IGR	0,051	4,36E-06	8,45E-03
cg06507186	15	22941822	CYFIP1	Body	-0,021	4,34E-06	8,45E-03
cg25384043	15	81295608	MESDC1	1stExon	-0,008	4,35E-06	8,45E-03
cg18293833	6	29600462	GABBR1	5'UTR	0,012	4,39E-06	8,49E-03
cg06910565	8	665236	ERICH1	Body	0,007	4,52E-06	8,72E-03
cg15880090	2	60627671		IGR	0,044	4,58E-06	8,73E-03
cg07756906	6	11537058	TMEM170B	TSS1500	0,04	4,57E-06	8,73E-03
cg10539298	7	6315828		IGR	0,021	4,58E-06	8,73E-03
cg00807189	8	120780022	TAF2	Body	-0,059	4,55E-06	8,73E-03
cg18250677	15	22980991	CYFIP1	Body	0,026	4,56E-06	8,73E-03
cg13953408	2	168993097	STK39	Body	-0,009	4,74E-06	8,87E-03
cg00460911	2	180872053	CWC22	TSS1500	-0,009	4,69E-06	8,87E-03
cg17564739	8	71980710		IGR	-0,023	4,78E-06	8,87E-03
cg13764850	9	94499729	ROR2	Body	-0,008	4,72E-06	8,87E-03
cg01389728	10	6620572	PRKCQ	5'UTR	0,055	4,74E-06	8,87E-03
cg11961590	10	114418962	VTI1A	Body	0,007	4,71E-06	8,87E-03
cg27112081	10	131342589	MGMT	Body	-0,013	4,78E-06	8,87E-03
cg24461814	12	52779281	KRT84	1stExon	-0,01	4,78E-06	8,87E-03
cg07142009	17	6656315		IGR	-0,06	4,73E-06	8,87E-03
cg11763417	19	8033897	ELAVL1	Body	-0,016	4,77E-06	8,87E-03
cg02062185	19	33762374		IGR	-0,013	4,79E-06	8,87E-03
cg02561103	7	115862891	TES	Body	-0,014	4,81E-06	8,88E-03
cg20913491	2	62081177	FAM161A	1stExon	0,004	4,83E-06	8,88E-03
cg04997558	17	80635674	RAB40B	Body	0,017	4,83E-06	8,88E-03
cg26189827	7	40338925	SUGCT	Body	0,037	4,86E-06	8,89E-03
cg02510471	12	54368639	HOXC11	Body	0,017	4,88E-06	8,89E-03
cg25456728	17	66255048	ARSG	TSS1500	-0,04	4,87E-06	8,89E-03
cg19559002	11	123044469	CLMP	Body	-0,008	4,91E-06	8,93E-03
cg01499518	7	75890022	SRRM3	Body	0,058	5,04E-06	9,15E-03
cg03738217	16	29159467		IGR	-0,033	5,06E-06	9,16E-03
cg12528597	11	57232556	RTN4RL2	Body	-0,015	5,10E-06	9,19E-03
cg17981006	12	91749400		IGR	0,019	5,09E-06	9,19E-03
cg20184509	12	76743649	BBS10	TSS1500	0,005	5,12E-06	9,19E-03
cg23858074	6	97730720	C6orf167	5'UTR	0,004	5,13E-06	9,19E-03
cg00994604	17	19600816	SLC47A2	Body	0,008	5,18E-06	9,27E-03
cg09435227	3	55505073	WNT5A	Body	-0,023	5,23E-06	9,28E-03
cg13027661	3	152292437		IGR	0,022	5,21E-06	9,28E-03
cg12643509	12	72527584		IGR	0,047	5,25E-06	9,28E-03
cg00456395	12	96641089	ELK3	Body	-0,019	5,23E-06	9,28E-03
cg00315781	12	110213263		IGR	-0,003	5,25E-06	9,28E-03
cg11469319	5	6667352	SRD5A1	Body	-0,008	5,29E-06	9,32E-03
cg06750177	3	172341525		IGR	0,052	5,38E-06	9,43E-03
cg18456782	15	28051715	OCA2	Body	-0,011	5,37E-06	9,43E-03
cg18087834	6	22220730		IGR	0,018	5,42E-06	9,45E-03
cg08551027	12	21589206	PYROXD1	TSS1500	0,042	5,43E-06	9,45E-03
cg06657852	16	86786582		IGR	-0,014	5,40E-06	9,45E-03
cg22668398	1	12703913	AADACL4	TSS1500	-0,01	5,46E-06	9,47E-03

cg23577562	4	3324940	RGS12	Body	-0,007	5,46E-06	9,47E-03
cg11060194	2	97526918	SEMA4C	Body	-0,025	5,50E-06	9,50E-03
cg26671261	2	220331822	SPEG	Body	0,012	5,51E-06	9,50E-03
cg03586682	19	45565422	CLASRP	ExonBnd	-0,008	5,52E-06	9,50E-03
cg07794500	1	121261369	LOC647121	Body	0,007	5,55E-06	9,51E-03
cg23732781	6	29595016	GABBR1	Body	0,05	5,61E-06	9,51E-03
cg26739697	7	76637493	3P1-PMS2P11	Body	-0,059	5,60E-06	9,51E-03
cg27160556	11	124181099	OR8D1	TSS1500	-0,028	5,61E-06	9,51E-03
cg11996798	12	50489021	SMARCD1	Body	0,005	5,58E-06	9,51E-03
cg23397168	15	81285343		IGR	-0,02	5,57E-06	9,51E-03
cg24759330	20	36662805	TTI1	TSS1500	0,018	5,56E-06	9,51E-03
cg01573293	1	20867822		IGR	-0,01	5,77E-06	9,51E-03
cg08207365	1	94609734		IGR	-0,025	5,73E-06	9,51E-03
cg20465207	1	155948698	ARHGEF2	TSS1500	0,004	5,80E-06	9,51E-03
cg03440037	4	108641553	PAPSS1	TSS200	0,009	5,71E-06	9,51E-03
cg03635649	5	41510801	PLCXD3	TSS200	-0,012	5,66E-06	9,51E-03
cg09918110	5	87798170		IGR	0,07	5,78E-06	9,51E-03
cg00703819	5	96210344	ERAP2	TSS1500	0,006	5,72E-06	9,51E-03
cg00432041	8	67341355	RRS1	5'UTR	0,012	5,67E-06	9,51E-03
cg26333159	11	119471437		IGR	-0,023	5,76E-06	9,51E-03
cg20467114	12	3365744	TSPAN9	Body	-0,009	5,67E-06	9,51E-03
cg10024987	16	89922043	SPIRE2	Body	-0,01	5,77E-06	9,51E-03
cg23148992	17	3558246	CTNS	Body	-0,006	5,74E-06	9,51E-03
cg12228229	17	7122261	DLG4	5'UTR	-0,011	5,70E-06	9,51E-03
cg21964793	19	2038527	MKNK2	Body	-0,015	5,75E-06	9,51E-03
cg07992453	1	35543507	ZMYM1	TSS1500	-0,008	5,83E-06	9,51E-03
cg14791221	5	14595490	FAM105A	Body	-0,003	5,83E-06	9,51E-03
cg14231749	17	50237249	CA10	5'UTR	-0,033	5,84E-06	9,51E-03
cg18888131	9	112172827	PTPN3	Body	-0,031	5,87E-06	9,54E-03
cg11829626	17	18811532	PRPSAP2	Body	-0,007	5,88E-06	9,55E-03
cg10769844	3	184290794	EPHB3	Body	-0,015	5,90E-06	9,55E-03
cg08217234	19	4042688		IGR	-0,015	5,92E-06	9,56E-03
cg22760668	14	69152424		IGR	0,007	5,94E-06	9,58E-03
cg17259245	3	51430028	RBM15B	1stExon	-0,02	6,03E-06	9,70E-03
cg09899933	1	183891130	RGL1	Body	0,007	6,07E-06	9,70E-03
cg13941292	15	78196623		IGR	-0,018	6,06E-06	9,70E-03
cg01720696	17	55521077	MSI2	Body	0,062	6,07E-06	9,70E-03
cg21418629	8	95651829	)C100288748	TSS200	-0,01	6,10E-06	9,72E-03
cg24503639	2	173173437		IGR	0,048	6,14E-06	9,74E-03
cg22563627	12	121210975	SPPL3	Body	-0,007	6,13E-06	9,74E-03
cg00703843	17	17632263	RAI1	5'UTR	-0,012	6,15E-06	9,75E-03
cg18940723	2	45837923	SRBD1	5'UTR	-0,009	6,17E-06	9,76E-03
cg27410351	1	97311333		IGR	0,045	6,22E-06	9,81E-03
cg05869585	16	8890233	TMEM186	Body	-0,015	6,26E-06	9,85E-03
cg05013420	1	246415964	SMYD3	Body	-0,033	6,35E-06	9,98E-03
cg10360845	3	191286105	LINCR-0002	Body	-0,027	6,47E-06	1,01E-02
cg01447495	6	128901776		IGR	-0,016	6,46E-06	1,01E-02
cg24042578	1	2066981	PRKCZ	5'UTR	0,033	6,60E-06	1,02E-02
cg23344338	4	151231429	LRBA	Body	0,007	6,60E-06	1,02E-02
cg15757838	5	143569897	KCTD16	5'UTR	0,048	6,59E-06	1,02E-02
cg08626830	11	113158946		IGR	-0,013	6,60E-06	1,02E-02
cg22467364	8	38758712	PLEKHA2	TSS200	-0,007	6,65E-06	1,03E-02
cg23760649	8	82958662		IGR	-0,019	6,71E-06	1,03E-02
cg15763776	9	117424357		IGR	0,034	6,72E-06	1,03E-02
cg06890317	11	884165	CHID1	ExonBnd	-0,017	6,76E-06	1,03E-02
cg24823998	11	66811737	MIR6860	TSS1500	-0,005	6,67E-06	1,03E-02
cg14679558	13	21652153		IGR	0,007	6,73E-06	1,03E-02
cg01329973	16	86912065		IGR	0,026	6,76E-06	1,03E-02
cg08020330	18	57084067		IGR	0,009	6,69E-06	1,03E-02
cg01360586	21	39285679	KCNJ6	5'UTR	0,037	6,68E-06	1,03E-02
cg27001818	22	33724826	LARGE	Body	0,035	6,74E-06	1,03E-02

cg21553700	3	195600410	TNK2	Body	-0,006	6,85E-06	1,03E-02
cg20723350	4	23891596	PPARGC1A	1stExon	0,026	6,84E-06	1,03E-02
cg11180069	7	131422872		IGR	-0,061	6,82E-06	1,03E-02
cg15895233	10	43616646	RET	Body	0,005	6,81E-06	1,03E-02
cg17249177	15	35225257	AQR	Body	-0,02	6,83E-06	1,03E-02
cg10190509	17	34308137	CCL16	Body	0,019	6,81E-06	1,03E-02
cg09921821	4	3371928	RGS12	Body	-0,008	6,90E-06	1,03E-02
cg16961816	7	96633530	DLX6AS	Body	-0,026	6,88E-06	1,03E-02
cg05929005	11	19542568	NAV2-AS4	Body	0,039	6,90E-06	1,03E-02
cg26201751	1	186788381		IGR	-0,023	6,95E-06	1,03E-02
cg26575164	1	151584257	SNX27	TSS1500	0,015	6,98E-06	1,04E-02
cg09493966	16	51165636		IGR	0,034	6,99E-06	1,04E-02
cg05894754	2	210675440	UNC80	Body	0,086	7,01E-06	1,04E-02
cg07305931	20	6604933		IGR	-0,006	7,02E-06	1,04E-02
cg18540614	1	43833945	ELOVL1	TSS1500	0,007	7,11E-06	1,04E-02
cg23203730	7	1950707	MAD1L1	Body	-0,011	7,10E-06	1,04E-02
cg09129067	8	103750904		IGR	0,033	7,12E-06	1,04E-02
cg02093006	10	32201942	ARHGAP12	5'UTR	0,008	7,09E-06	1,04E-02
cg22276046	17	45891090	OSBPL7	Body	-0,012	7,07E-06	1,04E-02
cg20818417	1	76082690		IGR	-0,019	7,13E-06	1,04E-02
cg14205239	20	24872950		IGR	-0,021	7,17E-06	1,04E-02
cg17902745	1	154226189	UBAP2L	Body	0,005	7,32E-06	1,05E-02
cg20893039	1	246117536	SMYD3	Body	-0,014	7,36E-06	1,05E-02
cg19441992	2	1568007		IGR	0,049	7,29E-06	1,05E-02
cg22088495	2	85804653	VAMP8	5'UTR	0,006	7,30E-06	1,05E-02
cg24882688	3	127353794	PODXL2	Body	-0,009	7,35E-06	1,05E-02
cg09638532	5	32694044		IGR	0,039	7,28E-06	1,05E-02
cg21923107	6	110545486	CDC40	Body	-0,009	7,25E-06	1,05E-02
cg01318963	10	98339870	TM9SF3	Body	0,03	7,29E-06	1,05E-02
cg03297660	11	116509416		IGR	0,037	7,27E-06	1,05E-02
cg14568338	15	99191797	IGF1R	TSS1500	0,01	7,33E-06	1,05E-02
cg25424659	19	11450592	RAB3D	TSS1500	0,025	7,33E-06	1,05E-02
cg27281030	19	54312881	NLRP12	Body	-0,018	7,20E-06	1,05E-02
cg20634498	2	88750864	FOXI3	Body	0,048	7,54E-06	1,05E-02
cg18889780	4	41258374	UCHL1	TSS1500	0,031	7,45E-06	1,05E-02
cg21575929	5	159344281	ADRA1B	1stExon	-0,017	7,53E-06	1,05E-02
cg09332710	8	67352040	ADHFE1	Body	0,039	7,52E-06	1,05E-02
cg05747357	11	1970665	MRPL23	Body	-0,016	7,48E-06	1,05E-02
cg07708947	14	64168265	SGPP1	Body	0,01	7,40E-06	1,05E-02
cg09576488	15	38276651		IGR	-0,014	7,52E-06	1,05E-02
cg17528768	15	59385298	RNF111	Body	-0,007	7,55E-06	1,05E-02
cg26080026	16	19516531	GDE1	Body	-0,006	7,48E-06	1,05E-02
cg24975211	17	38249266	NR1D1	3'UTR	0,013	7,43E-06	1,05E-02
cg22580900	17	50237646	CA10	TSS1500	-0,044	7,52E-06	1,05E-02
cg03037864	22	42916984	RRP7A	TSS1500	-0,025	7,45E-06	1,05E-02
cg27617060	22	47438629	TBC1D22A	Body	-0,015	7,48E-06	1,05E-02
cg10218605	7	158379132	PTPRN2	Body	-0,054	7,62E-06	1,05E-02
cg21418534	13	112800020		IGR	-0,024	7,62E-06	1,05E-02
cg03050195	1	27183371		IGR	-0,01	7,69E-06	1,05E-02
cg20786514	2	20189208	WDR35	Body	-0,019	7,70E-06	1,05E-02
cg06285619	2	85978355		IGR	0,042	7,74E-06	1,05E-02
cg19218358	2	235411933		IGR	-0,031	7,73E-06	1,05E-02
cg19769395	12	114680340		IGR	-0,007	7,68E-06	1,05E-02
cg04549697	14	69827589		IGR	0,038	7,74E-06	1,05E-02
cg01974973	15	76031920	DNM1P35	Body	0,01	7,71E-06	1,05E-02
cg11425201	19	40770176	AKT2	Body	-0,008	7,72E-06	1,05E-02
cg24733614	12	104990550	CHST11	Body	0,015	7,76E-06	1,05E-02
cg07373589	1	6189304	CHD5	Body	-0,015	7,80E-06	1,06E-02
cg19302347	2	236918009	AGAP1	Body	-0,018	7,84E-06	1,06E-02
cg15175985	5	122110695	SNX2	TSS200	0,007	7,86E-06	1,06E-02
cg04541854	7	42147789	GLI3	Body	0,009	7,87E-06	1,06E-02

cg25913970	12	2929960	ITFG2	Body	0,005	7,83E-06	1,06E-02
cg21834966	15	27109950		IGR	-0,008	7,86E-06	1,06E-02
cg21516443	11	3244486		IGR	0,035	7,91E-06	1,06E-02
cg13005191	22	31654801	LIMK2	Body	0,008	7,91E-06	1,06E-02
cg13112361	11	63258739	HRASLS5	TSS200	0,01	7,94E-06	1,06E-02
cg17048356	18	43678029	ATP5A1	Body	0,006	7,93E-06	1,06E-02
cg13064429	11	112161200		IGR	0,004	8,00E-06	1,06E-02
cg27162196	16	75183231	ZFP1	5'UTR	0,047	7,99E-06	1,06E-02
cg20224693	10	45360486		IGR	0,005	8,07E-06	1,07E-02
cg21926094	17	5018984	ZNF232	5'UTR	-0,07	8,06E-06	1,07E-02
cg10484566	17	78653711	RPTOR	Body	0,019	8,05E-06	1,07E-02
cg00666438	1	168464998	)C101928565	TSS200	0,008	8,17E-06	1,08E-02
cg27423627	3	69299355	FRMD4B	Body	0,045	8,18E-06	1,08E-02
cg26089958	5	111575752	)C101927023	Body	-0,013	8,15E-06	1,08E-02
cg22361050	10	48354460	ZNF488	TSS1500	-0,011	8,19E-06	1,08E-02
cg04427437	3	3081722	CNTN4	Body	-0,023	8,22E-06	1,08E-02
cg24327375	5	158871682		IGR	0,054	8,22E-06	1,08E-02
cg11720658	2	181846658	UBE2E3	5'UTR	-0,011	8,28E-06	1,08E-02
cg08272105	6	170399179		IGR	0,007	8,29E-06	1,08E-02
cg00185034	8	8610660		IGR	0,031	8,31E-06	1,08E-02
cg06520088	16	683852	WFIKKN1	Body	-0,008	8,33E-06	1,08E-02
cg17867420	3	68984818		IGR	0,04	8,39E-06	1,09E-02
cg23658987	1	175047784	TNN	Body	-0,185	8,46E-06	1,09E-02
cg06773397	6	70510879		IGR	0,01	8,44E-06	1,09E-02
cg01679206	7	73253049	WBSCR27	Body	0,06	8,44E-06	1,09E-02
cg11100113	8	82508781		IGR	0,019	8,45E-06	1,09E-02
cg00381736	2	33516681	LTBP1	Body	0,039	8,52E-06	1,09E-02
cg22459517	19	55587193	EPS8L1	TSS200	0,224	8,52E-06	1,09E-02
cg09408383	15	49726332	FGF7	Body	-0,006	8,57E-06	1,10E-02
cg15190738	8	20161380		IGR	0,008	8,60E-06	1,10E-02
cg00360846	14	105920403	MTA1	Body	-0,009	8,64E-06	1,10E-02
cg07577713	3	129983303	COL6A4P2	Body	0,013	8,73E-06	1,11E-02
cg06438343	11	66053295	YIF1A	Body	-0,017	8,75E-06	1,11E-02
cg00920424	3	127172519		IGR	-0,04	8,77E-06	1,11E-02
cg26452224	19	56595658		IGR	0,075	8,78E-06	1,11E-02
cg12398117	5	76474556		IGR	-0,005	8,83E-06	1,12E-02
cg09288844	8	23191340	LOXL2	Body	-0,019	8,92E-06	1,13E-02
cg23918976	15	52394247		IGR	0,009	8,98E-06	1,13E-02
cg04561782	5	661880	TPPP	3'UTR	0,01	9,03E-06	1,14E-02
cg00191110	9	68423958		IGR	0,037	9,09E-06	1,14E-02
cg18650669	17	10601739	ADPRM	5'UTR	-0,019	9,09E-06	1,14E-02
cg07146773	15	80466042	FAH	Body	0,014	9,12E-06	1,14E-02
cg26755729	1	91488005	ZNF644	TSS200	0,011	9,15E-06	1,14E-02
cg19754013	1	156252628	SMG5	TSS200	-0,006	9,20E-06	1,14E-02
cg27191564	3	97722598	GABRR3	Body	0,074	9,21E-06	1,14E-02
cg08017400	3	141203940		IGR	0,008	9,17E-06	1,14E-02
cg03000593	6	33283162	ZBTB22	Body	-0,035	9,24E-06	1,14E-02
cg05637265	8	144884124	SCRIB	Body	-0,009	9,24E-06	1,14E-02
cg07307789	12	4554809	FGF6	TSS200	-0,015	9,24E-06	1,14E-02
cg03288340	19	47615999	ZC3H4	5'UTR	0,005	9,28E-06	1,15E-02
cg00963163	14	55033662	SAMD4A	TSS1500	0,007	9,33E-06	1,15E-02
cg17423711	1	167190178	POU2F1	1stExon	0,005	9,39E-06	1,15E-02
cg07420502	2	216222060		IGR	-0,008	9,39E-06	1,15E-02
cg08751263	15	31547238		IGR	0,008	9,38E-06	1,15E-02
cg06400334	16	71436020		IGR	0,058	9,42E-06	1,15E-02
cg23964375	19	6433768	SLC25A41	1stExon	0,02	9,42E-06	1,15E-02
cg00035000	1	25255003	RUNX3	Body	-0,005	9,44E-06	1,15E-02
cg00469341	7	14031022	ETV1	5'UTR	0,007	9,47E-06	1,15E-02
cg12129367	4	38969100	TMEM156	3'UTR	0,009	9,55E-06	1,16E-02
cg04754260	16	88455670		IGR	0,032	9,53E-06	1,16E-02
cg22167400	17	26633978	FLJ40504	Body	-0,042	9,55E-06	1,16E-02

cg10692757	1	27215365	GPN2	Body	0,007	9,57E-06	1,16E-02
cg17691415	1	246476711	SMYD3	Body	-0,01	9,61E-06	1,16E-02
cg16664417	4	136237899		IGR	-0,024	9,63E-06	1,16E-02
cg03244496	7	134925373	STRA8	Body	-0,015	9,69E-06	1,17E-02
cg27390907	10	73481060	C10orf105	TSS1500	0,01	9,71E-06	1,17E-02
cg17468317	6	31620788	BAT3	TSS1500	-0,007	9,77E-06	1,17E-02
cg09254924	8	145218630	MROH1	5'UTR	-0,007	9,81E-06	1,18E-02
cg01440489	13	41593385	ELF1	5'UTR	-0,01	9,82E-06	1,18E-02
cg17916948	9	112906058	AKAP2	Body	0,028	9,86E-06	1,18E-02
cg17168630	19	44174930	PLAUR	TSS1500	0,024	9,87E-06	1,18E-02
cg12519470	12	59676888		IGR	-0,041	9,91E-06	1,18E-02
cg12196668	17	80774239	TBCD	Body	0,029	9,92E-06	1,18E-02
cg00152613	8	28314916	FBXO16	Body	0,063	9,96E-06	1,18E-02
cg09255748	19	40004995	SELV	TSS1500	0,034	9,96E-06	1,18E-02
cg18618206	17	754296	NXN	Body	0,026	1,00E-05	1,18E-02
cg06157732	1	61506847		IGR	-0,011	1,03E-05	1,19E-02
cg00708060	3	49208687	KLHDC8B	TSS1500	0,006	1,01E-05	1,19E-02
cg02456998	3	124293098	KALRN	Body	0,034	1,01E-05	1,19E-02
cg14015525	6	36295421	C6orf222	Body	0,043	1,01E-05	1,19E-02
cg26269295	7	84122234		IGR	-0,012	1,02E-05	1,19E-02
cg18561261	7	123284476		IGR	0,036	1,03E-05	1,19E-02
cg09667467	9	125030469	MRRF	5'UTR	0,017	1,02E-05	1,19E-02
cg03880642	10	105560568	SH3PXD2A	Body	0,055	1,03E-05	1,19E-02
cg23169946	11	44558561		IGR	-0,018	1,03E-05	1,19E-02
cg13001596	13	37572327	ALG5	Body	-0,027	1,02E-05	1,19E-02
cg13037841	14	70398036	SMOC1	Body	-0,018	1,02E-05	1,19E-02
cg15349620	16	57490384	COQ9	Body	0,005	1,02E-05	1,19E-02
cg09004999	17	46872075	TTLL6	TSS1500	0,02	1,02E-05	1,19E-02
cg05009512	17	74262278	FAM100B	Body	0,006	1,01E-05	1,19E-02
cg22656115	19	4440701	CHAF1A	Body	-0,007	1,01E-05	1,19E-02
cg01458219	19	53520353		IGR	0,042	1,03E-05	1,19E-02
cg17051523	22	17681506	CECR1	TSS1500	0,009	1,02E-05	1,19E-02
cg21026868	1	219742152		IGR	0,046	1,03E-05	1,19E-02
cg09099653	22	20307061	DGCR6L	Body	0,014	1,04E-05	1,19E-02
cg03872217	3	161235909		IGR	0,023	1,04E-05	1,19E-02
cg10586266	11	67199428	RPS6KB2	Body	-0,011	1,04E-05	1,19E-02
cg01334824	15	27787650		IGR	-0,012	1,04E-05	1,19E-02
cg10754697	17	1504809	SLC43A2	Body	-0,015	1,04E-05	1,19E-02
cg19901551	1	220128430	RNU5F-1	Body	0,019	1,05E-05	1,19E-02
cg20967739	1	50895827		IGR	-0,074	1,05E-05	1,20E-02
cg10958566	19	23084225		IGR	-0,007	1,05E-05	1,20E-02
cg01456575	4	150817949		IGR	-0,013	1,06E-05	1,20E-02
cg16202828	9	30732115		IGR	-0,009	1,06E-05	1,20E-02
cg23143371	11	27682015	BDNF	TSS1500	-0,037	1,06E-05	1,20E-02
cg05582340	12	113630080	C12orf52	3'UTR	0,009	1,06E-05	1,20E-02
cg11526943	15	42154836	SPTBN5	Body	-0,014	1,06E-05	1,20E-02
cg10188668	17	79880258	MAFG	3'UTR	0,011	1,06E-05	1,20E-02
cg02533155	11	95657676	MTMR2	TSS1500	-0,004	1,07E-05	1,20E-02
cg15785288	12	106451417		IGR	0,015	1,07E-05	1,20E-02
cg09322003	7	155584248		IGR	-0,053	1,08E-05	1,22E-02
cg02963973	6	35463625	TEAD3	5'UTR	0,012	1,09E-05	1,22E-02
cg22016654	1	63783524		IGR	-0,015	1,10E-05	1,22E-02
cg22727903	1	225507096	DNAH14	Body	-0,032	1,11E-05	1,22E-02
cg23454963	3	149689626	PFN2	TSS1500	-0,008	1,10E-05	1,22E-02
cg09293898	3	184543768	VPS8	Body	0,011	1,11E-05	1,22E-02
cg22111009	5	173416274	C5orf47	1stExon	0,004	1,11E-05	1,22E-02
cg24017912	6	17393521	CAP2	TSS1500	0,031	1,11E-05	1,22E-02
cg26062439	7	6577467	GRID2IP	Body	-0,011	1,09E-05	1,22E-02
cg23053506	7	158075705	PTPRN2	Body	-0,014	1,10E-05	1,22E-02
cg18449243	8	57819317		IGR	-0,009	1,10E-05	1,22E-02
cg18575406	10	5932529	ANKRD16	TSS1500	0,006	1,09E-05	1,22E-02

cg26699689	10	8287272		IGR	-0,005	1,10E-05	1,22E-02
cg10015444	14	62273629		IGR	0,009	1,10E-05	1,22E-02
cg12877372	15	80602300	LINC00927	Body	0,046	1,11E-05	1,22E-02
cg12062282	19	49524228		IGR	0,024	1,11E-05	1,22E-02
cg24050636	1	6050882	NPHP4	5'UTR	0,01	1,12E-05	1,22E-02
cg16530787	11	96038087	MAML2	Body	-0,013	1,12E-05	1,23E-02
cg00622318	10	130514131		IGR	0,052	1,13E-05	1,23E-02
cg27561431	4	890612	GAK	Body	-0,008	1,14E-05	1,23E-02
cg17100768	6	35117178		IGR	-0,01	1,13E-05	1,23E-02
cg16853241	19	38845191	CATSPERG	Body	-0,016	1,13E-05	1,23E-02
cg16264888	12	82752007	CCDC59	Body	-0,003	1,14E-05	1,24E-02
cg03328571	2	170214803	LRP2	Body	0,008	1,14E-05	1,24E-02
cg10552592	16	87743108	KLHDC4	Body	-0,007	1,15E-05	1,24E-02
cg09929164	17	48423123	XYLT2	TSS1500	0,011	1,15E-05	1,24E-02
cg26730653	2	87022189	CD8A	5'UTR	0,024	1,15E-05	1,24E-02
cg09800774	2	167005840	SCN1A	TSS200	0,023	1,16E-05	1,24E-02
cg01520586	3	50280404	GNAI2	Body	0,011	1,17E-05	1,24E-02
cg11040884	4	186458094	PDLIM3	TSS1500	0,061	1,17E-05	1,24E-02
cg25066207	6	56669999	DST	Body	0,052	1,17E-05	1,24E-02
cg20896300	7	33545220	BBS9	Body	0,004	1,16E-05	1,24E-02
cg14330140	8	84883227		IGR	0,015	1,17E-05	1,24E-02
cg10261735	8	101871481		IGR	0,061	1,15E-05	1,24E-02
cg00184240	12	122666660	LRRC43	5'UTR	0,015	1,16E-05	1,24E-02
cg10759591	17	77148972	HRNBP3	5'UTR	0,073	1,16E-05	1,24E-02
cg01315222	22	24289622		IGR	0,061	1,17E-05	1,24E-02
cg21266798	13	110433636	IRS2	Body	-0,02	1,17E-05	1,24E-02
cg19645861	2	16501343		IGR	0,032	1,18E-05	1,25E-02
cg07355632	14	64759104	ESR2	5'UTR	-0,012	1,18E-05	1,25E-02
cg08793061	5	72514669		IGR	0,019	1,18E-05	1,25E-02
cg04342698	1	156542440	IQGAP3	TSS200	0,013	1,18E-05	1,25E-02
cg14408367	8	82191962	FABP5	TSS1500	0,046	1,19E-05	1,25E-02
cg22066587	12	27314459		IGR	0,011	1,19E-05	1,25E-02
cg05918057	14	93372933		IGR	0,063	1,19E-05	1,25E-02
cg04553307	7	922775	C7orf20	Body	-0,019	1,19E-05	1,25E-02
cg13509838	17	46018764	PNPO	TSS200	0,01	1,20E-05	1,25E-02
cg14968834	18	37381479		IGR	-0,013	1,20E-05	1,25E-02
cg14458704	19	42441368		IGR	-0,013	1,20E-05	1,26E-02
cg16492341	10	26856105	APBB1IP	Body	-0,008	1,21E-05	1,26E-02
cg02452500	11	13161927		IGR	0,031	1,21E-05	1,26E-02
cg11706790	2	242750102	NEU4	TSS200	0,007	1,22E-05	1,27E-02
cg16087633	21	45702039		IGR	-0,013	1,22E-05	1,27E-02
cg27096087	12	120222488	CIT	Body	-0,008	1,22E-05	1,27E-02
cg21090813	16	50533054		IGR	0,029	1,23E-05	1,27E-02
cg26710931	8	17090860	CNOT7	Body	0,005	1,23E-05	1,27E-02
cg19749527	5	105259893		IGR	-0,017	1,23E-05	1,27E-02
cg14645880	9	134501604	RAPGEF1	Body	0,006	1,24E-05	1,27E-02
cg07943492	4	12060625		IGR	-0,014	1,24E-05	1,28E-02
cg08165784	12	107297238		IGR	-0,022	1,24E-05	1,28E-02
cg14374974	22	26895194	TFIP11	Body	-0,009	1,24E-05	1,28E-02
cg21832004	2	194712047		IGR	0,047	1,25E-05	1,28E-02
cg17107561	11	76501483	TSKU	5'UTR	0,047	1,25E-05	1,28E-02
cg07103124	10	49643086	MAPK8	3'UTR	0,012	1,25E-05	1,28E-02
cg21204904	1	206730441	RASSF5	Body	0,005	1,25E-05	1,28E-02
cg20418864	4	146571771	MMAA	Body	0,009	1,25E-05	1,28E-02
cg09858431	8	144432323	TOP1MT	5'UTR	-0,051	1,26E-05	1,28E-02
cg12497870	19	36210913	MLL4	Body	-0,015	1,26E-05	1,28E-02
cg09049982	20	32950073	ITCH	TSS1500	0,007	1,27E-05	1,29E-02
cg23881613	19	17462773	PLVAP	3'UTR	0,01	1,27E-05	1,29E-02
cg00290506	1	224804226	CNIH3	1stExon	0,006	1,27E-05	1,29E-02
cg18018043	3	155462063		IGR	0,005	1,28E-05	1,29E-02
cg02753027	19	39616041	PAK4	TSS1500	0,053	1,28E-05	1,29E-02

cg02835214	19	58125672	ZNF134	TSS200	0,004	1,28E-05	1,29E-02
cg07387813	3	40499408	RPL14	Body	0,023	1,28E-05	1,29E-02
cg24204949	3	64085259	RICKLE2-AS1	Body	-0,011	1,28E-05	1,29E-02
cg10351452	3	78955362	ROBO1	Body	-0,017	1,28E-05	1,29E-02
cg07184375	12	122469564	BCL7A	Body	-0,009	1,29E-05	1,29E-02
cg02933141	1	222722495	HHIPL2	TSS1500	-0,012	1,30E-05	1,30E-02
cg26467257	9	706307	KANK1	TSS1500	-0,076	1,30E-05	1,30E-02
cg23620712	11	67273646	PITPNM1	TSS1500	0,003	1,30E-05	1,30E-02
cg02254315	1	42800772	FOXJ3	TSS200	0,009	1,30E-05	1,30E-02
cg07984980	16	89898383	SPIRE2	Body	-0,02	1,30E-05	1,30E-02
cg13670878	2	11681803	GREB1	5'UTR	-0,029	1,31E-05	1,30E-02
cg12899842	2	32390937	SLC30A6	5'UTR	0,005	1,31E-05	1,30E-02
cg25862072	6	164520845		IGR	0,006	1,31E-05	1,30E-02
cg18561247	6	167198539	RPS6KA2	Body	-0,027	1,31E-05	1,30E-02
cg05047476	15	74212306	LOXL1-AS1	ExonBnd	-0,01	1,31E-05	1,30E-02
cg26426732	3	17449507	TBC1D5	Body	-0,008	1,33E-05	1,31E-02
cg01138087	3	141097568	ZBTB38	5'UTR	0,008	1,33E-05	1,31E-02
cg13475860	9	84371897		IGR	0,01	1,33E-05	1,31E-02
cg21213075	10	30880636		IGR	0,043	1,32E-05	1,31E-02
cg17655818	17	47902202	KAT7	Body	-0,036	1,33E-05	1,31E-02
cg24929136	18	12318427	TUBB6	5'UTR	0,047	1,33E-05	1,31E-02
cg17519749	2	2111648	MYT1L	5'UTR	0,019	1,33E-05	1,31E-02
cg13721515	8	74335868	STAU2-AS1	Body	-0,009	1,33E-05	1,31E-02
cg18585273	17	66197086		IGR	0,006	1,34E-05	1,31E-02
cg14254433	6	34482411	PACSIN1	5'UTR	0,06	1,35E-05	1,31E-02
cg20813462	7	2646259	IQCE	Body	-0,038	1,36E-05	1,31E-02
cg00956114	10	7625588	ITIH5	Body	-0,008	1,35E-05	1,31E-02
cg06793798	10	62538126	CDK1	TSS200	0,012	1,36E-05	1,31E-02
cg18139178	10	134997342	KNDC1	Body	0,015	1,35E-05	1,31E-02
cg11876492	12	93042545		IGR	0,021	1,34E-05	1,31E-02
cg22511564	16	46809514		IGR	-0,02	1,35E-05	1,31E-02
cg00182087	19	57679551	DUXA	TSS1500	0,066	1,35E-05	1,31E-02
cg15305780	20	33679819	TRPC4AP	Body	-0,011	1,35E-05	1,31E-02
cg13713832	11	66241284	PELI3	Body	-0,01	1,36E-05	1,31E-02
cg04740504	12	112608974	HECTD4	Body	0,006	1,36E-05	1,31E-02
cg01419469	17	8092488	C17orf59	1stExon	0,034	1,36E-05	1,31E-02
cg21077610	21	45502014	TRAPPC10	Body	0,008	1,37E-05	1,31E-02
cg09680704	1	150463904	MIR6878	TSS1500	-0,006	1,38E-05	1,32E-02
cg12359158	6	134210253	TCF21	TSS200	-0,034	1,37E-05	1,32E-02
cg02778245	7	130419514	KLF14	TSS1500	-0,015	1,38E-05	1,32E-02
cg02505956	7	157348266	PTPRN2	Body	0,01	1,37E-05	1,32E-02
cg07674570	11	65346152	EHBP1L1	Body	-0,022	1,37E-05	1,32E-02
cg05608261	11	122647924	UBASH3B	Body	-0,006	1,38E-05	1,32E-02
cg00401413	2	70196407	PCBP1-AS1	Body	-0,005	1,38E-05	1,32E-02
cg04845368	20	37263156	ARHGAP40	Body	0,034	1,38E-05	1,32E-02
cg11229343	2	242808427		IGR	-0,056	1,38E-05	1,32E-02
cg08645980	2	169984729	LRP2	3'UTR	-0,026	1,40E-05	1,32E-02
cg17686661	8	50538634		IGR	0,031	1,39E-05	1,32E-02
cg07119028	13	114777703	RASA3	Body	-0,015	1,40E-05	1,32E-02
cg10160332	14	106931455		IGR	0,041	1,39E-05	1,32E-02
cg22687413	1	11730430	FBXO6	Body	0,005	1,41E-05	1,33E-02
cg23528708	6	13814945	CCDC90A	TSS200	0,004	1,41E-05	1,33E-02
cg02378702	7	31099351	ADCYAP1R1	5'UTR	0,044	1,41E-05	1,33E-02
cg00599354	10	83580344		IGR	-0,009	1,41E-05	1,33E-02
cg22302675	7	157742562	PTPRN2	Body	-0,052	1,42E-05	1,33E-02
cg23527624	22	50609122	PANX2	TSS200	0,017	1,42E-05	1,33E-02
cg02713760	15	83953998	BNC1	TSS1500	-0,022	1,43E-05	1,34E-02
cg19400179	3	108321607	DZIP3	5'UTR	0,042	1,43E-05	1,34E-02
cg25720419	10	108626325	SORCS1	Body	0,045	1,43E-05	1,34E-02
cg05766064	18	19262576	ABHD3	Body	0,029	1,44E-05	1,34E-02
cg22424536	5	175792478	ARL10	TSS200	0,005	1,45E-05	1,35E-02

cg23462428	4	78928450		IGR	-0,011	1,45E-05	1,35E-02
cg03222672	9	125878106	C9orf45	TSS1500	-0,005	1,45E-05	1,35E-02
cg07713027	11	3862302	RHOG	TSS200	0,009	1,45E-05	1,35E-02
cg02374996	9	92220771	GADD45G	Body	0,005	1,46E-05	1,35E-02
cg03562766	1	23698478		IGR	-0,022	1,47E-05	1,35E-02
cg03987003	1	145516092	GNRHR2	TSS200	0,025	1,47E-05	1,35E-02
cg23205744	2	10507934	HPCAL1	TSS1500	-0,009	1,47E-05	1,35E-02
cg21556683	3	38592798	SCN5A	Body	-0,011	1,47E-05	1,35E-02
cg24620338	11	71498715	FAM86C	Body	0,004	1,47E-05	1,35E-02
cg24180480	17	79852289	ANAPC11	5'UTR	0,016	1,46E-05	1,35E-02
cg15494731	19	39398122	NFKBIB	Body	-0,013	1,47E-05	1,35E-02
cg00161715	3	72128806	LINC00877	Body	0,03	1,49E-05	1,37E-02
cg22980971	8	42148077	IKBKB	Body	0,01	1,49E-05	1,37E-02
cg26393930	17	65611272	PITPNC1	Body	-0,007	1,49E-05	1,37E-02
cg14529732	22	43306564	PACSIN2	Body	-0,012	1,49E-05	1,37E-02
cg07752348	3	46652499	)C100132146	TSS1500	-0,011	1,50E-05	1,37E-02
cg15822392	3	194605554		IGR	0,043	1,50E-05	1,37E-02
cg14858784	12	123579358	PITPNM2	5'UTR	-0,017	1,50E-05	1,37E-02
cg27014783	2	2712909		IGR	-0,033	1,51E-05	1,37E-02
cg08360197	2	178029001		IGR	0,042	1,51E-05	1,37E-02
cg09729182	3	121964928	CASR	5'UTR	0,007	1,51E-05	1,37E-02
cg20598211	5	142762454	NR3C1	Body	0,007	1,51E-05	1,37E-02
cg04157608	11	33269942		IGR	0,011	1,51E-05	1,37E-02
cg03853134	2	122494799	MKI67IP	TSS1500	0,022	1,53E-05	1,37E-02
cg15057325	2	197666930		IGR	0,035	1,53E-05	1,37E-02
cg02846343	3	149356486	WWTR1	Body	0,005	1,52E-05	1,37E-02
cg08667272	14	53307708		IGR	0,027	1,52E-05	1,37E-02
cg06946814	14	104194561	ZFYVE21	Body	-0,013	1,52E-05	1,37E-02
cg03093541	15	31453735	TRPM1	TSS1500	0,006	1,53E-05	1,37E-02
cg21140943	15	93631110	RGMA	Body	-0,004	1,53E-05	1,37E-02
cg00866531	16	2578056	AMDHD2	Body	-0,018	1,53E-05	1,37E-02
cg03423154	20	3675835	SIGLEC1	Body	-0,009	1,52E-05	1,37E-02
cg11635454	11	126173444	DCPS	TSS1500	-0,106	1,53E-05	1,37E-02
cg09741935	8	128149826		IGR	-0,011	1,54E-05	1,38E-02
cg19376973	2	42229025		IGR	0,004	1,54E-05	1,38E-02
cg13975093	7	872797	UNC84A	5'UTR	0,008	1,54E-05	1,38E-02
cg01382141	6	12151290	HIVEP1	Body	0,026	1,55E-05	1,38E-02
cg26797722	6	15432551	JARID2	Body	-0,021	1,55E-05	1,38E-02
cg27469271	7	28993397	TRIL	3'UTR	0,027	1,55E-05	1,38E-02
cg04890503	16	52058641		IGR	0,025	1,55E-05	1,38E-02
cg25847024	1	111991376	WDR77	Body	0,01	1,57E-05	1,38E-02
cg17485717	11	33081126	TCP11L1	Body	-0,095	1,56E-05	1,38E-02
cg11804940	12	122402696	WDR66	Body	-0,024	1,57E-05	1,38E-02
cg04001333	14	76045348	FLVCR2	1stExon	-0,07	1,57E-05	1,38E-02
cg15834145	14	91758390	CCDC88C	Body	-0,009	1,56E-05	1,38E-02
cg16635972	15	70994663	UACA	TSS200	0,007	1,57E-05	1,38E-02
cg23894163	16	29801976	KIF22	TSS1500	0,005	1,57E-05	1,38E-02
cg09741519	17	1810775		IGR	0,046	1,57E-05	1,38E-02
cg19193873	22	31556135	RNF185	TSS200	0,003	1,56E-05	1,38E-02
cg27396447	19	40561828	ZNF780B	5'UTR	0,005	1,57E-05	1,38E-02
cg11842501	18	46455341	SMAD7	Body	-0,004	1,58E-05	1,38E-02
cg05972616	1	89151276	PKN2-AS1	TSS1500	-0,008	1,59E-05	1,39E-02
cg13437786	1	244753513	C1orf101	Body	-0,013	1,59E-05	1,39E-02
cg14422922	1	247614727	OR2B11	1stExon	-0,011	1,59E-05	1,39E-02
cg22959667	2	109745614	SH3RF3	TSS1500	0,006	1,60E-05	1,39E-02
cg16142146	2	153056747		IGR	0,008	1,60E-05	1,39E-02
cg15540044	4	76439595	RCHY1	5'UTR	0,004	1,61E-05	1,39E-02
cg12663645	4	188122807		IGR	-0,026	1,61E-05	1,39E-02
cg05855208	7	31089142		IGR	0,041	1,60E-05	1,39E-02
cg26720961	8	143386260	TSNARE1	Body	-0,016	1,61E-05	1,39E-02
cg24689026	10	118387095	PNLIPRP2	Body	-0,063	1,61E-05	1,39E-02

cg07658646	12	11002197	PRR4	TSS200	0,045	1,59E-05	1,39E-02
cg26643813	12	119813750	CCDC60	Body	0,04	1,61E-05	1,39E-02
cg02565196	13	27883302		IGR	-0,036	1,61E-05	1,39E-02
cg07015014	14	91770418	CCDC88C	Body	-0,011	1,61E-05	1,39E-02
cg26027170	17	37394705		IGR	0,011	1,59E-05	1,39E-02
cg27005075	19	38942452	RYR1	Body	0,008	1,61E-05	1,39E-02
cg26285749	22	22288508	PPM1F	Body	-0,012	1,59E-05	1,39E-02
cg14083285	4	155850368		IGR	-0,013	1,62E-05	1,39E-02
cg12994248	8	121458287	MRPL13	TSS1500	0,061	1,62E-05	1,39E-02
cg01771065	14	102102407		IGR	0,034	1,62E-05	1,39E-02
cg01177907	16	3184694	ZNF213	TSS1500	0,006	1,62E-05	1,39E-02
cg16393107	1	175985758	RFWD2	Body	0,021	1,64E-05	1,39E-02
cg16508623	12	102271114	DRAM1	5'UTR	0,005	1,63E-05	1,39E-02
cg03524794	17	7750618	KDM6B	Body	-0,005	1,63E-05	1,39E-02
cg03071876	17	45929434	SP6	5'UTR	0,048	1,64E-05	1,39E-02
cg06423211	19	551401		IGR	0,096	1,63E-05	1,39E-02
cg07666312	19	17420143	DDA1	TSS200	-0,008	1,63E-05	1,39E-02
cg01627172	3	177434634	LINC00578	Body	-0,017	1,64E-05	1,39E-02
cg12537245	5	73935531	ENC1	5'UTR	-0,013	1,65E-05	1,40E-02
cg25585364	2	118846169	INSIG2	5'UTR	0,01	1,65E-05	1,40E-02
cg09630474	8	61495822	RAB2A	Body	-0,047	1,66E-05	1,40E-02
cg23216498	1	16202869	SPEN	Body	0,005	1,66E-05	1,40E-02
cg02658330	3	58653593	FAM3D	TSS1500	-0,011	1,67E-05	1,40E-02
cg25390199	17	18761479	PRPSAP2	TSS200	-0,01	1,66E-05	1,40E-02
cg02927961	19	11271097		IGR	0,012	1,67E-05	1,40E-02
cg05058272	2	237422930		IGR	-0,008	1,67E-05	1,40E-02
cg11809638	1	64449581	ROR1	Body	0,035	1,67E-05	1,40E-02
cg09886849	15	25235865		IGR	-0,022	1,67E-05	1,40E-02
cg22581191	4	14197865		IGR	0,019	1,67E-05	1,40E-02
cg06051411	17	73044991	KCTD2	Body	-0,007	1,68E-05	1,41E-02
cg19647370	12	26146815	RASSF8	5'UTR	-0,042	1,69E-05	1,41E-02
cg02789045	3	142644867		IGR	0,029	1,69E-05	1,41E-02
cg04930497	11	16848047	PLEKHA7	Body	-0,014	1,69E-05	1,41E-02
cg13202534	11	111170911	COLCA1	5'UTR	0,046	1,69E-05	1,41E-02
cg20863949	2	71213618	TEX261	3'UTR	0,016	1,70E-05	1,41E-02
cg22950970	11	67162822	RAD9A	Body	-0,009	1,70E-05	1,41E-02
cg02426584	22	25421509		IGR	-0,008	1,70E-05	1,41E-02
cg07415388	17	17717276	MIR33B	TSS200	-0,018	1,71E-05	1,41E-02
cg21864563	1	44584417	KLF17	TSS200	-0,006	1,71E-05	1,42E-02
cg25947311	3	14167113	CHCHD4	TSS1500	0,004	1,71E-05	1,42E-02
cg04775505	4	71114913	CSN3	Body	-0,05	1,71E-05	1,42E-02
cg03008269	11	13485254	BTBD10	TSS1500	-0,006	1,72E-05	1,42E-02
cg02418025	19	4584005		IGR	0,029	1,72E-05	1,42E-02
cg26023908	3	107601974	LOC285205	TSS200	0,007	1,73E-05	1,42E-02
cg22210246	1	247071424	AHCTF1	Body	-0,019	1,73E-05	1,42E-02
cg26661481	11	117855870	IL10RA	TSS1500	0,009	1,73E-05	1,42E-02
cg24237576	16	2285775	DNASE1L2	TSS1500	-0,019	1,73E-05	1,42E-02
cg19513940	18	67070120	DOK6	Body	0,018	1,74E-05	1,42E-02
cg23206822	1	19182586	TAS1R2	Body	0,046	1,74E-05	1,43E-02
cg05075536	17	37370444	STAC2	Body	0,025	1,74E-05	1,43E-02
cg24463664	8	38160704	WHSC1L1	Body	-0,026	1,75E-05	1,43E-02
cg25117062	2	10442836	HPCAL1	TSS1500	0,01	1,75E-05	1,43E-02
cg04310348	2	239186648	PER2	5'UTR	-0,021	1,76E-05	1,43E-02
cg25673474	4	75565779		IGR	0,009	1,76E-05	1,43E-02
cg14614693	5	114961897	MED7-TICAM2	TSS200	-0,008	1,77E-05	1,43E-02
cg14079656	6	168261473	MLLT4	Body	-0,014	1,76E-05	1,43E-02
cg06292146	17	19840295	AKAP10	Body	-0,007	1,76E-05	1,43E-02
cg07987191	19	46191614	SNRPD2	Body	-0,007	1,76E-05	1,43E-02
cg17344080	12	93101181	C12orf74	Body	0,008	1,77E-05	1,43E-02
cg13641012	5	491859	SLC9A3	Body	-0,013	1,77E-05	1,43E-02
cg09761351	10	70092558	PBLD	1stExon	0,006	1,78E-05	1,43E-02

cg02188838	16	16212171	ABCC1	Body	0,006	1,78E-05	1,44E-02
cg22758486	1	165415170	RXRG	TSS1500	0,044	1,78E-05	1,44E-02
cg13959088	3	13035351	IQSEC1	Body	0,004	1,79E-05	1,44E-02
cg13992911	5	1003504		IGR	0,046	1,80E-05	1,44E-02
cg15872103	2	114513914	SLC35F5	Body	0,004	1,80E-05	1,45E-02
cg16868350	19	55064373		IGR	-0,041	1,81E-05	1,45E-02
cg12164079	2	30143576	ALK	1stExon	-0,027	1,81E-05	1,45E-02
cg24000344	3	63883366	ATXN7	TSS1500	-0,01	1,81E-05	1,45E-02
cg10620395	5	1056468	SLC12A7	Body	-0,021	1,82E-05	1,45E-02
cg10430077	5	180648553		IGR	0,061	1,82E-05	1,45E-02
cg02156071	10	120101821	C10orf84	1stExon	0,01	1,82E-05	1,45E-02
cg01520985	12	75040836		IGR	0,042	1,82E-05	1,45E-02
cg12948972	16	85079428	KIAA0513	5'UTR	-0,016	1,82E-05	1,45E-02
cg07786995	1	162114714	NOS1AP	Body	0,023	1,82E-05	1,45E-02
cg16821558	1	40201703		IGR	0,043	1,83E-05	1,45E-02
cg08712625	19	920786	KISS1R	3'UTR	0,013	1,83E-05	1,45E-02
cg03705284	1	1051907	C1orf159	TSS200	0,003	1,84E-05	1,46E-02
cg24120028	20	35089018	DLGAP4	TSS1500	0,006	1,84E-05	1,46E-02
cg24549471	21	19165753	C21orf91	3'UTR	0,03	1,84E-05	1,46E-02
cg00694560	3	121707177	ILDR1	3'UTR	0,013	1,85E-05	1,46E-02
cg06901790	16	31712112	C16orf67	Body	0,009	1,85E-05	1,46E-02
cg01822071	16	35027026		IGR	0,02	1,85E-05	1,46E-02
cg02027653	13	98438857		IGR	0,02	1,86E-05	1,46E-02
cg11828462	16	57606713	ADGRG5	Body	-0,01	1,86E-05	1,46E-02
cg11964207	19	2199129	DOT1L	Body	-0,015	1,86E-05	1,46E-02
cg16131972	20	45282877	SLC13A3	5'UTR	-0,015	1,86E-05	1,46E-02
cg27033727	15	42470732	VPS39	Body	0,007	1,86E-05	1,46E-02
cg22554488	5	167000712	ODZ2	Body	-0,013	1,87E-05	1,47E-02
cg01960184	6	46185318		IGR	0,045	1,87E-05	1,47E-02
cg09430095	12	4646531	C12orf4	5'UTR	-0,01	1,88E-05	1,47E-02
cg05703471	15	72892326		IGR	-0,024	1,87E-05	1,47E-02
cg19732319	17	80630372	RAB40B	Body	-0,005	1,88E-05	1,47E-02
cg15207999	1	1021210	C1orf159	Body	-0,01	1,88E-05	1,47E-02
cg24474193	4	108662252		IGR	-0,015	1,89E-05	1,47E-02
cg23242017	7	97736138	LMTK2	TSS200	0,007	1,89E-05	1,47E-02
cg19352801	11	63971766	STIP1	3'UTR	-0,005	1,89E-05	1,47E-02
cg21508673	13	113818634	PROZ	Body	0,073	1,90E-05	1,47E-02
cg11891336	17	71152847		IGR	0,039	1,90E-05	1,47E-02
cg26571326	19	54986999		IGR	-0,011	1,89E-05	1,47E-02
cg06444395	8	128191022		IGR	0,037	1,90E-05	1,47E-02
cg17037149	11	57638798		IGR	0,014	1,90E-05	1,47E-02
cg12478874	15	80263632	BCL2A1	1stExon	-0,02	1,91E-05	1,47E-02
cg06517798	1	160121283	ATP1A4	TSS200	-0,01	1,91E-05	1,47E-02
cg20437603	4	15486033	CC2D2A	Body	-0,008	1,92E-05	1,48E-02
cg10031859	10	92627707		IGR	-0,009	1,92E-05	1,48E-02
cg03230017	22	31277462	OSBP2	Body	-0,018	1,93E-05	1,48E-02
cg27648270	19	10928172	MIR199A1	TSS200	-0,009	1,94E-05	1,49E-02
cg02268186	20	1291823	SDCBP2	Body	-0,007	1,94E-05	1,49E-02
cg16030751	13	20602090	ZMYM2	Body	-0,005	1,94E-05	1,49E-02
cg20465933	4	152376761	FAM160A1	5'UTR	0,05	1,95E-05	1,49E-02
cg20126720	8	145980942	ZNF251	5'UTR	0,005	1,95E-05	1,49E-02
cg15721475	17	5995146	WSCD1	Body	-0,008	1,95E-05	1,49E-02
cg15991072	1	67773404	IL12RB2	5'UTR	0,004	1,97E-05	1,49E-02
cg12894334	1	232736209		IGR	0,066	1,96E-05	1,49E-02
cg06478421	2	207687894		IGR	-0,005	1,96E-05	1,49E-02
cg08622280	7	72742188	TRIM50	TSS200	0,004	1,96E-05	1,49E-02
cg26313341	11	57901264	OR9Q1	5'UTR	0,046	1,96E-05	1,49E-02
cg06985153	15	90438687	AP3S2	TSS1500	-0,008	1,96E-05	1,49E-02
cg21443548	17	56190507		IGR	-0,004	1,96E-05	1,49E-02
cg00846166	19	10022875	OLFM2	Body	-0,022	1,95E-05	1,49E-02
cg23446377	4	102127231	PPP3CA	Body	-0,008	1,97E-05	1,49E-02

cg23896919	1	235819792	MIR5096	Body	0,012	1,97E-05	1,49E-02
cg09717406	6	36305133	C6orf222	TSS1500	0,036	1,97E-05	1,49E-02
cg24118715	10	70232072	DNA2	TSS200	0,039	1,97E-05	1,49E-02
cg17211404	6	46703422	PLA2G7	TSS1500	0,011	1,99E-05	1,50E-02
cg00271210	6	167070053	RPS6KA2	Body	-0,066	1,99E-05	1,50E-02
cg10469980	16	85660101	KIAA0182	5'UTR	-0,012	1,99E-05	1,50E-02
cg23017741	12	110559276		IGR	0,033	1,99E-05	1,50E-02
cg08522775	1	46686733	LURAP1	3'UTR	0,031	2,02E-05	1,50E-02
cg09445162	3	133186445	BFSP2	Body	0,011	2,02E-05	1,50E-02
cg15662654	3	194843063	XXYLT1	Body	-0,008	2,00E-05	1,50E-02
cg26210267	4	668877	ATP5I	TSS1500	0,022	2,02E-05	1,50E-02
cg19571266	6	34079664	GRM4	TSS1500	-0,03	2,02E-05	1,50E-02
cg07044458	7	4751984	FOXK1	Body	-0,021	2,02E-05	1,50E-02
cg04681554	7	27283409	EVX1	Body	0,007	2,02E-05	1,50E-02
cg01989084	8	125385995	TMEM65	TSS1500	0,01	2,01E-05	1,50E-02
cg15499112	10	35787572	CCNY	Body	0,014	2,01E-05	1,50E-02
cg27252467	13	19585665	LOC348021	Body	-0,056	2,02E-05	1,50E-02
cg12238567	17	11294684	SHISA6	Body	0,06	2,00E-05	1,50E-02
cg06212165	17	18291699	EVPLL	3'UTR	-0,021	2,00E-05	1,50E-02
cg25073464	18	67067278	DOK6	TSS1500	0,063	2,02E-05	1,50E-02
cg16004202	4	159689901	FNIP2	TSS1500	0,011	2,03E-05	1,51E-02
cg10847958	1	173961984	RC3H1	1stExon	0,007	2,04E-05	1,51E-02
cg17482237	1	236900382	ACTN2	Body	0,013	2,06E-05	1,51E-02
cg06236295	2	11674365	GREB1	1stExon	0,055	2,05E-05	1,51E-02
cg19763809	2	98703475	VWA3B	TSS200	0,013	2,05E-05	1,51E-02
cg02871925	3	50395650	TMEM115	1stExon	-0,006	2,05E-05	1,51E-02
cg03507977	3	129704114		IGR	-0,018	2,06E-05	1,51E-02
cg01198391	4	15376180	C1QTNF7	Body	0,006	2,05E-05	1,51E-02
cg07183637	6	31940692	STK19	Body	-0,06	2,05E-05	1,51E-02
cg10310756	6	112408973	TUBE1	TSS1500	-0,005	2,06E-05	1,51E-02
cg19666810	7	97822857	LMTK2	Body	0,008	2,06E-05	1,51E-02
cg25033364	11	17760602	KCNC1	Body	0,029	2,06E-05	1,51E-02
cg11132673	20	816121	FAM110A	TSS1500	0,056	2,06E-05	1,51E-02
cg02664390	1	179911884		IGR	0,029	2,07E-05	1,51E-02
cg16089483	7	100873366		IGR	-0,007	2,07E-05	1,51E-02
cg06609282	3	86283974		IGR	-0,013	2,08E-05	1,52E-02
cg19513876	17	17377803		IGR	0,018	2,08E-05	1,52E-02
cg16884241	19	42746143	GSK3A	Body	-0,004	2,08E-05	1,52E-02
cg23764158	1	21606057	ECE1	1stExon	0,006	2,09E-05	1,52E-02
cg06920486	2	99343994	MGAT4A	5'UTR	0,034	2,09E-05	1,52E-02
cg03795779	5	14731875	ANKH	Body	-0,006	2,09E-05	1,52E-02
cg17441396	11	2000940		IGR	-0,019	2,09E-05	1,52E-02
cg01139101	2	17492831		IGR	-0,012	2,10E-05	1,52E-02
cg01709493	14	101509391	MIR1185-1	Body	0,007	2,10E-05	1,52E-02
cg16157568	1	3655613	TP73-AS1	Body	0,011	2,11E-05	1,52E-02
cg22705016	2	27272692		IGR	-0,03	2,11E-05	1,52E-02
cg27619840	5	45262329	HCN1	Body	0,014	2,11E-05	1,52E-02
cg19233753	6	142410098	NMBR	TSS200	-0,011	2,12E-05	1,52E-02
cg09039561	7	84177905		IGR	-0,013	2,11E-05	1,52E-02
cg20739918	10	135206889	MTG1	TSS1500	0,007	2,11E-05	1,52E-02
cg12459514	2	23914071	KLHL29	Body	-0,015	2,12E-05	1,52E-02
cg19894264	6	30852079	DDR1	TSS1500	0,005	2,12E-05	1,52E-02
cg26687305	7	99905331	SPDYE3	5'UTR	-0,015	2,12E-05	1,52E-02
cg03575966	9	139443292	LINC01573	Body	-0,019	2,13E-05	1,53E-02
cg19963944	17	65045415	CACNG1	Body	0,044	2,13E-05	1,53E-02
cg02466397	19	38714092	DPF1	Body	-0,023	2,13E-05	1,53E-02
cg05986323	16	75498625	TMEM170A	1stExon	0,005	2,14E-05	1,53E-02
cg01332602	3	112738782	C3orf17	TSS1500	0,022	2,14E-05	1,53E-02
cg02102832	16	1581834	IFT140	Body	-0,008	2,14E-05	1,53E-02
cg15627410	19	40972132	SPTBN4	TSS1500	0,02	2,14E-05	1,53E-02
cg04087814	3	107943092		IGR	-0,016	2,15E-05	1,53E-02

cg04652889	16	11532168		IGR	0,026	2,15E-05	1,53E-02
cg16624456	2	219601973	TTLL4	5'UTR	0,013	2,15E-05	1,53E-02
cg13299268	8	113477467	CSMD3	Body	0,044	2,15E-05	1,53E-02
cg04563953	1	160312929	NCSTN	TSS200	0,007	2,16E-05	1,53E-02
cg04305974	10	48420532		IGR	-0,008	2,16E-05	1,53E-02
cg14226122	1	169863215	SCYL3	TSS200	-0,008	2,18E-05	1,54E-02
cg25967957	7	44646819	OGDH	5'UTR	0,004	2,17E-05	1,54E-02
cg21215818	9	126169333	DENND1A	Body	-0,016	2,18E-05	1,54E-02
cg14312894	12	57171980	HSD17B6	Body	0,005	2,17E-05	1,54E-02
cg07345874	14	21248717	RNASE6	TSS1500	0,037	2,18E-05	1,54E-02
cg10947302	5	137667064	CDC25C	5'UTR	-0,009	2,19E-05	1,54E-02
cg05642044	3	169940769	PRKCI	Body	0,008	2,20E-05	1,55E-02
cg00443946	16	86370962	LOC732275	Body	-0,055	2,20E-05	1,55E-02
cg15338227	2	3280765	TSSC1	Body	-0,009	2,21E-05	1,55E-02
cg06705986	10	86004888	RGR	1stExon	0,043	2,21E-05	1,55E-02
cg22335368	11	119044774	NLRX1	Body	-0,014	2,21E-05	1,55E-02
cg03271093	6	139012618		IGR	0,05	2,21E-05	1,55E-02
cg16927353	2	10442868	HPCAL1	TSS1500	0,004	2,23E-05	1,55E-02
cg07190966	4	24586472	DHX15	TSS1500	-0,007	2,23E-05	1,55E-02
cg14830402	10	6244686	PFKFB3	TSS200	0,004	2,23E-05	1,55E-02
cg05331573	11	56392210		IGR	0,006	2,22E-05	1,55E-02
cg08944880	14	79051760	NRXN3	5'UTR	-0,018	2,23E-05	1,55E-02
cg27609489	17	39325513	KRTAP4-3	TSS1500	-0,011	2,22E-05	1,55E-02
cg26349554	11	85522295	SYTL2	TSS200	0,011	2,24E-05	1,56E-02
cg04436724	2	1681494	PXDN	Body	-0,019	2,24E-05	1,56E-02
cg03245590	11	15329459		IGR	0,035	2,24E-05	1,56E-02
cg27479282	7	891084	SUN1	Body	0,007	2,24E-05	1,56E-02
cg18593303	6	16746693	ATXN1	5'UTR	0,027	2,25E-05	1,56E-02
cg06486344	9	33290885	SUGT1P1	Body	0,006	2,25E-05	1,56E-02
cg03412618	22	46481099	LOC400931	TSS1500	-0,012	2,25E-05	1,56E-02
cg21202824	3	115779189	LSAMP	Body	-0,037	2,27E-05	1,57E-02
cg01730970	1	27687074	MAP3K6	Body	0,008	2,28E-05	1,57E-02
cg03116258	7	27135214	HOXA1	1stExon	0,01	2,27E-05	1,57E-02
cg00282249	13	37005566	CCNA1	TSS1500	0,013	2,28E-05	1,57E-02
cg26741333	15	67932521	MAP2K5	Body	-0,012	2,27E-05	1,57E-02
cg01643006	18	13540734	LDLRAD4	Body	0,052	2,27E-05	1,57E-02
cg08781425	7	29382602	CHN2	Body	-0,018	2,29E-05	1,57E-02
cg24289524	13	39199366		IGR	-0,036	2,29E-05	1,57E-02
cg14249563	15	76492376	C15orf27	Body	0,012	2,29E-05	1,57E-02
cg17535895	20	56719651		IGR	0,039	2,29E-05	1,57E-02
cg21869951	1	43824422	CDC20	TSS1500	0,007	2,31E-05	1,57E-02
cg13839221	1	87745872		IGR	0,008	2,31E-05	1,57E-02
cg01586352	2	1871382	MYT1L	Body	0,037	2,30E-05	1,57E-02
cg15770012	6	168443352	KIF25	Body	-0,01	2,31E-05	1,57E-02
cg11171235	9	86595232	HNRNPK	5'UTR	0,005	2,30E-05	1,57E-02
cg25499748	9	139886815	C9orf142	TSS200	-0,004	2,31E-05	1,57E-02
cg00055369	11	108757287	DDX10	Body	0,009	2,30E-05	1,57E-02
cg05506899	19	29185732	)C100420587	Body	-0,025	2,31E-05	1,57E-02
cg18154214	16	74330809	PSMD7	5'UTR	0,005	2,31E-05	1,57E-02
cg17838664	1	90014462	LRRC8B	5'UTR	0,005	2,32E-05	1,58E-02
cg05043591	9	109040472		IGR	-0,019	2,32E-05	1,58E-02
cg09707587	7	73877151	GTF2IRD1	5'UTR	-0,03	2,33E-05	1,58E-02
cg12625464	6	139561673	TXLNB	3'UTR	0,029	2,34E-05	1,59E-02
cg16843436	16	17406298	XYLT1	Body	-0,006	2,34E-05	1,59E-02
cg10951590	8	4848439	CSMD1	Body	-0,062	2,35E-05	1,59E-02
cg23118964	6	79944830	HMGN3	TSS1500	-0,009	2,35E-05	1,59E-02
cg10207277	8	114449243	CSMD3	TSS200	-0,02	2,35E-05	1,59E-02
cg25167838	7	155605092	SHH	TSS200	-0,007	2,36E-05	1,59E-02
cg21218476	17	7117863	DLG4	Body	0,01	2,36E-05	1,59E-02
cg06234050	13	100089372		IGR	0,005	2,36E-05	1,59E-02
cg21606866	1	43735174	TMEM125	TSS1500	0,016	2,37E-05	1,59E-02

cg18486725	6	70508140	LMBRD1	TSS1500	-0,028	2,37E-05	1,59E-02
cg00553605	7	73149190	ABHD11-AS1	TSS1500	-0,009	2,37E-05	1,59E-02
cg18518256	17	48796788	LUC7L3	TSS200	0,004	2,37E-05	1,59E-02
cg13609167	4	2827061	SH3BP2	Body	-0,013	2,38E-05	1,59E-02
cg08044806	3	25365560		IGR	-0,019	2,38E-05	1,59E-02
cg27370261	5	177630999	HNRNPAB	TSS1500	0,006	2,39E-05	1,59E-02
cg05911799	6	32708899	HLA-DQA2	TSS1500	0,014	2,39E-05	1,59E-02
cg03988119	15	63797904	USP3	Body	0,006	2,38E-05	1,59E-02
cg06994834	7	34174956	BMPER	Body	-0,012	2,39E-05	1,59E-02
cg00306003	1	171640102		IGR	0,008	2,42E-05	1,60E-02
cg11948301	2	25862770	DTNB	Body	0,01	2,41E-05	1,60E-02
cg22183849	3	154042356	DHX36	TSS200	0,004	2,43E-05	1,60E-02
cg05003554	5	24488076	CDH10	Body	-0,01	2,42E-05	1,60E-02
cg20815819	6	2396932	GMDS-AS1	Body	-0,007	2,41E-05	1,60E-02
cg22202345	6	2985361		IGR	-0,014	2,43E-05	1,60E-02
cg08464208	6	27792307		IGR	-0,007	2,42E-05	1,60E-02
cg07895437	6	33282971	ZBTB22	Body	-0,047	2,43E-05	1,60E-02
cg23958704	8	103750999		IGR	0,051	2,39E-05	1,60E-02
cg13748640	9	102858493	ERP44	Body	-0,005	2,43E-05	1,60E-02
cg21415853	9	111718478	CTNNAL1	Body	-0,005	2,42E-05	1,60E-02
cg16928337	10	3234944		IGR	0,042	2,41E-05	1,60E-02
cg01452506	10	74114613	DNAJB12	1stExon	0,006	2,41E-05	1,60E-02
cg06768657	10	80222279		IGR	0,074	2,43E-05	1,60E-02
cg25896441	12	1616550		IGR	0,009	2,43E-05	1,60E-02
cg17020895	12	3423790		IGR	0,032	2,42E-05	1,60E-02
cg18132241	13	26042382	ATP8A2	Body	0,032	2,43E-05	1,60E-02
cg04740229	19	12251245	ZNF20	TSS200	-0,006	2,42E-05	1,60E-02
cg13684223	20	25471528	NINL	Body	-0,01	2,40E-05	1,60E-02
cg16090620	22	47197221	TBC1D22A	Body	-0,008	2,44E-05	1,60E-02
cg12937684	7	157451906	PTPRN2	Body	-0,018	2,44E-05	1,60E-02
cg01715299	1	111535465		IGR	0,032	2,45E-05	1,60E-02
cg12161007	5	177683033	COL23A1	Body	0,011	2,45E-05	1,60E-02
cg24591770	1	45082704	RNF220	Body	0,009	2,46E-05	1,60E-02
cg08065889	2	234121206		IGR	-0,005	2,46E-05	1,60E-02
cg26510634	3	123877804	KALRN	Body	-0,03	2,46E-05	1,60E-02
cg14692457	7	116554		IGR	0,008	2,46E-05	1,60E-02
cg23916104	11	7110083	RBMXL2	TSS200	-0,094	2,45E-05	1,60E-02
cg04124576	12	102055018	MYBPC1	Body	-0,016	2,46E-05	1,60E-02
cg11961401	22	18511180	FLJ41941	TSS1500	0,045	2,46E-05	1,60E-02
cg02867653	22	20100474	TRMT2A	Body	-0,015	2,45E-05	1,60E-02
cg13160333	1	245002976	COX20	Body	0,045	2,47E-05	1,60E-02
cg08768218	8	13372483	DLC1	TSS200	0,041	2,48E-05	1,61E-02
cg04503171	8	110346293	NUDCD1	1stExon	0,005	2,48E-05	1,61E-02
cg11476866	17	41920415		IGR	0,058	2,48E-05	1,61E-02
cg07857137	6	8579887	)C100506207	Body	-0,016	2,49E-05	1,61E-02
cg10891225	17	77771616	CBX8	TSS1500	0,008	2,50E-05	1,62E-02
cg03546668	8	55533785	RP1	Body	0,024	2,51E-05	1,62E-02
cg06099087	8	145254011	MROH1	ExonBnd	-0,016	2,51E-05	1,62E-02
cg00409049	2	118980919		IGR	-0,064	2,52E-05	1,62E-02
cg05918355	5	54515640		IGR	-0,017	2,52E-05	1,62E-02
cg23674189	19	5785241	PRR22	TSS1500	-0,008	2,51E-05	1,62E-02
cg10411581	14	101129598	LINC00523	Body	0,024	2,52E-05	1,62E-02
cg03619332	16	585859	SOLH	5'UTR	-0,019	2,52E-05	1,62E-02
cg25117895	6	5003999	RPP40	Body	0,01	2,54E-05	1,62E-02
cg08472444	9	138987320	NACC2	TSS200	0,009	2,53E-05	1,62E-02
cg18223876	12	7272965	C1RL-AS1	Body	-0,014	2,53E-05	1,62E-02
cg14411859	14	102829041	TECPR2	TSS1500	-0,014	2,54E-05	1,62E-02
cg25836232	18	12306837	TUBB6	TSS1500	0,061	2,54E-05	1,62E-02
cg14397690	15	60688948	ANXA2	5'UTR	0,032	2,54E-05	1,62E-02
cg18922874	14	24035805	AP1G2	Body	-0,006	2,55E-05	1,63E-02
cg09166893	17	27140005	FAM222B	TSS1500	-0,004	2,55E-05	1,63E-02

cg03157370	11	61525073	MYRF	Body	-0,013	2,57E-05	1,64E-02
cg20080845	22	50174170	BRD1	Body	-0,028	2,58E-05	1,64E-02
cg17395207	1	147082993	BCL9	5'UTR	0,009	2,59E-05	1,64E-02
cg24302310	3	193290518		IGR	-0,059	2,59E-05	1,64E-02
cg23054676	5	7869265	MTRR	1stExon	0,006	2,58E-05	1,64E-02
cg03454028	10	32217895	ARHGAP12	TSS200	0,005	2,59E-05	1,64E-02
cg18419351	11	126985971		IGR	0,009	2,59E-05	1,64E-02
cg14854901	5	72793969	BTF3	TSS1500	-0,012	2,60E-05	1,64E-02
cg14246568	6	105388153		IGR	0,027	2,60E-05	1,65E-02
cg17744997	6	167401611		IGR	0,046	2,61E-05	1,65E-02
cg16692998	19	19739173	LPAR2	TSS200	0,013	2,61E-05	1,65E-02
cg03647068	2	153480251	FMNL2	Body	0,015	2,62E-05	1,65E-02
cg18913798	15	41220123	DLL4	TSS1500	0,004	2,62E-05	1,65E-02
cg13829287	17	15414773	/P23C-CDRT4	Body	-0,012	2,62E-05	1,65E-02
cg11282105	1	76745328	ST6GALNAC3	Body	0,042	2,63E-05	1,66E-02
cg15396799	13	95953574	ABCC4	5'UTR	0,012	2,64E-05	1,66E-02
cg16775792	22	20748671	ZNF74	5'UTR	0,004	2,64E-05	1,66E-02
cg23046990	3	177340437	LINC00578	Body	0,006	2,64E-05	1,66E-02
cg10474063	5	169528168		IGR	0,008	2,64E-05	1,66E-02
cg19934863	1	111969519	OVGP1	Body	-0,016	2,65E-05	1,66E-02
cg10627136	5	130500762	HINT1	Body	0,004	2,65E-05	1,66E-02
cg11513563	5	141046296	ARAP3	Body	-0,009	2,65E-05	1,66E-02
cg02029844	7	100176334	LRCH4	Body	-0,009	2,65E-05	1,66E-02
cg17683143	9	90293233	DAPK1	Body	0,006	2,65E-05	1,66E-02
cg04359324	2	207116401		IGR	0,039	2,66E-05	1,66E-02
cg13332142	3	27575278		IGR	-0,009	2,66E-05	1,66E-02
cg19134233	6	56578373	RNU6-71P	Body	0,022	2,66E-05	1,66E-02
cg12168926	12	8973966	A2ML1	TSS1500	0,014	2,66E-05	1,66E-02
cg05074554	5	50262995		IGR	-0,016	2,67E-05	1,66E-02
cg04667664	16	30094818	PPP4C	Body	-0,021	2,67E-05	1,66E-02
cg20445283	19	33360599	SLC7A9	1stExon	-0,016	2,67E-05	1,66E-02
cg06156176	6	168860637	SMOC2	Body	-0,01	2,69E-05	1,67E-02
cg06202149	1	3605886	TP73	TSS1500	0,025	2,70E-05	1,67E-02
cg09175915	1	10683577	PEX14	Body	0,006	2,74E-05	1,67E-02
cg08029920	1	16070753	TMEM82	Body	-0,019	2,75E-05	1,67E-02
cg17625218	1	101577815		IGR	0,004	2,74E-05	1,67E-02
cg07199535	1	112901254		IGR	0,041	2,78E-05	1,67E-02
cg23899405	1	150721676	CTSS	Body	-0,012	2,69E-05	1,67E-02
cg22680451	1	160992367	F11R	TSS1500	-0,015	2,76E-05	1,67E-02
cg23166289	1	210001082	C1orf107	TSS1500	-0,075	2,78E-05	1,67E-02
cg06439547	1	230779251	COG2	Body	0,051	2,73E-05	1,67E-02
cg20271476	2	74596449	DCTN1	Body	-0,007	2,74E-05	1,67E-02
cg21579828	2	148601738	ACVR2A	TSS1500	0,007	2,73E-05	1,67E-02
cg26326264	2	177189676	MTX2	Body	-0,007	2,76E-05	1,67E-02
cg17854229	2	220019848	NHEJ1	Body	0,041	2,73E-05	1,67E-02
cg04539705	2	233384768		IGR	0,048	2,77E-05	1,67E-02
cg04696494	2	234620385	UGT1A5	TSS1500	-0,003	2,78E-05	1,67E-02
cg19467451	3	63884097	ATXN7	1stExon	0,007	2,78E-05	1,67E-02
cg14962136	4	103245785	SLC39A8	TSS1500	-0,005	2,71E-05	1,67E-02
cg07915271	4	184667781		IGR	-0,011	2,74E-05	1,67E-02
cg19034506	5	63525307	RNF180	Body	-0,051	2,72E-05	1,67E-02
cg24475062	6	32063394	TNXB	Body	-0,026	2,72E-05	1,67E-02
cg00570548	7	159533		IGR	0,008	2,77E-05	1,67E-02
cg09988421	7	150759464	SLC4A2	TSS1500	-0,014	2,78E-05	1,67E-02
cg24743574	8	100090791	VPS13B	Body	-0,011	2,71E-05	1,67E-02
cg01364755	10	1370694	ADARB2	Body	-0,009	2,78E-05	1,67E-02
cg11915224	11	72194963		IGR	-0,013	2,73E-05	1,67E-02
cg26695375	11	115039615		IGR	0,032	2,78E-05	1,67E-02
cg13528895	12	117135901		IGR	0,008	2,75E-05	1,67E-02
cg21604325	13	30062023	MTUS2	Body	-0,009	2,74E-05	1,67E-02
cg17507526	14	31637584	HECTD1	Body	-0,008	2,78E-05	1,67E-02

cg06195811	14	100011077	CCDC85C	Body	0,006	2,73E-05	1,67E-02
cg00776960	15	65688118	IGDCC4	Body	-0,053	2,77E-05	1,67E-02
cg02927074	15	80860549	ARNT2	Body	0,05	2,74E-05	1,67E-02
cg11547460	15	101178515	ASB7	Body	-0,011	2,72E-05	1,67E-02
cg26442633	16	1735466	HN1L	Body	-0,007	2,72E-05	1,67E-02
cg00522726	16	29806780	KIF22	5'UTR	0,006	2,72E-05	1,67E-02
cg03443590	17	78809852	RPTOR	Body	0,008	2,71E-05	1,67E-02
cg25964180	18	580334	CETN1	TSS200	-0,005	2,77E-05	1,67E-02
cg09174855	19	1592510	MBD3	Body	0,004	2,78E-05	1,67E-02
cg02254135	20	18341502		IGR	0,028	2,72E-05	1,67E-02
cg26187282	20	30734497	TM9SF4	Body	-0,009	2,75E-05	1,67E-02
cg08744476	20	32226532	CBFA2T2	Body	0,004	2,77E-05	1,67E-02
cg25505089	22	21331034	AIFM3	Body	-0,021	2,73E-05	1,67E-02
cg06212624	22	30942637	SEC14L6	1stExon	-0,007	2,74E-05	1,67E-02
cg13466487	5	10309051	CMBL	TSS1500	0,005	2,80E-05	1,67E-02
cg01819555	5	50791600		IGR	0,047	2,80E-05	1,67E-02
cg06440712	6	3089876	RIPK1	ExonBnd	-0,013	2,80E-05	1,67E-02
cg21576531	15	31490173		IGR	0,057	2,80E-05	1,67E-02
cg12838631	16	3023522	PKMYT1	Body	0,085	2,80E-05	1,67E-02
cg22284514	20	46586644		IGR	0,021	2,81E-05	1,67E-02
cg06212760	1	46664937	POMGNT1	Body	0,008	2,82E-05	1,67E-02
cg09233860	2	122043569	TFCP2L1	TSS1500	0,018	2,82E-05	1,67E-02
cg04785972	3	63263828	SYNPR	TSS200	-0,032	2,83E-05	1,67E-02
cg27152686	4	47645625	CORIN	Body	-0,025	2,82E-05	1,67E-02
cg08295661	10	127769903	ADAM12	Body	0,032	2,82E-05	1,67E-02
cg26973488	16	67450606	ZDHHC1	TSS1500	-0,013	2,82E-05	1,67E-02
cg18264803	20	33464359	ACSS2	Body	0,009	2,82E-05	1,67E-02
cg01910639	1	153507779	S100A6	Body	0,041	2,84E-05	1,67E-02
cg16113336	8	97744514	CPQ	5'UTR	-0,023	2,83E-05	1,67E-02
cg20133326	11	66617481	PC	Body	-0,006	2,83E-05	1,67E-02
cg09957135	11	123526101	SCN3B	TSS1500	0,038	2,83E-05	1,67E-02
cg10101352	13	30734052		IGR	0,007	2,84E-05	1,67E-02
cg07373946	16	7563521	RBFOX1	Body	-0,029	2,84E-05	1,67E-02
cg11812069	1	110627010		IGR	0,003	2,85E-05	1,67E-02
cg16023122	1	201708836	NAV1	TSS200	0,008	2,85E-05	1,67E-02
cg05426966	4	76556389	CDKL2	TSS1500	0,015	2,85E-05	1,67E-02
cg01306909	7	150676283	KCNH2	TSS1500	-0,005	2,85E-05	1,68E-02
cg06650475	20	10302885		IGR	-0,009	2,86E-05	1,68E-02
cg02870151	18	55991843	NEDD4L	Body	-0,005	2,87E-05	1,68E-02
cg00492055	3	138312356	CEP70	5'UTR	0,086	2,87E-05	1,68E-02
cg21607649	10	134884177		IGR	0,036	2,87E-05	1,68E-02
cg22207272	11	115375703	CADM1	TSS1500	-0,005	2,88E-05	1,68E-02
cg04878851	17	76778451	CYTH1	TSS200	0,014	2,88E-05	1,68E-02
cg04560163	5	73979408		IGR	0,02	2,88E-05	1,68E-02
cg02968844	1	884964	NOC2L	Body	-0,015	2,89E-05	1,68E-02
cg10952190	1	214725873	PTPN14	TSS1500	-0,034	2,89E-05	1,68E-02
cg12845952	3	167098119	ZBBX	TSS200	-0,004	2,89E-05	1,68E-02
cg25226555	5	98714340		IGR	0,066	2,88E-05	1,68E-02
cg27514933	14	24565765	PCK2	5'UTR	0,009	2,88E-05	1,68E-02
cg00084205	19	9485501	ZNF177	5'UTR	-0,036	2,89E-05	1,68E-02
cg04645534	8	23712550	STC1	TSS1500	-0,051	2,90E-05	1,68E-02
cg13347255	17	73726430	ITGB4	Body	-0,014	2,90E-05	1,68E-02
cg08455267	9	18500262	ADAMTSL1	Body	0,039	2,91E-05	1,69E-02
cg05443763	1	176501887	PAPPA2	5'UTR	0,041	2,93E-05	1,69E-02
cg13114549	14	20917403	OSGEP	Body	0,01	2,92E-05	1,69E-02
cg07435269	16	427561	TMEM8A	Body	0,004	2,92E-05	1,69E-02
cg04591032	17	46827458		IGR	0,026	2,93E-05	1,69E-02
cg20811072	17	78756839	RPTOR	Body	-0,007	2,92E-05	1,69E-02
cg12080886	21	42551951	PLAC4	5'UTR	-0,02	2,93E-05	1,69E-02
cg18587568	8	51926793		IGR	0,05	2,93E-05	1,69E-02
cg19394047	10	131223487		IGR	0,026	2,93E-05	1,69E-02

cg14162929	22	41939981	POLR3H	Body	0,004	2,94E-05	1,69E-02
cg00313184	1	113815402		IGR	0,008	2,94E-05	1,69E-02
cg08578313	1	155946507	ARHGEF2	Body	0,007	2,94E-05	1,69E-02
cg06875331	10	76729555	KAT6B	Body	0,009	2,95E-05	1,69E-02
cg16761329	5	134378113	C5orf66	5'UTR	0,043	2,96E-05	1,70E-02
cg26807301	16	22326380	POLR3E	Body	-0,01	2,96E-05	1,70E-02
cg11856182	19	35264148	ZNF599	TSS200	0,009	2,96E-05	1,70E-02
cg09637470	11	109963583	ZC3H12C	TSS1500	0,006	2,97E-05	1,70E-02
cg15709784	12	64845926	TBK1	TSS200	0,004	2,97E-05	1,70E-02
cg04935521	17	79098772	AATK	Body	-0,02	2,97E-05	1,70E-02
cg03095697	19	11781796		IGR	0,033	2,97E-05	1,70E-02
cg15080866	1	2705124		IGR	0,038	2,98E-05	1,70E-02
cg05237489	10	108623805	SORCS1	Body	-0,008	2,99E-05	1,71E-02
cg02581151	1	49616143	AGBL4	Body	-0,021	3,00E-05	1,71E-02
cg00118753	7	47802422	LINC00525	Body	0,009	3,00E-05	1,71E-02
cg05052869	11	134335356		IGR	-0,016	2,99E-05	1,71E-02
cg01559222	6	8436468	SLC35B3	TSS1500	-0,021	3,00E-05	1,71E-02
cg00953314	1	208257819	PLXNA2	Body	-0,015	3,01E-05	1,71E-02
cg03694423	6	114080463		IGR	-0,04	3,01E-05	1,71E-02
cg20574326	17	1582609	PRPF8	Body	-0,006	3,01E-05	1,71E-02
cg24578090	3	18158545	LOC339862	Body	0,044	3,02E-05	1,71E-02
cg10194060	4	68064683		IGR	0,05	3,01E-05	1,71E-02
cg12488207	10	135195153	PAOX	Body	-0,007	3,03E-05	1,71E-02
cg14361226	1	37310399	GRIK3	Body	0,03	3,05E-05	1,72E-02
cg06462964	6	28641757		IGR	0,004	3,06E-05	1,72E-02
cg03787603	7	101768610	CUX1	Body	0,007	3,05E-05	1,72E-02
cg11057082	12	52789558	KRT82	Body	-0,055	3,06E-05	1,72E-02
cg09189990	12	104350980	C12orf73	5'UTR	0,015	3,06E-05	1,72E-02
cg19948167	13	55819841		IGR	0,035	3,06E-05	1,72E-02
cg27582322	14	93002620	RIN3	Body	0,02	3,06E-05	1,72E-02
cg00863397	16	67215239	KIAA0895L	5'UTR	-0,016	3,06E-05	1,72E-02
cg08743881	17	26369049	NLK	TSS1500	0,011	3,05E-05	1,72E-02
cg02711726	17	80685570	FN3KRP	3'UTR	-0,008	3,04E-05	1,72E-02
cg18794488	22	32289716	DEPDC5	Body	0,005	3,04E-05	1,72E-02
cg15565180	1	247245211		IGR	0,012	3,07E-05	1,73E-02
cg02289322	5	176696395	NSD1	Body	-0,012	3,08E-05	1,73E-02
cg04356440	14	77271713	ANGEL1	Body	-0,009	3,09E-05	1,73E-02
cg26996244	7	131593640	)C101928782	TSS1500	0,028	3,10E-05	1,73E-02
cg10735689	18	21032577	RIOK3	TSS1500	-0,042	3,10E-05	1,73E-02
cg20576955	11	4606701	OR52I2	TSS1500	0,047	3,10E-05	1,73E-02
cg01017608	13	91627026		IGR	0,027	3,11E-05	1,74E-02
cg25694807	2	161329607	RBMS1	Body	0,012	3,11E-05	1,74E-02
cg19476792	1	70606641		IGR	0,006	3,12E-05	1,74E-02
cg24800074	12	120536977	RAB35	ExonBnd	-0,008	3,12E-05	1,74E-02
cg06707406	2	50570407	NRXN1	Body	-0,034	3,12E-05	1,74E-02
cg23560113	2	70141289	MXD1	TSS1500	0,009	3,14E-05	1,75E-02
cg26840260	8	145995978		IGR	0,016	3,14E-05	1,75E-02
cg00490648	16	83863902		IGR	0,005	3,15E-05	1,75E-02
cg26711184	3	82510133		IGR	0,008	3,16E-05	1,75E-02
cg16352072	1	910223	PLEKHN1	3'UTR	0,033	3,17E-05	1,75E-02
cg04362757	1	45197147		IGR	0,005	3,16E-05	1,75E-02
cg08882146	3	167600018		IGR	-0,011	3,16E-05	1,75E-02
cg07459252	14	37130212	PAX9	5'UTR	-0,012	3,17E-05	1,75E-02
cg04306007	2	161350288	RBMS1	5'UTR	0,005	3,17E-05	1,75E-02
cg24916396	5	72793696	BTF3	TSS1500	-0,052	3,17E-05	1,75E-02
cg04678892	2	147736419		IGR	-0,009	3,18E-05	1,76E-02
cg18411550	6	130339733	L3MBTL3	1stExon	0,004	3,19E-05	1,76E-02
cg18902238	2	226662251		IGR	0,054	3,20E-05	1,76E-02
cg02459951	1	165668044	ALDH9A1	TSS200	0,003	3,23E-05	1,77E-02
cg14966362	3	26388072		IGR	-0,006	3,22E-05	1,77E-02
cg05462905	5	54922354	SLC38A9	Body	-0,01	3,23E-05	1,77E-02

cg18149183	6	21441780		IGR	-0,03	3,23E-05	1,77E-02
cg00138325	6	42111924	C6orf132	TSS1500	0,008	3,21E-05	1,77E-02
cg04425788	10	131425198	MGMT	Body	0,018	3,23E-05	1,77E-02
cg12703042	11	111259829		IGR	0,037	3,23E-05	1,77E-02
cg14499074	12	109898292	KCTD10	Body	-0,007	3,23E-05	1,77E-02
cg00130181	13	100517033	CLYBL	Body	-0,013	3,22E-05	1,77E-02
cg23877213	14	23707185	RNF212B	1stExon	0,006	3,21E-05	1,77E-02
cg05842220	16	4735635	MGRN1	Body	-0,012	3,23E-05	1,77E-02
cg08109030	17	1189376	TUSC5	Body	-0,01	3,22E-05	1,77E-02
cg15681886	18	53212208	TCF4	Body	-0,009	3,24E-05	1,77E-02
cg16364693	20	36024650	SRC	Body	-0,007	3,23E-05	1,77E-02
cg02697702	10	62234080	ANK3	Body	-0,017	3,24E-05	1,77E-02
cg09610614	11	1104473		IGR	-0,009	3,24E-05	1,77E-02
cg05670240	2	135227167	TMEM163	Body	-0,016	3,25E-05	1,77E-02
cg09560438	3	10331548	GHRLOS	Body	-0,011	3,25E-05	1,77E-02
cg03476791	7	99067162		IGR	-0,07	3,25E-05	1,77E-02
cg19561055	1	246934409		IGR	0,005	3,27E-05	1,77E-02
cg07175433	4	21947400	KCNIP4	5'UTR	0,041	3,26E-05	1,77E-02
cg09758164	6	136865077	MAP7	Body	-0,028	3,27E-05	1,77E-02
cg13982366	9	138018566		IGR	-0,023	3,26E-05	1,77E-02
cg01312265	10	132930452	TCERG1L	Body	0,053	3,26E-05	1,77E-02
cg26931475	12	46743214		IGR	0,013	3,28E-05	1,77E-02
cg05543663	15	42372620	PLA2G4D	Body	-0,016	3,28E-05	1,77E-02
cg02619656	16	72460083		IGR	0,004	3,28E-05	1,77E-02
cg12580096	19	14017658	C19orf57	TSS1500	0,017	3,27E-05	1,77E-02
cg25039585	11	6703822	MRPL17	Body	0,006	3,28E-05	1,77E-02
cg25610029	2	68544328	CNRIP1	Body	0,007	3,29E-05	1,77E-02
cg26240566	10	120765114		IGR	0,043	3,29E-05	1,77E-02
cg16508645	15	40841990	C15orf57	Body	0,005	3,29E-05	1,77E-02
cg23441020	13	92993231	GPC5-AS2	Body	-0,043	3,30E-05	1,77E-02
cg22500501	10	73976536	ANAPC16	5'UTR	0,017	3,30E-05	1,77E-02
cg12552820	1	2231925	SKI	Body	0,004	3,32E-05	1,77E-02
cg07388756	1	88761053		IGR	0,005	3,31E-05	1,77E-02
cg08435936	2	190525813	ASNSD1	TSS1500	-0,007	3,32E-05	1,77E-02
cg19350970	2	238039148		IGR	-0,016	3,32E-05	1,77E-02
cg19622675	5	139683183	PFDN1	TSS1500	0,037	3,31E-05	1,77E-02
cg23709526	11	62675611		IGR	-0,012	3,32E-05	1,77E-02
cg03348405	11	122753328	C11orf63	1stExon	-0,008	3,31E-05	1,77E-02
cg08924023	13	31455173		IGR	0,008	3,32E-05	1,77E-02
cg03637821	17	29006869		IGR	0,012	3,32E-05	1,77E-02
cg23690480	21	46410455		IGR	-0,01	3,32E-05	1,77E-02
cg10403423	2	30410235		IGR	-0,02	3,34E-05	1,78E-02
cg03404709	2	242606531	ATG4B	Body	-0,018	3,33E-05	1,78E-02
cg06487103	6	2354089	GMDS-AS1	Body	-0,011	3,34E-05	1,78E-02
cg20788898	19	34193684	CHST8	Body	0,048	3,34E-05	1,78E-02
cg23604194	3	114344938	ZBTB20	TSS1500	-0,017	3,34E-05	1,78E-02
cg14757747	11	43846857	HSD17B12	Body	0,017	3,35E-05	1,78E-02
cg12444710	12	109540578	UNG	Body	0,018	3,35E-05	1,78E-02
cg22601058	10	12874207	LOC283070	TSS1500	0,009	3,35E-05	1,78E-02
cg26654702	20	19028668		IGR	0,046	3,35E-05	1,78E-02
cg23714415	8	56725731	TGS1	Body	-0,009	3,36E-05	1,78E-02
cg03085170	1	37783921		IGR	-0,014	3,38E-05	1,79E-02
cg17622053	8	116251556		IGR	-0,008	3,38E-05	1,79E-02
cg26092302	11	2277680		IGR	-0,035	3,38E-05	1,79E-02
cg20238921	12	8108380		IGR	0,033	3,41E-05	1,80E-02
cg17853833	22	31885141	EIF4ENIF1	5'UTR	0,006	3,41E-05	1,80E-02
cg02631196	1	3425761	MEGF6	Body	-0,015	3,43E-05	1,80E-02
cg23921342	1	34630923	CSMD2	Body	-0,009	3,44E-05	1,80E-02
cg08329648	1	76774252	ST6GALNAC3	Body	0,01	3,42E-05	1,80E-02
cg20770175	2	189839474	COL3A1	Body	-0,02	3,42E-05	1,80E-02
cg20974621	3	42815945	CCDC13	TSS1500	-0,006	3,42E-05	1,80E-02

cg05554592	3	107723149		IGR	0,006	3,44E-05	1,80E-02
cg06210240	4	72052544	SLC4A4	TSS1500	0,004	3,42E-05	1,80E-02
cg00125048	5	176023439	GPRIN1	3'UTR	-0,023	3,43E-05	1,80E-02
cg09376993	8	71314979	NCOA2	5'UTR	0,004	3,44E-05	1,80E-02
cg09703323	11	65197876		IGR	-0,01	3,44E-05	1,80E-02
cg10118210	13	108519554	FAM155A	TSS200	0,017	3,43E-05	1,80E-02
cg10705675	22	45351789	PHF21B	Body	0,01	3,44E-05	1,80E-02
cg16033376	19	47777635	PRR24	TSS1500	0,006	3,44E-05	1,80E-02
cg22335419	4	58357098		IGR	-0,025	3,45E-05	1,80E-02
cg21086902	7	4820767	KIAA0415	Body	0,008	3,45E-05	1,80E-02
cg01475734	2	220085125	ABCB6	TSS1500	-0,008	3,47E-05	1,80E-02
cg15351595	2	228653488		IGR	0,054	3,49E-05	1,80E-02
cg01012669	2	231822092		IGR	-0,023	3,48E-05	1,80E-02
cg02676380	4	122685587	PP12613	TSS200	-0,068	3,48E-05	1,80E-02
cg09890887	5	86563580	RASA1	TSS1500	0,017	3,47E-05	1,80E-02
cg21288091	8	105524569	LRP12	Body	0,009	3,48E-05	1,80E-02
cg13118835	9	100662669		IGR	0,008	3,47E-05	1,80E-02
cg16045811	9	128207786	MAPKAP1	Body	-0,009	3,48E-05	1,80E-02
cg25625670	11	57232742	RTN4RL2	Body	-0,025	3,47E-05	1,80E-02
cg08151654	11	62410183	GANAB	Body	-0,02	3,48E-05	1,80E-02
cg07617998	11	93396587	CEP295	5'UTR	0,009	3,49E-05	1,80E-02
cg07479864	15	72766497	ARIH1	TSS200	0,005	3,46E-05	1,80E-02
cg22994699	16	4412022	ꓛRO7-PAM16	Body	-0,016	3,48E-05	1,80E-02
cg15258847	18	5238219	C18orf18	TSS200	0,004	3,49E-05	1,80E-02
cg23106733	22	38325762	MICALL1	Body	-0,012	3,46E-05	1,80E-02
cg16236952	1	222344676		IGR	-0,035	3,50E-05	1,81E-02
cg02217176	10	133022707	TCERG1L	Body	-0,016	3,51E-05	1,81E-02
cg09012411	1	95167783		IGR	0,048	3,53E-05	1,82E-02
cg10135532	3	58477573	KCTD6	TSS1500	0,005	3,53E-05	1,82E-02
cg20656604	6	41800452	USP49	5'UTR	0,012	3,53E-05	1,82E-02
cg10983206	10	7564401		IGR	0,044	3,53E-05	1,82E-02
cg01776933	19	47851116	DHX34	TSS1500	0,011	3,52E-05	1,82E-02
cg21012871	20	42939451	FITM2	Body	0,006	3,54E-05	1,82E-02
cg24881558	5	1294198	TERT	Body	-0,016	3,56E-05	1,82E-02
cg11147740	9	35704339	TLN1	ExonBnd	-0,004	3,56E-05	1,82E-02
cg05689028	11	118086808	AMICA1	5'UTR	0,015	3,55E-05	1,82E-02
cg13830755	14	81904000		IGR	0,027	3,56E-05	1,82E-02
cg04569222	9	2239671		IGR	0,015	3,56E-05	1,82E-02
cg16450706	2	239988801	HDAC4	Body	-0,009	3,58E-05	1,82E-02
cg12202619	3	143570303		IGR	-0,013	3,57E-05	1,82E-02
cg13626798	6	132723376	MOXD1	TSS1500	0,042	3,58E-05	1,82E-02
cg20821028	9	138532149	GLT6D1	TSS1500	0,008	3,57E-05	1,82E-02
cg02727219	16	66087707		IGR	-0,039	3,58E-05	1,82E-02
cg08142347	19	42440001		IGR	0,042	3,57E-05	1,82E-02
cg00846400	22	36425210	RBM9	TSS1500	0,003	3,58E-05	1,82E-02
cg25140345	1	2440291	PANK4	Body	-0,005	3,59E-05	1,83E-02
cg01595273	2	44942982	CAMKMT	Body	-0,014	3,59E-05	1,83E-02
cg03089056	6	139139899	ECT2L	Body	0,028	3,59E-05	1,83E-02
cg05138464	19	34032363		IGR	0,038	3,59E-05	1,83E-02
cg13245575	8	134384819		IGR	-0,023	3,60E-05	1,83E-02
cg19394761	3	12805170	TMEM40	Body	0,038	3,61E-05	1,83E-02
cg13564064	9	126154367	DENND1A	Body	-0,011	3,60E-05	1,83E-02
cg03501666	12	99264415	ANKS1B	Body	0,016	3,61E-05	1,83E-02
cg01801868	3	51467418	VPRBP	Body	-0,005	3,64E-05	1,83E-02
cg11745271	4	17711566	FAM184B	Body	-0,037	3,62E-05	1,83E-02
cg16306115	6	24358306	KAAG1	1stExon	-0,013	3,64E-05	1,83E-02
cg24398822	6	41040505	NFYA	TSS1500	0,003	3,61E-05	1,83E-02
cg13401608	6	107223406	LOC553137	TSS1500	0,008	3,64E-05	1,83E-02
cg16213704	6	121840694		IGR	-0,016	3,64E-05	1,83E-02
cg25473596	7	141250973	AGK	TSS200	0,022	3,62E-05	1,83E-02
cg09851121	8	136465595		IGR	-0,013	3,63E-05	1,83E-02

cg13958495	9	138395346	MRPS2	Body	-0,005	3,64E-05	1,83E-02
cg06883551	11	18655919	SPTY2D1	1stExon	0,005	3,62E-05	1,83E-02
cg16644457	11	22359480	SLC17A6	TSS200	-0,012	3,63E-05	1,83E-02
cg12986485	11	78369307	ODZ4	Body	-0,006	3,63E-05	1,83E-02
cg14346316	11	125495727	CHEK1	TSS1500	0,006	3,62E-05	1,83E-02
cg08531399	12	58166635	METTL1	TSS1500	0,003	3,63E-05	1,83E-02
cg03591954	17	80964433	B3GNTL1	Body	0,006	3,64E-05	1,83E-02
cg20835656	10	61857489	ANK3	Body	-0,012	3,65E-05	1,83E-02
cg15253781	3	14203183	XPC	Body	-0,008	3,66E-05	1,83E-02
cg26613348	5	13648799		IGR	-0,02	3,67E-05	1,83E-02
cg04399875	16	878162		IGR	-0,027	3,66E-05	1,83E-02
cg09715862	14	21865632	SNORD8	TSS200	0,005	3,69E-05	1,84E-02
cg03741506	11	63764804	OTUB1	Body	-0,016	3,69E-05	1,84E-02
cg16422944	8	48188312	SPIDR	5'UTR	0,008	3,70E-05	1,85E-02
cg13984756	10	678263	DIP2C	Body	-0,009	3,70E-05	1,85E-02
cg10196290	1	212587951	TMEM206	Body	0,005	3,76E-05	1,85E-02
cg23362853	1	221983015		IGR	-0,021	3,76E-05	1,85E-02
cg11158729	2	97427027	CNNM4	1stExon	0,01	3,73E-05	1,85E-02
cg01984771	2	107189138		IGR	0,029	3,74E-05	1,85E-02
cg25568091	2	113032874	ZC3H6	TSS1500	0,004	3,77E-05	1,85E-02
cg18240059	4	3577354	LINC00955	TSS1500	0,027	3,74E-05	1,85E-02
cg01819982	4	81104878		IGR	0,01	3,75E-05	1,85E-02
cg21100518	6	29595002	GABBR1	Body	0,051	3,77E-05	1,85E-02
cg19293581	6	78645817		IGR	-0,019	3,73E-05	1,85E-02
cg26160086	7	157205545	DNAJB6	Body	-0,016	3,72E-05	1,85E-02
cg18674956	8	67023455		IGR	0,004	3,77E-05	1,85E-02
cg14266611	9	20478423	MLLT3	Body	0,017	3,74E-05	1,85E-02
cg14169945	9	81576285		IGR	0,075	3,75E-05	1,85E-02
cg02913168	9	123657310	PHF19	TSS200	0,008	3,72E-05	1,85E-02
cg10518327	10	77161702	C10orf41	Body	0,006	3,76E-05	1,85E-02
cg09688726	10	134562889	INPP5A	Body	-0,016	3,76E-05	1,85E-02
cg07613512	11	125199884	PKNOX2	5'UTR	0,053	3,74E-05	1,85E-02
cg11612617	12	102872937	IGF1	Body	0,024	3,72E-05	1,85E-02
cg03095244	12	115800623		IGR	-0,011	3,72E-05	1,85E-02
cg20765408	13	25085669	PARP4	5'UTR	0,038	3,73E-05	1,85E-02
cg13029429	14	94500569	OTUB2	Body	0,012	3,77E-05	1,85E-02
cg01551013	14	100203942		IGR	0,008	3,75E-05	1,85E-02
cg01181105	14	104165277	XRCC3	Body	-0,022	3,75E-05	1,85E-02
cg13998223	9	98389082		IGR	0,064	3,78E-05	1,85E-02
cg00997998	14	65150074		IGR	-0,02	3,78E-05	1,85E-02
cg01468907	16	84852577	CRISPLD2	TSS1500	-0,03	3,78E-05	1,86E-02
cg00471133	5	40551444		IGR	0,009	3,79E-05	1,86E-02
cg19506593	6	164443184		IGR	0,016	3,80E-05	1,86E-02
cg04053740	8	100168837	VPS13B	Body	0,009	3,79E-05	1,86E-02
cg07172280	9	140009455	DPP7	TSS1500	0,003	3,81E-05	1,86E-02
cg15154249	17	6656507		IGR	-0,037	3,80E-05	1,86E-02
cg13610835	19	39904138	PLEKHG2	5'UTR	0,009	3,80E-05	1,86E-02
cg02384897	22	30214219	ASCC2	Body	-0,008	3,80E-05	1,86E-02
cg19830147	21	47649323	MCM3APAS	Body	0,005	3,81E-05	1,86E-02
cg07562382	7	149129696	ZNF777	Body	0,006	3,82E-05	1,86E-02
cg16205325	3	48130383	MAP4	1stExon	0,012	3,82E-05	1,86E-02
cg25067153	11	58912240	FAM111A	TSS200	0,004	3,82E-05	1,86E-02
cg09533293	15	60689670	ANXA2	5'UTR	-0,011	3,83E-05	1,86E-02
cg01226171	9	107924816		IGR	0,006	3,83E-05	1,86E-02
cg20932849	1	161369236		IGR	0,004	3,84E-05	1,87E-02
cg15443403	3	9404422	THUMPD3	TSS1500	0,01	3,84E-05	1,87E-02
cg04528829	12	115133294		IGR	-0,019	3,84E-05	1,87E-02
cg19218505	11	8102016	TUB	TSS1500	-0,036	3,85E-05	1,87E-02
cg21908638	3	101497982	FAM55C	TSS1500	0,003	3,86E-05	1,87E-02
cg16884760	8	146233033		IGR	-0,02	3,86E-05	1,87E-02
cg00298481	11	27722063	BDNF	Body	0,004	3,86E-05	1,87E-02

cg06122864	6	29629187	MOG	Body	0,057	3,87E-05	1,87E-02
cg14017705	20	24754564		IGR	0,048	3,87E-05	1,87E-02
cg16443203	7	24459062		IGR	-0,007	3,88E-05	1,87E-02
cg18073142	1	185302226	GS1-279B7.1	Body	0,012	3,92E-05	1,87E-02
cg25994099	2	161322250	RBMS1	Body	-0,026	3,90E-05	1,87E-02
cg23144899	2	208994373	CRYGC	Body	-0,015	3,90E-05	1,87E-02
cg15845465	2	231451697		IGR	-0,035	3,92E-05	1,87E-02
cg01998801	4	2396368	ZFYVE28	Body	-0,016	3,91E-05	1,87E-02
cg12964657	4	3252413	C4orf44	Body	-0,015	3,90E-05	1,87E-02
cg19956606	4	3533644	LRPAP1	Body	0,005	3,90E-05	1,87E-02
cg20924858	6	129747513	LAMA2	Body	0,035	3,92E-05	1,87E-02
cg11246948	11	120581281	GRIK4	Body	0,045	3,89E-05	1,87E-02
cg05489292	12	112331187	MAPKAPK5	3'UTR	0,013	3,89E-05	1,87E-02
cg01257889	12	113230065	RPH3A	5'UTR	-0,06	3,91E-05	1,87E-02
cg16309970	13	114290596	TFDP1	Body	0,004	3,90E-05	1,87E-02
cg09788352	15	68522666	CLN6	TSS1500	0,022	3,88E-05	1,87E-02
cg09878473	15	92397206	SLCO3A1	1stExon	0,005	3,88E-05	1,87E-02
cg11459772	16	1730155	HN1L	Body	-0,017	3,91E-05	1,87E-02
cg07711336	20	22488591		IGR	0,011	3,90E-05	1,87E-02
cg10802541	5	77641721		IGR	0,012	3,93E-05	1,88E-02
cg21277452	6	28829340		IGR	-0,017	3,93E-05	1,88E-02
cg24879812	10	27735024		IGR	0,033	3,93E-05	1,88E-02
cg25219318	1	2527477	MMEL1	Body	0,008	3,96E-05	1,88E-02
cg13319825	2	85202220	KCMF1	Body	-0,006	3,95E-05	1,88E-02
cg26933447	3	162760728		IGR	0,04	3,94E-05	1,88E-02
cg23295955	4	26492123	CCKAR	TSS200	-0,017	3,96E-05	1,88E-02
cg12802784	6	3888501		IGR	0,019	3,96E-05	1,88E-02
cg22339837	11	63448596	RTN3	TSS1500	0,015	3,96E-05	1,88E-02
cg01965047	16	2140870	PKD1	Body	-0,032	3,95E-05	1,88E-02
cg14686309	17	9548019	USP43	TSS1500	0,003	3,95E-05	1,88E-02
cg12261170	22	30422977	MTMR3	3'UTR	-0,011	3,96E-05	1,88E-02
cg26338858	7	98788460	KPNA7	Body	0,032	3,97E-05	1,88E-02
cg15602054	2	170624685		IGR	-0,074	3,98E-05	1,88E-02
cg03725749	9	13141095	MPDZ	Body	0,025	3,98E-05	1,88E-02
cg06977911	9	124308284		IGR	0,006	3,98E-05	1,88E-02
cg03999020	16	88459356		IGR	0,03	3,98E-05	1,88E-02
cg20742075	18	21669758	TTC39C	Body	-0,008	3,99E-05	1,88E-02
cg22473312	19	28284823	LOC148189	Body	-0,003	3,99E-05	1,88E-02
cg18523042	5	140207388	PCDHA6	TSS200	-0,043	4,00E-05	1,89E-02
cg01219382	4	184328462		IGR	-0,006	4,01E-05	1,89E-02
cg13792025	7	66205470	RABGEF1	TSS200	0,005	4,01E-05	1,89E-02
cg15769486	2	85132558	TMSB10	TSS1500	0,006	4,03E-05	1,90E-02
cg25705589	2	240077384	HDAC4	Body	0,006	4,03E-05	1,90E-02
cg20528183	7	94284678	SGCE	Body	-0,008	4,03E-05	1,90E-02
cg04100147	1	43502282		IGR	0,012	4,04E-05	1,90E-02
cg05767990	5	119669499		IGR	-0,019	4,06E-05	1,91E-02
cg10040213	12	93328735		IGR	0,007	4,08E-05	1,91E-02
cg17510217	7	111024774	IMMP2L	Body	-0,018	4,09E-05	1,92E-02
cg14989668	16	57905351		IGR	0,01	4,09E-05	1,92E-02
cg23938645	17	78872563	RPTOR	Body	-0,017	4,09E-05	1,92E-02
cg00344888	15	58699976		IGR	-0,007	4,10E-05	1,92E-02
cg16008150	2	109231156	LIMS1	5'UTR	0,03	4,11E-05	1,92E-02
cg20840297	22	27044201		IGR	0,008	4,11E-05	1,92E-02
cg05237023	16	75182837	ZFP1	5'UTR	0,007	4,11E-05	1,92E-02
cg03075156	2	45986586	PRKCE	Body	-0,007	4,12E-05	1,92E-02
cg06909932	2	129104140		IGR	-0,014	4,13E-05	1,92E-02
cg14747322	5	173315714	CPEB4	5'UTR	-0,009	4,13E-05	1,92E-02
cg13426096	8	37887990	EIF4EBP1	TSS200	0,008	4,13E-05	1,92E-02
cg11280742	19	46445921	NOVA2	Body	0,037	4,13E-05	1,92E-02
cg01663052	14	31923667	C14orf126	Body	0,007	4,15E-05	1,93E-02
cg06951363	1	241683161	FH	TSS200	-0,013	4,15E-05	1,93E-02

cg21135086	2	109777962	SH3RF3	Body	0,007	4,16E-05	1,93E-02
cg21571055	22	36513305		IGR	-0,014	4,16E-05	1,93E-02
cg13225039	4	1722160	TMEM129	Body	0,006	4,17E-05	1,94E-02
cg05786241	17	7847306	CNTROB	Body	-0,014	4,17E-05	1,94E-02
cg21130286	3	193688638	LOC647323	Body	0,012	4,19E-05	1,94E-02
cg06525016	5	133473501	TCF7	Body	0,011	4,18E-05	1,94E-02
cg05219816	6	140982558		IGR	0,035	4,19E-05	1,94E-02
cg03242511	14	92789512	SLC24A4	TSS1500	0,006	4,19E-05	1,94E-02
cg23008112	16	1551673	TELO2	ExonBnd	-0,01	4,18E-05	1,94E-02
cg22224628	18	66381506	CCDC102B	TSS1500	-0,028	4,18E-05	1,94E-02
cg07147443	14	79933641	NRXN3	Body	-0,011	4,20E-05	1,94E-02
cg07661340	19	47354324	AP2S1	TSS200	-0,006	4,20E-05	1,94E-02
cg13216423	13	107220870	ARGLU1	TSS1500	-0,008	4,21E-05	1,94E-02
cg21811967	4	46939813	GABRA4	Body	0,06	4,21E-05	1,94E-02
cg14795708	1	93297732	RPL5	Body	0,005	4,22E-05	1,95E-02
cg06249747	14	69261882	C14orf181	3'UTR	-0,004	4,22E-05	1,95E-02
cg13416579	12	94648127	PLXNC1	Body	0,005	4,23E-05	1,95E-02
cg02868860	9	16624281	BNC2	Body	-0,019	4,24E-05	1,95E-02
cg00796973	1	21957811	RAP1GAP	Body	0,029	4,24E-05	1,95E-02
cg12906713	1	40889886		IGR	-0,006	4,25E-05	1,95E-02
cg18560110	7	47850152	PKD1L1	Body	-0,009	4,24E-05	1,95E-02
cg07862535	7	139043722	LUC7L2	TSS1500	0,009	4,25E-05	1,95E-02
cg23821003	12	131494244	GPR133	Body	-0,017	4,25E-05	1,95E-02
cg16450330	3	156392161	TIPARP-AS1	Body	0,003	4,27E-05	1,95E-02
cg14278989	5	71804672	ZNF366	TSS1500	-0,008	4,27E-05	1,95E-02
cg00078458	6	131019289		IGR	0,054	4,28E-05	1,95E-02
cg00430138	11	11688731		IGR	-0,019	4,26E-05	1,95E-02
cg02381317	11	65909761	PACS1	Body	0,009	4,28E-05	1,95E-02
cg23764129	11	113846017	HTR3A	1stExon	-0,018	4,27E-05	1,95E-02
cg01906801	17	38191472	MED24	Body	-0,027	4,28E-05	1,95E-02
cg07746245	17	79680420	SLC25A10	Body	-0,023	4,28E-05	1,95E-02
cg01612133	6	167317799		IGR	-0,049	4,28E-05	1,95E-02
cg18030372	4	2069750	NAT8L	3'UTR	-0,012	4,29E-05	1,96E-02
cg04585679	19	5044863	KDM4B	Body	-0,009	4,29E-05	1,96E-02
cg04675555	2	53700719		IGR	-0,021	4,30E-05	1,96E-02
cg04680907	7	134584245	CALD1	Body	0,005	4,31E-05	1,96E-02
cg04201335	7	144524837	TPK1	Body	-0,018	4,31E-05	1,96E-02
cg24426391	11	20384886	HTATIP2	TSS1500	0,123	4,31E-05	1,96E-02
cg06454894	2	88415702		IGR	-0,018	4,32E-05	1,96E-02
cg15710971	17	25621084	WSB1	TSS200	0,006	4,32E-05	1,96E-02
cg05004665	1	20800052		IGR	0,018	4,33E-05	1,96E-02
cg11544882	17	79388244	BAHCC1	Body	-0,009	4,33E-05	1,96E-02
cg22876643	1	68962318	DEPDC1	Body	0,01	4,34E-05	1,97E-02
cg19771625	6	2870674		IGR	-0,01	4,35E-05	1,97E-02
cg16863160	13	52598172	UTP14C	TSS1500	0,008	4,34E-05	1,97E-02
cg03775330	19	4682727	DPP9	Body	-0,017	4,35E-05	1,97E-02
cg11062956	16	2723328		IGR	0,013	4,36E-05	1,97E-02
cg19152802	5	109849887	MIR548F3	TSS1500	-0,022	4,37E-05	1,97E-02
cg09987444	8	68743080		IGR	0,028	4,37E-05	1,97E-02
cg09117417	1	75428569		IGR	0,046	4,39E-05	1,98E-02
cg07461182	5	106641822		IGR	0,032	4,39E-05	1,98E-02
cg18925236	6	168090500		IGR	0,022	4,38E-05	1,98E-02
cg24069444	10	125823413	CHST15	5'UTR	0,066	4,39E-05	1,98E-02
cg14047836	22	46165917	ATXN10	Body	-0,008	4,39E-05	1,98E-02
cg01051546	20	21106548	PLK1S1	TSS200	0,005	4,40E-05	1,98E-02
cg00267122	2	233469415	EFHD1	TSS1500	-0,011	4,40E-05	1,98E-02
cg08220944	3	25049424		IGR	0,018	4,41E-05	1,98E-02
cg01972118	4	182819201		IGR	-0,037	4,42E-05	1,98E-02
cg16582036	20	52796919		IGR	0,02	4,43E-05	1,99E-02
cg01666229	19	49372682	PLEKHA4	TSS1500	-0,01	4,43E-05	1,99E-02
cg10445080	6	167460377		IGR	0,031	4,43E-05	1,99E-02

cg14585261	1	17899250	ARHGEF10L	5'UTR	0,012	4,44E-05	1,99E-02
cg16581840	6	137114203	MAP3K5	TSS1500	0,005	4,44E-05	1,99E-02
cg21795850	21	18170094		IGR	0,051	4,45E-05	1,99E-02
cg03027037	20	23402404	NAPB	TSS1500	0,006	4,45E-05	1,99E-02
cg18453174	20	61662111		IGR	-0,026	4,46E-05	1,99E-02
cg12819298	1	41101999	RIMS3	Body	-0,019	4,47E-05	1,99E-02
cg09075163	1	157978579	KIRREL	Body	-0,007	4,47E-05	1,99E-02
cg06302321	7	100174737	LRCH4	Body	-0,011	4,48E-05	1,99E-02
cg26188218	17	25680444		IGR	-0,009	4,47E-05	1,99E-02
cg27313007	17	78617750	RPTOR	Body	-0,021	4,48E-05	1,99E-02
cg16115024	13	49136607		IGR	-0,005	4,48E-05	1,99E-02
cg26668837	11	8615356	STK33	1stExon	-0,012	4,49E-05	2,00E-02
cg11080731	12	94939916		IGR	0,018	4,49E-05	2,00E-02
cg19504616	8	71516499	TRAM1	Body	-0,019	4,50E-05	2,00E-02
cg02912790	19	45445491	APOC4	TSS200	-0,019	4,50E-05	2,00E-02
cg19269956	6	30524408	PRR3	TSS200	0,021	4,52E-05	2,01E-02
cg20506240	4	142023809	RNF150	Body	0,061	4,53E-05	2,01E-02
cg12253865	5	43602519	NNT	TSS1500	-0,007	4,53E-05	2,01E-02
cg13509731	2	204676448		IGR	0,032	4,54E-05	2,01E-02
cg07312312	1	109652455	C1orf194	Body	0,018	4,55E-05	2,01E-02
cg03541853	3	129408948	TMCC1	Body	-0,021	4,55E-05	2,01E-02
cg15045031	11	68163905	LRP5	5'UTR	-0,016	4,54E-05	2,01E-02
cg08140451	12	40564534		IGR	0,012	4,56E-05	2,01E-02
cg21328376	5	56664162		IGR	-0,009	4,56E-05	2,01E-02
cg08169311	11	69706622		IGR	0,048	4,56E-05	2,01E-02
cg06685111	6	30295466	HCG18	TSS1500	0,024	4,57E-05	2,01E-02
cg07665510	17	55952063	CUEDC1	Body	0,014	4,57E-05	2,01E-02
cg07597871	1	112162932	RAP1A	5'UTR	-0,003	4,60E-05	2,01E-02
cg15479189	1	184030983	TSEN15	Body	0,007	4,60E-05	2,01E-02
cg19899836	4	184668369		IGR	0,006	4,60E-05	2,01E-02
cg04734587	5	13810143	DNAH5	Body	-0,048	4,58E-05	2,01E-02
cg26932600	6	74230734	EEF1A1	1stExon	0,01	4,58E-05	2,01E-02
cg10682154	6	134561914	SGK1	Body	-0,006	4,58E-05	2,01E-02
cg07934856	10	6955209		IGR	0,073	4,58E-05	2,01E-02
cg08700546	11	47436675	SLC39A13	Body	-0,017	4,60E-05	2,01E-02
cg26786795	14	20344394	OR4K2	TSS200	0,037	4,60E-05	2,01E-02
cg15195008	15	42900947	STARD9	Body	-0,014	4,61E-05	2,01E-02
cg09635681	16	19729622	C16orf88	TSS200	-0,003	4,60E-05	2,01E-02
cg02448067	16	77932174	VAT1L	Body	-0,024	4,59E-05	2,01E-02
cg18706434	19	55857601	SUV420H2	Body	0,011	4,59E-05	2,01E-02
cg09161043	1	53068834	GPX7	Body	0,038	4,61E-05	2,02E-02
cg20304811	1	115124319	BCAS2	TSS200	-0,008	4,62E-05	2,02E-02
cg22109433	18	13472332	LDLRAD4	Body	0,041	4,63E-05	2,02E-02
cg25589905	19	41081360	SHKBP1	TSS1500	-0,01	4,63E-05	2,02E-02
cg15322667	1	1026255	C1orf159	Body	-0,015	4,64E-05	2,02E-02
cg03603234	2	20792663	HS1BP3-IT1	TSS1500	-0,011	4,64E-05	2,02E-02
cg06465175	7	29844768		IGR	0,029	4,64E-05	2,02E-02
cg27540736	2	28235811	BRE	Body	0,009	4,66E-05	2,02E-02
cg18364837	5	148737347	PCYOX1L	TSS1500	-0,014	4,66E-05	2,02E-02
cg20066627	20	21496713		IGR	-0,008	4,65E-05	2,02E-02
cg15996980	21	35445631	MRPS6	TSS200	0,006	4,65E-05	2,02E-02
cg00866123	7	2936698		IGR	0,009	4,67E-05	2,03E-02
cg04446786	8	143991594		IGR	0,039	4,67E-05	2,03E-02
cg24789562	11	27842648		IGR	-0,018	4,67E-05	2,03E-02
cg00582542	12	45564138		IGR	0,048	4,67E-05	2,03E-02
cg27261665	16	71265020	HYDIN	TSS1500	0,059	4,67E-05	2,03E-02
cg11494930	7	54688293		IGR	0,048	4,69E-05	2,03E-02
cg09196449	13	45451173		IGR	0,01	4,69E-05	2,03E-02
cg00522561	1	211797216		IGR	-0,03	4,69E-05	2,03E-02
cg22222922	6	161791922	PARK2	Body	0,031	4,69E-05	2,03E-02
cg18096939	14	101513572	MIR889	TSS1500	-0,018	4,70E-05	2,03E-02

cg09935440	7	135429446	FAM180A	Body	0,038	4,71E-05	2,03E-02
cg15644604	19	15443264		IGR	0,007	4,71E-05	2,03E-02
cg03181986	1	200591627		IGR	-0,032	4,71E-05	2,03E-02
cg27498980	10	6621174	PRKCQ-AS1	TSS1500	0,027	4,71E-05	2,03E-02
cg26755700	20	56689164		IGR	0,049	4,72E-05	2,03E-02
cg01580181	8	70744526	SLCO5A1	1stExon	-0,019	4,74E-05	2,04E-02
cg12872329	4	93536632	GRID2	Body	-0,019	4,74E-05	2,04E-02
cg26358139	5	149872600		IGR	-0,007	4,75E-05	2,04E-02
cg11707391	8	10001627	MSRA	Body	-0,014	4,75E-05	2,04E-02
cg23208353	3	126748542	PLXNA1	Body	-0,012	4,75E-05	2,04E-02
cg19726225	13	103109924		IGR	-0,005	4,76E-05	2,04E-02
cg03155428	1	3277493	PRDM16	Body	-0,017	4,77E-05	2,04E-02
cg07653058	10	88313467		IGR	-0,041	4,77E-05	2,04E-02
cg07326288	13	43651854	DNAJC15	Body	-0,016	4,77E-05	2,04E-02
cg00659605	14	102622352	WDR20	Body	-0,03	4,77E-05	2,04E-02
cg12643580	20	33606592	TRPC4AP	Body	-0,007	4,77E-05	2,04E-02
cg16914149	3	189873585		IGR	0,061	4,78E-05	2,04E-02
cg12870267	4	961136	DGKQ	ExonBnd	-0,013	4,78E-05	2,04E-02
cg20353857	5	131347831	ACSL6	TSS1500	0,008	4,78E-05	2,04E-02
cg11729174	13	95368297		IGR	0,03	4,78E-05	2,04E-02
cg11486821	11	75265714		IGR	-0,092	4,79E-05	2,04E-02
cg01369381	1	6450244	ACOT7	Body	0,007	4,80E-05	2,04E-02
cg23643526	1	119930673	HAO2	Body	0,052	4,80E-05	2,04E-02
cg11670584	3	120871756	STXBP5L	Body	0,059	4,80E-05	2,04E-02
cg19905050	19	54465557	CACNG8	TSS1500	0,016	4,80E-05	2,04E-02
cg11942541	20	44035138	DBNDD2	TSS200	0,004	4,80E-05	2,04E-02
cg23632906	1	180908561	KIAA1614	Body	0,017	4,81E-05	2,04E-02
cg22508905	4	20529994	MIR218-1	Body	-0,015	4,83E-05	2,05E-02
cg01175142	10	118387327	PNLIPRP2	Body	-0,042	4,82E-05	2,05E-02
cg08231326	18	63939369		IGR	0,04	4,83E-05	2,05E-02
cg00171166	20	35444275	C20orf117	Body	0,005	4,83E-05	2,05E-02
cg22760710	3	185083290	MAP3K13	5'UTR	0,005	4,84E-05	2,05E-02
cg01526553	12	56554264	MYL6	Body	0,033	4,85E-05	2,05E-02
cg08256017	12	125171143		IGR	0,006	4,85E-05	2,06E-02
cg01608175	1	9665010	TMEM201	3'UTR	-0,006	4,86E-05	2,06E-02
cg20086694	5	163722285		IGR	-0,007	4,86E-05	2,06E-02
cg11251399	1	3348571	PRDM16	Body	-0,01	4,87E-05	2,06E-02
cg15999067	7	77670191	MAGI2	Body	-0,015	4,87E-05	2,06E-02
cg26622110	17	47460554		IGR	0,006	4,87E-05	2,06E-02
cg18914967	12	122500697		IGR	-0,015	4,88E-05	2,06E-02
cg04693329	11	123995045	VWA5A	Body	-0,017	4,90E-05	2,07E-02
cg04833210	12	114309620	RBM19	Body	-0,007	4,90E-05	2,07E-02
cg07703623	20	48093800	KCNB1	Body	0,039	4,90E-05	2,07E-02
cg08688822	19	46105497	GPR4	TSS200	0,007	4,91E-05	2,07E-02
cg20481816	11	47448111	PSMC3	TSS200	-0,007	4,91E-05	2,07E-02
cg23239690	10	131505815	MGMT	Body	-0,01	4,92E-05	2,07E-02
cg02657292	7	138720909	ZC3HAV1L	TSS200	0,011	4,92E-05	2,07E-02
cg03402438	2	198365399	HSPD1	TSS1500	0,004	4,93E-05	2,07E-02
cg20530329	4	8160769	ABLIM2	TSS1500	0,005	4,94E-05	2,07E-02
cg03689992	13	40745362		IGR	0,012	4,94E-05	2,07E-02
cg25937046	18	21140643	NPC1	Body	-0,01	4,94E-05	2,07E-02
cg21025554	12	122063591	ORAI1	TSS1500	0,018	4,95E-05	2,07E-02
cg07604651	7	121944755	FEZF1	TSS200	0,017	4,95E-05	2,07E-02
cg03388984	4	152842957		IGR	0,036	4,97E-05	2,08E-02
cg25195965	5	73586748		IGR	0,023	4,96E-05	2,08E-02
cg00121876	6	166251659		IGR	-0,058	4,97E-05	2,08E-02
cg00950268	9	137849002		IGR	-0,015	4,97E-05	2,08E-02
cg15038331	15	74290466	PML	Body	-0,017	4,97E-05	2,08E-02
cg12472603	17	74705806	MXRA7	Body	0,005	4,97E-05	2,08E-02
cg02359728	2	17857849	SMC6	Body	-0,021	4,97E-05	2,08E-02
cg12592610	6	30164453	TRIM26	Body	0,008	4,98E-05	2,08E-02

cg16081687	8	845388	ERICH1-AS1	Body	-0,008	4,99E-05	2,08E-02
cg00720106	15	102189106	TM2D3	Body	-0,014	4,98E-05	2,08E-02
cg21460948	3	10992267		IGR	0,04	5,00E-05	2,08E-02
cg18590502	3	49203081	CCDC71	5'UTR	0,043	5,00E-05	2,08E-02
cg26670048	3	128399960		IGR	0,005	5,00E-05	2,08E-02
cg06047778	11	2596300	KCNQ1	Body	-0,012	4,99E-05	2,08E-02
cg19772114	6	28829321		IGR	-0,018	5,01E-05	2,08E-02
cg17301311	17	48641896	CACNA1G	Body	0,018	5,01E-05	2,08E-02
cg06710853	16	30510824	ITGAL	ExonBnd	-0,022	5,01E-05	2,08E-02
cg15293629	2	173995795	ZAK	Body	-0,009	5,02E-05	2,08E-02
cg03375002	7	73623876	LAT2	TSS1500	0,053	5,02E-05	2,08E-02
cg14441787	8	40811496		IGR	0,007	5,03E-05	2,09E-02
cg18667738	1	12630803	DHRS3	Body	0,012	5,04E-05	2,09E-02
cg25334860	17	1094299		IGR	-0,021	5,04E-05	2,09E-02
cg20240710	4	186586896	SORBS2	Body	-0,017	5,05E-05	2,09E-02
cg20335813	9	6842611	KDM4C	Body	0,011	5,05E-05	2,09E-02
cg12378790	3	21316163		IGR	-0,008	5,05E-05	2,09E-02
cg05221243	1	59971852	FGGY	Body	-0,009	5,06E-05	2,09E-02
cg03610364	20	40653415		IGR	0,054	5,06E-05	2,09E-02
cg24791271	10	2139706		IGR	-0,017	5,08E-05	2,09E-02
cg04186484	17	61966028		IGR	0,035	5,07E-05	2,09E-02
cg10951873	1	25254746	RUNX3	Body	0,008	5,08E-05	2,09E-02
cg11351809	17	14213798	HS3ST3B1	Body	-0,011	5,09E-05	2,10E-02
cg06856687	20	44934509		IGR	0,051	5,09E-05	2,10E-02
cg24124703	5	88122944	MEF2C	5'UTR	-0,01	5,12E-05	2,10E-02
cg11010680	22	50216963	BRD1	Body	-0,009	5,12E-05	2,10E-02
cg00278986	1	180832846	XPR1	ExonBnd	0,049	5,12E-05	2,10E-02
cg26212924	8	145150439	CYC1	Body	0,004	5,13E-05	2,11E-02
cg01834111	16	57085391	NLRC5	Body	-0,008	5,13E-05	2,11E-02
cg25629442	17	77070844	ENGASE	TSS200	0,017	5,13E-05	2,11E-02
cg02142098	22	38965659	DMC1	5'UTR	0,041	5,14E-05	2,11E-02
cg15195758	2	128529314	WDR33	5'UTR	-0,035	5,16E-05	2,11E-02
cg04513185	2	136876652	CXCR4	TSS1500	0,009	5,18E-05	2,11E-02
cg04298771	5	1337326	CLPTM1L	Body	0,006	5,17E-05	2,11E-02
cg03271162	9	14801827	FREM1	Body	-0,006	5,17E-05	2,11E-02
cg11788833	15	45459300		IGR	0,003	5,17E-05	2,11E-02
cg25990329	17	290476	FAM101B	3'UTR	-0,01	5,18E-05	2,11E-02
cg25512537	17	76250053		IGR	0,035	5,18E-05	2,11E-02
cg21737734	18	44236847	LOXHD1	Body	-0,012	5,16E-05	2,11E-02
cg05559640	19	35731617		IGR	0,052	5,15E-05	2,11E-02
cg03034820	20	42649372	TOX2	Body	0,022	5,17E-05	2,11E-02
cg22874598	22	17490761		IGR	0,063	5,17E-05	2,11E-02
cg21802374	3	146672661		IGR	0,028	5,19E-05	2,11E-02
cg02530375	11	69706831		IGR	0,04	5,19E-05	2,11E-02
cg02349914	17	2264538	SGSM2	Body	-0,011	5,20E-05	2,12E-02
cg26981637	14	101371792	MEG8	Body	-0,006	5,21E-05	2,12E-02
cg26181664	10	60272061	BICC1	TSS1500	-0,006	5,21E-05	2,12E-02
cg08280989	15	28363460	HERC2	Body	0,005	5,22E-05	2,12E-02
cg02326224	15	81474451		IGR	0,018	5,22E-05	2,12E-02
cg17752576	1	98336448	DPYD	Body	-0,006	5,23E-05	2,12E-02
cg06205640	5	171866874	SH3PXD2B	Body	0,011	5,23E-05	2,12E-02
cg13592931	5	140235596	PCDHA6	Body	-0,043	5,24E-05	2,12E-02
cg19640090	12	11975155	ETV6	Body	0,024	5,26E-05	2,13E-02
cg06385187	20	44098287	WFDC2	TSS200	0,026	5,26E-05	2,13E-02
cg11554771	1	6305590	HES3	3'UTR	0,005	5,27E-05	2,13E-02
cg17783135	3	194876491	C3orf21	Body	0,018	5,27E-05	2,13E-02
cg14372191	1	31467555	PUM1	Body	-0,013	5,28E-05	2,13E-02
cg19242560	8	124428896	WDYHV1	TSS200	0,011	5,28E-05	2,13E-02
cg19486507	11	67287418	CABP2	Body	-0,019	5,28E-05	2,13E-02
cg12559939	2	27858050	GPN1	Body	0,005	5,29E-05	2,13E-02
cg16974878	1	231622987		IGR	-0,049	5,30E-05	2,14E-02

cg07687608	12	112711764	HECTD4	Body	-0,005	5,31E-05	2,14E-02
cg13150534	17	79680935	SLC25A10	Body	-0,033	5,31E-05	2,14E-02
cg18247806	20	326496	NRSN2	TSS1500	-0,012	5,31E-05	2,14E-02
cg12463976	3	95618423		IGR	-0,022	5,32E-05	2,14E-02
cg05555928	11	63887634	MACROD1	Body	-0,018	5,33E-05	2,14E-02
cg05799906	15	73542085	NEO1	Body	0,044	5,32E-05	2,14E-02
cg25601547	2	64855072		IGR	-0,018	5,34E-05	2,14E-02
cg01132589	2	210444475	MAP2	1stExon	0,039	5,34E-05	2,14E-02
cg24866706	3	96338474		IGR	0,016	5,35E-05	2,14E-02
cg21887309	5	140209490	PCDHA6	Body	-0,028	5,34E-05	2,14E-02
cg22598098	6	167357717	RNASET2	Body	-0,012	5,35E-05	2,14E-02
cg17939931	8	138313657		IGR	-0,015	5,35E-05	2,14E-02
cg15817923	19	39881719	PAF1	TSS200	0,004	5,33E-05	2,14E-02
cg09651498	6	169860054	WDR27	Body	-0,025	5,35E-05	2,14E-02
cg14575899	15	52497340	MYO5C	Body	0,011	5,36E-05	2,14E-02
cg06178915	13	103498655	ERCC5	1stExon	-0,005	5,36E-05	2,14E-02
cg26612384	3	74581769		IGR	0,026	5,37E-05	2,15E-02
cg10019083	4	7985149	ABLIM2	Body	-0,02	5,38E-05	2,15E-02
cg00663739	1	235489969	ARID4B	Body	-0,01	5,39E-05	2,15E-02
cg18326783	10	102765364	LZTS2	Body	-0,013	5,39E-05	2,15E-02
cg01621716	2	11636147		IGR	0,035	5,39E-05	2,15E-02
cg19097150	7	2500200		IGR	-0,016	5,39E-05	2,15E-02
cg12523691	4	169717681	PALLD	Body	-0,016	5,41E-05	2,15E-02
cg16137853	11	102217577	BIRC2	TSS1500	-0,005	5,41E-05	2,15E-02
cg05860713	21	38092184	SIM2	Body	-0,006	5,42E-05	2,16E-02
cg27131703	4	147971312		IGR	0,022	5,43E-05	2,16E-02
cg08066214	12	132284672		IGR	-0,015	5,44E-05	2,16E-02
cg25943131	12	30848710	IPO8	5'UTR	-0,006	5,44E-05	2,16E-02
cg08495878	14	95027859	SERPINA4	5'UTR	0,02	5,45E-05	2,16E-02
cg03770138	9	136009651	RALGDS	Body	0,041	5,46E-05	2,17E-02
cg06484075	4	184393241		IGR	-0,033	5,47E-05	2,17E-02
cg10896616	5	1295267	TERT	TSS200	0,012	5,47E-05	2,17E-02
cg15184934	9	14180661	NFIB	1stExon	-0,016	5,46E-05	2,17E-02
cg12069423	10	15254709	FAM171A1	3'UTR	0,011	5,47E-05	2,17E-02
cg08357573	7	66858634		IGR	0,046	5,48E-05	2,17E-02
cg24127106	19	50183046	PRMT1	Body	-0,016	5,48E-05	2,17E-02
cg13523713	19	48614733	PLA2G4C	TSS1500	-0,02	5,49E-05	2,17E-02
cg12220285	12	670152	B4GALNT3	Body	-0,025	5,50E-05	2,17E-02
cg04938082	16	69156328	CHTF8	Body	0,007	5,50E-05	2,17E-02
cg14000461	5	32058012	PDZD2	Body	0,009	5,51E-05	2,17E-02
cg05854718	11	18548308	TSG101	Body	0,004	5,51E-05	2,17E-02
cg09907883	14	81999924	SEL1L	Body	0,003	5,51E-05	2,17E-02
cg11466857	20	13253578	ISM1	Body	0,008	5,51E-05	2,17E-02
cg16393012	12	15114387	ARHGDIB	5'UTR	-0,012	5,53E-05	2,17E-02
cg10262404	17	16283974	UBB	TSS1500	0,006	5,53E-05	2,17E-02
cg13457515	17	45934681	SP6	TSS1500	0,028	5,53E-05	2,17E-02
cg03187166	3	107807805	CD47	Body	-0,006	5,55E-05	2,18E-02
cg18605441	10	74033756	DDIT4	1stExon	0,005	5,55E-05	2,18E-02
cg13571388	1	26947729		IGR	0,003	5,55E-05	2,18E-02
cg08074528	22	50528586	MOV10L1	1stExon	0,024	5,57E-05	2,19E-02
cg01151118	18	75034488		IGR	0,032	5,57E-05	2,19E-02
cg19219364	3	164621774		IGR	-0,022	5,58E-05	2,19E-02
cg18953610	13	110787437		IGR	-0,014	5,58E-05	2,19E-02
cg25395545	6	167534955	CCR6	TSS1500	0,028	5,60E-05	2,19E-02
cg14233821	9	138012342	OLFM1	3'UTR	-0,018	5,61E-05	2,19E-02
cg09668058	13	42601138		IGR	0,009	5,61E-05	2,20E-02
cg06854253	4	142267605		IGR	0,021	5,62E-05	2,20E-02
cg20377955	7	20827825	SP8	TSS1500	-0,027	5,63E-05	2,20E-02
cg14488605	8	144822804		IGR	0,009	5,64E-05	2,20E-02
cg03866835	16	67562656	FAM65A	TSS200	0,004	5,64E-05	2,20E-02
cg25374512	9	26656405		IGR	0,048	5,65E-05	2,20E-02

cg02571201	8	73920559	TERF1	TSS1500	0,057	5,65E-05	2,20E-02
cg13763401	13	78774124	RNF219-AS1	Body	0,021	5,66E-05	2,21E-02
cg18750819	2	217342965	SMARCAL1	Body	0,005	5,69E-05	2,21E-02
cg20031659	6	114178324	MARCKS	TSS1500	-0,019	5,70E-05	2,21E-02
cg02968407	7	1660685		IGR	0,007	5,69E-05	2,21E-02
cg14158594	7	95951129	SLC25A13	Body	0,003	5,68E-05	2,21E-02
cg03604364	7	139705703	TBXAS1	Body	-0,02	5,69E-05	2,21E-02
cg04477940	9	108320289	FKTN	TSS200	0,008	5,69E-05	2,21E-02
cg11237734	11	27528142	BDNFOS	TSS1500	-0,005	5,70E-05	2,21E-02
cg09955683	13	31774536	B3GALTL	Body	0,007	5,69E-05	2,21E-02
cg23531968	13	90110573		IGR	0,041	5,69E-05	2,21E-02
cg26087735	14	104000447	TRMT61A	Body	-0,02	5,70E-05	2,21E-02
cg17867243	15	42371653	PLA2G4D	Body	-0,074	5,66E-05	2,21E-02
cg12838928	16	16112597	ABCC1	Body	-0,023	5,68E-05	2,21E-02
cg15194925	22	30987935	PES1	TSS200	-0,004	5,67E-05	2,21E-02
cg04626039	14	57159156		IGR	-0,031	5,71E-05	2,21E-02
cg15069758	1	110198731	GSTM4	Body	0,009	5,72E-05	2,21E-02
cg05004720	2	109605849	EDAR	TSS200	0,042	5,72E-05	2,21E-02
cg02909497	9	117174710	DFNB31	Body	-0,008	5,71E-05	2,21E-02
cg14851705	11	76020877		IGR	-0,01	5,72E-05	2,21E-02
cg18652121	3	179322868	MRPL47	TSS1500	-0,007	5,73E-05	2,21E-02
cg05255807	6	88411528	NCRNA00120	TSS1500	0,007	5,74E-05	2,21E-02
cg02856716	1	18993307	PAX7	Body	0,006	5,76E-05	2,21E-02
cg00907427	1	23668691	HNRNPR	5'UTR	-0,015	5,75E-05	2,21E-02
cg17031396	1	33226541	KIAA1522	Body	-0,017	5,75E-05	2,21E-02
cg07185843	5	74532852	ANKRD31	TSS200	0,005	5,75E-05	2,21E-02
cg03252258	7	97933035	BAIAP2L1	Body	0,005	5,76E-05	2,21E-02
cg00515437	15	83422206		IGR	-0,02	5,76E-05	2,21E-02
cg24613524	17	58363672	USP32	Body	-0,006	5,75E-05	2,21E-02
cg16604553	9	4489544	SLC1A1	TSS1500	-0,053	5,78E-05	2,22E-02
cg13737408	14	94393654	FAM181A	Body	0,037	5,78E-05	2,22E-02
cg18678763	11	4115507	RRM1	TSS1500	0,011	5,79E-05	2,22E-02
cg23318736	14	101779813		IGR	-0,053	5,79E-05	2,22E-02
cg09789791	14	52597044		IGR	0,019	5,80E-05	2,22E-02
cg07026599	16	79634736	MAF	TSS200	0,009	5,80E-05	2,22E-02
cg21527556	19	1984998		IGR	-0,024	5,80E-05	2,22E-02
cg25258033	6	167368657	RNASET2	Body	0,055	5,82E-05	2,22E-02
cg22499348	7	129289868	NRF1	5'UTR	-0,022	5,82E-05	2,22E-02
cg03823352	11	100001562	CNTN5	Body	-0,024	5,81E-05	2,22E-02
cg15879165	15	74903214	CLK3	5'UTR	-0,008	5,81E-05	2,22E-02
cg19404616	6	144801637	UTRN	Body	-0,007	5,83E-05	2,23E-02
cg08232895	19	47177742	PRKD2	3'UTR	-0,014	5,83E-05	2,23E-02
cg20255094	1	160771763	LY9	3'UTR	-0,014	5,84E-05	2,23E-02
cg10641499	22	35449356		IGR	0,011	5,85E-05	2,23E-02
cg05125875	15	40425922		IGR	0,01	5,86E-05	2,23E-02
cg00266388	10	134184547	LRRC27	Body	-0,01	5,86E-05	2,23E-02
cg08307963	1	147245485	GJA5	TSS200	-0,007	5,88E-05	2,23E-02
cg17284844	1	233743159		IGR	0,041	5,87E-05	2,23E-02
cg20411781	8	136676667		IGR	0,05	5,88E-05	2,23E-02
cg01281645	11	72751340	FCHSD2	Body	-0,008	5,88E-05	2,23E-02
cg03425615	12	133409657		IGR	-0,008	5,88E-05	2,23E-02
cg12321379	2	221758256		IGR	0,087	5,90E-05	2,23E-02
cg13510812	3	45429626	LARS2	TSS1500	0,059	5,89E-05	2,23E-02
cg09624733	3	191905708	FGF12	Body	0,044	5,91E-05	2,23E-02
cg25441269	9	127615686	WDR38	TSS200	0,005	5,89E-05	2,23E-02
cg07136909	11	71278894		IGR	0,121	5,90E-05	2,23E-02
cg08805208	12	131589169	GPR133	Body	-0,021	5,89E-05	2,23E-02
cg13437593	17	38210697	MED24	1stExon	0,003	5,90E-05	2,23E-02
cg06956230	17	42583527		IGR	0,019	5,90E-05	2,23E-02
cg14886198	19	40744336	AKT2	Body	-0,012	5,91E-05	2,23E-02
cg05141867	6	3075352		IGR	0,016	5,91E-05	2,23E-02

cg22608602	12	133235911	POLE	Body	-0,007	5,92E-05	2,23E-02
cg21698295	8	55466766		IGR	0,04	5,92E-05	2,24E-02
cg09187217	4	17586185	LAP3	Body	0,006	5,94E-05	2,24E-02
cg13158676	4	56251979	SRD5A3-AS1	TSS1500	-0,008	5,94E-05	2,24E-02
cg05717645	13	74727546		IGR	0,01	5,94E-05	2,24E-02
cg15431948	2	31138571	GALNT14	Body	0,024	5,98E-05	2,24E-02
cg16764807	6	20534632	CDKAL1	TSS200	0,003	5,97E-05	2,24E-02
cg25359978	7	156685986	LMBR1	TSS200	0,004	5,96E-05	2,24E-02
cg26967295	13	90206016	LINC00353	Body	-0,015	5,96E-05	2,24E-02
cg15545086	15	75941226	SNX33	TSS200	-0,015	5,96E-05	2,24E-02
cg03351248	15	100880064	ADAMTS17	Body	-0,034	5,97E-05	2,24E-02
cg04528771	17	27893087	ABHD15	Body	0,006	5,97E-05	2,24E-02
cg01153620	19	1828388	REXO1	Body	-0,021	5,96E-05	2,24E-02
cg15056793	20	35206859	TGIF2	5'UTR	0,007	5,98E-05	2,24E-02
cg17108237	11	94355875		IGR	-0,024	5,99E-05	2,25E-02
cg04567466	1	23894902		IGR	0,006	6,06E-05	2,25E-02
cg10193841	1	32203291	ADGRB2	Body	-0,008	6,06E-05	2,25E-02
cg26249174	1	61419988	NFIA-AS2	ExonBnd	-0,01	6,04E-05	2,25E-02
cg18560264	3	52324089	GLYCTK	Body	-0,007	6,01E-05	2,25E-02
cg16808912	3	99603495	MIR548G	Body	-0,051	6,01E-05	2,25E-02
cg02504489	5	150429587	TNIP1	Body	-0,013	6,03E-05	2,25E-02
cg11071615	6	30690438	TUBB	Body	-0,004	6,03E-05	2,25E-02
cg20338393	6	41323157		IGR	-0,034	6,06E-05	2,25E-02
cg27374734	6	150158148	LRP11	Body	0,005	6,01E-05	2,25E-02
cg23471905	7	84243859		IGR	0,049	6,02E-05	2,25E-02
cg13659034	7	143088584	EPHA1	Body	-0,01	6,03E-05	2,25E-02
cg12255284	8	67786401	MCMDC2	Body	-0,02	6,06E-05	2,25E-02
cg21594924	9	17811008		IGR	-0,026	6,02E-05	2,25E-02
cg19373670	9	93920550		IGR	0,008	6,05E-05	2,25E-02
cg06162995	9	140352098	NSMF	Body	-0,005	6,06E-05	2,25E-02
cg18977436	13	103054076	FGF14	5'UTR	-0,013	6,06E-05	2,25E-02
cg19635507	15	43713259	RNU6-28P	Body	-0,011	6,03E-05	2,25E-02
cg12196901	17	26630081	FLJ40504	Body	0,015	6,05E-05	2,25E-02
cg05806884	17	80052754	FASN	Body	-0,009	6,05E-05	2,25E-02
cg12216327	22	38292299		IGR	-0,007	6,03E-05	2,25E-02
cg13208614	1	110695039	SLC6A17	5'UTR	-0,033	6,07E-05	2,25E-02
cg25028766	6	158499155	SYNJ2	Body	0,007	6,07E-05	2,25E-02
cg02905206	6	168321999	MLLT4	Body	0,009	6,08E-05	2,25E-02
cg15778335	22	43011005	POLDIP3	TSS200	0,012	6,08E-05	2,25E-02
cg10397930	11	109959298		IGR	0,016	6,10E-05	2,26E-02
cg15370010	16	86990309		IGR	0,021	6,10E-05	2,26E-02
cg22034315	2	113953543	PSD4	Body	-0,012	6,14E-05	2,26E-02
cg22047910	2	196522755	SLC39A10	5'UTR	0,008	6,14E-05	2,26E-02
cg06725479	2	201937026	NDUFB3	5'UTR	0,01	6,13E-05	2,26E-02
cg18094414	3	125933236		IGR	0,044	6,11E-05	2,26E-02
cg25492814	4	47916879	NFXL1	TSS200	0,009	6,14E-05	2,26E-02
cg04964920	5	55162578	IL31RA	ExonBnd	-0,005	6,12E-05	2,26E-02
cg21948027	7	1809455		IGR	0,005	6,14E-05	2,26E-02
cg20761204	7	106433397		IGR	-0,009	6,14E-05	2,26E-02
cg22498240	8	8356560		IGR	0,024	6,13E-05	2,26E-02
cg11959771	9	101984403	ALG2	TSS200	0,003	6,12E-05	2,26E-02
cg13484341	10	70232027	DNA2	TSS200	0,016	6,11E-05	2,26E-02
cg02064275	17	39465713		IGR	0,027	6,11E-05	2,26E-02
cg10254317	17	80254303		IGR	0,008	6,13E-05	2,26E-02
cg24429613	21	47405667	COL6A1	Body	0,005	6,11E-05	2,26E-02
cg09309286	17	32960898	TMEM132E	Body	-0,021	6,15E-05	2,26E-02
cg03720511	17	4436362	SPNS2	Body	-0,016	6,16E-05	2,26E-02
cg18802946	15	25403253		IGR	0,053	6,17E-05	2,26E-02
cg20105647	3	154796045	MME	TSS1500	0,009	6,17E-05	2,27E-02
cg24358337	15	59665646	MYO1E	TSS1500	-0,025	6,18E-05	2,27E-02
cg09324461	1	48231258	TRABD2B	3'UTR	-0,012	6,19E-05	2,27E-02

cg04878489	6	43139942	SRF	Body	0,016	6,20E-05	2,27E-02
cg26159601	7	1248693		IGR	0,01	6,20E-05	2,27E-02
cg06540747	2	169966394		IGR	0,016	6,21E-05	2,27E-02
cg12861797	15	43585817	TGM7	Body	0,039	6,21E-05	2,27E-02
cg07891543	3	176762529	TBL1XR1	Body	-0,015	6,22E-05	2,27E-02
cg12432846	1	85741899	BCL10	Body	0,011	6,22E-05	2,27E-02
cg18716706	3	10276280	IRAK2	Body	-0,006	6,25E-05	2,28E-02
cg02608382	4	186127482		IGR	0,007	6,25E-05	2,28E-02
cg11712484	13	86594585		IGR	-0,014	6,24E-05	2,28E-02
cg10218519	16	59764694		IGR	-0,024	6,25E-05	2,28E-02
cg11985745	18	54314844		IGR	-0,01	6,24E-05	2,28E-02
cg01751472	19	18668571	KXD1	1stExon	0,003	6,24E-05	2,28E-02
cg08524197	16	188850	C16orf35	TSS200	0,004	6,26E-05	2,28E-02
cg15833634	17	17068185	MPRIP	Body	-0,012	6,25E-05	2,28E-02
cg04906879	2	171571842	LOC440925	TSS1500	0,003	6,26E-05	2,28E-02
cg21671020	6	30068493		IGR	0,009	6,27E-05	2,28E-02
cg09932762	6	137861989		IGR	0,014	6,27E-05	2,28E-02
cg16156925	22	37916286	CARD10	TSS1500	0,028	6,31E-05	2,29E-02
cg14290451	6	35436136	RPL10A	TSS200	0,005	6,32E-05	2,29E-02
cg24019429	16	57909782		IGR	0,012	6,32E-05	2,29E-02
cg14733958	17	26883828	PIGS	ExonBnd	0,007	6,32E-05	2,29E-02
cg13509756	9	89128406		IGR	0,013	6,34E-05	2,30E-02
cg09576223	22	39092757	JOSD1	Body	0,038	6,34E-05	2,30E-02
cg26365399	6	127587909	RNF146	TSS200	0,008	6,35E-05	2,30E-02
cg04867178	6	168503473		IGR	0,062	6,35E-05	2,30E-02
cg07717588	2	235907647	SH3BP4	5'UTR	-0,015	6,37E-05	2,30E-02
cg15019273	5	171620983	EFCAB9	TSS200	-0,014	6,37E-05	2,30E-02
cg09213102	16	71392373	CALB2	TSS1500	0,018	6,37E-05	2,30E-02
cg03816592	17	45886242	OSBPL7	Body	0,005	6,37E-05	2,30E-02
cg03048962	15	68658571	ITGA11	Body	-0,008	6,38E-05	2,30E-02
cg01525070	2	545407		IGR	0,012	6,40E-05	2,31E-02
cg01814991	6	147666788	STXBP5	Body	-0,02	6,40E-05	2,31E-02
cg07723340	8	13518960		IGR	-0,015	6,40E-05	2,31E-02
cg23385248	15	93579332		IGR	0,012	6,41E-05	2,31E-02
cg02705011	18	76752660	SALL3	Body	-0,033	6,41E-05	2,31E-02
cg11035216	3	50374929	RASSF1	TSS200	0,003	6,42E-05	2,31E-02
cg04495336	8	2419119		IGR	-0,04	6,43E-05	2,31E-02
cg15237899	9	79635402	FOXB2	1stExon	0,01	6,43E-05	2,31E-02
cg16407683	2	191394503	NEMP2	Body	-0,031	6,44E-05	2,32E-02
cg12817575	8	104426592	DCAF13	TSS1500	-0,027	6,44E-05	2,32E-02
cg14979079	3	17087078	PLCL2	Body	-0,005	6,45E-05	2,32E-02
cg20487572	1	45233219	KIF2C	3'UTR	-0,008	6,47E-05	2,32E-02
cg11339837	1	90967211		IGR	0,031	6,50E-05	2,32E-02
cg20232307	1	152539753	LCE3E	TSS1500	0,081	6,47E-05	2,32E-02
cg11290779	1	204092714	SOX13	Body	0,035	6,48E-05	2,32E-02
cg18326398	2	75328206	TACR1	Body	0,029	6,49E-05	2,32E-02
cg26931693	4	144280191	GAB1	Body	-0,016	6,48E-05	2,32E-02
cg03919533	12	57145872	PRIM1	Body	0,005	6,50E-05	2,32E-02
cg14886930	16	68298280	SLC7A6	TSS200	0,005	6,48E-05	2,32E-02
cg10370025	17	926264	ABR	Body	-0,007	6,48E-05	2,32E-02
cg26853458	17	9805074	RCVRN	Body	0,063	6,50E-05	2,32E-02
cg06968794	17	28403561	EFCAB5	Body	0,016	6,50E-05	2,32E-02
cg06579829	17	57970481	RPS6KB1	5'UTR	0,004	6,48E-05	2,32E-02
cg17241447	19	8201349	FBN3	Body	-0,01	6,49E-05	2,32E-02
cg13552832	9	18493888	ADAMTSL1	Body	0,007	6,51E-05	2,32E-02
cg21233902	9	20271083		IGR	-0,014	6,52E-05	2,32E-02
cg22561966	11	102139342		IGR	0,032	6,52E-05	2,32E-02
cg22005405	4	164676460	MARCH1	Body	0,012	6,54E-05	2,33E-02
cg23404479	8	58256345	)C101929488	Body	0,015	6,54E-05	2,33E-02
cg04931808	14	49274525		IGR	0,047	6,55E-05	2,33E-02
cg08895109	4	65871044	LOC401134	TSS1500	-0,018	6,56E-05	2,33E-02

cg20909752	15	72524565	PKM	TSS1500	0,02	6,56E-05	2,33E-02
cg06625777	16	30906395	BCL7C	TSS1500	-0,009	6,56E-05	2,33E-02
cg11451506	2	100210257	AFF3	Body	-0,023	6,59E-05	2,33E-02
cg09710279	5	118358672		IGR	0,035	6,58E-05	2,33E-02
cg13068216	6	158733981	TULP4	1stExon	-0,024	6,58E-05	2,33E-02
cg00135032	11	74382794		IGR	0,013	6,59E-05	2,33E-02
cg16359370	14	70379938	SMOC1	Body	-0,01	6,58E-05	2,33E-02
cg06185204	15	80351985	ZFAND6	TSS200	0,005	6,58E-05	2,33E-02
cg05312574	15	65281963	SPG21	TSS200	0,008	6,59E-05	2,33E-02
cg01906695	12	48399493	COL2A1	TSS1500	0,031	6,61E-05	2,34E-02
cg11097712	15	89750263		IGR	0,005	6,61E-05	2,34E-02
cg02583183	16	75450019	CFDP1	Body	-0,016	6,62E-05	2,34E-02
cg06639496	22	50749598		IGR	-0,018	6,62E-05	2,34E-02
cg03489181	10	72424633		IGR	0,023	6,63E-05	2,34E-02
cg00885365	2	215702120		IGR	0,036	6,63E-05	2,34E-02
cg06921552	11	69258203		IGR	0,004	6,66E-05	2,35E-02
cg23059366	17	19266654	B9D1	TSS1500	0,052	6,65E-05	2,35E-02
cg05238585	20	2690261	EBF4	Body	-0,036	6,65E-05	2,35E-02
cg20871579	1	1650919	CDK11B	5'UTR	0,005	6,67E-05	2,35E-02
cg20164652	11	8027831		IGR	0,047	6,66E-05	2,35E-02
cg22607922	12	111051092	TCTN1	TSS1500	0,007	6,67E-05	2,35E-02
cg03012280	15	41098255	ZFYVE19	TSS1500	-0,092	6,67E-05	2,35E-02
cg11928630	19	39649618	PAK4	5'UTR	0,039	6,68E-05	2,35E-02
cg14865394	2	215452484		IGR	-0,018	6,70E-05	2,35E-02
cg15914379	6	33195622		IGR	0,033	6,69E-05	2,35E-02
cg14123436	9	98544579		IGR	0,01	6,69E-05	2,35E-02
cg15061330	10	115580593		IGR	0,016	6,69E-05	2,35E-02
cg05427387	17	11523106	DNAH9	ExonBnd	-0,008	6,70E-05	2,35E-02
cg11841181	19	36423098		IGR	0,032	6,70E-05	2,35E-02
cg18769060	17	60198913		IGR	0,013	6,70E-05	2,35E-02
cg05996236	5	178286857	ZNF354B	TSS200	0,007	6,71E-05	2,35E-02
cg01900772	2	170820946	UBR3	Body	0,011	6,72E-05	2,35E-02
cg07998951	7	152456579	ACTR3B	TSS1500	0,039	6,72E-05	2,35E-02
cg23599976	7	156722536		IGR	0,036	6,74E-05	2,36E-02
cg13863103	9	98902324		IGR	0,014	6,74E-05	2,36E-02
cg23580358	19	14180843		IGR	-0,016	6,73E-05	2,36E-02
cg08774231	5	146258181	PPP2R2B	TSS200	0,004	6,75E-05	2,36E-02
cg17312370	20	60114063	CDH4	Body	-0,02	6,76E-05	2,36E-02
cg09111071	4	38665154	FLJ13197	Body	0,008	6,76E-05	2,36E-02
cg19424557	22	19515544		IGR	-0,016	6,77E-05	2,36E-02
cg09233112	1	12074638		IGR	0,017	6,78E-05	2,36E-02
cg24544889	1	177537910		IGR	-0,027	6,80E-05	2,37E-02
cg08453458	4	187491428		IGR	0,048	6,79E-05	2,37E-02
cg20332469	9	82773696		IGR	-0,013	6,80E-05	2,37E-02
cg03328727	9	92034102	SEMA4D	5'UTR	0,056	6,81E-05	2,37E-02
cg08272428	12	122037122		IGR	-0,007	6,81E-05	2,37E-02
cg02321871	13	50159138	RCBTB1	5'UTR	-0,008	6,81E-05	2,37E-02
cg25652672	16	90096456	GAS8-AS1	TSS200	-0,005	6,81E-05	2,37E-02
cg24439070	22	42977869	RRP7B	Body	0,005	6,81E-05	2,37E-02
cg11652095	21	38939404		IGR	-0,008	6,82E-05	2,37E-02
cg04723416	5	15616382	TD-2350J17.1	TSS1500	0,011	6,84E-05	2,37E-02
cg26894841	16	90016551	DEF8	5'UTR	-0,01	6,84E-05	2,37E-02
cg22211164	2	68615001	PLEK	Body	-0,017	6,85E-05	2,38E-02
cg26066474	18	76653676		IGR	0,042	6,85E-05	2,38E-02
cg04575478	17	76394126	PGS1	Body	-0,011	6,86E-05	2,38E-02
cg22598841	1	113615267	LRIG2	TSS1500	-0,011	6,88E-05	2,38E-02
cg00314248	2	132394574	LINC01087	TSS200	0,014	6,87E-05	2,38E-02
cg08581546	2	234732287	MROH2A	Body	-0,015	6,88E-05	2,38E-02
cg08550023	6	17393365	CAP2	TSS1500	0,034	6,87E-05	2,38E-02
cg00387552	10	7555607		IGR	0,006	6,88E-05	2,38E-02
cg14848862	12	95654404	VEZT	Body	-0,009	6,87E-05	2,38E-02

cg03564166	16	70380888	DDX19A	5'UTR	0,007	6,88E-05	2,38E-02
cg10458961	1	16448346		IGR	0,016	6,89E-05	2,38E-02
cg02585483	5	61602105	KIF2A	5'UTR	0,008	6,91E-05	2,38E-02
cg10974601	7	2287495	NUDT1	Body	-0,007	6,91E-05	2,38E-02
cg26328609	12	120425988		IGR	-0,032	6,92E-05	2,38E-02
cg07189550	1	179851385	TOR1AIP1	TSS200	0,003	6,98E-05	2,39E-02
cg26655295	2	677162	TMEM18	Body	0,004	6,96E-05	2,39E-02
cg21977467	3	19988404	RAB5A	TSS200	-0,007	6,93E-05	2,39E-02
cg24687806	6	168132832		IGR	-0,006	6,98E-05	2,39E-02
cg26796379	8	77766073	ZFHX4	Body	0,008	6,97E-05	2,39E-02
cg10231556	9	36401304	RNF38	TSS1500	0,008	6,98E-05	2,39E-02
cg24884648	10	95676572		IGR	-0,029	6,94E-05	2,39E-02
cg27351780	11	35639995	FJX1	1stExon	-0,007	6,95E-05	2,39E-02
cg02201489	12	4696656		IGR	-0,006	6,96E-05	2,39E-02
cg02645563	12	54610102		IGR	0,028	6,98E-05	2,39E-02
cg11555873	13	29102417		IGR	0,028	6,95E-05	2,39E-02
cg23416247	13	42535382	KIAA0564	TSS200	0,011	6,95E-05	2,39E-02
cg03923252	14	53019362	TXNDC16	TSS200	0,004	6,96E-05	2,39E-02
cg12069304	16	24787524	TNRC6A	Body	-0,008	6,95E-05	2,39E-02
cg11141437	17	2851165	RAP1GAP2	Body	-0,016	6,98E-05	2,39E-02
cg02652405	18	51094795	)C102724651	TSS200	0,041	6,98E-05	2,39E-02
cg03475229	19	57438933		IGR	0,032	6,97E-05	2,39E-02
cg14242447	20	32076804	CBFA2T2	TSS1500	-0,007	6,94E-05	2,39E-02
cg24847601	6	78176135		IGR	0,009	6,99E-05	2,39E-02
cg20735819	9	131971267		IGR	-0,01	6,99E-05	2,39E-02
cg02181349	4	186979601		IGR	0,05	7,00E-05	2,39E-02
cg01870995	12	3602546	PRMT8	Body	-0,013	7,00E-05	2,39E-02
cg24584367	1	156565633	GPATCH4	Body	0,013	7,06E-05	2,40E-02
cg23669186	3	122399420	PARP14	TSS1500	0,003	7,06E-05	2,40E-02
cg06507232	3	187838411		IGR	0,005	7,05E-05	2,40E-02
cg01589294	4	159082678	FAM198B	Body	-0,061	7,07E-05	2,40E-02
cg01881688	8	145897116		IGR	-0,032	7,05E-05	2,40E-02
cg14251471	9	23452127		IGR	-0,008	7,07E-05	2,40E-02
cg03983811	10	99400441	PI4K2A	TSS200	0,005	7,07E-05	2,40E-02
cg07326706	11	70808889	SHANK2	Body	-0,012	7,06E-05	2,40E-02
cg12429629	15	93152363		IGR	-0,022	7,05E-05	2,40E-02
cg17002108	19	12758083	MAN2B1	Body	-0,013	7,03E-05	2,40E-02
cg08326933	19	49967537	ALDH16A1	Body	-0,013	7,04E-05	2,40E-02
cg24787924	19	54231819	MIR518E	TSS1500	-0,016	7,06E-05	2,40E-02
cg23733739	8	97109610		IGR	0,03	7,08E-05	2,40E-02
cg09624538	1	179699342		IGR	0,012	7,08E-05	2,40E-02
cg09802076	12	85285441	SLC6A15	5'UTR	0,048	7,08E-05	2,40E-02
cg18861421	19	11450590	RAB3D	TSS1500	0,016	7,09E-05	2,40E-02
cg03548761	20	23754809		IGR	-0,012	7,09E-05	2,40E-02
cg27206407	17	76349089		IGR	0,01	7,10E-05	2,40E-02
cg08825146	2	84840595	DNAH6	Body	-0,022	7,12E-05	2,41E-02
cg15843385	1	155036091	EFNA4	TSS200	0,003	7,13E-05	2,41E-02
cg07758936	3	27525996	SLC4A7	TSS200	0,005	7,13E-05	2,41E-02
cg21568020	14	38028863		IGR	-0,011	7,15E-05	2,42E-02
cg18102084	12	106535424		IGR	0,037	7,17E-05	2,42E-02
cg15229570	10	117723600		IGR	0,028	7,18E-05	2,42E-02
cg17428913	1	36563557	COL8A2	Body	-0,009	7,19E-05	2,42E-02
cg01324000	16	71324258	FTSJD1	TSS1500	0,027	7,19E-05	2,42E-02
cg05099596	2	108604750	SLC5A7	Body	-0,023	7,20E-05	2,43E-02
cg25178565	10	22990128	PIP4K2A	Body	0,019	7,21E-05	2,43E-02
cg13853450	18	64271503	CDH19	TSS1500	0,012	7,21E-05	2,43E-02
cg02578961	22	46671616	TTC38	Body	-0,009	7,20E-05	2,43E-02
cg17411367	19	45525437	RELB	Body	-0,006	7,22E-05	2,43E-02
cg07404554	2	25570581		IGR	0,034	7,25E-05	2,43E-02
cg04332513	3	122135059	WDR5B	TSS200	-0,005	7,25E-05	2,43E-02
cg14414876	4	178365849		IGR	-0,035	7,26E-05	2,43E-02

cg09831641	5	82651164		IGR	-0,039	7,24E-05	2,43E-02
cg05348784	5	116184916		IGR	0,022	7,24E-05	2,43E-02
cg27585618	6	108093747	SCML4	5'UTR	-0,01	7,25E-05	2,43E-02
cg13895331	9	6418884	UHRF2	Body	-0,05	7,26E-05	2,43E-02
cg01077542	9	130649743	ST6GALNAC6	3'UTR	0,009	7,23E-05	2,43E-02
cg25157810	12	5107082		IGR	-0,016	7,26E-05	2,43E-02
cg09048510	16	86679454		IGR	0,026	7,24E-05	2,43E-02
cg27600804	16	89111748		IGR	0,037	7,27E-05	2,43E-02
cg05204587	17	3852656	ATP2A3	Body	-0,01	7,26E-05	2,43E-02
cg13518988	17	73401817	GRB2	TSS200	0,004	7,23E-05	2,43E-02
cg14545763	19	58920002	ZNF584	TSS200	0,004	7,26E-05	2,43E-02
cg20762897	7	132937989	EXOC4	Body	-0,003	7,27E-05	2,43E-02
cg17221506	5	131281504	MEIKIN	TSS200	0,014	7,31E-05	2,44E-02
cg22961699	15	77935488		IGR	-0,01	7,31E-05	2,44E-02
cg02084046	5	139027359	CXXC5	TSS1500	0,009	7,32E-05	2,44E-02
cg22751438	9	115333218	KIAA1958	5'UTR	-0,019	7,32E-05	2,44E-02
cg11995722	8	17548659	MTUS1	Body	-0,014	7,34E-05	2,44E-02
cg00856443	8	60127667		IGR	0,029	7,34E-05	2,44E-02
cg24776910	17	7117616	DLG4	Body	0,01	7,33E-05	2,44E-02
cg06598987	17	25846548	KSR1	5'UTR	0,008	7,34E-05	2,44E-02
cg27031373	5	52627604		IGR	0,042	7,35E-05	2,44E-02
cg09779589	5	54430727	CDC20B	Body	-0,021	7,36E-05	2,44E-02
cg22843111	5	79047136	CMYA5	Body	0,016	7,37E-05	2,44E-02
cg02389931	10	422076	DIP2C	Body	0,019	7,35E-05	2,44E-02
cg23175322	11	64765098	BATF2	TSS1500	-0,01	7,36E-05	2,44E-02
cg11858564	14	74182560	PNMA1	TSS1500	-0,01	7,37E-05	2,44E-02
cg02976190	17	74084167	EXOC7	Body	-0,008	7,36E-05	2,44E-02
cg22404151	19	58603088	ZSCAN18	Body	-0,013	7,36E-05	2,44E-02
cg08141342	1	45308976	PTCH2	TSS1500	-0,007	7,38E-05	2,45E-02
cg25199666	8	142933617		IGR	0,034	7,39E-05	2,45E-02
cg25428846	4	141107931		IGR	0,007	7,40E-05	2,45E-02
cg08285677	15	71371827		IGR	0,044	7,41E-05	2,45E-02
cg08551248	1	40255129	BMP8B	TSS1500	0,007	7,41E-05	2,45E-02
cg26051748	10	134651621		IGR	-0,017	7,42E-05	2,46E-02
cg03558515	11	133236244	OPCML	Body	0,048	7,43E-05	2,46E-02
cg22450693	17	48624483	SPATA20	TSS200	0,004	7,43E-05	2,46E-02
cg20499126	3	6433955		IGR	-0,02	7,46E-05	2,46E-02
cg08001520	3	128840660	RAB43	TSS200	0,021	7,45E-05	2,46E-02
cg10010780	4	187629520	FAT1	Body	0,008	7,46E-05	2,46E-02
cg01175698	5	28549424		IGR	-0,012	7,44E-05	2,46E-02
cg12322015	6	4136207	ECI2	TSS1500	0,019	7,46E-05	2,46E-02
cg21674088	9	735014	KANK1	Body	0,02	7,46E-05	2,46E-02
cg00415827	10	11728989		IGR	-0,031	7,44E-05	2,46E-02
cg03694279	10	43788993		IGR	0,051	7,45E-05	2,46E-02
cg11439475	15	52821378	MYO5A	TSS200	0,005	7,45E-05	2,46E-02
cg13420792	19	47364474		IGR	0,004	7,46E-05	2,46E-02
cg16976419	17	29592853	NF1	Body	-0,034	7,49E-05	2,46E-02
cg27607849	5	133389776		IGR	0,054	7,50E-05	2,46E-02
cg27066295	16	87778903	KLHDC4	Body	-0,022	7,51E-05	2,47E-02
cg17269633	17	42733600	C17orf104	TSS200	-0,026	7,51E-05	2,47E-02
cg05882969	1	38455985	SF3A3	TSS1500	0,005	7,53E-05	2,47E-02
cg19989750	3	129276211	PLXND1	Body	-0,019	7,53E-05	2,47E-02
cg13436451	6	168378673	HGC6.3	TSS1500	0,006	7,53E-05	2,47E-02
cg19397128	8	116681246	TRPS1	TSS200	-0,004	7,53E-05	2,47E-02
cg23747996	8	145723492	PPP1R16A	Body	-0,015	7,54E-05	2,47E-02
cg05346256	12	25959372		IGR	-0,01	7,52E-05	2,47E-02
cg12351768	13	113296192		IGR	-0,029	7,53E-05	2,47E-02
cg14138747	4	141825221	RNF150	Body	-0,013	7,55E-05	2,47E-02
cg22460725	7	51108584	COBL	Body	0,018	7,55E-05	2,47E-02
cg08452250	12	102266364		IGR	0,008	7,56E-05	2,47E-02
cg23670563	7	767237	PRKAR1B	TSS1500	0,007	7,58E-05	2,47E-02

cg03724006	11	62790783		IGR	0,006	7,57E-05	2,47E-02
cg14331765	11	121672049		IGR	0,036	7,58E-05	2,47E-02
cg04369874	15	44581627	CASC4	Body	-0,014	7,57E-05	2,47E-02
cg12426587	22	31265379	OSBP2	Body	-0,018	7,58E-05	2,47E-02
cg25827124	8	70312989		IGR	-0,01	7,59E-05	2,47E-02
cg19826848	11	20148170		IGR	-0,01	7,59E-05	2,47E-02
cg22922861	7	155287373		IGR	0,011	7,59E-05	2,47E-02
cg10817093	4	174089402	GALNT7	TSS1500	-0,003	7,60E-05	2,47E-02
cg12439608	4	39050213	KLHL5	5'UTR	0,046	7,61E-05	2,48E-02
cg02650000	9	130965465	DNM1	TSS200	0,007	7,61E-05	2,48E-02
cg22567250	3	112628051		IGR	-0,007	7,62E-05	2,48E-02
cg19379302	3	10857435	SLC6A11	TSS1500	-0,045	7,62E-05	2,48E-02
cg13173797	4	37969628	TBC1D1	Body	0,045	7,63E-05	2,48E-02
cg04851044	6	33638313	ITPR3	Body	-0,009	7,64E-05	2,48E-02
cg04119303	11	1660513	HCCA2	Body	0,039	7,65E-05	2,48E-02
cg06482656	8	33457681	DUSP26	TSS200	-0,03	7,66E-05	2,48E-02
cg10516516	3	54231340	CACNA2D3	Body	0,036	7,67E-05	2,49E-02
cg13274978	8	52320849	PXDNL	Body	-0,01	7,67E-05	2,49E-02
cg04623987	19	32816062		IGR	0,006	7,67E-05	2,49E-02
cg00069760	3	197035565		IGR	0,013	7,70E-05	2,49E-02
cg19082765	4	74059087	ANKRD17	Body	0,033	7,70E-05	2,49E-02
cg15183002	5	67513119	PIK3R1	5'UTR	-0,012	7,70E-05	2,49E-02
cg06575656	7	150809483	AGAP3	5'UTR	-0,029	7,69E-05	2,49E-02
cg00634483	8	49470964		IGR	0,068	7,70E-05	2,49E-02
cg04619450	15	58553983		IGR	0,005	7,69E-05	2,49E-02
cg23882790	19	21203004	ZNF430	TSS1500	0,078	7,69E-05	2,49E-02
cg14332346	11	18548210	TSG101	Body	-0,016	7,71E-05	2,49E-02
cg05833095	12	111015746	PPTC7	Body	0,013	7,71E-05	2,49E-02
cg19404812	4	170540609	CLCN3	TSS1500	0,035	7,72E-05	2,49E-02
cg13451481	1	234673929		IGR	0,007	7,72E-05	2,49E-02
cg11941455	11	72399575	ARAP1	ExonBnd	-0,019	7,72E-05	2,49E-02
cg16497732	8	141405143	TRAPPC9	Body	-0,006	7,74E-05	2,49E-02
cg02219125	14	89290504	TTC8	TSS1500	-0,005	7,74E-05	2,49E-02
cg14428237	1	198136929	NEK7	5'UTR	0,008	7,75E-05	2,49E-02
cg26038852	21	47640170	LSS	Body	-0,015	7,75E-05	2,49E-02
cg07535903	3	97594844	CRYBG3	TSS1500	-0,008	7,76E-05	2,49E-02
cg15525782	19	36210692	MLL4	Body	-0,014	7,76E-05	2,49E-02
cg06758132	19	49833652		IGR	0,005	7,76E-05	2,49E-02
cg11663975	1	202936448	CYB5R1	TSS200	0,008	7,77E-05	2,50E-02
cg05628436	22	39186607	DNAL4	5'UTR	-0,005	7,77E-05	2,50E-02
cg23439917	18	77221937	NFATC1	Body	-0,02	7,78E-05	2,50E-02
cg09266468	1	45241580	SNORD46	TSS1500	-0,005	7,88E-05	2,50E-02
cg21452447	2	1880131	MYT1L	Body	-0,018	7,84E-05	2,50E-02
cg20595215	3	8543014	LMCD1	TSS1500	0,013	7,87E-05	2,50E-02
cg04136727	3	127606259		IGR	0,037	7,81E-05	2,50E-02
cg13487184	3	183863572		IGR	0,011	7,81E-05	2,50E-02
cg17089729	4	40043204		IGR	0,006	7,87E-05	2,50E-02
cg25776255	4	147443144	SLC10A7	TSS200	0,004	7,81E-05	2,50E-02
cg19694371	5	149380223	HMGXB3	5'UTR	0,005	7,88E-05	2,50E-02
cg10050884	5	176830535	F12	Body	0,004	7,84E-05	2,50E-02
cg21531017	6	30159510	TRIM26	Body	-0,007	7,84E-05	2,50E-02
cg01367322	6	33283796	ZBTB22	Body	-0,019	7,85E-05	2,50E-02
cg21603716	8	12808039	KIAA1456	5'UTR	0,034	7,84E-05	2,50E-02
cg12792732	8	54674575	ATP6V1H	Body	0,032	7,80E-05	2,50E-02
cg10462600	8	73608394	KCNB2	Body	-0,02	7,87E-05	2,50E-02
cg13430300	8	73907269		IGR	0,017	7,87E-05	2,50E-02
cg22915154	11	68816030	TPCN2	TSS1500	0,014	7,85E-05	2,50E-02
cg13933023	11	116166109		IGR	-0,008	7,82E-05	2,50E-02
cg14654629	12	46121631	LOC400027	Body	0,006	7,88E-05	2,50E-02
cg12007863	12	96272432	CCDC38	Body	0,042	7,85E-05	2,50E-02
cg00244634	12	100661419	SCYL2	TSS200	0,011	7,87E-05	2,50E-02

cg18281527	13	51872358		IGR	0,008	7,79E-05	2,50E-02
cg05271910	13	100037488	UBAC2	Body	-0,007	7,81E-05	2,50E-02
cg10885791	15	57967914	GCOM1	Body	0,007	7,87E-05	2,50E-02
cg09236903	15	63353323	TPM1	Body	-0,007	7,85E-05	2,50E-02
cg10972686	18	47252789		IGR	0,014	7,83E-05	2,50E-02
cg13358109	19	49644284	PPFIA3	Body	0,036	7,86E-05	2,50E-02
cg06144080	20	33735161	EDEM2	TSS200	0,004	7,87E-05	2,50E-02
cg06951964	22	41840449	TOB2	5'UTR	-0,006	7,84E-05	2,50E-02
cg12430078	2	70121688	SNRNP27	Body	-0,019	7,89E-05	2,50E-02
cg23866762	3	4344834	SETMAR	TSS1500	0,052	7,89E-05	2,50E-02
cg23037777	20	55072651	C20orf43	Body	0,01	7,88E-05	2,50E-02
cg25324046	6	35889059	SRPK1	TSS200	0,003	7,90E-05	2,50E-02
cg11461359	20	45531200	EYA2	5'UTR	0,04	7,90E-05	2,50E-02
cg07014682	3	127207863		IGR	0,006	7,91E-05	2,50E-02
cg16629408	19	45924601	ERCC1	Body	0,009	7,91E-05	2,50E-02
cg12652115	1	180217433	LHX4	ExonBnd	-0,023	7,93E-05	2,51E-02
cg03848004	1	26107517	MAN1C1	Body	-0,015	7,95E-05	2,51E-02
cg12343639	17	5610303		IGR	0,016	7,95E-05	2,51E-02
cg05868183	17	48450068	MRPL27	Body	-0,003	7,95E-05	2,51E-02
cg14556787	10	1669329	ADARB2	Body	0,01	7,96E-05	2,51E-02
cg23340666	15	43623096	ADAL	5'UTR	0,011	7,96E-05	2,51E-02
cg04982343	13	74993258	LINC00381	TSS200	0,019	7,96E-05	2,51E-02
cg23810763	1	20441991	PLA2G2D	Body	-0,012	7,99E-05	2,52E-02
cg21231458	20	21485933		IGR	-0,01	7,99E-05	2,52E-02
cg23510153	10	38146293	ZNF248	5'UTR	-0,007	8,00E-05	2,52E-02
cg18848985	4	943690	TMEM175	Body	-0,004	8,01E-05	2,52E-02
cg12868544	6	31687926	LY6G6C	Body	-0,008	8,01E-05	2,52E-02
cg14482116	17	12690037		IGR	-0,046	8,01E-05	2,52E-02
cg08988357	6	81401251		IGR	-0,008	8,04E-05	2,52E-02
cg24082871	17	27333407	SEZ6	TSS1500	-0,012	8,03E-05	2,52E-02
cg06438276	19	56148093		IGR	-0,022	8,04E-05	2,52E-02
cg10494619	1	68748064		IGR	0,01	8,05E-05	2,53E-02
cg25024993	5	140248610	PCDHA7	Body	-0,043	8,06E-05	2,53E-02
cg00965566	10	17331997		IGR	0,005	8,06E-05	2,53E-02
cg12061236	6	151560886	AKAP12	TSS1500	0,026	8,08E-05	2,53E-02
cg00910893	15	78286548	LOC91450	Body	0,018	8,09E-05	2,53E-02
cg20805835	1	248005719	OR11L1	TSS1500	0,01	8,13E-05	2,54E-02
cg25221719	3	141319318	RASA2	Body	-0,013	8,12E-05	2,54E-02
cg27624327	5	510254	SLC9A3	Body	-0,008	8,13E-05	2,54E-02
cg07998216	8	70584832	SLCO5A1	3'UTR	0,036	8,12E-05	2,54E-02
cg17311081	1	22488606		IGR	0,005	8,18E-05	2,54E-02
cg27469518	1	90457601		IGR	-0,009	8,18E-05	2,54E-02
cg03311083	2	121698644	GLI2	Body	-0,011	8,14E-05	2,54E-02
cg18348936	3	15642978	HACL1	1stExon	0,006	8,15E-05	2,54E-02
cg10349898	5	43019006	LOC648987	Body	-0,006	8,17E-05	2,54E-02
cg17848407	5	126408806	FLJ44606	5'UTR	-0,059	8,15E-05	2,54E-02
cg01351925	5	141276282	LOC729080	TSS200	0,005	8,17E-05	2,54E-02
cg21099419	7	97844473		IGR	0,006	8,14E-05	2,54E-02
cg20074048	9	91933147	SECISBP2	TSS1500	0,005	8,16E-05	2,54E-02
cg17021559	10	75545355	ZSWIM8	TSS200	0,005	8,17E-05	2,54E-02
cg20554277	14	37506088	SLC25A21	Body	0,043	8,15E-05	2,54E-02
cg13277940	14	60558485	C14orf135	TSS200	-0,006	8,17E-05	2,54E-02
cg13309461	17	79664294	HGS	Body	-0,019	8,14E-05	2,54E-02
cg08844562	20	59599794		IGR	0,043	8,18E-05	2,54E-02
cg20487212	7	103272594	RELN	Body	0,05	8,18E-05	2,54E-02
cg06892501	11	5345105	OR51B2	1stExon	-0,009	8,19E-05	2,54E-02
cg11601923	1	78148301	ZZZ3	1stExon	0,002	8,19E-05	2,54E-02
cg04210361	1	193073866	GLRX2	Body	0,004	8,20E-05	2,54E-02
cg14638453	11	63804481	MACROD1	Body	0,015	8,20E-05	2,54E-02
cg25024210	18	2912429	EMILIN2	Body	-0,008	8,20E-05	2,54E-02
cg20226154	6	150346787	RAET1L	TSS200	-0,02	8,21E-05	2,55E-02

cg24523147	10	102421067		IGR	0,067	8,23E-05	2,55E-02
cg06481013	11	17573592	OTOG	Body	-0,012	8,23E-05	2,55E-02
cg01527805	13	24850703	SPATA13	5'UTR	-0,007	8,23E-05	2,55E-02
cg15051540	3	125933606		IGR	0,013	8,25E-05	2,55E-02
cg24767519	14	97090367		IGR	-0,016	8,24E-05	2,55E-02
cg19718090	20	36887672	LOC149684	TSS1500	-0,01	8,25E-05	2,55E-02
cg17879544	2	242842666	LINC01237	Body	-0,009	8,26E-05	2,55E-02
cg17943624	8	48424300	SPIDR	Body	-0,007	8,28E-05	2,56E-02
cg21463709	2	2124815	MYT1L	5'UTR	-0,037	8,29E-05	2,56E-02
cg19227145	7	7091125		IGR	0,038	8,29E-05	2,56E-02
cg04265090	1	44056769	PTPRF	Body	-0,009	8,36E-05	2,56E-02
cg03166702	1	92357392	TGFBR3	5'UTR	-0,051	8,34E-05	2,56E-02
cg00350652	1	109584526	WDR47	1stExon	0,007	8,34E-05	2,56E-02
cg25200909	1	203765349	ZBED6	TSS1500	0,003	8,34E-05	2,56E-02
cg01107905	1	209722136		IGR	0,023	8,33E-05	2,56E-02
cg16801535	3	9291372	SRGAP3	TSS200	0,01	8,32E-05	2,56E-02
cg10908447	5	153714203	GALNT10	Body	0,008	8,31E-05	2,56E-02
cg00491948	6	10839508	MAK	TSS1500	0,047	8,35E-05	2,56E-02
cg10045804	7	44621958	TMED4	TSS200	0,006	8,34E-05	2,56E-02
cg12430941	8	102860140	NCALD	5'UTR	-0,006	8,32E-05	2,56E-02
cg23965848	9	34989590	DNAJB5	TSS1500	0,004	8,35E-05	2,56E-02
cg12560896	11	32345151		IGR	0,033	8,35E-05	2,56E-02
cg15392722	11	62124488	ASRGL1	Body	0,006	8,33E-05	2,56E-02
cg26915158	11	130339200	ADAMTS15	Body	-0,007	8,33E-05	2,56E-02
cg22682953	12	112857226	PTPN11	Body	0,004	8,31E-05	2,56E-02
cg07219285	14	55154568	SAMD4A	Body	-0,015	8,30E-05	2,56E-02
cg04999058	16	73644907		IGR	0,027	8,33E-05	2,56E-02
cg17619218	17	74099818	EXOC7	TSS200	0,004	8,33E-05	2,56E-02
cg23767930	20	46365803	SULF2	Body	-0,015	8,34E-05	2,56E-02
cg16533812	16	87671094	JPH3	Body	-0,01	8,36E-05	2,56E-02
cg24299074	11	15133087	INSC	TSS1500	-0,009	8,37E-05	2,56E-02
cg08430157	9	38142075		IGR	-0,07	8,38E-05	2,56E-02
cg27500125	13	102126794	ITGBL1	5'UTR	-0,01	8,38E-05	2,56E-02
cg23442110	1	173607368	ANKRD45	Body	0,011	8,39E-05	2,56E-02
cg06005684	1	41902663		IGR	-0,008	8,40E-05	2,56E-02
cg09341892	1	203379814		IGR	0,052	8,41E-05	2,56E-02
cg12501142	2	11053801	KCNF1	1stExon	-0,007	8,41E-05	2,56E-02
cg12966662	5	90079705	ADGRV1	Body	0,004	8,41E-05	2,56E-02
cg01684562	6	80713632	TTK	TSS1500	-0,008	8,39E-05	2,56E-02
cg17701942	11	62575582	STX5	Body	0,022	8,40E-05	2,56E-02
cg10855961	17	9736776	GLP2R	Body	-0,005	8,41E-05	2,56E-02
cg09447681	9	132689547	FNBP1	Body	0,004	8,43E-05	2,57E-02
cg26361513	14	105821007	PACS2	Body	-0,012	8,43E-05	2,57E-02
cg14515506	9	14203578	NFIB	Body	-0,008	8,44E-05	2,57E-02
cg20231021	11	117197945	CEP164	TSS1500	-0,007	8,44E-05	2,57E-02
cg04227581	2	16059290	MYCNUT	TSS1500	-0,017	8,47E-05	2,57E-02
cg20916523	3	10184584	VHL	Body	0,066	8,46E-05	2,57E-02
cg15722404	3	12329242	PPARG	TSS1500	-0,009	8,45E-05	2,57E-02
cg26148059	6	33256755	PFDN6	TSS1500	0,006	8,46E-05	2,57E-02
cg02114831	7	100172801	LRCH4	Body	-0,013	8,47E-05	2,57E-02
cg10728452	11	133791257	IGSF9B	Body	-0,008	8,47E-05	2,57E-02
cg09861299	19	12223332	ZNF788	Body	0,012	8,47E-05	2,57E-02
cg20570458	3	124069933	KALRN	Body	0,043	8,48E-05	2,57E-02
cg00346286	14	45604338	FANCM	TSS1500	-0,009	8,49E-05	2,57E-02
cg27119212	7	2044678	MAD1L1	Body	-0,008	8,50E-05	2,57E-02
cg16490062	16	12211322	SNX29	Body	0,023	8,50E-05	2,57E-02
cg17050724	17	79008885	FLJ90757	TSS1500	0,015	8,49E-05	2,57E-02
cg01960233	22	40718732	TNRC6B	Body	0,006	8,50E-05	2,57E-02
cg09611656	7	156755775	NOM1	Body	0,007	8,50E-05	2,57E-02
cg04132263	13	79968174	RBM26	Body	0,063	8,54E-05	2,58E-02
cg03412953	6	69433703	ADGRB3	Body	-0,025	8,54E-05	2,58E-02

cg22688348	2	182514316	CERKL	Body	-0,014	8,55E-05	2,58E-02
cg21467066	7	150759490	SLC4A2	TSS1500	-0,006	8,55E-05	2,58E-02
cg13327846	15	52472389	GNB5	TSS1500	0,003	8,55E-05	2,58E-02
cg02449758	8	131172406	ASAP1	Body	-0,028	8,56E-05	2,58E-02
cg15990961	15	81937744		IGR	0,025	8,57E-05	2,58E-02
cg10630212	10	13236960	MCM10	Body	0,006	8,57E-05	2,58E-02
cg15921757	3	168864413	MECOM	TSS1500	0,01	8,59E-05	2,59E-02
cg27026368	1	203691921	ATP2B4	Body	0,005	8,63E-05	2,60E-02
cg26624732	5	70746330		IGR	-0,063	8,63E-05	2,60E-02
cg04523927	15	44449710	FRMD5	Body	-0,023	8,64E-05	2,60E-02
cg02477523	3	180456508		IGR	-0,018	8,65E-05	2,60E-02
cg18292238	3	184076589	CLCN2	ExonBnd	-0,014	8,65E-05	2,60E-02
cg04471171	1	34577639	CSMD2	Body	0,051	8,66E-05	2,60E-02
cg24391331	8	95648314		IGR	-0,038	8,67E-05	2,61E-02
cg03878722	16	76666860		IGR	-0,014	8,67E-05	2,61E-02
cg22046966	4	874380	GAK	Body	-0,014	8,69E-05	2,61E-02
cg08665844	4	113485177	C4orf21	Body	0,047	8,70E-05	2,61E-02
cg16956876	6	31746820	VARS	Body	-0,013	8,70E-05	2,61E-02
cg17760786	6	170259293		IGR	-0,017	8,69E-05	2,61E-02
cg26604148	13	25034271	PARP4	ExonBnd	0,028	8,70E-05	2,61E-02
cg26814747	18	77931743	PARD6G-AS1	Body	0,005	8,68E-05	2,61E-02
cg18680061	5	131705688	SLC22A5	1stExon	0,003	8,73E-05	2,61E-02
cg05000960	8	86133291	C8orf59	TSS1500	-0,021	8,73E-05	2,61E-02
cg07451926	11	124746772	ROBO3	Body	-0,007	8,73E-05	2,61E-02
cg07944405	15	37441051		IGR	-0,03	8,72E-05	2,61E-02
cg09744051	22	43115937	A4GALT	5'UTR	0,025	8,72E-05	2,61E-02
cg27499597	7	101413146		IGR	0,013	8,74E-05	2,61E-02
cg25510569	3	171506308	PLD1	5'UTR	0,011	8,74E-05	2,61E-02
cg17228206	14	76786903		IGR	0,079	8,77E-05	2,62E-02
cg06598196	1	150185923		IGR	0,011	8,79E-05	2,63E-02
cg26262962	1	155823199	GON4L	Body	0,006	8,80E-05	2,63E-02
cg01061909	2	81750913		IGR	-0,01	8,80E-05	2,63E-02
cg27430182	3	126195181	ZXDC	TSS1500	0,003	8,81E-05	2,63E-02
cg17691214	15	67840562	MAP2K5	TSS1500	0,005	8,81E-05	2,63E-02
cg10673265	6	33169975	SLC39A7	Body	-0,008	8,82E-05	2,63E-02
cg22988305	3	52240549	ALAS1	Body	-0,006	8,83E-05	2,63E-02
cg00501467	6	13015202	PHACTR1	Body	-0,02	8,83E-05	2,63E-02
cg00488298	2	176016944	ATF2	Body	-0,012	8,84E-05	2,63E-02
cg17014657	7	112106452	IFRD1	Body	0,009	8,84E-05	2,63E-02
cg15310387	8	142737232		IGR	0,006	8,84E-05	2,63E-02
cg04827747	7	134855666	C7orf49	TSS200	0,003	8,85E-05	2,63E-02
cg25261734	14	107114779		IGR	0,047	8,87E-05	2,63E-02
cg12553198	19	59066243	CHMP2A	1stExon	0,003	8,87E-05	2,63E-02
cg02856481	6	15512678	JARID2	Body	-0,01	8,88E-05	2,63E-02
cg06051716	6	169225783		IGR	0,009	8,88E-05	2,63E-02
cg00820621	13	83421906		IGR	-0,016	8,88E-05	2,63E-02
cg27018124	22	26879714	HPS4	1stExon	0,004	8,88E-05	2,63E-02
cg23244790	5	140811102	PCDHGA4	Body	-0,021	8,89E-05	2,64E-02
cg09538573	8	9952636	MSRA	Body	0,007	8,89E-05	2,64E-02
cg19662689	1	24829356	RCAN3AS	TSS1500	0,004	8,92E-05	2,64E-02
cg10383839	2	20251043	LAPTM4A	Body	0,003	8,93E-05	2,64E-02
cg18007559	5	1746649		IGR	-0,022	8,93E-05	2,64E-02
cg00825047	15	40226572	EIF2AK4	Body	0,007	8,92E-05	2,64E-02
cg21835874	17	2119457	SMG6	Body	0,033	8,91E-05	2,64E-02
cg01160311	17	42838541	ADAM11	Body	0,041	8,91E-05	2,64E-02
cg00778885	22	23236694	IGLL5	Body	-0,015	8,92E-05	2,64E-02
cg13288455	1	31386868		IGR	-0,008	8,94E-05	2,64E-02
cg02344868	19	39691009	NCCRP1	Body	0,022	8,96E-05	2,64E-02
cg12589798	20	44839734	CDH22	Body	-0,042	8,96E-05	2,64E-02
cg01099876	2	110371889	sept-10	TSS200	0,011	8,98E-05	2,65E-02
cg18489994	4	15235419		IGR	0,006	8,98E-05	2,65E-02

cg16581224	16	49783468	ZNF423	Body	-0,005	8,97E-05	2,65E-02
cg22752506	17	79895093	PYCR1	TSS200	-0,003	8,98E-05	2,65E-02
cg02228383	20	56934684	RAB22A	Body	0,006	8,98E-05	2,65E-02
cg09580409	4	183065519	MGC45800	Body	0,019	8,99E-05	2,65E-02
cg15849368	17	1811181		IGR	0,043	9,00E-05	2,65E-02
cg23896311	4	122791762	BBS7	TSS200	-0,007	9,01E-05	2,65E-02
cg21321814	3	145970378	PLSCR4	TSS1500	0,022	9,02E-05	2,65E-02
cg27438949	5	156782403	CYFIP2	Body	-0,012	9,02E-05	2,65E-02
cg15639860	7	100288433		IGR	-0,029	9,02E-05	2,65E-02
cg10650729	22	21978797		IGR	-0,014	9,02E-05	2,65E-02
cg03938375	5	75842553	IQGAP2	TSS1500	0,012	9,04E-05	2,65E-02
cg02888917	6	32940425	BRD2	5'UTR	0,005	9,04E-05	2,65E-02
cg02860203	22	42375285	sept-03	Body	-0,008	9,04E-05	2,65E-02
cg14384002	7	134881754	WDR91	Body	-0,015	9,05E-05	2,65E-02
cg08895440	1	36184853	C1orf216	TSS200	0,01	9,06E-05	2,65E-02
cg05048426	14	105843098	PACS2	Body	-0,029	9,06E-05	2,65E-02
cg00661753	20	2853149	PTPRA	5'UTR	0,004	9,07E-05	2,66E-02
cg04289040	6	27095113		IGR	0,008	9,08E-05	2,66E-02
cg04919519	14	103524989	CDC42BPB	TSS1500	0,018	9,08E-05	2,66E-02
cg17743012	15	29393777	APBA2	Body	0,02	9,08E-05	2,66E-02
cg04891733	21	35391280		IGR	-0,018	9,09E-05	2,66E-02
cg25754755	2	145089003	GTDC1	5'UTR	0,008	9,10E-05	2,66E-02
cg06446577	9	140687905	EHMT1	Body	-0,033	9,10E-05	2,66E-02
cg15898113	8	142532182		IGR	-0,025	9,11E-05	2,66E-02
cg00026341	6	49845521	CRISP1	TSS1500	0,052	9,11E-05	2,66E-02
cg24271538	3	12328290	PPARG	TSS1500	0,051	9,12E-05	2,66E-02
cg04311548	4	106481853	\RHGEF38-IT1	TSS1500	0,047	9,12E-05	2,66E-02
cg19629085	5	156668559	ITK	Body	0,006	9,12E-05	2,66E-02
cg00751463	13	28152851	LNX2	Body	-0,006	9,14E-05	2,66E-02
cg15729129	21	34407858		IGR	0,01	9,15E-05	2,67E-02
cg06559421	4	186697078	SORBS2	TSS200	-0,007	9,15E-05	2,67E-02
cg15300875	7	56131544	SUMF2	TSS1500	-0,046	9,16E-05	2,67E-02
cg08598287	10	43836083		IGR	-0,024	9,18E-05	2,67E-02
cg24030007	5	67433852		IGR	0,013	9,18E-05	2,67E-02
cg13953276	6	112375094	WISP3	TSS1500	0,025	9,19E-05	2,67E-02
cg15430883	1	11073233	TARDBP	5'UTR	0,006	9,26E-05	2,68E-02
cg25521012	1	27454165	SLC9A1	Body	-0,005	9,24E-05	2,68E-02
cg20031197	1	44711073	ERI3-IT1	TSS1500	-0,018	9,26E-05	2,68E-02
cg22568351	1	109203853	HENMT1	5'UTR	0,005	9,25E-05	2,68E-02
cg05373263	2	3063115		IGR	-0,057	9,22E-05	2,68E-02
cg03974193	2	102316442	MAP4K4	Body	0,037	9,23E-05	2,68E-02
cg26234414	3	20191055	KAT2B	Body	0,007	9,23E-05	2,68E-02
cg13943259	3	71992038		IGR	-0,023	9,21E-05	2,68E-02
cg23329372	5	178656269	ADAMTS2	Body	-0,017	9,25E-05	2,68E-02
cg10239429	8	6478624	MCPH1	Body	0,006	9,24E-05	2,68E-02
cg16431961	14	91806143	CCDC88C	Body	-0,013	9,25E-05	2,68E-02
cg06685590	16	15818816	NDE1	3'UTR	0,005	9,26E-05	2,68E-02
cg00108554	16	73157200		IGR	0,036	9,25E-05	2,68E-02
cg09683258	17	70429180		IGR	0,043	9,26E-05	2,68E-02
cg09565237	19	48637267	LIG1	Body	0,007	9,26E-05	2,68E-02
cg13469617	21	40119488	LINC00114	Body	0,032	9,25E-05	2,68E-02
cg13062935	11	19082514	MRGPRX2	TSS1500	-0,005	9,27E-05	2,68E-02
cg09033563	22	24373618	LOC391322	Body	0,01	9,27E-05	2,68E-02
cg13947987	2	70934491	ADD2	5'UTR	0,005	9,28E-05	2,68E-02
cg15975990	2	180871849	CWC22	TSS200	-0,014	9,30E-05	2,68E-02
cg07499182	13	33825496	STARD13	Body	0,075	9,29E-05	2,68E-02
cg24705093	15	83307727	CPEB1	5'UTR	0,033	9,29E-05	2,68E-02
cg01342226	4	141034510	MAML3	Body	0,027	9,30E-05	2,68E-02
cg23046671	6	112823117		IGR	0,024	9,30E-05	2,68E-02
cg02983495	4	122871876	TRPC3	Body	0,004	9,32E-05	2,68E-02
cg17260968	16	10746076	TEKT5	Body	0,012	9,32E-05	2,68E-02

cg14015996	17	28256508	EFCAB5	TSS1500	-0,004	9,33E-05	2,68E-02
cg06838151	1	111485674		IGR	0,032	9,34E-05	2,69E-02
cg02618140	2	37312106	GPATCH11	5'UTR	-0,007	9,34E-05	2,69E-02
cg16579049	4	57411074		IGR	0,023	9,34E-05	2,69E-02
cg14703796	7	143583145	TCAF1	TSS1500	0,012	9,35E-05	2,69E-02
cg16237788	22	30769573	CCDC157	ExonBnd	-0,019	9,35E-05	2,69E-02
cg23614108	3	168596412		IGR	0,03	9,36E-05	2,69E-02
cg02753749	10	23818762		IGR	-0,011	9,37E-05	2,69E-02
cg19500056	19	48281426	SEPW1	TSS1500	0,004	9,37E-05	2,69E-02
cg21211748	1	23858035	E2F2	TSS1500	0,005	9,40E-05	2,69E-02
cg11042063	2	173986862	ZAK	Body	-0,008	9,38E-05	2,69E-02
cg06130067	8	145734889	MFSD3	1stExon	0,004	9,40E-05	2,69E-02
cg25337227	12	72079796	TMEM19	TSS200	0,003	9,40E-05	2,69E-02
cg25718489	12	111077280	TCTN1	Body	-0,018	9,40E-05	2,69E-02
cg20358683	17	27468043	MYO18A	Body	0,004	9,40E-05	2,69E-02
cg12832519	17	38910212	KRT25	Body	0,018	9,40E-05	2,69E-02
cg17438661	19	33071683	PDCD5	TSS1500	0,008	9,39E-05	2,69E-02
cg21086156	19	54694904	MBOAT7	TSS1500	0,007	9,38E-05	2,69E-02
cg04809093	3	114862175	ZBTB20	5'UTR	-0,006	9,42E-05	2,69E-02
cg00506612	12	6673264	NOP2	Body	-0,028	9,42E-05	2,69E-02
cg09336277	16	1261666	CACNA1H	Body	-0,017	9,41E-05	2,69E-02
cg12110826	22	30774026	KIAA1656	TSS200	-0,013	9,42E-05	2,69E-02
cg01742680	3	152879841	RAP2B	TSS200	0,004	9,43E-05	2,69E-02
cg24090671	10	17686217	STAM	5'UTR	0,004	9,45E-05	2,70E-02
cg13570928	18	68664091		IGR	-0,01	9,45E-05	2,70E-02
cg05672801	22	38224528		IGR	-0,009	9,47E-05	2,70E-02
cg09416717	2	42996637	HAAO	Body	-0,01	9,48E-05	2,70E-02
cg11662221	15	39540037		IGR	0,01	9,49E-05	2,70E-02
cg14474363	8	29909179		IGR	-0,007	9,49E-05	2,70E-02
cg07384913	3	63637118	SNTN	TSS1500	-0,014	9,50E-05	2,70E-02
cg22046166	7	107771104	LAMB4	TSS1500	-0,006	9,51E-05	2,70E-02
cg09908110	11	63828713	MACROD1	Body	0,004	9,51E-05	2,70E-02
cg03403662	12	51420377	SLC11A2	TSS200	0,005	9,51E-05	2,70E-02
cg23280612	15	95397992	LOC440311	TSS1500	0,008	9,52E-05	2,70E-02
cg07398644	17	13332270		IGR	0,011	9,50E-05	2,70E-02
cg00163171	12	31963379		IGR	0,027	9,53E-05	2,71E-02
cg12004575	5	150003580	SYNPO	Body	0,026	9,54E-05	2,71E-02
cg19224070	5	65491435		IGR	-0,013	9,56E-05	2,71E-02
cg17514558	5	140621375	PCDHB19P	Body	-0,048	9,56E-05	2,71E-02
cg06534800	5	176931224	DOK3	Body	-0,016	9,55E-05	2,71E-02
cg20418529	6	166260012		IGR	-0,018	9,56E-05	2,71E-02
cg20054812	1	89357379	GTF2B	TSS200	-0,01	9,57E-05	2,71E-02
cg16525935	4	25397912	ANAPC4	Body	-0,008	9,57E-05	2,71E-02
cg23529561	10	23805745		IGR	-0,037	9,58E-05	2,71E-02
cg18445292	11	68610381	CPT1A	TSS1500	0,016	9,58E-05	2,71E-02
cg13162894	16	2079441	SLC9A3R2	Body	-0,019	9,59E-05	2,71E-02
cg10734102	2	225895015	DOCK10	Body	-0,049	9,59E-05	2,71E-02
cg12404044	2	26911734		IGR	0,039	9,60E-05	2,71E-02
cg07735571	22	39796170	MAP3K7IP1	Body	0,003	9,62E-05	2,72E-02
cg00919118	10	114886498	TCF7L2	Body	0,008	9,63E-05	2,72E-02
cg15168376	11	130405517		IGR	0,095	9,63E-05	2,72E-02
cg10786087	13	99092272	FARP1	Body	-0,005	9,65E-05	2,72E-02
cg12644885	1	154297985	ATP8B2	TSS200	0,011	9,67E-05	2,73E-02
cg16413842	6	170191082	C6orf122	Body	0,043	9,68E-05	2,73E-02
cg12552944	7	2353682	SNX8	Body	0,007	9,68E-05	2,73E-02
cg25270205	12	109208726	SSH1	Body	0,011	9,67E-05	2,73E-02
cg23431756	16	11836567	TXNDC11	1stExon	0,005	9,66E-05	2,73E-02
cg20882848	2	219483328	PLCD4	Body	-0,011	9,70E-05	2,73E-02
cg10661263	6	167508170		IGR	0,03	9,70E-05	2,73E-02
cg17703934	17	39136589	KRT40	Body	0,007	9,70E-05	2,73E-02
cg01788676	9	97021471	ZNF169	TSS200	0,007	9,70E-05	2,73E-02

cg16903025	8	124549553	FBXO32	Body	0,028	9,71E-05	2,73E-02
cg08548888	3	38180840	MYD88	Body	0,007	9,72E-05	2,73E-02
cg05092146	5	180577134		IGR	0,017	9,73E-05	2,73E-02
cg15359163	5	122429178	PRDM6	Body	-0,007	9,74E-05	2,73E-02
cg15844711	11	59435407	PATL1	Body	0,032	9,76E-05	2,73E-02
cg04665858	14	23297747	MRPL52	TSS1500	0,006	9,75E-05	2,73E-02
cg21759745	17	28672726		IGR	0,006	9,75E-05	2,73E-02
cg11041823	19	13884754	MRI1	3'UTR	0,02	9,75E-05	2,73E-02
cg25961432	19	48497784	ELSPBP1	TSS200	0,015	9,75E-05	2,73E-02
cg00159508	8	10556593	C8orf74	Body	-0,017	9,77E-05	2,74E-02
cg10219850	1	215373449	KCNK2	Body	0,054	9,78E-05	2,74E-02
cg01402764	8	138637966		IGR	-0,053	9,79E-05	2,74E-02
cg00376654	16	20360497	UMOD	Body	-0,049	9,79E-05	2,74E-02
cg06963151	4	108205101		IGR	0,047	9,80E-05	2,74E-02
cg24321686	5	37211931	C5orf42	Body	0,004	9,81E-05	2,74E-02
cg24890736	12	19549726		IGR	0,013	9,81E-05	2,74E-02
cg20796298	15	65687852	IGDCC4	Body	0,026	9,82E-05	2,74E-02
cg26228280	1	12514410	VPS13D	Body	0,012	9,88E-05	2,74E-02
cg16135795	2	275501	ACP1	Body	0,029	9,90E-05	2,74E-02
cg12387232	2	38763935		IGR	0,014	9,84E-05	2,74E-02
cg22989064	2	47004754		IGR	0,008	9,91E-05	2,74E-02
cg00996758	3	137486536		IGR	-0,014	9,87E-05	2,74E-02
cg11999525	3	138394199	PIK3CB	Body	-0,012	9,90E-05	2,74E-02
cg15430995	3	139008912		IGR	0,034	9,91E-05	2,74E-02
cg03537962	5	35219252	PRLR	5'UTR	-0,018	9,93E-05	2,74E-02
cg14253517	5	140810726	PCDHGA4	Body	-0,05	9,91E-05	2,74E-02
cg13629358	5	160112456	ATP10B	Body	0,036	9,93E-05	2,74E-02
cg21119165	6	56221436	RNU6-71P	Body	0,027	9,90E-05	2,74E-02
cg05410490	6	56825978	BEND6	5'UTR	0,043	9,93E-05	2,74E-02
cg15540764	7	36919658	ELMO1	Body	-0,012	9,86E-05	2,74E-02
cg19624555	7	95850447	MIR591	TSS1500	0,005	9,93E-05	2,74E-02
cg20618615	10	572067	DIP2C	Body	-0,007	9,84E-05	2,74E-02
cg07964275	10	11574278	USP6NL	1stExon	-0,045	9,90E-05	2,74E-02
cg16196602	11	65819332	SF3B2	TSS1500	0,008	9,92E-05	2,74E-02
cg12032489	11	66443637	RBM4B	Body	0,014	9,92E-05	2,74E-02
cg27590469	11	73007099	P2RY6	5'UTR	-0,005	9,89E-05	2,74E-02
cg16879115	12	7819180	APOBEC1	TSS1500	0,03	9,93E-05	2,74E-02
cg22151644	12	54444047	HOXC4	5'UTR	-0,03	9,88E-05	2,74E-02
cg02365078	12	72234032	TBC1D15	Body	0,007	9,86E-05	2,74E-02
cg01413632	12	76078445		IGR	-0,036	9,93E-05	2,74E-02
cg27638036	16	85624376		IGR	-0,008	9,85E-05	2,74E-02
cg19156170	17	70131300		IGR	0,048	9,84E-05	2,74E-02
cg26397500	18	33764857		IGR	0,007	9,89E-05	2,74E-02
cg11261509	18	56168083	ALPK2	Body	-0,019	9,90E-05	2,74E-02
cg09079395	19	50382406	TBC1D17	Body	0,006	9,86E-05	2,74E-02
cg17136126	19	57106501	ZNF71	TSS200	0,005	9,86E-05	2,74E-02
cg24834846	9	86264981		IGR	0,018	9,96E-05	2,75E-02
cg23357854	16	67283054	SLC9A5	1stExon	0,01	9,97E-05	2,75E-02
cg06023262	1	85230276		IGR	0,037	9,98E-05	2,75E-02
cg02628561	17	18061605	MYO15A	Body	0,008	9,98E-05	2,75E-02
cg22989941	1	154492645	TDRD10	Body	0,023	1,00E-04	2,76E-02
cg22425183	1	236372060	GPR137B	3'UTR	-0,01	1,00E-04	2,76E-02
cg12894947	4	6384908	PPP2R2C	Body	-0,018	1,00E-04	2,76E-02
cg23306829	3	111393685	PLCXD2	5'UTR	0,004	1,00E-04	2,76E-02
cg15069545	3	112710020	GTPBP8	1stExon	0,004	1,00E-04	2,76E-02
cg08627552	2	75746502	EVA1A	5'UTR	-0,011	1,00E-04	2,76E-02
cg18614139	14	83692140		IGR	-0,017	1,00E-04	2,76E-02
cg05062820	16	1098546		IGR	0,018	1,00E-04	2,76E-02
cg25550088	5	131573526		IGR	-0,01	1,00E-04	2,76E-02
cg06912197	6	159164511	SYTL3	Body	0,006	1,00E-04	2,76E-02
cg02525975	6	109819236	AKD1	Body	0,007	1,01E-04	2,76E-02

cg06401114	19	19627257	NDUFA13	Body	0,004	1,01E-04	2,76E-02
cg24849974	1	12031504	PLOD1	Body	-0,013	1,01E-04	2,76E-02
cg21924413	1	27272402	NUDC	Body	-0,01	1,01E-04	2,77E-02
cg20970152	1	186970760		IGR	0,033	1,01E-04	2,77E-02
cg07964553	4	113435544	NEUROG2	3'UTR	-0,011	1,01E-04	2,77E-02
cg16277096	7	134883132	WDR91	Body	0,006	1,01E-04	2,77E-02
cg18440480	8	110589698	SYBU	Body	-0,037	1,01E-04	2,77E-02
cg22812892	10	94333586	IDE	Body	0,005	1,01E-04	2,77E-02
cg27471384	11	28723777		IGR	0,034	1,01E-04	2,77E-02
cg08041992	21	33449118		IGR	0,04	1,01E-04	2,77E-02
cg00507823	16	71894108	ZNF821	Body	-0,011	1,01E-04	2,77E-02
cg09656364	6	69344263	ADGRB3	TSS1500	-0,021	1,01E-04	2,77E-02
cg14715518	15	69849751		IGR	-0,005	1,01E-04	2,77E-02
cg27401972	19	48958867	KCNJ14	TSS200	-0,01	1,01E-04	2,77E-02
cg15325982	15	77703520	PEAK1	5'UTR	0,024	1,02E-04	2,77E-02
cg25684142	3	27526881	SLC4A7	TSS1500	0,006	1,02E-04	2,77E-02
cg15018282	6	30181877	TRIM26	TSS1500	0,005	1,02E-04	2,77E-02
cg13916740	19	56904997	ZNF582	TSS200	0,005	1,02E-04	2,77E-02
cg01441988	22	45598886	C22orf9	Body	-0,015	1,02E-04	2,77E-02
cg06653784	3	49830623	CDHR4	Body	-0,013	1,02E-04	2,78E-02
cg14058180	1	40981224	EXO5	Body	0,008	1,02E-04	2,78E-02
cg12224388	2	545599		IGR	0,033	1,02E-04	2,78E-02
cg01989857	2	44001018	DYNC2LI1	TSS200	0,013	1,03E-04	2,78E-02
cg07181667	2	163157840	IFIH1	Body	-0,013	1,03E-04	2,78E-02
cg26618274	2	242693781	D2HGDH	Body	0,008	1,03E-04	2,78E-02
cg02520598	5	166406661		IGR	-0,006	1,02E-04	2,78E-02
cg14030158	6	25419323	LRRC16A	Body	-0,009	1,02E-04	2,78E-02
cg04214946	6	36651933	CDKN1A	Body	-0,005	1,02E-04	2,78E-02
cg23906191	7	1053629	C7orf50	Body	-0,016	1,02E-04	2,78E-02
cg20746880	7	139613177	TBXAS1	Body	0,019	1,02E-04	2,78E-02
cg14219752	8	30390375	RBPMS	Body	0,004	1,03E-04	2,78E-02
cg16899648	8	124599865		IGR	0,02	1,02E-04	2,78E-02
cg08421807	10	49867895		IGR	0,079	1,02E-04	2,78E-02
cg13497506	11	34127040	NAT10	TSS200	0,004	1,03E-04	2,78E-02
cg23615572	11	64086020	PRDX5	Body	-0,01	1,02E-04	2,78E-02
cg11727592	13	46916922	C13orf18	3'UTR	0,038	1,02E-04	2,78E-02
cg01768653	13	59310104		IGR	-0,009	1,02E-04	2,78E-02
cg21813383	15	33014021	GREM1	5'UTR	0,008	1,02E-04	2,78E-02
cg04413904	16	1088479		IGR	0,008	1,02E-04	2,78E-02
cg18478750	19	46057298	OPA3	Body	0,006	1,02E-04	2,78E-02
cg13853156	20	44441522	UBE2C	1stExon	0,007	1,02E-04	2,78E-02
cg21251385	20	52277050		IGR	-0,017	1,02E-04	2,78E-02
cg03769809	5	151020337		IGR	-0,01	1,03E-04	2,78E-02
cg15360181	3	143567492	SLC9A9	TSS200	0,045	1,03E-04	2,79E-02
cg10969521	21	36355537	RUNX1	Body	0,043	1,03E-04	2,79E-02
cg09976670	1	5934941	NPHP4	Body	-0,013	1,03E-04	2,79E-02
cg01844642	3	51989764	GPR62	1stExon	-0,028	1,03E-04	2,79E-02
cg09620585	3	175115181	NAALADL2	Body	-0,01	1,03E-04	2,79E-02
cg25142847	6	74075738		IGR	-0,008	1,03E-04	2,79E-02
cg27329780	7	146433739	CNTNAP2	Body	-0,016	1,03E-04	2,79E-02
cg10785054	9	130953221	CIZ1	5'UTR	0,011	1,03E-04	2,79E-02
cg14667005	11	7716535	OVCH2	Body	0,005	1,04E-04	2,79E-02
cg06067394	11	133789110	IGSF9B	Body	-0,078	1,03E-04	2,79E-02
cg22004988	17	31111281	MYO1D	Body	0,009	1,03E-04	2,79E-02
cg19843235	2	121309166		IGR	-0,027	1,04E-04	2,79E-02
cg16802855	16	86371130	LOC732275	Body	-0,058	1,04E-04	2,79E-02
cg19481727	14	107092283	MIR7641-2	TSS1500	-0,059	1,04E-04	2,79E-02
cg06029422	7	100453023	SLC12A9	Body	-0,01	1,04E-04	2,79E-02
cg26654675	8	137274357		IGR	-0,013	1,04E-04	2,79E-02
cg05254609	14	102687845	WDR20	Body	0,034	1,04E-04	2,79E-02
cg05345925	19	51485144	KLK7	Body	-0,022	1,04E-04	2,79E-02

cg13516390	1	6052785	KCNAB2	1stExon	-0,004	1,04E-04	2,79E-02
cg16359521	1	19535871	UBR4	Body	-0,008	1,04E-04	2,79E-02
cg14039463	1	212004169	LPGAT1	TSS200	0,003	1,04E-04	2,79E-02
cg25534294	2	155554931	KCNJ3	TSS200	0,008	1,04E-04	2,79E-02
cg08045042	11	1046454		IGR	0,058	1,04E-04	2,79E-02
cg25800682	19	38958452	RYR1	ExonBnd	-0,015	1,04E-04	2,79E-02
cg19559114	19	15375316	BRD4	Body	-0,011	1,04E-04	2,80E-02
cg04458139	8	92082487	OTUD6B	5'UTR	-0,004	1,04E-04	2,80E-02
cg01448944	6	35442288	TEAD3	3'UTR	-0,015	1,05E-04	2,80E-02
cg20866810	7	6621024	ZDHHC4	Body	0,007	1,05E-04	2,80E-02
cg22935551	19	35550654	)C100128675	Body	-0,004	1,05E-04	2,80E-02
cg04787785	16	19533038	GDE1	1stExon	0,005	1,05E-04	2,80E-02
cg13778336	1	13836752		IGR	-0,039	1,05E-04	2,80E-02
cg01281582	16	4782006	ANKS3	5'UTR	-0,005	1,05E-04	2,80E-02
cg00218999	12	13105149		IGR	0,008	1,05E-04	2,80E-02
cg07990541	17	72744669	SLC9A3R1	TSS200	0,006	1,05E-04	2,80E-02
cg18427905	2	232825958	DIS3L2	TSS1500	0,036	1,05E-04	2,80E-02
cg22097891	4	4405946	D4S234E	Body	-0,034	1,05E-04	2,80E-02
cg17632471	4	77610450	SHROOM3	Body	-0,005	1,05E-04	2,80E-02
cg12176595	7	152557625		IGR	0,015	1,05E-04	2,80E-02
cg13502403	12	1614380		IGR	0,046	1,05E-04	2,80E-02
cg13679619	19	30433015	URI1	TSS200	0,006	1,05E-04	2,80E-02
cg25270315	19	31869455		IGR	0,028	1,05E-04	2,80E-02
cg17879339	5	171199674		IGR	0,007	1,05E-04	2,80E-02
cg19012326	13	109851733	MYO16	Body	-0,024	1,05E-04	2,80E-02
cg25419523	6	24877624	FAM65B	5'UTR	0,011	1,05E-04	2,80E-02
cg17099954	7	128764517		IGR	0,011	1,05E-04	2,80E-02
cg00742738	14	104638843	KIF26A	Body	0,007	1,06E-04	2,81E-02
cg05043461	5	140735027	PCDHGA2	Body	-0,031	1,06E-04	2,81E-02
cg17002746	8	146074332		IGR	-0,006	1,06E-04	2,81E-02
cg11281513	9	34083909		IGR	0,006	1,06E-04	2,81E-02
cg04811887	9	100685180	C9orf156	TSS1500	-0,008	1,06E-04	2,81E-02
cg13576994	10	52220491	SGMS1	5'UTR	0,005	1,06E-04	2,81E-02
cg01032617	10	134789700	LINC01168	Body	-0,025	1,06E-04	2,81E-02
cg09876858	16	85855734		IGR	0,025	1,06E-04	2,81E-02
cg07584959	19	892937	MED16	5'UTR	0,006	1,06E-04	2,81E-02
cg14904295	19	57348529	PEG3	5'UTR	-0,043	1,06E-04	2,81E-02
cg14282721	20	2694093	EBF4	Body	0,027	1,06E-04	2,81E-02
cg12069856	16	89438504	ANKRD11	5'UTR	-0,014	1,06E-04	2,81E-02
cg07135540	5	176056414	EIF4E1B	TSS1500	0,05	1,06E-04	2,81E-02
cg05407582	15	85658860	PDE8A	Body	-0,015	1,06E-04	2,81E-02
cg22927302	3	50304463	SEMA3B	TSS1500	0,035	1,06E-04	2,81E-02
cg14297198	12	124886472	NCOR2	Body	-0,021	1,07E-04	2,82E-02
cg22703162	15	96885418		IGR	0,011	1,07E-04	2,82E-02
cg13416381	17	46090231		IGR	0,05	1,07E-04	2,82E-02
cg26302931	17	54897852	C17orf67	5'UTR	0,03	1,06E-04	2,82E-02
cg26558790	19	11640101	ECSIT	TSS200	-0,005	1,07E-04	2,82E-02
cg12682914	14	59197595		IGR	-0,027	1,07E-04	2,82E-02
cg13124342	22	47074659	GRAMD4	3'UTR	0,007	1,07E-04	2,82E-02
cg12608775	11	8680517	TRIM66	TSS200	-0,007	1,07E-04	2,82E-02
cg19228848	12	49463115	RHEBL1	Body	0,006	1,07E-04	2,82E-02
cg11844110	1	208042623		IGR	0,004	1,07E-04	2,82E-02
cg01502457	16	88934475	PABPN1L	TSS1500	-0,016	1,07E-04	2,82E-02
cg26335130	9	79074092	GCNT1	1stExon	0,009	1,07E-04	2,82E-02
cg17085710	7	98842834		IGR	0,032	1,07E-04	2,82E-02
cg17462978	7	150262075		IGR	0,023	1,07E-04	2,82E-02
cg27610545	12	73005050	TRHDE	Body	-0,027	1,07E-04	2,82E-02
cg09866598	16	4361610		IGR	0,044	1,07E-04	2,82E-02
cg19014295	6	30884928	VARS2	Body	-0,008	1,07E-04	2,83E-02
cg07931364	6	52227098	PAQR8	5'UTR	0,004	1,08E-04	2,83E-02
cg05161791	9	125987802	STRBP	Body	0,023	1,08E-04	2,83E-02

cg15825501	1	936610	HES4	TSS1500	0,009	1,08E-04	2,83E-02
cg05918682	3	127267038		IGR	0,005	1,08E-04	2,83E-02
cg04291430	17	48206283	SAMD14	5'UTR	-0,02	1,08E-04	2,83E-02
cg24517863	1	233601965		IGR	-0,013	1,08E-04	2,83E-02
cg15140619	3	128597795	ACAD9	TSS1500	0,007	1,08E-04	2,83E-02
cg16681085	6	4080051	FAM217A	TSS1500	0,006	1,08E-04	2,83E-02
cg25107609	10	126831774	CTBP2	5'UTR	-0,016	1,08E-04	2,83E-02
cg17432509	19	48104055		IGR	0,003	1,08E-04	2,83E-02
cg26293776	20	31048090	NOL4L	Body	-0,023	1,08E-04	2,83E-02
cg24968629	22	46770644	CELSR1	Body	0,025	1,08E-04	2,83E-02
cg03690133	11	1483833		IGR	-0,008	1,08E-04	2,84E-02
cg24840515	6	8097577	1E1-BLOC1S5	Body	0,008	1,08E-04	2,84E-02
cg01892727	3	3221728	CRBN	TSS1500	0,036	1,09E-04	2,84E-02
cg14022531	6	157910725	ZDHHC14	Body	-0,029	1,09E-04	2,84E-02
cg21021614	6	159282905		IGR	0,014	1,09E-04	2,84E-02
cg17191733	8	126416814		IGR	-0,016	1,09E-04	2,84E-02
cg12555036	10	127321776		IGR	-0,01	1,09E-04	2,84E-02
cg06014092	15	77924281	LINGO1	Body	0,034	1,09E-04	2,84E-02
cg11475454	17	19648853	ALDH3A1	1stExon	-0,031	1,09E-04	2,84E-02
cg24652994	7	122011261	CADPS2	Body	-0,013	1,09E-04	2,84E-02
cg12656692	6	31860317	EHMT2	Body	-0,006	1,09E-04	2,84E-02
cg07608848	2	1647185	PXDN	Body	0,006	1,09E-04	2,84E-02
cg01529804	6	47755325	OPN5	Body	-0,01	1,09E-04	2,84E-02
cg05623128	1	17318290	ATP13A2	Body	-0,008	1,09E-04	2,84E-02
cg05266155	13	44544993		IGR	-0,015	1,09E-04	2,85E-02
cg11197566	2	16246143		IGR	-0,007	1,09E-04	2,85E-02
cg04830357	5	140718339	PCDHGA1	Body	-0,05	1,09E-04	2,85E-02
cg18396176	19	13323320	CACNA1A	Body	-0,009	1,10E-04	2,85E-02
cg19304600	1	90048595	LRRC8B	Body	-0,013	1,10E-04	2,85E-02
cg06079067	12	101839861		IGR	0,009	1,10E-04	2,85E-02
cg24342013	13	48891544	RB1	Body	-0,045	1,10E-04	2,85E-02
cg23888634	11	62312268	AHNAK	5'UTR	0,023	1,10E-04	2,85E-02
cg27317150	6	110012180	AKD1	5'UTR	0,005	1,10E-04	2,85E-02
cg20571213	6	132891373	TAAR6	TSS200	-0,017	1,10E-04	2,85E-02
cg17892401	7	32529540	LSM5	Body	-0,005	1,10E-04	2,85E-02
cg23970570	8	119494183	SAMD12	Body	-0,009	1,10E-04	2,85E-02
cg24939483	10	31360234		IGR	0,009	1,10E-04	2,85E-02
cg13456197	16	86892396		IGR	-0,012	1,10E-04	2,85E-02
cg07833382	17	55055134	SCPEP1	TSS1500	0,003	1,10E-04	2,86E-02
cg21112259	12	53567307	CSAD	5'UTR	-0,011	1,10E-04	2,86E-02
cg08564027	20	61660810		IGR	-0,125	1,10E-04	2,86E-02
cg03058016	7	133981201	SLC35B4	ExonBnd	-0,008	1,11E-04	2,86E-02
cg16538471	7	20153546		IGR	0,034	1,11E-04	2,86E-02
cg22382455	15	42787814	SNAP23	TSS200	0,004	1,11E-04	2,86E-02
cg17253842	4	149347991	NR3C2	Body	-0,011	1,11E-04	2,86E-02
cg03217312	4	140713308	MAML3	Body	-0,014	1,11E-04	2,86E-02
cg03029595	17	12084204		IGR	-0,038	1,11E-04	2,86E-02
cg03180011	1	16955678	CROCCP2	Body	-0,034	1,11E-04	2,87E-02
cg04354317	1	165667545	ALDH9A1	Body	0,003	1,11E-04	2,87E-02
cg00678912	2	214106614		IGR	0,011	1,11E-04	2,87E-02
cg14641600	5	133483170	TCF7	3'UTR	-0,013	1,11E-04	2,87E-02
cg09632273	6	31696229	DDAH2	Body	0,009	1,11E-04	2,87E-02
cg12827134	7	4869581	RADIL	Body	0,015	1,11E-04	2,87E-02
cg06569139	8	101117949	RGS22	Body	0,014	1,11E-04	2,87E-02
cg24811740	8	145729537	GPT	5'UTR	-0,018	1,11E-04	2,87E-02
cg23324048	14	60794590		IGR	-0,003	1,11E-04	2,87E-02
cg18018581	15	68158422		IGR	0,007	1,11E-04	2,87E-02
cg23524184	17	48129754		IGR	0,004	1,11E-04	2,87E-02
cg20961045	17	55952128	CUEDC1	Body	0,014	1,11E-04	2,87E-02
cg02928184	22	47714258		IGR	0,063	1,11E-04	2,87E-02
cg20738735	5	13810195	DNAH5	Body	-0,047	1,11E-04	2,87E-02

cg23374270	7	2014604	MAD1L1	Body	-0,009	1,12E-04	2,87E-02
cg04906455	12	74931262	ATXN7L3B	TSS1500	-0,018	1,12E-04	2,87E-02
cg21285431	10	79789707	POLR3A	TSS1500	-0,011	1,12E-04	2,87E-02
cg25877869	15	58471936	AQP9	Body	0,053	1,12E-04	2,87E-02
cg03119731	19	54494658	CACNG6	TSS1500	0,014	1,12E-04	2,87E-02
cg27213600	5	114881367	FEM1C	TSS1500	-0,009	1,12E-04	2,87E-02
cg27099481	10	95361420	RBP4	TSS1500	0,027	1,12E-04	2,87E-02
cg26672952	5	34497525		IGR	0,028	1,12E-04	2,87E-02
cg15761414	19	35801014	MAG	Body	-0,023	1,12E-04	2,87E-02
cg08358683	5	43602380	NNT	TSS1500	0,012	1,12E-04	2,87E-02
cg19505296	11	82890671	PCF11	Body	0,009	1,12E-04	2,88E-02
cg16983759	18	77794750	C18orf22	Body	0,004	1,12E-04	2,88E-02
cg05462927	19	56660119	ZNF444	Body	0,007	1,12E-04	2,88E-02
cg27475074	6	28304640	ZSCAN31	TSS1500	-0,012	1,13E-04	2,88E-02
cg04591119	6	111197245	AMD1	5'UTR	0,006	1,13E-04	2,88E-02
cg21961270	1	3307177	PRDM16	Body	-0,012	1,13E-04	2,88E-02
cg14195027	1	21901685	ALPL	Body	0,038	1,13E-04	2,88E-02
cg00044354	1	67389884	MIER1	TSS1500	0,063	1,13E-04	2,88E-02
cg13562207	16	47887821	)C100507534	Body	0,051	1,13E-04	2,88E-02
cg16454099	7	92818323	HEPACAM2	3'UTR	-0,105	1,13E-04	2,88E-02
cg09418612	17	46019174	PNPO	1stExon	0,013	1,13E-04	2,88E-02
cg14663509	11	65592035		IGR	-0,007	1,13E-04	2,88E-02
cg02192677	1	3555737	WDR8	Body	-0,01	1,13E-04	2,88E-02
cg01928893	1	35826678	ZMYM4	Body	-0,005	1,13E-04	2,88E-02
cg27321949	4	3516692	LRPAP1	Body	-0,042	1,13E-04	2,88E-02
cg14699490	5	138949312	UBE2D2	5'UTR	-0,018	1,13E-04	2,88E-02
cg21436431	7	994059	ADAP1	1stExon	0,004	1,13E-04	2,88E-02
cg27159967	9	136539100	SARDH	Body	-0,023	1,13E-04	2,88E-02
cg04392073	1	60311151	HOOK1	Body	-0,008	1,13E-04	2,89E-02
cg06468863	9	139923554	C9orf139	5'UTR	0,007	1,13E-04	2,89E-02
cg08498383	2	198312034		IGR	-0,01	1,14E-04	2,89E-02
cg11800844	5	143469185		IGR	0,036	1,14E-04	2,89E-02
cg00598226	17	10099688	GAS7	Body	0,033	1,14E-04	2,89E-02
cg07931960	18	44098826	LOXHD1	Body	-0,025	1,14E-04	2,89E-02
cg03454456	19	2338773	SPPL2B	Body	-0,013	1,14E-04	2,89E-02
cg15677883	1	20658462	VWA5B1	Body	-0,006	1,14E-04	2,89E-02
cg22978941	12	109535460	UNG	5'UTR	0,005	1,14E-04	2,89E-02
cg22715945	9	26956769	IFT74	5'UTR	-0,007	1,14E-04	2,90E-02
cg10807643	14	55907417	TBPL2	TSS200	-0,047	1,14E-04	2,90E-02
cg10453998	20	6122280		IGR	0,009	1,14E-04	2,90E-02
cg08353146	1	205196756	TMCC2	TSS1500	-0,005	1,14E-04	2,90E-02
cg13365340	6	33245342	B3GALT4	1stExon	0,009	1,14E-04	2,90E-02
cg13365431	7	150767768	SLC4A2	Body	-0,015	1,14E-04	2,90E-02
cg14255089	10	180755	ZMYND11	5'UTR	0,004	1,14E-04	2,90E-02
cg16822853	11	47911523		IGR	-0,026	1,14E-04	2,90E-02
cg06751597	15	42788175	SNAP23	5'UTR	-0,013	1,14E-04	2,90E-02
cg23056979	6	30034518	PPP1R11	TSS1500	-0,015	1,15E-04	2,90E-02
cg27434984	4	147579200		IGR	0,035	1,15E-04	2,90E-02
cg01555907	9	32571504	NDUFB6	Body	-0,01	1,15E-04	2,90E-02
cg24586121	2	220377759	ACCN4	TSS1500	0,014	1,15E-04	2,90E-02
cg18044415	8	56013536	XKR4	TSS1500	-0,064	1,15E-04	2,90E-02
cg04684026	15	73867923	NPTN	Body	0,03	1,15E-04	2,90E-02
cg23359561	10	125824014	CHST15	5'UTR	0,046	1,15E-04	2,91E-02
cg22062068	11	7110074	RBMXL2	TSS200	-0,07	1,15E-04	2,91E-02
cg19198472	17	18136333	LLGL1	Body	-0,02	1,15E-04	2,91E-02
cg05298361	1	43523975		IGR	-0,013	1,15E-04	2,91E-02
cg01912547	20	33516613	GSS	3'UTR	-0,018	1,15E-04	2,91E-02
cg21409829	1	2850859		IGR	-0,01	1,16E-04	2,91E-02
cg01341891	1	78147727	ZZZ3	5'UTR	-0,011	1,16E-04	2,91E-02
cg21825743	2	208626879		IGR	0,004	1,16E-04	2,91E-02
cg00175579	6	33272215	TAPBP	Body	-0,009	1,16E-04	2,91E-02

cg07474324	16	28227095		IGR	-0,011	1,16E-04	2,91E-02
cg15282999	20	60124848	CDH4	Body	-0,006	1,16E-04	2,91E-02
cg17240760	1	111417994	CD53	5'UTR	-0,012	1,16E-04	2,91E-02
cg23409168	8	117101690		IGR	0,028	1,16E-04	2,91E-02
cg13216616	15	57037716		IGR	0,01	1,16E-04	2,91E-02
cg10605687	7	82764087	PCLO	Body	-0,024	1,16E-04	2,91E-02
cg03718241	6	3623988		IGR	0,032	1,16E-04	2,92E-02
cg19428160	11	17761718	KCNC1	Body	0,032	1,16E-04	2,92E-02
cg22302082	3	20081355	KAT2B	TSS200	0,011	1,16E-04	2,92E-02
cg07802710	5	140810260	PCDHGA4	Body	-0,028	1,16E-04	2,92E-02
cg27658314	7	157968629	PTPRN2	Body	-0,02	1,16E-04	2,92E-02
cg12018242	13	42711681	DGKH	TSS1500	0,013	1,16E-04	2,92E-02
cg11012837	20	60877805	ADRM1	TSS200	0,003	1,16E-04	2,92E-02
cg04501964	21	44161572	PDE9A	5'UTR	0,006	1,16E-04	2,92E-02
cg18521305	8	142316861		IGR	-0,01	1,17E-04	2,92E-02
cg03105348	10	50599620	DRGX	Body	0,039	1,17E-04	2,92E-02
cg08524179	10	3036185		IGR	0,007	1,17E-04	2,93E-02
cg01795810	5	151327739		IGR	0,044	1,17E-04	2,93E-02
cg20778660	6	62391103	KHDRBS2	Body	-0,014	1,17E-04	2,93E-02
cg14488288	6	169632788	THBS2	Body	-0,017	1,17E-04	2,93E-02
cg17953577	17	11501503	DNAH9	TSS1500	-0,013	1,17E-04	2,93E-02
cg12344206	19	58865401	A1BG-AS1	Body	-0,009	1,17E-04	2,93E-02
cg25127649	1	14075789	PRDM2	TSS200	0,005	1,17E-04	2,93E-02
cg06792358	6	24126324	NRSN1	TSS200	-0,006	1,17E-04	2,93E-02
cg25325125	10	71217644	TSPAN15	Body	0,008	1,17E-04	2,93E-02
cg10230831	13	35487202		IGR	-0,048	1,17E-04	2,93E-02
cg23195510	16	67678593	RLTPR	TSS1500	0,015	1,17E-04	2,93E-02
cg15041373	19	39577941	PAPL	Body	0,008	1,18E-04	2,93E-02
cg25996826	22	35743547	TOM1	3'UTR	-0,013	1,18E-04	2,93E-02
cg26719638	8	132056802		IGR	0,013	1,18E-04	2,93E-02
cg26485890	9	16869624	BNC2	Body	-0,02	1,18E-04	2,93E-02
cg16013727	1	232795714		IGR	0,017	1,18E-04	2,93E-02
cg08216099	2	1725761	PXDN	Body	0,037	1,18E-04	2,93E-02
cg23035058	10	133982861	JAKMIP3	3'UTR	-0,01	1,18E-04	2,93E-02
cg13035571	1	31997057		IGR	-0,015	1,18E-04	2,93E-02
cg05719742	10	77032059		IGR	-0,015	1,18E-04	2,94E-02
cg00911446	1	7520079	CAMTA1	Body	-0,013	1,18E-04	2,94E-02
cg21552104	8	9189178	LOC157273	Body	0,055	1,18E-04	2,94E-02
cg24385632	11	16838615	PLEKHA7	Body	-0,007	1,18E-04	2,94E-02
cg01645869	6	20210600	MBOAT1	Body	0,044	1,18E-04	2,94E-02
cg03148581	5	178396216		IGR	0,044	1,18E-04	2,94E-02
cg08970457	8	22526705	BIN3	TSS200	-0,006	1,18E-04	2,94E-02
cg21896553	8	22051581	BMP1	ExonBnd	-0,009	1,18E-04	2,94E-02
cg25572910	2	179319144	DFNB59	Body	-0,009	1,18E-04	2,94E-02
cg23346801	6	31915104	CFB	Body	0,027	1,19E-04	2,94E-02
cg01031587	1	110032394	ATXN7L2	Body	-0,007	1,19E-04	2,94E-02
cg20940853	12	57031651	BAZ2A	TSS1500	-0,039	1,19E-04	2,94E-02
cg08761885	19	57998848	ZNF419	TSS1500	0,039	1,19E-04	2,94E-02
cg12142346	2	3488593		IGR	-0,008	1,19E-04	2,94E-02
cg14450725	5	43020451		IGR	-0,006	1,19E-04	2,94E-02
cg02326566	5	140220686	PCDHA6	Body	-0,069	1,19E-04	2,94E-02
cg08757757	8	62200323	CLVS1	TSS1500	0,058	1,19E-04	2,94E-02
cg04622775	15	89442186	MFGE8	3'UTR	-0,007	1,19E-04	2,94E-02
cg18199495	1	19655472	PQLC2	3'UTR	-0,009	1,19E-04	2,94E-02
cg19615148	2	9645396	ADAM17	Body	0,009	1,19E-04	2,94E-02
cg06798115	3	45241685		IGR	0,051	1,19E-04	2,94E-02
cg04757012	6	33359817	KIFC1	Body	-0,003	1,19E-04	2,94E-02
cg22030047	11	8954541	C11orf16	1stExon	-0,02	1,19E-04	2,94E-02
cg21668988	14	64970665	ZBTB1	TSS1500	0,007	1,19E-04	2,95E-02
cg24139916	17	62222907	SNORD104	TSS1500	0,009	1,19E-04	2,95E-02
cg17265849	16	50340409	ADCY7	Body	-0,014	1,19E-04	2,95E-02

cg26602759	8	42015331	AP3M2	Body	0,013	1,20E-04	2,95E-02
cg07795931	12	119697414		IGR	0,006	1,19E-04	2,95E-02
cg17709391	13	101480751	NALCN-AS1	Body	-0,043	1,20E-04	2,95E-02
cg08855133	1	42404614	HIVEP3	Body	0,071	1,20E-04	2,95E-02
cg03915638	3	62356042	FEZF2	Body	0,005	1,20E-04	2,95E-02
cg23215600	3	134356086	KY	Body	0,012	1,20E-04	2,95E-02
cg14374463	15	81590933	IL16	5'UTR	0,029	1,20E-04	2,95E-02
cg27059537	7	13662497		IGR	-0,016	1,20E-04	2,95E-02
cg22175943	7	80329687		IGR	-0,012	1,20E-04	2,95E-02
cg02919030	12	88973461	KITLG	Body	-0,008	1,20E-04	2,95E-02
cg04361749	2	240980455		IGR	0,037	1,21E-04	2,96E-02
cg00480745	10	103578813	KCNIP2-AS1	TSS200	-0,003	1,20E-04	2,96E-02
cg27260462	10	131935477	GLRX3	Body	-0,013	1,20E-04	2,96E-02
cg11247438	13	106127569	DAOA-AS1	Body	0,039	1,20E-04	2,96E-02
cg05343105	16	29801371	KIF22	TSS1500	0,028	1,21E-04	2,96E-02
cg04197904	22	46318272	WNT7B	3'UTR	0,029	1,20E-04	2,96E-02
cg03631837	1	2237200	SKI	Body	-0,009	1,21E-04	2,96E-02
cg22029856	4	2276003	ZFYVE28	Body	-0,005	1,21E-04	2,96E-02
cg16160065	4	157493794		IGR	0,012	1,21E-04	2,96E-02
cg05500783	6	32410873	HLA-DRA	Body	0,029	1,21E-04	2,96E-02
cg23260840	7	80704698		IGR	-0,007	1,21E-04	2,96E-02
cg25678095	8	41833374	KAT6A	Body	-0,01	1,21E-04	2,96E-02
cg19115827	18	48649738		IGR	-0,023	1,21E-04	2,96E-02
cg10147942	20	5870450		IGR	-0,016	1,21E-04	2,96E-02
cg23598089	1	203652079	ATP2B4	5'UTR	0,027	1,21E-04	2,96E-02
cg08693344	16	56691562	MT1F	TSS1500	0,015	1,21E-04	2,96E-02
cg00804525	16	68943500	TMCO7	Body	-0,033	1,21E-04	2,96E-02
cg22795764	2	242138778	ANO7	Body	-0,013	1,21E-04	2,96E-02
cg02563625	4	165674997	LINC01207	TSS1500	0,021	1,21E-04	2,96E-02
cg27049372	5	175174026		IGR	0,047	1,21E-04	2,96E-02
cg19800435	7	27780026	TAX1BP1	5'UTR	-0,004	1,21E-04	2,96E-02
cg25875163	8	143763340	PSCA	Body	-0,015	1,21E-04	2,96E-02
cg14646676	10	32197260	ARHGAP12	1stExon	0,006	1,21E-04	2,96E-02
cg00953825	17	60757134	MRC2	Body	-0,012	1,21E-04	2,96E-02
cg25942111	9	36136796	GLIPR2	5'UTR	0,004	1,21E-04	2,96E-02
cg21533830	11	117301482	DSCAML1	Body	-0,009	1,21E-04	2,96E-02
cg16514063	1	223989873	TP53BP2	Body	-0,009	1,22E-04	2,96E-02
cg24197888	9	94186556	NFIL3	TSS1500	0,003	1,22E-04	2,96E-02
cg03090734	10	44525757		IGR	0,026	1,22E-04	2,96E-02
cg15843573	11	46375149	DGKZ	Body	-0,014	1,22E-04	2,96E-02
cg09839093	11	60244624		IGR	0,013	1,22E-04	2,96E-02
cg04864990	6	32168694	NOTCH4	Body	0,014	1,22E-04	2,96E-02
cg27503832	1	1301316		IGR	-0,025	1,22E-04	2,97E-02
cg22760420	6	44281348	AARS2	TSS1500	0,025	1,22E-04	2,97E-02
cg23048882	11	105480520	GRIA4	TSS1500	-0,029	1,22E-04	2,97E-02
cg03872502	16	1756007	MAPK8IP3	TSS1500	0,004	1,22E-04	2,97E-02
cg14398859	18	57455316		IGR	0,046	1,22E-04	2,97E-02
cg18929405	10	101105957	CNNM1	Body	0,005	1,22E-04	2,97E-02
cg05756446	1	65209545	RAVER2	TSS1500	0,031	1,23E-04	2,97E-02
cg09891761	1	169555674	F5	5'UTR	0,003	1,23E-04	2,97E-02
cg06370528	18	34972877	BRUNOL4	Body	0,058	1,23E-04	2,97E-02
cg14444103	4	147367017	SLC10A7	Body	-0,008	1,23E-04	2,97E-02
cg03885197	5	133353722		IGR	0,013	1,23E-04	2,97E-02
cg08274139	14	31484468	STRN3	Body	0,004	1,23E-04	2,98E-02
cg07697177	1	2125182	C1orf86	Body	-0,01	1,23E-04	2,98E-02
cg20291311	3	118891705	UPK1B	TSS1500	0,006	1,23E-04	2,98E-02
cg22146644	6	155184017		IGR	0,021	1,23E-04	2,98E-02
cg01784909	11	31269140		IGR	0,008	1,23E-04	2,98E-02
cg14998377	15	77961394	LINGO1	5'UTR	-0,019	1,23E-04	2,98E-02
cg16584092	5	180670374	GNB2L1	Body	-0,004	1,23E-04	2,98E-02
cg23677882	7	157281167		IGR	-0,015	1,23E-04	2,98E-02

cg15551981	8	28552181		IGR	0,044	1,23E-04	2,98E-02
cg13534450	8	75897297	CRISPLD1	5'UTR	-0,018	1,23E-04	2,98E-02
cg15671083	10	5406959	UCN3	TSS200	0,031	1,23E-04	2,98E-02
cg05376935	10	97748211	ENTPD1-AS1	Body	-0,012	1,23E-04	2,98E-02
cg09562240	19	44100382	ZNF576	TSS200	0,006	1,23E-04	2,98E-02
cg12385722	20	44539531	PLTP	5'UTR	-0,008	1,24E-04	2,98E-02
cg17244801	1	10758237	CASZ1	5'UTR	0,008	1,24E-04	2,98E-02
cg06678594	2	43573198	THADA	Body	-0,009	1,24E-04	2,98E-02
cg08687163	3	49940853	MST1R	1stExon	-0,021	1,24E-04	2,98E-02
cg00139683	20	896564	ANGPT4	1stExon	-0,015	1,24E-04	2,98E-02
cg19182014	20	33422404	HMGB3L1	TSS200	-0,013	1,24E-04	2,98E-02
cg26433368	1	18958594	PAX7	Body	0,016	1,24E-04	2,99E-02
cg26656139	12	124973211	NCOR2	Body	-0,022	1,24E-04	2,99E-02
cg14584359	16	76181877		IGR	0,037	1,24E-04	2,99E-02
cg07615087	21	38077347	SIM2	Body	0,014	1,24E-04	2,99E-02
cg07088388	11	109816936		IGR	-0,014	1,24E-04	2,99E-02
cg11945469	1	203013128	PPFIA4	Body	-0,008	1,24E-04	2,99E-02
cg11495719	11	124185113		IGR	0,021	1,24E-04	2,99E-02
cg04279396	1	227932553	SNAP47	Body	-0,014	1,24E-04	2,99E-02
cg07007773	12	52314610	ACVRL1	Body	-0,004	1,25E-04	2,99E-02
cg27313662	4	106395321	PPA2	TSS200	0,003	1,25E-04	2,99E-02
cg16192428	5	148067186		IGR	0,028	1,25E-04	2,99E-02
cg24630383	2	9537642	ASAP2	Body	0,009	1,25E-04	3,00E-02
cg01324745	1	45194991		IGR	-0,004	1,25E-04	3,00E-02
cg03470837	9	101251353	GABBR2	Body	-0,023	1,25E-04	3,00E-02
cg10655371	7	91749682	CYP51A1	Body	-0,026	1,26E-04	3,01E-02
cg21420674	1	2713926		IGR	0,03	1,26E-04	3,01E-02
cg13507492	2	74431332	MTHFD2	Body	-0,006	1,26E-04	3,01E-02
cg18371469	3	40954597		IGR	0,01	1,26E-04	3,01E-02
cg11085324	4	16901215	LDB2	TSS1500	-0,023	1,26E-04	3,01E-02
cg10214622	6	3321565	SLC22A23	Body	-0,032	1,26E-04	3,01E-02
cg04957628	6	137242738	SLC35D3	TSS1500	-0,006	1,26E-04	3,01E-02
cg03207121	7	1576174	MAFK	5'UTR	-0,018	1,26E-04	3,01E-02
cg17208577	11	66139335	SLC29A2	TSS200	0,006	1,26E-04	3,01E-02
cg05294593	11	66570615	C11orf80	Body	-0,013	1,26E-04	3,01E-02
cg05244948	11	118629624	DDX6	ExonBnd	-0,008	1,26E-04	3,01E-02
cg23979954	12	25801601	IFLTD1	TSS200	0,038	1,26E-04	3,01E-02
cg20099181	13	40646643		IGR	0,013	1,26E-04	3,01E-02
cg02687744	13	101132641	PCCA-AS1	Body	-0,007	1,26E-04	3,01E-02
cg12485783	17	46226930	SKAP1	Body	-0,024	1,26E-04	3,01E-02
cg08110485	20	55306190		IGR	0,048	1,26E-04	3,01E-02
cg03043822	22	25160098	TOP1P2	TSS1500	-0,07	1,26E-04	3,01E-02
cg03218909	1	156589008	HAPLN2	TSS200	0,008	1,26E-04	3,01E-02
cg01314544	2	23851841	KLHL29	Body	-0,055	1,27E-04	3,01E-02
cg25799994	2	166650699	GALNT3	5'UTR	0,004	1,26E-04	3,01E-02
cg00619363	6	113754685		IGR	0,018	1,27E-04	3,01E-02
cg03425163	13	24671500	SPATA13	Body	0,065	1,27E-04	3,01E-02
cg09497752	16	2986767	FLYWCH1	Body	-0,011	1,27E-04	3,01E-02
cg17417021	17	2942315		IGR	0,02	1,27E-04	3,01E-02
cg07876904	17	81049242	METRNL	Body	-0,007	1,27E-04	3,02E-02
cg10982668	7	30773423		IGR	-0,006	1,27E-04	3,02E-02
cg25382900	1	12148326	TNFRSF8	Body	0,042	1,27E-04	3,02E-02
cg20808089	6	11360140	NEDD9	5'UTR	-0,026	1,27E-04	3,02E-02
cg18922450	7	116192250	CAV1	Body	0,008	1,27E-04	3,02E-02
cg14026072	8	74331668	STAU2-AS1	TSS1500	-0,011	1,27E-04	3,02E-02
cg06093703	13	94979628	GPC6	Body	0,06	1,27E-04	3,02E-02
cg09262993	18	66861924		IGR	0,022	1,27E-04	3,02E-02
cg06780308	1	39265808		IGR	0,041	1,27E-04	3,02E-02
cg04222159	1	204981786	NFASC	Body	0,015	1,27E-04	3,02E-02
cg16999495	10	85995762	LRIT1	Body	0,008	1,27E-04	3,02E-02
cg01400476	6	18387809	RNF144B	1stExon	0,005	1,27E-04	3,02E-02

cg10234282	1	207975342	MIR29C	TSS200	0,009	1,28E-04	3,02E-02
cg21611868	3	135969116	PCCB	TSS200	0,004	1,28E-04	3,02E-02
cg03264349	11	45743877	)C100507384	Body	-0,051	1,28E-04	3,02E-02
cg10454268	11	59522542	STX3	TSS1500	0,005	1,28E-04	3,02E-02
cg07056155	14	90921668		IGR	-0,007	1,28E-04	3,02E-02
cg12153321	20	20678596	RALGAPA2	Body	0,005	1,28E-04	3,02E-02
cg11671946	7	141402884	KIAA1147	TSS1500	0,005	1,28E-04	3,02E-02
cg00688636	8	34032305		IGR	-0,039	1,28E-04	3,02E-02
cg13625370	9	139821646		IGR	-0,008	1,28E-04	3,02E-02
cg15635472	12	113659329	TPCN1	1stExon	0,013	1,28E-04	3,02E-02
cg12433713	10	131770585		IGR	-0,017	1,28E-04	3,03E-02
cg25315625	6	148700796	SASH1	Body	-0,012	1,28E-04	3,03E-02
cg09148021	1	78470417	DNAJB4	TSS1500	-0,027	1,28E-04	3,03E-02
cg11743336	3	23753985		IGR	0,012	1,28E-04	3,03E-02
cg14869487	5	1730143		IGR	-0,016	1,28E-04	3,03E-02
cg22776386	10	7605126	ITIH5	Body	-0,007	1,28E-04	3,03E-02
cg14290714	1	225966645	SRP9	Body	-0,016	1,29E-04	3,03E-02
cg01763754	5	139060476	CXXC5	Body	-0,011	1,29E-04	3,03E-02
cg12734386	15	76531223	ETFA	Body	-0,009	1,29E-04	3,04E-02
cg27353352	4	146054604		IGR	-0,044	1,29E-04	3,04E-02
cg01842444	10	112515252	RBM20	Body	-0,007	1,29E-04	3,04E-02
cg04304978	10	8809772		IGR	0,038	1,29E-04	3,04E-02
cg26086271	15	41913343		IGR	0,007	1,29E-04	3,04E-02
cg06268921	1	214158573		IGR	-0,01	1,30E-04	3,04E-02
cg05325631	4	113112222		IGR	0,005	1,29E-04	3,04E-02
cg20173654	5	150522585	ANXA6	TSS1500	0,019	1,30E-04	3,04E-02
cg10661376	10	134351180	INPP5A	TSS200	0,005	1,30E-04	3,04E-02
cg27449937	2	188847700		IGR	-0,014	1,30E-04	3,04E-02
cg07739966	3	183171199	LINC00888	Body	-0,026	1,30E-04	3,04E-02
cg14388783	9	103115158	TEX10	5'UTR	0,004	1,30E-04	3,04E-02
cg26751275	12	7033076	ATN1	TSS1500	0,013	1,30E-04	3,04E-02
cg05226048	1	90320315	LRRC8D	5'UTR	-0,004	1,30E-04	3,05E-02
cg13554903	9	133814759	FIBCD1	TSS1500	0,005	1,30E-04	3,05E-02
cg13500705	3	36805136		IGR	-0,046	1,30E-04	3,05E-02
cg17131560	1	64352886	ROR1	Body	0,033	1,30E-04	3,05E-02
cg17186700	1	109204812	HENMT1	TSS1500	-0,009	1,30E-04	3,05E-02
cg02944048	1	205113116	DSTYK	3'UTR	-0,007	1,30E-04	3,05E-02
cg14129735	6	30737007		IGR	-0,012	1,30E-04	3,05E-02
cg04084618	5	36607065	SLC1A3	TSS1500	-0,045	1,31E-04	3,06E-02
cg16043046	6	166205524		IGR	-0,006	1,31E-04	3,06E-02
cg20260034	8	145743952	LRRC14	5'UTR	0,025	1,31E-04	3,06E-02
cg16315060	13	30002471	MTUS2	TSS1500	-0,005	1,31E-04	3,06E-02
cg21465754	15	40986872	RAD51	TSS1500	0,003	1,31E-04	3,06E-02
cg03742890	19	13910592	ZSWIM4	Body	0,006	1,31E-04	3,06E-02
cg16524936	4	1340807	KIAA1530	TSS1500	0,003	1,31E-04	3,06E-02
cg01705575	7	152162042	)C100128822	Body	0,002	1,31E-04	3,06E-02
cg23963802	10	101874887		IGR	0,029	1,31E-04	3,06E-02
cg15939837	17	33020197		IGR	0,045	1,31E-04	3,06E-02
cg04674383	19	54481078	CACNG8	Body	-0,013	1,31E-04	3,06E-02
cg09197672	7	135434050	FAM180A	TSS1500	-0,012	1,32E-04	3,07E-02
cg15815730	19	52491114	ZNF350	TSS1500	0,003	1,32E-04	3,07E-02
cg04722168	1	26324647	PAFAH2	5'UTR	-0,003	1,32E-04	3,07E-02
cg04636128	12	30888017	CAPRIN2	Body	0,006	1,32E-04	3,07E-02
cg16496398	20	60884376	LAMA5	3'UTR	-0,015	1,32E-04	3,08E-02
cg27036456	18	55285747	NARS	Body	0,021	1,32E-04	3,08E-02
cg18576800	19	36430655	LRFN3	Body	-0,009	1,32E-04	3,08E-02
cg24711295	17	18820622	PRPSAP2	Body	0,025	1,33E-04	3,09E-02
cg15647195	16	1577092	IFT140	Body	0,003	1,33E-04	3,09E-02
cg25249613	9	120176056	ASTN2	Body	-0,008	1,33E-04	3,09E-02
cg07383210	12	44229886	TMEM117	TSS200	0,009	1,33E-04	3,09E-02
cg05731560	16	85568318		IGR	-0,027	1,33E-04	3,09E-02

cg16584178	21	22611577	NCAM2	Body	-0,016	1,33E-04	3,09E-02
cg23168251	2	47629743	MSH2	TSS1500	-0,018	1,33E-04	3,10E-02
cg04636069	4	7055967	TADA2B	Body	-0,015	1,33E-04	3,10E-02
cg18136963	6	139013146		IGR	0,182	1,33E-04	3,10E-02
cg14392677	9	139432990	NOTCH1	Body	0,041	1,33E-04	3,10E-02
cg15747747	22	34473573		IGR	0,038	1,34E-04	3,10E-02
cg14699376	5	37382801	WDR70	Body	0,008	1,34E-04	3,10E-02
cg03348792	12	53075482	KRT1	TSS1500	0,024	1,34E-04	3,10E-02
cg07437657	13	114220436		IGR	-0,012	1,34E-04	3,10E-02
cg01083620	3	195601109	TNK2	Body	-0,015	1,34E-04	3,10E-02
cg21868031	1	207925112	CD46	TSS1500	-0,009	1,34E-04	3,11E-02
cg06342490	2	42795193	MTA3	TSS1500	-0,013	1,34E-04	3,11E-02
cg01959980	2	73616832	ALMS1	Body	0,019	1,34E-04	3,11E-02
cg07912416	6	30860130	DDR1	Body	-0,023	1,34E-04	3,11E-02
cg14738823	12	15090958	ERP27	Body	0,031	1,34E-04	3,11E-02
cg07826858	13	46822839	LRRC63	Body	-0,02	1,34E-04	3,11E-02
cg01534677	2	190855319		IGR	0,016	1,34E-04	3,11E-02
cg00886875	3	106354325		IGR	-0,048	1,35E-04	3,11E-02
cg01396065	3	193789440		IGR	0,036	1,35E-04	3,11E-02
cg06047137	11	1936655		IGR	0,015	1,35E-04	3,11E-02
cg25530124	20	48180337	PTGIS	Body	0,011	1,35E-04	3,11E-02
cg03796229	7	122343649	RNF148	TSS1500	-0,012	1,35E-04	3,11E-02
cg02261018	10	89622396	PTEN	TSS1500	0,006	1,35E-04	3,11E-02
cg12060838	1	158028936	KIRREL	Body	-0,014	1,35E-04	3,11E-02
cg07160793	2	382090		IGR	-0,051	1,35E-04	3,11E-02
cg10079538	2	5217722		IGR	-0,026	1,35E-04	3,11E-02
cg10005427	2	158851505	UPP2	TSS200	-0,009	1,35E-04	3,11E-02
cg02943720	12	7036715	ATN1	5'UTR	0,009	1,35E-04	3,11E-02
cg06757796	2	69309365	ANTXR1	Body	0,008	1,35E-04	3,11E-02
cg03378525	7	5530946	FBXL18	Body	-0,008	1,35E-04	3,11E-02
cg03742460	20	60623639	TAF4	Body	-0,017	1,35E-04	3,11E-02
cg27048989	6	150397765		IGR	0,01	1,35E-04	3,12E-02
cg14132895	11	95433641		IGR	0,017	1,35E-04	3,12E-02
cg06005661	12	116973891	LINC00173	Body	0,008	1,36E-04	3,12E-02
cg26830233	15	65823086	PTPLAD1	Body	0,004	1,36E-04	3,12E-02
cg07693639	16	34989237	FLJ26245	Body	-0,026	1,36E-04	3,12E-02
cg01032946	16	30991033	SETD1A	Body	-0,007	1,36E-04	3,12E-02
cg10888923	9	137900384		IGR	-0,011	1,36E-04	3,13E-02
cg06853489	1	73531614		IGR	-0,076	1,36E-04	3,13E-02
cg19546795	5	117930623	)C102467225	TSS1500	-0,007	1,36E-04	3,13E-02
cg08067438	13	80054987	NDFIP2	TSS1500	-0,031	1,36E-04	3,13E-02
cg03774678	3	57134296	IL17RD	Body	-0,011	1,36E-04	3,13E-02
cg09068173	16	67815614	RANBP10	Body	-0,007	1,37E-04	3,13E-02
cg14767165	6	160183909	ACAT2	Body	-0,04	1,37E-04	3,13E-02
cg20951642	2	240509066		IGR	0,04	1,37E-04	3,13E-02
cg02610222	4	147560282	POU4F2	1stExon	-0,022	1,37E-04	3,13E-02
cg20745620	5	172203954		IGR	0,019	1,37E-04	3,13E-02
cg22615992	6	164093099		IGR	0,005	1,37E-04	3,13E-02
cg13467707	12	120739268	SIRT4	TSS1500	0,016	1,37E-04	3,13E-02
cg10225875	15	42458833	VPS39	ExonBnd	-0,006	1,37E-04	3,13E-02
cg01494399	16	81709890	CMIP	Body	-0,016	1,37E-04	3,13E-02
cg19544440	22	30655079		IGR	-0,013	1,37E-04	3,13E-02
cg17451688	1	234293071	SLC35F3	Body	0,006	1,37E-04	3,13E-02
cg21463554	2	495912		IGR	-0,008	1,37E-04	3,13E-02
cg02602700	2	18223165		IGR	0,03	1,37E-04	3,13E-02
cg13705873	12	117397003	FBXW8	Body	0,006	1,37E-04	3,13E-02
cg10361675	16	75300714	BCAR1	Body	-0,025	1,37E-04	3,13E-02
cg25490133	18	725716	YES1	Body	0,02	1,37E-04	3,13E-02
cg05877101	18	60194204	ZCCHC2	Body	-0,004	1,37E-04	3,13E-02
cg02818728	4	189079272		IGR	-0,016	1,38E-04	3,14E-02
cg24586958	3	113567010	GRAMD1C	Body	0,005	1,38E-04	3,14E-02

cg13803266	9	139937835	NPDC1	Body	-0,016	1,38E-04	3,14E-02
cg09256281	2	80069606	CTNNA2	Body	-0,009	1,38E-04	3,14E-02
cg02706018	7	27168780	HOXA4	3'UTR	-0,014	1,38E-04	3,14E-02
cg00584903	8	127633012		IGR	-0,008	1,38E-04	3,14E-02
cg24895637	3	79700740	ROBO1	5'UTR	0,045	1,38E-04	3,14E-02
cg21663536	7	148984568	ZNF783	Body	-0,008	1,38E-04	3,14E-02
cg00946472	8	59465599	SDCBP	TSS200	0,006	1,38E-04	3,14E-02
cg13675837	17	54910204	DGKE	TSS1500	0,04	1,38E-04	3,14E-02
cg20269954	18	27037507		IGR	0,125	1,38E-04	3,15E-02
cg24474772	15	61152779	RORA	Body	-0,024	1,38E-04	3,15E-02
cg24762501	19	51530751	KLK11	5'UTR	-0,05	1,38E-04	3,15E-02
cg26508239	10	29759299	SVIL	Body	0,011	1,39E-04	3,15E-02
cg13956986	2	217654653		IGR	-0,016	1,39E-04	3,15E-02
cg01305291	2	242003078	SNED1	Body	0,083	1,39E-04	3,15E-02
cg00754834	4	14843735	LINC00504	Body	-0,007	1,39E-04	3,15E-02
cg04774623	8	62563669	ASPH	ExonBnd	-0,031	1,39E-04	3,15E-02
cg01532187	8	145805577	KIAA1688	Body	0,015	1,39E-04	3,15E-02
cg21615638	10	50397072	C10orf128	TSS1500	-0,011	1,39E-04	3,15E-02
cg08658350	11	128366822	ETS1	Body	0,038	1,39E-04	3,15E-02
cg10625931	12	57632417	NDUFA4L2	5'UTR	0,005	1,39E-04	3,15E-02
cg03217568	19	16366744		IGR	-0,009	1,39E-04	3,15E-02
cg15581501	20	45980204	ZMYND8	5'UTR	-0,025	1,39E-04	3,15E-02
cg01812233	14	52538833		IGR	0,008	1,39E-04	3,15E-02
cg05892626	7	101375112		IGR	0,012	1,40E-04	3,16E-02
cg25934800	9	106193000		IGR	0,063	1,40E-04	3,16E-02
cg23810105	17	60457393	EFCAB3	Body	-0,013	1,40E-04	3,16E-02
cg09880254	4	141595018	TBC1D9	Body	-0,005	1,40E-04	3,16E-02
cg22488462	13	111336430	CARS2	Body	-0,008	1,40E-04	3,16E-02
cg27621931	13	24368124	MIPEP	Body	0,006	1,40E-04	3,16E-02
cg16775460	17	77984481	TBC1D16	Body	-0,012	1,40E-04	3,16E-02
cg25516529	2	220173617	PTPRN	5'UTR	-0,029	1,40E-04	3,16E-02
cg01720186	3	182970846	MCF2L2	Body	-0,01	1,40E-04	3,16E-02
cg19114154	9	134463859	RAPGEF1	Body	-0,005	1,40E-04	3,16E-02
cg26267011	14	103871225	MARK3	Body	-0,011	1,40E-04	3,16E-02
cg01252585	20	48999791		IGR	0,046	1,40E-04	3,16E-02
cg03027977	7	138916033	UBN2	TSS200	0,008	1,40E-04	3,16E-02
cg18644089	14	63159052		IGR	0,028	1,40E-04	3,16E-02
cg05782440	3	45268453	TMEM158	TSS1500	-0,05	1,40E-04	3,16E-02
cg12378722	13	45492232		IGR	0,004	1,40E-04	3,16E-02
cg06379883	2	223421805	SGPP2	Body	-0,027	1,41E-04	3,17E-02
cg21780440	11	9232315	DENND5A	Body	0,006	1,41E-04	3,17E-02
cg07774680	7	29127983	CPVL	Body	0,051	1,41E-04	3,17E-02
cg18852857	22	26118876	ADRBK2	3'UTR	-0,011	1,41E-04	3,17E-02
cg14534017	4	40738650		IGR	-0,019	1,41E-04	3,17E-02
cg21220551	9	127905013	SCAI	Body	0,016	1,41E-04	3,17E-02
cg01802062	16	29833153	PAGR1	3'UTR	0,065	1,41E-04	3,17E-02
cg19851413	17	9371130	STX8	Body	-0,008	1,41E-04	3,17E-02
cg22053068	7	156469314	RNF32	3'UTR	0,006	1,41E-04	3,17E-02
cg05901384	15	90210975	PLIN1	Body	-0,016	1,41E-04	3,17E-02
cg23607370	8	5528989		IGR	-0,026	1,41E-04	3,18E-02
cg26759515	16	67515027	ATP6V0D1	1stExon	0,009	1,42E-04	3,18E-02
cg04877889	15	65842018	HACD3	Body	0,006	1,42E-04	3,18E-02
cg13893480	22	39230137	NPTXR	Body	-0,018	1,42E-04	3,18E-02
cg10157234	6	91988964		IGR	0,038	1,42E-04	3,18E-02
cg15632423	20	61915241	ARFGAP1	ExonBnd	-0,025	1,42E-04	3,18E-02
cg25970230	4	147558544	POU4F2	TSS1500	0,013	1,42E-04	3,18E-02
cg10221434	1	11142599	EXOSC10	Body	0,008	1,42E-04	3,18E-02
cg06470804	5	180071930	FLT4	Body	-0,016	1,42E-04	3,19E-02
cg09418000	7	39056400	POU6F2	Body	-0,007	1,42E-04	3,19E-02
cg16833566	4	1656603	FAM53A	Body	0,007	1,42E-04	3,19E-02
cg09839355	9	133003333		IGR	0,005	1,42E-04	3,19E-02

cg17282204	22	18955907		IGR	0,024	1,42E-04	3,19E-02
cg05360883	9	5041151	JAK2	Body	0,009	1,43E-04	3,19E-02
cg11926937	17	39509300		IGR	0,009	1,43E-04	3,19E-02
cg03688665	18	48191491	MAPK4	5'UTR	0,053	1,43E-04	3,19E-02
cg07502045	22	45826894	RIBC2	Body	-0,01	1,43E-04	3,19E-02
cg25462573	14	106846184		IGR	-0,011	1,43E-04	3,19E-02
cg26280758	3	107430006	BBX	Body	0,009	1,43E-04	3,19E-02
cg20939322	14	53342622	FERMT2	Body	-0,016	1,43E-04	3,19E-02
cg11345288	16	57051060	NLRC5	1stExon	0,005	1,43E-04	3,19E-02
cg13689591	3	124239629	KALRN	Body	-0,032	1,43E-04	3,19E-02
cg14821322	11	216570	SIRT3	3'UTR	0,007	1,43E-04	3,19E-02
cg27540189	1	16819659	CROCCL2	TSS1500	0,018	1,43E-04	3,19E-02
cg11194883	9	139974977	UAP1L1	Body	-0,013	1,43E-04	3,19E-02
cg17156809	19	30989605	ZNF536	Body	-0,018	1,43E-04	3,19E-02
cg22550528	5	162992841		IGR	-0,015	1,43E-04	3,19E-02
cg04394271	19	57587592		IGR	0,027	1,43E-04	3,19E-02
cg25294104	7	135247694	NUP205	Body	0,004	1,44E-04	3,20E-02
cg08485958	10	134915608	ADGRA1	TSS200	0,032	1,44E-04	3,20E-02
cg23505823	12	123215524	GPR81	TSS1500	0,038	1,44E-04	3,20E-02
cg27607898	6	4136202	PECI	TSS1500	0,008	1,44E-04	3,20E-02
cg17595290	10	3881532		IGR	0,022	1,44E-04	3,20E-02
cg14338062	12	57883279	MARS	Body	-0,009	1,44E-04	3,20E-02
cg03613353	19	7695641	XAB2	TSS1500	0,061	1,44E-04	3,20E-02
cg19797304	14	24732234	TGM1	5'UTR	-0,007	1,44E-04	3,20E-02
cg07396182	8	139756299	COL22A1	Body	-0,026	1,44E-04	3,20E-02
cg12691689	4	38383876		IGR	-0,016	1,44E-04	3,20E-02
cg00983520	22	51017067	CPT1B	1stExon	-0,045	1,44E-04	3,20E-02
cg25758263	5	87546846	TMEM161B	Body	-0,015	1,44E-04	3,20E-02
cg22035501	5	112073398	APC	TSS200	0,007	1,44E-04	3,20E-02
cg15063310	19	48265140		IGR	0,007	1,44E-04	3,20E-02
cg26665274	20	6747409	BMP2	TSS1500	0,017	1,44E-04	3,20E-02
cg03215500	14	81371212	CEP128	Body	-0,007	1,45E-04	3,21E-02
cg18943482	17	72413983		IGR	-0,011	1,45E-04	3,21E-02
cg18822414	8	145738751	RECQL4	Body	-0,014	1,45E-04	3,21E-02
cg23398091	18	23806189	TAF4B	TSS1500	-0,004	1,45E-04	3,21E-02
cg15316925	19	16102012		IGR	-0,012	1,45E-04	3,21E-02
cg16000331	22	42230138	SREBF2	Body	-0,02	1,45E-04	3,21E-02
cg05454957	17	79906039	MYADML2	TSS1500	0,01	1,45E-04	3,21E-02
cg02673107	6	33138131	COL11A2	Body	0,026	1,45E-04	3,21E-02
cg09387982	8	28740897	INTS9	5'UTR	0,021	1,45E-04	3,21E-02
cg14313748	9	101498661	ANKS6	3'UTR	-0,018	1,45E-04	3,21E-02
cg20291363	1	31661978	NKAIN1	TSS1500	0,033	1,45E-04	3,21E-02
cg00844074	13	113701892	MCF2L	Body	0,006	1,45E-04	3,21E-02
cg06913958	1	85744641	BCL10	TSS1500	-0,007	1,46E-04	3,22E-02
cg19449377	17	39041199	KRT20	1stExon	0,015	1,46E-04	3,22E-02
cg18686547	2	153326947	FMNL2	Body	0,009	1,46E-04	3,22E-02
cg15593398	4	3076334	HTT	TSS200	0,005	1,46E-04	3,22E-02
cg07603382	7	93521153	TFPI2	TSS1500	0,052	1,46E-04	3,22E-02
cg04944524	17	54954220		IGR	0,007	1,46E-04	3,22E-02
cg01282871	17	12902156	ELAC2	Body	0,005	1,46E-04	3,22E-02
cg00179110	2	25942734		IGR	0,038	1,46E-04	3,22E-02
cg15244769	2	74791689	M1AP	Body	-0,009	1,46E-04	3,22E-02
cg03602667	19	1208924	STK11	Body	-0,01	1,46E-04	3,22E-02
cg14969542	3	100684923	ABI3BP	Body	0,012	1,46E-04	3,22E-02
cg11372312	7	4215172	SDK1	Body	-0,012	1,46E-04	3,22E-02
cg19851999	3	97540355		IGR	0,004	1,46E-04	3,22E-02
cg19925558	1	1072370		IGR	0,007	1,47E-04	3,23E-02
cg12430302	1	9424575	SPSB1	Body	-0,018	1,47E-04	3,23E-02
cg12753032	1	234742404	IRF2BP2	3'UTR	-0,005	1,47E-04	3,23E-02
cg03348475	4	57775757	REST	5'UTR	0,008	1,47E-04	3,23E-02
cg12136883	4	120613606		IGR	-0,028	1,47E-04	3,23E-02

cg01232941	6	23534838		IGR	0,028	1,47E-04	3,23E-02
cg02032966	6	34482493	PACSIN1	TSS200	0,099	1,47E-04	3,23E-02
cg17719337	7	155276050		IGR	-0,008	1,47E-04	3,23E-02
cg19234880	9	15476439	PSIP1	Body	0,009	1,47E-04	3,23E-02
cg22978342	10	97150059	SORBS1	Body	0,008	1,47E-04	3,23E-02
cg10320449	10	105396339	SH3PXD2A	Body	0,007	1,47E-04	3,23E-02
cg07538176	11	92807225		IGR	0,028	1,47E-04	3,23E-02
cg04456968	11	123055280	CLMP	Body	0,036	1,47E-04	3,23E-02
cg01493237	12	72451246		IGR	0,033	1,47E-04	3,23E-02
cg11983576	15	53090807		IGR	-0,01	1,47E-04	3,23E-02
cg01034217	17	13505307	HS3ST3A1	TSS200	0,009	1,47E-04	3,23E-02
cg10607812	22	38082319	NOL12	TSS200	-0,007	1,47E-04	3,23E-02
cg13010014	11	124740965	ROBO3	Body	0,031	1,48E-04	3,23E-02
cg14101380	11	70718575	SHANK2	Body	-0,015	1,48E-04	3,23E-02
cg07391700	4	186818155	SORBS2	5'UTR	0,027	1,48E-04	3,23E-02
cg02337436	10	131750033	EBF3	Body	0,007	1,48E-04	3,24E-02
cg08679951	3	116737690		IGR	0,025	1,48E-04	3,24E-02
cg09514185	10	1712988	ADARB2	Body	-0,042	1,48E-04	3,24E-02
cg18282392	4	174166207	GALNT7	Body	0,009	1,48E-04	3,24E-02
cg06306791	6	32854970		IGR	0,015	1,48E-04	3,24E-02
cg10971243	8	1326261		IGR	-0,012	1,48E-04	3,24E-02
cg10897031	8	141586957	AGO2	Body	-0,008	1,48E-04	3,24E-02
cg11243875	9	139443283	LINC01573	Body	-0,015	1,48E-04	3,24E-02
cg05202663	11	78788008	TENM4	5'UTR	-0,01	1,48E-04	3,24E-02
cg23684322	13	76363803	LMO7	5'UTR	0,005	1,48E-04	3,24E-02
cg16175822	19	6372283	ALKBH7	TSS200	0,042	1,48E-04	3,24E-02
cg09313485	19	40909312	PRX	Body	0,016	1,48E-04	3,24E-02
cg01393632	12	103344685		IGR	-0,006	1,49E-04	3,24E-02
cg05730092	14	101036470	BEGAIN	TSS1500	0,037	1,49E-04	3,24E-02
cg15582719	1	6296260	ICMT	TSS1500	0,003	1,49E-04	3,24E-02
cg13514129	1	39547527	MACF1	5'UTR	0,008	1,49E-04	3,24E-02
cg00455379	1	46033770	AKR1A1	Body	-0,015	1,50E-04	3,24E-02
cg10296191	1	156554147	TTC24	Body	-0,011	1,49E-04	3,24E-02
cg15055286	2	86422729	IMMT	1stExon	0,004	1,50E-04	3,24E-02
cg18069081	2	133182651	GPR39	Body	-0,024	1,49E-04	3,24E-02
cg21068545	2	135746585	MAP3K19	Body	-0,049	1,49E-04	3,24E-02
cg25888694	5	148737581	PCYOX1L	5'UTR	0,01	1,50E-04	3,24E-02
cg01259378	6	31759834	VARS	Body	-0,006	1,50E-04	3,24E-02
cg12820223	6	49533848		IGR	0,037	1,49E-04	3,24E-02
cg25338587	6	152562335	SYNE1	Body	0,017	1,49E-04	3,24E-02
cg20394376	6	156711764		IGR	-0,017	1,49E-04	3,24E-02
cg17026036	9	125703001	RABGAP1	TSS1500	-0,006	1,50E-04	3,24E-02
cg23515223	10	29845161	SVIL	5'UTR	-0,006	1,49E-04	3,24E-02
cg13342558	10	114043548	TECTB	1stExon	0,043	1,50E-04	3,24E-02
cg25269942	10	114106090	GUCY2GP	Body	-0,013	1,49E-04	3,24E-02
cg24143287	10	128209980	C10orf90	1stExon	0,029	1,49E-04	3,24E-02
cg03437695	11	63886786	MACROD1	Body	-0,013	1,49E-04	3,24E-02
cg09070195	11	121526797		IGR	0,003	1,49E-04	3,24E-02
cg18209212	12	130646256	FZD10	TSS1500	-0,01	1,49E-04	3,24E-02
cg20552028	14	49411005		IGR	-0,016	1,50E-04	3,24E-02
cg21728307	14	105398410	PLD4	Body	-0,009	1,49E-04	3,24E-02
cg02796251	16	31112679		IGR	0,012	1,49E-04	3,24E-02
cg00912407	16	84998665		IGR	-0,004	1,50E-04	3,24E-02
cg26458775	17	38167380		IGR	-0,007	1,50E-04	3,24E-02
cg07111678	18	60987770	BCL2	TSS1500	0,004	1,50E-04	3,24E-02
cg07611790	19	49240823	RASIP1	Body	-0,022	1,50E-04	3,24E-02
cg17237086	22	40814966	MKL1	Body	-0,024	1,49E-04	3,24E-02
cg04710198	22	47189600	TBC1D22A	Body	-0,006	1,50E-04	3,24E-02
cg18434588	5	80095132	MSH3	Body	0,014	1,50E-04	3,24E-02
cg13628594	2	47177456	TTC7A	Body	0,01	1,50E-04	3,24E-02
cg19265364	3	42672763	NKTR	Body	-0,005	1,50E-04	3,24E-02

cg04060943	6	134504523	SGK1	Body	-0,01	1,50E-04	3,24E-02
cg15211328	8	10297565		IGR	0,031	1,51E-04	3,24E-02
cg14184130	9	38437270		IGR	-0,016	1,51E-04	3,24E-02
cg21204870	9	112513325	PALM2	Body	-0,009	1,50E-04	3,24E-02
cg23271998	2	3260576	TSSC1	Body	-0,011	1,51E-04	3,24E-02
cg25680245	18	19284821	ABHD3	TSS200	0,013	1,51E-04	3,25E-02
cg14376490	4	185213277		IGR	-0,007	1,51E-04	3,25E-02
cg15547662	1	26324823	PAFAH2	TSS200	-0,004	1,51E-04	3,25E-02
cg10911062	8	39960940		IGR	0,048	1,51E-04	3,25E-02
cg11374891	16	67571220	FAM65A	TSS200	0,007	1,51E-04	3,25E-02
cg00169122	16	89488963	ANKRD11	5'UTR	0,031	1,51E-04	3,25E-02
cg19498110	22	17590091	IL17RA	Body	-0,014	1,51E-04	3,25E-02
cg06801922	7	131582388		IGR	-0,015	1,51E-04	3,25E-02
cg11608653	9	96407260	PHF2	Body	-0,015	1,51E-04	3,25E-02
cg21225824	16	812350	MSLN	5'UTR	-0,016	1,51E-04	3,25E-02
cg15735954	19	54972723	LENG8	3'UTR	-0,008	1,51E-04	3,25E-02
cg24391989	3	32010656	OSBPL10	Body	0,007	1,52E-04	3,26E-02
cg02967280	4	140224053	NDUFC1	TSS1500	0,012	1,52E-04	3,26E-02
cg15353589	1	209933361	TRAF3IP3	5'UTR	0,005	1,52E-04	3,26E-02
cg02175873	4	82964079		IGR	0,035	1,52E-04	3,26E-02
cg09407967	6	41375649		IGR	0,027	1,52E-04	3,26E-02
cg26528298	6	112375206	WISP3	TSS200	-0,011	1,52E-04	3,26E-02
cg23269985	12	131300142	STX2	Body	-0,005	1,52E-04	3,26E-02
cg01788932	14	104393771	TDRD9	TSS1500	0,02	1,52E-04	3,26E-02
cg13410002	20	20353861		IGR	0,034	1,52E-04	3,26E-02
cg26957988	22	30898256	SEC14L4	Body	0,022	1,52E-04	3,26E-02
cg15408111	5	180658789	TRIM41	Body	-0,006	1,52E-04	3,26E-02
cg11983739	6	25106269	CMAHP	Body	-0,019	1,52E-04	3,26E-02
cg09759637	2	11636201		IGR	0,065	1,53E-04	3,26E-02
cg09610987	3	49028278	P4HTM	Body	0,013	1,53E-04	3,26E-02
cg23042134	5	7749881	ADCY2	Body	0,014	1,52E-04	3,26E-02
cg24858738	6	13711938	RANBP9	TSS200	0,012	1,53E-04	3,26E-02
cg08074805	6	15077721		IGR	0,031	1,53E-04	3,26E-02
cg05108359	6	20280167		IGR	0,095	1,53E-04	3,26E-02
cg22915732	8	62200518	CLVS1	TSS200	-0,009	1,53E-04	3,26E-02
cg23857936	11	45946150	GYLTL1B	Body	-0,017	1,53E-04	3,26E-02
cg16602799	13	25254742	ATP12A	5'UTR	0,004	1,52E-04	3,26E-02
cg19513064	15	32966463	SCG5	Body	-0,011	1,53E-04	3,26E-02
cg25876840	17	41920477		IGR	0,035	1,53E-04	3,26E-02
cg06436508	17	56619769	C17orf47	Body	0,006	1,52E-04	3,26E-02
cg04355401	20	37075704	SNORA39	TSS1500	-0,004	1,53E-04	3,26E-02
cg07170231	20	5590750	?P5-1022P6.2	5'UTR	-0,006	1,53E-04	3,26E-02
cg02725037	1	209980957	IRF6	TSS1500	0,012	1,53E-04	3,26E-02
cg01000469	2	39347731	SOS1	TSS200	0,004	1,53E-04	3,26E-02
cg24168221	20	23587099	CST9	TSS1500	0,037	1,53E-04	3,26E-02
cg15676837	1	202137199	PTPRV	Body	-0,008	1,53E-04	3,26E-02
cg07536072	19	1227141	STK11	3'UTR	-0,009	1,53E-04	3,26E-02
cg04673466	19	19030860	DDX49	Body	0,007	1,53E-04	3,26E-02
cg08538869	1	19669284	CAPZB	Body	-0,009	1,54E-04	3,27E-02
cg27005421	7	103085016	SLC26A5	TSS1500	0,037	1,54E-04	3,27E-02
cg00824793	9	116102525	WDR31	1stExon	0,004	1,54E-04	3,27E-02
cg17978654	12	107350463	TMEM263	5'UTR	-0,012	1,54E-04	3,27E-02
cg14685131	17	26880453	UNC119	TSS1500	-0,008	1,54E-04	3,27E-02
cg03602297	2	30142128	ALK	Body	-0,047	1,54E-04	3,27E-02
cg16463165	16	2374474	ABCA3	Body	-0,018	1,54E-04	3,27E-02
cg17137324	10	86527253		IGR	-0,011	1,54E-04	3,27E-02
cg22109801	15	25479259	;NORD115-35	TSS200	-0,016	1,54E-04	3,27E-02
cg06197966	10	29948324	SVIL	5'UTR	0,019	1,54E-04	3,27E-02
cg08454744	1	245748620	KIF26B	Body	0,037	1,54E-04	3,27E-02
cg04419342	10	90284004	RNLS	Body	-0,005	1,54E-04	3,27E-02
cg08052899	13	111636713	LINC00431	Body	0,009	1,54E-04	3,27E-02

cg25388447	19	13975628		IGR	0,012	1,54E-04	3,27E-02
cg19256677	16	51847723		IGR	-0,032	1,54E-04	3,27E-02
cg21177958	1	37450610	GRIK3	Body	0,022	1,54E-04	3,27E-02
cg27424593	3	194168156	ATP13A3	Body	0,018	1,54E-04	3,27E-02
cg23016257	7	31377934	NEUROD6	Body	-0,007	1,54E-04	3,27E-02
cg15422585	12	110252596	TRPV4	1stExon	-0,007	1,55E-04	3,27E-02
cg11302688	1	26019432	MAN1C1	Body	0,021	1,55E-04	3,28E-02
cg18521771	9	117373239	C9orf91	TSS1500	0,021	1,55E-04	3,28E-02
cg06601203	10	112113375		IGR	0,039	1,55E-04	3,28E-02
cg13960778	2	124440686		IGR	-0,015	1,55E-04	3,28E-02
cg19354759	5	158128592	EBF1	Body	0,008	1,55E-04	3,28E-02
cg21063034	17	4917019	KIF1C	ExonBnd	-0,006	1,55E-04	3,28E-02
cg22031032	2	12217292	)C100506457	Body	0,007	1,55E-04	3,28E-02
cg04390141	3	191045992	CCDC50	TSS1500	0,039	1,55E-04	3,28E-02
cg27539480	7	27147084	HOXA3	3'UTR	0,006	1,55E-04	3,28E-02
cg13492331	10	14779377	FAM107B	Body	0,031	1,55E-04	3,28E-02
cg15797629	22	42813956	NFAM1	Body	-0,008	1,55E-04	3,28E-02
cg19403269	1	5569798		IGR	0,035	1,55E-04	3,28E-02
cg26632171	2	17997117	MSGN1	TSS1500	0,008	1,55E-04	3,28E-02
cg22052672	2	74663416	RTKN	Body	-0,01	1,55E-04	3,28E-02
cg01382804	6	25931017	SLC17A2	TSS200	0,009	1,56E-04	3,28E-02
cg25590798	15	49462514	GALK2	1stExon	-0,014	1,56E-04	3,28E-02
cg01681422	6	2684421	MYLK4	Body	-0,005	1,56E-04	3,29E-02
cg00847988	1	94463617	ABCA4	Body	0,025	1,57E-04	3,29E-02
cg07390685	3	63179588		IGR	-0,01	1,57E-04	3,29E-02
cg03680106	3	167338735	WDR49	5'UTR	-0,023	1,56E-04	3,29E-02
cg24266105	6	146351044	GRM1	Body	-0,024	1,56E-04	3,29E-02
cg01473249	6	166219132		IGR	-0,052	1,56E-04	3,29E-02
cg21399327	7	129285381	NRF1	5'UTR	0,006	1,56E-04	3,29E-02
cg14648877	12	124434035	CCDC92	5'UTR	-0,014	1,57E-04	3,29E-02
cg15077301	15	48738898	FBN1	ExonBnd	0,01	1,57E-04	3,29E-02
cg27408897	18	67136810	DOK6	Body	0,046	1,57E-04	3,29E-02
cg14642303	20	20672663	RALGAPA2	Body	-0,015	1,56E-04	3,29E-02
cg09337082	21	42792302	MX1	TSS200	-0,008	1,57E-04	3,29E-02
cg03601098	7	127228532	ARF5	1stExon	0,005	1,57E-04	3,29E-02
cg12934281	5	180565439		IGR	-0,067	1,57E-04	3,29E-02
cg09477090	6	27564060		IGR	-0,012	1,57E-04	3,29E-02
cg05482502	7	157340390	PTPRN2	Body	-0,013	1,57E-04	3,29E-02
cg05543585	8	27793566	SCARA5	Body	-0,02	1,57E-04	3,29E-02
cg16453673	12	105630771	APPL2	TSS1500	0,013	1,57E-04	3,29E-02
cg15764386	16	31470228	ARMC5	TSS200	0,009	1,57E-04	3,29E-02
cg20779181	6	40555323	LRFN2	TSS200	0,005	1,57E-04	3,29E-02
cg03117079	1	91488015	ZNF644	TSS1500	0,014	1,57E-04	3,29E-02
cg10759957	7	94294847	PEG10	Body	0,006	1,57E-04	3,29E-02
cg06892152	6	38670967	GLO1	TSS200	-0,006	1,57E-04	3,29E-02
cg20698170	15	42120362	JMJD7	1stExon	0,004	1,57E-04	3,30E-02
cg19138446	3	116018418	LSAMP	Body	-0,012	1,58E-04	3,30E-02
cg18030006	5	72672345		IGR	-0,007	1,58E-04	3,30E-02
cg19488149	6	89322387	RNGTT	3'UTR	0,013	1,58E-04	3,30E-02
cg05295233	19	3851244	ZFR2	Body	0,005	1,58E-04	3,30E-02
cg10042939	15	101147085	ASB7	5'UTR	-0,013	1,58E-04	3,30E-02
cg21999487	2	163010385	GCG	TSS1500	-0,032	1,58E-04	3,31E-02
cg16886861	16	87327167	)C101928682	TSS1500	-0,013	1,58E-04	3,31E-02
cg08870143	17	7312081	NLGN2	Body	-0,045	1,58E-04	3,31E-02
cg13860527	1	160068519	IGSF8	TSS200	0,009	1,58E-04	3,31E-02
cg08227227	10	1517151	ADARB2	Body	0,042	1,59E-04	3,31E-02
cg14784653	10	13545008	BEND7	TSS200	0,035	1,59E-04	3,31E-02
cg25342872	17	79175132	AZI1	Body	-0,007	1,59E-04	3,31E-02
cg18213268	8	8473069		IGR	0,024	1,59E-04	3,31E-02
cg05523930	8	87102286		IGR	0,01	1,59E-04	3,31E-02
cg13034391	10	89736294		IGR	-0,007	1,59E-04	3,31E-02

cg00308479	12	120904436	SRSF9	Body	-0,024	1,59E-04	3,31E-02
cg27560922	16	330267	ARHGDIG	TSS1500	0,011	1,59E-04	3,31E-02
cg08197943	17	67323709	ABCA5	TSS1500	0,004	1,59E-04	3,31E-02
cg13752382	22	24737646	SPECC1L	TSS1500	-0,006	1,59E-04	3,31E-02
cg14317399	1	90974850		IGR	-0,012	1,59E-04	3,31E-02
cg22169874	2	19273596		IGR	-0,019	1,59E-04	3,31E-02
cg21331360	10	81864356		IGR	0,026	1,59E-04	3,31E-02
cg13269832	21	26088773		IGR	0,033	1,59E-04	3,32E-02
cg09364770	2	70933416	ADD2	Body	-0,006	1,60E-04	3,32E-02
cg08003628	8	106332188	ZFPM2	Body	-0,043	1,60E-04	3,32E-02
cg12996758	17	3456376	TRPV3	Body	-0,017	1,60E-04	3,32E-02
cg23247450	8	30776429		IGR	0,059	1,60E-04	3,32E-02
cg19930057	18	19180767	ESCO1	TSS200	0,003	1,60E-04	3,32E-02
cg27079563	14	65570253	MAX	TSS1500	-0,011	1,60E-04	3,32E-02
cg18074820	15	99203739	IGF1R	Body	0,016	1,60E-04	3,32E-02
cg22079616	13	21141624	IFT88	5'UTR	0,005	1,60E-04	3,32E-02
cg23368579	18	519265		IGR	-0,008	1,60E-04	3,32E-02
cg23855063	17	68165638	KCNJ2	TSS200	-0,006	1,60E-04	3,32E-02
cg21587319	7	157633985	PTPRN2	Body	0,01	1,60E-04	3,32E-02
cg20037328	11	78003389	GAB2	5'UTR	-0,011	1,60E-04	3,32E-02
cg03220797	22	25506763	KIAA1671	Body	-0,006	1,60E-04	3,32E-02
cg05621516	6	99728990	C6orf168	3'UTR	0,019	1,60E-04	3,32E-02
cg07333568	1	236938353		IGR	0,034	1,61E-04	3,32E-02
cg27046189	17	41133424	ES3L-AARSD1	TSS1500	0,004	1,61E-04	3,32E-02
cg03029975	17	19280571	MAPK7	TSS1500	0,064	1,61E-04	3,33E-02
cg01543995	2	235893326	SH3BP4	5'UTR	-0,018	1,61E-04	3,33E-02
cg03820136	3	52231636	ALAS1	TSS1500	-0,024	1,61E-04	3,33E-02
cg24794325	10	13023778	CCDC3	Body	0,047	1,61E-04	3,33E-02
cg16840346	17	79826817	ARHGDIA	Body	-0,024	1,61E-04	3,33E-02
cg00742118	5	135359419		IGR	0,026	1,61E-04	3,33E-02
cg01364172	9	19228799		IGR	0,024	1,61E-04	3,33E-02
cg27504799	10	98502573		IGR	0,008	1,61E-04	3,33E-02
cg15151784	12	120877664	COX6A1	Body	0,007	1,61E-04	3,33E-02
cg16912386	17	9336502	STX8	Body	0,056	1,61E-04	3,33E-02
cg01199495	18	31019946	C18orf34	1stExon	0,043	1,61E-04	3,33E-02
cg10415968	3	175926854		IGR	-0,012	1,62E-04	3,33E-02
cg16380846	9	30773390		IGR	-0,022	1,62E-04	3,33E-02
cg17522727	11	794867	SLC25A22	Body	-0,006	1,62E-04	3,33E-02
cg03253191	3	127335771	MCM2	Body	-0,012	1,62E-04	3,34E-02
cg16513468	1	154476659	TDRD10	5'UTR	0,012	1,62E-04	3,34E-02
cg13838448	5	61870145	IPO11	Body	-0,015	1,62E-04	3,34E-02
cg17193808	12	54797509	ITGA5	Body	-0,01	1,62E-04	3,34E-02
cg21272919	15	25014966		IGR	0,061	1,62E-04	3,34E-02
cg17036014	16	46517128	ANKRD26P1	Body	-0,056	1,62E-04	3,34E-02
cg18302899	19	2038600	MKNK2	Body	-0,006	1,62E-04	3,34E-02
cg06670785	22	20073572	MIR1306	TSS200	0,005	1,62E-04	3,34E-02
cg16164062	14	53623525		IGR	-0,01	1,62E-04	3,34E-02
cg07288394	19	30205772	C19orf12	Body	0,006	1,62E-04	3,34E-02
cg26330326	2	62132771	COMMD1	TSS200	0,006	1,63E-04	3,34E-02
cg01154966	20	33680989	TRPC4AP	TSS1500	0,013	1,63E-04	3,34E-02
cg21221866	6	91186653		IGR	0,015	1,63E-04	3,34E-02
cg13802981	13	111409394		IGR	0,04	1,63E-04	3,34E-02
cg08225841	1	57104899		IGR	0,04	1,63E-04	3,34E-02
cg08210507	7	2109244	MAD1L1	Body	-0,01	1,63E-04	3,34E-02
cg18653386	12	1960381	CACNA2D4	Body	-0,012	1,63E-04	3,34E-02
cg24119481	19	16683605	SLC35E1	TSS1500	0,003	1,63E-04	3,34E-02
cg18670235	7	100862016	ZNHIT1	Body	0,021	1,63E-04	3,34E-02
cg12209275	19	34232225	CHST8	Body	0,05	1,63E-04	3,34E-02
cg01591132	22	45566896	NUP50	5'UTR	0,005	1,63E-04	3,35E-02
cg14140994	2	35226375		IGR	-0,038	1,63E-04	3,35E-02
cg23465295	5	20611854		IGR	0,032	1,63E-04	3,35E-02

cg04745561	6	31612138	BAG6	Body	-0,008	1,63E-04	3,35E-02
cg01007838	16	87247486		IGR	-0,012	1,64E-04	3,35E-02
cg14557787	19	50305342	AP2A1	Body	-0,005	1,64E-04	3,35E-02
cg15346715	15	63126894	TLN2	Body	0,005	1,64E-04	3,35E-02
cg00215017	10	134818683		IGR	0,01	1,64E-04	3,35E-02
cg07545037	12	54423920	HOXC4	5'UTR	-0,045	1,64E-04	3,35E-02
cg15972662	3	72897939	SHQ1	TSS1500	-0,005	1,64E-04	3,35E-02
cg04934402	1	3379775	ARHGEF16	Body	0,015	1,64E-04	3,35E-02
cg00113074	3	32345052	CMTM8	Body	0,036	1,64E-04	3,35E-02
cg19932904	3	121373149	HCLS1	Body	0,017	1,64E-04	3,35E-02
cg00493242	4	20660098		IGR	-0,021	1,64E-04	3,35E-02
cg26159428	9	110926162		IGR	0,014	1,64E-04	3,35E-02
cg15959715	10	26506713	GAD2	Body	0,012	1,64E-04	3,35E-02
cg22435982	17	42441259	FAM171A2	TSS200	0,004	1,64E-04	3,35E-02
cg04639335	2	25601659	DTNB	3'UTR	-0,021	1,64E-04	3,35E-02
cg01742249	4	6568120		IGR	0,04	1,65E-04	3,35E-02
cg24240172	4	184774470		IGR	0,04	1,65E-04	3,35E-02
cg06878278	6	112408704	TUBE1	1stExon	0,003	1,64E-04	3,35E-02
cg10458809	9	21975898	CDKN2A	TSS1500	-0,031	1,64E-04	3,35E-02
cg05487878	9	116355406	RGS3	TSS1500	0,032	1,65E-04	3,35E-02
cg07102435	11	102096771	YAP1	Body	0,01	1,65E-04	3,35E-02
cg08535171	13	44458022	C13orf31	Body	-0,004	1,64E-04	3,35E-02
cg03998119	14	88945315	PTPN21	Body	-0,009	1,65E-04	3,35E-02
cg02246235	20	37208754	ADIG	TSS1500	0,025	1,65E-04	3,35E-02
cg16931664	11	11169707		IGR	0,027	1,65E-04	3,35E-02
cg12356266	8	99984350		IGR	0,04	1,65E-04	3,35E-02
cg23206309	10	73571109	CDH23	TSS1500	0,005	1,65E-04	3,35E-02
cg11996652	11	70882283	SHANK2	5'UTR	-0,011	1,65E-04	3,35E-02
cg16660734	10	64960236	JMJD1C	ExonBnd	-0,006	1,65E-04	3,35E-02
cg15900011	19	36235235	U2AF1L4	Body	0,023	1,65E-04	3,35E-02
cg26005454	2	183904907	NCKAP1	TSS1500	-0,026	1,65E-04	3,36E-02
cg09970349	4	1711630	SLBP	Body	0,006	1,65E-04	3,36E-02
cg21747310	12	11709115		IGR	0,041	1,65E-04	3,36E-02
cg11217193	1	12538341	VPS13D	Body	0,016	1,66E-04	3,36E-02
cg09297334	8	71520303	TRAM1	Body	0,004	1,66E-04	3,36E-02
cg04066824	2	220493112	SLC4A3	Body	0,034	1,66E-04	3,37E-02
cg04723239	11	66816270	SYT12	3'UTR	-0,012	1,66E-04	3,37E-02
cg10196000	4	44163158		IGR	0,045	1,66E-04	3,37E-02
cg15312223	8	30560719	GSR	Body	0,009	1,66E-04	3,37E-02
cg17002677	8	72744974		IGR	0,013	1,66E-04	3,37E-02
cg15919561	9	126129601	CRB2	Body	-0,015	1,67E-04	3,37E-02
cg06007395	11	132182675	NTM	Body	-0,016	1,66E-04	3,37E-02
cg06901392	13	31736105	HSPH1	5'UTR	0,005	1,66E-04	3,37E-02
cg03051668	15	31212310	FAN1	Body	-0,021	1,66E-04	3,37E-02
cg05532093	18	77186817	NFATC1	Body	-0,014	1,66E-04	3,37E-02
cg00740822	19	49939611	SLC17A7	Body	0,017	1,67E-04	3,37E-02
cg01497154	20	3661910	ADAM33	Body	0,034	1,67E-04	3,37E-02
cg04611865	4	25162039	SEPSECS	1stExon	0,004	1,67E-04	3,37E-02
cg08862580	9	139715433	RABL6	Body	-0,021	1,67E-04	3,37E-02
cg07178553	1	2359658		IGR	-0,009	1,67E-04	3,37E-02
cg27124598	7	150940825	SMARCD3	Body	0,017	1,67E-04	3,37E-02
cg14467134	9	132636614	USP20	Body	-0,018	1,67E-04	3,37E-02
cg23637943	1	109969159	PSMA5	TSS200	0,006	1,67E-04	3,37E-02
cg26430382	1	228270029	ARF1	TSS1500	-0,005	1,67E-04	3,37E-02
cg19927707	3	133465063	TF	5'UTR	-0,014	1,68E-04	3,37E-02
cg06481350	4	4756928		IGR	0,03	1,67E-04	3,37E-02
cg09899557	4	23882563	PPARGC1A	Body	0,068	1,67E-04	3,37E-02
cg23615201	6	41467243		IGR	0,02	1,67E-04	3,37E-02
cg24363702	7	36819634		IGR	0,004	1,67E-04	3,37E-02
cg02389501	7	93757948		IGR	0,029	1,68E-04	3,37E-02
cg01595098	11	100557287	ARHGAP42	TSS1500	-0,008	1,67E-04	3,37E-02

cg14724899	11	122051844	LOC399959	Body	-0,012	1,68E-04	3,37E-02
cg25803423	14	23563820	C14orf119	TSS1500	-0,004	1,67E-04	3,37E-02
cg11654694	16	14415519		IGR	0,006	1,68E-04	3,37E-02
cg00264112	18	55504387		IGR	0,016	1,67E-04	3,37E-02
cg01740960	1	22969009	C1QC	TSS1500	0,024	1,68E-04	3,37E-02
cg16371949	3	81837752		IGR	-0,032	1,68E-04	3,37E-02
cg06748804	7	107515453		IGR	0,042	1,68E-04	3,37E-02
cg11773122	20	62026350		IGR	-0,027	1,68E-04	3,37E-02
cg23624671	1	63833285	ALG6	1stExon	0,003	1,68E-04	3,38E-02
cg15398569	5	157289431		IGR	0,034	1,68E-04	3,38E-02
cg13280063	6	100036136		IGR	0,025	1,68E-04	3,38E-02
cg17732347	6	143179316	HIVEP2	5'UTR	0,02	1,68E-04	3,38E-02
cg24678803	10	135378570	SYCE1	Body	0,028	1,68E-04	3,38E-02
cg25546539	11	6640996	TPP1	TSS1500	0,031	1,68E-04	3,38E-02
cg16054660	20	50481726		IGR	-0,011	1,69E-04	3,38E-02
cg24460369	12	109549041		IGR	0,036	1,69E-04	3,39E-02
cg09850632	1	229406661	RAB4A	TSS1500	0,014	1,69E-04	3,39E-02
cg20422819	5	1084425	SLC12A7	Body	-0,019	1,69E-04	3,39E-02
cg11070030	12	114232702		IGR	-0,057	1,69E-04	3,39E-02
cg11072367	14	77409055		IGR	-0,014	1,69E-04	3,39E-02
cg15333762	20	48802833	CEBPB-AS1	Body	0,004	1,69E-04	3,39E-02
cg19434960	21	25294672		IGR	0,03	1,69E-04	3,39E-02
cg23216152	9	16813986	BNC2	Body	-0,019	1,69E-04	3,39E-02
cg04093645	5	127872037	FBN2	Body	0,006	1,69E-04	3,39E-02
cg20796611	1	18152639	ACTL8	Body	-0,044	1,70E-04	3,39E-02
cg12177141	1	208635571		IGR	0,006	1,70E-04	3,39E-02
cg03034696	2	47400023	CALM2	Body	0,048	1,70E-04	3,39E-02
cg08373622	3	87036080	VGLL3	Body	-0,018	1,70E-04	3,39E-02
cg25669179	4	1351818	UVSSA	Body	-0,015	1,70E-04	3,39E-02
cg14266050	4	2845671	ADD1	5'UTR	0,01	1,70E-04	3,39E-02
cg19044219	9	118455939		IGR	0,024	1,70E-04	3,39E-02
cg13906939	11	131525464	NTM	Body	0,027	1,70E-04	3,39E-02
cg06536724	17	64544418	PRKCA	Body	0,01	1,70E-04	3,39E-02
cg03514258	17	72250129	TTYH2	Body	-0,012	1,70E-04	3,39E-02
cg06164973	16	87101534		IGR	-0,009	1,70E-04	3,39E-02
cg00589002	1	3606082	TP73	TSS1500	0,025	1,70E-04	3,39E-02
cg09774750	4	4261683		IGR	-0,038	1,70E-04	3,39E-02
cg04446724	7	157446432	PTPRN2	Body	0,024	1,70E-04	3,39E-02
cg15028083	11	32331230		IGR	0,041	1,70E-04	3,39E-02
cg08151651	13	110815855	COL4A1	Body	-0,064	1,70E-04	3,39E-02
cg26889572	2	106126690		IGR	0,01	1,70E-04	3,39E-02
cg27145339	7	135242290	NUP205	TSS1500	-0,073	1,70E-04	3,39E-02
cg07788469	10	134119433	STK32C	Body	-0,016	1,71E-04	3,39E-02
cg00672111	12	67752236		IGR	0,008	1,70E-04	3,39E-02
cg07772439	13	19453173		IGR	-0,019	1,70E-04	3,39E-02
cg02648298	13	113489411	ATP11A	Body	0,015	1,71E-04	3,39E-02
cg00634315	6	135051398		IGR	0,013	1,71E-04	3,39E-02
cg18176509	1	94305707		IGR	-0,006	1,71E-04	3,39E-02
cg05370693	9	706788	KANK1	TSS200	-0,006	1,71E-04	3,39E-02
cg05394561	17	39465682	KRTAP16-1	TSS200	0,027	1,71E-04	3,39E-02
cg02443061	11	1691234		IGR	0,043	1,71E-04	3,40E-02
cg15314149	11	108074227	NPAT	Body	-0,006	1,71E-04	3,40E-02
cg10284115	3	15841404	ANKRD28	Body	0,02	1,72E-04	3,40E-02
cg12430757	3	112353862	CCDC80	Body	0,031	1,72E-04	3,40E-02
cg02543863	19	53681314	ZNF665	Body	-0,006	1,72E-04	3,40E-02
cg10532647	2	119381315		IGR	-0,02	1,72E-04	3,41E-02
cg02007927	3	190252387	IL1RAP	5'UTR	0,024	1,72E-04	3,41E-02
cg09244489	4	89744743	FAM13A	TSS1500	-0,01	1,72E-04	3,41E-02
cg26425990	8	38416632		IGR	0,01	1,72E-04	3,41E-02
cg24927161	22	46176768	ATXN10	Body	-0,008	1,72E-04	3,41E-02
cg18827934	2	241732587	KIF1A	Body	-0,009	1,72E-04	3,41E-02

cg15964779	19	55709086	PTPRH	Body	0,034	1,72E-04	3,41E-02
cg20962239	1	152896755		IGR	0,062	1,72E-04	3,41E-02
cg01885559	2	8976797	KIDINS220	5'UTR	-0,027	1,72E-04	3,41E-02
cg15558658	10	70748649	KIAA1279	1stExon	0,005	1,73E-04	3,41E-02
cg01303769	1	40709795		IGR	-0,021	1,73E-04	3,42E-02
cg11355849	2	54903054		IGR	-0,006	1,73E-04	3,42E-02
cg18801303	2	107442467	ST6GAL2	Body	-0,005	1,73E-04	3,42E-02
cg24469729	7	27160520	HOXA3	5'UTR	0,03	1,73E-04	3,42E-02
cg14402917	7	50468187	IKZF1	Body	-0,018	1,73E-04	3,42E-02
cg14548871	10	102505330	PAX2	TSS200	0,003	1,73E-04	3,42E-02
cg03681383	12	25801522	IFLTD1	TSS200	0,046	1,73E-04	3,42E-02
cg26545584	12	44150219	PUS7L	5'UTR	-0,007	1,73E-04	3,42E-02
cg06196600	15	35985946	DPH6-AS1	Body	0,018	1,73E-04	3,42E-02
cg16311418	16	8830937	ABAT	Body	-0,012	1,73E-04	3,42E-02
cg12890447	19	36296641	PRODH2	Body	0,025	1,73E-04	3,42E-02
cg18798946	20	39946357		IGR	0,006	1,73E-04	3,42E-02
cg10681083	22	38427744	POLR2F	Body	-0,016	1,73E-04	3,42E-02
cg10064137	15	97058784		IGR	0,029	1,73E-04	3,42E-02
cg11052068	6	110501899	WASF1	TSS1500	0,002	1,74E-04	3,42E-02
cg25290553	2	129146270		IGR	0,037	1,74E-04	3,42E-02
cg09500171	4	42460818	ATP8A1	Body	-0,006	1,74E-04	3,42E-02
cg09030852	4	170629768	CLCN3	Body	-0,006	1,74E-04	3,42E-02
cg06024391	7	134345134	BPGM	5'UTR	-0,016	1,74E-04	3,43E-02
cg12446817	12	8835025	RIMKLB	Body	0,026	1,74E-04	3,43E-02
cg04016653	5	43420237		IGR	-0,008	1,74E-04	3,43E-02
cg12297676	6	1817714	GMDS	Body	-0,017	1,74E-04	3,43E-02
cg13361665	20	3189311	ITPA	TSS1500	-0,023	1,74E-04	3,43E-02
cg00579605	20	55309394		IGR	0,051	1,75E-04	3,43E-02
cg02456981	12	45773384	ANO6	Body	-0,004	1,75E-04	3,43E-02
cg16218089	1	150516304		IGR	-0,004	1,75E-04	3,44E-02
cg19252175	4	169240076	DDX60	TSS200	-0,011	1,75E-04	3,44E-02
cg17621966	20	33147743	MAP1LC3A	3'UTR	-0,016	1,75E-04	3,44E-02
cg08887581	1	16330716	C1orf64	TSS200	0,029	1,75E-04	3,44E-02
cg04259400	17	21160021		IGR	0,023	1,75E-04	3,44E-02
cg20433752	5	163076619		IGR	0,039	1,75E-04	3,44E-02
cg00553880	9	15511231	PSIP1	TSS1500	0,004	1,75E-04	3,44E-02
cg06458411	5	177002682		IGR	0,055	1,75E-04	3,44E-02
cg20250336	7	51545565		IGR	-0,01	1,75E-04	3,44E-02
cg18350939	13	113319969	ATP11AUN	5'UTR	-0,019	1,75E-04	3,44E-02
cg09508001	14	60431848	LRRC9	Body	-0,019	1,75E-04	3,44E-02
cg08248747	3	159725004	IL12A-AS1	Body	0,006	1,75E-04	3,44E-02
cg12360485	7	2174599	MAD1L1	Body	0,007	1,76E-04	3,44E-02
cg19057304	2	208684809		IGR	0,013	1,76E-04	3,44E-02
cg27233612	3	25824257	NGLY1	Body	-0,007	1,76E-04	3,44E-02
cg14514704	6	170713208	FAM120B	Body	-0,02	1,76E-04	3,44E-02
cg14210321	2	106509881	NCK2	3'UTR	0,005	1,76E-04	3,44E-02
cg16499402	3	149333648	WWTR1	Body	0,031	1,76E-04	3,44E-02
cg23350198	22	18893715	DGCR6	TSS200	0,007	1,76E-04	3,44E-02
cg17218045	1	26758308	DHDDS	TSS1500	-0,029	1,76E-04	3,44E-02
cg01121072	2	27579103	GTF3C2	5'UTR	0,004	1,76E-04	3,44E-02
cg01243072	2	43398171		IGR	0,029	1,76E-04	3,44E-02
cg04145764	6	158953257		IGR	-0,018	1,76E-04	3,44E-02
cg26388599	8	54752779	ATP6V1H	Body	-0,007	1,76E-04	3,44E-02
cg20184045	11	65339774	FAM89B	TSS200	0,003	1,76E-04	3,44E-02
cg11735079	12	85268215	SLC6A15	Body	-0,018	1,76E-04	3,44E-02
cg02957620	17	15903290	ZSWIM7	TSS1500	0,006	1,76E-04	3,44E-02
cg01759870	14	105936409	MTA1	Body	0,026	1,76E-04	3,44E-02
cg21486532	2	242735135	GAL3ST2	Body	0,046	1,77E-04	3,44E-02
cg27121758	19	3178742	S1PR4	TSS200	0,004	1,77E-04	3,44E-02
cg05291964	3	62457060	CADPS	Body	0,024	1,77E-04	3,45E-02
cg08481905	10	34424407	PARD3	Body	-0,009	1,77E-04	3,45E-02

cg21506790	11	5277210	HBG2	TSS1500	0,017	1,77E-04	3,45E-02
cg00818822	19	6002752	RFX2	Body	-0,01	1,77E-04	3,45E-02
cg06577885	12	97222154	CFAP54	Body	0,011	1,77E-04	3,45E-02
cg26692097	16	53738201	FTO	Body	0,009	1,77E-04	3,45E-02
cg17580267	17	79873971	SIRT7	Body	-0,007	1,77E-04	3,45E-02
cg03241502	19	7505149	ARHGEF18	5'UTR	-0,004	1,77E-04	3,45E-02
cg10225865	8	54605566		IGR	0,069	1,78E-04	3,45E-02
cg13681781	9	139102396	QSOX2	Body	0,004	1,78E-04	3,45E-02
cg13539803	13	110424782	IRS2	Body	-0,011	1,78E-04	3,45E-02
cg15886157	16	68894023	TANGO6	Body	0,005	1,78E-04	3,45E-02
cg17076431	6	49901607		IGR	0,019	1,78E-04	3,45E-02
cg09111065	18	57332428	CCBE1	Body	0,023	1,78E-04	3,45E-02
cg27657131	20	61847587	YTHDF1	TSS200	0,004	1,78E-04	3,45E-02
cg05947699	5	173345670	CPEB4	Body	-0,006	1,78E-04	3,46E-02
cg23160436	1	238753492		IGR	0,005	1,78E-04	3,46E-02
cg00765428	21	34638638	IL10RB	TSS200	0,006	1,78E-04	3,46E-02
cg12072904	4	146099544	OTUD4	5'UTR	-0,007	1,78E-04	3,46E-02
cg06205938	16	48656581	MIR5095	Body	0,033	1,78E-04	3,46E-02
cg21808526	22	28172750	MN1	Body	0,023	1,78E-04	3,46E-02
cg05822495	1	202244517	LGR6	Body	-0,006	1,78E-04	3,46E-02
cg07997076	13	21286934	IL17D	Body	0,006	1,78E-04	3,46E-02
cg00014118	1	1935561	KIAA1751	TSS1500	-0,035	1,79E-04	3,46E-02
cg00186842	1	41125900	RIMS3	5'UTR	0,006	1,79E-04	3,46E-02
cg13662578	15	63882292	USP3-AS1	Body	-0,009	1,79E-04	3,46E-02
cg15936886	12	83415024	TMTC2	Body	-0,01	1,79E-04	3,46E-02
cg13113599	16	595848	SOLH	5'UTR	-0,018	1,79E-04	3,46E-02
cg13975062	3	108308228	KIAA1524	5'UTR	0,005	1,79E-04	3,46E-02
cg18031902	14	105715860	BTBD6	Body	-0,018	1,79E-04	3,46E-02
cg14281482	17	79133201	AATK	Body	-0,008	1,79E-04	3,47E-02
cg15099879	1	1377656	VWA1	3'UTR	-0,012	1,79E-04	3,47E-02
cg00226984	2	239047305	KLHL30	TSS200	-0,018	1,79E-04	3,47E-02
cg19677181	5	153211415		IGR	0,032	1,80E-04	3,47E-02
cg19282987	2	197525023	CCDC150	Body	-0,016	1,80E-04	3,47E-02
cg11266874	3	93699352	ARL13B	Body	0,003	1,80E-04	3,47E-02
cg00841845	3	190971168		IGR	0,044	1,80E-04	3,47E-02
cg16393715	7	1948819	MAD1L1	Body	-0,024	1,80E-04	3,47E-02
cg07270153	7	19185260	FERD3L	TSS1500	0,029	1,80E-04	3,47E-02
cg17366563	8	144379157	ZNF696	3'UTR	-0,02	1,80E-04	3,47E-02
cg15434232	11	56187177	OR5R1	TSS1500	0,055	1,80E-04	3,47E-02
cg03991512	16	75150456	LDHD	Body	0,046	1,80E-04	3,47E-02
cg20909154	18	76361545		IGR	-0,031	1,80E-04	3,47E-02
cg19959820	19	28920279		IGR	-0,009	1,80E-04	3,47E-02
cg16733866	21	42792609	MX1	5'UTR	-0,022	1,80E-04	3,47E-02
cg11166580	6	120063560		IGR	-0,014	1,80E-04	3,48E-02
cg13734792	12	123563327	PITPNM2	5'UTR	0,013	1,80E-04	3,48E-02
cg11747695	13	111164966	COL4A2	3'UTR	-0,035	1,80E-04	3,48E-02
cg26613140	19	37157776	ZNF461	TSS200	0,004	1,80E-04	3,48E-02
cg13878066	1	15541799	TMEM51	Body	0,027	1,81E-04	3,48E-02
cg25540313	5	29930761		IGR	0,043	1,81E-04	3,48E-02
cg07335156	7	75947820		IGR	0,004	1,81E-04	3,48E-02
cg08579741	11	62374527	EML3	Body	-0,005	1,81E-04	3,48E-02
cg18888351	6	43464230	TJAP1	5'UTR	-0,013	1,81E-04	3,48E-02
cg15173119	10	85990383		IGR	0,013	1,81E-04	3,48E-02
cg02940515	15	22921400	CYFIP1	5'UTR	-0,019	1,81E-04	3,48E-02
cg20321719	9	134394131	POMT1	Body	-0,02	1,81E-04	3,48E-02
cg09754843	8	22464236	CCAR2	Body	-0,011	1,81E-04	3,48E-02
cg22053854	11	111944781	PIH1D2	1stExon	-0,003	1,81E-04	3,48E-02
cg15859475	16	15688843	KIAA0430	3'UTR	-0,019	1,81E-04	3,48E-02
cg24914355	2	176959229	HOXD13	Body	-0,018	1,81E-04	3,48E-02
cg09249970	2	191631354		IGR	-0,017	1,82E-04	3,48E-02
cg10864596	5	178487382	ZNF354C	TSS1500	0,004	1,82E-04	3,48E-02

cg21543536	6	116601288	TSPYL1	TSS200	0,006	1,82E-04	3,48E-02
cg26292232	10	74047853		IGR	0,036	1,82E-04	3,48E-02
cg18996702	16	80016330		IGR	-0,016	1,82E-04	3,48E-02
cg01807735	11	115861018		IGR	-0,045	1,82E-04	3,48E-02
cg11542063	2	178129680	NFE2L2	5'UTR	-0,006	1,82E-04	3,48E-02
cg18848955	20	38808985		IGR	-0,007	1,82E-04	3,48E-02
cg22509158	7	15727847	MEOX2-AS1	TSS200	-0,02	1,82E-04	3,48E-02
cg10848373	21	39047855	KCNJ6	Body	-0,045	1,82E-04	3,48E-02
cg08636535	21	45627358		IGR	0,014	1,82E-04	3,48E-02
cg14253461	2	105363367		IGR	-0,008	1,82E-04	3,49E-02
cg01243790	2	103379054	TMEM182	Body	0,006	1,82E-04	3,49E-02
cg15429472	2	136892168		IGR	0,037	1,82E-04	3,49E-02
cg02720998	2	239848054	FLJ43879	TSS200	0,028	1,82E-04	3,49E-02
cg20932944	16	50782796	CYLD	5'UTR	0,009	1,82E-04	3,49E-02
cg09729155	3	192960454	MGC2889	Body	-0,013	1,83E-04	3,49E-02
cg15286034	8	22414633	SORBS3	Body	-0,011	1,83E-04	3,49E-02
cg22496996	6	27471291		IGR	0,015	1,83E-04	3,49E-02
cg24293297	7	20831044		IGR	-0,022	1,83E-04	3,49E-02
cg13199780	5	155752946	SGCD	TSS1500	0,046	1,83E-04	3,49E-02
cg18379793	13	111868162	ARHGEF7	5'UTR	-0,007	1,83E-04	3,49E-02
cg14179354	16	58530614	NDRG4	Body	-0,026	1,83E-04	3,49E-02
cg14085495	1	204910606	NFASC	5'UTR	0,009	1,83E-04	3,49E-02
cg17987260	20	6747439	BMP2	TSS1500	0,026	1,83E-04	3,49E-02
cg06186330	17	46522513		IGR	0,004	1,83E-04	3,49E-02
cg22193150	22	27838621		IGR	-0,012	1,83E-04	3,50E-02
cg17816038	1	7123346	CAMTA1	Body	0,039	1,84E-04	3,50E-02
cg03194484	3	96983229	EPHA6	Body	-0,015	1,84E-04	3,50E-02
cg16809138	17	64672277	PRKCA	Body	-0,013	1,84E-04	3,50E-02
cg26435013	3	31574110	STT3B	TSS1500	0,008	1,84E-04	3,50E-02
cg18466804	5	110001068	TMEM232	Body	-0,014	1,84E-04	3,50E-02
cg27315170	6	31830937	NEU1	TSS1500	0,006	1,84E-04	3,50E-02
cg18843501	7	73273661		IGR	0,026	1,84E-04	3,50E-02
cg00383321	7	79707732		IGR	-0,031	1,84E-04	3,50E-02
cg16050442	8	145582714	SLC52A2	5'UTR	0,006	1,84E-04	3,50E-02
cg05077519	13	113347960	ATP11A	Body	-0,021	1,84E-04	3,50E-02
cg07238512	14	35746869	PSMA6	TSS1500	0,027	1,84E-04	3,50E-02
cg01822938	14	88456162	GALC	Body	-0,006	1,84E-04	3,50E-02
cg17977624	1	92912000		IGR	-0,009	1,85E-04	3,50E-02
cg19816778	9	77229115	RORB	Body	0,049	1,85E-04	3,50E-02
cg13599482	9	129453493	LMX1B	Body	-0,011	1,84E-04	3,50E-02
cg15889260	11	121891076		IGR	0,008	1,84E-04	3,50E-02
cg08826460	19	11201433	LDLR	Body	-0,006	1,85E-04	3,50E-02
cg02175741	2	219724891	WNT6	Body	0,011	1,85E-04	3,50E-02
cg16089415	15	62199811	VPS13C	Body	-0,009	1,85E-04	3,51E-02
cg02324558	8	59905025	TOX	Body	-0,034	1,85E-04	3,51E-02
cg01269795	6	26440101	BTN3A3	TSS1500	0,014	1,85E-04	3,51E-02
cg04272764	6	35738432		IGR	-0,009	1,85E-04	3,51E-02
cg17371070	8	125239977		IGR	0,022	1,85E-04	3,51E-02
cg01802493	12	130530121		IGR	0,014	1,85E-04	3,51E-02
cg22793860	7	98030482	BAIAP2L1	TSS200	0,02	1,86E-04	3,51E-02
cg08165592	17	16492455		IGR	0,007	1,86E-04	3,51E-02
cg15537082	8	9762300	MIR124-1	TSS1500	0,016	1,86E-04	3,52E-02
cg14500336	1	27492831		IGR	-0,006	1,86E-04	3,52E-02
cg26543816	4	38180741		IGR	0,01	1,86E-04	3,52E-02
cg12080812	11	65336212		IGR	-0,012	1,86E-04	3,52E-02
cg02245810	22	51017432	CPT1B	TSS1500	-0,02	1,86E-04	3,52E-02
cg04378518	20	60585259	TAF4	Body	0,005	1,86E-04	3,52E-02
cg12359759	14	103344876	TRAF3	Body	-0,009	1,86E-04	3,52E-02
cg02714356	15	90118981	C15orf42	1stExon	0,003	1,86E-04	3,52E-02
cg11465971	4	54966336	GSX2	5'UTR	0,012	1,87E-04	3,53E-02
cg00506625	16	88872689	CDT1	Body	-0,009	1,87E-04	3,53E-02

cg14004197	9	140216230	EXD3	Body	0,03	1,87E-04	3,53E-02
cg18603104	5	173760028		IGR	0,034	1,87E-04	3,53E-02
cg11059659	15	89925657	LINC00925	Body	0,01	1,87E-04	3,53E-02
cg16095353	8	66556852	MTFR1	TSS200	0,004	1,87E-04	3,53E-02
cg03331263	20	61146952	C20orf166	TSS1500	0,035	1,87E-04	3,53E-02
cg01129211	21	46832934	COL18A1	Body	0,028	1,87E-04	3,53E-02
cg26687638	12	45524776		IGR	0,01	1,87E-04	3,53E-02
cg08138956	20	37464900	PPP1R16B	Body	-0,01	1,87E-04	3,53E-02
cg25469212	1	31769620	ZCCHC17	TSS1500	0,003	1,87E-04	3,53E-02
cg14832490	1	20957761		IGR	0,029	1,88E-04	3,53E-02
cg18786718	16	2868001	PRSS21	Body	-0,036	1,88E-04	3,53E-02
cg03716591	5	1653609		IGR	0,016	1,88E-04	3,53E-02
cg09274864	14	55226964	SAMD4A	Body	-0,016	1,88E-04	3,54E-02
cg15957017	4	71571214	RUFY3	Body	0,003	1,88E-04	3,54E-02
cg21821103	1	3563485	WDR8	Body	-0,012	1,88E-04	3,54E-02
cg22272492	11	2925996	SLC22A18	Body	-0,012	1,88E-04	3,54E-02
cg04227885	17	2434802		IGR	0,052	1,88E-04	3,54E-02
cg16467919	11	68508621	MTL5	Body	-0,013	1,88E-04	3,54E-02
cg01649266	14	88952788	PTPN21	Body	-0,016	1,88E-04	3,54E-02
cg17074641	1	24575785		IGR	0,01	1,89E-04	3,54E-02
cg24706522	7	22160237	RAPGEF5	3'UTR	-0,006	1,89E-04	3,54E-02
cg25859835	8	49532875	)C101929217	TSS200	-0,022	1,89E-04	3,55E-02
cg08983190	9	2094330	SMARCA2	Body	0,025	1,89E-04	3,55E-02
cg24025815	13	50217565		IGR	0,039	1,89E-04	3,55E-02
cg02305651	19	5997013	RFX2	Body	-0,009	1,89E-04	3,55E-02
cg23816205	19	54931788	TTYH1	Body	0,049	1,89E-04	3,55E-02
cg00104108	20	53477283		IGR	0,039	1,89E-04	3,55E-02
cg27664182	13	115080391	ZNF828	5'UTR	0,008	1,89E-04	3,55E-02
cg16176495	2	193016788	TMEFF2	Body	0,03	1,89E-04	3,55E-02
cg05254651	4	3705147		IGR	0,054	1,90E-04	3,55E-02
cg15089077	1	55246867	TTC22	3'UTR	-0,052	1,90E-04	3,56E-02
cg17010657	5	125936146	PHAX	TSS1500	-0,008	1,90E-04	3,56E-02
cg21882990	6	111902385	RAF3IP2-AS1	Body	0,007	1,90E-04	3,56E-02
cg01002264	10	121137808	GRK5	Body	-0,007	1,90E-04	3,56E-02
cg10247071	17	8113965	AURKB	TSS200	0,008	1,90E-04	3,56E-02
cg23934072	1	200941706	KIF21B	3'UTR	-0,023	1,90E-04	3,56E-02
cg13041355	5	178622080	ADAMTS2	Body	-0,012	1,90E-04	3,56E-02
cg13126951	11	46336896	CREB3L1	Body	-0,005	1,90E-04	3,56E-02
cg21104040	13	112856976		IGR	-0,011	1,90E-04	3,56E-02
cg27003212	5	52210732	ITGA1	Body	0,044	1,90E-04	3,56E-02
cg07718811	19	2495052		IGR	0,006	1,90E-04	3,56E-02
cg23533100	1	107683503	NTNG1	5'UTR	-0,014	1,91E-04	3,56E-02
cg16655084	1	184005896	GLT25D2	Body	-0,011	1,91E-04	3,56E-02
cg08606504	1	184020263	TSEN15	TSS1500	0,015	1,91E-04	3,56E-02
cg02039606	1	198952654		IGR	0,006	1,91E-04	3,56E-02
cg13353050	3	32634406		IGR	0,009	1,91E-04	3,56E-02
cg00246486	3	112642487	CD200R1	3'UTR	-0,039	1,91E-04	3,56E-02
cg14516350	6	30166781	TRIM26	Body	-0,006	1,92E-04	3,56E-02
cg03616157	6	143363671		IGR	-0,02	1,91E-04	3,56E-02
cg02438481	6	168199400		IGR	0,053	1,91E-04	3,56E-02
cg09645743	7	117067136	ASZ1	Body	0,034	1,91E-04	3,56E-02
cg08701816	8	95278142		IGR	0,038	1,91E-04	3,56E-02
cg12376274	8	145841465	ARHGAP39	5'UTR	-0,015	1,91E-04	3,56E-02
cg20363058	9	140969982	CACNA1B	Body	0,03	1,91E-04	3,56E-02
cg21457273	12	51021403	DIP2B	Body	0,006	1,92E-04	3,56E-02
cg21218053	13	112620313		IGR	-0,019	1,91E-04	3,56E-02
cg05625341	14	50612578	SOS2	Body	0,007	1,91E-04	3,56E-02
cg15595739	17	63183451	RGS9	Body	0,006	1,91E-04	3,56E-02
cg01653752	17	79009041	FLJ90757	TSS1500	0,006	1,91E-04	3,56E-02
cg17088237	19	42797715	CIC	Body	-0,006	1,91E-04	3,56E-02
cg10898277	20	24821220		IGR	0,031	1,92E-04	3,56E-02

cg08849914	22	24978122		IGR	0,011	1,92E-04	3,56E-02
cg01037318	10	118648646	SHTN1	Body	0,035	1,92E-04	3,56E-02
cg14192858	13	53226935	SUGT1	5'UTR	0,005	1,92E-04	3,56E-02
cg21855360	6	257504		IGR	0,056	1,92E-04	3,56E-02
cg21675653	4	136045019		IGR	-0,017	1,92E-04	3,57E-02
cg00158401	10	127581141		IGR	-0,019	1,92E-04	3,57E-02
cg15650422	16	19896330	GPRC5B	TSS200	0,005	1,92E-04	3,57E-02
cg14532435	9	311917	DOCK8	Body	0,005	1,93E-04	3,57E-02
cg10621979	4	149365824		IGR	0,004	1,93E-04	3,57E-02
cg11048745	4	183847134		IGR	0,007	1,93E-04	3,57E-02
cg02006610	8	30889718	PURG	1stExon	-0,01	1,93E-04	3,57E-02
cg21824586	12	3213811	TSPAN9	5'UTR	-0,007	1,93E-04	3,57E-02
cg22835022	14	24707522	GMPR2	Body	-0,014	1,93E-04	3,57E-02
cg05754361	16	57209801	FAM192A	5'UTR	0,018	1,93E-04	3,57E-02
cg00651216	6	44213720	HSP90AB1	TSS1500	0,014	1,93E-04	3,58E-02
cg14872006	5	104246829		IGR	-0,024	1,93E-04	3,58E-02
cg01239112	7	77669957	MAGI2	Body	-0,056	1,93E-04	3,58E-02
cg21056542	15	75745592	SIN3A	5'UTR	0,002	1,93E-04	3,58E-02
cg26427750	16	56345466	GNAO1	Body	-0,01	1,93E-04	3,58E-02
cg14310162	22	32146344	C22orf30	TSS1500	0,003	1,93E-04	3,58E-02
cg04354805	1	27152792	ZDHHC18	TSS1500	0,007	1,94E-04	3,58E-02
cg19198913	2	129495073		IGR	-0,013	1,94E-04	3,58E-02
cg03031526	4	31070017	PCDH7	Body	-0,006	1,94E-04	3,58E-02
cg02848456	7	151006345		IGR	0,048	1,94E-04	3,58E-02
cg04714088	16	81478359	CMIP	TSS1500	-0,005	1,94E-04	3,58E-02
cg20211896	2	99543885	KIAA1211L	5'UTR	0,008	1,94E-04	3,58E-02
cg13654594	6	50684843	TFAP2D	Body	-0,045	1,94E-04	3,58E-02
cg19426944	12	48397225	COL2A1	Body	-0,009	1,94E-04	3,58E-02
cg00024516	4	123506194		IGR	0,032	1,94E-04	3,58E-02
cg23054364	1	38190177	EPHA10	Body	-0,009	1,95E-04	3,59E-02
cg24298107	1	163439561		IGR	-0,021	1,95E-04	3,59E-02
cg23915065	3	33759654	CLASP2	1stExon	0,003	1,95E-04	3,59E-02
cg14818451	3	53269296	TKT	Body	-0,046	1,95E-04	3,59E-02
cg09854553	4	57332968	SRP72	TSS1500	-0,019	1,95E-04	3,59E-02
cg26903096	4	183629432	TENM3	Body	-0,008	1,94E-04	3,59E-02
cg08903587	7	73097967	DNAJC30	TSS200	0,005	1,95E-04	3,59E-02
cg05063920	13	36045633	NBEA	Body	-0,027	1,95E-04	3,59E-02
cg10737847	14	80000354	NRXN3	Body	-0,01	1,95E-04	3,59E-02
cg23233468	17	37027923	LASP1	5'UTR	-0,003	1,95E-04	3,59E-02
cg21622831	22	25972277	ADRBK2	Body	0,007	1,94E-04	3,59E-02
cg07923758	3	32941766		IGR	0,004	1,95E-04	3,59E-02
cg21878148	13	70681276	KLHL1	Body	-0,046	1,95E-04	3,59E-02
cg15120426	15	77951471	LINGO1-AS2	TSS1500	-0,019	1,95E-04	3,59E-02
cg01096617	16	58569180	CNOT1	Body	-0,015	1,95E-04	3,59E-02
cg17931655	4	84519302	AGPAT9	Body	0,011	1,95E-04	3,59E-02
cg08290993	6	30654736	KIAA1949	5'UTR	0,005	1,95E-04	3,59E-02
cg06711448	12	118313657	KSR2	Body	0,039	1,95E-04	3,59E-02
cg08789539	19	16291535		IGR	0,005	1,95E-04	3,59E-02
cg02459827	5	108082637	FER	TSS1500	-0,018	1,95E-04	3,59E-02
cg10091135	16	83520096	CDH13	ExonBnd	-0,007	1,95E-04	3,59E-02
cg13675148	15	85324570	ZNF592	5'UTR	-0,007	1,96E-04	3,59E-02
cg07150925	3	49591642	BSN	TSS1500	-0,007	1,96E-04	3,59E-02
cg02871542	1	1489408	SSU72	Body	-0,021	1,96E-04	3,59E-02
cg07437198	1	15791795	CELA2A	Body	0,009	1,96E-04	3,59E-02
cg17436298	1	237947345	RYR2	Body	0,007	1,96E-04	3,59E-02
cg10128660	11	116509955		IGR	0,009	1,96E-04	3,59E-02
cg04403584	11	133681576	LOC646522	TSS1500	-0,017	1,96E-04	3,59E-02
cg27262702	16	88878803	APRT	TSS1500	0,009	1,96E-04	3,59E-02
cg18981477	20	6747166		IGR	0,055	1,96E-04	3,59E-02
cg14099664	3	32545349	CMTM6	TSS1500	-0,006	1,96E-04	3,59E-02
cg04926347	8	146052751	ZNF7	TSS200	0,012	1,96E-04	3,59E-02

cg03783060	10	11002051		IGR	0,012	1,96E-04	3,59E-02
cg24500676	11	4674156	OR51E1	Body	0,005	1,96E-04	3,59E-02
cg12842646	13	111290667	CARKD	Body	-0,02	1,96E-04	3,59E-02
cg01242309	20	325016		IGR	-0,026	1,96E-04	3,59E-02
cg27433764	10	87270441		IGR	-0,015	1,96E-04	3,59E-02
cg24904303	19	47617051	ZC3H4	TSS200	0,006	1,97E-04	3,60E-02
cg00808730	14	93215038	LGMN	TSS200	0,005	1,97E-04	3,60E-02
cg15891582	17	46810289		IGR	0,005	1,97E-04	3,60E-02
cg13304297	3	167968368	C3orf50	Body	-0,01	1,97E-04	3,60E-02
cg02955361	1	32479956	KHDRBS1	1stExon	0,003	1,97E-04	3,60E-02
cg03407782	2	104668320		IGR	-0,01	1,97E-04	3,60E-02
cg19869746	6	7107108	RREB1	TSS1500	0,003	1,97E-04	3,60E-02
cg00368973	17	30333964		IGR	0,027	1,98E-04	3,61E-02
cg20246707	1	154564029	ADAR	Body	0,005	1,98E-04	3,61E-02
cg14972576	5	97645526		IGR	0,038	1,98E-04	3,61E-02
cg17006443	11	70628938	SHANK2	Body	-0,014	1,98E-04	3,61E-02
cg14818464	15	90776461	CIB1	Body	0,007	1,98E-04	3,61E-02
cg13082666	4	70932281	CSN1S2AP	TSS1500	-0,03	1,98E-04	3,61E-02
cg19068479	11	2395948		IGR	-0,008	1,98E-04	3,61E-02
cg22721644	12	47427216		IGR	0,02	1,98E-04	3,61E-02
cg12871652	12	78581770	NAV3	Body	-0,063	1,98E-04	3,61E-02
cg06338290	12	109027870	SELPLG	TSS1500	-0,008	1,98E-04	3,61E-02
cg09125791	14	24707343	GMPR2	Body	-0,016	1,98E-04	3,61E-02
cg03248814	14	38069728	TTC6	5'UTR	0,021	1,98E-04	3,61E-02
cg13993945	1	202779497		IGR	0,02	1,99E-04	3,61E-02
cg08167638	7	79281930		IGR	0,027	1,99E-04	3,61E-02
cg27398640	15	77910606	LINGO1	Body	0,09	1,99E-04	3,61E-02
cg01374372	18	21082991	C18orf8	TSS1500	0,004	1,99E-04	3,61E-02
cg06879998	8	20112334	LZTS1	Body	0,046	1,99E-04	3,61E-02
cg17427302	5	132290039	AFF4	5'UTR	0,007	1,99E-04	3,62E-02
cg11672390	1	3157510	PRDM16	Body	-0,013	2,00E-04	3,62E-02
cg02769104	1	27333245	FAM46B	Body	-0,012	2,00E-04	3,62E-02
cg10147375	2	60689043	BCL11A	Body	0,006	1,99E-04	3,62E-02
cg10952176	3	52894493	110-MUSTN1	Body	-0,016	2,00E-04	3,62E-02
cg21688540	3	106234714		IGR	-0,013	1,99E-04	3,62E-02
cg23272908	4	511354	PIGG	Body	-0,019	1,99E-04	3,62E-02
cg14825221	4	83720052	SCD5	TSS200	0,006	1,99E-04	3,62E-02
cg18718984	6	110663610	C6orf186	Body	0,009	2,00E-04	3,62E-02
cg04516135	7	120589937	ING3	TSS1500	0,011	2,00E-04	3,62E-02
cg03628333	11	2214677		IGR	-0,02	2,00E-04	3,62E-02
cg11102819	11	74421983	CHRDL2	1stExon	0,025	2,00E-04	3,62E-02
cg09908426	11	82109410	)C101928989	Body	0,037	1,99E-04	3,62E-02
cg09460553	13	110521956		IGR	-0,079	1,99E-04	3,62E-02
cg12478809	15	75230753	COX5A	TSS1500	-0,01	1,99E-04	3,62E-02
cg08335199	17	76462855	DNAH17	Body	-0,018	1,99E-04	3,62E-02
cg12713893	18	19192271	SNRPD1	1stExon	-0,004	2,00E-04	3,62E-02
cg12996401	18	76537451		IGR	-0,012	2,00E-04	3,62E-02
cg01869360	2	88315450		IGR	0,026	2,00E-04	3,62E-02
cg02033302	14	35822554		IGR	-0,03	2,00E-04	3,62E-02
cg22805381	14	51760188		IGR	0,04	2,00E-04	3,62E-02
cg23672472	7	95951587	SLC25A13	TSS200	0,006	2,00E-04	3,62E-02
cg01121610	1	204043732	SOX13	5'UTR	0,009	2,00E-04	3,62E-02
cg05618934	4	1407592		IGR	-0,051	2,00E-04	3,62E-02
cg07465275	11	65375203	MAP3K11	Body	-0,013	2,00E-04	3,62E-02
cg15677194	19	51003982		IGR	-0,021	2,00E-04	3,62E-02
cg13702536	12	123215553	GPR81	TSS1500	0,032	2,01E-04	3,62E-02
cg10984870	10	51051319	PARG	Body	-0,009	2,01E-04	3,63E-02
cg00211087	17	73805065	UNK	Body	0,012	2,01E-04	3,63E-02
cg22346845	2	25014897	PTRHD1	Body	0,006	2,01E-04	3,63E-02
cg06816077	16	88513379		IGR	0,004	2,01E-04	3,63E-02
cg18563630	19	7699121	PCP2	TSS1500	0,023	2,01E-04	3,63E-02

cg19783675	5	132362235	ZCCHC10	5'UTR	0,004	2,01E-04	3,63E-02
cg06358566	2	88991375	RPIA	1stExon	0,005	2,01E-04	3,63E-02
cg23763424	2	172967795	DLX2	TSS1500	0,006	2,01E-04	3,63E-02
cg24774002	19	1816355	MIR1909	TSS200	-0,005	2,01E-04	3,63E-02
cg12468403	8	127621757		IGR	-0,01	2,02E-04	3,63E-02
cg20227471	2	25065550	ADCY3	Body	-0,007	2,02E-04	3,63E-02
cg08384373	4	186594836	SORBS2	Body	-0,019	2,02E-04	3,63E-02
cg19820328	10	125332630		IGR	0,037	2,02E-04	3,63E-02
cg26413781	12	71533526	TSPAN8	Body	0,01	2,02E-04	3,63E-02
cg14401904	14	101282417		IGR	0,009	2,02E-04	3,63E-02
cg22484417	11	117103196	PCSK7	5'UTR	0,004	2,02E-04	3,63E-02
cg05230522	2	61201907	PUS10	Body	-0,017	2,02E-04	3,64E-02
cg11531127	9	130478869	PTRH1	TSS1500	-0,012	2,02E-04	3,64E-02
cg21830005	10	129375290		IGR	0,038	2,02E-04	3,64E-02
cg12394010	11	102511692		IGR	0,011	2,02E-04	3,64E-02
cg18582260	13	25085301	PARP4	5'UTR	0,03	2,03E-04	3,64E-02
cg13848598	10	115804578	ADRB1	1stExon	-0,031	2,03E-04	3,64E-02
cg27177391	12	54020455	ATF7	TSS1500	-0,01	2,03E-04	3,64E-02
cg05867960	13	111366057	ING1	Body	0,002	2,03E-04	3,64E-02
cg21362986	1	38024945	DNALI1	ExonBnd	0,019	2,03E-04	3,64E-02
cg01079779	3	57176708	IL17RD	Body	0,029	2,03E-04	3,64E-02
cg01881265	17	934534	ABR	Body	-0,011	2,03E-04	3,64E-02
cg23123540	5	171711465	UBTD2	TSS1500	0,004	2,03E-04	3,64E-02
cg19377812	7	130353647	TSGA13	3'UTR	0,014	2,03E-04	3,65E-02
cg11293828	12	3598335		IGR	0,045	2,03E-04	3,65E-02
cg22888800	14	34599406		IGR	-0,015	2,03E-04	3,65E-02
cg02513379	16	27414281	IL21R	TSS200	0,039	2,03E-04	3,65E-02
cg09758422	1	109955015	PSMA5	Body	-0,011	2,04E-04	3,65E-02
cg10992704	1	162795932		IGR	-0,019	2,04E-04	3,65E-02
cg17024919	3	21792248	ZNF385D	Body	-0,053	2,04E-04	3,65E-02
cg06206987	6	130897017		IGR	0,021	2,04E-04	3,65E-02
cg12629509	10	132527438		IGR	-0,016	2,04E-04	3,65E-02
cg04257638	16	30710486	SRCAP	5'UTR	-0,005	2,04E-04	3,65E-02
cg18145340	17	57288349	C17orf71	1stExon	-0,009	2,04E-04	3,65E-02
cg06169597	19	619447	POLRMT	Body	0,013	2,04E-04	3,65E-02
cg05639624	19	50101012	PRR12	Body	0,005	2,04E-04	3,65E-02
cg00897115	8	145234268	HEATR7A	Body	-0,016	2,05E-04	3,65E-02
cg23258300	12	125693292		IGR	0,021	2,05E-04	3,65E-02
cg20866694	6	27181670		IGR	-0,041	2,05E-04	3,66E-02
cg11047257	16	86079139		IGR	0,022	2,05E-04	3,66E-02
cg19874783	4	78538529		IGR	0,018	2,05E-04	3,66E-02
cg09706180	15	50647709	GABPB1	TSS200	0,012	2,05E-04	3,66E-02
cg26625147	5	180215939		IGR	0,006	2,05E-04	3,67E-02
cg10066586	2	99398602		IGR	-0,012	2,06E-04	3,67E-02
cg15087803	3	86934063		IGR	-0,008	2,06E-04	3,67E-02
cg14263957	5	167904257		IGR	0,023	2,06E-04	3,67E-02
cg27213238	6	10530035	GCNT2	Body	0,072	2,06E-04	3,67E-02
cg02025177	6	168593649		IGR	-0,005	2,06E-04	3,67E-02
cg16556937	12	131869141		IGR	-0,026	2,06E-04	3,67E-02
cg03170625	16	82204964	MPHOSPH6	TSS1500	0,009	2,06E-04	3,67E-02
cg07086564	17	59371804	BCAS3	Body	-0,015	2,06E-04	3,67E-02
cg05505711	6	30714519		IGR	0,044	2,06E-04	3,67E-02
cg03075214	11	64867914	C11orf2	Body	-0,01	2,06E-04	3,67E-02
cg21967790	6	10430644	C6orf218	Body	0,051	2,06E-04	3,67E-02
cg03114157	9	96380636	PHF2	Body	-0,013	2,07E-04	3,68E-02
cg21614618	2	182145887	)C101927156	Body	0,053	2,07E-04	3,68E-02
cg01591898	22	38696032	CSNK1E	Body	-0,007	2,07E-04	3,68E-02
cg03321043	5	79950501	MSH3	1stExon	0,01	2,07E-04	3,68E-02
cg04614502	8	108433965	ANGPT1	Body	-0,023	2,07E-04	3,68E-02
cg12110244	7	104682137	KMT2E	Body	-0,012	2,07E-04	3,68E-02
cg24860560	8	143057294		IGR	0,02	2,07E-04	3,68E-02

cg08348496	15	89438671	HAPLN3	5'UTR	0,003	2,07E-04	3,68E-02
cg08231251	1	27114322	PIGV	TSS200	0,007	2,07E-04	3,68E-02
cg01438257	1	38134532		IGR	0,028	2,07E-04	3,68E-02
cg13129272	14	73602808	PSEN1	TSS1500	0,02	2,07E-04	3,68E-02
cg09659887	11	9406115	IPO7	TSS200	0,005	2,08E-04	3,68E-02
cg09340831	21	43518263	UMODL1	Body	0,062	2,08E-04	3,68E-02
cg13447174	18	43366882		IGR	-0,007	2,08E-04	3,69E-02
cg02786213	1	154539358	CHRNB2	TSS1500	0,028	2,08E-04	3,69E-02
cg10116453	1	200350606		IGR	0,007	2,08E-04	3,69E-02
cg00292905	21	33895894		IGR	0,006	2,08E-04	3,69E-02
cg02288964	1	967561	AGRN	Body	0,032	2,08E-04	3,69E-02
cg09039483	2	223617708		IGR	-0,011	2,08E-04	3,69E-02
cg12824877	1	2123747	FAAP20	Body	0,005	2,08E-04	3,69E-02
cg08990112	4	113558046	LARP7	TSS200	-0,017	2,08E-04	3,69E-02
cg09761604	19	34438962		IGR	0,034	2,08E-04	3,69E-02
cg20085956	12	56360499	CDK2	TSS200	0,007	2,09E-04	3,69E-02
cg16549396	13	68765417		IGR	-0,021	2,09E-04	3,69E-02
cg25338401	14	40382174		IGR	0,038	2,09E-04	3,69E-02
cg11122968	19	51727295	CD33	TSS1500	0,025	2,09E-04	3,69E-02
cg17406543	19	56057008	SBK3	TSS200	-0,007	2,09E-04	3,69E-02
cg17301506	20	32700554	EIF2S2	TSS1500	0,011	2,09E-04	3,69E-02
cg04642627	16	81944286	PLCG2	Body	0,005	2,09E-04	3,69E-02
cg07091154	11	70562728	SHANK2	Body	-0,008	2,09E-04	3,69E-02
cg18747379	15	86848336	AGBL1-AS1	Body	0,047	2,09E-04	3,69E-02
cg11861097	20	2794877	C20orf141	TSS1500	-0,01	2,09E-04	3,69E-02
cg13418782	7	100747594		IGR	0,031	2,09E-04	3,69E-02
cg19721012	15	65412217	PDCD7	Body	0,005	2,09E-04	3,70E-02
cg07811376	19	50980165	EMC10	Body	0,007	2,09E-04	3,70E-02
cg18361024	5	180661392	TRIM41	Body	-0,023	2,09E-04	3,70E-02
cg14214023	14	92226258		IGR	-0,014	2,09E-04	3,70E-02
cg06521051	17	78179311	CARD14	ExonBnd	-0,015	2,10E-04	3,70E-02
cg04132607	20	61041456	GATA5	Body	-0,017	2,10E-04	3,70E-02
cg01233392	1	85665444	SYDE2	Body	0,046	2,10E-04	3,70E-02
cg00518495	2	20625217		IGR	0,022	2,10E-04	3,70E-02
cg21947394	2	241260110		IGR	0,026	2,10E-04	3,70E-02
cg23251248	14	55907374	TBPL2	TSS200	-0,047	2,10E-04	3,70E-02
cg02740061	7	4828519	KIAA0415	Body	-0,01	2,11E-04	3,71E-02
cg07452879	17	71274436		IGR	0,015	2,11E-04	3,71E-02
cg06758644	1	12533120	VPS13D	Body	0,041	2,11E-04	3,71E-02
cg10759158	2	227418479		IGR	0,026	2,11E-04	3,71E-02
cg02001564	6	2920795		IGR	0,005	2,11E-04	3,71E-02
cg10604258	15	93588851	RGMA	Body	0,007	2,11E-04	3,71E-02
cg22515589	17	79426432	BAHCC1	Body	-0,028	2,11E-04	3,71E-02
cg06060327	1	190446974	LINC01351	TSS1500	-0,011	2,11E-04	3,71E-02
cg06156847	2	113672199	IL1F7	Body	-0,011	2,11E-04	3,71E-02
cg20410192	4	185746558	ACSL1	5'UTR	0,004	2,11E-04	3,71E-02
cg06193382	6	136571195	FAM54A	5'UTR	0,004	2,11E-04	3,71E-02
cg13830636	10	75503731	SEC24C	TSS1500	0,002	2,11E-04	3,71E-02
cg01722566	16	84975714		IGR	-0,019	2,11E-04	3,71E-02
cg04104780	20	18429695	DZANK1	ExonBnd	-0,007	2,11E-04	3,71E-02
cg20589463	19	48367851		IGR	0,01	2,12E-04	3,72E-02
cg11682700	3	114477956	ZBTB20	1stExon	-0,005	2,12E-04	3,72E-02
cg16946833	4	83214632		IGR	-0,014	2,12E-04	3,72E-02
cg03005767	5	11494492	CTNND2	Body	0,073	2,12E-04	3,72E-02
cg18229422	2	176958552	HOXD13	Body	-0,01	2,12E-04	3,72E-02
cg09296270	2	65087288		IGR	-0,028	2,13E-04	3,73E-02
cg20877312	3	156461894		IGR	-0,006	2,13E-04	3,73E-02
cg17216904	3	57262489	APPL1	Body	0,012	2,13E-04	3,73E-02
cg26074411	6	169568564		IGR	-0,007	2,13E-04	3,73E-02
cg00749291	11	12107626		IGR	-0,1	2,13E-04	3,73E-02
cg11940876	19	54694407	TSEN34	TSS1500	0,004	2,13E-04	3,73E-02

cg06743033	6	26549305		IGR	-0,007	2,13E-04	3,73E-02
cg05005422	7	101650989	CUX1	Body	0,015	2,13E-04	3,73E-02
cg14646861	9	78740278	PCSK5	Body	0,009	2,13E-04	3,73E-02
cg25647485	12	25144628		IGR	-0,01	2,13E-04	3,73E-02
cg23705098	7	1289853		IGR	0,031	2,13E-04	3,73E-02
cg19961945	19	7192407	INSR	Body	-0,01	2,13E-04	3,73E-02
cg21998745	3	26004406		IGR	-0,014	2,14E-04	3,73E-02
cg13687924	5	110569990	CAMK4	Body	0,016	2,14E-04	3,73E-02
cg07710907	8	6688074	XKR5	Body	-0,014	2,14E-04	3,74E-02
cg24041078	11	111383889	BTG4	TSS1500	0,004	2,14E-04	3,74E-02
cg12384499	15	89949617		IGR	0,014	2,14E-04	3,74E-02
cg15225701	5	108083455	FER	TSS200	0,014	2,14E-04	3,74E-02
cg18285692	12	6809758	PIANP	5'UTR	0,008	2,14E-04	3,74E-02
cg04993112	1	153030514	SPRR2A	TSS1500	0,032	2,14E-04	3,74E-02
cg10278318	2	235276581		IGR	0,008	2,14E-04	3,74E-02
cg16074109	12	124799011	FAM101A	Body	-0,017	2,14E-04	3,74E-02
cg22464067	16	88599027	ZFPM1	ExonBnd	-0,016	2,15E-04	3,74E-02
cg24378945	11	105947912	KBTBD3	5'UTR	-0,011	2,15E-04	3,75E-02
cg04805131	1	158978812	IFI16	TSS1500	0,005	2,15E-04	3,75E-02
cg14505104	3	30673950	TGFBR2	Body	-0,012	2,15E-04	3,75E-02
cg04001880	15	89876213	POLG	Body	-0,006	2,15E-04	3,75E-02
cg00077402	20	9597606	PAK7	Body	0,053	2,15E-04	3,75E-02
cg04282886	8	446890	TDRP	Body	-0,012	2,15E-04	3,75E-02
cg18613420	1	26670625	AIM1L	Body	-0,013	2,15E-04	3,75E-02
cg15224767	17	2496745	PAFAH1B1	TSS200	0,005	2,15E-04	3,75E-02
cg26835312	1	24513473	IL28RA	Body	0,02	2,16E-04	3,75E-02
cg01068240	1	43889011	KIAA0467	5'UTR	-0,009	2,16E-04	3,75E-02
cg24868645	5	137057949	KLHL3	TSS1500	0,014	2,16E-04	3,75E-02
cg01286964	10	74010595		IGR	-0,017	2,16E-04	3,75E-02
cg16621943	11	74914226	SLCO2B1	Body	-0,015	2,16E-04	3,75E-02
cg21854040	12	120121808		IGR	0,006	2,16E-04	3,75E-02
cg15456712	16	1705800	CRAMP1L	Body	-0,014	2,16E-04	3,75E-02
cg19561768	20	34171963	FER1L4	Body	-0,015	2,16E-04	3,75E-02
cg11740099	17	80279430	SECTM1	3'UTR	0,005	2,16E-04	3,75E-02
cg19974115	4	120222000	C4orf3	Body	-0,005	2,16E-04	3,75E-02
cg02167005	1	64013579	DLEU2L	TSS1500	-0,007	2,17E-04	3,75E-02
cg08569138	1	78470430	DNAJB4	TSS1500	-0,021	2,17E-04	3,75E-02
cg12168884	1	154999150	DCST2	Body	-0,011	2,17E-04	3,75E-02
cg17456047	3	108237840	MYH15	Body	0,031	2,16E-04	3,75E-02
cg11628282	3	179065624	MFN1	5'UTR	0,004	2,17E-04	3,75E-02
cg00422638	10	25466027	)C100128811	TSS1500	-0,008	2,17E-04	3,75E-02
cg01017197	10	102046482	BLOC1S2	TSS1500	0,007	2,17E-04	3,75E-02
cg16310045	10	114813758	TCF7L2	Body	-0,029	2,16E-04	3,75E-02
cg14878150	12	101673760	UTP20	TSS200	-0,004	2,16E-04	3,75E-02
cg01715525	14	104687182		IGR	-0,036	2,17E-04	3,75E-02
cg15886728	15	101817534	SELS	Body	0,003	2,17E-04	3,75E-02
cg12082397	19	1527146	PLK5	Body	0,056	2,16E-04	3,75E-02
cg18812257	20	48212528		IGR	0,034	2,17E-04	3,75E-02
cg21884354	14	105687060	BRF1	Body	0,042	2,17E-04	3,75E-02
cg05836523	13	111775045	ARHGEF7	Body	-0,004	2,17E-04	3,75E-02
cg06867916	14	69865653	SLC39A9	5'UTR	-0,003	2,17E-04	3,75E-02
cg08484992	16	88977278	CBFA2T3	5'UTR	0,057	2,17E-04	3,75E-02
cg06935052	22	24176449	SMARCB1	3'UTR	-0,014	2,17E-04	3,76E-02
cg20934138	22	18595307	TUBA8	5'UTR	0,019	2,17E-04	3,76E-02
cg20538228	17	79941335	ASPSCR1	Body	0,006	2,18E-04	3,76E-02
cg13453228	1	34925972		IGR	0,044	2,18E-04	3,76E-02
cg25957701	18	13419019	LDLRAD4	Body	0,005	2,18E-04	3,76E-02
cg11800342	9	113699234	LPAR1	Body	0,044	2,18E-04	3,76E-02
cg26824850	8	145107857	OPLAH	Body	-0,031	2,18E-04	3,76E-02
cg00764109	1	114697582	SYT6	TSS1500	-0,014	2,18E-04	3,77E-02
cg03625007	2	197478067		IGR	-0,026	2,18E-04	3,77E-02

cg17675088	5	93013224	FAM172A	Body	-0,016	2,18E-04	3,77E-02
cg21567971	6	164526833		IGR	-0,012	2,18E-04	3,77E-02
cg01677386	11	118938358	VPS11	TSS200	0,004	2,19E-04	3,77E-02
cg01691592	12	25801556	LMNTD1	TSS200	0,036	2,19E-04	3,77E-02
cg23763427	12	57878408		IGR	0,006	2,19E-04	3,77E-02
cg22153105	19	13941182	ZSWIM4	Body	-0,009	2,19E-04	3,77E-02
cg02757609	17	74552994		IGR	-0,036	2,19E-04	3,77E-02
cg16584696	10	134875826		IGR	0,011	2,19E-04	3,78E-02
cg10030833	17	65236030	HELZ	5'UTR	-0,025	2,19E-04	3,78E-02
cg10100800	19	13510044	CACNA1A	Body	0,036	2,19E-04	3,78E-02
cg16647243	14	64040326		IGR	0,009	2,20E-04	3,78E-02
cg09084892	4	187557837	FAT1	Body	0,007	2,20E-04	3,78E-02
cg16694455	5	14194251	TRIO	Body	0,009	2,20E-04	3,78E-02
cg04777383	5	177543666	N4BP3	5'UTR	0,003	2,20E-04	3,78E-02
cg07468402	14	70464107	SMOC1	Body	-0,006	2,20E-04	3,78E-02
cg19571972	20	36931430	BPI	TSS1500	0,037	2,20E-04	3,78E-02
cg04204874	3	16253377	GALNT15	Body	0,038	2,20E-04	3,78E-02
cg16891350	1	228800369	RHOU	Body	-0,007	2,20E-04	3,78E-02
cg18638793	12	108908854	FICD	TSS200	0,003	2,20E-04	3,79E-02
cg18450859	3	130743432	ASTE1	Body	0,007	2,21E-04	3,79E-02
cg13311357	4	48782334	FRYL	TSS200	0,008	2,21E-04	3,79E-02
cg16013223	8	56437691	XKR4	3'UTR	0,01	2,21E-04	3,79E-02
cg13950250	8	104430401	DCAF13	Body	0,006	2,21E-04	3,79E-02
cg22080035	6	167317118	RPS6KA2-AS1	TSS200	-0,007	2,21E-04	3,79E-02
cg06509933	10	5626595		IGR	-0,009	2,21E-04	3,79E-02
cg08649068	17	38512297	RARA	Body	-0,011	2,21E-04	3,79E-02
cg04536765	6	31621761	BAT3	TSS1500	-0,017	2,21E-04	3,79E-02
cg10615990	5	179050688	HNRNPH1	1stExon	0,004	2,21E-04	3,79E-02
cg21144084	13	76122506	UCHL3	TSS1500	-0,018	2,21E-04	3,79E-02
cg15462232	1	63788184	FOXD3	TSS1500	0,004	2,22E-04	3,80E-02
cg24769907	1	94147478	BCAR3	TSS1500	-0,008	2,22E-04	3,80E-02
cg10849854	2	11674557	GREB1	5'UTR	0,026	2,22E-04	3,80E-02
cg17647951	12	124008395	RILPL1	Body	0,008	2,22E-04	3,80E-02
cg09970483	15	64455548	PPIB	TSS200	0,004	2,21E-04	3,80E-02
cg09280095	15	93855060		IGR	-0,019	2,22E-04	3,80E-02
cg01240759	19	2507414		IGR	0,009	2,22E-04	3,80E-02
cg18785120	17	79899585	MYADML2	Body	0,023	2,22E-04	3,80E-02
cg17254176	7	151361260	PRKAG2	Body	-0,004	2,22E-04	3,80E-02
cg14821871	8	142264923	SLC45A4	TSS200	-0,028	2,22E-04	3,80E-02
cg13255969	9	136061521		IGR	-0,01	2,22E-04	3,80E-02
cg24868768	14	60406842	LRRC9	Body	0,051	2,22E-04	3,80E-02
cg00809917	6	161560605	AGPAT4	Body	-0,015	2,23E-04	3,81E-02
cg03953196	16	85814044	COX4NB	Body	-0,005	2,23E-04	3,81E-02
cg22926130	2	26418074	HADHA	Body	-0,01	2,23E-04	3,81E-02
cg02529111	1	21897469	ALPL	Body	-0,01	2,23E-04	3,81E-02
cg00085994	2	105702137	MRPS9	Body	0,026	2,23E-04	3,81E-02
cg16245761	9	98125856		IGR	0,036	2,23E-04	3,81E-02
cg07631650	7	17484093	KCCAT333	Body	-0,007	2,23E-04	3,81E-02
cg16309957	3	3222610	CRBN	TSS1500	-0,007	2,23E-04	3,81E-02
cg04230735	5	10077400		IGR	-0,007	2,23E-04	3,81E-02
cg03200747	6	80580285		IGR	-0,017	2,23E-04	3,81E-02
cg16934235	11	108423124	EXPH5	TSS200	-0,012	2,23E-04	3,81E-02
cg13453162	16	83029685	CDH13	5'UTR	-0,012	2,23E-04	3,81E-02
cg23464360	8	144513980	MAFA	TSS1500	0,034	2,23E-04	3,81E-02
cg01361290	2	144999454	GTDC1	5'UTR	0,025	2,24E-04	3,81E-02
cg20716653	5	65124049		IGR	0,014	2,24E-04	3,81E-02
cg04468543	5	77268331		IGR	-0,017	2,24E-04	3,81E-02
cg08284876	5	91970207		IGR	-0,007	2,24E-04	3,81E-02
cg13962347	6	5174647	LYRM4	Body	0,059	2,24E-04	3,81E-02
cg20522398	7	17316151		IGR	-0,013	2,24E-04	3,81E-02
cg27535142	7	150178401		IGR	0,035	2,24E-04	3,81E-02

cg01616489	12	133249980	POLE	Body	-0,015	2,24E-04	3,81E-02
cg14315198	17	26879866	UNC119	TSS1500	-0,024	2,24E-04	3,81E-02
cg25279605	2	128403143	GPR17	TSS1500	0,006	2,24E-04	3,81E-02
cg17455591	1	87379840	sept-15	5'UTR	-0,003	2,25E-04	3,82E-02
cg25700931	3	128039078	EEFSEC	Body	-0,006	2,25E-04	3,82E-02
cg11012643	3	185138857	MAP3K13	5'UTR	0,035	2,25E-04	3,82E-02
cg02788266	5	50694104		IGR	0,009	2,25E-04	3,82E-02
cg04428805	17	65239522	HELZ	5'UTR	-0,011	2,25E-04	3,82E-02
cg21349637	18	13218607	C18orf1	TSS200	-0,005	2,24E-04	3,82E-02
cg24977042	11	13738787	FAR1	Body	-0,006	2,25E-04	3,82E-02
cg09057327	14	101428404	SNORD114-7	TSS1500	0,025	2,25E-04	3,82E-02
cg16104006	17	1898874	RTN4RL1	Body	-0,006	2,25E-04	3,82E-02
cg25462858	10	105032988		IGR	0,022	2,25E-04	3,82E-02
cg19898108	12	54383692	HOXC10	3'UTR	-0,014	2,25E-04	3,82E-02
cg08307430	3	5163792	ARL8B	TSS200	-0,004	2,25E-04	3,82E-02
cg11867293	10	3972111		IGR	-0,005	2,25E-04	3,82E-02
cg04552235	10	112371252		IGR	-0,015	2,25E-04	3,82E-02
cg24012572	13	100569505	)C101927437	Body	0,017	2,25E-04	3,82E-02
cg03607648	2	234668930	UGT1A10	Body	-0,008	2,26E-04	3,82E-02
cg15773612	3	145618489		IGR	-0,031	2,26E-04	3,82E-02
cg15918355	6	29407689	OR10C1	TSS200	0,005	2,26E-04	3,82E-02
cg18087682	8	8239292	SGK223	1stExon	0,018	2,26E-04	3,82E-02
cg13880868	9	131285907	GLE1	Body	-0,011	2,26E-04	3,82E-02
cg25837813	11	67207973	CORO1B	Body	0,026	2,26E-04	3,82E-02
cg08864819	11	126197316	DCPS	Body	-0,01	2,26E-04	3,82E-02
cg01245880	22	30899870	SEC14L4	Body	0,045	2,26E-04	3,82E-02
cg00238570	2	110871644	MALL	Body	-0,008	2,26E-04	3,82E-02
cg02893019	6	57182421	PRIM2	1stExon	-0,005	2,26E-04	3,83E-02
cg06585307	16	1581015	IFT140	Body	-0,009	2,26E-04	3,83E-02
cg27176697	17	26734524	SLC46A1	TSS1500	0,007	2,26E-04	3,83E-02
cg18772668	15	66994542	SMAD6	TSS200	0,003	2,27E-04	3,83E-02
cg18318818	17	48640257	CACNA1G	Body	0,029	2,27E-04	3,83E-02
cg16330477	1	219920893		IGR	0,05	2,27E-04	3,83E-02
cg15899170	6	123014386	PKIB	5'UTR	0,011	2,27E-04	3,83E-02
cg01514353	22	46259643		IGR	0,031	2,27E-04	3,83E-02
cg10017626	2	65085243		IGR	-0,007	2,27E-04	3,83E-02
cg23103018	2	154800477	GALNT13	TSS1500	-0,017	2,27E-04	3,83E-02
cg18989174	7	149470820	ZNF467	TSS1500	0,004	2,27E-04	3,83E-02
cg19215541	16	86337686		IGR	-0,011	2,27E-04	3,83E-02
cg19675063	4	81975265	BMP3	3'UTR	-0,006	2,27E-04	3,83E-02
cg19835606	7	150778201	FASTK	TSS1500	0,006	2,27E-04	3,83E-02
cg01572872	8	125071131	FER1L6	Body	0,005	2,27E-04	3,83E-02
cg24617900	13	100436330	CLYBL	Body	-0,02	2,27E-04	3,83E-02
cg04318546	15	101147087	ASB7	5'UTR	0,023	2,28E-04	3,83E-02
cg21581038	2	128284637	IWS1	TSS1500	-0,004	2,28E-04	3,84E-02
cg16825643	16	88802743	FAM38A	Body	-0,012	2,28E-04	3,84E-02
cg10190686	16	19388171		IGR	-0,009	2,28E-04	3,84E-02
cg13419713	19	18059878		IGR	0,008	2,28E-04	3,84E-02
cg06946770	1	47139942	EFCAB14-AS1	Body	-0,006	2,28E-04	3,84E-02
cg12102573	17	27924969	ANKRD13B	Body	-0,01	2,28E-04	3,84E-02
cg11808871	1	1268942	TAS1R3	Body	-0,018	2,28E-04	3,84E-02
cg26198575	3	130912881	NEK11	Body	0,032	2,28E-04	3,84E-02
cg04424002	1	235099406	)C101927851	Body	-0,01	2,29E-04	3,84E-02
cg22733239	6	74059318		IGR	0,048	2,29E-04	3,84E-02
cg12510203	2	144915087	GTDC1	Body	0,009	2,29E-04	3,84E-02
cg21909090	9	79521848	PRUNE2	TSS1500	0,038	2,29E-04	3,85E-02
cg19132680	12	95449869	NR2C1	Body	0,009	2,29E-04	3,85E-02
cg15273874	2	234704600	MROH2A	ExonBnd	0,018	2,29E-04	3,85E-02
cg25845650	3	99254252		IGR	-0,007	2,29E-04	3,85E-02
cg21611187	4	38321009		IGR	-0,004	2,30E-04	3,85E-02
cg06675531	5	150019123	SYNPO	TSS1500	-0,007	2,30E-04	3,85E-02

cg18165649	5	153281200		IGR	0,038	2,29E-04	3,85E-02
cg07725318	6	30227326	HLA-L	TSS200	0,006	2,30E-04	3,85E-02
cg13716619	6	31829895	NEU1	Body	-0,019	2,30E-04	3,85E-02
cg04277890	7	110317028	IMMP2L	Body	-0,012	2,30E-04	3,85E-02
cg09981907	8	22022035	BMP1	TSS1500	0,004	2,29E-04	3,85E-02
cg22238152	13	21635828	LATS2	TSS200	0,009	2,30E-04	3,85E-02
cg07006158	16	70285910	EXOSC6	TSS200	0,006	2,30E-04	3,85E-02
cg17883035	19	45261808	BCL3	Body	-0,009	2,30E-04	3,85E-02
cg24379220	22	19196339	CLTCL1	Body	0,022	2,29E-04	3,85E-02
cg08716217	1	2483719	LOC115110	Body	0,004	2,30E-04	3,85E-02
cg14657532	2	219762543		IGR	-0,023	2,30E-04	3,85E-02
cg01893439	7	148581376	EZH2	5'UTR	0,003	2,30E-04	3,85E-02
cg10008353	7	35827622	SEPT7-AS1	Body	-0,035	2,31E-04	3,86E-02
cg05817476	10	3274619		IGR	-0,012	2,31E-04	3,86E-02
cg09648228	14	21737169	HNRNPC	5'UTR	-0,007	2,31E-04	3,86E-02
cg20532970	20	49220170	FAM65C	Body	-0,012	2,31E-04	3,86E-02
cg12585516	4	83123547		IGR	-0,016	2,31E-04	3,86E-02
cg10766599	15	70268721		IGR	0,035	2,31E-04	3,86E-02
cg17736252	1	78273462	FAM73A	Body	-0,018	2,31E-04	3,86E-02
cg15593507	4	182881898		IGR	-0,008	2,31E-04	3,86E-02
cg18716737	7	137661683	CREB3L2	Body	-0,014	2,31E-04	3,86E-02
cg24926364	13	114748575	RASA3	3'UTR	-0,009	2,31E-04	3,86E-02
cg22168087	7	27702803	HIBADH	TSS1500	0,055	2,31E-04	3,86E-02
cg21376058	1	9131028	SLC2A5	TSS1500	0,006	2,31E-04	3,86E-02
cg01195848	1	228466165	OBSCN	Body	-0,015	2,31E-04	3,86E-02
cg10045864	2	240036897	HDAC4	Body	-0,008	2,31E-04	3,86E-02
cg06417824	7	43959203	URGCP	5'UTR	-0,016	2,32E-04	3,86E-02
cg22753919	10	24928307	ARHGAP21	Body	-0,027	2,32E-04	3,87E-02
cg22656705	13	20207391	MPHOSPH8	TSS1500	-0,003	2,32E-04	3,87E-02
cg17986264	10	64576256	EGR2	TSS200	0,005	2,32E-04	3,87E-02
cg13311902	7	50773857	GRB10	5'UTR	-0,027	2,32E-04	3,87E-02
cg11087774	10	134730605		IGR	-0,017	2,32E-04	3,87E-02
cg05677741	14	75518249	MLH3	TSS200	0,007	2,32E-04	3,87E-02
cg00077606	5	37248894	C5orf42	5'UTR	0,009	2,32E-04	3,87E-02
cg24071544	6	13423721	GFOD1	Body	0,007	2,32E-04	3,87E-02
cg05170494	2	118845939	INSIG2	TSS200	0,005	2,33E-04	3,87E-02
cg01142721	10	85985448	LRIT2	TSS200	0,061	2,33E-04	3,87E-02
cg15989276	2	242063571	PASK	Body	0,017	2,33E-04	3,87E-02
cg21885107	14	73712289	PAPLN	Body	0,042	2,33E-04	3,87E-02
cg02334877	1	210484243		IGR	0,034	2,33E-04	3,88E-02
cg26656036	6	37026016		IGR	-0,016	2,33E-04	3,88E-02
cg19975209	20	35374653	NDRG3	TSS200	0,004	2,33E-04	3,88E-02
cg04593538	17	41740208	MEOX1	TSS1500	0,029	2,33E-04	3,88E-02
cg14801874	1	201265254	PKP1	Body	0,029	2,34E-04	3,88E-02
cg15933840	2	240123850	HDAC4	Body	0,017	2,34E-04	3,88E-02
cg15085633	6	158438072	SYNJ2	TSS200	-0,017	2,34E-04	3,88E-02
cg00525989	7	72936712	BAZ1B	TSS200	0,004	2,33E-04	3,88E-02
cg14996220	12	85673270	ALX1	TSS1500	-0,011	2,33E-04	3,88E-02
cg00594099	1	27893886	AHDC1	5'UTR	0,008	2,34E-04	3,88E-02
cg04203084	3	49131538	QRICH1	TSS200	-0,006	2,34E-04	3,88E-02
cg17493303	3	49450528	RHOA	TSS1500	-0,017	2,34E-04	3,88E-02
cg17566936	4	48271107	TEC	5'UTR	-0,006	2,34E-04	3,88E-02
cg02559239	5	139889642	ANKHD1	Body	0,025	2,34E-04	3,88E-02
cg19537820	1	120351812	REG4	5'UTR	-0,012	2,34E-04	3,88E-02
cg16423674	2	35635232		IGR	0,042	2,34E-04	3,88E-02
cg01286665	5	54525925		IGR	-0,05	2,35E-04	3,88E-02
cg26656672	6	164393351		IGR	-0,024	2,35E-04	3,88E-02
cg14250600	12	54121407	CALCOCO1	TSS200	-0,007	2,35E-04	3,88E-02
cg13201942	15	81344662		IGR	-0,025	2,34E-04	3,88E-02
cg07336438	16	1131466		IGR	0,036	2,35E-04	3,88E-02
cg10815411	11	7176226		IGR	0,036	2,35E-04	3,88E-02

cg07606115	11	130068175	ST14	Body	-0,021	2,35E-04	3,88E-02
cg23259633	9	130533850	SH2D3C	TSS1500	-0,006	2,35E-04	3,89E-02
cg02435191	3	194969372	XXYLT1	TSS1500	0,005	2,35E-04	3,89E-02
cg00622880	13	52379315	DHRS12	TSS1500	0,03	2,35E-04	3,89E-02
cg12460711	6	38140385	BTBD9	3'UTR	-0,011	2,35E-04	3,89E-02
cg10579012	1	22746853		IGR	0,032	2,35E-04	3,89E-02
cg01267262	2	219926062	IHH	TSS1500	0,006	2,35E-04	3,89E-02
cg17045651	3	159569504	SCHIP1	Body	0,027	2,35E-04	3,89E-02
cg14153356	6	10062223		IGR	0,034	2,35E-04	3,89E-02
cg19743522	12	113495566	DTX1	TSS200	0,018	2,35E-04	3,89E-02
cg10240725	12	97300803	NEDD1	TSS200	0,003	2,35E-04	3,89E-02
cg06677416	10	64495627		IGR	-0,011	2,35E-04	3,89E-02
cg14286790	6	45543042		IGR	-0,01	2,36E-04	3,89E-02
cg19185133	11	93474120	C11orf54	TSS1500	0,004	2,36E-04	3,89E-02
cg12368048	2	10441093		IGR	0,038	2,36E-04	3,89E-02
cg02262221	4	91047852	FAM190A	TSS1500	0,038	2,36E-04	3,89E-02
cg13329852	13	50265193	EBPL	Body	0,003	2,36E-04	3,89E-02
cg18060687	16	57109702	NLRC5	Body	0,016	2,36E-04	3,89E-02
cg12859213	7	30637735	GARS	Body	-0,005	2,36E-04	3,89E-02
cg05147195	6	33288734	DAXX	Body	-0,005	2,36E-04	3,89E-02
cg08760887	3	107658668		IGR	0,009	2,36E-04	3,89E-02
cg25370728	21	44106476	PDE9A	Body	0,006	2,36E-04	3,89E-02
cg03774868	2	115442503	DPP10	Body	0,02	2,36E-04	3,89E-02
cg15781725	2	153492558	FMNL2	Body	0,028	2,36E-04	3,89E-02
cg09870834	4	957817	DGKQ	Body	-0,007	2,36E-04	3,89E-02
cg00307191	12	75126292		IGR	-0,036	2,36E-04	3,89E-02
cg05950291	15	101309210		IGR	0,01	2,36E-04	3,89E-02
cg09587362	2	226139055		IGR	0,034	2,36E-04	3,89E-02
cg12921411	5	150004918	SYNPO	Body	0,029	2,37E-04	3,89E-02
cg26451835	6	117996421	NUS1	TSS200	-0,004	2,37E-04	3,89E-02
cg15326094	14	72785590	RGS6	5'UTR	0,03	2,37E-04	3,89E-02
cg12742140	17	42264438	TMUB2	1stExon	0,003	2,37E-04	3,89E-02
cg02011409	3	14697078	C3orf19	Body	0,005	2,37E-04	3,89E-02
cg22760523	15	72102618	NR2E3	TSS1500	-0,007	2,37E-04	3,89E-02
cg20523759	12	51788075	SLC4A8	5'UTR	0,043	2,37E-04	3,89E-02
cg00953352	12	56346910	DGKA	Body	0,013	2,37E-04	3,90E-02
cg21191592	9	95181961	CENPP	Body	0,007	2,38E-04	3,90E-02
cg26116103	12	51157776	ATF1	TSS200	0,004	2,38E-04	3,90E-02
cg07814076	18	19650235		IGR	-0,013	2,38E-04	3,90E-02
cg16521460	5	112156317	APC	Body	0,009	2,38E-04	3,90E-02
cg27610709	12	91576627	DCN	5'UTR	-0,019	2,38E-04	3,90E-02
cg02267483	5	140810404	PCDHGA4	Body	-0,039	2,38E-04	3,90E-02
cg00402074	6	46889742	GPR116	TSS200	0,007	2,38E-04	3,90E-02
cg02278697	12	129308719	SLC15A4	TSS200	0,003	2,38E-04	3,90E-02
cg18099817	19	11596394	ZNF653	Body	-0,015	2,38E-04	3,90E-02
cg08630533	20	55340857		IGR	0,036	2,38E-04	3,90E-02
cg11251215	1	45103933	RNF220	Body	-0,009	2,38E-04	3,90E-02
cg09652509	3	181252554		IGR	-0,004	2,38E-04	3,90E-02
cg19084252	1	228540694	OBSCN	Body	-0,008	2,38E-04	3,90E-02
cg20125559	2	240101314	HDAC4	Body	0,005	2,39E-04	3,90E-02
cg03325598	3	12994877	IQSEC1	Body	-0,009	2,39E-04	3,90E-02
cg17050526	5	167147735	TENM2	Body	0,027	2,38E-04	3,90E-02
cg07613405	2	178130312	NFE2L2	TSS1500	-0,013	2,39E-04	3,90E-02
cg09015385	6	126240277	NCOA7	TSS200	-0,003	2,39E-04	3,91E-02
cg10444530	8	52811374	PCMTD1	5'UTR	0,01	2,39E-04	3,91E-02
cg27412284	8	1462183	DLGAP2	5'UTR	0,013	2,39E-04	3,91E-02
cg02471319	12	4647845	RAD51AP1	TSS200	0,003	2,39E-04	3,91E-02
cg08871701	7	38453574	AMPH	Body	0,009	2,39E-04	3,91E-02
cg09507215	13	113348391	ATP11A	Body	-0,018	2,39E-04	3,91E-02
cg02803626	15	66972290	LINC01169	Body	-0,007	2,40E-04	3,91E-02
cg00819233	1	162601805	DDR2	TSS1500	0,005	2,40E-04	3,91E-02

cg06297413	4	2427374		IGR	-0,009	2,40E-04	3,91E-02
cg12224670	18	55891490	NEDD4L	Body	0,028	2,40E-04	3,91E-02
cg03578548	9	91267411	LOC286238	TSS1500	0,021	2,40E-04	3,91E-02
cg05455279	3	97109009	EPHA6	Body	0,041	2,40E-04	3,91E-02
cg23072559	19	55944231	SHISA7	3'UTR	0,035	2,40E-04	3,92E-02
cg14501895	1	27634872	WDTC1	3'UTR	-0,008	2,40E-04	3,92E-02
cg25626375	8	133072269	OC90	TSS1500	0,05	2,40E-04	3,92E-02
cg21238000	2	229476261		IGR	-0,024	2,40E-04	3,92E-02
cg15268456	8	144260671		IGR	0,046	2,41E-04	3,92E-02
cg03584094	10	35535915	CCNY	TSS200	0,007	2,41E-04	3,92E-02
cg26468036	13	52202515	WDFY2	Body	-0,014	2,41E-04	3,92E-02
cg19441462	15	101256329		IGR	-0,013	2,41E-04	3,92E-02
cg16779049	21	35828820	KCNE1	5'UTR	-0,009	2,41E-04	3,92E-02
cg15824312	1	78414376	FUBP1	3'UTR	0,019	2,41E-04	3,92E-02
cg02407647	2	48679415	PPP1R21	Body	-0,008	2,41E-04	3,92E-02
cg07811750	17	4890503	CAMTA2	5'UTR	0,004	2,41E-04	3,92E-02
cg02773250	7	75391678		IGR	0,035	2,41E-04	3,92E-02
cg19003257	10	134730870		IGR	-0,012	2,41E-04	3,92E-02
cg09038297	1	161130991	USP21	Body	-0,008	2,41E-04	3,92E-02
cg22843625	19	50979755	C19orf63	5'UTR	-0,005	2,41E-04	3,92E-02
cg27278476	3	42464252		IGR	-0,007	2,42E-04	3,93E-02
cg00493139	2	626713		IGR	-0,014	2,42E-04	3,93E-02
cg13394153	22	43634485	SCUBE1	Body	-0,01	2,42E-04	3,93E-02
cg14245910	7	129845377	TMEM209	TSS200	-0,005	2,42E-04	3,93E-02
cg13704271	1	32666264	CCDC28B	1stExon	0,005	2,43E-04	3,93E-02
cg25818214	3	64211155	PRICKLE2	TSS200	0,041	2,42E-04	3,93E-02
cg05110013	3	197091595		IGR	0,054	2,42E-04	3,93E-02
cg19463976	6	27173633		IGR	0,005	2,43E-04	3,93E-02
cg12939283	6	33044012	HLA-DPB1	Body	0,029	2,42E-04	3,93E-02
cg26565580	15	52433257	GNB5	Body	-0,013	2,42E-04	3,93E-02
cg03692867	17	78976336		IGR	0,003	2,42E-04	3,93E-02
cg13314173	18	62037312	LOC284294	Body	-0,01	2,42E-04	3,93E-02
cg19406914	19	5789520	DUS3L	Body	-0,007	2,43E-04	3,93E-02
cg08515910	19	14678306	NDUFB7	Body	-0,016	2,43E-04	3,93E-02
cg02796390	20	3726190	HSPA12B	Body	0,024	2,43E-04	3,93E-02
cg10858644	22	37345239		IGR	-0,011	2,42E-04	3,93E-02
cg14001429	7	152537589	ACTR3B	Body	-0,012	2,43E-04	3,93E-02
cg23222472	7	112135961		IGR	0,035	2,43E-04	3,93E-02
cg04615725	14	64653096	SYNE2	Body	-0,01	2,43E-04	3,93E-02
cg20823940	1	95007489	F3	TSS200	0,003	2,43E-04	3,93E-02
cg10228857	1	234232196	SLC35F3	Body	0,029	2,43E-04	3,93E-02
cg22137614	6	17909196	KIF13A	Body	-0,008	2,43E-04	3,93E-02
cg09358583	11	12211296	MICAL2	Body	0,035	2,43E-04	3,93E-02
cg00683285	14	28764188		IGR	-0,048	2,43E-04	3,93E-02
cg08714510	2	106755481	UXS1	TSS200	0,035	2,43E-04	3,93E-02
cg25205321	13	111096339	COL4A2	Body	0,041	2,43E-04	3,93E-02
cg11012585	21	16774722		IGR	-0,007	2,43E-04	3,93E-02
cg05458815	2	163040216	FAP	Body	-0,006	2,44E-04	3,94E-02
cg15578615	3	170074599	SKIL	TSS1500	0,006	2,44E-04	3,94E-02
cg06240412	20	52774697	CYP24A1	ExonBnd	0,006	2,44E-04	3,94E-02
cg01282432	14	57046857	C14orf101	Body	0,006	2,44E-04	3,94E-02
cg05646015	2	3697458		IGR	-0,014	2,44E-04	3,94E-02
cg23047271	3	64210991	PRICKLE2	1stExon	0,036	2,44E-04	3,94E-02
cg08405828	11	133236212	OPCML	Body	0,029	2,44E-04	3,94E-02
cg26228591	1	16054846	PLEKHM2	Body	-0,016	2,44E-04	3,94E-02
cg19216817	19	49950808	PIH1D1	Body	-0,019	2,44E-04	3,94E-02
cg22359668	1	247064037	AHCTF1	Body	0,008	2,45E-04	3,95E-02
cg03762541	3	111260252	CD96	TSS1500	0,005	2,45E-04	3,95E-02
cg08663733	6	161792028	PARK2	Body	0,004	2,45E-04	3,95E-02
cg12570013	19	57630160	USP29	TSS1500	0,028	2,45E-04	3,95E-02
cg06551785	1	23946588		IGR	0,034	2,45E-04	3,95E-02

cg08217798	9	134741723	MED27	Body	-0,012	2,45E-04	3,95E-02
cg19226619	1	184377004	C1orf21	5'UTR	-0,007	2,46E-04	3,95E-02
cg10768996	1	205236002	TMCC2	Body	-0,017	2,46E-04	3,95E-02
cg02522485	3	49531973	DAG1	5'UTR	0,011	2,46E-04	3,95E-02
cg07735790	6	34101545	GRM4	TSS200	0,025	2,46E-04	3,95E-02
cg07147204	6	168147405		IGR	-0,091	2,46E-04	3,95E-02
cg12164400	9	136214828	MED22	5'UTR	0,006	2,46E-04	3,95E-02
cg17270821	10	134362758	INPP5A	Body	0,034	2,46E-04	3,95E-02
cg01241241	15	33202135	FMN1	Body	-0,035	2,46E-04	3,95E-02
cg18475730	17	14683651		IGR	-0,031	2,46E-04	3,95E-02
cg02561259	3	52875714	110-MUSTN1	Body	0,003	2,46E-04	3,96E-02
cg23805962	4	152197649	PRSS48	TSS1500	0,01	2,46E-04	3,96E-02
cg08385375	5	34854712	TTC23L	Body	-0,033	2,46E-04	3,96E-02
cg10048935	14	53389849	FERMT2	Body	-0,01	2,46E-04	3,96E-02
cg03085549	16	75150819	LDHD	TSS200	0,066	2,47E-04	3,96E-02
cg20117497	21	47531238	COL6A2	5'UTR	-0,015	2,47E-04	3,96E-02
cg02421646	11	59943477	MS4A6A	Body	0,044	2,47E-04	3,96E-02
cg20808355	12	54990494		IGR	0,006	2,47E-04	3,96E-02
cg10366076	10	111723966	ADD3-AS1	Body	0,004	2,47E-04	3,96E-02
cg19081636	6	149469744		IGR	0,007	2,47E-04	3,96E-02
cg24212158	11	45275434	SYT13	Body	-0,009	2,47E-04	3,96E-02
cg25237400	12	132255332	SFSWAP	Body	0,018	2,47E-04	3,96E-02
cg22788591	1	39668623	MACF1	Body	-0,007	2,48E-04	3,97E-02
cg08571745	1	175065171	TNN	Body	0,025	2,48E-04	3,97E-02
cg25183214	2	222360426	EPHA4	Body	0,006	2,48E-04	3,97E-02
cg11289878	4	111121682		IGR	-0,008	2,48E-04	3,97E-02
cg20208206	7	8032590	GLCCI1	Body	-0,023	2,48E-04	3,97E-02
cg23804585	21	26983958		IGR	0,008	2,48E-04	3,97E-02
cg00881963	22	29131121	CHEK2	5'UTR	-0,007	2,48E-04	3,97E-02
cg22016779	2	230452311	DNER	Body	0,026	2,48E-04	3,97E-02
cg22736271	11	120113924	POU2F3	Body	0,025	2,48E-04	3,97E-02
cg05569169	12	110786730	ATP2A2	3'UTR	0,008	2,48E-04	3,97E-02
cg23357035	11	64677425	ATG2A	Body	-0,018	2,48E-04	3,97E-02
cg18131045	5	175594888	LOC643201	Body	0,035	2,49E-04	3,98E-02
cg17487612	20	36643563	TTI1	5'UTR	-0,011	2,49E-04	3,98E-02
cg22343980	1	168148117	TIPRL	TSS200	0,005	2,49E-04	3,98E-02
cg25106791	7	2962373	CARD11	Body	-0,008	2,49E-04	3,98E-02
cg06800840	12	121476549	OASL	Body	0,006	2,49E-04	3,98E-02
cg21715020	16	21610968	METTL9	5'UTR	0,004	2,49E-04	3,98E-02
cg08279861	17	73323741	GRB2	Body	-0,013	2,49E-04	3,98E-02
cg14627810	4	109090812	LEF1-AS1	Body	-0,041	2,49E-04	3,98E-02
cg27110790	19	35701964		IGR	0,019	2,49E-04	3,98E-02
cg10206380	16	57672032	GPR56	5'UTR	0,01	2,50E-04	3,99E-02
cg09519963	20	31467660		IGR	0,006	2,50E-04	3,99E-02
cg25228936	6	167815371		IGR	0,034	2,50E-04	3,99E-02
cg09650138	10	75669095		IGR	-0,004	2,50E-04	3,99E-02
cg10726868	11	12743980	TEAD1	5'UTR	0,037	2,50E-04	3,99E-02
cg25367758	19	588447	HCN2	TSS1500	0,014	2,50E-04	3,99E-02
cg00690544	19	36618552		IGR	0,02	2,50E-04	3,99E-02
cg01423722	22	50658614	TUBGCP6	Body	-0,016	2,50E-04	3,99E-02
cg03119233	4	7069300	GRPEL1	Body	0,006	2,51E-04	3,99E-02
cg06142043	6	8064743	MUTED	TSS200	0,011	2,51E-04	3,99E-02
cg21644009	6	28494863	GPX5	Body	-0,011	2,51E-04	3,99E-02
cg26937277	12	133003394		IGR	-0,007	2,51E-04	3,99E-02
cg06938319	16	79627831	MAF	3'UTR	-0,021	2,51E-04	3,99E-02
cg07643912	19	50819174	KCNC3	3'UTR	0,007	2,51E-04	3,99E-02
cg09718582	2	240751196		IGR	0,053	2,51E-04	3,99E-02
cg15497960	6	3196276	)C100507194	TSS1500	-0,012	2,51E-04	3,99E-02
cg04600018	7	2271814	MAD1L1	5'UTR	0,003	2,51E-04	3,99E-02
cg15258175	8	42578591	CHRNB3	Body	-0,023	2,51E-04	3,99E-02
cg08283712	11	118477728	PHLDB1	TSS1500	-0,012	2,51E-04	3,99E-02

cg10805684	1	161236074	PCP4L1	Body	-0,005	2,52E-04	3,99E-02
cg16675700	2	69240124	ANTXR1	TSS200	0,005	2,52E-04	3,99E-02
cg14876320	3	154463904		IGR	0,024	2,51E-04	3,99E-02
cg05868023	4	75230803	EREG	TSS200	0,017	2,52E-04	3,99E-02
cg15968342	7	158660249	WDR60	Body	-0,01	2,51E-04	3,99E-02
cg13011951	10	101736961	DNMBP	5'UTR	-0,012	2,51E-04	3,99E-02
cg26848002	11	9628760		IGR	0,006	2,51E-04	3,99E-02
cg10941822	14	92414151	FBLN5	TSS200	0,004	2,52E-04	3,99E-02
cg01515534	19	42415665		IGR	0,022	2,52E-04	3,99E-02
cg21226353	9	99419714		IGR	-0,013	2,52E-04	4,00E-02
cg11855803	17	36777874		IGR	0,025	2,52E-04	4,00E-02
cg17566238	2	240046071	HDAC4	Body	0,01	2,52E-04	4,00E-02
cg22753888	6	55038900	HCRTR2	TSS200	0,042	2,52E-04	4,00E-02
cg21582163	11	66112695	BRMS1	TSS200	-0,005	2,52E-04	4,00E-02
cg04946603	15	41221376	DLL4	TSS200	0,005	2,53E-04	4,00E-02
cg06944536	3	156391492	TIPARP	TSS1500	-0,008	2,53E-04	4,00E-02
cg18254881	14	93380612		IGR	0,013	2,53E-04	4,01E-02
cg11399834	8	14424410	SGCZ	Body	-0,017	2,53E-04	4,01E-02
cg14471064	9	139236663	GPSM1	Body	-0,017	2,53E-04	4,01E-02
cg08216792	13	108868060	LIG4	TSS1500	0,002	2,53E-04	4,01E-02
cg00867329	12	34539773		IGR	-0,012	2,54E-04	4,01E-02
cg00079891	16	122716	RHBDF1	TSS200	-0,005	2,54E-04	4,01E-02
cg00814065	16	2265398	PGP	TSS1500	0,005	2,54E-04	4,01E-02
cg26574621	1	3555038	WDR8	Body	0,005	2,54E-04	4,01E-02
cg08502797	1	216896593	ESRRG	1stExon	-0,006	2,54E-04	4,01E-02
cg22047487	5	3220947		IGR	-0,025	2,54E-04	4,01E-02
cg07478123	15	22933394	CYFIP1	Body	-0,016	2,54E-04	4,02E-02
cg21459123	19	42070397	CEACAM21	5'UTR	0,029	2,54E-04	4,02E-02
cg27011668	17	26350387		IGR	0,004	2,54E-04	4,02E-02
cg14994546	18	76446016		IGR	0,022	2,54E-04	4,02E-02
cg12253754	21	47743907	C21orf58	TSS200	0,004	2,54E-04	4,02E-02
cg12210725	3	113543986		IGR	0,042	2,54E-04	4,02E-02
cg16429080	2	72431433	EXOC6B	Body	-0,018	2,55E-04	4,02E-02
cg26196336	6	130702783	TMEM200A	5'UTR	0,018	2,55E-04	4,02E-02
cg15168294	8	143419464	TSNARE1	Body	-0,015	2,55E-04	4,02E-02
cg04117301	10	24783841	KIAA1217	Body	0,01	2,55E-04	4,02E-02
cg21081919	5	169730143		IGR	0,006	2,55E-04	4,03E-02
cg07159114	6	158763463	TULP4	Body	0,007	2,55E-04	4,03E-02
cg03965620	10	12238232	CDC123	5'UTR	0,005	2,55E-04	4,03E-02
cg08424446	12	133381374	GOLGA3	Body	-0,005	2,55E-04	4,03E-02
cg08407204	17	77723479		IGR	0,017	2,55E-04	4,03E-02
cg17981082	2	63815562	MDH1	TSS1500	0,02	2,56E-04	4,03E-02
cg07735147	6	6752385		IGR	-0,012	2,56E-04	4,03E-02
cg27497341	15	38173016		IGR	-0,034	2,56E-04	4,03E-02
cg02046994	22	26144121	MYO18B	5'UTR	-0,015	2,56E-04	4,03E-02
cg08631940	7	102499623	FBXL13	Body	0,019	2,56E-04	4,03E-02
cg07816247	1	15540152	TMEM51	5'UTR	0,037	2,56E-04	4,03E-02
cg17336442	8	48584942	SPIDR	Body	-0,008	2,56E-04	4,03E-02
cg25033715	16	50715445	SNX20	TSS200	0,014	2,56E-04	4,03E-02
cg10869669	17	73741289	ITGB4	Body	0,033	2,56E-04	4,03E-02
cg22497014	7	894009	UNC84A	Body	0,006	2,56E-04	4,03E-02
cg11784753	1	206853217		IGR	-0,025	2,57E-04	4,04E-02
cg24790350	2	28599685		IGR	0,011	2,57E-04	4,04E-02
cg16562342	1	109783503		IGR	-0,033	2,57E-04	4,04E-02
cg10042478	7	48009556	HUS1	Body	-0,023	2,57E-04	4,04E-02
cg21227694	17	53476305	MMD	Body	0,006	2,57E-04	4,04E-02
cg00114132	18	8657945		IGR	-0,01	2,57E-04	4,04E-02
cg16740886	1	9593025		IGR	-0,011	2,57E-04	4,04E-02
cg00022938	10	65133289	MIR1296	TSS1500	-0,028	2,57E-04	4,04E-02
cg11653516	6	168679967		IGR	0,018	2,57E-04	4,04E-02
cg18181674	1	25316863		IGR	-0,008	2,58E-04	4,04E-02

cg27390432	1	199874864		IGR	-0,043	2,58E-04	4,04E-02
cg05295696	4	8345406		IGR	-0,012	2,58E-04	4,04E-02
cg25923918	8	19535747	CSGALNACT1	Body	-0,008	2,58E-04	4,04E-02
cg23326075	9	112073589	EPB41L4B	Body	0,01	2,58E-04	4,04E-02
cg14039865	20	30326367	TPX2	TSS1500	-0,043	2,58E-04	4,04E-02
cg17053285	2	242009588	SNED1	Body	0,014	2,58E-04	4,05E-02
cg21994086	3	121264916	POLQ	TSS200	0,007	2,58E-04	4,05E-02
cg25026700	4	159690965	FNIP2	Body	-0,01	2,58E-04	4,05E-02
cg02639685	6	44213718	HSP90AB1	TSS1500	0,011	2,58E-04	4,05E-02
cg06071286	7	11490200	THSD7A	Body	0,048	2,58E-04	4,05E-02
cg14102434	14	57960985	C14orf105	TSS1500	0,011	2,58E-04	4,05E-02
cg01662083	16	79437712		IGR	-0,012	2,58E-04	4,05E-02
cg25790310	17	882609	NXN	1stExon	-0,004	2,58E-04	4,05E-02
cg09140929	4	7026341	TBC1D14	Body	-0,01	2,59E-04	4,05E-02
cg09439204	12	54090464		IGR	-0,031	2,59E-04	4,05E-02
cg02272761	7	30687058		IGR	0,009	2,59E-04	4,05E-02
cg16887521	10	76784798	KAT6B	Body	0,007	2,59E-04	4,05E-02
cg21045174	16	70323548	AARS	TSS200	-0,003	2,59E-04	4,05E-02
cg25921484	16	89988910	TUBB3	TSS1500	0,003	2,59E-04	4,05E-02
cg04924453	1	172987127		IGR	-0,009	2,59E-04	4,05E-02
cg21916600	21	43386354		IGR	0,005	2,59E-04	4,05E-02
cg23554506	7	93706476		IGR	-0,019	2,59E-04	4,05E-02
cg03255619	8	127579693		IGR	-0,009	2,59E-04	4,05E-02
cg14818776	22	46284202		IGR	-0,007	2,60E-04	4,05E-02
cg08685750	6	43713208		IGR	0,01	2,60E-04	4,05E-02
cg05858265	6	167370238	RNASET2	TSS200	0,004	2,60E-04	4,05E-02
cg03467897	13	60585970	DIAPH3	5'UTR	-0,013	2,60E-04	4,05E-02
cg13882748	16	5682258	MIR8065	TSS1500	0,036	2,60E-04	4,05E-02
cg01446372	21	30365398	RNF160	TSS200	0,004	2,60E-04	4,05E-02
cg09219813	3	152017017	MBNL1	TSS200	-0,007	2,60E-04	4,05E-02
cg23773006	11	71012846		IGR	0,023	2,60E-04	4,05E-02
cg17561788	22	20114683	RANBP1	3'UTR	0,023	2,60E-04	4,05E-02
cg17811228	5	139175116	PSD2	TSS1500	0,007	2,60E-04	4,05E-02
cg04555735	20	35402175	DSN1	5'UTR	0,004	2,60E-04	4,06E-02
cg04715245	1	154377091	IL6R	TSS1500	-0,004	2,61E-04	4,06E-02
cg06797322	2	151233798		IGR	-0,01	2,61E-04	4,06E-02
cg10034942	3	78657666	ROBO1	Body	-0,023	2,61E-04	4,06E-02
cg25261703	6	155510046	TIAM2	Body	0,041	2,61E-04	4,06E-02
cg26965639	7	142606734	TRPV5	Body	0,033	2,61E-04	4,06E-02
cg02188024	8	14718513	SGCZ	Body	-0,044	2,61E-04	4,06E-02
cg20661518	11	11953120	USP47	Body	-0,029	2,61E-04	4,06E-02
cg26833768	11	28112183	KIF18A	Body	0,011	2,61E-04	4,06E-02
cg20298878	19	16689203	MED26	ExonBnd	-0,008	2,61E-04	4,06E-02
cg05233047	2	122283880	CLASP1	Body	-0,018	2,61E-04	4,06E-02
cg24175289	4	77457089	SHROOM3	Body	0,005	2,61E-04	4,06E-02
cg06505010	5	1923653		IGR	-0,028	2,61E-04	4,06E-02
cg14263204	8	20131729		IGR	0,024	2,61E-04	4,06E-02
cg08327548	12	101187374	ANO4	TSS1500	0,053	2,61E-04	4,06E-02
cg10900663	14	74942544		IGR	0,041	2,61E-04	4,06E-02
cg01570128	22	35842551		IGR	-0,011	2,61E-04	4,06E-02
cg11192800	12	72057255	THAP2	TSS1500	0,011	2,61E-04	4,06E-02
cg11339680	1	25046407		IGR	-0,012	2,62E-04	4,06E-02
cg01187279	9	18566304	ADAMTSL1	Body	0,032	2,62E-04	4,06E-02
cg13533424	9	138057290		IGR	-0,038	2,62E-04	4,06E-02
cg11684930	10	135000940	KNDC1	Body	-0,012	2,62E-04	4,06E-02
cg24757159	3	99345675	MIR548G	Body	0,041	2,62E-04	4,06E-02
cg09706573	6	31936247	SKIV2L	Body	-0,005	2,62E-04	4,06E-02
cg00000765	1	230404313	GALNT2	Body	-0,006	2,62E-04	4,06E-02
cg16211299	21	38445247	PIGP	Body	0,005	2,62E-04	4,06E-02
cg05261759	9	130498309	TOR2A	TSS1500	-0,028	2,62E-04	4,07E-02
cg08650362	12	131298487	STX2	Body	-0,022	2,63E-04	4,07E-02

cg09588221	14	81088201	CEP128	Body	-0,034	2,62E-04	4,07E-02
cg14193561	17	7791158	CHD3	Body	0,002	2,63E-04	4,07E-02
cg08674847	17	41124249	ES3L-AARSD1	Body	-0,013	2,63E-04	4,07E-02
cg18174779	1	57822544	DAB1	5'UTR	-0,02	2,63E-04	4,07E-02
cg13359772	2	12880961	TRIB2	3'UTR	0,006	2,63E-04	4,07E-02
cg03731616	3	123603618	MYLK	TSS1500	-0,019	2,63E-04	4,07E-02
cg15103195	7	100797603	AP1S1	TSS200	0,006	2,63E-04	4,07E-02
cg05677724	12	131125565		IGR	-0,014	2,63E-04	4,07E-02
cg01486183	14	45930824		IGR	-0,015	2,63E-04	4,07E-02
cg21872037	17	6617470	SLC13A5	TSS1500	0,017	2,63E-04	4,07E-02
cg24509009	18	33993197	FHOD3	Body	0,007	2,63E-04	4,07E-02
cg15412857	21	46118468	TSPEAR	Body	-0,053	2,63E-04	4,07E-02
cg08246800	11	73356353	PLEKHB1	TSS1500	0,029	2,63E-04	4,07E-02
cg27449804	22	19951181	MIR4761	TSS200	-0,013	2,63E-04	4,07E-02
cg15760709	7	2040232	MAD1L1	Body	-0,008	2,64E-04	4,07E-02
cg07468956	17	80289701	SECTM1	5'UTR	-0,013	2,64E-04	4,07E-02
cg06697829	2	208633331	FZD5	Body	0,008	2,64E-04	4,07E-02
cg14311523	17	60747678	MRC2	Body	-0,013	2,64E-04	4,07E-02
cg08198590	20	23909512		IGR	0,021	2,64E-04	4,07E-02
cg07634154	2	110011475	SH3RF3	Body	-0,014	2,64E-04	4,07E-02
cg20303551	3	20089995	KAT2B	Body	-0,011	2,64E-04	4,07E-02
cg17355685	1	17487992		IGR	0,027	2,64E-04	4,07E-02
cg02978142	1	151584686	SNX27	1stExon	0,003	2,64E-04	4,07E-02
cg22039638	2	70996014	ADD2	TSS1500	0,046	2,64E-04	4,07E-02
cg26399254	4	177822250		IGR	-0,02	2,64E-04	4,07E-02
cg06161930	19	56633191	ZNF787	TSS1500	0,015	2,64E-04	4,07E-02
cg04456235	1	52762312	ZFYVE9	Body	0,052	2,65E-04	4,07E-02
cg20729976	1	211821638	)C105748977	Body	0,037	2,65E-04	4,07E-02
cg18656366	10	14996626	DCLRE1C	TSS200	-0,028	2,65E-04	4,07E-02
cg19513193	17	7123490	DLG4	TSS200	0,006	2,65E-04	4,07E-02
cg11047295	13	31039916	HMGB1	5'UTR	0,003	2,65E-04	4,08E-02
cg06353432	1	48398332	TRABD2B	Body	0,013	2,65E-04	4,08E-02
cg09066298	3	52302397	MIRLET7G	TSS200	0,013	2,65E-04	4,08E-02
cg27459021	5	555725		IGR	-0,005	2,65E-04	4,08E-02
cg13384849	5	173307471		IGR	-0,007	2,65E-04	4,08E-02
cg25138693	6	4447278		IGR	0,039	2,66E-04	4,08E-02
cg07701084	6	150067640	NUP43	5'UTR	0,005	2,66E-04	4,08E-02
cg16100486	6	167263401	RPS6KA2	Body	0,013	2,65E-04	4,08E-02
cg14178967	14	39583547	SIP1	1stExon	0,004	2,65E-04	4,08E-02
cg25194094	15	23077763	NIPA1	Body	-0,016	2,65E-04	4,08E-02
cg15743799	17	9805578	RCVRN	Body	0,039	2,65E-04	4,08E-02
cg16481791	17	72436362	GPRC5C	Body	0,013	2,65E-04	4,08E-02
cg10019329	21	42692748	FAM3B	Body	-0,006	2,66E-04	4,08E-02
cg05229229	4	3644703		IGR	0,034	2,66E-04	4,08E-02
cg03225664	1	64599338	ROR1	Body	-0,013	2,66E-04	4,08E-02
cg23561957	3	186382416	HRG	TSS1500	-0,041	2,66E-04	4,08E-02
cg26073987	4	6783056	KIAA0232	TSS1500	-0,004	2,66E-04	4,08E-02
cg10548506	11	72265397		IGR	0,035	2,66E-04	4,08E-02
cg19402603	10	3057951		IGR	-0,006	2,66E-04	4,08E-02
cg12254614	3	178831804		IGR	0,029	2,66E-04	4,08E-02
cg22235072	2	238708555	RBM44	5'UTR	-0,005	2,67E-04	4,08E-02
cg09981884	4	1720110	TMEM129	Body	-0,023	2,67E-04	4,08E-02
cg15835805	5	132162441	SHROOM1	TSS1500	0,028	2,67E-04	4,08E-02
cg13898564	9	78504994	PCSK5	TSS1500	-0,01	2,67E-04	4,08E-02
cg24311634	12	131519883	ADGRD1	Body	0,079	2,67E-04	4,08E-02
cg16769376	14	24685222	MDP1	1stExon	0,002	2,67E-04	4,08E-02
cg07878587	14	75058126	LTBP2	Body	0,03	2,66E-04	4,08E-02
cg15213313	15	91490052	UNC45A	Body	-0,01	2,66E-04	4,08E-02
cg01980562	19	1174207	SBNO2	1stExon	0,004	2,66E-04	4,08E-02
cg23181035	19	42593016	POU2F2	3'UTR	-0,013	2,66E-04	4,08E-02
cg10827485	10	134929797	GPR123	Body	0,023	2,67E-04	4,08E-02

cg23101910	13	96328925	DNAJC3	TSS1500	-0,008	2,67E-04	4,08E-02
cg06780892	7	20638177		IGR	0,01	2,67E-04	4,08E-02
cg14182954	7	110366936	IMMP2L	Body	-0,015	2,67E-04	4,09E-02
cg04865319	4	169791918	PALLD	5'UTR	0,027	2,68E-04	4,09E-02
cg25434807	6	107096374	QRSL1	Body	-0,01	2,68E-04	4,09E-02
cg04936554	7	47622718	TNS3	TSS1500	-0,011	2,68E-04	4,09E-02
cg13746655	7	99073174	ZNF789	5'UTR	-0,01	2,68E-04	4,09E-02
cg23885898	5	3027386		IGR	-0,006	2,68E-04	4,09E-02
cg23411614	2	102509317	MAP4K4	3'UTR	-0,009	2,68E-04	4,09E-02
cg13613976	7	1574413	MAFK	5'UTR	-0,011	2,68E-04	4,09E-02
cg25411987	20	61287678	SLCO4A1	5'UTR	-0,017	2,68E-04	4,09E-02
cg24462596	11	94706862	KDM4D	1stExon	0,011	2,69E-04	4,10E-02
cg04432430	3	50456112	CACNA2D2	Body	-0,015	2,69E-04	4,10E-02
cg02471179	17	76868514	TIMP2	Body	0,008	2,69E-04	4,10E-02
cg22189823	15	90815996		IGR	0,021	2,69E-04	4,10E-02
cg21519235	6	133930873	TARID	Body	0,04	2,69E-04	4,10E-02
cg15419210	11	107522568	ELMOD1	Body	0,018	2,69E-04	4,10E-02
cg05644049	1	11118880	SRM	Body	0,017	2,69E-04	4,10E-02
cg06690525	2	170430736	FASTKD1	TSS1500	0,022	2,69E-04	4,10E-02
cg00479908	19	46215637	FBXO46	Body	-0,009	2,69E-04	4,10E-02
cg21564780	19	2213874	DOT1L	Body	-0,029	2,69E-04	4,10E-02
cg09908745	8	123360240		IGR	0,044	2,70E-04	4,10E-02
cg13764858	20	14503777	MACROD2	Body	0,066	2,70E-04	4,10E-02
cg09798093	1	49325135	AGBL4	Body	0,029	2,70E-04	4,10E-02
cg10248128	2	29291558	C2orf71	Body	-0,006	2,70E-04	4,10E-02
cg02541099	2	241336014		IGR	-0,022	2,70E-04	4,10E-02
cg27122431	5	19988848	CDH18	TSS1500	0,027	2,70E-04	4,10E-02
cg18503080	11	118187537		IGR	0,003	2,70E-04	4,10E-02
cg03104293	11	132056886	NTM	Body	-0,045	2,70E-04	4,10E-02
cg19412478	22	26883640	SRRD	Body	0,005	2,70E-04	4,10E-02
cg12012381	8	1694654		IGR	-0,009	2,70E-04	4,11E-02
cg17587981	7	102988071	PSMC2	TSS200	-0,008	2,70E-04	4,11E-02
cg24390864	16	31471215	ARMC5	1stExon	0,003	2,70E-04	4,11E-02
cg20863668	17	73518382	TSEN54	Body	-0,014	2,70E-04	4,11E-02
cg05624445	18	24764599	CHST9	5'UTR	0,015	2,70E-04	4,11E-02
cg21243021	9	135513120	DDX31	Body	0,007	2,71E-04	4,11E-02
cg01600968	10	7629788	ITIH5	Body	-0,043	2,71E-04	4,11E-02
cg12899065	22	19710163	GP1BB	TSS1500	0,062	2,71E-04	4,11E-02
cg22247664	1	110199126	GSTM4	Body	-0,009	2,71E-04	4,11E-02
cg13688808	6	29760158	HCG4	Body	-0,018	2,71E-04	4,11E-02
cg26235292	4	99850587	EIF4E	5'UTR	0,003	2,71E-04	4,11E-02
cg26321462	5	10540223		IGR	0,006	2,71E-04	4,11E-02
cg07814629	12	49961959	MCRS1	TSS200	-0,008	2,71E-04	4,11E-02
cg00459997	12	130621721		IGR	-0,036	2,71E-04	4,11E-02
cg10256336	19	16999953	F2RL3	1stExon	0,007	2,71E-04	4,11E-02
cg17223947	18	30053693		IGR	-0,018	2,72E-04	4,11E-02
cg24520755	8	9996632	MSRA	Body	-0,006	2,72E-04	4,11E-02
cg14097135	19	4524928	PLIN5	Body	-0,019	2,72E-04	4,12E-02
cg12039766	2	109648496		IGR	-0,006	2,72E-04	4,12E-02
cg06521357	4	3486380	DOK7	Body	0,028	2,72E-04	4,12E-02
cg00312125	4	84030111	PLAC8	5'UTR	0,046	2,72E-04	4,12E-02
cg03147723	13	47470354	HTR2A	5'UTR	0,019	2,72E-04	4,12E-02
cg13195410	15	77900397		IGR	-0,019	2,72E-04	4,12E-02
cg22969950	17	8117574		IGR	-0,007	2,72E-04	4,12E-02
cg15550277	5	150385881		IGR	0,023	2,73E-04	4,12E-02
cg00308841	5	90790737		IGR	0,041	2,73E-04	4,12E-02
cg15613067	2	242053881	PASK	Body	-0,006	2,73E-04	4,13E-02
cg22184507	5	140710094	PCDHGA1	TSS200	-0,019	2,73E-04	4,13E-02
cg06350196	1	2435087	PLCH2	Body	0,008	2,73E-04	4,13E-02
cg18344938	5	67510873		IGR	0,004	2,73E-04	4,13E-02
cg04391175	15	77176539	SCAPER	TSS1500	0,014	2,73E-04	4,13E-02

cg23015354	3	64940669	.DAMTS9-AS2	Body	0,029	2,74E-04	4,13E-02
cg20913747	6	44695427		IGR	-0,011	2,74E-04	4,13E-02
cg21199659	9	133366752	ASS1	Body	-0,015	2,74E-04	4,13E-02
cg24824725	10	97453973	TCTN3	TSS200	0,005	2,74E-04	4,13E-02
cg04996921	15	68907224	CORO2B	Body	-0,014	2,74E-04	4,13E-02
cg03669175	19	57375856		IGR	-0,039	2,74E-04	4,13E-02
cg01800843	11	108092707	ATM	TSS1500	-0,002	2,74E-04	4,13E-02
cg02421553	16	2645410	PDPK1	Body	0,004	2,74E-04	4,13E-02
cg10591064	1	203296928		IGR	0,003	2,74E-04	4,13E-02
cg22540912	13	103498393	ERCC5	1stExon	0,003	2,74E-04	4,13E-02
cg17274827	1	94075555	BCAR3	Body	-0,009	2,75E-04	4,14E-02
cg07114634	2	118772300	CCDC93	TSS1500	-0,005	2,75E-04	4,14E-02
cg18867698	4	7396839	SORCS2	Body	0,009	2,75E-04	4,14E-02
cg02471248	6	152489392	MIR3163	Body	-0,009	2,75E-04	4,14E-02
cg07387044	8	145170347	KIAA1875	Body	-0,009	2,75E-04	4,14E-02
cg04208292	9	129622372	ZBTB34	TSS1500	-0,005	2,75E-04	4,14E-02
cg03696909	10	30448554		IGR	-0,018	2,74E-04	4,14E-02
cg04333273	10	103294488	BTRC	Body	0,005	2,75E-04	4,14E-02
cg20761290	11	60146039	MS4A7	5'UTR	0,008	2,75E-04	4,14E-02
cg00912757	13	46597943	ZC3H13	Body	-0,003	2,75E-04	4,14E-02
cg07632068	15	40179641	GPR176	Body	0,021	2,75E-04	4,14E-02
cg26535892	18	60738502		IGR	0,016	2,75E-04	4,14E-02
cg10642098	22	31689162	PIK3IP1	TSS1500	0,033	2,75E-04	4,14E-02
cg03072859	1	52483420		IGR	0,016	2,76E-04	4,14E-02
cg03990478	1	70819273	HHLA3	TSS1500	-0,027	2,76E-04	4,14E-02
cg24337809	1	203274882	BTG2	Body	0,003	2,76E-04	4,14E-02
cg18621661	8	141950472	PTK2	Body	-0,016	2,76E-04	4,14E-02
cg12100956	17	78086420	GAA	Body	-0,009	2,76E-04	4,14E-02
cg26141105	6	28214251	ZKSCAN4	Body	-0,005	2,76E-04	4,14E-02
cg18452817	11	8102237	TUB	TSS1500	-0,008	2,76E-04	4,14E-02
cg01643110	3	47619726	CSPG5	5'UTR	0,007	2,76E-04	4,15E-02
cg00050440	1	23571135		IGR	0,01	2,77E-04	4,15E-02
cg24553763	3	135455646		IGR	0,054	2,76E-04	4,15E-02
cg08450826	7	43599146	HECW1	Body	-0,007	2,76E-04	4,15E-02
cg06620731	12	108909559	FICD	5'UTR	-0,004	2,77E-04	4,15E-02
cg20433989	13	49683801	FNDC3A	Body	0,021	2,76E-04	4,15E-02
cg18514065	14	104165052	XRCC3	3'UTR	-0,014	2,76E-04	4,15E-02
cg08433755	18	21082800	C18orf8	TSS1500	0,033	2,77E-04	4,15E-02
cg01153131	6	114005956		IGR	0,042	2,77E-04	4,15E-02
cg12617538	9	69786570		IGR	-0,012	2,77E-04	4,15E-02
cg06296597	10	858900	LARP4B	Body	-0,017	2,77E-04	4,15E-02
cg05716075	16	88476249		IGR	0,008	2,77E-04	4,15E-02
cg16242690	8	78868991		IGR	-0,015	2,77E-04	4,15E-02
cg01967102	3	20145874	KAT2B	Body	-0,015	2,78E-04	4,15E-02
cg18604929	19	43198154		IGR	0,028	2,78E-04	4,15E-02
cg14834209	12	14876768		IGR	0,03	2,78E-04	4,16E-02
cg19906777	1	2023482	PRKCZ	Body	-0,011	2,78E-04	4,16E-02
cg18433402	5	135528954	LOC389332	TSS200	-0,005	2,78E-04	4,16E-02
cg02174156	1	4756792	AJAP1	Body	-0,011	2,78E-04	4,16E-02
cg11601112	2	183943319	DUSP19	5'UTR	-0,028	2,78E-04	4,16E-02
cg09373338	3	143695144	C3orf58	Body	-0,014	2,78E-04	4,16E-02
cg10312186	6	170403583		IGR	0,064	2,78E-04	4,16E-02
cg10675659	12	19283425	PLEKHA5	Body	-0,005	2,79E-04	4,16E-02
cg13452215	1	42630436	GUCA2A	TSS200	-0,009	2,79E-04	4,16E-02
cg06334363	9	108321115	FKTN	5'UTR	0,028	2,79E-04	4,17E-02
cg04665351	12	3000000	TULP3	TSS200	0,008	2,79E-04	4,17E-02
cg26730017	16	88358185		IGR	-0,017	2,79E-04	4,17E-02
cg07701909	1	116099209		IGR	-0,006	2,79E-04	4,17E-02
cg19730422	5	156718560	CYFIP2	Body	-0,035	2,80E-04	4,17E-02
cg09390414	11	2571525	KCNQ1	Body	-0,008	2,79E-04	4,17E-02
cg25636820	19	40424192	FCGBP	Body	-0,006	2,79E-04	4,17E-02

cg20110801	19	56014812	SSC5D	Body	-0,047	2,79E-04	4,17E-02
cg12661873	2	36761999	CRIM1	Body	0,005	2,80E-04	4,17E-02
cg23161505	7	150065239	REPIN1	TSS1500	0,004	2,80E-04	4,17E-02
cg10936796	2	20650757		IGR	0,005	2,80E-04	4,17E-02
cg23920151	12	121474312	OASL	Body	0,009	2,80E-04	4,17E-02
cg04058399	2	25481271	DNMT3A	Body	0,006	2,81E-04	4,18E-02
cg25635144	3	42814793	CCDC13	TSS200	-0,012	2,81E-04	4,18E-02
cg06147366	6	167411970	FGFR1OP	TSS1500	0,006	2,80E-04	4,18E-02
cg04240471	7	2420162	EIF3B	3'UTR	-0,007	2,81E-04	4,18E-02
cg24029091	7	132649982	CHCHD3	Body	-0,048	2,81E-04	4,18E-02
cg04900366	7	151301837	PRKAG2	Body	0,006	2,81E-04	4,18E-02
cg07705809	8	123790294		IGR	0,005	2,81E-04	4,18E-02
cg08326861	8	124750221	ANXA13	TSS1500	-0,021	2,81E-04	4,18E-02
cg14563732	8	143474839	TSNARE1	5'UTR	0,016	2,81E-04	4,18E-02
cg21196551	9	98173099		IGR	0,011	2,82E-04	4,18E-02
cg04065885	10	11381883		IGR	0,005	2,81E-04	4,18E-02
cg07680528	10	112168061		IGR	-0,017	2,81E-04	4,18E-02
cg26314240	11	45599468		IGR	0,036	2,81E-04	4,18E-02
cg08460710	11	61646959	MIR6746	TSS1500	-0,011	2,81E-04	4,18E-02
cg12869958	11	67017687	KDM2A	Body	-0,007	2,82E-04	4,18E-02
cg13449363	12	6982384	SPSB2	5'UTR	0,007	2,81E-04	4,18E-02
cg18842049	12	39305838		IGR	-0,014	2,80E-04	4,18E-02
cg16956999	12	133249241	POLE	Body	-0,004	2,81E-04	4,18E-02
cg05446493	14	93821404	UNC79	5'UTR	-0,021	2,81E-04	4,18E-02
cg13049261	14	99879199	SETD3	Body	0,029	2,82E-04	4,18E-02
cg21758314	14	103439105	CDC42BPB	Body	0,122	2,81E-04	4,18E-02
cg27248189	16	110305	RHBDF1	Body	-0,014	2,81E-04	4,18E-02
cg24942142	16	30041446	FAM57B	Body	-0,028	2,81E-04	4,18E-02
cg00979553	16	89124336		IGR	-0,013	2,82E-04	4,18E-02
cg22350777	19	40931943	SERTAD1	TSS200	0,005	2,81E-04	4,18E-02
cg17120273	10	88428026	LDB3	TSS1500	-0,009	2,82E-04	4,18E-02
cg25663478	16	6106901	RBFOX1	5'UTR	0,023	2,82E-04	4,18E-02
cg23715460	17	740917	NXN	Body	-0,005	2,82E-04	4,18E-02
cg13137376	6	30712221	IER3	1stExon	0,009	2,82E-04	4,18E-02
cg12982177	16	10962668		IGR	-0,011	2,82E-04	4,18E-02
cg05693308	10	63985934	RTKN2	Body	-0,012	2,82E-04	4,18E-02
cg17745090	4	138133531		IGR	-0,01	2,82E-04	4,18E-02
cg15718448	8	111930817		IGR	-0,012	2,83E-04	4,18E-02
cg11245333	1	220959931	MOSC1	TSS200	-0,007	2,83E-04	4,18E-02
cg17873919	5	134094447	DDX46	TSS200	0,005	2,83E-04	4,18E-02
cg05998089	6	29975141	HLA-J	Body	0,008	2,83E-04	4,19E-02
cg15098182	9	131534228	ZER1	TSS200	0,005	2,83E-04	4,19E-02
cg16224902	12	48513167	PFKM	Body	-0,017	2,83E-04	4,19E-02
cg23502926	14	104210385	PPP1R13B	Body	0,023	2,83E-04	4,19E-02
cg25314902	5	87971446	LOC645323	Body	0,005	2,83E-04	4,19E-02
cg03134886	13	20207718	MPHOSPH8	TSS200	0,003	2,83E-04	4,19E-02
cg18896834	12	131438783	ADGRD1	5'UTR	0,035	2,84E-04	4,19E-02
cg02983090	16	27437892	IL21R	TSS1500	0,003	2,84E-04	4,19E-02
cg06612122	16	59788951	LOC644649	Body	-0,008	2,84E-04	4,19E-02
cg01892317	1	246784780	CNST	Body	-0,004	2,84E-04	4,19E-02
cg12166570	14	74225642	C14orf43	5'UTR	0,005	2,84E-04	4,19E-02
cg22704351	2	241925548		IGR	-0,011	2,84E-04	4,19E-02
cg00058767	4	186934427		IGR	0,009	2,84E-04	4,19E-02
cg14537712	5	175579473	LOC643201	Body	0,035	2,84E-04	4,20E-02
cg16144447	1	36184773	C1orf216	1stExon	0,012	2,85E-04	4,20E-02
cg12094982	4	110449571	SEC24B	Body	-0,017	2,85E-04	4,20E-02
cg19107724	6	112760279		IGR	0,007	2,85E-04	4,20E-02
cg23670208	6	126967348		IGR	-0,008	2,85E-04	4,20E-02
cg07622201	7	49929836	VWC2	Body	-0,013	2,85E-04	4,20E-02
cg23421347	10	93338729	)C100188947	Body	-0,025	2,85E-04	4,20E-02
cg14849266	11	112325207		IGR	0,033	2,85E-04	4,20E-02

cg12178060	12	101800972	ARL1	Body	-0,005	2,85E-04	4,20E-02
cg24941600	14	22653259		IGR	0,038	2,85E-04	4,20E-02
cg10009236	16	128016	MPG	5'UTR	0,004	2,85E-04	4,20E-02
cg04735468	16	56389025	GNAO1	3'UTR	-0,019	2,85E-04	4,20E-02
cg25839877	17	76361930		IGR	-0,008	2,85E-04	4,20E-02
cg19598832	19	1646448	TCF3	Body	0,028	2,85E-04	4,20E-02
cg17611309	19	19233112	TMEM161A	Body	0,02	2,85E-04	4,20E-02
cg27463004	22	39716675	RPL3	TSS1500	-0,018	2,85E-04	4,20E-02
cg04900999	17	40610958	ATP6V0A1	1stExon	0,005	2,86E-04	4,20E-02
cg25278174	15	101972395	PCSK6	Body	0,017	2,86E-04	4,21E-02
cg14820441	17	58405210	USP32	Body	-0,013	2,86E-04	4,21E-02
cg17942978	2	57601109		IGR	-0,012	2,86E-04	4,21E-02
cg02321472	2	153224541	FMNL2	Body	-0,01	2,86E-04	4,21E-02
cg15628415	8	81003747	TPD52	Body	-0,021	2,87E-04	4,21E-02
cg25351263	15	89158396		IGR	0,008	2,87E-04	4,21E-02
cg04955351	17	1667921	SERPINF1	5'UTR	-0,011	2,87E-04	4,22E-02
cg14624314	5	145328877	SH3RF2	Body	-0,043	2,87E-04	4,22E-02
cg04447708	2	220432872	OBSL1	Body	-0,015	2,88E-04	4,22E-02
cg04069842	6	38814885	DNAH8	Body	0,015	2,88E-04	4,22E-02
cg21308062	13	111972898	C13orf16	TSS200	-0,018	2,88E-04	4,22E-02
cg12944017	18	264576	THOC1	Body	0,012	2,88E-04	4,22E-02
cg10587886	3	8361835	LMCD1-AS1	Body	0,051	2,88E-04	4,22E-02
cg16085986	8	56684895	TMEM68	5'UTR	0,008	2,88E-04	4,23E-02
cg03921796	9	129058523		IGR	0,015	2,88E-04	4,23E-02
cg23777302	11	69706954		IGR	0,044	2,88E-04	4,23E-02
cg18767612	9	82790394		IGR	0,013	2,88E-04	4,23E-02
cg10497845	1	19035763	PAX7	Body	-0,036	2,89E-04	4,23E-02
cg23657355	1	39038031		IGR	0,032	2,89E-04	4,23E-02
cg09162146	1	94703507	ARHGAP29	TSS1500	-0,01	2,89E-04	4,23E-02
cg23050873	2	234184376	ATG16L1	Body	0,009	2,89E-04	4,23E-02
cg08872632	2	242218975	HDLBP	5'UTR	-0,015	2,89E-04	4,23E-02
cg15995356	3	196966966	DLG1	Body	0,006	2,89E-04	4,23E-02
cg20452252	10	577236	DIP2C	Body	0,011	2,89E-04	4,23E-02
cg03647767	10	65001884	JMJD1C	Body	-0,008	2,89E-04	4,23E-02
cg16817237	11	60793675		IGR	0,036	2,89E-04	4,23E-02
cg25449930	22	40079505	CACNA1I	Body	0,018	2,89E-04	4,23E-02
cg14251596	22	41697184	ZC3H7B	TSS1500	-0,005	2,89E-04	4,23E-02
cg11916478	12	213776	IQSEC3	Body	0,007	2,89E-04	4,23E-02
cg14623518	17	79009114	FLJ90757	TSS1500	0,004	2,89E-04	4,23E-02
cg21627728	21	16335810	NRIP1	3'UTR	0,006	2,89E-04	4,23E-02
cg06536645	2	109230629	LIMS1	5'UTR	-0,006	2,90E-04	4,23E-02
cg08602190	10	128076260	ADAM12	Body	-0,01	2,89E-04	4,23E-02
cg08126654	12	50317816	)C101927292	Body	0,005	2,90E-04	4,23E-02
cg11950383	21	34400072	OLIG2	Body	0,015	2,90E-04	4,23E-02
cg00381514	1	20598179		IGR	0,026	2,91E-04	4,23E-02
cg13454346	1	89872856	LOC400759	TSS1500	0,024	2,91E-04	4,23E-02
cg05126036	1	155920658	ARHGEF2	Body	-0,016	2,91E-04	4,23E-02
cg21281234	1	212781775	ATF3	TSS1500	0,006	2,92E-04	4,23E-02
cg12055316	2	27601353	ZNF513	Body	-0,017	2,91E-04	4,23E-02
cg09860381	2	56243079	MIR217HG	Body	0,019	2,90E-04	4,23E-02
cg09464488	2	175547590	WIPF1	1stExon	0,006	2,90E-04	4,23E-02
cg27375330	3	15397117		IGR	-0,007	2,90E-04	4,23E-02
cg05669873	3	39149014	GORASP1	1stExon	0,003	2,92E-04	4,23E-02
cg00741295	3	164910562	SLITRK3	5'UTR	0,02	2,91E-04	4,23E-02
cg20985479	3	192233698	FGF12	Body	-0,027	2,91E-04	4,23E-02
cg03292921	4	575111		IGR	0,05	2,91E-04	4,23E-02
cg22826136	5	2683925		IGR	0,026	2,91E-04	4,23E-02
cg01621879	5	114847670		IGR	0,016	2,92E-04	4,23E-02
cg14644761	5	141082361		IGR	-0,004	2,91E-04	4,23E-02
cg22238600	7	77894058	MAGI2	Body	-0,008	2,90E-04	4,23E-02
cg00061203	8	59372090		IGR	0,034	2,91E-04	4,23E-02

cg16910293	8	140746578	TRAPPC9	Body	0,007	2,92E-04	4,23E-02
cg20772008	9	104227791		IGR	-0,011	2,91E-04	4,23E-02
cg06624369	9	114800463	MIR3134	Body	0,017	2,90E-04	4,23E-02
cg23558473	9	125667702	RC3H2	TSS200	-0,005	2,91E-04	4,23E-02
cg02727241	10	11196983	CELF2	Body	0,005	2,90E-04	4,23E-02
cg04420335	11	103800408	PDGFD	Body	0,028	2,92E-04	4,23E-02
cg20293035	12	76709502		IGR	-0,016	2,91E-04	4,23E-02
cg15054508	12	114434073		IGR	0,038	2,91E-04	4,23E-02
cg07298211	12	130717499		IGR	0,016	2,90E-04	4,23E-02
cg13959830	14	83107784		IGR	0,035	2,91E-04	4,23E-02
cg08069370	15	64387884	SNX1	TSS1500	0,015	2,91E-04	4,23E-02
cg26648725	15	81048839		IGR	0,006	2,92E-04	4,23E-02
cg19076690	16	4697774	MGRN1	Body	0,004	2,91E-04	4,23E-02
cg04958259	16	27781196	KIAA0556	ExonBnd	-0,015	2,91E-04	4,23E-02
cg27581721	16	31095566	POL3S	Body	-0,013	2,92E-04	4,23E-02
cg12914530	16	89500225	ANKRD11	5'UTR	-0,011	2,92E-04	4,23E-02
cg17842670	17	45925478	SP6	Body	0,033	2,91E-04	4,23E-02
cg06497674	19	3645760	PIP5K1C	Body	-0,015	2,91E-04	4,23E-02
cg14739799	20	50170861	NFATC2	5'UTR	0,009	2,90E-04	4,23E-02
cg00586889	20	50807767	ZFP64	Body	0,003	2,91E-04	4,23E-02
cg24168488	3	122863173	PDIA5	Body	0,006	2,92E-04	4,24E-02
cg16605240	9	130891728	PTGES2	TSS1500	0,025	2,92E-04	4,24E-02
cg10593292	3	195539133	MUC4	5'UTR	-0,033	2,93E-04	4,24E-02
cg26431815	8	668053	ERICH1	Body	-0,008	2,92E-04	4,24E-02
cg26989531	12	4383117	CCND2	1stExon	0,022	2,92E-04	4,24E-02
cg14391832	13	46287862	SPERT	Body	-0,016	2,93E-04	4,24E-02
cg21933664	13	113530259	ATP11A	Body	-0,014	2,93E-04	4,24E-02
cg12019265	15	36523650		IGR	-0,015	2,93E-04	4,24E-02
cg05610128	15	49187862	SHC4	Body	0,008	2,93E-04	4,24E-02
cg11775595	19	1453909	APC2	Body	0,053	2,93E-04	4,24E-02
cg13981319	19	10446556	ICAM3	Body	-0,005	2,93E-04	4,24E-02
cg13883479	9	574312	KANK1	5'UTR	0,041	2,93E-04	4,24E-02
cg18262805	12	69979195	CCT2	TSS1500	0,005	2,93E-04	4,24E-02
cg22496437	1	17900032	ARHGEF10L	5'UTR	0,037	2,93E-04	4,24E-02
cg27103937	19	348210		IGR	0,051	2,93E-04	4,24E-02
cg19927214	7	158336044	PTPRN2	Body	-0,008	2,93E-04	4,24E-02
cg15326535	8	1310764		IGR	0,005	2,93E-04	4,24E-02
cg24137599	12	81155811	LINC01490	TSS1500	-0,023	2,94E-04	4,24E-02
cg18281878	12	124376626	DNAH10	Body	-0,007	2,94E-04	4,24E-02
cg22161862	2	43205734		IGR	-0,014	2,94E-04	4,24E-02
cg10605404	2	242136184	ANO7	Body	0,009	2,94E-04	4,24E-02
cg20333288	8	94520572		IGR	0,008	2,94E-04	4,24E-02
cg19256819	9	37905463	SLC25A51	TSS1500	-0,003	2,94E-04	4,25E-02
cg01863011	1	12148346	TNFRSF8	5'UTR	0,032	2,94E-04	4,25E-02
cg11163337	1	222239086		IGR	-0,017	2,95E-04	4,25E-02
cg06748522	2	236760308	AGAP1	Body	0,012	2,95E-04	4,25E-02
cg06277849	4	186316674	ANKRD37	TSS1500	-0,008	2,94E-04	4,25E-02
cg14121971	6	29621375		IGR	0,006	2,95E-04	4,25E-02
cg01530633	8	16240840		IGR	-0,031	2,95E-04	4,25E-02
cg13890982	8	28655296	INTS9	Body	0,006	2,94E-04	4,25E-02
cg08492301	9	114675062	UGCG	Body	0,007	2,94E-04	4,25E-02
cg09865698	11	119597471	PVRL1	Body	0,026	2,95E-04	4,25E-02
cg26328502	13	31051417		IGR	0,005	2,95E-04	4,25E-02
cg18710985	20	16554249	KIF16B	TSS200	0,003	2,95E-04	4,25E-02
cg05888252	1	175857819		IGR	0,04	2,95E-04	4,25E-02
cg17067634	14	20504004	OR4K13	TSS1500	0,063	2,95E-04	4,25E-02
cg16939392	16	20817682	ERI2	Body	0,003	2,95E-04	4,25E-02
cg01738164	14	90147503		IGR	-0,006	2,95E-04	4,25E-02
cg03219831	4	57875130	POLR2B	Body	-0,028	2,95E-04	4,25E-02
cg01636258	4	81536423	C4orf22	Body	0,011	2,95E-04	4,25E-02
cg11227838	18	73944787		IGR	-0,017	2,95E-04	4,25E-02

cg05415051	1	221841208		IGR	0,006	2,96E-04	4,26E-02
cg25115034	5	21391623		IGR	-0,048	2,96E-04	4,26E-02
cg24088496	11	96071506	MAML2	Body	0,031	2,96E-04	4,26E-02
cg13611173	14	57857305	NAA30	5'UTR	0,006	2,96E-04	4,26E-02
cg05991685	16	2818793	SRRM2	Body	-0,016	2,96E-04	4,26E-02
cg25721516	17	42276931	ATXN7L3	TSS1500	0,006	2,96E-04	4,26E-02
cg07416590	1	177199034	FAM5B	Body	-0,011	2,97E-04	4,26E-02
cg14401476	2	28674256		IGR	0,014	2,97E-04	4,26E-02
cg26055923	2	150810538		IGR	0,016	2,97E-04	4,26E-02
cg02888364	2	242051960	PASK	Body	0,035	2,97E-04	4,26E-02
cg06355467	5	180876284		IGR	-0,008	2,97E-04	4,26E-02
cg12755815	7	107644570	LAMB1	TSS1500	-0,021	2,97E-04	4,26E-02
cg24890816	9	74638077		IGR	0,008	2,97E-04	4,26E-02
cg18621101	10	62494545		IGR	-0,006	2,97E-04	4,26E-02
cg16998336	10	63589943		IGR	0,006	2,97E-04	4,26E-02
cg12782524	14	105041382		IGR	-0,011	2,97E-04	4,26E-02
cg06970577	17	26898623	PIGS	1stExon	0,004	2,97E-04	4,26E-02
cg24901347	19	56057761	SBK3	TSS1500	0,026	2,97E-04	4,26E-02
cg17156388	3	129366778	TMCC1	3'UTR	-0,007	2,97E-04	4,26E-02
cg14543182	10	126615372		IGR	-0,019	2,97E-04	4,26E-02
cg20449494	1	177615954		IGR	-0,009	2,98E-04	4,26E-02
cg10414736	6	26521747	HCG11	TSS200	-0,006	2,98E-04	4,26E-02
cg02271811	6	137108883	MAP3K5	Body	-0,014	2,98E-04	4,26E-02
cg14266088	7	72845698		IGR	0,008	2,98E-04	4,26E-02
cg04156418	7	157293606		IGR	-0,017	2,98E-04	4,26E-02
cg12178835	14	69186571		IGR	0,021	2,98E-04	4,26E-02
cg14014715	15	52970892	FAM214A	TSS200	0,01	2,98E-04	4,26E-02
cg15995162	15	63929920	HERC1	Body	-0,01	2,98E-04	4,26E-02
cg05258477	6	170151703	C6orf70	TSS200	0,004	2,98E-04	4,26E-02
cg26635956	5	1194126		IGR	0,032	2,99E-04	4,27E-02
cg18354714	12	54891655	NCKAP1L	TSS1500	-0,003	2,99E-04	4,27E-02
cg13906416	19	45147440	PVR	1stExon	0,005	2,99E-04	4,27E-02
cg02674305	20	34359915	PHF20	TSS200	0,006	2,99E-04	4,27E-02
cg06609852	20	55445497		IGR	-0,016	2,99E-04	4,27E-02
cg12562012	5	158305034	EBF1	Body	0,01	2,99E-04	4,27E-02
cg21441575	10	6005869	IL15RA	Body	0,018	2,99E-04	4,27E-02
cg04059695	4	139787977		IGR	0,009	2,99E-04	4,27E-02
cg17120270	2	234368005	DGKD	Body	-0,013	2,99E-04	4,27E-02
cg09607229	3	44619924	ZNF167	Body	-0,012	2,99E-04	4,27E-02
cg24739864	6	4857484	CDYL	Body	-0,015	2,99E-04	4,27E-02
cg16964439	9	87287007	NTRK2	Body	-0,009	2,99E-04	4,27E-02
cg12232388	12	125027851		IGR	0,011	2,99E-04	4,27E-02
cg16682989	16	4987316	PPL	TSS200	0,004	3,00E-04	4,27E-02
cg00155314	1	244012398		IGR	0,004	3,00E-04	4,27E-02
cg10322825	1	198645295	PTPRC	Body	-0,007	3,00E-04	4,28E-02
cg07101432	8	140968284	TRAPPC9	Body	-0,019	3,00E-04	4,28E-02
cg10942227	12	67160208		IGR	-0,012	3,00E-04	4,28E-02
cg13507506	2	289147	FAM150B	TSS1500	-0,005	3,00E-04	4,28E-02
cg10460470	6	26313274		IGR	-0,003	3,00E-04	4,28E-02
cg05239872	7	102076730	ORAI2	5'UTR	-0,012	3,00E-04	4,28E-02
cg19784903	17	45786737	TBKBP1	Body	-0,004	3,00E-04	4,28E-02
cg09548718	16	17398605	XYLT1	Body	-0,021	3,01E-04	4,28E-02
cg09748973	4	1339675	KIAA1530	TSS1500	-0,019	3,01E-04	4,28E-02
cg21037362	5	72876476	UTP15	3'UTR	0,008	3,01E-04	4,28E-02
cg23460707	10	133558971		IGR	0,053	3,01E-04	4,28E-02
cg10422760	11	19263091	E2F8	5'UTR	0,009	3,01E-04	4,28E-02
cg27266530	12	111346507		IGR	-0,015	3,01E-04	4,29E-02
cg26048448	1	197879380		IGR	-0,028	3,01E-04	4,29E-02
cg22851415	1	112296918	C1orf183	Body	-0,006	3,02E-04	4,29E-02
cg11280087	5	128255842		IGR	-0,015	3,02E-04	4,29E-02
cg20712955	8	106810999	ZFPM2	Body	0,009	3,02E-04	4,29E-02

cg23733029	15	30110856	TJP1	Body	-0,011	3,02E-04	4,29E-02
cg14651910	7	105163122	PUS7	TSS1500	0,051	3,02E-04	4,29E-02
cg23131291	8	86162474	CA13	Body	0,004	3,02E-04	4,29E-02
cg17010241	1	43891291	SZT2	Body	-0,009	3,02E-04	4,29E-02
cg03793872	2	134770255		IGR	0,024	3,02E-04	4,29E-02
cg15147215	3	52552868	STAB1	Body	-0,008	3,02E-04	4,29E-02
cg23281602	6	74405609	)C101928489	Body	0,004	3,02E-04	4,29E-02
cg27115996	7	102741563	NAPEPLD	3'UTR	-0,017	3,02E-04	4,29E-02
cg04200607	10	72163475	EIF4EBP2	TSS1500	0,033	3,02E-04	4,29E-02
cg25503153	17	72894598		IGR	-0,011	3,02E-04	4,29E-02
cg15643724	16	2034532	GFER	Body	0,003	3,03E-04	4,30E-02
cg21827031	11	46958255	C11orf49	1stExon	0,009	3,03E-04	4,30E-02
cg17237111	4	114682438	CAMK2D	5'UTR	-0,011	3,03E-04	4,30E-02
cg06232675	3	184016689	PSMD2	TSS200	0,004	3,03E-04	4,30E-02
cg26554194	11	118871793	CCDC84	Body	-0,021	3,03E-04	4,30E-02
cg11185653	14	105669480		IGR	0,008	3,04E-04	4,30E-02
cg23667362	17	46019017	PNPO	5'UTR	0,007	3,04E-04	4,30E-02
cg14218444	2	147809697		IGR	0,014	3,04E-04	4,30E-02
cg21167157	12	123326461	HIP1R	Body	-0,028	3,04E-04	4,30E-02
cg15584851	9	20496496	MLLT3	Body	-0,021	3,05E-04	4,31E-02
cg14384178	13	111979098	C13orf16	Body	-0,015	3,05E-04	4,31E-02
cg01371498	15	49255686	SHC4	TSS200	0,008	3,05E-04	4,31E-02
cg10091408	17	35183637		IGR	0,043	3,05E-04	4,31E-02
cg17547408	4	71264948	PROL1	5'UTR	0,029	3,05E-04	4,31E-02
cg12464631	5	758136		IGR	0,029	3,05E-04	4,31E-02
cg01883759	7	28220576	JAZF1	TSS200	0,01	3,05E-04	4,31E-02
cg20544808	1	1099051		IGR	0,008	3,05E-04	4,31E-02
cg11564377	7	155584274		IGR	-0,038	3,05E-04	4,31E-02
cg05647571	8	109455664	EMC2	TSS200	0,01	3,05E-04	4,32E-02
cg05264985	19	36339236	NPHS1	Body	0,02	3,05E-04	4,32E-02
cg19328057	5	55289090	IL6ST	Body	0,029	3,06E-04	4,32E-02
cg23034244	12	57974745	KIF5A	ExonBnd	-0,017	3,06E-04	4,32E-02
cg23934145	18	65184858	DSEL	TSS1500	0,022	3,06E-04	4,32E-02
cg26302157	1	152747501	LCE1F	TSS1500	0,032	3,06E-04	4,32E-02
cg07058847	4	26219152		IGR	0,008	3,07E-04	4,32E-02
cg11065575	4	42403471	SHISA3	3'UTR	0,06	3,06E-04	4,32E-02
cg05938497	4	185939954	HELT	TSS200	0,016	3,06E-04	4,32E-02
cg10500461	5	149110563	PPARGC1B	Body	0,003	3,07E-04	4,32E-02
cg26242755	6	6624147	LOC285780	TSS1500	0,02	3,07E-04	4,32E-02
cg26786136	7	36233268	EEPD1	Body	-0,01	3,06E-04	4,32E-02
cg26150462	10	11206868	CUGBP2	TSS200	0,004	3,06E-04	4,32E-02
cg01606687	11	59368344	OSBP	ExonBnd	0,008	3,06E-04	4,32E-02
cg26341935	12	50677460	LIMA1	TSS200	0,006	3,06E-04	4,32E-02
cg22935821	12	53738700		IGR	0,003	3,06E-04	4,32E-02
cg27366994	19	5145824	KDM4B	Body	-0,013	3,06E-04	4,32E-02
cg27528247	22	51045178	MAPK8IP2	Body	-0,018	3,06E-04	4,32E-02
cg04190451	7	31122218	ADCYAP1R1	Body	-0,004	3,07E-04	4,32E-02
cg03348845	1	26402313		IGR	0,019	3,07E-04	4,32E-02
cg06940812	2	20553859		IGR	-0,008	3,07E-04	4,32E-02
cg11313065	3	46399396	CCR2	Body	0,005	3,07E-04	4,32E-02
cg20950856	10	80091516		IGR	-0,006	3,07E-04	4,32E-02
cg23719543	11	18373486	GTF2H1	Body	0,012	3,07E-04	4,32E-02
cg18116486	14	58667316	ACTR10	Body	-0,005	3,07E-04	4,32E-02
cg05084793	14	91977571	SMEK1	TSS1500	0,04	3,07E-04	4,32E-02
cg12015825	14	100602696	EVL	Body	-0,007	3,07E-04	4,32E-02
cg07967632	15	101601131	LRRK1	Body	-0,005	3,07E-04	4,32E-02
cg10575488	20	9378659	PLCB4	Body	0,017	3,07E-04	4,32E-02
cg13236649	20	55835020	BMP7	Body	0,044	3,07E-04	4,32E-02
cg06653543	22	34237443	LARGE	5'UTR	0,032	3,07E-04	4,32E-02
cg03002080	1	119461357	TBX15	Body	0,066	3,08E-04	4,32E-02
cg20136738	1	231114860	TTC13	TSS1500	0,009	3,08E-04	4,32E-02

cg15907278	3	31587250	STT3B	Body	-0,03	3,08E-04	4,32E-02
cg15024975	6	30685170	MDC1	1stExon	0,003	3,08E-04	4,32E-02
cg23026345	6	40400462	LRFN2	Body	0,005	3,08E-04	4,32E-02
cg04193905	6	115989126		IGR	-0,006	3,08E-04	4,32E-02
cg03992790	9	89937785		IGR	0,015	3,08E-04	4,32E-02
cg10853431	10	11574300	USP6NL	Body	-0,021	3,08E-04	4,32E-02
cg26866482	12	103351443	ASCL1	TSS200	-0,012	3,08E-04	4,32E-02
cg04498704	14	61681439		IGR	0,033	3,08E-04	4,32E-02
cg16943920	17	3631446	ITGAE	ExonBnd	0,004	3,08E-04	4,32E-02
cg07538777	19	5717584	LONP1	Body	-0,005	3,08E-04	4,32E-02
cg07129725	19	56915266	ZNF583	TSS1500	-0,006	3,08E-04	4,32E-02
cg03486411	16	3174915		IGR	0,006	3,08E-04	4,32E-02
cg06161779	21	25801042		IGR	-0,048	3,08E-04	4,32E-02
cg19900852	14	35451793	SRP54	TSS1500	0,006	3,08E-04	4,32E-02
cg19476058	19	59060359	TRIM28	Body	-0,007	3,08E-04	4,32E-02
cg23898184	1	192921219		IGR	0,054	3,09E-04	4,32E-02
cg10235961	3	48471218	PLXNB1	5'UTR	0,004	3,09E-04	4,32E-02
cg04111102	1	66153794		IGR	-0,011	3,09E-04	4,33E-02
cg24728949	5	106820169	EFNA5	Body	0,021	3,09E-04	4,33E-02
cg05618587	8	143473973	TSNARE1	5'UTR	0,01	3,09E-04	4,33E-02
cg02117132	10	114920853	TCF7L2	3'UTR	0,005	3,09E-04	4,33E-02
cg10463664	12	114232937		IGR	-0,066	3,09E-04	4,33E-02
cg10091053	16	89603535	SPG7	Body	-0,008	3,09E-04	4,33E-02
cg13934223	1	161711095		IGR	-0,029	3,09E-04	4,33E-02
cg09933021	2	48060153	FBXO11	Body	-0,016	3,10E-04	4,33E-02
cg00885708	5	139167076		IGR	0,01	3,09E-04	4,33E-02
cg22821709	10	34229048		IGR	0,015	3,10E-04	4,33E-02
cg10291250	17	48432287	XYLT2	Body	0,007	3,09E-04	4,33E-02
cg19018844	10	617607	DIP2C	Body	0,031	3,10E-04	4,33E-02
cg00644847	1	6787585		IGR	0,037	3,10E-04	4,33E-02
cg06037367	16	84075678	SLC38A8	1stExon	-0,018	3,10E-04	4,33E-02
cg07340145	16	86220038		IGR	-0,003	3,10E-04	4,33E-02
cg13061373	17	75327380	sept-09	5'UTR	0,024	3,10E-04	4,33E-02
cg08329647	19	42489545	ATP1A3	Body	-0,007	3,10E-04	4,33E-02
cg19584027	12	94529752		IGR	0,005	3,10E-04	4,33E-02
cg25778564	15	81425747	C15orf26	TSS1500	0,052	3,10E-04	4,33E-02
cg08598804	20	24115458		IGR	-0,013	3,10E-04	4,33E-02
cg21892385	1	186762438		IGR	0,052	3,10E-04	4,33E-02
cg23745864	18	77806288	C18orf22	3'UTR	-0,009	3,10E-04	4,33E-02
cg01442943	6	41215914	TREML2P	TSS1500	0,012	3,11E-04	4,33E-02
cg18538785	11	824762	PNPLA2	Body	-0,008	3,11E-04	4,33E-02
cg00712792	16	89921048	SPIRE2	Body	-0,005	3,11E-04	4,33E-02
cg19269426	1	235500468	GGPS1	Body	0,091	3,11E-04	4,34E-02
cg15375163	10	133947518	JAKMIP3	Body	0,012	3,11E-04	4,34E-02
cg02772605	1	28912323		IGR	0,015	3,11E-04	4,34E-02
cg02145222	1	30322306		IGR	-0,023	3,12E-04	4,34E-02
cg26663439	1	45475270	HECTD3	Body	-0,006	3,12E-04	4,34E-02
cg08701070	1	238770428		IGR	-0,048	3,12E-04	4,34E-02
cg01741344	2	86718091	KDM3A	Body	0,008	3,11E-04	4,34E-02
cg17726211	5	111527489	EPB41L4A	Body	0,014	3,11E-04	4,34E-02
cg11150306	11	76377950	LRRC32	5'UTR	0,011	3,12E-04	4,34E-02
cg08564527	16	67571988	FAM65A	5'UTR	-0,021	3,12E-04	4,34E-02
cg12405718	16	87801007	KLHDC4	TSS1500	-0,01	3,12E-04	4,34E-02
cg04487205	17	36997563	C17orf98	1stExon	-0,077	3,11E-04	4,34E-02
cg04041616	20	48403630		IGR	0,007	3,12E-04	4,34E-02
cg00770317	10	13424911		IGR	-0,016	3,12E-04	4,34E-02
cg10638044	1	110170914	AMPD2	Body	0,007	3,13E-04	4,34E-02
cg22649068	1	150552352	MCL1	TSS1500	0,005	3,12E-04	4,34E-02
cg18019663	1	208431081		IGR	-0,009	3,13E-04	4,34E-02
cg14325814	3	32139560		IGR	0,036	3,13E-04	4,34E-02
cg19008133	3	123124015	ADCY5	Body	-0,011	3,13E-04	4,34E-02

cg15776416	3	195529779	MUC4	Body	-0,008	3,13E-04	4,34E-02
cg14949231	4	184930888	STOX2	Body	0,011	3,13E-04	4,34E-02
cg19084075	6	26124329	HIST1H2AC	TSS200	0,006	3,13E-04	4,34E-02
cg22315844	6	69342841		IGR	0,045	3,13E-04	4,34E-02
cg05048917	6	107077187	QRSL1	TSS1500	0,036	3,13E-04	4,34E-02
cg23023378	6	144467340		IGR	-0,006	3,13E-04	4,34E-02
cg04221884	11	608381	PHRF1	Body	-0,016	3,13E-04	4,34E-02
cg16843162	11	17085300		IGR	-0,016	3,13E-04	4,34E-02
cg04836949	13	46699875		IGR	-0,044	3,13E-04	4,34E-02
cg01599539	14	23344910	LRP10	Body	-0,014	3,13E-04	4,34E-02
cg16270633	15	91256830		IGR	-0,011	3,13E-04	4,34E-02
cg15791361	22	32772257	LOC339666	TSS1500	0,01	3,12E-04	4,34E-02
cg16549994	22	36854045		IGR	-0,014	3,13E-04	4,34E-02
cg17708319	15	63926190	HERC1	Body	-0,007	3,13E-04	4,34E-02
cg09488511	22	37893410	CARD10	Body	0,009	3,13E-04	4,34E-02
cg27031506	7	128379115	CALU	TSS1500	0,004	3,14E-04	4,34E-02
cg15916804	10	75255500	PPP3CB	Body	0,004	3,14E-04	4,34E-02
cg11494123	11	66726233	PC	TSS1500	0,004	3,14E-04	4,34E-02
cg01655290	13	65104710		IGR	0,024	3,14E-04	4,34E-02
cg10025945	1	111682281	CEPT1	1stExon	-0,006	3,14E-04	4,34E-02
cg16038579	6	148572719		IGR	0,037	3,14E-04	4,34E-02
cg10920297	11	94803188	SFRS2B	1stExon	0,036	3,14E-04	4,34E-02
cg22775642	12	125332183	SCARB1	Body	-0,008	3,14E-04	4,34E-02
cg17772543	19	47925344		IGR	-0,008	3,14E-04	4,34E-02
cg22080282	18	72920932	ZADH2	1stExon	0,003	3,14E-04	4,34E-02
cg06012792	6	14857570		IGR	-0,013	3,14E-04	4,34E-02
cg02905169	11	76008349		IGR	-0,011	3,14E-04	4,34E-02
cg12098784	21	46782607		IGR	-0,008	3,14E-04	4,34E-02
cg03784956	1	228785238	DUSP5P	Body	-0,069	3,15E-04	4,35E-02
cg25609649	2	19547730		IGR	0,008	3,15E-04	4,35E-02
cg17796820	4	148708816	ARHGAP10	Body	-0,006	3,15E-04	4,35E-02
cg09498440	5	72415429	TMEM171	TSS1500	0,029	3,15E-04	4,35E-02
cg17813457	5	124512309	)C101927421	Body	-0,013	3,15E-04	4,35E-02
cg13635462	11	77790613	NDUFC2	Body	0,003	3,15E-04	4,35E-02
cg23712458	17	189002	RPH3AL	5'UTR	-0,028	3,15E-04	4,35E-02
cg10502303	2	16847831	FAM49A	TSS1500	0,041	3,15E-04	4,35E-02
cg07232225	2	3606306	RNASEH1-AS1	Body	0,004	3,16E-04	4,35E-02
cg06545665	13	99126686	STK24	Body	0,006	3,15E-04	4,35E-02
cg02681400	19	15375283	BRD4	Body	-0,022	3,15E-04	4,35E-02
cg12399224	20	62383456	ZBTB46	Body	0,016	3,16E-04	4,35E-02
cg20950655	1	153727168	INTS3	Body	-0,005	3,16E-04	4,35E-02
cg25350057	1	68629133	GPR177	Body	-0,007	3,16E-04	4,35E-02
cg06217450	3	142441654		IGR	0,005	3,16E-04	4,35E-02
cg11513546	5	38974566	RICTOR	Body	-0,007	3,16E-04	4,35E-02
cg03728425	17	80295545		IGR	-0,053	3,16E-04	4,35E-02
cg10019429	19	32836659	ZNF507	1stExon	-0,004	3,16E-04	4,35E-02
cg22833618	19	49658548	HRC	5'UTR	0,022	3,16E-04	4,35E-02
cg17952465	22	51016839	CPT1B	5'UTR	-0,058	3,16E-04	4,35E-02
cg23676348	2	12538123	)C100506457	Body	0,039	3,16E-04	4,36E-02
cg13392874	2	215493830		IGR	-0,012	3,17E-04	4,36E-02
cg11773558	19	23942126	ZNF681	TSS1500	0,016	3,17E-04	4,36E-02
cg17717091	12	120541617	RAB35	Body	0,005	3,17E-04	4,36E-02
cg26837952	17	80079810	CCDC57	Body	-0,016	3,17E-04	4,36E-02
cg03483142	8	87521078	FAM82B	TSS200	-0,005	3,17E-04	4,36E-02
cg25949033	10	3149760	PFKP	Body	-0,004	3,17E-04	4,36E-02
cg01194044	11	47486187		IGR	0,009	3,17E-04	4,36E-02
cg04838786	14	65821686		IGR	0,013	3,17E-04	4,36E-02
cg07950397	19	10022659	OLFM2	Body	0,028	3,17E-04	4,36E-02
cg11621113	19	12776725	MORG1	TSS1500	0,025	3,17E-04	4,36E-02
cg27133504	7	156917432		IGR	0,009	3,18E-04	4,36E-02
cg10273420	10	43953760	ZNF487	Body	0,009	3,18E-04	4,36E-02

cg01939681	5	160975024	GABRB2	5'UTR	-0,007	3,18E-04	4,36E-02
cg21798524	19	14828347	ZNF333	Body	-0,006	3,18E-04	4,36E-02
cg15864310	2	169805337	ABCB11	Body	0,046	3,18E-04	4,36E-02
cg02455013	11	119465987		IGR	-0,011	3,18E-04	4,36E-02
cg19753476	17	4803506	CHRNE	Body	-0,038	3,18E-04	4,36E-02
cg24861502	22	28628826	TTC28	Body	-0,016	3,18E-04	4,36E-02
cg10861135	9	14345348		IGR	-0,047	3,19E-04	4,37E-02
cg09621618	3	71117979	FOXP1	Body	-0,009	3,19E-04	4,37E-02
cg14485468	6	71553462	SMAP1	Body	-0,021	3,19E-04	4,37E-02
cg00690093	17	8166394	PFAS	Body	0,006	3,19E-04	4,37E-02
cg01476789	11	71146764	DHCR7	Body	-0,012	3,19E-04	4,37E-02
cg10440989	6	144540635		IGR	0,013	3,19E-04	4,38E-02
cg09514401	8	72988022	TRPA1	TSS1500	-0,014	3,20E-04	4,38E-02
cg18947446	12	117732155	NOS1	5'UTR	0,033	3,20E-04	4,38E-02
cg22803240	13	34296777		IGR	0,015	3,20E-04	4,38E-02
cg15573188	11	68673772	IGHMBP2	Body	0,005	3,20E-04	4,38E-02
cg08066790	12	115761694		IGR	0,016	3,20E-04	4,38E-02
cg07058058	12	29256578		IGR	-0,015	3,20E-04	4,38E-02
cg21777887	8	144951147	EPPK1	5'UTR	-0,024	3,21E-04	4,39E-02
cg27622679	3	181441978	SOX2OT	Body	-0,022	3,21E-04	4,39E-02
cg17129957	9	35920785		IGR	0,004	3,21E-04	4,39E-02
cg13251916	16	86377209	LINC00917	Body	0,056	3,21E-04	4,39E-02
cg11158629	4	9922462	SLC2A9	Body	0,007	3,21E-04	4,39E-02
cg20492200	14	64319715	SYNE2	1stExon	0,006	3,21E-04	4,39E-02
cg05505338	22	20024388	TANGO2	ExonBnd	-0,011	3,21E-04	4,39E-02
cg19180698	4	82471371		IGR	-0,012	3,21E-04	4,39E-02
cg10220031	6	12174943		IGR	-0,015	3,21E-04	4,39E-02
cg22816087	8	127516484		IGR	-0,02	3,21E-04	4,39E-02
cg26258845	2	207308087	ADAM23	TSS1500	0,003	3,22E-04	4,39E-02
cg06084371	17	1390318	MYO1C	TSS1500	0,006	3,21E-04	4,39E-02
cg09492774	1	117349215		IGR	0,022	3,22E-04	4,39E-02
cg14904272	3	126283883		IGR	0,011	3,22E-04	4,39E-02
cg20384759	12	116413613	MED13L	Body	-0,007	3,22E-04	4,39E-02
cg06280368	7	12663282	SCIN	Body	0,038	3,22E-04	4,39E-02
cg10941635	12	308722	SLC6A12	Body	-0,044	3,22E-04	4,39E-02
cg11556682	8	131611900		IGR	0,043	3,22E-04	4,39E-02
cg06185738	11	22359868	SLC17A6	5'UTR	-0,004	3,22E-04	4,39E-02
cg08064076	17	41639983		IGR	0,037	3,22E-04	4,39E-02
cg11053763	5	111527858	EPB41L4A	Body	0,006	3,23E-04	4,39E-02
cg24488099	7	6576558	GRID2IP	Body	0,009	3,23E-04	4,39E-02
cg17260918	10	115614141	NHLRC2	TSS1500	-0,01	3,23E-04	4,39E-02
cg10754668	17	35168702		IGR	0,046	3,23E-04	4,39E-02
cg03660365	17	79102708	AATK	Body	-0,016	3,23E-04	4,39E-02
cg00997647	20	17950965	SNX5	TSS1500	0,057	3,22E-04	4,39E-02
cg17172949	20	35419347	C20orf117	Body	0,01	3,23E-04	4,40E-02
cg20206644	20	49693799		IGR	0,033	3,23E-04	4,40E-02
cg16031515	1	205743344	RAB7L1	Body	0,068	3,23E-04	4,40E-02
cg05943122	8	23648352		IGR	0,008	3,23E-04	4,40E-02
cg17608570	10	130228545		IGR	-0,035	3,23E-04	4,40E-02
cg21044577	13	111364847	ING1	TSS1500	0,006	3,23E-04	4,40E-02
cg06138439	16	54973128		IGR	0,02	3,23E-04	4,40E-02
cg18135341	6	137341992	IL20RA	Body	0,02	3,24E-04	4,40E-02
cg00778920	11	60674370	PRPF19	TSS1500	0,003	3,24E-04	4,40E-02
cg13777545	15	63136962		IGR	0,021	3,24E-04	4,40E-02
cg18716076	5	50677808	ISL1	TSS1500	-0,015	3,24E-04	4,40E-02
cg14130908	9	6416274	UHRF2	Body	0,005	3,24E-04	4,40E-02
cg17597195	11	85339608	TMEM126B	TSS200	0,003	3,24E-04	4,40E-02
cg02833127	4	177116733	SPATA4	1stExon	0,005	3,24E-04	4,40E-02
cg17157851	5	38291643	EGFLAM-AS4	TSS1500	0,032	3,24E-04	4,40E-02
cg14567957	6	76204172	FILIP1	TSS1500	-0,027	3,24E-04	4,40E-02
cg03046204	11	106456733		IGR	0,029	3,24E-04	4,40E-02

cg22473427	22	32358655		IGR	0,013	3,24E-04	4,40E-02
cg14647379	5	171580730	STK10	Body	-0,056	3,25E-04	4,40E-02
cg04931655	16	27414210	IL21R	5'UTR	0,023	3,25E-04	4,40E-02
cg18231981	1	161016120	USF1	TSS1500	-0,003	3,25E-04	4,41E-02
cg26582230	4	3371652	RGS12	Body	0,006	3,25E-04	4,41E-02
cg10038867	1	21982511	RAP1GAP	5'UTR	0,017	3,25E-04	4,41E-02
cg23360332	1	22589385		IGR	0,029	3,25E-04	4,41E-02
cg00721915	3	45429647	LARS2	TSS1500	0,077	3,25E-04	4,41E-02
cg18853376	10	23633759	C10orf67	1stExon	0,016	3,25E-04	4,41E-02
cg27068297	22	50451975	IL17REL	TSS1500	0,057	3,25E-04	4,41E-02
cg06312789	3	126374304		IGR	0,028	3,25E-04	4,41E-02
cg07598755	16	30023028	DOC2A	TSS1500	-0,006	3,25E-04	4,41E-02
cg00792008	12	122189621	TMEM120B	Body	-0,037	3,26E-04	4,41E-02
cg19018435	1	183797630	RGL1	Body	-0,023	3,26E-04	4,41E-02
cg03101645	20	12989700	SPTLC3	5'UTR	-0,014	3,26E-04	4,41E-02
cg15465219	19	31210456		IGR	-0,018	3,26E-04	4,42E-02
cg05457490	7	155378460		IGR	-0,019	3,27E-04	4,42E-02
cg22254104	9	124991432	LHX6	TSS1500	-0,007	3,26E-04	4,42E-02
cg26748598	13	24091341		IGR	-0,006	3,26E-04	4,42E-02
cg12635386	17	10538871	MYH3	ExonBnd	-0,009	3,27E-04	4,42E-02
cg05521649	1	15721051	FHAD1	Body	0,039	3,27E-04	4,42E-02
cg05067847	1	226990401		IGR	0,007	3,27E-04	4,42E-02
cg15878792	3	53266479	TKT	Body	-0,019	3,27E-04	4,42E-02
cg23095642	1	52081938	OSBPL9	TSS1500	-0,019	3,27E-04	4,42E-02
cg01974292	9	96009796	WNK2	Body	-0,013	3,27E-04	4,42E-02
cg07614502	22	44351644	SAMM50	Body	0,003	3,27E-04	4,42E-02
cg16829306	1	21671508	ECE1	Body	0,028	3,27E-04	4,42E-02
cg09315329	13	21141066	IFT88	TSS200	0,011	3,27E-04	4,42E-02
cg17372923	20	34890822		IGR	-0,033	3,28E-04	4,42E-02
cg25094636	3	104221851		IGR	-0,012	3,28E-04	4,42E-02
cg09112514	4	55096230	PDGFRA	5'UTR	-0,014	3,28E-04	4,42E-02
cg08780496	6	152893787	SYNE1	Body	0,016	3,28E-04	4,42E-02
cg10422256	9	15135282		IGR	-0,004	3,28E-04	4,42E-02
cg19029418	9	102036562		IGR	0,006	3,28E-04	4,42E-02
cg06147728	3	43934530		IGR	0,013	3,28E-04	4,43E-02
cg06259158	6	36846157		IGR	0,007	3,28E-04	4,43E-02
cg13492245	9	130566157	FPGS	Body	0,012	3,28E-04	4,43E-02
cg07165637	16	28102779		IGR	-0,01	3,28E-04	4,43E-02
cg01825310	1	154972863		IGR	0,004	3,29E-04	4,43E-02
cg15199987	6	85821320		IGR	-0,01	3,29E-04	4,43E-02
cg04138721	8	11268948	AM167A-AS1	Body	0,017	3,29E-04	4,43E-02
cg06402833	8	129002466	PVT1	Body	-0,004	3,29E-04	4,43E-02
cg07056568	15	22436227		IGR	0,007	3,29E-04	4,43E-02
cg20013920	17	80809645	TBCD	Body	-0,009	3,29E-04	4,43E-02
cg12503473	12	94580426	PLXNC1	Body	0,015	3,29E-04	4,43E-02
cg21629505	13	36429710	DCLK1	1stExon	0,025	3,29E-04	4,43E-02
cg07729174	16	54199735		IGR	-0,009	3,29E-04	4,43E-02
cg22105335	2	85526054	TCF7L1	Body	0,004	3,29E-04	4,43E-02
cg17115402	17	72999571	CDR2L	Body	-0,012	3,29E-04	4,43E-02
cg04082532	1	115840767	NGF	5'UTR	0,041	3,30E-04	4,43E-02
cg10991031	2	241596051		IGR	0,031	3,30E-04	4,43E-02
cg03805782	3	124699654	HEG1	Body	-0,012	3,30E-04	4,43E-02
cg04363087	5	166952198	TENM2	Body	-0,006	3,30E-04	4,43E-02
cg23972767	7	4277473	SDK1	Body	-0,009	3,30E-04	4,43E-02
cg15454856	7	108168130	PNPLA8	TSS1500	-0,008	3,30E-04	4,43E-02
cg01263077	19	17316300	MYO9B	Body	0,016	3,30E-04	4,43E-02
cg23734418	13	61221316		IGR	0,056	3,30E-04	4,43E-02
cg21665814	4	161051260		IGR	-0,037	3,31E-04	4,44E-02
cg15110481	2	134277486	NCKAP5	5'UTR	-0,05	3,31E-04	4,44E-02
cg03929848	5	137057918	KLHL3	TSS1500	0,011	3,31E-04	4,44E-02
cg12834688	15	59107109	FAM63B	Body	-0,008	3,31E-04	4,44E-02

cg20366277	17	27454979	MYO18A	Body	-0,007	3,31E-04	4,44E-02
cg22169750	3	179689236	PEX5L	5'UTR	-0,008	3,31E-04	4,45E-02
cg04863377	15	45440142	DUOX1	Body	-0,011	3,31E-04	4,45E-02
cg22601577	19	14202476	SAMD1	TSS1500	-0,003	3,31E-04	4,45E-02
cg03753683	10	134791323		IGR	0,057	3,32E-04	4,45E-02
cg22805216	11	43381704	TTC17	Body	-0,033	3,32E-04	4,45E-02
cg13445279	6	24423344	MRS2	3'UTR	0,003	3,32E-04	4,45E-02
cg12894833	6	143296787	LINC01277	Body	0,017	3,32E-04	4,45E-02
cg17716986	10	116169561		IGR	0,021	3,32E-04	4,45E-02
cg15521034	11	124823390	CCDC15	TSS1500	-0,062	3,32E-04	4,45E-02
cg04421974	8	134224814	WISP1	Body	0,005	3,32E-04	4,45E-02
cg16660417	19	49104253	FAM83E	3'UTR	-0,013	3,32E-04	4,45E-02
cg15131647	1	153650884	NPR1	TSS1500	-0,03	3,33E-04	4,45E-02
cg05147764	1	226449708	LIN9	Body	0,007	3,33E-04	4,45E-02
cg27420677	2	10469241	HPCAL1	TSS1500	-0,007	3,33E-04	4,45E-02
cg23192899	2	55746287	CCDC104	TSS1500	0,003	3,33E-04	4,45E-02
cg11193423	2	67623159	ETAA1	TSS1500	-0,011	3,33E-04	4,45E-02
cg07452829	2	203879064	NBEAL1	TSS1500	-0,021	3,33E-04	4,45E-02
cg13755118	6	25761723	SLC17A4	5'UTR	-0,024	3,33E-04	4,45E-02
cg05488568	7	43695371	COA1	Body	-0,005	3,33E-04	4,45E-02
cg23997477	7	44325329	CAMK2B	Body	-0,005	3,33E-04	4,45E-02
cg27157522	7	55028778		IGR	-0,005	3,33E-04	4,45E-02
cg17456827	10	127812173	ADAM12	Body	-0,007	3,33E-04	4,45E-02
cg11421956	11	60689415	TMEM109	Body	-0,007	3,33E-04	4,45E-02
cg05637837	11	64574700	MEN1	Body	-0,012	3,33E-04	4,45E-02
cg23327896	11	73669290	DNAJB13	Body	-0,026	3,32E-04	4,45E-02
cg09692531	11	118436991	C11orf60	TSS1500	-0,003	3,33E-04	4,45E-02
cg26601267	15	61928177		IGR	0,009	3,33E-04	4,45E-02
cg21038819	17	73507788	CASKIN2	Body	-0,011	3,33E-04	4,45E-02
cg17499173	19	736285	PALM	Body	-0,018	3,33E-04	4,45E-02
cg13644262	4	6449564	PPP2R2C	Body	0,028	3,34E-04	4,45E-02
cg27659368	2	226525308		IGR	0,054	3,34E-04	4,45E-02
cg08233624	5	163774596		IGR	0,021	3,34E-04	4,45E-02
cg08429263	12	53553352	CSAD	Body	0,003	3,34E-04	4,45E-02
cg25635840	17	26898051	PIGS	Body	0,004	3,34E-04	4,45E-02
cg00870713	1	184722906	EDEM3	Body	-0,014	3,34E-04	4,45E-02
cg11737758	2	137931242	THSD7B	Body	0,041	3,34E-04	4,45E-02
cg08535850	8	55157423		IGR	0,022	3,34E-04	4,45E-02
cg08568425	10	78945446	KCNMA1	Body	0,013	3,34E-04	4,45E-02
cg02773974	12	62980871	MON2	Body	-0,007	3,34E-04	4,45E-02
cg03160005	14	51303308		IGR	0,007	3,34E-04	4,45E-02
cg24330297	22	22006397	MIR301B	TSS1500	0,003	3,34E-04	4,45E-02
cg16570995	6	37138163	PIM1	5'UTR	0,005	3,34E-04	4,45E-02
cg07809479	3	125820613	SLC41A3	TSS1500	-0,009	3,35E-04	4,46E-02
cg12012941	1	188676237		IGR	0,044	3,35E-04	4,46E-02
cg01163404	12	129822259	TMEM132D	Body	-0,014	3,35E-04	4,46E-02
cg01395831	10	13748597	FRMD4A	Body	0,012	3,35E-04	4,46E-02
cg26803305	18	43304452	SLC14A1	5'UTR	0,039	3,35E-04	4,46E-02
cg24455808	11	1977381	MRPL23	Body	-0,007	3,35E-04	4,46E-02
cg15124950	11	1272972	MUC5B	Body	-0,02	3,35E-04	4,46E-02
cg04206906	16	1732086	HN1L	Body	0,006	3,35E-04	4,46E-02
cg16232206	12	22486437	ST8SIA1	5'UTR	0,058	3,35E-04	4,46E-02
cg17216478	1	21616619	ECE1	1stExon	0,004	3,36E-04	4,46E-02
cg24443795	1	35252824		IGR	0,005	3,36E-04	4,47E-02
cg26166595	11	11998715	DKK3	Body	0,031	3,36E-04	4,47E-02
cg02399524	11	63258509	HRASLS5	1stExon	0,01	3,37E-04	4,47E-02
cg23070020	16	30578627		IGR	0,033	3,36E-04	4,47E-02
cg16456182	16	57844448	LOC388282	TSS200	-0,004	3,36E-04	4,47E-02
cg13239540	12	105656748		IGR	0,013	3,37E-04	4,47E-02
cg23982445	2	394333		IGR	0,025	3,37E-04	4,47E-02
cg09879168	2	149893581	LYPD6B	TSS1500	0,017	3,37E-04	4,47E-02

cg08309302	7	131891		IGR	-0,017	3,37E-04	4,47E-02
cg03727700	10	115614022	NHLRC2	TSS1500	0,003	3,37E-04	4,47E-02
cg21405039	11	67375423	C11orf72	TSS1500	0,008	3,37E-04	4,47E-02
cg06211255	11	65153842	FRMD8	TSS200	0,027	3,37E-04	4,48E-02
cg15793716	1	230472160	PGBD5	Body	0,032	3,38E-04	4,48E-02
cg03808418	9	2748746		IGR	-0,018	3,38E-04	4,48E-02
cg11931667	12	110151938	C12orf34	TSS1500	0,004	3,37E-04	4,48E-02
cg22883125	8	11540758		IGR	0,006	3,38E-04	4,48E-02
cg06096879	3	150421725	ERICH6-AS1	TSS200	-0,004	3,38E-04	4,48E-02
cg25289457	6	42832508	KIAA0240	Body	-0,008	3,38E-04	4,48E-02
cg20734092	10	22546132	)C100130992	Body	-0,051	3,38E-04	4,48E-02
cg20491103	10	121521886	INPP5F	Body	-0,026	3,38E-04	4,48E-02
cg21230007	14	64316569		IGR	0,006	3,38E-04	4,48E-02
cg15547759	7	2092420	MAD1L1	Body	-0,006	3,39E-04	4,48E-02
cg22630543	8	75425262		IGR	0,031	3,39E-04	4,48E-02
cg05338847	10	123914355	TACC2	5'UTR	0,043	3,39E-04	4,48E-02
cg07158473	1	43865857	SZT2	Body	0,017	3,39E-04	4,48E-02
cg00981330	6	114556847	)C101927768	Body	0,038	3,39E-04	4,48E-02
cg23896514	7	150103902	LOC728743	Body	0,021	3,39E-04	4,48E-02
cg10147044	15	75230572	COX5A	TSS200	0,002	3,39E-04	4,48E-02
cg07236207	1	897949	KLHL17	Body	0,01	3,39E-04	4,48E-02
cg26500016	5	95068252	RHOBTB3	Body	-0,013	3,39E-04	4,48E-02
cg02936526	1	22310686	CELA3B	ExonBnd	-0,021	3,40E-04	4,48E-02
cg03387111	3	101292956	PCNP	TSS200	-0,003	3,39E-04	4,48E-02
cg26589036	5	79603682		IGR	0,05	3,39E-04	4,48E-02
cg09271052	6	30977529	MUC22	5'UTR	0,053	3,40E-04	4,48E-02
cg24283684	6	136172947	PDE7B	1stExon	-0,013	3,40E-04	4,48E-02
cg02851435	6	156829146		IGR	0,006	3,40E-04	4,48E-02
cg13541456	9	136603618	SARDH	5'UTR	0,005	3,40E-04	4,48E-02
cg18207676	10	70782581		IGR	-0,013	3,40E-04	4,48E-02
cg14273027	13	70682736	KLHL1	TSS200	0,055	3,40E-04	4,48E-02
cg09895131	14	24629122	IRF9	TSS1500	-0,013	3,40E-04	4,48E-02
cg24260530	14	103344879	TRAF3	Body	-0,021	3,40E-04	4,48E-02
cg21465176	17	80358876	C17orf101	Body	-0,009	3,40E-04	4,48E-02
cg01379171	20	60970499	CABLES2	Body	-0,008	3,40E-04	4,48E-02
cg10994819	6	99399304		IGR	-0,025	3,40E-04	4,49E-02
cg16233192	10	123900239	TACC2	5'UTR	0,034	3,40E-04	4,49E-02
cg03667429	14	74181552	PNMA1	TSS1500	-0,007	3,40E-04	4,49E-02
cg18528621	17	60885154	MARCH10	TSS1500	-0,004	3,40E-04	4,49E-02
cg06454698	22	47031989	GRAMD4	Body	-0,012	3,40E-04	4,49E-02
cg11656478	2	38297759	CYP1B1	3'UTR	0,03	3,41E-04	4,49E-02
cg05927438	7	128695356	TNPO3	TSS200	-0,007	3,40E-04	4,49E-02
cg16851046	13	21291173	IL17D	Body	-0,007	3,40E-04	4,49E-02
cg23836455	16	67204226	NOL3	TSS200	0,003	3,40E-04	4,49E-02
cg18176307	12	387235		IGR	-0,004	3,41E-04	4,49E-02
cg27238955	1	198796384	MIR181A1HG	Body	-0,013	3,41E-04	4,49E-02
cg06019425	1	211666671	RD3	TSS1500	-0,014	3,41E-04	4,49E-02
cg24622439	2	212246986	ERBB4	3'UTR	-0,007	3,41E-04	4,49E-02
cg11583218	9	98919035		IGR	0,041	3,41E-04	4,49E-02
cg02850795	1	3549532	WDR8	Body	0,006	3,41E-04	4,49E-02
cg22027008	4	89444993	PIGY	TSS200	0,006	3,42E-04	4,49E-02
cg19139167	10	127824746	ADAM12	Body	-0,009	3,42E-04	4,49E-02
cg06308785	14	76618171	GPATCH2L	TSS200	-0,03	3,42E-04	4,49E-02
cg09572685	17	56293543	MKS1	Body	-0,006	3,42E-04	4,49E-02
cg07246325	20	30139533	HM13	Body	-0,007	3,42E-04	4,49E-02
cg18791979	20	32606766	RALY	5'UTR	0,006	3,41E-04	4,49E-02
cg19772199	21	39633699	KCNJ15	5'UTR	0,022	3,41E-04	4,49E-02
cg20829454	12	76630497		IGR	-0,01	3,42E-04	4,49E-02
cg13485248	2	2893732		IGR	0,007	3,42E-04	4,49E-02
cg02590750	6	30973562	MUC22	TSS200	0,006	3,42E-04	4,49E-02
cg22610784	8	33370693	C8orf41	1stExon	-0,005	3,42E-04	4,49E-02

cg20218843	12	104052107	STAB2	Body	0,049	3,42E-04	4,49E-02
cg27512832	16	15609068	C16orf45	Body	-0,008	3,42E-04	4,49E-02
cg16202615	2	43454763	ZFP36L2	TSS1500	0,005	3,43E-04	4,50E-02
cg01184315	17	4389850	SPNS3	Body	-0,009	3,43E-04	4,50E-02
cg04990472	14	69950043	FLJ44817	TSS1500	0,029	3,43E-04	4,50E-02
cg00914384	2	5593580		IGR	0,024	3,44E-04	4,50E-02
cg02152982	2	86667766	KDM3A	TSS1500	0,003	3,44E-04	4,50E-02
cg23258304	3	140456169		IGR	0,04	3,43E-04	4,50E-02
cg17968231	4	71019995	PRR27	5'UTR	-0,037	3,44E-04	4,50E-02
cg06073351	10	22625665		IGR	-0,007	3,43E-04	4,50E-02
cg18217594	12	9293085		IGR	-0,011	3,43E-04	4,50E-02
cg08782710	12	123497287	PITPNM2	ExonBnd	-0,011	3,44E-04	4,50E-02
cg01330096	13	112713372		IGR	-0,01	3,43E-04	4,50E-02
cg22530767	14	61787418	PRKCH	TSS1500	0,013	3,44E-04	4,50E-02
cg05566777	16	74782640	FA2H	Body	-0,01	3,44E-04	4,50E-02
cg18473137	17	30815034	CDK5R1	Body	0,02	3,43E-04	4,50E-02
cg10289051	19	7741937	MCEMP1	TSS200	-0,028	3,44E-04	4,50E-02
cg16404047	19	15308169	NOTCH3	Body	-0,004	3,43E-04	4,50E-02
cg01929087	20	34192501	FER1L4	Body	0,027	3,43E-04	4,50E-02
cg05096739	20	35124762	DLGAP4	Body	-0,008	3,43E-04	4,50E-02
cg23776282	22	41346522	RBX1	TSS1500	0,02	3,43E-04	4,50E-02
cg26611710	5	78907212	PAPD4	TSS1500	-0,02	3,44E-04	4,50E-02
cg15670585	16	21288557	CRYM	Body	0,028	3,44E-04	4,50E-02
cg16049537	20	19765838		IGR	0,015	3,44E-04	4,50E-02
cg06223370	12	31913198		IGR	-0,006	3,44E-04	4,50E-02
cg17259741	4	4291994	ZNF509	5'UTR	0,004	3,44E-04	4,50E-02
cg09635617	18	8959911		IGR	0,012	3,45E-04	4,50E-02
cg25468632	5	85913588	COX7C	TSS200	-0,011	3,45E-04	4,50E-02
cg25998967	6	111727497	REV3L	Body	0,056	3,45E-04	4,51E-02
cg14019951	3	174095086		IGR	-0,006	3,45E-04	4,51E-02
cg20584495	8	94973728		IGR	0,028	3,46E-04	4,52E-02
cg26589335	2	215896836	ABCA12	TSS200	0,018	3,46E-04	4,52E-02
cg12308861	2	224847533	SERPINE2	Body	-0,007	3,46E-04	4,52E-02
cg24867502	4	3486234	DOK7	TSS200	0,037	3,46E-04	4,52E-02
cg10444037	6	42531637	UBR2	TSS1500	-0,004	3,46E-04	4,52E-02
cg03733213	6	130346425	L3MBTL3	5'UTR	-0,005	3,46E-04	4,52E-02
cg03286098	8	42271413		IGR	0,007	3,46E-04	4,52E-02
cg14590027	9	119254997	ASTN2	Body	-0,01	3,46E-04	4,52E-02
cg13527143	16	87799610	KLHDC4	TSS200	0,004	3,46E-04	4,52E-02
cg08279444	1	202792366	MGAT4EP	Body	-0,007	3,47E-04	4,52E-02
cg05606089	5	77306302	AP3B1	Body	-0,007	3,47E-04	4,52E-02
cg04939692	5	306205	PDCD6	5'UTR	-0,007	3,47E-04	4,52E-02
cg11648304	22	48594011		IGR	0,006	3,47E-04	4,52E-02
cg10215242	20	61594271	SLC17A9	Body	-0,007	3,47E-04	4,52E-02
cg03799809	9	97403761	FBP1	TSS1500	0,004	3,47E-04	4,52E-02
cg07740063	11	126033141		IGR	-0,013	3,47E-04	4,53E-02
cg27474855	2	1991887	MYT1L	5'UTR	-0,009	3,48E-04	4,53E-02
cg02056921	1	901892	PLEKHN1	1stExon	0,006	3,48E-04	4,53E-02
cg06771188	6	27521477		IGR	-0,017	3,48E-04	4,53E-02
cg24631102	10	129349644	NPS	Body	-0,037	3,48E-04	4,53E-02
cg12152759	10	134811757		IGR	-0,005	3,48E-04	4,53E-02
cg26272064	16	75591276	TMEM231	TSS1500	-0,004	3,48E-04	4,53E-02
cg09351982	19	10016707	OLFM2	Body	-0,016	3,48E-04	4,53E-02
cg14821659	1	241765537	OPN3	Body	0,003	3,48E-04	4,53E-02
cg10673770	16	67245750	LRRC29	5'UTR	-0,009	3,48E-04	4,53E-02
cg01978669	1	52832116	CC2D1B	TSS1500	0,006	3,49E-04	4,53E-02
cg01025762	7	107531363	DLD	TSS1500	-0,003	3,49E-04	4,54E-02
cg16505464	13	37393242	RFXAP	TSS200	0,003	3,49E-04	4,54E-02
cg18536791	4	106854064	NPNT	Body	0,022	3,49E-04	4,54E-02
cg02657611	1	45814632	TESK2	Body	-0,01	3,50E-04	4,54E-02
cg11305249	3	136472342	STAG1	TSS1500	0,051	3,50E-04	4,54E-02

cg03062757	10	1226656		IGR	0,023	3,50E-04	4,54E-02
cg20868253	12	93965053	SOCS2	5'UTR	0,009	3,50E-04	4,54E-02
cg24685751	15	81648729	TMC3	Body	-0,01	3,50E-04	4,54E-02
cg08627327	17	79216928		IGR	-0,005	3,50E-04	4,54E-02
cg15274864	1	6508592	ESPN	Body	0,009	3,50E-04	4,54E-02
cg18242990	7	2884021	GNA12	TSS200	0,004	3,50E-04	4,54E-02
cg01220033	9	131419100	WDR34	5'UTR	0,004	3,50E-04	4,54E-02
cg15612485	12	22862518		IGR	0,041	3,50E-04	4,54E-02
cg17366016	16	50614342	NKD1	Body	0,007	3,50E-04	4,54E-02
cg15328005	3	43389621	SNRK	Body	0,006	3,50E-04	4,54E-02
cg03676026	7	107365731		IGR	-0,007	3,50E-04	4,54E-02
cg08833432	20	60719824	PSMA7	TSS1500	0,028	3,51E-04	4,55E-02
cg17619755	6	31760629	VARS	Body	0,043	3,51E-04	4,55E-02
cg03421507	12	109548872		IGR	0,046	3,52E-04	4,56E-02
cg19807520	5	178049773	CLK4	Body	0,006	3,52E-04	4,56E-02
cg02802916	9	138708887	CAMSAP1	Body	0,006	3,52E-04	4,56E-02
cg01092230	10	92979600	PCGF5	TSS1500	-0,028	3,52E-04	4,56E-02
cg18166300	11	44563830		IGR	-0,006	3,52E-04	4,56E-02
cg05413411	15	96227286		IGR	-0,012	3,52E-04	4,56E-02
cg16562793	20	58567932	CDH26	Body	0,031	3,52E-04	4,56E-02
cg01984450	5	110848444	STARD4	TSS200	0,007	3,52E-04	4,56E-02
cg04339240	16	75295370	BCAR1	Body	0,013	3,52E-04	4,56E-02
cg23395553	5	137290112	FAM13B	Body	0,037	3,53E-04	4,56E-02
cg25249293	19	51130408	SYT3	Body	-0,015	3,53E-04	4,56E-02
cg06589161	5	180618230		IGR	0,015	3,53E-04	4,56E-02
cg17044320	3	38180096	ACAA1	TSS1500	0,004	3,53E-04	4,56E-02
cg08977130	3	176915099	TBL1XR1	TSS200	0,011	3,53E-04	4,56E-02
cg17054360	7	91510326	MTERF	TSS1500	-0,015	3,53E-04	4,56E-02
cg00912526	10	35310849	CUL2	Body	-0,007	3,53E-04	4,56E-02
cg02687510	17	78118589	EIF4A3	Body	-0,009	3,53E-04	4,56E-02
cg20882194	22	26875404	HPS4	1stExon	-0,022	3,53E-04	4,56E-02
cg21614759	6	152128426	ESR1	TSS1500	-0,01	3,53E-04	4,57E-02
cg21074535	11	76410147	GUCY2E	Body	-0,013	3,53E-04	4,57E-02
cg11655627	13	27999466	GTF3A	Body	0,083	3,54E-04	4,57E-02
cg01637131	3	193853890	HES1	TSS200	-0,005	3,54E-04	4,57E-02
cg12919768	17	35179543		IGR	0,009	3,54E-04	4,57E-02
cg10865119	6	170190112	C6orf122	Body	0,017	3,54E-04	4,57E-02
cg10550776	6	1736920	GMDS	Body	0,014	3,54E-04	4,57E-02
cg21488538	2	98964436	CNGA3	5'UTR	-0,036	3,55E-04	4,58E-02
cg26976315	8	6423908	MCPH1	Body	-0,016	3,55E-04	4,58E-02
cg04252592	16	4387370	GLIS2	Body	-0,015	3,55E-04	4,58E-02
cg21853533	12	41501762		IGR	-0,018	3,55E-04	4,58E-02
cg15149615	3	33983771		IGR	-0,006	3,55E-04	4,58E-02
cg09498224	4	123379868		IGR	0,004	3,55E-04	4,58E-02
cg08761926	10	133664101		IGR	-0,021	3,55E-04	4,58E-02
cg26526953	19	1921199	SCAMP4	Body	-0,007	3,55E-04	4,58E-02
cg01317989	3	153838620	ARHGEF26	TSS1500	-0,043	3,55E-04	4,58E-02
cg05938958	1	248224887	OR2L3	1stExon	0,051	3,56E-04	4,58E-02
cg14874646	2	6466380		IGR	0,039	3,56E-04	4,58E-02
cg13553509	3	14061431	TPRXL	Body	-0,014	3,56E-04	4,58E-02
cg17312945	3	127284847		IGR	0,033	3,56E-04	4,58E-02
cg01964358	5	140566767	PCDHB9	TSS200	-0,014	3,56E-04	4,58E-02
cg09762252	7	71799220	CALN1	5'UTR	-0,007	3,56E-04	4,58E-02
cg19252956	9	273072	DOCK8	Body	-0,016	3,56E-04	4,58E-02
cg10574074	10	92922714		IGR	0,006	3,56E-04	4,58E-02
cg09258689	12	132853954	GALNT9	Body	0,034	3,56E-04	4,58E-02
cg26594377	14	90420975	EFCAB11	5'UTR	0,003	3,56E-04	4,58E-02
cg05189754	17	20173189	SPECC1	Body	-0,008	3,56E-04	4,58E-02
cg02810592	18	44525710	KATNAL2	TSS1500	-0,007	3,56E-04	4,58E-02
cg05889881	19	1626684	TCF3	Body	-0,02	3,56E-04	4,58E-02
cg08337835	19	43918868	TEX101	TSS200	0,005	3,56E-04	4,58E-02

cg27438200	19	57678903	DUXA	TSS200	0,069	3,56E-04	4,58E-02
cg14316339	22	38520900	PLA2G6	Body	-0,011	3,56E-04	4,58E-02
cg23729020	1	207925081	CD46	TSS1500	-0,006	3,57E-04	4,58E-02
cg07012660	6	3232396		IGR	-0,013	3,57E-04	4,58E-02
cg07330246	11	60585341		IGR	0,025	3,57E-04	4,58E-02
cg14580966	3	81699656	GBE1	Body	-0,008	3,57E-04	4,58E-02
cg23729050	2	208030732	KLF7	TSS200	-0,004	3,57E-04	4,58E-02
cg15300766	6	107077020	QRSL1	TSS1500	-0,016	3,57E-04	4,58E-02
cg26345216	11	117747030	FXYD6	5'UTR	-0,007	3,57E-04	4,58E-02
cg13609797	12	132106033		IGR	-0,015	3,57E-04	4,58E-02
cg10804438	3	51747196	GRM2	5'UTR	-0,005	3,58E-04	4,59E-02
cg27385636	3	11015492		IGR	0,01	3,58E-04	4,59E-02
cg25421941	10	11506312	USP6NL	Body	0,006	3,58E-04	4,59E-02
cg20169576	18	67070461	DOK6	Body	0,039	3,58E-04	4,59E-02
cg19590914	19	8028642	ELAVL1	Body	-0,023	3,58E-04	4,59E-02
cg15740518	3	149259182	WWTR1	Body	0,004	3,58E-04	4,59E-02
cg25089400	12	7651706	CD163	Body	0,031	3,58E-04	4,59E-02
cg25197240	10	134073863	STK32C	Body	-0,012	3,58E-04	4,59E-02
cg15719572	1	40506319	CAP1	1stExon	0,004	3,58E-04	4,59E-02
cg04734477	1	39788663	MACF1	ExonBnd	0,009	3,59E-04	4,59E-02
cg09024920	3	167172692	SERPINI2	Body	-0,008	3,59E-04	4,59E-02
cg01957946	9	130230645	LRSAM1	Body	-0,012	3,59E-04	4,59E-02
cg01043158	12	90908677		IGR	0,009	3,59E-04	4,59E-02
cg17018349	15	68136196		IGR	-0,021	3,59E-04	4,59E-02
cg06681193	18	29522728	KIAA1012	1stExon	0,004	3,59E-04	4,59E-02
cg02164262	22	44920214		IGR	0,045	3,59E-04	4,59E-02
cg26569985	1	62318010	INADL	Body	0,033	3,59E-04	4,60E-02
cg06394904	5	16617252	FAM134B	TSS200	0,005	3,59E-04	4,60E-02
cg03000869	10	72059799	LRRC20	3'UTR	-0,013	3,59E-04	4,60E-02
cg25218417	1	12374100	VPS13D	Body	0,009	3,61E-04	4,60E-02
cg20285609	1	41622030	SCMH1	5'UTR	0,005	3,60E-04	4,60E-02
cg24351658	1	42126724	HIVEP3	5'UTR	0,019	3,60E-04	4,60E-02
cg08123652	1	228353717	C1orf69	1stExon	0,003	3,60E-04	4,60E-02
cg04878973	3	49057761	NDUFAF3	TSS200	-0,003	3,60E-04	4,60E-02
cg25109019	3	181441345	SOX2OT	Body	-0,026	3,60E-04	4,60E-02
cg23748925	3	195466741		IGR	-0,006	3,60E-04	4,60E-02
cg21828007	5	65441131	SREK1	Body	-0,011	3,61E-04	4,60E-02
cg02256855	6	25084106	CMAHP	Body	-0,005	3,60E-04	4,60E-02
cg23845951	6	43139935	SRF	Body	0,009	3,61E-04	4,60E-02
cg22710544	6	44110317	TMEM63B	Body	-0,014	3,61E-04	4,60E-02
cg21075261	7	155633421		IGR	-0,009	3,60E-04	4,60E-02
cg14187229	9	137134853		IGR	-0,011	3,60E-04	4,60E-02
cg09654954	11	1368768		IGR	0,011	3,60E-04	4,60E-02
cg10249997	11	130318055	ADAMTS15	TSS1500	0,02	3,60E-04	4,60E-02
cg03475671	15	90317562		IGR	-0,013	3,60E-04	4,60E-02
cg16619995	16	88961842	CBFA2T3	Body	-0,006	3,60E-04	4,60E-02
cg23134318	22	25108105		IGR	0,008	3,61E-04	4,60E-02
cg27502912	22	51017001	CPT1B	1stExon	-0,056	3,61E-04	4,60E-02
cg16522719	5	86636497	RASA1	Body	-0,005	3,61E-04	4,60E-02
cg24639504	13	25875833	NUPL1	5'UTR	0,005	3,61E-04	4,60E-02
cg04668519	2	209087319		IGR	0,025	3,61E-04	4,60E-02
cg13076537	8	82192945	FABP5	Body	0,003	3,61E-04	4,60E-02
cg05562828	17	3906858		IGR	-0,009	3,61E-04	4,60E-02
cg08578305	12	25403937	KRAS	TSS200	0,003	3,61E-04	4,60E-02
cg22669633	4	54561536	)C100506444	TSS1500	-0,031	3,61E-04	4,60E-02
cg23012054	11	125325970	FEZ1	Body	0,019	3,61E-04	4,60E-02
cg10983639	16	23919746	PRKCB	Body	0,006	3,62E-04	4,60E-02
cg06193043	1	11908199	NPPA	TSS1500	0,052	3,62E-04	4,60E-02
cg10680793	1	23913713		IGR	-0,026	3,62E-04	4,60E-02
cg26163057	2	29038601	SPDYA	TSS1500	0,034	3,62E-04	4,60E-02
cg19083177	9	138391791	MRPS2	TSS1500	0,005	3,62E-04	4,60E-02

cg00252162	20	9813893	PAK7	5'UTR	-0,023	3,62E-04	4,60E-02
cg22600584	1	101094514	LINC01349	ExonBnd	-0,035	3,62E-04	4,60E-02
cg23373626	5	141031295	FCHSD1	TSS1500	0,008	3,62E-04	4,60E-02
cg16814576	19	39218661	ACTN4	ExonBnd	-0,023	3,62E-04	4,60E-02
cg24366702	19	58951778	ZNF132	TSS200	0,004	3,62E-04	4,60E-02
cg05875017	2	233390771	CHRND	TSS200	0,054	3,62E-04	4,60E-02
cg02052790	9	37662536	FRMPD1	5'UTR	0,009	3,63E-04	4,60E-02
cg13935310	11	107461639	ELMOD1	TSS200	0,005	3,63E-04	4,60E-02
cg13003673	8	122690114		IGR	-0,007	3,63E-04	4,61E-02
cg23883802	17	42290419	UBTF	Body	-0,017	3,63E-04	4,61E-02
cg12367415	6	33868187		IGR	-0,009	3,63E-04	4,61E-02
cg10166090	14	68162924	RDH11	TSS1500	-0,008	3,63E-04	4,61E-02
cg04154911	2	36725928	CRIM1	Body	0,01	3,63E-04	4,61E-02
cg24785373	2	12082591		IGR	0,004	3,63E-04	4,61E-02
cg05258914	6	83962736	ME1	Body	0,018	3,63E-04	4,61E-02
cg01016592	16	30615808	ZNF689	Body	-0,005	3,64E-04	4,61E-02
cg08004377	2	200231190	SATB2	Body	0,025	3,64E-04	4,61E-02
cg16459725	6	38092230	ZFAND3	Body	-0,009	3,64E-04	4,61E-02
cg13339886	21	32548426	TIAM1	Body	0,051	3,64E-04	4,61E-02
cg00608913	7	1132412	GPER1	Body	-0,007	3,64E-04	4,61E-02
cg12031031	12	103980882	STAB2	TSS200	0,013	3,64E-04	4,62E-02
cg01744019	16	215593	HBM	TSS1500	-0,025	3,64E-04	4,62E-02
cg11446126	17	79676206		IGR	-0,022	3,64E-04	4,62E-02
cg20784733	7	66205729	RABGEF1	1stExon	0,008	3,65E-04	4,62E-02
cg17527969	7	157367537	MIR153-2	TSS1500	-0,005	3,64E-04	4,62E-02
cg06761421	22	51043692	MAPK8IP2	Body	-0,021	3,65E-04	4,62E-02
cg06949673	1	56463319		IGR	0,032	3,65E-04	4,62E-02
cg06841846	17	28564094	SLC6A4	TSS1500	0,012	3,65E-04	4,62E-02
cg08790584	9	111017153		IGR	-0,006	3,65E-04	4,62E-02
cg05867925	6	26033854	HIST1H2AB	TSS200	-0,007	3,65E-04	4,62E-02
cg23406881	14	103241247		IGR	0,032	3,65E-04	4,62E-02
cg07659911	11	18481309	LDHAL6A	Body	-0,023	3,66E-04	4,62E-02
cg17532016	17	73100288	SLC16A5	Body	-0,01	3,66E-04	4,63E-02
cg04883714	20	4527714		IGR	-0,007	3,66E-04	4,63E-02
cg17122213	1	1564920	MIB2	Body	0,056	3,66E-04	4,63E-02
cg00471141	8	19796299	LPL	TSS1500	0,05	3,66E-04	4,63E-02
cg22277854	14	91681976	C14orf159	Body	-0,008	3,66E-04	4,63E-02
cg26492243	20	24179204	FLJ33581	TSS1500	-0,005	3,66E-04	4,63E-02
cg11390151	1	2387810		IGR	0,028	3,66E-04	4,63E-02
cg09070142	7	121532396	PTPRZ1	Body	0,037	3,66E-04	4,63E-02
cg01393991	1	41328487	CITED4	TSS1500	0,017	3,67E-04	4,63E-02
cg01938776	2	42596251		IGR	-0,019	3,67E-04	4,63E-02
cg12105556	3	151561366	ADACL2-AS1	Body	0,032	3,67E-04	4,63E-02
cg19354045	12	111051792	TCTN1	TSS200	0,004	3,67E-04	4,63E-02
cg19985477	13	78712201	RNF219-AS1	Body	-0,01	3,67E-04	4,63E-02
cg04834502	16	48644360	N4BP1	TSS1500	0,015	3,67E-04	4,63E-02
cg24053914	17	71852289		IGR	0,03	3,68E-04	4,64E-02
cg05045702	16	30616285	ZNF689	Body	-0,024	3,68E-04	4,64E-02
cg13140338	1	91317255		IGR	0,012	3,69E-04	4,64E-02
cg05847140	1	180384818	ACBD6	Body	-0,032	3,68E-04	4,64E-02
cg02616054	1	225652300		IGR	0,004	3,68E-04	4,64E-02
cg10899293	2	5506170		IGR	0,009	3,68E-04	4,64E-02
cg15088777	3	43147571	C3orf39	TSS200	-0,004	3,69E-04	4,64E-02
cg20159474	6	11968652		IGR	0,031	3,68E-04	4,64E-02
cg27270469	6	155964000		IGR	-0,013	3,68E-04	4,64E-02
cg07700175	9	95366306	CENPP	Body	-0,005	3,69E-04	4,64E-02
cg12519998	10	384286	DIP2C	Body	0,004	3,69E-04	4,64E-02
cg12074485	10	133036449	TCERG1L	Body	-0,013	3,68E-04	4,64E-02
cg10525349	11	117969714	TMPRSS4	ExonBnd	-0,007	3,69E-04	4,64E-02
cg09243384	11	122503929		IGR	0,05	3,68E-04	4,64E-02
cg13422161	12	52773842	KRT84	Body	-0,101	3,68E-04	4,64E-02

cg24742550	12	104873221	CHST11	Body	0,012	3,68E-04	4,64E-02
cg03228554	21	46308996	ITGB2	Body	-0,014	3,68E-04	4,64E-02
cg23762751	19	5809094		IGR	-0,013	3,69E-04	4,64E-02
cg02628881	19	34745219	KIAA0355	TSS1500	0,002	3,69E-04	4,64E-02
cg25920676	1	9990240	LZIC	3'UTR	0,004	3,70E-04	4,64E-02
cg02021304	1	15957014	DDI2	Body	0,004	3,70E-04	4,64E-02
cg06221609	1	152799138	LCE1A	TSS1500	0,021	3,71E-04	4,64E-02
cg24048361	1	224776790		IGR	0,005	3,70E-04	4,64E-02
cg11484289	2	7088460	RNF144A	5'UTR	0,006	3,69E-04	4,64E-02
cg26008643	2	25380006	EFR3B	3'UTR	0,023	3,70E-04	4,64E-02
cg06395942	3	134517972	EPHB1	Body	-0,011	3,70E-04	4,64E-02
cg16017429	5	1087252	SLC12A7	Body	-0,005	3,70E-04	4,64E-02
cg08295865	5	1790944		IGR	0,025	3,70E-04	4,64E-02
cg17557357	5	140729608	PCDHGA2	Body	-0,014	3,69E-04	4,64E-02
cg02424007	6	110967726	CDK19	Body	-0,007	3,70E-04	4,64E-02
cg15814481	6	138020063		IGR	-0,019	3,69E-04	4,64E-02
cg08974391	7	105401812	ATXN7L1	3'UTR	0,006	3,71E-04	4,64E-02
cg23982568	8	6118526		IGR	0,004	3,70E-04	4,64E-02
cg07662864	8	6470040	MCPH1	Body	0,016	3,70E-04	4,64E-02
cg00988777	8	143916152	GML	TSS200	-0,01	3,70E-04	4,64E-02
cg20653518	10	103539953		IGR	0,009	3,70E-04	4,64E-02
cg12335414	10	134359618	INPP5A	Body	-0,005	3,70E-04	4,64E-02
cg03744810	11	20295944		IGR	0,037	3,69E-04	4,64E-02
cg22364991	11	40150667	LRRC4C	Body	0,028	3,71E-04	4,64E-02
cg11333224	13	89278717		IGR	0,03	3,71E-04	4,64E-02
cg06030290	16	66747163		IGR	0,059	3,70E-04	4,64E-02
cg21515243	18	21033072	RIOK3	1stExon	0,005	3,71E-04	4,64E-02
cg13646565	19	21240978	ZNF430	3'UTR	0,008	3,69E-04	4,64E-02
cg00995241	20	8739884	PLCB1	Body	-0,005	3,70E-04	4,64E-02
cg03538429	15	45504828		IGR	0,025	3,71E-04	4,64E-02
cg09022552	16	28962119	NFATC2IP	TSS200	0,007	3,71E-04	4,65E-02
cg16463733	6	33422266	ZBTB9	TSS200	0,006	3,71E-04	4,65E-02
cg02112289	2	109818512	SH3RF3	Body	0,03	3,72E-04	4,65E-02
cg21230774	5	73980437	HEXB	TSS1500	0,004	3,72E-04	4,65E-02
cg15410753	7	2115808	MAD1L1	Body	-0,017	3,72E-04	4,65E-02
cg14257435	14	97165297		IGR	0,032	3,72E-04	4,65E-02
cg22930390	19	911815	C19orf22	Body	-0,033	3,72E-04	4,65E-02
cg19119995	20	33410514	NCOA6	5'UTR	0,018	3,72E-04	4,65E-02
cg18527588	16	72993161	ZFHX3	Body	-0,006	3,72E-04	4,65E-02
cg24231834	1	171182100		IGR	-0,017	3,72E-04	4,65E-02
cg03694868	2	62684297		IGR	0,009	3,72E-04	4,65E-02
cg04362419	3	138297086	CEP70	5'UTR	0,115	3,72E-04	4,65E-02
cg02165556	4	121684361	PRDM5	Body	0,009	3,72E-04	4,65E-02
cg05168220	11	6567122	DNHD1	Body	0,017	3,72E-04	4,65E-02
cg18750141	11	47652060	MTCH2	Body	0,01	3,72E-04	4,65E-02
cg22424078	12	121946599	KDM2B	Body	-0,015	3,72E-04	4,65E-02
cg25579251	1	180216614	LHX4	Body	-0,028	3,74E-04	4,65E-02
cg22189409	2	202609562	ALS2	Body	-0,023	3,73E-04	4,65E-02
cg05657325	2	222101137		IGR	-0,007	3,73E-04	4,65E-02
cg04075781	3	54154438		IGR	-0,041	3,74E-04	4,65E-02
cg05825997	4	3760692		IGR	-0,018	3,73E-04	4,65E-02
cg24989527	6	3259246	PSMG4	1stExon	0,004	3,74E-04	4,65E-02
cg18313096	6	43457113	TJAP1	TSS1500	-0,012	3,73E-04	4,65E-02
cg26141291	8	142201025	DENND3	Body	-0,012	3,73E-04	4,65E-02
cg13930766	9	122577345		IGR	0,039	3,74E-04	4,65E-02
cg05740071	10	35299094	CUL2	3'UTR	0,026	3,73E-04	4,65E-02
cg08436681	12	54115323	CALCOCO1	Body	-0,005	3,74E-04	4,65E-02
cg02986937	12	59314449	LRIG3	TSS1500	-0,02	3,74E-04	4,65E-02
cg11299763	13	86095869	LINC00351	Body	-0,017	3,73E-04	4,65E-02
cg11782260	14	20773481	TTC5	Body	0,026	3,74E-04	4,65E-02
cg13183539	19	3522577	FZR1	TSS1500	-0,007	3,73E-04	4,65E-02

cg01996091	19	4246782	CCDC94	TSS1500	0,011	3,73E-04	4,65E-02
cg13870198	19	10347419		IGR	0,038	3,73E-04	4,65E-02
cg07141702	19	44100719	ZNF576	1stExon	0,004	3,73E-04	4,65E-02
cg02222722	2	20101793	TTC32	TSS200	-0,005	3,74E-04	4,65E-02
cg25376969	3	183770030	HTR3C	TSS1500	0,012	3,74E-04	4,65E-02
cg15162597	7	73448316	ELN	Body	-0,01	3,74E-04	4,66E-02
cg02400760	12	50650711	LIMA1	5'UTR	-0,02	3,74E-04	4,66E-02
cg27403612	6	155231129		IGR	0,008	3,75E-04	4,66E-02
cg25282513	8	1267224		IGR	0,011	3,75E-04	4,66E-02
cg03927304	16	3017593	KREMEN2	Body	-0,03	3,75E-04	4,66E-02
cg17674418	4	8304222	HTRA3	ExonBnd	-0,017	3,75E-04	4,67E-02
cg07559745	3	64251919		IGR	0,033	3,76E-04	4,67E-02
cg04584833	17	1928794	RTN4RL1	TSS1500	0,004	3,76E-04	4,67E-02
cg16540393	19	37340210	ZNF345	TSS1500	-0,007	3,76E-04	4,67E-02
cg14404316	21	31589372	CLDN8	TSS1500	-0,013	3,76E-04	4,67E-02
cg07150165	4	24803285		IGR	0,006	3,76E-04	4,67E-02
cg13209845	12	94589513	PLXNC1	Body	-0,008	3,76E-04	4,67E-02
cg05162031	17	850367	NXN	Body	-0,009	3,76E-04	4,67E-02
cg03029893	10	75521906	SEC24C	Body	-0,013	3,77E-04	4,67E-02
cg21431729	2	24153049		IGR	0,012	3,77E-04	4,68E-02
cg15315515	6	45780821		IGR	-0,012	3,77E-04	4,68E-02
cg27001894	10	13022624	CCDC3	Body	0,075	3,77E-04	4,68E-02
cg24415154	12	57592326	LRP1	Body	-0,016	3,77E-04	4,68E-02
cg01783322	17	49243582	NME2	TSS200	0,003	3,77E-04	4,68E-02
cg24971036	3	125093764	ZNF148	5'UTR	0,008	3,77E-04	4,68E-02
cg04618386	2	8768440		IGR	0,007	3,78E-04	4,68E-02
cg20506071	2	217985565		IGR	0,054	3,78E-04	4,68E-02
cg14882720	3	156850191		IGR	0,006	3,78E-04	4,68E-02
cg11393549	4	7000195	TBC1D14	Body	-0,013	3,78E-04	4,68E-02
cg06792408	4	164476226	MARCH1	Body	-0,011	3,78E-04	4,68E-02
cg12350407	6	39508853	KIF6	Body	0,013	3,78E-04	4,68E-02
cg17284168	6	134491531	SGK1	Body	0,003	3,78E-04	4,68E-02
cg17436763	8	30601499	UBXN8	TSS200	-0,003	3,78E-04	4,68E-02
cg25558002	10	69893540	MYPN	Body	0,02	3,78E-04	4,68E-02
cg02618908	14	94596645	IFI27L2	TSS1500	0,007	3,78E-04	4,68E-02
cg16635578	12	118202284	KSR2	Body	0,028	3,78E-04	4,68E-02
cg07284707	3	15642677	BTD	TSS1500	-0,007	3,78E-04	4,68E-02
cg09696706	7	47479511	TNS3	5'UTR	-0,018	3,78E-04	4,68E-02
cg01105375	13	20748025		IGR	0,03	3,78E-04	4,68E-02
cg20949846	12	11087013	PRH2	3'UTR	0,005	3,78E-04	4,68E-02
cg09479489	12	51478729	CSRNP2	TSS1500	-0,006	3,79E-04	4,68E-02
cg09347218	16	25061029		IGR	0,016	3,79E-04	4,68E-02
cg00558218	16	70508887	FUK	Body	-0,017	3,79E-04	4,68E-02
cg27129763	1	90246190		IGR	0,011	3,79E-04	4,68E-02
cg14813458	2	3493699		IGR	0,011	3,79E-04	4,68E-02
cg13388277	7	63504673	ZNF727	TSS1500	0,057	3,79E-04	4,68E-02
cg14350114	9	115631083	SNX30	Body	0,004	3,79E-04	4,68E-02
cg22492847	11	70602173	SHANK2	Body	0,024	3,79E-04	4,68E-02
cg18013727	12	669455	B4GALNT3	Body	-0,025	3,79E-04	4,68E-02
cg14128195	12	19566172		IGR	0,008	3,79E-04	4,68E-02
cg07006769	12	131545880	ADGRD1	Body	0,024	3,79E-04	4,68E-02
cg13793718	13	25212248		IGR	-0,059	3,79E-04	4,68E-02
cg19433091	15	43426498	TMEM62	Body	0,029	3,79E-04	4,68E-02
cg13664848	22	24807317	[illegible]1L-ADORA2A	Body	-0,007	3,79E-04	4,68E-02
cg19566272	6	139695824	CITED2	TSS200	0,003	3,80E-04	4,68E-02
cg27619897	8	143700611		IGR	-0,015	3,80E-04	4,68E-02
cg26088680	14	95359766		IGR	0,059	3,80E-04	4,68E-02
cg27334938	18	77167042	NFATC1	Body	-0,008	3,80E-04	4,68E-02
cg00276752	17	35719639	ACACA	5'UTR	0,006	3,80E-04	4,68E-02
cg16443716	1	19330155		IGR	0,034	3,80E-04	4,68E-02
cg17495671	2	216613547		IGR	-0,009	3,80E-04	4,68E-02

cg23597430	20	36719030	RPRD1B	3'UTR	0,005	3,80E-04	4,68E-02
cg26554579	20	52108992	TSHZ2	3'UTR	0,031	3,80E-04	4,68E-02
cg14032709	1	164574402	PBX1	Body	-0,006	3,80E-04	4,69E-02
cg17661640	4	184504362		IGR	0,005	3,81E-04	4,69E-02
cg13678070	6	38670972	GLO1	TSS200	-0,005	3,81E-04	4,69E-02
cg03066914	6	133284503		IGR	0,039	3,81E-04	4,69E-02
cg04902710	11	63753765	OTUB1	5'UTR	0,004	3,80E-04	4,69E-02
cg24312460	1	40858062	SMAP2	TSS1500	0,005	3,81E-04	4,69E-02
cg15228268	10	3146741	PFKP	Body	0,018	3,81E-04	4,69E-02
cg02093647	2	148602395	ACVR2A	TSS200	0,005	3,81E-04	4,69E-02
cg11192141	7	2962304	CARD11	Body	-0,009	3,81E-04	4,69E-02
cg11761634	7	133236333	EXOC4	Body	0,01	3,81E-04	4,69E-02
cg07043857	15	51514698	CYP19A1	Body	-0,005	3,81E-04	4,69E-02
cg03924800	16	25042807		IGR	-0,004	3,81E-04	4,69E-02
cg20142224	16	57520176	DOK4	5'UTR	0,002	3,81E-04	4,69E-02
cg23098305	1	228112951	WNT9A	Body	-0,015	3,82E-04	4,69E-02
cg16906739	2	231773100	GPR55	3'UTR	-0,015	3,82E-04	4,69E-02
cg15066337	3	190105668	CLDN16	TSS200	-0,013	3,82E-04	4,69E-02
cg17285834	6	112364920		IGR	0,006	3,82E-04	4,69E-02
cg20952131	12	113670223	TPCN1	Body	-0,015	3,82E-04	4,69E-02
cg05162320	17	48389080		IGR	0,033	3,82E-04	4,69E-02
cg14607948	16	2746908	KCTD5	Body	-0,005	3,82E-04	4,69E-02
cg19287501	22	40084724	CACNA1I	3'UTR	0,023	3,82E-04	4,70E-02
cg07096654	1	6649036	ZBTB48	Body	-0,005	3,83E-04	4,70E-02
cg00316828	16	87985026	BANP	TSS200	0,003	3,83E-04	4,70E-02
cg06758681	16	89723958	SPATA33	TSS1500	0,005	3,83E-04	4,70E-02
cg11823729	6	55190909	GFRAL	TSS1500	0,036	3,83E-04	4,70E-02
cg26777557	4	76439826	RCHY1	TSS200	-0,005	3,84E-04	4,71E-02
cg27387908	18	67082858	DOK6	Body	-0,019	3,84E-04	4,71E-02
cg01642550	16	89098327		IGR	0,025	3,84E-04	4,71E-02
cg15325755	1	57455549		IGR	0,011	3,84E-04	4,71E-02
cg14571813	3	129062831		IGR	0,013	3,84E-04	4,71E-02
cg17142149	6	46293016	RCAN2	Body	0,007	3,84E-04	4,71E-02
cg25129640	10	18782829	CACNB2	Body	-0,008	3,84E-04	4,71E-02
cg25637792	2	68693494	FBXO48	5'UTR	0,005	3,85E-04	4,71E-02
cg02120470	1	67267129	INSL5	TSS200	0,005	3,85E-04	4,72E-02
cg06668300	2	95691755	MAL	Body	-0,006	3,85E-04	4,72E-02
cg09726239	3	119866176		IGR	0,02	3,85E-04	4,72E-02
cg02982198	10	27388890	ANKRD26	Body	0,003	3,85E-04	4,72E-02
cg12091708	5	140248390	PCDHA11	5'UTR	-0,017	3,85E-04	4,72E-02
cg01617164	2	200821026	C2orf47	Body	0,018	3,86E-04	4,72E-02
cg18784912	3	189176507		IGR	0,004	3,86E-04	4,72E-02
cg05402265	6	52926119	ICK	5'UTR	-0,005	3,86E-04	4,72E-02
cg16311192	7	91875427	KRIT1	TSS200	0,011	3,86E-04	4,72E-02
cg17663924	8	96516248	C8orf37-AS1	Body	-0,054	3,86E-04	4,72E-02
cg22722737	9	82187628	TLE4	5'UTR	0,004	3,86E-04	4,72E-02
cg16077048	9	129855889	ANGPTL2	Body	-0,007	3,86E-04	4,72E-02
cg13624321	11	122529755	UBASH3B	Body	-0,007	3,86E-04	4,72E-02
cg06261620	14	95047781	SERPINA5	1stExon	0,013	3,86E-04	4,72E-02
cg09863630	15	37189299	MEIS2	Body	0,022	3,86E-04	4,72E-02
cg11257766	18	22907319	ZNF521	5'UTR	0,026	3,86E-04	4,72E-02
cg05527034	4	155250680	DCHS2	Body	-0,007	3,86E-04	4,72E-02
cg16656526	7	143600473	TCAF1	TSS1500	-0,011	3,86E-04	4,72E-02
cg07338058	8	110657182	GOLSYN	TSS200	-0,003	3,86E-04	4,72E-02
cg25499769	12	128245397		IGR	0,015	3,87E-04	4,72E-02
cg06703304	16	68057150	DUS2L	TSS200	0,004	3,86E-04	4,72E-02
cg24536044	1	244816274	PPPDE1	TSS200	0,003	3,87E-04	4,72E-02
cg23792745	2	14415525	LINC00276	Body	-0,036	3,87E-04	4,72E-02
cg03732535	3	49171051	LAMB2	TSS1500	-0,027	3,87E-04	4,72E-02
cg15613012	6	28887742	TRIM27	Body	-0,007	3,87E-04	4,72E-02
cg03063857	6	29585617	GABBR1	Body	-0,006	3,87E-04	4,72E-02

cg05235844	9	111623979	ACTL7A	TSS1500	0,011	3,87E-04	4,72E-02
cg25899113	9	140098865	TMEM203	3'UTR	0,008	3,87E-04	4,72E-02
cg27100149	18	77196899	NFATC1	Body	-0,007	3,87E-04	4,72E-02
cg15817376	1	8228442		IGR	0,006	3,87E-04	4,72E-02
cg11805489	1	225662460		IGR	0,031	3,87E-04	4,72E-02
cg00528492	6	32408863	HLA-DRA	Body	0,037	3,87E-04	4,72E-02
cg11302533	3	197183662		IGR	-0,004	3,88E-04	4,72E-02
cg05866170	8	28118179		IGR	0,028	3,88E-04	4,72E-02
cg10484092	12	50362521		IGR	-0,014	3,88E-04	4,72E-02
cg17647582	17	77492364	RBFOX3	5'UTR	0,043	3,88E-04	4,72E-02
cg11672099	1	145548264	ANKRD35	TSS1500	0,055	3,88E-04	4,72E-02
cg01216025	9	100685034	C9orf156	TSS200	0,009	3,88E-04	4,72E-02
cg20947760	10	3392776	)C105376360	Body	0,024	3,88E-04	4,72E-02
cg09046979	16	28333134	SBK1	3'UTR	0,04	3,88E-04	4,72E-02
cg21438057	17	76556876	DNAH17	Body	0,004	3,88E-04	4,72E-02
cg06414885	18	59303257		IGR	-0,028	3,88E-04	4,72E-02
cg11765109	8	142449613	MROH5	Body	0,005	3,88E-04	4,72E-02
cg24032421	12	67689985	CAND1	Body	0,006	3,88E-04	4,72E-02
cg17921548	5	143678450	KCTD16	Body	0,027	3,88E-04	4,72E-02
cg13520026	9	97810769	C9orf3	Body	0,006	3,88E-04	4,72E-02
cg02263901	2	233739775	C2orf82	Body	-0,008	3,89E-04	4,72E-02
cg12124973	12	120740010	SIRT4	TSS200	0,014	3,89E-04	4,72E-02
cg04655510	20	21378703	NKX2-4	TSS1500	-0,021	3,89E-04	4,72E-02
cg14386848	20	37082122		IGR	0,007	3,89E-04	4,72E-02
cg02474731	2	150185860	LYPD6	TSS1500	-0,06	3,89E-04	4,73E-02
cg04071487	2	164067387		IGR	0,009	3,89E-04	4,73E-02
cg17269733	5	43041146	C5orf39	TSS1500	0,013	3,89E-04	4,73E-02
cg06685514	5	176896820	DBN1	Body	-0,016	3,89E-04	4,73E-02
cg08074767	6	168113741		IGR	0,016	3,89E-04	4,73E-02
cg05005432	11	6191109	OR52B2	1stExon	0,006	3,89E-04	4,73E-02
cg25092328	20	32308119	PXMP4	1stExon	0,003	3,89E-04	4,73E-02
cg02983619	2	233758508	NGEF	Body	-0,008	3,89E-04	4,73E-02
cg11962841	4	83295963	HNRNPD	TSS1500	-0,007	3,90E-04	4,73E-02
cg09795588	10	23479993	PTF1A	TSS1500	-0,016	3,90E-04	4,73E-02
cg17276120	11	4137105	RRM1	Body	0,004	3,90E-04	4,73E-02
cg13633560	11	76380921	LRRC32	5'UTR	0,029	3,90E-04	4,73E-02
cg10450336	16	22347383		IGR	-0,007	3,90E-04	4,73E-02
cg16115072	17	25938812	KSR1	Body	-0,019	3,90E-04	4,73E-02
cg09252331	1	15655927	FHAD1	Body	0,012	3,90E-04	4,73E-02
cg14255094	7	127730344	SND1	Body	-0,005	3,90E-04	4,73E-02
cg10203034	10	104162891	PSD	3'UTR	-0,016	3,91E-04	4,73E-02
cg23318812	12	66123127		IGR	0,011	3,91E-04	4,73E-02
cg04502647	12	123131858		IGR	-0,046	3,90E-04	4,73E-02
cg02324547	15	80454089	FAH	Body	-0,015	3,91E-04	4,73E-02
cg19096976	21	31721428	KRTAP23-1	TSS1500	-0,019	3,91E-04	4,73E-02
cg11754420	13	112979874		IGR	-0,05	3,91E-04	4,74E-02
cg23431697	17	16789912		IGR	-0,006	3,91E-04	4,74E-02
cg14401624	2	114037124	PAX8	TSS1500	-0,008	3,91E-04	4,74E-02
cg23106087	3	116164595	LSAMP	TSS1500	-0,027	3,91E-04	4,74E-02
cg26874591	11	66011899	PACS1	3'UTR	-0,006	3,91E-04	4,74E-02
cg06601071	16	85525842		IGR	0,033	3,91E-04	4,74E-02
cg25871008	21	43206631		IGR	0,023	3,92E-04	4,74E-02
cg20085738	3	134605173	EPHB1	Body	-0,011	3,92E-04	4,74E-02
cg18949300	6	167275778	RPS6KA2	TSS200	0,01	3,92E-04	4,74E-02
cg26784313	7	3983655	SDK1	Body	0,033	3,92E-04	4,74E-02
cg14193808	9	109440017		IGR	0,005	3,92E-04	4,74E-02
cg25686479	1	92737365	GLMN	Body	0,003	3,92E-04	4,74E-02
cg21413133	3	10400395	ATP2B2	Body	-0,007	3,92E-04	4,74E-02
cg10140849	4	76751599		IGR	-0,013	3,92E-04	4,74E-02
cg00918738	20	589310	TCF15	Body	0,014	3,92E-04	4,74E-02
cg16030471	2	239422060	LINC01107	Body	0,059	3,92E-04	4,74E-02

cg00389259	2	17968147		IGR	-0,007	3,93E-04	4,74E-02
cg20465832	1	48668317		IGR	0,036	3,93E-04	4,75E-02
cg11999269	5	82182048		IGR	0,053	3,93E-04	4,75E-02
cg24232378	7	106545895	PIK3CG	3'UTR	-0,016	3,93E-04	4,75E-02
cg22845525	12	57433450	MYO1A	Body	0,033	3,93E-04	4,75E-02
cg04871873	7	25164974	CYCS	TSS200	0,003	3,93E-04	4,75E-02
cg18951582	14	105207600	ADSSL1	Body	-0,006	3,93E-04	4,75E-02
cg16435591	15	86278795	AKAP13	ExonBnd	-0,01	3,93E-04	4,75E-02
cg20434811	3	150421723	FAM194A	1stExon	0,005	3,95E-04	4,75E-02
cg08415977	4	7463138	SORCS2	Body	-0,033	3,94E-04	4,75E-02
cg07205860	6	31833864	SLC44A4	Body	0,007	3,94E-04	4,75E-02
cg01905058	7	3037669	CARD11	5'UTR	0,005	3,95E-04	4,75E-02
cg11181435	7	134878393	WDR91	Body	-0,003	3,94E-04	4,75E-02
cg16268546	8	1651197	DLGAP2	3'UTR	-0,008	3,94E-04	4,75E-02
cg17279135	11	65151692	SLC25A45	TSS1500	-0,016	3,95E-04	4,75E-02
cg18732867	11	119040926	NLRX1	5'UTR	0,005	3,94E-04	4,75E-02
cg25703747	15	52416725	GNB5	Body	-0,009	3,94E-04	4,75E-02
cg08676614	16	77353810	ADAMTS18	Body	-0,009	3,95E-04	4,75E-02
cg07280845	17	21432361	C17orf51	3'UTR	-0,024	3,94E-04	4,75E-02
cg01154098	19	49810131	SLC6A16	Body	0,013	3,94E-04	4,75E-02
cg07799106	20	52493220	SUMO1P1	TSS1500	-0,011	3,95E-04	4,75E-02
cg01519897	5	153859049	HAND1	TSS1500	-0,01	3,95E-04	4,75E-02
cg08429337	7	22749617		IGR	0,01	3,95E-04	4,75E-02
cg08813321	7	66108590		IGR	0,007	3,95E-04	4,75E-02
cg01772563	7	95064399	PON2	TSS200	0,014	3,95E-04	4,75E-02
cg17061853	12	27091905	FGFR1OP2	5'UTR	-0,003	3,95E-04	4,75E-02
cg17162981	15	45248744	C15orf43	TSS200	0,011	3,95E-04	4,75E-02
cg08704743	19	5995403	)C100128568	Body	-0,007	3,95E-04	4,75E-02
cg20882782	22	17647292	CECR5	TSS1500	-0,009	3,95E-04	4,75E-02
cg19400821	11	115836746		IGR	0,01	3,95E-04	4,75E-02
cg17984720	1	52190638	OSBPL9	Body	-0,01	3,96E-04	4,75E-02
cg00180531	1	62941874	DOCK7	Body	-0,013	3,96E-04	4,75E-02
cg16079012	3	143099960	SLC9A9	Body	0,008	3,96E-04	4,75E-02
cg02752076	3	154622250		IGR	0,026	3,96E-04	4,75E-02
cg20353544	4	8200899	SH3TC1	TSS200	0,004	3,95E-04	4,75E-02
cg25644789	5	51303426		IGR	0,034	3,96E-04	4,75E-02
cg12272235	5	112311420	DCP2	TSS1500	0,011	3,96E-04	4,75E-02
cg26959945	7	20233217	MACC1	5'UTR	0,026	3,96E-04	4,75E-02
cg16952973	12	4253206		IGR	0,045	3,96E-04	4,75E-02
cg27145042	16	25026956	ARHGAP17	TSS1500	0,004	3,96E-04	4,75E-02
cg01251763	17	26303565		IGR	0,013	3,96E-04	4,75E-02
cg19134130	17	72830151	TMEM104	Body	-0,02	3,96E-04	4,75E-02
cg25443570	18	39886372	LINC00907	Body	0,019	3,96E-04	4,75E-02
cg09501333	1	113671664		IGR	-0,004	3,96E-04	4,75E-02
cg17706137	3	132062157	ACPP	Body	0,006	3,96E-04	4,75E-02
cg05846932	2	85431473	TCF7L1	Body	-0,012	3,97E-04	4,76E-02
cg00815583	8	28348123	FBXO16	TSS1500	-0,007	3,97E-04	4,76E-02
cg07265115	15	72639020	HEXA	Body	-0,01	3,97E-04	4,76E-02
cg04134809	9	135071870	NTNG2	Body	-0,006	3,97E-04	4,76E-02
cg11955974	5	71363693		IGR	0,024	3,97E-04	4,76E-02
cg23978800	9	97818542	C9orf3	Body	0,006	3,97E-04	4,76E-02
cg17064968	20	35271966	SLA2	5'UTR	0,008	3,97E-04	4,76E-02
cg00700389	20	62091149	KCNQ2	Body	-0,006	3,97E-04	4,76E-02
cg10343723	19	55919467	UBE2S	TSS200	0,004	3,97E-04	4,76E-02
cg16278838	3	29323531	RBMS3-AS3	TSS1500	0,021	3,97E-04	4,76E-02
cg06097357	14	35591625	PPP2R3C	TSS200	0,003	3,98E-04	4,76E-02
cg26771559	19	4102512	MAP2K2	Body	-0,01	3,98E-04	4,76E-02
cg16055614	1	150997133	PRUNE	5'UTR	-0,01	3,98E-04	4,76E-02
cg14676529	5	140235607	PCDHA6	Body	-0,048	3,98E-04	4,76E-02
cg06367102	7	1192688	ZFAND2A	3'UTR	-0,003	3,98E-04	4,76E-02
cg27337902	2	175581955		IGR	0,041	3,98E-04	4,76E-02

cg01687793	14	92040499	C14orf184	3'UTR	-0,003	3,98E-04	4,76E-02
cg22657044	20	48551982	RNF114	TSS1500	0,04	3,98E-04	4,76E-02
cg13349607	7	120962655		IGR	0,04	3,98E-04	4,76E-02
cg15567507	9	35057457	VCP	Body	-0,006	3,98E-04	4,76E-02
cg03133811	20	44842635	CDH22	Body	-0,014	3,98E-04	4,76E-02
cg14614102	22	43341739	PACSIN2	5'UTR	-0,012	3,98E-04	4,76E-02
cg00237589	12	32833447	DNM1L	5'UTR	-0,019	3,98E-04	4,76E-02
cg15067995	3	195485212	MUC4	Body	0,036	3,99E-04	4,76E-02
cg00377239	16	89549660	ANKRD11	Body	-0,016	3,99E-04	4,76E-02
cg05677600	20	5816049	C20orf196	Body	-0,005	3,99E-04	4,76E-02
cg02789519	1	231301989	TRIM67	Body	0,027	3,99E-04	4,76E-02
cg17297475	11	644218		IGR	-0,025	3,99E-04	4,76E-02
cg24820916	14	52313772		IGR	-0,003	3,99E-04	4,76E-02
cg05304806	14	89030280	ZC3H14	5'UTR	0,027	3,99E-04	4,76E-02
cg20135162	13	95927719	ABCC4	Body	-0,006	3,99E-04	4,76E-02
cg00921237	22	49496760		IGR	-0,036	3,99E-04	4,76E-02
cg08610630	15	33900982	RYR3	Body	0,034	4,00E-04	4,77E-02
cg07490201	15	86441382		IGR	-0,037	4,00E-04	4,77E-02
cg15596855	1	153970768	NUP210L	Body	-0,012	4,00E-04	4,77E-02
cg09635317	12	26800043	ITPR2	Body	-0,009	4,00E-04	4,77E-02
cg14576825	19	1676136		IGR	-0,046	4,00E-04	4,77E-02
cg08521684	3	195488725	MUC4	Body	-0,015	4,00E-04	4,77E-02
cg05231925	4	23768155		IGR	-0,015	4,00E-04	4,77E-02
cg21183606	11	36294277	COMMD9	3'UTR	-0,009	4,00E-04	4,77E-02
cg00622503	1	38680351	LINC01343	Body	-0,007	4,01E-04	4,77E-02
cg07457305	2	71118292		IGR	0,024	4,01E-04	4,78E-02
cg26224455	2	166477679	CSRNP3	Body	0,018	4,01E-04	4,78E-02
cg14843608	10	75416950	SYNPO2L	TSS1500	0,013	4,01E-04	4,78E-02
cg24313571	11	823352	PNPLA2	Body	-0,015	4,01E-04	4,78E-02
cg09317928	11	61716830	BEST1	TSS1500	0,008	4,01E-04	4,78E-02
cg18793412	13	38989650		IGR	0,017	4,01E-04	4,78E-02
cg11333576	19	424281	SHC2	Body	0,013	4,01E-04	4,78E-02
cg23413051	17	75557265		IGR	-0,01	4,02E-04	4,78E-02
cg26637046	21	44283481	WDR4	Body	-0,016	4,02E-04	4,78E-02
cg22627950	7	44622302	TMED4	TSS1500	0,004	4,02E-04	4,78E-02
cg16411863	14	76448962	TGFB3	TSS1500	0,004	4,02E-04	4,78E-02
cg10644879	4	89371416		IGR	0,008	4,02E-04	4,79E-02
cg15619125	9	139747228	MAMDC4	Body	-0,013	4,03E-04	4,79E-02
cg07053964	16	624366	PIGQ	Body	-0,013	4,03E-04	4,79E-02
cg20050268	16	81538348	CMIP	Body	-0,006	4,03E-04	4,79E-02
cg02154802	3	30975791		IGR	-0,057	4,03E-04	4,79E-02
cg07503392	1	59762523	FGGY	TSS200	0,003	4,04E-04	4,79E-02
cg25557534	3	137237384		IGR	-0,009	4,04E-04	4,79E-02
cg14549774	7	44240498	YKT6	TSS200	0,004	4,04E-04	4,79E-02
cg11017923	20	62401365	ZBTB46	Body	-0,003	4,04E-04	4,79E-02
cg12634245	6	10495364		IGR	0,023	4,04E-04	4,80E-02
cg05149676	12	53412779	EIF4B	ExonBnd	-0,005	4,04E-04	4,80E-02
cg00358323	2	97307693	FER1L5	TSS1500	-0,009	4,04E-04	4,80E-02
cg08477264	5	14485733	TRIO	Body	0,004	4,04E-04	4,80E-02
cg18541042	7	140227195	DENND2A	Body	-0,009	4,04E-04	4,80E-02
cg16843694	12	104188719	NT5DC3	Body	0,054	4,04E-04	4,80E-02
cg21732844	17	26732191	SLC46A1	Body	-0,008	4,05E-04	4,80E-02
cg11856036	14	100642219		IGR	-0,006	4,05E-04	4,80E-02
cg26505519	18	74182211	ZNF516	5'UTR	-0,021	4,05E-04	4,80E-02
cg12581474	1	44302667	ST3GAL3	Body	0,029	4,05E-04	4,81E-02
cg01804989	9	34666042		IGR	-0,003	4,05E-04	4,81E-02
cg16807523	5	63258434	HTR1A	TSS1500	-0,034	4,06E-04	4,81E-02
cg20641391	7	54624237	VSTM2A	Body	-0,02	4,06E-04	4,81E-02
cg07173540	12	1107567	ERC1	5'UTR	-0,022	4,06E-04	4,81E-02
cg27086900	14	101244542		IGR	0,006	4,06E-04	4,81E-02
cg01289020	16	71323706	FTSJD1	TSS200	-0,009	4,06E-04	4,81E-02

cg19391697	2	239008943	ESPNL	TSS200	0,053	4,06E-04	4,81E-02
cg01979481	7	158938780	VIPR2	TSS1500	-0,01	4,06E-04	4,81E-02
cg20240634	10	129322349		IGR	0,041	4,06E-04	4,81E-02
cg00300851	11	123892684	OR10G9	TSS1500	-0,02	4,06E-04	4,81E-02
cg01387945	10	31608209	ZEB1	Body	0,011	4,06E-04	4,81E-02
cg24805188	7	577882		IGR	-0,013	4,06E-04	4,81E-02
cg01570309	15	96769266	NR2F2-AS1	Body	0,038	4,06E-04	4,81E-02
cg07880064	19	44080720	PINLYP	TSS1500	-0,02	4,06E-04	4,81E-02
cg08749465	1	35325713	SMIM12	TSS1500	0,027	4,07E-04	4,81E-02
cg25642673	4	185353115	IRF2	5'UTR	0,011	4,07E-04	4,81E-02
cg08304516	13	30996569	LINC01058	TSS200	0,004	4,07E-04	4,81E-02
cg27435867	15	79140260		IGR	0,052	4,07E-04	4,81E-02
cg20625919	20	20210911	CFAP61	Body	-0,013	4,07E-04	4,81E-02
cg26536713	10	89788462		IGR	0,02	4,07E-04	4,81E-02
cg07618928	2	234183455	ATG16L1	Body	0,006	4,07E-04	4,81E-02
cg14666997	18	61547563		IGR	-0,009	4,07E-04	4,81E-02
cg10305094	2	170943045		IGR	-0,005	4,08E-04	4,81E-02
cg15946718	8	38123230	PPAPDC1B	Body	-0,005	4,08E-04	4,81E-02
cg20095485	12	78168505		IGR	-0,008	4,07E-04	4,81E-02
cg22543880	13	28712974	PAN3-AS1	Body	0,003	4,08E-04	4,81E-02
cg01214600	13	48506867	LINC00562	TSS200	-0,007	4,07E-04	4,81E-02
cg08691100	18	64417435		IGR	-0,01	4,07E-04	4,81E-02
cg14649096	11	129569608		IGR	0,008	4,08E-04	4,81E-02
cg25900312	12	110204235	AM222A-AS1	Body	0,007	4,08E-04	4,81E-02
cg20567785	12	129456496	GLT1D1	Body	0,075	4,08E-04	4,81E-02
cg18770678	3	49069678	QRICH1	Body	0,004	4,08E-04	4,81E-02
cg23278018	5	102682153		IGR	0,027	4,09E-04	4,81E-02
cg09842161	6	29598321	GABBR1	Body	0,01	4,09E-04	4,81E-02
cg06215670	6	33162506	RXRB	Body	-0,009	4,08E-04	4,81E-02
cg10734432	8	145180544		IGR	0,026	4,09E-04	4,81E-02
cg14448765	9	136729858	VAV2	Body	0,005	4,08E-04	4,81E-02
cg11660725	10	27389600	ANKRD26	TSS200	-0,002	4,09E-04	4,81E-02
cg02423452	16	19179456	SYT17	TSS200	0,008	4,09E-04	4,81E-02
cg11952457	17	74053137	SRP68	Body	-0,011	4,08E-04	4,81E-02
cg14202380	1	47070052	MKNK1	TSS200	0,003	4,09E-04	4,82E-02
cg06336792	10	35415599	CREM	TSS1500	0,005	4,09E-04	4,82E-02
cg15622519	14	103468278	CDC42BPB	Body	-0,019	4,09E-04	4,82E-02
cg00361159	1	167487871	CD247	TSS200	0,03	4,09E-04	4,82E-02
cg16657759	2	69536881		IGR	0,032	4,09E-04	4,82E-02
cg21614491	13	113506788	ATP11A	Body	0,01	4,09E-04	4,82E-02
cg06847237	16	55094729		IGR	-0,014	4,09E-04	4,82E-02
cg24363820	22	51016703	CPT1B	5'UTR	-0,05	4,09E-04	4,82E-02
cg06363136	7	140379020	ADCK2	Body	0,006	4,10E-04	4,82E-02
cg20327820	4	78979807	FRAS1	Body	-0,003	4,10E-04	4,82E-02
cg13689342	21	43722761		IGR	-0,011	4,10E-04	4,82E-02
cg23783229	8	49022400		IGR	-0,052	4,10E-04	4,82E-02
cg12241532	10	3162117	PFKP	Body	0,006	4,10E-04	4,82E-02
cg13067350	1	221274025		IGR	0,007	4,11E-04	4,82E-02
cg18606619	4	38565294		IGR	0,008	4,11E-04	4,82E-02
cg12338202	6	32047223	TNXB	Body	-0,006	4,11E-04	4,82E-02
cg20445657	6	36991164	FGD2	Body	-0,006	4,11E-04	4,82E-02
cg10859114	14	99732195	BCL11B	Body	0,05	4,11E-04	4,82E-02
cg17128299	19	18368636	KIAA1683	Body	-0,016	4,11E-04	4,82E-02
cg19605701	19	34003047	PEPD	Body	-0,008	4,11E-04	4,82E-02
cg10966137	22	27016930	CRYBA4	TSS1500	0,014	4,11E-04	4,82E-02
cg07811864	2	56007116		IGR	0,026	4,11E-04	4,83E-02
cg17846325	2	79168411		IGR	0,045	4,11E-04	4,83E-02
cg20284698	3	48956327	C3orf71	1stExon	0,013	4,11E-04	4,83E-02
cg25127992	3	194343060	TMEM44	Body	0,009	4,11E-04	4,83E-02
cg26896991	9	130984800	DNM1	Body	-0,006	4,11E-04	4,83E-02
cg19935055	11	66935121	KDM2A	Body	0,007	4,11E-04	4,83E-02

cg19649564	11	124932892	SLC37A2	TSS200	0,007	4,11E-04	4,83E-02
cg01740001	19	47579456	ZC3H4	Body	-0,023	4,11E-04	4,83E-02
cg04740931	11	63884779	FLRT1	Body	-0,008	4,12E-04	4,83E-02
cg04084655	9	140267453	EXD3	Body	-0,011	4,12E-04	4,83E-02
cg02932204	10	102773013	PDZD7	Body	0,09	4,12E-04	4,83E-02
cg23518166	1	65488760		IGR	-0,013	4,12E-04	4,83E-02
cg00362263	2	101179803	PDCL3	Body	0,003	4,12E-04	4,83E-02
cg10828721	18	2172050		IGR	-0,012	4,12E-04	4,83E-02
cg11378242	1	244999800	FAM36A	Body	-0,021	4,12E-04	4,83E-02
cg10073693	1	161590955		IGR	0,034	4,13E-04	4,83E-02
cg02745104	2	220071677	ZFAND2B	5'UTR	0,005	4,13E-04	4,83E-02
cg02829356	3	9833993	ARPC4	TSS1500	0,004	4,12E-04	4,83E-02
cg05570786	5	36600653		IGR	0,011	4,13E-04	4,83E-02
cg02934715	6	6306683	F13A1	Body	0,007	4,13E-04	4,83E-02
cg04282723	11	975233	AP2A2	Body	-0,015	4,13E-04	4,83E-02
cg08101955	11	112192015		IGR	0,012	4,13E-04	4,83E-02
cg16287284	12	101580136	SLC5A8	Body	0,058	4,13E-04	4,83E-02
cg02746150	12	121096453	CABP1	Body	-0,016	4,13E-04	4,83E-02
cg06981880	6	168333191	MLLT4	Body	0,005	4,13E-04	4,83E-02
cg01083505	7	116639595	ST7	Body	-0,007	4,13E-04	4,83E-02
cg20870668	7	2608671	IQCE	Body	-0,013	4,14E-04	4,84E-02
cg04117782	17	17875915	TOM1L2	TSS200	-0,006	4,14E-04	4,84E-02
cg11400539	20	5676824		IGR	-0,011	4,14E-04	4,84E-02
cg26353859	12	60081776	SLC16A7	TSS1500	-0,009	4,14E-04	4,84E-02
cg18496937	22	19702076	sept-05	1stExon	0,003	4,14E-04	4,84E-02
cg11619390	1	32931179	ZBTB8B	5'UTR	0,028	4,15E-04	4,84E-02
cg10453343	1	161049655	PVRL4	Body	-0,036	4,16E-04	4,84E-02
cg09042791	2	235904300	SH3BP4	5'UTR	-0,008	4,16E-04	4,84E-02
cg10833825	5	7869013	MTRR	TSS1500	0,005	4,16E-04	4,84E-02
cg03875000	5	103416633		IGR	0,038	4,16E-04	4,84E-02
cg01810346	6	169562727		IGR	-0,008	4,14E-04	4,84E-02
cg22514615	9	35829422	FAM221B	TSS1500	0,013	4,15E-04	4,84E-02
cg23908305	11	1958316	TNNT3	Body	-0,011	4,15E-04	4,84E-02
cg15105510	11	71934859	INPPL1	TSS1500	0,007	4,15E-04	4,84E-02
cg07212053	11	118230307	UBE4A	5'UTR	0,005	4,16E-04	4,84E-02
cg23454275	12	123189246	HCAR2	TSS1500	-0,017	4,15E-04	4,84E-02
cg13840419	13	97852983		IGR	-0,034	4,16E-04	4,84E-02
cg06667168	14	23564915	C14orf119	5'UTR	-0,015	4,15E-04	4,84E-02
cg18095720	14	50160168	KLHDC1	Body	0,006	4,14E-04	4,84E-02
cg13272552	14	55875634	KIAA0831	Body	-0,013	4,16E-04	4,84E-02
cg05863144	14	96830329	ATG2B	TSS1500	0,002	4,16E-04	4,84E-02
cg10058752	15	79290462	RASGRF1	Body	-0,006	4,15E-04	4,84E-02
cg17191813	16	3532548	NAA60	Body	-0,015	4,15E-04	4,84E-02
cg10380221	16	31075618	ZNF668	Body	0,017	4,15E-04	4,84E-02
cg17593330	19	2249032	AMH	TSS200	-0,013	4,14E-04	4,84E-02
cg01282080	19	15298078	NOTCH3	Body	-0,011	4,15E-04	4,84E-02
cg08337065	19	42873882	MEGF8	Body	-0,016	4,15E-04	4,84E-02
cg13093934	20	43356125	WISP2	3'UTR	-0,021	4,15E-04	4,84E-02
cg03913271	22	38302504	MICALL1	1stExon	0,004	4,15E-04	4,84E-02
cg09503608	6	31732516	C6orf26	3'UTR	0,015	4,16E-04	4,84E-02
cg04364453	2	10442941	HPCAL1	TSS1500	0,004	4,16E-04	4,84E-02
cg22760563	16	57163207	CPNE2	Body	0,022	4,16E-04	4,84E-02
cg24547673	2	75841339		IGR	0,022	4,16E-04	4,84E-02
cg21899091	11	791124	CEND1	TSS1500	-0,014	4,16E-04	4,84E-02
cg05220069	7	73149619	WBSCR26	Body	-0,012	4,16E-04	4,84E-02
cg04590313	2	182521378	CERKL	Body	0,007	4,17E-04	4,84E-02
cg03743580	4	4763753		IGR	0,034	4,17E-04	4,84E-02
cg15623846	4	46272848	GABRA2	Body	0,041	4,17E-04	4,84E-02
cg11327857	4	123747558	FGF2	TSS1500	-0,037	4,17E-04	4,84E-02
cg06450181	11	46524128	AMBRA1	Body	-0,009	4,17E-04	4,84E-02
cg21029505	16	83586029	CDH13	Body	0,031	4,17E-04	4,84E-02

cg11534441	1	18246271		IGR	0,066	4,17E-04	4,84E-02
cg08753890	8	29514014		IGR	-0,012	4,17E-04	4,84E-02
cg22036233	14	68283005	ZFYVE26	5'UTR	-0,004	4,17E-04	4,84E-02
cg04444591	15	31616179		IGR	0,007	4,17E-04	4,84E-02
cg15033031	17	48637941	CACNA1G	TSS1500	0,005	4,17E-04	4,84E-02
cg00145954	1	2076496	PRKCZ	Body	-0,03	4,18E-04	4,84E-02
cg03987192	1	26373407	SLC30A2	TSS1500	0,024	4,18E-04	4,84E-02
cg26077117	2	142568025	LRP1B	Body	0,029	4,18E-04	4,84E-02
cg09395732	3	10857456	SLC6A11	TSS1500	-0,029	4,18E-04	4,84E-02
cg13684013	3	106787885		IGR	0,019	4,18E-04	4,84E-02
cg22113651	4	128800634	PLK4	TSS1500	-0,021	4,18E-04	4,84E-02
cg20410114	6	3160502		IGR	0,029	4,17E-04	4,84E-02
cg20331241	6	32052409	TNXB	Body	-0,006	4,18E-04	4,84E-02
cg09305113	6	167263193	RPS6KA2	Body	-0,022	4,18E-04	4,84E-02
cg00908584	7	155024775		IGR	0,028	4,17E-04	4,84E-02
cg14564041	8	130554734	CCDC26	Body	-0,006	4,17E-04	4,84E-02
cg05996224	11	45868587	CRY2	TSS1500	-0,005	4,18E-04	4,84E-02
cg11013298	12	14927928	H2AFJ	1stExon	0,011	4,18E-04	4,84E-02
cg08643979	13	67946028	LINC00364	TSS1500	0,024	4,18E-04	4,84E-02
cg01219130	16	71680526	PHLPP2	3'UTR	-0,012	4,18E-04	4,84E-02
cg24605895	20	43094626	C20orf62	TSS1500	-0,015	4,18E-04	4,84E-02
cg12016968	22	38447387		IGR	0,005	4,18E-04	4,84E-02
cg16127845	7	1126423	GPER	TSS200	0,011	4,18E-04	4,84E-02
cg27206926	5	153825421	SAP30L	TSS200	0,004	4,19E-04	4,84E-02
cg04135242	1	53629086		IGR	-0,006	4,19E-04	4,84E-02
cg21596238	3	11684874	VGLL4	Body	0,007	4,19E-04	4,84E-02
cg01050965	5	177650821	AGXT2L2	Body	-0,015	4,19E-04	4,84E-02
cg15682473	14	50910682	MAP4K5	Body	0,036	4,19E-04	4,84E-02
cg24275088	3	49570136	DAG1	Body	-0,012	4,19E-04	4,85E-02
cg08783638	1	155904747	KIAA0907	TSS1500	-0,03	4,19E-04	4,85E-02
cg04585364	2	171628203		IGR	-0,021	4,19E-04	4,85E-02
cg21928307	7	36192369	EEPD1	TSS1500	0,007	4,19E-04	4,85E-02
cg14536347	2	115486631	DPP10	Body	-0,006	4,19E-04	4,85E-02
cg20443728	7	103085536	)C101927870	TSS200	0,065	4,20E-04	4,85E-02
cg07539443	12	114937328		IGR	0,04	4,20E-04	4,85E-02
cg12270320	13	52076252		IGR	-0,049	4,20E-04	4,85E-02
cg10770023	22	51016644	CPT1B	5'UTR	-0,055	4,20E-04	4,85E-02
cg10792923	4	103789516	UBE2D3	Body	0,017	4,20E-04	4,85E-02
cg00387133	6	71021740		IGR	-0,01	4,20E-04	4,85E-02
cg02633723	12	133173041		IGR	0,009	4,20E-04	4,85E-02
cg21691699	10	135099766	TUBGCP2	Body	-0,006	4,21E-04	4,86E-02
cg24538560	14	36873057		IGR	-0,009	4,21E-04	4,86E-02
cg16632096	6	33279574	TAPBP	Body	0,007	4,21E-04	4,86E-02
cg14126583	2	190446181	SLC40A1	TSS1500	0,006	4,21E-04	4,86E-02
cg00707739	3	23848132	UBE2E1	5'UTR	0,003	4,22E-04	4,86E-02
cg24660320	6	5004412	RPP40	TSS200	0,003	4,22E-04	4,86E-02
cg26371521	6	110222866		IGR	0,01	4,22E-04	4,86E-02
cg00994894	8	103795864		IGR	-0,016	4,22E-04	4,86E-02
cg10779909	9	706773	KANK1	TSS200	-0,005	4,22E-04	4,86E-02
cg22910493	12	110299688	GLTP	Body	-0,007	4,22E-04	4,86E-02
cg17928845	16	78109571		IGR	-0,018	4,22E-04	4,86E-02
cg03732506	17	73088155	SLC16A5	5'UTR	-0,014	4,22E-04	4,86E-02
cg14881567	19	16583117	EPS15L1	TSS1500	0,005	4,22E-04	4,86E-02
cg22887103	2	192458962		IGR	-0,02	4,22E-04	4,86E-02
cg15156367	1	92495004	EPHX4	TSS1500	-0,017	4,23E-04	4,86E-02
cg22238923	2	74781587	LOXL3	TSS1500	0,004	4,23E-04	4,86E-02
cg04560781	2	129511579		IGR	0,029	4,23E-04	4,86E-02
cg09207591	5	758277		IGR	0,036	4,23E-04	4,86E-02
cg09355865	7	151169755	RHEB	Body	-0,03	4,23E-04	4,86E-02
cg07294234	8	42911341	FNTA	TSS200	0,006	4,23E-04	4,86E-02
cg16406186	8	110988444	KCNV1	TSS1500	0,048	4,23E-04	4,86E-02

cg00320790	8	120587612	ENPP2	Body	-0,008	4,23E-04	4,86E-02
cg16983730	10	134106591	STK32C	Body	-0,016	4,23E-04	4,86E-02
cg25356935	11	119056076	PDZD3	TSS200	0,043	4,23E-04	4,86E-02
cg13025283	12	76896350	OSBPL8	5'UTR	-0,006	4,22E-04	4,86E-02
cg08277369	16	84538885	KIAA1609	TSS1500	0,046	4,23E-04	4,86E-02
cg09761040	19	13209981	LYL1	3'UTR	-0,012	4,23E-04	4,86E-02
cg17489534	19	45315572	BCAM	Body	-0,017	4,23E-04	4,86E-02
cg07975705	5	114961891	1ED7-TICAM2	TSS200	-0,003	4,23E-04	4,87E-02
cg01118506	2	122366951	CLASP1	5'UTR	0,013	4,24E-04	4,87E-02
cg23822289	1	151019274	C1orf56	TSS1500	-0,005	4,24E-04	4,87E-02
cg07040500	2	61362928	KIAA1841	3'UTR	-0,04	4,24E-04	4,87E-02
cg02357637	15	100268975	LYSMD4	3'UTR	-0,014	4,24E-04	4,87E-02
cg23937078	1	31246279		IGR	0,016	4,25E-04	4,87E-02
cg27258561	6	31275767		IGR	-0,077	4,24E-04	4,87E-02
cg18593219	8	145561333	SCRT1	TSS1500	0,004	4,24E-04	4,87E-02
cg24429836	16	75150744	LDHD	TSS200	0,062	4,24E-04	4,87E-02
cg17842130	17	723242	NXN	Body	-0,007	4,24E-04	4,87E-02
cg10358527	1	42809253		IGR	0,023	4,25E-04	4,87E-02
cg15797102	4	17158272		IGR	-0,007	4,25E-04	4,87E-02
cg12997282	17	43050496		IGR	-0,004	4,25E-04	4,87E-02
cg04794138	18	60750880		IGR	0,026	4,25E-04	4,87E-02
cg11976052	19	51411839	KLK4	Body	-0,035	4,25E-04	4,87E-02
cg10232889	1	9970257	CTNNBIP1	5'UTR	0,005	4,25E-04	4,87E-02
cg14334389	4	88451346	SPARCL1	TSS1500	0,018	4,25E-04	4,87E-02
cg13829980	4	113066590	C4orf32	1stExon	0,004	4,25E-04	4,87E-02
cg22136765	6	166519953		IGR	0,013	4,25E-04	4,87E-02
cg23142731	12	50899018	DIP2B	1stExon	0,007	4,25E-04	4,87E-02
cg00127036	5	176449346	ZNF346	TSS1500	0,013	4,25E-04	4,87E-02
cg12164992	7	4795022	FOXK1	Body	0,006	4,26E-04	4,87E-02
cg25929976	3	12328656	PPARG	TSS1500	0,05	4,26E-04	4,87E-02
cg03340191	12	111355992	MYL2	Body	0,02	4,26E-04	4,87E-02
cg09928813	16	30794679	ZNF629	Body	-0,006	4,26E-04	4,87E-02
cg24410956	2	179885708	CCDC141	Body	-0,014	4,26E-04	4,87E-02
cg25805368	7	1802235		IGR	-0,008	4,26E-04	4,88E-02
cg05002512	1	51425990	FAF1	TSS200	0,003	4,26E-04	4,88E-02
cg18750643	4	56663848		IGR	-0,006	4,26E-04	4,88E-02
cg11626052	7	157449311	PTPRN2	Body	-0,017	4,26E-04	4,88E-02
cg20803301	8	11155550	MTMR9	Body	0,009	4,26E-04	4,88E-02
cg16371477	14	24838828	NFATC4	Body	-0,004	4,26E-04	4,88E-02
cg06023946	1	175857528		IGR	0,011	4,27E-04	4,88E-02
cg24533917	1	203200291	CHIT1	TSS1500	0,022	4,27E-04	4,88E-02
cg10685531	1	3342726	PRDM16	Body	0,008	4,27E-04	4,88E-02
cg00528464	1	52498993	TXNDC12	Body	0,006	4,27E-04	4,88E-02
cg27010694	7	18336740	HDAC9	Body	0,012	4,27E-04	4,88E-02
cg20752420	7	150475993	)C100128542	Body	0,005	4,27E-04	4,88E-02
cg11102493	1	205140007	DSTYK	Body	0,004	4,27E-04	4,88E-02
cg02357046	4	1686926	FAM53A	TSS1500	-0,011	4,27E-04	4,88E-02
cg19339146	4	62397911	LPHN3	Body	0,016	4,27E-04	4,88E-02
cg00682007	5	140344745	PCDHA7	Body	0,016	4,27E-04	4,88E-02
cg05096490	17	18823319	PRPSAP2	Body	0,024	4,27E-04	4,88E-02
cg12950434	20	34580761	CNBD2	Body	-0,008	4,27E-04	4,88E-02
cg19115737	1	65838476	DNAJC6	Body	-0,041	4,28E-04	4,88E-02
cg25124952	22	31140102	OSBP2	Body	0,032	4,28E-04	4,88E-02
cg22350099	12	109459327	SVOP	TSS1500	0,046	4,28E-04	4,88E-02
cg06208824	14	53173887	PSMC6	TSS200	0,004	4,28E-04	4,88E-02
cg24435407	1	11909677		IGR	0,008	4,28E-04	4,88E-02
cg09576882	5	6714987	POLS	5'UTR	-0,005	4,28E-04	4,88E-02
cg17335701	5	96142712	ERAP1	5'UTR	-0,004	4,28E-04	4,88E-02
cg18691856	6	138358564		IGR	-0,004	4,28E-04	4,88E-02
cg16365842	6	170191057	C6orf122	Body	0,049	4,28E-04	4,88E-02
cg02100543	11	64879991	TM7SF2	Body	0,005	4,28E-04	4,88E-02

cg15596810	13	41554217	ELF1	Body	-0,007	4,28E-04	4,88E-02
cg16308770	3	27638017		IGR	-0,013	4,29E-04	4,89E-02
cg23534774	1	66036294	LEPR	Body	-0,041	4,29E-04	4,89E-02
cg11631334	3	196730272	MFI2	Body	0,01	4,29E-04	4,89E-02
cg05590686	5	66709752		IGR	0,008	4,29E-04	4,89E-02
cg05792022	13	41239732	FOXO1	1stExon	0,008	4,29E-04	4,89E-02
cg19572487	17	38476024	RARA	5'UTR	0,033	4,29E-04	4,89E-02
cg03934016	19	58111150	ZNF530	TSS200	-0,013	4,29E-04	4,89E-02
cg08864381	1	1590717	CDK11B	Body	0,003	4,30E-04	4,89E-02
cg14248235	2	26017996	ASXL2	Body	0,026	4,30E-04	4,89E-02
cg04008445	3	114788347	ZBTB20	5'UTR	0,004	4,30E-04	4,89E-02
cg23221504	2	171673110	GAD1	TSS200	-0,044	4,30E-04	4,89E-02
cg07169712	6	29571419	GABBR1	Body	-0,005	4,30E-04	4,89E-02
cg02450828	11	70264554	CTTN	Body	-0,016	4,30E-04	4,89E-02
cg23530906	12	114708303		IGR	0,046	4,30E-04	4,89E-02
cg07125278	16	67683757	RLTPR	Body	-0,012	4,30E-04	4,89E-02
cg02902854	17	3474736	TRPV1	Body	-0,015	4,30E-04	4,89E-02
cg12903174	1	240986655	RGS7	Body	-0,011	4,31E-04	4,89E-02
cg01569664	13	110434016	IRS2	Body	-0,01	4,31E-04	4,89E-02
cg21440088	18	19930963		IGR	-0,02	4,31E-04	4,89E-02
cg10214154	1	197694388	DENND1B	Body	-0,024	4,31E-04	4,90E-02
cg03495543	3	26690753	LRRC3B	5'UTR	-0,029	4,31E-04	4,90E-02
cg02229461	7	75185674	HIP1	Body	-0,01	4,31E-04	4,90E-02
cg04413193	1	244226143		IGR	-0,017	4,31E-04	4,90E-02
cg17814717	7	151512049	PRKAG2	TSS200	-0,011	4,32E-04	4,90E-02
cg03387353	8	61429246	RAB2A	TSS1500	-0,005	4,31E-04	4,90E-02
cg06730796	16	67271014	FHOD1	Body	-0,011	4,32E-04	4,90E-02
cg13076843	17	74475294	RHBDF2	Body	-0,008	4,31E-04	4,90E-02
cg27367526	7	89841692	STEAP2	5'UTR	0,039	4,32E-04	4,90E-02
cg01180167	11	84620643	DLG2	Body	-0,023	4,32E-04	4,90E-02
cg20037601	15	73652167	HCN4	Body	0,053	4,32E-04	4,90E-02
cg13117272	17	79681052	SLC25A10	Body	-0,016	4,32E-04	4,90E-02
cg08061100	7	134291373		IGR	-0,01	4,32E-04	4,90E-02
cg19284054	1	19025353	PAX7	Body	0,036	4,32E-04	4,90E-02
cg21785710	2	63662027	C2orf86	Body	-0,008	4,32E-04	4,90E-02
cg00516867	7	128417291	OPN1SW	TSS1500	0,031	4,32E-04	4,90E-02
cg25300094	8	23282956		IGR	0,03	4,32E-04	4,90E-02
cg01600529	2	60688991	BCL11A	Body	-0,011	4,33E-04	4,90E-02
cg02680669	8	40181105		IGR	0,034	4,33E-04	4,90E-02
cg19049344	2	166095825	SCN2A	TSS200	-0,004	4,33E-04	4,90E-02
cg03784611	3	196754501	MFI2	Body	-0,006	4,33E-04	4,90E-02
cg22339775	6	26224925	HIST1H3E	TSS1500	-0,038	4,33E-04	4,90E-02
cg12002139	6	158478872	SYNJ2	Body	-0,089	4,33E-04	4,90E-02
cg16024058	7	16570490	LRRC72	Body	0,017	4,33E-04	4,90E-02
cg09116008	9	130511667	SH2D3C	Body	-0,021	4,33E-04	4,90E-02
cg06132853	17	80157889	CCDC57	Body	-0,009	4,33E-04	4,90E-02
cg06716090	19	14251492		IGR	0,006	4,33E-04	4,90E-02
cg22623655	20	31478606		IGR	0,005	4,33E-04	4,90E-02
cg15101291	21	43502244	UMODL1	Body	-0,01	4,33E-04	4,90E-02
cg12469332	16	58283784	CCDC113	TSS200	0,008	4,34E-04	4,90E-02
cg07920503	13	25745406	FAM123A	1stExon	-0,013	4,34E-04	4,90E-02
cg04014586	1	245704710	KIF26B	Body	0,02	4,34E-04	4,91E-02
cg05386769	2	218681558	TNS1	Body	0,018	4,34E-04	4,91E-02
cg09876574	9	126561628	DENND1A	Body	-0,014	4,34E-04	4,91E-02
cg13632523	20	51117616		IGR	-0,006	4,34E-04	4,91E-02
cg00696685	8	144947335	EPPK1	1stExon	-0,017	4,34E-04	4,91E-02
cg09718810	5	179719756	MAPK9	TSS1500	0,003	4,34E-04	4,91E-02
cg12897945	19	639731	FGF22	TSS200	0,004	4,34E-04	4,91E-02
cg07638689	19	8204480	FBN3	Body	0,014	4,34E-04	4,91E-02
cg18960464	6	20653068	CDKAL1	Body	-0,012	4,35E-04	4,91E-02
cg07806820	11	69975284	ANO1	Body	-0,023	4,35E-04	4,91E-02

cg13598193	19	2799757	THOP1	Body	-0,016	4,35E-04	4,91E-02
cg15778089	3	35721037	ARPP-21	5'UTR	0,011	4,35E-04	4,91E-02
cg00190795	3	195599898	TNK2	Body	-0,016	4,35E-04	4,91E-02
cg10927093	1	181616634	CACNA1E	Body	0,03	4,35E-04	4,91E-02
cg13782200	7	148602593		IGR	-0,042	4,35E-04	4,91E-02
cg02958346	11	57425731	CLP1	5'UTR	0,008	4,35E-04	4,91E-02
cg06527531	6	160693578		IGR	0,007	4,35E-04	4,91E-02
cg20779092	8	145956688	ZNF251	Body	0,005	4,35E-04	4,91E-02
cg19542892	19	54694915	MBOAT7	TSS1500	0,006	4,36E-04	4,91E-02
cg05081374	2	108832664	LINC01594	Body	0,026	4,36E-04	4,91E-02
cg26548476	6	7441052		IGR	0,008	4,36E-04	4,91E-02
cg01797450	6	168134760		IGR	0,06	4,36E-04	4,91E-02
cg13685110	14	73834883	NUMB	5'UTR	0,012	4,36E-04	4,91E-02
cg02757802	16	4827792	sept-12	3'UTR	-0,022	4,36E-04	4,91E-02
cg26741954	19	48954979	GRWD1	Body	-0,007	4,36E-04	4,91E-02
cg19545348	1	211434133	RCOR3	Body	0,003	4,37E-04	4,92E-02
cg06206755	8	126444024	TRIB1	TSS1500	-0,005	4,37E-04	4,92E-02
cg26072173	11	1860224	TNNI2	TSS1500	-0,013	4,37E-04	4,92E-02
cg26291601	1	41170927	NFYC	5'UTR	0,013	4,37E-04	4,92E-02
cg17592968	2	161133255	RBMS1	Body	-0,004	4,37E-04	4,92E-02
cg14741143	3	156273315	SSR3	TSS1500	-0,06	4,38E-04	4,92E-02
cg07099915	4	122870599	TRPC3	Body	0,016	4,37E-04	4,92E-02
cg17304327	5	17929083		IGR	-0,013	4,38E-04	4,92E-02
cg00559081	6	56951467		IGR	-0,043	4,38E-04	4,92E-02
cg13332525	6	99949744	USP45	Body	-0,027	4,37E-04	4,92E-02
cg06904057	7	139498138	TBXAS1	5'UTR	0,035	4,37E-04	4,92E-02
cg11480684	9	25742548		IGR	-0,029	4,37E-04	4,92E-02
cg04250867	11	126081485	RPUSD4	1stExon	0,005	4,37E-04	4,92E-02
cg06658453	12	53580609	ZNF740	Body	0,006	4,37E-04	4,92E-02
cg02319883	12	102259350		IGR	-0,014	4,37E-04	4,92E-02
cg19788202	14	104941923		IGR	-0,007	4,38E-04	4,92E-02
cg25139934	8	17490421	PDGFRL	Body	-0,006	4,38E-04	4,92E-02
cg25946869	12	113796432	PLBD2	1stExon	0,01	4,38E-04	4,92E-02
cg15235798	13	112720244		IGR	-0,024	4,38E-04	4,92E-02
cg08661338	15	25321221	NORD116-12	TSS1500	0,042	4,38E-04	4,92E-02
cg00540222	12	47482450	PCED1B	5'UTR	0,021	4,38E-04	4,92E-02
cg02726137	5	140026622	NDUFA2	Body	0,023	4,38E-04	4,92E-02
cg19920898	5	171936462		IGR	0,024	4,38E-04	4,92E-02
cg22476550	20	37590555	DHX35	TSS1500	-0,006	4,38E-04	4,92E-02
cg13503253	12	51105029	DIP2B	Body	0,007	4,38E-04	4,92E-02
cg02882945	19	3272665	CELF5	Body	-0,029	4,38E-04	4,92E-02
cg25737788	1	15852955	DNAJC16	TSS1500	-0,004	4,39E-04	4,92E-02
cg15926342	5	171881757	SH3PXD2B	TSS1500	-0,004	4,39E-04	4,92E-02
cg11292771	19	34740773		IGR	-0,016	4,39E-04	4,93E-02
cg16950104	19	35648395	FXYD5	ExonBnd	-0,006	4,40E-04	4,93E-02
cg19047158	2	208080231		IGR	0,008	4,40E-04	4,93E-02
cg08476364	7	6370717	C7orf70	Body	-0,006	4,40E-04	4,93E-02
cg11833121	9	139685773	TMEM141	TSS200	0,01	4,40E-04	4,93E-02
cg02807450	11	95657743	MTMR2	TSS1500	-0,035	4,40E-04	4,93E-02
cg14730919	1	214139715	PROX1-AS1	Body	-0,008	4,40E-04	4,93E-02
cg19574906	10	70992431	OC101928994	TSS200	0,017	4,40E-04	4,93E-02
cg25137204	11	72975404	P2RY6	TSS200	-0,036	4,40E-04	4,93E-02
cg16914272	6	27806842	HIST1H2BN	1stExon	0,004	4,41E-04	4,93E-02
cg01503740	16	1445865		IGR	-0,014	4,41E-04	4,93E-02
cg03348978	17	36103230	HNF1B	Body	0,01	4,41E-04	4,93E-02
cg11885415	21	30715207	BACH1	3'UTR	0,063	4,41E-04	4,93E-02
cg14934821	9	139228820	GPSM1	Body	0,016	4,41E-04	4,93E-02
cg15523606	1	178120997	RASAL2	Body	0,022	4,41E-04	4,94E-02
cg13060454	2	111452572		IGR	0,023	4,41E-04	4,94E-02
cg11768886	4	5052795	STK32B	TSS1500	-0,006	4,41E-04	4,94E-02
cg24636907	4	178958666		IGR	0,031	4,41E-04	4,94E-02

cg20329111	3	50388124	CYB561D2	TSS200	0,004	4,41E-04	4,94E-02
cg11387741	3	58182558	DNASE1L3	Body	0,044	4,41E-04	4,94E-02
cg09437423	3	108541505	TRAT1	TSS200	0,056	4,42E-04	4,94E-02
cg07997455	8	10691934	PINX1	Body	-0,005	4,42E-04	4,94E-02
cg02947276	14	24731242	TGM1	Body	0,006	4,42E-04	4,94E-02
cg22045371	2	61244308	PEX13	TSS1500	0,012	4,42E-04	4,94E-02
cg06025827	5	169407696	FAM196B	1stExon	-0,045	4,42E-04	4,94E-02
cg19771599	1	11070084		IGR	0,005	4,42E-04	4,94E-02
cg16801686	1	145516446	GNRHR2	TSS1500	0,006	4,42E-04	4,94E-02
cg09263728	2	225451079	CUL3	TSS1500	-0,008	4,43E-04	4,94E-02
cg25692290	3	47844315	DHX30	TSS200	0,003	4,43E-04	4,94E-02
cg00671354	3	186819037		IGR	-0,009	4,42E-04	4,94E-02
cg05086944	4	43149680		IGR	0,041	4,43E-04	4,94E-02
cg21030557	5	142910936		IGR	0,02	4,43E-04	4,94E-02
cg06407284	5	176562036	NSD1	5'UTR	0,019	4,42E-04	4,94E-02
cg03844622	6	74071738	C6orf221	TSS1500	0,01	4,43E-04	4,94E-02
cg11202634	8	37184569		IGR	0,024	4,42E-04	4,94E-02
cg16746589	12	3566133		IGR	0,029	4,42E-04	4,94E-02
cg12393791	12	125863860	TMEM132B	Body	0,03	4,43E-04	4,94E-02
cg13769176	13	109280959	MYO16	TSS1500	-0,013	4,43E-04	4,94E-02
cg20451705	14	91744430	CCDC88C	Body	-0,012	4,43E-04	4,94E-02
cg03656996	15	40601261	PLCB2	TSS1500	-0,003	4,43E-04	4,94E-02
cg01288322	15	85259188	SEC11A	Body	0,006	4,42E-04	4,94E-02
cg14741666	17	34842576	ZNHIT3	1stExon	0,003	4,43E-04	4,94E-02
cg03359649	19	31397673		IGR	-0,016	4,43E-04	4,94E-02
cg13588826	21	47533197	COL6A2	Body	0,008	4,43E-04	4,94E-02
cg10477468	8	48872626	PRKDC	1stExon	0,005	4,43E-04	4,94E-02
cg14317075	3	46653653	)C100132146	TSS1500	-0,018	4,43E-04	4,94E-02
cg06517794	1	78180274	USP33	Body	-0,008	4,44E-04	4,94E-02
cg01375248	3	142724451	SR140	Body	-0,026	4,44E-04	4,94E-02
cg15829826	11	65153860	FRMD8	TSS200	0,006	4,44E-04	4,94E-02
cg18026626	15	78286614	LOC91450	TSS200	0,021	4,44E-04	4,94E-02
cg05057054	22	43716146	SCUBE1	Body	0,03	4,44E-04	4,94E-02
cg12145624	5	65891188	MAST4	TSS1500	0,04	4,44E-04	4,94E-02
cg24363180	2	51171657	NRXN1	Body	-0,013	4,45E-04	4,95E-02
cg17239185	1	19536960	UBR4	TSS1500	0,004	4,45E-04	4,95E-02
cg07582167	2	220333683	SPEG	Body	-0,006	4,45E-04	4,95E-02
cg11218324	3	47619690	CSPG5	5'UTR	0,002	4,46E-04	4,95E-02
cg10470200	5	106715906	EFNA5	3'UTR	0,004	4,46E-04	4,95E-02
cg00842665	5	122333956	SNX24	Body	-0,007	4,45E-04	4,95E-02
cg04623371	6	168768113		IGR	0,005	4,46E-04	4,95E-02
cg23848678	6	169116695		IGR	-0,007	4,45E-04	4,95E-02
cg20176371	8	1991815	MYOM2	TSS1500	-0,007	4,46E-04	4,95E-02
cg22207702	8	79593373	ZC2HC1A	Body	0,029	4,45E-04	4,95E-02
cg06693505	8	141057453	TRAPPC9	Body	0,044	4,46E-04	4,95E-02
cg24261644	18	19997864	CTAGE1	1stExon	-0,015	4,45E-04	4,95E-02
cg16946439	11	12131814	MICAL2	TSS1500	0,012	4,46E-04	4,95E-02
cg27271453	1	65699069		IGR	0,008	4,46E-04	4,95E-02
cg05644090	10	131826455		IGR	-0,012	4,46E-04	4,95E-02
cg22370694	1	228395660	OBSCN	TSS200	0,029	4,46E-04	4,95E-02
cg01247301	7	109237864		IGR	-0,023	4,46E-04	4,95E-02
cg17633422	1	149871093	BOLA1	TSS200	0,006	4,46E-04	4,95E-02
cg04251661	5	86410880	MIR4280	TSS200	0,036	4,47E-04	4,96E-02
cg03796591	6	56009431	COL21A1	Body	-0,013	4,47E-04	4,96E-02
cg09824023	12	52283224	ANKRD33	Body	-0,007	4,47E-04	4,96E-02
cg04577789	14	103377905		IGR	-0,012	4,47E-04	4,96E-02
cg01399860	9	136889061	NCRNA00094	TSS1500	0,031	4,47E-04	4,96E-02
cg22212700	11	74303682	POLD3	5'UTR	0,004	4,47E-04	4,96E-02
cg24352499	12	50360776		IGR	0,008	4,47E-04	4,96E-02
cg20006790	16	67650736	CTCF	Body	0,003	4,47E-04	4,96E-02
cg14145175	6	160183497	ACAT2	TSS200	0,004	4,47E-04	4,96E-02

cg18164357	11	77534497	C11orf67	5'UTR	0,008	4,47E-04	4,96E-02
cg12054575	13	34862475		IGR	-0,009	4,48E-04	4,96E-02
cg27022209	2	88876195	EIF2AK3	Body	0,003	4,48E-04	4,96E-02
cg21343498	5	32399118	ZFR	Body	-0,015	4,48E-04	4,96E-02
cg05947295	7	99555028		IGR	0,01	4,48E-04	4,96E-02
cg20018782	14	23317496		IGR	0,076	4,48E-04	4,96E-02
cg07007550	1	16011710	PLEKHM2	Body	0,012	4,48E-04	4,97E-02
cg11783944	22	25297885	SGSM1	Body	0,01	4,48E-04	4,97E-02
cg06759901	7	126446757	GRM8	Body	-0,021	4,49E-04	4,97E-02
cg08370757	16	84230590	ADAD2	3'UTR	-0,027	4,49E-04	4,97E-02
cg25809635	19	37341103	ZNF345	TSS200	0,003	4,49E-04	4,97E-02
cg13823218	3	182665652	DCUN1D1	Body	-0,01	4,49E-04	4,97E-02
cg02920383	7	150747107	ACCN3	Body	-0,005	4,49E-04	4,97E-02
cg17999327	22	22698078		IGR	-0,006	4,49E-04	4,97E-02
cg09973548	7	27208347	HOXA10-AS	TSS200	-0,013	4,50E-04	4,97E-02
cg13309702	8	144876577	SCRIB	Body	-0,016	4,50E-04	4,97E-02
cg24295609	10	131272536	MGMT	Body	0,019	4,50E-04	4,97E-02
cg16324121	3	9954273	IL17RE	Body	-0,021	4,50E-04	4,97E-02
cg18934605	4	74207894		IGR	-0,011	4,50E-04	4,97E-02
cg16985952	1	15068172	KIAA1026	Body	0,016	4,50E-04	4,98E-02
cg07045538	1	43914361	SZT2	Body	-0,005	4,50E-04	4,98E-02
cg06661542	10	858328	LARP4B	3'UTR	-0,014	4,50E-04	4,98E-02
cg17547152	12	132341427		IGR	-0,017	4,50E-04	4,98E-02
cg10318458	1	47407158	CYP4A11	TSS200	-0,032	4,51E-04	4,98E-02
cg24820828	3	133306214	CDV3	3'UTR	0,021	4,51E-04	4,98E-02
cg10825847	11	104894216	CASP5	TSS1500	0,03	4,51E-04	4,98E-02
cg14928992	4	190724127		IGR	0,03	4,51E-04	4,98E-02
cg23884358	7	184010		IGR	-0,008	4,51E-04	4,98E-02
cg24430147	1	115885042		IGR	0,004	4,51E-04	4,98E-02
cg19501345	7	73703695	CLIP2	TSS200	0,006	4,51E-04	4,98E-02
cg00177698	17	46018875	PNPO	TSS200	0,004	4,51E-04	4,98E-02
cg18063909	8	145215003	MROH1	5'UTR	-0,013	4,52E-04	4,98E-02
cg23009468	5	38061074		IGR	-0,01	4,52E-04	4,98E-02
cg10846125	5	108801973		IGR	-0,007	4,52E-04	4,98E-02
cg04822518	12	29936075	TMTC1	5'UTR	-0,011	4,52E-04	4,98E-02
cg26514117	19	13985553	MIR181D	TSS200	-0,007	4,52E-04	4,98E-02
cg14893128	20	32077794	CBFA2T2	TSS200	-0,006	4,52E-04	4,98E-02
cg18413948	6	41021017	APOBEC2	5'UTR	-0,007	4,52E-04	4,98E-02
cg04790574	1	6285067	ICMT	3'UTR	-0,014	4,52E-04	4,98E-02
cg15247625	2	234774830	MSL3L2	Body	0,012	4,52E-04	4,98E-02
cg15208393	20	60725081	SS18L1	5'UTR	-0,014	4,52E-04	4,98E-02
cg05730215	2	27435253	SLC5A6	TSS200	0,011	4,53E-04	4,99E-02
cg01089538	3	186745642	ST6GAL1	5'UTR	0,029	4,53E-04	4,99E-02
cg10546721	5	45252822		IGR	-0,016	4,53E-04	4,99E-02
cg16531955	12	69965254	FRS2	Body	0,02	4,53E-04	4,99E-02
cg27594758	14	78324122	ADCK1	Body	0,011	4,53E-04	4,99E-02
cg25430671	1	11714784	FBXO2	TSS200	-0,01	4,53E-04	4,99E-02
cg11415596	16	90039755	CENPBD1	TSS1500	0,011	4,53E-04	4,99E-02
cg15691256	1	32210421	ADGRB2	Body	-0,011	4,55E-04	4,99E-02
cg01224430	1	94293239	BCAR3	5'UTR	0,01	4,55E-04	4,99E-02
cg09946097	1	179187145	ABL2	Body	-0,035	4,54E-04	4,99E-02
cg00272709	1	203734167	LAX1	TSS200	0,018	4,55E-04	4,99E-02
cg23168390	3	113465001	ATP6V1A	TSS1500	0,004	4,54E-04	4,99E-02
cg07828145	3	133395097		IGR	0,039	4,54E-04	4,99E-02
cg04812891	6	45545177		IGR	-0,007	4,54E-04	4,99E-02
cg16524778	7	52568754		IGR	0,064	4,55E-04	4,99E-02
cg08951403	7	157960420	PTPRN2	Body	0,034	4,55E-04	4,99E-02
cg19456007	10	16046716		IGR	-0,045	4,55E-04	4,99E-02
cg07914282	10	126839947	CTBP2	5'UTR	-0,022	4,55E-04	4,99E-02
cg22773080	14	95999748	SNHG10	Body	-0,012	4,54E-04	4,99E-02
cg00806259	16	68265587	ESRP2	Body	-0,013	4,54E-04	4,99E-02

cg02643214	16	86309508	LINC01081	Body	-0,022	4,55E-04	4,99E-02
cg09215006	16	88354463		IGR	-0,058	4,54E-04	4,99E-02
cg05890616	17	41859201	C17orf105	Body	0,005	4,54E-04	4,99E-02
cg23439364	18	2875689	EMILIN2	Body	-0,024	4,54E-04	4,99E-02
cg26278116	19	9416669	ZNF699	TSS1500	-0,006	4,54E-04	4,99E-02
cg07810162	19	39235936	CAPN12	TSS1500	0,027	4,54E-04	4,99E-02
cg23651728	20	17511410	BFSP1	Body	0,008	4,54E-04	4,99E-02
cg01489519	20	61493186	TCFL5	TSS200	0,003	4,54E-04	4,99E-02
cg19151082	2	190682679	PMS1	5'UTR	-0,02	4,55E-04	4,99E-02
cg24694326	1	151162561	VPS72	1stExon	0,006	4,55E-04	4,99E-02
cg00433050	4	6296826	WFS1	Body	-0,007	4,55E-04	4,99E-02
cg08799997	6	167737937	TTLL2	TSS1500	-0,016	4,55E-04	4,99E-02
cg06741336	14	101696494		IGR	0,024	4,55E-04	4,99E-02
cg18956250	1	26798730	HMGN2	TSS200	0,003	4,56E-04	4,99E-02
cg12158389	1	38230244	EPHA10	Body	-0,016	4,56E-04	4,99E-02
cg12148243	16	88695031	ZC3H18	Body	-0,014	4,56E-04	4,99E-02
cg02089487	16	67472507	ATP6V0D1	Body	0,004	4,56E-04	4,99E-02
cg05592353	6	37607711	MDGA1	Body	-0,006	4,56E-04	4,99E-02
cg06433063	6	138572213	ARFGEF3	Body	-0,006	4,56E-04	4,99E-02
cg02573587	19	7504549	ARHGEF18	5'UTR	0,006	4,56E-04	5,00E-02
cg23585767	17	78519361	RPTOR	5'UTR	-0,019	4,56E-04	5,00E-02
cg15429629	4	186696632	SORBS2	5'UTR	-0,01	4,57E-04	5,00E-02
cg15234845	10	102496500		IGR	0,047	4,57E-04	5,00E-02
cg25042675	21	36666547		IGR	-0,006	4,57E-04	5,00E-02
cg15166900	3	69132709	ARL6IP5	TSS1500	0,038	4,57E-04	5,00E-02
cg25047094	2	182418171	CERKL	Body	-0,01	4,57E-04	5,00E-02
cg18411429	14	77270823	ANGEL1	Body	0,011	4,57E-04	5,00E-02
cg22635541	17	1808573		IGR	0,054	4,57E-04	5,00E-02
