# Supplementary material for: Epigenetic regulation of transcription factor binding motifs promotes Th1 response in Chagas disease cardiomyopathy
Source: Front Immunol. 2022 Aug 22;13:958200. doi: 10.3389/fimmu.2022.958200 (PMC9441916; doi:10.3389/fimmu.2022.958200)
Supplement: Supplementary Table 1 — Biological samples included in this study. [file DataSheet_1.zip › Supplementary Material/Supplementary Table 16.pdf]

Supplementary table 16. Gene ontology analysis based on diffrentially expressed genes between control and severe CCC and/or genes affected by at least one differentially methylated CpGs between tissue control and severe CCC and/or genes affected by at least one differentially methylated CpGs between blood asymptomatic and severe CCC samples.

ID	Term	Corrected pvalue	GOLevels	Nb. Genes	% Associated Genes	%Genes tissue RNA-seq	% Genes tisseu methylation	%Genes blood methylation	Genes tissue RNA-seq	Genes tissue methylation	Genes blood methylation
GO:0098609	cell-cell adhesion	6,33E-21	[3]	211,00	23,34	34,47	33,19	32,34	[ADAM8, AIF1, AKNA, BTLA, CARD11, CCDC88B, CCL19, CCR2, CD2, CD200R1, CD27, CD3E, CD4, CD40LG, CD5, CD6, CD74, CD80, CD84, CD86, CDKN2A, CEL, CRB2, CRTAM, CTLA4, DOCK8, EGR3, EMB, FGL2, FOXP3, FUT7, HLA-DMB, HLA-DPA1, HLA-DPB1, HLA-DRB1, ICOS, IFNG, IL18, IL7R, ITGA4, ITGB7, JAML, KLRK1, LAG3, LAX1, LCK, LEF1, MYB, NCKAP1L, NLRP3, NR4A3, P2RY12, PCDH20, PCDHA2, PIK3CG, PLA2G2D, PLEK, PRKCQ, PTAFR, PTPN22, PTPN6, PTPRC, RHOH, RUNX3, SASH3, SELE, SELL, SELPLG, SEMA4D, SYK, TESPA1, THY1, TIGIT, TNFAIP8L2, TNFSF13B, TNR, VNN1, WNT1, XCL1, XG, ZAP70]	[ABAT, ADA, ADGRL3, ADGRV1, AFDN, AKNA, ANK3, ANXA9, BCL2, BMP7, CCDC88B, CCR7, CD160, CD28, CD2AP, CD44, CD47, CD6, CDH17, CDH19, CDH2, CDHR2, COL19A1, CORO1A, CTLA4, CX3CL1, GATD3A, GCNT1, GRB2, HLA-A, HLA-DPA1, HLA-DPB1, IDO1, IRF1, ITGA8, ITGB5, JAK3, JAML, LAX1, LCK, LPP, MAP2K5, MBP, NCK2, NECTIN1, NEXN, NFAT5, NPTN, PCDH7, PCDH9, PCDHA6, PIEZO1, PKP4, PTPN2, PTPN22, PTPN6, PTPRC, PTPRF, PTPRM, RARA, RIPOR2, RUNX1, RUNX3, SEMA4D, STK10, TENM4, TESPA1, TGFB2, TJP2, TLN2, TNF, TNFAIP8L2, TNIP1, UBASH3B, VMP1, ZBTB16, ZC3H12A, ZMIZ1]	[ALOX15, ANK3, BTNL2, CARD11, CAV1, CD9, CDH13, CDH22, CDH4, CDSN, CELSR3, CLSTN2, CRB1, CXADR, DLG2, DSCAML1, DSG3, EGFR, FAT1, GCNT2, GNAS, HLA-DQB2, HLA-DRB1, HLA-G, IGSF21, IGSF9B, ITGB2, KIFC3, KIRREL3, LRRC7, LYN, MAGI1, MBL2, MBP, MYADM, NRXN3, PAK1, PAK4, PCDHA12, PCDHA13, PCDHA6, PCDHA7, PCDHA9, PCDHB13, PCDHB14, PCDHB15, PCDHB16, PCDHB18P, PCDHB2, PCDHB3, PCDHB4, PCDHB5, PCDHB6, PCDHB7, PCDHB8, PCDHGA10, PCDHGA2, PCDHGA4, PCDHGA6, PCDHGB1, PCDHGB3, PLPP3, PPP3CA, PRKCA, PRKCZ, PTPN22, PTPRT, RIPOR2, ROBO1, ROBO2, ROBO3, RUNX1, SCARF2, SDK1, SWAP70, UNC5D]
GO:0042110	T cell activation	8,05E-21	[4, 5]	142,00	27,84	53,53	32,35	14,12	[ADAM8, AIF1, APBB1IP, BTLA, BTN3A1, CAMK4, CARD11, CASP8, CCDC88B, CCL19, CCR2, CD1C, CD2, CD27, CD3D, CD3E, CD3G, CD4, CD40LG, CD5, CD6, CD74, CD80, CD86, CD8A, CD8B, CDKN2A, CLCF1, CLEC4D, CLEC4E, CRTAM, CTLA4, DOCK2, DOCK8, EGR3, EOMES, FGL2, FOXP3, FUT7, HLA-DMB, HLA-DOA, HLA-DPA1, HLA-DPB1, HLA-DRB1, ICOS, IFNG, IL18, IL7R, IRF4, ITK, JAML, KLRK1, LAG3, LAX1, LCK, LCP1, LEF1, LMO1, LY9, MYB, NCKAP1L, NLRP3, PIK3CG, PLA2G2D, PRDM1, PRKCQ, PTPN22, PTPN6, PTPRC, RHOH, RUNX3, SASH3, SEMA4A, SIT1, SLAMF6, SYK, TESPA1, THEMIS, THY1, TIGIT, TNFAIP8L2, TNFRSF18, TNFSF13B, TNFSF8, TOX, VNN1, WAS, WNT1, XCL1, ZAP70, ZNF683]	[ADA, APBB1IP, B2M, BATF, BCL2, CASP8, CCDC88B, CCND3, CCR7, CD160, CD28, CD3G, CD44, CD47, CD6, CDK6, CORO1A, CTLA4, FANCA, GATD3A, GRB2, GSN, HLA-A, HLA-DPA1, HLA-DPB1, IDO1, IRF1, JAK3, JAML, KIF13B, LAX1, LCK, LCP1, NCK2, NFATC2, PIK3CD, PSAP, PTPN2, PTPN22, PTPN6, PTPRC, RARA, RELB, RIPOR2, RORA, RUNX1, RUNX3, SLA2, STAT3, TESPA1, TNFAIP8L2, ZBTB16, ZC3H12A, ZEB1, ZMIZ1]	[BCL11B, BTNL2, CAMK4, CARD11, CAV1, CD151, CD8A, CXADR, FOXP1, HLA-DQB2, HLA-DRB1, HLA-G, LCP1, LYN, PAK1, PNKD, PPP3CA, PRKCZ, PTPN22, RIPOR2, RUNX1, TOX, TSC1, ZEB1]
GO:0002521	leukocyte differentiation	2,86E-17	[4, 5, 6, 7, 8]	143,00	25,63	51,85	32,72	15,43	[ADAM8, BLNK, BTK, C1QC, CALCR, CAMK4, CARD11, CASP8, CCL19, CCR2, CD2, CD27, CD3D, CD3E, CD3G, CD4, CD40LG, CD74, CD80, CD86, CD8A, CDKN2A, CLCF1, CLEC4D, CLEC4E, CR2, CRTAM, CSF1R, CTLA4, DOCK10, DOCK2, EGR3, EOMES, FCRL3, FGL2, FLT3, FOXP3, FUT7, GPR55, HCLS1, HLA-DOA, HLA-DRB1, IFNG, IKZF1, IL18, IL7R, INPP5D, IRF4, ITGA4, ITK, LAG3, LCK, LEF1, LRRC17, LY9, MS4A1, MYB, NCKAP1L, NLRP3, PLA2G2D, PRDM1, PTK2B, PTPN22, PTPN6, PTPRC, RHOH, RUNX3, SASH3, SEMA4A, SLAMF6, SLAMF8, SNX10, SYK, TESPA1, THEMIS, TNFRSF18, TNFSF8, TOX, TREM2, UBD, VNN1, WNT1, ZAP70, ZNF683]	[ADA, B2M, BATF, BATF3, BCL2, CASP8, CCR7, CD101, CD28, CD3G, CDH17, CDK6, CMTM7, CTLA4, FANCA, FARP2, FES, GAB2, GPR68, HDAC9, HLA-B, IL34, IRF1, JAK3, LCK, MED1, MITF, MMP14, NFATC2, PIK3CD, PSAP, PTK2B, PTPN2, PTPN22, PTPN6, PTPRC, PTPRJ, RARA, RELB, RORA, RUNX1, RUNX3, SBNO2, STAT3, TCF7L1, TESPA1, TNF, TSHR, UBASH3B, ZBTB16, ZC3H12A, ZEB1, ZMIZ1]	[BCL11B, CAMK4, CARD11, CARTPT, CD8A, CMTM7, FOXP1, GNAS, HLA-DQB2, HLA-DRB1, HLA-G, LYN, OSTM1, PNKD, PRKCA, PRKCZ, PTPN22, RB1, RUNX1, SIGLEC15, TESC, TNFRSF11A, TOX, TSC1, ZEB1]
GO:0030217	T cell differentiation	3,84E-17	[5, 6, 7, 8, 9, 10]	87,00	33,08	57,43	29,70	12,87	[ADAM8, CAMK4, CARD11, CCL19, CCR2, CD2, CD27, CD3D, CD3E, CD3G, CD4, CD74, CD80, CD86, CD8A, CDKN2A, CLEC4D, CLEC4E, CRTAM, CTLA4, DOCK2, EGR3, EOMES, FGL2, FOXP3, FUT7, HLA-DOA, IFNG, IL18, IL7R, IRF4, ITK, LAG3, LCK, LEF1, LY9, MYB, NCKAP1L, NLRP3, PLA2G2D, PRDM1, PTPN22, PTPRC, RHOH, RUNX3, SASH3, SEMA4A, SLAMF6, SYK, TESPA1, THEMIS, TNFRSF18, TNFSF8, TOX, VNN1, WNT1, ZAP70, ZNF683]	[ADA, B2M, BATF, BCL2, CCR7, CD28, CD3G, CDK6, CTLA4, FANCA, IRF1, JAK3, LCK, NFATC2, PIK3CD, PSAP, PTPN2, PTPN22, PTPRC, RARA, RELB, RORA, RUNX1, RUNX3, STAT3, TESPA1, ZBTB16, ZC3H12A, ZEB1, ZMIZ1]	[BCL11B, CAMK4, CARD11, CD8A, FOXP1, HLA-G, PNKD, PRKCZ, PTPN22, RUNX1, TOX, TSC1, ZEB1]
GO:0030098	lymphocyte differentiation	5,20E-17	[4, 5, 6, 7, 8, 9]	110,00	28,95	57,48	30,71	11,81	[ADAM8, BLNK, BTK, CAMK4, CARD11, CCL19, CCR2, CD2, CD27, CD3D, CD3E, CD3G, CD4, CD40LG, CD74, CD80, CD86, CD8A, CDKN2A, CLCF1, CLEC4D, CLEC4E, CR2, CRTAM, CTLA4, DOCK10, DOCK2, EGR3, EOMES, FCRL3, FGL2, FLT3, FOXP3, FUT7, HLA-DOA, IFNG, IKZF1, IL18, IL7R, INPP5D, IRF4, ITGA4, ITK, LAG3, LCK, LEF1, LY9, MS4A1, MYB, NCKAP1L, NLRP3, PLA2G2D, PRDM1, PTK2B, PTPN22, PTPN6, PTPRC, RHOH, RUNX3, SASH3, SEMA4A, SLAMF6, SLAMF8, SYK, TESPA1, THEMIS, TNFRSF18, TNFSF8, TOX, VNN1, WNT1, ZAP70, ZNF683]	[ADA, B2M, BATF, BCL2, CCR7, CD28, CD3G, CDH17, CDK6, CMTM7, CTLA4, FANCA, HDAC9, IRF1, JAK3, LCK, MMP14, NFATC2, PIK3CD, PSAP, PTK2B, PTPN2, PTPN22, PTPN6, PTPRC, PTPRJ, RARA, RELB, RORA, RUNX1, RUNX3, STAT3, TCF7L1, TESPA1, TSHR, ZBTB16, ZC3H12A, ZEB1, ZMIZ1]	[BCL11B, CAMK4, CARD11, CD8A, CMTM7, FOXP1, HLA-DQB2, HLA-G, PNKD, PRKCZ, PTPN22, RUNX1, TOX, TSC1, ZEB1]
GO:0002682	regulation of immune system process	2,49E-16	[2, 3]	326,00	18,70	49,60	32,44	17,96	[ADAM8, AIF1, AIM2, ANGPT1, BANK1, BLK, BLNK, BTK, BTLA, BTN3A1, BTN3A2, C1QA, C1QB, C1QC, C2, C8G, CAMK4, CARD11, CASP8, CCDC88B, CCL19, CCL24, CCR2, CD1B, CD1C, CD1E, CD2, CD200R1, CD244, CD247, CD27, CD3D, CD3E, CD3G, CD4, CD40LG, CD48, CD5, CD6, CD74, CD80, CD84, CD86, CD8A, CD8B, CD96, CDKN2A, CFP, CLCF1, CLEC1B, CLEC4D, CLEC4E, CLEC6A, CLNK, CNR1, CNR2, CR2, CRTAM, CSF1R, CTLA4, CXCL10, CXCL6, CXCR3, CYP11B1, DOCK8, EDN2, EGR3, EREG, FCN1, FCN3, FCRL3, FGL2, FGR, FOXP3, FUT7, FYB1, GBP5, GPR171, GPR55, GPSM3, H3C10, H3C11, H3C12, H3C7, HCLS1, HLA-DMB, HLA-DOA, HLA-DPA1, HLA-DPB1, HLA-DQA1, HLA-DRA, HLA-DRB1, HLA-DRB5, ICOS, IFNG, IKBKE, IL18, IL18RAP, IL7R, INPP5D, IRF4, ITGA4, ITGB7, ITK, JAML, KLRB1, KLRC1, KLRD1, KLRK1, LAG3, LAX1, LCK, LCP2, LEF1, LMO1, LPAL2, LRRC17, LST1, LTA, MIXL1, MOG, MS4A1, MUC19, MUC6, MYB, MYO1G, MZB1, NCKAP1L, NCR3, NLRP3, NLRP6, NR4A3, ORM2, P2RY12, PAX5, PILRA, PIM1, PLA2G2D, PLA2G7, PRDM1, PRKCQ, PTAFR, PTK2B, PTPN22, PTPN6, PTPRC, PYHIN1, RHEX, RHOH, RUNX3, SASH3, SELE, SELL, SH2D1A, SIT1, SLAMF6, SLAMF7, SLAMF8, STAP1, STAT1, SYK, TBC1D10C, TESPA1, THEMIS, THEMIS2, THY1, TIGIT, TLR6, TLR8, TNFAIP3, TNFAIP8L2, TNFRSF18, TNFSF13B, TNFSF8, TOX, TRAT1, TREM2, TSC22D3, VNN1, WAS, XCL1, XCL2, XG, ZAP70, ZNF683]	[ACTR3, ACVR1B, ADA, ADAM10, AIM2, ARRB2, B2M, BCL2, BIRC3, BPI, BTN3A3, CASP8, CCDC88B, CCM2, CCR7, CD101, CD160, CD28, CD38, CD3G, CD47, CD6, CDK6, COLEC11, COLEC12, CORO1A, CTLA4, CX3CL1, DOCK1, ELMO1, FANCA, FES, FYB1, GAB2, GATD3A, GBP1, GBP5, GPR68, GPRC5B, GPSM3, GRB2, GSN, HDAC1, HLA-A, HLA-B, HLA-DPA1, HLA-DPB1, HLA-DRA, IDO1, IL34, IRF1, JAK3, JAML, KLHL6, KLHL7, LAX1, LCK, LCP2, LIME1, LPXN, LRCH1, LTA, MED1, MITF, MMP14, MYO1G, NCK2, NCOR2, NFATC1, NFATC2, NLRC5, NMI, PCDHA6, PDE4B, PIK3CD, PLSCR1, PRKACB, PRKCE, PRKD2, PSMB8, PSMB9, PSMD9, PTK2B, PTPN1, PTPN2, PTPN22, PTPN6, PTPRC, PTPRJ, RARA, RELB, RIPOR2, RNF216, RORA, RUNX1, RUNX3, SLA2, SLAMF7, SLC7A2, STAT1, STAT3, STK10, TANK, TAPBPL, TBC1D10C, TCF7L1, TESPA1, TGFB2, TNF, TNFAIP3, TNFAIP8L2, TRAF3, UBASH3B, WIPF1, YAP1, ZBP1, ZBTB16, ZBTB20, ZC3H12A, ZEB1, ZMIZ1]	[ADORA1, AIM2, ALOX15, ARNT, BTNL2, CAMK4, CARD11, CARTPT, CAV1, CD8A, CD9, CHUK, COLEC11, CXADR, DOCK1, DRD2, DYSF, FOXP1, FYB1, GNAS, GPR31, HLA-DQB2, HLA-DRA, HLA-DRB1, HLA-G, HRG, ITGB2, KIR3DL2, KLK7, LILRA4, LYN, MAP3K1, MBL2, MEIS2, MMP12, MNDA, MTUS1, MUC2, MUC5B, MYH2, NFATC1, NLRC5, NR1H3, PAK1, PCDHA6, PDE4B, PNKD, PPP3CA, PRKCA, PRKCZ, PSMD7, PTPN22, PUS7, RB1, RIPOR2, RUNX1, SIGLEC15, SMAP1, SUSD4, SWAP70, TESC, TOX, TP73, TRAF2, VEGFC, WASL, ZEB1]
GO:0046649	lymphocyte activation	3,80E-16	[3, 4]	184,00	22,52	54,13	32,11	13,76	[ADAM8, AIF1, APBB1IP, BANK1, BLNK, BTK, BTLA, BTN3A1, CAMK4, CARD11, CASP8, CCDC88B, CCL19, CCR2, CD180, CD1C, CD2, CD244, CD27, CD3D, CD3E, CD3G, CD4, CD40LG, CD5, CD6, CD74, CD80, CD86, CD8A, CD8B, CDKN2A, CLCF1, CLEC4D, CLEC4E, CLNK, CR2, CRTAM, CTLA4, DOCK10, DOCK2, DOCK8, EGR3, EOMES, FCRL1, FCRL3, FCRLA, FGL2, FGR, FLT3, FOXP3, FUT7, GAPT, HLA-DMB, HLA-DOA, HLA-DPA1, HLA-DPB1, HLA-DRB1, ICOS, IFNG, IKZF1, IL18, IL7R, INPP5D, IRF4, ITGA4, ITK, JAML, KLRK1, LAG3, LAX1, LCK, LCP1, LEF1, LMO1, LST1, LY9, MS4A1, MYB, MZB1, NCKAP1L, NCR3, NLRP3, PIK3CG, PLA2G2D, PRDM1, PRF1, PRKCQ, PTK2B, PTPN22, PTPN6, PTPRC, RHOH, RUNX3, SASH3, SEMA4A, SIT1, SLAMF6, SLAMF7, SLAMF8, SYK, TBC1D10C, TESPA1, THEMIS, THY1, TIGIT, TNFAIP3, TNFAIP8L2, TNFRSF18, TNFSF13B, TNFSF8, TOX, VNN1, WAS, WNT1, XCL1, ZAP70, ZNF683]	[ADA, APBB1IP, B2M, BATF, BCL2, CASP8, CCDC88B, CCND3, CCR7, CD160, CD28, CD38, CD3G, CD44, CD47, CD6, CDH17, CDK6, CMTM7, CORO1A, CTLA4, FANCA, GATD3A, GRB2, GSN, HDAC9, HLA-A, HLA-DPA1, HLA-DPB1, IDO1, IL21R, IRF1, JAK3, JAML, KIF13B, LAX1, LCK, LCP1, MMP14, NCK2, NFATC2, PIK3CD, PRF1, PSAP, PTK2B, PTPN2, PTPN22, PTPN6, PTPRC, PTPRJ, RARA, RELB, RIPOR2, RORA, RUNX1, RUNX3, SLA2, SLAMF1, SLAMF7, STAT3, TBC1D10C, TCF7L1, TESPA1, TNFAIP3, TNFAIP8L2, TSHR, ZBTB16, ZC3H12A, ZEB1, ZMIZ1]	[BCL11B, BTNL2, CAMK4, CARD11, CAV1, CD151, CD8A, CHRNA4, CMTM7, CXADR, FOXP1, HLA-DQB2, HLA-DRB1, HLA-G, ITGB2, LCP1, LYN, MNDA, PAK1, PNKD, PPP3CA, PRDX1, PRKCZ, PTPN22, RIPOR2, RUNX1, SWAP70, TOX, TSC1, ZEB1]
GO:0045321	leukocyte activation	8,82E-15	[2, 3]	272,00	19,25	49,68	33,44	16,88	[ADAM8, AIF1, APBB1IP, BANK1, BIN2, BLNK, BTK, BTLA, BTN3A1, C1QA, CAMK4, CARD11, CASP8, CCDC88B, CCL19, CCR2, CD180, CD1C, CD2, CD244, CD27, CD3D, CD3E, CD3G, CD4, CD40LG, CD5, CD53, CD6, CD74, CD80, CD84, CD86, CD8A, CD8B, CDKN2A, CFP, CLCF1, CLEC12A, CLEC4D, CLEC4E, CLNK, CNR1, CNR2, CR2, CRTAM, CTLA4, CTSS, CXCL6, CYBB, DOCK10, DOCK2, DOCK8, EDN2, EGR3, EOMES, FCN1, FCRL1, FCRL3, FCRLA, FGL2, FGR, FLT3, FOXP3, FRMPD3, FUT7, GAPT, HLA-DMB, HLA-DOA, HLA-DPA1, HLA-DPB1, HLA-DRB1, ICOS, IFNG, IKZF1, IL18, IL18RAP, IL7R, INPP5D, IQGAP2, IRF4, ITGA4, ITK, JAML, KLRK1, LAG3, LAX1, LCK, LCP1, LCP2, LEF1, LMO1, LRMP, LST1, LY9, LYZ, MS4A1, MYB, MZB1, NAMPT, NCKAP1L, NCR3, NLRP3, NR4A3, ORM2, PIK3CG, PLA2G2D, PLAC8, PLAU, PRDM1, PRF1, PRKCQ, PTAFR, PTK2B, PTPN22, PTPN6, PTPRC, PTX3, RAB44, RHOH, RNASET2, RUNX3, SASH3, SELL, SELPLG, SEMA4A, SIT1, SLAMF6, SLAMF7, SLAMF8, SLC18A1, STAP1, SUCNR1, SYK, TBC1D10C, TESPA1, THEMIS, THY1, TIGIT, TLR6, TLR8, TNFAIP3, TNFAIP8L2, TNFRSF18, TNFSF13B, TNFSF8, TOX, TREM2, TXNDC5, UBD, VNN1, WAS, WNT1, XCL1, ZAP70, ZNF683]	[ADA, ADAM10, ADGRE5, APBB1IP, ASAH1, ATG7, B2M, BATF, BATF3, BCL2, BIN2, BPI, CAB39, CASP8, CCDC88B, CCND3, CCR7, CD160, CD28, CD38, CD3G, CD44, CD47, CD6, CDH17, CDK6, CMTM7, CORO1A, COTL1, CPNE3, CTLA4, CTSZ, CX3CL1, CYSTM1, EEF1A1, FANCA, FES, GAB2, GATD3A, GRB2, GSN, HDAC9, HLA-A, HLA-B, HLA-DPA1, HLA-DPB1, IDO1, IL21R, IRF1, JAK3, JAML, KCNAB2, KIF13B, LAX1, LCK, LCP1, LCP2, LPCAT1, MMP14, NCK2, NFATC2, PCDHA6, PIK3CD, PLSCR1, PRF1, PRKCE, PSAP, PTK2B, PTPN2, PTPN22, PTPN6, PTPRC, PTPRJ, PTPRN2, RARA, RELB, RIPOR2, RNASET2, RORA, RUNX1, RUNX3, S100A13, SBNO2, SLA2, SLAMF1, SLAMF7, SLC15A4, SLC7A2, STAT3, STK10, TBC1D10C, TCF7L1, TESPA1, TIMP2, TMC6, TNF, TNFAIP3, TNFAIP8L2, TRAF3, TSHR, UBR4, ZBTB16, ZC3H12A, ZEB1, ZMIZ1]	[AMPD3, ATP6V1D, BCL11B, BTNL2, CAMK4, CAMP, CARD11, CAV1, CD151, CD8A, CDK13, CHRNA4, CMTM7, CPLX2, CRISP3, CTSD, CXADR, DYSF, FOXP1, HLA-DQB2, HLA-DRB1, HLA-G, HYAL2, ITGB2, LCP1, LRRC7, LYN, LYZ, MMP12, MNDA, NR1H3, PAK1, PCDHA6, PDXK, PLAC8, PLPPR3, PNKD, PPP3CA, PRDX1, PRKCZ, PSMD7, PTPN22, PTPRN2, RAB37, RAB7A, RIPOR2, RUNX1, SLC15A4, SWAP70, TLR1, TOX, TSC1, ZEB1]
GO:0050863	regulation of T cell activation	3,76E-14	[5, 6, 7]	99,00	28,21	56,67	30,83	12,50	[ADAM8, AIF1, BTLA, CAMK4, CARD11, CCDC88B, CCL19, CCR2, CD2, CD27, CD3E, CD4, CD40LG, CD5, CD6, CD74, CD80, CD86, CDKN2A, CLCF1, CRTAM, CTLA4, DOCK8, EGR3, FGL2, FOXP3, HLA-DMB, HLA-DOA, HLA-DPA1, HLA-DPB1, HLA-DRB1, ICOS, IFNG, IL18, IL7R, IRF4, KLRK1, LAG3, LAX1, LCK, LEF1, LMO1, MYB, NCKAP1L, NLRP3, PLA2G2D, PRDM1, PRKCQ, PTPN22, PTPN6, PTPRC, RHOH, RUNX3, SASH3, SIT1, SYK, TESPA1, THY1, TIGIT, TNFAIP8L2, TNFRSF18, TNFSF13B, TNFSF8, TOX, VNN1, XCL1, ZAP70, ZNF683]	[ADA, CCDC88B, CCR7, CD160, CD28, CD47, CD6, CORO1A, CTLA4, FANCA, GATD3A, GRB2, GSN, HLA-A, HLA-DPA1, HLA-DPB1, IDO1, IRF1, JAK3, LAX1, LCK, NCK2, NFATC2, PTPN2, PTPN22, PTPN6, PTPRC, RARA, RIPOR2, RUNX1, RUNX3, TESPA1, TNFAIP8L2, ZBTB16, ZC3H12A, ZEB1, ZMIZ1]	[BTNL2, CAMK4, CARD11, CAV1, HLA-DQB2, HLA-DRB1, HLA-G, LYN, PAK1, PRKCZ, PTPN22, RIPOR2, RUNX1, TOX, ZEB1]
GO:1903039	positive regulation of leukocyte cell-cell adhesion	4,58E-14	[5, 6, 7]	78,00	31,84	56,52	31,52	11,96	[ADAM8, AIF1, BTLA, CARD11, CCDC88B, CCL19, CCR2, CD27, CD3E, CD4, CD40LG, CD5, CD6, CD74, CD80, CD86, CTLA4, DOCK8, EGR3, FOXP3, FUT7, HLA-DMB, HLA-DPA1, HLA-DPB1, ICOS, IFNG, IL18, IL7R, ITGA4, KLRK1, LCK, LEF1, MYB, NCKAP1L, NLRP3, NR4A3, PRKCQ, PTAFR, PTPN22, PTPN6, PTPRC, RHOH, RUNX3, SASH3, SELE, SYK, TESPA1, THY1, TNFSF13B, VNN1, XCL1, ZAP70]	[ADA, CCDC88B, CCR7, CD160, CD28, CD44, CD47, CD6, CORO1A, CTLA4, GATD3A, GRB2, HLA-A, HLA-DPA1, HLA-DPB1, JAK3, LCK, NCK2, NFAT5, PTPN22, PTPN6, PTPRC, RARA, RUNX1, RUNX3, TESPA1, TNF, ZBTB16, ZMIZ1]	[BTNL2, CARD11, CAV1, HLA-DQB2, HLA-G, ITGB2, LYN, PAK1, PRKCZ, PTPN22, RUNX1]

GO:0022409	positive regulation of cell-cell adhesion	6,73E-14	[4, 5, 6]	87,00	29,79	50,98	33,33	15,69	[ADAM8, AIF1, BTLA, CARD11, CCDC88B, CCL19, CCR2, CD27, CD3E, CD4, CD40LG, CD5, CD6, CD74, CD80, CD86, CTLA4, DOCK8, EGR3, FOXP3, FUT7, HLA-DMB, HLA-DPA1, HLA-DPB1, ICOS, IFNG, IL18, IL7R, ITGA4, KLRK1, LCK, LEF1, MYB, NCKAP1L, NLRP3, NR4A3, PRKCQ, PTAFR, PTPN22, PTPN6, PTPRC, RHOH, RUNX3, SASH3, SELE, SYK, TESPA1, THY1, TNFSF13B, VNN1, XCL1, ZAP70]	[ADA, AFDN, ANK3, BMP7, CCDC88B, CCR7, CD160, CD28, CD44, CD47, CD6, CORO1A, CTLA4, CX3CL1, GATD3A, GRB2, HLA-A, HLA-DPA1, HLA-DPB1, JAK3, LCK, NCK2, NFAT5, PIEZO1, PTPN22, PTPN6, PTPRC, RARA, RUNX1, RUNX3, TESPA1, TNF, ZBTB16, ZMIZ1]	[ALOX15, ANK3, BTNL2, CARD11, CAV1, GCNT2, HLA-DQB2, HLA-G, ITGB2, LYN, MAGI1, PAK1, PLPP3, PRKCZ, PTPN22, RUNX1]
GO:0030155	regulation of cell adhesion	1,34E-13	[3, 4]	167,00	22,12	41,24	38,14	20,62	[ADAM8, ADAMDEC1, AIF1, AKNA, ANGPT1, ANGPT2, APBB1IP, BTLA, CARD11, CCDC88B, CCL19, CCR2, CD27, CD3E, CD4, CD40LG, CD5, CD6, CD74, CD80, CD86, CDKN2A, CRTAM, CTLA4, CXCR3, CXCR4, CYTIP, DOCK8, EGR3, FGL2, FOXP3, FUT7, HLA-DMB, HLA-DPA1, HLA-DPB1, HLA-DRB1, ICOS, IFNG, IL18, IL7R, ITGA4, KLRK1, KNG1, LAG3, LAX1, LCK, LEF1, MYB, NCKAP1L, NLRP3, NR4A3, P2RY12, PIK3CG, PLA2G2D, PLAU, PLXNC1, PRKCQ, PTAFR, PTK2B, PTPN22, PTPN6, PTPRC, RHOH, RUNX3, SASH3, SELE, SEMA4D, SYK, TACSTD2, TESPA1, THY1, TIGIT, TNFAIP8L2, TNFRSF18, TNFSF13B, TNR, VNN1, WNT1, XCL1, ZAP70]	[ABAT, ACER2, ADA, ADAM10, AFDN, AKNA, ANK3, APBB1IP, BCL2, BMP7, CCDC88B, CCR7, CD160, CD28, CD44, CD47, CD6, CDK6, CORO1A, CORO1C, CTLA4, CX3CL1, CYTIP, DNM2, DOCK1, FES, GATD3A, GBP1, GRB2, GSK3B, GSN, HLA-A, HLA-DPA1, HLA-DPB1, IDO1, IRF1, JAK3, LAX1, LCK, LPXN, MAP2K5, MBP, MMP14, NCK2, NFAT5, PIEZO1, PKP4, PLXNC1, PRKCE, PRKD2, PTK2B, PTPN2, PTPN22, PTPN6, PTPRC, PTPRJ, RARA, RIN2, RIPOR2, RSU1, RUNX1, RUNX3, SEMA4D, SPOCK2, TESPA1, TGFB2, TGFBI, TNF, TNFAIP8L2, UBASH3B, WDPCP, ZBTB16, ZC3H12A, ZMIZ1]	[ALOX15, ANK3, BTNL2, CARD11, CAV1, CD9, CDH13, CDSN, DOCK1, DYSF, FMN1, GCNT2, HLA-DQB2, HLA-DRB1, HLA-G, HRG, ITGB2, LIMCH1, LYN, MAGI1, MBL2, MBP, MMP12, MYADM, MYF5, ONECUT2, PAK1, PDE3B, PLPP3, PPP3CA, PRKCA, PRKCZ, PTPN22, RIPOR2, RND1, RUNX1, SEMA5A, SWAP70, TESC, TSC1]
GO:0002684	positive regulation of immune system process	1,47E-13	[2, 3, 4]	235,00	19,70	50,00	33,09	16,91	[ADAM8, AIF1, AIM2, BLK, BLNK, BTK, BTLA, BTN3A1, BTN3A2, C1QA, C1QB, C1QC, C2, C8G, CARD11, CASP8, CCDC88B, CCL19, CCL24, CCR2, CD1B, CD1C, CD1E, CD2, CD244, CD247, CD27, CD3D, CD3E, CD3G, CD4, CD40LG, CD5, CD6, CD74, CD80, CD84, CD86, CFP, CLCF1, CLEC4D, CLEC4E, CLEC6A, CLNK, CNR1, CR2, CRTAM, CSF1R, CTLA4, CXCL10, CYP11B1, DOCK8, EDN2, EGR3, EREG, FCN1, FCN3, FCRL3, FGR, FOXP3, FUT7, FYB1, GBP5, GPSM3, HCLS1, HLA-DMB, HLA-DPA1, HLA-DPB1, HLA-DQA1, HLA-DRA, HLA-DRB1, HLA-DRB5, ICOS, IFNG, IKBKE, IL18, IL18RAP, IL7R, INPP5D, ITGA4, ITK, KLRK1, LAG3, LAX1, LCK, LCP2, LEF1, LTA, MOG, MS4A1, MUC19, MUC6, MYB, MYO1G, MZB1, NCKAP1L, NCR3, NLRP3, NR4A3, P2RY12, PAX5, PLA2G7, PRKCQ, PTAFR, PTK2B, PTPN22, PTPN6, PTPRC, PYHIN1, RHEX, RHOH, RUNX3, SASH3, SELE, SH2D1A, SLAMF6, STAP1, STAT1, SYK, TESPA1, THEMIS, THEMIS2, THY1, TLR6, TLR8, TNFRSF18, TNFSF13B, TOX, TRAT1, TREM2, VNN1, WAS, XCL1, XCL2, XG, ZAP70]	[ACTR3, ACVR1B, ADA, ADAM10, AIM2, B2M, BCL2, BTN3A3, CASP8, CCDC88B, CCR7, CD101, CD160, CD28, CD38, CD3G, CD47, CD6, COLEC11, CORO1A, CTLA4, CX3CL1, DOCK1, ELMO1, FES, FYB1, GAB2, GATD3A, GBP1, GBP5, GPR68, GPRC5B, GPSM3, GRB2, HLA-A, HLA-B, HLA-DPA1, HLA-DPB1, HLA-DRA, IDO1, IL34, JAK3, KLHL6, KLHL7, LAX1, LCK, LCP2, LIME1, LPXN, LTA, MED1, MMP14, MYO1G, NCK2, NCOR2, NFATC2, NLRC5, PDE4B, PIK3CD, PLSCR1, PRKACB, PRKCE, PRKD2, PSMB8, PSMB9, PSMD9, PTK2B, PTPN2, PTPN22, PTPN6, PTPRC, PTPRJ, RARA, RELB, RIPOR2, RORA, RUNX1, RUNX3, SLA2, STAT1, STAT3, TANK, TESPA1, TGFB2, TNF, WIPF1, ZBP1, ZBTB16, ZC3H12A, ZMIZ1]	[AIM2, ARNT, BTNL2, CARD11, CAV1, CHUK, COLEC11, DOCK1, DYSF, FOXP1, FYB1, GNAS, GPR31, HLA-DQB2, HLA-DRA, HLA-DRB1, HLA-G, HRG, ITGB2, KLK7, LYN, MBL2, MMP12, MNDA, MUC2, MUC5B, MYH2, NLRC5, PAK1, PDE4B, PNKD, PRKCA, PRKCZ, PSMD7, PTPN22, RB1, RIPOR2, RUNX1, SMAP1, SUSD4, SWAP70, TESC, TOX, TRAF2, VEGFC, WASL]
GO:0007159	leukocyte cell-cell adhesion	4,69E-13	[4]	100,00	27,03	54,62	34,45	10,92	[ADAM8, AIF1, BTLA, CARD11, CCDC88B, CCL19, CCR2, CD27, CD3E, CD4, CD40LG, CD5, CD6, CD74, CD80, CD86, CDKN2A, CRTAM, CTLA4, DOCK8, EGR3, FGL2, FOXP3, FUT7, HLA-DMB, HLA-DPA1, HLA-DPB1, HLA-DRB1, ICOS, IFNG, IL18, IL7R, ITGA4, ITGB7, KLRK1, LAG3, LAX1, LCK, LEF1, MYB, NCKAP1L, NLRP3, NR4A3, PLA2G2D, PRKCQ, PTAFR, PTPN22, PTPN6, PTPRC, RHOH, RUNX3, SASH3, SELE, SELL, SELPLG, SEMA4D, SYK, TESPA1, THY1, TIGIT, TNFAIP8L2, TNFSF13B, VNN1, XCL1, ZAP70]	[ADA, BMP7, CCDC88B, CCR7, CD160, CD28, CD44, CD47, CD6, CORO1A, CTLA4, GATD3A, GCNT1, GRB2, HLA-A, HLA-DPA1, HLA-DPB1, IDO1, IRF1, JAK3, LAX1, LCK, NCK2, NFAT5, PTPN2, PTPN22, PTPN6, PTPRC, RARA, RIPOR2, RUNX1, RUNX3, SEMA4D, STK10, TESPA1, TNF, TNFAIP8L2, TNIP1, ZBTB16, ZC3H12A, ZMIZ1]	[BTNL2, CARD11, CAV1, HLA-DQB2, HLA-DRB1, HLA-G, ITGB2, LYN, PAK1, PRKCZ, PTPN22, RIPOR2, RUNX1]
GO:0030097	hemopoiesis	4,96E-13	[4, 5, 6, 7]	198,00	20,58	47,03	34,25	18,72	[ADAM8, ANGPT1, BLNK, BTK, C1QC, CALCR, CAMK4, CARD11, CASP8, CCL19, CCR2, CD2, CD27, CD3D, CD3E, CD3G, CD4, CD40LG, CD74, CD80, CD86, CD8A, CDKN2A, CLCF1, CLEC1B, CLEC4D, CLEC4E, CR2, CRTAM, CSF1R, CTLA4, DOCK10, DOCK2, EGR3, EOMES, FCRL3, FGL2, FLT3, FOXP3, FUT7, GFI1, GPR171, GPR55, H3C10, H3C11, H3C12, H3C7, HCLS1, HLA-DOA, HLA-DRB1, IFNG, IKZF1, IL18, IL7R, INPP5D, IRF4, ITGA4, ITK, LAG3, LCK, LEF1, LMO1, LRRC17, LY9, MIXL1, MS4A1, MYB, NCKAP1L, NLRP3, NR4A3, PIM1, PLA2G2D, PLEK, PRDM1, PRKCQ, PTK2B, PTPN22, PTPN6, PTPRC, RHEX, RHOH, RTKN2, RUNX3, SASH3, SELPLG, SEMA4A, SLAMF6, SLAMF8, SNX10, STAT1, SYK, TESPA1, THEMIS, TNFRSF18, TNFSF8, TOX, TREM2, UBD, VNN1, WNT1, WNT10B, ZAP70, ZNF683]	[ACVR1B, ADA, B2M, BATF, BATF3, BCL2, CASP8, CCM2, CCR7, CD101, CD28, CD3G, CDH17, CDK6, CIAO3, CMTM7, CTLA4, DOCK1, ETV6, FANCA, FARP2, FES, GAB2, GATD3A, GPR68, HDAC1, HDAC9, HLA-B, HOXB3, HOXB7, IL34, IRF1, JAK3, KCNAB2, LCK, MED1, MFAP5, MITF, MMP14, NFATC2, PIK3CD, PIP4K2A, PSAP, PSMB8, PSMB9, PSMD9, PTK2B, PTPN2, PTPN22, PTPN6, PTPRC, PTPRJ, RARA, RELB, RORA, RUNX1, RUNX3, SBNO2, STAT1, STAT3, TCF7L1, TESPA1, TGFB2, TMEM190, TMEM91, TNF, TSHR, UBASH3B, YAP1, ZBTB16, ZC3H12A, ZEB1, ZMIZ1, ZNF160, ZNF385A]	[AGPAT5, ANLN, ARNT, ASXL1, BCL11B, CAMK4, CARD11, CARTPT, CD8A, CDK13, CMTM7, DOCK1, FOXP1, GNAS, HIPK2, HLA-DQB2, HLA-DRB1, HLA-G, HYAL2, KIRREL3, LYN, MEIS2, OSTM1, PNKD, PRKCA, PRKCZ, PSMD7, PTPN22, PTPRQ, PUS7, RB1, RUNX1, SIGLEC15, SMAP1, TESC, TNFRSF11A, TOX, TP73, TSC1, VPS33A, ZEB1]
GO:0022407	regulation of cell-cell adhesion	4,98E-13	[4, 5]	114,00	25,45	47,41	34,07	18,52	[ADAM8, AIF1, AKNA, BTLA, CARD11, CCDC88B, CCL19, CCR2, CD27, CD3E, CD4, CD40LG, CD5, CD6, CD74, CD80, CD86, CDKN2A, CRTAM, CTLA4, DOCK8, EGR3, FGL2, FOXP3, FUT7, HLA-DMB, HLA-DPA1, HLA-DPB1, HLA-DRB1, ICOS, IFNG, IL18, IL7R, ITGA4, KLRK1, LAG3, LAX1, LCK, LEF1, MYB, NCKAP1L, NLRP3, NR4A3, PLA2G2D, PRKCQ, PTAFR, PTPN22, PTPN6, PTPRC, RHOH, RUNX3, SASH3, SELE, SYK, TESPA1, THY1, TIGIT, TNFAIP8L2, TNFSF13B, TNR, VNN1, WNT1, XCL1, ZAP70]	[ABAT, ADA, AFDN, AKNA, ANK3, BMP7, CCDC88B, CCR7, CD160, CD28, CD44, CD47, CD6, CORO1A, CTLA4, CX3CL1, GATD3A, GRB2, HLA-A, HLA-DPA1, HLA-DPB1, IDO1, IRF1, JAK3, LAX1, LCK, MAP2K5, MBP, NCK2, NFAT5, PIEZO1, PTPN2, PTPN22, PTPN6, PTPRC, RARA, RIPOR2, RUNX1, RUNX3, TESPA1, TNF, TNFAIP8L2, UBASH3B, ZBTB16, ZC3H12A, ZMIZ1]	[ALOX15, ANK3, BTNL2, CARD11, CAV1, CD9, CDSN, GCNT2, HLA-DQB2, HLA-DRB1, HLA-G, ITGB2, LYN, MAGI1, MBL2, MBP, MYADM, PAK1, PLPP3, PRKCA, PRKCZ, PTPN22, RIPOR2, RUNX1, SWAP70]
GO:1902105	regulation of leukocyte differentiation	1,22E-12	[4, 5, 6, 7, 8, 9]	85,00	29,01	52,58	30,93	16,49	[ADAM8, BTK, C1QC, CAMK4, CARD11, CASP8, CCL19, CCR2, CD2, CD27, CD4, CD74, CD80, CD86, CDKN2A, CRTAM, CTLA4, EGR3, FCRL3, FGL2, FOXP3, GPR55, HCLS1, HLA-DOA, HLA-DRB1, IFNG, IL18, IL7R, INPP5D, IRF4, LAG3, LEF1, LRRC17, MYB, NCKAP1L, NLRP3, PRDM1, PTPN6, PTPRC, RHOH, RUNX3, SASH3, SLAMF8, SYK, TESPA1, TNFRSF18, TOX, TREM2, VNN1, ZAP70, ZNF683]	[ADA, CASP8, CD101, CD28, CDK6, CTLA4, FANCA, FES, GPR68, HLA-B, IL34, IRF1, JAK3, MITF, MMP14, NFATC2, PTPN2, PTPN6, PTPRC, RARA, RORA, RUNX1, RUNX3, TESPA1, TNF, UBASH3B, ZBTB16, ZC3H12A, ZEB1, ZMIZ1]	[CAMK4, CARD11, CARTPT, FOXP1, GNAS, HLA-DRB1, HLA-G, LYN, PRKCA, PRKCZ, RB1, RUNX1, SIGLEC15, TESC, TOX, ZEB1]
GO:0048534	hematopoietic or lymphoid organ development	2,00E-12	[3, 4, 5, 6]	203,00	20,20	46,67	34,67	18,67	[ADAM8, ANGPT1, BLNK, BTK, C1QC, CALCR, CAMK4, CARD11, CASP8, CCL19, CCR2, CD2, CD27, CD3D, CD3E, CD3G, CD4, CD40LG, CD74, CD80, CD86, CD8A, CDKN2A, CLCF1, CLEC1B, CLEC4D, CLEC4E, CR2, CRTAM, CSF1R, CTLA4, DOCK10, DOCK2, EGR3, EOMES, FCRL3, FGL2, FLT3, FOXP3, FUT7, GFI1, GPR171, GPR55, H3C10, H3C11, H3C12, H3C7, HCLS1, HLA-DOA, HLA-DRB1, IFNG, IKZF1, IL18, IL7R, INPP5D, IRF4, ITGA4, ITK, LAG3, LCK, LEF1, LMO1, LRRC17, LTA, LTB, LY9, MIXL1, MS4A1, MYB, NCKAP1L, NLRP3, NR4A3, PIM1, PLA2G2D, PLEK, PRDM1, PRKCQ, PTK2B, PTPN22, PTPN6, PTPRC, RHEX, RHOH, RTKN2, RUNX3, SASH3, SELPLG, SEMA4A, SLAMF6, SLAMF8, SNX10, STAT1, SYK, TESPA1, THEMIS, TNFRSF18, TNFSF8, TOX, TREM2, UBD, VNN1, WNT1, WNT10B, ZAP70, ZNF683]	[ACVR1B, ADA, B2M, BATF, BATF3, BCL2, CASP8, CCM2, CCR7, CD101, CD28, CD3G, CDH17, CDK6, CIAO3, CMTM7, CTLA4, DOCK1, ETV6, FANCA, FARP2, FES, GAB2, GATD3A, GPR68, HDAC1, HDAC9, HLA-B, HOXA3, HOXB3, HOXB7, IL34, IRF1, JAK3, JARID2, KCNAB2, LCK, LTA, MED1, MFAP5, MITF, MMP14, NFATC2, PIK3CD, PIP4K2A, PSAP, PSMB8, PSMB9, PSMD9, PTK2B, PTPN2, PTPN22, PTPN6, PTPRC, PTPRJ, RARA, RELB, RORA, RUNX1, RUNX3, SBNO2, STAT1, STAT3, TCF7L1, TESPA1, TGFB2, TMEM190, TMEM91, TNF, TSHR, UBASH3B, YAP1, ZBTB16, ZC3H12A, ZEB1, ZMIZ1, ZNF160, ZNF385A]	[AGPAT5, ANLN, ARNT, ASXL1, BCL11B, CAMK4, CARD11, CARTPT, CD8A, CDK13, CMTM7, DOCK1, FOXP1, GNAS, HIPK2, HLA-DQB2, HLA-DRB1, HLA-G, HYAL2, KIRREL3, LYN, MEIS2, OSTM1, PNKD, PRKCA, PRKCZ, PSMD7, PTPN22, PTPRQ, PUS7, RB1, RUNX1, SIGLEC15, SMAP1, TESC, TNFRSF11A, TOX, TP73, TSC1, TYR, VPS33A, ZEB1]
GO:1903037	regulation of leukocyte cell-cell adhesion	2,09E-12	[5, 6]	92,00	27,54	55,45	32,73	11,82	[ADAM8, AIF1, BTLA, CARD11, CCDC88B, CCL19, CCR2, CD27, CD3E, CD4, CD40LG, CD5, CD6, CD74, CD80, CD86, CDKN2A, CRTAM, CTLA4, DOCK8, EGR3, FGL2, FOXP3, FUT7, HLA-DMB, HLA-DPA1, HLA-DPB1, HLA-DRB1, ICOS, IFNG, IL18, IL7R, ITGA4, KLRK1, LAG3, LAX1, LCK, LEF1, MYB, NCKAP1L, NLRP3, NR4A3, PLA2G2D, PRKCQ, PTAFR, PTPN22, PTPN6, PTPRC, RHOH, RUNX3, SASH3, SELE, SYK, TESPA1, THY1, TIGIT, TNFAIP8L2, TNFSF13B, VNN1, XCL1, ZAP70]	[ADA, CCDC88B, CCR7, CD160, CD28, CD44, CD47, CD6, CORO1A, CTLA4, GATD3A, GRB2, HLA-A, HLA-DPA1, HLA-DPB1, IDO1, IRF1, JAK3, LAX1, LCK, NCK2, NFAT5, PTPN2, PTPN22, PTPN6, PTPRC, RARA, RIPOR2, RUNX1, RUNX3, TESPA1, TNF, TNFAIP8L2, ZBTB16, ZC3H12A, ZMIZ1]	[BTNL2, CARD11, CAV1, HLA-DQB2, HLA-DRB1, HLA-G, ITGB2, LYN, PAK1, PRKCZ, PTPN22, RIPOR2, RUNX1]
GO:0045785	positive regulation of cell adhesion	2,10E-11	[3, 4, 5]	111,00	24,50	44,19	36,43	19,38	[ADAM8, AIF1, ANGPT1, APBB1IP, BTLA, CARD11, CCDC88B, CCL19, CCR2, CD27, CD3E, CD4, CD40LG, CD5, CD6, CD74, CD80, CD86, CTLA4, DOCK8, EGR3, FOXP3, FUT7, HLA-DMB, HLA-DPA1, HLA-DPB1, ICOS, IFNG, IL18, IL7R, ITGA4, KLRK1, LCK, LEF1, MYB, NCKAP1L, NLRP3, NR4A3, P2RY12, PRKCQ, PTAFR, PTK2B, PTPN22, PTPN6, PTPRC, RHOH, RUNX3, SASH3, SELE, SYK, TESPA1, THY1, TNFRSF18, TNFSF13B, VNN1, XCL1, ZAP70]	[ADA, AFDN, ANK3, APBB1IP, BMP7, CCDC88B, CCR7, CD160, CD28, CD44, CD47, CD6, CDK6, CORO1A, CTLA4, CX3CL1, DNM2, DOCK1, GATD3A, GRB2, GSK3B, HLA-A, HLA-DPA1, HLA-DPB1, JAK3, LCK, NCK2, NFAT5, PIEZO1, PRKCE, PRKD2, PTK2B, PTPN22, PTPN6, PTPRC, PTPRJ, RARA, RIN2, RSU1, RUNX1, RUNX3, SPOCK2, TESPA1, TGFB2, TNF, ZBTB16, ZMIZ1]	[ALOX15, ANK3, BTNL2, CARD11, CAV1, CDH13, DOCK1, DYSF, FMN1, GCNT2, HLA-DQB2, HLA-G, HRG, ITGB2, LYN, MAGI1, MYADM, PAK1, PLPP3, PPP3CA, PRKCA, PRKCZ, PTPN22, RUNX1, TSC1]
GO:0046631	alpha-beta T cell activation	2,37E-11	[5, 6]	54,00	35,53	56,67	31,67	11,67	[CCL19, CCR2, CD3E, CD80, CD86, CRTAM, DOCK2, EOMES, FOXP3, FUT7, IFNG, IL18, IRF4, ITK, LEF1, LY9, MYB, NCKAP1L, NLRP3, PLA2G2D, PRDM1, PRKCQ, PTPN22, PTPRC, RUNX3, SASH3, SEMA4A, SLAMF6, SYK, TNFSF8, TOX, XCL1, ZAP70, ZNF683]	[ADA, BATF, BCL2, CD160, CD28, HLA-A, IRF1, JAK3, PSAP, PTPN22, PTPRC, RARA, RELB, RORA, RUNX1, RUNX3, STAT3, ZBTB16, ZC3H12A]	[BCL11B, FOXP1, HLA-DQB2, PRKCZ, PTPN22, RUNX1, TOX]
GO:0050870	positive regulation of T cell activation	2,68E-11	[5, 6, 7, 8]	69,00	30,67	56,63	31,33	12,05	[ADAM8, AIF1, BTLA, CARD11, CCDC88B, CCL19, CCR2, CD27, CD3E, CD4, CD40LG, CD5, CD6, CD74, CD80, CD86, CTLA4, DOCK8, EGR3, FOXP3, HLA-DMB, HLA-DPA1, HLA-DPB1, ICOS, IFNG, IL18, IL7R, KLRK1, LCK, LEF1, MYB, NCKAP1L, NLRP3, PRKCQ, PTPN22, PTPN6, PTPRC, RHOH, RUNX3, SASH3, SYK, TESPA1, THY1, TNFSF13B, VNN1, XCL1, ZAP70]	[ADA, CCDC88B, CCR7, CD160, CD28, CD47, CD6, CORO1A, CTLA4, GATD3A, GRB2, HLA-A, HLA-DPA1, HLA-DPB1, JAK3, LCK, NCK2, PTPN22, PTPN6, PTPRC, RARA, RUNX1, RUNX3, TESPA1, ZBTB16, ZMIZ1]	[BTNL2, CARD11, CAV1, HLA-DQB2, HLA-G, LYN, PAK1, PRKCZ, PTPN22, RUNX1]

GO:0002520	immune system development	4,79E-11	[2, 4, 5]	208,00	19,44	46,75	34,63	18,61	[ADAM8, ANGPT1, BLNK, BTK, C1QC, CALCR, CAMK4, CARD11, CASP8, CCL19, CCR2, CCR4, CD2, CD27, CD3D, CD3E, CD3G, CD4, CD40LG, CD74, CD80, CD86, CD8A, CDKN2A, CLCF1, CLEC1B, CLEC4D, CLEC4E, CR2, CRTAM, CSF1R, CTLA4, DOCK10, DOCK2, EGR3, EOMES, FCRL3, FGL2, FLT3, FOXP3, FUT7, GFI1, GPR171, GPR55, H3C10, H3C11, H3C12, H3C7, HCLS1, HLA-DOA, HLA-DRB1, ICOS, IFNG, IKZF1, IL18, IL7R, INPP5D, IRF4, ITGA4, ITK, LAG3, LCK, LEF1, LMO1, LRRC17, LTA, LTB, LY9, MIXL1, MS4A1, MYB, NCKAP1L, NLRP3, NR4A3, PIM1, PLA2G2D, PLEK, PRDM1, PRKCQ, PTK2B, PTPN22, PTPN6, PTPRC, RHEX, RHOH, RTKN2, RUNX3, SASH3, SELPLG, SEMA4A, SLAMF6, SLAMF8, SNX10, STAT1, SYK, TESPA1, THEMIS, TNFAIP3, TNFRSF18, TNFSF8, TOX, TREM2, UBD, VNN1, WNT1, WNT10B, ZAP70, ZNF683]	[ACVR1B, ADA, B2M, BATF, BATF3, BCL2, CASP8, CCM2, CCR7, CD101, CD28, CD3G, CDH17, CDK6, CIAO3, CMTM7, CTLA4, DOCK1, ETV6, FANCA, FARP2, FES, GAB2, GATD3A, GPR68, HDAC1, HDAC9, HLA-B, HOXA3, HOXB3, HOXB7, IDO1, IL34, IRF1, JAK3, JARID2, KCNAB2, LCK, LTA, MED1, MFAP5, MITF, MMP14, NFATC2, PIK3CD, PIP4K2A, PSAP, PSMB8, PSMB9, PSMD9, PTK2B, PTPN2, PTPN22, PTPN6, PTPRC, PTPRJ, RARA, RELB, RORA, RUNX1, RUNX3, SBNO2, STAT1, STAT3, TCF7L1, TESPA1, TGFB2, TMEM190, TMEM91, TNF, TNFAIP3, TSHR, UBASH3B, YAP1, ZBTB16, ZC3H12A, ZEB1, ZMIZ1, ZNF160, ZNF385A]	[AGPAT5, ANLN, ARNT, ASXL1, BCL11B, CAMK4, CARD11, CARTPT, CD8A, CDK13, CMTM7, DOCK1, FOXP1, GNAS, HIPK2, HLA-DQB2, HLA-DRB1, HLA-G, HYAL2, KIRREL3, LYN, MEIS2, OSTM1, PNKD, PRKCA, PRKCZ, PSMD7, PTPN22, PTPRQ, PUS7, RB1, RUNX1, SIGLEC15, SMAP1, SWAP70, TESC, TNFRSF11A, TOX, TP73, TSC1, TYR, VPS33A, ZEB1]
GO:0045580	regulation of T cell differentiation	1,37E-10	[6, 7, 8, 9, 10, 11]	53,00	34,64	61,29	27,42	11,29	[ADAM8, CAMK4, CARD11, CCL19, CCR2, CD2, CD27, CD74, CD80, CD86, CDKN2A, CRTAM, CTLA4, EGR3, FGL2, FOXP3, HLA-DOA, IFNG, IL18, IL7R, IRF4, LAG3, LEF1, MYB, NCKAP1L, NLRP3, PRDM1, PTPRC, RHOH, RUNX3, SASH3, SYK, TESPA1, TNFRSF18, TOX, VNN1, ZAP70, ZNF683]	[ADA, CD28, CTLA4, FANCA, IRF1, JAK3, NFATC2, PTPN2, PTPRC, RARA, RUNX1, RUNX3, TESPA1, ZBTB16, ZC3H12A, ZEB1, ZMIZ1]	[CAMK4, CARD11, HLA-G, PRKCZ, RUNX1, TOX, ZEB1]
GO:0045619	regulation of lymphocyte differentiation	1,98E-10	[5, 6, 7, 8, 9, 10]	59,00	32,24	62,32	27,54	10,14	[ADAM8, BTK, CAMK4, CARD11, CCL19, CCR2, CD2, CD27, CD74, CD80, CD86, CDKN2A, CRTAM, CTLA4, EGR3, FCRL3, FGL2, FOXP3, HLA-DOA, IFNG, IL18, IL7R, INPP5D, IRF4, LAG3, LEF1, MYB, NCKAP1L, NLRP3, PRDM1, PTPN6, PTPRC, RHOH, RUNX3, SASH3, SLAMF8, SYK, TESPA1, TNFRSF18, TOX, VNN1, ZAP70, ZNF683]	[ADA, CD28, CTLA4, FANCA, IRF1, JAK3, MMP14, NFATC2, PTPN2, PTPN6, PTPRC, RARA, RUNX1, RUNX3, TESPA1, ZBTB16, ZC3H12A, ZEB1, ZMIZ1]	[CAMK4, CARD11, HLA-G, PRKCZ, RUNX1, TOX, ZEB1]
GO:1903706	regulation of hemopoiesis	1,13E-09	[3, 4, 5, 6, 7, 8]	116,00	22,83	49,23	33,85	16,92	[ADAM8, BTK, C1QC, CAMK4, CARD11, CASP8, CCL19, CCR2, CD2, CD27, CD4, CD74, CD80, CD86, CDKN2A, CRTAM, CTLA4, EGR3, FCRL3, FGL2, FOXP3, GPR171, GPR55, H3C10, H3C11, H3C12, H3C7, HCLS1, HLA-DOA, HLA-DRB1, IFNG, IL18, IL7R, INPP5D, IRF4, LAG3, LEF1, LMO1, LRRC17, MIXL1, MYB, NCKAP1L, NLRP3, NR4A3, PIM1, PRDM1, PRKCQ, PTK2B, PTPN6, PTPRC, RHEX, RHOH, RUNX3, SASH3, SLAMF8, STAT1, SYK, TESPA1, TNFRSF18, TOX, TREM2, VNN1, ZAP70, ZNF683]	[ACVR1B, ADA, B2M, CASP8, CCM2, CD101, CD28, CDK6, CTLA4, FANCA, FES, GATD3A, GPR68, HDAC1, HLA-B, IL34, IRF1, JAK3, MED1, MITF, MMP14, NFATC2, PSMB8, PSMB9, PSMD9, PTK2B, PTPN2, PTPN6, PTPRC, RARA, RORA, RUNX1, RUNX3, STAT1, STAT3, TCF7L1, TESPA1, TNF, UBASH3B, YAP1, ZBTB16, ZC3H12A, ZEB1, ZMIZ1]	[ARNT, CAMK4, CARD11, CARTPT, FOXP1, GNAS, HLA-DRB1, HLA-G, LYN, MEIS2, PRKCA, PRKCZ, PSMD7, PUS7, RB1, RUNX1, SIGLEC15, SMAP1, TESC, TOX, TP73, ZEB1]
GO:0046632	alpha-beta T cell differentiation	1,31E-09	[6, 7, 8, 9, 10, 11]	42,00	38,18	57,78	31,11	11,11	[CCL19, CD80, CD86, EOMES, FOXP3, FUT7, IFNG, IL18, IRF4, ITK, LEF1, LY9, MYB, NCKAP1L, NLRP3, PLA2G2D, PRDM1, RUNX3, SASH3, SEMA4A, SLAMF6, SYK, TNFSF8, TOX, ZAP70, ZNF683]	[ADA, BATF, BCL2, IRF1, JAK3, PSAP, RARA, RELB, RORA, RUNX1, RUNX3, STAT3, ZBTB16, ZC3H12A]	[BCL11B, FOXP1, PRKCZ, RUNX1, TOX]
GO:0002694	regulation of leukocyte activation	7,56E-08	[3, 4, 5]	133,00	20,52	56,05	31,85	12,10	[ADAM8, AIF1, BANK1, BTK, BTLA, CAMK4, CARD11, CCDC88B, CCL19, CCR2, CD2, CD27, CD3E, CD4, CD40LG, CD5, CD6, CD74, CD80, CD84, CD86, CDKN2A, CLCF1, CLEC4D, CLNK, CNR1, CNR2, CRTAM, CTLA4, DOCK8, EGR3, FCRL3, FGL2, FGR, FOXP3, HLA-DMB, HLA-DOA, HLA-DPA1, HLA-DPB1, HLA-DRB1, ICOS, IFNG, IL18, IL7R, INPP5D, IRF4, KLRK1, LAG3, LAX1, LCK, LEF1, LMO1, LST1, MYB, MZB1, NCKAP1L, NLRP3, NR4A3, PLA2G2D, PRDM1, PRKCQ, PTAFR, PTPN22, PTPN6, PTPRC, RHOH, RUNX3, SASH3, SIT1, SLAMF8, STAP1, SYK, TBC1D10C, TESPA1, THY1, TIGIT, TLR6, TNFAIP3, TNFAIP8L2, TNFRSF18, TNFSF13B, TNFSF8, TOX, TREM2, VNN1, XCL1, ZAP70, ZNF683]	[ADA, BCL2, BPI, CCDC88B, CCR7, CD160, CD28, CD38, CD47, CD6, CORO1A, CTLA4, CX3CL1, FANCA, FES, GAB2, GATD3A, GRB2, GSN, HLA-A, HLA-DPA1, HLA-DPB1, IDO1, IRF1, JAK3, LAX1, LCK, MMP14, NCK2, NFATC2, PCDHA6, PLSCR1, PTPN2, PTPN22, PTPN6, PTPRC, RARA, RIPOR2, RORA, RUNX1, RUNX3, SLC7A2, TBC1D10C, TESPA1, TNFAIP3, TNFAIP8L2, ZBTB16, ZC3H12A, ZEB1, ZMIZ1]	[BTNL2, CAMK4, CARD11, CAV1, HLA-DQB2, HLA-DRB1, HLA-G, ITGB2, LYN, MNDA, NR1H3, PAK1, PCDHA6, PRKCZ, PTPN22, RIPOR2, RUNX1, TOX, ZEB1]
GO:1902107	positive regulation of leukocyte differentiation	1,92E-07	[4, 5, 6, 7, 8, 9, 10]	49,00	30,63	55,36	28,57	16,07	[ADAM8, BTK, CASP8, CCL19, CD27, CD4, CD74, CD80, CD86, EGR3, FOXP3, HCLS1, HLA-DRB1, IFNG, IL18, IL7R, INPP5D, LEF1, MYB, NCKAP1L, NLRP3, PTPRC, RHOH, RUNX3, SASH3, SYK, TESPA1, TOX, TREM2, VNN1, ZAP70]	[ADA, CASP8, CD101, FES, GPR68, IL34, MMP14, PTPRC, RARA, RORA, RUNX1, RUNX3, TESPA1, TNF, ZBTB16, ZMIZ1]	[GNAS, HLA-DRB1, HLA-G, PRKCA, PRKCZ, RB1, RUNX1, TESC, TOX]
GO:0050865	regulation of cell activation	2,31E-07	[3, 4]	139,00	19,97	54,60	31,90	13,50	[ADAM8, AIF1, BANK1, BTK, BTLA, CAMK4, CARD11, CCDC88B, CCL19, CCR2, CD2, CD27, CD3E, CD4, CD40LG, CD5, CD6, CD74, CD80, CD84, CD86, CDKN2A, CLCF1, CLEC4D, CLNK, CNR1, CNR2, CRTAM, CTLA4, DOCK8, EGR3, FCRL3, FGL2, FGR, FOXP3, HLA-DMB, HLA-DOA, HLA-DPA1, HLA-DPB1, HLA-DRB1, ICOS, IFNG, IL18, IL7R, INPP5D, IRF4, KLRK1, LAG3, LAX1, LCK, LEF1, LMO1, LST1, MYB, MZB1, NCKAP1L, NLRP3, NR4A3, PLA2G2D, PLEK, PRDM1, PRKCQ, PTAFR, PTPN22, PTPN6, PTPRC, RHOH, RUNX3, SASH3, SIT1, SLAMF8, STAP1, SYK, TBC1D10C, TESPA1, THY1, TIGIT, TLR6, TNFAIP3, TNFAIP8L2, TNFRSF18, TNFSF13B, TNFSF8, TOX, TREM2, VNN1, XCL1, ZAP70, ZNF683]	[ABAT, ADA, BCL2, BPI, CCDC88B, CCR7, CD160, CD28, CD38, CD47, CD6, CORO1A, CTLA4, CX3CL1, FANCA, FES, GAB2, GATD3A, GRB2, GSN, HLA-A, HLA-DPA1, HLA-DPB1, IDO1, IRF1, JAK3, LAX1, LCK, MMP14, NCK2, NFATC2, PCDHA6, PLSCR1, PTPN2, PTPN22, PTPN6, PTPRC, RARA, RIPOR2, RORA, RUNX1, RUNX3, SLC7A2, TBC1D10C, TESPA1, TNFAIP3, TNFAIP8L2, UBASH3B, ZBTB16, ZC3H12A, ZEB1, ZMIZ1]	[BTNL2, CAMK4, CARD11, CAV1, CD9, HLA-DQB2, HLA-DRB1, HLA-G, HRG, ITGB2, LYN, MNDA, NR1H3, PAK1, PCDHA6, PRKCA, PRKCZ, PTPN22, RIPOR2, RUNX1, TOX, ZEB1]
GO:0046635	positive regulation of alpha-beta T cell activation	5,14E-07	[6, 7, 8, 9]	28,00	42,42	57,58	30,30	12,12	[CCL19, CCR2, CD3E, CD80, CD86, FOXP3, IFNG, IL18, MYB, NCKAP1L, NLRP3, PRKCQ, PTPN22, PTPRC, RUNX3, SASH3, SYK, XCL1, ZAP70]	[ADA, CD160, CD28, HLA-A, PTPN22, PTPRC, RARA, RUNX1, RUNX3, ZBTB16]	[HLA-DQB2, PRKCZ, PTPN22, RUNX1]
GO:0046634	regulation of alpha-beta T cell activation	1,75E-06	[6, 7, 8]	35,00	35,00	57,50	32,50	10,00	[CCL19, CCR2, CD3E, CD80, CD86, CRTAM, FOXP3, IFNG, IL18, IRF4, MYB, NCKAP1L, NLRP3, PRDM1, PRKCQ, PTPN22, PTPRC, RUNX3, SASH3, SYK, XCL1, ZAP70, ZNF683]	[ADA, CD160, CD28, HLA-A, IRF1, JAK3, PTPN22, PTPRC, RARA, RUNX1, RUNX3, ZBTB16, ZC3H12A]	[HLA-DQB2, PRKCZ, PTPN22, RUNX1]
GO:1903708	positive regulation of hemopoiesis	1,93E-06	[3, 4, 5, 6, 7, 8, 9]	56,00	27,18	51,56	31,25	17,19	[ADAM8, BTK, CASP8, CCL19, CD27, CD4, CD74, CD80, CD86, EGR3, FOXP3, HCLS1, HLA-DRB1, IFNG, IL18, IL7R, INPP5D, LEF1, MYB, NCKAP1L, NLRP3, PTPRC, RHEX, RHOH, RUNX3, SASH3, STAT1, SYK, TESPA1, TOX, TREM2, VNN1, ZAP70]	[ACVR1B, ADA, CASP8, CD101, FES, GPR68, IL34, MED1, MMP14, PTPRC, RARA, RORA, RUNX1, RUNX3, STAT1, STAT3, TESPA1, TNF, ZBTB16, ZMIZ1]	[ARNT, GNAS, HLA-DRB1, HLA-G, PRKCA, PRKCZ, RB1, RUNX1, SMAP1, TESC, TOX]
GO:0051249	regulation of lymphocyte activation	2,14E-06	[4, 5, 6]	114,00	20,50	57,66	30,66	11,68	[ADAM8, AIF1, BANK1, BTK, BTLA, CAMK4, CARD11, CCDC88B, CCL19, CCR2, CD2, CD27, CD3E, CD4, CD40LG, CD5, CD6, CD74, CD80, CD86, CDKN2A, CLCF1, CLNK, CRTAM, CTLA4, DOCK8, EGR3, FCRL3, FGL2, FGR, FOXP3, HLA-DMB, HLA-DOA, HLA-DPA1, HLA-DPB1, HLA-DRB1, ICOS, IFNG, IL18, IL7R, INPP5D, IRF4, KLRK1, LAG3, LAX1, LCK, LEF1, LMO1, LST1, MYB, MZB1, NCKAP1L, NLRP3, PLA2G2D, PRDM1, PRKCQ, PTPN22, PTPN6, PTPRC, RHOH, RUNX3, SASH3, SIT1, SLAMF8, SYK, TBC1D10C, TESPA1, THY1, TIGIT, TNFAIP3, TNFAIP8L2, TNFRSF18, TNFSF13B, TNFSF8, TOX, VNN1, XCL1, ZAP70, ZNF683]	[ADA, BCL2, CCDC88B, CCR7, CD160, CD28, CD38, CD47, CD6, CORO1A, CTLA4, FANCA, GATD3A, GRB2, GSN, HLA-A, HLA-DPA1, HLA-DPB1, IDO1, IRF1, JAK3, LAX1, LCK, MMP14, NCK2, NFATC2, PTPN2, PTPN22, PTPN6, PTPRC, RARA, RIPOR2, RUNX1, RUNX3, TBC1D10C, TESPA1, TNFAIP3, TNFAIP8L2, ZBTB16, ZC3H12A, ZEB1, ZMIZ1]	[BTNL2, CAMK4, CARD11, CAV1, HLA-DQB2, HLA-DRB1, HLA-G, LYN, MNDA, PAK1, PRKCZ, PTPN22, RIPOR2, RUNX1, TOX, ZEB1]
GO:0043367	CD4-positive, alpha-beta T cell differentiation	9,89E-06	[7, 8, 9, 10, 11, 12]	29,00	37,18	59,38	28,13	12,50	[CCL19, CD80, CD86, FOXP3, FUT7, IFNG, IL18, IRF4, LEF1, LY9, MYB, NCKAP1L, NLRP3, PLA2G2D, RUNX3, SASH3, SEMA4A, SLAMF6, TOX]	[BATF, JAK3, RARA, RELB, RORA, RUNX1, RUNX3, STAT3, ZC3H12A]	[FOXP1, PRKCZ, RUNX1, TOX]
GO:0002292	T cell differentiation involved in immune response	1,32E-05	[6, 7, 8, 9, 10, 11]	27,00	38,57	62,96	25,93	11,11	[CCL19, CD80, CD86, CLEC4D, CLEC4E, EOMES, FGL2, FOXP3, IFNG, IL18, IRF4, LEF1, LY9, MYB, NLRP3, SEMA4A, SLAMF6]	[BATF, JAK3, RARA, RELB, RORA, STAT3, ZC3H12A]	[FOXP1, PRKCZ, TSC1]
GO:0070661	leukocyte proliferation	1,96E-05	[3]	75,00	22,80	63,22	27,59	9,20	[AIF1, BTK, BTN3A1, CARD11, CCDC88B, CCL19, CCR2, CD180, CD3E, CD4, CD40LG, CD6, CD74, CD80, CD86, CDKN2A, CLCF1, CR2, CRTAM, CSF1R, CTLA4, DOCK2, DOCK8, FCRL3, FLT3, FOXP3, GAPT, HLA-DMB, HLA-DPA1, HLA-DPB1, HLA-DRB1, IL18, IL5RA, IL7R, INPP5D, LEF1, LMO1, LST1, MS4A1, MZB1, NCKAP1L, PIK3CG, PLA2G2D, PRKCQ, PTPN22, PTPN6, PTPRC, SASH3, SYK, TNFAIP3, TNFSF13B, TNFSF8, TREM2, XCL1, ZAP70]	[ADA, BCL2, CCDC88B, CCND3, CD28, CD38, CD6, CORO1A, CTLA4, CX3CL1, GATD3A, HLA-A, HLA-DPA1, HLA-DPB1, IDO1, IL34, IRF1, JAK3, NCK2, NFATC2, PTPN22, PTPN6, PTPRC, TNFAIP3]	[BTNL2, CARD11, CD151, HLA-DRB1, HLA-G, LYN, MNDA, PTPN22]
GO:0046651	lymphocyte proliferation	3,02E-05	[4, 5]	69,00	23,23	63,75	26,25	10,00	[AIF1, BTK, BTN3A1, CARD11, CCDC88B, CCL19, CCR2, CD180, CD3E, CD4, CD40LG, CD6, CD74, CD80, CD86, CDKN2A, CLCF1, CR2, CRTAM, CTLA4, DOCK2, DOCK8, FCRL3, FLT3, FOXP3, GAPT, HLA-DMB, HLA-DPA1, HLA-DPB1, HLA-DRB1, IL18, IL7R, INPP5D, LEF1, LMO1, LST1, MS4A1, MZB1, NCKAP1L, PIK3CG, PLA2G2D, PRKCQ, PTPN22, PTPN6, PTPRC, SASH3, SYK, TNFSF13B, TNFSF8, XCL1, ZAP70]	[ADA, BCL2, CCDC88B, CCND3, CD28, CD38, CD6, CORO1A, CTLA4, GATD3A, HLA-A, HLA-DPA1, HLA-DPB1, IDO1, IRF1, JAK3, NCK2, NFATC2, PTPN22, PTPN6, PTPRC]	[BTNL2, CARD11, CD151, HLA-DRB1, HLA-G, LYN, MNDA, PTPN22]
GO:0035710	CD4-positive, alpha-beta T cell activation	3,56E-05	[6, 7]	32,00	33,33	60,00	28,57	11,43	[CCL19, CD80, CD86, FOXP3, FUT7, IFNG, IL18, IRF4, LEF1, LY9, MYB, NCKAP1L, NLRP3, PLA2G2D, PRKCQ, RUNX3, SASH3, SEMA4A, SLAMF6, TOX, XCL1]	[BATF, CD160, JAK3, RARA, RELB, RORA, RUNX1, RUNX3, STAT3, ZC3H12A]	[FOXP1, PRKCZ, RUNX1, TOX]
GO:0045621	positive regulation of lymphocyte differentiation	3,86E-05	[5, 6, 7, 8, 9, 10, 11]	34,00	32,08	66,67	23,08	10,26	[ADAM8, BTK, CCL19, CD27, CD74, CD80, CD86, EGR3, FOXP3, IFNG, IL18, IL7R, INPP5D, LEF1, MYB, NCKAP1L, NLRP3, PTPRC, RHOH, RUNX3, SASH3, SYK, TESPA1, TOX, VNN1, ZAP70]	[ADA, MMP14, PTPRC, RARA, RUNX1, RUNX3, TESPA1, ZBTB16, ZMIZ1]	[HLA-G, PRKCZ, RUNX1, TOX]
GO:0070665	positive regulation of leukocyte proliferation	8,02E-05	[4, 5, 6]	43,00	27,74	58,82	33,33	7,84	[AIF1, CARD11, CCDC88B, CCL19, CCR2, CD3E, CD4, CD40LG, CD6, CD74, CD80, CD86, CLCF1, CSF1R, FCRL3, FOXP3, HLA-DMB, HLA-DPA1, HLA-DPB1, IL18, IL5RA, NCKAP1L, PRKCQ, PTPN22, PTPRC, SASH3, SYK, TNFSF13B, XCL1, ZAP70]	[ADA, BCL2, CCDC88B, CD28, CD38, CD6, CORO1A, GATD3A, HLA-A, HLA-DPA1, HLA-DPB1, IL34, JAK3, NCK2, NFATC2, PTPN22, PTPRC]	[BTNL2, CARD11, LYN, PTPN22]
GO:0070663	regulation of leukocyte proliferation	1,04E-04	[4, 5]	60,00	23,81	59,72	30,56	9,72	[AIF1, BTK, CARD11, CCDC88B, CCL19, CCR2, CD3E, CD4, CD40LG, CD6, CD74, CD80, CD86, CDKN2A, CLCF1, CRTAM, CSF1R, CTLA4, FCRL3, FOXP3, HLA-DMB, HLA-DPA1, HLA-DPB1, HLA-DRB1, IL18, IL5RA, INPP5D, LMO1, LST1, MZB1, NCKAP1L, PLA2G2D, PRKCQ, PTPN22, PTPN6, PTPRC, SASH3, SYK, TNFAIP3, TNFSF13B, TNFSF8, XCL1, ZAP70]	[ADA, BCL2, CCDC88B, CD28, CD38, CD6, CORO1A, CTLA4, GATD3A, HLA-A, HLA-DPA1, HLA-DPB1, IDO1, IL34, IRF1, JAK3, NCK2, NFATC2, PTPN22, PTPN6, PTPRC, TNFAIP3]	[BTNL2, CARD11, HLA-DRB1, HLA-G, LYN, MNDA, PTPN22]
GO:0050670	regulation of lymphocyte proliferation	1,36E-04	[5, 6, 7]	56,00	24,35	59,70	29,85	10,45	[AIF1, BTK, CARD11, CCDC88B, CCL19, CCR2, CD3E, CD4, CD40LG, CD6, CD74, CD80, CD86, CDKN2A, CLCF1, CRTAM, CTLA4, FCRL3, FOXP3, HLA-DMB, HLA-DPA1, HLA-DPB1, HLA-DRB1, IL18, INPP5D, LMO1, LST1, MZB1, NCKAP1L, PLA2G2D, PRKCQ, PTPN22, PTPN6, PTPRC, SASH3, SYK, TNFSF13B, TNFSF8, XCL1, ZAP70]	[ADA, BCL2, CCDC88B, CD28, CD38, CD6, CORO1A, CTLA4, GATD3A, HLA-A, HLA-DPA1, HLA-DPB1, IDO1, IRF1, JAK3, NCK2, NFATC2, PTPN22, PTPN6, PTPRC]	[BTNL2, CARD11, HLA-DRB1, HLA-G, LYN, MNDA, PTPN22]
GO:0045582	positive regulation of T cell differentiation	2,25E-04	[6, 7, 8, 9, 10, 11, 12]	30,00	32,26	67,65	23,53	8,82	[ADAM8, CCL19, CD27, CD74, CD80, CD86, EGR3, FOXP3, IFNG, IL18, IL7R, LEF1, MYB, NCKAP1L, NLRP3, PTPRC, RHOH, RUNX3, SASH3, SYK, TESPA1, VNN1, ZAP70]	[ADA, PTPRC, RARA, RUNX1, RUNX3, TESPA1, ZBTB16, ZMIZ1]	[HLA-G, PRKCZ, RUNX1]
GO:0042102	positive regulation of T cell proliferation	2,30E-04	[6, 7, 8, 9]	32,00	31,07	62,50	30,00	7,50	[AIF1, CARD11, CCDC88B, CCL19, CCR2, CD3E, CD4, CD40LG, CD6, CD80, CD86, FOXP3, HLA-DMB, HLA-DPA1, HLA-DPB1, IL18, NCKAP1L, PRKCQ, PTPN22, PTPRC, SASH3, SYK, TNFSF13B, XCL1, ZAP70]	[CCDC88B, CD28, CD6, CORO1A, GATD3A, HLA-A, HLA-DPA1, HLA-DPB1, JAK3, NCK2, PTPN22, PTPRC]	[BTNL2, CARD11, PTPN22]
GO:0050671	positive regulation of lymphocyte proliferation	4,48E-04	[5, 6, 7, 8]	39,00	27,46	59,57	34,04	6,38	[AIF1, CARD11, CCDC88B, CCL19, CCR2, CD3E, CD4, CD40LG, CD6, CD74, CD80, CD86, CLCF1, FCRL3, FOXP3, HLA-DMB, HLA-DPA1, HLA-DPB1, IL18, NCKAP1L, PRKCQ, PTPN22, PTPRC, SASH3, SYK, TNFSF13B, XCL1, ZAP70]	[ADA, BCL2, CCDC88B, CD28, CD38, CD6, CORO1A, GATD3A, HLA-A, HLA-DPA1, HLA-DPB1, JAK3, NCK2, NFATC2, PTPN22, PTPRC]	[BTNL2, CARD11, PTPN22]
GO:0042098	T cell proliferation	4,98E-04	[5, 6]	50,00	24,51	62,30	27,87	9,84	[AIF1, BTN3A1, CARD11, CCDC88B, CCL19, CCR2, CD3E, CD4, CD40LG, CD6, CD80, CD86, CDKN2A, CLCF1, CRTAM, CTLA4, DOCK2, DOCK8, FOXP3, HLA-DMB, HLA-DPA1, HLA-DPB1, HLA-DRB1, IL18, LMO1, NCKAP1L, PIK3CG, PLA2G2D, PRKCQ, PTPN22, PTPN6, PTPRC, SASH3, SYK, TNFSF13B, TNFSF8, XCL1, ZAP70]	[CCDC88B, CCND3, CD28, CD6, CORO1A, CTLA4, GATD3A, HLA-A, HLA-DPA1, HLA-DPB1, IDO1, IRF1, JAK3, NCK2, PTPN22, PTPN6, PTPRC]	[BTNL2, CARD11, CD151, HLA-DRB1, HLA-G, PTPN22]
GO:0002293	alpha-beta T cell differentiation involved in immune response	5,53E-04	[7, 8, 9, 10, 11, 12]	23,00	36,51	60,87	30,43	8,70	[CCL19, CD80, CD86, EOMES, FOXP3, IFNG, IL18, IRF4, LEF1, LY9, MYB, NLRP3, SEMA4A, SLAMF6]	[BATF, JAK3, RARA, RELB, RORA, STAT3, ZC3H12A]	[FOXP1, PRKCZ]
GO:0002286	T cell activation involved in immune response	9,64E-04	[5, 6]	32,00	29,36	62,86	25,71	11,43	[APBB1IP, CCL19, CD1C, CD74, CD80, CD86, CLEC4D, CLEC4E, EOMES, FGL2, FOXP3, HLA-DMB, IFNG, IL18, IRF4, LCP1, LEF1, LY9, MYB, NLRP3, SEMA4A, SLAMF6]	[APBB1IP, BATF, JAK3, LCP1, RARA, RELB, RORA, STAT3, ZC3H12A]	[FOXP1, LCP1, PRKCZ, TSC1]
GO:0046637	regulation of alpha-beta T cell differentiation	1,05E-03	[7, 8, 9, 10, 11, 12]	23,00	35,38	64,00	28,00	8,00	[CCL19, CD80, CD86, FOXP3, IFNG, IL18, IRF4, MYB, NCKAP1L, NLRP3, PRDM1, RUNX3, SASH3, SYK, ZAP70, ZNF683]	[ADA, JAK3, RARA, RUNX1, RUNX3, ZBTB16, ZC3H12A]	[PRKCZ, RUNX1]

GO:0002250	adaptive immune response	1,08E-03	[3]	133,00	17,71	62,09	26,14	11,76	[BTK, BTLA, BTN3A1, BTN3A2, C1QA, C1QB, C1QC, C2, C8G, CAMK4, CCL19, CCR2, CD1B, CD1C, CD1E, CD244, CD247, CD27, CD3D, CD3E, CD3G, CD4, CD40LG, CD48, CD6, CD74, CD80, CD84, CD86, CD8A, CD8B, CLCF1, CLEC4D, CLEC6A, CR2, CRTAM, CTLA4, CTSS, CXCL10, EOMES, FGL2, FOXP3, FUT7, GAPT, HLA-DMA, HLA-DMB, HLA-DOA, HLA-DPA1, HLA-DPB1, HLA-DQA1, HLA-DRA, HLA-DRB1, HLA-DRB5, IFNG, IL18, IL18RAP, IL7R, INPP5D, IRF4, ITK, JCHAIN, KLRK1, LAG3, LAMP3, LAX1, LEF1, LTA, LY9, MCOLN2, MYO1G, NLRP3, NR4A3, PIK3CG, PRDM1, PRF1, PRKCQ, PTK2B, PTPN6, PTPRC, SASH3, SEMA4A, SH2D1A, SIT1, SLAMF6, SLAMF7, SYK, THEMIS, TLR8, TNFAIP3, TNFSF13B, TRAT1, WAS, XCL1, ZAP70, ZNF683]	[ADA, B2M, BATF, BTN3A3, CD160, CD28, CD3G, CD6, CTLA4, HLA-A, HLA-B, HLA-DPA1, HLA-DPB1, HLA-DRA, IRF1, JAK3, KLHL6, KLHL7, LAX1, LIME1, LTA, MYO1G, NCOR2, PIK3CD, PRF1, PRKD2, PTK2B, PTPN6, PTPRC, RELB, RORA, SLA2, SLAMF1, SLAMF7, STAT3, TANK, TAP1, TNF, TNFAIP3, ZC3H12A]	[ALOX15, CAMK4, CD8A, HLA-DQB2, HLA-DRA, HLA-DRB1, HLA-G, JCHAIN, LYN, MBL2, ORAI1, PNKD, PRKCZ, SUSD4, SWAP70, TNFRSF11A, TRAF2, TSC1]
GO:0042129	regulation of T cell proliferation	1,27E-03	[6, 7, 8]	44,00	25,14	61,82	29,09	9,09	[AIF1, CARD11, CCDC88B, CCL19, CCR2, CD3E, CD4, CD40LG, CD6, CD80, CD86, CDKN2A, CLCF1, CRTAM, CTLA4, FOXP3, HLA-DMB, HLA-DPA1, HLA-DPB1, HLA-DRB1, IL18, LMO1, NCKAP1L, PLA2G2D, PRKCQ, PTPN22, PTPN6, PTPRC, SASH3, SYK, TNFSF13B, TNFSF8, XCL1, ZAP70]	[CCDC88B, CD28, CD6, CORO1A, CTLA4, GATD3A, HLA-A, HLA-DPA1, HLA-DPB1, IDO1, IRF1, JAK3, NCK2, PTPN22, PTPN6, PTPRC]	[BTNL2, CARD11, HLA-DRB1, HLA-G, PTPN22]
GO:0050867	positive regulation of cell activation	1,68E-03	[3, 4, 5]	89,00	19,56	58,65	29,81	11,54	[ADAM8, AIF1, BTK, BTLA, CARD11, CCDC88B, CCL19, CCR2, CD2, CD27, CD3E, CD4, CD40LG, CD5, CD6, CD74, CD80, CD86, CLCF1, CLEC4D, CTLA4, DOCK8, EGR3, FCRL3, FGR, FOXP3, HLA-DMB, HLA-DPA1, HLA-DPB1, ICOS, IFNG, IL18, IL7R, INPP5D, KLRK1, LCK, LEF1, MYB, NCKAP1L, NLRP3, NR4A3, PLEK, PRKCQ, PTAFR, PTPN22, PTPN6, PTPRC, RHOH, RUNX3, SASH3, STAP1, SYK, TESPA1, THY1, TLR6, TNFSF13B, TOX, TREM2, VNN1, XCL1, ZAP70]	[ADA, BCL2, CCDC88B, CCR7, CD160, CD28, CD38, CD47, CD6, CORO1A, CTLA4, GAB2, GATD3A, GRB2, HLA-A, HLA-DPA1, HLA-DPB1, JAK3, LCK, MMP14, NCK2, NFATC2, PTPN22, PTPN6, PTPRC, RARA, RUNX1, RUNX3, TESPA1, ZBTB16, ZMIZ1]	[BTNL2, CARD11, CAV1, HLA-DQB2, HLA-G, ITGB2, LYN, PAK1, PRKCZ, PTPN22, RUNX1, TOX]
GO:0002285	lymphocyte activation involved in immune response	2,78E-03	[4, 5]	46,00	23,96	60,78	29,41	9,80	[APBB1IP, CCL19, CD180, CD1C, CD244, CD40LG, CD74, CD80, CD86, CLCF1, CLEC4D, CLEC4E, DOCK10, EOMES, FGL2, FOXP3, GAPT, HLA-DMB, IFNG, IL18, IRF4, LCP1, LEF1, LY9, MYB, NLRP3, PTK2B, PTPRC, SEMA4A, SLAMF6, ZNF683]	[ADA, APBB1IP, BATF, CD28, CDH17, CORO1A, JAK3, LCP1, PTK2B, PTPRC, RARA, RELB, RORA, STAT3, ZC3H12A]	[FOXP1, LCP1, PRKCZ, SWAP70, TSC1]
GO:0051251	positive regulation of lymphocyte activation	4,26E-03	[4, 5, 6, 7]	78,00	19,90	55,91	32,26	11,83	[ADAM8, AIF1, BTK, BTLA, CARD11, CCDC88B, CCL19, CCR2, CD27, CD3E, CD4, CD40LG, CD5, CD6, CD74, CD80, CD86, CLCF1, CTLA4, DOCK8, EGR3, FCRL3, FOXP3, HLA-DMB, HLA-DPA1, HLA-DPB1, ICOS, IFNG, IL18, IL7R, INPP5D, KLRK1, LCK, LEF1, MYB, NCKAP1L, NLRP3, PRKCQ, PTPN22, PTPN6, PTPRC, RHOH, RUNX3, SASH3, SYK, TESPA1, THY1, TNFSF13B, TOX, VNN1, XCL1, ZAP70]	[ADA, BCL2, CCDC88B, CCR7, CD160, CD28, CD38, CD47, CD6, CORO1A, CTLA4, GATD3A, GRB2, HLA-A, HLA-DPA1, HLA-DPB1, JAK3, LCK, MMP14, NCK2, NFATC2, PTPN22, PTPN6, PTPRC, RARA, RUNX1, RUNX3, TESPA1, ZBTB16, ZMIZ1]	[BTNL2, CARD11, CAV1, HLA-DQB2, HLA-G, LYN, PAK1, PRKCZ, PTPN22, RUNX1, TOX]
GO:0046638	positive regulation of alpha-beta T cell differentiation	4,82E-03	[7, 8, 9, 10, 11, 12, 13]	18,00	38,30	65,00	25,00	10,00	[CCL19, CD80, CD86, FOXP3, IFNG, IL18, MYB, NCKAP1L, NLRP3, RUNX3, SASH3, SYK, ZAP70]	[ADA, RARA, RUNX1, RUNX3, ZBTB16]	[PRKCZ, RUNX1]
GO:0032753	positive regulation of interleukin-4 production	4,90E-03	[4, 5, 6, 7, 8]	12,00	52,17	75,00	16,67	8,33	[CD3E, CD40LG, CD86, FOXP3, IRF4, NLRP3, PRKCQ, SASH3, SYK]	[CD28, RARA]	[PRKCZ]
GO:2000516	positive regulation of CD4-positive, alpha-beta T cell activation	6,37E-03	[7, 8, 9, 10]	15,00	42,86	80,00	13,33	6,67	[CCL19, CD80, CD86, FOXP3, IFNG, IL18, MYB, NCKAP1L, NLRP3, PRKCQ, SASH3, XCL1]	[CD160, RARA]	[PRKCZ]
GO:0002573	myeloid leukocyte differentiation	8,26E-03	[5, 6, 7, 8, 9]	50,00	22,52	35,19	40,74	24,07	[C1QC, CALCR, CAMK4, CASP8, CCL19, CD4, CD74, CSF1R, GPR55, HCLS1, HLA-DRB1, IFNG, INPP5D, IRF4, LEF1, LRRC17, SNX10, TREM2, UBD]	[BATF, BATF3, CASP8, CCR7, CD101, CDK6, FARP2, FES, GAB2, GPR68, IL34, MED1, MITF, PIK3CD, PTPN2, RARA, RELB, RORA, RUNX1, SBNO2, TNF, UBASH3B]	[CAMK4, CARTPT, FOXP1, GNAS, HLA-DRB1, LYN, OSTM1, PRKCA, RB1, RUNX1, SIGLEC15, TESC, TNFRSF11A]
GO:0002761	regulation of myeloid leukocyte differentiation	8,88E-03	[5, 6, 7, 8, 9, 10]	33,00	26,40	35,14	35,14	29,73	[C1QC, CAMK4, CASP8, CD4, CD74, GPR55, HCLS1, HLA-DRB1, IFNG, INPP5D, LEF1, LRRC17, TREM2]	[CASP8, CD101, CDK6, FES, GPR68, IL34, MITF, PTPN2, RARA, RORA, RUNX1, TNF, UBASH3B]	[CAMK4, CARTPT, FOXP1, GNAS, HLA-DRB1, LYN, PRKCA, RB1, RUNX1, SIGLEC15, TESC]
GO:0007162	negative regulation of cell adhesion	1,88E-02	[3, 4, 5]	61,00	20,54	38,16	38,16	23,68	[ADAMDEC1, AKNA, ANGPT1, ANGPT2, CD74, CD80, CD86, CDKN2A, CRTAM, CTLA4, FGL2, FOXP3, HLA-DRB1, KNG1, LAG3, LAX1, PLA2G2D, PLXNC1, PTPN22, PTPN6, PTPRC, RUNX3, SEMA4D, TACSTD2, TIGIT, TNFAIP8L2, TNR, WNT1, XCL1]	[ABAT, ACER2, ADAM10, AKNA, CORO1C, CTLA4, CX3CL1, GBP1, IDO1, IRF1, JAK3, LAX1, LPXN, MAP2K5, MBP, MMP14, PLXNC1, PTPN2, PTPN22, PTPN6, PTPRC, RIPOR2, RUNX1, RUNX3, SEMA4D, TGFBI, TNFAIP8L2, UBASH3B, ZC3H12A]	[CD9, CDH13, CDSN, GCNT2, HLA-DRB1, HLA-G, HRG, MBL2, MBP, MMP12, MYADM, PDE3B, PTPN22, RIPOR2, RND1, RUNX1, SEMA5A, SWAP70]
GO:2000514	regulation of CD4-positive, alpha-beta T cell activation	2,73E-02	[7, 8, 9]	20,00	32,26	63,64	27,27	9,09	[CCL19, CD80, CD86, FOXP3, IFNG, IL18, IRF4, MYB, NCKAP1L, NLRP3, PRKCQ, RUNX3, SASH3, XCL1]	[CD160, JAK3, RARA, RUNX1, RUNX3, ZC3H12A]	[PRKCZ, RUNX1]
GO:0045639	positive regulation of myeloid cell differentiation	2,95E-02	[4, 5, 6, 7, 8, 9, 10]	28,00	27,18	37,50	37,50	25,00	[CASP8, CD4, CD74, HCLS1, HLA-DRB1, IFNG, INPP5D, LEF1, NCKAP1L, RHEX, STAT1, TREM2]	[ACVR1B, CASP8, CD101, FES, GPR68, IL34, MED1, RORA, RUNX1, STAT1, STAT3, TNF]	[ARNT, GNAS, HLA-DRB1, PRKCA, RB1, RUNX1, SMAP1, TESC]
GO:0043370	regulation of CD4-positive, alpha-beta T cell differentiation	2,99E-02	[8, 9, 10, 11, 12, 13]	17,00	35,42	63,16	26,32	10,53	[CCL19, CD80, CD86, FOXP3, IFNG, IL18, IRF4, MYB, NCKAP1L, NLRP3, RUNX3, SASH3]	[JAK3, RARA, RUNX1, RUNX3, ZC3H12A]	[PRKCZ, RUNX1]
GO:0002763	positive regulation of myeloid leukocyte differentiation	4,52E-02	[5, 6, 7, 8, 9, 10, 11]	19,00	32,20	36,36	36,36	27,27	[CASP8, CD4, CD74, HCLS1, HLA-DRB1, IFNG, LEF1, TREM2]	[CASP8, CD101, FES, GPR68, IL34, RORA, RUNX1, TNF]	[GNAS, HLA-DRB1, PRKCA, RB1, RUNX1, TESC]
GO:0002682	regulation of immune system process	2,49E-16	[2, 3]	326,00	18,70	49,60	32,44	17,96	[ADAM8, AIF1, AIM2, ANGPT1, BANK1, BLK, BLNK, BTK, BTLA, BTN3A1, BTN3A2, C1QA, C1QB, C1QC, C2, C8G, CAMK4, CARD11, CASP8, CCDC88B, CCL19, CCL24, CCR2, CD1B, CD1C, CD1E, CD2, CD200R1, CD244, CD247, CD27, CD3D, CD3E, CD3G, CD4, CD40LG, CD48, CD5, CD6, CD74, CD80, CD84, CD86, CD8A, CD8B, CD96, CDKN2A, CFP, CLCF1, CLEC1B, CLEC4D, CLEC4E, CLEC6A, CLNK, CNR1, CNR2, CR2, CRTAM, CSF1R, CTLA4, CXCL10, CXCL6, CXCR3, CYP11B1, DOCK8, EDN2, EGR3, EREG, FCN1, FCN3, FCRL3, FGL2, FGR, FOXP3, FUT7, FYB1, GBP5, GPR171, GPR55, GPSM3, H3C10, H3C11, H3C12, H3C7, HCLS1, HLA-DMB, HLA-DOA, HLA-DPA1, HLA-DPB1, HLA-DQA1, HLA-DRA, HLA-DRB1, HLA-DRB5, ICOS, IFNG, IKBKE, IL18, IL18RAP, IL7R, INPP5D, IRF4, ITGA4, ITGB7, ITK, JAML, KLRB1, KLRC1, KLRD1, KLRK1, LAG3, LAX1, LCK, LCP2, LEF1, LMO1, LPAL2, LRRC17, LST1, LTA, MIXL1, MOG, MS4A1, MUC19, MUC6, MYB, MYO1G, MZB1, NCKAP1L, NCR3, NLRP3, NLRP6, NR4A3, ORM2, P2RY12, PAX5, PILRA, PIM1, PLA2G2D, PLA2G7, PRDM1, PRKCQ, PTAFR, PTK2B, PTPN22, PTPN6, PTPRC, PYHIN1, RHEX, RHOH, RUNX3, SASH3, SELE, SELL, SH2D1A, SIT1, SLAMF6, SLAMF7, SLAMF8, STAP1, STAT1, SYK, TBC1D10C, TESPA1, THEMIS, THEMIS2, THY1, TIGIT, TLR6, TLR8, TNFAIP3, TNFAIP8L2, TNFRSF18, TNFSF13B, TNFSF8, TOX, TRAT1, TREM2, TSC22D3, VNN1, WAS, XCL1, XCL2, XG, ZAP70, ZNF683]	[ACTR3, ACVR1B, ADA, ADAM10, AIM2, ARRB2, B2M, BCL2, BIRC3, BPI, BTN3A3, CASP8, CCDC88B, CCM2, CCR7, CD101, CD160, CD28, CD38, CD3G, CD47, CD6, CDK6, COLEC11, COLEC12, CORO1A, CTLA4, CX3CL1, DOCK1, ELMO1, FANCA, FES, FYB1, GAB2, GATD3A, GBP1, GBP5, GPR68, GPRC5B, GPSM3, GRB2, GSN, HDAC1, HLA-A, HLA-B, HLA-DPA1, HLA-DPB1, HLA-DRA, IDO1, IL34, IRF1, JAK3, JAML, KLHL6, KLHL7, LAX1, LCK, LCP2, LIME1, LPXN, LRCH1, LTA, MED1, MITF, MMP14, MYO1G, NCK2, NCOR2, NFATC1, NFATC2, NLRC5, NMI, PCDHA6, PDE4B, PIK3CD, PLSCR1, PRKACB, PRKCE, PRKD2, PSMB8, PSMB9, PSMD9, PTK2B, PTPN1, PTPN2, PTPN22, PTPN6, PTPRC, PTPRJ, RARA, RELB, RIPOR2, RNF216, RORA, RUNX1, RUNX3, SLA2, SLAMF7, SLC7A2, STAT1, STAT3, STK10, TANK, TAPBPL, TBC1D10C, TCF7L1, TESPA1, TGFB2, TNF, TNFAIP3, TNFAIP8L2, TRAF3, UBASH3B, WIPF1, YAP1, ZBP1, ZBTB16, ZBTB20, ZC3H12A, ZEB1, ZMIZ1]	[ADORA1, AIM2, ALOX15, ARNT, BTNL2, CAMK4, CARD11, CARTPT, CAV1, CD8A, CD9, CHUK, COLEC11, CXADR, DOCK1, DRD2, DYSF, FOXP1, FYB1, GNAS, GPR31, HLA-DQB2, HLA-DRA, HLA-DRB1, HLA-G, HRG, ITGB2, KIR3DL2, KLK7, LILRA4, LYN, MAP3K1, MBL2, MEIS2, MMP12, MNDA, MTUS1, MUC2, MUC5B, MYH2, NFATC1, NLRC5, NR1H3, PAK1, PCDHA6, PDE4B, PNKD, PPP3CA, PRKCA, PRKCZ, PSMD7, PTPN22, PUS7, RB1, RIPOR2, RUNX1, SIGLEC15, SMAP1, SUSD4, SWAP70, TESC, TOX, TP73, TRAF2, VEGFC, WASL, ZEB1]
GO:0048583	regulation of response to stimulus	5,38E-15	[2, 3]	694,00	15,33	32,33	38,22	29,45	[ABRA, ADAM8, AIF1, AIM2, AKNA, ANGPT1, ANGPT2, AOAH, ARAP2, AREG, ARHGAP15, ARHGAP30, ATF3, BANK1, BCL2L14, BLK, BLNK, BMPR1B, BTK, BTLA, BTN3A1, BTN3A2, C1QA, C1QB, C1QC, C2, C8G, CALCR, CARD11, CASP8, CCL19, CCL24, CCR2, CCR4, CD180, CD1B, CD1C, CD1E, CD200R1, CD247, CD27, CD3D, CD3E, CD3G, CD4, CD40LG, CD48, CD6, CD74, CD80, CD84, CD86, CD8A, CD8B, CD96, CDKN2A, CEL, CFP, CHRDL1, CISH, CLCF1, CLEC1B, CLEC4D, CLEC4E, CLEC6A, CLNK, CNR1, CNR2, CR2, CRABP2, CRB2, CRTAM, CSF1R, CTLA4, CXCL10, CXCL6, CXCR4, CYP11B1, DAPK1, DEPDC1B, DRD1, DUSP2, DUSP8, EDN2, ELF3, EREG, ERFE, FASLG, FCN1, FCN3, FCRL3, FFAR4, FGD2, FGD3, FGL2, FGR, FLT3, FOLR2, FOXP3, FRZB, FUT7, FYB1, GBP5, GDF6, GFI1, GPR174, GPR55, GPRC5A, GPSM3, HCLS1, HLA-DMB, HLA-DPA1, HLA-DPB1, HLA-DQA1, HLA-DRA, HLA-DRB1, HLA-DRB5, ICOS, IER3, IFNG, IKBKE, IL10RA, IL18, IL18RAP, IL26, IL7R, INPP5D, IRF4, ITGA4, ITGB7, ITK, JAML, JPH1, KLRB1, KLRC1, KLRD1, KLRK1, KNG1, LAG3, LAX1, LCK, LCP1, LCP2, LEF1, LMCD1, LPAL2, LTA, LY86, LYPD6B, MAPK13, MOG, MS4A1, MSX1, MUC19, MUC6, MUSK, MYB, MYO1G, MZB1, NAMPT, NCF1, NCKAP1L, NCR3, NLRP3, NLRP6, NPFFR2, NPPC, NPTX2, NR4A2, NR4A3, NUP210, P2RY10, P2RY12, PAX5, PIK3CG, PILRA, PIM2, PLA2G2D, PLA2G7, PLAU, PLEK, PRKCQ, PROK1, PSCA, PSD4, PTAFR, PTK2B, PTPN22, PTPN6, PTPRC, PYHIN1, RAB3B, RAD9B, RGS1, RGS10, RGS18, RHOH, RNASE6, RTKN2, SASH3, SELE, SELL, SEMA4A, SEMA4D, SH2D1A, SLA, SLAMF6, SLAMF7, SLAMF8, SMPDL3B, SPINK1, STAP1, STAT1, SUCNR1, SYK, TAGAP, TBC1D10C, TESPA1, THEMIS, THEMIS2, THY1, TLR10, TLR6, TLR8, TNFAIP3, TNFAIP8L2, TNFRSF18, TNFSF13B, TNIP3, TNMD, TNR, TRABD2A, TRAF1, TRAT1, TREM2, TSPAN8, UBD, VNN1, VWC2L, WAS, WNT1, WNT10B, XCL1, XCL2, ZAP70, ZBED2, ZNF683]	[ABAT, ACAA2, ACTR3, ACVR1B, ADA, ADAM10, ADAMTS12, ADGRV1, AGAP2, AIM2, AKAP13, AKAP6, AKAP9, AKNA, ANKS1A, AOAH, ARHGAP25, ARHGAP26, ARHGEF3, ARRB2, ATG7, AUTS2, B2M, BABAM2, BCAR3, BCL2, BCL2L14, BDNF, BICD1, BIRC3, BMP7, BMPR1A, BTN3A3, C12orf66, C6orf89, CACNA1A, CAMK2G, CASP8, CAVIN4, CC2D1A, CCDC88C, CCM2, CCND3, CCR7, CD160, CD28, CD2AP, CD38, CD3G, CD44, CD47, CD6, CDC42SE2, CDH2, CDK6, CFLAR, CGNL1, CHN2, CHST11, CHSY1, CLEC16A, COLEC11, COLEC12, CTLA4, CX3CL1, CYREN, DAPK1, DDAH1, DEDD2, DIXDC1, DNM2, DOCK1, DUSP16, EDEM1, EHMT1, ELMO1, ETFA, F2R, FAM13A, FANCA, FBXO5, FES, FYB1, GAB2, GATD3A, GBP1, GBP5, GHR, GLIPR2, GNAQ, GNG7, GPR68, GPRC5B, GPSM3, GPX4, GRB2, GRIK5, GSK3B, GSN, HDAC1, HDAC9, HLA-A, HLA-B, HLA-DPA1, HLA-DPB1, HLA-DRA, HMGA1, HOMER1, HOMER2, HTT, IDO1, IGF1R, IGFBP4, IL17RA, IL34, INAVA, IQSEC1, IQSEC3, IRF1, ITGA8, ITPR1, JAK3, JAML, JARID2, KCNA2, KCTD11, KLB, KLHL6, KLHL7, KSR1, LAX1, LCK, LCP1, LCP2, LGR4, LIME1, LITAF, LPXN, LTA, LTBP1, MAML3, MAP2K2, MAP2K5, MAP4K5, MARK3, MCC, MCPH1, MDFIC, MEAK7, MED1, MGLL, MIER1, MIOS, MMP14, MRTFA, MTNR1B, MVB12B, MYO1G, MYO9B, NCK2, NCOR2, NEO1, NETO1, NFAT5, NFATC1, NFATC2, NLRC5, NMI, NOS1AP	[ABCC1, ABCC8, ABRA, ADCY5, ADORA1, ADRA1B, ADRB3, AFAP1, AIM2, AKAP12, AKAP13, AKAP9, ALK, ALOX15, AMER3, ANKS1A, ANKS1B, ARHGAP24, ARHGAP26, ARHGAP32, ARHGAP44, ARHGEF10, ARHGEF3, ARNT, ASXL1, BCAR3, BCLAF1, BPIFB1, BTNL2, CACNG2, CAMKMT, CARD11, CARD8, CARTPT, CASP5, CAV1, CD8A, CD9, CDH13, CELF4, CHRNA4, CHSY1, CHUK, COLEC11, CTTN, CXADR, DCC, DCN, DENND2B, DLG2, DMPK, DOCK1, DRAXIN, DRD2, DYSF, EGFR, EPS15L1, ERBB4, ESR2, EYA2, F10, FBN2, FBXO32, FGF7, FGF9, FMN2, FNDC4, FOXH1, FOXP1, FYB1, GCNT2, GDF6, GFRAL, GNAS, GNAT1, GNG4, GNG7, GPR161, GPR31, GREM2, GRIK2, GRIN2A, GRK1, HGF, HIP1, HIPK2, HLA-DQB2, HLA-DRA, HLA-DRB1, HLA-G, HRG, HYAL2, IGF1R, IGSF9B, ITGB2, ITPR1, KCNK2, KIF7, KIR3DL2, KLK7, LCP1, LDLRAD4, LILRA4, LIMD1, LMO3, LPAR6, LRRC7, LYN, MAGI1, MAGI2, MAML2, MAP3K1, MAP3K5, MAPRE2, MBL2, MCC, MEF2A, MIER1, MMP12, MNDA, MTUS1, MUC2, MUC5B, MYADM, MYH2, MYLK, NACC2, NCOR1, NDRG4, NFATC1, NGF, NLRC5, NOS1AP, NR1H3, NUP93, NXN, ONECUT2, P2RX2, PAK1, PCDHA6, PCSK9, PDE11A, PDE3B, PDE4B, PDE6B, PIAS2, PLCE1, PLD3, PLPP3, PNKD, PPARGC1A, PPP3CA, PRDX1, PRKAA1, PRKAG2, PRKAR2B, PRKCA, PRKCZ, PRKN, PSD2, PSMD7, PTPN22, PTPRT, RAB7A, RALGAPA2, RAMP1, RB1, RCAN2, RFX4, RGS12, RGS3, RIMS1, RIPOR2, RNASEH2B, RNF13, RNF14, RNF43, ROBO1, ROBO2, RSPO1, RUNX1, SALL3, SCEL, SEMA5A, SHANK1, SHISA6, SHOC2, SKI, SLC6A2, SMAD6, SMYD2, SOX17, SOX7, SRI, ST18, STARD13, STK25, STRN3, SUSD4, SWAP70, SYT17, TAF2, TIAM1, TIAM2, TLR1, TNFRSF11A, TP73, TPX2, TRAF2, TRAF7, TRIM63, TRIM68, TSC1, TUB, USP8, VEGFC, VSNL1, WASL, WDFY1, WFS1, WNK2, WNT16, WRAP53, ZEB1, ZNF653]

GO:0007166	cell surface receptor signaling pathway	1,84E-14	[3, 4, 5]	540,00	16,08	33,61	39,93	26,46	[ADGRG6, ADORA3, AIM2, ANGPT1, ANGPT2, AREG, ATF3, BCL2L14, BLK, BLNK, BMPR1B, BTK, BTLA, BTN3A1, BTN3A2, CALCR, CARD11, CASP8, CCL19, CCL24, CCR2, CCR3, CCR4, CCR5, CCR8, CD180, CD2, CD200R1, CD247, CD27, CD3D, CD3E, CD3G, CD4, CD40LG, CD6, CD74, CD80, CD86, CD8A, CD8B, CHRDL1, CISH, CLCF1, CLEC1B, CLEC4D, CLEC4E, CLEC6A, CLNK, CR2, CRB2, CSF1R, CSF2RA, CTLA4, CXCL10, CXCL11, CXCL5, CXCL6, CXCL9, CXCR3, CXCR4, CXCR6, CYBB, CYSLTR1, DAPK1, DEPDC1B, DOK2, DRD1, EDN2, ELF3, EPHA6, EREG, ERFE, FAM83B, FASLG, FCN1, FCRL3, FGR, FLT3, FOLR2, FOXP3, FRZB, FUT7, FYB1, GALNT3, GDF6, GFI1, GFRA2, GPR171, GPR34, GPRC5A, H3C10, H3C11, H3C12, H3C7, HCLS1, HLA-DPA1, HLA-DPB1, HLA-DQA1, HLA-DRA, HLA-DRB1, HLA-DRB5, IFNG, IKBKE, IL10RA, IL12RB2, IL18, IL18RAP, IL22, IL26, IL2RG, IL5RA, IL7R, IL9R, INPP5D, IRF4, IRF5, ITGA4, ITGB7, ITK, KLRB1, KLRC1, KLRD1, KLRG1, KLRK1, LAG3, LAX1, LCK, LCP1, LCP2, LEF1, LTA, LTB, LY86, LYPD6B, MOG, MS4A1, MSX1, MUC19, MUC6, MUSK, MYO1G, MZB1, NCF1, NCKAP1L, NCR3, NELL2, NPPB, NPPC, NPTX2, NR4A2, NR4A3, OASL, P2RY10, P2RY12, P2RY13, P2RY2, PAX5, PIM1, PLEK, PLXNC1, PRKCQ, PTAFR, PTK2B, PTPN22, PTPN6, PTPRC, RHEX, SDC1, SEMA4A, SEMA4D, SH2D2A, SH2D6, SLA, STAP1, STAT1, SYK, TESPA1, THEMIS, THEMIS2, THY1, TNFAIP3, TNFRSF11B, TNFRSF18, TNFSF13B, TNFSF8, TNMD, TRABD2A, TRAF1, TRAT1, TREM2, UBD, VWC2L, WAS, WNT1, WNT10A, WNT10B, XCL1, XCL2, XCR1, ZAP70, ZBED2]	[ABAT, ACTR3, ACVR1B, ADA, ADAM10, ADAMTS12, ADGRE5, ADGRL3, ADGRL4, ADGRV1, ADORA3, AGAP2, AGO2, AIM2, AKAP9, ANKS1A, ANXA9, AP3S1, ARRB2, ATOH8, ATP6V0A2, ATP6V1B2, B2M, BATF, BCAR3, BCL2, BCL2L14, BCL9, BDNF, BIRC3, BMP7, BMPR1A, BPI, BTN3A3, CAMK2G, CASP8, CCDC88C, CCM2, CCND3, CCR7, CD101, CD160, CD28, CD38, CD3G, CD44, CD47, CD6, CDH17, CDH2, CDK6, CFLAR, CHRNA1, CHST11, CHSY1, CLEC14A, COL4A2, CORO1A, CPNE3, CTLA4, CX3CL1, CXCL11, DAPK1, DEDD2, DIABLO, DIXDC1, DNM2, DOCK1, DOK2, DST, ELMO1, EPHA10, ETFA, EVL, F13A1, F2R, FANCA, FARP2, FES, FYB1, GAB2, GABRA5, GATD3A, GBP1, GFRA2, GHR, GMDS, GNAQ, GPRC5B, GRB2, GRIK5, GRM7, GSK3B, HDAC1, HLA-A, HLA-B, HLA-DPA1, HLA-DPB1, HLA-DRA, HNRNPF, HOMER1, HOMER2, HTT, IGF1R, IGFBP4, IL11RA, IL12RB2, IL17RA, IL19, IL21R, IL34, INSYN2A, IRF1, IRF2, ITGA10, ITGA8, ITGA9, ITGB5, ITGB6, JAK3, KLB, KLHL6, KLHL7, LAX1, LCK, LCP1, LCP2, LGR4, LIME1, LPXN, LTA, LTBP1, MAML3, MAP2K5, MARK2, MCC, MDFIC, MED1, MITF, MMP14, MRTFA, MVB12B, MYO1G, MYO9B, NCF4, NCK2, NCOR2, NEO1, NETO1, NFATC1, NFATC2, NLRC5, NMI, NPR2, NPTN, NTRK2, OASL, OPRM1, PARD3, PDCD10, PDE4B, PDZD2, PHF14, PIGU, PIK3CD, PLCE1, PLSCR1, PLXNC1, POLR2A, PRICKLE1, PRKACB	[ADAP1, ADCY5, ADGRA1, ADORA1, AGO2, AIM2, AKAP9, ALK, ALOX15, AMER3, ANKS1A, ANKS1B, ARNT, ATP6V0D1, ATP6V1D, BCAR3, BOC, BTNL2, C2CD5, CACNG2, CARD11, CARD8, CAV1, CD8A, CDH13, CELF4, CELSR3, CHRNA4, CHSY1, CHUK, COL4A2, CPZ, CTTN, DCC, DCN, DISP3, DLG2, DMPK, DNER, DOCK1, DRAXIN, DRD2, DST, EGFR, EPS15L1, ERBB4, EYA2, FBN2, FGF12, FGF7, FGF9, FOXH1, FOXP1, FYB1, GABBR1, GABRA5, GABRG3, GCNT2, GDF6, GFRAL, GP6, GPR161, GREM2, GRIK2, GRIN2A, GRM7, GRM8, HGF, HIP1, HIPK2, HLA-DQB2, HLA-DRA, HLA-DRB1, HLA-G, HRG, HYAL2, IGF1R, IGSF9B, ITGB2, JADE2, KCNA5, KIF7, LCP1, LDLRAD4, LILRA4, LIMD1, LRRC7, LYN, MAGI1, MAGI2, MAML2, MAP3K1, MCC, MMP12, MNDA, MUC2, MUC5B, MYH2, NDRG4, NEURL1B, NFATC1, NGF, NLRC5, NR1H3, NUP93, NXN, ONECUT2, P2RX2, PAK1, PDE4B, PDE6B, PLCE1, PLPP3, PPARGC1A, PPP3CA, PRKAA1, PRKCA, PRKCZ, PRKN, PSMD7, PTPN22, PTPRT, RAB7A, RB1, RFX4, RIMS1, RNF43, ROBO1, ROBO2, RSPO1, RUNX1, SALL3, SCEL, SEMA5A, SHANK1, SHISA6, SHOC2, SKI, SLC17A7, SMAD1, SMAD6, SOX17, SOX7, SST, ST18, TBXT, TIA1, TIAM1, TNFRSF11A, TRAF2, TRIM68, TSC1, UNC5D, USP8, VEGFC, WASL, WNK2, WNT16, ZEB1]
GO:0007165	signal transduction	2,84E-14	[2, 3, 4]	933,00	14,39	29,89	38,75	31,35	[ABRA, ADAM8, ADGRG6, ADORA3, AIF1, AIM2, ANGPT1, ANGPT2, APBB1IP, ARAP2, AREG, ARHGAP15, ARHGAP30, ATF3, BANK1, BCL2L14, BHLHA15, BLK, BLNK, BMPR1B, BTG2, BTK, BTLA, BTN3A1, BTN3A2, CALCR, CAMK4, CARD11, CASP8, CCL19, CCL24, CCR2, CCR3, CCR4, CCR5, CCR8, CD180, CD2, CD200R1, CD244, CD247, CD27, CD3D, CD3E, CD3G, CD4, CD40LG, CD5, CD53, CD6, CD74, CD80, CD86, CD8A, CD8B, CDKN2A, CHRDL1, CISH, CLCF1, CLEC1B, CLEC4D, CLEC4E, CLEC6A, CLNK, CNR1, CNR2, CORO2A, CR2, CRABP2, CRB2, CREM, CSF1R, CSF2RA, CTLA4, CTSS, CXCL10, CXCL11, CXCL5, CXCL6, CXCL9, CXCR3, CXCR4, CXCR6, CYBB, CYSLTR1, DAPK1, DAPP1, DEPDC1B, DOCK10, DOCK2, DOCK8, DOK2, DRD1, DTHD1, DUSP2, DUSP8, E2F8, EDN2, ELF3, EPHA6, EREG, ERFE, FAM83B, FASLG, FCN1, FCRL3, FFAR4, FGD2, FGD3, FGR, FIBIN, FLT3, FOLR2, FOXP3, FRZB, FUT7, FYB1, GABBR2, GALNT3, GDF6, GFI1, GFRA2, GPR141, GPR150, GPR171, GPR174, GPR19, GPR34, GPR55, GPR83, GPRC5A, H3C10, H3C11, H3C12, H3C7, HCLS1, HLA-DPA1, HLA-DPB1, HLA-DQA1, HLA-DRA, HLA-DRB1, HLA-DRB5, ICOS, IER3, IFNG, IKBKE, IL10RA, IL12RB2, IL18, IL18RAP, IL22, IL26, IL2RG, IL5RA, IL7R, IL9R, INPP5D, IQGAP2, IRF4, IRF5, ITGA4, ITGB7, ITK, JPH1, KLF5, KLRB1, KLRC1, KLRC2, KLRD1, KLRG1, KLRK1, KNG1, LAG3, LAX1, LCK, LCP1, LCP2, LEF1, LMCD1, LRRC63, LTA, LTB, LY86, LYPD6B, MACC1, MAPK13, MCOLN2, MCOLN3, MOG, MS4A1, MSX1, MUC19, MUC6, MUSK, MYO1G, MZB1, NAMPT, NCF1, NCKAP1L, NCR3, NELL2, NLRP3, NLRP6, NMUR1, NPFFR2, NPPB, NPPC, NPTX2, NR4A1, NR4A2, NR4A3, NSG1, OASL, OPN5, OR2I1P, OXGR1, P2RY10, P2RY12, P2RY13, P2RY2, PARP8, PAX5, PIK3C2G, PIK3CG, PILRA, PIM1, PIM2, PLAU, PLCH2, PLEK, PLPPR4, PLXNC1, PNOC, PRKCQ, PROK1, PSCA, PSD4, PTAFR, PTGFR, PTK2B, PTPN22, PTPN6, PTPRC, PYHIN1, RAB39B, RAB3B, RAB3C, RAB44, RAD9B, RASSF6, RGS1, RGS10, RGS18, RHEX, RHOH, RLN2, RNASE6, RTKN2, SDC1, SELE, SEMA4A, SEMA4D, SH2D2A, SH2D6, SIT1, SLA, SMPDL3B, SPINK1, STAP1, STAT1, STK17B, SUCNR1, SYK, TAGAP, TBC1D10C, TESPA1, THEMIS, THEMIS2, THY1, TIFAB, TLR10, TLR6, TLR8, TNFAIP3, TNFRSF11B, TNFRSF18, TNFSF13B, TNFSF8, TNIP3, TNMD, TRABD2A, TRAF1, TRAT1, TREM2, UBD, VNN1, VWC2L, WAS, WNT1, WNT10A, WNT10B, XCL1, XCL2, XCR1, ZAP70, ZBED2, ZNF385B]	[ABAT, ACAA2, ACER2, ACTR3, ACVR1B, ADA, ADAM10, ADAMTS12, ADCY1, ADGRE5, ADGRL3, ADGRL4, ADGRV1, ADORA3, AFDN, AGAP2, AGO2, AGRN, AIM2, AKAP13, AKAP6, AKAP9, ANK3, ANKS1A, ANXA9, AP3S1, APBB1IP, ARHGAP25, ARHGAP26, ARHGEF10L, ARHGEF3, ARRB2, ATG10, ATOH8, ATP6V0A2, ATP6V1B2, AUTS2, B2M, BABAM2, BATF, BCAR3, BCL2, BCL2L14, BCL9, BDNF, BICD1, BIRC3, BMP7, BMPR1A, BPI, BTN3A3, C12orf66, C6orf89, CAB39, CACNA1A, CAMK2G, CAP2, CASP8, CAVIN4, CC2D1A, CCDC88C, CCM2, CCND3, CCR7, CD101, CD160, CD28, CD2AP, CD38, CD3G, CD44, CD47, CD6, CDC42BPB, CDC42SE2, CDH17, CDH2, CDK6, CFLAR, CGNL1, CHN2, CHRNA1, CHST11, CHSY1, CLEC14A, CLEC16A, CMTM7, COL15A1, COL4A2, COLEC12, CORO1A, CORO1C, CPNE3, CTLA4, CX3CL1, CXCL11, DAPK1, DDAH1, DEDD2, DERL1, DGKA, DIABLO, DIXDC1, DNM2, DOCK1, DOCK3, DOK2, DST, DTNA, DUSP16, EDEM1, EHMT1, ELK3, ELMO1, EPHA10, ETFA, EVL, EXT1, F13A1, F2R, FAM13A, FANCA, FARP2, FES, FEZ2, FYB1, GAB2, GABRA5, GATD3A, GBP1, GCOM1, GFRA2, GHR, GLIPR2, GMDS, GNAQ, GNG7, GPR25, GPR6, GPR68, GPRC5B, GRB2, GRIK5, GRK3, GRM7, GSK3B, GSN, HDAC1, HDAC9, HDGFL3, HLA-A, HLA-B, HLA-DPA1, HLA-DPB1, HLA-DRA, HNRNPF, HOMER1, HOMER2, HTT, IGF1R, IGFBP4, IL11RA	[ABCA4, ABRA, ADAP1, ADCY5, ADGRA1, ADORA1, ADRA1B, ADRB3, AFAP1, AGO2, AIM2, AKAP12, AKAP13, AKAP9, ALDH1A2, ALK, ALOX15, AMER3, ANK3, ANKS1A, ANKS1B, ARHGAP24, ARHGAP26, ARHGAP32, ARHGAP44, ARHGEF10, ARHGEF3, ARNT, ASIC2, ASXL1, ATP1B1, ATP2A3, ATP6V0D1, ATP6V1D, BCAR3, BCL11B, BCLAF1, BOC, BPIFB1, BTNL2, C2CD5, CABP2, CACNG2, CAMK4, CAMKMT, CARD11, CARD8, CARTPT, CASKIN1, CASP5, CAV1, CD8A, CDC42EP3, CDH13, CELF4, CELSR3, CHRNA4, CHSY1, CHUK, CMTM7, COL4A2, CPZ, CTTN, DCC, DCN, DENND2B, DISP3, DLG2, DMPK, DNER, DOCK1, DRAXIN, DRD2, DST, DTHD1, EGFR, EPS15L1, ERBB4, ESR2, ESRRB, ESRRG, EYA2, F10, FBN2, FGF12, FGF14, FGF7, FGF9, FMN2, FOXH1, FOXP1, FYB1, GABBR1, GABRA5, GABRG3, GABRR3, GCNT2, GDF6, GFRAL, GNAS, GNAT1, GNB4, GNG4, GNG7, GP6, GPR15, GPR161, GPR31, GPSM2, GREM2, GRIK2, GRIN2A, GRK1, GRM7, GRM8, HGF, HIP1, HIPK2, HLA-DQB2, HLA-DRA, HLA-DRB1, HLA-G, HRG, HTR3B, HTR5A, HTR7, HUNK, HYAL2, IGF1R, IGSF9B, IMPA2, INPP5A, ITGB2, ITPR1, JADE2, KCNA5, KCNK2, KIF7, LCP1, LDLRAD4, LILRA4, LIMD1, LMO3, LPAR6, LRRC63, LRRC7, LYN, MACC1, MAGI1, MAGI2, MAML2, MAP3K1, MAP3K5, MAPRE2, MAST4, MBL2, MBP, MC2R, MC3R, MCC, MEF2A, METRN, MIER1, MMP12, MNDA, MUC2, MUC5B, MYADM, MYH2, NACC2, NCOR1, NDRG4, NEURL1B, NFATC1, NGF, NLRC5, NOS1AP, NR1H3, NR2F1, NRXN3, NUP93, NXN, NYAP2, ONECUT2, OR10A2, OR1J4, OR1N2, OR2A14, OR2B3, OR2W1, OR52A5, OR52E6, OR5P2, OR5T2, OR6P1, P2RX2, PAK1, PAK4, PARP8, PCDHA6, PCP4, PCSK9, PDE11A, PDE1A, PDE3B, PDE4B, PDE4C, PDE6B, PDE7A, PDE8B, PDZD8, PGR, PIAS2, PIK3C2G, PITPNC1, PLCE1, PLEKHB1, PLEKHH3, PLPP2, PLPP3, PLPPR3, PNKD, PNOC, PPARGC1A, PPP2R5A, PPP3CA, PRDX1, PRKAA1, PRKAG2, PRKAR2B, PRKCA, PRKCZ, PRKN, PSD2, PSMD7, PSTPIP1, PTPN22, PTPRT, RAB35, RAB37, RAB7A, RALGAPA2, RAMP1, RARB, RASAL2, RASGEF1C, RASSF6, RB1, RCAN2, REM2, RFX4, RGS12, RGS3, RIMS1, RIPOR2, RND1, RNF13, RNF14, RNF43, ROBO1, ROBO2, RORB, RPS6KA2, RSPO1, RUNX1, RXRA, SALL3, SAV1, SCEL, SEMA5A, SGCD, SHANK1, SHISA6, SHOC2, SKI, SLC17A7, SLC6A2, SMAD1, SMAD6, SMYD2, SORCS1, SORCS3, SOX17, SOX7, SRGN, SRI, SST, ST18, STARD13, STK25, STRN3, TAF2, TAS1R2, TBXT, TEAD1, TIA1, TIAM1, TIAM2, TLR1, TNFRSF11A, TNS3, TP73, TPX2, TRAF2, TRAF7
GO:0002684	positive regulation of immune system process	1,47E-13	[2, 3, 4]	235,00	19,70	50,00	33,09	16,91	[ADAM8, AIF1, AIM2, BLK, BLNK, BTK, BTLA, BTN3A1, BTN3A2, C1QA, C1QB, C1QC, C2, C8G, CARD11, CASP8, CCDC88B, CCL19, CCL24, CCR2, CD1B, CD1C, CD1E, CD2, CD244, CD247, CD27, CD3D, CD3E, CD3G, CD4, CD40LG, CD5, CD6, CD74, CD80, CD84, CD86, CFP, CLCF1, CLEC4D, CLEC4E, CLEC6A, CLNK, CNR1, CR2, CRTAM, CSF1R, CTLA4, CXCL10, CYP11B1, DOCK8, EDN2, EGR3, EREG, FCN1, FCN3, FCRL3, FGR, FOXP3, FUT7, FYB1, GBP5, GPSM3, HCLS1, HLA-DMB, HLA-DPA1, HLA-DPB1, HLA-DQA1, HLA-DRA, HLA-DRB1, HLA-DRB5, ICOS, IFNG, IKBKE, IL18, IL18RAP, IL7R, INPP5D, ITGA4, ITK, KLRK1, LAG3, LAX1, LCK, LCP2, LEF1, LTA, MOG, MS4A1, MUC19, MUC6, MYB, MYO1G, MZB1, NCKAP1L, NCR3, NLRP3, NR4A3, P2RY12, PAX5, PLA2G7, PRKCQ, PTAFR, PTK2B, PTPN22, PTPN6, PTPRC, PYHIN1, RHEX, RHOH, RUNX3, SASH3, SELE, SH2D1A, SLAMF6, STAP1, STAT1, SYK, TESPA1, THEMIS, THEMIS2, THY1, TLR6, TLR8, TNFRSF18, TNFSF13B, TOX, TRAT1, TREM2, VNN1, WAS, XCL1, XCL2, XG, ZAP70]	[ACTR3, ACVR1B, ADA, ADAM10, AIM2, B2M, BCL2, BTN3A3, CASP8, CCDC88B, CCR7, CD101, CD160, CD28, CD38, CD3G, CD47, CD6, COLEC11, CORO1A, CTLA4, CX3CL1, DOCK1, ELMO1, FES, FYB1, GAB2, GATD3A, GBP1, GBP5, GPR68, GPRC5B, GPSM3, GRB2, HLA-A, HLA-B, HLA-DPA1, HLA-DPB1, HLA-DRA, IDO1, IL34, JAK3, KLHL6, KLHL7, LAX1, LCK, LCP2, LIME1, LPXN, LTA, MED1, MMP14, MYO1G, NCK2, NCOR2, NFATC2, NLRC5, PDE4B, PIK3CD, PLSCR1, PRKACB, PRKCE, PRKD2, PSMB8, PSMB9, PSMD9, PTK2B, PTPN2, PTPN22, PTPN6, PTPRC, PTPRJ, RARA, RELB, RIPOR2, RORA, RUNX1, RUNX3, SLA2, STAT1, STAT3, TANK, TESPA1, TGFB2, TNF, WIPF1, ZBP1, ZBTB16, ZC3H12A, ZMIZ1]	[AIM2, ARNT, BTNL2, CARD11, CAV1, CHUK, COLEC11, DOCK1, DYSF, FOXP1, FYB1, GNAS, GPR31, HLA-DQB2, HLA-DRA, HLA-DRB1, HLA-G, HRG, ITGB2, KLK7, LYN, MBL2, MMP12, MNDA, MUC2, MUC5B, MYH2, NLRC5, PAK1, PDE4B, PNKD, PRKCA, PRKCZ, PSMD7, PTPN22, RB1, RIPOR2, RUNX1, SMAP1, SUSD4, SWAP70, TESC, TOX, TRAF2, VEGFC, WASL]
GO:0035556	intracellular signal transduction	2,97E-11	[3, 4, 5]	477,00	15,91	30,15	41,79	28,05	[ABRA, ADAM8, ADGRG6, AIF1, ANGPT1, ARAP2, AREG, ARHGAP15, ARHGAP30, ATF3, BANK1, BHLHA15, BLK, BLNK, BTG2, BTK, CALCR, CAMK4, CARD11, CASP8, CCL19, CCL24, CCR2, CCR3, CCR4, CCR5, CCR8, CD200R1, CD27, CD3E, CD4, CD40LG, CD74, CD80, CD86, CDKN2A, CISH, CLEC6A, CLNK, CNR1, CNR2, CORO2A, CSF1R, CSF2RA, CXCL10, CXCL11, CXCL9, CXCR3, CXCR4, CXCR6, DAPK1, DEPDC1B, DOCK10, DOCK2, DOCK8, DOK2, DRD1, DUSP2, DUSP8, E2F8, EDN2, EREG, FASLG, FCRL3, FFAR4, FGD2, FGD3, FGR, FIBIN, FLT3, GDF6, GFRA2, GPR174, GPR55, HCLS1, HLA-DRB1, ICOS, IER3, IKBKE, IL18, IL26, IL2RG, IL5RA, INPP5D, ITK, JPH1, KLRG1, LAX1, LCK, LCP1, LCP2, LMCD1, MAPK13, MCOLN2, MCOLN3, MSX1, MUSK, NCF1, NLRP3, NLRP6, NMUR1, NPFFR2, NPPB, NPPC, NR4A3, P2RY10, P2RY12, PIK3C2G, PIK3CG, PIM2, PLCH2, PLEK, PRKCQ, PROK1, PSCA, PSD4, PTAFR, PTGFR, PTK2B, PTPN22, PTPN6, PTPRC, PYHIN1, RAB39B, RAB3B, RAB3C, RAB44, RAD9B, RHOH, RNASE6, RTKN2, SELE, SEMA4D, SH2D6, SPINK1, STAT1, STK17B, SYK, TAGAP, TBC1D10C, TIFAB, TLR6, TLR8, TNFAIP3, TNFSF13B, TNIP3, TRAF1, TRAT1, TREM2, UBD, VNN1, WAS, WNT1, XCL1, XCL2, XCR1, ZAP70, ZNF385B]	[ACER2, ADA, ADCY1, ADGRE5, ADGRL3, ADGRL4, ADGRV1, AGAP2, AKAP13, AKAP6, ARHGAP25, ARHGAP26, ARHGEF3, ARRB2, AUTS2, BABAM2, BATF, BCAR3, BCL2, BIRC3, BMP7, C12orf66, C6orf89, CAB39, CACNA1A, CASP8, CAVIN4, CC2D1A, CCDC88C, CCM2, CCR7, CD160, CD28, CD2AP, CD44, CDC42BPB, CDC42SE2, CDH2, CFLAR, CGNL1, CHN2, CLEC16A, CX3CL1, CXCL11, DAPK1, DDAH1, DEDD2, DGKA, DIABLO, DNM2, DOCK1, DOCK3, DOK2, DUSP16, EHMT1, ELMO1, ETFA, F2R, FAM13A, FARP2, GAB2, GBP1, GCOM1, GFRA2, GHR, GLIPR2, GNAQ, GPR6, GPRC5B, GRB2, GRIK5, GSK3B, GSN, HDAC1, HDAC9, HOMER2, HTT, IGF1R, IGFBP4, IL34, INAVA, IQSEC1, IQSEC3, ITPR1, ITPR2, JAK3, KCNC2, KCNH1, KCTD11, KIAA0355, KLB, KSR1, LAX1, LCK, LCP1, LCP2, LGR4, LIME1, LITAF, MAP2K2, MAP2K5, MAP4K5, MARK2, MARK3, MAST2, MAST4, MBP, MDFIC, MEAK7, MED1, MIER1, MIOS, MRVI1, MTNR1B, MYO9B, NCK2, NFAT5, NFATC1, NFATC2, NLRC5, NOS1AP, NPR2, NPTN, NSMCE1, NTRK2, OPRM1, PCDHA6, PDCD10, PDE9A, PDZD2, PHF20, PHLPP2, PIGU, PIK3CD, PIP4K2A, PIP5K1B, PLCE1, PPP1R13L, PPP2R5C, PPP3CC, PRKACB, PRKAG2, PRKAR1B, PRKCE, PRKD2, PRODH, PSAP, PSMB8, PSMB9, PSMD9, PTK2B, PTPN1, PTPN2, PTPN22, PTPN6, PTPRC, PTPRJ, RAB13, RAB2A, RAB30, RAB34, RALB, RASA3, RASSF3, RASSF5, RELB, RFFL, RGS3	[ABRA, ADCY5, ADORA1, ADRA1B, ADRB3, AKAP12, AKAP13, ALK, ALOX15, ANKS1B, ARHGAP24, ARHGAP26, ARHGAP32, ARHGAP44, ARHGEF10, ARHGEF3, ATP1B1, ATP2A3, BCAR3, BCL11B, BCLAF1, CAMK4, CARD11, CARD8, CARTPT, CAV1, CDC42EP3, CDH13, CHUK, DCN, DENND2B, DLG2, DMPK, DOCK1, DRD2, EGFR, ERBB4, F10, FGF7, FGF9, FMN2, GCNT2, GDF6, GFRAL, GNAS, GPR161, GPSM2, GRIK2, GRIN2A, HGF, HIP1, HIPK2, HLA-DRB1, HLA-G, HTR5A, HUNK, HYAL2, IGF1R, INPP5A, ITPR1, LCP1, LIMD1, LMO3, LPAR6, LRRC7, LYN, MAGI2, MAP3K1, MAP3K5, MAPRE2, MAST4, MBL2, MBP, MC2R, MC3R, MEF2A, MIER1, MYADM, NACC2, NCOR1, NDRG4, NFATC1, NGF, NLRC5, NOS1AP, NYAP2, P2RX2, PAK1, PAK4, PCDHA6, PDE11A, PDE3B, PDZD8, PIK3C2G, PLCE1, PPP3CA, PRDX1, PRKAA1, PRKAG2, PRKAR2B, PRKCA, PRKCZ, PRKN, PSD2, PSMD7, PTPN22, RAB35, RAB37, RAB7A, RALGAPA2, RAMP1, RASAL2, RASGEF1C, RB1, RCAN2, RGS3, RIPOR2, RND1, RNF13, ROBO1, RPS6KA2, SAV1, SEMA5A, SGCD, SHOC2, SLC6A2, SMAD1, SMYD2, SRI, STARD13, STK25, TAF2, TEAD1, TIAM1, TIAM2, TNFRSF11A, TNS3, TP73, TPX2, TRAF2, TRAF7, TSC1, USP8, WFS1, WNK2, WNK4, WNT16]

GO:0048584	positive regulation of response to stimulus	9,87E-11	[2, 3, 4]	420,00	16,22	35,26	37,82	26,92	[ABRA, ADAM8, AIF1, AIM2, ANGPT1, AREG, ATF3, BANK1, BLK, BLNK, BMPR1B, BTK, BTN3A1, BTN3A2, C1QA, C1QB, C1QC, C2, C8G, CALCR, CARD11, CASP8, CCL19, CCL24, CCR2, CCR4, CD180, CD1B, CD1C, CD1E, CD247, CD27, CD3D, CD3E, CD3G, CD4, CD40LG, CD6, CD74, CD80, CD86, CDKN2A, CFP, CLCF1, CLEC4D, CLEC4E, CLEC6A, CLNK, CNR1, CR2, CRB2, CRTAM, CSF1R, CTLA4, CXCL10, CXCR4, CYP11B1, DEPDC1B, EDN2, ELF3, EREG, ERFE, FASLG, FCN1, FCN3, FCRL3, FFAR4, FGD2, FGR, FLT3, FOXP3, FYB1, GBP5, GDF6, GFI1, GPR174, GPR55, GPSM3, HCLS1, HLA-DMB, HLA-DPA1, HLA-DPB1, HLA-DQA1, HLA-DRA, HLA-DRB1, HLA-DRB5, ICOS, IFNG, IKBKE, IL10RA, IL18, IL18RAP, IL26, IL7R, INPP5D, ITK, KLRK1, LAG3, LAX1, LCK, LCP1, LCP2, LMCD1, LTA, LY86, MAPK13, MOG, MS4A1, MSX1, MUC19, MUC6, MUSK, MYB, MYO1G, NCF1, NCKAP1L, NCR3, NLRP3, NPPC, NR4A3, P2RY10, P2RY12, PAX5, PIK3CG, PIM2, PLA2G7, PLEK, PRKCQ, PROK1, PTAFR, PTK2B, PTPN22, PTPN6, PTPRC, PYHIN1, RAB3B, RTKN2, SASH3, SEMA4D, SH2D1A, SLAMF6, STAP1, SUCNR1, SYK, TESPA1, THEMIS, THEMIS2, THY1, TLR10, TLR6, TLR8, TNFAIP3, TNFRSF18, TNFSF13B, TRAF1, TRAT1, TREM2, UBD, WAS, WNT1, WNT10B, XCL1, XCL2, ZAP70, ZBED2]	[ABAT, ACTR3, ACVR1B, ADA, ADAM10, ADGRV1, AGAP2, AIM2, AKAP13, AKAP6, ARHGEF3, ARRB2, AUTS2, B2M, BABAM2, BCAR3, BCL2, BDNF, BIRC3, BMP7, BMPR1A, BTN3A3, CASP8, CC2D1A, CCM2, CCR7, CD160, CD28, CD38, CD3G, CD44, CD47, CD6, CDH2, CFLAR, CHSY1, CLEC16A, COLEC11, CTLA4, CX3CL1, DEDD2, DIXDC1, DOCK1, EDEM1, ELMO1, ETFA, F2R, FYB1, GAB2, GATD3A, GBP1, GBP5, GHR, GLIPR2, GPR68, GPRC5B, GPSM3, GRB2, GSK3B, GSN, HLA-A, HLA-B, HLA-DPA1, HLA-DPB1, HLA-DRA, HMGA1, HOMER1, HTT, IDO1, IGF1R, IGFBP4, IL17RA, IL34, INAVA, ITGA8, KLB, KLHL6, KLHL7, KSR1, LAX1, LCK, LCP1, LCP2, LGR4, LIME1, LITAF, LPXN, LTA, MAML3, MAP2K2, MAP2K5, MAP4K5, MDFIC, MED1, MIER1, MIOS, MRTFA, MYO1G, NCK2, NCOR2, NEO1, NETO1, NFAT5, NFATC2, NLRC5, NOS1AP, NPR2, NPTN, NSMCE1, NTRK2, OPRM1, PDCD10, PDE4B, PDZD2, PIGU, PIK3CD, PLCE1, PLSCR1, PPP3CC, PRKACB, PRKCE, PRKD2, PSAP, PSMB8, PSMB9, PSMD9, PTK2B, PTPN1, PTPN2, PTPN22, PTPN6, PTPRC, PTPRJ, RAB34, RARA, RECK, RELB, RIPOR2, RORA, RPTOR, RRAGC, RUNX1, RWDD3, S100A13, SEMA4D, SIK3, SLA2, SLAMF1, SMARCA4, SPIDR, SSTR4, STAT3, TANK, TERT, TESPA1, TGFB2, TIMP2, TMEM108, TNF, TNFAIP3, TNFSF10, TNIP1, TP53BP2, TRAF1, TRAF3, TSPAN5, TTC23, UBE3A, WIPF1, WWOX, YAP1, ZBP1	[ABCC1, ABRA, ADORA1, ADRA1B, ADRB3, AIM2, AKAP12, AKAP13, ALK, ALOX15, ARHGEF3, ARNT, ASXL1, BCAR3, BCLAF1, BTNL2, CACNG2, CARD11, CARTPT, CAV1, CDH13, CHSY1, CHUK, COLEC11, DCN, DENND2B, DOCK1, DRD2, DYSF, EGFR, ERBB4, EYA2, F10, FGF7, FGF9, FMN2, FOXP1, FYB1, GCNT2, GDF6, GFRAL, GNAS, GPR31, HGF, HIP1, HIPK2, HLA-DQB2, HLA-DRA, HLA-DRB1, HLA-G, HRG, HYAL2, IGF1R, IGSF9B, ITGB2, KCNK2, KIF7, KLK7, LCP1, LMO3, LPAR6, LYN, MAML2, MAP3K1, MAP3K5, MAPRE2, MBL2, MIER1, MMP12, MNDA, MUC2, MUC5B, MYH2, MYLK, NACC2, NDRG4, NGF, NLRC5, NOS1AP, NR1H3, NUP93, P2RX2, PAK1, PDE4B, PLCE1, PLPP3, PNKD, PPP3CA, PRKAA1, PRKCA, PRKCZ, PRKN, PSMD7, PTPN22, RIMS1, RIPOR2, RNF13, ROBO1, ROBO2, RSPO1, RUNX1, SCEL, SEMA5A, SHANK1, SHOC2, SKI, SLC6A2, SRI, ST18, STK25, SUSD4, SWAP70, TIAM1, TLR1, TNFRSF11A, TP73, TRAF2, TRAF7, USP8, VEGFC, VSNL1, WASL, WDFY1, WNK2, WNT16, WRAP53]
GO:0050778	positive regulation of immune response	8,30E-10	[3, 4, 5]	170,00	20,07	48,99	33,33	17,68	[ADAM8, AIM2, BLK, BLNK, BTK, BTN3A1, BTN3A2, C1QA, C1QB, C1QC, C2, C8G, CARD11, CCL19, CCR2, CD1B, CD1C, CD1E, CD247, CD3D, CD3E, CD3G, CD4, CD74, CD80, CD86, CFP, CLCF1, CLEC4D, CLEC4E, CLEC6A, CLNK, CNR1, CR2, CRTAM, CTLA4, CXCL10, CYP11B1, EREG, FCN1, FCN3, FCRL3, FGR, FOXP3, FYB1, GBP5, HLA-DMB, HLA-DPA1, HLA-DPB1, HLA-DQA1, HLA-DRA, HLA-DRB1, HLA-DRB5, IFNG, IKBKE, IL18, IL18RAP, INPP5D, ITK, KLRK1, LAG3, LAX1, LCK, LCP2, LTA, MOG, MS4A1, MUC19, MUC6, MYB, MYO1G, NCKAP1L, NCR3, NLRP3, NR4A3, PAX5, PRKCQ, PTAFR, PTPN22, PTPN6, PTPRC, PYHIN1, SASH3, SH2D1A, SLAMF6, STAP1, SYK, TESPA1, THEMIS, THEMIS2, THY1, TLR8, TNFSF13B, TRAT1, WAS, XCL1, ZAP70]	[ACTR3, ADA, AIM2, B2M, BCL2, BTN3A3, CCR7, CD160, CD28, CD38, CD3G, CD47, COLEC11, CTLA4, DOCK1, ELMO1, FYB1, GAB2, GBP1, GBP5, GPRC5B, GRB2, HLA-A, HLA-B, HLA-DPA1, HLA-DPB1, HLA-DRA, IDO1, KLHL6, KLHL7, LAX1, LCK, LCP2, LIME1, LPXN, LTA, MED1, MYO1G, NCOR2, NFATC2, NLRC5, PDE4B, PIK3CD, PLSCR1, PRKACB, PRKCE, PRKD2, PSMB8, PSMB9, PSMD9, PTPN2, PTPN22, PTPN6, PTPRC, PTPRJ, RARA, RELB, RUNX1, SLA2, TANK, TESPA1, TGFB2, TNF, WIPF1, ZBP1, ZC3H12A]	[AIM2, BTNL2, CARD11, CHUK, COLEC11, DOCK1, FOXP1, FYB1, GPR31, HLA-DQB2, HLA-DRA, HLA-DRB1, HLA-G, HRG, ITGB2, KLK7, LYN, MBL2, MMP12, MNDA, MUC2, MUC5B, MYH2, NLRC5, PAK1, PDE4B, PNKD, PRKCA, PRKCZ, PSMD7, PTPN22, RUNX1, SUSD4, TRAF2, WASL]
GO:0010646	regulation of cell communication	1,08E-09	[3, 4]	572,00	15,07	26,32	39,49	34,19	[ABRA, ADAM8, AIF1, AIM2, ANGPT1, ARAP2, AREG, ARHGAP15, ARHGAP30, ATF3, BANK1, BCL2L14, BLK, BMPR1B, CACNA1E, CALCR, CARD11, CARD17, CASP8, CCL19, CCL24, CCR2, CD180, CD2, CD200R1, CD244, CD27, CD3E, CD4, CD40LG, CD74, CD80, CD86, CEL, CHRDL1, CISH, CLCF1, CLEC4E, CLEC6A, CNR1, CNR2, CRABP2, CRB2, CRTAM, CSF1R, CXCR4, DAPK1, DEPDC1B, DRD1, DUSP2, DUSP8, ELAVL4, ELF3, EREG, ERFE, FASLG, FCN1, FCRL3, FFAR4, FGD2, FGD3, FGR, FLT3, FOLR2, FOXP3, FRZB, FUT7, GDF6, GFI1, GPR174, GPR55, GPRC5A, HCLS1, HLA-DRB1, ICOS, IER3, IFNG, IKBKE, IL10RA, IL18, IL26, IL7R, INPP5D, IRF4, JPH1, KCNJ10, KMO, LAX1, LCK, LCP1, LEF1, LMCD1, LPAL2, LY86, LYPD6B, MSX1, MUSK, MYB, MZB1, NCF1, NLRP3, NLRP6, NPFFR2, NPPC, NPTX2, NR4A2, NSG1, ORM2, P2RY10, P2RY12, PAX5, PIK3CG, PIM2, PLAU, PLEK, PRKCQ, PROK1, PSCA, PSD4, PTK2B, PTPN22, PTPN6, PTPRC, PYHIN1, RAB3B, RAD9B, RGS1, RGS10, RGS18, RHOH, RNASE6, RTKN2, SEMA4D, SLA, SMPDL3B, SPINK1, STAP1, STAT1, SYK, TAGAP, TBC1D10C, TESPA1, THY1, TLR10, TLR6, TLR8, TNFAIP3, TNFRSF18, TNIP3, TNMD, TNR, TRABD2A, TRAF1, TRAT1, TREM2, UBD, VNN1, VWC2L, WNT1, WNT10B, XCL1, XCL2, ZAP70, ZBED2]	[ABAT, ACAA2, ACVR1B, ADA, ADAM10, ADAMTS12, ADCY1, ADGRV1, AGAP2, AIM2, AKAP13, AKAP6, AKAP9, ANK3, ANKS1A, ARHGAP25, ARHGAP26, ARHGEF3, ARRB2, AUTS2, BCAR3, BCL2, BCL2L14, BDNF, BICD1, BIRC3, BMP7, BMPR1A, BTBD9, C12orf66, C6orf89, CACNA1A, CACNB2, CASP8, CAVIN4, CC2D1A, CCDC88C, CCM2, CCND3, CCR7, CD160, CD28, CD2AP, CD38, CD44, CD47, CDC42SE2, CDH2, CFLAR, CGNL1, CHN2, CHST11, CHSY1, CLEC16A, CX3CL1, DAPK1, DEDD2, DIXDC1, DNM2, DUSP16, EHMT1, ETFA, F2R, FAM13A, FANCA, GATD3A, GBP1, GHR, GLIPR2, GNAQ, GNG7, GPR68, GPRC5B, GRB2, GRIK5, GRM7, GSK3B, GSN, HDAC1, HDAC9, HOMER1, HOMER2, HTT, IGF1R, IGFBP4, IL17RA, IL34, INAVA, IQSEC1, IQSEC3, IRF1, ITGA8, ITPR1, ITPR2, JAK3, KCNA2, KCNC2, KCTD11, KLB, KSR1, LAX1, LCK, LCP1, LGR4, LIME1, LITAF, LPXN, LTBP1, MAML3, MAP2K2, MAP2K5, MAP4K5, MARK3, MBP, MCC, MDFIC, MED1, MGLL, MIER1, MIOS, MMP14, MRTFA, MTNR1B, MVB12B, MYO9B, NCK2, NCOR2, NEO1, NETO1, NFAT5, NFATC1, NLRC5, NOS1AP, NPR2, NPTN, NTRK2, OPRM1, PCDHA6, PDCD10, PDZD2, PHF14, PHF20, PHLPP2, PIGBOS1, PIGU, PIK3CD, PIP4K2A, PIP5K1B, PLCE1, PLSCR1, PPP1R13L, PPP2R5C, PPP3CC, PRICKLE1, PRKACB, PRKAG2, PRKAR1B, PRKCE, PRKD2, PSAP, PSMB8, PSMB9, PSMD9, PTK2B, PTPN1, PTPN2, PTPN22, PTPN6	[ABCC8, ABRA, ADCY5, ADORA1, ADRA1B, ADRB3, AFAP1, AIM2, AKAP12, AKAP13, AKAP9, ALK, ALOX15, AMER3, ANK3, ANKS1A, ANKS1B, ARHGAP24, ARHGAP26, ARHGAP32, ARHGAP44, ARHGEF10, ARHGEF3, ARNT, ASXL1, BCAR3, BCHE, BCLAF1, BPIFB1, BTNL2, CACNG2, CALB2, CAMKMT, CARD11, CARD8, CARTPT, CAV1, CDH13, CELF4, CHRNA4, CHSY1, CHUK, CLSTN2, CNTNAP4, CPLX2, CTTN, CXADR, DCC, DCN, DENND2B, DLG2, DMPK, DRAXIN, DRD2, EGFR, EPS15L1, ERBB4, ESR2, EYA2, F10, FBN2, FGF12, FGF14, FGF7, FGF9, FOXH1, FOXP1, GCNT2, GDF6, GFRAL, GNAS, GNAT1, GNG4, GNG7, GPR161, GREM2, GRIK2, GRIN2A, GRK1, GRM7, GRM8, HGF, HIP1, HIPK2, HLA-DRB1, HLA-G, HRG, HYAL2, IGF1R, IGSF9B, ITPR1, KCNA5, KCNG2, KCNS3, KDM5B, KIF7, LCP1, LDLRAD4, LILRA4, LIMD1, LMO3, LPAR6, LRRC7, LYN, MAGI2, MAML2, MAP3K1, MAP3K5, MAPRE2, MBL2, MBP, MCC, MIER1, MMP12, MYADM, NACC2, NCOR1, NDRG4, NFATC1, NGF, NLRC5, NOS1AP, NR1H3, NRXN3, NUP93, NXN, ONECUT2, P2RX2, PAK1, PCDHA6, PCSK9, PDE11A, PDE3B, PDE6B, PFKM, PIAS2, PLCE1, PLPP3, PNKD, PPARGC1A, PPP3CA, PRDX1, PRKAA1, PRKAG2, PRKAR2B, PRKCA, PRKCZ, PRKN, PSD2, PSMD7, PTPN22, PTPRT, RAB7A, RALGAPA2, RAMP1, RB1, RCAN2, RFX4, RGS12, RGS3, RIMS1, RIPOR2, RNF13, RNF14, RNF43, ROBO1, ROBO2, RSPO1, RUNX1, SALL3, SCEL, SEMA5A, SEPTIN9, SHANK1, SHISA6, SHOC2, SKI, SLC6A2, SMAD6, SMYD2, SORCS3, SOX17, SOX7, SRGN, SRI, ST18, STARD13, STK25, STRN3, STXBP5L, SYT17, TAF2, TIAM1, TIAM2, TLR1, TNFRSF11A, TP73, TPX2, TRAF2, TRAF7, TRIM68, TSC1, TUB, TYMP, USP8, VEGFC, VSNL1, WDFY1, WFS1, WNK2, WNT16, ZEB1, ZNF653]
GO:0023051	regulation of signaling	1,67E-09	[2, 3]	576,00	15,01	26,20	39,30	34,50	[ABRA, ADAM8, AIF1, AIM2, ANGPT1, ARAP2, AREG, ARHGAP15, ARHGAP30, ATF3, BANK1, BCL2L14, BLK, BMPR1B, CACNA1E, CALCR, CARD11, CARD17, CASP8, CCL19, CCL24, CCR2, CD180, CD2, CD200R1, CD244, CD27, CD3E, CD4, CD40LG, CD74, CD80, CD86, CEL, CHRDL1, CISH, CLCF1, CLEC4E, CLEC6A, CNR1, CNR2, CRABP2, CRB2, CRTAM, CSF1R, CXCR4, DAPK1, DEPDC1B, DRD1, DUSP2, DUSP8, ELAVL4, ELF3, EREG, ERFE, FASLG, FCN1, FCRL3, FFAR4, FGD2, FGD3, FGR, FLT3, FOLR2, FOXP3, FRZB, FUT7, GDF6, GFI1, GPR174, GPR55, GPRC5A, HCLS1, HLA-DRB1, ICOS, IER3, IFNG, IKBKE, IL10RA, IL18, IL26, IL7R, INPP5D, IRF4, JPH1, KCNJ10, KMO, LAX1, LCK, LCP1, LEF1, LMCD1, LPAL2, LY86, LYPD6B, MSX1, MUSK, MYB, MZB1, NCF1, NLRP3, NLRP6, NPFFR2, NPPC, NPTX2, NR4A2, NSG1, ORM2, P2RY10, P2RY12, PAX5, PIK3CG, PIM2, PLAU, PLEK, PRKCQ, PROK1, PSCA, PSD4, PTK2B, PTPN22, PTPN6, PTPRC, PYHIN1, RAB3B, RAD9B, RGS1, RGS10, RGS18, RHOH, RNASE6, RTKN2, SEMA4D, SLA, SMPDL3B, SPINK1, STAP1, STAT1, SYK, TAGAP, TBC1D10C, TESPA1, THY1, TLR10, TLR6, TLR8, TNFAIP3, TNFRSF18, TNIP3, TNMD, TNR, TRABD2A, TRAF1, TRAT1, TREM2, UBD, VNN1, VWC2L, WNT1, WNT10B, XCL1, XCL2, ZAP70, ZBED2]	[ABAT, ACAA2, ACVR1B, ADA, ADAM10, ADAMTS12, ADCY1, ADGRV1, AGAP2, AIM2, AKAP13, AKAP6, AKAP9, ANKS1A, ARHGAP25, ARHGAP26, ARHGEF3, ARRB2, AUTS2, BCAR3, BCL2, BCL2L14, BDNF, BICD1, BIRC3, BMP7, BMPR1A, BTBD9, C12orf66, C6orf89, CACNA1A, CACNB2, CASP8, CAVIN4, CC2D1A, CCDC88C, CCM2, CCND3, CCR7, CD160, CD28, CD2AP, CD38, CD44, CD47, CDC42SE2, CDH2, CFLAR, CGNL1, CHN2, CHST11, CHSY1, CLEC16A, CX3CL1, DAPK1, DEDD2, DIXDC1, DNM2, DUSP16, ECE1, EHMT1, ETFA, F2R, FAM13A, FANCA, GATD3A, GBP1, GHR, GLIPR2, GNAQ, GNG7, GPR68, GPRC5B, GRB2, GRIK5, GRM7, GSK3B, GSN, HDAC1, HDAC9, HOMER1, HOMER2, HTT, IGF1R, IGFBP4, IL17RA, IL34, INAVA, IQSEC1, IQSEC3, IRF1, ITGA8, ITPR1, ITPR2, JAK3, KCNA2, KCNC2, KCTD11, KLB, KSR1, LAX1, LCK, LCP1, LGR4, LIME1, LITAF, LPXN, LTBP1, MAML3, MAP2K2, MAP2K5, MAP4K5, MARK3, MBP, MCC, MDFIC, MED1, MGLL, MIER1, MIOS, MMP14, MRTFA, MTNR1B, MVB12B, MYO9B, NCK2, NCOR2, NEO1, NETO1, NFAT5, NFATC1, NLRC5, NOS1AP, NPR2, NPTN, NTRK2, OPRM1, PCDHA6, PDCD10, PDZD2, PHF14, PHF20, PHLPP2, PIGBOS1, PIGU, PIK3CD, PIP4K2A, PIP5K1B, PLCE1, PLSCR1, PPP1R13L, PPP2R5C, PPP3CC, PRICKLE1, PRKACB, PRKAG2, PRKAR1B, PRKCE, PRKD2, PSAP, PSMB8, PSMB9, PSMD9, PTK2B, PTPN1	[ABCC8, ABRA, ADCY5, ADORA1, ADRA1B, ADRB3, AFAP1, AIM2, AKAP12, AKAP13, AKAP9, ALK, ALOX15, AMER3, ANKS1A, ANKS1B, ARHGAP24, ARHGAP26, ARHGAP32, ARHGAP44, ARHGEF10, ARHGEF3, ARNT, ASXL1, ATP1B1, ATP2A3, ATP2B2, BCAR3, BCHE, BCLAF1, BPIFB1, BTNL2, CACNG2, CALB2, CAMKMT, CARD11, CARD8, CARTPT, CAV1, CDH13, CELF4, CHRNA4, CHSY1, CHUK, CLSTN2, CNTNAP4, CPLX2, CTTN, CXADR, DCC, DCN, DENND2B, DLG2, DMPK, DRAXIN, DRD2, EGFR, EPS15L1, ERBB4, ESR2, EYA2, F10, FBN2, FGF12, FGF14, FGF7, FGF9, FOXH1, FOXP1, GCNT2, GDF6, GFRAL, GNAS, GNAT1, GNG4, GNG7, GPR161, GREM2, GRIK2, GRIN2A, GRK1, GRM7, GRM8, HGF, HIP1, HIPK2, HLA-DRB1, HLA-G, HRG, HYAL2, IGF1R, IGSF9B, ITPR1, KCNA5, KCNG2, KCNS3, KDM5B, KIF7, LCP1, LDLRAD4, LILRA4, LIMD1, LMO3, LPAR6, LRRC7, LYN, MAGI2, MAML2, MAP3K1, MAP3K5, MAPRE2, MBL2, MBP, MCC, MIER1, MMP12, MYADM, NACC2, NCOR1, NDRG4, NFATC1, NGF, NLRC5, NOS1AP, NR1H3, NRXN3, NUP93, NXN, ONECUT2, P2RX2, PAK1, PCDHA6, PCSK9, PDE11A, PDE3B, PDE6B, PFKM, PIAS2, PLCE1, PLPP3, PNKD, PPARGC1A, PPP3CA, PRDX1, PRKAA1, PRKAG2, PRKAR2B, PRKCA, PRKCZ, PRKN, PSD2, PSMD7, PTPN22, PTPRT, RAB7A, RALGAPA2, RAMP1, RB1, RCAN2, RFX4, RGS12, RGS3, RIMS1, RIPOR2, RNF13, RNF14, RNF43, ROBO1, ROBO2, RSPO1, RUNX1, SALL3, SCEL, SEMA5A, SEPTIN9, SHANK1, SHISA6, SHOC2, SKI, SLC6A2, SMAD6, SMYD2, SORCS3, SOX17, SOX7, SRGN, SRI, ST18, STARD13, STIM1, STK25, STRN3, STXBP5L, SYT17, TAF2, TIAM1, TIAM2, TLR1, TNFRSF11A, TP73, TPX2, TRAF2, TRAF7, TRIM68, TSC1, TUB, TYMP, USP8, VEGFC, VSNL1, WDFY1, WFS1, WNK2, WNT16, ZEB1, ZNF653]

GO:0009966	regulation of signal transduction	2,57E-08	[3, 4, 5]	499,00	15,17	25,51	41,22	33,27	[ABRA, ADAM8, ANGPT1, ARAP2, AREG, ARHGAP15, ARHGAP30, ATF3, BANK1, BCL2L14, BLK, BMPR1B, CALCR, CARD11, CASP8, CCL19, CCL24, CCR2, CD180, CD27, CD3E, CD4, CD40LG, CD74, CD80, CD86, CHRDL1, CISH, CLCF1, CLEC6A, CRABP2, CRB2, CSF1R, CXCR4, DAPK1, DEPDC1B, DUSP2, DUSP8, ELF3, EREG, ERFE, FASLG, FCRL3, FFAR4, FGD2, FGD3, FGR, FLT3, FOLR2, FOXP3, FRZB, FUT7, GDF6, GFI1, GPR174, GPR55, GPRC5A, HCLS1, HLA-DRB1, ICOS, IER3, IFNG, IKBKE, IL10RA, IL18, IL26, IL7R, INPP5D, IRF4, JPH1, LAX1, LCK, LEF1, LMCD1, LY86, LYPD6B, MSX1, MUSK, MZB1, NCF1, NLRP3, NLRP6, NPFFR2, NPPC, NPTX2, NR4A2, P2RY10, P2RY12, PAX5, PIK3CG, PIM2, PLAU, PLEK, PRKCQ, PROK1, PSCA, PSD4, PTK2B, PTPN22, PTPN6, PTPRC, PYHIN1, RAD9B, RGS1, RGS10, RGS18, RHOH, RNASE6, RTKN2, SEMA4D, SLA, SMPDL3B, SPINK1, STAP1, STAT1, SYK, TAGAP, TBC1D10C, TESPA1, THY1, TLR6, TNFAIP3, TNFRSF18, TNIP3, TNMD, TRABD2A, TRAF1, TRAT1, TREM2, UBD, VNN1, VWC2L, WNT1, WNT10B, XCL1, XCL2, ZAP70, ZBED2]	[ABAT, ACAA2, ACVR1B, ADA, ADAM10, ADAMTS12, ADGRV1, AGAP2, AKAP13, AKAP6, AKAP9, ANKS1A, ARHGAP25, ARHGAP26, ARHGEF3, ARRB2, AUTS2, BCAR3, BCL2, BCL2L14, BDNF, BICD1, BIRC3, BMP7, BMPR1A, C12orf66, C6orf89, CASP8, CAVIN4, CC2D1A, CCDC88C, CCM2, CCND3, CCR7, CD160, CD28, CD2AP, CD44, CD47, CDC42SE2, CDH2, CFLAR, CGNL1, CHN2, CHST11, CHSY1, CLEC16A, CX3CL1, DAPK1, DEDD2, DIXDC1, DNM2, DUSP16, EHMT1, ETFA, F2R, FAM13A, FANCA, GATD3A, GBP1, GHR, GLIPR2, GNAQ, GNG7, GPRC5B, GRB2, GRIK5, GSK3B, GSN, HDAC1, HDAC9, HOMER1, HOMER2, HTT, IGF1R, IGFBP4, IL34, INAVA, IQSEC1, IQSEC3, IRF1, ITGA8, ITPR1, JAK3, KCTD11, KLB, KSR1, LAX1, LCK, LGR4, LIME1, LITAF, LPXN, LTBP1, MAML3, MAP2K2, MAP2K5, MAP4K5, MARK3, MCC, MDFIC, MED1, MGLL, MIER1, MIOS, MMP14, MRTFA, MTNR1B, MVB12B, MYO9B, NCK2, NCOR2, NEO1, NETO1, NFAT5, NFATC1, NLRC5, NOS1AP, NPR2, NPTN, NTRK2, OPRM1, PDCD10, PDZD2, PHF14, PHF20, PHLPP2, PIGBOS1, PIGU, PIK3CD, PIP4K2A, PIP5K1B, PLCE1, PLSCR1, PPP1R13L, PPP2R5C, PPP3CC, PRICKLE1, PRKACB, PRKAG2, PRKAR1B, PRKCE, PRKD2, PSAP, PSMB8, PSMB9, PSMD9, PTK2B, PTPN1, PTPN2, PTPN22, PTPN6, PTPRC, PTPRJ, PXN, RAB34, RASA3, RBMS3, RECK, RFFL, RGS3, RIPOR2, RNF213, RORA, RPA3, RPTOR, RRAGC	[ABRA, ADORA1, ADRA1B, ADRB3, AFAP1, AKAP12, AKAP13, AKAP9, ALK, ALOX15, AMER3, ANKS1A, ANKS1B, ARHGAP24, ARHGAP26, ARHGAP32, ARHGAP44, ARHGEF10, ARHGEF3, ARNT, ASXL1, BCAR3, BCLAF1, BPIFB1, BTNL2, CACNG2, CAMKMT, CARD11, CARD8, CARTPT, CAV1, CDH13, CELF4, CHSY1, CHUK, CTTN, DCC, DCN, DENND2B, DLG2, DMPK, DRAXIN, DRD2, EGFR, EPS15L1, ERBB4, ESR2, EYA2, F10, FBN2, FGF7, FGF9, FOXH1, FOXP1, GCNT2, GDF6, GFRAL, GNAS, GNAT1, GNG4, GNG7, GPR161, GREM2, GRIK2, GRIN2A, GRK1, HGF, HIP1, HIPK2, HLA-DRB1, HLA-G, HRG, HYAL2, IGF1R, IGSF9B, ITPR1, KIF7, LDLRAD4, LILRA4, LIMD1, LMO3, LPAR6, LRRC7, LYN, MAGI2, MAML2, MAP3K1, MAP3K5, MAPRE2, MCC, MIER1, MMP12, MYADM, NACC2, NCOR1, NDRG4, NFATC1, NGF, NLRC5, NOS1AP, NR1H3, NUP93, NXN, ONECUT2, P2RX2, PAK1, PCSK9, PDE11A, PDE3B, PDE6B, PIAS2, PLCE1, PLPP3, PPARGC1A, PPP3CA, PRDX1, PRKAA1, PRKAG2, PRKAR2B, PRKCA, PRKCZ, PRKN, PSD2, PSMD7, PTPN22, PTPRT, RAB7A, RALGAPA2, RAMP1, RB1, RCAN2, RFX4, RGS12, RGS3, RIMS1, RIPOR2, RNF13, RNF14, RNF43, ROBO1, ROBO2, RSPO1, RUNX1, SALL3, SCEL, SEMA5A, SHANK1, SHISA6, SHOC2, SKI, SLC6A2, SMAD6, SMYD2, SOX17, SOX7, SRI, ST18, STARD13, STK25, STRN3, TAF2, TIAM1, TIAM2, TLR1, TNFRSF11A, TP73, TPX2, TRAF2, TRAF7, TRIM68, TSC1, TUB, USP8, VEGFC, WDFY1, WFS1, WNK2, WNT16, ZEB1, ZNF653]
GO:0050790	regulation of catalytic activity	3,75E-06	[3]	389,00	15,30	27,29	39,68	33,03	[ADAM8, ADORA3, AIM2, ANGPT1, ARAP2, AREG, ARHGAP15, ARHGAP30, CARD17, CASP8, CCL19, CCL24, CCR2, CD27, CD4, CD40LG, CD74, CDC20B, CDKN2A, CISH, CNR1, CNR2, CRB2, CSF1R, CXCL10, CXCR4, DAPK1, DEPDC1B, DOCK10, DOCK2, DOCK8, DRD1, DUSP2, DUSP8, EDN2, EPHA6, EREG, FASLG, FCRL3, FGD2, FGD3, FGR, FLT3, GABBR2, GBA3, GFI1, GPR55, GPRC5A, GPSM3, GZMA, GZMK, HLA-DRB1, IFNG, IKBKE, IL18, IQGAP2, ITK, KLRK1, KNG1, LAMP3, LAX1, LCK, LCP1, LCP2, LEF1, LPAL2, MID1IP1, MUSK, NCF1, NCF1B, NCF1C, NCKAP1L, NEK5, NLRP3, NMUR1, NPFFR2, NR4A1, NR4A2, OASL, P2RY12, P2RY13, PARP8, PIK3CG, PLAAT4, PLEK, PLXNC1, PPP1R42, PPP2R2B, PRKCQ, PROK1, PTAFR, PTK2B, PTPN22, PTPN6, PTPRC, PTX3, RARRES1, RGS1, RGS10, RGS18, RHOH, RLN2, SELE, SEMA4D, SH2D1A, SLAMF8, SMAP2, SPINK1, STAP1, SYK, TAGAP, TBC1D10C, TFPI2, THY1, TLR6, TNFAIP3, WAS, XCL1, XCL2]	[ACAP1, ACER2, ADCY1, ADGRV1, ADORA3, AFDN, AGAP1, AGAP2, AGRN, AIM2, AKAP13, AKAP9, ALS2CL, ANXA9, ARHGAP25, ARHGAP26, ARHGEF10L, ARRB2, ASAP2, BCAR3, BCL2, BDNF, BICD1, BIRC3, BMP7, C6orf89, CAB39, CABP1, CACNA1A, CAP2, CASP8, CCND3, CCR7, CD44, CDC25B, CDK5RAP1, CFLAR, CHN2, CORO1C, CX3CL1, DAPK1, DBNDD2, DDAH1, DIABLO, DNM2, DOCK1, DOCK3, DPEP1, DUSP16, EEF1A1, EPHA10, ETFA, F2R, FAM13A, FBXO5, FRY, GHR, GNAQ, GPRC5B, GPSM3, GRM7, GSK3B, GSN, HDAC1, HDAC9, HTT, IGF1R, IL34, INAVA, IQSEC1, KSR1, LAX1, LCK, LCP1, LCP2, LGR4, LIME1, LIPC, LRCH1, MAP2K2, MAP2K5, MAP4K5, MARK2, MBP, MCPH1, MDFIC, MRTFA, MYO9B, NCAPH, NCF4, NCK2, NLRC5, NOS1AP, NTRK2, OASL, PARP4, PCDHA6, PCIF1, PDCD10, PDZD2, PHACTR1, PKP4, PLAAT4, PLCE1, PLEKHG6, PLSCR1, PLXNC1, PPP1R3B, PPP2R5C, PRKACB, PRKAG2, PRKAR1B, PRKCE, PRKD2, PRKRIP1, PRPSAP2, PSAP, PSMB8, PSMB9, PTK2B, PTPN1, PTPN2, PTPN22, PTPN6, PTPRC, PTPRJ, PTPRN2, RALB, RASA3, RECK, RFFL, RGS3, RIN2, RIN3, RNF180, RNH1, RORA, RPTOR, RSU1, SEMA4D, SET, SFI1, SGK1, SGSM3, SH2D4A, SH3BP1, SIPA1L1, SLA2, SMG6, SNX9, SPOCK2, SRGAP3, STAT3, STK10, TANK, TBC1D10C, TBC1D2B, TERT, TGFB2, TIMP2, TNF, TNFAIP3, TNFAIP8, TNFSF10, TNIP1	[ACAP2, ADAP1, ADCY5, ADORA1, ADRB3, AGAP1, AIM2, AKAP13, AKAP9, ALK, ANKS1B, ARHGAP24, ARHGAP26, ARHGAP32, ARHGAP44, ARHGEF10, ATP1B1, ATP2A3, BCAR3, C3P1, CARD8, CARTPT, CAV1, CCDC8, CCNG1, CCNP, CDK5R2, CST5, CTSD, DLG2, DMPK, DNAJC24, DOCK1, DOK7, DPM3, DRD2, EGFR, ELL, ERBB4, GFRAL, GNAS, GNAT1, GPSM2, GRIN2A, GRM7, HGF, HIP1, HLA-DRB1, HMBOX1, HRG, HSPA2, HYAL2, IGF1R, ITGB2, LCP1, LYN, MAGI2, MAP2, MAP3K1, MAP3K5, MAPRE2, MBL2, MBP, MTRR, NGF, NLRC5, NOS1AP, NOSTRIN, NR1H3, OAZ1, ORAI1, PAK1, PAK4, PARP8, PCDHA6, PCIF1, PHACTR1, PHACTR3, PLCE1, PNKD, PPP1R3B, PPP2R3A, PPP2R5A, PPP3CA, PPP6R2, PRKAA1, PRKAG2, PRKAR2B, PRKCA, PRKCZ, PRKN, PTPN22, PTPRN2, PTPRQ, PTPRT, RABGAP1L, RALGAPA2, RASAL2, RB1, RCAN2, RGS12, RGS3, RIMS1, RNH1, ROBO1, SERINC2, SERINC5, SERPINA10, SERPINA4, SHOC2, SKI, SLC6A2, SMAP1, SOX7, ST18, STARD13, STIM1, STK25, STXBP5L, TBC1D1, TBC1D17, TBC1D19, TBC1D5, TBC1D8, TERF1, TESC, TFPI2, TIAM1, TIAM2, TMEM132D, TNFAIP8, TNFRSF11A, TP73, TPX2, TRAF2, TRAF7, TSC1, UMODL1, USP6NL, VAC14, VEGFC, WASL, WRAP53, ZCCHC9]
GO:0010647	positive regulation of cell communication	3,08E-05	[3, 4, 5]	316,00	15,57	29,77	40,17	30,06	[ABRA, ADAM8, AIF1, AIM2, ANGPT1, AREG, ATF3, BANK1, BLK, BMPR1B, CALCR, CARD11, CASP8, CCL19, CCL24, CCR2, CD180, CD2, CD244, CD27, CD3E, CD4, CD40LG, CD74, CD80, CD86, CLCF1, CLEC4E, CLEC6A, CNR1, CRB2, CRTAM, CSF1R, CXCR4, DEPDC1B, DRD1, ELF3, EREG, ERFE, FASLG, FCN1, FCRL3, FFAR4, FGD2, FGR, FLT3, GDF6, GFI1, GPR174, GPR55, HCLS1, HLA-DRB1, ICOS, IFNG, IKBKE, IL10RA, IL18, IL26, IL7R, KMO, LCK, LCP1, LMCD1, LY86, MSX1, MUSK, MYB, NCF1, NLRP3, NPPC, NSG1, ORM2, P2RY10, P2RY12, PIK3CG, PIM2, PROK1, PTK2B, PTPN22, PTPN6, PTPRC, PYHIN1, RAB3B, RTKN2, SEMA4D, STAP1, SYK, TESPA1, TLR6, TLR8, TNFAIP3, TNFRSF18, TNR, TRAF1, TRAT1, TREM2, UBD, WNT1, WNT10B, XCL1, XCL2, ZAP70, ZBED2]	[ABAT, ACVR1B, ADA, ADCY1, ADGRV1, AGAP2, AIM2, AKAP13, AKAP6, ANK3, ARHGEF3, ARRB2, AUTS2, BCAR3, BCL2, BDNF, BIRC3, BMP7, BMPR1A, CACNB2, CASP8, CC2D1A, CCM2, CCR7, CD160, CD28, CD38, CD44, CDH2, CFLAR, CHSY1, CLEC16A, CX3CL1, DEDD2, DIXDC1, ETFA, F2R, GATD3A, GHR, GLIPR2, GPR68, GPRC5B, GRB2, GRIK5, GSK3B, GSN, HOMER1, HTT, IGF1R, IGFBP4, IL17RA, IL34, INAVA, ITGA8, KLB, KSR1, LCK, LCP1, LGR4, LITAF, MAML3, MAP2K2, MAP2K5, MAP4K5, MBP, MDFIC, MED1, MIER1, MIOS, MRTFA, MTNR1B, NCK2, NEO1, NETO1, NFAT5, NLRC5, NOS1AP, NPR2, NPTN, NTRK2, OPRM1, PDCD10, PDZD2, PIGU, PIK3CD, PLCE1, PPP3CC, PRKCE, PRKD2, PSAP, PSMB8, PSMB9, PSMD9, PTK2B, PTPN1, PTPN2, PTPN22, PTPN6, PTPRC, PTPRJ, RAB34, RECK, RORA, RPTOR, RRAGC, RWDD3, S100A13, SEMA4D, SIK3, SLAMF1, SLC4A8, SMARCA4, SQSTM1, SSTR4, STAT3, TANK, TERT, TESPA1, TGFB2, TIMP2, TMEM108, TNF, TNFAIP3, TNFSF10, TP53BP2, TRAF1, TRAF3, TSHZ3, TSPAN5, TTC23, UBE3A, WWOX, YAP1, ZBP1, ZC3H12A, ZC3H3, ZMIZ1, ZNF385A, ZNF423]	[ABRA, ADORA1, ADRA1B, ADRB3, AIM2, AKAP12, AKAP13, ALK, ALOX15, ANK3, ARHGEF3, ARNT, ASXL1, BCAR3, BCLAF1, BTNL2, CACNG2, CALB2, CARD11, CARD8, CARTPT, CAV1, CDH13, CHSY1, CHUK, CLSTN2, DCN, DENND2B, DRD2, EGFR, ERBB4, F10, FGF7, FGF9, FOXP1, GCNT2, GDF6, GFRAL, GNAS, GRIK2, GRIN2A, HGF, HIP1, HIPK2, HLA-DRB1, HYAL2, IGF1R, IGSF9B, KIF7, LCP1, LMO3, LPAR6, LYN, MAML2, MAP3K1, MAP3K5, MAPRE2, MBL2, MBP, MIER1, MMP12, NACC2, NDRG4, NGF, NLRC5, NOS1AP, NR1H3, NUP93, P2RX2, PAK1, PFKM, PLCE1, PPP3CA, PRKAA1, PRKCA, PRKCZ, PRKN, PSMD7, PTPN22, RIMS1, RNF13, ROBO1, ROBO2, RSPO1, SCEL, SEMA5A, SHANK1, SHOC2, SKI, SLC6A2, SRI, ST18, STK25, TIAM1, TLR1, TNFRSF11A, TP73, TRAF2, TRAF7, USP8, VSNL1, WDFY1, WNK2, WNT16]
GO:0023056	positive regulation of signaling	4,94E-05	[2, 3, 4]	316,00	15,51	29,86	40,29	29,86	[ABRA, ADAM8, AIF1, AIM2, ANGPT1, AREG, ATF3, BANK1, BLK, BMPR1B, CALCR, CARD11, CASP8, CCL19, CCL24, CCR2, CD180, CD2, CD244, CD27, CD3E, CD4, CD40LG, CD74, CD80, CD86, CLCF1, CLEC4E, CLEC6A, CNR1, CRB2, CRTAM, CSF1R, CXCR4, DEPDC1B, DRD1, ELF3, EREG, ERFE, FASLG, FCN1, FCRL3, FFAR4, FGD2, FGR, FLT3, GDF6, GFI1, GPR174, GPR55, HCLS1, HLA-DRB1, ICOS, IFNG, IKBKE, IL10RA, IL18, IL26, IL7R, KMO, LCK, LCP1, LMCD1, LY86, MSX1, MUSK, MYB, NCF1, NLRP3, NPPC, NSG1, ORM2, P2RY10, P2RY12, PIK3CG, PIM2, PROK1, PTK2B, PTPN22, PTPN6, PTPRC, PYHIN1, RAB3B, RTKN2, SEMA4D, STAP1, SYK, TESPA1, TLR6, TLR8, TNFAIP3, TNFRSF18, TNR, TRAF1, TRAT1, TREM2, UBD, WNT1, WNT10B, XCL1, XCL2, ZAP70, ZBED2]	[ABAT, ACVR1B, ADA, ADCY1, ADGRV1, AGAP2, AIM2, AKAP13, AKAP6, ARHGEF3, ARRB2, AUTS2, BCAR3, BCL2, BDNF, BIRC3, BMP7, BMPR1A, CACNB2, CASP8, CC2D1A, CCM2, CCR7, CD160, CD28, CD38, CD44, CDH2, CFLAR, CHSY1, CLEC16A, CX3CL1, DEDD2, DIXDC1, ECE1, ETFA, F2R, GATD3A, GHR, GLIPR2, GPR68, GPRC5B, GRB2, GRIK5, GSK3B, GSN, HOMER1, HTT, IGF1R, IGFBP4, IL17RA, IL34, INAVA, ITGA8, KLB, KSR1, LCK, LCP1, LGR4, LITAF, MAML3, MAP2K2, MAP2K5, MAP4K5, MBP, MDFIC, MED1, MIER1, MIOS, MRTFA, MTNR1B, NCK2, NEO1, NETO1, NFAT5, NLRC5, NOS1AP, NPR2, NPTN, NTRK2, OPRM1, PDCD10, PDZD2, PIGU, PIK3CD, PLCE1, PPP3CC, PRKCE, PRKD2, PSAP, PSMB8, PSMB9, PSMD9, PTK2B, PTPN1, PTPN2, PTPN22, PTPN6, PTPRC, PTPRJ, RAB34, RECK, RORA, RPTOR, RRAGC, RWDD3, S100A13, SEMA4D, SIK3, SLAMF1, SLC4A8, SMARCA4, SQSTM1, SSTR4, STAT3, TANK, TERT, TESPA1, TGFB2, TIMP2, TMEM108, TNF, TNFAIP3, TNFSF10, TP53BP2, TRAF1, TRAF3, TSHZ3, TSPAN5, TTC23, UBE3A, WWOX, YAP1, ZBP1, ZC3H12A, ZC3H3, ZMIZ1, ZNF385A, ZNF423]	[ABRA, ADORA1, ADRA1B, ADRB3, AIM2, AKAP12, AKAP13, ALK, ALOX15, ARHGEF3, ARNT, ASXL1, BCAR3, BCLAF1, BTNL2, CACNG2, CALB2, CARD11, CARD8, CARTPT, CAV1, CDH13, CHSY1, CHUK, CLSTN2, DCN, DENND2B, DRD2, EGFR, ERBB4, F10, FGF7, FGF9, FOXP1, GCNT2, GDF6, GFRAL, GNAS, GRIK2, GRIN2A, HGF, HIP1, HIPK2, HLA-DRB1, HYAL2, IGF1R, IGSF9B, KIF7, LCP1, LMO3, LPAR6, LYN, MAML2, MAP3K1, MAP3K5, MAPRE2, MBL2, MBP, MIER1, MMP12, NACC2, NDRG4, NGF, NLRC5, NOS1AP, NR1H3, NUP93, P2RX2, PAK1, PFKM, PLCE1, PPP3CA, PRKAA1, PRKCA, PRKCZ, PRKN, PSMD7, PTPN22, RIMS1, RNF13, ROBO1, ROBO2, RSPO1, SCEL, SEMA5A, SHANK1, SHOC2, SKI, SLC6A2, SRI, ST18, STK25, TIAM1, TLR1, TNFRSF11A, TP73, TRAF2, TRAF7, USP8, VSNL1, WDFY1, WNK2, WNT16]

GO:1902531	regulation of intracellular signal transduction	4,95E-05	[4, 5, 6]	300,00	15,64	27,36	42,55	30,09	[ABRA, ADAM8, ANGPT1, ARAP2, AREG, ARHGAP15, ARHGAP30, ATF3, BANK1, CALCR, CARD11, CASP8, CCL19, CCL24, CD27, CD3E, CD4, CD40LG, CD74, CD80, CD86, CLEC6A, CSF1R, CXCR4, DEPDC1B, DUSP2, DUSP8, EREG, FASLG, FCRL3, FFAR4, FGD2, FGD3, FGR, FLT3, GDF6, GPR174, GPR55, HCLS1, HLA-DRB1, ICOS, IER3, IKBKE, IL18, IL26, JPH1, LAX1, LCK, LMCD1, MSX1, MUSK, NCF1, NLRP3, NLRP6, NPFFR2, NPPC, P2RY10, P2RY12, PIK3CG, PIM2, PLEK, PROK1, PSCA, PSD4, PTK2B, PTPN22, PTPN6, PTPRC, PYHIN1, RAD9B, RHOH, RNASE6, RTKN2, SEMA4D, SPINK1, STAT1, SYK, TAGAP, TBC1D10C, TLR6, TNFAIP3, TNIP3, TRAF1, TRAT1, TREM2, UBD, VNN1, XCL1, XCL2, ZAP70]	[ADA, ADGRV1, AGAP2, AKAP13, AKAP6, ARHGAP25, ARHGAP26, ARHGEF3, ARRB2, AUTS2, BCAR3, BCL2, BIRC3, BMP7, C12orf66, CASP8, CAVIN4, CC2D1A, CCM2, CCR7, CD28, CD2AP, CD44, CDC42SE2, CDH2, CFLAR, CGNL1, CHN2, CLEC16A, CX3CL1, DNM2, DUSP16, EHMT1, ETFA, F2R, FAM13A, GBP1, GHR, GLIPR2, GPRC5B, GRB2, GRIK5, GSK3B, HDAC1, HDAC9, HOMER2, HTT, IGF1R, IGFBP4, IL34, INAVA, IQSEC1, IQSEC3, ITPR1, KCTD11, KLB, KSR1, LAX1, LCK, LIME1, LITAF, MAP2K2, MAP2K5, MAP4K5, MARK3, MDFIC, MIER1, MIOS, MTNR1B, MYO9B, NCK2, NFAT5, NOS1AP, NPR2, NPTN, NTRK2, OPRM1, PDCD10, PDZD2, PHF20, PHLPP2, PIGU, PIK3CD, PIP4K2A, PIP5K1B, PLCE1, PPP1R13L, PPP2R5C, PRKAG2, PRKAR1B, PRKCE, PRKD2, PSAP, PTK2B, PTPN1, PTPN2, PTPN22, PTPN6, PTPRC, PTPRJ, RASA3, RFFL, RGS3, RIPOR2, RORA, RPA3, RPTOR, RRAGC, S100A13, SEMA4D, SETD9, SGMS1, SGSM3, SH3BP1, SIK3, SIPA1L1, SLA2, SLAMF1, SQSTM1, SRGAP3, SSTR4, STAT1, TANK, TBC1D10C, TGFB2, TIMP2, TNF, TNFAIP3, TNFSF10, TNIP1, TP53BP2, TRAF1, TRAF3, TRIO, TTI1, UACA, UBE3A, VRK2, ZC3H12A, ZNF385A]	[ABRA, ADORA1, ADRA1B, ADRB3, AKAP12, AKAP13, ALK, ALOX15, ANKS1B, ARHGAP24, ARHGAP26, ARHGAP32, ARHGAP44, ARHGEF10, ARHGEF3, BCAR3, BCLAF1, CARD11, CARD8, CARTPT, CAV1, CDH13, CHUK, DCN, DENND2B, DRD2, EGFR, ERBB4, F10, FGF7, FGF9, GCNT2, GDF6, GFRAL, GNAS, GRIK2, HGF, HIP1, HIPK2, HLA-DRB1, HLA-G, HYAL2, IGF1R, ITPR1, LIMD1, LMO3, LPAR6, LYN, MAGI2, MAP3K1, MAP3K5, MAPRE2, MIER1, MYADM, NACC2, NCOR1, NDRG4, NGF, NOS1AP, P2RX2, PAK1, PDE11A, PDE3B, PLCE1, PPP3CA, PRDX1, PRKAA1, PRKAG2, PRKAR2B, PRKCA, PRKCZ, PRKN, PSD2, PTPN22, RALGAPA2, RCAN2, RGS3, RIPOR2, RNF13, ROBO1, SEMA5A, SHOC2, SLC6A2, SMYD2, SRI, STARD13, STK25, TAF2, TIAM1, TIAM2, TNFRSF11A, TP73, TPX2, TRAF2, TRAF7, TSC1, WFS1, WNK2, WNT16]
GO:0023014	signal transduction by protein phosphorylation	1,71E-04	[3, 4, 5, 7, 8]	171,00	17,22	28,19	38,83	32,98	[ADAM8, ANGPT1, AREG, ATF3, BANK1, CALCR, CCL19, CCL24, CCR5, CD27, CD4, CD40LG, CD74, CLEC1B, CSF1R, CSF2RA, CXCR4, DUSP2, DUSP8, EREG, FCRL3, FFAR4, FGD2, FLT3, GDF6, GFRA2, GPR55, HLA-DRB1, IL18, IL26, IL2RG, IL5RA, LAX1, MAPK13, MUSK, NCF1, NLRP3, NLRP6, NPFFR2, PIK3CG, PROK1, PSCA, PTK2B, PTPN22, PTPN6, PTPRC, SYK, TBC1D10C, TLR6, TRAF1, TREM2, XCL1, XCL2]	[AKAP13, ARRB2, BCAR3, BMP7, C6orf89, CAB39, CCM2, CCR7, CD44, CDH2, CFLAR, CX3CL1, DUSP16, ETFA, F2R, GBP1, GFRA2, GHR, GLIPR2, GRB2, GRIK5, IGF1R, IGFBP4, IL34, INAVA, JAK3, KCTD11, KLB, KSR1, LAX1, LIME1, MAP2K2, MAP2K5, MAP4K5, MARK3, MBP, MDFIC, MED1, NPTN, NTRK2, OPRM1, PDCD10, PDZD2, PLCE1, PRKCE, PRKD2, PSAP, PSMB8, PSMB9, PSMD9, PTK2B, PTPN1, PTPN2, PTPN22, PTPN6, PTPRC, PTPRJ, RASA3, RGS3, RORA, SLAMF1, SSTR4, STK10, TANK, TBC1D10C, TGFB2, TIMP2, TNF, TNIP1, TRAF1, TRAF3, VRK2, ZC3H12A]	[ADORA1, ADRA1B, ADRB3, AKAP12, AKAP13, ALK, ALOX15, ANKS1B, BCAR3, CARTPT, CAV1, CHUK, DENND2B, DLG2, DRD2, EGFR, ERBB4, FGF7, FGF9, GCNT2, GDF6, GFRAL, GRIK2, HGF, HIPK2, HLA-DRB1, HYAL2, IGF1R, LMO3, LRRC7, LYN, MAP3K1, MAP3K5, MBL2, MBP, MEF2A, NCOR1, NDRG4, NGF, PAK1, PAK4, PLCE1, PPP3CA, PRDX1, PRKAA1, PRKCA, PRKCZ, PRKN, PSMD7, PTPN22, RASAL2, RGS3, ROBO1, SMAD1, STK25, TIAM1, TNFRSF11A, TP73, TRAF2, TRAF7, WNK2, WNT16]
GO:0043408	regulation of MAPK cascade	1,84E-04	[5, 6, 7, 8, 9, 10]	140,00	18,04	27,74	39,35	32,90	[ADAM8, ANGPT1, AREG, ATF3, BANK1, CALCR, CCL19, CCL24, CD27, CD4, CD40LG, CD74, CSF1R, CXCR4, DUSP2, DUSP8, FCRL3, FFAR4, FGD2, FLT3, GDF6, GPR55, HLA-DRB1, IL26, LAX1, MUSK, NCF1, NLRP6, NPFFR2, PIK3CG, PROK1, PSCA, PTK2B, PTPN22, PTPN6, PTPRC, SYK, TBC1D10C, TLR6, TRAF1, TREM2, XCL1, XCL2]	[AKAP13, ARRB2, BCAR3, BMP7, CCM2, CCR7, CD44, CDH2, CFLAR, CX3CL1, DUSP16, ETFA, F2R, GBP1, GHR, GLIPR2, GRB2, GRIK5, IGF1R, IGFBP4, IL34, INAVA, KCTD11, KLB, KSR1, LAX1, LIME1, MAP2K2, MAP2K5, MAP4K5, MDFIC, NPTN, NTRK2, OPRM1, PDCD10, PDZD2, PLCE1, PRKCE, PRKD2, PSAP, PTK2B, PTPN1, PTPN2, PTPN22, PTPN6, PTPRC, PTPRJ, RGS3, RORA, SLAMF1, SSTR4, TANK, TBC1D10C, TGFB2, TIMP2, TNF, TNIP1, TRAF1, TRAF3, VRK2, ZC3H12A]	[ADORA1, ADRA1B, ADRB3, AKAP12, AKAP13, ALK, ALOX15, ANKS1B, BCAR3, CARTPT, CAV1, DENND2B, DRD2, EGFR, ERBB4, FGF9, GCNT2, GDF6, GFRAL, GRIK2, HGF, HIPK2, HLA-DRB1, HYAL2, IGF1R, LMO3, LYN, MAP3K1, MAP3K5, NCOR1, NDRG4, NGF, PAK1, PLCE1, PPP3CA, PRDX1, PRKAA1, PRKCA, PRKCZ, PRKN, PTPN22, RGS3, ROBO1, STK25, TIAM1, TNFRSF11A, TP73, TRAF2, TRAF7, WNK2, WNT16]
GO:1902533	positive regulation of intracellular signal transduction	1,90E-04	[4, 5, 6, 7]	187,00	16,91	30,43	39,13	30,43	[ABRA, ADAM8, ANGPT1, AREG, BANK1, CALCR, CARD11, CASP8, CCL19, CCL24, CD27, CD3E, CD4, CD40LG, CD74, CD80, CD86, CLEC6A, CSF1R, CXCR4, EREG, FASLG, FCRL3, FFAR4, FGD2, FGR, FLT3, GDF6, GPR174, GPR55, HCLS1, HLA-DRB1, ICOS, IKBKE, IL18, IL26, LCK, LMCD1, MSX1, MUSK, NCF1, NPPC, P2RY10, P2RY12, PIK3CG, PIM2, PROK1, PTK2B, PTPN22, PTPN6, PTPRC, PYHIN1, RTKN2, SEMA4D, SYK, TLR6, TRAF1, TRAT1, TREM2, UBD, XCL1, XCL2, ZAP70]	[ADA, ADGRV1, AGAP2, AKAP13, AKAP6, ARHGEF3, ARRB2, AUTS2, BCAR3, BCL2, BIRC3, CASP8, CC2D1A, CCM2, CCR7, CD28, CD44, CDH2, CFLAR, CLEC16A, CX3CL1, ETFA, F2R, GHR, GLIPR2, GPRC5B, GRB2, HTT, IGF1R, IGFBP4, IL34, INAVA, KLB, KSR1, LCK, LITAF, MAP2K2, MAP2K5, MAP4K5, MDFIC, MIER1, MIOS, NCK2, NFAT5, NOS1AP, NPR2, NPTN, NTRK2, OPRM1, PDCD10, PIGU, PIK3CD, PLCE1, PRKCE, PRKD2, PSAP, PTK2B, PTPN1, PTPN2, PTPN22, PTPN6, PTPRC, PTPRJ, RORA, RPTOR, RRAGC, S100A13, SEMA4D, SIK3, SLAMF1, SSTR4, TANK, TGFB2, TIMP2, TNF, TNFSF10, TRAF1, TRAF3, UBE3A, ZC3H12A, ZNF385A]	[ABRA, ADORA1, ADRA1B, ADRB3, AKAP12, AKAP13, ALK, ALOX15, ARHGEF3, BCAR3, BCLAF1, CARD11, CARTPT, CAV1, CDH13, CHUK, DCN, DENND2B, DRD2, EGFR, ERBB4, F10, FGF7, FGF9, GCNT2, GDF6, GFRAL, GNAS, HGF, HIP1, HIPK2, HLA-DRB1, IGF1R, LPAR6, LYN, MAP3K1, MAP3K5, MAPRE2, MIER1, NACC2, NDRG4, NGF, NOS1AP, P2RX2, PAK1, PLCE1, PRKAA1, PRKCA, PRKCZ, PRKN, PTPN22, RNF13, ROBO1, SEMA5A, SHOC2, SLC6A2, STK25, TIAM1, TNFRSF11A, TP73, TRAF2, TRAF7, WNT16]
GO:0043085	positive regulation of catalytic activity	3,52E-04	[4]	250,00	15,84	27,27	38,55	34,18	[ADAM8, AIM2, ANGPT1, ARAP2, AREG, ARHGAP15, ARHGAP30, CASP8, CCL19, CCL24, CD4, CD40LG, CD74, CDC20B, CDKN2A, CSF1R, CXCR4, DAPK1, DEPDC1B, DOCK10, DOCK2, DOCK8, EDN2, EPHA6, EREG, FASLG, FCRL3, FGD2, FGR, FLT3, GBA3, GPR55, GPRC5A, HLA-DRB1, IFNG, IL18, ITK, LCK, LCP2, LPAL2, MID1IP1, MUSK, NCF1, NCF1B, NCF1C, NCKAP1L, NEK5, NLRP3, NMUR1, NR4A2, P2RY12, PARP8, PIK3CG, PLAAT4, PLEK, PRKCQ, PROK1, PTAFR, PTK2B, PTPRC, RGS1, RGS10, RGS18, SELE, SEMA4D, SH2D1A, SMAP2, STAP1, SYK, TAGAP, TBC1D10C, THY1, TLR6, XCL1, XCL2]	[ACAP1, ACER2, ADCY1, AFDN, AGAP1, AGAP2, AGRN, AIM2, AKAP13, ALS2CL, ARHGAP25, ARHGAP26, ARHGEF10L, ASAP2, BCAR3, BCL2, BDNF, C6orf89, CAB39, CACNA1A, CASP8, CCND3, CCR7, CD44, CDC25B, CFLAR, CHN2, CORO1C, CX3CL1, DAPK1, DIABLO, DOCK1, DOCK3, EPHA10, ETFA, F2R, FAM13A, GHR, GNAQ, GPRC5B, GSK3B, GSN, IGF1R, IL34, INAVA, IQSEC1, KSR1, LCK, LCP2, MAP2K2, MAP2K5, MAP4K5, MARK2, MBP, MDFIC, MRTFA, MYO9B, NCAPH, NCF4, NOS1AP, NTRK2, PDCD10, PDZD2, PKP4, PLAAT4, PLCE1, PLEKHG6, PLSCR1, PPP2R5C, PRKACB, PRKAG2, PRKAR1B, PRKCE, PRKD2, PSAP, PTK2B, PTPN1, PTPRC, RALB, RASA3, RGS3, RIN2, RIN3, RORA, RPTOR, RSU1, SEMA4D, SGSM3, SH3BP1, SIPA1L1, SNX9, SRGAP3, STAT3, STK10, TANK, TBC1D10C, TBC1D2B, TERT, TGFB2, TIMP2, TNF, TNFSF10, TNIP1, UACA, UBE2L3, USP6NL]	[ACAP2, ADAP1, ADCY5, ADORA1, AGAP1, AIM2, AKAP13, ALK, ARHGAP24, ARHGAP26, ARHGAP32, ARHGAP44, ARHGEF10, ATP1B1, ATP2A3, BCAR3, CARD8, CARTPT, CAV1, CDK5R2, CTSD, DNAJC24, DOCK1, DOK7, DPM3, DRD2, EGFR, ERBB4, GFRAL, GNAS, GNAT1, GRIN2A, HGF, HIP1, HLA-DRB1, HMBOX1, HSPA2, IGF1R, ITGB2, LYN, MAGI2, MAP3K1, MAP3K5, MAPRE2, MBL2, MBP, NGF, NOS1AP, NR1H3, ORAI1, PAK1, PAK4, PARP8, PLCE1, PPP2R5A, PPP3CA, PRKAA1, PRKAG2, PRKAR2B, PRKCA, PRKCZ, RABGAP1L, RALGAPA2, RASAL2, RGS12, RGS3, ROBO1, SERINC2, SERINC5, SLC6A2, SMAP1, SOX7, ST18, STARD13, STIM1, STK25, STXBP5L, TBC1D1, TBC1D17, TBC1D19, TBC1D5, TBC1D8, TIAM1, TIAM2, TNFRSF11A, TP73, TPX2, TRAF2, TRAF7, TSC1, USP6NL, VAC14, VEGFC, WRAP53]
GO:0000165	MAPK cascade	3,93E-04	[4, 5, 6, 8, 9]	167,00	17,15	28,26	38,59	33,15	[ADAM8, ANGPT1, AREG, ATF3, BANK1, CALCR, CCL19, CCL24, CCR5, CD27, CD4, CD40LG, CD74, CSF1R, CSF2RA, CXCR4, DUSP2, DUSP8, EREG, FCRL3, FFAR4, FGD2, FLT3, GDF6, GFRA2, GPR55, HLA-DRB1, IL18, IL26, IL2RG, IL5RA, LAX1, MAPK13, MUSK, NCF1, NLRP3, NLRP6, NPFFR2, PIK3CG, PROK1, PSCA, PTK2B, PTPN22, PTPN6, PTPRC, SYK, TBC1D10C, TLR6, TRAF1, TREM2, XCL1, XCL2]	[AKAP13, ARRB2, BCAR3, BMP7, C6orf89, CCM2, CCR7, CD44, CDH2, CFLAR, CX3CL1, DUSP16, ETFA, F2R, GBP1, GFRA2, GHR, GLIPR2, GRB2, GRIK5, IGF1R, IGFBP4, IL34, INAVA, JAK3, KCTD11, KLB, KSR1, LAX1, LIME1, MAP2K2, MAP2K5, MAP4K5, MARK3, MBP, MDFIC, MED1, NPTN, NTRK2, OPRM1, PDCD10, PDZD2, PLCE1, PRKCE, PRKD2, PSAP, PSMB8, PSMB9, PSMD9, PTK2B, PTPN1, PTPN2, PTPN22, PTPN6, PTPRC, PTPRJ, RASA3, RGS3, RORA, SLAMF1, SSTR4, TANK, TBC1D10C, TGFB2, TIMP2, TNF, TNIP1, TRAF1, TRAF3, VRK2, ZC3H12A]	[ADORA1, ADRA1B, ADRB3, AKAP12, AKAP13, ALK, ALOX15, ANKS1B, BCAR3, CARTPT, CAV1, CHUK, DENND2B, DLG2, DRD2, EGFR, ERBB4, FGF7, FGF9, GCNT2, GDF6, GFRAL, GRIK2, HGF, HIPK2, HLA-DRB1, HYAL2, IGF1R, LMO3, LRRC7, LYN, MAP3K1, MAP3K5, MBL2, MBP, MEF2A, NCOR1, NDRG4, NGF, PAK1, PLCE1, PPP3CA, PRDX1, PRKAA1, PRKCA, PRKCZ, PRKN, PSMD7, PTPN22, RASAL2, RGS3, ROBO1, SMAD1, STK25, TIAM1, TNFRSF11A, TP73, TRAF2, TRAF7, WNK2, WNT16]

GO:0009967	positive regulation of signal transduction	5,06E-04 [3, 4, 5, 6]	274,00	15,51	28,19	41,61	30,20 [ABRA, ADAM8, ANGPT1, AREG, ATF3, BANK1, BMPR1B, CALCR, CARD11, CASP8, CCL19, CCL24, CCR2, CD180, CD27, CD3E, CD4, CD40LG, CD74, CD80, CD86, CLCF1, CLEC6A, CRB2, CSF1R, CXCR4, DEPDC1B, ELF3, EREG, ERFE, FASLG, FCRL3, FFAR4, FGD2, FGR, FLT3, GDF6, GFI1, GPR174, GPR55, HCLS1, HLA-DRB1, ICOS, IFNG, IKBKE, IL10RA, IL18, IL26, IL7R, LCK, LMCD1, LY86, MSX1, MUSK, NCF1, NPPC, P2RY10, P2RY12, PIK3CG, PIM2, PROK1, PTK2B, PTPN22, PTPN6, PTPRC, PYHIN1, RTKN2, SEMA4D, STAP1, SYK, TESPA1, TLR6, TNFAIP3, TNFRSF18, TRAF1, TRAT1, TREM2, UBD, WNT1, WNT10B, XCL1, XCL2, ZAP70, ZBED2]	[ABAT, ACVR1B, ADA, ADGRV1, AGAP2, AKAP13, AKAP6, ARHGEF3, ARRB2, AUTS2, BCAR3, BCL2, BDNF, BIRC3, BMP7, BMPR1A, CASP8, CC2D1A, CCM2, CCR7, CD28, CD44, CDH2, CFLAR, CHSY1, CLEC16A, CX3CL1, DEDD2, DIXDC1, ETFA, F2R, GATD3A, GHR, GLIPR2, GPRC5B, GRB2, GSK3B, GSN, HOMER1, HTT, IGF1R, IGFBP4, IL34, INAVA, ITGA8, KLB, KSR1, LCK, LGR4, LITAF, MAML3, MAP2K2, MAP2K5, MAP4K5, MDFIC, MED1, MIER1, MIOS, MRTFA, NCK2, NEO1, NETO1, NFAT5, NLRC5, NOS1AP, NPR2, NPTN, NTRK2, OPRM1, PDCD10, PDZD2, PIGU, PIK3CD, PLCE1, PPP3CC, PRKCE, PRKD2, PSAP, PSMB8, PSMB9, PSMD9, PTK2B, PTPN1, PTPN2, PTPN22, PTPN6, PTPRC, PTPRJ, RAB34, RECK, RORA, RPTOR, RRAGC, RWDD3, S100A13, SEMA4D, SIK3, SLAMF1, SMARCA4, SSTR4, STAT3, TANK, TERT, TESPA1, TGFB2, TIMP2, TMEM108, TNF, TNFAIP3, TNFSF10, TP53BP2, TRAF1, TRAF3, TSPAN5, TTC23, UBE3A, WWOX, YAP1, ZBP1, ZC3H12A, ZC3H3, ZMIZ1, ZNF385A, ZNF423]	[ABRA, ADORA1, ADRA1B, ADRB3, AKAP12, AKAP13, ALK, ALOX15, ARHGEF3, ARNT, ASXL1, BCAR3, BCLAF1, CACNG2, CARD11, CARTPT, CAV1, CDH13, CHSY1, CHUK, DCN, DENND2B, DRD2, EGFR, ERBB4, F10, FGF7, FGF9, FOXP1, GCNT2, GDF6, GFRAL, GNAS, HGF, HIP1, HIPK2, HLA-DRB1, HYAL2, IGF1R, IGSF9B, KIF7, LMO3, LPAR6, LYN, MAML2, MAP3K1, MAP3K5, MAPRE2, MIER1, MMP12, NACC2, NDRG4, NGF, NLRC5, NOS1AP, NR1H3, NUP93, P2RX2, PAK1, PLCE1, PPP3CA, PRKAA1, PRKCA, PRKCZ, PRKN, PSMD7, PTPN22, RIMS1, RNF13, ROBO1, ROBO2, RSPO1, SCEL, SEMA5A, SHANK1, SHOC2, SKI, SLC6A2, ST18, STK25, TIAM1, TLR1, TNFRSF11A, TP73, TRAF2, TRAF7, USP8, WDFY1, WNK2, WNT16]
GO:0006468	protein phosphorylation	1,03E-03 [6, 7]	317,00	15,03	27,92	41,31	30,77 [ADAM8, AIF1, ANGPT1, AREG, ATF3, BANK1, BLK, BMPR1B, BTK, CALCR, CAMK4, CCL19, CCL24, CCR5, CD27, CD3E, CD4, CD40LG, CD74, CD80, CDKN2A, CLCF1, CLEC1B, CSF1R, CSF2RA, CXCL10, CXCR4, DAPK1, DRD1, DUSP2, DUSP8, EPHA6, EREG, FCRL3, FFAR4, FGD2, FGR, FLT3, GDF6, GFRA2, GPR55, GPRC5A, HCLS1, HLA-DRB1, IFNG, IKBKE, IL12RB2, IL18, IL26, IL2RG, IL5RA, ITK, LAX1, LCK, LCP2, MAPK13, MUSK, MYO3B, NCF1, NCKAP1L, NEK5, NELL2, NLRP3, NLRP6, NPFFR2, NR4A3, PARP8, PIK3CG, PIM1, PIM2, PRKCQ, PROK1, PSCA, PTK2B, PTPN22, PTPN6, PTPRC, RUNX3, SEMA4D, SH2D1A, SLA, SPINK1, STAP1, STK17B, STYK1, SYK, TBC1D10C, THY1, TLR6, TLR8, TNFAIP3, TNFRSF18, TRAF1, TREM2, WNT1, XCL1, XCL2, ZAP70]	[ACVR1B, ADAM10, ADCY1, AGAP2, AKAP13, AKAP9, ARRB2, BCAR3, BCL2, BDNF, BMP7, BMPR1A, C6orf89, CAB39, CAMK1G, CAMK2G, CCDC88C, CCM2, CCND3, CCR7, CD44, CDC25B, CDC42BPB, CDH2, CDK2AP1, CDK5RAP1, CDK6, CFLAR, COLEC11, CORO1C, CPNE3, CTDSPL, CX3CL1, DAPK1, DBNDD2, DOCK3, DUSP16, EEF2K, EPHA10, ETFA, F2R, FES, GAK, GATD3A, GBP1, GFRA2, GHR, GLIPR2, GNAQ, GPRC5B, GRB2, GRIK5, GRK3, GSK3B, GTF2H3, HTT, IGF1R, IGFBP4, IL12RB2, IL34, INAVA, JAK3, KCTD11, KLB, KSR1, LAX1, LCK, LCP2, LIME1, LTBP1, MAP2K2, MAP2K5, MAP4K5, MARK2, MARK3, MAST2, MAST4, MBP, MDFIC, MED1, NCK2, NIBAN1, NPR2, NPTN, NTRK2, OPRM1, PARD3, PDCD10, PDZD2, PIK3CD, PLCE1, PLK5, PPP2R5C, PRKACB, PRKAG2, PRKAR1B, PRKCE, PRKD2, PRKRIP1, PSAP, PSMB8, PSMB9, PSMD9, PTK2B, PTPN1, PTPN2, PTPN22, PTPN6, PTPRC, PTPRJ, RALB, RARA, RASA3, RGS3, RORA, RPS6KA2, RPTOR, RUNX3, SEMA4D, SGK1, SIK3, SLA, SLAMF1, SLCO3A1, SNX9, SQSTM1, SSTR4, STAT3, STK10, STK38L, TANK, TBC1D10C, TGFB2, TIMP2, TNF, TNFAIP3, TNIP1, TNK2, TRAF1, TRAF3, TRIO, UBASH3B, VRK2, WDFY2, ZC3H12A]	[ADCK2, ADCY5, ADORA1, ADRA1B, ADRB3, AKAP12, AKAP13, AKAP9, ALK, ALOX15, ANKS1B, BCAR3, CAMK4, CAMP, CARTPT, CAV1, CCNG1, CCNP, CDK13, CDK5R2, CELSR3, CHUK, COLEC11, DENND2B, DLG2, DMPK, DOK7, DRD2, EGFR, ERBB4, FGF7, FGF9, GCNT2, GDF6, GFRAL, GRIK2, GRK1, HGF, HIPK2, HLA-DRB1, HRG, HSPA2, HTATIP2, HUNK, HYAL2, IGF1R, ITGB2, LDLRAD4, LIMCH1, LMO3, LRRC7, LYN, MAP3K1, MAP3K5, MAST4, MBL2, MBP, MEF2A, MYADM, MYLK, NCOR1, NDRG4, NGF, PAK1, PAK4, PARP8, PLCE1, PLK5, PLPP3, PPARGC1A, PPP2R5A, PPP3CA, PRDX1, PRKAA1, PRKAG2, PRKAR2B, PRKCA, PRKCZ, PRKN, PSMD7, PTPN22, PTPRQ, PTPRT, RASAL2, RB1, RGS3, ROBO1, RPS6KA2, RSPO1, SMAD1, SMAD6, SPEG, ST3GAL1, STK25, TESC, TIAM1, TNFRSF11A, TOP1, TP73, TPX2, TRAF2, TRAF7, TSC1, VEGFC, WDFY2, WNK2, WNK4, WNT16]
GO:0023057	negative regulation of signaling	1,03E-03 [2, 3, 4]	239,00	15,74	23,35	41,25	35,41 [ANGPT1, AREG, ATF3, BANK1, CARD17, CASP8, CD200R1, CD3E, CD74, CHRDL1, CISH, CNR1, CNR2, DRD1, DUSP2, DUSP8, EREG, FASLG, FCRL3, FFAR4, FOXP3, FRZB, GPRC5A, INPP5D, IRF4, LAX1, LPAL2, NLRP3, NLRP6, NR4A2, P2RY12, PLEK, PRKCQ, PSCA, PTK2B, PTPN22, PTPN6, PTPRC, RGS1, RGS10, RGS18, RHOH, RTKN2, SMPDL3B, SPINK1, STAP1, STAT1, TBC1D10C, THY1, TLR6, TLR8, TNFAIP3, TNIP3, TNMD, TNR, TRABD2A, TRAT1, VNN1, VWC2L, WNT1]	[ABAT, ACAA2, ADA, ADAMTS12, AGAP2, ARHGAP25, ARRB2, BCL2, BDNF, BICD1, BMP7, C12orf66, C6orf89, CASP8, CCDC88C, CCM2, CCR7, CD160, CD2AP, CD38, CD44, CD47, CDH2, CFLAR, CGNL1, CHST11, CX3CL1, DNM2, DUSP16, F2R, GBP1, GRB2, GRIK5, GSK3B, HDAC1, HDAC9, HOMER2, HTT, IGF1R, IGFBP4, ITPR1, LAX1, LGR4, LITAF, LPXN, LTBP1, MAP2K5, MARK3, MCC, MMP14, MRTFA, MTNR1B, NCK2, NCOR2, NFATC1, NLRC5, OPRM1, PCDHA6, PDZD2, PHF14, PHLPP2, PPP2R5C, PRICKLE1, PRKACB, PRKD2, PSMB8, PSMB9, PSMD9, PTK2B, PTPN1, PTPN2, PTPN22, PTPN6, PTPRC, PTPRJ, RASA3, RBMS3, RFFL, RGS3, RIPOR2, RNF213, RORA, SH3BP1, SLA2, SLAMF1, SLIT1, SMARCA4, SSTR4, STAT1, TANK, TBC1D10C, TERT, TGFB2, TIMP2, TNF, TNFAIP3, TNFSF10, TNIP1, TNS2, TRABD2B, UACA, UBASH3B, WWOX, YAP1, ZC3H12A, ZNF385A]	[ABCC8, ADORA1, ANKS1B, ARHGAP24, ARHGAP44, ASXL1, BCHE, BPIFB1, BTNL2, CARD8, CARTPT, CAV1, CELF4, CTTN, DCC, DCN, DRAXIN, DRD2, EGFR, EPS15L1, EYA2, FBN2, FGF9, FOXH1, FOXP1, GFRAL, GPR161, GREM2, GRIK2, HGF, HIPK2, HLA-G, HRG, HYAL2, IGF1R, ITPR1, KIF7, LDLRAD4, LILRA4, LIMD1, LMO3, LYN, MAGI2, MCC, MMP12, MYADM, NCOR1, NDRG4, NFATC1, NLRC5, NR1H3, NXN, ONECUT2, PCDHA6, PCSK9, PDE11A, PDE3B, PIAS2, PNKD, PPP3CA, PRKAA1, PRKCA, PRKCZ, PRKN, PSMD7, PTPN22, PTPRT, RAB7A, RB1, RFX4, RGS12, RGS3, RIPOR2, RNF43, ROBO1, SALL3, SHISA6, SKI, SMAD6, SORCS3, SOX17, SRGN, SRI, STRN3, STXBP5L, TRAF2, TSC1, VSNL1, WFS1, WNK2, ZNF653]
GO:0043410	positive regulation of MAPK cascade	1,42E-03 [5, 6, 7, 8, 9, 10, 11]	108,00	18,59	28,57	38,66	32,77 [ADAM8, ANGPT1, AREG, BANK1, CALCR, CCL19, CCL24, CD27, CD4, CD40LG, CD74, CSF1R, CXCR4, FCRL3, FFAR4, FGD2, FLT3, GDF6, GPR55, HLA-DRB1, IL26, MUSK, NCF1, PIK3CG, PROK1, PTK2B, PTPN22, PTPRC, SYK, TLR6, TRAF1, TREM2, XCL1, XCL2]	[AKAP13, ARRB2, BCAR3, CCM2, CCR7, CD44, CDH2, CFLAR, CX3CL1, ETFA, F2R, GHR, GLIPR2, IGF1R, IGFBP4, IL34, INAVA, KLB, KSR1, MAP2K2, MAP2K5, MAP4K5, MDFIC, NPTN, NTRK2, OPRM1, PDCD10, PLCE1, PRKCE, PRKD2, PSAP, PTK2B, PTPN1, PTPN22, PTPRC, PTPRJ, RORA, SLAMF1, SSTR4, TANK, TGFB2, TIMP2, TNF, TRAF1, TRAF3, ZC3H12A]	[ADORA1, ADRA1B, ADRB3, AKAP12, AKAP13, ALK, ALOX15, BCAR3, CARTPT, DENND2B, DRD2, EGFR, ERBB4, FGF9, GCNT2, GDF6, GFRAL, HGF, HIPK2, HLA-DRB1, IGF1R, MAP3K1, MAP3K5, NDRG4, NGF, PAK1, PLCE1, PRKAA1, PRKCA, PRKCZ, PTPN22, ROBO1, STK25, TIAM1, TNFRSF11A, TP73, TRAF2, TRAF7, WNT16]
GO:0010648	negative regulation of cell communication	1,93E-03 [3, 4, 5]	237,00	15,64	23,14	41,57	35,29 [ANGPT1, AREG, ATF3, BANK1, CARD17, CASP8, CD200R1, CD3E, CD74, CHRDL1, CISH, CNR1, CNR2, DRD1, DUSP2, DUSP8, EREG, FASLG, FCRL3, FFAR4, FOXP3, FRZB, GPRC5A, INPP5D, IRF4, LAX1, LPAL2, NLRP3, NLRP6, NR4A2, P2RY12, PLEK, PRKCQ, PSCA, PTK2B, PTPN22, PTPN6, PTPRC, RGS1, RGS10, RGS18, RHOH, RTKN2, SMPDL3B, SPINK1, STAP1, STAT1, TBC1D10C, THY1, TLR6, TLR8, TNFAIP3, TNIP3, TNMD, TNR, TRABD2A, VNN1, VWC2L, WNT1]	[ABAT, ACAA2, ADA, ADAMTS12, AGAP2, ARHGAP25, ARRB2, BCL2, BDNF, BICD1, BMP7, C12orf66, C6orf89, CASP8, CCDC88C, CCM2, CCR7, CD160, CD2AP, CD38, CD44, CD47, CDH2, CFLAR, CGNL1, CHST11, CX3CL1, DNM2, DUSP16, F2R, GBP1, GRB2, GRIK5, GSK3B, HDAC1, HDAC9, HOMER2, HTT, IGF1R, IGFBP4, ITPR1, LAX1, LGR4, LITAF, LPXN, LTBP1, MAP2K5, MARK3, MCC, MMP14, MRTFA, MTNR1B, NCK2, NCOR2, NFATC1, NLRC5, OPRM1, PCDHA6, PDZD2, PHF14, PHLPP2, PPP2R5C, PRICKLE1, PRKACB, PRKD2, PSMB8, PSMB9, PSMD9, PTK2B, PTPN1, PTPN2, PTPN22, PTPN6, PTPRC, PTPRJ, RASA3, RBMS3, RFFL, RGS3, RIPOR2, RNF213, RORA, SH3BP1, SLA2, SLAMF1, SLIT1, SMARCA4, SSTR4, STAT1, TANK, TBC1D10C, TERT, TGFB2, TIMP2, TNF, TNFAIP3, TNFSF10, TNIP1, TNS2, TRABD2B, UACA, UBASH3B, WWOX, YAP1, ZC3H12A, ZNF385A]	[ABCC8, ADORA1, ANKS1B, ARHGAP24, ARHGAP44, ASXL1, BCHE, BPIFB1, BTNL2, CARD8, CARTPT, CAV1, CELF4, CTTN, DCC, DCN, DRAXIN, DRD2, EGFR, EPS15L1, EYA2, FBN2, FGF9, FOXH1, FOXP1, GFRAL, GPR161, GREM2, GRIK2, HGF, HIPK2, HLA-G, HRG, HYAL2, IGF1R, ITPR1, KIF7, LDLRAD4, LILRA4, LIMD1, LMO3, LYN, MAGI2, MCC, MMP12, MYADM, NCOR1, NDRG4, NFATC1, NLRC5, NR1H3, NXN, ONECUT2, PCDHA6, PDE11A, PDE3B, PIAS2, PNKD, PPP3CA, PRKAA1, PRKCA, PRKCZ, PRKN, PSMD7, PTPN22, PTPRT, RAB7A, RB1, RFX4, RGS12, RGS3, RIPOR2, RNF43, ROBO1, SALL3, SHISA6, SKI, SMAD6, SORCS3, SOX17, SRGN, SRI, STRN3, STXBP5L, TRAF2, TSC1, VSNL1, WFS1, WNK2, ZNF653]
GO:0001932	regulation of protein phosphorylation	9,73E-03 [6, 7, 8]	237,00	15,35	27,31	40,77	31,92 [ADAM8, AIF1, ANGPT1, AREG, ATF3, BANK1, CALCR, CCL19, CCL24, CD27, CD3E, CD4, CD40LG, CD74, CD80, CDKN2A, CLCF1, CSF1R, CXCL10, CXCR4, DRD1, DUSP2, DUSP8, EREG, FCRL3, FFAR4, FGD2, FGR, FLT3, GDF6, GFRA2, GPR55, GPRC5A, HCLS1, HLA-DRB1, IFNG, IL18, IL26, LAX1, LCP2, MUSK, NCF1, NCKAP1L, NLRP3, NLRP6, NPFFR2, NR4A3, PARP8, PIK3CG, PROK1, PSCA, PTK2B, PTPN22, PTPN6, PTPRC, SEMA4D, SH2D1A, SPINK1, STAP1, SYK, TBC1D10C, THY1, TLR6, TLR8, TNFAIP3, TNFRSF18, TRAF1, TREM2, WNT1, XCL1, XCL2]	[ACVR1B, ADCY1, AGAP2, AKAP13, AKAP9, ARRB2, BCAR3, BCL2, BDNF, BMP7, BMPR1A, CAB39, CCDC88C, CCM2, CCND3, CCR7, CD44, CDC25B, CDH2, CDK2AP1, CDK5RAP1, CFLAR, CORO1C, CTDSPL, CX3CL1, DBNDD2, DOCK3, DUSP16, EEF2K, ETFA, F2R, GATD3A, GBP1, GFRA2, GHR, GLIPR2, GNAQ, GPRC5B, GRB2, GRIK5, HTT, IGF1R, IGFBP4, IL34, INAVA, KCTD11, KLB, KSR1, LAX1, LCP2, LIME1, MAP2K2, MAP2K5, MAP4K5, MARK2, MDFIC, NCK2, NIBAN1, NPTN, NTRK2, OPRM1, PARD3, PDCD10, PDZD2, PLCE1, PPP2R5C, PRKACB, PRKAG2, PRKAR1B, PRKCE, PRKD2, PRKRIP1, PSAP, PTK2B, PTPN1, PTPN2, PTPN22, PTPN6, PTPRC, PTPRJ, RALB, RGS3, RORA, RPTOR, SEMA4D, SLAMF1, SLCO3A1, SNX9, SQSTM1, SSTR4, STAT3, STK10, TANK, TBC1D10C, TGFB2, TIMP2, TNF, TNFAIP3, TNIP1, TNK2, TRAF1, TRAF3, UBASH3B, VRK2, WDFY2, ZC3H12A]	[ADCY5, ADORA1, ADRA1B, ADRB3, AKAP12, AKAP13, AKAP9, ALK, ALOX15, ANKS1B, BCAR3, CAMP, CARTPT, CAV1, CCNG1, CCNP, CDK5R2, CELSR3, DENND2B, DOK7, DRD2, EGFR, ERBB4, FGF7, FGF9, GCNT2, GDF6, GFRAL, GRIK2, HGF, HIPK2, HLA-DRB1, HRG, HSPA2, HYAL2, IGF1R, ITGB2, LDLRAD4, LIMCH1, LMO3, LYN, MAP3K1, MAP3K5, MYADM, NCOR1, NDRG4, NGF, PAK1, PAK4, PARP8, PLCE1, PLPP3, PPARGC1A, PPP2R5A, PPP3CA, PRDX1, PRKAA1, PRKAG2, PRKAR2B, PRKCA, PRKCZ, PRKN, PTPN22, PTPRQ, PTPRT, RB1, RGS3, ROBO1, RSPO1, SMAD6, STK25, TESC, TIAM1, TNFRSF11A, TP73, TPX2, TRAF2, TRAF7, TSC1, VEGFC, WDFY2, WNK2, WNT16]

GO:0019220	regulation of phosphate metabolic process	1,09E-02 [5, 6]	287,00	14,86	26,67	40,00	33,33	[ADAM8, AIF1, ANGPT1, AREG, ATF3, BANK1, CALCR, CCL19, CCL24, CD244, CD27, CD3E, CD4, CD40LG, CD74, CD80, CDCA2, CDKN2A, CISH, CLCF1, CSF1R, CXCL10, CXCR4, DRD1, DUSP2, DUSP8, EPHA6, EREG, FCRL3, FFAR4, FGD2, FGR, FLT3, GDF6, GFRA2, GPR55, GPRC5A, HCLS1, HLA-DRB1, IFNG, IL18, IL26, LAX1, LCP2, MUSK, NCF1, NCKAP1L, NLRP3, NLRP6, NPFFR2, NR4A3, NUP210, P2RY12, PARP8, PFKFB4, PIK3CG, PLEK, PPP1R42, PPP2R2B, PROK1, PSCA, PTAFR, PTK2B, PTPN22, PTPN6, PTPRC, RHOH, SEMA4D, SH2D1A, SLAMF8, SPINK1, STAP1, SYK, TBC1D10C, THY1, TLR6, TLR8, TNFAIP3, TNFRSF18, TRAF1, TREM2, WNT1, XCL1, XCL2]	[ACVR1B, ADCY1, AGAP2, AKAP13, AKAP9, ARRB2, BCAR3, BCL2, BDNF, BMP7, BMPR1A, CAB39, CACNA1A, CAPN2, CCDC88C, CCM2, CCND3, CCR7, CD44, CDC25B, CDH2, CDK2AP1, CDK5RAP1, CFLAR, CORO1C, CTDSPL, CX3CL1, DBNDD2, DOCK3, DUSP16, EEF1A1, EEF2K, EPHA10, ETFA, F2R, GATD3A, GBP1, GFRA2, GHR, GLIPR2, GNAQ, GPRC5B, GRB2, GRIK5, GSK3B, HTT, IGF1R, IGFBP4, IL34, INAVA, KCTD11, KLB, KSR1, LAX1, LCP2, LIME1, LPCAT1, MAP2K2, MAP2K5, MAP4K5, MARK2, MCPH1, MDFIC, MRTFA, NCK2, NIBAN1, NLRC5, NPTN, NTRK2, NUP153, OPRM1, PARD3, PCIF1, PDCD10, PDZD2, PHACTR1, PLCE1, PPP1R3B, PPP2R5C, PRKACB, PRKAG2, PRKAR1B, PRKCE, PRKD2, PRKRIP1, PSAP, PTK2B, PTPN1, PTPN2, PTPN22, PTPN6, PTPRC, PTPRJ, RALB, RGS3, RORA, RPTOR, SEMA4D, SET, SFI1, SH2D4A, SLA2, SLAMF1, SLC25A12, SLCO3A1, SMG6, SNX9, SQSTM1, SSTR4, STAT3, STK10, TANK, TBC1D10C, TGFB2, TIMP2, TNF, TNFAIP3, TNIP1, TNK2, TRAF1, TRAF3, UBASH3B, VRK2, WDFY2, ZBTB20, ZC3H12A]	[ADCY5, ADORA1, ADRA1B, ADRB3, AKAP12, AKAP13, AKAP9, ALK, ALOX15, ANKS1B, ARNT, BCAR3, CAMP, CARTPT, CAV1, CCDC8, CCNG1, CCNP, CDCA2, CDK5R2, CELSR3, DENND2B, DLG2, DMPK, DOK7, DRD2, EGFR, ELL, ERBB4, ESRRB, FGF7, FGF9, GCNT2, GDF6, GFRAL, GRIK2, HGF, HIPK2, HLA-DRB1, HRG, HSPA2, HYAL2, IGF1R, ITGB2, LDLRAD4, LIMCH1, LMO3, LYN, MAGI2, MAP3K1, MAP3K5, MYADM, NCOR1, NDRG4, NGF, NLRC5, NUP93, PAK1, PAK4, PARP8, PCIF1, PHACTR1, PHACTR3, PLCE1, PLPP3, PPARGC1A, PPP1R3B, PPP2R3A, PPP2R5A, PPP3CA, PPP6R2, PRDX1, PRKAA1, PRKAG2, PRKAR2B, PRKCA, PRKCZ, PRKN, PTPN22, PTPRQ, PTPRT, RB1, RCAN2, RGS3, ROBO1, RSPO1, SHOC2, SMAD6, STK25, SWAP70, TESC, TIAM1, TMEM132D, TNFRSF11A, TP73, TPX2, TRAF2, TRAF7, TSC1, VAC14, VEGFC, WDFY2, WNK2, WNT16, ZCCHC9]
GO:0016301	kinase activity	2,79E-02 [6]	236,00	15,12	27,27	41,67	31,06	[ADAM8, ANGPT1, AREG, BLK, BMPR1B, BTK, CAMK4, CARD11, CCL19, CD4, CD40LG, CD74, CDKN2A, CERKL, CISH, CSF1R, CXCL10, CXCR4, DAPK1, DUSP2, DUSP8, EPHA6, EREG, FGD2, FGR, FLT3, GPRC5A, HKDC1, HLA-DRB1, IFNG, IKBKE, IL18, ITK, LAX1, LCK, LCP2, MAPK13, MUSK, MYO3B, NCF1, NCKAP1L, NEK5, NELL2, NLRP3, NLRP6, NPFFR2, NR4A3, P2RY12, PARP8, PFKFB4, PIK3C2G, PIK3CG, PIM1, PIM2, PRKCQ, PROK1, PTK2B, PTPN22, PTPN6, PTPRC, RHOH, SH2D1A, SLA, SLAMF8, STAP1, STK17B, STYK1, SYK, THY1, TLR6, TNFAIP3, ZAP70]	[ACVR1B, ADCY1, AGAP2, AKAP13, AKAP9, BDNF, BMP7, BMPR1A, CAB39, CAMK1G, CAMK2G, CCND3, CCR7, CD44, CDC25B, CDC42BPB, CDK5RAP1, CDK6, COLEC11, COLEC12, CORO1C, CPNE3, DAPK1, DBNDD2, DCK, DGKA, DOCK3, DUSP16, EEF1A1, EEF2K, EPHA10, ETFA, F2R, FES, FGGY, GAK, GHR, GNAQ, GPRC5B, GRK3, GSK3B, GTF2H3, HTT, IGF1R, IL34, INAVA, JAK3, KSR1, LAX1, LCK, LCP2, LIME1, LTBP1, MAP2K2, MAP2K5, MAP4K5, MARK2, MARK3, MAST2, MAST4, MCPH1, MDFIC, NCK2, NLRC5, NME7, NPR2, NTRK2, PDCD10, PIK3CD, PIP4K2A, PIP5K1B, PLCE1, PLK5, PRKACB, PRKAG2, PRKAR1B, PRKCE, PRKD2, PRKRIP1, PTK2B, PTPN1, PTPN2, PTPN22, PTPN6, PTPRC, PTPRJ, RALB, RGS3, RORA, RPS6KA2, RPTOR, SGK1, SGMS1, SIK3, SLA, SLA2, SNX9, SQSTM1, STK10, STK38L, TANK, TGFB2, TJP2, TK2, TNF, TNFAIP3, TNK2, TRIO, UBASH3B, VRK2]	[ADCK2, ADCY5, ADORA1, AK8, AKAP13, AKAP9, ALK, ANKS1B, CAMK4, CARD11, CARTPT, CAV1, CCNG1, CCNP, CDK13, CDK5R2, CHUK, COLEC11, DCK, DLG2, DMPK, DOK7, DRD2, EGFR, ERBB4, GFRAL, GK5, GRK1, HGF, HIPK2, HLA-DRB1, HTATIP2, HUNK, HYAL2, IGF1R, KCNA5, LYN, MAGI2, MAP3K1, MAP3K5, MAST4, MYLK, NGF, NLRC5, PAK1, PAK4, PARP8, PDXK, PFKM, PIK3C2G, PLCE1, PLK5, PPP2R5A, PPP3CA, PRKAA1, PRKAG2, PRKAR2B, PRKCA, PRKCZ, PRKN, PTPN22, PTPRQ, PTPRT, RB1, RGS3, ROBO1, RPS6KA2, SPEG, STK25, TESC, TIAM1, TNFRSF11A, TOP1, TP73, TPX2, TRAF2, TRAF7, TSC1, VAC14, VEGFC, WNK2, WNK4]
GO:0031399	regulation of protein modification process	3,14E-02 [5, 6, 7]	290,00	14,66	24,92	42,59	32,49	[ADAM8, AIF1, ANGPT1, AREG, ATF3, BANK1, CALCR, CCL19, CCL24, CD27, CD3E, CD4, CD40LG, CD74, CD80, CDC20B, CDCA2, CDKN2A, CLCF1, CSF1R, CXCL10, CXCR4, DRD1, DUSP2, DUSP8, EREG, FCRL3, FFAR4, FGD2, FGR, FLT3, FOXP3, GDF6, GFI1, GFRA2, GPR55, GPRC5A, HCLS1, HLA-DRB1, IFNG, IL18, IL26, LAX1, LCP2, MUSK, MYB, NCF1, NCKAP1L, NLRP3, NLRP6, NPFFR2, NR4A3, PARP8, PAX5, PIK3CG, PPP2R2B, PROK1, PSCA, PTK2B, PTPN22, PTPN6, PTPRC, SEMA4D, SH2D1A, SPINK1, STAP1, SYK, TBC1D10C, THY1, TLR6, TLR8, TNFAIP3, TNFRSF18, TRABD2A, TRAF1, TREM2, WNT1, XCL1, XCL2]	[ACER2, ACVR1B, ADCY1, AGAP2, AKAP13, AKAP9, ARRB2, ATG10, ATG7, AUTS2, BCAR3, BCL2, BDNF, BIRC3, BMP7, BMPR1A, C6orf89, CAB39, CCDC88C, CCM2, CCND3, CCR7, CD44, CDC25B, CDH2, CDK2AP1, CDK5RAP1, CDYL, CFLAR, CORO1C, CTDSPL, CX3CL1, DBNDD2, DERL1, DOCK3, DUSP16, EEF2K, ETFA, F2R, FBXO5, FRY, GATD3A, GBP1, GFRA2, GHR, GLIPR2, GNAQ, GPRC5B, GRB2, GRIK5, GSK3B, HTT, IGF1R, IGFBP4, IL34, INAVA, JARID2, KCTD11, KLB, KSR1, LAX1, LCP2, LIME1, MAP2K2, MAP2K5, MAP4K5, MARK2, MDFIC, MRTFA, NCK2, NIBAN1, NMI, NOS1AP, NPTN, NTRK2, OPRM1, PARD3, PDCD10, PDZD2, PHACTR1, PLCE1, PPP1R3B, PPP2R5C, PRICKLE1, PRKACB, PRKAG2, PRKAR1B, PRKCE, PRKD2, PRKRIP1, PSAP, PTK2B, PTPN1, PTPN2, PTPN22, PTPN6, PTPRC, PTPRJ, RALB, RASSF3, RASSF5, RCHY1, RGS3, RNF180, RORA, RPTOR, RWDD3, SEMA4D, SET, SLAMF1, SLCO3A1, SNX9, SQSTM1, SSTR4, STAT3, STK10, TANK, TBC1D10C, TGFB2, TIMP2, TNF, TNFAIP3, TNIP1, TNK2, TRABD2B, TRAF1, TRAF3, UBASH3B, UBE2L3, UBE3A, VRK2, WDFY2, WDR70, ZC3H12A, ZMIZ1]	[ADCY5, ADORA1, ADRA1B, ADRB3, AKAP12, AKAP13, AKAP9, ALK, ALOX15, ANKS1B, ARNT, BCAR3, CAMP, CARTPT, CAV1, CCNG1, CCNP, CDCA2, CDK5R2, CELSR3, DENND2B, DMPK, DOK7, DRD2, DYSF, EGFR, ERBB4, FGF7, FGF9, GCNT2, GDF6, GFRAL, GRIK2, HGF, HIPK2, HLA-DRB1, HRG, HSPA2, HYAL2, IGF1R, ITGB2, JDP2, LDLRAD4, LIMCH1, LMO3, LYN, MAGI2, MAP3K1, MAP3K5, MYADM, N4BP1, NCOR1, NDRG4, NGF, NOS1AP, NXN, PAK1, PAK4, PARP8, PHACTR1, PHACTR3, PLCE1, PLPP3, PPARGC1A, PPP1R3B, PPP2R3A, PPP2R5A, PPP3CA, PPP6R2, PRDX1, PRKAA1, PRKAG2, PRKAR2B, PRKCA, PRKCZ, PRKN, PTPN22, PTPRQ, PTPRT, RAMP1, RB1, RCAN2, RGS3, ROBO1, RSPO1, SHOC2, SKI, SMAD6, STK25, SWAP70, TESC, TIAM1, TNFRSF11A, TP73, TPX2, TRAF2, TRAF7, TSC1, VEGFC, WDFY2, WFS1, WNK2, WNT16]
GO:0031401	positive regulation of protein modification process	3,18E-02 [5, 6, 7, 8]	205,00	15,40	26,34	43,30	30,36	[ADAM8, AIF1, ANGPT1, AREG, BANK1, CALCR, CCL19, CCL24, CD27, CD3E, CD4, CD40LG, CD74, CD80, CDC20B, CDCA2, CDKN2A, CLCF1, CSF1R, CXCR4, EREG, FCRL3, FFAR4, FGD2, FLT3, FOXP3, GDF6, GPR55, GPRC5A, HCLS1, HLA-DRB1, IFNG, IL18, IL26, LCP2, MUSK, MYB, NCF1, NCKAP1L, NLRP3, NR4A3, PARP8, PIK3CG, PROK1, PTK2B, PTPN22, PTPRC, SEMA4D, SH2D1A, STAP1, SYK, TLR6, TNFRSF18, TRABD2A, TRAF1, TREM2, WNT1, XCL1, XCL2]	[ACVR1B, ADCY1, AGAP2, AKAP13, AKAP9, ARRB2, ATG10, ATG7, AUTS2, BCAR3, BCL2, BDNF, BIRC3, BMP7, BMPR1A, C6orf89, CAB39, CCM2, CCND3, CCR7, CD44, CDC25B, CDH2, CDK2AP1, CFLAR, CX3CL1, DERL1, DOCK3, ETFA, F2R, GATD3A, GHR, GLIPR2, GPRC5B, IGF1R, IGFBP4, IL34, INAVA, JARID2, KLB, KSR1, LCP2, MAP2K2, MAP2K5, MAP4K5, MARK2, MDFIC, NIBAN1, NMI, NOS1AP, NPTN, NTRK2, OPRM1, PDCD10, PDZD2, PLCE1, PRICKLE1, PRKACB, PRKAG2, PRKAR1B, PRKCE, PRKD2, PSAP, PTK2B, PTPN1, PTPN22, PTPRC, PTPRJ, RALB, RASSF3, RASSF5, RCHY1, RNF180, RORA, RPTOR, RWDD3, SEMA4D, SLAMF1, SLCO3A1, SNX9, SQSTM1, SSTR4, STAT3, STK10, TANK, TGFB2, TIMP2, TNF, TNIP1, TNK2, TRABD2B, TRAF1, TRAF3, UBE2L3, UBE3A, WDFY2, ZC3H12A]	[ADCY5, ADORA1, ADRA1B, ADRB3, AKAP12, AKAP13, AKAP9, ALK, ALOX15, ARNT, BCAR3, CAMP, CARTPT, CAV1, CDCA2, CDK5R2, DENND2B, DOK7, DRD2, EGFR, ERBB4, FGF7, FGF9, GCNT2, GDF6, GFRAL, HGF, HIPK2, HLA-DRB1, HSPA2, IGF1R, JDP2, LIMCH1, LYN, MAGI2, MAP3K1, MAP3K5, NDRG4, NGF, NOS1AP, PAK1, PAK4, PARP8, PLCE1, PPARGC1A, PPP2R5A, PPP3CA, PRKAA1, PRKAG2, PRKAR2B, PRKCA, PRKCZ, PRKN, PTPN22, RAMP1, ROBO1, RSPO1, STK25, TIAM1, TNFRSF11A, TP73, TPX2, TRAF2, TRAF7, VEGFC, WDFY2, WFS1, WNT16]
GO:0001934	positive regulation of protein phosphorylation	4,15E-02 [6, 7, 8, 9]	172,00	15,81	28,04	41,27	30,69	[ADAM8, AIF1, ANGPT1, AREG, BANK1, CALCR, CCL19, CCL24, CD27, CD3E, CD4, CD40LG, CD74, CD80, CLCF1, CSF1R, CXCR4, EREG, FCRL3, FFAR4, FGD2, FLT3, GDF6, GPR55, GPRC5A, HCLS1, HLA-DRB1, IFNG, IL18, IL26, LCP2, MUSK, NCF1, NCKAP1L, NLRP3, NR4A3, PARP8, PIK3CG, PROK1, PTK2B, PTPN22, PTPRC, SEMA4D, SH2D1A, STAP1, SYK, TLR6, TNFRSF18, TRAF1, TREM2, WNT1, XCL1, XCL2]	[ACVR1B, ADCY1, AGAP2, AKAP13, AKAP9, ARRB2, BCAR3, BCL2, BDNF, BMP7, BMPR1A, CAB39, CCM2, CCND3, CCR7, CD44, CDC25B, CDH2, CDK2AP1, CFLAR, CX3CL1, DOCK3, ETFA, F2R, GATD3A, GHR, GLIPR2, GPRC5B, IGF1R, IGFBP4, IL34, INAVA, KLB, KSR1, LCP2, MAP2K2, MAP2K5, MAP4K5, MARK2, MDFIC, NIBAN1, NPTN, NTRK2, OPRM1, PDCD10, PDZD2, PLCE1, PRKACB, PRKAG2, PRKAR1B, PRKCE, PRKD2, PSAP, PTK2B, PTPN1, PTPN22, PTPRC, PTPRJ, RALB, RORA, RPTOR, SEMA4D, SLAMF1, SLCO3A1, SNX9, SQSTM1, SSTR4, STAT3, STK10, TANK, TGFB2, TIMP2, TNF, TNK2, TRAF1, TRAF3, WDFY2, ZC3H12A]	[ADCY5, ADORA1, ADRA1B, ADRB3, AKAP12, AKAP13, AKAP9, ALK, ALOX15, BCAR3, CAMP, CARTPT, CAV1, CDK5R2, DENND2B, DOK7, DRD2, EGFR, ERBB4, FGF7, FGF9, GCNT2, GDF6, GFRAL, HGF, HIPK2, HLA-DRB1, HSPA2, IGF1R, LIMCH1, LYN, MAP3K1, MAP3K5, NDRG4, NGF, PAK1, PAK4, PARP8, PLCE1, PPP3CA, PRKAA1, PRKAG2, PRKAR2B, PRKCA, PRKCZ, PTPN22, ROBO1, RSPO1, STK25, TIAM1, TNFRSF11A, TP73, TPX2, TRAF2, TRAF7, VEGFC, WDFY2, WNT16]
GO:0030155	regulation of cell adhesion	1,34E-13 [3, 4]	167,00	22,12	41,24	38,14	20,62	[ADAM8, ADAMDEC1, AIF1, AKNA, ANGPT1, ANGPT2, APBB1IP, BTLA, CARD11, CCDC88B, CCL19, CCR2, CD27, CD3E, CD4, CD40LG, CD5, CD6, CD74, CD80, CD86, CDKN2A, CRTAM, CTLA4, CXCR3, CXCR4, CYTIP, DOCK8, EGR3, FGL2, FOXP3, FUT7, HLA-DMB, HLA-DPA1, HLA-DPB1, HLA-DRB1, ICOS, IFNG, IL18, IL7R, ITGA4, KLRK1, KNG1, LAG3, LAX1, LCK, LEF1, MYB, NCKAP1L, NLRP3, NR4A3, P2RY12, PIK3CG, PLA2G2D, PLAU, PLXNC1, PRKCQ, PTAFR, PTK2B, PTPN22, PTPN6, PTPRC, RHOH, RUNX3, SASH3, SELE, SEMA4D, SYK, TACSTD2, TESPA1, THY1, TIGIT, TNFAIP8L2, TNFRSF18, TNFSF13B, TNR, VNN1, WNT1, XCL1, ZAP70]	[ABAT, ACER2, ADA, ADAM10, AFDN, AKNA, ANK3, APBB1IP, BCL2, BMP7, CCDC88B, CCR7, CD160, CD28, CD44, CD47, CD6, CDK6, CORO1A, CORO1C, CTLA4, CX3CL1, CYTIP, DNM2, DOCK1, FES, GATD3A, GBP1, GRB2, GSK3B, GSN, HLA-A, HLA-DPA1, HLA-DPB1, IDO1, IRF1, JAK3, LAX1, LCK, LPXN, MAP2K5, MBP, MMP14, NCK2, NFAT5, PIEZO1, PKP4, PLXNC1, PRKCE, PRKD2, PTK2B, PTPN2, PTPN22, PTPN6, PTPRC, PTPRJ, RARA, RIN2, RIPOR2, RSU1, RUNX1, RUNX3, SEMA4D, SPOCK2, TESPA1, TGFB2, TGFBI, TNF, TNFAIP8L2, UBASH3B, WDPCP, ZBTB16, ZC3H12A, ZMIZ1]	[ALOX15, ANK3, BTNL2, CARD11, CAV1, CD9, CDH13, CDSN, DOCK1, DYSF, FMN1, GCNT2, HLA-DQB2, HLA-DRB1, HLA-G, HRG, ITGB2, LIMCH1, LYN, MAGI1, MBL2, MBP, MMP12, MYADM, MYF5, ONECUT2, PAK1, PDE3B, PLPP3, PPP3CA, PRKCA, PRKCZ, PTPN22, RIPOR2, RND1, RUNX1, SEMA5A, SWAP70, TESC, TSC1]

GO:0002683	negative regulation of immune system process	1,05E-04	[2, 3, 4]	95,00	20,26	45,05	37,84	17,12	[ANGPT1, BANK1, BTK, C1QC, CCR2, CD200R1, CD74, CD80, CD84, CD86, CD96, CDKN2A, CLNK, CNR1, CNR2, CRTAM, CTLA4, FCRL3, FGL2, FGR, FOXP3, GPR171, GPR55, HLA-DOA, HLA-DRB1, IL7R, INPP5D, KLRK1, LAG3, LAX1, LPAL2, LRRC17, LST1, MIXL1, NLRP6, PLA2G2D, PTK2B, PTPN22, PTPN6, PTPRC, RUNX3, SLAMF8, STAP1, TBC1D10C, THY1, TIGIT, TNFAIP3, TNFAIP8L2, TSC22D3, XCL1]	[ADA, ARRB2, BPI, CD160, CD47, CDK6, CTLA4, CX3CL1, GATD3A, GBP1, GPR68, HLA-A, HLA-B, IDO1, IRF1, JAK3, LAX1, LPXN, LRCH1, NLRC5, NMI, PCDHA6, PTK2B, PTPN2, PTPN22, PTPN6, PTPRC, PTPRJ, RARA, RIPOR2, RUNX1, RUNX3, SLA2, TAPBPL, TBC1D10C, TGFB2, TNF, TNFAIP3, TNFAIP8L2, UBASH3B, ZBTB16, ZC3H12A]	[ADORA1, ALOX15, BTNL2, CARTPT, DRD2, HLA-DRB1, HLA-G, LYN, MEIS2, MMP12, MNDA, NLRC5, NR1H3, PCDHA6, PTPN22, RIPOR2, RUNX1, SUSD4, WASL]
GO:0002695	negative regulation of leukocyte activation	7,22E-03	[3, 4, 5, 6]	45,00	23,56	52,54	32,20	15,25	[BANK1, BTK, CCR2, CD74, CD80, CD84, CD86, CDKN2A, CLNK, CNR1, CNR2, CRTAM, CTLA4, FGL2, FGR, FOXP3, HLA-DRB1, INPP5D, LAG3, LAX1, LST1, PLA2G2D, PTPN22, PTPN6, PTPRC, RUNX3, TBC1D10C, TIGIT, TNFAIP3, TNFAIP8L2, XCL1]	[BPI, CTLA4, CX3CL1, IDO1, IRF1, JAK3, LAX1, PCDHA6, PTPN2, PTPN22, PTPN6, PTPRC, RIPOR2, RUNX1, RUNX3, TBC1D10C, TNFAIP3, TNFAIP8L2, ZC3H12A]	[HLA-DRB1, HLA-G, LYN, MNDA, NR1H3, PCDHA6, PTPN22, RIPOR2, RUNX1]
GO:0050866	negative regulation of cell activation	1,45E-02	[3, 4, 5]	48,00	22,54	50,00	33,87	16,13	[BANK1, BTK, CCR2, CD74, CD80, CD84, CD86, CDKN2A, CLNK, CNR1, CNR2, CRTAM, CTLA4, FGL2, FGR, FOXP3, HLA-DRB1, INPP5D, LAG3, LAX1, LST1, PLA2G2D, PTPN22, PTPN6, PTPRC, RUNX3, TBC1D10C, TIGIT, TNFAIP3, TNFAIP8L2, XCL1]	[ABAT, BPI, CTLA4, CX3CL1, IDO1, IRF1, JAK3, LAX1, PCDHA6, PTPN2, PTPN22, PTPN6, PTPRC, RIPOR2, RUNX1, RUNX3, TBC1D10C, TNFAIP3, TNFAIP8L2, UBASH3B, ZC3H12A]	[CD9, HLA-DRB1, HLA-G, LYN, MNDA, NR1H3, PCDHA6, PTPN22, RIPOR2, RUNX1]
GO:0007162	negative regulation of cell adhesion	1,88E-02	[3, 4, 5]	61,00	20,54	38,16	38,16	23,68	[ADAMDEC1, AKNA, ANGPT1, ANGPT2, CD74, CD80, CD86, CDKN2A, CRTAM, CTLA4, FGL2, FOXP3, HLA-DRB1, KNG1, LAG3, LAX1, PLA2G2D, PLXNC1, PTPN22, PTPN6, PTPRC, RUNX3, SEMA4D, TACSTD2, TIGIT, TNFAIP8L2, TNR, WNT1, XCL1]	[ABAT, ACER2, ADAM10, AKNA, CORO1C, CTLA4, CX3CL1, GBP1, IDO1, IRF1, JAK3, LAX1, LPXN, MAP2K5, MBP, MMP14, PLXNC1, PTPN2, PTPN22, PTPN6, PTPRC, RIPOR2, RUNX1, RUNX3, SEMA4D, TGFBI, TNFAIP8L2, UBASH3B, ZC3H12A]	[CD9, CDH13, CDSN, GCNT2, HLA-DRB1, HLA-G, HRG, MBL2, MBP, MMP12, MYADM, PDE3B, PTPN22, RIPOR2, RND1, RUNX1, SEMA5A, SWAP70]
GO:0042110	T cell activation	8,05E-21	[4, 5]	142,00	27,84	53,53	32,35	14,12	[ADAM8, AIF1, APBB1IP, BTLA, BTN3A1, CAMK4, CARD11, CASP8, CCDC88B, CCL19, CCR2, CD1C, CD2, CD27, CD3D, CD3E, CD3G, CD4, CD40LG, CD5, CD6, CD74, CD80, CD86, CD8A, CD8B, CDKN2A, CLCF1, CLEC4D, CLEC4E, CRTAM, CTLA4, DOCK2, DOCK8, EGR3, EOMES, FGL2, FOXP3, FUT7, HLA-DMB, HLA-DOA, HLA-DPA1, HLA-DPB1, HLA-DRB1, ICOS, IFNG, IL18, IL7R, IRF4, ITK, JAML, KLRK1, LAG3, LAX1, LCK, LCP1, LEF1, LMO1, LY9, MYB, NCKAP1L, NLRP3, PIK3CG, PLA2G2D, PRDM1, PRKCQ, PTPN22, PTPN6, PTPRC, RHOH, RUNX3, SASH3, SEMA4A, SIT1, SLAMF6, SYK, TESPA1, THEMIS, THY1, TIGIT, TNFAIP8L2, TNFRSF18, TNFSF13B, TNFSF8, TOX, VNN1, WAS, WNT1, XCL1, ZAP70, ZNF683]	[ADA, APBB1IP, B2M, BATF, BCL2, CASP8, CCDC88B, CCND3, CCR7, CD160, CD28, CD3G, CD44, CD47, CD6, CDK6, CORO1A, CTLA4, FANCA, GATD3A, GRB2, GSN, HLA-A, HLA-DPA1, HLA-DPB1, IDO1, IRF1, JAK3, JAML, KIF13B, LAX1, LCK, LCP1, NCK2, NFATC2, PIK3CD, PSAP, PTPN2, PTPN22, PTPN6, PTPRC, RARA, RELB, RIPOR2, RORA, RUNX1, RUNX3, SLA2, STAT3, TESPA1, TNFAIP8L2, ZBTB16, ZC3H12A, ZEB1, ZMIZ1]	[BCL11B, BTNL2, CAMK4, CARD11, CAV1, CD151, CD8A, CXADR, FOXP1, HLA-DQB2, HLA-DRB1, HLA-G, LCP1, LYN, PAK1, PNKD, PPP3CA, PRKCZ, PTPN22, RIPOR2, RUNX1, TOX, TSC1, ZEB1]
GO:0046649	lymphocyte activation	3,80E-16	[3, 4]	184,00	22,52	54,13	32,11	13,76	[ADAM8, AIF1, APBB1IP, BANK1, BLNK, BTK, BTLA, BTN3A1, CAMK4, CARD11, CASP8, CCDC88B, CCL19, CCR2, CD180, CD1C, CD2, CD244, CD27, CD3D, CD3E, CD3G, CD4, CD40LG, CD5, CD6, CD74, CD80, CD86, CD8A, CD8B, CDKN2A, CLCF1, CLEC4D, CLEC4E, CLNK, CR2, CRTAM, CTLA4, DOCK10, DOCK2, DOCK8, EGR3, EOMES, FCRL1, FCRL3, FCRLA, FGL2, FGR, FLT3, FOXP3, FUT7, GAPT, HLA-DMB, HLA-DOA, HLA-DPA1, HLA-DPB1, HLA-DRB1, ICOS, IFNG, IKZF1, IL18, IL7R, INPP5D, IRF4, ITGA4, ITK, JAML, KLRK1, LAG3, LAX1, LCK, LCP1, LEF1, LMO1, LST1, LY9, MS4A1, MYB, MZB1, NCKAP1L, NCR3, NLRP3, PIK3CG, PLA2G2D, PRDM1, PRF1, PRKCQ, PTK2B, PTPN22, PTPN6, PTPRC, RHOH, RUNX3, SASH3, SEMA4A, SIT1, SLAMF6, SLAMF7, SLAMF8, SYK, TBC1D10C, TESPA1, THEMIS, THY1, TIGIT, TNFAIP3, TNFAIP8L2, TNFRSF18, TNFSF13B, TNFSF8, TOX, VNN1, WAS, WNT1, XCL1, ZAP70, ZNF683]	[ADA, APBB1IP, B2M, BATF, BCL2, CASP8, CCDC88B, CCND3, CCR7, CD160, CD28, CD38, CD3G, CD44, CD47, CD6, CDH17, CDK6, CMTM7, CORO1A, CTLA4, FANCA, GATD3A, GRB2, GSN, HDAC9, HLA-A, HLA-DPA1, HLA-DPB1, IDO1, IL21R, IRF1, JAK3, JAML, KIF13B, LAX1, LCK, LCP1, MMP14, NCK2, NFATC2, PIK3CD, PRF1, PSAP, PTK2B, PTPN2, PTPN22, PTPN6, PTPRC, PTPRJ, RARA, RELB, RIPOR2, RORA, RUNX1, RUNX3, SLA2, SLAMF1, SLAMF7, STAT3, TBC1D10C, TCF7L1, TESPA1, TNFAIP3, TNFAIP8L2, TSHR, ZBTB16, ZC3H12A, ZEB1, ZMIZ1]	[BCL11B, BTNL2, CAMK4, CARD11, CAV1, CD151, CD8A, CHRNA4, CMTM7, CXADR, FOXP1, HLA-DQB2, HLA-DRB1, HLA-G, ITGB2, LCP1, LYN, MNDA, PAK1, PNKD, PPP3CA, PRDX1, PRKCZ, PTPN22, RIPOR2, RUNX1, SWAP70, TOX, TSC1, ZEB1]
GO:0002684	positive regulation of immune system process	1,47E-13	[2, 3, 4]	235,00	19,70	50,00	33,09	16,91	[ADAM8, AIF1, AIM2, BLK, BLNK, BTK, BTLA, BTN3A1, BTN3A2, C1QA, C1QB, C1QC, C2, C8G, CARD11, CASP8, CCDC88B, CCL19, CCL24, CCR2, CD1B, CD1C, CD1E, CD2, CD244, CD247, CD27, CD3D, CD3E, CD3G, CD4, CD40LG, CD5, CD6, CD74, CD80, CD84, CD86, CFP, CLCF1, CLEC4D, CLEC4E, CLEC6A, CLNK, CNR1, CR2, CRTAM, CSF1R, CTLA4, CXCL10, CYP11B1, DOCK8, EDN2, EGR3, EREG, FCN1, FCN3, FCRL3, FGR, FOXP3, FUT7, FYB1, GBP5, GPSM3, HCLS1, HLA-DMB, HLA-DPA1, HLA-DPB1, HLA-DQA1, HLA-DRA, HLA-DRB1, HLA-DRB5, ICOS, IFNG, IKBKE, IL18, IL18RAP, IL7R, INPP5D, ITGA4, ITK, KLRK1, LAG3, LAX1, LCK, LCP2, LEF1, LTA, MOG, MS4A1, MUC19, MUC6, MYB, MYO1G, MZB1, NCKAP1L, NCR3, NLRP3, NR4A3, P2RY12, PAX5, PLA2G7, PRKCQ, PTAFR, PTK2B, PTPN22, PTPN6, PTPRC, PYHIN1, RHEX, RHOH, RUNX3, SASH3, SELE, SH2D1A, SLAMF6, STAP1, STAT1, SYK, TESPA1, THEMIS, THEMIS2, THY1, TLR6, TLR8, TNFRSF18, TNFSF13B, TOX, TRAT1, TREM2, VNN1, WAS, XCL1, XCL2, XG, ZAP70]	[ACTR3, ACVR1B, ADA, ADAM10, AIM2, B2M, BCL2, BTN3A3, CASP8, CCDC88B, CCR7, CD101, CD160, CD28, CD38, CD3G, CD47, CD6, COLEC11, CORO1A, CTLA4, CX3CL1, DOCK1, ELMO1, FES, FYB1, GAB2, GATD3A, GBP1, GBP5, GPR68, GPRC5B, GPSM3, GRB2, HLA-A, HLA-B, HLA-DPA1, HLA-DPB1, HLA-DRA, IDO1, IL34, JAK3, KLHL6, KLHL7, LAX1, LCK, LCP2, LIME1, LPXN, LTA, MED1, MMP14, MYO1G, NCK2, NCOR2, NFATC2, NLRC5, PDE4B, PIK3CD, PLSCR1, PRKACB, PRKCE, PRKD2, PSMB8, PSMB9, PSMD9, PTK2B, PTPN2, PTPN22, PTPN6, PTPRC, PTPRJ, RARA, RELB, RIPOR2, RORA, RUNX1, RUNX3, SLA2, STAT1, STAT3, TANK, TESPA1, TGFB2, TNF, WIPF1, ZBP1, ZBTB16, ZC3H12A, ZMIZ1]	[AIM2, ARNT, BTNL2, CARD11, CAV1, CHUK, COLEC11, DOCK1, DYSF, FOXP1, FYB1, GNAS, GPR31, HLA-DQB2, HLA-DRA, HLA-DRB1, HLA-G, HRG, ITGB2, KLK7, LYN, MBL2, MMP12, MNDA, MUC2, MUC5B, MYH2, NLRC5, PAK1, PDE4B, PNKD, PRKCA, PRKCZ, PSMD7, PTPN22, RB1, RIPOR2, RUNX1, SMAP1, SUSD4, SWAP70, TESC, TOX, TRAF2, VEGFC, WASL]
GO:0050776	regulation of immune response	7,47E-13	[3, 4]	218,00	19,91	50,79	31,35	17,86	[ADAM8, AIM2, ANGPT1, BLK, BLNK, BTK, BTLA, BTN3A1, BTN3A2, C1QA, C1QB, C1QC, C2, C8G, CARD11, CCL19, CCR2, CD1B, CD1C, CD1E, CD200R1, CD247, CD3D, CD3E, CD3G, CD4, CD40LG, CD48, CD74, CD80, CD84, CD86, CD8A, CD8B, CD96, CFP, CLCF1, CLEC1B, CLEC4D, CLEC4E, CLEC6A, CLNK, CNR1, CR2, CRTAM, CTLA4, CXCL10, CYP11B1, EREG, FCN1, FCN3, FCRL3, FGL2, FGR, FOXP3, FUT7, FYB1, GBP5, HLA-DMB, HLA-DPA1, HLA-DPB1, HLA-DQA1, HLA-DRA, HLA-DRB1, HLA-DRB5, IFNG, IKBKE, IL18, IL18RAP, IL7R, INPP5D, IRF4, ITGA4, ITGB7, ITK, JAML, KLRB1, KLRC1, KLRD1, KLRK1, LAG3, LAX1, LCK, LCP2, LPAL2, LTA, MOG, MS4A1, MUC19, MUC6, MYB, MYO1G, NCKAP1L, NCR3, NLRP3, NLRP6, NR4A3, PAX5, PILRA, PLA2G2D, PRKCQ, PTAFR, PTPN22, PTPN6, PTPRC, PYHIN1, SASH3, SELL, SH2D1A, SLAMF6, SLAMF7, SLAMF8, STAP1, STAT1, SYK, TESPA1, THEMIS, THEMIS2, THY1, TLR8, TNFAIP3, TNFSF13B, TRAT1, TREM2, WAS, XCL1, ZAP70, ZNF683]	[ACTR3, ADA, AIM2, ARRB2, B2M, BCL2, BIRC3, BTN3A3, CCR7, CD160, CD28, CD38, CD3G, CD47, COLEC11, COLEC12, CTLA4, DOCK1, ELMO1, FES, FYB1, GAB2, GBP1, GBP5, GPRC5B, GRB2, HLA-A, HLA-B, HLA-DPA1, HLA-DPB1, HLA-DRA, IDO1, IRF1, JAK3, JAML, KLHL6, KLHL7, LAX1, LCK, LCP2, LIME1, LPXN, LTA, MED1, MYO1G, NCOR2, NFATC1, NFATC2, NLRC5, NMI, PDE4B, PIK3CD, PLSCR1, PRKACB, PRKCE, PRKD2, PSMB8, PSMB9, PSMD9, PTPN1, PTPN2, PTPN22, PTPN6, PTPRC, PTPRJ, RARA, RELB, RUNX1, SLA2, SLAMF7, STAT1, TANK, TESPA1, TGFB2, TNF, TNFAIP3, WIPF1, ZBP1, ZC3H12A]	[AIM2, ALOX15, BTNL2, CARD11, CD8A, CHUK, COLEC11, CXADR, DOCK1, DRD2, FOXP1, FYB1, GPR31, HLA-DQB2, HLA-DRA, HLA-DRB1, HLA-G, HRG, ITGB2, KIR3DL2, KLK7, LILRA4, LYN, MAP3K1, MBL2, MMP12, MNDA, MUC2, MUC5B, MYH2, NFATC1, NLRC5, NR1H3, PAK1, PDE4B, PNKD, PPP3CA, PRKCA, PRKCZ, PSMD7, PTPN22, RUNX1, SUSD4, TRAF2, WASL]
GO:0050778	positive regulation of immune response	8,30E-10	[3, 4, 5]	170,00	20,07	48,99	33,33	17,68	[ADAM8, AIM2, BLK, BLNK, BTK, BTN3A1, BTN3A2, C1QA, C1QB, C1QC, C2, C8G, CARD11, CCL19, CCR2, CD1B, CD1C, CD1E, CD247, CD3D, CD3E, CD3G, CD4, CD74, CD80, CD86, CFP, CLCF1, CLEC4D, CLEC4E, CLEC6A, CLNK, CNR1, CR2, CRTAM, CTLA4, CXCL10, CYP11B1, EREG, FCN1, FCN3, FCRL3, FGR, FOXP3, FYB1, GBP5, HLA-DMB, HLA-DPA1, HLA-DPB1, HLA-DQA1, HLA-DRA, HLA-DRB1, HLA-DRB5, IFNG, IKBKE, IL18, IL18RAP, INPP5D, ITK, KLRK1, LAG3, LAX1, LCK, LCP2, LTA, MOG, MS4A1, MUC19, MUC6, MYB, MYO1G, NCKAP1L, NCR3, NLRP3, NR4A3, PAX5, PRKCQ, PTAFR, PTPN22, PTPN6, PTPRC, PYHIN1, SASH3, SH2D1A, SLAMF6, STAP1, SYK, TESPA1, THEMIS, THEMIS2, THY1, TLR8, TNFSF13B, TRAT1, WAS, XCL1, ZAP70]	[ACTR3, ADA, AIM2, B2M, BCL2, BTN3A3, CCR7, CD160, CD28, CD38, CD3G, CD47, COLEC11, CTLA4, DOCK1, ELMO1, FYB1, GAB2, GBP1, GBP5, GPRC5B, GRB2, HLA-A, HLA-B, HLA-DPA1, HLA-DPB1, HLA-DRA, IDO1, KLHL6, KLHL7, LAX1, LCK, LCP2, LIME1, LPXN, LTA, MED1, MYO1G, NCOR2, NFATC2, NLRC5, PDE4B, PIK3CD, PLSCR1, PRKACB, PRKCE, PRKD2, PSMB8, PSMB9, PSMD9, PTPN2, PTPN22, PTPN6, PTPRC, PTPRJ, RARA, RELB, RUNX1, SLA2, TANK, TESPA1, TGFB2, TNF, WIPF1, ZBP1, ZC3H12A]	[AIM2, BTNL2, CARD11, CHUK, COLEC11, DOCK1, FOXP1, FYB1, GPR31, HLA-DQB2, HLA-DRA, HLA-DRB1, HLA-G, HRG, ITGB2, KLK7, LYN, MBL2, MMP12, MNDA, MUC2, MUC5B, MYH2, NLRC5, PAK1, PDE4B, PNKD, PRKCA, PRKCZ, PSMD7, PTPN22, RUNX1, SUSD4, TRAF2, WASL]
GO:0002250	adaptive immune response	1,08E-03	[3]	133,00	17,71	62,09	26,14	11,76	[BTK, BTLA, BTN3A1, BTN3A2, C1QA, C1QB, C1QC, C2, C8G, CAMK4, CCL19, CCR2, CD1B, CD1C, CD1E, CD244, CD247, CD27, CD3D, CD3E, CD3G, CD4, CD40LG, CD48, CD6, CD74, CD80, CD84, CD86, CD8A, CD8B, CLCF1, CLEC4D, CLEC6A, CR2, CRTAM, CTLA4, CTSS, CXCL10, EOMES, FGL2, FOXP3, FUT7, GAPT, HLA-DMA, HLA-DMB, HLA-DOA, HLA-DPA1, HLA-DPB1, HLA-DQA1, HLA-DRA, HLA-DRB1, HLA-DRB5, IFNG, IL18, IL18RAP, IL7R, INPP5D, IRF4, ITK, JCHAIN, KLRK1, LAG3, LAMP3, LAX1, LEF1, LTA, LY9, MCOLN2, MYO1G, NLRP3, NR4A3, PIK3CG, PRDM1, PRF1, PRKCQ, PTK2B, PTPN6, PTPRC, SASH3, SEMA4A, SH2D1A, SIT1, SLAMF6, SLAMF7, SYK, THEMIS, TLR8, TNFAIP3, TNFSF13B, TRAT1, WAS, XCL1, ZAP70, ZNF683]	[ADA, B2M, BATF, BTN3A3, CD160, CD28, CD3G, CD6, CTLA4, HLA-A, HLA-B, HLA-DPA1, HLA-DPB1, HLA-DRA, IRF1, JAK3, KLHL6, KLHL7, LAX1, LIME1, LTA, MYO1G, NCOR2, PIK3CD, PRF1, PRKD2, PTK2B, PTPN6, PTPRC, RELB, RORA, SLA2, SLAMF1, SLAMF7, STAT3, TANK, TAP1, TNF, TNFAIP3, ZC3H12A]	[ALOX15, CAMK4, CD8A, HLA-DQB2, HLA-DRA, HLA-DRB1, HLA-G, JCHAIN, LYN, MBL2, ORAI1, PNKD, PRKCZ, SUSD4, SWAP70, TNFRSF11A, TRAF2, TSC1]
GO:0002699	positive regulation of immune effector process	2,17E-03	[3, 4, 5]	56,00	22,67	61,67	25,00	13,33	[BTK, CCL19, CCR2, CD1B, CD1C, CD1E, CD244, CD74, CD80, CD84, CD86, CLCF1, CLNK, CRTAM, FGR, FOXP3, HLA-DMB, IFNG, IL18, IL18RAP, KLRK1, LAG3, LTA, MYB, MZB1, NCR3, NLRP3, NR4A3, PTAFR, PTPN22, PTPRC, SASH3, SH2D1A, SLAMF6, STAP1, SYK, XCL1]	[B2M, CD160, CD28, FES, GAB2, GPRC5B, HLA-A, HLA-B, LTA, PTPN22, PTPRC, PTPRJ, RARA, TANK, TNF]	[HLA-G, ITGB2, KLK7, LYN, PNKD, PRKCZ, PTPN22, TRAF2]
GO:0002703	regulation of leukocyte mediated immunity	8,26E-03	[4, 5]	50,00	22,52	60,38	26,42	13,21	[BTK, CCR2, CD1B, CD1C, CD1E, CD84, CD96, CLCF1, CLNK, CRTAM, CXCL6, FGR, FOXP3, FUT7, IL18, IL18RAP, IL7R, KLRK1, LAG3, LTA, NCR3, NLRP3, PTAFR, PTPN6, PTPRC, SASH3, SH2D1A, SLAMF6, STAP1, SYK, WAS, XCL1]	[ARRB2, B2M, CD160, CD28, FES, GAB2, HLA-A, HLA-B, JAK3, LTA, PTPN6, PTPRC, TANK, TNF]	[HLA-G, ITGB2, LYN, PNKD, PRKCZ, SUSD4, TRAF2]
GO:0002708	positive regulation of lymphocyte mediated immunity	1,36E-02	[5, 6, 7]	31,00	26,72	60,61	27,27	12,12	[BTK, CD1B, CD1C, CD1E, CLCF1, CLNK, CRTAM, FOXP3, IL18, IL18RAP, KLRK1, LAG3, LTA, NCR3, NLRP3, PTPRC, SASH3, SH2D1A, SLAMF6, XCL1]	[B2M, CD160, CD28, HLA-A, HLA-B, LTA, PTPRC, TANK, TNF]	[HLA-G, PNKD, PRKCZ, TRAF2]
GO:0031343	positive regulation of cell killing	1,68E-02	[3, 4, 5]	22,00	31,43	66,67	25,00	8,33	[CD1B, CD1C, CD1E, CRTAM, IFNG, IL18RAP, KLRK1, LAG3, NCR3, PRF1, PTPRC, SH2D1A, SLAMF6, STAP1, SYK, XCL1]	[B2M, CD160, HLA-A, HLA-B, PRF1, PTPRC]	[HLA-G, PNKD]
GO:0002705	positive regulation of leukocyte mediated immunity	1,82E-02	[4, 5, 6]	37,00	24,50	61,54	25,64	12,82	[BTK, CD1B, CD1C, CD1E, CLCF1, CLNK, CRTAM, FGR, FOXP3, IL18, IL18RAP, KLRK1, LAG3, LTA, NCR3, NLRP3, PTAFR, PTPRC, SASH3, SH2D1A, SLAMF6, STAP1, SYK, XCL1]	[B2M, CD160, CD28, GAB2, HLA-A, HLA-B, LTA, PTPRC, TANK, TNF]	[HLA-G, ITGB2, PNKD, PRKCZ, TRAF2]
GO:0002228	natural killer cell mediated immunity	2,14E-02	[4, 5, 6, 7]	21,00	31,82	60,87	30,43	8,70	[CD96, CLNK, CRTAM, IL18, IL18RAP, KLRC2, KLRD1, KLRK1, LAG3, NCR3, PTPN6, SH2D1A, SLAMF6, SLAMF7]	[ARRB2, CD160, CORO1A, HLA-A, HLA-B, PTPN6, SLAMF7]	[HLA-G, PRDX1]
GO:0002706	regulation of lymphocyte mediated immunity	2,19E-02	[5, 6]	39,00	23,93	61,90	26,19	11,90	[BTK, CCR2, CD1B, CD1C, CD1E, CD96, CLCF1, CLNK, CRTAM, FOXP3, FUT7, IL18, IL18RAP, IL7R, KLRK1, LAG3, LTA, NCR3, NLRP3, PTPN6, PTPRC, SASH3, SH2D1A, SLAMF6, WAS, XCL1]	[ARRB2, B2M, CD160, CD28, HLA-A, HLA-B, LTA, PTPN6, PTPRC, TANK, TNF]	[HLA-G, PNKD, PRKCZ, SUSD4, TRAF2]
GO:0002819	regulation of adaptive immune response	2,37E-02	[4, 5]	42,00	23,08	52,17	32,61	15,22	[BTK, CCL19, CCR2, CD1B, CD1C, CD1E, CD4, CD48, CD80, CLCF1, FOXP3, FUT7, IL18, IL7R, LTA, NLRP3, PRKCQ, PTPN6, PTPRC, SASH3, TNFAIP3, TNFSF13B, WAS, XCL1]	[ADA, B2M, CD160, CD28, HLA-A, HLA-B, IRF1, JAK3, LTA, PTPN6, PTPRC, TANK, TNF, TNFAIP3, ZC3H12A]	[ALOX15, HLA-DQB2, HLA-G, PNKD, PRKCZ, SUSD4, TRAF2]
GO:0002697	regulation of immune effector process	3,25E-02	[3, 4]	92,00	18,11	62,38	26,73	10,89	[AIM2, ANGPT1, BTK, C1QA, C1QB, C1QC, C2, C8G, CCL19, CCR2, CD1B, CD1C, CD1E, CD244, CD40LG, CD74, CD80, CD84, CD86, CD96, CFP, CLCF1, CLNK, CR2, CRTAM, CXCL10, CXCL6, CYP11B1, FCRL3, FGL2, FGR, FOXP3, FUT7, HLA-DMB, IFNG, IL18, IL18RAP, IL7R, IRF4, KLRK1, LAG3, LPAL2, LTA, MYB, MZB1, NCR3, NLRP3, NR4A3, PTAFR, PTPN22, PTPN6, PTPRC, SASH3, SH2D1A, SLAMF6, SLAMF8, STAP1, STAT1, SYK, TNFAIP3, WAS, XCL1, ZNF683]	[AIM2, ARRB2, B2M, BIRC3, CD160, CD28, CD47, FES, GAB2, GPRC5B, HLA-A, HLA-B, JAK3, LTA, PTPN22, PTPN6, PTPRC, PTPRJ, RARA, RNF216, STAT1, TANK, TGFB2, TNF, TNFAIP3, TRAF3, ZC3H12A]	[AIM2, HLA-G, ITGB2, KLK7, LYN, MMP12, PNKD, PRKCZ, PTPN22, SUSD4, TRAF2]
GO:0001912	positive regulation of leukocyte mediated cytotoxicity	4,52E-02	[4, 5, 6, 7]	19,00	32,20	65,00	25,00	10,00	[CD1B, CD1C, CD1E, CRTAM, IL18RAP, KLRK1, LAG3, NCR3, PTPRC, SH2D1A, SLAMF6, STAP1, XCL1]	[B2M, CD160, HLA-A, HLA-B, PTPRC]	[HLA-G, PNKD]

GO:0045321	leukocyte activation	8,82E-15	[2, 3]	272,00	19,25	49,68	33,44	16,88	[ADAM8, AIF1, APBB1IP, BANK1, BIN2, BLNK, BTK, BTLA, BTN3A1, C1QA, CAMK4, CARD11, CASP8, CCDC88B, CCL19, CCR2, CD180, CD1C, CD2, CD244, CD27, CD3D, CD3E, CD3G, CD4, CD40LG, CD5, CD53, CD6, CD74, CD80, CD84, CD86, CD8A, CD8B, CDKN2A, CFP, CLCF1, CLEC12A, CLEC4D, CLEC4E, CLNK, CNR1, CNR2, CR2, CRTAM, CTLA4, CTSS, CXCL6, CYBB, DOCK10, DOCK2, DOCK8, EDN2, EGR3, EOMES, FCN1, FCRL1, FCRL3, FCRLA, FGL2, FGR, FLT3, FOXP3, FRMPD3, FUT7, GAPT, HLA-DMB, HLA-DOA, HLA-DPA1, HLA-DPB1, HLA-DRB1, ICOS, IFNG, IKZF1, IL18, IL18RAP, IL7R, INPP5D, IQGAP2, IRF4, ITGA4, ITK, JAML, KLRK1, LAG3, LAX1, LCK, LCP1, LCP2, LEF1, LMO1, LRMP, LST1, LY9, LYZ, MS4A1, MYB, MZB1, NAMPT, NCKAP1L, NCR3, NLRP3, NR4A3, ORM2, PIK3CG, PLA2G2D, PLAC8, PLAU, PRDM1, PRF1, PRKCQ, PTAFR, PTK2B, PTPN22, PTPN6, PTPRC, PTX3, RAB44, RHOH, RNASET2, RUNX3, SASH3, SELL, SELPLG, SEMA4A, SIT1, SLAMF6, SLAMF7, SLAMF8, SLC18A1, STAP1, SUCNR1, SYK, TBC1D10C, TESPA1, THEMIS, THY1, TIGIT, TLR6, TLR8, TNFAIP3, TNFAIP8L2, TNFRSF18, TNFSF13B, TNFSF8, TOX, TREM2, TXNDC5, UBD, VNN1, WAS, WNT1, XCL1, ZAP70, ZNF683]	[ADA, ADAM10, ADGRE5, APBB1IP, ASAH1, ATG7, B2M, BATF, BATF3, BCL2, BIN2, BPI, CAB39, CASP8, CCDC88B, CCND3, CCR7, CD160, CD28, CD38, CD3G, CD44, CD47, CD6, CDH17, CDK6, CMTM7, CORO1A, COTL1, CPNE3, CTLA4, CTSZ, CX3CL1, CYSTM1, EEF1A1, FANCA, FES, GAB2, GATD3A, GRB2, GSN, HDAC9, HLA-A, HLA-B, HLA-DPA1, HLA-DPB1, IDO1, IL21R, IRF1, JAK3, JAML, KCNAB2, KIF13B, LAX1, LCK, LCP1, LCP2, LPCAT1, MMP14, NCK2, NFATC2, PCDHA6, PIK3CD, PLSCR1, PRF1, PRKCE, PSAP, PTK2B, PTPN2, PTPN22, PTPN6, PTPRC, PTPRJ, PTPRN2, RARA, RELB, RIPOR2, RNASET2, RORA, RUNX1, RUNX3, S100A13, SBNO2, SLA2, SLAMF1, SLAMF7, SLC15A4, SLC7A2, STAT3, STK10, TBC1D10C, TCF7L1, TESPA1, TIMP2, TMC6, TNF, TNFAIP3, TNFAIP8L2, TRAF3, TSHR, UBR4, ZBTB16, ZC3H12A, ZEB1, ZMIZ1]	[AMPD3, ATP6V1D, BCL11B, BTNL2, CAMK4, CAMP, CARD11, CAV1, CD151, CD8A, CDK13, CHRNA4, CMTM7, CPLX2, CRISP3, CTSD, CXADR, DYSF, FOXP1, HLA-DQB2, HLA-DRB1, HLA-G, HYAL2, ITGB2, LCP1, LRRC7, LYN, LYZ, MMP12, MNDA, NR1H3, PAK1, PCDHA6, PDXK, PLAC8, PLPPR3, PNKD, PPP3CA, PRDX1, PRKCZ, PSMD7, PTPN22, PTPRN2, RAB37, RAB7A, RIPOR2, RUNX1, SLC15A4, SWAP70, TLR1, TOX, TSC1, ZEB1]
GO:0002366	leukocyte activation involved in immune response	5,07E-03	[3, 4]	134,00	17,25	45,58	36,05	18,37	[ADAM8, APBB1IP, BIN2, BTK, CCL19, CCR2, CD180, CD1C, CD244, CD40LG, CD53, CD74, CD80, CD84, CD86, CFP, CLCF1, CLEC12A, CLEC4D, CLEC4E, CTSS, CYBB, DOCK10, DOCK2, EOMES, FCN1, FGL2, FGR, FOXP3, FRMPD3, GAPT, HLA-DMB, IFNG, IL18, IQGAP2, IRF4, LCP1, LEF1, LRMP, LY9, LYZ, MYB, NCKAP1L, NLRP3, NR4A3, ORM2, PIK3CG, PLAC8, PLAU, PTAFR, PTK2B, PTPN6, PTPRC, PTX3, RAB44, RNASET2, SELL, SEMA4A, SLAMF6, SLC18A1, SUCNR1, SYK, TBC1D10C, TREM2, TXNDC5, VNN1, ZNF683]	[ADA, ADAM10, ADGRE5, APBB1IP, ASAH1, ATG7, B2M, BATF, BIN2, BPI, CAB39, CD28, CD44, CD47, CDH17, CORO1A, COTL1, CPNE3, CTSZ, CYSTM1, EEF1A1, FES, GAB2, GSN, HLA-B, JAK3, KCNAB2, LCP1, LPCAT1, PIK3CD, PRKCE, PSAP, PTK2B, PTPN6, PTPRC, PTPRJ, PTPRN2, RARA, RELB, RNASET2, RORA, S100A13, SBNO2, SLAMF1, SLC15A4, STAT3, STK10, TBC1D10C, TIMP2, TMC6, TRAF3, UBR4, ZC3H12A]	[AMPD3, ATP6V1D, CAMP, CDK13, CPLX2, CRISP3, CTSD, DYSF, FOXP1, ITGB2, LCP1, LRRC7, LYN, LYZ, MMP12, MNDA, PDXK, PLAC8, PLPPR3, PRKCZ, PSMD7, PTPRN2, RAB37, RAB7A, SLC15A4, SWAP70, TSC1]
GO:0002274	myeloid leukocyte activation	6,66E-03	[3, 4]	125,00	17,41	43,07	36,50	20,44	[ADAM8, AIF1, BIN2, BTK, C1QA, CAMK4, CCR2, CD2, CD53, CD74, CD84, CFP, CLEC12A, CLEC4D, CNR1, CNR2, CTSS, CXCL6, CYBB, DOCK2, EDN2, FCN1, FGL2, FGR, FRMPD3, IFNG, IL18, IL18RAP, IQGAP2, IRF4, LCP2, LRMP, LYZ, NAMPT, NCKAP1L, NR4A3, ORM2, PIK3CG, PLAC8, PLAU, PTAFR, PTPN6, PTPRC, PTX3, RAB44, RHOH, RNASET2, SELL, SLC18A1, STAP1, SUCNR1, SYK, TBC1D10C, TLR6, TLR8, TREM2, TXNDC5, UBD, VNN1]	[ADAM10, ADGRE5, ASAH1, ATG7, B2M, BATF, BATF3, BIN2, BPI, CAB39, CD44, CD47, COTL1, CPNE3, CTSZ, CX3CL1, CYSTM1, EEF1A1, FES, GAB2, GSN, HLA-B, KCNAB2, LCP2, LPCAT1, PCDHA6, PIK3CD, PLSCR1, PRKCE, PSAP, PTPN6, PTPRC, PTPRJ, PTPRN2, RELB, RNASET2, RORA, S100A13, SBNO2, SLAMF1, SLC15A4, SLC7A2, STK10, TBC1D10C, TIMP2, TMC6, TNF, TRAF3, UBR4, ZC3H12A]	[AMPD3, ATP6V1D, CAMK4, CAMP, CDK13, CPLX2, CRISP3, CTSD, DYSF, FOXP1, HYAL2, ITGB2, LRRC7, LYN, LYZ, MMP12, MNDA, NR1H3, PCDHA6, PDXK, PLAC8, PLPPR3, PSMD7, PTPRN2, RAB37, RAB7A, SLC15A4, TLR1]
GO:0032940	secretion by cell	3,67E-02	[3, 5]	251,00	14,93	32,61	36,23	31,16	[ADAM8, AIF1, AIM2, ANGPT1, BANK1, BIN2, BLK, BTK, BTN3A1, BTN3A2, CACNA1E, CARD11, CARD17, CCL19, CCR2, CD2, CD200R1, CD244, CD40LG, CD53, CD84, CFP, CLEC12A, CLEC4D, CLEC4E, CLEC6A, CNR1, CRTAM, CSF1R, CTSS, CTSW, CYBB, DOCK2, DRD1, FCN1, FFAR4, FGL2, FGR, FOXP3, FRMPD3, GBP5, HLA-DRB1, IFNG, IL26, IQGAP2, KLF5, KLRG1, KMO, KNG1, LCP1, LCP2, LPAL2, LRMP, LYZ, MCOLN2, MYB, MYO1G, NCKAP1L, NLRP3, NR4A3, ORM2, P2RY12, PIK3CG, PLAC8, PLAU, PLEK, PTAFR, PTPN22, PTPN6, PTPRC, PTX3, RAB3B, RAB3C, RAB44, RNASET2, SDC1, SELL, SLC18A1, SUCNR1, SYK, SYTL1, TBC1D10C, TLR10, TLR6, TLR8, TNFAIP3, TNFSF13B, TREM2, TXNDC5, VNN1]	[ABAT, ADAM10, ADCY1, ADGRE5, AIM2, ASAH1, ATG7, B2M, BDNF, BIN2, BPI, CAB39, CACNA1A, CACNB2, CAMK2G, CBLN4, CCM2, CCR7, CD160, CD2AP, CD38, CD44, CD47, CORO1A, COTL1, CPNE3, CTSZ, CX3CL1, CYSTM1, EEF1A1, EXOC6B, EXPH5, F13A1, F2R, FES, GAB2, GBP1, GBP5, GNPTAB, GPR68, GRIK5, GRM7, GSK3B, GSN, HDAC9, HLA-B, IL17RA, ITGB6, ITPR1, ITPR2, KCNA2, KCNAB2, KCNC2, KCTD11, LCP1, LCP2, LGR4, LPCAT1, MBP, MICAL2, MTNR1B, MYO1G, NTRK2, OPRM1, PCDH7, PIK3CD, PPFIA1, PPFIA4, PRKCE, PSAP, PSMD9, PTPN22, PTPN6, PTPRC, PTPRJ, PTPRN2, RAB13, RALB, RNASET2, S100A13, SEPTIN9, SLC15A4, SLC1A2, SLC1A5, SLC4A8, STK10, STX11, SYN3, TANK, TBC1D10C, TGFB2, TIMP2, TMC6, TNF, TNFAIP3, TRAF3, TSPOAP1, UBE2Z, UBR4, ZC3H12A]	[ABCA12, ABCC8, ADCY5, ADORA1, AIM2, AKAP12, AMPD3, ARHGAP44, ATP6V1D, BTNL2, CADPS2, CAMP, CARD11, CARD8, CARTPT, CD9, CDK13, CDK5R2, CHRNA4, CHUK, CPLX2, CRISP3, CTAGE1, CTSD, DRD2, DYSF, EGFR, EXOC4, FGF7, FOXP1, FRMD4A, GNAS, GRM7, HGF, HLA-DRB1, HRG, HYAL2, ITGB2, ITPR1, KCNA5, KCNG2, KCNS3, KDM5B, LCP1, LRRC7, LYN, LYZ, MBL2, MBP, MMP12, MNDA, NR1H3, NRXN3, PAK1, PDXK, PFKM, PLAC8, PLPPR3, PNKD, PPP3CA, PRKCA, PRKCZ, PRKN, PSMD7, PTPN22, PTPRN2, RAB37, RAB7A, RIMS1, SEPTIN9, SERPINA4, SLC15A4, SLC17A7, SLC1A7, SRGN, SRI, STXBP5L, SYT17, TBX3, TIAM1, TLR1, TNFRSF11A, TRAF2, VEGFC, VPS33A, VSNL1]
GO:0042110	T cell activation	8,05E-21	[4, 5]	142,00	27,84	53,53	32,35	14,12	[ADAM8, AIF1, APBB1IP, BTLA, BTN3A1, CAMK4, CARD11, CASP8, CCDC88B, CCL19, CCR2, CD1C, CD2, CD27, CD3D, CD3E, CD3G, CD4, CD40LG, CD5, CD6, CD74, CD80, CD86, CD8A, CD8B, CDKN2A, CLCF1, CLEC4D, CLEC4E, CRTAM, CTLA4, DOCK2, DOCK8, EGR3, EOMES, FGL2, FOXP3, FUT7, HLA-DMB, HLA-DOA, HLA-DPA1, HLA-DPB1, HLA-DRB1, ICOS, IFNG, IL18, IL7R, IRF4, ITK, JAML, KLRK1, LAG3, LAX1, LCK, LCP1, LEF1, LMO1, LY9, MYB, NCKAP1L, NLRP3, PIK3CG, PLA2G2D, PRDM1, PRKCQ, PTPN22, PTPN6, PTPRC, RHOH, RUNX3, SASH3, SEMA4A, SIT1, SLAMF6, SYK, TESPA1, THEMIS, THY1, TIGIT, TNFAIP8L2, TNFRSF18, TNFSF13B, TNFSF8, TOX, VNN1, WAS, WNT1, XCL1, ZAP70, ZNF683]	[ADA, APBB1IP, B2M, BATF, BCL2, CASP8, CCDC88B, CCND3, CCR7, CD160, CD28, CD3G, CD44, CD47, CD6, CDK6, CORO1A, CTLA4, FANCA, GATD3A, GRB2, GSN, HLA-A, HLA-DPA1, HLA-DPB1, IDO1, IRF1, JAK3, JAML, KIF13B, LAX1, LCK, LCP1, NCK2, NFATC2, PIK3CD, PSAP, PTPN2, PTPN22, PTPN6, PTPRC, RARA, RELB, RIPOR2, RORA, RUNX1, RUNX3, SLA2, STAT3, TESPA1, TNFAIP8L2, ZBTB16, ZC3H12A, ZEB1, ZMIZ1]	[BCL11B, BTNL2, CAMK4, CARD11, CAV1, CD151, CD8A, CXADR, FOXP1, HLA-DQB2, HLA-DRB1, HLA-G, LCP1, LYN, PAK1, PNKD, PPP3CA, PRKCZ, PTPN22, RIPOR2, RUNX1, TOX, TSC1, ZEB1]
GO:0002521	leukocyte differentiation	2,86E-17	[4, 5, 6, 7, 8]	143,00	25,63	51,85	32,72	15,43	[ADAM8, BLNK, BTK, C1QC, CALCR, CAMK4, CARD11, CASP8, CCL19, CCR2, CD2, CD27, CD3D, CD3E, CD3G, CD4, CD40LG, CD74, CD80, CD86, CD8A, CDKN2A, CLCF1, CLEC4D, CLEC4E, CR2, CRTAM, CSF1R, CTLA4, DOCK10, DOCK2, EGR3, EOMES, FCRL3, FGL2, FLT3, FOXP3, FUT7, GPR55, HCLS1, HLA-DOA, HLA-DRB1, IFNG, IKZF1, IL18, IL7R, INPP5D, IRF4, ITGA4, ITK, LAG3, LCK, LEF1, LRRC17, LY9, MS4A1, MYB, NCKAP1L, NLRP3, PLA2G2D, PRDM1, PTK2B, PTPN22, PTPN6, PTPRC, RHOH, RUNX3, SASH3, SEMA4A, SLAMF6, SLAMF8, SNX10, SYK, TESPA1, THEMIS, TNFRSF18, TNFSF8, TOX, TREM2, UBD, VNN1, WNT1, ZAP70, ZNF683]	[ADA, B2M, BATF, BATF3, BCL2, CASP8, CCR7, CD101, CD28, CD3G, CDH17, CDK6, CMTM7, CTLA4, FANCA, FARP2, FES, GAB2, GPR68, HDAC9, HLA-B, IL34, IRF1, JAK3, LCK, MED1, MITF, MMP14, NFATC2, PIK3CD, PSAP, PTK2B, PTPN2, PTPN22, PTPN6, PTPRC, PTPRJ, RARA, RELB, RORA, RUNX1, RUNX3, SBNO2, STAT3, TCF7L1, TESPA1, TNF, TSHR, UBASH3B, ZBTB16, ZC3H12A, ZEB1, ZMIZ1]	[BCL11B, CAMK4, CARD11, CARTPT, CD8A, CMTM7, FOXP1, GNAS, HLA-DQB2, HLA-DRB1, HLA-G, LYN, OSTM1, PNKD, PRKCA, PRKCZ, PTPN22, RB1, RUNX1, SIGLEC15, TESC, TNFRSF11A, TOX, TSC1, ZEB1]
GO:0030098	lymphocyte differentiation	5,20E-17	[4, 5, 6, 7, 8, 9]	110,00	28,95	57,48	30,71	11,81	[ADAM8, BLNK, BTK, CAMK4, CARD11, CCL19, CCR2, CD2, CD27, CD3D, CD3E, CD3G, CD4, CD40LG, CD74, CD80, CD86, CD8A, CDKN2A, CLCF1, CLEC4D, CLEC4E, CR2, CRTAM, CTLA4, DOCK10, DOCK2, EGR3, EOMES, FCRL3, FGL2, FLT3, FOXP3, FUT7, HLA-DOA, IFNG, IKZF1, IL18, IL7R, INPP5D, IRF4, ITGA4, ITK, LAG3, LCK, LEF1, LY9, MS4A1, MYB, NCKAP1L, NLRP3, PLA2G2D, PRDM1, PTK2B, PTPN22, PTPN6, PTPRC, RHOH, RUNX3, SASH3, SEMA4A, SLAMF6, SLAMF8, SYK, TESPA1, THEMIS, TNFRSF18, TNFSF8, TOX, VNN1, WNT1, ZAP70, ZNF683]	[ADA, B2M, BATF, BCL2, CCR7, CD28, CD3G, CDH17, CDK6, CMTM7, CTLA4, FANCA, HDAC9, IRF1, JAK3, LCK, MMP14, NFATC2, PIK3CD, PSAP, PTK2B, PTPN2, PTPN22, PTPN6, PTPRC, PTPRJ, RARA, RELB, RORA, RUNX1, RUNX3, STAT3, TCF7L1, TESPA1, TSHR, ZBTB16, ZC3H12A, ZEB1, ZMIZ1]	[BCL11B, CAMK4, CARD11, CD8A, CMTM7, FOXP1, HLA-DQB2, HLA-G, PNKD, PRKCZ, PTPN22, RUNX1, TOX, TSC1, ZEB1]
GO:0002682	regulation of immune system process	2,49E-16	[2, 3]	326,00	18,70	49,60	32,44	17,96	[ADAM8, AIF1, AIM2, ANGPT1, BANK1, BLK, BLNK, BTK, BTLA, BTN3A1, BTN3A2, C1QA, C1QB, C1QC, C2, C8G, CAMK4, CARD11, CASP8, CCDC88B, CCL19, CCL24, CCR2, CD1B, CD1C, CD1E, CD2, CD200R1, CD244, CD247, CD27, CD3D, CD3E, CD3G, CD4, CD40LG, CD48, CD5, CD6, CD74, CD80, CD84, CD86, CD8A, CD8B, CD96, CDKN2A, CFP, CLCF1, CLEC1B, CLEC4D, CLEC4E, CLEC6A, CLNK, CNR1, CNR2, CR2, CRTAM, CSF1R, CTLA4, CXCL10, CXCL6, CXCR3, CYP11B1, DOCK8, EDN2, EGR3, EREG, FCN1, FCN3, FCRL3, FGL2, FGR, FOXP3, FUT7, FYB1, GBP5, GPR171, GPR55, GPSM3, H3C10, H3C11, H3C12, H3C7, HCLS1, HLA-DMB, HLA-DOA, HLA-DPA1, HLA-DPB1, HLA-DQA1, HLA-DRA, HLA-DRB1, HLA-DRB5, ICOS, IFNG, IKBKE, IL18, IL18RAP, IL7R, INPP5D, IRF4, ITGA4, ITGB7, ITK, JAML, KLRB1, KLRC1, KLRD1, KLRK1, LAG3, LAX1, LCK, LCP2, LEF1, LMO1, LPAL2, LRRC17, LST1, LTA, MIXL1, MOG, MS4A1, MUC19, MUC6, MYB, MYO1G, MZB1, NCKAP1L, NCR3, NLRP3, NLRP6, NR4A3, ORM2, P2RY12, PAX5, PILRA, PIM1, PLA2G2D, PLA2G7, PRDM1, PRKCQ, PTAFR, PTK2B, PTPN22, PTPN6, PTPRC, PYHIN1, RHEX, RHOH, RUNX3, SASH3, SELE, SELL, SH2D1A, SIT1, SLAMF6, SLAMF7, SLAMF8, STAP1, STAT1, SYK, TBC1D10C, TESPA1, THEMIS, THEMIS2, THY1, TIGIT, TLR6, TLR8, TNFAIP3, TNFAIP8L2, TNFRSF18, TNFSF13B, TNFSF8, TOX, TRAT1, TREM2, TSC22D3, VNN1, WAS, XCL1, XCL2, XG, ZAP70, ZNF683]	[ACTR3, ACVR1B, ADA, ADAM10, AIM2, ARRB2, B2M, BCL2, BIRC3, BPI, BTN3A3, CASP8, CCDC88B, CCM2, CCR7, CD101, CD160, CD28, CD38, CD3G, CD47, CD6, CDK6, COLEC11, COLEC12, CORO1A, CTLA4, CX3CL1, DOCK1, ELMO1, FANCA, FES, FYB1, GAB2, GATD3A, GBP1, GBP5, GPR68, GPRC5B, GPSM3, GRB2, GSN, HDAC1, HLA-A, HLA-B, HLA-DPA1, HLA-DPB1, HLA-DRA, IDO1, IL34, IRF1, JAK3, JAML, KLHL6, KLHL7, LAX1, LCK, LCP2, LIME1, LPXN, LRCH1, LTA, MED1, MITF, MMP14, MYO1G, NCK2, NCOR2, NFATC1, NFATC2, NLRC5, NMI, PCDHA6, PDE4B, PIK3CD, PLSCR1, PRKACB, PRKCE, PRKD2, PSMB8, PSMB9, PSMD9, PTK2B, PTPN1, PTPN2, PTPN22, PTPN6, PTPRC, PTPRJ, RARA, RELB, RIPOR2, RNF216, RORA, RUNX1, RUNX3, SLA2, SLAMF7, SLC7A2, STAT1, STAT3, STK10, TANK, TAPBPL, TBC1D10C, TCF7L1, TESPA1, TGFB2, TNF, TNFAIP3, TNFAIP8L2, TRAF3, UBASH3B, WIPF1, YAP1, ZBP1, ZBTB16, ZBTB20, ZC3H12A, ZEB1, ZMIZ1]	[ADORA1, AIM2, ALOX15, ARNT, BTNL2, CAMK4, CARD11, CARTPT, CAV1, CD8A, CD9, CHUK, COLEC11, CXADR, DOCK1, DRD2, DYSF, FOXP1, FYB1, GNAS, GPR31, HLA-DQB2, HLA-DRA, HLA-DRB1, HLA-G, HRG, ITGB2, KIR3DL2, KLK7, LILRA4, LYN, MAP3K1, MBL2, MEIS2, MMP12, MNDA, MTUS1, MUC2, MUC5B, MYH2, NFATC1, NLRC5, NR1H3, PAK1, PCDHA6, PDE4B, PNKD, PPP3CA, PRKCA, PRKCZ, PSMD7, PTPN22, PUS7, RB1, RIPOR2, RUNX1, SIGLEC15, SMAP1, SUSD4, SWAP70, TESC, TOX, TP73, TRAF2, VEGFC, WASL, ZEB1]
GO:0046649	lymphocyte activation	3,80E-16	[3, 4]	184,00	22,52	54,13	32,11	13,76	[ADAM8, AIF1, APBB1IP, BANK1, BLNK, BTK, BTLA, BTN3A1, CAMK4, CARD11, CASP8, CCDC88B, CCL19, CCR2, CD180, CD1C, CD2, CD244, CD27, CD3D, CD3E, CD3G, CD4, CD40LG, CD5, CD6, CD74, CD80, CD86, CD8A, CD8B, CDKN2A, CLCF1, CLEC4D, CLEC4E, CLNK, CR2, CRTAM, CTLA4, DOCK10, DOCK2, DOCK8, EGR3, EOMES, FCRL1, FCRL3, FCRLA, FGL2, FGR, FLT3, FOXP3, FUT7, GAPT, HLA-DMB, HLA-DOA, HLA-DPA1, HLA-DPB1, HLA-DRB1, ICOS, IFNG, IKZF1, IL18, IL7R, INPP5D, IRF4, ITGA4, ITK, JAML, KLRK1, LAG3, LAX1, LCK, LCP1, LEF1, LMO1, LST1, LY9, MS4A1, MYB, MZB1, NCKAP1L, NCR3, NLRP3, PIK3CG, PLA2G2D, PRDM1, PRF1, PRKCQ, PTK2B, PTPN22, PTPN6, PTPRC, RHOH, RUNX3, SASH3, SEMA4A, SIT1, SLAMF6, SLAMF7, SLAMF8, SYK, TBC1D10C, TESPA1, THEMIS, THY1, TIGIT, TNFAIP3, TNFAIP8L2, TNFRSF18, TNFSF13B, TNFSF8, TOX, VNN1, WAS, WNT1, XCL1, ZAP70, ZNF683]	[ADA, APBB1IP, B2M, BATF, BCL2, CASP8, CCDC88B, CCND3, CCR7, CD160, CD28, CD38, CD3G, CD44, CD47, CD6, CDH17, CDK6, CMTM7, CORO1A, CTLA4, FANCA, GATD3A, GRB2, GSN, HDAC9, HLA-A, HLA-DPA1, HLA-DPB1, IDO1, IL21R, IRF1, JAK3, JAML, KIF13B, LAX1, LCK, LCP1, MMP14, NCK2, NFATC2, PIK3CD, PRF1, PSAP, PTK2B, PTPN2, PTPN22, PTPN6, PTPRC, PTPRJ, RARA, RELB, RIPOR2, RORA, RUNX1, RUNX3, SLA2, SLAMF1, SLAMF7, STAT3, TBC1D10C, TCF7L1, TESPA1, TNFAIP3, TNFAIP8L2, TSHR, ZBTB16, ZC3H12A, ZEB1, ZMIZ1]	[BCL11B, BTNL2, CAMK4, CARD11, CAV1, CD151, CD8A, CHRNA4, CMTM7, CXADR, FOXP1, HLA-DQB2, HLA-DRB1, HLA-G, ITGB2, LCP1, LYN, MNDA, PAK1, PNKD, PPP3CA, PRDX1, PRKCZ, PTPN22, RIPOR2, RUNX1, SWAP70, TOX, TSC1, ZEB1]

GO:0045321	leukocyte activation	8,82E-15	[2, 3]	272,00	19,25	49,68	33,44	16,88	[ADAM8, AIF1, APBB1IP, BANK1, BIN2, BLNK, BTK, BTLA, BTN3A1, C1QA, CAMK4, CARD11, CASP8, CCDC88B, CCL19, CCR2, CD180, CD1C, CD2, CD244, CD27, CD3D, CD3E, CD3G, CD4, CD40LG, CD5, CD53, CD6, CD74, CD80, CD84, CD86, CD8A, CD8B, CDKN2A, CFP, CLCF1, CLEC12A, CLEC4D, CLEC4E, CLNK, CNR1, CNR2, CR2, CRTAM, CTLA4, CTSS, CXCL6, CYBB, DOCK10, DOCK2, DOCK8, EDN2, EGR3, EOMES, FCN1, FCRL1, FCRL3, FCRLA, FGL2, FGR, FLT3, FOXP3, FRMPD3, FUT7, GAPT, HLA-DMB, HLA-DOA, HLA-DPA1, HLA-DPB1, HLA-DRB1, ICOS, IFNG, IKZF1, IL18, IL18RAP, IL7R, INPP5D, IQGAP2, IRF4, ITGA4, ITK, JAML, KLRK1, LAG3, LAX1, LCK, LCP1, LCP2, LEF1, LMO1, LRMP, LST1, LY9, LYZ, MS4A1, MYB, MZB1, NAMPT, NCKAP1L, NCR3, NLRP3, NR4A3, ORM2, PIK3CG, PLA2G2D, PLAC8, PLAU, PRDM1, PRF1, PRKCQ, PTAFR, PTK2B, PTPN22, PTPN6, PTPRC, PTX3, RAB44, RHOH, RNASET2, RUNX3, SASH3, SELL, SELPLG, SEMA4A, SIT1, SLAMF6, SLAMF7, SLAMF8, SLC18A1, STAP1, SUCNR1, SYK, TBC1D10C, TESPA1, THEMIS, THY1, TIGIT, TLR6, TLR8, TNFAIP3, TNFAIP8L2, TNFRSF18, TNFSF13B, TNFSF8, TOX, TREM2, TXNDC5, UBD, VNN1, WAS, WNT1, XCL1, ZAP70, ZNF683]	[ADA, ADAM10, ADGRE5, APBB1IP, ASAH1, ATG7, B2M, BATF, BATF3, BCL2, BIN2, BPI, CAB39, CASP8, CCDC88B, CCND3, CCR7, CD160, CD28, CD38, CD3G, CD44, CD47, CD6, CDH17, CDK6, CMTM7, CORO1A, COTL1, CPNE3, CTLA4, CTSZ, CX3CL1, CYSTM1, EEF1A1, FANCA, FES, GAB2, GATD3A, GRB2, GSN, HDAC9, HLA-A, HLA-B, HLA-DPA1, HLA-DPB1, IDO1, IL21R, IRF1, JAK3, JAML, KCNAB2, KIF13B, LAX1, LCK, LCP1, LCP2, LPCAT1, MMP14, NCK2, NFATC2, PCDHA6, PIK3CD, PLSCR1, PRF1, PRKCE, PSAP, PTK2B, PTPN2, PTPN22, PTPN6, PTPRC, PTPRJ, PTPRN2, RARA, RELB, RIPOR2, RNASET2, RORA, RUNX1, RUNX3, S100A13, SBNO2, SLA2, SLAMF1, SLAMF7, SLC15A4, SLC7A2, STAT3, STK10, TBC1D10C, TCF7L1, TESPA1, TIMP2, TMC6, TNF, TNFAIP3, TNFAIP8L2, TRAF3, TSHR, UBR4, ZBTB16, ZC3H12A, ZEB1, ZMIZ1]	[AMPD3, ATP6V1D, BCL11B, BTNL2, CAMK4, CAMP, CARD11, CAV1, CD151, CD8A, CDK13, CHRNA4, CMTM7, CPLX2, CRISP3, CTSD, CXADR, DYSF, FOXP1, HLA-DQB2, HLA-DRB1, HLA-G, HYAL2, ITGB2, LCP1, LRRC7, LYN, LYZ, MMP12, MNDA, NR1H3, PAK1, PCDHA6, PDXK, PLAC8, PLPPR3, PNKD, PPP3CA, PRDX1, PRKCZ, PSMD7, PTPN22, PTPRN2, RAB37, RAB7A, RIPOR2, RUNX1, SLC15A4, SWAP70, TLR1, TOX, TSC1, ZEB1]
GO:0002684	positive regulation of immune system process	1,47E-13	[2, 3, 4]	235,00	19,70	50,00	33,09	16,91	[ADAM8, AIF1, AIM2, BLK, BLNK, BTK, BTLA, BTN3A1, BTN3A2, C1QA, C1QB, C1QC, C2, C8G, CARD11, CASP8, CCDC88B, CCL19, CCL24, CCR2, CD1B, CD1C, CD1E, CD2, CD244, CD247, CD27, CD3D, CD3E, CD3G, CD4, CD40LG, CD5, CD6, CD74, CD80, CD84, CD86, CFP, CLCF1, CLEC4D, CLEC4E, CLEC6A, CLNK, CNR1, CR2, CRTAM, CSF1R, CTLA4, CXCL10, CYP11B1, DOCK8, EDN2, EGR3, EREG, FCN1, FCN3, FCRL3, FGR, FOXP3, FUT7, FYB1, GBP5, GPSM3, HCLS1, HLA-DMB, HLA-DPA1, HLA-DPB1, HLA-DQA1, HLA-DRA, HLA-DRB1, HLA-DRB5, ICOS, IFNG, IKBKE, IL18, IL18RAP, IL7R, INPP5D, ITGA4, ITK, KLRK1, LAG3, LAX1, LCK, LCP2, LEF1, LTA, MOG, MS4A1, MUC19, MUC6, MYB, MYO1G, MZB1, NCKAP1L, NCR3, NLRP3, NR4A3, P2RY12, PAX5, PLA2G7, PRKCQ, PTAFR, PTK2B, PTPN22, PTPN6, PTPRC, PYHIN1, RHEX, RHOH, RUNX3, SASH3, SELE, SH2D1A, SLAMF6, STAP1, STAT1, SYK, TESPA1, THEMIS, THEMIS2, THY1, TLR6, TLR8, TNFRSF18, TNFSF13B, TOX, TRAT1, TREM2, VNN1, WAS, XCL1, XCL2, XG, ZAP70]	[ACTR3, ACVR1B, ADA, ADAM10, AIM2, B2M, BCL2, BTN3A3, CASP8, CCDC88B, CCR7, CD101, CD160, CD28, CD38, CD3G, CD47, CD6, COLEC11, CORO1A, CTLA4, CX3CL1, DOCK1, ELMO1, FES, FYB1, GAB2, GATD3A, GBP1, GBP5, GPR68, GPRC5B, GPSM3, GRB2, HLA-A, HLA-B, HLA-DPA1, HLA-DPB1, HLA-DRA, IDO1, IL34, JAK3, KLHL6, KLHL7, LAX1, LCK, LCP2, LIME1, LPXN, LTA, MED1, MMP14, MYO1G, NCK2, NCOR2, NFATC2, NLRC5, PDE4B, PIK3CD, PLSCR1, PRKACB, PRKCE, PRKD2, PSMB8, PSMB9, PSMD9, PTK2B, PTPN2, PTPN22, PTPN6, PTPRC, PTPRJ, RARA, RELB, RIPOR2, RORA, RUNX1, RUNX3, SLA2, STAT1, STAT3, TANK, TESPA1, TGFB2, TNF, WIPF1, ZBP1, ZBTB16, ZC3H12A, ZMIZ1]	[AIM2, ARNT, BTNL2, CARD11, CAV1, CHUK, COLEC11, DOCK1, DYSF, FOXP1, FYB1, GNAS, GPR31, HLA-DQB2, HLA-DRA, HLA-DRB1, HLA-G, HRG, ITGB2, KLK7, LYN, MBL2, MMP12, MNDA, MUC2, MUC5B, MYH2, NLRC5, PAK1, PDE4B, PNKD, PRKCA, PRKCZ, PSMD7, PTPN22, RB1, RIPOR2, RUNX1, SMAP1, SUSD4, SWAP70, TESC, TOX, TRAF2, VEGFC, WASL]
GO:0030097	hemopoiesis	4,96E-13	[4, 5, 6, 7]	198,00	20,58	47,03	34,25	18,72	[ADAM8, ANGPT1, BLNK, BTK, C1QC, CALCR, CAMK4, CARD11, CASP8, CCL19, CCR2, CD2, CD27, CD3D, CD3E, CD3G, CD4, CD40LG, CD74, CD80, CD86, CD8A, CDKN2A, CLCF1, CLEC1B, CLEC4D, CLEC4E, CR2, CRTAM, CSF1R, CTLA4, DOCK10, DOCK2, EGR3, EOMES, FCRL3, FGL2, FLT3, FOXP3, FUT7, GFI1, GPR171, GPR55, H3C10, H3C11, H3C12, H3C7, HCLS1, HLA-DOA, HLA-DRB1, IFNG, IKZF1, IL18, IL7R, INPP5D, IRF4, ITGA4, ITK, LAG3, LCK, LEF1, LMO1, LRRC17, LY9, MIXL1, MS4A1, MYB, NCKAP1L, NLRP3, NR4A3, PIM1, PLA2G2D, PLEK, PRDM1, PRKCQ, PTK2B, PTPN22, PTPN6, PTPRC, RHEX, RHOH, RTKN2, RUNX3, SASH3, SELPLG, SEMA4A, SLAMF6, SLAMF8, SNX10, STAT1, SYK, TESPA1, THEMIS, TNFRSF18, TNFSF8, TOX, TREM2, UBD, VNN1, WNT1, WNT10B, ZAP70, ZNF683]	[ACVR1B, ADA, B2M, BATF, BATF3, BCL2, CASP8, CCM2, CCR7, CD101, CD28, CD3G, CDH17, CDK6, CIAO3, CMTM7, CTLA4, DOCK1, ETV6, FANCA, FARP2, FES, GAB2, GATD3A, GPR68, HDAC1, HDAC9, HLA-B, HOXB3, HOXB7, IL34, IRF1, JAK3, KCNAB2, LCK, MED1, MFAP5, MITF, MMP14, NFATC2, PIK3CD, PIP4K2A, PSAP, PSMB8, PSMB9, PSMD9, PTK2B, PTPN2, PTPN22, PTPN6, PTPRC, PTPRJ, RARA, RELB, RORA, RUNX1, RUNX3, SBNO2, STAT1, STAT3, TCF7L1, TESPA1, TGFB2, TMEM190, TMEM91, TNF, TSHR, UBASH3B, YAP1, ZBTB16, ZC3H12A, ZEB1, ZMIZ1, ZNF160, ZNF385A]	[AGPAT5, ANLN, ARNT, ASXL1, BCL11B, CAMK4, CARD11, CARTPT, CD8A, CDK13, CMTM7, DOCK1, FOXP1, GNAS, HIPK2, HLA-DQB2, HLA-DRB1, HLA-G, HYAL2, KIRREL3, LYN, MEIS2, OSTM1, PNKD, PRKCA, PRKCZ, PSMD7, PTPN22, PTPRQ, PUS7, RB1, RUNX1, SIGLEC15, SMAP1, TESC, TNFRSF11A, TOX, TP73, TSC1, VPS33A, ZEB1]
GO:0050776	regulation of immune response	7,47E-13	[3, 4]	218,00	19,91	50,79	31,35	17,86	[ADAM8, AIM2, ANGPT1, BLK, BLNK, BTK, BTLA, BTN3A1, BTN3A2, C1QA, C1QB, C1QC, C2, C8G, CARD11, CCL19, CCR2, CD1B, CD1C, CD1E, CD200R1, CD247, CD3D, CD3E, CD3G, CD4, CD40LG, CD48, CD74, CD80, CD84, CD86, CD8A, CD8B, CD96, CFP, CLCF1, CLEC1B, CLEC4D, CLEC4E, CLEC6A, CLNK, CNR1, CR2, CRTAM, CTLA4, CXCL10, CYP11B1, EREG, FCN1, FCN3, FCRL3, FGL2, FGR, FOXP3, FUT7, FYB1, GBP5, HLA-DMB, HLA-DPA1, HLA-DPB1, HLA-DQA1, HLA-DRA, HLA-DRB1, HLA-DRB5, IFNG, IKBKE, IL18, IL18RAP, IL7R, INPP5D, IRF4, ITGA4, ITGB7, ITK, JAML, KLRB1, KLRC1, KLRD1, KLRK1, LAG3, LAX1, LCK, LCP2, LPAL2, LTA, MOG, MS4A1, MUC19, MUC6, MYB, MYO1G, NCKAP1L, NCR3, NLRP3, NLRP6, NR4A3, PAX5, PILRA, PLA2G2D, PRKCQ, PTAFR, PTPN22, PTPN6, PTPRC, PYHIN1, SASH3, SELL, SH2D1A, SLAMF6, SLAMF7, SLAMF8, STAP1, STAT1, SYK, TESPA1, THEMIS, THEMIS2, THY1, TLR8, TNFAIP3, TNFSF13B, TRAT1, TREM2, WAS, XCL1, ZAP70, ZNF683]	[ACTR3, ADA, AIM2, ARRB2, B2M, BCL2, BIRC3, BTN3A3, CCR7, CD160, CD28, CD38, CD3G, CD47, COLEC11, COLEC12, CTLA4, DOCK1, ELMO1, FES, FYB1, GAB2, GBP1, GBP5, GPRC5B, GRB2, HLA-A, HLA-B, HLA-DPA1, HLA-DPB1, HLA-DRA, IDO1, IRF1, JAK3, JAML, KLHL6, KLHL7, LAX1, LCK, LCP2, LIME1, LPXN, LTA, MED1, MYO1G, NCOR2, NFATC1, NFATC2, NLRC5, NMI, PDE4B, PIK3CD, PLSCR1, PRKACB, PRKCE, PRKD2, PSMB8, PSMB9, PSMD9, PTPN1, PTPN2, PTPN22, PTPN6, PTPRC, PTPRJ, RARA, RELB, RUNX1, SLA2, SLAMF7, STAT1, TANK, TESPA1, TGFB2, TNF, TNFAIP3, WIPF1, ZBP1, ZC3H12A]	[AIM2, ALOX15, BTNL2, CARD11, CD8A, CHUK, COLEC11, CXADR, DOCK1, DRD2, FOXP1, FYB1, GPR31, HLA-DQB2, HLA-DRA, HLA-DRB1, HLA-G, HRG, ITGB2, KIR3DL2, KLK7, LILRA4, LYN, MAP3K1, MBL2, MMP12, MNDA, MUC2, MUC5B, MYH2, NFATC1, NLRC5, NR1H3, PAK1, PDE4B, PNKD, PPP3CA, PRKCA, PRKCZ, PSMD7, PTPN22, RUNX1, SUSD4, TRAF2, WASL]
GO:0048534	hematopoietic or lymphoid organ development	2,00E-12	[3, 4, 5, 6]	203,00	20,20	46,67	34,67	18,67	[ADAM8, ANGPT1, BLNK, BTK, C1QC, CALCR, CAMK4, CARD11, CASP8, CCL19, CCR2, CD2, CD27, CD3D, CD3E, CD3G, CD4, CD40LG, CD74, CD80, CD86, CD8A, CDKN2A, CLCF1, CLEC1B, CLEC4D, CLEC4E, CR2, CRTAM, CSF1R, CTLA4, DOCK10, DOCK2, EGR3, EOMES, FCRL3, FGL2, FLT3, FOXP3, FUT7, GFI1, GPR171, GPR55, H3C10, H3C11, H3C12, H3C7, HCLS1, HLA-DOA, HLA-DRB1, IFNG, IKZF1, IL18, IL7R, INPP5D, IRF4, ITGA4, ITK, LAG3, LCK, LEF1, LMO1, LRRC17, LTA, LTB, LY9, MIXL1, MS4A1, MYB, NCKAP1L, NLRP3, NR4A3, PIM1, PLA2G2D, PLEK, PRDM1, PRKCQ, PTK2B, PTPN22, PTPN6, PTPRC, RHEX, RHOH, RTKN2, RUNX3, SASH3, SELPLG, SEMA4A, SLAMF6, SLAMF8, SNX10, STAT1, SYK, TESPA1, THEMIS, TNFRSF18, TNFSF8, TOX, TREM2, UBD, VNN1, WNT1, WNT10B, ZAP70, ZNF683]	[ACVR1B, ADA, B2M, BATF, BATF3, BCL2, CASP8, CCM2, CCR7, CD101, CD28, CD3G, CDH17, CDK6, CIAO3, CMTM7, CTLA4, DOCK1, ETV6, FANCA, FARP2, FES, GAB2, GATD3A, GPR68, HDAC1, HDAC9, HLA-B, HOXA3, HOXB3, HOXB7, IL34, IRF1, JAK3, JARID2, KCNAB2, LCK, LTA, MED1, MFAP5, MITF, MMP14, NFATC2, PIK3CD, PIP4K2A, PSAP, PSMB8, PSMB9, PSMD9, PTK2B, PTPN2, PTPN22, PTPN6, PTPRC, PTPRJ, RARA, RELB, RORA, RUNX1, RUNX3, SBNO2, STAT1, STAT3, TCF7L1, TESPA1, TGFB2, TMEM190, TMEM91, TNF, TSHR, UBASH3B, YAP1, ZBTB16, ZC3H12A, ZEB1, ZMIZ1, ZNF160, ZNF385A]	[AGPAT5, ANLN, ARNT, ASXL1, BCL11B, CAMK4, CARD11, CARTPT, CD8A, CDK13, CMTM7, DOCK1, FOXP1, GNAS, HIPK2, HLA-DQB2, HLA-DRB1, HLA-G, HYAL2, KIRREL3, LYN, MEIS2, OSTM1, PNKD, PRKCA, PRKCZ, PSMD7, PTPN22, PTPRQ, PUS7, RB1, RUNX1, SIGLEC15, SMAP1, TESC, TNFRSF11A, TOX, TP73, TSC1, TYR, VPS33A, ZEB1]
GO:0002520	immune system development	4,79E-11	[2, 4, 5]	208,00	19,44	46,75	34,63	18,61	[ADAM8, ANGPT1, BLNK, BTK, C1QC, CALCR, CAMK4, CARD11, CASP8, CCL19, CCR2, CCR4, CD2, CD27, CD3D, CD3E, CD3G, CD4, CD40LG, CD74, CD80, CD86, CD8A, CDKN2A, CLCF1, CLEC1B, CLEC4D, CLEC4E, CR2, CRTAM, CSF1R, CTLA4, DOCK10, DOCK2, EGR3, EOMES, FCRL3, FGL2, FLT3, FOXP3, FUT7, GFI1, GPR171, GPR55, H3C10, H3C11, H3C12, H3C7, HCLS1, HLA-DOA, HLA-DRB1, ICOS, IFNG, IKZF1, IL18, IL7R, INPP5D, IRF4, ITGA4, ITK, LAG3, LCK, LEF1, LMO1, LRRC17, LTA, LTB, LY9, MIXL1, MS4A1, MYB, NCKAP1L, NLRP3, NR4A3, PIM1, PLA2G2D, PLEK, PRDM1, PRKCQ, PTK2B, PTPN22, PTPN6, PTPRC, RHEX, RHOH, RTKN2, RUNX3, SASH3, SELPLG, SEMA4A, SLAMF6, SLAMF8, SNX10, STAT1, SYK, TESPA1, THEMIS, TNFAIP3, TNFRSF18, TNFSF8, TOX, TREM2, UBD, VNN1, WNT1, WNT10B, ZAP70, ZNF683]	[ACVR1B, ADA, B2M, BATF, BATF3, BCL2, CASP8, CCM2, CCR7, CD101, CD28, CD3G, CDH17, CDK6, CIAO3, CMTM7, CTLA4, DOCK1, ETV6, FANCA, FARP2, FES, GAB2, GATD3A, GPR68, HDAC1, HDAC9, HLA-B, HOXA3, HOXB3, HOXB7, IDO1, IL34, IRF1, JAK3, JARID2, KCNAB2, LCK, LTA, MED1, MFAP5, MITF, MMP14, NFATC2, PIK3CD, PIP4K2A, PSAP, PSMB8, PSMB9, PSMD9, PTK2B, PTPN2, PTPN22, PTPN6, PTPRC, PTPRJ, RARA, RELB, RORA, RUNX1, RUNX3, SBNO2, STAT1, STAT3, TCF7L1, TESPA1, TGFB2, TMEM190, TMEM91, TNF, TNFAIP3, TSHR, UBASH3B, YAP1, ZBTB16, ZC3H12A, ZEB1, ZMIZ1, ZNF160, ZNF385A]	[AGPAT5, ANLN, ARNT, ASXL1, BCL11B, CAMK4, CARD11, CARTPT, CD8A, CDK13, CMTM7, DOCK1, FOXP1, GNAS, HIPK2, HLA-DQB2, HLA-DRB1, HLA-G, HYAL2, KIRREL3, LYN, MEIS2, OSTM1, PNKD, PRKCA, PRKCZ, PSMD7, PTPN22, PTPRQ, PUS7, RB1, RUNX1, SIGLEC15, SMAP1, SWAP70, TESC, TNFRSF11A, TOX, TP73, TSC1, TYR, VPS33A, ZEB1]

GO:0048584	positive regulation of response to stimulus	9,87E-11	[2, 3, 4]	420,00	16,22	35,26	37,82	26,92	[ABRA, ADAM8, AIF1, AIM2, ANGPT1, AREG, ATF3, BANK1, BLK, BLNK, BMPR1B, BTK, BTN3A1, BTN3A2, C1QA, C1QB, C1QC, C2, C8G, CALCR, CARD11, CASP8, CCL19, CCL24, CCR2, CCR4, CD180, CD1B, CD1C, CD1E, CD247, CD27, CD3D, CD3E, CD3G, CD4, CD40LG, CD6, CD74, CD80, CD86, CDKN2A, CFP, CLCF1, CLEC4D, CLEC4E, CLEC6A, CLNK, CNR1, CR2, CRB2, CRTAM, CSF1R, CTLA4, CXCL10, CXCR4, CYP11B1, DEPDC1B, EDN2, ELF3, EREG, ERFE, FASLG, FCN1, FCN3, FCRL3, FFAR4, FGD2, FGR, FLT3, FOXP3, FYB1, GBP5, GDF6, GFI1, GPR174, GPR55, GPSM3, HCLS1, HLA-DMB, HLA-DPA1, HLA-DPB1, HLA-DQA1, HLA-DRA, HLA-DRB1, HLA-DRB5, ICOS, IFNG, IKBKE, IL10RA, IL18, IL18RAP, IL26, IL7R, INPP5D, ITK, KLRK1, LAG3, LAX1, LCK, LCP1, LCP2, LMCD1, LTA, LY86, MAPK13, MOG, MS4A1, MSX1, MUC19, MUC6, MUSK, MYB, MYO1G, NCF1, NCKAP1L, NCR3, NLRP3, NPPC, NR4A3, P2RY10, P2RY12, PAX5, PIK3CG, PIM2, PLA2G7, PLEK, PRKCQ, PROK1, PTAFR, PTK2B, PTPN22, PTPN6, PTPRC, PYHIN1, RAB3B, RTKN2, SASH3, SEMA4D, SH2D1A, SLAMF6, STAP1, SUCNR1, SYK, TESPA1, THEMIS, THEMIS2, THY1, TLR10, TLR6, TLR8, TNFAIP3, TNFRSF18, TNFSF13B, TRAF1, TRAT1, TREM2, UBD, WAS, WNT1, WNT10B, XCL1, XCL2, ZAP70, ZBED2]	[ABAT, ACTR3, ACVR1B, ADA, ADAM10, ADGRV1, AGAP2, AIM2, AKAP13, AKAP6, ARHGEF3, ARRB2, AUTS2, B2M, BABAM2, BCAR3, BCL2, BDNF, BIRC3, BMP7, BMPR1A, BTN3A3, CASP8, CC2D1A, CCM2, CCR7, CD160, CD28, CD38, CD3G, CD44, CD47, CD6, CDH2, CFLAR, CHSY1, CLEC16A, COLEC11, CTLA4, CX3CL1, DEDD2, DIXDC1, DOCK1, EDEM1, ELMO1, ETFA, F2R, FYB1, GAB2, GATD3A, GBP1, GBP5, GHR, GLIPR2, GPR68, GPRC5B, GPSM3, GRB2, GSK3B, GSN, HLA-A, HLA-B, HLA-DPA1, HLA-DPB1, HLA-DRA, HMGA1, HOMER1, HTT, IDO1, IGF1R, IGFBP4, IL17RA, IL34, INAVA, ITGA8, KLB, KLHL6, KLHL7, KSR1, LAX1, LCK, LCP1, LCP2, LGR4, LIME1, LITAF, LPXN, LTA, MAML3, MAP2K2, MAP2K5, MAP4K5, MDFIC, MED1, MIER1, MIOS, MRTFA, MYO1G, NCK2, NCOR2, NEO1, NETO1, NFAT5, NFATC2, NLRC5, NOS1AP, NPR2, NPTN, NSMCE1, NTRK2, OPRM1, PDCD10, PDE4B, PDZD2, PIGU, PIK3CD, PLCE1, PLSCR1, PPP3CC, PRKACB, PRKCE, PRKD2, PSAP, PSMB8, PSMB9, PSMD9, PTK2B, PTPN1, PTPN2, PTPN22, PTPN6, PTPRC, PTPRJ, RAB34, RARA, RECK, RELB, RIPOR2, RORA, RPTOR, RRAGC, RUNX1, RWDD3, S100A13, SEMA4D, SIK3, SLA2, SLAMF1, SMARCA4, SPIDR, SSTR4, STAT3, TANK, TERT, TESPA1, TGFB2, TIMP2, TMEM108, TNF, TNFAIP3, TNFSF10, TNIP1, TP53BP2, TRAF1, TRAF3, TSPAN5, TTC23, UBE3A, WIPF1, WWOX, YAP1, ZBP1	[ABCC1, ABRA, ADORA1, ADRA1B, ADRB3, AIM2, AKAP12, AKAP13, ALK, ALOX15, ARHGEF3, ARNT, ASXL1, BCAR3, BCLAF1, BTNL2, CACNG2, CARD11, CARTPT, CAV1, CDH13, CHSY1, CHUK, COLEC11, DCN, DENND2B, DOCK1, DRD2, DYSF, EGFR, ERBB4, EYA2, F10, FGF7, FGF9, FMN2, FOXP1, FYB1, GCNT2, GDF6, GFRAL, GNAS, GPR31, HGF, HIP1, HIPK2, HLA-DQB2, HLA-DRA, HLA-DRB1, HLA-G, HRG, HYAL2, IGF1R, IGSF9B, ITGB2, KCNK2, KIF7, KLK7, LCP1, LMO3, LPAR6, LYN, MAML2, MAP3K1, MAP3K5, MAPRE2, MBL2, MIER1, MMP12, MNDA, MUC2, MUC5B, MYH2, MYLK, NACC2, NDRG4, NGF, NLRC5, NOS1AP, NR1H3, NUP93, P2RX2, PAK1, PDE4B, PLCE1, PLPP3, PNKD, PPP3CA, PRKAA1, PRKCA, PRKCZ, PRKN, PSMD7, PTPN22, RIMS1, RIPOR2, RNF13, ROBO1, ROBO2, RSPO1, RUNX1, SCEL, SEMA5A, SHANK1, SHOC2, SKI, SLC6A2, SRI, ST18, STK25, SUSD4, SWAP70, TIAM1, TLR1, TNFRSF11A, TP73, TRAF2, TRAF7, USP8, VEGFC, VSNL1, WASL, WDFY1, WNK2, WNT16, WRAP53]
GO:0050778	positive regulation of immune response	8,30E-10	[3, 4, 5]	170,00	20,07	48,99	33,33	17,68	[ADAM8, AIM2, BLK, BLNK, BTK, BTN3A1, BTN3A2, C1QA, C1QB, C1QC, C2, C8G, CARD11, CCL19, CCR2, CD1B, CD1C, CD1E, CD247, CD3D, CD3E, CD3G, CD4, CD74, CD80, CD86, CFP, CLCF1, CLEC4D, CLEC4E, CLEC6A, CLNK, CNR1, CR2, CRTAM, CTLA4, CXCL10, CYP11B1, EREG, FCN1, FCN3, FCRL3, FGR, FOXP3, FYB1, GBP5, HLA-DMB, HLA-DPA1, HLA-DPB1, HLA-DQA1, HLA-DRA, HLA-DRB1, HLA-DRB5, IFNG, IKBKE, IL18, IL18RAP, INPP5D, ITK, KLRK1, LAG3, LAX1, LCK, LCP2, LTA, MOG, MS4A1, MUC19, MUC6, MYB, MYO1G, NCKAP1L, NCR3, NLRP3, NR4A3, PAX5, PRKCQ, PTAFR, PTPN22, PTPN6, PTPRC, PYHIN1, SASH3, SH2D1A, SLAMF6, STAP1, SYK, TESPA1, THEMIS, THEMIS2, THY1, TLR8, TNFSF13B, TRAT1, WAS, XCL1, ZAP70]	[ACTR3, ADA, AIM2, B2M, BCL2, BTN3A3, CCR7, CD160, CD28, CD38, CD3G, CD47, COLEC11, CTLA4, DOCK1, ELMO1, FYB1, GAB2, GBP1, GBP5, GPRC5B, GRB2, HLA-A, HLA-B, HLA-DPA1, HLA-DPB1, HLA-DRA, IDO1, KLHL6, KLHL7, LAX1, LCK, LCP2, LIME1, LPXN, LTA, MED1, MYO1G, NCOR2, NFATC2, NLRC5, PDE4B, PIK3CD, PLSCR1, PRKACB, PRKCE, PRKD2, PSMB8, PSMB9, PSMD9, PTPN2, PTPN22, PTPN6, PTPRC, PTPRJ, RARA, RELB, RUNX1, SLA2, TANK, TESPA1, TGFB2, TNF, WIPF1, ZBP1, ZC3H12A]	[AIM2, BTNL2, CARD11, CHUK, COLEC11, DOCK1, FOXP1, FYB1, GPR31, HLA-DQB2, HLA-DRA, HLA-DRB1, HLA-G, HRG, ITGB2, KLK7, LYN, MBL2, MMP12, MNDA, MUC2, MUC5B, MYH2, NLRC5, PAK1, PDE4B, PNKD, PRKCA, PRKCZ, PSMD7, PTPN22, RUNX1, SUSD4, TRAF2, WASL]
GO:0002694	regulation of leukocyte activation	7,56E-08	[3, 4, 5]	133,00	20,52	56,05	31,85	12,10	[ADAM8, AIF1, BANK1, BTK, BTLA, CAMK4, CARD11, CCDC88B, CCL19, CCR2, CD2, CD27, CD3E, CD4, CD40LG, CD5, CD6, CD74, CD80, CD84, CD86, CDKN2A, CLCF1, CLEC4D, CLNK, CNR1, CNR2, CRTAM, CTLA4, DOCK8, EGR3, FCRL3, FGL2, FGR, FOXP3, HLA-DMB, HLA-DOA, HLA-DPA1, HLA-DPB1, HLA-DRB1, ICOS, IFNG, IL18, IL7R, INPP5D, IRF4, KLRK1, LAG3, LAX1, LCK, LEF1, LMO1, LST1, MYB, MZB1, NCKAP1L, NLRP3, NR4A3, PLA2G2D, PRDM1, PRKCQ, PTAFR, PTPN22, PTPN6, PTPRC, RHOH, RUNX3, SASH3, SIT1, SLAMF8, STAP1, SYK, TBC1D10C, TESPA1, THY1, TIGIT, TLR6, TNFAIP3, TNFAIP8L2, TNFRSF18, TNFSF13B, TNFSF8, TOX, TREM2, VNN1, XCL1, ZAP70, ZNF683]	[ADA, BCL2, BPI, CCDC88B, CCR7, CD160, CD28, CD38, CD47, CD6, CORO1A, CTLA4, CX3CL1, FANCA, FES, GAB2, GATD3A, GRB2, GSN, HLA-A, HLA-DPA1, HLA-DPB1, IDO1, IRF1, JAK3, LAX1, LCK, MMP14, NCK2, NFATC2, PCDHA6, PLSCR1, PTPN2, PTPN22, PTPN6, PTPRC, RARA, RIPOR2, RORA, RUNX1, RUNX3, SLC7A2, TBC1D10C, TESPA1, TNFAIP3, TNFAIP8L2, ZBTB16, ZC3H12A, ZEB1, ZMIZ1]	[BTNL2, CAMK4, CARD11, CAV1, HLA-DQB2, HLA-DRB1, HLA-G, ITGB2, LYN, MNDA, NR1H3, PAK1, PCDHA6, PRKCZ, PTPN22, RIPOR2, RUNX1, TOX, ZEB1]
GO:0050865	regulation of cell activation	2,31E-07	[3, 4]	139,00	19,97	54,60	31,90	13,50	[ADAM8, AIF1, BANK1, BTK, BTLA, CAMK4, CARD11, CCDC88B, CCL19, CCR2, CD2, CD27, CD3E, CD4, CD40LG, CD5, CD6, CD74, CD80, CD84, CD86, CDKN2A, CLCF1, CLEC4D, CLNK, CNR1, CNR2, CRTAM, CTLA4, DOCK8, EGR3, FCRL3, FGL2, FGR, FOXP3, HLA-DMB, HLA-DOA, HLA-DPA1, HLA-DPB1, HLA-DRB1, ICOS, IFNG, IL18, IL7R, INPP5D, IRF4, KLRK1, LAG3, LAX1, LCK, LEF1, LMO1, LST1, MYB, MZB1, NCKAP1L, NLRP3, NR4A3, PLA2G2D, PLEK, PRDM1, PRKCQ, PTAFR, PTPN22, PTPN6, PTPRC, RHOH, RUNX3, SASH3, SIT1, SLAMF8, STAP1, SYK, TBC1D10C, TESPA1, THY1, TIGIT, TLR6, TNFAIP3, TNFAIP8L2, TNFRSF18, TNFSF13B, TNFSF8, TOX, TREM2, VNN1, XCL1, ZAP70, ZNF683]	[ABAT, ADA, BCL2, BPI, CCDC88B, CCR7, CD160, CD28, CD38, CD47, CD6, CORO1A, CTLA4, CX3CL1, FANCA, FES, GAB2, GATD3A, GRB2, GSN, HLA-A, HLA-DPA1, HLA-DPB1, IDO1, IRF1, JAK3, LAX1, LCK, MMP14, NCK2, NFATC2, PCDHA6, PLSCR1, PTPN2, PTPN22, PTPN6, PTPRC, RARA, RIPOR2, RORA, RUNX1, RUNX3, SLC7A2, TBC1D10C, TESPA1, TNFAIP3, TNFAIP8L2, UBASH3B, ZBTB16, ZC3H12A, ZEB1, ZMIZ1]	[BTNL2, CAMK4, CARD11, CAV1, CD9, HLA-DQB2, HLA-DRB1, HLA-G, HRG, ITGB2, LYN, MNDA, NR1H3, PAK1, PCDHA6, PRKCA, PRKCZ, PTPN22, RIPOR2, RUNX1, TOX, ZEB1]
GO:0051249	regulation of lymphocyte activation	2,14E-06	[4, 5, 6]	114,00	20,50	57,66	30,66	11,68	[ADAM8, AIF1, BANK1, BTK, BTLA, CAMK4, CARD11, CCDC88B, CCL19, CCR2, CD2, CD27, CD3E, CD4, CD40LG, CD5, CD6, CD74, CD80, CD86, CDKN2A, CLCF1, CLNK, CRTAM, CTLA4, DOCK8, EGR3, FCRL3, FGL2, FGR, FOXP3, HLA-DMB, HLA-DOA, HLA-DPA1, HLA-DPB1, HLA-DRB1, ICOS, IFNG, IL18, IL7R, INPP5D, IRF4, KLRK1, LAG3, LAX1, LCK, LEF1, LMO1, LST1, MYB, MZB1, NCKAP1L, NLRP3, PLA2G2D, PRDM1, PRKCQ, PTPN22, PTPN6, PTPRC, RHOH, RUNX3, SASH3, SIT1, SLAMF8, SYK, TBC1D10C, TESPA1, THY1, TIGIT, TNFAIP3, TNFAIP8L2, TNFRSF18, TNFSF13B, TNFSF8, TOX, VNN1, XCL1, ZAP70, ZNF683]	[ADA, BCL2, CCDC88B, CCR7, CD160, CD28, CD38, CD47, CD6, CORO1A, CTLA4, FANCA, GATD3A, GRB2, GSN, HLA-A, HLA-DPA1, HLA-DPB1, IDO1, IRF1, JAK3, LAX1, LCK, MMP14, NCK2, NFATC2, PTPN2, PTPN22, PTPN6, PTPRC, RARA, RIPOR2, RUNX1, RUNX3, TBC1D10C, TESPA1, TNFAIP3, TNFAIP8L2, ZBTB16, ZC3H12A, ZEB1, ZMIZ1]	[BTNL2, CAMK4, CARD11, CAV1, HLA-DQB2, HLA-DRB1, HLA-G, LYN, MNDA, PAK1, PRKCZ, PTPN22, RIPOR2, RUNX1, TOX, ZEB1]
GO:0050852	T cell receptor signaling pathway	4,30E-06	[6, 7, 8, 9, 10]	58,00	26,24	44,74	40,79	14,47	[BTN3A1, BTN3A2, CARD11, CD247, CD3D, CD3E, CD3G, CD4, CTLA4, FOXP3, FYB1, HLA-DPA1, HLA-DPB1, HLA-DQA1, HLA-DRA, HLA-DRB1, HLA-DRB5, INPP5D, ITK, LCK, LCP2, MOG, NR4A3, PRKCQ, PTPN22, PTPN6, PTPRC, TESPA1, THEMIS, THEMIS2, THY1, TRAT1, WAS, ZAP70]	[ADA, BTN3A3, CCR7, CD160, CD28, CD3G, CTLA4, FYB1, GBP1, HLA-A, HLA-DPA1, HLA-DPB1, HLA-DRA, LCK, LCP2, LIME1, NCOR2, PDE4B, PIK3CD, PRKD2, PSMB8, PSMB9, PSMD9, PTPN2, PTPN22, PTPN6, PTPRC, PTPRJ, SLA2, TESPA1, ZC3H12A]	[BTNL2, CARD11, CHUK, FYB1, HLA-DQB2, HLA-DRA, HLA-DRB1, PAK1, PDE4B, PSMD7, PTPN22]
GO:0002768	immune response-regulating cell surface receptor signaling pathway	6,83E-06	[4, 5, 6]	112,00	20,25	42,22	38,52	19,26	[BLK, BLNK, BTK, BTLA, BTN3A1, BTN3A2, CARD11, CD200R1, CD247, CD3D, CD3E, CD3G, CD4, CLEC4D, CLEC4E, CLEC6A, CR2, CTLA4, FCN1, FCRL3, FGR, FOXP3, FYB1, HLA-DPA1, HLA-DPB1, HLA-DQA1, HLA-DRA, HLA-DRB1, HLA-DRB5, INPP5D, ITK, KLRK1, LAX1, LCK, LCP2, MOG, MS4A1, MUC19, MUC6, MYO1G, NCKAP1L, NCR3, NR4A3, PAX5, PRKCQ, PTPN22, PTPN6, PTPRC, STAP1, SYK, TESPA1, THEMIS, THEMIS2, THY1, TRAT1, WAS, ZAP70]	[ACTR3, ADA, BCL2, BTN3A3, CCR7, CD160, CD28, CD38, CD3G, CD47, CTLA4, DOCK1, ELMO1, FYB1, GAB2, GBP1, GRB2, HLA-A, HLA-DPA1, HLA-DPB1, HLA-DRA, KLHL6, KLHL7, LAX1, LCK, LCP2, LIME1, LPXN, MYO1G, NCOR2, NFATC1, NFATC2, PDE4B, PIK3CD, PLSCR1, PRKACB, PRKCE, PRKD2, PSMB8, PSMB9, PSMD9, PTPN2, PTPN22, PTPN6, PTPRC, PTPRJ, RELB, RUNX1, SLA2, TESPA1, WIPF1, ZC3H12A]	[BTNL2, CARD11, CHUK, DOCK1, FOXP1, FYB1, HLA-DQB2, HLA-DRA, HLA-DRB1, HLA-G, LILRA4, LYN, MAP3K1, MNDA, MUC2, MUC5B, MYH2, NFATC1, PAK1, PDE4B, PPP3CA, PRKCA, PSMD7, PTPN22, RUNX1, WASL]
GO:0050851	antigen receptor-mediated signaling pathway	5,11E-05	[5, 6, 7, 8, 9]	78,00	21,91	44,90	39,80	15,31	[BLK, BLNK, BTK, BTN3A1, BTN3A2, CARD11, CD247, CD3D, CD3E, CD3G, CD4, CTLA4, FCRL3, FOXP3, FYB1, HLA-DPA1, HLA-DPB1, HLA-DQA1, HLA-DRA, HLA-DRB1, HLA-DRB5, INPP5D, ITK, LAX1, LCK, LCP2, MOG, MS4A1, NCKAP1L, NR4A3, PAX5, PRKCQ, PTPN22, PTPN6, PTPRC, STAP1, SYK, TESPA1, THEMIS, THEMIS2, THY1, TRAT1, WAS, ZAP70]	[ADA, BCL2, BTN3A3, CCR7, CD160, CD28, CD38, CD3G, CTLA4, FYB1, GBP1, HLA-A, HLA-DPA1, HLA-DPB1, HLA-DRA, KLHL6, KLHL7, LAX1, LCK, LCP2, LIME1, LPXN, NCOR2, NFATC2, PDE4B, PIK3CD, PRKD2, PSMB8, PSMB9, PSMD9, PTPN2, PTPN22, PTPN6, PTPRC, PTPRJ, RUNX1, SLA2, TESPA1, ZC3H12A]	[BTNL2, CARD11, CHUK, FOXP1, FYB1, HLA-DQB2, HLA-DRA, HLA-DRB1, LYN, MNDA, PAK1, PDE4B, PSMD7, PTPN22, RUNX1]
GO:0002429	immune response-activating cell surface receptor signaling pathway	6,68E-05	[4, 5, 6, 7, 8]	104,00	19,96	43,65	39,68	16,67	[BLK, BLNK, BTK, BTN3A1, BTN3A2, CARD11, CD247, CD3D, CD3E, CD3G, CD4, CLEC4D, CLEC4E, CLEC6A, CR2, CTLA4, FCN1, FCRL3, FGR, FOXP3, FYB1, HLA-DPA1, HLA-DPB1, HLA-DQA1, HLA-DRA, HLA-DRB1, HLA-DRB5, INPP5D, ITK, KLRK1, LAX1, LCK, LCP2, MOG, MS4A1, MUC19, MUC6, MYO1G, NCKAP1L, NCR3, NR4A3, PAX5, PRKCQ, PTPN22, PTPN6, PTPRC, STAP1, SYK, TESPA1, THEMIS, THEMIS2, THY1, TRAT1, WAS, ZAP70]	[ACTR3, ADA, BCL2, BTN3A3, CCR7, CD160, CD28, CD38, CD3G, CD47, CTLA4, DOCK1, ELMO1, FYB1, GBP1, GRB2, HLA-A, HLA-DPA1, HLA-DPB1, HLA-DRA, KLHL6, KLHL7, LAX1, LCK, LCP2, LIME1, LPXN, MYO1G, NCOR2, NFATC2, PDE4B, PIK3CD, PLSCR1, PRKACB, PRKCE, PRKD2, PSMB8, PSMB9, PSMD9, PTPN2, PTPN22, PTPN6, PTPRC, PTPRJ, RELB, RUNX1, SLA2, TESPA1, WIPF1, ZC3H12A]	[BTNL2, CARD11, CHUK, DOCK1, FOXP1, FYB1, HLA-DQB2, HLA-DRA, HLA-DRB1, LYN, MNDA, MUC2, MUC5B, MYH2, PAK1, PDE4B, PRKCA, PSMD7, PTPN22, RUNX1, WASL]
GO:0002253	activation of immune response	1,08E-04	[2, 4, 5, 6]	118,00	19,00	46,15	36,36	17,48	[AIM2, BLK, BLNK, BTK, BTN3A1, BTN3A2, C1QA, C1QB, C1QC, C2, C8G, CARD11, CD247, CD3D, CD3E, CD3G, CD4, CFP, CLEC4D, CLEC4E, CLEC6A, CR2, CTLA4, CXCL10, CYP11B1, FCN1, FCN3, FCRL3, FGR, FOXP3, FYB1, HLA-DPA1, HLA-DPB1, HLA-DQA1, HLA-DRA, HLA-DRB1, HLA-DRB5, INPP5D, ITK, KLRK1, LAX1, LCK, LCP2, MOG, MS4A1, MUC19, MUC6, MYO1G, NCKAP1L, NCR3, NR4A3, PAX5, PRKCQ, PTPN22, PTPN6, PTPRC, PYHIN1, STAP1, SYK, TESPA1, THEMIS, THEMIS2, THY1, TRAT1, WAS, ZAP70]	[ACTR3, ADA, AIM2, BCL2, BTN3A3, CCR7, CD160, CD28, CD38, CD3G, CD47, COLEC11, CTLA4, DOCK1, ELMO1, FYB1, GBP1, GRB2, HLA-A, HLA-DPA1, HLA-DPB1, HLA-DRA, KLHL6, KLHL7, LAX1, LCK, LCP2, LIME1, LPXN, MYO1G, NCOR2, NFATC2, PDE4B, PIK3CD, PLSCR1, PRKACB, PRKCE, PRKD2, PSMB8, PSMB9, PSMD9, PTPN2, PTPN22, PTPN6, PTPRC, PTPRJ, RELB, RUNX1, SLA2, TESPA1, WIPF1, ZC3H12A]	[AIM2, BTNL2, CARD11, CHUK, COLEC11, DOCK1, FOXP1, FYB1, HLA-DQB2, HLA-DRA, HLA-DRB1, LYN, MBL2, MNDA, MUC2, MUC5B, MYH2, PAK1, PDE4B, PRKCA, PSMD7, PTPN22, RUNX1, SUSD4, WASL]
GO:0002250	adaptive immune response	1,08E-03	[3]	133,00	17,71	62,09	26,14	11,76	[BTK, BTLA, BTN3A1, BTN3A2, C1QA, C1QB, C1QC, C2, C8G, CAMK4, CCL19, CCR2, CD1B, CD1C, CD1E, CD244, CD247, CD27, CD3D, CD3E, CD3G, CD4, CD40LG, CD48, CD6, CD74, CD80, CD84, CD86, CD8A, CD8B, CLCF1, CLEC4D, CLEC6A, CR2, CRTAM, CTLA4, CTSS, CXCL10, EOMES, FGL2, FOXP3, FUT7, GAPT, HLA-DMA, HLA-DMB, HLA-DOA, HLA-DPA1, HLA-DPB1, HLA-DQA1, HLA-DRA, HLA-DRB1, HLA-DRB5, IFNG, IL18, IL18RAP, IL7R, INPP5D, IRF4, ITK, JCHAIN, KLRK1, LAG3, LAMP3, LAX1, LEF1, LTA, LY9, MCOLN2, MYO1G, NLRP3, NR4A3, PIK3CG, PRDM1, PRF1, PRKCQ, PTK2B, PTPN6, PTPRC, SASH3, SEMA4A, SH2D1A, SIT1, SLAMF6, SLAMF7, SYK, THEMIS, TLR8, TNFAIP3, TNFSF13B, TRAT1, WAS, XCL1, ZAP70, ZNF683]	[ADA, B2M, BATF, BTN3A3, CD160, CD28, CD3G, CD6, CTLA4, HLA-A, HLA-B, HLA-DPA1, HLA-DPB1, HLA-DRA, IRF1, JAK3, KLHL6, KLHL7, LAX1, LIME1, LTA, MYO1G, NCOR2, PIK3CD, PRF1, PRKD2, PTK2B, PTPN6, PTPRC, RELB, RORA, SLA2, SLAMF1, SLAMF7, STAT3, TANK, TAP1, TNF, TNFAIP3, ZC3H12A]	[ALOX15, CAMK4, CD8A, HLA-DQB2, HLA-DRA, HLA-DRB1, HLA-G, JCHAIN, LYN, MBL2, ORAI1, PNKD, PRKCZ, SUSD4, SWAP70, TNFRSF11A, TRAF2, TSC1]

GO:0050867	positive regulation of cell activation	1,68E-03	[3, 4, 5]	89,00	19,56	58,65	29,81	11,54	[ADAM8, AIF1, BTK, BTLA, CARD11, CCDC88B, CCL19, CCR2, CD2, CD27, CD3E, CD4, CD40LG, CD5, CD6, CD74, CD80, CD86, CLCF1, CLEC4D, CTLA4, DOCK8, EGR3, FCRL3, FGR, FOXP3, HLA-DMB, HLA-DPA1, HLA-DPB1, ICOS, IFNG, IL18, IL7R, INPP5D, KLRK1, LCK, LEF1, MYB, NCKAP1L, NLRP3, NR4A3, PLEK, PRKCQ, PTAFR, PTPN22, PTPN6, PTPRC, RHOH, RUNX3, SASH3, STAP1, SYK, TESPA1, THY1, TLR6, TNFSF13B, TOX, TREM2, VNN1, XCL1, ZAP70]	[ADA, BCL2, CCDC88B, CCR7, CD160, CD28, CD38, CD47, CD6, CORO1A, CTLA4, GAB2, GATD3A, GRB2, HLA-A, HLA-DPA1, HLA-DPB1, JAK3, LCK, MMP14, NCK2, NFATC2, PTPN22, PTPN6, PTPRC, RARA, RUNX1, RUNX3, TESPA1, ZBTB16, ZMIZ1]	[BTNL2, CARD11, CAV1, HLA-DQB2, HLA-G, ITGB2, LYN, PAK1, PRKCZ, PTPN22, RUNX1, TOX]
GO:0002366	leukocyte activation involved in immune response	5,07E-03	[3, 4]	134,00	17,25	45,58	36,05	18,37	[ADAM8, APBB1IP, BIN2, BTK, CCL19, CCR2, CD180, CD1C, CD244, CD40LG, CD53, CD74, CD80, CD84, CD86, CFP, CLCF1, CLEC12A, CLEC4D, CLEC4E, CTSS, CYBB, DOCK10, DOCK2, EOMES, FCN1, FGL2, FGR, FOXP3, FRMPD3, GAPT, HLA-DMB, IFNG, IL18, IQGAP2, IRF4, LCP1, LEF1, LRMP, LY9, LYZ, MYB, NCKAP1L, NLRP3, NR4A3, ORM2, PIK3CG, PLAC8, PLAU, PTAFR, PTK2B, PTPN6, PTPRC, PTX3, RAB44, RNASET2, SELL, SEMA4A, SLAMF6, SLC18A1, SUCNR1, SYK, TBC1D10C, TREM2, TXNDC5, VNN1, ZNF683]	[ADA, ADAM10, ADGRE5, APBB1IP, ASAH1, ATG7, B2M, BATF, BIN2, BPI, CAB39, CD28, CD44, CD47, CDH17, CORO1A, COTL1, CPNE3, CTSZ, CYSTM1, EEF1A1, FES, GAB2, GSN, HLA-B, JAK3, KCNAB2, LCP1, LPCAT1, PIK3CD, PRKCE, PSAP, PTK2B, PTPN6, PTPRC, PTPRJ, PTPRN2, RARA, RELB, RNASET2, RORA, S100A13, SBNO2, SLAMF1, SLC15A4, STAT3, STK10, TBC1D10C, TIMP2, TMC6, TRAF3, UBR4, ZC3H12A]	[AMPD3, ATP6V1D, CAMP, CDK13, CPLX2, CRISP3, CTSD, DYSF, FOXP1, ITGB2, LCP1, LRRC7, LYN, LYZ, MMP12, MNDA, PDXK, PLAC8, PLPPR3, PRKCZ, PSMD7, PTPRN2, RAB37, RAB7A, SLC15A4, SWAP70, TSC1]
GO:0002274	myeloid leukocyte activation	6,66E-03	[3, 4]	125,00	17,41	43,07	36,50	20,44	[ADAM8, AIF1, BIN2, BTK, C1QA, CAMK4, CCR2, CD2, CD53, CD74, CD84, CFP, CLEC12A, CLEC4D, CNR1, CNR2, CTSS, CXCL6, CYBB, DOCK2, EDN2, FCN1, FGL2, FGR, FRMPD3, IFNG, IL18, IL18RAP, IQGAP2, IRF4, LCP2, LRMP, LYZ, NAMPT, NCKAP1L, NR4A3, ORM2, PIK3CG, PLAC8, PLAU, PTAFR, PTPN6, PTPRC, PTX3, RAB44, RHOH, RNASET2, SELL, SLC18A1, STAP1, SUCNR1, SYK, TBC1D10C, TLR6, TLR8, TREM2, TXNDC5, UBD, VNN1]	[ADAM10, ADGRE5, ASAH1, ATG7, B2M, BATF, BATF3, BIN2, BPI, CAB39, CD44, CD47, COTL1, CPNE3, CTSZ, CX3CL1, CYSTM1, EEF1A1, FES, GAB2, GSN, HLA-B, KCNAB2, LCP2, LPCAT1, PCDHA6, PIK3CD, PLSCR1, PRKCE, PSAP, PTPN6, PTPRC, PTPRJ, PTPRN2, RELB, RNASET2, RORA, S100A13, SBNO2, SLAMF1, SLC15A4, SLC7A2, STK10, TBC1D10C, TIMP2, TMC6, TNF, TRAF3, UBR4, ZC3H12A]	[AMPD3, ATP6V1D, CAMK4, CAMP, CDK13, CPLX2, CRISP3, CTSD, DYSF, FOXP1, HYAL2, ITGB2, LRRC7, LYN, LYZ, MMP12, MNDA, NR1H3, PCDHA6, PDXK, PLAC8, PLPPR3, PSMD7, PTPRN2, RAB37, RAB7A, SLC15A4, TLR1]
GO:0002697	regulation of immune effector process	3,25E-02	[3, 4]	92,00	18,11	62,38	26,73	10,89	[AIM2, ANGPT1, BTK, C1QA, C1QB, C1QC, C2, C8G, CCL19, CCR2, CD1B, CD1C, CD1E, CD244, CD40LG, CD74, CD80, CD84, CD86, CD96, CFP, CLCF1, CLNK, CR2, CRTAM, CXCL10, CXCL6, CYP11B1, FCRL3, FGL2, FGR, FOXP3, FUT7, HLA-DMB, IFNG, IL18, IL18RAP, IL7R, IRF4, KLRK1, LAG3, LPAL2, LTA, MYB, MZB1, NCR3, NLRP3, NR4A3, PTAFR, PTPN22, PTPN6, PTPRC, SASH3, SH2D1A, SLAMF6, SLAMF8, STAP1, STAT1, SYK, TNFAIP3, WAS, XCL1, ZNF683]	[AIM2, ARRB2, B2M, BIRC3, CD160, CD28, CD47, FES, GAB2, GPRC5B, HLA-A, HLA-B, JAK3, LTA, PTPN22, PTPN6, PTPRC, PTPRJ, RARA, RNF216, STAT1, TANK, TGFB2, TNF, TNFAIP3, TRAF3, ZC3H12A]	[AIM2, HLA-G, ITGB2, KLK7, LYN, MMP12, PNKD, PRKCZ, PTPN22, SUSD4, TRAF2]
GO:0001816	cytokine production	1,96E-12	[2, 5]	181,00	20,95	44,98	36,84	18,18	[ADAM8, AIF1, AIM2, ANGPT1, BANK1, BTK, BTN3A1, BTN3A2, CARD11, CARD17, CASP8, CCDC88B, CCL19, CCR2, CD2, CD200R1, CD244, CD247, CD3E, CD4, CD40LG, CD6, CD74, CD80, CD84, CD86, CD96, CEL, CLCF1, CLEC4E, CLEC6A, CLNK, CRTAM, CSF1R, CXCL6, CYBB, EOMES, EREG, FCN1, FFAR4, FGR, FOXP3, GBP5, GPSM3, HLA-DPA1, HLA-DPB1, HLA-DRB1, IFNG, IKBKE, IL12RB2, IL18, IL18RAP, IL26, IL5RA, INPP5D, IRF4, IRF5, ITK, KLRK1, LAG3, LCP1, LCP2, LEF1, LPAL2, LTA, LTB, LY9, MAPK13, MCOLN2, MOG, MYB, NCKAP1L, NLRP3, NR4A3, ORM2, PIK3CG, PRKCQ, PTAFR, PTPN22, PTPN6, PTPRC, PYHIN1, SASH3, SLAMF6, STAT1, SUCNR1, SYK, TIGIT, TLR10, TLR6, TLR8, TNFAIP3, TREM2, XCL1]	[AIM2, ARRB2, B2M, BATF, BIRC3, BPI, BTN3A3, CAPN2, CASP8, CCDC88B, CCM2, CCR7, CD160, CD28, CD2AP, CD47, CD6, CX3CL1, F2R, GBP1, GBP5, GPRC5B, GPSM3, HDAC9, HLA-A, HLA-B, HLA-DPA1, HLA-DPB1, HOMER2, IDO1, IL12RB2, IL17RA, INAVA, IRF1, ITGB6, JAK3, LCP1, LCP2, LGR4, LITAF, LRRFIP1, LTA, LTBP1, MAP2K5, MAST2, MBP, NFAT5, NFATC1, NFATC2, NLRC5, NMI, NPTN, PDE4B, PDZD2, PIK3CD, PRKD2, PTPN22, PTPN6, PTPRC, PTPRJ, RARA, RELB, RNF216, RORA, RUNX1, S100A13, SLAMF1, STAT1, STAT3, TANK, TGFB2, TNF, TNFAIP3, TRAF3, ZBP1, ZBTB20, ZC3H12A]	[ABCC8, AIM2, AKAP12, ARNT, BTNL2, CARD11, CARD8, CHUK, DRD2, DYSF, FOXP1, HGF, HLA-DRB1, HLA-G, HYAL2, LCP1, LILRA4, LRRFIP1, LYN, MAGI1, MBL2, MBP, MMP12, MNDA, NAV3, NFATC1, NLRC5, NLRP4, PDE4B, PLD3, PNKD, PRKCZ, PTPN22, RUNX1, SRGN, TIA1, TLR1, TRAF2]
GO:0001817	regulation of cytokine production	3,75E-11	[3, 4, 5, 6]	165,00	21,02	45,03	36,65	18,32	[ADAM8, AIF1, AIM2, ANGPT1, BANK1, BTK, BTN3A1, BTN3A2, CARD11, CARD17, CASP8, CCDC88B, CCL19, CCR2, CD2, CD200R1, CD244, CD3E, CD4, CD40LG, CD6, CD74, CD80, CD84, CD86, CD96, CLCF1, CLEC4E, CLEC6A, CLNK, CRTAM, CSF1R, CXCL6, CYBB, EREG, FCN1, FFAR4, FGR, FOXP3, GBP5, GPSM3, HLA-DPA1, HLA-DPB1, HLA-DRB1, IFNG, IKBKE, IL12RB2, IL18, IL26, IL5RA, INPP5D, IRF4, IRF5, KLRK1, LAG3, LCP1, LEF1, LPAL2, LTA, LTB, LY9, MAPK13, MCOLN2, MOG, MYB, NCKAP1L, NLRP3, NR4A3, ORM2, PRKCQ, PTAFR, PTPN22, PTPN6, PTPRC, PYHIN1, SASH3, SLAMF6, STAT1, SYK, TIGIT, TLR10, TLR6, TLR8, TNFAIP3, TREM2, XCL1]	[AIM2, ARRB2, B2M, BIRC3, BPI, BTN3A3, CAPN2, CASP8, CCDC88B, CCM2, CCR7, CD160, CD28, CD2AP, CD47, CD6, CX3CL1, F2R, GBP1, GBP5, GPRC5B, GPSM3, HDAC9, HLA-A, HLA-B, HLA-DPA1, HLA-DPB1, HOMER2, IDO1, IL12RB2, IL17RA, INAVA, IRF1, ITGB6, JAK3, LCP1, LGR4, LITAF, LRRFIP1, LTA, LTBP1, MAP2K5, MAST2, MBP, NLRC5, NMI, NPTN, PDE4B, PDZD2, PRKD2, PTPN22, PTPN6, PTPRC, PTPRJ, RARA, RELB, RNF216, RORA, RUNX1, SLAMF1, STAT1, STAT3, TANK, TGFB2, TNF, TNFAIP3, TRAF3, ZBP1, ZBTB20, ZC3H12A]	[ABCC8, AIM2, AKAP12, ARNT, BTNL2, CARD11, CARD8, CHUK, DRD2, FOXP1, HGF, HLA-DRB1, HLA-G, HYAL2, LCP1, LILRA4, LRRFIP1, LYN, MAGI1, MBL2, MBP, MMP12, MNDA, NAV3, NLRC5, NLRP4, PDE4B, PLD3, PRKCZ, PTPN22, RUNX1, SRGN, TIA1, TLR1, TRAF2]
GO:0001819	positive regulation of cytokine production	1,77E-06	[3, 4, 5, 6, 7]	106,00	21,07	49,22	32,03	18,75	[ADAM8, AIF1, AIM2, CARD11, CASP8, CCDC88B, CCL19, CCR2, CD2, CD244, CD3E, CD4, CD40LG, CD6, CD74, CD80, CD86, CLEC4E, CLEC6A, CLNK, CRTAM, CSF1R, CYBB, EREG, FCN1, FGR, FOXP3, GBP5, GPSM3, HLA-DPA1, HLA-DPB1, IFNG, IL12RB2, IL18, IL26, IRF4, IRF5, KLRK1, LCP1, LPAL2, LTA, LTB, LY9, MAPK13, MCOLN2, MYB, NLRP3, NR4A3, ORM2, PRKCQ, PTAFR, PTPN22, PTPRC, PYHIN1, SASH3, SLAMF6, STAT1, SYK, TIGIT, TLR6, TLR8, TREM2, XCL1]	[AIM2, B2M, BIRC3, CASP8, CCDC88B, CCM2, CCR7, CD160, CD28, CD6, CX3CL1, F2R, GBP5, GPRC5B, GPSM3, HLA-A, HLA-DPA1, HLA-DPB1, IDO1, IL12RB2, IL17RA, INAVA, IRF1, LCP1, LRRFIP1, LTA, MBP, PDE4B, PRKD2, PTPN22, PTPRC, PTPRJ, RARA, RORA, RUNX1, STAT1, STAT3, TANK, TNF, ZBP1, ZBTB20]	[ABCC8, AIM2, AKAP12, ARNT, BTNL2, CARD11, CARD8, CHUK, DRD2, HGF, HLA-G, HYAL2, LCP1, LRRFIP1, MBL2, MBP, MMP12, MNDA, PDE4B, PRKCZ, PTPN22, RUNX1, TLR1, TRAF2]
GO:0050663	cytokine secretion	6,92E-05	[3, 4, 5, 6, 7, 8, 9]	64,00	23,44	47,37	26,32	26,32	[AIF1, AIM2, ANGPT1, BANK1, BTN3A1, BTN3A2, CARD11, CARD17, CCL19, CD2, CD200R1, CD244, CLEC4E, CLEC6A, CRTAM, CSF1R, FCN1, FFAR4, FGR, FOXP3, GBP5, HLA-DRB1, IFNG, IL26, LCP1, LCP2, LPAL2, MCOLN2, NLRP3, ORM2, PTPN22, SYK, TLR10, TLR6, TLR8, TNFAIP3]	[AIM2, CCM2, CCR7, CD160, CX3CL1, F2R, GBP1, GBP5, HDAC9, IL17RA, ITGB6, LCP1, LCP2, LGR4, MBP, PTPN22, S100A13, TNF, TNFAIP3, ZC3H12A]	[AIM2, AKAP12, BTNL2, CARD11, CARD8, CHUK, DRD2, DYSF, FOXP1, HLA-DRB1, HYAL2, LCP1, LYN, MBL2, MBP, MMP12, PRKCZ, PTPN22, SRGN, TLR1]
GO:0001817	regulation of cytokine production	3,75E-11	[3, 4, 5, 6]	165,00	21,02	45,03	36,65	18,32	[ADAM8, AIF1, AIM2, ANGPT1, BANK1, BTK, BTN3A1, BTN3A2, CARD11, CARD17, CASP8, CCDC88B, CCL19, CCR2, CD2, CD200R1, CD244, CD3E, CD4, CD40LG, CD6, CD74, CD80, CD84, CD86, CD96, CLCF1, CLEC4E, CLEC6A, CLNK, CRTAM, CSF1R, CXCL6, CYBB, EREG, FCN1, FFAR4, FGR, FOXP3, GBP5, GPSM3, HLA-DPA1, HLA-DPB1, HLA-DRB1, IFNG, IKBKE, IL12RB2, IL18, IL26, IL5RA, INPP5D, IRF4, IRF5, KLRK1, LAG3, LCP1, LEF1, LPAL2, LTA, LTB, LY9, MAPK13, MCOLN2, MOG, MYB, NCKAP1L, NLRP3, NR4A3, ORM2, PRKCQ, PTAFR, PTPN22, PTPN6, PTPRC, PYHIN1, SASH3, SLAMF6, STAT1, SYK, TIGIT, TLR10, TLR6, TLR8, TNFAIP3, TREM2, XCL1]	[AIM2, ARRB2, B2M, BIRC3, BPI, BTN3A3, CAPN2, CASP8, CCDC88B, CCM2, CCR7, CD160, CD28, CD2AP, CD47, CD6, CX3CL1, F2R, GBP1, GBP5, GPRC5B, GPSM3, HDAC9, HLA-A, HLA-B, HLA-DPA1, HLA-DPB1, HOMER2, IDO1, IL12RB2, IL17RA, INAVA, IRF1, ITGB6, JAK3, LCP1, LGR4, LITAF, LRRFIP1, LTA, LTBP1, MAP2K5, MAST2, MBP, NLRC5, NMI, NPTN, PDE4B, PDZD2, PRKD2, PTPN22, PTPN6, PTPRC, PTPRJ, RARA, RELB, RNF216, RORA, RUNX1, SLAMF1, STAT1, STAT3, TANK, TGFB2, TNF, TNFAIP3, TRAF3, ZBP1, ZBTB20, ZC3H12A]	[ABCC8, AIM2, AKAP12, ARNT, BTNL2, CARD11, CARD8, CHUK, DRD2, FOXP1, HGF, HLA-DRB1, HLA-G, HYAL2, LCP1, LILRA4, LRRFIP1, LYN, MAGI1, MBL2, MBP, MMP12, MNDA, NAV3, NLRC5, NLRP4, PDE4B, PLD3, PRKCZ, PTPN22, RUNX1, SRGN, TIA1, TLR1, TRAF2]
GO:0050663	cytokine secretion	6,92E-05	[3, 4, 5, 6, 7, 8, 9]	64,00	23,44	47,37	26,32	26,32	[AIF1, AIM2, ANGPT1, BANK1, BTN3A1, BTN3A2, CARD11, CARD17, CCL19, CD2, CD200R1, CD244, CLEC4E, CLEC6A, CRTAM, CSF1R, FCN1, FFAR4, FGR, FOXP3, GBP5, HLA-DRB1, IFNG, IL26, LCP1, LCP2, LPAL2, MCOLN2, NLRP3, ORM2, PTPN22, SYK, TLR10, TLR6, TLR8, TNFAIP3]	[AIM2, CCM2, CCR7, CD160, CX3CL1, F2R, GBP1, GBP5, HDAC9, IL17RA, ITGB6, LCP1, LCP2, LGR4, MBP, PTPN22, S100A13, TNF, TNFAIP3, ZC3H12A]	[AIM2, AKAP12, BTNL2, CARD11, CARD8, CHUK, DRD2, DYSF, FOXP1, HLA-DRB1, HYAL2, LCP1, LYN, MBL2, MBP, MMP12, PRKCZ, PTPN22, SRGN, TLR1]
GO:0051049	regulation of transport	5,15E-04	[3, 4]	308,00	15,22	27,41	32,83	39,76	[ADAM8, AIF1, AIM2, ANGPT1, ANO9, BANK1, BLK, C2, CACNA1E, CALCR, CARD17, CASP8, CCL19, CCR2, CD2, CD200R1, CD244, CD4, CD40LG, CD74, CD84, CLCNKA, CLEC4E, CLEC6A, CNR1, CRTAM, CSF1R, CTSS, CXCL10, CXCL11, CXCL9, CXCR4, CYBB, DAPK1, DOCK2, DRD1, ERFE, FCN1, FCRL3, FFAR4, FGR, FOXP3, HCLS1, HCN1, HLA-DRB1, IFNG, IL26, IL2RG, JPH1, KCNA3, KCNJ10, KCNQ5, KMO, LCP1, LPAL2, LRRC38, MYB, NCKAP1L, NLRP3, NLRP6, NPPB, NR4A3, ORM2, P2RY12, P2RY2, PIK3CG, PIM1, PTAFR, PTK2B, PTPN22, PTPN6, PTPRC, PTX3, RAB3B, RAB3C, SDC1, SELE, SLC31A2, SPINK1, STAP1, SYK, TCAF2, THY1, TIFAB, TLR10, TLR6, TLR8, TNFAIP3, TRAT1, TREM2, XCL1]	[ABAT, ACAA2, ADA, ADCY1, AIM2, AKAP6, AKAP9, ANK3, ARRB2, ATXN2, B2M, BCL2, BICD1, BTBD9, CAB39, CABP1, CACNA1A, CACNB2, CAMK2G, CASP8, CCM2, CCR7, CD160, CD2AP, CD38, CD47, CDH2, CORO1A, CX3CL1, CXCL11, DAPK1, DENND5B, DNM2, DPP6, EDEM1, EEF2K, EXPH5, F2R, FES, GAB2, GBP1, GCC2, GPR68, GRIK5, GRM7, GSK3B, HDAC9, HECW2, HOMER1, HOMER2, HTT, IL17RA, ITPR1, ITPR2, KCNA2, KCNAB2, KCNC2, KCNG3, KCNH1, KCNIP1, KCNIP4, KCNQ1, KCTD11, LCP1, LGR4, LIME1, MAP2K2, MBP, MDFIC, MTNR1B, NETO1, NOS1AP, NUP153, OPRM1, PDCD10, PDE4B, PPP1CC, PPP3CC, PRKAG2, PRKAR1B, PRKCE, PRKD2, PSMD9, PTK2B, PTPN1, PTPN22, PTPN6, PTPRC, PTPRJ, RAB13, RALB, RUFY3, SEPTIN9, SGIP1, SGK1, SLC1A2, SLC4A8, SSTR4, TANK, TERT, TGFB2, TNF, TNFAIP3, TNK2, TP53BP2, TRIM46, UBASH3B, UBE2L3, ZC3H12A]	[ABCA12, ABCC8, ADCY5, ADORA1, AIM2, AKAP12, AKAP9, ALOX15, ANK3, ANKRD13B, ARHGAP44, ASIC2, ATP1B1, BTNL2, C11orf65, C2CD5, CACNA1S, CACNA2D3, CACNG2, CADPS2, CARD8, CARTPT, CATSPER2, CAV1, CD151, CDH13, CDK5R2, CHRNA4, CHUK, COMT, CPLX2, CYP4A11, DISP3, DLG2, DMPK, DNM3, DPP6, DRD2, DYSF, EGFR, FGF12, FGF14, FOXP1, FRMD4A, GNAS, GRIN2A, GRM7, HIP1, HLA-DRB1, HSPA2, HYAL2, ITGB2, ITPR1, KCNA5, KCNB2, KCND2, KCNG2, KCNJ15, KCNJ3, KCNK15, KCNMA1, KCNS3, KDM5B, LCP1, LRRC55, LRRC7, LYN, MAGI1, MAGI2, MAP2, MBL2, MBP, MEF2A, MFGE8, MMP12, MYLK, NDRG4, NKAIN2, NKAIN3, NOS1AP, NR1H3, NRXN3, NUMB, OAZ1, ORAI1, OSBPL6, P2RX2, PAK1, PCSK9, PDE4B, PFKM, PNKD, PPARGC1A, PPP3CA, PRKAA1, PRKAG2, PRKCA, PRKCZ, PRKN, PTPN22, RAB7A, REM2, RIMS1, RSPO1, SCN3B, SEPTIN9, SHANK1, SHISA6, SLC17A7, SLC30A5, SMAP1, SRGN, SRI, STIM1, STXBP5L, SYT17, TBC1D5, TCAF1, TESC, TIAM1, TLR1, TNFRSF11A, TP73, TRAF2, TUB, USP36, VEGFC, VSNL1, WASL, WFS1, WNK2, WNK4]
GO:0050707	regulation of cytokine secretion	1,39E-03	[3, 4, 5, 6, 7, 8, 9, 10]	55,00	23,01	46,88	25,00	28,12	[AIF1, AIM2, ANGPT1, BANK1, CARD17, CCL19, CD2, CD200R1, CD244, CLEC4E, CLEC6A, CRTAM, CSF1R, FCN1, FFAR4, FGR, FOXP3, HLA-DRB1, IFNG, IL26, LCP1, LPAL2, NLRP3, ORM2, PTPN22, SYK, TLR10, TLR6, TLR8, TNFAIP3]	[AIM2, CCM2, CCR7, CD160, CX3CL1, F2R, GBP1, HDAC9, IL17RA, LCP1, LGR4, MBP, PTPN22, TNF, TNFAIP3, ZC3H12A]	[AIM2, AKAP12, BTNL2, CARD8, CHUK, DRD2, FOXP1, HLA-DRB1, HYAL2, LCP1, LYN, MBL2, MBP, MMP12, PRKCZ, PTPN22, SRGN, TLR1]
GO:0071692	protein localization to extracellular region	4,63E-03	[4]	120,00	17,73	35,82	31,34	32,84	[ADAM8, AIF1, AIM2, ANGPT1, BANK1, BLK, BTN3A1, BTN3A2, CACNA1E, CARD11, CARD17, CCL19, CD2, CD200R1, CD244, CD40LG, CLEC4E, CLEC6A, CNR1, CRTAM, CSF1R, FCN1, FFAR4, FGR, FOXP3, GBP5, HLA-DRB1, IFNG, IL26, KLRG1, LCP1, LCP2, LPAL2, MCOLN2, NLRP3, ORM2, PLEK, PTPN22, RAB3B, RAB3C, SUCNR1, SYK, TLR10, TLR6, TLR8, TNFAIP3, TNFSF13B, TREM2]	[ABAT, AIM2, CACNA1A, CAMK2G, CBLN4, CCM2, CCR7, CD160, CD2AP, CD38, CX3CL1, EXPH5, F2R, GBP1, GBP5, GNPTAB, GPR68, HDAC9, IL17RA, ITGB6, ITPR1, ITPR2, KCNC2, LCP1, LCP2, LGR4, LTBP1, MBP, MTNR1B, OPRM1, PRKCE, PSAP, PSMD9, PTPN22, PTPRN2, RAB13, S100A13, TANK, TGFB2, TNF, TNFAIP3, ZC3H12A]	[ABCC8, ADCY5, AIM2, AKAP12, BTNL2, CARD11, CARD8, CARTPT, CHUK, CTAGE1, DRD2, DYSF, EGFR, FBN2, FOXP1, FRMD4A, GNAS, HLA-DRB1, HYAL2, ITPR1, KCNA5, KCNG2, KCNS3, LCP1, LYN, MBL2, MBP, MMP12, NR1H3, PFKM, PPP3CA, PRKCA, PRKCZ, PRKN, PTPN22, PTPRN2, SRGN, SRI, STXBP5L, TIAM1, TLR1, TRAF2, VEGFC, VSNL1]

GO:0009306	protein secretion	5,41E-03	[4, 5, 6, 7, 8]	118,00	17,69	36,36	31,06	32,58	[ADAM8, AIF1, AIM2, ANGPT1, BANK1, BLK, BTN3A1, BTN3A2, CACNA1E, CARD11, CARD17, CCL19, CD2, CD200R1, CD244, CD40LG, CLEC4E, CLEC6A, CNR1, CRTAM, CSF1R, FCN1, FFAR4, FGR, FOXP3, GBP5, HLA-DRB1, IFNG, IL26, KLRG1, LCP1, LCP2, LPAL2, MCOLN2, NLRP3, ORM2, PLEK, PTPN22, RAB3B, RAB3C, SUCNR1, SYK, TLR10, TLR6, TLR8, TNFAIP3, TNFSF13B, TREM2]	[ABAT, AIM2, CACNA1A, CAMK2G, CBLN4, CCM2, CCR7, CD160, CD2AP, CD38, CX3CL1, EXPH5, F2R, GBP1, GBP5, GNPTAB, GPR68, HDAC9, IL17RA, ITGB6, ITPR1, ITPR2, KCNC2, LCP1, LCP2, LGR4, MBP, MTNR1B, OPRM1, PRKCE, PSAP, PSMD9, PTPN22, PTPRN2, RAB13, S100A13, TANK, TGFB2, TNF, TNFAIP3, ZC3H12A]	[ABCC8, ADCY5, AIM2, AKAP12, BTNL2, CARD11, CARD8, CARTPT, CHUK, CTAGE1, DRD2, DYSF, EGFR, FOXP1, FRMD4A, GNAS, HLA-DRB1, HYAL2, ITPR1, KCNA5, KCNG2, KCNS3, LCP1, LYN, MBL2, MBP, MMP12, NR1H3, PFKM, PPP3CA, PRKCA, PRKCZ, PRKN, PTPN22, PTPRN2, SRGN, SRI, STXBP5L, TIAM1, TLR1, TRAF2, VEGFC, VSNL1]
GO:0002274	myeloid leukocyte activation	6,66E-03	[3, 4]	125,00	17,41	43,07	36,50	20,44	[ADAM8, AIF1, BIN2, BTK, C1QA, CAMK4, CCR2, CD2, CD53, CD74, CD84, CFP, CLEC12A, CLEC4D, CNR1, CNR2, CTSS, CXCL6, CYBB, DOCK2, EDN2, FCN1, FGL2, FGR, FRMPD3, IFNG, IL18, IL18RAP, IQGAP2, IRF4, LCP2, LRMP, LYZ, NAMPT, NCKAP1L, NR4A3, ORM2, PIK3CG, PLAC8, PLAU, PTAFR, PTPN6, PTPRC, PTX3, RAB44, RHOH, RNASET2, SELL, SLC18A1, STAP1, SUCNR1, SYK, TBC1D10C, TLR6, TLR8, TREM2, TXNDC5, UBD, VNN1]	[ADAM10, ADGRE5, ASAH1, ATG7, B2M, BATF, BATF3, BIN2, BPI, CAB39, CD44, CD47, COTL1, CPNE3, CTSZ, CX3CL1, CYSTM1, EEF1A1, FES, GAB2, GSN, HLA-B, KCNAB2, LCP2, LPCAT1, PCDHA6, PIK3CD, PLSCR1, PRKCE, PSAP, PTPN6, PTPRC, PTPRJ, PTPRN2, RELB, RNASET2, RORA, S100A13, SBNO2, SLAMF1, SLC15A4, SLC7A2, STK10, TBC1D10C, TIMP2, TMC6, TNF, TRAF3, UBR4, ZC3H12A]	[AMPD3, ATP6V1D, CAMK4, CAMP, CDK13, CPLX2, CRISP3, CTSD, DYSF, FOXP1, HYAL2, ITGB2, LRRC7, LYN, LYZ, MMP12, MNDA, NR1H3, PCDHA6, PDXK, PLAC8, PLPPR3, PSMD7, PTPRN2, RAB37, RAB7A, SLC15A4, TLR1]
GO:0050708	regulation of protein secretion	1,35E-02	[4, 5, 6, 7, 8, 9]	96,00	18,22	33,96	29,25	36,79	[ADAM8, AIF1, AIM2, ANGPT1, BANK1, BLK, CACNA1E, CARD17, CCL19, CD2, CD200R1, CD244, CD40LG, CLEC4E, CLEC6A, CNR1, CRTAM, CSF1R, FCN1, FFAR4, FGR, FOXP3, HLA-DRB1, IFNG, IL26, LCP1, LPAL2, NLRP3, ORM2, PTPN22, SYK, TLR10, TLR6, TLR8, TNFAIP3, TREM2]	[ABAT, AIM2, CACNA1A, CCM2, CCR7, CD160, CD2AP, CD38, CX3CL1, EXPH5, F2R, GBP1, GPR68, HDAC9, IL17RA, ITPR1, ITPR2, KCNC2, LCP1, LGR4, MBP, MTNR1B, OPRM1, PRKCE, PSMD9, PTPN22, TANK, TGFB2, TNF, TNFAIP3, ZC3H12A]	[ABCC8, ADCY5, AIM2, AKAP12, BTNL2, CARD8, CARTPT, CHUK, DRD2, EGFR, FOXP1, FRMD4A, GNAS, HLA-DRB1, HYAL2, ITPR1, KCNA5, KCNG2, KCNS3, LCP1, LYN, MBL2, MBP, MMP12, NR1H3, PFKM, PPP3CA, PRKCA, PRKCZ, PRKN, PTPN22, SRGN, SRI, STXBP5L, TIAM1, TLR1, TRAF2, VEGFC, VSNL1]
GO:0046903	secretion	1,59E-02	[4]	273,00	14,92	33,56	35,57	30,87	[ABCB11, ADAM8, AIF1, AIM2, ANGPT1, AQP5, BANK1, BIN2, BLK, BTK, BTN3A1, BTN3A2, CACNA1E, CARD11, CARD17, CCL19, CCR2, CD2, CD200R1, CD244, CD40LG, CD53, CD74, CD84, CEL, CFP, CLEC12A, CLEC4D, CLEC4E, CLEC6A, CNR1, CRTAM, CSF1R, CTSS, CTSW, CYBB, DOCK2, DRD1, FCN1, FFAR4, FGL2, FGR, FOXP3, FRMPD3, GBP5, HLA-DRB1, IFNG, IL26, IQGAP2, KLF5, KLRG1, KMO, KNG1, LCP1, LCP2, LPAL2, LRMP, LYZ, MCOLN2, MYB, MYO1G, NCKAP1L, NLRP3, NLRP6, NPPB, NR4A3, ORM2, P2RY12, P2RY2, PIK3CG, PLA2G2D, PLAC8, PLAU, PLEK, PTAFR, PTPN22, PTPN6, PTPRC, PTX3, RAB3B, RAB3C, RAB44, RNASET2, SDC1, SELL, SLC18A1, SNX10, SUCNR1, SYK, SYTL1, TBC1D10C, TIFAB, TLR10, TLR6, TLR8, TNFAIP3, TNFSF13B, TREM2, TXNDC5, VNN1]	[ABAT, ADA, ADAM10, ADCY1, ADGRE5, AIM2, ASAH1, ATG7, B2M, BDNF, BIN2, BPI, CAB39, CACNA1A, CACNB2, CAMK2G, CBLN4, CCM2, CCR7, CD160, CD2AP, CD38, CD44, CD47, CORO1A, COTL1, CPNE3, CTSZ, CX3CL1, CYSTM1, EEF1A1, EXOC6B, EXPH5, F13A1, F2R, FES, GAB2, GBP1, GBP5, GNPTAB, GPR68, GRIK5, GRM7, GSK3B, GSN, HDAC9, HLA-B, IL17RA, ITGB6, ITPR1, ITPR2, KCNA2, KCNAB2, KCNC2, KCNQ1, KCTD11, LCP1, LCP2, LGR4, LPCAT1, MBP, MED1, MICAL2, MTNR1B, MYO1G, NCOR2, NTRK2, OPRM1, PCDH7, PIK3CD, PPFIA1, PPFIA4, PRKCE, PSAP, PSMD9, PTPN22, PTPN6, PTPRC, PTPRJ, PTPRN2, RAB13, RALB, RNASET2, S100A13, SEPTIN9, SGK1, SLC15A4, SLC1A2, SLC1A5, SLC4A8, SSTR4, STK10, STX11, SYN3, TANK, TBC1D10C, TGFB2, TIMP2, TMC6, TNF, TNFAIP3, TRAF3, TSPOAP1, UBE2Z, UBR4, ZC3H12A]	[ABCA12, ABCC8, ADCY5, ADORA1, AIM2, AKAP12, AMPD3, ARHGAP44, ATP6V1D, BTNL2, CADPS2, CAMP, CARD11, CARD8, CARTPT, CAV1, CD9, CDK13, CDK5R2, CHRNA4, CHUK, COMT, CPLX2, CRISP3, CTAGE1, CTSD, CYP4A11, DRD2, DYSF, EGFR, ERBB4, EXOC4, FGF7, FOXP1, FRMD4A, GNAS, GRM7, HGF, HLA-DRB1, HRG, HYAL2, ITGB2, ITPR1, KCNA5, KCNG2, KCNS3, KDM5B, LCP1, LRRC7, LYN, LYZ, MBL2, MBP, MMP12, MNDA, NR1H3, NRXN3, PAK1, PDXK, PFKM, PLAC8, PLPPR3, PNKD, PPP3CA, PRKCA, PRKCZ, PRKN, PSMD7, PTPN22, PTPRN2, RAB37, RAB7A, RIMS1, SEPTIN9, SERPINA4, SLC15A4, SLC17A7, SLC1A7, SLC9A4, SRGN, SRI, STXBP5L, SYT17, TBX3, TIAM1, TLR1, TNFRSF11A, TRAF2, VEGFC, VPS33A, VSNL1, WNK4]
GO:0002792	negative regulation of peptide secretion	3,41E-02	[4, 5, 6, 7, 8, 9]	35,00	24,48	34,21	34,21	31,58	[ANGPT1, BANK1, CARD17, CD200R1, CD74, FFAR4, FOXP3, LPAL2, NLRP3, PTPN22, TLR6, TLR8, TNFAIP3]	[CCR7, CX3CL1, F2R, GBP1, HDAC9, LGR4, MTNR1B, OPRM1, PSMD9, PTPN22, TNF, TNFAIP3, ZC3H12A]	[ABCC8, CARD8, CARTPT, DRD2, FRMD4A, NR1H3, PPP3CA, PRKN, PTPN22, SRGN, STXBP5L, VSNL1]
GO:0032940	secretion by cell	3,67E-02	[3, 5]	251,00	14,93	32,61	36,23	31,16	[ADAM8, AIF1, AIM2, ANGPT1, BANK1, BIN2, BLK, BTK, BTN3A1, BTN3A2, CACNA1E, CARD11, CARD17, CCL19, CCR2, CD2, CD200R1, CD244, CD40LG, CD53, CD84, CFP, CLEC12A, CLEC4D, CLEC4E, CLEC6A, CNR1, CRTAM, CSF1R, CTSS, CTSW, CYBB, DOCK2, DRD1, FCN1, FFAR4, FGL2, FGR, FOXP3, FRMPD3, GBP5, HLA-DRB1, IFNG, IL26, IQGAP2, KLF5, KLRG1, KMO, KNG1, LCP1, LCP2, LPAL2, LRMP, LYZ, MCOLN2, MYB, MYO1G, NCKAP1L, NLRP3, NR4A3, ORM2, P2RY12, PIK3CG, PLAC8, PLAU, PLEK, PTAFR, PTPN22, PTPN6, PTPRC, PTX3, RAB3B, RAB3C, RAB44, RNASET2, SDC1, SELL, SLC18A1, SUCNR1, SYK, SYTL1, TBC1D10C, TLR10, TLR6, TLR8, TNFAIP3, TNFSF13B, TREM2, TXNDC5, VNN1]	[ABAT, ADAM10, ADCY1, ADGRE5, AIM2, ASAH1, ATG7, B2M, BDNF, BIN2, BPI, CAB39, CACNA1A, CACNB2, CAMK2G, CBLN4, CCM2, CCR7, CD160, CD2AP, CD38, CD44, CD47, CORO1A, COTL1, CPNE3, CTSZ, CX3CL1, CYSTM1, EEF1A1, EXOC6B, EXPH5, F13A1, F2R, FES, GAB2, GBP1, GBP5, GNPTAB, GPR68, GRIK5, GRM7, GSK3B, GSN, HDAC9, HLA-B, IL17RA, ITGB6, ITPR1, ITPR2, KCNA2, KCNAB2, KCNC2, KCTD11, LCP1, LCP2, LGR4, LPCAT1, MBP, MICAL2, MTNR1B, MYO1G, NTRK2, OPRM1, PCDH7, PIK3CD, PPFIA1, PPFIA4, PRKCE, PSAP, PSMD9, PTPN22, PTPN6, PTPRC, PTPRJ, PTPRN2, RAB13, RALB, RNASET2, S100A13, SEPTIN9, SLC15A4, SLC1A2, SLC1A5, SLC4A8, STK10, STX11, SYN3, TANK, TBC1D10C, TGFB2, TIMP2, TMC6, TNF, TNFAIP3, TRAF3, TSPOAP1, UBE2Z, UBR4, ZC3H12A]	[ABCA12, ABCC8, ADCY5, ADORA1, AIM2, AKAP12, AMPD3, ARHGAP44, ATP6V1D, BTNL2, CADPS2, CAMP, CARD11, CARD8, CARTPT, CD9, CDK13, CDK5R2, CHRNA4, CHUK, CPLX2, CRISP3, CTAGE1, CTSD, DRD2, DYSF, EGFR, EXOC4, FGF7, FOXP1, FRMD4A, GNAS, GRM7, HGF, HLA-DRB1, HRG, HYAL2, ITGB2, ITPR1, KCNA5, KCNG2, KCNS3, KDM5B, LCP1, LRRC7, LYN, LYZ, MBL2, MBP, MMP12, MNDA, NR1H3, NRXN3, PAK1, PDXK, PFKM, PLAC8, PLPPR3, PNKD, PPP3CA, PRKCA, PRKCZ, PRKN, PSMD7, PTPN22, PTPRN2, RAB37, RAB7A, RIMS1, SEPTIN9, SERPINA4, SLC15A4, SLC17A7, SLC1A7, SRGN, SRI, STXBP5L, SYT17, TBX3, TIAM1, TLR1, TNFRSF11A, TRAF2, VEGFC, VPS33A, VSNL1]
GO:0002790	peptide secretion	3,91E-02	[5, 6, 7]	120,00	16,97	36,57	30,60	32,84	[ADAM8, AIF1, AIM2, ANGPT1, BANK1, BLK, BTN3A1, BTN3A2, CACNA1E, CARD11, CARD17, CCL19, CD2, CD200R1, CD244, CD40LG, CD74, CLEC4E, CLEC6A, CNR1, CRTAM, CSF1R, FCN1, FFAR4, FGR, FOXP3, GBP5, HLA-DRB1, IFNG, IL26, KLRG1, LCP1, LCP2, LPAL2, MCOLN2, NLRP3, ORM2, PLEK, PTPN22, RAB3B, RAB3C, SUCNR1, SYK, TLR10, TLR6, TLR8, TNFAIP3, TNFSF13B, TREM2]	[ABAT, AIM2, CACNA1A, CAMK2G, CBLN4, CCM2, CCR7, CD160, CD2AP, CD38, CX3CL1, EXPH5, F2R, GBP1, GBP5, GNPTAB, GPR68, HDAC9, IL17RA, ITGB6, ITPR1, ITPR2, KCNC2, LCP1, LCP2, LGR4, MBP, MTNR1B, OPRM1, PRKCE, PSAP, PSMD9, PTPN22, PTPRN2, RAB13, S100A13, TANK, TGFB2, TNF, TNFAIP3, ZC3H12A]	[ABCC8, ADCY5, ADORA1, AIM2, AKAP12, BTNL2, CARD11, CARD8, CARTPT, CHUK, CTAGE1, DRD2, DYSF, EGFR, FOXP1, FRMD4A, GNAS, HLA-DRB1, HYAL2, ITPR1, KCNA5, KCNG2, KCNS3, LCP1, LYN, MBL2, MBP, MMP12, NR1H3, PFKM, PPP3CA, PRKCA, PRKCZ, PRKN, PTPN22, PTPRN2, SRGN, SRI, STXBP5L, TIAM1, TLR1, TRAF2, VEGFC, VSNL1]
GO:0140352	export from cell	4,75E-02	[2, 4]	257,00	14,83	31,91	36,52	31,56	[ADAM8, AIF1, AIM2, ANGPT1, BANK1, BIN2, BLK, BTK, BTN3A1, BTN3A2, CACNA1E, CARD11, CARD17, CCL19, CCR2, CD2, CD200R1, CD244, CD40LG, CD53, CD84, CFP, CLEC12A, CLEC4D, CLEC4E, CLEC6A, CNR1, CRTAM, CSF1R, CTSS, CTSW, CYBB, DOCK2, DRD1, FCN1, FFAR4, FGL2, FGR, FOXP3, FRMPD3, GBP5, HLA-DRB1, IFNG, IL26, IQGAP2, KLF5, KLRG1, KMO, KNG1, LCP1, LCP2, LPAL2, LRMP, LYZ, MCOLN2, MYB, MYO1G, NCKAP1L, NLRP3, NR4A3, ORM2, P2RY12, PIK3CG, PLAC8, PLAU, PLEK, PTAFR, PTPN22, PTPN6, PTPRC, PTX3, RAB3B, RAB3C, RAB44, RNASET2, SDC1, SELL, SLC18A1, SUCNR1, SYK, SYTL1, TBC1D10C, TLR10, TLR6, TLR8, TNFAIP3, TNFSF13B, TREM2, TXNDC5, VNN1]	[ABAT, ABCG2, ADAM10, ADCY1, ADGRE5, AIM2, ASAH1, ATG7, B2M, BDNF, BIN2, BPI, CAB39, CACNA1A, CACNB2, CAMK2G, CBLN4, CCM2, CCR7, CD160, CD2AP, CD38, CD44, CD47, CORO1A, COTL1, CPNE3, CTSZ, CX3CL1, CYSTM1, EEF1A1, EXOC6B, EXPH5, F13A1, F2R, FES, GAB2, GBP1, GBP5, GNPTAB, GPR68, GRIK5, GRM7, GSK3B, GSN, HDAC9, HLA-B, IL17RA, ITGB6, ITPR1, ITPR2, KCNA2, KCNAB2, KCNC2, KCNQ1, KCTD11, LCP1, LCP2, LGR4, LPCAT1, MBP, MICAL2, MTNR1B, MYO1G, NTRK2, OPRM1, PCDH7, PIK3CD, PPFIA1, PPFIA4, PRKCE, PSAP, PSMD9, PTPN22, PTPN6, PTPRC, PTPRJ, PTPRN2, RAB13, RALB, RNASET2, S100A13, SEPTIN9, SLC15A4, SLC17A3, SLC1A2, SLC1A5, SLC4A8, STK10, STX11, SYN3, TANK, TBC1D10C, TGFB2, TIMP2, TMC6, TNF, TNFAIP3, TRAF3, TSPOAP1, UBE2Z, UBR4, ZC3H12A]	[ABCA12, ABCC1, ABCC8, ADCY5, ADORA1, AIM2, AKAP12, AMPD3, ARHGAP44, ATP1B1, ATP6V1D, BTNL2, CADPS2, CAMP, CARD11, CARD8, CARTPT, CD9, CDK13, CDK5R2, CHRNA4, CHUK, CPLX2, CRISP3, CTAGE1, CTSD, DRD2, DYSF, EGFR, EXOC4, FGF7, FOXP1, FRMD4A, GNAS, GRM7, HGF, HLA-DRB1, HRG, HYAL2, ITGB2, ITPR1, KCNA5, KCNG2, KCNS3, KCNT2, KDM5B, LCP1, LRRC7, LYN, LYZ, MBL2, MBP, MMP12, MNDA, NR1H3, NRXN3, PAK1, PDXK, PFKM, PLAC8, PLPPR3, PNKD, PPP3CA, PRKCA, PRKCZ, PRKN, PSMD7, PTPN22, PTPRN2, RAB37, RAB7A, RIMS1, SEPTIN9, SERPINA4, SLC15A4, SLC17A7, SLC1A7, SRGN, SRI, STXBP5L, SYT17, TBX3, TIAM1, TLR1, TNFRSF11A, TRAF2, VEGFC, VPS33A, VSNL1]
GO:0006952	defense response	8,67E-09	[3]	309,00	16,80	46,89	32,77	20,34	[ADAM8, ADORA3, AIF1, AIM2, AKNA, AOAH, BLNK, BMPR1B, BTK, C1QA, C1QB, C1QC, C2, C8G, CAMK4, CARD11, CCDC88B, CCL19, CCL24, CCR2, CCR3, CCR4, CCR5, CD180, CD200R1, CD244, CD4, CD40LG, CD48, CD6, CD74, CD84, CD96, CEL, CFP, CLEC1B, CLEC4D, CLEC4E, CLEC6A, CLNK, CNR1, CNR2, CR2, CRTAM, CSF1R, CXCL10, CXCL11, CXCL5, CXCL6, CXCL9, CXCR3, CXCR4, CXCR6, CYBB, CYSLTR1, DAPK1, DRD1, ELF3, EREG, FASLG, FCMR, FCN1, FCN3, FFAR4, FGL2, FGR, FOLR2, FOSL1, FOXP3, FUT7, GBP5, GBP6, GNLY, GPSM3, HLA-DPA1, HLA-DPB1, HLA-DQA1, HLA-DRA, HLA-DRB1, HLA-DRB5, IFNG, IKBKE, IL18, IL18RAP, IL22, IL5RA, IRF4, IRF5, ITK, JCHAIN, KLRC2, KLRC3, KLRD1, KLRG1, KLRK1, KNG1, LAG3, LCP1, LPAL2, LTA, LY75, LY86, LY9, LYZ, MAPK13, MCOLN2, MPEG1, MRC1, MS4A1, MUC19, MUC6, NAMPT, NCF1, NCR3, NLRP3, NLRP6, OASL, ORM2, PIK3CG, PLA2G2D, PLA2G7, PLAC8, PRDM1, PRF1, PRKCQ, PTAFR, PTGFR, PTK2B, PTPN22, PTPN6, PTPRC, PTPRCAP, PTX3, PYHIN1, RNASE6, RNASET2, SDC1, SELE, SH2D1A, SLAMF6, SLAMF7, SLAMF8, SMPDL3B, SP140, STAP1, STAT1, SUCNR1, SYK, THEMIS2, TLR10, TLR6, TLR8, TNFAIP3, TNFAIP8L2, TNFSF8, TNIP3, TRAT1, TREM2, TRIM14, UBD, VNN1, WAS, XCL1, XCL2, XCR1, ZNF683]	[ACTR3, ADA, ADAMTS12, ADCY1, ADGRE5, ADORA3, AIM2, AKNA, AOAH, ARRB2, ATG7, B2M, BATF, BCL2, BDNF, BIRC3, BPI, CAMK2G, CCDC88B, CCM2, CCR7, CD160, CD28, CD44, CD47, CD6, COLEC11, COLEC12, CORO1A, COTL1, CX3CL1, CXCL11, DAPK1, DPEP1, EVL, F2R, FANCA, GABRA5, GBP1, GBP5, GPR68, GPRC5B, GPSM3, GPX4, GRIK5, GSN, HDAC9, HLA-A, HLA-B, HLA-DPA1, HLA-DPB1, HLA-DRA, IDO1, IGFBP4, IL17RA, IL34, INAVA, IRF1, IRF2, ITGB6, JAK3, LCP1, LGR4, LSP1, LTA, MCPH1, MED1, MGLL, MPEG1, NLRC5, NMI, OASL, OPRM1, PARP4, PCDHA6, PIK3CD, PLGRKT, PLK5, PLSCR1, PRF1, PRKACB, PSMB8, PSMB9, PSMD9, PSTPIP1, PTK2B, PTPN1, PTPN2, PTPN22, PTPN6, PTPRC, PTPRCAP, RBMS3, RELB, RNASET2, RNF166, RNF216, RORA, RTP4, SBNO2, SGMS1, SLAMF1, SLAMF7, SLC7A2, SP140, STAT1, STAT3, STX8, TAP1, TNF, TNFAIP3, TNFAIP8L2, TNIP1, TRAF3, ZBP1, ZC3H12A]	[ABCC1, ACER3, ADORA1, AIM2, ALOX15, ASIC4, BPIFB1, CAMK4, CAMP, CARD11, CASP5, CHST1, CHUK, COLEC11, CRISP3, DEFB118, DRD2, EGFR, FNDC4, FOXP1, GABRA5, GPR31, GRIK2, HGF, HLA-DQB2, HLA-DRA, HLA-DRB1, HLA-G, HRG, HYAL2, ITGB2, JCHAIN, KIR3DL2, KLK7, LCP1, LGALS8, LILRA4, LYN, LYZ, MAGI1, MAP3K5, MBL2, MMP12, MNDA, MUC2, MUC5B, NFRKB, NLRC5, NLRP4, NR1H3, PAK1, PCDHA6, PLAC8, PLD3, PLK5, PLPPR3, PNKD, PRDX1, PRKCA, PRKCZ, PSMD7, PSTPIP1, PTPN22, RB1, SERINC5, SIGLEC15, SMAD1, SUSD4, TLR1, TNFRSF11A, TRIM4, TRIM68]

GO:0006954	inflammatory response	9,10E-09	[4]	160,00	19,90	50,56	31,11	18,33	[ADAM8, ADORA3, AIF1, AIM2, AKNA, AOAH, BLNK, BMPR1B, BTK, C1QA, CAMK4, CCL19, CCL24, CCR2, CCR3, CCR4, CCR5, CD180, CD200R1, CD40LG, CD6, CD96, CEL, CNR1, CNR2, CSF1R, CXCL10, CXCL11, CXCL5, CXCL6, CXCL9, CXCR3, CXCR4, CXCR6, CYBB, CYSLTR1, ELF3, FFAR4, FOLR2, FOXP3, FUT7, GBP5, GPSM3, HLA-DRB1, IFNG, IL18, IL18RAP, IL22, IL5RA, KLRG1, KNG1, LCP1, LPAL2, LTA, LY75, LY86, LYZ, MAPK13, MS4A1, NAMPT, NCR3, NLRP3, NLRP6, ORM2, PIK3CG, PLA2G2D, PLA2G7, PRKCQ, PTAFR, PTGFR, PTPRC, PTX3, SDC1, SELE, SLAMF8, SMPDL3B, STAP1, SUCNR1, SYK, THEMIS2, TLR10, TLR6, TLR8, TNFAIP3, TNFAIP8L2, TNIP3, TREM2, VNN1, XCL1, XCL2, XCR1]	[ADA, ADAMTS12, ADCY1, ADGRE5, ADORA3, AIM2, AKNA, AOAH, BIRC3, CCM2, CCR7, CD28, CD44, CD47, CD6, CX3CL1, CXCL11, DPEP1, F2R, FANCA, GBP5, GPR68, GPRC5B, GPSM3, GPX4, HDAC9, IDO1, IGFBP4, IL17RA, IL34, ITGB6, LCP1, LTA, MCPH1, MGLL, NMI, OPRM1, PARP4, PCDHA6, PIK3CD, PLGRKT, PLSCR1, PSTPIP1, PTPN2, PTPRC, RELB, RORA, SBNO2, SGMS1, SLC7A2, STAT3, TNF, TNFAIP3, TNFAIP8L2, TNIP1, ZC3H12A]	[ABCC1, ACER3, ADORA1, AIM2, ALOX15, CAMK4, CASP5, CHST1, CHUK, EGFR, FNDC4, FOXP1, GPR31, HGF, HLA-DRB1, HYAL2, ITGB2, LCP1, LYN, LYZ, MAGI1, MBL2, NFRKB, NLRP4, NR1H3, PCDHA6, PLD3, PRKCZ, PSTPIP1, RB1, SMAD1, TLR1, TNFRSF11A]
GO:0032101	regulation of response to external stimulus	9,38E-06	[3, 4]	191,00	17,47	42,79	31,63	25,58	[ADAM8, AIF1, AIM2, AKNA, ANGPT2, AOAH, BTK, CARD11, CCL19, CCL24, CCR2, CCR4, CD180, CD200R1, CD6, CD74, CD96, CEL, CLEC4D, CLEC4E, CLEC6A, CLNK, CNR1, CNR2, CRTAM, CSF1R, CXCL10, CXCL6, CXCR4, DRD1, EDN2, EREG, FCN1, FFAR4, FGL2, FGR, FOXP3, FUT7, GBP5, GPSM3, HLA-DRB1, IFNG, IKBKE, IL18, IL18RAP, KLRK1, KNG1, LAG3, LCP1, LTA, LY86, MAPK13, MUC19, MUC6, NCKAP1L, NCR3, NLRP3, NLRP6, NR4A3, P2RY12, PIK3CG, PLA2G2D, PLA2G7, PLAU, PLEK, PRKCQ, PTK2B, PTPN22, PTPN6, PTPRC, PYHIN1, SELE, SEMA4A, SEMA4D, SH2D1A, SLAMF6, SLAMF8, SMPDL3B, STAP1, STAT1, SUCNR1, SYK, TLR10, TLR6, TLR8, TNFAIP3, TNFAIP8L2, TNR, TREM2, TSPAN8, XCL1, XCL2]	[ABAT, ADA, ADAM10, ADAMTS12, AIM2, AKNA, AOAH, ARRB2, BIRC3, CCM2, CCR7, CD160, CD28, CD47, CD6, CX3CL1, F2R, FANCA, GBP5, GPRC5B, GPSM3, GPX4, HLA-A, HLA-B, IDO1, IL17RA, IL34, IRF1, LCP1, LTA, MCPH1, MED1, MGLL, NLRC5, NMI, OPRM1, PCDHA6, PLSCR1, PRKACB, PRKD2, PSMB8, PSMB9, PSMD9, PTK2B, PTPN1, PTPN2, PTPN22, PTPN6, PTPRC, PTPRF, RELB, RIPOR2, RNF216, RORA, SBNO2, SEMA4D, SLC7A2, STAT1, STAT3, TNF, TNFAIP3, TNFAIP8L2, TNIP1, TRAF3, UBASH3B, ZBP1, ZC3H12A, ZNF160]	[ABCC1, ABCC8, ADORA1, AIM2, ALOX15, CAMKMT, CARD11, CARD8, CARTPT, CASP5, CAV1, CD9, CDH13, CHUK, DRD2, DYSF, EGFR, FNDC4, FOXP1, GNAT1, GPR31, GRK1, HGF, HLA-DRB1, HLA-G, HRG, HYAL2, KLK7, LCP1, LYN, MAGI1, MMP12, MNDA, MTUS1, MUC2, MUC5B, NLRC5, NR1H3, PAK1, PCDHA6, PDE6B, PLD3, PRKCA, PSMD7, PTPN22, RB1, RIPOR2, ROBO1, ROBO2, SEMA5A, SUSD4, SWAP70, TIAM1, TNFRSF11A, VEGFC]
GO:0031347	regulation of defense response	1,42E-05	[4, 5]	136,00	18,92	42,04	35,67	22,29	[ADAM8, AIM2, AKNA, AOAH, BTK, CARD11, CCL24, CCR2, CD200R1, CD6, CD96, CLEC4D, CLEC4E, CLEC6A, CLNK, CNR1, CNR2, CRTAM, CXCL6, EREG, FCN1, FFAR4, FGL2, FGR, FOXP3, FUT7, GBP5, GPSM3, HLA-DRB1, IFNG, IKBKE, IL18, IL18RAP, KLRK1, LAG3, LCP1, LTA, MAPK13, MUC19, MUC6, NCR3, NLRP3, NLRP6, PIK3CG, PLA2G2D, PLA2G7, PTPN22, PTPN6, PTPRC, PYHIN1, SELE, SH2D1A, SLAMF6, SLAMF8, SMPDL3B, STAP1, STAT1, SUCNR1, SYK, TLR10, TLR6, TLR8, TNFAIP3, TNFAIP8L2, TREM2, XCL1]	[ADA, ADAMTS12, AIM2, AKNA, AOAH, ARRB2, BIRC3, CCM2, CCR7, CD160, CD28, CD47, CD6, CX3CL1, FANCA, GBP5, GPRC5B, GPSM3, GPX4, HLA-A, HLA-B, IDO1, IL17RA, IRF1, LCP1, LTA, MCPH1, MED1, MGLL, NLRC5, NMI, PCDHA6, PLSCR1, PRKACB, PSMB8, PSMB9, PSMD9, PTPN1, PTPN2, PTPN22, PTPN6, PTPRC, RELB, RNF216, RORA, SBNO2, SLC7A2, STAT1, STAT3, TNF, TNFAIP3, TNFAIP8L2, TNIP1, TRAF3, ZBP1, ZC3H12A]	[ABCC1, ADORA1, AIM2, ALOX15, CARD11, CASP5, CHUK, DRD2, EGFR, FNDC4, FOXP1, GPR31, HGF, HLA-DRB1, HLA-G, HYAL2, KLK7, LCP1, LYN, MAGI1, MMP12, MNDA, MUC2, MUC5B, NLRC5, NR1H3, PAK1, PCDHA6, PLD3, PRKCA, PSMD7, PTPN22, RB1, SUSD4, TNFRSF11A]
GO:0051707	response to other organism	3,19E-05	[2, 4]	265,00	16,10	45,42	33,66	20,92	[ADAM8, ADAMTS9, AIF1, AIM2, BANK1, BTK, C1QA, C1QB, C1QC, C2, C8G, CARD11, CARD17, CASP8, CCDC88B, CCL19, CCL24, CCR4, CCR5, CD180, CD244, CD4, CD6, CD80, CD84, CD86, CD96, CFP, CLEC4D, CLEC4E, CLEC6A, CLNK, CNR1, CNR2, CR2, CRTAM, CSF1R, CXCL10, CXCL11, CXCL5, CXCL6, CXCL9, CXCR4, CYBB, DAPK1, EREG, FASLG, FCN1, FCN3, FER1L6, FGL2, FGR, FOSL1, FOXP3, GBP5, GBP6, GFI1, GNLY, GPM6A, GZMA, HLA-DPA1, HLA-DPB1, HLA-DQA1, HLA-DRA, HLA-DRB1, HLA-DRB5, IFNG, IKBKE, IL10RA, IL12RB2, IL18, IL18RAP, IRF4, IRF5, JCHAIN, KLRC2, KLRD1, KLRG1, KLRK1, KMO, LAG3, LCP1, LTA, LY86, LY9, LYZ, MCOLN2, MPEG1, MRC1, MS4A1, MUC19, MUC6, NCF1, NCR3, NLRP3, NLRP6, NPPC, NR4A1, NUGGC, OASL, PIK3CG, PIM2, PLAC8, PRDM1, PRF1, PTAFR, PTGFR, PTPN22, PTPN6, PTPRC, PTX3, PYHIN1, RGS1, RNASE6, RNASET2, SELE, SH2D1A, SLAMF6, SLAMF7, SLAMF8, SMPDL3B, STAP1, STAT1, SYK, TLR10, TLR6, TLR8, TNFAIP3, TNFAIP8L2, TNFSF8, TNIP3, TREM2, TRIM14, UBD, VNN1, WAS, XCL1, XCL2, ZNF683]	[ACTR3, AIM2, ARRB2, ATG7, B2M, BATF, BATF3, BCL2, BIRC3, BPI, CAMK2G, CAPN2, CASP8, CCDC88B, CCR7, CD160, CD44, CD47, CD6, CDK6, CFLAR, COLEC11, COLEC12, CORO1A, COTL1, CUBN, CX3CL1, CXCL11, DAPK1, EVL, F2R, GBP1, GBP5, GSN, HLA-A, HLA-B, HLA-DPA1, HLA-DPB1, HLA-DRA, HMGA1, IDO1, IL12RB2, IL17RA, IL34, INAVA, IRF1, IRF2, JAK3, KCTD11, KLHL6, KLHL7, LCP1, LGR4, LITAF, LTA, MACROD2, MED1, MPEG1, NCOR2, NEXN, NFIB, NLRC5, NMI, OASL, OPRM1, PCDHA6, PDE4B, PIK3CD, PLSCR1, PRF1, PRKACB, PRKCE, PSMB8, PSMB9, PSMD9, PSTPIP1, PTPN1, PTPN2, PTPN22, PTPN6, PTPRC, RARA, RELB, RNASET2, RNF166, RNF216, RTP4, SBNO2, SGMS1, SLAMF1, SLAMF7, STAT1, STOX2, STX8, TMEM229B, TNF, TNFAIP3, TNFAIP8L2, TRAF3, UPF1, WIPF1, ZBP1, ZC3H12A]	[ABCC8, AIM2, AKAP12, BPIFB1, CAMP, CARD11, CARD8, CAV1, CHUK, COLEC11, COMT, CRISP3, DCN, DEFB118, DRD2, FOXP1, GPM6A, GPR31, HLA-DQB2, HLA-DRA, HLA-DRB1, HLA-G, HRG, HYAL2, IFI44, JCHAIN, KLK7, LCP1, LGALS8, LILRA4, LYN, LYZ, MACROD2, MAP3K5, MBL2, MMP12, MNDA, MUC2, MUC5B, NLRC5, NR1H3, PAK1, PCDHA6, PDE4B, PLAC8, PLPPR3, PNKD, PPARGC1A, PRDX1, PRKCA, PSMD7, PSTPIP1, PTPN22, SERINC5, SIGLEC15, SLC17A5, SMAD6, STOX2, SUSD4, TLR1, TNFRSF11A, TRIM4, TRIM68, WASL]
GO:0032103	positive regulation of response to external stimulus	6,15E-04	[3, 4, 5]	104,00	19,08	48,28	28,45	23,28	[ADAM8, AIF1, AIM2, BTK, CARD11, CCL19, CCL24, CCR2, CCR4, CD180, CD6, CD74, CLEC4D, CLEC4E, CLEC6A, CLNK, CNR1, CRTAM, CSF1R, CXCL10, CXCR4, EDN2, EREG, FCN1, GBP5, GPSM3, IFNG, IKBKE, IL18, IL18RAP, KLRK1, LAG3, LCP1, LTA, LY86, MAPK13, MUC19, MUC6, NCKAP1L, NCR3, P2RY12, PIK3CG, PLA2G7, PTK2B, PYHIN1, SH2D1A, SLAMF6, STAP1, SUCNR1, SYK, TLR10, TLR6, TLR8, TREM2, XCL1, XCL2]	[ADAM10, AIM2, CCM2, CCR7, CD160, CD28, CD47, CD6, CX3CL1, GBP5, GPRC5B, GPSM3, IDO1, IL17RA, IL34, LCP1, LTA, MED1, NLRC5, OPRM1, PLSCR1, PRKACB, PRKD2, PSMB8, PSMB9, PSMD9, PTK2B, RELB, RIPOR2, STAT3, TNF, TNIP1, ZBP1]	[ABCC1, AIM2, CARD11, CDH13, CHUK, DYSF, EGFR, HLA-G, HRG, HYAL2, KLK7, LCP1, LYN, MMP12, MNDA, MUC2, MUC5B, NLRC5, PAK1, PRKCA, PSMD7, RIPOR2, SEMA5A, SWAP70, TIAM1, TNFRSF11A, VEGFC]
GO:0050727	regulation of inflammatory response	1,30E-03	[4, 5, 6]	78,00	20,47	41,76	37,36	20,88	[ADAM8, AKNA, AOAH, BTK, CCL24, CCR2, CD200R1, CD6, CNR1, CNR2, FFAR4, FOXP3, FUT7, GBP5, GPSM3, HLA-DRB1, IFNG, IL18, LCP1, LTA, MAPK13, NLRP3, NLRP6, PIK3CG, PLA2G2D, PLA2G7, PTPRC, SELE, SLAMF8, SMPDL3B, STAP1, SUCNR1, TLR10, TLR6, TNFAIP3, TNFAIP8L2, TREM2, XCL1]	[ADA, ADAMTS12, AKNA, AOAH, BIRC3, CCM2, CCR7, CD28, CD47, CD6, CX3CL1, FANCA, GBP5, GPRC5B, GPSM3, GPX4, IDO1, IL17RA, LCP1, LTA, MCPH1, MGLL, PCDHA6, PTPN2, PTPRC, RORA, SBNO2, SLC7A2, STAT3, TNF, TNFAIP3, TNFAIP8L2, TNIP1, ZC3H12A]	[ABCC1, ADORA1, ALOX15, CASP5, EGFR, FNDC4, FOXP1, GPR31, HGF, HLA-DRB1, HYAL2, LCP1, LYN, MAGI1, NR1H3, PCDHA6, PLD3, RB1, TNFRSF11A]
GO:0080134	regulation of response to stress	1,92E-03	[3, 4]	241,00	15,61	35,71	39,10	25,19	[ADAM8, AIM2, AKNA, AOAH, BTK, CARD11, CCL19, CCL24, CCR2, CD200R1, CD27, CD40LG, CD6, CD74, CD96, CDKN2A, CEL, CLEC4D, CLEC4E, CLEC6A, CLNK, CNR1, CNR2, CRTAM, CXCL6, CXCR4, DEPDC1B, EREG, FCN1, FFAR4, FGD2, FGL2, FGR, FOXP3, FUT7, GBP5, GDF6, GPSM3, HLA-DRB1, IER3, IFNG, IKBKE, IL18, IL18RAP, IL26, KLRK1, KNG1, LAG3, LCP1, LTA, MAPK13, MSX1, MUC19, MUC6, NAMPT, NCF1, NCR3, NLRP3, NLRP6, NR4A3, NUP210, PIK3CG, PLA2G2D, PLA2G7, PLAU, PLEK, PRKCQ, PTK2B, PTPN22, PTPN6, PTPRC, PYHIN1, SELE, SH2D1A, SLAMF6, SLAMF8, SMPDL3B, STAP1, STAT1, SUCNR1, SYK, THY1, TLR10, TLR6, TLR8, TNFAIP3, TNFAIP8L2, TNR, TRAF1, TREM2, TSPAN8, VNN1, WAS, WNT1, XCL1]	[ABAT, ADA, ADAMTS12, AIM2, AKNA, AOAH, ARRB2, ATG7, B2M, BABAM2, BCL2, BIRC3, BMP7, BMPR1A, CAMK2G, CCM2, CCR7, CD160, CD28, CD44, CD47, CD6, CDK6, CX3CL1, CYREN, DDAH1, EDEM1, F2R, FANCA, FBXO5, GBP5, GPRC5B, GPSM3, GPX4, GRIK5, GSK3B, HLA-A, HLA-B, HMGA1, IDO1, IGF1R, IL17RA, INAVA, IRF1, LCP1, LTA, MAP2K2, MAP4K5, MCPH1, MDFIC, MEAK7, MED1, MGLL, NCK2, NLRC5, NMI, NSMCE1, NUP153, OPRM1, PCDHA6, PDCD10, PIGBOS1, PLSCR1, PRKACB, PRKCE, PSAP, PSMB8, PSMB9, PSMD9, PTK2B, PTPN1, PTPN2, PTPN22, PTPN6, PTPRC, PTPRF, RELB, RNF216, RORA, RPA3, RPTOR, RWDD3, SBNO2, SLAMF1, SLC7A2, SPIDR, STAT1, STAT3, TANK, TERT, TGFB2, TNF, TNFAIP3, TNFAIP8L2, TNIP1, TRAF1, TRAF3, UBASH3B, WDR70, ZBP1, ZC3H12A, ZNF160, ZNF365, ZNF385A]	[ABCC1, ABCC8, ADORA1, AIM2, ALOX15, ANKS1B, BCLAF1, CARD11, CASP5, CAV1, CD9, CHUK, DRD2, EGFR, EYA2, FMN2, FNDC4, FOXP1, GDF6, GPR31, GRIK2, HGF, HIPK2, HLA-DRB1, HLA-G, HRG, HYAL2, IGF1R, KCNK2, KLK7, LCP1, LYN, MAGI1, MAP3K5, MMP12, MNDA, MUC2, MUC5B, MYLK, NACC2, NCOR1, NLRC5, NR1H3, NUP93, PAK1, PCDHA6, PLD3, PLPP3, PPP3CA, PRDX1, PRKCA, PRKN, PSMD7, PTPN22, RB1, RNASEH2B, RNF13, SMYD2, STK25, SUSD4, TIAM1, TNFRSF11A, TRAF2, TSC1, WFS1, WNT16, WRAP53]
GO:0031349	positive regulation of defense response	9,64E-03	[3, 4, 5, 6]	77,00	19,59	47,13	31,03	21,84	[ADAM8, AIM2, BTK, CARD11, CCL24, CCR2, CD6, CLEC4D, CLEC4E, CLEC6A, CLNK, CNR1, CRTAM, EREG, FCN1, GBP5, GPSM3, IFNG, IKBKE, IL18, IL18RAP, KLRK1, LAG3, LCP1, LTA, MAPK13, MUC19, MUC6, NCR3, PIK3CG, PLA2G7, PYHIN1, SH2D1A, SLAMF6, STAP1, SUCNR1, SYK, TLR10, TLR6, TLR8, TREM2]	[AIM2, CCM2, CCR7, CD160, CD28, CD47, CD6, CX3CL1, GBP5, GPRC5B, GPSM3, IDO1, IL17RA, LCP1, LTA, MED1, NLRC5, PLSCR1, PRKACB, PSMB8, PSMB9, PSMD9, RELB, STAT3, TNF, TNIP1, ZBP1]	[ABCC1, AIM2, CARD11, CHUK, EGFR, HLA-G, HYAL2, KLK7, LCP1, LYN, MMP12, MNDA, MUC2, MUC5B, NLRC5, PAK1, PRKCA, PSMD7, TNFRSF11A]
GO:0045087	innate immune response	2,30E-02	[3, 4, 5, 6]	164,00	16,09	49,74	30,69	19,58	[ADAM8, AIF1, AIM2, BTK, C1QA, C1QB, C1QC, C2, C8G, CARD11, CCL19, CCL24, CD180, CD244, CD6, CD84, CD96, CFP, CLEC4D, CLEC4E, CLEC6A, CLNK, CR2, CRTAM, CSF1R, CXCL10, CYBB, DAPK1, EREG, FASLG, FCN1, FCN3, FGR, GBP5, GBP6, HLA-DPA1, HLA-DPB1, HLA-DQA1, HLA-DRA, HLA-DRB1, HLA-DRB5, IFNG, IKBKE, IL18, IL18RAP, IRF4, IRF5, JCHAIN, KLRC2, KLRD1, KLRG1, KLRK1, LAG3, LY86, LY9, MCOLN2, MPEG1, MRC1, MUC19, MUC6, NCF1, NCR3, NLRP3, NLRP6, OASL, PIK3CG, PRDM1, PTAFR, PTPN22, PTPN6, PTX3, PYHIN1, RNASE6, RNASET2, SH2D1A, SLAMF6, SLAMF7, SLAMF8, SMPDL3B, STAT1, SYK, TLR10, TLR6, TLR8, TNFAIP3, TNFAIP8L2, TREM2, TRIM14, UBD, VNN1, WAS, XCL1, XCL2, ZNF683]	[ACTR3, AIM2, ARRB2, B2M, BIRC3, BPI, CAMK2G, CD160, CD44, CD47, CD6, COLEC11, COLEC12, CORO1A, CX3CL1, DAPK1, EVL, GBP1, GBP5, GSN, HLA-A, HLA-B, HLA-DPA1, HLA-DPB1, HLA-DRA, IL34, INAVA, IRF1, IRF2, JAK3, LGR4, MED1, MPEG1, NLRC5, NMI, OASL, PIK3CD, PLSCR1, PRKACB, PSMB8, PSMB9, PSMD9, PSTPIP1, PTPN1, PTPN2, PTPN22, PTPN6, RELB, RNASET2, RNF166, SLAMF1, SLAMF7, STAT1, STX8, TNFAIP3, TNFAIP8L2, TRAF3, ZBP1]	[AIM2, BPIFB1, CAMP, CARD11, CHUK, COLEC11, CRISP3, DEFB118, DRD2, HLA-DQB2, HLA-DRA, HLA-DRB1, HLA-G, JCHAIN, LILRA4, LYN, MAP3K5, MBL2, MMP12, MNDA, MUC2, MUC5B, NLRC5, NR1H3, PAK1, PNKD, PRDX1, PRKCA, PSMD7, PSTPIP1, PTPN22, SERINC5, SIGLEC15, SUSD4, TLR1, TRIM4, TRIM68]
GO:0098742	cell-cell adhesion via plasma-membrane adhesion molecules	5,40E-08	[4]	73,00	25,98	12,99	25,97	61,04	[CD6, CD84, CRB2, CRTAM, EMB, JAML, PCDH20, PCDHA2, SELE, SELL]	[ADGRL3, CD6, CDH17, CDH19, CDH2, CDHR2, CX3CL1, JAML, MAP2K5, MBP, NECTIN1, NEXN, NPTN, PCDH7, PCDH9, PCDHA6, PTPRF, PTPRM, TENM4, TGFB2]	[CDH13, CDH22, CDH4, CELSR3, CLSTN2, CRB1, CXADR, DSCAML1, DSG3, FAT1, IGSF21, IGSF9B, ITGB2, KIRREL3, MBL2, MBP, MYADM, PCDHA12, PCDHA13, PCDHA6, PCDHA7, PCDHA9, PCDHB13, PCDHB14, PCDHB15, PCDHB16, PCDHB18P, PCDHB2, PCDHB3, PCDHB4, PCDHB5, PCDHB6, PCDHB7, PCDHB8, PCDHGA10, PCDHGA2, PCDHGA4, PCDHGA6, PCDHGB1, PCDHGB3, PTPRT, ROBO1, ROBO2, ROBO3, SCARF2, SDK1, UNC5D]
GO:0045058	T cell selection	4,81E-06	[2, 6, 7, 8, 9, 10, 11]	23,00	45,10	59,26	29,63	11,11	[CARD11, CD3D, CD3E, CD3G, CD4, CD74, DOCK2, FOXP3, IRF4, LY9, PTPRC, SLAMF6, SYK, THEMIS, TOX, ZAP70]	[BATF, BCL2, CCR7, CD28, CD3G, PTPN2, PTPRC, STAT3]	[BCL11B, CARD11, TOX]
GO:0033077	T cell differentiation in thymus	6,10E-05	[6, 7, 8, 9, 10, 11]	28,00	35,44	51,43	31,43	17,14	[ADAM8, CAMK4, CARD11, CD3D, CD3E, CD3G, CD74, CDKN2A, DOCK2, EGR3, FOXP3, IL7R, PTPRC, TESPA1, TOX, VNN1, WNT1, ZAP70]	[ADA, B2M, BCL2, CCR7, CD28, CD3G, CDK6, PTPN2, PTPRC, TESPA1, ZEB1]	[BCL11B, CAMK4, CARD11, PNKD, TOX, ZEB1]
GO:0043368	positive T cell selection	1,03E-04	[3, 7, 8, 9, 10, 11, 12]	18,00	47,37	61,90	28,57	9,52	[CD3D, CD3E, CD3G, CD74, DOCK2, FOXP3, IRF4, LY9, PTPRC, SLAMF6, THEMIS, TOX, ZAP70]	[BATF, BCL2, CD3G, PTPN2, PTPRC, STAT3]	[BCL11B, TOX]
GO:0045061	thymic T cell selection	4,90E-03	[3, 7, 8, 9, 10, 11, 12]	12,00	52,17	56,25	31,25	12,50	[CARD11, CD3D, CD3E, CD3G, CD74, DOCK2, PTPRC, TOX, ZAP70]	[CCR7, CD28, CD3G, PTPN2, PTPRC]	[CARD11, TOX]
GO:0045059	positive thymic T cell selection	2,02E-02	[4, 8, 9, 10, 11, 12, 13]	9,00	60,00	66,67	25,00	8,33	[CD3D, CD3E, CD3G, CD74, DOCK2, PTPRC, TOX, ZAP70]	[CD3G, PTPN2, PTPRC]	[TOX]
GO:0043383	negative T cell selection	2,15E-02	[3, 7, 8, 9, 10, 11, 12]	8,00	66,67	66,67	33,33	0,00	[CD3E, CD74, DOCK2, PTPRC, THEMIS, ZAP70]	[CCR7, CD28, PTPRC]	[]
GO:0033081	regulation of T cell differentiation in thymus	3,83E-02	[7, 8, 9, 10, 11, 12]	12,00	44,44	56,25	25,00	18,75	[ADAM8, CAMK4, CDKN2A, EGR3, FOXP3, IL7R, TESPA1, TOX, VNN1]	[ADA, PTPN2, TESPA1, ZEB1]	[CAMK4, TOX, ZEB1]
GO:0032663	regulation of interleukin-2 production	4,82E-06	[4, 5, 6, 7]	25,00	42,37	46,67	33,33	20,00	[CARD11, CCR2, CD3E, CD4, CD80, CD86, FOXP3, IRF4, LAG3, PRKCQ, PTPRC, SASH3, TNFAIP3, XCL1]	[CD28, GBP1, HDAC9, HOMER2, PDE4B, PRKD2, PTPRC, RUNX1, TANK, TNFAIP3]	[BTNL2, CARD11, NAV3, PDE4B, RUNX1, TRAF2]
GO:0032623	interleukin-2 production	1,32E-05	[3, 6]	27,00	38,57	50,00	31,25	18,75	[CARD11, CCR2, CD247, CD3E, CD4, CD80, CD86, FOXP3, IL18, IRF4, LAG3, PRKCQ, PTPRC, SASH3, TNFAIP3, XCL1]	[CD28, GBP1, HDAC9, HOMER2, PDE4B, PRKD2, PTPRC, RUNX1, TANK, TNFAIP3]	[BTNL2, CARD11, NAV3, PDE4B, RUNX1, TRAF2]
GO:0032743	positive regulation of interleukin-2 production	8,84E-05	[4, 5, 6, 7, 8]	17,00	50,00	47,62	28,57	23,81	[CARD11, CCR2, CD3E, CD4, CD80, CD86, IRF4, PRKCQ, PTPRC, SASH3]	[CD28, PDE4B, PRKD2, PTPRC, RUNX1, TANK]	[BTNL2, CARD11, PDE4B, RUNX1, TRAF2]

GO:0042108	positive regulation of cytokine biosynthetic process	2,84E-03	[4, 5, 6, 7, 8]	25,00	32,05	70,37	18,52	11,11	[CARD11, CCR2, CD3E, CD4, CD74, CD80, CD86, CYBB, EREG, IFNG, IRF4, LPAL2, LTB, PRKCQ, PTAFR, PTPRC, SYK, TLR6, TLR8]	[CD28, IRF1, PTPRC, STAT3, TNF]	[AKAP12, CARD11, TLR1]
GO:0042094	interleukin-2 biosynthetic process	4,90E-03	[4, 5, 6, 7]	12,00	52,17	78,57	14,29	7,14	[CARD11, CD3E, CD4, CD80, CD86, FOXP3, IL18, IRF4, LAG3, PRKCQ, PTPRC]	[CD28, PTPRC]	[CARD11]
GO:0032753	positive regulation of interleukin-4 production	4,90E-03	[4, 5, 6, 7, 8]	12,00	52,17	75,00	16,67	8,33	[CD3E, CD40LG, CD86, FOXP3, IRF4, NLRP3, PRKCQ, SASH3, SYK]	[CD28, RARA]	[PRKCZ]
GO:0042035	regulation of cytokine biosynthetic process	6,28E-03	[4, 5, 6, 7]	32,00	27,35	64,71	23,53	11,76	[CARD11, CCR2, CD3E, CD4, CD74, CD80, CD86, CYBB, EREG, FOXP3, IFNG, INPP5D, IRF4, LAG3, LPAL2, LTB, PRKCQ, PTAFR. PTPRC. SYK. TLR6. TLR8]	[CD28, IRF1, MAP2K5, MAST2, NMI, PTPRC, STAT3. TNF]	[AKAP12, CARD11, TIA1, TLR1]
GO:0045076	regulation of interleukin-2 biosynthetic process	6,59E-03	[5, 6, 7, 8]	11,00	55,00	76,92	15,38	7,69	[CARD11, CD3E, CD4, CD80, CD86, FOXP3, IRF4, LAG3, PRKCQ, PTPRC]	[CD28, PTPRC]	[CARD11]
GO:0045086	positive regulation of interleukin-2 biosynthetic process	9,20E-03	[5, 6, 7, 8, 9]	9,00	64,29	72,73	18,18	9,09	[CARD11, CD3E, CD4, CD80, CD86, IRF4, PRKCQ, PTPRC]	[CD28, PTPRC]	[CARD11]
GO:0042089	cytokine biosynthetic process	1,60E-02	[3, 5, 6]	33,00	26,19	65,71	22,86	11,43	[CARD11, CCR2, CD3E, CD4, CD74, CD80, CD86, CYBB, EREG, FOXP3, IFNG, IL18, INPP5D, IRF4, LAG3, LPAL2, LTB, PRKCQ. PTAFR. PTPRC. SYK. TLR6. TLR8]	[CD28, IRF1, MAP2K5, MAST2, NMI, PTPRC, STAT3. TNF]	[AKAP12, CARD11, TIA1, TLR1]
GO:0034097	response to cytokine	2,10E-09	[4]	231,00	18,23	42,15	39,85	18,01	[AIF1, AIM2, ANGPT1, BTK, CASP8, CCL19, CCL24, CCR2, CCR3, CCR4, CCR5, CCR8, CD27, CD4, CD40LG, CD74, CD80, CD86, CISH, CLCF1, CSF1R, CSF2RA, CXCL10, CXCL11, CXCL5, CXCL6, CXCL9, CXCR3, CXCR4, CXCR6, DAPK1, DOCK8, EDN2, EREG, FASLG, FLT3, FOSL1, GBP5, GBP6, GFI1, GFPT2, GFRA2, GLDC, H3C10, H3C11, H3C12, H3C7, HCLS1, HLA-DPA1, HLA-DPB1, HLA-DQA1, HLA-DRA, HLA-DRB1, HLA-DRB5, IFNG, IKBKE, IL10RA, IL12RB2, IL18, IL18RAP, IL22, IL26, IL2RG, IL5RA, IL7R, IL9R, INPP5D, IRF4, IRF5, ITGA4, KLF5, KMO, LAMP3, LCP1, LEF1, LTA, LTB, MAPK13, MRC1, NELL2, NR4A3, OASL, PIM1, PTAFR, PTK2B, PTPN6, PTPN7, PTPRC, PYHIN1, RHEX, RUFY4, SDC1, SELE, SELPLG, SNX10, STAP1, STAT1, SYK, TNFAIP3, TNFRSF11B, TNFRSF18, TNFSF13B, TNFSF8, TRAF1, TREM2, UBD, WAS, XCL1, XCL2, XCR1]	[ACTR3, ADAM10, ADAMTS12, AFF3, AIM2, AKAP6, ASAH1, B2M, BATF, BCL2, BIRC3, CAMK2G, CAPN2, CASP8, CCM2, CCR7, CD38, CD44, CD47, CORO1A, CX3CL1, CXCL11, DAPK1, ETFA, EVL, F13A1, GAB2, GBP1, GBP5, GFRA2, GHR, GRB2, GSK3B, GSN, HLA-A, HLA-B, HLA-DPA1, HLA-DPB1, HLA-DRA, HNRNPF, IL11RA, IL12RB2, IL17RA, IL19, IL21R, IL34, IRF1, IRF2, JAK3, JARID2, KAT6B, LAPTM5, LCP1, LRCH1, LTA, MAP2K5, MED1, NFAT5, NLRC5, NMI, NMNAT3, NPR2, OASL, OPRM1, PDCD10, PIK3CD, PLSCR1, PSMB8, PSMB9, PSMD9, PTK2B, PTPN1, PTPN2, PTPN6, PTPN7, PTPRC, RARA, RELB, RFFL, RIPOR2, RORA, RUNX1, SBNO2, SGMS1, SLIT1, SMARCA4, SPOCK2, SQSTM1, SSTR1, STAT1, STAT3, STX8, TANK, TIMP2, TNF, TNFAIP3, TRAF1, TRAF3, UGCG, UPF1, VRK2, ZBP1, ZC3H12A, ZEB1]	[AFF3, AIM2, AKAP12, ALDH1A2, ALOX15, BCLAF1, CAMP, CARD8, CAV1, CHUK, FOXH1, GFPT2, GFRAL, GREM2, HGF, HLA-DQB2, HLA-DRA, HLA-DRB1, HLA-G, HYAL2, ITGB2, KCNA5, KDM5B, LCP1, MAP3K5, MMP12, MNDA, MSC, MYNN, NLRC5, NR1H3, PPARGC1A, PRKCA, PRKN, PSMD7, PTPRT, RIPOR2, ROBO1, RUNX1, SOX17, ST18, TNFRSF11A, TRAF2, TRIM63, TRIM68, VEGFC, ZEB1]
GO:0071345	cellular response to cytokine stimulus	5,20E-09	[5]	216,00	18,38	43,27	38,78	17,96	[AIF1, AIM2, ANGPT1, BTK, CASP8, CCL19, CCL24, CCR2, CCR3, CCR4, CCR5, CCR8, CD27, CD4, CD40LG, CD74, CD80, CD86, CISH, CLCF1, CSF1R, CSF2RA, CXCL10, CXCL11, CXCL5, CXCL6, CXCL9, CXCR3, CXCR4, CXCR6, DAPK1, DOCK8, EDN2, EREG, FASLG, FLT3, GBP5, GBP6, GFI1, GFPT2, GFRA2, GLDC, H3C10, H3C11, H3C12, H3C7, HCLS1, HLA-DPA1, HLA-DPB1, HLA-DQA1, HLA-DRA, HLA-DRB1, HLA-DRB5, IFNG, IKBKE, IL10RA, IL12RB2, IL18, IL18RAP, IL22, IL26, IL2RG, IL5RA, IL7R, IL9R, INPP5D, IRF4, IRF5, ITGA4, KLF5, KMO, LCP1, LEF1, LTA, LTB, MAPK13, MRC1, NELL2, NR4A3, OASL, PIM1, PTAFR, PTK2B, PTPN6, PTPN7, PTPRC, PYHIN1, RHEX, RUFY4, SDC1, SELPLG, SNX10, STAP1, STAT1, SYK, TNFAIP3, TNFRSF11B, TNFRSF18, TNFSF13B, TNFSF8, TRAF1, TREM2, WAS, XCL1, XCL2, XCR1]	[ACTR3, ADAMTS12, AIM2, AKAP6, ASAH1, B2M, BATF, BCL2, BIRC3, CAMK2G, CAPN2, CASP8, CCM2, CCR7, CD44, CD47, CORO1A, CX3CL1, CXCL11, DAPK1, ETFA, EVL, F13A1, GAB2, GBP1, GBP5, GFRA2, GHR, GRB2, GSK3B, GSN, HLA-A, HLA-B, HLA-DPA1, HLA-DPB1, HLA-DRA, HNRNPF, IL11RA, IL12RB2, IL17RA, IL19, IL21R, IL34, IRF1, IRF2, JAK3, JARID2, KAT6B, LAPTM5, LCP1, LRCH1, LTA, MED1, NFAT5, NLRC5, NMI, NPR2, OASL, OPRM1, PDCD10, PIK3CD, PSMB8, PSMB9, PSMD9, PTK2B, PTPN1, PTPN2, PTPN6, PTPN7, PTPRC, RFFL, RIPOR2, RORA, RUNX1, SBNO2, SGMS1, SLIT1, SMARCA4, SPOCK2, SQSTM1, SSTR1, STAT1, STAT3, STX8, TANK, TNF, TNFAIP3, TRAF1, TRAF3, UGCG, UPF1, VRK2, ZBP1, ZC3H12A, ZEB1]	[AIM2, AKAP12, ALOX15, BCLAF1, CAMP, CARD8, CAV1, CHUK, FOXH1, GFPT2, GFRAL, GREM2, HGF, HLA-DQB2, HLA-DRA, HLA-DRB1, HLA-G, HYAL2, ITGB2, KCNA5, KDM5B, LCP1, MAP3K5, MMP12, MNDA, MSC, MYNN, NLRC5, NR1H3, PPARGC1A, PRKCA, PRKN, PSMD7, PTPRT, RIPOR2, ROBO1, RUNX1, SOX17, ST18, TNFRSF11A, TRAF2, TRIM68, VEGFC, ZEB1]
GO:0071310	cellular response to organic substance	5,43E-09	[4]	452,00	15,58	32,86	38,91	28,23	[AIF1, AIM2, ANGPT1, ANGPT2, AREG, ATF3, BHLHA15, BMPR1B, BTK, CARD17, CASP8, CCL19, CCL24, CCR2, CCR3, CCR4, CCR5, CCR8, CD180, CD200R1, CD27, CD4, CD40LG, CD6, CD74, CD80, CD86, CHRDL1, CISH, CLCF1, CRB2, CSF1R, CSF2RA, CTSS, CXCL10, CXCL11, CXCL5, CXCL6, CXCL9, CXCR3, CXCR4, CXCR6, CYBB, CYP11B1, DAPK1, DDC, DOCK8, DRD1, EDN2, EGR3, ELAVL4, EREG, ERFE, FASLG, FLT3, FOLR2, FUT7, GALNT3, GBP5, GBP6, GDF6, GFI1, GFPT2, GFRA2, GLDC, GPR150, GRAMD1B, GRAMD1C, H3C10, H3C11, H3C12, H3C7, HCLS1, HCN1, HLA-DPA1, HLA-DPB1, HLA-DQA1, HLA-DRA, HLA-DRB1, HLA-DRB5, HSPA7, IFNG, IKBKE, IL10RA, IL12RB2, IL18, IL18RAP, IL22, IL26, IL2RG, IL5RA, IL7R, IL9R, INPP5D, IRF4, IRF5, ITGA4, KLF5, KLRK1, KMO, LCP1, LEF1, LTA, LTB, LY86, MAPK13, MRC1, MSX1, MYB, MZB1, NAMPT, NELL2, NLRP3, NPFFR2, NR4A1, NR4A2, NR4A3, NSG1, NUGGC, OASL, P2RY12, P2RY13, P2RY2, PARP8, PIK3CG, PIM1, PRKCQ, PTAFR, PTGFR, PTK2B, PTPN22, PTPN6, PTPN7, PTPRC, PYHIN1, RGS10, RHEX, RUFY4, SDC1, SELPLG, SNX10, STAP1, STAT1, SYK, TLR6, TNFAIP3, TNFRSF11B, TNFRSF18, TNFSF13B, TNFSF8, TNIP3, TNMD, TRAF1, TREM2, UGT3A2, VWC2L, WAS, WNT1, WNT10A, WNT10B, XCL1, XCL2, XCR1]	[ACTR3, ACVR1B, ADAMTS12, ADCY1, AGRN, AIM2, AKAP13, AKAP6, AKAP9, AP3S1, ARRB2, ASAH1, ATG7, ATP6V0A2, ATP6V1B2, B2M, BATF, BCAR3, BCL2, BDNF, BIRC3, BMP7, BMPR1A, BPI, CACNA1A, CAMK2G, CAPN2, CASP7, CASP8, CCM2, CCND3, CCR7, CD44, CD47, CD6, CFLAR, CHST11, COL4A2, COLEC12, CORO1A, CPNE3, CX3CL1, CXCL11, DAPK1, DERL1, DNM2, DNMT3A, EDEM1, EEF1A1, EEF2K, ETFA, EVL, F13A1, FES, GAB2, GATD3A, GBP1, GBP5, GFRA2, GHR, GNAQ, GPR68, GRB2, GRIK5, GSK3B, GSN, HDAC1, HDAC9, HLA-A, HLA-B, HLA-DPA1, HLA-DPB1, HLA-DRA, HNRNPF, IGF1R, IL11RA, IL12RB2, IL17RA, IL19, IL21R, IL34, IRF1, IRF2, ITGA8, ITGB5, ITPR2, JAK3, JARID2, KAT6B, KCNQ1, KCTD11, KLB, LAPTM5, LCP1, LGR4, LITAF, LPIN1, LPIN2, LRCH1, LTA, LTBP1, MAP2K5, MED1, NCK2, NCOR2, NEO1, NFAT5, NLRC5, NMI, NPR2, NPTN, NR3C1, NSG2, NTRK2, OASL, OPRM1, PARD3, PDCD10, PDE4B, PDIA6, PDZD2, PIGBOS1, PIGU, PIK3CD, PLK5, POLR2A, PRKACB, PRKAR1B, PRKCE, PRKD2, PSAP, PSMB8, PSMB9, PSMD9, PTK2B, PTPN1, PTPN2, PTPN22, PTPN6, PTPN7, PTPRC, PTPRK, PTPRN2, PXN, RAB13, RALB, RARA, RFFL, RIPOR2, RORA, RPS6KA2, RPTOR, RRAGC, RUNX1, RXRA, SATB2, SBNO2, SGMS1, SLIT1, SMARCA4, SPIDR, SPOCK2, SQSTM1, SSTR1, SSTR4, STAT1, STAT3, STX8, TANK, TCF4, TGFB2, THRB, TMEM108,	[ABCC1, ABCC8, ACAP2, ADCY5, AIM2, AKAP12, AKAP13, AKAP9, ALDH1A2, ALK, ALOX15, AQP8, ARNT, ATP6V0D1, ATP6V1D, BCAR3, BCLAF1, C2CD5, CACNA1S, CAMP, CARD8, CAV1, CHRNA4, CHUK, COL4A2, COL5A2, CPEB1, DCN, DNAJB12, DRD2, EGFR, ERBB4, ESR2, ESRRB, ESRRG, FBN2, FBXO32, FGF12, FGF7, FGF9, FOXH1, FOXP1, GCNT2, GDF6, GFPT2, GFRAL, GNAS, GREM2, HGF, HIPK2, HLA-DQB2, HLA-DRA, HLA-DRB1, HLA-G, HRG, HSPA2, HTR3B, HTR5A, HTR7, HYAL2, IGF1R, ITGB2, KCNA5, KDM5B, LCP1, LDLRAD4, LMO3, LYN, MAGI2, MAP2, MAP3K5, MMP12, MNDA, MSC, MYNN, NCOR1, NGF, NLRC5, NR1H3, NR2F1, ONECUT2, OR5T2, PACRG, PAK1, PAK4, PARP8, PCSK9, PDE3B, PDE4B, PGR, PIAS2, PLK5, PPARGC1A, PPP3CA, PRKAA1, PRKAR2B, PRKCA, PRKCZ, PRKN, PSMD7, PTPN22, PTPRN2, PTPRT, RAB35, RAMP1, RARB, RB1, RIPOR2, RNF14, ROBO1, ROBO2, RORB, RPS6KA2, RUNX1, RXRA, SHOC2, SKI, SMAD1, SMAD6, SOX17, SOX5, SRI, SST, ST18, STMN2, STRN3, TBXT, TESC, TIA1, TIAM1, TNFRSF11A, TRAF2, TRIM68, TSC1, UMODL1, USP8, VEGFC, VSNL1, WFS1, ZEB1]
GO:0010033	response to organic substance	8,72E-08	[3]	526,00	14,92	32,29	38,92	28,80	[AIF1, AIM2, AMPD1, ANGPT1, ANGPT2, AREG, ATF3, BHLHA15, BMPR1B, BTG2, BTK, CALCR, CARD17, CASP8, CCL19, CCL24, CCR2, CCR3, CCR4, CCR5, CCR8, CD180, CD200R1, CD27, CD4, CD40LG, CD6, CD74, CD80, CD86, CD96, CHRDL1, CISH, CLCF1, CNR1, CNR2, CRB2, CSF1R, CSF2RA, CTSS, CXCL10, CXCL11, CXCL5, CXCL6, CXCL9, CXCR3, CXCR4, CXCR6, CYBB, CYP11B1, DAPK1, DDC, DNAJB5, DOCK8, DRD1, EDN2, EGR3, ELAVL4, EREG, ERFE, ERP27, FASLG, FIBIN, FLT3, FOLR2, FOSL1, FUT7, GALNT3, GBP5, GBP6, GDF6, GFI1, GFPT2, GFRA2, GLDC, GPR150, GPR83, GRAMD1B, GRAMD1C, H3C10, H3C11, H3C12, H3C7, HCLS1, HCN1, HLA-DPA1, HLA-DPB1, HLA-DQA1, HLA-DRA, HLA-DRB1, HLA-DRB5, HSPA7, IFNG, IKBKE, IL10RA, IL12RB2, IL18, IL18RAP, IL22, IL26, IL2RG, IL5RA, IL7R, IL9R, INPP5D, IRF4, IRF5, ITGA4, KLF5, KLRG1, KLRK1, KMO, LAMP3, LCP1, LEF1, LPAL2, LTA, LTB, LY86, MAPK13, MRC1, MSX1, MUSK, MYB, MZB1, NAMPT, NELL2, NLRP3, NPFFR2, NPPC, NR4A1, NR4A2, NR4A3, NSG1, NUGGC, OASL, P2RY12, P2RY13, P2RY2, PARP8, PIK3CG, PIM1, PRKCQ, PTAFR, PTGFR, PTK2B, PTPN22, PTPN6, PTPN7, PTPRC, PYHIN1, RGS10, RHEX, RUFY4, RUNX3, SDC1, SELE, SELL, SELPLG, SH2D2A, SNX10, STAP1, STAT1, SYK, TBXAS1, TLR6, TNFAIP3, TNFRSF11B, TNFRSF18, TNFSF13B, TNFSF8, TNIP3, TNMD, TRAF1, TREM2, UBD, UGT3A2, VWC2L, WAS, WNT1, WNT10A, WNT10B, XCL1, XCL2, XCR1]	[ABAT, ACER2, ACTR3, ACVR1B, ADA, ADAM10, ADAMTS12, ADCY1, ADGRL3, AFF3, AGRN, AIM2, AKAP13, AKAP6, AKAP9, AP3S1, ARRB2, ASAH1, ATG7, ATP6V0A2, ATP6V1B2, B2M, BATF, BCAR3, BCL2, BDNF, BIRC3, BMP7, BMPR1A, BPI, CAB39, CACNA1A, CAMK2G, CAPN2, CASP7, CASP8, CCM2, CCND3, CCR7, CD38, CD44, CD47, CD6, CFLAR, CHST11, COL4A2, COLEC12, CORO1A, CPNE3, CX3CL1, CXCL11, DAPK1, DERL1, DNM2, DNMT3A, DPEP1, EDEM1, EEF1A1, EEF2K, ETFA, EVL, F13A1, F2R, FES, GAB2, GATD3A, GBP1, GBP5, GCNT1, GFRA2, GHR, GNAQ, GPR68, GPX4, GRB2, GRIK5, GSK3B, GSN, HDAC1, HDAC9, HLA-A, HLA-B, HLA-DPA1, HLA-DPB1, HLA-DRA, HNRNPF, HOMER1, HOMER2, IDO1, IGF1R, IL11RA, IL12RB2, IL17RA, IL19, IL21R, IL34, INAVA, IRF1, IRF2, ITGA8, ITGB5, ITPR2, JAK3, JARID2, KAT6B, KCNC2, KCNQ1, KCTD11, KLB, LAPTM5, LCP1, LGR4, LITAF, LPIN1, LPIN2, LRCH1, LTA, LTBP1, MAN1B1, MAP2K5, MAP4K5, MEAK7, MED1, MMP14, NCK2, NCOR2, NEO1, NFAT5, NLRC5, NMI, NMNAT3, NPR2, NPTN, NR3C1, NSG2, NTRK2, OASL, OPRM1, PARD3, PCDHA6, PDCD10, PDE4B, PDIA6, PDZD2, PIGBOS1, PIGU, PIK3CD, PLK5, PLSCR1, POLR2A, PRKACB, PRKAR1B, PRKCE, PRKD2, PSAP, PSMB8, PSMB9, PSMD9, PTK2B, PTPN1, PTPN2, PTPN22, PTPN6, PTPN7, PTPRC, PTPRK, PTPRN2, PXN, RAB13, RALB, RARA, RELB, RFFL	[ABCC1, ABCC8, ACAP2, ADCY5, AFF3, AIM2, AKAP12, AKAP13, AKAP9, ALDH1A2, ALK, ALOX15, AQP8, ARNT, ASXL1, ATP6V0D1, ATP6V1D, BCAR3, BCHE, BCLAF1, C2CD5, CACNA1S, CAMP, CARD8, CATSPER2, CAV1, CD9, CDH13, CHRNA4, CHUK, COL4A2, COL5A2, COMT, CPEB1, DCN, DNAJB12, DRD2, EGFR, ERBB4, ESR2, ESRRB, ESRRG, FBN2, FBXO32, FGF12, FGF7, FGF9, FNDC4, FOXH1, FOXP1, GCNT2, GDF6, GFPT2, GFRAL, GNAS, GPR31, GREM2, GRIN2A, HGF, HIPK2, HLA-DQB2, HLA-DRA, HLA-DRB1, HLA-G, HRG, HSPA2, HTR3B, HTR5A, HTR7, HYAL2, IGF1R, ITGB2, KCNA5, KCNMA1, KDM5B, LCP1, LDLRAD4, LMO3, LYN, MAGI2, MAP2, MAP3K5, MEIS2, MMP12, MNDA, MSC, MYNN, NCOR1, NGF, NLRC5, NR1H3, NR2F1, ONECUT2, OR5T2, P2RX2, PACRG, PAK1, PAK4, PARP8, PAX4, PCDHA6, PCDHB16, PCSK9, PDE3B, PDE4B, PGR, PIAS2, PLK5, PPARGC1A, PPP3CA, PRKAA1, PRKAR2B, PRKCA, PRKCZ, PRKN, PSMD7, PTPN22, PTPRN2, PTPRT, RAB35, RAMP1, RARB, RB1, RIPOR2, RNF14, ROBO1, ROBO2, RORB, RPS6KA2, RUNX1, RXRA, SDK1, SHOC2, SKI, SMAD1, SMAD6, SNRPN, SOX17, SOX5, SRI, SST, ST18, STMN2, STRN3, TAF2, TBXT, TESC, TIA1, TIAM1, TLR1, TNFRSF11A, TP73, TRAF2, TRIM63, TRIM68, TSC1, TUB, TYR, UMODL1, UNC79, USP8, VEGFC, VSNL1, WFS1, ZEB1]

GO:0070887	cellular response to chemical stimulus	1,42E-06	[3]	521,00	14,69	31,24	39,44	29,32	[ADAM8, AIF1, AIM2, ANGPT1, ANGPT2, AQP5, AREG, ATF3, B3GAT1, BHLHA15, BIN2, BMPR1B, BTK, CARD17, CASP8, CCL19, CCL24, CCR2, CCR3, CCR4, CCR5, CCR8, CD180, CD200R1, CD27, CD4, CD40LG, CD6, CD69, CD74, CD80, CD86, CHRDL1, CISH, CLCF1, CNR2, CRB2, CSF1R, CSF2RA, CTSS, CXCL10, CXCL11, CXCL5, CXCL6, CXCL9, CXCR3, CXCR4, CXCR6, CYBB, CYP11B1, DAPK1, DDC, DOCK8, DRD1, EDN2, EGR3, ELAVL4, EREG, ERFE, FASLG, FLT3, FOLR2, FUT7, GALNT3, GBP5, GBP6, GDF6, GFI1, GFPT2, GFRA2, GLDC, GPR150, GPSM3, GRAMD1B, GRAMD1C, H3C10, H3C11, H3C12, H3C7, HCLS1, HCN1, HLA-DPA1, HLA-DPB1, HLA-DQA1, HLA-DRA, HLA-DRB1, HLA-DRB5, HSPA7, IFNG, IKBKE, IL10RA, IL12RB2, IL18, IL18RAP, IL22, IL26, IL2RG, IL5RA, IL7R, IL9R, INPP5D, IPCEF1, IRF4, IRF5, ITGA4, JAML, KLF5, KLRK1, KMO, LCP1, LEF1, LTA, LTB, LY86, MAPK13, MRC1, MSX1, MT1HL1, MYB, MZB1, NAMPT, NCF1, NCKAP1L, NELL2, NLRP3, NME8, NPFFR2, NR4A1, NR4A2, NR4A3, NSG1, NUGGC, OASL, P2RY12, P2RY13, P2RY2, PARP8, PIK3CG, PIM1, PLA2G7, PRKCQ, PTAFR, PTGFR, PTK2B, PTPN22, PTPN6, PTPN7, PTPRC, PYHIN1, RGS10, RHEX, RUFY4, SDC1, SELPLG, SLAMF8, SNX10, STAP1, STAT1, SYK, TLR6, TNFAIP3, TNFRSF11B, TNFRSF18, TNFSF13B, TNFSF8, TNIP3, TNMD, TRAF1, TREM2, UGT3A2, VNN1, VWC2L, WAS, WNT1, WNT10A, WNT10B, XCL1, XCL2, XCR1]	[ABCG2, ACAA2, ACER2, ACTR3, ACVR1B, ADAM10, ADAMTS12, ADCY1, ADGRV1, AGRN, AIM2, AKAP13, AKAP6, AKAP9, ANK3, AP3S1, ARRB2, ASAH1, ATG7, ATP6V0A2, ATP6V1B2, B2M, BATF, BCAR3, BCL2, BDNF, BIN2, BIRC3, BMP7, BMPR1A, BPI, CAB39, CACNA1A, CAMK2G, CAPN2, CASP7, CASP8, CCM2, CCND3, CCR7, CD44, CD47, CD6, CD69, CFLAR, CHST11, COL4A2, COLEC12, CORO1A, CPNE3, CPNE7, CX3CL1, CXCL11, DAPK1, DDAH1, DERL1, DIABLO, DNM2, DNMT3A, DPEP1, EDEM1, EEF1A1, EEF2K, ETFA, ETV5, EVL, F13A1, FES, GAB2, GATD3A, GBP1, GBP5, GFRA2, GHR, GNAQ, GPR68, GPSM3, GPX4, GRB2, GRIK5, GSK3B, GSN, GSTO2, HDAC1, HDAC9, HLA-A, HLA-B, HLA-DPA1, HLA-DPB1, HLA-DRA, HNRNPF, IGF1R, IL11RA, IL12RB2, IL17RA, IL19, IL21R, IL34, IPCEF1, IRF1, IRF2, ITGA8, ITGA9, ITGB5, ITPR2, JAK3, JAML, JARID2, KAT6B, KCNC2, KCNH1, KCNQ1, KCTD11, KLB, LAPTM5, LCP1, LGR4, LITAF, LPIN1, LPIN2, LRCH1, LTA, LTBP1, MAP2K5, MEAK7, MED1, MYT1, NCF4, NCK2, NCOR2, NEO1, NFAT5, NLRC5, NMI, NPR2, NPTN, NR3C1, NSG2, NTRK2, OASL, OPRM1, PARD3, PCDHA6, PDCD10, PDE4B, PDIA6, PDZD2, PHLPP2, PIGBOS1, PIGU, PIK3CD, PLK5, POLR2A, PRKACB, PRKAR1B, PRKCE, PRKD2, PRODH, PSAP, PSMB8, PSMB9, PSMD9, PTK2B, PTPN1, PTPN2, PTPN22, PTPN6, PTPN7, PTPRC, PTPRK, PTPRN2, PXN, RAB13, RALB, RARA [illegible]	[ABCC1, ABCC8, ACAP2, ADCY5, AIM2, AKAP12, AKAP13, AKAP9, ALDH1A2, ALK, ALOX15, ANK3, AQP8, ARNT, ATP6V0D1, ATP6V1D, BCAR3, BCHE, BCLAF1, C2CD5, CACNA1S, CAMP, CARD8, CAV1, CHRNA4, CHUK, COL4A2, COL5A2, CPEB1, CPNE4, CXADR, DCN, DLG2, DNAJB12, DRD2, DYSF, EGFR, ERBB4, ESR2, ESRRB, ESRRG, FBN2, FBXO32, FGF12, FGF7, FGF9, FMN2, FOXH1, FOXP1, GCNT2, GDF6, GFPT2, GFRAL, GNAS, GREM2, HGF, HIPK2, HLA-DQB2, HLA-DRA, HLA-DRB1, HLA-G, HRG, HSPA2, HTR3B, HTR5A, HTR7, HYAL2, IGF1R, ITGB2, KCNA5, KCND2, KCNK2, KCNK3, KDM5B, LCP1, LDLRAD4, LIMD1, LMO3, LYN, MAGI2, MAP2, MAP3K5, MAPRE2, MEF2A, MMP12, MNDA, MSC, MTUS1, MYLK, MYNN, NCOR1, NGF, NLRC5, NR1H3, NR2F1, NXN, ONECUT2, OR5T2, P2RX2, PACRG, PAK1, PAK4, PARP8, PCDHA6, PCSK9, PDE3B, PDE4B, PGR, PIAS2, PLK5, PPARGC1A, PPP3CA, PRDX1, PRKAA1, PRKAR2B, PRKCA, PRKCZ, PRKN, PSMD7, PTPN22, PTPRN2, PTPRT, RAB35, RAMP1, RARB, RB1, RIPOR2, RNF14, ROBO1, ROBO2, ROBO3, RORB, RPS6KA2, RUNX1, RXRA, SEMA5A, SHOC2, SKI, SLC38A1, SLC6A2, SMAD1, SMAD6, SOX17, SOX5, SRI, SRXN1, SST, ST18, STK25, STMN2, STRN3, SWAP70, SYT17, TBXT, TESC, TIA1, TIAM1, TNFRSF11A, TRAF2, TRIM68, TSC1, UMODL1, USP8, VEGFC, VSNL1, WFS1, WNT16, ZEB1]
GO:0019221	cytokine-mediated signaling pathway	7,38E-06	[4, 5, 6]	154,00	18,51	47,19	37,64	15,17	[AIM2, ANGPT1, CASP8, CCL19, CCL24, CCR2, CCR3, CCR4, CCR5, CCR8, CD27, CD4, CD40LG, CD74, CD80, CD86, CISH, CLCF1, CSF1R, CSF2RA, CXCL10, CXCL11, CXCL5, CXCL6, CXCL9, CXCR3, CXCR4, CXCR6, EDN2, EREG, FASLG, FLT3, GFI1, GFRA2, H3C10, H3C11, H3C12, H3C7, HLA-DPA1, HLA-DPB1, HLA-DQA1, HLA-DRA, HLA-DRB1, HLA-DRB5, IFNG, IKBKE, IL10RA, IL12RB2, IL18, IL18RAP, IL22, IL26, IL2RG, IL5RA, IL7R, IL9R, INPP5D, IRF4, IRF5, LCP1, LTA, LTB, NR4A3, OASL, PIM1, PTAFR, PTK2B, PTPN6, PTPRC, RHEX, SDC1, STAP1, STAT1, SYK, TNFAIP3, TNFRSF11B, TNFRSF18, TNFSF13B, TNFSF8, TRAF1, TREM2, XCL1, XCL2, XCR1]	[AIM2, B2M, BATF, BCL2, BIRC3, CAMK2G, CASP8, CCM2, CCR7, CD44, CX3CL1, CXCL11, ETFA, F13A1, GAB2, GBP1, GFRA2, GHR, GRB2, HLA-A, HLA-B, HLA-DPA1, HLA-DPB1, HLA-DRA, HNRNPF, IL11RA, IL12RB2, IL17RA, IL19, IL21R, IL34, IRF1, IRF2, JAK3, LCP1, LTA, MED1, NLRC5, NMI, OASL, OPRM1, PIK3CD, PSMB8, PSMB9, PSMD9, PTK2B, PTPN1, PTPN2, PTPN6, PTPRC, RFFL, RORA, RUNX1, SLIT1, SMARCA4, SQSTM1, STAT1, STAT3, TANK, TNF, TNFAIP3, TRAF1, TRAF3, UGCG, VRK2, ZBP1, ZEB1]	[AIM2, ALOX15, CARD8, CAV1, CHUK, GFRAL, GREM2, HGF, HLA-DQB2, HLA-DRA, HLA-DRB1, HLA-G, ITGB2, LCP1, MMP12, NLRC5, NR1H3, PRKCA, PRKN, PSMD7, ROBO1, RUNX1, ST18, TNFRSF11A, TRAF2, TRIM68, ZEB1]
GO:1901700	response to oxygen-containing compound	3,17E-03	[3]	278,00	15,15	31,56	39,20	29,24	[AIF1, ANGPT2, AREG, BTG2, BTK, CALCR, CARD17, CASP8, CCL19, CCR5, CD180, CD27, CD4, CD6, CD80, CD86, CD96, CNR1, CNR2, CXCL10, CXCL11, CXCL5, CXCL6, CXCL9, CYBB, CYP11B1, DAPK1, DRD1, ELAVL4, EREG, ERFE, FASLG, FIBIN, FOLR2, FOSL1, FUT7, GFI1, GLDC, GRAMD1B, GRAMD1C, HCN1, HLA-DRB1, IL10RA, IL12RB2, IL18, IL18RAP, IRF5, ITGA4, KLF5, KLRG1, KLRK1, KMO, LCP1, LPAL2, LTA, LY86, MAPK13, MRC1, MUSK, MYB, MZB1, NAMPT, NCF1, NLRP3, NME8, NPPC, NR4A1, NR4A2, NR4A3, NSG1, NUGGC, P2RY12, P2RY2, PIK3CG, PIM1, PRKCQ, PTAFR, PTGFR, PTK2B, PTPN22, RGS10, SDC1, SELE, SELL, SH2D2A, STAP1, STAT1, TBXAS1, TLR6, TNFAIP3, TNIP3, TREM2, UGT3A2, WNT1, WNT10B]	[ABAT, ACER2, ADA, ADCY1, ADGRL3, AGRN, AKAP6, AKAP9, AP3S1, ARRB2, ATG7, ATP6V0A2, ATP6V1B2, BCAR3, BCL2, BMP7, BPI, CACNA1A, CAPN2, CASP8, CCND3, CCR7, CD38, CD6, CFLAR, COLEC12, CX3CL1, CXCL11, DAPK1, DNM2, DNMT3A, DPEP1, EDEM1, EEF2K, F2R, FES, GCNT1, GHR, GNAQ, GPR68, GPX4, GRB2, GRIK5, GSK3B, GSN, HDAC9, HOMER1, HOMER2, IDO1, IGF1R, IL12RB2, INAVA, ITPR2, JAK3, KCNC2, KCNQ1, KCTD11, LCP1, LITAF, LPIN1, LPIN2, LTA, MAN1B1, MAP4K5, MEAK7, MED1, NCOR2, NR3C1, NSG2, NTRK2, OPRM1, PCDHA6, PDCD10, PDE4B, PIGU, PRKACB, PRKAR1B, PRKCE, PRKD2, PSAP, PTK2B, PTPN1, PTPN2, PTPN22, PTPRK, PTPRN2, PXN, RAB13, RARA, RORA, RPS6KA2, RPTOR, RRAGC, RXRA, SBNO2, SCAP, SGMS1, SLC1A2, SLIT1, SMARCA4, SPIDR, SSTR1, STAT1, STAT3, TANK, TGFB2, TNF, TNFAIP3, TNFSF10, TNS2, TSHR, UBE3A, UPF1, XRN1, YAP1, ZBTB20, ZC3H12A, ZEB1]	[ABCC1, ABCC8, ADCY5, AKAP12, AKAP9, ALDH1A2, ALK, AQP8, ASXL1, ATP6V0D1, ATP6V1D, BCAR3, BCHE, C2CD5, CAMP, CARD8, CATSPER2, CAV1, CD9, CHRNA4, CHUK, COL5A2, COMT, CPEB1, DCN, DRD2, EGFR, FBXO32, FOXP1, GNAS, GRIN2A, HGF, HLA-DRB1, HTR3B, HTR5A, HTR7, HYAL2, IGF1R, KCNA5, KCNMA1, KPNA4, LCP1, LYN, MAP3K5, MMP12, NR1H3, OR5T2, P2RX2, PAK1, PAX4, PCDHA6, PCDHB16, PCSK9, PDE3B, PDE4B, PPARGC1A, PPP3CA, PRDX1, PRKAA1, PRKAR2B, PRKCA, PRKCZ, PRKN, PTPN22, PTPRN2, RARB, RORB, RPS6KA2, RXRA, SDK1, SLC6A2, SMAD6, SRI, SST, STK25, STRN3, TBXT, TESC, TIAM1, TNFRSF11A, TRAF2, TSC1, TYR, UMODL1, UNC79, USP8, VSNL1, ZEB1]
GO:0032673	regulation of interleukin-4 production	2,37E-03	[4, 5, 6, 7]	14,00	48,28	73,33	13,33	13,33	[CD3E, CD40LG, CD86, FOXP3, HLA-DRB1, IRF4, LEF1, NLRP3, PRKCQ, SASH3, SYK]	[CD28, RARA]	[HLA-DRB1, PRKCZ]
GO:0032753	positive regulation of interleukin-4 production	4,90E-03	[4, 5, 6, 7, 8]	12,00	52,17	75,00	16,67	8,33	[CD3E, CD40LG, CD86, FOXP3, IRF4, NLRP3, PRKCQ, SASH3, SYK]	[CD28, RARA]	[PRKCZ]
GO:2000516	positive regulation of CD4-positive, alpha-beta T cell activation	6,37E-03	[7, 8, 9, 10]	15,00	42,86	80,00	13,33	6,67	[CCL19, CD80, CD86, FOXP3, IFNG, IL18, MYB, NCKAP1L, NLRP3, PRKCQ, SASH3, XCL1]	[CD160, RARA]	[PRKCZ]
GO:0045076	regulation of interleukin-2 biosynthetic process	6,59E-03	[5, 6, 7, 8]	11,00	55,00	76,92	15,38	7,69	[CARD11, CD3E, CD4, CD80, CD86, FOXP3, IRF4, LAG3, PRKCQ, PTPRC]	[CD28, PTPRC]	[CARD11]
GO:0032633	interleukin-4 production	1,44E-02	[3, 6]	15,00	40,54	75,00	12,50	12,50	[CD3E, CD40LG, CD86, FOXP3, HLA-DRB1, IRF4, ITK, LEF1, NLRP3, PRKCQ, SASH3, SYK]	[CD28, RARA]	[HLA-DRB1, PRKCZ]
GO:0050854	regulation of antigen receptor-mediated signaling pathway	1,45E-04	[4, 5, 6, 7, 8, 9, 10]	25,00	36,76	36,36	45,45	18,18	[BLK, CARD11, FCRL3, LCK, PAX5, PTPN22, PTPN6, PTPRC, STAP1, TESPA1, THY1, TRAT1]	[ADA, CCR7, CD160, GBP1, LCK, LPXN, PRKD2, PTPN2, PTPN22, PTPN6, PTPRC, PTPRJ, RUNX1, SLA2, TESPA1]	[BTNL2, CARD11, FOXP1, LYN, PTPN22, RUNX1]
GO:0050856	regulation of T cell receptor signaling pathway	3,60E-02	[5, 6, 7, 8, 9, 10, 11]	16,00	36,36	31,82	54,55	13,64	[CARD11, LCK, PTPN22, PTPN6, TESPA1, THY1, TRAT1]	[ADA, CCR7, CD160, GBP1, LCK, PRKD2, PTPN2, PTPN22, PTPN6, PTPRJ, SLA2, TESPA1]	[BTNL2, CARD11, PTPN22]
GO:0051093	negative regulation of developmental process	2,57E-02	[2, 3, 4]	169,00	15,99	27,37	37,99	34,64	[ADAMTS9, ANGPT2, AREG, ASCL2, BHLHA15, C1QC, CCR2, CD74, CDKN2A, CTLA4, CXCL10, EAF2, EREG, FASLG, FGL2, FOXP3, FRZB, GFI1, GLIS1, GPR171, GPR55, HOPX, IFNG, IL18, INPP5D, LAG3, LEF1, LRRC17, MIXL1, MSX1, NAMPT, NPPB, NPPC, P2RY12, PLAC8, PTK2B, RUNX3, SEMA4A, SEMA4D, SLC18A1, STAP1, STAT1, TACSTD2, THY1, TNFRSF11B, TNMD, TNR, WNT1, WNT10B]	[ADAMTS12, ADGRV1, B2M, BCL2, BDNF, BMP7, BMPR1A, CD160, CD38, CDK6, CFLAR, COL4A2, COL5A1, CORO1C, CRMP1, CTLA4, CTSZ, CX3CL1, DIXDC1, DPEP1, FBXO5, GAK, GATD3A, GBP1, GPR68, GSK3B, HDAC9, IRF1, JAK3, JARID2, MAP2K5, MED1, NFATC1, NFATC2, NFIB, NPR2, PDCD10, PHF14, PRICKLE1, PRKACB, PTK2B, PTPN2, PTPRM, RARA, RORA, RUFY3, RUNX1, RUNX3, SEMA4D, SLIT1, SRGAP3, STAT1, STAT3, TCF4, TERT, TGFB2, THRB, TNF, TRIM46, TRIO, UBASH3B, UBE3A, YAP1, ZBTB16, ZC3H12A, ZEB1, ZHX2, ZNF365]	[ABCC8, ASXL1, C11orf65, CARTPT, CAV1, CDK13, CDSN, COL4A2, COL5A2, CXADR, DCC, DCN, DISP3, DNM3, DRAXIN, EGFR, ERBB4, ESRRB, FGF9, GNAS, GPR161, HLA-G, HRG, JDP2, KCNK2, LDLRAD4, LIMD1, LINGO1, LYN, MAP2, MEIS2, NFATC1, NR1H3, NR2F1, PAK1, PDE3B, PLAC8, PPARGC1A, PPP3CA, PRKCA, PRKN, RARB, RB1, RFX4, ROBO1, ROBO2, RORB, RUNX1, SAV1, SEMA5A, SKI, SMAD6, SOX17, SRGN, STARD13, STMN2, TBX3, THBS2, TP73, TSC1, VEGFC, ZEB1]
GO:0031295	T cell costimulation	5,37E-03	[3, 6, 7, 8, 9]	21,00	34,43	56,00	28,00	16,00	[BTLA, CARD11, CCL19, CD3E, CD40LG, CD5, CD80, CD86, CTLA4, ICOS, KLRK1, LCK, PTPN6, TNFSF13B]	[CCR7, CD160, CD28, CTLA4, GRB2, LCK, PTPN6]	[CARD11, CAV1, LYN, PAK1]
GO:0060333	interferon-gamma-mediated signaling pathway	6,96E-03	[5, 6, 7, 8, 9]	28,00	28,87	33,33	47,22	19,44	[HLA-DPA1, HLA-DPB1, HLA-DQA1, HLA-DRA, HLA-DRB1, HLA-DRB5, IFNG, IRF4, IRF5, OASL, PTAFR, STAT1]	[B2M, CAMK2G, CD44, GBP1, HLA-A, HLA-B, HLA-DPA1, HLA-DPB1, HLA-DRA, IRF1, IRF2, MED1, NLRC5, NMI, OASL, PTPN2, STAT1]	[HLA-DQB2, HLA-DRA, HLA-DRB1, HLA-G, NLRC5, NR1H3, TRIM68]
GO:0014068	positive regulation of phosphatidylinositol 3-kinase signaling	4,65E-02	[5, 6, 7, 8, 9, 10]	27,00	27,00	33,33	46,67	20,00	[ANGPT1, FGR, FLT3, HCLS1, IL18, MUSK, NCF1, PIK3CG, PTPN6, SEMA4D]	[AGAP2, CCM2, CD28, F2R, IGF1R, NTRK2, PIGU, PIK3CD, PTPN6, RORA, SEMA4D, TGFB2, TNF, UBE3A]	[DCN, ERBB4, HGF, IGF1R, LYN, WNT16]
GO:0036037	CD8-positive, alpha-beta T cell activation	3,83E-02	[6, 7]	12,00	44,44	47,06	35,29	17,65	[CRTAM, EOMES, NCKAP1L, PTPN22, RUNX3, TNFSF8, TOX, XCL1]	[BCL2, HLA-A, IRF1, PTPN22, RUNX1, RUNX3]	[PTPN22, RUNX1, TOX]
GO:0072676	lymphocyte migration	1,19E-02	[3, 5, 6]	32,00	26,45	63,89	27,78	8,33	[ADAM8, AIF1, CCL19, CCL24, CCR2, CD200R1, CRTAM, CXCL10, CXCL11, CXCR3, DOCK8, FUT7, ITGA4, ITGB7, KLF5, KLRK1, MYO1G, PIK3CG, PTK2B, XCL1, XCL2, XG, ZAP70]	[ADAM10, CCR7, CX3CL1, CXCL11, LRCH1, MYO1G, PIK3CD, PTK2B, RIPOR2, STK10]	[GPR15, RIPOR2, WASL]
GO:0032609	interferon-gamma production	1,19E-02	[3, 6]	32,00	26,45	60,00	32,50	7,50	[BTN3A1, BTN3A2, CCR2, CD2, CD244, CD3E, CD96, CRTAM, EOMES, FOXP3, HLA-DPA1, HLA-DPB1, HLA-DRB1, IL12RB2, IL18, IL18RAP, ITK, KLRK1, LTA, PTPN22, SASH3, SLAMF6, TLR8, XCL1]	[CCR7, CD160, CD47, HLA-A, HLA-DPA1, HLA-DPB1, IL12RB2, LTA, PDE4B, PTPN22, RARA, TNF, ZC3H12A]	[HLA-DRB1, PDE4B, PTPN22]
GO:0045066	regulatory T cell differentiation	3,41E-02	[6, 7, 8, 9, 10, 11]	14,00	40,00	47,06	35,29	17,65	[CTLA4, FOXP3, FUT7, IFNG, LAG3, PLA2G2D, TNFRSF18, TOX]	[CD28, CTLA4, FANCA, IRF1, NFATC2, RUNX1]	[HLA-G, RUNX1, TOX]
GO:0002396	MHC protein complex assembly	3,12E-02	[7]	6,00	85,71	44,44	22,22	33,33	[HLA-DMA, HLA-DMB, HLA-DRA, HLA-DRB1]	[HLA-DRA, TAPBPL]	[HLA-DRA, HLA-DRB1, TAPBP]
GO:0006968	cellular defense response	1,59E-02	[4]	20,00	33,33	72,73	13,64	13,64	[CCR2, CCR3, CCR5, CXCL9, FCMR, FOSL1, GNLY, ITK, KLRC2, KLRC3, KLRG1, NCF1, PRF1, PTK2B, SH2D1A, TRAT1]	[LSP1, PRF1, PTK2B]	[HLA-G, KIR3DL2, MNDA]
GO:1990869	cellular response to chemokine	2,14E-02	[6]	28,00	28,00	67,74	25,81	6,45	[CCL19, CCL24, CCR2, CCR3, CCR4, CCR5, CCR8, CXCL10, CXCL11, CXCL5, CXCL6, CXCL9, CXCR3, CXCR4, CXCR6, DOCK8, PTK2B, TREM2, XCL1, XCL2, XCR1]	[CCR7, CX3CL1, CXCL11, LRCH1, PTK2B, RIPOR2, SLIT1, ZC3H12A]	[RIPOR2, ROBO1]
GO:0004896	cytokine receptor activity	2,14E-02	[5, 6, 7]	28,00	28,00	70,00	26,67	3,33	[CCR2, CCR3, CCR4, CCR5, CCR8, CD4, CD74, CSF2RA, CXCR3, CXCR4, CXCR6, FLT3, GFRA2, IL10RA, IL12RB2, IL18RAP, IL2RG, IL5RA, IL7R, IL9R, XCR1]	[CCR7, CD44, GFRA2, GHR, IL11RA, IL12RB2, IL17RA, IL21R]	[GFRAL]
GO:0032675	regulation of interleukin-6 production	2,30E-02	[4, 5, 6, 7]	37,00	24,18	43,18	40,91	15,91	[AIF1, BANK1, CD200R1, CD74, EREG, FOXP3, IFNG, INPP5D, LCP1, MAPK13, NCKAP1L, PTAFR, PTPN22, PTPN6, SYK, TLR6, TLR8, TNFAIP3, TREM2]	[ARRB2, BPI, CAPN2, CD47, CX3CL1, F2R, HLA-B, INAVA, LCP1, MBP, PTPN22, PTPN6, SLAMF1, STAT3, TNF, TNFAIP3, ZBTB20, ZC3H12A]	[HGF, HYAL2, LCP1, MBL2, MBP, PTPN22, TLR1]
GO:0032635	interleukin-6 production	2,91E-02	[3, 6]	39,00	23,49	45,65	39,13	15,22	[AIF1, BANK1, CD200R1, CD74, EREG, FOXP3, IFNG, IL18, IL18RAP, INPP5D, LCP1, MAPK13, NCKAP1L, PTAFR, PTPN22, PTPN6, SYK, TLR6, TLR8, TNFAIP3, TREM2]	[ARRB2, BPI, CAPN2, CD47, CX3CL1, F2R, HLA-B, INAVA, LCP1, MBP, PTPN22, PTPN6, SLAMF1, STAT3, TNF, TNFAIP3, ZBTB20, ZC3H12A]	[HGF, HYAL2, LCP1, MBL2, MBP, PTPN22, TLR1]

GO:0051239	regulation of multicellular organismal process	9,06E-16	[2, 3]	553,00	16,21	29,67	38,20	32,13	[ADAM8, ADAMTS9, ADORA3, AIF1, AIM2, ANGPT1, ANGPT2, AREG, ASCL2, BANK1, BHLHE40, BMPR1B, BTK, BTN3A1, BTN3A2, C1QC, CALCR, CAMK4, CARD11, CARD17, CASP8, CCDC88B, CCL19, CCL24, CCR2, CCR3, CD2, CD200R1, CD244, CD27, CD3E, CD4, CD40LG, CD6, CD74, CD80, CD84, CD86, CD96, CDKN2A, CEL, CLCF1, CLEC4E, CLEC6A, CLNK, CNR1, CRABP2, CRB2, CRTAM, CSF1R, CTLA4, CXCL10, CXCL6, CXCR4, CYBB, EAF2, EDN2, EGR3, ELAVL4, EREG, FASLG, FCN1, FCRL3, FFAR4, FGL2, FGR, FOXP3, FRZB, GBP5, GDF6, GFI1, GPR171, GPR55, GPSM3, H3C10, H3C11, H3C12, H3C7, HCLS1, HLA-DOA, HLA-DPA1, HLA-DPB1, HLA-DRB1, IFNG, IKBKE, IL12RB2, IL18, IL26, IL5RA, IL7R, INPP5D, IRF4, IRF5, KLHL41, KLRK1, KNG1, LAG3, LCP1, LEF1, LMCD1, LMO1, LPAL2, LRRC17, LTA, LTB, LY9, MAPK13, MCOLN2, MIXL1, MOG, MSX1, MUSK, MYB, MYCL, NCKAP1L, NELL2, NLRP3, NLRP6, NPPB, NPPC, NPTX2, NR4A2, NR4A3, ORM2, P2RY12, P2RY2, PIK3CG, PIM1, PLAAT4, PLAC8, PLAU, PLEK, PLXNC1, PRDM1, PRKCQ, PROK1, PTAFR, PTK2B, PTPN22, PTPN6, PTPRC, PYHIN1, RHEX, RHOH, RLN2, RUNX3, SASH3, SEMA4A, SEMA4D, SLAMF6, SLAMF8, STAP1, STAT1, SYK, TACSTD2, TBXAS1, TESPA1, THY1, TIFAB, TIGIT, TLR10, TLR6, TLR8, TNFAIP3, TNFRSF11B, TNFRSF18, TNMD, TNR, TOX, TREM2, TSPAN8, UCP2, VNN1, VWC2L, WNT1, WNT10A, WNT10B, XCL1, ZAP70, ZNF683, ZNF804A]	[ABAT, ACVR1B, ADA, ADAM10, ADAMTS12, ADGRL3, ADGRV1, ADORA3, AFDN, AGAP2, AGO2, AGRN, AIM2, AKAP6, AKAP9, ARRB2, ATOH8, B2M, BASP1, BCL2, BDNF, BIRC3, BMP7, BMPR1A, BPI, BTN3A3, C21orf91, CACNA1A, CACNB2, CAMK2G, CAPN2, CASP8, CCDC88B, CCM2, CCR7, CD101, CD160, CD28, CD2AP, CD38, CD47, CD6, CDH2, CDK5RAP1, CDK6, CELF1, CELF2, CFLAR, CHSY1, COL4A2, COL5A1, CORO1C, CRMP1, CTLA4, CTSZ, CUX1, CX3CL1, DDAH1, DIXDC1, DNM2, DOCK1, DPEP1, ECE1, EEF2K, EHMT1, ETV5, EVL, F2R, FANCA, FBXO5, FES, GAK, GATD3A, GBP1, GBP5, GBX2, GHR, GLIPR2, GPR68, GPRC5B, GPSM3, GSK3B, HDAC1, HDAC9, HECW2, HLA-A, HLA-B, HLA-DPA1, HLA-DPB1, HOMER1, HOMER2, HOXB3, HOXB7, HSPB6, IDO1, IGF1R, IL12RB2, IL17RA, IL34, INAVA, IQSEC1, IRF1, ITGB6, ITPR1, ITPR2, JAK3, JARID2, KCNA2, KCNIP1, KCNIP4, KCNQ1, KCTD11, KDM2B, KIF13B, LCP1, LGR4, LITAF, LPIN1, LRRFIP1, LRRN3, LTA, LTBP1, MAFG, MAP2K5, MAP6, MARK2, MAST2, MBP, MCC, MED1, MGLL, MITF, MMP14, MTG1, MTNR1B, NECTIN1, NETO1, NFATC1, NFATC2, NFIB, NLRC5, NMI, NOS1AP, NPR2, NPTN, NTRK2, OLFM1, OPRM1, PARD3, PDCD10, PDE4B, PDE9A, PDZD2, PHACTR1, PHF14, PIGU, PLAAT4, PLCE1, PLK5, PLXNC1, PPP1CC, PRICKLE1, PRKACB, PRKCE, PRKD2, PROM1, PSMB8, PSMB9, PSMD9, PTK2B, PTPN2	[ABCC8, ADORA1, ADRA1B, ADRB3, AGO2, AIM2, AKAP12, AKAP9, ALK, ARHGAP44, ARNT, ASIC2, ATP1B1, ATP2A3, ATP2B2, BCL11B, BTNL2, CACNA1S, CACNA2D3, CACNG2, CAMK4, CAMP, CARD11, CARD8, CARTPT, CAV1, CD9, CDH4, CDSN, CELF4, CELSR3, CHODL, CHSY1, CHUK, CLSTN2, COBL, COL4A2, COL5A2, COMT, CSMD3, CTTN, CXADR, DCC, DCN, DISP3, DMPK, DNM3, DOCK1, DRAXIN, DRD2, EGFR, ERBB4, ESRRB, ESRRG, FBN2, FBXO32, FGF12, FGF7, FGF9, FOXP1, FTO, GCNT2, GDF6, GNAS, GPR161, HGF, HIPK2, HLA-DRB1, HLA-G, HRG, HTATIP2, HYAL2, IGF1R, IGSF9B, ITGB2, ITPR1, JADE2, KCNA5, KCNB2, KCND2, KCNG2, KCNJ3, KCNK15, KCNK2, KCNK3, KCNMA1, KCNMB2, KDM5B, LCP1, LDLRAD4, LILRA4, LIMD1, LINGO1, LRRC7, LRRFIP1, LYN, MAGI1, MAGI2, MAP2, MAP6, MAPRE2, MBL2, MBP, MC3R, MCC, MEF2A, MEIS2, METRN, MMP12, MNDA, MYADM, MYF5, NAV3, NDRG4, NFATC1, NGF, NLRC5, NLRP4, NOS1AP, NR1H3, NR2F1, NUMB, OPALIN, PAK1, PAK4, PCP4, PCSK9, PDE3B, PDE4B, PHACTR1, PLAC8, PLCE1, PLD3, PLK5, PLPP3, PPARGC1A, PPP3CA, PRKAA1, PRKCA, PRKCZ, PRKN, PSMD7, PTPN22, PUS7, RARB, RB1, RBFOX1, RFX4, RIMS1, RNH1, ROBO1, ROBO2, RORB, RTN4IP1, RUNX1, RXRA, SAV1, SCN3B, SDK1, SEMA5A, SHANK1, SHISA6, SIGLEC15, SKI, SLITRK6, SMAD1, SMAD6, SMAP1, SOX17, SOX5, SRGN, SRI, STARD13, STIM1, STK25, STMN2, SYT17, TBX3, TESC, THBS2, TIA1, TIAM1, TIAM2, TLR1, TNFRSF11A, TOX, TP73, TRAF2, TRIM63, TSC1, TYMP, UMODL1, UNC5D, VEGFC, WNK4, ZEB1]
GO:0048731	system development	2,12E-12	[3, 4]	762,00	14,72	25,24	37,44	37,32	[ADAM8, ADAMTS4, ADAMTS9, ADGRG6, AIF1, AKNA, ANGPT1, ANGPT2, AQP5, AREG, ASCL2, ATF3, BFSP2, BHLHE22, BHLHE40, BICDL1, BLNK, BMPR1B, BTG2, BTK, C1QA, C1QB, C1QC, CALCR, CAMK4, CARD11, CASP8, CCL19, CCL24, CCR2, CCR3, CCR4, CD2, CD27, CD3D, CD3E, CD3G, CD4, CD40LG, CD74, CD80, CD86, CD8A, CDKN2A, CEL, CHRDL1, CHRDL2, CLCF1, CLEC1B, CLEC4D, CLEC4E, CNR1, COL9A2, CR2, CRABP2, CRB2, CRTAM, CSF1R, CTLA4, CXCL10, CXCR3, CXCR4, CYBB, DOCK10, DOCK2, DOK2, DRC1, DRD1, E2F8, EAF2, EDN2, EGR3, ELAVL4, ELF3, EMB, EOMES, EPHA6, EREG, FASLG, FCRL3, FGL2, FGR, FLT3, FOLR2, FOSL1, FOXP3, FRZB, FUT7, GDF6, GFI1, GFRA2, GPM6A, GPR171, GPR55, GRXCR2, H3C10, H3C11, H3C12, H3C7, HCLS1, HCN1, HLA-DOA, HLA-DRB1, ICOS, IFNG, IKZF1, IL18, IL7R, INPP5D, IRF4, ITGA4, ITK, JPH1, KCNJ10, KLF5, KLHL41, KRT85, LAG3, LCK, LCP1, LEF1, LIPM, LMO1, LPAL2, LRRC17, LST1, LTA, LTB, LY9, MCOLN3, MIXL1, MOG, MS4A1, MSX1, MUSK, MYB, MYCL, MYO3B, NCKAP1L, NELL2, NLRP3, NPPB, NPPC, NR4A1, NR4A2, NR4A3, OPN5, P2RY12, PAX5, PCDHA2, PIK3CG, PIM1, PLA2G2D, PLAAT4, PLEK, PLPPR4, PLXNC1, PRDM1, PRKCQ, PROK1, PTK2B, PTPN22, PTPN6, PTPRC, PTX3, RHEX, RHOH, RLN2, RNASE6, RTKN2, RUNX3, SASH3, SDC1, SELPLG, SEMA4A, SEMA4D, SH2D2A, SLAMF6, SLAMF8, SNX10, SPEF2, ST14, STAP1, STAT1, SYK, TACSTD2, TESPA1, TEX11, THEMIS, THY1, TIFAB, TNFAIP3, TNFRSF11B, TNFRSF18, TNFSF8, TNMD, TNR, TOX, TREM2, UBD, UGT8, VNN1, VWC2L, WNT1, WNT10A, WNT10B, ZAP70, ZNF683, ZNF804A]	[ABAT, ABT1, ACVR1B, ADA, ADAM10, ADAMTS12, ADCY1, ADGRL3, ADGRV1, AFDN, AGAP2, AGO2, AGRN, AKAP13, AKAP6, AKNA, ANGPTL2, ANK3, ANKRD11, ANKS1A, ARHGAP26, ARRB2, ASAH1, ATOH8, ATXN1, AUTS2, B2M, BASP1, BATF, BATF3, BCAR3, BCL2, BCL9, BDNF, BICDL1, BMP7, BMPR1A, C21orf91, CACNA1A, CAMK2G, CASP7, CASP8, CAVIN4, CBLN4, CCM2, CCR7, CD101, CD160, CD28, CD38, CD3G, CD44, CDH17, CDH2, CDK5RAP1, CDK6, CFLAR, CHRNA1, CHST11, CHSY1, CIAO3, CLEC14A, CLMP, CMTM7, COL15A1, COL19A1, COL4A2, COL5A1, COLEC12, CORO1C, CPLANE1, CRMP1, CSGALNACT1, CTLA4, CTSZ, CUX1, CX3CL1, DBX1, DDAH1, DIXDC1, DNASE1L2, DNM2, DNMT3A, DOCK1, DOK2, DPEP1, DYM, ECE1, EEF2K, ELK3, ENPEP, EPHA10, ETV5, ETV6, EVL, EXPH5, EXT1, FANCA, FARP2, FBXO5, FES, FEZ2, FOXK1, FRY, GAB2, GABRA5, GAK, GATD3A, GBX2, GCNT1, GFRA2, GHR, GLIPR2, GPD2, GPR68, GPRC5B, GRB2, GSK3B, GSN, HDAC1, HDAC9, HDGFL3, HECA, HECW2, HERC1, HIVEP3, HLA-B, HOMER1, HOXA3, HOXA4, HOXB1, HOXB3, HOXB7, HSD11B1, HSPB6, IDO1, IL34, IQSEC1, IRF1, IRX1, IRX2, ITGA8, JAK3, JARID2, KCNA2, KCNAB2, KCNC2, KCNQ1, KCTD11, KDM2B, KIAA1217, KIF13B, KLHL1, LCK, LCP1, LGR4, LPCAT1, LPIN1, LRRN3, LSAMP, LTA, LTBP1, MACROD2, MAP2K5, MAP6, MARK2, MBP, MCPH1	[ABCA12, ABCC8, ACER3, ADAMTS2, ADORA1, AGO2, AGPAT5, AK8, AKAP13, ALDH1A2, ALK, ALOX15, ANK3, ANKS1A, ANLN, ARHGAP24, ARHGAP26, ARHGAP44, ARHGEF10, ARNT, ASIC2, ASXL1, ATP2B2, ATP6V0D1, ATXN1, BCAR3, BCAS1, BCHE, BCL11B, BFSP1, BOC, CA10, CAMK4, CAMP, CARD11, CARTPT, CASP5, CAV1, CD8A, CD9, CDH13, CDH22, CDH4, CDK13, CDK5R2, CDSN, CELF4, CELSR3, CERS3, CHODL, CHST8, CHSY1, CLN8, CLSTN2, CMTM7, CNTNAP2, COBL, COL4A2, COL5A2, CPLX2, CRB1, CRTAC1, CSMD3, CTTN, CXADR, CYP4A11, DCC, DCN, DISP3, DNER, DNM3, DOCK1, DPF1, DRAXIN, DRD2, DSCAML1, DSG3, DYSF, EGFR, ENPEP, ERBB4, ESRRB, EVPL, EXOC4, FAT1, FBN2, FGF12, FGF14, FGF7, FGF9, FMN1, FOXH1, FOXP1, FTO, GABRA5, GBA2, GCNT2, GDF6, GFRAL, GNAS, GNAT1, GNB4, GPM6A, GPR161, GREB1L, GREM2, GRIN2A, HGF, HID1, HIPK2, HLA-DQB2, HLA-DRB1, HLA-G, HOXC9, HRG, HTATIP2, HTR5A, HYAL2, IGSF21, IGSF9B, IRX2, ITGB2, JADE2, KCNA5, KCNK2, KCNK3, KDM5B, KIAA1217, KIRREL3, KLK13, KRT3, LARGE1, LCE2A, LCE3E, LCP1, LDLRAD4, LGALS8, LHX6, LHX8, LINGO1, LRRC7, LYN, MACROD2, MAGI2, MAP2, MAP6, MAPRE2, MBL2, MBP, MC2R, MEF2A, MEIS2, METRN, MFGE8, MMP12, MSC, MXRA8, MYF5, MYH15, MYLK, MYOM2, NAV3, NDRG4, NEBL, NFATC1, NGF, NPHS2, NR2F1, NRXN3, NTM, NUMB, NXN, NYAP2, OLIG3, ONECUT2, OPALIN, ORAI1, OSTM1, P2RX2, PAK1, PAK4, PAX4, PCDHA6, PCDHA7, PCDHB13, PCDHB14, PCDHB15, PCDHB16, PCDHB2, PCDHB3, PCDHB4, PCDHB5, PCDHB6, PCP4, PCSK9, PDE3B, PDE6B, PGR, PHACTR1, PHF2, PIGT, PLCE1, PLK5, PNKD, POU6F2, PPARGC1A, PPHLN1, PPP3CA, PRDM13, PRDX1, PRKAA1, PRKCA, PRKCZ, PRKN, PSMD7, PTPN22, PTPRQ, PUS7, RAB35, RAMP1, RARB, RB1, RBFOX1, RBFOX3, RBM20, RERE, RFX4, RIMS1, RIPOR2, RND1, RNH1, ROBO1, ROBO2, ROBO3, RORB, RPS6KA2, RTN4IP1, RUNX1, RXRA, SALL3, SAV1, SCEL, SCN3B, SDK1, SEMA5A, SERINC5, SETDB2, SGCD, SGCZ, SHANK1, SIGLEC15, SKI, SLC12A5, SLC17A7, SLC17A8, SLC38A1, SLC38A10, SLC4A7, SLITRK6, SMAD1, SMAD6, SMAP1, SMYD2, SOX17, SOX5, SPEG, SRD5A2, SRGN, SRI, SRRM4, STARD13, STIM1, STK25, STMN2, STOX2, SWAP70, SYNE2, SYT17, TACC3, TBX3, TBXT, TCHH, TEAD1, TESC, THBS2, THSD7A, TIAM1, TIAM2, TMEM126A, TNFRSF11A, TNS3, TOX, TP73, TSC1, TUB, TYMP, TYR, UMODL1, UNC5D, USH1G, VEGFC, VPS33A
GO:0007275	multicellular organism development	8,86E-11	[2, 3]	826,00	14,30	24,41	37,46	38,13	[ADAM8, ADAMTS4, ADAMTS9, ADGRG6, AIF1, AKNA, ANGPT1, ANGPT2, AQP5, AREG, ASCL2, ATF3, BFSP2, BHLHE22, BHLHE40, BICDL1, BLNK, BMPR1B, BTG2, BTK, C1QA, C1QB, C1QC, CALCR, CAMK4, CARD11, CASP8, CCL19, CCL24, CCR2, CCR3, CCR4, CD2, CD27, CD3D, CD3E, CD3G, CD4, CD40LG, CD74, CD80, CD86, CD8A, CDKN2A, CEL, CHRDL1, CHRDL2, CLCF1, CLEC1B, CLEC4D, CLEC4E, CNR1, COL9A2, CR2, CRABP2, CRB2, CREM, CRTAM, CSF1R, CTLA4, CXCL10, CXCR3, CXCR4, CYBB, DDC, DOCK10, DOCK2, DOK2, DRC1, DRD1, DUSP2, E2F8, EAF2, EDN2, EGR3, ELAVL4, ELF3, EMB, EOMES, EPHA6, EREG, FASLG, FCRL3, FGL2, FGR, FLT3, FOLR2, FOSL1, FOXP3, FRZB, FUT7, GDF6, GFI1, GFRA2, GPM6A, GPR171, GPR55, GRXCR2, H3C10, H3C11, H3C12, H3C7, HCLS1, HCN1, HLA-DOA, HLA-DRB1, HOPX, HORMAD1, ICOS, IFNG, IKZF1, IL18, IL7R, INPP5D, IRF4, ITGA4, ITK, JPH1, KCNJ10, KLF5, KLHL41, KRT85, LAG3, LCK, LCP1, LEF1, LIPM, LMO1, LPAL2, LRRC17, LST1, LTA, LTB, LY9, MCOLN3, MIXL1, MOG, MS4A1, MSX1, MUSK, MYB, MYCL, MYO3B, NCKAP1L, NELL2, NLRP14, NLRP3, NME8, NPPB, NPPC, NR4A1, NR4A2, NR4A3, OPN5, P2RY12, PAX5, PCDHA2, PIK3CG, PIM1, PLA2G2D, PLAAT4, PLEK, PLPPR4, PLXNC1, PRDM1, PRKCQ, PROK1, PTK2B, PTPN22, PTPN6, PTPRC, PTX3, RHEX, RHOH, RLN2, RNASE6, RTKN2, RUNX3, SASH3, SDC1, SELPLG, SEMA4A, SEMA4D, SH2D2A, SLAMF6, SLAMF8, SNX10, SPEF2, SPIC, ST14, STAP1, STAT1, SYK, TACSTD2, TENT5C, TESPA1, TEX11, THEMIS, THY1, TIFAB, TNFAIP3, TNFRSF11B, TNFRSF18, TNFSF8, TNMD, TNR, TOX, TREM2, TRERF1, UBD, UGT8, VNN1, VWC2L, WNT1, WNT10A, WNT10B, ZAP70, ZNF683, ZNF804A]	[ABAT, ABT1, ACVR1B, ADA, ADAM10, ADAMTS12, ADCY1, ADGRL3, ADGRV1, AFDN, AFF3, AGAP2, AGO2, AGRN, AKAP13, AKAP6, AKNA, ANGPTL2, ANK3, ANKRD11, ANKS1A, ARHGAP26, ARRB2, ASAH1, ATOH8, ATXN1, AUTS2, B2M, BASP1, BATF, BATF3, BCAR3, BCL2, BCL9, BDNF, BICDL1, BMP7, BMPR1A, BTN3A3, C21orf91, CACNA1A, CAMK2G, CAPN2, CASP7, CASP8, CAVIN4, CBLN4, CCM2, CCR7, CD101, CD160, CD28, CD38, CD3G, CD44, CDH17, CDH19, CDH2, CDK5RAP1, CDK6, CELF1, CFDP1, CFLAR, CHRNA1, CHST11, CHSY1, CIAO3, CLEC14A, CLMP, CMTM7, COL15A1, COL19A1, COL4A2, COL5A1, COLEC11, COLEC12, CORO1C, CPLANE1, CRMP1, CSGALNACT1, CTLA4, CTSZ, CUX1, CX3CL1, DACH1, DBX1, DDAH1, DIXDC1, DNASE1L2, DNM2, DNMT3A, DOCK1, DOK2, DPEP1, DYM, ECE1, EEF2K, EHMT1, ELK3, ENPEP, EPHA10, ETV5, ETV6, EVL, EXPH5, EXT1, FANCA, FARP2, FBXO5, FES, FEZ2, FOXK1, FRY, GAB2, GABRA5, GAK, GATD3A, GBX2, GCNT1, GFRA2, GHR, GLIPR2, GPD2, GPR68, GPRC5B, GPX4, GRB2, GSK3B, GSN, HDAC1, HDAC9, HDGFL3, HECA, HECW2, HERC1, HIVEP3, HLA-B, HOMER1, HOXA3, HOXA4, HOXB1, HOXB3, HOXB7, HSD11B1, HSPB6, IDO1, IGF1R, IL34, IQSEC1, IRF1, IRX1, IRX2, ITGA8, ITGB5, ITPR1, JAK3, JARID2, KCNA2, KCNAB2, KCNC2, KCNQ1, KCTD11, KDM2B, KIAA1217, KIF13B, KLHL1, LCK, LCP1, LGR4, LMBR1	[ABCA12, ABCC8, ACER3, ADAMTS2, ADORA1, AFF3, AGO2, AGPAT5, AK8, AKAP13, ALDH1A2, ALK, ALOX15, ANK3, ANKS1A, ANKS1B, ANLN, ARHGAP24, ARHGAP26, ARHGAP44, ARHGEF10, ARNT, ASIC2, ASXL1, ASZ1, ATP2B2, ATP6V0D1, ATXN1, BCAR3, BCAS1, BCHE, BCL11B, BFSP1, BOC, CA10, CAMK4, CAMP, CARD11, CARTPT, CASP5, CATSPER2, CAV1, CD8A, CD9, CDH13, CDH22, CDH4, CDK13, CDK5R2, CDSN, CELF4, CELSR3, CERS3, CHODL, CHST8, CHSY1, CLN8, CLSTN2, CMTM7, CNTNAP2, COBL, COL4A2, COL5A2, COLEC11, CPLX2, CRB1, CRTAC1, CSMD3, CTTN, CXADR, CYP4A11, DCC, DCN, DISP3, DNER, DNM3, DOCK1, DPF1, DRAXIN, DRD2, DSCAML1, DSG3, DYSF, EGFR, ELL, ENPEP, ERBB4, ESRRB, EVPL, EXOC4, EYA2, FAT1, FBN2, FGF12, FGF14, FGF7, FGF9, FMN1, FMN2, FOXH1, FOXP1, FTO, GABRA5, GBA2, GCNT2, GDF6, GFRAL, GNAS, GNAT1, GNB4, GPM6A, GPR161, GREB1L, GREM2, GRIN2A, HGF, HID1, HIPK2, HLA-DQB2, HLA-DRB1, HLA-G, HOXC9, HRG, HTATIP2, HTR5A, HUNK, HYAL2, IGF1R, IGSF21, IGSF9B, IRX2, ITGB2, ITPR1, JADE2, KCNA5, KCNK2, KCNK3, KDM5B, KIAA1217, KIRREL3, KLK13, KRT3, LARGE1, LCE2A, LCE3E, LCP1, LDLRAD4, LGALS8, LHX6, LHX8, LINGO1, LPAR6, LRRC7, LYN, MACROD2, MAGI2, MAP2, MAP6, MAPRE2, MBL2, MBP, MC2R, MEF2A, MEIS2, METRN, MFGE8, MMP12, MNS1, MSC, MXRA8, MYADM, MYBPHL, MYCBPAP, MYF5, MYH15, MYLK, MYOM2, NAV3, NDRG4, NEBL, NECAB1, NFATC1, NGF, NPHS2, NR1H3, NR2F1, NRXN3, NTM, NUMB, NXN, NYAP2, OLIG3, ONECUT2, OPALIN, ORAI1, OSTM1, P2RX2, PAK1, PAK4, PAX4, PCDHA6, PCDHA7, PCDHB13, PCDHB14, PCDHB15, PCDHB16, PCDHB2, PCDHB3, PCDHB4, PCDHB5, PCDHB6, PCP4, PCSK9, PDE3B, PDE6B, PGR, PHACTR1, PHF2, PIGT, PIWIL3, PLCE1, PLD6, PLEKHB1, PLK5, PNKD, POU6F2, PPARGC1A, PPDPFL, PPHLN1, PPP2R3A, PPP3CA, PRDM13, PRDX1, PRKAA1, PRKCA, PRKCZ, PRKN, PSMD7, PTPN22, PTPRQ, PUS7, RAB35, RAMP1, RARB, RB1, RBFOX1, RBFOX3, RBM20, RBM46, RERE, RFX4, RIMS1, RIPOR2, RNASEH2B, RND1, RNF43, RNH1, ROBO1, ROBO2, ROBO3, RORB, RPS6KA2, RTN4IP1, RUNX1, RXRA, SALL3, SAV1, SCEL, SCN3B, SDK1, SEMA5A, SERINC5, SETDB2, SGCD, SGCZ, SHANK1, SIGLEC15, SKI, SLC12A5, SLC17A7, SLC17A8, SLC38A1, SLC38A10, SLC4A7, SLITRK6, SMAD1, SMAD6, SMAP1, SMYD2, SOX17, SOX5, SOX7, SPATA16, SPEG, SRD5A2, SRGN, SRI, SRRM4, STARD13, STIM1, STK25
GO:0007399	nervous system development	5,09E-09	[4, 5]	411,00	15,89	18,37	41,50	40,14	[ADGRG6, AIF1, AKNA, AREG, ASCL2, BHLHE22, BHLHE40, BICDL1, BMPR1B, BTG2, C1QA, CCR2, CCR4, CD3E, CEL, CHRDL1, CLCF1, CNR1, CRABP2, CRB2, CSF1R, CXCR4, DOCK10, DOK2, DRD1, EGR3, ELAVL4, EMB, EOMES, EPHA6, FOLR2, GDF6, GFI1, GFRA2, GPM6A, GRXCR2, HCLS1, HCN1, IFNG, ITGA4, KCNJ10, LEF1, LST1, LTA, MCOLN3, MOG, MSX1, MUSK, MYB, MYCL, NCKAP1L, NELL2, NPPC, NR4A2, NR4A3, P2RY12, PAX5, PCDHA2, PLPPR4, PLXNC1, PRDM1, PRKCQ, PTK2B, RNASE6, RUNX3, SEMA4A, SEMA4D, SPEF2, ST14, STAP1, THY1, TIFAB, TNR, TOX, TREM2, UGT8, VWC2L, WNT1, WNT10A, WNT10B, ZNF804A]	[ABAT, ABT1, ACVR1B, ADAM10, ADCY1, ADGRL3, ADGRV1, AFDN, AGRN, AKNA, ANK3, ANKS1A, ARHGAP26, ARRB2, ATOH8, ATXN1, AUTS2, B2M, BASP1, BCL2, BDNF, BICDL1, BMP7, BMPR1A, C21orf91, CACNA1A, CAMK2G, CBLN4, CD38, CDH2, CDK5RAP1, CDK6, CFLAR, COLEC12, CORO1C, CPLANE1, CRMP1, CTSZ, CUX1, CX3CL1, DBX1, DIXDC1, DNM2, DNMT3A, DOK2, DPEP1, EEF2K, EPHA10, ETV5, ETV6, EVL, EXT1, FARP2, FES, FEZ2, FRY, GAB2, GABRA5, GAK, GATD3A, GBX2, GFRA2, GPRC5B, GRB2, GSK3B, GSN, HDAC1, HDAC9, HDGFL3, HECW2, HERC1, HOXA3, HOXB1, HOXB3, IL34, IQSEC1, IRX1, IRX2, ITGA8, JARID2, KCNA2, KCNC2, KCTD11, KDM2B, KIF13B, KLHL1, LRRN3, LSAMP, LTA, MACROD2, MAP6, MARK2, MBP, MCPH1, MDGA2, MED1, MMP14, MRTFA, MTHFD1L, MYT1, NAV1, NCK2, NECTIN1, NEO1, NEXN, NFIB, NPTN, NTRK2, OLFM1, OPRM1, PARD3, PCDHA6, PDZD2, PHACTR1, PHLPP2, PIGU, PIK3CD, PLK5, PLXNC1, POU6F1, PPP1CC, PPP3CC, PRICKLE1, PRKACB, PRKD2, PROM1, PSAP, PTK2B, PTPRF, PTPRK, PTPRM, RAB13, RARA, RBFOX1, RECK, RERE, RIPOR2, RORA, RUFY3, RUNX1, RUNX3, SALL4, SATB2, SEMA4D, SGK1, SIPA1L1, SLC1A2, SLC25A12, SLC6A11, SLIT1, SMARCA4, SPEN, SPOCK2, SRGAP3, SSTR1, SSTR4, STAT3, TCF4, TCF7L1, TENM4, TERT, TG, TGFB2, THRB, TIMP2, TMEM108, TNF, TRIM46, TRIO, TSHR	[ABCC8, ACER3, ADORA1, AK8, ALDH1A2, ALK, ANK3, ANKS1A, ARHGAP26, ARHGAP44, ARHGEF10, ASIC2, ATP2B2, ATP6V0D1, ATXN1, BCAS1, BCHE, BCL11B, BOC, CA10, CASP5, CD9, CDH22, CDH4, CDK5R2, CELSR3, CHODL, CHST8, CLN8, CLSTN2, CNTNAP2, COBL, CPLX2, CRB1, CRTAC1, CSMD3, CTTN, DCC, DISP3, DNER, DNM3, DPF1, DRAXIN, DRD2, DSCAML1, EGFR, ERBB4, FGF12, FGF14, FGF9, GABRA5, GBA2, GDF6, GFRAL, GNAT1, GNB4, GPM6A, GPR161, GRIN2A, HGF, HIPK2, HTR5A, IGSF21, IGSF9B, IRX2, JADE2, KCNK3, KIRREL3, LHX6, LHX8, LINGO1, LRRC7, LYN, MACROD2, MAGI2, MAP2, MAP6, MAPRE2, MBL2, MBP, MEF2A, MEIS2, METRN, MMP12, MXRA8, NAV3, NDRG4, NGF, NR2F1, NRXN3, NTM, NUMB, NYAP2, OLIG3, ONECUT2, OPALIN, PAK1, PAK4, PCDHA6, PCDHA7, PCDHB13, PCDHB14, PCDHB15, PCDHB16, PCDHB2, PCDHB3, PCDHB4, PCDHB5, PCDHB6, PCP4, PCSK9, PHACTR1, PIGT, PLK5, POU6F2, PPARGC1A, PPP3CA, PRDM13, PRKCA, PRKCZ, PRKN, PTPRQ, RAB35, RARB, RB1, RBFOX1, RBFOX3, RERE, RFX4, RIMS1, RIPOR2, RND1, ROBO1, ROBO2, ROBO3, RORB, RTN4IP1, RUNX1, SALL3, SCN3B, SDK1, SEMA5A, SERINC5, SHANK1, SKI, SLC12A5, SLC17A7, SLC17A8, SLC4A7, SLITRK6, SMAD1, SOX17, SOX5, SRRM4, STK25, STMN2, SYNE2, SYT17, TACC3, TBX3, TBXT, THBS2, TIAM1, TIAM2, TMEM126A, TOX, TP73, TSC1, TYMP, UNC5D, USH1G, VEGFC, WASL, WNT16, ZEB1, ZIC4, ZNF430]

GO:0048699	generation of neurons	6,45E-09	[5, 6, 7]	285,00	17,15	21,10	43,18	35,71	[AKNA, AREG, ASCL2, BHLHE40, BICDL1, BMPR1B, BTG2, C1QA, CCR2, CCR4, CD3E, CLCF1, CNR1, CRABP2, CSF1R, CXCR4, DOCK10, DOK2, DRD1, ELAVL4, EMB, EOMES, EPHA6, FOLR2, GDF6, GFI1, GFRA2, GPM6A, GRXCR2, HCLS1, HCN1, IFNG, ITGA4, LEF1, LST1, LTA, MCOLN3, MUSK, MYB, MYCL, NCKAP1L, NELL2, NPPC, NR4A2, NR4A3, P2RY12, PLPPR4, PLXNC1, PRDM1, PRKCQ, PTK2B, RUNX3, SEMA4A, SEMA4D, STAP1, THY1, TNR, TOX, TREM2, UGT8, VWC2L, WNT1, WNT10A, WNT10B, ZNF804A]	[ABT1, ADAM10, ADCY1, ADGRL3, ADGRV1, AFDN, AGRN, AKNA, ANK3, ANKS1A, AUTS2, B2M, BCL2, BDNF, BICDL1, BMP7, BMPR1A, C21orf91, CACNA1A, CAMK2G, CD38, CDH2, CDK5RAP1, CDK6, CFLAR, CRMP1, CTSZ, CUX1, CX3CL1, DBX1, DIXDC1, DNM2, DNMT3A, DOK2, DPEP1, EEF2K, EPHA10, ETV5, EVL, EXT1, FARP2, FES, FEZ2, FRY, GAB2, GABRA5, GAK, GATD3A, GBX2, GFRA2, GPRC5B, GRB2, GSK3B, HDAC1, HDAC9, HDGFL3, HECW2, HERC1, HOXB3, IL34, IQSEC1, IRX1, IRX2, KCTD11, KIF13B, KLHL1, LTA, MAP6, MARK2, MDGA2, MED1, MRTFA, NAV1, NCK2, NECTIN1, NEO1, NEXN, NFIB, NPTN, NTRK2, OLFM1, OPRM1, PARD3, PDZD2, PHACTR1, PIGU, PIK3CD, PLK5, PLXNC1, PPP1CC, PROM1, PSAP, PTK2B, PTPRF, PTPRK, PTPRM, RAB13, RARA, RERE, RIPOR2, RORA, RUFY3, RUNX1, RUNX3, SATB2, SEMA4D, SGK1, SIPA1L1, SLIT1, SPEN, SRGAP3, STAT3, TCF4, TCF7L1, TENM4, TERT, TGFB2, THRB, TIMP2, TMEM108, TNF, TRIM46, TRIO, TSHR, UBE3A, UGCG, UST, WDPCP, YAP1, ZEB1, ZHX2, ZMIZ1, ZNF365]	[ABCC8, ALDH1A2, ALK, ANK3, ANKS1A, ARHGAP44, ATP2B2, BCHE, BCL11B, BOC, CDH4, CDK5R2, CELSR3, CHODL, CNTNAP2, COBL, CRB1, CRTAC1, CSMD3, CTTN, DCC, DISP3, DNER, DNM3, DRAXIN, DRD2, DSCAML1, EGFR, ERBB4, GABRA5, GBA2, GDF6, GNAT1, GPM6A, HGF, HIPK2, IRX2, JADE2, KIRREL3, LHX6, LHX8, LINGO1, LRRC7, LYN, MAGI2, MAP2, MAP6, MAPRE2, MEF2A, METRN, MMP12, NDRG4, NGF, NR2F1, NRXN3, NTM, NUMB, NYAP2, OLIG3, ONECUT2, OPALIN, PAK1, PAK4, PCP4, PCSK9, PHACTR1, PIGT, PLK5, PPP3CA, PRKCA, PRKCZ, PRKN, PTPRQ, RAB35, RARB, RB1, RERE, RIMS1, RIPOR2, RND1, ROBO1, ROBO2, ROBO3, RORB, RTN4IP1, RUNX1, SALL3, SDK1, SEMA5A, SHANK1, SKI, SLC12A5, SLC4A7, SLITRK6, SOX5, SRRM4, STK25, STMN2, SYT17, TIAM1, TIAM2, TOX, TP73, TSC1, UNC5D, USH1G, VEGFC, WASL, WNT16, ZEB1]
GO:0045595	regulation of cell differentiation	8,65E-09	[3, 4]	328,00	16,57	30,45	37,43	32,12	[ADAM8, ADAMTS9, AREG, ASCL2, BHLHA15, BHLHE40, BMPR1B, BTK, C1QC, CAMK4, CARD11, CASP8, CCL19, CCR2, CD2, CD27, CD4, CD53, CD74, CD80, CD86, CDKN2A, CLCF1, CNR1, CRABP2, CRB2, CRTAM, CTLA4, CXCL10, CXCL9, CXCR4, EGR3, ELAVL4, EREG, FCRL3, FFAR4, FGL2, FOXP3, FRZB, GDF6, GFI1, GLIS1, GPR171, GPR55, H3C10, H3C11, H3C12, H3C7, HCLS1, HLA-DOA, HLA-DRB1, HOPX, IFNG, IL18, IL7R, INPP5D, IRF4, KLF5, KLHL41, LAG3, LCP1, LEF1, LMO1, LRRC17, LTA, MEDAG, MIXL1, MSX1, MUSK, MYB, MYCL, NCKAP1L, NEK5, NLRP3, NPPC, NR4A3, P2RY12, PIM1, PLAAT4, PLXNC1, PRDM1, PRKCQ, PTK2B, PTPN6, PTPRC, RHEX, RHOH, RUNX3, SASH3, SEMA4A, SEMA4D, SLAMF8, STAP1, STAT1, SYK, TACSTD2, TESPA1, THY1, TNFRSF18, TNR, TOX, TREM2, VNN1, VWC2L, WNT1, WNT10B, ZAP70, ZNF683, ZNF804A]	[ACVR1B, ADA, ADAM10, ADAMTS12, ADGRV1, AFDN, AKAP13, AKAP6, ARRB2, ATOH8, B2M, BCL2, BDNF, BMP7, BMPR1A, C21orf91, CACNA1A, CAMK2G, CAPN2, CASP8, CCM2, CD101, CD28, CD38, CDH2, CDK5RAP1, CDK6, CFLAR, COL5A1, CORO1C, CRMP1, CTLA4, CTSZ, CUX1, CX3CL1, DIXDC1, DNM2, DOCK1, DPEP1, EEF2K, ETV5, FANCA, FBXO5, FES, GAK, GATD3A, GBP1, GHR, GLIPR2, GPR68, GPRC5B, GSK3B, HDAC1, HDAC9, HECW2, HLA-B, HOXB3, IL34, IRF1, JAK3, KCTD11, KIF13B, LCP1, LTA, MAFG, MAP6, MARK2, MED1, MITF, MMP14, NFATC1, NFATC2, NPR2, NPTN, NTRK2, OLFM1, OPRM1, PDZD2, PHACTR1, PLAAT4, PLK5, PLXNC1, PPP1CC, PPP1R13L, PRICKLE1, PROM1, PSMB8, PSMB9, PSMD9, PTK2B, PTPN2, PTPN6, PTPRC, PTPRF, RARA, RBFOX1, RECK, RIN2, RIPOR2, RORA, RUFY3, RUNX1, RUNX3, SEMA4D, SIPA1L1, SPEN, SPOCK2, SRGAP3, ST7, STAT1, STAT3, TCF4, TCF7L1, TENM4, TERT, TESPA1, TGFB2, THRB, TIMP2, TNF, TRIM46, TRIO, UBASH3B, UBE3A, UST, WDFY2, YAP1, ZBTB16, ZC3H12A, ZEB1, ZHX2, ZMIZ1, ZNF365, ZNF385A]	[ABCC8, AKAP13, ALK, ARHGAP44, ARNT, ASIC2, ASXL1, BCL11B, BOC, CAMK4, CARD11, CARTPT, CAV1, CDH4, CDK13, CDSN, CHODL, COBL, COL5A2, CSMD3, CTTN, DCC, DISP3, DMPK, DNM3, DOCK1, DRAXIN, DRD2, EGFR, ERBB4, ESRRB, FBN2, FGF9, FOXP1, FTO, GCNT2, GDF6, GNAS, HGF, HLA-DRB1, HLA-G, JADE2, JDP2, LCP1, LDLRAD4, LIMD1, LINGO1, LMO3, LRRC7, LYN, MAGI2, MAP2, MAP3K5, MAP6, MEF2A, MEIS2, METRN, MMP12, MYADM, MYF5, NDRG4, NFATC1, NGF, NR1H3, NR2F1, NUMB, OPALIN, PAK1, PAX4, PCP4, PHACTR1, PLEKHB1, PLK5, PPP3CA, PRKCA, PRKCZ, PRKN, PSMD7, PTPRQ, PUS7, RARB, RB1, RBFOX1, RIMS1, RIPOR2, ROBO1, ROBO2, RORB, RTN4IP1, RUNX1, SAV1, SDK1, SEMA5A, SHANK1, SIGLEC15, SKI, SMAD1, SMAD6, SMAP1, SOX17, SOX5, STK25, STMN2, SYT17, TBX3, TESC, TIAM1, TIAM2, TOX, TP73, TSC1, UNC5D, VEGFC, WDFY2, ZEB1]
GO:2000026	regulation of multicellular organismal development	1,01E-08	[3, 4]	360,00	16,22	28,72	39,23	32,05	[ADAM8, ADAMTS9, ANGPT2, AREG, ASCL2, BHLHE40, BMPR1B, BTK, C1QC, CAMK4, CARD11, CASP8, CCL19, CCL24, CCR2, CCR3, CD2, CD27, CD3E, CD4, CD74, CD80, CD86, CDKN2A, CLCF1, CNR1, CRABP2, CRB2, CRTAM, CTLA4, CXCL10, CXCR4, CYBB, EAF2, EGR3, ELAVL4, FASLG, FCRL3, FGL2, FOXP3, FRZB, GDF6, GFI1, GPR171, GPR55, H3C10, H3C11, H3C12, H3C7, HCLS1, HLA-DOA, HLA-DRB1, IFNG, IL18, IL7R, INPP5D, IRF4, KLHL41, LAG3, LEF1, LMO1, LRRC17, LTA, MIXL1, MSX1, MUSK, MYB, MYCL, NCKAP1L, NLRP3, NPPB, NR4A3, P2RY12, PIM1, PLAAT4, PLXNC1, PRDM1, PRKCQ, PROK1, PTK2B, PTPN6, PTPRC, RHEX, RHOH, RLN2, RUNX3, SASH3, SEMA4A, SEMA4D, SLAMF8, STAP1, STAT1, SYK, TACSTD2, TESPA1, THY1, TIFAB, TNFAIP3, TNFRSF11B, TNFRSF18, TNMD, TNR, TOX, TREM2, VNN1, VWC2L, WNT1, WNT10A, WNT10B, ZAP70, ZNF683, ZNF804A]	[ACVR1B, ADA, ADAM10, ADAMTS12, ADGRL3, ADGRV1, AFDN, AGAP2, AGO2, AGRN, AKAP6, ARRB2, ATOH8, B2M, BASP1, BCL2, BDNF, BMP7, BMPR1A, C21orf91, CACNA1A, CAMK2G, CASP8, CCM2, CD101, CD160, CD28, CD38, CDH2, CDK5RAP1, CDK6, CFLAR, COL4A2, COL5A1, CRMP1, CTLA4, CTSZ, CUX1, CX3CL1, DDAH1, DIXDC1, DNM2, DPEP1, EEF2K, EHMT1, ETV5, FANCA, FBXO5, FES, GAK, GATD3A, GBX2, GLIPR2, GPR68, GPRC5B, GSK3B, HDAC1, HDAC9, HECW2, HLA-B, HOXB3, HOXB7, HSPB6, IDO1, IL34, IRF1, JAK3, JARID2, KCTD11, KIF13B, LGR4, LRRN3, LTA, MAFG, MAP2K5, MAP6, MARK2, MBP, MED1, MITF, MMP14, NECTIN1, NFATC1, NFATC2, NFIB, NPTN, NTRK2, OLFM1, OPRM1, PARD3, PDCD10, PDZD2, PHACTR1, PHF14, PIGU, PLAAT4, PLK5, PLXNC1, PPP1CC, PRICKLE1, PRKACB, PRKD2, PROM1, PSMB8, PSMB9, PSMD9, PTK2B, PTPN2, PTPN6, PTPRC, PTPRF, PTPRM, RARA, RBFOX1, RECK, RIN2, RNH1, RORA, RUFY3, RUNX1, RUNX3, RXRA, SEMA4D, SIPA1L1, SLC25A12, SLIT1, SPEN, SRGAP3, STAT1, STAT3, TCF4, TCF7L1, TENM4, TERT, TESPA1, TG, TGFB2, THRB, TIMP2, TNF, TNFAIP3, TRIM46, UBASH3B, UBE3A, UST, WDPCP, YAP1, ZBTB16, ZC3H12A, ZEB1, ZHX2, ZMIZ1, ZNF365]	[ABCC8, AGO2, ALK, ARHGAP44, ARNT, ASIC2, BCL11B, CAMK4, CAMP, CARD11, CARTPT, CAV1, CDH4, CDSN, CELF4, CELSR3, CHODL, CLSTN2, COBL, COL4A2, COL5A2, CSMD3, CTTN, CXADR, DCC, DCN, DISP3, DNM3, DRAXIN, DRD2, EGFR, ERBB4, FBN2, FGF7, FGF9, FOXP1, GCNT2, GDF6, GNAS, GPR161, HGF, HIPK2, HLA-DRB1, HLA-G, HRG, HTATIP2, ITGB2, JADE2, KCNA5, KCNK2, KDM5B, LDLRAD4, LINGO1, LRRC7, LYN, MAGI2, MAP2, MAP6, MBL2, MBP, MEIS2, METRN, MMP12, MYADM, MYF5, NDRG4, NFATC1, NGF, NR2F1, NUMB, OPALIN, PAK1, PAK4, PCP4, PDE3B, PHACTR1, PLK5, PPP3CA, PRKAA1, PRKCA, PRKCZ, PRKN, PSMD7, PUS7, RARB, RB1, RBFOX1, RFX4, RIMS1, RNH1, ROBO1, ROBO2, RTN4IP1, RUNX1, RXRA, SAV1, SDK1, SEMA5A, SHANK1, SIGLEC15, SKI, SLITRK6, SMAD1, SMAP1, SOX17, SOX5, SRGN, STARD13, STIM1, STK25, STMN2, SYT17, TBX3, TESC, THBS2, TIAM1, TIAM2, TOX, TP73, TSC1, TYMP, UMODL1, UNC5D, VEGFC, ZEB1]
GO:0050793	regulation of developmental process	1,09E-08	[2, 3]	438,00	15,60	28,63	38,53	32,84	[ADAM8, ADAMTS9, ANGPT2, AREG, ARHGAP15, ASCL2, BHLHA15, BHLHE40, BMPR1B, BTK, C1QC, CAMK4, CARD11, CASP8, CCL19, CCL24, CCR2, CCR3, CD2, CD27, CD3E, CD4, CD53, CD74, CD80, CD86, CDKN2A, CLCF1, CNR1, CRABP2, CRB2, CRTAM, CSF1R, CTLA4, CXCL10, CXCL9, CXCR4, CYBB, EAF2, EGR3, ELAVL4, EREG, ERMN, FASLG, FCRL3, FFAR4, FGD2, FGD3, FGL2, FGR, FOXP3, FRZB, GDF6, GFI1, GLIS1, GPR171, GPR55, H3C10, H3C11, H3C12, H3C7, HCLS1, HLA-DOA, HLA-DRB1, HOPX, IFNG, IL18, IL7R, INPP5D, IRF4, KLF5, KLHL41, LAG3, LCP1, LEF1, LMO1, LRRC17, LST1, LTA, MEDAG, MIXL1, MSX1, MUSK, MYB, MYCL, NAMPT, NCKAP1L, NEK5, NLRP3, NPPB, NPPC, NR4A3, P2RY12, PIM1, PLAAT4, PLAC8, PLXNC1, PRDM1, PRKCQ, PROK1, PTK2B, PTPN6, PTPRC, RHEX, RHOH, RLN2, RUNX3, SASH3, SEMA4A, SEMA4D, SLAMF8, SLC18A1, STAP1, STAT1, SYK, TACSTD2, TESPA1, THY1, TIFAB, TNFAIP3, TNFRSF11B, TNFRSF18, TNFSF13B, TNMD, TNR, TOX, TREM2, VNN1, VWC2L, WAS, WNT1, WNT10A, WNT10B, ZAP70, ZNF683, ZNF804A]	[ACVR1B, ADA, ADAM10, ADAMTS12, ADGRL3, ADGRV1, AFDN, AGAP2, AGO2, AGRN, AKAP13, AKAP6, ARRB2, ATOH8, B2M, BASP1, BCL2, BDNF, BMP7, BMPR1A, C21orf91, CACNA1A, CAMK2G, CAPN2, CAPZB, CASP8, CCM2, CD101, CD160, CD28, CD38, CD44, CDC42SE2, CDH2, CDK5RAP1, CDK6, CELF1, CFDP1, CFLAR, COL4A2, COL5A1, CORO1A, CORO1C, CRMP1, CTLA4, CTSZ, CUX1, CX3CL1, DDAH1, DIXDC1, DNM2, DOCK1, DPEP1, EEF2K, EHMT1, ETV5, FANCA, FBXO5, FES, GAK, GATD3A, GBP1, GBX2, GHR, GLIPR2, GPR68, GPRC5B, GSK3B, HDAC1, HDAC9, HECW2, HLA-B, HMGA1, HOXB3, HOXB7, HSPB6, IDO1, IL34, IRF1, JAK3, JARID2, KCTD11, KDM2B, KIF13B, LCP1, LGR4, LRRN3, LTA, MAFG, MAP2K5, MAP6, MARK2, MBP, MED1, MITF, MMP14, NECTIN1, NFATC1, NFATC2, NFIB, NPR2, NPTN, NTRK2, OLFM1, OPRM1, PALMD, PARD3, PARVB, PDCD10, PDZD2, PHACTR1, PHF14, PIGU, PLAAT4, PLK5, PLXNC1, PPP1CC, PPP1R13L, PRICKLE1, PRKACB, PRKD2, PROM1, PSMB8, PSMB9, PSMD9, PTK2B, PTPN2, PTPN6, PTPRC, PTPRF, PTPRM, RARA, RBFOX1, RECK, RIN2, RIPOR2, RNH1, RORA, RUFY3, RUNX1, RUNX3, RXRA, S100A13, SEMA4D, SIPA1L1, SLC25A12, SLIT1, SPEN, SPOCK2, SRGAP3, ST7, STAT1, STAT3, TCF4, TCF7L1, TENM4, TERT, TESPA1, TG, TGFB2, THRB, TIMP2, TMEM135, TNF, TNFAIP3, TRIM46, TRIO,	[ABCC8, AGO2, AKAP13, ALK, ARHGAP44, ARNT, ASIC2, ASXL1, BCL11B, BOC, C11orf65, CAMK4, CAMP, CARD11, CARTPT, CAV1, CDC42EP3, CDH4, CDK13, CDSN, CELF4, CELSR3, CHODL, CLSTN2, COBL, COL4A2, COL5A2, CSMD3, CTTN, CXADR, DCC, DCN, DISP3, DMPK, DNM3, DOCK1, DRAXIN, DRD2, EGFR, ERBB4, ESRRB, FAM171A1, FBN2, FGF7, FGF9, FOXP1, FTO, GCNT2, GDF6, GNAS, GPR161, HGF, HIPK2, HLA-DRB1, HLA-G, HRG, HTATIP2, ITGB2, JADE2, JDP2, KCNA5, KCNK2, KDM5B, LCP1, LDLRAD4, LIMD1, LINGO1, LMO3, LRRC7, LYN, MAGI2, MAP2, MAP3K5, MAP6, MBL2, MBP, MEF2A, MEIS2, METRN, MMP12, MYADM, MYF5, NDRG4, NFATC1, NGF, NR1H3, NR2F1, NUMB, OPALIN, PAK1, PAK4, PALM2AKAP2, PAX4, PCP4, PDE3B, PDZD8, PHACTR1, PLAC8, PLD6, PLEKHB1, PLK5, PPARGC1A, PPP3CA, PRKAA1, PRKCA, PRKCZ, PRKN, PSMD7, PTPRQ, PUS7, RARB, RB1, RBFOX1, RFX4, RIMS1, RIPOR2, RNH1, ROBO1, ROBO2, RORB, RTN4IP1, RUNX1, RXRA, SAV1, SDK1, SEMA5A, SHANK1, SIGLEC15, SKI, SLITRK6, SMAD1, SMAD6, SMAP1, SOX17, SOX5, SRGN, STARD13, STIM1, STK25, STMN2, SYT17, TBX3, TESC, THBS2, TIAM1, TIAM2, TOX, TP73, TSC1, TYMP, UMODL1, UNC5D, VEGFC, WASL, WDFY2, ZEB1]

GO:0030154	cell differentiation	1,16E-08	[3]	652,00	14,56	28,75	36,54	34,70	[ADAM8, ADAMTS9, ADGRG6, AKNA, ANGPT1, ANGPT2, AREG, ASCL2, ATF3, BFSP2, BHLHA15, BHLHE22, BHLHE40, BICDL1, BLK, BLNK, BMPR1B, BTG2, BTK, C1QA, C1QC, CALCR, CAMK4, CARD11, CASP8, CCL19, CCR2, CCR4, CD2, CD27, CD3D, CD3E, CD3G, CD4, CD40LG, CD53, CD74, CD80, CD86, CD8A, CDKN2A, CFAP54, CHRDL1, CHRDL2, CLCF1, CLEC1B, CLEC4D, CLEC4E, CNR1, CR2, CRABP2, CRB2, CREM, CRTAM, CSF1R, CTLA4, CXCL10, CXCL9, CXCR4, DHRS9, DOCK10, DOCK2, DOK2, DRD1, E2F8, EGR3, ELAVL4, ELF3, EMB, EOMES, EPHA6, EREG, FASLG, FCRL3, FCRLA, FFAR4, FGL2, FGR, FLT3, FOLR2, FOXP3, FRZB, FUT7, GDF6, GFI1, GFRA2, GLIS1, GPM6A, GPR171, GPR55, GRXCR2, H3C10, H3C11, H3C12, H3C7, HCLS1, HCN1, HLA-DOA, HLA-DRB1, HOPX, HORMAD1, IFNG, IKZF1, IL18, IL7R, INPP5D, IRF4, ITGA4, ITGB7, ITK, KCNJ10, KLF5, KLHL41, KLRK1, KRT85, LAG3, LCK, LCP1, LEF1, LIPM, LMO1, LRRC17, LST1, LTA, LY9, M1AP, MCOLN3, MEDAG, MIXL1, MREG, MS4A1, MSX1, MUSK, MYB, MYCL, NAP1L3, NCKAP1L, NEK5, NELL2, NLRP14, NLRP3, NME8, NPPC, NR4A1, NR4A2, NR4A3, P2RY12, PAX5, PIM1, PLA2G2D, PLAAT4, PLAC8, PLEK, PLPPR4, PLXNC1, PRDM1, PRKCQ, PTK2B, PTPN22, PTPN6, PTPRC, PTX3, RHEX, RHOH, RUNX3, SASH3, SDC1, SEMA4A, SEMA4D, SH2D2A, SLA, SLAMF6, SLAMF8, SNX10, SOAT2, SPEF2, SPIC, SPINK1, ST14, STAP1, STAT1, SYK, TACSTD2, TESPA1, THEMIS, THY1, TNFRSF18, TNFSF8, TNMD, TNR, TOX, TREM2, UBD, UGT8, VNN1, VSIG1, VWC2L, WNT1, WNT10A, WNT10B, ZAP70, ZNF683, ZNF804A]	[ABT1, ACVR1B, ADA, ADAM10, ADAMTS12, ADCY1, ADGRL3, ADGRV1, AFDN, AFF4, AGRN, AKAP13, AKAP6, AKNA, ANK3, ANKS1A, ARMC2, ARRB2, ASAH1, ATOH8, AUTS2, B2M, BASP1, BATF, BATF3, BCL2, BCL9, BDNF, BICDL1, BMP7, BMPR1A, C21orf91, CACNA1A, CAMK2G, CAPN2, CASP7, CASP8, CAVIN4, CCDC36, CCDC88C, CCM2, CCR7, CD101, CD28, CD38, CD3G, CDC25B, CDH17, CDH2, CDHR2, CDK5RAP1, CDK6, CDYL, CELF1, CFLAR, CHST11, CHSY1, CIAO3, CMTM7, COL15A1, COL19A1, COL4A2, COL5A1, CORO1C, CRMP1, CTLA4, CTSZ, CUX1, CX3CL1, DBX1, DIXDC1, DNASE1L2, DNM2, DNMT3A, DOCK1, DOK2, DPEP1, EEF2K, ELK3, EPHA10, ETV5, ETV6, EVL, EXPH5, EXT1, FANCA, FARP2, FBXO5, FES, FEZ2, FOXK1, FRY, GAB2, GABRA5, GAK, GATD3A, GBP1, GBX2, GFRA2, GHR, GLIPR2, GPR68, GPRC5B, GRB2, GSK3B, GSN, HDAC1, HDAC9, HDGFL3, HECW2, HERC1, HIVEP3, HLA-B, HOMER1, HOXB3, HOXB7, IL34, IQSEC1, IRF1, IRX1, IRX2, ITGA8, ITGB5, JAK3, JARID2, KCNAB2, KCNH1, KCTD11, KIF13B, KLHL1, LCK, LCP1, LGR4, LTA, MAFG, MAP6, MARK2, MAST2, MDGA2, MED1, METTL8, MITF, MMP14, MRTFA, MYT1, NAV1, NCK2, NECTIN1, NEO1, NEXN, NFATC1, NFATC2, NFIB, NPR2, NPTN, NTRK2, OLFM1, OPRM1, PARD3, PARVB, PDZD2, PHACTR1, PIGU, PIK3CD, PIP4K2A, PKP4, PLAAT4, PLK5, PLXNC1, POU6F1, PPP1CC, PPP1R13L	[ABCA12, ABCC8, AGPAT5, AKAP13, ALDH1A2, ALK, ANK3, ANKS1A, ANLN, ARHGAP24, ARHGAP44, ARHGEF10, ARNT, ASIC2, ASXL1, ASZ1, ATP2B2, BCHE, BCL11B, BFSP1, BOC, BRDT, CAMK4, CAPN9, CARD11, CARTPT, CATSPER2, CAV1, CD8A, CD9, CDH4, CDK13, CDK5R2, CDSN, CELF4, CELSR3, CERS3, CHODL, CHSY1, CHUK, CMTM7, CNTNAP2, COBL, COL4A2, COL5A2, CPLX2, CRB1, CRTAC1, CSMD3, CTTN, CXADR, DCC, DISP3, DMPK, DNER, DNM3, DOCK1, DRAXIN, DRD2, DSCAML1, DSG3, DYSF, EGFR, ERBB4, ESRRB, EVPL, EYA2, FAT1, FBN2, FGF9, FMN2, FOXP1, FTO, GABRA5, GBA2, GCNT2, GDF6, GNAS, GNAT1, GPM6A, GRIN2A, HGF, HID1, HIP1, HIPK2, HLA-DQB2, HLA-DRB1, HLA-G, HSPA2, HTATIP2, HYAL2, IRX2, ITGB2, JADE2, JDP2, KDM5B, KIRREL3, KLK13, KRT3, LCE2A, LCE3E, LCP1, LDLRAD4, LHX6, LHX8, LIMD1, LINGO1, LMO3, LRRC7, LYN, M1AP, MAGI2, MAP2, MAP3K5, MAP6, MAPRE2, MBL2, MEF2A, MEIS2, METRN, MMP12, MXRA8, MYADM, MYCBPAP, MYF5, MYOM2, NAV3, NDRG4, NEBL, NFATC1, NGF, NPHS2, NR1H3, NR2F1, NRXN3, NTM, NUMB, NXN, NYAP2, OLIG3, ONECUT2, OPALIN, OSTM1, P2RX2, PACRG, PAK1, PAK4, PAX4, PCP4, PCSK9, PGR, PHACTR1, PIGT, PIWIL3, PLAC8, PLD3, PLD6, PLEKHB1, PLK5, PNKD, POU6F2, PPARGC1A, PPDPFL, PPHLN1, PPP3CA, PRDM13, PRKCA, PRKCZ, PRKN, PSMD7, PTPN22, PTPRQ, PUS7, RAB35, RARB, RB1, RBFOX1, RBM46, RERE, RHCG, RIMS1, RIPOR2, RND1, RNF151, ROBO1, ROBO2, ROBO3, RORB, RPS6KA2, RTN4IP1, RUNX1, RXRA, SALL3, SAV1, SCEL, SDK1, SEMA5A, SGCD, SGCZ, SHANK1, SHCBP1L, SIGLEC15, SKI, SLC12A5, SLC4A7, SLC9A4, SLITRK6, SMAD1, SMAD6, SMAP1, SOX17, SOX5, SOX7, SPAG16, SPATA16, SPEG, SRD5A2, SRRM4, STIM1, STK25, STMN2, SYNE2, SYT17, TBX3, TBXT, TCHH, TDRD9, TESC, THSD7A, TIAM1, TIAM2, TNFRSF11A, TOX, TP73, TPGS1, TSC1, TYMP, UNC5D, USH1G, VEGFC, VPS33A, WASL, WDFY2, WNT16, ZEB1]
GO:0022008	neurogenesis	1,43E-08	[4, 5, 6]	298,00	16,86	21,18	42,37	36,45	[ADGRG6, AKNA, AREG, ASCL2, BHLHE22, BHLHE40, BICDL1, BMPR1B, BTG2, C1QA, CCR2, CCR4, CD3E, CLCF1, CNR1, CRABP2, CSF1R, CXCR4, DOCK10, DOK2, DRD1, ELAVL4, EMB, EOMES, EPHA6, FOLR2, GDF6, GFI1, GFRA2, GPM6A, GRXCR2, HCLS1, HCN1, IFNG, ITGA4, KCNJ10, LEF1, LST1, LTA, MCOLN3, MUSK, MYB, MYCL, NCKAP1L, NELL2, NPPC, NR4A2, NR4A3, P2RY12, PLPPR4, PLXNC1, PRDM1, PRKCQ, PTK2B, RUNX3, SEMA4A, SEMA4D, STAP1, THY1, TNR, TOX, TREM2, UGT8, VWC2L, WNT1, WNT10A, WNT10B, ZNF804A]	[ABT1, ADAM10, ADCY1, ADGRL3, ADGRV1, AFDN, AGRN, AKNA, ANK3, ANKS1A, AUTS2, B2M, BCL2, BDNF, BICDL1, BMP7, BMPR1A, C21orf91, CACNA1A, CAMK2G, CD38, CDH2, CDK5RAP1, CDK6, CFLAR, CRMP1, CTSZ, CUX1, CX3CL1, DBX1, DIXDC1, DNM2, DNMT3A, DOK2, DPEP1, EEF2K, EPHA10, ETV5, ETV6, EVL, EXT1, FARP2, FES, FEZ2, FRY, GAB2, GABRA5, GAK, GATD3A, GBX2, GFRA2, GPRC5B, GRB2, GSK3B, GSN, HDAC1, HDAC9, HDGFL3, HECW2, HERC1, HOXB3, IL34, IQSEC1, IRX1, IRX2, KCTD11, KIF13B, KLHL1, LTA, MAP6, MARK2, MDGA2, MED1, MMP14, MRTFA, NAV1, NCK2, NECTIN1, NEO1, NEXN, NFIB, NPTN, NTRK2, OLFM1, OPRM1, PARD3, PDZD2, PHACTR1, PIGU, PIK3CD, PLK5, PLXNC1, PPP1CC, PROM1, PSAP, PTK2B, PTPRF, PTPRK, PTPRM, RAB13, RARA, RERE, RIPOR2, RORA, RUFY3, RUNX1, RUNX3, SATB2, SEMA4D, SGK1, SIPA1L1, SLIT1, SPEN, SRGAP3, STAT3, TCF4, TCF7L1, TENM4, TERT, TGFB2, THRB, TIMP2, TMEM108, TNF, TRIM46, TRIO, TSHR, UBE3A, UGCG, UST, WDPCP, YAP1, ZEB1, ZHX2, ZMIZ1, ZNF365]	[ABCC8, ALDH1A2, ALK, ANK3, ANKS1A, ARHGAP44, ARHGEF10, ATP2B2, BCHE, BCL11B, BOC, CD9, CDH4, CDK5R2, CELSR3, CHODL, CNTNAP2, COBL, CRB1, CRTAC1, CSMD3, CTTN, DCC, DISP3, DNER, DNM3, DRAXIN, DRD2, DSCAML1, EGFR, ERBB4, GABRA5, GBA2, GDF6, GNAT1, GPM6A, GRIN2A, HGF, HIPK2, IRX2, JADE2, KIRREL3, LHX6, LHX8, LINGO1, LRRC7, LYN, MAGI2, MAP2, MAP6, MAPRE2, MEF2A, METRN, MMP12, MXRA8, NAV3, NDRG4, NGF, NR2F1, NRXN3, NTM, NUMB, NYAP2, OLIG3, ONECUT2, OPALIN, PAK1, PAK4, PCP4, PCSK9, PHACTR1, PIGT, PLK5, PPP3CA, PRDM13, PRKCA, PRKCZ, PRKN, PTPRQ, RAB35, RARB, RB1, RERE, RIMS1, RIPOR2, RND1, ROBO1, ROBO2, ROBO3, RORB, RTN4IP1, RUNX1, SALL3, SDK1, SEMA5A, SHANK1, SKI, SLC12A5, SLC4A7, SLITRK6, SOX5, SRRM4, STK25, STMN2, SYNE2, SYT17, TIAM1, TIAM2, TOX, TP73, TSC1, UNC5D, USH1G, VEGFC, WASL, WNT16, ZEB1]
GO:0051240	positive regulation of multicellular organismal process	1,25E-07	[2, 3, 4]	313,00	16,38	33,05	37,01	29,94	[ADAM8, AIF1, AIM2, ANGPT1, ANGPT2, AREG, BMPR1B, BTK, CARD11, CASP8, CCDC88B, CCL19, CCL24, CCR2, CCR3, CD2, CD244, CD27, CD3E, CD4, CD40LG, CD6, CD74, CD80, CD86, CLCF1, CLEC4E, CLEC6A, CLNK, CNR1, CRABP2, CRB2, CRTAM, CSF1R, CXCR4, CYBB, EDN2, EGR3, ELAVL4, EREG, FCN1, FFAR4, FGR, FOXP3, GBP5, GDF6, GPSM3, HCLS1, HLA-DPA1, HLA-DPB1, HLA-DRB1, IFNG, IL12RB2, IL18, IL26, IL7R, INPP5D, IRF4, IRF5, KLRK1, LCP1, LEF1, LPAL2, LTA, LTB, LY9, MAPK13, MCOLN2, MUSK, MYB, NCKAP1L, NELL2, NLRP3, NPPB, NPPC, NR4A3, ORM2, P2RY12, P2RY2, PIM1, PLAAT4, PLAC8, PLEK, PLXNC1, PRKCQ, PTAFR, PTK2B, PTPN22, PTPRC, PYHIN1, RHEX, RHOH, RLN2, RUNX3, SASH3, SEMA4A, SEMA4D, SLAMF6, STAT1, SYK, TBXAS1, TESPA1, TIGIT, TLR6, TLR8, TNFAIP3, TNR, TOX, TREM2, UCP2, VNN1, VWC2L, WNT1, WNT10B, XCL1, ZAP70, ZNF804A]	[ABAT, ACVR1B, ADA, ADGRL3, ADGRV1, AGAP2, AGO2, AGRN, AIM2, AKAP6, ARRB2, ATOH8, B2M, BASP1, BCL2, BDNF, BIRC3, BMP7, BMPR1A, C21orf91, CASP8, CCDC88B, CCM2, CCR7, CD101, CD160, CD28, CD38, CD6, CELF1, CFLAR, CUX1, CX3CL1, DDAH1, DOCK1, EEF2K, EHMT1, ETV5, F2R, FBXO5, FES, GATD3A, GBP5, GHR, GLIPR2, GPR68, GPRC5B, GPSM3, HDAC1, HDAC9, HLA-A, HLA-DPA1, HLA-DPB1, HOXB7, HSPB6, IDO1, IGF1R, IL12RB2, IL17RA, IL34, INAVA, IQSEC1, IRF1, KCNQ1, KCTD11, KDM2B, LCP1, LGR4, LPIN1, LRRFIP1, LRRN3, LTA, MAP6, MARK2, MBP, MED1, MMP14, MTNR1B, NETO1, NOS1AP, NPTN, NTRK2, OLFM1, OPRM1, PARD3, PDE4B, PDE9A, PDZD2, PIGU, PLAAT4, PLK5, PLXNC1, PPP1CC, PRKCE, PRKD2, PROM1, PTK2B, PTPN22, PTPRC, PTPRJ, RARA, RIN2, RORA, RUFY3, RUNX1, RUNX3, SEMA4D, SLC25A12, SPEN, STAT1, STAT3, TANK, TCF4, TCF7L1, TENM4, TERT, TESPA1, TGFB2, TIMP2, TNF, TNFAIP3, TSHR, UCP2, YAP1, ZBP1, ZBTB16, ZBTB20, ZC3H12A, ZEB1, ZMIZ1, ZNF365]	[ABCC8, ADRA1B, ADRB3, AGO2, AIM2, AKAP12, ALK, ARNT, ASIC2, BTNL2, CAMP, CARD11, CARD8, CARTPT, CAV1, CDH4, CHODL, CHUK, CLSTN2, COBL, CTTN, DOCK1, DRD2, EGFR, ERBB4, ESRRB, ESRRG, FBN2, FGF7, FGF9, FOXP1, GCNT2, GDF6, GNAS, HGF, HIPK2, HLA-DRB1, HLA-G, HRG, HYAL2, IGF1R, IGSF9B, ITGB2, JADE2, KCNA5, KDM5B, LCP1, LRRC7, LRRFIP1, LYN, MAGI2, MAP6, MAPRE2, MBL2, MBP, MEF2A, METRN, MMP12, MNDA, MYF5, NDRG4, NGF, NOS1AP, NUMB, PAK1, PAK4, PCP4, PDE4B, PLAC8, PLK5, PLPP3, PPARGC1A, PPP3CA, PRKAA1, PRKCA, PRKCZ, PRKN, PTPN22, RARB, RB1, RIMS1, ROBO1, ROBO2, RUNX1, SCN3B, SEMA5A, SHANK1, SLITRK6, SMAD1, SMAP1, SOX5, STIM1, STK25, STMN2, SYT17, TESC, THBS2, TIAM1, TIAM2, TLR1, TNFRSF11A, TOX, TP73, TRAF2, VEGFC, ZEB1]
GO:0030182	neuron differentiation	8,12E-07	[4, 6, 7, 8]	252,00	16,88	19,41	43,59	37,00	[AREG, ASCL2, BICDL1, BMPR1B, BTG2, C1QA, CD3E, CNR1, CRABP2, CSF1R, CXCR4, DOCK10, DOK2, DRD1, ELAVL4, EMB, EOMES, EPHA6, FOLR2, GDF6, GFI1, GFRA2, GPM6A, GRXCR2, HCLS1, HCN1, ITGA4, LST1, MCOLN3, MUSK, MYCL, NCKAP1L, NELL2, NPPC, NR4A2, NR4A3, PLPPR4, PLXNC1, PRDM1, PRKCQ, PTK2B, RUNX3, SEMA4A, SEMA4D, THY1, TNR, TOX, UGT8, VWC2L, WNT1, WNT10A, WNT10B, ZNF804A]	[ABT1, ADAM10, ADCY1, ADGRV1, AGRN, ANK3, ANKS1A, AUTS2, B2M, BCL2, BDNF, BICDL1, BMP7, C21orf91, CACNA1A, CAMK2G, CD38, CDH2, CDK5RAP1, CFLAR, CRMP1, CTSZ, CUX1, CX3CL1, DBX1, DIXDC1, DNM2, DNMT3A, DOK2, DPEP1, EEF2K, EPHA10, ETV5, EVL, EXT1, FARP2, FES, FEZ2, FRY, GAB2, GABRA5, GAK, GATD3A, GBX2, GFRA2, GPRC5B, GRB2, GSK3B, HDAC1, HDAC9, HDGFL3, HECW2, HERC1, IQSEC1, IRX1, IRX2, KCTD11, KIF13B, KLHL1, MAP6, MARK2, MDGA2, MED1, MRTFA, NCK2, NECTIN1, NEO1, NEXN, NFIB, NPTN, NTRK2, OLFM1, PARD3, PDZD2, PHACTR1, PIGU, PIK3CD, PLK5, PLXNC1, PPP1CC, PROM1, PSAP, PTK2B, PTPRF, PTPRK, PTPRM, RAB13, RARA, RERE, RIPOR2, RORA, RUFY3, RUNX1, RUNX3, SATB2, SEMA4D, SGK1, SIPA1L1, SLIT1, SRGAP3, STAT3, TCF4, TCF7L1, TENM4, TGFB2, THRB, TIMP2, TMEM108, TRIM46, TRIO, TSHR, UBE3A, UGCG, UST, WDPCP, ZEB1, ZHX2, ZMIZ1, ZNF365]	[ALDH1A2, ALK, ANK3, ANKS1A, ARHGAP44, ATP2B2, BCL11B, BOC, CDH4, CELSR3, CHODL, CNTNAP2, COBL, CRB1, CRTAC1, CSMD3, CTTN, DCC, DISP3, DNM3, DRAXIN, DRD2, DSCAML1, EGFR, ERBB4, GABRA5, GBA2, GDF6, GNAT1, GPM6A, HGF, HIPK2, IRX2, JADE2, KIRREL3, LHX6, LHX8, LINGO1, LRRC7, LYN, MAGI2, MAP2, MAP6, MAPRE2, MEF2A, METRN, NDRG4, NGF, NR2F1, NRXN3, NTM, NUMB, NYAP2, OLIG3, ONECUT2, PAK1, PAK4, PCP4, PCSK9, PHACTR1, PIGT, PLK5, PPP3CA, PRKCA, PRKCZ, PRKN, PTPRQ, RAB35, RARB, RB1, RERE, RIMS1, RIPOR2, RND1, ROBO1, ROBO2, ROBO3, RORB, RTN4IP1, RUNX1, SALL3, SDK1, SEMA5A, SHANK1, SLC12A5, SLC4A7, SLITRK6, SRRM4, STK25, STMN2, SYT17, TIAM1, TIAM2, TOX, TP73, TSC1, UNC5D, USH1G, WASL, WNT16, ZEB1]

GO:0048468	cell development	9,64E-07	[3, 4]	362,00	15,67	21,99	41,69	36,32	[ADGRG6, ANGPT2, AREG, ASCL2, BFSP2, BHLHA15, BHLHE40, BICDL1, BMPR1B, BTG2, BTK, C1QA, CCL19, CCR2, CD3E, CLCF1, CLEC1B, CNR1, CRABP2, CSF1R, CXCR4, DOCK10, DOK2, DRD1, ELAVL4, EMB, EPHA6, EREG, FASLG, FOLR2, FRZB, GDF6, GFI1, GFRA2, GPM6A, GRXCR2, HCLS1, HCN1, HORMAD1, IFNG, ITGA4, ITGB7, KCNJ10, KLF5, KLHL41, LEF1, LST1, LTA, MUSK, MYB, MYCL, NCKAP1L, NELL2, NPPC, NR4A2, NR4A3, P2RY12, PLPPR4, PLXNC1, PRDM1, PRKCQ, PTK2B, PTPN6, PTPRC, PTX3, RHEX, RUNX3, SDC1, SEMA4A, SEMA4D, SLAMF8, SPEF2, SPINK1, ST14, STAP1, TACSTD2, THY1, TNMD, TNR, TOX, TREM2, UGT8, VSIG1, VWC2L, WNT10B, ZNF804A]	[ADAM10, ADCY1, ADGRV1, AFDN, AFF4, AGRN, AKAP13, AKAP6, ANK3, ANKS1A, ARMC2, AUTS2, B2M, BCL2, BDNF, BICDL1, BMP7, BMPR1A, C21orf91, CACNA1A, CAMK2G, CCDC36, CCDC88C, CCM2, CD38, CDC25B, CDH2, CDK5RAP1, CDK6, CDYL, CELF1, CFLAR, CHST11, CHSY1, CORO1C, CRMP1, CTSZ, CUX1, CX3CL1, DIXDC1, DNASE1L2, DNM2, DOCK1, DOK2, DPEP1, EEF2K, EPHA10, ETV5, EVL, EXPH5, EXT1, FARP2, FBXO5, FES, FEZ2, FRY, GAB2, GABRA5, GAK, GATD3A, GBP1, GBX2, GFRA2, GPR68, GPRC5B, GRB2, GSK3B, GSN, HDAC1, HDAC9, HDGFL3, HECW2, HERC1, HOMER1, HOXB3, IL34, IQSEC1, IRX1, IRX2, ITGA8, KCTD11, KIF13B, KLHL1, LTA, MAP6, MARK2, MED1, MRTFA, NCK2, NECTIN1, NEO1, NEXN, NFATC2, NFIB, NPR2, NPTN, NTRK2, OLFM1, OPRM1, PARD3, PARVB, PDZD2, PHACTR1, PIGU, PIK3CD, PIP4K2A, PLK5, PLXNC1, PPP1CC, PSAP, PTK2B, PTPN6, PTPRC, PTPRF, PTPRK, PTPRM, PXN, RAB13, RARA, RECK, RERE, RIPOR2, RORA, RPS6KA2, RSPH6A, RUFY3, RUNX1, RUNX3, SATB2, SEMA4D, SGCD, SGK1, SIPA1L1, SLIT1, SPEN, SRGAP3, STAT3, STRBP, TCF4, TCF7L1, TDRD5, TENM4, TERT, TGFB2, THRB, TIMP2, TJP2, TMEM108, TNF, TRIM46, TRIO, TSHR, UBE3A, UGCG, UST, WDPCP, YAP1, ZBTB16, ZEB1, ZHX2, ZMIZ1, ZNF365, ZNF385A]	[ABCC8, AKAP13, ALDH1A2, ALK, ANK3, ANKS1A, ARHGAP44, ARHGEF10, ASIC2, ASXL1, BCL11B, BFSP1, BOC, CAPN9, CATSPER2, CD9, CDH4, CDSN, CELF4, CELSR3, CHODL, CHSY1, CNTNAP2, COBL, CRB1, CRTAC1, CSMD3, CTTN, CXADR, DCC, DISP3, DNER, DNM3, DOCK1, DRAXIN, DRD2, DSCAML1, DYSF, EGFR, ERBB4, FAT1, FMN2, FOXP1, GABRA5, GBA2, GDF6, GNAT1, GPM6A, HGF, HID1, HSPA2, IRX2, JADE2, KIRREL3, LHX6, LHX8, LIMD1, LINGO1, LRRC7, LYN, MAGI2, MAP2, MAP6, MAPRE2, MBL2, MEF2A, METRN, MMP12, MXRA8, MYADM, MYF5, MYOM2, NDRG4, NEBL, NGF, NPHS2, NR2F1, NRXN3, NTM, NUMB, NYAP2, ONECUT2, OPALIN, P2RX2, PACRG, PAK1, PAK4, PCP4, PGR, PHACTR1, PLD6, PLK5, PPP3CA, PRKCA, PRKCZ, PRKN, PTPRQ, RAB35, RARB, RB1, RERE, RIMS1, RIPOR2, RND1, ROBO1, ROBO2, ROBO3, RORB, RPS6KA2, RTN4IP1, RUNX1, SALL3, SDK1, SEMA5A, SGCD, SGCZ, SHANK1, SIGLEC15, SKI, SLC12A5, SLC4A7, SLC9A4, SLITRK6, SOX17, SPAG16, SRRM4, STK25, STMN2, SYT17, TBX3, TIAM1, TIAM2, TOX, TP73, TPGS1, TSC1, UNC5D, USH1G, VEGFC, VPS33A, WASL, ZEB1]
GO:0048513	animal organ development	1,61E-06	[3, 4, 5]	560,00	14,52	28,57	37,60	33,83	[ADAM8, ADAMTS9, ADGRG6, AIF1, AKNA, ANGPT1, ANGPT2, AQP5, AREG, ASCL2, ATF3, BFSP2, BLNK, BMPR1B, BTG2, BTK, C1QB, C1QC, CALCR, CAMK4, CARD11, CASP8, CCL19, CCR2, CD2, CD27, CD3D, CD3E, CD3G, CD4, CD40LG, CD74, CD80, CD86, CD8A, CDKN2A, CEL, CHRDL1, CHRDL2, CLCF1, CLEC1B, CLEC4D, CLEC4E, CR2, CRB2, CRTAM, CSF1R, CTLA4, CXCL10, CXCR4, DOCK10, DOCK2, DRC1, DRD1, E2F8, EAF2, EDN2, EGR3, ELF3, EOMES, EREG, FASLG, FCRL3, FGL2, FGR, FLT3, FOLR2, FOSL1, FOXP3, FRZB, FUT7, GDF6, GFI1, GPM6A, GPR171, GPR55, GRXCR2, H3C10, H3C11, H3C12, H3C7, HCLS1, HCN1, HLA-DOA, HLA-DRB1, IFNG, IKZF1, IL18, IL7R, INPP5D, IRF4, ITGA4, ITK, JPH1, KLF5, KLHL41, KRT85, LAG3, LCK, LCP1, LEF1, LIPM, LMO1, LPAL2, LRRC17, LTA, LTB, LY9, MCOLN3, MIXL1, MS4A1, MSX1, MYB, MYCL, MYO3B, NCKAP1L, NELL2, NLRP3, NPPC, NR4A1, NR4A2, NR4A3, OPN5, P2RY12, PAX5, PIM1, PLA2G2D, PLAAT4, PLEK, PLPPR4, PRDM1, PRKCQ, PTK2B, PTPN22, PTPN6, PTPRC, PTX3, RHEX, RHOH, RNASE6, RTKN2, RUNX3, SASH3, SDC1, SELPLG, SEMA4A, SEMA4D, SLAMF6, SLAMF8, SNX10, SPEF2, ST14, STAT1, SYK, TACSTD2, TESPA1, TEX11, THEMIS, THY1, TIFAB, TNFAIP3, TNFRSF11B, TNFRSF18, TNFSF8, TNR, TOX, TREM2, UBD, VNN1, WNT1, WNT10A, WNT10B, ZAP70, ZNF683]	[ABAT, ABT1, ACVR1B, ADA, ADAM10, ADAMTS12, ADCY1, ADGRL3, ADGRV1, AFDN, AGAP2, AGRN, AKAP13, AKAP6, AKNA, ANKRD11, ARRB2, ASAH1, ATXN1, B2M, BASP1, BATF, BATF3, BCAR3, BCL2, BCL9, BDNF, BMP7, BMPR1A, C21orf91, CACNA1A, CASP7, CASP8, CAVIN4, CCM2, CCR7, CD101, CD28, CD3G, CD44, CDH17, CDH2, CDK5RAP1, CDK6, CFLAR, CHRNA1, CHST11, CHSY1, CIAO3, CMTM7, COL19A1, COL5A1, COLEC12, CORO1C, CPLANE1, CSGALNACT1, CTLA4, CTSZ, DBX1, DIXDC1, DNASE1L2, DNM2, DOCK1, DPEP1, DYM, ECE1, ENPEP, ETV5, ETV6, EVL, EXPH5, EXT1, FANCA, FARP2, FBXO5, FES, FOXK1, GAB2, GABRA5, GATD3A, GBX2, GCNT1, GHR, GLIPR2, GPD2, GPR68, GRB2, GSK3B, HDAC1, HDAC9, HECA, HERC1, HIVEP3, HLA-B, HOMER1, HOXA3, HOXA4, HOXB1, HOXB3, HOXB7, HSD11B1, IL34, IRF1, IRX1, ITGA8, JAK3, JARID2, KCNAB2, KCNC2, KCNQ1, KCTD11, KDM2B, KLHL1, LCK, LCP1, LGR4, LPCAT1, LPIN1, LTA, LTBP1, MACROD2, MAP2K5, MBP, MCPH1, MDGA2, MED1, METTL8, MFAP5, MICAL2, MITF, MMP14, MRTFA, MTHFD1L, MTNR1B, NCOR2, NECTIN1, NFATC1, NFATC2, NFIB, NPR2, NTRK2, OLFM1, PDLIM2, PDZD2, PHACTR1, PHF14, PHLPP2, PIK3CD, PIP4K2A, PKD1L3, PKP4, PLAAT4, PLCE1, POU6F1, PPP1R13L, PPP3CC, PRICKLE1, PRKD2, PROM1, PRPSAP2, PSAP, PSMB8, PSMB9, PSMD9	[ABCA12, ADAMTS2, AGPAT5, AK8, AKAP13, ALDH1A2, ALK, ALOX15, ANLN, ARNT, ASXL1, ATP6V0D1, ATXN1, BCAR3, BCL11B, BFSP1, CA10, CAMK4, CARD11, CARTPT, CASP5, CAV1, CD8A, CD9, CDH22, CDK13, CDK5R2, CDSN, CELF4, CELSR3, CERS3, CHODL, CHSY1, CMTM7, CNTNAP2, COBL, COL5A2, CRB1, CRTAC1, CXADR, CYP4A11, DCC, DCN, DNER, DOCK1, DRAXIN, DRD2, DSCAML1, DSG3, EGFR, ENPEP, ERBB4, ESRRB, EVPL, EXOC4, FAT1, FBN2, FGF12, FGF7, FGF9, FMN1, FOXH1, FOXP1, FTO, GABRA5, GCNT2, GDF6, GNAS, GNAT1, GNB4, GPM6A, GREB1L, GREM2, GRIN2A, HGF, HID1, HIPK2, HLA-DQB2, HLA-DRB1, HLA-G, HOXC9, HTR5A, HYAL2, KCNA5, KCNK2, KCNK3, KDM5B, KIRREL3, KLK13, KRT3, LARGE1, LCE2A, LCE3E, LCP1, LDLRAD4, LHX6, LHX8, LYN, MACROD2, MAGI2, MAPRE2, MBL2, MBP, MC2R, MEF2A, MEIS2, MMP12, MSC, MYF5, MYH15, MYLK, MYOM2, NDRG4, NEBL, NFATC1, NPHS2, NUMB, OLIG3, ONECUT2, ORAI1, OSTM1, P2RX2, PAK1, PAX4, PCSK9, PDE6B, PGR, PHACTR1, PHF2, PLCE1, PNKD, PPARGC1A, PPHLN1, PPP3CA, PRKAA1, PRKCA, PRKCZ, PSMD7, PTPN22, PTPRQ, PUS7, RARB, RB1, RBFOX1, RBM20, RERE, RFX4, RIPOR2, ROBO1, ROBO2, RORB, RPS6KA2, RUNX1, RXRA, SALL3, SAV1, SCEL, SDK1, SEMA5A, SETDB2, SGCD, SGCZ, SHANK1, SIGLEC15, SKI, SLC17A7, SLC17A8, SLC38A10, SLC4A7, SLITRK6, SMAD1, SMAD6, SMAP1, SMYD2, SOX17, SOX5, SPEG, SRD5A2, SRGN, SRI, STIM1, STOX2, SYNE2, TACC3, TBX3, TBXT, TCHH, TEAD1, TESC, TIAM1, TNFRSF11A, TNS3, TOX, TP73, TSC1, TUB, TYR, UMODL1, USH1G, VEGFC, VPS33A, WFS1, WNK4, WNT16, ZEB1, ZNF430]
GO:0009653	anatomical structure morphogenesis	7,64E-06	[2, 3]	431,00	14,95	22,56	41,65	35,79	[ADAM8, ADAMTS9, ADGRG6, ANGPT1, ANGPT2, AQP5, AREG, ARHGAP15, BMPR1B, CCL24, CCR2, CCR3, CD53, CEL, CLEC1B, CPM, CRABP2, CRB2, CSF1R, CXCL10, CXCL9, CXCR3, CXCR4, CYBB, DOCK10, DOK2, DUSP2, E2F8, EGR3, ELAVL4, ELF3, EMB, EOMES, EPHA6, EREG, ERMN, FASLG, FGD2, FGD3, FGR, FOLR2, FRZB, GFRA2, GPM6A, GRXCR2, HCLS1, HCN1, HOPX, IER3, IL18, IL7R, ITGA4, ITGB7, KLF5, KLHL41, LEF1, LST1, MIXL1, MSX1, MYO3B, NCKAP1L, NELL2, NPPB, NPPC, NR4A1, NR4A2, NR4A3, OPN5, PAX5, PIK3CG, PIM1, PLPPR4, PLXNC1, PRDM1, PRKCQ, PROK1, PTK2B, PTPN6, RLN2, SDC1, SEMA4A, SEMA4D, SH2D2A, SLC18A1, SNX10, SPEF2, ST14, STAT1, SYK, TACSTD2, THY1, TIFAB, TNFAIP3, TNFRSF11B, TNFSF13B, TNMD, TNR, TREM2, UGT8, VSIG1, WAS, WNT1, WNT10A, WNT10B]	[ADA, ADAM10, ADAMTS12, ADCY1, AFDN, AFF3, AGO2, AGRN, AKAP13, ANGPTL2, ANK3, ANKRD11, ARRB2, ATOH8, AUTS2, BASP1, BCAR3, BCL2, BDNF, BICD1, BMP7, BMPR1A, CACNA1A, CAP2, CAPN2, CAPZB, CCDC88C, CCM2, CD160, CD44, CDC42SE2, CDH17, CDH19, CDH2, CFDP1, CFLAR, CHST11, CHSY1, CLEC14A, COL15A1, COL19A1, COL4A2, COL5A1, CORO1A, CORO1C, CPLANE1, CRISPLD1, CRMP1, CSGALNACT1, CTSZ, CUX1, CX3CL1, DDAH1, DNM2, DOCK1, DOK2, ECE1, EEF2K, ELK3, ENPEP, EPHA10, ETV5, EVL, EXT1, F2R, FES, FEZ2, FRY, GAB2, GATD3A, GBP1, GBX2, GCNT1, GFRA2, GHR, GRB2, GSK3B, HDAC1, HDAC9, HECW2, HERC1, HOXA3, HOXA4, HOXB1, HOXB3, HOXB7, HSPB6, IRX1, ITGA8, ITGB5, KCNA2, KCNH1, KDM2B, KIF13B, KLHL6, KLHL7, LGR4, LMBR1, MAP2K5, MAP6, MARK2, MBP, MED1, MFAP5, MICAL2, MMP14, MRTFA, MTHFD1L, NECTIN1, NEO1, NEXN, NFATC1, NFATC2, NFIB, NPTN, NTRK2, OLFM1, PALMD, PARD3, PARVB, PDCD10, PHACTR1, PIGU, PIK3CD, PLXNC1, PPP1R13L, PRICKLE1, PRKACB, PRKD2, PROM1, PSAP, PSMB8, PSMB9, PSMD9, PTK2B, PTPN6, PTPRM, PXN, RARA, RECK, RERE, RIN2, RIPOR2, RNF213, RNH1, RORA, RUFY3, RUNX1, RXRA, S100A13, SALL4, SATB2, SBNO2, SEMA4D, SGCD, SGK1, SH3BP1, SIPA1L1, SLC12A6, SLC38A1, SLIT1, SRGAP3, STAT1, STAT3, TCF4, TENM4, TERT	[ABCC8, AFF3, AGO2, AKAP13, ALDH1A2, ANK3, ARHGAP24, ARHGAP44, ASTN2, ASXL1, BCAR3, BCL11B, BOC, C11orf65, CAMP, CAPN9, CAV1, CD151, CD9, CDC42EP3, CDH13, CDH4, CDK5R2, CDSN, CELSR3, CHODL, CHSY1, CHUK, CNTNAP2, COBL, COL4A2, COL5A2, CRB1, CTTN, DCC, DCN, DNM3, DOCK1, DRAXIN, DRD2, DSCAML1, DYSF, EGFR, ENPEP, ERBB4, EXOC4, EYA2, FAM171A1, FAT1, FBN2, FGF7, FGF9, FMN1, FOXH1, FOXP1, GNAS, GNAT1, GPM6A, GREB1L, GREM2, HGF, HIPK2, HLA-G, HOXC9, HRG, HTATIP2, HYAL2, ITGB2, JADE2, KCNA5, KDM5B, KIRREL3, LGALS8, LHX8, LIMD1, LINGO1, MAGI2, MAP2, MAP6, MAPRE2, MBL2, MBP, MEF2A, MEIS2, METRN, MFGE8, MMP12, MYADM, MYF5, MYLK, MYOM2, NDRG4, NEBL, NFATC1, NGF, NRXN3, NUMB, NYAP2, ONECUT2, PAK1, PAK4, PALM2AKAP2, PAX4, PDE3B, PDZD8, PGR, PHACTR1, PLD6, PPARGC1A, PPP3CA, PRKCA, PRKCZ, PRKN, PSMD7, PTPRQ, RAMP1, RARB, RB1, RBM46, RERE, RIMS1, RIPOR2, RNH1, ROBO1, ROBO2, ROBO3, RORB, RUNX1, RXRA, SALL3, SAV1, SDK1, SEMA5A, SETDB2, SGCD, SHANK1, SKI, SLC38A1, SLITRK6, SMAD1, SMAD6, SOX17, SOX5, SOX7, STARD13, STIM1, STK25, SYT17, TBX3, TBXT, THBS2, THSD7A, TIAM1, TIAM2, TM4SF1, TSC1, TYMP, UNC5D, USH1G, VEGFC, VPS33A, WASL, WNK4, WNT16, ZEB1]
GO:0045597	positive regulation of cell differentiation	1,07E-05	[3, 4, 5]	186,00	17,53	31,25	37,02	31,73	[ADAM8, ADAMTS9, AREG, BMPR1B, BTK, CASP8, CCL19, CCR2, CD27, CD4, CD53, CD74, CD80, CD86, CLCF1, CNR1, CRABP2, CRB2, CXCL9, CXCR4, EGR3, ELAVL4, FFAR4, FOXP3, FRZB, GDF6, HCLS1, HLA-DRB1, IFNG, IL18, IL7R, INPP5D, KLF5, LCP1, LEF1, LTA, MEDAG, MUSK, MYB, NCKAP1L, NEK5, NLRP3, NPPC, P2RY12, PIM1, PLAAT4, PLXNC1, PTK2B, PTPRC, RHEX, RHOH, RUNX3, SASH3, SEMA4D, STAT1, SYK, TACSTD2, TESPA1, TOX, TREM2, VNN1, VWC2L, WNT10B, ZAP70, ZNF804A]	[ACVR1B, ADA, AKAP6, ARRB2, ATOH8, BCL2, BDNF, BMP7, BMPR1A, C21orf91, CAPN2, CASP8, CD101, CDH2, CFLAR, CUX1, CX3CL1, DNM2, DOCK1, EEF2K, ETV5, FBXO5, FES, GATD3A, GHR, GLIPR2, GPR68, GPRC5B, HDAC1, IL34, KCTD11, LCP1, LTA, MAP6, MARK2, MED1, MMP14, NFATC2, NPTN, NTRK2, OLFM1, OPRM1, PDZD2, PLAAT4, PLK5, PLXNC1, PPP1CC, PPP1R13L, PROM1, PTK2B, PTPRC, RARA, RIN2, RIPOR2, RORA, RUFY3, RUNX1, RUNX3, SEMA4D, SPEN, STAT1, STAT3, TCF4, TCF7L1, TENM4, TERT, TESPA1, TGFB2, TIMP2, TNF, WDFY2, ZBTB16, ZC3H12A, ZEB1, ZMIZ1, ZNF365, ZNF385A]	[ALK, ARNT, ASIC2, BOC, CDH4, CHODL, COBL, CTTN, DOCK1, DRD2, FBN2, GCNT2, GDF6, GNAS, HGF, HLA-DRB1, HLA-G, JADE2, LCP1, LMO3, LRRC7, LYN, MAGI2, MAP3K5, MAP6, MEF2A, METRN, MMP12, MYADM, MYF5, NDRG4, NGF, NUMB, PAK1, PAX4, PCP4, PLK5, PPP3CA, PRKCA, PRKCZ, PRKN, RARB, RB1, RIMS1, RIPOR2, ROBO1, ROBO2, RUNX1, SAV1, SEMA5A, SHANK1, SMAD1, SMAP1, SOX17, SOX5, STK25, STMN2, SYT17, TESC, TIAM1, TIAM2, TOX, TP73, VEGFC, WDFY2, ZEB1]

GO:0051094	positive regulation of developmental process	4,50E-05	[2, 3, 4]	241,00	16,31	29,06	38,87	32,08	[ADAM8, ADAMTS9, ANGPT2, AREG, BMPR1B, BTK, CASP8, CCL19, CCL24, CCR2, CCR3, CD27, CD3E, CD4, CD53, CD74, CD80, CD86, CDKN2A, CLCF1, CNR1, CRABP2, CRB2, CXCL9, CXCR4, CYBB, EGR3, ELAVL4, FFAR4, FOXP3, FRZB, GDF6, HCLS1, HLA-DRB1, IFNG, IL18, IL7R, INPP5D, KLF5, LCP1, LEF1, LTA, MEDAG, MIXL1, MUSK, MYB, NCKAP1L, NEK5, NLRP3, NPPC, P2RY12, PIM1, PLAAT4, PLXNC1, PTK2B, PTPRC, RHEX, RHOH, RLN2, RUNX3, SASH3, SEMA4A, SEMA4D, STAT1, SYK, TACSTD2, TESPA1, TNFAIP3, TNFSF13B, TOX, TREM2, VNN1, VWC2L, WNT1, WNT10B, ZAP70, ZNF804A]	[ACVR1B, ADA, ADGRL3, ADGRV1, AGAP2, AGO2, AGRN, AKAP6, ARRB2, ATOH8, B2M, BASP1, BCL2, BDNF, BMP7, BMPR1A, C21orf91, CAPN2, CASP8, CD101, CD28, CDH2, CELF1, CFLAR, CORO1C, CUX1, CX3CL1, DDAH1, DNM2, DOCK1, EEF2K, ETV5, FBXO5, FES, GATD3A, GHR, GLIPR2, GPR68, GPRC5B, HDAC1, HDAC9, HMGA1, HOXB7, HSPB6, IDO1, IL34, KCTD11, KDM2B, LCP1, LGR4, LRRN3, LTA, MAP6, MARK2, MED1, MMP14, NFATC2, NPTN, NTRK2, OLFM1, OPRM1, PARD3, PDZD2, PIGU, PLAAT4, PLK5, PLXNC1, PPP1CC, PPP1R13L, PRKD2, PROM1, PTK2B, PTPRC, RARA, RIN2, RIPOR2, RORA, RUFY3, RUNX1, RUNX3, SEMA4D, SLC25A12, SPEN, STAT1, STAT3, TCF4, TCF7L1, TENM4, TERT, TESPA1, TGFB2, TIMP2, TNF, TNFAIP3, TSHR, WDFY2, YAP1, ZBTB16, ZC3H12A, ZEB1, ZMIZ1, ZNF365, ZNF385A]	[AGO2, ALK, ARNT, ASIC2, BOC, CAMP, CDH4, CHODL, CLSTN2, COBL, CTTN, DCN, DOCK1, DRD2, ERBB4, ESRRB, FBN2, FGF7, FGF9, GCNT2, GDF6, GNAS, HGF, HIPK2, HLA-DRB1, HLA-G, ITGB2, JADE2, KCNA5, KDM5B, LCP1, LMO3, LRRC7, LYN, MAGI2, MAP3K5, MAP6, MEF2A, METRN, MMP12, MYADM, MYF5, NDRG4, NGF, NUMB, PAK1, PAK4, PAX4, PCP4, PLD6, PLK5, PPARGC1A, PPP3CA, PRKAA1, PRKCA, PRKCZ, PRKN, RARB, RB1, RIMS1, RIPOR2, ROBO1, ROBO2, RUNX1, SAV1, SEMA5A, SHANK1, SLITRK6, SMAD1, SMAP1, SOX17, SOX5, STIM1, STK25, STMN2, SYT17, TESC, THBS2, TIAM1, TIAM2, TOX, TP73, VEGFC, WDFY2, ZEB1]
GO:0048666	neuron development	9,16E-05	[4, 5, 7, 8, 9]	204,00	16,74	19,82	41,44	38,74	[AREG, BICDL1, BMPR1B, BTG2, C1QA, CD3E, CNR1, CRABP2, CSF1R, CXCR4, DOCK10, DOK2, DRD1, ELAVL4, EMB, EPHA6, FOLR2, GFI1, GFRA2, GPM6A, GRXCR2, HCLS1, HCN1, ITGA4, LST1, MUSK, NCKAP1L, NELL2, NPPC, NR4A2, NR4A3, PLPPR4, PLXNC1, PRDM1, PRKCQ, PTK2B, RUNX3, SEMA4A, SEMA4D, THY1, TNR, TOX, UGT8, ZNF804A]	[ADAM10, ADCY1, ADGRV1, AGRN, ANK3, ANKS1A, AUTS2, B2M, BCL2, BDNF, BICDL1, BMP7, C21orf91, CACNA1A, CAMK2G, CD38, CDH2, CFLAR, CRMP1, CTSZ, CUX1, CX3CL1, DNM2, DOK2, EEF2K, EPHA10, EVL, EXT1, FARP2, FES, FEZ2, FRY, GAB2, GABRA5, GAK, GATD3A, GBX2, GFRA2, GRB2, GSK3B, HDGFL3, HECW2, HERC1, IQSEC1, KIF13B, KLHL1, MAP6, MARK2, MRTFA, NCK2, NECTIN1, NEO1, NEXN, NFIB, NPTN, NTRK2, OLFM1, PARD3, PHACTR1, PIGU, PIK3CD, PLK5, PLXNC1, PTK2B, PTPRF, PTPRK, PTPRM, RAB13, RERE, RIPOR2, RORA, RUFY3, RUNX1, RUNX3, SEMA4D, SGK1, SIPA1L1, SLIT1, SRGAP3, TENM4, TGFB2, THRB, TMEM108, TRIM46, TRIO, TSHR, UBE3A, UGCG, UST, WDPCP, ZMIZ1, ZNF365]	[ALK, ANK3, ANKS1A, ARHGAP44, BCL11B, BOC, CDH4, CELSR3, CHODL, CNTNAP2, COBL, CRB1, CRTAC1, CSMD3, CTTN, DCC, DNM3, DRAXIN, DRD2, DSCAML1, EGFR, GABRA5, GBA2, GNAT1, GPM6A, HGF, JADE2, KIRREL3, LHX6, LHX8, LINGO1, LRRC7, LYN, MAGI2, MAP2, MAP6, MAPRE2, MEF2A, METRN, NDRG4, NGF, NR2F1, NRXN3, NTM, NUMB, NYAP2, ONECUT2, PAK1, PAK4, PHACTR1, PLK5, PPP3CA, PRKCA, PRKCZ, PRKN, PTPRQ, RAB35, RB1, RERE, RIMS1, RIPOR2, RND1, ROBO1, ROBO2, ROBO3, RORB, RTN4IP1, RUNX1, SALL3, SDK1, SEMA5A, SHANK1, SLC12A5, SLC4A7, SLITRK6, SRRM4, STK25, STMN2, SYT17, TIAM1, TIAM2, TOX, TSC1, UNC5D, USH1G, WASL]
GO:0000902	cell morphogenesis	5,77E-04	[3, 4]	188,00	16,61	24,38	42,29	33,33	[ADAM8, AREG, ARHGAP15, BMPR1B, CCL24, CEL, CLEC1B, CRABP2, CSF1R, CXCR4, DOCK10, DOK2, ELAVL4, EMB, EPHA6, ERMN, FGD2, FGD3, FGR, GFRA2, GPM6A, GRXCR2, HCLS1, IL7R, ITGA4, ITGB7, LEF1, LST1, MSX1, NCKAP1L, NELL2, NPPC, NR4A2, NR4A3, PLPPR4, PLXNC1, PRKCQ, PTK2B, PTPN6, SEMA4A, SEMA4D, ST14, TACSTD2, THY1, TNMD, TNR, UGT8, VSIG1, WAS]	[ADAM10, ADCY1, AGRN, ANK3, AUTS2, BCL2, BDNF, BMP7, CACNA1A, CAP2, CAPZB, CCDC88C, CD44, CDC42SE2, CDH17, CDH19, CDH2, CFDP1, CORO1A, CORO1C, CRMP1, CUX1, DNM2, DOCK1, DOK2, EEF2K, EPHA10, EVL, EXT1, FES, FEZ2, FRY, GAB2, GATD3A, GBP1, GBX2, GFRA2, GRB2, GSK3B, HECW2, ITGA8, KIF13B, MAP6, MARK2, MED1, NECTIN1, NEO1, NEXN, NFIB, NPTN, NTRK2, OLFM1, PALMD, PARD3, PARVB, PHACTR1, PIGU, PIK3CD, PLXNC1, PTK2B, PTPN6, PTPRM, PXN, RERE, RIPOR2, RUFY3, S100A13, SEMA4D, SGK1, SIPA1L1, SLIT1, SRGAP3, TGFB2, TMEM108, TRAF3, TRIM46, TRIO, UBE3A, UST, WDPCP, WIPF1, YAP1, ZMYM2, ZNF365, ZNF385A]	[ANK3, ARHGAP44, ASXL1, BCL11B, BOC, CDC42EP3, CDH13, CDH4, CELSR3, CHODL, CNTNAP2, COBL, CRB1, CTTN, DCC, DNM3, DOCK1, DRAXIN, DRD2, DSCAML1, EGFR, FAM171A1, FAT1, GPM6A, HGF, ITGB2, JADE2, KIRREL3, LIMD1, LINGO1, MAP2, MAP6, MBL2, MEF2A, METRN, MYADM, NGF, NRXN3, NUMB, NYAP2, PAK1, PALM2AKAP2, PDZD8, PHACTR1, PPP3CA, PRKCA, PRKCZ, PRKN, PTPRQ, RB1, RERE, RIMS1, RIPOR2, ROBO1, ROBO2, ROBO3, SEMA5A, SHANK1, SLITRK6, SOX17, STK25, SYT17, TIAM1, TIAM2, UNC5D, VPS33A, WASL]
GO:0120036	plasma membrane bounded cell projection organization	1,79E-03	[5]	252,00	15,53	21,48	41,85	36,67	[AIF1, AREG, BICDL1, BMPR1B, BTG2, CCL19, CD3E, CFAP54, CNR1, CRABP2, CSF1R, CXCR4, DNAH8, DOCK10, DOK2, DRC1, ELAVL4, EMB, EPHA6, FGD2, FGD3, FOLR2, GFI1, GFRA2, GPM6A, GRXCR2, HCLS1, ITGA4, KLF5, KLHL41, LST1, MUSK, NCKAP1L, NELL2, NME8, NPPC, NR4A2, NR4A3, P2RY12, PLEK, PLPPR4, PLXNC1, PRKCQ, PTK2B, SEMA4A, SEMA4D, SNX10, SPEF2, STAP1, TACSTD2, THY1, TNR, TOX, TREM2, UGT8, WAS, WNT1, ZNF804A]	[ACTR3, ADAM10, ADCY1, ADGRV1, AGRN, AKAP9, ANK3, ARMC2, AUTS2, B2M, BCL2, BDNF, BICDL1, BMP7, C21orf91, CACNA1A, CAMK2G, CAPZB, CCR7, CD2AP, CD38, CD44, CDH2, CDHR2, CEP135, CEP250, CFLAR, CLUAP1, CORO1A, CORO1C, CPLANE1, CRMP1, CTSZ, CUX1, CX3CL1, DNM2, DOK2, EEF2K, ENTR1, EPHA10, EVL, EXT1, FES, FEZ2, FRY, GAB2, GAK, GATD3A, GBX2, GFRA2, GRB2, GSK3B, GSN, HDGFL3, HECW2, HERC1, HOMER1, HTT, IGF1R, IQSEC1, KIF13B, KLHL1, MAP6, MARK2, MRTFA, NCK2, NECTIN1, NEO1, NEXN, NFIB, NPTN, NTRK2, OLFM1, PARD3, PARVB, PHACTR1, PIGU, PIK3CD, PLCE1, PLK5, PLXNC1, PRKD2, PTK2B, PTPRF, PTPRK, PTPRM, RAB13, RERE, RIPOR2, RORA, RSPH6A, RSPH9, RTTN, RUFY3, SAXO1, SEMA4D, SEPTIN9, SFI1, SGK1, SIPA1L1, SLIT1, SPATA13, SRGAP3, TCTN2, TMEM108, TRIM46, TRIO, TSHR, UBE3A, UST, WDPCP, WRAP73, ZNF365]	[ABLIM2, AKAP9, ALK, ANK3, ANLN, ARHGAP24, ARHGAP44, ATP6V0D1, ATP6V1D, BCL11B, BOC, CAV1, CDC42EP3, CDH13, CDH4, CELSR3, CFAP46, CFAP61, CHODL, CNTNAP2, COBL, CRTAC1, CSMD3, CTTN, DCC, DNAJB13, DNM3, DRAXIN, DRD2, DSCAML1, EGFR, GPM6A, HGF, HRG, IGF1R, JADE2, KIRREL3, LINGO1, LRRC7, LYN, MAGI2, MAP2, MAP6, MAPRE1, MAPRE2, MEF2A, METRN, MNS1, MYLK, NAV3, NDRG4, NGF, NR2F1, NRXN3, NUMB, NYAP2, ONECUT2, PAK1, PAK4, PHACTR1, PLCE1, PLK5, PPP3CA, PRKAR2B, PRKCA, PRKCZ, PRKN, RAB35, RB1, RERE, RFX4, RIMS1, RIPOR2, ROBO1, ROBO2, ROBO3, RTN4IP1, SDK1, SEMA5A, SEPTIN9, SHANK1, SLC12A5, SLITRK6, SPAG1, SPAG16, STK25, STMN2, SYNE2, SYT17, TIAM1, TIAM2, TOX, TPGS1, TSC1, TTLL8, TUB, UNC5D, USH1G, WASL]
GO:0030030	cell projection organization	3,58E-03	[4]	255,00	15,33	21,61	42,12	36,26	[AIF1, AREG, BICDL1, BMPR1B, BTG2, CCL19, CD3E, CFAP54, CNR1, CRABP2, CSF1R, CXCR4, DNAH8, DOCK10, DOK2, DRC1, ELAVL4, EMB, EPHA6, ERMN, FGD2, FGD3, FOLR2, GFI1, GFRA2, GPM6A, GRXCR2, HCLS1, ITGA4, KLF5, KLHL41, LST1, MUSK, NCKAP1L, NELL2, NME8, NPPC, NR4A2, NR4A3, P2RY12, PLEK, PLPPR4, PLXNC1, PRKCQ, PTK2B, SEMA4A, SEMA4D, SNX10, SPEF2, STAP1, TACSTD2, THY1, TNR, TOX, TREM2, UGT8, WAS, WNT1, ZNF804A]	[ACTR3, ADAM10, ADCY1, ADGRV1, AGRN, AKAP9, ANK3, ARMC2, AUTS2, B2M, BCL2, BDNF, BICDL1, BMP7, C21orf91, CACNA1A, CAMK2G, CAPZB, CCR7, CD2AP, CD38, CD44, CDH2, CDHR2, CEP135, CEP250, CFLAR, CLUAP1, CORO1A, CORO1C, CPLANE1, CRMP1, CTSZ, CUX1, CX3CL1, DNM2, DOK2, EEF2K, ENTR1, EPHA10, EVL, EXT1, FES, FEZ2, FRY, GAB2, GAK, GATD3A, GBX2, GFRA2, GRB2, GSK3B, GSN, HDGFL3, HECW2, HERC1, HOMER1, HTT, IGF1R, IQSEC1, ITGA8, KIF13B, KLHL1, MAP6, MARK2, MRTFA, NCK2, NECTIN1, NEO1, NEXN, NFIB, NPTN, NTRK2, OLFM1, PARD3, PARVB, PHACTR1, PIGU, PIK3CD, PLCE1, PLK5, PLXNC1, PRKD2, PTK2B, PTPRF, PTPRK, PTPRM, RAB13, RAB34, RERE, RIPOR2, RORA, RSPH6A, RSPH9, RTTN, RUFY3, SAXO1, SEMA4D, SEPTIN9, SFI1, SGK1, SIPA1L1, SLIT1, SPATA13, SRGAP3, TCTN2, TMEM108, TRIM46, TRIO, TSHR, UBE3A, UST, WDPCP, WRAP73, ZNF365]	[ABLIM2, AKAP9, ALK, ANK3, ANLN, ARHGAP24, ARHGAP44, ATP6V0D1, ATP6V1D, BCL11B, BOC, CAV1, CDC42EP3, CDH13, CDH4, CELSR3, CFAP46, CFAP61, CHODL, CNTNAP2, COBL, CRTAC1, CSMD3, CTTN, DCC, DNAJB13, DNM3, DRAXIN, DRD2, DSCAML1, EGFR, GPM6A, HGF, HRG, IGF1R, JADE2, KIRREL3, LINGO1, LRRC7, LYN, MAGI2, MAP2, MAP6, MAPRE1, MAPRE2, MEF2A, METRN, MNS1, MYLK, NAV3, NDRG4, NGF, NR2F1, NRXN3, NUMB, NYAP2, ONECUT2, PAK1, PAK4, PHACTR1, PLCE1, PLK5, PPP3CA, PRKAR2B, PRKCA, PRKCZ, PRKN, RAB35, RB1, RERE, RFX4, RIMS1, RIPOR2, ROBO1, ROBO2, ROBO3, RTN4IP1, SDK1, SEMA5A, SEPTIN9, SHANK1, SLC12A5, SLITRK6, SPAG1, SPAG16, STK25, STMN2, SYNE2, SYT17, TIAM1, TIAM2, TOX, TPGS1, TSC1, TTLL8, TUB, UNC5D, USH1G, WASL]
GO:0031175	neuron projection development	6,40E-03	[5, 6, 8, 9, 10]	175,00	16,23	20,63	42,86	36,51	[AREG, BICDL1, BMPR1B, BTG2, CD3E, CNR1, CRABP2, CSF1R, CXCR4, DOCK10, DOK2, ELAVL4, EMB, EPHA6, FOLR2, GFI1, GFRA2, GPM6A, GRXCR2, HCLS1, ITGA4, LST1, MUSK, NCKAP1L, NELL2, NPPC, NR4A2, NR4A3, PLPPR4, PLXNC1, PRKCQ, PTK2B, SEMA4A, SEMA4D, THY1, TNR, TOX, UGT8, ZNF804A]	[ADAM10, ADCY1, ADGRV1, AGRN, ANK3, AUTS2, B2M, BCL2, BDNF, BICDL1, BMP7, C21orf91, CACNA1A, CAMK2G, CD38, CDH2, CFLAR, CRMP1, CTSZ, CUX1, CX3CL1, DNM2, DOK2, EEF2K, EPHA10, EVL, EXT1, FES, FEZ2, FRY, GAB2, GAK, GATD3A, GBX2, GFRA2, GRB2, GSK3B, HDGFL3, HECW2, HERC1, IQSEC1, KIF13B, KLHL1, MAP6, MARK2, MRTFA, NCK2, NECTIN1, NEO1, NEXN, NFIB, NPTN, NTRK2, OLFM1, PARD3, PHACTR1, PIGU, PIK3CD, PLK5, PLXNC1, PTK2B, PTPRF, PTPRK, PTPRM, RAB13, RERE, RIPOR2, RORA, RUFY3, SEMA4D, SGK1, SIPA1L1, SLIT1, SRGAP3, TMEM108, TRIM46, TRIO, TSHR, UBE3A, UST, ZNF365]	[ALK, ANK3, ARHGAP44, BCL11B, BOC, CDH4, CELSR3, CHODL, CNTNAP2, COBL, CRTAC1, CSMD3, CTTN, DCC, DNM3, DRAXIN, DRD2, DSCAML1, EGFR, GPM6A, HGF, JADE2, KIRREL3, LINGO1, LRRC7, LYN, MAGI2, MAP2, MAP6, MEF2A, METRN, NDRG4, NGF, NR2F1, NRXN3, NUMB, NYAP2, PAK1, PAK4, PHACTR1, PLK5, PPP3CA, PRKCA, PRKCZ, PRKN, RAB35, RB1, RERE, RIMS1, RIPOR2, ROBO1, ROBO2, ROBO3, RTN4IP1, SDK1, SEMA5A, SHANK1, SLC12A5, SLITRK6, STK25, STMN2, SYT17, TIAM1, TIAM2, TOX, TSC1, UNC5D, USH1G, WASL]

GO:0022603	regulation of anatomical structure morphogenesis	1,20E-02	[3, 4]	187,00	15,91	21,78	44,55	33,66	[ADAMTS9, ANGPT2, AREG, ARHGAP15, CCL24, CCR2, CCR3, CD53, CRABP2, CRB2, CSF1R, CXCL10, CXCL9, CXCR4, CYBB, ERMN, FASLG, FGD2, FGD3, FGR, HCLS1, LST1, MSX1, NPPB, PIM1, PLXNC1, PROK1, PTK2B, RLN2, SEMA4A, SEMA4D, SLC18A1, STAT1, TACSTD2, THY1, TNFAIP3, TNFRSF11B, TNFSF13B, TNMD, TNR, TREM2, WAS, WNT1, WNT10A]	[ADA, ADAM10, ADAMTS12, AGO2, AKAP13, ARRB2, BASP1, BCL2, BDNF, BMP7, BMPR1A, CACNA1A, CAPN2, CAPZB, CD160, CD44, CDC42SE2, CDH2, CFDP1, CFLAR, COL4A2, COL5A1, CORO1A, CORO1C, CUX1, CX3CL1, DDAH1, DNM2, DOCK1, EEF2K, ETV5, FES, GATD3A, GBP1, GSK3B, HDAC9, HECW2, HOXB7, HSPB6, KIF13B, LGR4, MAP2K5, MAP6, MARK2, MBP, MED1, NFATC2, NFIB, NTRK2, OLFM1, PALMD, PARVB, PDCD10, PIGU, PLXNC1, PRICKLE1, PRKD2, PSMB8, PSMB9, PSMD9, PTK2B, PTPRM, RECK, RIN2, RIPOR2, RNH1, RORA, RUFY3, RUNX1, RXRA, S100A13, SEMA4D, SIPA1L1, STAT1, STAT3, TCF4, TENM4, TERT, TGFB2, THRB, TMEM135, TNF, TNFAIP3, TRIM46, UBE3A, UST, WDPCP, WIPF1, ZC3H12A, ZMYM2]	[ABCC8, AGO2, AKAP13, ARHGAP44, C11orf65, CAMP, CDC42EP3, CDH4, CELSR3, CHODL, COL4A2, COL5A2, CTTN, DCC, DCN, DNM3, DOCK1, DRAXIN, FAM171A1, FGF7, FOXP1, HGF, HIPK2, HLA-G, HRG, HTATIP2, ITGB2, KCNA5, LIMD1, LINGO1, MAGI2, MAP2, MAP6, MBL2, MBP, METRN, MYADM, MYF5, NGF, PAK1, PAK4, PALM2AKAP2, PDE3B, PDZD8, PLD6, PPARGC1A, PPP3CA, PRKCA, PRKN, PSMD7, RIMS1, RIPOR2, RNH1, ROBO1, ROBO2, RUNX1, RXRA, SEMA5A, SMAD1, STARD13, STIM1, STK25, SYT17, THBS2, TIAM1, TIAM2, VEGFC, WASL]
GO:0051960	regulation of nervous system development	2,27E-02	[4, 5, 6]	164,00	16,11	16,09	46,55	37,36	[ASCL2, BHLHE40, CCR2, CLCF1, CNR1, CRABP2, CXCR4, ELAVL4, GDF6, GFI1, HCLS1, IFNG, LTA, MUSK, MYB, MYCL, P2RY12, PLXNC1, PTK2B, SEMA4A, SEMA4D, STAP1, THY1, TNR, TOX, TREM2, VWC2L, ZNF804A]	[ADAM10, ADGRL3, AFDN, AGRN, B2M, BCL2, BDNF, BMP7, BMPR1A, C21orf91, CACNA1A, CAMK2G, CD38, CDH2, CDK5RAP1, CFLAR, CRMP1, CTSZ, CUX1, CX3CL1, DIXDC1, DNM2, DPEP1, EEF2K, ETV5, FES, GAK, GATD3A, GBX2, GPRC5B, GSK3B, HDAC1, HECW2, HOXB3, IL34, KCTD11, KIF13B, LRRN3, LTA, MAP6, MARK2, MED1, NECTIN1, NPTN, NTRK2, OLFM1, OPRM1, PARD3, PHACTR1, PLK5, PLXNC1, PPP1CC, PRKACB, PTK2B, PTPRF, RARA, RECK, RORA, RUFY3, SEMA4D, SIPA1L1, SLC25A12, SLIT1, SPEN, SRGAP3, STAT3, TCF4, TCF7L1, TENM4, TERT, TG, THRB, TIMP2, TNF, TRIM46, UBE3A, UST, YAP1, ZEB1, ZHX2, ZNF365]	[ABCC8, ALK, ARHGAP44, ASIC2, BCL11B, CDH4, CHODL, CLSTN2, COBL, CSMD3, CTTN, DCC, DISP3, DNM3, DRAXIN, DRD2, ERBB4, GDF6, GPR161, HGF, JADE2, LINGO1, LRRC7, LYN, MAGI2, MAP2, MAP6, METRN, MMP12, NDRG4, NGF, NR2F1, NUMB, OPALIN, PAK1, PCP4, PHACTR1, PLK5, PPP3CA, PRKCA, PRKN, RARB, RFX4, RIMS1, ROBO1, ROBO2, RTN4IP1, SDK1, SEMA5A, SHANK1, SKI, SLITRK6, STK25, STMN2, SYT17, THBS2, TIAM1, TIAM2, TOX, TP73, TSC1, TYMP, UNC5D, VEGFC, ZEB1]
GO:0031344	regulation of cell projection organization	4,02E-02	[4, 5]	126,00	16,78	19,12	41,91	38,97	[CCL19, CNR1, CRABP2, CXCR4, ELAVL4, ERMN, GFI1, GPM6A, HCLS1, KLF5, KLHL41, MUSK, P2RY12, PLXNC1, PTK2B, SEMA4A, SEMA4D, STAP1, TACSTD2, THY1, TNR, TOX, TREM2, WAS, WNT1, ZNF804A]	[ACTR3, ADAM10, AGRN, AUTS2, B2M, BDNF, BMP7, C21orf91, CACNA1A, CAMK2G, CAPZB, CCR7, CD38, CD44, CDH2, CDHR2, CEP135, CFLAR, CORO1C, CRMP1, CTSZ, CUX1, CX3CL1, DNM2, EEF2K, ENTR1, EVL, FES, GAK, GSK3B, HECW2, HOMER1, HTT, KIF13B, MAP6, MARK2, NPTN, NTRK2, OLFM1, PLCE1, PLK5, PLXNC1, PTK2B, PTPRF, RIPOR2, RORA, RUFY3, SAXO1, SEMA4D, SEPTIN9, SIPA1L1, TRIM46, UBE3A, UST, WDPCP, WRAP73, ZNF365]	[ALK, ANLN, ARHGAP24, ARHGAP44, CAV1, CDC42EP3, CDH4, CHODL, COBL, CSMD3, CTTN, DCC, DNM3, DRAXIN, GPM6A, HGF, HRG, LINGO1, LRRC7, LYN, MAGI2, MAP2, MAP6, MAPRE2, METRN, MNS1, NAV3, NDRG4, NGF, NR2F1, PAK1, PLCE1, PLK5, PPP3CA, PRKN, RIMS1, RIPOR2, ROBO1, ROBO2, RTN4IP1, SDK1, SEMA5A, SEPTIN9, SHANK1, STK25, STMN2, SYNE2, SYT17, TIAM1, TIAM2, TOX, TSC1, WASL]
GO:0050793	regulation of developmental process	1,09E-08	[2, 3]	438,00	15,60	28,63	38,53	32,84	[ADAM8, ADAMTS9, ANGPT2, AREG, ARHGAP15, ASCL2, BHLHA15, BHLHE40, BMPR1B, BTK, C1QC, CAMK4, CARD11, CASP8, CCL19, CCL24, CCR2, CCR3, CD2, CD27, CD3E, CD4, CD53, CD74, CD80, CD86, CDKN2A, CLCF1, CNR1, CRABP2, CRB2, CRTAM, CSF1R, CTLA4, CXCL10, CXCL9, CXCR4, CYBB, EAF2, EGR3, ELAVL4, EREG, ERMN, FASLG, FCRL3, FFAR4, FGD2, FGD3, FGL2, FGR, FOXP3, FRZB, GDF6, GFI1, GLIS1, GPR171, GPR55, H3C10, H3C11, H3C12, H3C7, HCLS1, HLA-DOA, HLA-DRB1, HOPX, IFNG, IL18, IL7R, INPP5D, IRF4, KLF5, KLHL41, LAG3, LCP1, LEF1, LMO1, LRRC17, LST1, LTA, MEDAG, MIXL1, MSX1, MUSK, MYB, MYCL, NAMPT, NCKAP1L, NEK5, NLRP3, NPPB, NPPC, NR4A3, P2RY12, PIM1, PLAAT4, PLAC8, PLXNC1, PRDM1, PRKCQ, PROK1, PTK2B, PTPN6, PTPRC, RHEX, RHOH, RLN2, RUNX3, SASH3, SEMA4A, SEMA4D, SLAMF8, SLC18A1, STAP1, STAT1, SYK, TACSTD2, TESPA1, THY1, TIFAB, TNFAIP3, TNFRSF11B, TNFRSF18, TNFSF13B, TNMD, TNR, TOX, TREM2, VNN1, VWC2L, WAS, WNT1, WNT10A, WNT10B, ZAP70, ZNF683, ZNF804A]	[ACVR1B, ADA, ADAM10, ADAMTS12, ADGRL3, ADGRV1, AFDN, AGAP2, AGO2, AGRN, AKAP13, AKAP6, ARRB2, ATOH8, B2M, BASP1, BCL2, BDNF, BMP7, BMPR1A, C21orf91, CACNA1A, CAMK2G, CAPN2, CAPZB, CASP8, CCM2, CD101, CD160, CD28, CD38, CD44, CDC42SE2, CDH2, CDK5RAP1, CDK6, CELF1, CFDP1, CFLAR, COL4A2, COL5A1, CORO1A, CORO1C, CRMP1, CTLA4, CTSZ, CUX1, CX3CL1, DDAH1, DIXDC1, DNM2, DOCK1, DPEP1, EEF2K, EHMT1, ETV5, FANCA, FBXO5, FES, GAK, GATD3A, GBP1, GBX2, GHR, GLIPR2, GPR68, GPRC5B, GSK3B, HDAC1, HDAC9, HECW2, HLA-B, HMGA1, HOXB3, HOXB7, HSPB6, IDO1, IL34, IRF1, JAK3, JARID2, KCTD11, KDM2B, KIF13B, LCP1, LGR4, LRRN3, LTA, MAFG, MAP2K5, MAP6, MARK2, MBP, MED1, MITF, MMP14, NECTIN1, NFATC1, NFATC2, NFIB, NPR2, NPTN, NTRK2, OLFM1, OPRM1, PALMD, PARD3, PARVB, PDCD10, PDZD2, PHACTR1, PHF14, PIGU, PLAAT4, PLK5, PLXNC1, PPP1CC, PPP1R13L, PRICKLE1, PRKACB, PRKD2, PROM1, PSMB8, PSMB9, PSMD9, PTK2B, PTPN2, PTPN6, PTPRC, PTPRF, PTPRM, RARA, RBFOX1, RECK, RIN2, RIPOR2, RNH1, RORA, RUFY3, RUNX1, RUNX3, RXRA, S100A13, SEMA4D, SIPA1L1, SLC25A12, SLIT1, SPEN, SPOCK2, SRGAP3, ST7, STAT1, STAT3, TCF4, TCF7L1, TENM4, TERT, TESPA1, TG, TGFB2, THRB, TIMP2, TMEM135, TNF, TNFAIP3, TRIM46, TRIO	[ABCC8, AGO2, AKAP13, ALK, ARHGAP44, ARNT, ASIC2, ASXL1, BCL11B, BOC, C11orf65, CAMK4, CAMP, CARD11, CARTPT, CAV1, CDC42EP3, CDH4, CDK13, CDSN, CELF4, CELSR3, CHODL, CLSTN2, COBL, COL4A2, COL5A2, CSMD3, CTTN, CXADR, DCC, DCN, DISP3, DMPK, DNM3, DOCK1, DRAXIN, DRD2, EGFR, ERBB4, ESRRB, FAM171A1, FBN2, FGF7, FGF9, FOXP1, FTO, GCNT2, GDF6, GNAS, GPR161, HGF, HIPK2, HLA-DRB1, HLA-G, HRG, HTATIP2, ITGB2, JADE2, JDP2, KCNA5, KCNK2, KDM5B, LCP1, LDLRAD4, LIMD1, LINGO1, LMO3, LRRC7, LYN, MAGI2, MAP2, MAP3K5, MAP6, MBL2, MBP, MEF2A, MEIS2, METRN, MMP12, MYADM, MYF5, NDRG4, NFATC1, NGF, NR1H3, NR2F1, NUMB, OPALIN, PAK1, PAK4, PALM2AKAP2, PAX4, PCP4, PDE3B, PDZD8, PHACTR1, PLAC8, PLD6, PLEKHB1, PLK5, PPARGC1A, PPP3CA, PRKAA1, PRKCA, PRKCZ, PRKN, PSMD7, PTPRQ, PUS7, RARB, RB1, RBFOX1, RFX4, RIMS1, RIPOR2, RNH1, ROBO1, ROBO2, RORB, RTN4IP1, RUNX1, RXRA, SAV1, SDK1, SEMA5A, SHANK1, SIGLEC15, SKI, SLITRK6, SMAD1, SMAD6, SMAP1, SOX17, SOX5, SRGN, STARD13, STIM1, STK25, STMN2, SYT17, TBX3, TESC, THBS2, TIAM1, TIAM2, TOX, TP73, TSC1, TYMP, UMODL1, UNC5D, VEGFC, WASL, WDFY2, ZEB1]
GO:0009653	anatomical structure morphogenesis	7,64E-06	[2, 3]	431,00	14,95	22,56	41,65	35,79	[ADAM8, ADAMTS9, ADGRG6, ANGPT1, ANGPT2, AQP5, AREG, ARHGAP15, BMPR1B, CCL24, CCR2, CCR3, CD53, CEL, CLEC1B, CPM, CRABP2, CRB2, CSF1R, CXCL10, CXCL9, CXCR3, CXCR4, CYBB, DOCK10, DOK2, DUSP2, E2F8, EGR3, ELAVL4, ELF3, EMB, EOMES, EPHA6, EREG, ERMN, FASLG, FGD2, FGD3, FGR, FOLR2, FRZB, GFRA2, GPM6A, GRXCR2, HCLS1, HCN1, HOPX, IER3, IL18, IL7R, ITGA4, ITGB7, KLF5, KLHL41, LEF1, LST1, MIXL1, MSX1, MYO3B, NCKAP1L, NELL2, NPPB, NPPC, NR4A1, NR4A2, NR4A3, OPN5, PAX5, PIK3CG, PIM1, PLPPR4, PLXNC1, PRDM1, PRKCQ, PROK1, PTK2B, PTPN6, RLN2, SDC1, SEMA4A, SEMA4D, SH2D2A, SLC18A1, SNX10, SPEF2, ST14, STAT1, SYK, TACSTD2, THY1, TIFAB, TNFAIP3, TNFRSF11B, TNFSF13B, TNMD, TNR, TREM2, UGT8, VSIG1, WAS, WNT1, WNT10A, WNT10B]	[ADA, ADAM10, ADAMTS12, ADCY1, AFDN, AFF3, AGO2, AGRN, AKAP13, ANGPTL2, ANK3, ANKRD11, ARRB2, ATOH8, AUTS2, BASP1, BCAR3, BCL2, BDNF, BICD1, BMP7, BMPR1A, CACNA1A, CAP2, CAPN2, CAPZB, CCDC88C, CCM2, CD160, CD44, CDC42SE2, CDH17, CDH19, CDH2, CFDP1, CFLAR, CHST11, CHSY1, CLEC14A, COL15A1, COL19A1, COL4A2, COL5A1, CORO1A, CORO1C, CPLANE1, CRISPLD1, CRMP1, CSGALNACT1, CTSZ, CUX1, CX3CL1, DDAH1, DNM2, DOCK1, DOK2, ECE1, EEF2K, ELK3, ENPEP, EPHA10, ETV5, EVL, EXT1, F2R, FES, FEZ2, FRY, GAB2, GATD3A, GBP1, GBX2, GCNT1, GFRA2, GHR, GRB2, GSK3B, HDAC1, HDAC9, HECW2, HERC1, HOXA3, HOXA4, HOXB1, HOXB3, HOXB7, HSPB6, IRX1, ITGA8, ITGB5, KCNA2, KCNH1, KDM2B, KIF13B, KLHL6, KLHL7, LGR4, LMBR1, MAP2K5, MAP6, MARK2, MBP, MED1, MFAP5, MICAL2, MMP14, MRTFA, MTHFD1L, NECTIN1, NEO1, NEXN, NFATC1, NFATC2, NFIB, NPTN, NTRK2, OLFM1, PALMD, PARD3, PARVB, PDCD10, PHACTR1, PIGU, PIK3CD, PLXNC1, PPP1R13L, PRICKLE1, PRKACB, PRKD2, PROM1, PSAP, PSMB8, PSMB9, PSMD9, PTK2B, PTPN6, PTPRM, PXN, RARA, RECK, RERE, RIN2, RIPOR2, RNF213, RNH1, RORA, RUFY3, RUNX1, RXRA, S100A13, SALL4, SATB2, SBNO2, SEMA4D, SGCD, SGK1, SH3BP1, SIPA1L1, SLC12A6, SLC38A1, SLIT1, SRGAP3, STAT1, STAT3, TCF4, TENM4, TERT	[ABCC8, AFF3, AGO2, AKAP13, ALDH1A2, ANK3, ARHGAP24, ARHGAP44, ASTN2, ASXL1, BCAR3, BCL11B, BOC, C11orf65, CAMP, CAPN9, CAV1, CD151, CD9, CDC42EP3, CDH13, CDH4, CDK5R2, CDSN, CELSR3, CHODL, CHSY1, CHUK, CNTNAP2, COBL, COL4A2, COL5A2, CRB1, CTTN, DCC, DCN, DNM3, DOCK1, DRAXIN, DRD2, DSCAML1, DYSF, EGFR, ENPEP, ERBB4, EXOC4, EYA2, FAM171A1, FAT1, FBN2, FGF7, FGF9, FMN1, FOXH1, FOXP1, GNAS, GNAT1, GPM6A, GREB1L, GREM2, HGF, HIPK2, HLA-G, HOXC9, HRG, HTATIP2, HYAL2, ITGB2, JADE2, KCNA5, KDM5B, KIRREL3, LGALS8, LHX8, LIMD1, LINGO1, MAGI2, MAP2, MAP6, MAPRE2, MBL2, MBP, MEF2A, MEIS2, METRN, MFGE8, MMP12, MYADM, MYF5, MYLK, MYOM2, NDRG4, NEBL, NFATC1, NGF, NRXN3, NUMB, NYAP2, ONECUT2, PAK1, PAK4, PALM2AKAP2, PAX4, PDE3B, PDZD8, PGR, PHACTR1, PLD6, PPARGC1A, PPP3CA, PRKCA, PRKCZ, PRKN, PSMD7, PTPRQ, RAMP1, RARB, RB1, RBM46, RERE, RIMS1, RIPOR2, RNH1, ROBO1, ROBO2, ROBO3, RORB, RUNX1, RXRA, SALL3, SAV1, SDK1, SEMA5A, SETDB2, SGCD, SHANK1, SKI, SLC38A1, SLITRK6, SMAD1, SMAD6, SOX17, SOX5, SOX7, STARD13, STIM1, STK25, SYT17, TBX3, TBXT, THBS2, THSD7A, TIAM1, TIAM2, TM4SF1, TSC1, TYMP, UNC5D, USH1G, VEGFC, VPS33A, WASL, WNK4, WNT16, ZEB1]
GO:0000902	cell morphogenesis	5,77E-04	[3, 4]	188,00	16,61	24,38	42,29	33,33	[ADAM8, AREG, ARHGAP15, BMPR1B, CCL24, CEL, CLEC1B, CRABP2, CSF1R, CXCR4, DOCK10, DOK2, ELAVL4, EMB, EPHA6, ERMN, FGD2, FGD3, FGR, GFRA2, GPM6A, GRXCR2, HCLS1, IL7R, ITGA4, ITGB7, LEF1, LST1, MSX1, NCKAP1L, NELL2, NPPC, NR4A2, NR4A3, PLPPR4, PLXNC1, PRKCQ, PTK2B, PTPN6, SEMA4A, SEMA4D, ST14, TACSTD2, THY1, TNMD, TNR, UGT8, VSIG1, WAS]	[ADAM10, ADCY1, AGRN, ANK3, AUTS2, BCL2, BDNF, BMP7, CACNA1A, CAP2, CAPZB, CCDC88C, CD44, CDC42SE2, CDH17, CDH19, CDH2, CFDP1, CORO1A, CORO1C, CRMP1, CUX1, DNM2, DOCK1, DOK2, EEF2K, EPHA10, EVL, EXT1, FES, FEZ2, FRY, GAB2, GATD3A, GBP1, GBX2, GFRA2, GRB2, GSK3B, HECW2, ITGA8, KIF13B, MAP6, MARK2, MED1, NECTIN1, NEO1, NEXN, NFIB, NPTN, NTRK2, OLFM1, PALMD, PARD3, PARVB, PHACTR1, PIGU, PIK3CD, PLXNC1, PTK2B, PTPN6, PTPRM, PXN, RERE, RIPOR2, RUFY3, S100A13, SEMA4D, SGK1, SIPA1L1, SLIT1, SRGAP3, TGFB2, TMEM108, TRAF3, TRIM46, TRIO, UBE3A, UST, WDPCP, WIPF1, YAP1, ZMYM2, ZNF365, ZNF385A]	[ANK3, ARHGAP44, ASXL1, BCL11B, BOC, CDC42EP3, CDH13, CDH4, CELSR3, CHODL, CNTNAP2, COBL, CRB1, CTTN, DCC, DNM3, DOCK1, DRAXIN, DRD2, DSCAML1, EGFR, FAM171A1, FAT1, GPM6A, HGF, ITGB2, JADE2, KIRREL3, LIMD1, LINGO1, MAP2, MAP6, MBL2, MEF2A, METRN, MYADM, NGF, NRXN3, NUMB, NYAP2, PAK1, PALM2AKAP2, PDZD8, PHACTR1, PPP3CA, PRKCA, PRKCZ, PRKN, PTPRQ, RB1, RERE, RIMS1, RIPOR2, ROBO1, ROBO2, ROBO3, SEMA5A, SHANK1, SLITRK6, SOX17, STK25, SYT17, TIAM1, TIAM2, UNC5D, VPS33A, WASL]

GO:0048646	anatomical structure formation involved in morphogenesis	8,64E-03 [2, 3, 4]	189,00	15,94	28,36	37,81	33,83	[ADAM8, ADAMTS9, ADGRG6, ANGPT1, ANGPT2, AREG, CCL24, CCR2, CCR3, CD53, CEL, CLEC1B, CRB2, CXCL10, CXCL9, CXCR3, CXCR4, CYBB, DUSP2, E2F8, EGR3, EOMES, EREG, FASLG, FOLR2, HOPX, IL18, ITGA4, KLF5, KLHL41, LEF1, MIXL1, MSX1, NELL2, NPPB, NR4A1, NR4A3, PIK3CG, PIM1, PROK1, PTK2B, PTPN6, RLN2, SEMA4A, SH2D2A, ST14, STAT1, SYK, THY1, TIFAB, TNFAIP3, TNFSF13B, TNMD, TREM2, UGT8, WNT1, WNT10B]	[ADA, AGO2, AKAP13, ANGPTL2, ATOH8, BMP7, BMPR1A, CACNA1A, CAPN2, CD160, CFLAR, CLEC14A, COL15A1, COL4A2, COL5A1, CX3CL1, DDAH1, ELK3, ENPEP, GATD3A, GBX2, GRB2, HDAC1, HDAC9, HERC1, HOXA3, HOXB1, HOXB3, HSPB6, IRX1, ITGA8, ITGB5, KCNH1, KDM2B, KLHL6, KLHL7, MAP2K5, MED1, MMP14, MTHFD1L, NECTIN1, NFATC2, NFIB, OLFM1, PDCD10, PIGU, PRICKLE1, PRKACB, PRKD2, PROM1, PSAP, PTK2B, PTPN6, PTPRM, RARA, RECK, RIPOR2, RNF213, RNH1, RORA, RUNX1, SALL4, SBNO2, SLC12A6, SLC38A1, STAT1, STAT3, TCF4, TENM4, TERT, TGFB2, TGFBI, TNF, TNFAIP3, ZC3H12A, ZNF385A]	[ABCC8, AGO2, AKAP13, ALDH1A2, ARHGAP24, CAMP, CAPN9, CAV1, CD9, CDH13, CDK5R2, COBL, COL4A2, COL5A2, CRB1, DCN, DYSF, ENPEP, EXOC4, EYA2, FBN2, FGF9, FMN1, FOXH1, GNAS, HGF, HIPK2, HLA-G, HRG, HTATIP2, ITGB2, KCNA5, MEF2A, MFGE8, MYF5, MYOM2, NEBL, NRXN3, PAK4, PDE3B, PPP3CA, PRKCA, RAMP1, RBM46, RIPOR2, RNH1, ROBO1, ROBO2, RUNX1, SDK1, SEMA5A, SKI, SLC38A1, SMAD1, SOX17, SOX7, STARD13, STIM1, TBX3, TBXT, THBS2, THSD7A, TM4SF1, TSC1, TYMP, VEGFC, VPS33A, WNT16]
GO:0022603	regulation of anatomical structure morphogenesis	1,20E-02 [3, 4]	187,00	15,91	21,78	44,55	33,66	[ADAMTS9, ANGPT2, AREG, ARHGAP15, CCL24, CCR2, CCR3, CD53, CRABP2, CRB2, CSF1R, CXCL10, CXCL9, CXCR4, CYBB, ERMN, FASLG, FGD2, FGD3, FGR, HCLS1, LST1, MSX1, NPPB, PIM1, PLXNC1, PROK1, PTK2B, RLN2, SEMA4A, SEMA4D, SLC18A1, STAT1, TACSTD2, THY1, TNFAIP3, TNFRSF11B, TNFSF13B, TNMD, TNR, TREM2, WAS, WNT1, WNT10A]	[ADA, ADAM10, ADAMTS12, AGO2, AKAP13, ARRB2, BASP1, BCL2, BDNF, BMP7, BMPR1A, CACNA1A, CAPN2, CAPZB, CD160, CD44, CDC42SE2, CDH2, CFDP1, CFLAR, COL4A2, COL5A1, CORO1A, CORO1C, CUX1, CX3CL1, DDAH1, DNM2, DOCK1, EEF2K, ETV5, FES, GATD3A, GBP1, GSK3B, HDAC9, HECW2, HOXB7, HSPB6, KIF13B, LGR4, MAP2K5, MAP6, MARK2, MBP, MED1, NFATC2, NFIB, NTRK2, OLFM1, PALMD, PARVB, PDCD10, PIGU, PLXNC1, PRICKLE1, PRKD2, PSMB8, PSMB9, PSMD9, PTK2B, PTPRM, RECK, RIN2, RIPOR2, RNH1, RORA, RUFY3, RUNX1, RXRA, S100A13, SEMA4D, SIPA1L1, STAT1, STAT3, TCF4, TENM4, TERT, TGFB2, THRB, TMEM135, TNF, TNFAIP3, TRIM46, UBE3A, UST, WDPCP, WIPF1, ZC3H12A, ZMYM2]	[ABCC8, AGO2, AKAP13, ARHGAP44, C11orf65, CAMP, CDC42EP3, CDH4, CELSR3, CHODL, COL4A2, COL5A2, CTTN, DCC, DCN, DNM3, DOCK1, DRAXIN, FAM171A1, FGF7, FOXP1, HGF, HIPK2, HLA-G, HRG, HTATIP2, ITGB2, KCNA5, LIMD1, LINGO1, MAGI2, MAP2, MAP6, MBL2, MBP, METRN, MYADM, MYF5, NGF, PAK1, PAK4, PALM2AKAP2, PDE3B, PDZD8, PLD6, PPARGC1A, PPP3CA, PRKCA, PRKN, PSMD7, RIMS1, RIPOR2, RNH1, ROBO1, ROBO2, RUNX1, RXRA, SEMA5A, SMAD1, STARD13, STIM1, STK25, SYT17, THBS2, TIAM1, TIAM2, VEGFC, WASL]
GO:0009653	anatomical structure morphogenesis	7,64E-06 [2, 3]	431,00	14,95	22,56	41,65	35,79	[ADAM8, ADAMTS9, ADGRG6, ANGPT1, ANGPT2, AQP5, AREG, ARHGAP15, BMPR1B, CCL24, CCR2, CCR3, CD53, CEL, CLEC1B, CPM, CRABP2, CRB2, CSF1R, CXCL10, CXCL9, CXCR3, CXCR4, CYBB, DOCK10, DOK2, DUSP2, E2F8, EGR3, ELAVL4, ELF3, EMB, EOMES, EPHA6, EREG, ERMN, FASLG, FGD2, FGD3, FGR, FOLR2, FRZB, GFRA2, GPM6A, GRXCR2, HCLS1, HCN1, HOPX, IER3, IL18, IL7R, ITGA4, ITGB7, KLF5, KLHL41, LEF1, LST1, MIXL1, MSX1, MYO3B, NCKAP1L, NELL2, NPPB, NPPC, NR4A1, NR4A2, NR4A3, OPN5, PAX5, PIK3CG, PIM1, PLPPR4, PLXNC1, PRDM1, PRKCQ, PROK1, PTK2B, PTPN6, RLN2, SDC1, SEMA4A, SEMA4D, SH2D2A, SLC18A1, SNX10, SPEF2, ST14, STAT1, SYK, TACSTD2, THY1, TIFAB, TNFAIP3, TNFRSF11B, TNFSF13B, TNMD, TNR, TREM2, UGT8, VSIG1, WAS, WNT1, WNT10A, WNT10B]	[ADA, ADAM10, ADAMTS12, ADCY1, AFDN, AFF3, AGO2, AGRN, AKAP13, ANGPTL2, ANK3, ANKRD11, ARRB2, ATOH8, AUTS2, BASP1, BCAR3, BCL2, BDNF, BICD1, BMP7, BMPR1A, CACNA1A, CAP2, CAPN2, CAPZB, CCDC88C, CCM2, CD160, CD44, CDC42SE2, CDH17, CDH19, CDH2, CFDP1, CFLAR, CHST11, CHSY1, CLEC14A, COL15A1, COL19A1, COL4A2, COL5A1, CORO1A, CORO1C, CPLANE1, CRISPLD1, CRMP1, CSGALNACT1, CTSZ, CUX1, CX3CL1, DDAH1, DNM2, DOCK1, DOK2, ECE1, EEF2K, ELK3, ENPEP, EPHA10, ETV5, EVL, EXT1, F2R, FES, FEZ2, FRY, GAB2, GATD3A, GBP1, GBX2, GCNT1, GFRA2, GHR, GRB2, GSK3B, HDAC1, HDAC9, HECW2, HERC1, HOXA3, HOXA4, HOXB1, HOXB3, HOXB7, HSPB6, IRX1, ITGA8, ITGB5, KCNA2, KCNH1, KDM2B, KIF13B, KLHL6, KLHL7, LGR4, LMBR1, MAP2K5, MAP6, MARK2, MBP, MED1, MFAP5, MICAL2, MMP14, MRTFA, MTHFD1L, NECTIN1, NEO1, NEXN, NFATC1, NFATC2, NFIB, NPTN, NTRK2, OLFM1, PALMD, PARD3, PARVB, PDCD10, PHACTR1, PIGU, PIK3CD, PLXNC1, PPP1R13L, PRICKLE1, PRKACB, PRKD2, PROM1, PSAP, PSMB8, PSMB9, PSMD9, PTK2B, PTPN6, PTPRM, PXN, RARA, RECK, RERE, RIN2, RIPOR2, RNF213, RNH1, RORA, RUFY3, RUNX1, RXRA, S100A13, SALL4, SATB2, SBNO2, SEMA4D, SGCD, SGK1, SH3BP1, SIPA1L1, SLC12A6, SLC38A1, SLIT1, SRGAP3, STAT1, STAT3, TCF4, TENM4, TERT [illegible]	[ABCC8, AFF3, AGO2, AKAP13, ALDH1A2, ANK3, ARHGAP24, ARHGAP44, ASTN2, ASXL1, BCAR3, BCL11B, BOC, C11orf65, CAMP, CAPN9, CAV1, CD151, CD9, CDC42EP3, CDH13, CDH4, CDK5R2, CDSN, CELSR3, CHODL, CHSY1, CHUK, CNTNAP2, COBL, COL4A2, COL5A2, CRB1, CTTN, DCC, DCN, DNM3, DOCK1, DRAXIN, DRD2, DSCAML1, DYSF, EGFR, ENPEP, ERBB4, EXOC4, EYA2, FAM171A1, FAT1, FBN2, FGF7, FGF9, FMN1, FOXH1, FOXP1, GNAS, GNAT1, GPM6A, GREB1L, GREM2, HGF, HIPK2, HLA-G, HOXC9, HRG, HTATIP2, HYAL2, ITGB2, JADE2, KCNA5, KDM5B, KIRREL3, LGALS8, LHX8, LIMD1, LINGO1, MAGI2, MAP2, MAP6, MAPRE2, MBL2, MBP, MEF2A, MEIS2, METRN, MFGE8, MMP12, MYADM, MYF5, MYLK, MYOM2, NDRG4, NEBL, NFATC1, NGF, NRXN3, NUMB, NYAP2, ONECUT2, PAK1, PAK4, PALM2AKAP2, PAX4, PDE3B, PDZD8, PGR, PHACTR1, PLD6, PPARGC1A, PPP3CA, PRKCA, PRKCZ, PRKN, PSMD7, PTPRQ, RAMP1, RARB, RB1, RBM46, RERE, RIMS1, RIPOR2, RNH1, ROBO1, ROBO2, ROBO3, RORB, RUNX1, RXRA, SALL3, SAV1, SDK1, SEMA5A, SETDB2, SGCD, SHANK1, SKI, SLC38A1, SLITRK6, SMAD1, SMAD6, SOX17, SOX5, SOX7, STARD13, STIM1, STK25, SYT17, TBX3, TBXT, THBS2, THSD7A, TIAM1, TIAM2, TM4SF1, TSC1, TYMP, UNC5D, USH1G, VEGFC, VPS33A, WASL, WNK4, WNT16, ZEB1]
GO:0072358	cardiovascular system development	2,37E-03 [4, 5, 6]	136,00	17,39	25,17	40,82	34,01	[ADAM8, ADAMTS9, ANGPT1, ANGPT2, CCL24, CCR2, CCR3, CEL, CRB2, CXCL10, CXCR3, CXCR4, CYBB, E2F8, EGR3, EREG, FASLG, FOLR2, FOSL1, IL18, KLF5, LEF1, NELL2, NPPB, NR4A1, PIK3CG, PRDM1, PROK1, PTK2B, RLN2, SEMA4A, SH2D2A, STAT1, SYK, THY1, TNFAIP3, TNMD]	[ADAM10, AGO2, ANGPTL2, BMP7, BMPR1A, CCM2, CD160, CDH2, CFLAR, CLEC14A, COL15A1, COL4A2, COL5A1, CPLANE1, CX3CL1, DDAH1, DNM2, ELK3, ENPEP, GATD3A, GBX2, HDAC9, HOXA3, HOXB3, HSPB6, KCNQ1, LTBP1, MAP2K5, MED1, MMP14, NFATC1, NFATC2, NTRK2, PDCD10, PIGU, PRICKLE1, PRKD2, PTK2B, PTPRM, RECK, RIN2, RNF213, RNH1, RORA, RUNX1, SGCD, SLC12A6, SLC38A1, STAT1, STAT3, TCF4, TERT, TGFB2, TGFBI, TNF, TNFAIP3, WDPCP, YAP1, ZC3H12A, ZMIZ1]	[ABCC8, AGO2, ALDH1A2, ARHGAP24, CAMP, CAV1, CDH13, COL4A2, DCN, DYSF, ENPEP, FGF9, FOXH1, HGF, HIPK2, HLA-G, HRG, HTATIP2, ITGB2, KCNA5, LGALS8, MFGE8, MYLK, NFATC1, NRXN3, NXN, PAK4, PDE3B, PPP3CA, PRKCA, RAMP1, RNH1, ROBO1, ROBO2, RUNX1, SEMA5A, SGCD, SLC38A1, SMAD1, SMAD6, SOX17, SPEG, STARD13, STIM1, TBX3, TBXT, THBS2, THSD7A, TYMP, VEGFC]
GO:0035239	tube morphogenesis	2,66E-03 [3, 4, 5]	154,00	16,90	25,45	40,00	34,55	[ADAM8, ADAMTS9, ANGPT1, ANGPT2, AREG, CCL24, CCR2, CCR3, CEL, CSF1R, CXCL10, CXCR3, CXCR4, CYBB, E2F8, EGR3, EOMES, EREG, FASLG, FOLR2, IL18, KLF5, LEF1, NELL2, NPPB, NR4A1, NR4A3, PIK3CG, PRDM1, PROK1, PTK2B, RLN2, SEMA4A, SH2D2A, ST14, STAT1, SYK, TACSTD2, THY1, TNFAIP3, TNMD, WNT1]	[ADAMTS12, AGO2, ANGPTL2, ATOH8, BCL2, BMP7, BMPR1A, CCM2, CD160, CDH2, CLEC14A, COL15A1, COL4A2, CTSZ, CX3CL1, DDAH1, ELK3, ENPEP, ETV5, GATD3A, GBX2, HDAC9, HOXA3, HOXB3, HOXB7, HSPB6, IRX1, KDM2B, LGR4, MAP2K5, MED1, MICAL2, MMP14, MTHFD1L, NFIB, NTRK2, PDCD10, PIGU, PRICKLE1, PRKACB, PRKD2, PTK2B, PTPRM, RARA, RECK, RIN2, RNF213, RNH1, RORA, RUNX1, SALL4, SGCD, SLC12A6, SLC38A1, STAT1, STAT3, TCF4, TERT, TGFB2, TGFBI, TNF, TNFAIP3, YAP1, ZC3H12A, ZEB1, ZMIZ1]	[ABCC8, AGO2, ARHGAP24, CAMP, CAV1, CDH13, COBL, COL4A2, DCN, DYSF, EGFR, ENPEP, FGF9, FMN1, FOXH1, FOXP1, GREB1L, HGF, HIPK2, HLA-G, HRG, HTATIP2, ITGB2, KCNA5, KDM5B, MFGE8, MYLK, NDRG4, NRXN3, PAK1, PAK4, PDE3B, PGR, PPP3CA, PRKCA, RAMP1, RNH1, ROBO1, RUNX1, SEMA5A, SETDB2, SGCD, SKI, SLC38A1, SMAD1, SOX17, STARD13, STIM1, TBX3, TBXT, THBS2, THSD7A, TSC1, TYMP, VEGFC, WNK4, ZEB1]
GO:0001525	angiogenesis	5,65E-03 [3, 4, 5, 6, 7, 8, 9, 10]	101,00	18,36	30,00	35,45	34,55	[ADAM8, ADAMTS9, ANGPT1, ANGPT2, CCL24, CCR2, CCR3, CEL, CXCL10, CXCR3, CXCR4, CYBB, E2F8, EGR3, EREG, FASLG, IL18, KLF5, LEF1, NELL2, NPPB, NR4A1, PIK3CG, PROK1, PTK2B, RLN2, SEMA4A, SH2D2A, STAT1, SYK, THY1, TNFAIP3, TNMD]	[AGO2, ANGPTL2, CD160, CLEC14A, COL15A1, COL4A2, CX3CL1, DDAH1, ELK3, ENPEP, GBX2, HDAC9, HOXA3, HOXB3, HSPB6, MAP2K5, MED1, MMP14, PDCD10, PIGU, PRKD2, PTK2B, PTPRM, RECK, RNF213, RNH1, RORA, RUNX1, SLC12A6, SLC38A1, STAT1, STAT3, TCF4, TERT, TGFB2, TGFBI, TNF, TNFAIP3, ZC3H12A]	[ABCC8, AGO2, ARHGAP24, CAMP, CAV1, CDH13, COL4A2, DCN, DYSF, ENPEP, FGF9, HGF, HIPK2, HLA-G, HRG, HTATIP2, ITGB2, KCNA5, MFGE8, NRXN3, PAK4, PDE3B, PPP3CA, PRKCA, RAMP1, RNH1, ROBO1, RUNX1, SEMA5A, SLC38A1, SMAD1, SOX17, STARD13, STIM1, THBS2, THSD7A, TYMP, VEGFC]
GO:0048514	blood vessel morphogenesis	7,62E-03 [4, 5, 6, 7, 8, 9]	115,00	17,67	28,00	38,40	33,60	[ADAM8, ADAMTS9, ANGPT1, ANGPT2, CCL24, CCR2, CCR3, CEL, CXCL10, CXCR3, CXCR4, CYBB, E2F8, EGR3, EREG, FASLG, FOLR2, IL18, KLF5, LEF1, NELL2, NPPB, NR4A1, PIK3CG, PRDM1, PROK1, PTK2B, RLN2, SEMA4A, SH2D2A, STAT1, SYK, THY1, TNFAIP3, TNMD]	[AGO2, ANGPTL2, BMPR1A, CCM2, CD160, CDH2, CLEC14A, COL15A1, COL4A2, CX3CL1, DDAH1, ELK3, ENPEP, GATD3A, GBX2, HDAC9, HOXA3, HOXB3, HSPB6, MAP2K5, MED1, MMP14, NTRK2, PDCD10, PIGU, PRKD2, PTK2B, PTPRM, RECK, RIN2, RNF213, RNH1, RORA, RUNX1, SGCD, SLC12A6, SLC38A1, STAT1, STAT3, TCF4, TERT, TGFB2, TGFBI, TNF, TNFAIP3, YAP1, ZC3H12A, ZMIZ1]	[ABCC8, AGO2, ARHGAP24, CAMP, CAV1, CDH13, COL4A2, DCN, DYSF, ENPEP, FGF9, FOXH1, HGF, HIPK2, HLA-G, HRG, HTATIP2, ITGB2, KCNA5, MFGE8, MYLK, NRXN3, PAK4, PDE3B, PPP3CA, PRKCA, RAMP1, RNH1, ROBO1, RUNX1, SEMA5A, SGCD, SLC38A1, SMAD1, SOX17, STARD13, STIM1, TBXT, THBS2, THSD7A, TYMP, VEGFC]
GO:0048646	anatomical structure formation involved in morphogenesis	8,64E-03 [2, 3, 4]	189,00	15,94	28,36	37,81	33,83	[ADAM8, ADAMTS9, ADGRG6, ANGPT1, ANGPT2, AREG, CCL24, CCR2, CCR3, CD53, CEL, CLEC1B, CRB2, CXCL10, CXCL9, CXCR3, CXCR4, CYBB, DUSP2, E2F8, EGR3, EOMES, EREG, FASLG, FOLR2, HOPX, IL18, ITGA4, KLF5, KLHL41, LEF1, MIXL1, MSX1, NELL2, NPPB, NR4A1, NR4A3, PIK3CG, PIM1, PROK1, PTK2B, PTPN6, RLN2, SEMA4A, SH2D2A, ST14, STAT1, SYK, THY1, TIFAB, TNFAIP3, TNFSF13B, TNMD, TREM2, UGT8, WNT1, WNT10B]	[ADA, AGO2, AKAP13, ANGPTL2, ATOH8, BMP7, BMPR1A, CACNA1A, CAPN2, CD160, CFLAR, CLEC14A, COL15A1, COL4A2, COL5A1, CX3CL1, DDAH1, ELK3, ENPEP, GATD3A, GBX2, GRB2, HDAC1, HDAC9, HERC1, HOXA3, HOXB1, HOXB3, HSPB6, IRX1, ITGA8, ITGB5, KCNH1, KDM2B, KLHL6, KLHL7, MAP2K5, MED1, MMP14, MTHFD1L, NECTIN1, NFATC2, NFIB, OLFM1, PDCD10, PIGU, PRICKLE1, PRKACB, PRKD2, PROM1, PSAP, PTK2B, PTPN6, PTPRM, RARA, RECK, RIPOR2, RNF213, RNH1, RORA, RUNX1, SALL4, SBNO2, SLC12A6, SLC38A1, STAT1, STAT3, TCF4, TENM4, TERT, TGFB2, TGFBI, TNF, TNFAIP3, ZC3H12A, ZNF385A]	[ABCC8, AGO2, AKAP13, ALDH1A2, ARHGAP24, CAMP, CAPN9, CAV1, CD9, CDH13, CDK5R2, COBL, COL4A2, COL5A2, CRB1, DCN, DYSF, ENPEP, EXOC4, EYA2, FBN2, FGF9, FMN1, FOXH1, GNAS, HGF, HIPK2, HLA-G, HRG, HTATIP2, ITGB2, KCNA5, MEF2A, MFGE8, MYF5, MYOM2, NEBL, NRXN3, PAK4, PDE3B, PPP3CA, PRKCA, RAMP1, RBM46, RIPOR2, RNH1, ROBO1, ROBO2, RUNX1, SDK1, SEMA5A, SKI, SLC38A1, SMAD1, SOX17, SOX7, STARD13, STIM1, TBX3, TBXT, THBS2, THSD7A, TM4SF1, TSC1, TYMP, VEGFC, VPS33A, WNT16]

GO:0072359	circulatory system development	1,10E-02	[4, 5]	186,00	15,97	21,39	40,30	38,31	[ADAM8, ADAMTS9, ADGRG6, ANGPT1, ANGPT2, CCL24, CCR2, CCR3, CEL, CRB2, CXCL10, CXCR3, CXCR4, CYBB, DRC1, E2F8, EGR3, EOMES, EREG, FASLG, FOLR2, FOSL1, IL18, KLF5, LEF1, MIXL1, MSX1, NELL2, NPPB, NR4A1, PIK3CG, PIM1, PRDM1, PROK1, PTK2B, RLN2, SEMA4A, SH2D2A, STAT1, SYK, THY1, TNFAIP3, TNMD]	[ADAM10, AGO2, AKAP13, AKAP6, ANGPTL2, ARRB2, BASP1, BMP7, BMPR1A, CASP7, CCM2, CD160, CDH2, CFLAR, CLEC14A, COL15A1, COL4A2, COL5A1, CPLANE1, CX3CL1, DDAH1, DNM2, DPEP1, ECE1, ELK3, ENPEP, GATD3A, GBX2, HDAC9, HOXA3, HOXB3, HSPB6, JARID2, KCNQ1, LTBP1, MAP2K5, MED1, MICAL2, MMP14, NFATC1, NFATC2, NTRK2, OLFM1, PDCD10, PDLIM2, PDZD2, PIGU, PLCE1, POU6F1, PPP1R13L, PRICKLE1, PRKD2, PTK2B, PTPRM, RARA, RECK, RIN2, RNF213, RNH1, RORA, RPS6KA2, RUNX1, RXRA, SALL4, SGCD, SLC12A6, SLC38A1, SLIT1, STAT1, STAT3, TCF4, TENM4, TERT, TGFB2, TGFBI, TNF, TNFAIP3, WDPCP, YAP1, ZC3H12A, ZMIZ1]	[ABCC8, AGO2, AKAP13, ALDH1A2, ARHGAP24, ASXL1, CAMP, CAV1, CDH13, COL4A2, CXADR, DCN, DYSF, EGFR, ENPEP, ERBB4, FGF12, FGF9, FOXH1, GREB1L, HGF, HIPK2, HLA-G, HRG, HTATIP2, ITGB2, KCNA5, KCNK2, LGALS8, MEF2A, MFGE8, MYLK, MYOM2, NDRG4, NEBL, NFATC1, NRXN3, NXN, PAK1, PAK4, PDE3B, PLCE1, PPP3CA, PRKCA, RAMP1, RARB, RB1, RBM20, RNH1, ROBO1, ROBO2, RPS6KA2, RUNX1, RXRA, SAV1, SEMA5A, SETDB2, SGCD, SGCZ, SLC38A1, SMAD1, SMAD6, SMYD2, SOX17, SPEG, SRI, STARD13, STIM1, TBX3, TBXT, THBS2, THSD7A, TP73, TSC1, TYMP, VEGFC, WNT16]
GO:0001568	blood vessel development	1,21E-02	[3, 5, 6, 7, 8]	127,00	17,16	26,28	40,15	33,58	[ADAM8, ADAMTS9, ANGPT1, ANGPT2, CCL24, CCR2, CCR3, CEL, CXCL10, CXCR3, CXCR4, CYBB, E2F8, EGR3, EREG, FASLG, FOLR2, FOSL1, IL18, KLF5, LEF1, NELL2, NPPB, NR4A1, PIK3CG, PRDM1, PROK1, PTK2B, RLN2, SEMA4A, SH2D2A, STAT1, SYK, THY1, TNFAIP3, TNMD]	[AGO2, ANGPTL2, BMP7, BMPR1A, CCM2, CD160, CDH2, CFLAR, CLEC14A, COL15A1, COL4A2, COL5A1, CPLANE1, CX3CL1, DDAH1, DNM2, ELK3, ENPEP, GATD3A, GBX2, HDAC9, HOXA3, HOXB3, HSPB6, LTBP1, MAP2K5, MED1, MMP14, NTRK2, PDCD10, PIGU, PRICKLE1, PRKD2, PTK2B, PTPRM, RECK, RIN2, RNF213, RNH1, RORA, RUNX1, SGCD, SLC12A6, SLC38A1, STAT1, STAT3, TCF4, TERT, TGFB2, TGFBI, TNF, TNFAIP3, YAP1, ZC3H12A, ZMIZ1]	[ABCC8, AGO2, ALDH1A2, ARHGAP24, CAMP, CAV1, CDH13, COL4A2, DCN, DYSF, ENPEP, FGF9, FOXH1, HGF, HIPK2, HLA-G, HRG, HTATIP2, ITGB2, KCNA5, MFGE8, MYLK, NRXN3, PAK4, PDE3B, PPP3CA, PRKCA, RAMP1, RNH1, ROBO1, ROBO2, RUNX1, SEMA5A, SGCD, SLC38A1, SMAD1, SMAD6, SOX17, STARD13, STIM1, TBX3, TBXT, THBS2, THSD7A, TYMP, VEGFC]
GO:0001944	vasculature development	1,47E-02	[4, 5, 6, 7]	131,00	16,95	25,35	40,85	33,80	[ADAM8, ADAMTS9, ANGPT1, ANGPT2, CCL24, CCR2, CCR3, CEL, CXCL10, CXCR3, CXCR4, CYBB, E2F8, EGR3, EREG, FASLG, FOLR2, FOSL1, IL18, KLF5, LEF1, NELL2, NPPB, NR4A1, PIK3CG, PRDM1, PROK1, PTK2B, RLN2, SEMA4A, SH2D2A, STAT1, SYK, THY1, TNFAIP3, TNMD]	[ADAM10, AGO2, ANGPTL2, BMP7, BMPR1A, CCM2, CD160, CDH2, CFLAR, CLEC14A, COL15A1, COL4A2, COL5A1, CPLANE1, CX3CL1, DDAH1, DNM2, ELK3, ENPEP, GATD3A, GBX2, HDAC9, HOXA3, HOXB3, HSPB6, LTBP1, MAP2K5, MED1, MMP14, NFATC1, NFATC2, NTRK2, PDCD10, PIGU, PRICKLE1, PRKD2, PTK2B, PTPRM, RECK, RIN2, RNF213, RNH1, RORA, RUNX1, SGCD, SLC12A6, SLC38A1, STAT1, STAT3, TCF4, TERT, TGFB2, TGFBI, TNF, TNFAIP3, YAP1, ZC3H12A, ZMIZ1]	[ABCC8, AGO2, ALDH1A2, ARHGAP24, CAMP, CAV1, CDH13, COL4A2, DCN, DYSF, ENPEP, FGF9, FOXH1, HGF, HIPK2, HLA-G, HRG, HTATIP2, ITGB2, KCNA5, LGALS8, MFGE8, MYLK, NFATC1, NRXN3, PAK4, PDE3B, PPP3CA, PRKCA, RAMP1, RNH1, ROBO1, ROBO2, RUNX1, SEMA5A, SGCD, SLC38A1, SMAD1, SMAD6, SOX17, STARD13, STIM1, TBX3, TBXT, THBS2, THSD7A, TYMP, VEGFC]
GO:0035295	tube development	2,01E-02	[3, 4]	180,00	15,90	23,83	39,90	36,27	[ADAM8, ADAMTS9, ANGPT1, ANGPT2, AREG, CCL24, CCR2, CCR3, CEL, CSF1R, CXCL10, CXCR3, CXCR4, CYBB, E2F8, EDN2, EGR3, EOMES, EREG, FASLG, FOLR2, IL18, KLF5, LEF1, MIXL1, NELL2, NPPB, NR4A1, NR4A3, PIK3CG, PRDM1, PROK1, PTK2B, PTPN6, RLN2, SDC1, SEMA4A, SH2D2A, ST14, STAT1, SYK, TACSTD2, THY1, TNFAIP3, TNMD, WNT1]	[ADA, ADAMTS12, AGO2, ANGPTL2, ATOH8, BASP1, BCL2, BDNF, BMP7, BMPR1A, CCM2, CD160, CDH2, CLEC14A, CLMP, COL15A1, COL4A2, CTSZ, CX3CL1, DDAH1, ELK3, ENPEP, ETV5, GATD3A, GBX2, HDAC9, HECA, HOXA3, HOXB3, HOXB7, HSD11B1, HSPB6, IRX1, KDM2B, LGR4, MAP2K5, MED1, MICAL2, MMP14, MTHFD1L, NFIB, NTRK2, PDCD10, PHF14, PIGU, PRICKLE1, PRKACB, PRKD2, PROM1, PTK2B, PTPN6, PTPRM, RARA, RECK, RIN2, RNF213, RNH1, RORA, RUNX1, SALL4, SGCD, SLC12A6, SLC38A1, STAT1, STAT3, TCF4, TERT, TGFB2, TGFBI, THRB, TNF, TNFAIP3, TNS3, YAP1, ZC3H12A, ZEB1, ZMIZ1]	[ABCA12, ABCC8, ADAMTS2, AGO2, ALDH1A2, ARHGAP24, ASXL1, CAMP, CAV1, CDH13, COBL, COL4A2, DCN, DYSF, EGFR, ENPEP, FGF7, FGF9, FMN1, FOXH1, FOXP1, GPR161, GREB1L, HGF, HIPK2, HLA-G, HRG, HTATIP2, ITGB2, KCNA5, KDM5B, MFGE8, MMP12, MYLK, NDRG4, NRXN3, PAK1, PAK4, PDE3B, PGR, PPP3CA, PRKCA, RAMP1, RARB, RB1, RNH1, ROBO1, ROBO2, RUNX1, SAV1, SEMA5A, SETDB2, SGCD, SKI, SLC38A1, SMAD1, SMAD6, SOX17, STARD13, STIM1, TBX3, TBXT, THBS2, THSD7A, TNS3, TSC1, TYMP, VEGFC, WNK4, ZEB1]
GO:0032879	regulation of localization	8,31E-07	[2, 3]	451,00	15,09	26,49	36,34	37,17	[ADAM8, ADAMTS9, ADORA3, AIF1, AIM2, ANGPT1, ANGPT2, ANO9, AREG, BANK1, BLK, C2, CACNA1E, CALCR, CARD17, CASP8, CCL19, CCL24, CCR2, CCR5, CD2, CD200R1, CD244, CD247, CD4, CD40LG, CD74, CD84, CLCNKA, CLEC4E, CLEC6A, CNR1, CRTAM, CSF1R, CTSS, CXCL10, CXCL11, CXCL9, CXCR3, CXCR4, CYBB, DAPK1, DOCK10, DOCK2, DOCK8, DRC1, DRD1, EDN2, EREG, ERFE, FASLG, FCN1, FCRL3, FFAR4, FGR, FOXP3, FUT7, GPSM3, HCLS1, HCN1, HLA-DRB1, IFNG, IKBKE, IL26, IL2RG, ITGA4, JPH1, KCNA3, KCNJ10, KCNQ5, KLRK1, KMO, LCK, LCP1, LEF1, LPAL2, LRRC38, MCOLN2, MCOLN3, MYB, NCKAP1L, NELL2, NLRP3, NLRP6, NPPB, NR4A3, ORM2, P2RY12, P2RY2, PIK3CG, PIM1, PLA2G7, PLAU, PLCH2, PLXNC1, PTAFR, PTK2B, PTPN22, PTPN6, PTPRC, PTX3, PYHIN1, RAB3B, RAB3C, SDC1, SELE, SEMA4A, SEMA4D, SLAMF8, SLC31A2, SPINK1, STAP1, SYK, TACSTD2, TCAF2, THY1, TIFAB, TLR10, TLR6, TLR8, TNFAIP3, TNFRSF18, TRAT1, TREM2, WAS, XCL1, XCL2, XCR1, XG]	[ABAT, ACAA2, ACVR1B, ADA, ADAM10, ADCY1, ADORA3, AFDN, AGO2, AIM2, AKAP6, AKAP9, ANK3, ARRB2, ATOH8, ATXN2, B2M, BCL2, BDNF, BICD1, BMP7, BMPR1A, BTBD9, CAB39, CABP1, CACNA1A, CACNB2, CAMK2G, CASP8, CCM2, CCR7, CD160, CD2AP, CD38, CD47, CDH2, CDK6, CEP135, CEP250, CORO1A, CORO1C, CPNE3, CX3CL1, CXCL11, DACH1, DAPK1, DENND5B, DNM2, DOCK1, DPEP1, DPP6, EDEM1, EEF2K, ENTR1, EVL, EXPH5, F2R, FBXO5, FES, GAB2, GBP1, GCC2, GLIPR2, GPR68, GPSM3, GRIK5, GRM7, GSK3B, GSN, HDAC9, HECW2, HOMER1, HOMER2, HTT, IGF1R, IL17RA, IL34, IQSEC1, ITPR1, ITPR2, KCNA2, KCNAB2, KCNC2, KCNG3, KCNH1, KCNIP1, KCNIP4, KCNQ1, KCTD11, LCK, LCP1, LGR4, LIME1, LRCH1, MAP2K2, MAP2K5, MBP, MCC, MDFIC, MEAK7, MITF, MMP14, MTNR1B, NETO1, NEXN, NOS1AP, NPTN, NUP153, OPRM1, PARD3, PDCD10, PDE4B, PDZD2, PHACTR1, PIGU, PIK3CD, PLCE1, PLIN3, PLXNC1, PPFIA1, PPP1CC, PPP3CC, PRKAG2, PRKAR1B, PRKCE, PRKD2, PSMD9, PTK2B, PTPN1, PTPN2, PTPN22, PTPN6, PTPRC, PTPRJ, PTPRK, PTPRM, RAB13, RALB, RASA3, RASSF3, RASSF5, RECK, RFFL, RIN2, RIPOR2, RORA, RUFY3, SEMA4D, SEPTIN9, SGIP1, SGK1, SH3BP1, SLC1A2, SLC4A8, SPATA13, SPIDR, SPOCK2, SQSTM1, SRGAP3, SSH2, SSTR4, STAT3, STK10, STX8, TANK, TERT, TGFB2, TNF, TNFAIP3, TNIP1, [illegible]	[ABCA12, ABCC8, ABI3, ADCY5, ADORA1, AGO2, AIM2, AKAP12, AKAP9, ALOX15, ANK3, ANKRD13B, ARHGAP44, ASIC2, ASTN2, ASXL1, ATP1B1, BTNL2, C11orf65, C2CD5, CACNA1S, CACNA2D3, CACNG2, CADPS2, CARD8, CARTPT, CATSPER2, CAV1, CD151, CD9, CDH13, CDK5R2, CELSR3, CHRNA4, CHUK, COMT, CPLX2, CTSD, CYP4A11, DCN, DISP3, DLG2, DMPK, DNM3, DOCK1, DPP6, DRD2, DYSF, EGFR, EPB41L2, ERBB4, F10, FGF12, FGF14, FGF7, FGF9, FOXP1, FRMD4A, FTO, GCNT2, GNAS, GPSM2, GRIN2A, GRM7, HGF, HIP1, HLA-DRB1, HRG, HSPA2, HYAL2, IGF1R, ITGB2, ITPR1, KCNA5, KCNB2, KCND2, KCNG2, KCNJ15, KCNJ3, KCNK15, KCNMA1, KCNS3, KDM5B, KIAA1614, LCP1, LDLRAD4, LIMCH1, LRRC55, LRRC7, LYN, MAGI1, MAGI2, MAP2, MAPRE1, MAPRE2, MBL2, MBP, MCC, MEF2A, MFGE8, MMP12, MTUS1, MYADM, MYLK, NAV3, NDRG4, NKAIN2, NKAIN3, NOS1AP, NR1H3, NRXN3, NUMB, OAZ1, ONECUT2, ORAI1, OSBPL6, P2RX2, PAK1, PCSK9, PDE4B, PFKM, PHACTR1, PLCE1, PLPP3, PNKD, PPARGC1A, PPP2R5A, PPP3CA, PRKAA1, PRKAG2, PRKCA, PRKCZ, PRKN, PTPN22, PTPRT, RAB7A, RABGAP1L, RB1, REM2, RIMS1, RIPOR2, ROBO1, RSPO1, SCN3B, SEMA5A, SEPTIN9, SHANK1, SHISA6, SLC17A7, SLC30A5, SMAP1, SRGN, SRI, SST, STARD13, STIM1, STXBP5L, SWAP70, SYNE2, SYT17, TBC1D1, TBC1D5, TCAF1, TERF1, TESC, TIAM1, TLR1, TNFRSF11A, TP73, TRAF2, TUB, UNC5D, USP36, USP8, VEGFC, VSNL1, WASL, WFS1, WNK2, WNK4, WRAP53]
GO:0051049	regulation of transport	5,15E-04	[3, 4]	308,00	15,22	27,41	32,83	39,76	[ADAM8, AIF1, AIM2, ANGPT1, ANO9, BANK1, BLK, C2, CACNA1E, CALCR, CARD17, CASP8, CCL19, CCR2, CD2, CD200R1, CD244, CD4, CD40LG, CD74, CD84, CLCNKA, CLEC4E, CLEC6A, CNR1, CRTAM, CSF1R, CTSS, CXCL10, CXCL11, CXCL9, CXCR4, CYBB, DAPK1, DOCK2, DRD1, ERFE, FCN1, FCRL3, FFAR4, FGR, FOXP3, HCLS1, HCN1, HLA-DRB1, IFNG, IL26, IL2RG, JPH1, KCNA3, KCNJ10, KCNQ5, KMO, LCP1, LPAL2, LRRC38, MYB, NCKAP1L, NLRP3, NLRP6, NPPB, NR4A3, ORM2, P2RY12, P2RY2, PIK3CG, PIM1, PTAFR, PTK2B, PTPN22, PTPN6, PTPRC, PTX3, RAB3B, RAB3C, SDC1, SELE, SLC31A2, SPINK1, STAP1, SYK, TCAF2, THY1, TIFAB, TLR10, TLR6, TLR8, TNFAIP3, TRAT1, TREM2, XCL1]	[ABAT, ACAA2, ADA, ADCY1, AIM2, AKAP6, AKAP9, ANK3, ARRB2, ATXN2, B2M, BCL2, BICD1, BTBD9, CAB39, CABP1, CACNA1A, CACNB2, CAMK2G, CASP8, CCM2, CCR7, CD160, CD2AP, CD38, CD47, CDH2, CORO1A, CX3CL1, CXCL11, DAPK1, DENND5B, DNM2, DPP6, EDEM1, EEF2K, EXPH5, F2R, FES, GAB2, GBP1, GCC2, GPR68, GRIK5, GRM7, GSK3B, HDAC9, HECW2, HOMER1, HOMER2, HTT, IL17RA, ITPR1, ITPR2, KCNA2, KCNAB2, KCNC2, KCNG3, KCNH1, KCNIP1, KCNIP4, KCNQ1, KCTD11, LCP1, LGR4, LIME1, MAP2K2, MBP, MDFIC, MTNR1B, NETO1, NOS1AP, NUP153, OPRM1, PDCD10, PDE4B, PPP1CC, PPP3CC, PRKAG2, PRKAR1B, PRKCE, PRKD2, PSMD9, PTK2B, PTPN1, PTPN22, PTPN6, PTPRC, PTPRJ, RAB13, RALB, RUFY3, SEPTIN9, SGIP1, SGK1, SLC1A2, SLC4A8, SSTR4, TANK, TERT, TGFB2, TNF, TNFAIP3, TNK2, TP53BP2, TRIM46, UBASH3B, UBE2L3, ZC3H12A]	[ABCA12, ABCC8, ADCY5, ADORA1, AIM2, AKAP12, AKAP9, ALOX15, ANK3, ANKRD13B, ARHGAP44, ASIC2, ATP1B1, BTNL2, C11orf65, C2CD5, CACNA1S, CACNA2D3, CACNG2, CADPS2, CARD8, CARTPT, CATSPER2, CAV1, CD151, CDH13, CDK5R2, CHRNA4, CHUK, COMT, CPLX2, CYP4A11, DISP3, DLG2, DMPK, DNM3, DPP6, DRD2, DYSF, EGFR, FGF12, FGF14, FOXP1, FRMD4A, GNAS, GRIN2A, GRM7, HIP1, HLA-DRB1, HSPA2, HYAL2, ITGB2, ITPR1, KCNA5, KCNB2, KCND2, KCNG2, KCNJ15, KCNJ3, KCNK15, KCNMA1, KCNS3, KDM5B, LCP1, LRRC55, LRRC7, LYN, MAGI1, MAGI2, MAP2, MBL2, MBP, MEF2A, MFGE8, MMP12, MYLK, NDRG4, NKAIN2, NKAIN3, NOS1AP, NR1H3, NRXN3, NUMB, OAZ1, ORAI1, OSBPL6, P2RX2, PAK1, PCSK9, PDE4B, PFKM, PNKD, PPARGC1A, PPP3CA, PRKAA1, PRKAG2, PRKCA, PRKCZ, PRKN, PTPN22, RAB7A, REM2, RIMS1, RSPO1, SCN3B, SEPTIN9, SHANK1, SHISA6, SLC17A7, SLC30A5, SMAP1, SRGN, SRI, STIM1, STXBP5L, SYT17, TBC1D5, TCAF1, TESC, TIAM1, TLR1, TNFRSF11A, TP73, TRAF2, TUB, USP36, VEGFC, VSNL1, WASL, WFS1, WNK2, WNK4]
GO:0043269	regulation of ion transport	5,25E-04	[4, 5]	134,00	17,94	28,08	30,14	41,78	[ANO9, CACNA1E, CALCR, CCR2, CD4, CD84, CLCNKA, CNR1, CTSS, CXCL10, CXCL11, CXCL9, CXCR4, CYBB, DAPK1, DRD1, ERFE, FCRL3, HCN1, IFNG, JPH1, KCNA3, KCNJ10, KCNQ5, KMO, LRRC38, NLRP3, P2RY12, PIK3CG, PTAFR, PTK2B, PTPN22, PTPN6, RAB3B, SLC31A2, SPINK1, SYK, TCAF2, THY1, TREM2, XCL1]	[ABAT, AKAP6, AKAP9, ANK3, ARRB2, B2M, BCL2, CAB39, CACNA1A, CACNB2, CAMK2G, CORO1A, CX3CL1, CXCL11, DAPK1, DNM2, DPP6, F2R, GRM7, HECW2, HOMER1, HOMER2, HTT, KCNA2, KCNAB2, KCNC2, KCNG3, KCNH1, KCNIP1, KCNIP4, KCNQ1, LIME1, MTNR1B, NETO1, NOS1AP, OPRM1, PDE4B, PRKCE, PRKD2, PTK2B, PTPN22, PTPN6, SSTR4, UBASH3B]	[ABCC8, ADORA1, AKAP9, ANK3, ASIC2, ATP1B1, CACNA1S, CACNA2D3, CACNG2, CATSPER2, CAV1, CHRNA4, CYP4A11, DLG2, DMPK, DPP6, DRD2, DYSF, FGF12, FGF14, GRIN2A, GRM7, HSPA2, KCNA5, KCNB2, KCND2, KCNG2, KCNJ15, KCNJ3, KCNK15, KCNMA1, KCNS3, LRRC55, LRRC7, LYN, MYLK, NKAIN2, NKAIN3, NOS1AP, ORAI1, P2RX2, PCSK9, PDE4B, PPARGC1A, PRKCA, PRKN, PTPN22, REM2, SCN3B, SHANK1, SHISA6, SLC30A5, SRI, STIM1, SYT17, TCAF1, TESC, TNFRSF11A, WFS1, WNK2, WNK4]

GO:0051050	positive regulation of transport	1,16E-02	[2, 3, 4, 5]	176,00	16,09	31,05	30,53	38,42	[ADAM8, AIF1, AIM2, ANGPT1, BLK, C2, CALCR, CASP8, CCL19, CCR2, CD2, CD244, CD4, CLEC4E, CLEC6A, CNR1, CRTAM, CSF1R, CTSS, CXCL10, CXCL11, CXCL9, DOCK2, DRD1, ERFE, FCN1, FFAR4, FGR, HCLS1, HLA-DRB1, IFNG, IL26, IL2RG, KMO, LCP1, LPAL2, LRRC38, MYB, NCKAP1L, NLRP3, NPPB, NR4A3, ORM2, P2RY12, P2RY2, PTAFR, PTPN22, PTPRC, PTX3, RAB3B, SDC1, SELE, STAP1, SYK, TCAF2, THY1, TLR8, TREM2, XCL1]	[ABAT, AIM2, AKAP6, AKAP9, ANK3, ARRB2, B2M, BCL2, BICD1, CACNA1A, CACNB2, CASP8, CCM2, CD160, CD2AP, CD38, CD47, CDH2, CX3CL1, CXCL11, DENND5B, DNM2, EDEM1, EEF2K, EXPH5, F2R, GAB2, GPR68, GSK3B, HOMER1, HTT, IL17RA, KCNC2, KCNQ1, LCP1, MBP, NOS1AP, PDCD10, PPP3CC, PRKCE, PRKD2, PSMD9, PTPN22, PTPRC, PTPRJ, RUFY3, SGIP1, SGK1, SLC1A2, SLC4A8, SSTR4, TERT, TGFB2, TNF, TP53BP2, TRIM46, UBE2L3, ZC3H12A]	[ABCA12, ABCC8, ADORA1, AIM2, AKAP12, AKAP9, ANK3, ATP1B1, BTNL2, C2CD5, CACNG2, CADPS2, CARD8, CARTPT, CAV1, CD151, CDK5R2, CHUK, CYP4A11, DNM3, DRD2, EGFR, FGF12, FGF14, FRMD4A, HIP1, HLA-DRB1, HSPA2, HYAL2, ITGB2, LCP1, LRRC55, MAGI1, MAGI2, MAP2, MBL2, MBP, MEF2A, MFGE8, MMP12, MYLK, NOS1AP, NR1H3, OAZ1, ORAI1, P2RX2, PAK1, PCSK9, PFKM, PPP3CA, PRKAA1, PRKCA, PRKCZ, PRKN, PTPN22, RAB7A, SCN3B, SRI, STIM1, TBC1D5, TCAF1, TESC, TLR1, TNFRSF11A, TP73, TUB, USP36, VEGFC, VSNL1, WASL, WFS1, WNK2, WNK4]
GO:0046903	secretion	1,59E-02	[4]	273,00	14,92	33,56	35,57	30,87	[ABCB11, ADAM8, AIF1, AIM2, ANGPT1, AQP5, BANK1, BIN2, BLK, BTK, BTN3A1, BTN3A2, CACNA1E, CARD11, CARD17, CCL19, CCR2, CD2, CD200R1, CD244, CD40LG, CD53, CD74, CD84, CEL, CFP, CLEC12A, CLEC4D, CLEC4E, CLEC6A, CNR1, CRTAM, CSF1R, CTSS, CTSW, CYBB, DOCK2, DRD1, FCN1, FFAR4, FGL2, FGR, FOXP3, FRMPD3, GBP5, HLA-DRB1, IFNG, IL26, IQGAP2, KLF5, KLRG1, KMO, KNG1, LCP1, LCP2, LPAL2, LRMP, LYZ, MCOLN2, MYB, MYO1G, NCKAP1L, NLRP3, NLRP6, NPPB, NR4A3, ORM2, P2RY12, P2RY2, PIK3CG, PLA2G2D, PLAC8, PLAU, PLEK, PTAFR, PTPN22, PTPN6, PTPRC, PTX3, RAB3B, RAB3C, RAB44, RNASET2, SDC1, SELL, SLC18A1, SNX10, SUCNR1, SYK, SYTL1, TBC1D10C, TIFAB, TLR10, TLR6, TLR8, TNFAIP3, TNFSF13B, TREM2, TXNDC5, VNN1]	[ABAT, ADA, ADAM10, ADCY1, ADGRE5, AIM2, ASAH1, ATG7, B2M, BDNF, BIN2, BPI, CAB39, CACNA1A, CACNB2, CAMK2G, CBLN4, CCM2, CCR7, CD160, CD2AP, CD38, CD44, CD47, CORO1A, COTL1, CPNE3, CTSZ, CX3CL1, CYSTM1, EEF1A1, EXOC6B, EXPH5, F13A1, F2R, FES, GAB2, GBP1, GBP5, GNPTAB, GPR68, GRIK5, GRM7, GSK3B, GSN, HDAC9, HLA-B, IL17RA, ITGB6, ITPR1, ITPR2, KCNA2, KCNAB2, KCNC2, KCNQ1, KCTD11, LCP1, LCP2, LGR4, LPCAT1, MBP, MED1, MICAL2, MTNR1B, MYO1G, NCOR2, NTRK2, OPRM1, PCDH7, PIK3CD, PPFIA1, PPFIA4, PRKCE, PSAP, PSMD9, PTPN22, PTPN6, PTPRC, PTPRJ, PTPRN2, RAB13, RALB, RNASET2, S100A13, SEPTIN9, SGK1, SLC15A4, SLC1A2, SLC1A5, SLC4A8, SSTR4, STK10, STX11, SYN3, TANK, TBC1D10C, TGFB2, TIMP2, TMC6, TNF, TNFAIP3, TRAF3, TSPOAP1, UBE2Z, UBR4, ZC3H12A]	[ABCA12, ABCC8, ADCY5, ADORA1, AIM2, AKAP12, AMPD3, ARHGAP44, ATP6V1D, BTNL2, CADPS2, CAMP, CARD11, CARD8, CARTPT, CAV1, CD9, CDK13, CDK5R2, CHRNA4, CHUK, COMT, CPLX2, CRISP3, CTAGE1, CTSD, CYP4A11, DRD2, DYSF, EGFR, ERBB4, EXOC4, FGF7, FOXP1, FRMD4A, GNAS, GRM7, HGF, HLA-DRB1, HRG, HYAL2, ITGB2, ITPR1, KCNA5, KCNG2, KCNS3, KDM5B, LCP1, LRRC7, LYN, LYZ, MBL2, MBP, MMP12, MNDA, NR1H3, NRXN3, PAK1, PDXK, PFKM, PLAC8, PLPPR3, PNKD, PPP3CA, PRKCA, PRKCZ, PRKN, PSMD7, PTPN22, PTPRN2, RAB37, RAB7A, RIMS1, SEPTIN9, SERPINA4, SLC15A4, SLC17A7, SLC1A7, SLC9A4, SRGN, SRI, STXBP5L, SYT17, TBX3, TIAM1, TLR1, TNFRSF11A, TRAF2, VEGFC, VPS33A, VSNL1, WNK4]
GO:0030001	metal ion transport	6,39E-08	[6]	186,00	18,64	22,77	35,64	41,58	[ABCB11, ATP1B4, BHLHA15, CACNA1E, CALCR, CCL19, CCR5, CD4, CD84, CNR1, CP, CXCL10, CXCL11, CXCL9, CXCR4, CYSLTR1, DRD1, FASLG, FCRL3, GPM6A, HCN1, IFNG, JPH1, KCNA3, KCNJ10, KCNQ5, LCK, LRRC38, MCOLN2, MCOLN3, MS4A1, NMUR1, P2RY12, PIK3CG, PLCH2, PTK2B, PTPN22, PTPN6, PTPRC, SLC18A1, SLC31A2, SPINK1, THY1, TREM2, XCL1, XCR1]	[AKAP6, AKAP9, ANK3, ANXA9, ARRB2, ATP6V0A2, ATP6V1B2, B2M, BCL2, CAB39, CACHD1, CACNA1A, CACNB2, CAMK2G, CCR7, CORO1A, CX3CL1, CXCL11, DNM2, DPP6, F2R, FAM155A, GRIK5, HECW2, HOMER1, HOMER2, HTT, ITPR1, ITPR2, KCNA2, KCNAB2, KCNC2, KCNG3, KCNH1, KCNIP1, KCNIP4, KCNK9, KCNQ1, LCK, LIME1, MICU2, MTNR1B, NECTIN1, NETO1, NOS1AP, OPRM1, PDE4B, PKD1L3, PLCE1, PRKCE, PRKD2, PTK2B, PTPN22, PTPN6, PTPRC, RASA3, SGK1, SLC12A6, SLC12A8, SLC13A4, SLC17A3, SLC1A2, SLC20A2, SLC22A3, SLC28A3, SLC38A1, SLC38A11, SLC4A8, SLC6A11, SLC9A9, TSPOAP1, UBASH3B]	[ABCC8, ADORA1, AKAP9, ANK3, ASIC2, ASIC4, ATP1B1, ATP2A3, ATP2B2, ATP6V0D1, ATP6V1D, CACNA1S, CACNA2D3, CACNG2, CATSPER2, CAV1, CHRNA4, DMPK, DPP6, DRD2, DYSF, FGF12, FGF14, FLVCR2, GPM6A, GRIK2, GRIN2A, HRG, HSPA2, ITPR1, KCNA5, KCNB2, KCND2, KCNG2, KCNJ15, KCNJ3, KCNK15, KCNK2, KCNK3, KCNMA1, KCNMB2, KCNS3, KCNT2, LRRC55, LYN, MYLK, NKAIN2, NKAIN3, NOS1AP, ORAI1, P2RX2, PCSK9, PDE4B, PLCE1, PPP3CA, PRKCA, PRKCZ, PTPN22, RAMP1, REM2, SCARA5, SCN3B, SLC12A5, SLC12A7, SLC17A7, SLC17A8, SLC30A5, SLC38A1, SLC38A10, SLC38A8, SLC4A7, SLC5A11, SLC6A12, SLC6A2, SLC9A3, SLC9A4, SRI, STIM1, TESC, TMPRSS3, TSC1, WFS1, WNK2, WNK4]
GO:0006812	cation transport	6,94E-07	[5]	224,00	17,34	23,36	34,84	41,80	[ABCB11, ATP1B4, BHLHA15, BTK, CACNA1E, CALCR, CALHM6, CCL19, CCR2, CCR5, CD4, CD84, CNR1, CP, CTSS, CXCL10, CXCL11, CXCL9, CXCR4, CYSLTR1, DAPK1, DRD1, FASLG, FCRL3, GPM6A, HCN1, IFNG, JPH1, KCNA3, KCNJ10, KCNQ5, LCK, LRRC38, MCOLN2, MCOLN3, MS4A1, NMUR1, P2RY12, PIK3CG, PLCH2, PTK2B, PTPN22, PTPN6, PTPRC, RAB3B, SLC18A1, SLC25A48, SLC31A2, SLC36A2, SPINK1, SYK, THY1, TREM2, UCP2, UNC80, XCL1, XCR1]	[ABAT, ABCG2, ADA, AKAP6, AKAP9, ANK3, ANXA9, ARRB2, ATP6V0A2, ATP6V1B2, B2M, BCL2, CAB39, CACHD1, CACNA1A, CACNB2, CALHM4, CAMK2G, CCR7, CHRNA1, CORO1A, CX3CL1, CXCL11, DAPK1, DNM2, DPP6, F2R, FAM155A, GRIK5, HECW2, HOMER1, HOMER2, HTT, ITPR1, ITPR2, KCNA2, KCNAB2, KCNC2, KCNG3, KCNH1, KCNIP1, KCNIP4, KCNK9, KCNQ1, LCK, LIME1, MICU2, MTNR1B, NECTIN1, NETO1, NOS1AP, OPRM1, PDE4B, PIEZO1, PKD1L3, PLCE1, PRKCE, PRKD2, PTK2B, PTPN22, PTPN6, PTPRC, RASA3, SGK1, SLC12A6, SLC12A8, SLC13A4, SLC15A4, SLC17A3, SLC1A2, SLC1A5, SLC20A2, SLC22A3, SLC28A3, SLC38A1, SLC38A11, SLC4A8, SLC6A11, SLC7A10, SLC7A2, SLC9A9, TSPOAP1, UBASH3B, UCP2, UNC80]	[ABCC8, ADORA1, AKAP9, ANK3, ASIC2, ASIC4, ATP1B1, ATP2A3, ATP2B2, ATP6V0D1, ATP6V1D, CACNA1S, CACNA2D3, CACNG2, CATSPER2, CAV1, CHRNA4, CLCN3, COX10, DLG2, DMAC2L, DMPK, DPP6, DRD2, DYSF, FGF12, FGF14, FLVCR2, GPM6A, GRIK2, GRIN2A, HRG, HSPA2, HTR3B, ITPR1, KCNA5, KCNB2, KCND2, KCNG2, KCNJ15, KCNJ3, KCNK15, KCNK2, KCNK3, KCNMA1, KCNMB2, KCNS3, KCNT2, LRRC55, LRRC7, LYN, MYLK, NKAIN2, NKAIN3, NOS1AP, ORAI1, P2RX2, PCSK9, PDE4B, PLCE1, PPARGC1A, PPP3CA, PRKCA, PRKCZ, PRKN, PTPN22, RAMP1, REM2, RHCG, SCARA5, SCN3B, SFXN2, SHANK1, SHISA6, SLC12A5, SLC12A7, SLC15A4, SLC17A5, SLC17A7, SLC17A8, SLC25A2, SLC25A42, SLC30A5, SLC38A1, SLC38A10, SLC38A8, SLC4A7, SLC5A11, SLC6A12, SLC6A2, SLC9A3, SLC9A4, SRI, STIM1, SYT17, TESC, TMCO3, TMPRSS3, TSC1, WFS1, WNK2, WNK4]
GO:0098655	cation transmembrane transport	1,05E-06	[4, 6]	187,00	18,03	21,74	36,71	41,55	[ABCB11, ATP1B4, BHLHA15, CACNA1E, CALCR, CALHM6, CCL19, CCR2, CCR5, CNR1, CTSS, CXCL10, CXCL11, CXCL9, DAPK1, DRD1, FASLG, GPM6A, HCN1, IFNG, JPH1, KCNA3, KCNJ10, KCNQ5, LCK, LRRC38, MCOLN2, MCOLN3, P2RY12, PIK3CG, PLCH2, PTK2B, PTPN22, PTPN6, PTPRC, SLC18A1, SLC25A48, SLC31A2, SLC36A2, THY1, TREM2, UCP2, UNC80, XCL1, XCR1]	[ABCG2, AKAP6, AKAP9, ANK3, ANXA9, ATP6V0A2, ATP6V1B2, CAB39, CACHD1, CACNA1A, CACNB2, CALHM4, CCR7, CHRNA1, CORO1A, CX3CL1, CXCL11, DAPK1, DNM2, DPP6, F2R, FAM155A, GRIK5, HECW2, HOMER1, HTT, ITPR1, ITPR2, KCNA2, KCNAB2, KCNC2, KCNG3, KCNH1, KCNIP1, KCNIP4, KCNK9, KCNQ1, LCK, LIME1, MICU2, MTNR1B, NETO1, NOS1AP, OPRM1, PDE4B, PIEZO1, PKD1L3, PLCE1, PRKCE, PRKD2, PTK2B, PTPN22, PTPN6, PTPRC, RASA3, SGK1, SLC12A6, SLC12A8, SLC13A4, SLC15A4, SLC17A3, SLC1A2, SLC1A5, SLC20A2, SLC22A3, SLC28A3, SLC38A1, SLC4A8, SLC6A11, SLC7A10, SLC7A2, SLC9A9, TSPOAP1, UBASH3B, UCP2, UNC80]	[ABCC8, AKAP9, ANK3, ASIC2, ASIC4, ATP1B1, ATP2A3, ATP2B2, ATP6V0D1, ATP6V1D, CACNA1S, CACNA2D3, CACNG2, CATSPER2, CAV1, CHRNA4, CLCN3, COX10, DLG2, DMAC2L, DPP6, DRD2, DYSF, FGF12, FGF14, GPM6A, GRIK2, GRIN2A, HSPA2, HTR3B, ITPR1, KCNA5, KCNB2, KCND2, KCNG2, KCNJ15, KCNJ3, KCNK15, KCNK2, KCNK3, KCNMA1, KCNMB2, KCNS3, KCNT2, LRRC55, LRRC7, LYN, NOS1AP, ORAI1, P2RX2, PCSK9, PDE4B, PLCE1, PPARGC1A, PRKCA, PRKCZ, PTPN22, REM2, RHCG, SCARA5, SCN3B, SFXN2, SHANK1, SHISA6, SLC12A5, SLC12A7, SLC15A4, SLC17A5, SLC17A7, SLC25A2, SLC25A42, SLC30A5, SLC38A1, SLC4A7, SLC5A11, SLC6A12, SLC6A2, SLC9A3, SLC9A4, SRI, STIM1, TESC, TMCO3, TMPRSS3, WNK2, WNK4]
GO:0034220	ion transmembrane transport	7,49E-06	[3, 5]	226,00	16,85	22,98	35,08	41,94	[ABCB11, ANO9, ATP1B4, BHLHA15, CACNA1E, CALCR, CALHM6, CCL19, CCR2, CCR5, CLCNKA, CNR1, CTSS, CXCL10, CXCL11, CXCL9, CYBB, DAPK1, DRD1, EMB, FASLG, FOLR2, GPM6A, HCN1, IFNG, JPH1, KCNA3, KCNJ10, KCNQ5, LCK, LRRC38, MCOLN2, MCOLN3, MID1IP1, NR4A2, P2RY12, PIK3CG, PLCH2, PTAFR, PTK2B, PTPN22, PTPN6, PTPRC, SLC16A9, SLC18A1, SLC25A48, SLC31A2, SLC36A2, SLCO5A1, TCAF2, THY1, TREM2, TSC22D3, UCP2, UNC80, XCL1, XCR1]	[ABCG2, AKAP6, AKAP9, ANK3, ANXA9, ATP6V0A2, ATP6V1B2, CAB39, CACHD1, CACNA1A, CACNB2, CALHM4, CCR7, CHRNA1, CORO1A, CX3CL1, CXCL11, DAPK1, DNM2, DPP6, F2R, FAM155A, GABRA5, GRIK5, HECW2, HOMER1, HTT, ITPR1, ITPR2, KCNA2, KCNAB2, KCNC2, KCNG3, KCNH1, KCNIP1, KCNIP4, KCNK9, KCNQ1, LCK, LIME1, MICU2, MTNR1B, NETO1, NOS1AP, OPRM1, PDE4B, PHLPP2, PIEZO1, PKD1L3, PLCE1, PRKAG2, PRKCE, PRKD2, PTK2B, PTPN22, PTPN6, PTPRC, RASA3, SGK1, SLC12A6, SLC12A8, SLC13A4, SLC15A4, SLC16A12, SLC17A3, SLC1A2, SLC1A5, SLC20A2, SLC22A3, SLC25A12, SLC25A32, SLC28A3, SLC38A1, SLC38A11, SLC43A2, SLC4A8, SLC6A11, SLC7A10, SLC7A2, SLC9A9, SLCO3A1, STX8, TMC6, TSPOAP1, UBASH3B, UCP2, UNC80]	[ABCC1, ABCC8, AKAP9, ANK3, ANO2, ANO4, ASIC2, ASIC4, ATP1B1, ATP2A3, ATP2B2, ATP6V0D1, ATP6V1D, CACNA1S, CACNA2D3, CACNG2, CATSPER2, CAV1, CHRNA4, CLCN3, CLCN7, COX10, DLG2, DMAC2L, DPP6, DRD2, DYSF, FGF12, FGF14, GABRA5, GABRG3, GABRR3, GPM6A, GRIK2, GRIN2A, HSPA2, HTR3B, ITPR1, KCNA5, KCNB2, KCND2, KCNG2, KCNJ15, KCNJ3, KCNK15, KCNK2, KCNK3, KCNMA1, KCNMB2, KCNS3, KCNT2, LRRC55, LRRC7, LYN, NOS1AP, ORAI1, OSTM1, P2RX2, PCSK9, PDE4B, PLCE1, PPARGC1A, PRKAG2, PRKCA, PRKCZ, PTPN22, REM2, RHCG, SCARA5, SCN3B, SFXN2, SHANK1, SHISA6, SLC12A5, SLC12A7, SLC15A4, SLC16A4, SLC17A5, SLC17A7, SLC17A8, SLC1A7, SLC25A18, SLC25A2, SLC25A42, SLC30A5, SLC38A1, SLC38A10, SLC38A8, SLC4A7, SLC5A11, SLC6A12, SLC6A2, SLC9A3, SLC9A4, SRI, STIM1, TCAF1, TESC, TMC5, TMCO3, TMPRSS3, UNC79, WNK2, WNK4]
GO:0098662	inorganic cation transmembrane transport	1,25E-05	[5, 7]	161,00	18,11	21,23	36,87	41,90	[ABCB11, BHLHA15, CACNA1E, CALCR, CCL19, CCR5, CNR1, CXCL10, CXCL11, CXCL9, DRD1, FASLG, GPM6A, HCN1, IFNG, JPH1, KCNA3, KCNJ10, KCNQ5, LCK, LRRC38, MCOLN2, MCOLN3, P2RY12, PIK3CG, PLCH2, PTK2B, PTPN22, PTPN6, PTPRC, SLC18A1, SLC31A2, SLC36A2, THY1, TREM2, UCP2, XCL1, XCR1]	[AKAP6, AKAP9, ANK3, ANXA9, ATP6V0A2, ATP6V1B2, CAB39, CACHD1, CACNA1A, CACNB2, CCR7, CORO1A, CX3CL1, CXCL11, DNM2, DPP6, F2R, FAM155A, GRIK5, HECW2, HTT, ITPR1, ITPR2, KCNA2, KCNAB2, KCNC2, KCNG3, KCNH1, KCNIP1, KCNIP4, KCNK9, KCNQ1, LCK, LIME1, MICU2, MTNR1B, NETO1, NOS1AP, OPRM1, PDE4B, PKD1L3, PLCE1, PRKCE, PRKD2, PTK2B, PTPN22, PTPN6, PTPRC, RASA3, SGK1, SLC12A6, SLC12A8, SLC13A4, SLC15A4, SLC17A3, SLC1A2, SLC20A2, SLC22A3, SLC28A3, SLC38A1, SLC4A8, SLC6A11, SLC9A9, TSPOAP1, UBASH3B, UCP2]	[ABCC8, AKAP9, ANK3, ASIC2, ASIC4, ATP1B1, ATP2A3, ATP2B2, ATP6V0D1, ATP6V1D, CACNA1S, CACNA2D3, CACNG2, CATSPER2, CAV1, CLCN3, COX10, DMAC2L, DPP6, DRD2, DYSF, FGF12, FGF14, GPM6A, GRIK2, GRIN2A, HSPA2, ITPR1, KCNA5, KCNB2, KCND2, KCNG2, KCNJ15, KCNJ3, KCNK15, KCNK2, KCNK3, KCNMA1, KCNMB2, KCNS3, KCNT2, LRRC55, LYN, NOS1AP, ORAI1, PCSK9, PDE4B, PLCE1, PRKCA, PRKCZ, PTPN22, REM2, RHCG, SCARA5, SCN3B, SLC12A5, SLC12A7, SLC15A4, SLC17A5, SLC17A7, SLC30A5, SLC38A1, SLC4A7, SLC5A11, SLC6A12, SLC6A2, SLC9A3, SLC9A4, SRI, STIM1, TESC, TMCO3, TMPRSS3, WNK2, WNK4]

GO:0006811	ion transport	1,71E-05	[4]	289,00	15,93	23,96	35,14	40,89	[ABCB11, ANO9, ATP10B, ATP1B4, BHLHA15, BTK, CACNA1E, CALCR, CALHM6, CCL19, CCR2, CCR5, CD4, CD84, CLCNKA, CNR1, CP, CTSS, CXCL10, CXCL11, CXCL9, CXCR4, CYBB, CYSLTR1, DAPK1, DRD1, EMB, ERFE, FASLG, FCRL3, FOLR2, GPM6A, HCN1, IFNG, JPH1, KCNA3, KCNJ10, KCNQ5, KMO, LCK, LPAL2, LRRC38, MCOLN2, MCOLN3, MID1IP1, MS4A1, NLRP3, NMUR1, NR4A2, P2RY12, PIK3CG, PLA2G2D, PLCH2, PTAFR, PTK2B, PTPN22, PTPN6, PTPRC, RAB3B, SLC16A9, SLC18A1, SLC25A48, SLC31A2, SLC36A2, SLCO5A1, SPINK1, SYK, TCAF2, THY1, TREM2, TSC22D3, UCP2, UNC80, XCL1, XCR1]	[ABAT, ABCG2, ADA, AKAP6, AKAP9, ANK3, ANXA9, ARRB2, ATP6V0A2, ATP6V1B2, ATP9B, B2M, BCL2, BDNF, CA12, CAB39, CACHD1, CACNA1A, CACNB2, CALHM4, CAMK2G, CCR7, CHRNA1, CLNS1A, CORO1A, CX3CL1, CXCL11, DAPK1, DNM2, DPP6, F2R, FAM155A, GABRA5, GRIK5, GRM7, HECW2, HOMER1, HOMER2, HTT, ITPR1, ITPR2, KCNA2, KCNAB2, KCNC2, KCNG3, KCNH1, KCNIP1, KCNIP4, KCNK9, KCNQ1, LCK, LIME1, MICU2, MTNR1B, NECTIN1, NETO1, NOS1AP, NTRK2, OPRM1, PDE4B, PHLPP2, PIEZO1, PKD1L3, PLCE1, PLSCR1, PPFIA1, PPFIA4, PRKAG2, PRKCE, PRKD2, PSAP, PTK2B, PTPN22, PTPN6, PTPRC, RASA3, RXRA, SGK1, SLC12A6, SLC12A8, SLC13A4, SLC15A4, SLC16A12, SLC17A3, SLC1A2, SLC1A5, SLC20A2, SLC22A3, SLC25A12, SLC25A32, SLC28A3, SLC37A3, SLC38A1, SLC38A11, SLC43A2, SLC4A8, SLC6A11, SLC7A10, SLC7A2, SLC9A9, SLCO3A1, SSTR4, STX8, TG, TMC6, TSPOAP1, UBASH3B, UCP2, UNC80, XKR9]	[ABCA12, ABCA4, ABCC1, ABCC8, ADORA1, AKAP9, ANK3, ANO2, ANO4, ASIC2, ASIC4, ATP1B1, ATP2A3, ATP2B2, ATP6V0D1, ATP6V1D, CACNA1S, CACNA2D3, CACNG2, CATSPER2, CAV1, CHRNA4, CLCN3, CLCN7, COX10, CYB5R2, CYP4A11, DLG2, DMAC2L, DMPK, DPP6, DRD2, DYSF, FGF12, FGF14, FLVCR2, GABRA5, GABRG3, GABRR3, GPM6A, GRIK2, GRIN2A, GRM7, HRG, HSPA2, HTR3B, ITPR1, KCNA5, KCNB2, KCND2, KCNG2, KCNJ15, KCNJ3, KCNK15, KCNK2, KCNK3, KCNMA1, KCNMB2, KCNS3, KCNT2, LRRC55, LRRC7, LYN, MYLK, NKAIN2, NKAIN3, NOS1AP, ORAI1, OSTM1, P2RX2, PCSK9, PDE4B, PITPNC1, PLCE1, PLSCR2, PPARGC1A, PPP3CA, PRKAG2, PRKCA, PRKCZ, PRKN, PTPN22, RAMP1, REM2, RHCG, RIMS1, RXRA, SCARA5, SCN3B, SFXN2, SHANK1, SHISA6, SLC12A5, SLC12A7, SLC15A4, SLC16A4, SLC17A5, SLC17A7, SLC17A8, SLC1A7, SLC25A18, SLC25A2, SLC25A42, SLC30A5, SLC38A1, SLC38A10, SLC38A8, SLC4A7, SLC5A11, SLC6A12, SLC6A2, SLC9A3, SLC9A4, SRI, STIM1, SYT17, TCAF1, TESC, TMC5, TMCO3, TMPRSS3, TNFRSF11A, TOMM40, TSC1, UNC79, WFS1, WNK2, WNK4]
GO:0098660	inorganic ion transmembrane transport	5,74E-05	[4, 6]	171,00	17,48	21,58	35,79	42,63	[ABCB11, ANO9, BHLHA15, CACNA1E, CALCR, CCL19, CCR5, CLCNKA, CNR1, CXCL10, CXCL11, CXCL9, DRD1, FASLG, GPM6A, HCN1, IFNG, JPH1, KCNA3, KCNJ10, KCNQ5, LCK, LRRC38, MCOLN2, MCOLN3, P2RY12, PIK3CG, PLCH2, PTAFR, PTK2B, PTPN22, PTPN6, PTPRC, SLC18A1, SLC31A2, SLC36A2, THY1, TREM2, UCP2, XCL1, XCR1]	[AKAP6, AKAP9, ANK3, ANXA9, ATP6V0A2, ATP6V1B2, CAB39, CACHD1, CACNA1A, CACNB2, CCR7, CORO1A, CX3CL1, CXCL11, DNM2, DPP6, F2R, FAM155A, GABRA5, GRIK5, HECW2, HTT, ITPR1, ITPR2, KCNA2, KCNAB2, KCNC2, KCNG3, KCNH1, KCNIP1, KCNIP4, KCNK9, KCNQ1, LCK, LIME1, MICU2, MTNR1B, NETO1, NOS1AP, OPRM1, PDE4B, PKD1L3, PLCE1, PRKCE, PRKD2, PTK2B, PTPN22, PTPN6, PTPRC, RASA3, SGK1, SLC12A6, SLC12A8, SLC13A4, SLC15A4, SLC17A3, SLC1A2, SLC20A2, SLC22A3, SLC28A3, SLC38A1, SLC4A8, SLC6A11, SLC9A9, STX8, TSPOAP1, UBASH3B, UCP2]	[ABCC8, AKAP9, ANK3, ANO2, ANO4, ASIC2, ASIC4, ATP1B1, ATP2A3, ATP2B2, ATP6V0D1, ATP6V1D, CACNA1S, CACNA2D3, CACNG2, CATSPER2, CAV1, CLCN3, CLCN7, COX10, DMAC2L, DPP6, DRD2, DYSF, FGF12, FGF14, GABRA5, GABRG3, GABRR3, GPM6A, GRIK2, GRIN2A, HSPA2, ITPR1, KCNA5, KCNB2, KCND2, KCNG2, KCNJ15, KCNJ3, KCNK15, KCNK2, KCNK3, KCNMA1, KCNMB2, KCNS3, KCNT2, LRRC55, LYN, NOS1AP, ORAI1, PCSK9, PDE4B, PLCE1, PRKCA, PRKCZ, PTPN22, REM2, RHCG, SCARA5, SCN3B, SLC12A5, SLC12A7, SLC15A4, SLC17A5, SLC17A7, SLC30A5, SLC38A1, SLC4A7, SLC5A11, SLC6A12, SLC6A2, SLC9A3, SLC9A4, SRI, STIM1, TESC, TMCO3, TMPRSS3, WNK2, WNK4]
GO:0006813	potassium ion transport	1,30E-04	[7]	62,00	23,40	14,93	37,31	47,76	[ATP1B4, DRD1, HCN1, KCNA3, KCNJ10, KCNQ5, LRRC38, P2RY12, PTK2B, TREM2]	[AKAP6, AKAP9, ANK3, CAB39, DNM2, DPP6, GRIK5, KCNA2, KCNAB2, KCNC2, KCNG3, KCNH1, KCNIP1, KCNIP4, KCNK9, KCNQ1, MTNR1B, NETO1, NOS1AP, PRKD2, PTK2B, SGK1, SLC12A6, SLC12A8, SLC9A9]	[ABCC8, ADORA1, AKAP9, ANK3, ATP1B1, CAV1, DPP6, DRD2, GRIK2, KCNA5, KCNB2, KCND2, KCNG2, KCNJ15, KCNJ3, KCNK15, KCNK2, KCNK3, KCNMA1, KCNMB2, KCNS3, KCNT2, LRRC55, NOS1AP, PRKCZ, SLC12A5, SLC12A7, SLC9A3, SLC9A4, TSC1, WNK2, WNK4]
GO:0022836	gated channel activity	1,49E-04	[6, 8]	96,00	20,04	18,45	33,01	48,54	[ANO9, CACNA1E, CCR2, CLCNKA, CNR1, CYBB, DAPK1, HCN1, IFNG, JPH1, KCNA3, KCNJ10, KCNQ5, LRRC38, MCOLN2, MCOLN3, PTAFR, PTK2B, TREM2]	[AKAP6, ANK3, CACHD1, CACNA1A, CACNB2, CHRNA1, DAPK1, FAM155A, GABRA5, GRIK5, HTT, ITPR1, ITPR2, KCNA2, KCNAB2, KCNC2, KCNG3, KCNH1, KCNIP1, KCNIP4, KCNK9, KCNQ1, MTNR1B, NETO1, NOS1AP, OPRM1, PDE4B, PIEZO1, PRKD2, PTK2B, RASA3, SLC17A3, TMC6, TSPOAP1]	[ABCC8, ANK3, ANO2, ANO4, ASIC2, CACNA1S, CACNA2D3, CACNG2, CATSPER2, CAV1, CHRNA4, CLCN3, CLCN7, DLG2, DRD2, DYSF, FGF14, GABRA5, GABRG3, GABRR3, GRIK2, GRIN2A, HTR3B, ITPR1, KCNA5, KCNB2, KCND2, KCNG2, KCNJ15, KCNJ3, KCNK2, KCNK3, KCNMA1, KCNMB2, KCNS3, KCNT2, LRRC55, LRRC7, NOS1AP, P2RX2, PDE4B, PPARGC1A, PRKCA, REM2, SCN3B, SHANK1, SHISA6, SLC17A7, SRI, TMC5]
GO:0022843	voltage-gated cation channel activity	1,51E-04	[6, 7, 8, 9, 10, 11]	56,00	24,24	19,67	32,79	47,54	[CACNA1E, CCR2, CNR1, DAPK1, HCN1, IFNG, KCNA3, KCNJ10, KCNQ5, LRRC38, PTK2B, TREM2]	[AKAP6, ANK3, CACHD1, CACNA1A, CACNB2, DAPK1, KCNA2, KCNAB2, KCNC2, KCNG3, KCNH1, KCNK9, KCNQ1, MTNR1B, NOS1AP, OPRM1, PDE4B, PRKD2, PTK2B, TSPOAP1]	[ABCC8, ANK3, CACNA1S, CACNA2D3, CACNG2, CAV1, DLG2, DRD2, DYSF, FGF14, GRIN2A, KCNA5, KCNB2, KCND2, KCNG2, KCNJ15, KCNJ3, KCNK2, KCNK3, KCNMA1, KCNS3, KCNT2, LRRC55, LRRC7, NOS1AP, PDE4B, PPARGC1A, REM2, SRI]
GO:0015318	inorganic molecular entity transmembrane transporter activity	1,53E-04	[4, 6]	185,00	16,99	15,66	35,86	48,48	[ABCB11, ANO9, AQP5, ATP1B4, CACNA1E, CALHM6, CCR2, CLCNKA, CNR1, CTSS, DAPK1, GPM6A, HCN1, IFNG, JPH1, KCNA3, KCNJ10, KCNQ5, LRRC38, MCOLN2, MCOLN3, NR4A2, PTAFR, PTK2B, SLC16A9, SLC18A1, SLC31A2, SLC36A2, TCAF2, TREM2, UNC80]	[ABCG2, AKAP6, AKAP9, ANK3, ANXA9, ATP6V0A2, ATP6V1B2, CAB39, CACHD1, CACNA1A, CACNB2, CALHM4, CHRNA1, DAPK1, DNM2, DPP6, FAM155A, GABRA5, GRIK5, HECW2, HOMER1, HTT, ITPR1, ITPR2, KCNA2, KCNAB2, KCNC2, KCNG3, KCNH1, KCNIP1, KCNIP4, KCNK9, KCNQ1, MTNR1B, NETO1, NOS1AP, OPRM1, PDE4B, PHLPP2, PIEZO1, PKD1L3, PRKCE, PRKD2, PTK2B, RASA3, SGK1, SLC12A6, SLC12A8, SLC13A4, SLC15A4, SLC16A12, SLC17A3, SLC1A2, SLC1A5, SLC20A2, SLC22A3, SLC25A12, SLC25A32, SLC28A3, SLC38A1, SLC38A11, SLC43A2, SLC4A8, SLC6A11, SLC7A10, SLC7A2, SLC9A9, STX8, TMC6, TSPOAP1, UNC80]	[ABCC8, AKAP9, ANK3, ANO2, ANO4, AQP8, ASIC2, ASIC4, ATP1B1, ATP2A3, ATP2B2, ATP6V0D1, ATP6V1D, CACNA1S, CACNA2D3, CACNG2, CATSPER2, CAV1, CHRNA4, CLCN3, CLCN7, COX10, DLG2, DMAC2L, DPP6, DRD2, DYSF, FGF12, FGF14, GABRA5, GABRG3, GABRR3, GPM6A, GRIK2, GRIN2A, HSPA2, HTR3B, ITPR1, KCNA5, KCNB2, KCND2, KCNG2, KCNJ15, KCNJ3, KCNK15, KCNK2, KCNK3, KCNMA1, KCNMB2, KCNS3, KCNT2, LRRC55, LRRC7, NOS1AP, ORAI1, P2RX2, PCSK9, PDE4B, PPARGC1A, PRKCA, PRKCZ, REM2, RHCG, SCN3B, SFXN2, SHANK1, SHISA6, SLC12A5, SLC12A7, SLC15A4, SLC16A4, SLC17A5, SLC17A7, SLC17A8, SLC1A7, SLC25A18, SLC25A2, SLC30A5, SLC38A1, SLC38A10, SLC38A8, SLC4A7, SLC5A11, SLC6A12, SLC6A2, SLC9A3, SLC9A4, SRI, STIM1, TCAF1, TESC, TMC5, TMCO3, TMPRSS3, WNK2, WNK4]
GO:0008324	cation transmembrane transporter activity	1,56E-04	[5, 7]	157,00	17,60	14,79	36,69	48,52	[ABCB11, ATP1B4, CACNA1E, CALHM6, CCR2, CNR1, CTSS, DAPK1, GPM6A, HCN1, IFNG, JPH1, KCNA3, KCNJ10, KCNQ5, LRRC38, MCOLN2, MCOLN3, PTK2B, SLC18A1, SLC25A48, SLC31A2, SLC36A2, TREM2, UNC80]	[ABCG2, AKAP6, AKAP9, ANK3, ANXA9, ATP6V0A2, ATP6V1B2, CAB39, CACHD1, CACNA1A, CACNB2, CALHM4, CHRNA1, DAPK1, DNM2, DPP6, FAM155A, GRIK5, HECW2, HOMER1, HTT, ITPR1, ITPR2, KCNA2, KCNAB2, KCNC2, KCNG3, KCNH1, KCNIP1, KCNIP4, KCNK9, KCNQ1, MTNR1B, NETO1, NOS1AP, OPRM1, PDE4B, PIEZO1, PKD1L3, PRKCE, PRKD2, PTK2B, RASA3, SGK1, SLC12A6, SLC12A8, SLC13A4, SLC15A4, SLC17A3, SLC1A2, SLC1A5, SLC20A2, SLC22A3, SLC28A3, SLC38A1, SLC4A8, SLC6A11, SLC7A10, SLC7A2, SLC9A9, TSPOAP1, UNC80]	[ABCC8, AKAP9, ANK3, ASIC2, ASIC4, ATP1B1, ATP2A3, ATP2B2, ATP6V0D1, ATP6V1D, CACNA1S, CACNA2D3, CACNG2, CATSPER2, CAV1, CHRNA4, CLCN3, COX10, DLG2, DMAC2L, DPP6, DRD2, DYSF, FGF12, FGF14, GPM6A, GRIK2, GRIN2A, HSPA2, HTR3B, ITPR1, KCNA5, KCNB2, KCND2, KCNG2, KCNJ15, KCNJ3, KCNK15, KCNK2, KCNK3, KCNMA1, KCNMB2, KCNS3, KCNT2, LRRC55, LRRC7, NOS1AP, ORAI1, P2RX2, PCSK9, PDE4B, PPARGC1A, PRKCA, PRKCZ, REM2, RHCG, SCN3B, SFXN2, SHANK1, SHISA6, SLC12A5, SLC12A7, SLC15A4, SLC17A5, SLC17A7, SLC25A2, SLC25A42, SLC30A5, SLC38A1, SLC4A7, SLC5A11, SLC6A12, SLC6A2, SLC9A3, SLC9A4, SRI, STIM1, TESC, TMCO3, TMPRSS3, WNK2, WNK4]
GO:0071805	potassium ion transmembrane transport	1,68E-04	[6, 8]	56,00	24,14	11,67	40,00	48,33	[HCN1, KCNA3, KCNJ10, KCNQ5, LRRC38, P2RY12, TREM2]	[AKAP6, AKAP9, ANK3, CAB39, DNM2, DPP6, GRIK5, KCNA2, KCNAB2, KCNC2, KCNG3, KCNH1, KCNIP1, KCNIP4, KCNK9, KCNQ1, MTNR1B, NETO1, NOS1AP, PRKD2, SGK1, SLC12A6, SLC12A8, SLC9A9]	[ABCC8, AKAP9, ANK3, ATP1B1, CAV1, DPP6, GRIK2, KCNA5, KCNB2, KCND2, KCNG2, KCNJ15, KCNJ3, KCNK15, KCNK2, KCNK3, KCNMA1, KCNMB2, KCNS3, KCNT2, LRRC55, NOS1AP, PRKCZ, SLC12A5, SLC12A7, SLC9A3, SLC9A4, WNK2, WNK4]
GO:0034765	regulation of ion transmembrane transport	1,72E-04	[4, 5, 6]	101,00	19,73	25,89	32,14	41,96	[ANO9, CACNA1E, CALCR, CCR2, CLCNKA, CTSS, CXCL10, CXCL11, CXCL9, CYBB, DAPK1, DRD1, HCN1, IFNG, JPH1, KCNA3, KCNJ10, KCNQ5, LRRC38, PIK3CG, PTAFR, PTK2B, PTPN22, PTPN6, SLC31A2, TCAF2, THY1, TREM2, XCL1]	[AKAP6, AKAP9, ANK3, CAB39, CACNA1A, CACNB2, CORO1A, CX3CL1, CXCL11, DAPK1, DNM2, DPP6, F2R, HECW2, HOMER1, HTT, KCNA2, KCNAB2, KCNC2, KCNG3, KCNH1, KCNIP1, KCNIP4, KCNQ1, LIME1, MTNR1B, NETO1, NOS1AP, OPRM1, PDE4B, PRKCE, PRKD2, PTK2B, PTPN22, PTPN6, UBASH3B]	[ABCC8, AKAP9, ANK3, ASIC2, ATP1B1, CACNA1S, CACNA2D3, CACNG2, CATSPER2, CAV1, DLG2, DPP6, DRD2, DYSF, FGF12, FGF14, GRIN2A, HSPA2, KCNA5, KCNB2, KCND2, KCNG2, KCNJ15, KCNJ3, KCNK15, KCNMA1, KCNS3, LRRC55, LRRC7, LYN, NOS1AP, PCSK9, PDE4B, PPARGC1A, PRKCA, PTPN22, REM2, SCN3B, SHANK1, SHISA6, SLC30A5, SRI, STIM1, TCAF1, TESC, WNK2, WNK4]
GO:0005244	voltage-gated ion channel activity	1,86E-04	[5, 7, 8, 10]	66,00	22,68	21,13	32,39	46,48	[CACNA1E, CCR2, CLCNKA, CNR1, CYBB, DAPK1, HCN1, IFNG, KCNA3, KCNJ10, KCNQ5, LRRC38, PTAFR, PTK2B, TREM2]	[AKAP6, ANK3, CACHD1, CACNA1A, CACNB2, DAPK1, KCNA2, KCNAB2, KCNC2, KCNG3, KCNH1, KCNIP1, KCNIP4, KCNK9, KCNQ1, MTNR1B, NOS1AP, OPRM1, PDE4B, PRKD2, PTK2B, SLC17A3, TSPOAP1]	[ABCC8, ANK3, CACNA1S, CACNA2D3, CACNG2, CATSPER2, CAV1, CLCN3, CLCN7, DLG2, DRD2, DYSF, FGF14, GRIN2A, KCNA5, KCNB2, KCND2, KCNG2, KCNJ15, KCNJ3, KCNK2, KCNK3, KCNMA1, KCNS3, KCNT2, LRRC55, LRRC7, NOS1AP, PDE4B, PPARGC1A, REM2, SCN3B, SRI]
GO:0005267	potassium channel activity	1,91E-04	[7, 8, 9, 10, 11]	45,00	26,47	12,24	38,78	48,98	[HCN1, KCNA3, KCNJ10, KCNQ5, LRRC38, TREM2]	[AKAP6, AKAP9, ANK3, DPP6, GRIK5, KCNA2, KCNAB2, KCNC2, KCNG3, KCNH1, KCNIP1, KCNIP4, KCNK9, KCNQ1, MTNR1B, NETO1, NOS1AP. PRKD2. SGK1]	[ABCC8, AKAP9, ANK3, CAV1, DPP6, GRIK2, KCNA5, KCNB2, KCND2, KCNG2, KCNJ15, KCNJ3, KCNK15, KCNK2, KCNK3, KCNMA1, KCNMB2, KCNS3, KCNT2, LRRC55, NOS1AP, PRKCZ, WNK2, WNK4]
GO:0046873	metal ion transmembrane transporter activity	2,01E-04	[6, 7, 8, 9]	117,00	18,90	12,00	38,40	49,60	[ABCB11, CACNA1E, CNR1, GPM6A, HCN1, JPH1, KCNA3, KCNJ10, KCNQ5, LRRC38, MCOLN2, MCOLN3, SLC18A1, SLC31A2, TREM2]	[AKAP6, AKAP9, ANK3, ANXA9, CAB39, CACHD1, CACNA1A, CACNB2, DNM2, DPP6, FAM155A, GRIK5, HECW2, HTT, ITPR1, ITPR2, KCNA2, KCNAB2, KCNC2, KCNG3, KCNH1, KCNIP1, KCNIP4, KCNK9, KCNQ1, MTNR1B, NETO1, NOS1AP, OPRM1, PDE4B, PKD1L3, PRKCE, PRKD2, RASA3, SGK1, SLC12A6, SLC12A8, SLC13A4, SLC17A3, SLC1A2, SLC20A2, SLC22A3, SLC28A3, SLC38A1, SLC4A8, SLC6A11, SLC9A9, TSPOAP1]	[ABCC8, AKAP9, ANK3, ASIC2, ASIC4, ATP1B1, ATP2A3, ATP2B2, CACNA1S, CACNA2D3, CACNG2, CATSPER2, CAV1, DPP6, DRD2, DYSF, FGF12, FGF14, GPM6A, GRIK2, GRIN2A, HSPA2, ITPR1, KCNA5, KCNB2, KCND2, KCNG2, KCNJ15, KCNJ3, KCNK15, KCNK2, KCNK3, KCNMA1, KCNMB2, KCNS3, KCNT2, LRRC55, NOS1AP, ORAI1, PCSK9, PDE4B, PRKCA, PRKCZ, REM2, SCN3B, SLC12A5, SLC12A7, SLC17A7, SLC30A5, SLC38A1, SLC4A7, SLC5A11, SLC6A12, SLC6A2, SLC9A3, SLC9A4, SRI, STIM1, TESC, TMPRSS3, WNK2, WNK4]

GO:0022890	inorganic cation transmembrane transporter activity	3,53E-04	[5, 6, 7, 8]	149,00	17,57	14,91	36,02	49,07	[ABCB11, ATP1B4, CACNA1E, CALHM6, CCR2, CNR1, CTSS, DAPK1, GPM6A, HCN1, IFNG, JPH1, KCNA3, KCNJ10, KCNQ5, LRRC38, MCOLN2, MCOLN3, PTK2B, SLC18A1, SLC31A2, SLC36A2, TREM2, UNC80]	[AKAP6, AKAP9, ANK3, ANXA9, ATP6V0A2, ATP6V1B2, CAB39, CACHD1, CACNA1A, CACNB2, CALHM4, CHRNA1, DAPK1, DNM2, DPP6, FAM155A, GRIK5, HECW2, HOMER1, HTT, ITPR1, ITPR2, KCNA2, KCNAB2, KCNC2, KCNG3, KCNH1, KCNIP1, KCNIP4, KCNK9, KCNQ1, MTNR1B, NETO1, NOS1AP, OPRM1, PDE4B, PIEZO1, PKD1L3, PRKCE, PRKD2, PTK2B, RASA3, SGK1, SLC12A6, SLC12A8, SLC13A4, SLC15A4, SLC17A3, SLC1A2, SLC20A2, SLC22A3, SLC28A3, SLC38A1, SLC4A8, SLC6A11, SLC9A9, TSPOAP1, UNC80]	[ABCC8, AKAP9, ANK3, ASIC2, ASIC4, ATP1B1, ATP2A3, ATP2B2, ATP6V0D1, ATP6V1D, CACNA1S, CACNA2D3, CACNG2, CATSPER2, CAV1, CHRNA4, CLCN3, COX10, DLG2, DMAC2L, DPP6, DRD2, DYSF, FGF12, FGF14, GPM6A, GRIK2, GRIN2A, HSPA2, HTR3B, ITPR1, KCNA5, KCNB2, KCND2, KCNG2, KCNJ15, KCNJ3, KCNK15, KCNK2, KCNK3, KCNMA1, KCNMB2, KCNS3, KCNT2, LRRC55, LRRC7, NOS1AP, ORAI1, P2RX2, PCSK9, PDE4B, PPARGC1A, PRKCA, PRKCZ, REM2, RHCG, SCN3B, SHANK1, SHISA6, SLC12A5, SLC12A7, SLC15A4, SLC17A5, SLC17A7, SLC30A5, SLC38A1, SLC4A7, SLC5A11, SLC6A12, SLC6A2, SLC9A3, SLC9A4, SRI, STIM1, TESC, TMCO3, TMPRSS3, WNK2, WNK4]
GO:0005249	voltage-gated potassium channel activity	3,77E-04	[7, 8, 9, 10, 11, 12]	32,00	30,48	17,65	35,29	47,06	[HCN1, KCNA3, KCNJ10, KCNQ5, LRRC38, TREM2]	[AKAP6, ANK3, KCNA2, KCNAB2, KCNC2, KCNG3, KCNH1, KCNK9, KCNQ1, MTNR1B, NOS1AP, PRKD2]	[ABCC8, ANK3, CAV1, KCNA5, KCNB2, KCND2, KCNG2, KCNJ15, KCNJ3, KCNK2, KCNK3, KCNMA1, KCNS3, KCNT2, LRRC55, NOS1AP]
GO:0051049	regulation of transport	5,15E-04	[3, 4]	308,00	15,22	27,41	32,83	39,76	[ADAM8, AIF1, AIM2, ANGPT1, ANO9, BANK1, BLK, C2, CACNA1E, CALCR, CARD17, CASP8, CCL19, CCR2, CD2, CD200R1, CD244, CD4, CD40LG, CD74, CD84, CLCNKA, CLEC4E, CLEC6A, CNR1, CRTAM, CSF1R, CTSS, CXCL10, CXCL11, CXCL9, CXCR4, CYBB, DAPK1, DOCK2, DRD1, ERFE, FCN1, FCRL3, FFAR4, FGR, FOXP3, HCLS1, HCN1, HLA-DRB1, IFNG, IL26, IL2RG, JPH1, KCNA3, KCNJ10, KCNQ5, KMO, LCP1, LPAL2, LRRC38, MYB, NCKAP1L, NLRP3, NLRP6, NPPB, NR4A3, ORM2, P2RY12, P2RY2, PIK3CG, PIM1, PTAFR, PTK2B, PTPN22, PTPN6, PTPRC, PTX3, RAB3B, RAB3C, SDC1, SELE, SLC31A2, SPINK1, STAP1, SYK, TCAF2, THY1, TIFAB, TLR10, TLR6, TLR8, TNFAIP3, TRAT1, TREM2, XCL1]	[ABAT, ACAA2, ADA, ADCY1, AIM2, AKAP6, AKAP9, ANK3, ARRB2, ATXN2, B2M, BCL2, BICD1, BTBD9, CAB39, CABP1, CACNA1A, CACNB2, CAMK2G, CASP8, CCM2, CCR7, CD160, CD2AP, CD38, CD47, CDH2, CORO1A, CX3CL1, CXCL11, DAPK1, DENND5B, DNM2, DPP6, EDEM1, EEF2K, EXPH5, F2R, FES, GAB2, GBP1, GCC2, GPR68, GRIK5, GRM7, GSK3B, HDAC9, HECW2, HOMER1, HOMER2, HTT, IL17RA, ITPR1, ITPR2, KCNA2, KCNAB2, KCNC2, KCNG3, KCNH1, KCNIP1, KCNIP4, KCNQ1, KCTD11, LCP1, LGR4, LIME1, MAP2K2, MBP, MDFIC, MTNR1B, NETO1, NOS1AP, NUP153, OPRM1, PDCD10, PDE4B, PPP1CC, PPP3CC, PRKAG2, PRKAR1B, PRKCE, PRKD2, PSMD9, PTK2B, PTPN1, PTPN22, PTPN6, PTPRC, PTPRJ, RAB13, RALB, RUFY3, SEPTIN9, SGIP1, SGK1, SLC1A2, SLC4A8, SSTR4, TANK, TERT, TGFB2, TNF, TNFAIP3, TNK2, TP53BP2, TRIM46, UBASH3B, UBE2L3, ZC3H12A]	[ABCA12, ABCC8, ADCY5, ADORA1, AIM2, AKAP12, AKAP9, ALOX15, ANK3, ANKRD13B, ARHGAP44, ASIC2, ATP1B1, BTNL2, C11orf65, C2CD5, CACNA1S, CACNA2D3, CACNG2, CADPS2, CARD8, CARTPT, CATSPER2, CAV1, CD151, CDH13, CDK5R2, CHRNA4, CHUK, COMT, CPLX2, CYP4A11, DISP3, DLG2, DMPK, DNM3, DPP6, DRD2, DYSF, EGFR, FGF12, FGF14, FOXP1, FRMD4A, GNAS, GRIN2A, GRM7, HIP1, HLA-DRB1, HSPA2, HYAL2, ITGB2, ITPR1, KCNA5, KCNB2, KCND2, KCNG2, KCNJ15, KCNJ3, KCNK15, KCNMA1, KCNS3, KDM5B, LCP1, LRRC55, LRRC7, LYN, MAGI1, MAGI2, MAP2, MBL2, MBP, MEF2A, MFGE8, MMP12, MYLK, NDRG4, NKAIN2, NKAIN3, NOS1AP, NR1H3, NRXN3, NUMB, OAZ1, ORAI1, OSBPL6, P2RX2, PAK1, PCSK9, PDE4B, PFKM, PNKD, PPARGC1A, PPP3CA, PRKAA1, PRKAG2, PRKCA, PRKCZ, PRKN, PTPN22, RAB7A, REM2, RIMS1, RSPO1, SCN3B, SEPTIN9, SHANK1, SHISA6, SLC17A7, SLC30A5, SMAP1, SRGN, SRI, STIM1, STXBP5L, SYT17, TBC1D5, TCAF1, TESC, TIAM1, TLR1, TNFRSF11A, TP73, TRAF2, TUB, USP36, VEGFC, VSNL1, WASL, WFS1, WNK2, WNK4]
GO:0043269	regulation of ion transport	5,25E-04	[4, 5]	134,00	17,94	28,08	30,14	41,78	[ANO9, CACNA1E, CALCR, CCR2, CD4, CD84, CLCNKA, CNR1, CTSS, CXCL10, CXCL11, CXCL9, CXCR4, CYBB, DAPK1, DRD1, ERFE, FCRL3, HCN1, IFNG, JPH1, KCNA3, KCNJ10, KCNQ5, KMO, LRRC38, NLRP3, P2RY12, PIK3CG, PTAFR, PTK2B, PTPN22, PTPN6, RAB3B, SLC31A2, SPINK1, SYK, TCAF2, THY1, TREM2, XCL1]	[ABAT, AKAP6, AKAP9, ANK3, ARRB2, B2M, BCL2, CAB39, CACNA1A, CACNB2, CAMK2G, CORO1A, CX3CL1, CXCL11, DAPK1, DNM2, DPP6, F2R, GRM7, HECW2, HOMER1, HOMER2, HTT, KCNA2, KCNAB2, KCNC2, KCNG3, KCNH1, KCNIP1, KCNIP4, KCNQ1, LIME1, MTNR1B, NETO1, NOS1AP, OPRM1, PDE4B, PRKCE, PRKD2, PTK2B, PTPN22, PTPN6, SSTR4, UBASH3B]	[ABCC8, ADORA1, AKAP9, ANK3, ASIC2, ATP1B1, CACNA1S, CACNA2D3, CACNG2, CATSPER2, CAV1, CHRNA4, CYP4A11, DLG2, DMPK, DPP6, DRD2, DYSF, FGF12, FGF14, GRIN2A, GRM7, HSPA2, KCNA5, KCNB2, KCND2, KCNG2, KCNJ15, KCNJ3, KCNK15, KCNMA1, KCNS3, LRRC55, LRRC7, LYN, MYLK, NKAIN2, NKAIN3, NOS1AP, ORAI1, P2RX2, PCSK9, PDE4B, PPARGC1A, PRKCA, PRKN, PTPN22, REM2, SCN3B, SHANK1, SHISA6, SLC30A5, SRI, STIM1, SYT17, TCAF1, TESC, TNFRSF11A, WFS1, WNK2, WNK4]
GO:0034762	regulation of transmembrane transport	6,24E-04	[3, 4, 5]	113,00	18,65	26,40	32,80	40,80	[ANO9, CACNA1E, CALCR, CCR2, CLCNKA, CTSS, CXCL10, CXCL11, CXCL9, CYBB, DAPK1, DRD1, ERFE, FFAR4, HCN1, IFNG, JPH1, KCNA3, KCNJ10, KCNQ5, LRRC38, NR4A3, PIK3CG, PIM1, PTAFR, PTK2B, PTPN22, PTPN6, SLC31A2, TCAF2, THY1, TREM2, XCL1]	[AKAP6, AKAP9, ANK3, BCL2, CAB39, CACNA1A, CACNB2, CORO1A, CX3CL1, CXCL11, DAPK1, DNM2, DPP6, F2R, HECW2, HOMER1, HTT, KCNA2, KCNAB2, KCNC2, KCNG3, KCNH1, KCNIP1, KCNIP4, KCNQ1, LIME1, MTNR1B, NETO1, NOS1AP, OPRM1, PDE4B, PRKAG2, PRKCE, PRKD2, PTK2B, PTPN22, PTPN6, SLC1A2, TERT, TNF, UBASH3B]	[ABCC8, AKAP9, ANK3, ASIC2, ATP1B1, C2CD5, CACNA1S, CACNA2D3, CACNG2, CATSPER2, CAV1, DLG2, DPP6, DRD2, DYSF, FGF12, FGF14, GRIN2A, HSPA2, KCNA5, KCNB2, KCND2, KCNG2, KCNJ15, KCNJ3, KCNK15, KCNMA1, KCNS3, LRRC55, LRRC7, LYN, MEF2A, NOS1AP, OAZ1, PCSK9, PDE4B, PPARGC1A, PRKAG2, PRKCA, PTPN22, REM2, SCN3B, SHANK1, SHISA6, SLC30A5, SRI, STIM1, TCAF1, TESC, WNK2, WNK4]
GO:0015075	ion transmembrane transporter activity	8,33E-04	[4, 6]	189,00	16,54	16,34	35,64	48,02	[ABCB11, ANO9, ATP1B4, CACNA1E, CALHM6, CCR2, CLCNKA, CNR1, CTSS, CYBB, DAPK1, GPM6A, HCN1, IFNG, JPH1, KCNA3, KCNJ10, KCNQ5, LRRC38, MCOLN2, MCOLN3, NR4A2, PTAFR, PTK2B, SLC16A9, SLC18A1, SLC25A48, SLC31A2, SLC36A2, SLCO5A1, TCAF2, TREM2, UNC80]	[ABCG2, AKAP6, AKAP9, ANK3, ANXA9, ATP6V0A2, ATP6V1B2, CAB39, CACHD1, CACNA1A, CACNB2, CALHM4, CHRNA1, DAPK1, DNM2, DPP6, FAM155A, GABRA5, GRIK5, HECW2, HOMER1, HTT, ITPR1, ITPR2, KCNA2, KCNAB2, KCNC2, KCNG3, KCNH1, KCNIP1, KCNIP4, KCNK9, KCNQ1, MTNR1B, NETO1, NOS1AP, OPRM1, PDE4B, PHLPP2, PIEZO1, PKD1L3, PRKCE, PRKD2, PTK2B, RASA3, SGK1, SLC12A6, SLC12A8, SLC13A4, SLC15A4, SLC16A12, SLC17A3, SLC1A2, SLC1A5, SLC20A2, SLC22A3, SLC25A12, SLC25A32, SLC28A3, SLC38A1, SLC38A11, SLC43A2, SLC4A8, SLC6A11, SLC7A10, SLC7A2, SLC9A9, SLCO3A1, STX8, TMC6, TSPOAP1, UNC80]	[ABCC1, ABCC8, AKAP9, ANK3, ANO2, ANO4, ASIC2, ASIC4, ATP1B1, ATP2A3, ATP2B2, ATP6V0D1, ATP6V1D, CACNA1S, CACNA2D3, CACNG2, CATSPER2, CAV1, CHRNA4, CLCN3, CLCN7, COX10, DLG2, DMAC2L, DPP6, DRD2, DYSF, FGF12, FGF14, GABRA5, GABRG3, GABRR3, GPM6A, GRIK2, GRIN2A, HSPA2, HTR3B, ITPR1, KCNA5, KCNB2, KCND2, KCNG2, KCNJ15, KCNJ3, KCNK15, KCNK2, KCNK3, KCNMA1, KCNMB2, KCNS3, KCNT2, LRRC55, LRRC7, NOS1AP, ORAI1, P2RX2, PCSK9, PDE4B, PPARGC1A, PRKCA, PRKCZ, REM2, RHCG, SCN3B, SFXN2, SHANK1, SHISA6, SLC12A5, SLC12A7, SLC15A4, SLC16A4, SLC17A5, SLC17A7, SLC17A8, SLC1A7, SLC25A18, SLC25A2, SLC25A42, SLC30A5, SLC38A1, SLC38A10, SLC38A8, SLC4A7, SLC5A11, SLC6A12, SLC6A2, SLC9A3, SLC9A4, SRI, STIM1, TCAF1, TESC, TMC5, TMCO3, TMPRSS3, WNK2, WNK4]
GO:0005216	ion channel activity	1,52E-03	[5, 6, 7, 8]	120,00	18,05	17,56	33,59	48,85	[ANO9, CACNA1E, CALHM6, CCR2, CLCNKA, CNR1, CTSS, DAPK1, GPM6A, HCN1, IFNG, JPH1, KCNA3, KCNJ10, KCNQ5, LRRC38, MCOLN2, MCOLN3, PTAFR, PTK2B, TCAF2, TREM2, UNC80]	[AKAP6, AKAP9, ANK3, ANXA9, CACHD1, CACNA1A, CACNB2, CALHM4, CHRNA1, DAPK1, DPP6, FAM155A, GABRA5, GRIK5, HOMER1, HTT, ITPR1, ITPR2, KCNA2, KCNAB2, KCNC2, KCNG3, KCNH1, KCNIP1, KCNIP4, KCNK9, KCNQ1, MTNR1B, NETO1, NOS1AP, OPRM1, PDE4B, PHLPP2, PIEZO1, PKD1L3, PRKD2, PTK2B, RASA3, SGK1, SLC17A3, STX8, TMC6, TSPOAP1, UNC80]	[ABCC8, AKAP9, ANK3, ANO2, ANO4, ASIC2, ASIC4, CACNA1S, CACNA2D3, CACNG2, CATSPER2, CAV1, CHRNA4, CLCN3, CLCN7, DLG2, DPP6, DRD2, DYSF, FGF12, FGF14, GABRA5, GABRG3, GABRR3, GPM6A, GRIK2, GRIN2A, HTR3B, ITPR1, KCNA5, KCNB2, KCND2, KCNG2, KCNJ15, KCNJ3, KCNK15, KCNK2, KCNK3, KCNMA1, KCNMB2, KCNS3, KCNT2, LRRC55, LRRC7, NOS1AP, ORAI1, P2RX2, PCSK9, PDE4B, PPARGC1A, PRKCA, PRKCZ, REM2, SCN3B, SHANK1, SHISA6, SLC17A7, SRI, STIM1, TCAF1, TMC5, TMPRSS3, WNK2, WNK4]
GO:0015267	channel activity	3,88E-03	[5, 7]	129,00	17,46	18,44	32,62	48,94	[ANO9, AQP5, CACNA1E, CALHM6, CCR2, CLCNKA, CNR1, CTSS, CYBB, DAPK1, GPM6A, HCN1, IFNG, JPH1, KCNA3, KCNJ10, KCNQ5, LRRC38, MCOLN2, MCOLN3, PRF1, PTAFR, PTK2B, TCAF2, TREM2, UNC80]	[AKAP6, AKAP9, ANK3, ANXA9, BCL2, CACHD1, CACNA1A, CACNB2, CALHM4, CHRNA1, DAPK1, DPP6, FAM155A, GABRA5, GRIK5, HOMER1, HTT, ITPR1, ITPR2, KCNA2, KCNAB2, KCNC2, KCNG3, KCNH1, KCNIP1, KCNIP4, KCNK9, KCNQ1, MTNR1B, NETO1, NOS1AP, OPRM1, PDE4B, PHLPP2, PIEZO1, PKD1L3, PRF1, PRKD2, PTK2B, RASA3, SGK1, SLC17A3, STX8, TMC6, TSPOAP1, UNC80]	[ABCC8, AKAP9, ANK3, ANO2, ANO4, AQP8, ASIC2, ASIC4, CABP2, CACNA1S, CACNA2D3, CACNG2, CATSPER2, CAV1, CHRNA4, CLCN3, CLCN7, DLG2, DPP6, DRD2, DYSF, FGF12, FGF14, GABRA5, GABRG3, GABRR3, GJA3, GPM6A, GRIK2, GRIN2A, HTR3B, ITPR1, KCNA5, KCNB2, KCND2, KCNG2, KCNJ15, KCNJ3, KCNK15, KCNK2, KCNK3, KCNMA1, KCNMB2, KCNS3, KCNT2, LRRC55, LRRC7, NOS1AP, NRXN3, ORAI1, P2RX2, PCSK9, PDE4B, PPARGC1A, PRKCA, PRKCZ, REM2, SCN3B, SHANK1, SHISA6, SLC17A7, SRI, STIM1, TCAF1, TMC5, TMPRSS3, TOMM40, WNK2, WNK4]
GO:0005261	cation channel activity	4,42E-03	[6, 7, 8, 9]	102,00	18,41	16,96	34,82	48,21	[CACNA1E, CALHM6, CCR2, CNR1, CTSS, DAPK1, GPM6A, HCN1, IFNG, JPH1, KCNA3, KCNJ10, KCNQ5, LRRC38, MCOLN2, MCOLN3, PTK2B, TREM2, UNC80]	[AKAP6, AKAP9, ANK3, ANXA9, CACHD1, CACNA1A, CACNB2, CALHM4, CHRNA1, DAPK1, DPP6, FAM155A, GRIK5, HOMER1, HTT, ITPR1, ITPR2, KCNA2, KCNAB2, KCNC2, KCNG3, KCNH1, KCNIP1, KCNIP4, KCNK9, KCNQ1, MTNR1B, NETO1, NOS1AP, OPRM1, PDE4B, PIEZO1, PKD1L3, PRKD2, PTK2B, RASA3, SGK1, TSPOAP1, UNC80]	[ABCC8, AKAP9, ANK3, ASIC2, ASIC4, CACNA1S, CACNA2D3, CACNG2, CATSPER2, CAV1, CHRNA4, DLG2, DPP6, DRD2, DYSF, FGF12, FGF14, GPM6A, GRIK2, GRIN2A, HTR3B, ITPR1, KCNA5, KCNB2, KCND2, KCNG2, KCNJ15, KCNJ3, KCNK15, KCNK2, KCNK3, KCNMA1, KCNMB2, KCNS3, KCNT2, LRRC55, LRRC7, NOS1AP, ORAI1, P2RX2, PCSK9, PDE4B, PPARGC1A, PRKCA, PRKCZ, REM2, SCN3B, SHANK1, SHISA6, SRI, STIM1, TMPRSS3, WNK2, WNK4]
GO:1904062	regulation of cation transmembrane transport	5,16E-03	[5, 6, 7]	74,00	20,00	22,35	38,82	38,82	[CALCR, CCR2, CTSS, CXCL10, CXCL11, CXCL9, DAPK1, DRD1, IFNG, JPH1, LRRC38, PIK3CG, PTK2B, PTPN22, PTPN6, SLC31A2, THY1, TREM2, XCL1]	[AKAP6, AKAP9, ANK3, CAB39, CACNA1A, CACNB2, CORO1A, CX3CL1, CXCL11, DAPK1, DNM2, DPP6, F2R, HECW2, HOMER1, HTT, KCNAB2, KCNC2, KCNIP1, KCNIP4, KCNQ1, LIME1, MTNR1B, NETO1, NOS1AP, OPRM1, PDE4B, PRKCE, PRKD2, PTK2B, PTPN22, PTPN6, UBASH3B]	[ABCC8, AKAP9, ANK3, ATP1B1, CACNG2, CAV1, DLG2, DPP6, DRD2, DYSF, FGF12, FGF14, GRIN2A, HSPA2, LRRC55, LRRC7, LYN, NOS1AP, PCSK9, PDE4B, PPARGC1A, PRKCA, PTPN22, REM2, SCN3B, SHANK1, SHISA6, SLC30A5, SRI, STIM1, TESC, WNK2, WNK4]
GO:0010959	regulation of metal ion transport	6,94E-03	[5, 6, 7]	82,00	19,39	23,91	39,13	36,96	[CALCR, CD4, CD84, CXCL10, CXCL11, CXCL9, CXCR4, DRD1, FCRL3, IFNG, JPH1, LRRC38, P2RY12, PIK3CG, PTK2B, PTPN22, PTPN6, SLC31A2, SPINK1, THY1, TREM2, XCL1]	[AKAP6, AKAP9, ANK3, ARRB2, B2M, BCL2, CAB39, CACNA1A, CACNB2, CAMK2G, CORO1A, CX3CL1, CXCL11, DNM2, DPP6, F2R, HECW2, HOMER1, HOMER2, HTT, KCNAB2, KCNC2, KCNIP1, KCNIP4, KCNQ1, LIME1, MTNR1B, NETO1, NOS1AP, PDE4B, PRKCE, PRKD2, PTK2B, PTPN22, PTPN6, UBASH3B]	[ABCC8, ADORA1, AKAP9, ANK3, ATP1B1, CAV1, DMPK, DPP6, DRD2, DYSF, FGF12, FGF14, HSPA2, KCNA5, LRRC55, LYN, MYLK, NKAIN2, NKAIN3, NOS1AP, ORAI1, P2RX2, PCSK9, PDE4B, PRKCA, PTPN22, REM2, SCN3B, SRI, STIM1, TESC, WFS1, WNK2, WNK4]
GO:0015672	monovalent inorganic cation transport	9,18E-03	[6]	109,00	17,87	11,97	35,04	52,99	[ABCB11, ATP1B4, DRD1, HCN1, KCNA3, KCNJ10, KCNQ5, LRRC38, P2RY12, PTK2B, SLC18A1, SLC36A2, TREM2, UCP2]	[AKAP6, AKAP9, ANK3, ATP6V0A2, ATP6V1B2, CAB39, DNM2, DPP6, GRIK5, HECW2, KCNA2, KCNAB2, KCNC2, KCNG3, KCNH1, KCNIP1, KCNIP4, KCNK9, KCNQ1, MTNR1B, NETO1, NOS1AP, PRKCE, PRKD2, PTK2B, SGK1, SLC12A6, SLC12A8, SLC13A4, SLC15A4, SLC17A3, SLC1A2, SLC20A2, SLC22A3, SLC28A3, SLC38A1, SLC38A11, SLC4A8, SLC6A11, SLC9A9, UCP2]	[ABCC8, ADORA1, AKAP9, ANK3, ASIC2, ASIC4, ATP1B1, ATP2A3, ATP6V0D1, ATP6V1D, CAV1, CLCN3, COX10, DMAC2L, DMPK, DPP6, DRD2, FGF12, GRIK2, KCNA5, KCNB2, KCND2, KCNG2, KCNJ15, KCNJ3, KCNK15, KCNK2, KCNK3, KCNMA1, KCNMB2, KCNS3, KCNT2, LRRC55, NKAIN2, NKAIN3, NOS1AP, PCSK9, PRKCZ, RHCG, SCN3B, SLC12A5, SLC12A7, SLC15A4, SLC17A5, SLC17A7, SLC17A8, SLC30A5, SLC38A1, SLC38A10, SLC38A8, SLC4A7, SLC5A11, SLC6A12, SLC6A2, SLC9A3, SLC9A4, TESC, TMCO3, TMPRSS3, TSC1, WNK2, WNK4]

GO:0055085	transmembrane transport	1,13E-02	[2, 4]	265,00	15,03	22,15	35,29	42,56	[ABCB11, ANO9, AQP5, ATP1B4, BHLHA15, CACNA1E, CALCR, CALHM6, CCL19, CCR2, CCR5, CLCNKA, CNR1, CTSS, CXCL10, CXCL11, CXCL9, CYBB, DAPK1, DRD1, EMB, ERFE, FASLG, FFAR4, FOLR2, GPM6A, HCN1, IFNG, JPH1, KCNA3, KCNJ10, KCNQ5, LCK, LRRC38, MCOLN2, MCOLN3, MID1IP1, NR4A2, NR4A3, P2RY12, PIK3CG, PIM1, PLCH2, PRF1, PTAFR, PTK2B, PTPN22, PTPN6, PTPRC, SIDT1, SLC16A9, SLC18A1, SLC25A48, SLC31A2, SLC36A2, SLCO5A1, TCAF2, THY1, TREM2, TSC22D3, UCP2, UNC80, XCL1, XCR1]	[ABCB10, ABCG2, AKAP6, AKAP9, ANK3, ANXA9, ATP6V0A2, ATP6V1B2, BCL2, CAB39, CACHD1, CACNA1A, CACNB2, CALHM4, CCR7, CDH17, CHRNA1, CORO1A, CX3CL1, CXCL11, DAPK1, DERL1, DNM2, DPP6, F2R, FAM155A, GABRA5, GRIK5, HECW2, HOMER1, HTT, ITPR1, ITPR2, KCNA2, KCNAB2, KCNC2, KCNG3, KCNH1, KCNIP1, KCNIP4, KCNK9, KCNQ1, LCK, LIME1, MICU2, MTNR1B, NETO1, NOS1AP, OPRM1, PDE4B, PEX14, PHLPP2, PIEZO1, PKD1L3, PLCE1, PRF1, PRKAG2, PRKCE, PRKD2, PSMB8, PSMB9, PSMD9, PTK2B, PTPN22, PTPN6, PTPRC, RASA3, SGK1, SLC12A6, SLC12A8, SLC13A4, SLC15A4, SLC16A12, SLC17A3, SLC1A2, SLC1A5, SLC20A2, SLC22A3, SLC25A12, SLC25A32, SLC28A3, SLC37A3, SLC38A1, SLC38A11, SLC43A2, SLC49A4, SLC4A8, SLC6A11, SLC7A10, SLC7A2, SLC9A9, SLCO3A1, STX8, TAP1, TERT, TMC6, TMEM241, TNF, TSPOAP1, UBASH3B, UCP2, UNC80]	[ABCA12, ABCA4, ABCC1, ABCC8, AKAP9, ANK3, ANO2, ANO4, AQP8, ASIC2, ASIC4, ATP1B1, ATP2A3, ATP2B2, ATP6V0D1, ATP6V1D, C2CD5, CABP2, CACNA1S, CACNA2D3, CACNG2, CATSPER2, CAV1, CHRNA4, CLCN3, CLCN7, COX10, DLG2, DMAC2L, DPP6, DRD2, DYSF, FGF12, FGF14, FLVCR2, GABRA5, GABRG3, GABRR3, GJA3, GPM6A, GRIK2, GRIN2A, HSPA2, HTR3B, ITPR1, KCNA5, KCNB2, KCND2, KCNG2, KCNJ15, KCNJ3, KCNK15, KCNK2, KCNK3, KCNMA1, KCNMB2, KCNS3, KCNT2, LRRC55, LRRC7, LYN, MEF2A, NOS1AP, NRXN3, OAZ1, ORAI1, OSTM1, P2RX2, PCSK9, PDE4B, PEX14, PEX19, PLCE1, PPARGC1A, PRKAG2, PRKCA, PRKCZ, PSMD7, PTPN22, REM2, RHCG, SCARA5, SCN3B, SFXN2, SHANK1, SHISA6, SLC12A5, SLC12A7, SLC15A4, SLC16A4, SLC17A5, SLC17A7, SLC17A8, SLC1A7, SLC25A18, SLC25A2, SLC25A42, SLC29A3, SLC30A5, SLC35F4, SLC38A1, SLC38A10, SLC38A8, SLC4A7, SLC5A11, SLC6A12, SLC6A2, SLC9A3, SLC9A4, SPNS3, SRI, STIM1, TAPBP, TCAF1, TESC, TMC5, TMCO3, TMPRSS3, TOMM40, TSC1, UNC79, WNK2, WNK4]
GO:0015077	monovalent inorganic cation transmembrane transporter activity	1,31E-02	[6, 7, 8, 9]	94,00	18,40	10,00	38,00	52,00	[ABCB11, ATP1B4, HCN1, KCNA3, KCNJ10, KCNQ5, LRRC38, SLC18A1, SLC36A2, TREM2]	[AKAP6, AKAP9, ANK3, ATP6V0A2, ATP6V1B2, CAB39, DNM2, DPP6, GRIK5, HECW2, KCNA2, KCNAB2, KCNC2, KCNG3, KCNH1, KCNIP1, KCNIP4, KCNK9, KCNQ1, MTNR1B, NETO1, NOS1AP, PRKCE, PRKD2, SGK1, SLC12A6, SLC12A8, SLC13A4, SLC15A4, SLC17A3, SLC1A2, SLC20A2, SLC22A3, SLC28A3, SLC38A1, SLC4A8, SLC6A11, SLC9A9]	[ABCC8, AKAP9, ANK3, ASIC2, ASIC4, ATP1B1, ATP2A3, ATP6V0D1, ATP6V1D, CAV1, CLCN3, COX10, DMAC2L, DPP6, FGF12, GRIK2, KCNA5, KCNB2, KCND2, KCNG2, KCNJ15, KCNJ3, KCNK15, KCNK2, KCNK3, KCNMA1, KCNMB2, KCNS3, KCNT2, LRRC55, NOS1AP, PCSK9, PRKCZ, RHCG, SCN3B, SLC12A5, SLC12A7, SLC15A4, SLC17A5, SLC17A7, SLC38A1, SLC4A7, SLC5A11, SLC6A12, SLC6A2, SLC9A3, SLC9A4, TESC, TMCO3, TMPRSS3, WNK2, WNK4]
GO:0022857	transmembrane transporter activity	1,57E-02	[3, 5]	213,00	15,49	16,67	35,53	47,81	[ABCB11, ANO9, AQP5, ATP1B4, CACNA1E, CALHM6, CCR2, CLCNKA, CNR1, CTSS, CYBB, DAPK1, GPM6A, HCN1, IFNG, JPH1, KCNA3, KCNJ10, KCNQ5, LRRC38, MCOLN2, MCOLN3, NR4A2, PIM1, PRF1, PTAFR, PTK2B, SIDT1, SLC16A9, SLC18A1, SLC25A48, SLC31A2, SLC36A2, SLCO5A1, TCAF2, TREM2, UCP2, UNC80]	[ABCB10, ABCG2, AKAP6, AKAP9, ANK3, ANXA9, ATP6V0A2, ATP6V1B2, BCL2, CAB39, CACHD1, CACNA1A, CACNB2, CALHM4, CDH17, CHRNA1, DAPK1, DNM2, DPP6, FAM155A, GABRA5, GRIK5, HECW2, HOMER1, HTT, ITPR1, ITPR2, KCNA2, KCNAB2, KCNC2, KCNG3, KCNH1, KCNIP1, KCNIP4, KCNK9, KCNQ1, MTNR1B, NETO1, NOS1AP, OPRM1, PDE4B, PHLPP2, PIEZO1, PKD1L3, PRF1, PRKCE, PRKD2, PTK2B, RASA3, SGK1, SLC12A6, SLC12A8, SLC13A4, SLC15A4, SLC16A12, SLC17A3, SLC1A2, SLC1A5, SLC20A2, SLC22A3, SLC25A12, SLC25A32, SLC28A3, SLC37A3, SLC38A1, SLC38A11, SLC43A2, SLC49A4, SLC4A8, SLC6A11, SLC7A10, SLC7A2, SLC9A9, SLCO3A1, STX8, TAP1, TMC6, TMEM241, TSPOAP1, UCP2, UNC80]	[ABCA12, ABCA4, ABCC1, ABCC8, AKAP9, ANK3, ANO2, ANO4, AQP8, ASIC2, ASIC4, ATP1B1, ATP2A3, ATP2B2, ATP6V0D1, ATP6V1D, CABP2, CACNA1S, CACNA2D3, CACNG2, CATSPER2, CAV1, CHRNA4, CLCN3, CLCN7, COX10, DLG2, DMAC2L, DPP6, DRD2, DYSF, FGF12, FGF14, FLVCR2, GABRA5, GABRG3, GABRR3, GJA3, GPM6A, GRIK2, GRIN2A, HSPA2, HTR3B, ITPR1, KCNA5, KCNB2, KCND2, KCNG2, KCNJ15, KCNJ3, KCNK15, KCNK2, KCNK3, KCNMA1, KCNMB2, KCNS3, KCNT2, LRRC55, LRRC7, NOS1AP, NRXN3, ORAI1, P2RX2, PCSK9, PDE4B, PPARGC1A, PRKCA, PRKCZ, REM2, RHCG, SCN3B, SFXN2, SHANK1, SHISA6, SLC12A5, SLC12A7, SLC15A4, SLC16A4, SLC17A5, SLC17A7, SLC17A8, SLC1A7, SLC25A18, SLC25A2, SLC25A42, SLC29A3, SLC30A5, SLC35F4, SLC38A1, SLC38A10, SLC38A8, SLC4A7, SLC5A11, SLC6A12, SLC6A2, SLC9A3, SLC9A4, SPNS3, SRI, STIM1, TAPBP, TCAF1, TESC, TMC5, TMCO3, TMPRSS3, TOMM40, WNK2, WNK4]
GO:0055080	cation homeostasis	6,09E-04	[6]	140,00	17,70	35,10	31,13	33,77	[CALCR, CCL19, CCR2, CCR3, CCR4, CCR5, CCR8, CD200R1, CD4, CD52, CNR1, CP, CXCL10, CXCL11, CXCL9, CXCR3, CXCR4, CXCR6, CXorf21, CYSLTR1, DRD1, EDN2, ERFE, FASLG, FATE1, FFAR4, GPR174, GPR55, IFNG, JPH1, KCNJ10, KNG1, LCK, MCOLN2, MCOLN3, MS4A1, MT1HL1, NLRP3, NPPB, P2RY10, PIK3CG, PLCH2, PTGFR, PTK2B, PTPN6, PTPRC, SLAMF8, SLC31A2, SNX10, THY1, UNC80, XCL1, XCR1]	[ABCG2, AKAP6, ANK3, ATP6V0A2, ATP6V1B2, B2M, BCL2, BTBD9, C19orf12, CACNA1A, CACNB2, CCR7, CD38, CD52, CORO1A, CX3CL1, CXCL11, F2R, FAM155A, GPR6, GRIK5, HTT, ITPR1, ITPR2, LCK, LIME1, MAFG, MICU2, MTNR1B, NEO1, NPTN, PLCE1, PRKCE, PRKD2, PTK2B, PTPN6, PTPRC, RASA3, SGCD, SLC12A6, SLC12A8, SLC4A8, SLC9A9, TSPOAP1, UBASH3B, UBE3A, UNC80]	[ADCY5, ADORA1, ADRA1B, ANK3, ATP1B1, ATP2A3, ATP2B2, ATP6V0D1, ATP6V1D, CALB2, CAV1, CLCN3, COMT, CYP4A11, CYP4F12, DMPK, DMXL1, DRD2, EGFR, GRIK2, GRIN2A, HYAL2, ITPR1, KCNA5, KCNK3, KCNMA1, LPAR6, LYN, MC3R, P2RX2, PDE6B, PDZD8, PLCE1, PRKCA, PRKN, RAB7A, RHCG, SCARA5, SGCD, SLC12A5, SLC12A7, SLC30A5, SLC4A7, SLC9A3, SLC9A4, SRI, STIM1, SWAP70, TMPRSS3, VPS33A, WFS1]
GO:0098771	inorganic ion homeostasis	1,01E-03	[6]	141,00	17,54	35,10	31,13	33,77	[CALCR, CCL19, CCR2, CCR3, CCR4, CCR5, CCR8, CD200R1, CD4, CD52, CNR1, CP, CXCL10, CXCL11, CXCL9, CXCR3, CXCR4, CXCR6, CXorf21, CYSLTR1, DRD1, EDN2, ERFE, FASLG, FATE1, FFAR4, GPR174, GPR55, IFNG, JPH1, KCNJ10, KNG1, LCK, MCOLN2, MCOLN3, MS4A1, MT1HL1, NLRP3, NPPB, P2RY10, PIK3CG, PLCH2, PTGFR, PTK2B, PTPN6, PTPRC, SLAMF8, SLC31A2, SNX10, TBXAS1, THY1, XCL1, XCR1]	[ABCG2, AKAP6, ANK3, ATP6V0A2, ATP6V1B2, B2M, BCL2, BTBD9, C19orf12, CA12, CACNA1A, CACNB2, CCR7, CD38, CD52, CORO1A, CX3CL1, CXCL11, F2R, FAM155A, GPR6, GRIK5, HTT, ITPR1, ITPR2, LCK, LIME1, MAFG, MICU2, MTNR1B, NEO1, NPTN, PLCE1, PRKCE, PRKD2, PTK2B, PTPN6, PTPRC, RASA3, SGCD, SLC12A6, SLC12A8, SLC4A8, SLC9A9, TSPOAP1, UBASH3B, UBE3A]	[ADCY5, ADORA1, ADRA1B, ANK3, ATP1B1, ATP2A3, ATP2B2, ATP6V0D1, ATP6V1D, CALB2, CAV1, CLCN3, COMT, CYP4A11, CYP4F12, DMPK, DMXL1, DRD2, EGFR, GRIK2, GRIN2A, HYAL2, ITPR1, KCNA5, KCNK3, KCNMA1, LPAR6, LYN, MC3R, P2RX2, PDE6B, PDZD8, PLCE1, PRKCA, PRKN, RAB7A, RHCG, SCARA5, SGCD, SLC12A5, SLC12A7, SLC30A5, SLC4A7, SLC9A3, SLC9A4, SRI, STIM1, SWAP70, TMPRSS3, VPS33A, WFS1]
GO:0042592	homeostatic process	1,01E-03	[3]	317,00	15,05	31,10	34,88	34,01	[ADAM8, ANGPT1, BHLHA15, CALCR, CARD11, CCL19, CCR2, CCR3, CCR4, CCR5, CCR8, CD200R1, CD4, CD52, CD74, CEL, CNR1, CP, CSF1R, CXCL10, CXCL11, CXCL6, CXCL9, CXCR3, CXCR4, CXCR6, CXorf21, CYBB, CYP11B1, CYSLTR1, DOCK10, DRD1, EDN2, ERFE, FASLG, FATE1, FFAR4, FLT3, FOXP3, GAPT, GPR174, GPR55, GRAMD1B, HCLS1, HKDC1, HLA-DRB1, IFNG, IKZF1, IL18, IL7R, INPP5D, IRF4, JCHAIN, JPH1, KCNJ10, KNG1, LCK, LCP1, LMO1, LPAL2, LYZ, MCOLN2, MCOLN3, MS4A1, MT1HL1, MUC6, NCF1, NCKAP1L, NELL2, NLRP3, NME8, NPPB, NR4A3, P2RY10, P2RY2, PIK3CG, PLAC8, PLCH2, PRKCQ, PTGFR, PTK2B, PTPN6, PTPRC, RHEX, SASH3, SIT1, SLAMF8, SLC31A2, SNX10, SOAT2, STAT1, SUCNR1, SYK, TBXAS1, THY1, TNFAIP3, TNFRSF11B, TNFSF13B, TRERF1, TSC22D3, TXNDC5, UCP2, UNC80, VSIG1, WNT10B, XCL1, XCR1]	[ABAT, ABCG2, ACOXL, ACVR1B, ADA, ADCY1, ADGRV1, AFDN, AKAP6, ANK3, ANKRD11, ATP6V0A2, ATP6V1B2, B2M, BCL2, BTBD9, C19orf12, CA12, CACNA1A, CACNB2, CCR7, CD38, CD52, CDH2, CDK6, CHRNA1, CIAO3, CLNS1A, CORO1A, CUBN, CX3CL1, CXCL11, DCLRE1B, DEDD2, EHMT1, F2R, FAM155A, FGGY, FOXK1, GPR6, GPR68, GRIK5, HDAC9, HOMER1, HOMER2, HTT, IGF1R, INAVA, ITPR1, ITPR2, JAK3, LCK, LCP1, LGR4, LIME1, LIPC, LPCAT1, LPIN1, MAFG, MAP4K5, MBP, MED1, MICU2, MTNR1B, MYT1, NCF4, NEO1, NPTN, NXNL2, ORMDL1, PDE4B, PDIA6, PIK3CD, PLCE1, PPP1R13L, PRKACB, PRKAR1B, PRKCE, PRKD2, PROM1, PTK2B, PTPN2, PTPN6, PTPRC, PTPRN2, RASA3, RORA, RPA3, SGCD, SGIP1, SLC12A6, SLC12A8, SLC4A8, SLC9A9, SMARCA4, SMG6, STAT1, STAT3, TERT, TJP2, TM6SF2, TNF, TNFAIP3, TNIP1, TNS2, TRERF1, TSHR, TSPOAP1, TXNDC11, UBASH3B, UBE3A, UCP2, UGCG, UNC80, UPF1, XRN1, YAP1, ZBTB20, ZNF365, ZNF423]	[ABCA12, ABCA4, ABCC8, ADCY5, ADORA1, ADRA1B, ADRB3, AMPD3, ANK3, ARNT, ASXL1, ATP1B1, ATP2A3, ATP2B2, ATP6V0D1, ATP6V1D, CALB2, CARD11, CARTPT, CAV1, CLCN3, COMT, CRB1, CSMD1, CXADR, CYP4A11, CYP4F12, DCN, DISP3, DMPK, DMXL1, DRD2, EGFR, ESRRB, ESRRG, FGF12, FTO, GCNT2, GNAS, GRIK2, GRIN2A, HIPK2, HLA-DRB1, HMBOX1, HYAL2, IGF1R, ITPR1, JCHAIN, KCNA5, KCNK3, KCNMA1, LARGE1, LCP1, LPAR6, LYN, LYZ, MAGI1, MAPRE2, MBL2, MBP, MC3R, MUC2, NR1H3, NXN, OIT3, P2RX2, PCSK9, PDE4B, PDE6B, PDZD8, PFKM, PLAC8, PLCE1, PPARGC1A, PPP3CA, PRDX1, PRKAA1, PRKAR2B, PRKCA, PRKN, PTPRN2, RAB7A, RB1, RHCG, SCARA5, SCN3B, SGCD, SGCZ, SIGLEC15, SLC12A5, SLC12A7, SLC17A7, SLC30A5, SLC4A7, SLC6A2, SLC9A3, SLC9A4, SMAD1, SMAP1, SRI, STIM1, STN1, STXBP5L, SWAP70, TERF1, TIAM1, TMPRSS3, TNFRSF11A, TUB, TXNDC11, USH1G, VPS33A, VSNL1, WFS1, WNK2, WNK4, WRAP53]
GO:0051480	regulation of cytosolic calcium ion concentration	1,09E-03	[8, 10]	81,00	20,25	46,07	32,58	21,35	[CALCR, CCL19, CCR2, CCR3, CCR4, CCR5, CCR8, CD200R1, CD4, CD52, CNR1, CXCL10, CXCL11, CXCL9, CXCR3, CXCR4, CXCR6, CYSLTR1, DRD1, EDN2, FASLG, FFAR4, GPR174, GPR55, JPH1, KNG1, LCK, MCOLN2, MCOLN3, MS4A1, NLRP3, P2RY10, PIK3CG, PLCH2, PTGFR, PTK2B, PTPN6, PTPRC, THY1, XCL1, XCR1]	[AKAP6, BCL2, CACNA1A, CACNB2, CCR7, CD38, CD52, CORO1A, CX3CL1, CXCL11, F2R, FAM155A, GPR6, HTT, ITPR1, ITPR2, LCK, LIME1, MTNR1B, NPTN, PLCE1, PRKCE, PRKD2, PTK2B, PTPN6, PTPRC, RASA3, TSPOAP1, UBASH3B]	[ADCY5, ADORA1, ADRA1B, ATP2B2, CALB2, CAV1, DRD2, GRIN2A, ITPR1, KCNA5, KCNK3, LPAR6, LYN, P2RX2, PDE6B, PLCE1, PRKCA, SRI, SWAP70]
GO:0050801	ion homeostasis	4,22E-03	[5]	149,00	16,87	34,38	30,00	35,63	[CALCR, CCL19, CCR2, CCR3, CCR4, CCR5, CCR8, CD200R1, CD4, CD52, CNR1, CP, CXCL10, CXCL11, CXCL9, CXCR3, CXCR4, CXCR6, CXorf21, CYSLTR1, DRD1, EDN2, ERFE, FASLG, FATE1, FFAR4, GPR174, GPR55, IFNG, JPH1, KCNJ10, KNG1, LCK, MCOLN2, MCOLN3, MS4A1, MT1HL1, NLRP3, NPPB, P2RY10, P2RY2, PIK3CG, PLCH2, PTGFR, PTK2B, PTPN6, PTPRC, SLAMF8, SLC31A2, SNX10, TBXAS1, THY1, UNC80, XCL1, XCR1]	[ABCG2, AKAP6, ANK3, ATP6V0A2, ATP6V1B2, B2M, BCL2, BTBD9, C19orf12, CA12, CACNA1A, CACNB2, CCR7, CD38, CD52, CORO1A, CX3CL1, CXCL11, F2R, FAM155A, GPR6, GRIK5, HTT, ITPR1, ITPR2, LCK, LIME1, MAFG, MICU2, MTNR1B, NEO1, NPTN, PLCE1, PRKCE, PRKD2, PTK2B, PTPN6, PTPRC, RASA3, SGCD, SLC12A6, SLC12A8, SLC4A8, SLC9A9, TSPOAP1, UBASH3B, UBE3A, UNC80]	[ADCY5, ADORA1, ADRA1B, ANK3, ATP1B1, ATP2A3, ATP2B2, ATP6V0D1, ATP6V1D, CALB2, CAV1, CLCN3, COMT, CYP4A11, CYP4F12, DCN, DMPK, DMXL1, DRD2, EGFR, FGF12, GRIK2, GRIN2A, HYAL2, ITPR1, KCNA5, KCNK3, KCNMA1, LPAR6, LYN, MC3R, P2RX2, PDE6B, PDZD8, PLCE1, PRKAA1, PRKCA, PRKN, RAB7A, RHCG, SCARA5, SCN3B, SGCD, SLC12A5, SLC12A7, SLC30A5, SLC4A7, SLC9A3, SLC9A4, SRI, STIM1, SWAP70, TMPRSS3, VPS33A, WFS1, WNK2, WNK4]
GO:0060402	calcium ion transport into cytosol	5,76E-03	[4, 10, 12]	44,00	23,78	41,18	41,18	17,65	[CALCR, CCL19, CCR5, CD4, CXCL10, CXCL11, CXCL9, DRD1, FASLG, JPH1, LCK, MCOLN2, MCOLN3, MS4A1, PLCH2, PTK2B, PTPN6, PTPRC, THY1, XCL1, XCR1]	[AKAP6, BCL2, CCR7, CORO1A, CX3CL1, CXCL11, F2R, FAM155A, HTT, ITPR1, ITPR2, LCK, LIME1, PLCE1, PRKCE, PRKD2, PTK2B, PTPN6, PTPRC, RASA3, UBASH3B]	[CAV1, DRD2, GRIN2A, ITPR1, LYN, P2RX2, PLCE1, PRKCA, SRI]
GO:0051209	release of sequestered calcium ion into cytosol	5,78E-03	[4, 5, 6, 7, 8, 10, 11, 12, 13, 14]	36,00	26,09	41,86	44,19	13,95	[CCL19, CCR5, CXCL10, CXCL11, CXCL9, DRD1, FASLG, JPH1, LCK, MCOLN2, MCOLN3, PLCH2, PTK2B, PTPN6, PTPRC, THY1, XCL1, XCR1]	[AKAP6, CCR7, CORO1A, CX3CL1, CXCL11, F2R, HTT, ITPR1, ITPR2, LCK, LIME1, PLCE1, PRKCE, PRKD2, PTK2B, PTPN6, PTPRC, RASA3, UBASH3B]	[DRD2, ITPR1, LYN, PLCE1, PRKCA, SRI]
GO:0055065	metal ion homeostasis	1,05E-02	[7]	123,00	17,32	37,59	30,83	31,58	[CALCR, CCL19, CCR2, CCR3, CCR4, CCR5, CCR8, CD200R1, CD4, CD52, CNR1, CP, CXCL10, CXCL11, CXCL9, CXCR3, CXCR4, CXCR6, CYSLTR1, DRD1, EDN2, ERFE, FASLG, FATE1, FFAR4, GPR174, GPR55, IFNG, JPH1, KCNJ10, KNG1, LCK, MCOLN2, MCOLN3, MS4A1, MT1HL1, NLRP3, NPPB, P2RY10, PIK3CG, PLCH2, PTGFR, PTK2B, PTPN6, PTPRC, SLC31A2, SNX10, THY1, XCL1, XCR1]	[ABCG2, AKAP6, ANK3, ATP6V0A2, B2M, BCL2, BTBD9, C19orf12, CACNA1A, CACNB2, CCR7, CD38, CD52, CORO1A, CX3CL1, CXCL11, F2R, FAM155A, GPR6, GRIK5, HTT, ITPR1, ITPR2, LCK, LIME1, MICU2, MTNR1B, NEO1, NPTN, PLCE1, PRKCE, PRKD2, PTK2B, PTPN6, PTPRC, RASA3, SGCD, SLC12A6, SLC12A8, TSPOAP1, UBASH3B]	[ADCY5, ADORA1, ADRA1B, ANK3, ATP1B1, ATP2A3, ATP2B2, ATP6V0D1, CALB2, CAV1, COMT, CYP4A11, CYP4F12, DMPK, DRD2, EGFR, GRIK2, GRIN2A, HYAL2, ITPR1, KCNA5, KCNK3, KCNMA1, LPAR6, LYN, MC3R, P2RX2, PDE6B, PDZD8, PLCE1, PRKCA, PRKN, SCARA5, SGCD, SLC12A5, SLC12A7, SLC30A5, SRI, STIM1, SWAP70, TMPRSS3, WFS1]

GO:0055074	calcium ion homeostasis	1,49E-02	[8]	94,00	18,25	41,75	32,04	26,21	[CALCR, CCL19, CCR2, CCR3, CCR4, CCR5, CCR8, CD200R1, CD4, CD52, CNR1, CXCL10, CXCL11, CXCL9, CXCR3, CXCR4, CXCR6, CYSLTR1, DRD1, EDN2, FASLG, FATE1, FFAR4, GPR174, GPR55, JPH1, KNG1, LCK, MCOLN2, MCOLN3, MS4A1, NLRP3, P2RY10, PIK3CG, PLCH2, PTGFR, PTK2B, PTPN6, PTPRC, SNX10, THY1, XCL1, XCR1]	[AKAP6, BCL2, C19orf12, CACNA1A, CACNB2, CCR7, CD38, CD52, CORO1A, CX3CL1, CXCL11, F2R, FAM155A, GPR6, GRIK5, HTT, ITPR1, ITPR2, LCK, LIME1, MICU2, MTNR1B, NPTN, PLCE1, PRKCE, PRKD2, PTK2B, PTPN6, PTPRC, RASA3, SGCD, TSPOAP1, UBASH3B]	[ADCY5, ADORA1, ADRA1B, ATP1B1, ATP2A3, ATP2B2, CALB2, CAV1, DMPK, DRD2, GRIK2, GRIN2A, ITPR1, KCNA5, KCNK3, LPAR6, LYN, P2RX2, PDE6B, PDZD8, PLCE1, PRKCA, SGCD, SRI, STIM1, SWAP70, WFS1]
GO:0051208	sequestering of calcium ion	1,92E-02	[3, 4, 5, 8, 10]	36,00	25,00	41,86	44,19	13,95	[CCL19, CCR5, CXCL10, CXCL11, CXCL9, DRD1, FASLG, JPH1, LCK, MCOLN2, MCOLN3, PLCH2, PTK2B, PTPN6, PTPRC, THY1, XCL1, XCR1]	[AKAP6, CCR7, CORO1A, CX3CL1, CXCL11, F2R, HTT, ITPR1, ITPR2, LCK, LIME1, PLCE1, PRKCE, PRKD2, PTK2B, PTPN6, PTPRC, RASA3, UBASH3B]	[DRD2, ITPR1, LYN, PLCE1, PRKCA, SRI]
GO:0097553	calcium ion transmembrane import into cytosol	1,93E-02	[5, 7, 9, 10, 11, 13]	39,00	24,22	41,30	43,48	15,22	[CALCR, CCL19, CCR5, CXCL10, CXCL11, CXCL9, DRD1, FASLG, JPH1, LCK, MCOLN2, MCOLN3, PLCH2, PTK2B, PTPN6, PTPRC, THY1, XCL1, XCR1]	[AKAP6, CCR7, CORO1A, CX3CL1, CXCL11, F2R, FAM155A, HTT, ITPR1, ITPR2, LCK, LIME1, PLCE1, PRKCE, PRKD2, PTK2B, PTPN6, PTPRC, RASA3, UBASH3B]	[DRD2, GRIN2A, ITPR1, LYN, PLCE1, PRKCA, SRI]
GO:0006874	cellular calcium ion homeostasis	2,15E-02	[7, 9]	92,00	18,29	42,00	32,00	26,00	[CALCR, CCL19, CCR2, CCR3, CCR4, CCR5, CCR8, CD200R1, CD4, CD52, CNR1, CXCL10, CXCL11, CXCL9, CXCR3, CXCR4, CXCR6, CYSLTR1, DRD1, EDN2, FASLG, FATE1, FFAR4, GPR174, GPR55, JPH1, KNG1, LCK, MCOLN2, MCOLN3, MS4A1, NLRP3, P2RY10, PIK3CG, PLCH2, PTGFR, PTK2B, PTPN6, PTPRC, THY1, XCL1, XCR1]	[AKAP6, BCL2, C19orf12, CACNA1A, CACNB2, CCR7, CD38, CD52, CORO1A, CX3CL1, CXCL11, F2R, FAM155A, GPR6, GRIK5, HTT, ITPR1, ITPR2, LCK, LIME1, MICU2, MTNR1B, NPTN, PLCE1, PRKCE, PRKD2, PTK2B, PTPN6, PTPRC, RASA3, TSPOAP1, UBASH3B]	[ADCY5, ADORA1, ADRA1B, ATP1B1, ATP2A3, ATP2B2, CALB2, CAV1, DMPK, DRD2, GRIK2, GRIN2A, ITPR1, KCNA5, KCNK3, LPAR6, LYN, P2RX2, PDE6B, PDZD8, PLCE1, PRKCA, SRI, STIM1, SWAP70, WFS1]
GO:0048878	chemical homeostasis	2,76E-02	[4]	199,00	15,52	30,56	33,80	35,65	[BHLHA15, CALCR, CCL19, CCR2, CCR3, CCR4, CCR5, CCR8, CD200R1, CD4, CD52, CNR1, CP, CXCL10, CXCL11, CXCL9, CXCR3, CXCR4, CXCR6, CXorf21, CYP11B1, CYSLTR1, DRD1, EDN2, ERFE, FASLG, FATE1, FFAR4, GPR174, GPR55, GRAMD1B, HKDC1, HLA-DRB1, IFNG, IL18, JPH1, KCNJ10, KNG1, LCK, LCP1, LPAL2, MCOLN2, MCOLN3, MS4A1, MT1HL1, NLRP3, NPPB, P2RY10, P2RY2, PIK3CG, PLCH2, PTGFR, PTK2B, PTPN6, PTPRC, SLAMF8, SLC31A2, SNX10, SOAT2, STAT1, SUCNR1, TBXAS1, THY1, UNC80, XCL1, XCR1]	[ABCG2, ACOXL, ADCY1, AKAP6, ANK3, ATP6V0A2, ATP6V1B2, B2M, BCL2, BTBD9, C19orf12, CA12, CACNA1A, CACNB2, CCR7, CD38, CD52, CIAO3, CORO1A, CX3CL1, CXCL11, F2R, FAM155A, FOXK1, GPR6, GPR68, GRIK5, HDAC9, HOMER1, HOMER2, HTT, IGF1R, ITPR1, ITPR2, LCK, LCP1, LIME1, LIPC, LPCAT1, MAFG, MAP4K5, MICU2, MTNR1B, NEO1, NPTN, ORMDL1, PLCE1, PRKACB, PRKAR1B, PRKCE, PRKD2, PTK2B, PTPN2, PTPN6, PTPRC, PTPRN2, RASA3, RORA, SGCD, SLC12A6, SLC12A8, SLC4A8, SLC9A9, SMARCA4, STAT1, STAT3, TM6SF2, TSPOAP1, UBASH3B, UBE3A, UGCG, UNC80, ZBTB20]	[ABCA12, ABCC8, ADCY5, ADORA1, ADRA1B, ANK3, ATP1B1, ATP2A3, ATP2B2, ATP6V0D1, ATP6V1D, CALB2, CARTPT, CAV1, CLCN3, COMT, CSMD1, CYP4A11, CYP4F12, DCN, DISP3, DMPK, DMXL1, DRD2, EGFR, FGF12, GNAS, GRIK2, GRIN2A, HLA-DRB1, HYAL2, IGF1R, ITPR1, KCNA5, KCNK3, KCNMA1, LCP1, LPAR6, LYN, MC3R, NR1H3, OIT3, P2RX2, PCSK9, PDE6B, PDZD8, PFKM, PLCE1, PPARGC1A, PPP3CA, PRKAA1, PRKAR2B, PRKCA, PRKN, PTPRN2, RAB7A, RHCG, SCARA5, SCN3B, SGCD, SLC12A5, SLC12A7, SLC30A5, SLC4A7, SLC9A3, SLC9A4, SRI, STIM1, STXBP5L, SWAP70, TIAM1, TMPRSS3, VPS33A, VSNL1, WFS1, WNK2, WNK4]
GO:0072507	divalent inorganic cation homeostasis	3,18E-02	[7]	99,00	17,74	40,37	31,19	28,44	[CALCR, CCL19, CCR2, CCR3, CCR4, CCR5, CCR8, CD200R1, CD4, CD52, CNR1, CXCL10, CXCL11, CXCL9, CXCR3, CXCR4, CXCR6, CYSLTR1, DRD1, EDN2, FASLG, FATE1, FFAR4, GPR174, GPR55, JPH1, KNG1, LCK, MCOLN2, MCOLN3, MS4A1, MT1HL1, NLRP3, P2RY10, PIK3CG, PLCH2, PTGFR, PTK2B, PTPN6, PTPRC, SNX10, THY1, XCL1, XCR1]	[AKAP6, ANK3, BCL2, C19orf12, CACNA1A, CACNB2, CCR7, CD38, CD52, CORO1A, CX3CL1, CXCL11, F2R, FAM155A, GPR6, GRIK5, HTT, ITPR1, ITPR2, LCK, LIME1, MICU2, MTNR1B, NPTN, PLCE1, PRKCE, PRKD2, PTK2B, PTPN6, PTPRC, RASA3, SGCD, TSPOAP1, UBASH3B]	[ADCY5, ADORA1, ADRA1B, ANK3, ATP1B1, ATP2A3, ATP2B2, CALB2, CAV1, DMPK, DRD2, EGFR, GRIK2, GRIN2A, ITPR1, KCNA5, KCNK3, LPAR6, LYN, P2RX2, PDE6B, PDZD8, PLCE1, PRKCA, PRKN, SGCD, SLC30A5, SRI, STIM1, SWAP70, WFS1]
GO:0006873	cellular ion homeostasis	3,97E-02	[4, 6]	123,00	16,87	38,93	29,77	31,30	[CALCR, CCL19, CCR2, CCR3, CCR4, CCR5, CCR8, CD200R1, CD4, CD52, CNR1, CP, CXCL10, CXCL11, CXCL9, CXCR3, CXCR4, CXCR6, CXorf21, CYSLTR1, DRD1, EDN2, ERFE, FASLG, FATE1, FFAR4, GPR174, GPR55, IFNG, JPH1, KNG1, LCK, MCOLN2, MCOLN3, MS4A1, MT1HL1, NLRP3, P2RY10, P2RY2, PIK3CG, PLCH2, PTGFR, PTK2B, PTPN6, PTPRC, SLAMF8, SLC31A2, TBXAS1, THY1, XCL1, XCR1]	[ABCG2, AKAP6, ATP6V0A2, ATP6V1B2, BCL2, C19orf12, CACNA1A, CACNB2, CCR7, CD38, CD52, CORO1A, CX3CL1, CXCL11, F2R, FAM155A, GPR6, GRIK5, HTT, ITPR1, ITPR2, LCK, LIME1, MAFG, MICU2, MTNR1B, NPTN, PLCE1, PRKCE, PRKD2, PTK2B, PTPN6, PTPRC, RASA3, SLC4A8, SLC9A9, TSPOAP1, UBASH3B, UBE3A]	[ADCY5, ADORA1, ADRA1B, ATP1B1, ATP2A3, ATP2B2, ATP6V0D1, ATP6V1D, CALB2, CAV1, CLCN3, DMPK, DMXL1, DRD2, GRIK2, GRIN2A, ITPR1, KCNA5, KCNK3, KCNMA1, LPAR6, LYN, P2RX2, PDE6B, PDZD8, PLCE1, PRKCA, RAB7A, RHCG, SCARA5, SLC12A5, SLC30A5, SLC4A7, SLC9A3, SLC9A4, SRI, STIM1, SWAP70, TMPRSS3, VPS33A, WFS1]
