# Supplementary material for: Epigenetic regulation of transcription factor binding motifs promotes Th1 response in Chagas disease cardiomyopathy
Source: Front Immunol. 2022 Aug 22;13:958200. doi: 10.3389/fimmu.2022.958200 (PMC9441916; doi:10.3389/fimmu.2022.958200)
Supplement: Supplementary Table 1 — Biological samples included in this study. [file DataSheet_1.zip › Supplementary Material/Supplementary Table 17.pdf]

**Supplementary table 17.** Gene ontology analysis based on genes affected by at least one differentially methylated CpGs between blood moderate CCC samples and blood severe CCC samples.

| ID | Term | Corrected pvalue | Level | Nb. Genes | % Associated Genes | Associated Genes Found |
|---|---|---|---|---|---|---|
| GO:0006958 | complement activation, classical pathway | 5,32E-06 | [4, 5, 6, 7, 8] | 5,00 | 3,03 | [C1QC, CD46, HNRNPC, IGLL5, PSMA7] |
| GO:0004984 | olfactory receptor activity | 6,07E-19 | [6, 8] | 14,00 | 3,23 | [OR10C1, OR10G9, OR11L1, OR2B11, OR2L3, OR4K13, OR4K2, OR51B2, OR51E1, OR52B2, OR52I2, OR5R1, OR8D1, OR9Q1] |
| GO:0006910 | phagocytosis, recognition | 4,43E-02 | [3, 4, 6] | 4,00 | 3,85 | [IGLL5, MFGE8, SCARB1, TUB] |
| GO:0050907 | detection of chemical stimulus involved in sensory perception | 1,44E-18 | [4, 6] | 19,00 | 3,94 | [ASIC3, OR10C1, OR10G9, OR11L1, OR2B11, OR2L3, OR4K13, OR4K2, OR51B2, OR51E1, OR52B2, OR52I2, OR5R1, OR8D1, OR9Q1, TAS1R2, TAS1R3, TRPA1, TRPV1] |
| GO:0007608 | sensory perception of smell | 1,23E-16 | [6] | 20,00 | 4,31 | [ADCY3, BBS4, NCAM2, OR10C1, OR10G9, OR11L1, OR2B11, OR2L3, OR4K13, OR4K2, OR51B2, OR51E1, OR52B2, OR52I2, OR5R1, OR8D1, OR9Q1, SLC24A4, TTC8, UBR3] |
| GO:0006956 | complement activation | 8,17E-06 | [3, 4, 5, 6, 7] | 9,00 | 4,39 | [C1QC, CD46, CFB, CPNE2, HNRNPC, IGLL5, KRT1, PIKFYVE, PSMA7] |
| GO:0002455 | humoral immune response mediated by circulating immunoglobulin | 9,05E-05 | [4, 7] | 8,00 | 4,42 | [BCL3, C1QC, CD46, GNAO1, HNRNPC, IGLL5, PSMA7, PTPRC] |
| GO:0002455 | humoral immune response mediated by circulating immunoglobulin | 9,05E-05 | [4, 7] | 8,00 | 4,42 | [BCL3, C1QC, CD46, GNAO1, HNRNPC, IGLL5, PSMA7, PTPRC] |
| GO:0030449 | regulation of complement activation | 2,11E-02 | [4, 5, 6, 7, 8] | 6,00 | 4,76 | [C1QC, CD46, CFB, CPNE2, HNRNPC, PSMA7] |
| GO:0009593 | detection of chemical stimulus | 1,83E-16 | [3] | 26,00 | 5,00 | TAS1R3, TGFB3, TRPA1, TRPV1] |
| GO:0007606 | sensory perception of chemical stimulus | 3,42E-17 | [5] | 27,00 | 5,01 | TAS1R3, TRPA1, TRPV1, TTC8, UBR3] |
| GO:0050906 | detection of stimulus involved in sensory perception | 9,24E-15 | [3, 5] | 32,00 | 5,84 | [ADGRV1, ANO1, ASIC3, ATP8A2, BEST1, CACNA2D4, CXCR4, EPHB1, HTR2A, OR10C1, OR10G9, OR11L1, OR2B11, OR2L3, OR4K13, OR4K2, OR51B2, OR51E1, OR52B2, OR52I2, OR5R1, OR8D1, OR9Q1, PDZD7, SCN1A, SERPINE2, TAS1R2, TAS1R3, TRPA1, TRPV1, WDR47, WHRN] |
| GO:0042742 | defense response to bacterium | 6,53E-09 | [4, 5, 6] | 23,00 | 6,08 | [ADAM17, ANKRD17, BCL3, BPI, CHAMP1, DLC1, FOXP1, GSDMB, IGLL5, IL6R, KLK7, MYD88, PLA2G6, PLAC8, PPP1R11, PXDN, RNASE6, SSC5D, STAB1, STAB2, SYT12, TBK1, WFDC2] |
| GO:0006959 | humoral immune response | 3,95E-07 | [3] | 30,00 | 7,35 | PSMA7, PTPRC, RNASE6, SPNS2, ST6GAL1, WFDC2] |
| GO:0019724 | B cell mediated immunity | 3,16E-02 | [5] | 23,00 | 8,78 | [BCL10, BCL3, BCL6, C1QC, CCR6, CD46, ERCC1, EXOSC6, GNAO1, HNRNPC, HSPD1, IGLL5, KMT5C, LIG4, MSH2, MYD88, POU2F2, PSMA7, PTPRC, SHLD1, SLA2, THOC1, UNG] |
| GO:0019724 | B cell mediated immunity | 3,16E-02 | [5] | 23,00 | 8,78 | [BCL10, BCL3, BCL6, C1QC, CCR6, CD46, ERCC1, EXOSC6, GNAO1, HNRNPC, HSPD1, IGLL5, KMT5C, LIG4, MSH2, MYD88, POU2F2, PSMA7, PTPRC, SHLD1, SLA2, THOC1, UNG] |
| GO:0002449 | lymphocyte mediated immunity | 4,80E-03 | [4] | 40,00 | 9,88 | [BCL10, BCL3, BCL6, BTN3A3, C1QC, CADM1, CCR2, CCR6, CD46, CD8A, CD96, CLEC4G, DENND1B, DLG1, ERCC1, EXOSC6, FZD5, GNAO1, HMGB1, HNRNPC, HSPD1, IGLL5, IL31RA, KMT5C, LIG4, MSH2, MYD88, POU2F2, PPP3CB, PRKCZ, PSMA7, PTPRC, PVR, SHLD1, SLA2, SMAD7, THOC1, TUBB, UNG, ZBTB1] |
| GO:0002250 | adaptive immune response | 2,32E-07 | [3] | 75,00 | 9,99 | [ADAM17, ADCY7, BCL10, BCL3, BCL6, BTN3A3, C1QC, CAMK4, CCR2, CCR6, CD247, CD46, CD8A, CLEC4G, CTSS, DCLRE1C, DENND1B, DLG1, EIF2AK4, ERAP1, ERAP2, ERCC1, EXOSC6, FZD5, GNAO1, HLA-DPB1, HLA-DQA2, HLA-DRA, HMGB1, HNRNPC, HSPD1, IGLL5, IL31RA, IL6ST, ITK, JAK2, KMT5C, LAT2, LAX1, LIG4, LOXL3, LY9, MEF2C, MSH2, MYD88, NCOR2, ORAI1, OTUB1, PIK3CG, POU2F2, PPP3CB, PRKCB, PRKCQ, PRKCZ, PRKD2, PSMA7, PTPRC, PVR, RC3H1, RC3H2, RELB, RHBDF2, RORA, RORC, SHLD1, SKAP1, SLA2, SMAD7, TAP2, THOC1, |
| GO:0004930 | G protein-coupled receptor activity | 3,14E-08 | [4, 5, 6] | 92,00 | 10,34 | [ADCYAP1R1, ADGRA1, ADGRB2, ADGRB3, ADGRD1, ADGRF5, ADGRG1, ADGRG5, ADGRL3, ADGRV1, ADRA1B, ADRB1, CASR, CCKAR, CCR2, CCR6, CD200R1, CELSR1, CHRM4, CXCR4, DHX15, DHX34, F2RL3, FZD10, FZD5, GABBR1, GABBR2, GLP2R, GNRHR2, GPER1, GPR161, GPR17, GPR176, GPR39, GPR4, GPR55, GPR62, GPRC5B, GPRC5C, GRIK3, GRM1, GRM2, GRM4, GRM8, HCAR1, HCAR2, HCRTR2, HTR1A, HTR2A, KISS1R, LPAR1, LPAR2, MRGPRX2, MTNR1B, NMBR, NPBWR2, NPR1, NPRL3, OPN1SW, OPN3, OPN5, OR10C1, OR10G9, OR11L1, |
| GO:0048018 | receptor ligand activity | 4,69E-02 | [3, 4, 5, 6] | 62,00 | 11,65 | [ADA2, AMH, BDNF, BDNF-AS, BMP1, BMP2, BMP3, BMP7, BMP8B, BTC, CCL16, CCN6, CMTM8, CSPG5, EFNA5, ENC1, ENHO, EREG, FAM3B, FAM3D, FBN1, FGF12, FGF14, FGF2, FGF22, FGF6, FGF7, GCG, GFER, GMFB, GNAI2, GREM1, GUCA2A, HMGB1, IGF1, IL16, IL17D, IL37, INSL5, KITLG, LRPAP1, MACC1, MEN1, METRNL, NGF, NPPA, PARD3, PDGFD, SECTM1, SEMA3B, |
| GO:0045087 | innate immune response | 1,92E-04 | [3, 4, 5, 6] | 126,00 | 12,37 | [ADAR, AMH, ANKHD1, ANKRD17, APPL1, APPL2, ARHGEF2, ARHGEF7, ASS1, BCL10, BIRC2, BPI, BTRC, C1QC, CADM1, CAMK2B, CAMK2D, CARD11, CCL16, CD46, CD47, CD96, CFB, CHAMP1, CHID1, CNOT7, CYLD, DAPK1, DDX60, DHX36, ECSIT, EPRS1, ERAP1, EREG, EVL, F12, GNAO1, GPER1, HLA-DPB1, HLA-DQA2, HLA-DRA, HMGB1, HNRNPC, HOOK1, HSP90AB1, ICAM3, IFI16, IFIH1, IFNLR1, IGF2BP1, IGLL5, IKBKB, IL1RAP, IPO7, IRF2, IRF6, IRF9, ITCH, JAK2, KIF16B, KRAS, KRT1, LY9, MAP3K5, MST1R, MUC13, MUC4, MUC5B, MX1, MYD88, MYO1C, MYO1E, NDUFA2, NLRC5, NLRX1, OASL, OTUD4, PARP14, PDPK1, PIK3CG, PIKFYVE, PLCG2, PLD4, PML, POLR3A, POLR3E, POLR3H, PPARG, PRKCA, PRKDC, PSMA5, PSMA6, PSMA7, |
| GO:0009617 | response to bacterium | 7,30E-03 | [3, 5] | 102,00 | 12,47 | [ABR, ADAM17, AKAP12, ALPL, AMH, ANKRD17, ASS1, ATP2A2, BAIAP2L1, BCL10, BCL3, BMP2, BPI, CAV1, CCDC80, CD47, CD96, CDK19, CHAMP1, CHIT1, CUBN, DCN, DLC1, ERAP1, F3, FER, FER1L6, FOXP1, FZD5, GFI1, GSDMB, HMGB1, HSPD1, IGLL5, IL10RA, IL12RB2, IL37, IL6R, IRAK2, JAK2, KLK7, LPL, LSM5, LY6G6C, MACROD2, MAPK8, MEF2C, MYD88, NAALADL2, NCOR2, NDUFA2, NFIB, NFKBIB, NLRC5, NOS1, NOTCH1, NR1D1, PAF1, PCDHA6, PIKFYVE, PLA2G6, PLAC8, PLCG2, PLSCR4, PNLIPRP2, PPARGC1A, PPP1R11, PRKCA, PRKCE, PRLR, PRPF8, PXDN, RAB29, RARA, REG4, RHOA, RNASE6, SASH1, SBNO2, SCARB1, SELENOS, SGMS1, SLC22A5, SMAD6, SRC, SSC5D, STAB1, STAB2, STOX2, SYT12, TBK1, TF, TJP1, TMED7-TICAM2, |
| GO:0098542 | defense response to other organism | 9,10E-05 | [3, 4, 5] | 159,00 | 12,79 | [ADAM17, ADAR, AGBL4, AMH, ANKHD1, ANKRD17, APOBEC1, APPL1, APPL2, ARHGEF2, ARHGEF7, ASS1, ATG16L1, BATF2, BCL10, BCL2, BCL3, BIRC2, BPI, BTRC, C1QC, CADM1, CAMK2B, CAMK2D, CARD11, CCL16, CD46, CD47, CD96, CFB, CHAMP1, CHID1, CNOT7, CYLD, DAPK1, DDIT4, DDX60, DHX36, DLC1, DNAJC3, ECSIT, EIF2AK4, EPRS1, ERAP1, EREG, EVL, EXOC7, F12, FAM111A, FOXP1, GNAO1, GPER1, GSDMB, HLA-DPB1, HLA-DQA2, HLA-DRA, HMGB1, HNRNPC, HOOK1, HRG, HSP90AB1, ICAM3, IFI16, IFIH1, IFNLR1, IGF2BP1, IGLL5, IKBKB, IL10RB, IL17RA, IL1RAP, IL31RA, IL6R, ILF3, IPO7, IRF2, IRF6, IRF9, ITCH, JAK2, KIF16B, KLK7, KRAS, KRT1, LRSAM1, LY9, MAP3K5, MST1R, MUC13, MUC4, MUC5B, MX1, MYD88, MYO1C, MYO1E, NDUFA2, NLRC5, NLRX1, OASL, OTUD4, PARP14, PDPK1, PIK3CG, PIKFYVE, PLA2G6, PLAC8, PLCG2, PLD4, PML, POLR3A, POLR3E, POLR3H, PPARG, PPP1R11, PRKCA, PRKDC, PSMA5, |
| GO:0007600 | sensory perception | 4,69E-02 | [4] | 143,00 | 13,61 | [ABCA4, ACP3, ADCY3, ADGRV1, ANO1, ASIC3, ATP2B2, ATP2B4, ATP8A2, ATXN7, BBS10, BBS4, BBS7, BBS9, BEST1, BFSP2, BTBD9, CABP1, CABP2, CACNA2D4, CACNB2, CCDC50, CCR2, CDH23, CHRNB2, CLN6, CNGA3, CNNM4, CNTN5, COL11A2, COL18A1, COL2A1, CRYBA4, CRYBG3, CRYGC, CRYM, CXCR4, CYP1B1, DHRS3, DLG2, DLL4, DRGX, EPHB1, ESPN, ESPNL, EYA4, FABP5, FAM161A, FBXO11, GABRB2, GNAI2, GPRIN1, GRM1, GRM8, HEXB, HOXA1, HTR2A, ITPR3, KCNE1, KCNQ1, LAMB2, LOXHD1, LRIG2, LRP2, MIPEP, MME, MRGPRX2, MYO15A, MYO1A, MYO5A, NCAM2, NECTIN4, NLGN2, NR2E3, OLFM2, OPA3, OPN1SW, OPN5, OPRPN, OR10C1, OR10G9, OR11L1, OR2B11, OR2L3, OR4K13, OR4K2, OR51B2, OR51E1, OR52B2, OR52I2, OR5R1, OR8D1, OR9Q1, OTOG, PAX2, PCARE, PCDHA6, PDZD7, PJVK, PLCB2, POU4F2, POU6F2, PRR4, RBP4, RCVRN, RD3, RETREG1, RGR, RGS9, RIPOR2, ROR1, RORA, RORB, |
| GO:0051707 | response to other organism | 7,29E-03 | [2, 4] | 235,00 | 14,28 | [ABR, ADAM17, ADAR, AGBL4, AKAP12, ALPL, AMH, ANKHD1, ANKRD17, AP1S1, APOBEC1, APPL1, APPL2, ARF1, ARHGEF2, ARHGEF7, ASS1, ATG16L1, ATP2A2, BAIAP2L1, BATF2, BCL10, BCL2, BCL3, BIRC2, BMP2, BPI, BTRC, C1QC, CADM1, CAMK2B, CAMK2D, CARD11, CAV1, CCDC80, CCL16, CD46, CD47, CD96, CDK19, CFB, CHAMP1, CHID1, CHIT1, CNOT7, CST9, CUBN, CXCR4, CYLD, DAPK1, DCLK1, DCN, DDIT4, DDX60, DHX36, DLC1, DNAJC3, ECSIT, EIF2AK4, EPRS1, ERAP1, EREG, EVL, EXOC7, F12, F3, FAM111A, FER, FER1L6, FOXP1, FZD5, GFI1, GNAO1, GPER1, GSDMB, HLA-DPB1, HLA-DQA2, HLA-DRA, HMGB1, HMGN2, HNRNPC, HOOK1, HRG, HSP90AB1, HSPD1, ICAM3, IFI16, IFIH1, IFNLR1, IGF2BP1, IGLL5, IKBKB, IL10RA, IL10RB, IL12RB2, IL17RA, IL1RAP, IL31RA, IL37, IL6R, ILF3, IPO7, IRAK2, IRF2, IRF6, IRF9, ITCH, JAK2, KIF16B, KLK7, KRAS, KRT1, LPL, LRSAM1, LSM5, LY6G6C, LY9, MACROD2, MAP3K5, MAPK8, MEF2C, MST1R, MUC13, MUC4, MUC5B, MX1, MYD88, MYO1C, MYO1E, NAALADL2, NCOR2, NDUFA2, NFIB, NFKBIB, NLRC5, NLRX1, NOS1, NOTCH1, NR1D1, OASL, OTUD4, PAF1, PARP14, PCDHA6, PDPK1, PIK3CG, PIKFYVE, PLA2G6, PLAC8, PLCG2, PLD4, PLSCR4, PML, PNLIPRP2, POLR3A, POLR3E, POLR3H, PPARG, PPARGC1A, PPP1R11, PPP2R3C, PRKCA, PRKCE, PRKDC, PRLR, PRPF8, PSMA5, PSMA6, PSMA7, PSMC2, PSMC3, PSMC6, PSMD2, PSMD7, PTGES2, PTPN11, PTPRC, PUM1, PVR, PXDN, RAB29, RAB43, RARA, REG4, RELB, RHOA, RIOK3, RNASE6, |
| GO:0006952 | defense response | 1,36E-02 | [3] | 269,00 | 14,63 | [ABCC1, ABR, ADAM17, ADAR, ADCY7, AGBL4, ALS2, AMH, ANKHD1, ANKRD17, ANO6, AP3B1, APOBEC1, APPL1, APPL2, ARHGEF2, ARHGEF7, ASIC4, ASS1, ATG16L1, ATM, BATF2, BCL10, BCL2, BCL3, BCL6, BDNF, BDNF-AS, BIRC2, BMP2, BPI, BRD4, BTRC, C1QC, CADM1, CAMK2B, CAMK2D, CAMK4, CARD11, CASP5, CCL16, CCN4, CCR2, CCR6, CD163, CD200R1, CD46, CD47, CD96, CDK19, CFB, CHAMP1, CHID1, CNOT7, CTNNBIP1, CXCR4, CYLD, CYP19A1, DAPK1, DDIT4, DDX60, DHX34, DHX36, DLC1, DNAJC3, DNASE1L3, DUOX1, ECSIT, EIF2AK4, EIF4E, EPRS1, ERAP1, EREG, ESR1, ETS1, EVL, EXOC7, F11R, F12, F3, FAM111A, FEM1C, FOXP1, GARS1, GNAO1, GPER1, GPR17, GPR4, GPRC5B, GSDMB, HDAC4, HDAC9, HLA-DPB1, HLA-DQA2, HLA-DRA, HMGB1, HNRNPC, HOOK1, HRG, HSP90AB1, HSPD1, HTR1A, ICAM3, IFI16, IFIH1, IFNLR1, IGF1, IGF2BP1, IGLL5, IKBKB, IL10RB, IL16, IL17D, IL17RA, IL17RE, IL1RAP, IL31RA, IL37, IL6R, IL6ST, ILF3, IPO7, IRAK2, IRF2, IRF6, IRF9, ISL1, ITCH, ITGAL, ITGB2, ITK, JAK2, KDM6B, KIF16B, KLK7, KRAS, KRT1, LDLR, LOXL3, LPL, LRP1, LRSAM1, LY9, MAP3K5, MAPK7, MAPK8IP2, MCPH1, MEF2C, MEN1, METRNL, MST1R, MUC13, MUC4, MUC5B, MX1, MYD88, MYO1C, MYO1E, NAPEPLD, NDUFA2, NFAM1, NFATC4, NFE2L2, NLRC5, NLRP12, NLRX1, NOTCH1, NPPA, NR1D1, OASL, OTUD4, PARP14, PARP4, PCDHA6, PDPK1, PIK3CG, PIKFYVE, PLA2G2D, PLA2G4C, PLA2G6, PLA2G7, PLAC8, PLCG2, PLD4, PLK5, PML, PNLIPRP2, PNMA1, POLR3A, POLR3E, POLR3H, PPARG, PPP1R11, PRDX5, PRKCA, PRKCQ, PRKCZ, PRKDC, PSEN1, PSMA5, PSMA6, PSMA7, PSMC2, PSMC3, PSMC6, PSMD2, PSMD7, PTGIS, PTPN11, PTPRC, PUM1, PVR, PXDN, |
| GO:0080090 | regulation of primary metabolic process | 3,68E-02 | [3, 4] | 1281,00 | 20,16 | AGRN, AHCTF1, AHNAK, AKAP12, AKAP13, AKT2, ALAS1, ALK, ALKAL2, ALS2, ALX1, ALX4, AMBRA1, AMH, ANGPT1, ANGPT4, ANKRD31, ANKRD33, ANKS1B, ANTXR1, ANXA2, AP2A1, APC, APC2, APOBEC1, APPL2, ARF1, ARHGEF10L, ARHGEF2, ARHGEF7, ARID2, ARID4B, ARIH1, ARL6IP5, ARNT2, ARNTL, ASCC2, ASCL1, ASPH, ASPSCR1, ASXL2, ATF1, ATF2, ATF3, ATF7, ATG14, ATG4B, ATM, ATN1, ATP13A2, ATP1A3, ATP1A4, ATP2A3, ATP2B4, ATXN1, ATXN7L3, AURKB, BABAM2, BACH1, BAG6, BANP, BATF2, BAZ1B, BAZ2A, BBS4, BBS7, BBX, BCAR3, BCAS3, BCL10, BCL11A, BCL11B, BCL2, BCL3, BCL6, BCL7A, BCL9, BDNF, BDNF-AS, BEND6, BHLHE40, BICRAL, BIRC2, BMP2, BMP3, BMP7, BMP8B, BMPER, BNC1, BNC2, BRD1, BRD2, BRD4, BRF1, BRMS1, BRSK2, BTC, BTG2, BTRC, C1orf61, CACNA1A, CALCOCO1, CALM2, CAMK2D, CAMK4, CAMTA1, CAMTA2, CAND1, CAPRIN2, CARD10, CARD11, CARD14, CASR, CASZ1, CAV1, CBFA2T2, CBFA2T3, CBX8, CC2D1B, CCAR2, CCBE1, CCDC3, CCDC88C, CCL16, CCNA1, CCND2, CCNL1, CCNY, CCR2, CCT2, CD33, CDC123, CDC20, CDC20B, CDC25B, CDC25C, CDH13, CDK1, CDK11B, CDK2, CDK5R1, CDKN1A, CDKN2A, CDT1, CDYL, CELF2, CELF4, CELF5, CEP290, CEP295, CEP43, CHAMP1, CHCHD3, CHD3, CHD5, CHEK1, CHEK2, CHRNB2, CHTF8, CIB1, CIC, CITED2, CITED4, CIZ1, CLCN2, CLK3, CLK4, CLN6, CNOT1, CNOT7, COMMD1, COP1, CPEB1, CPEB4, CPT1A, CRB2, CREB3L1, CREB3L2, CREB5, CREM, CRIM1, CRY2, CRYM, CSNK1E, CSRNP2, CSRNP3, CST9, CTBP2, CTCF, CTNNBIP1, CUL2, CUL3, CUX1, CWC22, CXCR4, CXXC5, CYCS, CYFIP1, CYFIP2, CYLD, CYP1B1, CYP4A11, CYP51A1, CYREN, DAB1, DAG1, DAPK1, DAXX, DBNDD2, DCAF1, DCN, DCP2, DCUN1D1, DDA1, DDIT4, DDR2, DDX49, DEPDC1, DESI2, DGKQ, DGKZ, DHCR7, DHX34, DHX36, DIPK2A, DKK3, DLC1, DLG1, DLG4, DLL1, DLL4, DLX2, DNAJB5, DNAJB6, DNAJC15, DNAJC3, DNAJC30, DNM1L, DNMT3A, DNPEP, DOCK7, DOK4, DOK7, DOT1L, DPF1, DRGX, DSTYK, DTX1, DUS2, DUSP19, DUSP26, DUXA, E2F2, E2F8, EBF1, EBF3, EBF4, EDAR, EDEM2, EEF1A1, EEFSEC, EFNA5, EGR2, EHF, EHMT1, EHMT2, EIF2AK3, EIF2AK4, EIF2S2, EIF3B, EIF4A3, EIF4B, EIF4E, EIF4E1B, EIF4EBP1, EIF4EBP2, EIF4ENIF1, ELAVL1, ELF1, ELK3, ELOA, EMC10, ENC1, ENHO, ENPP2, EPHA1, EPHA4, EPHB1, EPRS1, ERBB4, ERC1, ERCC1, ERCC8, EREG, ESR1, ESR2, ESRP2, ESRRG, ETAA1, ETFA, ETS1, ETV1, ETV6, EVX1, EXOSC10, EXOSC6, EYA2, EYA4, EZH2, F12, F3, FABP5, FAF1, FAM161A, FAM172A, FAM220A, FANCM, FASN, FASTK, FASTKD1, FBLN5, FBP1, FBXO2, FBXW8, FEM1C, FER, FERD3L, FEZF1, FEZF2, FGD2, FGF2, FGF7, FHOD1, FITM2, FKTN, FLT4, FNIP2, FNTA, FOXB2, FOXD3, FOXI3, FOXJ3, FOXK1, FOXO1, FOXP1, FRS2, FTO, FUBP1, FZD10, FZD5, FZR1, GABPB1, GADD45G, GARS1, GAS7, GATA5, GCG, GCNT2, GFI1, GFRAL, GGPS1, GLE1, GLI2, GLI3, GLIPR2, GLIS2, GLMN, GLO1, GLRX2, GMFB, GNA12, GNAI2, GON4L, GORASP1, GPER1, GPR55, GPRC5B, GPRC5C, GPRIN1, GPT, GRAMD4, GRB2, GREM1, GRM1, GRM4, GSK3A, GSX2, GTF2B, GTF2H1, GTF2IRD1, GUCA2A, H2AC4, H2AC6, H2AJ, H3C6, H3C8, HACD3, HAND1, HCAR2, HCLS1, HDAC4, HDAC9, HECW1, HEG1, HELT, HES1, HES3, HES4, HEXB, HGS, HHATL, HINT1, HIP1, HIP1R, HIVEP1, HIVEP3, HMBOX1, HMGB1, HMGN3, HNF1B, HNRNPAB, HNRNPC, HNRNPD, HNRNPH1, HNRNPK, HNRNPR, HOXA1, HOXA3, HOXA4, HOXC10, HOXC11, HOXC4, HOXD13, HRC, HRG, HSD17B4, HSP90AB1, HSPD1, HTATIP2, HTR1A, HTR2A, HTT, HUS1, IDE, IFI16, IFRD1, IFT74, IGF1, IGF1R, IGF2BP1, IGHMBP2, IHH, IKBKB, IKZF1, IL1RAP, IL31RA, IL37, IL6R, IL6ST, ILF3, ING1, ING3, INKA2, INPP5F, INSIG2, INSR, IPO7, IQGAP3, IRAK2, IRF2, IRF2BP2, IRF6, IRF9, IRS2, ISL1, ITCH, ITGA1, ITGA5, ITGB2, ITIH5, IWS1, JAK2, JARID2, JAZF1, JMJD1C, KALRN, KAT2B, KAT6A, KAT6B, KAT7, KCNK2, KDM2A, KDM2B, KDM3A, KDM4B, KDM4C, KDM4D, KDM6B, KHDRBS1, KHDRBS2, KIDINS220, KIRREL1, KITLG, KLF14, KLF17, KLF7, KMT2D, KMT2E, KMT5C, KNDC1, KPNA7, KRAS, KSR1, L3MBTL3, LARP4B, LARP7, LATS2, LAX1, LDB2, LDLR, LDLRAD4, LEPR, LGMN, LHX4, LHX6, LHX8, LIMS1, LIN9, LLGL1, LMBR1, LMCD1, LMO7, LMTK2, LMX1B, LONP1, LOXL2, LOXL3, LPAR1, LPCAT1, LPGAT1, LRIG2, LRP1, LRP2, LRP5, LRRC14, LRRK1, LSS, LTN1, LYL1, MACC1, MAF, MAFA, MAFG, MAFK, MAGI2, MAK, MAL, MAML2, MAML3, MAP2K2, MAP2K5, MAP3K11, MAP3K13, MAP3K19, MAP3K2, MAP3K20, MAP3K5, MAP3K6, MAP4K4, MAP4K5, MAPK7, MAPK8, MAPK8IP2, MAPK8IP3, MAPK9, MAPKAP1, MAPKAPK5, MARS1, MAX, MBD3, MBNL1, MCM2, MCPH1, MCRS1, ME1, MECOM, MED13L, MED16, MED22, MED24, MED26, MED27, MEF2C, MEIOC, MEIS2, MELTF, MEN1, MEOX1, MGMT, MICAL2, MIDEAS, MIER1, MKNK1, MKNK2, MLLT3, MMD, MN1, MPHOSPH8, MRPL13, MRPL23, MRTFA, MSANTD1, MSGN1, MSH2, MSH3, MSI2, MSL3P1, MST1R, MTA1, MTA3, MTERF1, MTG1, MTMR2, MTMR3, MTMR9, MXD1, MYD88, MYO1C, MYRF, MYT1L, N4BP1, NACC2, NAT10, NCK2, NCKAP1L, NCOA2, NCOA6, NCOA7, NCOR2, NCSTN, NDFIP2, NDRG4, NDUFA13, NEDD4L, NEDD9, NEK7, NEUROD6, NEUROG2, NF1, NFAM1, NFATC1, NFATC2, NFATC2IP, NFATC4, NFE2L2, NFIB, NFIL3, NFKBIB, NFXL1, NFYA, NFYC, NGF, NKD1, NKX2-4, NLK, NLRC5, NLRP12, NME2, NNT, NOC2L, NOL3, NOP2, NOS1, NOS1AP, NOTCH1, NOTCH3, NOTCH4, NOVA2, NPAT, NPNT, NPPA, NPTN, NR1D1, NR2C1, NR2E3, NR3C1, NR3C2, NRF1, NRIP1, NRXN1, NSD1, NSD3, NSMF, NTRK2, NUP205, NUP43, NUP50, NUP58, NXN, OASL, OLIG2, OPA3, OPRPN, OSBPL7, OSBPL8, OTUB1, OTUD4, P2RY6, PABPC1L, PAF1, PAFAH1B1, PAGR1, PAK4, PAK5, PAOX, PAPLN, PARD3, PARG, PARP14, PARP4, PASK, PAX2, PAX7, PAX8, PAX9, PBLD, PBX1, PCGF5, PDCD5, PDCD6, PDCL3, PDE8A, PDGFD, PDGFRA, PDLIM1, PDPK1, PDZD2, PDZD3, PEG3, PER2, PEX14, PFDN1, PFKFB3, PFKM, PFN2, PGP, PHACTR1, PHF19, PHF2, PHF20, |

| | | | | | |
|---|---|---|---|---|---|
| GO:0080090 | regulation of primary metabolic process | 3,68E-02 [3, 4] | 1281,00 | 20,16 | AGRN, AHCTF1, AHNAK, AKAP12, AKAP13, AKT2, ALAS1, ALK, ALKAL2, ALS2, ALX1, ALX4, AMBRA1, AMH, ANGPT1, ANGPT4, ANKRD31, ANKRD33, ANKS1B, ANTXR1, ANXA2, AP2A1, APC, APC2, APOBEC1, APPL2, ARF1, ARHGEF10L, ARHGEF2, ARHGEF7, ARID2, ARID4B, ARIH1, ARL6IP5, ARNT2, ARNTL, ASCC2, ASCL1, ASPH, ASPSCR1, ASXL2, ATF1, ATF2, ATF3, ATF7, ATG14, ATG4B, ATM, ATN1, ATP13A2, ATP1A3, ATP1A4, ATP2A3, ATP2B4, ATXN1, ATXN7L3, AURKB, BABAM2, BACH1, BAG6, BANP, BATF2, BAZ1B, BAZ2A, BBS4, BBS7, BBX, BCAR3, BCAS3, BCL10, BCL11A, BCL11B, BCL2, BCL3, BCL6, BCL7A, BCL9, BDNF, BDNF-AS, BEND6, BHLHE40, BICRAL, BIRC2, BMP2, BMP3, BMP7, BMP8B, BMPER, BNC1, BNC2, BRD1, BRD2, BRD4, BRF1, BRMS1, BRSK2, BTC, BTG2, BTRC, C1orf61, CACNA1A, CALCOCO1, CALM2, CAMK2D, CAMK4, CAMTA1, CAMTA2, CAND1, CAPRIN2, CARD10, CARD11, CARD14, CASR, CASZ1, CAV1, CBFA2T2, CBFA2T3, CBX8, CC2D1B, CCAR2, CCBE1, CCDC3, CCDC88C, CCL16, CCNA1, CCND2, CCNL1, CCNY, CCR2, CCT2, CD33, CDC123, CDC20, CDC20B, CDC25B, CDC25C, CDH13, CDK1, CDK11B, CDK2, CDK5R1, CDKN1A, CDKN2A, CDT1, CDYL, CELF2, CELF4, CELF5, CEP290, CEP295, CEP43, CHAMP1, CHCHD3, CHD3, CHD5, CHEK1, CHEK2, CHRNB2, CHTF8, CIB1, CIC, CITED2, CITED4, CIZ1, CLCN2, CLK3, CLK4, CLN6, CNOT1, CNOT7, COMMD1, COP1, CPEB1, CPEB4, CPT1A, CRB2, CREB3L1, CREB3L2, CREB5, CREM, CRIM1, CRY2, CRYM, CSNK1E, CSRNP2, CSRNP3, CST9, CTBP2, CTCF, CTNNBIP1, CUL2, CUL3, CUX1, CWC22, CXCR4, CXXC5, CYCS, CYFIP1, CYFIP2, CYLD, CYP1B1, CYP4A11, CYP51A1, CYREN, DAB1, DAG1, DAPK1, DAXX, DBNDD2, DCAF1, DCN, DCP2, DCUN1D1, DDA1, DDIT4, DDR2, DDX49, DEPDC1, DESI2, DGKQ, DGKZ, DHCR7, DHX34, DHX36, DIPK2A, DKK3, DLC1, DLG1, DLG4, DLL1, DLL4, DLX2, DNAJB5, DNAJB6, DNAJC15, DNAJC3, DNAJC30, DNM1L, DNMT3A, DNPEP, DOCK7, DOK4, DOK7, DOT1L, DPF1, DRGX, DSTYK, DTX1, DUS2, DUSP19, DUSP26, DUXA, E2F2, E2F8, EBF1, EBF3, EBF4, EDAR, EDEM2, EEF1A1, EEFSEC, EFNA5, EGR2, EHF, EHMT1, EHMT2, EIF2AK3, EIF2AK4, EIF2S2, EIF3B, EIF4A3, EIF4B, EIF4E, EIF4E1B, EIF4EBP1, EIF4EBP2, EIF4ENIF1, ELAVL1, ELF1, ELK3, ELOA, EMC10, ENC1, ENHO, ENPP2, EPHA1, EPHA4, EPHB1, EPRS1, ERBB4, ERC1, ERCC1, ERCC8, EREG, ESR1, ESR2, ESRP2, ESRRG, ETAA1, ETFA, ETS1, ETV1, ETV6, EVX1, EXOSC10, EXOSC6, EYA2, EYA4, EZH2, F12, F3, FABP5, FAF1, FAM161A, FAM172A, FAM220A, FANCM, FASN, FASTK, FASTKD1, FBLN5, FBP1, FBXO2, FBXW8, FEM1C, FER, FERD3L, FEZF1, FEZF2, FGD2, FGF2, FGF7, FHOD1, FITM2, FKTN, FLT4, FNIP2, FNTA, FOXB2, FOXD3, FOXI3, FOXJ3, FOXK1, FOXO1, FOXP1, FRS2, FTO, FUBP1, FZD10, FZD5, FZR1, GABPB1, GADD45G, GARS1, GAS7, GATA5, GCG, GCNT2, GFI1, GFRAL, GGPS1, GLE1, GLI2, GLI3, GLIPR2, GLIS2, GLMN, GLO1, GLRX2, GMFB, GNA12, GNAI2, GON4L, GORASP1, GPER1, GPR55, GPRC5B, GPRC5C, GPRIN1, GPT, GRAMD4, GRB2, GREM1, GRM1, GRM4, GSK3A, GSX2, GTF2B, GTF2H1, GTF2IRD1, GUCA2A, H2AC4, H2AC6, H2AJ, H3C6, H3C8, HACD3, HAND1, HCAR2, HCLS1, HDAC4, HDAC9, HECW1, HEG1, HELT, HES1, HES3, HES4, HEXB, HGS, HHATL, HINT1, HIP1, HIP1R, HIVEP1, HIVEP3, HMBOX1, HMGB1, HMGN3, HNF1B, HNRNPAB, HNRNPC, HNRNPD, HNRNPH1, HNRNPK, HNRNPR, HOXA1, HOXA3, HOXA4, HOXC10, HOXC11, HOXC4, HOXD13, HRC, HRG, HSD17B4, HSP90AB1, HSPD1, HTATIP2, HTR1A, HTR2A, HTT, HUS1, IDE, IFI16, IFRD1, IFT74, IGF1, IGF1R, IGF2BP1, IGHMBP2, IHH, IKBKB, IKZF1, IL1RAP, IL31RA, IL37, IL6R, IL6ST, ILF3, ING1, ING3, INKA2, INPP5F, INSIG2, INSR, IPO7, IQGAP3, IRAK2, IRF2, IRF2BP2, IRF6, IRF9, IRS2, ISL1, ITCH, ITGA1, ITGA5, ITGB2, ITIH5, IWS1, JAK2, JARID2, JAZF1, JMJD1C, KALRN, KAT2B, KAT6A, KAT6B, KAT7, KCNK2, KDM2A, KDM2B, KDM3A, KDM4B, KDM4C, KDM4D, KDM6B, KHDRBS1, KHDRBS2, KIDINS220, KIRREL1, KITLG, KLF14, KLF17, KLF7, KMT2D, KMT2E, KMT5C, KNDC1, KPNA7, KRAS, KSR1, L3MBTL3, LARP4B, LARP7, LATS2, LAX1, LDB2, LDLR, LDLRAD4, LEPR, LGMN, LHX4, LHX6, LHX8, LIMS1, LIN9, LLGL1, LMBR1, LMCD1, LMO7, LMTK2, LMX1B, LONP1, LOXL2, LOXL3, LPAR1, LPCAT1, LPGAT1, LRIG2, LRP1, LRP2, LRP5, LRRC14, LRRK1, LSS, LTN1, LYL1, MACC1, MAF, MAFA, MAFG, MAFK, MAGI2, MAK, MAL, MAML2, MAML3, MAP2K2, MAP2K5, MAP3K11, MAP3K13, MAP3K19, MAP3K2, MAP3K20, MAP3K5, MAP3K6, MAP4K4, MAP4K5, MAPK7, MAPK8, MAPK8IP2, MAPK8IP3, MAPK9, MAPKAP1, MAPKAPK5, MARS1, MAX, MBD3, MBNL1, MCM2, MCPH1, MCRS1, ME1, MECOM, MED13L, MED16, MED22, MED24, MED26, MED27, MEF2C, MEIOC, MEIS2, MELTF, MEN1, MEOX1, MGMT, MICAL2, MIDEAS, MIER1, MKNK1, MKNK2, MLLT3, MMD, MN1, MPHOSPH8, MRPL13, MRPL23, MRTFA, MSANTD1, MSGN1, MSH2, MSH3, MSI2, MSL3P1, MST1R, MTA1, MTA3, MTERF1, MTG1, MTMR2, MTMR3, MTMR9, MXD1, MYD88, MYO1C, MYRF, MYT1L, N4BP1, NACC2, NAT10, NCK2, NCKAP1L, NCOA2, NCOA6, NCOA7, NCOR2, NCSTN, NDFIP2, NDRG4, NDUFA13, NEDD4L, NEDD9, NEK7, NEUROD6, NEUROG2, NF1, NFAM1, NFATC1, NFATC2, NFATC2IP, NFATC4, NFE2L2, NFIB, NFIL3, NFKBIB, NFXL1, NFYA, NFYC, NGF, NKD1, NKX2-4, NLK, NLRC5, NLRP12, NME2, NNT, NOC2L, NOL3, NOP2, NOS1, NOS1AP, NOTCH1, NOTCH3, NOTCH4, NOVA2, NPAT, NPNT, NPPA, NPTN, NR1D1, NR2C1, NR2E3, NR3C1, NR3C2, NRF1, NRIP1, NRXN1, NSD1, NSD3, NSMF, NTRK2, NUP205, NUP43, NUP50, NUP58, NXN, OASL, OLIG2, OPA3, OPRPN, OSBPL7, OSBPL8, OTUB1, OTUD4, P2RY6, PABPC1L, PAF1, PAFAH1B1, PAGR1, PAK4, PAK5, PAOX, PAPLN, PARD3, PARG, PARP14, PARP4, PASK, PAX2, PAX7, PAX8, PAX9, PBLD, PBX1, PCGF5, PDCD5, PDCD6, PDCL3, PDE8A, PDGFD, PDGFRA, PDLIM1, PDPK1, PDZD2, PDZD3, PEG3, PER2, PEX14, PFDN1, PFKFB3, PFKM, PFN2, PGP, PHACTR1, PHF19, PHF2, PHF20, |
| GO:0019222 | regulation of metabolic process | 2,96E-03 [2, 3] | 1471,00 | 20,17 | AFDN, AFF3, AFF4, AGBL4, AGO2, AGRN, AHCTF1, AHNAK, AKAP12, AKAP13, AKT2, ALAS1, ALK, ALKAL2, ALMS1, ALPK2, ALS2, ALX1, ALX4, AMBRA1, AMH, ANGPT1, ANGPT4, ANK3, ANKRD13B, ANKRD17, ANKRD31, ANKRD33, ANKS1B, ANTXR1, ANXA2, AP2A1, APBA2, APC, APC2, APOBEC1, APPL1, APPL2, ARAP1, ARF1, ARFGEF3, ARHGEF10L, ARHGEF2, ARHGEF7, ARID2, ARID4B, ARIH1, ARL6IP5, ARNT2, ARNTL, ASCC2, ASCL1, ASPH, ASPSCR1, ASS1, ASXL2, ASZ1, ATF1, ATF2, ATF3, ATF7, ATG14, ATG16L1, ATG4B, ATM, ATN1, ATP13A2, ATP1A3, ATP1A4, ATP2A3, ATP2B4, ATP6V0A1, ATP6V0D1, ATP6V1A, ATP6V1H, ATXN1, ATXN7L3, ATXN7L3B, AURKB, BABAM2, BACH1, BAG6, BANP, BATF2, BAZ1B, BAZ2A, BBS4, BBS7, BBX, BCAR3, BCAS3, BCL10, BCL11A, BCL11B, BCL2, BCL3, BCL6, BCL7A, BCL9, BDNF, BDNF-AS, BEND6, BHLHE40, BICRAL, BIRC2, BMP2, BMP3, BMP7, BMP8B, BMPER, BNC1, BNC2, BPI, BRD1, BRD2, BRD4, BRF1, BRMS1, BRSK2, BTBD10, BTC, BTG2, BTN3A3, BTRC, C1orf61, CACNA1A, CADM1, CALCOCO1, CALM2, CAMK2D, CAMK4, CAMTA1, CAMTA2, CAND1, CAPRIN2, CARD10, CARD11, CARD14, CASR, CASZ1, CAV1, CBFA2T2, CBFA2T3, CBX8, CC2D1B, CCAR2, CCBE1, CCDC3, CCDC88C, CCL16, CCN4, CCN6, CCNA1, CCND2, CCNL1, CCNY, CCR2, CCT2, CD200R1, CD33, CD46, CD47, CD96, CDC123, CDC20, CDC20B, CDC25B, CDC25C, CDH13, CDK1, CDK11B, CDK2, CDK5R1, CDKN1A, CDKN2A, CDT1, CDYL, CELF2, CELF4, CELF5, CELSR1, CEP290, CEP295, CEP43, CHAMP1, CHCHD3, CHD3, CHD5, CHEK1, CHEK2, CHID1, CHRNB2, CHTF8, CIB1, CIC, CILK1, CITED2, CITED4, CIZ1, CLCN2, CLK3, CLK4, CLN6, CLP1, CLYBL, CNOT1, CNOT7, CNRIP1, CNST, COL2A1, COMMD1, COP1, CPEB1, CPEB4, CPT1A, CRB2, CREB3L1, CREB3L2, CREB5, CREM, CRIM1, CRIP1, CRY2, CRYM, CSNK1E, CSRNP2, CSRNP3, CST9, CTBP2, CTCF, CTNNBIP1, CTNS, CTTN, CUL2, CUL3, CUX1, CWC22, CXCR4, CXXC5, CYCS, CYFIP1, CYFIP2, CYLD, CYP1B1, CYP4A11, CYP51A1, CYREN, DAB1, DAG1, DAPK1, DAXX, DBNDD2, DCAF1, DCN, DCP2, DCPS, DCUN1D1, DDA1, DDAH2, DDIT4, DDR1, DDR2, DDX49, DDX6, DDX60, DEPDC1, DEPDC5, DESI2, DGKQ, DGKZ, DHCR7, DHX34, DHX36, DIPK2A, DIS3L2, DKK3, DLC1, DLG1, DLG2, DLG4, DLL1, DLL4, DLX2, DNA2, DNAJB5, DNAJB6, DNAJC15, DNAJC3, DNAJC30, DNM1L, DNMT3A, DNPEP, DOCK7, DOK4, DOK7, DOT1L, DPF1, DRAM1, DRGX, DSTYK, DTX1, DUS2, DUSP19, DUSP26, DUXA, E2F2, E2F8, EBF1, EBF3, EBF4, ECE1, ECSIT, EDAR, EDEM2, EEF1A1, EEFSEC, EFNA5, EGR2, EHF, EHMT1, EHMT2, EIF2AK3, EIF2AK4, EIF2S2, EIF3B, EIF4A3, EIF4B, EIF4E, EIF4E1B, EIF4EBP1, EIF4EBP2, EIF4ENIF1, ELAVL1, ELF1, ELK3, ELOA, EMC10, ENC1, ENHO, ENPP2, EPB41L4B, EPHA1, EPHA10, EPHA4, EPHA6, EPHB1, EPHB3, EPRS1, ERBB4, ERC1, ERCC1, ERCC8, EREG, ESCO1, ESR1, ESR2, ESRP2, ESRRG, ETAA1, ETFA, ETS1, ETV1, ETV6, EVX1, EXOC4, EXOC7, EXOSC10, EXOSC6, EYA2, EYA4, EZH2, F11R, F12, F3, FABP5, FAF1, FAM161A, FAM172A, FAM220A, FANCM, FAP, FARP1, FASN, FASTK, FASTKD1, FBLN5, FBP1, FBXO2, FBXW8, FEM1C, FER, FERD3L, FEZ1, FEZF1, FEZF2, FGD2, FGF2, FGF7, FH, FHOD1, FITM2, FKTN, FLT4, FNIP2, FNTA, FOXB2, FOXD3, FOXI3, FOXJ3, FOXK1, FOXO1, FOXP1, FRMD8, FRS2, FTO, FUBP1, FUT8, FZD10, FZD5, FZR1, GABPB1, GADD45G, GARS1, GAS7, GATA5, GCG, GCNT2, GFI1, GFRAL, GGPS1, GLE1, GLI2, GLI3, GLIPR2, GLIS2, GLMN, GLO1, GLRX2, GMFB, GNA12, GNAI2, GON4L, GORASP1, GPER1, GPR137B, GPR55, GPRC5B, GPRC5C, GPRIN1, GPSM1, GPT, GRAMD4, GRB10, GRB2, GREM1, GRM1, GRM4, GSG1L, GSK3A, GSX2, GTF2B, GTF2H1, GTF2IRD1, GUCA2A, H2AC4, H2AC6, H2AJ, H3C6, H3C8, HACD3, HADHA, HAND1, HCAR2, HCLS1, HDAC4, HDAC9, HECW1, HEG1, HELT, HELZ, HENMT1, HERC1, HES1, HES3, HES4, HEXB, HGS, HHATL, HINT1, HIP1, HIP1R, HIVEP1, HIVEP3, HLA-DPB1, HMBOX1, HMGB1, HMGN3, HNF1B, HNRNPAB, HNRNPC, HNRNPD, HNRNPH1, HNRNPK, HNRNPR, HOOK1, HOXA1, HOXA3, HOXA4, HOXC10, HOXC11, HOXC4, HOXD13, HRC, HRG, HSD17B4, HSP90AB1, HSPD1, HSPH1, HTATIP2, HTR1A, HTR2A, HTT, HUS1, IDE, IFI16, IFIH1, IFRD1, IFT74, IFT88, IGF1, IGF1R, IGF2BP1, IGHMBP2, IHH, IKBKB, IKZF1, IL10RA, IL12RB2, IL16, IL17D, IL17RA, IL1RAP, IL31RA, IL37, IL6R, IL6ST, ILF3, ING1, ING3, INKA2, INPP5F, INPPL1, INSIG2, INSR, IPO7, IPO8, IQGAP3, IRAK2, IRF2, IRF2BP2, IRF6, IRF9, IRS2, ISL1, ITCH, ITGA1, ITGA5, ITGB2, ITIH5, IWS1, JAK2, JARID2, JAZF1, JMJD1C, KALRN, KAT2B, KAT6A, KAT6B, KAT7, KCNK2, KCNQ1, KDM2A, KDM2B, KDM3A, KDM4B, KDM4C, KDM4D, KDM6B, KHDRBS1, KHDRBS2, KIDINS220, KIF16B, KIF25, KIF2A, KIRREL1, KITLG, KLF14, KLF17, KLF7, KMT2D, KMT2E, KMT5C, KNDC1, KPNA7, KRAS, KSR1, KSR2, L3MBTL3, LARP4B, LARP7, LATS2, LAX1, LDB2, LDLR, LDLRAD4, LEPR, LGMN, LHX4, LHX6, LHX8, LIMK2, LIMS1, LIN9, LLGL1, LMBR1, LMCD1, LMO7, LMTK2, LMX1B, LONP1, LOXL2, LOXL3, LPAR1, LPAR2, LPCAT1, LPGAT1, LPL, LRIG2, LRIG3, LRP1, LRP2, LRP5, LRPAP1, LRRC14, LRRC32, LRRK1, LRSAM1, LSM2, LSM5, LSS, LTBP1, LTN1, LURAP1, LY9, LYL1, LZTS1, MACC1, MAF, MAFA, MAFG, MAFK, MAGI2, MAK, MAL, MAML2, MAML3, MAP2K2, MAP2K5, MAP3K11, MAP3K13, MAP3K19, MAP3K2, MAP3K20, MAP3K5, MAP3K6, MAP4K4, MAP4K5, MAPK4, MAPK7, MAPK8, MAPK8IP2, MAPK8IP3, MAPK9, MAPKAP1, MAPKAPK5, MARF1, MARS1, MAX, MBD3, MBNL1, MCL1, MCM2, MCPH1, MCRS1, ME1, MECOM, MED13L, MED16, MED22, MED24, MED26, MED27, MEF2C, MEGF8, MEIOC, MEIS2, MELTF, MEN1, MEOX1, MGMT, MICAL2, MIDEAS, MIER1, MKNK1, MKNK2, MLLT3, MMD, MN1, MOG, MOV10L1, MPHOSPH8, MRPL13, MRPL23, MRTFA, MSANTD1, MSGN1, MSH2, MSH3, MSI2, MSL3P1, MST1R, MTA1, MTA3, MTERF1, MTG1, MTMR2, MTMR3, MTMR9, MXD1, MYD88, MYO1C, MYO1D, MYO1E, MYRF, MYT1L, N4BP1, NACC2, |
| GO:0031323 | regulation of cellular metabolic process | 1,34E-03 [3, 4] | 1338,00 | 20,38 | AGO2, AGRN, AHCTF1, AHNAK, AKAP12, AKAP13, AKT2, ALK, ALKAL2, ALS2, ALX1, ALX4, AMBRA1, AMH, ANGPT1, ANGPT4, ANKRD13B, ANKRD17, ANKRD31, ANKRD33, ANKS1B, ANTXR1, ANXA2, AP2A1, APC, APOBEC1, APPL1, APPL2, ARAP1, ARF1, ARFGEF3, ARHGEF10L, ARHGEF2, ARHGEF7, ARID2, ARID4B, ARIH1, ARL6IP5, ARNT2, ARNTL, ASCC2, ASCL1, ASPH, ASPSCR1, ASS1, ASXL2, ATF1, ATF2, ATF3, ATF7, ATG14, ATG16L1, ATM, ATN1, ATP13A2, ATP1A3, ATP1A4, ATP2A3, ATP2B4, ATP6V0A1, ATP6V0D1, ATP6V1A, ATP6V1H, ATXN1, ATXN7L3, AURKB, BABAM2, BACH1, BAG6, BANP, BATF2, BAZ1B, BAZ2A, BBS7, BBX, BCAR3, BCAS3, BCL10, BCL11A, BCL11B, BCL2, BCL3, BCL6, BCL7A, BCL9, BDNF, BDNF-AS, BEND6, BHLHE40, BICRAL, BIRC2, BMP2, BMP3, BMP7, BMP8B, BMPER, BNC1, BNC2, BRD1, BRD2, BRD4, BRF1, BRMS1, BRSK2, BTBD10, BTC, BTG2, BTRC, C1orf61, CACNA1A, CALCOCO1, CALM2, CAMK2D, CAMK4, CAMTA1, CAMTA2, CAND1, CAPRIN2, CARD10, CARD11, CARD14, CASR, CASZ1, CAV1, CBFA2T2, CBFA2T3, CBX8, CC2D1B, CCAR2, CCBE1, CCDC88C, CCL16, CCN6, CCNA1, CCND2, CCNL1, CCNY, CCR2, CCT2, CD33, CD47, CDC123, CDC20, CDC20B, CDC25B, CDC25C, CDH13, CDK1, CDK11B, CDK2, CDK5R1, CDKN1A, CDKN2A, CDT1, CDYL, CELF2, CELF4, CELF5, CEP290, CEP295, CEP43, CHAMP1, CHCHD3, CHD3, CHD5, CHEK1, CHEK2, CHRNB2, CHTF8, CIB1, CIC, CITED2, CITED4, CIZ1, CLCN2, CLK3, CLK4, CLN6, CLYBL, CNOT1, CNOT7, CNST, COMMD1, COP1, CPEB1, CPEB4, CPT1A, CRB2, CREB3L1, CREB3L2, CREB5, CREM, CRIM1, CRY2, CRYM, CSNK1E, CSRNP2, CSRNP3, CST9, CTBP2, CTCF, CTNNBIP1, CTNS, CTTN, CUL2, CUL3, CUX1, CWC22, CXCR4, CXXC5, CYCS, CYFIP1, CYFIP2, CYLD, CYP1B1, CYREN, DAB1, DAG1, DAPK1, DAXX, DBNDD2, DCAF1, DCN, DCP2, DCUN1D1, DDA1, DDAH2, DDIT4, DDR1, DDR2, DDX49, DEPDC1, DEPDC5, DESI2, DGKQ, DGKZ, DHX34, DHX36, DIPK2A, DKK3, DLC1, DLG1, DLG2, DLG4, DLL1, DLL4, DLX2, DNA2, DNAJB5, DNAJB6, DNAJC15, DNAJC3, DNAJC30, DNM1L, DNMT3A, DNPEP, DOCK7, DOK4, DOK7, DOT1L, DPF1, DRAM1, DRGX, DSTYK, DTX1, DUS2, DUSP19, DUSP26, DUXA, E2F2, E2F8, EBF1, EBF3, EBF4, ECE1, EDAR, EDEM2, EEF1A1, EEFSEC, EFNA5, EGR2, EHF, EHMT1, EHMT2, EIF2AK3, EIF2AK4, EIF2S2, EIF3B, EIF4A3, EIF4B, EIF4E, EIF4E1B, EIF4EBP1, EIF4EBP2, EIF4ENIF1, ELAVL1, ELF1, ELK3, ELOA, EMC10, ENC1, ENPP2, EPHA1, EPHA10, EPHA4, EPHA6, EPHB1, EPHB3, EPRS1, ERBB4, ERC1, ERCC1, ERCC8, EREG, ESCO1, ESR1, ESR2, ESRP2, ESRRG, ETAA1, ETFA, ETS1, ETV1, ETV6, EVX1, EXOC4, EXOC7, EXOSC10, EXOSC6, EYA2, EYA4, EZH2, F12, F3, FABP5, FAF1, FAM161A, FAM172A, FAM220A, FANCM, FARP1, FASN, FASTK, FASTKD1, FBLN5, FBP1, FBXO2, FBXW8, FEM1C, FER, FERD3L, FEZ1, FEZF1, FEZF2, FGD2, FGF2, FGF7, FHOD1, FITM2, FKTN, FLT4, FNIP2, FNTA, FOXB2, FOXD3, FOXI3, FOXJ3, FOXK1, FOXO1, FOXP1, FRS2, FTO, FUBP1, FZD10, FZD5, FZR1, GABPB1, GADD45G, GARS1, GAS7, GATA5, GCG, GCNT2, GFI1, GFRAL, GLE1, GLI2, GLI3, GLIPR2, GLIS2, GLMN, GLO1, GLRX2, GMFB, GNA12, GNAI2, GON4L, GORASP1, GPER1, GPR137B, GPR55, GPRC5B, GPRC5C, GPRIN1, GPSM1, GPT, GRAMD4, GRB10, GRB2, GREM1, GRM1, GRM4, GSG1L, GSK3A, GSX2, GTF2B, GTF2H1, GTF2IRD1, GUCA2A, H2AC4, H2AC6, H2AJ, H3C6, H3C8, HACD3, HAND1, HCLS1, HDAC4, HDAC9, HEG1, HELT, HERC1, HES1, HES3, HES4, HEXB, HGS, HHATL, HINT1, HIP1, HIP1R, HIVEP1, HIVEP3, HMBOX1, HMGB1, HMGN3, HNF1B, HNRNPAB, HNRNPC, HNRNPD, HNRNPH1, HNRNPK, HNRNPR, HOXA1, HOXA3, HOXA4, HOXC10, HOXC11, HOXC4, HOXD13, HRC, HRG, HSD17B4, HSP90AB1, HSPD1, HTATIP2, HTR1A, HTR2A, HTT, HUS1, IDE, IFI16, IFRD1, IFT74, IFT88, IGF1, IGF1R, IGF2BP1, IGHMBP2, IHH, IKBKB, IKZF1, IL10RA, IL1RAP, IL31RA, IL37, IL6R, IL6ST, ILF3, ING1, ING3, INKA2, INPP5F, INSIG2, INSR, IPO7, IQGAP3, IRAK2, IRF2, IRF2BP2, IRF6, IRF9, IRS2, ISL1, ITCH, ITGA1, ITGA5, ITGB2, ITIH5, IWS1, JAK2, JARID2, JAZF1, JMJD1C, KALRN, KAT2B, KAT6A, KAT6B, KAT7, KCNK2, KDM2A, KDM2B, KDM3A, KDM4B, KDM4C, KDM4D, KDM6B, KHDRBS1, KHDRBS2, KIDINS220, KIF16B, KIF25, KIF2A, KIRREL1, KITLG, KLF14, KLF17, KLF7, KMT2D, KMT2E, KMT5C, KNDC1, KPNA7, KRAS, KSR1, L3MBTL3, LARP4B, LARP7, LATS2, LAX1, LDB2, LDLR, LDLRAD4, LEPR, LGMN, LHX4, LHX6, LHX8, LIMK2, LIMS1, LIN9, LLGL1, LMCD1, LMO7, LMTK2, LMX1B, LONP1, LOXL2, LOXL3, LPAR1, LPCAT1, LPGAT1, LRIG2, LRIG3, LRP1, LRP2, LRP5, LRPAP1, LRRC14, LRRK1, LRSAM1, LTN1, LYL1, LZTS1, MACC1, MAF, MAFA, MAFG, MAFK, MAGI2, MAK, MAL, MAML2, MAML3, MAP2K2, MAP2K5, MAP3K11, MAP3K13, MAP3K19, MAP3K2, MAP3K20, MAP3K5, MAP3K6, MAP4K4, MAP4K5, MAPK7, MAPK8, MAPK8IP2, MAPK8IP3, MAPK9, MAPKAP1, MAPKAPK5, MARF1, MARS1, MAX, MBD3, MBNL1, MCL1, MCM2, MCPH1, MCRS1, MECOM, MED13L, MED16, MED22, MED24, MED26, MED27, MEF2C, MEIOC, MEIS2, MELTF, MEN1, MEOX1, MGMT, MICAL2, MIDEAS, MIER1, MKNK1, MKNK2, MLLT3, MMD, MN1, MPHOSPH8, MRPL13, MRPL23, MRTFA, MSANTD1, MSGN1, MSH2, MSH3, MSI2, MSL3P1, MST1R, MTA1, MTA3, MTERF1, MTG1, MTMR2, MTMR3, MTMR9, MXD1, MYD88, MYO1C, MYO1D, MYRF, MYT1L, N4BP1, NACC2, NAT10, NCK2, NCKAP1L, NCOA2, NCOA6, NCOA7, NCOR2, NCSTN, NDFIP2, NDRG4, NDUFA13, NEDD4L, NEDD9, NEK7, NEUROD6, NEUROG2, NF1, NFAM1, NFATC1, NFATC2, NFATC2IP, NFATC4, NFE2L2, NFIB, NFIL3, NFKBIB, NFXL1, NFYA, NFYC, NGF, NKX2-4, NLK, NLRC5, NLRP12, NME2, NNT, NOC2L, NOL3, NOP2, NOS1, NOS1AP, NOTCH1, NOTCH3, NOTCH4, NOVA2, NPAT, NPC1, NPNT, NPPA, NPRL3, NPTN, NR1D1, NR2C1, NR2E3, NR3C1, NR3C2, NRF1, NRIP1, NRXN1, NSD1, NSD3, NSG1, NSMF, NTRK2, NUMB, NUP205, NUP43, NUP50, NUP58, NXN, OASL, OLIG2, OPRPN, OSBPL7, OSBPL8, |

| | | | | | |
|---|---|---|---|---|---|
| GO:0048519 | negative regulation of biological process | 2,94E-04 [1, 2, 3] | 1191,00 | 20,71 | ADGRV1, ADRB1, AEBP2, AFDN, AGBL4, AGO2, AJAP1, AKT2, ALPK2, ALX1, AMBRA1, AMER2, AMH, ANGPT1, ANGPT4, ANK3, ANKRD13B, ANKRD17, ANKRD26, ANKRD33, ANKS1B, ANTXR1, ANXA2, ANXA6, AP2A1, APC, APC2, APOBEC1, APPL1, APPL2, ARAP1, ARF1, ARFGEF3, ARHGAP10, ARHGAP12, ARHGAP21, ARHGAP42, ARHGDIA, ARHGDIB, ARHGDIG, ARHGEF18, ARHGEF2, ARHGEF7, ARID2, ARL6IP5, ARNT2, ARNTL, ASAP1, ASCL1, ASPH, ASS1, ASTN2, ASZ1, ATF2, ATF3, ATG14, ATM, ATN1, ATP13A2, ATP1A3, ATP1A4, ATP2A2, ATP2B4, ATP5F1A, ATP8A2, ATXN1, ATXN7, AURKB, BABAM2, BACH1, BAG6, BANP, BAZ2A, BBS4, BCAS3, BCL10, BCL11A, BCL11B, BCL2, BCL2A1, BCL3, BCL6, BCL7A, BDNF, BDNF-AS, BEND6, BHLHE40, BICC1, BIRC2, BMERB1, BMP2, BMP7, BMPER, BPI, BRINP2, BRMS1, BTBD10, BTC, BTG2, BTG4, BTRC, C1QC, C4orf3, CABP1, CACNA1A, CALM2, CAMK2D, CAMSAP1, CAPRIN2, CAPZB, CARD10, CARD14, CARMIL1, CARMIL2, CASP2, CAV1, CBFA2T2, CBFA2T3, CBX8, CC2D1B, CCAR2, CCDC3, CCDC88C, CCN4, CCN5, CCN6, CCND2, CCR2, CD200R1, CD33, CD46, CD47, CD96, CDC123, CDC20, CDC25C, CDH13, CDK1, CDK2, CDK5R1, CDKN1A, CDKN2A, CDT1, CDYL, CELF4, CEND1, CEP43, CFDP1, CHCHD3, CHD5, CHEK1, CHEK2, CHID1, CHMP2A, CHRNB2, CHST11, CIB1, CIC, CITED2, CLASP1, CLASP2, CLEC4G, CLP1, CNOT1, CNOT7, CNST, CNTN4, COL18A1, COL2A1, COL3A1, COL4A2, COMMD1, CORO1B, CORO2B, CPEB1, CPEB4, CRB2, CRBN, CREB3L1, CREM, CRIM1, CRY2, CRYM, CSNK1A1L, CSRNP2, CSRNP3, CST9, CTBP2, CTCF, CTNNA2, CTNNBIP1, CTNS, CTTN, CUL3, CUX1, CXXC5, CYFIP1, CYLD, CYP19A1, CYP1B1, CYP51A1, CYREN, DAB1, DAG1, DAPK1, DAXX, DBNDD2, DCAF1, DCLK1, DCN, DCP2, DCPS, DDAH2, DDIT4, DDR1, DDX6, DENND5A, DEPDC1, DEPDC5, DGKQ, DGKZ, DHRS3, DHX34, DHX36, DIPK2A, DIS3L2, DKK3, DLC1, DLG1, DLG2, DLG4, DLL1, DLL4, DLX2, DNA2, DNAJB5, DNAJB6, DNAJC15, DNAJC3, DNM1, DNM1L, DNMT3A, DNPEP, DOCK7, DOCK8, DOT1L, DPF1, DSTYK, DTX1, DUS2, DUSP19, DUSP26, E2F2, E2F8, EFNA5, EHMT1, EHMT2, EIF2AK3, EIF2AK4, EIF4A3, EIF4E, EIF4EBP1, EIF4EBP2, EIF4ENIF1, ELAVL1, ELF1, ELK3, ENC1, ENHO, EPHA1, EPHA4, EPHB1, EPPK1, EPRS1, EPS15L1, ERBB4, ERC1, ERCC1, ERCC5, EREG, ESPN, ESR1, ESR2, ETAA1, ETS1, ETV6, EVL, EXOSC10, EXOSC6, EYA2, EYA4, EZH2, F11R, F12, F2RL3, FABP5, FAF1, FAM111A, FAM172A, FAM220A, FAM3D, FAM89B, FAP, FARP1, FASN, FASTKD1, FBLN5, FBN1, FBN2, FBP1, FBXO2, FBXO6, FEM1C, FER, FERD3L, FEZ1, FEZF1, FEZF2, FGF12, FGF2, FHOD3, FICD, FKTN, FLT4, FNIP2, FOXD3, FOXK1, FOXO1, FOXP1, FRMD4A, FRMD5, FRS2, FTO, FXYD5, FZD10, FZD5, FZR1, GABRB2, GAK, GARS1, GAS7, GATA5, GCG, GCNT2, GFI1, GFRAL, GJA5, GLI2, GLI3, GLIS2, GLMN, GLO1, GLRX3, GMFB, GML, GNAI2, GNB5, GON4L, GORASP1, GPER1, GPR137B, GPR161, GPR17, GPR4, GPR55, GPRIN1, GPSM1, GRAMD4, GRB10, GRB2, GREM1, GRID2, GRID2IP, GRIK3, GRK5, GSK3A, H2AC4, H2AC6, H2AJ, H3C6, H3C8, HADHA, HAND1, HCAR2, HCLS1, HDAC4, HDAC9, HECW1, HEG1, HELT, HELZ, HENMT1, HERC1, HES1, HES3, HES4, HGS, HHATL, HIP1R, HIVEP1, HMBOX1, HMGB1, HNF1B, HNRNPC, HNRNPD, HNRNPK, HNRNPR, HOXC10, HRC, HRG, HSP90AB1, HSPD1, HTATIP2, HTR2A, HTRA3, HTT, HUS1, IDE, IER3, IFI16, IFIH1, IFNLR1, IFRD1, IFT74, IGF1, IGF1R, IGF2BP1, IGHMBP2, IHH, IKBKB, IKZF1, IL10RA, IL17D, IL17RD, IL31RA, IL37, IL6ST, ILF3, ING1, ING3, INKA2, INPP5F, INPPL1, INSIG2, INTS3, IPO7, IPO8, IQGAP2, IQGAP3, IRAK2, IRF2, IRF2BP2, IRF6, IRS2, ISL1, ISM1, ITCH, ITFG2, ITGA1, ITGA5, ITGB2, ITIH5, ITPR2, ITPR3, JAK2, JARID2, JAZF1, JPH3, KALRN, KANK1, KANK3, KAT2B, KAT6A, KAT6B, KAT7, KCNB1, KCNE1, KCNH2, KCNK2, KCNMA1, KCNQ1, KCTD10, KCTD6, KDM2A, KDM2B, KDM3A, KDM4B, KDM4C, KDM4D, KHDRBS1, KIF25, KIF26A, KIF2A, KIRREL1, KITLG, KLF7, KMT2D, KMT2E, KPNA7, KRAS, KREMEN2, KRIT1, KRT1, L3MBTL3, LATS2, LAX1, LDB2, LDLR, LDLRAD4, LEPR, LGMN, LHX4, LIG4, LIMA1, LIMS1, LINGO1, LLGL1, LMCD1, LMTK2, LONP1, LOXL2, LOXL3, LPAR1, LPCAT1, LPL, LRIG2, LRIG3, LRP1, LRP2, LRP5, LRPAP1, LRRC14, LRRC32, LRRK1, LRSAM1, LSM2, LSM5, LTBP1, LTBP2, LZTS1, LZTS2, MAD1L1, MAF, MAFK, MAG, MAGI2, MAK, MAP2, MAP2K2, MAP2K5, MAP3K20, MAP4, MAP4K4, MAP4K5, MAPK7, MAPK8, MAPK8IP2, MAPKAP1, MAPKAPK5, MARF1, MARK3, MAX, MBD3, MCL1, MCM2, MCPH1, MDC1, MEAK7, MECOM, MEF2C, MEGF8, MEIOC, MEIS2, MELTF, MEN1, METRNL, MGMT, MGRN1, MIER1, MLLT3, MOV10L1, MPHOSPH8, MRPL13, MRPL23, MRTFA, MSH2, MSH3, MSL3P1, MT1F, MTA1, MTA3, MTMR2, MTMR9, MTNR1B, MTRNR2L4, MX1, MXD1, MYD88, MYL2, MYO16, MYO18A, MYO1C, MYO1D, MYO5A, MYT1L, N4BP1, NACC2, NAPEPLD, NAT10, NAV3, NCK2, NCKAP1L, NCOA2, NCOA7, NCOR2, NDFIP2, NDRG3, NDRG4, NDUFA13, NDUFA2, NEDD4L, NEK11, NEPRO, NF1, NFATC1, NFATC2, NFATC4, NFE2L2, NFIB, NFIL3, NFKBIB, NGEF, NGF, NKD1, NLK, NLRC5, NLRP12, NLRX1, NME2, NNT, NOC2L, NOL3, NOS1, NOTCH1, NOTCH3, NOTCH4, NOVA2, NPAT, NPC1, NPHP4, NPPA, NPR1, NPRL3, NPTN, NR1D1, NR2C1, NR2E3, NR3C1, NRIP1, NRXN1, NSD1, NTRK2, NUMB, NUP205, NUP43, NUP50, NUP58, NXN, OASL, OLFM1, OLIG2, OPRPN, OSBPL8, OTUB1, OTUD4, OVGP1, P2RY6, PACSIN1, PACSIN2, PAF1, PAFAH1B1, PAFAH2, PAK4, PAK5, PALM, PAPLN, PARD3, PARP14, PASK, PATL1, PAX2, PAX7, PAX8, PAX9, PBLD, PBX1, PC, PCDHA6, PDCD5, PDCD6, PDCL3, PDE8A, PDGFRA, PDPK1, PDZD2, PDZD3, PEG10, PEG3, PELI3, PER2, PEX14, PFN2, PGP, PHACTR1, PHF19, PHF2, PHLPP2, PIH1D1, PIK3CG, PIK3IP1, PIK3R1, PIM1, PINX1, PKD1, PKIB, PKMYT1, PKP1, PLA2G2D, PLA2G6, PLAC8, PLAUR, PLCB1, PLCB2, PLCB4, PLCD4, PLCE1, PLCG2, PLCH2, PLCL2, PLEK, PLEKHN1, PLIN5, PLXNA1, PLXNA2, PLXNB1, |
| GO:0043412 | macromolecule modification | 3,46E-03 [4] | 960,00 | 20,86 | AGBL4, AKAP12, AKAP13, AKT2, ALG2, ALG5, ALG6, ALK, ALKAL2, ALPK2, ALS2, AMH, ANAPC11, ANAPC16, ANAPC4, ANGPT1, ANGPT4, ANKRD16, ANKS1B, ANTXR1, ANXA2, APC, APOBEC1, APOBEC2, ARHGEF2, ARHGEF7, ARID4B, ARIH1, ARL6IP5, ARNTL, ASB7, ASCC2, ASPH, ASPSCR1, ASXL2, ASZ1, ATAT1, ATF2, ATF3, ATG14, ATG16L1, ATG4B, ATM, ATP13A2, ATP2B4, ATXN7, ATXN7L3, AURKB, B3GALT4, B3GLCT, B3GNTL1, BABAM2, BAG6, BAZ1B, BAZ2A, BCAR3, BCL10, BCL11A, BCL2, BCL6, BDNF, BDNF-AS, BIRC2, BMP2, BMP3, BMP7, BMP8B, BMPER, BRD1, BRD4, BRMS1, BRSK2, BTBD6, BTC, BTG2, BTRC, C10orf90, CALM2, CALU, CAMK2B, CAMK2D, CAMK4, CAMKMT, CAMTA1, CAND1, CAPRIN2, CARD10, CARD14, CASR, CAV1, CBX8, CCAR2, CCDC88C, CCL16, CCNA1, CCND2, CCNL1, CCNY, CD33, CDC20, CDC20B, CDC25B, CDC25C, CDC42BPB, CDK1, CDK11B, CDK19, CDK2, CDK5R1, CDKAL1, CDKL2, CDKN1A, CDKN2A, CDYL, CEP295, CEP43, CHAMP1, CHCHD4, CHD5, CHEK1, CHEK2, CIB1, CILK1, CIT, CLASP1, CLK3, CLK4, CMTR2, CNOT7, COL3A1, COMMD1, COP1, CRBN, CRIM1, CRY2, CSNK1A1L, CSNK1E, CSNK1G3, CTCF, CUL2, CUL3, CXCR4, CYCS, CYLD, DAB1, DAG1, DAPK1, DAXX, DBNDD2, DCAF1, DCAF13, DCLK1, DCN, DCUN1D1, DDA1, DDIT4, DDR1, DDR2, DESI2, DGKQ, DHDDS, DIPK2A, DLC1, DLD, DLG1, DLG2, DLG4, DNAJC3, DNAJC6, DNM1L, DNMT3A, DOCK7, DOK4, DOK7, DOT1L, DPF1, DSTYK, DTX1, DUS2, DUS3L, DUSP19, DUSP26, DZIP3, EDAR, EDEM2, EDEM3, EFNA4, EFNA5, EGR2, EHMT1, EHMT2, EIF2AK3, EIF2AK4, EIPR1, EMC10, ENC1, ENGASE, ENPP2, EPHA1, EPHA10, EPHA4, EPHA6, EPHB1, EPHB3, ERBB4, ERC1, ERCC8, EREG, ESCO1, ESR1, ETAA1, ETFA, EVA1A, EXOSC6, EYA2, EYA4, EZH2, F13A1, F3, F5, FAF1, FAM161A, FAM220A, FAM86C1, FANCM, FASN, FASTK, FASTKD1, FBN1, FBXL13, FBXL18, FBXO11, FBXO2, FBXO32, FBXO6, FBXW8, FEM1C, FER, FEZF2, FGD2, FGF2, FGF22, FGF6, FGF7, FICD, FKTN, FLT4, FN3KRP, FNIP2, FNTA, FOXK1, FOXO1, FRS2, FTO, FUT8, FZD10, FZD5, FZR1, GAD1, GADD45G, GAK, GALNT10, GALNT13, GALNT14, GALNT15, GALNT2, GALNT3, GALNT7, GALNT9, GARS1, GCG, GCNT1, GCNT2, GFI1, GFRAL, GLIPR2, GLMN, GLYCTK, GMFB, GNAI2, GORASP1, GPER1, GPR55, GPRC5B, GPRC5C, GPRIN1, GRB10, GRB2, GREM1, GRK3, GRK5, GRM1, GRM4, GSK3A, GTF2B, GTF2H1, H2BC15, HACD3, HCLS1, HDAC4, HDAC9, HECTD1, HECTD3, HECTD4, HECW1, HEG1, HENMT1, HERC1, HERC2, HES1, HGS, HHATL, HMGB1, HRC, HRG, HS3ST3B1, HSP90AB1, HTATIP2, HTR2A, HTT, HUS1, ICMT, IFIH1, IGF1, IGF1R, IKBKB, IL12RB2, IL17RD, IL31RA, IL6R, IL6ST, ILF3, ING3, INKA2, INPP5F, INSR, IPO7, IQGAP3, IRAK2, IRF2BP2, IRS2, ISL1, ITCH, ITGA1, ITGA5, ITGB2, ITK, IWS1, JAK2, JARID2, JMJD1C, JMJD7, JOSD1, KALRN, KAT2B, KAT6A, KAT6B, KAT7, KCMF1, KCTD10, KCTD6, KDM2A, KDM2B, KDM3A, KDM4B, KDM4C, KDM4D, KDM6B, KIDINS220, KIRREL1, KITLG, KLHL17, KLHL3, KLHL5, KMT2D, KMT2E, KMT5C, KNDC1, KPNA7, KRAS, KRT1, KSR1, KSR2, LAMB1, LAMB2, LARGE1, LARGE2, LARP7, LATS2, LAX1, LDB2, LDHD, LDLRAD4, LEPR, LHPP, LIMK2, LIN9, LLGL1, LMO7, LMTK2, LONP1, LOXL2, LOXL3, LPAR1, LRP1, LRP5, LRRC8B, LRRC8D, LRRK1, LRSAM1, LTBP1, LTN1, MACROD1, MACROD2, MAGI2, MAK, MAN1C1, MAN2B1, MAP2K2, MAP2K5, MAP3K11, MAP3K13, MAP3K19, MAP3K2, MAP3K20, MAP3K5, MAP3K6, MAP4K4, MAP4K5, MAPK4, MAPK7, MAPK8, MAPK8IP2, MAPK8IP3, MAPK9, MAPKAP1, MAPKAPK5, MARCHF1, MARCHF10, MARK3, MAST4, MBD3, MCM2, MCRS1, MDP1, MECOM, MED22, MED24, MED27, MEF2C, MELTF, MEN1, METTL1, METTL22, MFGE8, MFNG, MGAT4A, MGMT, MGRN1, MIB2, MIER1, MINDY2, MKNK1, MKNK2, MMD, MOV10L1, MPG, MPHOSPH8, MRPL23, MRTFA, MSL3P1, MSLN, MSRA, MST1R, MTA1, MTA3, MTMR2, MTMR3, MTMR9, MTRR, MUC13, MUC4, MUC5B, MYD88, MYH3, MYLK, MYLK4, MYO1C, N4BP1, NAA30, NAA60, NAT10, NCCRP1, NCK2, NCKAP1L, NCOA7, NDFIP2, NDRG4, NEDD4L, NEDD9, NEK11, NEK7, NF1, NFE2L2, NGF, NGLY1, NKTR, NLK, NLRP12, NME2, NNT, NOC2L, NOP2, NOS1, NOS1AP, NOTCH1, NPC1, NPHS1, NPNT, NPPA, NPR1, NPTN, NRXN1, NSD1, NSD3, NSMF, NTRK2, NUP205, NUP43, NUP50, NUP58, NUS1, NXN, OBSCN, OBSL1, OGDH, OPN1SW, OPN3, OPN5, OSBPL8, OSGEP, OTUB1, OTUB2, OTUD4, OTUD6B, P2RY6, P4HTM, PAF1, PAFAH1B1, PAGR1, PAK4, PAK5, PARD3, PARG, PARP14, PARP4, PASK, PAX7, PBLD, PCGF5, PCMTD1, PCNP, PDCD6, PDCL3, PDE8A, PDGFD, PDGFRA, PDGFRL, PDPK1, PDZD2, PEAK1, PELI3, PER2, PEX13, PEX14, PFDN1, PFN2, PGP, PHACTR1, PHF19, PHF2, PHF20, PHLPP2, PIGG, PIGP, PIGQ, PIGS, PIGV, PIGY, PIH1D1, PIK3CB, PIK3CG, PIK3R1, PIK3R5, PIM1, PINX1, PKD1, PKIB, PKMYT1, PLA2G6, PLAUR, PLCB1, PLCE1, PLCL2, PLK4, PLK5, PLOD1, PLVAP, PML, PNPLA2, POLG, POMGNT1, POMGNT2, POMT1, POU4F2, PPA2, PPARGC1A, PPIB, PPM1F, PPP1R11, PPP1R16A, PPP1R16B, PPP2R2B, PPP2R2C, PPP2R3C, PPP3CA, PPP3CB, PPP4C, PPTC7, PRAG1, PRDM16, PRDM5, PRKAG2, PRKAR1B, PRKCA, PRKCB, PRKCE, PRKCH, PRKCI, PRKCQ, PRKCZ, PRKD2, PRKDC, PRKN, PRLR, PRMT1, PRMT8, PRPF19, PSCA, PSEN1, PSMA5, PSMA6, PSMA7, PSMC2, PSMC3, PSMC6, PSMD2, PSMD7, PTEN, PTK2, PTPN11, PTPN14, PTPN21, PTPN3, PTPRA, PTPRC, PTPRF, PTPRH, PTPRN, PTPRN2, PTPRO, PTPRZ1, PUS10, PUS7, PUS7L, RAB2A, RAB3D, RAB40B, RAB43, RAB5A, RACK1, RAD51, RANBP9, RAP1A, RAP2B, RAPGEF1, RARA, RASA1, |
| GO:0006464 | cellular protein modification process | 5,06E-03 [5, 6] | 916,00 | 20,90 | ALG2, ALG5, ALG6, ALK, ALKAL2, ALPK2, ALS2, AMH, ANAPC11, ANAPC16, ANAPC4, ANGPT1, ANGPT4, ANKS1B, ANTXR1, ANXA2, APC, ARHGEF2, ARHGEF7, ARID4B, ARIH1, ARL6IP5, ARNTL, ASB7, ASPH, ASPSCR1, ASXL2, ATAT1, ATF2, ATF3, ATG14, ATG16L1, ATG4B, ATM, ATP13A2, ATP2B4, ATXN7, ATXN7L3, AURKB, B3GALT4, B3GLCT, B3GNTL1, BABAM2, BAG6, BAZ1B, BAZ2A, BCAR3, BCL10, BCL11A, BCL2, BCL6, BDNF, BDNF-AS, BIRC2, BMP2, BMP3, BMP7, BMP8B, BMPER, BRD1, BRD4, BRMS1, BRSK2, BTBD6, BTC, BTG2, BTRC, C10orf90, CALM2, CALU, CAMK2B, CAMK2D, CAMK4, CAMKMT, CAMTA1, CAND1, CAPRIN2, CARD10, CARD14, CASR, CAV1, CBX8, CCAR2, CCDC88C, CCL16, CCNA1, CCND2, CCNL1, CCNY, CD33, CDC20, CDC20B, CDC25B, CDC25C, CDC42BPB, CDK1, CDK11B, CDK19, CDK2, CDK5R1, CDKL2, CDKN1A, CDKN2A, CDYL, CEP295, CEP43, CHAMP1, CHCHD4, CHD5, CHEK1, CHEK2, CIB1, CILK1, CIT, CLASP1, CLK3, CLK4, CNOT7, COL3A1, COMMD1, COP1, CRBN, CRIM1, CRY2, CSNK1A1L, CSNK1E, CSNK1G3, CTCF, CUL2, CUL3, CXCR4, CYCS, CYLD, DAB1, DAG1, DAPK1, DAXX, DBNDD2, DCAF1, DCAF13, DCLK1, DCN, DCUN1D1, DDA1, DDIT4, DDR1, DDR2, DESI2, DGKQ, DHDDS, DIPK2A, DLC1, DLD, DLG1, DLG2, DLG4, DNAJC3, DNAJC6, DNM1L, DOCK7, DOK4, DOK7, DOT1L, DPF1, DSTYK, DTX1, DUS2, DUSP19, DUSP26, DZIP3, EDAR, EDEM2, EDEM3, EFNA4, EFNA5, EGR2, EHMT1, EHMT2, EIF2AK3, EIF2AK4, EIPR1, EMC10, ENC1, ENGASE, ENPP2, EPHA1, EPHA10, EPHA4, EPHA6, EPHB1, EPHB3, ERBB4, ERC1, ERCC8, EREG, ESCO1, ESR1, ETAA1, ETFA, EVA1A, EYA2, EYA4, EZH2, F13A1, F3, F5, FAF1, FAM161A, FAM220A, FAM86C1, FANCM, FASN, FASTK, FASTKD1, FBN1, FBXL13, FBXL18, FBXO11, FBXO2, FBXO32, FBXO6, FBXW8, FEM1C, FER, FEZF2, FGD2, FGF2, FGF22, FGF6, FGF7, FICD, FKTN, FLT4, FN3KRP, FNIP2, FNTA, FOXK1, FOXO1, FRS2, FUT8, FZD10, FZD5, FZR1, GAD1, GADD45G, GAK, GALNT10, GALNT13, GALNT14, GALNT15, GALNT2, GALNT3, GALNT7, GALNT9, GARS1, GCG, GCNT1, GCNT2, GFI1, GFRAL, GLIPR2, GLMN, GLYCTK, GMFB, GNAI2, GORASP1, GPER1, GPR55, GPRC5B, GPRC5C, GPRIN1, GRB10, GRB2, GREM1, GRK3, GRK5, GRM1, GRM4, GSK3A, GTF2B, GTF2H1, H2BC15, HACD3, HCLS1, HDAC4, HDAC9, HECTD1, HECTD3, HECTD4, HECW1, HEG1, HERC1, HERC2, HES1, HGS, HHATL, HMGB1, HRC, HRG, HS3ST3B1, HSP90AB1, HTATIP2, HTR2A, HTT, HUS1, ICMT, IFIH1, IGF1, IGF1R, IKBKB, IL12RB2, IL17RD, IL31RA, IL6R, IL6ST, ILF3, ING3, INKA2, INPP5F, INSR, IPO7, IQGAP3, IRAK2, IRF2BP2, IRS2, ISL1, ITCH, ITGA1, ITGA5, ITGB2, ITK, IWS1, JAK2, JARID2, JMJD1C, JMJD7, JOSD1, KALRN, KAT2B, KAT6A, KAT6B, KAT7, KCMF1, KCTD10, KCTD6, KDM2A, KDM2B, KDM3A, KDM4B, KDM4C, KDM4D, KDM6B, KIDINS220, KIRREL1, KITLG, KLHL17, KLHL3, KLHL5, KMT2D, KMT2E, KMT5C, KNDC1, KPNA7, KRAS, KRT1, KSR1, KSR2, LAMB1, LAMB2, LARGE1, LARGE2, LATS2, LAX1, LDB2, LDHD, LDLRAD4, LEPR, LHPP, LIMK2, LLGL1, LMO7, LMTK2, LONP1, LOXL2, LOXL3, LPAR1, LRP1, LRP5, LRRC8B, LRRC8D, LRRK1, LRSAM1, LTBP1, LTN1, MACROD1, MACROD2, MAGI2, MAK, MAN1C1, MAN2B1, MAP2K2, MAP2K5, MAP3K11, MAP3K13, MAP3K19, MAP3K2, MAP3K20, MAP3K5, MAP3K6, MAP4K4, MAP4K5, MAPK4, MAPK7, MAPK8, MAPK8IP2, MAPK8IP3, MAPK9, MAPKAP1, MAPKAPK5, MARCHF1, MARCHF10, MARK3, MAST4, MBD3, MCM2, MCRS1, MDP1, MECOM, MED22, MED24, MED27, MEF2C, MELTF, MEN1, METTL22, MFGE8, MFNG, MGAT4A, MGRN1, MIB2, MIER1, MINDY2, MKNK1, MKNK2, MMD, MRPL23, MRTFA, MSL3P1, MSLN, MSRA, MST1R, MTA1, MTA3, MTMR2, MTMR3, MTMR9, MUC13, MUC4, MUC5B, MYD88, MYH3, MYLK, MYLK4, MYO1C, N4BP1, NAA30, NAA60, NCCRP1, NCK2, NCKAP1L, NCOA7, NDFIP2, NDRG4, NEDD4L, NEDD9, NEK11, NEK7, NF1, NFE2L2, NGF, NGLY1, NKTR, NLK, NLRP12, NME2, NNT, NOC2L, NOS1, NOS1AP, NOTCH1, NPC1, NPHS1, NPNT, NPPA, NPR1, NPTN, NRXN1, NSD1, NSD3, NSMF, NTRK2, NUP205, NUP43, NUP50, NUP58, NUS1, NXN, OBSCN, OBSL1, OGDH, OPN1SW, OPN3, OPN5, OSBPL8, OTUB1, OTUB2, OTUD4, OTUD6B, P2RY6, P4HTM, PAF1, PAFAH1B1, PAGR1, PAK4, PAK5, PARD3, PARG, PARP14, PARP4, PASK, PAX7, PBLD, PCGF5, PCMTD1, PCNP, PDCD6, PDCL3, PDE8A, PDGFD, PDGFRA, PDGFRL, PDPK1, PDZD2, PEAK1, PELI3, PER2, PEX13, PEX14, PFDN1, PFN2, PGP, PHACTR1, PHF19, PHF2, PHF20, PHLPP2, PIGG, PIGP, PIGQ, PIGS, PIGV, PIGY, PIH1D1, PIK3CB, PIK3CG, PIK3R1, PIK3R5, PIM1, PINX1, PKD1, PKIB, PKMYT1, PLA2G6, PLAUR, PLCB1, PLCE1, PLCL2, PLK4, PLK5, PLOD1, PLVAP, PML, PNPLA2, POLG, POMGNT1, POMGNT2, POMT1, POU4F2, PPA2, PPARGC1A, PPIB, PPM1F, PPP1R11, PPP1R16A, PPP1R16B, PPP2R2B, PPP2R2C, PPP2R3C, PPP3CA, PPP3CB, PPP4C, PPTC7, PRAG1, PRDM16, PRDM5, PRKAG2, PRKAR1B, PRKCA, PRKCB, PRKCE, PRKCH, PRKCI, PRKCQ, PRKCZ, PRKD2, PRKDC, PRKN, PRLR, PRMT1, PRMT8, PRPF19, PSCA, PSEN1, PSMA5, PSMA6, PSMA7, PSMC2, PSMC3, PSMC6, PSMD2, PSMD7, PTEN, PTK2, PTPN11, PTPN14, PTPN21, PTPN3, PTPRA, PTPRC, PTPRF, PTPRH, PTPRN, PTPRN2, PTPRO, PTPRZ1, RAB2A, RAB3D, RAB40B, RAB43, RAB5A, RACK1, RAD51, RANBP9, RAP1A, RAP2B, RAPGEF1, RARA, RASA1, RASA2, RASA3, RASAL2, RASGRF1, RASGRP4, RASIP1, RASSF5, RB1, |

| | | | | | |
|---|---|---|---|---|---|
| GO:0048583 | regulation of response to stimulus | 6,87E-04 [2, 3] | 953,00 | 21,05 | AKR1C2, ALK, ALKAL2, ALPK2, ALS2, AMER2, AMH, ANGPT1, ANKRD17, ANKS1A, ANKS1B, ANO1, ANO6, ANTXR1, ANXA2, APC, APC2, APPL1, APPL2, ARAP1, ARAP3, ARFGAP1, ARFGEF3, ARHGAP10, ARHGAP12, ARHGAP17, ARHGAP21, ARHGAP29, ARHGAP39, ARHGAP40, ARHGAP42, ARHGAP45, ARHGDIA, ARHGDIB, ARHGDIG, ARHGEF16, ARHGEF18, ARHGEF2, ARHGEF26, ARHGEF7, ARID2, ARL6IP5, ARNTL, ARPC4, ASCL1, ASXL2, ATF3, ATM, ATP2A2, ATP2B4, AURKB, BABAM2, BAG6, BAIAP2L1, BANP, BBS4, BCAR1, BCAR3, BCL10, BCL2, BCL2L14, BCL3, BCL6, BDNF, BDNF-AS, BEGAIN, BEND6, BICC1, BIRC2, BMP2, BMP3, BMP7, BMP8B, BMPER, BRD1, BRD4, BRSK2, BTC, BTN3A3, BTRC, C12orf4, C1QC, CACNA1A, CACNG8, CADM1, CALM2, CAMK2B, CAMK2D, CAMKMT, CAMTA1, CAPRIN2, CARD10, CARD11, CARD14, CASP2, CASP5, CASR, CAV1, CBFA2T2, CBX8, CCAR2, CCBE1, CCDC3, CCDC88C, CCL16, CCN4, CCNY, CCR2, CCR6, CD200R1, CD247, CD33, CD46, CD47, CD8A, CD96, CDH13, CDK1, CDK19, CDK2, CDK5R1, CDKN1A, CDKN2A, CELA2A, CELF4, CFB, CHD3, CHD5, CHEK1, CHEK2, CHID1, CHN2, CHRNB2, CHST11, CIB1, CITED2, CLASP1, CLASP2, CLEC4G, CMAHP, CNIH3, CNOT1, CNOT7, CNRIP1, COL2A1, COL3A1, CORO1B, CORO2B, CPNE2, CRB2, CREB3L1, CRY2, CSNK1A1L, CSNK1E, CSNK1G3, CTBP2, CTNNA2, CTNNBIP1, CTNND2, CTTN, CUL3, CXCR4, CXXC5, CYFIP1, CYFIP2, CYLD, CYP19A1, CYP1B1, CYREN, CYTH1, DAB1, DAG1, DAPK1, DAXX, DCN, DDIT4, DDX60, DENND1A, DENND1B, DEPDC5, DGKQ, DGKZ, DHRS3, DHX36, DIPK2A, DKK3, DLC1, DLG1, DLG2, DLG4, DLL1, DLL4, DLX2, DNA2, DNAJB6, DNAJC3, DNASE1L3, DNM1, DNM1L, DOK4, DOT1L, DSTYK, DTX1, DUOX1, DUSP19, DUSP26, EDAR, EDEM2, EEF1E1-BLOC1S5, EFHD1, EFNA5, EGR2, EHMT1, EHMT2, EIF2AK3, EIF2AK4, EIF4A3, ELF1, ELMO1, EMC10, ENHO, EPHA4, EPHB1, EPPK1, EPS15L1, EPS8L1, ERAP1, ERBB4, ERCC1, ERCC8, EREG, ESR1, ESR2, ETAA1, ETFA, ETS1, EXOSC6, EYA2, EYA4, EZH2, F11R, F12, F2RL3, F3, FABP5, FAF1, FAM110A, FAM13A, FAM13B, FAM89B, FAP, FASN, FBLN5, FBN1, FBN2, FBP1, FBXO32, FEM1C, FER, FEZF2, FGD2, FGF2, FGF22, FGF6, FGF7, FICD, FKTN, FLT4, FNIP2, FNTA, FOXO1, FOXP1, FRMPD1, FRS2, FUT8, FZD10, FZD5, GAB1, GAB2, GABRA4, GADD45G, GATA5, GCG, GCNT2, GFI1, GFRAL, GLI2, GLI3, GLIPR2, GLIS2, GLMN, GLRX2, GLRX3, GNA12, GNAI2, GNAO1, GPC6, GPER1, GPR137B, GPR161, GPR17, GPR4, GPR55, GPR62, GPRC5B, GPRIN1, GRAMD4, GRB10, GRB2, GREM1, GRIA4, GRID2, GRK5, GRM1, GRM2, GRM4, GSG1L, GSK3A, GSX2, GTF2IRD1, HACD3, HCLS1, HDAC4, HDAC9, HECW1, HEG1, HES1, HGS, HINT1, HIP1, HIP1R, HLA-DPB1, HLA-DQA2, HLA-DRA, HMGB1, HMGN3, HNF1B, HNRNPC, HNRNPK, HOOK1, HRC, HRG, HSP90AB1, HSPD1, HSPH1, HTR2A, HTRA3, HTT, HUS1, ICAM3, IER3, IFI16, IFIH1, IFNLR1, IFT140, IGF1, IGF1R, IGF2BP1, IGLL5, IGSF9B, IHH, IKBKB, IL10RA, IL10RB, IL16, IL17D, IL17RA, IL17RD, IL20RA, IL31RA, IL37, IL6R, IL6ST, ING3, INPP5F, INSIG2, INSR, IQGAP3, IQSEC1, IQSEC3, IRAK2, IRS2, ISL1, ITCH, ITFG2, ITGA1, ITGA5, ITGAL, ITGB2, ITK, JAK2, JAML, JARID2, JPH3, KALRN, KANK1, KAT2B, KAT6A, KAT7, KCNK2, KCTD10, KCTD16, KCTD6, KDM4D, KHDRBS1, KIDINS220, KIF26A, KITLG, KLF14, KLF7, KLK7, KMT2D, KMT5C, KRAS, KREMEN2, KRT1, KSR1, LAT2, LATS2, LAX1, LDLR, LDLRAD4, LGMN, LGR6, LIMS1, LLGL1, LMCD1, LOXL3, LPAR1, LPL, LRIG2, LRP1, LRP2, LRP5, LRPAP1, LRRC14, LRRK1, LRSAM1, LTBP1, LURAP1, LYPD6, LYPD6B, LZTS2, MACF1, MAGI2, MAL, MAML2, MAML3, MAP2K2, MAP2K5, MAP3K11, MAP3K13, MAP3K2, MAP3K20, MAP3K5, MAP3K6, MAP4K4, MAP4K5, MAPK7, MAPK8, MAPK8IP2, MAPK8IP3, MAPK9, MAPKAP1, MAPKAPK5, MARK3, MBD3, MCF2L, MCL1, MCPH1, MEAK7, MECOM, MEF2C, MEGF8, MEN1, METRNL, MFNG, MGMT, MGRN1, MIB2, MIER1, MKS1, MLLT3, MOG, MRPL23, MRTFA, MSH2, MST1R, MTMR2, MTNR1B, MTRNR2L4, MTUS1, MUC13, MUC4, MUC5B, MYD88, MYLK, MYO1C, MYO1E, MYO5A, MYO9B, NACC2, NAPEPLD, NCK2, NCKAP1, NCKAP1L, NCOA7, NCOR2, NDFIP2, NDRG4, NDUFA13, NDUFA2, NEO1, NEPRO, NF1, NFAM1, NFATC1, NFATC2, NFATC4, NFE2L2, NGEF, NGF, NKD1, NLGN2, NLK, NLRC5, NLRP12, NLRX1, NOC2L, NOL3, NOP2, NOS1, NOS1AP, NOTCH1, NOTCH3, NOTCH4, NPDC1, NPHP4, NPNT, NPPA, NPR1, NPRL3, NPTN, NPTXR, NR1D1, NR2C1, NR3C2, NRXN1, NTRK2, NUP205, NUP43, NUP50, NUP58, NXN, OBSCN, OSBPL8, OTUB1, OTUD4, P2RY6, PAFAH1B1, PAGR1, PAK5, PALM, PARG, PARP14, PBLD, PCDHA6, PCSK6, PDCD5, PDCD6, PDE8A, PDE9A, PDGFD, PDGFRA, PDPK1, PDZD2, PDZD3, PEG10, PELI3, PER2, PEX5L, PHF20, PHLPP2, PIANP, PIH1D1, PIK3CB, PIK3CG, PIK3IP1, PIK3R1, PIK3R5, PIKFYVE, PIP4K2A, PIP5K1C, PLA2G2D, PLA2G6, PLA2G7, PLAUR, PLCB1, PLCE1, PLCG2, PLCL2, PLD4, PLEK, PLEKHA4, PLEKHG2, PLIN5, PLXNB1, PML, POLQ, POU4F2, PPARG, PPARGC1A, PPM1F, PPP1R13B, PPP1R16B, PPP2R3C, PPP3CA, PPP3CB, PPP4C, PRAG1, PRDM16, PRKAG2, PRKAR1B, PRKCA, PRKCB, PRKCE, PRKCH, PRKCI, PRKCQ, PRKCZ, PRKD2, PRKDC, PRKN, PRLR, PRMT1, PSCA, PSD, PSD2, PSD3, PSD4, PSEN1, PSMA5, PSMA6, PSMA7, PSMC2, PSMC3, PSMC6, PSMD2, PSMD7, PTCH2, PTEN, PTGES2, PTGIS, PTK2, PTPN11, PTPN3, PTPRC, PTPRF, PTPRO, PUM1, PVR, PXDN, PYCR1, RAB29, RACK1, RAD51, RAD51AP1, RAD9A, RALGAPA2, RANBP9, RAP1A, RAP1GAP, RAP1GAP2, RAPGEF1, RARA, RASA1, RASA2, RASA3, RASGRF1, RASGRP4, RASIP1, RB1, RBPMS, |
| GO:0048518 | positive regulation of biological process | 2,79E-08 [1, 2, 3] | 1382,00 | 21,07 | ADCY5, ADCY7, ADCYAP1R1, ADD1, ADGRB2, ADGRB3, ADGRF5, ADGRG1, ADGRL3, ADGRV1, ADIG, ADRA1B, ADRB1, ADRM1, AFDN, AGBL4, AGO2, AGRN, AKAP12, AKAP13, AKR1C2, AKT2, ALDH3A1, ALK, ALKAL2, ALMS1, ALS2, ALX1, ALX4, AMBRA1, AMH, ANAPC11, ANAPC4, ANGPT1, ANGPT4, ANK3, ANKRD17, ANKRD31, ANO1, ANO6, ANTXR1, ANXA2, AP2A1, AP3B1, APBB1IP, APC, APC2, APOBEC1, APOC4, APPL1, APPL2, ARAP1, ARF1, ARHGDIA, ARHGEF10L, ARHGEF16, ARHGEF18, ARHGEF2, ARHGEF26, ARHGEF7, ARID2, ARID4B, ARIH1, ARL6IP5, ARNT2, ARNTL, ARPC4, ASAP1, ASCL1, ASPH, ASPSCR1, ASS1, ASXL2, ATAT1, ATF1, ATF2, ATF3, ATG14, ATG16L1, ATG4B, ATM, ATP13A2, ATP1A3, ATP1A4, ATP2A2, ATP2A3, ATP2B4, ATP5F1A, ATP8A1, ATP8A2, ATXN7L3, AURKB, BABAM2, BACH1, BAG6, BAIAP2L1, BANP, BAZ1B, BBS4, BBS7, BCAR1, BCAR3, BCAS3, BCL10, BCL11A, BCL11B, BCL2, BCL3, BCL6, BCL9, BDNF, BDNF-AS, BEND6, BICRAL, BIRC2, BLOC1S5, BMP1, BMP2, BMP3, BMP7, BMP8B, BMPER, BNC1, BRD1, BRD4, BRF1, BRINP2, BRMS1, BTBD10, BTC, BTG2, BTN3A3, BTRC, C12orf4, C1QC, C1orf61, C21orf91, CACNA1A, CACNA1G, CACNA1H, CACNA1I, CACNA2D2, CACNB2, CADM1, CADPS, CADPS2, CALB2, CALCOCO1, CALM2, CAMK2B, CAMK2D, CAMK4, CAMTA1, CAMTA2, CAND1, CAPRIN2, CARD10, CARD11, CARD14, CARMIL1, CARMIL2, CASP2, CASR, CASZ1, CAV1, CBFA2T2, CBFA2T3, CBX8, CCAR2, CCBE1, CCDC3, CCDC80, CCL16, CCN4, CCND2, CCNY, CCR2, CCR6, CCT2, CD247, CD33, CD46, CD47, CD53, CDC123, CDC20, CDC20B, CDC25B, CDC25C, CDH13, CDH4, CDK1, CDK19, CDK2, CDK5R1, CDKN1A, CDKN2A, CDT1, CELF4, CEP131, CEP290, CEP295, CEP43, CFB, CHAMP1, CHD5, CHEK1, CHEK2, CHMP2A, CHRNB2, CHTF8, CIB1, CIP2A, CIT, CITED2, CITED4, CIZ1, CLASP1, CLASP2, CLN6, CLTCL1, CLYBL, CNOT1, CNOT7, CNST, COBL, COL18A1, COL3A1, COMMD1, COP1, CORO1B, CORO2B, CPEB1, CPNE2, CPNE5, CPT1A, CRB2, CRBN, CREB3L1, CREB3L2, CREB5, CREM, CSNK1E, CSNK1G3, CSPG5, CSRNP2, CSRNP3, CTBP2, CTCF, CTNNA2, CTNNBIP1, CTSS, CTTN, CUL3, CUX1, CXCR4, CXXC5, CYCS, CYFIP1, CYFIP2, CYLD, CYP19A1, CYP1B1, CYP4A11, DAB1, DAG1, DAPK1, DAXX, DBN1, DCN, DCTN1, DCUN1D1, DDA1, DDAH2, DDHD2, DDIT4, DDR1, DDR2, DDX49, DDX60, DEF8, DENND1B, DGKQ, DGKZ, DHDDS, DHX15, DHX34, DHX36, DIPK2A, DLC1, DLG1, DLG4, DLL1, DLL4, DLX2, DNA2, DNAJC3, DNM1, DNM1L, DNMT3A, DNPEP, DOCK7, DOCK8, DOK4, DOK7, DOT1L, DPF1, DPP10, DSTYK, DTX1, DUOX1, DUSP19, DUSP26, E2F2, E2F8, EBF1, EBF3, EBF4, ECE1, EDAR, EDEM2, EEF1E1-BLOC1S5, EEPD1, EFNA5, EGR2, EHF, EHMT1, EIF2AK3, EIF2AK4, EIF4A3, EIF4E, EIF4EBP1, EIPR1, ELAVL1, ELF1, ELK3, ELMO1, EMC10, ENC1, ENHO, ENPP2, EPB41L4B, EPHA1, EPHA10, EPHA4, EPHA6, EPHB1, EPHB3, EPS8L1, ERAP1, ERBB4, ERC1, ERCC1, ERCC5, ERCC8, EREG, ESPN, ESR1, ESR2, ESRP2, ESRRG, ETAA1, ETFA, ETS1, ETV1, ETV6, EVL, EVX1, EXOC7, EXOSC6, EXPH5, EYA2, EYA4, EZH2, F11R, F12, F2RL3, F3, FABP5, FAF1, FAM110A, FAM161A, FAM89B, FANCM, FAP, FASN, FBLN5, FBN2, FBXW8, FCHSD1, FCHSD2, FER, FEZ1, FEZF1, FEZF2, FGD2, FGF12, FGF14, FGF2, FGF22, FGF6, FGF7, FH, FHOD1, FITM2, FLRT1, FLT4, FMN1, FNIP2, FNTA, FOXD3, FOXJ3, FOXK1, FOXO1, FOXP1, FRMD4A, FRMD5, FRMD8, FRS2, FTO, FUBP1, FZD10, FZD5, FZR1, GAB1, GAB2, GABPB1, GADD45G, GARS1, GATA5, GCG, GCNT2, GFI1, GFRAL, GJA5, GLI2, GLI3, GLIPR2, GLIS2, GLMN, GLP2R, GMFB, GML, GNAI2, GNAO1, GORASP1, GPER1, GPR137B, GPR17, GPR4, GPR55, GPR62, GPRC5B, GPRC5C, GPRIN1, GPSM1, GPT, GRAMD4, GRB10, GRB2, GREM1, GRID2, GRK5, GRM1, GRM4, GSK3A, GSX2, GTF2B, GTF2H1, GUCA2A, HACD3, HADHA, HAND1, HCAR2, HCLS1, HDAC4, HDAC9, HECW1, HEG1, HELT, HES1, HES3, HEXB, HGS, HINT1, HIP1, HIP1R, HIVEP1, HIVEP3, HLA-DPB1, HLA-DQA2, HLA-DRA, HMBOX1, HMGB1, HMGN3, HNF1B, HNRNPAB, HNRNPC, HNRNPD, HNRNPK, HNRNPR, HOOK1, HOXA1, HOXA3, HOXA4, HOXC10, HOXC11, HOXC4, HOXD13, HPS4, HRC, HRG, HSD17B12, HSD17B4, HSP90AB1, HSPD1, HSPH1, HTATIP2, HTR1A, HTR2A, HTR3A, HTT, ICAM3, IDE, IFI16, IFIH1, IFT74, IGF1, IGF1R, IGF2BP1, IGLL5, IGSF9B, IHH, IKBKB, IKZF1, IL10RA, IL10RB, IL12RB2, IL15RA, IL16, IL17D, IL17RA, IL1RAP, IL20RA, IL31RA, IL37, IL6R, IL6ST, ILDR1, ILF3, ING1, ING3, INPP5F, INSIG2, INSL5, INSR, IQGAP2, IQGAP3, IQSEC1, IRAK2, IRF2, IRF6, IRS2, ISL1, ITCH, ITGA1, ITGA5, ITGB2, ITK, ITPR3, JAK2, JAML, JARID2, KALRN, KANK1, KAT2B, KAT6A, KAT6B, KAT7, KCNB1, KCNC1, KCNE1, KCNH2, KCNJ2, KCNK2, KCNMA1, KCNQ1, KDM2B, KDM3A, KDM4C, KDM4D, KDM6B, KHDRBS1, KIDINS220, KIF26B, KIF2A, KIRREL1, KITLG, KLF14, KLF17, KLF7, KLK7, KMT2D, KMT2E, KMT5C, KNDC1, KPNA7, KRAS, KRT1, KSR1, KSR2, LAMA2, LAMB1, LARP4B, LARP7, LAT2, LATS2, LAX1, LDB2, LDLR, LEPR, LGMN, LGR6, LHX4, LHX6, LHX8, LIG4, LIMS1, LLGL1, LMCD1, LMO7, LMTK2, LMX1B, LONP1, LOXL2, LOXL3, LPAR1, LPCAT1, LPGAT1, LPL, LRCH4, LRIG2, LRP1, LRP2, LRP5, LRPAP1, LRRC32, LRRK1, LRSAM1, LTBP2, LURAP1, LY9, LYL1, LYPD6, LZTS1, MACC1, MACF1, MAF, MAFA, MAFG, MAG, MAGI2, MAK, MAL, MAML2, MAML3, MAP2, MAP2K2, MAP2K5, MAP3K11, MAP3K13, MAP3K19, MAP3K2, MAP3K20, MAP3K5, MAP3K6, MAP4K4, MAP4K5, MAP6, MAPK7, MAPK8, MAPK8IP2, MAPK8IP3, MAPK9, MAPKAP1, MAPKAPK5, MARS1, MAX, MCF2L, MCL1, MCRS1, MEAK7, MECOM, MED16, MED22, MED24, MED26, MED27, MEF2C, MEGF8, MEIOC, MEIS2, MELTF, MEN1, MEOX1, METRNL, MFGE8, MFNG, MGMT, MIB2, MICAL2, MIER1, MIPEP, MLLT3, MMD, MME, MOG, MPHOSPH8, MRGPRX2, MRPL23, MRTFA, MSANTD1, MSGN1, MSH2, MST1R, MTA1, MTA3, MTCH2, MTMR2, MTMR9, MTNR1B, MUC13, MUC4, MUC5B, MYD88, MYLK, MYO18A, MYO1C, MYO1E, MYRF, NACC2, |
| GO:0048523 | negative regulation of cellular process | 1,01E-05 [2, 3, 4] | 1095,00 | 21,17 | AGO2, AJAP1, AKT2, ALPK2, ALX1, AMBRA1, AMER2, AMH, ANGPT1, ANGPT4, ANK3, ANKRD13B, ANKRD17, ANKRD26, ANKRD33, ANKS1B, ANTXR1, ANXA2, ANXA6, AP2A1, APC, APC2, APOBEC1, APPL1, APPL2, ARAP1, ARF1, ARFGEF3, ARHGAP10, ARHGAP12, ARHGAP21, ARHGAP42, ARHGDIA, ARHGDIB, ARHGDIG, ARHGEF18, ARHGEF2, ARHGEF7, ARID2, ARL6IP5, ARNT2, ARNTL, ASAP1, ASCL1, ASPH, ASS1, ASTN2, ATF2, ATF3, ATG14, ATM, ATN1, ATP13A2, ATP1A3, ATP1A4, ATP2B4, ATP5F1A, ATP8A2, ATXN1, ATXN7, AURKB, BABAM2, BACH1, BAG6, BAZ2A, BBS4, BCAS3, BCL10, BCL11A, BCL11B, BCL2, BCL2A1, BCL3, BCL6, BCL7A, BDNF, BDNF-AS, BEND6, BHLHE40, BICC1, BIRC2, BMERB1, BMP2, BMP7, BMPER, BPI, BRINP2, BRMS1, BTBD10, BTC, BTG2, BTG4, BTRC, C1QC, C4orf3, CABP1, CACNA1A, CALM2, CAMK2D, CAMSAP1, CAPRIN2, CAPZB, CARD10, CARD14, CARMIL1, CARMIL2, CASP2, CAV1, CBFA2T2, CBFA2T3, CBX8, CC2D1B, CCAR2, CCDC3, CCDC88C, CCN4, CCN5, CCN6, CCND2, CCR2, CD200R1, CD33, CD46, CD47, CDC123, CDC20, CDC25C, CDH13, CDK1, CDK2, CDK5R1, CDKN1A, CDKN2A, CDT1, CDYL, CELF4, CEND1, CEP43, CFDP1, CHCHD3, CHD5, CHEK1, CHEK2, CHMP2A, CHST11, CIB1, CIC, CITED2, CLASP1, CLASP2, CLEC4G, CNOT1, CNOT7, CNST, CNTN4, COL18A1, COL2A1, COL3A1, COMMD1, CORO1B, CORO2B, CPEB1, CPEB4, CRB2, CRBN, CREB3L1, CREM, CRIM1, CRY2, CRYM, CSNK1A1L, CSRNP2, CSRNP3, CST9, CTBP2, CTCF, CTNNA2, CTNNBIP1, CTNS, CTTN, CUL3, CUX1, CXXC5, CYFIP1, CYLD, CYP19A1, CYP1B1, CYP51A1, CYREN, DAB1, DAG1, DAPK1, DAXX, DBNDD2, DCAF1, DCLK1, DCN, DCP2, DCPS, DDAH2, DDIT4, DDR1, DDX6, DENND5A, DEPDC1, DEPDC5, DGKQ, DGKZ, DHRS3, DHX34, DHX36, DIPK2A, DIS3L2, DKK3, DLC1, DLG1, DLG2, DLG4, DLL1, DLL4, DLX2, DNA2, DNAJB5, DNAJB6, DNAJC15, DNAJC3, DNM1, DNM1L, DNMT3A, DNPEP, DOCK8, DOT1L, DPF1, DSTYK, DTX1, DUS2, DUSP19, DUSP26, E2F2, E2F8, EFNA5, EHMT1, EHMT2, EIF2AK3, EIF2AK4, EIF4A3, EIF4E, EIF4EBP1, EIF4EBP2, EIF4ENIF1, ELAVL1, ELF1, ELK3, ENC1, ENHO, EPHA1, EPHA4, EPHB1, EPPK1, EPRS1, EPS15L1, ERBB4, ERC1, ERCC1, ERCC5, EREG, ESPN, ESR1, ESR2, ETAA1, ETS1, ETV6, EVL, EXOSC10, EYA2, EYA4, EZH2, F11R, F2RL3, FABP5, FAM172A, FAM220A, FAM3D, FAM89B, FAP, FARP1, FASN, FBLN5, FBN1, FBN2, FBP1, FBXO2, FBXO6, FER, FERD3L, FEZ1, FEZF1, FEZF2, FGF12, FGF2, FHOD3, FKTN, FLT4, FNIP2, FOXD3, FOXK1, FOXO1, FOXP1, FRMD4A, FRMD5, FRS2, FTO, FXYD5, FZD5, FZR1, GABRB2, GAK, GARS1, GAS7, GCG, GCNT2, GFI1, GFRAL, GLI2, GLI3, GLIS2, GLMN, GLO1, GMFB, GML, GNAI2, GNB5, GON4L, GORASP1, GPER1, GPR137B, GPR161, GPR55, GPRIN1, GRAMD4, GRB10, GRB2, GREM1, GRID2, GRID2IP, GRIK3, GRK5, GSK3A, H2AC4, H2AC6, H2AJ, H3C6, H3C8, HADHA, HAND1, HCLS1, HDAC4, HDAC9, HECW1, HEG1, HELT, HERC1, HES1, HES3, HES4, HGS, HHATL, HIP1R, HIVEP1, HMBOX1, HMGB1, HNF1B, HNRNPC, HNRNPD, HNRNPK, HNRNPR, HRC, HRG, HSP90AB1, HSPD1, HTATIP2, HTR2A, HTRA3, HTT, HUS1, IDE, IER3, IFI16, IFNLR1, IFRD1, IFT74, IGF1, IGF1R, IGF2BP1, IGHMBP2, IHH, IKBKB, IKZF1, IL10RA, IL17RD, IL31RA, IL37, IL6ST, ILF3, ING1, ING3, INKA2, INPP5F, INPPL1, INSIG2, INTS3, IPO7, IRF2, IRF2BP2, IRF6, IRS2, ISL1, ITCH, ITFG2, ITGA1, ITGA5, ITGB2, ITIH5, ITPR2, ITPR3, JAK2, JARID2, JAZF1, JPH3, KALRN, KANK1, KANK3, KAT2B, KAT6A, KAT6B, KAT7, KCNB1, KCNE1, KCNH2, KCNK2, KCTD10, KCTD6, KDM2A, KDM2B, KDM3A, KDM4B, KDM4C, KDM4D, KHDRBS1, KIF25, KIF26A, KIF2A, KIRREL1, KITLG, KLF7, KMT2D, KMT2E, KRAS, KREMEN2, KRIT1, L3MBTL3, LATS2, LAX1, LDB2, LDLR, LDLRAD4, LEPR, LGMN, LHX4, LIG4, LIMA1, LIMS1, LINGO1, LLGL1, LMCD1, LMTK2, LONP1, LOXL2, LOXL3, LPAR1, LPCAT1, LPL, LRIG2, LRIG3, LRP1, LRP2, LRP5, LRPAP1, LRRC14, LRRC32, LRRK1, LRSAM1, LTBP1, LTBP2, LZTS1, LZTS2, MAD1L1, MAF, MAFK, MAG, MAGI2, MAK, MAP2, MAP2K5, MAP3K20, MAP4, MAP4K4, MAP4K5, MAPK7, MAPK8, MAPK8IP2, MAPKAP1, MAPKAPK5, MARF1, MARK3, MAX, MBD3, MCL1, MCM2, MCPH1, MDC1, MEAK7, MECOM, MEF2C, MEGF8, MEIOC, MEIS2, MELTF, MEN1, MGMT, MGRN1, MIER1, MLLT3, MPHOSPH8, MRPL13, MRPL23, MRTFA, MSH2, MSH3, MSL3P1, MTA1, MTA3, MTMR2, MTMR9, MTNR1B, MTRNR2L4, MXD1, MYD88, MYL2, MYO16, MYO18A, MYO1C, MYO1D, MYO5A, MYT1L, N4BP1, NACC2, NAT10, NAV3, NCK2, NCKAP1L, NCOA2, NCOA7, NCOR2, NDRG3, NDRG4, NDUFA13, NDUFA2, NEDD4L, NEK11, NEPRO, NF1, NFATC1, NFATC2, NFATC4, NFE2L2, NFIB, NFIL3, NGEF, NGF, NKD1, NLK, NLRC5, NLRP12, NLRX1, NME2, NNT, NOC2L, NOL3, NOS1, NOTCH1, NOTCH3, NOTCH4, NPAT, NPC1, NPHP4, NPPA, NPR1, NPRL3, NR1D1, NR2C1, NR2E3, NR3C1, NRIP1, NRXN1, NSD1, NTRK2, NUMB, NXN, OLIG2, OPRPN, OSBPL8, OTUB1, OTUD4, OVGP1, P2RY6, PACSIN1, PACSIN2, PAF1, PAFAH1B1, PAFAH2, PAK4, PAK5, PALM, PAPLN, PARD3, PARP14, PASK, PAX2, PAX7, PAX8, PAX9, PBLD, PBX1, PCDHA6, PDCD5, PDCD6, PDCL3, PDE8A, PDGFRA, PDPK1, PDZD2, PDZD3, PEG10, PEG3, PELI3, PER2, PEX14, PFN2, PGP, PHACTR1, PHF19, PHF2, PHLPP2, PIH1D1, PIK3CG, PIK3IP1, PIK3R1, PIM1, PINX1, PKD1, PKIB, PKMYT1, PKP1, PLA2G2D, PLA2G6, PLAC8, PLAUR, PLCB1, PLCB2, PLCB4, PLCD4, PLCE1, PLCG2, PLCH2, PLCL2, PLEK, PLEKHN1, PLIN5, PLXNA1, PLXNA2, PLXNB1, PLXNC1, PLXND1, PML, POLQ, POU2F1, POU4F2, PPARG, PPARGC1A, PPARGC1B, PPFIA1, PPM1F, PPP1R11, PPP1R13B, PPP1R16B, PPP3CA, PPP3CB, PRAG1, PRDM16, PRDM2, PRDM5, PRDM6, PRDX5, PRKAG2, PRKAR1B, PRKCA, PRKCB, PRKCE, PRKCH, PRKCI, PRKCQ, PRKCZ, PRKD2, PRKDC, PRKN, PRLR, PRMT1, PRPF19, PSCA, PSEN1, PSMA5, PSMA6, PSMA7, PSMC2, PSMC3, PSMC6, PSMD2, PSMD7, PSME3IP1, PTCH2, PTEN, PTGIS, PTK2, PTPN11, PTPN14, PTPN3, PTPRA, PTPRC, PTPRH, |

| | | | | | |
|---|---|---|---|---|---|
| GO:0044085 | cellular component biogenesis | 6,71E-03 [3] | 716,00 | 21,33 | ALS2, AMBRA1, AMH, ANGPT1, ANGPT4, ANK3, ANKRD28, ANKS1B, ANO6, ANTXR1, ANXA2, AP2S1, APC, APOBEC1, APOC4, APPL2, ARAP1, ARF1, ARHGAP40, ARHGEF10L, ARHGEF18, ARHGEF2, ARHGEF26, ARHGEF7, ARL13B, ARPC4, ASAP1, ATAT1, ATF1, ATG14, ATG16L1, ATG2A, ATG2B, ATM, ATP13A2, ATP6V0D1, ATXN10, AURKB, B9D1, BAG6, BAIAP2L1, BAZ2A, BBS10, BBS4, BBS7, BBS9, BCAS3, BCL11B, BCL2, BCL9, BDNF, BDNF-AS, BIN3, BIRC2, BMP1, BMP7, BRD2, BRF1, BSN, C10orf90, CACNA1A, CADPS2, CAMSAP1, CAND1, CAPZB, CARD10, CARMIL1, CARMIL2, CAV1, CC2D2A, CCDC113, CCDC13, CCDC28B, CCT2, CD47, CDC123, CDC20, CDH10, CDH13, CDH18, CDH19, CDH23, CDH26, CDH4, CDK1, CDK2, CDK5R1, CDKN2A, CDT1, CELF2, CELF4, CELF5, CENPP, CEP131, CEP164, CEP290, CEP295, CEP43, CEP70, CETN1, CFAP46, CFAP54, CFAP74, CHAF1A, CHCHD4, CHEK2, CHMP2A, CHRNB2, CILK1, CLASP1, CLASP2, CLDN16, CLDN8, CLP1, CLYBL, CNIH3, CNOT1, CNOT7, CNTN5, CNTROB, COA1, COBL, CORO1B, CORO2B, CORO7-PAM16, COX20, CPLANE1, CRBN, CSNK1E, CTCF, CTNNA2, CTNNBIP1, CTNND2, CTTN, CUL3, CYFIP1, CYFIP2, CYLD, DAG1, DARS1, DAXX, DBN1, DCAF13, DCTN1, DDX10, DDX31, DDX49, DDX6, DEF8, DGKQ, DHX30, DIAPH3, DLC1, DLG1, DLG4, DMC1, DNA2, DNAH17, DNAH5, DNAH8, DNAJB12, DNAJB13, DNAJB6, DNAJC15, DNER, DNM1L, DNMT3A, DST, DYNC2LI1, ECSIT, EFNA5, EHMT2, EIF2S2, EIF3B, EIF4A3, EIF4B, EIF4EBP1, ELAVL1, ELN, EPHA1, EPHB1, EPHB3, EPPK1, EPRS1, EPS8L1, ERBB4, ERCC1, ERCC5, ESPN, ESPNL, ESR1, EVL, EXOSC10, EXOSC6, F11R, F5, FAF1, FAM161A, FAM171A1, FAM172A, FARP1, FARP2, FASN, FBLN5, FCHSD1, FCHSD2, FER, FERMT2, FEZ1, FGD2, FHOD1, FHOD3, FITM2, FLRT1, FMN1, FNIP2, FXYD5, FZD5, FZR1, GABRA2, GABRB2, GAK, GARS1, GEMIN2, GJA5, GLRX3, GMFB, GORASP1, GPC6, GPRIN1, GRB2, GREM1, GRID2, GRWD1, GTF2B, GTF2H1, GTF3A, H2BC15, H3C6, H3C8, HAPLN2, HCLS1, HCN1, HDAC4, HDAC9, HEG1, HES1, HGS, HIP1, HIP1R, HLA-DRA, HMGB1, HNF1B, HNRNPC, HOOK1, HPS4, HRG, HSP90AB1, HSPD1, HTT, HYDIN, IBA57, IFT140, IFT46, IFT74, IFT88, IHH, IKBKB, IL1RAP, IMMP2L, INPP5F, INPPL1, IQGAP2, ISL1, ITGA5, ITGB4, JAK2, JHY, KANK1, KANK3, KAT2B, KAT6A, KAT6B, KCNB1, KCNB2, KCNC1, KCNC3, KCNF1, KCNJ2, KCNV1, KCTD10, KCTD14, KCTD16, KCTD2, KCTD5, KCTD6, KHDC4, KIF25, KIF2A, KIFC1, KIRREL1, KMT2D, KRT1, LAMA5, LAMB1, LAMB2, LAMB4, LDB2, LDB3, LDLRAD4, LIMA1, LIMS1, LIN9, LLGL1, LONP1, LPAR1, LRCH4, LRFN3, LRP1, LRSAM1, LSM2, LUC7L2, LUC7L3, LYRM4, LZTS1, LZTS2, M1AP, MACF1, MAGI2, MAK, MAP1LC3A, MAP2, MAP4, MAP4K4, MAPK8IP2, MAPK9, MARCKS, MAX, MBD3, MCM2, MCMDC2, MDGA1, MEF2C, MEIOC, MEN1, MIPEP, MKS1, MLH3, MPHOSPH6, MPHOSPH8, MRPS2, MTMR2, MTMR3, MYH3, MYL2, MYLK, MYO1A, MYO1C, MYO1E, MYOM2, MYPN, NAA60, NACC2, NAPB, NAT10, NAV3, NCK2, NCKAP1, NCKAP1L, NDE1, NDUFA13, NDUFA2, NDUFAF3, NDUFB3, NDUFB6, NDUFB7, NDUFC1, NDUFC2, NECTIN1, NEDD1, NEDD9, NEK7, NFASC, NIFK, NINL, NLGN2, NOC2L, NOL3, NOM1, NOP2, NOTCH1, NPC1, NPHP4, NPHS1, NPRL3, NPTN, NRXN1, NRXN3, NSG1, NTNG2, NTRK2, NUP205, OBSCN, OBSL1, OTUD6B, PACS2, PACSIN2, PAF1, PAFAH1B1, PALM, PARD3, PATJ, PATL1, PCDHB9, PCLO, PCNT, PDCD6, PDGFRA, PDLIM1, PDPK1, PEAK1, PES1, PEX14, PFDN6, PFKM, PFKP, PFN2, PHACTR1, PHF2, PHLDB1, PIEZO1, PIH1D1, PIH1D2, PIK3R1, PIKFYVE, PIP4K2A, PIP5K1C, PKD1, PKP1, PLA2G6, PLCE1, PLD1, PLEK, PLEKHA7, PLEKHG2, PLK4, PLXNB1, PLXND1, PML, POLQ, PPARGC1A, PPFIA1, PPM1F, PPP1R16B, PPP3CA, PRKCA, PRKCE, PRKCH, PRKCI, PRKCZ, PRKD2, PRKDC, PRKN, PRLR, PRMT1, PRMT8, PRPF19, PRPF8, PRUNE1, PSEN1, PSIP1, PSMC2, PSMC3, PSMC6, PSMG4, PTEN, PTK2, PTPN11, PTPRO, PXDN, RAB29, RAB35, RAB5A, RACK1, RAD51, RANBP9, RAP1A, RAP1GAP, RAPGEF1, RASA1, RASIP1, RB1, RBFA, RBIS, RBX1, RC3H1, RCL1, REST, RFX2, RHOA, RHOG, RHOU, |
| GO:0051649 | establishment of localization in cell | 2,58E-02 [3] | 634,00 | 21,36 | ANO1, ANO6, ANXA2, AP1G2, AP1S1, AP2A1, AP2A2, AP2S1, AP3B1, AP3M2, AP3S2, AP5Z1, APBA2, APPL1, APPL2, APRT, ARF1, ARF5, ARFGAP1, ARHGAP21, ARHGAP45, ARHGEF2, ARL1, ARL8B, ARNTL, ASPSCR1, ATG14, ATM, ATP11A, ATP13A2, ATP1A3, ATP1A4, ATP2A2, ATP2A3, ATP2B4, ATP5F1A, ATP5ME, ATP6V0A1, ATP8A1, ATXN1, AURKB, BBS4, BBS7, BCAS3, BCL2, BLOC1S2, BLOC1S5, BPI, BRSK2, BSN, BTBD9, C12orf4, CABP1, CACNA1A, CACNA1B, CACNB2, CADPS, CADPS2, CALM2, CAMK2D, CAND1, CAP1, CAPZB, CAV1, CCDC88C, CCDC93, CCR2, CCT2, CD33, CD47, CD53, CDC40, CDK1, CDK5R1, CDKN1A, CDT1, CEACAM21, CEP131, CEP290, CHAMP1, CHCHD4, CHID1, CHIT1, CHMP2A, CHRNB2, CHRNB3, CIB1, CILK1, CLASP1, CLASP2, CLTCL1, CMTM6, CNIH3, COG2, COMMD9, CORO7-PAM16, CPT1A, CPT1B, CREB3L2, CRISPLD2, CRY2, CSPG5, CTAGE1, CTBP2, CTSS, CTTN, CUL3, CYB5R1, CYC1, CYFIP1, DCLK1, DCTN1, DDHD2, DDX19A, DENND1A, DENND1B, DENND2A, DENND3, DENND5A, DESI2, DGKQ, DLC1, DLG1, DLG2, DNAJC15, DNAJC3, DNAJC30, DNAJC6, DNM1, DNM1L, DOC2A, DOCK7, DOK3, DPP7, DSN1, DST, DYNC2LI1, ECI2, EDEM2, EEF1A1, EFNA5, EGR2, EIF4A3, EIF4E, EIF4ENIF1, EIPR1, ELAVL1, EPS15L1, ERC1, ERGIC3, ERP44, EXOC4, EXPH5, F13A1, F2RL3, F3, F5, FABP5, FAM110A, FAM155A, FAM53A, FER, FEZ1, FGF14, FGF2, FHOD1, FZD5, FZR1, GAA, GAB2, GAD1, GAD2, GAK, GCG, GEMIN2, GJA5, GLE1, GLI3, GORASP1, GPER1, GPRIN1, GRM4, GRPEL1, GSG1L, GSK3A, HACL1, HAO2, HCLS1, HERC2, HEXB, HGS, HM13, HMGB1, HMGN3, HOOK1, HPS4, HRC, HRG, HSD17B4, HSP90AB1, HSPD1, HTATIP2, HTR2A, HTT, ICMT, IDE, IFT140, IFT46, IFT74, IFT88, IGF1, IMMP2L, ING1, INPP5F, IPO11, IPO7, IPO8, IQGAP2, ITGAL, ITGB2, ITPR2, ITPR3, IWS1, JAK2, JPH3, KCMF1, KCNAB2, KCNB1, KCNE1, KCNH2, KCNJ2, KCNJ3, KCNQ1, KHDRBS1, KIF13A, KIF16B, KIF18A, KIF1A, KIF1C, KIF22, KIF25, KIF2C, KIF5A, KIFC1, KLF7, KPNA7, KRT1, LAT2, LDLR, LIN7B, LLGL1, LMTK2, LPCAT1, LRP1, LRP5, LTBP2, LZTS1, LZTS2, MAD1L1, MAGI2, MAK, MAN2B1, MAP2, MAP2K2, MAP4, MAP4K5, MAP6, MAPK8, MAPK8IP3, MCEMP1, MCPH1, MEF2C, MEIOC, MGRN1, MICALL1, MIPEP, MME, MON2, MRGPRX2, MRPL13, MRPL23, MTMR2, MX1, MYO15A, MYO1A, MYO1C, MYO1D, MYO1E, MYO5A, MYO5C, NAPB, NCKAP1L, NCOA2, NCSTN, NDE1, NDRG4, NDUFA13, NDUFA2, NDUFC2, NEU1, NF1, NFAM1, NFASC, NHLRC2, NLGN2, NME2, NOL3, NOS1, NOS1AP, NPC1, NPPA, NR1D1, NRXN1, NRXN3, NSG1, NUMB, NUP205, NUP43, NUP50, NUP58, NUS1, OSBP, P2RY6, PACSIN1, PAFAH1B1, PAOX, PARD3, PCDH7, PCLO, PCNT, PCYOX1L, PDCD5, PDCD6, PDPK1, PER2, PEX13, PEX14, PEX5L, PFN2, PHAX, PI4K2A, PIK3CG, PIK3R1, PIKFYVE, PINX1, PIP5K1C, PKD1, PKM, PKP1, PLA2G6, PLAC8, PLAUR, PLCB1, PLCB2, PLCB4, PLCD4, PLCE1, PLCG2, PLCH2, PLCL2, PLD1, PLEK, PML, POLDIP3, PPFIA1, PPFIA3, PPFIA4, PPP1R13B, PPP3CA, PPP3CB, PRDX5, PRKAG2, PRKAR1B, PRKCA, PRKCB, PRKCE, PRKCI, PRKCZ, PRKD2, PRKN, PROZ, PSEN1, PSIP1, PSMA5, PSMC2, PSMC3, PSMD2, PSMD7, PTEN, PTGES2, PTK2, PTPN11, PTPN14, PTPRC, PTPRN, PTPRN2, PXDN, RAB22A, RAB29, RAB2A, RAB35, RAB3D, RAB40B, RAB43, RAB4A, RAB5A, RABGAP1, RABGEF1, RANBP1, RAP1A, RAP2B, RASA3, RBM15B, RBM26, RHOA, RHOBTB3, RHOG, RHOU, RILPL1, RIMS3, RIPOR1, RITA1, RNASET2, RPH3A, RPH3AL, RPL10A, RPL14, RPL3, RPL5, RRS1, RUFY3, RYR1, RYR2, RYR3, SAMM50, SCG5, SCN5A, SCRIB, SCYL2, SDCBP, SDCBP2, SEC24B, SEC24C, SEL1L, SELENOS, SEPTIN5, SEPTIN9, SERPINA4, SERPINB6, SGPP1, SGPP2, SGSM1, SGSM2, SH2D3C, SHH, SIRT4, |
| GO:0006996 | organelle organization | 1,01E-04 [4] | 893,00 | 21,39 | ALS2, AMBRA1, ANAPC11, ANAPC4, ANK3, ANKRD28, ANKRD31, ANTXR1, ANXA2, AP3B1, APC, APC2, APOBEC1, ARAP1, ARAP3, ARF1, ARFGEF3, ARHGAP10, ARHGAP12, ARHGAP17, ARHGAP21, ARHGAP40, ARHGDIA, ARHGDIB, ARHGEF10L, ARHGEF18, ARHGEF2, ARHGEF7, ARID2, ARID4B, ARL1, ARL13B, ARPC4, ASAP1, ASZ1, ATAT1, ATF2, ATG14, ATG16L1, ATG2A, ATG2B, ATM, ATP13A2, ATP2A2, ATP5F1A, ATP5ME, ATP6V0A1, ATP6V0D1, ATP6V1A, ATP6V1H, ATP8A2, ATP8B2, ATXN10, ATXN7, ATXN7L3, AURKB, B9D1, BABAM2, BAG6, BAIAP2L1, BANP, BAZ1B, BAZ2A, BBS10, BBS4, BBS7, BBS9, BCAR1, BCAS3, BCL11B, BCL2, BCL6, BFSP1, BFSP2, BIN3, BLOC1S2, BLOC1S5, BMERB1, BMP7, BRD1, BRD2, BRD4, BRMS1, BRSK2, BTC, C10orf90, C11orf80, CALM2, CAMK2D, CAMKMT, CAMSAP1, CAP1, CAP2, CAPZB, CARMIL1, CARMIL2, CAV1, CBX8, CC2D2A, CCAR2, CCDC113, CCDC13, CCDC28B, CCDC88C, CCNA1, CCT2, CD47, CDC20, CDC25B, CDC25C, CDC42BPB, CDK1, CDK2, CDK5R1, CDKN2A, CDT1, CELSR1, CENPP, CEP131, CEP164, CEP290, CEP295, CEP43, CEP70, CETN1, CFAP46, CFAP54, CFAP61, CFAP74, CHAF1A, CHAMP1, CHCHD3, CHCHD4, CHD3, CHD5, CHEK1, CHEK2, CHMP2A, CHTF8, CIB1, CILK1, CIT, CLASP1, CLASP2, CLCN3, CLIP2, CLN6, CLVS1, CNIH3, CNOT1, CNOT7, CNTROB, COA1, COBL, COG2, CORO1B, CORO2B, CORO7-PAM16, COX20, CPLANE1, CSNK1E, CSPG5, CTCF, CTNNA2, CTTN, CUL3, CYCS, CYFIP1, CYLD, DAG1, DAXX, DBN1, DCAF1, DCLRE1C, DCN, DCP2, DCTN1, DDHD2, DDX6, DHDDS, DHX30, DHX36, DIAPH3, DIS3L2, DLC1, DLG1, DLG2, DLG4, DMC1, DNA2, DNAH17, DNAH5, DNAH8, DNAJB13, DNAJB6, DNAJC15, DNM1, DNM1L, DNMT3A, DOCK7, DOT1L, DPF1, DSN1, DST, DYNC2LI1, ECI2, ECSIT, EFCAB11, EFNA5, EHBP1L1, EHMT1, EHMT2, EIF2AK3, ELMO1, ELN, EML3, EPB41L4A, EPB41L4B, EPHA1, EPPK1, ERBB4, ERCC1, ERCC8, EREG, ESCO1, ESPN, ESPNL, ESR1, ETS1, EVL, EVPLL, EXOSC10, EYA2, EYA4, EZH2, F11R, F2RL3, F3, F5, FAM161A, FAM171A1, FAM172A, FANCM, FARP1, FARP2, FAT1, FBXW8, FCHSD1, FCHSD2, FER, FEZ1, FEZF2, FGD2, FGF7, FHOD1, FHOD3, FITM2, FMN1, FMNL2, FNIP2, FRMD5, FRMD6, FZD10, FZD5, FZR1, GAA, GAB1, GABPB1, GAK, GCG, GET4, GFI1, GINS4, GMFB, GOLGA5, GORASP1, GPER1, GRB2, GRID2, GRPEL1, GRWD1, GSK3A, GTF2B, GTF2H1, H2AC4, H2AC6, H2AJ, H2BC15, H3C6, H3C8, HACL1, HAO2, HCLS1, HDAC4, HDAC9, HEPACAM2, HEXB, HGS, HIP1, HIP1R, HMBOX1, HMGB1, HMGN3, HNRNPC, HNRNPD, HOOK1, HPS4, HRG, HSD17B4, HSP90AB1, HSPD1, HTT, HUS1, HYDIN, IDE, IFT140, IFT46, IFT74, IFT88, IGF1, IGHMBP2, IKBKB, IKZF1, IL1RAP, IMMP2L, IMMT, ING1, ING3, INPPL1, INSR, IQGAP2, IQGAP3, IQSEC1, IQSEC3, ISL1, ITPA, IWS1, JAK2, JARID2, JHY, JMJD1C, KALRN, KANK1, KANK3, KAT2B, KAT6A, KAT6B, KAT7, KATNAL2, KDM2A, KDM2B, KDM3A, KDM4B, KDM4C, KDM4D, KDM6B, KIAA1614, KIF13A, KIF18A, KIF22, KIF25, KIF2A, KIF2C, KIFC1, KIRREL1, KIZ, KLHDC8B, KLHL1, KLHL17, KMT2D, KMT2E, KMT5C, KPNA7, KRAS, KRT20, KRT25, KRT84, L3MBTL3, LAMA5, LDB2, LDB3, LIG4, LIMA1, LIMK2, LLGL1, LONP1, LOXL2, LPAR1, LRIG3, LRP1, LRP5, LRSAM1, LURAP1, LZTS1, LZTS2, M1AP, MACF1, MAD1L1, MAK, MAP1LC3A, MAP2, MAP2K2, MAP3K20, MAP4, MAP6, MAP7, MAPK8, MAPK9, MAPKAP1, MAPKAPK5, MARCKS, MARF1, MARK3, MAST4, MBD3, MCM2, MCMDC2, MCPH1, MCRS1, MECOM, MED22, MED24, MEF2C, MEIKIN, MEIOC, MEN1, MFN1, MICAL2, MICAL3, MICALL1, MIER1, MIGA1, MIPEP, MKS1, MLH3, MLLT3, MOV10L1, MPHOSPH8, MPRIP, MRPS2, MRRF, MRTFA, MSH2, MSH3, MSL3P1, MTA1, MTA3, MTERF1, MTFR1, MTFR2, MTMR3, MTX2, MX1, MYH3, MYL2, MYO15A, MYO18A, MYO1A, MYO1C, MYO1D, MYO1E, MYO5A, MYO5C, MYOM2, MYPN, NAA60, NAT10, NAV1, NAV3, NCK2, NCKAP1, NCKAP1L, NCKAP5, NDE1, NDUFA13, NDUFA2, NDUFAF3, NDUFB3, NDUFB6, NDUFB7, NDUFC1, NDUFC2, NEDD1, NEDD9, NEK11, NEK7, NF1, NINL, NLGN2, NOC2L, NOL3, NOP2, NOS1, NOS1AP, NOTCH1, NPC1, NPHP4, NPHS1, NPRL3, NR3C1, NRF1, NRXN1, NSD1, NSD3, NTNG2, NUP205, OBSCN, OBSL1, OPA3, OTUB1, PABPC1L, PACS2, PACSIN1, PACSIN2, PAF1, PAFAH1B1, PAGR1, PAK4, PAK5, PALLD, PALM, PAOX, PARD3, PARG, PATL1, PAX7, PCGF5, PCLO, PCNT, PDCD5, PDCD6, PDCL3, PDGFRA, PDLIM1, PDLIM3, PDPK1, PDZD2, PER2, PES1, PEX13, PEX14, PEX5L, PFN2, PHACTR1, PHF19, PHF2, PHF20, PHLDB1, PI4K2A, PIH1D1, PIK3R1, PIKFYVE, PINX1, PIP4K2A, PIP5K1C, PKD1, PKIB, PKMYT1, PKP1, PLA2G6, PLAUR, PLCB1, PLCE1, PLEK, PLEKHG2, PLEKHM2, PLIN5, PLK4, PLXNB1, PML, PNPLA2, POLD3, POLE, POLQ, POLRMT, PPARGC1A, PPARGC1B, PPFIA1, PPL, PPM1F, PPP1R13B, PPP2R3C, PRDM16, PRDM5, PRDM6, PRIM1, PRIM2, PRKCA, PRKCB, PRKCE, PRKCI, PRKCQ, PRKCZ, PRKD2, PRKDC, PRKN, PRMT1, PRMT8, PRUNE1, PSEN1, PTEN, PTGES2, PTK2, PTPRA, PTPRN, PXDN, PXDNL, RAB22A, RAB29, RAB2A, RAB35, RAB43, RAB5A, RAD51, RANBP1, RANBP9, RASA1, RASSF1, RASSF8, RB1, RBX1, RC3H1, RCBTB1, RECQL4, RELN, REST, RETREG1, RFLNA, RFLNB, RFX2, RHOA, RHOBTB3, RHOG, RHOU, |
| GO:0006996 | organelle organization | 1,01E-04 [4] | 893,00 | 21,39 | ALS2, AMBRA1, ANAPC11, ANAPC4, ANK3, ANKRD28, ANKRD31, ANTXR1, ANXA2, AP3B1, APC, APC2, APOBEC1, ARAP1, ARAP3, ARF1, ARFGEF3, ARHGAP10, ARHGAP12, ARHGAP17, ARHGAP21, ARHGAP40, ARHGDIA, ARHGDIB, ARHGEF10L, ARHGEF18, ARHGEF2, ARHGEF7, ARID2, ARID4B, ARL1, ARL13B, ARPC4, ASAP1, ASZ1, ATAT1, ATF2, ATG14, ATG16L1, ATG2A, ATG2B, ATM, ATP13A2, ATP2A2, ATP5F1A, ATP5ME, ATP6V0A1, ATP6V0D1, ATP6V1A, ATP6V1H, ATP8A2, ATP8B2, ATXN10, ATXN7, ATXN7L3, AURKB, B9D1, BABAM2, BAG6, BAIAP2L1, BANP, BAZ1B, BAZ2A, BBS10, BBS4, BBS7, BBS9, BCAR1, BCAS3, BCL11B, BCL2, BCL6, BFSP1, BFSP2, BIN3, BLOC1S2, BLOC1S5, BMERB1, BMP7, BRD1, BRD2, BRD4, BRMS1, BRSK2, BTC, C10orf90, C11orf80, CALM2, CAMK2D, CAMKMT, CAMSAP1, CAP1, CAP2, CAPZB, CARMIL1, CARMIL2, CAV1, CBX8, CC2D2A, CCAR2, CCDC113, CCDC13, CCDC28B, CCDC88C, CCNA1, CCT2, CD47, CDC20, CDC25B, CDC25C, CDC42BPB, CDK1, CDK2, CDK5R1, CDKN2A, CDT1, CELSR1, CENPP, CEP131, CEP164, CEP290, CEP295, CEP43, CEP70, CETN1, CFAP46, CFAP54, CFAP61, CFAP74, CHAF1A, CHAMP1, CHCHD3, CHCHD4, CHD3, CHD5, CHEK1, CHEK2, CHMP2A, CHTF8, CIB1, CILK1, CIT, CLASP1, CLASP2, CLCN3, CLIP2, CLN6, CLVS1, CNIH3, CNOT1, CNOT7, CNTROB, COA1, COBL, COG2, CORO1B, CORO2B, CORO7-PAM16, COX20, CPLANE1, CSNK1E, CSPG5, CTCF, CTNNA2, CTTN, CUL3, CYCS, CYFIP1, CYLD, DAG1, DAXX, DBN1, DCAF1, DCLRE1C, DCN, DCP2, DCTN1, DDHD2, DDX6, DHDDS, DHX30, DHX36, DIAPH3, DIS3L2, DLC1, DLG1, DLG2, DLG4, DMC1, DNA2, DNAH17, DNAH5, DNAH8, DNAJB13, DNAJB6, DNAJC15, DNM1, DNM1L, DNMT3A, DOCK7, DOT1L, DPF1, DSN1, DST, DYNC2LI1, ECI2, ECSIT, EFCAB11, EFNA5, EHBP1L1, EHMT1, EHMT2, EIF2AK3, ELMO1, ELN, EML3, EPB41L4A, EPB41L4B, EPHA1, EPPK1, ERBB4, ERCC1, ERCC8, EREG, ESCO1, ESPN, ESPNL, ESR1, ETS1, EVL, EVPLL, EXOSC10, EYA2, EYA4, EZH2, F11R, F2RL3, F3, F5, FAM161A, FAM171A1, FAM172A, FANCM, FARP1, FARP2, FAT1, FBXW8, FCHSD1, FCHSD2, FER, FEZ1, FEZF2, FGD2, FGF7, FHOD1, FHOD3, FITM2, FMN1, FMNL2, FNIP2, FRMD5, FRMD6, FZD10, FZD5, FZR1, GAA, GAB1, GABPB1, GAK, GCG, GET4, GFI1, GINS4, GMFB, GOLGA5, GORASP1, GPER1, GRB2, GRID2, GRPEL1, GRWD1, GSK3A, GTF2B, GTF2H1, H2AC4, H2AC6, H2AJ, H2BC15, H3C6, H3C8, HACL1, HAO2, HCLS1, HDAC4, HDAC9, HEPACAM2, HEXB, HGS, HIP1, HIP1R, HMBOX1, HMGB1, HMGN3, HNRNPC, HNRNPD, HOOK1, HPS4, HRG, HSD17B4, HSP90AB1, HSPD1, HTT, HUS1, HYDIN, IDE, IFT140, IFT46, IFT74, IFT88, IGF1, IGHMBP2, IKBKB, IKZF1, IL1RAP, IMMP2L, IMMT, ING1, ING3, INPPL1, INSR, IQGAP2, IQGAP3, IQSEC1, IQSEC3, ISL1, ITPA, IWS1, JAK2, JARID2, JHY, JMJD1C, KALRN, KANK1, KANK3, KAT2B, KAT6A, KAT6B, KAT7, KATNAL2, KDM2A, KDM2B, KDM3A, KDM4B, KDM4C, KDM4D, KDM6B, KIAA1614, KIF13A, KIF18A, KIF22, KIF25, KIF2A, KIF2C, KIFC1, KIRREL1, KIZ, KLHDC8B, KLHL1, KLHL17, KMT2D, KMT2E, KMT5C, KPNA7, KRAS, KRT20, KRT25, KRT84, L3MBTL3, LAMA5, LDB2, LDB3, LIG4, LIMA1, LIMK2, LLGL1, LONP1, LOXL2, LPAR1, LRIG3, LRP1, LRP5, LRSAM1, LURAP1, LZTS1, LZTS2, M1AP, MACF1, MAD1L1, MAK, MAP1LC3A, MAP2, MAP2K2, MAP3K20, MAP4, MAP6, MAP7, MAPK8, MAPK9, MAPKAP1, MAPKAPK5, MARCKS, MARF1, MARK3, MAST4, MBD3, MCM2, MCMDC2, MCPH1, MCRS1, MECOM, MED22, MED24, MEF2C, MEIKIN, MEIOC, MEN1, MFN1, MICAL2, MICAL3, MICALL1, MIER1, MIGA1, MIPEP, MKS1, MLH3, MLLT3, MOV10L1, MPHOSPH8, MPRIP, MRPS2, MRRF, MRTFA, MSH2, MSH3, MSL3P1, MTA1, MTA3, MTERF1, MTFR1, MTFR2, MTMR3, MTX2, MX1, MYH3, MYL2, MYO15A, MYO18A, MYO1A, MYO1C, MYO1D, MYO1E, MYO5A, MYO5C, MYOM2, MYPN, NAA60, NAT10, NAV1, NAV3, NCK2, NCKAP1, NCKAP1L, NCKAP5, NDE1, NDUFA13, NDUFA2, NDUFAF3, NDUFB3, NDUFB6, NDUFB7, NDUFC1, NDUFC2, NEDD1, NEDD9, NEK11, NEK7, NF1, NINL, NLGN2, NOC2L, NOL3, NOP2, NOS1, NOS1AP, NOTCH1, NPC1, NPHP4, NPHS1, NPRL3, NR3C1, NRF1, NRXN1, NSD1, NSD3, NTNG2, NUP205, OBSCN, OBSL1, OPA3, OTUB1, PABPC1L, PACS2, PACSIN1, PACSIN2, PAF1, PAFAH1B1, PAGR1, PAK4, PAK5, PALLD, PALM, PAOX, PARD3, PARG, PATL1, PAX7, PCGF5, PCLO, PCNT, PDCD5, PDCD6, PDCL3, PDGFRA, PDLIM1, PDLIM3, PDPK1, PDZD2, PER2, PES1, PEX13, PEX14, PEX5L, PFN2, PHACTR1, PHF19, PHF2, PHF20, PHLDB1, PI4K2A, PIH1D1, PIK3R1, PIKFYVE, PINX1, PIP4K2A, PIP5K1C, PKD1, PKIB, PKMYT1, PKP1, PLA2G6, PLAUR, PLCB1, PLCE1, PLEK, PLEKHG2, PLEKHM2, PLIN5, PLK4, PLXNB1, PML, PNPLA2, POLD3, POLE, POLQ, POLRMT, PPARGC1A, PPARGC1B, PPFIA1, PPL, PPM1F, PPP1R13B, PPP2R3C, PRDM16, PRDM5, PRDM6, PRIM1, PRIM2, PRKCA, PRKCB, PRKCE, PRKCI, PRKCQ, PRKCZ, PRKD2, PRKDC, PRKN, PRMT1, PRMT8, PRUNE1, PSEN1, PTEN, PTGES2, PTK2, PTPRA, PTPRN, PXDN, PXDNL, RAB22A, RAB29, RAB2A, RAB35, RAB43, RAB5A, RAD51, RANBP1, RANBP9, RASA1, RASSF1, RASSF8, RB1, RBX1, RC3H1, RCBTB1, RECQL4, RELN, REST, RETREG1, RFLNA, RFLNB, RFX2, RHOA, RHOBTB3, RHOG, RHOU, |

| | | | | | | |
|---|---|---|---|---|---|---|
| GO:0032268 | regulation of cellular protein metabolic process | 3,52E-02 | [4, 5, 6] | 605,00 | 21,42 | ANGPT4, ANKS1B, ANTXR1, ANXA2, APC, ARHGEF2, ARHGEF7, ARIH1, ARL6IP5, ARNTL, ASPH, ASPSCR1, ATF3, ATG14, ATM, ATP13A2, ATP2A3, ATP2B4, BAG6, BBS7, BCAR3, BCL10, BCL2, BCL3, BCL6, BDNF, BDNF-AS, BIRC2, BMP2, BMP3, BMP7, BMP8B, BMPER, BRD4, BRMS1, BRSK2, BTC, BTG2, BTRC, CALM2, CAMK2D, CAMTA1, CAPRIN2, CARD10, CARD14, CASR, CAV1, CBFA2T3, CCAR2, CCBE1, CCDC88C, CCL16, CCNA1, CCND2, CCNL1, CCNY, CD33, CDC123, CDC20, CDC20B, CDC25B, CDC25C, CDK1, CDK5R1, CDKN1A, CDKN2A, CDYL, CELF4, CEP295, CEP43, CHAMP1, CHEK1, CHEK2, CIB1, CLN6, CNOT1, CNOT7, COMMD1, COP1, CPEB1, CPEB4, CRB2, CRIM1, CRY2, CSNK1E, CST9, CTCF, CUL3, CXCR4, CYCS, CYFIP1, CYFIP2, CYLD, DAB1, DAG1, DAPK1, DAXX, DBNDD2, DCUN1D1, DDA1, DDIT4, DDR2, DESI2, DGKQ, DHX36, DIPK2A, DLC1, DLG1, DLG4, DNAJB6, DNAJC3, DNM1L, DNPEP, DOCK7, DOK4, DOK7, DSTYK, DUS2, DUSP19, DUSP26, EDAR, EDEM2, EEF1A1, EEFSEC, EFNA5, EHMT2, EIF2AK3, EIF2AK4, EIF2S2, EIF3B, EIF4A3, EIF4B, EIF4E, EIF4E1B, EIF4EBP1, EIF4EBP2, EIF4ENIF1, ELAVL1, ELOA, EMC10, ENC1, ENPP2, EPHA1, EPHA4, EPHB1, EPRS1, ERBB4, EREG, ETAA1, ETFA, EZH2, F12, F3, FAF1, FAM161A, FANCM, FASN, FBXO2, FBXW8, FEM1C, FER, FGD2, FGF2, FGF7, FKTN, FLT4, FNIP2, FNTA, FOXO1, FRS2, FTO, FZD10, FZD5, FZR1, GADD45G, GCG, GCNT2, GFI1, GFRAL, GLE1, GLIPR2, GLMN, GMFB, GNA12, GNAI2, GORASP1, GPER1, GPR55, GPRC5B, GPRC5C, GPRIN1, GRAMD4, GRB2, GREM1, GRM1, GRM4, GSK3A, GTF2H1, HACD3, HCLS1, HDAC4, HEG1, HES1, HGS, HHATL, HIP1, HIP1R, HMGB1, HNRNPD, HNRNPR, HRC, HRG, HSP90AB1, HSPD1, HTR2A, HTT, HUS1, IDE, IFI16, IGF1, IGF1R, IGF2BP1, IKBKB, IL31RA, IL6R, IL6ST, ILF3, ING3, INKA2, INPP5F, INSR, IPO7, IQGAP3, IRAK2, ISL1, ITCH, ITGA1, ITGA5, ITGB2, ITIH5, IWS1, JAK2, JARID2, KAT2B, KAT7, KDM3A, KDM4B, KDM4C, KDM4D, KHDRBS1, KIDINS220, KIRREL1, KITLG, KMT2E, KNDC1, KPNA7, KRAS, KSR1, LARP4B, LATS2, LAX1, LDLR, LDLRAD4, LEPR, LGMN, LLGL1, LMTK2, LPAR1, LRIG2, LRP1, LRP2, LRP5, LRRK1, LTN1, MAGI2, MAL, MAP2K2, MAP2K5, MAP3K11, MAP3K13, MAP3K19, MAP3K2, MAP3K20, MAP3K5, MAP3K6, MAP4K4, MAP4K5, MAPK8, MAPK8IP2, MAPK8IP3, MAPK9, MAPKAP1, MAPKAPK5, MCM2, MECOM, MELTF, MEN1, MKNK1, MKNK2, MMD, MRPL13, MRPL23, MRTFA, MST1R, MTA1, MTG1, MYD88, MYO1C, N4BP1, NAT10, NCK2, NCKAP1L, NCOA7, NDFIP2, NDRG4, NDUFA13, NEDD9, NF1, NFE2L2, NGF, NLRP12, NNT, NOC2L, NOL3, NOS1, NOS1AP, NOTCH1, NOTCH4, NPNT, NPPA, NPTN, NRXN1, NSD1, NSD3, NSMF, NTRK2, NXN, OPRPN, OSBPL7, OSBPL8, OTUB1, OTUD4, P2RY6, PAF1, PAFAH1B1, PAK4, PAK5, PAPLN, PARD3, PARG, PARP14, PASK, PAX2, PAX7, PBLD, PDCD5, PDCD6, PDCL3, PDE8A, PDGFD, PDGFRA, PDPK1, PDZD2, PER2, PFN2, PHACTR1, PHF19, PIH1D1, PIK3CB, PIK3CG, PIK3R5, PINX1, PKD1, PKIB, PKMYT1, PLA2G6, PLAUR, PLCB1, PLCE1, PLCL2, PLD1, PLEKHN1, PML, POLDIP3, POMT1, PPARG, PPARGC1A, PPM1F, PPP1R11, PPP1R16A, PPP1R16B, PPP2R2B, PPP2R2C, PPP2R3C, PPP3CA, PRDX5, PRKAG2, PRKAR1B, PRKCA, PRKCE, PRKCZ, PRKD2, PRKDC, PRKN, PRLR, PRMT1, PSCA, PSEN1, PSMC2, PSME3IP1, PTEN, PTK2, PTPN11, PTPN3, PTPRA, PTPRC, PTPRH, PTPRO, PUM1, PUS7, PZP, RACK1, RAD51, RANBP9, RAP1A, RAP2B, RAPGEF1, RARA, RASIP1, RASSF5, RB1, RBM20, RBM4B, RBPMS, RBX1, RC3H1, RCAN2, RCHY1, RELN, REST, RET, |
| GO:0032268 | regulation of cellular protein metabolic process | 3,52E-02 | [4, 5, 6] | 605,00 | 21,42 | ANGPT4, ANKS1B, ANTXR1, ANXA2, APC, ARHGEF2, ARHGEF7, ARIH1, ARL6IP5, ARNTL, ASPH, ASPSCR1, ATF3, ATG14, ATM, ATP13A2, ATP2A3, ATP2B4, BAG6, BBS7, BCAR3, BCL10, BCL2, BCL3, BCL6, BDNF, BDNF-AS, BIRC2, BMP2, BMP3, BMP7, BMP8B, BMPER, BRD4, BRMS1, BRSK2, BTC, BTG2, BTRC, CALM2, CAMK2D, CAMTA1, CAPRIN2, CARD10, CARD14, CASR, CAV1, CBFA2T3, CCAR2, CCBE1, CCDC88C, CCL16, CCNA1, CCND2, CCNL1, CCNY, CD33, CDC123, CDC20, CDC20B, CDC25B, CDC25C, CDK1, CDK5R1, CDKN1A, CDKN2A, CDYL, CELF4, CEP295, CEP43, CHAMP1, CHEK1, CHEK2, CIB1, CLN6, CNOT1, CNOT7, COMMD1, COP1, CPEB1, CPEB4, CRB2, CRIM1, CRY2, CSNK1E, CST9, CTCF, CUL3, CXCR4, CYCS, CYFIP1, CYFIP2, CYLD, DAB1, DAG1, DAPK1, DAXX, DBNDD2, DCUN1D1, DDA1, DDIT4, DDR2, DESI2, DGKQ, DHX36, DIPK2A, DLC1, DLG1, DLG4, DNAJB6, DNAJC3, DNM1L, DNPEP, DOCK7, DOK4, DOK7, DSTYK, DUS2, DUSP19, DUSP26, EDAR, EDEM2, EEF1A1, EEFSEC, EFNA5, EHMT2, EIF2AK3, EIF2AK4, EIF2S2, EIF3B, EIF4A3, EIF4B, EIF4E, EIF4E1B, EIF4EBP1, EIF4EBP2, EIF4ENIF1, ELAVL1, ELOA, EMC10, ENC1, ENPP2, EPHA1, EPHA4, EPHB1, EPRS1, ERBB4, EREG, ETAA1, ETFA, EZH2, F12, F3, FAF1, FAM161A, FANCM, FASN, FBXO2, FBXW8, FEM1C, FER, FGD2, FGF2, FGF7, FKTN, FLT4, FNIP2, FNTA, FOXO1, FRS2, FTO, FZD10, FZD5, FZR1, GADD45G, GCG, GCNT2, GFI1, GFRAL, GLE1, GLIPR2, GLMN, GMFB, GNA12, GNAI2, GORASP1, GPER1, GPR55, GPRC5B, GPRC5C, GPRIN1, GRAMD4, GRB2, GREM1, GRM1, GRM4, GSK3A, GTF2H1, HACD3, HCLS1, HDAC4, HEG1, HES1, HGS, HHATL, HIP1, HIP1R, HMGB1, HNRNPD, HNRNPR, HRC, HRG, HSP90AB1, HSPD1, HTR2A, HTT, HUS1, IDE, IFI16, IGF1, IGF1R, IGF2BP1, IKBKB, IL31RA, IL6R, IL6ST, ILF3, ING3, INKA2, INPP5F, INSR, IPO7, IQGAP3, IRAK2, ISL1, ITCH, ITGA1, ITGA5, ITGB2, ITIH5, IWS1, JAK2, JARID2, KAT2B, KAT7, KDM3A, KDM4B, KDM4C, KDM4D, KHDRBS1, KIDINS220, KIRREL1, KITLG, KMT2E, KNDC1, KPNA7, KRAS, KSR1, LARP4B, LATS2, LAX1, LDLR, LDLRAD4, LEPR, LGMN, LLGL1, LMTK2, LPAR1, LRIG2, LRP1, LRP2, LRP5, LRRK1, LTN1, MAGI2, MAL, MAP2K2, MAP2K5, MAP3K11, MAP3K13, MAP3K19, MAP3K2, MAP3K20, MAP3K5, MAP3K6, MAP4K4, MAP4K5, MAPK8, MAPK8IP2, MAPK8IP3, MAPK9, MAPKAP1, MAPKAPK5, MCM2, MECOM, MELTF, MEN1, MKNK1, MKNK2, MMD, MRPL13, MRPL23, MRTFA, MST1R, MTA1, MTG1, MYD88, MYO1C, N4BP1, NAT10, NCK2, NCKAP1L, NCOA7, NDFIP2, NDRG4, NDUFA13, NEDD9, NF1, NFE2L2, NGF, NLRP12, NNT, NOC2L, NOL3, NOS1, NOS1AP, NOTCH1, NOTCH4, NPNT, NPPA, NPTN, NRXN1, NSD1, NSD3, NSMF, NTRK2, NXN, OPRPN, OSBPL7, OSBPL8, OTUB1, OTUD4, P2RY6, PAF1, PAFAH1B1, PAK4, PAK5, PAPLN, PARD3, PARG, PARP14, PASK, PAX2, PAX7, PBLD, PDCD5, PDCD6, PDCL3, PDE8A, PDGFD, PDGFRA, PDPK1, PDZD2, PER2, PFN2, PHACTR1, PHF19, PIH1D1, PIK3CB, PIK3CG, PIK3R5, PINX1, PKD1, PKIB, PKMYT1, PLA2G6, PLAUR, PLCB1, PLCE1, PLCL2, PLD1, PLEKHN1, PML, POLDIP3, POMT1, PPARG, PPARGC1A, PPM1F, PPP1R11, PPP1R16A, PPP1R16B, PPP2R2B, PPP2R2C, PPP2R3C, PPP3CA, PRDX5, PRKAG2, PRKAR1B, PRKCA, PRKCE, PRKCZ, PRKD2, PRKDC, PRKN, PRLR, PRMT1, PSCA, PSEN1, PSMC2, PSME3IP1, PTEN, PTK2, PTPN11, PTPN3, PTPRA, PTPRC, PTPRH, PTPRO, PUM1, PUS7, PZP, RACK1, RAD51, RANBP9, RAP1A, RAP2B, RAPGEF1, RARA, RASIP1, RASSF5, RB1, RBM20, RBM4B, RBPMS, RBX1, RC3H1, RCAN2, RCHY1, RELN, REST, RET, |
| GO:0048522 | positive regulation of cellular process | 1,89E-09 | [2, 3, 4] | 1278,00 | 21,43 | ADCYAP1R1, ADD1, ADGRB2, ADGRB3, ADGRF5, ADGRG1, ADGRL3, ADGRV1, ADIG, ADRA1B, ADRM1, AFDN, AGBL4, AGO2, AGRN, AKAP12, AKAP13, AKR1C2, AKT2, ALDH3A1, ALK, ALKAL2, ALS2, ALX1, ALX4, AMBRA1, AMH, ANAPC11, ANAPC4, ANGPT1, ANGPT4, ANK3, ANKRD17, ANKRD31, ANO1, ANO6, ANTXR1, ANXA2, AP2A1, AP3B1, APBB1IP, APC, APOBEC1, APPL1, APPL2, ARAP1, ARF1, ARHGDIA, ARHGEF10L, ARHGEF16, ARHGEF18, ARHGEF2, ARHGEF26, ARHGEF7, ARID2, ARID4B, ARIH1, ARL6IP5, ARNT2, ARNTL, ARPC4, ASAP1, ASCL1, ASPH, ASPSCR1, ASS1, ASXL2, ATAT1, ATF1, ATF2, ATF3, ATG14, ATG16L1, ATM, ATP13A2, ATP2A3, ATP2B4, ATP5F1A, ATP8A1, ATP8A2, ATXN7L3, AURKB, BABAM2, BACH1, BAG6, BAIAP2L1, BANP, BBS4, BBS7, BCAR1, BCAR3, BCAS3, BCL10, BCL11B, BCL2, BCL3, BCL6, BCL9, BDNF, BDNF-AS, BEND6, BICRAL, BIRC2, BLOC1S5, BMP2, BMP3, BMP7, BMP8B, BMPER, BNC1, BRD1, BRD4, BRF1, BRINP2, BRMS1, BTBD10, BTC, BTG2, BTRC, C1orf61, C21orf91, CACNA1A, CACNA1G, CACNA1H, CACNA1I, CACNB2, CADM1, CADPS, CADPS2, CALB2, CALCOCO1, CALM2, CAMK2B, CAMK2D, CAMK4, CAMTA1, CAMTA2, CAND1, CAPRIN2, CARD10, CARD11, CARD14, CARMIL1, CARMIL2, CASP2, CASR, CASZ1, CAV1, CBFA2T2, CBFA2T3, CBX8, CCAR2, CCBE1, CCDC3, CCDC80, CCL16, CCN4, CCND2, CCNY, CCR2, CCR6, CCT2, CD247, CD33, CD46, CD47, CD53, CDC123, CDC20, CDC20B, CDC25B, CDC25C, CDH13, CDH4, CDK1, CDK19, CDK2, CDK5R1, CDKN1A, CDKN2A, CDT1, CELF4, CEP131, CEP290, CEP295, CEP43, CHAMP1, CHD5, CHEK1, CHEK2, CHMP2A, CHRNB2, CHTF8, CIB1, CIP2A, CIT, CITED2, CITED4, CIZ1, CLASP1, CLASP2, CLN6, CLTCL1, CLYBL, CNOT1, CNOT7, CNST, COBL, COL18A1, COL3A1, COMMD1, COP1, CORO1B, CPEB1, CPNE5, CPT1A, CRB2, CRBN, CREB3L1, CREB3L2, CREB5, CREM, CSNK1E, CSNK1G3, CSPG5, CSRNP2, CSRNP3, CTBP2, CTCF, CTNNA2, CTNNBIP1, CTSS, CTTN, CUL3, CUX1, CXCR4, CXXC5, CYCS, CYFIP1, CYFIP2, CYLD, CYP19A1, CYP1B1, DAB1, DAG1, DAPK1, DAXX, DBN1, DCN, DCTN1, DCUN1D1, DDA1, DDAH2, DDHD2, DDIT4, DDR1, DDR2, DDX49, DDX60, DEF8, DGKQ, DGKZ, DHDDS, DHX34, DHX36, DIPK2A, DLC1, DLG1, DLG4, DLL1, DLL4, DLX2, DNA2, DNAJC3, DNM1, DNM1L, DNMT3A, DNPEP, DOCK7, DOCK8, DOK4, DOK7, DOT1L, DPF1, DPP10, DSTYK, DTX1, DUOX1, DUSP19, DUSP26, E2F2, E2F8, EBF1, EBF3, EBF4, ECE1, EDAR, EDEM2, EEF1E1-BLOC1S5, EFNA5, EGR2, EHF, EIF2AK3, EIF2AK4, EIF4A3, EIF4E, EIF4EBP1, EIPR1, ELAVL1, ELF1, ELK3, EMC10, ENC1, ENHO, ENPP2, EPB41L4B, EPHA1, EPHA10, EPHA4, EPHA6, EPHB1, EPHB3, EPS8L1, ERBB4, ERCC1, ERCC5, ERCC8, EREG, ESPN, ESR1, ESR2, ESRP2, ESRRG, ETAA1, ETFA, ETS1, ETV1, ETV6, EVL, EVX1, EXOC7, EXOSC6, EXPH5, EYA2, EYA4, EZH2, F11R, F12, F2RL3, F3, FABP5, FAF1, FAM110A, FAM161A, FAM89B, FANCM, FAP, FASN, FBLN5, FBN2, FBXW8, FCHSD1, FCHSD2, FER, FEZ1, FEZF1, FEZF2, FGD2, FGF14, FGF2, FGF22, FGF6, FGF7, FHOD1, FLRT1, FLT4, FMN1, FNIP2, FNTA, FOXD3, FOXJ3, FOXK1, FOXO1, FOXP1, FRMD4A, FRMD5, FRMD8, FRS2, FTO, FUBP1, FZD10, FZD5, FZR1, GAB1, GAB2, GABPB1, GADD45G, GARS1, GATA5, GCG, GCNT2, GFI1, GFRAL, GJA5, GLI2, GLI3, GLIPR2, GLIS2, GLMN, GLP2R, GMFB, GML, GNAI2, GNAO1, GORASP1, GPER1, GPR137B, GPR17, GPR4, GPR55, GPR62, GPRC5B, GPRC5C, GPRIN1, GPSM1, GPT, GRAMD4, GRB10, GRB2, GREM1, GRID2, GRK5, GRM1, GRM4, GSK3A, GSX2, GTF2B, GTF2H1, GUCA2A, HACD3, HAND1, HCAR2, HCLS1, HDAC4, HDAC9, HELT, HES1, HES3, HEXB, HGS, HINT1, HIP1, HIP1R, HIVEP1, HIVEP3, HLA-DPB1, HMBOX1, HMGB1, HMGN3, HNF1B, HNRNPAB, HNRNPC, HNRNPD, HNRNPK, HNRNPR, HOOK1, HOXA1, HOXA3, HOXA4, HOXC10, HOXC11, HOXC4, HOXD13, HPS4, HRG, HSD17B12, HSD17B4, HSP90AB1, HSPD1, HSPH1, HTATIP2, HTR1A, HTR2A, HTR3A, HTT, IFI16, IFIH1, IFT74, IGF1, IGF1R, IGF2BP1, IGLL5, IGSF9B, IHH, IKBKB, IKZF1, IL10RA, IL10RB, IL12RB2, IL15RA, IL16, IL17RA, IL1RAP, IL20RA, IL31RA, IL37, IL6R, IL6ST, ILDR1, ILF3, ING1, ING3, INPP5F, INSIG2, INSR, IQGAP2, IQGAP3, IQSEC1, IRAK2, IRF2, IRF6, IRS2, ISL1, ITCH, ITGA1, ITGA5, ITGB2, ITPR3, JAK2, JAML, JARID2, KALRN, KANK1, KAT2B, KAT6A, KAT6B, KAT7, KCNB1, KCNC1, KCNE1, KCNH2, KCNJ2, KCNK2, KCNMA1, KCNQ1, KDM2B, KDM3A, KDM4C, KDM4D, KDM6B, KHDRBS1, KIDINS220, KIF26B, KIF2A, KIRREL1, KITLG, KLF14, KLF17, KLF7, KMT2D, KMT2E, KMT5C, KNDC1, KPNA7, KRAS, KSR1, LAMA2, LAMB1, LARP4B, LARP7, LATS2, LDB2, LDLR, LEPR, LGMN, LGR6, LHX4, LHX6, LHX8, LIG4, LIMS1, LLGL1, LMCD1, LMO7, LMTK2, LMX1B, LONP1, LOXL2, LOXL3, LPAR1, LPGAT1, LPL, LRCH4, LRIG2, LRP1, LRP2, LRP5, LRPAP1, LRRK1, LRSAM1, LTBP2, LURAP1, LYL1, LYPD6, LZTS1, MACC1, MACF1, MAF, MAFA, MAFG, MAG, MAGI2, MAK, MAL, MAML2, MAML3, MAP2, MAP2K2, MAP2K5, MAP3K11, MAP3K13, MAP3K19, MAP3K2, MAP3K20, MAP3K5, MAP3K6, MAP4K4, MAP4K5, MAP6, MAPK7, MAPK8, MAPK8IP2, MAPK8IP3, MAPK9, MAPKAP1, MAPKAPK5, MARS1, MAX, MCF2L, MCL1, MCRS1, MEAK7, MECOM, MED16, MED22, MED24, MED26, MED27, MEF2C, MEGF8, MEIOC, MEIS2, MELTF, MEN1, MEOX1, METRNL, MFGE8, MFNG, MGMT, MIB2, MICAL2, MIER1, MIPEP, MLLT3, MMD, MME, MPHOSPH8, MRGPRX2, MRPL23, MRTFA, MSANTD1, MSGN1, MSH2, MST1R, MTA1, MTA3, MTCH2, MTMR2, MTMR9, MTNR1B, MYD88, MYLK, MYO18A, MYO1C, MYO1E, MYRF, NACC2, NAPEPLD, NAT10, NAV3, NCCRP1, NCK2, NCKAP1, NCKAP1L, NCOA2, NCOA6, NCOA7, NCSTN, NDFIP2, NDRG4, NDUFA13, NDUFA2, NEDD4L, NEDD9, NEK7, NEO1, NEPRO, NEUROD6, NF1, NFAM1, NFATC1, NFATC2, NFATC2IP, NFATC4, NFE2L2, NFIB, NFIL3, NFKBIB, NFYA, NFYC, NGEF, NGF, NKD1, NKX2-4, NLGN2, NLRC5, NLRP12, NME2, NNT, NOL3, NOP2, NOS1, NOS1AP, NOTCH1, NOTCH3, |
| GO:0006796 | phosphate-containing compound metabolic process | 1,21E-03 | [4] | 750,00 | 21,47 | ADCY5, ADCY7, ADCYAP1R1, ADGRF5, ADRA1B, ADSS1, AGK, AGPAT4, AK9, AKAP12, AKAP13, AKR1A1, AKT2, ALK, ALKAL2, ALPK2, ALPL, ALS2, AMBRA1, AMH, AMPD2, ANGPT1, ANGPT4, ANKS1B, ANTXR1, ANXA2, APC, APRT, ARF1, ARFGEF3, ARHGEF2, ARHGEF7, ARL6IP5, ATF3, ATG14, ATM, ATP13A2, ATP1A3, ATP1A4, ATP2B4, ATP5F1A, ATP5ME, AURKB, BAZ1B, BCAR3, BCL10, BCL2, BDNF, BDNF-AS, BMP2, BMP3, BMP7, BMP8B, BMPER, BPGM, BRD4, BRSK2, BTBD10, BTC, BTRC, CACNA1A, CALM2, CAMK2B, CAMK2D, CAMK4, CAMTA1, CAPRIN2, CARD10, CARD11, CARD14, CASR, CAV1, CBFA2T3, CCDC88C, CCL16, CCNA1, CCND2, CCNL1, CCNY, CD33, CDC25B, CDC25C, CDC42BPB, CDK1, CDK11B, CDK19, CDK2, CDK5R1, CDKL2, CDKN1A, CDKN2A, CEP43, CEPT1, CERKL, CHAMP1, CHEK1, CHEK2, CIB1, CILK1, CIT, CLASP1, CLK3, CLK4, CLP1, CNOT7, CNST, COQ9, COX5A, COX6A1, COX7C, CRIM1, CRY2, CSNK1A1L, CSNK1E, CSNK1G3, CSRNP2, CSRNP3, CUL3, CXCR4, CYC1, CYCS, CYLD, DAB1, DAG1, DAPK1, DAXX, DBNDD2, DCAF1, DCLK1, DDHD2, DDIT4, DDR1, DDR2, DGKA, DGKD, DGKE, DGKH, DGKQ, DGKZ, DHDDS, DHX34, DIPK2A, DLC1, DLD, DLG1, DLG2, DLG4, DNAJC15, DNAJC3, DNAJC30, DNAJC6, DOCK7, DOK4, DOK7, DSTYK, DUS2, DUSP19, DUSP26, EDAR, EEF1A1, EFNA4, EFNA5, EFR3B, EIF2AK3, EIF2AK4, ELOVL1, EMC10, ENPP2, EPHA1, EPHA10, EPHA4, EPHA6, EPHB1, EPHB3, EPS8L1, ERBB4, ERC1, EREG, ETAA1, ETFA, EYA2, EYA4, EZH2, F3, FABP5, FAF1, FAM220A, FAR1, FARP1, FASN, FASTK, FASTKD1, FBP1, FCSK, FER, FGD2, FGF2, FGF22, FGF6, FGF7, FGGY, FITM2, FKTN, FLT4, FN3KRP, FNIP2, FOXK1, FOXO1, FRS2, FZD10, FZD5, GADD45G, GAK, GALK2, GARS1, GCG, GCNT2, GDE1, GFRAL, GGPS1, GLIPR2, GLMN, GLYCTK, GMFB, GMPR2, GNAI2, GPER1, GPR55, GPRC5B, GPRC5C, GPRIN1, GRB10, GRB2, GREM1, GRK3, GRK5, GRM1, GRM4, GSK3A, GTF2H1, GUCA2A, HAAO, HACD3, HADHA, HCLS1, HDAC4, HDHD5, HEG1, HES1, HEXB, HGS, HINT1, HMGB1, HOOK1, HRC, HRG, HSD17B12, HSD17B4, HSP90AB1, HTATIP2, HTR2A, HTT, HUS1, IDNK, IGF1, IGF1R, IKBKB, IL12RB2, IL17RD, IL31RA, IL6R, IL6ST, ILF3, INKA2, INPP5A, INPP5F, INPPL1, INSR, IPO7, IQGAP3, IRAK2, IRS2, ISL1, ITCH, ITGA1, ITGA5, ITGB2, ITK, ITPA, JAK2, KALRN, KAT2B, KDM4D, KIDINS220, KIF2A, KIRREL1, KITLG, KNDC1, KRAS, KSR1, KSR2, LATS2, LAX1, LDB2, LDHD, LDLR, LDLRAD4, LEPR, LHPP, LIMK2, LLGL1, LMTK2, LONP1, LPAR1, LPCAT1, LPGAT1, LPL, LRP1, LRP5, LRRC8B, LRRC8D, LRRK1, LTBP1, MAGI2, MAK, MAP2K2, MAP2K5, MAP3K11, MAP3K13, MAP3K19, MAP3K2, MAP3K20, MAP3K5, MAP3K6, MAP4K4, MAP4K5, MAPK4, MAPK7, MAPK8, MAPK8IP2, MAPK8IP3, MAPK9, MAPKAP1, MAPKAPK5, MARF1, MARK3, MAST4, MBOAT1, MBOAT7, MCM2, MCPH1, MDP1, ME1, MECOM, MEF2C, MEN1, MFN1, MKNK1, MKNK2, MMD, MRTFA, MSH2, MST1R, MTMR2, MTMR3, MTMR9, MYD88, MYH3, MYLK, MYLK4, MYO1D, NAPEPLD, NAXD, NCK2, NCKAP1L, NDRG4, NDUFA13, NDUFA2, NDUFB3, NDUFB6, NDUFB7, NDUFC1, NDUFC2, NEDD9, NEK11, NEK7, NF1, NGF, NLK, NLRC5, NLRP12, NME2, NNT, NOS1, NOTCH1, NPHS1, NPNT, NPPA, NPR1, NPTN, NRXN1, NSD1, NSMF, NT5DC3, NTRK2, NUDT1, NUP205, NUP43, NUP50, NUP58, NUS1, OBSCN, OC90, OGDH, OSBPL10, OSBPL8, OXSM, P2RY6, PAFAH1B1, PAK4, PAK5, PANK4, PAPSS1, PARD3, PARP14, PASK, PBLD, PCNX4, PDCL3, PDE7B, PDE8A, PDE9A, PDGFD, PDGFRA, PDGFRL, PDPK1, PDZD2, PDZD3, PEAK1, PFAS, PFKFB3, PFKM, PFKP, PFN2, PGP, PGS1, PHACTR1, PHLPP2, PI4K2A, PIGG, PIGP, PIGQ, PIGS, PIGV, PIGY, PIH1D1, PIK3CB, PIK3CG, PIK3IP1, PIK3R1, PIK3R5, PIKFYVE, PIM1, PIP4K2A, PIP5K1C, PITPNM1, PITPNM2, PKD1, PKIB, PKM, PKMYT1, PLA2G2D, PLA2G4C, PLA2G4D, PLA2G6, PLA2G7, PLAAT5, PLAUR, PLCB1, PLCB2, PLCB4, PLCD4, PLCE1, PLCG2, PLCH2, PLCL2, PLD1, PLD4, PLEK, PLEKHA2, PLEKHA4, PLK4, PLK5, PLPP5, PLVAP, PML, PNLIPRP2, PNPLA8, PNPO, POLG, POU4F2, PPA2, PPARGC1A, PPARGC1B, PPM1F, PPP1R11, PPP1R16A, PPP1R16B, PPP2R2B, PPP2R2C, PPP2R3C, PPP3CA, PPP3CB, PPP4C, PPTC7, PRAG1, PRDX5, PRKAG2, PRKAR1B, PRKCA, PRKCB, PRKCE, PRKCH, PRKCI, PRKCQ, PRKCZ, PRKD2, |

| | | | | | |
|---|---|---|---|---|---|
| GO:0006793 | phosphorus metabolic process | 7,85E-04 [3] | 757,00 | 21,51 | ADCY5, ADCY7, ADCYAP1R1, ADGRF5, ADRA1B, ADSS1, AGK, AGPAT4, AK9, AKAP12, AKAP13, AKR1A1, AKT2, ALK, ALKAL2, ALPK2, ALPL, ALS2, AMBRA1, AMDHD2, AMH, AMPD2, ANGPT1, ANGPT4, ANKS1B, ANTXR1, ANXA2, APC, APRT, ARF1, ARFGEF3, ARHGEF2, ARHGEF7, ARL6IP5, ATF3, ATG14, ATM, ATP13A2, ATP1A3, ATP1A4, ATP2B4, ATP5F1A, ATP5ME, AURKB, BAZ1B, BCAR3, BCL10, BCL2, BDNF, BDNF-AS, BMP2, BMP3, BMP7, BMP8B, BMPER, BPGM, BRD4, BRSK2, BTBD10, BTC, BTRC, CACNA1A, CALM2, CAMK2B, CAMK2D, CAMK4, CAMTA1, CAPRIN2, CARD10, CARD11, CARD14, CASR, CAV1, CBFA2T3, CCDC88C, CCL16, CCNA1, CCND2, CCNL1, CCNY, CD33, CDC25B, CDC25C, CDC42BPB, CDK1, CDK11B, CDK19, CDK2, CDK5R1, CDKL2, CDKN1A, CDKN2A, CEP43, CEPT1, CERKL, CHAMP1, CHEK1, CHEK2, CIB1, CILK1, CIT, CLASP1, CLK3, CLK4, CLP1, CNOT7, CNST, COQ9, COX5A, COX6A1, COX7C, CRIM1, CRY2, CSGALNACT1, CSNK1A1L, CSNK1E, CSNK1G3, CSRNP2, CSRNP3, CUL3, CXCR4, CYC1, CYCS, CYLD, DAB1, DAG1, DAPK1, DAXX, DBNDD2, DCAF1, DCLK1, DDHD2, DDIT4, DDR1, DDR2, DGKA, DGKD, DGKE, DGKH, DGKQ, DGKZ, DHDDS, DHX34, DIPK2A, DLC1, DLD, DLG1, DLG2, DLG4, DNAJC15, DNAJC3, DNAJC30, DNAJC6, DOCK7, DOK4, DOK7, DSTYK, DUS2, DUSP19, DUSP26, EDAR, EEF1A1, EFNA4, EFNA5, EFR3B, EIF2AK3, EIF2AK4, ELOVL1, EMC10, ENPP2, EPHA1, EPHA10, EPHA4, EPHA6, EPHB1, EPHB3, EPS8L1, ERBB4, ERC1, EREG, ETAA1, ETFA, EYA2, EYA4, EZH2, F3, FABP5, FAF1, FAM220A, FAR1, FARP1, FASN, FASTK, FASTKD1, FBP1, FCSK, FER, FGD2, FGF2, FGF22, FGF6, FGF7, FGGY, FITM2, FKTN, FLT4, FN3KRP, FNIP2, FOXK1, FOXO1, FRS2, FUT8, FZD10, FZD5, GADD45G, GAK, GALK2, GARS1, GCG, GCNT2, GDE1, GFRAL, GGPS1, GLIPR2, GLMN, GLYCTK, GMDS, GMFB, GMPR2, GNAI2, GPER1, GPR55, GPRC5B, GPRC5C, GPRIN1, GRB10, GRB2, GREM1, GRK3, GRK5, GRM1, GRM4, GSK3A, GTF2H1, GUCA2A, HAAO, HACD3, HADHA, HCLS1, HDAC4, HDHD5, HEG1, HES1, HEXB, HGS, HINT1, HMGB1, HOOK1, HRC, HRG, HSD17B12, HSD17B4, HSP90AB1, HTATIP2, HTR2A, HTT, HUS1, IDNK, IGF1, IGF1R, IKBKB, IL12RB2, IL17RD, IL31RA, IL6R, IL6ST, ILF3, INKA2, INPP5A, INPP5F, INPPL1, INSR, IPO7, IQGAP3, IRAK2, IRS2, ISL1, ITCH, ITGA1, ITGA5, ITGB2, ITK, ITPA, JAK2, KALRN, KAT2B, KDM4D, KIDINS220, KIF2A, KIRREL1, KITLG, KNDC1, KRAS, KSR1, KSR2, LATS2, LAX1, LDB2, LDHD, LDLR, LDLRAD4, LEPR, LHPP, LIMK2, LLGL1, LMTK2, LONP1, LPAR1, LPCAT1, LPGAT1, LPL, LRP1, LRP5, LRRC8B, LRRC8D, LRRK1, LTBP1, MAGI2, MAK, MAP2K2, MAP2K5, MAP3K11, MAP3K13, MAP3K19, MAP3K2, MAP3K20, MAP3K5, MAP3K6, MAP4K4, MAP4K5, MAPK4, MAPK7, MAPK8, MAPK8IP2, MAPK8IP3, MAPK9, MAPKAP1, MAPKAPK5, MARF1, MARK3, MAST4, MBOAT1, MBOAT7, MCM2, MCPH1, MDP1, ME1, MECOM, MEF2C, MEN1, MFN1, MKNK1, MKNK2, MMD, MRTFA, MSH2, MST1R, MTMR2, MTMR3, MTMR9, MYD88, MYH3, MYLK, MYLK4, MYO1D, NAPEPLD, NAXD, NCK2, NCKAP1L, NDRG4, NDUFA13, NDUFA2, NDUFB3, NDUFB6, NDUFB7, NDUFC1, NDUFC2, NEDD9, NEK11, NEK7, NF1, NGF, NLK, NLRC5, NLRP12, NME2, NNT, NOS1, NOTCH1, NPHS1, NPNT, NPPA, NPR1, NPTN, NRXN1, NSD1, NSMF, NT5DC3, NTRK2, NUDT1, NUP205, NUP43, NUP50, NUP58, NUS1, OBSCN, OC90, OGDH, OSBPL10, OSBPL8, OXSM, P2RY6, PAFAH1B1, PAK4, PAK5, PANK4, PAPSS1, PARD3, PARP14, PASK, PBLD, PCNX4, PDCL3, PDE7B, PDE8A, PDE9A, PDGFD, PDGFRA, PDGFRL, PDPK1, PDZD2, PDZD3, PEAK1, PFAS, PFKFB3, PFKM, PFKP, PFN2, PGP, PGS1, PHACTR1, PHLPP2, PI4K2A, PIGG, PIGP, PIGQ, PIGS, PIGV, PIGY, PIH1D1, PIK3CB, PIK3CG, PIK3IP1, PIK3R1, PIK3R5, PIKFYVE, PIM1, PIP4K2A, PIP5K1C, PITPNM1, PITPNM2, PKD1, PKIB, PKM, PKMYT1, PLA2G2D, PLA2G4C, PLA2G4D, PLA2G6, PLA2G7, PLAAT5, PLAUR, PLCB1, PLCB2, PLCB4, PLCD4, PLCE1, PLCG2, PLCH2, PLCL2, PLD1, PLD4, PLEK, PLEKHA2, PLEKHA4, PLK4, PLK5, PLPP5, PLVAP, PML, PNLIPRP2, PNPLA8, PNPO, POLG, POU4F2, PPA2, PPARGC1A, PPARGC1B, PPM1F, PPP1R11, PPP1R16A, PPP1R16B, PPP2R2B, PPP2R2C, PPP2R3C, PPP3CA, PPP3CB, PPP4C, PPTC7, PRAG1, PRDX5, PRKAG2, PRKAR1B, PRKCA, PRKCB, PRKCE, PRKCH, PRKCI, |
| GO:0048513 | animal organ development | 3,59E-05 [3, 4, 5] | 835,00 | 21,65 | AKAP13, AKT2, ALAS1, ALK, ALPK2, ALPL, ALX1, ALX4, AMH, ANGPT1, ANKH, ANKRD11, ANKRD33, ANO6, ANXA2, ANXA6, AP2A1, AP2A2, AP2S1, AP3B1, APRT, ARHGEF7, ARID2, ARID4B, ARL13B, ARNT2, ARNTL, ASCL1, ASS1, ASXL2, ATAT1, ATF2, ATF3, ATG16L1, ATM, ATP2B4, ATP6V0D1, ATP8A2, ATXN1, B9D1, BAG6, BATF2, BBS4, BBS7, BBX, BCAR3, BCL11B, BCL2, BCL2A1, BCL3, BCL6, BCL9, BDNF, BDNF-AS, BFSP1, BFSP2, BICC1, BIN3, BIRC2, BMERB1, BMP1, BMP2, BMP3, BMP7, BMP8B, BMPER, BNC2, BPGM, BRD1, BTG2, BTRC, C1QC, C21orf91, CA10, CACNA1A, CACNA1H, CALM2, CAMK4, CARD11, CASP2, CASP5, CASR, CAV1, CBFA2T3, CC2D2A, CCBE1, CCDC85C, CCKAR, CCN4, CCR2, CCR6, CD46, CD8A, CDH22, CDH23, CDK1, CDK5R1, CDKN1A, CDKN2A, CELA2A, CELF4, CELSR1, CEND1, CEP290, CHD5, CHRDL2, CHRNB2, CHRND, CHST11, CIB1, CIC, CITED2, CLASP1, CLASP2, CLCN2, CLP1, CNNM4, CNRIP1, CNTN4, CNTNAP2, COBL, COL11A2, COL18A1, COL2A1, COL3A1, COL4A1, COL6A1, COL6A2, COL8A2, CPLANE1, CRB2, CREB3L2, CRIP1, CRISPLD2, CRYBA4, CRYBG3, CRYGC, CSGALNACT1, CSN3, CSNK1E, CTNNA2, CTNNBIP1, CTNS, CUL3, CXCR4, CYLD, CYP19A1, CYP1B1, CYP4A11, DAB1, DAG1, DCAF1, DCHS2, DCLK1, DCLRE1C, DCN, DCTN1, DDIT4, DDR1, DDR2, DGCR6, DGCR6L, DHCR7, DHRS3, DHX15, DHX36, DIPK2A, DKK3, DLC1, DLG1, DLL1, DLL4, DLX2, DMC1, DNAH5, DNAJB6, DNAJC30, DNASE1L2, DNER, DOCK10, DOCK7, DRGX, DSCAML1, DTX1, E2F8, ECE1, EDAR, EFNA4, EGR2, EIF2AK3, EIF2S2, EIF4A3, EIF4E, EIF4EBP1, ELN, ELOVL1, EPHA4, EPHB1, EPHB3, ERBB4, ERCC1, EREG, ESR1, ESRP2, ETS1, ETV1, ETV6, EVL, EVX1, EXOC4, EXPH5, EYA4, EZH2, F3, FA2H, FAM172A, FARP2, FASN, FAT1, FBN1, FBN2, FBXW8, FEZ1, FEZF1, FEZF2, FGF12, FGF2, FGF6, FGF7, FHOD3, FJX1, FKTN, FLT4, FMN1, FNDC3A, FOXK1, FOXO1, FOXP1, FPGS, FRAS1, FREM1, FRS2, FTO, FZD5, FZR1, GAA, GAB2, GABRB2, GARS1, GATA5, GCNT1, GCNT2, GFER, GFI1, GJA5, GLI2, GLI3, GLIPR2, GLIS2, GLO1, GNAI2, GNAO1, GON4L, GPC6, GPER1, GPR137B, GPR4, GPR55, GPRIN1, GRB2, GREM1, GRID2, GSK3A, GSX2, H3C6, H3C8, HAND1, HCLS1, HCN1, HDAC4, HDAC9, HECTD1, HEG1, HERC1, HES1, HES3, HIVEP3, HMGB1, HNF1B, HNRNPAB, HNRNPD, HOXA1, HOXA3, HOXA4, HOXC10, HOXC11, HOXC4, HOXD13, HSD17B4, HSP90AB1, HYDIN, IFI16, IFT140, IFT74, IFT88, IGF1, IGF2BP1, IGSF8, IHH, IKZF1, IL17D, IL17RD, IL31RA, IL6R, IMMP2L, ING3, INPPL1, INSIG2, INSR, IQGAP3, IRF2BP2, IRF6, IRS2, ISL1, ITCH, ITFG2, ITGA11, ITGB4, ITK, JAK2, JARID2, JHY, KALRN, KAT2B, KAT6A, KAT7, KCNAB2, KCNC1, KCNK2, KCNQ1, KDM2B, KDM6B, KIF18A, KIF1A, KIF26B, KIF2A, KITLG, KLF7, KLHL1, KLHL17, KLHL3, KLK4, KMT2D, KMT2E, KNDC1, KRAS, KRT1, KRT20, KRT25, KRT40, KRT82, KRT84, KRTAP16-1, KRTAP23-1, KRTAP4-3, L3MBTL3, LAMA2, LAMA5, LAMB1, LAMB2, LAMB4, LARGE1, LCE1A, LCE1F, LCE3E, LDB2, LDB3, LDLRAD4, LEPR, LHX4, LHX6, LHX8, LIG4, LIMK2, LONP1, LOXL2, LOXL3, LPAR1, LPCAT1, LRIG3, LRP1, LRP10, LRP2, LRP5, LRRK1, LTBP1, LTBP2, LY9, LYL1, LZTS1, LZTS2, MACROD2, MAD1L1, MAF, MAGI2, MAP2K5, MAPK8IP3, MAPKAP1, MAX, MBD3, MBOAT7, MCM2, MCPH1, MDGA1, MECOM, MEF2C, MEGF8, MEIS2, MEN1, MEOX1, MFNG, MGMT, MICAL2, MIPEP, MKNK2, MKS1, MLLT3, MME, MRPL13, MRTFA, MSH2, MSLN, MTNR1B, MYH15, MYH3, MYL2, MYL6, MYLK, MYO15A, MYO16, MYO18B, MYO1D, MYO1E, MYO5A, MYOM2, NCKAP1L, NCOA6, NCOR2, NCSTN, NDE1, NDRG4, NECTIN1, NEUROD6, NF1, NFAM1, NFATC1, NFATC2, NFATC4, NFE2L2, NFIB, NHEJ1, NKD1, NME2, NOM1, NOTCH1, NOTCH3, NOTCH4, NPHP4, NPHS1, NPNT, NPPA, NPRL3, NR2E3, NRIP1, NRXN1, NTNG1, NTNG2, NTRK2, NUDT1, NUMB, NUP210L, OBSL1, OC90, OGDH, OLFM1, OLIG2, OPN5, ORAI1, P4HTM, PAF1, PAFAH1B1, PALLD, PAPPA2, PAX2, PAX7, PAX8, PAX9, PBLD, PBX1, PCK2, PCSK5, PCSK6, PDCD6, PDGFD, PDGFRA, PDLIM1, PDLIM3, PDPK1, PDZD2, PDZD7, PEG10, PEX13, PFKFB3, PHACTR1, PHF2, PHLDB1, PHLPP2, PIK3R1, PIM1, PIP4K2A, PITPNM1, PKD1, PKM, PKP1, PLA2G2D, PLCB1, PLCE1, PLCG2, PLCL2, PLD4, PLEK, PLEKHA4, PLK4, PLXNA1, PLXNA2, PLXNB1, PLXND1, PML, POLE, POU2F2, POU4F2, PPARG, PPARGC1A, PPARGC1B, PPIB, PPL, PPP2R3C, PPP3CA, PPP3CB, PRDM16, PRICKLE2, PRKCA, PRKCB, PRKCH, PRKCI, PRKCQ, PRKCZ, PRKD2, PRKDC, PRLR, PRMT1, PRPSAP2, PSEN1, PSMA5, PSMA6, PSMA7, PSMC2, PSMC3, PSMC6, PSMD2, PSMD7, PTCH2, PTEN, PTF1A, PTGIS, PTK2, PTPN11, PTPN3, PTPRC, PTPRN, PTPRO, PTPRZ1, PUS7, RAP1A, RAP1GAP, RARA, RASGRP4, RB1, RBFOX1, RBFOX2, RBM20, RBP4, RC3H1, RC3H2, RD3, RELB, RELN, REST, RET, RFLNA, RFLNB, RHEB, RHOA, RHOBTB3, RIPK1, RIPOR2, |
| GO:0048513 | animal organ development | 3,59E-05 [3, 4, 5] | 835,00 | 21,65 | AKAP13, AKT2, ALAS1, ALK, ALPK2, ALPL, ALX1, ALX4, AMH, ANGPT1, ANKH, ANKRD11, ANKRD33, ANO6, ANXA2, ANXA6, AP2A1, AP2A2, AP2S1, AP3B1, APRT, ARHGEF7, ARID2, ARID4B, ARL13B, ARNT2, ARNTL, ASCL1, ASS1, ASXL2, ATAT1, ATF2, ATF3, ATG16L1, ATM, ATP2B4, ATP6V0D1, ATP8A2, ATXN1, B9D1, BAG6, BATF2, BBS4, BBS7, BBX, BCAR3, BCL11B, BCL2, BCL2A1, BCL3, BCL6, BCL9, BDNF, BDNF-AS, BFSP1, BFSP2, BICC1, BIN3, BIRC2, BMERB1, BMP1, BMP2, BMP3, BMP7, BMP8B, BMPER, BNC2, BPGM, BRD1, BTG2, BTRC, C1QC, C21orf91, CA10, CACNA1A, CACNA1H, CALM2, CAMK4, CARD11, CASP2, CASP5, CASR, CAV1, CBFA2T3, CC2D2A, CCBE1, CCDC85C, CCKAR, CCN4, CCR2, CCR6, CD46, CD8A, CDH22, CDH23, CDK1, CDK5R1, CDKN1A, CDKN2A, CELA2A, CELF4, CELSR1, CEND1, CEP290, CHD5, CHRDL2, CHRNB2, CHRND, CHST11, CIB1, CIC, CITED2, CLASP1, CLASP2, CLCN2, CLP1, CNNM4, CNRIP1, CNTN4, CNTNAP2, COBL, COL11A2, COL18A1, COL2A1, COL3A1, COL4A1, COL6A1, COL6A2, COL8A2, CPLANE1, CRB2, CREB3L2, CRIP1, CRISPLD2, CRYBA4, CRYBG3, CRYGC, CSGALNACT1, CSN3, CSNK1E, CTNNA2, CTNNBIP1, CTNS, CUL3, CXCR4, CYLD, CYP19A1, CYP1B1, CYP4A11, DAB1, DAG1, DCAF1, DCHS2, DCLK1, DCLRE1C, DCN, DCTN1, DDIT4, DDR1, DDR2, DGCR6, DGCR6L, DHCR7, DHRS3, DHX15, DHX36, DIPK2A, DKK3, DLC1, DLG1, DLL1, DLL4, DLX2, DMC1, DNAH5, DNAJB6, DNAJC30, DNASE1L2, DNER, DOCK10, DOCK7, DRGX, DSCAML1, DTX1, E2F8, ECE1, EDAR, EFNA4, EGR2, EIF2AK3, EIF2S2, EIF4A3, EIF4E, EIF4EBP1, ELN, ELOVL1, EPHA4, EPHB1, EPHB3, ERBB4, ERCC1, EREG, ESR1, ESRP2, ETS1, ETV1, ETV6, EVL, EVX1, EXOC4, EXPH5, EYA4, EZH2, F3, FA2H, FAM172A, FARP2, FASN, FAT1, FBN1, FBN2, FBXW8, FEZ1, FEZF1, FEZF2, FGF12, FGF2, FGF6, FGF7, FHOD3, FJX1, FKTN, FLT4, FMN1, FNDC3A, FOXK1, FOXO1, FOXP1, FPGS, FRAS1, FREM1, FRS2, FTO, FZD5, FZR1, GAA, GAB2, GABRB2, GARS1, GATA5, GCNT1, GCNT2, GFER, GFI1, GJA5, GLI2, GLI3, GLIPR2, GLIS2, GLO1, GNAI2, GNAO1, GON4L, GPC6, GPER1, GPR137B, GPR4, GPR55, GPRIN1, GRB2, GREM1, GRID2, GSK3A, GSX2, H3C6, H3C8, HAND1, HCLS1, HCN1, HDAC4, HDAC9, HECTD1, HEG1, HERC1, HES1, HES3, HIVEP3, HMGB1, HNF1B, HNRNPAB, HNRNPD, HOXA1, HOXA3, HOXA4, HOXC10, HOXC11, HOXC4, HOXD13, HSD17B4, HSP90AB1, HYDIN, IFI16, IFT140, IFT74, IFT88, IGF1, IGF2BP1, IGSF8, IHH, IKZF1, IL17D, IL17RD, IL31RA, IL6R, IMMP2L, ING3, INPPL1, INSIG2, INSR, IQGAP3, IRF2BP2, IRF6, IRS2, ISL1, ITCH, ITFG2, ITGA11, ITGB4, ITK, JAK2, JARID2, JHY, KALRN, KAT2B, KAT6A, KAT7, KCNAB2, KCNC1, KCNK2, KCNQ1, KDM2B, KDM6B, KIF18A, KIF1A, KIF26B, KIF2A, KITLG, KLF7, KLHL1, KLHL17, KLHL3, KLK4, KMT2D, KMT2E, KNDC1, KRAS, KRT1, KRT20, KRT25, KRT40, KRT82, KRT84, KRTAP16-1, KRTAP23-1, KRTAP4-3, L3MBTL3, LAMA2, LAMA5, LAMB1, LAMB2, LAMB4, LARGE1, LCE1A, LCE1F, LCE3E, LDB2, LDB3, LDLRAD4, LEPR, LHX4, LHX6, LHX8, LIG4, LIMK2, LONP1, LOXL2, LOXL3, LPAR1, LPCAT1, LRIG3, LRP1, LRP10, LRP2, LRP5, LRRK1, LTBP1, LTBP2, LY9, LYL1, LZTS1, LZTS2, MACROD2, MAD1L1, MAF, MAGI2, MAP2K5, MAPK8IP3, MAPKAP1, MAX, MBD3, MBOAT7, MCM2, MCPH1, MDGA1, MECOM, MEF2C, MEGF8, MEIS2, MEN1, MEOX1, MFNG, MGMT, MICAL2, MIPEP, MKNK2, MKS1, MLLT3, MME, MRPL13, MRTFA, MSH2, MSLN, MTNR1B, MYH15, MYH3, MYL2, MYL6, MYLK, MYO15A, MYO16, MYO18B, MYO1D, MYO1E, MYO5A, MYOM2, NCKAP1L, NCOA6, NCOR2, NCSTN, NDE1, NDRG4, NECTIN1, NEUROD6, NF1, NFAM1, NFATC1, NFATC2, NFATC4, NFE2L2, NFIB, NHEJ1, NKD1, NME2, NOM1, NOTCH1, NOTCH3, NOTCH4, NPHP4, NPHS1, NPNT, NPPA, NPRL3, NR2E3, NRIP1, NRXN1, NTNG1, NTNG2, NTRK2, NUDT1, NUMB, NUP210L, OBSL1, OC90, OGDH, OLFM1, OLIG2, OPN5, ORAI1, P4HTM, PAF1, PAFAH1B1, PALLD, PAPPA2, PAX2, PAX7, PAX8, PAX9, PBLD, PBX1, PCK2, PCSK5, PCSK6, PDCD6, PDGFD, PDGFRA, PDLIM1, PDLIM3, PDPK1, PDZD2, PDZD7, PEG10, PEX13, PFKFB3, PHACTR1, PHF2, PHLDB1, PHLPP2, PIK3R1, PIM1, PIP4K2A, PITPNM1, PKD1, PKM, PKP1, PLA2G2D, PLCB1, PLCE1, PLCG2, PLCL2, PLD4, PLEK, PLEKHA4, PLK4, PLXNA1, PLXNA2, PLXNB1, PLXND1, PML, POLE, POU2F2, POU4F2, PPARG, PPARGC1A, PPARGC1B, PPIB, PPL, PPP2R3C, PPP3CA, PPP3CB, PRDM16, PRICKLE2, PRKCA, PRKCB, PRKCH, PRKCI, PRKCQ, PRKCZ, PRKD2, PRKDC, PRLR, PRMT1, PRPSAP2, PSEN1, PSMA5, PSMA6, PSMA7, PSMC2, PSMC3, PSMC6, PSMD2, PSMD7, PTCH2, PTEN, PTF1A, PTGIS, PTK2, PTPN11, PTPN3, PTPRC, PTPRN, PTPRO, PTPRZ1, PUS7, RAP1A, RAP1GAP, RARA, RASGRP4, RB1, RBFOX1, RBFOX2, RBM20, RBP4, RC3H1, RC3H2, RD3, RELB, RELN, REST, RET, RFLNA, RFLNB, RHEB, RHOA, RHOBTB3, RIPK1, RIPOR2, |

| | | | | | |
|---|---|---|---|---|---|
| GO:0016043 | cellular component organization | 1,32E-14 [3] | 1447,00 | 21,75 | ADAMTS2, ADAR, ADCK1, ADD1, ADD2, ADGRB2, ADGRB3, ADGRL3, ADGRV1, ADRM1, AEBP2, AFDN, AGK, AGO2, AGRN, AHCTF1, AKAP13, AKT2, ALAS1, ALDH9A1, ALK, ALKAL2, ALKBH7, ALMS1, ALS2, AMBRA1, AMH, AMPH, ANAPC11, ANAPC4, ANGPT1, ANGPT4, ANK3, ANKRD13B, ANKRD28, ANKRD31, ANKS1B, ANO4, ANO6, ANO7, ANTXR1, ANXA2, ANXA6, AP2A1, AP2A2, AP2S1, AP3B1, APC, APC2, APOBEC1, APOC4, APPL1, APPL2, ARAP1, ARAP3, ARF1, ARFGAP1, ARFGEF3, ARHGAP10, ARHGAP12, ARHGAP17, ARHGAP21, ARHGAP39, ARHGAP40, ARHGDIA, ARHGDIB, ARHGEF10L, ARHGEF18, ARHGEF2, ARHGEF26, ARHGEF7, ARID2, ARID4B, ARL1, ARL13B, ARPC4, ASAP1, ASPH, ASZ1, ATAT1, ATF1, ATF2, ATG14, ATG16L1, ATG2A, ATG2B, ATM, ATP10B, ATP11A, ATP13A2, ATP1A3, ATP2A2, ATP5F1A, ATP5ME, ATP6V0A1, ATP6V0D1, ATP6V1A, ATP6V1H, ATP8A1, ATP8A2, ATP8B2, ATP9B, ATXN10, ATXN7, ATXN7L3, AURKB, B9D1, BABAM2, BAG6, BAIAP2L1, BANP, BAZ1B, BAZ2A, BBS10, BBS4, BBS7, BBS9, BCAR1, BCAS3, BCL11A, BCL11B, BCL2, BCL3, BCL6, BCL9, BDNF, BDNF-AS, BFSP1, BFSP2, BIN3, BIRC2, BLOC1S2, BLOC1S5, BMERB1, BMP1, BMP7, BRD1, BRD2, BRD4, BRF1, BRMS1, BRSK2, BSN, BTBD9, BTC, BTG2, C10orf90, C11orf80, C1QC, C21orf91, CACNA1A, CACNA2D2, CACNB2, CADM1, CADPS2, CALM2, CAMK2B, CAMK2D, CAMKMT, CAMSAP1, CAND1, CAP1, CAP2, CAPRIN2, CAPZB, CARD10, CARMIL1, CARMIL2, CAV1, CBFA2T2, CBX8, CC2D2A, CCAR2, CCDC113, CCDC13, CCDC28B, CCDC80, CCDC88C, CCKAR, CCNA1, CCT2, CD47, CD53, CDC123, CDC20, CDC25B, CDC25C, CDC42BPB, CDH10, CDH13, CDH18, CDH19, CDH23, CDH26, CDH4, CDK1, CDK11B, CDK2, CDK5R1, CDKN1A, CDKN2A, CDT1, CELF2, CELF4, CELF5, CELSR1, CENPP, CEP131, CEP164, CEP290, CEP295, CEP43, CEP70, CETN1, CFAP46, CFAP54, CFAP61, CFAP74, CHAF1A, CHAMP1, CHCHD3, CHCHD4, CHD3, CHD5, CHEK1, CHEK2, CHMP2A, CHRNB2, CHTF8, CIB1, CILK1, CIT, CLASP1, CLASP2, CLCN3, CLDN16, CLDN8, CLIP2, CLN6, CLP1, CLTCL1, CLVS1, CLYBL, CNIH3, CNOT1, CNOT7, CNTN4, CNTN5, CNTNAP2, CNTROB, COA1, COBL, COG2, COL11A2, COL18A1, COL22A1, COL23A1, COL2A1, COL3A1, COL4A1, COL4A2, COL6A1, COL6A2, COL8A2, CORO1B, CORO2B, CORO7-PAM16, COX20, CPLANE1, CPNE5, CRBN, CREB3L1, CREB3L2, CRIM1, CRISP1, CRISPLD2, CSGALNACT1, CSMD3, CSNK1E, CSPG5, CTBP2, CTCF, CTNNA2, CTNNBIP1, CTNND2, CTSS, CTTN, CUL3, CUX1, CXCR4, CYCS, CYFIP1, CYFIP2, CYLD, CYP1B1, DAB1, DAG1, DARS1, DAXX, DBN1, DCAF1, DCLK1, DCLRE1C, DCN, DCP2, DCTN1, DDHD2, DDIT4, DDR1, DDR2, DDX49, DDX6, DEF8, DENND5A, DGKQ, DHDDS, DHX30, DHX36, DIAPH3, DIS3L2, DLC1, DLG1, DLG2, DLG4, DLL1, DMC1, DNA2, DNAH17, DNAH5, DNAH8, DNAH9, DNAJB12, DNAJB13, DNAJB6, DNAJC15, DNAJC6, DNASE1L3, DNER, DNM1, DNM1L, DNMT3A, DOCK10, DOCK7, DOK4, DOK6, DOT1L, DPF1, DRGX, DSCAML1, DSN1, DST, DYNC2LI1, ECI2, ECSIT, EFCAB11, EFHD1, EFNA4, EFNA5, EGR2, EHBP1L1, EHMT1, EHMT2, EIF2AK3, EIF2AK4, EIF2S2, EIF3B, EIF4B, EIF4EBP1, ELAVL1, ELMO1, ELN, EML3, ENC1, ENPP2, EPB41L4A, EPB41L4B, EPHA1, EPHA10, EPHA4, EPHA6, EPHB1, EPHB3, EPPK1, EPRS1, EPS15L1, EPS8L1, ERBB4, ERCC1, ERCC5, ERCC8, EREG, ESCO1, ESPN, ESPNL, ESR1, ESR2, ETS1, ETV1, ETV6, EVL, EVPLL, EXOC4, EXOSC10, EYA2, EYA4, EZH2, F11R, F2RL3, F3, F5, FA2H, FAF1, FAM161A, FAM171A1, FAM172A, FANCM, FAP, FARP1, FARP2, FASN, FAT1, FBLN5, FBN1, FBN2, FBP1, FBXW8, FCHSD1, FCHSD2, FER, FER1L4, FER1L5, FER1L6, FERMT2, FEZ1, FEZF1, FEZF2, FGD2, FGF2, FGF7, FHOD1, FHOD3, FITM2, FLRT1, FMN1, FMNL2, FNBP1, FNIP2, FNTA, FRMD5, FRMD6, FRS2, FRYL, FXYD5, FZD10, FZD5, FZR1, GAA, GAB1, GAB2, GABPB1, GABRA2, GABRB2, GAK, GARS1, GAS7, GCG, GEMIN2, GET4, GFI1, GINS4, GJA5, GLE1, GLI2, GLI3, GLMN, GLRX3, GLTP, GMFB, GOLGA5, GORASP1, GPC6, GPER1, GPRIN1, GRAMD1C, GRB10, GRB2, GREM1, GRID2, GRPEL1, GRWD1, GSG1L, GSK3A, GTF2B, GTF2H1, H2AC4, H2AC6, H2AJ, H2BC15, H3C6, H3C8, HACL1, HAO2, HAPLN2, HCLS1, HCN1, HDAC4, HDAC9, HECW1, HEG1, HEPACAM2, HERC1, HES1, HEXB, HGS, HIP1, HIP1R, HLA-DRA, HMBOX1, HMGB1, HMGN3, HNF1B, HNRNPC, HNRNPD, HNRNPK, HOOK1, HPS4, HRG, HSD17B12, HSD17B4, HSP90AB1, HSPD1, HTT, HUS1, HYDIN, IBA57, ICAM3, IDE, IFRD1, IFT140, IFT46, IFT74, IFT88, IGF1, IGF1R, IGHMBP2, IGLL5, IGSF9B, IHH, IKBKB, IKZF1, IL10RA, IL1RAP, IMMP2L, IMMT, ING1, ING3, INPP5F, INPPL1, INSR, IQGAP2, IQGAP3, IQSEC1, IQSEC3, IRS2, ISL1, ITCH, ITGA1, ITGA11, ITGA5, ITGAE, ITGAL, ITGB2, ITGB4, ITPA, IWS1, JAK2, JARID2, JHY, JMJD1C, KALRN, KANK1, KANK3, KAT2B, KAT6A, KAT6B, KAT7, KATNAL2, KCNB1, KCNB2, KCNC1, KCNC3, KCNF1, KCNJ2, KCNMA1, KCNV1, KCTD10, KCTD14, KCTD16, KCTD2, KCTD5, KCTD6, KDM2A, KDM2B, KDM3A, KDM4B, KDM4C, KDM4D, KDM6B, KHDC4, KIAA1614, KIDINS220, KIF13A, KIF18A, KIF1A, KIF22, KIF25, KIF26A, KIF2A, KIF2C, KIF5A, KIFC1, KIRREL1, KIZ, KLF7, KLHDC8B, KLHL1, KLHL17, KLK4, KLK7, KMT2D, KMT2E, KMT5C, KNDC1, KPNA7, KRAS, KRT1, KRT20, KRT25, KRT84, L3MBTL3, LAMA2, LAMA5, LAMB1, LAMB2, LAMB4, LDB2, LDB3, LDLR, LDLRAD4, LGMN, LGR6, LHX4, LIG4, LIMA1, LIMK2, LIMS1, LIN9, LINGO1, LLGL1, LONP1, LOXL2, LOXL3, LPAR1, LPL, LRCH4, LRFN2, LRFN3, LRIG2, LRIG3, LRP1, LRP12, LRP2, LRP5, LRPAP1, LRRC4C, LRSAM1, LSM2, LTBP2, LUC7L2, LUC7L3, LURAP1, LYRM4, LZTS1, LZTS2, M1AP, MACF1, MAD1L1, MAG, MAGI2, MAK, MAL, MAP1LC3A, MAP2, MAP2K2, MAP2K5, MAP3K13, MAP3K20, MAP4, MAP4K4, MAP6, MAP7, MAPK7, MAPK8, MAPK8IP2, MAPK8IP3, MAPK9, MAPKAP1, MAPKAPK5, MARCKS, MARF1, MARK3, MAST4, MAX, MBD3, MCM2, MCMDC2, MCPH1, MCRS1, MDGA1, MECOM, MED22, MED24, MEF2C, MEGF8, MEIKIN, |
| GO:0006366 | transcription by RNA polymerase II | 1,91E-02 [6, 7, 8, 9, 10] | 531,00 | 21,79 | ATN1, ATP2B4, ATXN1, AURKB, BACH1, BATF2, BAZ1B, BBS7, BBX, BCAS3, BCL11A, BCL11B, BCL3, BCL6, BCL9, BHLHE40, BMP2, BMP7, BNC1, BNC2, BRD2, BRD4, BRMS1, BTF3, BTG2, BTN3A3, C1orf61, CALCOCO1, CAMK2D, CAMTA1, CAMTA2, CAND1, CAPRIN2, CAV1, CBFA2T2, CBX8, CC2D1B, CCNL1, CDH13, CDK1, CDK2, CDKN1A, CDKN2A, CHCHD3, CHD3, CHD5, CHEK1, CIC, CITED2, CITED4, CLP1, CNOT1, CNOT7, CREB3L1, CREB3L2, CREB5, CREM, CRY2, CRYM, CSRNP2, CSRNP3, CTBP2, CTCF, CTNNBIP1, CUL2, CUL3, CUX1, CXXC5, CYFIP1, DCAF1, DCN, DGKQ, DHX36, DLG1, DLL1, DLL4, DLX2, DNAJB5, DNMT3A, DOT1L, DPF1, DRGX, DTX1, DUSP26, DUXA, E2F2, E2F8, EBF1, EBF3, EBF4, EGR2, EHF, EHMT1, EHMT2, ELF1, ELK3, ELOA, ERCC1, ESR1, ESR2, ESRRG, ETS1, ETV1, ETV6, EVX1, EZH2, FAM220A, FBLN5, FBP1, FERD3L, FEZF1, FEZF2, FGF2, FHOD1, FNIP2, FOXB2, FOXD3, FOXI3, FOXJ3, FOXK1, FOXO1, FOXP1, FUBP1, FZD5, GABPB1, GARS1, GATA5, GFI1, GLI2, GLI3, GLIS2, GLO1, GON4L, GPER1, GPRIN1, GREM1, GSK3A, GSX2, GTF2B, GTF2H1, GTF2IRD1, HAND1, HCLS1, HDAC4, HDAC9, HELT, HES1, HES3, HES4, HEXB, HIVEP1, HMBOX1, HMGB1, HMGN3, HNF1B, HNRNPK, HOXA1, HOXA3, HOXA4, HOXC10, HOXC11, HOXC4, HOXD13, HSD17B4, HSPD1, HTATIP2, IFI16, IFRD1, IFT74, IGF1, IGHMBP2, IHH, IKBKB, IKZF1, INSIG2, INTS1, INTS3, INTS9, IRF2, IRF2BP2, IRF6, IRF9, ISL1, IWS1, JARID2, JAZF1, KALRN, KAT2B, KAT6A, KAT6B, KAT7, KDM2B, KDM3A, KDM4C, KDM6B, KHDRBS1, KLF14, KLF17, KLF7, KMT2D, LARP7, LDB2, LHX4, LHX6, LHX8, LIN9, LMCD1, LMO7, LMX1B, LOXL2, LRP5, LYL1, MACC1, MAF, MAFA, MAFG, MAFK, MAML2, MAML3, MAP2K5, MAPK7, MAX, MBD3, MCM2, MCPH1, MCRS1, MECOM, MED13L, MED16, MED22, MED24, MED26, MED27, MEF2C, MEIS2, MELTF, MEN1, MEOX1, MICAL2, MIDEAS, MIER1, MLLT3, MRPL23, MRTFA, MSGN1, MTA1, MTA3, MXD1, MYO1C, MYRF, MYT1L, NACC2, NCK2, NCOA2, NCOA6, NCOA7, NCOR2, NEDD4L, NEUROD6, NEUROG2, NFATC1, NFATC2, NFATC2IP, NFATC4, NFE2L2, NFIB, NFIL3, NFXL1, NFYA, NFYC, NKX2-4, NLRC5, NME2, NOC2L, NOP2, NOS1, NOTCH1, NOTCH3, NOTCH4, NPNT, NPPA, NR1D1, NR2C1, NR2E3, NR3C1, NR3C2, NRF1, NRIP1, NSD1, OLIG2, PAF1, PAGR1, PAX2, PAX7, PAX8, PAX9, PBX1, PCF11, PCGF5, PDLIM1, PDZD2, PEG3, PER2, PFKM, PHAX, PHF19, PHF20, PHRF1, PIK3R1, PKD1, PKMYT1, PKNOX2, PLAC8, PLXND1, POLR2B, POLR2F, POU2F1, POU2F2, POU2F3, POU4F2, POU6F2, PPARG, PPARGC1A, PPARGC1B, PPP3CA, PPP3CB, PRDM16, PRDM2, PRDM5, PRDM6, PRKCB, PRKD2, PRKDC, PRKN, PSEN1, PSMA5, PSMA6, PSMA7, PSMC2, PSMC3, PSMC6, PSMD2, PSMD7, PTEN, PTF1A, PTPRN, PURG, QRICH1, RAI1, RARA, RB1, RBX1, RCOR3, RELB, REST, RFX2, RFXAP, RGMA, RHOA, RIPK1, RITA1, RNF111, RNGTT, RORA, RORB, RORC, RPRD1B, RREB1, RUNX1, RUNX3, RXRB, RXRG, SATB2, SBNO2, SCRT1, SEMA4D, SERTAD1, |
| GO:0030154 | cell differentiation | 1,35E-08 [3] | 984,00 | 21,98 | AFDN, AFF4, AGBL4, AGRN, AJAP1, AKAP13, AKR1C2, AKT2, ALAS1, ALK, ALKAL2, ALPK2, ALPL, ALS2, ALX1, AMBRA1, AMH, ANGPT1, ANK3, ANKRD17, ANKRD26, ANKRD33, ANKS1A, ANTXR1, ANXA13, ANXA2, ANXA6, AP2A1, AP3B1, APC, APC2, APPL2, ARF1, ARHGDIA, ARHGEF2, ARHGEF26, ARHGEF7, ARID4B, ARL13B, ARNTL, ASAP1, ASCL1, ASXL2, ASZ1, ATAT1, ATF1, ATF2, ATF3, ATM, ATP2A2, ATP2B2, ATP2B4, ATP8A2, ATXN10, B9D1, BAG6, BATF2, BBS4, BBS7, BBS9, BCL11A, BCL11B, BCL2, BCL3, BCL6, BCL9, BDNF, BDNF-AS, BEND6, BFSP1, BFSP2, BHLHE40, BIN3, BIRC2, BLOC1S2, BLOC1S5, BMERB1, BMP1, BMP2, BMP3, BMP7, BMP8B, BNC1, BPGM, BRD1, BRINP2, BRSK2, BTBD6, BTC, BTG2, BTG4, C1QC, C21orf91, CACNA1A, CACNA1H, CACNA2D2, CADM1, CAMK2B, CAMK4, CAMSAP1, CAND1, CAPRIN2, CARD11, CARMIL1, CASP2, CASZ1, CATSPERG, CAV1, CBFA2T2, CBFA2T3, CCDC3, CCDC88C, CCKAR, CCN4, CCR2, CCR6, CD46, CD53, CD8A, CDC20, CDC25B, CDH23, CDH4, CDK1, CDK5R1, CDKN1A, CDKN2A, CDYL, CELA2A, CELF4, CELSR1, CEND1, CEP131, CEP290, CFAP54, CHD5, CHEK2, CHRDL2, CHRNB2, CHST11, CIB1, CIT, CITED2, CLASP1, CLASP2, CLCN2, CNTN4, CNTNAP2, COBL, COL11A2, COL18A1, COL2A1, COL3A1, COL4A1, COL4A2, COL6A1, COL6A2, CPNE5, CPT1A, CRB2, CREB3L1, CREB3L2, CREM, CSMD3, CSNK1E, CSPG5, CTBP2, CTNNA2, CTNNBIP1, CTNND2, CTTN, CUL3, CUX1, CXCR4, CYFIP1, CYFIP2, CYLD, CYP24A1, DAB1, DAG1, DBN1, DCAF1, DCLK1, DCLRE1C, DDIT4, DDR1, DDR2, DDX6, DENND5A, DHCR7, DHDDS, DHX15, DHX36, DLD, DLG4, DLL1, DLL4, DLX2, DMC1, DNAJB6, DNASE1L2, DNASE1L3, DNER, DNM1L, DNMT3A, DOCK10, DOCK7, DOK4, DOK6, DRGX, DSCAML1, DTX1, E2F8, EDAR, EFCAB9, EFHD1, EFNA4, EFNA5, EGR2, EHF, EHMT2, EIF2AK3, EIF2AK4, EIF4E, EIF4ENIF1, ELF1, ELK3, ELSPBP1, ENC1, EPHA1, EPHA10, EPHA4, EPHA6, EPHB1, EPHB3, ERAP1, ERBB4, ERCC1, EREG, ESR1, ETS1, ETV1, ETV6, EVL, EVX1, EXPH5, EYA2, EYA4, EZH2, F11R, F3, FA2H, FAM120B, FAM172A, FARP1, FARP2, FASN, FAT1, FBN1, FBN2, FBXW8, FER, FER1L5, FERD3L, FERMT2, FEZ1, FEZF1, FEZF2, FGF2, FGF22, FGF6, FHOD3, FLRT1, FNDC3A, FOXB2, FOXD3, FOXI3, FOXK1, FOXO1, FOXP1, FRMD6, FRS2, FRYL, FTO, FZD10, FZD5, FZR1, GAB1, GAB2, GABRB2, GADD45G, GAK, GARS1, GAS7, GATA5, GCNT2, GFI1, GLI2, GLI3, GLIPR2, GLIS2, GLMN, GLO1, GLRX2, GNA12, GNAO1, GON4L, GORASP1, GPER1, GPR137B, GPR17, GPR4, GPR55, GPRC5B, GPRIN1, GPSM1, GRB10, GRB2, GREM1, GRID2, GRK5, GSK3A, GSX2, H3C6, H3C8, HAND1, HAPLN2, HCLS1, HCN1, HDAC4, HDAC9, HECW1, HEG1, HELT, HERC1, HES1, HES3, HES4, HEXB, HIP1, HIVEP3, HMGB1, HNF1B, HNRNPAB, HNRNPC, HOOK1, HOXC10, HPS4, HSD17B4, HSP90AB1, HTATIP2, HTR2A, HYDIN, IFI16, IFRD1, IFT140, IFT74, IFT88, IGF1, IHH, IKBKB, IKZF1, IL17RD, IL31RA, IL6R, IL6ST, ING3, INPP5F, INSC, IQSEC1, IRF2BP2, IRF6, IRS2, ISL1, ITCH, ITFG2, ITGA1, ITGA11, ITGA5, ITGB2, ITGB4, ITK, JAK2, JARID2, KALRN, KANK1, KAT2B, KAT6A, KAT7, KCNAB2, KDM3A, KDM4C, KDM6B, KIAA1522, KIDINS220, KIF1A, KIF2A, KIF5A, KIFBP, KITLG, KLF7, KLHL1, KMT2D, KMT2E, KNDC1, KRAS, KRT1, KRT20, KRT25, KRT40, KRT82, KRT84, KRTAP16-1, KRTAP23-1, KRTAP4-3, L3MBTL3, LAMA2, LAMA5, LAMB1, LAMB2, LAMB4, LCE1A, LCE1F, LCE3E, LDB2, LDB3, LDHD, LDLR, LDLRAD4, LEPR, LGR6, LHX4, LHX6, LHX8, LIG4, LIMS1, LINGO1, LLGL1, LMX1B, LONP1, LOXL2, LOXL3, LPAR1, LPL, LRIG2, LRP1, LRP12, LRP2, LRP5, LRRC4C, LRRK1, LTBP2, LY9, LYL1, LZTS1, M1AP, MACF1, MAF, MAFG, MAG, MAGI2, MAK, MAL, MAP2, MAP3K13, MAP3K20, MAP3K5, MAP4, MAP4K4, MAP6, MAPK7, MAPK8IP2, MAPK8IP3, MAPK9, MARF1, MBNL1, MBOAT7, MCL1, MCMDC2, MDGA1, MECOM, MEF2C, MEGF8, MEIOC, MEIS2, MELTF, MEN1, MEOX1, METRNL, MFNG, MGMT, MICALL1, MKS1, MLLT3, MMD, MME, MOV10L1, MRC2, MRTFA, MSGN1, MSH2, MSI2, MTMR2, MYD88, MYH3, MYL2, MYO16, MYO18B, MYO1E, MYO5A, MYOM2, MYPN, MYRF, MYT1L, NAPEPLD, NAV1, NAV3, NCAM2, NCK2, NCKAP1, NCKAP1L, NCOA2, NCOA6, NCSTN, NDE1, NDRG3, NDRG4, NECTIN1, NEDD4L, NEDD9, NEO1, NEPRO, NEUROD6, NEUROG2, NF1, NFAM1, NFASC, NFATC1, NFATC2, NFATC4, NFE2L2, NFIB, NGEF, NGF, NHEJ1, NKD1, NKX2-4, NME2, NOL3, NOS1, NOTCH1, NOTCH3, NOTCH4, NPHP4, NPHS1, NPNT, NPPA, NPTN, NR1D1, NR2C1, NR2E3, NRXN1, NRXN3, NSMF, NTM, NTNG1, NTNG2, NTRK2, NUMB, NUP210L, NUS1, NXN, OBSCN, OBSL1, OCA2, OGDH, OLFM1, OLFM2, OLIG2, OPCML, OSBP2, OSBPL8, P4HTM, PABPC1L, PACSIN1, PAF1, PAFAH1B1, PAK4, PALLD, PAQR8, PARD3, PAX2, PAX7, PAX8, PBLD, PBX1, PCARE, PCK2, PCSK6, PDCD6, PDGFRA, PDPK1, PDZD2, PDZD7, PEAK1, PEG10, PER2, PEX13, PHACTR1, PHF19, PHLDB1, PIH1D1, PIK3CB, PIK3R1, PIM1, PIP4K2A, PKMYT1, PKP1, PLA2G2D, PLAC8, PLCB1, PLCG2, PLCL2, PLD4, PLEK, PLEKHB1, PLK4, PLK5, PLXNA1, PLXNA2, PLXNB1, PLXNC1, PLXND1, PML, POMGNT2, POU2F1, POU2F2, POU2F3, POU4F2, POU6F2, PPARG, PPARGC1A, PPARGC1B, PPL, PPP1R16B, PPP2R3C, PPP3CA, PPP3CB, PRAG1, PRDM16, PRDM5, PRDM6, PRKCA, PRKCB, PRKCH, PRKCI, PRKCQ, PRKCZ, PRKDC, PRKN, PRLR, PRMT1, PRPF19, PRUNE1, PSD, PSEN1, PSMA5, PSMA6, PSMA7, PSMC2, PSMC3, PSMC6, PSMD2, PSMD7, PTCH2, PTEN, PTF1A, PTK2, PTPN11, PTPRA, PTPRC, PTPRF, PTPRO, PTPRZ1, PUM1, PUS7, PXDN, PXDNL, RAB29, RAB35, RADIL, RANBP1, RANBP9, |

| | | | | | | |
|---|---|---|---|---|---|---|
| GO:0030154 | cell differentiation | 1,35E-08 | [3] | 984,00 | 21,98 | AFDN, AFF4, AGBL4, AGRN, AJAP1, AKAP13, AKR1C2, AKT2, ALAS1, ALK, ALKAL2, ALPK2, ALPL, ALS2, ALX1, AMBRA1, AMH, ANGPT1, ANK3, ANKRD17, ANKRD26, ANKRD33, ANKS1A, ANTXR1, ANXA13, ANXA2, ANXA6, AP2A1, AP3B1, APC, APC2, APPL2, ARF1, ARHGDIA, ARHGEF2, ARHGEF26, ARHGEF7, ARID4B, ARL13B, ARNTL, ASAP1, ASCL1, ASXL2, ASZ1, ATAT1, ATF1, ATF2, ATF3, ATM, ATP2A2, ATP2B2, ATP2B4, ATP8A2, ATXN10, B9D1, BAG6, BATF2, BBS4, BBS7, BBS9, BCL11A, BCL11B, BCL2, BCL3, BCL6, BCL9, BDNF, BDNF-AS, BEND6, BFSP1, BFSP2, BHLHE40, BIN3, BIRC2, BLOC1S2, BLOC1S5, BMERB1, BMP1, BMP2, BMP3, BMP7, BMP8B, BNC1, BPGM, BRD1, BRINP2, BRSK2, BTBD6, BTC, BTG2, BTG4, C1QC, C21orf91, CACNA1A, CACNA1H, CACNA2D2, CADM1, CAMK2B, CAMK4, CAMSAP1, CAND1, CAPRIN2, CARD11, CARMIL1, CASP2, CASZ1, CATSPERG, CAV1, CBFA2T2, CBFA2T3, CCDC3, CCDC88C, CCKAR, CCN4, CCR2, CCR6, CD46, CD53, CD8A, CDC20, CDC25B, CDH23, CDH4, CDK1, CDK5R1, CDKN1A, CDKN2A, CDYL, CELA2A, CELF4, CELSR1, CEND1, CEP131, CEP290, CFAP54, CHD5, CHEK2, CHRDL2, CHRNB2, CHST11, CIB1, CIT, CITED2, CLASP1, CLASP2, CLCN2, CNTN4, CNTNAP2, COBL, COL11A2, COL18A1, COL2A1, COL3A1, COL4A1, COL4A2, COL6A1, COL6A2, CPNE5, CPT1A, CRB2, CREB3L1, CREB3L2, CREM, CSMD3, CSNK1E, CSPG5, CTBP2, CTNNA2, CTNNBIP1, CTNND2, CTTN, CUL3, CUX1, CXCR4, CYFIP1, CYFIP2, CYLD, CYP24A1, DAB1, DAG1, DBN1, DCAF1, DCLK1, DCLRE1C, DDIT4, DDR1, DDR2, DDX6, DENND5A, DHCR7, DHDDS, DHX15, DHX36, DLD, DLG4, DLL1, DLL4, DLX2, DMC1, DNAJB6, DNASE1L2, DNASE1L3, DNER, DNM1L, DNMT3A, DOCK10, DOCK7, DOK4, DOK6, DRGX, DSCAML1, DTX1, E2F8, EDAR, EFCAB9, EFHD1, EFNA4, EFNA5, EGR2, EHF, EHMT2, EIF2AK3, EIF2AK4, EIF4E, EIF4ENIF1, ELF1, ELK3, ELSPBP1, ENC1, EPHA1, EPHA10, EPHA4, EPHA6, EPHB1, EPHB3, ERAP1, ERBB4, ERCC1, EREG, ESR1, ETS1, ETV1, ETV6, EVL, EVX1, EXPH5, EYA2, EYA4, EZH2, F11R, F3, FA2H, FAM120B, FAM172A, FARP1, FARP2, FASN, FAT1, FBN1, FBN2, FBXW8, FER, FER1L5, FERD3L, FERMT2, FEZ1, FEZF1, FEZF2, FGF2, FGF22, FGF6, FHOD3, FLRT1, FNDC3A, FOXB2, FOXD3, FOXI3, FOXK1, FOXO1, FOXP1, FRMD6, FRS2, FRYL, FTO, FZD10, FZD5, FZR1, GAB1, GAB2, GABRB2, GADD45G, GAK, GARS1, GAS7, GATA5, GCNT2, GFI1, GLI2, GLI3, GLIPR2, GLIS2, GLMN, GLO1, GLRX2, GNA12, GNAO1, GON4L, GORASP1, GPER1, GPR137B, GPR17, GPR4, GPR55, GPRC5B, GPRIN1, GPSM1, GRB10, GRB2, GREM1, GRID2, GRK5, GSK3A, GSX2, H3C6, H3C8, HAND1, HAPLN2, HCLS1, HCN1, HDAC4, HDAC9, HECW1, HEG1, HELT, HERC1, HES1, HES3, HES4, HEXB, HIP1, HIVEP3, HMGB1, HNF1B, HNRNPAB, HNRNPC, HOOK1, HOXC10, HPS4, HSD17B4, HSP90AB1, HTATIP2, HTR2A, HYDIN, IFI16, IFRD1, IFT140, IFT74, IFT88, IGF1, IHH, IKBKB, IKZF1, IL17RD, IL31RA, IL6R, IL6ST, ING3, INPP5F, INSC, IQSEC1, IRF2BP2, IRF6, IRS2, ISL1, ITCH, ITFG2, ITGA1, ITGA11, ITGA5, ITGB2, ITGB4, ITK, JAK2, JARID2, KALRN, KANK1, KAT2B, KAT6A, KAT7, KCNAB2, KDM3A, KDM4C, KDM6B, KIAA1522, KIDINS220, KIF1A, KIF2A, KIF5A, KIFBP, KITLG, KLF7, KLHL1, KMT2D, KMT2E, KNDC1, KRAS, KRT1, KRT20, KRT25, KRT40, KRT82, KRT84, KRTAP16-1, KRTAP23-1, KRTAP4-3, L3MBTL3, LAMA2, LAMA5, LAMB1, LAMB2, LAMB4, LCE1A, LCE1F, LCE3E, LDB2, LDB3, LDHD, LDLR, LDLRAD4, LEPR, LGR6, LHX4, LHX6, LHX8, LIG4, LIMS1, LINGO1, LLGL1, LMX1B, LONP1, LOXL2, LOXL3, LPAR1, LPL, LRIG2, LRP1, LRP12, LRP2, LRP5, LRRC4C, LRRK1, LTBP2, LY9, LYL1, LZTS1, M1AP, MACF1, MAF, MAFG, MAG, MAGI2, MAK, MAL, MAP2, MAP3K13, MAP3K20, MAP3K5, MAP4, MAP4K4, MAP6, MAPK7, MAPK8IP2, MAPK8IP3, MAPK9, MARF1, MBNL1, MBOAT7, MCL1, MCMDC2, MDGA1, MECOM, MEF2C, MEGF8, MEIOC, MEIS2, MELTF, MEN1, MEOX1, METRNL, MFNG, MGMT, MICALL1, MKS1, MLLT3, MMD, MME, MOV10L1, MRC2, MRTFA, MSGN1, MSH2, MSI2, MTMR2, MYD88, MYH3, MYL2, MYO16, MYO18B, MYO1E, MYO5A, MYOM2, MYPN, MYRF, MYT1L, NAPEPLD, NAV1, NAV3, NCAM2, NCK2, NCKAP1, NCKAP1L, NCOA2, NCOA6, NCSTN, NDE1, NDRG3, NDRG4, NECTIN1, NEDD4L, NEDD9, NEO1, NEPRO, NEUROD6, NEUROG2, NF1, NFAM1, NFASC, NFATC1, NFATC2, NFATC4, NFE2L2, NFIB, NGEF, NGF, NHEJ1, NKD1, NKX2-4, NME2, NOL3, NOS1, NOTCH1, NOTCH3, NOTCH4, NPHP4, NPHS1, NPNT, NPPA, NPTN, NR1D1, NR2C1, NR2E3, NRXN1, NRXN3, NSMF, NTM, NTNG1, NTNG2, NTRK2, NUMB, NUP210L, NUS1, NXN, OBSCN, OBSL1, OCA2, OGDH, OLFM1, OLFM2, OLIG2, OPCML, OSBP2, OSBPL8, P4HTM, PABPC1L, PACSIN1, PAF1, PAFAH1B1, PAK4, PALLD, PAQR8, PARD3, PAX2, PAX7, PAX8, PBLD, PBX1, PCARE, PCK2, PCSK6, PDCD6, PDGFRA, PDPK1, PDZD2, PDZD7, PEAK1, PEG10, PER2, PEX13, PHACTR1, PHF19, PHLDB1, PIH1D1, PIK3CB, PIK3R1, PIM1, PIP4K2A, PKMYT1, PKP1, PLA2G2D, PLAC8, PLCB1, PLCG2, PLCL2, PLD4, PLEK, PLEKHB1, PLK4, PLK5, PLXNA1, PLXNA2, PLXNB1, PLXNC1, PLXND1, PML, POMGNT2, POU2F1, POU2F2, POU2F3, POU4F2, POU6F2, PPARG, PPARGC1A, PPARGC1B, PPL, PPP1R16B, PPP2R3C, PPP3CA, PPP3CB, PRAG1, PRDM16, PRDM5, PRDM6, PRKCA, PRKCB, PRKCH, PRKCI, PRKCQ, PRKCZ, PRKDC, PRKN, PRLR, PRMT1, PRPF19, PRUNE1, PSD, PSEN1, PSMA5, PSMA6, PSMA7, PSMC2, PSMC3, PSMC6, PSMD2, PSMD7, PTCH2, PTEN, PTF1A, PTK2, PTPN11, PTPRA, PTPRC, PTPRF, PTPRO, PTPRZ1, PUM1, PUS7, PXDN, PXDNL, RAB29, RAB35, RADIL, RANBP1, RANBP9, |
| GO:0009891 | positive regulation of biosynthetic process | 1,78E-02 | [3, 4, 5] | 486,00 | 22,00 | ARNTL, ASCL1, ASS1, ASXL2, ATF1, ATF2, ATF3, ATM, ATXN7L3, AURKB, BACH1, BANP, BCAR3, BCAS3, BCL10, BCL11B, BCL3, BCL9, BICRAL, BIRC2, BMP2, BMP7, BRD1, BRD4, BRF1, BTRC, C1orf61, CALCOCO1, CAMK4, CAMTA1, CAMTA2, CAND1, CAPRIN2, CARD11, CASZ1, CBX8, CCAR2, CCDC3, CCR2, CCT2, CDC123, CDH13, CDK1, CDK2, CDKN2A, CDT1, CEP290, CHEK1, CHEK2, CHTF8, CITED2, CITED4, CIZ1, CNOT7, CREB3L1, CREB3L2, CREB5, CREM, CSRNP2, CSRNP3, CTBP2, CTCF, CYFIP1, DAXX, DCN, DDAH2, DHX34, DHX36, DLL1, DLX2, DNA2, DNAJC3, DOT1L, DPF1, DTX1, E2F2, E2F8, EBF1, EBF3, EBF4, EGR2, EHF, EIF2AK3, EIF2AK4, EIF4A3, ELAVL1, ELF1, ELK3, ERBB4, ERCC1, EREG, ESR1, ESR2, ESRRG, ETS1, ETV1, ETV6, EVX1, F3, FAF1, FBLN5, FEZF1, FEZF2, FGF2, FGF7, FHOD1, FOXD3, FOXJ3, FOXK1, FOXO1, FUBP1, FZD5, FZR1, GABPB1, GARS1, GATA5, GCG, GFI1, GLI2, GLI3, GLIS2, GLMN, GPER1, GPRIN1, GPT, GREM1, GSK3A, GTF2B, GTF2H1, GUCA2A, HAND1, HCLS1, HDAC4, HELT, HES1, HES3, HEXB, HIVEP1, HIVEP3, HMBOX1, HMGB1, HMGN3, HNF1B, HNRNPAB, HNRNPD, HNRNPK, HOXA1, HOXA3, HOXA4, HOXC10, HOXC11, HOXC4, HOXD13, HSD17B4, HSP90AB1, HSPH1, HTATIP2, HTR2A, IFI16, IFT74, IGF1, IHH, IKBKB, IKZF1, IL31RA, ILF3, ING1, ING3, INSIG2, INSR, IRF2, IRF6, IRS2, ISL1, ITGB2, JAK2, KAT2B, KAT6A, KAT6B, KAT7, KDM3A, KDM6B, KHDRBS1, KLF14, KLF17, KLF7, KMT2D, KMT2E, LARP4B, LARP7, LDB2, LDLR, LHX4, LHX6, LHX8, LMO7, LMX1B, LONP1, LPGAT1, LRP5, LYL1, MACC1, MAF, MAFA, MAFG, MAK, MAML2, MAML3, MAP2K2, MAP2K5, MAP3K2, MAP3K5, MAPK7, MAPKAPK5, MARS1, MAX, MCRS1, MECOM, MED16, MED22, MED24, MED26, MED27, MEF2C, MEIS2, MELTF, MEN1, MEOX1, MICAL2, MLLT3, MRTFA, MSANTD1, MSGN1, MTA1, MTA3, MYD88, MYRF, NAT10, NCK2, NCOA2, NCOA6, NCOA7, NCSTN, NEK7, NEUROD6, NFATC1, NFATC2, NFATC2IP, NFATC4, NFE2L2, NFIB, NFIL3, NFKBIB, NFYA, NFYC, NKX2-4, NLRC5, NLRP12, NME2, NOS1, NOS1AP, NOTCH1, NOTCH3, NOTCH4, NPAT, NPNT, NR1D1, NR2E3, NR3C1, NRF1, NRIP1, NSD1, NSD3, P2RY6, PABPC1L, PAF1, PAGR1, PAOX, PARD3, PASK, PAX2, PAX7, PAX8, PAX9, PBX1, PCGF5, PDLIM1, PDZD2, PEG3, PER2, PFKM, PHF2, PIH1D1, PIK3R1, PIM1, PKD1, PKIB, PLA2G6, PLAC8, PLCB1, PLD1, PLIN5, PLXND1, PML, POLDIP3, POMT1, POU2F1, POU2F2, POU2F3, POU4F2, POU6F2, PPARG, PPARGC1A, PPARGC1B, PPP3CA, PPP3CB, PRDM16, PRDM2, PRKCB, PRKCQ, PRKD2, PRKDC, PRKN, PRMT1, PSEN1, PSMC2, PSMC3, PSMC6, PTF1A, PTGES2, PTPRC, PTPRN, RACK1, RAI1, RARA, RB1, RBPMS, RCVRN, RELB, REST, RET, RFX2, RFXAP, RGMA, RHOA, RHOG, RIPK1, RNF111, ROR2, RORA, RORB, RORC, RPL5, RPRD1B, RPS6KB1, RPS6KB2, |
| GO:0010557 | positive regulation of macromolecule biosynthetic process | 3,46E-02 | [4, 5, 6] | 456,00 | 22,01 | ATF3, ATM, ATXN7L3, AURKB, BACH1, BANP, BCAR3, BCAS3, BCL10, BCL11B, BCL3, BCL9, BICRAL, BIRC2, BMP2, BMP7, BRD1, BRD4, BRF1, BTRC, C1orf61, CALCOCO1, CAMK4, CAMTA1, CAMTA2, CAND1, CAPRIN2, CARD11, CASZ1, CCAR2, CCR2, CCT2, CDC123, CDH13, CDK1, CDK2, CDKN2A, CDT1, CEP290, CHEK1, CHEK2, CHTF8, CITED2, CITED4, CIZ1, CNOT7, CREB3L1, CREB3L2, CREB5, CREM, CSRNP2, CSRNP3, CTBP2, CTCF, CYFIP1, DAXX, DCN, DHX36, DLL1, DLX2, DNA2, DNAJC3, DOT1L, DPF1, DTX1, E2F2, E2F8, EBF1, EBF3, EBF4, EGR2, EHF, EIF2AK3, EIF2AK4, EIF4A3, ELAVL1, ELF1, ELK3, ERBB4, ERCC1, EREG, ESR1, ESR2, ESRRG, ETS1, ETV1, ETV6, EVX1, F3, FAF1, FBLN5, FEZF1, FEZF2, FGF2, FGF7, FHOD1, FOXD3, FOXJ3, FOXK1, FOXO1, FUBP1, FZD5, FZR1, GABPB1, GARS1, GATA5, GFI1, GLI2, GLI3, GLIS2, GLMN, GPER1, GPRIN1, GREM1, GSK3A, GTF2B, GTF2H1, HAND1, HCLS1, HDAC4, HELT, HES1, HES3, HEXB, HIVEP1, HIVEP3, HMBOX1, HMGB1, HMGN3, HNF1B, HNRNPAB, HNRNPD, HNRNPK, HOXA1, HOXA3, HOXA4, HOXC10, HOXC11, HOXC4, HOXD13, HSD17B4, HSP90AB1, HSPH1, HTATIP2, IFI16, IFT74, IGF1, IHH, IKBKB, IKZF1, IL31RA, ILF3, ING1, ING3, INSIG2, INSR, IRF2, IRF6, IRS2, ISL1, JAK2, KAT2B, KAT6A, KAT6B, KAT7, KDM3A, KDM6B, KHDRBS1, KLF14, KLF17, KLF7, KMT2D, KMT2E, LARP4B, LARP7, LDB2, LHX4, LHX6, LHX8, LMO7, LMX1B, LONP1, LRP5, LYL1, MACC1, MAF, MAFA, MAFG, MAK, MAML2, MAML3, MAP2K2, MAP2K5, MAP3K2, MAP3K5, MAPK7, MAPKAPK5, MARS1, MAX, MCRS1, MECOM, MED16, MED22, MED24, MED26, MED27, MEF2C, MEIS2, MELTF, MEN1, MEOX1, MICAL2, MLLT3, MRTFA, MSANTD1, MSGN1, MTA1, MTA3, MYD88, MYRF, NAT10, NCK2, NCOA2, NCOA6, NCOA7, NCSTN, NEK7, NEUROD6, NFATC1, NFATC2, NFATC2IP, NFATC4, NFE2L2, NFIB, NFIL3, NFKBIB, NFYA, NFYC, NKX2-4, NLRC5, NLRP12, NME2, NOS1, NOTCH1, NOTCH3, NOTCH4, NPAT, NPNT, NR1D1, NR2E3, NR3C1, NRF1, NRIP1, NSD1, NSD3, PABPC1L, PAF1, PAGR1, PASK, PAX2, PAX7, PAX8, PAX9, PBX1, PCGF5, PDLIM1, PDZD2, PEG3, PER2, PFKM, PHF2, PIH1D1, PIK3R1, PIM1, PKD1, PKIB, PLAC8, PLCB1, PLD1, PLXND1, PML, POLDIP3, POMT1, POU2F1, POU2F2, POU2F3, POU4F2, POU6F2, PPARG, PPARGC1A, PPARGC1B, PPP3CA, PPP3CB, PRDM16, PRDM2, PRKCB, PRKCQ, PRKD2, PRKDC, PRKN, PRMT1, PSEN1, PSMC2, PSMC3, PSMC6, PTF1A, PTGES2, PTPRC, PTPRN, RAI1, RARA, RB1, RBPMS, RELB, REST, RET, RFX2, RFXAP, RGMA, RHOA, RHOG, RIPK1, RNF111, ROR2, RORA, RORB, RORC, RPL5, RPRD1B, RPS6KB1, RPS6KB2, RPTOR, |
| GO:0007267 | cell-cell signaling | 3,90E-02 | [2, 3] | 448,00 | 22,01 | ARHGEF7, ARNTL, ATP2A2, BCL9, BDNF, BDNF-AS, BEGAIN, BICC1, BMP2, BMP3, BRSK2, BSN, BTBD9, BTRC, CACNA1A, CACNA1B, CACNA1E, CACNA1G, CACNA2D2, CACNB2, CADM1, CADPS, CADPS2, CALB2, CALCOCO1, CAMK2B, CAPRIN2, CARD11, CASR, CAV1, CCAR2, CCDC88C, CCKAR, CCL16, CCN4, CCN5, CCN6, CCNY, CCR2, CD200R1, CD33, CDC20, CDK5R1, CELA2A, CELF4, CELSR1, CHRM4, CHRNB2, CHRNB3, CHRND, CHRNE, CMAHP, CNIH3, CNTN4, CPT1A, CRY2, CSNK1A1L, CSNK1E, CSNK1G3, CSPG5, CTBP2, CTNNBIP1, CTNND2, CUL3, CYFIP1, CYLD, CYP19A1, DAG1, DBN1, DGKE, DGKZ, DHX15, DHX34, DHX36, DKK3, DLG1, DLG2, DLG4, DLGAP2, DLL1, DNM1, DNM1L, DOC2A, DTNB, EFNA4, EFNA5, EGR2, EIF2AK4, EIF4A3, EIF4E, EIF4EBP2, EIPR1, ENHO, EPHA4, EPHB1, EREG, ESR1, ESR2, EXOC4, FABP5, FAM3B, FAM3D, FARP1, FAT1, FCHSD1, FCHSD2, FERMT2, FGF12, FGF14, FGF2, FGF7, FJX1, FNTA, FOXO1, FOXP1, FRMD8, FZD10, FZD5, FZR1, GABBR1, GABBR2, GABRA2, GABRA4, GABRB2, GABRR3, GAD1, GAD2, GCG, GJA5, GLI2, GLI3, GLMN, GNAI2, GNAO1, GPC6, GPER1, GPR176, GPRC5B, GPRIN1, GRB10, GRB2, GREM1, GRIA4, GRID2, GRID2IP, GRIK3, GRIK4, GRK5, GRM1, GRM2, GRM4, GRM8, GSK3A, HADHA, HCAR2, HCN2, HCN4, HCRTR2, HDAC9, HECW1, HES1, HMGB1, HMGN3, HNF1B, HOOK1, HSPD1, HTR1A, HTR2A, HTR3A, HTR3C, IFIH1, IGSF9B, IHH, IL16, IL17RA, IL1RAP, IL37, ILDR1, IRS2, ISL1, ITGB2, ITPR2, ITPR3, JAK2, JPH3, KALRN, KANK1, KCMF1, KCNB1, KCNJ3, KCNQ1, KCNQ2, KIF5A, KLF7, KRAS, KREMEN2, LAMA2, LATS2, LGMN, LGR6, LIN7B, LLGL1, LPL, LRFN2, LRP1, LRP5, LRRC32, LRRC4C, LRRK1, LYPD6, LYPD6B, LZTS1, LZTS2, MACF1, MAFA, MAGI2, MAPK8IP2, MEF2C, MKS1, MLLT3, MME, MTMR2, MTNR1B, MYO5A, NAPB, NCSTN, NDUFA2, NF1, NFATC1, NFATC4, NGF, NKD1, NLGN2, NLK, NLRP12, NOL3, NOS1, NOTCH1, NPHP4, NPPA, NPS, NPTN, NPTXR, NR1D1, NRXN1, NRXN3, NSG1, NSMF, NTNG1, NTNG2, NTRK2, NXN, PACSIN2, PAF1, PAFAH1B1, PANX2, PARD3, PASK, PAX8, PCDHA6, PCDHB9, PCLO, PCSK5, PDE7B, PDZD2, PER2, PFKM, PFN2, PIP5K1C, PKD1, PLA2G6, PLCB1, PLCB2, PLCB4, PLCG2, PLCL2, PLEKHA4, PML, PPFIA1, PPFIA3, PPFIA4, PPP1R11, PPP3CA, PPP3CB, PRICKLE2, PRKAR1B, PRKCA, PRKCB, PRKCE, PRKCZ, PRKD2, PRKN, PSEN1, PSMA5, PSMA6, PSMA7, PSMC2, PSMC3, PSMC6, PSMD2, PSMD7, PTEN, PTPN11, PTPRA, PTPRN, PTPRN2, PTPRO, RAB43, RAB5A, RACK1, RAP1A, RAPGEF1, RARA, RASGRF1, RBP4, RBX1, RELN, REST, RHOA, RIMS3, RNF146, RNF220, ROR1, ROR2, RORA, |
| GO:0031328 | positive regulation of cellular biosynthetic process | 1,57E-02 | [4, 5, 6] | 480,00 | 22,04 | ARNTL, ASCL1, ASS1, ASXL2, ATF1, ATF2, ATF3, ATM, ATXN7L3, AURKB, BACH1, BANP, BCAR3, BCAS3, BCL10, BCL11B, BCL3, BCL9, BICRAL, BIRC2, BMP2, BMP7, BRD1, BRD4, BRF1, BTRC, C1orf61, CALCOCO1, CAMK4, CAMTA1, CAMTA2, CAND1, CAPRIN2, CARD11, CASZ1, CCAR2, CCR2, CCT2, CDC123, CDH13, CDK1, CDK2, CDKN2A, CDT1, CEP290, CHEK1, CHEK2, CHTF8, CITED2, CITED4, CIZ1, CNOT7, CREB3L1, CREB3L2, CREB5, CREM, CSRNP2, CSRNP3, CTBP2, CTCF, CYFIP1, DAXX, DCN, DDAH2, DHX34, DHX36, DLL1, DLX2, DNA2, DNAJC3, DOT1L, DPF1, DTX1, E2F2, E2F8, EBF1, EBF3, EBF4, EGR2, EHF, EIF2AK3, EIF2AK4, EIF4A3, ELAVL1, ELF1, ELK3, ERBB4, ERCC1, EREG, ESR1, ESR2, ESRRG, ETS1, ETV1, ETV6, EVX1, F3, FAF1, FBLN5, FEZF1, FEZF2, FGF2, FGF7, FHOD1, FOXD3, FOXJ3, FOXK1, FOXO1, FUBP1, FZD5, FZR1, GABPB1, GARS1, GATA5, GCG, GFI1, GLI2, GLI3, GLIS2, GLMN, GPER1, GPRIN1, GPT, GREM1, GSK3A, GTF2B, GTF2H1, GUCA2A, HAND1, HCLS1, HDAC4, HELT, HES1, HES3, HEXB, HIVEP1, HIVEP3, HMBOX1, HMGB1, HMGN3, HNF1B, HNRNPAB, HNRNPD, HNRNPK, HOXA1, HOXA3, HOXA4, HOXC10, HOXC11, HOXC4, HOXD13, HSD17B4, HSP90AB1, HTATIP2, HTR2A, IFI16, IFT74, IGF1, IHH, IKBKB, IKZF1, IL31RA, ILF3, ING1, ING3, INSIG2, INSR, IRF2, IRF6, IRS2, ISL1, ITGB2, JAK2, KAT2B, KAT6A, KAT6B, KAT7, KDM3A, KDM6B, KHDRBS1, KLF14, KLF17, KLF7, KMT2D, KMT2E, LARP4B, LARP7, LDB2, LDLR, LHX4, LHX6, LHX8, LMO7, LMX1B, LONP1, LPGAT1, LRP5, LYL1, MACC1, MAF, MAFA, MAFG, MAK, MAML2, MAML3, MAP2K2, MAP2K5, MAP3K2, MAP3K5, MAPK7, MAPKAPK5, MARS1, MAX, MCRS1, MECOM, MED16, MED22, MED24, MED26, MED27, MEF2C, MEIS2, MELTF, MEN1, MEOX1, MICAL2, MLLT3, MRTFA, MSANTD1, MSGN1, MTA1, MTA3, MYD88, MYRF, NAT10, NCK2, NCOA2, NCOA6, NCOA7, NCSTN, NEK7, NEUROD6, NFATC1, NFATC2, NFATC2IP, NFATC4, NFE2L2, NFIB, NFIL3, NFKBIB, NFYA, NFYC, NKX2-4, NLRC5, NME2, NOS1, NOS1AP, NOTCH1, NOTCH3, NOTCH4, NPAT, NPNT, NR1D1, NR2E3, NR3C1, NRF1, NRIP1, NSD1, NSD3, P2RY6, PABPC1L, PAF1, PAGR1, PAOX, PARD3, PASK, PAX2, PAX7, PAX8, PAX9, PBX1, PCGF5, PDLIM1, PDZD2, PEG3, PER2, PFKM, PHF2, PIH1D1, PIK3R1, PIM1, PKD1, PKIB, PLA2G6, PLAC8, PLCB1, PLD1, PLIN5, PLXND1, PML, POLDIP3, POMT1, POU2F1, POU2F2, POU2F3, POU4F2, POU6F2, PPARG, PPARGC1A, PPARGC1B, PPP3CA, PPP3CB, PRDM16, PRDM2, PRKCB, PRKCQ, PRKD2, PRKDC, PRKN, PRMT1, PSEN1, PSMC2, PSMC3, PSMC6, PTF1A, PTGES2, PTPRC, PTPRN, RACK1, RAI1, RARA, RB1, RBPMS, RCVRN, RELB, REST, RET, RFX2, RFXAP, RGMA, RHOA, RHOG, RIPK1, RNF111, ROR2, RORA, RORB, RORC, RPL5, RPRD1B, RPS6KB1, RPS6KB2, RPTOR, RPUSD4, RREB1, |

| | | | | | |
|---|---|---|---|---|---|
| | | | | 22,05 | ALS2, AMBRA1, AMH, ANGPT1, ANGPT4, ANK3, ANKRD28, ANKS1B, ANO6, ANTXR1, ANXA2, AP2S1, APC, APOBEC1, APOC4, APPL2, ARAP1, ARF1, ARHGAP40, ARHGEF10L, ARHGEF18, ARHGEF2, ARHGEF26, ARHGEF7, ARL13B, ARPC4, ASAP1, ATAT1, ATF1, ATG14, ATG16L1, ATG2A, ATG2B, ATM, ATP6V0D1, ATXN10, AURKB, B9D1, BAG6, BAIAP2L1, BAZ2A, BBS10, BBS4, BBS7, BBS9, BCAS3, BCL11B, BCL2, BCL9, BDNF, BDNF-AS, BIN3, BIRC2, BMP1, BMP7, BRD2, BRF1, BSN, C10orf90, CACNA1A, CADPS2, CAMSAP1, CAND1, CAPZB, CARD10, CARMIL1, CARMIL2, CAV1, CC2D2A, CCDC113, CCDC13, CCDC28B, CCT2, CD47, CDC123, CDC20, CDH10, CDH13, CDH18, CDH19, CDH23, CDH26, CDH4, CDK1, CDK2, CDK5R1, CDKN2A, CDT1, CELF2, CELF4, CELF5, CENPP, CEP131, CEP164, CEP290, CEP295, CEP43, CEP70, CETN1, CFAP46, CFAP54, CFAP74, CHAF1A, CHCHD4, CHEK2, CHMP2A, CHRNB2, CILK1, CLASP1, CLASP2, CLDN16, CLDN8, CLP1, CLYBL, CNIH3, CNOT1, CNOT7, CNTN5, CNTROB, COA1, COBL, CORO1B, CORO2B, CORO7-PAM16, COX20, CPLANE1, CRBN, CSNK1E, CTCF, CTNNA2, CTNNBIP1, CTNND2, CTTN, CUL3, CYFIP1, CYFIP2, CYLD, DAG1, DARS1, DAXX, DBN1, DCTN1, DDX6, DEF8, DGKQ, DHX30, DIAPH3, DLC1, DLG1, DLG4, DMC1, DNA2, DNAH17, DNAH5, DNAH8, DNAJB12, DNAJB13, DNAJB6, DNAJC15, DNER, DNM1L, DNMT3A, DST, DYNC2LI1, ECSIT, EFNA5, EHMT2, EIF2S2, EIF3B, EIF4B, EIF4EBP1, ELAVL1, ELN, EPHA1, EPHB1, EPHB3, EPPK1, EPRS1, EPS8L1, ERBB4, ERCC1, ERCC5, ESPN, ESPNL, ESR1, EVL, F11R, F5, FAF1, FAM161A, FAM171A1, FAM172A, FARP1, FARP2, FASN, FBLN5, FCHSD1, FCHSD2, FER, FERMT2, FEZ1, FGD2, FHOD1, FHOD3, FITM2, FLRT1, FMN1, FNIP2, FXYD5, FZD5, FZR1, GABRA2, GABRB2, GAK, GARS1, GEMIN2, GJA5, GLRX3, GMFB, GORASP1, GPC6, GPRIN1, GRB2, GREM1, GRID2, GRWD1, GTF2B, GTF2H1, H2BC15, H3C6, H3C8, HAPLN2, HCLS1, HCN1, HDAC4, HDAC9, HEG1, HES1, HGS, HIP1, HIP1R, HLA-DRA, HMGB1, HNF1B, HNRNPC, HOOK1, HPS4, HRG, HSP90AB1, HSPD1, HTT, HYDIN, IBA57, IFT140, IFT46, IFT74, IFT88, IHH, IKBKB, IL1RAP, IMMP2L, INPP5F, INPPL1, IQGAP2, ISL1, ITGA5, ITGB4, JAK2, JHY, KANK1, KANK3, KAT2B, KAT6A, KAT6B, KCNB1, KCNB2, KCNC1, KCNC3, KCNF1, KCNJ2, KCNV1, KCTD10, KCTD14, KCTD16, KCTD2, KCTD5, KCTD6, KHDC4, KIF25, KIF2A, KIFC1, KIRREL1, KMT2D, KRT1, LAMA5, LAMB1, LAMB2, LAMB4, LDB2, LDB3, LDLRAD4, LIMA1, LIMS1, LIN9, LLGL1, LONP1, LPAR1, LRCH4, LRFN3, LRP1, LRSAM1, LSM2, LUC7L2, LUC7L3, LYRM4, LZTS1, LZTS2, M1AP, MACF1, MAGI2, MAK, MAP1LC3A, MAP2, MAP4, MAP4K4, MAPK8IP2, MAPK9, MARCKS, MAX, MBD3, MCM2, MCMDC2, MDGA1, MEF2C, MEIOC, MEN1, MIPEP, MKS1, MLH3, MPHOSPH6, MPHOSPH8, MRPS2, MTMR2, MTMR3, MYH3, MYL2, MYLK, MYO1A, MYO1C, MYO1E, MYOM2, MYPN, NAA60, NACC2, NAPB, NAV3, NCK2, NCKAP1, NCKAP1L, NDE1, NDUFA13, NDUFA2, NDUFAF3, NDUFB3, NDUFB6, NDUFB7, NDUFC1, NDUFC2, NECTIN1, NEDD1, NEDD9, NEK7, NFASC, NIFK, NINL, NLGN2, NOC2L, NOL3, NOP2, NOTCH1, NPC1, NPHP4, NPHS1, NPRL3, NPTN, NRXN1, NRXN3, NSG1, NTNG2, NTRK2, NUP205, OBSCN, OBSL1, OTUD6B, PACS2, PACSIN2, PAF1, PAFAH1B1, PALM, PARD3, PATJ, PATL1, PCDHB9, PCLO, PCNT, PDCD6, PDGFRA, PDLIM1, PDPK1, PEAK1, PEX14, PFDN6, PFKM, PFKP, PFN2, PHACTR1, PHF2, PHLDB1, PIEZO1, PIH1D1, PIH1D2, PIK3R1, PIKFYVE, PIP4K2A, PIP5K1C, PKD1, PKP1, PLCE1, PLD1, PLEK, PLEKHA7, PLEKHG2, PLK4, PLXNB1, PLXND1, PML, POLQ, PPARGC1A, PPFIA1, PPM1F, PPP1R16B, PRKCA, PRKCE, PRKCH, PRKCI, PRKCZ, PRKD2, PRKDC, PRKN, PRLR, PRMT1, PRMT8, PRPF19, PRPF8, PRUNE1, PSEN1, PSIP1, PSMC2, PSMC3, PSMC6, PSMG4, PTEN, PTK2, PTPN11, PTPRO, PXDN, RAB29, RAB35, RAB5A, RACK1, RAD51, RANBP9, RAP1A, RAP1GAP, RAPGEF1, RASA1, RASIP1, RB1, RBX1, RC3H1, RFX2, RHOA, RHOG, RHOU, RICTOR, RIOK3, RIPK1, RIPOR2, RO60, ROCK2, RP1, RPL3, RPL5, RRM1, RRP7A, RRP7BP, RRS1, RTKN, RTN3, RUNX1, RYR1, |
| GO:0022607 | cellular component assembly | 5,67E-05 [4] | 687,00 | | |
| | | | | 22,07 | ANTXR1, APPL2, ARAP1, ARHGEF7, ARL6IP5, ARNTL, ASCL1, ASXL2, ATF3, ATM, BCAR3, BCL10, BCL2, BDNF, BDNF-AS, BIRC2, BMP2, BMP3, BMP7, BMP8B, BMPER, BRD4, BTC, CACNB2, CADM1, CALB2, CALM2, CAMTA1, CAPRIN2, CARD10, CARD11, CARD14, CASP2, CASR, CAV1, CCAR2, CCBE1, CCL16, CCN4, CCR2, CDH13, CDK1, CHD5, CHRNB2, CIB1, CITED2, COL3A1, CRB2, CSNK1E, CSNK1G3, CTBP2, CXCR4, CXXC5, CYFIP1, CYFIP2, CYLD, CYP19A1, CYP1B1, DAXX, DCN, DDX60, DHX36, DLG4, DLL1, DLL4, DNM1L, DOK4, DSTYK, DUSP19, EDAR, EEF1E1-BLOC1S5, EIF2AK4, EMC10, ENHO, EPHA4, ERBB4, EREG, ESR1, ETFA, EZH2, F11R, F2RL3, F3, FABP5, FAF1, FAM110A, FASN, FGD2, FGF2, FGF22, FGF6, FGF7, FLT4, FOXP1, FRMD8, FRS2, FZD10, FZD5, GAB1, GADD45G, GATA5, GCG, GCNT2, GFI1, GFRAL, GJA5, GLIPR2, GLMN, GNAI2, GPER1, GPR137B, GPR17, GPR4, GPR55, GPR62, GPRC5B, GPRIN1, GRB10, GRB2, GREM1, GRM1, GRM4, GSK3A, GSX2, HACD3, HCAR2, HCLS1, HES1, HINT1, HIP1, HIP1R, HMGB1, HOOK1, HSP90AB1, HSPD1, HTR2A, HTT, IFIH1, IGF1, IGF1R, IGF2BP1, IGSF9B, IHH, IKBKB, IL10RA, IL10RB, IL16, IL17RA, IL1RAP, IL20RA, IL31RA, IL37, IL6R, IL6ST, ILDR1, ING3, INSR, IQGAP3, IRAK2, IRS2, ISL1, ITGA1, ITGA5, ITPR3, JAK2, KALRN, KANK1, KAT2B, KCNB1, KIDINS220, KITLG, KLF14, KMT2D, KRAS, KSR1, LAMA2, LGMN, LGR6, LIMS1, LLGL1, LMCD1, LOXL3, LPAR1, LPL, LRP1, LRP2, LRPAP1, LRRK1, LURAP1, LYPD6, MACF1, MAL, MAML2, MAML3, MAP2K2, MAP2K5, MAP3K11, MAP3K13, MAP3K2, MAP3K20, MAP3K5, MAP3K6, MAP4K4, MAP4K5, MAPK8, MAPK8IP2, MAPK8IP3, MAPK9, MAPKAPK5, MCL1, MEN1, MFNG, MIB2, MIER1, MLLT3, MME, MRPL23, MRTFA, MST1R, MTNR1B, MYD88, MYO1C, MYO1E, NACC2, NCK2, NCSTN, NDFIP2, NDRG4, NDUFA2, NEO1, NEPRO, NF1, NFAM1, NFATC4, NGF, NKD1, NLGN2, NLRC5, NLRP12, NOL3, NOS1, NOS1AP, NOTCH1, NOTCH4, NPNT, NPPA, NPR1, NPS, NPTN, NR2C1, NR3C2, NRXN1, NSG1, NTRK2, OSBPL8, P2RY6, PAFAH1B1, PAGR1, PANX2, PARP14, PDCD5, PDE8A, PDGFD, PDGFRA, PDZD2, PFKM, PIH1D1, PIK3CB, PIK3CG, PIK3R1, PIK3R5, PLA2G6, PLAUR, PLCB1, PLCE1, PLEKHA4, PLXNB1, PML, PPP1R13B, PPP3CA, PPP3CB, PRAG1, PRKCA, PRKCB, PRKCE, PRKCH, PRKCI, PRKCZ, PRKD2, PRKN, PRLR, PRMT1, PSEN1, PSMA5, PSMA6, PSMA7, PSMC2, PSMC3, PSMC6, PSMD2, PSMD7, PTEN, PTGIS, PTK2, PTPN11, PTPRC, PUM1, RAB29, RACK1, RAD9A, RAP1A, RAPGEF1, RASGRF1, RASGRP4, RBP4, RBPMS, RC3H1, RC3H2, RELN, RET, RGS9, RHEB, RHOA, RHOG, RICTOR, RIMS3, |
| GO:0010647 | positive regulation of cell communication | 2,92E-02 [3, 4, 5] | 448,00 | | |
| | | | | 22,08 | ANXA2, APPL2, ARAP1, ARHGEF7, ARL6IP5, ARNTL, ASCL1, ASXL2, ATF3, ATM, BCAR3, BCL10, BCL2, BDNF, BDNF-AS, BIRC2, BMP2, BMP3, BMP7, BMP8B, BMPER, BRD4, BTC, CACNB2, CADM1, CALB2, CALM2, CAMTA1, CAPRIN2, CARD10, CARD11, CARD14, CASP2, CASR, CAV1, CCAR2, CCBE1, CCL16, CCN4, CCR2, CDH13, CDK1, CHD5, CHRNB2, CIB1, CITED2, COL3A1, CRB2, CSNK1E, CSNK1G3, CTBP2, CXCR4, CXXC5, CYFIP1, CYFIP2, CYLD, CYP19A1, CYP1B1, DAXX, DCN, DDX60, DHX36, DLG4, DLL1, DLL4, DNM1L, DOK4, DSTYK, DUSP19, ECE1, EDAR, EEF1E1-BLOC1S5, EIF2AK4, EMC10, ENHO, EPHA4, ERBB4, EREG, ESR1, ETFA, EZH2, F11R, F2RL3, F3, FABP5, FAF1, FAM110A, FASN, FGD2, FGF2, FGF22, FGF6, FGF7, FLT4, FOXP1, FRMD8, FRS2, FZD10, FZD5, GAB1, GADD45G, GATA5, GCG, GCNT2, GFI1, GFRAL, GLIPR2, GLMN, GNAI2, GPER1, GPR137B, GPR17, GPR4, GPR55, GPR62, GPRC5B, GPRIN1, GRB10, GRB2, GREM1, GRM1, GRM4, GSK3A, GSX2, HACD3, HCAR2, HCLS1, HES1, HINT1, HIP1, HIP1R, HMGB1, HOOK1, HSP90AB1, HSPD1, HTR2A, HTT, IFIH1, IGF1, IGF1R, IGF2BP1, IGSF9B, IHH, IKBKB, IL10RA, IL10RB, IL16, IL17RA, IL1RAP, IL20RA, IL31RA, IL37, IL6R, IL6ST, ILDR1, ING3, INPP5F, INSR, IQGAP3, IRAK2, IRS2, ISL1, ITGA1, ITGA5, ITPR3, JAK2, KALRN, KANK1, KAT2B, KCNB1, KIDINS220, KITLG, KLF14, KMT2D, KRAS, KSR1, LAMA2, LGMN, LGR6, LIMS1, LLGL1, LMCD1, LOXL3, LPAR1, LPL, LRP1, LRP2, LRPAP1, LRRK1, LURAP1, LYPD6, MACF1, MAL, MAML2, MAML3, MAP2K2, MAP2K5, MAP3K11, MAP3K13, MAP3K2, MAP3K20, MAP3K5, MAP3K6, MAP4K4, MAP4K5, MAPK8, MAPK8IP2, MAPK8IP3, MAPK9, MAPKAPK5, MCL1, MEN1, MFNG, MIB2, MIER1, MLLT3, MME, MRPL23, MRTFA, MST1R, MTNR1B, MYD88, MYO1C, MYO1E, NACC2, NCK2, NCSTN, NDFIP2, NDRG4, NDUFA2, NEO1, NEPRO, NF1, NFAM1, NFATC4, NGF, NKD1, NLGN2, NLRC5, NLRP12, NOL3, NOS1, NOS1AP, NOTCH1, NOTCH4, NPNT, NPPA, NPR1, NPS, NPTN, NR2C1, NR3C2, NRXN1, NSG1, NTRK2, OSBPL8, P2RY6, PAFAH1B1, PAGR1, PANX2, PARP14, PDCD5, PDE8A, PDGFD, PDGFRA, PDZD2, PFKM, PIH1D1, PIK3CB, PIK3CG, PIK3R1, PIK3R5, PLA2G6, PLAUR, PLCB1, PLCE1, PLEKHA4, PLXNB1, PML, PPP1R13B, PPP3CA, PPP3CB, PRAG1, PRKCA, PRKCB, PRKCE, PRKCH, PRKCI, PRKCZ, PRKD2, PRKN, PRLR, PRMT1, PSEN1, PSMA5, PSMA6, PSMA7, PSMC2, PSMC3, PSMC6, PSMD2, PSMD7, PTEN, PTGIS, PTK2, PTPN11, PTPRC, PUM1, RAB29, RACK1, RAD9A, RAP1A, RAPGEF1, RASGRF1, RASGRP4, RBP4, RBPMS, RC3H1, RC3H2, RELN, RET, RGS9, RHEB, RHOA, RHOG, RICTOR, RIMS3, |
| GO:0023056 | positive regulation of signaling | 2,63E-02 [2, 3, 4] | 450,00 | | |
| | | | | 22,08 | ATXN1, AURKB, BACH1, BATF2, BAZ1B, BBS7, BBX, BCAS3, BCL11A, BCL11B, BCL3, BCL6, BCL9, BHLHE40, BMP2, BMP7, BNC1, BNC2, BRD2, BRD4, BRMS1, BTG2, C1orf61, CALCOCO1, CAMK2D, CAMTA1, CAMTA2, CAND1, CAPRIN2, CAV1, CBFA2T2, CBX8, CC2D1B, CCNL1, CDH13, CDK2, CDKN1A, CDKN2A, CHCHD3, CHD3, CHD5, CHEK1, CIC, CITED2, CITED4, CNOT1, CNOT7, CREB3L1, CREB3L2, CREB5, CREM, CRY2, CRYM, CSRNP2, CSRNP3, CTBP2, CTCF, CTNNBIP1, CUL2, CUL3, CUX1, CXXC5, CYFIP1, DCAF1, DCN, DGKQ, DHX36, DLG1, DLL1, DLL4, DLX2, DNAJB5, DNMT3A, DOT1L, DPF1, DRGX, DUSP26, DUXA, E2F2, E2F8, EBF1, EBF3, EBF4, EGR2, EHF, EHMT1, EHMT2, ELF1, ELK3, ELOA, ERCC1, ESR1, ESR2, ESRRG, ETS1, ETV1, ETV6, EVX1, EZH2, FAM220A, FBLN5, FBP1, FERD3L, FEZF1, FEZF2, FGF2, FHOD1, FNIP2, FOXB2, FOXD3, FOXI3, FOXJ3, FOXK1, FOXO1, FOXP1, FUBP1, FZD5, GABPB1, GARS1, GATA5, GFI1, GLI2, GLI3, GLIS2, GLO1, GON4L, GPER1, GPRIN1, GREM1, GSK3A, GSX2, GTF2B, GTF2H1, GTF2IRD1, HAND1, HCLS1, HDAC4, HDAC9, HELT, HES1, HES3, HES4, HEXB, HIVEP1, HMBOX1, HMGB1, HMGN3, HNF1B, HNRNPK, HOXA1, HOXA3, HOXA4, HOXC10, HOXC11, HOXC4, HOXD13, HSD17B4, HSPD1, HTATIP2, IFI16, IFRD1, IFT74, IGF1, IGHMBP2, IHH, IKBKB, IKZF1, INSIG2, IRF2, IRF2BP2, IRF6, IRF9, ISL1, JARID2, JAZF1, KALRN, KAT2B, KAT6A, KAT6B, KAT7, KDM2B, KDM3A, KDM4C, KDM6B, KHDRBS1, KLF14, KLF17, KLF7, KMT2D, LARP7, LDB2, LHX4, LHX6, LHX8, LIN9, LMCD1, LMO7, LMX1B, LOXL2, LRP5, LYL1, MACC1, MAF, MAFA, MAFG, MAFK, MAML2, MAML3, MAP2K5, MAPK7, MAX, MBD3, MCM2, MCPH1, MCRS1, MECOM, MED13L, MED16, MED22, MED26, MED27, MEF2C, MEIS2, MELTF, MEN1, MEOX1, MICAL2, MIDEAS, MIER1, MLLT3, MRPL23, MRTFA, MSGN1, MTA1, MTA3, MXD1, MYRF, MYT1L, NACC2, NCK2, NCOA2, NCOA6, NCOA7, NCOR2, NEDD4L, NEUROD6, NEUROG2, NFATC1, NFATC2, NFATC2IP, NFATC4, NFE2L2, NFIB, NFIL3, NFXL1, NFYA, NFYC, NKX2-4, NLRC5, NME2, NOC2L, NOP2, NOS1, NOTCH1, NOTCH3, NOTCH4, NPNT, NR1D1, NR2C1, NR2E3, NR3C1, NR3C2, NRF1, NRIP1, NSD1, OLIG2, PAF1, PAGR1, PAX2, PAX7, PAX8, PAX9, PBX1, PCGF5, PDLIM1, PDZD2, PEG3, PER2, PFKM, PHF19, PHF20, PIK3R1, PKD1, PKMYT1, PKNOX2, PLAC8, PLXND1, POU2F1, POU2F2, POU2F3, POU4F2, POU6F2, PPARG, PPARGC1A, PPARGC1B, PPP3CA, PPP3CB, PRDM16, PRDM2, PRDM5, PRDM6, PRKCB, PRKD2, PRKDC, PRKN, PSEN1, PSMA5, PSMA6, PSMA7, PSMC2, PSMC3, PSMC6, PSMD2, PSMD7, PTF1A, PTPRN, PURG, QRICH1, RAI1, RARA, RB1, RBX1, RCOR3, RELB, REST, RFX2, RFXAP, RGMA, RHOA, RIPK1, RITA1, RNF111, RORA, RORB, RORC, RPRD1B, RREB1, RUNX1, RUNX3, RXRB, RXRG, SATB2, SBNO2, SCRT1, |
| GO:0006357 | regulation of transcription by RNA polymerase II | 7,11E-03 [6, 7, 8, 9, 10, 11] | 505,00 | | |
| GO:0009893 | positive regulation of metabolic process | 5,37E-08 [2, 3, 4] | 905,00 | 22,09 | AGRN, AKAP12, AKAP13, AKT2, ALK, ALKAL2, ALMS1, ALS2, ALX1, ALX4, AMBRA1, AMH, ANGPT1, ANGPT4, ANK3, ANKRD17, ANKRD31, ANTXR1, ANXA2, APC, APC2, APOBEC1, APPL1, APPL2, ARAP1, ARHGEF10L, ARHGEF2, ARHGEF7, ARID2, ARID4B, ARIH1, ARL6IP5, ARNT2, ARNTL, ASCL1, ASPH, ASPSCR1, ASS1, ASXL2, ATF1, ATF2, ATF3, ATG14, ATG16L1, ATG4B, ATM, ATP13A2, ATP2A3, ATP2B4, ATXN7L3, AURKB, BABAM2, BACH1, BAG6, BANP, BAZ1B, BBS7, BCAR3, BCAS3, BCL10, BCL11A, BCL11B, BCL2, BCL3, BCL6, BCL9, BDNF, BDNF-AS, BICRAL, BIRC2, BMP2, BMP3, BMP7, BMP8B, BMPER, BRD1, BRD4, BRF1, BRMS1, BTBD10, BTC, BTG2, BTRC, C1orf61, CADM1, CALCOCO1, CALM2, CAMK4, CAMTA1, CAMTA2, CAND1, CAPRIN2, CARD10, CARD11, CARD14, CASR, CASZ1, CAV1, CBFA2T3, CBX8, CCAR2, CCBE1, CCDC3, CCL16, CCND2, CCNY, CCR2, CCT2, CD33, CD46, CDC123, CDC20, CDC20B, CDC25B, CDH13, CDK1, CDK2, CDK5R1, CDKN1A, CDKN2A, CDT1, CELF4, CEP290, CEP295, CHAMP1, CHEK1, CHEK2, CHTF8, CIB1, CITED2, CITED4, CIZ1, CLN6, CLYBL, CNOT1, CNOT7, COMMD1, COP1, CPEB1, CPT1A, CREB3L1, CREB3L2, CREB5, CREM, CSNK1E, CSRNP2, CSRNP3, CTBP2, CTCF, CUL3, CXCR4, CYCS, CYFIP1, CYFIP2, CYP1B1, DAB1, DAG1, DAPK1, DAXX, DCN, DCUN1D1, DDA1, DDAH2, DDR1, DDR2, DDX60, DGKQ, DGKZ, DHX34, DHX36, DIPK2A, DLC1, DLG1, DLG4, DLL1, DLL4, DLX2, DNA2, DNAJC3, DNPEP, DOCK7, DOK4, DOK7, DOT1L, DPF1, DSTYK, DTX1, DUSP19, DUSP26, E2F2, E2F8, EBF1, EBF3, EBF4, ECE1, EDAR, EDEM2, EFNA5, EGR2, EHF, EHMT1, EIF2AK3, EIF2AK4, EIF4A3, ELAVL1, ELF1, ELK3, EMC10, ENPP2, EPB41L4B, EPHA1, EPHA10, EPHA4, EPHA6, EPHB1, EPHB3, ERBB4, ERCC1, ERCC8, EREG, ESR1, ESR2, ESRRG, ETAA1, ETFA, ETS1, ETV1, ETV6, EVX1, EXOSC6, EYA2, EYA4, EZH2, F12, F3, FABP5, FAF1, FAM161A, FANCM, FASN, FBLN5, FBXW8, FEZF1, FEZF2, FGD2, FGF2, FGF7, FH, FHOD1, FLT4, FNIP2, FNTA, FOXD3, FOXJ3, FOXK1, FOXO1, FRMD8, FRS2, FTO, FUBP1, FZD10, FZD5, FZR1, GABPB1, GADD45G, GARS1, GATA5, GCG, GCNT2, GFI1, GFRAL, GLI2, GLI3, GLIPR2, GLIS2, GLMN, GNAI2, GORASP1, GPER1, GPR55, GPRC5B, GPRC5C, GPRIN1, GPSM1, GPT, GRAMD4, GRB10, GRB2, GREM1, GRM1, GRM4, GSK3A, GTF2B, GTF2H1, GUCA2A, HACD3, HADHA, HAND1, HCLS1, HDAC4, HECW1, HEG1, HELT, HES1, HES3, HEXB, HGS, HIP1, HIP1R, HIVEP1, HIVEP3, HLA-DPB1, HMBOX1, HMGB1, HMGN3, HNF1B, HNRNPAB, HNRNPC, HNRNPD, HNRNPK, HNRNPR, HOOK1, HOXA1, HOXA3, HOXA4, HOXC10, HOXC11, HOXC4, HOXD13, HSD17B4, HSP90AB1, HSPD1, HSPH1, HTATIP2, HTR2A, HTT, IDE, IFI16, IFIH1, IFT74, IGF1, IGF1R, IGF2BP1, IHH, IKBKB, IKZF1, IL12RB2, IL16, IL17D, IL17RA, IL1RAP, IL31RA, IL37, IL6R, IL6ST, ILF3, ING1, ING3, INPP5F, INSIG2, INSR, IQGAP3, IRAK2, IRF2, IRF6, IRS2, ISL1, ITCH, ITGA1, ITGA5, ITGB2, JAK2, JARID2, KAT2B, KAT6A, KAT6B, KAT7, KDM3A, KDM4C, KDM4D, KDM6B, KHDRBS1, KIDINS220, KIF2A, KITLG, KLF14, KLF17, KLF7, KMT2D, KMT2E, KMT5C, KNDC1, KPNA7, KRAS, KSR1, KSR2, LARP4B, LARP7, LDB2, LDLR, LEPR, LGMN, LHX4, LHX6, LHX8, LIMS1, LLGL1, LMO7, LMTK2, LMX1B, LONP1, LPAR1, LPCAT1, LPGAT1, LPL, LRP1, LRP2, LRP5, LRRC32, LRRK1, LRSAM1, LURAP1, LY9, LYL1, MACC1, MAF, MAFA, MAFG, MAGI2, MAK, MAL, MAML2, MAML3, MAP2K2, MAP2K5, MAP3K11, MAP3K13, MAP3K19, MAP3K2, MAP3K20, MAP3K5, MAP3K6, MAP4K4, MAP4K5, MAPK7, MAPK8, MAPK8IP2, MAPK8IP3, MAPK9, MAPKAP1, MAPKAPK5, MARS1, MAX, MCRS1, MECOM, MED16, MED22, MED24, MED26, MED27, MEF2C, MEIOC, MEIS2, MELTF, MEN1, MEOX1, MGMT, MICAL2, MLLT3, MMD, MPHOSPH8, MRPL23, MRTFA, MSANTD1, MSGN1, MSH2, MST1R, MTA1, MTA3, MTMR9, MYD88, MYO1C, MYO1E, MYRF, NAT10, NCK2, NCKAP1L, NCOA2, NCOA6, NCOA7, NCOR2, NCSTN, NDFIP2, NDRG4, NDUFA13, NDUFA2, NEDD4L, NEDD9, NEK7, NEUROD6, NFAM1, NFATC1, NFATC2, NFATC2IP, NFATC4, NFE2L2, NFIB, NFIL3, NFKBIB, NFYA, NFYC, NGF, NKD1, NKX2-4, NLRC5, NLRP12, NLRX1, NME2, NNT, NOS1, NOS1AP, NOTCH1, NOTCH3, NOTCH4, NPAT, NPNT, NPRL3, NPTN, NR1D1, NR2E3, NR3C1, NRF1, NRIP1, NRXN1, NSD1, NSD3, NSG1, NSMF, NTRK2, NUS1, OLFM1, OSBPL7, OSBPL8, OTUD4, P2RY6, PABPC1L, PAF1, PAGR1, PAK4, PAK5, PANX2, PAOX, PARD3, PARP14, PASK, PAX2, PAX7, PAX8, PAX9, PBX1, PCGF5, PDCD5, PDCD6, PDCL3, PDE8A, PDGFD, PDGFRA, PDLIM1, PDPK1, PDZD2, PEG3, PER2, PFKFB3, PFKM, PFN2, PHF19, PHF2, PIH1D1, PIK3CB, PIK3CG, PIK3R1, PIK3R5, PIM1, PIP4K2A, PKD1, PKIB, PKP1, PLA2G6, PLAC8, PLAUR, PLCB1, PLCE1, PLCG2, PLD1, PLEK, PLEKHN1, PLIN5, PLXND1, PML, PNPLA2, POLDIP3, POLR2F, POLR3A, POLR3E, POLR3H, POMT1, POU2F1, POU2F2, POU2F3, POU4F2, POU6F2, PPARG, PPARGC1A, PPARGC1B, PPM1F, PPP1R16B, PPP2R3C, PPP3CA, PPP3CB, PRDM16, PRDM2, PRDX5, PRKAG2, PRKAR1B, PRKCA, PRKCB, PRKCE, PRKCQ, PRKCZ, PRKD2, PRKDC, PRKN, PRLR, PRMT1, PRPF19, PSEN1, PSMC2, PSMC3, PSMC6, PTEN, PTF1A, PTGES2, PTK2, PTPN11, PTPRA, PTPRC, PTPRN, PUM1, RAB29, RACK1, |

| | | | | | | |
|---|---|---|---|---|---|---|
| GO:0009893 | positive regulation of metabolic process | 5,37E-08 | [2, 3, 4] | 905,00 | 22,09 | AGRN, AKAP12, AKAP13, AKT2, ALK, ALKAL2, ALMS1, ALS2, ALX1, ALX4, AMBRA1, AMH, ANGPT1, ANGPT4, ANK3, ANKRD17, ANKRD31, ANTXR1, ANXA2, APC, APC2, APOBEC1, APPL1, APPL2, ARAP1, ARHGEF10L, ARHGEF2, ARHGEF7, ARID2, ARID4B, ARIH1, ARL6IP5, ARNT2, ARNTL, ASCL1, ASPH, ASPSCR1, ASS1, ASXL2, ATF1, ATF2, ATF3, ATG14, ATG16L1, ATG4B, ATM, ATP13A2, ATP2A3, ATP2B4, ATXN7L3, AURKB, BABAM2, BACH1, BAG6, BANP, BAZ1B, BBS7, BCAR3, BCAS3, BCL10, BCL11A, BCL11B, BCL2, BCL3, BCL6, BCL9, BDNF, BDNF-AS, BICRAL, BIRC2, BMP2, BMP3, BMP7, BMP8B, BMPER, BRD1, BRD4, BRF1, BRMS1, BTBD10, BTC, BTG2, BTRC, C1orf61, CADM1, CALCOCO1, CALM2, CAMK4, CAMTA1, CAMTA2, CAND1, CAPRIN2, CARD10, CARD11, CARD14, CASR, CASZ1, CAV1, CBFA2T3, CBX8, CCAR2, CCBE1, CCDC3, CCL16, CCND2, CCNY, CCR2, CCT2, CD33, CD46, CDC123, CDC20, CDC20B, CDC25B, CDH13, CDK1, CDK2, CDK5R1, CDKN1A, CDKN2A, CDT1, CELF4, CEP290, CEP295, CHAMP1, CHEK1, CHEK2, CHTF8, CIB1, CITED2, CITED4, CIZ1, CLN6, CLYBL, CNOT1, CNOT7, COMMD1, COP1, CPEB1, CPT1A, CREB3L1, CREB3L2, CREB5, CREM, CSNK1E, CSRNP2, CSRNP3, CTBP2, CTCF, CUL3, CXCR4, CYCS, CYFIP1, CYFIP2, CYP1B1, DAB1, DAG1, DAPK1, DAXX, DCN, DCUN1D1, DDA1, DDAH2, DDR1, DDR2, DDX60, DGKQ, DGKZ, DHX34, DHX36, DIPK2A, DLC1, DLG1, DLG4, DLL1, DLL4, DLX2, DNA2, DNAJC3, DNPEP, DOCK7, DOK4, DOK7, DOT1L, DPF1, DSTYK, DTX1, DUSP19, DUSP26, E2F2, E2F8, EBF1, EBF3, EBF4, ECE1, EDAR, EDEM2, EFNA5, EGR2, EHF, EHMT1, EIF2AK3, EIF2AK4, EIF4A3, ELAVL1, ELF1, ELK3, EMC10, ENPP2, EPB41L4B, EPHA1, EPHA10, EPHA4, EPHA6, EPHB1, EPHB3, ERBB4, ERCC1, ERCC8, EREG, ESR1, ESR2, ESRRG, ETAA1, ETFA, ETS1, ETV1, ETV6, EVX1, EXOSC6, EYA2, EYA4, EZH2, F12, F3, FABP5, FAF1, FAM161A, FANCM, FASN, FBLN5, FBXW8, FEZF1, FEZF2, FGD2, FGF2, FGF7, FH, FHOD1, FLT4, FNIP2, FNTA, FOXD3, FOXJ3, FOXK1, FOXO1, FRMD8, FRS2, FTO, FUBP1, FZD10, FZD5, FZR1, GABPB1, GADD45G, GARS1, GATA5, GCG, GCNT2, GFI1, GFRAL, GLI2, GLI3, GLIPR2, GLIS2, GLMN, GNAI2, GORASP1, GPER1, GPR55, GPRC5B, GPRC5C, GPRIN1, GPSM1, GPT, GRAMD4, GRB10, GRB2, GREM1, GRM1, GRM4, GSK3A, GTF2B, GTF2H1, GUCA2A, HACD3, HADHA, HAND1, HCLS1, HDAC4, HECW1, HEG1, HELT, HES1, HES3, HEXB, HGS, HIP1, HIP1R, HIVEP1, HIVEP3, HLA-DPB1, HMBOX1, HMGB1, HMGN3, HNF1B, HNRNPAB, HNRNPC, HNRNPD, HNRNPK, HNRNPR, HOOK1, HOXA1, HOXA3, HOXA4, HOXC10, HOXC11, HOXC4, HOXD13, HSD17B4, HSP90AB1, HSPD1, HSPH1, HTATIP2, HTR2A, HTT, IDE, IFI16, IFIH1, IFT74, IGF1, IGF1R, IGF2BP1, IHH, IKBKB, IKZF1, IL12RB2, IL16, IL17D, IL17RA, IL1RAP, IL31RA, IL37, IL6R, IL6ST, ILF3, ING1, ING3, INPP5F, INSIG2, INSR, IQGAP3, IRAK2, IRF2, IRF6, IRS2, ISL1, ITCH, ITGA1, ITGA5, ITGB2, JAK2, JARID2, KAT2B, KAT6A, KAT6B, KAT7, KDM3A, KDM4C, KDM4D, KDM6B, KHDRBS1, KIDINS220, KIF2A, KITLG, KLF14, KLF17, KLF7, KMT2D, KMT2E, KMT5C, KNDC1, KPNA7, KRAS, KSR1, KSR2, LARP4B, LARP7, LDB2, LDLR, LEPR, LGMN, LHX4, LHX6, LHX8, LIMS1, LLGL1, LMO7, LMTK2, LMX1B, LONP1, LPAR1, LPCAT1, LPGAT1, LPL, LRP1, LRP2, LRP5, LRRC32, LRRK1, LRSAM1, LURAP1, LY9, LYL1, MACC1, MAF, MAFA, MAFG, MAGI2, MAK, MAL, MAML2, MAML3, MAP2K2, MAP2K5, MAP3K11, MAP3K13, MAP3K19, MAP3K2, MAP3K20, MAP3K5, MAP3K6, MAP4K4, MAP4K5, MAPK7, MAPK8, MAPK8IP2, MAPK8IP3, MAPK9, MAPKAP1, MAPKAPK5, MARS1, MAX, MCRS1, MECOM, MED16, MED22, MED24, MED26, MED27, MEF2C, MEIOC, MEIS2, MELTF, MEN1, MEOX1, MGMT, MICAL2, MLLT3, MMD, MPHOSPH8, MRPL23, MRTFA, MSANTD1, MSGN1, MSH2, MST1R, MTA1, MTA3, MTMR9, MYD88, MYO1C, MYO1E, MYRF, NAT10, NCK2, NCKAP1L, NCOA2, NCOA6, NCOA7, NCOR2, NCSTN, NDFIP2, NDRG4, NDUFA13, NDUFA2, NEDD4L, NEDD9, NEK7, NEUROD6, NFAM1, NFATC1, NFATC2, NFATC2IP, NFATC4, NFE2L2, NFIB, NFIL3, NFKBIB, NFYA, NFYC, NGF, NKD1, NKX2-4, NLRC5, NLRP12, NLRX1, NME2, NNT, NOS1, NOS1AP, NOTCH1, NOTCH3, NOTCH4, NPAT, NPNT, NPRL3, NPTN, NR1D1, NR2E3, NR3C1, NRF1, NRIP1, NRXN1, NSD1, NSD3, NSG1, NSMF, NTRK2, NUS1, OLFM1, OSBPL7, OSBPL8, OTUD4, P2RY6, PABPC1L, PAF1, PAGR1, PAK4, PAK5, PANX2, PAOX, PARD3, PARP14, PASK, PAX2, PAX7, PAX8, PAX9, PBX1, PCGF5, PDCD5, PDCD6, PDCL3, PDE8A, PDGFD, PDGFRA, PDLIM1, PDPK1, PDZD2, PEG3, PER2, PFKFB3, PFKM, PFN2, PHF19, PHF2, PIH1D1, PIK3CB, PIK3CG, PIK3R1, PIK3R5, PIM1, PIP4K2A, PKD1, PKIB, PKP1, PLA2G6, PLAC8, PLAUR, PLCB1, PLCE1, PLCG2, PLD1, PLEK, PLEKHN1, PLIN5, PLXND1, PML, PNPLA2, POLDIP3, POLR2F, POLR3A, POLR3E, POLR3H, POMT1, POU2F1, POU2F2, POU2F3, POU4F2, POU6F2, PPARG, PPARGC1A, PPARGC1B, PPM1F, PPP1R16B, PPP2R3C, PPP3CA, PPP3CB, PRDM16, PRDM2, PRDX5, PRKAG2, PRKAR1B, PRKCA, PRKCB, PRKCE, PRKCQ, PRKCZ, PRKD2, PRKDC, PRKN, PRLR, PRMT1, PRPF19, PSEN1, PSMC2, PSMC3, PSMC6, PTEN, PTF1A, PTGES2, PTK2, PTPN11, PTPRA, PTPRC, PTPRN, PUM1, RAB29, RACK1, |
| GO:0016310 | phosphorylation | 5,32E-04 | [5] | 572,00 | 22,20 | ALS2, AMBRA1, AMH, ANGPT1, ANGPT4, ANKS1B, ANTXR1, ANXA2, APC, ARHGEF2, ARHGEF7, ARL6IP5, ATF3, ATG14, ATM, ATP13A2, ATP2B4, ATP5F1A, ATP5ME, AURKB, BAZ1B, BCAR3, BCL10, BCL2, BDNF, BDNF-AS, BMP2, BMP3, BMP7, BMP8B, BMPER, BPGM, BRD4, BRSK2, BTBD10, BTC, BTRC, CALM2, CAMK2B, CAMK2D, CAMK4, CAPRIN2, CARD10, CARD11, CARD14, CASR, CAV1, CBFA2T3, CCDC88C, CCL16, CCNA1, CCND2, CCNL1, CCNY, CDC25B, CDC25C, CDC42BPB, CDK1, CDK11B, CDK19, CDK2, CDK5R1, CDKL2, CDKN1A, CDKN2A, CEP43, CERKL, CHAMP1, CHEK1, CHEK2, CIB1, CILK1, CIT, CLASP1, CLK3, CLK4, CLP1, CNOT7, COQ9, COX5A, COX6A1, COX7C, CRIM1, CSNK1A1L, CSNK1E, CSNK1G3, CUL3, CXCR4, CYC1, CYCS, CYLD, DAB1, DAG1, DAPK1, DAXX, DBNDD2, DCAF1, DCLK1, DDIT4, DDR1, DDR2, DGKA, DGKD, DGKE, DGKH, DGKQ, DGKZ, DHDDS, DIPK2A, DLD, DLG1, DLG2, DLG4, DNAJC15, DNAJC3, DNAJC30, DOCK7, DOK4, DOK7, DSTYK, DUS2, DUSP19, DUSP26, EDAR, EEF1A1, EFNA4, EFNA5, EFR3B, EIF2AK3, EIF2AK4, EMC10, ENPP2, EPHA1, EPHA10, EPHA4, EPHA6, EPHB1, EPHB3, EPS8L1, ERBB4, ERC1, EREG, ETAA1, ETFA, EZH2, F3, FAF1, FASN, FASTK, FASTKD1, FBP1, FCSK, FER, FGD2, FGF2, FGF22, FGF6, FGF7, FGGY, FKTN, FLT4, FN3KRP, FNIP2, FOXK1, FOXO1, FRS2, FZD10, FZD5, GADD45G, GAK, GALK2, GARS1, GCG, GCNT2, GFRAL, GLIPR2, GLMN, GLYCTK, GMFB, GNAI2, GPER1, GPR55, GPRC5B, GPRC5C, GPRIN1, GRB10, GRB2, GREM1, GRK3, GRK5, GRM1, GRM4, GSK3A, GTF2H1, HACD3, HCLS1, HDAC4, HEG1, HES1, HGS, HMGB1, HOOK1, HRC, HRG, HSP90AB1, HTATIP2, HTR2A, HTT, HUS1, IDNK, IGF1, IGF1R, IKBKB, IL12RB2, IL17RD, IL31RA, IL6R, IL6ST, ILF3, INKA2, INPP5F, INSR, IPO7, IQGAP3, IRAK2, IRS2, ISL1, ITCH, ITGA1, ITGA5, ITGB2, ITK, JAK2, KALRN, KAT2B, KDM4D, KIDINS220, KIF2A, KIRREL1, KITLG, KNDC1, KRAS, KSR1, KSR2, LATS2, LAX1, LDB2, LDHD, LDLRAD4, LEPR, LIMK2, LLGL1, LMTK2, LONP1, LPAR1, LRP1, LRP5, LRRK1, LTBP1, MAGI2, MAK, MAP2K2, MAP2K5, MAP3K11, MAP3K13, MAP3K19, MAP3K2, MAP3K20, MAP3K5, MAP3K6, MAP4K4, MAP4K5, MAPK4, MAPK7, MAPK8, MAPK8IP2, MAPK8IP3, MAPK9, MAPKAP1, MAPKAPK5, MARK3, MAST4, MCM2, MCPH1, MECOM, MEF2C, MEN1, MKNK1, MKNK2, MMD, MSH2, MST1R, MYD88, MYLK, MYLK4, NCK2, NCKAP1L, NDRG4, NDUFA13, NDUFA2, NDUFB3, NDUFB6, NDUFB7, NDUFC1, NDUFC2, NEDD9, NEK11, NEK7, NF1, NGF, NLK, NLRC5, NLRP12, NME2, NNT, NOS1, NOTCH1, NPHS1, NPNT, NPPA, NPR1, NPTN, NRXN1, NSD1, NTRK2, NUP205, NUP43, NUP50, NUP58, OBSCN, OGDH, OSBPL8, P2RY6, PAFAH1B1, PAK4, PAK5, PANK4, PAPSS1, PARD3, PARP14, PASK, PBLD, PDCL3, PDE8A, PDGFD, PDGFRA, PDGFRL, PDPK1, PDZD2, PEAK1, PFKFB3, PFKM, PFKP, PFN2, PI4K2A, PIH1D1, PIK3CB, PIK3CG, PIK3IP1, PIK3R1, PIK3R5, PIKFYVE, PIM1, PIP4K2A, PIP5K1C, PKD1, PKIB, PKM, PKMYT1, PLA2G6, PLAUR, PLCB1, PLCE1, PLCL2, PLK4, PLK5, PLVAP, PML, POU4F2, PPARGC1A, PPARGC1B, PPM1F, PPP2R3C, PPP3CA, PPP3CB, PRAG1, PRKAG2, PRKAR1B, PRKCA, PRKCB, PRKCE, PRKCH, PRKCI, PRKCQ, PRKCZ, PRKD2, PRKDC, PRKN, PRLR, PRMT1, PSCA, PSEN1, PSMA5, PSMA6, PSMA7, PSMC2, PSMC3, PSMC6, PSMD2, PSMD7, PTEN, PTK2, PTPN11, PTPRA, PTPRC, PTPRH, PTPRO, |
| GO:0010604 | positive regulation of macromolecule metabolic process | 1,00E-07 | [3, 4, 5] | 842,00 | 22,22 | ALX1, ALX4, AMH, ANGPT1, ANGPT4, ANK3, ANKRD17, ANKRD31, ANTXR1, ANXA2, APC, APC2, APOBEC1, APPL1, ARAP1, ARHGEF10L, ARHGEF2, ARHGEF7, ARID2, ARID4B, ARIH1, ARL6IP5, ARNT2, ARNTL, ASCL1, ASPH, ASPSCR1, ASXL2, ATF1, ATF2, ATF3, ATG14, ATG4B, ATM, ATP13A2, ATP2A3, ATP2B4, ATXN7L3, AURKB, BABAM2, BACH1, BAG6, BANP, BAZ1B, BBS7, BCAR3, BCAS3, BCL10, BCL11A, BCL11B, BCL2, BCL3, BCL6, BCL9, BDNF, BDNF-AS, BICRAL, BIRC2, BMP2, BMP3, BMP7, BMP8B, BMPER, BRD1, BRD4, BRF1, BRMS1, BTC, BTG2, BTRC, C1orf61, CADM1, CALCOCO1, CALM2, CAMK4, CAMTA1, CAMTA2, CAND1, CAPRIN2, CARD10, CARD11, CARD14, CASR, CASZ1, CAV1, CBFA2T3, CBX8, CCAR2, CCBE1, CCL16, CCND2, CCNY, CCR2, CCT2, CD33, CD46, CDC123, CDC20, CDC20B, CDC25B, CDH13, CDK1, CDK2, CDK5R1, CDKN1A, CDKN2A, CDT1, CELF4, CEP290, CEP295, CHAMP1, CHEK1, CHEK2, CHTF8, CIB1, CITED2, CITED4, CIZ1, CLN6, CNOT1, CNOT7, COMMD1, COP1, CPEB1, CREB3L1, CREB3L2, CREB5, CREM, CSNK1E, CSRNP2, CSRNP3, CTBP2, CTCF, CUL3, CXCR4, CYCS, CYFIP1, CYFIP2, CYP1B1, DAB1, DAG1, DAPK1, DAXX, DCN, DCUN1D1, DDA1, DDR2, DDX60, DGKQ, DHX34, DHX36, DIPK2A, DLC1, DLG1, DLG4, DLL1, DLL4, DLX2, DNA2, DNAJC3, DNPEP, DOCK7, DOK4, DOK7, DOT1L, DPF1, DSTYK, DTX1, DUSP19, DUSP26, E2F2, E2F8, EBF1, EBF3, EBF4, ECE1, EDAR, EDEM2, EFNA5, EGR2, EHF, EIF2AK3, EIF2AK4, EIF4A3, ELAVL1, ELF1, ELK3, EMC10, ENPP2, EPB41L4B, EPHA4, ERBB4, ERCC1, ERCC8, EREG, ESR1, ESR2, ESRRG, ETAA1, ETFA, ETS1, ETV1, ETV6, EVX1, EXOSC6, EYA2, EYA4, EZH2, F12, F3, FAF1, FAM161A, FANCM, FASN, FBLN5, FBXW8, FEZF1, FEZF2, FGD2, FGF2, FGF7, FHOD1, FLT4, FNIP2, FNTA, FOXD3, FOXJ3, FOXK1, FOXO1, FRMD8, FRS2, FTO, FUBP1, FZD10, FZD5, FZR1, GABPB1, GADD45G, GARS1, GATA5, GCG, GCNT2, GFI1, GFRAL, GLI2, GLI3, GLIPR2, GLIS2, GLMN, GNAI2, GORASP1, GPER1, GPR55, GPRC5B, GPRC5C, GPRIN1, GRAMD4, GREM1, GRM1, GRM4, GSK3A, GTF2B, GTF2H1, HACD3, HAND1, HCLS1, HDAC4, HECW1, HEG1, HELT, HES1, HES3, HEXB, HGS, HIP1, HIP1R, HIVEP1, HIVEP3, HLA-DPB1, HMBOX1, HMGB1, HMGN3, HNF1B, HNRNPAB, HNRNPC, HNRNPD, HNRNPK, HNRNPR, HOOK1, HOXA1, HOXA3, HOXA4, HOXC10, HOXC11, HOXC4, HOXD13, HSD17B4, HSP90AB1, HSPD1, HSPH1, HTATIP2, HTR2A, IDE, IFI16, IFIH1, IFT74, IGF1, IGF1R, IGF2BP1, IHH, IKBKB, IKZF1, IL12RB2, IL16, IL17D, IL17RA, IL1RAP, IL31RA, IL37, IL6R, IL6ST, ILF3, ING1, ING3, INPP5F, INSIG2, INSR, IQGAP3, IRAK2, IRF2, IRF6, IRS2, ISL1, ITCH, ITGA1, ITGA5, JAK2, JARID2, KAT2B, KAT6A, KAT6B, KAT7, KDM3A, KDM4C, KDM4D, KDM6B, KHDRBS1, KIDINS220, KITLG, KLF14, KLF17, KLF7, KMT2D, KMT2E, KMT5C, KNDC1, KPNA7, KRAS, KSR1, LARP4B, LARP7, LDB2, LDLR, LEPR, LGMN, LHX4, LHX6, LHX8, LIMS1, LLGL1, LMO7, LMX1B, LONP1, LPAR1, LPCAT1, LPL, LRP1, LRP2, LRP5, LRRC32, LRRK1, LURAP1, LY9, LYL1, MACC1, MAF, MAFA, MAFG, MAGI2, MAK, MAL, MAML2, MAML3, MAP2K2, MAP2K5, MAP3K11, MAP3K13, MAP3K19, MAP3K2, MAP3K20, MAP3K5, MAP3K6, MAP4K4, MAP4K5, MAPK7, MAPK8, MAPK8IP2, MAPK8IP3, MAPK9, MAPKAP1, MAPKAPK5, MARS1, MAX, MCRS1, MECOM, MED16, MED22, MED24, MED26, MED27, MEF2C, MEIOC, MEIS2, MELTF, MEN1, MEOX1, MGMT, MICAL2, MLLT3, MMD, MPHOSPH8, MRPL23, MRTFA, MSANTD1, MSGN1, MSH2, MST1R, MTA1, MTA3, MYD88, MYO1C, MYO1E, MYRF, NAT10, NCK2, NCKAP1L, NCOA2, NCOA6, NCOA7, NCOR2, NCSTN, NDFIP2, NDRG4, NDUFA13, NDUFA2, NEDD4L, NEDD9, NEK7, NEUROD6, NFAM1, NFATC1, NFATC2, NFATC2IP, NFATC4, NFE2L2, NFIB, NFIL3, NFKBIB, NFYA, NFYC, NGF, NKD1, NKX2-4, NLRC5, NLRP12, NLRX1, NME2, NOS1, NOS1AP, NOTCH1, NOTCH3, NOTCH4, NPAT, NPNT, NPTN, NR1D1, NR2E3, NR3C1, NRF1, NRIP1, NRXN1, NSD1, NSD3, NSG1, NSMF, NTRK2, OLFM1, OSBPL7, OSBPL8, OTUD4, P2RY6, PABPC1L, PAF1, PAGR1, PAK4, PAK5, PANX2, PARP14, PASK, PAX2, PAX7, PAX8, PAX9, PBX1, PCGF5, PDCD5, PDCD6, PDCL3, PDE8A, PDGFD, PDGFRA, PDLIM1, PDPK1, PDZD2, PEG3, PER2, PFKM, PFN2, PHF19, PHF2, PIH1D1, PIK3CB, PIK3CG, PIK3R1, PIK3R5, PIM1, PKD1, PKIB, PKP1, PLA2G6, PLAC8, PLAUR, PLCB1, PLCE1, PLCG2, PLD1, PLEKHN1, PLXND1, PML, POLDIP3, POLR2F, POLR3A, POLR3E, POLR3H, POMT1, POU2F1, POU2F2, POU2F3, POU4F2, POU6F2, PPARG, PPARGC1A, PPARGC1B, PPM1F, PPP1R16B, PPP2R3C, PPP3CA, PPP3CB, PRDM16, PRDM2, PRDX5, PRKAG2, PRKAR1B, PRKCA, PRKCB, PRKCE, PRKCQ, PRKCZ, PRKD2, PRKDC, PRKN, PRLR, PRMT1, PRPF19, PSEN1, PSMC2, PSMC3, PSMC6, PTEN, PTF1A, PTGES2, PTK2, PTPN11, PTPRA, PTPRC, PTPRN, PUM1, RAB29, RACK1, RAD51, RAI1, RAP1A, RAP2B, |
| GO:0010604 | positive regulation of macromolecule metabolic process | 1,00E-07 | [3, 4, 5] | 842,00 | 22,22 | ALX1, ALX4, AMH, ANGPT1, ANGPT4, ANK3, ANKRD17, ANKRD31, ANTXR1, ANXA2, APC, APC2, APOBEC1, APPL1, ARAP1, ARHGEF10L, ARHGEF2, ARHGEF7, ARID2, ARID4B, ARIH1, ARL6IP5, ARNT2, ARNTL, ASCL1, ASPH, ASPSCR1, ASXL2, ATF1, ATF2, ATF3, ATG14, ATG4B, ATM, ATP13A2, ATP2A3, ATP2B4, ATXN7L3, AURKB, BABAM2, BACH1, BAG6, BANP, BAZ1B, BBS7, BCAR3, BCAS3, BCL10, BCL11A, BCL11B, BCL2, BCL3, BCL6, BCL9, BDNF, BDNF-AS, BICRAL, BIRC2, BMP2, BMP3, BMP7, BMP8B, BMPER, BRD1, BRD4, BRF1, BRMS1, BTC, BTG2, BTRC, C1orf61, CADM1, CALCOCO1, CALM2, CAMK4, CAMTA1, CAMTA2, CAND1, CAPRIN2, CARD10, CARD11, CARD14, CASR, CASZ1, CAV1, CBFA2T3, CBX8, CCAR2, CCBE1, CCL16, CCND2, CCNY, CCR2, CCT2, CD33, CD46, CDC123, CDC20, CDC20B, CDC25B, CDH13, CDK1, CDK2, CDK5R1, CDKN1A, CDKN2A, CDT1, CELF4, CEP290, CEP295, CHAMP1, CHEK1, CHEK2, CHTF8, CIB1, CITED2, CITED4, CIZ1, CLN6, CNOT1, CNOT7, COMMD1, COP1, CPEB1, CREB3L1, CREB3L2, CREB5, CREM, CSNK1E, CSRNP2, CSRNP3, CTBP2, CTCF, CUL3, CXCR4, CYCS, CYFIP1, CYFIP2, CYP1B1, DAB1, DAG1, DAPK1, DAXX, DCN, DCUN1D1, DDA1, DDR2, DDX60, DGKQ, DHX34, DHX36, DIPK2A, DLC1, DLG1, DLG4, DLL1, DLL4, DLX2, DNA2, DNAJC3, DNPEP, DOCK7, DOK4, DOK7, DOT1L, DPF1, DSTYK, DTX1, DUSP19, DUSP26, E2F2, E2F8, EBF1, EBF3, EBF4, ECE1, EDAR, EDEM2, EFNA5, EGR2, EHF, EIF2AK3, EIF2AK4, EIF4A3, ELAVL1, ELF1, ELK3, EMC10, ENPP2, EPB41L4B, EPHA4, ERBB4, ERCC1, ERCC8, EREG, ESR1, ESR2, ESRRG, ETAA1, ETFA, ETS1, ETV1, ETV6, EVX1, EXOSC6, EYA2, EYA4, EZH2, F12, F3, FAF1, FAM161A, FANCM, FASN, FBLN5, FBXW8, FEZF1, FEZF2, FGD2, FGF2, FGF7, FHOD1, FLT4, FNIP2, FNTA, FOXD3, FOXJ3, FOXK1, FOXO1, FRMD8, FRS2, FTO, FUBP1, FZD10, FZD5, FZR1, GABPB1, GADD45G, GARS1, GATA5, GCG, GCNT2, GFI1, GFRAL, GLI2, GLI3, GLIPR2, GLIS2, GLMN, GNAI2, GORASP1, GPER1, GPR55, GPRC5B, GPRC5C, GPRIN1, GRAMD4, GREM1, GRM1, GRM4, GSK3A, GTF2B, GTF2H1, HACD3, HAND1, HCLS1, HDAC4, HECW1, HEG1, HELT, HES1, HES3, HEXB, HGS, HIP1, HIP1R, HIVEP1, HIVEP3, HLA-DPB1, HMBOX1, HMGB1, HMGN3, HNF1B, HNRNPAB, HNRNPC, HNRNPD, HNRNPK, HNRNPR, HOOK1, HOXA1, HOXA3, HOXA4, HOXC10, HOXC11, HOXC4, HOXD13, HSD17B4, HSP90AB1, HSPD1, HSPH1, HTATIP2, HTR2A, IDE, IFI16, IFIH1, IFT74, IGF1, IGF1R, IGF2BP1, IHH, IKBKB, IKZF1, IL12RB2, IL16, IL17D, IL17RA, IL1RAP, IL31RA, IL37, IL6R, IL6ST, ILF3, ING1, ING3, INPP5F, INSIG2, INSR, IQGAP3, IRAK2, IRF2, IRF6, IRS2, ISL1, ITCH, ITGA1, ITGA5, JAK2, JARID2, KAT2B, KAT6A, KAT6B, KAT7, KDM3A, KDM4C, KDM4D, KDM6B, KHDRBS1, KIDINS220, KITLG, KLF14, KLF17, KLF7, KMT2D, KMT2E, KMT5C, KNDC1, KPNA7, KRAS, KSR1, LARP4B, LARP7, LDB2, LDLR, LEPR, LGMN, LHX4, LHX6, LHX8, LIMS1, LLGL1, LMO7, LMX1B, LONP1, LPAR1, LPCAT1, LPL, LRP1, LRP2, LRP5, LRRC32, LRRK1, LURAP1, LY9, LYL1, MACC1, MAF, MAFA, MAFG, MAGI2, MAK, MAL, MAML2, MAML3, MAP2K2, MAP2K5, MAP3K11, MAP3K13, MAP3K19, MAP3K2, MAP3K20, MAP3K5, MAP3K6, MAP4K4, MAP4K5, MAPK7, MAPK8, MAPK8IP2, MAPK8IP3, MAPK9, MAPKAP1, MAPKAPK5, MARS1, MAX, MCRS1, MECOM, MED16, MED22, MED24, MED26, MED27, MEF2C, MEIOC, MEIS2, MELTF, MEN1, MEOX1, MGMT, MICAL2, MLLT3, MMD, MPHOSPH8, MRPL23, MRTFA, MSANTD1, MSGN1, MSH2, MST1R, MTA1, MTA3, MYD88, MYO1C, MYO1E, MYRF, NAT10, NCK2, NCKAP1L, NCOA2, NCOA6, NCOA7, NCOR2, NCSTN, NDFIP2, NDRG4, NDUFA13, NDUFA2, NEDD4L, NEDD9, NEK7, NEUROD6, NFAM1, NFATC1, NFATC2, NFATC2IP, NFATC4, NFE2L2, NFIB, NFIL3, NFKBIB, NFYA, NFYC, NGF, NKD1, NKX2-4, NLRC5, NLRP12, NLRX1, NME2, NOS1, NOS1AP, NOTCH1, NOTCH3, NOTCH4, NPAT, NPNT, NPTN, NR1D1, NR2E3, NR3C1, NRF1, NRIP1, NRXN1, NSD1, NSD3, NSG1, NSMF, NTRK2, OLFM1, OSBPL7, OSBPL8, OTUD4, P2RY6, PABPC1L, PAF1, PAGR1, PAK4, PAK5, PANX2, PARP14, PASK, PAX2, PAX7, PAX8, PAX9, PBX1, PCGF5, PDCD5, PDCD6, PDCL3, PDE8A, PDGFD, PDGFRA, PDLIM1, PDPK1, PDZD2, PEG3, PER2, PFKM, PFN2, PHF19, PHF2, PIH1D1, PIK3CB, PIK3CG, PIK3R1, PIK3R5, PIM1, PKD1, PKIB, PKP1, PLA2G6, PLAC8, PLAUR, PLCB1, PLCE1, PLCG2, PLD1, PLEKHN1, PLXND1, PML, POLDIP3, POLR2F, POLR3A, POLR3E, POLR3H, POMT1, POU2F1, POU2F2, POU2F3, POU4F2, POU6F2, PPARG, PPARGC1A, PPARGC1B, PPM1F, PPP1R16B, PPP2R3C, PPP3CA, PPP3CB, PRDM16, PRDM2, PRDX5, PRKAG2, PRKAR1B, PRKCA, PRKCB, PRKCE, PRKCQ, PRKCZ, PRKD2, PRKDC, PRKN, PRLR, PRMT1, PRPF19, PSEN1, PSMC2, PSMC3, PSMC6, PTEN, PTF1A, PTGES2, PTK2, PTPN11, PTPRA, PTPRC, PTPRN, PUM1, RAB29, RACK1, RAD51, RAI1, RAP1A, RAP2B, |

| | | | | | | |
|---|---|---|---|---|---|---|
| GO:0048870 | cell motility | 4,86E-02 | [2, 3] | 400,00 | 22,22 | APPL2, ARHGDIA, ARHGDIB, ARHGEF16, ARHGEF2, ARHGEF7, ARID2, ARL13B, ASCL1, ATM, ATP1A4, ATP2B4, ATP5F1A, ATP8A1, BBS4, BCAR1, BCAS3, BCL11B, BCL2, BIN3, BMERB1, BMP2, BMP7, BMPER, BTC, CACNA1I, CAMK2B, CAP1, CARD10, CARMIL1, CARMIL2, CAV1, CCBE1, CCKAR, CCL16, CCN4, CCR2, CCR6, CD200R1, CD47, CDC42BPB, CDH13, CDK1, CDK5R1, CEACAM21, CELSR1, CEND1, CEP43, CFAP46, CFAP54, CIB1, CITED2, CLASP1, CLASP2, COL18A1, COL3A1, CORO1B, CRB2, CTNNA2, CTTN, CUL3, CXCR4, CYP19A1, CYP1B1, DAB1, DAG1, DCLK1, DCN, DDIT4, DDR1, DDR2, DGKZ, DHX34, DLC1, DLL4, DNAH17, DNAH5, DNAH8, DNER, DNM1L, DOCK10, DOCK7, DOCK8, DRGX, DST, DUOX1, EFCAB9, ELMO1, EMC10, ENC1, ENPP2, EPB41L4B, EPHA1, EPHA4, EPHB1, EPHB3, EPPK1, ERBB4, EREG, ETS1, EVL, F11R, F3, FAM110A, FAM89B, FAP, FAT1, FER, FEZF1, FEZF2, FGF2, FGF7, FLT4, FMNL2, FOXP1, FRMD5, FUT8, FZR1, GAB1, GCNT1, GCNT2, GLI3, GLIPR2, GLMN, GNA12, GNAI2, GPC6, GPER1, GRB10, GRB2, GREM1, GSK3A, GSX2, HDAC4, HDAC9, HES1, HEXB, HMGB1, HRG, IGF1, IGF1R, IGSF8, IL16, IL17RA, IL6R, ING3, INPP5F, INSR, IQSEC1, IRS2, ISL1, ITGA1, ITGA11, ITGA5, ITGAL, ITGB2, ITGB4, ITGBL1, JAK2, JAML, KANK1, KIF2A, KITLG, KRIT1, LAMA2, LAMA5, LAMB1, LAMB2, LAMB4, LDB2, LDLRAD4, LGMN, LGR6, LHX6, LIMA1, LOXL2, LPAR1, LRIG2, LRP1, LRP12, LRP5, LURAP1, MACF1, MAG, MAGI2, MAP2K5, MAP4K4, MAPK8IP3, MBOAT7, MDGA1, MEAK7, MEF2C, MEGF8, MEN1, MRTFA, MTUS1, MYD88, MYLK, MYO18A, MYO1C, MYO1E, NARS1, NAV1, NAV3, NCK2, NCKAP1, NCKAP1L, NDE1, NDRG4, NEDD9, NF1, NFATC2, NFE2L2, NKD1, NLRP12, NOL3, NOTCH1, NPHP4, NSMF, NTNG1, NTNG2, NTRK2, NUMB, NUS1, OGDH, OSBPL8, P2RY6, PAFAH1B1, PAK4, PAK5, PALLD, PBLD, PCDHA6, PDCD6, PDGFD, PDGFRA, PDLIM1, PDPK1, PDZD2, PEAK1, PEX13, PFN2, PHACTR1, PIK3CB, PIK3CG, PIK3R1, PIP5K1C, PLA2G7, PLCB1, PLCG2, PLD1, PLTP, PLVAP, PLXNA1, PLXNA2, PLXNB1, PLXNC1, PLXND1, PML, PODXL2, POMGNT2, PPARG, PPARGC1A, PPM1F, PPP3CA, PRAG1, PRKCA, PRKCE, PRKCI, PRKCQ, PRKCZ, PRKD2, PSEN1, PTEN, PTGES2, PTK2, PTPN11, PTPRC, |
| GO:0009888 | tissue development | 4,62E-03 | [3] | 487,00 | 22,25 | ANO6, ANXA6, AP2A1, AP2A2, AP2S1, AP3B1, ARHGAP12, ARHGEF26, ARID2, ARID4B, ARL13B, ARNTL, ASCL1, ASTN2, ATF2, ATF3, ATM, ATP2A2, B9D1, BBS4, BBS7, BCL10, BCL11B, BCL2, BCL9, BDNF, BDNF-AS, BFSP1, BFSP2, BIN3, BMP1, BMP2, BMP3, BMP7, BMP8B, BMPER, BNC1, BNC2, BRD2, BRSK2, BTG2, BTRC, CARMIL2, CASR, CAV1, CBFA2T2, CC2D2A, CCDC88C, CCN4, CDK1, CDKN1A, CELA2A, CELSR1, CEP290, CHRDL2, CHRND, CHST11, CITED2, CLASP1, CLASP2, CNNM4, CNRIP1, COBL, COL11A2, COL18A1, COL2A1, COL3A1, COL4A1, COL4A2, COL6A1, COL6A2, CPLANE1, CPQ, CPT1A, CRB2, CREB3L2, CRIP1, CSGALNACT1, CTNNBIP1, CXCR4, CYP1B1, DAG1, DCN, DDR1, DDR2, DHX15, DIPK2A, DLC1, DLG1, DLL1, DLL4, DLX2, DNASE1L2, DNER, E2F8, EDAR, EGR2, EHF, EIF2AK3, ELN, EPHA4, EPHB1, EPPK1, ERBB4, EREG, ESR1, ESRP2, EVPLL, EXOC4, EXPH5, EYA2, EZH2, F11R, FA2H, FABP5, FAM172A, FASN, FAT1, FBN2, FBXW8, FERD3L, FGF2, FGF6, FGF7, FHOD3, FMN1, FNDC3A, FOXO1, FOXP1, FRAS1, FRMD6, FRS2, FTO, FZD5, FZR1, GAA, GARS1, GATA5, GCNT1, GCNT2, GJA5, GLI2, GLI3, GLIPR2, GLMN, GPC6, GPR161, GPR4, GRB2, GREM1, GSK3A, HAND1, HAPLN2, HDAC4, HDAC9, HEG1, HES1, HES3, HIVEP3, HNF1B, HNRNPAB, HOXA3, HOXC11, HOXC4, HOXD13, HSD17B4, HYDIN, IFRD1, IFT140, IFT74, IGF1, IGSF8, IHH, IKBKB, IKZF1, IL17RD, IL31RA, IL6R, INSR, IQGAP3, IRF6, ISL1, ITGA5, ITGB2, ITGB4, JAK2, JARID2, KCNK2, KDM2B, KDM6B, KIF16B, KIF26B, KITLG, KLF7, KLHL3, KLK4, KLK7, KRAS, KRT1, KRT20, KRT25, KRT40, KRT82, KRT84, KRTAP16-1, KRTAP23-1, KRTAP4-3, LAMA2, LAMA5, LAMB1, LAMB2, LAMB4, LARGE1, LCE1A, LCE1F, LCE3E, LDB2, LDLRAD4, LGR6, LONP1, LOXL2, LOXL3, LRP2, LRP5, LTBP2, LZTS2, MAF, MAFG, MAGI2, MBD3, MEF2C, MEGF8, MEN1, MEOX1, MGMT, MICAL2, MKS1, MLLT3, MRTFA, MSGN1, MYD88, MYH15, MYL2, MYL6, MYLK, MYO18B, MYO1E, MYO5A, MYOM2, NDRG4, NECTIN1, NF1, NFATC4, NFIB, NKD1, NME2, NOL3, NOM1, NOTCH1, NOTCH4, NPHS1, NPNT, NPPA, NPRL3, NTNG1, NTNG2, NUP210L, OBSL1, OC90, OLFM1, ORAI1, PAF1, PAFAH1B1, PALLD, PAX2, PAX7, PAX8, PAX9, PBLD, PBX1, PCK2, PCSK6, PDCD6, PDGFD, PDGFRA, PDPK1, PDZD2, PDZD7, PHLDB1, PIH1D1, PIM1, PKD1, PKM, PKP1, PLEKHA4, PLK4, PLOD1, PLXNA1, PLXNA2, PLXND1, PML, POU2F3, POU4F2, PPARG, PPARGC1A, PPL, PPP1R16B, PPP3CA, PRICKLE2, PRKCA, PRKCH, PRKD2, PRKDC, PRLR, PSEN1, PSMA5, PSMA6, PSMA7, PSMC2, PSMC3, PSMC6, PSMD2, PSMD7, PTCH2, PTEN, PTF1A, PTGIS, PTPN11, PTPRC, PTPRO, PUS7, RAP1A, RAPGEF1, RARA, RASIP1, RB1, RBFOX1, RBP4, RET, RFLNA, RFLNB, RGMA, RHBDF2, RHEB, RHOA, RILPL1, RIPK1, RIPOR2, ROBO1, ROCK2, ROR1, ROR2, RORA, RORC, RREB1, RUNX1, |
| GO:0007275 | multicellular organism development | 3,65E-16 | [2, 3] | 1289,00 | 22,32 | ADGRB3, ADGRF5, ADGRG1, ADGRL3, ADGRV1, AFDN, AFF3, AGBL4, AGO2, AGRN, AJAP1, AKAP13, AKT2, ALAS1, ALG5, ALK, ALKAL2, ALPK2, ALPL, ALS2, ALX1, ALX4, AMBRA1, AMH, ANGPT1, ANGPT4, ANGPTL2, ANK3, ANKH, ANKRD11, ANKRD17, ANKRD33, ANKS1A, ANKS1B, ANKS6, ANO1, ANO6, ANTXR1, ANXA2, ANXA6, AP2A1, AP2A2, AP2S1, AP3B1, APBA2, APC, APC2, APPL2, APRT, ARF1, ARHGDIA, ARHGDIB, ARHGDIG, ARHGEF2, ARHGEF7, ARID2, ARID4B, ARL13B, ARNT2, ARNTL, ASAP1, ASCL1, ASPH, ASS1, ASXL2, ASZ1, ATAT1, ATF1, ATF2, ATF3, ATG16L1, ATM, ATN1, ATP11A, ATP2B2, ATP2B4, ATP6V0D1, ATP8A2, ATXN1, ATXN10, AURKB, B9D1, BAG6, BANP, BATF2, BBS4, BBS7, BBX, BCAR3, BCAS3, BCL10, BCL11A, BCL11B, BCL2, BCL2A1, BCL3, BCL6, BCL9, BDNF, BDNF-AS, BEND6, BFSP1, BFSP2, BHLHE40, BICC1, BIN3, BIRC2, BLOC1S2, BLOC1S5, BMERB1, BMP1, BMP2, BMP3, BMP7, BMP8B, BMPER, BNC2, BPGM, BRD1, BRD2, BRINP2, BRSK2, BSN, BTBD6, BTD, BTF3, BTG2, BTG4, BTN3A3, BTRC, C19orf57, C1QC, C21orf91, CA10, CACNA1A, CACNA1H, CADM1, CALM2, CAMK2B, CAMK4, CAMSAP1, CAPRIN2, CARD10, CARD11, CASP2, CASP5, CASR, CASZ1, CATSPERG, CAV1, CBFA2T2, CBFA2T3, CC2D2A, CCBE1, CCDC85C, CCKAR, CCN4, CCN6, CCR2, CCR6, CD46, CD8A, CDC20, CDH10, CDH13, CDH18, CDH19, CDH22, CDH23, CDH26, CDH4, CDK1, CDK5R1, CDKN1A, CDKN2A, CELA2A, CELF4, CELSR1, CEND1, CEP131, CEP290, CFDP1, CHAMP1, CHD5, CHEK1, CHRDL2, CHRNB2, CHRND, CHST11, CHST8, CIB1, CIC, CILK1, CIT, CITED2, CLASP1, CLASP2, CLCN2, CLMP, CLP1, CMIP, CMTM8, CNNM4, CNOT1, CNRIP1, CNTN4, CNTN5, CNTNAP2, COBL, COL11A2, COL18A1, COL2A1, COL3A1, COL4A1, COL4A2, COL6A1, COL6A2, COL8A2, CPLANE1, CPNE5, CRB2, CREB3L1, CREB3L2, CREM, CRIM1, CRIP1, CRISPLD2, CRYBA4, CRYBG3, CRYGC, CSGALNACT1, CSMD3, CSN3, CSNK1E, CSPG5, CTNNA2, CTNNBIP1, CTNND2, CTNS, CTTN, CUL3, CUX1, CXCR4, CYFIP1, CYFIP2, CYLD, CYP19A1, CYP1B1, CYP4A11, DAB1, DAG1, DBN1, DCAF1, DCHS2, DCLK1, DCLRE1C, DCN, DCTN1, DDIT4, DDR1, DDR2, DDX6, DENND5A, DGCR6, DGCR6L, DGKD, DHCR7, DHDDS, DHRS3, DHX15, DHX30, DHX35, DHX36, DIPK2A, DKK3, DLC1, DLD, DLG1, DLG4, DLL1, DLL4, DLX2, DMC1, DNAH5, DNAJB6, DNAJC30, DNASE1L2, DNER, DNM1L, DNMT3A, DOC2A, DOCK10, DOCK7, DOK4, DOK6, DPF1, DRGX, DSCAML1, DTX1, DUOX1, DYNC2LI1, E2F2, E2F8, EBF1, EBF3, EBF4, ECE1, EDAR, EFHD1, EFNA4, EFNA5, EGR2, EHF, EHMT1, EHMT2, EIF2AK3, EIF2AK4, EIF2S2, EIF4A3, EIF4E, EIF4EBP1, EIF4ENIF1, ELK3, ELN, ELOVL1, EMC10, ENC1, ENPP2, EPHA1, EPHA10, EPHA4, EPHA6, EPHB1, EPHB3, ERAP1, ERBB4, ERC1, ERCC1, EREG, ERMARD, ESR1, ESRP2, ETS1, ETV1, ETV6, EVL, EVX1, EXOC4, EXOSC6, EXPH5, EYA2, EYA4, EZH2, F11R, F3, FA2H, FAM172A, FAP, FARP1, FARP2, FASN, FAT1, FBLN5, FBN1, FBN2, FBXW8, FERD3L, FEZ1, FEZF1, FEZF2, FGF12, FGF14, FGF2, FGF6, FGF7, FHOD3, FJX1, FKTN, FLRT1, FLT4, FMN1, FNDC3A, FOXD3, FOXK1, FOXO1, FOXP1, FPGS, FRAS1, FREM1, FRS2, FRYL, FTO, FUT8, FZD10, FZD5, FZR1, GAA, GAB1, GAB2, GABRA2, GABRA4, GABRB2, GADD45G, GAK, GALC, GARS1, GAS7, GATA5, GCNT1, GCNT2, GFER, GFI1, GFRAL, GINS4, GJA5, GLI2, GLI3, GLIPR2, GLIS2, GLMN, GLO1, GMFB, GNA12, GNAI2, GNAO1, GON4L, GORASP1, GPC6, GPER1, GPR137B, GPR161, GPR17, GPR4, GPR55, GPRC5B, GPRIN1, GPSM1, GRB10, GRB2, GREM1, GRID2, GSK3A, GSS, GSX2, GTF2IRD1, H3C6, H3C8, HAND1, HAPLN2, HAPLN3, HCLS1, HCN1, HDAC4, HDAC9, HECTD1, HECW1, HEG1, HELT, HERC1, HES1, HES3, HES4, HEXB, HGS, HIP1R, HIVEP3, HM13, HMGB1, HNF1B, HNRNPAB, HNRNPD, HOOK1, HOXA1, HOXA3, HOXA4, HOXC10, HOXC11, HOXC4, HOXD13, HRG, HSD17B4, HSP90AB1, HSPD1, HTATIP2, HUS1, HYDIN, IDE, IFI16, IFRD1, IFT140, IFT74, IFT88, IGF1, IGF1R, IGF2BP1, IGSF8, IGSF9B, IHH, IKBKB, IKZF1, IL17D, IL17RD, IL1RAP, IL31RA, IL6R, IL6ST, IMMP2L, ING3, INPP5F, INPPL1, INSC, INSIG2, INSR, IQCE, IQGAP3, IQSEC1, IRF2BP2, IRF6, IRS2, ISL1, ISM1, ITCH, ITFG2, ITGA1, ITGA11, ITGA5, ITGB2, ITGB4, ITK, JAK2, JARID2, JHY, KALRN, KANK1, KAT2B, KAT6A, KAT7, KCNAB2, KCNC1, KCNK2, KCNQ1, KCNQ2, KDM2B, KDM4C, KDM6B, KIAA1217, KIDINS220, KIF16B, KIF18A, KIF1A, KIF26A, KIF26B, KIF2A, KIF5A, KIFBP, KITLG, KLF7, KLHL1, KLHL17, KLHL3, KLK4, KMT2D, KMT2E, KMT5C, KNDC1, KPNA7, KRAS, KREMEN2, KRIT1, KRT1, KRT20, KRT25, KRT40, KRT82, KRT84, KRTAP16-1, KRTAP23-1, KRTAP4-3, L3MBTL3, LAMA2, LAMA5, LAMB1, LAMB2, LAMB4, LARGE1, LCE1A, LCE1F, LCE3E, LDB2, LDB3, LDHD, LDLR, LDLRAD4, LEPR, LGR6, LHX4, LHX6, LHX8, LIG4, LIMK2, LINGO1, LLGL1, LMBR1, LMTK2, LMX1B, LONP1, LOXL2, LOXL3, LPAR1, LPCAT1, LRCH4, LRFN3, LRIG2, LRIG3, LRP1, LRP10, LRP12, LRP2, LRP5, LRRC4C, LRRK1, LSAMP, LTBP1, LTBP2, LY9, LYL1, LZTS1, LZTS2, MACF1, MACROD2, MAD1L1, MAF, MAFG, MAFK, MAG, MAGI2, MAK, MAL, MALL, MAP2, MAP2K5, MAP3K13, MAP3K20, MAP4, MAP4K4, MAP6, MAPK7, MAPK8IP2, MAPK8IP3, MAPKAP1, MAX, MBD3, MBNL1, MBOAT7, MCL1, MCM2, MCPH1, MDGA1, MECOM, MEF2C, MEGF8, MEIS2, MELTF, MEN1, MEOX1, MFGE8, MFNG, MGMT, MICAL2, MICALL1, MIPEP, MKNK2, MKS1, MLLT3, MMD, MME, MN1, MOG, MOV10L1, MRPL13, MRTFA, MSGN1, MSH2, MSH3, MSLN, MST1R, MTMR2, MTNR1B, MYH15, MYH3, MYL2, MYL6, MYLK, MYO15A, MYO16, MYO18B, MYO1D, MYO1E, MYO5A, MYOM2, MYPN, MYRF, MYT1L, N4BP3, NAV1, NAV3, NCAM2, NCK2, NCKAP1, NCKAP1L, NCOA2, NCOA6, NCOR2, NCSTN, NDE1, NDRG4, NDUFA2, NECTIN1, NEDD4L, NEO1, NEPRO, NEUROD6, NEUROG2, NF1, NFAM1, NFASC, NFATC1, NFATC2, NFATC4, NFE2L2, NFIB, NGEF, NGF, NHEJ1, NKD1, NKX2-4, NLGN2, NME2, NOL3, NOM1, NOS1, NOTCH1, NOTCH3, NOTCH4, NPAT, NPHP4, NPHS1, NPNT, NPPA, NPR1, |
| GO:0007275 | multicellular organism development | 3,65E-16 | [2, 3] | 1289,00 | 22,32 | ADGRB3, ADGRF5, ADGRG1, ADGRL3, ADGRV1, AFDN, AFF3, AGBL4, AGO2, AGRN, AJAP1, AKAP13, AKT2, ALAS1, ALG5, ALK, ALKAL2, ALPK2, ALPL, ALS2, ALX1, ALX4, AMBRA1, AMH, ANGPT1, ANGPT4, ANGPTL2, ANK3, ANKH, ANKRD11, ANKRD17, ANKRD33, ANKS1A, ANKS1B, ANKS6, ANO1, ANO6, ANTXR1, ANXA2, ANXA6, AP2A1, AP2A2, AP2S1, AP3B1, APBA2, APC, APC2, APPL2, APRT, ARF1, ARHGDIA, ARHGDIB, ARHGDIG, ARHGEF2, ARHGEF7, ARID2, ARID4B, ARL13B, ARNT2, ARNTL, ASAP1, ASCL1, ASPH, ASS1, ASXL2, ASZ1, ATAT1, ATF1, ATF2, ATF3, ATG16L1, ATM, ATN1, ATP11A, ATP2B2, ATP2B4, ATP6V0D1, ATP8A2, ATXN1, ATXN10, AURKB, B9D1, BAG6, BANP, BATF2, BBS4, BBS7, BBX, BCAR3, BCAS3, BCL10, BCL11A, BCL11B, BCL2, BCL2A1, BCL3, BCL6, BCL9, BDNF, BDNF-AS, BEND6, BFSP1, BFSP2, BHLHE40, BICC1, BIN3, BIRC2, BLOC1S2, BLOC1S5, BMERB1, BMP1, BMP2, BMP3, BMP7, BMP8B, BMPER, BNC2, BPGM, BRD1, BRD2, BRINP2, BRSK2, BSN, BTBD6, BTD, BTF3, BTG2, BTG4, BTN3A3, BTRC, C19orf57, C1QC, C21orf91, CA10, CACNA1A, CACNA1H, CADM1, CALM2, CAMK2B, CAMK4, CAMSAP1, CAPRIN2, CARD10, CARD11, CASP2, CASP5, CASR, CASZ1, CATSPERG, CAV1, CBFA2T2, CBFA2T3, CC2D2A, CCBE1, CCDC85C, CCKAR, CCN4, CCN6, CCR2, CCR6, CD46, CD8A, CDC20, CDH10, CDH13, CDH18, CDH19, CDH22, CDH23, CDH26, CDH4, CDK1, CDK5R1, CDKN1A, CDKN2A, CELA2A, CELF4, CELSR1, CEND1, CEP131, CEP290, CFDP1, CHAMP1, CHD5, CHEK1, CHRDL2, CHRNB2, CHRND, CHST11, CHST8, CIB1, CIC, CILK1, CIT, CITED2, CLASP1, CLASP2, CLCN2, CLMP, CLP1, CMIP, CMTM8, CNNM4, CNOT1, CNRIP1, CNTN4, CNTN5, CNTNAP2, COBL, COL11A2, COL18A1, COL2A1, COL3A1, COL4A1, COL4A2, COL6A1, COL6A2, COL8A2, CPLANE1, CPNE5, CRB2, CREB3L1, CREB3L2, CREM, CRIM1, CRIP1, CRISPLD2, CRYBA4, CRYBG3, CRYGC, CSGALNACT1, CSMD3, CSN3, CSNK1E, CSPG5, CTNNA2, CTNNBIP1, CTNND2, CTNS, CTTN, CUL3, CUX1, CXCR4, CYFIP1, CYFIP2, CYLD, CYP19A1, CYP1B1, CYP4A11, DAB1, DAG1, DBN1, DCAF1, DCHS2, DCLK1, DCLRE1C, DCN, DCTN1, DDIT4, DDR1, DDR2, DDX6, DENND5A, DGCR6, DGCR6L, DGKD, DHCR7, DHDDS, DHRS3, DHX15, DHX30, DHX35, DHX36, DIPK2A, DKK3, DLC1, DLD, DLG1, DLG4, DLL1, DLL4, DLX2, DMC1, DNAH5, DNAJB6, DNAJC30, DNASE1L2, DNER, DNM1L, DNMT3A, DOC2A, DOCK10, DOCK7, DOK4, DOK6, DPF1, DRGX, DSCAML1, DTX1, DUOX1, DYNC2LI1, E2F2, E2F8, EBF1, EBF3, EBF4, ECE1, EDAR, EFHD1, EFNA4, EFNA5, EGR2, EHF, EHMT1, EHMT2, EIF2AK3, EIF2AK4, EIF2S2, EIF4A3, EIF4E, EIF4EBP1, EIF4ENIF1, ELK3, ELN, ELOVL1, EMC10, ENC1, ENPP2, EPHA1, EPHA10, EPHA4, EPHA6, EPHB1, EPHB3, ERAP1, ERBB4, ERC1, ERCC1, EREG, ERMARD, ESR1, ESRP2, ETS1, ETV1, ETV6, EVL, EVX1, EXOC4, EXOSC6, EXPH5, EYA2, EYA4, EZH2, F11R, F3, FA2H, FAM172A, FAP, FARP1, FARP2, FASN, FAT1, FBLN5, FBN1, FBN2, FBXW8, FERD3L, FEZ1, FEZF1, FEZF2, FGF12, FGF14, FGF2, FGF6, FGF7, FHOD3, FJX1, FKTN, FLRT1, FLT4, FMN1, FNDC3A, FOXD3, FOXK1, FOXO1, FOXP1, FPGS, FRAS1, FREM1, FRS2, FRYL, FTO, FUT8, FZD10, FZD5, FZR1, GAA, GAB1, GAB2, GABRA2, GABRA4, GABRB2, GADD45G, GAK, GALC, GARS1, GAS7, GATA5, GCNT1, GCNT2, GFER, GFI1, GFRAL, GINS4, GJA5, GLI2, GLI3, GLIPR2, GLIS2, GLMN, GLO1, GMFB, GNA12, GNAI2, GNAO1, GON4L, GORASP1, GPC6, GPER1, GPR137B, GPR161, GPR17, GPR4, GPR55, GPRC5B, GPRIN1, GPSM1, GRB10, GRB2, GREM1, GRID2, GSK3A, GSS, GSX2, GTF2IRD1, H3C6, H3C8, HAND1, HAPLN2, HAPLN3, HCLS1, HCN1, HDAC4, HDAC9, HECTD1, HECW1, HEG1, HELT, HERC1, HES1, HES3, HES4, HEXB, HGS, HIP1R, HIVEP3, HM13, HMGB1, HNF1B, HNRNPAB, HNRNPD, HOOK1, HOXA1, HOXA3, HOXA4, HOXC10, HOXC11, HOXC4, HOXD13, HRG, HSD17B4, HSP90AB1, HSPD1, HTATIP2, HUS1, HYDIN, IDE, IFI16, IFRD1, IFT140, IFT74, IFT88, IGF1, IGF1R, IGF2BP1, IGSF8, IGSF9B, IHH, IKBKB, IKZF1, IL17D, IL17RD, IL1RAP, IL31RA, IL6R, IL6ST, IMMP2L, ING3, INPP5F, INPPL1, INSC, INSIG2, INSR, IQCE, IQGAP3, IQSEC1, IRF2BP2, IRF6, IRS2, ISL1, ISM1, ITCH, ITFG2, ITGA1, ITGA11, ITGA5, ITGB2, ITGB4, ITK, JAK2, JARID2, JHY, KALRN, KANK1, KAT2B, KAT6A, KAT7, KCNAB2, KCNC1, KCNK2, KCNQ1, KCNQ2, KDM2B, KDM4C, KDM6B, KIAA1217, KIDINS220, KIF16B, KIF18A, KIF1A, KIF26A, KIF26B, KIF2A, KIF5A, KIFBP, KITLG, KLF7, KLHL1, KLHL17, KLHL3, KLK4, KMT2D, KMT2E, KMT5C, KNDC1, KPNA7, KRAS, KREMEN2, KRIT1, KRT1, KRT20, KRT25, KRT40, KRT82, KRT84, KRTAP16-1, KRTAP23-1, KRTAP4-3, L3MBTL3, LAMA2, LAMA5, LAMB1, LAMB2, LAMB4, LARGE1, LCE1A, LCE1F, LCE3E, LDB2, LDB3, LDHD, LDLR, LDLRAD4, LEPR, LGR6, LHX4, LHX6, LHX8, LIG4, LIMK2, LINGO1, LLGL1, LMBR1, LMTK2, LMX1B, LONP1, LOXL2, LOXL3, LPAR1, LPCAT1, LRCH4, LRFN3, LRIG2, LRIG3, LRP1, LRP10, LRP12, LRP2, LRP5, LRRC4C, LRRK1, LSAMP, LTBP1, LTBP2, LY9, LYL1, LZTS1, LZTS2, MACF1, MACROD2, MAD1L1, MAF, MAFG, MAFK, MAG, MAGI2, MAK, MAL, MALL, MAP2, MAP2K5, MAP3K13, MAP3K20, MAP4, MAP4K4, MAP6, MAPK7, MAPK8IP2, MAPK8IP3, MAPKAP1, MAX, MBD3, MBNL1, MBOAT7, MCL1, MCM2, MCPH1, MDGA1, MECOM, MEF2C, MEGF8, MEIS2, MELTF, MEN1, MEOX1, MFGE8, MFNG, MGMT, MICAL2, MICALL1, MIPEP, MKNK2, MKS1, MLLT3, MMD, MME, MN1, MOG, MOV10L1, MRPL13, MRTFA, MSGN1, MSH2, MSH3, MSLN, MST1R, MTMR2, MTNR1B, MYH15, MYH3, MYL2, MYL6, MYLK, MYO15A, MYO16, MYO18B, MYO1D, MYO1E, MYO5A, MYOM2, MYPN, MYRF, MYT1L, N4BP3, NAV1, NAV3, NCAM2, NCK2, NCKAP1, NCKAP1L, NCOA2, NCOA6, NCOR2, NCSTN, NDE1, NDRG4, NDUFA2, NECTIN1, NEDD4L, NEO1, NEPRO, NEUROD6, NEUROG2, NF1, NFAM1, NFASC, NFATC1, NFATC2, NFATC4, NFE2L2, NFIB, NGEF, NGF, NHEJ1, NKD1, NKX2-4, NLGN2, NME2, NOL3, NOM1, NOS1, NOTCH1, NOTCH3, NOTCH4, NPAT, NPHP4, NPHS1, NPNT, NPPA, NPR1, |

| | | | | | | |
|---|---|---|---|---|---|---|
| GO:0032879 | regulation of localization | 5,10E-06 | [2, 3] | 670,00 | 22,42 | ANK3, ANKRD13B, ANO1, ANO6, ANXA2, ANXA6, AP2A1, AP2S1, APBA2, APC, APOC4, APPL1, APPL2, ARAP1, ARF1, ARFGAP1, ARHGDIA, ARHGDIB, ARHGDIG, ARHGEF16, ARHGEF2, ARHGEF7, ARID2, ARL6IP5, ARNTL, ASPSCR1, ASTN2, ASXL2, ATM, ATP13A2, ATP1A3, ATP1A4, ATP2A2, ATP2B4, ATP5F1A, ATP8A1, ATP8A2, AURKB, B9D1, BBS4, BCAR1, BCAS3, BCL2, BCL6, BDNF, BDNF-AS, BEST1, BMERB1, BMP2, BMP7, BMPER, BRSK2, BSN, BTBD9, BTC, C12orf4, C4orf3, CABP1, CACNA1A, CACNA1B, CACNA1E, CACNA1G, CACNA1H, CACNA1I, CACNA2D2, CACNA2D3, CACNA2D4, CACNB2, CACNG1, CACNG6, CACNG8, CADM1, CADPS, CADPS2, CALM2, CAMK2B, CAMK2D, CARD10, CARMIL1, CARMIL2, CASR, CAV1, CCBE1, CCKAR, CCN4, CCR2, CCR6, CCT2, CD200R1, CD247, CD33, CD47, CDH13, CDK1, CDK5R1, CDT1, CELA2A, CEP131, CEP290, CEP295, CEP43, CHMP2A, CHRNB2, CHRNB3, CIB1, CITED2, CLASP1, CLASP2, CLCN2, CLTCL1, CNIH3, CNST, COL18A1, COL3A1, COMMD1, CORIN, CORO1B, CORO2B, CPT1A, CRBN, CRY2, CSNK1E, CSPG5, CTNNA2, CTSS, CXCR4, CYFIP1, CYLD, CYP19A1, CYP1B1, CYP4A11, CYP51A1, DAG1, DAPK1, DBN1, DCLK1, DCN, DCP2, DDR2, DGKQ, DHX36, DLC1, DLG1, DLG2, DLG4, DLL1, DLL4, DNAJB6, DNAJC30, DNAJC6, DNM1, DNM1L, DOC2A, DOCK10, DOCK7, DOCK8, DPP10, DUOX1, EDEM2, EEPD1, EFNA5, EIF2AK3, EIPR1, EMC10, ENC1, ENHO, ENPP2, EPB41L4B, EPHA1, EPPK1, EPRS1, ERBB4, EREG, ETS1, EVL, EXOC7, EXOSC10, EXPH5, F2RL3, F3, FABP5, FAM110A, FAM3D, FAM89B, FER, FEZ1, FEZF2, FGF12, FGF14, FGF2, FGF7, FITM2, FLT4, FOXP1, FRMD4A, FRMD5, FRMD8, FTO, FXYD5, FXYD6, FZD5, FZR1, GAB1, GAB2, GCG, GCNT2, GJA5, GLI3, GLIPR2, GLIS2, GLMN, GNA12, GNAI2, GNB5, GPC6, GPER1, GPR137B, GPRIN1, GRB10, GREM1, GRIA4, GSG1L, GSK3A, GSX2, HADHA, HCAR2, HCLS1, HCN1, HCN2, HCN4, HDAC4, HDAC9, HECW1, HGS, HIP1, HIP1R, HMGB1, HMGN3, HNRNPC, HNRNPK, HOOK1, HPS4, HRC, HRG, HSP90AB1, HSPD1, HTR1A, HTR2A, HTR3A, HTT, IFIH1, IGF1, IGF1R, IL15RA, IL16, IL17RA, IL1RAP, IL37, IL6R, ILDR1, INPP5F, INSR, IQSEC1, IRS2, ISL1, ITGA5, ITGB2, ITPR2, ITPR3, IWS1, JAK2, JPH3, KALRN, KANK1, KAT7, KCNAB2, KCNB1, KCNB2, KCNC1, KCNC3, KCNE1, KCNF1, KCNH2, KCNIP4, KCNJ14, KCNJ15, KCNJ2, KCNJ3, KCNJ6, KCNMA1, KCNQ1, KCNQ2, KCNV1, KHDRBS1, KIAA1614, KIF2A, KITLG, KLF7, KRIT1, KRT20, LAMA2, LAMA5, LAMB1, LARP7, LDB2, LDLRAD4, LEPR, LGMN, LGR6, LLGL1, LONP1, LPAR1, LPL, LRIG2, LRP1, LRP5, LRPAP1, LRRC32, LRSAM1, LZTS1, LZTS2, MACF1, MAD1L1, MAGI2, MAP2, MAP2K2, MAP2K5, MAP4K4, MAPK8, MAPK8IP2, MAPK8IP3, MCRS1, MEAK7, MEF2C, MEGF8, MFGE8, MMD, MTMR2, MTNR1B, MTUS1, MYD88, MYLK, MYO18A, MYO1C, MYO1E, MYO5A, NALCN, NAPB, NAV3, NCKAP1L, NDFIP2, NDRG4, NDUFA2, NEDD4L, NEDD9, NF1, NFE2L2, NKAIN1, NKD1, NLGN2, NLRP12, NOL3, NOS1, NOS1AP, NOTCH1, NPHS1, NPPA, NPR1, NPTN, NR1D1, NRXN1, NRXN3, NSMF, NTNG1, NTNG2, NUMB, NUP58, NUS1, ORAI1, OSBPL8, P2RY6, PACSIN1, PACSIN2, PANX2, PARD3, PASK, PAX8, PBLD, PCLO, PCNT, PDCD5, PDCD6, PDGFD, PDGFRA, PDPK1, PDZD2, PER2, PFKM, PFN2, PHACTR1, PIK3CB, PIK3CG, PIK3R1, PIM1, PINX1, PLA2G6, PLA2G7, PLCB1, PLCB2, PLCB4, PLCD4, PLCE1, PLCG2, PLCH2, PLCL2, PLD1, PLEKHM2, PLIN5, PLTP, PLVAP, PLXNA1, PLXNA2, PLXNB1, PLXNC1, PLXND1, PML, PNPLA2, POU4F2, PPARG, PPARGC1A, PPFIA1, PPM1F, PPP1R11, PPP1R13B, PPP3CA, PPP3CB, PRAG1, PRKAG2, PRKAR1B, PRKCA, PRKCB, PRKCE, PRKCH, PRKCI, PRKCZ, PRKD2, PRKN, PSEN1, PTEN, PTGES2, PTK2, PTPN11, PTPN14, PTPN3, PTPRC, PTPRO, RAB29, RAB3D, RAB43, RAB4A, RAB5A, RACK1, RAP1A, RAP2B, RAPGEF1, RASA3, RASGRF1, RASSF5, RB1, RBM26, RBP4, RCVRN, RELN, REST, RET, RGS9, RHBDF1, RHBDF2, |
| GO:0048585 | negative regulation of response to stimulus | 2,45E-02 | [2, 3, 4] | 393,00 | 22,44 | ARL6IP5, ARNTL, ATF3, ATM, ATP2B4, BBS4, BCL2, BCL3, BCL6, BDNF, BDNF-AS, BEND6, BICC1, BMP2, BMP7, BMPER, BTC, BTRC, CALM2, CAV1, CBFA2T2, CCAR2, CCDC3, CCDC88C, CCR2, CD200R1, CD46, CD47, CD96, CDKN1A, CELF4, CHEK2, CHID1, CHST11, CIB1, CLASP1, CLASP2, CLEC4G, CNOT1, CNOT7, COL2A1, COL3A1, CORO1B, CREB3L1, CRY2, CSNK1A1L, CTNNA2, CTNNBIP1, CTTN, CUL3, CYLD, CYP19A1, CYREN, DAB1, DAG1, DCN, DDIT4, DEPDC5, DGKZ, DHRS3, DKK3, DLC1, DLG1, DLL1, DLL4, DLX2, DNAJC3, DNM1, DUSP19, DUSP26, EIF4A3, ELF1, ENHO, EPHA4, EPPK1, EPS15L1, ERCC1, EREG, ESR1, ETS1, EYA2, EYA4, EZH2, F12, FAM89B, FAP, FASN, FBN1, FBN2, FBP1, FEM1C, FER, FGF2, FKTN, FNIP2, FOXO1, FOXP1, GFRAL, GLI2, GLI3, GLIS2, GLMN, GNAI2, GPER1, GPR161, GPR17, GPRIN1, GRAMD4, GRB10, GRB2, GREM1, GRID2, GSK3A, HDAC9, HECW1, HEG1, HGS, HRG, HTRA3, HTT, IFI16, IGF1, IGF1R, IL17RD, ING3, INPP5F, INSIG2, ISL1, ITCH, ITFG2, ITGA1, KANK1, KCTD10, KCTD6, KIF26A, KLF7, KREMEN2, KRT1, LATS2, LAX1, LDLR, LDLRAD4, LGMN, LOXL3, LPAR1, LPL, LRIG2, LRP1, LRP2, LRPAP1, LRRC14, LTBP1, LZTS2, MAGI2, MAP2K5, MAPK7, MAPK8IP2, MAPKAP1, MAPKAPK5, MARK3, MCL1, MEAK7, MECOM, MEGF8, MEN1, METRNL, MGRN1, MLLT3, MRTFA, MTMR2, MTNR1B, MYO1C, NCK2, NCOA7, NCOR2, NDRG4, NDUFA13, NDUFA2, NF1, NFATC1, NFATC4, NFE2L2, NKD1, NLK, NLRC5, NLRP12, NLRX1, NOC2L, NOL3, NOTCH1, NOTCH3, NPHP4, NPPA, NPRL3, NR1D1, NRXN1, NXN, OTUB1, OTUD4, PAFAH1B1, PAK5, PALM, PARP14, PBLD, PCDHA6, PDCD6, PDE8A, PDGFRA, PDPK1, PDZD2, PDZD3, PEG10, PELI3, PHLPP2, PIH1D1, PIK3IP1, PLAUR, PLCL2, PLEK, PLIN5, POLQ, PPARG, PPP3CB, PRDM16, PRKCA, PRKCB, PRKCQ, PRKCZ, PRKD2, PRKDC, PRKN, PSCA, PSEN1, PSMA5, PSMA6, PSMA7, PSMC2, PSMC3, PSMC6, PSMD2, PSMD7, PTCH2, PTEN, PTGIS, PTPN11, PTPN3, PTPRC, PTPRO, PXDN, PYCR1, RACK1, RANBP9, RAPGEF1, RASA1, RASA2, RASA3, RASIP1, RB1, RBX1, RC3H1, RC3H2, REST, RGMA, RGS12, RGS22, RGS3, RGS6, RGS7, RGS9, RHOA, RIOK3, RIPK1, RIPOR2, RITA1, ROBO1, ROR2, RORA, RPH3AL, RPS6KB1, RTKN2, RTN4RL1, SALL3, SCAI, |
| GO:0019220 | regulation of phosphate metabolic process | 5,74E-03 | [5, 6] | 434,00 | 22,48 | ANKS1B, ANXA2, APC, ARF1, ARFGEF3, ARHGEF2, ARHGEF7, ARL6IP5, ATF3, ATG14, ATM, ATP2B4, BCAR3, BCL10, BCL2, BDNF, BDNF-AS, BMP2, BMP3, BMP7, BMP8B, BMPER, BRD4, BTBD10, BTC, CACNA1A, CALM2, CAMTA1, CAPRIN2, CARD10, CARD14, CASR, CAV1, CBFA2T3, CCDC88C, CCL16, CCNA1, CCND2, CCNL1, CCNY, CD33, CDC25B, CDC25C, CDK1, CDK5R1, CDKN1A, CDKN2A, CEP43, CHAMP1, CHEK2, CIB1, CNOT7, CNST, CRY2, CSRNP2, CSRNP3, CXCR4, CYLD, DAB1, DAG1, DAXX, DBNDD2, DDIT4, DDR1, DDR2, DGKQ, DGKZ, DHX34, DIPK2A, DLC1, DLG1, DLG2, DLG4, DNAJC15, DNAJC3, DNAJC30, DOCK7, DOK4, DOK7, DSTYK, DUS2, DUSP19, DUSP26, EDAR, EEF1A1, EFNA5, EIF2AK3, EIF2AK4, EMC10, ENPP2, EPHA1, EPHA10, EPHA4, EPHA6, EPHB1, EPHB3, ERBB4, EREG, ETAA1, ETFA, EZH2, F3, FAF1, FARP1, FASN, FBP1, FER, FGD2, FGF2, FGF7, FKTN, FLT4, FNIP2, FOXO1, FRS2, FZD10, FZD5, GADD45G, GCG, GCNT2, GFRAL, GLIPR2, GLMN, GMFB, GNAI2, GPER1, GPR55, GPRC5B, GPRC5C, GRB10, GRB2, GREM1, GRM1, GRM4, GSK3A, GTF2H1, GUCA2A, HACD3, HCLS1, HDAC4, HEG1, HES1, HGS, HMGB1, HRC, HRG, HSP90AB1, HTR2A, HTT, HUS1, IGF1, IGF1R, IKBKB, IL31RA, IL6R, IL6ST, INKA2, INPP5F, INSR, IPO7, IQGAP3, IRAK2, IRS2, ISL1, ITCH, ITGA1, ITGA5, ITGB2, JAK2, KAT2B, KDM4D, KIDINS220, KIRREL1, KITLG, KNDC1, KRAS, KSR1, LATS2, LAX1, LDB2, LDLR, LDLRAD4, LEPR, LIMK2, LLGL1, LMTK2, LPAR1, LPCAT1, LRP1, LRP5, LRRK1, MAGI2, MAP2K2, MAP2K5, MAP3K11, MAP3K13, MAP3K19, MAP3K2, MAP3K20, MAP3K5, MAP3K6, MAP4K4, MAP4K5, MAPK8IP2, MAPK8IP3, MAPKAP1, MAPKAPK5, MARF1, MCM2, MCPH1, MECOM, MEF2C, MEN1, MMD, MRTFA, MST1R, MTMR2, MTMR3, MTMR9, MYD88, MYO1D, NCK2, NCKAP1L, NDRG4, NEDD9, NF1, NGF, NLRC5, NLRP12, NNT, NOS1, NOTCH1, NPNT, NPPA, NPTN, NRXN1, NSD1, NSMF, NTRK2, NUP205, NUP43, NUP50, NUP58, OSBPL8, P2RY6, PAFAH1B1, PAK4, PAK5, PARD3, PARP14, PBLD, PDCL3, PDE8A, PDGFD, PDGFRA, PDPK1, PDZD2, PDZD3, PFKFB3, PFN2, PHACTR1, PIH1D1, PIK3CB, PIK3CG, PIK3IP1, PIK3R1, PIK3R5, PKD1, PKIB, PKMYT1, PLA2G6, PLAUR, PLCB1, PLCE1, PLCL2, PLEK, PML, PPARGC1A, PPARGC1B, PPM1F, PPP1R11, PPP1R16A, PPP1R16B, PPP2R2B, PPP2R2C, PPP2R3C, PPP3CA, PRKAG2, PRKAR1B, PRKCA, PRKCE, PRKCZ, PRKD2, PRKDC, PRKN, PRLR, PRMT1, PSCA, PSEN1, PTEN, PTK2, PTPN11, PTPRA, PTPRC, PTPRH, PTPRO, RACK1, RAD51, RANBP9, RAP1A, RAP2B, RAPGEF1, RASIP1, RB1, RBM26, RBPMS, RCAN2, RCVRN, RD3, RELN, RET, RGS3, RHBDF2, RHOA, |
| GO:0051254 | positive regulation of RNA metabolic process | 8,63E-03 | [4, 5, 6, 7, 8] | 420,00 | 22,50 | BCAS3, BCL10, BCL11B, BCL3, BCL9, BICRAL, BIRC2, BMP2, BMP7, BRD1, BRD4, BRF1, BTG2, BTRC, C1orf61, CALCOCO1, CAMK4, CAMTA1, CAMTA2, CAND1, CAPRIN2, CASZ1, CCAR2, CDH13, CDK2, CDKN2A, CELF4, CEP290, CHEK1, CHEK2, CITED2, CITED4, CNOT1, CNOT7, CPEB1, CREB3L1, CREB3L2, CREB5, CREM, CSRNP2, CSRNP3, CTBP2, CTCF, CYFIP1, DAXX, DCN, DHX36, DLL1, DLX2, DOT1L, DPF1, DTX1, E2F2, E2F8, EBF1, EBF3, EBF4, EGR2, EHF, EIF2AK3, ELF1, ELK3, ERBB4, ERCC1, ESR1, ESR2, ESRRG, ETS1, ETV1, ETV6, EVX1, F3, FBLN5, FEZF1, FEZF2, FGF2, FGF7, FHOD1, FOXD3, FOXJ3, FOXK1, FOXO1, FTO, FUBP1, FZD5, FZR1, GABPB1, GARS1, GATA5, GFI1, GLI2, GLI3, GLIS2, GPER1, GPRIN1, GREM1, GSK3A, GTF2B, GTF2H1, HAND1, HCLS1, HDAC4, HELT, HES1, HES3, HEXB, HIVEP1, HIVEP3, HMBOX1, HMGB1, HMGN3, HNF1B, HNRNPAB, HNRNPD, HNRNPK, HNRNPR, HOXA1, HOXA3, HOXA4, HOXC10, HOXC11, HOXC4, HOXD13, HSD17B4, HTATIP2, IFI16, IFT74, IGF1, IHH, IKBKB, IKZF1, IL31RA, ILF3, ING1, ING3, INSIG2, INSR, IRF2, IRF6, ISL1, KAT2B, KAT6A, KAT6B, KAT7, KDM3A, KDM6B, KLF14, KLF17, KLF7, KMT2D, KMT2E, LARP7, LDB2, LHX4, LHX6, LHX8, LMO7, LMX1B, LONP1, LRP5, LYL1, MACC1, MAF, MAFA, MAFG, MAK, MAML2, MAML3, MAP2K2, MAP2K5, MAP3K2, MAP3K5, MAPK7, MARS1, MAX, MCRS1, MECOM, MED16, MED22, MED24, MED26, MED27, MEF2C, MEIS2, MELTF, MEN1, MEOX1, MICAL2, MLLT3, MRTFA, MSANTD1, MSGN1, MTA1, MTA3, MYRF, NCK2, NCOA2, NCOA6, NCOA7, NEUROD6, NFATC1, NFATC2, NFATC2IP, NFATC4, NFE2L2, NFIB, NFIL3, NFKBIB, NFYA, NFYC, NKX2-4, NLRC5, NME2, NOS1, NOTCH1, NOTCH3, NOTCH4, NPAT, NPNT, NR1D1, NR2E3, NR3C1, NRF1, NRIP1, NSD1, NSD3, PABPC1L, PAF1, PAGR1, PAX2, PAX7, PAX8, PAX9, PBX1, PCGF5, PDLIM1, PDZD2, PEG3, PER2, PFKM, PHF2, PIH1D1, PIK3R1, PIM1, PKD1, PLAC8, PLCB1, PLEKHN1, PLXND1, PML, POU2F1, POU2F2, POU2F3, POU4F2, POU6F2, PPARG, PPARGC1A, PPARGC1B, PPP3CA, PPP3CB, PRDM16, PRDM2, PRDX5, PRKCB, PRKD2, PRKDC, PRKN, PRPF19, PSEN1, PSMC2, PSMC3, PSMC6, PTF1A, PTGES2, PTPRN, PUM1, RAI1, RARA, RB1, RBM20, RBPMS, RC3H1, RELB, REST, RET, RFX2, RFXAP, RGMA, RHOG, RIPK1, RNF111, ROCK2, ROR2, RORA, RORB, RORC, RPRD1B, RPTOR, RREB1, RUNX1, RUNX3, RXRB, RXRG, SATB2, SBNO2, SERTAD1, SETD3, SHH, SIM2, SIN3A, |
| GO:0009967 | positive regulation of signal transduction | 1,08E-02 | [3, 4, 5, 6] | 399,00 | 22,58 | ARAP1, ARHGEF7, ARL6IP5, ARNTL, ASCL1, ASXL2, ATF3, ATM, BCAR3, BCL10, BCL2, BDNF, BDNF-AS, BIRC2, BMP2, BMP3, BMP7, BMP8B, BMPER, BRD4, BTC, CALM2, CAMTA1, CAPRIN2, CARD10, CARD11, CARD14, CASP2, CASR, CAV1, CCAR2, CCBE1, CCL16, CCN4, CCR2, CDH13, CDK1, CHD5, CIB1, CITED2, COL3A1, CRB2, CSNK1E, CSNK1G3, CTBP2, CXCR4, CXXC5, CYFIP1, CYFIP2, CYLD, CYP1B1, DAXX, DCN, DDX60, DHX36, DLG4, DLL1, DLL4, DNM1L, DOK4, DSTYK, DUSP19, EDAR, EEF1E1-BLOC1S5, EMC10, ENHO, EPHA4, ERBB4, EREG, ESR1, ETFA, EZH2, F11R, F2RL3, F3, FABP5, FAF1, FAM110A, FASN, FGD2, FGF2, FGF22, FGF6, FGF7, FLT4, FOXP1, FRS2, FZD10, FZD5, GAB1, GADD45G, GATA5, GCG, GCNT2, GFI1, GFRAL, GLIPR2, GNAI2, GPER1, GPR137B, GPR17, GPR4, GPR55, GPR62, GPRC5B, GPRIN1, GRB10, GRB2, GREM1, GRM1, GRM4, GSK3A, GSX2, HACD3, HCLS1, HES1, HINT1, HIP1, HIP1R, HMGB1, HSP90AB1, HTR2A, HTT, IGF1, IGF1R, IGF2BP1, IGSF9B, IHH, IKBKB, IL10RA, IL10RB, IL20RA, IL31RA, IL37, IL6R, IL6ST, ING3, INSR, IQGAP3, IRAK2, IRS2, ISL1, ITGA1, ITGA5, JAK2, KALRN, KANK1, KAT2B, KIDINS220, KITLG, KLF14, KMT2D, KRAS, KSR1, LGR6, LIMS1, LLGL1, LMCD1, LOXL3, LPAR1, LRP1, LRP2, LRPAP1, LRRK1, LURAP1, LYPD6, MACF1, MAL, MAML2, MAML3, MAP2K2, MAP2K5, MAP3K11, MAP3K13, MAP3K2, MAP3K20, MAP3K5, MAP3K6, MAP4K4, MAP4K5, MAPK8, MAPK8IP2, MAPK8IP3, MAPK9, MAPKAPK5, MCL1, MEN1, MFNG, MIB2, MIER1, MLLT3, MRPL23, MRTFA, MST1R, MYD88, MYO1C, MYO1E, NACC2, NCK2, NDFIP2, NDRG4, NDUFA2, NEO1, NEPRO, NF1, NFAM1, NFATC4, NGF, NKD1, NLGN2, NLRC5, NLRP12, NOL3, NOS1, NOS1AP, NOTCH1, NOTCH4, NPNT, NPPA, NPR1, NPTN, NR2C1, NR3C2, NRXN1, NTRK2, OSBPL8, P2RY6, PAFAH1B1, PAGR1, PARP14, PDCD5, PDE8A, PDGFD, PDGFRA, PDZD2, PIH1D1, PIK3CB, PIK3CG, PIK3R1, PIK3R5, PLA2G6, PLAUR, PLCB1, PLCE1, PLEKHA4, PLXNB1, PML, PPP1R13B, PPP3CA, PRAG1, PRKCA, PRKCB, PRKCE, PRKCH, PRKCI, PRKCZ, PRKD2, PRKN, PRLR, PRMT1, PSEN1, PSMA5, PSMA6, PSMA7, PSMC2, PSMC3, PSMC6, PSMD2, PSMD7, PTEN, PTGIS, PTK2, PTPN11, PTPRC, PUM1, RAB29, RACK1, RAD9A, RAP1A, RAPGEF1, RASGRF1, RASGRP4, RBPMS, RC3H1, RC3H2, RELN, RET, RGS9, RHEB, RHOA, RHOG, RICTOR, RIPK1, RNF111, RNF146, RNF220, ROBO1, ROCK2, ROR1, ROR2, RORA, RPTOR, RTKN2, SASH1, |
| GO:0009967 | positive regulation of signal transduction | 1,08E-02 | [3, 4, 5, 6] | 399,00 | 22,58 | ARAP1, ARHGEF7, ARL6IP5, ARNTL, ASCL1, ASXL2, ATF3, ATM, BCAR3, BCL10, BCL2, BDNF, BDNF-AS, BIRC2, BMP2, BMP3, BMP7, BMP8B, BMPER, BRD4, BTC, CALM2, CAMTA1, CAPRIN2, CARD10, CARD11, CARD14, CASP2, CASR, CAV1, CCAR2, CCBE1, CCL16, CCN4, CCR2, CDH13, CDK1, CHD5, CIB1, CITED2, COL3A1, CRB2, CSNK1E, CSNK1G3, CTBP2, CXCR4, CXXC5, CYFIP1, CYFIP2, CYLD, CYP1B1, DAXX, DCN, DDX60, DHX36, DLG4, DLL1, DLL4, DNM1L, DOK4, DSTYK, DUSP19, EDAR, EEF1E1-BLOC1S5, EMC10, ENHO, EPHA4, ERBB4, EREG, ESR1, ETFA, EZH2, F11R, F2RL3, F3, FABP5, FAF1, FAM110A, FASN, FGD2, FGF2, FGF22, FGF6, FGF7, FLT4, FOXP1, FRS2, FZD10, FZD5, GAB1, GADD45G, GATA5, GCG, GCNT2, GFI1, GFRAL, GLIPR2, GNAI2, GPER1, GPR137B, GPR17, GPR4, GPR55, GPR62, GPRC5B, GPRIN1, GRB10, GRB2, GREM1, GRM1, GRM4, GSK3A, GSX2, HACD3, HCLS1, HES1, HINT1, HIP1, HIP1R, HMGB1, HSP90AB1, HTR2A, HTT, IGF1, IGF1R, IGF2BP1, IGSF9B, IHH, IKBKB, IL10RA, IL10RB, IL20RA, IL31RA, IL37, IL6R, IL6ST, ING3, INSR, IQGAP3, IRAK2, IRS2, ISL1, ITGA1, ITGA5, JAK2, KALRN, KANK1, KAT2B, KIDINS220, KITLG, KLF14, KMT2D, KRAS, KSR1, LGR6, LIMS1, LLGL1, LMCD1, LOXL3, LPAR1, LRP1, LRP2, LRPAP1, LRRK1, LURAP1, LYPD6, MACF1, MAL, MAML2, MAML3, MAP2K2, MAP2K5, MAP3K11, MAP3K13, MAP3K2, MAP3K20, MAP3K5, MAP3K6, MAP4K4, MAP4K5, MAPK8, MAPK8IP2, MAPK8IP3, MAPK9, MAPKAPK5, MCL1, MEN1, MFNG, MIB2, MIER1, MLLT3, MRPL23, MRTFA, MST1R, MYD88, MYO1C, MYO1E, NACC2, NCK2, NDFIP2, NDRG4, NDUFA2, NEO1, NEPRO, NF1, NFAM1, NFATC4, NGF, NKD1, NLGN2, NLRC5, NLRP12, NOL3, NOS1, NOS1AP, NOTCH1, NOTCH4, NPNT, NPPA, NPR1, NPTN, NR2C1, NR3C2, NRXN1, NTRK2, OSBPL8, P2RY6, PAFAH1B1, PAGR1, PARP14, PDCD5, PDE8A, PDGFD, PDGFRA, PDZD2, PIH1D1, PIK3CB, PIK3CG, PIK3R1, PIK3R5, PLA2G6, PLAUR, PLCB1, PLCE1, PLEKHA4, PLXNB1, PML, PPP1R13B, PPP3CA, PRAG1, PRKCA, PRKCB, PRKCE, PRKCH, PRKCI, PRKCZ, PRKD2, PRKN, PRLR, PRMT1, PSEN1, PSMA5, PSMA6, PSMA7, PSMC2, PSMC3, PSMC6, PSMD2, PSMD7, PTEN, PTGIS, PTK2, PTPN11, PTPRC, PUM1, RAB29, RACK1, RAD9A, RAP1A, RAPGEF1, RASGRF1, RASGRP4, RBPMS, RC3H1, RC3H2, RELN, RET, RGS9, RHEB, RHOA, RHOG, RICTOR, RIPK1, RNF111, RNF146, RNF220, ROBO1, ROCK2, ROR1, ROR2, RORA, RPTOR, RTKN2, SASH1, |
| GO:1903508 | positive regulation of nucleic acid-templated transcription | 9,93E-03 | [6, 7, 8, 9, 10] | 402,00 | 22,58 | BCAS3, BCL10, BCL11B, BCL3, BCL9, BICRAL, BIRC2, BMP2, BMP7, BRD1, BRD4, BRF1, BTRC, C1orf61, CALCOCO1, CAMK4, CAMTA1, CAMTA2, CAND1, CAPRIN2, CASZ1, CCAR2, CDH13, CDK2, CDKN2A, CEP290, CHEK1, CHEK2, CITED2, CITED4, CNOT7, CREB3L1, CREB3L2, CREB5, CREM, CSRNP2, CSRNP3, CTBP2, CTCF, CYFIP1, DAXX, DCN, DHX36, DLL1, DLX2, DOT1L, DPF1, DTX1, E2F2, E2F8, EBF1, EBF3, EBF4, EGR2, EHF, EIF2AK3, ELF1, ELK3, ERBB4, ERCC1, ESR1, ESR2, ESRRG, ETS1, ETV1, ETV6, EVX1, F3, FBLN5, FEZF1, FEZF2, FGF2, FGF7, FHOD1, FOXD3, FOXJ3, FOXK1, FOXO1, FUBP1, FZD5, FZR1, GABPB1, GARS1, GATA5, GFI1, GLI2, GLI3, GLIS2, GPER1, GPRIN1, GREM1, GSK3A, GTF2B, GTF2H1, HAND1, HCLS1, HDAC4, HELT, HES1, HES3, HEXB, HIVEP1, HIVEP3, HMBOX1, HMGB1, HMGN3, HNF1B, HNRNPAB, HNRNPD, HNRNPK, HOXA1, HOXA3, HOXA4, HOXC10, HOXC11, HOXC4, HOXD13, HSD17B4, HTATIP2, IFI16, IFT74, IGF1, IHH, IKBKB, IKZF1, IL31RA, ILF3, ING1, ING3, INSIG2, INSR, IRF2, IRF6, ISL1, KAT2B, KAT6A, KAT6B, KAT7, KDM3A, KDM6B, KLF14, KLF17, KLF7, KMT2D, KMT2E, LARP7, LDB2, LHX4, LHX6, LHX8, LMO7, LMX1B, LONP1, LRP5, LYL1, MACC1, MAF, MAFA, MAFG, MAK, MAML2, MAML3, MAP2K2, MAP2K5, MAP3K2, MAP3K5, MAPK7, MARS1, MAX, MCRS1, MECOM, MED16, MED22, MED24, MED26, MED27, MEF2C, MEIS2, MELTF, MEN1, MEOX1, MICAL2, MLLT3, MRTFA, MSANTD1, MSGN1, MTA1, MTA3, MYRF, NCK2, NCOA2, NCOA6, NCOA7, NEUROD6, NFATC1, NFATC2, NFATC2IP, NFATC4, NFE2L2, NFIB, NFIL3, NFKBIB, NFYA, NFYC, NKX2-4, NLRC5, NME2, NOS1, NOTCH1, NOTCH3, NOTCH4, NPAT, NPNT, NR1D1, NR2E3, NR3C1, NRF1, NRIP1, NSD1, NSD3, PABPC1L, PAF1, PAGR1, PAX2, PAX7, PAX8, PAX9, PBX1, PCGF5, PDLIM1, PDZD2, PEG3, PER2, PFKM, PHF2, PIH1D1, PIK3R1, PIM1, PKD1, PLAC8, PLCB1, PLXND1, PML, POU2F1, POU2F2, POU2F3, POU4F2, POU6F2, PPARG, PPARGC1A, PPARGC1B, PPP3CA, PPP3CB, PRDM16, PRDM2, PRKCB, PRKD2, PRKDC, PRKN, PSEN1, PSMC2, PSMC3, PSMC6, PTF1A, PTGES2, PTPRN, RAI1, RARA, RB1, RBPMS, RELB, REST, RET, RFX2, RFXAP, RGMA, RHOG, RIPK1, RNF111, ROR2, RORA, RORB, RORC, RPRD1B, RPTOR, RREB1, |

| | | | | | |
|---|---|---|---|---|---|
| GO:0045935 | positive regulation of nucleobase-containing compound metabolic process | 1,41E-03 [4, 5, 6] | 462,00 | 22,60 | ATF2, ATF3, ATM, ATXN7L3, AURKB, BABAM2, BACH1, BANP, BCAS3, BCL10, BCL11B, BCL3, BCL9, BICRAL, BIRC2, BMP2, BMP7, BRD1, BRD4, BRF1, BTG2, BTRC, C1orf61, CALCOCO1, CAMK4, CAMTA1, CAMTA2, CAND1, CAPRIN2, CASZ1, CBX8, CCAR2, CCT2, CDH13, CDK2, CDKN2A, CELF4, CEP290, CHEK1, CHEK2, CHTF8, CITED2, CITED4, CIZ1, CNOT1, CNOT7, CPEB1, CREB3L1, CREB3L2, CREB5, CREM, CSRNP2, CSRNP3, CTBP2, CTCF, CYFIP1, DAXX, DCN, DHX36, DLL1, DLX2, DOT1L, DPF1, DTX1, E2F2, E2F8, EBF1, EBF3, EBF4, EGR2, EHF, EIF2AK3, ELF1, ELK3, ERBB4, ERCC1, ERCC8, ESR1, ESR2, ESRRG, ETS1, ETV1, ETV6, EVX1, EXOSC6, EYA2, EYA4, F3, FBLN5, FEZF1, FEZF2, FGF2, FGF7, FHOD1, FOXD3, FOXJ3, FOXK1, FOXO1, FTO, FUBP1, FZD5, FZR1, GABPB1, GARS1, GATA5, GFI1, GLI2, GLI3, GLIS2, GPER1, GPRIN1, GREM1, GSK3A, GTF2B, GTF2H1, GUCA2A, HAND1, HCLS1, HDAC4, HELT, HES1, HES3, HEXB, HIVEP1, HIVEP3, HMBOX1, HMGB1, HMGN3, HNF1B, HNRNPAB, HNRNPD, HNRNPK, HNRNPR, HOXA1, HOXA3, HOXA4, HOXC10, HOXC11, HOXC4, HOXD13, HSD17B4, HSP90AB1, HTATIP2, HTR2A, IFI16, IFT74, IGF1, IHH, IKBKB, IKZF1, IL31RA, ILF3, ING1, ING3, INSIG2, INSR, IRF2, IRF6, ISL1, KAT2B, KAT6A, KAT6B, KAT7, KDM3A, KDM4D, KDM6B, KLF14, KLF17, KLF7, KMT2D, KMT2E, KMT5C, LARP7, LDB2, LHX4, LHX6, LHX8, LMO7, LMX1B, LONP1, LRP5, LYL1, MACC1, MAF, MAFA, MAFG, MAK, MAML2, MAML3, MAP2K2, MAP2K5, MAP3K2, MAP3K5, MAPK7, MAPKAPK5, MARS1, MAX, MCRS1, MECOM, MED16, MED22, MED24, MED26, MED27, MEF2C, MEIS2, MELTF, MEN1, MEOX1, MGMT, MICAL2, MLLT3, MRTFA, MSANTD1, MSGN1, MSH2, MTA1, MTA3, MYRF, NCK2, NCOA2, NCOA6, NCOA7, NEK7, NEUROD6, NFATC1, NFATC2, NFATC2IP, NFATC4, NFE2L2, NFIB, NFIL3, NFKBIB, NFYA, NFYC, NKX2-4, NLRC5, NME2, NOS1, NOTCH1, NOTCH3, NOTCH4, NPAT, NPNT, NR1D1, NR2E3, NR3C1, NRF1, NRIP1, NSD1, NSD3, OTUD4, PABPC1L, PAF1, PAGR1, PAX2, PAX7, PAX8, PAX9, PBX1, PCGF5, PDLIM1, PDZD2, PEG3, PER2, PFKFB3, PFKM, PHF2, PIH1D1, PIK3R1, PIM1, PKD1, PKIB, PLAC8, PLCB1, PLEKHN1, PLXND1, PML, POU2F1, POU2F2, POU2F3, POU4F2, POU6F2, PPARG, PPARGC1A, PPARGC1B, PPP3CA, PPP3CB, PRDM16, PRDM2, PRDX5, PRKCB, PRKCQ, PRKD2, PRKDC, PRKN, PRPF19, PSEN1, PSMC2, PSMC3, PSMC6, PTF1A, PTGES2, PTPRC, PTPRN, PUM1, RAD51, RAI1, RARA, RB1, RBM20, RBPMS, RC3H1, RCVRN, RELB, REST, RET, RFX2, RFXAP, RGMA, RHOG, RIPK1, RNF111, ROCK2, ROR2, RORA, RORB, RORC, RPRD1B, RPTOR, RREB1, RUNX1, RUNX3, RXRB, |
| GO:0048731 | system development | 6,13E-16 [3, 4] | 1171,00 | 22,61 | ADGRL3, ADGRV1, AFDN, AGBL4, AGO2, AGRN, AJAP1, AKAP13, AKT2, ALAS1, ALK, ALKAL2, ALPK2, ALPL, ALS2, ALX1, ALX4, AMBRA1, AMH, ANGPT1, ANGPT4, ANGPTL2, ANK3, ANKH, ANKRD11, ANKRD17, ANKRD33, ANKS1A, ANO6, ANTXR1, ANXA2, ANXA6, AP2A1, AP2A2, AP2S1, AP3B1, APBA2, APPL2, APRT, ARF1, ARHGDIA, ARHGEF2, ARHGEF7, ARID2, ARID4B, ARL13B, ARNT2, ARNTL, ASAP1, ASCL1, ASS1, ASXL2, ATAT1, ATF1, ATF2, ATF3, ATG16L1, ATM, ATN1, ATP2B2, ATP2B4, ATP6V0D1, ATP8A2, ATXN1, ATXN10, B9D1, BAG6, BATF2, BBS4, BBS7, BBX, BCAR3, BCAS3, BCL10, BCL11A, BCL11B, BCL2, BCL2A1, BCL3, BCL6, BCL9, BDNF, BDNF-AS, BEND6, BFSP1, BFSP2, BHLHE40, BICC1, BIN3, BIRC2, BLOC1S2, BLOC1S5, BMERB1, BMP1, BMP2, BMP3, BMP7, BMP8B, BMPER, BNC2, BPGM, BRD1, BRD2, BRINP2, BRSK2, BSN, BTBD6, BTD, BTG2, BTG4, BTRC, C1QC, C21orf91, CA10, CACNA1A, CACNA1H, CALM2, CAMK2B, CAMK4, CAMSAP1, CAPRIN2, CARD10, CARD11, CASP2, CASP5, CASR, CASZ1, CAV1, CBFA2T2, CBFA2T3, CC2D2A, CCBE1, CCDC85C, CCKAR, CCN4, CCN6, CCR2, CCR6, CD46, CD8A, CDC20, CDH13, CDH22, CDH23, CDH4, CDK1, CDK5R1, CDKN1A, CDKN2A, CELA2A, CELF4, CELSR1, CEND1, CEP290, CHAMP1, CHD5, CHRDL2, CHRNB2, CHRND, CHST11, CHST8, CIB1, CIC, CIT, CITED2, CLASP1, CLASP2, CLCN2, CLMP, CLP1, CMTM8, CNNM4, CNRIP1, CNTN4, CNTN5, CNTNAP2, COBL, COL11A2, COL18A1, COL2A1, COL3A1, COL4A1, COL4A2, COL6A1, COL6A2, COL8A2, CPLANE1, CPNE5, CRB2, CREB3L1, CREB3L2, CRIM1, CRIP1, CRISPLD2, CRYBA4, CRYBG3, CRYGC, CSGALNACT1, CSMD3, CSN3, CSNK1E, CSPG5, CTNNA2, CTNNBIP1, CTNND2, CTNS, CTTN, CUL3, CUX1, CXCR4, CYFIP1, CYFIP2, CYLD, CYP19A1, CYP1B1, CYP4A11, DAB1, DAG1, DBN1, DCAF1, DCHS2, DCLK1, DCLRE1C, DCN, DCTN1, DDIT4, DDR1, DDR2, DDX6, DENND5A, DGCR6, DGCR6L, DHCR7, DHDDS, DHRS3, DHX15, DHX30, DHX36, DIPK2A, DKK3, DLC1, DLG1, DLG4, DLL1, DLL4, DLX2, DMC1, DNAH5, DNAJB6, DNAJC30, DNASE1L2, DNER, DNM1L, DNMT3A, DOC2A, DOCK10, DOCK7, DOK4, DOK6, DPF1, DRGX, DSCAML1, DTX1, E2F2, E2F8, ECE1, EDAR, EFHD1, EFNA4, EFNA5, EGR2, EHMT2, EIF2AK3, EIF2AK4, EIF2S2, EIF4A3, EIF4E, EIF4EBP1, EIF4ENIF1, ELK3, ELN, ELOVL1, EMC10, ENC1, ENPP2, EPHA1, EPHA10, EPHA4, EPHA6, EPHB1, EPHB3, ERAP1, ERBB4, ERCC1, EREG, ESR1, ESRP2, ETS1, ETV1, ETV6, EVL, EVX1, EXOC4, EXOSC6, EXPH5, EYA4, EZH2, F3, FA2H, FAM172A, FAP, FARP1, FARP2, FASN, FAT1, FBLN5, FBN1, FBN2, FBXW8, FERD3L, FEZ1, FEZF1, FEZF2, FGF12, FGF14, FGF2, FGF6, FGF7, FHOD3, FJX1, FKTN, FLRT1, FLT4, FMN1, FNDC3A, FOXK1, FOXO1, FOXP1, FPGS, FRAS1, FREM1, FRS2, FRYL, FTO, FZD10, FZD5, FZR1, GAA, GAB1, GAB2, GABRA2, GABRA4, GABRB2, GAK, GALC, GARS1, GAS7, GATA5, GCNT1, GCNT2, GFER, GFI1, GFRAL, GJA5, GLI2, GLI3, GLIPR2, GLIS2, GLMN, GLO1, GMFB, GNAI2, GNAO1, GON4L, GORASP1, GPC6, GPER1, GPR137B, GPR161, GPR17, GPR4, GPR55, GPRC5B, GPRIN1, GPSM1, GRB10, GRB2, GREM1, GRID2, GSK3A, GSS, GSX2, H3C6, H3C8, HAND1, HAPLN2, HAPLN3, HCLS1, HCN1, HDAC4, HDAC9, HECTD1, HECW1, HEG1, HELT, HERC1, HES1, HES3, HES4, HEXB, HGS, HIP1R, HIVEP3, HMGB1, HNF1B, HNRNPAB, HNRNPD, HOXA1, HOXA3, HOXA4, HOXC10, HOXC11, HOXC4, HOXD13, HRG, HSD17B4, HSP90AB1, HSPD1, HTATIP2, HYDIN, IFI16, IFRD1, IFT140, IFT74, IFT88, IGF1, IGF2BP1, IGSF8, IGSF9B, IHH, IKZF1, IL17D, IL17RD, IL1RAP, IL31RA, IL6R, IL6ST, IMMP2L, ING3, INPP5F, INPPL1, INSC, INSIG2, INSR, IQGAP3, IQSEC1, IRF2BP2, IRF6, IRS2, ISL1, ISM1, ITCH, ITFG2, ITGA1, ITGA11, ITGA5, ITGB2, ITGB4, ITK, JAK2, JARID2, JHY, KALRN, KANK1, KAT2B, KAT6A, KAT7, KCNAB2, KCNC1, KCNK2, KCNQ1, KCNQ2, KDM2B, KDM4C, KDM6B, KIAA1217, KIDINS220, KIF18A, KIF1A, KIF26A, KIF26B, KIF2A, KIF5A, KIFBP, KITLG, KLF7, KLHL1, KLHL17, KLHL3, KLK4, KMT2D, KMT2E, KMT5C, KNDC1, KRAS, KRIT1, KRT1, KRT20, KRT25, KRT40, KRT82, KRT84, KRTAP16-1, KRTAP23-1, KRTAP4-3, L3MBTL3, LAMA2, LAMA5, LAMB1, LAMB2, LAMB4, LARGE1, LCE1A, LCE1F, LCE3E, LDB2, LDB3, LDLR, LDLRAD4, LEPR, LGR6, LHX4, LHX6, LHX8, LIG4, LIMK2, LINGO1, LLGL1, LMX1B, LONP1, LOXL2, LOXL3, LPAR1, LPCAT1, LRCH4, LRFN3, LRIG2, LRIG3, LRP1, LRP10, LRP12, LRP2, LRP5, LRRC4C, LRRK1, LSAMP, LTBP1, LTBP2, LY9, LYL1, LZTS1, LZTS2, MACF1, MACROD2, MAD1L1, MAF, MAFK, MAG, MAGI2, MAL, MALL, MAP2, MAP2K5, MAP3K13, MAP4, MAP4K4, MAP6, MAPK7, MAPK8IP2, MAPK8IP3, MAPKAP1, MAX, MBD3, MBNL1, MBOAT7, MCM2, MCPH1, MDGA1, MECOM, MEF2C, MEGF8, MEIS2, MELTF, MEN1, MEOX1, MFGE8, MFNG, MGMT, MICAL2, MICALL1, MIPEP, MKNK2, MKS1, MLLT3, MMD, MME, MOG, MRPL13, MRTFA, MSH2, MSH3, MSLN, MTMR2, MTNR1B, MYH15, MYH3, MYL2, MYL6, MYLK, MYO15A, MYO16, MYO18B, MYO1D, MYO1E, MYO5A, MYOM2, MYPN, MYRF, MYT1L, N4BP3, NAV1, NAV3, NCAM2, NCK2, NCKAP1, NCKAP1L, NCOA2, NCOA6, NCOR2, NCSTN, NDE1, NDRG4, NECTIN1, NEDD4L, NEO1, NEPRO, NEUROD6, NEUROG2, NF1, NFAM1, NFASC, NFATC1, NFATC2, NFATC4, NFE2L2, NFIB, NGEF, NGF, NHEJ1, NKD1, NLGN2, NME2, NOL3, NOM1, NOS1, NOTCH1, NOTCH3, NOTCH4, NPHP4, NPHS1, NPNT, NPPA, NPR1, NPRL3, NPTN, NR1D1, NR2E3, NRIP1, NRSN1, NRSN2, NRXN1, NRXN3, NSMF, NTM, NTNG1, NTNG2, NTRK2, NUDT1, NUMB, NUP210L, NUS1, NXN, OBSL1, OC90, OGDH, OLFM1, OLIG2, OPCML, OPN5, ORAI1, P4HTM, PACSIN1, PAF1, PAFAH1B1, PAK4, PALLD, PALM, PAPPA2, PAPSS1, PARD3, PAX2, PAX7, PAX8, PAX9, PBLD, PBX1, PCARE, PCDHA11, PCDHA6, PCDHA7, PCDHB9, PCK2, PCLO, PCSK5, PCSK6, PDCD6, PDCL3, PDGFD, PDGFRA, PDLIM1, PDLIM3, PDPK1, PDZD2, PDZD7, PEG10, PER2, PEX13, PFKFB3, PHACTR1, PHF2, PHLDB1, PHLPP2, PIK3CB, PIK3CG, PIK3R1, PIKFYVE, |
| GO:0048731 | system development | 6,13E-16 [3, 4] | 1171,00 | 22,61 | ADGRL3, ADGRV1, AFDN, AGBL4, AGO2, AGRN, AJAP1, AKAP13, AKT2, ALAS1, ALK, ALKAL2, ALPK2, ALPL, ALS2, ALX1, ALX4, AMBRA1, AMH, ANGPT1, ANGPT4, ANGPTL2, ANK3, ANKH, ANKRD11, ANKRD17, ANKRD33, ANKS1A, ANO6, ANTXR1, ANXA2, ANXA6, AP2A1, AP2A2, AP2S1, AP3B1, APBA2, APPL2, APRT, ARF1, ARHGDIA, ARHGEF2, ARHGEF7, ARID2, ARID4B, ARL13B, ARNT2, ARNTL, ASAP1, ASCL1, ASS1, ASXL2, ATAT1, ATF1, ATF2, ATF3, ATG16L1, ATM, ATN1, ATP2B2, ATP2B4, ATP6V0D1, ATP8A2, ATXN1, ATXN10, B9D1, BAG6, BATF2, BBS4, BBS7, BBX, BCAR3, BCAS3, BCL10, BCL11A, BCL11B, BCL2, BCL2A1, BCL3, BCL6, BCL9, BDNF, BDNF-AS, BEND6, BFSP1, BFSP2, BHLHE40, BICC1, BIN3, BIRC2, BLOC1S2, BLOC1S5, BMERB1, BMP1, BMP2, BMP3, BMP7, BMP8B, BMPER, BNC2, BPGM, BRD1, BRD2, BRINP2, BRSK2, BSN, BTBD6, BTD, BTG2, BTG4, BTRC, C1QC, C21orf91, CA10, CACNA1A, CACNA1H, CALM2, CAMK2B, CAMK4, CAMSAP1, CAPRIN2, CARD10, CARD11, CASP2, CASP5, CASR, CASZ1, CAV1, CBFA2T2, CBFA2T3, CC2D2A, CCBE1, CCDC85C, CCKAR, CCN4, CCN6, CCR2, CCR6, CD46, CD8A, CDC20, CDH13, CDH22, CDH23, CDH4, CDK1, CDK5R1, CDKN1A, CDKN2A, CELA2A, CELF4, CELSR1, CEND1, CEP290, CHAMP1, CHD5, CHRDL2, CHRNB2, CHRND, CHST11, CHST8, CIB1, CIC, CIT, CITED2, CLASP1, CLASP2, CLCN2, CLMP, CLP1, CMTM8, CNNM4, CNRIP1, CNTN4, CNTN5, CNTNAP2, COBL, COL11A2, COL18A1, COL2A1, COL3A1, COL4A1, COL4A2, COL6A1, COL6A2, COL8A2, CPLANE1, CPNE5, CRB2, CREB3L1, CREB3L2, CRIM1, CRIP1, CRISPLD2, CRYBA4, CRYBG3, CRYGC, CSGALNACT1, CSMD3, CSN3, CSNK1E, CSPG5, CTNNA2, CTNNBIP1, CTNND2, CTNS, CTTN, CUL3, CUX1, CXCR4, CYFIP1, CYFIP2, CYLD, CYP19A1, CYP1B1, CYP4A11, DAB1, DAG1, DBN1, DCAF1, DCHS2, DCLK1, DCLRE1C, DCN, DCTN1, DDIT4, DDR1, DDR2, DDX6, DENND5A, DGCR6, DGCR6L, DHCR7, DHDDS, DHRS3, DHX15, DHX30, DHX36, DIPK2A, DKK3, DLC1, DLG1, DLG4, DLL1, DLL4, DLX2, DMC1, DNAH5, DNAJB6, DNAJC30, DNASE1L2, DNER, DNM1L, DNMT3A, DOC2A, DOCK10, DOCK7, DOK4, DOK6, DPF1, DRGX, DSCAML1, DTX1, E2F2, E2F8, ECE1, EDAR, EFHD1, EFNA4, EFNA5, EGR2, EHMT2, EIF2AK3, EIF2AK4, EIF2S2, EIF4A3, EIF4E, EIF4EBP1, EIF4ENIF1, ELK3, ELN, ELOVL1, EMC10, ENC1, ENPP2, EPHA1, EPHA10, EPHA4, EPHA6, EPHB1, EPHB3, ERAP1, ERBB4, ERCC1, EREG, ESR1, ESRP2, ETS1, ETV1, ETV6, EVL, EVX1, EXOC4, EXOSC6, EXPH5, EYA4, EZH2, F3, FA2H, FAM172A, FAP, FARP1, FARP2, FASN, FAT1, FBLN5, FBN1, FBN2, FBXW8, FERD3L, FEZ1, FEZF1, FEZF2, FGF12, FGF14, FGF2, FGF6, FGF7, FHOD3, FJX1, FKTN, FLRT1, FLT4, FMN1, FNDC3A, FOXK1, FOXO1, FOXP1, FPGS, FRAS1, FREM1, FRS2, FRYL, FTO, FZD10, FZD5, FZR1, GAA, GAB1, GAB2, GABRA2, GABRA4, GABRB2, GAK, GALC, GARS1, GAS7, GATA5, GCNT1, GCNT2, GFER, GFI1, GFRAL, GJA5, GLI2, GLI3, GLIPR2, GLIS2, GLMN, GLO1, GMFB, GNAI2, GNAO1, GON4L, GORASP1, GPC6, GPER1, GPR137B, GPR161, GPR17, GPR4, GPR55, GPRC5B, GPRIN1, GPSM1, GRB10, GRB2, GREM1, GRID2, GSK3A, GSS, GSX2, H3C6, H3C8, HAND1, HAPLN2, HAPLN3, HCLS1, HCN1, HDAC4, HDAC9, HECTD1, HECW1, HEG1, HELT, HERC1, HES1, HES3, HES4, HEXB, HGS, HIP1R, HIVEP3, HMGB1, HNF1B, HNRNPAB, HNRNPD, HOXA1, HOXA3, HOXA4, HOXC10, HOXC11, HOXC4, HOXD13, HRG, HSD17B4, HSP90AB1, HSPD1, HTATIP2, HYDIN, IFI16, IFRD1, IFT140, IFT74, IFT88, IGF1, IGF2BP1, IGSF8, IGSF9B, IHH, IKZF1, IL17D, IL17RD, IL1RAP, IL31RA, IL6R, IL6ST, IMMP2L, ING3, INPP5F, INPPL1, INSC, INSIG2, INSR, IQGAP3, IQSEC1, IRF2BP2, IRF6, IRS2, ISL1, ISM1, ITCH, ITFG2, ITGA1, ITGA11, ITGA5, ITGB2, ITGB4, ITK, JAK2, JARID2, JHY, KALRN, KANK1, KAT2B, KAT6A, KAT7, KCNAB2, KCNC1, KCNK2, KCNQ1, KCNQ2, KDM2B, KDM4C, KDM6B, KIAA1217, KIDINS220, KIF18A, KIF1A, KIF26A, KIF26B, KIF2A, KIF5A, KIFBP, KITLG, KLF7, KLHL1, KLHL17, KLHL3, KLK4, KMT2D, KMT2E, KMT5C, KNDC1, KRAS, KRIT1, KRT1, KRT20, KRT25, KRT40, KRT82, KRT84, KRTAP16-1, KRTAP23-1, KRTAP4-3, L3MBTL3, LAMA2, LAMA5, LAMB1, LAMB2, LAMB4, LARGE1, LCE1A, LCE1F, LCE3E, LDB2, LDB3, LDLR, LDLRAD4, LEPR, LGR6, LHX4, LHX6, LHX8, LIG4, LIMK2, LINGO1, LLGL1, LMX1B, LONP1, LOXL2, LOXL3, LPAR1, LPCAT1, LRCH4, LRFN3, LRIG2, LRIG3, LRP1, LRP10, LRP12, LRP2, LRP5, LRRC4C, LRRK1, LSAMP, LTBP1, LTBP2, LY9, LYL1, LZTS1, LZTS2, MACF1, MACROD2, MAD1L1, MAF, MAFK, MAG, MAGI2, MAL, MALL, MAP2, MAP2K5, MAP3K13, MAP4, MAP4K4, MAP6, MAPK7, MAPK8IP2, MAPK8IP3, MAPKAP1, MAX, MBD3, MBNL1, MBOAT7, MCM2, MCPH1, MDGA1, MECOM, MEF2C, MEGF8, MEIS2, MELTF, MEN1, MEOX1, MFGE8, MFNG, MGMT, MICAL2, MICALL1, MIPEP, MKNK2, MKS1, MLLT3, MMD, MME, MOG, MRPL13, MRTFA, MSH2, MSH3, MSLN, MTMR2, MTNR1B, MYH15, MYH3, MYL2, MYL6, MYLK, MYO15A, MYO16, MYO18B, MYO1D, MYO1E, MYO5A, MYOM2, MYPN, MYRF, MYT1L, N4BP3, NAV1, NAV3, NCAM2, NCK2, NCKAP1, NCKAP1L, NCOA2, NCOA6, NCOR2, NCSTN, NDE1, NDRG4, NECTIN1, NEDD4L, NEO1, NEPRO, NEUROD6, NEUROG2, NF1, NFAM1, NFASC, NFATC1, NFATC2, NFATC4, NFE2L2, NFIB, NGEF, NGF, NHEJ1, NKD1, NLGN2, NME2, NOL3, NOM1, NOS1, NOTCH1, NOTCH3, NOTCH4, NPHP4, NPHS1, NPNT, NPPA, NPR1, NPRL3, NPTN, NR1D1, NR2E3, NRIP1, NRSN1, NRSN2, NRXN1, NRXN3, NSMF, NTM, NTNG1, NTNG2, NTRK2, NUDT1, NUMB, NUP210L, NUS1, NXN, OBSL1, OC90, OGDH, OLFM1, OLIG2, OPCML, OPN5, ORAI1, P4HTM, PACSIN1, PAF1, PAFAH1B1, PAK4, PALLD, PALM, PAPPA2, PAPSS1, PARD3, PAX2, PAX7, PAX8, PAX9, PBLD, PBX1, PCARE, PCDHA11, PCDHA6, PCDHA7, PCDHB9, PCK2, PCLO, PCSK5, PCSK6, PDCD6, PDCL3, PDGFD, PDGFRA, PDLIM1, PDLIM3, PDPK1, PDZD2, PDZD7, PEG10, PER2, PEX13, PFKFB3, PHACTR1, PHF2, PHLDB1, PHLPP2, PIK3CB, PIK3CG, PIK3R1, PIKFYVE, |

| | | | | | | |
|---|---|---|---|---|---|---|
| GO:0048731 | system development | 6,13E-16 | [3, 4] | 1171,00 | 22,61 | ADGRL3, ADGRV1, AFDN, AGBL4, AGO2, AGRN, AJAP1, AKAP13, AKT2, ALAS1, ALK, ALKAL2, ALPK2, ALPL, ALS2, ALX1, ALX4, AMBRA1, AMH, ANGPT1, ANGPT4, ANGPTL2, ANK3, ANKH, ANKRD11, ANKRD17, ANKRD33, ANKS1A, ANO6, ANTXR1, ANXA2, ANXA6, AP2A1, AP2A2, AP2S1, AP3B1, APBA2, APPL2, APRT, ARF1, ARHGDIA, ARHGEF2, ARHGEF7, ARID2, ARID4B, ARL13B, ARNT2, ARNTL, ASAP1, ASCL1, ASS1, ASXL2, ATAT1, ATF1, ATF2, ATF3, ATG16L1, ATM, ATN1, ATP2B2, ATP2B4, ATP6V0D1, ATP8A2, ATXN1, ATXN10, B9D1, BAG6, BATF2, BBS4, BBS7, BBX, BCAR3, BCAS3, BCL10, BCL11A, BCL11B, BCL2, BCL2A1, BCL3, BCL6, BCL9, BDNF, BDNF-AS, BEND6, BFSP1, BFSP2, BHLHE40, BICC1, BIN3, BIRC2, BLOC1S2, BLOC1S5, BMERB1, BMP1, BMP2, BMP3, BMP7, BMP8B, BMPER, BNC2, BPGM, BRD1, BRD2, BRINP2, BRSK2, BSN, BTBD6, BTD, BTG2, BTG4, BTRC, C1QC, C21orf91, CA10, CACNA1A, CACNA1H, CALM2, CAMK2B, CAMK4, CAMSAP1, CAPRIN2, CARD10, CARD11, CASP2, CASP5, CASR, CASZ1, CAV1, CBFA2T2, CBFA2T3, CC2D2A, CCBE1, CCDC85C, CCKAR, CCN4, CCN6, CCR2, CCR6, CD46, CD8A, CDC20, CDH13, CDH22, CDH23, CDH4, CDK1, CDK5R1, CDKN1A, CDKN2A, CELA2A, CELF4, CELSR1, CEND1, CEP290, CHAMP1, CHD5, CHRDL2, CHRNB2, CHRND, CHST11, CHST8, CIB1, CIC, CIT, CITED2, CLASP1, CLASP2, CLCN2, CLMP, CLP1, CMTM8, CNNM4, CNRIP1, CNTN4, CNTN5, CNTNAP2, COBL, COL11A2, COL18A1, COL2A1, COL3A1, COL4A1, COL4A2, COL6A1, COL6A2, COL8A2, CPLANE1, CPNE5, CRB2, CREB3L1, CREB3L2, CRIM1, CRIP1, CRISPLD2, CRYBA4, CRYBG3, CRYGC, CSGALNACT1, CSMD3, CSN3, CSNK1E, CSPG5, CTNNA2, CTNNBIP1, CTNND2, CTNS, CTTN, CUL3, CUX1, CXCR4, CYFIP1, CYFIP2, CYLD, CYP19A1, CYP1B1, CYP4A11, DAB1, DAG1, DBN1, DCAF1, DCHS2, DCLK1, DCLRE1C, DCN, DCTN1, DDIT4, DDR1, DDR2, DDX6, DENND5A, DGCR6, DGCR6L, DHCR7, DHDDS, DHRS3, DHX15, DHX30, DHX36, DIPK2A, DKK3, DLC1, DLG1, DLG4, DLL1, DLL4, DLX2, DMC1, DNAH5, DNAJB6, DNAJC30, DNASE1L2, DNER, DNM1L, DNMT3A, DOC2A, DOCK10, DOCK7, DOK4, DOK6, DPF1, DRGX, DSCAML1, DTX1, E2F2, E2F8, ECE1, EDAR, EFHD1, EFNA4, EFNA5, EGR2, EHMT2, EIF2AK3, EIF2AK4, EIF2S2, EIF4A3, EIF4E, EIF4EBP1, EIF4ENIF1, ELK3, ELN, ELOVL1, EMC10, ENC1, ENPP2, EPHA1, EPHA10, EPHA4, EPHA6, EPHB1, EPHB3, ERAP1, ERBB4, ERCC1, EREG, ESR1, ESRP2, ETS1, ETV1, ETV6, EVL, EVX1, EXOC4, EXOSC6, EXPH5, EYA4, EZH2, F3, FA2H, FAM172A, FAP, FARP1, FARP2, FASN, FAT1, FBLN5, FBN1, FBN2, FBXW8, FERD3L, FEZ1, FEZF1, FEZF2, FGF12, FGF14, FGF2, FGF6, FGF7, FHOD3, FJX1, FKTN, FLRT1, FLT4, FMN1, FNDC3A, FOXK1, FOXO1, FOXP1, FPGS, FRAS1, FREM1, FRS2, FRYL, FTO, FZD10, FZD5, FZR1, GAA, GAB1, GAB2, GABRA2, GABRA4, GABRB2, GAK, GALC, GARS1, GAS7, GATA5, GCNT1, GCNT2, GFER, GFI1, GFRAL, GJA5, GLI2, GLI3, GLIPR2, GLIS2, GLMN, GLO1, GMFB, GNAI2, GNAO1, GON4L, GORASP1, GPC6, GPER1, GPR137B, GPR161, GPR17, GPR4, GPR55, GPRC5B, GPRIN1, GPSM1, GRB10, GRB2, GREM1, GRID2, GSK3A, GSS, GSX2, H3C6, H3C8, HAND1, HAPLN2, HAPLN3, HCLS1, HCN1, HDAC4, HDAC9, HECTD1, HECW1, HEG1, HELT, HERC1, HES1, HES3, HES4, HEXB, HGS, HIP1R, HIVEP3, HMGB1, HNF1B, HNRNPAB, HNRNPD, HOXA1, HOXA3, HOXA4, HOXC10, HOXC11, HOXC4, HOXD13, HRG, HSD17B4, HSP90AB1, HSPD1, HTATIP2, HYDIN, IFI16, IFRD1, IFT140, IFT74, IFT88, IGF1, IGF2BP1, IGSF8, IGSF9B, IHH, IKZF1, IL17D, IL17RD, IL1RAP, IL31RA, IL6R, IL6ST, IMMP2L, ING3, INPP5F, INPPL1, INSC, INSIG2, INSR, IQGAP3, IQSEC1, IRF2BP2, IRF6, IRS2, ISL1, ISM1, ITCH, ITFG2, ITGA1, ITGA11, ITGA5, ITGB2, ITGB4, ITK, JAK2, JARID2, JHY, KALRN, KANK1, KAT2B, KAT6A, KAT7, KCNAB2, KCNC1, KCNK2, KCNQ1, KCNQ2, KDM2B, KDM4C, KDM6B, KIAA1217, KIDINS220, KIF18A, KIF1A, KIF26A, KIF26B, KIF2A, KIF5A, KIFBP, KITLG, KLF7, KLHL1, KLHL17, KLHL3, KLK4, KMT2D, KMT2E, KMT5C, KNDC1, KRAS, KRIT1, KRT1, KRT20, KRT25, KRT40, KRT82, KRT84, KRTAP16-1, KRTAP23-1, KRTAP4-3, L3MBTL3, LAMA2, LAMA5, LAMB1, LAMB2, LAMB4, LARGE1, LCE1A, LCE1F, LCE3E, LDB2, LDB3, LDLR, LDLRAD4, LEPR, LGR6, LHX4, LHX6, LHX8, LIG4, LIMK2, LINGO1, LLGL1, LMX1B, LONP1, LOXL2, LOXL3, LPAR1, LPCAT1, LRCH4, LRFN3, LRIG2, LRIG3, LRP1, LRP10, LRP12, LRP2, LRP5, LRRC4C, LRRK1, LSAMP, LTBP1, LTBP2, LY9, LYL1, LZTS1, LZTS2, MACF1, MACROD2, MAD1L1, MAF, MAFK, MAG, MAGI2, MAL, MALL, MAP2, MAP2K5, MAP3K13, MAP4, MAP4K4, MAP6, MAPK7, MAPK8IP2, MAPK8IP3, MAPKAP1, MAX, MBD3, MBNL1, MBOAT7, MCM2, MCPH1, MDGA1, MECOM, MEF2C, MEGF8, MEIS2, MELTF, MEN1, MEOX1, MFGE8, MFNG, MGMT, MICAL2, MICALL1, MIPEP, MKNK2, MKS1, MLLT3, MMD, MME, MOG, MRPL13, MRTFA, MSH2, MSH3, MSLN, MTMR2, MTNR1B, MYH15, MYH3, MYL2, MYL6, MYLK, MYO15A, MYO16, MYO18B, MYO1D, MYO1E, MYO5A, MYOM2, MYPN, MYRF, MYT1L, N4BP3, NAV1, NAV3, NCAM2, NCK2, NCKAP1, NCKAP1L, NCOA2, NCOA6, NCOR2, NCSTN, NDE1, NDRG4, NECTIN1, NEDD4L, NEO1, NEPRO, NEUROD6, NEUROG2, NF1, NFAM1, NFASC, NFATC1, NFATC2, NFATC4, NFE2L2, NFIB, NGEF, NGF, NHEJ1, NKD1, NLGN2, NME2, NOL3, NOM1, NOS1, NOTCH1, NOTCH3, NOTCH4, NPHP4, NPHS1, NPNT, NPPA, NPR1, NPRL3, NPTN, NR1D1, NR2E3, NRIP1, NRSN1, NRSN2, NRXN1, NRXN3, NSMF, NTM, NTNG1, NTNG2, NTRK2, NUDT1, NUMB, NUP210L, NUS1, NXN, OBSL1, OC90, OGDH, OLFM1, OLIG2, OPCML, OPN5, ORAI1, P4HTM, PACSIN1, PAF1, PAFAH1B1, PAK4, PALLD, PALM, PAPPA2, PAPSS1, PARD3, PAX2, PAX7, PAX8, PAX9, PBLD, PBX1, PCARE, PCDHA11, PCDHA6, PCDHA7, PCDHB9, PCK2, PCLO, PCSK5, PCSK6, PDCD6, PDCL3, PDGFD, PDGFRA, PDLIM1, PDLIM3, PDPK1, PDZD2, PDZD7, PEG10, PER2, PEX13, PFKFB3, PHACTR1, PHF2, PHLDB1, PHLPP2, PIK3CB, PIK3CG, PIK3R1, PIKFYVE, |
| GO:0045893 | positive regulation of transcription, DNA-templated | 1,23E-02 | [5, 6, 7, 8, 9, 10, 11] | 381,00 | 22,68 | BCL11B, BCL3, BCL9, BICRAL, BMP2, BMP7, BRD1, BRD4, BRF1, BTRC, C1orf61, CALCOCO1, CAMK4, CAMTA1, CAMTA2, CAND1, CAPRIN2, CASZ1, CDH13, CDK2, CDKN2A, CEP290, CHEK1, CHEK2, CITED2, CITED4, CNOT7, CREB3L1, CREB3L2, CREB5, CREM, CSRNP2, CSRNP3, CTBP2, CTCF, DCN, DHX36, DLL1, DLX2, DOT1L, DPF1, E2F2, E2F8, EBF1, EBF3, EBF4, EGR2, EHF, EIF2AK3, ELF1, ELK3, ERBB4, ERCC1, ESR1, ESR2, ESRRG, ETS1, ETV1, ETV6, EVX1, F3, FBLN5, FEZF1, FEZF2, FGF2, FGF7, FHOD1, FOXD3, FOXJ3, FOXK1, FOXO1, FUBP1, FZD5, FZR1, GABPB1, GARS1, GATA5, GFI1, GLI2, GLI3, GLIS2, GPER1, GPRIN1, GREM1, GSK3A, GTF2B, GTF2H1, HAND1, HCLS1, HDAC4, HELT, HES1, HES3, HEXB, HIVEP1, HIVEP3, HMBOX1, HMGB1, HMGN3, HNF1B, HNRNPAB, HNRNPD, HNRNPK, HOXA1, HOXA3, HOXA4, HOXC10, HOXC11, HOXC4, HOXD13, HSD17B4, HTATIP2, IFI16, IFT74, IGF1, IHH, IKBKB, IKZF1, IL31RA, ILF3, ING1, ING3, INSIG2, INSR, IRF2, IRF6, ISL1, KAT2B, KAT6A, KAT6B, KAT7, KDM3A, KDM6B, KLF14, KLF17, KLF7, KMT2D, KMT2E, LARP7, LDB2, LHX4, LHX6, LHX8, LMO7, LMX1B, LONP1, LRP5, LYL1, MACC1, MAF, MAFA, MAFG, MAML2, MAML3, MAP2K2, MAP2K5, MAP3K2, MAP3K5, MAPK7, MARS1, MAX, MCRS1, MECOM, MED16, MED22, MED24, MEF2C, MEIS2, MELTF, MEN1, MEOX1, MICAL2, MLLT3, MRTFA, MSANTD1, MSGN1, MYRF, NCK2, NCOA2, NCOA6, NCOA7, NEUROD6, NFATC1, NFATC2, NFATC2IP, NFATC4, NFE2L2, NFIB, NFIL3, NFYA, NFYC, NKX2-4, NLRC5, NME2, NOS1, NOTCH1, NOTCH3, NOTCH4, NPAT, NPNT, NR1D1, NR2E3, NR3C1, NRF1, NRIP1, NSD1, NSD3, PABPC1L, PAF1, PAGR1, PAX2, PAX7, PAX8, PAX9, PBX1, PCGF5, PDZD2, PEG3, PFKM, PHF2, PIH1D1, PIK3R1, PIM1, PKD1, PLAC8, PLCB1, PLXND1, PML, POU2F1, POU2F2, POU2F3, POU4F2, POU6F2, PPARG, PPARGC1A, PPARGC1B, PPP3CA, PPP3CB, PRDM16, PRDM2, PRKD2, PRKDC, PRKN, PSEN1, PSMC2, PSMC3, PSMC6, PTF1A, PTGES2, PTPRN, RAI1, RARA, RB1, RELB, REST, RET, RFX2, RFXAP, RGMA, RHOG, RIPK1, RNF111, ROR2, RORA, RORB, RORC, RPRD1B, RPTOR, RREB1, RUNX1, RUNX3, RXRB, RXRG, SATB2, SBNO2, SERTAD1, SETD3, SHH, SIM2, SIN3A, SIRT7, SKAP1, SKI, SLC40A1, SLC9A1, SMAD3, SMAD6, SMAD7, |
| GO:0051173 | positive regulation of nitrogen compound metabolic process | 8,76E-09 | [3, 4, 5] | 778,00 | 22,70 | AMH, ANGPT1, ANGPT4, ANKRD31, ANTXR1, ANXA2, APC, APC2, APOBEC1, ARHGEF10L, ARHGEF2, ARHGEF7, ARID2, ARID4B, ARIH1, ARL6IP5, ARNT2, ARNTL, ASCL1, ASPH, ASPSCR1, ASS1, ASXL2, ATF1, ATF2, ATF3, ATG14, ATG4B, ATM, ATP2A3, ATP2B4, ATXN7L3, AURKB, BABAM2, BACH1, BAG6, BANP, BBS7, BCAR3, BCAS3, BCL10, BCL11B, BCL2, BCL3, BCL6, BCL9, BDNF, BDNF-AS, BICRAL, BIRC2, BMP2, BMP3, BMP7, BMP8B, BMPER, BRD1, BRD4, BRF1, BRMS1, BTC, BTG2, BTRC, C1orf61, CALCOCO1, CALM2, CAMK4, CAMTA1, CAMTA2, CAND1, CAPRIN2, CARD10, CARD11, CARD14, CASR, CASZ1, CAV1, CBFA2T3, CBX8, CCAR2, CCBE1, CCL16, CCND2, CCNY, CCR2, CCT2, CD33, CDC123, CDC20, CDC20B, CDC25B, CDH13, CDK1, CDK2, CDK5R1, CDKN1A, CDKN2A, CELF4, CEP290, CEP295, CHAMP1, CHEK1, CHEK2, CHTF8, CIB1, CITED2, CITED4, CIZ1, CLN6, CLYBL, CNOT1, CNOT7, COMMD1, COP1, CPEB1, CREB3L1, CREB3L2, CREB5, CREM, CSNK1E, CSRNP2, CSRNP3, CTBP2, CTCF, CUL3, CXCR4, CYCS, CYFIP1, CYFIP2, DAB1, DAG1, DAPK1, DAXX, DCN, DCUN1D1, DDA1, DDAH2, DDR2, DGKQ, DHX36, DIPK2A, DLC1, DLG1, DLG4, DLL1, DLX2, DNAJC3, DNPEP, DOCK7, DOK4, DOK7, DOT1L, DPF1, DSTYK, DTX1, DUSP19, DUSP26, E2F2, E2F8, EBF1, EBF3, EBF4, EDAR, EDEM2, EFNA5, EGR2, EHF, EIF2AK3, EIF2AK4, EIF4A3, ELAVL1, ELF1, ELK3, EMC10, ENPP2, EPHA4, ERBB4, ERCC1, ERCC8, EREG, ESR1, ESR2, ESRRG, ETAA1, ETFA, ETS1, ETV1, ETV6, EVX1, EXOSC6, EYA2, EYA4, EZH2, F12, F3, FAF1, FAM161A, FANCM, FASN, FBLN5, FBXW8, FEZF1, FEZF2, FGD2, FGF2, FGF7, FHOD1, FLT4, FNIP2, FNTA, FOXD3, FOXJ3, FOXK1, FOXO1, FRS2, FTO, FUBP1, FZD10, FZD5, FZR1, GABPB1, GADD45G, GARS1, GATA5, GCG, GCNT2, GFI1, GFRAL, GLI2, GLI3, GLIPR2, GLIS2, GLMN, GNAI2, GORASP1, GPER1, GPR55, GPRC5B, GPRC5C, GPRIN1, GRAMD4, GREM1, GRM1, GRM4, GSK3A, GTF2B, GTF2H1, GUCA2A, HACD3, HAND1, HCLS1, HDAC4, HECW1, HELT, HES1, HES3, HEXB, HIP1, HIP1R, HIVEP1, HIVEP3, HMBOX1, HMGB1, HMGN3, HNF1B, HNRNPAB, HNRNPD, HNRNPK, HNRNPR, HOXA1, HOXA3, HOXA4, HOXC10, HOXC11, HOXC4, HOXD13, HSD17B4, HSP90AB1, HSPD1, HTATIP2, HTR2A, IDE, IFI16, IFT74, IGF1, IGF1R, IHH, IKBKB, IKZF1, IL31RA, IL6R, IL6ST, ILF3, ING1, ING3, INSIG2, INSR, IQGAP3, IRAK2, IRF2, IRF6, ISL1, ITCH, ITGA1, ITGA5, ITGB2, JAK2, JARID2, KAT2B, KAT6A, KAT6B, KAT7, KDM3A, KDM4D, KDM6B, KHDRBS1, KIDINS220, KITLG, KLF14, KLF17, KLF7, KMT2D, KMT2E, KMT5C, KNDC1, KPNA7, KRAS, KSR1, LARP4B, LARP7, LDB2, LDLR, LEPR, LGMN, LHX4, LHX6, LHX8, LLGL1, LMO7, LMX1B, LONP1, LPAR1, LPCAT1, LRP1, LRP2, LRP5, LRRK1, LYL1, MACC1, MAF, MAFA, MAFG, MAGI2, MAK, MAL, MAML2, MAML3, MAP2K2, MAP2K5, MAP3K11, MAP3K13, MAP3K19, MAP3K2, MAP3K20, MAP3K5, MAP3K6, MAP4K4, MAP4K5, MAPK7, MAPK8, MAPK8IP2, MAPK8IP3, MAPK9, MAPKAP1, MAPKAPK5, MARS1, MAX, MCRS1, MECOM, MED16, MED22, MED24, MED26, MED27, MEF2C, MEIS2, MELTF, MEN1, MEOX1, MGMT, MICAL2, MLLT3, MMD, MRTFA, MSANTD1, MSGN1, MSH2, MST1R, MTA1, MTA3, MYD88, MYO1C, MYRF, NAT10, NCK2, NCKAP1L, NCOA2, NCOA6, NCOA7, NCSTN, NDFIP2, NDRG4, NDUFA13, NEDD4L, NEDD9, NEK7, NEUROD6, NFATC1, NFATC2, NFATC2IP, NFATC4, NFE2L2, NFIB, NFIL3, NFKBIB, NFYA, NFYC, NGF, NKD1, NKX2-4, NLRC5, NLRP12, NME2, NOS1, NOS1AP, NOTCH1, NOTCH3, NOTCH4, NPAT, NPNT, NPTN, NR1D1, NR2E3, NR3C1, NRF1, NRIP1, NRXN1, NSD1, NSD3, NSMF, NTRK2, OSBPL7, OSBPL8, OTUD4, P2RY6, PABPC1L, PAF1, PAGR1, PAK4, PAK5, PAOX, PARP14, PASK, PAX2, PAX7, PAX8, PAX9, PBX1, PCGF5, PDCD5, PDCD6, PDE8A, PDGFD, PDGFRA, PDLIM1, PDPK1, PDZD2, PEG3, PER2, PFKFB3, PFKM, PFN2, PHF19, PHF2, PIH1D1, PIK3CB, PIK3CG, PIK3R1, PIK3R5, PIM1, PKD1, PKIB, PLA2G6, PLAC8, PLAUR, PLCB1, PLCE1, PLD1, PLEKHN1, PLXND1, PML, POLDIP3, POMT1, POU2F1, POU2F2, POU2F3, POU4F2, POU6F2, PPARG, PPARGC1A, PPARGC1B, PPM1F, PPP1R16B, PPP2R3C, PPP3CA, PPP3CB, PRDM16, PRDM2, PRDX5, PRKAG2, PRKAR1B, PRKCA, PRKCB, PRKCE, PRKCQ, PRKCZ, PRKD2, PRKDC, PRKN, PRLR, PRMT1, PRPF19, PSEN1, PSMC2, PSMC3, PSMC6, PTEN, PTF1A, PTGES2, PTK2, PTPN11, PTPRA, PTPRC, PTPRN, PUM1, RACK1, RAD51, RAI1, RAP1A, RAP2B, RAPGEF1, RARA, RASSF5, RB1, RBM20, RBPMS, RBX1, RC3H1, RCHY1, |
| GO:0051173 | positive regulation of nitrogen compound metabolic process | 8,76E-09 | [3, 4, 5] | 778,00 | 22,70 | AMH, ANGPT1, ANGPT4, ANKRD31, ANTXR1, ANXA2, APC, APC2, APOBEC1, ARHGEF10L, ARHGEF2, ARHGEF7, ARID2, ARID4B, ARIH1, ARL6IP5, ARNT2, ARNTL, ASCL1, ASPH, ASPSCR1, ASS1, ASXL2, ATF1, ATF2, ATF3, ATG14, ATG4B, ATM, ATP2A3, ATP2B4, ATXN7L3, AURKB, BABAM2, BACH1, BAG6, BANP, BBS7, BCAR3, BCAS3, BCL10, BCL11B, BCL2, BCL3, BCL6, BCL9, BDNF, BDNF-AS, BICRAL, BIRC2, BMP2, BMP3, BMP7, BMP8B, BMPER, BRD1, BRD4, BRF1, BRMS1, BTC, BTG2, BTRC, C1orf61, CALCOCO1, CALM2, CAMK4, CAMTA1, CAMTA2, CAND1, CAPRIN2, CARD10, CARD11, CARD14, CASR, CASZ1, CAV1, CBFA2T3, CBX8, CCAR2, CCBE1, CCL16, CCND2, CCNY, CCR2, CCT2, CD33, CDC123, CDC20, CDC20B, CDC25B, CDH13, CDK1, CDK2, CDK5R1, CDKN1A, CDKN2A, CELF4, CEP290, CEP295, CHAMP1, CHEK1, CHEK2, CHTF8, CIB1, CITED2, CITED4, CIZ1, CLN6, CLYBL, CNOT1, CNOT7, COMMD1, COP1, CPEB1, CREB3L1, CREB3L2, CREB5, CREM, CSNK1E, CSRNP2, CSRNP3, CTBP2, CTCF, CUL3, CXCR4, CYCS, CYFIP1, CYFIP2, DAB1, DAG1, DAPK1, DAXX, DCN, DCUN1D1, DDA1, DDAH2, DDR2, DGKQ, DHX36, DIPK2A, DLC1, DLG1, DLG4, DLL1, DLX2, DNAJC3, DNPEP, DOCK7, DOK4, DOK7, DOT1L, DPF1, DSTYK, DTX1, DUSP19, DUSP26, E2F2, E2F8, EBF1, EBF3, EBF4, EDAR, EDEM2, EFNA5, EGR2, EHF, EIF2AK3, EIF2AK4, EIF4A3, ELAVL1, ELF1, ELK3, EMC10, ENPP2, EPHA4, ERBB4, ERCC1, ERCC8, EREG, ESR1, ESR2, ESRRG, ETAA1, ETFA, ETS1, ETV1, ETV6, EVX1, EXOSC6, EYA2, EYA4, EZH2, F12, F3, FAF1, FAM161A, FANCM, FASN, FBLN5, FBXW8, FEZF1, FEZF2, FGD2, FGF2, FGF7, FHOD1, FLT4, FNIP2, FNTA, FOXD3, FOXJ3, FOXK1, FOXO1, FRS2, FTO, FUBP1, FZD10, FZD5, FZR1, GABPB1, GADD45G, GARS1, GATA5, GCG, GCNT2, GFI1, GFRAL, GLI2, GLI3, GLIPR2, GLIS2, GLMN, GNAI2, GORASP1, GPER1, GPR55, GPRC5B, GPRC5C, GPRIN1, GRAMD4, GREM1, GRM1, GRM4, GSK3A, GTF2B, GTF2H1, GUCA2A, HACD3, HAND1, HCLS1, HDAC4, HECW1, HELT, HES1, HES3, HEXB, HIP1, HIP1R, HIVEP1, HIVEP3, HMBOX1, HMGB1, HMGN3, HNF1B, HNRNPAB, HNRNPD, HNRNPK, HNRNPR, HOXA1, HOXA3, HOXA4, HOXC10, HOXC11, HOXC4, HOXD13, HSD17B4, HSP90AB1, HSPD1, HTATIP2, HTR2A, IDE, IFI16, IFT74, IGF1, IGF1R, IHH, IKBKB, IKZF1, IL31RA, IL6R, IL6ST, ILF3, ING1, ING3, INSIG2, INSR, IQGAP3, IRAK2, IRF2, IRF6, ISL1, ITCH, ITGA1, ITGA5, ITGB2, JAK2, JARID2, KAT2B, KAT6A, KAT6B, KAT7, KDM3A, KDM4D, KDM6B, KHDRBS1, KIDINS220, KITLG, KLF14, KLF17, KLF7, KMT2D, KMT2E, KMT5C, KNDC1, KPNA7, KRAS, KSR1, LARP4B, LARP7, LDB2, LDLR, LEPR, LGMN, LHX4, LHX6, LHX8, LLGL1, LMO7, LMX1B, LONP1, LPAR1, LPCAT1, LRP1, LRP2, LRP5, LRRK1, LYL1, MACC1, MAF, MAFA, MAFG, MAGI2, MAK, MAL, MAML2, MAML3, MAP2K2, MAP2K5, MAP3K11, MAP3K13, MAP3K19, MAP3K2, MAP3K20, MAP3K5, MAP3K6, MAP4K4, MAP4K5, MAPK7, MAPK8, MAPK8IP2, MAPK8IP3, MAPK9, MAPKAP1, MAPKAPK5, MARS1, MAX, MCRS1, MECOM, MED16, MED22, MED24, MED26, MED27, MEF2C, MEIS2, MELTF, MEN1, MEOX1, MGMT, MICAL2, MLLT3, MMD, MRTFA, MSANTD1, MSGN1, MSH2, MST1R, MTA1, MTA3, MYD88, MYO1C, MYRF, NAT10, NCK2, NCKAP1L, NCOA2, NCOA6, NCOA7, NCSTN, NDFIP2, NDRG4, NDUFA13, NEDD4L, NEDD9, NEK7, NEUROD6, NFATC1, NFATC2, NFATC2IP, NFATC4, NFE2L2, NFIB, NFIL3, NFKBIB, NFYA, NFYC, NGF, NKD1, NKX2-4, NLRC5, NLRP12, NME2, NOS1, NOS1AP, NOTCH1, NOTCH3, NOTCH4, NPAT, NPNT, NPTN, NR1D1, NR2E3, NR3C1, NRF1, NRIP1, NRXN1, NSD1, NSD3, NSMF, NTRK2, OSBPL7, OSBPL8, OTUD4, P2RY6, PABPC1L, PAF1, PAGR1, PAK4, PAK5, PAOX, PARP14, PASK, PAX2, PAX7, PAX8, PAX9, PBX1, PCGF5, PDCD5, PDCD6, PDE8A, PDGFD, PDGFRA, PDLIM1, PDPK1, PDZD2, PEG3, PER2, PFKFB3, PFKM, PFN2, PHF19, PHF2, PIH1D1, PIK3CB, PIK3CG, PIK3R1, PIK3R5, PIM1, PKD1, PKIB, PLA2G6, PLAC8, PLAUR, PLCB1, PLCE1, PLD1, PLEKHN1, PLXND1, PML, POLDIP3, POMT1, POU2F1, POU2F2, POU2F3, POU4F2, POU6F2, PPARG, PPARGC1A, PPARGC1B, PPM1F, PPP1R16B, PPP2R3C, PPP3CA, PPP3CB, PRDM16, PRDM2, PRDX5, PRKAG2, PRKAR1B, PRKCA, PRKCB, PRKCE, PRKCQ, PRKCZ, PRKD2, PRKDC, PRKN, PRLR, PRMT1, PRPF19, PSEN1, PSMC2, PSMC3, PSMC6, PTEN, PTF1A, PTGES2, PTK2, PTPN11, PTPRA, PTPRC, PTPRN, PUM1, RACK1, RAD51, RAI1, RAP1A, RAP2B, RAPGEF1, RARA, RASSF5, RB1, RBM20, RBPMS, RBX1, RC3H1, RCHY1, |

| | | | | | |
|---|---|---|---|---|---|
| GO:0031399 | regulation of protein modification process | 1,14E-03 [5, 6, 7] | 449,00 | 22,70 | ARHGEF7, ARL6IP5, ARNTL, ASPSCR1, ATF3, ATG14, ATM, ATP2B4, BCAR3, BCL10, BCL2, BCL6, BDNF, BDNF-AS, BIRC2, BMP2, BMP3, BMP7, BMP8B, BMPER, BRD4, BRMS1, BTC, BTRC, CALM2, CAMK2D, CAMTA1, CAPRIN2, CARD10, CARD14, CASR, CAV1, CCAR2, CCDC88C, CCL16, CCNA1, CCND2, CCNL1, CCNY, CD33, CDC20, CDC20B, CDC25B, CDC25C, CDK1, CDK5R1, CDKN1A, CDKN2A, CDYL, CEP295, CEP43, CHAMP1, CHEK1, CHEK2, CIB1, CNOT7, COMMD1, CRY2, CTCF, CUL3, CXCR4, CYLD, DAB1, DAG1, DAXX, DBNDD2, DCUN1D1, DDIT4, DDR2, DGKQ, DIPK2A, DLC1, DLG1, DLG4, DNAJC3, DNM1L, DOCK7, DOK4, DOK7, DSTYK, DUS2, DUSP19, DUSP26, EDAR, EFNA5, EHMT2, EIF2AK3, EIF2AK4, EMC10, ENPP2, EPHA1, EPHA4, EPHB1, ERBB4, EREG, ETAA1, ETFA, EZH2, F3, FAF1, FAM161A, FANCM, FASN, FBXO2, FEM1C, FER, FGD2, FGF2, FGF7, FKTN, FLT4, FNIP2, FNTA, FOXO1, FRS2, FZD10, FZD5, FZR1, GADD45G, GCG, GCNT2, GFI1, GFRAL, GLIPR2, GLMN, GMFB, GNAI2, GORASP1, GPER1, GPR55, GPRC5B, GPRC5C, GRB2, GREM1, GRM1, GRM4, GSK3A, GTF2H1, HACD3, HCLS1, HDAC4, HEG1, HES1, HGS, HHATL, HMGB1, HRC, HRG, HSP90AB1, HTR2A, HTT, HUS1, IGF1, IGF1R, IKBKB, IL31RA, IL6R, IL6ST, ING3, INKA2, INPP5F, INSR, IPO7, IQGAP3, IRAK2, ISL1, ITCH, ITGA1, ITGA5, ITGB2, IWS1, JAK2, JARID2, KAT2B, KAT7, KDM3A, KDM4B, KDM4C, KDM4D, KIDINS220, KIRREL1, KITLG, KMT2E, KNDC1, KPNA7, KRAS, KSR1, LATS2, LAX1, LDLRAD4, LEPR, LLGL1, LMTK2, LPAR1, LRP1, LRP5, LRRK1, MAGI2, MAP2K2, MAP2K5, MAP3K11, MAP3K13, MAP3K19, MAP3K2, MAP3K20, MAP3K5, MAP3K6, MAP4K4, MAP4K5, MAPK8, MAPK8IP2, MAPK8IP3, MAPK9, MAPKAP1, MAPKAPK5, MCM2, MECOM, MEN1, MMD, MRPL23, MRTFA, MST1R, MTA1, MYD88, MYO1C, N4BP1, NCK2, NCKAP1L, NCOA7, NDFIP2, NDRG4, NEDD9, NF1, NGF, NLRP12, NNT, NOC2L, NOS1, NOS1AP, NOTCH1, NPNT, NPPA, NPTN, NRXN1, NSD1, NSD3, NSMF, NTRK2, NXN, OSBPL8, OTUB1, OTUD4, P2RY6, PAF1, PAFAH1B1, PAK4, PAK5, PARD3, PARG, PARP14, PAX7, PBLD, PDCD6, PDCL3, PDE8A, PDGFD, PDGFRA, PDPK1, PDZD2, PER2, PFN2, PHACTR1, PHF19, PIH1D1, PIK3CB, PIK3CG, PIK3R5, PINX1, PKD1, PKIB, PKMYT1, PLA2G6, PLAUR, PLCB1, PLCE1, PLCL2, PML, POMT1, PPARGC1A, PPM1F, PPP1R11, PPP1R16A, PPP1R16B, PPP2R2B, PPP2R2C, PPP2R3C, PPP3CA, PRKAG2, PRKAR1B, PRKCA, PRKCE, PRKCZ, PRKD2, PRKDC, PRKN, PRLR, PRMT1, PSCA, PSEN1, PTEN, PTK2, PTPN11, PTPRA, PTPRC, PTPRH, PTPRO, RACK1, RAD51, RANBP9, RAP1A, RAP2B, RAPGEF1, RASIP1, RASSF5, RB1, |
| GO:0031325 | positive regulation of cellular metabolic process | 1,43E-09 [3, 4, 5] | 819,00 | 22,71 | ALK, ALKAL2, ALS2, ALX1, ALX4, AMBRA1, AMH, ANGPT1, ANGPT4, ANKRD31, ANTXR1, ANXA2, APOBEC1, APPL1, ARAP1, ARHGEF10L, ARHGEF2, ARHGEF7, ARID2, ARID4B, ARIH1, ARL6IP5, ARNT2, ARNTL, ASCL1, ASPH, ASPSCR1, ASS1, ASXL2, ATF1, ATF2, ATF3, ATG14, ATG16L1, ATM, ATP2A3, ATP2B4, ATXN7L3, AURKB, BABAM2, BACH1, BAG6, BANP, BBS7, BCAR3, BCAS3, BCL10, BCL11B, BCL2, BCL3, BCL6, BCL9, BDNF, BDNF-AS, BICRAL, BIRC2, BMP2, BMP3, BMP7, BMP8B, BMPER, BRD1, BRD4, BRF1, BRMS1, BTBD10, BTC, BTG2, BTRC, C1orf61, CALCOCO1, CALM2, CAMK4, CAMTA1, CAMTA2, CAND1, CAPRIN2, CARD10, CARD11, CARD14, CASR, CASZ1, CAV1, CBFA2T3, CBX8, CCAR2, CCBE1, CCL16, CCND2, CCNY, CCR2, CCT2, CD33, CDC123, CDC20, CDC20B, CDC25B, CDH13, CDK1, CDK2, CDK5R1, CDKN1A, CDKN2A, CDT1, CELF4, CEP290, CEP295, CHAMP1, CHEK1, CHEK2, CHTF8, CIB1, CITED2, CITED4, CIZ1, CLN6, CLYBL, CNOT1, CNOT7, COMMD1, COP1, CPEB1, CPT1A, CREB3L1, CREB3L2, CREB5, CREM, CSNK1E, CSRNP2, CSRNP3, CTBP2, CTCF, CUL3, CXCR4, CYCS, CYFIP1, CYFIP2, DAB1, DAG1, DAPK1, DAXX, DCN, DCUN1D1, DDA1, DDAH2, DDR1, DDR2, DGKQ, DGKZ, DHX34, DHX36, DIPK2A, DLC1, DLG1, DLG4, DLL1, DLX2, DNA2, DNAJC3, DNPEP, DOCK7, DOK4, DOK7, DOT1L, DPF1, DSTYK, DTX1, DUSP19, DUSP26, E2F2, E2F8, EBF1, EBF3, EBF4, ECE1, EDAR, EDEM2, EFNA5, EGR2, EHF, EIF2AK3, EIF2AK4, EIF4A3, ELAVL1, ELF1, ELK3, EMC10, ENPP2, EPHA1, EPHA10, EPHA4, EPHA6, EPHB1, EPHB3, ERBB4, ERCC1, ERCC8, EREG, ESR1, ESR2, ESRRG, ETAA1, ETFA, ETS1, ETV1, ETV6, EVX1, EXOSC6, EYA2, EYA4, EZH2, F12, F3, FAF1, FAM161A, FANCM, FASN, FBLN5, FBXW8, FEZF1, FEZF2, FGD2, FGF2, FGF7, FHOD1, FLT4, FNIP2, FNTA, FOXD3, FOXJ3, FOXK1, FOXO1, FRS2, FTO, FUBP1, FZD10, FZD5, FZR1, GABPB1, GADD45G, GARS1, GATA5, GCG, GCNT2, GFI1, GFRAL, GLI2, GLI3, GLIPR2, GLIS2, GLMN, GNAI2, GORASP1, GPER1, GPR55, GPRC5B, GPRC5C, GPRIN1, GPSM1, GPT, GRAMD4, GRB2, GREM1, GRM1, GRM4, GSK3A, GTF2B, GTF2H1, GUCA2A, HACD3, HAND1, HCLS1, HDAC4, HELT, HES1, HES3, HEXB, HIP1, HIP1R, HIVEP1, HIVEP3, HMBOX1, HMGB1, HMGN3, HNF1B, HNRNPAB, HNRNPD, HNRNPK, HNRNPR, HOXA1, HOXA3, HOXA4, HOXC10, HOXC11, HOXC4, HOXD13, HSD17B4, HSP90AB1, HSPD1, HTATIP2, HTR2A, HTT, IFI16, IFT74, IGF1, IGF1R, IHH, IKBKB, IKZF1, IL31RA, IL6R, IL6ST, ILF3, ING1, ING3, INPP5F, INSIG2, INSR, IQGAP3, IRAK2, IRF2, IRF6, IRS2, ISL1, ITCH, ITGA1, ITGA5, ITGB2, JAK2, JARID2, KAT2B, KAT6A, KAT6B, KAT7, KDM3A, KDM4D, KDM6B, KHDRBS1, KIDINS220, KIF2A, KITLG, KLF14, KLF17, KLF7, KMT2D, KMT2E, KMT5C, KNDC1, KPNA7, KRAS, KSR1, LARP4B, LARP7, LDB2, LDLR, LEPR, LGMN, LHX4, LHX6, LHX8, LLGL1, LMO7, LMTK2, LMX1B, LONP1, LPAR1, LPGAT1, LRP1, LRP2, LRP5, LRRK1, LRSAM1, LYL1, MACC1, MAF, MAFA, MAFG, MAGI2, MAK, MAL, MAML2, MAML3, MAP2K2, MAP2K5, MAP3K11, MAP3K13, MAP3K19, MAP3K2, MAP3K20, MAP3K5, MAP3K6, MAP4K4, MAP4K5, MAPK7, MAPK8IP2, MAPK8IP3, MAPK9, MAPKAP1, MAPKAPK5, MARS1, MAX, MCRS1, MECOM, MED16, MED22, MED24, MED26, MED27, MEF2C, MEIS2, MELTF, MEN1, MEOX1, MGMT, MICAL2, MLLT3, MMD, MRTFA, MSANTD1, MSGN1, MSH2, MST1R, MTA1, MTA3, MTMR9, MYD88, MYO1C, MYRF, NAT10, NCK2, NCKAP1L, NCOA2, NCOA6, NCOA7, NCSTN, NDFIP2, NDRG4, NDUFA13, NEDD9, NEK7, NEUROD6, NFATC1, NFATC2, NFATC2IP, NFATC4, NFE2L2, NFIB, NFIL3, NFKBIB, NFYA, NFYC, NGF, NKX2-4, NLRC5, NLRP12, NME2, NNT, NOS1, NOS1AP, NOTCH1, NOTCH3, NOTCH4, NPAT, NPNT, NPTN, NR1D1, NR2E3, NR3C1, NRF1, NRIP1, NRXN1, NSD1, NSD3, NSG1, NSMF, NTRK2, OSBPL7, OSBPL8, OTUD4, P2RY6, PABPC1L, PAF1, PAGR1, PAK4, PAK5, PAOX, PARD3, PARP14, PASK, PAX2, PAX7, PAX8, PAX9, PBX1, PCGF5, PDCD5, PDCD6, PDE8A, PDGFD, PDGFRA, PDLIM1, PDPK1, PDZD2, PEG3, PER2, PFKFB3, PFKM, PFN2, PHF19, PHF2, PIH1D1, PIK3CB, PIK3CG, PIK3R1, PIK3R5, PIM1, PIP4K2A, PKD1, PKIB, PLA2G6, PLAC8, PLAUR, PLCB1, PLCE1, PLCG2, PLD1, PLEK, PLEKHN1, PLIN5, PLXND1, PML, PNPLA2, POLDIP3, POMT1, POU2F1, POU2F2, POU2F3, POU4F2, POU6F2, PPARG, PPARGC1A, PPARGC1B, PPM1F, PPP1R16B, PPP2R3C, PPP3CA, PPP3CB, PRDM16, PRDM2, PRDX5, PRKAG2, PRKAR1B, PRKCA, PRKCB, PRKCE, PRKCQ, PRKCZ, PRKD2, PRKDC, PRKN, PRLR, PRMT1, PRPF19, PSEN1, PSMC2, PSMC3, PSMC6, PTEN, PTF1A, PTGES2, PTK2, PTPN11, PTPRA, PTPRC, PTPRN, PUM1, RAB29, RACK1, RAD51, RAI1, RAP1A, RAP2B, RAPGEF1, |
| GO:0031325 | positive regulation of cellular metabolic process | 1,43E-09 [3, 4, 5] | 819,00 | 22,71 | ALK, ALKAL2, ALS2, ALX1, ALX4, AMBRA1, AMH, ANGPT1, ANGPT4, ANKRD31, ANTXR1, ANXA2, APOBEC1, APPL1, ARAP1, ARHGEF10L, ARHGEF2, ARHGEF7, ARID2, ARID4B, ARIH1, ARL6IP5, ARNT2, ARNTL, ASCL1, ASPH, ASPSCR1, ASS1, ASXL2, ATF1, ATF2, ATF3, ATG14, ATG16L1, ATM, ATP2A3, ATP2B4, ATXN7L3, AURKB, BABAM2, BACH1, BAG6, BANP, BBS7, BCAR3, BCAS3, BCL10, BCL11B, BCL2, BCL3, BCL6, BCL9, BDNF, BDNF-AS, BICRAL, BIRC2, BMP2, BMP3, BMP7, BMP8B, BMPER, BRD1, BRD4, BRF1, BRMS1, BTBD10, BTC, BTG2, BTRC, C1orf61, CALCOCO1, CALM2, CAMK4, CAMTA1, CAMTA2, CAND1, CAPRIN2, CARD10, CARD11, CARD14, CASR, CASZ1, CAV1, CBFA2T3, CBX8, CCAR2, CCBE1, CCL16, CCND2, CCNY, CCR2, CCT2, CD33, CDC123, CDC20, CDC20B, CDC25B, CDH13, CDK1, CDK2, CDK5R1, CDKN1A, CDKN2A, CDT1, CELF4, CEP290, CEP295, CHAMP1, CHEK1, CHEK2, CHTF8, CIB1, CITED2, CITED4, CIZ1, CLN6, CLYBL, CNOT1, CNOT7, COMMD1, COP1, CPEB1, CPT1A, CREB3L1, CREB3L2, CREB5, CREM, CSNK1E, CSRNP2, CSRNP3, CTBP2, CTCF, CUL3, CXCR4, CYCS, CYFIP1, CYFIP2, DAB1, DAG1, DAPK1, DAXX, DCN, DCUN1D1, DDA1, DDAH2, DDR1, DDR2, DGKQ, DGKZ, DHX34, DHX36, DIPK2A, DLC1, DLG1, DLG4, DLL1, DLX2, DNA2, DNAJC3, DNPEP, DOCK7, DOK4, DOK7, DOT1L, DPF1, DSTYK, DTX1, DUSP19, DUSP26, E2F2, E2F8, EBF1, EBF3, EBF4, ECE1, EDAR, EDEM2, EFNA5, EGR2, EHF, EIF2AK3, EIF2AK4, EIF4A3, ELAVL1, ELF1, ELK3, EMC10, ENPP2, EPHA1, EPHA10, EPHA4, EPHA6, EPHB1, EPHB3, ERBB4, ERCC1, ERCC8, EREG, ESR1, ESR2, ESRRG, ETAA1, ETFA, ETS1, ETV1, ETV6, EVX1, EXOSC6, EYA2, EYA4, EZH2, F12, F3, FAF1, FAM161A, FANCM, FASN, FBLN5, FBXW8, FEZF1, FEZF2, FGD2, FGF2, FGF7, FHOD1, FLT4, FNIP2, FNTA, FOXD3, FOXJ3, FOXK1, FOXO1, FRS2, FTO, FUBP1, FZD10, FZD5, FZR1, GABPB1, GADD45G, GARS1, GATA5, GCG, GCNT2, GFI1, GFRAL, GLI2, GLI3, GLIPR2, GLIS2, GLMN, GNAI2, GORASP1, GPER1, GPR55, GPRC5B, GPRC5C, GPRIN1, GPSM1, GPT, GRAMD4, GRB2, GREM1, GRM1, GRM4, GSK3A, GTF2B, GTF2H1, GUCA2A, HACD3, HAND1, HCLS1, HDAC4, HELT, HES1, HES3, HEXB, HIP1, HIP1R, HIVEP1, HIVEP3, HMBOX1, HMGB1, HMGN3, HNF1B, HNRNPAB, HNRNPD, HNRNPK, HNRNPR, HOXA1, HOXA3, HOXA4, HOXC10, HOXC11, HOXC4, HOXD13, HSD17B4, HSP90AB1, HSPD1, HTATIP2, HTR2A, HTT, IFI16, IFT74, IGF1, IGF1R, IHH, IKBKB, IKZF1, IL31RA, IL6R, IL6ST, ILF3, ING1, ING3, INPP5F, INSIG2, INSR, IQGAP3, IRAK2, IRF2, IRF6, IRS2, ISL1, ITCH, ITGA1, ITGA5, ITGB2, JAK2, JARID2, KAT2B, KAT6A, KAT6B, KAT7, KDM3A, KDM4D, KDM6B, KHDRBS1, KIDINS220, KIF2A, KITLG, KLF14, KLF17, KLF7, KMT2D, KMT2E, KMT5C, KNDC1, KPNA7, KRAS, KSR1, LARP4B, LARP7, LDB2, LDLR, LEPR, LGMN, LHX4, LHX6, LHX8, LLGL1, LMO7, LMTK2, LMX1B, LONP1, LPAR1, LPGAT1, LRP1, LRP2, LRP5, LRRK1, LRSAM1, LYL1, MACC1, MAF, MAFA, MAFG, MAGI2, MAK, MAL, MAML2, MAML3, MAP2K2, MAP2K5, MAP3K11, MAP3K13, MAP3K19, MAP3K2, MAP3K20, MAP3K5, MAP3K6, MAP4K4, MAP4K5, MAPK7, MAPK8IP2, MAPK8IP3, MAPK9, MAPKAP1, MAPKAPK5, MARS1, MAX, MCRS1, MECOM, MED16, MED22, MED24, MED26, MED27, MEF2C, MEIS2, MELTF, MEN1, MEOX1, MGMT, MICAL2, MLLT3, MMD, MRTFA, MSANTD1, MSGN1, MSH2, MST1R, MTA1, MTA3, MTMR9, MYD88, MYO1C, MYRF, NAT10, NCK2, NCKAP1L, NCOA2, NCOA6, NCOA7, NCSTN, NDFIP2, NDRG4, NDUFA13, NEDD9, NEK7, NEUROD6, NFATC1, NFATC2, NFATC2IP, NFATC4, NFE2L2, NFIB, NFIL3, NFKBIB, NFYA, NFYC, NGF, NKX2-4, NLRC5, NLRP12, NME2, NNT, NOS1, NOS1AP, NOTCH1, NOTCH3, NOTCH4, NPAT, NPNT, NPTN, NR1D1, NR2E3, NR3C1, NRF1, NRIP1, NRXN1, NSD1, NSD3, NSG1, NSMF, NTRK2, OSBPL7, OSBPL8, OTUD4, P2RY6, PABPC1L, PAF1, PAGR1, PAK4, PAK5, PAOX, PARD3, PARP14, PASK, PAX2, PAX7, PAX8, PAX9, PBX1, PCGF5, PDCD5, PDCD6, PDE8A, PDGFD, PDGFRA, PDLIM1, PDPK1, PDZD2, PEG3, PER2, PFKFB3, PFKM, PFN2, PHF19, PHF2, PIH1D1, PIK3CB, PIK3CG, PIK3R1, PIK3R5, PIM1, PIP4K2A, PKD1, PKIB, PLA2G6, PLAC8, PLAUR, PLCB1, PLCE1, PLCG2, PLD1, PLEK, PLEKHN1, PLIN5, PLXND1, PML, PNPLA2, POLDIP3, POMT1, POU2F1, POU2F2, POU2F3, POU4F2, POU6F2, PPARG, PPARGC1A, PPARGC1B, PPM1F, PPP1R16B, PPP2R3C, PPP3CA, PPP3CB, PRDM16, PRDM2, PRDX5, PRKAG2, PRKAR1B, PRKCA, PRKCB, PRKCE, PRKCQ, PRKCZ, PRKD2, PRKDC, PRKN, PRLR, PRMT1, PRPF19, PSEN1, PSMC2, PSMC3, PSMC6, PTEN, PTF1A, PTGES2, PTK2, PTPN11, PTPRA, PTPRC, PTPRN, PUM1, RAB29, RACK1, RAD51, RAI1, RAP1A, RAP2B, RAPGEF1, |
| GO:0006811 | ion transport | 4,05E-03 [4] | 412,00 | 22,71 | ANXA2, ANXA6, AQP9, ARF1, ARL6IP5, ASIC3, ASIC4, ASPH, ATP10B, ATP11A, ATP12A, ATP13A2, ATP13A3, ATP1A3, ATP1A4, ATP2A2, ATP2A3, ATP2B2, ATP2B4, ATP5F1A, ATP5ME, ATP6V0A1, ATP6V0D1, ATP6V1A, ATP6V1H, ATP8A1, ATP8A2, ATP8B2, ATP9B, BCL2, BDNF, BDNF-AS, BEST1, C4orf3, CA13, CACNA1A, CACNA1B, CACNA1E, CACNA1G, CACNA1H, CACNA1I, CACNA2D2, CACNA2D3, CACNA2D4, CACNB2, CACNG1, CACNG6, CACNG8, CALM2, CAMK2B, CAMK2D, CASR, CAV1, CCR2, CD33, CDH23, CDK2, CHRNB2, CHRNB3, CHRND, CHRNE, CLCN2, CLCN3, CLDN16, CNGA3, CNIH3, CNNM1, CNNM4, COMMD1, COMMD9, COX5A, COX6A1, COX7C, CPT1A, CPT1B, CRBN, CRY2, CTNS, CTSS, CXCR4, CYB5R1, CYC1, CYP4A11, DAPK1, DLG1, DLG2, DLG4, DNAJC30, DNM1L, DPP10, EPRS1, F2RL3, F3, FABP5, FAM155A, FGF12, FGF14, FGF2, FLVCR2, FXYD5, FXYD6, GABRA2, GABRA4, GABRB2, GABRR3, GCG, GJA5, GLTP, GNAI2, GNB5, GPER1, GPRIN1, GRIA4, GRID2, GRIK3, GRIK4, GRM2, GSG1L, HCN1, HCN2, HCN4, HECW1, HRC, HRG, HTR1A, HTR2A, HTR3A, HTR3C, HTT, IL16, IRS2, ITPR2, ITPR3, JPH3, KCNAB2, KCNB1, KCNB2, KCNC1, KCNC3, KCNE1, KCNF1, KCNH2, KCNIP4, KCNJ14, KCNJ15, KCNJ2, KCNJ3, KCNJ6, KCNK2, KCNMA1, KCNQ1, KCNQ2, KCNV1, KLHL3, LASP1, LDLR, LLGL1, LMTK2, LRP2, LRRC8B, LRRC8D, MAPK8IP2, MCUR1, MEF2C, MELTF, MFSD3, MRS2, MTNR1B, MYLK, MYO5A, NALCN, NCOA2, NDFIP2, NDUFA4L2, NECTIN1, NEDD4L, NF1, NIPA1, NKAIN1, NLGN2, NNT, NOL3, NOS1, NOS1AP, NPPA, NRXN1, NTRK2, OC90, OCA2, ORAI1, ORAI2, OSBPL10, OSBPL8, P2RY6, PACC1, PANX2, PDPK1, PDZD3, PER2, PHLPP2, PIEZO1, PIK3CG, PITPNC1, PITPNM1, PITPNM2, PKD1, PKD1L1, PLA2G2D, PLA2G6, PLCB1, PLCB2, PLCB4, PLCD4, PLCE1, PLCG2, PLCH2, PLCL2, PLSCR4, PLTP, PML, PNPLA8, PPARG, PPARGC1A, PPFIA1, PPFIA3, PPFIA4, PPP3CA, PRKAG2, PRKCA, PRKCB, PRKCE, PRKCZ, PRKD2, PRKN, PSEN1, PTEN, PTPN3, PTPRC, RAB43, RASA1, RASA3, RASGRF1, RCVRN, RELN, RGS9, RHOA, RIPK1, RPS6KB1, RYR1, RYR2, RYR3, S100A6, SCARA5, SCARB1, SCN1A, SCN2A, SCN3B, SCN5A, SERPINE2, SGK1, SHANK2, SHISA6, SHISA7, SHISA9, SLC10A7, SLC11A2, SLC12A7, SLC12A9, SLC13A3, SLC13A5, SLC15A4, SLC16A5, SLC16A7, SLC17A2, SLC17A4, SLC17A6, SLC17A7, SLC17A9, SLC1A1, SLC1A3, SLC1A7, SLC22A18, SLC22A23, SLC22A5, SLC24A4, SLC25A10, SLC25A13, SLC25A21, SLC25A22, SLC25A41, SLC25A45, SLC26A5, SLC2A9, SLC30A2, SLC30A6, SLC35B3, SLC35B4, SLC37A2, SLC38A8, SLC38A9, SLC39A10, |
| GO:0009966 | regulation of signal transduction | 9,63E-09 [3, 4, 5] | 750,00 | 22,80 | ANGPT1, ANKRD17, ANKS1A, ANKS1B, ANTXR1, APC, APC2, APPL1, APPL2, ARAP1, ARAP3, ARFGAP1, ARFGEF3, ARHGAP10, ARHGAP12, ARHGAP17, ARHGAP21, ARHGAP29, ARHGAP39, ARHGAP40, ARHGAP42, ARHGAP45, ARHGDIA, ARHGDIB, ARHGDIG, ARHGEF16, ARHGEF18, ARHGEF2, ARHGEF26, ARHGEF7, ARL6IP5, ARNTL, ASCL1, ASXL2, ATF3, ATM, ATP2B4, AURKB, BAIAP2L1, BANP, BCAR3, BCL10, BCL2, BCL2L14, BCL3, BCL6, BDNF, BDNF-AS, BEGAIN, BEND6, BICC1, BIRC2, BMP2, BMP3, BMP7, BMP8B, BMPER, BRD1, BRD4, BTC, BTRC, CACNG8, CALM2, CAMK2D, CAMKMT, CAMTA1, CAPRIN2, CARD10, CARD11, CARD14, CASP2, CASR, CAV1, CBFA2T2, CCAR2, CCBE1, CCDC3, CCDC88C, CCL16, CCN4, CCNY, CCR2, CD46, CD47, CDH13, CDK1, CDK2, CDK5R1, CELF4, CHD3, CHD5, CHEK1, CHEK2, CHN2, CHST11, CIB1, CITED2, CMAHP, CNIH3, CNOT1, CNOT7, CNRIP1, COL2A1, COL3A1, CRB2, CREB3L1, CRY2, CSNK1A1L, CSNK1E, CSNK1G3, CTBP2, CTNNBIP1, CTNND2, CTTN, CUL3, CXCR4, CXXC5, CYFIP1, CYFIP2, CYLD, CYP1B1, CYTH1, DAB1, DAG1, DAPK1, DAXX, DCN, DDIT4, DDX60, DENND1A, DEPDC5, DGKQ, DGKZ, DHRS3, DHX36, DIPK2A, DKK3, DLC1, DLG1, DLG2, DLG4, DLL1, DLL4, DLX2, DNA2, DNM1, DNM1L, DOK4, DOT1L, DSTYK, DTX1, DUSP19, DUSP26, EDAR, EEF1E1-BLOC1S5, EGR2, EHMT1, EHMT2, EIF2AK3, EIF4A3, ELF1, EMC10, ENHO, EPHA4, EPHB1, EPS15L1, EPS8L1, ERBB4, EREG, ESR1, ESR2, ETFA, EYA2, EYA4, EZH2, F11R, F2RL3, F3, FABP5, FAF1, FAM110A, FAM13A, FAM13B, FAM89B, FASN, FBN1, FBN2, FBP1, FER, FGD2, FGF2, FGF22, FGF6, FGF7, FICD, FKTN, FLT4, FNIP2, FNTA, FOXO1, FOXP1, FRMPD1, FRS2, FZD10, FZD5, GAB1, GADD45G, GATA5, GCG, GCNT2, GFI1, GFRAL, GLI2, GLI3, GLIPR2, GLIS2, GLRX2, GNA12, GNAI2, GPC6, GPER1, GPR137B, GPR161, GPR17, GPR4, GPR55, GPR62, GPRC5B, GPRIN1, GRAMD4, GRB10, GRB2, GREM1, GRIA4, GRK5, GRM1, GRM2, GRM4, GSG1L, GSK3A, GSX2, HACD3, HCLS1, HDAC9, HECW1, HEG1, HES1, HGS, HINT1, HIP1, HIP1R, HMGB1, HNF1B, HNRNPK, HRC, HRG, HSP90AB1, HTR2A, HTRA3, HTT, HUS1, IER3, IFT140, IGF1, IGF1R, IGF2BP1, IGSF9B, IHH, IKBKB, IL10RA, IL10RB, IL17RD, IL20RA, IL31RA, IL37, IL6R, IL6ST, ING3, INPP5F, INSIG2, INSR, IQGAP3, IQSEC1, IQSEC3, IRAK2, IRS2, ISL1, ITCH, ITFG2, ITGA1, ITGA5, JAK2, JPH3, KALRN, KANK1, KAT2B, KAT6A, KCTD10, KCTD16, KCTD6, KIDINS220, KIF26A, KITLG, KLF14, KMT2D, KRAS, KREMEN2, KSR1, LATS2, LAX1, LDLRAD4, LGMN, LGR6, LIMS1, LLGL1, LMCD1, LOXL3, LPAR1, LRIG2, LRP1, LRP2, LRPAP1, LRRC14, LRRK1, LTBP1, LURAP1, LYPD6, LYPD6B, LZTS2, MACF1, MAGI2, MAL, MAML2, MAML3, MAP2K2, MAP2K5, MAP3K11, MAP3K13, MAP3K2, MAP3K20, MAP3K5, MAP3K6, MAP4K4, MAP4K5, MAPK7, MAPK8, MAPK8IP2, MAPK8IP3, MAPK9, MAPKAP1, MAPKAPK5, MARK3, MBD3, MCF2L, MCL1, MECOM, MEF2C, MEGF8, MEN1, MFNG, MGRN1, MIB2, MIER1, MKS1, MLLT3, MRPL23, MRTFA, MST1R, MTMR2, MTNR1B, MTRNR2L4, MUC4, MYD88, MYO1C, MYO1E, MYO5A, MYO9B, NACC2, NCK2, NCOR2, NDFIP2, NDRG4, NDUFA13, NDUFA2, NEO1, NEPRO, NF1, NFAM1, NFATC1, NFATC4, NFE2L2, NGEF, NGF, NKD1, NLGN2, NLK, NLRC5, NLRP12, NLRX1, NOC2L, NOL3, NOP2, NOS1, NOS1AP, NOTCH1, NOTCH3, NOTCH4, NPHP4, NPNT, NPPA, NPR1, NPRL3, NPTN, NPTXR, NR1D1, NR2C1, NR3C2, NRXN1, NTRK2, NXN, OBSCN, OSBPL8, OTUD4, P2RY6, PAFAH1B1, PAGR1, PAK5, PALM, PARP14, PBLD, PCSK6, PDCD5, PDCD6, PDE8A, PDGFD, PDGFRA, PDPK1, PDZD2, PDZD3, PEG10, PELI3, PEX5L, PHF20, PHLPP2, PIH1D1, PIK3CB, PIK3CG, PIK3IP1, PIK3R1, PIK3R5, PIP4K2A, PIP5K1C, PLA2G6, PLAUR, PLCB1, PLCE1, PLCL2, PLEK, PLEKHA4, PLEKHG2, PLIN5, PLXNB1, PML, POU4F2, PPARG, PPARGC1A, PPP1R13B, PPP1R16B, PPP3CA, PRAG1, PRDM16, PRKAG2, PRKAR1B, PRKCA, PRKCB, PRKCE, PRKCH, PRKCI, PRKCQ, PRKCZ, PRKD2, PRKN, PRLR, PRMT1, PSCA, PSD, PSD2, PSD3, PSD4, PSEN1, PSMA5, PSMA6, PSMA7, PSMC2, PSMC3, PSMC6, PSMD2, PSMD7, PTCH2, PTEN, PTGES2, PTGIS, PTK2, PTPN11, PTPN3, PTPRC, PTPRO, PUM1, PXDN, RAB29, RACK1, RAD9A, RALGAPA2, RANBP9, RAP1A, RAP1GAP, RAP1GAP2, RAPGEF1, RASA1, RASA2, RASA3, |

| | | | | | | |
|---|---|---|---|---|---|---|
| GO:0051247 | positive regulation of protein metabolic process | 1,78E-03 | [4, 5, 6] | 419,00 | 22,81 | APC2, ARHGEF2, ARHGEF7, ARIH1, ARL6IP5, ARNTL, ASPH, ASPSCR1, ATG14, ATG4B, ATM, ATP2A3, ATP2B4, BAG6, BBS7, BCAR3, BCL10, BCL2, BCL3, BCL6, BDNF, BDNF-AS, BIRC2, BMP2, BMP3, BMP7, BMP8B, BMPER, BRD4, BRMS1, BTC, BTRC, CALM2, CAMTA1, CAPRIN2, CARD10, CARD11, CARD14, CASR, CAV1, CBFA2T3, CCBE1, CCL16, CCND2, CCNY, CCR2, CD33, CDC123, CDC20, CDC20B, CDC25B, CDK1, CDK5R1, CDKN1A, CDKN2A, CEP295, CHAMP1, CHEK2, CIB1, CLN6, COMMD1, COP1, CSNK1E, CUL3, CXCR4, CYCS, CYFIP2, DAB1, DAG1, DAPK1, DAXX, DCUN1D1, DDA1, DDR2, DGKQ, DHX36, DIPK2A, DLC1, DLG1, DLG4, DNAJC3, DNPEP, DOCK7, DOK4, DOK7, DSTYK, DUSP19, DUSP26, EDAR, EDEM2, EFNA5, EIF2AK3, EIF2AK4, EIF4A3, ELAVL1, EMC10, ENPP2, EPHA4, ERBB4, EREG, ETAA1, ETFA, EZH2, F12, F3, FAF1, FAM161A, FANCM, FASN, FBXW8, FGD2, FGF2, FGF7, FLT4, FNIP2, FNTA, FOXO1, FRS2, FZD10, FZD5, FZR1, GADD45G, GCG, GCNT2, GFRAL, GLIPR2, GLMN, GNAI2, GORASP1, GPER1, GPR55, GPRC5B, GPRC5C, GPRIN1, GRAMD4, GREM1, GRM1, GRM4, GSK3A, HACD3, HCLS1, HDAC4, HECW1, HES1, HIP1, HIP1R, HMGB1, HNRNPD, HNRNPK, HSP90AB1, HSPD1, HTR2A, IDE, IFI16, IGF1, IGF1R, IL31RA, IL6R, IL6ST, ING3, INSR, IQGAP3, IRAK2, ISL1, ITCH, ITGA1, ITGA5, JAK2, JARID2, KAT7, KHDRBS1, KIDINS220, KITLG, KMT2E, KNDC1, KPNA7, KRAS, KSR1, LARP4B, LDB2, LDLR, LEPR, LGMN, LLGL1, LPAR1, LPCAT1, LRP1, LRP2, LRRK1, MAGI2, MAL, MAP2K2, MAP2K5, MAP3K11, MAP3K13, MAP3K19, MAP3K2, MAP3K20, MAP3K5, MAP3K6, MAP4K4, MAP4K5, MAPK7, MAPK8, MAPK8IP2, MAPK8IP3, MAPK9, MAPKAP1, MAPKAPK5, MELTF, MMD, MRTFA, MST1R, MTA1, MYD88, MYO1C, NAT10, NCK2, NCKAP1L, NCSTN, NDFIP2, NDRG4, NDUFA13, NEDD4L, NEDD9, NFE2L2, NGF, NKD1, NLRP12, NOS1, NOS1AP, NOTCH1, NPNT, NPTN, NRXN1, NSD3, NSMF, NTRK2, OSBPL7, OSBPL8, P2RY6, PAF1, PAK4, PAK5, PARP14, PASK, PAX7, PDCD5, PDCD6, PDE8A, PDGFD, PDGFRA, PDPK1, PDZD2, PFN2, PHF19, PIH1D1, PIK3CB, PIK3CG, PIK3R5, PKD1, PLA2G6, PLAUR, PLCB1, PLCE1, PLD1, PML, POLDIP3, POMT1, PPARG, PPARGC1A, PPM1F, PPP1R16B, PPP2R3C, PPP3CA, PRKAG2, PRKAR1B, PRKCA, PRKCE, PRKCQ, PRKCZ, PRKD2, PRKDC, PRKN, PRLR, PRMT1, PSEN1, PSMC2, PTEN, PTK2, PTPN11, PTPRA, PTPRC, RACK1, RAP1A, RAP2B, RAPGEF1, |
| GO:0051247 | positive regulation of protein metabolic process | 1,78E-03 | [4, 5, 6] | 419,00 | 22,81 | APC2, ARHGEF2, ARHGEF7, ARIH1, ARL6IP5, ARNTL, ASPH, ASPSCR1, ATG14, ATG4B, ATM, ATP2A3, ATP2B4, BAG6, BBS7, BCAR3, BCL10, BCL2, BCL3, BCL6, BDNF, BDNF-AS, BIRC2, BMP2, BMP3, BMP7, BMP8B, BMPER, BRD4, BRMS1, BTC, BTRC, CALM2, CAMTA1, CAPRIN2, CARD10, CARD11, CARD14, CASR, CAV1, CBFA2T3, CCBE1, CCL16, CCND2, CCNY, CCR2, CD33, CDC123, CDC20, CDC20B, CDC25B, CDK1, CDK5R1, CDKN1A, CDKN2A, CEP295, CHAMP1, CHEK2, CIB1, CLN6, COMMD1, COP1, CSNK1E, CUL3, CXCR4, CYCS, CYFIP2, DAB1, DAG1, DAPK1, DAXX, DCUN1D1, DDA1, DDR2, DGKQ, DHX36, DIPK2A, DLC1, DLG1, DLG4, DNAJC3, DNPEP, DOCK7, DOK4, DOK7, DSTYK, DUSP19, DUSP26, EDAR, EDEM2, EFNA5, EIF2AK3, EIF2AK4, EIF4A3, ELAVL1, EMC10, ENPP2, EPHA4, ERBB4, EREG, ETAA1, ETFA, EZH2, F12, F3, FAF1, FAM161A, FANCM, FASN, FBXW8, FGD2, FGF2, FGF7, FLT4, FNIP2, FNTA, FOXO1, FRS2, FZD10, FZD5, FZR1, GADD45G, GCG, GCNT2, GFRAL, GLIPR2, GLMN, GNAI2, GORASP1, GPER1, GPR55, GPRC5B, GPRC5C, GPRIN1, GRAMD4, GREM1, GRM1, GRM4, GSK3A, HACD3, HCLS1, HDAC4, HECW1, HES1, HIP1, HIP1R, HMGB1, HNRNPD, HNRNPK, HSP90AB1, HSPD1, HTR2A, IDE, IFI16, IGF1, IGF1R, IL31RA, IL6R, IL6ST, ING3, INSR, IQGAP3, IRAK2, ISL1, ITCH, ITGA1, ITGA5, JAK2, JARID2, KAT7, KHDRBS1, KIDINS220, KITLG, KMT2E, KNDC1, KPNA7, KRAS, KSR1, LARP4B, LDB2, LDLR, LEPR, LGMN, LLGL1, LPAR1, LPCAT1, LRP1, LRP2, LRRK1, MAGI2, MAL, MAP2K2, MAP2K5, MAP3K11, MAP3K13, MAP3K19, MAP3K2, MAP3K20, MAP3K5, MAP3K6, MAP4K4, MAP4K5, MAPK7, MAPK8, MAPK8IP2, MAPK8IP3, MAPK9, MAPKAP1, MAPKAPK5, MELTF, MMD, MRTFA, MST1R, MTA1, MYD88, MYO1C, NAT10, NCK2, NCKAP1L, NCSTN, NDFIP2, NDRG4, NDUFA13, NEDD4L, NEDD9, NFE2L2, NGF, NKD1, NLRP12, NOS1, NOS1AP, NOTCH1, NPNT, NPTN, NRXN1, NSD3, NSMF, NTRK2, OSBPL7, OSBPL8, P2RY6, PAF1, PAK4, PAK5, PARP14, PASK, PAX7, PDCD5, PDCD6, PDE8A, PDGFD, PDGFRA, PDPK1, PDZD2, PFN2, PHF19, PIH1D1, PIK3CB, PIK3CG, PIK3R5, PKD1, PLA2G6, PLAUR, PLCB1, PLCE1, PLD1, PML, POLDIP3, POMT1, PPARG, PPARGC1A, PPM1F, PPP1R16B, PPP2R3C, PPP3CA, PRKAG2, PRKAR1B, PRKCA, PRKCE, PRKCQ, PRKCZ, PRKD2, PRKDC, PRKN, PRLR, PRMT1, PSEN1, PSMC2, PTEN, PTK2, PTPN11, PTPRA, PTPRC, RACK1, RAP1A, RAP2B, RAPGEF1, |
| GO:0016477 | cell migration | 8,63E-03 | [3, 4] | 370,00 | 22,83 | ARHGDIA, ARHGDIB, ARHGEF16, ARHGEF2, ARHGEF7, ARID2, ARL13B, ASCL1, ATM, ATP2B4, ATP5F1A, ATP8A1, BBS4, BCAR1, BCAS3, BCL11B, BCL2, BIN3, BMERB1, BMP2, BMP7, BMPER, CAMK2B, CAP1, CARD10, CARMIL1, CARMIL2, CAV1, CCBE1, CCKAR, CCL16, CCN4, CCR2, CCR6, CD200R1, CD47, CDC42BPB, CDH13, CDK1, CDK5R1, CEACAM21, CELSR1, CEND1, CEP43, CIB1, CITED2, CLASP1, CLASP2, COL18A1, COL3A1, CORO1B, CRB2, CTNNA2, CTTN, CUL3, CXCR4, CYP19A1, CYP1B1, DAB1, DAG1, DCLK1, DCN, DDIT4, DDR1, DDR2, DGKZ, DHX34, DLC1, DLL4, DNER, DNM1L, DOCK10, DOCK7, DOCK8, DRGX, ELMO1, EMC10, ENC1, ENPP2, EPB41L4B, EPHA1, EPHA4, EPHB1, EPHB3, EPPK1, ERBB4, ETS1, EVL, F11R, F3, FAM110A, FAM89B, FAP, FAT1, FER, FEZF1, FEZF2, FGF2, FGF7, FLT4, FMNL2, FOXP1, FRMD5, FUT8, FZR1, GAB1, GCNT1, GCNT2, GLI3, GLIPR2, GLMN, GNA12, GNAI2, GPC6, GPER1, GRB10, GRB2, GREM1, GSK3A, GSX2, HDAC4, HDAC9, HES1, HEXB, HMGB1, HRG, IGF1, IGF1R, IL16, IL17RA, IL6R, INSR, IQSEC1, IRS2, ISL1, ITGA1, ITGA11, ITGA5, ITGAL, ITGB2, ITGB4, ITGBL1, JAK2, JAML, KANK1, KIF2A, KITLG, KRIT1, LAMA2, LAMA5, LAMB1, LAMB2, LAMB4, LDB2, LDLRAD4, LGMN, LGR6, LHX6, LIMA1, LOXL2, LPAR1, LRIG2, LRP1, LRP12, LRP5, LURAP1, MACF1, MAG, MAGI2, MAP2K5, MAP4K4, MAPK8IP3, MBOAT7, MDGA1, MEAK7, MEF2C, MEGF8, MEN1, MRTFA, MTUS1, MYD88, MYLK, MYO18A, MYO1C, MYO1E, NARS1, NAV1, NAV3, NCK2, NCKAP1, NCKAP1L, NDE1, NDRG4, NEDD9, NF1, NFATC2, NFE2L2, NLRP12, NOL3, NOTCH1, NSMF, NTNG1, NTNG2, NTRK2, NUMB, NUS1, OGDH, OSBPL8, P2RY6, PAFAH1B1, PAK4, PAK5, PALLD, PBLD, PCDHA6, PDCD6, PDGFD, PDGFRA, PDLIM1, PDPK1, PEAK1, PEX13, PFN2, PHACTR1, PIK3CB, PIK3CG, PIK3R1, PIP5K1C, PLA2G7, PLCB1, PLCG2, PLVAP, PLXNA1, PLXNA2, PLXNB1, PLXNC1, PLXND1, PML, PODXL2, POMGNT2, PPARG, PPARGC1A, PPM1F, PPP3CA, PRAG1, PRKCA, PRKCE, PRKCI, PRKCQ, PRKCZ, PRKD2, PSEN1, PTEN, PTGES2, PTK2, PTPN11, PTPRC, PTPRF, PTPRO, PXDN, RACK1, RAP2B, RBFOX2, RELN, RET, RHBDF1, RHOA, RHOG, RHOU, RIPOR1, RIPOR2, ROBO1, ROCK2, ROR2, RORA, RREB1, RUFY3, SASH1, SATB2, SCAI, |
| GO:0050793 | regulation of developmental process | 4,99E-07 | [2, 3] | 641,00 | 22,84 | ALKAL2, ALPK2, ALX1, AMH, ANGPT4, ANKH, ANKRD17, ANKRD26, ANO6, ANXA2, AP2A1, AP2A2, AP2S1, AP3B1, APC, APC2, APPL2, ARAP1, ARF1, ARHGDIA, ARHGDIB, ARHGEF18, ARHGEF2, ARHGEF7, ARNTL, ASAP1, ASCL1, ASXL2, ATAT1, ATF1, ATF2, ATP2B4, ATP8A2, BAG6, BBS4, BCL11A, BCL11B, BCL2, BCL6, BDNF, BDNF-AS, BEND6, BHLHE40, BIRC2, BLOC1S5, BMERB1, BMP1, BMP2, BMP7, BMPER, BNC1, BRD1, BRINP2, BRSK2, BTC, C1QC, C21orf91, CACNA1A, CACNA2D2, CAMK2B, CAMK4, CAMSAP1, CAPRIN2, CAPZB, CARD10, CARD11, CARMIL1, CASZ1, CAV1, CBFA2T2, CCBE1, CCDC3, CCN4, CCN6, CCR2, CD46, CD53, CDC20, CDH4, CDK1, CDK5R1, CDKN1A, CDKN2A, CELF4, CELSR1, CEND1, CFDP1, CHAMP1, CHEK2, CHRNB2, CIB1, CITED2, CLASP1, CLASP2, CNOT1, CNTN4, COBL, COL3A1, COL4A2, CORO1B, CPNE5, CRB2, CREB3L1, CREB3L2, CSMD3, CSNK1E, CSPG5, CTNNA2, CTNNBIP1, CTTN, CUX1, CXCR4, CYFIP1, CYLD, CYP1B1, DAB1, DAG1, DBN1, DCN, DDHD2, DDR2, DDX6, DENND5A, DHX36, DLC1, DLG1, DLG4, DLL1, DLL4, DLX2, DNM1L, DNMBP, DOCK7, DTX1, E2F2, EFNA5, EGR2, EHMT1, EIF2AK3, EIF2AK4, EIF4E, EIF4ENIF1, ELAVL1, EMC10, ENC1, ENPP2, EPHA1, EPHA4, EPHB1, EPHB3, EPPK1, ERAP1, ERBB4, EREG, ESR1, ETS1, EXOSC6, EZH2, F11R, F3, FAM171A1, FARP1, FBLN5, FBN1, FBN2, FBXW8, FERD3L, FERMT2, FEZ1, FEZF1, FEZF2, FGD2, FGF2, FGF7, FITM2, FLRT1, FLT4, FMNL2, FOXO1, FOXP1, FRS2, FTO, FZD5, FZR1, GAB1, GAK, GARS1, GATA5, GCNT2, GFI1, GLI2, GLI3, GLIPR2, GNA12, GORASP1, GPC6, GPER1, GPR137B, GPR161, GPR4, GPR55, GPRC5B, GPRIN1, GREM1, GRID2, GSK3A, GSX2, H3C6, H3C8, HCLS1, HDAC4, HDAC9, HECW1, HELT, HES1, HES3, HES4, HEXB, HGS, HMGB1, HNF1B, HOXC11, HOXD13, HRG, HSP90AB1, HTATIP2, HTR2A, IFRD1, IGF1, IHH, IKBKB, IL17D, IL17RD, IL1RAP, IL6R, IL6ST, ING3, INPP5F, INSR, IQGAP3, ISL1, ISM1, ITCH, ITGA5, ITGB2, JAK2, JARID2, KALRN, KANK1, KAT2B, KAT7, KCNK2, KDM2B, KDM3A, KDM4C, KIDINS220, KIF1A, KIF2A, KITLG, KLF7, KMT2D, KMT2E, KMT5C, KNDC1, KRAS, KRIT1, KRT1, KRT84, LAMA2, LAMA5, LATS2, LDB2, LDLR, LDLRAD4, LGMN, LIG4, LIMS1, LINGO1, LLGL1, LONP1, LOXL2, LOXL3, LPAR1, LPL, LRCH4, LRFN3, LRIG2, LRP1, LRP12, LRP2, LRP5, LRRC4C, LTBP2, LZTS1, MACF1, MAF, MAFG, MAG, MAGI2, MAP2, MAP2K5, MAP3K13, MAP3K5, MAP6, MAPK7, MAPK8IP3, MAPK9, MDGA1, MEF2C, MEGF8, MEIS2, MELTF, MEN1, METRNL, MKS1, MLLT3, MMD, MME, MSH2, MTMR2, MYL12B, MYRF, NAPEPLD, NCKAP1L, NCOA2, NDRG4, NECTIN1, NEDD4L, NEDD9, NEPRO, NF1, NFAM1, NFATC1, NFATC2, NFATC4, NFE2L2, NFIB, NGEF, NGF, NKD1, NLGN2, NME2, NOL3, NOS1, NOTCH1, NOTCH3, NOTCH4, NPNT, NPR1, NPTN, NR1D1, NRXN1, NSMF, NTNG1, NTNG2, NTRK2, NUMB, OBSL1, OLFM1, OLFM2, OLIG2, P4HTM, PACSIN1, PAF1, PAFAH1B1, PAK4, PALM, PALM2AKAP2, PARD3, PAX2, PAX7, PAX8, PAX9, PBLD, PBX1, PDCD6, PDCL3, PDGFD, PDGFRA, PDPK1, PDZD2, PER2, PHACTR1, PHLDB1, PIK3R1, PIM1, PKM, PLAC8, PLCB1, PLEKHA4, PLEKHB1, PLK5, PLXNA1, PLXNA2, PLXNB1, PLXNC1, PLXND1, PML, POU4F2, PPARG, PPARGC1A, PPARGC1B, PPIB, PPP1R16B, PPP2R3C, PPP3CA, PRAG1, PRDM16, PRDM6, PRICKLE2, PRKCA, PRKCB, PRKCH, PRKCI, PRKCQ, PRKCZ, PRKD2, PRKDC, PRKN, PRLR, PRMT1, PRPF19, PRUNE1, PSEN1, PSMA5, PSMA6, PSMA7, PSMC2, PSMC3, PSMC6, PSMD2, PSMD7, PTCH2, PTEN, PTF1A, PTGIS, PTK2, PTPN11, PTPRC, PTPRF, PTPRO, PTPRZ1, PUS7, QRICH1, RAB29, RACK1, RAI1, RANBP1, RAP1A, RAP1GAP, RAP1GAP2, RAPGEF1, RARA, RASA1, RB1, RBFOX1, RBFOX2, RBM19, RBP4, RC3H1, |
| GO:0050793 | regulation of developmental process | 4,99E-07 | [2, 3] | 641,00 | 22,84 | ALKAL2, ALPK2, ALX1, AMH, ANGPT4, ANKH, ANKRD17, ANKRD26, ANO6, ANXA2, AP2A1, AP2A2, AP2S1, AP3B1, APC, APC2, APPL2, ARAP1, ARF1, ARHGDIA, ARHGDIB, ARHGEF18, ARHGEF2, ARHGEF7, ARNTL, ASAP1, ASCL1, ASXL2, ATAT1, ATF1, ATF2, ATP2B4, ATP8A2, BAG6, BBS4, BCL11A, BCL11B, BCL2, BCL6, BDNF, BDNF-AS, BEND6, BHLHE40, BIRC2, BLOC1S5, BMERB1, BMP1, BMP2, BMP7, BMPER, BNC1, BRD1, BRINP2, BRSK2, BTC, C1QC, C21orf91, CACNA1A, CACNA2D2, CAMK2B, CAMK4, CAMSAP1, CAPRIN2, CAPZB, CARD10, CARD11, CARMIL1, CASZ1, CAV1, CBFA2T2, CCBE1, CCDC3, CCN4, CCN6, CCR2, CD46, CD53, CDC20, CDH4, CDK1, CDK5R1, CDKN1A, CDKN2A, CELF4, CELSR1, CEND1, CFDP1, CHAMP1, CHEK2, CHRNB2, CIB1, CITED2, CLASP1, CLASP2, CNOT1, CNTN4, COBL, COL3A1, COL4A2, CORO1B, CPNE5, CRB2, CREB3L1, CREB3L2, CSMD3, CSNK1E, CSPG5, CTNNA2, CTNNBIP1, CTTN, CUX1, CXCR4, CYFIP1, CYLD, CYP1B1, DAB1, DAG1, DBN1, DCN, DDHD2, DDR2, DDX6, DENND5A, DHX36, DLC1, DLG1, DLG4, DLL1, DLL4, DLX2, DNM1L, DNMBP, DOCK7, DTX1, E2F2, EFNA5, EGR2, EHMT1, EIF2AK3, EIF2AK4, EIF4E, EIF4ENIF1, ELAVL1, EMC10, ENC1, ENPP2, EPHA1, EPHA4, EPHB1, EPHB3, EPPK1, ERAP1, ERBB4, EREG, ESR1, ETS1, EXOSC6, EZH2, F11R, F3, FAM171A1, FARP1, FBLN5, FBN1, FBN2, FBXW8, FERD3L, FERMT2, FEZ1, FEZF1, FEZF2, FGD2, FGF2, FGF7, FITM2, FLRT1, FLT4, FMNL2, FOXO1, FOXP1, FRS2, FTO, FZD5, FZR1, GAB1, GAK, GARS1, GATA5, GCNT2, GFI1, GLI2, GLI3, GLIPR2, GNA12, GORASP1, GPC6, GPER1, GPR137B, GPR161, GPR4, GPR55, GPRC5B, GPRIN1, GREM1, GRID2, GSK3A, GSX2, H3C6, H3C8, HCLS1, HDAC4, HDAC9, HECW1, HELT, HES1, HES3, HES4, HEXB, HGS, HMGB1, HNF1B, HOXC11, HOXD13, HRG, HSP90AB1, HTATIP2, HTR2A, IFRD1, IGF1, IHH, IKBKB, IL17D, IL17RD, IL1RAP, IL6R, IL6ST, ING3, INPP5F, INSR, IQGAP3, ISL1, ISM1, ITCH, ITGA5, ITGB2, JAK2, JARID2, KALRN, KANK1, KAT2B, KAT7, KCNK2, KDM2B, KDM3A, KDM4C, KIDINS220, KIF1A, KIF2A, KITLG, KLF7, KMT2D, KMT2E, KMT5C, KNDC1, KRAS, KRIT1, KRT1, KRT84, LAMA2, LAMA5, LATS2, LDB2, LDLR, LDLRAD4, LGMN, LIG4, LIMS1, LINGO1, LLGL1, LONP1, LOXL2, LOXL3, LPAR1, LPL, LRCH4, LRFN3, LRIG2, LRP1, LRP12, LRP2, LRP5, LRRC4C, LTBP2, LZTS1, MACF1, MAF, MAFG, MAG, MAGI2, MAP2, MAP2K5, MAP3K13, MAP3K5, MAP6, MAPK7, MAPK8IP3, MAPK9, MDGA1, MEF2C, MEGF8, MEIS2, MELTF, MEN1, METRNL, MKS1, MLLT3, MMD, MME, MSH2, MTMR2, MYL12B, MYRF, NAPEPLD, NCKAP1L, NCOA2, NDRG4, NECTIN1, NEDD4L, NEDD9, NEPRO, NF1, NFAM1, NFATC1, NFATC2, NFATC4, NFE2L2, NFIB, NGEF, NGF, NKD1, NLGN2, NME2, NOL3, NOS1, NOTCH1, NOTCH3, NOTCH4, NPNT, NPR1, NPTN, NR1D1, NRXN1, NSMF, NTNG1, NTNG2, NTRK2, NUMB, OBSL1, OLFM1, OLFM2, OLIG2, P4HTM, PACSIN1, PAF1, PAFAH1B1, PAK4, PALM, PALM2AKAP2, PARD3, PAX2, PAX7, PAX8, PAX9, PBLD, PBX1, PDCD6, PDCL3, PDGFD, PDGFRA, PDPK1, PDZD2, PER2, PHACTR1, PHLDB1, PIK3R1, PIM1, PKM, PLAC8, PLCB1, PLEKHA4, PLEKHB1, PLK5, PLXNA1, PLXNA2, PLXNB1, PLXNC1, PLXND1, PML, POU4F2, PPARG, PPARGC1A, PPARGC1B, PPIB, PPP1R16B, PPP2R3C, PPP3CA, PRAG1, PRDM16, PRDM6, PRICKLE2, PRKCA, PRKCB, PRKCH, PRKCI, PRKCQ, PRKCZ, PRKD2, PRKDC, PRKN, PRLR, PRMT1, PRPF19, PRUNE1, PSEN1, PSMA5, PSMA6, PSMA7, PSMC2, PSMC3, PSMC6, PSMD2, PSMD7, PTCH2, PTEN, PTF1A, PTGIS, PTK2, PTPN11, PTPRC, PTPRF, PTPRO, PTPRZ1, PUS7, QRICH1, RAB29, RACK1, RAI1, RANBP1, RAP1A, RAP1GAP, RAP1GAP2, RAPGEF1, RARA, RASA1, RB1, RBFOX1, RBFOX2, RBM19, RBP4, RC3H1, |
| GO:0050793 | regulation of developmental process | 4,99E-07 | [2, 3] | 641,00 | 22,84 | ALKAL2, ALPK2, ALX1, AMH, ANGPT4, ANKH, ANKRD17, ANKRD26, ANO6, ANXA2, AP2A1, AP2A2, AP2S1, AP3B1, APC, APC2, APPL2, ARAP1, ARF1, ARHGDIA, ARHGDIB, ARHGEF18, ARHGEF2, ARHGEF7, ARNTL, ASAP1, ASCL1, ASXL2, ATAT1, ATF1, ATF2, ATP2B4, ATP8A2, BAG6, BBS4, BCL11A, BCL11B, BCL2, BCL6, BDNF, BDNF-AS, BEND6, BHLHE40, BIRC2, BLOC1S5, BMERB1, BMP1, BMP2, BMP7, BMPER, BNC1, BRD1, BRINP2, BRSK2, BTC, C1QC, C21orf91, CACNA1A, CACNA2D2, CAMK2B, CAMK4, CAMSAP1, CAPRIN2, CAPZB, CARD10, CARD11, CARMIL1, CASZ1, CAV1, CBFA2T2, CCBE1, CCDC3, CCN4, CCN6, CCR2, CD46, CD53, CDC20, CDH4, CDK1, CDK5R1, CDKN1A, CDKN2A, CELF4, CELSR1, CEND1, CFDP1, CHAMP1, CHEK2, CHRNB2, CIB1, CITED2, CLASP1, CLASP2, CNOT1, CNTN4, COBL, COL3A1, COL4A2, CORO1B, CPNE5, CRB2, CREB3L1, CREB3L2, CSMD3, CSNK1E, CSPG5, CTNNA2, CTNNBIP1, CTTN, CUX1, CXCR4, CYFIP1, CYLD, CYP1B1, DAB1, DAG1, DBN1, DCN, DDHD2, DDR2, DDX6, DENND5A, DHX36, DLC1, DLG1, DLG4, DLL1, DLL4, DLX2, DNM1L, DNMBP, DOCK7, DTX1, E2F2, EFNA5, EGR2, EHMT1, EIF2AK3, EIF2AK4, EIF4E, EIF4ENIF1, ELAVL1, EMC10, ENC1, ENPP2, EPHA1, EPHA4, EPHB1, EPHB3, EPPK1, ERAP1, ERBB4, EREG, ESR1, ETS1, EXOSC6, EZH2, F11R, F3, FAM171A1, FARP1, FBLN5, FBN1, FBN2, FBXW8, FERD3L, FERMT2, FEZ1, FEZF1, FEZF2, FGD2, FGF2, FGF7, FITM2, FLRT1, FLT4, FMNL2, FOXO1, FOXP1, FRS2, FTO, FZD5, FZR1, GAB1, GAK, GARS1, GATA5, GCNT2, GFI1, GLI2, GLI3, GLIPR2, GNA12, GORASP1, GPC6, GPER1, GPR137B, GPR161, GPR4, GPR55, GPRC5B, GPRIN1, GREM1, GRID2, GSK3A, GSX2, H3C6, H3C8, HCLS1, HDAC4, HDAC9, HECW1, HELT, HES1, HES3, HES4, HEXB, HGS, HMGB1, HNF1B, HOXC11, HOXD13, HRG, HSP90AB1, HTATIP2, HTR2A, IFRD1, IGF1, IHH, IKBKB, IL17D, IL17RD, IL1RAP, IL6R, IL6ST, ING3, INPP5F, INSR, IQGAP3, ISL1, ISM1, ITCH, ITGA5, ITGB2, JAK2, JARID2, KALRN, KANK1, KAT2B, KAT7, KCNK2, KDM2B, KDM3A, KDM4C, KIDINS220, KIF1A, KIF2A, KITLG, KLF7, KMT2D, KMT2E, KMT5C, KNDC1, KRAS, KRIT1, KRT1, KRT84, LAMA2, LAMA5, LATS2, LDB2, LDLR, LDLRAD4, LGMN, LIG4, LIMS1, LINGO1, LLGL1, LONP1, LOXL2, LOXL3, LPAR1, LPL, LRCH4, LRFN3, LRIG2, LRP1, LRP12, LRP2, LRP5, LRRC4C, LTBP2, LZTS1, MACF1, MAF, MAFG, MAG, MAGI2, MAP2, MAP2K5, MAP3K13, MAP3K5, MAP6, MAPK7, MAPK8IP3, MAPK9, MDGA1, MEF2C, MEGF8, MEIS2, MELTF, MEN1, METRNL, MKS1, MLLT3, MMD, MME, MSH2, MTMR2, MYL12B, MYRF, NAPEPLD, NCKAP1L, NCOA2, NDRG4, NECTIN1, NEDD4L, NEDD9, NEPRO, NF1, NFAM1, NFATC1, NFATC2, NFATC4, NFE2L2, NFIB, NGEF, NGF, NKD1, NLGN2, NME2, NOL3, NOS1, NOTCH1, NOTCH3, NOTCH4, NPNT, NPR1, NPTN, NR1D1, NRXN1, NSMF, NTNG1, NTNG2, NTRK2, NUMB, OBSL1, OLFM1, OLFM2, OLIG2, P4HTM, PACSIN1, PAF1, PAFAH1B1, PAK4, PALM, PALM2AKAP2, PARD3, PAX2, PAX7, PAX8, PAX9, PBLD, PBX1, PDCD6, PDCL3, PDGFD, PDGFRA, PDPK1, PDZD2, PER2, PHACTR1, PHLDB1, PIK3R1, PIM1, PKM, PLAC8, PLCB1, PLEKHA4, PLEKHB1, PLK5, PLXNA1, PLXNA2, PLXNB1, PLXNC1, PLXND1, PML, POU4F2, PPARG, PPARGC1A, PPARGC1B, PPIB, PPP1R16B, PPP2R3C, PPP3CA, PRAG1, PRDM16, PRDM6, PRICKLE2, PRKCA, PRKCB, PRKCH, PRKCI, PRKCQ, PRKCZ, PRKD2, PRKDC, PRKN, PRLR, PRMT1, PRPF19, PRUNE1, PSEN1, PSMA5, PSMA6, PSMA7, PSMC2, PSMC3, PSMC6, PSMD2, PSMD7, PTCH2, PTEN, PTF1A, PTGIS, PTK2, PTPN11, PTPRC, PTPRF, PTPRO, PTPRZ1, PUS7, QRICH1, RAB29, RACK1, RAI1, RANBP1, RAP1A, RAP1GAP, RAP1GAP2, RAPGEF1, RARA, RASA1, RB1, RBFOX1, RBFOX2, RBM19, RBP4, RC3H1, |

| | | | | | | |
|---|---|---|---|---|---|---|
| GO:0033043 | regulation of organelle organization | 4,98E-02 | [4, 5] | 317,00 | 22,84 | ARHGDIB, ARHGEF10L, ARHGEF18, ARHGEF2, ARHGEF7, ARPC4, ASAP1, ATAT1, ATM, ATP13A2, ATXN7, AURKB, BAIAP2L1, BBS4, BCAS3, BCL2, BCL6, BMERB1, BMP7, BRD4, BTC, C10orf90, CAMK2D, CAMSAP1, CAPZB, CARMIL1, CARMIL2, CCT2, CD47, CDC20, CDC25C, CDK5R1, CDT1, CELSR1, CEP131, CEP295, CEP70, CHEK1, CHMP2A, CIB1, CLASP1, CLASP2, CNOT1, CNTROB, CORO1B, CORO2B, CTCF, CTNNA2, CTTN, CUL3, CYFIP1, CYLD, DBN1, DCN, DCP2, DCTN1, DDHD2, DHX36, DLC1, DLG1, DNM1L, DYNC2LI1, EFNA5, EHMT2, ELN, EPHA1, ERCC1, EREG, ESPN, EVL, EXOSC10, F11R, FCHSD1, FCHSD2, FER, FEZ1, FHOD1, FHOD3, FMN1, FNIP2, FZD10, FZD5, FZR1, GCG, GFI1, GMFB, GPER1, GRB2, GRID2, GSK3A, HCLS1, HIP1R, HMBOX1, HNRNPC, HNRNPD, HPS4, HRG, HTT, IFT140, IFT88, IGF1, IL1RAP, ING3, INSR, IQGAP2, IQGAP3, ISL1, IWS1, JARID2, KANK1, KANK3, KAT2B, KAT7, KDM3A, KDM4B, KDM4C, KDM4D, KIF18A, KIF25, KIF2A, KIRREL1, KMT2E, KPNA7, LIG4, LIMA1, LPAR1, LRP1, LRP5, LRSAM1, LZTS1, MAD1L1, MAK, MAP2, MAP2K2, MAP4, MAP6, MAPK8, MAPK9, MAPKAPK5, MCM2, MCPH1, MEF2C, MIER1, MLLT3, MPHOSPH8, MTMR3, MYO1C, MYO1E, NAT10, NAV3, NCK2, NCKAP1, NCKAP1L, NEK7, NOC2L, NOL3, NOS1, NPHS1, NPRL3, NRXN1, NSD1, NSD3, OBSL1, OTUB1, PAF1, PAFAH1B1, PARG, PAX7, PDCD5, PDGFRA, PDZD2, PFN2, PHF19, PHF2, PHLDB1, PIH1D1, PIK3R1, PIKFYVE, PINX1, PIP4K2A, PKD1, PKIB, PKMYT1, PLA2G6, PLAUR, PLCB1, PLEK, PLEKHG2, PLK4, PLXNB1, PML, PPARGC1A, PPFIA1, PPM1F, PPP1R13B, PRKCB, PRKCE, PRKCQ, PRKD2, PRKN, PRUNE1, PTK2, PXDN, RAB5A, RANBP1, RASA1, RASSF1, RASSF8, RB1, RHOA, RHOBTB3, RHOG, RHOU, RICTOR, RNASE6, ROCK2, RP1, RPS6KA2, SCIN, SDCBP, SEC24B, SEPTIN9, SETD1A, SETD5, SETMAR, SH3PXD2B, SIN3A, SIRT4, SIRT7, |
| GO:0042592 | homeostatic process | 1,53E-04 | [3] | 482,00 | 22,88 | ALAS1, ALMS1, AMH, AMPD2, ANGPT1, ANK3, ANKRD11, ANO1, ANO6, ANTXR1, ANXA6, APC, APOC4, APPL2, AQP9, ARF1, ARID2, ARNTL, ASPSCR1, ATM, ATP12A, ATP13A2, ATP13A3, ATP1A3, ATP1A4, ATP2A2, ATP2A3, ATP2B2, ATP2B4, ATP6V0A1, ATP6V0D1, ATP6V1A, ATP6V1H, AURKB, BBS10, BBS4, BCL10, BCL2, BCL6, BPGM, BRD1, BRSK2, BTBD9, C19orf12, CACNA1A, CACNA1G, CACNB2, CALB2, CALM2, CAMK2D, CAND1, CARD11, CASR, CAV1, CCN4, CCR2, CCR6, CCT2, CD200R1, CDH23, CITED2, CLCN3, CLDN16, CLN6, CNNM4, COL11A2, COL2A1, COMMD1, COMMD9, CORIN, CRY2, CSMD1, CUBN, CXCR4, CYLD, CYP4A11, DCLRE1C, DCN, DCP2, DCTN1, DEF8, DHX36, DLD, DLG4, DLL1, DNA2, DNAJC16, DOCK10, DOCK7, EFNA5, EHMT1, ELOVL1, ENC1, ERCC1, ERP44, ESR1, ESRRG, ETS1, EXOSC10, EZH2, F11R, F2RL3, F3, FA2H, FABP5, FAM155A, FAM3B, FASN, FGF12, FGF2, FGGY, FH, FITM2, FOXK1, FOXO1, FTO, GAA, GADD45G, GARS1, GCG, GCNT2, GJA5, GLRX2, GLRX3, GNAI2, GPER1, GPR137B, GPR17, GPR4, GPR55, GPRIN1, GRB10, GRM1, GRM2, GSR, HAAO, HACD3, HADHA, HCAR2, HCLS1, HCN4, HCRTR2, HDAC9, HECTD4, HEXB, HMBOX1, HMGB1, HMGN3, HNRNPC, HNRNPD, HOOK1, HOXC10, HRC, HSP90AB1, HTR2A, HTT, HUS1, IDE, IGF1R, IHH, IKZF1, IL20RA, IL31RA, IMMT, INSR, IRS2, ITPR2, ITPR3, JAK2, JPH3, KAT7, KCNB1, KCNH2, KCNJ2, KCNMA1, KDM3A, KDM6B, KIF2A, KITLG, KLF7, KLHL3, KMT2E, KRAS, KRIT1, KRT1, KSR2, L3MBTL3, LAMA2, LARGE1, LARGE2, LDB2, LDHD, LDLR, LEPR, LIMA1, LLGL1, LPAR1, LPAR2, LPCAT1, LPL, LRP1, LRP5, LRRK1, LTBP2, MAFG, MAK, MALL, MAP4K5, MAPKAPK5, MCL1, MCUR1, MEF2C, MELTF, MEN1, METRNL, MKS1, MT1F, MTNR1B, MUC13, MUC4, MYO5A, NAPEPLD, NAT10, NCKAP1L, NCSTN, NECTIN4, NEDD4L, NEK7, NEO1, NF1, NFE2L2, NHLRC2, NME2, NNT, NOL3, NOS1, NOTCH1, NOVA2, NPC1, NPHP4, NPR1, NPRL3, NPTN, NR1D1, NUS1, NXN, OPRPN, P2RY6, P4HTM, PACS2, PASK, PAX2, PBLD, PCK2, PDGFRA, PDIA5, PDPK1, PER2, PFKM, PIH1D1, PIK3CB, PIK3CG, PIK3R1, PINX1, PKD1, PKIB, PLA2G6, PLAC8, PLCB1, PLCB2, PLCB4, PLCD4, PLCE1, PLCG2, PLCH2, PLCL2, PML, PNPLA2, PNPLA8, POLD3, POLE, POLG, PPARG, PPARGC1A, PPARGC1B, PPP2R3C, PPP3CA, PPP3CB, PRDM16, PRDX5, PRIM1, PRIM2, PRKAR1B, PRKCA, PRKCB, PRKCE, PRKCQ, PRKD2, PRKDC, PRKN, PRLR, PRMT1, PRR4, PSEN1, PTEN, PTGES2, PTPN11, PTPN3, PTPRC, PTPRN, PTPRN2, QSOX2, RAB3D, RAB43, RACK1, RAD51, RALY, RASA3, RB1, RBFOX2, RBP4, RC3H1, RC3H2, RECQL4, RHEB, RORA, RP1, RPH3AL, RYR1, RYR2, RYR3, S1PR4, SCARA5, SCARB1, SCN3B, SCN5A, SELENOS, SERPINF1, SGCD, SGCZ, SIN3A, SKIL, SLC10A7, SLC11A2, SLC12A7, SLC12A9, SLC17A7, |
| GO:0051239 | regulation of multicellular organismal process | 6,53E-10 | [2, 3] | 782,00 | 22,93 | AGRN, AJAP1, AKAP12, ALK, ALKAL2, ALMS1, ALPK2, ALX1, AMH, ANGPT1, ANGPT4, ANKH, ANKRD17, ANO6, ANXA2, ANXA6, AP2A1, AP2A2, AP2S1, AP3B1, APC, APPL1, APPL2, ARF1, ARHGAP42, ARHGDIA, ARHGDIB, ARHGEF2, ARHGEF7, ARNTL, ASAP1, ASCL1, ASPH, ATF1, ATF2, ATP1A3, ATP1A4, ATP2A2, ATP2A3, ATP2B2, ATP2B4, ATP5F1A, ATP8A2, BAG6, BBS4, BCAR1, BCAS3, BCL10, BCL11A, BCL11B, BCL2, BCL3, BCL6, BDNF, BDNF-AS, BEGAIN, BEND6, BHLHE40, BIRC2, BMERB1, BMP1, BMP2, BMP7, BMPER, BNC1, BPI, BRD1, BRINP2, BRSK2, BTN3A3, C1QC, C21orf91, CACNA1A, CACNA1B, CACNA1G, CACNA1H, CACNA2D2, CACNA2D3, CACNA2D4, CACNB2, CACNG1, CACNG6, CACNG8, CADM1, CALM2, CAMK2B, CAMK2D, CAMK4, CAPRIN2, CARD10, CARD11, CASR, CASZ1, CAV1, CBFA2T2, CCBE1, CCN4, CCN6, CCR2, CCR6, CD200R1, CD33, CD46, CD47, CD96, CDC20, CDH4, CDK1, CDK5R1, CDKN2A, CELA2A, CELF2, CELF4, CELSR1, CEND1, CHAMP1, CHID1, CHRNB2, CIB1, CITED2, CLASP1, CLASP2, CNOT1, CNTN4, COBL, COL3A1, COL4A2, CORIN, CORO2B, CPNE5, CRB2, CREB3L1, CREB3L2, CRY2, CSMD3, CSNK1E, CTNNA2, CTNNBIP1, CTTN, CUX1, CXCR4, CYFIP1, CYLD, CYP1B1, CYP51A1, DAB1, DAG1, DBN1, DCN, DDR2, DDX6, DDX60, DEF8, DENND5A, DHRS3, DHX15, DHX34, DHX36, DLG1, DLG4, DLL1, DLL4, DLX2, DNM1L, DOCK7, DTX1, E2F2, ECE1, EFNA5, EGR2, EHMT1, EIF2AK3, EIF2AK4, EIF4A3, EIF4E, EIF4ENIF1, ELAVL1, ELF1, EMC10, ENC1, ENPP2, EPB41L4B, EPHA1, EPHA4, EPHB1, EPHB3, EPPK1, ERAP1, ERBB4, EREG, ESR1, ESRRG, ETS1, EVL, EXOSC6, EZH2, F11R, F12, F3, FA2H, FABP5, FAP, FARP1, FBLN5, FBN1, FBN2, FBXO32, FBXW8, FERD3L, FEZ1, FEZF1, FEZF2, FGF12, FGF2, FGF7, FH, FLRT1, FLT4, FOXO1, FOXP1, FRMD8, FRS2, FTO, FXYD6, FZD5, GAA, GAB1, GADD45G, GAK, GARS1, GATA5, GCNT2, GFI1, GJA5, GLI2, GLI3, GLIPR2, GLMN, GLRX3, GNAI2, GNAO1, GORASP1, GPC6, GPER1, GPR137B, GPR161, GPR4, GPR55, GPRC5B, GPRIN1, GRB10, GREM1, GRID2, GRM1, GSK3A, GSX2, GTF2IRD1, H3C6, H3C8, HADHA, HCAR2, HCLS1, HCN4, HCRTR2, HDAC4, HDAC9, HECW1, HEG1, HELT, HES1, HES3, HES4, HGS, HIP1R, HLA-DPB1, HMGB1, HNF1B, HNRNPK, HOOK1, HOXC10, HOXC11, HOXD13, HRC, HRG, HSP90AB1, HSPD1, HTATIP2, HTR2A, IFI16, IFIH1, IFRD1, IGF1, IGF1R, IGF2BP1, IGSF9B, IHH, IKBKB, IL12RB2, IL16, IL17D, IL17RA, IL17RD, IL1RAP, IL20RA, IL37, IL6R, IL6ST, INPP5F, INSR, IQGAP3, IQSEC1, ISL1, ISM1, ITCH, ITGA5, ITGB2, ITPR2, ITPR3, JAK2, JARID2, KALRN, KANK1, KAT2B, KAT7, KCNB2, KCNE1, KCNH2, KCNIP4, KCNJ14, KCNJ2, KCNJ3, KCNK2, KCNMA1, KCNQ1, KDM2B, KDM3A, KDM4C, KDM6B, KIDINS220, KIF1A, KIF2A, KITLG, KLF7, KMT2D, KMT2E, KMT5C, KNDC1, KREMEN2, KRIT1, KRT1, KRT84, KSR2, LAMA2, LAMA5, LATS2, LDB2, LDLR, LDLRAD4, LEPR, LGMN, LIG4, LINGO1, LLGL1, LMCD1, LONP1, LOXL2, LOXL3, LPAR1, LPL, LRCH4, LRFN3, LRIG2, LRP1, LRP2, LRP5, LRPAP1, LRRC32, LRRC4C, LTBP1, LTBP2, LURAP1, LY9, LZTS1, MACF1, MAF, MAFG, MAG, MAGI2, MAP2, MAP2K5, MAP3K13, MAP4K4, MAP6, MAPK7, MAPK8IP3, MDGA1, MEF2C, MEGF8, MEIS2, MEN1, MKS1, MLLT3, MMD, MME, MOG, MSH2, MTG1, MTMR2, MTNR1B, MYD88, MYL2, MYO1C, MYRF, NAV3, NCKAP1L, NCOA2, NDRG4, NDUFA2, NECTIN1, NEDD4L, NEPRO, NF1, NFAM1, NFATC1, NFATC2, NFATC4, NFE2L2, NFIB, NGEF, NGF, NKD1, NLGN2, NLRC5, NLRP12, NLRX1, NME2, NOL3, NOS1, NOS1AP, NOTCH1, NOTCH3, NOTCH4, NOVA2, NPHS1, NPNT, NPPA, NPR1, NPRL3, NPS, NPTN, NPTXR, NR1D1, NRXN1, NSMF, NTNG1, NTNG2, NTRK2, NUMB, NUS1, OBSL1, OLFM1, OLIG2, OPRPN, P4HTM, PACSIN1, PAF1, PAFAH1B1, PAK4, PANX2, PARD3, PASK, PAX2, PAX8, PAX9, PBLD, PBX1, PDCD6, PDCL3, PDE9A, PDGFD, PDGFRA, PDPK1, PDZD2, PER2, PFN2, PHACTR1, PHLDB1, PIK3CG, PIK3R1, PIM1, PKM, PLAC8, PLAUR, PLCB1, PLCE1, PLCG2, PLCL2, PLD4, PLEK, PLEKHA4, PLK5, PLXNA1, PLXNA2, PLXNB1, PLXNC1, PLXND1, PML, POLR2F, POLR3A, POLR3E, POLR3H, POU4F2, PPARG, PPARGC1A, PPARGC1B, PPIB, PPM1F, PPP1R11, PPP1R16B, PPP2R3C, PPP3CA, PRAG1, PRDM16, PRICKLE2, PRKCA, PRKCB, PRKCE, PRKCH, PRKCI, PRKCQ, PRKCZ, PRKD2, PRKDC, PRKN, PRLR, PRMT1, PRPF19, PRUNE1, PSEN1, PSMA5, PSMA6, PSMA7, PSMC2, PSMC3, PSMC6, PSMD2, PSMD7, PTCH2, PTEN, PTF1A, PTGIS, PTK2, PTPN11, PTPRC, PTPRF, PTPRO, |
| GO:0010648 | negative regulation of cell communication | 9,90E-03 | [3, 4, 5] | 348,00 | 22,97 | BCL6, BDNF, BDNF-AS, BEND6, BICC1, BMP2, BMP7, BMPER, BTC, BTRC, CALM2, CAV1, CBFA2T2, CCAR2, CCDC3, CCDC88C, CD200R1, CD46, CD47, CELF4, CHST11, CIB1, CNOT1, CNOT7, COL2A1, CREB3L1, CRY2, CSNK1A1L, CTNNBIP1, CTTN, CUL3, CYLD, DAB1, DAG1, DCN, DDIT4, DEPDC5, DGKZ, DHRS3, DKK3, DLC1, DLG1, DLL1, DLL4, DLX2, DNM1, DUSP19, DUSP26, EIF4A3, ELF1, ENHO, EPS15L1, EREG, ESR1, EYA2, EYA4, EZH2, FAM3D, FAM89B, FASN, FBN1, FBN2, FBP1, FGF2, FKTN, FNIP2, FOXO1, FOXP1, GFRAL, GLI2, GLI3, GLIS2, GNAI2, GPER1, GPR161, GRAMD4, GRB10, GRB2, GREM1, GRID2, GRID2IP, GRIK3, GSK3A, HADHA, HDAC9, HECW1, HEG1, HGS, HRG, HTR2A, HTRA3, HTT, IGF1, IGF1R, IL17RD, IL37, ING3, INPP5F, INSIG2, ISL1, ITCH, ITFG2, ITGA1, KALRN, KANK1, KCNB1, KCTD10, KCTD6, KIF26A, KLF7, KREMEN2, LATS2, LAX1, LDLRAD4, LGMN, LPAR1, LRP1, LRP2, LRPAP1, LRRC14, LRRC32, LTBP1, LZTS2, MAGI2, MAP2K5, MAPK7, MAPK8IP2, MAPKAP1, MAPKAPK5, MARK3, MCL1, MECOM, MEGF8, MEN1, MGRN1, MLLT3, MRTFA, MTMR2, MTNR1B, NCK2, NCOR2, NDRG4, NDUFA13, NDUFA2, NF1, NFATC1, NFATC4, NFE2L2, NKD1, NLK, NLRC5, NLRP12, NLRX1, NOC2L, NOL3, NOTCH1, NOTCH3, NPHP4, NPPA, NPRL3, NR1D1, NXN, OTUD4, PAFAH1B1, PAK5, PALM, PARP14, PBLD, PCDHA6, PDCD6, PDPK1, PDZD2, PDZD3, PEG10, PELI3, PHLPP2, PIH1D1, PIK3IP1, PLA2G6, PLAUR, PLCL2, PLEK, PLIN5, PML, PPARG, PPP1R11, PPP3CA, PRDM16, PRKCA, PRKCB, PRKCQ, PRKCZ, PRKD2, PRKN, PSCA, PSEN1, PSMA5, PSMA6, PSMA7, PSMC2, PSMC3, PSMC6, PSMD2, PSMD7, PTCH2, PTEN, PTPN11, PTPN3, PTPRC, PTPRO, PXDN, RACK1, RANBP9, RAP1A, RAPGEF1, RASA1, RASA2, RASA3, RASIP1, RB1, RBX1, REST, RGS12, RGS22, RGS3, RGS6, RGS7, RGS9, RHOA, RIOK3, RIPK1, RIPOR2, RITA1, ROBO1, ROR2, RORA, RPH3AL, RPS6KB1, RTKN2, SALL3, SCAI, SCYL2, SELENOS, SERPINE2, SH3BP4, SH3RF2, SHANK2, SHH, SHISA6, SIRT3, SIRT4, SKI, SKIL, SLA2, SLC24A4, SLC6A4, SLC9A3R1, |
| GO:0023057 | negative regulation of signaling | 8,57E-03 | [2, 3, 4] | 349,00 | 22,99 | BCL6, BDNF, BDNF-AS, BEND6, BICC1, BMP2, BMP7, BMPER, BTC, BTRC, CALM2, CAV1, CBFA2T2, CCAR2, CCDC3, CCDC88C, CD200R1, CD46, CD47, CELF4, CHST11, CIB1, CNOT1, CNOT7, COL2A1, CREB3L1, CRY2, CSNK1A1L, CTNNBIP1, CTTN, CUL3, CYLD, DAB1, DAG1, DCN, DDIT4, DEPDC5, DGKZ, DHRS3, DKK3, DLC1, DLG1, DLL1, DLL4, DLX2, DNM1, DUSP19, DUSP26, EIF4A3, ELF1, ENHO, EPS15L1, EREG, ESR1, EYA2, EYA4, EZH2, FAM3D, FAM89B, FASN, FBN1, FBN2, FBP1, FGF2, FKTN, FNIP2, FOXO1, FOXP1, GFRAL, GLI2, GLI3, GLIS2, GNAI2, GPER1, GPR161, GRAMD4, GRB10, GRB2, GREM1, GRID2, GRID2IP, GRIK3, GSK3A, HADHA, HDAC9, HECW1, HEG1, HGS, HRG, HTR2A, HTRA3, HTT, IGF1, IGF1R, IL17RD, IL37, ING3, INPP5F, INSIG2, ISL1, ITCH, ITFG2, ITGA1, KALRN, KANK1, KCNB1, KCTD10, KCTD6, KIF26A, KLF7, KREMEN2, LATS2, LAX1, LDLRAD4, LGMN, LPAR1, LRP1, LRP2, LRPAP1, LRRC14, LRRC32, LTBP1, LZTS2, MAGI2, MAP2K5, MAPK7, MAPK8IP2, MAPKAP1, MAPKAPK5, MARK3, MCL1, MECOM, MEGF8, MEN1, MGRN1, MLLT3, MRTFA, MTMR2, MTNR1B, NCK2, NCOR2, NDRG4, NDUFA13, NDUFA2, NF1, NFATC1, NFATC4, NFE2L2, NKD1, NLK, NLRC5, NLRP12, NLRX1, NOC2L, NOL3, NOTCH1, NOTCH3, NPHP4, NPPA, NPRL3, NR1D1, NXN, OTUD4, PAFAH1B1, PAK5, PALM, PARP14, PBLD, PCDHA6, PDCD6, PDPK1, PDZD2, PDZD3, PEG10, PELI3, PHLPP2, PIH1D1, PIK3IP1, PLA2G6, PLAUR, PLCL2, PLEK, PLIN5, PML, PPARG, PPP1R11, PPP3CA, PRDM16, PRKCA, PRKCB, PRKCQ, PRKCZ, PRKD2, PRKN, PSCA, PSEN1, PSMA5, PSMA6, PSMA7, PSMC2, PSMC3, PSMC6, PSMD2, PSMD7, PTCH2, PTEN, PTPN11, PTPN3, PTPRC, PTPRO, PXDN, RACK1, RANBP9, RAP1A, RAPGEF1, RASA1, RASA2, RASA3, RASIP1, RB1, RBX1, REST, RGS12, RGS22, RGS3, RGS6, RGS7, RGS9, RHOA, RIOK3, RIPK1, RIPOR2, RITA1, ROBO1, ROR2, RORA, RPH3AL, RPS6KB1, RTKN2, SALL3, SCAI, SCYL2, SELENOS, SERPINE2, SH3BP4, SH3RF2, SHANK2, SHH, SHISA6, SIRT3, SIRT4, SKI, SKIL, SLA2, SLC24A4, SLC6A4, SLC9A3R1, |
| GO:0010646 | regulation of cell communication | 4,72E-12 | [3, 4] | 873,00 | 23,00 | AMER2, AMH, ANGPT1, ANK3, ANKRD17, ANKS1A, ANKS1B, ANO1, ANTXR1, APBA2, APC, APC2, APPL1, APPL2, ARAP1, ARAP3, ARF1, ARFGAP1, ARFGEF3, ARHGAP10, ARHGAP12, ARHGAP17, ARHGAP21, ARHGAP29, ARHGAP39, ARHGAP40, ARHGAP42, ARHGAP45, ARHGDIA, ARHGDIB, ARHGDIG, ARHGEF16, ARHGEF18, ARHGEF2, ARHGEF26, ARHGEF7, ARL6IP5, ARNTL, ASCL1, ASXL2, ATF3, ATM, ATP2A2, ATP2B4, AURKB, BAIAP2L1, BANP, BCAR3, BCL10, BCL2, BCL2L14, BCL3, BCL6, BDNF, BDNF-AS, BEGAIN, BEND6, BICC1, BIRC2, BMP2, BMP3, BMP7, BMP8B, BMPER, BRD1, BRD4, BRSK2, BTBD9, BTC, BTRC, CACNA1A, CACNA1B, CACNA1E, CACNA2D2, CACNB2, CACNG8, CADM1, CALB2, CALM2, CAMK2B, CAMK2D, CAMKMT, CAMTA1, CAPRIN2, CARD10, CARD11, CARD14, CASP2, CASR, CAV1, CBFA2T2, CCAR2, CCBE1, CCDC3, CCDC88C, CCKAR, CCL16, CCN4, CCNY, CCR2, CD200R1, CD46, CD47, CDC20, CDH13, CDK1, CDK2, CDK5R1, CELA2A, CELF4, CHD3, CHD5, CHEK1, CHEK2, CHN2, CHRNB2, CHRNB3, CHST11, CIB1, CITED2, CMAHP, CNIH3, CNOT1, CNOT7, CNRIP1, CNTN4, COL2A1, COL3A1, CPT1A, CRB2, CREB3L1, CRY2, CSNK1A1L, CSNK1E, CSNK1G3, CSPG5, CTBP2, CTNNBIP1, CTNND2, CTTN, CUL3, CXCR4, CXXC5, CYFIP1, CYFIP2, CYLD, CYP19A1, CYP1B1, CYTH1, DAB1, DAG1, DAPK1, DAXX, DBN1, DCN, DDIT4, DDX60, DENND1A, DEPDC5, DGKE, DGKQ, DGKZ, DHRS3, DHX15, DHX34, DHX36, DIPK2A, DKK3, DLC1, DLG1, DLG2, DLG4, DLL1, DLL4, DLX2, DNA2, DNM1, DNM1L, DOK4, DOT1L, DSTYK, DTX1, DUSP19, DUSP26, EDAR, EEF1E1-BLOC1S5, EFNA5, EGR2, EHMT1, EHMT2, EIF2AK3, EIF2AK4, EIF4A3, EIF4E, EIF4EBP2, EIPR1, ELF1, EMC10, ENHO, EPHA4, EPHB1, EPS15L1, EPS8L1, ERBB4, EREG, ESR1, ESR2, ETFA, EYA2, EYA4, EZH2, F11R, F2RL3, F3, FABP5, FAF1, FAM110A, FAM13A, FAM13B, FAM3D, FAM89B, FASN, FBN1, FBN2, FBP1, FER, FGD2, FGF12, FGF14, FGF2, FGF22, FGF6, FGF7, FICD, FKTN, FLT4, FNIP2, FNTA, FOXO1, FOXP1, FRMD8, FRMPD1, FRS2, FZD10, FZD5, FZR1, GAB1, GADD45G, GATA5, GCG, GCNT2, GFI1, GFRAL, GJA5, GLI2, GLI3, GLIPR2, GLIS2, GLMN, GLRX2, GNA12, GNAI2, GPC6, GPER1, GPR137B, GPR161, GPR17, GPR4, GPR55, GPR62, GPRC5B, GPRIN1, GRAMD4, GRB10, GRB2, GREM1, GRIA4, GRID2, GRID2IP, GRIK3, GRIK4, GRK5, GRM1, GRM2, GRM4, GRM8, GSG1L, GSK3A, GSX2, HACD3, HADHA, HCAR2, HCLS1, HDAC9, HECW1, HEG1, HES1, HGS, HINT1, HIP1, HIP1R, HMGB1, HMGN3, HNF1B, HNRNPK, HOOK1, HRC, HRG, HSP90AB1, HSPD1, HTR1A, HTR2A, HTRA3, HTT, HUS1, IER3, IFIH1, IFT140, IGF1, IGF1R, IGF2BP1, IGSF9B, IHH, IKBKB, IL10RA, IL10RB, IL16, IL17RA, IL17RD, IL1RAP, IL20RA, IL31RA, IL37, IL6R, IL6ST, ILDR1, ING3, INPP5F, INSIG2, INSR, IQGAP3, IQSEC1, IQSEC3, IRAK2, IRS2, ISL1, ITCH, ITFG2, ITGA1, ITGA5, ITPR2, ITPR3, JAK2, JPH3, KALRN, KANK1, KAT2B, KAT6A, KCNB1, KCTD10, KCTD16, KCTD6, KIDINS220, KIF26A, KITLG, KLF14, KLF7, KMT2D, KRAS, KREMEN2, KSR1, LAMA2, LATS2, LAX1, LDLRAD4, LGMN, LGR6, LIMS1, LLGL1, LMCD1, LOXL3, LPAR1, LPL, LRFN2, LRIG2, LRP1, LRP2, LRP5, LRPAP1, LRRC14, LRRC32, LRRC4C, LRRK1, LTBP1, LURAP1, LYPD6, LYPD6B, LZTS1, LZTS2, MACF1, MAGI2, MAL, MAML2, MAML3, MAP2K2, MAP2K5, MAP3K11, MAP3K13, MAP3K2, MAP3K20, MAP3K5, MAP3K6, MAP4K4, MAP4K5, MAPK7, MAPK8, MAPK8IP2, MAPK8IP3, MAPK9, MAPKAP1, MAPKAPK5, MARK3, MBD3, MCF2L, MCL1, MECOM, MEF2C, MEGF8, MEN1, MFNG, MGRN1, MIB2, MIER1, MKS1, MLLT3, MME, MRPL23, MRTFA, MST1R, MTMR2, MTNR1B, MTRNR2L4, MUC4, MYD88, MYO1C, MYO1E, MYO5A, MYO9B, NACC2, NAPB, NCK2, NCOR2, NCSTN, NDFIP2, NDRG4, NDUFA13, NDUFA2, NEO1, NEPRO, NF1, NFAM1, NFATC1, NFATC4, NFE2L2, NGEF, NGF, NKD1, NLGN2, NLK, NLRC5, NLRP12, NLRX1, NOC2L, NOL3, NOP2, NOS1, NOS1AP, NOTCH1, NOTCH3, NOTCH4, NPHP4, NPNT, NPPA, NPR1, NPRL3, NPS, NPTN, NPTXR, NR1D1, NR2C1, NR3C2, NRXN1, NRXN3, NSG1, NSMF, NTNG1, NTNG2, NTRK2, NXN, OBSCN, OSBPL8, OTUD4, P2RY6, PACSIN2, PAFAH1B1, PAGR1, PAK5, PALM, PANX2, PARP14, PASK, PAX8, PBLD, PCDHA6, PCSK6, PDCD5, PDCD6, PDE8A, PDGFD, PDGFRA, PDPK1, PDZD2, PDZD3, PEG10, PELI3, PER2, PEX5L, PFKM, PFN2, PHF20, PHLPP2, PIH1D1, PIK3CB, PIK3CG, PIK3IP1, PIK3R1, PIK3R5, PIP4K2A, PIP5K1C, PLA2G6, PLAUR, PLCB1, PLCB4, PLCE1, PLCL2, PLEK, PLEKHA4, PLEKHG2, PLIN5, PLXNB1, PML, POU4F2, PPARG, PPARGC1A, PPFIA3, PPP1R11, PPP1R13B, PPP1R16B, PPP3CA, PPP3CB, PRAG1, PRDM16, PRKAG2, PRKAR1B, PRKCA, PRKCB, PRKCE, PRKCH, PRKCI, PRKCQ, PRKCZ, PRKD2, PRKN, PRLR, PRMT1, PSCA, PSD, PSD2, PSD3, PSD4, PSEN1, PSMA5, PSMA6, PSMA7, PSMC2, PSMC3, PSMC6, PSMD2, PSMD7, PTCH2, PTEN, PTGES2, PTGIS, PTK2, PTPN11, PTPN3, PTPRA, PTPRC, PTPRO, PUM1, PXDN, RAB29, RAB5A, RACK1, RAD9A, RALGAPA2, RANBP9, RAP1A, RAP1GAP, RAP1GAP2, RAPGEF1, RARA, RASA1, RASA2, |

| | | | | | | |
|---|---|---|---|---|---|---|
| GO:0032270 | positive regulation of cellular protein metabolic process | 1,55E-03 | [4, 5, 6, 7] | 394,00 | 23,01 | ARHGEF2, ARHGEF7, ARIH1, ARL6IP5, ARNTL, ASPH, ASPSCR1, ATG14, ATM, ATP2A3, ATP2B4, BAG6, BBS7, BCAR3, BCL10, BCL2, BCL3, BCL6, BDNF, BDNF-AS, BIRC2, BMP2, BMP3, BMP7, BMP8B, BMPER, BRD4, BRMS1, BTC, BTRC, CALM2, CAMTA1, CAPRIN2, CARD10, CARD14, CASR, CAV1, CBFA2T3, CCBE1, CCL16, CCND2, CCNY, CD33, CDC123, CDC20, CDC20B, CDC25B, CDK1, CDK5R1, CDKN1A, CDKN2A, CEP295, CHAMP1, CHEK2, CIB1, CLN6, COMMD1, COP1, CSNK1E, CUL3, CXCR4, CYCS, CYFIP2, DAB1, DAG1, DAPK1, DAXX, DCUN1D1, DDA1, DDR2, DGKQ, DHX36, DIPK2A, DLC1, DLG1, DLG4, DNAJC3, DNPEP, DOCK7, DOK4, DOK7, DSTYK, DUSP19, DUSP26, EDAR, EDEM2, EFNA5, EIF2AK3, EIF2AK4, EIF4A3, ELAVL1, EMC10, ENPP2, EPHA4, ERBB4, EREG, ETAA1, ETFA, EZH2, F12, F3, FAF1, FAM161A, FANCM, FASN, FBXW8, FGD2, FGF2, FGF7, FLT4, FNIP2, FNTA, FRS2, FZD10, FZD5, FZR1, GADD45G, GCG, GCNT2, GFRAL, GLIPR2, GNAI2, GORASP1, GPER1, GPR55, GPRC5B, GPRC5C, GPRIN1, GRAMD4, GREM1, GRM1, GRM4, GSK3A, HACD3, HCLS1, HDAC4, HES1, HIP1, HIP1R, HMGB1, HNRNPD, HSP90AB1, HSPD1, HTR2A, IFI16, IGF1, IGF1R, IL31RA, IL6R, IL6ST, ING3, INSR, IQGAP3, IRAK2, ISL1, ITGA1, ITGA5, JAK2, JARID2, KAT7, KHDRBS1, KIDINS220, KITLG, KMT2E, KNDC1, KPNA7, KRAS, KSR1, LARP4B, LDLR, LEPR, LGMN, LLGL1, LPAR1, LRP1, LRP2, LRRK1, MAGI2, MAL, MAP2K2, MAP2K5, MAP3K11, MAP3K13, MAP3K19, MAP3K2, MAP3K20, MAP3K5, MAP3K6, MAP4K4, MAP4K5, MAPK8IP2, MAPK8IP3, MAPK9, MAPKAP1, MAPKAPK5, MELTF, MMD, MRTFA, MST1R, MTA1, MYD88, MYO1C, NAT10, NCK2, NCKAP1L, NDFIP2, NDRG4, NDUFA13, NEDD9, NFE2L2, NGF, NLRP12, NOS1, NOS1AP, NOTCH1, NPNT, NPTN, NRXN1, NSD3, NSMF, NTRK2, OSBPL7, OSBPL8, P2RY6, PAF1, PAK4, PAK5, PARP14, PASK, PAX7, PDCD5, PDCD6, PDE8A, PDGFD, PDGFRA, PDPK1, PDZD2, PFN2, PHF19, PIH1D1, PIK3CB, PIK3CG, PIK3R5, PKD1, PLA2G6, PLAUR, PLCB1, PLCE1, PLD1, PML, POLDIP3, POMT1, PPARG, PPARGC1A, PPM1F, PPP1R16B, PPP2R3C, PPP3CA, PRKAG2, PRKAR1B, PRKCA, PRKCE, PRKCZ, PRKD2, PRKDC, PRKN, PRLR, PRMT1, PSEN1, PSMC2, PTEN, PTK2, PTPN11, PTPRA, PTPRC, RACK1, RAP1A, |
| GO:0032270 | positive regulation of cellular protein metabolic process | 1,55E-03 | [4, 5, 6, 7] | 394,00 | 23,01 | ARHGEF2, ARHGEF7, ARIH1, ARL6IP5, ARNTL, ASPH, ASPSCR1, ATG14, ATM, ATP2A3, ATP2B4, BAG6, BBS7, BCAR3, BCL10, BCL2, BCL3, BCL6, BDNF, BDNF-AS, BIRC2, BMP2, BMP3, BMP7, BMP8B, BMPER, BRD4, BRMS1, BTC, BTRC, CALM2, CAMTA1, CAPRIN2, CARD10, CARD14, CASR, CAV1, CBFA2T3, CCBE1, CCL16, CCND2, CCNY, CD33, CDC123, CDC20, CDC20B, CDC25B, CDK1, CDK5R1, CDKN1A, CDKN2A, CEP295, CHAMP1, CHEK2, CIB1, CLN6, COMMD1, COP1, CSNK1E, CUL3, CXCR4, CYCS, CYFIP2, DAB1, DAG1, DAPK1, DAXX, DCUN1D1, DDA1, DDR2, DGKQ, DHX36, DIPK2A, DLC1, DLG1, DLG4, DNAJC3, DNPEP, DOCK7, DOK4, DOK7, DSTYK, DUSP19, DUSP26, EDAR, EDEM2, EFNA5, EIF2AK3, EIF2AK4, EIF4A3, ELAVL1, EMC10, ENPP2, EPHA4, ERBB4, EREG, ETAA1, ETFA, EZH2, F12, F3, FAF1, FAM161A, FANCM, FASN, FBXW8, FGD2, FGF2, FGF7, FLT4, FNIP2, FNTA, FRS2, FZD10, FZD5, FZR1, GADD45G, GCG, GCNT2, GFRAL, GLIPR2, GNAI2, GORASP1, GPER1, GPR55, GPRC5B, GPRC5C, GPRIN1, GRAMD4, GREM1, GRM1, GRM4, GSK3A, HACD3, HCLS1, HDAC4, HES1, HIP1, HIP1R, HMGB1, HNRNPD, HSP90AB1, HSPD1, HTR2A, IFI16, IGF1, IGF1R, IL31RA, IL6R, IL6ST, ING3, INSR, IQGAP3, IRAK2, ISL1, ITGA1, ITGA5, JAK2, JARID2, KAT7, KHDRBS1, KIDINS220, KITLG, KMT2E, KNDC1, KPNA7, KRAS, KSR1, LARP4B, LDLR, LEPR, LGMN, LLGL1, LPAR1, LRP1, LRP2, LRRK1, MAGI2, MAL, MAP2K2, MAP2K5, MAP3K11, MAP3K13, MAP3K19, MAP3K2, MAP3K20, MAP3K5, MAP3K6, MAP4K4, MAP4K5, MAPK8IP2, MAPK8IP3, MAPK9, MAPKAP1, MAPKAPK5, MELTF, MMD, MRTFA, MST1R, MTA1, MYD88, MYO1C, NAT10, NCK2, NCKAP1L, NDFIP2, NDRG4, NDUFA13, NEDD9, NFE2L2, NGF, NLRP12, NOS1, NOS1AP, NOTCH1, NPNT, NPTN, NRXN1, NSD3, NSMF, NTRK2, OSBPL7, OSBPL8, P2RY6, PAF1, PAK4, PAK5, PARP14, PASK, PAX7, PDCD5, PDCD6, PDE8A, PDGFD, PDGFRA, PDPK1, PDZD2, PFN2, PHF19, PIH1D1, PIK3CB, PIK3CG, PIK3R5, PKD1, PLA2G6, PLAUR, PLCB1, PLCE1, PLD1, PML, POLDIP3, POMT1, PPARG, PPARGC1A, PPM1F, PPP1R16B, PPP2R3C, PPP3CA, PRKAG2, PRKAR1B, PRKCA, PRKCE, PRKCZ, PRKD2, PRKDC, PRKN, PRLR, PRMT1, PSEN1, PSMC2, PTEN, PTK2, PTPN11, PTPRA, PTPRC, RACK1, RAP1A, |
| GO:0045595 | regulation of cell differentiation | 1,10E-04 | [3, 4] | 457,00 | 23,09 | APPL2, ARF1, ARHGDIA, ARHGEF2, ARHGEF7, ARNTL, ASAP1, ASCL1, ASXL2, ATAT1, ATF1, ATP8A2, BCL11A, BCL11B, BCL2, BCL6, BDNF, BDNF-AS, BEND6, BHLHE40, BIRC2, BLOC1S5, BMERB1, BMP2, BMP7, BNC1, BRD1, BRINP2, BRSK2, BTC, C1QC, C21orf91, CACNA1A, CAMK2B, CAMK4, CAPRIN2, CARD11, CARMIL1, CASZ1, CAV1, CBFA2T2, CCDC3, CCN4, CCR2, CD46, CD53, CDC20, CDH4, CDK1, CDK5R1, CDKN2A, CHEK2, CHRNB2, CIB1, CLASP1, CLASP2, CNTN4, COBL, COL3A1, CPNE5, CRB2, CREB3L2, CSMD3, CSNK1E, CSPG5, CTNNA2, CTNNBIP1, CTTN, CUX1, CXCR4, CYFIP1, CYLD, DAB1, DAG1, DBN1, DDR2, DDX6, DENND5A, DHX36, DLG4, DLL1, DLL4, DLX2, DNM1L, DOCK7, DTX1, EFNA5, EGR2, EIF2AK4, EIF4E, EIF4ENIF1, ENC1, EPHA4, EPHB1, EPHB3, ERBB4, EREG, ETS1, EZH2, F11R, FBN1, FBN2, FBXW8, FERD3L, FEZ1, FEZF1, FEZF2, FGF2, FOXO1, FOXP1, FRS2, FTO, GAK, GARS1, GATA5, GCNT2, GFI1, GLI2, GLI3, GLIPR2, GORASP1, GPER1, GPR137B, GPR55, GPRC5B, GPRIN1, GREM1, GRID2, GSK3A, GSX2, H3C6, H3C8, HCLS1, HDAC4, HDAC9, HECW1, HELT, HES1, HES3, HES4, HMGB1, HSP90AB1, HTR2A, IFRD1, IGF1, IHH, IKBKB, IL17RD, IL6R, IL6ST, INPP5F, ISL1, ITCH, JAK2, KALRN, KANK1, KAT2B, KAT7, KDM3A, KDM4C, KIDINS220, KIF1A, KITLG, KLF7, KMT2D, KMT2E, KNDC1, KRAS, KRT84, LDB2, LDLR, LDLRAD4, LIG4, LIMS1, LINGO1, LLGL1, LONP1, LOXL2, LOXL3, LPAR1, LPL, LRIG2, LRP1, LRP12, LRP2, LRP5, LRRC4C, LTBP2, LZTS1, MACF1, MAF, MAFG, MAG, MAGI2, MAP2, MAP3K13, MAP3K5, MAP6, MAPK8IP3, MAPK9, MEF2C, MEGF8, MEIS2, MELTF, MEN1, METRNL, MMD, MME, NAPEPLD, NCKAP1L, NCOA2, NDRG4, NEDD4L, NEDD9, NEPRO, NF1, NFAM1, NFATC1, NFATC2, NFATC4, NFE2L2, NGEF, NGF, NME2, NOL3, NOS1, NOTCH1, NOTCH3, NOTCH4, NPNT, NPTN, NR1D1, NSMF, NTNG1, NTNG2, NTRK2, NUMB, OBSL1, OLFM1, OLFM2, OLIG2, P4HTM, PACSIN1, PAF1, PAFAH1B1, PAX2, PAX7, PAX8, PBLD, PBX1, PDGFRA, PDZD2, PER2, PHACTR1, PHLDB1, PIK3R1, PIM1, PLCB1, PLEKHB1, PLK5, PLXNA1, PLXNA2, PLXNB1, PLXNC1, PLXND1, POU4F2, PPARG, PPARGC1B, PPP2R3C, PPP3CA, PRAG1, PRDM16, PRDM6, PRKCA, PRKCB, PRKCH, PRKCI, PRKCQ, PRKCZ, PRKDC, PRKN, PRLR, PRMT1, PRPF19, PRUNE1, PSEN1, PSMA5, PSMA6, PSMA7, PSMC2, PSMC3, PSMC6, PSMD2, PSMD7, PTCH2, PTEN, PTK2, PTPN11, PTPRC, PTPRF, PTPRO, PTPRZ1, PUS7, RAB29, RANBP1, RAP1A, RAP1GAP, RAP1GAP2, RAPGEF1, RARA, RB1, RBFOX1, RBFOX2, RC3H1, RC3H2, RELN, REST, RET, RFLNA, RFLNB, RGMA, RHEB, RHOA, RIPK1, RIPOR2, ROBO1, |
| GO:0045595 | regulation of cell differentiation | 1,10E-04 | [3, 4] | 457,00 | 23,09 | APPL2, ARF1, ARHGDIA, ARHGEF2, ARHGEF7, ARNTL, ASAP1, ASCL1, ASXL2, ATAT1, ATF1, ATP8A2, BCL11A, BCL11B, BCL2, BCL6, BDNF, BDNF-AS, BEND6, BHLHE40, BIRC2, BLOC1S5, BMERB1, BMP2, BMP7, BNC1, BRD1, BRINP2, BRSK2, BTC, C1QC, C21orf91, CACNA1A, CAMK2B, CAMK4, CAPRIN2, CARD11, CARMIL1, CASZ1, CAV1, CBFA2T2, CCDC3, CCN4, CCR2, CD46, CD53, CDC20, CDH4, CDK1, CDK5R1, CDKN2A, CHEK2, CHRNB2, CIB1, CLASP1, CLASP2, CNTN4, COBL, COL3A1, CPNE5, CRB2, CREB3L2, CSMD3, CSNK1E, CSPG5, CTNNA2, CTNNBIP1, CTTN, CUX1, CXCR4, CYFIP1, CYLD, DAB1, DAG1, DBN1, DDR2, DDX6, DENND5A, DHX36, DLG4, DLL1, DLL4, DLX2, DNM1L, DOCK7, DTX1, EFNA5, EGR2, EIF2AK4, EIF4E, EIF4ENIF1, ENC1, EPHA4, EPHB1, EPHB3, ERBB4, EREG, ETS1, EZH2, F11R, FBN1, FBN2, FBXW8, FERD3L, FEZ1, FEZF1, FEZF2, FGF2, FOXO1, FOXP1, FRS2, FTO, GAK, GARS1, GATA5, GCNT2, GFI1, GLI2, GLI3, GLIPR2, GORASP1, GPER1, GPR137B, GPR55, GPRC5B, GPRIN1, GREM1, GRID2, GSK3A, GSX2, H3C6, H3C8, HCLS1, HDAC4, HDAC9, HECW1, HELT, HES1, HES3, HES4, HMGB1, HSP90AB1, HTR2A, IFRD1, IGF1, IHH, IKBKB, IL17RD, IL6R, IL6ST, INPP5F, ISL1, ITCH, JAK2, KALRN, KANK1, KAT2B, KAT7, KDM3A, KDM4C, KIDINS220, KIF1A, KITLG, KLF7, KMT2D, KMT2E, KNDC1, KRAS, KRT84, LDB2, LDLR, LDLRAD4, LIG4, LIMS1, LINGO1, LLGL1, LONP1, LOXL2, LOXL3, LPAR1, LPL, LRIG2, LRP1, LRP12, LRP2, LRP5, LRRC4C, LTBP2, LZTS1, MACF1, MAF, MAFG, MAG, MAGI2, MAP2, MAP3K13, MAP3K5, MAP6, MAPK8IP3, MAPK9, MEF2C, MEGF8, MEIS2, MELTF, MEN1, METRNL, MMD, MME, NAPEPLD, NCKAP1L, NCOA2, NDRG4, NEDD4L, NEDD9, NEPRO, NF1, NFAM1, NFATC1, NFATC2, NFATC4, NFE2L2, NGEF, NGF, NME2, NOL3, NOS1, NOTCH1, NOTCH3, NOTCH4, NPNT, NPTN, NR1D1, NSMF, NTNG1, NTNG2, NTRK2, NUMB, OBSL1, OLFM1, OLFM2, OLIG2, P4HTM, PACSIN1, PAF1, PAFAH1B1, PAX2, PAX7, PAX8, PBLD, PBX1, PDGFRA, PDZD2, PER2, PHACTR1, PHLDB1, PIK3R1, PIM1, PLCB1, PLEKHB1, PLK5, PLXNA1, PLXNA2, PLXNB1, PLXNC1, PLXND1, POU4F2, PPARG, PPARGC1B, PPP2R3C, PPP3CA, PRAG1, PRDM16, PRDM6, PRKCA, PRKCB, PRKCH, PRKCI, PRKCQ, PRKCZ, PRKDC, PRKN, PRLR, PRMT1, PRPF19, PRUNE1, PSEN1, PSMA5, PSMA6, PSMA7, PSMC2, PSMC3, PSMC6, PSMD2, PSMD7, PTCH2, PTEN, PTK2, PTPN11, PTPRC, PTPRF, PTPRO, PTPRZ1, PUS7, RAB29, RANBP1, RAP1A, RAP1GAP, RAP1GAP2, RAPGEF1, RARA, RB1, RBFOX1, RBFOX2, RC3H1, RC3H2, RELN, REST, RET, RFLNA, RFLNB, RGMA, RHEB, RHOA, RIPK1, RIPOR2, ROBO1, |
| GO:0055085 | transmembrane transport | 4,73E-04 | [2, 4] | 408,00 | 23,14 | ANXA6, APPL1, APPL2, AQP9, ARF1, ARL6IP5, ASIC3, ASIC4, ASPH, ASPSCR1, ATP12A, ATP13A2, ATP1A3, ATP1A4, ATP2A2, ATP2A3, ATP2B2, ATP2B4, ATP5F1A, ATP5ME, ATP6V0A1, ATP6V0D1, ATP6V1A, ATP6V1H, ATP8A1, ATP8B2, BCL2, BEST1, C4orf3, CABP2, CACNA1A, CACNA1B, CACNA1E, CACNA1G, CACNA1H, CACNA1I, CACNA2D2, CACNA2D3, CACNA2D4, CACNB2, CACNG1, CACNG6, CACNG8, CALM2, CAMK2D, CASR, CAV1, CCR2, CHCHD4, CHRNB2, CHRNB3, CHRND, CHRNE, CLCN2, CLCN3, CLDN16, CLTCL1, CNGA3, CNIH3, CNNM1, CNNM4, COMMD1, COX5A, COX6A1, COX7C, CPT1A, CPT1B, CRBN, CRISP1, CSN3, CTNS, CTSS, CYC1, DAPK1, DLG1, DLG2, DLG4, DNAJC15, DNAJC30, DPP10, EIF2S2, F2RL3, FABP5, FAM155A, FGF12, FGF14, FGF2, FLVCR2, FXYD5, FXYD6, GABRA2, GABRA4, GABRB2, GABRR3, GJA5, GNAI2, GNB5, GPER1, GPRIN1, GRB10, GRIA4, GRID2, GRIK3, GRIK4, GRM2, GRPEL1, GSG1L, GSK3A, HCN1, HCN2, HCN4, HECW1, HRC, HSPD1, HTR2A, HTR3A, HTR3C, HTT, IGF1, INAFM1, INSR, IRS2, ITPR2, ITPR3, JPH3, KCNAB2, KCNB1, KCNB2, KCNC1, KCNC3, KCNE1, KCNF1, KCNH2, KCNIP4, KCNJ14, KCNJ15, KCNJ2, KCNJ3, KCNJ6, KCNK2, KCNMA1, KCNQ1, KCNQ2, KCNV1, KIF2A, LASP1, LLGL1, LMBRD1, LONP1, LRP2, LRRC8B, LRRC8D, MAPK8IP2, MCL1, MCUR1, MEF2C, MFSD3, MIPEP, MRS2, MTNR1B, MYO5A, NALCN, NDUFA4L2, NEDD4L, NFE2L2, NIPA1, NLGN2, NNT, NOL3, NOS1, NOS1AP, NPPA, NRXN1, NRXN3, OCA2, ORAI1, ORAI2, OSBPL8, P2RY6, PACC1, PANX2, PDPK1, PDZD3, PER2, PEX13, PEX14, PEX5L, PHLPP2, PIEZO1, PIK3CG, PIK3R1, PIM1, PKD1, PKD1L1, PLA2G6, PLCB1, PLCB2, PLCB4, PLCD4, PLCE1, PLCG2, PLCH2, PLCL2, POU4F2, PPARGC1A, PRKAG2, PRKCA, PRKCB, PRKCE, PRKCI, PRKCZ, PRKD2, PSEN1, PSMA5, PSMA6, PSMA7, PSMC2, PSMC3, PSMC6, PSMD2, PSMD7, PTEN, PTPN11, PTPN3, PTPRC, RACK1, RAP1A, RASA1, RASA3, RASGRF1, RBP4, RELN, RGS9, RIPK1, RNF185, RYR1, RYR2, RYR3, S100A6, SCARA5, SCARB1, SCN1A, SCN2A, SCN3B, SCN5A, SEL1L, SELENOS, SGK1, SHANK2, SHISA6, SHISA7, SHISA9, SLC10A7, SLC11A2, SLC12A7, SLC12A9, SLC13A3, SLC13A5, SLC14A1, SLC15A4, SLC16A5, SLC16A7, SLC17A2, SLC17A4, SLC17A6, SLC17A7, SLC17A9, SLC1A1, SLC1A3, SLC1A7, SLC22A18, SLC22A23, SLC22A5, SLC24A4, SLC25A10, SLC25A13, SLC25A21, SLC25A22, SLC25A41, SLC25A45, SLC26A5, SLC29A2, SLC2A5, SLC2A9, SLC30A2, SLC30A6, SLC35B3, SLC35B4, SLC35D3, SLC35E1, SLC37A2, SLC38A8, SLC38A9, SLC39A10, SLC39A13, SLC39A7, SLC39A8, SLC39A9, SLC40A1, |
| GO:0023051 | regulation of signaling | 4,07E-13 | [2, 3] | 888,00 | 23,14 | AMER2, AMH, ANGPT1, ANKRD17, ANKS1A, ANKS1B, ANO1, ANTXR1, ANXA2, APBA2, APC, APC2, APPL1, APPL2, ARAP1, ARAP3, ARF1, ARFGAP1, ARFGEF3, ARHGAP10, ARHGAP12, ARHGAP17, ARHGAP21, ARHGAP29, ARHGAP39, ARHGAP40, ARHGAP42, ARHGAP45, ARHGDIA, ARHGDIB, ARHGDIG, ARHGEF16, ARHGEF18, ARHGEF2, ARHGEF26, ARHGEF7, ARL6IP5, ARNTL, ASCL1, ASPH, ASXL2, ATF3, ATM, ATP1A3, ATP1A4, ATP2A2, ATP2A3, ATP2B2, ATP2B4, AURKB, BAIAP2L1, BANP, BCAR3, BCL10, BCL2, BCL2L14, BCL3, BCL6, BDNF, BDNF-AS, BEGAIN, BEND6, BICC1, BIRC2, BMP2, BMP3, BMP7, BMP8B, BMPER, BRD1, BRD4, BRSK2, BTBD9, BTC, BTRC, CACNA1A, CACNA1B, CACNA1E, CACNA2D2, CACNB2, CACNG8, CADM1, CALB2, CALM2, CAMK2B, CAMK2D, CAMKMT, CAMTA1, CAPRIN2, CARD10, CARD11, CARD14, CASP2, CASR, CAV1, CBFA2T2, CCAR2, CCBE1, CCDC3, CCDC88C, CCKAR, CCL16, CCN4, CCNY, CCR2, CD200R1, CD46, CD47, CDC20, CDH13, CDK1, CDK2, CDK5R1, CELA2A, CELF4, CHD3, CHD5, CHEK1, CHEK2, CHN2, CHRNB2, CHRNB3, CHST11, CIB1, CITED2, CMAHP, CNIH3, CNOT1, CNOT7, CNRIP1, CNTN4, COL2A1, COL3A1, CORIN, CPT1A, CRB2, CREB3L1, CRY2, CSNK1A1L, CSNK1E, CSNK1G3, CSPG5, CTBP2, CTNNBIP1, CTNND2, CTTN, CUL3, CXCR4, CXXC5, CYFIP1, CYFIP2, CYLD, CYP19A1, CYP1B1, CYTH1, DAB1, DAG1, DAPK1, DAXX, DBN1, DCN, DDIT4, DDX60, DENND1A, DEPDC5, DGKE, DGKQ, DGKZ, DHRS3, DHX15, DHX34, DHX36, DIPK2A, DKK3, DLC1, DLG1, DLG2, DLG4, DLL1, DLL4, DLX2, DNA2, DNM1, DNM1L, DOK4, DOT1L, DSTYK, DTX1, DUSP19, DUSP26, ECE1, EDAR, EEF1E1-BLOC1S5, EFNA5, EGR2, EHMT1, EHMT2, EIF2AK3, EIF2AK4, EIF4A3, EIF4E, EIF4EBP2, EIPR1, ELF1, EMC10, ENHO, EPHA4, EPHB1, EPS15L1, EPS8L1, ERBB4, EREG, ESR1, ESR2, ETFA, EYA2, EYA4, EZH2, F11R, F2RL3, F3, FABP5, FAF1, FAM110A, FAM13A, FAM13B, FAM3D, FAM89B, FASN, FBN1, FBN2, FBP1, FER, FGD2, FGF12, FGF14, FGF2, FGF22, FGF6, FGF7, FICD, FKTN, FLT4, FNIP2, FNTA, FOXO1, FOXP1, FRMD8, FRMPD1, FRS2, FXYD6, FZD10, FZD5, FZR1, GAB1, GADD45G, GATA5, GCG, GCNT2, GFI1, GFRAL, GJA5, GLI2, GLI3, GLIPR2, GLIS2, GLMN, GLRX2, GNA12, GNAI2, GPC6, GPER1, GPR137B, GPR161, GPR17, GPR4, GPR55, GPR62, GPRC5B, GPRIN1, GRAMD4, GRB10, GRB2, GREM1, GRIA4, GRID2, GRID2IP, GRIK3, GRIK4, GRK5, GRM1, GRM2, GRM4, GRM8, GSG1L, GSK3A, GSX2, HACD3, HADHA, HCAR2, HCLS1, HDAC9, HECW1, HEG1, HES1, HGS, HINT1, HIP1, HIP1R, HMGB1, HMGN3, HNF1B, HNRNPK, HOOK1, HRC, HRG, HSP90AB1, HSPD1, HTR1A, HTR2A, HTRA3, HTT, HUS1, IER3, IFIH1, IFT140, IGF1, IGF1R, IGF2BP1, IGSF9B, IHH, IKBKB, IL10RA, IL10RB, IL16, IL17RA, IL17RD, IL1RAP, IL20RA, IL31RA, IL37, IL6R, IL6ST, ILDR1, ING3, INPP5F, INSIG2, INSR, IQGAP3, IQSEC1, IQSEC3, IRAK2, IRS2, ISL1, ITCH, ITFG2, ITGA1, ITGA5, ITPR2, ITPR3, JAK2, JPH3, KALRN, KANK1, KAT2B, KAT6A, KCNB1, KCTD10, KCTD16, KCTD6, KIDINS220, KIF16B, KIF26A, KITLG, KLF14, KLF7, KMT2D, KRAS, KREMEN2, KSR1, LAMA2, LATS2, LAX1, LDLRAD4, LGMN, LGR6, LIMS1, LLGL1, LMCD1, LMO7, LOXL3, LPAR1, LPL, LRFN2, LRIG2, LRP1, LRP2, LRP5, LRPAP1, LRRC14, LRRC32, LRRC4C, LRRK1, LTBP1, LURAP1, LYPD6, LYPD6B, LZTS1, LZTS2, MACF1, MAGI2, MAL, MAML2, MAML3, MAP2K2, MAP2K5, MAP3K11, MAP3K13, MAP3K2, MAP3K20, MAP3K5, MAP3K6, MAP4K4, MAP4K5, MAPK7, MAPK8, MAPK8IP2, MAPK8IP3, MAPK9, MAPKAP1, MAPKAPK5, MARK3, MBD3, MCF2L, MCL1, MECOM, MEF2C, MEGF8, MEN1, MFNG, MGRN1, MIB2, MIER1, MKS1, MLLT3, MME, MRPL23, MRTFA, MST1R, MTMR2, MTNR1B, MTRNR2L4, MUC4, MYD88, MYO1C, MYO1E, MYO5A, MYO9B, NACC2, NAPB, NCK2, NCOR2, NCSTN, NDFIP2, NDRG4, NDUFA13, NDUFA2, NEO1, NEPRO, NF1, NFAM1, NFATC1, NFATC4, NFE2L2, NGEF, NGF, NKD1, NLGN2, NLK, NLRC5, NLRP12, NLRX1, NOC2L, NOL3, NOP2, NOS1, NOS1AP, NOTCH1, NOTCH3, NOTCH4, NPHP4, NPNT, NPPA, NPR1, NPRL3, NPS, NPTN, NPTXR, NR1D1, NR2C1, NR3C2, NRXN1, NRXN3, NSG1, NSMF, NTNG1, NTNG2, NTRK2, NXN, OBSCN, OSBPL8, OTUD4, P2RY6, PACSIN2, PAFAH1B1, PAGR1, PAK5, PALM, PANX2, PARP14, PASK, PAX8, PBLD, PCDHA6, PCSK6, PDCD5, PDCD6, PDE8A, PDGFD, PDGFRA, PDPK1, PDZD2, PDZD3, PEG10, PELI3, PER2, PEX5L, PFKM, PFN2, PHF20, PHLPP2, PIH1D1, PIK3CB, PIK3CG, PIK3IP1, PIK3R1, PIK3R5, PIP4K2A, PIP5K1C, PLA2G6, PLAUR, PLCB1, PLCB4, PLCE1, PLCL2, PLEK, PLEKHA4, PLEKHG2, PLIN5, PLXNB1, PML, POU4F2, PPARG, PPARGC1A, PPFIA3, PPP1R11, PPP1R13B, PPP1R16B, PPP3CA, PPP3CB, PRAG1, PRDM16, PRKAG2, PRKAR1B, PRKCA, PRKCB, PRKCE, PRKCH, PRKCI, PRKCQ, PRKCZ, PRKD2, PRKN, PRLR, PRMT1, PSCA, PSD, PSD2, PSD3, PSD4, PSEN1, PSMA5, PSMA6, PSMA7, PSMC2, PSMC3, PSMC6, PSMD2, PSMD7, PTCH2, PTEN, PTGES2, PTGIS, PTK2, PTPN11, PTPN3, PTPRA, PTPRC, PTPRO, PUM1, PXDN, RAB29, RAB5A, RACK1, RAD9A, RALGAPA2, RANBP9, RAP1A, RAP1GAP, RAP1GAP2, RAPGEF1, RARA, RASA1, RASA2, RASA3, RASGRF1, RASGRP4, RASIP1, RB1, RBP4, RBPMS, RBX1, RC3H1, RC3H2, RCAN2, RELN, |

| | | | | | |
|---|---|---|---|---|---|
| GO:0050790 | regulation of catalytic activity | 3,06E-07 [3] | 589,00 | 23,17 | AKT2, ALK, ALKAL2, ALS2, AMBRA1, AMH, ANGPT1, ANGPT4, ANKS1B, ANTXR1, ANXA2, APC, APC2, APOBEC1, ARAP1, ARAP3, ARF1, ARFGAP1, ARFGEF3, ARHGAP10, ARHGAP12, ARHGAP17, ARHGAP21, ARHGAP29, ARHGAP39, ARHGAP40, ARHGAP42, ARHGAP45, ARHGDIA, ARHGDIB, ARHGDIG, ARHGEF10L, ARHGEF16, ARHGEF7, ARL1, ARL6IP5, ASAP1, ASAP2, ASPH, ATG14, ATP13A2, ATP2A3, ATP2B4, ATP6V1H, AURKB, BBS4, BCAR3, BCAS3, BCL10, BCL2, BCL6, BDNF, BDNF-AS, BIRC2, BMP1, BMP2, BMP7, BRSK2, BTC, BTRC, CABP1, CACNA1A, CALM2, CAMK2D, CAND1, CAP1, CAP2, CARD10, CARD14, CASR, CAV1, CBX8, CCAR2, CCL16, CCNA1, CCND2, CCNL1, CCNY, CCR2, CCT2, CD33, CDC20, CDC20B, CDC25B, CDC25C, CDK1, CDK5R1, CDKN1A, CDKN2A, CEP43, CHN2, CHTF8, CIB1, CNST, COX6A1, CPNE2, CRB2, CRIM1, CRY2, CSRNP2, CSRNP3, CST9, CXCR4, CYCS, CYFIP2, DAB1, DAG1, DAPK1, DAXX, DBNDD2, DCUN1D1, DDAH2, DDR1, DDR2, DENND1A, DENND1B, DEPDC1, DEPDC5, DGKQ, DGKZ, DIPK2A, DLC1, DLG1, DLG2, DLG4, DNAJB4, DNAJB6, DNAJC15, DNAJC3, DNAL4, DNM1L, DNPEP, DOCK10, DOCK7, DOCK8, DOK7, DSTYK, DUS2, DUSP19, DUSP26, ECSIT, EEF1A1, EFCAB11, EFNA5, EGR2, EIF2AK3, ELMOD1, EPHA1, EPHA10, EPHA4, EPHA6, EPHB1, EPHB3, ERBB4, EREG, ESR1, ETAA1, ETFA, EZH2, F11R, F3, FAF1, FAM13A, FAM13B, FARP1, FASN, FEM1C, FGD2, FGF2, FICD, FLT4, FNIP2, FNTA, FRS2, FZD10, FZD5, FZR1, GABBR2, GADD45G, GCG, GFI1, GFRAL, GLMN, GMFB, GNAI2, GNB5, GORASP1, GPER1, GPR137B, GPR55, GPRC5B, GPRC5C, GPRIN1, GPSM1, GRAMD4, GREM1, GRM1, GRM2, GRM4, GRPEL1, GSK3A, GTF2H1, GUCA2A, HACD3, HDAC9, HEG1, HGS, HIP1, HIP1R, HMBOX1, HMGB1, HNRNPD, HNRNPR, HRG, HSP90AB1, HSPD1, HSPH1, HTR2A, HTT, IFI16, IGF1, IGF1R, IKBKB, IL6R, INKA2, INSR, IPO7, IQGAP2, IQGAP3, IQSEC1, IRAK2, IRS2, ITGA1, ITGB2, ITIH5, ITK, JAK2, KALRN, KAT2B, KIDINS220, KITLG, KRAS, KRIT1, KSR1, LATS2, LAX1, LDB2, LEPR, LGMN, LIMS1, LLGL1, LMTK2, LPAR1, LPAR2, LPL, LRP1, LRP5, MAGI2, MAL, MAP2, MAP2K2, MAP2K5, MAP3K11, MAP3K13, MAP3K19, MAP3K2, MAP3K20, MAP3K5, MAP3K6, MAP4K4, MAP4K5, MAPK7, MAPK8, MAPK8IP3, MAPKAP1, MAPKAPK5, MARF1, MCM2, MCPH1, MEF2C, MEN1, MGMT, MMD, MRPL23, MRTFA, MSH2, MSH3, MST1R, MTMR9, MTRR, MYO1D, MYO9B, NCK2, NCKAP1L, NCSTN, NDE1, NDUFA13, NEDD9, NEK7, NF1, NGEF, NGF, NHEJ1, NLRC5, NLRP12, NOL3, NOS1, NOS1AP, NOTCH1, NPNT, NPPA, NPRL3, NRXN1, NTRK2, NUS1, OASL, OPRPN, ORAI1, OSBPL8, P2RY6, PAFAH1B1, PAK4, PAK5, PALM, PAPLN, PARP4, PAX2, PCDHA6, PCP2, PCSK5, PCSK6, PDCD5, PDCD6, PDGFD, PDGFRA, PDPK1, PDZD2, PDZD3, PFN2, PHACTR1, PIH1D1, PIK3CB, PIK3CG, PIK3IP1, PIK3R1, PIK3R5, PINLYP, PINX1, PKD1, PKIB, PKMYT1, PLAUR, PLCB1, PLCB2, PLCE1, PLEK, PLIN5, PLXNA1, PLXNA2, PLXNB1, PLXNC1, PLXND1, PML, PPARG, PPARGC1B, PPM1F, PPP1R11, PPP1R16A, PPP1R16B, PPP2R2B, PPP2R2C, PPP2R3C, PPP3CA, PPP4C, PRDX5, PRKAG2, PRKAR1B, PRKCA, PRKCE, PRKCQ, PRKCZ, PRKD2, PRKN, PRLR, PRPSAP2, PSEN1, PSMD2, PTEN, PTK2, PTPN11, PTPRA, PTPRC, PTPRH, PTPRN2, PTPRO, PZP, RAB4A, RABGAP1, RACK1, RALGAPA2, RALGDS, RANBP1, RAP1A, RAP1GAP, RAP1GAP2, RAP2B, RAPGEF1, RASA1, RASA2, RASA3, RASAL2, RASGRF1, RASGRP4, RASIP1, RB1, RBM26, RCAN2, RCVRN, RD3, RELN, REST, RET, RGMA, RGS12, RGS3, RGS6, RGS7, RGS9, RHOA, RHOG, |
| GO:0050790 | regulation of catalytic activity | 3,06E-07 [3] | 589,00 | 23,17 | AKT2, ALK, ALKAL2, ALS2, AMBRA1, AMH, ANGPT1, ANGPT4, ANKS1B, ANTXR1, ANXA2, APC, APC2, APOBEC1, ARAP1, ARAP3, ARF1, ARFGAP1, ARFGEF3, ARHGAP10, ARHGAP12, ARHGAP17, ARHGAP21, ARHGAP29, ARHGAP39, ARHGAP40, ARHGAP42, ARHGAP45, ARHGDIA, ARHGDIB, ARHGDIG, ARHGEF10L, ARHGEF16, ARHGEF7, ARL1, ARL6IP5, ASAP1, ASAP2, ASPH, ATG14, ATP13A2, ATP2A3, ATP2B4, ATP6V1H, AURKB, BBS4, BCAR3, BCAS3, BCL10, BCL2, BCL6, BDNF, BDNF-AS, BIRC2, BMP1, BMP2, BMP7, BRSK2, BTC, BTRC, CABP1, CACNA1A, CALM2, CAMK2D, CAND1, CAP1, CAP2, CARD10, CARD14, CASR, CAV1, CBX8, CCAR2, CCL16, CCNA1, CCND2, CCNL1, CCNY, CCR2, CCT2, CD33, CDC20, CDC20B, CDC25B, CDC25C, CDK1, CDK5R1, CDKN1A, CDKN2A, CEP43, CHN2, CHTF8, CIB1, CNST, COX6A1, CPNE2, CRB2, CRIM1, CRY2, CSRNP2, CSRNP3, CST9, CXCR4, CYCS, CYFIP2, DAB1, DAG1, DAPK1, DAXX, DBNDD2, DCUN1D1, DDAH2, DDR1, DDR2, DENND1A, DENND1B, DEPDC1, DEPDC5, DGKQ, DGKZ, DIPK2A, DLC1, DLG1, DLG2, DLG4, DNAJB4, DNAJB6, DNAJC15, DNAJC3, DNAL4, DNM1L, DNPEP, DOCK10, DOCK7, DOCK8, DOK7, DSTYK, DUS2, DUSP19, DUSP26, ECSIT, EEF1A1, EFCAB11, EFNA5, EGR2, EIF2AK3, ELMOD1, EPHA1, EPHA10, EPHA4, EPHA6, EPHB1, EPHB3, ERBB4, EREG, ESR1, ETAA1, ETFA, EZH2, F11R, F3, FAF1, FAM13A, FAM13B, FARP1, FASN, FEM1C, FGD2, FGF2, FICD, FLT4, FNIP2, FNTA, FRS2, FZD10, FZD5, FZR1, GABBR2, GADD45G, GCG, GFI1, GFRAL, GLMN, GMFB, GNAI2, GNB5, GORASP1, GPER1, GPR137B, GPR55, GPRC5B, GPRC5C, GPRIN1, GPSM1, GRAMD4, GREM1, GRM1, GRM2, GRM4, GRPEL1, GSK3A, GTF2H1, GUCA2A, HACD3, HDAC9, HEG1, HGS, HIP1, HIP1R, HMBOX1, HMGB1, HNRNPD, HNRNPR, HRG, HSP90AB1, HSPD1, HSPH1, HTR2A, HTT, IFI16, IGF1, IGF1R, IKBKB, IL6R, INKA2, INSR, IPO7, IQGAP2, IQGAP3, IQSEC1, IRAK2, IRS2, ITGA1, ITGB2, ITIH5, ITK, JAK2, KALRN, KAT2B, KIDINS220, KITLG, KRAS, KRIT1, KSR1, LATS2, LAX1, LDB2, LEPR, LGMN, LIMS1, LLGL1, LMTK2, LPAR1, LPAR2, LPL, LRP1, LRP5, MAGI2, MAL, MAP2, MAP2K2, MAP2K5, MAP3K11, MAP3K13, MAP3K19, MAP3K2, MAP3K20, MAP3K5, MAP3K6, MAP4K4, MAP4K5, MAPK7, MAPK8, MAPK8IP3, MAPKAP1, MAPKAPK5, MARF1, MCM2, MCPH1, MEF2C, MEN1, MGMT, MMD, MRPL23, MRTFA, MSH2, MSH3, MST1R, MTMR9, MTRR, MYO1D, MYO9B, NCK2, NCKAP1L, NCSTN, NDE1, NDUFA13, NEDD9, NEK7, NF1, NGEF, NGF, NHEJ1, NLRC5, NLRP12, NOL3, NOS1, NOS1AP, NOTCH1, NPNT, NPPA, NPRL3, NRXN1, NTRK2, NUS1, OASL, OPRPN, ORAI1, OSBPL8, P2RY6, PAFAH1B1, PAK4, PAK5, PALM, PAPLN, PARP4, PAX2, PCDHA6, PCP2, PCSK5, PCSK6, PDCD5, PDCD6, PDGFD, PDGFRA, PDPK1, PDZD2, PDZD3, PFN2, PHACTR1, PIH1D1, PIK3CB, PIK3CG, PIK3IP1, PIK3R1, PIK3R5, PINLYP, PINX1, PKD1, PKIB, PKMYT1, PLAUR, PLCB1, PLCB2, PLCE1, PLEK, PLIN5, PLXNA1, PLXNA2, PLXNB1, PLXNC1, PLXND1, PML, PPARG, PPARGC1B, PPM1F, PPP1R11, PPP1R16A, PPP1R16B, PPP2R2B, PPP2R2C, PPP2R3C, PPP3CA, PPP4C, PRDX5, PRKAG2, PRKAR1B, PRKCA, PRKCE, PRKCQ, PRKCZ, PRKD2, PRKN, PRLR, PRPSAP2, PSEN1, PSMD2, PTEN, PTK2, PTPN11, PTPRA, PTPRC, PTPRH, PTPRN2, PTPRO, PZP, RAB4A, RABGAP1, RACK1, RALGAPA2, RALGDS, RANBP1, RAP1A, RAP1GAP, RAP1GAP2, RAP2B, RAPGEF1, RASA1, RASA2, RASA3, RASAL2, RASGRF1, RASGRP4, RASIP1, RB1, RBM26, RCAN2, RCVRN, RD3, RELN, REST, RET, RGMA, RGS12, RGS3, RGS6, RGS7, RGS9, RHOA, RHOG, |
| GO:0006468 | protein phosphorylation | 1,63E-05 [6, 7] | 489,00 | 23,19 | ANGPT1, ANGPT4, ANKS1B, ANTXR1, ANXA2, APC, ARHGEF2, ARHGEF7, ARL6IP5, ATF3, ATG14, ATM, ATP13A2, ATP2B4, AURKB, BAZ1B, BCAR3, BCL2, BDNF, BDNF-AS, BMP2, BMP3, BMP7, BMP8B, BMPER, BRD4, BRSK2, BTC, BTRC, CALM2, CAMK2B, CAMK2D, CAMK4, CAPRIN2, CARD10, CARD14, CASR, CAV1, CCDC88C, CCL16, CCNA1, CCND2, CCNL1, CCNY, CDC25B, CDC25C, CDC42BPB, CDK1, CDK11B, CDK19, CDK2, CDK5R1, CDKL2, CDKN1A, CDKN2A, CEP43, CHAMP1, CHEK1, CHEK2, CIB1, CILK1, CIT, CLASP1, CLK3, CLK4, CNOT7, CRIM1, CSNK1A1L, CSNK1E, CSNK1G3, CUL3, CXCR4, CYLD, DAB1, DAG1, DAPK1, DAXX, DBNDD2, DCAF1, DCLK1, DDIT4, DDR1, DDR2, DGKQ, DHDDS, DIPK2A, DLG1, DLG2, DLG4, DNAJC3, DOCK7, DOK4, DOK7, DSTYK, DUS2, DUSP19, DUSP26, EDAR, EFNA4, EFNA5, EIF2AK3, EIF2AK4, EMC10, ENPP2, EPHA1, EPHA10, EPHA4, EPHA6, EPHB1, EPHB3, ERBB4, ERC1, EREG, ETAA1, ETFA, EZH2, F3, FAF1, FASN, FASTK, FASTKD1, FER, FGD2, FGF2, FGF22, FGF6, FGF7, FKTN, FLT4, FNIP2, FOXO1, FRS2, FZD10, FZD5, GADD45G, GAK, GARS1, GCG, GCNT2, GFRAL, GLIPR2, GLYCTK, GMFB, GNAI2, GPER1, GPR55, GPRC5B, GPRC5C, GPRIN1, GRB10, GRB2, GREM1, GRK3, GRK5, GRM1, GRM4, GSK3A, GTF2H1, HACD3, HCLS1, HEG1, HES1, HGS, HMGB1, HRC, HRG, HSP90AB1, HTATIP2, HTR2A, HTT, HUS1, IGF1, IGF1R, IKBKB, IL12RB2, IL17RD, IL31RA, IL6R, IL6ST, ILF3, INKA2, INPP5F, INSR, IPO7, IQGAP3, IRAK2, IRS2, ISL1, ITCH, ITGA1, ITGA5, ITGB2, ITK, JAK2, KALRN, KAT2B, KDM4D, KIDINS220, KIRREL1, KITLG, KNDC1, KRAS, KSR1, KSR2, LATS2, LAX1, LDLRAD4, LEPR, LIMK2, LLGL1, LMTK2, LONP1, LPAR1, LRP1, LRP5, LRRK1, LTBP1, MAK, MAP2K2, MAP2K5, MAP3K11, MAP3K13, MAP3K19, MAP3K2, MAP3K20, MAP3K5, MAP3K6, MAP4K4, MAP4K5, MAPK4, MAPK7, MAPK8, MAPK8IP2, MAPK8IP3, MAPK9, MAPKAP1, MAPKAPK5, MARK3, MAST4, MCM2, MECOM, MEF2C, MEN1, MKNK1, MKNK2, MMD, MST1R, MYD88, MYLK, MYLK4, NCK2, NCKAP1L, NDRG4, NEDD9, NEK11, NEK7, NF1, NGF, NLK, NLRP12, NME2, NNT, NOS1, NOTCH1, NPHS1, NPNT, NPPA, NPR1, NPTN, NRXN1, NSD1, NTRK2, OBSCN, OSBPL8, P2RY6, PAFAH1B1, PAK4, PAK5, PARD3, PARP14, PASK, PBLD, PDCL3, PDE8A, PDGFD, PDGFRA, PDGFRL, PDPK1, PDZD2, PEAK1, PFN2, PIH1D1, PIK3CB, PIK3CG, PIK3R1, PIK3R5, PIM1, PKD1, PKIB, PKMYT1, PLA2G6, PLAUR, PLCB1, PLCE1, PLCL2, PLK4, PLK5, PLVAP, PML, POU4F2, PPARGC1A, PPM1F, PPP2R3C, PPP3CA, PPP3CB, PRAG1, PRKAG2, PRKAR1B, PRKCA, PRKCB, PRKCE, PRKCH, PRKCI, PRKCQ, PRKCZ, PRKD2, PRKDC, PRKN, PRLR, PRMT1, PSCA, PSEN1, PSMA5, PSMA6, PSMA7, PSMC2, PSMC3, PSMC6, PSMD2, PSMD7, PTEN, PTK2, PTPN11, PTPRA, PTPRC, PTPRH, PTPRO, RACK1, RAD51, RANBP9, |
| GO:0022857 | transmembrane transporter activity | 1,03E-02 [3, 5] | 319,00 | 23,20 | ATP13A2, ATP1A3, ATP1A4, ATP2A2, ATP2A3, ATP2B2, ATP2B4, ATP5F1A, ATP5ME, ATP6V0A1, ATP6V0D1, ATP6V1A, ATP6V1H, ATP8A1, BCL2, BEST1, C4orf3, CABP2, CACNA1A, CACNA1B, CACNA1E, CACNA1G, CACNA1H, CACNA1I, CACNA2D2, CACNA2D3, CACNA2D4, CACNB2, CACNG1, CACNG6, CACNG8, CALM2, CAMK2D, CAV1, CCR2, CHRNB2, CHRNB3, CHRND, CHRNE, CLCN2, CLCN3, CLDN16, CNGA3, CNIH3, CNNM1, CNNM4, COMMD1, COX5A, COX6A1, COX7C, CRISP1, CTNS, CTSS, DAPK1, DLG1, DLG2, DLG4, DPP10, FAM155A, FGF12, FGF14, FLVCR2, FXYD5, FXYD6, GABRA2, GABRA4, GABRB2, GABRR3, GJA5, GNB5, GPRIN1, GRIA4, GRID2, GRIK3, GRIK4, GRM2, GSG1L, HCN1, HCN2, HCN4, HECW1, HRC, HTR3A, HTR3C, HTT, INAFM1, ITPR2, ITPR3, JPH3, KCNAB2, KCNB1, KCNB2, KCNC1, KCNC3, KCNE1, KCNF1, KCNH2, KCNIP4, KCNJ14, KCNJ15, KCNJ2, KCNJ3, KCNJ6, KCNK2, KCNMA1, KCNQ1, KCNQ2, KCNV1, LASP1, LLGL1, LMBRD1, LONP1, LRRC8B, LRRC8D, MAPK8IP2, MCL1, MEF2C, MFSD3, MIPEP, MRS2, MTNR1B, MYO5A, NALCN, NDUFA4L2, NEDD4L, NIPA1, NLGN2, NNT, NOL3, NOS1, NOS1AP, NPPA, NRXN1, NRXN3, OCA2, ORAI1, ORAI2, P2RY6, PACC1, PANX2, PDZD3, PHLPP2, PIEZO1, PIM1, PKD1, PKD1L1, PLA2G6, PLCG2, PPARGC1A, PRKCA, PRKCB, PRKCE, PRKCZ, PRKD2, PSEN1, PTEN, PTPN3, RACK1, RASA1, RASA3, RASGRF1, RBP4, RELN, RGS9, RIPK1, RYR1, RYR2, RYR3, S100A6, SCN1A, SCN2A, SCN3B, SCN5A, SGK1, SHANK2, SHISA6, SHISA7, SHISA9, SLC10A7, SLC11A2, SLC12A7, SLC12A9, SLC13A3, SLC13A5, SLC14A1, SLC15A4, SLC16A5, SLC16A7, SLC17A2, SLC17A4, SLC17A6, SLC17A7, SLC17A9, SLC1A1, SLC1A3, SLC1A7, SLC22A18, SLC22A23, SLC22A5, SLC24A4, SLC25A10, SLC25A13, SLC25A21, SLC25A22, SLC25A41, SLC25A45, SLC26A5, SLC29A2, SLC2A5, SLC2A9, SLC30A2, SLC30A6, SLC35B3, SLC35B4, SLC35D3, SLC35E1, SLC37A2, SLC38A8, SLC38A9, SLC39A10, SLC39A13, SLC39A7, SLC39A8, SLC39A9, SLC40A1, SLC41A2, SLC41A3, SLC43A2, SLC44A4, SLC45A4, SLC46A1, SLC47A2, |
| GO:0071495 | cellular response to endogenous stimulus | 2,07E-03 [3] | 357,00 | 23,26 | ARHGEF18, ARHGEF2, ARNTL, ASS1, ATP1A3, ATP1A4, ATP2B4, ATP6V0A1, ATP6V0D1, ATP6V1A, ATP6V1H, BAIAP2L1, BBS4, BCAR1, BCAR3, BCL11A, BDNF, BDNF-AS, BMP2, BMP3, BMP7, BMP8B, BMPER, CACNA1A, CACNA1H, CALCOCO1, CASR, CAV1, CCKAR, CD200R1, CDK5R1, CHAMP1, CHEK2, CHRM4, CHRNB2, CHST11, CIB1, CITED2, CNOT1, COL2A1, COL3A1, COL4A1, COL4A2, COL6A1, CORO1B, CPEB1, CPEB4, CRB2, CREB3L1, CRY2, CTSS, CYFIP1, DAXX, DDIT4, DHX15, DHX34, DKK3, DLL4, DNMT3A, DSTYK, EEF1A1, EFNA5, EHMT2, EIF4A3, EIF4E, EIF4EBP1, EIF4EBP2, ENHO, EPHA4, EPRS1, ERBB4, ESR1, ESR2, ESRP2, ESRRG, EZH2, F11R, FAM89B, FBN1, FBN2, FBXO32, FER, FERMT2, FGF12, FGF2, FGF22, FGF6, FGF7, FLRT1, FNTA, FOXO1, FOXP1, FRS2, FUT8, FZR1, GAB1, GABRB2, GALNT3, GARS1, GATA5, GCG, GCNT2, GLP2R, GNAI2, GNAO1, GNB5, GNRHR2, GPER1, GPT, GRB10, GRB2, GREM1, GSK3A, HCN1, HCN2, HCN4, HCRTR2, HDAC4, HDAC9, HES1, HIVEP1, HNRNPD, HNRNPH1, HSP90AB1, HTR1A, HTR2A, HTR3A, HTR3C, HTRA3, IDE, IGF1, IGF1R, IL17RD, ING3, INSIG2, INSR, IRS2, ISL1, ITPR2, JAK2, KANK1, KAT2B, KCNE1, KCNQ1, KCTD6, KDM3A, KIDINS220, KIF16B, KIF18A, KMT2D, LATS2, LDLRAD4, LEPR, LGMN, LIMS1, LPL, LRP1, LRP2, LRRC32, LTBP1, LTBP2, MAGI2, MAPK7, MARS1, MAX, MEF2C, MEGF8, MEN1, MICALL1, MRPL23, MYO1C, MYO1E, MYO5A, NCOA2, NCOR2, NCSTN, NEO1, NFE2L2, NGF, NLK, NOTCH1, NPC1, NPNT, NPTN, NR1D1, NR2C1, NR2E3, NR3C1, NR3C2, NRIP1, NRXN1, NSG1, NSMF, NTRK2, OSBPL8, P2RY6, PAGR1, PALM, PAQR8, PARD3, PAX2, PAX8, PBLD, PCK2, PCSK6, PDCD5, PDE8A, PDGFD, PDGFRA, PDGFRL, PDPK1, PDZD2, PEG10, PIK3CG, PIK3R1, PKM, PLCB1, PML, POLR2B, POLR2F, POU4F2, PPARG, PPARGC1A, PPARGC1B, PPP3CA, PRDM16, PRKAR1B, PRKCA, PRKCB, PRKCE, PRKCI, PRKCQ, PRKCZ, PRKD2, PRKDC, PRKN, PRLR, PSEN1, PTEN, PTK2, PTPN11, PTPRA, PXDN, RAB35, RAD51, RAP1A, RAP1GAP, RAPGEF1, RARA, RB1, RBFOX2, REST, |
| GO:0048646 | anatomical structure formation involved in morphogenesis | 4,01E-02 [2, 3, 4] | 276,00 | 23,27 | [ACTN2, ACVRL1, ADAM12, ADAM17, ADGRB2, ADGRB3, ADGRG1, AGO2, AKAP13, ALX1, ANGPT1, ANGPT4, ANGPTL2, ANXA2, ATF2, ATM, ATP2B4, ATP8A2, BBS4, BCAS3, BCL10, BCL3, BCL6, BMERB1, BMP2, BMP7, BMPER, BRD2, CACNA1A, CACNA1H, CARD10, CAV1, CC2D2A, CCBE1, CCN6, CCR2, CD53, CDH13, CDK5R1, CELSR1, CEND1, CEP290, CHAMP1, CIB1, CITED2, CLASP1, CLASP2, CNNM4, COBL, COL18A1, COL2A1, COL4A1, COL4A2, COL6A1, COL8A2, CRB2, CREB3L1, CUL3, CXCR4, CYP1B1, DAG1, DCN, DLC1, DLL1, DLL4, E2F2, E2F8, EDAR, EGR2, EIF2AK3, ELK3, EMC10, ENPP2, EPHA1, EPHB1, EPHB3, ERAP1, ERCC1, EREG, ETS1, EXOC4, EYA2, F3, FAP, FBLN5, FBN2, FER1L5, FGF2, FGF6, FHOD3, FJX1, FLT4, FMN1, FOXO1, FRS2, FZD5, GAB1, GARS1, GATA5, GJA5, GLI2, GLI3, GLMN, GPR4, GRB2, GREM1, GRID2, HAND1, HDAC9, HERC1, HES1, HGS, HNF1B, HOXA1, HOXA3, HOXC11, HRG, HTATIP2, IHH, ISL1, ISM1, ITGA5, ITGB2, ITGB4, KDM2B, KDM4C, KDM6B, KIF16B, KIF26B, KIF2A, KLK4, KNDC1, KRIT1, KRT1, LAMB1, LDB3, LEPR, LONP1, LOXL2, LRP2, MAP2K5, MAPK7, MBOAT7, MEF2C, MEN1, MEOX1, MFGE8, MFNG, MKS1, MSGN1, MTMR2, MYH3, MYL2, MYOM2, MYPN, NECTIN1, NF1, NFASC, NFATC2, NFATC4, NFE2L2, NFIB, NKD1, NOS1, NOTCH1, NOTCH3, NOTCH4, NPHS1, NPR1, NRXN1, NRXN3, NUS1, OBSCN, OBSL1, OLFM1, PAF1, PAFAH1B1, PAK4, PAX2, PAX8, PDCD6, PDCL3, PDGFRA, PDPK1, PHLDB1, PIK3CG, PIKFYVE, PIM1, PKM, PLXNA2, PLXND1, PML, PPARG, PPARGC1B, PPP1R16B, PPP3CA, PPP3CB, PRKCA, PRKCB, PRKD2, PRKDC, PSEN1, PSMA7, PTEN, PTF1A, PTGIS, PTK2, PTPN11, PTPN14, RARA, RASIP1, RBP4, RC3H1, |
| GO:0048646 | anatomical structure formation involved in morphogenesis | 4,01E-02 [2, 3, 4] | 276,00 | 23,27 | [ACTN2, ACVRL1, ADAM12, ADAM17, ADGRB2, ADGRB3, ADGRG1, AGO2, AKAP13, ALX1, ANGPT1, ANGPT4, ANGPTL2, ANXA2, ATF2, ATM, ATP2B4, ATP8A2, BBS4, BCAS3, BCL10, BCL3, BCL6, BMERB1, BMP2, BMP7, BMPER, BRD2, CACNA1A, CACNA1H, CARD10, CAV1, CC2D2A, CCBE1, CCN6, CCR2, CD53, CDH13, CDK5R1, CELSR1, CEND1, CEP290, CHAMP1, CIB1, CITED2, CLASP1, CLASP2, CNNM4, COBL, COL18A1, COL2A1, COL4A1, COL4A2, COL6A1, COL8A2, CRB2, CREB3L1, CUL3, CXCR4, CYP1B1, DAG1, DCN, DLC1, DLL1, DLL4, E2F2, E2F8, EDAR, EGR2, EIF2AK3, ELK3, EMC10, ENPP2, EPHA1, EPHB1, EPHB3, ERAP1, ERCC1, EREG, ETS1, EXOC4, EYA2, F3, FAP, FBLN5, FBN2, FER1L5, FGF2, FGF6, FHOD3, FJX1, FLT4, FMN1, FOXO1, FRS2, FZD5, GAB1, GARS1, GATA5, GJA5, GLI2, GLI3, GLMN, GPR4, GRB2, GREM1, GRID2, HAND1, HDAC9, HERC1, HES1, HGS, HNF1B, HOXA1, HOXA3, HOXC11, HRG, HTATIP2, IHH, ISL1, ISM1, ITGA5, ITGB2, ITGB4, KDM2B, KDM4C, KDM6B, KIF16B, KIF26B, KIF2A, KLK4, KNDC1, KRIT1, KRT1, LAMB1, LDB3, LEPR, LONP1, LOXL2, LRP2, MAP2K5, MAPK7, MBOAT7, MEF2C, MEN1, MEOX1, MFGE8, MFNG, MKS1, MSGN1, MTMR2, MYH3, MYL2, MYOM2, MYPN, NECTIN1, NF1, NFASC, NFATC2, NFATC4, NFE2L2, NFIB, NKD1, NOS1, NOTCH1, NOTCH3, NOTCH4, NPHS1, NPR1, NRXN1, NRXN3, NUS1, OBSCN, OBSL1, OLFM1, PAF1, PAFAH1B1, PAK4, PAX2, PAX8, PDCD6, PDCL3, PDGFRA, PDPK1, PHLDB1, PIK3CG, PIKFYVE, PIM1, PKM, PLXNA2, PLXND1, PML, PPARG, PPARGC1B, PPP1R16B, PPP3CA, PPP3CB, PRKCA, PRKCB, PRKD2, PRKDC, PSEN1, PSMA7, PTEN, PTF1A, PTGIS, PTK2, PTPN11, PTPN14, RARA, RASIP1, RBP4, RC3H1, |

| | | | | | | |
|---|---|---|---|---|---|---|
| GO:0035556 | intracellular signal transduction | 8,29E-10 | [3, 4, 5] | 698,00 | 23,28 | AKT2, ALK, ALKAL2, ALS2, AMH, ANGPT1, ANKRD17, ANKS1B, ANTXR1, ARAP1, ARAP3, ARFGAP1, ARFGEF3, ARHGAP10, ARHGAP12, ARHGAP17, ARHGAP21, ARHGAP29, ARHGAP39, ARHGAP40, ARHGAP42, ARHGAP45, ARHGDIA, ARHGDIB, ARHGDIG, ARHGEF16, ARHGEF18, ARHGEF2, ARHGEF26, ARHGEF7, ARL6IP5, ARNTL, ASB7, ATF3, ATM, ATP2A2, ATP2A3, ATP2B4, AURKB, BABAM2, BAG6, BANP, BCAR3, BCL10, BCL11B, BCL2, BCL2A1, BCL3, BCL6, BIRC2, BMP2, BMP7, BMPER, BRD1, BRD4, BRSK2, BTC, BTG2, BTRC, CACNA1A, CADPS, CALM2, CAMK2D, CAMK4, CAMTA1, CARD10, CARD11, CARD14, CASP2, CASR, CAV1, CCAR2, CCDC88C, CCL16, CCR2, CCR6, CD200R1, CDC25C, CDC42BPB, CDH13, CDK1, CDK2, CDK5R1, CDKN1A, CDKN2A, CELSR1, CHD3, CHD5, CHEK1, CHEK2, CHN2, CIB1, CILK1, CIT, CLASP1, CNOT1, CNOT7, CNRIP1, COL3A1, CREB3L1, CRIP1, CTNNAL1, CUL2, CUL3, CXCR4, CXXC5, CYFIP1, CYLD, CYP1B1, CYTH1, DAB1, DAG1, DAPK1, DAXX, DCLK1, DCN, DDAH2, DDIT4, DDX60, DEF8, DENND1A, DEPDC1, DEPDC5, DGKA, DGKD, DGKE, DGKH, DGKQ, DGKZ, DHDDS, DHX34, DHX36, DIPK2A, DLC1, DLG1, DLG2, DLG4, DNA2, DNM1L, DNMBP, DOCK10, DOCK7, DOCK8, DOK3, DOK4, DSTYK, DUSP19, DUSP26, E2F2, E2F8, EDAR, EEF1E1-BLOC1S5, EFCAB11, EGR2, EHMT1, EHMT2, EIF2AK3, EIF4EBP1, EIF4EBP2, ELMO1, EMC10, EPHA4, EPHB1, EPS8L1, ERBB4, ERC1, EREG, ESR1, ETFA, EXOC7, EZH2, F11R, F2RL3, F3, FAM110A, FAM13A, FAM13B, FARP2, FASN, FBP1, FER, FGD2, FGF2, FGF22, FGF6, FGF7, FKTN, FLT4, FNIP2, FOXO1, FRS2, FZD10, FZD5, FZR1, GAB1, GAB2, GADD45G, GARS1, GCG, GCNT2, GCOM1, GFRAL, GLIPR2, GML, GNA12, GNAI2, GPER1, GPR137B, GPR161, GPR17, GPR4, GPR55, GPR62, GPRC5B, GPRIN1, GRB10, GRB2, GREM1, GRM1, GRM2, GRM4, GSK3A, HACD3, HCLS1, HDAC4, HDAC9, HEG1, HGS, HINT1, HIP1, HMGB1, HNRNPK, HRC, HSP90AB1, HTR2A, HTT, HUS1, IER3, IFI16, IFIH1, IGF1, IGF1R, IKBKB, IL17RD, IL20RA, IL31RA, IL37, ING3, INPP5A, INPP5F, INSR, IQGAP3, IQSEC1, IQSEC3, IRAK2, IRS2, ITCH, ITFG2, ITGA1, ITK, ITPR2, ITPR3, JAK2, JPH3, KALRN, KANK1, KAT6A, KAT7, KCTD10, KIAA0355, KIDINS220, KITLG, KNDC1, KRAS, KRIT1, KSR1, KSR2, LAT2, LATS2, LAX1, LIMS1, LLGL1, LMCD1, LPAR1, LPAR2, LRP1, LRP2, LRPAP1, LRRK1, LURAP1, MAFA, MAGI2, MAK, MAP2K2, MAP2K5, MAP3K11, MAP3K13, MAP3K19, MAP3K2, MAP3K20, MAP3K5, MAP3K6, MAP4K4, MAP4K5, MAPK4, MAPK7, MAPK8, MAPK8IP2, MAPK8IP3, MAPK9, MAPKAP1, MAPKAPK5, MARK3, MAST4, MBD3, MCF2L, MCL1, MEAK7, MECOM, MEF2C, MEN1, MGRN1, MIB2, MIER1, MKNK1, MKNK2, MRPL23, MSH2, MST1R, MTNR1B, MYD88, MYO16, MYO5A, MYO9B, NACC2, NCALD, NCK2, NCKAP1, NCOA2, NDFIP2, NDRG4, NDUFA13, NDUFA2, NEK11, NF1, NFAM1, NFATC1, NFATC2, NFATC4, NFE2L2, NGEF, NGF, NLK, NLRC5, NLRP12, NLRX1, NME2, NOC2L, NOL3, NOP2, NOS1, NOS1AP, NOTCH1, NPHP4, NPHS1, NPNT, NPPA, NPR1, NPRL3, NPTN, NR1D1, NR3C2, NRXN1, NTRK2, OBSCN, OSBPL8, P2RY6, PAFAH1B1, PAK4, PAK5, PASK, PATJ, PAX2, PCDHA6, PCLO, PDCD6, PDE8A, PDE9A, PDGFD, PDGFRA, PDPK1, PDZD2, PDZD3, PEX5L, PHF20, PHLPP2, PIH1D1, PIK3CB, PIK3CG, PIK3IP1, PIK3R1, PIK3R5, PIKFYVE, PIP4K2A, PIP5K1C, PITPNM2, PLA2G4C, PLA2G6, PLAUR, PLCB1, PLCB2, PLCB4, PLCD4, PLCE1, PLCG2, PLCH2, PLCL2, PLD1, PLEK, PLEKHG2, PLVAP, PLXNB1, PML, POU4F2, PPM1F, PPP1R13B, PPP1R16B, PPP3CA, PPP3CB, PPP4C, PRAG1, PRKAG2, PRKAR1B, PRKCA, PRKCB, PRKCE, PRKCH, PRKCI, PRKCQ, PRKCZ, PRKD2, PRKDC, PRKN, PRMT1, PRPF19, PRSS33, PSCA, PSD, PSD2, PSD3, PSD4, PSEN1, PSMA5, PSMA6, PSMA7, PSMC2, PSMC3, PSMC6, PSMD2, PSMD7, PTEN, PTGES2, PTK2, PTPN11, PTPRA, PTPRC, PUM1, RAB22A, RAB29, RAB2A, RAB35, RAB3D, RAB40B, RAB43, RAB4A, RAB5A, RACK1, RAD9A, RALGAPA2, RALGDS, RANBP9, RAP1A, RAP1GAP, RAP1GAP2, RAP2B, RAPGEF1, RAPGEF5, RASA1, RASA2, RASA3, RASAL2, RASGRF1, RASGRP4, RASIP1, RASSF1, |
| GO:0031401 | positive regulation of protein modification process | 7,40E-03 | [5, 6, 7, 8] | 311,00 | 23,37 | ARNTL, ASPSCR1, ATG14, ATM, ATP2B4, BCAR3, BCL10, BCL2, BCL6, BDNF, BDNF-AS, BIRC2, BMP2, BMP3, BMP7, BMP8B, BMPER, BRD4, BRMS1, BTC, BTRC, CALM2, CAMTA1, CAPRIN2, CARD10, CARD14, CASR, CAV1, CCL16, CCND2, CCNY, CD33, CDC20, CDC20B, CDC25B, CDK1, CDK5R1, CDKN1A, CDKN2A, CEP295, CHAMP1, CHEK2, CIB1, COMMD1, CUL3, CXCR4, DAB1, DAG1, DAXX, DCUN1D1, DDR2, DGKQ, DIPK2A, DLC1, DLG1, DLG4, DOCK7, DOK4, DOK7, DSTYK, DUSP19, DUSP26, EDAR, EFNA5, EMC10, ENPP2, EPHA4, ERBB4, EREG, ETAA1, ETFA, EZH2, F3, FAM161A, FANCM, FASN, FGD2, FGF2, FGF7, FLT4, FNIP2, FNTA, FRS2, FZD10, FZD5, FZR1, GADD45G, GCG, GCNT2, GFRAL, GLIPR2, GNAI2, GORASP1, GPER1, GPR55, GPRC5B, GPRC5C, GREM1, GRM1, GRM4, GSK3A, HACD3, HCLS1, HDAC4, HES1, HMGB1, HSP90AB1, HTR2A, IGF1, IGF1R, IL31RA, IL6R, IL6ST, ING3, INSR, IQGAP3, IRAK2, ISL1, ITGA1, ITGA5, JAK2, JARID2, KAT7, KIDINS220, KITLG, KMT2E, KNDC1, KPNA7, KRAS, KSR1, LEPR, LLGL1, LPAR1, LRP1, LRRK1, MAGI2, MAP2K2, MAP2K5, MAP3K11, MAP3K13, MAP3K19, MAP3K2, MAP3K20, MAP3K5, MAP3K6, MAP4K4, MAP4K5, MAPK8IP2, MAPK8IP3, MAPK9, MAPKAP1, MAPKAPK5, MMD, MST1R, MTA1, MYD88, MYO1C, NCKAP1L, NDFIP2, NDRG4, NEDD9, NGF, NOS1, NOS1AP, NOTCH1, NPNT, NPTN, NRXN1, NSD3, NSMF, NTRK2, OSBPL8, P2RY6, PAF1, PAK4, PAK5, PARP14, PAX7, PDCD6, PDE8A, PDGFD, PDGFRA, PDPK1, PDZD2, PFN2, PHF19, PIH1D1, PIK3CB, PIK3CG, PIK3R5, PKD1, PLA2G6, PLAUR, PLCB1, PLCE1, POMT1, PPARGC1A, PPP1R16B, PPP2R3C, PPP3CA, PRKAG2, PRKAR1B, PRKCA, PRKCE, PRKCZ, PRKD2, PRKN, PRLR, PRMT1, PSEN1, PTEN, PTK2, PTPN11, PTPRA, PTPRC, RACK1, RAP1A, RAP2B, RAPGEF1, RASSF5, RBPMS, RBX1, RCHY1, RELN, RET, RHBDF2, RHOA, RICTOR, RIPK1, RNF111, RNF180, |
| GO:0031401 | positive regulation of protein modification process | 7,40E-03 | [5, 6, 7, 8] | 311,00 | 23,37 | ARNTL, ASPSCR1, ATG14, ATM, ATP2B4, BCAR3, BCL10, BCL2, BCL6, BDNF, BDNF-AS, BIRC2, BMP2, BMP3, BMP7, BMP8B, BMPER, BRD4, BRMS1, BTC, BTRC, CALM2, CAMTA1, CAPRIN2, CARD10, CARD14, CASR, CAV1, CCL16, CCND2, CCNY, CD33, CDC20, CDC20B, CDC25B, CDK1, CDK5R1, CDKN1A, CDKN2A, CEP295, CHAMP1, CHEK2, CIB1, COMMD1, CUL3, CXCR4, DAB1, DAG1, DAXX, DCUN1D1, DDR2, DGKQ, DIPK2A, DLC1, DLG1, DLG4, DOCK7, DOK4, DOK7, DSTYK, DUSP19, DUSP26, EDAR, EFNA5, EMC10, ENPP2, EPHA4, ERBB4, EREG, ETAA1, ETFA, EZH2, F3, FAM161A, FANCM, FASN, FGD2, FGF2, FGF7, FLT4, FNIP2, FNTA, FRS2, FZD10, FZD5, FZR1, GADD45G, GCG, GCNT2, GFRAL, GLIPR2, GNAI2, GORASP1, GPER1, GPR55, GPRC5B, GPRC5C, GREM1, GRM1, GRM4, GSK3A, HACD3, HCLS1, HDAC4, HES1, HMGB1, HSP90AB1, HTR2A, IGF1, IGF1R, IL31RA, IL6R, IL6ST, ING3, INSR, IQGAP3, IRAK2, ISL1, ITGA1, ITGA5, JAK2, JARID2, KAT7, KIDINS220, KITLG, KMT2E, KNDC1, KPNA7, KRAS, KSR1, LEPR, LLGL1, LPAR1, LRP1, LRRK1, MAGI2, MAP2K2, MAP2K5, MAP3K11, MAP3K13, MAP3K19, MAP3K2, MAP3K20, MAP3K5, MAP3K6, MAP4K4, MAP4K5, MAPK8IP2, MAPK8IP3, MAPK9, MAPKAP1, MAPKAPK5, MMD, MST1R, MTA1, MYD88, MYO1C, NCKAP1L, NDFIP2, NDRG4, NEDD9, NGF, NOS1, NOS1AP, NOTCH1, NPNT, NPTN, NRXN1, NSD3, NSMF, NTRK2, OSBPL8, P2RY6, PAF1, PAK4, PAK5, PARP14, PAX7, PDCD6, PDE8A, PDGFD, PDGFRA, PDPK1, PDZD2, PFN2, PHF19, PIH1D1, PIK3CB, PIK3CG, PIK3R5, PKD1, PLA2G6, PLAUR, PLCB1, PLCE1, POMT1, PPARGC1A, PPP1R16B, PPP2R3C, PPP3CA, PRKAG2, PRKAR1B, PRKCA, PRKCE, PRKCZ, PRKD2, PRKN, PRLR, PRMT1, PSEN1, PTEN, PTK2, PTPN11, PTPRA, PTPRC, RACK1, RAP1A, RAP2B, RAPGEF1, RASSF5, RBPMS, RBX1, RCHY1, RELN, RET, RHBDF2, RHOA, RICTOR, RIPK1, RNF111, RNF180, |
| GO:0051336 | regulation of hydrolase activity | 4,98E-03 | [4] | 321,00 | 23,40 | ARHGAP12, ARHGAP17, ARHGAP21, ARHGAP29, ARHGAP39, ARHGAP40, ARHGAP42, ARHGAP45, ARHGDIA, ARHGDIB, ARHGDIG, ARHGEF10L, ARHGEF16, ARHGEF7, ARL1, ARL6IP5, ASAP1, ASAP2, ASPH, ATP13A2, ATP2A3, BBS4, BCAR3, BCAS3, BCL10, BCL6, BDNF, BDNF-AS, BIRC2, BMP2, BRSK2, CACNA1A, CALM2, CAMK2D, CASR, CAV1, CCL16, CD33, CDKN2A, CHN2, CNST, CRB2, CRIM1, CRY2, CSRNP2, CSRNP3, CST9, CYCS, CYFIP2, DAPK1, DENND1A, DENND1B, DEPDC1, DEPDC5, DLC1, DLG2, DNAJB4, DNAJB6, DNAJC15, DNAL4, DNM1L, DNPEP, DOCK10, DOCK7, DOCK8, EFNA5, EGR2, EIF2AK3, ELMOD1, EPHA1, EPHA4, EPHB3, ESR1, EZH2, F11R, F3, FAM13A, FAM13B, FARP1, FASN, FGD2, FGF2, FICD, FNTA, FZD10, GNAI2, GNB5, GPER1, GPR137B, GPR55, GPRIN1, GPSM1, GRAMD4, HACD3, HDAC9, HIP1, HIP1R, HMGB1, HRG, HSP90AB1, HSPD1, HTR2A, HTT, IFI16, IKBKB, IQGAP2, IQGAP3, IQSEC1, ITGA1, ITIH5, ITK, JAK2, KALRN, LEPR, LGMN, LIMS1, LLGL1, LMTK2, LPAR1, LPAR2, LPL, LRP1, MAGI2, MAL, MAP2, MAP2K5, MAP3K5, MAP4K4, MAPK7, MAPK8, MARF1, MCM2, MEF2C, MGMT, MRTFA, MSH2, MSH3, MTMR9, MYO1D, MYO9B, NCKAP1L, NDE1, NDUFA13, NEDD9, NF1, NGEF, NGF, NLRP12, NOL3, NOS1, NPNT, NPRL3, NTRK2, OASL, OPRPN, P2RY6, PAFAH1B1, PAPLN, PAX2, PCSK5, PCSK6, PDCD5, PDCD6, PDGFRA, PDPK1, PDZD2, PFN2, PHACTR1, PIH1D1, PKMYT1, PLAUR, PLCB1, PLCB2, PLEK, PLIN5, PLXNA1, PLXNA2, PLXNB1, PLXNC1, PLXND1, PML, PPARG, PPARGC1B, PPM1F, PPP1R11, PPP1R16A, PPP1R16B, PPP2R2B, PPP2R2C, PPP3CA, PRDX5, PRKCZ, PRKD2, PTK2, PTPRA, PTPRC, PTPRN2, PZP, RAB4A, RABGAP1, RACK1, RALGAPA2, RANBP1, RAP1A, RAP1GAP, RAP1GAP2, RAPGEF1, RASA1, RASA2, RASA3, RASAL2, RASGRF1, RASGRP4, RASIP1, RBM26, RCAN2, REST, RGMA, RGS12, RGS3, RGS6, RGS7, RGS9, RHOA, RHOG, RICTOR, RIN3, RIPK1, ROBO1, ROCK2, RTKN, S1PR4, SCRIB, SEMA4D, SERPINA4, SERPINA5, SERPINB6, SERPINE2, SERPINF1, SERPINI1, |
| GO:0051336 | regulation of hydrolase activity | 4,98E-03 | [4] | 321,00 | 23,40 | ARHGAP12, ARHGAP17, ARHGAP21, ARHGAP29, ARHGAP39, ARHGAP40, ARHGAP42, ARHGAP45, ARHGDIA, ARHGDIB, ARHGDIG, ARHGEF10L, ARHGEF16, ARHGEF7, ARL1, ARL6IP5, ASAP1, ASAP2, ASPH, ATP13A2, ATP2A3, BBS4, BCAR3, BCAS3, BCL10, BCL6, BDNF, BDNF-AS, BIRC2, BMP2, BRSK2, CACNA1A, CALM2, CAMK2D, CASR, CAV1, CCL16, CD33, CDKN2A, CHN2, CNST, CRB2, CRIM1, CRY2, CSRNP2, CSRNP3, CST9, CYCS, CYFIP2, DAPK1, DENND1A, DENND1B, DEPDC1, DEPDC5, DLC1, DLG2, DNAJB4, DNAJB6, DNAJC15, DNAL4, DNM1L, DNPEP, DOCK10, DOCK7, DOCK8, EFNA5, EGR2, EIF2AK3, ELMOD1, EPHA1, EPHA4, EPHB3, ESR1, EZH2, F11R, F3, FAM13A, FAM13B, FARP1, FASN, FGD2, FGF2, FICD, FNTA, FZD10, GNAI2, GNB5, GPER1, GPR137B, GPR55, GPRIN1, GPSM1, GRAMD4, HACD3, HDAC9, HIP1, HIP1R, HMGB1, HRG, HSP90AB1, HSPD1, HTR2A, HTT, IFI16, IKBKB, IQGAP2, IQGAP3, IQSEC1, ITGA1, ITIH5, ITK, JAK2, KALRN, LEPR, LGMN, LIMS1, LLGL1, LMTK2, LPAR1, LPAR2, LPL, LRP1, MAGI2, MAL, MAP2, MAP2K5, MAP3K5, MAP4K4, MAPK7, MAPK8, MARF1, MCM2, MEF2C, MGMT, MRTFA, MSH2, MSH3, MTMR9, MYO1D, MYO9B, NCKAP1L, NDE1, NDUFA13, NEDD9, NF1, NGEF, NGF, NLRP12, NOL3, NOS1, NPNT, NPRL3, NTRK2, OASL, OPRPN, P2RY6, PAFAH1B1, PAPLN, PAX2, PCSK5, PCSK6, PDCD5, PDCD6, PDGFRA, PDPK1, PDZD2, PFN2, PHACTR1, PIH1D1, PKMYT1, PLAUR, PLCB1, PLCB2, PLEK, PLIN5, PLXNA1, PLXNA2, PLXNB1, PLXNC1, PLXND1, PML, PPARG, PPARGC1B, PPM1F, PPP1R11, PPP1R16A, PPP1R16B, PPP2R2B, PPP2R2C, PPP3CA, PRDX5, PRKCZ, PRKD2, PTK2, PTPRA, PTPRC, PTPRN2, PZP, RAB4A, RABGAP1, RACK1, RALGAPA2, RANBP1, RAP1A, RAP1GAP, RAP1GAP2, RAPGEF1, RASA1, RASA2, RASA3, RASAL2, RASGRF1, RASGRP4, RASIP1, RBM26, RCAN2, REST, RGMA, RGS12, RGS3, RGS6, RGS7, RGS9, RHOA, RHOG, RICTOR, RIN3, RIPK1, ROBO1, ROCK2, RTKN, S1PR4, SCRIB, SEMA4D, SERPINA4, SERPINA5, SERPINB6, SERPINE2, SERPINF1, SERPINI1, |
| GO:0009968 | negative regulation of signal transduction | 5,06E-03 | [3, 4, 5, 6] | 316,00 | 23,41 | BEND6, BICC1, BMP2, BMP7, BMPER, BTC, BTRC, CALM2, CAV1, CBFA2T2, CCAR2, CCDC3, CCDC88C, CD46, CD47, CELF4, CHST11, CIB1, CNOT1, CNOT7, COL2A1, CREB3L1, CRY2, CSNK1A1L, CTNNBIP1, CTTN, CUL3, CYLD, DAB1, DAG1, DCN, DDIT4, DEPDC5, DGKZ, DHRS3, DKK3, DLC1, DLG1, DLL1, DLL4, DLX2, DNM1, DUSP19, DUSP26, EIF4A3, ELF1, ENHO, EPS15L1, EREG, ESR1, EYA2, EYA4, EZH2, FAM89B, FASN, FBN1, FBN2, FBP1, FGF2, FKTN, FNIP2, FOXO1, FOXP1, GFRAL, GLI2, GLI3, GLIS2, GNAI2, GPER1, GPR161, GRAMD4, GRB10, GRB2, GREM1, GSK3A, HDAC9, HECW1, HEG1, HGS, HRG, HTRA3, HTT, IGF1, IGF1R, IL17RD, ING3, INPP5F, INSIG2, ISL1, ITCH, ITFG2, ITGA1, KANK1, KCTD10, KCTD6, KIF26A, KREMEN2, LATS2, LAX1, LDLRAD4, LGMN, LPAR1, LRP1, LRP2, LRPAP1, LRRC14, LTBP1, LZTS2, MAGI2, MAP2K5, MAPK7, MAPK8IP2, MAPKAP1, MAPKAPK5, MARK3, MCL1, MECOM, MEGF8, MEN1, MGRN1, MLLT3, MRTFA, MTMR2, MTNR1B, NCK2, NCOR2, NDRG4, NDUFA13, NDUFA2, NF1, NFATC1, NFATC4, NFE2L2, NKD1, NLK, NLRC5, NLRP12, NLRX1, NOC2L, NOL3, NOTCH1, NOTCH3, NPHP4, NPPA, NPRL3, NR1D1, NXN, OTUD4, PAFAH1B1, PAK5, PALM, PARP14, PBLD, PDCD6, PDPK1, PDZD2, PDZD3, PEG10, PELI3, PHLPP2, PIH1D1, PIK3IP1, PLAUR, PLCL2, PLEK, PLIN5, PPARG, PRDM16, PRKCA, PRKCB, PRKCQ, PRKCZ, PRKD2, PRKN, PSCA, PSEN1, PSMA5, PSMA6, PSMA7, PSMC2, PSMC3, PSMC6, PSMD2, PSMD7, PTCH2, PTEN, PTPN3, PTPRC, PTPRO, PXDN, RACK1, RANBP9, RAPGEF1, RASA1, RASA2, RASA3, RASIP1, RB1, RBX1, RGS12, RGS22, RGS3, RGS6, RGS7, RGS9, RHOA, RIOK3, RIPK1, RIPOR2, RITA1, ROBO1, ROR2, RORA, RPH3AL, RPS6KB1, RTKN2, SALL3, SCAI, SCYL2, SELENOS, SERPINE2, SH3BP4, SH3RF2, SHH, SHISA6, SIRT3, SKI, SKIL, SLA2, SLC6A4, SLC9A3R1, SMAD3, SMAD6, SMAD7, |
| GO:1902531 | regulation of intracellular signal transduction | 1,14E-05 | [4, 5, 6] | 451,00 | 23,51 | ARFGEF3, ARHGAP10, ARHGAP12, ARHGAP17, ARHGAP21, ARHGAP29, ARHGAP39, ARHGAP40, ARHGAP42, ARHGAP45, ARHGDIA, ARHGDIB, ARHGDIG, ARHGEF16, ARHGEF18, ARHGEF2, ARHGEF26, ARHGEF7, ARL6IP5, ARNTL, ATF3, ATM, ATP2B4, AURKB, BANP, BCAR3, BCL10, BCL2, BCL3, BCL6, BIRC2, BMP2, BMP7, BMPER, BRD1, BRD4, BTC, BTRC, CALM2, CAMK2D, CAMTA1, CARD10, CARD11, CARD14, CASR, CAV1, CCAR2, CCL16, CDH13, CDK1, CDK2, CDK5R1, CHD3, CHD5, CHEK1, CHEK2, CHN2, CIB1, COL3A1, CREB3L1, CUL3, CXCR4, CXXC5, CYLD, CYTH1, DAG1, DAXX, DCN, DDIT4, DDX60, DENND1A, DEPDC5, DGKQ, DHX36, DIPK2A, DLC1, DLG1, DNA2, DNM1L, DOK4, DSTYK, DUSP19, DUSP26, EDAR, EEF1E1-BLOC1S5, EGR2, EHMT1, EHMT2, EIF2AK3, EMC10, EPHA4, EPHB1, EPS8L1, ERBB4, EREG, ESR1, ETFA, EZH2, F11R, F2RL3, F3, FAM110A, FAM13A, FAM13B, FASN, FBP1, FGD2, FGF2, FGF22, FGF6, FGF7, FKTN, FLT4, FNIP2, FOXO1, FRS2, FZD10, FZD5, GAB1, GADD45G, GCG, GCNT2, GFRAL, GLIPR2, GNA12, GNAI2, GPER1, GPR137B, GPR17, GPR4, GPR55, GPR62, GPRC5B, GRB2, GREM1, GRM1, GRM2, GRM4, GSK3A, HACD3, HCLS1, HDAC9, HEG1, HGS, HINT1, HIP1, HMGB1, HNRNPK, HRC, HSP90AB1, HTR2A, HTT, HUS1, IER3, IGF1, IGF1R, IKBKB, IL20RA, IL37, ING3, INPP5F, INSR, IQGAP3, IQSEC1, IQSEC3, IRAK2, IRS2, ITCH, ITFG2, ITGA1, JAK2, JPH3, KALRN, KANK1, KAT6A, KCTD10, KIDINS220, KITLG, KRAS, KSR1, LAX1, LIMS1, LMCD1, LPAR1, LRP1, LRP2, LRPAP1, LRRK1, LURAP1, MAGI2, MAP2K2, MAP2K5, MAP3K11, MAP3K13, MAP3K2, MAP3K20, MAP3K5, MAP3K6, MAP4K4, MAP4K5, MAPK7, MAPK8IP2, MAPK8IP3, MAPKAP1, MAPKAPK5, MARK3, MBD3, MCF2L, MCL1, MECOM, MEN1, MGRN1, MIB2, MIER1, MRPL23, MST1R, MTNR1B, MYD88, MYO5A, MYO9B, NACC2, NCK2, NDFIP2, NDRG4, NDUFA13, NDUFA2, NF1, NFE2L2, NGEF, NGF, NLRP12, NLRX1, NOC2L, NOL3, NOP2, NOS1, NOS1AP, NOTCH1, NPNT, NPPA, NPR1, NPRL3, NPTN, NR1D1, NR3C2, NRXN1, NTRK2, OBSCN, OSBPL8, P2RY6, PAFAH1B1, PDCD6, PDE8A, PDGFD, PDGFRA, PDPK1, PDZD2, PDZD3, PEX5L, PHF20, PHLPP2, PIH1D1, PIK3CB, PIK3CG, PIK3IP1, PIK3R1, PIK3R5, PIP4K2A, PIP5K1C, PLA2G6, PLAUR, PLCB1, PLCE1, PLEK, PLEKHG2, PLXNB1, PML, POU4F2, PPP1R13B, PPP1R16B, PPP3CA, PRAG1, PRKAG2, PRKAR1B, PRKCA, PRKCB, PRKCE, PRKCZ, PRKD2, PRKN, PRMT1, PSCA, PSD, PSD2, PSD3, PSD4, PSEN1, PTEN, PTGES2, PTK2, PTPN11, PTPRC, PUM1, RACK1, RAD9A, RALGAPA2, RANBP9, RAP1A, RAP1GAP, RAP1GAP2, RAPGEF1, RASA1, |
| GO:2000026 | regulation of multicellular organismal development | 3,00E-07 | [3, 4] | 523,00 | 23,57 | AP2A2, AP2S1, AP3B1, APPL2, ARF1, ARHGDIA, ARHGDIB, ARHGEF2, ARHGEF7, ARNTL, ASAP1, ASCL1, ATF1, ATF2, ATP2B4, ATP8A2, BAG6, BCL11A, BCL11B, BCL2, BCL6, BDNF, BDNF-AS, BEND6, BHLHE40, BMERB1, BMP1, BMP2, BMP7, BMPER, BRD1, BRINP2, BRSK2, C1QC, C21orf91, CACNA1A, CAMK2B, CAMK4, CAPRIN2, CARD10, CARD11, CASZ1, CAV1, CBFA2T2, CCBE1, CCN4, CCN6, CCR2, CD46, CDC20, CDH4, CDK1, CDK5R1, CDKN2A, CELF4, CELSR1, CEND1, CHAMP1, CHRNB2, CIB1, CITED2, CLASP1, CLASP2, CNTN4, COBL, COL3A1, COL4A2, CPNE5, CRB2, CREB3L1, CREB3L2, CSMD3, CSNK1E, CTNNA2, CTNNBIP1, CTTN, CUX1, CXCR4, CYFIP1, CYLD, CYP1B1, DAB1, DAG1, DBN1, DCN, DDR2, DDX6, DENND5A, DHX36, DLG1, DLG4, DLL1, DLL4, DLX2, DNM1L, DOCK7, DTX1, E2F2, EFNA5, EGR2, EHMT1, EIF2AK3, EIF2AK4, EIF4E, EIF4ENIF1, EMC10, ENC1, ENPP2, EPHA1, EPHA4, EPHB1, EPHB3, ERAP1, ERBB4, ESR1, ETS1, EXOSC6, EZH2, F11R, F3, FARP1, FBLN5, FBN1, FBN2, FBXW8, FERD3L, FEZ1, FEZF1, FEZF2, FGF2, FGF7, FLRT1, FOXP1, FRS2, FZD5, GAB1, GAK, GARS1, GATA5, GCNT2, GFI1, GLI2, GLI3, GLIPR2, GORASP1, GPC6, GPER1, GPR137B, GPR161, GPR4, GPR55, GPRC5B, GPRIN1, GREM1, GRID2, GSK3A, GSX2, H3C6, H3C8, HCLS1, HDAC4, HDAC9, HECW1, HELT, HES1, HES3, HES4, HGS, HMGB1, HNF1B, HOXC11, HOXD13, HRG, HTATIP2, IFRD1, IGF1, IHH, IKBKB, IL17D, IL17RD, IL1RAP, IL6R, IL6ST, INPP5F, INSR, IQGAP3, ISL1, ISM1, ITCH, ITGA5, ITGB2, JARID2, KALRN, KANK1, KAT2B, KAT7, KCNK2, KDM4C, KIDINS220, KIF1A, KIF2A, KITLG, KLF7, KMT2D, KMT2E, KMT5C, KNDC1, KRIT1, KRT1, KRT84, LAMA2, LAMA5, LDB2, LDLR, LDLRAD4, LIG4, LINGO1, LLGL1, LONP1, LOXL2, LOXL3, LPAR1, LRCH4, LRFN3, LRIG2, LRP1, LRP2, LRRC4C, LTBP2, LZTS1, MACF1, MAF, MAFG, MAG, MAGI2, MAP2, MAP2K5, MAP3K13, MAP6, MAPK7, MAPK8IP3, MDGA1, MEF2C, MEGF8, MEIS2, MKS1, MLLT3, MMD, MME, MSH2, MTMR2, MYRF, NCKAP1L, NCOA2, NDRG4, NECTIN1, NEDD4L, NEPRO, NF1, NFAM1, NFATC1, NFATC2, NFATC4, NFE2L2, NFIB, NGEF, NGF, NKD1, NLGN2, NME2, NOL3, NOS1, NOTCH1, NOTCH3, NOTCH4, NPR1, NPTN, NR1D1, NRXN1, NSMF, NTNG1, NTNG2, NTRK2, NUMB, OBSL1, OLFM1, OLIG2, P4HTM, PACSIN1, PAF1, PAFAH1B1, PAK4, PARD3, PAX2, PAX8, PAX9, PBLD, PBX1, PDCD6, PDCL3, PDGFD, PDPK1, PDZD2, PER2, PHACTR1, PHLDB1, PIK3R1, PIM1, PKM, PLCB1, PLEKHA4, PLK5, PLXNA1, PLXNA2, PLXNB1, PLXNC1, PLXND1, PML, POU4F2, PPARG, PPARGC1B, PPP1R16B, PPP2R3C, PPP3CA, PRAG1, PRICKLE2, PRKCA, PRKCB, PRKCH, PRKCI, PRKCQ, PRKCZ, PRKD2, PRKDC, PRKN, PRLR, PRMT1, PRPF19, PRUNE1, PSEN1, PSMA5, PSMA6, PSMA7, PSMC2, PSMC3, PSMC6, PSMD2, PSMD7, PTCH2, PTEN, PTF1A, PTGIS, PTK2, PTPN11, PTPRC, PTPRF, PTPRO, PTPRZ1, PUS7, RAB29, RACK1, RANBP1, RAP1A, RAP1GAP, RAP1GAP2, RAPGEF1, RARA, RB1, RBFOX1, RBFOX2, RBM19, RBP4, |

| | | | | | | |
|---|---|---|---|---|---|---|
| GO:0006812 | cation transport | 3,74E-03 | [5] | 305,00 | 23,61 | ATP2B2, ATP2B4, ATP5F1A, ATP5ME, ATP6V0A1, ATP6V0D1, ATP6V1A, ATP6V1H, ATP8A1, BCL2, BEST1, C4orf3, CACNA1A, CACNA1B, CACNA1E, CACNA1G, CACNA1H, CACNA1I, CACNA2D2, CACNA2D3, CACNA2D4, CACNB2, CACNG1, CACNG6, CACNG8, CALM2, CAMK2B, CAMK2D, CASR, CAV1, CCR2, CD33, CDH23, CDK2, CHRNB2, CHRNB3, CHRND, CHRNE, CLCN3, CLDN16, CNGA3, CNIH3, CNNM4, COMMD1, COMMD9, COX5A, COX6A1, COX7C, CTSS, CXCR4, CYC1, DAPK1, DLG1, DLG2, DLG4, DNAJC30, DNM1L, DPP10, F2RL3, F3, FAM155A, FGF12, FGF14, FGF2, FLVCR2, FXYD5, FXYD6, GCG, GJA5, GNAI2, GNB5, GPER1, GPRIN1, GRIA4, GRIK3, GRIK4, GSG1L, HCN1, HCN2, HCN4, HECW1, HRC, HRG, HTR1A, HTR2A, HTR3A, HTR3C, HTT, IL16, ITPR2, ITPR3, JPH3, KCNAB2, KCNB1, KCNB2, KCNC1, KCNC3, KCNE1, KCNF1, KCNH2, KCNIP4, KCNJ14, KCNJ15, KCNJ2, KCNJ3, KCNJ6, KCNK2, KCNMA1, KCNQ1, KCNQ2, KCNV1, KLHL3, LLGL1, LMTK2, LRP2, MAPK8IP2, MCUR1, MEF2C, MELTF, MFSD3, MRS2, MTNR1B, MYLK, MYO5A, NALCN, NDFIP2, NDUFA4L2, NECTIN1, NEDD4L, NIPA1, NKAIN1, NLGN2, NNT, NOL3, NOS1, NOS1AP, NPPA, NRXN1, OCA2, ORAI1, ORAI2, P2RY6, PANX2, PDPK1, PIEZO1, PIK3CG, PKD1, PKD1L1, PLA2G6, PLCB1, PLCB2, PLCB4, PLCD4, PLCE1, PLCG2, PLCH2, PLCL2, PML, PPARGC1A, PPP3CA, PRKCA, PRKCB, PRKCE, PRKCZ, PRKD2, PRKN, PSEN1, PTEN, PTPN3, PTPRC, RAB43, RASA1, RASA3, RASGRF1, RCVRN, RELN, RGS9, RHOA, RYR1, RYR2, RYR3, SCARA5, SCN1A, SCN2A, SCN3B, SCN5A, SERPINE2, SGK1, SHANK2, SHISA6, SHISA7, SHISA9, SLC10A7, SLC11A2, SLC12A7, SLC12A9, SLC13A3, SLC13A5, SLC15A4, SLC17A2, SLC17A4, SLC17A6, SLC17A7, SLC1A1, SLC1A3, SLC22A18, SLC22A5, SLC24A4, SLC25A45, SLC2A9, SLC30A2, SLC30A6, SLC38A8, SLC38A9, SLC39A10, SLC39A13, SLC39A7, |
| GO:0051094 | positive regulation of developmental process | 5,34E-04 | [2, 3, 4] | 349,00 | 23,61 | ARHGEF2, ARHGEF7, ARNTL, ASCL1, ASXL2, ATF1, ATP8A2, BBS4, BCL2, BCL6, BDNF, BDNF-AS, BEND6, BLOC1S5, BMP1, BMP2, BMP7, BMPER, BNC1, BRD1, BRINP2, BTC, C21orf91, CACNA2D2, CAMK2B, CAPRIN2, CARMIL1, CBFA2T2, CCBE1, CCDC3, CCN4, CCR2, CD46, CD53, CDC20, CDH4, CDK1, CDKN2A, CHAMP1, CHEK2, CIB1, CITED2, CLASP1, CLASP2, COBL, CORO1B, CPNE5, CRB2, CREB3L2, CSNK1E, CSPG5, CTNNA2, CTNNBIP1, CTTN, CUX1, CXCR4, CYFIP1, CYLD, CYP1B1, DAB1, DAG1, DBN1, DCN, DDHD2, DDR2, DHX36, DLG4, DLL1, DLX2, DNM1L, EFNA5, EGR2, EMC10, ENC1, ENPP2, EPHA1, EPHA4, EPHB1, EPHB3, ERAP1, ERBB4, ETS1, EXOSC6, EZH2, F11R, F3, FBN2, FBXW8, FEZ1, FEZF1, FEZF2, FGF2, FGF7, FLRT1, FRS2, GAB1, GARS1, GATA5, GCNT2, GLI2, GLI3, GLIPR2, GPER1, GPRC5B, GREM1, GRID2, GSX2, HCLS1, HDAC9, HES1, HMGB1, HOXC11, HSP90AB1, HTR2A, IGF1, IHH, IL1RAP, IL6R, IL6ST, INSR, IQGAP3, ISL1, ITGA5, ITGB2, JAK2, KALRN, KAT7, KDM2B, KDM4C, KIDINS220, KIF2A, KITLG, KMT5C, KRAS, LIG4, LIMS1, LLGL1, LONP1, LOXL2, LPAR1, LPL, LRCH4, LRP1, LRP2, LRP5, LTBP2, LZTS1, MACF1, MAG, MAGI2, MAP3K13, MAP3K5, MAP6, MAPK8IP3, MAPK9, MEF2C, MEGF8, METRNL, MMD, MME, MSH2, MYRF, NAPEPLD, NCKAP1L, NCOA2, NDRG4, NEDD4L, NEDD9, NFATC2, NFE2L2, NGF, NLGN2, NME2, NOTCH1, NPNT, NPTN, NRXN1, NSMF, NTRK2, NUMB, OBSL1, OLFM1, OLFM2, OLIG2, PACSIN1, PAFAH1B1, PAK4, PARD3, PAX2, PAX8, PDCD6, PDCL3, PDGFD, PDPK1, PDZD2, PHLDB1, PIM1, PKM, PLCB1, PLK5, PLXNA1, PLXNA2, PLXNB1, PLXNC1, PLXND1, POU4F2, PPARG, PPARGC1A, PPARGC1B, PPIB, PPP1R16B, PPP2R3C, PPP3CA, PRDM16, PRKCA, PRKCB, PRKCH, PRKCI, PRKCZ, PRKD2, PRKDC, PRKN, PRMT1, PRPF19, PSEN1, PSMA7, PTCH2, PTEN, PTGIS, PTPRC, PTPRZ1, RACK1, RANBP1, RAP1A, RAPGEF1, RARA, RB1, RBM19, RELN, REST, RET, RGMA, RHEB, RHOA, RIPK1, RIPOR2, ROBO1, ROR1, ROR2, RORA, RREB1, RUFY3, RUNX1, RUNX3, SASH1, SCIN, SDCBP, |
| GO:0051240 | positive regulation of multicellular organismal process | 1,55E-06 | [2, 3, 4] | 455,00 | 23,81 | ANGPT4, ANKRD17, ANO6, ANXA2, AP2A1, AP3B1, APC, APPL1, APPL2, ARF1, ARHGDIA, ARHGEF2, ARHGEF7, ARNTL, ASCL1, ATF1, ATF2, ATP1A3, ATP1A4, ATP2A2, ATP5F1A, ATP8A2, BBS4, BCAR1, BCAS3, BCL10, BCL2, BCL3, BCL6, BDNF, BDNF-AS, BEND6, BIRC2, BMP1, BMP2, BMP7, BMPER, BNC1, BRD1, BRINP2, C21orf91, CACNA2D2, CADM1, CAMK2B, CAMK2D, CAPRIN2, CARD11, CASR, CAV1, CBFA2T2, CCBE1, CCN4, CCR2, CCR6, CD46, CDC20, CDH4, CDK1, CHAMP1, CIB1, CITED2, CLASP1, CLASP2, COBL, CPNE5, CRB2, CREB3L2, CSNK1E, CTNNBIP1, CTTN, CUX1, CXCR4, CYFIP1, CYLD, CYP1B1, DAB1, DAG1, DBN1, DDR2, DDX60, DEF8, DHX15, DHX34, DHX36, DLG4, DLL1, DLX2, DNM1L, EFNA5, EGR2, EHMT1, EIF2AK3, EMC10, ENC1, ENPP2, EPB41L4B, EPHA1, EPHA4, EPHB1, EPHB3, ERAP1, ERBB4, EREG, ESRRG, ETS1, EXOSC6, EZH2, F11R, F12, F3, FABP5, FBN2, FBXW8, FEZ1, FEZF1, FEZF2, FGF2, FGF7, FH, FLRT1, FLT4, FOXP1, FRMD8, FRS2, FZD5, GAB1, GADD45G, GARS1, GATA5, GCNT2, GJA5, GLI2, GLI3, GLIPR2, GLMN, GNAI2, GPER1, GPRC5B, GPRIN1, GRB10, GREM1, GRID2, GSK3A, GSX2, HADHA, HCAR2, HCLS1, HDAC4, HDAC9, HEG1, HES1, HLA-DPB1, HMGB1, HOOK1, HOXC11, HRC, HRG, HSPD1, HTR2A, IFI16, IFIH1, IGF1, IGF1R, IGF2BP1, IGSF9B, IHH, IL12RB2, IL16, IL17D, IL17RA, IL1RAP, IL6R, IL6ST, IQGAP3, IQSEC1, ISL1, ITGA5, ITGB2, JAK2, KALRN, KAT7, KCNQ1, KDM2B, KDM3A, KDM4C, KDM6B, KIDINS220, KIF2A, KITLG, KMT5C, KSR2, LDLR, LEPR, LGMN, LIG4, LLGL1, LONP1, LOXL2, LPAR1, LPL, LRCH4, LRP1, LRP2, LRP5, LRPAP1, LTBP2, LURAP1, LY9, LZTS1, MACF1, MAG, MAGI2, MAP3K13, MAP4K4, MAP6, MAPK8IP3, MEF2C, MEGF8, MMD, MME, MSH2, MTNR1B, MYD88, MYRF, NCKAP1L, NCOA2, NDRG4, NDUFA2, NEDD4L, NFAM1, NFATC4, NFE2L2, NGF, NLGN2, NLRP12, NLRX1, NME2, NOS1, NOS1AP, NOTCH1, NPNT, NPPA, NPR1, NPS, NPTN, NRXN1, NSMF, NTRK2, NUMB, NUS1, OBSL1, OLFM1, OLIG2, PACSIN1, PAFAH1B1, PAK4, PANX2, PARD3, PAX2, PAX8, PDCD6, PDCL3, PDE9A, PDGFD, PDPK1, PDZD2, PER2, PHLDB1, PIK3R1, PIM1, PKM, PLAC8, PLCB1, PLCG2, PLEK, PLK5, PLXNA1, PLXNA2, PLXNB1, PLXNC1, PLXND1, POLR2F, POLR3A, POLR3E, POLR3H, POU4F2, PPARG, PPARGC1A, PPARGC1B, PPIB, PPM1F, PPP1R16B, PPP2R3C, PPP3CA, PRDM16, PRKCA, PRKCB, PRKCE, PRKCH, PRKCI, PRKCQ, PRKCZ, PRKD2, PRKDC, PRKN, PRLR, PRMT1, PRPF19, PSEN1, PSMA7, PTEN, PTGIS, PTK2, PTPN11, PTPRC, PTPRZ1, PUM1, RACK1, RANBP1, RAP1A, RAPGEF1, RARA, RB1, RBM19, RELN, REST, RET, RGMA, RHEB, RHOA, |
| GO:0034220 | ion transmembrane transport | 7,13E-04 | [3, 5] | 320,00 | 23,86 | ATP12A, ATP13A2, ATP1A3, ATP1A4, ATP2A2, ATP2A3, ATP2B2, ATP2B4, ATP5F1A, ATP5ME, ATP6V0A1, ATP6V0D1, ATP6V1A, ATP6V1H, ATP8A1, ATP8B2, BEST1, C4orf3, CACNA1A, CACNA1B, CACNA1E, CACNA1G, CACNA1H, CACNA1I, CACNA2D2, CACNA2D3, CACNA2D4, CACNB2, CACNG1, CACNG6, CACNG8, CALM2, CAMK2D, CASR, CAV1, CCR2, CHRNB2, CHRNB3, CHRND, CHRNE, CLCN2, CLCN3, CLDN16, CNGA3, CNIH3, CNNM4, COMMD1, COX5A, COX6A1, COX7C, CPT1A, CPT1B, CRBN, CTNS, CTSS, CYC1, DAPK1, DLG1, DLG2, DLG4, DNAJC30, DPP10, F2RL3, FAM155A, FGF12, FGF14, FGF2, FXYD5, FXYD6, GABRA2, GABRA4, GABRB2, GABRR3, GJA5, GNB5, GPER1, GPRIN1, GRIA4, GRID2, GRIK3, GRIK4, GSG1L, HCN1, HCN2, HCN4, HECW1, HRC, HTR2A, HTR3A, HTR3C, HTT, IRS2, ITPR2, ITPR3, JPH3, KCNAB2, KCNB1, KCNB2, KCNC1, KCNC3, KCNE1, KCNF1, KCNH2, KCNIP4, KCNJ14, KCNJ15, KCNJ2, KCNJ3, KCNJ6, KCNK2, KCNMA1, KCNQ1, KCNQ2, KCNV1, LASP1, LLGL1, LRP2, LRRC8B, LRRC8D, MAPK8IP2, MCUR1, MEF2C, MFSD3, MRS2, MTNR1B, MYO5A, NALCN, NDUFA4L2, NEDD4L, NIPA1, NLGN2, NNT, NOL3, NOS1, NOS1AP, NPPA, NRXN1, OCA2, ORAI1, ORAI2, P2RY6, PACC1, PDPK1, PER2, PHLPP2, PIEZO1, PIK3CG, PKD1, PKD1L1, PLA2G6, PLCB1, PLCB2, PLCB4, PLCD4, PLCE1, PLCG2, PLCH2, PLCL2, PPARGC1A, PRKAG2, PRKCA, PRKCE, PRKCZ, PRKD2, PSEN1, PTEN, PTPN3, PTPRC, RASA1, RASA3, RASGRF1, RELN, RGS9, RIPK1, RYR1, RYR2, RYR3, S100A6, SCARA5, SCN1A, SCN2A, SCN3B, SCN5A, SGK1, SHANK2, SHISA6, SHISA7, SHISA9, SLC10A7, SLC11A2, SLC12A7, SLC12A9, SLC13A3, SLC13A5, SLC15A4, SLC16A5, SLC16A7, SLC17A2, SLC17A4, SLC17A6, SLC17A7, SLC1A1, SLC1A3, SLC1A7, SLC22A5, SLC24A4, SLC25A10, SLC25A13, SLC25A21, SLC25A22, SLC25A41, SLC25A45, SLC26A5, SLC2A9, SLC30A2, SLC30A6, SLC35B3, SLC35B4, SLC37A2, SLC38A8, SLC38A9, SLC39A10, SLC39A13, SLC39A7, SLC39A8, SLC39A9, SLC40A1, SLC41A2, SLC41A3, SLC43A2, SLC44A4, SLC45A4, SLC46A1, |
| GO:0009790 | embryo development | 5,92E-03 | [3, 4] | 271,00 | 23,90 | [ABR, ACVR2A, ACVRL1, ADD1, ADGRF5, AFF3, ALS2, ALX1, ALX4, AMBRA1, ANGPT1, ANKRD11, ANKS6, APBA2, ARHGDIG, ARID2, ARL13B, ARNT2, ATM, ATP11A, ATP8A2, B9D1, BAG6, BBS4, BBS7, BCL10, BMP2, BMP7, BRD2, BTF3, BTN3A3, CC2D2A, CDK1, CDK5R1, CELF4, CELSR1, CEP290, CHEK1, CHST11, CITED2, CLASP1, CLASP2, CMIP, COBL, COL2A1, COL4A2, COL6A1, CPLANE1, CRB2, CUL3, DAG1, DBN1, DHX35, DHX36, DLC1, DLD, DLG1, DLL1, DLL4, DLX2, DNAJB6, DNMT3A, DSCAML1, E2F8, ECE1, EHMT1, EIF2S2, EIF4A3, ERBB4, ERCC1, EVX1, EXOC4, EYA2, FBN1, FBN2, FBXW8, FERD3L, FGF2, FOXD3, FRAS1, FRS2, FUT8, FZD5, GARS1, GINS4, GJA5, GLI2, GLI3, GLMN, GNA12, GPR161, GRB2, GREM1, HAND1, HEG1, HES1, HES3, HM13, HNF1B, HOXA1, HOXA3, HOXA4, HOXC10, HOXC11, HOXC4, HOXD13, HUS1, IFT140, IGF1, IHH, INSIG2, INSR, ISL1, ITGA5, ITGB2, ITGB4, KDM2B, KDM4C, KDM6B, KIAA1217, KIDINS220, KIF16B, KIFBP, KITLG, KPNA7, LAMA2, LAMA5, LAMB1, LDHD, LIG4, LMBR1, LMX1B, LOXL3, LRIG3, LRP2, LRP5, MAFG, MAP2K5, MAP3K20, MAPK7, MAPK8IP3, MBD3, MBNL1, MEF2C, MEGF8, MEIS2, MEOX1, MFNG, MICAL2, MKS1, MSGN1, MSH2, MYH3, MYO15A, MYO18B, MYO1E, NDRG4, NDUFA2, NF1, NFE2L2, NKD1, NOTCH1, NPAT, NXN, PAF1, PAFAH1B1, PAX2, PAX7, PAX8, PBX1, PCSK5, PCSK6, PDCD6, PDGFRA, PDZD7, PHLDB1, PKD1, PLCB1, PLK4, PLXNA2, POLE, PPP3CA, PRKCA, PRKD2, PRKDC, PRMT1, PRPF19, PSEN1, |
| GO:0051128 | regulation of cellular component organization | 2,32E-10 | [3, 4] | 610,00 | 23,91 | ANKS1B, ANO6, ANTXR1, ANXA2, AP2A1, AP2S1, APC, APC2, APOBEC1, APPL1, APPL2, ARAP1, ARF1, ARFGAP1, ARHGAP17, ARHGAP40, ARHGDIA, ARHGDIB, ARHGEF10L, ARHGEF18, ARHGEF2, ARHGEF7, ARPC4, ASAP1, ASPH, ATAT1, ATF1, ATM, ATP13A2, ATP8A1, ATP8A2, ATXN7, AURKB, BAIAP2L1, BBS10, BBS4, BCAR1, BCAS3, BCL11A, BCL2, BCL6, BDNF, BDNF-AS, BIN3, BIRC2, BMERB1, BMP7, BRD4, BRSK2, BTBD9, BTC, C10orf90, C21orf91, CACNA1A, CAMK2B, CAMK2D, CAMSAP1, CAND1, CAPRIN2, CAPZB, CARMIL1, CARMIL2, CAV1, CBFA2T2, CCAR2, CCT2, CD47, CD53, CDC20, CDC25C, CDH13, CDH4, CDK11B, CDK5R1, CDKN1A, CDKN2A, CDT1, CELSR1, CEP131, CEP295, CEP43, CEP70, CHEK1, CHMP2A, CHRNB2, CIB1, CLASP1, CLASP2, CNOT1, CNTROB, COBL, CORO1B, CORO2B, CPNE5, CRBN, CREB3L2, CRIM1, CSMD3, CTCF, CTNNA2, CTNNBIP1, CTTN, CUL3, CUX1, CXCR4, CYFIP1, CYLD, DAB1, DAG1, DBN1, DCN, DCP2, DCTN1, DDHD2, DDR1, DDR2, DDX49, DEF8, DENND5A, DGKQ, DHX36, DLC1, DLG1, DLG4, DLL1, DNAJB6, DNAJC15, DNAJC6, DNM1, DNM1L, DYNC2LI1, EFNA5, EGR2, EHMT2, EIF4EBP1, ELN, ENC1, ENPP2, EPHA1, EPHA4, EPHB1, EPHB3, EPS8L1, ERCC1, ERCC5, EREG, ESPN, ESR1, ESR2, ETS1, EVL, EXOSC10, EZH2, F11R, F3, FAF1, FAP, FARP1, FARP2, FBLN5, FBP1, FBXW8, FCHSD1, FCHSD2, FER, FEZ1, FEZF2, FHOD1, FHOD3, FLRT1, FMN1, FNIP2, FZD10, FZD5, FZR1, GAK, GCG, GFI1, GLE1, GLMN, GMFB, GORASP1, GPC6, GPER1, GPRIN1, GRB2, GREM1, GRID2, GSG1L, GSK3A, GTF2H1, HCLS1, HDAC4, HECW1, HIP1, HIP1R, HMBOX1, HMGB1, HNRNPC, HNRNPD, HNRNPK, HPS4, HRG, HTT, IFRD1, IFT140, IFT88, IGF1, IGF1R, IKBKB, IL10RA, IL1RAP, ING1, ING3, INPP5F, INSR, IQGAP2, IQGAP3, IQSEC1, ISL1, ITCH, IWS1, JARID2, KALRN, KANK1, KANK3, KAT2B, KAT7, KDM2B, KDM3A, KDM4B, KDM4C, KDM4D, KIDINS220, KIF18A, KIF1A, KIF25, KIF26A, KIF2A, KIRREL1, KMT2E, KNDC1, KPNA7, LDB2, LDLR, LDLRAD4, LIG4, LIMA1, LIMS1, LINGO1, LLGL1, LPAR1, LRCH4, LRFN2, LRFN3, LRIG2, LRP1, LRP5, LRPAP1, LRRC4C, LRSAM1, LZTS1, MACF1, MAD1L1, MAG, MAGI2, MAK, MAP2, MAP2K2, MAP2K5, MAP3K13, MAP4, MAP4K4, MAP6, MAPK8, MAPK8IP2, MAPK9, MAPKAPK5, MCM2, MCPH1, MDGA1, MEF2C, MEGF8, MELTF, MIER1, MLLT3, MPHOSPH8, MTMR2, MTMR3, MYL2, MYO1C, MYO1E, NAPB, NAT10, NAV3, NCK2, NCKAP1, NCKAP1L, NCOA2, NDRG3, NDRG4, NDUFA13, NDUFA2, NECTIN1, NEDD4L, NEK7, NFATC2, NFATC4, NFE2L2, NGEF, NGF, NLGN2, NME2, NOC2L, NOL3, NOS1, NOTCH1, NPHP4, NPHS1, NPPA, NPR1, NPRL3, NPTN, NRXN1, NSD1, NSD3, NSMF, NTNG1, NTNG2, NTRK2, NUMB, OBSL1, OLFM1, OTUB1, PACSIN1, PACSIN2, PAF1, PAFAH1B1, PAK4, PAK5, PALM, PAPPA2, PARG, PAX7, PDCD5, PDGFRA, PDZD2, PEAK1, PFN2, PHF19, PHF2, PHLDB1, PIEZO1, PIH1D1, PIK3CB, PIK3R1, PIKFYVE, PINX1, PIP4K2A, PKD1, PKIB, PKMYT1, PLA2G6, PLAUR, PLCB1, PLCE1, PLCG2, PLD1, PLEK, PLEKHG2, PLEKHM2, PLK4, PLK5, PLXNA1, PLXNA2, PLXNB1, PLXNC1, PLXND1, PML, POU4F2, PPARG, PPARGC1A, PPFIA1, PPM1F, PPP1R13B, PPP1R16B, PPP3CA, PPP3CB, PRAG1, PRKCA, PRKCB, PRKCE, PRKCH, PRKCI, PRKCQ, PRKCZ, PRKD2, PRKN, PRUNE1, PSEN1, PSMC2, PSMC3, PSMC6, PTCH2, PTEN, PTK2, PTPN11, PTPRF, PTPRO, PXDN, RAB29, RAB4A, RAB5A, RACK1, RANBP1, RAP1A, RAP1GAP, RAP1GAP2, RAPGEF1, RASA1, RASIP1, RASSF1, RASSF8, RB1, RBX1, RELN, RET, RGMA, RHOA, RHOBTB3, RHOG, RHOU, RICTOR, RIOK3, |
| GO:0060341 | regulation of cellular localization | 8,12E-03 | [3, 4] | 260,00 | 23,94 | [ABCA12, ABR, ACTN2, ADCY5, ADCYAP1R1, AKT2, ANGPT1, ANK3, ANO1, ANXA2, ANXA6, APBA2, APC, APPL1, ARF1, ARHGEF16, ASTN2, ATP13A2, ATP2A2, ATP2B4, AURKB, BCAS3, BCL2, BDNF, BDNF-AS, BRSK2, BSN, BTBD9, C12orf4, CABP1, CACNA1A, CACNB2, CALM2, CAMK2D, CARD10, CAV1, CCR2, CCT2, CD247, CDK1, CDK5R1, CDT1, CEP131, CEP290, CHRNB2, CHRNB3, CIB1, CNST, COMMD1, CSNK1E, CSPG5, CYFIP1, CYLD, DAG1, DCLK1, DGKQ, DHX36, DLG1, DNAJC30, DNM1, DNM1L, DPP10, EDEM2, EFNA5, EIF2AK3, EIPR1, ERBB4, F2RL3, FER, FEZ1, FGF14, FGF2, FZD5, FZR1, GAB2, GCG, GJA5, GLI3, GLIS2, GPC6, GPER1, GPR137B, GPRIN1, GSG1L, GSK3A, HCLS1, HMGN3, HPS4, HRC, HSP90AB1, HTR2A, HTT, INPP5F, ITGB2, ITPR2, ITPR3, IWS1, JAK2, JPH3, KAT7, KCNAB2, KCNB1, KCNE1, KCNH2, KCNQ1, KHDRBS1, KIAA1614, KLF7, LARP7, LRIG2, LRP1, LRP5, LRPAP1, LZTS1, LZTS2, MAD1L1, MAGI2, MAP2, MAP2K2, MAPK8, MCRS1, MEAK7, MEF2C, MTMR2, MYO1C, MYO1E, MYO5A, NAPB, NDRG4, NEDD4L, NF1, NLGN2, NOL3, NOS1, NOS1AP, NPPA, NPTN, NR1D1, NRXN1, NRXN3, NUMB, NUP58, NUS1, P2RY6, PARD3, PCNT, PDCD5, PDPK1, PDZD2, PER2, PFN2, PIK3R1, PINX1, PLA2G6, PLCB1, PLCB2, PLCB4, PLCD4, PLCE1, PLCG2, PLCH2, PLCL2, PLD1, PML, PPFIA1, PPM1F, PPP1R13B, PPP3CB, PRKAR1B, PRKCA, PRKCB, PRKCE, PRKCH, PRKCI, PRKCZ, PRKD2, PRKN, PSEN1, PTEN, PTGES2, PTPN11, PTPN14, PTPRC, RAB29, RAB43, RAB5A, RACK1, |
| GO:0030001 | metal ion transport | 1,35E-02 | [6] | 240,00 | 24,05 | [ABCB11, ABCB6, ACTN2, ACTN4, ADCYAP1R1, AHNAK, ANK3, ANO6, ANXA2, ANXA6, ARF1, ASIC3, ASIC4, ATP12A, ATP1A3, ATP1A4, ATP2A2, ATP2A3, ATP2B2, ATP2B4, ATP6V0A1, ATP6V0D1, ATP6V1A, ATP6V1H, BCL2, BEST1, C4orf3, CACNA1A, CACNA1B, CACNA1E, CACNA1G, CACNA1H, CACNA1I, CACNA2D2, CACNA2D3, CACNA2D4, CACNB2, CACNG1, CACNG6, CACNG8, CALM2, CAMK2B, CAMK2D, CASR, CAV1, CD33, CDH23, CDK2, CHRNB2, CLDN16, CNNM4, COMMD1, COMMD9, CXCR4, DLG1, DNM1L, DPP10, F2RL3, F3, FAM155A, FGF12, FGF14, FGF2, FLVCR2, FXYD5, FXYD6, GCG, GJA5, GNAI2, GNB5, GPER1, GPRIN1, GRIK3, GRIK4, HCN1, HCN2, HCN4, HECW1, HRC, HRG, HTR2A, HTT, IL16, ITPR2, ITPR3, JPH3, KCNAB2, KCNB1, KCNB2, KCNC1, KCNC3, KCNE1, KCNF1, KCNH2, KCNIP4, KCNJ14, KCNJ15, KCNJ2, KCNJ3, KCNJ6, KCNK2, KCNMA1, KCNQ1, KCNQ2, KCNV1, KLHL3, LMTK2, LRP2, MCUR1, MELTF, MRS2, MTNR1B, MYLK, MYO5A, NALCN, NDFIP2, NECTIN1, NEDD4L, NIPA1, NKAIN1, NOL3, NOS1, NOS1AP, NPPA, ORAI1, ORAI2, P2RY6, PDPK1, PIK3CG, PKD1, PKD1L1, PLA2G6, PLCB1, PLCB2, PLCB4, PLCD4, PLCE1, PLCG2, PLCH2, PLCL2, PML, PPP3CA, PRKCA, PRKCB, PRKCE, PRKCZ, PRKD2, PSEN1, PTEN, PTPN3, PTPRC, RAB43, RASA1, RASA3, RCVRN, RGS9, RHOA, RYR1, RYR2, RYR3, SCARA5, SCN1A, SCN2A, SCN3B, SCN5A, SERPINE2, SGK1, SLC10A7, SLC11A2, SLC12A7, SLC12A9, SLC13A3, SLC13A5, SLC17A2, SLC17A4, SLC17A6, SLC17A7, SLC1A1, |
| GO:0098655 | cation transmembrane transport | 7,39E-03 | [4, 6] | 250,00 | 24,11 | [ABCB11, ABCB6, ACTN2, ACTN4, AHNAK, ANK3, ANO1, ANO6, ANXA2, ANXA6, ARF1, ASIC3, ASIC4, ATP12A, ATP13A2, ATP1A3, ATP1A4, ATP2A2, ATP2A3, ATP2B2, ATP2B4, ATP5F1A, ATP5ME, ATP6V0A1, ATP6V0D1, ATP6V1A, ATP6V1H, ATP8A1, C4orf3, CACNA1A, CACNA1B, CACNA1E, CACNA1G, CACNA1H, CACNA1I, CACNA2D2, CACNA2D3, CACNA2D4, CACNB2, CACNG1, CACNG6, CACNG8, CALM2, CAMK2D, CAV1, CCR2, CHRNB2, CHRNB3, CHRND, CHRNE, CLCN3, CLDN16, CNGA3, CNIH3, CNNM4, COMMD1, COX5A, COX6A1, COX7C, CTSS, CYC1, DAPK1, DLG1, DLG2, DLG4, DNAJC30, DPP10, F2RL3, FAM155A, FGF12, FGF14, FGF2, FXYD5, FXYD6, GNB5, GPER1, GPRIN1, GRIA4, GRIK3, GRIK4, GSG1L, HCN1, HCN2, HCN4, HECW1, HRC, HTR2A, HTR3A, HTR3C, HTT, ITPR2, ITPR3, JPH3, KCNAB2, KCNB1, KCNB2, KCNC1, KCNC3, KCNE1, KCNF1, KCNH2, KCNIP4, KCNJ14, KCNJ15, KCNJ2, KCNJ3, KCNJ6, KCNK2, KCNMA1, KCNQ1, KCNQ2, KCNV1, LLGL1, MAPK8IP2, MCUR1, MEF2C, MFSD3, MRS2, MTNR1B, MYO5A, NALCN, NDUFA4L2, NEDD4L, NIPA1, NLGN2, NNT, NOL3, NOS1, NOS1AP, NPPA, NRXN1, OCA2, ORAI1, ORAI2, P2RY6, PDPK1, PIEZO1, PIK3CG, PKD1, PKD1L1, PLA2G6, PLCB1, PLCB2, PLCB4, PLCD4, PLCE1, PLCG2, PLCH2, PLCL2, PPARGC1A, PRKCA, PRKCE, PRKCZ, PRKD2, PSEN1, PTEN, PTPN3, PTPRC, RASA1, RASA3, RASGRF1, RELN, RGS9, RYR1, RYR2, RYR3, SCARA5, SCN1A, SCN2A, SCN3B, SCN5A, SGK1, SHANK2, SHISA6, SHISA7, SHISA9, SLC11A2, SLC12A7, SLC12A9, SLC13A3, SLC13A5, SLC15A4, SLC17A2, SLC17A4, SLC17A7, SLC1A1, SLC1A3, SLC22A5, SLC24A4, SLC25A45, SLC2A9, SLC30A2, SLC30A6, SLC38A9, |

| | | | | | | |
|---|---|---|---|---|---|---|
| GO:0009653 | anatomical structure morphogenesis | 2,67E-13 | [2, 3] | 695,00 | 24,12 | ALX4, AMH, ANGPT1, ANGPT4, ANGPTL2, ANK3, ANKRD11, ANTXR1, ANXA2, ANXA6, AP2A1, AP2A2, AP2S1, AP3B1, ARAP1, ARHGAP12, ARHGDIA, ARHGEF18, ARHGEF2, ARHGEF26, ARHGEF7, ARID2, ARL13B, ASPH, ASTN2, ASXL2, ATF2, ATM, ATP2B4, ATP8A2, B9D1, BBS4, BBS7, BCAR3, BCAS3, BCL10, BCL11A, BCL11B, BCL2, BCL3, BCL6, BDNF, BDNF-AS, BIN3, BMERB1, BMP1, BMP2, BMP7, BMPER, BRD2, BRSK2, BTRC, CACNA1A, CACNA1H, CAMK2B, CAMSAP1, CAP1, CAP2, CAPRIN2, CAPZB, CARD10, CARMIL1, CARMIL2, CASR, CAV1, CC2D2A, CCBE1, CCDC88C, CCKAR, CCN6, CCR2, CD53, CDH10, CDH13, CDH18, CDH19, CDH23, CDH26, CDH4, CDK5R1, CELSR1, CEND1, CEP290, CFDP1, CHAMP1, CHRNB2, CHST11, CIB1, CITED2, CLASP1, CLASP2, CLTCL1, CNNM4, CNRIP1, CNTN4, CNTNAP2, COBL, COL11A2, COL18A1, COL2A1, COL3A1, COL4A1, COL4A2, COL6A1, COL6A2, COL8A2, CORO1B, CPLANE1, CPNE5, CRB2, CREB3L1, CRIP1, CRISPLD1, CRISPLD2, CSGALNACT1, CSPG5, CTNNA2, CTNNBIP1, CTNND2, CTTN, CUL3, CUX1, CXCR4, CYFIP1, CYFIP2, CYP1B1, DAB1, DAG1, DBN1, DCLK1, DCN, DDHD2, DDR1, DGCR6, DGCR6L, DHRS3, DHX15, DHX36, DKK3, DLC1, DLD, DLG1, DLG4, DLL1, DLL4, DLX2, DNAJB6, DNM1L, DNMBP, DOCK10, DOCK7, DOK4, DOK6, DRGX, DSCAML1, E2F2, E2F8, ECE1, EDAR, EFNA4, EFNA5, EGR2, EIF2AK3, EIF2AK4, EIF4A3, ELK3, ELN, EMC10, ENPP2, EPHA1, EPHA10, EPHA4, EPHA6, EPHB1, EPHB3, ERAP1, ERBB4, ERCC1, EREG, ESR1, ESRP2, ETS1, ETV1, EVL, EXOC4, EYA2, EYA4, F11R, F3, FAM171A1, FAP, FARP1, FAT1, FBLN5, FBN1, FBN2, FBN3, FBXW8, FER, FER1L5, FERMT2, FEZ1, FEZF1, FEZF2, FGD2, FGF2, FGF6, FGF7, FHOD3, FITM2, FJX1, FLRT1, FLT4, FMN1, FMNL2, FOXB2, FOXD3, FOXI3, FOXO1, FOXP1, FRAS1, FREM1, FRMD6, FRS2, FRYL, FZD5, FZR1, GAA, GAB1, GAB2, GARS1, GAS7, GATA5, GCNT1, GJA5, GLI2, GLI3, GLMN, GNA12, GORASP1, GPC6, GPR4, GPRIN1, GRB10, GRB2, GREM1, GRID2, GSK3A, GSX2, HAND1, HCLS1, HCN1, HDAC9, HECW1, HEG1, HERC1, HES1, HES3, HEXB, HGS, HNF1B, HOXA1, HOXA3, HOXA4, HOXC10, HOXC11, HOXC4, HOXD13, HRG, HSP90AB1, HTATIP2, IER3, IFRD1, IFT140, IHH, IMMT, INPPL1, INSIG2, INSR, IQCE, IRS2, ISL1, ISM1, ITGA1, ITGA5, ITGB2, ITGB4, KALRN, KANK1, KDM2B, KDM4C, KDM6B, KIDINS220, KIF16B, KIF1A, KIF26B, KIF2A, KIF5A, KLF7, KLHL3, KLK4, KNDC1, KRAS, KRIT1, KRT1, KRT25, LAMA2, LAMA5, LAMB1, LAMB2, LAMB4, LDB3, LDHD, LDLR, LEPR, LGR6, LHX4, LHX8, LIG1, LIMS1, LINGO1, LLGL1, LMBR1, LONP1, LOXL2, LPAR1, LRIG3, LRP1, LRP2, LRP5, LRRC4C, LTBP2, LZTS1, LZTS2, MACF1, MAG, MAGI2, MAP2, MAP2K5, MAP3K13, MAP3K20, MAP4K4, MAP6, MAPK7, MAPK8IP2, MAPK8IP3, MBNL1, MBOAT7, MEF2C, MEGF8, MEIS2, MELTF, MEN1, MEOX1, MFGE8, MFNG, MICAL2, MKS1, MLLT3, MRTFA, MSGN1, MTMR2, MYH3, MYL12B, MYL2, MYLK, MYO15A, MYO16, MYO18B, MYO1E, MYO5A, MYOM2, MYPN, NCKAP1, NCKAP1L, NCOA2, NDRG4, NECTIN1, NEDD4L, NEDD9, NEO1, NF1, NFASC, NFATC1, NFATC2, NFATC4, NFE2L2, NFIB, NGEF, NGF, NKD1, NOL3, NOS1, NOTCH1, NOTCH3, NOTCH4, NPHS1, NPNT, NPR1, NPRL3, NPTN, NR2E3, NRXN1, NRXN3, NSMF, NTNG1, NTNG2, NTRK2, NUMB, NUS1, OBSCN, OBSL1, OLFM1, OPA3, OPN5, PACSIN1, PACSIN2, PAF1, PAFAH1B1, PAK4, PALLD, PALM, PALM2AKAP2, PAPPA2, PARD3, PAX2, PAX7, PAX8, PAX9, PBX1, PCSK5, PDCD6, PDCL3, PDGFRA, PDPK1, PDZD7, PEAK1, PHACTR1, PHLDB1, PIK3CB, PIK3CG, PIK3R1, PIKFYVE, PIM1, PKD1, PKM, PLEKHA4, PLXNA1, PLXNA2, PLXNB1, PLXNC1, PLXND1, PML, POU4F2, PPARG, PPARGC1A, PPARGC1B, PPP1R16B, PPP3CA, PPP3CB, PRAG1, PRICKLE2, PRKCA, PRKCB, PRKCI, PRKCQ, PRKCZ, PRKD2, PRKDC, PRKN, PSEN1, PSMA5, PSMA6, PSMA7, PSMC2, PSMC3, PSMC6, PSMD2, PSMD7, PTEN, PTF1A, PTGIS, PTK2, PTPN11, PTPN14, |
| GO:0009653 | anatomical structure morphogenesis | 2,67E-13 | [2, 3] | 695,00 | 24,12 | ALX4, AMH, ANGPT1, ANGPT4, ANGPTL2, ANK3, ANKRD11, ANTXR1, ANXA2, ANXA6, AP2A1, AP2A2, AP2S1, AP3B1, ARAP1, ARHGAP12, ARHGDIA, ARHGEF18, ARHGEF2, ARHGEF26, ARHGEF7, ARID2, ARL13B, ASPH, ASTN2, ASXL2, ATF2, ATM, ATP2B4, ATP8A2, B9D1, BBS4, BBS7, BCAR3, BCAS3, BCL10, BCL11A, BCL11B, BCL2, BCL3, BCL6, BDNF, BDNF-AS, BIN3, BMERB1, BMP1, BMP2, BMP7, BMPER, BRD2, BRSK2, BTRC, CACNA1A, CACNA1H, CAMK2B, CAMSAP1, CAP1, CAP2, CAPRIN2, CAPZB, CARD10, CARMIL1, CARMIL2, CASR, CAV1, CC2D2A, CCBE1, CCDC88C, CCKAR, CCN6, CCR2, CD53, CDH10, CDH13, CDH18, CDH19, CDH23, CDH26, CDH4, CDK5R1, CELSR1, CEND1, CEP290, CFDP1, CHAMP1, CHRNB2, CHST11, CIB1, CITED2, CLASP1, CLASP2, CLTCL1, CNNM4, CNRIP1, CNTN4, CNTNAP2, COBL, COL11A2, COL18A1, COL2A1, COL3A1, COL4A1, COL4A2, COL6A1, COL6A2, COL8A2, CORO1B, CPLANE1, CPNE5, CRB2, CREB3L1, CRIP1, CRISPLD1, CRISPLD2, CSGALNACT1, CSPG5, CTNNA2, CTNNBIP1, CTNND2, CTTN, CUL3, CUX1, CXCR4, CYFIP1, CYFIP2, CYP1B1, DAB1, DAG1, DBN1, DCLK1, DCN, DDHD2, DDR1, DGCR6, DGCR6L, DHRS3, DHX15, DHX36, DKK3, DLC1, DLD, DLG1, DLG4, DLL1, DLL4, DLX2, DNAJB6, DNM1L, DNMBP, DOCK10, DOCK7, DOK4, DOK6, DRGX, DSCAML1, E2F2, E2F8, ECE1, EDAR, EFNA4, EFNA5, EGR2, EIF2AK3, EIF2AK4, EIF4A3, ELK3, ELN, EMC10, ENPP2, EPHA1, EPHA10, EPHA4, EPHA6, EPHB1, EPHB3, ERAP1, ERBB4, ERCC1, EREG, ESR1, ESRP2, ETS1, ETV1, EVL, EXOC4, EYA2, EYA4, F11R, F3, FAM171A1, FAP, FARP1, FAT1, FBLN5, FBN1, FBN2, FBN3, FBXW8, FER, FER1L5, FERMT2, FEZ1, FEZF1, FEZF2, FGD2, FGF2, FGF6, FGF7, FHOD3, FITM2, FJX1, FLRT1, FLT4, FMN1, FMNL2, FOXB2, FOXD3, FOXI3, FOXO1, FOXP1, FRAS1, FREM1, FRMD6, FRS2, FRYL, FZD5, FZR1, GAA, GAB1, GAB2, GARS1, GAS7, GATA5, GCNT1, GJA5, GLI2, GLI3, GLMN, GNA12, GORASP1, GPC6, GPR4, GPRIN1, GRB10, GRB2, GREM1, GRID2, GSK3A, GSX2, HAND1, HCLS1, HCN1, HDAC9, HECW1, HEG1, HERC1, HES1, HES3, HEXB, HGS, HNF1B, HOXA1, HOXA3, HOXA4, HOXC10, HOXC11, HOXC4, HOXD13, HRG, HSP90AB1, HTATIP2, IER3, IFRD1, IFT140, IHH, IMMT, INPPL1, INSIG2, INSR, IQCE, IRS2, ISL1, ISM1, ITGA1, ITGA5, ITGB2, ITGB4, KALRN, KANK1, KDM2B, KDM4C, KDM6B, KIDINS220, KIF16B, KIF1A, KIF26B, KIF2A, KIF5A, KLF7, KLHL3, KLK4, KNDC1, KRAS, KRIT1, KRT1, KRT25, LAMA2, LAMA5, LAMB1, LAMB2, LAMB4, LDB3, LDHD, LDLR, LEPR, LGR6, LHX4, LHX8, LIG1, LIMS1, LINGO1, LLGL1, LMBR1, LONP1, LOXL2, LPAR1, LRIG3, LRP1, LRP2, LRP5, LRRC4C, LTBP2, LZTS1, LZTS2, MACF1, MAG, MAGI2, MAP2, MAP2K5, MAP3K13, MAP3K20, MAP4K4, MAP6, MAPK7, MAPK8IP2, MAPK8IP3, MBNL1, MBOAT7, MEF2C, MEGF8, MEIS2, MELTF, MEN1, MEOX1, MFGE8, MFNG, MICAL2, MKS1, MLLT3, MRTFA, MSGN1, MTMR2, MYH3, MYL12B, MYL2, MYLK, MYO15A, MYO16, MYO18B, MYO1E, MYO5A, MYOM2, MYPN, NCKAP1, NCKAP1L, NCOA2, NDRG4, NECTIN1, NEDD4L, NEDD9, NEO1, NF1, NFASC, NFATC1, NFATC2, NFATC4, NFE2L2, NFIB, NGEF, NGF, NKD1, NOL3, NOS1, NOTCH1, NOTCH3, NOTCH4, NPHS1, NPNT, NPR1, NPRL3, NPTN, NR2E3, NRXN1, NRXN3, NSMF, NTNG1, NTNG2, NTRK2, NUMB, NUS1, OBSCN, OBSL1, OLFM1, OPA3, OPN5, PACSIN1, PACSIN2, PAF1, PAFAH1B1, PAK4, PALLD, PALM, PALM2AKAP2, PAPPA2, PARD3, PAX2, PAX7, PAX8, PAX9, PBX1, PCSK5, PDCD6, PDCL3, PDGFRA, PDPK1, PDZD7, PEAK1, PHACTR1, PHLDB1, PIK3CB, PIK3CG, PIK3R1, PIKFYVE, PIM1, PKD1, PKM, PLEKHA4, PLXNA1, PLXNA2, PLXNB1, PLXNC1, PLXND1, PML, POU4F2, PPARG, PPARGC1A, PPARGC1B, PPP1R16B, PPP3CA, PPP3CB, PRAG1, PRICKLE2, PRKCA, PRKCB, PRKCI, PRKCQ, PRKCZ, PRKD2, PRKDC, PRKN, PSEN1, PSMA5, PSMA6, PSMA7, PSMC2, PSMC3, PSMC6, PSMD2, PSMD7, PTEN, PTF1A, PTGIS, PTK2, PTPN11, PTPN14, |
| GO:0009653 | anatomical structure morphogenesis | 2,67E-13 | [2, 3] | 695,00 | 24,12 | ALX4, AMH, ANGPT1, ANGPT4, ANGPTL2, ANK3, ANKRD11, ANTXR1, ANXA2, ANXA6, AP2A1, AP2A2, AP2S1, AP3B1, ARAP1, ARHGAP12, ARHGDIA, ARHGEF18, ARHGEF2, ARHGEF26, ARHGEF7, ARID2, ARL13B, ASPH, ASTN2, ASXL2, ATF2, ATM, ATP2B4, ATP8A2, B9D1, BBS4, BBS7, BCAR3, BCAS3, BCL10, BCL11A, BCL11B, BCL2, BCL3, BCL6, BDNF, BDNF-AS, BIN3, BMERB1, BMP1, BMP2, BMP7, BMPER, BRD2, BRSK2, BTRC, CACNA1A, CACNA1H, CAMK2B, CAMSAP1, CAP1, CAP2, CAPRIN2, CAPZB, CARD10, CARMIL1, CARMIL2, CASR, CAV1, CC2D2A, CCBE1, CCDC88C, CCKAR, CCN6, CCR2, CD53, CDH10, CDH13, CDH18, CDH19, CDH23, CDH26, CDH4, CDK5R1, CELSR1, CEND1, CEP290, CFDP1, CHAMP1, CHRNB2, CHST11, CIB1, CITED2, CLASP1, CLASP2, CLTCL1, CNNM4, CNRIP1, CNTN4, CNTNAP2, COBL, COL11A2, COL18A1, COL2A1, COL3A1, COL4A1, COL4A2, COL6A1, COL6A2, COL8A2, CORO1B, CPLANE1, CPNE5, CRB2, CREB3L1, CRIP1, CRISPLD1, CRISPLD2, CSGALNACT1, CSPG5, CTNNA2, CTNNBIP1, CTNND2, CTTN, CUL3, CUX1, CXCR4, CYFIP1, CYFIP2, CYP1B1, DAB1, DAG1, DBN1, DCLK1, DCN, DDHD2, DDR1, DGCR6, DGCR6L, DHRS3, DHX15, DHX36, DKK3, DLC1, DLD, DLG1, DLG4, DLL1, DLL4, DLX2, DNAJB6, DNM1L, DNMBP, DOCK10, DOCK7, DOK4, DOK6, DRGX, DSCAML1, E2F2, E2F8, ECE1, EDAR, EFNA4, EFNA5, EGR2, EIF2AK3, EIF2AK4, EIF4A3, ELK3, ELN, EMC10, ENPP2, EPHA1, EPHA10, EPHA4, EPHA6, EPHB1, EPHB3, ERAP1, ERBB4, ERCC1, EREG, ESR1, ESRP2, ETS1, ETV1, EVL, EXOC4, EYA2, EYA4, F11R, F3, FAM171A1, FAP, FARP1, FAT1, FBLN5, FBN1, FBN2, FBN3, FBXW8, FER, FER1L5, FERMT2, FEZ1, FEZF1, FEZF2, FGD2, FGF2, FGF6, FGF7, FHOD3, FITM2, FJX1, FLRT1, FLT4, FMN1, FMNL2, FOXB2, FOXD3, FOXI3, FOXO1, FOXP1, FRAS1, FREM1, FRMD6, FRS2, FRYL, FZD5, FZR1, GAA, GAB1, GAB2, GARS1, GAS7, GATA5, GCNT1, GJA5, GLI2, GLI3, GLMN, GNA12, GORASP1, GPC6, GPR4, GPRIN1, GRB10, GRB2, GREM1, GRID2, GSK3A, GSX2, HAND1, HCLS1, HCN1, HDAC9, HECW1, HEG1, HERC1, HES1, HES3, HEXB, HGS, HNF1B, HOXA1, HOXA3, HOXA4, HOXC10, HOXC11, HOXC4, HOXD13, HRG, HSP90AB1, HTATIP2, IER3, IFRD1, IFT140, IHH, IMMT, INPPL1, INSIG2, INSR, IQCE, IRS2, ISL1, ISM1, ITGA1, ITGA5, ITGB2, ITGB4, KALRN, KANK1, KDM2B, KDM4C, KDM6B, KIDINS220, KIF16B, KIF1A, KIF26B, KIF2A, KIF5A, KLF7, KLHL3, KLK4, KNDC1, KRAS, KRIT1, KRT1, KRT25, LAMA2, LAMA5, LAMB1, LAMB2, LAMB4, LDB3, LDHD, LDLR, LEPR, LGR6, LHX4, LHX8, LIG1, LIMS1, LINGO1, LLGL1, LMBR1, LONP1, LOXL2, LPAR1, LRIG3, LRP1, LRP2, LRP5, LRRC4C, LTBP2, LZTS1, LZTS2, MACF1, MAG, MAGI2, MAP2, MAP2K5, MAP3K13, MAP3K20, MAP4K4, MAP6, MAPK7, MAPK8IP2, MAPK8IP3, MBNL1, MBOAT7, MEF2C, MEGF8, MEIS2, MELTF, MEN1, MEOX1, MFGE8, MFNG, MICAL2, MKS1, MLLT3, MRTFA, MSGN1, MTMR2, MYH3, MYL12B, MYL2, MYLK, MYO15A, MYO16, MYO18B, MYO1E, MYO5A, MYOM2, MYPN, NCKAP1, NCKAP1L, NCOA2, NDRG4, NECTIN1, NEDD4L, NEDD9, NEO1, NF1, NFASC, NFATC1, NFATC2, NFATC4, NFE2L2, NFIB, NGEF, NGF, NKD1, NOL3, NOS1, NOTCH1, NOTCH3, NOTCH4, NPHS1, NPNT, NPR1, NPRL3, NPTN, NR2E3, NRXN1, NRXN3, NSMF, NTNG1, NTNG2, NTRK2, NUMB, NUS1, OBSCN, OBSL1, OLFM1, OPA3, OPN5, PACSIN1, PACSIN2, PAF1, PAFAH1B1, PAK4, PALLD, PALM, PALM2AKAP2, PAPPA2, PARD3, PAX2, PAX7, PAX8, PAX9, PBX1, PCSK5, PDCD6, PDCL3, PDGFRA, PDPK1, PDZD7, PEAK1, PHACTR1, PHLDB1, PIK3CB, PIK3CG, PIK3R1, PIKFYVE, PIM1, PKD1, PKM, PLEKHA4, PLXNA1, PLXNA2, PLXNB1, PLXNC1, PLXND1, PML, POU4F2, PPARG, PPARGC1A, PPARGC1B, PPP1R16B, PPP3CA, PPP3CB, PRAG1, PRICKLE2, PRKCA, PRKCB, PRKCI, PRKCQ, PRKCZ, PRKD2, PRKDC, PRKN, PSEN1, PSMA5, PSMA6, PSMA7, PSMC2, PSMC3, PSMC6, PSMD2, PSMD7, PTEN, PTF1A, PTGIS, PTK2, PTPN11, PTPN14, |
| GO:0009653 | anatomical structure morphogenesis | 2,67E-13 | [2, 3] | 695,00 | 24,12 | ALX4, AMH, ANGPT1, ANGPT4, ANGPTL2, ANK3, ANKRD11, ANTXR1, ANXA2, ANXA6, AP2A1, AP2A2, AP2S1, AP3B1, ARAP1, ARHGAP12, ARHGDIA, ARHGEF18, ARHGEF2, ARHGEF26, ARHGEF7, ARID2, ARL13B, ASPH, ASTN2, ASXL2, ATF2, ATM, ATP2B4, ATP8A2, B9D1, BBS4, BBS7, BCAR3, BCAS3, BCL10, BCL11A, BCL11B, BCL2, BCL3, BCL6, BDNF, BDNF-AS, BIN3, BMERB1, BMP1, BMP2, BMP7, BMPER, BRD2, BRSK2, BTRC, CACNA1A, CACNA1H, CAMK2B, CAMSAP1, CAP1, CAP2, CAPRIN2, CAPZB, CARD10, CARMIL1, CARMIL2, CASR, CAV1, CC2D2A, CCBE1, CCDC88C, CCKAR, CCN6, CCR2, CD53, CDH10, CDH13, CDH18, CDH19, CDH23, CDH26, CDH4, CDK5R1, CELSR1, CEND1, CEP290, CFDP1, CHAMP1, CHRNB2, CHST11, CIB1, CITED2, CLASP1, CLASP2, CLTCL1, CNNM4, CNRIP1, CNTN4, CNTNAP2, COBL, COL11A2, COL18A1, COL2A1, COL3A1, COL4A1, COL4A2, COL6A1, COL6A2, COL8A2, CORO1B, CPLANE1, CPNE5, CRB2, CREB3L1, CRIP1, CRISPLD1, CRISPLD2, CSGALNACT1, CSPG5, CTNNA2, CTNNBIP1, CTNND2, CTTN, CUL3, CUX1, CXCR4, CYFIP1, CYFIP2, CYP1B1, DAB1, DAG1, DBN1, DCLK1, DCN, DDHD2, DDR1, DGCR6, DGCR6L, DHRS3, DHX15, DHX36, DKK3, DLC1, DLD, DLG1, DLG4, DLL1, DLL4, DLX2, DNAJB6, DNM1L, DNMBP, DOCK10, DOCK7, DOK4, DOK6, DRGX, DSCAML1, E2F2, E2F8, ECE1, EDAR, EFNA4, EFNA5, EGR2, EIF2AK3, EIF2AK4, EIF4A3, ELK3, ELN, EMC10, ENPP2, EPHA1, EPHA10, EPHA4, EPHA6, EPHB1, EPHB3, ERAP1, ERBB4, ERCC1, EREG, ESR1, ESRP2, ETS1, ETV1, EVL, EXOC4, EYA2, EYA4, F11R, F3, FAM171A1, FAP, FARP1, FAT1, FBLN5, FBN1, FBN2, FBN3, FBXW8, FER, FER1L5, FERMT2, FEZ1, FEZF1, FEZF2, FGD2, FGF2, FGF6, FGF7, FHOD3, FITM2, FJX1, FLRT1, FLT4, FMN1, FMNL2, FOXB2, FOXD3, FOXI3, FOXO1, FOXP1, FRAS1, FREM1, FRMD6, FRS2, FRYL, FZD5, FZR1, GAA, GAB1, GAB2, GARS1, GAS7, GATA5, GCNT1, GJA5, GLI2, GLI3, GLMN, GNA12, GORASP1, GPC6, GPR4, GPRIN1, GRB10, GRB2, GREM1, GRID2, GSK3A, GSX2, HAND1, HCLS1, HCN1, HDAC9, HECW1, HEG1, HERC1, HES1, HES3, HEXB, HGS, HNF1B, HOXA1, HOXA3, HOXA4, HOXC10, HOXC11, HOXC4, HOXD13, HRG, HSP90AB1, HTATIP2, IER3, IFRD1, IFT140, IHH, IMMT, INPPL1, INSIG2, INSR, IQCE, IRS2, ISL1, ISM1, ITGA1, ITGA5, ITGB2, ITGB4, KALRN, KANK1, KDM2B, KDM4C, KDM6B, KIDINS220, KIF16B, KIF1A, KIF26B, KIF2A, KIF5A, KLF7, KLHL3, KLK4, KNDC1, KRAS, KRIT1, KRT1, KRT25, LAMA2, LAMA5, LAMB1, LAMB2, LAMB4, LDB3, LDHD, LDLR, LEPR, LGR6, LHX4, LHX8, LIG1, LIMS1, LINGO1, LLGL1, LMBR1, LONP1, LOXL2, LPAR1, LRIG3, LRP1, LRP2, LRP5, LRRC4C, LTBP2, LZTS1, LZTS2, MACF1, MAG, MAGI2, MAP2, MAP2K5, MAP3K13, MAP3K20, MAP4K4, MAP6, MAPK7, MAPK8IP2, MAPK8IP3, MBNL1, MBOAT7, MEF2C, MEGF8, MEIS2, MELTF, MEN1, MEOX1, MFGE8, MFNG, MICAL2, MKS1, MLLT3, MRTFA, MSGN1, MTMR2, MYH3, MYL12B, MYL2, MYLK, MYO15A, MYO16, MYO18B, MYO1E, MYO5A, MYOM2, MYPN, NCKAP1, NCKAP1L, NCOA2, NDRG4, NECTIN1, NEDD4L, NEDD9, NEO1, NF1, NFASC, NFATC1, NFATC2, NFATC4, NFE2L2, NFIB, NGEF, NGF, NKD1, NOL3, NOS1, NOTCH1, NOTCH3, NOTCH4, NPHS1, NPNT, NPR1, NPRL3, NPTN, NR2E3, NRXN1, NRXN3, NSMF, NTNG1, NTNG2, NTRK2, NUMB, NUS1, OBSCN, OBSL1, OLFM1, OPA3, OPN5, PACSIN1, PACSIN2, PAF1, PAFAH1B1, PAK4, PALLD, PALM, PALM2AKAP2, PAPPA2, PARD3, PAX2, PAX7, PAX8, PAX9, PBX1, PCSK5, PDCD6, PDCL3, PDGFRA, PDPK1, PDZD7, PEAK1, PHACTR1, PHLDB1, PIK3CB, PIK3CG, PIK3R1, PIKFYVE, PIM1, PKD1, PKM, PLEKHA4, PLXNA1, PLXNA2, PLXNB1, PLXNC1, PLXND1, PML, POU4F2, PPARG, PPARGC1A, PPARGC1B, PPP1R16B, PPP3CA, PPP3CB, PRAG1, PRICKLE2, PRKCA, PRKCB, PRKCI, PRKCQ, PRKCZ, PRKD2, PRKDC, PRKN, PSEN1, PSMA5, PSMA6, PSMA7, PSMC2, PSMC3, PSMC6, PSMD2, PSMD7, PTEN, PTF1A, PTGIS, PTK2, PTPN11, PTPN14, |
| GO:0072359 | circulatory system development | 7,33E-04 | [4, 5] | 283,00 | 24,29 | [ACTN2, ACVRL1, ADAM12, ADGRB2, ADGRB3, ADGRF5, ADGRG1, AGO2, AKAP13, ALPK2, ANGPT1, ANGPT4, ANGPTL2, ANKRD17, ANTXR1, ANXA2, ARID2, ARL13B, ASCL1, ATF2, ATM, ATP2B4, B9D1, BBS4, BBS7, BCAS3, BICC1, BMP2, BMP7, BMPER, CARD10, CAV1, CC2D2A, CCBE1, CCN6, CCR2, CDH13, CDK1, CDKN1A, CHAMP1, CIB1, CITED2, CNRIP1, COL18A1, COL2A1, COL3A1, COL4A1, COL4A2, COL8A2, CPLANE1, CRB2, CREB3L1, CRIP1, CXCR4, CYP1B1, DAG1, DCN, DHCR7, DHRS3, DHX36, DIPK2A, DLC1, DLL1, DLL4, DNAH5, E2F2, E2F8, ECE1, EIF2AK3, ELK3, ELN, EMC10, ENPP2, EPHA1, EPHB1, EPHB3, ERAP1, ERBB4, EREG, ETS1, F3, FAP, FBLN5, FBN1, FBXW8, FGF12, FGF2, FGF6, FHOD3, FLT4, FOXO1, FRS2, FZD5, GAA, GAB1, GARS1, GATA5, GJA5, GLI2, GLI3, GLMN, GPER1, GPR4, GREM1, GSK3A, HAND1, HDAC9, HECTD1, HEG1, HES1, HGS, HOXA1, HOXA3, HRG, HTATIP2, IFT140, IFT74, IGF1, IHH, IL6R, IMMP2L, INSR, ISL1, ISM1, ITGA5, ITGB2, JARID2, KAT2B, KCNK2, KCNQ1, KDM6B, KIF2A, KRIT1, KRT1, LDB3, LDLR, LEPR, LONP1, LOXL2, LRP1, LRP2, LRP5, LTBP1, LYL1, MAP2K5, MAPK7, MBD3, MEF2C, MEGF8, MEN1, MFGE8, MICAL2, MKS1, MYL2, MYLK, MYO18B, MYO1E, MYOM2, NCOA6, NDRG4, NF1, NFATC1, NFATC2, NFATC4, NFE2L2, NOTCH1, NOTCH3, NOTCH4, NPPA, NPR1, NPRL3, NRXN1, NRXN3, NTRK2, NUS1, NXN, OBSL1, OLFM1, PAK4, PAX8, PCSK5, PDCD6, PDCL3, PDGFD, PDGFRA, PDLIM1, PDLIM3, PDPK1, PDZD2, PIK3CG, PIM1, PKD1, PKM, PLCE1, PLXND1, PML, POU4F2, PPARG, PPP1R16B, PPP3CA, PPP3CB, PRKCA, PRKCB, PRKD2, PRKDC, PSEN1, PSMA7, PTEN, PTGIS, PTK2, PTPN11, PTPN14, RAP1A, |
| GO:2000145 | regulation of cell motility | 5,68E-03 | [3, 4, 5] | 241,00 | 24,29 | [ABL2, ABR, ACTN4, ACVRL1, ADAM17, ADGRG1, AFDN, AGO2, AKAP12, AKT2, AMH, ANGPT1, ANGPT4, ANO6, APC, APPL1, APPL2, ARHGDIA, ARHGDIB, ARHGEF2, ARHGEF7, ARID2, ATM, ATP2B4, ATP5F1A, ATP8A1, BBS4, BCAR1, BCAS3, BCL2, BMERB1, BMP2, BMP7, BMPER, BTC, CAMK2B, CARD10, CARMIL1, CARMIL2, CAV1, CCBE1, CCN4, CCR2, CCR6, CD200R1, CDH13, CEP43, CIB1, CITED2, CLASP1, CLASP2, COL18A1, COL3A1, CORO1B, CTNNA2, CXCR4, CYP19A1, CYP1B1, DAG1, DCN, DDR2, DLC1, DLL4, DNM1L, DOCK10, DOCK7, DOCK8, DUOX1, EMC10, ENC1, ENPP2, EPB41L4B, EPHA1, EPPK1, ERBB4, EREG, ETS1, EVL, F3, FAM110A, FAM89B, FER, FGF2, FGF7, FLT4, FOXP1, FRMD5, FZR1, GAB1, GCNT2, GLIPR2, GNA12, GNAI2, GPER1, GREM1, GSX2, HDAC4, HDAC9, HMGB1, HRG, IGF1, IGF1R, IL6R, INPP5F, INSR, IQSEC1, IRS2, ITGA5, JAK2, KANK1, KIF2A, KITLG, KRIT1, LAMA2, LAMA5, LAMB1, LDB2, LDLRAD4, LGMN, LGR6, LPAR1, LRIG2, LRP1, MACF1, MAGI2, MAP2K5, MAP4K4, MAPK8IP3, MEAK7, MEF2C, MTUS1, MYD88, MYLK, MYO1C, MYO1E, NAV3, NCKAP1L, NDRG4, NEDD9, NF1, NFE2L2, NKD1, NOTCH1, NSMF, NTNG1, NTNG2, NUMB, NUS1, OSBPL8, P2RY6, PBLD, PDCD6, PDGFD, PDGFRA, PDPK1, PDZD2, PFN2, PHACTR1, PIK3R1, PLA2G7, PLCB1, PLCG2, PLVAP, PLXNA1, PLXNA2, PLXNB1, PLXNC1, PLXND1, PPARG, PPARGC1A, PPM1F, PPP3CA, PRAG1, PRKCA, PRKCE, PRKD2, PTEN, PTK2, PTPRC, RACK1, RAP2B, RELN, RET, RHOA, RHOG, RIPOR1, |

| | | | | | | |
|---|---|---|---|---|---|---|
| GO:0044093 | positive regulation of molecular function | 1,99E-08 | [3] | 475,00 | 24,31 | ALK, ALKAL2, ALS2, AMBRA1, AMH, ANGPT1, ANGPT4, ANK3, ANTXR1, ANXA2, APC2, APOBEC1, ARAP1, ARAP3, ARF1, ARFGAP1, ARHGAP10, ARHGAP12, ARHGAP17, ARHGAP21, ARHGAP29, ARHGAP39, ARHGAP40, ARHGAP42, ARHGAP45, ARHGDIA, ARHGDIB, ARHGDIG, ARHGEF10L, ARHGEF16, ARHGEF2, ARHGEF7, ARL1, ARL6IP5, ASAP1, ASAP2, ASPH, ATF2, ATG14, ATP2A3, ATP2B4, AURKB, BCAR3, BCAS3, BCL10, BCL2, BDNF, BDNF-AS, BMP2, BTC, BTRC, CACNA1A, CACNB2, CALM2, CAPRIN2, CARD10, CARD11, CARD14, CASR, CAV1, CCL16, CCND2, CCNY, CCR2, CCT2, CD33, CDC20, CDC20B, CDC25B, CDK1, CDK5R1, CDKN1A, CDKN2A, CDT1, CHN2, CHTF8, CIB1, CTBP2, CTSS, CXCR4, CYCS, CYFIP2, DAB1, DAG1, DAPK1, DAXX, DCUN1D1, DDR1, DDR2, DENND1A, DENND1B, DEPDC1, DEPDC5, DGKQ, DGKZ, DIPK2A, DLC1, DLG1, DLG4, DNAJB4, DNAJB6, DNAJC15, DNAL4, DNM1L, DNPEP, DOCK10, DOCK7, DOCK8, DOK7, DSTYK, DUSP19, EFNA5, EGR2, EIF2AK3, ELMOD1, EPHA1, EPHA10, EPHA4, EPHA6, EPHB1, EPHB3, ERBB4, ERC1, EREG, ESR1, ESR2, ETAA1, ETFA, EZH2, F11R, F3, FAM13A, FAM13B, FASN, FER, FGD2, FGF14, FGF2, FLT4, FNTA, FRS2, FZD10, FZD5, FZR1, GADD45G, GCG, GFRAL, GMFB, GNAI2, GNB5, GORASP1, GPER1, GPR55, GPRC5B, GPRC5C, GPRIN1, GPSM1, GRAMD4, GREM1, GRM1, GRM4, GSK3A, GTF2B, GUCA2A, HACD3, HCLS1, HDAC4, HES1, HIP1, HIP1R, HMBOX1, HMGB1, HMGN3, HNRNPD, HOXA3, HSP90AB1, HSPD1, HTR2A, HTR3A, HTT, IDE, IFI16, IGF1, IGF1R, IKBKB, IL1RAP, IL6R, INSR, IQGAP3, IQSEC1, IRAK2, ISL1, ITGA1, ITGB2, ITK, JAK2, KALRN, KCNC1, KDM4D, KIDINS220, KITLG, KRAS, KSR1, LGMN, LIMS1, LLGL1, LMTK2, LPAR1, LPAR2, LRP1, LRP5, MAGI2, MAL, MAP2K2, MAP2K5, MAP3K11, MAP3K13, MAP3K19, MAP3K2, MAP3K20, MAP3K5, MAP3K6, MAP4K4, MAP4K5, MAPK8, MAPK8IP3, MAPKAP1, MAPKAPK5, MARK3, MEF2C, MEN1, MFNG, MMD, MRTFA, MSH2, MSH3, MST1R, MTMR9, MYD88, MYO9B, NCKAP1L, NCSTN, NDUFA13, NEDD9, NEK7, NEUROG2, NF1, NFAM1, NGF, NHEJ1, NLRP12, NOL3, NOS1, NOS1AP, NPNT, NPPA, NPRL3, NRXN1, NTRK2, NUS1, ORAI1, OSBPL8, P2RY6, PAK4, PAK5, PDCD5, PDCD6, PDGFD, PDGFRA, PDPK1, PDZD2, PFN2, PIH1D1, PIK3CB, PIK3CG, PIK3R5, PINX1, PKD1, PKIB, PLAUR, PLCB1, PLCB2, PLCE1, PLCG2, PLCL2, PLEK, PLIN5, PLXNB1, PLXND1, PML, POU4F2, PPARG, PPARGC1A, PPARGC1B, PPM1F, PPP2R3C, PPP3CA, PRKAG2, PRKAR1B, PRKCA, PRKCB, PRKCE, PRKCH, PRKCI, PRKCQ, PRKCZ, PRKD2, PRKN, PRLR, PSEN1, PSMA6, PTEN, PTK2, PTPN11, PTPRA, PTPRC, RAB4A, RABGAP1, RACK1, RALGAPA2, RANBP1, RAP1A, RAP1GAP, RAP1GAP2, RAPGEF1, RARA, RASA1, RASA2, RASA3, RASAL2, RASGRF1, RASGRP4, RB1, RCVRN, RELN, REST, RET, |
| GO:0044093 | positive regulation of molecular function | 1,99E-08 | [3] | 475,00 | 24,31 | ALK, ALKAL2, ALS2, AMBRA1, AMH, ANGPT1, ANGPT4, ANK3, ANTXR1, ANXA2, APC2, APOBEC1, ARAP1, ARAP3, ARF1, ARFGAP1, ARHGAP10, ARHGAP12, ARHGAP17, ARHGAP21, ARHGAP29, ARHGAP39, ARHGAP40, ARHGAP42, ARHGAP45, ARHGDIA, ARHGDIB, ARHGDIG, ARHGEF10L, ARHGEF16, ARHGEF2, ARHGEF7, ARL1, ARL6IP5, ASAP1, ASAP2, ASPH, ATF2, ATG14, ATP2A3, ATP2B4, AURKB, BCAR3, BCAS3, BCL10, BCL2, BDNF, BDNF-AS, BMP2, BTC, BTRC, CACNA1A, CACNB2, CALM2, CAPRIN2, CARD10, CARD11, CARD14, CASR, CAV1, CCL16, CCND2, CCNY, CCR2, CCT2, CD33, CDC20, CDC20B, CDC25B, CDK1, CDK5R1, CDKN1A, CDKN2A, CDT1, CHN2, CHTF8, CIB1, CTBP2, CTSS, CXCR4, CYCS, CYFIP2, DAB1, DAG1, DAPK1, DAXX, DCUN1D1, DDR1, DDR2, DENND1A, DENND1B, DEPDC1, DEPDC5, DGKQ, DGKZ, DIPK2A, DLC1, DLG1, DLG4, DNAJB4, DNAJB6, DNAJC15, DNAL4, DNM1L, DNPEP, DOCK10, DOCK7, DOCK8, DOK7, DSTYK, DUSP19, EFNA5, EGR2, EIF2AK3, ELMOD1, EPHA1, EPHA10, EPHA4, EPHA6, EPHB1, EPHB3, ERBB4, ERC1, EREG, ESR1, ESR2, ETAA1, ETFA, EZH2, F11R, F3, FAM13A, FAM13B, FASN, FER, FGD2, FGF14, FGF2, FLT4, FNTA, FRS2, FZD10, FZD5, FZR1, GADD45G, GCG, GFRAL, GMFB, GNAI2, GNB5, GORASP1, GPER1, GPR55, GPRC5B, GPRC5C, GPRIN1, GPSM1, GRAMD4, GREM1, GRM1, GRM4, GSK3A, GTF2B, GUCA2A, HACD3, HCLS1, HDAC4, HES1, HIP1, HIP1R, HMBOX1, HMGB1, HMGN3, HNRNPD, HOXA3, HSP90AB1, HSPD1, HTR2A, HTR3A, HTT, IDE, IFI16, IGF1, IGF1R, IKBKB, IL1RAP, IL6R, INSR, IQGAP3, IQSEC1, IRAK2, ISL1, ITGA1, ITGB2, ITK, JAK2, KALRN, KCNC1, KDM4D, KIDINS220, KITLG, KRAS, KSR1, LGMN, LIMS1, LLGL1, LMTK2, LPAR1, LPAR2, LRP1, LRP5, MAGI2, MAL, MAP2K2, MAP2K5, MAP3K11, MAP3K13, MAP3K19, MAP3K2, MAP3K20, MAP3K5, MAP3K6, MAP4K4, MAP4K5, MAPK8, MAPK8IP3, MAPKAP1, MAPKAPK5, MARK3, MEF2C, MEN1, MFNG, MMD, MRTFA, MSH2, MSH3, MST1R, MTMR9, MYD88, MYO9B, NCKAP1L, NCSTN, NDUFA13, NEDD9, NEK7, NEUROG2, NF1, NFAM1, NGF, NHEJ1, NLRP12, NOL3, NOS1, NOS1AP, NPNT, NPPA, NPRL3, NRXN1, NTRK2, NUS1, ORAI1, OSBPL8, P2RY6, PAK4, PAK5, PDCD5, PDCD6, PDGFD, PDGFRA, PDPK1, PDZD2, PFN2, PIH1D1, PIK3CB, PIK3CG, PIK3R5, PINX1, PKD1, PKIB, PLAUR, PLCB1, PLCB2, PLCE1, PLCG2, PLCL2, PLEK, PLIN5, PLXNB1, PLXND1, PML, POU4F2, PPARG, PPARGC1A, PPARGC1B, PPM1F, PPP2R3C, PPP3CA, PRKAG2, PRKAR1B, PRKCA, PRKCB, PRKCE, PRKCH, PRKCI, PRKCQ, PRKCZ, PRKD2, PRKN, PRLR, PSEN1, PSMA6, PTEN, PTK2, PTPN11, PTPRA, PTPRC, RAB4A, RABGAP1, RACK1, RALGAPA2, RANBP1, RAP1A, RAP1GAP, RAP1GAP2, RAPGEF1, RARA, RASA1, RASA2, RASA3, RASAL2, RASGRF1, RASGRP4, RB1, RCVRN, RELN, REST, RET, |
| GO:0015318 | inorganic molecular entity transmembrane transporter activity | 1,67E-03 | [4, 6] | 265,00 | 24,33 | [ABCB11, ACTN2, ACTN4, AHNAK, ANK3, ANKH, ANO1, ANO4, ANO6, ANO7, ANXA2, ANXA6, AQP9, ASIC3, ASIC4, ATP12A, ATP13A2, ATP1A3, ATP1A4, ATP2A2, ATP2A3, ATP2B2, ATP2B4, ATP5F1A, ATP5ME, ATP6V0A1, ATP6V0D1, ATP6V1A, ATP6V1H, ATP8A1, BEST1, C4orf3, CACNA1A, CACNA1B, CACNA1E, CACNA1G, CACNA1H, CACNA1I, CACNA2D2, CACNA2D3, CACNA2D4, CACNB2, CACNG1, CACNG6, CACNG8, CALM2, CAMK2D, CAV1, CCR2, CHRNB2, CHRNB3, CHRND, CHRNE, CLCN2, CLCN3, CLDN16, CNGA3, CNIH3, CNNM4, COMMD1, COX5A, COX6A1, COX7C, CTNS, CTSS, DAPK1, DLG1, DLG2, DLG4, DPP10, FAM155A, FGF12, FGF14, FXYD5, FXYD6, GABRA2, GABRA4, GABRB2, GABRR3, GNB5, GPRIN1, GRIA4, GRID2, GRIK3, GRIK4, GSG1L, HCN1, HCN2, HCN4, HECW1, HRC, HTR3A, HTR3C, HTT, ITPR2, ITPR3, JPH3, KCNAB2, KCNB1, KCNB2, KCNC1, KCNC3, KCNE1, KCNF1, KCNH2, KCNIP4, KCNJ14, KCNJ15, KCNJ2, KCNJ3, KCNJ6, KCNK2, KCNMA1, KCNQ1, KCNQ2, KCNV1, LLGL1, LRRC8B, LRRC8D, MAPK8IP2, MEF2C, MFSD3, MIPEP, MRS2, MTNR1B, MYO5A, NALCN, NDUFA4L2, NEDD4L, NIPA1, NLGN2, NNT, NOL3, NOS1, NOS1AP, NPPA, NRXN1, OCA2, ORAI1, ORAI2, P2RY6, PACC1, PHLPP2, PIEZO1, PKD1, PKD1L1, PLA2G6, PLCG2, PPARGC1A, PRKCA, PRKCE, PRKCZ, PRKD2, PSEN1, PTEN, PTPN3, RASA1, RASA3, RASGRF1, RBP4, RELN, RGS9, RYR1, RYR2, RYR3, SCN1A, SCN2A, SCN3B, SCN5A, SGK1, SHANK2, SHISA6, SHISA7, SHISA9, SLC10A7, SLC11A2, SLC12A7, SLC12A9, SLC13A3, SLC13A5, SLC14A1, SLC15A4, SLC16A5, SLC16A7, SLC17A2, SLC17A4, SLC17A6, SLC17A7, SLC1A1, SLC1A3, SLC1A7, SLC24A4, SLC25A10, |
| GO:0015075 | ion transmembrane transporter activity | 5,74E-04 | [4, 6] | 279,00 | 24,41 | ATP2B2, ATP2B4, ATP5F1A, ATP5ME, ATP6V0A1, ATP6V0D1, ATP6V1A, ATP6V1H, ATP8A1, BEST1, C4orf3, CACNA1A, CACNA1B, CACNA1E, CACNA1G, CACNA1H, CACNA1I, CACNA2D2, CACNA2D3, CACNA2D4, CACNB2, CACNG1, CACNG6, CACNG8, CALM2, CAMK2D, CAV1, CCR2, CHRNB2, CHRNB3, CHRND, CHRNE, CLCN2, CLCN3, CLDN16, CNGA3, CNIH3, CNNM4, COMMD1, COX5A, COX6A1, COX7C, CTNS, CTSS, DAPK1, DLG1, DLG2, DLG4, DPP10, FAM155A, FGF12, FGF14, FXYD5, FXYD6, GABRA2, GABRA4, GABRB2, GABRR3, GNB5, GPRIN1, GRIA4, GRID2, GRIK3, GRIK4, GSG1L, HCN1, HCN2, HCN4, HECW1, HRC, HTR3A, HTR3C, HTT, ITPR2, ITPR3, JPH3, KCNAB2, KCNB1, KCNB2, KCNC1, KCNC3, KCNE1, KCNF1, KCNH2, KCNIP4, KCNJ14, KCNJ15, KCNJ2, KCNJ3, KCNJ6, KCNK2, KCNMA1, KCNQ1, KCNQ2, KCNV1, LASP1, LLGL1, LRRC8B, LRRC8D, MAPK8IP2, MEF2C, MFSD3, MRS2, MTNR1B, MYO5A, NALCN, NDUFA4L2, NEDD4L, NIPA1, NLGN2, NNT, NOL3, NOS1, NOS1AP, NPPA, NRXN1, OCA2, ORAI1, ORAI2, P2RY6, PACC1, PHLPP2, PIEZO1, PKD1, PKD1L1, PLA2G6, PLCG2, PPARGC1A, PRKCA, PRKCE, PRKCZ, PRKD2, PSEN1, PTEN, PTPN3, RASA1, RASA3, RASGRF1, RELN, RGS9, RIPK1, RYR1, RYR2, RYR3, S100A6, SCN1A, SCN2A, SCN3B, SCN5A, SGK1, SHANK2, SHISA6, SHISA7, SHISA9, SLC10A7, SLC11A2, SLC12A7, SLC12A9, SLC13A3, SLC13A5, SLC15A4, SLC16A5, SLC16A7, SLC17A2, SLC17A4, SLC17A6, SLC17A7, SLC1A1, SLC1A3, SLC1A7, SLC22A5, SLC24A4, SLC25A10, SLC25A13, SLC25A21, SLC25A22, SLC25A41, SLC25A45, SLC26A5, SLC2A9, SLC30A2, SLC30A6, SLC35B3, SLC35B4, SLC37A2, SLC38A8, SLC38A9, SLC39A10, SLC39A13, SLC39A7, SLC39A8, SLC39A9, SLC40A1, SLC41A2, SLC41A3, SLC43A2, SLC44A4, SLC45A4, SLC46A1, SLC4A2, SLC4A3, SLC4A4, SLC4A7, SLC4A8, SLC52A2, SLC5A6, SLC5A7, |
| GO:0040012 | regulation of locomotion | 1,70E-03 | [2, 3] | 253,00 | 24,47 | [ABL2, ABR, ACTN4, ACVRL1, ADAM17, ADGRG1, AFDN, AGO2, AKAP12, AKT2, AMH, ANGPT1, ANGPT4, ANO6, APC, APPL1, APPL2, ARHGDIA, ARHGDIB, ARHGEF2, ARHGEF7, ARID2, ATM, ATP2B4, ATP5F1A, ATP8A1, BBS4, BCAR1, BCAS3, BCL2, BMERB1, BMP2, BMP7, BMPER, BTC, CAMK2B, CARD10, CARMIL1, CARMIL2, CASR, CAV1, CCBE1, CCN4, CCR2, CCR6, CD200R1, CDH13, CEP43, CHRM4, CIB1, CITED2, CLASP1, CLASP2, COL18A1, COL3A1, CORO1B, CTNNA2, CXCR4, CYP19A1, CYP1B1, DAG1, DCN, DDR2, DLC1, DLL4, DNM1L, DOCK10, DOCK7, DOCK8, DUOX1, EMC10, ENC1, ENPP2, EPB41L4B, EPHA1, EPPK1, ERBB4, EREG, ETS1, EVL, F3, FAM110A, FAM89B, FER, FEZF2, FGF2, FGF7, FLT4, FOXP1, FRMD5, FZR1, GAB1, GCNT2, GLIPR2, GNA12, GNAI2, GPER1, GREM1, GSX2, HDAC4, HDAC9, HMGB1, HRG, IGF1, IGF1R, IL16, IL6R, INPP5F, INSR, IQSEC1, IRS2, ITGA5, JAK2, KANK1, KIF2A, KITLG, KRIT1, LAMA2, LAMA5, LAMB1, LDB2, LDLRAD4, LGMN, LGR6, LPAR1, LRIG2, LRP1, MACF1, MAGI2, MAP2K5, MAP4K4, MAPK8IP3, MEAK7, MEF2C, MEGF8, MEN1, MTUS1, MYD88, MYLK, MYO1C, MYO1E, NAV3, NCKAP1L, NDRG4, NEDD9, NF1, NFE2L2, NKD1, NOTCH1, NSMF, NTNG1, NTNG2, NUMB, NUS1, OSBPL8, P2RY6, PBLD, PDCD6, PDGFD, PDGFRA, PDPK1, PDZD2, PFN2, PHACTR1, PIK3R1, PLA2G7, PLCB1, PLCG2, PLVAP, PLXNA1, PLXNA2, PLXNB1, PLXNC1, PLXND1, POU4F2, PPARG, PPARGC1A, PPM1F, PPP3CA, PRAG1, PRKCA, PRKCE, PRKD2, PTEN, PTK2, |
| GO:0035295 | tube development | 5,06E-04 | [3, 4] | 277,00 | 24,47 | [ABCA12, ACVRL1, ADAM12, ADAMTS2, ADGRB2, ADGRB3, ADGRF5, ADGRG1, AGO2, ALX1, ALX4, AMBRA1, ANGPT1, ANGPT4, ANGPTL2, ANXA2, AP3B1, ARID2, ARL13B, ASCL1, ASS1, ATF2, ATP2B4, BAG6, BBS4, BBS7, BCAS3, BCL10, BCL2, BDNF, BDNF-AS, BMP2, BMP7, BMPER, BRD2, BTRC, CARD10, CASR, CAV1, CBFA2T2, CC2D2A, CCBE1, CCN6, CCR2, CDH13, CDKN1A, CELSR1, CEP290, CHAMP1, CIB1, CIC, CITED2, CLMP, COBL, COL18A1, COL2A1, COL3A1, COL4A1, COL4A2, COL8A2, CREB3L1, CRISPLD2, CTNNBIP1, CXCR4, CYP1B1, DAB1, DAG1, DCN, DDR1, DHCR7, DHX15, DLC1, DLG1, DLL1, DLL4, E2F2, E2F8, EDAR, EIF2AK3, EIF4E, EIF4EBP1, ELK3, EMC10, ENPP2, EPHA1, EPHA4, EPHB1, EPHB3, ERAP1, EREG, ESR1, ESRP2, ETS1, F3, FAP, FBLN5, FERD3L, FGF2, FGF6, FGF7, FLT4, FMN1, FOXP1, FZD5, GAB1, GARS1, GATA5, GJA5, GLI2, GLI3, GLMN, GPR161, GPR4, GREM1, HAND1, HDAC9, HEG1, HES1, HES3, HGS, HNF1B, HOXA1, HOXA3, HOXD13, HRG, HTATIP2, IFT140, IHH, ING3, ISL1, ISM1, ITGA5, ITGB2, KDM2B, KIF18A, KIF26B, KIF2A, KLHL3, KRAS, KRIT1, KRT1, LAMA5, LDLR, LEPR, LOXL2, LOXL3, LRP1, LRP2, LRP5, LTBP2, LZTS2, MAP2K5, MAPK7, MAPK8IP3, MEF2C, MEGF8, MEN1, MFGE8, MICAL2, MKS1, MME, MYLK, MYO18B, MYO1E, NDRG4, NF1, NFATC4, NFE2L2, NFIB, NOTCH1, NOTCH3, NOTCH4, NPNT, NPR1, NPRL3, NRXN1, NRXN3, NTRK2, NUS1, PAK4, PAX2, PAX7, PAX8, PBX1, PCSK5, PDCD6, PDCL3, PDGFRA, PDPK1, PIK3CG, PKD1, PKM, PLXNA2, PLXND1, PML, PPARG, PPP1R16B, PPP3CA, PRKCA, PRKCB, PRKD2, PSEN1, PSMA7, PTEN, PTGIS, PTK2, RAP1A, RARA, RASA1, RASIP1, RB1, RBP4, RC3H2, RET, RGMA, |
| GO:0051270 | regulation of cellular component movement | 6,81E-04 | [3, 4] | 266,00 | 24,54 | [ABL2, ABR, ACTN4, ACVRL1, ADAM17, ADGRG1, AFDN, AGO2, AKAP12, AKT2, AMH, ANGPT1, ANGPT4, ANO6, APC, APPL1, APPL2, ARAP1, ARHGDIA, ARHGDIB, ARHGEF2, ARHGEF7, ARID2, ATM, ATP1A3, ATP1A4, ATP2A2, ATP2B4, ATP5F1A, ATP8A1, BBS4, BCAR1, BCAS3, BCL2, BCL6, BMERB1, BMP2, BMP7, BMPER, BTC, CAMK2B, CAMK2D, CARD10, CARMIL1, CARMIL2, CAV1, CCBE1, CCN4, CCR2, CCR6, CD200R1, CDH13, CEP43, CIB1, CITED2, CLASP1, CLASP2, COL18A1, COL3A1, CORO1B, CTNNA2, CXCR4, CYP19A1, CYP1B1, DAG1, DCN, DDR2, DLC1, DLG1, DLL4, DNM1L, DOCK10, DOCK7, DOCK8, DUOX1, EMC10, ENC1, ENPP2, EPB41L4B, EPHA1, EPPK1, ERBB4, EREG, ETS1, EVL, F3, FAM110A, FAM89B, FER, FEZ1, FEZF2, FGF2, FGF7, FLT4, FOXP1, FRMD5, FZR1, GAB1, GCNT2, GJA5, GLIPR2, GNA12, GNAI2, GPER1, GREM1, GSX2, HCN4, HDAC4, HDAC9, HMGB1, HRG, IGF1, IGF1R, IL6R, INPP5F, INSR, IQSEC1, IRS2, ITGA5, JAK2, KANK1, KCNJ2, KIF2A, KITLG, KRIT1, LAMA2, LAMA5, LAMB1, LDB2, LDLRAD4, LGMN, LGR6, LPAR1, LRIG2, LRP1, LZTS1, MACF1, MAGI2, MAP2, MAP2K5, MAP4K4, MAPK8IP3, MEAK7, MEF2C, MEGF8, MTUS1, MYD88, MYLK, MYO1C, MYO1E, NAV3, NCKAP1L, NDRG4, NEDD9, NF1, NFE2L2, NKD1, NOS1AP, NOTCH1, NSMF, NTNG1, NTNG2, NUMB, NUS1, OSBPL8, P2RY6, PBLD, PDCD6, PDGFD, PDGFRA, PDPK1, PDZD2, PFN2, PHACTR1, PIK3R1, PLA2G7, PLCB1, PLCG2, PLVAP, PLXNA1, PLXNA2, PLXNB1, PLXNC1, PLXND1, POU4F2, PPARG, PPARGC1A, PPM1F, PPP3CA, PRAG1, PRKCA, PRKCE, PRKD2, PTEN, PTK2, PTPRC, PTPRO, RACK1, RAP2B, RELN, RET, RHOA, RHOG, RIPOR1, RIPOR2, ROBO1, ROCK2, |
| GO:0001944 | vasculature development | 3,69E-02 | [4, 5, 6, 7] | 190,00 | 24,58 | [ACVRL1, ADAM12, ADGRB2, ADGRB3, ADGRF5, ADGRG1, AGO2, ANGPT1, ANGPT4, ANGPTL2, ANKRD17, ANTXR1, ANXA2, ARID2, ATF2, ATP2B4, B9D1, BCAS3, BMP7, BMPER, CARD10, CAV1, CCBE1, CCN6, CCR2, CDH13, CHAMP1, CIB1, CITED2, COL18A1, COL3A1, COL4A1, COL4A2, COL8A2, CPLANE1, CREB3L1, CXCR4, CYP1B1, DAG1, DCN, DHCR7, DLL1, DLL4, E2F2, E2F8, EIF2AK3, ELK3, EMC10, ENPP2, EPHA1, EPHB1, EPHB3, ERAP1, EREG, ETS1, F3, FAP, FBLN5, FBXW8, FGF2, FGF6, FLT4, FOXO1, FZD5, GAB1, GARS1, GJA5, GLI3, GLMN, GPER1, GPR4, GREM1, HAND1, HDAC9, HECTD1, HEG1, HES1, HGS, HOXA1, HOXA3, HRG, HTATIP2, IHH, IL6R, IMMP2L, ISL1, ISM1, ITGA5, ITGB2, KIF2A, KRIT1, KRT1, LDLR, LEPR, LOXL2, LRP1, LRP2, LRP5, LTBP1, LYL1, MAP2K5, MAPK7, MEF2C, MEGF8, MEN1, MFGE8, MYLK, MYO18B, MYO1E, NF1, NFATC1, NFATC2, NFATC4, NFE2L2, NOTCH1, NOTCH3, NOTCH4, NPR1, NPRL3, NRXN1, NRXN3, NTRK2, NUS1, PAK4, PCSK5, PDCD6, PDCL3, PDGFD, PDGFRA, PDPK1, PIK3CG, PKD1, PKM, PLXND1, PML, PPARG, PPP1R16B, PPP3CA, PPP3CB, PRKCA, PRKCB, PRKD2, |
| GO:0030334 | regulation of cell migration | 4,10E-03 | [4, 5, 6] | 228,00 | 24,60 | [ABR, ACTN4, ACVRL1, ADAM17, ADGRG1, AFDN, AGO2, AKAP12, AKT2, AMH, ANGPT1, ANGPT4, ANO6, APC, APPL1, APPL2, ARHGDIA, ARHGDIB, ARHGEF2, ARHGEF7, ARID2, ATM, ATP2B4, ATP5F1A, ATP8A1, BCAR1, BCAS3, BCL2, BMERB1, BMP2, BMP7, BMPER, CAMK2B, CARD10, CARMIL1, CARMIL2, CAV1, CCBE1, CCN4, CCR2, CCR6, CD200R1, CDH13, CEP43, CIB1, CITED2, CLASP1, CLASP2, COL18A1, COL3A1, CORO1B, CTNNA2, CXCR4, CYP19A1, CYP1B1, DAG1, DCN, DDR2, DLC1, DLL4, DNM1L, DOCK10, DOCK7, DOCK8, EMC10, ENC1, ENPP2, EPB41L4B, EPHA1, EPPK1, ERBB4, ETS1, EVL, F3, FAM110A, FAM89B, FER, FGF2, FGF7, FLT4, FOXP1, FRMD5, FZR1, GAB1, GCNT2, GLIPR2, GNA12, GNAI2, GPER1, GREM1, GSX2, HDAC4, HDAC9, HMGB1, HRG, IGF1, IGF1R, IL6R, INSR, IQSEC1, IRS2, ITGA5, JAK2, KANK1, KIF2A, KITLG, KRIT1, LAMA2, LAMA5, LAMB1, LDB2, LDLRAD4, LGMN, LGR6, LPAR1, LRIG2, LRP1, MACF1, MAGI2, MAP2K5, MAP4K4, MAPK8IP3, MEAK7, MEF2C, MTUS1, MYD88, MYLK, MYO1C, MYO1E, NAV3, NCKAP1L, NDRG4, NEDD9, NF1, NFE2L2, NOTCH1, NSMF, NTNG1, NTNG2, NUMB, NUS1, OSBPL8, P2RY6, PBLD, PDCD6, PDGFD, PDGFRA, PDPK1, PFN2, PHACTR1, PIK3R1, PLA2G7, PLCB1, PLCG2, PLVAP, PLXNA1, PLXNA2, PLXNB1, PLXNC1, PLXND1, PPARG, |
| GO:0045597 | positive regulation of cell differentiation | 7,92E-04 | [3, 4, 5] | 261,00 | 24,60 | [AAMDC, ABL2, ACVR2A, ACVRL1, ADD1, ADIG, ALK, ALKAL2, ALX1, AP2A1, AP3B1, ARF1, ARHGDIA, ARHGEF2, ARHGEF7, ARNTL, ASCL1, ASXL2, ATF1, ATP8A2, BCL2, BCL6, BDNF, BDNF-AS, BEND6, BLOC1S5, BMP2, BMP7, BNC1, BRD1, BRINP2, BTC, C21orf91, CAMK2B, CAPRIN2, CARMIL1, CBFA2T2, CCDC3, CCN4, CCR2, CD46, CD53, CDH4, CHEK2, CIB1, COBL, CPNE5, CRB2, CREB3L2, CSNK1E, CSPG5, CTNNA2, CTNNBIP1, CTTN, CUX1, CXCR4, CYFIP1, CYLD, DAB1, DAG1, DBN1, DDR2, DHX36, DLG4, DLX2, DNM1L, EFNA5, EGR2, ENC1, EPHA4, ETS1, EZH2, F11R, FBN2, FBXW8, FEZ1, FEZF1, FEZF2, FGF2, GARS1, GATA5, GCNT2, GLI2, GLI3, GLIPR2, GPER1, GPRC5B, GREM1, GSX2, HCLS1, HES1, HMGB1, HSP90AB1, HTR2A, IGF1, IHH, IL6R, IL6ST, ISL1, JAK2, KALRN, KAT7, KDM4C, KIDINS220, KITLG, LIG4, LIMS1, LLGL1, LONP1, LOXL2, LPAR1, LPL, LRP1, LRP2, LRP5, LTBP2, LZTS1, MACF1, MAG, MAGI2, MAP3K13, MAP3K5, MAP6, MAPK8IP3, MAPK9, MEF2C, MEGF8, METRNL, MMD, MME, NAPEPLD, NCKAP1L, NCOA2, NDRG4, NEDD4L, NEDD9, NFATC2, NFE2L2, NGF, NME2, NOTCH1, NPNT, NPTN, NSMF, NTRK2, NUMB, OBSL1, OLFM1, OLFM2, OLIG2, PACSIN1, PAFAH1B1, PAX2, PAX8, PDZD2, PIM1, PLCB1, PLK5, PLXNA1, PLXNA2, PLXNB1, PLXNC1, PLXND1, POU4F2, PPARG, PPARGC1B, PPP2R3C, PPP3CA, PRDM16, PRKCA, PRKCH, PRKCI, PRKCZ, PRKDC, PRKN, PRMT1, PRPF19, PSEN1, PTCH2, PTEN, PTPRC, PTPRZ1, RANBP1, RAP1A, RAPGEF1, RARA, RB1, |

| | | | | | |
|---|---|---|---|---|---|
| GO:0051338 | regulation of transferase activity | 3,92E-04 [4] | 269,00 | 24,63 | [ACSL1, ADAM17, ADAR, ADCY2, ADCY3, ADCY5, ADCY7, AKAP13, ALK, ALKAL2, ALS2, AMBRA1, AMH, ANGPT1, ANGPT4, ANKS1B, APC, ARHGEF7, ATG14, ATP2B4, AURKB, BCL10, BDNF, BDNF-AS, BMP2, BMP7, BTC, BTRC, CALM2, CARD10, CARD14, CAV1, CCNA1, CCND2, CCNL1, CCNY, CCT2, CDC20, CDC20B, CDC25B, CDC25C, CDK1, CDK5R1, CDKN1A, CDKN2A, CEP43, CHTF8, CIB1, CXCR4, DAB1, DAG1, DAXX, DBNDD2, DCUN1D1, DDR1, DDR2, DGKQ, DGKZ, DIPK2A, DLG1, DLG4, DNAJC3, DNM1L, DOK7, DSTYK, DUS2, DUSP19, DUSP26, EEF1A1, EFNA5, EPHA1, EPHA10, EPHA4, EPHA6, EPHB1, EPHB3, ERBB4, EREG, ETAA1, ETFA, EZH2, F3, FAF1, FEM1C, FGD2, FGF2, FLT4, FRS2, FZD10, FZD5, FZR1, GADD45G, GCG, GFRAL, GMFB, GORASP1, GPRC5B, GPRC5C, GREM1, GRM1, GRM4, GSK3A, GTF2H1, HACD3, HEG1, HGS, HMBOX1, HNRNPD, HSP90AB1, HTR2A, HTT, IGF1, IGF1R, IL6R, INKA2, INSR, IPO7, IQGAP3, IRAK2, IRS2, ITGA1, JAK2, KAT2B, KIDINS220, KITLG, KRAS, KSR1, LATS2, LAX1, LDB2, LLGL1, LMTK2, LPAR1, LRP5, MAP2K2, MAP2K5, MAP3K11, MAP3K13, MAP3K19, MAP3K2, MAP3K20, MAP3K5, MAP3K6, MAP4K4, MAP4K5, MAPK8IP3, MAPKAP1, MAPKAPK5, MCM2, MCPH1, MEN1, MMD, MRPL23, MST1R, NCK2, NCKAP1L, NEDD9, NEK7, NF1, NGF, NLRC5, NPPA, NRXN1, NTRK2, OSBPL8, PAK4, PAK5, PARP4, PDGFD, PDGFRA, PDPK1, PDZD2, PIH1D1, PIK3CB, PIK3CG, PIK3IP1, PIK3R1, PIK3R5, PINX1, PKD1, PKIB, PKMYT1, PLCE1, PPARG, PPM1F, PPP2R3C, PPP3CA, PRKAG2, PRKAR1B, PRKCA, PRKCQ, PRKCZ, PRKD2, PRKN, PRLR, PSEN1, PTEN, PTK2, PTPN11, PTPRC, PTPRH, PTPRO, RACK1, RAP1A, RAP2B, RAPGEF1, RASIP1, RB1, RELN, |
| GO:0030155 | regulation of cell adhesion | 3,70E-02 [3, 4] | 186,00 | 24,64 | [ABAT, ABI3BP, ABL2, ACTN4, ACVRL1, ADAMTS18, ADGRG1, AFDN, AJAP1, ANGPT1, ANK3, AP3B1, APBB1IP, ARHGDIA, ARHGDIB, ARHGDIG, ARHGEF7, ASS1, ATM, BCAS3, BCL10, BCL2, BCL6, BMP2, BMP7, CARD11, CARMIL1, CAV1, CCDC80, CCR2, CD46, CD47, CDH13, CDKN2A, CELA2A, CIB1, CITED2, CLASP1, CLASP2, CLEC4G, CORO2B, CSPG5, CXCR4, CYLD, CYP1B1, CYTH1, DAB1, DAG1, DDR1, DLC1, DLG1, DLL1, DOCK8, DTX1, DUSP26, EFNA5, ENC1, EPB41L4B, EPHA1, EPHB3, ETS1, F11R, FAF1, FMN1, FRMD5, FXYD5, FZR1, GCNT2, GLI2, GLI3, GLMN, GNAO1, GPR4, GRB2, GREM1, HES1, HLA-DPB1, HMGB1, HRG, HSD17B12, HSPD1, HSPH1, IFT74, IGF1, IHH, IL6ST, ITGA5, ITGB2, JAK2, KANK1, KIF26B, LAMA2, LAMA5, LAX1, LDB2, LIMS1, LMO7, LOXL3, LRFN3, LRP1, LRRC32, MACF1, MAD1L1, MAP2K5, MAP4K4, MAPK7, MDGA1, MELTF, MEN1, MIPEP, NCK2, NCKAP1L, NEDD9, NF1, NOTCH1, NOTCH4, NPNT, PDPK1, PEAK1, PIEZO1, PIK3CB, PIK3CG, PIK3R1, PKD1, PLA2G2D, PLAUR, PLEKHA2, PLXNA1, PLXNA2, PLXNB1, PLXNC1, PLXND1, PML, PPM1F, PPP3CA, PRKCA, PRKCE, PRKCQ, PRKCZ, PRKD2, |
| GO:0045859 | regulation of protein kinase activity | 9,95E-03 [6, 7, 8, 9] | 212,00 | 24,65 | [ACSL1, ADAM17, ADAR, ADCY2, ADCY3, ADCY5, ADCY7, AKAP13, ALK, ALKAL2, ALS2, AMH, ANGPT1, ANGPT4, ANKS1B, APC, ARHGEF7, ATP2B4, BDNF, BDNF-AS, BMP2, BMP7, BTC, CALM2, CARD10, CARD14, CAV1, CCNA1, CCND2, CCNL1, CCNY, CDC25B, CDC25C, CDK1, CDK5R1, CDKN1A, CDKN2A, CEP43, CIB1, CXCR4, DAB1, DAG1, DAXX, DBNDD2, DDR2, DGKQ, DIPK2A, DLG1, DLG4, DNAJC3, DOK7, DUS2, DUSP19, DUSP26, EFNA5, EPHA1, EPHA4, EREG, ETAA1, ETFA, EZH2, F3, FAF1, FGD2, FGF2, FRS2, FZD10, FZD5, GADD45G, GCG, GFRAL, GMFB, GPRC5B, GPRC5C, GREM1, GRM1, GRM4, GTF2H1, HACD3, HEG1, HGS, HSP90AB1, HTR2A, HTT, IGF1, IGF1R, IL6R, INKA2, INSR, IPO7, IQGAP3, IRAK2, ITGA1, JAK2, KAT2B, KIDINS220, KITLG, KRAS, KSR1, LATS2, LAX1, LLGL1, LPAR1, LRP5, MAP2K2, MAP2K5, MAP3K11, MAP3K13, MAP3K19, MAP3K2, MAP3K20, MAP3K5, MAP3K6, MAP4K4, MAP4K5, MAPK8IP3, MAPKAP1, MAPKAPK5, MCM2, MEN1, MMD, MST1R, NCK2, NCKAP1L, NEDD9, NF1, NGF, NPPA, NRXN1, NTRK2, OSBPL8, PAK4, PAK5, PDGFD, PDPK1, PIH1D1, PIK3CB, PIK3CG, PIK3R5, PKD1, PKIB, PKMYT1, PLCE1, PPM1F, PPP2R3C, PPP3CA, PRKAG2, PRKAR1B, PRKCA, PRKCZ, PRKD2, PRLR, PSEN1, PTEN, PTK2, PTPN11, PTPRC, PTPRH, PTPRO, RACK1, RAP1A, |
| GO:0022603 | regulation of anatomical structure morphogenesis | 9,71E-05 [3, 4] | 290,00 | 24,68 | [ABR, ACTN4, ACVRL1, ADAM12, ADCK1, ADGRB2, ADGRB3, AGO2, AJAP1, AKAP13, ANGPT4, AP2A1, AP2A2, AP2S1, ARAP1, ARHGDIA, ARHGEF18, ARHGEF7, ATF2, ATP2B4, BCL11A, BCL2, BCL6, BDNF, BDNF-AS, BMP2, BMP7, BMPER, BRSK2, CACNA1A, CAMK2B, CAMSAP1, CAPRIN2, CAPZB, CARD10, CARMIL1, CCBE1, CCN6, CCR2, CD53, CDH4, CDK5R1, CELSR1, CFDP1, CHAMP1, CHRNB2, CIB1, CITED2, CLASP1, CLASP2, COL4A2, CORO1B, CPNE5, CRB2, CREB3L1, CSPG5, CTTN, CUX1, CXCR4, CYFIP1, CYP1B1, DAB1, DAG1, DBN1, DCN, DDHD2, DHX36, DLC1, DLG1, DLG4, DLL1, DLL4, DNM1L, DNMBP, E2F2, EFNA5, EGR2, EMC10, ENPP2, EPHA1, EPHA4, EPHB3, ERAP1, ESR1, ETS1, F11R, F3, FAM171A1, FBLN5, FBXW8, FERMT2, FEZF2, FGD2, FGF2, FGF7, FITM2, FLT4, FMNL2, FOXP1, FRS2, FZD5, GAB1, GARS1, GATA5, GNA12, GORASP1, GPC6, GPR4, GPRIN1, GREM1, HCLS1, HDAC9, HECW1, HES1, HEXB, HGS, HNF1B, HOXC11, HOXD13, HRG, HTATIP2, IFRD1, ISL1, ISM1, ITGA5, ITGB2, KALRN, KANK1, KIF1A, KIF2A, KNDC1, KRIT1, KRT1, LIMS1, LINGO1, LLGL1, LONP1, LPAR1, LRP1, LRRC4C, LZTS1, MACF1, MAG, MAGI2, MAP2, MAP2K5, MAP3K13, MAP6, MAPK7, MEF2C, MEGF8, MELTF, MKS1, MLLT3, MYL12B, NCOA2, NEDD4L, NEDD9, NF1, NFATC2, NFATC4, NFE2L2, NFIB, NGEF, NGF, NKD1, NOL3, NOTCH1, NPR1, NSMF, NTNG1, NTNG2, NTRK2, OBSL1, OLFM1, PAFAH1B1, PAK4, PALM, PALM2AKAP2, PAX2, PAX8, PAX9, PDCD6, PDCL3, PDPK1, PHLDB1, PIM1, PKM, PLEKHA4, PLXNA1, PLXNA2, PLXNB1, PLXNC1, PLXND1, PML, POU4F2, PPARG, PPARGC1A, PPP1R16B, PPP3CA, PRAG1, PRICKLE2, PRKCA, PRKCB, PRKD2, PRKDC, PRKN, PSEN1, |
| GO:0022603 | regulation of anatomical structure morphogenesis | 9,71E-05 [3, 4] | 290,00 | 24,68 | [ABR, ACTN4, ACVRL1, ADAM12, ADCK1, ADGRB2, ADGRB3, AGO2, AJAP1, AKAP13, ANGPT4, AP2A1, AP2A2, AP2S1, ARAP1, ARHGDIA, ARHGEF18, ARHGEF7, ATF2, ATP2B4, BCL11A, BCL2, BCL6, BDNF, BDNF-AS, BMP2, BMP7, BMPER, BRSK2, CACNA1A, CAMK2B, CAMSAP1, CAPRIN2, CAPZB, CARD10, CARMIL1, CCBE1, CCN6, CCR2, CD53, CDH4, CDK5R1, CELSR1, CFDP1, CHAMP1, CHRNB2, CIB1, CITED2, CLASP1, CLASP2, COL4A2, CORO1B, CPNE5, CRB2, CREB3L1, CSPG5, CTTN, CUX1, CXCR4, CYFIP1, CYP1B1, DAB1, DAG1, DBN1, DCN, DDHD2, DHX36, DLC1, DLG1, DLG4, DLL1, DLL4, DNM1L, DNMBP, E2F2, EFNA5, EGR2, EMC10, ENPP2, EPHA1, EPHA4, EPHB3, ERAP1, ESR1, ETS1, F11R, F3, FAM171A1, FBLN5, FBXW8, FERMT2, FEZF2, FGD2, FGF2, FGF7, FITM2, FLT4, FMNL2, FOXP1, FRS2, FZD5, GAB1, GARS1, GATA5, GNA12, GORASP1, GPC6, GPR4, GPRIN1, GREM1, HCLS1, HDAC9, HECW1, HES1, HEXB, HGS, HNF1B, HOXC11, HOXD13, HRG, HTATIP2, IFRD1, ISL1, ISM1, ITGA5, ITGB2, KALRN, KANK1, KIF1A, KIF2A, KNDC1, KRIT1, KRT1, LIMS1, LINGO1, LLGL1, LONP1, LPAR1, LRP1, LRRC4C, LZTS1, MACF1, MAG, MAGI2, MAP2, MAP2K5, MAP3K13, MAP6, MAPK7, MEF2C, MEGF8, MELTF, MKS1, MLLT3, MYL12B, NCOA2, NEDD4L, NEDD9, NF1, NFATC2, NFATC4, NFE2L2, NFIB, NGEF, NGF, NKD1, NOL3, NOTCH1, NPR1, NSMF, NTNG1, NTNG2, NTRK2, OBSL1, OLFM1, PAFAH1B1, PAK4, PALM, PALM2AKAP2, PAX2, PAX8, PAX9, PDCD6, PDCL3, PDPK1, PHLDB1, PIM1, PKM, PLEKHA4, PLXNA1, PLXNA2, PLXNB1, PLXNC1, PLXND1, PML, POU4F2, PPARG, PPARGC1A, PPP1R16B, PPP3CA, PRAG1, PRICKLE2, PRKCA, PRKCB, PRKD2, PRKDC, PRKN, PSEN1, |
| GO:0007417 | central nervous system development | 1,97E-04 [4, 5, 6] | 278,00 | 24,69 | [AARS1, AATK, ABAT, ABCB6, ABR, ADAM23, ADGRG1, ADGRL3, AFDN, AGBL4, ALK, ARL13B, ARNT2, ASCL1, ATAT1, ATG16L1, ATM, ATN1, ATP2B4, ATP6V0D1, ATXN1, BAG6, BBS4, BBS7, BCL11B, BCL2, BCL2A1, BMERB1, BMP2, BMP7, BTD, BTG2, C21orf91, CA10, CACNA1A, CALM2, CASP2, CASP5, CCDC85C, CCKAR, CDH22, CDK5R1, CELSR1, CEND1, CEP290, CHD5, CHRNB2, CHST8, CIC, CITED2, CLASP1, CLP1, CNTN4, CNTNAP2, COL2A1, COL3A1, COL4A1, CPLANE1, CSNK1E, CTNNA2, CTNS, CXCR4, DAB1, DAG1, DCLK1, DCTN1, DDIT4, DHX30, DLC1, DLL1, DLL4, DLX2, DNAH5, DNAJC30, DNER, DOCK7, DRGX, DSCAML1, EGR2, EPHA4, EPHB1, EPHB3, ERBB4, ETS1, EVX1, EZH2, FA2H, FEZ1, FEZF1, FEZF2, FPGS, FRS2, FZR1, GABRA4, GARS1, GLI2, GLI3, GLIS2, GPR17, GPRIN1, GRID2, GSX2, HAPLN2, HAPLN3, HELT, HERC1, HES1, HES3, HNF1B, HNRNPD, HOXC10, HSP90AB1, HYDIN, IGF2BP1, IL6ST, IMMP2L, IRS2, ISL1, JARID2, JHY, KALRN, KCNC1, KDM2B, KDM6B, KIF1A, KLHL1, KLHL17, KNDC1, KRAS, LAMB1, LAMB2, LDLR, LHX4, LHX6, LHX8, LIG4, LOXL3, LPAR1, LRP1, LRP2, LZTS1, MACROD2, MAG, MAL, MAP2, MAPK8IP3, MAPKAP1, MBD3, MBOAT7, MCPH1, MDGA1, MEIS2, MELTF, MEN1, MOG, MRTFA, MYO16, MYO1D, MYRF, NCKAP1, NCOA6, NCSTN, NDE1, NDRG4, NEUROD6, NF1, NFIB, NHEJ1, NOTCH1, NOTCH3, NR1D1, NRXN1, NTRK2, NUMB, OGDH, OLIG2, PAFAH1B1, PAX2, PAX7, PAX8, PBX1, PEX13, PFKFB3, PHACTR1, PHLPP2, PITPNM1, PKD1, PLCB1, PLXNA2, POU6F2, PPARG, PPARGC1A, PPP3CA, PRKD2, PRKDC, PRKN, PRPF19, PSEN1, PTEN, PTF1A, PTK2, PTPN11, PTPRZ1, RARA, RBFOX2, RELN, RHEB, RHOA, RNASE6, |
| GO:0007417 | central nervous system development | 1,97E-04 [4, 5, 6] | 278,00 | 24,69 | [AARS1, AATK, ABAT, ABCB6, ABR, ADAM23, ADGRG1, ADGRL3, AFDN, AGBL4, ALK, ARL13B, ARNT2, ASCL1, ATAT1, ATG16L1, ATM, ATN1, ATP2B4, ATP6V0D1, ATXN1, BAG6, BBS4, BBS7, BCL11B, BCL2, BCL2A1, BMERB1, BMP2, BMP7, BTD, BTG2, C21orf91, CA10, CACNA1A, CALM2, CASP2, CASP5, CCDC85C, CCKAR, CDH22, CDK5R1, CELSR1, CEND1, CEP290, CHD5, CHRNB2, CHST8, CIC, CITED2, CLASP1, CLP1, CNTN4, CNTNAP2, COL2A1, COL3A1, COL4A1, CPLANE1, CSNK1E, CTNNA2, CTNS, CXCR4, DAB1, DAG1, DCLK1, DCTN1, DDIT4, DHX30, DLC1, DLL1, DLL4, DLX2, DNAH5, DNAJC30, DNER, DOCK7, DRGX, DSCAML1, EGR2, EPHA4, EPHB1, EPHB3, ERBB4, ETS1, EVX1, EZH2, FA2H, FEZ1, FEZF1, FEZF2, FPGS, FRS2, FZR1, GABRA4, GARS1, GLI2, GLI3, GLIS2, GPR17, GPRIN1, GRID2, GSX2, HAPLN2, HAPLN3, HELT, HERC1, HES1, HES3, HNF1B, HNRNPD, HOXC10, HSP90AB1, HYDIN, IGF2BP1, IL6ST, IMMP2L, IRS2, ISL1, JARID2, JHY, KALRN, KCNC1, KDM2B, KDM6B, KIF1A, KLHL1, KLHL17, KNDC1, KRAS, LAMB1, LAMB2, LDLR, LHX4, LHX6, LHX8, LIG4, LOXL3, LPAR1, LRP1, LRP2, LZTS1, MACROD2, MAG, MAL, MAP2, MAPK8IP3, MAPKAP1, MBD3, MBOAT7, MCPH1, MDGA1, MEIS2, MELTF, MEN1, MOG, MRTFA, MYO16, MYO1D, MYRF, NCKAP1, NCOA6, NCSTN, NDE1, NDRG4, NEUROD6, NF1, NFIB, NHEJ1, NOTCH1, NOTCH3, NR1D1, NRXN1, NTRK2, NUMB, OGDH, OLIG2, PAFAH1B1, PAX2, PAX7, PAX8, PBX1, PEX13, PFKFB3, PHACTR1, PHLPP2, PITPNM1, PKD1, PLCB1, PLXNA2, POU6F2, PPARG, PPARGC1A, PPP3CA, PRKD2, PRKDC, PRKN, PRPF19, PSEN1, PTEN, PTF1A, PTK2, PTPN11, PTPRZ1, RARA, RBFOX2, RELN, RHEB, RHOA, RNASE6, |
| GO:0043549 | regulation of kinase activity | 1,55E-03 [5, 7, 8] | 240,00 | 24,69 | [ACSL1, ADAM17, ADAR, ADCY2, ADCY3, ADCY5, ADCY7, AKAP13, ALK, ALKAL2, ALS2, AMBRA1, AMH, ANGPT1, ANGPT4, ANKS1B, APC, ARHGEF7, ATG14, ATP2B4, BCL10, BDNF, BDNF-AS, BMP2, BMP7, BTC, CALM2, CARD10, CARD14, CAV1, CCNA1, CCND2, CCNL1, CCNY, CDC25B, CDC25C, CDK1, CDK5R1, CDKN1A, CDKN2A, CEP43, CIB1, CXCR4, DAB1, DAG1, DAXX, DBNDD2, DDR1, DDR2, DGKQ, DGKZ, DIPK2A, DLG1, DLG4, DNAJC3, DOK7, DSTYK, DUS2, DUSP19, DUSP26, EEF1A1, EFNA5, EPHA1, EPHA10, EPHA4, EPHA6, EPHB1, EPHB3, ERBB4, EREG, ETAA1, ETFA, EZH2, F3, FAF1, FGD2, FGF2, FLT4, FRS2, FZD10, FZD5, GADD45G, GCG, GFRAL, GMFB, GPRC5B, GPRC5C, GREM1, GRM1, GRM4, GTF2H1, HACD3, HEG1, HGS, HSP90AB1, HTR2A, HTT, IGF1, IGF1R, IL6R, INKA2, INSR, IPO7, IQGAP3, IRAK2, IRS2, ITGA1, JAK2, KAT2B, KIDINS220, KITLG, KRAS, KSR1, LATS2, LAX1, LDB2, LLGL1, LMTK2, LPAR1, LRP5, MAP2K2, MAP2K5, MAP3K11, MAP3K13, MAP3K19, MAP3K2, MAP3K20, MAP3K5, MAP3K6, MAP4K4, MAP4K5, MAPK8IP3, MAPKAP1, MAPKAPK5, MCM2, MCPH1, MEN1, MMD, MST1R, NCK2, NCKAP1L, NEDD9, NF1, NGF, NLRC5, NPPA, NRXN1, NTRK2, OSBPL8, PAK4, PAK5, PDGFD, PDGFRA, PDPK1, PIH1D1, PIK3CB, PIK3CG, PIK3IP1, PIK3R1, PIK3R5, PKD1, PKIB, PKMYT1, PLCE1, PPM1F, PPP2R3C, PPP3CA, PRKAG2, PRKAR1B, PRKCA, PRKCZ, PRKD2, PRKN, PRLR, PSEN1, PTEN, PTK2, PTPN11, PTPRC, PTPRH, PTPRO, RACK1, RAP1A, RAP2B, RAPGEF1, |
| GO:0043085 | positive regulation of catalytic activity | 2,44E-07 [4] | 390,00 | 24,71 | AMH, ANGPT1, ANGPT4, ANTXR1, APC2, APOBEC1, ARAP1, ARAP3, ARF1, ARFGAP1, ARHGAP10, ARHGAP12, ARHGAP17, ARHGAP21, ARHGAP29, ARHGAP39, ARHGAP40, ARHGAP42, ARHGAP45, ARHGDIA, ARHGDIB, ARHGDIG, ARHGEF10L, ARHGEF16, ARHGEF7, ARL1, ARL6IP5, ASAP1, ASAP2, ASPH, ATG14, ATP2A3, ATP2B4, AURKB, BCAR3, BCAS3, BCL10, BCL2, BDNF, BDNF-AS, BMP2, BTC, BTRC, CACNA1A, CALM2, CARD10, CARD14, CASR, CAV1, CCL16, CCND2, CCNY, CCT2, CD33, CDC20, CDC20B, CDC25B, CDK1, CDK5R1, CDKN1A, CDKN2A, CHN2, CHTF8, CIB1, CXCR4, CYCS, CYFIP2, DAB1, DAG1, DAPK1, DAXX, DCUN1D1, DDR1, DDR2, DENND1A, DENND1B, DEPDC1, DEPDC5, DGKQ, DGKZ, DIPK2A, DLC1, DLG1, DLG4, DNAJB4, DNAJB6, DNAJC15, DNAL4, DNM1L, DNPEP, DOCK10, DOCK7, DOCK8, DOK7, DSTYK, DUSP19, EFNA5, EGR2, EIF2AK3, ELMOD1, EPHA1, EPHA10, EPHA4, EPHA6, EPHB1, EPHB3, ERBB4, EREG, ESR1, ETAA1, ETFA, EZH2, F11R, F3, FAM13A, FAM13B, FASN, FGD2, FGF2, FLT4, FNTA, FRS2, FZD10, FZD5, FZR1, GADD45G, GCG, GFRAL, GMFB, GNAI2, GNB5, GORASP1, GPER1, GPR55, GPRC5B, GPRC5C, GPRIN1, GRAMD4, GREM1, GRM1, GRM4, GSK3A, GUCA2A, HACD3, HIP1, HIP1R, HMBOX1, HMGB1, HNRNPD, HSP90AB1, HSPD1, HTR2A, IFI16, IGF1, IGF1R, IL6R, INSR, IQGAP3, IQSEC1, IRAK2, ITGA1, ITGB2, ITK, JAK2, KALRN, KIDINS220, KITLG, KRAS, KSR1, LGMN, LIMS1, LLGL1, LMTK2, LPAR1, LPAR2, MAGI2, MAL, MAP2K2, MAP2K5, MAP3K11, MAP3K13, MAP3K19, MAP3K2, MAP3K20, MAP3K5, MAP3K6, MAP4K4, MAP4K5, MAPK8, MAPK8IP3, MAPKAP1, MAPKAPK5, MEF2C, MMD, MRTFA, MSH2, MSH3, MST1R, MTMR9, MYO9B, NCKAP1L, NCSTN, NDUFA13, NEDD9, NEK7, NF1, NGF, NHEJ1, NLRP12, NOS1, NOS1AP, NPNT, NPRL3, NRXN1, NTRK2, NUS1, ORAI1, OSBPL8, P2RY6, PAK4, PAK5, PDCD5, PDCD6, PDGFD, PDGFRA, PDPK1, PDZD2, PFN2, PIH1D1, PIK3CB, PIK3CG, PIK3R5, PKD1, PKIB, PLCB1, PLCB2, PLCE1, PLEK, PLIN5, PLXNB1, PML, PPARG, PPARGC1B, PPM1F, PPP2R3C, PPP3CA, PRKAG2, PRKAR1B, PRKCA, PRKCE, PRKCQ, PRKCZ, PRKD2, PRLR, PSEN1, PTEN, PTK2, PTPN11, PTPRA, PTPRC, RAB4A, RABGAP1, RACK1, RALGAPA2, RANBP1, RAP1A, RAP1GAP, RAP1GAP2, RAPGEF1, RASA1, RASA2, RASA3, RASAL2, RASGRF1, RASGRP4, RCVRN, RELN, REST, RET, RGMA, RGS12, RGS3, RGS6, RGS7, RGS9, RHOA, RHOG, |
| GO:0043085 | positive regulation of catalytic activity | 2,44E-07 [4] | 390,00 | 24,71 | AMH, ANGPT1, ANGPT4, ANTXR1, APC2, APOBEC1, ARAP1, ARAP3, ARF1, ARFGAP1, ARHGAP10, ARHGAP12, ARHGAP17, ARHGAP21, ARHGAP29, ARHGAP39, ARHGAP40, ARHGAP42, ARHGAP45, ARHGDIA, ARHGDIB, ARHGDIG, ARHGEF10L, ARHGEF16, ARHGEF7, ARL1, ARL6IP5, ASAP1, ASAP2, ASPH, ATG14, ATP2A3, ATP2B4, AURKB, BCAR3, BCAS3, BCL10, BCL2, BDNF, BDNF-AS, BMP2, BTC, BTRC, CACNA1A, CALM2, CARD10, CARD14, CASR, CAV1, CCL16, CCND2, CCNY, CCT2, CD33, CDC20, CDC20B, CDC25B, CDK1, CDK5R1, CDKN1A, CDKN2A, CHN2, CHTF8, CIB1, CXCR4, CYCS, CYFIP2, DAB1, DAG1, DAPK1, DAXX, DCUN1D1, DDR1, DDR2, DENND1A, DENND1B, DEPDC1, DEPDC5, DGKQ, DGKZ, DIPK2A, DLC1, DLG1, DLG4, DNAJB4, DNAJB6, DNAJC15, DNAL4, DNM1L, DNPEP, DOCK10, DOCK7, DOCK8, DOK7, DSTYK, DUSP19, EFNA5, EGR2, EIF2AK3, ELMOD1, EPHA1, EPHA10, EPHA4, EPHA6, EPHB1, EPHB3, ERBB4, EREG, ESR1, ETAA1, ETFA, EZH2, F11R, F3, FAM13A, FAM13B, FASN, FGD2, FGF2, FLT4, FNTA, FRS2, FZD10, FZD5, FZR1, GADD45G, GCG, GFRAL, GMFB, GNAI2, GNB5, GORASP1, GPER1, GPR55, GPRC5B, GPRC5C, GPRIN1, GRAMD4, GREM1, GRM1, GRM4, GSK3A, GUCA2A, HACD3, HIP1, HIP1R, HMBOX1, HMGB1, HNRNPD, HSP90AB1, HSPD1, HTR2A, IFI16, IGF1, IGF1R, IL6R, INSR, IQGAP3, IQSEC1, IRAK2, ITGA1, ITGB2, ITK, JAK2, KALRN, KIDINS220, KITLG, KRAS, KSR1, LGMN, LIMS1, LLGL1, LMTK2, LPAR1, LPAR2, MAGI2, MAL, MAP2K2, MAP2K5, MAP3K11, MAP3K13, MAP3K19, MAP3K2, MAP3K20, MAP3K5, MAP3K6, MAP4K4, MAP4K5, MAPK8, MAPK8IP3, MAPKAP1, MAPKAPK5, MEF2C, MMD, MRTFA, MSH2, MSH3, MST1R, MTMR9, MYO9B, NCKAP1L, NCSTN, NDUFA13, NEDD9, NEK7, NF1, NGF, NHEJ1, NLRP12, NOS1, NOS1AP, NPNT, NPRL3, NRXN1, NTRK2, NUS1, ORAI1, OSBPL8, P2RY6, PAK4, PAK5, PDCD5, PDCD6, PDGFD, PDGFRA, PDPK1, PDZD2, PFN2, PIH1D1, PIK3CB, PIK3CG, PIK3R5, PKD1, PKIB, PLCB1, PLCB2, PLCE1, PLEK, PLIN5, PLXNB1, PML, PPARG, PPARGC1B, PPM1F, PPP2R3C, PPP3CA, PRKAG2, PRKAR1B, PRKCA, PRKCE, PRKCQ, PRKCZ, PRKD2, PRLR, PSEN1, PTEN, PTK2, PTPN11, PTPRA, PTPRC, RAB4A, RABGAP1, RACK1, RALGAPA2, RANBP1, RAP1A, RAP1GAP, RAP1GAP2, RAPGEF1, RASA1, RASA2, RASA3, RASAL2, RASGRF1, RASGRP4, RCVRN, RELN, REST, RET, RGMA, RGS12, RGS3, RGS6, RGS7, RGS9, RHOA, RHOG, |
| GO:0007420 | brain development | 7,39E-03 [4, 5, 6, 7] | 207,00 | 24,79 | [AARS1, AATK, ABAT, ABCB6, ABR, ADGRG1, ADGRL3, AFDN, ALK, ARL13B, ARNT2, ASCL1, ATAT1, ATG16L1, ATM, ATP2B4, ATP6V0D1, ATXN1, BAG6, BBS4, BBS7, BCL11B, BCL2, BCL2A1, BMERB1, BMP2, BMP7, BTG2, C21orf91, CA10, CACNA1A, CALM2, CASP2, CASP5, CCDC85C, CCKAR, CDH22, CDK5R1, CEND1, CEP290, CHD5, CHRNB2, CIC, CLASP1, CLP1, CNTN4, CNTNAP2, COL3A1, COL4A1, CPLANE1, CSNK1E, CTNNA2, CTNS, CXCR4, DAB1, DCLK1, DDIT4, DLC1, DLL1, DLX2, DNAH5, DNAJC30, DOCK7, DSCAML1, EGR2, EPHB3, ERBB4, ETS1, EZH2, FEZ1, FEZF1, FEZF2, FPGS, FRS2, FZR1, GARS1, GLI2, GLI3, GPRIN1, GRID2, GSX2, HERC1, HES1, HES3, HNF1B, HNRNPD, HYDIN, IGF2BP1, IMMP2L, IRS2, ISL1, JHY, KALRN, KCNC1, KDM2B, KDM6B, KIF1A, KLHL1, KLHL17, KNDC1, KRAS, LAMB1, LHX6, LHX8, LPAR1, LRP1, LRP2, LZTS1, MACROD2, MAPK8IP3, MAPKAP1, MBD3, MBOAT7, MCPH1, MDGA1, MEIS2, MEN1, MRTFA, MYO16, MYO1D, NCOA6, NCSTN, NDE1, NDRG4, NEUROD6, NF1, NFIB, NOTCH1, NOTCH3, NRXN1, NTRK2, NUMB, OGDH, OLIG2, PAFAH1B1, PAX2, PBX1, PEX13, PFKFB3, PHACTR1, PHLPP2, PITPNM1, PLCB1, PLXNA2, PPARGC1A, PPP3CA, PRKDC, PSEN1, PTEN, PTF1A, PTPN11, RARA, RBFOX2, RELN, RHOA, RNASE6, ROBO1, RORA, RRM1, RTN4RL1, |

| | | | | | | |
|---|---|---|---|---|---|---|
| GO:0035239 | tube morphogenesis | 2,64E-03 | [3, 4, 5] | 226,00 | 24,81 | [ACVRL1, ADAM12, ADGRB2, ADGRB3, ADGRF5, ADGRG1, AGO2, ALX1, ANGPT1, ANGPT4, ANGPTL2, ANXA2, ARID2, ARL13B, ATF2, ATP2B4, BBS4, BBS7, BCAS3, BCL10, BCL2, BMP2, BMP7, BMPER, BRD2, BTRC, CARD10, CASR, CAV1, CC2D2A, CCBE1, CCN6, CCR2, CDH13, CELSR1, CEP290, CHAMP1, CIB1, CITED2, COBL, COL18A1, COL3A1, COL4A1, COL4A2, COL8A2, CREB3L1, CTNNBIP1, CXCR4, CYP1B1, DAG1, DCN, DDR1, DLC1, DLG1, DLL1, DLL4, E2F2, E2F8, EDAR, EIF2AK3, ELK3, EMC10, ENPP2, EPHA1, EPHA4, EPHB1, EPHB3, ERAP1, EREG, ESR1, ESRP2, ETS1, F3, FAP, FBLN5, FGF2, FGF6, FLT4, FMN1, FOXP1, FZD5, GAB1, GARS1, GJA5, GLI2, GLI3, GLMN, GPR4, GREM1, HAND1, HDAC9, HEG1, HES1, HGS, HNF1B, HOXA1, HOXA3, HOXD13, HRG, HTATIP2, IHH, ISL1, ISM1, ITGA5, ITGB2, KDM2B, KIF26B, KIF2A, KLHL3, KRAS, KRIT1, KRT1, LAMA5, LDLR, LEPR, LOXL2, LRP1, LRP2, LRP5, LZTS2, MAP2K5, MAPK7, MEF2C, MEGF8, MEN1, MFGE8, MICAL2, MKS1, MYLK, MYO18B, MYO1E, NDRG4, NF1, NFATC4, NFE2L2, NFIB, NOTCH1, NOTCH3, NOTCH4, NPNT, NPR1, NPRL3, NRXN1, NRXN3, NTRK2, NUS1, PAK4, PAX2, PAX8, PBX1, PDCD6, PDCL3, PDGFRA, PDPK1, PIK3CG, PKD1, PKM, PLXND1, PML, PPARG, PPP1R16B, PPP3CA, PRKCA, PRKCB, PRKD2, PSEN1, PSMA7, PTEN, PTGIS, PTK2, |
| GO:1905114 | cell surface receptor signaling pathway involved in cell-cell signaling | 2,73E-02 | [3, 4, 5, 6] | 182,00 | 24,86 | [ABAT, ADRB1, AGO2, ALPK2, AMER2, AP2A1, AP2A2, AP2S1, APC, APC2, ARNTL, BCL9, BDNF, BDNF-AS, BEGAIN, BICC1, BMP2, BTRC, CALCOCO1, CAPRIN2, CAV1, CCAR2, CCDC88C, CCN4, CCNY, CELF4, CELSR1, CHRM4, CHRNB2, CHRNB3, CHRND, CHRNE, CMAHP, CSNK1A1L, CSNK1E, CSNK1G3, CTNNBIP1, CTNND2, CUL3, CYLD, DKK3, DLG4, EIF4A3, ESR1, FERMT2, FNTA, FOXO1, FZD10, FZD5, GABBR1, GABRA2, GABRA4, GABRB2, GLI2, GLI3, GNAO1, GPC6, GPRC5B, GPRIN1, GRB10, GREM1, GRIA4, GRID2, GRIK3, GRIK4, GRK5, GRM1, GSK3A, HECW1, HNF1B, HTR3A, IGSF9B, ISL1, KANK1, KREMEN2, LATS2, LGR6, LLGL1, LRP1, LRP5, LRRK1, LYPD6, LYPD6B, LZTS2, MACF1, MAGI2, MAPK8IP2, MEF2C, MKS1, MLLT3, MTMR2, NFATC1, NFATC4, NKD1, NLGN2, NLK, NOTCH1, NPHP4, NPTXR, NRXN1, NXN, PAF1, PDZD2, PKD1, PLCB1, PLCB2, PLCG2, PLEKHA4, PPP3CA, PPP3CB, PRICKLE2, PRKCZ, PRKD2, PRKN, PSEN1, PSMA5, PSMA6, PSMA7, PSMC2, PSMC3, PSMC6, PSMD2, PSMD7, PTEN, PTPRO, RAB5A, RACK1, RAPGEF1, RBX1, RELN, RHOA, RNF146, RNF220, ROR1, ROR2, |
| GO:0022803 | passive transmembrane transporter activity | 2,43E-02 | [4, 6] | 184,00 | 24,86 | [ACTN2, AHNAK, ANK3, ANO1, ANO4, ANO6, ANO7, ANXA2, ANXA6, AQP9, ASIC3, ASIC4, ATP2B4, ATP5F1A, ATP5ME, BCL2, BEST1, CABP2, CACNA1A, CACNA1B, CACNA1E, CACNA1G, CACNA1H, CACNA1I, CACNA2D2, CACNA2D3, CACNA2D4, CACNB2, CACNG1, CACNG6, CACNG8, CALM2, CAMK2D, CAV1, CCR2, CHRNB2, CHRNB3, CHRND, CHRNE, CLCN2, CLCN3, CNGA3, CNIH3, COMMD1, CRISP1, CTSS, DAPK1, DLG1, DLG2, DLG4, DPP10, FAM155A, FGF12, FGF14, FXYD5, FXYD6, GABRA2, GABRA4, GABRB2, GABRR3, GJA5, GNB5, GPRIN1, GRIA4, GRID2, GRIK3, GRIK4, GRM2, GSG1L, HCN1, HCN2, HCN4, HRC, HTR3A, HTR3C, HTT, INAFM1, ITPR2, ITPR3, JPH3, KCNAB2, KCNB1, KCNB2, KCNC1, KCNC3, KCNE1, KCNF1, KCNH2, KCNIP4, KCNJ14, KCNJ15, KCNJ2, KCNJ3, KCNJ6, KCNK2, KCNMA1, KCNQ1, KCNQ2, KCNV1, LLGL1, LRRC8B, LRRC8D, MAPK8IP2, MEF2C, MIPEP, MTNR1B, MYO5A, NALCN, NEDD4L, NLGN2, NOL3, NOS1, NOS1AP, NPPA, NRXN1, NRXN3, ORAI1, ORAI2, P2RY6, PACC1, PANX2, PDZD3, PHLPP2, PIEZO1, PKD1, PKD1L1, PLA2G6, PLCG2, PPARGC1A, PRKCA, PRKCB, |
| GO:0070848 | response to growth factor | 1,71E-02 | [4] | 190,00 | 24,87 | [ACVR2A, ACVRL1, ADAM17, AMH, APPL1, APPL2, ARHGEF18, ASCL1, ATP2B4, BCAR1, BDNF, BDNF-AS, BMP2, BMP3, BMP7, BMP8B, BMPER, CASR, CAV1, CCBE1, CHST11, CIB1, CITED2, COL2A1, COL3A1, COL4A2, CORO1B, CRB2, CREB3L1, CYFIP1, CYFIP2, DCN, DDIT4, DKK3, DLL1, DLL4, DSTYK, EEF1A1, EIF4A3, ERBB4, ESRP2, F11R, FAM89B, FBN1, FBN2, FER, FERMT2, FEZ1, FGF12, FGF2, FGF22, FGF6, FGF7, FLRT1, FLT4, FNTA, FRS2, FUT8, GAB1, GALNT3, GARS1, GATA5, GCNT2, GRB10, GRB2, GREM1, HES1, HGS, HIVEP1, HNRNPH1, HRG, HSP90AB1, HTR3A, HTRA3, IL17RD, ING3, INSR, ITGA5, KCNC1, KIDINS220, KIF16B, LDLRAD4, LGMN, LIMS1, LRP2, LRRC32, LTBP1, LTBP2, LZTS1, MAGI2, MAP2K5, MAPK7, MARS1, MEF2C, MEGF8, MEIS2, MEN1, MICALL1, MYO1C, MYO1E, NEO1, NGF, NLK, NOS1, NOTCH1, NPNT, NPTN, NR3C1, NRXN1, NTRK2, NUS1, PARD3, PAX2, PAX9, PBLD, PCSK6, PDCD5, PDCD6, PDE8A, PDGFD, PDGFRA, PDGFRL, PDPK1, PDZD2, PEG10, PLK5, PML, POLR2B, POLR2F, PPARGC1A, PPP3CA, PRDM16, PRKCB, PRKCZ, PRKD2, PTEN, PTK2, PTPN11, PXDN, RAB35, RACK1, |
| GO:0007167 | enzyme linked receptor protein signaling pathway | 1,01E-04 | [4, 5, 6] | 276,00 | 24,89 | [ACVR2A, ACVRL1, ADAM17, AKT2, ALK, AMH, ANGPT1, ANGPT4, ANKS1A, ANKS1B, AP2A1, AP2A2, AP2S1, APC, APPL1, APPL2, ARAP1, ARHGEF18, ARHGEF7, ARPC4, ATP6V0A1, ATP6V0D1, ATP6V1A, ATP6V1H, BAIAP2L1, BCAR1, BCAR3, BDNF, BDNF-AS, BMP2, BMP3, BMP7, BMP8B, BMPER, BTC, CAV1, CCBE1, CD8A, CDH13, CDK5R1, CHST11, CITED2, COL3A1, COL4A1, COL4A2, CRB2, CREB3L1, CRIM1, CYFIP1, CYFIP2, DCN, DDIT4, DDR1, DDR2, DGKD, DGKQ, DKK3, DLL1, DNM1, DOK4, DSTYK, EFNA4, EFNA5, EGR2, EIF2AK3, EIF4EBP1, EIF4EBP2, ELMO1, ENHO, EPHA1, EPHA10, EPHA4, EPHA6, EPHB1, EPHB3, EPS15L1, ERBB4, EREG, ESRP2, F11R, F3, FAM89B, FBN1, FBN2, FER, FERMT2, FGF12, FGF2, FGF22, FGF6, FGF7, FLRT1, FLT4, FNTA, FOXO1, FRS2, FUT8, GAB1, GAB2, GALNT3, GARS1, GCNT2, GFRAL, GNAI2, GPER1, GPRIN1, GRB10, GRB2, GREM1, GSK3A, HES1, HGS, HIP1, HIP1R, HIVEP1, HNRNPH1, HRG, HSP90AB1, HTRA3, IDE, IGF1, IGF1R, IL17RD, IL31RA, IL6ST, ING3, INSR, IRS2, ITGA1, ITGA5, JAK2, KALRN, KANK1, KIF16B, LDLRAD4, LGMN, LMTK2, LRIG2, LRP1, LRP2, LRRC32, LTBP1, LTBP2, MAGI2, MEGF8, MEN1, MST1R, MYO1C, MYO1E, NCK2, NCKAP1, NCKAP1L, NCSTN, NDRG4, NEDD9, NEO1, NFATC4, NGEF, NGF, NLK, NOTCH1, NPNT, NPPA, NPR1, NPTN, NRXN1, NTRK2, NUS1, OSBPL8, PARD3, PBLD, PCSK6, PDCD6, PDGFD, PDGFRA, PDGFRL, PDPK1, PDZD2, PDZD3, PEG10, PIK3CB, PIK3R1, PLAUR, PLCB1, PLCE1, PML, POLR2B, POLR2F, PPP3CA, PRDM16, PRKCA, PRKCB, PRKCQ, PRKCZ, PRKD2, PRLR, PSEN1, PTEN, PTK2, PTPN11, PTPN3, PTPRA, PTPRF, PXDN, RAPGEF1, RASA1, RASGRP4, RBFOX2, |
| GO:0072358 | cardiovascular system development | 1,10E-02 | [4, 5, 6] | 195,00 | 24,94 | [ACVRL1, ADAM12, ADGRB2, ADGRB3, ADGRF5, ADGRG1, AGO2, ANGPT1, ANGPT4, ANGPTL2, ANKRD17, ANTXR1, ANXA2, ARID2, ATF2, ATP2B4, B9D1, BCAS3, BMP7, BMPER, CARD10, CAV1, CCBE1, CCN6, CCR2, CDH13, CHAMP1, CIB1, CITED2, COL18A1, COL3A1, COL4A1, COL4A2, COL8A2, CPLANE1, CRB2, CREB3L1, CXCR4, CYP1B1, DAG1, DCN, DHCR7, DLL1, DLL4, E2F2, E2F8, EIF2AK3, ELK3, EMC10, ENPP2, EPHA1, EPHB1, EPHB3, ERAP1, EREG, ETS1, F3, FAP, FBLN5, FBXW8, FGF2, FGF6, FLT4, FOXO1, FZD5, GAB1, GARS1, GJA5, GLI3, GLMN, GPER1, GPR4, GREM1, HAND1, HDAC9, HECTD1, HEG1, HES1, HGS, HOXA1, HOXA3, HRG, HTATIP2, IHH, IL6R, IMMP2L, ISL1, ISM1, ITGA5, ITGB2, KCNQ1, KIF2A, KRIT1, KRT1, LDLR, LEPR, LOXL2, LRP1, LRP2, LRP5, LTBP1, LYL1, MAP2K5, MAPK7, MEF2C, MEGF8, MEN1, MFGE8, MYLK, MYO18B, MYO1E, NF1, NFATC1, NFATC2, NFATC4, NFE2L2, NOTCH1, NOTCH3, NOTCH4, NPR1, NPRL3, NRXN1, NRXN3, NTRK2, NUS1, NXN, PAK4, PCSK5, PDCD6, PDCL3, PDGFD, PDGFRA, PDPK1, PIK3CG, PKD1, PKM, PLXND1, PML, PPARG, PPP1R16B, PPP3CA, PPP3CB, |
| GO:0016301 | kinase activity | 4,92E-08 | [6] | 390,00 | 24,98 | ANGPT1, ANGPT4, ANKS1B, ANTXR1, APC, ARHGEF7, ATG14, ATM, ATP2B4, AURKB, BAZ1B, BCL10, BDNF, BDNF-AS, BMP2, BMP7, BRD4, BRSK2, BTC, CALM2, CAMK2B, CAMK2D, CAMK4, CARD10, CARD11, CARD14, CAV1, CCNA1, CCND2, CCNL1, CCNY, CDC25B, CDC25C, CDC42BPB, CDK1, CDK11B, CDK19, CDK2, CDK5R1, CDKL2, CDKN1A, CDKN2A, CEP43, CERKL, CHEK1, CHEK2, CIB1, CILK1, CIT, CLASP1, CLK3, CLK4, CLP1, CRIM1, CSNK1A1L, CSNK1E, CSNK1G3, CXCR4, DAB1, DAG1, DAPK1, DAXX, DBNDD2, DCAF1, DCLK1, DDR1, DDR2, DGKA, DGKD, DGKE, DGKH, DGKQ, DGKZ, DHDDS, DIPK2A, DLG1, DLG2, DLG4, DNAJC3, DOK7, DSTYK, DUS2, DUSP19, DUSP26, EEF1A1, EFNA4, EFNA5, EIF2AK3, EIF2AK4, EPHA1, EPHA10, EPHA4, EPHA6, EPHB1, EPHB3, EPS8L1, ERBB4, EREG, ETAA1, ETFA, EZH2, F3, FAF1, FASTK, FASTKD1, FCSK, FER, FGD2, FGF2, FGGY, FLT4, FN3KRP, FRS2, FZD10, FZD5, GADD45G, GAK, GALK2, GCG, GFRAL, GLYCTK, GMFB, GPRC5B, GPRC5C, GREM1, GRK3, GRK5, GRM1, GRM4, GSK3A, GTF2H1, HACD3, HEG1, HGS, HOOK1, HSP90AB1, HTATIP2, HTR2A, HTT, IDNK, IGF1, IGF1R, IKBKB, IL6R, INKA2, INSR, IPO7, IQGAP3, IRAK2, IRS2, ITGA1, ITK, JAK2, KALRN, KAT2B, KIDINS220, KIF2A, KITLG, KRAS, KSR1, KSR2, LATS2, LAX1, LDB2, LIMK2, LLGL1, LMTK2, LONP1, LPAR1, LRP5, LRRK1, LTBP1, MAGI2, MAK, MAP2K2, MAP2K5, MAP3K11, MAP3K13, MAP3K19, MAP3K2, MAP3K20, MAP3K5, MAP3K6, MAP4K4, MAP4K5, MAPK4, MAPK7, MAPK8, MAPK8IP3, MAPK9, MAPKAP1, MAPKAPK5, MARK3, MAST4, MCM2, MCPH1, MEN1, MKNK1, MKNK2, MMD, MST1R, MYLK, MYLK4, NCK2, NCKAP1L, NEDD9, NEK11, NEK7, NF1, NGF, NLK, NLRC5, NME2, NPPA, NPR1, NRXN1, NTRK2, OBSCN, OSBPL8, PAK4, PAK5, PANK4, PAPSS1, PASK, PDGFD, PDGFRA, PDGFRL, PDPK1, PEAK1, PFKFB3, PFKM, PFKP, PI4K2A, PIH1D1, PIK3CB, PIK3CG, PIK3IP1, PIK3R1, PIK3R5, PIKFYVE, PIM1, PIP4K2A, PIP5K1C, PKD1, PKIB, PKM, PKMYT1, PLCE1, PLK4, PLK5, PPM1F, PPP2R3C, PPP3CA, PRAG1, PRKAG2, PRKAR1B, PRKCA, PRKCB, PRKCE, PRKCH, PRKCI, PRKCQ, PRKCZ, PRKD2, PRKDC, PRKN, PRLR, PSEN1, PTEN, PTK2, PTPN11, PTPRC, PTPRH, PTPRO, RACK1, RAP1A, RAP2B, RAPGEF1, RASIP1, |
| GO:0071363 | cellular response to growth factor stimulus | 1,75E-02 | [5] | 183,00 | 25,00 | [ACVR2A, ACVRL1, ADAM17, AMH, APPL1, APPL2, ARHGEF18, ATP2B4, BCAR1, BDNF, BDNF-AS, BMP2, BMP3, BMP7, BMP8B, BMPER, CASR, CAV1, CCBE1, CHST11, CIB1, CITED2, COL2A1, COL3A1, COL4A2, CORO1B, CRB2, CREB3L1, CYFIP1, CYFIP2, DCN, DDIT4, DKK3, DLL1, DLL4, DSTYK, EEF1A1, EIF4A3, ERBB4, ESRP2, F11R, FAM89B, FBN1, FBN2, FERMT2, FEZ1, FGF12, FGF2, FGF22, FGF6, FGF7, FLRT1, FLT4, FNTA, FRS2, FUT8, GAB1, GALNT3, GARS1, GATA5, GCNT2, GRB10, GRB2, GREM1, HES1, HGS, HIVEP1, HNRNPH1, HRG, HSP90AB1, HTR3A, HTRA3, IL17RD, ING3, INSR, ITGA5, KIDINS220, KIF16B, LDLRAD4, LGMN, LIMS1, LRP2, LRRC32, LTBP1, LTBP2, LZTS1, MAGI2, MAP2K5, MAPK7, MARS1, MEF2C, MEGF8, MEN1, MICALL1, MYO1C, MYO1E, NEO1, NGF, NLK, NOS1, NOTCH1, NPNT, NPTN, NR3C1, NRXN1, NTRK2, NUS1, PARD3, PAX2, PAX9, PBLD, PCSK6, PDCD5, PDCD6, PDE8A, PDGFD, PDGFRA, PDGFRL, PDPK1, PDZD2, PEG10, PLK5, PML, POLR2B, POLR2F, PPARGC1A, PPP3CA, PRDM16, PRKCB, PRKCZ, PRKD2, PTEN, PTK2, PTPN11, PXDN, RAB35, RACK1, RAP1A, RAP1GAP, RAPGEF1, |
| GO:0005216 | ion channel activity | 3,40E-02 | [5, 6, 7, 8] | 167,00 | 25,11 | [ACTN2, AHNAK, ANK3, ANO1, ANO4, ANO6, ANO7, ANXA2, ANXA6, ASIC3, ASIC4, ATP2B4, ATP5F1A, ATP5ME, BEST1, CACNA1A, CACNA1B, CACNA1E, CACNA1G, CACNA1H, CACNA1I, CACNA2D2, CACNA2D3, CACNA2D4, CACNB2, CACNG1, CACNG6, CACNG8, CALM2, CAMK2D, CAV1, CCR2, CHRNB2, CHRNB3, CHRND, CHRNE, CLCN2, CLCN3, CNGA3, CNIH3, COMMD1, CTSS, DAPK1, DLG1, DLG2, DLG4, DPP10, FAM155A, FGF12, FGF14, FXYD5, FXYD6, GABRA2, GABRA4, GABRB2, GABRR3, GNB5, GPRIN1, GRIA4, GRID2, GRIK3, GRIK4, GSG1L, HCN1, HCN2, HCN4, HRC, HTR3A, HTR3C, HTT, ITPR2, ITPR3, JPH3, KCNAB2, KCNB1, KCNB2, KCNC1, KCNC3, KCNE1, KCNF1, KCNH2, KCNIP4, KCNJ14, KCNJ15, KCNJ2, KCNJ3, KCNJ6, KCNK2, KCNMA1, KCNQ1, KCNQ2, KCNV1, LLGL1, LRRC8B, LRRC8D, MAPK8IP2, MEF2C, MTNR1B, MYO5A, NALCN, NEDD4L, NLGN2, NOL3, NOS1, NOS1AP, NPPA, NRXN1, ORAI1, ORAI2, P2RY6, PACC1, PHLPP2, PIEZO1, PKD1, PKD1L1, PLA2G6, PLCG2, PPARGC1A, PRKCA, PRKCZ, PRKD2, PSEN1, PTPN3, RASA1, RASA3, RASGRF1, RELN, RGS9, RYR1, RYR2, RYR3, SCN1A, SCN2A, |
| GO:0060322 | head development | 9,11E-04 | [3] | 222,00 | 25,17 | [AARS1, AATK, ABAT, ABCB6, ABR, ADGRG1, ADGRL3, AFDN, ALK, ANKRD11, ARL13B, ARNT2, ASCL1, ASPH, ATAT1, ATG16L1, ATM, ATP2B4, ATP6V0D1, ATXN1, BAG6, BBS4, BBS7, BCL11B, BCL2, BCL2A1, BMERB1, BMP2, BMP7, BTG2, C21orf91, CA10, CACNA1A, CALM2, CASP2, CASP5, CCDC85C, CCKAR, CDH22, CDK5R1, CEND1, CEP290, CHD5, CHRNB2, CIC, CLASP1, CLP1, CNTN4, CNTNAP2, COL2A1, COL3A1, COL4A1, CPLANE1, CRISPLD1, CRISPLD2, CSNK1E, CTNNA2, CTNS, CXCR4, DAB1, DCLK1, DDIT4, DDX10, DLC1, DLL1, DLX2, DNAH5, DNAJC30, DOCK7, DSCAML1, EGR2, EPHB3, ERBB4, ETS1, EZH2, FEZ1, FEZF1, FEZF2, FPGS, FRS2, FZR1, GARS1, GLI2, GLI3, GPRIN1, GRID2, GSX2, HERC1, HES1, HES3, HNF1B, HNRNPD, HYDIN, IGF2BP1, IHH, IMMP2L, IRS2, ISL1, JHY, KALRN, KCNC1, KDM2B, KDM6B, KIF1A, KLHL1, KLHL17, KNDC1, KRAS, LAMB1, LHX6, LHX8, LIMK2, LPAR1, LRP1, LRP2, LZTS1, MACROD2, MAPK8IP3, MAPKAP1, MBD3, MBOAT7, MCPH1, MDGA1, MEIS2, MEN1, MKS1, MRTFA, MYH3, MYO16, MYO1D, NCOA6, NCSTN, NDE1, NDRG4, NEUROD6, NF1, NFIB, NOTCH1, NOTCH3, NRXN1, NTRK2, NUMB, OGDH, OLIG2, PAFAH1B1, PAX2, PAX9, PBX1, PDGFRA, PEX13, PFAS, PFKFB3, PHACTR1, PHLPP2, PITPNM1, PLCB1, PLXNA2, PPARGC1A, PPP3CA, |
| GO:0007169 | transmembrane receptor protein tyrosine kinase signaling pathway | 4,37E-03 | [5, 6, 7] | 193,00 | 25,26 | [ADAM17, AKT2, ALK, ANGPT1, ANGPT4, ANKS1A, ANKS1B, AP2A1, AP2A2, AP2S1, APC, APPL1, ARAP1, ARHGEF7, ARPC4, ATP6V0A1, ATP6V0D1, ATP6V1A, ATP6V1H, BAIAP2L1, BCAR1, BCAR3, BDNF, BDNF-AS, BMP2, BTC, CCBE1, CD8A, CDH13, CDK5R1, COL4A1, COL4A2, CREB3L1, CRIM1, CYFIP1, CYFIP2, DCN, DDIT4, DDR1, DDR2, DGKD, DGKQ, DLL1, DNM1, DOK4, DSTYK, EFNA4, EFNA5, EGR2, EIF2AK3, EIF4EBP1, EIF4EBP2, ELMO1, ENHO, EPHA1, EPHA10, EPHA4, EPHA6, EPHB1, EPHB3, EPS15L1, ERBB4, EREG, ESRP2, F3, FER, FGF12, FGF2, FGF22, FGF6, FGF7, FLRT1, FLT4, FOXO1, FRS2, GAB1, GAB2, GALNT3, GFRAL, GNAI2, GPER1, GPRIN1, GRB10, GRB2, GREM1, GSK3A, HGS, HIP1, HIP1R, HNRNPH1, HRG, IDE, IGF1, IGF1R, IL31RA, INSR, IRS2, ITGA1, ITGA5, JAK2, KALRN, KANK1, KIF16B, LGMN, LMTK2, LRIG2, LRP1, MST1R, MYO1C, MYO1E, NCK2, NCKAP1, NCKAP1L, NCSTN, NDRG4, NEDD9, NFATC4, NGEF, NGF, NPTN, NRXN1, NTRK2, NUS1, OSBPL8, PDCD6, PDGFD, PDGFRA, PDGFRL, PDPK1, PIK3CB, PIK3R1, PLAUR, PLCB1, PLCE1, POLR2B, POLR2F, PRKCA, PRKCB, PRKCQ, |
| GO:0008324 | cation transmembrane transporter activity | 4,08E-04 | [5, 7] | 226,00 | 25,34 | [ABCB11, ACTN2, ACTN4, AHNAK, ANK3, ANO1, ANO6, ANXA2, ANXA6, ASIC3, ASIC4, ATP12A, ATP13A2, ATP1A3, ATP1A4, ATP2A2, ATP2A3, ATP2B2, ATP2B4, ATP5F1A, ATP5ME, ATP6V0A1, ATP6V0D1, ATP6V1A, ATP6V1H, ATP8A1, C4orf3, CACNA1A, CACNA1B, CACNA1E, CACNA1G, CACNA1H, CACNA1I, CACNA2D2, CACNA2D3, CACNA2D4, CACNB2, CACNG1, CACNG6, CACNG8, CALM2, CAMK2D, CAV1, CCR2, CHRNB2, CHRNB3, CHRND, CHRNE, CLCN3, CLDN16, CNGA3, CNIH3, CNNM4, COMMD1, COX5A, COX6A1, COX7C, CTSS, DAPK1, DLG1, DLG2, DLG4, DPP10, FAM155A, FGF12, FGF14, FXYD5, FXYD6, GNB5, GPRIN1, GRIA4, GRIK3, GRIK4, GSG1L, HCN1, HCN2, HCN4, HECW1, HRC, HTR3A, HTR3C, HTT, ITPR2, ITPR3, JPH3, KCNAB2, KCNB1, KCNB2, KCNC1, KCNC3, KCNE1, KCNF1, KCNH2, KCNIP4, KCNJ14, KCNJ15, KCNJ2, KCNJ3, KCNJ6, KCNK2, KCNMA1, KCNQ1, KCNQ2, KCNV1, LLGL1, MAPK8IP2, MEF2C, MFSD3, MRS2, MTNR1B, MYO5A, NALCN, NDUFA4L2, NEDD4L, NIPA1, NLGN2, NNT, NOL3, NOS1, NOS1AP, NPPA, NRXN1, OCA2, ORAI1, ORAI2, P2RY6, PIEZO1, PKD1, PKD1L1, PLA2G6, PLCG2, PPARGC1A, PRKCA, PRKCE, PRKCZ, PRKD2, PSEN1, PTEN, PTPN3, RASA1, RASA3, RASGRF1, RELN, RGS9, RYR1, RYR2, RYR3, SCN1A, SCN2A, SCN3B, SCN5A, SGK1, SHANK2, SHISA6, SHISA7, SHISA9, SLC11A2, SLC12A7, SLC12A9, SLC13A3, SLC13A5, SLC15A4, SLC17A2, SLC17A4, SLC17A7, SLC1A1, SLC1A3, SLC22A5, SLC24A4, SLC25A45, SLC2A9, SLC30A2, |
| GO:0007010 | cytoskeleton organization | 1,27E-08 | [5] | 373,00 | 25,43 | ARHGAP12, ARHGAP17, ARHGAP21, ARHGAP40, ARHGDIA, ARHGDIB, ARHGEF10L, ARHGEF18, ARHGEF2, ARHGEF7, ARPC4, ATAT1, ATP8A2, ATXN7, AURKB, BAIAP2L1, BBS4, BCAR1, BCAS3, BCL2, BCL6, BFSP1, BFSP2, BIN3, BMERB1, BRSK2, C10orf90, CALM2, CAMSAP1, CAP1, CAP2, CAPZB, CARMIL1, CARMIL2, CC2D2A, CCDC13, CCDC88C, CD47, CDC20, CDC42BPB, CDK1, CDK2, CDK5R1, CELSR1, CEP131, CEP295, CEP43, CEP70, CETN1, CFAP46, CFAP74, CHD3, CHEK1, CHEK2, CHMP2A, CIB1, CIT, CLASP1, CLASP2, CLIP2, CNTROB, COBL, CORO1B, CORO2B, CORO7-PAM16, CSPG5, CTNNA2, CTTN, CUL3, CYFIP1, CYLD, DAG1, DBN1, DCTN1, DHDDS, DIAPH3, DLC1, DLG1, DNAH17, DNAH5, DNAH8, DNAJB13, DNAJB6, DOCK7, DST, EFCAB11, EFNA5, EHBP1L1, ELMO1, ELN, EML3, EPB41L4A, EPB41L4B, EPHA1, EPPK1, ESPN, ESPNL, EVL, EVPLL, F11R, F3, FAM171A1, FARP1, FARP2, FAT1, FCHSD1, FCHSD2, FER, FGD2, FGF7, FHOD1, FHOD3, FITM2, FMN1, FMNL2, FRMD5, FRMD6, FZD10, GAB1, GMFB, GRB2, HCLS1, HEPACAM2, HIP1, HIP1R, HOOK1, HRG, HTT, HYDIN, IKBKB, INPPL1, IQGAP2, IQGAP3, IQSEC1, IQSEC3, JAK2, JHY, KANK1, KANK3, KAT2B, KATNAL2, KIAA1614, KIF18A, KIF25, KIF2A, KIF2C, KIFC1, KIRREL1, KIZ, KLHL1, KLHL17, KRAS, KRT20, KRT25, KRT84, LDB3, LIMA1, LIMK2, LLGL1, LPAR1, LRP1, LURAP1, LZTS2, MACF1, MAP2, MAP3K20, MAP4, MAP6, MAP7, MAPKAP1, MARCKS, MARK3, MAST4, MCPH1, MEF2C, MICAL2, MICAL3, MICALL1, MPRIP, MRTFA, MYH3, MYL2, MYO15A, MYO18A, MYO1A, MYO1C, MYO1D, MYO1E, MYO5A, MYO5C, MYOM2, MYPN, NAV1, NAV3, NCK2, NCKAP1, NCKAP1L, NCKAP5, NDE1, NEDD9, NEK7, NF1, NINL, NOL3, NOS1AP, NPHP4, NPHS1, OBSCN, OBSL1, PACSIN1, PACSIN2, PAFAH1B1, PAK4, PAK5, PALLD, PALM, PARD3, PCLO, PCNT, PDCL3, PDGFRA, PDLIM1, PDLIM3, PDPK1, PEX14, PFN2, PHACTR1, PHLDB1, PIK3R1, PIP5K1C, PKD1, PKP1, PLCE1, PLEK, PLEKHG2, PLK4, PLXNB1, PPARGC1B, PPFIA1, PPL, PPM1F, PPP2R3C, PRKCE, PRKCI, PRKCZ, PRKD2, PRKN, PRUNE1, PTK2, PTPRA, PXDN, RANBP1, RANBP9, RASA1, RASSF1, RASSF8, RFLNA, RFLNB, RHOA, RHOBTB3, RHOG, RHOU, RICTOR, ROCK2, RP1, RTKN, RUFY3, SAMD14, SCIN, SDCBP, |

| | | | | | | |
|---|---|---|---|---|---|---|
| GO:0007010 | cytoskeleton organization | 1,27E-08 | [5] | 373,00 | 25,43 | ARHGAP12, ARHGAP17, ARHGAP21, ARHGAP40, ARHGDIA, ARHGDIB, ARHGEF10L, ARHGEF18, ARHGEF2, ARHGEF7, ARPC4, ATAT1, ATP8A2, ATXN7, AURKB, BAIAP2L1, BBS4, BCAR1, BCAS3, BCL2, BCL6, BFSP1, BFSP2, BIN3, BMERB1, BRSK2, C10orf90, CALM2, CAMSAP1, CAP1, CAP2, CAPZB, CARMIL1, CARMIL2, CC2D2A, CCDC13, CCDC88C, CD47, CDC20, CDC42BPB, CDK1, CDK2, CDK5R1, CELSR1, CEP131, CEP295, CEP43, CEP70, CETN1, CFAP46, CFAP74, CHD3, CHEK1, CHEK2, CHMP2A, CIB1, CIT, CLASP1, CLASP2, CLIP2, CNTROB, COBL, CORO1B, CORO2B, CORO7-PAM16, CSPG5, CTNNA2, CTTN, CUL3, CYFIP1, CYLD, DAG1, DBN1, DCTN1, DHDDS, DIAPH3, DLC1, DLG1, DNAH17, DNAH5, DNAH8, DNAJB13, DNAJB6, DOCK7, DST, EFCAB11, EFNA5, EHBP1L1, ELMO1, ELN, EML3, EPB41L4A, EPB41L4B, EPHA1, EPPK1, ESPN, ESPNL, EVL, EVPLL, F11R, F3, FAM171A1, FARP1, FARP2, FAT1, FCHSD1, FCHSD2, FER, FGD2, FGF7, FHOD1, FHOD3, FITM2, FMN1, FMNL2, FRMD5, FRMD6, FZD10, GAB1, GMFB, GRB2, HCLS1, HEPACAM2, HIP1, HIP1R, HOOK1, HRG, HTT, HYDIN, IKBKB, INPPL1, IQGAP2, IQGAP3, IQSEC1, IQSEC3, JAK2, JHY, KANK1, KANK3, KAT2B, KATNAL2, KIAA1614, KIF18A, KIF25, KIF2A, KIF2C, KIFC1, KIRREL1, KIZ, KLHL1, KLHL17, KRAS, KRT20, KRT25, KRT84, LDB3, LIMA1, LIMK2, LLGL1, LPAR1, LRP1, LURAP1, LZTS2, MACF1, MAP2, MAP3K20, MAP4, MAP6, MAP7, MAPKAP1, MARCKS, MARK3, MAST4, MCPH1, MEF2C, MICAL2, MICAL3, MICALL1, MPRIP, MRTFA, MYH3, MYL2, MYO15A, MYO18A, MYO1A, MYO1C, MYO1D, MYO1E, MYO5A, MYO5C, MYOM2, MYPN, NAV1, NAV3, NCK2, NCKAP1, NCKAP1L, NCKAP5, NDE1, NEDD9, NEK7, NF1, NINL, NOL3, NOS1AP, NPHP4, NPHS1, OBSCN, OBSL1, PACSIN1, PACSIN2, PAFAH1B1, PAK4, PAK5, PALLD, PALM, PARD3, PCLO, PCNT, PDCL3, PDGFRA, PDLIM1, PDLIM3, PDPK1, PEX14, PFN2, PHACTR1, PHLDB1, PIK3R1, PIP5K1C, PKD1, PKP1, PLCE1, PLEK, PLEKHG2, PLK4, PLXNB1, PPARGC1B, PPFIA1, PPL, PPM1F, PPP2R3C, PRKCE, PRKCI, PRKCZ, PRKD2, PRKN, PRUNE1, PTK2, PTPRA, PXDN, RANBP1, RANBP9, RASA1, RASSF1, RASSF8, RFLNA, RFLNB, RHOA, RHOBTB3, RHOG, RHOU, RICTOR, ROCK2, RP1, RTKN, RUFY3, SAMD14, SCIN, SDCBP, |
| GO:0007010 | cytoskeleton organization | 1,27E-08 | [5] | 373,00 | 25,43 | ARHGAP12, ARHGAP17, ARHGAP21, ARHGAP40, ARHGDIA, ARHGDIB, ARHGEF10L, ARHGEF18, ARHGEF2, ARHGEF7, ARPC4, ATAT1, ATP8A2, ATXN7, AURKB, BAIAP2L1, BBS4, BCAR1, BCAS3, BCL2, BCL6, BFSP1, BFSP2, BIN3, BMERB1, BRSK2, C10orf90, CALM2, CAMSAP1, CAP1, CAP2, CAPZB, CARMIL1, CARMIL2, CC2D2A, CCDC13, CCDC88C, CD47, CDC20, CDC42BPB, CDK1, CDK2, CDK5R1, CELSR1, CEP131, CEP295, CEP43, CEP70, CETN1, CFAP46, CFAP74, CHD3, CHEK1, CHEK2, CHMP2A, CIB1, CIT, CLASP1, CLASP2, CLIP2, CNTROB, COBL, CORO1B, CORO2B, CORO7-PAM16, CSPG5, CTNNA2, CTTN, CUL3, CYFIP1, CYLD, DAG1, DBN1, DCTN1, DHDDS, DIAPH3, DLC1, DLG1, DNAH17, DNAH5, DNAH8, DNAJB13, DNAJB6, DOCK7, DST, EFCAB11, EFNA5, EHBP1L1, ELMO1, ELN, EML3, EPB41L4A, EPB41L4B, EPHA1, EPPK1, ESPN, ESPNL, EVL, EVPLL, F11R, F3, FAM171A1, FARP1, FARP2, FAT1, FCHSD1, FCHSD2, FER, FGD2, FGF7, FHOD1, FHOD3, FITM2, FMN1, FMNL2, FRMD5, FRMD6, FZD10, GAB1, GMFB, GRB2, HCLS1, HEPACAM2, HIP1, HIP1R, HOOK1, HRG, HTT, HYDIN, IKBKB, INPPL1, IQGAP2, IQGAP3, IQSEC1, IQSEC3, JAK2, JHY, KANK1, KANK3, KAT2B, KATNAL2, KIAA1614, KIF18A, KIF25, KIF2A, KIF2C, KIFC1, KIRREL1, KIZ, KLHL1, KLHL17, KRAS, KRT20, KRT25, KRT84, LDB3, LIMA1, LIMK2, LLGL1, LPAR1, LRP1, LURAP1, LZTS2, MACF1, MAP2, MAP3K20, MAP4, MAP6, MAP7, MAPKAP1, MARCKS, MARK3, MAST4, MCPH1, MEF2C, MICAL2, MICAL3, MICALL1, MPRIP, MRTFA, MYH3, MYL2, MYO15A, MYO18A, MYO1A, MYO1C, MYO1D, MYO1E, MYO5A, MYO5C, MYOM2, MYPN, NAV1, NAV3, NCK2, NCKAP1, NCKAP1L, NCKAP5, NDE1, NEDD9, NEK7, NF1, NINL, NOL3, NOS1AP, NPHP4, NPHS1, OBSCN, OBSL1, PACSIN1, PACSIN2, PAFAH1B1, PAK4, PAK5, PALLD, PALM, PARD3, PCLO, PCNT, PDCL3, PDGFRA, PDLIM1, PDLIM3, PDPK1, PEX14, PFN2, PHACTR1, PHLDB1, PIK3R1, PIP5K1C, PKD1, PKP1, PLCE1, PLEK, PLEKHG2, PLK4, PLXNB1, PPARGC1B, PPFIA1, PPL, PPM1F, PPP2R3C, PRKCE, PRKCI, PRKCZ, PRKD2, PRKN, PRUNE1, PTK2, PTPRA, PXDN, RANBP1, RANBP9, RASA1, RASSF1, RASSF8, RFLNA, RFLNB, RHOA, RHOBTB3, RHOG, RHOU, RICTOR, ROCK2, RP1, RTKN, RUFY3, SAMD14, SCIN, SDCBP, |
| GO:0044057 | regulation of system process | 2,14E-02 | [3, 4] | 161,00 | 25,43 | [ABAT, ABCG8, ACP3, ADRA1B, ADRB1, ANXA6, ARHGAP42, ASPH, ATP1A3, ATP1A4, ATP2A2, ATP2A3, ATP2B2, ATP2B4, BEGAIN, CACNA1B, CACNA1G, CACNA1H, CACNA2D2, CACNA2D3, CACNA2D4, CACNB2, CACNG1, CACNG6, CACNG8, CALM2, CAMK2B, CAMK2D, CASR, CAV1, CELF2, CELF4, CORIN, CORO2B, CRY2, CTTN, DAG1, DHX15, DHX34, DLG1, DLG4, ECE1, EGR2, EIF2AK3, EIF4A3, FABP5, FBXO32, FGF12, FOXO1, FTO, FXYD6, GAA, GATA5, GJA5, GLRX3, GNAI2, GNAO1, GPER1, GPRIN1, GRM1, GSK3A, GSX2, GTF2IRD1, HCAR2, HCN4, HDAC4, HIP1R, HRC, HTR2A, IGF1, IGSF9B, IL6ST, ITPR2, ITPR3, JAK2, JARID2, KCNB2, KCNE1, KCNH2, KCNIP4, KCNJ14, KCNJ2, KCNJ3, KCNMA1, KCNQ1, LLGL1, LMCD1, MAG, MTG1, MTMR2, MTNR1B, MYL2, MYRF, NLGN2, NOS1, NOS1AP, NOTCH1, NPHS1, NPNT, NPPA, NPR1, NPTXR, NRXN1, OPRPN, PARD3, PDE9A, PDZD2, PER2, PIK3CG, PLCE1, PPARGC1A, PPP3CA, |
| GO:0044087 | regulation of cellular component biogenesis | 1,52E-05 | [3, 4] | 266,00 | 25,50 | [ACTR3B, ACVRL1, ADD1, ADD2, ADGRB2, ADGRB3, ADGRL3, AGRN, AKAP13, ALMS1, ANKS1B, ANTXR1, APC, APOBEC1, ARAP1, ARF1, ARHGAP40, ARHGEF10L, ARHGEF18, ARHGEF2, ARHGEF7, ARPC4, ASAP1, ATAT1, ATM, ATP13A2, BAIAP2L1, BBS10, BBS4, BCAS3, BDNF, BDNF-AS, BIN3, BIRC2, BMP7, C10orf90, CAMSAP1, CAND1, CAPZB, CARMIL1, CARMIL2, CAV1, CD47, CDK5R1, CDT1, CEP295, CHMP2A, CHRNB2, CLASP1, CLASP2, CNOT1, CNTROB, COBL, CORO1B, CORO2B, CRBN, CTNNA2, CTNNBIP1, CTTN, CYFIP1, CYLD, DAG1, DBN1, DCTN1, DEF8, DLC1, DLG1, DNAJB6, DNAJC15, DYNC2LI1, EFNA5, EHMT2, EIF4EBP1, ELN, EPHA1, EPHB1, EPHB3, EPS8L1, ERCC1, ERCC5, ESPN, ESR1, EVL, F11R, FAF1, FARP1, FARP2, FCHSD1, FCHSD2, FER, FEZ1, FHOD1, FHOD3, FLRT1, FMN1, FNIP2, FZD5, GMFB, GPC6, GPRIN1, GRB2, GREM1, GRID2, GTF2H1, HCLS1, HDAC4, HGS, HIP1R, HMGB1, HRG, HTT, IFT140, IFT88, IKBKB, IL1RAP, IQGAP2, ISL1, KANK1, KANK3, KAT2B, KIRREL1, LDB2, LDLRAD4, LIMS1, LPAR1, LRCH4, LRFN3, LRP1, LRSAM1, LZTS1, MACF1, MAK, MAP2, MAP4, MAP4K4, MAPK9, MDGA1, MEF2C, MPHOSPH8, MTMR3, MYO1C, MYO1E, NAPB, NAV3, NCK2, NCKAP1, NCKAP1L, NECTIN1, NLGN2, NOTCH1, NPHP4, NPHS1, NPRL3, NRXN1, NTNG2, NTRK2, PALM, PEAK1, PFN2, PHF2, PHLDB1, PIEZO1, PIH1D1, PIK3R1, PIKFYVE, PIP4K2A, PLA2G6, PLCE1, PLD1, PLEK, PLEKHG2, PLK4, PPFIA1, PPM1F, PPP1R16B, PPP3CA, PRKCA, PRKCE, PRKCH, PRKCZ, PRKN, PRUNE1, PSMC2, PSMC3, PSMC6, PTEN, PTK2, PTPN11, PXDN, RAB5A, RACK1, RAP1A, RAP1GAP, RAPGEF1, RASA1, RASIP1, RB1, RBX1, REST, RHOA, RHOG, RICTOR, RIOK3, RIPOR2, |
| GO:0007423 | sensory organ development | 1,79E-02 | [4, 5, 6] | 159,00 | 25,56 | [ABR, ACVRL1, ADAMTS18, ADGRV1, ASCL1, ATP2B4, ATP8A2, B9D1, BBS4, BBS7, BCAR3, BCL11B, BCL2, BDNF, BDNF-AS, BFSP1, BFSP2, BMP2, BMP7, BMPER, BNC2, CACNA1A, CASP2, CC2D2A, CDH23, CELF4, CELSR1, CEP290, CITED2, CLCN2, COL2A1, COL4A1, COL8A2, CRYBA4, CRYBG3, CRYGC, CTNS, CYP1B1, DDR1, DLG1, DLL1, DLL4, DLX2, ECE1, EPHA4, EPHB1, EYA4, FAT1, FBN1, FBN2, FGF2, FJX1, FRS2, FZD5, FZR1, GABRB2, GLI2, GLI3, HCN1, HES1, HOXA1, IFT140, IFT88, IHH, INSIG2, KCNK2, KCNQ1, KDM2B, LAMB2, LIMK2, LPCAT1, LRIG3, LRP10, LRP5, MAF, MAX, MCM2, MEIS2, MIPEP, MKS1, MTNR1B, MYH15, MYO15A, MYOM2, NECTIN1, NF1, NKD1, NOTCH1, NPHP4, NR2E3, NTRK2, OC90, OPN5, PAFAH1B1, PAX2, PAX8, PBX1, PDGFRA, PDZD7, POU4F2, PRDM16, PRKCI, PSEN1, PTF1A, PTPN11, RARA, RBP4, RD3, RET, RIPOR2, ROR1, ROR2, RORA, RORB, RP1, RRM1, SCRIB, SDK1, SEC24B, SERPINF1, |
| GO:0007399 | nervous system development | 4,32E-19 | [4, 5] | 662,00 | 25,59 | AP2A1, APBA2, APPL2, ARF1, ARHGDIA, ARHGEF2, ARHGEF7, ARL13B, ARNT2, ARNTL, ASAP1, ASCL1, ATAT1, ATF1, ATG16L1, ATM, ATN1, ATP2B2, ATP2B4, ATP6V0D1, ATP8A2, ATXN1, ATXN10, BAG6, BBS4, BBS7, BCL10, BCL11A, BCL11B, BCL2, BCL2A1, BCL6, BDNF, BDNF-AS, BEND6, BHLHE40, BLOC1S2, BLOC1S5, BMERB1, BMP2, BMP7, BRD2, BRINP2, BRSK2, BSN, BTBD6, BTD, BTG2, BTG4, C21orf91, CA10, CACNA1A, CALM2, CAMK2B, CAMSAP1, CAPRIN2, CASP2, CASP5, CASZ1, CBFA2T2, CC2D2A, CCDC85C, CCKAR, CCR2, CDC20, CDH22, CDH23, CDH4, CDK1, CDK5R1, CELSR1, CEND1, CEP290, CHD5, CHRNB2, CHST8, CIB1, CIC, CIT, CITED2, CLASP1, CLP1, CMTM8, CNTN4, CNTN5, CNTNAP2, COBL, COL2A1, COL3A1, COL4A1, CPLANE1, CPNE5, CRB2, CREB3L2, CRIM1, CSMD3, CSNK1E, CSPG5, CTNNA2, CTNND2, CTNS, CTTN, CUX1, CXCR4, CYFIP1, CYFIP2, DAB1, DAG1, DBN1, DCLK1, DCTN1, DDIT4, DDR1, DDX6, DENND5A, DHDDS, DHX30, DHX36, DLC1, DLG1, DLG4, DLL1, DLL4, DLX2, DNAH5, DNAJC30, DNER, DNM1L, DNMT3A, DOC2A, DOCK10, DOCK7, DOK4, DOK6, DPF1, DRGX, DSCAML1, DTX1, EFHD1, EFNA4, EFNA5, EGR2, EHMT2, EIF2AK3, EIF2AK4, EIF4E, EIF4ENIF1, ENC1, EPHA1, EPHA10, EPHA4, EPHA6, EPHB1, EPHB3, ERBB4, ETS1, ETV1, ETV6, EVL, EVX1, EZH2, FA2H, FARP1, FARP2, FBXW8, FERD3L, FEZ1, FEZF1, FEZF2, FGF12, FGF14, FGF2, FKTN, FLRT1, FPGS, FRS2, FRYL, FZD10, FZD5, FZR1, GAB1, GAB2, GABRA2, GABRA4, GABRB2, GAK, GALC, GARS1, GAS7, GFI1, GFRAL, GLI2, GLI3, GLIS2, GLMN, GMFB, GORASP1, GPC6, GPER1, GPR161, GPR17, GPRC5B, GPRIN1, GPSM1, GRB10, GRB2, GRID2, GSK3A, GSS, GSX2, HAPLN2, HAPLN3, HCLS1, HCN1, HDAC4, HDAC9, HECW1, HELT, HERC1, HES1, HES3, HES4, HEXB, HMGB1, HNF1B, HNRNPD, HOXA1, HOXA3, HOXC10, HSP90AB1, HYDIN, IFRD1, IFT140, IFT88, IGF2BP1, IGSF8, IGSF9B, IHH, IL1RAP, IL6ST, IMMP2L, INPP5F, INSC, IQSEC1, IRS2, ISL1, ITGA1, JAK2, JARID2, JHY, KALRN, KANK1, KCNC1, KCNQ2, KDM2B, KDM4C, KDM6B, KIDINS220, KIF1A, KIF26A, KIF2A, KIF5A, KIFBP, KLF7, KLHL1, KLHL17, KNDC1, KRAS, LAMA2, LAMB1, LAMB2, LDB2, LDLR, LEPR, LGR6, LHX4, LHX6, LHX8, LIG4, LINGO1, LLGL1, LMX1B, LOXL3, LPAR1, LRCH4, LRFN3, LRIG2, LRP1, LRP12, LRP2, LRRC4C, LSAMP, LZTS1, MACF1, MACROD2, MAFK, MAG, MAGI2, MAL, MALL, MAP2, MAP3K13, MAP4, MAP4K4, MAP6, MAPK7, MAPK8IP2, MAPK8IP3, MAPKAP1, MBD3, MBNL1, MBOAT7, MCPH1, MDGA1, MEF2C, MEGF8, MEIS2, MELTF, MEN1, MICALL1, MKS1, MMD, MME, MOG, MRTFA, MTMR2, MYO16, MYO1D, MYO5A, MYPN, MYRF, MYT1L, N4BP3, NAV1, NAV3, NCAM2, NCK2, NCKAP1, NCKAP1L, NCOA2, NCOA6, NCSTN, NDE1, NDRG4, NECTIN1, NEDD4L, NEO1, NEPRO, NEUROD6, NEUROG2, NF1, NFASC, NFATC4, NFE2L2, NFIB, NGEF, NGF, NHEJ1, NKD1, NLGN2, NME2, NOL3, NOS1, NOTCH1, NOTCH3, NPHP4, NPTN, NR1D1, NR2E3, NRSN1, NRSN2, NRXN1, NRXN3, NSMF, NTM, NTNG1, NTNG2, NTRK2, NUMB, OBSL1, OGDH, OLFM1, OLIG2, OPCML, PACSIN1, PAFAH1B1, PAK4, PALLD, PALM, PARD3, PAX2, PAX7, PAX8, PBX1, PCARE, PCDHA11, PCDHA6, PCDHA7, PCDHB9, PCLO, PDZD2, PDZD7, PER2, PEX13, PFKFB3, PHACTR1, PHLPP2, PIK3CB, PIK3R1, PIKFYVE, PITPNM1, PKD1, PKMYT1, PLCB1, PLK5, PLXNA1, PLXNA2, PLXNB1, PLXNC1, PLXND1, POMGNT2, POU4F2, POU6F2, PPARG, PPARGC1A, PPP3CA, PPP3CB, PRAG1, PRDM16, PRDM5, PRDM6, PRKCA, PRKCH, PRKCI, PRKCQ, PRKCZ, PRKD2, PRKDC, PRKN, PRMT1, PRPF19, PRUNE1, PRX, PSD, PSEN1, PTEN, PTF1A, PTK2, PTPN11, PTPRA, PTPRF, PTPRO, PTPRZ1, RAB29, RAB35, RANBP1, RANBP9, RAP1A, RAP1GAP, RAP1GAP2, RAPGEF1, |
| GO:0007399 | nervous system development | 4,32E-19 | [4, 5] | 662,00 | 25,59 | AP2A1, APBA2, APPL2, ARF1, ARHGDIA, ARHGEF2, ARHGEF7, ARL13B, ARNT2, ARNTL, ASAP1, ASCL1, ATAT1, ATF1, ATG16L1, ATM, ATN1, ATP2B2, ATP2B4, ATP6V0D1, ATP8A2, ATXN1, ATXN10, BAG6, BBS4, BBS7, BCL10, BCL11A, BCL11B, BCL2, BCL2A1, BCL6, BDNF, BDNF-AS, BEND6, BHLHE40, BLOC1S2, BLOC1S5, BMERB1, BMP2, BMP7, BRD2, BRINP2, BRSK2, BSN, BTBD6, BTD, BTG2, BTG4, C21orf91, CA10, CACNA1A, CALM2, CAMK2B, CAMSAP1, CAPRIN2, CASP2, CASP5, CASZ1, CBFA2T2, CC2D2A, CCDC85C, CCKAR, CCR2, CDC20, CDH22, CDH23, CDH4, CDK1, CDK5R1, CELSR1, CEND1, CEP290, CHD5, CHRNB2, CHST8, CIB1, CIC, CIT, CITED2, CLASP1, CLP1, CMTM8, CNTN4, CNTN5, CNTNAP2, COBL, COL2A1, COL3A1, COL4A1, CPLANE1, CPNE5, CRB2, CREB3L2, CRIM1, CSMD3, CSNK1E, CSPG5, CTNNA2, CTNND2, CTNS, CTTN, CUX1, CXCR4, CYFIP1, CYFIP2, DAB1, DAG1, DBN1, DCLK1, DCTN1, DDIT4, DDR1, DDX6, DENND5A, DHDDS, DHX30, DHX36, DLC1, DLG1, DLG4, DLL1, DLL4, DLX2, DNAH5, DNAJC30, DNER, DNM1L, DNMT3A, DOC2A, DOCK10, DOCK7, DOK4, DOK6, DPF1, DRGX, DSCAML1, DTX1, EFHD1, EFNA4, EFNA5, EGR2, EHMT2, EIF2AK3, EIF2AK4, EIF4E, EIF4ENIF1, ENC1, EPHA1, EPHA10, EPHA4, EPHA6, EPHB1, EPHB3, ERBB4, ETS1, ETV1, ETV6, EVL, EVX1, EZH2, FA2H, FARP1, FARP2, FBXW8, FERD3L, FEZ1, FEZF1, FEZF2, FGF12, FGF14, FGF2, FKTN, FLRT1, FPGS, FRS2, FRYL, FZD10, FZD5, FZR1, GAB1, GAB2, GABRA2, GABRA4, GABRB2, GAK, GALC, GARS1, GAS7, GFI1, GFRAL, GLI2, GLI3, GLIS2, GLMN, GMFB, GORASP1, GPC6, GPER1, GPR161, GPR17, GPRC5B, GPRIN1, GPSM1, GRB10, GRB2, GRID2, GSK3A, GSS, GSX2, HAPLN2, HAPLN3, HCLS1, HCN1, HDAC4, HDAC9, HECW1, HELT, HERC1, HES1, HES3, HES4, HEXB, HMGB1, HNF1B, HNRNPD, HOXA1, HOXA3, HOXC10, HSP90AB1, HYDIN, IFRD1, IFT140, IFT88, IGF2BP1, IGSF8, IGSF9B, IHH, IL1RAP, IL6ST, IMMP2L, INPP5F, INSC, IQSEC1, IRS2, ISL1, ITGA1, JAK2, JARID2, JHY, KALRN, KANK1, KCNC1, KCNQ2, KDM2B, KDM4C, KDM6B, KIDINS220, KIF1A, KIF26A, KIF2A, KIF5A, KIFBP, KLF7, KLHL1, KLHL17, KNDC1, KRAS, LAMA2, LAMB1, LAMB2, LDB2, LDLR, LEPR, LGR6, LHX4, LHX6, LHX8, LIG4, LINGO1, LLGL1, LMX1B, LOXL3, LPAR1, LRCH4, LRFN3, LRIG2, LRP1, LRP12, LRP2, LRRC4C, LSAMP, LZTS1, MACF1, MACROD2, MAFK, MAG, MAGI2, MAL, MALL, MAP2, MAP3K13, MAP4, MAP4K4, MAP6, MAPK7, MAPK8IP2, MAPK8IP3, MAPKAP1, MBD3, MBNL1, MBOAT7, MCPH1, MDGA1, MEF2C, MEGF8, MEIS2, MELTF, MEN1, MICALL1, MKS1, MMD, MME, MOG, MRTFA, MTMR2, MYO16, MYO1D, MYO5A, MYPN, MYRF, MYT1L, N4BP3, NAV1, NAV3, NCAM2, NCK2, NCKAP1, NCKAP1L, NCOA2, NCOA6, NCSTN, NDE1, NDRG4, NECTIN1, NEDD4L, NEO1, NEPRO, NEUROD6, NEUROG2, NF1, NFASC, NFATC4, NFE2L2, NFIB, NGEF, NGF, NHEJ1, NKD1, NLGN2, NME2, NOL3, NOS1, NOTCH1, NOTCH3, NPHP4, NPTN, NR1D1, NR2E3, NRSN1, NRSN2, NRXN1, NRXN3, NSMF, NTM, NTNG1, NTNG2, NTRK2, NUMB, OBSL1, OGDH, OLFM1, OLIG2, OPCML, PACSIN1, PAFAH1B1, PAK4, PALLD, PALM, PARD3, PAX2, PAX7, PAX8, PBX1, PCARE, PCDHA11, PCDHA6, PCDHA7, PCDHB9, PCLO, PDZD2, PDZD7, PER2, PEX13, PFKFB3, PHACTR1, PHLPP2, PIK3CB, PIK3R1, PIKFYVE, PITPNM1, PKD1, PKMYT1, PLCB1, PLK5, PLXNA1, PLXNA2, PLXNB1, PLXNC1, PLXND1, POMGNT2, POU4F2, POU6F2, PPARG, PPARGC1A, PPP3CA, PPP3CB, PRAG1, PRDM16, PRDM5, PRDM6, PRKCA, PRKCH, PRKCI, PRKCQ, PRKCZ, PRKD2, PRKDC, PRKN, PRMT1, PRPF19, PRUNE1, PRX, PSD, PSEN1, PTEN, PTF1A, PTK2, PTPN11, PTPRA, PTPRF, PTPRO, PTPRZ1, RAB29, RAB35, RANBP1, RANBP9, RAP1A, RAP1GAP, RAP1GAP2, RAPGEF1, |
| GO:0007399 | nervous system development | 4,32E-19 | [4, 5] | 662,00 | 25,59 | AP2A1, APBA2, APPL2, ARF1, ARHGDIA, ARHGEF2, ARHGEF7, ARL13B, ARNT2, ARNTL, ASAP1, ASCL1, ATAT1, ATF1, ATG16L1, ATM, ATN1, ATP2B2, ATP2B4, ATP6V0D1, ATP8A2, ATXN1, ATXN10, BAG6, BBS4, BBS7, BCL10, BCL11A, BCL11B, BCL2, BCL2A1, BCL6, BDNF, BDNF-AS, BEND6, BHLHE40, BLOC1S2, BLOC1S5, BMERB1, BMP2, BMP7, BRD2, BRINP2, BRSK2, BSN, BTBD6, BTD, BTG2, BTG4, C21orf91, CA10, CACNA1A, CALM2, CAMK2B, CAMSAP1, CAPRIN2, CASP2, CASP5, CASZ1, CBFA2T2, CC2D2A, CCDC85C, CCKAR, CCR2, CDC20, CDH22, CDH23, CDH4, CDK1, CDK5R1, CELSR1, CEND1, CEP290, CHD5, CHRNB2, CHST8, CIB1, CIC, CIT, CITED2, CLASP1, CLP1, CMTM8, CNTN4, CNTN5, CNTNAP2, COBL, COL2A1, COL3A1, COL4A1, CPLANE1, CPNE5, CRB2, CREB3L2, CRIM1, CSMD3, CSNK1E, CSPG5, CTNNA2, CTNND2, CTNS, CTTN, CUX1, CXCR4, CYFIP1, CYFIP2, DAB1, DAG1, DBN1, DCLK1, DCTN1, DDIT4, DDR1, DDX6, DENND5A, DHDDS, DHX30, DHX36, DLC1, DLG1, DLG4, DLL1, DLL4, DLX2, DNAH5, DNAJC30, DNER, DNM1L, DNMT3A, DOC2A, DOCK10, DOCK7, DOK4, DOK6, DPF1, DRGX, DSCAML1, DTX1, EFHD1, EFNA4, EFNA5, EGR2, EHMT2, EIF2AK3, EIF2AK4, EIF4E, EIF4ENIF1, ENC1, EPHA1, EPHA10, EPHA4, EPHA6, EPHB1, EPHB3, ERBB4, ETS1, ETV1, ETV6, EVL, EVX1, EZH2, FA2H, FARP1, FARP2, FBXW8, FERD3L, FEZ1, FEZF1, FEZF2, FGF12, FGF14, FGF2, FKTN, FLRT1, FPGS, FRS2, FRYL, FZD10, FZD5, FZR1, GAB1, GAB2, GABRA2, GABRA4, GABRB2, GAK, GALC, GARS1, GAS7, GFI1, GFRAL, GLI2, GLI3, GLIS2, GLMN, GMFB, GORASP1, GPC6, GPER1, GPR161, GPR17, GPRC5B, GPRIN1, GPSM1, GRB10, GRB2, GRID2, GSK3A, GSS, GSX2, HAPLN2, HAPLN3, HCLS1, HCN1, HDAC4, HDAC9, HECW1, HELT, HERC1, HES1, HES3, HES4, HEXB, HMGB1, HNF1B, HNRNPD, HOXA1, HOXA3, HOXC10, HSP90AB1, HYDIN, IFRD1, IFT140, IFT88, IGF2BP1, IGSF8, IGSF9B, IHH, IL1RAP, IL6ST, IMMP2L, INPP5F, INSC, IQSEC1, IRS2, ISL1, ITGA1, JAK2, JARID2, JHY, KALRN, KANK1, KCNC1, KCNQ2, KDM2B, KDM4C, KDM6B, KIDINS220, KIF1A, KIF26A, KIF2A, KIF5A, KIFBP, KLF7, KLHL1, KLHL17, KNDC1, KRAS, LAMA2, LAMB1, LAMB2, LDB2, LDLR, LEPR, LGR6, LHX4, LHX6, LHX8, LIG4, LINGO1, LLGL1, LMX1B, LOXL3, LPAR1, LRCH4, LRFN3, LRIG2, LRP1, LRP12, LRP2, LRRC4C, LSAMP, LZTS1, MACF1, MACROD2, MAFK, MAG, MAGI2, MAL, MALL, MAP2, MAP3K13, MAP4, MAP4K4, MAP6, MAPK7, MAPK8IP2, MAPK8IP3, MAPKAP1, MBD3, MBNL1, MBOAT7, MCPH1, MDGA1, MEF2C, MEGF8, MEIS2, MELTF, MEN1, MICALL1, MKS1, MMD, MME, MOG, MRTFA, MTMR2, MYO16, MYO1D, MYO5A, MYPN, MYRF, MYT1L, N4BP3, NAV1, NAV3, NCAM2, NCK2, NCKAP1, NCKAP1L, NCOA2, NCOA6, NCSTN, NDE1, NDRG4, NECTIN1, NEDD4L, NEO1, NEPRO, NEUROD6, NEUROG2, NF1, NFASC, NFATC4, NFE2L2, NFIB, NGEF, NGF, NHEJ1, NKD1, NLGN2, NME2, NOL3, NOS1, NOTCH1, NOTCH3, NPHP4, NPTN, NR1D1, NR2E3, NRSN1, NRSN2, NRXN1, NRXN3, NSMF, NTM, NTNG1, NTNG2, NTRK2, NUMB, OBSL1, OGDH, OLFM1, OLIG2, OPCML, PACSIN1, PAFAH1B1, PAK4, PALLD, PALM, PARD3, PAX2, PAX7, PAX8, PBX1, PCARE, PCDHA11, PCDHA6, PCDHA7, PCDHB9, PCLO, PDZD2, PDZD7, PER2, PEX13, PFKFB3, PHACTR1, PHLPP2, PIK3CB, PIK3R1, PIKFYVE, PITPNM1, PKD1, PKMYT1, PLCB1, PLK5, PLXNA1, PLXNA2, PLXNB1, PLXNC1, PLXND1, POMGNT2, POU4F2, POU6F2, PPARG, PPARGC1A, PPP3CA, PPP3CB, PRAG1, PRDM16, PRDM5, PRDM6, PRKCA, PRKCH, PRKCI, PRKCQ, PRKCZ, PRKD2, PRKDC, PRKN, PRMT1, PRPF19, PRUNE1, PRX, PSD, PSEN1, PTEN, PTF1A, PTK2, PTPN11, PTPRA, PTPRF, PTPRO, PTPRZ1, RAB29, RAB35, RANBP1, RANBP9, RAP1A, RAP1GAP, RAP1GAP2, RAPGEF1, |

| | | | | | |
|---|---|---|---|---|---|
| GO:0051347 | positive regulation of transferase activity | 1,47E-03 [5] | 194,00 | 25,63 | [ACSL1, ADAM17, ADCY2, ADCY3, ADCY5, ADCY7, AKAP13, ALK, ALKAL2, ALS2, AMBRA1, AMH, ANGPT1, ANGPT4, ARHGEF7, ATG14, ATP2B4, AURKB, BCL10, BDNF, BDNF-AS, BMP2, BTC, BTRC, CALM2, CARD10, CARD14, CCND2, CCNY, CCT2, CDC20, CDC20B, CDC25B, CDK1, CDK5R1, CDKN1A, CHTF8, CIB1, CXCR4, DAB1, DAG1, DAXX, DCUN1D1, DDR1, DDR2, DGKQ, DGKZ, DIPK2A, DLG1, DLG4, DOK7, DSTYK, DUSP19, EFNA5, EPHA1, EPHA10, EPHA4, EPHA6, EPHB1, EPHB3, ERBB4, EREG, ETAA1, ETFA, EZH2, F3, FGD2, FGF2, FLT4, FRS2, FZD10, FZD5, FZR1, GADD45G, GCG, GFRAL, GORASP1, GPRC5B, GPRC5C, GREM1, GRM1, GRM4, GSK3A, HACD3, HMBOX1, HNRNPD, HSP90AB1, HTR2A, IGF1, IGF1R, IL6R, INSR, IQGAP3, IRAK2, ITGA1, JAK2, KIDINS220, KITLG, KRAS, KSR1, LLGL1, LMTK2, LPAR1, MAP2K2, MAP2K5, MAP3K11, MAP3K13, MAP3K19, MAP3K2, MAP3K20, MAP3K5, MAP3K6, MAP4K4, MAP4K5, MAPK8IP3, MAPKAP1, MAPKAPK5, MMD, MST1R, NCKAP1L, NEDD9, NEK7, NGF, NRXN1, NTRK2, OSBPL8, PAK4, PAK5, PDGFD, PDGFRA, PDPK1, PDZD2, PIH1D1, PIK3CB, PIK3CG, PIK3R5, |
| GO:0051130 | positive regulation of cellular component organization | 3,07E-08 [3, 4, 5] | 335,00 | 25,77 | ARAP1, ARF1, ARHGDIA, ARHGEF10L, ARHGEF7, ARPC4, ASAP1, ATAT1, ATF1, ATM, ATP8A1, ATP8A2, AURKB, BAIAP2L1, BBS4, BCAS3, BCL2, BCL6, BDNF, BDNF-AS, BMP7, BRD4, BTC, C21orf91, CAMK2B, CAND1, CAPRIN2, CARMIL1, CARMIL2, CAV1, CBFA2T2, CCT2, CD47, CD53, CDC20, CDH4, CDK5R1, CDT1, CEP295, CLASP1, CLASP2, CNOT1, COBL, CORO1B, CPNE5, CRBN, CREB3L2, CTNNA2, CTTN, CUL3, CUX1, CXCR4, CYFIP1, DAG1, DBN1, DCN, DCTN1, DDHD2, DDR2, DEF8, DHX36, DLG1, DLG4, DLL1, DNM1, DNM1L, EFNA5, ENC1, ENPP2, EPHA1, EPHA4, EPHB1, EPHB3, EPS8L1, ERCC1, ERCC5, EREG, ESPN, ESR1, EVL, EZH2, F3, FAF1, FBXW8, FCHSD1, FCHSD2, FER, FEZ1, FHOD1, FLRT1, FMN1, FNIP2, FZD5, GCG, GMFB, GPER1, GRB2, GREM1, GRID2, GSK3A, GTF2H1, HCLS1, HDAC4, HIP1, HIP1R, HMBOX1, HNRNPD, HNRNPK, HPS4, HRG, HTT, IGF1, IGF1R, IL1RAP, ING3, INSR, IQGAP2, IQSEC1, ISL1, JARID2, KALRN, KAT7, KIDINS220, KIF2A, KIRREL1, KMT2E, KPNA7, LIG4, LIMS1, LLGL1, LPAR1, LRCH4, LRP1, LRP5, LRSAM1, LZTS1, MACF1, MAGI2, MAP3K13, MAP4K4, MAP6, MAPK8, MAPK8IP2, MAPK9, MAPKAPK5, MEGF8, MELTF, MIER1, MPHOSPH8, MYO1C, MYO1E, NAV3, NCK2, NCKAP1, NCKAP1L, NCOA2, NDRG4, NDUFA2, NEDD4L, NEK7, NFATC2, NFE2L2, NGF, NLGN2, NME2, NOS1, NPHP4, NPHS1, NPTN, NRXN1, NSD3, NTRK2, OBSL1, PACSIN1, PAF1, PAFAH1B1, PALM, PAX7, PDCD5, PFN2, PHF19, PHLDB1, PIEZO1, PIH1D1, PIP4K2A, PKIB, PLA2G6, PLAUR, PLCB1, PLCE1, PLCG2, PLEK, PLEKHM2, PLK4, PLK5, PLXNA1, PLXNA2, PLXNB1, PLXNC1, PLXND1, PML, POU4F2, PPARG, PPARGC1A, PPM1F, PPP1R13B, PPP3CA, PRKCB, PRKCE, PRKCI, PRKCQ, PRKD2, PRKN, PSEN1, PSMC2, PSMC3, PSMC6, PXDN, RACK1, RANBP1, RAP1A, RAPGEF1, RASIP1, RB1, RBX1, RELN, RET, RGMA, RHOA, RHOU, RICTOR, RIPOR2, ROBO1, ROCK2, ROR1, ROR2, RORA, RP1, RREB1, RUFY3, SCIN, SCYL2, SDCBP, SEMA4D, SEPTIN9, SERPINF1, SERPINI1, SH3PXD2B, SHANK2, SHTN1, SIN3A, SKAP1, SKIL, SLC6A4, SLITRK3, |
| GO:0051130 | positive regulation of cellular component organization | 3,07E-08 [3, 4, 5] | 335,00 | 25,77 | ARAP1, ARF1, ARHGDIA, ARHGEF10L, ARHGEF7, ARPC4, ASAP1, ATAT1, ATF1, ATM, ATP8A1, ATP8A2, AURKB, BAIAP2L1, BBS4, BCAS3, BCL2, BCL6, BDNF, BDNF-AS, BMP7, BRD4, BTC, C21orf91, CAMK2B, CAND1, CAPRIN2, CARMIL1, CARMIL2, CAV1, CBFA2T2, CCT2, CD47, CD53, CDC20, CDH4, CDK5R1, CDT1, CEP295, CLASP1, CLASP2, CNOT1, COBL, CORO1B, CPNE5, CRBN, CREB3L2, CTNNA2, CTTN, CUL3, CUX1, CXCR4, CYFIP1, DAG1, DBN1, DCN, DCTN1, DDHD2, DDR2, DEF8, DHX36, DLG1, DLG4, DLL1, DNM1, DNM1L, EFNA5, ENC1, ENPP2, EPHA1, EPHA4, EPHB1, EPHB3, EPS8L1, ERCC1, ERCC5, EREG, ESPN, ESR1, EVL, EZH2, F3, FAF1, FBXW8, FCHSD1, FCHSD2, FER, FEZ1, FHOD1, FLRT1, FMN1, FNIP2, FZD5, GCG, GMFB, GPER1, GRB2, GREM1, GRID2, GSK3A, GTF2H1, HCLS1, HDAC4, HIP1, HIP1R, HMBOX1, HNRNPD, HNRNPK, HPS4, HRG, HTT, IGF1, IGF1R, IL1RAP, ING3, INSR, IQGAP2, IQSEC1, ISL1, JARID2, KALRN, KAT7, KIDINS220, KIF2A, KIRREL1, KMT2E, KPNA7, LIG4, LIMS1, LLGL1, LPAR1, LRCH4, LRP1, LRP5, LRSAM1, LZTS1, MACF1, MAGI2, MAP3K13, MAP4K4, MAP6, MAPK8, MAPK8IP2, MAPK9, MAPKAPK5, MEGF8, MELTF, MIER1, MPHOSPH8, MYO1C, MYO1E, NAV3, NCK2, NCKAP1, NCKAP1L, NCOA2, NDRG4, NDUFA2, NEDD4L, NEK7, NFATC2, NFE2L2, NGF, NLGN2, NME2, NOS1, NPHP4, NPHS1, NPTN, NRXN1, NSD3, NTRK2, OBSL1, PACSIN1, PAF1, PAFAH1B1, PALM, PAX7, PDCD5, PFN2, PHF19, PHLDB1, PIEZO1, PIH1D1, PIP4K2A, PKIB, PLA2G6, PLAUR, PLCB1, PLCE1, PLCG2, PLEK, PLEKHM2, PLK4, PLK5, PLXNA1, PLXNA2, PLXNB1, PLXNC1, PLXND1, PML, POU4F2, PPARG, PPARGC1A, PPM1F, PPP1R13B, PPP3CA, PRKCB, PRKCE, PRKCI, PRKCQ, PRKD2, PRKN, PSEN1, PSMC2, PSMC3, PSMC6, PXDN, RACK1, RANBP1, RAP1A, RAPGEF1, RASIP1, RB1, RBX1, RELN, RET, RGMA, RHOA, RHOU, RICTOR, RIPOR2, ROBO1, ROCK2, ROR1, ROR2, RORA, RP1, RREB1, RUFY3, SCIN, SCYL2, SDCBP, SEMA4D, SEPTIN9, SERPINF1, SERPINI1, SH3PXD2B, SHANK2, SHTN1, SIN3A, SKAP1, SKIL, SLC6A4, SLITRK3, |
| GO:0022890 | inorganic cation transmembrane transporter activity | 1,52E-04 [5, 6, 7, 8] | 219,00 | 25,83 | [ABCB11, ACTN2, ACTN4, AHNAK, ANK3, ANO1, ANO6, ANXA2, ANXA6, ASIC3, ASIC4, ATP12A, ATP13A2, ATP1A3, ATP1A4, ATP2A2, ATP2A3, ATP2B2, ATP2B4, ATP5F1A, ATP5ME, ATP6V0A1, ATP6V0D1, ATP6V1A, ATP6V1H, ATP8A1, C4orf3, CACNA1A, CACNA1B, CACNA1E, CACNA1G, CACNA1H, CACNA1I, CACNA2D2, CACNA2D3, CACNA2D4, CACNB2, CACNG1, CACNG6, CACNG8, CALM2, CAMK2D, CAV1, CCR2, CHRNB2, CHRNB3, CHRND, CHRNE, CLCN3, CLDN16, CNGA3, CNIH3, CNNM4, COMMD1, COX5A, COX6A1, COX7C, CTSS, DAPK1, DLG1, DLG2, DLG4, DPP10, FAM155A, FGF12, FGF14, FXYD5, FXYD6, GNB5, GPRIN1, GRIA4, GRIK3, GRIK4, GSG1L, HCN1, HCN2, HCN4, HECW1, HRC, HTR3A, HTR3C, HTT, ITPR2, ITPR3, JPH3, KCNAB2, KCNB1, KCNB2, KCNC1, KCNC3, KCNE1, KCNF1, KCNH2, KCNIP4, KCNJ14, KCNJ15, KCNJ2, KCNJ3, KCNJ6, KCNK2, KCNMA1, KCNQ1, KCNQ2, KCNV1, LLGL1, MAPK8IP2, MEF2C, MFSD3, MRS2, MTNR1B, MYO5A, NALCN, NDUFA4L2, NEDD4L, NIPA1, NLGN2, NNT, NOL3, NOS1, NOS1AP, NPPA, NRXN1, ORAI1, ORAI2, P2RY6, PIEZO1, PKD1, PKD1L1, PLA2G6, PLCG2, PPARGC1A, PRKCA, PRKCE, PRKCZ, PRKD2, PSEN1, PTEN, PTPN3, RASA1, RASA3, RASGRF1, RELN, RGS9, RYR1, RYR2, RYR3, SCN1A, SCN2A, SCN3B, SCN5A, SGK1, SHANK2, SHISA6, SHISA7, SHISA9, SLC11A2, SLC12A7, SLC12A9, SLC13A3, SLC13A5, SLC15A4, SLC17A2, SLC17A4, SLC17A7, SLC1A1, SLC1A3, SLC24A4, SLC2A9, SLC30A2, SLC30A6, SLC39A10, SLC39A13, |
| GO:0048468 | cell development | 1,02E-17 [3, 4] | 597,00 | 25,84 | ANXA2, ANXA6, AP2A1, APPL2, ARF1, ARHGDIA, ARHGEF2, ARHGEF26, ARHGEF7, ARID4B, ARL13B, ARNTL, ASAP1, ASCL1, ATAT1, ATF1, ATM, ATP2A2, ATP8A2, ATXN10, BBS4, BCL11A, BCL11B, BCL2, BCL6, BDNF, BDNF-AS, BEND6, BFSP1, BFSP2, BHLHE40, BIN3, BLOC1S2, BLOC1S5, BMERB1, BMP2, BMP7, BNC1, BPGM, BRD1, BRINP2, BRSK2, BTG2, C21orf91, CACNA1A, CACNA2D2, CAMK2B, CAMSAP1, CAPRIN2, CARMIL1, CASP2, CASZ1, CBFA2T2, CCDC88C, CCKAR, CCR2, CCR6, CDC20, CDC25B, CDH23, CDH4, CDK1, CDK5R1, CDKN1A, CDYL, CELF4, CEP290, CHD5, CHRNB2, CHST11, CIB1, CITED2, CNTN4, CNTNAP2, COBL, COL18A1, COL3A1, COL6A1, COL6A2, CPNE5, CREB3L2, CSMD3, CSNK1E, CSPG5, CTNNA2, CTNND2, CTTN, CUL3, CUX1, CXCR4, CYFIP1, CYFIP2, DAB1, DAG1, DBN1, DCLK1, DDR1, DDX6, DENND5A, DHX15, DHX36, DLD, DLG4, DLL1, DLL4, DLX2, DMC1, DNASE1L2, DNASE1L3, DNER, DNM1L, DOCK10, DOCK7, DOK4, DOK6, DRGX, DSCAML1, DTX1, EFCAB9, EFHD1, EFNA4, EFNA5, EGR2, EHMT2, EIF2AK3, EIF2AK4, EIF4E, EIF4ENIF1, ELSPBP1, ENC1, EPHA1, EPHA10, EPHA4, EPHA6, EPHB1, EPHB3, ERBB4, ERCC1, EREG, ESR1, ETV1, EVL, EXPH5, EZH2, F11R, FA2H, FAM172A, FARP1, FARP2, FASN, FAT1, FBN1, FBXW8, FER, FERD3L, FERMT2, FEZ1, FEZF1, FEZF2, FHOD3, FLRT1, FNDC3A, FOXP1, FRMD6, FRS2, FRYL, FZD5, FZR1, GAB1, GAB2, GABRB2, GAK, GAS7, GFI1, GLI2, GLI3, GORASP1, GPER1, GPR4, GPRC5B, GPRIN1, GRB10, GRB2, GRID2, GSK3A, GSX2, HAPLN2, HCLS1, HCN1, HDAC4, HDAC9, HECW1, HEG1, HELT, HERC1, HES1, HES3, HES4, HEXB, HMGB1, HOOK1, HSD17B4, HSP90AB1, HYDIN, IFRD1, IFT140, IFT74, IFT88, IGF1, IHH, IKBKB, IL6ST, ING3, INPP5F, IQSEC1, IRF6, IRS2, ISL1, ITGA1, JAK2, KALRN, KANK1, KDM3A, KDM4C, KIDINS220, KIF1A, KIF5A, KITLG, KLF7, KLHL1, KMT2D, KNDC1, KRAS, L3MBTL3, LAMA2, LAMA5, LAMB1, LAMB2, LAMB4, LDB3, LDHD, LDLR, LGR6, LHX4, LHX6, LHX8, LIG4, LIMS1, LINGO1, LLGL1, LPAR1, LRIG2, LRP1, LRP12, LRP2, LRP5, LRRC4C, LRRK1, LZTS1, MACF1, MAF, MAG, MAGI2, MAP2, MAP3K13, MAP4, MAP4K4, MAP6, MAPK7, MAPK8IP2, MAPK8IP3, MARF1, MCMDC2, MEF2C, MEGF8, MEIOC, MELTF, MEN1, MICALL1, MKS1, MMD, MME, MOV10L1, MRTFA, MSH2, MSI2, MTMR2, MYD88, MYH3, MYL2, MYO16, MYO18B, MYO1E, MYOM2, MYPN, MYRF, MYT1L, NCAM2, NCK2, NCKAP1, NCKAP1L, NCOA2, NCSTN, NDRG4, NECTIN1, NEDD4L, NEDD9, NEO1, NEPRO, NF1, NFASC, NFATC2, NFATC4, NFE2L2, NFIB, NGEF, NGF, NME2, NOL3, NOS1, NOTCH1, NOTCH3, NOTCH4, NPHP4, NPHS1, NPPA, NPTN, NR1D1, NR2E3, NRXN1, NRXN3, NSMF, NTM, NTNG1, NTNG2, NTRK2, NUMB, NUP210L, OBSCN, OBSL1, OCA2, OGDH, OLFM1, OLIG2, OPCML, OSBP2, PABPC1L, PACSIN1, PAFAH1B1, PAK4, PALLD, PAQR8, PARD3, PAX2, PBX1, PCARE, PDCD6, PDGFRA, PDPK1, PDZD2, PDZD7, PEAK1, PER2, PHACTR1, PIK3CB, PIK3R1, PIP4K2A, PLK5, PLXNA1, PLXNA2, PLXNB1, PLXNC1, PLXND1, POU2F2, POU4F2, PPARG, PPP1R16B, PPP3CA, PPP3CB, PRAG1, PRKCA, PRKCH, PRKCI, PRKCQ, PRKCZ, PRKDC, PRKN, PRMT1, PRPF19, PRUNE1, PSD, PSEN1, PTEN, PTK2, PTPN11, PTPRA, PTPRC, PTPRF, PTPRO, PTPRZ1, PXDN, RAB29, RAB35, RADIL, RANBP1, RANBP9, RAP1A, RAP1GAP, RAP1GAP2, RAPGEF1, RARA, RASGRF1, RB1, RBFOX2, RELN, REST, |
| GO:0048468 | cell development | 1,02E-17 [3, 4] | 597,00 | 25,84 | ANXA2, ANXA6, AP2A1, APPL2, ARF1, ARHGDIA, ARHGEF2, ARHGEF26, ARHGEF7, ARID4B, ARL13B, ARNTL, ASAP1, ASCL1, ATAT1, ATF1, ATM, ATP2A2, ATP8A2, ATXN10, BBS4, BCL11A, BCL11B, BCL2, BCL6, BDNF, BDNF-AS, BEND6, BFSP1, BFSP2, BHLHE40, BIN3, BLOC1S2, BLOC1S5, BMERB1, BMP2, BMP7, BNC1, BPGM, BRD1, BRINP2, BRSK2, BTG2, C21orf91, CACNA1A, CACNA2D2, CAMK2B, CAMSAP1, CAPRIN2, CARMIL1, CASP2, CASZ1, CBFA2T2, CCDC88C, CCKAR, CCR2, CCR6, CDC20, CDC25B, CDH23, CDH4, CDK1, CDK5R1, CDKN1A, CDYL, CELF4, CEP290, CHD5, CHRNB2, CHST11, CIB1, CITED2, CNTN4, CNTNAP2, COBL, COL18A1, COL3A1, COL6A1, COL6A2, CPNE5, CREB3L2, CSMD3, CSNK1E, CSPG5, CTNNA2, CTNND2, CTTN, CUL3, CUX1, CXCR4, CYFIP1, CYFIP2, DAB1, DAG1, DBN1, DCLK1, DDR1, DDX6, DENND5A, DHX15, DHX36, DLD, DLG4, DLL1, DLL4, DLX2, DMC1, DNASE1L2, DNASE1L3, DNER, DNM1L, DOCK10, DOCK7, DOK4, DOK6, DRGX, DSCAML1, DTX1, EFCAB9, EFHD1, EFNA4, EFNA5, EGR2, EHMT2, EIF2AK3, EIF2AK4, EIF4E, EIF4ENIF1, ELSPBP1, ENC1, EPHA1, EPHA10, EPHA4, EPHA6, EPHB1, EPHB3, ERBB4, ERCC1, EREG, ESR1, ETV1, EVL, EXPH5, EZH2, F11R, FA2H, FAM172A, FARP1, FARP2, FASN, FAT1, FBN1, FBXW8, FER, FERD3L, FERMT2, FEZ1, FEZF1, FEZF2, FHOD3, FLRT1, FNDC3A, FOXP1, FRMD6, FRS2, FRYL, FZD5, FZR1, GAB1, GAB2, GABRB2, GAK, GAS7, GFI1, GLI2, GLI3, GORASP1, GPER1, GPR4, GPRC5B, GPRIN1, GRB10, GRB2, GRID2, GSK3A, GSX2, HAPLN2, HCLS1, HCN1, HDAC4, HDAC9, HECW1, HEG1, HELT, HERC1, HES1, HES3, HES4, HEXB, HMGB1, HOOK1, HSD17B4, HSP90AB1, HYDIN, IFRD1, IFT140, IFT74, IFT88, IGF1, IHH, IKBKB, IL6ST, ING3, INPP5F, IQSEC1, IRF6, IRS2, ISL1, ITGA1, JAK2, KALRN, KANK1, KDM3A, KDM4C, KIDINS220, KIF1A, KIF5A, KITLG, KLF7, KLHL1, KMT2D, KNDC1, KRAS, L3MBTL3, LAMA2, LAMA5, LAMB1, LAMB2, LAMB4, LDB3, LDHD, LDLR, LGR6, LHX4, LHX6, LHX8, LIG4, LIMS1, LINGO1, LLGL1, LPAR1, LRIG2, LRP1, LRP12, LRP2, LRP5, LRRC4C, LRRK1, LZTS1, MACF1, MAF, MAG, MAGI2, MAP2, MAP3K13, MAP4, MAP4K4, MAP6, MAPK7, MAPK8IP2, MAPK8IP3, MARF1, MCMDC2, MEF2C, MEGF8, MEIOC, MELTF, MEN1, MICALL1, MKS1, MMD, MME, MOV10L1, MRTFA, MSH2, MSI2, MTMR2, MYD88, MYH3, MYL2, MYO16, MYO18B, MYO1E, MYOM2, MYPN, MYRF, MYT1L, NCAM2, NCK2, NCKAP1, NCKAP1L, NCOA2, NCSTN, NDRG4, NECTIN1, NEDD4L, NEDD9, NEO1, NEPRO, NF1, NFASC, NFATC2, NFATC4, NFE2L2, NFIB, NGEF, NGF, NME2, NOL3, NOS1, NOTCH1, NOTCH3, NOTCH4, NPHP4, NPHS1, NPPA, NPTN, NR1D1, NR2E3, NRXN1, NRXN3, NSMF, NTM, NTNG1, NTNG2, NTRK2, NUMB, NUP210L, OBSCN, OBSL1, OCA2, OGDH, OLFM1, OLIG2, OPCML, OSBP2, PABPC1L, PACSIN1, PAFAH1B1, PAK4, PALLD, PAQR8, PARD3, PAX2, PBX1, PCARE, PDCD6, PDGFRA, PDPK1, PDZD2, PDZD7, PEAK1, PER2, PHACTR1, PIK3CB, PIK3R1, PIP4K2A, PLK5, PLXNA1, PLXNA2, PLXNB1, PLXNC1, PLXND1, POU2F2, POU4F2, PPARG, PPP1R16B, PPP3CA, PPP3CB, PRAG1, PRKCA, PRKCH, PRKCI, PRKCQ, PRKCZ, PRKDC, PRKN, PRMT1, PRPF19, PRUNE1, PSD, PSEN1, PTEN, PTK2, PTPN11, PTPRA, PTPRC, PTPRF, PTPRO, PTPRZ1, PXDN, RAB29, RAB35, RADIL, RANBP1, RANBP9, RAP1A, RAP1GAP, RAP1GAP2, RAPGEF1, RARA, RASGRF1, RB1, RBFOX2, RELN, REST, |
| GO:0048468 | cell development | 1,02E-17 [3, 4] | 597,00 | 25,84 | ANXA2, ANXA6, AP2A1, APPL2, ARF1, ARHGDIA, ARHGEF2, ARHGEF26, ARHGEF7, ARID4B, ARL13B, ARNTL, ASAP1, ASCL1, ATAT1, ATF1, ATM, ATP2A2, ATP8A2, ATXN10, BBS4, BCL11A, BCL11B, BCL2, BCL6, BDNF, BDNF-AS, BEND6, BFSP1, BFSP2, BHLHE40, BIN3, BLOC1S2, BLOC1S5, BMERB1, BMP2, BMP7, BNC1, BPGM, BRD1, BRINP2, BRSK2, BTG2, C21orf91, CACNA1A, CACNA2D2, CAMK2B, CAMSAP1, CAPRIN2, CARMIL1, CASP2, CASZ1, CBFA2T2, CCDC88C, CCKAR, CCR2, CCR6, CDC20, CDC25B, CDH23, CDH4, CDK1, CDK5R1, CDKN1A, CDYL, CELF4, CEP290, CHD5, CHRNB2, CHST11, CIB1, CITED2, CNTN4, CNTNAP2, COBL, COL18A1, COL3A1, COL6A1, COL6A2, CPNE5, CREB3L2, CSMD3, CSNK1E, CSPG5, CTNNA2, CTNND2, CTTN, CUL3, CUX1, CXCR4, CYFIP1, CYFIP2, DAB1, DAG1, DBN1, DCLK1, DDR1, DDX6, DENND5A, DHX15, DHX36, DLD, DLG4, DLL1, DLL4, DLX2, DMC1, DNASE1L2, DNASE1L3, DNER, DNM1L, DOCK10, DOCK7, DOK4, DOK6, DRGX, DSCAML1, DTX1, EFCAB9, EFHD1, EFNA4, EFNA5, EGR2, EHMT2, EIF2AK3, EIF2AK4, EIF4E, EIF4ENIF1, ELSPBP1, ENC1, EPHA1, EPHA10, EPHA4, EPHA6, EPHB1, EPHB3, ERBB4, ERCC1, EREG, ESR1, ETV1, EVL, EXPH5, EZH2, F11R, FA2H, FAM172A, FARP1, FARP2, FASN, FAT1, FBN1, FBXW8, FER, FERD3L, FERMT2, FEZ1, FEZF1, FEZF2, FHOD3, FLRT1, FNDC3A, FOXP1, FRMD6, FRS2, FRYL, FZD5, FZR1, GAB1, GAB2, GABRB2, GAK, GAS7, GFI1, GLI2, GLI3, GORASP1, GPER1, GPR4, GPRC5B, GPRIN1, GRB10, GRB2, GRID2, GSK3A, GSX2, HAPLN2, HCLS1, HCN1, HDAC4, HDAC9, HECW1, HEG1, HELT, HERC1, HES1, HES3, HES4, HEXB, HMGB1, HOOK1, HSD17B4, HSP90AB1, HYDIN, IFRD1, IFT140, IFT74, IFT88, IGF1, IHH, IKBKB, IL6ST, ING3, INPP5F, IQSEC1, IRF6, IRS2, ISL1, ITGA1, JAK2, KALRN, KANK1, KDM3A, KDM4C, KIDINS220, KIF1A, KIF5A, KITLG, KLF7, KLHL1, KMT2D, KNDC1, KRAS, L3MBTL3, LAMA2, LAMA5, LAMB1, LAMB2, LAMB4, LDB3, LDHD, LDLR, LGR6, LHX4, LHX6, LHX8, LIG4, LIMS1, LINGO1, LLGL1, LPAR1, LRIG2, LRP1, LRP12, LRP2, LRP5, LRRC4C, LRRK1, LZTS1, MACF1, MAF, MAG, MAGI2, MAP2, MAP3K13, MAP4, MAP4K4, MAP6, MAPK7, MAPK8IP2, MAPK8IP3, MARF1, MCMDC2, MEF2C, MEGF8, MEIOC, MELTF, MEN1, MICALL1, MKS1, MMD, MME, MOV10L1, MRTFA, MSH2, MSI2, MTMR2, MYD88, MYH3, MYL2, MYO16, MYO18B, MYO1E, MYOM2, MYPN, MYRF, MYT1L, NCAM2, NCK2, NCKAP1, NCKAP1L, NCOA2, NCSTN, NDRG4, NECTIN1, NEDD4L, NEDD9, NEO1, NEPRO, NF1, NFASC, NFATC2, NFATC4, NFE2L2, NFIB, NGEF, NGF, NME2, NOL3, NOS1, NOTCH1, NOTCH3, NOTCH4, NPHP4, NPHS1, NPPA, NPTN, NR1D1, NR2E3, NRXN1, NRXN3, NSMF, NTM, NTNG1, NTNG2, NTRK2, NUMB, NUP210L, OBSCN, OBSL1, OCA2, OGDH, OLFM1, OLIG2, OPCML, OSBP2, PABPC1L, PACSIN1, PAFAH1B1, PAK4, PALLD, PAQR8, PARD3, PAX2, PBX1, PCARE, PDCD6, PDGFRA, PDPK1, PDZD2, PDZD7, PEAK1, PER2, PHACTR1, PIK3CB, PIK3R1, PIP4K2A, PLK5, PLXNA1, PLXNA2, PLXNB1, PLXNC1, PLXND1, POU2F2, POU4F2, PPARG, PPP1R16B, PPP3CA, PPP3CB, PRAG1, PRKCA, PRKCH, PRKCI, PRKCQ, PRKCZ, PRKDC, PRKN, PRMT1, PRPF19, PRUNE1, PSD, PSEN1, PTEN, PTK2, PTPN11, PTPRA, PTPRC, PTPRF, PTPRO, PTPRZ1, PXDN, RAB29, RAB35, RADIL, RANBP1, RANBP9, RAP1A, RAP1GAP, RAP1GAP2, RAPGEF1, RARA, RASGRF1, RB1, RBFOX2, RELN, REST, |
| GO:0033674 | positive regulation of kinase activity | 3,23E-03 [6, 7, 8, 9] | 173,00 | 25,86 | [ACSL1, ADAM17, ADCY2, ADCY3, ADCY5, ADCY7, AKAP13, ALK, ALKAL2, ALS2, AMBRA1, AMH, ANGPT1, ANGPT4, ARHGEF7, ATG14, ATP2B4, BCL10, BDNF, BDNF-AS, BMP2, BTC, CALM2, CARD10, CARD14, CCND2, CCNY, CDC25B, CDK1, CDK5R1, CDKN1A, CIB1, CXCR4, DAB1, DAG1, DAXX, DDR1, DDR2, DGKQ, DGKZ, DIPK2A, DLG1, DLG4, DOK7, DSTYK, DUSP19, EFNA5, EPHA1, EPHA10, EPHA4, EPHA6, EPHB1, EPHB3, ERBB4, EREG, ETAA1, ETFA, EZH2, F3, FGD2, FGF2, FLT4, FRS2, FZD10, FZD5, GADD45G, GCG, GFRAL, GPRC5B, GPRC5C, GREM1, GRM1, GRM4, HACD3, HSP90AB1, HTR2A, IGF1, IGF1R, IL6R, INSR, IQGAP3, IRAK2, ITGA1, JAK2, KIDINS220, KITLG, KRAS, KSR1, LLGL1, LMTK2, LPAR1, MAP2K2, MAP2K5, MAP3K11, MAP3K13, MAP3K19, MAP3K2, MAP3K20, MAP3K5, MAP3K6, MAP4K4, MAP4K5, MAPK8IP3, MAPKAP1, MAPKAPK5, MMD, MST1R, NCKAP1L, NEDD9, NGF, NRXN1, NTRK2, OSBPL8, PAK4, PAK5, PDGFD, PDGFRA, PDPK1, PIH1D1, PIK3CB, PIK3CG, PIK3R5, PKD1, PLCE1, PPP2R3C, PPP3CA, PRKAG2, PRKAR1B, PRKCA, PRKCZ, PRKD2, PRLR, PSEN1, PTK2, PTPN11, PTPRC, |
| GO:0060627 | regulation of vesicle-mediated transport | 1,59E-02 [3, 4, 5] | 152,00 | 25,89 | [ABL2, ABR, ACTN2, ACTN4, AKT2, ANGPT1, ANKRD13B, ANO6, ANXA2, AP2A1, AP2S1, APBA2, APPL1, APPL2, ARF1, ARFGAP1, ATP13A2, ATP2A2, BRSK2, BSN, BTBD9, C12orf4, CACNA1A, CACNA1G, CACNA1H, CACNA1I, CACNB2, CADPS, CADPS2, CAV1, CCR2, CD47, CDH13, CDK5R1, CHMP2A, CHRNB2, CHRNB3, CLASP1, CLASP2, CNST, CSPG5, CYFIP1, DGKQ, DLG4, DLL1, DNAJC6, DNM1, DNM1L, DOC2A, EIPR1, EXPH5, F3, FER, FGF14, GAB2, GNAI2, GREM1, GSG1L, HGS, HIP1, HIP1R, HMGB1, HNRNPC, HNRNPK, HTR2A, IL15RA, INPP5F, INSR, ITGB2, KCNB1, LLGL1, LRP1, LRPAP1, LRSAM1, MAGI2, MAP2K2, MAPK8IP2, MFGE8, MTMR2, MYO18A, NAPB, NCKAP1L, NDRG4, NDUFA2, NEDD4L, NLGN2, NOL3, NOTCH1, NPPA, NRXN1, NRXN3, NUMB, PACSIN1, PACSIN2, PCLO, PDPK1, PFN2, PIK3CB, PLA2G6, PLCG2, PLD1, PPARG, PPP3CA, PPP3CB, PRKAR1B, PRKCB, PRKN, PTEN, PTPRC, RAB29, RAB3D, RAB43, RAB4A, RAB5A, |

| | | | | | | |
|---|---|---|---|---|---|---|
| GO:0004672 | protein kinase activity | 8,12E-09 | [7, 8] | 338,00 | 26,00 | ANTXR1, APC, ARHGEF7, ATM, ATP2B4, AURKB, BAZ1B, BDNF, BDNF-AS, BMP2, BMP7, BRD4, BRSK2, BTC, CALM2, CAMK2B, CAMK2D, CAMK4, CARD10, CARD14, CAV1, CCNA1, CCND2, CCNL1, CCNY, CDC25B, CDC25C, CDC42BPB, CDK1, CDK11B, CDK19, CDK2, CDK5R1, CDKL2, CDKN1A, CDKN2A, CEP43, CHEK1, CHEK2, CIB1, CILK1, CIT, CLASP1, CLK3, CLK4, CRIM1, CSNK1A1L, CSNK1E, CSNK1G3, CXCR4, DAB1, DAG1, DAPK1, DAXX, DBNDD2, DCAF1, DCLK1, DDR1, DDR2, DGKQ, DHDDS, DIPK2A, DLG1, DLG4, DNAJC3, DOK7, DSTYK, DUS2, DUSP19, DUSP26, EFNA4, EFNA5, EIF2AK3, EIF2AK4, EPHA1, EPHA10, EPHA4, EPHA6, EPHB1, EPHB3, ERBB4, EREG, ETAA1, ETFA, EZH2, F3, FAF1, FASTK, FASTKD1, FER, FGD2, FGF2, FLT4, FRS2, FZD10, FZD5, GADD45G, GAK, GCG, GFRAL, GMFB, GPRC5B, GPRC5C, GREM1, GRK3, GRK5, GRM1, GRM4, GSK3A, GTF2H1, HACD3, HEG1, HGS, HSP90AB1, HTATIP2, HTR2A, HTT, IGF1, IGF1R, IKBKB, IL6R, INKA2, INSR, IPO7, IQGAP3, IRAK2, ITGA1, ITK, JAK2, KALRN, KAT2B, KIDINS220, KITLG, KRAS, KSR1, KSR2, LATS2, LAX1, LIMK2, LLGL1, LMTK2, LONP1, LPAR1, LRP5, LRRK1, LTBP1, MAK, MAP2K2, MAP2K5, MAP3K11, MAP3K13, MAP3K19, MAP3K2, MAP3K20, MAP3K5, MAP3K6, MAP4K4, MAP4K5, MAPK4, MAPK7, MAPK8, MAPK8IP3, MAPK9, MAPKAP1, MAPKAPK5, MARK3, MAST4, MCM2, MEN1, MKNK1, MKNK2, MMD, MST1R, MYLK, MYLK4, NCK2, NCKAP1L, NEDD9, NEK11, NEK7, NF1, NGF, NLK, NME2, NPPA, NPR1, NRXN1, NTRK2, OBSCN, OSBPL8, PAK4, PAK5, PASK, PDGFD, PDGFRA, PDGFRL, PDPK1, PEAK1, PIH1D1, PIK3CB, PIK3CG, PIK3R5, PIM1, PKD1, PKIB, PKMYT1, PLCE1, PLK4, PLK5, PPM1F, PPP2R3C, PPP3CA, PRAG1, PRKAG2, PRKAR1B, PRKCA, PRKCB, PRKCE, PRKCH, PRKCI, PRKCQ, PRKCZ, PRKD2, PRKDC, PRLR, PSEN1, PTEN, PTK2, PTPN11, PTPRC, PTPRH, PTPRO, RACK1, RAP1A, RAP2B, RAPGEF1, RASIP1, RB1, RELN, RET, RGS3, RHBDF2, RHOA, RICTOR, RIOK3, RIPK1, ROBO1, ROCK2, ROR1, ROR2, RORA, RPS6KA2, RPS6KB1, RPS6KB2, RPTOR, SASH1, |
| GO:0034762 | regulation of transmembrane transport | 6,20E-03 | [3, 4, 5] | 158,00 | 26,07 | [ACSL6, ACTN2, ACTN4, AHNAK, AKT2, ANK3, ANO6, APPL1, APPL2, ARF1, ARL6IP5, ASPSCR1, ATP2B4, BCL2, C4orf3, CACNA1A, CACNA1B, CACNA1E, CACNA1G, CACNA1H, CACNA1I, CACNA2D2, CACNA2D3, CACNA2D4, CACNB2, CACNG1, CACNG6, CACNG8, CALM2, CAMK2D, CAV1, CCR2, CLCN2, CLTCL1, CNIH3, COMMD1, CRBN, CTSS, DAPK1, DLG1, DLG2, DLG4, DNAJC30, DPP10, F2RL3, FABP5, FGF12, FGF14, FXYD5, FXYD6, GJA5, GNAI2, GNB5, GPER1, GPRIN1, GRB10, GRIA4, GSG1L, GSK3A, HCN1, HCN2, HCN4, HECW1, HRC, HTR3A, HTT, IGF1, INSR, IRS2, JPH3, KCNAB2, KCNB1, KCNB2, KCNC1, KCNC3, KCNE1, KCNF1, KCNH2, KCNIP4, KCNJ14, KCNJ15, KCNJ2, KCNJ3, KCNJ6, KCNMA1, KCNQ1, KCNQ2, KCNV1, KIF2A, LLGL1, LONP1, MAPK8IP2, MEF2C, MTNR1B, MYO5A, NALCN, NEDD4L, NFE2L2, NLGN2, NOL3, NOS1, NOS1AP, NPPA, NRXN1, OSBPL8, P2RY6, PDPK1, PER2, PIK3CG, PIK3R1, PIM1, |
| GO:0044089 | positive regulation of cellular component biogenesis | 7,78E-03 | [3, 4, 5] | 154,00 | 26,10 | [ACTR3B, ADGRB2, ADGRB3, ADGRL3, AGRN, ANTXR1, APC, ARAP1, ARF1, ARHGEF10L, ARPC4, ASAP1, ATAT1, ATM, ATP13A2, BAIAP2L1, BBS4, BCAS3, BDNF, BDNF-AS, BMP7, CAND1, CARMIL1, CARMIL2, CAV1, CD47, CDK5R1, CDT1, CEP295, CHMP2A, CLASP1, CLASP2, CNOT1, COBL, CORO1B, CRBN, CTNNA2, CTTN, CYFIP1, DAG1, DCTN1, DEF8, DLG1, EFNA5, EPHA1, EPHB1, EPHB3, EPS8L1, ERCC1, ERCC5, ESPN, ESR1, EVL, FAF1, FCHSD1, FCHSD2, FER, FHOD1, FLRT1, FMN1, FNIP2, GMFB, GRB2, GRID2, GTF2H1, HDAC4, HGS, HIP1R, HRG, HTT, IL1RAP, IQGAP2, KIRREL1, LDB2, LIMS1, LPAR1, LRCH4, LRSAM1, MAP4K4, MAPK9, MPHOSPH8, MYO1C, MYO1E, NAV3, NCK2, NCKAP1, NCKAP1L, NLGN2, NPHP4, NPHS1, NRXN1, NTRK2, PALM, PFN2, PHLDB1, PIEZO1, PIH1D1, PIP4K2A, PLCE1, PLEK, PLK4, PPM1F, PRKCA, PRKCE, PSMC2, PSMC3, PSMC6, PXDN, RACK1, RASIP1, RBX1, RHOA, RICTOR, RIPOR2, ROCK2, RP1, |
| GO:0007268 | chemical synaptic transmission | 1,51E-04 | [6, 7] | 206,00 | 26,11 | [ABAT, ABR, ADRB1, AKAP12, ALS2, AMPH, APBA2, ARF1, ATP2A2, BDNF, BDNF-AS, BEGAIN, BSN, BTBD9, CACNA1A, CACNA1B, CACNA1E, CACNA1G, CACNA2D2, CACNB2, CADPS, CADPS2, CALB2, CAMK2B, CCR2, CD200R1, CDC20, CELF4, CHRM4, CHRNB2, CHRNB3, CHRND, CHRNE, CNIH3, CNTN4, CSPG5, CTBP2, CYFIP1, DBN1, DGKE, DGKZ, DHX15, DHX34, DLG1, DLG2, DLG4, DLGAP2, DNM1, DNM1L, DOC2A, EGR2, EIF2AK4, EIF4A3, EIF4E, EIF4EBP2, EPHA4, EPHB1, EXOC4, FCHSD1, FCHSD2, FGF12, FGF14, FNTA, FZR1, GABBR1, GABBR2, GABRA2, GABRA4, GABRB2, GABRR3, GAD1, GAD2, GNAI2, GPER1, GPR176, GPRIN1, GRIA4, GRID2, GRID2IP, GRIK3, GRIK4, GRM1, GRM2, GRM4, GRM8, GSK3A, HCRTR2, HTR1A, HTR2A, HTR3A, HTR3C, IGSF9B, ITPR3, JAK2, JPH3, KCNB1, KCNQ2, KIF5A, KRAS, LAMA2, LGMN, LIN7B, LLGL1, LRFN2, LRRC4C, LYPD6, LYPD6B, LZTS1, MAPK8IP2, MEF2C, MME, MTMR2, MTNR1B, MYO5A, NAPB, NCSTN, NF1, NFATC4, NGF, NLGN2, NOL3, NPS, NPTN, NPTXR, NRXN1, NRXN3, NSG1, NSMF, NTNG1, NTNG2, NTRK2, PACSIN2, PAFAH1B1, PCDHA6, PCDHB9, PCLO, PDE7B, PFN2, PIP5K1C, PLA2G6, PLCB1, PLCB4, PLCL2, PPFIA1, PPFIA3, PPFIA4, PPP3CA, PPP3CB, PRKAR1B, PRKCA, PRKCB, PRKCE, PRKCZ, PRKN, PSEN1, PTEN, PTPRA, |
| GO:0030030 | cell projection organization | 8,47E-13 | [4] | 435,00 | 26,16 | ATF1, ATP1A3, ATP6V0D1, ATP8A2, ATXN10, B9D1, BBS10, BBS4, BBS7, BBS9, BCAS3, BCL11A, BCL11B, BCL2, BDNF, BDNF-AS, BIN3, BLOC1S2, BLOC1S5, BMP7, BRSK2, BTG2, C21orf91, CACNA1A, CAMK2B, CAMSAP1, CAPRIN2, CAPZB, CARMIL1, CARMIL2, CAV1, CBFA2T2, CC2D2A, CCDC113, CCDC13, CCDC28B, CCKAR, CDC20, CDH13, CDH23, CDH4, CDK1, CDK5R1, CEP131, CEP164, CEP290, CEP43, CEP70, CFAP46, CFAP54, CFAP61, CFAP74, CHRNB2, CIB1, CILK1, CLASP1, CNTN4, CNTNAP2, CNTROB, COBL, CORO1B, CPLANE1, CPNE5, CREB3L2, CSMD3, CSNK1E, CSPG5, CTNNA2, CTNND2, CTTN, CUL3, CUX1, CXCR4, CYFIP1, CYFIP2, CYLD, DAB1, DAG1, DBN1, DCLK1, DCTN1, DDR1, DEF8, DENND5A, DHX36, DLG4, DNAH17, DNAH5, DNAH8, DNAH9, DNAJB13, DNM1L, DOCK10, DOCK7, DOK4, DOK6, DRGX, DSCAML1, DYNC2LI1, EFHD1, EFNA4, EFNA5, EGR2, EIF2AK4, ENC1, ENPP2, EPHA1, EPHA10, EPHA4, EPHA6, EPHB1, EPHB3, EPS8L1, ESPN, ETV1, EVL, EZH2, FAM161A, FARP1, FBXW8, FER, FEZ1, FEZF1, FEZF2, FGD2, FLRT1, FRS2, FRYL, FXYD5, FZR1, GAB1, GAB2, GAK, GAS7, GFI1, GLI2, GLI3, GORASP1, GPRIN1, GRB10, GRB2, GRID2, GSK3A, HCLS1, HDAC4, HECW1, HERC1, HES1, HMGB1, HRG, HSP90AB1, HTT, HYDIN, IFRD1, IFT140, IFT46, IFT74, IFT88, IGF1R, INPP5F, INPPL1, INSR, IQSEC1, IRS2, ISL1, ITGA1, JAK2, JHY, KALRN, KANK1, KIDINS220, KIF1A, KIF5A, KLF7, KLHL1, KNDC1, LAMA2, LAMA5, LAMB1, LAMB2, LGMN, LGR6, LHX4, LIMA1, LINGO1, LLGL1, LPAR1, LRIG2, LRP1, LRP12, LRP2, LRRC4C, LZTS1, MACF1, MAG, MAGI2, MAK, MAP2, MAP3K13, MAP4, MAP4K4, MAP6, MAPK7, MAPK8IP2, MAPK8IP3, MEF2C, MEGF8, MICALL1, MKS1, MRTFA, MTMR2, MYLK, MYO16, MYO1A, MYPN, NAV3, NCAM2, NCK2, NCKAP1, NCKAP1L, NCOA2, NDE1, NDRG4, NECTIN1, NEDD1, NEDD4L, NEO1, NFASC, NFATC4, NFE2L2, NFIB, NGEF, NGF, NINL, NME2, NOL3, NOTCH1, NPHP4, NPTN, NRXN1, NRXN3, NSMF, NTNG1, NTNG2, NTRK2, NUMB, OBSL1, OLFM1, PACSIN1, PACSIN2, PAFAH1B1, PAK4, PALLD, PALM, PARD3, PAX2, PCNT, PDZD7, PFN2, PHACTR1, PIK3CB, PIK3R1, PLCE1, PLD1, PLEK, PLK4, PLK5, PLXNA1, PLXNA2, PLXNB1, PLXNC1, PLXND1, POU4F2, PPP1R16B, PPP3CA, PPP3CB, PRAG1, PRKCA, PRKCI, PRKCQ, PRKCZ, PRKD2, PRKN, PRMT1, PSD, PSEN1, PTEN, PTK2, PTPN11, PTPRA, PTPRF, PTPRO, PTPRZ1, RAB29, RAB35, RAB5A, RANBP1, RANBP9, RAP1A, RAP1GAP, RAP1GAP2, RAPGEF1, RASGRF1, RB1, RBFOX2, RELN, RET, RFX2, RGMA, RHOA, RHOBTB3, RHOG, RHOU, RIPOR2, RO60, ROBO1, ROBO3, ROR1, ROR2, RORA, RP1, |
| GO:0005261 | cation channel activity | 1,47E-02 | [6, 7, 8, 9] | 145,00 | 26,17 | [ACTN2, AHNAK, ANK3, ANO1, ANO6, ANXA2, ANXA6, ASIC3, ASIC4, ATP2B4, ATP5F1A, ATP5ME, CACNA1A, CACNA1B, CACNA1E, CACNA1G, CACNA1H, CACNA1I, CACNA2D2, CACNA2D3, CACNA2D4, CACNB2, CACNG1, CACNG6, CACNG8, CALM2, CAMK2D, CAV1, CCR2, CHRNB2, CHRNB3, CHRND, CHRNE, CNGA3, CNIH3, COMMD1, CTSS, DAPK1, DLG1, DLG2, DLG4, DPP10, FAM155A, FGF12, FGF14, FXYD5, FXYD6, GNB5, GPRIN1, GRIA4, GRIK3, GRIK4, GSG1L, HCN1, HCN2, HCN4, HRC, HTR3A, HTR3C, HTT, ITPR2, ITPR3, JPH3, KCNAB2, KCNB1, KCNB2, KCNC1, KCNC3, KCNE1, KCNF1, KCNH2, KCNIP4, KCNJ14, KCNJ15, KCNJ2, KCNJ3, KCNJ6, KCNK2, KCNMA1, KCNQ1, KCNQ2, KCNV1, LLGL1, MAPK8IP2, MEF2C, MTNR1B, MYO5A, NALCN, NEDD4L, NLGN2, NOL3, NOS1, NOS1AP, NPPA, NRXN1, ORAI1, ORAI2, P2RY6, PIEZO1, PKD1, PKD1L1, PLA2G6, PLCG2, PPARGC1A, PRKCA, PRKCZ, PRKD2, PSEN1, PTPN3, RASA1, RASA3, |
| GO:0099536 | synaptic signaling | 5,43E-05 | [3, 4] | 215,00 | 26,22 | [ABAT, ABR, ADRB1, AGRN, AKAP12, ALS2, AMPH, APBA2, ARF1, ATP2A2, BDNF, BDNF-AS, BEGAIN, BSN, BTBD9, CACNA1A, CACNA1B, CACNA1E, CACNA1G, CACNA2D2, CACNB2, CADPS, CADPS2, CALB2, CAMK2B, CCR2, CD200R1, CDC20, CDK5R1, CELF4, CHRM4, CHRNB2, CHRNB3, CHRND, CHRNE, CNIH3, CNTN4, CSPG5, CTBP2, CYFIP1, DAG1, DBN1, DGKE, DGKZ, DHX15, DHX34, DLG1, DLG2, DLG4, DLGAP2, DNM1, DNM1L, DOC2A, DTNB, EGR2, EIF2AK4, EIF4A3, EIF4E, EIF4EBP2, EPHA4, EPHB1, EXOC4, FABP5, FARP1, FCHSD1, FCHSD2, FGF12, FGF14, FNTA, FZR1, GABBR1, GABBR2, GABRA2, GABRA4, GABRB2, GABRR3, GAD1, GAD2, GNAI2, GPER1, GPR176, GPRIN1, GRIA4, GRID2, GRID2IP, GRIK3, GRIK4, GRM1, GRM2, GRM4, GRM8, GSK3A, HCRTR2, HTR1A, HTR2A, HTR3A, HTR3C, IGSF9B, IL1RAP, ITPR3, JAK2, JPH3, KCMF1, KCNB1, KCNQ2, KIF5A, KRAS, LAMA2, LGMN, LIN7B, LLGL1, LRFN2, LRRC4C, LYPD6, LYPD6B, LZTS1, MAPK8IP2, MEF2C, MME, MTMR2, MTNR1B, MYO5A, NAPB, NCSTN, NF1, NFATC4, NGF, NLGN2, NOL3, NOS1, NPS, NPTN, NPTXR, NRXN1, NRXN3, NSG1, NSMF, NTNG1, NTNG2, NTRK2, PACSIN2, PAFAH1B1, PCDHA6, PCDHB9, PCLO, PDE7B, PFN2, PIP5K1C, PLA2G6, PLCB1, PLCB4, PLCL2, PPFIA1, PPFIA3, PPFIA4, PPP3CA, PPP3CB, PRKAR1B, PRKCA, |
| GO:0045860 | positive regulation of protein kinase activity | 6,05E-03 | [7, 8, 9, 10] | 155,00 | 26,23 | [ACSL1, ADAM17, ADCY2, ADCY3, ADCY5, ADCY7, AKAP13, ALK, ALKAL2, ALS2, AMH, ANGPT1, ANGPT4, ARHGEF7, ATP2B4, BDNF, BDNF-AS, BMP2, BTC, CALM2, CARD10, CARD14, CCND2, CCNY, CDC25B, CDK1, CDK5R1, CDKN1A, CIB1, CXCR4, DAB1, DAG1, DAXX, DDR2, DGKQ, DIPK2A, DLG1, DLG4, DOK7, DUSP19, EFNA5, EPHA4, EREG, ETAA1, ETFA, EZH2, F3, FGD2, FGF2, FRS2, FZD10, FZD5, GADD45G, GCG, GFRAL, GPRC5B, GPRC5C, GREM1, GRM1, GRM4, HACD3, HSP90AB1, HTR2A, IGF1, IGF1R, IL6R, INSR, IQGAP3, IRAK2, ITGA1, JAK2, KIDINS220, KITLG, KRAS, KSR1, LLGL1, LPAR1, MAP2K2, MAP2K5, MAP3K11, MAP3K13, MAP3K19, MAP3K2, MAP3K20, MAP3K5, MAP3K6, MAP4K4, MAP4K5, MAPK8IP3, MAPKAP1, MAPKAPK5, MMD, MST1R, NCKAP1L, NEDD9, NGF, NRXN1, NTRK2, OSBPL8, PAK4, PAK5, PDGFD, PDPK1, PIH1D1, PIK3CB, PIK3CG, PIK3R5, PKD1, PLCE1, PPP2R3C, PPP3CA, PRKAG2, PRKAR1B, |
| GO:0099537 | trans-synaptic signaling | 3,78E-05 | [4, 5] | 211,00 | 26,38 | [ABAT, ABR, ADRB1, AKAP12, ALS2, AMPH, APBA2, ARF1, ATP2A2, BDNF, BDNF-AS, BEGAIN, BSN, BTBD9, CACNA1A, CACNA1B, CACNA1E, CACNA1G, CACNA2D2, CACNB2, CADPS, CADPS2, CALB2, CAMK2B, CCR2, CD200R1, CDC20, CELF4, CHRM4, CHRNB2, CHRNB3, CHRND, CHRNE, CNIH3, CNTN4, CSPG5, CTBP2, CYFIP1, DAG1, DBN1, DGKE, DGKZ, DHX15, DHX34, DLG1, DLG2, DLG4, DLGAP2, DNM1, DNM1L, DOC2A, EGR2, EIF2AK4, EIF4A3, EIF4E, EIF4EBP2, EPHA4, EPHB1, EXOC4, FABP5, FARP1, FCHSD1, FCHSD2, FGF12, FGF14, FNTA, FZR1, GABBR1, GABBR2, GABRA2, GABRA4, GABRB2, GABRR3, GAD1, GAD2, GNAI2, GPER1, GPR176, GPRIN1, GRIA4, GRID2, GRID2IP, GRIK3, GRIK4, GRM1, GRM2, GRM4, GRM8, GSK3A, HCRTR2, HTR1A, HTR2A, HTR3A, HTR3C, IGSF9B, IL1RAP, ITPR3, JAK2, JPH3, KCNB1, KCNQ2, KIF5A, KRAS, LAMA2, LGMN, LIN7B, LLGL1, LRFN2, LRRC4C, LYPD6, LYPD6B, LZTS1, MAPK8IP2, MEF2C, MME, MTMR2, MTNR1B, MYO5A, NAPB, NCSTN, NF1, NFATC4, NGF, NLGN2, NOL3, NOS1, NPS, NPTN, NPTXR, NRXN1, NRXN3, NSG1, NSMF, NTNG1, NTNG2, NTRK2, PACSIN2, PAFAH1B1, PCDHA6, PCDHB9, PCLO, PDE7B, PFN2, PIP5K1C, PLA2G6, PLCB1, PLCB4, PLCL2, PPFIA1, PPFIA3, PPFIA4, PPP3CA, PPP3CB, PRKAR1B, PRKCA, PRKCB, PRKCE, |
| GO:0120036 | plasma membrane bounded cell projection organization | 2,20E-13 | [5] | 429,00 | 26,43 | ATF1, ATP1A3, ATP6V0D1, ATP8A2, ATXN10, B9D1, BBS10, BBS4, BBS7, BBS9, BCAS3, BCL11A, BCL11B, BCL2, BDNF, BDNF-AS, BIN3, BLOC1S2, BLOC1S5, BMP7, BRSK2, BTG2, C21orf91, CACNA1A, CAMK2B, CAMSAP1, CAPRIN2, CAPZB, CARMIL1, CARMIL2, CAV1, CBFA2T2, CC2D2A, CCDC113, CCDC13, CCDC28B, CCKAR, CDC20, CDH13, CDH23, CDH4, CDK1, CDK5R1, CEP131, CEP164, CEP290, CEP43, CEP70, CFAP46, CFAP54, CFAP61, CFAP74, CHRNB2, CIB1, CILK1, CLASP1, CNTN4, CNTNAP2, CNTROB, COBL, CORO1B, CPLANE1, CPNE5, CREB3L2, CSMD3, CSNK1E, CTNNA2, CTNND2, CTTN, CUX1, CXCR4, CYFIP1, CYFIP2, CYLD, DAB1, DAG1, DBN1, DCLK1, DCTN1, DDR1, DEF8, DENND5A, DHX36, DLG4, DNAH17, DNAH5, DNAH8, DNAJB13, DNM1L, DOCK10, DOCK7, DOK4, DOK6, DRGX, DSCAML1, DYNC2LI1, EFHD1, EFNA4, EFNA5, EGR2, EIF2AK4, ENC1, ENPP2, EPHA1, EPHA10, EPHA4, EPHA6, EPHB1, EPHB3, EPS8L1, ESPN, ETV1, EVL, EZH2, FAM161A, FARP1, FBXW8, FER, FEZ1, FEZF1, FEZF2, FGD2, FLRT1, FRS2, FRYL, FXYD5, FZR1, GAB1, GAB2, GAK, GAS7, GFI1, GLI2, GLI3, GORASP1, GPRIN1, GRB10, GRB2, GRID2, GSK3A, HCLS1, HDAC4, HECW1, HERC1, HES1, HMGB1, HRG, HSP90AB1, HTT, HYDIN, IFRD1, IFT140, IFT46, IFT74, IFT88, IGF1R, INPP5F, INPPL1, INSR, IQSEC1, IRS2, ISL1, ITGA1, JAK2, JHY, KALRN, KANK1, KIDINS220, KIF1A, KIF5A, KLF7, KLHL1, KNDC1, LAMA2, LAMA5, LAMB1, LAMB2, LGMN, LGR6, LHX4, LIMA1, LINGO1, LLGL1, LPAR1, LRIG2, LRP1, LRP12, LRP2, LRRC4C, LZTS1, MACF1, MAG, MAGI2, MAK, MAP2, MAP3K13, MAP4, MAP4K4, MAP6, MAPK7, MAPK8IP2, MAPK8IP3, MEF2C, MEGF8, MICALL1, MKS1, MRTFA, MTMR2, MYLK, MYO16, MYO1A, MYPN, NAV3, NCAM2, NCK2, NCKAP1, NCKAP1L, NCOA2, NDE1, NDRG4, NECTIN1, NEDD1, NEDD4L, NEO1, NFASC, NFATC4, NFE2L2, NFIB, NGEF, NGF, NINL, NME2, NOL3, NOTCH1, NPHP4, NPTN, NRXN1, NRXN3, NSMF, NTNG1, NTNG2, NTRK2, NUMB, OBSL1, OLFM1, PACSIN1, PAFAH1B1, PAK4, PALLD, PALM, PARD3, PAX2, PCNT, PDZD7, PFN2, PHACTR1, PIK3CB, PIK3R1, PLCE1, PLD1, PLEK, PLK4, PLK5, PLXNA1, PLXNA2, PLXNB1, PLXNC1, PLXND1, POU4F2, PPP1R16B, PPP3CA, PPP3CB, PRAG1, PRKCA, PRKCI, PRKCQ, PRKCZ, PRKD2, PRKN, PRMT1, PSD, PSEN1, PTEN, PTK2, PTPN11, PTPRA, PTPRF, PTPRO, PTPRZ1, RAB29, RAB35, RAB5A, RANBP1, RANBP9, RAP1A, RAP1GAP, RAP1GAP2, RAPGEF1, |
| GO:0022836 | gated channel activity | 2,88E-02 | [6, 8] | 127,00 | 26,51 | [ACTN2, AHNAK, ANK3, ANO1, ANO4, ANO6, ANO7, ANXA6, ASIC3, CACNA1A, CACNA1B, CACNA1E, CACNA1G, CACNA1H, CACNA1I, CACNA2D2, CACNA2D3, CACNA2D4, CACNB2, CACNG1, CACNG6, CACNG8, CALM2, CAMK2D, CAV1, CCR2, CHRNB2, CHRNB3, CHRND, CHRNE, CLCN2, CLCN3, CNGA3, CNIH3, DAPK1, DLG1, DLG2, DLG4, FAM155A, FGF14, GABRA2, GABRA4, GABRB2, GABRR3, GNB5, GPRIN1, GRIA4, GRID2, GRIK3, GRIK4, GSG1L, HCN1, HCN2, HCN4, HRC, HTR3A, HTR3C, HTT, ITPR2, ITPR3, JPH3, KCNAB2, KCNB1, KCNB2, KCNC1, KCNC3, KCNE1, KCNF1, KCNH2, KCNIP4, KCNJ14, KCNJ15, KCNJ2, KCNJ3, KCNJ6, KCNK2, KCNMA1, KCNQ1, KCNQ2, KCNV1, LLGL1, MAPK8IP2, MEF2C, MTNR1B, MYO5A, NALCN, NLGN2, NOL3, NOS1, NOS1AP, NPPA, NRXN1, P2RY6, PACC1, PIEZO1, PPARGC1A, PRKCA, PRKD2, RASA3, RASGRF1, RELN, RGS9, RYR1, RYR2, RYR3, SCN1A, SCN2A, SCN3B, SCN5A, SHANK2, SHISA6, |
| GO:0004674 | protein serine/threonine kinase activity | 1,12E-06 | [8, 9] | 244,00 | 26,64 | [AATK, ABR, ACSL1, ACVR2A, ACVRL1, ADAM17, ADCK1, ADCK2, AKAP13, AKT2, ALK, ALPK2, ALS2, AMH, ANKS1B, ANTXR1, APC, ARHGEF7, ATM, ATP2B4, AURKB, BMP2, BMP7, BRD4, BRSK2, CALM2, CAMK2B, CAMK2D, CAMK4, CAV1, CCNA1, CCND2, CCNL1, CCNY, CDC25C, CDC42BPB, CDK1, CDK11B, CDK19, CDK2, CDK5R1, CDKL2, CDKN1A, CDKN2A, CHEK1, CHEK2, CIB1, CILK1, CIT, CLASP1, CLK3, CLK4, CSNK1A1L, CSNK1E, CSNK1G3, CXCR4, DAPK1, DAXX, DCAF1, DCLK1, DHDDS, DIPK2A, DSTYK, DUSP19, EIF2AK3, EIF2AK4, EPHA4, ETAA1, ETFA, EZH2, F3, FASTK, FGD2, FGF2, FRS2, FZD10, FZD5, GADD45G, GAK, GFRAL, GRK3, GRK5, GRM1, GRM4, GSK3A, GTF2H1, HACD3, HEG1, HGS, HSP90AB1, HTATIP2, HTR2A, HTT, IGF1, IGF1R, IKBKB, INKA2, INSR, IPO7, IQGAP3, IRAK2, ITGA1, JAK2, KALRN, KAT2B, KIDINS220, KITLG, KRAS, KSR1, KSR2, LATS2, LAX1, LIMK2, LMTK2, LONP1, LPAR1, LRP5, LRRK1, LTBP1, MAK, MAP2K2, MAP2K5, MAP3K11, MAP3K13, MAP3K19, MAP3K2, MAP3K20, MAP3K5, MAP3K6, MAP4K4, MAP4K5, MAPK4, MAPK7, MAPK8, MAPK8IP3, MAPK9, MAPKAPK5, MARK3, MAST4, MCM2, MEN1, MKNK1, MKNK2, MST1R, MYLK, MYLK4, NEK11, NEK7, NF1, NGF, NLK, NME2, NPPA, NRXN1, OBSCN, PAK4, PAK5, PASK, PDGFD, PDPK1, PIH1D1, PIK3CB, PIK3CG, PIK3R5, PIM1, PKD1, PKIB, PKMYT1, PLCE1, PLK4, PRKAG2, PRKAR1B, PRKCA, PRKCB, PRKCE, PRKCH, PRKCI, PRKCQ, PRKCZ, PRKD2, PRKDC, PSEN1, PTEN, PTPN11, PTPRC, RAPGEF1, RASIP1, |
| GO:0046873 | metal ion transmembrane transporter activity | 9,10E-04 | [6, 7, 8, 9] | 165,00 | 26,66 | [ABCB11, ACTN2, ACTN4, AHNAK, ANK3, ANXA2, ANXA6, ASIC3, ASIC4, ATP12A, ATP1A3, ATP1A4, ATP2A2, ATP2A3, ATP2B2, ATP2B4, C4orf3, CACNA1A, CACNA1B, CACNA1E, CACNA1G, CACNA1H, CACNA1I, CACNA2D2, CACNA2D3, CACNA2D4, CACNB2, CACNG1, CACNG6, CACNG8, CALM2, CAMK2D, CAV1, CLDN16, CNNM4, COMMD1, DLG1, DPP10, FAM155A, FGF12, FGF14, FXYD5, FXYD6, GNB5, GPRIN1, GRIK3, GRIK4, HCN1, HCN2, HCN4, HECW1, HRC, HTT, ITPR2, ITPR3, JPH3, KCNAB2, KCNB1, KCNB2, KCNC1, KCNC3, KCNE1, KCNF1, KCNH2, KCNIP4, KCNJ14, KCNJ15, KCNJ2, KCNJ3, KCNJ6, KCNK2, KCNMA1, KCNQ1, KCNQ2, KCNV1, MRS2, MTNR1B, MYO5A, NALCN, NEDD4L, NIPA1, NOS1, NOS1AP, NPPA, ORAI1, ORAI2, P2RY6, PKD1, PKD1L1, PLA2G6, PLCG2, PRKCA, PRKCE, PRKCZ, PRKD2, PSEN1, PTEN, PTPN3, RASA1, RASA3, RYR1, RYR2, RYR3, SCN1A, SCN2A, SCN3B, SCN5A, SGK1, SLC11A2, SLC12A7, SLC12A9, SLC13A3, SLC13A5, SLC17A2, SLC17A4, SLC17A7, SLC1A1, SLC1A3, SLC24A4, SLC30A2, SLC30A6, SLC39A10, SLC39A13, SLC39A7, SLC39A8, SLC39A9, SLC40A1, SLC4A4, |

| | | | | | | |
|---|---|---|---|---|---|---|
| GO:0051345 | positive regulation of hydrolase activity | 6,84E-06 | [5] | 217,00 | 26,72 | [ABL2, ABR, ADAP1, ADCYAP1R1, ADGRB3, ADRB1, ADRM1, AFDN, AGAP1, AGAP3, AGRN, AKT2, ALS2, ANTXR1, APC2, ARAP1, ARAP3, ARFGAP1, ARHGAP10, ARHGAP12, ARHGAP17, ARHGAP21, ARHGAP29, ARHGAP39, ARHGAP40, ARHGAP42, ARHGAP45, ARHGDIA, ARHGDIB, ARHGDIG, ARHGEF10L, ARHGEF16, ARHGEF7, ARL1, ARL6IP5, ASAP1, ASAP2, ASPH, ATP2A3, BCAR3, BCAS3, BCL10, BDNF, BDNF-AS, BMP2, CACNA1A, CALM2, CASR, CAV1, CCL16, CD33, CDKN2A, CHN2, CYCS, CYFIP2, DAPK1, DENND1A, DENND1B, DEPDC1, DEPDC5, DLC1, DNAJB4, DNAJB6, DNAJC15, DNAL4, DNM1L, DNPEP, DOCK10, DOCK7, DOCK8, EGR2, EIF2AK3, ELMOD1, EPHA1, EPHA4, ESR1, EZH2, F11R, F3, FAM13A, FAM13B, FASN, FGF2, FNTA, FZD10, GNB5, GPER1, GPR55, GPRIN1, GRAMD4, HACD3, HIP1, HIP1R, HMGB1, HSP90AB1, HSPD1, HTR2A, IFI16, IQSEC1, ITGA1, ITK, JAK2, KALRN, LGMN, LIMS1, LLGL1, LPAR1, LPAR2, MAGI2, MAL, MAP3K5, MAP4K4, MAPK8, MEF2C, MRTFA, MSH2, MSH3, MTMR9, MYO9B, NCKAP1L, NDUFA13, NEDD9, NF1, NGF, NLRP12, NPNT, NPRL3, NTRK2, P2RY6, PDCD5, PDCD6, PDGFRA, PDPK1, PDZD2, PFN2, PLCB1, PLCB2, PLEK, PLIN5, PLXNB1, PML, PPARG, PPARGC1B, PPM1F, PPP3CA, PRKCZ, PRKD2, PTPRA, PTPRC, RAB4A, RABGAP1, RACK1, |
| GO:0051345 | positive regulation of hydrolase activity | 6,84E-06 | [5] | 217,00 | 26,72 | [ABL2, ABR, ADAP1, ADCYAP1R1, ADGRB3, ADRB1, ADRM1, AFDN, AGAP1, AGAP3, AGRN, AKT2, ALS2, ANTXR1, APC2, ARAP1, ARAP3, ARFGAP1, ARHGAP10, ARHGAP12, ARHGAP17, ARHGAP21, ARHGAP29, ARHGAP39, ARHGAP40, ARHGAP42, ARHGAP45, ARHGDIA, ARHGDIB, ARHGDIG, ARHGEF10L, ARHGEF16, ARHGEF7, ARL1, ARL6IP5, ASAP1, ASAP2, ASPH, ATP2A3, BCAR3, BCAS3, BCL10, BDNF, BDNF-AS, BMP2, CACNA1A, CALM2, CASR, CAV1, CCL16, CD33, CDKN2A, CHN2, CYCS, CYFIP2, DAPK1, DENND1A, DENND1B, DEPDC1, DEPDC5, DLC1, DNAJB4, DNAJB6, DNAJC15, DNAL4, DNM1L, DNPEP, DOCK10, DOCK7, DOCK8, EGR2, EIF2AK3, ELMOD1, EPHA1, EPHA4, ESR1, EZH2, F11R, F3, FAM13A, FAM13B, FASN, FGF2, FNTA, FZD10, GNB5, GPER1, GPR55, GPRIN1, GRAMD4, HACD3, HIP1, HIP1R, HMGB1, HSP90AB1, HSPD1, HTR2A, IFI16, IQSEC1, ITGA1, ITK, JAK2, KALRN, LGMN, LIMS1, LLGL1, LPAR1, LPAR2, MAGI2, MAL, MAP3K5, MAP4K4, MAPK8, MEF2C, MRTFA, MSH2, MSH3, MTMR9, MYO9B, NCKAP1L, NDUFA13, NEDD9, NF1, NGF, NLRP12, NPNT, NPRL3, NTRK2, P2RY6, PDCD5, PDCD6, PDGFRA, PDPK1, PDZD2, PFN2, PLCB1, PLCB2, PLEK, PLIN5, PLXNB1, PML, PPARG, PPARGC1B, PPM1F, PPP3CA, PRKCZ, PRKD2, PTPRA, PTPRC, RAB4A, RABGAP1, RACK1, |
| GO:0001654 | eye development | 3,49E-02 | [5, 6, 7] | 111,00 | 27,07 | [ACVRL1, ADAMTS18, ATP2B4, ATP8A2, B9D1, BBS4, BBS7, BCAR3, BCL11B, BCL2, BDNF, BDNF-AS, BFSP1, BFSP2, BMP7, CACNA1A, CASP2, CC2D2A, CELF4, CEP290, CITED2, CLCN2, COL4A1, COL8A2, CRYBA4, CRYBG3, CRYGC, CTNS, CYP1B1, DLG1, DLL1, DLL4, DLX2, EPHB1, FAT1, FBN1, FBN2, FGF2, FJX1, FRS2, FZD5, FZR1, GLI3, HCN1, IFT140, IHH, KDM2B, LAMB2, LIMK2, LPCAT1, LRP5, MAF, MAX, MEIS2, MIPEP, MTNR1B, MYH15, MYOM2, NECTIN1, NF1, NKD1, NPHP4, NR2E3, NTRK2, OPN5, PAX2, PBX1, PDGFRA, POU4F2, PRKCI, PSEN1, PTF1A, RARA, RBP4, RD3, RET, RORB, RP1, RRM1, SDK1, SERPINF1, SH3PXD2B, SHH, SKI, SKIL, SLC17A6, SLC17A7, SLC4A4, SMAD3, SMARCD3, SMOC1, SOS1, SRF, STK19, TENM3, TENT2, |
| GO:0022008 | neurogenesis | 9,08E-18 | [4, 5, 6] | 479,00 | 27,11 | ATAT1, ATF1, ATP2B2, ATP2B4, ATP8A2, ATXN10, BBS4, BCL11A, BCL11B, BCL2, BCL6, BDNF, BDNF-AS, BEND6, BHLHE40, BLOC1S2, BLOC1S5, BMERB1, BMP2, BMP7, BRINP2, BRSK2, BTBD6, BTG2, BTG4, C21orf91, CACNA1A, CAMK2B, CAMSAP1, CAPRIN2, CASZ1, CBFA2T2, CCKAR, CCR2, CDC20, CDH23, CDH4, CDK1, CDK5R1, CELSR1, CEND1, CEP290, CHD5, CHRNB2, CIB1, CIT, CNTN4, CNTNAP2, COBL, COL3A1, CPNE5, CREB3L2, CSMD3, CSNK1E, CSPG5, CTNNA2, CTNND2, CTTN, CUX1, CXCR4, CYFIP1, CYFIP2, DAB1, DAG1, DBN1, DCLK1, DDIT4, DDR1, DDX6, DENND5A, DHDDS, DHX36, DLG4, DLL1, DLL4, DLX2, DNER, DNM1L, DNMT3A, DOCK10, DOCK7, DOK4, DOK6, DRGX, DSCAML1, DTX1, EFHD1, EFNA4, EFNA5, EGR2, EHMT2, EIF2AK4, EIF4E, EIF4ENIF1, ENC1, EPHA1, EPHA10, EPHA4, EPHA6, EPHB1, EPHB3, ERBB4, ETV1, ETV6, EVL, EVX1, EZH2, FA2H, FARP1, FARP2, FBXW8, FERD3L, FEZ1, FEZF1, FEZF2, FLRT1, FRS2, FRYL, FZD10, FZD5, FZR1, GAB1, GAB2, GABRB2, GAK, GAS7, GFI1, GLI2, GLI3, GORASP1, GPER1, GPR17, GPRC5B, GPRIN1, GRB10, GRB2, GRID2, GSK3A, GSX2, HCLS1, HCN1, HDAC9, HECW1, HELT, HERC1, HES1, HES3, HES4, HEXB, HMGB1, HOXC10, HSP90AB1, IFRD1, IFT140, IFT88, IHH, IL6ST, INPP5F, IQSEC1, IRS2, ISL1, ITGA1, JAK2, KALRN, KANK1, KDM4C, KIDINS220, KIF1A, KIF5A, KLF7, KLHL1, KNDC1, KRAS, LAMA2, LAMB1, LAMB2, LDB2, LDLR, LEPR, LGR6, LHX4, LHX6, LHX8, LIG4, LINGO1, LLGL1, LMX1B, LPAR1, LRIG2, LRP1, LRP12, LRP2, LRRC4C, LZTS1, MACF1, MAG, MAGI2, MAP2, MAP3K13, MAP4, MAP4K4, MAP6, MAPK7, MAPK8IP2, MAPK8IP3, MBOAT7, MDGA1, MEF2C, MEGF8, MICALL1, MKS1, MMD, MME, MRTFA, MTMR2, MYO16, MYPN, MYRF, MYT1L, NAV1, NAV3, NCAM2, NCK2, NCKAP1, NCKAP1L, NCOA2, NCSTN, NDE1, NDRG4, NECTIN1, NEDD4L, NEO1, NEPRO, NEUROG2, NF1, NFASC, NFATC4, NFE2L2, NFIB, NGEF, NGF, NKD1, NME2, NOL3, NOS1, NOTCH1, NOTCH3, NPHP4, NPTN, NR1D1, NR2E3, NRXN1, NRXN3, NSMF, NTM, NTNG1, NTNG2, NTRK2, NUMB, OBSL1, OGDH, OLFM1, OLIG2, OPCML, PACSIN1, PAFAH1B1, PAK4, PALLD, PARD3, PAX2, PAX7, PBX1, PCARE, PDZD2, PDZD7, PER2, PEX13, PHACTR1, PIK3CB, PIK3R1, PLK5, PLXNA1, PLXNA2, PLXNB1, PLXNC1, PLXND1, POMGNT2, POU4F2, PPARG, PPP3CA, PPP3CB, PRAG1, PRDM16, PRDM5, PRDM6, PRKCA, PRKCH, PRKCI, PRKCQ, PRKCZ, PRKN, PRMT1, PRPF19, PRUNE1, PSD, PSEN1, PTEN, PTF1A, PTK2, PTPN11, PTPRA, PTPRF, PTPRO, PTPRZ1, RAB29, RAB35, RANBP1, RANBP9, RAP1A, RAP1GAP, RAP1GAP2, RAPGEF1, RARA, RASGRF1, RB1, RBFOX2, RELN, REST, RET, RGMA, RHEB, RHOA, RHOBTB3, RHOG, RIPOR2, RITA1, ROBO1, ROBO3, ROR1, ROR2, RORA, RORB, RP1, RTN4RL1, RTN4RL2, RUFY3, RUNX1, RUNX3, |
| GO:0022008 | neurogenesis | 9,08E-18 | [4, 5, 6] | 479,00 | 27,11 | ATAT1, ATF1, ATP2B2, ATP2B4, ATP8A2, ATXN10, BBS4, BCL11A, BCL11B, BCL2, BCL6, BDNF, BDNF-AS, BEND6, BHLHE40, BLOC1S2, BLOC1S5, BMERB1, BMP2, BMP7, BRINP2, BRSK2, BTBD6, BTG2, BTG4, C21orf91, CACNA1A, CAMK2B, CAMSAP1, CAPRIN2, CASZ1, CBFA2T2, CCKAR, CCR2, CDC20, CDH23, CDH4, CDK1, CDK5R1, CELSR1, CEND1, CEP290, CHD5, CHRNB2, CIB1, CIT, CNTN4, CNTNAP2, COBL, COL3A1, CPNE5, CREB3L2, CSMD3, CSNK1E, CSPG5, CTNNA2, CTNND2, CTTN, CUX1, CXCR4, CYFIP1, CYFIP2, DAB1, DAG1, DBN1, DCLK1, DDIT4, DDR1, DDX6, DENND5A, DHDDS, DHX36, DLG4, DLL1, DLL4, DLX2, DNER, DNM1L, DNMT3A, DOCK10, DOCK7, DOK4, DOK6, DRGX, DSCAML1, DTX1, EFHD1, EFNA4, EFNA5, EGR2, EHMT2, EIF2AK4, EIF4E, EIF4ENIF1, ENC1, EPHA1, EPHA10, EPHA4, EPHA6, EPHB1, EPHB3, ERBB4, ETV1, ETV6, EVL, EVX1, EZH2, FA2H, FARP1, FARP2, FBXW8, FERD3L, FEZ1, FEZF1, FEZF2, FLRT1, FRS2, FRYL, FZD10, FZD5, FZR1, GAB1, GAB2, GABRB2, GAK, GAS7, GFI1, GLI2, GLI3, GORASP1, GPER1, GPR17, GPRC5B, GPRIN1, GRB10, GRB2, GRID2, GSK3A, GSX2, HCLS1, HCN1, HDAC9, HECW1, HELT, HERC1, HES1, HES3, HES4, HEXB, HMGB1, HOXC10, HSP90AB1, IFRD1, IFT140, IFT88, IHH, IL6ST, INPP5F, IQSEC1, IRS2, ISL1, ITGA1, JAK2, KALRN, KANK1, KDM4C, KIDINS220, KIF1A, KIF5A, KLF7, KLHL1, KNDC1, KRAS, LAMA2, LAMB1, LAMB2, LDB2, LDLR, LEPR, LGR6, LHX4, LHX6, LHX8, LIG4, LINGO1, LLGL1, LMX1B, LPAR1, LRIG2, LRP1, LRP12, LRP2, LRRC4C, LZTS1, MACF1, MAG, MAGI2, MAP2, MAP3K13, MAP4, MAP4K4, MAP6, MAPK7, MAPK8IP2, MAPK8IP3, MBOAT7, MDGA1, MEF2C, MEGF8, MICALL1, MKS1, MMD, MME, MRTFA, MTMR2, MYO16, MYPN, MYRF, MYT1L, NAV1, NAV3, NCAM2, NCK2, NCKAP1, NCKAP1L, NCOA2, NCSTN, NDE1, NDRG4, NECTIN1, NEDD4L, NEO1, NEPRO, NEUROG2, NF1, NFASC, NFATC4, NFE2L2, NFIB, NGEF, NGF, NKD1, NME2, NOL3, NOS1, NOTCH1, NOTCH3, NPHP4, NPTN, NR1D1, NR2E3, NRXN1, NRXN3, NSMF, NTM, NTNG1, NTNG2, NTRK2, NUMB, OBSL1, OGDH, OLFM1, OLIG2, OPCML, PACSIN1, PAFAH1B1, PAK4, PALLD, PARD3, PAX2, PAX7, PBX1, PCARE, PDZD2, PDZD7, PER2, PEX13, PHACTR1, PIK3CB, PIK3R1, PLK5, PLXNA1, PLXNA2, PLXNB1, PLXNC1, PLXND1, POMGNT2, POU4F2, PPARG, PPP3CA, PPP3CB, PRAG1, PRDM16, PRDM5, PRDM6, PRKCA, PRKCH, PRKCI, PRKCQ, PRKCZ, PRKN, PRMT1, PRPF19, PRUNE1, PSD, PSEN1, PTEN, PTF1A, PTK2, PTPN11, PTPRA, PTPRF, PTPRO, PTPRZ1, RAB29, RAB35, RANBP1, RANBP9, RAP1A, RAP1GAP, RAP1GAP2, RAPGEF1, RARA, RASGRF1, RB1, RBFOX2, RELN, REST, RET, RGMA, RHEB, RHOA, RHOBTB3, RHOG, RIPOR2, RITA1, ROBO1, ROBO3, ROR1, ROR2, RORA, RORB, RP1, RTN4RL1, RTN4RL2, RUFY3, RUNX1, RUNX3, |
| GO:0048880 | sensory system development | 2,44E-02 | [4, 5] | 114,00 | 27,14 | [ACVRL1, ADAMTS18, ATP2B4, ATP8A2, B9D1, BBS4, BBS7, BCAR3, BCL11B, BCL2, BDNF, BDNF-AS, BFSP1, BFSP2, BMP7, CACNA1A, CASP2, CC2D2A, CELF4, CEP290, CITED2, CLCN2, COL4A1, COL8A2, CRYBA4, CRYBG3, CRYGC, CTNS, CYP1B1, DLG1, DLL1, DLL4, DLX2, EPHB1, FAT1, FBN1, FBN2, FGF2, FJX1, FRS2, FZD5, FZR1, GLI3, HCN1, IFT140, IHH, ISL1, KDM2B, LAMB2, LIMK2, LPCAT1, LRP5, MAF, MAX, MEIS2, MIPEP, MTNR1B, MYH15, MYOM2, NECTIN1, NF1, NKD1, NPHP4, NR2E3, NTRK2, OPN5, PAX2, PBX1, PDGFRA, POU4F2, PRKCI, PSEN1, PTF1A, RARA, RBP4, RD3, RET, RORB, RP1, RRM1, SDK1, SERPINF1, SH3PXD2B, SHH, SKI, SKIL, SLC17A6, SLC17A7, SLC44A4, SLC4A4, SMAD3, SMARCD3, SMOC1, SOS1, SRF, STK19, |
| GO:0060284 | regulation of cell development | 3,92E-09 | [4, 5] | 280,00 | 27,18 | [ABL2, ACTN4, ADD1, ADGRB3, ADGRG1, AFDN, AKAP13, ALK, ALKAL2, AP2A1, APPL2, ARF1, ARHGDIA, ARHGEF2, ARHGEF7, ARNTL, ASAP1, ASCL1, ATF1, ATP8A2, BCL11A, BCL11B, BCL2, BCL6, BDNF, BDNF-AS, BEND6, BHLHE40, BMERB1, BMP2, BMP7, BNC1, BRINP2, BRSK2, C21orf91, CACNA1A, CAMK2B, CAPRIN2, CARMIL1, CASZ1, CBFA2T2, CCR2, CDC20, CDH4, CDK1, CDK5R1, CHRNB2, CIB1, CNTN4, COBL, COL3A1, CPNE5, CREB3L2, CSMD3, CSNK1E, CSPG5, CTNNA2, CTTN, CUX1, CXCR4, CYFIP1, DAB1, DAG1, DBN1, DDX6, DENND5A, DHX36, DLG4, DLL1, DLL4, DLX2, DNM1L, DOCK7, DTX1, EFNA5, EGR2, EIF2AK4, EIF4E, EIF4ENIF1, ENC1, EPHA4, EPHB3, ERBB4, EZH2, F11R, FBN1, FBXW8, FERD3L, FEZ1, FEZF1, FEZF2, GAK, GFI1, GLI2, GLI3, GORASP1, GPER1, GPRC5B, GPRIN1, GRID2, GSK3A, GSX2, HCLS1, HDAC4, HDAC9, HECW1, HELT, HES1, HES3, HES4, IFRD1, IGF1, IKBKB, IL6ST, INPP5F, ISL1, KALRN, KANK1, KDM4C, KIDINS220, KIF1A, KNDC1, LDLR, LIG4, LIMS1, LINGO1, LLGL1, LPAR1, LRIG2, LRP1, LRP2, LRRC4C, LZTS1, MACF1, MAG, MAGI2, MAP2, MAP3K13, MAP6, MAPK8IP3, MEF2C, MEGF8, MELTF, MMD, MME, NCOA2, NDRG4, NEDD4L, NEDD9, NEPRO, NF1, NFATC4, NFE2L2, NGEF, NGF, NME2, NOL3, NOS1, NOTCH1, NOTCH3, NPTN, NR1D1, NSMF, NTNG1, NTNG2, NTRK2, NUMB, OBSL1, OLFM1, OLIG2, PACSIN1, PAFAH1B1, PBX1, PDZD2, PER2, PHACTR1, PLK5, PLXNA1, PLXNA2, PLXNB1, PLXNC1, PLXND1, POU4F2, PPARG, PPP3CA, PRAG1, PRKCH, PRKCI, PRKDC, PRKN, PRPF19, PRUNE1, PSEN1, PTEN, PTK2, PTPRF, PTPRO, PTPRZ1, RAB29, RANBP1, RAP1A, RAP1GAP, RAP1GAP2, RAPGEF1, RARA, RELN, REST, RET, RFLNA, |
| GO:0043547 | positive regulation of GTPase activity | 1,71E-02 | [6] | 117,00 | 27,21 | [ABR, ADAP1, ADGRB3, ADRB1, AFDN, AGAP1, AGAP3, AGRN, AKT2, ALS2, APC2, ARAP1, ARAP3, ARFGAP1, ARHGAP10, ARHGAP12, ARHGAP17, ARHGAP21, ARHGAP29, ARHGAP39, ARHGAP40, ARHGAP42, ARHGAP45, ARHGDIA, ARHGDIB, ARHGDIG, ARHGEF10L, ARHGEF16, ARHGEF7, ASAP1, ASAP2, BCAR3, BCAS3, CCL16, CHN2, DENND1A, DENND1B, DEPDC1, DEPDC5, DLC1, DNM1L, DOCK10, DOCK7, DOCK8, EGR2, ELMOD1, EPHA1, EZH2, F11R, FAM13A, FAM13B, FZD10, GNB5, HACD3, IQSEC1, KALRN, LIMS1, LLGL1, MAP4K4, MYO9B, NCKAP1L, NEDD9, NF1, NPRL3, PDZD2, PLCB1, PLXNB1, RABGAP1, RACK1, RALGAPA2, RANBP1, RAP1A, RAP1GAP, RAP1GAP2, RAPGEF1, RASA1, RASA2, RASA3, RASAL2, RASGRF1, |
| GO:0060589 | nucleoside-triphosphatase regulator activity | 4,98E-02 | [5] | 100,00 | 27,47 | [ABR, ADAP1, ADGRB3, AGAP1, AGAP3, ALS2, ARAP1, ARAP3, ARFGAP1, ARHGAP10, ARHGAP12, ARHGAP17, ARHGAP21, ARHGAP29, ARHGAP39, ARHGAP40, ARHGAP42, ARHGAP45, ARHGDIA, ARHGDIB, ARHGDIG, ARHGEF10L, ASAP1, ASAP2, BMP1, BRSK2, CHN2, DEPDC1, DEPDC5, DLC1, DNAJB4, DNAJB6, DNAJC15, DNM1L, EGR2, ELMOD1, FAM13A, FAM13B, FNIP2, GNB5, GPSM1, GRPEL1, HACD3, HSPH1, IPO7, IQGAP2, KRIT1, LLGL1, MYO9B, NCKAP1L, NF1, NPRL3, PCP2, PLCB1, PLXNB1, RAB4A, RABGAP1, RALGAPA2, RALGDS, RANBP1, RAP1GAP, RAP1GAP2, RASA1, RASA2, RASA3, RASAL2, RGS12, RGS3, RGS6, RGS7, RGS9, RIN3, RTKN, SGSM1, SGSM2, SH3BP4, SMAP1, SMAP2, SMOC2, SOS1, SRGAP3, STARD13, STXBP5, |
| GO:0034329 | cell junction assembly | 3,00E-03 | [5] | 129,00 | 27,51 | [ACTN2, ACTN4, ACVRL1, ADD2, ADGRB2, ADGRB3, ADGRL3, AFDN, AGRN, APC, ARHGEF7, BCAS3, BCL2, BDNF, BDNF-AS, BSN, CACNA1A, CAV1, CDH10, CDH13, CDH18, CDH19, CDH23, CDH26, CDH4, CHRNB2, CLASP1, CLASP2, CLDN16, CLDN8, CNTN5, CORO2B, CTNND2, CTTN, DLC1, DLG1, DNER, DST, EFNA5, EPHB1, EPHB3, ERBB4, F11R, FARP1, FERMT2, FLRT1, FMN1, FZD5, FZR1, GABRA2, GABRB2, GJA5, GPC6, GPRIN1, GREM1, GRID2, HDAC9, HEG1, HRG, IKBKB, IL1RAP, ITGA5, ITGB4, LDB2, LIMS1, LRCH4, LRFN3, LRP1, MACF1, MAGI2, MAP4K4, MDGA1, MEF2C, MYO1C, MYO1E, NECTIN1, NFASC, NLGN2, NPHP4, NPTN, NRXN1, NRXN3, NTNG2, NTRK2, PARD3, PATJ, PCDHB9, PCLO, PDPK1, PEAK1, PIP5K1C, PKP1, PLXNB1, |
| GO:0034330 | cell junction organization | 9,25E-07 | [4] | 211,00 | 27,51 | [ACTL8, ACTN2, ACTN4, ACVRL1, ADD1, ADD2, ADGRB2, ADGRB3, ADGRL3, AFDN, AGRN, ALS2, ANK3, APC, ARF1, ARHGAP39, ARHGEF7, BCAS3, BCL2, BDNF, BDNF-AS, BSN, C1QC, CACNA1A, CACNA2D2, CACNB2, CADM1, CAMK2B, CAPRIN2, CAV1, CDC20, CDH10, CDH13, CDH18, CDH19, CDH23, CDH26, CDH4, CDK5R1, CHRNB2, CLASP1, CLASP2, CLDN16, CLDN8, CNTN5, COL4A1, CORO2B, CTBP2, CTNNA2, CTNND2, CTTN, CYFIP1, DAG1, DBN1, DCTN1, DHX36, DLC1, DLG1, DLG2, DLG4, DNER, DNM1L, DOCK10, DST, EFNA5, EPHA4, EPHB1, EPHB3, ERBB4, F11R, FARP1, FERMT2, FLRT1, FMN1, FNTA, FZD5, FZR1, GABRA2, GABRB2, GJA5, GPC6, GPRIN1, GREM1, GRID2, HCLS1, HDAC9, HEG1, HRG, IGF1R, IGSF9B, IKBKB, IL10RA, IL1RAP, INSR, IQSEC1, ITGA5, ITGB4, KALRN, KIF1A, LAMB2, LDB2, LGMN, LIMS1, LLGL1, LRCH4, LRFN2, LRFN3, LRP1, LRRC4C, MACF1, MAGI2, MAP4K4, MDGA1, MEF2C, MTMR2, MYO1C, MYO1E, MYO5A, NECTIN1, NECTIN4, NFASC, NFATC4, NGEF, NLGN2, NOL3, NOS1AP, NPHP4, NPTN, NRXN1, NRXN3, NTNG1, NTNG2, NTRK2, NUMB, PAFAH1B1, PALM, PARD3, PATJ, PCDHB9, PCLO, PDPK1, PDZD2, PEAK1, PFN2, PIP5K1C, PKP1, PLEKHA7, PLXNB1, PLXND1, PPFIA1, PPFIA3, PPFIA4, PPM1F, PRKCA, PRKCH, PRKCI, PSEN1, PTEN, PTK2, PTPRF, PTPRO, |
| GO:0048699 | generation of neurons | 2,47E-18 | [5, 6, 7] | 458,00 | 27,56 | ATAT1, ATF1, ATP2B2, ATP2B4, ATP8A2, ATXN10, BBS4, BCL11A, BCL11B, BCL2, BCL6, BDNF, BDNF-AS, BEND6, BHLHE40, BLOC1S2, BLOC1S5, BMERB1, BMP2, BMP7, BRINP2, BRSK2, BTG2, BTG4, C21orf91, CACNA1A, CAMK2B, CAMSAP1, CAPRIN2, CASZ1, CBFA2T2, CCKAR, CCR2, CDC20, CDH23, CDH4, CDK1, CDK5R1, CELSR1, CEND1, CEP290, CHD5, CHRNB2, CIB1, CIT, CNTN4, CNTNAP2, COBL, COL3A1, CPNE5, CREB3L2, CSMD3, CSNK1E, CTNNA2, CTNND2, CTTN, CUX1, CXCR4, CYFIP1, CYFIP2, DAB1, DAG1, DBN1, DCLK1, DDIT4, DDR1, DDX6, DENND5A, DHDDS, DHX36, DLG4, DLL1, DLL4, DLX2, DNER, DNM1L, DNMT3A, DOCK10, DOCK7, DOK4, DOK6, DRGX, DSCAML1, DTX1, EFHD1, EFNA4, EFNA5, EGR2, EHMT2, EIF2AK4, EIF4E, EIF4ENIF1, ENC1, EPHA1, EPHA10, EPHA4, EPHA6, EPHB1, EPHB3, ERBB4, ETV1, EVL, EVX1, EZH2, FARP1, FARP2, FBXW8, FERD3L, FEZ1, FEZF1, FEZF2, FLRT1, FRS2, FRYL, FZD10, FZD5, FZR1, GAB1, GAB2, GABRB2, GAK, GAS7, GFI1, GLI2, GLI3, GORASP1, GPER1, GPRC5B, GPRIN1, GRB10, GRB2, GRID2, GSK3A, GSX2, HCLS1, HCN1, HDAC9, HECW1, HELT, HERC1, HES1, HES3, HES4, HMGB1, HOXC10, HSP90AB1, IFRD1, IFT140, IFT88, IHH, IL6ST, INPP5F, IQSEC1, IRS2, ISL1, ITGA1, JAK2, KALRN, KANK1, KDM4C, KIDINS220, KIF1A, KIF5A, KLF7, KLHL1, KNDC1, LAMA2, LAMB1, LAMB2, LDB2, LDLR, LGR6, LHX4, LHX6, LHX8, LIG4, LINGO1, LLGL1, LMX1B, LPAR1, LRIG2, LRP1, LRP12, LRP2, LRRC4C, LZTS1, MACF1, MAG, MAGI2, MAP2, MAP3K13, MAP4, MAP4K4, MAP6, MAPK7, MAPK8IP2, MAPK8IP3, MDGA1, MEF2C, MEGF8, MICALL1, MKS1, MMD, MME, MRTFA, MTMR2, MYO16, MYPN, MYT1L, NAV1, NCAM2, NCK2, NCKAP1, NCKAP1L, NCOA2, NDE1, NDRG4, NECTIN1, NEDD4L, NEO1, NEPRO, NEUROG2, NF1, NFASC, NFATC4, NFE2L2, NFIB, NGEF, NGF, NKD1, NME2, NOL3, NOS1, NOTCH1, NOTCH3, NPHP4, NPTN, NR1D1, NR2E3, NRXN1, NRXN3, NSMF, NTM, NTNG1, NTNG2, NTRK2, NUMB, OBSL1, OGDH, OLFM1, OLIG2, OPCML, PACSIN1, PAFAH1B1, PAK4, PALLD, PARD3, PAX2, PAX7, PBX1, PCARE, PDZD2, PDZD7, PER2, PEX13, PHACTR1, PIK3CB, PIK3R1, PLK5, PLXNA1, PLXNA2, PLXNB1, PLXNC1, PLXND1, POMGNT2, POU4F2, PPARG, PPP3CA, PPP3CB, PRAG1, PRKCA, PRKCH, PRKCI, PRKCQ, PRKCZ, PRKN, PRMT1, PRPF19, PRUNE1, PSD, PSEN1, PTEN, PTF1A, PTK2, PTPN11, PTPRA, PTPRF, PTPRO, PTPRZ1, RAB29, RAB35, RANBP1, RANBP9, RAP1A, RAP1GAP, RAP1GAP2, RAPGEF1, RARA, RASGRF1, RB1, RBFOX2, RELN, REST, RET, RGMA, RHEB, RHOA, RHOBTB3, RHOG, RIPOR2, ROBO1, ROBO3, ROR1, ROR2, RORA, RORB, RP1, |

| | | | | | | |
|---|---|---|---|---|---|---|
| GO:0048699 | generation of neurons | 2,47E-18 | [5, 6, 7] | 458,00 | 27,56 | ATAT1, ATF1, ATP2B2, ATP2B4, ATP8A2, ATXN10, BBS4, BCL11A, BCL11B, BCL2, BCL6, BDNF, BDNF-AS, BEND6, BHLHE40, BLOC1S2, BLOC1S5, BMERB1, BMP2, BMP7, BRINP2, BRSK2, BTG2, BTG4, C21orf91, CACNA1A, CAMK2B, CAMSAP1, CAPRIN2, CASZ1, CBFA2T2, CCKAR, CCR2, CDC20, CDH23, CDH4, CDK1, CDK5R1, CELSR1, CEND1, CEP290, CHD5, CHRNB2, CIB1, CIT, CNTN4, CNTNAP2, COBL, COL3A1, CPNE5, CREB3L2, CSMD3, CSNK1E, CTNNA2, CTNND2, CTTN, CUX1, CXCR4, CYFIP1, CYFIP2, DAB1, DAG1, DBN1, DCLK1, DDIT4, DDR1, DDX6, DENND5A, DHDDS, DHX36, DLG4, DLL1, DLL4, DLX2, DNER, DNM1L, DNMT3A, DOCK10, DOCK7, DOK4, DOK6, DRGX, DSCAML1, DTX1, EFHD1, EFNA4, EFNA5, EGR2, EHMT2, EIF2AK4, EIF4E, EIF4ENIF1, ENC1, EPHA1, EPHA10, EPHA4, EPHA6, EPHB1, EPHB3, ERBB4, ETV1, EVL, EVX1, EZH2, FARP1, FARP2, FBXW8, FERD3L, FEZ1, FEZF1, FEZF2, FLRT1, FRS2, FRYL, FZD10, FZD5, FZR1, GAB1, GAB2, GABRB2, GAK, GAS7, GFI1, GLI2, GLI3, GORASP1, GPER1, GPRC5B, GPRIN1, GRB10, GRB2, GRID2, GSK3A, GSX2, HCLS1, HCN1, HDAC9, HECW1, HELT, HERC1, HES1, HES3, HES4, HMGB1, HOXC10, HSP90AB1, IFRD1, IFT140, IFT88, IHH, IL6ST, INPP5F, IQSEC1, IRS2, ISL1, ITGA1, JAK2, KALRN, KANK1, KDM4C, KIDINS220, KIF1A, KIF5A, KLF7, KLHL1, KNDC1, LAMA2, LAMB1, LAMB2, LDB2, LDLR, LGR6, LHX4, LHX6, LHX8, LIG4, LINGO1, LLGL1, LMX1B, LPAR1, LRIG2, LRP1, LRP12, LRP2, LRRC4C, LZTS1, MACF1, MAG, MAGI2, MAP2, MAP3K13, MAP4, MAP4K4, MAP6, MAPK7, MAPK8IP2, MAPK8IP3, MDGA1, MEF2C, MEGF8, MICALL1, MKS1, MMD, MME, MRTFA, MTMR2, MYO16, MYPN, MYT1L, NAV1, NCAM2, NCK2, NCKAP1, NCKAP1L, NCOA2, NDE1, NDRG4, NECTIN1, NEDD4L, NEO1, NEPRO, NEUROG2, NF1, NFASC, NFATC4, NFE2L2, NFIB, NGEF, NGF, NKD1, NME2, NOL3, NOS1, NOTCH1, NOTCH3, NPHP4, NPTN, NR1D1, NR2E3, NRXN1, NRXN3, NSMF, NTM, NTNG1, NTNG2, NTRK2, NUMB, OBSL1, OGDH, OLFM1, OLIG2, OPCML, PACSIN1, PAFAH1B1, PAK4, PALLD, PARD3, PAX2, PAX7, PBX1, PCARE, PDZD2, PDZD7, PER2, PEX13, PHACTR1, PIK3CB, PIK3R1, PLK5, PLXNA1, PLXNA2, PLXNB1, PLXNC1, PLXND1, POMGNT2, POU4F2, PPARG, PPP3CA, PPP3CB, PRAG1, PRKCA, PRKCH, PRKCI, PRKCQ, PRKCZ, PRKN, PRMT1, PRPF19, PRUNE1, PSD, PSEN1, PTEN, PTF1A, PTK2, PTPN11, PTPRA, PTPRF, PTPRO, PTPRZ1, RAB29, RAB35, RANBP1, RANBP9, RAP1A, RAP1GAP, RAP1GAP2, RAPGEF1, RARA, RASGRF1, RB1, RBFOX2, RELN, REST, RET, RGMA, RHEB, RHOA, RHOBTB3, RHOG, RIPOR2, ROBO1, ROBO3, ROR1, ROR2, RORA, RORB, RP1, |
| GO:1902903 | regulation of supramolecular fiber organization | 1,76E-02 | [4, 5] | 109,00 | 27,59 | [ACTN2, ACTR3B, ADD1, ADD2, AKAP13, ALMS1, APC, APC2, ARAP1, ARF1, ARHGAP40, ARHGEF10L, ARHGEF18, ARHGEF2, ARHGEF7, ARPC4, ATXN7, BAIAP2L1, BBS4, BMERB1, CAMSAP1, CAPZB, CARMIL1, CARMIL2, CD47, CDK5R1, CIB1, CLASP1, CLASP2, CORO1B, CORO2B, CTNNA2, CTTN, CYFIP1, DBN1, DCTN1, DLC1, DLG1, ELN, EPHA1, EVL, F11R, FCHSD1, FCHSD2, FER, FHOD1, FHOD3, FMN1, GMFB, GRB2, HCLS1, HIP1R, IQGAP2, KANK1, KANK3, KIRREL1, LDLR, LIMA1, LPAR1, MAP2, MEF2C, MYO1C, MYO1E, NAV3, NCK2, NCKAP1, NCKAP1L, NPHS1, PFN2, PIK3R1, PLEK, PLEKHG2, PPFIA1, PPM1F, PRKCE, PRKN, PRUNE1, PSEN1, PXDN, RASA1, RB1, RHOA, RICTOR, ROCK2, SCIN, SH3PXD2B, SLC9A1, SMAD3, SORBS3, SPIRE2, |
| GO:0018209 | peptidyl-serine modification | 3,84E-02 | [7, 8] | 101,00 | 27,60 | [AKT2, AMH, ANGPT1, ANTXR1, ATM, ATP2B4, AURKB, BCAR3, BCL2, BDNF, BDNF-AS, BRSK2, CAMK2D, CAMK4, CAPRIN2, CAV1, CDK1, CDK2, CDK5R1, CHEK2, CLASP1, CSNK1A1L, CSNK1E, CSNK1G3, DCLK1, DCN, DDIT4, DGKQ, DOCK7, EIF2AK3, FNIP2, GALNT13, GALNT15, GALNT2, GALNT3, GCG, GSK3A, HCLS1, HRC, HSP90AB1, IKBKB, INPP5F, LATS2, LMTK2, MAP2K2, MAP3K13, MAPK7, MAPK8, MAPK9, MAPKAP1, MAPKAPK5, MARK3, MAST4, MKNK1, MKNK2, NCK2, NGF, NLK, NOS1, NRXN1, NSD1, NTRK2, PDPK1, PFN2, PKD1, PLCL2, PPM1F, PRKCA, PRKCB, PRKCE, PRKCH, PRKCI, PRKCQ, PRKCZ, PRKD2, PRKDC, PTEN, RACK1, RET, RICTOR, RIPK1, ROCK2, RPS6KA2, RPS6KB1, RPTOR, SBK1, SGK1, SH2D3C, SMAD7, SMYD3, |
| GO:0043010 | camera-type eye development | 4,23E-02 | [6, 7, 8] | 99,00 | 27,65 | [ACVRL1, ATP2B4, ATP8A2, B9D1, BBS4, BCAR3, BCL11B, BFSP1, BFSP2, BMP7, CACNA1A, CASP2, CC2D2A, CELF4, CITED2, CLCN2, COL4A1, COL8A2, CRYBA4, CRYBG3, CRYGC, CTNS, CYP1B1, DLG1, DLL1, DLL4, DLX2, EPHB1, FAT1, FBN1, FBN2, FGF2, FJX1, FRS2, FZD5, FZR1, GLI3, HCN1, IFT140, IHH, KDM2B, LAMB2, LIMK2, LPCAT1, LRP5, MAF, MAX, MIPEP, MTNR1B, MYH15, MYOM2, NECTIN1, NF1, NPHP4, NR2E3, NTRK2, OPN5, PAX2, PDGFRA, POU4F2, PSEN1, PTF1A, RARA, RBP4, RD3, RET, RORB, RP1, RRM1, SDK1, SERPINF1, SHH, SKI, SKIL, |
| GO:0022604 | regulation of cell morphogenesis | 3,03E-04 | [4, 5] | 148,00 | 27,66 | [ACTN4, ADGRB3, ARAP1, ARHGDIA, ARHGEF18, ARHGEF7, BCL11A, BDNF, BDNF-AS, BRSK2, CACNA1A, CAMK2B, CAMSAP1, CAPRIN2, CAPZB, CARMIL1, CDH4, CDK5R1, CFDP1, CHRNB2, CIB1, CORO1B, CPNE5, CSPG5, CTTN, CUX1, CXCR4, CYFIP1, DAB1, DAG1, DBN1, DHX36, DLC1, DLG1, DLG4, DNM1L, DNMBP, EFNA5, EGR2, ENPP2, EPHA4, EPHB3, F11R, FAM171A1, FBXW8, FERMT2, FEZF2, FGD2, FITM2, FMNL2, GNA12, GORASP1, GPRIN1, HCLS1, HECW1, HEXB, IFRD1, ITGB2, KALRN, KANK1, KIF1A, KNDC1, LIMS1, LINGO1, LLGL1, LPAR1, LRP1, LRRC4C, LZTS1, MACF1, MAG, MAP2, MAP3K13, MAP6, MEGF8, MELTF, MYL12B, NCOA2, NEDD4L, NEDD9, NFATC4, NGEF, NGF, NOL3, NSMF, NTNG1, NTNG2, NTRK2, OBSL1, OLFM1, PAFAH1B1, PALM, PALM2AKAP2, PLXNA1, PLXNA2, PLXNB1, PLXNC1, PLXND1, POU4F2, PPP3CA, PRAG1, PRKDC, PRKN, PSEN1, PTEN, PTK2, PTPRO, QRICH1, RASA1, RELN, RET, RGMA, |
| GO:0030182 | neuron differentiation | 1,97E-17 | [4, 6, 7, 8] | 417,00 | 27,93 | ATXN10, BBS4, BCL11A, BCL11B, BCL2, BCL6, BDNF, BDNF-AS, BEND6, BLOC1S2, BLOC1S5, BMP2, BMP7, BRINP2, BRSK2, BTG2, BTG4, C21orf91, CACNA1A, CAMK2B, CAMSAP1, CAPRIN2, CASZ1, CBFA2T2, CCKAR, CDC20, CDH23, CDH4, CDK5R1, CEND1, CEP290, CHD5, CHRNB2, CIB1, CNTN4, CNTNAP2, COBL, CPNE5, CREB3L2, CSMD3, CSNK1E, CTNNA2, CTNND2, CTTN, CUX1, CXCR4, CYFIP1, CYFIP2, DAB1, DAG1, DBN1, DCLK1, DDIT4, DDR1, DDX6, DENND5A, DHX36, DLG4, DLL1, DLL4, DLX2, DNM1L, DNMT3A, DOCK10, DOCK7, DOK4, DOK6, DRGX, DSCAML1, DTX1, EFHD1, EFNA4, EFNA5, EGR2, EHMT2, EIF2AK4, EIF4E, EIF4ENIF1, ENC1, EPHA1, EPHA10, EPHA4, EPHA6, EPHB1, EPHB3, ERBB4, ETV1, EVL, EVX1, EZH2, FARP1, FARP2, FBXW8, FEZ1, FEZF1, FEZF2, FLRT1, FRS2, FRYL, FZD10, FZD5, FZR1, GAB1, GAB2, GABRB2, GAK, GAS7, GFI1, GLI2, GLI3, GORASP1, GPRC5B, GPRIN1, GRB10, GRB2, GRID2, GSK3A, GSX2, HCLS1, HCN1, HDAC9, HECW1, HELT, HERC1, HES1, HES3, HES4, HMGB1, HOXC10, HSP90AB1, IFRD1, IFT140, IFT88, IHH, INPP5F, IQSEC1, IRS2, ISL1, ITGA1, JAK2, KALRN, KANK1, KDM4C, KIDINS220, KIF1A, KIF5A, KLF7, KLHL1, KNDC1, LAMA2, LAMB1, LAMB2, LDB2, LGR6, LHX4, LHX6, LHX8, LINGO1, LLGL1, LMX1B, LPAR1, LRIG2, LRP1, LRP12, LRP2, LRRC4C, LZTS1, MACF1, MAG, MAGI2, MAP2, MAP3K13, MAP4, MAP4K4, MAP6, MAPK7, MAPK8IP2, MAPK8IP3, MDGA1, MEF2C, MEGF8, MICALL1, MKS1, MMD, MRTFA, MTMR2, MYO16, MYPN, MYT1L, NCAM2, NCK2, NCKAP1, NCKAP1L, NCOA2, NDRG4, NECTIN1, NEDD4L, NEO1, NEPRO, NEUROG2, NFASC, NFATC4, NFE2L2, NFIB, NGEF, NGF, NKD1, NME2, NOL3, NOTCH1, NOTCH3, NPHP4, NPTN, NR2E3, NRXN1, NRXN3, NSMF, NTM, NTNG1, NTNG2, NTRK2, NUMB, OBSL1, OGDH, OLFM1, OLIG2, OPCML, PACSIN1, PAFAH1B1, PAK4, PALLD, PARD3, PAX2, PAX7, PBX1, PCARE, PDZD2, PDZD7, PHACTR1, PIK3CB, PIK3R1, PLK5, PLXNA1, PLXNA2, PLXNB1, PLXNC1, PLXND1, POU4F2, PPP3CA, PPP3CB, PRAG1, PRKCA, PRKCI, PRKCQ, PRKCZ, PRKN, PRMT1, PRPF19, PSD, PSEN1, PTEN, PTF1A, PTK2, PTPN11, PTPRA, PTPRF, PTPRO, PTPRZ1, RAB29, RAB35, RANBP1, RANBP9, RAP1A, RAP1GAP, RAP1GAP2, RAPGEF1, RARA, RASGRF1, RB1, RBFOX2, RELN, REST, RET, RGMA, RHOA, RHOBTB3, RHOG, RIPOR2, ROBO1, ROBO3, ROR1, ROR2, RORA, RORB, RP1, RTN4RL1, RTN4RL2, RUFY3, RUNX1, RUNX3, S100A6, SALL3, |
| GO:0050808 | synapse organization | 7,00E-04 | [5] | 132,00 | 27,97 | [ACTL8, ADD2, ADGRB2, ADGRB3, ADGRL3, AGRN, ALS2, ANK3, ARF1, ARHGAP39, ARHGEF7, BDNF, BDNF-AS, BSN, C1QC, CACNA1A, CACNA2D2, CACNB2, CAMK2B, CAPRIN2, CDC20, CDK5R1, CHRNB2, CNTN5, COL4A1, CTBP2, CTNNA2, CTNND2, CTTN, CYFIP1, DAG1, DBN1, DCTN1, DHX36, DLG1, DLG2, DLG4, DNER, DNM1L, DOCK10, EFNA5, EPHA4, EPHB1, EPHB3, ERBB4, FARP1, FLRT1, FNTA, FZD5, FZR1, GABRA2, GABRB2, GPC6, GPRIN1, GRID2, HCLS1, IGF1R, IGSF9B, IL10RA, IL1RAP, INSR, KALRN, KIF1A, LAMB2, LGMN, LLGL1, LRCH4, LRFN2, LRFN3, LRRC4C, MAGI2, MDGA1, MEF2C, MTMR2, MYO5A, NECTIN1, NFASC, NFATC4, NGEF, NLGN2, NOL3, NOS1AP, NPTN, NRXN1, NRXN3, NTNG1, NTNG2, NTRK2, PAFAH1B1, PALM, |
| GO:0007264 | small GTPase mediated signal transduction | 1,15E-05 | [4, 5, 6] | 169,00 | 28,07 | [ABL2, ABR, ADCYAP1R1, ADGRG1, AKAP13, ALS2, ARAP1, ARAP3, ARFGAP1, ARFGEF3, ARHGAP10, ARHGAP12, ARHGAP17, ARHGAP21, ARHGAP29, ARHGAP39, ARHGAP40, ARHGAP42, ARHGAP45, ARHGDIA, ARHGDIB, ARHGDIG, ARHGEF16, ARHGEF18, ARHGEF2, ARHGEF26, ARHGEF7, BCAR3, BCL11B, BCL6, CCDC88C, CDH13, CDK2, CDKN1A, CDKN2A, CELSR1, CHN2, COL3A1, CTNNAL1, CUL3, CYFIP1, CYTH1, DAB1, DENND1A, DLC1, DOCK10, DOCK7, DOCK8, DOK3, EGR2, ELMO1, EPS8L1, ETFA, F11R, F2RL3, FAM13A, FAM13B, FARP2, FBP1, FGD2, FGF2, GNA12, GPR17, GPR4, GPR55, GRB2, HACD3, HEG1, IGF1, IQGAP3, IQSEC1, IQSEC3, IRS2, JAK2, KALRN, KANK1, KCTD10, KIAA0355, KITLG, KNDC1, KRAS, KRIT1, KSR1, KSR2, LPAR1, MAP4K4, MAPKAP1, MAPKAPK5, MCF2L, MYO9B, NCKAP1, NF1, NGEF, NGF, NOTCH1, OBSCN, PLCE1, PLD1, PLEKHG2, PRAG1, PSD, PSD2, PSD3, PSD4, PTGES2, RAB22A, RAB29, RAB2A, RAB35, RAB3D, RAB40B, RAB43, RAB4A, RAB5A, RALGAPA2, RALGDS, RAP1A, RAP1GAP, RAP1GAP2, RAP2B, RAPGEF1, RAPGEF5, RASA1, RASA2, RASA3, RASGRF1, RASGRP4, |
| GO:0010720 | positive regulation of cell development | 1,01E-05 | [4, 5, 6] | 171,00 | 28,08 | [ABL2, ADD1, ALK, ALKAL2, AP2A1, ARF1, ARHGDIA, ARHGEF2, ARHGEF7, ASCL1, ATF1, ATP8A2, BCL2, BCL6, BDNF, BDNF-AS, BEND6, BMP2, BMP7, BNC1, BRINP2, C21orf91, CAMK2B, CAPRIN2, CARMIL1, CBFA2T2, CCR2, CDH4, CIB1, COBL, CPNE5, CREB3L2, CSNK1E, CSPG5, CTTN, CUX1, CXCR4, CYFIP1, DAB1, DAG1, DBN1, DHX36, DLG4, DLX2, DNM1L, EFNA5, EGR2, ENC1, EPHA4, EZH2, F11R, FBXW8, FEZ1, FEZF1, FEZF2, GLI2, GLI3, GPER1, GPRC5B, GSX2, HCLS1, HES1, IGF1, IL6ST, KALRN, KDM4C, KIDINS220, LIG4, LIMS1, LLGL1, LPAR1, LRP1, LRP2, LZTS1, MACF1, MAG, MAGI2, MAP3K13, MAP6, MAPK8IP3, MEF2C, MEGF8, MMD, MME, NCOA2, NDRG4, NEDD4L, NEDD9, NFE2L2, NGF, NME2, NOTCH1, NPTN, NSMF, NTRK2, NUMB, OBSL1, OLIG2, PACSIN1, PAFAH1B1, PDZD2, PLK5, PLXNA1, PLXNA2, PLXNB1, PLXNC1, PLXND1, POU4F2, PPARG, PRKCH, PRKCI, PRKDC, PRKN, PRPF19, PSEN1, PTEN, PTPRZ1, RANBP1, RAP1A, RAPGEF1, RARA, RELN, REST, RET, RGMA, RHEB, RHOA, ROBO1, ROR1, ROR2, RORA, RREB1, RUFY3, SEMA4D, SERPINE2, SERPINF1, SERPINI1, SHANK2, SHH, SHTN1, |
| GO:0099177 | regulation of trans-synaptic signaling | 2,64E-04 | [3, 4, 5, 6] | 139,00 | 28,08 | [ABAT, ABR, AKAP12, APBA2, ARF1, ATP2A2, BDNF, BDNF-AS, BEGAIN, BTBD9, CACNA1A, CACNA1B, CACNA2D2, CACNB2, CALB2, CAMK2B, CCR2, CDC20, CELF4, CHRNB2, CHRNB3, CNTN4, CSPG5, CYFIP1, DBN1, DGKE, DGKZ, DHX15, DHX34, DLG4, DNM1, DNM1L, EGR2, EIF2AK4, EIF4A3, EIF4E, EIF4EBP2, EPHA4, EPHB1, FABP5, FGF14, FZR1, GNAI2, GPER1, GPRIN1, GRIA4, GRID2, GRID2IP, GRIK3, GRIK4, GRM1, GRM2, GRM4, GRM8, HTR2A, IGSF9B, ITPR3, JAK2, JPH3, KCNB1, KRAS, LAMA2, LGMN, LLGL1, LRFN2, LRRC4C, LZTS1, MAPK8IP2, MEF2C, MME, MTMR2, NAPB, NCSTN, NF1, NFATC4, NGF, NLGN2, NOL3, NPS, NPTN, NPTXR, NRXN1, NRXN3, NSG1, NSMF, NTNG1, NTNG2, NTRK2, PACSIN2, PCDHA6, PFN2, PLA2G6, PLCB1, PLCB4, PLCL2, PPFIA3, PPP3CA, PPP3CB, PRKAR1B, PRKCA, PRKCB, PRKCE, PRKCZ, PRKN, PSEN1, PTEN, PTPRA, RAB5A, RAP1A, RARA, RASGRF1, RELN, RIMS3, ROR2, RORA, SEPTIN5, |
| GO:0030695 | GTPase regulator activity | 4,34E-02 | [6] | 91,00 | 28,09 | [ABR, ADAP1, ADGRB3, AGAP1, AGAP3, ALS2, ARAP1, ARAP3, ARFGAP1, ARHGAP10, ARHGAP12, ARHGAP17, ARHGAP21, ARHGAP29, ARHGAP39, ARHGAP40, ARHGAP42, ARHGAP45, ARHGDIA, ARHGDIB, ARHGDIG, ARHGEF10L, ASAP1, ASAP2, BMP1, CHN2, DEPDC1, DEPDC5, DLC1, DNM1L, EGR2, ELMOD1, FAM13A, FAM13B, GNB5, GPSM1, HACD3, IPO7, IQGAP2, KRIT1, LLGL1, MYO9B, NCKAP1L, NF1, NPRL3, PCP2, PLCB1, PLXNB1, RABGAP1, RALGAPA2, RALGDS, RANBP1, RAP1GAP, RAP1GAP2, RASA1, RASA2, RASA3, RASAL2, RGS12, RGS3, RGS6, RGS7, RGS9, RIN3, RTKN, SGSM1, SGSM2, SH3BP4, SMAP1, SMAP2, SMOC2, SOS1, SRGAP3, STARD13, STXBP5, STXBP5L, SYDE2, TBC1D1, TBC1D14, TBC1D15, TBC1D16, |
| GO:0007265 | Ras protein signal transduction | 1,85E-03 | [5, 6, 7] | 120,00 | 28,10 | [ABL2, ADGRG1, AKAP13, ALS2, ARFGAP1, ARFGEF3, ARHGAP17, ARHGAP29, ARHGAP42, ARHGDIA, ARHGDIB, ARHGDIG, ARHGEF18, ARHGEF2, BCL11B, BCL6, CDH13, CDK2, CDKN1A, CDKN2A, CELSR1, COL3A1, CTNNAL1, CUL3, CYFIP1, CYTH1, DENND1A, DLC1, DOK3, ELMO1, EPS8L1, ETFA, F11R, F2RL3, FARP2, FBP1, FGF2, GNA12, GPR17, GPR4, GPR55, GRB2, HACD3, HEG1, IGF1, IQGAP3, IQSEC1, IQSEC3, IRS2, JAK2, KALRN, KANK1, KCTD10, KIAA0355, KITLG, KRAS, KSR1, KSR2, LPAR1, MAP4K4, MAPKAP1, MAPKAPK5, MYO9B, NCKAP1, NF1, NGF, NOTCH1, PLCE1, PLD1, PRAG1, PSD, PSD2, PSD3, PSD4, PTGES2, RAB22A, RAB29, RAB2A, RAB35, RAB3D, RAB40B, RAB43, RAB4A, RAB5A, RALGDS, RAP1A, RAP2B, RAPGEF1, |
| GO:0051960 | regulation of nervous system development | 8,72E-12 | [4, 5, 6] | 288,00 | 28,29 | [ABL2, ADGRB2, ADGRB3, ADGRG1, ADGRL3, AFDN, AGRN, ALK, ALKAL2, AP2A1, APPL2, ARF1, ARHGDIA, ARHGEF2, ARHGEF7, ARNTL, ASAP1, ASCL1, ATF1, ATP8A2, BCL11A, BCL11B, BCL2, BCL6, BDNF, BDNF-AS, BEND6, BHLHE40, BMERB1, BMP2, BMP7, BRINP2, BRSK2, C21orf91, CACNA1A, CAMK2B, CAPRIN2, CASZ1, CBFA2T2, CCR2, CDC20, CDH4, CDK1, CDK5R1, CELSR1, CEND1, CHRNB2, CIB1, CNTN4, COBL, COL3A1, CPNE5, CREB3L2, CSMD3, CSNK1E, CTNNA2, CTTN, CUX1, CXCR4, CYFIP1, DAB1, DAG1, DBN1, DDX6, DENND5A, DHX36, DLG1, DLG4, DLL1, DLL4, DLX2, DNM1L, DOCK7, DTX1, EFNA5, EGR2, EIF2AK3, EIF2AK4, EIF4E, EIF4ENIF1, ENC1, EPHA4, EPHB1, EPHB3, ERBB4, EZH2, FARP1, FBXW8, FERD3L, FEZ1, FEZF1, FEZF2, FLRT1, GAK, GFI1, GLI2, GLI3, GORASP1, GPC6, GPER1, GPR161, GPRC5B, GPRIN1, GRID2, GSK3A, GSX2, HCLS1, HECW1, HELT, HES1, HES3, HES4, IFRD1, IL1RAP, IL6ST, INPP5F, ISL1, KALRN, KANK1, KDM4C, KIDINS220, KIF1A, KNDC1, LDLR, LIG4, LINGO1, LLGL1, LPAR1, LRCH4, LRFN3, LRIG2, LRP1, LRP2, LRRC4C, LZTS1, MACF1, MAG, MAGI2, MAP2, MAP3K13, MAP6, MAPK8IP3, MDGA1, MEF2C, MEGF8, MKS1, MMD, MME, MTMR2, MYRF, NCOA2, NDRG4, NECTIN1, NEDD4L, NEPRO, NF1, NFATC4, NFE2L2, NGEF, NGF, NLGN2, NME2, NOL3, NOS1, NOTCH1, NOTCH3, NPTN, NR1D1, NRXN1, NSMF, NTNG1, NTNG2, NTRK2, NUMB, OBSL1, OLFM1, OLIG2, PACSIN1, PAFAH1B1, PARD3, PAX2, PBX1, PER2, PHACTR1, PLK5, PLXNA1, PLXNA2, PLXNB1, PLXNC1, PLXND1, POU4F2, PPARG, PPP3CA, PRAG1, PRKCA, PRKCH, PRKCI, PRKN, PRPF19, PRUNE1, PSEN1, PTEN, PTK2, PTPRF, PTPRO, PTPRZ1, RAB29, RANBP1, |
| GO:0120035 | regulation of plasma membrane bounded cell projection organization | 6,61E-08 | [5, 6] | 210,00 | 28,30 | [ABL2, ADGRB3, AGRN, ALK, ALKAL2, AP2A1, APC, ARAP1, ARF1, ARHGDIA, ARHGEF7, ASAP1, ATF1, ATP8A2, BBS4, BCAS3, BCL11A, BDNF, BDNF-AS, BIN3, BMP7, BRSK2, C21orf91, CACNA1A, CAMK2B, CAPRIN2, CAPZB, CARMIL1, CARMIL2, CAV1, CBFA2T2, CDC20, CDH4, CDK5R1, CHRNB2, CIB1, CNTROB, COBL, CORO1B, CPNE5, CREB3L2, CSMD3, CTNNA2, CTTN, CUX1, CXCR4, CYFIP1, CYLD, DAB1, DBN1, DEF8, DENND5A, DHX36, DLG4, DNM1L, DYNC2LI1, EFNA5, EGR2, ENC1, ENPP2, EPHA4, EPHB3, EPS8L1, ESPN, EVL, EZH2, FBXW8, FER, FEZ1, FEZF2, GAK, GFI1, GORASP1, GPRIN1, GRID2, GSK3A, HCLS1, HDAC4, HECW1, HRG, HTT, IFRD1, IFT140, IFT88, INPP5F, KALRN, KANK1, KIDINS220, KIF1A, KNDC1, LINGO1, LLGL1, LPAR1, LRIG2, LRP1, LRRC4C, LZTS1, MACF1, MAG, MAGI2, MAK, MAP2, MAP3K13, MAP4, MAP6, MEF2C, MEGF8, NAV3, NCKAP1, NCOA2, NDRG4, NEDD4L, NFATC4, NFE2L2, NGEF, NGF, NME2, NOL3, NPTN, NRXN1, NSMF, NTNG1, NTNG2, NTRK2, OBSL1, OLFM1, PACSIN1, PAFAH1B1, PALM, PFN2, PLCE1, PLD1, PLK5, PLXNA1, PLXNA2, PLXNB1, PLXNC1, PLXND1, POU4F2, PPP1R16B, PPP3CA, PRAG1, PRKCI, PRKN, PSEN1, PTEN, PTK2, PTPRF, PTPRO, RAB29, RAB5A, RANBP1, RAP1A, RAP1GAP, RAP1GAP2, RAPGEF1, RELN, RET, RGMA, RHOA, RHOG, |
| GO:0032970 | regulation of actin filament-based process | 1,61E-03 | [3, 4] | 120,00 | 28,30 | [ABL2, ACTN2, ACTR3B, ADD1, ADD2, AKAP13, ALMS1, ARAP1, ARF1, ARHGAP17, ARHGAP40, ARHGDIA, ARHGDIB, ARHGEF10L, ARHGEF18, ARHGEF7, ARPC4, ATP1A3, ATP1A4, ATP2A2, BAIAP2L1, BBS4, BCAS3, CAMK2D, CAPZB, CARMIL1, CARMIL2, CAV1, CD47, CDK5R1, CELSR1, CLASP1, CLASP2, CORO1B, CORO2B, CTNNA2, CTTN, CYFIP1, DBN1, DLC1, DLG1, EFNA5, ELN, EPHA1, EVL, F11R, FCHSD1, FCHSD2, FER, FHOD1, FHOD3, FMN1, FRMD6, FZD10, GJA5, GMFB, GRB2, HCLS1, HCN4, HIP1R, HRG, IQGAP2, IQGAP3, KANK1, KANK3, KCNJ2, KIRREL1, LIMA1, LPAR1, LRP1, MEF2C, MYO1C, MYO1E, NCK2, NCKAP1, NCKAP1L, NOS1AP, NPHS1, PDGFRA, PFN2, PIK3R1, PLEK, PLEKHG2, PPFIA1, PPM1F, PRKCE, PRKN, PXDN, RASA1, RHOA, |
| GO:0018105 | peptidyl-serine phosphorylation | 1,80E-02 | [7, 8, 9] | 96,00 | 28,32 | [AKT2, AMH, ANGPT1, ANTXR1, ATM, ATP2B4, AURKB, BCAR3, BCL2, BDNF, BDNF-AS, BRSK2, CAMK2D, CAMK4, CAPRIN2, CAV1, CDK1, CDK2, CDK5R1, CHEK2, CLASP1, CSNK1A1L, CSNK1E, CSNK1G3, DCLK1, DDIT4, DGKQ, DOCK7, EIF2AK3, FNIP2, GCG, GSK3A, HCLS1, HRC, HSP90AB1, IKBKB, INPP5F, LATS2, LMTK2, MAP2K2, MAP3K13, MAPK7, MAPK8, MAPK9, MAPKAP1, MAPKAPK5, MARK3, MAST4, MKNK1, MKNK2, NCK2, NGF, NLK, NOS1, NRXN1, NSD1, NTRK2, PDPK1, PFN2, PKD1, PLCL2, PPM1F, PRKCA, PRKCB, PRKCE, PRKCH, PRKCI, |
| GO:0032956 | regulation of actin cytoskeleton organization | 5,51E-03 | [4, 5, 6, 7] | 107,00 | 28,38 | [ABL2, ACTN2, ACTR3B, ADD1, ADD2, AKAP13, ALMS1, ARAP1, ARF1, ARHGAP17, ARHGAP40, ARHGDIA, ARHGDIB, ARHGEF10L, ARHGEF18, ARHGEF7, ARPC4, BAIAP2L1, BBS4, BCAS3, CAPZB, CARMIL1, CARMIL2, CD47, CDK5R1, CELSR1, CLASP1, CLASP2, CORO1B, CORO2B, CTNNA2, CTTN, CYFIP1, DBN1, DLC1, DLG1, EFNA5, ELN, EPHA1, EVL, F11R, FCHSD1, FCHSD2, FER, FHOD1, FHOD3, FMN1, FZD10, GMFB, GRB2, HCLS1, HIP1R, HRG, IQGAP2, IQGAP3, KANK1, KANK3, KIRREL1, LIMA1, LPAR1, LRP1, MEF2C, MYO1C, MYO1E, NCK2, NCKAP1, NCKAP1L, NPHS1, PDGFRA, PFN2, PIK3R1, PLEK, PLEKHG2, PPFIA1, PPM1F, PRKCE, PRKN, PXDN, RASA1, RHOA, RHOBTB3, RHOG, RHOU, RICTOR, ROCK2, SCIN, SH3PXD2B, SLC9A1, SMAD3, |
| GO:0043087 | regulation of GTPase activity | 6,76E-05 | [5] | 144,00 | 28,46 | [ABR, ADAP1, ADGRB3, ADRB1, AFDN, AGAP1, AGAP3, AGRN, AKT2, ALS2, APC2, ARAP1, ARAP3, ARFGAP1, ARHGAP10, ARHGAP12, ARHGAP17, ARHGAP21, ARHGAP29, ARHGAP39, ARHGAP40, ARHGAP42, ARHGAP45, ARHGDIA, ARHGDIB, ARHGDIG, ARHGEF10L, ARHGEF16, ARHGEF7, ASAP1, ASAP2, BBS4, BCAR3, BCAS3, BCL6, CCL16, CHN2, DENND1A, DENND1B, DEPDC1, DEPDC5, DLC1, DNM1L, DOCK10, DOCK7, DOCK8, EFNA5, EGR2, ELMOD1, EPHA1, EPHA4, EPHB3, EZH2, F11R, FAM13A, FAM13B, FGD2, FICD, FZD10, GNB5, GPR137B, GPSM1, HACD3, IQGAP2, IQGAP3, IQSEC1, KALRN, LIMS1, LLGL1, MAP4K4, MYO9B, NCKAP1L, NEDD9, NF1, NGEF, NPRL3, NTRK2, PAFAH1B1, PDZD2, PLCB1, PLXNA1, PLXNA2, PLXNB1, PLXNC1, PLXND1, PTK2, PTPRN2, RABGAP1, RACK1, RALGAPA2, RANBP1, RAP1A, RAP1GAP, RAP1GAP2, RAPGEF1, RASA1, RASA2, RASA3, RASAL2, RASGRF1, RASIP1, RGMA, RGS12, RGS3, |

| | | | | | | |
|---|---|---|---|---|---|---|
| GO:0000902 | cell morphogenesis | 2,73E-14 | [3, 4] | 323,00 | 28,53 | BDNF-AS, BIN3, BMP7, BRSK2, CACNA1A, CAMK2B, CAMSAP1, CAP1, CAP2, CAPRIN2, CAPZB, CARMIL1, CCDC88C, CCKAR, CDH10, CDH13, CDH18, CDH19, CDH23, CDH26, CDH4, CDK5R1, CFDP1, CHRNB2, CIB1, CNTN4, CNTNAP2, COBL, COL18A1, COL6A1, COL6A2, CORO1B, CPNE5, CSPG5, CTNNA2, CTNND2, CTTN, CUL3, CUX1, CXCR4, CYFIP1, CYFIP2, DAB1, DAG1, DBN1, DCLK1, DDR1, DHX15, DHX36, DLC1, DLG1, DLG4, DNM1L, DNMBP, DOCK10, DOCK7, DOK4, DOK6, DRGX, DSCAML1, EFNA4, EFNA5, EGR2, EIF2AK4, ENPP2, EPHA1, EPHA10, EPHA4, EPHA6, EPHB1, EPHB3, ETV1, EVL, F11R, FAM171A1, FARP1, FAT1, FBXW8, FER, FERMT2, FEZ1, FEZF1, FEZF2, FGD2, FITM2, FLRT1, FMNL2, FRMD6, FRS2, FRYL, FZR1, GAB1, GAB2, GAS7, GLI2, GLI3, GNA12, GORASP1, GPRIN1, GRB10, GRB2, GREM1, HCLS1, HECW1, HEG1, HES1, HEXB, HSP90AB1, IFRD1, IHH, IRS2, ISL1, ITGA1, ITGB2, KALRN, KANK1, KIDINS220, KIF1A, KIF5A, KLF7, KNDC1, LAMA2, LAMA5, LAMB1, LAMB2, LAMB4, LGR6, LHX4, LIMS1, LINGO1, LLGL1, LPAR1, LRP1, LRP2, LRRC4C, LZTS1, MACF1, MAG, MAP2, MAP3K13, MAP4K4, MAP6, MAPK7, MAPK8IP2, MAPK8IP3, MEF2C, MEGF8, MELTF, MYL12B, MYO16, MYPN, NCKAP1, NCKAP1L, NCOA2, NECTIN1, NEDD4L, NEDD9, NEO1, NFASC, NFATC4, NFIB, NGEF, NGF, NOL3, NOTCH1, NOTCH4, NPTN, NRXN1, NRXN3, NSMF, NTNG1, NTNG2, NTRK2, NUMB, OBSL1, OLFM1, PACSIN1, PACSIN2, PAFAH1B1, PALLD, PALM, PALM2AKAP2, PARD3, PAX2, PDZD7, PEAK1, PHACTR1, PIK3CB, PIK3R1, PLXNA1, PLXNA2, PLXNB1, PLXNC1, PLXND1, POU4F2, PPP3CA, PPP3CB, PRAG1, PRKCA, PRKCQ, PRKCZ, PRKDC, PRKN, PSEN1, PTEN, PTK2, PTPN11, PTPRA, PTPRO, PTPRZ1, PXDN, QRICH1, RADIL, RANBP9, RAP1GAP, RASA1, RB1, RBFOX2, RELN, RET, RGMA, RHOA, RHOBTB3, RHOG, RHOU, RILPL1, RIPOR2, ROBO1, |
| GO:0000902 | cell morphogenesis | 2,73E-14 | [3, 4] | 323,00 | 28,53 | BDNF-AS, BIN3, BMP7, BRSK2, CACNA1A, CAMK2B, CAMSAP1, CAP1, CAP2, CAPRIN2, CAPZB, CARMIL1, CCDC88C, CCKAR, CDH10, CDH13, CDH18, CDH19, CDH23, CDH26, CDH4, CDK5R1, CFDP1, CHRNB2, CIB1, CNTN4, CNTNAP2, COBL, COL18A1, COL6A1, COL6A2, CORO1B, CPNE5, CSPG5, CTNNA2, CTNND2, CTTN, CUL3, CUX1, CXCR4, CYFIP1, CYFIP2, DAB1, DAG1, DBN1, DCLK1, DDR1, DHX15, DHX36, DLC1, DLG1, DLG4, DNM1L, DNMBP, DOCK10, DOCK7, DOK4, DOK6, DRGX, DSCAML1, EFNA4, EFNA5, EGR2, EIF2AK4, ENPP2, EPHA1, EPHA10, EPHA4, EPHA6, EPHB1, EPHB3, ETV1, EVL, F11R, FAM171A1, FARP1, FAT1, FBXW8, FER, FERMT2, FEZ1, FEZF1, FEZF2, FGD2, FITM2, FLRT1, FMNL2, FRMD6, FRS2, FRYL, FZR1, GAB1, GAB2, GAS7, GLI2, GLI3, GNA12, GORASP1, GPRIN1, GRB10, GRB2, GREM1, HCLS1, HECW1, HEG1, HES1, HEXB, HSP90AB1, IFRD1, IHH, IRS2, ISL1, ITGA1, ITGB2, KALRN, KANK1, KIDINS220, KIF1A, KIF5A, KLF7, KNDC1, LAMA2, LAMA5, LAMB1, LAMB2, LAMB4, LGR6, LHX4, LIMS1, LINGO1, LLGL1, LPAR1, LRP1, LRP2, LRRC4C, LZTS1, MACF1, MAG, MAP2, MAP3K13, MAP4K4, MAP6, MAPK7, MAPK8IP2, MAPK8IP3, MEF2C, MEGF8, MELTF, MYL12B, MYO16, MYPN, NCKAP1, NCKAP1L, NCOA2, NECTIN1, NEDD4L, NEDD9, NEO1, NFASC, NFATC4, NFIB, NGEF, NGF, NOL3, NOTCH1, NOTCH4, NPTN, NRXN1, NRXN3, NSMF, NTNG1, NTNG2, NTRK2, NUMB, OBSL1, OLFM1, PACSIN1, PACSIN2, PAFAH1B1, PALLD, PALM, PALM2AKAP2, PARD3, PAX2, PDZD7, PEAK1, PHACTR1, PIK3CB, PIK3R1, PLXNA1, PLXNA2, PLXNB1, PLXNC1, PLXND1, POU4F2, PPP3CA, PPP3CB, PRAG1, PRKCA, PRKCQ, PRKCZ, PRKDC, PRKN, PSEN1, PTEN, PTK2, PTPN11, PTPRA, PTPRO, PTPRZ1, PXDN, QRICH1, RADIL, RANBP9, RAP1GAP, RASA1, RB1, RBFOX2, RELN, RET, RGMA, RHOA, RHOBTB3, RHOG, RHOU, RILPL1, RIPOR2, ROBO1, |
| GO:0050767 | regulation of neurogenesis | 1,03E-10 | [5, 6, 7, 8] | 256,00 | 28,54 | [ABL2, ADGRB3, ADGRG1, AFDN, ALK, ALKAL2, AP2A1, APPL2, ARF1, ARHGDIA, ARHGEF2, ARHGEF7, ARNTL, ASAP1, ASCL1, ATF1, ATP8A2, BCL11A, BCL11B, BCL2, BCL6, BDNF, BDNF-AS, BEND6, BHLHE40, BMERB1, BMP2, BMP7, BRINP2, BRSK2, C21orf91, CACNA1A, CAMK2B, CAPRIN2, CASZ1, CBFA2T2, CCR2, CDC20, CDH4, CDK1, CDK5R1, CHRNB2, CIB1, CNTN4, COBL, COL3A1, CPNE5, CREB3L2, CSMD3, CSNK1E, CTNNA2, CTTN, CUX1, CXCR4, CYFIP1, DAB1, DAG1, DBN1, DDX6, DENND5A, DHX36, DLG4, DLL1, DLL4, DLX2, DNM1L, DOCK7, DTX1, EFNA5, EGR2, EIF2AK4, EIF4E, EIF4ENIF1, ENC1, EPHA4, EPHB3, ERBB4, EZH2, FBXW8, FERD3L, FEZ1, FEZF1, FEZF2, GAK, GFI1, GLI2, GLI3, GORASP1, GPER1, GPRC5B, GPRIN1, GRID2, GSK3A, GSX2, HCLS1, HECW1, HELT, HES1, HES3, HES4, IFRD1, IL6ST, INPP5F, ISL1, KALRN, KANK1, KDM4C, KIDINS220, KIF1A, KNDC1, LDLR, LIG4, LINGO1, LLGL1, LPAR1, LRIG2, LRP1, LRP2, LRRC4C, LZTS1, MACF1, MAG, MAGI2, MAP2, MAP3K13, MAP6, MAPK8IP3, MEF2C, MEGF8, MMD, MME, NCOA2, NDRG4, NEDD4L, NEPRO, NF1, NFATC4, NFE2L2, NGEF, NGF, NME2, NOL3, NOS1, NOTCH1, NOTCH3, NPTN, NR1D1, NSMF, NTNG1, NTNG2, NTRK2, NUMB, OBSL1, OLFM1, OLIG2, PACSIN1, PAFAH1B1, PBX1, PER2, PHACTR1, PLK5, PLXNA1, PLXNA2, PLXNB1, PLXNC1, PLXND1, POU4F2, PPARG, PPP3CA, PRAG1, PRKCH, PRKCI, PRKN, PRPF19, PRUNE1, PSEN1, PTEN, PTK2, PTPRF, PTPRO, PTPRZ1, RAB29, RANBP1, RAP1A, RAP1GAP, |
| GO:0048666 | neuron development | 1,44E-15 | [4, 5, 7, 8, 9] | 348,00 | 28,55 | BCL2, BDNF, BDNF-AS, BLOC1S2, BLOC1S5, BMP7, BRSK2, BTG2, C21orf91, CACNA1A, CAMK2B, CAMSAP1, CAPRIN2, CBFA2T2, CCKAR, CDC20, CDH23, CDH4, CDK5R1, CEP290, CHRNB2, CIB1, CNTN4, CNTNAP2, COBL, CPNE5, CREB3L2, CSMD3, CTNNA2, CTNND2, CTTN, CUX1, CXCR4, CYFIP1, CYFIP2, DAB1, DAG1, DBN1, DCLK1, DDR1, DENND5A, DHX36, DLG4, DNM1L, DOCK10, DOCK7, DOK4, DOK6, DRGX, DSCAML1, EFHD1, EFNA4, EFNA5, EGR2, EIF2AK4, ENC1, EPHA1, EPHA10, EPHA4, EPHA6, EPHB1, EPHB3, ETV1, EVL, EZH2, FARP1, FARP2, FBXW8, FEZ1, FEZF1, FEZF2, FLRT1, FRS2, FRYL, FZR1, GAB1, GAB2, GABRB2, GAK, GAS7, GFI1, GLI2, GLI3, GORASP1, GPRIN1, GRB10, GRB2, GRID2, GSK3A, HCLS1, HCN1, HECW1, HERC1, HES1, HMGB1, HSP90AB1, IFRD1, IFT140, IFT88, IHH, INPP5F, IQSEC1, IRS2, ISL1, ITGA1, JAK2, KALRN, KANK1, KIDINS220, KIF1A, KIF5A, KLF7, KLHL1, KNDC1, LAMA2, LAMB1, LAMB2, LGR6, LHX4, LHX6, LHX8, LINGO1, LLGL1, LPAR1, LRIG2, LRP1, LRP12, LRP2, LRRC4C, LZTS1, MACF1, MAG, MAGI2, MAP2, MAP3K13, MAP4, MAP4K4, MAP6, MAPK7, MAPK8IP2, MAPK8IP3, MEF2C, MEGF8, MICALL1, MKS1, MRTFA, MTMR2, MYO16, MYPN, MYT1L, NCAM2, NCK2, NCKAP1, NCKAP1L, NCOA2, NDRG4, NECTIN1, NEDD4L, NEO1, NFASC, NFATC4, NFE2L2, NFIB, NGEF, NGF, NME2, NOL3, NOTCH1, NPHP4, NPTN, NR2E3, NRXN1, NRXN3, NSMF, NTM, NTNG1, NTNG2, NTRK2, NUMB, OBSL1, OGDH, OLFM1, OPCML, PACSIN1, PAFAH1B1, PAK4, PALLD, PARD3, PAX2, PBX1, PCARE, PDZD7, PHACTR1, PIK3CB, PIK3R1, PLK5, PLXNA1, PLXNA2, PLXNB1, PLXNC1, PLXND1, POU4F2, PPP3CA, PPP3CB, PRAG1, PRKCA, PRKCI, PRKCQ, PRKCZ, PRKN, PRMT1, PSD, PSEN1, PTEN, PTK2, PTPN11, PTPRA, PTPRF, PTPRO, PTPRZ1, RAB29, RAB35, RANBP1, RANBP9, RAP1A, RAP1GAP, RAP1GAP2, RAPGEF1, RASGRF1, RB1, RBFOX2, RELN, RET, RGMA, RHOA, RHOBTB3, RHOG, RIPOR2, ROBO1, ROBO3, ROR1, ROR2, RORA, RORB, RP1, RTN4RL1, RTN4RL2, RUFY3, RUNX1, RUNX3, S100A6, SALL3, SAMD14, SCRIB, SCYL3, |
| GO:0031175 | neuron projection development | 1,58E-13 | [5, 6, 8, 9, 10] | 308,00 | 28,57 | BLOC1S5, BMP7, BRSK2, BTG2, C21orf91, CACNA1A, CAMK2B, CAMSAP1, CAPRIN2, CBFA2T2, CCKAR, CDC20, CDH23, CDH4, CDK5R1, CHRNB2, CIB1, CNTN4, CNTNAP2, COBL, CPNE5, CREB3L2, CSMD3, CTNNA2, CTNND2, CTTN, CUX1, CXCR4, CYFIP1, CYFIP2, DAB1, DAG1, DBN1, DCLK1, DDR1, DENND5A, DHX36, DLG4, DNM1L, DOCK10, DOCK7, DOK4, DOK6, DRGX, DSCAML1, EFHD1, EFNA4, EFNA5, EGR2, EIF2AK4, ENC1, EPHA1, EPHA10, EPHA4, EPHA6, EPHB1, EPHB3, ETV1, EVL, EZH2, FARP1, FBXW8, FEZ1, FEZF1, FEZF2, FLRT1, FRS2, FRYL, FZR1, GAB1, GAB2, GAK, GAS7, GFI1, GLI2, GLI3, GORASP1, GPRIN1, GRB10, GRB2, GRID2, GSK3A, HCLS1, HECW1, HERC1, HES1, HMGB1, HSP90AB1, IFRD1, IFT88, INPP5F, IQSEC1, IRS2, ISL1, ITGA1, JAK2, KALRN, KANK1, KIDINS220, KIF1A, KIF5A, KLF7, KLHL1, KNDC1, LAMA2, LAMB1, LAMB2, LGR6, LHX4, LINGO1, LLGL1, LPAR1, LRIG2, LRP1, LRP12, LRP2, LRRC4C, LZTS1, MACF1, MAG, MAGI2, MAP2, MAP3K13, MAP4, MAP4K4, MAP6, MAPK7, MAPK8IP2, MAPK8IP3, MEF2C, MEGF8, MICALL1, MKS1, MRTFA, MYO16, MYPN, NCAM2, NCK2, NCKAP1, NCKAP1L, NCOA2, NDRG4, NECTIN1, NEDD4L, NEO1, NFASC, NFATC4, NFE2L2, NFIB, NGEF, NGF, NME2, NOL3, NOTCH1, NPTN, NRXN1, NRXN3, NSMF, NTNG1, NTNG2, NTRK2, NUMB, OBSL1, OLFM1, PACSIN1, PAFAH1B1, PAK4, PALLD, PARD3, PAX2, PDZD7, PHACTR1, PIK3CB, PIK3R1, PLK5, PLXNA1, PLXNA2, PLXNB1, PLXNC1, PLXND1, POU4F2, PPP3CA, PPP3CB, PRAG1, PRKCA, PRKCI, PRKCQ, PRKCZ, PRKN, PRMT1, PSD, PSEN1, PTEN, PTK2, PTPN11, PTPRA, PTPRF, PTPRO, PTPRZ1, RAB29, RAB35, RANBP1, RANBP9, RAP1A, RAP1GAP, RAP1GAP2, RAPGEF1, RASGRF1, RB1, RBFOX2, RELN, RET, RGMA, RHOA, RHOBTB3, RHOG, RIPOR2, ROBO1, ROBO3, ROR1, ROR2, RORA, RTN4RL1, RTN4RL2, RUFY3, S100A6, SAMD14, SCRIB, |
| GO:0097435 | supramolecular fiber organization | 7,54E-09 | [4] | 215,00 | 28,70 | [ACTN2, ACTN4, ACTR3B, ADAMTS2, ADD1, ADD2, AKAP13, ALMS1, ANXA2, APC, APC2, ARAP1, ARF1, ARHGAP12, ARHGAP17, ARHGAP40, ARHGEF10L, ARHGEF18, ARHGEF2, ARHGEF7, ARPC4, ATXN7, BAIAP2L1, BBS4, BCAR1, BCL2, BFSP1, BFSP2, BIN3, BMERB1, CAMSAP1, CAP1, CAP2, CAPZB, CARMIL1, CARMIL2, CCDC13, CCDC88C, CD47, CDK5R1, CDKN2A, CIB1, CLASP1, CLASP2, CLIP2, COBL, COL11A2, COL2A1, COL3A1, CORO1B, CORO2B, CORO7-PAM16, CTNNA2, CTTN, CUL3, CYFIP1, CYP1B1, DBN1, DCTN1, DDR2, DIAPH3, DLC1, DLG1, DNAJB6, DST, ELMO1, ELN, EPHA1, EPPK1, ESPN, ESPNL, EVL, F11R, F3, FAM171A1, FAT1, FBLN5, FCHSD1, FCHSD2, FER, FHOD1, FHOD3, FMN1, GMFB, GRB2, GREM1, HCLS1, HIP1, HIP1R, HOOK1, HSP90AB1, INPPL1, IQGAP2, JAK2, KANK1, KANK3, KATNAL2, KIF18A, KIF2A, KIF2C, KIRREL1, KRT20, KRT25, LDB3, LDLR, LIMA1, LIMK2, LOXL2, LOXL3, LPAR1, LTBP2, MAP2, MARCKS, MEF2C, MICAL2, MICAL3, MPRIP, MYH3, MYL2, MYO15A, MYO1A, MYO1C, MYO1D, MYO1E, MYO5A, MYO5C, MYOM2, MYPN, NAV3, NCK2, NCKAP1, NCKAP1L, NCKAP5, NDE1, NEDD9, NF1, NPHS1, OBSCN, OBSL1, PACSIN1, PAFAH1B1, PDGFRA, PDLIM1, PDLIM3, PFN2, PHACTR1, PIK3R1, PKP1, PLEK, PLEKHG2, PPARGC1B, PPFIA1, PPM1F, PRKCE, PRKCI, PRKN, |
| GO:0097435 | supramolecular fiber organization | 7,54E-09 | [4] | 215,00 | 28,70 | [ACTN2, ACTN4, ACTR3B, ADAMTS2, ADD1, ADD2, AKAP13, ALMS1, ANXA2, APC, APC2, ARAP1, ARF1, ARHGAP12, ARHGAP17, ARHGAP40, ARHGEF10L, ARHGEF18, ARHGEF2, ARHGEF7, ARPC4, ATXN7, BAIAP2L1, BBS4, BCAR1, BCL2, BFSP1, BFSP2, BIN3, BMERB1, CAMSAP1, CAP1, CAP2, CAPZB, CARMIL1, CARMIL2, CCDC13, CCDC88C, CD47, CDK5R1, CDKN2A, CIB1, CLASP1, CLASP2, CLIP2, COBL, COL11A2, COL2A1, COL3A1, CORO1B, CORO2B, CORO7-PAM16, CTNNA2, CTTN, CUL3, CYFIP1, CYP1B1, DBN1, DCTN1, DDR2, DIAPH3, DLC1, DLG1, DNAJB6, DST, ELMO1, ELN, EPHA1, EPPK1, ESPN, ESPNL, EVL, F11R, F3, FAM171A1, FAT1, FBLN5, FCHSD1, FCHSD2, FER, FHOD1, FHOD3, FMN1, GMFB, GRB2, GREM1, HCLS1, HIP1, HIP1R, HOOK1, HSP90AB1, INPPL1, IQGAP2, JAK2, KANK1, KANK3, KATNAL2, KIF18A, KIF2A, KIF2C, KIRREL1, KRT20, KRT25, LDB3, LDLR, LIMA1, LIMK2, LOXL2, LOXL3, LPAR1, LTBP2, MAP2, MARCKS, MEF2C, MICAL2, MICAL3, MPRIP, MYH3, MYL2, MYO15A, MYO1A, MYO1C, MYO1D, MYO1E, MYO5A, MYO5C, MYOM2, MYPN, NAV3, NCK2, NCKAP1, NCKAP1L, NCKAP5, NDE1, NEDD9, NF1, NPHS1, OBSCN, OBSL1, PACSIN1, PAFAH1B1, PDGFRA, PDLIM1, PDLIM3, PFN2, PHACTR1, PIK3R1, PKP1, PLEK, PLEKHG2, PPARGC1B, PPFIA1, PPM1F, PRKCE, PRKCI, PRKN, |
| GO:0032989 | cellular component morphogenesis | 9,92E-11 | [3, 4] | 246,00 | 28,84 | [ACTN2, ADGRB3, AGRN, AKAP13, ALS2, ANK3, ARHGDIA, ARHGEF7, ATP8A2, BCL11A, BCL11B, BCL2, BDNF, BDNF-AS, BMP7, BRSK2, CACNA1A, CAMK2B, CAPRIN2, CCKAR, CDH4, CDK5R1, CHRNB2, CLASP1, CLASP2, CNTN4, CNTNAP2, COBL, CORO1B, CPNE5, CSPG5, CTNNA2, CTNND2, CTTN, CUX1, CXCR4, CYFIP1, CYFIP2, DAB1, DAG1, DBN1, DCLK1, DDR1, DHX36, DLG4, DNM1L, DOCK10, DOCK7, DOK4, DOK6, DRGX, DSCAML1, EFNA4, EFNA5, EGR2, EIF2AK4, ENPP2, EPHA1, EPHA10, EPHA4, EPHA6, EPHB1, EPHB3, ETV1, EVL, FARP1, FBXW8, FEZ1, FEZF1, FEZF2, FHOD3, FLRT1, FRS2, GAB1, GAB2, GAS7, GLI2, GLI3, GORASP1, GPRIN1, GRB10, GRB2, HCLS1, HECW1, HSP90AB1, IFRD1, IHH, IMMT, IRS2, ISL1, ITGA1, KALRN, KANK1, KIDINS220, KIF1A, KIF5A, KLF7, KNDC1, LAMA2, LAMB2, LDB3, LGR6, LHX4, LINGO1, LLGL1, LRP1, LRP2, LRRC4C, LZTS1, MACF1, MAG, MAP2, MAP3K13, MAP4K4, MAP6, MAPK7, MAPK8IP2, MAPK8IP3, MEF2C, MEGF8, MTMR2, MYH3, MYL2, MYO16, MYOM2, MYPN, NCKAP1, NCKAP1L, NCOA2, NECTIN1, NEDD4L, NEO1, NFASC, NFATC4, NFIB, NGEF, NGF, NOL3, NOTCH1, NPTN, NRXN1, NRXN3, NSMF, NTNG1, NTNG2, NTRK2, NUMB, OBSCN, OBSL1, OLFM1, OPA3, PACSIN1, PACSIN2, PAFAH1B1, PALLD, PARD3, PAX2, PDGFRA, PHACTR1, PHLDB1, PIK3CB, PIK3R1, PIKFYVE, PLXNA1, PLXNA2, PLXNB1, PLXNC1, PLXND1, POU4F2, PPP3CA, PPP3CB, PRKCA, PRKCQ, PRKCZ, PRKN, PSEN1, PTEN, PTK2, PTPN11, PTPRA, |
| GO:0010975 | regulation of neuron projection development | 3,56E-06 | [6, 7, 8, 9, 10, 11] | 160,00 | 28,88 | [ABL2, ADGRB3, ALK, ALKAL2, AP2A1, ARF1, ARHGDIA, ARHGEF7, ASAP1, ATF1, ATP8A2, BCL11A, BDNF, BDNF-AS, BMP7, BRSK2, C21orf91, CACNA1A, CAMK2B, CAPRIN2, CBFA2T2, CDC20, CDH4, CDK5R1, CHRNB2, CIB1, COBL, CPNE5, CREB3L2, CSMD3, CTNNA2, CTTN, CUX1, CXCR4, CYFIP1, DAB1, DBN1, DENND5A, DHX36, DLG4, DNM1L, EFNA5, EGR2, ENC1, EPHA4, EPHB3, EZH2, FBXW8, FEZ1, FEZF2, GAK, GFI1, GORASP1, GPRIN1, GRID2, GSK3A, HCLS1, HECW1, IFRD1, INPP5F, KALRN, KANK1, KIDINS220, KIF1A, KNDC1, LINGO1, LLGL1, LPAR1, LRIG2, LRP1, LRRC4C, LZTS1, MACF1, MAG, MAGI2, MAP2, MAP3K13, MAP6, MEF2C, MEGF8, NCOA2, NDRG4, NEDD4L, NFATC4, NFE2L2, NGEF, NGF, NME2, NOL3, NPTN, NSMF, NTNG1, NTNG2, NTRK2, OBSL1, OLFM1, PACSIN1, PAFAH1B1, PLK5, PLXNA1, PLXNA2, PLXNB1, PLXNC1, PLXND1, POU4F2, PPP3CA, PRAG1, PRKCI, PRKN, PSEN1, PTEN, PTK2, PTPRF, |
| GO:0010769 | regulation of cell morphogenesis involved in differentiation | 6,82E-03 | [5, 6] | 96,00 | 29,00 | [ACTN4, ADGRB3, ARHGDIA, ARHGEF7, BDNF, BDNF-AS, BRSK2, CACNA1A, CAMK2B, CAPRIN2, CARMIL1, CDH4, CDK5R1, CHRNB2, CIB1, CSPG5, CTTN, CUX1, CYFIP1, DAB1, DBN1, DHX36, DNM1L, EFNA5, EGR2, EPHA4, EPHB3, FBXW8, FEZF2, GORASP1, GPRIN1, HCLS1, HECW1, IFRD1, KALRN, KANK1, KIF1A, KNDC1, LIMS1, LINGO1, LRP1, LRRC4C, LZTS1, MACF1, MAG, MAP2, MAP3K13, MAP6, MEGF8, MELTF, NEDD4L, NEDD9, NFATC4, NGEF, NGF, NOL3, NSMF, NTRK2, OBSL1, OLFM1, PAFAH1B1, PLXNA1, PLXNA2, PLXNB1, PLXNC1, PLXND1, |
| GO:0021537 | telencephalon development | 2,56E-02 | [3, 4, 6, 7, 8, 9] | 83,00 | 29,02 | [ADGRG1, AFDN, ALK, ARL13B, ASCL1, ATAT1, ATG16L1, ATP2B4, BBS4, BCL11B, BCL2A1, BMERB1, BMP2, BTG2, CCDC85C, CDK5R1, CNTNAP2, COL3A1, CXCR4, DAB1, DLX2, DNAH5, EPHB3, ERBB4, EZH2, FEZ1, FEZF1, FEZF2, GLI3, GPRIN1, GSX2, HERC1, HES1, IGF2BP1, KDM2B, KDM6B, LAMB1, LHX6, LPAR1, LRP1, LZTS1, MBOAT7, MCPH1, NDE1, NEUROD6, NF1, NFIB, NTRK2, NUMB, OGDH, PAFAH1B1, PEX13, PHACTR1, PHLPP2, PLCB1, PSEN1, PTEN, RARA, RELN, RHOA, ROBO1, RTN4RL1, RTN4RL2, SALL3, SCN5A, SECISBP2, SHH, SKI, SRD5A1, |
| GO:0031589 | cell-substrate adhesion | 1,29E-03 | [3] | 109,00 | 29,07 | [ABI3BP, ACTN2, ACTN4, ACVRL1, AJAP1, ANGPT1, ANGPT4, ANTXR1, ARHGEF7, BCAM, BCAS3, BCL2, BCL6, CARMIL1, CCDC80, CD96, CDH13, CDKN2A, CIB1, CLASP1, CLASP2, COL3A1, CORO2B, CSPG5, CTTN, DAG1, DDR1, DLC1, EFNA5, ENC1, EPHA1, EPHB1, EPHB3, FBLN5, FER, FERMT2, FMN1, FREM1, GCNT2, GREM1, HRG, HSD17B12, ITGA1, ITGA11, ITGA5, ITGAL, ITGB2, ITGB4, ITGBL1, JAK2, KANK1, LAMA5, LAMB1, LAMB2, LAMB4, LDB2, LIMS1, LRP1, MACF1, MAP4K4, MELTF, MEN1, MSLN, MUC4, NEDD9, NF1, NOTCH1, NPNT, PDPK1, PEAK1, PIK3CB, PIK3R1, PKD1, PLEKHA2, PPFIA1, PPM1F, PPP3CA, PRKCE, PRKCZ, PTEN, PTK2, PTPRO, PXDN, RADIL, RASA1, RHOA, ROCK2, RREB1, SGCE, SIGLEC1, SKAP1, SLC9A1, SMAD3, |
| GO:0031346 | positive regulation of cell projection organization | 5,64E-05 | [4, 5, 6] | 126,00 | 29,44 | [ABL2, AGRN, ALK, ALKAL2, AP2A1, APC, ARAP1, ARF1, ARHGDIA, ARHGEF7, ATF1, ATP8A2, BBS4, BCAS3, BDNF, BDNF-AS, BMP7, C21orf91, CAMK2B, CAPRIN2, CARMIL1, CARMIL2, CBFA2T2, CDH4, COBL, CORO1B, CPNE5, CREB3L2, CTTN, CUX1, CXCR4, CYFIP1, DBN1, DEF8, DHX36, DLG4, DNM1L, EFNA5, ENC1, ENPP2, EPHA4, EPS8L1, ESPN, EZH2, FBXW8, FEZ1, HCLS1, HDAC4, HTT, KALRN, KIDINS220, LLGL1, LPAR1, LRP1, LZTS1, MACF1, MAGI2, MAP3K13, MAP6, MEGF8, NAV3, NCKAP1, NCOA2, NDRG4, NEDD4L, NFE2L2, NGF, NME2, NPTN, NTRK2, OBSL1, PACSIN1, PAFAH1B1, PALM, PLCE1, PLK5, PLXNA1, PLXNA2, PLXNB1, PLXNC1, PLXND1, POU4F2, PRKCI, PRKN, PSEN1, RANBP1, RAP1A, RAPGEF1, RELN, RET, RGMA, |
| GO:0031346 | positive regulation of cell projection organization | 5,64E-05 | [4, 5, 6] | 126,00 | 29,44 | [ABL2, AGRN, ALK, ALKAL2, AP2A1, APC, ARAP1, ARF1, ARHGDIA, ARHGEF7, ATF1, ATP8A2, BBS4, BCAS3, BDNF, BDNF-AS, BMP7, C21orf91, CAMK2B, CAPRIN2, CARMIL1, CARMIL2, CBFA2T2, CDH4, COBL, CORO1B, CPNE5, CREB3L2, CTTN, CUX1, CXCR4, CYFIP1, DBN1, DEF8, DHX36, DLG4, DNM1L, EFNA5, ENC1, ENPP2, EPHA4, EPS8L1, ESPN, EZH2, FBXW8, FEZ1, HCLS1, HDAC4, HTT, KALRN, KIDINS220, LLGL1, LPAR1, LRP1, LZTS1, MACF1, MAGI2, MAP3K13, MAP6, MEGF8, NAV3, NCKAP1, NCOA2, NDRG4, NEDD4L, NFE2L2, NGF, NME2, NPTN, NTRK2, OBSL1, PACSIN1, PAFAH1B1, PALM, PLCE1, PLK5, PLXNA1, PLXNA2, PLXNB1, PLXNC1, PLXND1, POU4F2, PRKCI, PRKN, PSEN1, RANBP1, RAP1A, RAPGEF1, RELN, RET, RGMA, |
| GO:0045664 | regulation of neuron differentiation | 5,84E-10 | [5, 6, 7, 8, 9] | 212,00 | 29,53 | [ABL2, ADGRB3, ALK, ALKAL2, AP2A1, ARF1, ARHGDIA, ARHGEF2, ARHGEF7, ASAP1, ASCL1, ATF1, ATP8A2, BCL11A, BCL11B, BCL2, BCL6, BDNF, BDNF-AS, BEND6, BMP2, BMP7, BRINP2, BRSK2, C21orf91, CACNA1A, CAMK2B, CAPRIN2, CASZ1, CBFA2T2, CDC20, CDH4, CDK5R1, CHRNB2, CIB1, CNTN4, COBL, CPNE5, CREB3L2, CSMD3, CSNK1E, CTNNA2, CTTN, CUX1, CXCR4, CYFIP1, DAB1, DBN1, DDX6, DENND5A, DHX36, DLG4, DLL1, DLX2, DNM1L, DTX1, EFNA5, EGR2, EIF2AK4, EIF4E, EIF4ENIF1, ENC1, EPHA4, EPHB3, EZH2, FBXW8, FEZ1, FEZF1, FEZF2, GAK, GFI1, GLI2, GLI3, GORASP1, GPRC5B, GPRIN1, GRID2, GSK3A, HCLS1, HECW1, HES1, HES3, HES4, IFRD1, INPP5F, ISL1, KALRN, KANK1, KDM4C, KIDINS220, KIF1A, KNDC1, LINGO1, LLGL1, LPAR1, LRIG2, LRP1, LRRC4C, LZTS1, MACF1, MAG, MAGI2, MAP2, MAP3K13, MAP6, MAPK8IP3, MEF2C, MEGF8, MMD, NCOA2, NDRG4, NEDD4L, NEPRO, NFATC4, NFE2L2, NGEF, NGF, NME2, NOL3, NOTCH1, NOTCH3, NPTN, NSMF, NTNG1, NTNG2, NTRK2, OBSL1, OLFM1, OLIG2, PACSIN1, PAFAH1B1, PBX1, PLK5, PLXNA1, PLXNA2, PLXNB1, PLXNC1, PLXND1, POU4F2, PPP3CA, PRAG1, PRKCI, PRKN, PRPF19, PSEN1, PTEN, PTK2, PTPRF, PTPRO, RAB29, RANBP1, RAP1A, RAP1GAP, RAP1GAP2, RAPGEF1, RARA, RELN, REST, RET, RGMA, RHOA, |

| | | | | | | |
|---|---|---|---|---|---|---|
| GO:0051962 | positive regulation of nervous system development | 6,81E-08 | [3, 4, 5, 6, 7] | 176,00 | 29,53 | [ABL2, ADGRB2, ADGRB3, ADGRL3, AGRN, ALK, ALKAL2, AP2A1, ARF1, ARHGDIA, ARHGEF2, ARHGEF7, ASCL1, ATF1, ATP8A2, BCL2, BCL6, BDNF, BDNF-AS, BEND6, BMP2, BMP7, BRINP2, C21orf91, CAMK2B, CAPRIN2, CBFA2T2, CCR2, CDC20, CDH4, COBL, CPNE5, CREB3L2, CSNK1E, CTTN, CUX1, CXCR4, CYFIP1, DAB1, DAG1, DBN1, DHX36, DLG4, DLX2, DNM1L, EFNA5, EGR2, ENC1, EPHA4, EPHB1, EPHB3, EZH2, FBXW8, FEZ1, FEZF1, FEZF2, FLRT1, GLI2, GLI3, GPER1, GPRC5B, GRID2, GSX2, HCLS1, HES1, IL1RAP, IL6ST, KALRN, KDM4C, KIDINS220, LIG4, LLGL1, LPAR1, LRCH4, LRP1, LRP2, LZTS1, MACF1, MAG, MAGI2, MAP3K13, MAP6, MAPK8IP3, MEF2C, MEGF8, MMD, MME, MYRF, NCOA2, NDRG4, NEDD4L, NFE2L2, NGF, NLGN2, NME2, NOTCH1, NPTN, NRXN1, NSMF, NTRK2, NUMB, OBSL1, OLIG2, PACSIN1, PAFAH1B1, PARD3, PAX2, PLK5, PLXNA1, PLXNA2, PLXNB1, PLXNC1, PLXND1, POU4F2, PPARG, PRKCH, PRKCI, PRKN, PRPF19, PSEN1, PTEN, PTPRZ1, RANBP1, RAP1A, RAPGEF1, RARA, RELN, REST, RET, RGMA, RHEB, RHOA, ROBO1, ROR1, ROR2, RORA, RUFY3, SEMA4D, SERPINE2, SERPINF1, |
| GO:0000904 | cell morphogenesis involved in differentiation | 1,09E-11 | [4, 5] | 240,00 | 29,56 | [ACTN4, ADGRB3, AGRN, ALS2, ANK3, ANTXR1, ARHGDIA, ARHGEF26, ARHGEF7, ARL13B, ATP8A2, BCL11B, BCL2, BDNF, BDNF-AS, BMP7, BRSK2, CACNA1A, CAMK2B, CAPRIN2, CARMIL1, CCDC88C, CCKAR, CDH4, CDK5R1, CHRNB2, CIB1, CNTN4, COBL, COL18A1, COL6A1, COL6A2, CSPG5, CTNNA2, CTNND2, CTTN, CUL3, CUX1, CXCR4, CYFIP1, CYFIP2, DAB1, DAG1, DBN1, DCLK1, DHX15, DHX36, DLG4, DNM1L, DOCK10, DOCK7, DOK4, DOK6, DRGX, DSCAML1, EFNA4, EFNA5, EGR2, EPHA1, EPHA10, EPHA4, EPHA6, EPHB1, EPHB3, ETV1, EVL, FARP1, FAT1, FBXW8, FER, FERMT2, FEZ1, FEZF1, FEZF2, FRMD6, FRS2, GAB1, GAB2, GLI2, GLI3, GORASP1, GPRIN1, GRB10, GRB2, HCLS1, HECW1, HEG1, HES1, HSP90AB1, IFRD1, IHH, IRS2, ISL1, KALRN, KANK1, KIDINS220, KIF1A, KIF5A, KLF7, KNDC1, LAMA2, LAMA5, LAMB1, LAMB2, LAMB4, LGR6, LHX4, LIMS1, LINGO1, LLGL1, LRP1, LRRC4C, LZTS1, MACF1, MAG, MAP2, MAP3K13, MAP6, MAPK7, MAPK8IP2, MAPK8IP3, MEF2C, MEGF8, MELTF, MYPN, NECTIN1, NEDD4L, NEDD9, NEO1, NFASC, NFATC4, NFIB, NGEF, NGF, NOL3, NOTCH1, NOTCH4, NPTN, NRXN1, NRXN3, NSMF, NTNG1, NTNG2, NTRK2, NUMB, OBSL1, OLFM1, PAFAH1B1, PALLD, PARD3, PAX2, PDZD7, PEAK1, PHACTR1, PIK3CB, PIK3R1, PLXNA1, PLXNA2, PLXNB1, PLXNC1, PLXND1, POU4F2, PPP3CA, PPP3CB, PRKCA, PRKCQ, PRKDC, PSEN1, PTEN, PTK2, PTPN11, PTPRA, PTPRO, PTPRZ1, PXDN, RADIL, RANBP9, RAP1GAP, RB1, RBFOX2, RELN, RET, RGMA, RHOA, |
| GO:0061564 | axon development | 1,41E-07 | [6, 7, 9, 10, 11] | 168,00 | 29,68 | [AGRN, ALS2, ANK3, ARHGDIA, ARHGEF7, ATP8A2, BCL11B, BCL2, BDNF, BDNF-AS, BMP7, BRSK2, CACNA1A, CCKAR, CDH4, CDK5R1, CHRNB2, CNTN4, COBL, CTNNA2, CTTN, CXCR4, CYFIP1, CYFIP2, DAB1, DAG1, DBN1, DCLK1, DDR1, DOCK7, DOK4, DOK6, DRGX, DSCAML1, EFNA4, EFNA5, EGR2, EPHA1, EPHA10, EPHA4, EPHA6, EPHB1, EPHB3, ETV1, EVL, FEZ1, FEZF1, FEZF2, FRS2, GAB1, GAB2, GLI2, GLI3, GPRIN1, GRB10, GRB2, HCLS1, HSP90AB1, IFRD1, INPP5F, IRS2, ISL1, JAK2, KIF5A, KLF7, LAMA2, LAMB2, LGR6, LHX4, LINGO1, LLGL1, LRIG2, LRP1, LRRC4C, LZTS1, MACF1, MAG, MAP2, MAP3K13, MAP6, MAPK7, MAPK8IP3, MEGF8, MYPN, NCAM2, NECTIN1, NEO1, NFASC, NFIB, NGF, NOTCH1, NPTN, NRXN1, NRXN3, NTNG1, NTNG2, NTRK2, NUMB, OLFM1, PAFAH1B1, PALLD, PARD3, PAX2, PIK3CB, PIK3R1, PLXNA1, PLXNA2, PLXNB1, PLXNC1, PLXND1, POU4F2, PPP3CB, PRKCA, PRKCQ, PSEN1, PTEN, PTK2, PTPN11, PTPRA, PTPRF, PTPRO, PTPRZ1, RANBP9, RAP1GAP, RELN, RET, RGMA, RHOA, RHOBTB3, RHOG, ROBO1, ROBO3, RTN4RL1, RTN4RL2, RUFY3, S100A6, SEMA3B, SEMA4C, |
| GO:0032990 | cell part morphogenesis | 5,25E-11 | [4, 5] | 224,00 | 29,71 | [ADGRB3, AGRN, ALS2, ANK3, ARHGDIA, ARHGEF7, ATP8A2, BCL11A, BCL11B, BCL2, BDNF, BDNF-AS, BMP7, BRSK2, CACNA1A, CAMK2B, CAPRIN2, CCKAR, CDH4, CDK5R1, CHRNB2, CNTN4, CNTNAP2, COBL, CORO1B, CPNE5, CSPG5, CTNNA2, CTNND2, CTTN, CUX1, CXCR4, CYFIP1, CYFIP2, DAB1, DAG1, DBN1, DCLK1, DDR1, DHX36, DLG4, DNM1L, DOCK10, DOCK7, DOK4, DOK6, DRGX, DSCAML1, EFNA4, EFNA5, EGR2, EIF2AK4, ENPP2, EPHA1, EPHA10, EPHA4, EPHA6, EPHB1, EPHB3, ETV1, EVL, FARP1, FBXW8, FEZ1, FEZF1, FEZF2, FLRT1, FRS2, GAB1, GAB2, GAS7, GLI2, GLI3, GORASP1, GPRIN1, GRB10, GRB2, HCLS1, HECW1, HSP90AB1, IFRD1, IMMT, IRS2, ISL1, ITGA1, KALRN, KANK1, KIDINS220, KIF1A, KIF5A, KLF7, KNDC1, LAMA2, LAMB2, LGR6, LHX4, LINGO1, LLGL1, LRP1, LRP2, LRRC4C, LZTS1, MACF1, MAG, MAP2, MAP3K13, MAP4K4, MAP6, MAPK7, MAPK8IP2, MAPK8IP3, MEGF8, MYO16, MYPN, NCKAP1, NCKAP1L, NCOA2, NECTIN1, NEDD4L, NEO1, NFASC, NFATC4, NFIB, NGEF, NGF, NOL3, NOTCH1, NPTN, NRXN1, NRXN3, NSMF, NTNG1, NTNG2, NTRK2, NUMB, OBSL1, OLFM1, OPA3, PACSIN1, PACSIN2, PAFAH1B1, PALLD, PARD3, PAX2, PHACTR1, PIK3CB, PIK3R1, PLXNA1, PLXNA2, PLXNB1, PLXNC1, PLXND1, POU4F2, PPP3CA, PPP3CB, PRKCA, PRKCQ, PRKCZ, PRKN, |
| GO:0008154 | actin polymerization or depolymerization | 4,87E-02 | [5, 6, 8] | 70,00 | 29,79 | [ACTN2, ACTR3B, ADD1, ADD2, ARF1, ARHGAP40, ARHGEF7, ARPC4, BAIAP2L1, BBS4, CAP1, CAP2, CAPZB, CARMIL1, CARMIL2, COBL, CORO1B, CORO7-PAM16, CTNNA2, CTTN, CYFIP1, DBN1, DIAPH3, DLG1, ELN, EVL, FCHSD1, FCHSD2, FER, FHOD3, FMN1, GMFB, GRB2, HCLS1, HIP1R, IQGAP2, JAK2, KANK1, KANK3, KIRREL1, LIMA1, MICAL2, MICAL3, MYO1C, MYO1E, NCK2, NCKAP1, NCKAP1L, NPHS1, PFN2, PLEK, PLEKHG2, PRKCE, RASA1, RHOA, RICTOR, SCIN, SPIRE2, SPTBN4, SPTBN5, SSH1, SVIL, TMSB10, TRAF3, TRIM27, TTC17, VPS72, WASF1, |
| GO:0050769 | positive regulation of neurogenesis | 4,34E-07 | [4, 5, 6, 7, 8, 9] | 157,00 | 29,79 | [ABL2, ALK, ALKAL2, AP2A1, ARF1, ARHGDIA, ARHGEF2, ARHGEF7, ASCL1, ATF1, ATP8A2, BCL2, BCL6, BDNF, BDNF-AS, BEND6, BMP2, BMP7, BRINP2, C21orf91, CAMK2B, CAPRIN2, CBFA2T2, CCR2, CDH4, COBL, CPNE5, CREB3L2, CSNK1E, CTTN, CUX1, CXCR4, CYFIP1, DAB1, DAG1, DBN1, DHX36, DLG4, DLX2, DNM1L, EFNA5, EGR2, ENC1, EPHA4, EZH2, FBXW8, FEZ1, FEZF1, FEZF2, GLI2, GLI3, GPER1, GPRC5B, GSX2, HCLS1, HES1, IL6ST, KALRN, KDM4C, KIDINS220, LIG4, LLGL1, LPAR1, LRP1, LRP2, LZTS1, MACF1, MAG, MAGI2, MAP3K13, MAP6, MAPK8IP3, MEF2C, MEGF8, MMD, MME, NCOA2, NDRG4, NEDD4L, NFE2L2, NGF, NME2, NOTCH1, NPTN, NSMF, NTRK2, NUMB, OBSL1, OLIG2, PACSIN1, PAFAH1B1, PLK5, PLXNA1, PLXNA2, PLXNB1, PLXNC1, PLXND1, POU4F2, PPARG, PRKCH, PRKCI, PRKN, PRPF19, PSEN1, PTEN, PTPRZ1, RANBP1, RAP1A, RAPGEF1, RARA, RELN, REST, RET, RGMA, RHEB, RHOA, ROBO1, |
| GO:0048812 | neuron projection morphogenesis | 7,36E-11 | [6, 7, 8, 9, 10, 11] | 214,00 | 29,97 | [ADGRB3, AGRN, ALS2, ANK3, ARHGDIA, ARHGEF7, ATP8A2, BCL11A, BCL11B, BCL2, BDNF, BDNF-AS, BMP7, BRSK2, CACNA1A, CAMK2B, CAPRIN2, CCKAR, CDH4, CDK5R1, CHRNB2, CNTN4, CNTNAP2, COBL, CPNE5, CTNNA2, CTNND2, CTTN, CUX1, CXCR4, CYFIP1, CYFIP2, DAB1, DAG1, DBN1, DCLK1, DDR1, DHX36, DLG4, DNM1L, DOCK10, DOCK7, DOK4, DOK6, DRGX, DSCAML1, EFNA4, EFNA5, EGR2, EIF2AK4, EPHA1, EPHA10, EPHA4, EPHA6, EPHB1, EPHB3, ETV1, EVL, FARP1, FBXW8, FEZ1, FEZF1, FEZF2, FLRT1, FRS2, GAB1, GAB2, GAS7, GLI2, GLI3, GORASP1, GPRIN1, GRB10, GRB2, HCLS1, HECW1, HSP90AB1, IFRD1, IRS2, ISL1, ITGA1, KALRN, KIDINS220, KIF1A, KIF5A, KLF7, KNDC1, LAMA2, LAMB2, LGR6, LHX4, LINGO1, LLGL1, LRP1, LRP2, LRRC4C, LZTS1, MACF1, MAG, MAP2, MAP3K13, MAP4K4, MAP6, MAPK7, MAPK8IP2, MAPK8IP3, MEGF8, MYO16, MYPN, NCKAP1, NCKAP1L, NCOA2, NECTIN1, NEDD4L, NEO1, NFASC, NFATC4, NFIB, NGEF, NGF, NOL3, NOTCH1, NPTN, NRXN1, NRXN3, NSMF, NTNG1, NTNG2, NTRK2, NUMB, OBSL1, OLFM1, PACSIN1, PAFAH1B1, PALLD, PARD3, PAX2, PHACTR1, PIK3CB, PIK3R1, PLXNA1, PLXNA2, PLXNB1, PLXNC1, PLXND1, POU4F2, PPP3CA, PPP3CB, PRKCA, PRKCQ, PRKCZ, PRKN, PSEN1, PTEN, PTK2, PTPN11, PTPRA, PTPRO, PTPRZ1, RANBP9, |
| GO:0007160 | cell-matrix adhesion | 2,80E-02 | [4] | 72,00 | 30,00 | [ACTN2, ACVRL1, AJAP1, ARHGEF7, BCAM, BCAS3, BCL2, BCL6, CD96, CDH13, CDKN2A, CIB1, CLASP1, CLASP2, COL3A1, CORO2B, CTTN, DAG1, DDR1, DLC1, EFNA5, ENC1, EPHA1, FBLN5, FERMT2, FMN1, FREM1, GREM1, HRG, ITGA1, ITGA11, ITGAL, ITGB2, ITGB4, ITGBL1, LDB2, LIMS1, LRP1, MACF1, MAP4K4, MSLN, MUC4, NF1, NPNT, PDPK1, PEAK1, PIK3CB, PIK3R1, PKD1, PLEKHA2, PPFIA1, PPM1F, PRKCZ, PTEN, PTK2, RASA1, RHOA, ROCK2, SGCE, SIGLEC1, SKAP1, SLC9A1, SMAD3, SNED1, SORBS1, SRC, SRF, TESK2, TIAM1, TNN, UTRN, WDPCP] |
| GO:0035725 | sodium ion transmembrane transport | 3,74E-02 | [6, 8] | 69,00 | 30,13 | [ABCB11, ACTN4, ANK3, ANO6, ARF1, ASIC3, ASIC4, ATP12A, ATP1A3, ATP1A4, ATP2B4, CACNA1G, CACNA1H, CACNA1I, CAMK2D, CNNM4, COMMD1, DLG1, FGF12, FXYD5, FXYD6, GRIK3, GRIK4, HCN1, HCN2, HCN4, HECW1, NALCN, NEDD4L, NOS1, PRKCE, PRKD2, PTPN3, SCN1A, SCN2A, SCN3B, SCN5A, SGK1, SLC13A3, SLC13A5, SLC17A2, SLC17A4, SLC17A7, SLC1A1, SLC1A3, SLC24A4, SLC4A4, SLC4A7, SLC4A8, SLC5A6, SLC5A7, SLC5A8, SLC6A11, SLC6A12, SLC6A15, SLC6A16, SLC6A17, SLC6A4, SLC9A1, SLC9A3, SLC9A3R1, SLC9A4, SLC9A5, SLC9A9, |
| GO:0120039 | plasma membrane bounded cell projection morphogenesis | 1,22E-11 | [5, 6, 7] | 220,00 | 30,22 | [ADGRB3, AGRN, ALS2, ANK3, ARHGDIA, ARHGEF7, ATP8A2, BCL11A, BCL11B, BCL2, BDNF, BDNF-AS, BMP7, BRSK2, CACNA1A, CAMK2B, CAPRIN2, CCKAR, CDH4, CDK5R1, CHRNB2, CNTN4, CNTNAP2, COBL, CORO1B, CPNE5, CTNNA2, CTNND2, CTTN, CUX1, CXCR4, CYFIP1, CYFIP2, DAB1, DAG1, DBN1, DCLK1, DDR1, DHX36, DLG4, DNM1L, DOCK10, DOCK7, DOK4, DOK6, DRGX, DSCAML1, EFNA4, EFNA5, EGR2, EIF2AK4, ENPP2, EPHA1, EPHA10, EPHA4, EPHA6, EPHB1, EPHB3, ETV1, EVL, FARP1, FBXW8, FEZ1, FEZF1, FEZF2, FLRT1, FRS2, GAB1, GAB2, GAS7, GLI2, GLI3, GORASP1, GPRIN1, GRB10, GRB2, HCLS1, HECW1, HSP90AB1, IFRD1, IRS2, ISL1, ITGA1, KALRN, KANK1, KIDINS220, KIF1A, KIF5A, KLF7, KNDC1, LAMA2, LAMB2, LGR6, LHX4, LINGO1, LLGL1, LRP1, LRP2, LRRC4C, LZTS1, MACF1, MAG, MAP2, MAP3K13, MAP4K4, MAP6, MAPK7, MAPK8IP2, MAPK8IP3, MEGF8, MYO16, MYPN, NCKAP1, NCKAP1L, NCOA2, NECTIN1, NEDD4L, NEO1, NFASC, NFATC4, NFIB, NGEF, NGF, NOL3, NOTCH1, NPTN, NRXN1, NRXN3, NSMF, NTNG1, NTNG2, NTRK2, NUMB, OBSL1, OLFM1, PACSIN1, PAFAH1B1, PALLD, PARD3, PAX2, PHACTR1, PIK3CB, PIK3R1, PLXNA1, PLXNA2, PLXNB1, PLXNC1, PLXND1, POU4F2, PPP3CA, PPP3CB, PRKCA, PRKCQ, PRKCZ, PRKN, PSEN1, PTEN, PTK2, PTPN11, |
| GO:0110053 | regulation of actin filament organization | 2,37E-03 | [5, 6, 7, 8] | 89,00 | 30,27 | [ACTN2, ACTR3B, ADD1, ADD2, ALMS1, ARAP1, ARF1, ARHGAP40, ARHGEF10L, ARHGEF18, ARHGEF7, ARPC4, BAIAP2L1, BBS4, CAPZB, CARMIL1, CARMIL2, CD47, CLASP1, CLASP2, CORO1B, CORO2B, CTNNA2, CTTN, CYFIP1, DBN1, DLC1, DLG1, ELN, EPHA1, EVL, F11R, FCHSD1, FCHSD2, FER, FHOD1, FHOD3, FMN1, GMFB, GRB2, HCLS1, HIP1R, IQGAP2, KANK1, KANK3, KIRREL1, LIMA1, LPAR1, MYO1C, MYO1E, NCK2, NCKAP1, NCKAP1L, NPHS1, PFN2, PIK3R1, PLEK, PLEKHG2, PPFIA1, PPM1F, PRKCE, PRKN, PXDN, RASA1, RHOA, RICTOR, ROCK2, |
| GO:0048858 | cell projection morphogenesis | 6,80E-12 | [4, 5, 6] | 222,00 | 30,29 | [ADGRB3, AGRN, ALS2, ANK3, ARHGDIA, ARHGEF7, ATP8A2, BCL11A, BCL11B, BCL2, BDNF, BDNF-AS, BMP7, BRSK2, CACNA1A, CAMK2B, CAPRIN2, CCKAR, CDH4, CDK5R1, CHRNB2, CNTN4, CNTNAP2, COBL, CORO1B, CPNE5, CSPG5, CTNNA2, CTNND2, CTTN, CUX1, CXCR4, CYFIP1, CYFIP2, DAB1, DAG1, DBN1, DCLK1, DDR1, DHX36, DLG4, DNM1L, DOCK10, DOCK7, DOK4, DOK6, DRGX, DSCAML1, EFNA4, EFNA5, EGR2, EIF2AK4, ENPP2, EPHA1, EPHA10, EPHA4, EPHA6, EPHB1, EPHB3, ETV1, EVL, FARP1, FBXW8, FEZ1, FEZF1, FEZF2, FLRT1, FRS2, GAB1, GAB2, GAS7, GLI2, GLI3, GORASP1, GPRIN1, GRB10, GRB2, HCLS1, HECW1, HSP90AB1, IFRD1, IRS2, ISL1, ITGA1, KALRN, KANK1, KIDINS220, KIF1A, KIF5A, KLF7, KNDC1, LAMA2, LAMB2, LGR6, LHX4, LINGO1, LLGL1, LRP1, LRP2, LRRC4C, LZTS1, MACF1, MAG, MAP2, MAP3K13, MAP4K4, MAP6, MAPK7, MAPK8IP2, MAPK8IP3, MEGF8, MYO16, MYPN, NCKAP1, NCKAP1L, NCOA2, NECTIN1, NEDD4L, NEO1, NFASC, NFATC4, NFIB, NGEF, NGF, NOL3, NOTCH1, NPTN, NRXN1, NRXN3, NSMF, NTNG1, NTNG2, NTRK2, NUMB, OBSL1, OLFM1, PACSIN1, PACSIN2, PAFAH1B1, PALLD, PARD3, PAX2, PHACTR1, PIK3CB, PIK3R1, PLXNA1, PLXNA2, PLXNB1, PLXNC1, PLXND1, POU4F2, PPP3CA, PPP3CB, PRKCA, PRKCQ, PRKCZ, PRKN, PSEN1, PTEN, |
| GO:0030036 | actin cytoskeleton organization | 3,17E-12 | [3, 6] | 225,00 | 30,32 | [ABL2, ABLIM2, ABR, ACTL8, ACTN2, ACTN4, ACTR3B, ADD1, ADD2, AKAP13, ALMS1, ANTXR1, ARAP1, ARF1, ARFGEF3, ARHGAP12, ARHGAP17, ARHGAP40, ARHGDIA, ARHGDIB, ARHGEF10L, ARHGEF18, ARHGEF2, ARHGEF7, ARPC4, BAIAP2L1, BBS4, BCAR1, BCAS3, BCL2, BCL6, BIN3, BRSK2, CAP1, CAP2, CAPZB, CARMIL1, CARMIL2, CD47, CDC42BPB, CDK5R1, CELSR1, CIT, CLASP1, CLASP2, COBL, CORO1B, CORO2B, CORO7-PAM16, CTNNA2, CTTN, CUL3, CYFIP1, DBN1, DHDDS, DIAPH3, DLC1, DLG1, DNAJB6, EFNA5, EHBP1L1, ELMO1, ELN, EPB41L4A, EPB41L4B, EPHA1, ESPN, ESPNL, EVL, F11R, F3, FAM171A1, FARP1, FARP2, FAT1, FCHSD1, FCHSD2, FER, FGD2, FGF7, FHOD1, FHOD3, FMN1, FMNL2, FRMD5, FRMD6, FZD10, GAB1, GMFB, GRB2, HCLS1, HIP1, HIP1R, HRG, IKBKB, INPPL1, IQGAP2, IQGAP3, IQSEC1, IQSEC3, JAK2, KANK1, KANK3, KIRREL1, KLHL1, KLHL17, KRAS, LDB3, LIMA1, LIMK2, LLGL1, LPAR1, LRP1, LURAP1, MAPKAP1, MARCKS, MEF2C, MICAL2, MICAL3, MICALL1, MPRIP, MRTFA, MYH3, MYL2, MYO15A, MYO18A, MYO1A, MYO1C, MYO1D, MYO1E, MYO5A, MYO5C, MYOM2, MYPN, NCK2, NCKAP1, NCKAP1L, NEDD9, NF1, NOS1AP, NPHP4, NPHS1, OBSCN, OBSL1, PACSIN1, PACSIN2, PAFAH1B1, PALLD, PDCL3, PDGFRA, PDLIM1, PDLIM3, PDPK1, PFN2, PHACTR1, PIK3R1, PIP5K1C, PLEK, PLEKHG2, PPARGC1B, PPFIA1, PPM1F, PRKCE, PRKCI, PRKN, PXDN, RASA1, RFLNA, RFLNB, RHOA, RHOBTB3, RHOG, RHOU, RICTOR, ROCK2, RTKN, RUFY3, |
| GO:0030036 | actin cytoskeleton organization | 3,17E-12 | [3, 6] | 225,00 | 30,32 | [ABL2, ABLIM2, ABR, ACTL8, ACTN2, ACTN4, ACTR3B, ADD1, ADD2, AKAP13, ALMS1, ANTXR1, ARAP1, ARF1, ARFGEF3, ARHGAP12, ARHGAP17, ARHGAP40, ARHGDIA, ARHGDIB, ARHGEF10L, ARHGEF18, ARHGEF2, ARHGEF7, ARPC4, BAIAP2L1, BBS4, BCAR1, BCAS3, BCL2, BCL6, BIN3, BRSK2, CAP1, CAP2, CAPZB, CARMIL1, CARMIL2, CD47, CDC42BPB, CDK5R1, CELSR1, CIT, CLASP1, CLASP2, COBL, CORO1B, CORO2B, CORO7-PAM16, CTNNA2, CTTN, CUL3, CYFIP1, DBN1, DHDDS, DIAPH3, DLC1, DLG1, DNAJB6, EFNA5, EHBP1L1, ELMO1, ELN, EPB41L4A, EPB41L4B, EPHA1, ESPN, ESPNL, EVL, F11R, F3, FAM171A1, FARP1, FARP2, FAT1, FCHSD1, FCHSD2, FER, FGD2, FGF7, FHOD1, FHOD3, FMN1, FMNL2, FRMD5, FRMD6, FZD10, GAB1, GMFB, GRB2, HCLS1, HIP1, HIP1R, HRG, IKBKB, INPPL1, IQGAP2, IQGAP3, IQSEC1, IQSEC3, JAK2, KANK1, KANK3, KIRREL1, KLHL1, KLHL17, KRAS, LDB3, LIMA1, LIMK2, LLGL1, LPAR1, LRP1, LURAP1, MAPKAP1, MARCKS, MEF2C, MICAL2, MICAL3, MICALL1, MPRIP, MRTFA, MYH3, MYL2, MYO15A, MYO18A, MYO1A, MYO1C, MYO1D, MYO1E, MYO5A, MYO5C, MYOM2, MYPN, NCK2, NCKAP1, NCKAP1L, NEDD9, NF1, NOS1AP, NPHP4, NPHS1, OBSCN, OBSL1, PACSIN1, PACSIN2, PAFAH1B1, PALLD, PDCL3, PDGFRA, PDLIM1, PDLIM3, PDPK1, PFN2, PHACTR1, PIK3R1, PIP5K1C, PLEK, PLEKHG2, PPARGC1B, PPFIA1, PPM1F, PRKCE, PRKCI, PRKN, PXDN, RASA1, RFLNA, RFLNB, RHOA, RHOBTB3, RHOG, RHOU, RICTOR, ROCK2, RTKN, RUFY3, |
| GO:0007409 | axonogenesis | 4,28E-08 | [6, 7, 8, 9, 10, 11, 12] | 158,00 | 30,50 | [AGRN, ALS2, ANK3, ARHGDIA, ARHGEF7, ATP8A2, BCL11B, BCL2, BDNF, BDNF-AS, BMP7, BRSK2, CACNA1A, CCKAR, CDH4, CDK5R1, CHRNB2, CNTN4, COBL, CTNNA2, CTTN, CXCR4, CYFIP1, CYFIP2, DAB1, DAG1, DBN1, DCLK1, DOCK7, DOK4, DOK6, DRGX, DSCAML1, EFNA4, EFNA5, EGR2, EPHA1, EPHA10, EPHA4, EPHA6, EPHB1, EPHB3, ETV1, EVL, FEZ1, FEZF1, FEZF2, FRS2, GAB1, GAB2, GLI2, GLI3, GPRIN1, GRB10, GRB2, HCLS1, HSP90AB1, IFRD1, IRS2, ISL1, KIF5A, KLF7, LAMA2, LAMB2, LGR6, LHX4, LINGO1, LLGL1, LRP1, LRRC4C, LZTS1, MACF1, MAG, MAP2, MAP3K13, MAP6, MAPK7, MAPK8IP3, MEGF8, MYPN, NECTIN1, NEO1, NFASC, NFIB, NGF, NOTCH1, NPTN, NRXN1, NRXN3, NTNG1, NTNG2, NTRK2, NUMB, OLFM1, PAFAH1B1, PALLD, PARD3, PAX2, PIK3CB, PIK3R1, PLXNA1, PLXNA2, PLXNB1, PLXNC1, PLXND1, POU4F2, PPP3CB, PRKCA, PRKCQ, PSEN1, PTEN, PTK2, PTPN11, PTPRA, PTPRO, PTPRZ1, |
| GO:0031032 | actomyosin structure organization | 4,87E-02 | [4, 7] | 64,00 | 30,62 | [ACTN2, AKAP13, ALMS1, ARAP1, ARHGEF10L, ARHGEF18, BBS4, CARMIL1, CD47, CDC42BPB, CIT, CLASP1, CLASP2, CORO2B, CUL3, DHDDS, DLC1, ELN, EPB41L4A, EPB41L4B, EPHA1, EVL, F11R, FAM171A1, FHOD1, FHOD3, FRMD5, FRMD6, LDB3, LPAR1, LURAP1, MEF2C, MYH3, MYL2, MYO18A, MYOM2, MYPN, OBSCN, OBSL1, PDGFRA, PDLIM1, PFN2, PHACTR1, PIK3R1, |
| GO:0010976 | positive regulation of neuron projection development | 2,89E-04 | [5, 6, 7, 8, 9, 10, 11, 12] | 98,00 | 30,72 | [ABL2, ALK, ALKAL2, AP2A1, ARF1, ARHGDIA, ARHGEF7, ATF1, ATP8A2, BDNF, BDNF-AS, BMP7, C21orf91, CAMK2B, CAPRIN2, CBFA2T2, CDH4, COBL, CPNE5, CREB3L2, CTTN, CUX1, CXCR4, CYFIP1, DBN1, DHX36, DLG4, DNM1L, EFNA5, ENC1, EPHA4, EZH2, FBXW8, FEZ1, HCLS1, KALRN, KIDINS220, LLGL1, LPAR1, LRP1, LZTS1, MACF1, MAGI2, MAP3K13, MAP6, MEGF8, NCOA2, NDRG4, NEDD4L, NFE2L2, NGF, NME2, NPTN, NTRK2, OBSL1, PACSIN1, PAFAH1B1, PLK5, PLXNA1, PLXNA2, PLXNB1, PLXNC1, PLXND1, POU4F2, PRKCI, PRKN, PSEN1, RANBP1, |
| GO:0005085 | guanyl-nucleotide exchange factor activity | 9,35E-03 | [4] | 75,00 | 30,74 | [ABR, ADRB1, AKAP13, ALS2, ARFGEF3, ARHGEF10L, ARHGEF16, ARHGEF18, ARHGEF2, ARHGEF26, ARHGEF7, BCAR3, BMP1, CCDC88C, CYTH1, DENND11, DENND1A, DENND1B, DENND2A, DENND3, DENND5A, DNMBP, DOCK10, DOCK7, DOCK8, ECT2L, ELMO1, EPHA4, EPS8L1, ETFA, FARP1, FARP2, FGD2, GPSM1, HERC1, HERC2, HPS4, IQSEC1, IQSEC3, KALRN, KNDC1, MCF2L, MCF2L2, NGEF, OBSCN, PCP2, PLCD4, PLCE1, PLEKHG2, PLEKHG7, PSD, PSD2, PSD3, PSD4, PTGES2, RABGEF1, RALGDS, RAP1A, RAPGEF1, RAPGEF5, RASGRF1, |
| GO:0015276 | ligand-gated ion channel activity | 6,33E-03 | [6, 7, 8, 9, 10] | 73,00 | 31,06 | [ACTN2, ANXA6, ASIC3, CACNG8, CALM2, CAMK2D, CAV1, CCR2, CHRNB2, CHRNB3, CHRND, CHRNE, CNGA3, CNIH3, DAPK1, DLG1, DLG2, DLG4, GABRA2, GABRA4, GABRB2, GABRR3, GPRIN1, GRIA4, GRID2, GRIK3, GRIK4, GSG1L, HCN1, HCN2, HCN4, HRC, HTR3A, HTR3C, HTT, ITPR2, ITPR3, JPH3, KCNH2, KCNJ14, KCNJ15, KCNJ2, KCNJ3, KCNJ6, LLGL1, MAPK8IP2, MEF2C, MYO5A, NLGN2, NOL3, NOS1, NRXN1, P2RY6, PPARGC1A, PRKCA, PRKD2, RASA3, RASGRF1, RELN, RGS9, RYR1, RYR2, RYR3, SHANK2, SHISA6, SHISA7, SHISA9, SLC17A7, |
| GO:0048667 | cell morphogenesis involved in neuron differentiation | 3,68E-12 | [5, 6, 8, 9, 10] | 202,00 | 31,22 | [ADGRB3, AGRN, ALS2, ANK3, ARHGDIA, ARHGEF7, ATP8A2, BCL11B, BCL2, BDNF, BDNF-AS, BMP7, BRSK2, CACNA1A, CAMK2B, CAPRIN2, CCKAR, CDH4, CDK5R1, CHRNB2, CNTN4, COBL, CTNNA2, CTNND2, CTTN, CUX1, CXCR4, CYFIP1, CYFIP2, DAB1, DAG1, DBN1, DCLK1, DHX36, DLG4, DNM1L, DOCK10, DOCK7, DOK4, DOK6, DRGX, DSCAML1, EFNA4, EFNA5, EGR2, EPHA1, EPHA10, EPHA4, EPHA6, EPHB1, EPHB3, ETV1, EVL, FARP1, FBXW8, FEZ1, FEZF1, FEZF2, FRS2, GAB1, GAB2, GLI2, GLI3, GORASP1, GPRIN1, GRB10, GRB2, HCLS1, HECW1, HES1, HSP90AB1, IFRD1, IRS2, ISL1, KALRN, KIDINS220, KIF1A, KIF5A, KLF7, KNDC1, LAMA2, LAMB2, LGR6, LHX4, LINGO1, LLGL1, LRP1, LRRC4C, LZTS1, MACF1, MAG, MAP2, MAP3K13, MAP6, MAPK7, MAPK8IP2, MAPK8IP3, MEF2C, MEGF8, MYPN, NECTIN1, NEDD4L, NEO1, NFASC, NFATC4, NFIB, NGEF, NGF, NOL3, NOTCH1, NPTN, NRXN1, NRXN3, NSMF, NTNG1, NTNG2, NTRK2, NUMB, OBSL1, OLFM1, PAFAH1B1, PALLD, PARD3, PAX2, PDZD7, PHACTR1, PIK3CB, PIK3R1, PLXNA1, PLXNA2, PLXNB1, PLXNC1, PLXND1, POU4F2, PPP3CA, PPP3CB, PRKCA, PRKCQ, PSEN1, PTEN, PTK2, PTPN11, PTPRA, PTPRO, PTPRZ1, RANBP9, RAP1GAP, RB1, RBFOX2, RELN, RET, RGMA, RHOA, RHOBTB3, RHOG, RIPOR2, ROBO1, ROBO3, RUFY3, S100A6, SCRIB, |

| | | | | | | |
|---|---|---|---|---|---|---|
| GO:0051056 | regulation of small GTPase mediated signal transduction | 1,86E-05 | [5, 6, 7] | 109,00 | 31,23 | [ABL2, ABR, ADCYAP1R1, ADGRG1, AKAP13, ALS2, ARAP1, ARAP3, ARFGAP1, ARFGEF3, ARHGAP10, ARHGAP12, ARHGAP17, ARHGAP21, ARHGAP29, ARHGAP39, ARHGAP40, ARHGAP42, ARHGAP45, ARHGDIA, ARHGDIB, ARHGDIG, ARHGEF16, ARHGEF18, ARHGEF2, ARHGEF26, ARHGEF7, BCL6, CHN2, COL3A1, CUL3, CYTH1, DENND1A, DLC1, EGR2, EPS8L1, ETFA, F11R, F2RL3, FAM13A, FAM13B, FBP1, FGD2, GPR17, GPR4, GPR55, GRB2, HEG1, IGF1, IQSEC1, IQSEC3, IRS2, JAK2, KALRN, KANK1, KCTD10, KITLG, KRAS, LPAR1, MAP4K4, MAPKAP1, MCF2L, MYO9B, NF1, NGEF, NGF, NOTCH1, OBSCN, PLCE1, PLEKHG2, PRAG1, PSD, PSD2, PSD3, PSD4, PTGES2, RALGAPA2, RAP1GAP, RAP1GAP2, RAPGEF1, RASA1, RASA2, RASA3, RASGRF1, |
| GO:0045666 | positive regulation of neuron differentiation | 3,50E-07 | [5, 6, 7, 8, 9, 10] | 130,00 | 31,40 | [ABL2, ALK, ALKAL2, AP2A1, ARF1, ARHGDIA, ARHGEF2, ARHGEF7, ASCL1, ATF1, ATP8A2, BCL2, BCL6, BDNF, BDNF-AS, BEND6, BMP2, BMP7, BRINP2, C21orf91, CAMK2B, CAPRIN2, CBFA2T2, CDH4, COBL, CPNE5, CREB3L2, CSNK1E, CTTN, CUX1, CXCR4, CYFIP1, DAB1, DBN1, DHX36, DLG4, DLX2, DNM1L, EFNA5, ENC1, EPHA4, EZH2, FBXW8, FEZ1, FEZF1, FEZF2, GLI2, GPRC5B, HCLS1, KALRN, KDM4C, KIDINS220, LLGL1, LPAR1, LRP1, LZTS1, MACF1, MAGI2, MAP3K13, MAP6, MAPK8IP3, MEF2C, MEGF8, MMD, NCOA2, NDRG4, NEDD4L, NFE2L2, NGF, NME2, NPTN, NTRK2, OBSL1, PACSIN1, PAFAH1B1, PLK5, PLXNA1, PLXNA2, PLXNB1, PLXNC1, PLXND1, POU4F2, PRKCI, PRKN, PRPF19, PSEN1, PTEN, RANBP1, RAP1A, RAPGEF1, RARA, |
| GO:0016358 | dendrite development | 1,38E-04 | [3, 6, 7, 9, 10, 11] | 89,00 | 31,90 | [ADGRB3, AGRN, ALK, ARF1, ARHGEF7, ASAP1, BBS4, BCL11A, BDNF, BDNF-AS, BMP7, C21orf91, CACNA1A, CAMK2B, CAPRIN2, CDC20, CDK5R1, CHRNB2, COBL, CSMD3, CTNNA2, CTNND2, CTTN, CUX1, CYFIP1, DAB1, DBN1, DCLK1, DHX36, DLG4, DNM1L, DOCK10, EPHA4, EPHB1, EPHB3, EZH2, FARP1, FBXW8, FEZF2, FLRT1, GORASP1, GPRIN1, GSK3A, HCLS1, HECW1, IQSEC1, KALRN, KIDINS220, KIF1A, KLF7, KLHL1, KNDC1, LLGL1, LPAR1, LZTS1, MAP2, MAP6, MAPK8IP2, MEF2C, NCK2, NCOA2, NEDD4L, NFATC4, NGEF, NOL3, NSMF, OBSL1, |
| GO:0007015 | actin filament organization | 2,79E-09 | [4, 5, 7] | 147,00 | 32,17 | [ACTN2, ACTN4, ACTR3B, ADD1, ADD2, ALMS1, ARAP1, ARF1, ARHGAP12, ARHGAP17, ARHGAP40, ARHGEF10L, ARHGEF18, ARHGEF2, ARHGEF7, ARPC4, BAIAP2L1, BBS4, BCAR1, BCL2, BIN3, CAP1, CAP2, CAPZB, CARMIL1, CARMIL2, CD47, CLASP1, CLASP2, COBL, CORO1B, CORO2B, CORO7-PAM16, CTNNA2, CTTN, CUL3, CYFIP1, DBN1, DIAPH3, DLC1, DLG1, ELMO1, ELN, EPHA1, ESPN, ESPNL, EVL, F11R, F3, FAM171A1, FAT1, FCHSD1, FCHSD2, FER, FHOD1, FHOD3, FMN1, GMFB, GRB2, HCLS1, HIP1, HIP1R, INPPL1, IQGAP2, JAK2, KANK1, KANK3, KIRREL1, LIMA1, LPAR1, MARCKS, MICAL2, MICAL3, MPRIP, MYO15A, MYO1A, MYO1C, MYO1D, MYO1E, MYO5A, MYO5C, MYOM2, NCK2, NCKAP1, NCKAP1L, NEDD9, NPHS1, PACSIN1, PDLIM1, PDLIM3, PFN2, PHACTR1, PIK3R1, PLEK, PLEKHG2, PPARGC1B, PPFIA1, PPM1F, PRKCE, PRKCI, PRKN, PXDN, RASA1, RFLNA, RFLNB, RHOA, RHOBTB3, RHOG, RHOU, RICTOR, ROCK2, |
| GO:0007015 | actin filament organization | 2,79E-09 | [4, 5, 7] | 147,00 | 32,17 | [ACTN2, ACTN4, ACTR3B, ADD1, ADD2, ALMS1, ARAP1, ARF1, ARHGAP12, ARHGAP17, ARHGAP40, ARHGEF10L, ARHGEF18, ARHGEF2, ARHGEF7, ARPC4, BAIAP2L1, BBS4, BCAR1, BCL2, BIN3, CAP1, CAP2, CAPZB, CARMIL1, CARMIL2, CD47, CLASP1, CLASP2, COBL, CORO1B, CORO2B, CORO7-PAM16, CTNNA2, CTTN, CUL3, CYFIP1, DBN1, DIAPH3, DLC1, DLG1, ELMO1, ELN, EPHA1, ESPN, ESPNL, EVL, F11R, F3, FAM171A1, FAT1, FCHSD1, FCHSD2, FER, FHOD1, FHOD3, FMN1, GMFB, GRB2, HCLS1, HIP1, HIP1R, INPPL1, IQGAP2, JAK2, KANK1, KANK3, KIRREL1, LIMA1, LPAR1, MARCKS, MICAL2, MICAL3, MPRIP, MYO15A, MYO1A, MYO1C, MYO1D, MYO1E, MYO5A, MYO5C, MYOM2, NCK2, NCKAP1, NCKAP1L, NEDD9, NPHS1, PACSIN1, PDLIM1, PDLIM3, PFN2, PHACTR1, PIK3R1, PLEK, PLEKHG2, PPARGC1B, PPFIA1, PPM1F, PRKCE, PRKCI, PRKN, PXDN, RASA1, RFLNA, RFLNB, RHOA, RHOBTB3, RHOG, RHOU, RICTOR, ROCK2, |
| GO:0099094 | ligand-gated cation channel activity | 4,72E-03 | [7, 8, 9, 10, 11] | 66,00 | 32,20 | [ACTN2, ASIC3, CACNG8, CALM2, CAMK2D, CAV1, CCR2, CHRNB2, CHRNB3, CHRND, CHRNE, CNGA3, CNIH3, DAPK1, DLG1, DLG2, DLG4, GPRIN1, GRIA4, GRIK3, GRIK4, GSG1L, HCN1, HCN2, HCN4, HRC, HTR3A, HTR3C, HTT, ITPR2, ITPR3, JPH3, KCNH2, KCNJ14, KCNJ15, KCNJ2, KCNJ3, KCNJ6, LLGL1, MAPK8IP2, MEF2C, MYO5A, NLGN2, NOL3, NOS1, NRXN1, P2RY6, |
| GO:0046578 | regulation of Ras protein signal transduction | 3,97E-03 | [6, 7, 8] | 68,00 | 32,23 | [ABL2, ADGRG1, AKAP13, ALS2, ARFGAP1, ARFGEF3, ARHGAP17, ARHGAP42, ARHGDIA, ARHGDIB, ARHGEF18, ARHGEF2, BCL6, COL3A1, CUL3, CYTH1, DENND1A, DLC1, EPS8L1, ETFA, F11R, F2RL3, FBP1, GPR17, GPR4, GPR55, GRB2, HEG1, IGF1, IQSEC1, IQSEC3, IRS2, JAK2, KALRN, KANK1, KCTD10, KITLG, KRAS, LPAR1, MAP4K4, MAPKAP1, MYO9B, NF1, NGF, NOTCH1, PLCE1, PRAG1, PSD, PSD2, PSD3, PSD4, PTGES2, RAPGEF1, RASA1, RASA2, RASA3, RASGRF1, RASGRP4, RASIP1, RIPOR2, ROBO1, SCAI, STARD13, STK19, SYNPO2L, TIMP2, TRIM67, |
| GO:0007411 | axon guidance | 6,36E-06 | [4, 5, 6, 7, 8, 9, 10, 11, 12, 13] | 99,00 | 32,57 | [AGRN, BCL11B, BDNF, BDNF-AS, BMP7, CDH4, CDK5R1, CNTN4, CXCR4, CYFIP1, CYFIP2, DAB1, DAG1, DOK4, DOK6, DRGX, DSCAML1, EFNA4, EFNA5, EGR2, EPHA1, EPHA10, EPHA4, EPHA6, EPHB1, EPHB3, ETV1, EVL, FEZ1, FEZF1, FEZF2, FRS2, GAB1, GAB2, GLI2, GLI3, GRB10, GRB2, IRS2, ISL1, KIF5A, KLF7, LAMA2, LAMB2, LGR6, LHX4, LRP1, LZTS1, MAPK7, MAPK8IP3, MEGF8, MYPN, NECTIN1, NEO1, NFASC, NFIB, NPTN, NRXN1, NRXN3, PALLD, PIK3CB, PIK3R1, PLXNA1, PLXNA2, PLXNB1, PLXNC1, PLXND1, POU4F2, PRKCA, PRKCQ, PTK2, |
| GO:0007613 | memory | 4,22E-02 | [3, 6] | 48,00 | 33,57 | [ATXN1, BDNF, BDNF-AS, BTBD9, CAMK4, CHRNB2, CIC, CTNS, DHX15, DHX34, EHMT2, EIF2AK4, EIF4EBP2, GNAI2, GPRIN1, HTR2A, INSR, ITGA5, ITPR3, JPH3, KALRN, KCNK2, LDLR, LGMN, LRCH4, NFATC4, NGF, NOL3, PAK5, PLA2G6, PLCB1, PPP3CB, PRKCZ, PSEN1, PTEN, RASGRF1, RELN, SCN2A, SERPINF1, SGK1, SHANK2, SHISA7, SLC17A7, SLC24A4, SLC6A4, SRF, |
| GO:0051017 | actin filament bundle assembly | 5,78E-03 | [5, 6, 7, 9] | 56,00 | 33,94 | [ACTN4, ADD1, ADD2, ALMS1, ARAP1, ARHGEF10L, ARHGEF18, BAIAP2L1, BBS4, CARMIL1, CD47, CLASP1, CLASP2, CORO1B, CORO2B, CUL3, DLC1, ELN, EPHA1, ESPN, ESPNL, EVL, F11R, FAM171A1, FHOD1, LIMA1, LPAR1, MARCKS, NEDD9, PDLIM1, PFN2, PHACTR1, PIK3R1, PLEK, PPFIA1, PPM1F, PRKN, PXDN, RHOA, ROCK2, SH3PXD2B, SHROOM1, SLC9A1, SMAD3, |
| GO:0050773 | regulation of dendrite development | 2,35E-03 | [4, 7, 8, 9, 10, 11, 12] | 57,00 | 34,34 | [ADGRB3, ALK, ARF1, ARHGEF7, ASAP1, BCL11A, BMP7, C21orf91, CAMK2B, CAPRIN2, CDC20, CDK5R1, CHRNB2, COBL, CSMD3, CTTN, CUX1, CYFIP1, DBN1, DHX36, DNM1L, EPHA4, EZH2, FBXW8, GORASP1, GPRIN1, GSK3A, HCLS1, HECW1, KALRN, KIF1A, KNDC1, LPAR1, LZTS1, MEF2C, NEDD4L, NFATC4, NGEF, NOL3, NSMF, OBSL1, PACSIN1, PAFAH1B1, PPP3CA, PSEN1, |
| GO:0048813 | dendrite morphogenesis | 3,99E-03 | [4, 6, 7, 8, 9, 10, 11, 12] | 55,00 | 34,38 | [ADGRB3, ARHGEF7, CACNA1A, CAMK2B, CAPRIN2, CDK5R1, CHRNB2, CTNNA2, CTNND2, CTTN, CUX1, DBN1, DCLK1, DHX36, DLG4, DNM1L, DOCK10, EPHA4, EPHB1, EPHB3, FARP1, FBXW8, GORASP1, GPRIN1, HCLS1, HECW1, KALRN, KIDINS220, KIF1A, KLF7, KNDC1, LLGL1, LZTS1, MAP2, MAP6, MAPK8IP2, NEDD4L, NFATC4, NGEF, NOL3, NSMF, OBSL1, PAFAH1B1, |
| GO:0061572 | actin filament bundle organization | 8,52E-04 | [5, 6, 8] | 59,00 | 35,12 | [ACTN4, ADD1, ADD2, ALMS1, ARAP1, ARHGEF10L, ARHGEF18, BAIAP2L1, BBS4, CARMIL1, CD47, CLASP1, CLASP2, CORO1B, CORO2B, CUL3, DLC1, ELN, EPHA1, ESPN, ESPNL, EVL, F11R, FAM171A1, FHOD1, LIMA1, LPAR1, MARCKS, NEDD9, PDLIM1, PFN2, PHACTR1, PIK3R1, PLEK, PPFIA1, PPM1F, PRKN, PXDN, RFLNA, RFLNB, RHOA, ROCK2, SH3PXD2B, SHROOM1, SHTN1, |
| GO:0030010 | establishment of cell polarity | 8,36E-04 | [3] | 55,00 | 35,71 | [ACTR10, ALPK2, ARHGEF2, BCAS3, BRSK2, CLASP1, CYTH1, DCTN1, DOCK7, DOCK8, EPHB1, FAM89B, FEZ1, FRMD4A, FRMD4B, HES1, HSP90AB1, HTT, KANK1, KIF25, KIF26B, KRIT1, LLGL1, LZTS1, MAP2, MAP4, MCPH1, MYO18A, NDE1, PAFAH1B1, PARD3, PKD1, PRKCI, PRKCZ, PTGES2, PTK2, RACK1, RHOA, RICTOR, RIPOR2, ROCK2, RUFY3, SCRIB, SHH, SHTN1, |
| GO:0043149 | stress fiber assembly | 1,58E-02 | [5, 7, 8, 9, 11] | 41,00 | 36,61 | [ALMS1, ARAP1, ARHGEF10L, ARHGEF18, BBS4, CARMIL1, CD47, CLASP1, CLASP2, CORO2B, CUL3, DLC1, ELN, EPHA1, EVL, F11R, FAM171A1, FHOD1, LPAR1, PDLIM1, PFN2, PHACTR1, PIK3R1, PPFIA1, PPM1F, PXDN, RHOA, ROCK2, SH3PXD2B, SLC9A1, SMAD3, SORBS1, SORBS3, SRC, SRF, SYNPO2L, TGFB3, TJP1, TMEFF2, TPM1, TTC8] |
