# Supplementary material for: Epigenetic regulation of transcription factor binding motifs promotes Th1 response in Chagas disease cardiomyopathy
Source: Front Immunol. 2022 Aug 22;13:958200. doi: 10.3389/fimmu.2022.958200 (PMC9441916; doi:10.3389/fimmu.2022.958200)
Supplement: Supplementary Table 1 — Biological samples included in this study. [file DataSheet_1.zip › Supplementary Material/Supplementary Table 2.pdf]

Supplementary table 2. Cell lines description.

Cell line	Description
CHRF28811	Acute megakaryoblastic leukemia
AMLBLAST	Acute Myeloid Leukemia
AMLPZ12	Acute Myeloid Leukemia
TSU1621MT	AML cell lines
AC16	Cardiomyocyte
BCELL	B lymphocyte
CA46	B lymphocyte
NAMALWA	B lymphocyte
P4936	B lymphocyte
RAJI	B lymphocyte
RAMOS	B lymphocyte
RCK8	B lymphocyte
FARAGE	B lymphocyte
GM06990	B lymphocyte
GM10248	B lymphocyte
GM10266	B lymphocyte
GM10847	B lymphocyte
GM12801	B lymphocyte
GM12864	B lymphocyte
GM12865	B lymphocyte
GM12866	B lymphocyte
GM12867	B lymphocyte
GM12868	B lymphocyte
GM12869	B lymphocyte
GM12870	B lymphocyte

GM12872	B lymphocyte
GM12873	B lymphocyte
GM12874	B lymphocyte
GM12875	B lymphocyte
GM12878	B lymphocyte
GM12891	B lymphocyte
GM12892	B lymphocyte
GM13976	B lymphocyte
GM13977	B lymphocyte
GM15510	B lymphocyte
GM18505	B lymphocyte
GM18526	B lymphocyte
GM18951	B lymphocyte
GM19099	B lymphocyte
GM19193	B lymphocyte
GM19238	B lymphocyte
GM19239	B lymphocyte
GM19240	B lymphocyte
GM20000	B lymphocyte
LYMPHOBLASTOID	B lymphocyte
MUTUL	B lymphocyte
U2932	B lymphocyte
LP1	B lymphocyte
ME1	B lymphocyte
SUDHL10	B lymphocyte
SUDHL2	B lymphocyte
SUDHL4	B lymphocyte
SUDHL5	B lymphocyte

SUDHL6	B lymphocyte
DOHH2	B lymphocyte
GRANTA519	B lymphocyte
HBL1	B lymphocyte
KARPAS422	B lymphocyte
MM1S	B lymphocyte
OCILY10	B lymphocyte
OCILY19	B lymphocyte
OCILY1	B lymphocyte
OCILY3	B lymphocyte
OCILY7	B lymphocyte
OE33	B lymphocyte
PFEIFFER	B lymphocyte
WSUDLCL2	B lymphocyte
BC3	B lymphocyte
BCBL1	B lymphocyte
CD4	T lymphocyte
CD8	T lymphocyte
DND41	T lymphocyte
CUTLL1	T lymphocyte
RPMI8402	T lymphocyte
CCRFCEM	T lymphocyte
PRIMA2	T lymphocyte
PRIMA5	T lymphocyte
JURKAT	T lymphocyte
HPBALL	T lymphocyte
NALM6	Lymphocyte-like (myeloid leukemia)
MACROPHAGE	Macrophage

MV411	Macrophage
KOPTK1	T lymphocyte
LOUCY	T lymphocyte
