# Supplementary material for: Epigenetic regulation of transcription factor binding motifs promotes Th1 response in Chagas disease cardiomyopathy
Source: Front Immunol. 2022 Aug 22;13:958200. doi: 10.3389/fimmu.2022.958200 (PMC9441916; doi:10.3389/fimmu.2022.958200)
Supplement: Supplementary Table 1 — Biological samples included in this study. [file DataSheet_1.zip › Supplementary Material/Supplementary Table 3.pdf]

Supplementary table 3. List of unique differentially expressed genes identified by RNA-seq between 8 end stage CCC heart tissue samples and 6 heart tissue samples obtained from organ donors.

Gene id	Gene Name	Gene description	Fold Change	Log2 Fold Change	pvalue	Corrected pvalue
ENSG00000270641	TSIX	TSIX transcript, XIST antisense RNA	3104.19	11.6	1.37E-08	8.49E-07
ENSG00000226777	KIAA0125	family with sequence similarity 30 member A	1552.09	10.6	1.25E-20	7.15E-18
ENSG00000116748	AMPD1	adenosine monophosphate deaminase 1	1200.98	10.23	1.76E-18	6.78E-16
ENSG00000254709	IGLL5	immunoglobulin lambda like polypeptide 5	1074.91	10.07	3.85E-19	1.72E-16
ENSG00000121895	TMEM156	transmembrane protein 156	1002.93	9.97	5.03E-20	2.58E-17
ENSG00000231486	AC096579.7		739.29	9.53	4.43E-17	1.30E-14
ENSG00000136573	BLK	BLK proto-oncogene, Src family tyrosine kinase	704.28	9.46	1.04E-14	2.05E-12
ENSG00000268864	CTB-167G5.5	solute carrier family 7 (cationic amino acid transporter, y+ system), member 2 (SLC7A2) pseudogene	-552.56	-9.11	3.75E-09	2.75E-07
ENSG00000109684	CLNK	cytokine dependent hematopoietic cell linker	501.46	8.97	2.37E-20	1.30E-17
ENSG00000169435	RASSF6	Ras association domain family member 6	433.53	8.76	3.70E-13	5.97E-11
ENSG00000248713	RP11-766F14.2	chromosome 4 open reading frame 54	-404.5	-8.66	3.22E-16	8.28E-14
ENSG00000172752	COL6A5	collagen type VI alpha 5 chain	401.71	8.65	4.63E-07	1.89E-05
ENSG00000163435	ELF3	E74 like ETS transcription factor 3	-330.84	-8.37	5.32E-18	1.90E-15
ENSG00000229807	XIST	X inactive specific transcript	290.02	8.18	4.31E-12	5.55E-10
ENSG00000176083	ZNF683	zinc finger protein 683	276.28	8.11	6.27E-18	2.22E-15

ENSG00000246084	CTD-2506J14.1	long intergenic non-protein coding RNA 2325	274.37	8.1	5.67E-11	5.88E-09
ENSG00000158485	CD1B	CD1b molecule	263.2	8.04	3.82E-09	2.79E-07
ENSG00000143297	FCRL5	Fc receptor like 5	261.38	8.03	6.43E-33	1.98E-29
ENSG00000205846	CLEC6A	C-type lectin domain containing 6A	202.25	7.66	8.62E-08	4.44E-06
ENSG00000056291	NPFFR2	neuropeptide FF receptor 2	199.47	7.64	2.41E-10	2.23E-08
ENSG00000188822	CNR2	cannabinoid receptor 2	181.02	7.5	7.10E-08	3.72E-06
ENSG00000117215	PLA2G2D	phospholipase A2 group IID	165.42	7.37	1.29E-10	1.27E-08
ENSG00000128438	TBC1D27	TBC1 domain family member 27, pseudogene	165.42	7.37	2.79E-09	2.08E-07
ENSG00000249318	AC010468.2	novel transcript	162.02	7.34	2.30E-08	1.38E-06
ENSG00000132465	IGJ	joining chain of multimeric IgA and IgM	148.06	7.21	3.32E-34	1.46E-30
ENSG00000251408	RP11-586D19.2	novel transcript	142.02	7.15	4.26E-08	2.35E-06
ENSG00000235621	LINC00494	long intergenic non-protein coding RNA 494	141.04	7.14	7.54E-09	4.99E-07
ENSG00000255183	RP11-720D4.3	long intergenic non-protein coding RNA 2711	139.1	7.12	1.62E-07	7.63E-06
ENSG00000170819	BFSP2	beaded filament structural protein 2	128.89	7.01	1.64E-08	1.00E-06
ENSG00000256039	RP11-291B21.2	long intergenic non-protein coding RNA 2446	128	7	4.07E-32	1.14E-28
ENSG00000183625	CCR3	C-C motif chemokine receptor 3	126.24	6.98	1.15E-06	4.19E-05
ENSG00000259731	RP11-326N17.1	novel transcript	123.64	6.95	9.03E-08	4.60E-06
ENSG00000091181	IL5RA	interleukin 5 receptor subunit alpha	112.99	6.82	1.35E-13	2.29E-11
ENSG00000196092	PAX5	paired box 5	109.9	6.78	1.13E-09	9.23E-08
ENSG00000204110	RP1-153P14.8	long intergenic non-protein coding RNA 2520	109.14	6.77	7.58E-07	2.94E-05
ENSG00000116147	TNR	tenascin R	108.38	6.76	4.48E-07	1.85E-05
ENSG00000188282	RUFY4	RUN and FYVE domain containing 4	95.67	6.58	1.34E-10	1.30E-08
ENSG00000258379	RP11-204N11.2	novel transcript	95.01	6.57	6.11E-07	2.44E-05
ENSG00000039068	CDH1	cadherin 1	93.05	6.54	5.85E-07	2.35E-05
ENSG00000105369	CD79A	CD79a molecule	91.14	6.51	2.42E-26	3.11E-23
ENSG00000240754	RP11-38J22.6		88.65	6.47	6.85E-07	2.70E-05
ENSG00000189233	NUGGC	nuclear GTPase, germinal center associated	86.22	6.43	3.42E-36	2.11E-32

ENSG00000134028	ADAMDEC1	ADAM like decysin 1	84.45	6.4	2.19E-07	9.82E-06
ENSG00000160882	CYP11B1	cytochrome P450 family 11 subfamily B member 1	83.29	6.38	6.23E-06	1.80E-04
ENSG00000249201	CTD-3080P12.3	uncharacterized LOC101928857	79.34	6.31	2.10E-07	9.45E-06
ENSG00000138755	CXCL9	C-X-C motif chemokine ligand 9	78.25	6.29	3.56E-20	1.89E-17
ENSG00000135443	KRT85	keratin 85	76.64	6.26	8.14E-07	3.13E-05
ENSG00000266088	RP5-1028K7.2	novel transcript	70.52	6.14	3.52E-05	7.44E-04
ENSG00000256155	RP11-277P12.9	long intergenic non-protein coding RNA 2598	68.12	6.09	6.78E-07	2.68E-05
ENSG00000226025	LGALS17A	galectin 14 pseudogene	67.65	6.08	3.24E-08	1.85E-06
ENSG00000146285	SCML4	Scm polycomb group protein like 4	66.72	6.06	5.47E-34	2.11E-30
ENSG00000230943	RP11-367G18.1	long intergenic non-protein coding RNA 2541	63.12	5.98	1.09E-05	2.87E-04
ENSG00000227920	RP1-153P14.5	novel transcript	62.68	5.97	3.94E-05	8.14E-04
ENSG00000255833	TIFAB	TIFA inhibitor	59.71	5.9	5.10E-12	6.52E-10
ENSG00000259343	RP11-761I4.3	TMC3 antisense RNA 1	59.71	5.9	8.68E-06	2.37E-04
ENSG00000180537	RNF182	ring finger protein 182	59.71	5.9	2.27E-05	5.16E-04
ENSG00000186265	BTLA	B and T lymphocyte associated	58.89	5.88	1.59E-17	5.01E-15
ENSG00000169213	RAB3B	RAB3B, member RAS oncogene family	58.89	5.88	1.54E-09	1.21E-07
ENSG00000265714	AL122127.3		58.08	5.86	1.58E-05	3.84E-04
ENSG00000230146	SEPHS1P4	selenophosphate synthetase 1 pseudogene 4	57.28	5.84	8.00E-06	2.21E-04
ENSG00000249993	BFSP2-AS1	BFSP2 antisense RNA 1	56.1	5.81	3.12E-05	6.72E-04
ENSG00000122787	AKR1D1	aldo-keto reductase family 1 member D1	54.95	5.78	1.29E-04	2.17E-03
ENSG00000258512	RP11-796G6.2	long intergenic non-protein coding RNA 239	54.57	5.77	8.06E-06	2.23E-04
ENSG00000254650	RP11-665E10.5	MT-CYB pseudogene 41	54.57	5.77	3.90E-05	8.07E-04
ENSG00000230006	ANKRD36BP2	ankyrin repeat domain 36B pseudogene 2	53.45	5.74	3.98E-35	2.04E-31
ENSG00000225720	RP4-742C19.12	apolipoprotein B mRNA editing enzyme, catalytic polypeptide-like 3 (APOBEC3) family pseudogene	53.08	5.73	5.74E-05	1.12E-03
ENSG00000174453	VWC2L	von Willebrand factor C domain containing 2 like	52.71	5.72	3.39E-06	1.07E-04

ENSG00000267178	PHF5CP	PHD finger protein 5C pseudogene	52.71	5.72	1.67E-04	2.65E-03
ENSG00000112812	PRSS16	serine protease 16	51.63	5.69	2.46E-05	5.51E-04
ENSG00000115884	SDC1	syndecan 1	50.56	5.66	9.40E-39	2.90E-34
ENSG00000110777	POU2AF1	POU class 2 homeobox associating factor 1	50.56	5.66	1.23E-29	2.36E-26
ENSG00000264781	hsa-mir-4537	microRNA 4537	49.52	5.63	1.56E-05	3.82E-04
ENSG00000205592	MUC19	mucin 19, oligomeric	48.84	5.61	4.88E-05	9.73E-04
ENSG00000153789	FAM92B	CBY1 interacting BAR domain containing 2	48.84	5.61	2.67E-04	3.85E-03
ENSG00000105717	PBX4	PBX homeobox 4	47.84	5.58	9.13E-15	1.83E-12
ENSG00000238121	LINC00426	long intergenic non-protein coding RNA 426	47.5	5.57	3.65E-27	5.11E-24
ENSG00000244618	RN7SL334P	RNA, 7SL, cytoplasmic 334, pseudogene	47.18	5.56	1.32E-04	2.20E-03
ENSG00000215764	KIR2DL2		47.18	5.56	2.78E-04	3.98E-03
ENSG00000196684	HSH2D	hematopoietic SH2 domain containing	46.21	5.53	2.06E-27	3.35E-24
ENSG00000228168	HNRNPA1P21	heterogeneous nuclear ribonucleoprotein A1 pseudogene 21	46.21	5.53	1.29E-04	2.17E-03
ENSG00000234663	AC104820.2	long intergenic non-protein coding RNA 1934	42.81	5.42	2.10E-18	7.87E-16
ENSG00000244649	CTD-2377D24.6	long intergenic non-protein coding RNA 2086	42.81	5.42	3.28E-05	7.03E-04
ENSG00000122043	LINC00544	long intergenic non-protein coding RNA 544	42.81	5.42	6.61E-05	1.25E-03
ENSG00000235366	LINC01055	long intergenic non-protein coding RNA 1055	42.22	5.4	1.19E-04	2.02E-03
ENSG00000233093	LINC00892	long intergenic non-protein coding RNA 892	41.93	5.39	2.29E-15	5.15E-13
ENSG00000241577	RP11-523O18.7	novel transcript, sense intronic to LRRC18	41.07	5.36	6.15E-05	1.18E-03
ENSG00000255354	RP11-148O21.2		41.07	5.36	8.99E-05	1.61E-03
ENSG00000166105	GLB1L3	galactosidase beta 1 like 3	39.4	5.3	1.76E-07	8.14E-06
ENSG00000253980	CTB-4E7.1	novel transcript	39.12	5.29	3.66E-07	1.55E-05
ENSG00000260719	AC009133.17	novel transcript, antisense to C16orf54	38.59	5.27	4.73E-08	2.58E-06

ENSG00000244432	RPL39P28	ribosomal protein L39 pseudogene 28	38.59	5.27	1.66E-04	2.63E-03
ENSG00000166211	SPIC	Spi-C transcription factor	38.59	5.27	3.16E-04	4.40E-03
ENSG00000127318	IL22	interleukin 22	38.05	5.25	1.31E-04	2.19E-03
ENSG00000220517	ASS1P1	argininosuccinate synthetase 1 pseudogene 1	36.76	5.2	9.85E-11	9.86E-09
ENSG00000204475	NCR3	natural cytotoxicity triggering receptor 3	36.76	5.2	1.31E-10	1.27E-08
ENSG00000215506	TPTE2P4	transmembrane phosphoinositide 3-phosphatase and tensin homolog 2 pseudogene 4	-36.76	-5.2	3.54E-06	1.11E-04
ENSG00000150556	LYPD6B	LY6/PLAUR domain containing 6B	36.76	5.2	2.07E-04	3.13E-03
ENSG00000104970	KIR3DX1	killer cell immunoglobulin like receptor, three Ig domains X1 (pseudogene)	36.5	5.19	2.24E-07	1.00E-05
ENSG00000226659	RP11-137H2.4	TSPAN14 antisense RNA 1	36.25	5.18	9.19E-05	1.64E-03
ENSG00000203416	FAM32B	family with sequence similarity 32 member B, pseudogene	36.25	5.18	1.40E-04	2.31E-03
ENSG00000204969	PCDHA2	protocadherin alpha 2	36.25	5.18	1.69E-04	2.68E-03
ENSG00000170476	MZB1	marginal zone B and B1 cell specific protein	35.75	5.16	1.10E-37	8.50E-34
ENSG00000225079	FTH1P22	ferritin heavy chain 1 pseudogene 22	35.75	5.16	3.93E-10	3.52E-08
ENSG00000143185	XCL2	X-C motif chemokine ligand 2	35.51	5.15	5.09E-10	4.47E-08
ENSG00000152766	ANKRD22	ankyrin repeat domain 22	34.54	5.11	4.19E-09	3.05E-07
ENSG00000261786	RP4-555D20.2	novel transcript	34.54	5.11	9.57E-04	1.01E-02
ENSG00000156738	MS4A1	membrane spanning 4-domains A1	34.3	5.1	6.86E-15	1.40E-12
ENSG00000132704	FCRL2	Fc receptor like 2	34.3	5.1	5.62E-09	3.88E-07
ENSG00000162739	SLAMF6	SLAM family member 6	33.59	5.07	3.53E-12	4.67E-10
ENSG00000168671	UGT3A2	UDP glycosyltransferase family 3 member A2	33.13	5.05	7.11E-04	8.11E-03
ENSG00000237301	RP4-680D5.2	novel transcript	32.9	5.04	1.31E-04	2.19E-03
ENSG00000141293	SKAP1	src kinase associated phosphoprotein 1	32.67	5.03	1.32E-17	4.28E-15
ENSG00000168421	RHOH	ras homolog family member H	32.45	5.02	6.60E-26	7.82E-23

ENSG00000152969	JAKMIP1	janus kinase and microtubule interacting protein 1	32.45	5.02	3.07E-11	3.35E-09
ENSG00000243772	KIR2DL3	killer cell immunoglobulin like receptor, two Ig domains and long cytoplasmic tail 3	32.45	5.02	7.72E-04	8.61E-03
ENSG00000235576	AC092580.4	long intergenic non-protein coding RNA 1871	32.22	5.01	7.04E-13	1.05E-10
ENSG00000223511	RP13-297E16.4	novel transcript	32	5	6.30E-07	2.50E-05
ENSG00000013725	CD6	CD6 molecule	31.78	4.99	1.99E-15	4.50E-13
ENSG00000205809	KLRC2	killer cell lectin like receptor C2	31.78	4.99	8.57E-13	1.25E-10
ENSG00000258869	RP11-204N11.1	long intergenic non-protein coding RNA 2312	31.78	4.99	4.19E-12	5.42E-10
ENSG00000123977	DAW1	dynein assembly factor with WD repeats 1	-31.56	-4.98	2.12E-05	4.89E-04
ENSG00000181036	FCRL6	Fc receptor like 6	31.34	4.97	2.29E-38	3.52E-34
ENSG00000181143	MUC16	mucin 16, cell surface associated	31.12	4.96	5.39E-12	6.84E-10
ENSG00000167618	LAIR2	leukocyte associated immunoglobulin like receptor 2	31.12	4.96	1.05E-03	1.09E-02
ENSG00000237286	AC004906.3	CARD11 antisense RNA 1	30.91	4.95	5.57E-04	6.76E-03
ENSG00000100450	GZMH	granzyme H	30.06	4.91	1.19E-09	9.63E-08
ENSG00000026751	SLAMF7	SLAM family member 7	29.65	4.89	1.70E-14	3.20E-12
ENSG00000078081	LAMP3	lysosomal associated membrane protein 3	29.45	4.88	1.45E-07	6.95E-06
ENSG00000173578	XCR1	X-C motif chemokine receptor 1	29.04	4.86	2.72E-11	3.00E-09
ENSG00000251309	RP11-498M5.2	novel transcript	29.04	4.86	3.89E-04	5.17E-03
ENSG00000234680	VN2R3P	vomeronasal 2 receptor 3, pseudogene	-28.44	-4.83	6.60E-05	1.25E-03
ENSG00000226667	RP11-292F22.5		28.44	4.83	4.12E-04	5.39E-03
ENSG00000122188	LAX1	lymphocyte transmembrane adaptor 1	28.25	4.82	1.06E-24	1.02E-21
ENSG00000255391	CTD-2028E8.1	high-mobility group box 1 (HMGB1) pseudogene	28.25	4.82	4.62E-07	1.89E-05
ENSG00000153283	CD96	CD96 molecule	27.86	4.8	6.76E-14	1.20E-11
ENSG00000172673	THEMIS	thymocyte selection associated	27.28	4.77	1.06E-20	6.24E-18
ENSG00000222179	RN7SKP26	RN7SK pseudogene 26	27.1	4.76	6.89E-04	7.95E-03
ENSG00000177494	ZBED2	zinc finger BED-type containing 2	26.91	4.75	5.35E-08	2.86E-06
ENSG00000113263	ITK	IL2 inducible T cell kinase	26.35	4.72	3.66E-30	8.68E-27

ENSG00000060140	STYK1	serine/threonine/tyrosine kinase 1	26.35	4.72	7.88E-17	2.21E-14
ENSG00000176320	RP11-404O13.5	novel transcript	26.17	4.71	3.45E-07	1.47E-05
ENSG00000248969	CTD-2113L7.1	novel transcript, antisense to SPEF2	26.17	4.71	4.79E-04	6.05E-03
ENSG00000127152	BCL11B	BAF chromatin remodeling complex subunit BCL11B	25.99	4.7	2.26E-25	2.32E-22
ENSG00000259278	RP11-62C7.2	novel transcript	25.81	4.69	1.20E-07	5.89E-06
ENSG00000254965	RP11-113K21.2	C1D nuclear receptor corepressor pseudogene 5	25.81	4.69	4.38E-04	5.63E-03
ENSG00000122224	LY9	lymphocyte antigen 9	25.63	4.68	9.44E-26	1.04E-22
ENSG00000137078	SIT1	signaling threshold regulating transmembrane adaptor 1	25.63	4.68	7.32E-16	1.75E-13
ENSG00000100298	APOBEC3H	apolipoprotein B mRNA editing enzyme catalytic subunit 3H	25.11	4.65	2.11E-11	2.42E-09
ENSG00000264198	RP11-94L15.2		24.76	4.63	6.09E-23	4.81E-20
ENSG00000224523	RP13-52K8.1	pseudogene similar to part of SLC9A2 (solute carrier family 9 (sodium\/hydrogen exchanger), isoform 2)	24.76	4.63	6.01E-04	7.14E-03
ENSG00000176769	TCERG1L	transcription elongation regulator 1 like	24.76	4.63	6.52E-04	7.60E-03
ENSG00000272763	RP11-357H14.17	novel transcript	24.76	4.63	8.74E-04	9.46E-03
ENSG00000172724	CCL19	C-C motif chemokine ligand 19	24.59	4.62	1.90E-10	1.79E-08
ENSG00000254510	RP11-867G23.10	novel transcript	-24.42	-4.61	8.89E-09	5.76E-07
ENSG00000124203	ZNF831	zinc finger protein 831	24.25	4.6	1.06E-22	8.19E-20
ENSG00000231764	DLX6-AS1	DLX6 antisense RNA 1	24.25	4.6	1.35E-03	1.32E-02
ENSG00000127129	EDN2	endothelin 2	-23.92	-4.58	2.51E-08	1.48E-06
ENSG00000162676	GFI1	growth factor independent 1 transcriptional repressor	23.75	4.57	8.03E-22	5.50E-19
ENSG00000168685	IL7R	interleukin 7 receptor	23.75	4.57	7.81E-16	1.82E-13
ENSG00000244720	RP11-402J7.2	NT5C3A pseudogene 2	23.75	4.57	3.97E-11	4.20E-09
ENSG00000167077	MEI1	meiotic double-stranded break formation protein 1	23.43	4.55	6.81E-30	1.50E-26
ENSG00000168081	PNOC	prepronociceptin	22.94	4.52	5.97E-07	2.39E-05
ENSG00000226003	RP11-312J18.3	peptidylprolyl isomerase A pseudogene 37	22.94	4.52	8.31E-04	9.09E-03
ENSG00000124334	IL9R	interleukin 9 receptor	22.78	4.51	1.93E-08	1.17E-06

ENSG00000188820	FAM26F	calcium homeostasis modulator family member 6	22.63	4.5	8.38E-07	3.21E-05
ENSG00000188011	CXXC11	receptor transporter protein 5 (putative)	22.63	4.5	9.25E-07	3.49E-05
ENSG00000158488	CD1E	CD1e molecule	22.32	4.48	3.82E-06	1.18E-04
ENSG00000161405	IKZF3	IKAROS family zinc finger 3	22.16	4.47	2.42E-12	3.33E-10
ENSG00000263787	RP11-456D7.1	SKAP1 antisense RNA 1	22.16	4.47	2.55E-06	8.42E-05
ENSG00000259803	SLC22A31	solute carrier family 22 member 31	22.16	4.47	1.75E-03	1.60E-02
ENSG00000233261	LINC00264	family with sequence similarity 238 member A	22.16	4.47	1.93E-03	1.73E-02
ENSG00000230539	AOAH-IT1	AOAH intronic transcript 1	22.01	4.46	6.27E-11	6.44E-09
ENSG00000253701	AL928768.3		22.01	4.46	3.72E-06	1.16E-04
ENSG00000198851	CD3E	CD3e molecule	21.71	4.44	4.57E-19	1.98E-16
ENSG00000269919	RP1-134E15.3	novel transcript, sense intronic to PRDM1	21.71	4.44	1.28E-12	1.83E-10
ENSG00000161570	CCL5		21.71	4.44	6.65E-12	8.36E-10
ENSG00000167476	JSRP1	junctional sarcoplasmic reticulum protein 1	21.71	4.44	2.17E-09	1.65E-07
ENSG00000216054	AC019201.1		21.71	4.44	9.08E-06	2.46E-04
ENSG00000180535	BHLHA15	basic helix-loop-helix family member a15	21.26	4.41	3.97E-10	3.55E-08
ENSG00000182183	FAM159A	shisa like 2A	21.11	4.4	3.77E-12	4.97E-10
ENSG00000253364	RP11-731F5.2	chronic obstructive pulmonary disease associated lncRNA 1	21.11	4.4	9.07E-04	9.74E-03
ENSG00000236481	AC002331.1	long intergenic non-protein coding RNA 2195	20.97	4.39	1.38E-03	1.33E-02
ENSG00000181215	C4orf50	chromosome 4 open reading frame 50	20.82	4.38	1.06E-08	6.76E-07
ENSG00000173239	LIPM	lipase family member M	20.82	4.38	8.24E-06	2.27E-04
ENSG00000224805	LINC00853	long intergenic non-protein coding RNA 853	20.82	4.38	1.48E-03	1.41E-02
ENSG00000144406	UNC80	unc-80 homolog, NALCN channel complex subunit	20.68	4.37	1.44E-11	1.68E-09
ENSG00000073861	TBX21	T-box transcription factor 21	20.25	4.34	2.41E-27	3.72E-24
ENSG00000189238	LINC00943	long intergenic non-protein coding RNA 943	20.25	4.34	1.17E-13	2.01E-11
ENSG00000227039	ITGB2-AS1	ITGB2 antisense RNA 1	20.11	4.33	7.97E-19	3.27E-16

ENSG00000163519	TRAT1	T cell receptor associated transmembrane adaptor 1	20.11		4.33	7.40E-17	2.11E-14
ENSG00000124256	ZBP1	Z-DNA binding protein 1	20.11		4.33	2.70E-16	7.12E-14
ENSG00000229754	CXCR2P1	C-X-C motif chemokine receptor 2 pseudogene 1	20.11		4.33	4.37E-08	2.40E-06
ENSG00000153563	CD8A	CD8a molecule	19.97		4.32	1.24E-23	1.09E-20
ENSG00000140284	SLC27A2	solute carrier family 27 member 2	19.97		4.32	8.35E-08	4.33E-06
ENSG00000213434	VTI1BP2	vesicle transport through interaction with t-SNAREs 1B pseudogene 2	19.7		4.3	7.70E-04	8.60E-03
ENSG00000196934	RIMBP3B		19.7		4.3	1.32E-03	1.29E-02
ENSG00000153898	MCOLN2	mucolipin TRP cation channel 2	19.56		4.29	4.28E-18	1.55E-15
ENSG00000135925	WNT10A	Wnt family member 10A	19.43		4.28	8.67E-08	4.45E-06
ENSG00000227678	RP11-73O6.3	novel transcript, antisense to L3MBTL3	19.43		4.28	1.20E-05	3.08E-04
ENSG00000122223	CD244	CD244 molecule	19.29		4.27	2.55E-19	1.16E-16
ENSG00000116824	CD2	CD2 molecule	19.29		4.27	5.00E-19	2.11E-16
ENSG00000183542	KLRC4	killer cell lectin like receptor C4	19.29		4.27	2.67E-11	2.97E-09
ENSG00000267311	RP11-99A1.2	novel transcript, sense intronic to RAB27B	19.16		4.26	1.31E-03	1.29E-02
ENSG00000005844	ITGAL	integrin subunit alpha L	18.9		4.24	9.01E-26	1.03E-22
ENSG00000187323	DCC	DCC netrin 1 receptor	18.9		4.24	1.31E-05	3.30E-04
ENSG00000172116	CD8B	CD8b molecule	18.77		4.23	4.67E-11	4.87E-09
ENSG00000240787	RP11-615J4.4	pseudogene similar to part of poliovirus receptor-related 2 (herpesvirus entry mediator) BPVRL2	18.64		4.22	1.09E-06	4.03E-05
ENSG00000259307	PLCB2-AS1	PLCB2 antisense RNA 1	18.64		4.22	1.17E-03	1.18E-02
ENSG00000159618	GPR114	adhesion G protein-coupled receptor G5	18.38		4.2	4.65E-14	8.42E-12
ENSG00000065675	PRKCQ	protein kinase C theta	18.25		4.19	7.27E-25	7.23E-22
ENSG00000197616	MYH6	myosin heavy chain 6		-18	-4.17	2.54E-15	5.64E-13
ENSG00000119508	NR4A3	nuclear receptor subfamily 4 group A member 3		-18	-4.17	7.64E-12	9.53E-10
ENSG00000251215	GOLGA5P1	golgin A5 pseudogene 1	17.88		4.16	4.98E-09	3.54E-07
ENSG00000224557	HLA-DPB2	major histocompatibility complex, class II, DP beta 2 (pseudogene)	17.63		4.14	1.59E-05	3.88E-04

ENSG00000102245	CD40LG	CD40 ligand	17.51	4.13	3.37E-17	9.98E-15
ENSG00000227145	IL21-AS1	IL21 antisense RNA 1	17.51	4.13	2.41E-05	5.42E-04
ENSG00000238685	ACA64		17.51	4.13	1.71E-03	1.57E-02
ENSG00000164287	CDC20B	cell division cycle 20B	17.39	4.12	2.69E-03	2.22E-02
ENSG00000205021	CCL3L1		17.27	4.11	1.55E-05	3.78E-04
ENSG00000167286	CD3D	CD3d molecule	17.15	4.1	3.91E-16	9.96E-14
ENSG00000249948	GBA3	glucosylceramidase beta 3 (gene/pseudogene)	17.15	4.1	3.09E-05	6.66E-04
ENSG00000263264	CTB-133G6.1		17.03	4.09	5.53E-15	1.14E-12
ENSG00000245164	LINC00861	long intergenic non-protein coding RNA 861	16.91	4.08	2.82E-09	2.10E-07
ENSG00000232812	RP11-459K23.2	long intergenic non-protein coding RNA 1717	16.8	4.07	2.59E-03	2.16E-02
ENSG00000213809	KLRK1	killer cell lectin like receptor K1	16.68	4.06	1.92E-10	1.80E-08
ENSG00000110848	CD69	CD69 molecule	16.56	4.05	3.72E-23	3.19E-20
ENSG00000163599	CTLA4	cytotoxic T-lymphocyte associated protein 4	16.56	4.05	1.25E-16	3.37E-14
ENSG00000134545	KLRC1	killer cell lectin like receptor C1	16.56	4.05	2.40E-14	4.43E-12
ENSG00000179934	CCR8	C-C motif chemokine receptor 8	16.56	4.05	2.66E-04	3.84E-03
ENSG00000154451	GBP5	guanylate binding protein 5	16.45	4.04	5.20E-10	4.55E-08
ENSG00000169436	COL22A1	collagen type XXII alpha 1 chain	16.45	4.04	6.12E-08	3.23E-06
ENSG00000213886	UBD	ubiquitin D	16.34	4.03	4.82E-05	9.65E-04
ENSG00000205890	RP11-473M20.5	novel transcript, antisense to CCDC64B	16.34	4.03	2.00E-03	1.78E-02
ENSG00000177272	KCNA3	potassium voltage-gated channel subfamily A member 3	16.22	4.02	1.57E-17	4.99E-15
ENSG00000256128	LINC00944	long intergenic non-protein coding RNA 944	16.22	4.02	3.49E-11	3.75E-09
ENSG00000139193	CD27	CD27 molecule	16.11	4.01	1.45E-18	5.64E-16
ENSG00000125084	WNT1	Wnt family member 1	16.11	4.01	9.54E-06	2.58E-04
ENSG00000173988	LRRC63	leucine rich repeat containing 63	16	4	2.04E-06	6.99E-05
ENSG00000160654	CD3G	CD3g molecule	15.89	3.99	1.10E-19	5.45E-17
ENSG00000186810	CXCR3	C-X-C motif chemokine receptor 3	15.89	3.99	1.97E-17	6.08E-15
ENSG00000155961	RAB39B	RAB39B, member RAS oncogene family	15.89	3.99	2.97E-15	6.50E-13

ENSG00000111537	IFNG	interferon gamma	15.89	3.99	4.22E-09	3.05E-07
ENSG00000174123	TLR10	toll like receptor 10	15.89	3.99	7.99E-08	4.15E-06
ENSG00000229228	LINC00582	long intergenic non-protein coding RNA 582	15.89	3.99	5.11E-06	1.51E-04
ENSG00000117560	FASLG	Fas ligand	15.78	3.98	6.69E-12	8.38E-10
ENSG00000229405	AC092580.1	proteasome (prosome, macropain) subunit, beta type, 1 (PSMB1) pseudogene	15.78	3.98	6.85E-10	5.84E-08
ENSG00000177455	CD19	CD19 molecule	15.78	3.98	1.07E-07	5.30E-06
ENSG00000101842	VSIG1	V-set and immunoglobulin domain containing 1	15.78	3.98	3.39E-05	7.21E-04
ENSG00000103522	IL21R	interleukin 21 receptor	15.67	3.97	3.23E-13	5.25E-11
ENSG00000262400	RP11-191A15.1	calcium activated nucleotidase 1 (CANT1) pseudogene	15.67	3.97	2.84E-03	2.33E-02
ENSG00000160185	UBASH3A	ubiquitin associated and SH3 domain containing A	15.56	3.96	6.86E-14	1.22E-11
ENSG00000164512	ANKRD55	ankyrin repeat domain 55	15.45	3.95	1.80E-07	8.32E-06
ENSG00000129277	CCL4		15.35	3.94	1.07E-20	6.24E-18
ENSG00000034053	APBA2	amyloid beta precursor protein binding family A member 2	15.35	3.94	3.99E-18	1.46E-15
ENSG00000007908	SELE	selectin E	-15.35	-3.94	4.27E-06	1.30E-04
ENSG00000227159	DDX11L16	DEAD/H-box helicase 11 like 16 (pseudogene)	15.35	3.94	2.22E-03	1.92E-02
ENSG00000167653	PSCA	prostate stem cell antigen	15.35	3.94	2.51E-03	2.11E-02
ENSG00000262823	RP13-580F15.2	novel transcript, antisense to SPNS3	15.24	3.93	9.96E-06	2.67E-04
ENSG00000251149	MTND5P5	MT-ND5 pseudogene 5	15.24	3.93	3.15E-03	2.51E-02
ENSG00000166592	RRAD	RRAD, Ras related glycolysis inhibitor and calcium channel regulator	-15.14	-3.92	9.17E-19	3.72E-16
ENSG00000089012	SIRPG	signal regulatory protein gamma	15.03	3.91	4.61E-12	5.92E-10
ENSG00000184613	NELL2	neural EGFL like 2	15.03	3.91	7.68E-07	2.98E-05
ENSG00000115085	ZAP70	zeta chain of T cell receptor associated protein kinase 70	14.93	3.9	8.77E-29	1.59E-25
ENSG00000160593	AMICA1	junction adhesion molecule like	14.93	3.9	3.61E-27	5.11E-24
ENSG00000166523	CLEC4E	C-type lectin domain family 4 member E	14.93	3.9	6.36E-18	2.23E-15
ENSG00000243536	ANTXRLP1		14.93	3.9	5.61E-08	2.98E-06

ENSG00000215044	AHCYP1		14.93	3.9	3.71E-06	1.16E-04
ENSG00000015413	DPEP1	dipeptidase 1	14.93	3.9	3.74E-05	7.80E-04
ENSG00000137265	IRF4	interferon regulatory factor 4	14.83	3.89	7.35E-22	5.15E-19
ENSG00000245648	RP11-277P12.20	KLRK1 antisense RNA 1	14.83	3.89	7.64E-13	1.13E-10
ENSG00000085265	FCN1	ficolin 1	14.83	3.89	6.80E-10	5.82E-08
ENSG00000153064	BANK1	B cell scaffold protein with ankyrin repeats 1	14.72	3.88	1.59E-10	1.52E-08
ENSG00000136250	AOAH	acyloxyacyl hydrolase	14.62	3.87	1.51E-22	1.13E-19
ENSG00000226557	TRAF6P1	TNF receptor associated factor 6 pseudogene 1	14.62	3.87	2.66E-03	2.20E-02
ENSG00000109943	CRTAM	cytotoxic and regulatory T cell molecule	14.52	3.86	3.08E-13	5.02E-11
ENSG00000117322	CR2	complement C3d receptor 2	14.52	3.86	7.93E-03	4.91E-02
ENSG00000255776	RP11-436I9.3	VDAC2 pseudogene 2	14.42	3.85	1.76E-06	6.09E-05
ENSG00000228314	CYP4F29P	cytochrome P450 family 4 subfamily F member 29, pseudogene	14.32	3.84	4.82E-05	9.65E-04
ENSG00000147168	IL2RG	interleukin 2 receptor subunit gamma	14.22	3.83	1.67E-22	1.22E-19
ENSG00000240403	KIR3DL2	killer cell immunoglobulin like receptor, three Ig domains and long cytoplasmic tail 2	14.22	3.83	3.33E-03	2.61E-02
ENSG00000186481	ANKRD20A5P	ankyrin repeat domain 20 family member A5, pseudogene	14.12	3.82	3.76E-11	4.02E-09
ENSG00000205810	KLRC3	killer cell lectin like receptor C3	14.12	3.82	1.15E-06	4.21E-05
ENSG00000174885	NLRP6	NLR family pyrin domain containing 6	13.93	3.8	3.04E-11	3.34E-09
ENSG00000100351	GRAP2	GRB2 related adaptor protein 2	13.74	3.78	4.53E-20	2.37E-17
ENSG00000272279	RP11-157J24.2	novel transcript	13.74	3.78	4.53E-04	5.80E-03
ENSG00000078589	P2RY10	P2Y receptor family member 10	13.64	3.77	2.22E-13	3.70E-11
ENSG00000204659	CBY3	chibby family member 3	13.55	3.76	3.60E-03	2.76E-02
ENSG00000226681	AC020595.1	novel transcript	13.55	3.76	4.12E-03	3.05E-02
ENSG00000235300	AC090627.1	SKAP1 antisense RNA 2	13.45	3.75	6.99E-10	5.95E-08
ENSG00000174946	GPR171	G protein-coupled receptor 171	13.36	3.74	1.64E-19	7.77E-17
ENSG00000205784	ARRDC5	arrestin domain containing 5	13.36	3.74	1.84E-10	1.75E-08
ENSG00000169245	CXCL10	C-X-C motif chemokine ligand 10	13.36	3.74	1.09E-06	4.01E-05
ENSG00000240505	TNFRSF13B	TNF receptor superfamily member 13B	13.27	3.73	6.75E-06	1.92E-04

ENSG00000228058	RP11-552D4.1	long intergenic non-protein coding RNA 1736	13.27	3.73	4.10E-03	3.03E-02
ENSG00000243836	WDR86-AS1	WDR86 antisense RNA 1	13.09	3.71	2.64E-05	5.84E-04
ENSG00000198286	CARD11	caspase recruitment domain family member 11	13	3.7	3.95E-16	9.98E-14
ENSG00000145649	GZMA	granzyme A	13	3.7	2.10E-13	3.54E-11
ENSG00000240535	CTD-2313F11.1	novel transcript, antisense to GZMK	13	3.7	3.91E-06	1.20E-04
ENSG00000007264	MATK	megakaryocyte-associated tyrosine kinase	12.82	3.68	1.66E-21	1.09E-18
ENSG00000134539	KLRD1	killer cell lectin like receptor D1	12.82	3.68	1.31E-20	7.31E-18
ENSG00000187912	CLEC17A	C-type lectin domain containing 17A	12.82	3.68	2.15E-04	3.24E-03
ENSG00000163508	EOMES	eomesodermin	12.73	3.67	8.27E-13	1.21E-10
ENSG00000020633	RUNX3	RUNX family transcription factor 3	12.55	3.65	1.71E-25	1.82E-22
ENSG00000109956	B3GAT1	beta-1,3-glucuronyltransferase 1	12.47	3.64	2.66E-11	2.97E-09
ENSG00000175779	C15orf53	long intergenic non-protein coding RNA 2694	12.47	3.64	7.76E-07	3.00E-05
ENSG00000100385	IL2RB	interleukin 2 receptor subunit beta	12.3	3.62	1.25E-18	5.00E-16
ENSG00000160856	FCRL3	Fc receptor like 3	12.3	3.62	3.87E-08	2.15E-06
ENSG00000242258	LINC00996	long intergenic non-protein coding RNA 996	12.3	3.62	1.96E-07	8.93E-06
ENSG00000105366	SIGLEC8	sialic acid binding Ig like lectin 8	12.3	3.62	1.33E-05	3.35E-04
ENSG00000121966	CXCR4	C-X-C motif chemokine receptor 4	12.21	3.61	1.50E-26	2.01E-23
ENSG00000090382	LYZ	lysozyme	12.13	3.6	7.66E-17	2.17E-14
ENSG00000265787	CYP4F35P	cytochrome P450 family 4 subfamily F member 35, pseudogene	12.13	3.6	6.17E-05	1.19E-03
ENSG00000171954	CYP4F22	cytochrome P450 family 4 subfamily F member 22	11.96	3.58	8.21E-06	2.26E-04
ENSG00000117091	CD48	CD48 molecule	11.88	3.57	1.20E-17	4.03E-15
ENSG00000176092	AIM1L	crystallin beta-gamma domain containing 2	11.88	3.57	1.52E-05	3.72E-04
ENSG00000255819	KLRC4-KLRK1	KLRC4-KLRK1 readthrough	11.79	3.56	8.62E-08	4.44E-06
ENSG00000132437	DDC	dopa decarboxylase	11.79	3.56	4.56E-03	3.28E-02

ENSG00000182866	LCK	LCK proto-oncogene, Src family tyrosine kinase	11.71	3.55	1.39E-17	4.45E-15
ENSG00000172543	CTSW	cathepsin W	11.63	3.54	3.83E-38	3.93E-34
ENSG00000057657	PRDM1	PR/SET domain 1	11.63	3.54	2.48E-31	6.37E-28
ENSG00000173762	CD7	CD7 molecule	11.63	3.54	6.44E-15	1.32E-12
ENSG00000241490	RP11-553L6.2	novel transcript, antisense to ZNF80	11.63	3.54	6.80E-05	1.28E-03
ENSG00000237988	OR2I1P	olfactory receptor family 2 subfamily I member 1 pseudogene	11.55	3.53	6.93E-05	1.30E-03
ENSG00000259242	AC002306.1	novel transcript, antisense to PBX4	11.47	3.52	5.15E-05	1.02E-03
ENSG00000105374	NKG7	natural killer cell granule protein 7	11.39	3.51	1.74E-21	1.12E-18
ENSG00000183813	CCR4	C-C motif chemokine receptor 4	11.39	3.51	1.02E-13	1.77E-11
ENSG00000164483	SAMD3	sterile alpha motif domain containing 3	11.31	3.5	5.45E-21	3.29E-18
ENSG00000197540	GZMM	granzyme M	11.31	3.5	4.36E-13	6.81E-11
ENSG00000158517	NCF1	neutrophil cytosolic factor 1	11.24	3.49	2.44E-21	1.53E-18
ENSG00000259834	RP11-284N8.3	novel transcript	11.24	3.49	4.14E-21	2.55E-18
ENSG00000178773	CPNE7	copine 7	11.24	3.49	1.12E-05	2.92E-04
ENSG00000161798	AQP5	aquaporin 5	-11.16	-3.48	4.05E-06	1.24E-04
ENSG00000101144	BMP7	bone morphogenetic protein 7	-11.16	-3.48	5.12E-06	1.51E-04
ENSG00000186188	FFAR4	free fatty acid receptor 4	11.16	3.48	2.35E-05	5.32E-04
ENSG00000164588	HCN1	hyperpolarization activated cyclic nucleotide gated potassium channel 1	-11.16	-3.48	6.16E-04	7.27E-03
ENSG00000134242	PTPN22	protein tyrosine phosphatase non-receptor type 22	11.08	3.47	1.11E-21	7.42E-19
ENSG00000122025	FLT3	fms related receptor tyrosine kinase 3	11.08	3.47	1.26E-06	4.54E-05
ENSG00000222086	AC010609.1		11.08	3.47	4.19E-04	5.46E-03
ENSG00000228427	RP5-1091N2.9	novel transcript	11	3.46	3.64E-05	7.64E-04
ENSG00000179593	ALOX15B	arachidonate 15-lipoxygenase type B	10.93	3.45	4.72E-05	9.50E-04
ENSG00000241525	AC108004.3	novel transcript, antisense to C17orf97	10.93	3.45	1.58E-04	2.54E-03
ENSG00000185352	HS6ST3	heparan sulfate 6-O-sulfotransferase 3	-10.93	-3.45	3.27E-04	4.51E-03

ENSG00000175463	TBC1D10C	TBC1 domain family member 10C	10.85	3.44	9.04E-30	1.86E-26
ENSG00000131401	NAPSB	napsin B aspartic peptidase, pseudogene	10.85	3.44	1.14E-14	2.21E-12
ENSG00000035720	STAP1	signal transducing adaptor family member 1	10.85	3.44	1.18E-06	4.27E-05
ENSG00000197153	HIST1H3J	H3 clustered histone 12	10.85	3.44	4.11E-06	1.26E-04
ENSG00000175857	GAPT	GRB2 binding adaptor protein, transmembrane	10.7	3.42	4.96E-10	4.37E-08
ENSG00000204165	CXorf65	chromosome X open reading frame 65	10.7	3.42	6.34E-05	1.21E-03
ENSG00000139626	ITGB7	integrin subunit beta 7	10.63	3.41	1.30E-33	4.46E-30
ENSG00000207939	MIR223		10.63	3.41	1.23E-05	3.13E-04
ENSG00000185811	IKZF1	IKAROS family zinc finger 1	10.56	3.4	9.31E-24	8.44E-21
ENSG00000157303	SUSD3	sushi domain containing 3	10.56	3.4	4.20E-13	6.63E-11
ENSG00000050730	TNIP3	TNFAIP3 interacting protein 3	10.48	3.39	7.81E-08	4.06E-06
ENSG00000152495	CAMK4	calcium/calmodulin dependent protein kinase IV	10.41	3.38	3.41E-20	1.84E-17
ENSG00000163564	PYHIN1	pyrin and HIN domain family member 1	10.41	3.38	3.00E-12	4.06E-10
ENSG00000272053	RP11-367G6.3		10.34	3.37	1.52E-05	3.72E-04
ENSG00000272264	RP11-92K15.3	novel transcript	10.34	3.37	1.21E-04	2.05E-03
ENSG00000205020	CCL4L1		10.34	3.37	7.02E-04	8.05E-03
ENSG00000073734	ABCB11	ATP binding cassette subfamily B member 11	10.34	3.37	2.51E-03	2.11E-02
ENSG00000185101	ANO9	anoctamin 9	10.2	3.35	4.51E-19	1.98E-16
ENSG00000110448	CD5	CD5 molecule	10.2	3.35	1.55E-16	4.15E-14
ENSG00000104814	MAP4K1	mitogen-activated protein kinase kinase kinase kinase 1	10.13	3.34	4.14E-26	5.10E-23
ENSG00000236790	LINC00299	long intergenic non-protein coding RNA 299	10.06	3.33	1.48E-07	7.05E-06
ENSG00000251301	RP11-81H14.2	long intergenic non-protein coding RNA 2384	10.06	3.33	1.52E-07	7.20E-06
ENSG00000253522	MIR146A	MIR3142 host gene	10.06	3.33	2.24E-07	1.00E-05
ENSG00000253535	RP11-624C23.1	novel transcript	9.99	3.32	7.60E-05	1.41E-03
ENSG00000199377	RNU5F-1	RNA, U5F small nuclear 1	9.99	3.32	2.71E-04	3.89E-03
ENSG00000250421	RP11-83M16.6	novel transcript	9.85	3.3	1.82E-05	4.33E-04
ENSG00000135697	BCMO1	beta-carotene oxygenase 1	9.85	3.3	2.29E-04	3.41E-03

ENSG00000258535	RP11-280K24.4	novel transcript	9.85	3.3	5.89E-03	3.95E-02
ENSG00000240219	RP11-430C7.5	novel transcript	9.78	3.29	9.83E-07	3.67E-05
ENSG00000250829	RP11-11N5.1	novel transcript	9.78	3.29	7.18E-05	1.34E-03
ENSG00000177984	LCN15	lipocalin 15	-9.78	-3.29	2.26E-04	3.37E-03
ENSG00000088340	FER1L4	fer-1 like family member 4 (pseudogene)	9.71	3.28	9.79E-13	1.42E-10
ENSG00000113088	GZMK	granzyme K	9.65	3.27	7.76E-09	5.13E-07
ENSG00000127324	TSPAN8	tetraspanin 8	9.65	3.27	3.48E-05	7.37E-04
ENSG00000256582	RP11-75L1.1	long intergenic non-protein coding RNA 2390	9.65	3.27	2.15E-04	3.23E-03
ENSG00000187808	SOWAHD	sosondowah ankyrin repeat domain family member D	9.65	3.27	2.65E-04	3.82E-03
ENSG00000232871	SEC1P	secretory blood group 1, pseudogene	9.65	3.27	3.21E-04	4.45E-03
ENSG00000126264	HCST	hematopoietic cell signal transducer	9.51	3.25	3.61E-22	2.59E-19
ENSG00000231128	RP5-1073O3.2	novel transcript, antisense to PTPN22	9.51	3.25	2.33E-04	3.46E-03
ENSG00000253686	CTB-43E15.3	long intergenic non-protein coding RNA 1484	9.51	3.25	4.09E-04	5.37E-03
ENSG00000115165	CYTIP	cytohesin 1 interacting protein	9.45	3.24	1.54E-19	7.39E-17
ENSG00000070190	DAPP1	dual adaptor of phosphotyrosine and 3-phosphoinositides 1	9.45	3.24	7.41E-16	1.76E-13
ENSG00000197057	DTHD1	death domain containing 1	9.45	3.24	2.24E-11	2.52E-09
ENSG00000203876	RP11-451M19.3	ADD3 antisense RNA 1	9.45	3.24	5.31E-05	1.05E-03
ENSG00000230747	AC021188.4	novel transcript	9.38	3.23	1.26E-09	1.01E-07
ENSG00000102970	CCL17	C-C motif chemokine ligand 17	9.38	3.23	4.03E-05	8.30E-04
ENSG00000199592	RNA5SP321	RNA, 5S ribosomal pseudogene 321	9.38	3.23	4.16E-04	5.43E-03
ENSG00000182487	NCF1B	neutrophil cytosolic factor 1B pseudogene	9.32	3.22	3.19E-16	8.27E-14
ENSG00000086300	SNX10	sorting nexin 10	9.32	3.22	5.50E-14	9.84E-12
ENSG00000160791	CCR5	C-C motif chemokine receptor 5	9.32	3.22	3.46E-12	4.60E-10
ENSG00000235785	AL109767.1	novel transcript	9.32	3.22	1.19E-04	2.02E-03
ENSG00000070915	SLC12A3	solute carrier family 12 member 3	9.32	3.22	1.16E-03	1.18E-02
ENSG00000225783	MIAT	myocardial infarction associated transcript	9.25	3.21	4.72E-19	2.02E-16

ENSG00000255026	RP11-326C3.2	novel transcript	9.25	3.21	9.86E-08	4.94E-06
ENSG00000235833	AC159540.14	coiled-coil domain containing 144C (CCDC144C) pseudogene	9.25	3.21	6.51E-06	1.87E-04
ENSG00000259772	RP11-16E12.2	novel transcript	9.19	3.2	1.36E-09	1.08E-07
ENSG00000163568	AIM2	absent in melanoma 2	9.19	3.2	5.74E-09	3.95E-07
ENSG00000100346	CACNA1I	calcium voltage-gated channel subunit alpha1 I	9.13	3.19	5.66E-07	2.27E-05
ENSG00000266999	AC015849.16		9.06	3.18	2.89E-04	4.09E-03
ENSG00000176919	C8G	complement C8 gamma chain	-9	-3.17	1.26E-12	1.81E-10
ENSG00000183347	GBP6	guanylate binding protein family member 6	9	3.17	1.19E-05	3.07E-04
ENSG00000102096	PIM2	Pim-2 proto-oncogene, serine/threonine kinase	8.94	3.16	6.19E-24	5.78E-21
ENSG00000167895	TMC8	transmembrane channel like 8	8.94	3.16	5.16E-23	4.19E-20
ENSG00000082074	FYB	FYN binding protein 1	8.94	3.16	9.56E-18	3.27E-15
ENSG00000100453	GZMB	granzyme B	8.94	3.16	2.87E-12	3.91E-10
ENSG00000225460	RP13-93L13.1	ubiquitin-conjugating enzyme E2 pseudogene	8.94	3.16	1.39E-05	3.47E-04
ENSG00000270933	CTD-2227E11.1	novel transcript	8.94	3.16	2.54E-04	3.71E-03
ENSG00000124875	CXCL6	C-X-C motif chemokine ligand 6	-8.94	-3.16	1.87E-03	1.70E-02
ENSG00000164691	TAGAP	T cell activation RhoGTPase activating protein	8.88	3.15	1.00E-17	3.40E-15
ENSG00000158714	SLAMF8	SLAM family member 8	8.88	3.15	5.42E-14	9.77E-12
ENSG00000204655	MOG	myelin oligodendrocyte glycoprotein	-8.88	-3.15	1.19E-11	1.44E-09
ENSG00000227507	LTB	lymphotoxin beta	8.88	3.15	5.38E-09	3.77E-07
ENSG00000022556	NLRP2	NLR family pyrin domain containing 2	8.88	3.15	2.23E-05	5.10E-04
ENSG00000223750	SIRPB3P	signal regulatory protein beta 3, pseudogene	8.88	3.15	1.58E-03	1.48E-02
ENSG00000214787	MS4A4E	membrane spanning 4-domains A4E	8.82	3.14	1.86E-19	8.69E-17
ENSG00000213402	PTPRCAP	protein tyrosine phosphatase receptor type C associated protein	8.82	3.14	1.26E-17	4.18E-15
ENSG00000074706	IPCEF1	interaction protein for cytohesin exchange factors 1	8.82	3.14	2.30E-17	7.01E-15
ENSG00000149527	PLCH2	phospholipase C eta 2	8.82	3.14	3.77E-13	6.05E-11

ENSG00000048462	TNFRSF17	TNF receptor superfamily member 17	8.82	3.14	6.11E-06	1.77E-04
ENSG00000147138	GPR174	G protein-coupled receptor 174	8.82	3.14	1.93E-05	4.54E-04
ENSG00000265154	MIR151B	microRNA 151b	8.82	3.14	1.18E-04	2.01E-03
ENSG00000118322	ATP10B	ATPase phospholipid transporting 10B (putative)	8.75	3.13	3.67E-10	3.32E-08
ENSG00000165682	CLEC1B	C-type lectin domain family 1 member B	8.75	3.13	3.85E-05	8.01E-04
ENSG00000259342	RP11-519G16.5	novel transcript, antisense to C15orf48	-8.75	-3.13	6.30E-05	1.20E-03
ENSG00000236928	RP11-792A8.1	GrpE-like 1, mitochondrial (E. coli) (GRPEL1) pseudogene	8.75	3.13	4.51E-04	5.77E-03
ENSG00000169442	CD52	CD52 molecule	8.69	3.12	1.31E-17	4.28E-15
ENSG00000188305	C19orf35	PEAK family member 3	8.69	3.12	3.81E-13	6.09E-11
ENSG00000235994	RP3-470B24.5	novel transcript	-8.69	-3.12	5.12E-06	1.51E-04
ENSG00000179840	C1orf200	PIK3CD antisense RNA 1	8.69	3.12	2.99E-04	4.21E-03
ENSG00000261635	RP11-618N24.1	tropomyosin 3 (TPM3) pseudogene	-8.63	-3.11	3.59E-06	1.13E-04
ENSG00000252105	RNU1-143P	RNA, U1 small nuclear 143, pseudogene	8.63	3.11	1.80E-04	2.82E-03
ENSG00000171759	PAH	phenylalanine hydroxylase	8.63	3.11	4.85E-04	6.10E-03
ENSG00000180644	PRF1	perforin 1	8.57	3.1	5.88E-28	1.01E-24
ENSG00000121807	CCR2	C-C motif chemokine receptor 2	8.51	3.09	8.93E-17	2.48E-14
ENSG00000132185	FCRLA	Fc receptor like A	8.51	3.09	2.18E-06	7.39E-05
ENSG00000183918	SH2D1A	SH2 domain containing 1A	8.46	3.08	1.51E-08	9.26E-07
ENSG00000147889	CDKN2A	cyclin dependent kinase inhibitor 2A	8.46	3.08	1.54E-06	5.39E-05
ENSG00000162374	ELAVL4	ELAV like RNA binding protein 4	8.4	3.07	5.19E-04	6.44E-03
ENSG00000254872	RP13-870H17.3	long intergenic non-protein coding RNA 2688	8.4	3.07	8.19E-04	8.98E-03
ENSG00000185905	C16orf54	chromosome 16 open reading frame 54	8.34	3.06	2.90E-18	1.08E-15
ENSG00000178199	ZC3H12D	zinc finger CCCH-type containing 12D	8.34	3.06	7.76E-16	1.82E-13
ENSG00000041353	RAB27B	RAB27B, member RAS oncogene family	8.34	3.06	1.36E-11	1.60E-09
ENSG00000196533	C1orf186		8.34	3.06	1.59E-09	1.24E-07

ENSG00000006555	TTC22	tetratricopeptide repeat domain 22	8.34	3.06	2.86E-09	2.13E-07
ENSG00000160183	TMPRSS3	transmembrane serine protease 3	8.34	3.06	1.45E-04	2.37E-03
ENSG00000263155	MYZAP	myocardial zonula adherens protein	-8.28	-3.05	1.27E-14	2.44E-12
ENSG00000102962	CCL22	C-C motif chemokine ligand 22	8.28	3.05	4.68E-06	1.40E-04
ENSG00000142512	SIGLEC10	sialic acid binding Ig like lectin 10	8.28	3.05	1.86E-05	4.40E-04
ENSG00000224904	RP5-934G17.6	SBF1 pseudogene 2	-8.28	-3.05	8.71E-04	9.44E-03
ENSG00000090104	RGS1	regulator of G protein signaling 1	8.22	3.04	1.97E-12	2.75E-10
ENSG00000164089	ETNPPL	ethanolamine-phosphate phospho-lyase	-8.22	-3.04	2.72E-11	3.00E-09
ENSG00000253702	RP11-567J20.1	long intergenic non-protein coding RNA 2847	8.22	3.04	1.84E-04	2.86E-03
ENSG00000150637	CD226	CD226 molecule	8.17	3.03	3.14E-15	6.73E-13
ENSG00000220008	LINGO3	leucine rich repeat and Ig domain containing 3	8.17	3.03	1.05E-08	6.74E-07
ENSG00000174429	ABRA	actin binding Rho activating protein	-8.17	-3.03	1.76E-06	6.09E-05
ENSG00000188869	TMC3	transmembrane channel like 3	8.17	3.03	1.36E-05	3.39E-04
ENSG00000223695	RP4-633O19__A.1	MYH9 divergent transcript	-8.17	-3.03	1.49E-05	3.67E-04
ENSG00000186335	SLC36A2	solute carrier family 36 member 2	-8.17	-3.03	4.53E-05	9.19E-04
ENSG00000229162	RP11-84D1.1	RUNX3 antisense RNA 1	8.17	3.03	5.68E-04	6.84E-03
ENSG00000158481	CD1C	CD1c molecule	8.11	3.02	2.51E-08	1.48E-06
ENSG00000013588	GPRC5A	G protein-coupled receptor class C group 5 member A	-8.11	-3.02	5.44E-07	2.20E-05
ENSG00000234299	CDK2AP2P1		8.11	3.02	8.06E-04	8.89E-03
ENSG00000259277	RP13-126C7.1	novel transcript	8.11	3.02	1.26E-03	1.25E-02
ENSG00000243323	PTPRVP	protein tyrosine phosphatase receptor type V, pseudogene	8	3	1.98E-07	8.99E-06
ENSG00000123901	GPR83	G protein-coupled receptor 83	8	3	2.53E-06	8.38E-05
ENSG00000135898	GPR55	G protein-coupled receptor 55	7.94	2.99	1.36E-05	3.40E-04
ENSG00000163534	FCRL1	Fc receptor like 1	7.89	2.98	3.02E-06	9.70E-05
ENSG00000079263	SP140	SP140 nuclear body protein	7.84	2.97	6.10E-20	3.08E-17
ENSG00000198821	CD247	CD247 molecule	7.84	2.97	1.95E-12	2.73E-10

ENSG00000125910	S1PR4	sphingosine-1-phosphate receptor 4	7.84	2.97	1.31E-11	1.57E-09
ENSG00000272908	RP11-121A8.1	novel transcript	7.84	2.97	2.31E-07	1.03E-05
ENSG00000156475	PPP2R2B	protein phosphatase 2 regulatory subunit Bbeta	7.84	2.97	1.24E-05	3.15E-04
ENSG00000088002	SULT2B1	sulfotransferase family 2B member 1	7.84	2.97	4.06E-04	5.34E-03
ENSG00000255987	RP11-1094M14.4	TOMM20 pseudogene 2	7.78	2.96	1.32E-09	1.05E-07
ENSG00000108691	CCL2	C-C motif chemokine ligand 2	-7.73	-2.95	4.48E-05	9.12E-04
ENSG00000267554	RP11-686D22.10	RAB32, member RAS oncogene family (RAB32) pseudogene	7.67	2.94	4.90E-09	3.51E-07
ENSG00000228655	AC096558.1	novel transcript, antisense to ARHGAP15	7.62	2.93	3.82E-10	3.44E-08
ENSG00000243544	RN7SL172P	RNA, 7SL, cytoplasmic 172, pseudogene	7.62	2.93	1.72E-05	4.14E-04
ENSG00000118849	RARRES1	retinoic acid receptor responder 1	-7.62	-2.93	2.00E-05	4.67E-04
ENSG00000100427	MLC1	modulator of VRAC current 1	7.62	2.93	2.85E-04	4.04E-03
ENSG00000089692	LAG3	lymphocyte activating 3	7.57	2.92	7.12E-09	4.74E-07
ENSG00000101057	MYBL2	MYB proto-oncogene like 2	7.57	2.92	2.45E-05	5.49E-04
ENSG00000184557	SOCS3	suppressor of cytokine signaling 3	-7.57	-2.92	6.76E-05	1.27E-03
ENSG00000182489	XKRX	XK related X-linked	7.57	2.92	1.85E-04	2.87E-03
ENSG00000232953	HSPA8P18	heat shock protein family A (Hsp70) member 8 pseudogene 18	7.57	2.92	9.29E-04	9.90E-03
ENSG00000163273	NPPC	natriuretic peptide C	-7.52	-2.91	5.66E-06	1.65E-04
ENSG00000229367	HMGN2P19	high mobility group nucleosomal binding domain 2 pseudogene 19	-7.52	-2.91	1.75E-03	1.60E-02
ENSG00000260217	RP11-809F4.3	novel transcript	7.46	2.9	1.43E-03	1.37E-02
ENSG00000136492	BRIP1	BRCA1 interacting helicase 1	7.41	2.89	1.08E-08	6.89E-07
ENSG00000267046	RP11-1094M14.9	E2F transcription factor 3 pseudogene 1	7.41	2.89	1.90E-07	8.73E-06
ENSG00000259686	HNRNPA1P71	heterogeneous nuclear ribonucleoprotein A1 pseudogene 71	7.41	2.89	3.72E-06	1.16E-04
ENSG00000146070	PLA2G7	phospholipase A2 group VII	7.36	2.88	1.32E-07	6.44E-06

ENSG00000166091	CMTM5	CKLF like MARVEL transmembrane domain containing 5	-7.36	-2.88	4.02E-05	8.29E-04
ENSG00000268027	AC006129.2	novel transcript, sense intronic to CEACAM21	7.31	2.87	5.30E-10	4.63E-08
ENSG00000165178	NCF1C	neutrophil cytosolic factor 1C pseudogene	7.31	2.87	2.06E-09	1.57E-07
ENSG00000009790	TRAF3IP3	TRAF3 interacting protein 3	7.26	2.86	4.60E-15	9.58E-13
ENSG00000096996	IL12RB1	interleukin 12 receptor subunit beta 1	7.26	2.86	1.03E-09	8.59E-08
ENSG00000228601	RPL39P	ribosomal protein L39 pseudogene	7.26	2.86	3.58E-07	1.52E-05
ENSG00000237593	RP11-317B3.2	ribosomal protein L17 (RPL17) pseudogene	7.21	2.85	1.95E-05	4.57E-04
ENSG00000158077	NLRP14	NLR family pyrin domain containing 14	7.21	2.85	1.60E-04	2.57E-03
ENSG00000272282	RP11-222K16.2	long intergenic non-protein coding RNA 2084	7.21	2.85	1.66E-04	2.63E-03
ENSG00000226675	RP11-666A1.3		7.21	2.85	1.18E-03	1.19E-02
ENSG00000101082	SLA2	Src like adaptor 2	7.16	2.84	1.83E-14	3.41E-12
ENSG00000198502	HLA-DRB5	major histocompatibility complex, class II, DR beta 5	7.16	2.84	4.47E-06	1.34E-04
ENSG00000261315	LARP4P	LARP4 pseudogene	-7.16	-2.84	1.24E-05	3.15E-04
ENSG00000101892	ATP1B4	ATPase Na+/K+ transporting family member beta 4	7.16	2.84	7.75E-04	8.63E-03
ENSG00000250290	CTC-820M8.1	non-SMC condensin I complex subunit G pseudogene 1	7.16	2.84	1.05E-03	1.09E-02
ENSG00000124721	DNAH8	dynein axonemal heavy chain 8	7.11	2.83	1.12E-06	4.09E-05
ENSG00000225792	AC004540.4	SNX10 antisense RNA 1	7.11	2.83	2.13E-03	1.87E-02
ENSG00000213262	AL365331.2	VDAC2 pseudogene 3	7.06	2.82	8.26E-04	9.05E-03
ENSG00000254815	RP11-496I9.1	LMNTD2 antisense RNA 1	7.06	2.82	1.16E-03	1.18E-02
ENSG00000130475	FCHO1	FCH and mu domain containing endocytic adaptor 1	7.01	2.81	2.58E-13	4.28E-11
ENSG00000183742	MACC1	MET transcriptional regulator MACC1	7.01	2.81	4.23E-08	2.34E-06
ENSG00000198216	CACNA1E	calcium voltage-gated channel subunit alpha1 E	-7.01	-2.81	1.34E-06	4.75E-05
ENSG00000261008	AC004158.2	long intergenic non-protein coding RNA 1572	7.01	2.81	1.03E-04	1.79E-03

ENSG00000240487	RP11-553L6.3	FAM214B pseudogene 1	7.01	2.81	1.11E-04	1.91E-03
ENSG00000169248	CXCL11	C-X-C motif chemokine ligand 11	7.01	2.81	4.37E-04	5.63E-03
ENSG00000174255	ZNF80	zinc finger protein 80	7.01	2.81	6.69E-04	7.75E-03
ENSG00000135127	CCDC64	BICD family like cargo adaptor 1	6.96	2.8	1.32E-10	1.28E-08
ENSG00000167780	SOAT2	sterol O-acyltransferase 2	6.96	2.8	1.55E-03	1.46E-02
ENSG00000239636	RP4-728D4.2	TFAP2E antisense RNA 1	6.92	2.79	1.29E-06	4.62E-05
ENSG00000166501	PRKCB	protein kinase C beta	6.87	2.78	3.09E-15	6.70E-13
ENSG00000197471	SPN	sialophorin	6.87	2.78	1.06E-14	2.07E-12
ENSG00000167208	SNX20	sorting nexin 20	6.87	2.78	1.96E-14	3.64E-12
ENSG00000231758	AC092652.1	ARHGAP15 antisense RNA 1	6.87	2.78	4.61E-09	3.33E-07
ENSG00000163600	ICOS	inducible T cell costimulator	6.87	2.78	2.64E-04	3.82E-03
ENSG00000229757	RP4-738P11.4	ribosomal protein L7a (RPL7A) pseudogene	6.87	2.78	8.07E-04	8.89E-03
ENSG00000224689	ZNF812	zinc finger protein 812, pseudogene	6.82	2.77	4.91E-16	1.22E-13
ENSG00000143851	PTPN7	protein tyrosine phosphatase non-receptor type 7	6.82	2.77	2.58E-12	3.53E-10
ENSG00000215559	ANKRD20A11P	ankyrin repeat domain 20 family member A11, pseudogene	6.82	2.77	5.62E-08	2.98E-06
ENSG00000110934	BIN2	bridging integrator 2	6.77	2.76	5.12E-17	1.48E-14
ENSG00000023892	DEF6	DEF6 guanine nucleotide exchange factor	6.77	2.76	4.76E-16	1.19E-13
ENSG00000173200	PARP15	poly(ADP-ribose) polymerase family member 15	6.77	2.76	4.77E-13	7.39E-11
ENSG00000166278	C2	complement C2	6.77	2.76	5.62E-13	8.65E-11
ENSG00000156127	BATF	basic leucine zipper ATF-like transcription factor	6.77	2.76	6.98E-09	4.66E-07
ENSG00000253651	SOD1P3	superoxide dismutase 1 pseudogene 3	6.77	2.76	7.07E-04	8.09E-03
ENSG00000087589	CASS4	Cas scaffold protein family member 4	6.73	2.75	4.74E-23	3.95E-20
ENSG00000168334	XIRP1	xin actin binding repeat containing 1	-6.73	-2.75	1.51E-11	1.76E-09
ENSG00000237943	PRKCQ-AS1	PRKCQ antisense RNA 1	6.73	2.75	2.86E-08	1.66E-06
ENSG00000187862	TTC24	tetratricopeptide repeat domain 24	6.73	2.75	5.59E-06	1.64E-04
ENSG00000197991	PCDH20	novel protein	-6.68	-2.74	3.91E-13	6.21E-11

ENSG00000225528	RP3-370M22.8	novel protein similar to translation machinery associated 7 homolog (S. cerevisiae) TMA7	6.68	2.74	7.73E-05	1.42E-03
ENSG00000159904	ZNF890P	zinc finger protein 890, pseudogene	6.68	2.74	2.56E-03	2.15E-02
ENSG00000185112	FAM43A	family with sequence similarity 43 member A	-6.63	-2.73	1.52E-10	1.46E-08
ENSG00000257284	RP11-190J23.1	novel transcript, antisense to ARHGAP15	6.63	2.73	7.70E-08	4.02E-06
ENSG00000182611	HIST1H2AJ		6.63	2.73	1.08E-07	5.37E-06
ENSG00000241717	VWFP1	von Willebrand factor pseudogene 1	6.63	2.73	1.40E-04	2.31E-03
ENSG00000235192	AC009495.2	novel transcript	6.63	2.73	3.77E-04	5.05E-03
ENSG00000261218	RP11-960L18.1	novel transcript	6.63	2.73	1.21E-03	1.21E-02
ENSG00000248599	RP11-302J23.1	uncharacterized LOC441374	6.59	2.72	3.59E-07	1.52E-05
ENSG00000232979	AC092580.2	pseudogene similar to part of proteasome (prosome, macropain) subunit, beta type, 1 (PSMB1)	6.59	2.72	1.81E-05	4.32E-04
ENSG00000150625	GPM6A	glycoprotein M6A	6.59	2.72	1.92E-05	4.53E-04
ENSG00000247193	RP11-431M7.3	novel transcript, antisense to CENTD1	6.59	2.72	1.40E-03	1.36E-02
ENSG00000116852	KIF21B	kinesin family member 21B	6.54	2.71	5.15E-17	1.48E-14
ENSG00000111796	KLRB1	killer cell lectin like receptor B1	6.54	2.71	8.00E-07	3.08E-05
ENSG00000237567	RP3-359N14.2	long intergenic non-protein coding RNA 2836	6.54	2.71	2.77E-03	2.28E-02
ENSG00000151883	PARP8	poly(ADP-ribose) polymerase family member 8	6.5	2.7	7.34E-19	3.06E-16
ENSG00000095585	BLNK	B cell linker	6.5	2.7	9.06E-17	2.49E-14
ENSG00000146192	FGD2	FYVE, RhoGEF and PH domain containing 2	6.5	2.7	5.98E-16	1.45E-13
ENSG00000167261	DPEP2	dipeptidase 2	6.5	2.7	2.08E-12	2.87E-10
ENSG00000143452	HORMAD1	HORMA domain containing 1	6.5	2.7	1.02E-03	1.06E-02
ENSG00000159753	RLTPR	capping protein regulator and myosin 1 linker 2	6.45	2.69	3.08E-13	5.02E-11
ENSG00000271856	RP11-861A13.4	long intergenic non-protein coding RNA 1215	6.45	2.69	8.79E-05	1.58E-03

ENSG00000104848	KCNA7	potassium voltage-gated channel subfamily A member 7	-6.45	-2.69	4.67E-04	5.93E-03
ENSG00000229474	PATL2	PAT1 homolog 2	6.41	2.68	8.37E-12	1.04E-09
ENSG00000261191	RP11-16L14.2	novel transcript, antisense to C15orf41	-6.41	-2.68	8.84E-06	2.40E-04
ENSG00000165383	LRRC18	leucine rich repeat containing 18	6.36	2.67	4.12E-07	1.72E-05
ENSG00000181291	TMEM132E	transmembrane protein 132E	6.36	2.67	1.26E-04	2.12E-03
ENSG00000081237	PTPRC	protein tyrosine phosphatase receptor type C	6.32	2.66	2.48E-15	5.54E-13
ENSG00000171476	HOPX	HOP homeobox	-6.32	-2.66	1.40E-09	1.11E-07
ENSG00000143184	XCL1	X-C motif chemokine ligand 1	6.32	2.66	2.10E-06	7.16E-05
ENSG00000156510	HKDC1	hexokinase domain containing 1	6.32	2.66	1.21E-05	3.11E-04
ENSG00000260582	TPST2P1	tyrosylprotein sulfotransferase 2 pseudogene 1	-6.32	-2.66	3.55E-05	7.49E-04
ENSG00000225039	LINC01058	long intergenic non-protein coding RNA 1058	6.32	2.66	1.56E-03	1.46E-02
ENSG00000253603	CTA-397H3.3	competing endogenous lncRNA 3 for miR-645	-6.32	-2.66	3.42E-03	2.66E-02
ENSG00000115232	ITGA4	integrin subunit alpha 4	6.28	2.65	5.40E-16	1.33E-13
ENSG00000223414	LINC00473		-6.28	-2.65	7.21E-09	4.79E-07
ENSG00000237940	AC093642.3	long intergenic non-protein coding RNA 1238	6.28	2.65	1.23E-06	4.45E-05
ENSG00000177669	MBOAT4	membrane bound O-acyltransferase domain containing 4	6.28	2.65	2.94E-05	6.40E-04
ENSG00000259598	RP11-275I4.1	novel transcript, antisense to RASGRP1	6.28	2.65	3.09E-04	4.32E-03
ENSG00000121053	EPX	eosinophil peroxidase	6.28	2.65	6.10E-04	7.22E-03
ENSG00000185792	NLRP9	NLR family pyrin domain containing 9	6.28	2.65	1.33E-03	1.30E-02
ENSG00000148702	HABP2	hyaluronan binding protein 2	-6.23	-2.64	4.75E-05	9.53E-04
ENSG00000230175	RP11-466F5.3	ribosomal protein S13 (RPS13) pseudogene	6.23	2.64	1.08E-03	1.11E-02
ENSG00000218991	CCNG1P1	cyclin G1 pseudogene 1	6.23	2.64	3.03E-03	2.44E-02
ENSG00000196735	HLA-DQA1	major histocompatibility complex, class II, DQ alpha 1	6.19	2.63	2.61E-10	2.40E-08
ENSG00000105246	EBI3	Epstein-Barr virus induced 3	6.19	2.63	2.16E-08	1.30E-06

ENSG00000266705	MIR4437	microRNA 4437	6.19	2.63	2.12E-03	1.87E-02
ENSG00000077984	CST7	cystatin F	6.15	2.62	6.85E-16	1.65E-13
ENSG00000237840	FAM21FP	family with sequence similarity 21 member F, pseudogene	6.15	2.62	1.94E-05	4.56E-04
ENSG00000130487	KLHDC7B	kelch domain containing 7B	6.15	2.62	3.99E-05	8.25E-04
ENSG00000054938	CHRDL2	chordin like 2	-6.15	-2.62	8.33E-05	1.52E-03
ENSG00000100985	MMP9	matrix metallopeptidase 9	6.15	2.62	2.84E-04	4.03E-03
ENSG00000111536	IL26	interleukin 26	6.15	2.62	3.08E-04	4.31E-03
ENSG00000139970	RTN1	reticulon 1	6.11	2.61	8.84E-15	1.78E-12
ENSG00000185760	KCNQ5	potassium voltage-gated channel subfamily Q member 5	6.11	2.61	7.72E-05	1.42E-03
ENSG00000066294	CD84	CD84 molecule	6.06	2.6	4.62E-14	8.42E-12
ENSG00000105967	TFEC	transcription factor EC	6.06	2.6	3.23E-12	4.31E-10
ENSG00000205436	EXOC3L4	exocyst complex component 3 like 4	6.06	2.6	8.63E-08	4.44E-06
ENSG00000271725	RP11-761I4.4	novel transcript	6.06	2.6	1.90E-04	2.93E-03
ENSG00000254900	RP11-152H18.4	novel transcript	6.06	2.6	2.78E-04	3.97E-03
ENSG00000147234	FRMPD3	FERM and PDZ domain containing 3	6.02	2.59	4.54E-07	1.87E-05
ENSG00000226979	LTA	lymphotoxin alpha	6.02	2.59	1.26E-03	1.25E-02
ENSG00000188452	CERKL	ceramide kinase like	5.98	2.58	1.43E-11	1.67E-09
ENSG00000102802	MEDAG	mesenteric estrogen dependent adipogenesis	-5.98	-2.58	2.41E-08	1.43E-06
ENSG00000110876	SELPLG	selectin P ligand	5.94	2.57	2.08E-18	7.87E-16
ENSG00000267369	RP11-1094M14.8	TATA-box binding protein associated factor 5 like pseudogene 1	5.94	2.57	5.28E-09	3.72E-07
ENSG00000073737	DHRS9	dehydrogenase/reductase 9	5.94	2.57	1.66E-08	1.01E-06
ENSG00000215863	LINC01138		5.94	2.57	2.64E-03	2.19E-02
ENSG00000135905	DOCK10	dedicator of cytokinesis 10	5.9	2.56	3.23E-17	9.65E-15
ENSG00000128340	RAC2	Rac family small GTPase 2	5.9	2.56	5.75E-13	8.81E-11
ENSG00000132514	CLEC10A	C-type lectin domain containing 10A	5.9	2.56	6.96E-13	1.04E-10
ENSG00000105122	RASAL3	RAS protein activator like 3	5.9	2.56	4.05E-12	5.29E-10
ENSG00000236278	PEBP1P3	phosphatidylethanolamine binding protein 1 pseudogene 3	5.9	2.56	2.44E-07	1.08E-05
ENSG00000162772	ATF3	activating transcription factor 3	-5.9	-2.56	1.75E-05	4.19E-04
ENSG00000254205	RP11-92K15.1	novel transcript	5.9	2.56	9.60E-04	1.02E-02

ENSG00000257829	RP11-845M18.6	novel transcript, antisense to KRT86	5.9	2.56	1.64E-03	1.52E-02
ENSG00000106948	AKNA	AT-hook transcription factor	5.86	2.55	6.51E-18	2.25E-15
ENSG00000112799	LY86	lymphocyte antigen 86	5.86	2.55	9.17E-09	5.92E-07
ENSG00000261573	RP11-553K8.5	novel transcript	5.86	2.55	5.47E-07	2.20E-05
ENSG00000272899	RP11-309L24.9	ATP6V1F neighbor	-5.86	-2.55	3.82E-05	7.96E-04
ENSG00000250061	RP11-541P9.3		-5.86	-2.55	1.55E-04	2.51E-03
ENSG00000227403	AC009299.3	long intergenic non-protein coding RNA 1806	5.86	2.55	5.46E-04	6.66E-03
ENSG00000188596	C12orf55	cilia and flagella associated protein 54	5.82	2.54	1.23E-13	2.10E-11
ENSG00000137801	THBS1	thrombospondin 1	-5.82	-2.54	1.27E-06	4.57E-05
ENSG00000071909	MYO3B	myosin IIIB	5.82	2.54	2.07E-05	4.78E-04
ENSG00000269404	SPIB	Spi-B transcription factor	5.82	2.54	1.77E-03	1.62E-02
ENSG00000140368	PSTPIP1	proline-serine-threonine phosphatase interacting protein 1	5.78	2.53	1.67E-14	3.18E-12
ENSG00000205744	DENND1C	DENN domain containing 1C	5.78	2.53	3.16E-11	3.42E-09
ENSG00000181631	P2RY13	purinergic receptor P2Y13	5.78	2.53	1.87E-09	1.43E-07
ENSG00000273259	RP11-986E7.7	novel protein	-5.78	-2.53	1.93E-04	2.97E-03
ENSG00000221299	Z83826.1		-5.78	-2.53	2.20E-04	3.29E-03
ENSG00000240007	RP6-206I17.4		5.78	2.53	5.62E-04	6.79E-03
ENSG00000221476	MIR1827	microRNA 1827	5.78	2.53	2.16E-03	1.89E-02
ENSG00000077420	APBB1IP	amyloid beta precursor protein binding family B member 1 interacting protein	5.74	2.52	1.53E-12	2.16E-10
ENSG00000130176	CNN1	calponin 1	-5.74	-2.52	8.68E-09	5.64E-07
ENSG00000187037	GPR141	G protein-coupled receptor 141	5.74	2.52	4.86E-06	1.44E-04
ENSG00000172322	CLEC12A	C-type lectin domain family 12 member A	5.74	2.52	6.00E-06	1.74E-04
ENSG00000177807	KCNJ10	potassium inwardly rectifying channel subfamily J member 10	5.74	2.52	1.65E-04	2.63E-03
ENSG00000137101	CD72	CD72 molecule	5.7	2.51	6.91E-07	2.72E-05
ENSG00000064547	LPAR2	lysophosphatidic acid receptor 2	5.66	2.5	2.08E-11	2.40E-09
ENSG00000101916	TLR8	toll like receptor 8	5.66	2.5	2.09E-08	1.26E-06
ENSG00000104972	LILRB1	leukocyte immunoglobulin like receptor B1	5.66	2.5	3.29E-08	1.88E-06

ENSG00000267293	RP11-8H2.1	U3 small nucleolar RNA-associated protein 18 homolog, pseudogene	5.66	2.5	6.52E-05	1.24E-03
ENSG00000264386	MIR4513	microRNA 4513	5.66	2.5	3.54E-03	2.74E-02
ENSG00000180096	SEPT1	septin 1	5.62	2.49	6.73E-09	4.52E-07
ENSG00000189350	FAM179A	TOG array regulator of axonemal microtubules 2	-5.62	-2.49	3.42E-05	7.26E-04
ENSG00000226751	AF127936.5		5.58	2.48	7.05E-06	1.99E-04
ENSG00000252985	SNORD116	Small nucleolar RNA SNORD116	5.58	2.48	8.50E-05	1.54E-03
ENSG00000236474	GCNT1P1	glucosaminyl (N-acetyl) transferase 1 pseudogene 1	5.58	2.48	1.26E-03	1.25E-02
ENSG00000229990	RP11-574K11.8	novel transcript, antisense to CAMK2G	5.58	2.48	1.34E-03	1.31E-02
ENSG00000204252	HLA-DOA	major histocompatibility complex, class II, DO alpha	5.54	2.47	1.94E-17	6.02E-15
ENSG00000232815	LINC00537	double homeobox 4 like 50 (pseudogene)	5.54	2.47	1.09E-09	8.97E-08
ENSG00000184292	TACSTD2	tumor associated calcium signal transducer 2	5.54	2.47	4.06E-05	8.35E-04
ENSG00000212456	RNVU1-13		5.54	2.47	1.05E-04	1.83E-03
ENSG00000110324	IL10RA	interleukin 10 receptor subunit alpha	5.5	2.46	8.33E-16	1.93E-13
ENSG00000140968	IRF8	interferon regulatory factor 8	5.5	2.46	7.89E-13	1.16E-10
ENSG00000152229	PSTPIP2	proline-serine-threonine phosphatase interacting protein 2	5.5	2.46	3.94E-11	4.20E-09
ENSG00000117009	KMO	kynurenine 3-monooxygenase	5.5	2.46	1.80E-07	8.32E-06
ENSG00000256937	RP11-436I9.5	keratin 17 pseudogene 8	5.5	2.46	5.88E-07	2.36E-05
ENSG00000226287	TMEM191A	transmembrane protein 191A (pseudogene)	5.5	2.46	4.61E-06	1.38E-04
ENSG00000147570	DNAJC5B	DnaJ heat shock protein family (Hsp40) member C5 beta	5.5	2.46	3.35E-03	2.63E-02
ENSG00000255080	RP11-1082L8.3		5.5	2.46	4.01E-03	2.99E-02
ENSG00000143119	CD53	CD53 molecule	5.46	2.45	9.06E-12	1.12E-09
ENSG00000136167	LCP1	lymphocyte cytosolic protein 1	5.46	2.45	3.74E-10	3.37E-08
ENSG00000186891	TNFRSF18	TNF receptor superfamily member 18	5.46	2.45	1.25E-06	4.50E-05

ENSG00000250654	RP11-834C11.7	novel pseudogene	5.46	2.45	1.51E-03	1.43E-02
ENSG00000269641	CTB-167G5.6		-5.46	-2.45	2.71E-03	2.23E-02
ENSG00000184845	DRD1	dopamine receptor D1	-5.46	-2.45	4.93E-03	3.48E-02
ENSG00000107099	DOCK8	dedicator of cytokinesis 8	5.43	2.44	1.94E-15	4.42E-13
ENSG00000161929	SCIMP	SLP adaptor and CSK interacting membrane protein	5.39	2.43	9.25E-12	1.13E-09
ENSG00000160255	ITGB2	integrin subunit beta 2	5.39	2.43	9.88E-11	9.86E-09
ENSG00000232687	RPL12P9	ribosomal protein L12 pseudogene 9	5.39	2.43	3.98E-03	2.98E-02
ENSG00000155926	SLA	Src like adaptor	5.35	2.42	6.91E-13	1.04E-10
ENSG00000132518	GUCY2D	guanylate cyclase 2D, retinal	5.35	2.42	9.69E-04	1.02E-02
ENSG00000135749	PCNXL2	pecanex 2	5.31	2.41	2.84E-16	7.40E-14
ENSG00000134516	DOCK2	dedicator of cytokinesis 2	5.31	2.41	4.10E-15	8.65E-13
ENSG00000172794	RAB37	RAB37, member RAS oncogene family	5.31	2.41	2.74E-10	2.51E-08
ENSG00000108370	RGS9	regulator of G protein signaling 9	5.31	2.41	4.91E-08	2.66E-06
ENSG00000227777	RP4-738P11.3	ribosomal protein L7a (RPL7A) pseudogene	5.28	2.4	7.60E-04	8.52E-03
ENSG00000180448	HMHA1	Rho GTPase activating protein 45	5.24	2.39	1.67E-16	4.42E-14
ENSG00000010610	CD4	CD4 molecule	5.24	2.39	1.56E-13	2.65E-11
ENSG00000089847	ANKRD24	ankyrin repeat domain 24	5.24	2.39	2.87E-08	1.66E-06
ENSG00000128011	LRFN1	leucine rich repeat and fibronectin type III domain containing 1	5.24	2.39	8.17E-06	2.25E-04
ENSG00000230362	RP11-809F4.2	actin gamma 1 pseudogene 23	5.24	2.39	2.91E-05	6.35E-04
ENSG00000231621	AC013264.2	ANKRD44 antisense RNA 1	5.24	2.39	1.19E-04	2.03E-03
ENSG00000234965	SHISA8	shisa family member 8	-5.24	-2.39	3.95E-03	2.96E-02
ENSG00000145287	PLAC8	placenta associated 8	5.21	2.38	1.12E-09	9.23E-08
ENSG00000007129	CEACAM21	CEA cell adhesion molecule 21	5.21	2.38	4.14E-08	2.29E-06
ENSG00000271631	RP11-408O19.5	novel transcript, antisense to SLC46A2	5.21	2.38	2.50E-03	2.10E-02
ENSG00000122122	SASH3	SAM and SH3 domain containing 3	5.17	2.37	3.36E-11	3.62E-09
ENSG00000115523	GNLY	granulysin	5.17	2.37	6.69E-11	6.85E-09
ENSG00000185338	SOCS1	suppressor of cytokine signaling 1	5.17	2.37	4.20E-09	3.05E-07
ENSG00000165175	MID1IP1	MID1 interacting protein 1	-5.17	-2.37	7.88E-09	5.20E-07

ENSG00000175505	CLCF1	cardiotrophin like cytokine factor 1	-5.17	-2.37	3.60E-06	1.13E-04
ENSG00000254449	SF3A3P2	splicing factor 3a, subunit 3 pseudogene 2	-5.17	-2.37	6.06E-05	1.17E-03
ENSG00000187398	LUZP2	leucine zipper protein 2	5.17	2.37	7.68E-04	8.59E-03
ENSG00000214514	KRT42P	keratin 42, pseudogene	5.17	2.37	2.56E-03	2.14E-02
ENSG00000267539	RP11-138H8.7		5.17	2.37	3.72E-03	2.83E-02
ENSG00000138964	PARVG	parvin gamma	5.13	2.36	1.05E-14	2.06E-12
ENSG00000185862	EVI2B	ecotropic viral integration site 2B	5.13	2.36	3.11E-12	4.18E-10
ENSG00000185522	C11orf35	lamin tail domain containing 2	5.13	2.36	2.59E-05	5.75E-04
ENSG00000165621	OXGR1	oxoglutarate receptor 1	-5.13	-2.36	4.77E-04	6.04E-03
ENSG00000248568	KRT8P48	keratin 8 pseudogene 48	5.13	2.36	9.46E-04	1.01E-02
ENSG00000167634	NLRP7	NLR family pyrin domain containing 7	5.13	2.36	2.66E-03	2.20E-02
ENSG00000237499	RP11-356I2.4	wound and keratinocyte migration associated lncRNA 2	5.1	2.35	6.18E-12	7.81E-10
ENSG00000102879	CORO1A	coronin 1A	5.1	2.35	3.12E-11	3.40E-09
ENSG00000075886	TUBA3D	tubulin alpha 3d	-5.1	-2.35	1.11E-05	2.91E-04
ENSG00000228278	ORM2	orosomucoid 2	5.1	2.35	7.69E-05	1.42E-03
ENSG00000248529	RP11-2O17.2	novel transcript	5.1	2.35	1.11E-03	1.14E-02
ENSG00000244998	CTD-3064M3.4	novel transcript, antisense to PTP4A3	-5.06	-2.34	1.30E-08	8.11E-07
ENSG00000205045	SLFN12L	schlafen family member 12 like	5.06	2.34	2.73E-08	1.60E-06
ENSG00000136541	ERMN	ermin	5.06	2.34	3.39E-08	1.92E-06
ENSG00000246526	RP11-539L10.2	long intergenic non-protein coding RNA 2481	5.06	2.34	3.24E-06	1.03E-04
ENSG00000175643	RMI2	RecQ mediated genome instability 2	5.06	2.34	5.02E-06	1.49E-04
ENSG00000261208	RP11-452D12.1	novel Pumilio domain-containing protein KIAA0020 pseudogene	5.06	2.34	6.57E-06	1.88E-04
ENSG00000156966	B3GNT7	UDP-GlcNAc:betaGal beta-1,3-N-acetylglucosaminyltransferase 7	-5.06	-2.34	1.29E-05	3.26E-04
ENSG00000187479	C11orf96	chromosome 11 open reading frame 96	-5.03	-2.33	1.06E-08	6.77E-07
ENSG00000103056	SMPD3	sphingomyelin phosphodiesterase 3	5.03	2.33	8.94E-08	4.57E-06

ENSG00000269220	LINC00528	long intergenic non-protein coding RNA 528	5.03	2.33	4.05E-06	1.24E-04
ENSG00000165171	WBSCR27	methyltransferase like 27	5.03	2.33	1.86E-05	4.40E-04
ENSG00000253669	KB-1732A1.1	growth arrest associated lncRNA 1	-5.03	-2.33	3.51E-05	7.43E-04
ENSG00000261439	CTD-2050B12.2	GNAO1 antisense RNA 1	5.03	2.33	1.20E-04	2.03E-03
ENSG00000225978	HAR1A	highly accelerated region 1A	5.03	2.33	5.28E-04	6.52E-03
ENSG00000124935	SCGB1D2	secretoglobin family 1D member 2	-5.03	-2.33	1.29E-03	1.27E-02
ENSG00000254838	GVINP1	GTPase, very large interferon inducible pseudogene 1	4.99	2.32	6.95E-13	1.04E-10
ENSG00000139438	FAM222A	family with sequence similarity 222 member A	-4.99	-2.32	3.81E-09	2.79E-07
ENSG00000258733	CTD-2341M24.1	long intergenic non-protein coding RNA 2328	4.99	2.32	3.51E-08	1.97E-06
ENSG00000231933	CTA-125H2.2	MYO18B antisense RNA 1	-4.99	-2.32	3.98E-07	1.67E-05
ENSG00000236467	RP11-443A13.5	KCNMA1 antisense RNA 1	4.99	2.32	2.77E-05	6.10E-04
ENSG00000147378	FATE1	fetal and adult testis expressed 1	4.99	2.32	1.25E-04	2.11E-03
ENSG00000064787	BCAS1	brain enriched myelin associated protein 1	4.99	2.32	2.48E-03	2.09E-02
ENSG00000214093	RP11-247I13.3	ribosomal protein S18 (RPS18) pseudogene	4.99	2.32	3.28E-03	2.59E-02
ENSG00000170571	EMB	embigin	4.96	2.31	1.17E-19	5.74E-17
ENSG00000168546	GFRA2	GDNF family receptor alpha 2	4.96	2.31	2.23E-19	1.03E-16
ENSG00000183484	GPR132	G protein-coupled receptor 132	4.96	2.31	3.14E-12	4.20E-10
ENSG00000106952	TNFSF8	TNF superfamily member 8	4.96	2.31	5.62E-09	3.88E-07
ENSG00000229677	RP11-383F6.1	ribosomal protein L17 (RPL17) pseudogene	4.96	2.31	1.44E-05	3.57E-04
ENSG00000257289	RP11-611O2.6	guanine nucleotide binding protein, alpha transducing 3 (GNAT3) pseudogene	4.96	2.31	4.59E-05	9.29E-04
ENSG00000260487	RP11-297C4.3	novel transcript, antisense to ITGAL	4.96	2.31	4.16E-03	3.06E-02
ENSG00000128815	WDFY4	WDFY family member 4	4.92	2.3	1.54E-11	1.78E-09
ENSG00000261054	RP11-6O2.4	novel transcript, antisense to SYNM	-4.92	-2.3	4.70E-06	1.40E-04
ENSG00000118513	MYB	MYB proto-oncogene, transcription factor	4.92	2.3	2.68E-04	3.86E-03

ENSG00000230399	RBBP8P1	RBBP8 pseudogene 1	4.92	2.3	3.29E-04	4.53E-03
ENSG00000227681	RP11-307P5.1	novel transcript	4.92	2.3	2.77E-03	2.27E-02
ENSG00000247774	PCED1B-AS1	PCED1B antisense RNA 1	4.89	2.29	2.90E-09	2.15E-07
ENSG00000204767	FAM196B	inhibitory synaptic factor family member 2B	4.89	2.29	1.13E-08	7.13E-07
ENSG00000260727	SLC7A5P1	solute carrier family 7 member 5 pseudogene 1	4.89	2.29	1.61E-07	7.60E-06
ENSG00000222057	RNU4-62P	RNA, U4 small nuclear 62, pseudogene	4.89	2.29	2.67E-05	5.91E-04
ENSG00000261618	RP11-79H23.3	long intergenic non-protein coding RNA 2605	4.89	2.29	1.12E-03	1.14E-02
ENSG00000180353	HCLS1	hematopoietic cell-specific Lyn substrate 1	4.86	2.28	5.98E-16	1.45E-13
ENSG00000135426	TESPA1	thymocyte expressed, positive selection associated 1	4.86	2.28	5.93E-08	3.13E-06
ENSG00000264925	Z98949.1		-4.86	-2.28	2.96E-05	6.45E-04
ENSG00000159307	SCUBE1	signal peptide, CUB domain and EGF like domain containing 1	4.86	2.28	2.39E-04	3.53E-03
ENSG00000251891	RNU7-79P	RNA, U7 small nuclear 79 pseudogene	-4.86	-2.28	6.92E-04	7.98E-03
ENSG00000225342	AC079630.4	LRRK2 divergent transcript	4.86	2.28	1.26E-03	1.25E-02
ENSG00000196550	FAM72A	family with sequence similarity 72 member A	4.82	2.27	7.56E-07	2.94E-05
ENSG00000183831	ANKRD45	ankyrin repeat domain 45	4.82	2.27	5.14E-04	6.38E-03
ENSG00000216009	MIR874	microRNA 874	4.82	2.27	2.12E-03	1.86E-02
ENSG00000261292	RP11-389G6.3	novel transcript	4.82	2.27	3.83E-03	2.89E-02
ENSG00000168071	CCDC88B	coiled-coil domain containing 88B	4.79	2.26	1.14E-16	3.11E-14
ENSG00000172349	IL16	interleukin 16	4.79	2.26	3.97E-15	8.45E-13
ENSG00000184661	CDCA2	cell division cycle associated 2	-4.79	-2.26	7.28E-07	2.84E-05
ENSG00000213071	LPAL2	lipoprotein(a) like 2, pseudogene	4.79	2.26	7.59E-06	2.12E-04
ENSG00000124343	XG	Xg glycoprotein (Xg blood group)	4.79	2.26	3.74E-05	7.81E-04
ENSG00000263806	AL592188.3		4.79	2.26	7.62E-04	8.54E-03
ENSG00000244620	AL122127.25	novel transcript	4.79	2.26	4.19E-03	3.08E-02
ENSG00000125637	PSD4	pleckstrin and Sec7 domain containing 4	4.76	2.25	3.95E-11	4.20E-09

ENSG00000054219	LY75	lymphocyte antigen 75	4.76	2.25	4.31E-10	3.83E-08
ENSG00000123358	NR4A1	nuclear receptor subfamily 4 group A member 1	-4.76	-2.25	2.04E-07	9.24E-06
ENSG00000267230	RP11-376M2.2		-4.76	-2.25	6.78E-04	7.83E-03
ENSG00000263642	MIR4802	microRNA 4802	4.76	2.25	4.33E-03	3.16E-02
ENSG00000112297	AIM1	crystallin beta-gamma domain containing 1	4.72	2.24	4.59E-15	9.58E-13
ENSG00000242052	RP11-190C22.1	ribosomal protein L10 pseudogene 7	-4.72	-2.24	2.58E-07	1.13E-05
ENSG00000172653	C17orf66		4.72	2.24	3.24E-04	4.48E-03
ENSG00000164266	SPINK1	serine peptidase inhibitor Kazal type 1	4.72	2.24	6.28E-04	7.39E-03
ENSG00000236334	PPIAL4G	peptidylprolyl isomerase A like 4G	4.72	2.24	2.10E-03	1.86E-02
ENSG00000223865	HLA-DPB1	major histocompatibility complex, class II, DP beta 1	4.69	2.23	5.96E-13	9.09E-11
ENSG00000106789	CORO2A	coronin 2A	4.69	2.23	1.14E-11	1.39E-09
ENSG00000106785	TRIM14	tripartite motif containing 14	4.69	2.23	3.33E-11	3.60E-09
ENSG00000053524	MCF2L2	MCF.2 cell line derived transforming sequence-like 2	4.69	2.23	4.42E-07	1.82E-05
ENSG00000124713	GNMT	glycine N-methyltransferase	-4.69	-2.23	4.56E-07	1.87E-05
ENSG00000182010	RTKN2	rhotekin 2	4.69	2.23	2.43E-06	8.09E-05
ENSG00000250603	CTC-228N24.2	novel transcript	-4.69	-2.23	6.01E-04	7.14E-03
ENSG00000161905	ALOX15	arachidonate 15-lipoxygenase	4.69	2.23	1.30E-03	1.28E-02
ENSG00000035499	DEPDC1B	DEP domain containing 1B	4.69	2.23	3.32E-03	2.61E-02
ENSG00000010671	BTK	Bruton tyrosine kinase	4.66	2.22	8.40E-12	1.04E-09
ENSG00000197146	AL133458.1		4.66	2.22	7.04E-06	1.99E-04
ENSG00000198223	CSF2RA	colony stimulating factor 2 receptor subunit alpha	4.63	2.21	5.13E-12	6.54E-10
ENSG00000167984	NLRC3	NLR family CARD domain containing 3	4.63	2.21	1.32E-11	1.57E-09
ENSG00000198846	TOX	thymocyte selection associated high mobility group box	4.63	2.21	7.34E-11	7.49E-09
ENSG00000137571	SLCO5A1	solute carrier organic anion transporter family member 5A1	-4.63	-2.21	9.85E-11	9.86E-09
ENSG00000133321	RARRES3	phospholipase A and acyltransferase 4	4.63	2.21	5.46E-08	2.91E-06

ENSG00000265148	BZRAP1-AS1	TSPOAP1, SUPT4H1 and RNF43 antisense RNA 1	4.63	2.21	9.32E-06	2.52E-04
ENSG00000153234	NR4A2	nuclear receptor subfamily 4 group A member 2	-4.63	-2.21	4.17E-04	5.43E-03
ENSG00000196374	HIST1H2BM		4.63	2.21	6.28E-04	7.39E-03
ENSG00000174332	GLIS1	GLIS family zinc finger 1	4.63	2.21	1.13E-03	1.16E-02
ENSG00000056558	TRAF1	TNF receptor associated factor 1	4.59	2.2	1.70E-14	3.20E-12
ENSG00000141506	PIK3R5	phosphoinositide-3-kinase regulatory subunit 5	4.59	2.2	1.35E-11	1.60E-09
ENSG00000232867	RP11-179D22.1	ribosomal protein L36a (RPL36A) pseudogene	-4.59	-2.2	9.15E-05	1.64E-03
ENSG00000237914	RP11-77C3.3	SIRPG antisense RNA 1	4.59	2.2	1.56E-04	2.52E-03
ENSG00000136928	GABBR2	gamma-aminobutyric acid type B receptor subunit 2	-4.59	-2.2	1.65E-04	2.62E-03
ENSG00000175592	FOSL1	FOS like 1, AP-1 transcription factor subunit	-4.59	-2.2	9.28E-04	9.90E-03
ENSG00000238113	RP11-262H14.1	long intergenic non-protein coding RNA 1410	4.56	2.19	1.69E-09	1.30E-07
ENSG00000233098	RP11-344E13.3	CCDC144NL antisense RNA 1	4.56	2.19	2.88E-08	1.66E-06
ENSG00000138795	LEF1	lymphoid enhancer binding factor 1	4.56	2.19	9.13E-08	4.63E-06
ENSG00000164761	TNFRSF11B	TNF receptor superfamily member 11b	-4.56	-2.19	1.38E-07	6.70E-06
ENSG00000182557	SPNS3	sphingolipid transporter 3 (putative)	4.56	2.19	7.70E-06	2.15E-04
ENSG00000189430	NCR1	natural cytotoxicity triggering receptor 1	4.56	2.19	1.54E-05	3.77E-04
ENSG00000143320	CRABP2	cellular retinoic acid binding protein 2	4.56	2.19	2.62E-05	5.82E-04
ENSG00000273142	RP11-458F8.4	long intergenic non-protein coding RNA 2604	4.56	2.19	1.07E-04	1.85E-03
ENSG00000256443	RP11-794G24.1	novel transcript	-4.56	-2.19	7.83E-04	8.69E-03
ENSG00000204936	CD177	CD177 molecule	-4.56	-2.19	3.70E-03	2.82E-02
ENSG00000036565	SLC18A1	solute carrier family 18 member A1	4.56	2.19	5.91E-03	3.96E-02
ENSG00000084070	SMAP2	small ArfGAP2	4.53	2.18	2.93E-15	6.45E-13
ENSG00000145088	EAF2	ELL associated factor 2	4.53	2.18	8.30E-14	1.45E-11
ENSG00000171659	GPR34	G protein-coupled receptor 34	4.53	2.18	1.10E-13	1.90E-11

ENSG00000260401	RP11-800A3.4	novel transcript, overlapping to P2RY2	-4.53	-2.18	2.68E-13	4.42E-11
ENSG00000258929	RP11-58E21.3		4.53	2.18	5.68E-06	1.65E-04
ENSG00000133246	PRAM1	PML-RARA regulated adaptor molecule 1	4.53	2.18	2.44E-05	5.48E-04
ENSG00000124882	EREG	epiregulin	-4.53	-2.18	4.27E-03	3.13E-02
ENSG00000133874	RNF122	ring finger protein 122	-4.5	-2.17	5.30E-09	3.72E-07
ENSG00000223804	RP6-206I17.1	Poly	4.5	2.17	5.59E-09	3.88E-07
ENSG00000249713	CTD-2236F14.1	novel transcript	4.5	2.17	1.05E-05	2.79E-04
ENSG00000178445	GLDC	glycine decarboxylase	4.5	2.17	4.06E-05	8.36E-04
ENSG00000166589	CDH16	cadherin 16	-4.5	-2.17	4.47E-04	5.74E-03
ENSG00000229871	RP4-710M16.1	ribosomal protein SA pseudogene 20	-4.5	-2.17	1.36E-03	1.32E-02
ENSG00000136867	SLC31A2	solute carrier family 31 member 2	4.47	2.16	6.16E-09	4.18E-07
ENSG00000180828	BHLHE22	basic helix-loop-helix family member e22	4.47	2.16	5.21E-07	2.11E-05
ENSG00000237484	AP000476.1	long intergenic non-protein coding RNA 1684	4.47	2.16	3.50E-06	1.10E-04
ENSG00000257277	RP11-434H14.1	novel transcript, antisense to ARHGAP15	4.47	2.16	6.56E-06	1.88E-04
ENSG00000222371	RN7SKP202	RN7SK pseudogene 202	-4.47	-2.16	1.73E-04	2.72E-03
ENSG00000234425	RP11-528G1.2	novel transcript	4.47	2.16	3.53E-03	2.73E-02
ENSG00000206875	RNU6-761P	RNA, U6 small nuclear 761, pseudogene	4.47	2.16	3.54E-03	2.73E-02
ENSG00000259415	RP11-7M10.2	novel transcript	4.47	2.16	3.70E-03	2.81E-02
ENSG00000154252	GAL3ST2	galactose-3-O-sulfotransferase 2	4.47	2.16	4.77E-03	3.40E-02
ENSG00000175591	P2RY2	purinergic receptor P2Y2	-4.44	-2.15	4.44E-13	6.91E-11
ENSG00000183508	FAM46C	terminal nucleotidyltransferase 5C	4.44	2.15	2.13E-10	1.99E-08
ENSG00000015285	WAS	WASP actin nucleation promoting factor	4.44	2.15	4.23E-10	3.76E-08
ENSG00000203813	HIST1H3H		4.44	2.15	1.22E-09	9.88E-08
ENSG00000124575	HIST1H1D	H1.3 linker histone, cluster member	4.44	2.15	1.82E-09	1.40E-07
ENSG00000231858	AC067945.4	STAT4 antisense RNA 1	4.44	2.15	4.28E-06	1.30E-04
ENSG00000265055	AC145343.2	novel transcript	-4.44	-2.15	4.01E-03	2.99E-02

ENSG00000187904	AC097382.5	novel transcript, sense overlapping TBC1D14	4.44	2.15	4.41E-03	3.20E-02
ENSG00000120498	TEX11	testis expressed 11	4.44	2.15	4.85E-03	3.44E-02
ENSG00000251889	RNU4-49P	RNA, U4 small nuclear 49, pseudogene	4.44	2.15	5.77E-03	3.88E-02
ENSG00000168824	NSG1	neuronal vesicle trafficking associated 1	-4.41	-2.14	1.16E-08	7.32E-07
ENSG00000121594	CD80	CD80 molecule	4.41	2.14	7.19E-06	2.02E-04
ENSG00000222389	RNU2-28P	RNA, U2 small nuclear 28, pseudogene	-4.41	-2.14	1.06E-04	1.84E-03
ENSG00000112232	KHDRBS2	KH RNA binding domain containing, signal transduction associated 2	4.41	2.14	2.83E-04	4.02E-03
ENSG00000267246	RP11-798G7.7	novel transcript	4.41	2.14	9.18E-04	9.82E-03
ENSG00000253525	CTD-2114J12.1	ceruloplasmin pseudogene	4.41	2.14	3.07E-03	2.46E-02
ENSG00000249988	RP11-669M16.1	novel transcript	4.41	2.14	4.63E-03	3.32E-02
ENSG00000145569	FAM105A	OTU deubiquitinase with linear linkage specificity like	4.38	2.13	1.43E-18	5.64E-16
ENSG00000110077	MS4A6A	membrane spanning 4-domains A6A	4.38	2.13	4.29E-13	6.74E-11
ENSG00000104894	CD37	CD37 molecule	4.38	2.13	2.23E-11	2.52E-09
ENSG00000150681	RGS18	regulator of G protein signaling 18	4.38	2.13	6.77E-10	5.81E-08
ENSG00000147443	DOK2	docking protein 2	4.38	2.13	1.22E-08	7.63E-07
ENSG00000261616	RP11-6O2.3	novel transcript, antisense to TTC23	-4.38	-2.13	1.29E-08	8.04E-07
ENSG00000118242	MREG	melanoregulin	4.38	2.13	9.75E-07	3.65E-05
ENSG00000116990	MYCL	MYCL proto-oncogene, bHLH transcription factor	4.38	2.13	8.46E-06	2.32E-04
ENSG00000186074	CD300LF	CD300 molecule like family member f	4.38	2.13	2.71E-05	5.99E-04
ENSG00000258912	RP11-1079H9.1	long intergenic non-protein coding RNA 2316	4.38	2.13	6.12E-04	7.23E-03
ENSG00000250614	AC007078.4	novel transcript, antisense to DTX2	4.38	2.13	4.57E-03	3.29E-02
ENSG00000118308	LRMP	inositol 1,4,5-triphosphate receptor associated 2	4.35	2.12	1.26E-12	1.81E-10

ENSG00000121933	ADORA3	transmembrane and immunoglobulin domain containing 3	4.35	2.12	2.58E-10	2.37E-08
ENSG00000236946	HNRNPA1P70	heterogeneous nuclear ribonucleoprotein A1 pseudogene 70	4.35	2.12	6.95E-06	1.97E-04
ENSG00000257640	RP11-570L15.2		4.35	2.12	1.72E-05	4.14E-04
ENSG00000230035	RP11-174G17	IGSF21 antisense RNA 1	4.35	2.12	2.72E-03	2.24E-02
ENSG00000230649	AC024084.1	novel transcript	4.35	2.12	4.57E-03	3.29E-02
ENSG00000075884	ARHGAP15	Rho GTPase activating protein 15	4.32	2.11	7.77E-15	1.58E-12
ENSG00000124496	TRERF1	transcriptional regulating factor 1	4.32	2.11	6.47E-13	9.82E-11
ENSG00000043462	LCP2	lymphocyte cytosolic protein 2	4.32	2.11	4.07E-11	4.26E-09
ENSG00000182572	HIST1H3I		4.32	2.11	3.21E-07	1.38E-05
ENSG00000145832	SLC25A48	solute carrier family 25 member 48	-4.32	-2.11	2.91E-03	2.36E-02
ENSG00000255780	RP11-1029F8.1	chromosome 3 open reading frame 19 (C3orf19) pseudogene	4.32	2.11	3.17E-03	2.52E-02
ENSG00000127084	FGD3	FYVE, RhoGEF and PH domain containing 3	4.29	2.1	9.97E-15	1.98E-12
ENSG00000103490	PYCARD	PYD and CARD domain containing	4.29	2.1	2.52E-10	2.32E-08
ENSG00000102575	ACP5	acid phosphatase 5, tartrate resistant	4.29	2.1	3.46E-07	1.48E-05
ENSG00000188610	FAM72B	family with sequence similarity 72 member B	4.29	2.1	1.19E-04	2.02E-03
ENSG00000162888	C1orf147	chromosome 1 open reading frame 147	4.29	2.1	3.58E-04	4.83E-03
ENSG00000256708	RP11-444B24.2	mitochondrial ribosomal protein L21 (MRPL21) pseudogene	4.29	2.1	2.23E-03	1.93E-02
ENSG00000272799	RP11-474N24.6	novel transcript	-4.29	-2.1	4.32E-03	3.15E-02
ENSG00000241211	IQCJ-SCHIP1-AS1	IQCJ-SCHIP1 readthrough antisense RNA 1	-4.26	-2.09	2.56E-06	8.44E-05
ENSG00000152292	SH2D6	SH2 domain containing 6	-4.26	-2.09	1.68E-03	1.55E-02
ENSG00000228991	RP11-318K12.1		4.26	2.09	4.61E-03	3.30E-02
ENSG00000118503	TNFAIP3	TNF alpha induced protein 3	4.23	2.08	3.01E-12	4.06E-10

ENSG00000123338	NCKAP1L	NCK associated protein 1 like	4.23	2.08	3.68E-10	3.33E-08
ENSG00000099958	DERL3	derlin 3	4.23	2.08	9.02E-08	4.60E-06
ENSG00000235522	AC009505.2	novel transcript	4.23	2.08	2.13E-06	7.25E-05
ENSG00000230606	AC159540.1	novel transcript	4.23	2.08	2.85E-06	9.26E-05
ENSG00000233264	AC006042.8	peptidylprolyl isomerase (cyclophilin)-like 4 (PPIL4) pseudogene	4.23	2.08	6.24E-06	1.80E-04
ENSG00000236423	LINC01134	long intergenic non-protein coding RNA 1134	-4.23	-2.08	1.73E-04	2.73E-03
ENSG00000224743	TEX26-AS1	TEX26 antisense RNA 1	-4.23	-2.08	1.19E-03	1.20E-02
ENSG00000258181	RP11-493L12.4	novel transcript	4.23	2.08	1.69E-03	1.56E-02
ENSG00000120937	NPPB	natriuretic peptide B	4.23	2.08	2.69E-03	2.22E-02
ENSG00000259984	RP11-335G20.7	novel pseudogene	4.23	2.08	2.80E-03	2.30E-02
ENSG00000197262	CCL4L2		4.23	2.08	5.63E-03	3.83E-02
ENSG00000120280	CXorf21	TLR adaptor interacting with endolysosomal SLC15A4	4.2	2.07	1.79E-06	6.17E-05
ENSG00000172901	AQPEP	laeverin	4.2	2.07	9.77E-06	2.63E-04
ENSG00000244681	MTHFD2P1	methylenetetrahydrofolate dehydrogenase (NADP+ dependent) 2, methenyltetrahydrofolate cyclohydrolase pseudogene 1	4.2	2.07	3.08E-04	4.32E-03
ENSG00000176049	JAKMIP2	janus kinase and microtubule interacting protein 2	4.17	2.06	3.11E-08	1.79E-06
ENSG00000105851	PIK3CG	phosphatidylinositol-4,5-bisphosphate 3-kinase catalytic subunit gamma	4.14	2.05	2.21E-13	3.70E-11
ENSG00000157514	TSC22D3	TSC22 domain family member 3	4.14	2.05	9.89E-11	9.86E-09
ENSG00000239713	APOBEC3G	apolipoprotein B mRNA editing enzyme catalytic subunit 3G	4.14	2.05	7.15E-10	6.07E-08
ENSG00000091490	SEL1L3	SEL1L family member 3	4.14	2.05	7.67E-10	6.49E-08
ENSG00000187151	ANGPTL5	angiopoietin like 5	4.14	2.05	1.45E-06	5.10E-05
ENSG00000004948	CALCR	calcitonin receptor	4.14	2.05	4.30E-04	5.56E-03
ENSG00000139144	PIK3C2G	phosphatidylinositol-4-phosphate 3-kinase catalytic subunit type 2 gamma	4.14	2.05	7.35E-04	8.29E-03
ENSG00000260249	RP11-401P9.5	novel transcript, antisense to SNX20	4.14	2.05	1.17E-03	1.18E-02

ENSG00000132182	NUP210	nucleoporin 210	4.11	2.04	1.32E-08	8.25E-07
ENSG00000255141	HNRNPA1P76	heterogeneous nuclear ribonucleoprotein A1 pseudogene 76	4.11	2.04	3.73E-05	7.80E-04
ENSG00000231799	RP13-93L13.2	proliferation-associated 2G4 pseudogene 6	4.11	2.04	4.57E-05	9.26E-04
ENSG00000240767	RN7SL288P	RNA, 7SL, cytoplasmic 288, pseudogene	4.11	2.04	1.64E-03	1.52E-02
ENSG00000229899	AC084290.2	pseudogene similar to part of the cytochrome c oxidase subunit I (COX1)	4.11	2.04	2.95E-03	2.39E-02
ENSG00000186517	ARHGAP30	Rho GTPase activating protein 30	4.08	2.03	5.03E-11	5.24E-09
ENSG00000265206	MIR142	novel transcript, MIR142 host	4.08	2.03	1.10E-06	4.05E-05
ENSG00000231734	RP6-206I17.2		4.08	2.03	3.41E-03	2.66E-02
ENSG00000127585	FBXL16	F-box and leucine rich repeat protein 16	4.06	2.02	6.07E-07	2.43E-05
ENSG00000227155	RP11-165F24.3	DOCK8 antisense RNA 2	4.06	2.02	6.36E-07	2.52E-05
ENSG00000171596	NMUR1	neuromedin U receptor 1	4.06	2.02	2.10E-05	4.85E-04
ENSG00000258875	CTD-2547L24.3	novel transcript, antisense to GPR68	4.06	2.02	1.09E-04	1.88E-03
ENSG00000240710	RP11-430C7.4	novel transcript	4.06	2.02	1.10E-04	1.90E-03
ENSG00000250771	RP11-153M7.3	toll-like receptor 2 pseudogene	4.06	2.02	2.57E-04	3.74E-03
ENSG00000183150	GPR19	G protein-coupled receptor 19	4.06	2.02	1.43E-03	1.38E-02
ENSG00000154227	CERS3	ceramide synthase 3	4.06	2.02	2.32E-03	1.99E-02
ENSG00000196126	HLA-DRB1	major histocompatibility complex, class II, DR beta 1	4.03	2.01	1.33E-10	1.29E-08
ENSG00000188404	SELL	selectin L	4.03	2.01	2.82E-06	9.18E-05
ENSG00000118432	CNR1	cannabinoid receptor 1	4.03	2.01	9.91E-06	2.66E-04
ENSG00000255414	LINC01059		-4.03	-2.01	5.83E-05	1.14E-03
ENSG00000183734	ASCL2	achaete-scute family bHLH transcription factor 2	4.03	2.01	4.76E-04	6.03E-03
ENSG00000118492	ADGB	androglobin	4.03	2.01	3.78E-03	2.86E-02
ENSG00000229647	AC007879.7	myocardin-induced smooth muscle lncRNA, inducer of differentiation	4.03	2.01	5.73E-03	3.87E-02
ENSG00000204472	AIF1	allograft inflammatory factor 1	4	2	1.18E-07	5.81E-06
ENSG00000214212	C19orf38	chromosome 19 open reading frame 38	4	2	1.19E-05	3.07E-04

ENSG00000243710	WDR65	cilia and flagella associated protein 57	4	2	2.40E-04	3.53E-03
ENSG00000170396	ZNF804A	zinc finger protein 804A	4	2	6.10E-04	7.22E-03
ENSG00000232487	RASA3-IT1	RASA3 intronic transcript 1	4	2	4.36E-03	3.17E-02
ENSG00000122872	ARL4P	ADP ribosylation factor like GTPase 4A pseudogene 1	4	2	4.57E-03	3.28E-02
ENSG00000139998	RAB15	RAB15, member RAS oncogene family	-3.97	-1.99	6.24E-11	6.43E-09
ENSG00000180061	TMEM150B	transmembrane protein 150B	3.97	1.99	2.49E-08	1.47E-06
ENSG00000080031	PTPRH	protein tyrosine phosphatase receptor type H	-3.97	-1.99	2.72E-07	1.19E-05
ENSG00000116299	KIAA1324	endosome-lysosome associated apoptosis and autophagy regulator 1	3.97	1.99	9.96E-06	2.67E-04
ENSG00000233858	AC026904.1	long intergenic non-protein coding RNA 2599	3.97	1.99	8.86E-05	1.59E-03
ENSG00000236499	LINC00896	long intergenic non-protein coding RNA 896	3.97	1.99	1.04E-04	1.82E-03
ENSG00000114529	C3orf52	chromosome 3 open reading frame 52	-3.97	-1.99	1.11E-04	1.90E-03
ENSG00000255163	HSPE1P18	heat shock protein family E (Hsp10) member 1 pseudogene 18	3.97	1.99	5.31E-04	6.54E-03
ENSG00000255587	RAB44	RAB44, member RAS oncogene family	3.97	1.99	7.45E-04	8.39E-03
ENSG00000214814	FER1L6	fer-1 like family member 6	-3.97	-1.99	1.83E-03	1.67E-02
ENSG00000197629	MPEG1	macrophage expressed 1	3.94	1.98	1.01E-11	1.24E-09
ENSG00000099625	C19orf26	CACN subunit beta associated regulatory protein	-3.94	-1.98	1.14E-09	9.32E-08
ENSG00000255308	RP11-428C19.4	CSRP3 and E2F8 antisense RNA 1	-3.94	-1.98	6.43E-06	1.84E-04
ENSG00000237133	AC020594.5		-3.94	-1.98	5.93E-05	1.15E-03
ENSG00000260997	RP4-647J21.1	novel transcript, overlapping MYO1G	3.94	1.98	1.30E-04	2.18E-03
ENSG00000158859	ADAMTS4	ADAM metallopeptidase with thrombospondin type 1 motif 4	-3.94	-1.98	2.92E-04	4.12E-03
ENSG00000000005	TNMD	tenomodulin	3.94	1.98	1.74E-03	1.60E-02
ENSG00000125740	FOSB	FosB proto-oncogene, AP-1 transcription factor subunit	-3.94	-1.98	3.85E-03	2.90E-02

ENSG00000204287	HLA-DRA	major histocompatibility complex, class II, DR alpha	3.92	1.97	6.54E-09	4.42E-07
ENSG00000163131	CTSS	cathepsin S	3.92	1.97	1.60E-08	9.84E-07
ENSG00000166428	PLD4	phospholipase D family member 4	3.92	1.97	7.78E-06	2.16E-04
ENSG00000165923	AGBL2	AGBL carboxypeptidase 2	3.92	1.97	1.88E-04	2.92E-03
ENSG00000169884	WNT10B	Wnt family member 10B	3.92	1.97	2.83E-04	4.02E-03
ENSG00000255221	CARD17	caspase recruitment domain family member 17	3.92	1.97	6.14E-03	4.07E-02
ENSG00000272211	RP11-347P5.1	novel transcript, antisense to STK17B	3.89	1.96	3.94E-09	2.87E-07
ENSG00000154096	THY1	Thy-1 cell surface antigen	3.89	1.96	4.46E-06	1.34E-04
ENSG00000139055	ERP27	endoplasmic reticulum protein 27	3.89	1.96	5.11E-06	1.51E-04
ENSG00000168447	SCNN1B	sodium channel epithelial 1 subunit beta	3.89	1.96	2.13E-05	4.91E-04
ENSG00000204928	GRXCR2	glutaredoxin and cysteine rich domain containing 2	-3.89	-1.96	5.75E-05	1.12E-03
ENSG00000240404	RP11-142L1.3	SET nuclear oncogene (SET) pseudogene	-3.89	-1.96	2.51E-04	3.68E-03
ENSG00000058335	RASGRF1	Ras protein specific guanine nucleotide releasing factor 1	3.89	1.96	1.51E-03	1.44E-02
ENSG00000261996	CTC-281F24.1	novel transcript	3.89	1.96	6.43E-03	4.21E-02
ENSG00000162777	DENND2D	DENN domain containing 2D	3.86	1.95	5.62E-10	4.87E-08
ENSG00000273237	CTB-119C2.1		-3.86	-1.95	1.76E-07	8.14E-06
ENSG00000134061	CD180	CD180 molecule	3.86	1.95	9.39E-07	3.54E-05
ENSG00000115956	PLEK	pleckstrin	3.86	1.95	2.21E-06	7.46E-05
ENSG00000255733	IFNG-AS1	IFNG antisense RNA 1	3.86	1.95	4.00E-05	8.26E-04
ENSG00000109511	ANXA10	annexin A10	3.86	1.95	9.34E-04	9.94E-03
ENSG00000240474	RN7SL116P	RNA, 7SL, cytoplasmic 116, pseudogene	3.86	1.95	2.27E-03	1.96E-02
ENSG00000182578	CSF1R	colony stimulating factor 1 receptor	3.84	1.94	3.11E-15	6.71E-13
ENSG00000242574	HLA-DMB	major histocompatibility complex, class II, DM beta	3.84	1.94	9.86E-11	9.86E-09
ENSG00000100767	PAPLN	papilin, proteoglycan like sulfated glycoprotein	3.84	1.94	3.13E-09	2.31E-07
ENSG00000173918	C1QTNF1	C1q and TNF related 1	-3.84	-1.94	6.60E-09	4.45E-07
ENSG00000254419	RP11-261P9.4	novel transcript	3.84	1.94	1.97E-05	4.61E-04

ENSG00000112299	VNN1	vanin 1	3.84	1.94	1.06E-04	1.84E-03
ENSG00000140297	GCNT3	glucosaminyl (N-acetyl) transferase 3, mucin type	-3.84	-1.94	7.33E-04	8.28E-03
ENSG00000103449	SALL1	spalt like transcription factor 1	-3.84	-1.94	4.98E-03	3.49E-02
ENSG00000232909	RP3-510O8.4	novel transcript	3.84	1.94	5.67E-03	3.84E-02
ENSG00000134107	BHLHE40	basic helix-loop-helix family member e40	-3.81	-1.93	1.48E-07	7.05E-06
ENSG00000258987	RP11-131H24.4	novel transcript, antisense to ASB2	-3.81	-1.93	1.06E-05	2.82E-04
ENSG00000167094	TTC16	tetratricopeptide repeat domain 16	3.81	1.93	1.10E-05	2.90E-04
ENSG00000256316	HIST1H3F		3.81	1.93	2.73E-05	6.02E-04
ENSG00000250651	PABPC1P7	poly(A) binding protein cytoplasmic 1 pseudogene 7	3.81	1.93	1.58E-04	2.54E-03
ENSG00000259727	RP11-1069G10.2	novel transcript	-3.81	-1.93	7.24E-04	8.20E-03
ENSG00000272023	CTC-350I8.1	AFF4 divergent transcript	3.81	1.93	5.70E-03	3.85E-02
ENSG00000072818	ACAP1	ArfGAP with coiled-coil, ankyrin repeat and PH domains 1	3.78	1.92	2.76E-14	5.07E-12
ENSG00000142347	MYO1F	myosin IF	3.78	1.92	4.93E-09	3.51E-07
ENSG00000273447	AC004067.5	novel transcript, antisense to CASP6	3.78	1.92	2.81E-06	9.18E-05
ENSG00000171848	RRM2	ribonucleotide reductase regulatory subunit M2	3.78	1.92	7.56E-04	8.50E-03
ENSG00000272275	RP11-791G15.2	novel transcript	-3.78	-1.92	9.86E-04	1.03E-02
ENSG00000227531	RP11-202G18.1	novel transcript	3.78	1.92	1.21E-03	1.21E-02
ENSG00000228499	TMSB10P1	thymosin beta 10 pseudogene 1	3.78	1.92	5.42E-03	3.72E-02
ENSG00000145703	IQGAP2	IQ motif containing GTPase activating protein 2	3.76	1.91	3.20E-17	9.65E-15
ENSG00000028277	POU2F2	POU class 2 homeobox 2	3.76	1.91	2.22E-08	1.34E-06
ENSG00000126759	CFP	complement factor properdin	3.76	1.91	9.19E-07	3.48E-05
ENSG00000139194	RBP5	retinol binding protein 5	3.76	1.91	1.46E-06	5.14E-05
ENSG00000091879	ANGPT2	angiopoietin 2	-3.76	-1.91	1.85E-06	6.36E-05
ENSG00000152932	RAB3C	RAB3C, member RAS oncogene family	3.76	1.91	1.14E-03	1.16E-02
ENSG00000198010	DLGAP2	DLG associated protein 2	3.76	1.91	3.43E-03	2.67E-02
ENSG00000121281	ADCY7	adenylate cyclase 7	3.73	1.9	1.43E-14	2.75E-12
ENSG00000143110	C1orf162	chromosome 1 open reading frame 162	3.73	1.9	2.68E-09	2.00E-07

ENSG00000102554	KLF5	Kruppel like factor 5	-3.73	-1.9	3.73E-07	1.58E-05
ENSG00000204482	LST1	leukocyte specific transcript 1	3.73	1.9	1.33E-05	3.34E-04
ENSG00000226337	RP11-274B18.4	TMEM252 divergent transcript	-3.73	-1.9	4.47E-03	3.23E-02
ENSG00000143125	PROK1	prokineticin 1	-3.73	-1.9	4.49E-03	3.24E-02
ENSG00000137841	PLCB2	phospholipase C beta 2	3.71	1.89	1.51E-15	3.47E-13
ENSG00000120899	PTK2B	protein tyrosine kinase 2 beta	3.71	1.89	8.53E-13	1.25E-10
ENSG00000239474	KLHL41	kelch like family member 41	-3.71	-1.89	8.76E-10	7.34E-08
ENSG00000181847	TIGIT	T cell immunoreceptor with Ig and ITIM domains	3.71	1.89	8.89E-06	2.41E-04
ENSG00000259792	RP11-114H24.6	novel transcript, antisense to TBC1D2B	3.71	1.89	6.81E-05	1.28E-03
ENSG00000226091	LINC00937	long intergenic non-protein coding RNA 937	3.71	1.89	2.29E-04	3.41E-03
ENSG00000162873	KLHDC8A	kelch domain containing 8A	3.71	1.89	6.26E-04	7.37E-03
ENSG00000268758	EMR4P	adhesion G protein-coupled receptor E4, pseudogene	3.71	1.89	9.92E-04	1.04E-02
ENSG00000159374	M1AP	meiosis 1 associated protein	3.71	1.89	1.53E-03	1.45E-02
ENSG00000238266	LINC00707	long intergenic non-protein coding RNA 707	3.71	1.89	2.07E-03	1.83E-02
ENSG00000229914	RP11-404O13.4	ribosomal protein S10 (RPS10) pseudogene	3.71	1.89	3.72E-03	2.83E-02
ENSG00000162998	FRZB	frizzled related protein	3.68	1.88	9.61E-07	3.61E-05
ENSG00000163606	CD200R1	CD200 receptor 1	3.68	1.88	2.19E-06	7.43E-05
ENSG00000246375	RP11-10L7.1	PPM1K divergent transcript	3.68	1.88	2.22E-06	7.47E-05
ENSG00000266017	MIR4477A	microRNA 4477b	3.68	1.88	1.06E-05	2.82E-04
ENSG00000105352	CEACAM4	CEA cell adhesion molecule 4	3.68	1.88	2.49E-05	5.57E-04
ENSG00000137747	TMPRSS13	transmembrane serine protease 13	3.68	1.88	5.15E-05	1.02E-03
ENSG00000256262	USP30-AS1	USP30 antisense RNA 1	3.68	1.88	3.36E-04	4.60E-03
ENSG00000133466	C1QTNF6	C1q and TNF related 6	3.66	1.87	2.36E-10	2.19E-08
ENSG00000047365	ARAP2	ArfGAP with RhoGAP domain, ankyrin repeat and PH domain 2	3.66	1.87	1.07E-09	8.86E-08
ENSG00000135678	CPM	carboxypeptidase M	3.66	1.87	1.33E-08	8.25E-07
ENSG00000251628	RP11-371M22.1		-3.66	-1.87	2.28E-07	1.02E-05
ENSG00000142748	FCN3	ficolin 3	-3.66	-1.87	2.38E-07	1.06E-05
ENSG00000168229	PTGDR	prostaglandin D2 receptor	3.66	1.87	7.55E-06	2.11E-04
ENSG00000161643	SIGLEC16	sialic acid binding Ig like lectin 16	3.66	1.87	1.22E-05	3.13E-04

ENSG00000258268	RP11-570L15.1		3.66	1.87	2.32E-05	5.26E-04
ENSG00000256540	RP11-598F7.6	novel transcript	-3.66	-1.87	3.32E-05	7.09E-04
ENSG00000012124	CD22	CD22 molecule	3.66	1.87	7.86E-05	1.45E-03
ENSG00000109321	AREG	amphiregulin	-3.66	-1.87	3.27E-03	2.58E-02
ENSG00000254491	RP11-145O15.2	nuclear transcription factor Y, gamma (NFYC) pseudogene	-3.66	-1.87	8.12E-03	4.99E-02
ENSG00000136490	LIMD2	LIM domain containing 2	3.63	1.86	1.35E-08	8.32E-07
ENSG00000149418	ST14	ST14 transmembrane serine protease matriptase	3.63	1.86	1.46E-08	8.98E-07
ENSG00000115415	STAT1	signal transducer and activator of transcription 1	3.63	1.86	2.81E-08	1.64E-06
ENSG00000016391	CHDH	choline dehydrogenase	-3.63	-1.86	1.22E-07	6.00E-06
ENSG00000196189	SEMA4A	semaphorin 4A	3.63	1.86	5.27E-07	2.13E-05
ENSG00000169413	RNASE6	ribonuclease A family member k6	3.63	1.86	7.90E-07	3.05E-05
ENSG00000102524	TNFSF13B	TNF superfamily member 13b	3.63	1.86	1.79E-06	6.17E-05
ENSG00000240888	RP13-635I23.3	novel transcript	3.63	1.86	5.20E-05	1.03E-03
ENSG00000258539	RP11-12J10.3	novel transcript, METTL10-FAM53B readthrough	3.63	1.86	9.61E-05	1.70E-03
ENSG00000163638	ADAMTS9	ADAM metallopeptidase with thrombospondin type 1 motif 9	-3.63	-1.86	2.81E-04	4.00E-03
ENSG00000227158	AC073621.2	ligand of numb-protein X 1, E3 ubiquitin protein ligase pseudogene	-3.63	-1.86	4.49E-03	3.24E-02
ENSG00000030304	MUSK	muscle associated receptor tyrosine kinase	3.61	1.85	1.20E-11	1.45E-09
ENSG00000100100	PIK3IP1	phosphoinositide-3-kinase interacting protein 1	3.61	1.85	5.21E-09	3.67E-07
ENSG00000213654	GPSM3	G protein signaling modulator 3	3.61	1.85	9.17E-09	5.92E-07
ENSG00000170379	FAM115C	TRPM8 channel associated factor 2	3.61	1.85	2.94E-08	1.69E-06
ENSG00000272782	RP4-607J23.2		-3.61	-1.85	1.49E-07	7.06E-06
ENSG00000261270	RP11-325K4.3	novel transcript, sense intronic to HERPUD1	3.61	1.85	1.64E-07	7.64E-06
ENSG00000264663	KRT8P34	keratin 8 pseudogene 34	3.61	1.85	1.62E-03	1.51E-02
ENSG00000157856	DRC1	dynein regulatory complex subunit 1	-3.61	-1.85	3.16E-03	2.52E-02
ENSG00000231528	FAM225A	family with sequence similarity 225 member A	3.61	1.85	4.56E-03	3.28E-02

ENSG00000225407	CTD-2384B11.2	novel transcript, antisense to F2R	3.61	1.85	4.94E-03	3.48E-02
ENSG00000107014	RLN2	relaxin 2	3.61	1.85	6.02E-03	4.01E-02
ENSG00000237638	AC007386.2	long intergenic non-protein coding RNA 2245	3.61	1.85	6.80E-03	4.38E-02
ENSG00000171643	S100Z	S100 calcium binding protein Z	3.61	1.85	7.41E-03	4.67E-02
ENSG00000072858	SIDT1	SID1 transmembrane family member 1	3.58	1.84	1.28E-11	1.54E-09
ENSG00000173372	C1QA	complement C1q A chain	3.58	1.84	2.82E-08	1.64E-06
ENSG00000162894	FAIM3	Fc fragment of IgM receptor	3.58	1.84	3.28E-08	1.87E-06
ENSG00000136286	MYO1G	myosin IG	3.58	1.84	1.46E-06	5.14E-05
ENSG00000137331	IER3	immediate early response 3	-3.58	-1.84	4.78E-06	1.42E-04
ENSG00000180539	C9orf139	long intergenic non-protein coding RNA 2908	3.58	1.84	1.84E-05	4.37E-04
ENSG00000216802	RP11-390P2.2	mitochondrial carrier homolog 1 (MTCH1) pseudogene	3.58	1.84	2.29E-05	5.20E-04
ENSG00000197459	HIST1H2BH		3.58	1.84	3.61E-04	4.87E-03
ENSG00000248268	CTC-499J9.1	novel transcript	3.58	1.84	6.32E-04	7.41E-03
ENSG00000265768	MIR4506	microRNA 4506	-3.58	-1.84	2.26E-03	1.95E-02
ENSG00000163735	CXCL5	C-X-C motif chemokine ligand 5	-3.58	-1.84	6.18E-03	4.09E-02
ENSG00000223491	RP3-328E19.4		3.58	1.84	7.45E-03	4.69E-02
ENSG00000163154	TNFAIP8L2	TNF alpha induced protein 8 like 2	3.56	1.83	2.44E-06	8.13E-05
ENSG00000256817	TPT1P12	tumor protein, translationally-controlled 1 pseudogene 12	3.56	1.83	5.64E-04	6.81E-03
ENSG00000128342	LIF	LIF interleukin 6 family cytokine	-3.56	-1.83	1.23E-03	1.23E-02
ENSG00000264773	MIR4420	microRNA 4420	3.56	1.83	6.95E-03	4.45E-02
ENSG00000114737	CISH	cytokine inducible SH2 containing protein	-3.53	-1.82	5.60E-08	2.98E-06
ENSG00000155307	SAMSN1	SAM domain, SH3 domain and nuclear localization signals 1	3.53	1.82	1.15E-06	4.20E-05
ENSG00000253925	CTB-178M22.1	novel transcript	-3.53	-1.82	1.72E-05	4.14E-04
ENSG00000267074	RP11-1094M14.5	novel transcript, sense intronic SLFN12L	3.53	1.82	2.05E-04	3.11E-03
ENSG00000168143	FAM83B	family with sequence similarity 83 member B	-3.53	-1.82	7.10E-04	8.11E-03
ENSG00000172460	PRSS30P	serine protease 30, pseudogene	3.53	1.82	1.85E-03	1.68E-02

ENSG00000124249	KCNK15	potassium two pore domain channel subfamily K member 15	-3.53	-1.82	2.76E-03	2.27E-02
ENSG00000081320	STK17B	serine/threonine kinase 17b	3.51	1.81	2.03E-12	2.81E-10
ENSG00000186854	TRABD2A	TraB domain containing 2A	3.51	1.81	1.44E-07	6.94E-06
ENSG00000151651	ADAM8	ADAM metallopeptidase domain 8	3.51	1.81	3.50E-06	1.10E-04
ENSG00000069399	BCL3	BCL3 transcription coactivator	-3.51	-1.81	7.54E-06	2.11E-04
ENSG00000177452	RP4-597J3.1	ribosomal protein L19 (RPL19) pseudogene	-3.51	-1.81	9.15E-05	1.64E-03
ENSG00000167971	CASKIN1	CASK interacting protein 1	-3.51	-1.81	1.02E-04	1.79E-03
ENSG00000167483	FAM129C	niban apoptosis regulator 3	3.51	1.81	2.13E-03	1.87E-02
ENSG00000179242	CDH4	cadherin 4	-3.51	-1.81	2.39E-03	2.04E-02
ENSG00000149488	TMC2	transmembrane channel like 2	3.51	1.81	4.44E-03	3.22E-02
ENSG00000199157	MIR208A	microRNA 208a	-3.51	-1.81	5.58E-03	3.81E-02
ENSG00000243811	APOBEC3D	apolipoprotein B mRNA editing enzyme catalytic subunit 3D	3.48	1.8	1.29E-09	1.04E-07
ENSG00000026297	RNASET2	ribonuclease T2	3.48	1.8	1.39E-09	1.10E-07
ENSG00000081985	IL12RB2	interleukin 12 receptor subunit beta 2	3.48	1.8	1.30E-06	4.64E-05
ENSG00000128606	LRRC17	leucine rich repeat containing 17	3.48	1.8	2.80E-06	9.14E-05
ENSG00000122861	PLAU	plasminogen activator, urokinase	-3.48	-1.8	4.20E-06	1.28E-04
ENSG00000229644	NAMPTL	nicotinamide phosphoribosyltransferase pseudogene 1	-3.48	-1.8	1.05E-05	2.79E-04
ENSG00000115607	IL18RAP	interleukin 18 receptor accessory protein	3.48	1.8	2.25E-05	5.13E-04
ENSG00000185022	MAFF	MAF bZIP transcription factor F	-3.48	-1.8	2.45E-05	5.49E-04
ENSG00000188015	S100A3	S100 calcium binding protein A3	-3.48	-1.8	5.52E-04	6.71E-03
ENSG00000237512	UNC5B-AS1	UNC5B antisense RNA 1	-3.46	-1.79	8.51E-08	4.41E-06
ENSG00000253978	CTB-178M22.2	uncharacterized LOC101927862	-3.46	-1.79	1.44E-04	2.36E-03
ENSG00000248466	RP11-640B6.1	pseudogene similar to part of LIM and senescent cell antigen-like domains 2 (LIMS2)	3.46	1.79	1.90E-04	2.93E-03
ENSG00000270127	RP11-526I2.5	novel transcript	3.46	1.79	2.92E-04	4.12E-03

ENSG00000261471	RP11-61F12.1	novel transcript, antisense to COTL1	3.46	1.79	5.43E-04	6.65E-03
ENSG00000196361	ELAVL3	ELAV like RNA binding protein 3	-3.46	-1.79	5.84E-04	6.99E-03
ENSG00000241319	SETP6	SET pseudogene 6	-3.46	-1.79	9.05E-04	9.73E-03
ENSG00000226807	MROH5	maestro heat like repeat family member 5 (gene/pseudogene)	-3.46	-1.79	2.16E-03	1.89E-02
ENSG00000249721	RP11-83M16.4	ribosomal protein L23a (RPL23A) pseudogene	3.46	1.79	2.56E-03	2.14E-02
ENSG00000109674	NEIL3	nei like DNA glycosylase 3	3.46	1.79	3.49E-03	2.71E-02
ENSG00000229727	AC013460.1	novel transcript	3.46	1.79	3.53E-03	2.73E-02
ENSG00000239465	RP11-330L19.2	ribosomal protein L21 (RPL21) pseudogene	3.46	1.79	4.07E-03	3.02E-02
ENSG00000240210	RP11-204K16.1	ribosomal protein L7a (RPL7A) pseudogene	3.46	1.79	4.55E-03	3.28E-02
ENSG00000234996	RP11-480I12.7	actin gamma 1 pseudogene 25	3.46	1.79	7.21E-03	4.58E-02
ENSG00000162711	NLRP3	NLR family pyrin domain containing 3	3.43	1.78	5.83E-10	5.03E-08
ENSG00000172215	CXCR6	C-X-C motif chemokine receptor 6	3.43	1.78	4.73E-09	3.40E-07
ENSG00000235568	NFAM1	NFAT activating protein with ITAM motif 1	3.43	1.78	2.99E-07	1.29E-05
ENSG00000166825	ANPEP	alanyl aminopeptidase, membrane	3.43	1.78	6.96E-07	2.74E-05
ENSG00000203497	PDCD4-AS1	PDCD4 antisense RNA 1	3.43	1.78	1.28E-06	4.57E-05
ENSG00000047457	CP	ceruloplasmin	3.43	1.78	1.54E-06	5.39E-05
ENSG00000260018	RP11-505K9.1	MBTPS1 divergent transcript	-3.43	-1.78	1.53E-04	2.48E-03
ENSG00000272825	LL21NC02-1C16.2	novel transcript	3.43	1.78	1.84E-03	1.67E-02
ENSG00000205663	RP11-706O15.5		3.43	1.78	2.18E-03	1.90E-02
ENSG00000136040	PLXNC1	plexin C1	3.41	1.77	6.99E-14	1.23E-11
ENSG00000180999	C1orf105	chromosome 1 open reading frame 105	-3.41	-1.77	5.62E-09	3.88E-07
ENSG00000137193	PIM1	Pim-1 proto-oncogene, serine/threonine kinase	-3.41	-1.77	1.62E-07	7.63E-06
ENSG00000120262	CCDC170	coiled-coil domain containing 170	3.41	1.77	1.91E-07	8.76E-06
ENSG00000139187	KLRG1	killer cell lectin like receptor G1	3.41	1.77	4.41E-07	1.82E-05
ENSG00000197168	NEK5	NIMA related kinase 5	3.41	1.77	1.51E-06	5.30E-05

ENSG00000228784	LINC00954	long intergenic non-protein coding RNA 954	3.41	1.77	5.06E-06	1.50E-04
ENSG00000269345	VN1R85P	vomeronasal 1 receptor 85 pseudogene	3.41	1.77	4.13E-05	8.47E-04
ENSG00000228056	CFL1P3	cofilin 1 pseudogene 3	-3.41	-1.77	8.60E-05	1.56E-03
ENSG00000161944	ASGR2	asialoglycoprotein receptor 2	3.41	1.77	2.70E-04	3.89E-03
ENSG00000260953	RP11-426C22.6	novel transcript	-3.41	-1.77	4.03E-04	5.31E-03
ENSG00000250137	RP11-380P13.1	novel transcript, antisense to PPARGC1A	-3.41	-1.77	7.19E-04	8.17E-03
ENSG00000178125	PPP1R42	protein phosphatase 1 regulatory subunit 42	-3.41	-1.77	1.78E-03	1.63E-02
ENSG00000270723	RP11-401N16.1	ribosomal protein L23a pseudogene 92	3.41	1.77	6.92E-03	4.44E-02
ENSG00000026950	BTN3A1	butyrophilin subfamily 3 member A1	3.39	1.76	1.19E-10	1.17E-08
ENSG00000074370	ATP2A3	ATPase sarcoplasmic/endoplasmic reticulum Ca2+ transporting 3	3.39	1.76	4.38E-10	3.88E-08
ENSG00000019582	CD74	CD74 molecule	3.39	1.76	5.47E-09	3.82E-07
ENSG00000168016	TRANK1	tetratricopeptide repeat and ankyrin repeat containing 1	3.39	1.76	3.76E-08	2.10E-06
ENSG00000154188	ANGPT1	angiopoietin 1	3.39	1.76	2.63E-07	1.15E-05
ENSG00000163013	FBXO41	F-box protein 41	3.39	1.76	1.41E-06	4.98E-05
ENSG00000196668	LINC00173	long intergenic non-protein coding RNA 173	3.39	1.76	1.43E-04	2.35E-03
ENSG00000231633	LINC00283		3.39	1.76	1.89E-04	2.92E-03
ENSG00000128383	APOBEC3A	apolipoprotein B mRNA editing enzyme catalytic subunit 3A	3.39	1.76	1.97E-04	3.01E-03
ENSG00000186510	CLCNKA	chloride voltage-gated channel Ka	-3.39	-1.76	4.95E-04	6.20E-03
ENSG00000243225	RP11-7F17.1	ribosomal protein, large, P1 (RPLP1) pseudogene	-3.39	-1.76	1.60E-03	1.49E-02
ENSG00000221743	Z95152.1		3.39	1.76	3.83E-03	2.89E-02
ENSG00000255650	FAM222A-AS1	FAM222A antisense RNA 1	-3.39	-1.76	4.25E-03	3.12E-02
ENSG00000104369	JPH1	junctophilin 1	-3.36	-1.75	1.02E-09	8.52E-08
ENSG00000169403	PTAFR	platelet activating factor receptor	3.36	1.75	3.79E-07	1.60E-05
ENSG00000101938	CHRDL1	chordin like 1	3.36	1.75	2.56E-05	5.70E-04
ENSG00000228956	AC144521.1	SATB1 antisense RNA 1	3.36	1.75	5.91E-05	1.15E-03

ENSG00000203815	FAM231D		3.36	1.75	8.43E-05	1.54E-03
ENSG00000234883	MIR155HG	MIR155 host gene	3.36	1.75	1.19E-04	2.02E-03
ENSG00000170835	CEL	carboxyl ester lipase	-3.36	-1.75	2.76E-04	3.95E-03
ENSG00000228548	ITPKB-AS1	ITPKB antisense RNA 1	3.36	1.75	5.75E-04	6.91E-03
ENSG00000236320	SLFN14	schlafen family member 14	3.36	1.75	7.58E-04	8.51E-03
ENSG00000228403	RP11-563N6.6	novel transcript	3.36	1.75	1.43E-03	1.38E-02
ENSG00000166407	LMO1	LIM domain only 1	-3.36	-1.75	2.91E-03	2.37E-02
ENSG00000103187	COTL1	coactosin like F-actin binding protein 1	3.34	1.74	2.16E-06	7.35E-05
ENSG00000177508	IRX3	iroquois homeobox 3	-3.34	-1.74	3.29E-06	1.05E-04
ENSG00000085514	PILRA	paired immunoglobin like type 2 receptor alpha	3.34	1.74	4.27E-06	1.30E-04
ENSG00000156500	FAM122C	PABIR family member 3	-3.34	-1.74	2.38E-05	5.37E-04
ENSG00000197705	KLHL14	kelch like family member 14	3.34	1.74	1.18E-03	1.19E-02
ENSG00000185527	PDE6G	phosphodiesterase 6G	3.34	1.74	3.83E-03	2.89E-02
ENSG00000267474	CTC-548K16.6	novel transcript, sense intronic to PKN1	3.34	1.74	4.40E-03	3.20E-02
ENSG00000127951	FGL2	fibrinogen like 2	3.32	1.73	2.34E-09	1.77E-07
ENSG00000231389	HLA-DPA1	major histocompatibility complex, class II, DP alpha 1	3.32	1.73	3.51E-07	1.49E-05
ENSG00000150782	IL18	interleukin 18	3.32	1.73	4.25E-07	1.77E-05
ENSG00000169313	P2RY12	purinergic receptor P2Y12	3.32	1.73	4.37E-06	1.33E-04
ENSG00000231752	EMBP1	embigin pseudogene 1	3.32	1.73	2.62E-04	3.80E-03
ENSG00000049768	FOXP3	forkhead box P3	3.32	1.73	5.29E-04	6.53E-03
ENSG00000131355	EMR3	adhesion G protein-coupled receptor E3	3.32	1.73	5.75E-03	3.88E-02
ENSG00000135248	FAM71F1	family with sequence similarity 71 member F1	-3.32	-1.73	6.22E-03	4.11E-02
ENSG00000255189	GLYATL1P1	glycine-N-acyltransferase like 1 pseudogene 1	3.32	1.73	7.49E-03	4.71E-02
ENSG00000196730	DAPK1	death associated protein kinase 1	3.29	1.72	2.19E-09	1.66E-07
ENSG00000111679	PTPN6	protein tyrosine phosphatase non-receptor type 6	3.29	1.72	4.26E-08	2.35E-06
ENSG00000235777	DPYD-AS2	DPYD antisense RNA 2	3.29	1.72	3.12E-06	1.00E-04
ENSG00000229124	VIM-AS1	VIM antisense RNA 1	3.29	1.72	5.37E-06	1.58E-04
ENSG00000108932	SLC16A6	solute carrier family 16 member 6	3.29	1.72	1.03E-04	1.80E-03

ENSG00000259630	CTD-2262B20.1	fatty acid binding protein 5 pseudogene 9	-3.29	-1.72	1.17E-04	2.00E-03
ENSG00000240498	CDKN2B-AS1	CDKN2B antisense RNA 1	3.29	1.72	5.48E-04	6.69E-03
ENSG00000254254	RP11-17A4.2	PENK antisense RNA 1	3.29	1.72	7.09E-04	8.10E-03
ENSG00000261787	TCF24	transcription factor 24	-3.29	-1.72	7.17E-04	8.15E-03
ENSG00000130812	ANGPTL6	angiopoietin like 6	3.29	1.72	8.62E-04	9.36E-03
ENSG00000229870	RP11-507K13.6	ribosomal protein L21 pseudogene 89	3.29	1.72	4.65E-03	3.33E-02
ENSG00000102854	MSLN	mesothelin	3.29	1.72	6.53E-03	4.25E-02
ENSG00000065413	ANKRD44	ankyrin repeat domain 44	3.27	1.71	1.58E-12	2.22E-10
ENSG00000250588	IQCJ-SCHIP1		-3.27	-1.71	1.72E-07	7.99E-06
ENSG00000156711	MAPK13	mitogen-activated protein kinase 13	3.27	1.71	9.73E-07	3.64E-05
ENSG00000237424	FOXD2-AS1	FOXD2 adjacent opposite strand RNA 1	3.27	1.71	8.75E-05	1.58E-03
ENSG00000230188	RP11-405L18.4	ribosomal protein L21 (RPL21) pseudogene	3.27	1.71	1.67E-03	1.54E-02
ENSG00000178015	GPR150	G protein-coupled receptor 150	3.27	1.71	6.71E-03	4.34E-02
ENSG00000042980	ADAM28	ADAM metallopeptidase domain 28	3.25	1.7	1.21E-10	1.19E-08
ENSG00000187764	SEMA4D	semaphorin 4D	3.25	1.7	1.90E-10	1.79E-08
ENSG00000071282	LMCD1	LIM and cysteine rich domains 1	-3.25	-1.7	8.03E-06	2.22E-04
ENSG00000140090	SLC24A4	solute carrier family 24 member 4	3.25	1.7	2.32E-05	5.27E-04
ENSG00000249816	LINC00964	long intergenic non-protein coding RNA 964	-3.25	-1.7	3.21E-05	6.87E-04
ENSG00000239332	LINC01119	long intergenic non-protein coding RNA 1119	-3.25	-1.7	2.53E-04	3.69E-03
ENSG00000197249	SERPINA1	serpin family A member 1	3.25	1.7	7.86E-04	8.72E-03
ENSG00000258676	RP11-386M24.3	novel transcript	-3.25	-1.7	3.34E-03	2.62E-02
ENSG00000267096	CTD-2537I9.13	novel transcript	3.25	1.7	5.03E-03	3.52E-02
ENSG00000223668	EEF1A1P24	eukaryotic translation elongation factor 1 alpha 1 pseudogene 24	3.25	1.7	7.10E-03	4.53E-02
ENSG00000165025	SYK	spleen associated tyrosine kinase	3.23	1.69	4.01E-11	4.22E-09
ENSG00000108846	ABCC3	ATP binding cassette subfamily C member 3	3.23	1.69	7.32E-07	2.86E-05

ENSG00000188176	SMTNL2	smoothelin like 2	-3.23	-1.69	4.40E-06	1.33E-04
ENSG00000198829	SUCNR1	succinate receptor 1	3.23	1.69	5.17E-05	1.02E-03
ENSG00000230537	RP11-305L7.1	novel transcript	3.23	1.69	3.80E-04	5.07E-03
ENSG00000156466	GDF6	growth differentiation factor 6	3.23	1.69	4.85E-04	6.10E-03
ENSG00000233968	RP11-354E11.2	novel transcript, antisense to C10orf112	3.23	1.69	3.45E-03	2.69E-02
ENSG00000174607	UGT8	UDP glycosyltransferase 8	3.23	1.69	4.18E-03	3.08E-02
ENSG00000124818	OPN5	opsin 5	-3.23	-1.69	7.27E-03	4.60E-02
ENSG00000089639	GMIP	GEM interacting protein	3.2	1.68	9.20E-08	4.65E-06
ENSG00000129673	AANAT	aralkylamine N-acetyltransferase	3.2	1.68	2.86E-05	6.26E-04
ENSG00000151164	RAD9B	RAD9 checkpoint clamp component B	-3.2	-1.68	2.06E-03	1.82E-02
ENSG00000109758	HGFAC	HGF activator	-3.2	-1.68	5.67E-03	3.84E-02
ENSG00000146530	VWDE	von Willebrand factor D and EGF domains	3.2	1.68	6.80E-03	4.38E-02
ENSG00000076944	STXBP2	syntaxin binding protein 2	3.18	1.67	2.75E-05	6.04E-04
ENSG00000130844	ZNF331	zinc finger protein 331	-3.18	-1.67	4.24E-05	8.69E-04
ENSG00000196747	HIST1H2AI	H2A clustered histone 13	3.18	1.67	6.87E-04	7.92E-03
ENSG00000112414	GPR126	adhesion G protein-coupled receptor G6	3.16	1.66	1.01E-10	1.00E-08
ENSG00000142185	TRPM2	transient receptor potential cation channel subfamily M member 2	3.16	1.66	4.87E-09	3.49E-07
ENSG00000183748	MRC1L1		3.16	1.66	2.83E-08	1.64E-06
ENSG00000173369	C1QB	complement C1q B chain	3.16	1.66	9.48E-06	2.56E-04
ENSG00000000938	FGR	FGR proto-oncogene, Src family tyrosine kinase	3.16	1.66	6.70E-05	1.26E-03
ENSG00000184956	MUC6	mucin 6, oligomeric mucus/gel-forming	3.16	1.66	1.30E-04	2.17E-03
ENSG00000006075	CCL3		3.16	1.66	2.81E-03	2.31E-02
ENSG00000142765	SYTL1	synaptotagmin like 1	3.14	1.65	7.88E-10	6.65E-08
ENSG00000177602	GSG2	histone H3 associated protein kinase	3.14	1.65	5.61E-06	1.64E-04
ENSG00000105825	TFPI2	tissue factor pathway inhibitor 2	-3.14	-1.65	1.46E-05	3.61E-04
ENSG00000186310	NAP1L3	nucleosome assembly protein 1 like 3	3.14	1.65	3.07E-05	6.63E-04
ENSG00000153446	C16orf89	chromosome 16 open reading frame 89	3.14	1.65	9.63E-05	1.70E-03

ENSG00000254923	RP11-1236K1.8	deubiquitinating enzyme 3 pseudogene	3.14	1.65	1.56E-03	1.47E-02
ENSG00000229854	RP11-524G24.2	novel transcript	-3.14	-1.65	3.53E-03	2.73E-02
ENSG00000235880	RP11-59O6.3	novel transcript	3.14	1.65	6.62E-03	4.30E-02
ENSG00000172345	STARD5	StAR related lipid transfer domain containing 5	3.12	1.64	1.79E-10	1.70E-08
ENSG00000049089	COL9A2	collagen type IX alpha 2 chain	3.12	1.64	3.27E-07	1.40E-05
ENSG00000175567	UCP2	uncoupling protein 2	3.12	1.64	4.18E-07	1.74E-05
ENSG00000055732	MCOLN3	mucolipin TRP cation channel 3	3.12	1.64	1.11E-06	4.05E-05
ENSG00000125384	PTGER2	prostaglandin E receptor 2	3.12	1.64	3.20E-04	4.44E-03
ENSG00000168350	DEGS2	delta 4-desaturase, sphingolipid 2	3.12	1.64	4.10E-04	5.37E-03
ENSG00000229417	NPM1P25	nucleophosmin 1 pseudogene 25	3.12	1.64	3.68E-03	2.81E-02
ENSG00000074410	CA12	carbonic anhydrase 12	3.12	1.64	4.88E-03	3.45E-02
ENSG00000165457	FOLR2	folate receptor beta	3.1	1.63	2.82E-08	1.64E-06
ENSG00000239264	TXNDC5	thioredoxin domain containing 5	3.1	1.63	1.40E-06	4.93E-05
ENSG00000258926	RP11-47I22.1	novel transcript, antisense to PRKCH	3.1	1.63	2.95E-06	9.53E-05
ENSG00000152582	SPEF2	sperm flagellar 2	3.1	1.63	1.34E-05	3.36E-04
ENSG00000261488	RP11-757F18.5	TGF-beta induced lncRNA	-3.1	-1.63	2.07E-04	3.13E-03
ENSG00000178752	FAM132B	erythroferrone	-3.1	-1.63	4.20E-04	5.47E-03
ENSG00000266378	RP11-214O1.3	novel transcript	3.1	1.63	1.26E-03	1.25E-02
ENSG00000248791	CTD-2165H16.3	pleckstrin homology domain containing, family A (phosphoinositide binding specific) member 1 (PLEKHA1) pseudogene	3.1	1.63	1.53E-03	1.45E-02
ENSG00000254750	CASP1P2	caspase 1 pseudogene 2	3.1	1.63	2.01E-03	1.79E-02
ENSG00000179388	EGR3	early growth response 3	-3.1	-1.63	2.57E-03	2.15E-02
ENSG00000144331	ZNF385B	zinc finger protein 385B	3.1	1.63	2.80E-03	2.30E-02
ENSG00000241163	LINC00877	long intergenic non-protein coding RNA 877	3.1	1.63	3.08E-03	2.47E-02
ENSG00000187994	RINL	Ras and Rab interactor like	3.07	1.62	5.57E-10	4.84E-08
ENSG00000129173	E2F8	E2F transcription factor 8	-3.07	-1.62	1.08E-08	6.87E-07
ENSG00000148908	RGS10	regulator of G protein signaling 10	3.07	1.62	1.63E-07	7.64E-06
ENSG00000177464	GPR4	G protein-coupled receptor 4	-3.07	-1.62	4.99E-07	2.03E-05
ENSG00000143466	IKBKE		3.07	1.62	1.10E-05	2.90E-04

ENSG00000114013	CD86	CD86 molecule	3.07	1.62	3.47E-05	7.37E-04
ENSG00000165449	SLC16A9	solute carrier family 16 member 9	3.07	1.62	1.86E-04	2.88E-03
ENSG00000198374	HIST1H2AL		3.07	1.62	1.87E-04	2.90E-03
ENSG00000125735	TNFSF14	TNF superfamily member 14	3.07	1.62	2.23E-04	3.33E-03
ENSG00000188761	BCL2L15	BCL2 like 15	3.07	1.62	7.92E-04	8.77E-03
ENSG00000250994	AC005355.1	novel transcript	-3.07	-1.62	1.07E-03	1.11E-02
ENSG00000219249	AMZ2P2	AMZ2 pseudogene 2	3.07	1.62	2.53E-03	2.12E-02
ENSG00000104081	BMF	Bcl2 modifying factor	3.05	1.61	4.58E-10	4.04E-08
ENSG00000251603	RP11-164P12.4		-3.05	-1.61	1.15E-09	9.38E-08
ENSG00000225217	HSPA7	heat shock protein family A (Hsp70) member 7 (pseudogene)	3.05	1.61	8.64E-09	5.63E-07
ENSG00000173715	C11orf80	chromosome 11 open reading frame 80	3.05	1.61	2.83E-08	1.64E-06
ENSG00000151575	TEX9	testis expressed 9	3.05	1.61	1.34E-07	6.55E-06
ENSG00000105835	NAMPT	nicotinamide phosphoribosyltransferase	-3.05	-1.61	3.16E-06	1.01E-04
ENSG00000186818	LILRB4	leukocyte immunoglobulin like receptor B4	3.05	1.61	1.29E-05	3.27E-04
ENSG00000140675	SLC5A2	solute carrier family 5 member 2	3.05	1.61	1.44E-04	2.37E-03
ENSG00000201957	SNORA25		3.05	1.61	3.73E-04	5.00E-03
ENSG00000166527	CLEC4D	C-type lectin domain family 4 member D	3.05	1.61	5.23E-04	6.47E-03
ENSG00000255836	RP11-157G21.2	M-phase phosphoprotein 8 (MPHOSPH8) pseudogene	3.05	1.61	5.70E-04	6.86E-03
ENSG00000121380	BCL2L14	BCL2 like 14	3.05	1.61	1.65E-03	1.53E-02
ENSG00000272668	RP11-190A12.8	novel transcript, antisense to VSIG8	3.05	1.61	2.83E-03	2.32E-02
ENSG00000259513	CYCSP38	CYCS pseudogene 38	3.05	1.61	3.76E-03	2.85E-02
ENSG00000163661	PTX3	pentraxin 3	-3.05	-1.61	4.25E-03	3.12E-02
ENSG00000206965	RNU6-5P	RNA, U6 small nuclear 5, pseudogene	3.05	1.61	5.50E-03	3.76E-02
ENSG00000015133	CCDC88C	coiled-coil domain containing 88C	3.03	1.6	1.67E-09	1.30E-07
ENSG00000174130	TLR6	toll like receptor 6	3.03	1.6	1.67E-09	1.30E-07

ENSG00000168918	INPP5D	inositol polyphosphate-5-phosphatase D	3.03	1.6	6.47E-09	4.38E-07
ENSG00000186470	BTN3A2	butyrophilin subfamily 3 member A2	3.03	1.6	7.74E-08	4.03E-06
ENSG00000155465	SLC7A7	solute carrier family 7 member 7	3.03	1.6	1.05E-06	3.89E-05
ENSG00000163132	MSX1	msh homeobox 1	-3.03	-1.6	2.61E-06	8.59E-05
ENSG00000130164	LDLR	low density lipoprotein receptor	-3.03	-1.6	3.58E-05	7.55E-04
ENSG00000223949	RP11-24J23.2	ROR1 antisense RNA 1	-3.03	-1.6	5.07E-04	6.32E-03
ENSG00000271538	RP11-326I11.4	long intergenic non-protein coding RNA 2427	3.03	1.6	5.18E-04	6.43E-03
ENSG00000232464	CTA-125H2.1	novel transcript	-3.03	-1.6	7.80E-04	8.67E-03
ENSG00000270659	RP11-105N14.1	novel transcript	3.03	1.6	1.66E-03	1.54E-02
ENSG00000106236	NPTX2	neuronal pentraxin 2	-3.03	-1.6	2.45E-03	2.07E-02
ENSG00000162494	LRRC38	leucine rich repeat containing 38	-3.03	-1.6	5.12E-03	3.56E-02
ENSG00000200735	RNY4P8		3.03	1.6	7.04E-03	4.49E-02
ENSG00000130755	GMFG	glia maturation factor gamma	3.01	1.59	2.87E-08	1.66E-06
ENSG00000167613	LAIR1	leukocyte associated immunoglobulin like receptor 1	3.01	1.59	1.79E-07	8.28E-06
ENSG00000117600	LPPR4	phospholipid phosphatase related 4	3.01	1.59	6.93E-06	1.96E-04
ENSG00000122420	PTGFR	prostaglandin F receptor	3.01	1.59	7.75E-06	2.16E-04
ENSG00000141968	VAV1	vav guanine nucleotide exchange factor 1	3.01	1.59	8.27E-06	2.27E-04
ENSG00000257221	RP11-689B22.2	novel transcript, antisense to SELPLG	3.01	1.59	1.59E-04	2.55E-03
ENSG00000138696	BMPR1B	bone morphogenetic protein receptor type 1B	-3.01	-1.59	1.77E-04	2.77E-03
ENSG00000121898	CPXM2	carboxypeptidase X, M14 family member 2	3.01	1.59	1.79E-04	2.81E-03
ENSG00000238005	RP11-443B7.1	novel transcript	3.01	1.59	4.93E-03	3.48E-02
ENSG00000204257	HLA-DMA	major histocompatibility complex, class II, DM alpha	2.99	1.58	5.61E-09	3.88E-07
ENSG00000130775	THEMIS2	thymocyte selection associated family member 2	2.99	1.58	7.05E-07	2.77E-05
ENSG00000158050	DUSP2	dual specificity phosphatase 2	2.99	1.58	2.78E-06	9.10E-05
ENSG00000256720	RP11-436I9.6	BTG anti-proliferation factor 1 pseudogene 1	2.99	1.58	7.46E-04	8.40E-03

ENSG00000223946	RP11-533O20.2	novel transcript	2.99	1.58	1.54E-03	1.46E-02
ENSG00000263293	RP11-290H9.4	EFCAB13 divergent transcript	-2.99	-1.58	2.13E-03	1.87E-02
ENSG00000241158	ADAMTS9-AS1	ADAMTS9 antisense RNA 1	-2.99	-1.58	2.16E-03	1.89E-02
ENSG00000235532	LINC00402	long intergenic non-protein coding RNA 402	2.99	1.58	2.41E-03	2.05E-02
ENSG00000064012	CASP8	caspase 8	2.97	1.57	1.79E-09	1.38E-07
ENSG00000095794	CREM	cAMP responsive element modulator	-2.97	-1.57	4.11E-08	2.28E-06
ENSG00000179715	PCED1B	PC-esterase domain containing 1B	2.97	1.57	2.36E-06	7.90E-05
ENSG00000027869	SH2D2A	SH2 domain containing 2A	2.97	1.57	5.81E-06	1.68E-04
ENSG00000148204	CRB2	crumbs cell polarity complex component 2	2.97	1.57	1.93E-04	2.97E-03
ENSG00000226237	RP11-276H19.1	GAS1 adjacent regulatory RNA	2.97	1.57	9.82E-04	1.03E-02
ENSG00000100678	SLC8A3	solute carrier family 8 member A3	2.97	1.57	2.42E-03	2.05E-02
ENSG00000130768	SMPDL3B	sphingomyelin phosphodiesterase acid like 3B	2.97	1.57	3.24E-03	2.56E-02
ENSG00000185155	MIXL1	Mix paired-like homeobox	2.97	1.57	3.61E-03	2.76E-02
ENSG00000166831	RBPMS2	RNA binding protein, mRNA processing factor 2	-2.95	-1.56	8.45E-11	8.59E-09
ENSG00000271913	RP1-111C20.4	TAGAP antisense RNA 1	2.95	1.56	1.54E-09	1.21E-07
ENSG00000173198	CYSLTR1	cysteinyl leukotriene receptor 1	2.95	1.56	1.21E-08	7.58E-07
ENSG00000011590	ZBTB32	zinc finger and BTB domain containing 32	2.95	1.56	1.05E-07	5.21E-06
ENSG00000235999	RP11-403I13.8		2.95	1.56	1.23E-05	3.13E-04
ENSG00000115339	GALNT3	polypeptide N-acetylgalactosaminyltransferase 3	2.95	1.56	1.46E-05	3.61E-04
ENSG00000162174	ASRGL1	asparaginase and isoaspartyl peptidase 1	-2.95	-1.56	1.29E-04	2.17E-03
ENSG00000104783	KCNN4	potassium calcium-activated channel subfamily N member 4	2.95	1.56	1.29E-04	2.17E-03
ENSG00000230322	RP3-323N1.2	novel transcript	2.95	1.56	1.36E-04	2.26E-03
ENSG00000239482	RP11-90K6.1	novel transcript	-2.95	-1.56	2.50E-04	3.66E-03
ENSG00000227218	RP11-203J24.8	novel transcript	2.95	1.56	3.31E-04	4.55E-03
ENSG00000140678	ITGAX	integrin subunit alpha X	2.95	1.56	1.20E-03	1.20E-02
ENSG00000267312	RP11-1094M14.7	adenylosuccinate synthase (ADSS) pseudogene	2.95	1.56	2.60E-03	2.17E-02

ENSG00000266389	CTB-41I6.1	PIK3R5 divergent transcript	2.95	1.56	3.68E-03	2.80E-02
ENSG00000251450	CTC-459I6.1	RASGRF2 antisense RNA 1	-2.95	-1.56	5.13E-03	3.56E-02
ENSG00000143196	DPT	dermatopontin	2.93	1.55	7.04E-09	4.69E-07
ENSG00000261971	RP11-473M20.7	MMP25 antisense RNA 1	2.93	1.55	1.95E-05	4.57E-04
ENSG00000099860	GADD45B	growth arrest and DNA damage inducible beta	-2.93	-1.55	2.65E-05	5.87E-04
ENSG00000214851	LINC00612	long intergenic non-protein coding RNA 612	2.93	1.55	9.30E-05	1.66E-03
ENSG00000135114	OASL	2-5-oligoadenylate synthetase like	2.93	1.55	3.91E-04	5.19E-03
ENSG00000080224	EPHA6	EPH receptor A6	2.93	1.55	4.08E-04	5.36E-03
ENSG00000259236	CTD-2611K5.5	golgin A8 family member V, pseudogene	2.93	1.55	5.22E-04	6.46E-03
ENSG00000238528	snoU13		2.93	1.55	5.41E-04	6.64E-03
ENSG00000271151	RP11-394I13.2	novel transcript	2.93	1.55	1.87E-03	1.69E-02
ENSG00000179083	FAM133A	family with sequence similarity 133 member A	2.93	1.55	3.16E-03	2.52E-02
ENSG00000236039	AC019117.2	long intergenic non-protein coding RNA 2889	-2.93	-1.55	5.29E-03	3.65E-02
ENSG00000113889	KNG1	kininogen 1	-2.93	-1.55	5.77E-03	3.88E-02
ENSG00000128604	IRF5	interferon regulatory factor 5	2.91	1.54	2.25E-08	1.35E-06
ENSG00000138439	FAM117B	family with sequence similarity 117 member B	2.91	1.54	1.29E-07	6.30E-06
ENSG00000088827	SIGLEC1	sialic acid binding Ig like lectin 1	2.91	1.54	2.21E-06	7.44E-05
ENSG00000198417	MT1F	metallothionein 1F	2.91	1.54	3.02E-06	9.70E-05
ENSG00000159388	BTG2	BTG anti-proliferation factor 2	-2.91	-1.54	1.30E-05	3.27E-04
ENSG00000165168	CYBB	cytochrome b-245 beta chain	2.91	1.54	3.04E-05	6.58E-04
ENSG00000176971	FIBIN	fin bud initiation factor homolog	2.91	1.54	5.43E-05	1.07E-03
ENSG00000005381	MPO	myeloperoxidase	2.91	1.54	1.02E-03	1.06E-02
ENSG00000242651	RN7SL862P	RNA, 7SL, cytoplasmic 862, pseudogene	2.91	1.54	5.14E-03	3.57E-02
ENSG00000244921	CTB-36O1.7	MT-CYB pseudogene 18	-2.89	-1.53	1.55E-06	5.39E-05
ENSG00000131459	GFPT2	glutamine-fructose-6-phosphate transaminase 2	-2.89	-1.53	7.38E-05	1.37E-03
ENSG00000095970	TREM2	triggering receptor expressed on myeloid cells 2	2.89	1.53	9.80E-05	1.72E-03
ENSG00000225496	AC104651.2	RTRAF pseudogene 1	2.89	1.53	1.48E-03	1.41E-02
ENSG00000214894	LINC00243	long intergenic non-protein coding RNA 243	2.89	1.53	2.90E-03	2.36E-02

ENSG00000151023	ENKUR	enkurin, TRPC channel interacting protein	2.89	1.53	3.26E-03	2.58E-02
ENSG00000125144	MT1G	metallothionein 1G	2.89	1.53	6.32E-03	4.16E-02
ENSG00000100629	CEP128	centrosomal protein 128	2.87	1.52	2.54E-09	1.91E-07
ENSG00000137094	DNAJB5	DnaJ heat shock protein family (Hsp40) member B5	-2.87	-1.52	8.22E-09	5.41E-07
ENSG00000167470	MIDN	midnolin	-2.87	-1.52	9.70E-07	3.64E-05
ENSG00000110079	MS4A4A	membrane spanning 4-domains A4A	2.87	1.52	4.75E-06	1.42E-04
ENSG00000258810	RP11-219E7.1	novel transcript, antisense to RNASE1	2.87	1.52	2.25E-04	3.35E-03
ENSG00000136449	MYCBPAP	MYCBP associated protein	2.87	1.52	4.02E-04	5.30E-03
ENSG00000180549	FUT7	fucosyltransferase 7	2.87	1.52	1.66E-03	1.54E-02
ENSG00000253519	AC106801.1	novel transcript	-2.87	-1.52	2.86E-03	2.34E-02
ENSG00000065320	NTN1	netrin 1	-2.85	-1.51	1.06E-09	8.82E-08
ENSG00000244682	FCGR2C	Fc fragment of IgG receptor IIc (gene/pseudogene)	2.85	1.51	1.26E-09	1.02E-07
ENSG00000198865	CCDC152	coiled-coil domain containing 152	2.85	1.51	1.54E-09	1.21E-07
ENSG00000184545	DUSP8	dual specificity phosphatase 8	-2.85	-1.51	5.91E-09	4.04E-07
ENSG00000059377	TBXAS1	thromboxane A synthase 1	2.85	1.51	5.99E-09	4.08E-07
ENSG00000159189	C1QC	complement C1q C chain	2.85	1.51	7.50E-06	2.10E-04
ENSG00000105383	CD33	CD33 molecule	2.85	1.51	1.67E-05	4.04E-04
ENSG00000178075	GRAMD1C	GRAM domain containing 1C	2.85	1.51	3.21E-05	6.87E-04
ENSG00000248986	RP11-774O3.1		-2.85	-1.51	1.15E-04	1.97E-03
ENSG00000260806	RP11-872J21.3	PAPOLA divergent transcript	-2.85	-1.51	1.07E-03	1.10E-02
ENSG00000104974	LILRA1	leukocyte immunoglobulin like receptor A1	2.85	1.51	1.31E-03	1.29E-02
ENSG00000254362	RP11-14I17.3	novel transcript	2.85	1.51	1.54E-03	1.45E-02
ENSG00000227227	AC017101.10	novel transcript	-2.85	-1.51	1.63E-03	1.52E-02
ENSG00000086288	NME8	NME/NM23 family member 8	2.85	1.51	2.11E-03	1.86E-02
ENSG00000114268	PFKFB4	6-phosphofructo-2-kinase/fructose-2,6-biphosphatase 4	2.83	1.5	1.39E-07	6.73E-06
ENSG00000244020	MT1HL1	metallothionein 1H like 1	-2.83	-1.5	2.87E-04	4.07E-03
ENSG00000227630	LINC01132	long intergenic non-protein coding RNA 1132	-2.83	-1.5	5.26E-04	6.51E-03
ENSG00000254503	CTD-2521M24.4	high-mobility group box 3 (HMGB3) pseudogene	2.83	1.5	6.12E-04	7.23E-03

ENSG00000106178	CCL24	C-C motif chemokine ligand 24	2.83	1.5	5.30E-03	3.66E-02
