# Supplementary material for: Epigenetic regulation of transcription factor binding motifs promotes Th1 response in Chagas disease cardiomyopathy
Source: Front Immunol. 2022 Aug 22;13:958200. doi: 10.3389/fimmu.2022.958200 (PMC9441916; doi:10.3389/fimmu.2022.958200)
Supplement: Supplementary Table 1 — Biological samples included in this study. [file DataSheet_1.zip › Supplementary Material/Supplementary Table 4.pdf]

Supplementary table 4. Statistics on coding sequences detected by RNA-seq and on differentially expressed genes.

Gene type	Nb. All genes	Percentage (All genes)	Nb. DEGs	Percentage (DEG)	Chisq square pvalue	Chisq square corrected pvalue
**protein_coding**	18899	41.94	893	63.38	2.10E-21	3.36E-20
**pseudogene**	9565	21.22	155	11	6.20E-15	4.96E-14
**lincRNA**	5312	11.79	146	10.36	1.60E-01	2.56E-01
**antisense**	4499	9.98	132	9.37	5.20E-01	7.56E-01
**miRNA**	1705	3.78	19	1.35	4.90E-06	1.96E-05
**misc_RNA**	1455	3.23	8	0.57	5.10E-08	2.72E-07
**snRNA**	1150	2.55	9	0.64	1.20E-05	3.84E-05
**snoRNA**	922	2.05	7	0.5	8.10E-05	2.16E-04
**sense_intronic**	678	1.5	14	0.99	1.50E-01	2.56E-01
**processed_transcript**	404	0.9	14	0.99	8.20E-01	9.83E-01
**rRNA**	235	0.52	1	0.07	3.20E-02	6.40E-02
**sense_overlapping**	175	0.39	5	0.35	1.00E+00	1.00E+00
**polymorphic_pseudogene**	31	0.07	5	0.35	9.40E-04	2.15E-03
**Mt_tRNA**	22	0.05	0	0	8.40E-01	9.83E-01
**3prime_overlapping_ncrna**	12	0.03	1	0.07	8.60E-01	9.83E-01
**Mt_rRNA**	2	0	0	0	1.00E+00	1.00E+00
