# Supplementary material for: Epigenetic regulation of transcription factor binding motifs promotes Th1 response in Chagas disease cardiomyopathy
Source: Front Immunol. 2022 Aug 22;13:958200. doi: 10.3389/fimmu.2022.958200 (PMC9441916; doi:10.3389/fimmu.2022.958200)
Supplement: Supplementary Table 1 — Biological samples included in this study. [file DataSheet_1.zip › Supplementary Material/Supplementary Table 5.pdf]

Supplementary table 5. List of unique differentially expressed genes identified by RNA-seq between 8 DCM heart tissue samples and 6 heart tissue samples obtained from organ donors.

Gene id	Gene Name	Fold Change	Log2 Fold Change	pvalue	Corrected pvalue	DE in CCC
ENSG00000213934	HBG1	14631,01	13,84	3,71E-23	1,54E-21	No DEG
ENSG00000255633	MTRNR2L9	-1376,82	-10,43	5,13E-40	6,90E-38	No DEG
ENSG00000210077	MT-TV	-852,73	-9,74	1,03E-19	3,06E-18	No DEG
ENSG00000231501	MTND4LP1	-659,81	-9,37	4,59E-17	1,06E-15	No DEG
ENSG00000210082	MT-RNR2	-580,47	-9,18	1,15E-146	1,18E-142	No DEG
ENSG00000210049	MT-TF	-510,03	-8,99	1,23E-23	5,37E-22	No DEG
ENSG00000269028	MTRNR2L12	-506,79	-8,99	1,92E-202	5,90E-198	No DEG
ENSG00000211459	MT-RNR1	-489,48	-8,94	1,09E-128	7,74E-125	No DEG
ENSG00000271043	MTRNR2L2	-441,96	-8,79	1,26E-128	7,74E-125	No DEG
ENSG00000163435	ELF3	-404,95	-8,66	1,11E-09	1,12E-08	DOWN
ENSG00000248713	RP11-766F14.2	-380,88	-8,57	3,97E-16	8,27E-15	DOWN
ENSG00000213937	CLDN9	-332,72	-8,38	3,06E-10	3,30E-09	No DEG
ENSG00000249072	RP11-777B9.5	-304,25	-8,25	6,58E-09	5,95E-08	No DEG
ENSG00000166589	CDH16	-216,07	-7,76	5,01E-07	3,42E-06	DOWN
ENSG00000255823	MTRNR2L8	-165,74	-7,37	4,14E-106	1,27E-102	No DEG
ENSG00000196565	HBG2	163,19	7,35	2,57E-18	6,68E-17	No DEG
ENSG00000256045	MTRNR2L10	-157,59	-7,30	7,26E-17	1,64E-15	No DEG
ENSG00000174521	TTC9B	-151,25	-7,24	4,37E-06	2,55E-05	No DEG
ENSG00000264063	MIR3687	131,82	7,04	3,00E-99	8,36E-96	No DEG
ENSG00000269496	AC007919.2	-118,85	-6,89	6,23E-07	4,17E-06	No DEG
ENSG00000129437	KLK14	-115,88	-6,86	3,12E-05	1,57E-04	No DEG
ENSG00000141750	STAC2	-105,00	-6,71	5,51E-05	2,65E-04	No DEG
ENSG00000227758	HCG9P5	-101,35	-6,66	2,53E-04	1,07E-03	No DEG
ENSG00000267795	SMIM22	-100,01	-6,64	2,07E-04	8,87E-04	No DEG
ENSG00000223722	RP11-467L13.5	-95,53	-6,58	4,07E-06	2,39E-05	No DEG
ENSG00000265830	AL592188.7	86,50	6,43	9,17E-83	1,41E-79	No DEG
ENSG00000198555	RP11-598D12.4	-84,93	-6,41	2,52E-04	1,06E-03	No DEG
ENSG00000266219	AL592188.8	84,31	6,40	6,77E-21	2,28E-19	No DEG

ENSG00000135248	FAM71F1	-83,70	-6,39	1,56E-05	8,30E-05	DOWN
ENSG00000232623	AP000266.7	-78,81	-6,30	1,64E-05	8,73E-05	No DEG
ENSG00000236888	RPS20P5	-73,31	-6,20	1,07E-03	3,94E-03	No DEG
ENSG00000255843	AP000593.7	-71,14	-6,15	1,28E-04	5,70E-04	No DEG
ENSG00000236060	HSPB1P1	-70,03	-6,13	2,55E-12	3,59E-11	No DEG
ENSG00000229807	XIST	65,83	6,04	2,41E-06	1,46E-05	UP
ENSG00000184106	TREML3P	-65,53	-6,03	1,03E-03	3,80E-03	No DEG
ENSG00000103089	FA2H	-62,11	-5,96	8,64E-05	3,98E-04	No DEG
ENSG00000180344	AL035460.1	-61,15	-5,93	1,57E-03	5,52E-03	No DEG
ENSG00000254684	RP11-882I15.1	-60,85	-5,93	4,15E-04	1,67E-03	No DEG
ENSG00000270160	RP11-264E20.2	-58,96	-5,88	1,59E-03	5,59E-03	No DEG
ENSG00000130612	CYP2G1P	-56,51	-5,82	1,79E-03	6,23E-03	No DEG
ENSG00000267523	CTD-2537I9.12	-49,89	-5,64	2,34E-03	7,93E-03	No DEG
ENSG00000135346	CGA	-49,48	-5,63	2,99E-03	9,87E-03	No DEG
ENSG00000264201	MIR4701	-48,52	-5,60	7,37E-04	2,81E-03	No DEG
ENSG00000139330	KERA	47,91	5,58	1,76E-04	7,65E-04	No DEG
ENSG00000255650	FAM222A-AS1	-45,94	-5,52	8,87E-09	7,91E-08	DOWN
ENSG00000180537	RNF182	41,92	5,39	2,14E-04	9,14E-04	UP
ENSG00000264462	MIR3648	41,76	5,38	1,62E-32	1,35E-30	No DEG
ENSG00000121895	TMEM156	40,95	5,36	1,16E-04	5,23E-04	UP
ENSG00000226958	CTD-2328D6.1	40,71	5,35	3,69E-05	1,84E-04	No DEG
ENSG00000230216	HSPB1P2	-40,36	-5,33	1,94E-03	6,69E-03	No DEG
ENSG00000185742	C11orf87	34,77	5,12	1,31E-03	4,72E-03	No DEG
ENSG00000145040	UCN2	-34,43	-5,11	1,12E-03	4,10E-03	No DEG
ENSG00000270672	MTRNR2L6	-34,12	-5,09	1,99E-13	3,19E-12	No DEG
ENSG00000008056	SYN1	-33,60	-5,07	2,57E-35	2,51E-33	No DEG
ENSG00000224904	RP5-934G17.6	-33,22	-5,05	1,83E-05	9,65E-05	DOWN
ENSG00000163958	ZDHHC19	-32,30	-5,01	6,76E-03	2,02E-02	No DEG
ENSG00000240096	RP11-85G20.1	-32,28	-5,01	1,00E-02	2,86E-02	No DEG
ENSG00000256618	MTRNR2L1	-31,81	-4,99	2,16E-25	1,07E-23	No DEG
ENSG00000236844	AC091633.2	31,16	4,96	2,02E-03	6,95E-03	No DEG
ENSG00000215506	TPTE2P4	-30,48	-4,93	3,31E-05	1,66E-04	DOWN

ENSG00000205869	KRTAP5-1	-28,51	-4,83	8,32E-09	7,45E-08	No DEG
ENSG00000205592	MUC19	28,13	4,81	1,11E-03	4,05E-03	UP
ENSG00000250863	RP11-663P9.1	27,30	4,77	2,64E-03	8,82E-03	No DEG
ENSG00000204969	PCDHA2	27,27	4,77	1,28E-03	4,61E-03	UP
ENSG00000259547	CYCSP2	-27,01	-4,76	9,78E-03	2,79E-02	No DEG
ENSG00000256633	RP11-169D4.2	-26,98	-4,75	5,43E-03	1,67E-02	No DEG
ENSG00000236510	AC011284.3	-26,69	-4,74	8,72E-03	2,52E-02	No DEG
ENSG00000212961	HNRNPA1P40	-26,48	-4,73	5,20E-03	1,60E-02	No DEG
ENSG00000265525	AL592188.5	26,29	4,72	7,14E-07	4,73E-06	No DEG
ENSG00000232346	SC22CB-1E7.1	26,07	4,70	5,81E-04	2,26E-03	No DEG
ENSG00000188536	HBA2	25,95	4,70	2,67E-13	4,22E-12	No DEG
ENSG00000233473	RAD1P2	25,65	4,68	1,04E-03	3,83E-03	No DEG
ENSG00000243910	TUBA4B	-25,37	-4,66	1,20E-07	9,07E-07	No DEG
ENSG00000270190	RP11-803D5.4	-25,18	-4,65	4,43E-07	3,05E-06	No DEG
ENSG00000266141	MIR2909	25,15	4,65	8,09E-04	3,06E-03	No DEG
ENSG00000233005	AC067959.1	24,63	4,62	1,80E-03	6,26E-03	No DEG
ENSG00000239705	RP11-65N13.8	-24,46	-4,61	9,84E-03	2,81E-02	No DEG
ENSG00000273274	ZBTB8B	24,39	4,61	2,78E-03	9,24E-03	No DEG
ENSG00000206172	HBA1	24,01	4,59	3,53E-14	6,11E-13	No DEG
ENSG00000212340	RNU6-739P	-23,82	-4,57	1,09E-02	3,06E-02	No DEG
ENSG00000250282	RP5-875H18.4	-23,17	-4,53	2,62E-06	1,58E-05	No DEG
ENSG00000227815	RP11-195C7.3	23,08	4,53	8,82E-04	3,31E-03	No DEG
ENSG00000254966	RP11-1081L13.4	-22,82	-4,51	9,44E-03	2,71E-02	No DEG
ENSG00000228974	AC006483.5	-22,77	-4,51	1,13E-02	3,18E-02	No DEG
ENSG00000265396	MIR3128	22,26	4,48	8,23E-04	3,11E-03	No DEG
ENSG00000198744	RP5-857K21.11	-22,14	-4,47	9,69E-79	1,10E-75	No DEG
ENSG00000168334	XIRP1	-22,04	-4,46	3,55E-27	2,06E-25	DOWN
ENSG00000259343	RP11-761I4.3	21,96	4,46	2,52E-03	8,47E-03	UP
ENSG00000265511	RP11-524F11.1	-21,95	-4,46	1,59E-06	9,93E-06	No DEG
ENSG00000175592	FOSL1	-21,79	-4,45	1,83E-07	1,34E-06	DOWN
ENSG00000237575	PYY2	-21,70	-4,44	1,27E-02	3,51E-02	No DEG
ENSG00000264827	AL592188.4	21,63	4,44	2,70E-28	1,70E-26	No DEG

ENSG00000069399	BCL3	-20,63	-4,37	8,14E-24	3,62E-22	DOWN
ENSG00000108691	CCL2	-19,90	-4,31	9,53E-10	9,69E-09	DOWN
ENSG00000254248	RP11-320N21.2	-19,81	-4,31	1,43E-02	3,89E-02	No DEG
ENSG00000263624	RP11-45M22.3	-19,68	-4,30	1,05E-19	3,12E-18	No DEG
ENSG00000127129	EDN2	-19,58	-4,29	8,82E-05	4,05E-04	DOWN
ENSG00000089169	RPH3A	-19,57	-4,29	7,58E-04	2,88E-03	No DEG
ENSG00000240404	RP11-142L1.3	-19,50	-4,29	1,17E-10	1,33E-09	DOWN
ENSG00000266658	RNA28S5	19,44	4,28	6,84E-40	9,05E-38	No DEG
ENSG00000142089	IFITM3	-19,38	-4,28	2,66E-47	5,88E-45	No DEG
ENSG00000126010	GRPR	-19,15	-4,26	9,84E-10	9,98E-09	No DEG
ENSG00000271303	SRXN1	18,99	4,25	6,49E-22	2,39E-20	No DEG
ENSG00000225892	RP11-384K6.2	-18,94	-4,24	1,76E-10	1,96E-09	No DEG
ENSG00000256443	RP11-794G24.1	-18,88	-4,24	7,49E-08	5,84E-07	DOWN
ENSG00000229344	RP5-857K21.7	-18,87	-4,24	8,21E-41	1,16E-38	No DEG
ENSG00000259342	RP11-519G16.5	-18,83	-4,24	1,78E-05	9,37E-05	DOWN
ENSG00000186907	RTN4RL2	-18,76	-4,23	8,80E-09	7,86E-08	No DEG
ENSG00000201998	SNORA23	18,61	4,22	3,90E-50	1,12E-47	No DEG
ENSG00000230873	STMND1	-18,60	-4,22	1,08E-02	3,05E-02	No DEG
ENSG00000233496	SYNJ2-IT1	-18,30	-4,19	2,98E-04	1,24E-03	No DEG
ENSG00000224769	AC069213.1	-18,27	-4,19	1,74E-09	1,71E-08	No DEG
ENSG00000253339	RP11-434I12.3	-18,19	-4,19	1,55E-02	4,15E-02	No DEG
ENSG00000244056	RN7SL417P	-18,03	-4,17	1,95E-08	1,66E-07	No DEG
ENSG00000264925	Z98949.1	-18,01	-4,17	3,83E-08	3,11E-07	DOWN
ENSG00000230715	RP11-274B21.4	-17,86	-4,16	5,45E-09	5,00E-08	No DEG
ENSG00000259570	RP11-671M22.4	-17,43	-4,12	4,67E-10	4,94E-09	No DEG
ENSG00000272543	MIR4787	17,36	4,12	4,62E-05	2,25E-04	No DEG
ENSG00000251062	CTD-2218G20.1	17,35	4,12	2,20E-03	7,49E-03	No DEG
ENSG00000267883	PRED60	-17,21	-4,11	3,30E-04	1,36E-03	No DEG
ENSG00000250835	LSM3P4	-17,19	-4,10	4,27E-05	2,09E-04	No DEG
ENSG00000177464	GPR4	-17,08	-4,09	1,89E-23	8,10E-22	DOWN
ENSG00000226659	RP11-137H2.4	17,03	4,09	3,09E-03	1,01E-02	UP
ENSG00000268864	CTB-167G5.5	-16,95	-4,08	3,95E-05	1,95E-04	DOWN

ENSG00000202159	RNU6-742P	16,92	4,08	2,82E-03	9,36E-03	No DEG
ENSG00000148734	NPFFR1	-16,85	-4,07	1,70E-02	4,50E-02	No DEG
ENSG00000261193	RP11-863P13.5	-16,81	-4,07	1,75E-02	4,62E-02	No DEG
ENSG00000243302	RP11-274B21.2	-16,75	-4,07	8,06E-25	3,83E-23	No DEG
ENSG00000104848	KCNA7	-16,73	-4,06	9,94E-05	4,52E-04	DOWN
ENSG00000204533	AC024580.1	-16,72	-4,06	1,76E-04	7,67E-04	No DEG
ENSG00000254510	RP11-867G23.10	-16,66	-4,06	2,01E-07	1,47E-06	DOWN
ENSG00000139438	FAM222A	-16,59	-4,05	1,60E-13	2,60E-12	DOWN
ENSG00000225972	MTND1P23	-16,57	-4,05	1,51E-04	6,64E-04	No DEG
ENSG00000265768	MIR4506	-16,23	-4,02	1,42E-06	8,95E-06	DOWN
ENSG00000197616	MYH6	-16,15	-4,01	1,18E-11	1,53E-10	DOWN
ENSG00000254851	RP11-109L13.1	-16,14	-4,01	1,61E-07	1,19E-06	No DEG
ENSG00000231587	SNORD62B	-16,05	-4,00	1,56E-05	8,30E-05	No DEG
ENSG00000273259	RP11-986E7.7	-15,95	-4,00	6,52E-06	3,69E-05	DOWN
ENSG00000272872	LL22NC03-N14H11.1	15,85	3,99	4,48E-03	1,41E-02	No DEG
ENSG00000267782	RP11-799D4.3	-15,81	-3,98	4,18E-04	1,68E-03	No DEG
ENSG00000229854	RP11-524G24.2	-15,72	-3,97	2,04E-05	1,06E-04	DOWN
ENSG00000212864	RNF208	-15,65	-3,97	7,73E-13	1,16E-11	No DEG
ENSG00000237291	RP11-782C8.4	-15,61	-3,96	5,31E-06	3,05E-05	No DEG
ENSG00000172156	CCL11	-15,61	-3,96	4,57E-09	4,23E-08	No DEG
ENSG00000124507	PACSIN1	-15,54	-3,96	1,99E-21	7,01E-20	No DEG
ENSG00000265579	RP11-713C5.1	15,54	3,96	5,68E-03	1,74E-02	No DEG
ENSG00000260689	HNRNPA3P11	-15,50	-3,95	1,65E-11	2,10E-10	No DEG
ENSG00000248527	MTATP6P1	-15,46	-3,95	1,95E-85	3,32E-82	No DEG
ENSG00000237337	RP11-567C20.2	15,41	3,95	3,09E-03	1,01E-02	No DEG
ENSG00000255710	RP11-667M19.9	-15,34	-3,94	6,69E-15	1,25E-13	No DEG
ENSG00000260401	RP11-800A3.4	-15,12	-3,92	1,25E-32	1,05E-30	DOWN
ENSG00000222121	AL512290.1	15,00	3,91	3,05E-03	1,00E-02	No DEG
ENSG00000109684	CLNK	14,95	3,90	1,42E-04	6,29E-04	UP
ENSG00000157654	PALM2-AKAP2	14,88	3,90	9,51E-09	8,44E-08	No DEG
ENSG00000257531	RP3-405J10.2	-14,87	-3,89	3,63E-04	1,48E-03	No DEG
ENSG00000272060	RNA18S5	14,80	3,89	1,62E-20	5,24E-19	No DEG

ENSG00000254131	RP11-1007J8.1	-14,72	-3,88	9,08E-08	6,98E-07	No DEG
ENSG00000244734	HBB	14,71	3,88	1,30E-08	1,13E-07	No DEG
ENSG00000212254	RNU6-124P	14,68	3,88	3,78E-03	1,21E-02	No DEG
ENSG00000271272	RP11-510P12.1	14,62	3,87	4,36E-03	1,37E-02	No DEG
ENSG00000256540	RP11-598F7.6	-14,56	-3,86	4,09E-14	7,01E-13	DOWN
ENSG00000089327	FXYD5	-14,56	-3,86	3,68E-35	3,57E-33	No DEG
ENSG00000162763	LRRC52	-14,43	-3,85	2,45E-04	1,04E-03	No DEG
ENSG00000101892	ATP1B4	14,37	3,85	1,71E-04	7,47E-04	UP
ENSG00000265807	AL592188.6	14,30	3,84	7,08E-08	5,54E-07	No DEG
ENSG00000249780	RP11-352E6.2	-14,27	-3,84	6,41E-08	5,05E-07	No DEG
ENSG00000268896	RP11-256I23.1	-14,25	-3,83	1,29E-04	5,75E-04	No DEG
ENSG00000156966	B3GNT7	-14,17	-3,82	1,29E-10	1,46E-09	DOWN
ENSG00000271537	RP11-365F18.6	14,14	3,82	6,51E-03	1,96E-02	No DEG
ENSG00000259683	RP11-182J1.14	-14,07	-3,81	1,08E-18	2,94E-17	No DEG
ENSG00000128342	LIF	-14,02	-3,81	1,79E-07	1,31E-06	DOWN
ENSG00000183615	FAM167B	-13,92	-3,80	6,43E-13	9,74E-12	No DEG
ENSG00000124216	SNAI1	-13,92	-3,80	8,23E-06	4,59E-05	No DEG
ENSG00000056291	NPFFR2	13,91	3,80	6,73E-03	2,01E-02	UP
ENSG00000256995	RP11-114G22.1	-13,79	-3,79	2,15E-03	7,34E-03	No DEG
ENSG00000134115	CNTN6	13,78	3,78	2,71E-04	1,13E-03	No DEG
ENSG00000166091	CMTM5	-13,72	-3,78	2,93E-07	2,08E-06	DOWN
ENSG00000142669	SH3BGRL3	-13,70	-3,78	1,43E-34	1,35E-32	No DEG
ENSG00000243859	RPL5P17	13,68	3,77	1,15E-06	7,38E-06	No DEG
ENSG00000232867	RP11-179D22.1	-13,64	-3,77	6,14E-06	3,49E-05	DOWN
ENSG00000103257	SLC7A5	-13,59	-3,76	3,41E-26	1,80E-24	No DEG
ENSG00000168528	SERINC2	-13,56	-3,76	1,66E-15	3,27E-14	No DEG
ENSG00000144406	UNC80	13,51	3,76	9,13E-06	5,06E-05	UP
ENSG00000159840	ZYX	-13,35	-3,74	3,86E-44	6,73E-42	No DEG
ENSG00000204936	CD177	-13,35	-3,74	9,47E-05	4,32E-04	DOWN
ENSG00000114737	CISH	-13,25	-3,73	2,91E-13	4,58E-12	DOWN
ENSG00000139915	MDGA2	13,23	3,73	7,17E-03	2,13E-02	No DEG
ENSG00000259602	RP11-138E16.1	13,16	3,72	4,99E-03	1,55E-02	No DEG

ENSG00000232081	LARGE-IT1	-13,12	-3,71	1,65E-05	8,78E-05	No DEG
ENSG00000227512	RP11-413M3.4	-13,12	-3,71	9,66E-07	6,26E-06	No DEG
ENSG00000269653	CTB-102L5.7	-13,04	-3,71	3,04E-05	1,54E-04	No DEG
ENSG00000103145	HCFC1R1	-12,97	-3,70	2,24E-75	2,22E-72	No DEG
ENSG00000179820	MYADM	-12,93	-3,69	4,25E-32	3,47E-30	No DEG
ENSG00000128283	CDC42EP1	-12,91	-3,69	1,65E-31	1,28E-29	No DEG
ENSG00000258987	RP11-131H24.4	-12,90	-3,69	1,57E-12	2,27E-11	DOWN
ENSG00000228166	MTND1P11	-12,89	-3,69	2,64E-07	1,89E-06	No DEG
ENSG00000168062	BATF2	-12,88	-3,69	2,70E-15	5,21E-14	No DEG
ENSG00000257681	RP11-341G23.4	12,83	3,68	6,77E-03	2,02E-02	No DEG
ENSG00000198888	MT-ND1	-12,81	-3,68	2,75E-45	5,25E-43	No DEG
ENSG00000184545	DUSP8	-12,75	-3,67	8,00E-41	1,14E-38	DOWN
ENSG00000109846	CRYAB	-12,72	-3,67	1,15E-175	1,76E-171	No DEG
ENSG00000267650	CTD-2553C6.1	-12,69	-3,67	2,37E-04	1,00E-03	No DEG
ENSG00000198816	ZNF358	-12,69	-3,67	6,24E-89	1,20E-85	No DEG
ENSG00000172935	MRGPRF	-12,62	-3,66	3,01E-19	8,63E-18	No DEG
ENSG00000250230	RP11-855O10.2	-12,62	-3,66	1,11E-03	4,07E-03	No DEG
ENSG00000241352	RP11-392P7.1	12,60	3,65	3,52E-05	1,76E-04	No DEG
ENSG00000254449	SF3A3P2	-12,54	-3,65	7,69E-08	5,98E-07	DOWN
ENSG00000228737	AC008781.7	-12,46	-3,64	1,20E-03	4,36E-03	No DEG
ENSG00000269396	AC187652.1	-12,41	-3,63	8,05E-04	3,05E-03	No DEG
ENSG00000167173	C15orf39	-12,40	-3,63	4,79E-53	1,62E-50	No DEG
ENSG00000139540	SLC39A5	-12,39	-3,63	4,83E-04	1,92E-03	No DEG
ENSG00000177725	AC105206.1	-12,36	-3,63	1,18E-04	5,31E-04	No DEG
ENSG00000118849	RARRES1	-12,34	-3,63	1,97E-07	1,44E-06	DOWN
ENSG00000198712	MT-CO2	-12,33	-3,62	2,60E-45	4,99E-43	No DEG
ENSG00000176919	C8G	-12,28	-3,62	3,89E-16	8,14E-15	DOWN
ENSG00000250421	RP11-83M16.6	12,23	3,61	2,08E-05	1,08E-04	UP
ENSG00000203643	AC012456.3	12,21	3,61	5,95E-03	1,81E-02	No DEG
ENSG00000243679	RP11-274B21.3	-12,21	-3,61	3,68E-20	1,15E-18	No DEG
ENSG00000127324	TSPAN8	12,21	3,61	9,34E-09	8,31E-08	UP
ENSG00000260582	TPST2P1	-12,11	-3,60	7,63E-06	4,27E-05	DOWN

ENSG00000261635	RP11-618N24.1	-12,09	-3,60	2,17E-06	1,32E-05	DOWN
ENSG00000271959	CTD-3064M3.7	-12,08	-3,59	1,53E-12	2,23E-11	No DEG
ENSG00000075702	WDR62	-12,08	-3,59	4,87E-17	1,12E-15	No DEG
ENSG00000168961	LGALS9	-12,05	-3,59	1,63E-25	8,19E-24	No DEG
ENSG00000131759	RARA	-12,04	-3,59	9,53E-45	1,75E-42	No DEG
ENSG00000270210	RP11-373D23.3	-12,00	-3,58	2,62E-04	1,10E-03	No DEG
ENSG00000254810	RP11-672A2.4	-11,94	-3,58	8,89E-07	5,80E-06	No DEG
ENSG00000123358	NR4A1	-11,88	-3,57	2,14E-07	1,55E-06	DOWN
ENSG00000264572	MIR4296	-11,75	-3,56	2,13E-04	9,09E-04	No DEG
ENSG00000158955	WNT9B	-11,75	-3,55	4,18E-06	2,44E-05	No DEG
ENSG00000207780	MIR648	-11,74	-3,55	2,94E-04	1,22E-03	No DEG
ENSG00000133874	RNF122	-11,73	-3,55	1,51E-18	4,01E-17	DOWN
ENSG00000000005	TNMD	11,72	3,55	2,31E-05	1,19E-04	UP
ENSG00000210151	MT-TS1	11,69	3,55	3,21E-36	3,42E-34	No DEG
ENSG00000166592	RRAD	-11,66	-3,54	2,16E-12	3,07E-11	DOWN
ENSG00000137404	NRM	-11,64	-3,54	6,55E-23	2,65E-21	No DEG
ENSG00000228288	PCAT6	-11,58	-3,53	1,61E-04	7,07E-04	No DEG
ENSG00000231052	RP11-91N2.3	-11,52	-3,53	2,25E-03	7,65E-03	No DEG
ENSG00000156885	COX6A2	-11,49	-3,52	2,13E-67	1,67E-64	No DEG
ENSG00000105205	CLC	11,48	3,52	1,03E-02	2,91E-02	No DEG
ENSG00000185519	FAM131C	-11,47	-3,52	2,35E-09	2,26E-08	No DEG
ENSG00000188681	TEKT4P2	-11,44	-3,52	1,45E-10	1,63E-09	No DEG
ENSG00000223609	HBD	11,43	3,51	7,73E-07	5,09E-06	No DEG
ENSG00000196517	SLC6A9	-11,40	-3,51	1,80E-23	7,73E-22	No DEG
ENSG00000255202	RP4-541C22.5	-11,40	-3,51	4,50E-03	1,41E-02	No DEG
ENSG00000265078	RN7SL664P	11,38	3,51	6,87E-07	4,57E-06	No DEG
ENSG00000139998	RAB15	-11,34	-3,50	3,25E-23	1,37E-21	DOWN
ENSG00000183644	C11orf88	11,32	3,50	7,18E-03	2,13E-02	No DEG
ENSG00000126368	NR1D1	-11,29	-3,50	3,79E-17	8,84E-16	No DEG
ENSG00000266447	AC046143.2	11,28	3,50	2,50E-05	1,28E-04	No DEG
ENSG00000102007	PLP2	-11,26	-3,49	8,74E-46	1,73E-43	No DEG
ENSG00000225356	RP11-433O3.1	-11,09	-3,47	8,62E-04	3,24E-03	No DEG

ENSG00000223695	RP4-633O19__A.1	-11,08	-3,47	8,06E-06	4,50E-05	DOWN
ENSG00000265139	RP11-227G15.2	11,07	3,47	2,40E-15	4,67E-14	No DEG
ENSG00000111199	TRPV4	-11,05	-3,47	1,10E-08	9,66E-08	No DEG
ENSG00000177447	CBX3P1	11,00	3,46	9,34E-03	2,68E-02	No DEG
ENSG00000105131	EPHX3	-10,97	-3,46	1,60E-04	7,00E-04	No DEG
ENSG00000258751	RP11-2G1.1	10,95	3,45	1,02E-02	2,89E-02	No DEG
ENSG00000205177	C11orf91	-10,87	-3,44	1,53E-06	9,54E-06	No DEG
ENSG00000144230	GPR17	-10,80	-3,43	5,99E-18	1,51E-16	No DEG
ENSG00000214354	AC133644.3	-10,80	-3,43	5,52E-05	2,65E-04	No DEG
ENSG00000220157	HNRNPA1P12	-10,79	-3,43	2,19E-04	9,32E-04	No DEG
ENSG00000272799	RP11-474N24.6	-10,78	-3,43	3,58E-03	1,15E-02	DOWN
ENSG00000231888	MTND5P15	-10,75	-3,43	7,08E-04	2,71E-03	No DEG
ENSG00000238359	snoU13	10,73	3,42	3,88E-06	2,28E-05	No DEG
ENSG00000250274	CTB-114C7.4	-10,69	-3,42	1,28E-03	4,61E-03	No DEG
ENSG00000198899	MT-ATP6	-10,69	-3,42	4,67E-56	2,02E-53	No DEG
ENSG00000137496	IL18BP	-10,69	-3,42	4,18E-28	2,60E-26	No DEG
ENSG00000243477	NAT6	-10,66	-3,41	1,06E-44	1,93E-42	No DEG
ENSG00000178821	TMEM52	-10,63	-3,41	8,10E-08	6,27E-07	No DEG
ENSG00000213133	PPP1R14BP5	-10,51	-3,39	4,07E-04	1,64E-03	No DEG
ENSG00000256694	RP11-598F7.5	-10,48	-3,39	7,68E-12	1,02E-10	No DEG
ENSG00000144290	SLC4A10	10,45	3,38	1,81E-04	7,85E-04	No DEG
ENSG00000185201	IFITM2	-10,33	-3,37	9,14E-27	5,10E-25	No DEG
ENSG00000213132	AC022498.1	-10,28	-3,36	1,28E-10	1,45E-09	No DEG
ENSG00000201747	RNU6-534P	10,28	3,36	7,50E-03	2,22E-02	No DEG
ENSG00000227959	RP11-276H7.2	-10,27	-3,36	3,35E-07	2,35E-06	No DEG
ENSG00000137166	FOXP4	-10,22	-3,35	2,02E-48	4,80E-46	No DEG
ENSG00000236423	LINC01134	-10,19	-3,35	7,46E-07	4,93E-06	DOWN
ENSG00000250710	OR7E99P	-10,19	-3,35	2,28E-03	7,74E-03	No DEG
ENSG00000260953	RP11-426C22.6	-10,12	-3,34	2,34E-06	1,42E-05	DOWN
ENSG00000187151	ANGPTL5	10,11	3,34	3,96E-18	1,02E-16	UP
ENSG00000228539	RP11-526K17.2	-10,10	-3,34	1,95E-09	1,90E-08	No DEG
ENSG00000270488	RP11-245G13.1	-10,09	-3,34	1,82E-05	9,56E-05	No DEG

ENSG00000130382	MLLT1	-10,08	-3,33	6,86E-81	8,77E-78	No DEG
ENSG00000141741	MIEN1	-10,03	-3,33	1,80E-40	2,46E-38	No DEG
ENSG00000149798	CDC42EP2	-10,00	-3,32	2,50E-20	7,94E-19	No DEG
ENSG00000137331	IER3	-9,99	-3,32	1,18E-10	1,35E-09	DOWN
ENSG00000229604	MTATP8P2	-9,98	-3,32	2,05E-10	2,26E-09	No DEG
ENSG00000270457	RP11-467C18.1	-9,98	-3,32	1,92E-04	8,27E-04	No DEG
ENSG00000244230	RN7SL151P	9,90	3,31	8,10E-04	3,06E-03	No DEG
ENSG00000259910	RP11-616M22.2	-9,87	-3,30	6,42E-04	2,48E-03	No DEG
ENSG00000115155	OTOF	-9,87	-3,30	5,19E-04	2,04E-03	No DEG
ENSG00000124564	SLC17A3	9,80	3,29	8,33E-05	3,85E-04	No DEG
ENSG00000264590	RN7SL512P	-9,76	-3,29	5,01E-03	1,55E-02	No DEG
ENSG00000264585	MIR4449	9,66	3,27	3,57E-04	1,46E-03	No DEG
ENSG00000135144	DTX1	-9,65	-3,27	5,85E-13	8,91E-12	No DEG
ENSG00000183386	FHL3	-9,64	-3,27	2,31E-19	6,65E-18	No DEG
ENSG00000255148	RP4-791M13.4	-9,64	-3,27	1,29E-14	2,34E-13	No DEG
ENSG00000197905	TEAD4	-9,58	-3,26	2,07E-18	5,42E-17	No DEG
ENSG00000162430	SEPN1	-9,58	-3,26	3,08E-58	1,47E-55	No DEG
ENSG00000207764	MIR133A2	-9,56	-3,26	3,45E-05	1,72E-04	No DEG
ENSG00000166987	MBD6	-9,56	-3,26	8,51E-54	3,11E-51	No DEG
ENSG00000272078	RP4-734G22.3	-9,55	-3,26	1,32E-18	3,54E-17	No DEG
ENSG00000264676	RN7SL204P	9,54	3,25	1,06E-02	3,00E-02	No DEG
ENSG00000106211	HSPB1	-9,51	-3,25	1,23E-37	1,43E-35	No DEG
ENSG00000218208	RP11-367G18.2	9,49	3,25	4,48E-04	1,79E-03	No DEG
ENSG00000101335	MYL9	-9,49	-3,25	5,40E-79	6,37E-76	No DEG
ENSG00000125534	PPDPF	-9,49	-3,25	1,03E-30	7,57E-29	No DEG
ENSG00000175505	CLCF1	-9,48	-3,25	6,22E-07	4,17E-06	DOWN
ENSG00000270659	RP11-105N14.1	9,48	3,24	2,20E-09	2,13E-08	UP
ENSG00000241627	UBQLN4P1	-9,46	-3,24	2,78E-04	1,16E-03	No DEG
ENSG00000231933	CTA-125H2.2	-9,45	-3,24	1,21E-08	1,06E-07	DOWN
ENSG00000207638	MIR99A	9,45	3,24	1,07E-05	5,87E-05	No DEG
ENSG00000110400	PVRL1	-9,44	-3,24	3,35E-34	3,10E-32	No DEG
ENSG00000168490	PHYHIP	-9,43	-3,24	5,07E-19	1,42E-17	No DEG

ENSG00000124772	CPNE5	-9,40	-3,23	1,00E-13	1,65E-12	No DEG
ENSG00000107317	PTGDS	-9,37	-3,23	4,29E-30	3,02E-28	No DEG
ENSG00000179262	RAD23A	-9,35	-3,23	4,32E-122	1,66E-118	No DEG
ENSG00000203392	AC105020.1	-9,33	-3,22	2,44E-07	1,75E-06	No DEG
ENSG00000200708	RN7SKP93	-9,31	-3,22	1,25E-03	4,52E-03	No DEG
ENSG00000173065	FAM222B	-9,28	-3,21	5,90E-49	1,47E-46	No DEG
ENSG00000064300	NGFR	-9,27	-3,21	4,85E-14	8,24E-13	No DEG
ENSG00000111676	ATN1	-9,24	-3,21	1,49E-51	4,65E-49	No DEG
ENSG00000237075	RP11-497H16.2	-9,24	-3,21	6,02E-09	5,49E-08	No DEG
ENSG00000176087	SLC35A4	-9,23	-3,21	1,57E-80	1,93E-77	No DEG
ENSG00000262966	RP11-85B7.2	-9,17	-3,20	2,50E-07	1,79E-06	No DEG
ENSG00000228056	CFL1P3	-9,17	-3,20	4,82E-09	4,46E-08	DOWN
ENSG00000102265	TIMP1	-9,14	-3,19	1,56E-20	5,06E-19	No DEG
ENSG00000197604	AC022532.1	-9,13	-3,19	4,60E-06	2,67E-05	No DEG
ENSG00000224956	RP11-206L10.1	-9,11	-3,19	6,55E-06	3,70E-05	No DEG
ENSG00000224051	GLTPD1	-9,11	-3,19	1,20E-36	1,30E-34	No DEG
ENSG00000139572	GPR84	-9,06	-3,18	5,36E-04	2,11E-03	No DEG
ENSG00000239815	RP11-309L24.4	-9,05	-3,18	3,88E-05	1,92E-04	No DEG
ENSG00000134107	BHLHE40	-9,02	-3,17	1,38E-09	1,38E-08	DOWN
ENSG00000224298	AC069363.1	-9,01	-3,17	1,21E-04	5,42E-04	No DEG
ENSG00000258388	PPT2-EGFL8	-9,00	-3,17	5,65E-05	2,70E-04	No DEG
ENSG00000156009	MAGEA8	-8,99	-3,17	3,42E-03	1,11E-02	No DEG
ENSG00000111181	SLC6A12	-8,98	-3,17	2,94E-06	1,76E-05	No DEG
ENSG00000250290	CTC-820M8.1	8,97	3,17	4,46E-04	1,79E-03	UP
ENSG00000124343	XG	8,93	3,16	1,97E-08	1,68E-07	UP
ENSG00000079432	CIC	-8,91	-3,16	2,20E-64	1,65E-61	No DEG
ENSG00000259699	HMGB1P8	-8,91	-3,16	2,16E-07	1,57E-06	No DEG
ENSG00000173918	C1QTNF1	-8,90	-3,15	3,80E-20	1,18E-18	DOWN
ENSG00000141738	GRB7	-8,87	-3,15	5,35E-06	3,07E-05	No DEG
ENSG00000123999	INHA	-8,83	-3,14	4,28E-12	5,87E-11	No DEG
ENSG00000109758	HGFAC	-8,83	-3,14	6,94E-04	2,66E-03	DOWN
ENSG00000273487	RP4-621B10.8	8,82	3,14	5,54E-06	3,17E-05	No DEG

ENSG00000264007	RP11-68I3.10	-8,82	-3,14	7,83E-09	7,02E-08	No DEG
ENSG00000141965	FEM1A	8,80	3,14	2,02E-22	7,74E-21	No DEG
ENSG00000225401	TGIF2P1	-8,80	-3,14	9,03E-05	4,14E-04	No DEG
ENSG00000134590	FAM127A	-8,79	-3,14	7,61E-83	1,23E-79	No DEG
ENSG00000143469	SYT14	8,75	3,13	1,21E-02	3,36E-02	No DEG
ENSG00000167553	TUBA1C	-8,75	-3,13	1,81E-17	4,38E-16	No DEG
ENSG00000173801	JUP	-8,74	-3,13	1,87E-71	1,59E-68	No DEG
ENSG00000251889	RNU4-49P	8,73	3,13	3,23E-04	1,33E-03	UP
ENSG00000196182	STK40	-8,73	-3,13	1,21E-71	1,06E-68	No DEG
ENSG00000230965	SNX18P13	8,73	3,13	1,21E-02	3,37E-02	No DEG
ENSG00000198938	MT-CO3	-8,70	-3,12	3,85E-48	8,94E-46	No DEG
ENSG00000117266	CDK18	-8,70	-3,12	6,22E-55	2,51E-52	No DEG
ENSG00000182175	RGMA	-8,69	-3,12	1,44E-31	1,13E-29	No DEG
ENSG00000251402	FAM90A25P	-8,69	-3,12	1,15E-03	4,20E-03	No DEG
ENSG00000250899	RP11-253E3.3	-8,69	-3,12	9,79E-17	2,18E-15	No DEG
ENSG00000261207	LA16c-361A3.3	-8,66	-3,11	1,03E-08	9,11E-08	No DEG
ENSG00000223162	RNA5SP280	8,62	3,11	1,27E-02	3,51E-02	No DEG
ENSG00000164855	TMEM184A	-8,62	-3,11	3,89E-11	4,72E-10	No DEG
ENSG00000249274	PDLIM1P4	-8,58	-3,10	5,85E-12	7,90E-11	No DEG
ENSG00000272843	RP11-313P13.5	-8,56	-3,10	1,46E-06	9,17E-06	No DEG
ENSG00000213199	ASIC3	-8,55	-3,10	1,05E-19	3,11E-18	No DEG
ENSG00000267356	RP11-411B10.3	8,52	3,09	3,90E-04	1,58E-03	No DEG
ENSG00000252481	SCARNA13	8,52	3,09	6,73E-32	5,41E-30	No DEG
ENSG00000261959	RP11-893F2.14	-8,51	-3,09	4,76E-04	1,89E-03	No DEG
ENSG00000244146	RP11-366M4.2	8,50	3,09	1,30E-02	3,57E-02	No DEG
ENSG00000228948	SLC25A6P5	8,49	3,09	1,23E-03	4,45E-03	No DEG
ENSG00000198727	MT-CYB	-8,46	-3,08	4,80E-50	1,34E-47	No DEG
ENSG00000215873	FEN1P1	-8,45	-3,08	1,37E-03	4,88E-03	No DEG
ENSG00000175274	TP53I11	-8,44	-3,08	3,06E-56	1,34E-53	No DEG
ENSG00000186567	CEACAM19	-8,43	-3,07	1,33E-20	4,33E-19	No DEG
ENSG00000239797	RPL21P39	8,42	3,07	1,70E-04	7,44E-04	No DEG
ENSG00000176547	OR4C3	-8,41	-3,07	4,55E-03	1,43E-02	No DEG

ENSG00000137193	PIM1	-8,41	-3,07	3,73E-13	5,79E-12	DOWN
ENSG00000140564	FURIN	-8,40	-3,07	2,71E-44	4,77E-42	No DEG
ENSG00000136274	NACAD	-8,40	-3,07	4,04E-19	1,14E-17	No DEG
ENSG00000124713	GNMT	-8,39	-3,07	1,00E-08	8,86E-08	DOWN
ENSG00000175591	P2RY2	-8,39	-3,07	1,50E-23	6,51E-22	DOWN
ENSG00000181029	TRAPPC5	8,39	3,07	2,08E-04	8,89E-04	No DEG
ENSG00000231884	NDUFB1P1	8,37	3,07	1,25E-03	4,52E-03	No DEG
ENSG00000212204	RNA5SP91	-8,36	-3,06	9,19E-04	3,43E-03	No DEG
ENSG00000224805	LINC00853	8,35	3,06	1,27E-02	3,52E-02	UP
ENSG00000104973	MED25	-8,32	-3,06	6,19E-59	3,11E-56	No DEG
ENSG00000148840	PPRC1	-8,31	-3,06	1,05E-25	5,38E-24	No DEG
ENSG00000131477	RAMP2	-8,31	-3,05	1,49E-22	5,82E-21	No DEG
ENSG00000101670	LIPG	-8,31	-3,05	1,17E-06	7,45E-06	No DEG
ENSG00000242540	AC010729.1	8,26	3,05	1,73E-03	6,04E-03	No DEG
ENSG00000100884	CPNE6	-8,25	-3,04	3,90E-04	1,58E-03	No DEG
ENSG00000099814	CEP170B	-8,23	-3,04	2,79E-58	1,36E-55	No DEG
ENSG00000239776	AC079949.1	8,22	3,04	3,26E-23	1,37E-21	No DEG
ENSG00000178719	GRINA	-8,22	-3,04	6,85E-61	3,97E-58	No DEG
ENSG00000117013	KCNQ4	-8,20	-3,03	1,46E-17	3,56E-16	No DEG
ENSG00000198685	C3orf27	-8,18	-3,03	1,10E-03	4,02E-03	No DEG
ENSG00000255213	NPM1P35	8,17	3,03	1,41E-02	3,84E-02	No DEG
ENSG00000219186	FTH1P19	-8,13	-3,02	1,80E-03	6,25E-03	No DEG
ENSG00000177984	LCN15	-8,13	-3,02	1,51E-03	5,36E-03	DOWN
ENSG00000225893	RP11-6J24.3	8,10	3,02	3,82E-04	1,55E-03	No DEG
ENSG00000133619	KRBA1	-8,09	-3,02	1,88E-18	4,96E-17	No DEG
ENSG00000188015	S100A3	-8,08	-3,02	3,99E-05	1,97E-04	DOWN
ENSG00000135097	MSI1	-8,06	-3,01	1,50E-27	8,95E-26	No DEG
ENSG00000169436	COL22A1	8,05	3,01	2,76E-06	1,66E-05	UP
ENSG00000272279	RP11-157J24.2	8,05	3,01	1,81E-03	6,28E-03	UP
ENSG00000162761	LMX1A	8,05	3,01	3,06E-04	1,27E-03	No DEG
ENSG00000258948	KRT8P1	-8,02	-3,00	1,21E-06	7,72E-06	No DEG
ENSG00000090339	ICAM1	-8,02	-3,00	3,12E-15	6,00E-14	No DEG

ENSG00000239332	LINC01119	-8,01	-3,00	8,69E-06	4,83E-05	DOWN
ENSG00000146112	PPP1R18	-8,00	-3,00	5,77E-21	1,96E-19	No DEG
ENSG00000158859	ADAMTS4	-8,00	-3,00	3,75E-06	2,21E-05	DOWN
ENSG00000226763	SRRM5	-8,00	-3,00	4,22E-05	2,07E-04	No DEG
ENSG00000130590	SAMD10	-7,98	-3,00	6,07E-15	1,13E-13	No DEG
ENSG00000254401	RP11-179A10.1	-7,97	-2,99	6,83E-04	2,62E-03	No DEG
ENSG00000273237	CTB-119C2.1	-7,96	-2,99	9,48E-13	1,41E-11	DOWN
ENSG00000005882	PDK2	-7,96	-2,99	5,03E-45	9,42E-43	No DEG
ENSG00000271113	RP11-159H10.4	-7,94	-2,99	2,56E-05	1,31E-04	No DEG
ENSG00000258170	RP11-263K4.5	7,93	2,99	1,40E-02	3,81E-02	No DEG
ENSG00000222889	RN7SKP29	-7,93	-2,99	1,06E-06	6,81E-06	No DEG
ENSG00000253519	AC106801.1	-7,93	-2,99	9,84E-07	6,37E-06	DOWN
ENSG00000218991	CCNG1P1	7,92	2,98	1,04E-03	3,84E-03	UP
ENSG00000108509	CAMTA2	-7,89	-2,98	2,25E-92	4,93E-89	No DEG
ENSG00000225485	ARHGAP23	-7,88	-2,98	4,07E-54	1,51E-51	No DEG
ENSG00000272899	RP11-309L24.9	-7,88	-2,98	4,77E-04	1,90E-03	DOWN
ENSG00000226421	SLC25A5P5	-7,87	-2,98	1,11E-03	4,05E-03	No DEG
ENSG00000264553	MIR4257	-7,87	-2,98	6,63E-03	1,99E-02	No DEG
ENSG00000240849	TMEM189	-7,85	-2,97	1,87E-46	3,79E-44	No DEG
ENSG00000186335	SLC36A2	-7,85	-2,97	6,03E-05	2,87E-04	DOWN
ENSG00000261315	LARP4P	-7,84	-2,97	2,16E-05	1,12E-04	DOWN
ENSG00000223336	RNU2-6P	7,82	2,97	2,36E-15	4,59E-14	No DEG
ENSG00000179083	FAM133A	7,81	2,97	4,44E-04	1,78E-03	UP
ENSG00000256609	RP11-64B16.4	-7,81	-2,96	4,36E-12	5,97E-11	No DEG
ENSG00000204396	VWA7	-7,80	-2,96	1,97E-24	9,12E-23	No DEG
ENSG00000235012	AF121897.4	-7,78	-2,96	1,74E-06	1,08E-05	No DEG
ENSG00000160741	CRTC2	-7,78	-2,96	1,71E-42	2,65E-40	No DEG
ENSG00000205584	AC005488.11	-7,78	-2,96	1,04E-03	3,85E-03	No DEG
ENSG00000143631	FLG	-7,78	-2,96	4,57E-18	1,16E-16	No DEG
ENSG00000226676	RP11-589B3.6	-7,77	-2,96	1,85E-03	6,41E-03	No DEG
ENSG00000196136	SERPINA3	-7,76	-2,96	2,19E-06	1,34E-05	No DEG
ENSG00000175264	CHST1	-7,74	-2,95	1,46E-18	3,89E-17	No DEG

ENSG00000174903	RAB1B	-7,73	-2,95	2,06E-81	2,87E-78	No DEG
ENSG00000102312	PORCN	-7,72	-2,95	2,04E-39	2,59E-37	No DEG
ENSG00000163191	S100A11	-7,72	-2,95	1,67E-22	6,48E-21	No DEG
ENSG00000125841	NRSN2	-7,72	-2,95	2,53E-35	2,49E-33	No DEG
ENSG00000239697	TNFSF12	-7,72	-2,95	7,06E-47	1,49E-44	No DEG
ENSG00000234175	RP11-730A19.9	-7,71	-2,95	1,49E-11	1,91E-10	No DEG
ENSG00000214491	SEC14L6	7,71	2,95	2,82E-03	9,35E-03	No DEG
ENSG00000233968	RP11-354E11.2	7,70	2,94	2,36E-05	1,22E-04	UP
ENSG00000198763	MT-ND2	-7,69	-2,94	9,05E-44	1,49E-41	No DEG
ENSG00000113916	BCL6	-7,69	-2,94	1,24E-18	3,34E-17	No DEG
ENSG00000104859	CLASRP	-7,65	-2,94	6,31E-40	8,38E-38	No DEG
ENSG00000261625	RP11-554A11.4	-7,64	-2,93	1,24E-06	7,87E-06	No DEG
ENSG00000108518	PFN1	-7,60	-2,93	1,06E-53	3,83E-51	No DEG
ENSG00000160972	PPP1R16A	-7,60	-2,93	4,67E-34	4,29E-32	No DEG
ENSG00000013588	GPRC5A	-7,59	-2,92	5,21E-05	2,51E-04	DOWN
ENSG00000205336	GPR56	-7,58	-2,92	6,68E-30	4,65E-28	No DEG
ENSG00000268592	RP11-244K5.8	7,56	2,92	4,82E-12	6,57E-11	No DEG
ENSG00000230149	RP3-508I15.19	-7,55	-2,92	1,25E-04	5,57E-04	No DEG
ENSG00000168140	VASN	-7,52	-2,91	1,98E-13	3,17E-12	No DEG
ENSG00000198886	MT-ND4	-7,52	-2,91	4,98E-49	1,25E-46	No DEG
ENSG00000164082	GRM2	-7,50	-2,91	1,33E-05	7,19E-05	No DEG
ENSG00000237851	RP1-67K17.4	-7,49	-2,90	3,12E-05	1,57E-04	No DEG
ENSG00000112033	PPARD	-7,49	-2,90	1,97E-51	6,03E-49	No DEG
ENSG00000260871	CTD-2373J6.1	7,48	2,90	1,45E-04	6,38E-04	No DEG
ENSG00000157613	CREB3L1	-7,48	-2,90	2,62E-26	1,40E-24	No DEG
ENSG00000229037	RP11-272G22.3	7,47	2,90	1,07E-03	3,95E-03	No DEG
ENSG00000214617	SLC6A10P	-7,47	-2,90	6,95E-06	3,91E-05	No DEG
ENSG00000205041	CTC-425O23.2	-7,47	-2,90	8,01E-07	5,26E-06	No DEG
ENSG00000132693	CRP	-7,46	-2,90	8,85E-03	2,56E-02	No DEG
ENSG00000260300	RP11-505K9.4	-7,46	-2,90	5,58E-06	3,19E-05	No DEG
ENSG00000224722	PRNCR1	-7,46	-2,90	7,47E-05	3,49E-04	No DEG
ENSG00000229782	AC118754.4	-7,45	-2,90	8,23E-05	3,81E-04	No DEG

ENSG00000267493	CIRBP-AS1	-7,45	-2,90	1,41E-11	1,82E-10	No DEG
ENSG00000222346	RNA5SP237	7,44	2,89	2,56E-03	8,59E-03	No DEG
ENSG00000104853	CLPTM1	-7,43	-2,89	3,76E-95	9,61E-92	No DEG
ENSG00000230581	RP11-390F4.8	-7,43	-2,89	6,44E-10	6,70E-09	No DEG
ENSG00000241106	HLA-DOB	-7,43	-2,89	1,12E-04	5,06E-04	No DEG
ENSG00000137726	FXYD6	-7,42	-2,89	1,93E-55	7,98E-53	No DEG
ENSG00000247627	MTND4P12	-7,42	-2,89	2,46E-17	5,85E-16	No DEG
ENSG00000167595	C19orf55	-7,41	-2,89	3,81E-34	3,52E-32	No DEG
ENSG00000171992	SYNPO	-7,41	-2,89	5,38E-50	1,49E-47	No DEG
ENSG00000175130	MARCKSL1	-7,40	-2,89	1,44E-17	3,51E-16	No DEG
ENSG00000267427	CTC-503J8.6	-7,38	-2,88	1,83E-45	3,55E-43	No DEG
ENSG00000204469	PRRC2A	-7,37	-2,88	1,25E-126	6,41E-123	No DEG
ENSG00000258675	RP11-299L17.3	7,36	2,88	1,04E-03	3,85E-03	No DEG
ENSG00000267579	RP11-126O1.2	-7,36	-2,88	1,04E-03	3,83E-03	No DEG
ENSG00000258943	RP11-696D21.2	-7,36	-2,88	3,82E-04	1,55E-03	No DEG
ENSG00000005486	RHBDD2	-7,34	-2,88	3,37E-60	1,85E-57	No DEG
ENSG00000271882	KB-1410C5.5	7,33	2,87	1,31E-04	5,84E-04	No DEG
ENSG00000215750	AC145212.1	-7,33	-2,87	1,32E-07	9,90E-07	No DEG
ENSG00000167100	SAMD14	-7,33	-2,87	6,69E-19	1,86E-17	No DEG
ENSG00000128016	ZFP36	-7,32	-2,87	8,46E-08	6,54E-07	No DEG
ENSG00000234161	PABPC5-AS1	7,32	2,87	2,31E-03	7,84E-03	No DEG
ENSG00000154146	NRGN	-7,30	-2,87	2,98E-14	5,19E-13	No DEG
ENSG00000235370	DNM1P51	-7,29	-2,87	3,43E-11	4,20E-10	No DEG
ENSG00000110697	PITPNM1	-7,28	-2,86	1,05E-48	2,55E-46	No DEG
ENSG00000229065	RP11-80I15.4	-7,28	-2,86	5,57E-07	3,76E-06	No DEG
ENSG00000203950	FAM127B	-7,28	-2,86	1,56E-35	1,56E-33	No DEG
ENSG00000188199	NUTM2B	-7,28	-2,86	3,29E-07	2,31E-06	No DEG
ENSG00000160401	C9orf117	-7,27	-2,86	7,37E-09	6,63E-08	No DEG
ENSG00000245330	KB-1471A8.1	7,27	2,86	1,15E-03	4,21E-03	No DEG
ENSG00000266097	MIR5192	-7,25	-2,86	1,05E-03	3,87E-03	No DEG
ENSG00000264604	AL158069.1	7,25	2,86	4,56E-05	2,22E-04	No DEG
ENSG00000188599	NPIPP1	-7,24	-2,86	1,14E-12	1,68E-11	No DEG

ENSG00000261582	RP4-614O4.11	-7,24	-2,86	1,01E-06	6,50E-06	No DEG
ENSG00000199523	RNA5SP226	-7,24	-2,86	1,39E-03	4,95E-03	No DEG
ENSG00000250790	RP11-46H11.3	7,24	2,86	5,58E-04	2,18E-03	No DEG
ENSG00000143382	ADAMTSL4	-7,23	-2,85	8,96E-37	9,85E-35	No DEG
ENSG00000181773	GPR3	-7,23	-2,85	9,52E-06	5,26E-05	No DEG
ENSG00000212549	RNA5SP354	7,20	2,85	1,43E-02	3,88E-02	No DEG
ENSG00000272405	RP11-284F21.10	-7,19	-2,85	7,43E-04	2,83E-03	No DEG
ENSG00000231942	HNRNPA1P36	-7,19	-2,85	4,65E-04	1,85E-03	No DEG
ENSG00000238342	snoU13	7,17	2,84	2,20E-03	7,49E-03	UP
ENSG00000090975	PITPNM2	-7,16	-2,84	2,37E-38	2,87E-36	No DEG
ENSG00000225442	MPRIP-AS1	-7,15	-2,84	6,18E-07	4,15E-06	No DEG
ENSG00000235245	RP11-122K13.12	-7,13	-2,83	5,22E-07	3,54E-06	No DEG
ENSG00000175898	CTD-2369P2.2	-7,13	-2,83	5,44E-13	8,30E-12	No DEG
ENSG00000240350	AC017002.1	-7,12	-2,83	3,05E-06	1,83E-05	No DEG
ENSG00000272010	CTD-3025N20.3	7,11	2,83	4,19E-10	4,46E-09	No DEG
ENSG00000203876	RP11-451M19.3	7,10	2,83	3,98E-04	1,61E-03	UP
ENSG00000173264	GPR137	-7,10	-2,83	2,14E-41	3,09E-39	No DEG
ENSG00000100243	CYB5R3	-7,10	-2,83	4,21E-58	1,99E-55	No DEG
ENSG00000011451	WIZ	-7,10	-2,83	1,15E-124	5,03E-121	No DEG
ENSG00000228261	RP11-127L20.3	-7,09	-2,83	1,77E-08	1,52E-07	No DEG
ENSG00000157734	SNX22	-7,09	-2,83	4,94E-14	8,38E-13	No DEG
ENSG00000130176	CNN1	-7,06	-2,82	1,49E-08	1,30E-07	DOWN
ENSG00000100139	MICALL1	-7,05	-2,82	1,47E-36	1,59E-34	No DEG
ENSG00000172757	CFL1	-7,04	-2,82	5,46E-51	1,61E-48	No DEG
ENSG00000242435	UPK3BP1	-7,03	-2,81	3,98E-03	1,27E-02	No DEG
ENSG00000248027	CTD-2383M3.1	7,01	2,81	4,85E-05	2,35E-04	No DEG
ENSG00000213015	ZNF580	-7,01	-2,81	4,24E-26	2,22E-24	No DEG
ENSG00000253223	RP11-409C19.2	-7,01	-2,81	1,60E-05	8,51E-05	No DEG
ENSG00000218416	AC110619.2	-6,98	-2,80	1,35E-05	7,28E-05	No DEG
ENSG00000167930	ITFG3	-6,97	-2,80	4,94E-47	1,07E-44	No DEG
ENSG00000167566	NCKAP5L	-6,97	-2,80	7,46E-86	1,35E-82	No DEG
ENSG00000199815	SNORA43	6,97	2,80	3,15E-03	1,03E-02	No DEG

ENSG00000253838	RP11-44K6.2	-6,97	-2,80	4,47E-03	1,40E-02	No DEG
ENSG00000269545	CTD-3138B18.4	-6,96	-2,80	1,20E-03	4,35E-03	No DEG
ENSG00000249158	PCDHA11	6,94	2,79	4,23E-04	1,70E-03	No DEG
ENSG00000108679	LGALS3BP	-6,93	-2,79	4,28E-50	1,21E-47	No DEG
ENSG00000105193	RPS16	-6,93	-2,79	7,63E-28	4,62E-26	No DEG
ENSG00000090924	PLEKHG2	-6,90	-2,79	4,56E-24	2,06E-22	No DEG
ENSG00000167797	CDK2AP2	-6,89	-2,79	1,45E-39	1,84E-37	No DEG
ENSG00000156500	FAM122C	-6,88	-2,78	1,15E-09	1,15E-08	DOWN
ENSG00000204310	AGPAT1	-6,87	-2,78	7,37E-40	9,70E-38	No DEG
ENSG00000054938	CHRDL2	-6,85	-2,78	2,90E-04	1,21E-03	DOWN
ENSG00000126705	AHDC1	-6,84	-2,77	2,87E-53	9,91E-51	No DEG
ENSG00000223882	ABCC5-AS1	-6,84	-2,77	4,58E-05	2,23E-04	No DEG
ENSG00000135451	TROAP	-6,83	-2,77	5,98E-04	2,32E-03	No DEG
ENSG00000236397	DDX11L2	-6,83	-2,77	1,69E-08	1,45E-07	No DEG
ENSG00000258311	RP11-644F5.10	-6,82	-2,77	2,11E-07	1,53E-06	No DEG
ENSG00000063245	EPN1	-6,81	-2,77	1,46E-49	3,84E-47	No DEG
ENSG00000167182	SP2	-6,80	-2,77	2,05E-43	3,32E-41	No DEG
ENSG00000003249	DBNDD1	-6,80	-2,77	3,65E-11	4,44E-10	No DEG
ENSG00000080603	SRCAP	-6,80	-2,77	2,98E-73	2,77E-70	No DEG
ENSG00000233799	AC139887.4	-6,80	-2,76	1,11E-05	6,05E-05	No DEG
ENSG00000153443	UBALD1	-6,80	-2,76	5,94E-32	4,80E-30	No DEG
ENSG00000209082	MT-TL1	-6,79	-2,76	2,53E-08	2,12E-07	No DEG
ENSG00000100949	RABGGTA	-6,79	-2,76	3,26E-60	1,82E-57	No DEG
ENSG00000089351	GRAMD1A	-6,79	-2,76	1,43E-37	1,65E-35	No DEG
ENSG00000132481	TRIM47	-6,77	-2,76	1,29E-15	2,57E-14	No DEG
ENSG00000255414	LINC01059	-6,75	-2,75	2,30E-06	1,40E-05	DOWN
ENSG00000142677	IL22RA1	-6,75	-2,75	1,07E-07	8,14E-07	No DEG
ENSG00000197324	LRP10	-6,74	-2,75	3,82E-63	2,60E-60	No DEG
ENSG00000126353	CCR7	-6,74	-2,75	3,00E-06	1,79E-05	No DEG
ENSG00000205275	CSPG4P9	-6,74	-2,75	2,21E-06	1,35E-05	No DEG
ENSG00000258794	RP11-313F23.5	-6,73	-2,75	1,98E-04	8,51E-04	No DEG
ENSG00000258986	TMEM179	-6,73	-2,75	1,25E-05	6,79E-05	No DEG

ENSG00000232177	MTND4P24	-6,72	-2,75	1,49E-09	1,48E-08	No DEG
ENSG00000137077	CCL21	-6,71	-2,75	1,55E-05	8,28E-05	No DEG
ENSG00000113749	HRH2	-6,71	-2,75	5,08E-41	7,28E-39	No DEG
ENSG00000226009	KCNIP2-AS1	-6,70	-2,75	7,51E-05	3,50E-04	No DEG
ENSG00000137801	THBS1	-6,69	-2,74	1,96E-06	1,20E-05	DOWN
ENSG00000186174	BCL9L	-6,69	-2,74	4,63E-36	4,89E-34	No DEG
ENSG00000225342	AC079630.4	6,69	2,74	3,14E-04	1,30E-03	UP
ENSG00000108352	RAPGEFL1	-6,69	-2,74	1,63E-16	3,53E-15	No DEG
ENSG00000061337	LZTS1	-6,67	-2,74	8,90E-18	2,22E-16	No DEG
ENSG00000141574	SECTM1	-6,67	-2,74	4,12E-13	6,36E-12	No DEG
ENSG00000188322	SBK1	-6,67	-2,74	2,03E-09	1,98E-08	No DEG
ENSG00000214248	AC010336.1	-6,66	-2,74	7,54E-03	2,23E-02	No DEG
ENSG00000169246	NPIPB3	-6,66	-2,74	2,73E-11	3,39E-10	No DEG
ENSG00000252343	RNU2-34P	-6,66	-2,74	9,74E-03	2,78E-02	No DEG
ENSG00000240418	CTD-2013N17.1	-6,65	-2,73	2,04E-03	6,99E-03	No DEG
ENSG00000267161	AC005943.5	-6,65	-2,73	1,51E-07	1,12E-06	No DEG
ENSG00000149679	CABLES2	-6,64	-2,73	9,08E-31	6,76E-29	No DEG
ENSG00000254719	RP11-351I24.3	6,64	2,73	1,15E-03	4,19E-03	No DEG
ENSG00000125898	FAM110A	-6,64	-2,73	2,31E-10	2,54E-09	No DEG
ENSG00000198792	TMEM184B	-6,63	-2,73	1,81E-37	2,08E-35	No DEG
ENSG00000243449	C4orf48	6,61	2,73	3,07E-06	1,83E-05	No DEG
ENSG00000251768	RNA5SP217	6,60	2,72	9,92E-04	3,68E-03	No DEG
ENSG00000185633	NDUFA4L2	-6,58	-2,72	1,71E-33	1,54E-31	No DEG
ENSG00000166444	ST5	-6,57	-2,72	1,66E-40	2,29E-38	No DEG
ENSG00000163814	CDCP1	-6,57	-2,71	3,81E-07	2,65E-06	No DEG
ENSG00000232456	RP11-5P18.10	-6,56	-2,71	2,41E-03	8,13E-03	No DEG
ENSG00000115568	ZNF142	-6,56	-2,71	8,83E-45	1,63E-42	No DEG
ENSG00000164867	NOS3	-6,56	-2,71	1,29E-14	2,34E-13	No DEG
ENSG00000120937	NPPB	6,56	2,71	1,56E-03	5,50E-03	UP
ENSG00000176349	AC110781.3	-6,55	-2,71	3,08E-05	1,56E-04	No DEG
ENSG00000148337	CIZ1	-6,55	-2,71	6,33E-45	1,18E-42	No DEG
ENSG00000222371	RN7SKP202	-6,55	-2,71	1,24E-05	6,71E-05	DOWN

ENSG00000116604	MEF2D	-6,54	-2,71	4,65E-57	2,10E-54	No DEG
ENSG00000260018	RP11-505K9.1	-6,53	-2,71	7,61E-07	5,02E-06	DOWN
ENSG00000113070	HBEGF	-6,53	-2,71	1,07E-11	1,40E-10	No DEG
ENSG00000164266	SPINK1	6,51	2,70	4,93E-04	1,95E-03	UP
ENSG00000167680	SEMA6B	-6,50	-2,70	4,24E-15	8,05E-14	No DEG
ENSG00000264345	RP11-958F21.1	6,50	2,70	6,19E-04	2,40E-03	No DEG
ENSG00000248936	RP11-36B15.1	-6,50	-2,70	4,95E-04	1,96E-03	No DEG
ENSG00000253807	LINC01170	6,47	2,69	5,75E-04	2,24E-03	No DEG
ENSG00000267534	S1PR2	-6,46	-2,69	1,35E-09	1,35E-08	No DEG
ENSG00000124155	PIGT	-6,46	-2,69	5,44E-55	2,23E-52	No DEG
ENSG00000241335	RNA5-8S5	6,46	2,69	2,27E-06	1,38E-05	No DEG
ENSG00000231920	NEBL-AS1	6,45	2,69	1,32E-03	4,73E-03	No DEG
ENSG00000229827	AC093899.3	-6,45	-2,69	7,24E-03	2,15E-02	No DEG
ENSG00000170835	CEL	-6,44	-2,69	6,96E-07	4,63E-06	DOWN
ENSG00000232871	SEC1P	6,44	2,69	3,57E-03	1,15E-02	UP
ENSG00000142675	CNKSR1	-6,44	-2,69	1,41E-09	1,40E-08	No DEG
ENSG00000168268	NT5DC2	-6,44	-2,69	3,98E-24	1,81E-22	No DEG
ENSG00000162738	VANGL2	-6,43	-2,68	1,93E-27	1,13E-25	No DEG
ENSG00000126243	LRFN3	-6,43	-2,68	2,08E-35	2,06E-33	No DEG
ENSG00000227182	VN1R28P	-6,42	-2,68	6,65E-03	1,99E-02	No DEG
ENSG00000135074	ADAM19	-6,42	-2,68	8,91E-16	1,80E-14	No DEG
ENSG00000154035	C17orf103	-6,42	-2,68	2,94E-44	5,15E-42	No DEG
ENSG00000230928	RP11-34A14.3	-6,41	-2,68	8,62E-04	3,24E-03	No DEG
ENSG00000205100	HSP90AA4P	6,41	2,68	1,68E-05	8,89E-05	No DEG
ENSG00000119737	GPR75	-6,40	-2,68	1,13E-10	1,30E-09	No DEG
ENSG00000064932	SBNO2	-6,40	-2,68	8,28E-15	1,53E-13	No DEG
ENSG00000177105	RHOG	-6,38	-2,67	5,69E-32	4,61E-30	No DEG
ENSG00000243696	RP5-966M1.6	-6,38	-2,67	2,99E-04	1,24E-03	No DEG
ENSG00000111321	LTBR	-6,38	-2,67	2,76E-23	1,17E-21	No DEG
ENSG00000179813	FAM216B	6,37	2,67	2,76E-06	1,66E-05	No DEG
ENSG00000261272	MUC22	-6,37	-2,67	1,02E-05	5,59E-05	No DEG
ENSG00000160877	NACC1	-6,37	-2,67	5,35E-56	2,28E-53	No DEG

ENSG00000078061	ARAF	-6,35	-2,67	1,73E-75	1,77E-72	No DEG
ENSG00000125207	PIWIL1	6,35	2,67	5,85E-03	1,78E-02	No DEG
ENSG00000240764	PCDHGC5	-6,35	-2,67	4,06E-17	9,43E-16	No DEG
ENSG00000167900	TK1	-6,34	-2,67	1,89E-10	2,10E-09	No DEG
ENSG00000265260	RN7SL74P	6,34	2,66	1,69E-02	4,48E-02	No DEG
ENSG00000176320	RP11-404O13.5	6,33	2,66	1,94E-03	6,68E-03	UP
ENSG00000188662	HILS1	-6,29	-2,65	1,12E-06	7,18E-06	No DEG
ENSG00000116649	SRM	-6,29	-2,65	1,95E-51	6,03E-49	No DEG
ENSG00000119508	NR4A3	-6,29	-2,65	5,31E-05	2,56E-04	DOWN
ENSG00000130173	C19orf80	-6,28	-2,65	1,30E-07	9,74E-07	No DEG
ENSG00000237447	CDC27P2	6,28	2,65	1,69E-02	4,48E-02	No DEG
ENSG00000224629	RP5-1142J19.2	-6,28	-2,65	1,80E-03	6,26E-03	No DEG
ENSG00000260781	ARHGAP23P1	-6,28	-2,65	3,25E-07	2,29E-06	No DEG
ENSG00000181652	ATG9B	-6,27	-2,65	6,80E-07	4,53E-06	No DEG
ENSG00000196843	ARID5A	-6,26	-2,65	3,75E-14	6,46E-13	No DEG
ENSG00000236772	RP5-1184F4.5	-6,26	-2,65	9,68E-07	6,27E-06	No DEG
ENSG00000104885	DOT1L	-6,25	-2,64	6,74E-22	2,46E-20	No DEG
ENSG00000050820	BCAR1	-6,25	-2,64	2,25E-32	1,87E-30	No DEG
ENSG00000243151	AL592188.1	6,25	2,64	1,44E-12	2,09E-11	No DEG
ENSG00000167965	MLST8	-6,25	-2,64	4,31E-46	8,69E-44	No DEG
ENSG00000154188	ANGPT1	6,21	2,63	1,47E-14	2,65E-13	UP
ENSG00000088836	SLC4A11	-6,20	-2,63	8,44E-05	3,90E-04	No DEG
ENSG00000228789	HCG22	-6,20	-2,63	1,12E-03	4,10E-03	No DEG
ENSG00000269620	AL590560.1	-6,20	-2,63	5,06E-03	1,57E-02	No DEG
ENSG00000128594	LRRC4	-6,19	-2,63	1,87E-06	1,15E-05	No DEG
ENSG00000269345	VN1R85P	6,18	2,63	1,71E-09	1,68E-08	UP
ENSG00000105520	DKFZP761J1410	-6,18	-2,63	5,52E-21	1,88E-19	No DEG
ENSG00000235994	RP3-470B24.5	-6,17	-2,62	5,21E-04	2,05E-03	DOWN
ENSG00000228436	RP5-864K19.4	-6,16	-2,62	2,44E-14	4,28E-13	No DEG
ENSG00000109066	TMEM104	-6,16	-2,62	6,66E-42	9,82E-40	No DEG
ENSG00000104856	RELB	-6,16	-2,62	4,72E-11	5,65E-10	No DEG
ENSG00000169136	ATF5	-6,16	-2,62	4,00E-15	7,61E-14	No DEG

ENSG00000160396	HIPK4	-6,16	-2,62	3,24E-06	1,93E-05	No DEG
ENSG00000197692	CBX3P7	6,16	2,62	1,76E-03	6,14E-03	No DEG
ENSG00000132024	CC2D1A	-6,15	-2,62	9,89E-64	7,06E-61	No DEG
ENSG00000137309	HMGA1	-6,14	-2,62	1,17E-54	4,59E-52	No DEG
ENSG00000262678	RP5-1050D4.4	-6,14	-2,62	3,53E-03	1,14E-02	No DEG
ENSG00000259635	AC100830.3	-6,14	-2,62	1,90E-04	8,19E-04	No DEG
ENSG00000272349	RP11-397O8.7	6,14	2,62	2,95E-06	1,77E-05	No DEG
ENSG00000226498	RP11-182B22.2	-6,12	-2,61	1,06E-07	8,05E-07	No DEG
ENSG00000122515	ZMIZ2	-6,12	-2,61	2,48E-40	3,35E-38	No DEG
ENSG00000233328	PFN1P1	-6,11	-2,61	4,05E-04	1,64E-03	No DEG
ENSG00000183831	ANKRD45	6,11	2,61	7,03E-04	2,69E-03	UP
ENSG00000151575	TEX9	6,11	2,61	3,05E-11	3,76E-10	UP
ENSG00000168282	MGAT2	6,10	2,61	5,04E-10	5,30E-09	No DEG
ENSG00000136574	GATA4	-6,09	-2,61	1,02E-30	7,53E-29	No DEG
ENSG00000179134	SAMD4B	-6,09	-2,61	2,62E-42	3,97E-40	No DEG
ENSG00000099381	SETD1A	-6,09	-2,61	2,17E-41	3,13E-39	No DEG
ENSG00000241781	AL161626.1	6,09	2,61	5,49E-04	2,15E-03	No DEG
ENSG00000201433	RNU6-335P	6,09	2,61	2,24E-03	7,64E-03	No DEG
ENSG00000171282	RP11-1055B8.7	-6,09	-2,61	7,06E-38	8,33E-36	No DEG
ENSG00000264376	RN7SL613P	6,08	2,60	8,28E-05	3,83E-04	No DEG
ENSG00000091181	IL5RA	6,08	2,60	2,50E-03	8,40E-03	UP
ENSG00000159871	LYPD5	-6,08	-2,60	5,56E-07	3,75E-06	No DEG
ENSG00000132003	ZSWIM4	-6,08	-2,60	6,72E-18	1,69E-16	No DEG
ENSG00000090372	STRN4	-6,08	-2,60	1,55E-58	7,66E-56	No DEG
ENSG00000268593	CTD-2611O12.6	-6,06	-2,60	5,36E-04	2,10E-03	No DEG
ENSG00000198720	ANKRD13B	-6,05	-2,60	4,12E-30	2,91E-28	No DEG
ENSG00000125538	IL1B	-6,03	-2,59	7,90E-05	3,67E-04	No DEG
ENSG00000185133	INPP5J	-6,02	-2,59	1,14E-23	4,99E-22	No DEG
ENSG00000125319	C17orf53	-6,01	-2,59	6,39E-07	4,27E-06	No DEG
ENSG00000263902	RN7SL437P	6,01	2,59	4,44E-03	1,40E-02	No DEG
ENSG00000221676	RNU6ATAC	6,00	2,59	2,41E-03	8,14E-03	No DEG
ENSG00000161798	AQP5	-6,00	-2,58	2,18E-04	9,30E-04	DOWN

ENSG00000228544	CCDC183-AS1	-6,00	-2,58	5,13E-08	4,10E-07	No DEG
ENSG00000177542	SLC25A22	-5,99	-2,58	9,98E-18	2,47E-16	No DEG
ENSG00000127824	TUBA4A	-5,99	-2,58	1,52E-33	1,37E-31	No DEG
ENSG00000250673	RP11-6L6.2	-5,98	-2,58	4,19E-03	1,33E-02	No DEG
ENSG00000258521	RP11-638I2.9	-5,97	-2,58	8,57E-05	3,95E-04	No DEG
ENSG00000264392	AC002477.1	5,96	2,58	1,01E-03	3,73E-03	No DEG
ENSG00000267375	CTB-186G2.4	-5,96	-2,57	2,80E-04	1,17E-03	No DEG
ENSG00000263105	RP11-95P2.3	-5,95	-2,57	4,01E-08	3,25E-07	No DEG
ENSG00000260145	RP11-322D14.2	-5,95	-2,57	1,74E-03	6,09E-03	No DEG
ENSG00000170209	ANKK1	-5,95	-2,57	4,27E-03	1,35E-02	No DEG
ENSG00000270378	RP11-108K3.5	-5,95	-2,57	1,11E-02	3,12E-02	No DEG
ENSG00000196739	COL27A1	-5,94	-2,57	3,65E-48	8,55E-46	No DEG
ENSG00000132471	WBP2	-5,94	-2,57	1,35E-49	3,61E-47	No DEG
ENSG00000152284	TCF7L1	-5,93	-2,57	6,46E-25	3,10E-23	No DEG
ENSG00000229081	LINC01165	-5,93	-2,57	7,94E-05	3,69E-04	No DEG
ENSG00000188522	FAM83G	-5,93	-2,57	3,09E-11	3,80E-10	No DEG
ENSG00000225873	LINC00694	-5,93	-2,57	3,41E-05	1,71E-04	No DEG
ENSG00000103855	CD276	-5,92	-2,57	1,02E-25	5,21E-24	No DEG
ENSG00000165621	OXGR1	-5,91	-2,56	3,36E-04	1,38E-03	DOWN
ENSG00000213088	DARC	-5,91	-2,56	5,02E-09	4,63E-08	No DEG
ENSG00000185033	SEMA4B	-5,90	-2,56	1,15E-18	3,12E-17	No DEG
ENSG00000142235	LMTK3	-5,90	-2,56	2,59E-05	1,32E-04	No DEG
ENSG00000147065	MSN	-5,89	-2,56	1,71E-24	7,96E-23	No DEG
ENSG00000171631	P2RY6	-5,88	-2,56	3,84E-09	3,60E-08	No DEG
ENSG00000137216	TMEM63B	-5,88	-2,56	4,82E-48	1,11E-45	No DEG
ENSG00000116521	SCAMP3	-5,88	-2,56	5,10E-59	2,65E-56	No DEG
ENSG00000180089	TMEM86B	-5,87	-2,55	1,32E-03	4,72E-03	No DEG
ENSG00000006432	MAP3K9	-5,87	-2,55	2,45E-20	7,81E-19	No DEG
ENSG00000187808	SOWAHD	5,87	2,55	1,20E-03	4,36E-03	UP
ENSG00000103148	NPRL3	-5,86	-2,55	2,88E-25	1,42E-23	No DEG
ENSG00000179611	DGKZP1	-5,85	-2,55	4,24E-08	3,43E-07	No DEG
ENSG00000270808	RP11-574K11.27	-5,84	-2,55	1,68E-03	5,88E-03	No DEG

ENSG00000174669	SLC29A2	-5,84	-2,55	6,22E-21	2,11E-19	No DEG
ENSG00000172346	CSDC2	-5,84	-2,55	1,22E-26	6,71E-25	No DEG
ENSG00000104969	SGTA	-5,84	-2,55	5,65E-76	5,98E-73	No DEG
ENSG00000199883	RN7SKP90	-5,83	-2,54	1,54E-61	9,11E-59	No DEG
ENSG00000128606	LRRC17	5,83	2,54	7,94E-12	1,05E-10	UP
ENSG00000153234	NR4A2	-5,82	-2,54	1,81E-04	7,86E-04	DOWN
ENSG00000006062	MAP3K14	-5,81	-2,54	6,38E-23	2,60E-21	No DEG
ENSG00000234810	RP11-466L17.1	5,81	2,54	1,79E-04	7,79E-04	No DEG
ENSG00000148400	NOTCH1	-5,79	-2,53	1,07E-40	1,50E-38	No DEG
ENSG00000268171	AC068620.1	5,78	2,53	2,05E-03	7,03E-03	No DEG
ENSG00000161664	ASB16	-5,78	-2,53	6,50E-22	2,39E-20	No DEG
ENSG00000181092	ADIPOQ	5,78	2,53	3,18E-03	1,04E-02	No DEG
ENSG00000119777	TMEM214	-5,78	-2,53	5,26E-46	1,05E-43	No DEG
ENSG00000268520	CTD-2616J11.10	-5,77	-2,53	8,08E-05	3,75E-04	No DEG
ENSG00000135390	ATP5G2	-5,77	-2,53	1,05E-68	8,51E-66	No DEG
ENSG00000270066	SCARNA2	5,76	2,53	7,91E-52	2,58E-49	No DEG
ENSG00000142627	EPHA2	-5,76	-2,53	1,14E-18	3,08E-17	No DEG
ENSG00000254521	SIGLEC12	-5,75	-2,52	4,11E-03	1,30E-02	No DEG
ENSG00000199290	Y_RNA	-5,75	-2,52	6,06E-03	1,84E-02	No DEG
ENSG00000232810	TNF	-5,75	-2,52	6,38E-06	3,61E-05	No DEG
ENSG00000225193	RPS12P26	-5,75	-2,52	2,00E-05	1,04E-04	No DEG
ENSG00000177452	RP4-597J3.1	-5,75	-2,52	2,06E-07	1,50E-06	DOWN
ENSG00000234648	AL162151.3	5,75	2,52	2,81E-05	1,43E-04	No DEG
ENSG00000197136	PCNXL3	-5,75	-2,52	8,06E-60	4,34E-57	No DEG
ENSG00000160678	S100A1	-5,74	-2,52	1,23E-18	3,33E-17	No DEG
ENSG00000256721	CACNA1C-IT3	-5,74	-2,52	7,34E-12	9,76E-11	No DEG
ENSG00000089159	PXN	-5,74	-2,52	2,82E-51	8,47E-49	No DEG
ENSG00000269788	RP11-399F4.2	-5,74	-2,52	2,67E-04	1,12E-03	No DEG
ENSG00000183479	TREX2	-5,74	-2,52	9,19E-05	4,21E-04	No DEG
ENSG00000077984	CST7	-5,73	-2,52	1,37E-11	1,76E-10	UP
ENSG00000105568	PPP2R1A	-5,73	-2,52	6,68E-73	6,03E-70	No DEG
ENSG00000210196	MT-TP	-5,73	-2,52	9,55E-21	3,17E-19	No DEG

ENSG00000207087	RNU6-242P	5,73	2,52	7,45E-04	2,84E-03	No DEG
ENSG00000198925	ATG9A	-5,73	-2,52	4,11E-53	1,40E-50	No DEG
ENSG00000243279	PRAF2	-5,73	-2,52	2,88E-12	4,02E-11	No DEG
ENSG00000117407	ARTN	-5,73	-2,52	8,59E-05	3,96E-04	No DEG
ENSG00000228019	RP11-166O4.4	-5,71	-2,51	9,97E-04	3,69E-03	No DEG
ENSG00000166523	CLEC4E	5,71	2,51	3,17E-08	2,61E-07	UP
ENSG00000177728	KIAA0195	-5,71	-2,51	7,17E-62	4,40E-59	No DEG
ENSG00000227778	XXyac-YX155B6.2	-5,70	-2,51	1,39E-04	6,15E-04	No DEG
ENSG00000257702	LBX2-AS1	-5,70	-2,51	1,08E-07	8,23E-07	No DEG
ENSG00000257289	RP11-611O2.6	5,70	2,51	1,35E-05	7,29E-05	UP
ENSG00000110046	ATG2A	-5,69	-2,51	2,36E-77	2,59E-74	No DEG
ENSG00000104946	TBC1D17	-5,69	-2,51	9,01E-34	8,18E-32	No DEG
ENSG00000207062	SNORA15	5,69	2,51	5,36E-03	1,65E-02	No DEG
ENSG00000255992	RP11-417L19.4	-5,68	-2,51	2,82E-03	9,35E-03	No DEG
ENSG00000253859	RP11-157I4.4	5,68	2,51	1,57E-03	5,55E-03	No DEG
ENSG00000184584	TMEM173	-5,68	-2,50	1,05E-26	5,79E-25	No DEG
ENSG00000197106	SLC6A17	-5,67	-2,50	1,17E-03	4,25E-03	No DEG
ENSG00000182704	TSKU	-5,67	-2,50	1,25E-18	3,37E-17	No DEG
ENSG00000273402	RP5-855D21.2	5,67	2,50	9,17E-08	7,04E-07	No DEG
ENSG00000259364	RP11-64K12.9	-5,67	-2,50	3,90E-03	1,25E-02	No DEG
ENSG00000197932	F8A1	-5,67	-2,50	1,55E-12	2,25E-11	No DEG
ENSG00000272068	RP11-284F21.9	-5,67	-2,50	2,99E-03	9,85E-03	No DEG
ENSG00000108262	GIT1	-5,66	-2,50	2,31E-67	1,77E-64	No DEG
ENSG00000158850	B4GALT3	-5,66	-2,50	2,05E-48	4,84E-46	No DEG
ENSG00000179772	FOXS1	-5,66	-2,50	1,19E-09	1,20E-08	No DEG
ENSG00000214160	ALG3	-5,66	-2,50	1,11E-26	6,14E-25	No DEG
ENSG00000225265	RP11-378J18.3	5,65	2,50	3,07E-08	2,53E-07	No DEG
ENSG00000197483	ZNF628	-5,65	-2,50	4,43E-20	1,37E-18	No DEG
ENSG00000204463	BAG6	-5,65	-2,50	5,73E-115	1,95E-111	No DEG
ENSG00000268433	RP11-420K14.2	5,65	2,50	2,36E-06	1,43E-05	No DEG
ENSG00000161180	CCDC116	-5,65	-2,50	9,06E-04	3,39E-03	No DEG
ENSG00000232464	CTA-125H2.1	-5,64	-2,50	4,56E-06	2,65E-05	DOWN

ENSG00000201988	Y_RNA	-5,64	-2,50	4,62E-03	1,45E-02	No DEG
ENSG00000258611	RP11-368J22.2	-5,63	-2,49	5,59E-03	1,71E-02	No DEG
ENSG00000250138	RP11-848G14.5	-5,63	-2,49	1,97E-21	6,95E-20	No DEG
ENSG00000179348	GATA2	-5,63	-2,49	1,37E-23	5,95E-22	No DEG
ENSG00000167987	VPS37C	-5,63	-2,49	1,39E-39	1,77E-37	No DEG
ENSG00000198198	SZT2	-5,63	-2,49	3,69E-62	2,31E-59	No DEG
ENSG00000019582	CD74	-5,62	-2,49	1,21E-26	6,66E-25	UP
ENSG00000232545	KB-318B8.7	-5,62	-2,49	7,87E-14	1,31E-12	No DEG
ENSG00000243978	RGAG1	-5,60	-2,49	2,27E-14	4,00E-13	No DEG
ENSG00000137204	SLC22A7	-5,60	-2,49	3,64E-04	1,48E-03	No DEG
ENSG00000215208	KRT18P60	-5,60	-2,48	4,52E-11	5,43E-10	No DEG
ENSG00000146083	RNF44	-5,59	-2,48	6,58E-34	6,01E-32	No DEG
ENSG00000141905	NFIC	-5,59	-2,48	1,14E-45	2,23E-43	No DEG
ENSG00000105649	RAB3A	-5,59	-2,48	1,17E-08	1,02E-07	No DEG
ENSG00000257270	RP11-521B24.5	-5,58	-2,48	5,95E-06	3,39E-05	No DEG
ENSG00000235832	CNN2P3	-5,58	-2,48	1,10E-03	4,04E-03	No DEG
ENSG00000176909	MAMSTR	-5,58	-2,48	2,08E-07	1,51E-06	No DEG
ENSG00000245748	RP11-367J11.2	-5,57	-2,48	1,19E-05	6,48E-05	No DEG
ENSG00000162526	TSSK3	-5,57	-2,48	1,32E-06	8,37E-06	No DEG
ENSG00000137507	LRRC32	-5,57	-2,48	9,69E-16	1,95E-14	No DEG
ENSG00000253669	KB-1732A1.1	-5,57	-2,48	1,10E-05	6,01E-05	DOWN
ENSG00000148344	PTGES	-5,56	-2,48	6,07E-04	2,36E-03	No DEG
ENSG00000261488	RP11-757F18.5	-5,56	-2,47	1,67E-06	1,04E-05	DOWN
ENSG00000198216	CACNA1E	-5,55	-2,47	7,91E-05	3,68E-04	DOWN
ENSG00000222845	RN7SKP42	5,55	2,47	1,52E-03	5,38E-03	No DEG
ENSG00000126005	MMP24-AS1	-5,55	-2,47	1,37E-30	9,95E-29	No DEG
ENSG00000249661	TNRC18P1	-5,54	-2,47	1,74E-12	2,50E-11	No DEG
ENSG00000141150	RASL10B	-5,54	-2,47	1,32E-25	6,65E-24	No DEG
ENSG00000212747	FAM127C	-5,54	-2,47	1,13E-27	6,74E-26	No DEG
ENSG00000212907	MT-ND4L	-5,54	-2,47	1,09E-35	1,11E-33	No DEG
ENSG00000136383	ALPK3	-5,54	-2,47	2,50E-33	2,23E-31	No DEG
ENSG00000136848	DAB2IP	-5,54	-2,47	1,34E-35	1,35E-33	No DEG

ENSG00000109062	SLC9A3R1	-5,54	-2,47	3,64E-13	5,65E-12	No DEG
ENSG00000156886	ITGAD	-5,53	-2,47	6,56E-10	6,83E-09	No DEG
ENSG00000198840	MT-ND3	-5,53	-2,47	1,14E-25	5,82E-24	No DEG
ENSG00000263089	RP11-166P13.4	-5,53	-2,47	1,17E-15	2,34E-14	No DEG
ENSG00000231925	TAPBP	-5,52	-2,47	2,24E-60	1,27E-57	No DEG
ENSG00000162650	ATXN7L2	-5,52	-2,46	3,23E-18	8,34E-17	No DEG
ENSG00000267385	CTB-50L17.14	-5,52	-2,46	1,03E-04	4,67E-04	No DEG
ENSG00000130164	LDLR	-5,51	-2,46	3,73E-09	3,50E-08	DOWN
ENSG00000199237	RNU6-834P	5,51	2,46	6,45E-03	1,94E-02	No DEG
ENSG00000108292	MLLT6	-5,49	-2,46	2,04E-89	4,18E-86	No DEG
ENSG00000263126	CTC-479C5.10	-5,49	-2,46	1,75E-04	7,61E-04	No DEG
ENSG00000214787	MS4A4E	5,49	2,46	2,25E-18	5,88E-17	UP
ENSG00000186704	DTX2P1	-5,49	-2,46	3,40E-10	3,65E-09	No DEG
ENSG00000143434	SEMA6C	-5,48	-2,46	8,99E-38	1,05E-35	No DEG
ENSG00000007866	TEAD3	-5,48	-2,45	1,12E-48	2,70E-46	No DEG
ENSG00000248986	RP11-774O3.1	-5,48	-2,45	2,30E-06	1,40E-05	DOWN
ENSG00000006704	GTF2IRD1	-5,48	-2,45	1,97E-29	1,33E-27	No DEG
ENSG00000106404	CLDN15	-5,47	-2,45	1,03E-11	1,35E-10	No DEG
ENSG00000187583	PLEKHN1	-5,47	-2,45	1,50E-05	8,02E-05	No DEG
ENSG00000011347	SYT7	-5,47	-2,45	4,93E-25	2,39E-23	No DEG
ENSG00000140527	WDR93	5,46	2,45	4,20E-03	1,33E-02	No DEG
ENSG00000141522	ARHGDIA	-5,46	-2,45	2,92E-26	1,56E-24	No DEG
ENSG00000087903	RFX2	-5,46	-2,45	7,13E-13	1,08E-11	No DEG
ENSG00000272849	RP11-347I19.8	-5,45	-2,45	2,71E-05	1,38E-04	No DEG
ENSG00000139151	PLCZ1	5,45	2,45	1,75E-05	9,26E-05	No DEG
ENSG00000227188	RP5-1104E15.6	-5,45	-2,45	1,29E-03	4,62E-03	No DEG
ENSG00000177303	CASKIN2	-5,44	-2,44	1,90E-41	2,77E-39	No DEG
ENSG00000184923	NUTM2A	-5,44	-2,44	2,45E-17	5,82E-16	No DEG
ENSG00000075426	FOSL2	-5,44	-2,44	5,96E-12	8,05E-11	No DEG
ENSG00000105723	GSK3A	-5,44	-2,44	3,75E-39	4,64E-37	No DEG
ENSG00000241400	RP11-764I5.1	-5,43	-2,44	1,99E-04	8,55E-04	No DEG
ENSG00000183570	PCBP3	-5,42	-2,44	9,19E-07	5,98E-06	No DEG

ENSG00000186310	NAP1L3	5,42	2,44	2,71E-08	2,26E-07	UP
ENSG00000172081	MOB3A	-5,41	-2,44	1,52E-42	2,37E-40	No DEG
ENSG00000108821	COL1A1	-5,41	-2,43	5,69E-06	3,25E-05	No DEG
ENSG00000076826	CAMSAP3	-5,40	-2,43	3,89E-04	1,58E-03	No DEG
ENSG00000140682	TGFB1I1	-5,40	-2,43	4,68E-20	1,44E-18	No DEG
ENSG00000070444	MNT	-5,40	-2,43	5,55E-23	2,28E-21	No DEG
ENSG00000056661	PCGF2	-5,40	-2,43	4,70E-19	1,32E-17	No DEG
ENSG00000052749	RRP12	-5,40	-2,43	7,46E-30	5,17E-28	No DEG
ENSG00000072310	SREBF1	-5,39	-2,43	8,22E-23	3,29E-21	No DEG
ENSG00000250479	CHCHD10	-5,38	-2,43	2,08E-44	3,72E-42	No DEG
ENSG00000117984	CTSD	-5,38	-2,43	6,78E-53	2,26E-50	No DEG
ENSG00000272597	RP11-446H18.6	5,38	2,43	1,52E-03	5,39E-03	No DEG
ENSG00000248107	CTC-339D2.1	5,38	2,43	3,45E-04	1,41E-03	No DEG
ENSG00000264274	MIR4799	5,37	2,42	7,96E-05	3,70E-04	No DEG
ENSG00000253925	CTB-178M22.1	-5,37	-2,42	7,71E-08	6,00E-07	DOWN
ENSG00000229046	HMGN1P2	-5,37	-2,42	2,32E-04	9,85E-04	No DEG
ENSG00000227741	RP11-536C5.7	-5,36	-2,42	6,62E-05	3,13E-04	No DEG
ENSG00000107140	TESK1	-5,36	-2,42	1,54E-22	6,01E-21	No DEG
ENSG00000261054	RP11-6O2.4	-5,36	-2,42	2,23E-05	1,15E-04	DOWN
ENSG00000100302	RASD2	-5,36	-2,42	1,67E-14	2,99E-13	No DEG
ENSG00000130158	DOCK6	-5,35	-2,42	1,10E-49	2,98E-47	No DEG
ENSG00000231252	RP11-436K8.1	5,35	2,42	2,19E-04	9,32E-04	No DEG
ENSG00000237584	RAC1P4	-5,35	-2,42	2,94E-03	9,71E-03	No DEG
ENSG00000269937	RP11-20I23.8	-5,35	-2,42	7,19E-04	2,75E-03	No DEG
ENSG00000243365	RN7SL278P	-5,34	-2,42	1,12E-02	3,13E-02	No DEG
ENSG00000263603	CTD-2349P21.5	-5,34	-2,42	7,17E-03	2,13E-02	No DEG
ENSG00000144063	MALL	-5,33	-2,41	1,34E-07	1,00E-06	No DEG
ENSG00000157856	DRC1	-5,32	-2,41	5,22E-04	2,05E-03	DOWN
ENSG00000124191	TOX2	-5,32	-2,41	7,36E-08	5,74E-07	No DEG
ENSG00000269487	CTB-174O21.2	-5,32	-2,41	7,45E-04	2,84E-03	No DEG
ENSG00000254414	RP11-182J1.1	5,31	2,41	1,14E-05	6,20E-05	No DEG
ENSG00000129467	ADCY4	-5,31	-2,41	2,60E-29	1,74E-27	No DEG

ENSG00000162298	SYVN1	-5,30	-2,41	1,36E-54	5,29E-52	No DEG
ENSG00000171206	TRIM8	-5,29	-2,40	1,04E-24	4,90E-23	No DEG
ENSG00000178217	SH2D4B	-5,29	-2,40	1,24E-03	4,46E-03	No DEG
ENSG00000225978	HAR1A	5,28	2,40	2,75E-04	1,15E-03	UP
ENSG00000140497	SCAMP2	-5,28	-2,40	6,57E-63	4,39E-60	No DEG
ENSG00000222872	RNU4-78P	-5,28	-2,40	2,98E-03	9,83E-03	No DEG
ENSG00000135253	KCP	-5,28	-2,40	1,47E-05	7,89E-05	No DEG
ENSG00000199121	MIR26B	-5,28	-2,40	3,45E-03	1,12E-02	No DEG
ENSG00000136997	MYC	-5,28	-2,40	4,54E-10	4,82E-09	No DEG
ENSG00000198026	ZNF335	-5,28	-2,40	1,08E-35	1,11E-33	No DEG
ENSG00000171067	C11orf24	-5,27	-2,40	6,02E-23	2,46E-21	No DEG
ENSG00000090020	SLC9A1	-5,27	-2,40	1,02E-20	3,38E-19	No DEG
ENSG00000203879	GDI1	-5,27	-2,40	6,89E-44	1,16E-41	No DEG
ENSG00000139144	PIK3C2G	5,27	2,40	1,95E-04	8,41E-04	UP
ENSG00000187116	LILRA5	-5,26	-2,40	7,46E-06	4,18E-05	No DEG
ENSG00000106991	ENG	-5,26	-2,39	1,12E-24	5,26E-23	No DEG
ENSG00000167371	PRRT2	-5,25	-2,39	1,21E-12	1,79E-11	No DEG
ENSG00000077150	NFKB2	-5,25	-2,39	1,62E-13	2,63E-12	No DEG
ENSG00000185112	FAM43A	-5,25	-2,39	3,20E-06	1,91E-05	DOWN
ENSG00000235241	RP11-108M9.5	5,24	2,39	6,06E-03	1,84E-02	No DEG
ENSG00000188706	ZDHHC9	-5,24	-2,39	7,27E-23	2,93E-21	No DEG
ENSG00000272789	RP11-286H15.1	-5,23	-2,39	2,61E-04	1,10E-03	No DEG
ENSG00000216197	AP001623.1	-5,23	-2,39	2,36E-08	1,99E-07	No DEG
ENSG00000272941	RP11-134L10.1	-5,22	-2,39	1,34E-05	7,24E-05	No DEG
ENSG00000196453	ZNF777	-5,22	-2,38	2,56E-28	1,62E-26	No DEG
ENSG00000167874	TMEM88	-5,22	-2,38	8,15E-24	3,62E-22	No DEG
ENSG00000134780	DAGLA	-5,22	-2,38	1,32E-21	4,72E-20	No DEG
ENSG00000270415	DPPA3P3	5,22	2,38	7,02E-05	3,30E-04	No DEG
ENSG00000249669	MIR143HG	-5,21	-2,38	5,03E-17	1,15E-15	No DEG
ENSG00000224997	AL049840.1	-5,21	-2,38	1,53E-05	8,14E-05	No DEG
ENSG00000099797	TECR	-5,21	-2,38	9,32E-40	1,22E-37	No DEG
ENSG00000179899	PHC1P1	-5,20	-2,38	9,05E-15	1,67E-13	No DEG

ENSG00000079308	TNS1	-5,19	-2,38	9,63E-28	5,78E-26	No DEG
ENSG00000256347	OR8R1P	-5,19	-2,38	1,32E-02	3,62E-02	No DEG
ENSG00000204052	LRRC73	-5,19	-2,37	1,42E-07	1,06E-06	No DEG
ENSG00000267896	AC018766.4	-5,19	-2,37	8,66E-04	3,26E-03	No DEG
ENSG00000077312	SNRPA	-5,18	-2,37	8,10E-33	6,93E-31	No DEG
ENSG00000143858	SYT2	-5,18	-2,37	8,71E-12	1,15E-10	No DEG
ENSG00000143126	CELSR2	-5,18	-2,37	1,32E-16	2,87E-15	No DEG
ENSG00000233098	RP11-344E13.3	5,18	2,37	1,72E-09	1,70E-08	UP
ENSG00000178860	MSC	-5,17	-2,37	5,98E-09	5,45E-08	No DEG
ENSG00000242242	PVRL3-AS1	5,17	2,37	1,41E-03	5,03E-03	No DEG
ENSG00000095777	MYO3A	5,17	2,37	1,95E-04	8,39E-04	No DEG
ENSG00000250061	RP11-541P9.3	-5,17	-2,37	2,80E-04	1,17E-03	DOWN
ENSG00000242060	RPS3AP49	5,17	2,37	1,97E-04	8,49E-04	No DEG
ENSG00000218175	AC016739.2	-5,17	-2,37	4,55E-19	1,28E-17	No DEG
ENSG00000054356	PTPRN	-5,17	-2,37	5,43E-04	2,13E-03	No DEG
ENSG00000147144	CCDC120	-5,16	-2,37	4,07E-08	3,30E-07	No DEG
ENSG00000204277	RP11-219G17.4	-5,16	-2,37	8,06E-03	2,36E-02	No DEG
ENSG00000215840	RP11-122G18.7	-5,16	-2,37	3,98E-05	1,96E-04	No DEG
ENSG00000252469	RNU7-160P	-5,16	-2,37	1,78E-03	6,19E-03	No DEG
ENSG00000224689	ZNF812	5,16	2,37	1,11E-12	1,64E-11	UP
ENSG00000068323	TFE3	-5,15	-2,37	1,11E-20	3,66E-19	No DEG
ENSG00000099625	C19orf26	-5,15	-2,36	1,01E-10	1,16E-09	DOWN
ENSG00000228570	NUTM2E	-5,15	-2,36	7,22E-04	2,75E-03	No DEG
ENSG00000149131	SERPING1	-5,14	-2,36	4,50E-24	2,04E-22	No DEG
ENSG00000259094	RP11-77A13.1	-5,13	-2,36	4,15E-03	1,32E-02	No DEG
ENSG00000224551	HMGB3P21	-5,13	-2,36	1,84E-03	6,39E-03	No DEG
ENSG00000072071	LPHN1	-5,13	-2,36	3,83E-32	3,14E-30	No DEG
ENSG00000262601	CTC-786C10.1	-5,13	-2,36	2,39E-13	3,79E-12	No DEG
ENSG00000105063	PPP6R1	-5,13	-2,36	4,57E-51	1,36E-48	No DEG
ENSG00000251791	SCARNA6	5,13	2,36	6,10E-40	8,14E-38	No DEG
ENSG00000132005	RFX1	-5,12	-2,36	5,92E-30	4,13E-28	No DEG
ENSG00000159166	LAD1	-5,12	-2,36	9,96E-07	6,44E-06	No DEG

ENSG00000143333	RGS16	-5,12	-2,36	3,87E-05	1,92E-04	No DEG
ENSG00000134765	DSC1	5,12	2,36	1,69E-09	1,67E-08	No DEG
ENSG00000131203	IDO1	-5,12	-2,36	3,02E-03	9,94E-03	No DEG
ENSG00000161202	DVL3	-5,11	-2,35	6,79E-58	3,15E-55	No DEG
ENSG00000178752	FAM132B	-5,11	-2,35	1,66E-05	8,81E-05	DOWN
ENSG00000229367	HMGN2P19	-5,11	-2,35	4,74E-03	1,48E-02	DOWN
ENSG00000187904	AC097382.5	5,11	2,35	4,87E-03	1,52E-02	UP
ENSG00000173039	RELA	-5,11	-2,35	1,13E-29	7,72E-28	No DEG
ENSG00000231485	RP4-535B20.1	5,10	2,35	1,41E-05	7,57E-05	No DEG
ENSG00000269936	MIR145	-5,10	-2,35	1,22E-12	1,80E-11	No DEG
ENSG00000163874	ZC3H12A	-5,09	-2,35	8,08E-10	8,31E-09	No DEG
ENSG00000030582	GRN	-5,08	-2,35	1,20E-40	1,66E-38	No DEG
ENSG00000198435	NRARP	-5,08	-2,35	4,05E-11	4,90E-10	No DEG
ENSG00000188026	RILPL1	-5,08	-2,35	2,28E-34	2,13E-32	No DEG
ENSG00000272658	LTB4R2	-5,08	-2,34	1,37E-08	1,19E-07	No DEG
ENSG00000273327	OR6L2P	-5,08	-2,34	3,80E-03	1,22E-02	No DEG
ENSG00000140961	OSGIN1	-5,07	-2,34	8,79E-11	1,01E-09	No DEG
ENSG00000265296	FEM1AP2	-5,07	-2,34	4,05E-05	1,99E-04	No DEG
ENSG00000225190	PLEKHM1	-5,07	-2,34	4,69E-38	5,60E-36	No DEG
ENSG00000186265	BTLA	5,07	2,34	7,36E-05	3,44E-04	UP
ENSG00000202538	RNU4-2	5,06	2,34	1,43E-06	8,97E-06	No DEG
ENSG00000251497	RP11-197N18.7	-5,06	-2,34	1,50E-06	9,37E-06	No DEG
ENSG00000109079	TNFAIP1	-5,06	-2,34	9,83E-24	4,35E-22	No DEG
ENSG00000112787	FBRSL1	-5,06	-2,34	7,90E-49	1,94E-46	No DEG
ENSG00000154025	SLC5A10	-5,06	-2,34	9,82E-05	4,47E-04	No DEG
ENSG00000204673	AKT1S1	-5,06	-2,34	4,35E-31	3,30E-29	No DEG
ENSG00000176454	LPCAT4	-5,05	-2,34	2,16E-19	6,23E-18	No DEG
ENSG00000244921	CTB-36O1.7	-5,05	-2,34	2,03E-09	1,98E-08	DOWN
ENSG00000158481	CD1C	5,05	2,34	5,41E-07	3,67E-06	UP
ENSG00000204348	DXO	-5,05	-2,34	2,70E-27	1,58E-25	No DEG
ENSG00000178188	SH2B1	-5,05	-2,34	7,06E-43	1,12E-40	No DEG
ENSG00000120896	SORBS3	-5,05	-2,34	2,63E-35	2,56E-33	No DEG

ENSG00000237683	AL627309.1	-5,05	-2,34	2,91E-04	1,21E-03	No DEG
ENSG00000229871	RP4-710M16.1	-5,04	-2,33	9,67E-04	3,60E-03	DOWN
ENSG00000088888	MAVS	-5,04	-2,33	7,03E-37	7,79E-35	No DEG
ENSG00000133110	POSTN	5,04	2,33	8,21E-06	4,58E-05	No DEG
ENSG00000077454	LRCH4	-5,04	-2,33	3,56E-28	2,22E-26	No DEG
ENSG00000065717	TLE2	-5,04	-2,33	6,61E-56	2,78E-53	No DEG
ENSG00000114923	SLC4A3	-5,04	-2,33	2,44E-33	2,18E-31	No DEG
ENSG00000105738	SIPA1L3	-5,04	-2,33	2,58E-47	5,73E-45	No DEG
ENSG00000239157	snoU13	-5,04	-2,33	6,23E-04	2,41E-03	No DEG
ENSG00000172534	HCFC1	-5,03	-2,33	4,71E-71	3,90E-68	No DEG
ENSG00000118898	PPL	-5,03	-2,33	1,29E-15	2,56E-14	No DEG
ENSG00000185291	IL3RA	-5,03	-2,33	5,49E-12	7,45E-11	No DEG
ENSG00000241360	PDXP	5,02	2,33	1,02E-09	1,03E-08	No DEG
ENSG00000100626	GALNT16	-5,02	-2,33	1,36E-27	8,09E-26	No DEG
ENSG00000249170	RP11-1J11.1	-5,02	-2,33	7,62E-05	3,55E-04	No DEG
ENSG00000215237	RP11-54D18.2	5,01	2,33	8,71E-05	4,00E-04	No DEG
ENSG00000102858	MGRN1	-5,01	-2,33	2,50E-30	1,80E-28	No DEG
ENSG00000115163	CENPA	5,01	2,33	1,42E-03	5,05E-03	No DEG
ENSG00000207725	MIR222	5,00	2,32	7,20E-03	2,14E-02	No DEG
ENSG00000100867	DHRS2	-5,00	-2,32	3,13E-04	1,29E-03	No DEG
ENSG00000131462	TUBG1	-5,00	-2,32	9,00E-41	1,27E-38	No DEG
ENSG00000263931	RP11-180P8.1	5,00	2,32	3,39E-08	2,77E-07	No DEG
ENSG00000169992	NLGN2	-5,00	-2,32	1,24E-13	2,03E-12	No DEG
ENSG00000239602	RP11-449H3.1	-5,00	-2,32	1,05E-03	3,88E-03	No DEG
ENSG00000160446	ZDHHC12	-5,00	-2,32	7,94E-21	2,66E-19	No DEG
ENSG00000259865	RP11-488L18.10	4,99	2,32	3,32E-14	5,76E-13	No DEG
ENSG00000205476	CCDC85C	-4,99	-2,32	3,71E-14	6,39E-13	No DEG
ENSG00000140323	DISP2	-4,99	-2,32	3,17E-06	1,89E-05	No DEG
ENSG00000101276	SLC52A3	-4,99	-2,32	5,58E-10	5,85E-09	No DEG
ENSG00000242052	RP11-190C22.1	-4,98	-2,32	2,13E-07	1,54E-06	DOWN
ENSG00000108557	RAI1	-4,98	-2,32	2,98E-57	1,37E-54	No DEG
ENSG00000170290	SLN	4,98	2,31	4,36E-06	2,54E-05	No DEG

ENSG00000090534	THPO	-4,97	-2,31	1,41E-04	6,25E-04	No DEG
ENSG00000259424	RP11-35O15.1	-4,97	-2,31	7,52E-04	2,86E-03	No DEG
ENSG00000125505	MBOAT7	-4,97	-2,31	6,06E-26	3,16E-24	No DEG
ENSG00000146205	ANO7	-4,96	-2,31	8,47E-12	1,12E-10	No DEG
ENSG00000203280	CTA-221G9.11	-4,96	-2,31	3,44E-11	4,21E-10	No DEG
ENSG00000267473	AC005789.11	-4,96	-2,31	3,52E-03	1,14E-02	No DEG
ENSG00000252284	SNORD28	-4,95	-2,31	5,03E-03	1,56E-02	No DEG
ENSG00000244998	CTD-3064M3.4	-4,95	-2,31	1,08E-07	8,21E-07	DOWN
ENSG00000100197	CYP2D6	-4,94	-2,31	6,70E-16	1,37E-14	No DEG
ENSG00000258551	RP11-661P17.1	4,94	2,30	3,27E-04	1,35E-03	No DEG
ENSG00000267194	RP1-193H18.2	4,94	2,30	1,36E-07	1,01E-06	No DEG
ENSG00000156466	GDF6	4,94	2,30	1,50E-05	8,02E-05	UP
ENSG00000168824	NSG1	-4,94	-2,30	2,19E-07	1,58E-06	DOWN
ENSG00000220553	RPL5P19	4,94	2,30	2,60E-03	8,69E-03	No DEG
ENSG00000155961	RAB39B	4,93	2,30	1,93E-06	1,19E-05	UP
ENSG00000164054	SHISA5	-4,93	-2,30	4,59E-44	7,87E-42	No DEG
ENSG00000175643	RMI2	4,93	2,30	4,26E-06	2,49E-05	UP
ENSG00000269899	RP11-589N15.2	-4,93	-2,30	3,27E-05	1,65E-04	No DEG
ENSG00000269515	AL137026.1	-4,93	-2,30	8,89E-03	2,57E-02	No DEG
ENSG00000096070	BRPF3	-4,93	-2,30	9,55E-44	1,57E-41	No DEG
ENSG00000130175	PRKCSH	-4,92	-2,30	1,76E-49	4,57E-47	No DEG
ENSG00000116039	ATP6V1B1	-4,92	-2,30	5,72E-05	2,74E-04	No DEG
ENSG00000234449	RP11-706O15.3	-4,92	-2,30	1,07E-04	4,84E-04	No DEG
ENSG00000198844	ARHGEF15	-4,92	-2,30	1,34E-35	1,35E-33	No DEG
ENSG00000182379	NXPH4	-4,92	-2,30	2,45E-05	1,26E-04	No DEG
ENSG00000272953	RP11-1275H24.2	4,91	2,30	4,63E-03	1,45E-02	No DEG
ENSG00000272056	RP11-503P10.1	-4,91	-2,30	2,95E-04	1,23E-03	No DEG
ENSG00000089486	CDIP1	-4,91	-2,30	1,94E-37	2,22E-35	No DEG
ENSG00000179862	CITED4	-4,91	-2,29	3,09E-09	2,93E-08	No DEG
ENSG00000119242	CCDC92	-4,91	-2,29	5,39E-52	1,78E-49	No DEG
ENSG00000161999	JMJD8	-4,91	-2,29	1,47E-38	1,80E-36	No DEG
ENSG00000234506	RP11-274B18.2	-4,91	-2,29	3,67E-03	1,18E-02	No DEG

ENSG00000133055	MYBPH	-4,91	-2,29	7,83E-04	2,97E-03	No DEG
ENSG00000168488	ATXN2L	-4,90	-2,29	5,80E-43	9,22E-41	No DEG
ENSG00000111077	TENC1	-4,90	-2,29	1,07E-39	1,38E-37	No DEG
ENSG00000145016	KIAA0226	-4,90	-2,29	1,03E-19	3,07E-18	No DEG
ENSG00000169994	MYO7B	-4,90	-2,29	2,12E-11	2,66E-10	No DEG
ENSG00000271579	RP11-116D17.3	4,90	2,29	2,16E-03	7,37E-03	No DEG
ENSG00000087087	SRRT	-4,90	-2,29	3,80E-45	7,16E-43	No DEG
ENSG00000157570	TSPAN18	-4,89	-2,29	2,31E-45	4,46E-43	No DEG
ENSG00000203563	AC138951.1	-4,89	-2,29	4,54E-03	1,42E-02	No DEG
ENSG00000169783	LINGO1	-4,89	-2,29	1,00E-07	7,64E-07	No DEG
ENSG00000104897	SF3A2	-4,89	-2,29	2,92E-39	3,66E-37	No DEG
ENSG00000242419	PCDHGC4	-4,88	-2,29	1,36E-16	2,96E-15	No DEG
ENSG00000185875	THNSL1	4,88	2,29	4,06E-13	6,29E-12	No DEG
ENSG00000108798	ABI3	-4,88	-2,29	7,93E-16	1,61E-14	No DEG
ENSG00000250771	RP11-153M7.3	4,88	2,29	9,15E-06	5,07E-05	UP
ENSG00000143842	SOX13	-4,88	-2,29	1,22E-26	6,68E-25	No DEG
ENSG00000270903	HNRNPA3P9	-4,88	-2,29	1,60E-06	9,97E-06	No DEG
ENSG00000080845	DLGAP4	-4,88	-2,29	6,10E-47	1,30E-44	No DEG
ENSG00000105609	LILRB5	-4,88	-2,29	2,19E-09	2,12E-08	No DEG
ENSG00000230287	RP11-305E17.4	-4,88	-2,29	2,33E-03	7,91E-03	No DEG
ENSG00000105364	MRPL4	-4,87	-2,29	1,70E-37	1,96E-35	No DEG
ENSG00000260091	RP11-33B1.4	-4,86	-2,28	1,43E-03	5,08E-03	No DEG
ENSG00000213171	LINGO4	-4,86	-2,28	1,24E-05	6,73E-05	No DEG
ENSG00000252010	SCARNA5	4,86	2,28	1,77E-35	1,77E-33	No DEG
ENSG00000266998	RP11-936I5.1	-4,86	-2,28	7,14E-05	3,35E-04	No DEG
ENSG00000146453	PNLDC1	-4,86	-2,28	4,55E-04	1,82E-03	No DEG
ENSG00000264736	BDP1P	4,85	2,28	4,57E-03	1,43E-02	No DEG
ENSG00000159388	BTG2	-4,85	-2,28	6,01E-06	3,42E-05	DOWN
ENSG00000149050	ZNF214	4,85	2,28	5,35E-18	1,36E-16	No DEG
ENSG00000255176	AP002954.3	-4,85	-2,28	2,11E-08	1,79E-07	No DEG
ENSG00000152292	SH2D6	-4,85	-2,28	1,57E-03	5,54E-03	DOWN
ENSG00000198804	MT-CO1	-4,84	-2,28	7,52E-20	2,28E-18	No DEG

ENSG00000226744	AC079781.5	4,84	2,28	4,79E-03	1,49E-02	No DEG
ENSG00000270124	RP11-118F19.1	-4,84	-2,27	2,86E-04	1,19E-03	No DEG
ENSG00000226180	AC010536.1	-4,84	-2,27	1,18E-04	5,28E-04	No DEG
ENSG00000229180	GS1-124K5.11	-4,83	-2,27	9,39E-13	1,40E-11	No DEG
ENSG00000172375	C2CD2L	-4,83	-2,27	3,53E-26	1,86E-24	No DEG
ENSG00000183793	NPIPA5	-4,83	-2,27	7,66E-04	2,91E-03	No DEG
ENSG00000105722	ERF	-4,83	-2,27	3,80E-25	1,86E-23	No DEG
ENSG00000158480	SPATA2	-4,82	-2,27	3,04E-24	1,39E-22	No DEG
ENSG00000236254	MTND4P14	-4,82	-2,27	1,90E-04	8,19E-04	No DEG
ENSG00000260144	RP11-361M10.3	-4,82	-2,27	4,13E-05	2,03E-04	No DEG
ENSG00000141985	SH3GL1	-4,82	-2,27	1,25E-20	4,10E-19	No DEG
ENSG00000272782	RP4-607J23.2	-4,81	-2,27	1,24E-07	9,35E-07	DOWN
ENSG00000127666	TICAM1	-4,81	-2,27	2,30E-18	6,01E-17	No DEG
ENSG00000203943	SAMD13	4,81	2,27	1,83E-08	1,57E-07	No DEG
ENSG00000262833	RP11-28G8.1	-4,81	-2,27	3,18E-06	1,90E-05	No DEG
ENSG00000156860	FBRS	-4,81	-2,27	8,80E-52	2,84E-49	No DEG
ENSG00000269402	AC116407.2	-4,81	-2,27	4,86E-03	1,51E-02	No DEG
ENSG00000269690	AC096677.1	-4,81	-2,27	1,22E-19	3,58E-18	No DEG
ENSG00000105409	ATP1A3	-4,81	-2,26	1,75E-18	4,64E-17	No DEG
ENSG00000270012	LL0XNC01-7P3.1	-4,80	-2,26	5,37E-12	7,30E-11	No DEG
ENSG00000095321	CRAT	-4,80	-2,26	2,62E-59	1,39E-56	No DEG
ENSG00000205636	LINC00583	4,80	2,26	3,99E-03	1,27E-02	No DEG
ENSG00000001617	SEMA3F	-4,80	-2,26	1,37E-18	3,66E-17	No DEG
ENSG00000184967	NOC4L	-4,79	-2,26	2,20E-20	7,04E-19	No DEG
ENSG00000248161	RP11-499E18.1	4,79	2,26	5,42E-03	1,67E-02	No DEG
ENSG00000224959	AC017002.2	-4,79	-2,26	2,72E-06	1,64E-05	No DEG
ENSG00000250571	GLI4	-4,79	-2,26	5,64E-22	2,08E-20	No DEG
ENSG00000272636	DOC2B	-4,79	-2,26	2,99E-13	4,69E-12	No DEG
ENSG00000183444	OR7E38P	-4,79	-2,26	1,02E-13	1,69E-12	No DEG
ENSG00000207612	MIR604	-4,79	-2,26	2,02E-05	1,05E-04	No DEG
ENSG00000230712	AP000354.4	-4,78	-2,26	2,12E-04	9,07E-04	No DEG
ENSG00000249540	RP11-789L4.1	-4,78	-2,26	4,34E-03	1,37E-02	No DEG

ENSG00000065989	PDE4A	-4,78	-2,26	5,37E-21	1,84E-19	No DEG
ENSG00000180447	GAS1	4,78	2,26	5,54E-28	3,42E-26	No DEG
ENSG00000149091	DGKZ	-4,78	-2,26	1,75E-32	1,46E-30	No DEG
ENSG00000158796	DEDD	-4,78	-2,26	3,55E-39	4,41E-37	No DEG
ENSG00000103168	TAF1C	-4,78	-2,26	3,55E-24	1,62E-22	No DEG
ENSG00000171101	SIGLEC17P	-4,78	-2,26	4,28E-03	1,35E-02	No DEG
ENSG00000125971	DYNLRB1	-4,78	-2,26	3,03E-62	1,94E-59	No DEG
ENSG00000255375	RP1-65P5.5	-4,78	-2,26	3,91E-03	1,25E-02	No DEG
ENSG00000130762	ARHGEF16	-4,78	-2,26	1,07E-07	8,12E-07	No DEG
ENSG00000261600	RP11-575H3.1	4,77	2,25	3,46E-03	1,12E-02	No DEG
ENSG00000120457	KCNJ5	-4,76	-2,25	1,21E-21	4,35E-20	No DEG
ENSG00000036672	USP2	-4,76	-2,25	1,60E-38	1,95E-36	No DEG
ENSG00000217684	RPS3AP24	4,76	2,25	4,48E-04	1,79E-03	No DEG
ENSG00000173557	C2orf70	-4,76	-2,25	1,04E-02	2,94E-02	No DEG
ENSG00000145681	HAPLN1	4,76	2,25	3,99E-04	1,61E-03	No DEG
ENSG00000130479	MAP1S	-4,75	-2,25	7,42E-30	5,15E-28	No DEG
ENSG00000254995	STX16-NPEPL1	-4,75	-2,25	8,73E-08	6,74E-07	No DEG
ENSG00000238541	snoU13	-4,75	-2,25	1,22E-06	7,75E-06	No DEG
ENSG00000140297	GCNT3	-4,75	-2,25	9,38E-04	3,50E-03	DOWN
ENSG00000139514	SLC7A1	-4,75	-2,25	6,07E-17	1,38E-15	No DEG
ENSG00000165495	PKNOX2	-4,75	-2,25	1,17E-17	2,87E-16	No DEG
ENSG00000099864	PALM	-4,75	-2,25	9,19E-21	3,06E-19	No DEG
ENSG00000138031	ADCY3	-4,75	-2,25	4,55E-23	1,88E-21	No DEG
ENSG00000143632	ACTA1	-4,73	-2,24	1,59E-09	1,57E-08	No DEG
ENSG00000158863	FAM160B2	-4,73	-2,24	1,75E-54	6,62E-52	No DEG
ENSG00000107521	HPS1	-4,73	-2,24	4,28E-37	4,79E-35	No DEG
ENSG00000134917	ADAMTS8	-4,73	-2,24	1,95E-06	1,20E-05	No DEG
ENSG00000073737	DHRS9	4,72	2,24	3,44E-05	1,72E-04	UP
ENSG00000271538	RP11-326I11.4	4,72	2,24	4,84E-06	2,80E-05	UP
ENSG00000135723	FHOD1	-4,72	-2,24	8,03E-25	3,82E-23	No DEG
ENSG00000127948	POR	-4,72	-2,24	3,39E-16	7,14E-15	No DEG
ENSG00000198150	AC135178.1	-4,72	-2,24	1,08E-21	3,90E-20	No DEG

ENSG00000184371	CSF1	-4,72	-2,24	8,32E-21	2,78E-19	No DEG
ENSG00000211584	SLC48A1	-4,72	-2,24	3,07E-49	7,79E-47	No DEG
ENSG00000207554	MIR647	-4,71	-2,24	1,86E-03	6,45E-03	No DEG
ENSG00000160271	RALGDS	-4,71	-2,24	6,72E-22	2,46E-20	No DEG
ENSG00000177666	PNPLA2	-4,71	-2,24	3,94E-50	1,12E-47	No DEG
ENSG00000261534	RP11-244O19.1	-4,71	-2,24	8,40E-14	1,40E-12	No DEG
ENSG00000102034	ELF4	-4,71	-2,23	5,69E-12	7,71E-11	No DEG
ENSG00000146013	GFRA3	-4,71	-2,23	2,20E-05	1,14E-04	No DEG
ENSG00000221264	MIR1284	4,70	2,23	4,98E-03	1,55E-02	No DEG
ENSG00000212443	SNORA53	4,70	2,23	1,53E-11	1,96E-10	No DEG
ENSG00000225630	MTND2P28	-4,70	-2,23	2,07E-17	5,00E-16	No DEG
ENSG00000250120	PCDHA10	4,70	2,23	4,24E-04	1,70E-03	No DEG
ENSG00000113889	KNG1	-4,70	-2,23	3,52E-04	1,44E-03	DOWN
ENSG00000127124	HIVEP3	-4,69	-2,23	1,33E-17	3,26E-16	No DEG
ENSG00000171476	HOPX	-4,69	-2,23	9,28E-04	3,46E-03	DOWN
ENSG00000252873	SNORD112	4,69	2,23	4,54E-03	1,42E-02	No DEG
ENSG00000177595	PIDD	-4,69	-2,23	2,27E-18	5,92E-17	No DEG
ENSG00000104881	PPP1R13L	-4,69	-2,23	7,09E-26	3,68E-24	No DEG
ENSG00000258448	RP11-109N23.5	-4,69	-2,23	9,13E-04	3,41E-03	No DEG
ENSG00000233929	MT1XP1	-4,68	-2,23	1,17E-03	4,27E-03	No DEG
ENSG00000237133	AC020594.5	-4,68	-2,23	1,03E-04	4,66E-04	DOWN
ENSG00000181409	AATK	-4,68	-2,23	2,26E-13	3,60E-12	No DEG
ENSG00000185761	ADAMTSL5	-4,68	-2,23	1,56E-18	4,14E-17	No DEG
ENSG00000140464	PML	-4,68	-2,23	7,70E-35	7,36E-33	No DEG
ENSG00000102760	RGCC	4,67	2,22	1,25E-14	2,27E-13	No DEG
ENSG00000117713	ARID1A	-4,67	-2,22	2,05E-42	3,17E-40	No DEG
ENSG00000239475	HYDIN2	-4,67	-2,22	3,01E-03	9,92E-03	No DEG
ENSG00000126461	SCAF1	-4,66	-2,22	1,86E-62	1,22E-59	No DEG
ENSG00000272784	RP11-335L23.5	4,66	2,22	3,25E-03	1,06E-02	No DEG
ENSG00000205746	RP11-1212A22.1	-4,66	-2,22	8,45E-17	1,89E-15	No DEG
ENSG00000171236	LRG1	-4,66	-2,22	6,16E-04	2,39E-03	No DEG
ENSG00000250994	AC005355.1	-4,66	-2,22	3,83E-04	1,55E-03	DOWN

ENSG00000198865	CCDC152	4,66	2,22	8,64E-22	3,13E-20	UP
ENSG00000180422	LINC00304	-4,65	-2,22	4,74E-03	1,48E-02	No DEG
ENSG00000232056	AC092687.4	-4,65	-2,22	2,71E-05	1,38E-04	No DEG
ENSG00000181009	OR52N5	-4,65	-2,22	7,41E-03	2,19E-02	No DEG
ENSG00000244953	RP11-613D13.8	-4,65	-2,22	4,09E-05	2,01E-04	No DEG
ENSG00000103495	MAZ	-4,65	-2,22	1,26E-20	4,11E-19	No DEG
ENSG00000100604	CHGA	-4,64	-2,21	1,04E-04	4,70E-04	No DEG
ENSG00000233631	RP11-457M11.2	4,63	2,21	3,08E-06	1,84E-05	No DEG
ENSG00000102870	ZNF629	-4,63	-2,21	4,85E-33	4,26E-31	No DEG
ENSG00000075399	VPS9D1	-4,63	-2,21	1,26E-32	1,06E-30	No DEG
ENSG00000215218	UBE2QL1	-4,63	-2,21	4,22E-27	2,44E-25	No DEG
ENSG00000163825	RTP3	-4,63	-2,21	2,36E-04	1,00E-03	No DEG
ENSG00000138764	CCNG2	4,63	2,21	6,06E-15	1,13E-13	No DEG
ENSG00000169258	GPRIN1	-4,63	-2,21	2,79E-07	1,99E-06	No DEG
ENSG00000113721	PDGFRB	-4,62	-2,21	2,38E-23	1,01E-21	No DEG
ENSG00000106683	LIMK1	-4,62	-2,21	1,66E-25	8,32E-24	No DEG
ENSG00000253958	CLDN23	4,62	2,21	1,44E-03	5,11E-03	No DEG
ENSG00000236304	AP001189.4	-4,62	-2,21	2,14E-06	1,31E-05	No DEG
ENSG00000130669	PAK4	-4,62	-2,21	6,41E-22	2,36E-20	No DEG
ENSG00000103507	BCKDK	-4,62	-2,21	1,03E-61	6,20E-59	No DEG
ENSG00000214402	LCNL1	-4,62	-2,21	1,32E-06	8,34E-06	No DEG
ENSG00000254254	RP11-17A4.2	4,62	2,21	1,31E-04	5,83E-04	UP
ENSG00000261399	LA16c-329F2.1	-4,61	-2,21	4,19E-03	1,33E-02	No DEG
ENSG00000196535	MYO18A	-4,60	-2,20	3,76E-47	8,18E-45	No DEG
ENSG00000178772	CPN2	-4,60	-2,20	2,80E-03	9,29E-03	No DEG
ENSG00000007047	MARK4	-4,60	-2,20	1,10E-51	3,51E-49	No DEG
ENSG00000258753	RP11-794A8.1	-4,60	-2,20	5,11E-04	2,02E-03	No DEG
ENSG00000258429	PDF	4,60	2,20	1,57E-17	3,83E-16	No DEG
ENSG00000204899	MZT1	4,60	2,20	1,78E-31	1,38E-29	No DEG
ENSG00000142784	WDTC1	-4,59	-2,20	8,55E-55	3,41E-52	No DEG
ENSG00000105220	GPI	-4,59	-2,20	2,70E-82	3,94E-79	No DEG
ENSG00000240733	RN7SL502P	-4,59	-2,20	9,22E-04	3,44E-03	No DEG

ENSG00000138834	MAPK8IP3	-4,58	-2,20	1,12E-39	1,45E-37	No DEG
ENSG00000064545	TMEM161A	-4,58	-2,20	1,76E-22	6,78E-21	No DEG
ENSG00000124920	MYRF	-4,58	-2,19	1,56E-11	1,99E-10	No DEG
ENSG00000132382	MYBBP1A	-4,58	-2,19	6,04E-41	8,62E-39	No DEG
ENSG00000173988	LRRC63	4,58	2,19	4,12E-03	1,31E-02	UP
ENSG00000250731	TPM3P6	-4,58	-2,19	7,72E-05	3,60E-04	No DEG
ENSG00000213860	RPL21P75	4,57	2,19	2,37E-03	8,00E-03	No DEG
ENSG00000160392	C19orf47	-4,57	-2,19	1,33E-27	7,96E-26	No DEG
ENSG00000234026	RP11-310E22.4	-4,57	-2,19	1,58E-03	5,55E-03	No DEG
ENSG00000175662	TOM1L2	-4,57	-2,19	7,41E-27	4,16E-25	No DEG
ENSG00000143028	SYPL2	-4,57	-2,19	5,79E-28	3,57E-26	No DEG
ENSG00000099904	ZDHHC8	-4,57	-2,19	8,03E-38	9,44E-36	No DEG
ENSG00000198837	DENND4B	-4,57	-2,19	1,31E-53	4,69E-51	No DEG
ENSG00000188176	SMTNL2	-4,57	-2,19	1,33E-07	9,93E-07	DOWN
ENSG00000099365	STX1B	-4,56	-2,19	1,23E-04	5,49E-04	No DEG
ENSG00000182809	CRIP2	-4,56	-2,19	2,51E-38	3,02E-36	No DEG
ENSG00000105556	MIER2	-4,56	-2,19	8,59E-23	3,43E-21	No DEG
ENSG00000166831	RBPMS2	-4,56	-2,19	1,18E-23	5,16E-22	DOWN
ENSG00000166960	CCDC178	4,55	2,19	4,87E-03	1,52E-02	No DEG
ENSG00000183018	SPNS2	-4,55	-2,19	7,43E-13	1,12E-11	No DEG
ENSG00000133065	SLC41A1	-4,55	-2,19	4,14E-30	2,92E-28	No DEG
ENSG00000183682	BMP8A	-4,55	-2,19	3,73E-07	2,60E-06	No DEG
ENSG00000213928	IRF9	-4,55	-2,19	3,67E-14	6,33E-13	No DEG
ENSG00000253702	RP11-567J20.1	4,55	2,18	4,93E-03	1,53E-02	UP
ENSG00000188384	CSPG4P8	-4,54	-2,18	6,28E-07	4,21E-06	No DEG
ENSG00000132361	CLUH	-4,54	-2,18	2,64E-37	3,00E-35	No DEG
ENSG00000177045	SIX5	-4,54	-2,18	6,40E-13	9,70E-12	No DEG
ENSG00000149564	ESAM	-4,53	-2,18	1,56E-23	6,75E-22	No DEG
ENSG00000198563	DDX39B	-4,53	-2,18	2,63E-24	1,21E-22	No DEG
ENSG00000155066	PROM2	-4,52	-2,18	6,35E-07	4,25E-06	No DEG
ENSG00000227242	NBPF13P	-4,52	-2,18	1,63E-19	4,75E-18	No DEG
ENSG00000258531	BANF1P1	-4,52	-2,18	3,55E-04	1,45E-03	No DEG

ENSG00000129244	ATP1B2	-4,52	-2,18	3,52E-11	4,30E-10	No DEG
ENSG00000181074	OR52N4	-4,52	-2,18	7,60E-04	2,89E-03	No DEG
ENSG00000237232	ZNF295-AS1	-4,52	-2,18	7,98E-04	3,02E-03	No DEG
ENSG00000133069	TMCC2	-4,52	-2,18	7,18E-09	6,47E-08	No DEG
ENSG00000196557	CACNA1H	-4,52	-2,17	8,82E-19	2,42E-17	No DEG
ENSG00000244226	ILF2P1	-4,51	-2,17	8,38E-09	7,50E-08	No DEG
ENSG00000158158	CNNM4	-4,51	-2,17	7,12E-26	3,68E-24	No DEG
ENSG00000164442	CITED2	-4,51	-2,17	2,95E-15	5,67E-14	No DEG
ENSG00000173581	CCDC106	-4,51	-2,17	1,30E-21	4,68E-20	No DEG
ENSG00000141028	CDRT15P1	-4,51	-2,17	6,82E-04	2,62E-03	No DEG
ENSG00000234147	RP3-460G2.2	4,51	2,17	5,26E-05	2,54E-04	No DEG
ENSG00000167470	MIDN	-4,51	-2,17	2,67E-10	2,90E-09	DOWN
ENSG00000210100	MT-TI	-4,50	-2,17	4,27E-03	1,35E-02	No DEG
ENSG00000261340	RP11-215H22.1	4,50	2,17	5,62E-07	3,79E-06	No DEG
ENSG00000070614	NDST1	-4,50	-2,17	5,50E-95	1,30E-91	No DEG
ENSG00000132613	MTSS1L	-4,49	-2,17	5,47E-22	2,02E-20	No DEG
ENSG00000162728	KCNJ9	-4,49	-2,17	6,49E-03	1,95E-02	No DEG
ENSG00000269963	RP11-73M18.9	-4,49	-2,17	2,64E-03	8,82E-03	No DEG
ENSG00000107731	UNC5B	-4,49	-2,17	2,44E-18	6,35E-17	No DEG
ENSG00000213123	TCTEX1D2	4,49	2,17	4,39E-04	1,76E-03	No DEG
ENSG00000135744	AGT	-4,48	-2,16	2,22E-24	1,02E-22	No DEG
ENSG00000259411	HNRNPA1P45	-4,48	-2,16	1,41E-03	5,03E-03	No DEG
ENSG00000105700	KXD1	-4,48	-2,16	7,48E-47	1,57E-44	No DEG
ENSG00000267230	RP11-376M2.2	-4,48	-2,16	9,00E-04	3,37E-03	DOWN
ENSG00000156587	UBE2L6	-4,48	-2,16	2,70E-20	8,53E-19	No DEG
ENSG00000174429	ABRA	-4,48	-2,16	2,79E-04	1,16E-03	DOWN
ENSG00000074181	NOTCH3	-4,48	-2,16	1,88E-43	3,06E-41	No DEG
ENSG00000164061	BSN	-4,47	-2,16	5,20E-08	4,16E-07	No DEG
ENSG00000177732	SOX12	-4,47	-2,16	1,06E-19	3,13E-18	No DEG
ENSG00000269148	AC092301.3	-4,47	-2,16	3,15E-03	1,03E-02	No DEG
ENSG00000270110	RP5-1139B12.4	-4,47	-2,16	1,99E-08	1,70E-07	No DEG
ENSG00000127418	FGFRL1	-4,47	-2,16	2,69E-29	1,80E-27	No DEG

ENSG00000114554	PLXNA1	-4,46	-2,16	9,74E-43	1,53E-40	No DEG
ENSG00000122420	PTGFR	4,46	2,16	8,63E-08	6,66E-07	UP
ENSG00000224743	TEX26-AS1	-4,46	-2,16	1,49E-04	6,56E-04	DOWN
ENSG00000244585	RPL12P33	-4,46	-2,16	1,72E-05	9,11E-05	No DEG
ENSG00000252813	AC108861.1	-4,46	-2,16	3,79E-03	1,21E-02	No DEG
ENSG00000257038	RP11-800A3.7	-4,46	-2,16	6,56E-05	3,10E-04	No DEG
ENSG00000105639	JAK3	-4,46	-2,16	1,41E-07	1,05E-06	No DEG
ENSG00000182492	BGN	-4,45	-2,16	5,72E-09	5,23E-08	No DEG
ENSG00000260884	AC009120.5	-4,45	-2,16	4,38E-07	3,02E-06	No DEG
ENSG00000103197	TSC2	-4,45	-2,15	5,82E-33	5,09E-31	No DEG
ENSG00000019102	VSIG2	-4,44	-2,15	4,91E-08	3,94E-07	No DEG
ENSG00000183458	RP11-958N24.1	-4,44	-2,15	3,62E-28	2,26E-26	No DEG
ENSG00000188566	NDOR1	-4,44	-2,15	3,13E-26	1,67E-24	No DEG
ENSG00000186462	NAP1L2	4,44	2,15	1,05E-09	1,07E-08	No DEG
ENSG00000116337	AMPD2	-4,44	-2,15	7,18E-21	2,41E-19	No DEG
ENSG00000174500	GCSAM	-4,44	-2,15	2,04E-04	8,77E-04	No DEG
ENSG00000142178	SIK1	-4,44	-2,15	1,71E-04	7,48E-04	No DEG
ENSG00000130748	TMEM160	4,44	2,15	6,21E-23	2,54E-21	No DEG
ENSG00000070814	TCOF1	-4,43	-2,15	6,07E-59	3,11E-56	No DEG
ENSG00000266941	AC104532.3	-4,43	-2,15	8,48E-03	2,46E-02	No DEG
ENSG00000130202	PVRL2	-4,43	-2,15	2,79E-19	8,01E-18	No DEG
ENSG00000127616	SMARCA4	-4,43	-2,15	1,67E-63	1,17E-60	No DEG
ENSG00000013306	SLC25A39	-4,43	-2,15	2,16E-25	1,07E-23	No DEG
ENSG00000160326	SLC2A6	-4,43	-2,15	3,33E-13	5,20E-12	No DEG
ENSG00000261614	YBX3P1	-4,42	-2,14	7,83E-05	3,64E-04	No DEG
ENSG00000151917	BEND6	4,41	2,14	1,60E-06	9,98E-06	No DEG
ENSG00000260276	RP11-77H9.2	-4,41	-2,14	2,91E-09	2,77E-08	No DEG
ENSG00000169019	COMMD8	4,41	2,14	7,18E-42	1,05E-39	No DEG
ENSG00000139329	LUM	4,41	2,14	3,57E-10	3,82E-09	No DEG
ENSG00000170906	NDUFA3	-4,41	-2,14	1,12E-29	7,72E-28	No DEG
ENSG00000269397	CTB-92J24.2	4,41	2,14	2,68E-03	8,93E-03	No DEG
ENSG00000106635	BCL7B	-4,40	-2,14	4,45E-34	4,10E-32	No DEG

ENSG00000112759	SLC29A1	-4,40	-2,14	1,33E-26	7,24E-25	No DEG
ENSG00000253864	AC131025.8	-4,40	-2,14	8,80E-11	1,02E-09	No DEG
ENSG00000111664	GNB3	-4,40	-2,14	5,31E-17	1,21E-15	No DEG
ENSG00000198853	RUSC2	-4,40	-2,14	2,01E-53	7,07E-51	No DEG
ENSG00000263975	CTB-58E17.9	-4,40	-2,14	6,89E-03	2,05E-02	No DEG
ENSG00000186510	CLCNKA	-4,40	-2,14	8,04E-05	3,73E-04	DOWN
ENSG00000204624	PTCHD2	-4,40	-2,14	2,74E-03	9,12E-03	No DEG
ENSG00000007541	PIGQ	-4,39	-2,13	1,29E-28	8,32E-27	No DEG
ENSG00000235573	RP3-412A9.12	-4,39	-2,13	9,88E-03	2,82E-02	No DEG
ENSG00000224094	RPS24P8	4,39	2,13	5,89E-27	3,35E-25	No DEG
ENSG00000236364	RP11-525G13.2	-4,38	-2,13	1,07E-03	3,95E-03	No DEG
ENSG00000154736	ADAMTS5	4,38	2,13	4,66E-19	1,31E-17	No DEG
ENSG00000085185	BCORL1	-4,38	-2,13	3,16E-17	7,44E-16	No DEG
ENSG00000233930	KRTAP5-AS1	-4,38	-2,13	1,32E-05	7,14E-05	No DEG
ENSG00000119669	IRF2BPL	-4,38	-2,13	8,36E-16	1,70E-14	No DEG
ENSG00000220008	LINGO3	4,38	2,13	5,48E-06	3,14E-05	UP
ENSG00000202290	RNA5SP37	-4,37	-2,13	2,91E-05	1,47E-04	No DEG
ENSG00000080854	IGSF9B	-4,37	-2,13	1,18E-11	1,52E-10	No DEG
ENSG00000268816	CTD-3093M3.1	4,37	2,13	1,88E-05	9,86E-05	No DEG
ENSG00000143373	ZNF687	-4,37	-2,13	7,66E-51	2,22E-48	No DEG
ENSG00000241732	RP11-38P22.2	4,37	2,13	9,78E-12	1,28E-10	No DEG
ENSG00000197183	C20orf112	-4,37	-2,13	1,42E-16	3,09E-15	No DEG
ENSG00000259395	RP11-475A13.2	-4,37	-2,13	2,74E-03	9,11E-03	No DEG
ENSG00000221968	FADS3	-4,37	-2,13	3,55E-17	8,31E-16	No DEG
ENSG00000111012	CYP27B1	-4,37	-2,13	9,52E-04	3,54E-03	No DEG
ENSG00000250564	RP11-215P8.4	-4,37	-2,13	1,14E-02	3,19E-02	No DEG
ENSG00000088881	EBF4	-4,37	-2,13	1,83E-12	2,62E-11	No DEG
ENSG00000253270	RP11-1105O14.1	-4,37	-2,13	4,42E-08	3,57E-07	No DEG
ENSG00000257545	RP11-144F15.1	-4,37	-2,13	2,14E-03	7,32E-03	No DEG
ENSG00000266591	AC022537.1	4,36	2,13	4,27E-06	2,49E-05	No DEG
ENSG00000061273	HDAC7	-4,36	-2,13	2,81E-33	2,50E-31	No DEG
ENSG00000138074	SLC5A6	-4,36	-2,13	9,13E-20	2,74E-18	No DEG

ENSG00000239857	GET4	4,36	2,13	1,80E-07	1,32E-06	No DEG
ENSG00000112511	PHF1	-4,36	-2,12	8,01E-33	6,86E-31	No DEG
ENSG00000224598	RPS5P2	-4,36	-2,12	7,67E-04	2,91E-03	No DEG
ENSG00000235833	AC159540.14	4,36	2,12	4,04E-03	1,29E-02	UP
ENSG00000122678	POLM	-4,36	-2,12	2,66E-36	2,86E-34	No DEG
ENSG00000180616	SSTR2	-4,36	-2,12	6,74E-07	4,49E-06	No DEG
ENSG00000165271	NOL6	-4,36	-2,12	7,84E-28	4,74E-26	No DEG
ENSG00000231633	LINC00283	4,36	2,12	1,17E-05	6,35E-05	UP
ENSG00000017483	SLC38A5	-4,35	-2,12	9,52E-07	6,18E-06	No DEG
ENSG00000160094	ZNF362	-4,35	-2,12	9,15E-31	6,79E-29	No DEG
ENSG00000243716	NPIPB5	-4,35	-2,12	8,04E-09	7,21E-08	No DEG
ENSG00000272275	RP11-791G15.2	-4,35	-2,12	1,02E-03	3,78E-03	DOWN
ENSG00000167491	GATAD2A	-4,34	-2,12	3,28E-23	1,37E-21	No DEG
ENSG00000261924	CTD-2561B21.5	-4,34	-2,12	3,12E-05	1,58E-04	No DEG
ENSG00000105953	OGDH	-4,34	-2,12	4,20E-44	7,26E-42	No DEG
ENSG00000126464	PRR12	-4,34	-2,12	1,05E-12	1,56E-11	No DEG
ENSG00000200065	Y_RNA	4,34	2,12	2,93E-03	9,67E-03	No DEG
ENSG00000004948	CALCR	4,34	2,12	1,40E-03	5,00E-03	UP
ENSG00000213398	LCAT	-4,34	-2,12	3,71E-25	1,82E-23	No DEG
ENSG00000077044	DGKD	-4,34	-2,12	7,57E-12	1,00E-10	No DEG
ENSG00000106330	MOSPD3	-4,34	-2,12	2,93E-22	1,11E-20	No DEG
ENSG00000107738	C10orf54	-4,33	-2,12	1,35E-28	8,68E-27	No DEG
ENSG00000177337	DLGAP1-AS1	-4,33	-2,12	1,26E-11	1,62E-10	No DEG
ENSG00000259013	RP11-1017G21.3	-4,33	-2,11	1,71E-03	5,98E-03	No DEG
ENSG00000102882	MAPK3	-4,33	-2,11	9,79E-40	1,27E-37	No DEG
ENSG00000233478	RP1-187B23.1	-4,33	-2,11	1,15E-04	5,18E-04	No DEG
ENSG00000144579	CTDSP1	-4,32	-2,11	1,17E-74	1,12E-71	No DEG
ENSG00000129925	TMEM8A	-4,32	-2,11	7,12E-31	5,37E-29	No DEG
ENSG00000207808	MIR27A	-4,32	-2,11	5,85E-03	1,78E-02	No DEG
ENSG00000204657	OR2H2	-4,32	-2,11	8,68E-03	2,51E-02	No DEG
ENSG00000230175	RP11-466F5.3	4,32	2,11	5,33E-03	1,64E-02	UP
ENSG00000166292	TMEM100	4,32	2,11	8,34E-10	8,54E-09	No DEG

ENSG00000160447	PKN3	-4,32	-2,11	1,20E-18	3,25E-17	No DEG
ENSG00000160932	LY6E	-4,32	-2,11	5,74E-18	1,45E-16	No DEG
ENSG00000241527	CA15P1	-4,32	-2,11	2,01E-03	6,90E-03	No DEG
ENSG00000262222	RP11-876N24.4	-4,32	-2,11	2,04E-06	1,25E-05	No DEG
ENSG00000142611	PRDM16	-4,32	-2,11	4,21E-37	4,73E-35	No DEG
ENSG00000159363	ATP13A2	-4,32	-2,11	7,35E-29	4,80E-27	No DEG
ENSG00000131398	KCNC3	-4,31	-2,11	3,29E-10	3,55E-09	No DEG
ENSG00000066336	SPI1	-4,31	-2,11	1,51E-09	1,49E-08	No DEG
ENSG00000224973	LARGE-AS1	-4,31	-2,11	4,88E-06	2,82E-05	No DEG
ENSG00000261474	RP11-452L6.1	-4,31	-2,11	1,55E-07	1,15E-06	No DEG
ENSG00000137699	TRIM29	-4,31	-2,11	4,80E-03	1,50E-02	No DEG
ENSG00000255851	AC061975.9	-4,31	-2,11	1,08E-02	3,04E-02	No DEG
ENSG00000263155	MYZAP	-4,30	-2,11	5,43E-07	3,67E-06	DOWN
ENSG00000187244	BCAM	-4,30	-2,10	6,17E-32	4,97E-30	No DEG
ENSG00000198088	NUP62CL	4,30	2,10	1,10E-08	9,66E-08	No DEG
ENSG00000088899	LZTS3	-4,30	-2,10	3,85E-14	6,62E-13	No DEG
ENSG00000007384	RHBDF1	-4,30	-2,10	2,04E-31	1,57E-29	No DEG
ENSG00000188643	S100A16	-4,30	-2,10	3,30E-22	1,24E-20	No DEG
ENSG00000104472	CHRAC1	-4,29	-2,10	2,78E-21	9,70E-20	No DEG
ENSG00000143294	PRCC	-4,29	-2,10	5,96E-81	7,95E-78	No DEG
ENSG00000197283	SYNGAP1	-4,28	-2,10	5,52E-21	1,88E-19	No DEG
ENSG00000172250	SERHL	-4,28	-2,10	8,53E-05	3,93E-04	No DEG
ENSG00000086300	SNX10	4,27	2,10	2,00E-18	5,25E-17	UP
ENSG00000003137	CYP26B1	-4,27	-2,10	2,06E-05	1,07E-04	No DEG
ENSG00000188086	PRSS45	-4,27	-2,10	9,20E-07	5,99E-06	No DEG
ENSG00000197182	FLJ27365	-4,27	-2,09	1,89E-14	3,37E-13	No DEG
ENSG00000204564	C6orf136	-4,27	-2,09	3,14E-23	1,32E-21	No DEG
ENSG00000164638	SLC29A4	-4,27	-2,09	2,83E-05	1,44E-04	No DEG
ENSG00000176971	FIBIN	4,27	2,09	2,59E-08	2,16E-07	UP
ENSG00000197301	RP11-366L20.2	4,27	2,09	3,42E-04	1,40E-03	No DEG
ENSG00000143590	EFNA3	-4,27	-2,09	5,76E-03	1,76E-02	No DEG
ENSG00000186407	CD300E	-4,27	-2,09	5,94E-04	2,31E-03	No DEG

ENSG00000254884	RP11-682B13.2	-4,26	-2,09	6,51E-04	2,51E-03	No DEG
ENSG00000259689	ABCB10P1	4,26	2,09	2,97E-03	9,79E-03	No DEG
ENSG00000268049	CTD-2619J13.9	-4,26	-2,09	2,04E-03	7,01E-03	No DEG
ENSG00000115194	SLC30A3	-4,25	-2,09	9,87E-18	2,45E-16	No DEG
ENSG00000064601	CTSA	-4,25	-2,09	3,00E-42	4,53E-40	No DEG
ENSG00000067182	TNFRSF1A	-4,25	-2,09	4,89E-14	8,30E-13	No DEG
ENSG00000250444	CCT5P1	-4,24	-2,08	1,27E-07	9,53E-07	No DEG
ENSG00000187840	EIF4EBP1	-4,24	-2,08	1,36E-14	2,47E-13	No DEG
ENSG00000204619	PPP1R11	-4,24	-2,08	3,32E-42	5,00E-40	No DEG
ENSG00000259438	CTD-2650P22.1	4,24	2,08	8,94E-03	2,58E-02	No DEG
ENSG00000179364	PACS2	-4,24	-2,08	1,43E-49	3,77E-47	No DEG
ENSG00000165272	AQP3	-4,23	-2,08	4,54E-07	3,12E-06	No DEG
ENSG00000155363	MOV10	-4,23	-2,08	8,76E-29	5,68E-27	No DEG
ENSG00000165175	MID1IP1	-4,22	-2,08	2,92E-07	2,07E-06	DOWN
ENSG00000007376	RPUSD1	-4,22	-2,08	3,26E-22	1,23E-20	No DEG
ENSG00000130702	LAMA5	-4,22	-2,08	1,96E-49	5,06E-47	No DEG
ENSG00000179101	RP11-349N19.2	-4,22	-2,08	4,46E-05	2,18E-04	No DEG
ENSG00000265744	MIR4427	-4,22	-2,08	8,58E-03	2,49E-02	No DEG
ENSG00000115756	HPCAL1	-4,22	-2,08	9,78E-33	8,29E-31	No DEG
ENSG00000173930	SLCO4C1	4,22	2,08	8,65E-05	3,98E-04	No DEG
ENSG00000178498	DTX3	-4,22	-2,08	1,08E-32	9,12E-31	No DEG
ENSG00000214820	MPRIPP1	-4,22	-2,08	3,46E-07	2,42E-06	No DEG
ENSG00000088038	CNOT3	-4,21	-2,08	1,16E-22	4,56E-21	No DEG
ENSG00000179242	CDH4	-4,21	-2,08	2,04E-03	7,01E-03	DOWN
ENSG00000168040	FADD	4,21	2,07	6,26E-31	4,73E-29	No DEG
ENSG00000267461	RP11-120M18.5	-4,21	-2,07	1,01E-03	3,75E-03	No DEG
ENSG00000140511	HAPLN3	-4,21	-2,07	1,92E-08	1,64E-07	No DEG
ENSG00000235316	DUSP8P5	-4,21	-2,07	1,41E-14	2,55E-13	No DEG
ENSG00000072163	LIMS2	-4,21	-2,07	4,07E-30	2,88E-28	No DEG
ENSG00000101224	CDC25B	-4,21	-2,07	5,65E-11	6,69E-10	No DEG
ENSG00000196923	PDLIM7	-4,21	-2,07	2,65E-15	5,13E-14	No DEG
ENSG00000262777	RP11-818O24.3	-4,21	-2,07	9,32E-04	3,48E-03	No DEG

ENSG00000253228	NRBF2P4	-4,21	-2,07	9,82E-03	2,80E-02	No DEG
ENSG00000148702	HABP2	-4,21	-2,07	1,58E-03	5,58E-03	DOWN
ENSG00000125744	RTN2	-4,21	-2,07	3,14E-12	4,37E-11	No DEG
ENSG00000212123	PRR22	-4,20	-2,07	4,32E-04	1,73E-03	No DEG
ENSG00000184271	POU6F1	-4,20	-2,07	2,28E-26	1,23E-24	No DEG
ENSG00000180525	PRR26	-4,20	-2,07	3,73E-07	2,59E-06	No DEG
ENSG00000146090	RASGEF1C	-4,20	-2,07	1,04E-04	4,73E-04	No DEG
ENSG00000171659	GPR34	4,19	2,07	1,23E-16	2,71E-15	UP
ENSG00000161970	RPL26	4,19	2,07	2,76E-30	1,98E-28	No DEG
ENSG00000117122	MFAP2	-4,19	-2,07	4,53E-11	5,44E-10	No DEG
ENSG00000260349	RP11-473I1.5	-4,19	-2,07	2,92E-25	1,44E-23	No DEG
ENSG00000078814	MYH7B	-4,18	-2,07	5,60E-47	1,20E-44	No DEG
ENSG00000138316	ADAMTS14	-4,18	-2,06	1,53E-06	9,58E-06	No DEG
ENSG00000197168	NEK5	4,18	2,06	1,72E-07	1,26E-06	UP
ENSG00000160796	NBEAL2	-4,18	-2,06	2,18E-39	2,75E-37	No DEG
ENSG00000187513	GJA4	-4,18	-2,06	1,08E-22	4,27E-21	No DEG
ENSG00000222973	RNU2-25P	4,18	2,06	8,71E-05	4,00E-04	No DEG
ENSG00000236039	AC019117.2	-4,18	-2,06	3,68E-03	1,18E-02	DOWN
ENSG00000150625	GPM6A	4,17	2,06	3,48E-03	1,13E-02	UP
ENSG00000122025	FLT3	4,17	2,06	3,62E-05	1,80E-04	UP
ENSG00000250777	RP13-884E18.4	4,17	2,06	4,92E-03	1,53E-02	No DEG
ENSG00000160785	SLC25A44	-4,17	-2,06	5,76E-36	6,04E-34	No DEG
ENSG00000253330	RP11-697N18.3	-4,17	-2,06	1,47E-06	9,21E-06	No DEG
ENSG00000129173	E2F8	-4,17	-2,06	1,94E-14	3,45E-13	DOWN
ENSG00000135312	HTR1B	-4,17	-2,06	7,45E-03	2,20E-02	No DEG
ENSG00000137221	TJAP1	-4,17	-2,06	1,68E-56	7,48E-54	No DEG
ENSG00000105732	ZNF574	-4,17	-2,06	2,35E-26	1,26E-24	No DEG
ENSG00000223542	RP1-283K11.3	-4,17	-2,06	6,40E-04	2,47E-03	No DEG
ENSG00000205271	CSPG4P10	-4,16	-2,06	8,84E-06	4,91E-05	No DEG
ENSG00000253159	PCDHGA12	-4,16	-2,06	8,13E-25	3,86E-23	No DEG
ENSG00000177679	SRRM3	-4,16	-2,06	2,36E-13	3,74E-12	No DEG
ENSG00000270083	RP1-257I20.14	-4,16	-2,05	3,31E-06	1,97E-05	No DEG

ENSG00000096433	ITPR3	-4,15	-2,05	2,15E-20	6,88E-19	No DEG
ENSG00000161642	ZNF385A	-4,15	-2,05	6,10E-11	7,20E-10	No DEG
ENSG00000221044	U3	-4,15	-2,05	4,95E-03	1,54E-02	No DEG
ENSG00000183638	RP1L1	-4,15	-2,05	1,59E-03	5,59E-03	No DEG
ENSG00000255585	RP11-188C12.2	-4,15	-2,05	1,86E-03	6,46E-03	No DEG
ENSG00000172828	CES3	-4,14	-2,05	3,70E-05	1,84E-04	No DEG
ENSG00000132359	RAP1GAP2	-4,14	-2,05	8,02E-17	1,80E-15	No DEG
ENSG00000183114	FAM43B	-4,14	-2,05	5,08E-06	2,92E-05	No DEG
ENSG00000101084	C20orf24	4,14	2,05	7,53E-34	6,85E-32	No DEG
ENSG00000225373	WASH5P	-4,14	-2,05	3,77E-07	2,62E-06	No DEG
ENSG00000110719	TCIRG1	-4,14	-2,05	7,02E-19	1,94E-17	No DEG
ENSG00000122359	ANXA11	-4,13	-2,05	3,02E-45	5,71E-43	No DEG
ENSG00000236472	AC002401.1	-4,13	-2,05	1,10E-02	3,09E-02	No DEG
ENSG00000101134	DOK5	4,13	2,05	8,29E-11	9,61E-10	No DEG
ENSG00000134259	NGF	-4,13	-2,05	1,41E-06	8,89E-06	No DEG
ENSG00000164850	GPER1	-4,13	-2,04	1,72E-08	1,48E-07	No DEG
ENSG00000185792	NLRP9	4,13	2,04	6,55E-03	1,97E-02	UP
ENSG00000196549	MME	4,12	2,04	2,28E-14	4,01E-13	No DEG
ENSG00000239686	RP11-665C16.1	-4,12	-2,04	3,66E-06	2,16E-05	No DEG
ENSG00000105323	HNRNPUL1	-4,12	-2,04	4,13E-35	3,97E-33	No DEG
ENSG00000230578	RP13-228J13.5	4,12	2,04	2,92E-03	9,67E-03	No DEG
ENSG00000265489	RP11-1090M7.1	-4,12	-2,04	3,99E-07	2,76E-06	No DEG
ENSG00000181830	SLC35C1	-4,12	-2,04	3,74E-14	6,45E-13	No DEG
ENSG00000262006	RP11-700H6.4	-4,12	-2,04	5,58E-03	1,71E-02	No DEG
ENSG00000273193	RP11-523G9.3	4,12	2,04	8,68E-05	4,00E-04	No DEG
ENSG00000272821	CTA-384D8.36	-4,12	-2,04	4,58E-06	2,66E-05	No DEG
ENSG00000181616	OR52H1	-4,12	-2,04	1,40E-03	4,99E-03	No DEG
ENSG00000123416	TUBA1B	-4,12	-2,04	1,75E-19	5,09E-18	No DEG
ENSG00000163630	SYNPR	4,12	2,04	9,77E-04	3,63E-03	No DEG
ENSG00000172831	CES2	-4,12	-2,04	3,62E-17	8,47E-16	No DEG
ENSG00000081842	PCDHA6	4,11	2,04	5,58E-03	1,71E-02	No DEG
ENSG00000153446	C16orf89	4,11	2,04	5,04E-07	3,43E-06	UP

ENSG00000212282	RNU6-578P	4,11	2,04	6,16E-03	1,86E-02	No DEG
ENSG00000134684	YARS	-4,11	-2,04	2,88E-21	1,00E-19	No DEG
ENSG00000164880	INTS1	-4,10	-2,04	1,20E-48	2,89E-46	No DEG
ENSG00000126247	CAPNS1	-4,10	-2,04	5,28E-30	3,69E-28	No DEG
ENSG00000161714	PLCD3	-4,10	-2,04	1,06E-20	3,50E-19	No DEG
ENSG00000271761	XXbac-BPGBPG55C20	4,10	2,03	4,53E-03	1,42E-02	No DEG
ENSG00000171130	ATP6V0E2	-4,10	-2,03	1,45E-18	3,87E-17	No DEG
ENSG00000186103	ARGFX	-4,10	-2,03	9,25E-03	2,66E-02	No DEG
ENSG00000225091	SNORA71A	-4,09	-2,03	5,36E-03	1,65E-02	No DEG
ENSG00000267242	AC069278.4	4,09	2,03	3,69E-05	1,84E-04	No DEG
ENSG00000141639	MAPK4	-4,09	-2,03	1,92E-07	1,40E-06	No DEG
ENSG00000234771	RP11-395P17.3	-4,09	-2,03	1,15E-12	1,70E-11	No DEG
ENSG00000049283	EPN3	-4,09	-2,03	1,11E-06	7,12E-06	No DEG
ENSG00000186452	TMPRSS12	-4,09	-2,03	1,17E-02	3,27E-02	No DEG
ENSG00000220349	RP3-431A14.4	-4,09	-2,03	9,70E-03	2,77E-02	No DEG
ENSG00000236567	RP11-39K24.7	-4,08	-2,03	2,35E-05	1,21E-04	No DEG
ENSG00000174996	KLC2	-4,08	-2,03	2,51E-28	1,59E-26	No DEG
ENSG00000111247	RAD51AP1	4,08	2,03	4,52E-07	3,10E-06	No DEG
ENSG00000163638	ADAMTS9	-4,08	-2,03	3,86E-05	1,91E-04	DOWN
ENSG00000100439	ABHD4	-4,08	-2,03	2,31E-17	5,52E-16	No DEG
ENSG00000198077	CYP2A7	-4,08	-2,03	6,13E-03	1,85E-02	No DEG
ENSG00000176490	DIRAS1	-4,08	-2,03	1,26E-16	2,76E-15	No DEG
ENSG00000110047	EHD1	-4,08	-2,03	3,82E-27	2,21E-25	No DEG
ENSG00000104805	NUCB1	-4,08	-2,03	1,04E-44	1,89E-42	No DEG
ENSG00000181001	OR52N1	-4,07	-2,03	3,69E-03	1,18E-02	No DEG
ENSG00000204301	NOTCH4	-4,07	-2,03	4,26E-20	1,32E-18	No DEG
ENSG00000253944	RP11-156K13.1	4,07	2,03	7,90E-12	1,05E-10	No DEG
ENSG00000011422	PLAUR	-4,07	-2,03	1,16E-07	8,80E-07	No DEG
ENSG00000154133	ROBO4	-4,07	-2,03	2,02E-21	7,12E-20	No DEG
ENSG00000205791	LOH12CR2	4,07	2,02	7,94E-08	6,16E-07	No DEG
ENSG00000255197	RP11-750H9.5	-4,07	-2,02	7,22E-05	3,39E-04	No DEG
ENSG00000024422	EHD2	-4,07	-2,02	1,96E-36	2,12E-34	No DEG

ENSG00000273058	RP11-385F5.5	-4,07	-2,02	3,43E-06	2,03E-05	No DEG
ENSG00000227070	RP11-191G24.1	-4,07	-2,02	9,82E-04	3,64E-03	No DEG
ENSG00000103932	RPAP1	-4,06	-2,02	1,51E-22	5,89E-21	No DEG
ENSG00000272235	RP11-22L13.1	-4,06	-2,02	3,05E-08	2,52E-07	No DEG
ENSG00000231327	AC016700.5	-4,06	-2,02	2,16E-03	7,37E-03	No DEG
ENSG00000226942	IL9RP3	-4,06	-2,02	1,02E-02	2,90E-02	No DEG
ENSG00000215946	MIR941-1	-4,06	-2,02	5,71E-03	1,74E-02	No DEG
ENSG00000225183	RP4-758J24.4	4,06	2,02	6,94E-03	2,07E-02	No DEG
ENSG00000223905	RP11-335E14.1	-4,05	-2,02	2,59E-04	1,09E-03	No DEG
ENSG00000260139	CSPG4P13	-4,05	-2,02	6,55E-07	4,37E-06	No DEG
ENSG00000227076	RP11-4C20.4	-4,05	-2,02	6,94E-05	3,27E-04	No DEG
ENSG00000055070	SZRD1	-4,05	-2,02	1,31E-49	3,52E-47	No DEG
ENSG00000271936	RP11-443B20.1	-4,05	-2,02	9,37E-04	3,49E-03	No DEG
ENSG00000162174	ASRGL1	-4,05	-2,02	1,35E-06	8,52E-06	DOWN
ENSG00000076108	BAZ2A	-4,05	-2,02	7,54E-28	4,57E-26	No DEG
ENSG00000263335	AF001548.5	-4,05	-2,02	1,92E-03	6,64E-03	No DEG
ENSG00000136295	TTYH3	-4,05	-2,02	2,14E-14	3,78E-13	No DEG
ENSG00000232381	OR52U1P	-4,04	-2,02	1,03E-03	3,80E-03	No DEG
ENSG00000253200	RP11-582J16.5	-4,04	-2,01	2,33E-07	1,68E-06	No DEG
ENSG00000235878	AP001468.1	-4,04	-2,01	1,07E-06	6,89E-06	No DEG
ENSG00000256616	RP11-815J4.6	4,04	2,01	1,75E-05	9,27E-05	No DEG
ENSG00000111886	GABRR2	-4,04	-2,01	8,42E-05	3,89E-04	No DEG
ENSG00000267686	RP11-795H16.3	-4,03	-2,01	2,60E-03	8,69E-03	No DEG
ENSG00000235027	AC068580.6	-4,03	-2,01	9,59E-11	1,10E-09	No DEG
ENSG00000128274	A4GALT	-4,03	-2,01	4,08E-25	1,99E-23	No DEG
ENSG00000113389	NPR3	4,03	2,01	3,20E-07	2,26E-06	No DEG
ENSG00000105726	ATP13A1	-4,03	-2,01	2,48E-35	2,44E-33	No DEG
ENSG00000235902	RP11-626E13.1	-4,02	-2,01	1,32E-07	9,86E-07	No DEG
ENSG00000037897	METTL1	-4,02	-2,01	2,44E-09	2,35E-08	No DEG
ENSG00000249096	RP11-290F5.1	-4,02	-2,01	4,68E-07	3,21E-06	No DEG
ENSG00000210112	MT-TM	-4,02	-2,01	1,40E-03	4,98E-03	No DEG
ENSG00000237786	GFOD1-AS1	-4,02	-2,01	1,28E-05	6,93E-05	No DEG

ENSG00000181017	OR56B2P	-4,02	-2,01	5,65E-03	1,73E-02	No DEG
ENSG00000228063	RP11-135J2.4	4,02	2,01	2,28E-06	1,39E-05	No DEG
ENSG00000176692	FOXC2	-4,02	-2,01	5,90E-05	2,81E-04	No DEG
ENSG00000183307	CECR6	-4,02	-2,01	1,81E-07	1,32E-06	No DEG
ENSG00000104427	ZC2HC1A	4,02	2,01	3,07E-13	4,82E-12	No DEG
ENSG00000253007	SNORA76	-4,02	-2,01	3,84E-05	1,90E-04	No DEG
ENSG00000166333	ILK	-4,02	-2,01	1,77E-08	1,52E-07	No DEG
ENSG00000175220	ARHGAP1	-4,02	-2,01	7,40E-17	1,67E-15	No DEG
ENSG00000099956	SMARCB1	-4,02	-2,01	1,54E-34	1,44E-32	No DEG
ENSG00000213216	RP11-355O1.7	4,02	2,01	1,03E-04	4,69E-04	No DEG
ENSG00000068976	PYGM	-4,01	-2,01	6,25E-12	8,40E-11	No DEG
ENSG00000121005	CRISPLD1	4,01	2,00	2,10E-04	9,01E-04	No DEG
ENSG00000273156	RP11-127B20.2	4,01	2,00	5,06E-06	2,91E-05	No DEG
ENSG00000128591	FLNC	-4,01	-2,00	1,87E-11	2,36E-10	No DEG
ENSG00000237513	RP11-325F22.2	-4,01	-2,00	3,99E-03	1,27E-02	No DEG
ENSG00000253824	KB-173C10.2	4,01	2,00	1,55E-03	5,46E-03	No DEG
ENSG00000120318	ARAP3	-4,01	-2,00	4,13E-39	5,09E-37	No DEG
ENSG00000259020	RP11-529H20.3	-4,01	-2,00	4,23E-04	1,70E-03	No DEG
ENSG00000114529	C3orf52	-4,01	-2,00	2,57E-04	1,08E-03	DOWN
ENSG00000176170	SPHK1	-4,00	-2,00	1,82E-04	7,89E-04	No DEG
ENSG00000241973	PI4KA	-4,00	-2,00	2,90E-37	3,28E-35	No DEG
ENSG00000185885	IFITM1	-4,00	-2,00	5,70E-15	1,07E-13	No DEG
ENSG00000243055	GK-AS1	-4,00	-2,00	8,46E-06	4,71E-05	No DEG
ENSG00000165449	SLC16A9	4,00	2,00	3,11E-06	1,85E-05	UP
ENSG00000201027	RN7SKP107	-4,00	-2,00	6,03E-12	8,14E-11	No DEG
ENSG00000129667	RHBDF2	-4,00	-2,00	1,08E-15	2,15E-14	No DEG
ENSG00000198715	C1orf85	-3,99	-2,00	1,89E-15	3,70E-14	No DEG
ENSG00000065054	SLC9A3R2	-3,99	-2,00	8,48E-16	1,72E-14	No DEG
ENSG00000250635	CTD-3224K15.2	-3,99	-2,00	6,22E-03	1,88E-02	No DEG
ENSG00000141867	BRD4	-3,99	-2,00	1,10E-34	1,04E-32	No DEG
ENSG00000170417	TMEM182	3,99	2,00	1,01E-23	4,45E-22	No DEG
ENSG00000231172	AC007099.1	-3,99	-2,00	1,10E-02	3,10E-02	No DEG

ENSG00000136689	IL1RN	-3,99	-2,00	7,90E-03	2,32E-02	No DEG
ENSG00000111424	VDR	-3,99	-1,99	7,94E-05	3,69E-04	No DEG
ENSG00000226383	AC093375.1	3,99	1,99	9,11E-04	3,41E-03	No DEG
ENSG00000227184	EPPK1	-3,99	-1,99	1,65E-09	1,63E-08	No DEG
ENSG00000124479	NDP	3,98	1,99	7,73E-03	2,27E-02	No DEG
ENSG00000185634	SHC4	3,98	1,99	3,77E-12	5,20E-11	No DEG
ENSG00000175206	NPPA	3,98	1,99	6,28E-03	1,90E-02	No DEG
ENSG00000205238	SPDYE2	-3,98	-1,99	2,81E-08	2,33E-07	No DEG
ENSG00000253406	AC012613.2	-3,98	-1,99	5,44E-14	9,18E-13	No DEG
ENSG00000068354	TBC1D25	-3,97	-1,99	7,30E-32	5,83E-30	No DEG
ENSG00000231050	RP1-140A9.1	-3,97	-1,99	3,16E-03	1,03E-02	No DEG
ENSG00000100241	SBF1	-3,97	-1,99	6,59E-21	2,23E-19	No DEG
ENSG00000124664	SPDEF	-3,97	-1,99	9,16E-04	3,42E-03	No DEG
ENSG00000063180	CA11	-3,97	-1,99	4,16E-08	3,37E-07	No DEG
ENSG00000130844	ZNF331	-3,97	-1,99	5,49E-07	3,71E-06	DOWN
ENSG00000073008	PVR	-3,96	-1,99	4,84E-13	7,45E-12	No DEG
ENSG00000224901	RP11-448G4.4	3,96	1,99	9,12E-04	3,41E-03	No DEG
ENSG00000135094	SDS	-3,96	-1,99	3,84E-04	1,56E-03	No DEG
ENSG00000095370	SH2D3C	-3,96	-1,99	1,82E-16	3,92E-15	No DEG
ENSG00000199157	MIR208A	-3,96	-1,99	7,95E-03	2,33E-02	DOWN
ENSG00000108963	DPH1	-3,96	-1,99	1,05E-35	1,08E-33	No DEG
ENSG00000126107	HECTD3	-3,96	-1,99	2,20E-36	2,37E-34	No DEG
ENSG00000153347	FAM81B	3,96	1,99	3,15E-03	1,03E-02	No DEG
ENSG00000201839	SNORD114-3	3,96	1,99	7,25E-03	2,15E-02	No DEG
ENSG00000168256	NKIRAS2	-3,96	-1,98	5,53E-64	4,04E-61	No DEG
ENSG00000261366	MANEA-AS1	3,96	1,98	3,78E-13	5,87E-12	No DEG
ENSG00000228211	HYALP1	3,95	1,98	8,22E-03	2,40E-02	No DEG
ENSG00000119574	ZBTB45	-3,95	-1,98	6,40E-23	2,60E-21	No DEG
ENSG00000185504	C17orf70	-3,95	-1,98	1,30E-39	1,67E-37	No DEG
ENSG00000124935	SCGB1D2	-3,95	-1,98	3,17E-03	1,04E-02	DOWN
ENSG00000131480	AOC2	-3,95	-1,98	2,42E-04	1,02E-03	No DEG
ENSG00000153815	CMIP	-3,94	-1,98	1,90E-13	3,06E-12	No DEG

ENSG00000198885	ITPRIPL1	-3,94	-1,98	3,99E-09	3,73E-08	No DEG
ENSG00000270419	CAHM	3,94	1,98	4,41E-09	4,10E-08	No DEG
ENSG00000097007	ABL1	-3,94	-1,98	6,84E-28	4,17E-26	No DEG
ENSG00000179921	GPBAR1	-3,94	-1,98	3,05E-08	2,52E-07	No DEG
ENSG00000170458	CD14	-3,94	-1,98	7,89E-08	6,13E-07	No DEG
ENSG00000109072	VTN	-3,94	-1,98	1,84E-09	1,81E-08	No DEG
ENSG00000084463	WBP11	-3,94	-1,98	8,22E-48	1,87E-45	No DEG
ENSG00000259335	HNRNPMP1	-3,94	-1,98	6,27E-05	2,97E-04	No DEG
ENSG00000244180	AL592188.2	3,93	1,98	1,00E-03	3,72E-03	No DEG
ENSG00000205578	POM121B	-3,93	-1,98	2,17E-06	1,33E-05	No DEG
ENSG00000157216	SSBP3	-3,93	-1,98	5,53E-28	3,42E-26	No DEG
ENSG00000227487	NCAM1-AS1	-3,93	-1,98	3,49E-03	1,13E-02	No DEG
ENSG00000147799	ARHGAP39	-3,93	-1,97	6,41E-08	5,05E-07	No DEG
ENSG00000172803	SNX32	-3,93	-1,97	8,46E-06	4,71E-05	No DEG
ENSG00000235940	MTND1P21	-3,93	-1,97	4,33E-10	4,60E-09	No DEG
ENSG00000123405	NFE2	-3,93	-1,97	2,19E-03	7,47E-03	No DEG
ENSG00000179277	MEIS3P1	-3,93	-1,97	1,48E-24	6,93E-23	No DEG
ENSG00000266946	MRPL37P1	-3,93	-1,97	1,50E-04	6,60E-04	No DEG
ENSG00000006071	ABCC8	-3,92	-1,97	3,30E-06	1,96E-05	No DEG
ENSG00000267941	AC008267.1	3,92	1,97	5,22E-03	1,61E-02	No DEG
ENSG00000167962	ZNF598	-3,92	-1,97	1,34E-22	5,25E-21	No DEG
ENSG00000161638	ITGA5	-3,92	-1,97	5,24E-10	5,51E-09	No DEG
ENSG00000176340	COX8A	-3,92	-1,97	4,41E-25	2,15E-23	No DEG
ENSG00000104812	GYS1	-3,92	-1,97	7,53E-28	4,57E-26	No DEG
ENSG00000126903	SLC10A3	-3,92	-1,97	5,44E-21	1,86E-19	No DEG
ENSG00000075624	ACTB	-3,92	-1,97	9,06E-16	1,83E-14	No DEG
ENSG00000099985	OSM	-3,91	-1,97	1,54E-03	5,44E-03	No DEG
ENSG00000132819	RBM38	-3,91	-1,97	3,65E-19	1,04E-17	No DEG
ENSG00000133067	LGR6	-3,90	-1,97	2,41E-08	2,03E-07	No DEG
ENSG00000138162	TACC2	-3,90	-1,97	2,64E-24	1,21E-22	No DEG
ENSG00000169715	MT1E	3,90	1,97	6,47E-11	7,62E-10	No DEG
ENSG00000214192	UBE2V1P2	-3,90	-1,96	5,13E-05	2,47E-04	No DEG

ENSG00000087258	GNAO1	-3,90	-1,96	3,95E-09	3,69E-08	No DEG
ENSG00000165458	INPPL1	-3,90	-1,96	9,70E-38	1,13E-35	No DEG
ENSG00000136908	DPM2	-3,90	-1,96	1,17E-19	3,44E-18	No DEG
ENSG00000233231	HNRNPA1P49	-3,90	-1,96	6,75E-08	5,29E-07	No DEG
ENSG00000125434	SLC25A35	-3,90	-1,96	4,29E-06	2,50E-05	No DEG
ENSG00000196547	MAN2A2	-3,90	-1,96	9,69E-19	2,65E-17	No DEG
ENSG00000170439	METTL7B	-3,90	-1,96	3,29E-08	2,70E-07	No DEG
ENSG00000177051	FBXO46	-3,89	-1,96	1,49E-30	1,08E-28	No DEG
ENSG00000108823	SGCA	-3,89	-1,96	2,06E-22	7,85E-21	No DEG
ENSG00000261147	RP11-697E2.6	-3,89	-1,96	1,14E-02	3,20E-02	No DEG
ENSG00000118137	APOA1	3,89	1,96	1,79E-06	1,11E-05	No DEG
ENSG00000138379	MSTN	3,89	1,96	3,57E-04	1,46E-03	No DEG
ENSG00000203397	RP13-36G14.3	-3,89	-1,96	4,79E-05	2,33E-04	No DEG
ENSG00000106809	OGN	3,89	1,96	6,80E-07	4,53E-06	No DEG
ENSG00000143569	UBAP2L	-3,89	-1,96	5,53E-51	1,62E-48	No DEG
ENSG00000184557	SOCS3	-3,89	-1,96	6,05E-03	1,83E-02	DOWN
ENSG00000197935	ZNF311	3,89	1,96	6,86E-08	5,37E-07	No DEG
ENSG00000221995	TIAF1	-3,89	-1,96	2,20E-10	2,41E-09	No DEG
ENSG00000249649	MRPS33P2	-3,89	-1,96	3,97E-03	1,27E-02	No DEG
ENSG00000173641	HSPB7	-3,89	-1,96	5,73E-40	7,68E-38	No DEG
ENSG00000227887	RPS26P13	-3,88	-1,96	9,02E-04	3,38E-03	No DEG
ENSG00000023171	GRAMD1B	-3,88	-1,96	2,89E-05	1,47E-04	No DEG
ENSG00000265817	FSBP	3,88	1,96	1,55E-06	9,70E-06	No DEG
ENSG00000261221	ZNF865	-3,88	-1,96	2,13E-26	1,15E-24	No DEG
ENSG00000143368	SF3B4	-3,88	-1,96	2,97E-29	1,98E-27	No DEG
ENSG00000130402	ACTN4	-3,88	-1,96	1,04E-22	4,12E-21	No DEG
ENSG00000005884	ITGA3	-3,88	-1,96	8,32E-24	3,69E-22	No DEG
ENSG00000014164	ZC3H3	-3,88	-1,96	3,10E-22	1,17E-20	No DEG
ENSG00000164897	TMUB1	-3,88	-1,96	2,04E-22	7,81E-21	No DEG
ENSG00000117408	IPO13	-3,88	-1,95	1,76E-35	1,76E-33	No DEG
ENSG00000139718	SETD1B	-3,88	-1,95	6,50E-33	5,65E-31	No DEG
ENSG00000229120	CYCSP4	-3,88	-1,95	4,35E-03	1,37E-02	No DEG

ENSG00000188760	TMEM198	-3,87	-1,95	6,95E-11	8,14E-10	No DEG
ENSG00000197405	C5AR1	-3,87	-1,95	1,68E-07	1,24E-06	No DEG
ENSG00000156453	PCDH1	-3,87	-1,95	8,00E-19	2,20E-17	No DEG
ENSG00000101255	TRIB3	-3,87	-1,95	8,41E-05	3,88E-04	No DEG
ENSG00000141349	G6PC3	-3,87	-1,95	5,21E-20	1,60E-18	No DEG
ENSG00000077238	IL4R	-3,86	-1,95	6,04E-09	5,50E-08	No DEG
ENSG00000258858	RP11-982M15.5	-3,86	-1,95	2,48E-04	1,05E-03	No DEG
ENSG00000107021	TBC1D13	-3,86	-1,95	8,09E-31	6,04E-29	No DEG
ENSG00000233021	RP11-490E15.2	-3,86	-1,95	2,02E-03	6,95E-03	No DEG
ENSG00000125447	GGA3	-3,86	-1,95	3,17E-36	3,39E-34	No DEG
ENSG00000173327	MAP3K11	-3,86	-1,95	2,65E-25	1,31E-23	No DEG
ENSG00000236842	RP11-399K21.10	3,86	1,95	1,15E-02	3,22E-02	No DEG
ENSG00000176945	MUC20	-3,86	-1,95	2,09E-05	1,09E-04	No DEG
ENSG00000229222	KRT18P4	-3,86	-1,95	1,19E-05	6,47E-05	No DEG
ENSG00000204839	MROH6	-3,86	-1,95	4,21E-05	2,07E-04	No DEG
ENSG00000111641	NOP2	-3,86	-1,95	3,17E-20	9,95E-19	No DEG
ENSG00000109047	RCVRN	-3,86	-1,95	2,87E-03	9,50E-03	No DEG
ENSG00000259291	RP11-617F23.1	-3,85	-1,95	2,22E-10	2,44E-09	No DEG
ENSG00000187775	DNAH17	-3,85	-1,95	1,57E-10	1,77E-09	No DEG
ENSG00000110675	ELMOD1	3,85	1,95	3,99E-05	1,97E-04	No DEG
ENSG00000182109	RP11-69E11.4	-3,85	-1,95	7,34E-06	4,12E-05	No DEG
ENSG00000229235	RP11-8L18.3	-3,85	-1,95	1,36E-03	4,85E-03	No DEG
ENSG00000151929	BAG3	-3,85	-1,94	2,48E-29	1,67E-27	No DEG
ENSG00000241231	RP11-275H4.1	3,85	1,94	8,90E-03	2,57E-02	No DEG
ENSG00000037042	TUBG2	-3,85	-1,94	1,24E-21	4,45E-20	No DEG
ENSG00000167524	SGK494	-3,85	-1,94	6,64E-04	2,55E-03	No DEG
ENSG00000136802	LRRC8A	-3,85	-1,94	3,02E-17	7,11E-16	No DEG
ENSG00000163909	HEYL	-3,84	-1,94	1,08E-09	1,09E-08	No DEG
ENSG00000204882	GPR20	-3,84	-1,94	1,27E-08	1,11E-07	No DEG
ENSG00000170190	SLC16A5	-3,84	-1,94	2,12E-13	3,39E-12	No DEG
ENSG00000136699	SMPD4	-3,84	-1,94	2,42E-38	2,92E-36	No DEG
ENSG00000119227	PIGZ	-3,84	-1,94	1,86E-14	3,31E-13	No DEG

ENSG00000260460	RP11-284F21.8	-3,84	-1,94	5,58E-04	2,18E-03	No DEG
ENSG00000186019	AC084219.4	3,84	1,94	8,05E-05	3,73E-04	No DEG
ENSG00000119900	OGFRL1	3,84	1,94	1,16E-23	5,08E-22	No DEG
ENSG00000149925	ALDOA	-3,84	-1,94	1,30E-34	1,23E-32	No DEG
ENSG00000253298	AC008703.1	-3,83	-1,94	5,72E-03	1,75E-02	No DEG
ENSG00000116525	TRIM62	-3,83	-1,94	3,46E-19	9,84E-18	No DEG
ENSG00000251441	RTEL1P1	-3,83	-1,94	1,17E-07	8,85E-07	No DEG
ENSG00000170075	GPR37L1	-3,83	-1,94	2,40E-06	1,46E-05	No DEG
ENSG00000171722	C1orf111	-3,83	-1,94	1,20E-03	4,36E-03	No DEG
ENSG00000005238	FAM214B	-3,83	-1,94	3,99E-23	1,66E-21	No DEG
ENSG00000133048	CHI3L1	-3,83	-1,94	6,06E-03	1,84E-02	No DEG
ENSG00000161558	TMEM143	-3,83	-1,94	1,04E-16	2,31E-15	No DEG
ENSG00000177700	POLR2L	-3,83	-1,94	3,39E-26	1,79E-24	No DEG
ENSG00000160803	UBQLN4	-3,83	-1,94	1,18E-35	1,20E-33	No DEG
ENSG00000221157	AC009196.1	3,83	1,94	8,22E-03	2,40E-02	No DEG
ENSG00000166311	SMPD1	-3,82	-1,94	3,04E-24	1,39E-22	No DEG
ENSG00000112137	PHACTR1	-3,82	-1,93	5,35E-13	8,18E-12	No DEG
ENSG00000213212	NCLP1	-3,82	-1,93	1,12E-03	4,10E-03	No DEG
ENSG00000140332	TLE3	-3,82	-1,93	3,20E-14	5,56E-13	No DEG
ENSG00000105223	PLD3	-3,82	-1,93	9,30E-40	1,22E-37	No DEG
ENSG00000165802	NSMF	-3,82	-1,93	1,87E-20	6,02E-19	No DEG
ENSG00000106348	IMPDH1	-3,82	-1,93	1,30E-21	4,67E-20	No DEG
ENSG00000019144	PHLDB1	-3,82	-1,93	3,17E-26	1,69E-24	No DEG
ENSG00000167861	HID1	-3,82	-1,93	2,40E-12	3,40E-11	No DEG
ENSG00000179855	GIPC3	-3,81	-1,93	1,20E-08	1,05E-07	No DEG
ENSG00000172738	TMEM217	-3,81	-1,93	1,09E-04	4,93E-04	No DEG
ENSG00000169385	RNASE2	-3,81	-1,93	4,10E-03	1,30E-02	No DEG
ENSG00000182481	KPNA2	-3,81	-1,93	3,51E-10	3,76E-09	No DEG
ENSG00000238278	ALG1L6P	-3,81	-1,93	7,33E-05	3,43E-04	No DEG
ENSG00000161847	RAVER1	-3,81	-1,93	3,55E-22	1,33E-20	No DEG
ENSG00000269430	LRRC3DN	-3,81	-1,93	4,68E-08	3,76E-07	No DEG
ENSG00000223401	RP11-211G3.2	-3,81	-1,93	4,07E-05	2,00E-04	No DEG

ENSG00000261512	RP11-46D6.1	-3,81	-1,93	1,19E-07	9,01E-07	No DEG
ENSG00000147231	CXorf57	3,81	1,93	1,85E-05	9,74E-05	No DEG
ENSG00000135437	RDH5	-3,80	-1,92	6,09E-05	2,90E-04	No DEG
ENSG00000207609	MIR491	3,80	1,92	8,58E-05	3,95E-04	No DEG
ENSG00000101986	ABCD1	-3,79	-1,92	1,18E-35	1,20E-33	No DEG
ENSG00000102554	KLF5	-3,79	-1,92	2,82E-07	2,00E-06	DOWN
ENSG00000168453	HR	-3,79	-1,92	1,94E-06	1,19E-05	No DEG
ENSG00000063176	SPHK2	-3,79	-1,92	5,17E-15	9,74E-14	No DEG
ENSG00000196961	AP2A1	-3,79	-1,92	1,67E-46	3,43E-44	No DEG
ENSG00000138622	HCN4	-3,79	-1,92	2,85E-15	5,49E-14	No DEG
ENSG00000271075	RP11-589M4.4	-3,79	-1,92	5,20E-05	2,51E-04	No DEG
ENSG00000241458	RPL7P19	3,79	1,92	1,99E-03	6,86E-03	No DEG
ENSG00000184489	PTP4A3	-3,78	-1,92	5,34E-23	2,20E-21	No DEG
ENSG00000204209	DAXX	-3,78	-1,92	1,81E-35	1,80E-33	No DEG
ENSG00000223633	RP11-143A22.1	3,78	1,92	2,85E-04	1,19E-03	No DEG
ENSG00000116035	VAX2	-3,78	-1,92	1,45E-03	5,16E-03	No DEG
ENSG00000215835	RP11-9L18.2	-3,78	-1,92	7,93E-07	5,21E-06	No DEG
ENSG00000270184	RP11-568J23.5	-3,78	-1,92	4,27E-05	2,09E-04	No DEG
ENSG00000261884	CTC-479C5.12	-3,78	-1,92	8,92E-09	7,95E-08	No DEG
ENSG00000136378	ADAMTS7	-3,78	-1,92	2,14E-11	2,68E-10	No DEG
ENSG00000216285	RP11-490H24.5	-3,78	-1,92	6,19E-06	3,51E-05	No DEG
ENSG00000160999	SH2B2	-3,77	-1,92	2,77E-04	1,16E-03	No DEG
ENSG00000248626	GAPDHP40	-3,77	-1,92	1,71E-04	7,47E-04	No DEG
ENSG00000158008	EXTL1	-3,77	-1,91	4,27E-06	2,49E-05	No DEG
ENSG00000237721	AF064858.11	-3,77	-1,91	1,19E-02	3,32E-02	No DEG
ENSG00000273199	AP000692.10	3,77	1,91	7,40E-03	2,19E-02	No DEG
ENSG00000175785	PRIMA1	-3,77	-1,91	1,79E-08	1,54E-07	No DEG
ENSG00000258968	RP11-829H16.5	-3,77	-1,91	7,01E-05	3,29E-04	No DEG
ENSG00000255825	RP5-1154L15.1	-3,77	-1,91	6,37E-03	1,92E-02	No DEG
ENSG00000239279	RN7SL184P	-3,76	-1,91	4,67E-03	1,46E-02	No DEG
ENSG00000144837	PLA1A	-3,76	-1,91	3,01E-04	1,25E-03	No DEG
ENSG00000185475	TMEM179B	-3,76	-1,91	8,74E-26	4,49E-24	No DEG

ENSG00000224843	LINC00240	3,76	1,91	2,00E-05	1,04E-04	No DEG
ENSG00000167971	CASKIN1	-3,76	-1,91	4,31E-05	2,11E-04	DOWN
ENSG00000251593	MSNP1	-3,76	-1,91	3,68E-03	1,18E-02	No DEG
ENSG00000076604	TRAF4	-3,76	-1,91	1,17E-06	7,44E-06	No DEG
ENSG00000269900	RMRP	3,76	1,91	4,57E-24	2,06E-22	No DEG
ENSG00000170485	NPAS2	-3,76	-1,91	5,64E-29	3,71E-27	No DEG
ENSG00000229523	RP11-7G23.4	-3,76	-1,91	1,17E-02	3,27E-02	No DEG
ENSG00000021300	PLEKHB1	-3,76	-1,91	1,22E-10	1,39E-09	No DEG
ENSG00000198786	MT-ND5	-3,75	-1,91	7,05E-21	2,37E-19	No DEG
ENSG00000099917	MED15	-3,75	-1,91	9,72E-32	7,73E-30	No DEG
ENSG00000260898	ADPGK-AS1	-3,75	-1,91	3,46E-05	1,73E-04	No DEG
ENSG00000246627	CACNA1C-AS1	-3,75	-1,91	4,62E-05	2,25E-04	No DEG
ENSG00000270093	AP000473.8	3,75	1,91	1,12E-03	4,11E-03	No DEG
ENSG00000244509	APOBEC3C	-3,75	-1,91	5,16E-15	9,73E-14	No DEG
ENSG00000257675	RP11-641A6.9	3,75	1,91	2,63E-03	8,79E-03	No DEG
ENSG00000204959	ARHGEF34P	-3,75	-1,91	1,06E-05	5,83E-05	No DEG
ENSG00000145506	NKD2	-3,75	-1,91	1,67E-06	1,04E-05	No DEG
ENSG00000121104	FAM117A	-3,75	-1,91	3,13E-12	4,36E-11	No DEG
ENSG00000167548	KMT2D	-3,75	-1,91	1,89E-22	7,27E-21	No DEG
ENSG00000143369	ECM1	-3,75	-1,91	1,28E-11	1,66E-10	No DEG
ENSG00000225695	HNRNPA1P35	-3,75	-1,91	5,70E-04	2,23E-03	No DEG
ENSG00000269641	CTB-167G5.6	-3,74	-1,90	6,63E-03	1,99E-02	DOWN
ENSG00000007908	SELE	-3,74	-1,90	7,60E-03	2,24E-02	DOWN
ENSG00000180155	LYNX1	-3,74	-1,90	1,72E-22	6,65E-21	No DEG
ENSG00000227910	RP11-73B2.6	3,74	1,90	4,68E-03	1,47E-02	No DEG
ENSG00000100345	MYH9	-3,74	-1,90	8,72E-18	2,17E-16	No DEG
ENSG00000233611	AC079135.1	-3,74	-1,90	1,25E-06	7,94E-06	No DEG
ENSG00000180610	ZBTB12P1	-3,74	-1,90	6,01E-04	2,33E-03	No DEG
ENSG00000214022	REPIN1	-3,74	-1,90	4,14E-33	3,65E-31	No DEG
ENSG00000141456	PELP1	-3,74	-1,90	2,51E-27	1,47E-25	No DEG
ENSG00000271437	RP11-295B17.6	-3,73	-1,90	2,81E-04	1,17E-03	No DEG
ENSG00000157870	FAM213B	-3,73	-1,90	1,38E-12	2,02E-11	No DEG

ENSG00000113083	LOX	3,73	1,90	6,89E-11	8,07E-10	No DEG
ENSG00000182950	ODF3L1	-3,73	-1,90	8,80E-03	2,54E-02	No DEG
ENSG00000100206	DMC1	3,73	1,90	1,44E-05	7,71E-05	No DEG
ENSG00000231852	CYP21A2	-3,73	-1,90	8,41E-04	3,17E-03	No DEG
ENSG00000225349	RP11-820K3.2	-3,73	-1,90	2,28E-03	7,74E-03	No DEG
ENSG00000172350	ABCG4	-3,72	-1,90	1,50E-05	8,03E-05	No DEG
ENSG00000152137	HSPB8	-3,72	-1,90	2,79E-54	1,04E-51	No DEG
ENSG00000137094	DNAJB5	-3,72	-1,90	1,66E-16	3,60E-15	DOWN
ENSG00000229472	RP1-64K7.4	-3,72	-1,90	4,85E-04	1,92E-03	No DEG
ENSG00000086015	MAST2	-3,72	-1,90	2,34E-40	3,17E-38	No DEG
ENSG00000082482	KCNK2	3,72	1,90	9,22E-04	3,44E-03	No DEG
ENSG00000075826	SEC31B	-3,72	-1,90	1,88E-24	8,71E-23	No DEG
ENSG00000214185	XPOTP1	3,72	1,89	1,24E-03	4,49E-03	No DEG
ENSG00000238086	PPP1R26P1	-3,72	-1,89	3,33E-08	2,73E-07	No DEG
ENSG00000228110	ST13P19	3,72	1,89	6,39E-03	1,93E-02	No DEG
ENSG00000173546	CSPG4	-3,72	-1,89	5,22E-09	4,80E-08	No DEG
ENSG00000121764	HCRTR1	-3,72	-1,89	3,98E-06	2,33E-05	No DEG
ENSG00000160753	RUSC1	-3,72	-1,89	4,47E-28	2,77E-26	No DEG
ENSG00000072195	SPEG	-3,72	-1,89	6,11E-27	3,45E-25	No DEG
ENSG00000183654	MARCH11	3,72	1,89	3,26E-06	1,94E-05	No DEG
ENSG00000231081	RP4-760C5.3	-3,72	-1,89	6,69E-04	2,57E-03	No DEG
ENSG00000162104	ADCY9	-3,72	-1,89	5,96E-29	3,92E-27	No DEG
ENSG00000237328	RAI1-AS1	-3,72	-1,89	2,67E-03	8,89E-03	No DEG
ENSG00000134470	IL15RA	-3,71	-1,89	3,05E-10	3,30E-09	No DEG
ENSG00000183873	SCN5A	-3,71	-1,89	1,95E-22	7,48E-21	No DEG
ENSG00000006210	CX3CL1	-3,71	-1,89	8,72E-16	1,76E-14	No DEG
ENSG00000200087	SNORA73B	3,71	1,89	6,33E-18	1,60E-16	No DEG
ENSG00000228232	GAPDHP1	-3,71	-1,89	9,99E-31	7,39E-29	No DEG
ENSG00000163710	PCOLCE2	3,71	1,89	2,16E-14	3,82E-13	No DEG
ENSG00000226237	RP11-276H19.1	3,71	1,89	8,25E-05	3,82E-04	UP
ENSG00000071282	LMCD1	-3,71	-1,89	1,12E-06	7,20E-06	DOWN
ENSG00000090920	FCGBP	-3,70	-1,89	1,23E-03	4,46E-03	No DEG

ENSG00000227158	AC073621.2	-3,70	-1,89	5,44E-03	1,67E-02	DOWN
ENSG00000140320	BAHD1	-3,70	-1,89	2,47E-27	1,45E-25	No DEG
ENSG00000182319	SGK223	-3,70	-1,89	1,49E-12	2,16E-11	No DEG
ENSG00000144043	TEX261	-3,70	-1,89	7,65E-44	1,28E-41	No DEG
ENSG00000172269	DPAGT1	-3,70	-1,89	8,21E-20	2,48E-18	No DEG
ENSG00000248394	FOSL1P1	-3,70	-1,89	7,83E-03	2,30E-02	No DEG
ENSG00000229116	RP11-20J15.3	-3,70	-1,89	2,62E-11	3,26E-10	No DEG
ENSG00000196381	ZNF781	3,70	1,89	7,15E-11	8,36E-10	No DEG
ENSG00000238344	SNORD126	3,70	1,89	1,13E-02	3,18E-02	No DEG
ENSG00000112561	TFEB	-3,70	-1,89	9,49E-29	6,13E-27	No DEG
ENSG00000179941	BBS10	3,70	1,89	5,17E-17	1,18E-15	No DEG
ENSG00000249175	CTC-484P3.3	-3,70	-1,89	3,89E-05	1,93E-04	No DEG
ENSG00000188677	PARVB	-3,69	-1,89	1,51E-13	2,46E-12	No DEG
ENSG00000080986	NDC80	3,69	1,89	2,33E-06	1,41E-05	No DEG
ENSG00000079313	REXO1	-3,69	-1,88	2,31E-34	2,15E-32	No DEG
ENSG00000160445	ZER1	-3,69	-1,88	7,67E-36	7,98E-34	No DEG
ENSG00000188257	PLA2G2A	-3,69	-1,88	2,95E-03	9,74E-03	No DEG
ENSG00000100226	GTPBP1	-3,69	-1,88	4,00E-30	2,84E-28	No DEG
ENSG00000136928	GABBR2	-3,69	-1,88	7,27E-05	3,41E-04	DOWN
ENSG00000166716	ZNF592	-3,69	-1,88	5,03E-43	8,04E-41	No DEG
ENSG00000267080	ASB16-AS1	-3,69	-1,88	1,48E-12	2,15E-11	No DEG
ENSG00000185432	METTL7A	3,69	1,88	4,38E-10	4,66E-09	No DEG
ENSG00000225663	FAM195B	-3,68	-1,88	3,52E-15	6,74E-14	No DEG
ENSG00000236233	RP11-12A20.7	-3,68	-1,88	5,39E-04	2,11E-03	No DEG
ENSG00000201554	Y_RNA	3,68	1,88	1,66E-03	5,82E-03	No DEG
ENSG00000203647	AC106827.1	-3,68	-1,88	9,83E-04	3,65E-03	No DEG
ENSG00000197061	HIST1H4C	3,68	1,88	1,95E-14	3,46E-13	No DEG
ENSG00000250397	RP11-1391J7.1	-3,68	-1,88	2,01E-03	6,92E-03	No DEG
ENSG00000090238	YPEL3	-3,68	-1,88	1,03E-17	2,53E-16	No DEG
ENSG00000200253	RNU6-529P	3,68	1,88	1,46E-05	7,84E-05	No DEG
ENSG00000015285	WAS	-3,68	-1,88	1,68E-09	1,66E-08	UP
ENSG00000134575	ACP2	-3,68	-1,88	2,89E-28	1,82E-26	No DEG

ENSG00000259726	CSPG4P11	-3,68	-1,88	4,49E-07	3,08E-06	No DEG
ENSG00000087266	SH3BP2	-3,67	-1,88	1,75E-17	4,24E-16	No DEG
ENSG00000201390	RNU6-1141P	3,67	1,88	3,52E-03	1,14E-02	No DEG
ENSG00000109991	P2RX3	-3,67	-1,88	1,05E-04	4,77E-04	No DEG
ENSG00000111665	CDCA3	-3,67	-1,88	1,09E-08	9,58E-08	No DEG
ENSG00000255974	CYP2A6	-3,67	-1,88	1,98E-03	6,81E-03	No DEG
ENSG00000205250	E2F4	-3,67	-1,88	4,62E-30	3,24E-28	No DEG
ENSG00000138166	DUSP5	-3,67	-1,88	6,12E-06	3,48E-05	No DEG
ENSG00000237994	RP1-146A15.1	3,67	1,87	1,06E-03	3,90E-03	No DEG
ENSG00000177764	ZCCHC3	3,67	1,87	2,40E-10	2,63E-09	No DEG
ENSG00000168389	MFSD2A	-3,66	-1,87	2,49E-05	1,28E-04	No DEG
ENSG00000100092	SH3BP1	-3,66	-1,87	3,39E-06	2,01E-05	No DEG
ENSG00000072518	MARK2	-3,66	-1,87	2,02E-49	5,16E-47	No DEG
ENSG00000100079	LGALS2	-3,66	-1,87	2,93E-05	1,49E-04	No DEG
ENSG00000184682	C11orf89	-3,66	-1,87	4,17E-06	2,44E-05	No DEG
ENSG00000100299	ARSA	-3,66	-1,87	7,33E-25	3,50E-23	No DEG
ENSG00000164050	PLXNB1	-3,66	-1,87	9,22E-29	5,97E-27	No DEG
ENSG00000151164	RAD9B	-3,66	-1,87	1,64E-04	7,17E-04	DOWN
ENSG00000166689	PLEKHA7	-3,66	-1,87	4,91E-10	5,17E-09	No DEG
ENSG00000263813	MIR3679	-3,66	-1,87	2,48E-03	8,34E-03	No DEG
ENSG00000112299	VNN1	3,65	1,87	3,74E-05	1,86E-04	UP
ENSG00000142655	PEX14	-3,65	-1,87	3,98E-25	1,94E-23	No DEG
ENSG00000162877	PM20D1	-3,65	-1,87	1,89E-06	1,17E-05	No DEG
ENSG00000269836	CTD-3032J10.4	-3,65	-1,87	5,40E-03	1,66E-02	No DEG
ENSG00000007933	FMO3	3,65	1,87	4,68E-06	2,71E-05	No DEG
ENSG00000162551	ALPL	-3,65	-1,87	8,14E-11	9,45E-10	No DEG
ENSG00000261633	RP11-405F3.5	-3,65	-1,87	8,31E-03	2,42E-02	No DEG
ENSG00000268751	SCGB1B2P	-3,65	-1,87	3,80E-03	1,22E-02	No DEG
ENSG00000124249	KCNK15	-3,65	-1,87	3,40E-03	1,10E-02	DOWN
ENSG00000253576	GS1-5L10.1	3,65	1,87	2,93E-05	1,49E-04	No DEG
ENSG00000205595	AREGB	-3,65	-1,87	5,11E-03	1,58E-02	No DEG
ENSG00000141576	RNF157	-3,64	-1,86	2,95E-12	4,12E-11	No DEG

ENSG00000058404	CAMK2B	-3,64	-1,86	7,17E-18	1,80E-16	No DEG
ENSG00000106333	PCOLCE	-3,64	-1,86	2,87E-09	2,73E-08	No DEG
ENSG00000241764	AC002467.7	3,64	1,86	1,81E-11	2,29E-10	No DEG
ENSG00000133895	MEN1	-3,64	-1,86	1,03E-28	6,66E-27	No DEG
ENSG00000250669	RP11-83M16.3	3,64	1,86	4,33E-03	1,37E-02	No DEG
ENSG00000005206	SPPL2B	-3,64	-1,86	7,93E-29	5,17E-27	No DEG
ENSG00000162458	FBLIM1	-3,64	-1,86	1,24E-14	2,26E-13	No DEG
ENSG00000270640	RP11-373D23.2	-3,64	-1,86	4,94E-05	2,39E-04	No DEG
ENSG00000269102	CTD-2525I3.5	-3,63	-1,86	2,82E-03	9,34E-03	No DEG
ENSG00000237586	RP11-383J24.2	-3,63	-1,86	7,96E-08	6,17E-07	No DEG
ENSG00000245148	ARAP1-AS2	-3,63	-1,86	5,00E-05	2,42E-04	No DEG
ENSG00000173334	TRIB1	-3,63	-1,86	6,63E-08	5,21E-07	No DEG
ENSG00000253978	CTB-178M22.2	-3,63	-1,86	2,63E-04	1,11E-03	DOWN
ENSG00000223724	RAD17P2	-3,63	-1,86	3,05E-04	1,26E-03	No DEG
ENSG00000111644	ACRBP	-3,63	-1,86	2,34E-05	1,20E-04	No DEG
ENSG00000204338	CYP21A1P	-3,63	-1,86	2,12E-03	7,24E-03	No DEG
ENSG00000251860	snoU13	-3,63	-1,86	1,35E-02	3,70E-02	No DEG
ENSG00000142453	CARM1	-3,63	-1,86	1,87E-27	1,10E-25	No DEG
ENSG00000165914	TTC7B	-3,62	-1,86	1,45E-54	5,55E-52	No DEG
ENSG00000064687	ABCA7	-3,62	-1,86	6,36E-11	7,50E-10	No DEG
ENSG00000231646	AC008174.3	3,62	1,86	5,72E-05	2,74E-04	No DEG
ENSG00000105402	NAPA	-3,62	-1,86	2,70E-44	4,77E-42	No DEG
ENSG00000186111	PIP5K1C	-3,62	-1,86	2,44E-39	3,06E-37	No DEG
ENSG00000205565	RP11-497H16.6	-3,62	-1,86	2,74E-04	1,15E-03	No DEG
ENSG00000259224	SLC35G6	-3,62	-1,86	6,08E-03	1,84E-02	No DEG
ENSG00000241439	RP11-666A20.3	-3,62	-1,86	6,53E-03	1,96E-02	No DEG
ENSG00000262020	RP11-66H6.3	-3,62	-1,85	4,03E-03	1,28E-02	No DEG
ENSG00000182791	CCDC87	-3,62	-1,85	2,93E-03	9,68E-03	No DEG
ENSG00000271529	CICP14	-3,62	-1,85	3,18E-15	6,12E-14	No DEG
ENSG00000167615	LENG8	-3,61	-1,85	1,15E-20	3,77E-19	No DEG
ENSG00000076201	PTPN23	-3,61	-1,85	2,57E-30	1,84E-28	No DEG
ENSG00000109089	CDR2L	-3,61	-1,85	2,49E-11	3,10E-10	No DEG

ENSG00000267381	CTD-2086O20.3	3,61	1,85	3,36E-17	7,89E-16	No DEG
ENSG00000270764	CTB-152G17.5	-3,61	-1,85	1,22E-02	3,38E-02	No DEG
ENSG00000089505	CMTM1	-3,61	-1,85	8,58E-05	3,96E-04	No DEG
ENSG00000177803	RP11-606P2.1	-3,61	-1,85	7,73E-03	2,27E-02	No DEG
ENSG00000260367	RP11-264B17.4	-3,61	-1,85	1,26E-02	3,50E-02	No DEG
ENSG00000177943	MAMDC4	-3,60	-1,85	2,35E-17	5,60E-16	No DEG
ENSG00000250722	SEPP1	3,60	1,85	2,41E-23	1,02E-21	No DEG
ENSG00000131459	GFPT2	-3,60	-1,85	7,05E-06	3,96E-05	DOWN
ENSG00000164742	ADCY1	-3,60	-1,85	1,87E-18	4,92E-17	No DEG
ENSG00000074219	TEAD2	-3,60	-1,85	4,77E-16	9,85E-15	No DEG
ENSG00000160867	FGFR4	-3,60	-1,85	4,11E-04	1,66E-03	No DEG
ENSG00000054967	RELT	-3,60	-1,85	2,36E-09	2,27E-08	No DEG
ENSG00000143340	FAM163A	-3,60	-1,85	8,89E-04	3,33E-03	No DEG
ENSG00000123146	CD97	-3,60	-1,85	5,06E-16	1,04E-14	No DEG
ENSG00000273002	RP11-336K24.12	-3,60	-1,85	3,07E-05	1,55E-04	No DEG
ENSG00000087237	CETP	-3,60	-1,85	7,91E-05	3,68E-04	No DEG
ENSG00000271699	SNX29P2	-3,59	-1,85	2,09E-03	7,15E-03	No DEG
ENSG00000242539	AC007620.3	3,59	1,85	3,20E-08	2,63E-07	No DEG
ENSG00000226159	RP11-478K7.2	3,59	1,85	1,28E-03	4,61E-03	No DEG
ENSG00000270441	RP11-694I15.7	-3,59	-1,84	2,42E-05	1,24E-04	No DEG
ENSG00000212464	SNORA12	3,59	1,84	3,13E-23	1,32E-21	No DEG
ENSG00000175520	UBQLN3	-3,59	-1,84	5,42E-04	2,12E-03	No DEG
ENSG00000213763	ACTBP2	-3,59	-1,84	8,35E-03	2,43E-02	No DEG
ENSG00000239213	RP11-85F14.5	3,59	1,84	3,46E-06	2,04E-05	No DEG
ENSG00000213828	AC017028.1	-3,59	-1,84	1,79E-03	6,24E-03	No DEG
ENSG00000205212	CCDC144NL	3,59	1,84	5,67E-03	1,73E-02	No DEG
ENSG00000176055	MBLAC2	3,59	1,84	2,23E-10	2,44E-09	No DEG
ENSG00000235290	HLA-W	-3,59	-1,84	4,73E-05	2,30E-04	No DEG
ENSG00000141068	KSR1	-3,59	-1,84	1,82E-10	2,02E-09	No DEG
ENSG00000250548	RP11-47I22.2	-3,58	-1,84	1,21E-04	5,41E-04	No DEG
ENSG00000241158	ADAMTS9-AS1	-3,58	-1,84	1,84E-05	9,67E-05	DOWN
ENSG00000140853	NLRC5	-3,58	-1,84	2,26E-11	2,83E-10	No DEG

ENSG00000050327	ARHGEF5	-3,58	-1,84	8,35E-08	6,47E-07	No DEG
ENSG00000126003	PLAGL2	-3,58	-1,84	2,58E-25	1,27E-23	No DEG
ENSG00000127083	OMD	3,58	1,84	1,24E-10	1,41E-09	No DEG
ENSG00000271424	RP11-647O20.1	-3,57	-1,84	1,15E-03	4,18E-03	No DEG
ENSG00000006712	PAF1	-3,57	-1,84	2,72E-53	9,48E-51	No DEG
ENSG00000239151	RNU7-195P	-3,57	-1,84	7,35E-03	2,18E-02	No DEG
ENSG00000260686	CTB-36H16.2	3,57	1,84	1,98E-09	1,93E-08	No DEG
ENSG00000023902	PLEKHO1	-3,57	-1,84	8,30E-10	8,51E-09	No DEG
ENSG00000169989	TIGD4	3,57	1,84	7,33E-07	4,85E-06	No DEG
ENSG00000269919	RP1-134E15.3	3,57	1,84	2,36E-03	7,99E-03	UP
ENSG00000271499	CTC-559E9.9	-3,57	-1,84	1,64E-04	7,19E-04	No DEG
ENSG00000188869	TMC3	3,57	1,84	2,31E-03	7,84E-03	UP
ENSG00000249364	RP11-434D9.1	3,57	1,84	1,32E-03	4,73E-03	No DEG
ENSG00000175221	MED16	-3,57	-1,84	1,18E-44	2,13E-42	No DEG
ENSG00000267504	RP11-845C23.2	-3,57	-1,84	1,91E-03	6,61E-03	No DEG
ENSG00000251692	PTX4	-3,57	-1,84	1,28E-02	3,53E-02	No DEG
ENSG00000106113	CRHR2	-3,57	-1,83	1,30E-04	5,77E-04	No DEG
ENSG00000248256	OCIAD1-AS1	3,57	1,83	4,07E-05	2,00E-04	No DEG
ENSG00000078804	TP53INP2	-3,57	-1,83	3,94E-17	9,16E-16	No DEG
ENSG00000162881	OXER1	-3,57	-1,83	9,77E-06	5,39E-05	No DEG
ENSG00000169507	SLC38A11	3,56	1,83	8,90E-07	5,80E-06	No DEG
ENSG00000271146	RP11-479F13.1	-3,56	-1,83	2,66E-03	8,89E-03	No DEG
ENSG00000105429	MEGF8	-3,56	-1,83	6,39E-23	2,60E-21	No DEG
ENSG00000143167	GPA33	-3,56	-1,83	2,68E-04	1,12E-03	No DEG
ENSG00000260660	RP11-69H7.2	-3,56	-1,83	3,42E-03	1,11E-02	No DEG
ENSG00000222068	RN7SKP154	-3,56	-1,83	4,29E-03	1,36E-02	No DEG
ENSG00000122861	PLAU	-3,56	-1,83	4,88E-06	2,82E-05	DOWN
ENSG00000148396	SEC16A	-3,56	-1,83	1,48E-29	1,01E-27	No DEG
ENSG00000122986	HVCN1	-3,56	-1,83	2,42E-11	3,02E-10	No DEG
ENSG00000230399	RBBP8P1	3,56	1,83	2,38E-03	8,04E-03	UP
ENSG00000169221	TBC1D10B	-3,56	-1,83	2,26E-26	1,22E-24	No DEG
ENSG00000110900	TSPAN11	-3,56	-1,83	6,80E-06	3,83E-05	No DEG

ENSG00000130723	PRRC2B	-3,55	-1,83	3,66E-47	8,01E-45	No DEG
ENSG00000167136	ENDOG	3,55	1,83	1,82E-09	1,79E-08	No DEG
ENSG00000162517	PEF1	-3,55	-1,83	2,78E-29	1,85E-27	No DEG
ENSG00000229951	AC104695.3	-3,55	-1,83	1,09E-06	7,01E-06	No DEG
ENSG00000134070	IRAK2	-3,55	-1,83	2,79E-08	2,32E-07	No DEG
ENSG00000269048	Z98049.1	-3,55	-1,83	1,57E-05	8,36E-05	No DEG
ENSG00000273100	RP11-302L19.3	-3,55	-1,83	3,64E-03	1,17E-02	No DEG
ENSG00000233330	RP1-12G14.7	3,55	1,83	6,78E-08	5,31E-07	No DEG
ENSG00000159164	SV2A	-3,55	-1,83	6,02E-06	3,43E-05	No DEG
ENSG00000262410	RP11-388C12.8	-3,55	-1,83	1,45E-04	6,39E-04	No DEG
ENSG00000176399	DMRTA1	3,54	1,83	1,45E-06	9,13E-06	No DEG
ENSG00000167772	ANGPTL4	-3,54	-1,83	5,70E-04	2,22E-03	No DEG
ENSG00000132589	FLOT2	-3,54	-1,83	5,02E-33	4,40E-31	No DEG
ENSG00000179832	MROH1	-3,54	-1,83	1,52E-27	9,03E-26	No DEG
ENSG00000153140	CETN3	3,54	1,82	9,13E-10	9,30E-09	No DEG
ENSG00000111752	PHC1	-3,54	-1,82	5,02E-37	5,60E-35	No DEG
ENSG00000182934	SRPR	-3,54	-1,82	2,49E-43	4,00E-41	No DEG
ENSG00000105270	CLIP3	-3,54	-1,82	5,11E-08	4,09E-07	No DEG
ENSG00000139083	ETV6	-3,54	-1,82	5,00E-13	7,67E-12	No DEG
ENSG00000151208	DLG5	-3,54	-1,82	3,37E-18	8,68E-17	No DEG
ENSG00000214290	COLCA2	-3,54	-1,82	3,34E-03	1,09E-02	No DEG
ENSG00000271225	RP11-460N11.3	-3,53	-1,82	8,06E-04	3,05E-03	No DEG
ENSG00000228925	AC016722.4	-3,53	-1,82	6,27E-05	2,98E-04	No DEG
ENSG00000248113	RP11-791G16.4	3,53	1,82	1,23E-03	4,46E-03	No DEG
ENSG00000099875	MKNK2	-3,53	-1,82	8,37E-15	1,55E-13	No DEG
ENSG00000169314	C22orf15	-3,53	-1,82	6,81E-05	3,21E-04	No DEG
ENSG00000136866	ZFP37	3,53	1,82	2,58E-17	6,10E-16	No DEG
ENSG00000187144	SPATA21	-3,53	-1,82	2,40E-04	1,02E-03	No DEG
ENSG00000008517	IL32	-3,53	-1,82	7,91E-07	5,20E-06	No DEG
ENSG00000272216	LL22NC03-2H8.5	3,53	1,82	5,04E-07	3,43E-06	No DEG
ENSG00000205744	DENND1C	-3,53	-1,82	3,48E-05	1,74E-04	UP
ENSG00000146070	PLA2G7	3,53	1,82	4,82E-04	1,91E-03	UP

ENSG00000120586	MRC1	3,53	1,82	6,97E-07	4,63E-06	No DEG
ENSG00000213923	CSNK1E	-3,53	-1,82	7,59E-46	1,51E-43	No DEG
ENSG00000105699	LSR	-3,53	-1,82	2,27E-12	3,22E-11	No DEG
ENSG00000105204	DYRK1B	-3,52	-1,82	9,47E-11	1,09E-09	No DEG
ENSG00000227145	IL21-AS1	3,52	1,82	1,15E-02	3,21E-02	UP
ENSG00000204580	DDR1	-3,52	-1,82	5,57E-19	1,56E-17	No DEG
ENSG00000240466	RN7SL331P	-3,52	-1,82	2,65E-03	8,83E-03	No DEG
ENSG00000142039	CCDC97	-3,52	-1,82	9,81E-36	1,02E-33	No DEG
ENSG00000232368	FTLP2	-3,52	-1,82	7,95E-10	8,19E-09	No DEG
ENSG00000173846	PLK3	-3,52	-1,82	4,17E-06	2,44E-05	No DEG
ENSG00000180357	ZNF609	-3,52	-1,81	6,55E-33	5,67E-31	No DEG
ENSG00000161277	THAP8	-3,52	-1,81	1,73E-04	7,55E-04	No DEG
ENSG00000235802	HCFC1-AS1	-3,51	-1,81	7,58E-03	2,23E-02	No DEG
ENSG00000162231	NXF1	-3,51	-1,81	5,11E-27	2,92E-25	No DEG
ENSG00000143630	HCN3	-3,51	-1,81	1,02E-06	6,59E-06	No DEG
ENSG00000224259	LINC01133	3,51	1,81	7,09E-03	2,11E-02	No DEG
ENSG00000182389	CACNB4	3,51	1,81	4,92E-09	4,55E-08	No DEG
ENSG00000061938	TNK2	-3,51	-1,81	2,01E-11	2,54E-10	No DEG
ENSG00000258930	RP5-1163L11.2	-3,51	-1,81	4,82E-07	3,30E-06	No DEG
ENSG00000131831	RAI2	-3,51	-1,81	1,06E-20	3,50E-19	No DEG
ENSG00000133454	MYO18B	-3,51	-1,81	3,37E-20	1,05E-18	No DEG
ENSG00000111252	SH2B3	-3,51	-1,81	2,00E-12	2,85E-11	No DEG
ENSG00000011021	CLCN6	-3,51	-1,81	3,25E-24	1,48E-22	No DEG
ENSG00000102898	NUTF2	-3,50	-1,81	8,03E-39	9,85E-37	No DEG
ENSG00000260400	RP11-119F7.5	3,50	1,81	1,23E-04	5,52E-04	No DEG
ENSG00000224877	C17orf89	3,50	1,81	5,88E-24	2,63E-22	No DEG
ENSG00000221986	MYBPHL	-3,50	-1,81	4,40E-03	1,39E-02	No DEG
ENSG00000155970	MICU3	3,50	1,81	3,69E-24	1,68E-22	No DEG
ENSG00000134815	DHX34	-3,50	-1,81	1,15E-23	5,04E-22	No DEG
ENSG00000215559	ANKRD20A11P	3,50	1,81	2,01E-03	6,92E-03	UP
ENSG00000253485	PCDHGA5	-3,50	-1,81	3,21E-17	7,55E-16	No DEG
ENSG00000261191	RP11-16L14.2	-3,50	-1,81	2,27E-03	7,70E-03	DOWN

ENSG00000072110	ACTN1	-3,50	-1,81	1,12E-12	1,65E-11	No DEG
ENSG00000138483	CCDC54	3,50	1,81	2,56E-05	1,31E-04	No DEG
ENSG00000133056	PIK3C2B	-3,50	-1,81	5,26E-16	1,08E-14	No DEG
ENSG00000070423	RNF126	-3,50	-1,81	6,82E-21	2,30E-19	No DEG
ENSG00000224186	C5orf66	-3,50	-1,81	1,28E-09	1,28E-08	No DEG
ENSG00000178055	PRSS42	-3,50	-1,81	3,52E-06	2,08E-05	No DEG
ENSG00000121101	TEX14	-3,50	-1,81	2,85E-04	1,19E-03	No DEG
ENSG00000205544	TMEM256	3,49	1,80	2,41E-15	4,68E-14	No DEG
ENSG00000271347	RP11-701H24.7	3,49	1,80	1,23E-30	9,03E-29	No DEG
ENSG00000093100	XXbac-B461K10.4	-3,49	-1,80	5,39E-04	2,11E-03	No DEG
ENSG00000100146	SOX10	-3,49	-1,80	4,08E-05	2,01E-04	No DEG
ENSG00000138769	CDKL2	3,49	1,80	3,15E-04	1,30E-03	No DEG
ENSG00000198892	SHISA4	-3,49	-1,80	1,07E-13	1,77E-12	No DEG
ENSG00000232684	ATP11A-AS1	-3,49	-1,80	2,66E-04	1,12E-03	No DEG
ENSG00000172382	PRSS27	-3,49	-1,80	3,89E-03	1,24E-02	No DEG
ENSG00000187838	TMEM256-PLSCR3	-3,49	-1,80	8,31E-03	2,42E-02	No DEG
ENSG00000132510	KDM6B	-3,49	-1,80	1,87E-23	8,03E-22	No DEG
ENSG00000055118	KCNH2	-3,49	-1,80	5,82E-18	1,47E-16	No DEG
ENSG00000225234	TRAPPC12-AS1	-3,48	-1,80	6,71E-06	3,79E-05	No DEG
ENSG00000255031	RP11-802E16.3	-3,48	-1,80	5,75E-04	2,24E-03	No DEG
ENSG00000261655	CTD-3064M3.3	-3,48	-1,80	6,16E-04	2,39E-03	No DEG
ENSG00000114353	GNAI2	-3,48	-1,80	1,57E-25	7,89E-24	No DEG
ENSG00000164889	SLC4A2	-3,48	-1,80	3,66E-17	8,55E-16	No DEG
ENSG00000198858	R3HDM4	-3,48	-1,80	9,55E-15	1,76E-13	No DEG
ENSG00000106351	AGFG2	-3,48	-1,80	6,15E-20	1,87E-18	No DEG
ENSG00000204314	PRRT1	-3,48	-1,80	1,05E-03	3,86E-03	No DEG
ENSG00000126603	GLIS2	-3,48	-1,80	1,30E-18	3,49E-17	No DEG
ENSG00000060656	PTPRU	-3,48	-1,80	3,93E-17	9,14E-16	No DEG
ENSG00000175820	CCDC168	3,47	1,80	4,84E-05	2,35E-04	No DEG
ENSG00000260248	RP11-143K11.1	-3,47	-1,80	2,38E-03	8,05E-03	No DEG
ENSG00000157978	LDLRAP1	-3,47	-1,80	4,69E-25	2,28E-23	No DEG
ENSG00000256167	ATF4P4	-3,47	-1,80	9,51E-06	5,25E-05	No DEG

ENSG00000185274	WBSCR17	-3,47	-1,80	8,44E-10	8,64E-09	No DEG
ENSG00000249019	RP11-539G18.1	-3,47	-1,80	3,10E-04	1,28E-03	No DEG
ENSG00000124762	CDKN1A	-3,47	-1,80	1,47E-07	1,09E-06	No DEG
ENSG00000148331	ASB6	-3,47	-1,80	2,83E-31	2,17E-29	No DEG
ENSG00000259582	RP11-461F11.3	3,47	1,80	9,78E-03	2,79E-02	No DEG
ENSG00000109501	WFS1	-3,47	-1,80	1,32E-14	2,40E-13	No DEG
ENSG00000096092	TMEM14A	3,47	1,80	9,33E-20	2,80E-18	No DEG
ENSG00000264940	SNORD3C	3,47	1,80	1,02E-03	3,78E-03	No DEG
ENSG00000033327	GAB2	-3,47	-1,80	2,74E-27	1,60E-25	No DEG
ENSG00000270102	RP11-498E2.8	-3,47	-1,79	4,73E-03	1,48E-02	No DEG
ENSG00000160410	SHKBP1	-3,47	-1,79	1,16E-25	5,90E-24	No DEG
ENSG00000189058	APOD	-3,47	-1,79	9,87E-07	6,39E-06	No DEG
ENSG00000215184	RP11-8L18.2	-3,47	-1,79	6,93E-04	2,66E-03	No DEG
ENSG00000227500	SCAMP4	-3,47	-1,79	1,66E-17	4,05E-16	No DEG
ENSG00000010704	HFE	3,47	1,79	1,84E-17	4,44E-16	No DEG
ENSG00000257258	RP11-946L16.1	3,46	1,79	9,73E-03	2,78E-02	No DEG
ENSG00000145888	GLRA1	-3,46	-1,79	2,25E-03	7,65E-03	No DEG
ENSG00000137818	RPLP1	-3,46	-1,79	3,52E-36	3,74E-34	No DEG
ENSG00000053702	NRIP2	-3,46	-1,79	1,46E-17	3,56E-16	No DEG
ENSG00000108840	HDAC5	-3,46	-1,79	1,85E-29	1,25E-27	No DEG
ENSG00000094914	AAAS	-3,46	-1,79	3,84E-27	2,22E-25	No DEG
ENSG00000269605	Z97053.1	-3,46	-1,79	5,57E-03	1,71E-02	No DEG
ENSG00000252616	AC079140.1	-3,46	-1,79	1,23E-02	3,42E-02	No DEG
ENSG00000101337	TM9SF4	-3,45	-1,79	1,41E-40	1,94E-38	No DEG
ENSG00000181638	ZFP41	-3,45	-1,79	3,83E-14	6,58E-13	No DEG
ENSG00000196700	ZNF512B	-3,45	-1,79	9,49E-33	8,06E-31	No DEG
ENSG00000236285	NPM1P8	-3,45	-1,79	2,43E-07	1,75E-06	No DEG
ENSG00000065491	TBC1D22B	-3,45	-1,79	1,34E-20	4,36E-19	No DEG
ENSG00000144596	GRIP2	-3,45	-1,79	3,25E-12	4,51E-11	No DEG
ENSG00000205517	RGL3	-3,45	-1,79	1,94E-05	1,02E-04	No DEG
ENSG00000091073	DTX2	-3,45	-1,79	6,31E-21	2,13E-19	No DEG
ENSG00000225032	RP11-228B15.4	-3,45	-1,78	2,10E-13	3,37E-12	No DEG

ENSG00000145901	TNIP1	-3,45	-1,78	7,07E-26	3,67E-24	No DEG
ENSG00000223703	AC027612.4	-3,44	-1,78	7,01E-03	2,09E-02	No DEG
ENSG00000103335	PIEZO1	-3,44	-1,78	6,14E-19	1,71E-17	No DEG
ENSG00000151552	QDPR	3,44	1,78	4,58E-11	5,49E-10	No DEG
ENSG00000174292	TNK1	-3,44	-1,78	4,59E-13	7,07E-12	No DEG
ENSG00000267279	RP11-879F14.2	3,44	1,78	4,36E-07	3,00E-06	No DEG
ENSG00000178971	CTC1	-3,44	-1,78	8,70E-23	3,47E-21	No DEG
ENSG00000238286	SLC35E1P1	-3,44	-1,78	6,42E-06	3,64E-05	No DEG
ENSG00000175518	UBQLNL	-3,44	-1,78	6,19E-04	2,40E-03	No DEG
ENSG00000148444	COMMD3	3,44	1,78	1,93E-15	3,78E-14	No DEG
ENSG00000271264	RP11-558F24.6	3,44	1,78	1,38E-02	3,78E-02	No DEG
ENSG00000254552	XIRP2-AS1	3,44	1,78	1,75E-04	7,61E-04	No DEG
ENSG00000181350	FAM211A	-3,44	-1,78	1,57E-08	1,36E-07	No DEG
ENSG00000160013	PTGIR	-3,44	-1,78	7,63E-05	3,56E-04	No DEG
ENSG00000142149	HUNK	-3,44	-1,78	9,43E-05	4,30E-04	No DEG
ENSG00000100258	LMF2	-3,43	-1,78	2,23E-35	2,20E-33	No DEG
ENSG00000268457	AL160175.1	-3,43	-1,78	9,21E-07	5,99E-06	No DEG
ENSG00000164877	MICALL2	-3,43	-1,78	1,27E-08	1,11E-07	No DEG
ENSG00000161914	ZNF653	-3,43	-1,78	1,01E-04	4,59E-04	No DEG
ENSG00000272864	RP11-17E13.2	3,43	1,78	5,08E-03	1,57E-02	No DEG
ENSG00000239306	RBM14	-3,43	-1,78	1,22E-15	2,42E-14	No DEG
ENSG00000164283	ESM1	3,43	1,78	2,21E-03	7,52E-03	No DEG
ENSG00000261342	AC006538.1	-3,43	-1,78	4,22E-04	1,70E-03	No DEG
ENSG00000112214	FHL5	3,43	1,78	2,40E-08	2,02E-07	No DEG
ENSG00000234167	TCEB2P4	-3,43	-1,78	7,31E-03	2,16E-02	No DEG
ENSG00000172548	NIPAL4	-3,43	-1,78	5,83E-04	2,27E-03	No DEG
ENSG00000107105	ELAVL2	3,43	1,78	1,21E-02	3,38E-02	No DEG
ENSG00000258951	KRT18P7	-3,43	-1,78	7,56E-06	4,24E-05	No DEG
ENSG00000179241	LDLRAD3	-3,43	-1,78	2,75E-10	2,99E-09	No DEG
ENSG00000166889	PATL1	-3,42	-1,78	2,26E-42	3,45E-40	No DEG
ENSG00000160284	SPATC1L	-3,42	-1,77	1,47E-06	9,20E-06	No DEG
ENSG00000268155	AL161450.1	3,42	1,77	5,07E-04	2,00E-03	No DEG

ENSG00000231956	HNRNPA1P9	-3,42	-1,77	1,45E-02	3,93E-02	No DEG
ENSG00000185803	SLC52A2	-3,42	-1,77	1,17E-12	1,72E-11	No DEG
ENSG00000188807	TMEM201	-3,42	-1,77	5,30E-27	3,02E-25	No DEG
ENSG00000075886	TUBA3D	-3,42	-1,77	1,32E-03	4,75E-03	DOWN
ENSG00000182873	RP11-181G12.2	-3,42	-1,77	1,48E-05	7,91E-05	No DEG
ENSG00000123989	CHPF	-3,42	-1,77	2,53E-17	6,01E-16	No DEG
ENSG00000152117	AC093838.4	-3,41	-1,77	3,83E-09	3,59E-08	No DEG
ENSG00000188389	PDCD1	-3,41	-1,77	1,00E-08	8,88E-08	No DEG
ENSG00000226625	RP11-290L7.2	-3,41	-1,77	6,71E-03	2,01E-02	No DEG
ENSG00000236180	RP11-163G10.4	3,41	1,77	9,27E-03	2,66E-02	No DEG
ENSG00000172432	GTPBP2	-3,41	-1,77	1,36E-30	9,95E-29	No DEG
ENSG00000179526	SHARPIN	-3,41	-1,77	1,83E-30	1,32E-28	No DEG
ENSG00000228218	ATF4P3	-3,41	-1,77	2,87E-05	1,46E-04	No DEG
ENSG00000130940	CASZ1	-3,41	-1,77	8,08E-31	6,04E-29	No DEG
ENSG00000198416	CTD-2173L22.4	3,41	1,77	1,51E-07	1,12E-06	No DEG
ENSG00000254221	PCDHGB1	-3,41	-1,77	4,19E-14	7,16E-13	No DEG
ENSG00000272142	RP11-428J1.5	-3,40	-1,77	1,01E-03	3,74E-03	No DEG
ENSG00000131669	NINJ1	-3,40	-1,77	9,39E-14	1,56E-12	No DEG
ENSG00000236013	RP3-332B22.1	3,40	1,77	3,53E-09	3,32E-08	No DEG
ENSG00000269352	AC018766.5	-3,40	-1,77	8,80E-06	4,89E-05	No DEG
ENSG00000162391	FAM151A	-3,40	-1,77	4,53E-10	4,81E-09	No DEG
ENSG00000235280	MCF2L-AS1	-3,40	-1,77	1,26E-03	4,56E-03	No DEG
ENSG00000272717	RP11-342I1.2	-3,40	-1,77	5,91E-04	2,30E-03	No DEG
ENSG00000169710	FASN	-3,40	-1,77	1,12E-11	1,45E-10	No DEG
ENSG00000177508	IRX3	-3,40	-1,77	1,83E-07	1,34E-06	DOWN
ENSG00000252009	SNORD112	3,40	1,77	6,87E-03	2,05E-02	No DEG
ENSG00000232043	RP4-530I15.9	-3,40	-1,77	3,57E-07	2,49E-06	No DEG
ENSG00000221886	C5orf54	3,40	1,77	6,31E-12	8,46E-11	No DEG
ENSG00000171159	C9orf16	-3,40	-1,77	6,61E-38	7,83E-36	No DEG
ENSG00000183340	JRKL	3,40	1,76	5,27E-20	1,61E-18	No DEG
ENSG00000118432	CNR1	3,40	1,76	1,05E-06	6,77E-06	UP
ENSG00000012223	LTF	-3,40	-1,76	3,67E-07	2,56E-06	No DEG

ENSG00000232803	RP11-93B14.5	-3,40	-1,76	3,76E-03	1,20E-02	No DEG
ENSG00000069188	SDK2	-3,39	-1,76	1,77E-07	1,30E-06	No DEG
ENSG00000224940	PRRT4	-3,39	-1,76	7,58E-04	2,88E-03	No DEG
ENSG00000186889	TMEM17	3,39	1,76	8,03E-10	8,26E-09	No DEG
ENSG00000100647	KIAA0247	-3,39	-1,76	1,48E-20	4,81E-19	No DEG
ENSG00000126391	FRMD8	-3,39	-1,76	1,98E-13	3,18E-12	No DEG
ENSG00000140067	FAM181A	-3,39	-1,76	5,81E-03	1,77E-02	No DEG
ENSG00000101187	SLCO4A1	-3,39	-1,76	4,67E-04	1,86E-03	No DEG
ENSG00000244242	IFITM10	-3,39	-1,76	6,33E-04	2,45E-03	No DEG
ENSG00000106665	CLIP2	-3,39	-1,76	3,44E-14	5,95E-13	No DEG
ENSG00000151640	DPYSL4	-3,39	-1,76	2,02E-09	1,97E-08	No DEG
ENSG00000223972	DDX11L1	-3,39	-1,76	1,12E-02	3,15E-02	No DEG
ENSG00000175229	GAL3ST3	-3,39	-1,76	2,02E-04	8,67E-04	No DEG
ENSG00000167994	RAB3IL1	-3,39	-1,76	3,73E-16	7,82E-15	No DEG
ENSG00000186575	NF2	-3,39	-1,76	1,31E-46	2,74E-44	No DEG
ENSG00000212579	SNORA40	3,39	1,76	4,72E-04	1,88E-03	No DEG
ENSG00000189343	RPS2P46	3,38	1,76	1,76E-13	2,85E-12	No DEG
ENSG00000269293	ZSCAN16-AS1	3,38	1,76	6,14E-13	9,32E-12	No DEG
ENSG00000076924	XAB2	-3,38	-1,76	7,05E-28	4,29E-26	No DEG
ENSG00000247877	CTD-2001C12.1	3,38	1,76	3,60E-03	1,16E-02	No DEG
ENSG00000254328	CTC-308K20.4	-3,38	-1,76	1,01E-02	2,87E-02	No DEG
ENSG00000165682	CLEC1B	3,38	1,76	5,27E-03	1,63E-02	UP
ENSG00000241878	PISD	-3,38	-1,76	1,44E-17	3,52E-16	No DEG
ENSG00000207574	MIR661	-3,37	-1,75	9,97E-03	2,84E-02	No DEG
ENSG00000130749	ZC3H4	-3,37	-1,75	9,46E-34	8,54E-32	No DEG
ENSG00000144589	STK11IP	-3,37	-1,75	7,14E-37	7,88E-35	No DEG
ENSG00000156574	NODAL	-3,37	-1,75	8,46E-04	3,18E-03	No DEG
ENSG00000262576	PCDHGA4	-3,37	-1,75	4,53E-21	1,56E-19	No DEG
ENSG00000221988	PPT2	-3,37	-1,75	6,60E-23	2,67E-21	No DEG
ENSG00000177103	DSCAML1	-3,37	-1,75	6,63E-11	7,79E-10	No DEG
ENSG00000238041	RP11-59K5.1	-3,37	-1,75	1,00E-02	2,86E-02	No DEG
ENSG00000258708	SLC25A21-AS1	3,37	1,75	5,11E-05	2,47E-04	No DEG

ENSG00000230316	FEZF1-AS1	3,37	1,75	2,45E-03	8,26E-03	No DEG
ENSG00000140854	KATNB1	-3,37	-1,75	1,55E-16	3,37E-15	No DEG
ENSG00000246375	RP11-10L7.1	3,37	1,75	3,29E-05	1,66E-04	UP
ENSG00000101220	C20orf27	-3,37	-1,75	4,32E-14	7,37E-13	No DEG
ENSG00000213699	SLC35F6	-3,37	-1,75	1,71E-30	1,24E-28	No DEG
ENSG00000221247	AC136704.1	-3,36	-1,75	1,56E-02	4,18E-02	No DEG
ENSG00000170323	FABP4	3,36	1,75	1,69E-10	1,89E-09	No DEG
ENSG00000206646	Y_RNA	-3,36	-1,75	9,45E-03	2,71E-02	No DEG
ENSG00000159335	PTMS	-3,36	-1,75	2,17E-21	7,61E-20	No DEG
ENSG00000236182	RP11-297K7.1	-3,36	-1,75	9,31E-03	2,67E-02	No DEG
ENSG00000159842	ABR	-3,36	-1,75	6,93E-21	2,33E-19	No DEG
ENSG00000178307	TMEM11	-3,36	-1,75	5,41E-14	9,15E-13	No DEG
ENSG00000109163	GNRHR	3,36	1,75	1,10E-05	6,04E-05	No DEG
ENSG00000185359	HGS	-3,36	-1,75	3,73E-35	3,61E-33	No DEG
ENSG00000170385	SLC30A1	3,36	1,75	1,01E-11	1,32E-10	No DEG
ENSG00000197584	KCNMB2	3,36	1,75	5,70E-07	3,84E-06	No DEG
ENSG00000263499	RP11-118E18.4	-3,36	-1,75	8,65E-04	3,25E-03	No DEG
ENSG00000157557	ETS2	-3,36	-1,75	8,59E-09	7,68E-08	No DEG
ENSG00000106366	SERPINE1	-3,36	-1,75	9,41E-03	2,70E-02	No DEG
ENSG00000262903	RP11-235E17.6	-3,36	-1,75	1,64E-06	1,02E-05	No DEG
ENSG00000100350	FOXRED2	-3,36	-1,75	1,65E-10	1,84E-09	No DEG
ENSG00000141540	TTYH2	-3,36	-1,75	2,56E-12	3,60E-11	No DEG
ENSG00000160062	ZBTB8A	3,36	1,75	3,78E-14	6,50E-13	No DEG
ENSG00000160404	TOR2A	-3,35	-1,75	3,04E-07	2,15E-06	No DEG
ENSG00000182836	PLCXD3	3,35	1,75	2,71E-10	2,95E-09	No DEG
ENSG00000210156	MT-TK	-3,35	-1,75	5,69E-03	1,74E-02	No DEG
ENSG00000149577	SIDT2	-3,35	-1,75	2,45E-19	7,04E-18	No DEG
ENSG00000100311	PDGFB	-3,35	-1,75	1,34E-16	2,93E-15	No DEG
ENSG00000100403	ZC3H7B	-3,35	-1,74	5,33E-34	4,88E-32	No DEG
ENSG00000171223	JUNB	-3,35	-1,74	1,29E-03	4,66E-03	No DEG
ENSG00000108523	RNF167	-3,35	-1,74	7,20E-27	4,04E-25	No DEG
ENSG00000066322	ELOVL1	-3,35	-1,74	4,84E-14	8,23E-13	No DEG

ENSG00000183751	TBL3	-3,35	-1,74	2,54E-32	2,09E-30	No DEG
ENSG00000186918	ZNF395	-3,35	-1,74	1,31E-16	2,87E-15	No DEG
ENSG00000229739	RP11-295K2.3	3,35	1,74	1,10E-03	4,04E-03	No DEG
ENSG00000102103	PQBP1	-3,35	-1,74	3,36E-20	1,05E-18	No DEG
ENSG00000211445	GPX3	-3,35	-1,74	1,22E-25	6,18E-24	No DEG
ENSG00000261008	AC004158.2	3,35	1,74	6,39E-03	1,92E-02	UP
ENSG00000127399	LRRC61	-3,35	-1,74	4,57E-08	3,68E-07	No DEG
ENSG00000166925	TSC22D4	-3,35	-1,74	2,20E-23	9,40E-22	No DEG
ENSG00000172000	ZNF556	3,34	1,74	1,31E-02	3,60E-02	No DEG
ENSG00000186583	SPATC1	-3,34	-1,74	2,55E-04	1,07E-03	No DEG
ENSG00000183153	GJD3	3,34	1,74	1,26E-02	3,47E-02	No DEG
ENSG00000254909	RP11-110I1.5	-3,34	-1,74	1,91E-04	8,26E-04	No DEG
ENSG00000085872	CHERP	-3,34	-1,74	3,89E-22	1,45E-20	No DEG
ENSG00000070182	SPTB	-3,34	-1,74	3,59E-19	1,02E-17	No DEG
ENSG00000268279	RP11-434D12.1	-3,34	-1,74	6,36E-05	3,01E-04	No DEG
ENSG00000189367	KIAA0408	3,34	1,74	1,98E-03	6,82E-03	No DEG
ENSG00000073734	ABCB11	3,34	1,74	8,06E-03	2,36E-02	UP
ENSG00000164970	FAM219A	-3,34	-1,74	4,12E-31	3,14E-29	No DEG
ENSG00000248275	TRIM52-AS1	-3,34	-1,74	2,40E-07	1,73E-06	No DEG
ENSG00000204160	ZDHHC18	-3,34	-1,74	1,59E-18	4,22E-17	No DEG
ENSG00000256268	RP11-221N13.3	3,34	1,74	3,56E-03	1,15E-02	No DEG
ENSG00000204771	CTD-3232M19.2	-3,34	-1,74	1,97E-03	6,80E-03	No DEG
ENSG00000065320	NTN1	-3,34	-1,74	1,73E-06	1,07E-05	DOWN
ENSG00000184343	SRPK3	-3,33	-1,74	9,26E-15	1,71E-13	No DEG
ENSG00000133243	BTBD2	-3,33	-1,74	5,01E-31	3,80E-29	No DEG
ENSG00000122140	MRPS2	-3,33	-1,74	2,31E-28	1,46E-26	No DEG
ENSG00000005249	PRKAR2B	3,33	1,74	1,72E-12	2,48E-11	No DEG
ENSG00000139645	ANKRD52	-3,33	-1,74	1,69E-44	3,03E-42	No DEG
ENSG00000236466	RP11-795J1.1	3,33	1,74	3,52E-06	2,08E-05	No DEG
ENSG00000242719	RN7SL806P	-3,33	-1,74	1,35E-02	3,69E-02	No DEG
ENSG00000181444	ZNF467	-3,33	-1,74	2,78E-09	2,65E-08	No DEG
ENSG00000153130	SCOC	3,33	1,74	6,16E-21	2,09E-19	No DEG

ENSG00000105137	SYDE1	-3,33	-1,74	6,94E-44	1,16E-41	No DEG
ENSG00000165507	C10orf10	-3,33	-1,74	3,03E-06	1,81E-05	No DEG
ENSG00000188827	SLX4	-3,33	-1,73	1,09E-21	3,92E-20	No DEG
ENSG00000148841	ITPRIP	-3,33	-1,73	2,58E-09	2,47E-08	No DEG
ENSG00000258040	AC005086.3	-3,33	-1,73	1,24E-02	3,45E-02	No DEG
ENSG00000196169	KIF19	-3,33	-1,73	3,44E-03	1,12E-02	No DEG
ENSG00000167978	SRRM2	-3,33	-1,73	1,00E-26	5,56E-25	No DEG
ENSG00000267541	RP11-380M21.2	-3,33	-1,73	4,05E-07	2,80E-06	No DEG
ENSG00000207863	MIR125B2	3,33	1,73	9,42E-03	2,70E-02	No DEG
ENSG00000266969	RP11-773H22.4	3,32	1,73	7,81E-03	2,29E-02	No DEG
ENSG00000076555	ACACB	-3,32	-1,73	6,39E-25	3,07E-23	No DEG
ENSG00000128346	C22orf23	-3,32	-1,73	4,48E-18	1,14E-16	No DEG
ENSG00000083454	P2RX5	-3,32	-1,73	3,15E-09	2,98E-08	No DEG
ENSG00000183337	BCOR	-3,32	-1,73	4,65E-17	1,07E-15	No DEG
ENSG00000106823	ECM2	3,32	1,73	8,18E-10	8,40E-09	No DEG
ENSG00000170312	CDK1	3,32	1,73	3,33E-06	1,98E-05	No DEG
ENSG00000256282	RP11-504G3.4	-3,32	-1,73	3,16E-04	1,31E-03	No DEG
ENSG00000235532	LINC00402	3,32	1,73	1,45E-03	5,15E-03	UP
ENSG00000223518	CSNK1A1P1	-3,32	-1,73	6,43E-06	3,64E-05	No DEG
ENSG00000198959	TGM2	-3,32	-1,73	1,32E-11	1,70E-10	No DEG
ENSG00000265053	RN7SL321P	3,32	1,73	1,39E-02	3,80E-02	No DEG
ENSG00000267326	AC015849.14	3,32	1,73	1,25E-02	3,46E-02	No DEG
ENSG00000203709	C1orf132	-3,32	-1,73	1,07E-11	1,40E-10	No DEG
ENSG00000114867	EIF4G1	-3,32	-1,73	1,45E-47	3,27E-45	No DEG
ENSG00000266980	RP11-552F3.4	-3,31	-1,73	1,26E-02	3,48E-02	No DEG
ENSG00000099994	SUSD2	-3,31	-1,73	4,36E-05	2,13E-04	No DEG
ENSG00000132017	DCAF15	-3,31	-1,73	9,90E-23	3,93E-21	No DEG
ENSG00000142798	HSPG2	-3,31	-1,73	3,93E-18	1,01E-16	No DEG
ENSG00000160767	FAM189B	-3,31	-1,73	2,04E-17	4,94E-16	No DEG
ENSG00000251229	RP11-503N18.5	-3,31	-1,73	1,67E-03	5,87E-03	No DEG
ENSG00000183506	PI4KAP2	-3,31	-1,73	1,52E-12	2,21E-11	No DEG
ENSG00000272308	RP11-231G3.1	3,31	1,73	4,82E-03	1,50E-02	No DEG

ENSG00000106819	ASPN	3,31	1,73	1,19E-06	7,59E-06	No DEG
ENSG00000072062	PRKACA	-3,31	-1,73	1,69E-30	1,22E-28	No DEG
ENSG00000218226	TATDN2P2	-3,30	-1,72	5,12E-07	3,48E-06	No DEG
ENSG00000271811	RP1-79C4.4	-3,30	-1,72	1,79E-06	1,11E-05	No DEG
ENSG00000197122	SRC	-3,30	-1,72	7,19E-12	9,56E-11	No DEG
ENSG00000038358	EDC4	-3,30	-1,72	2,10E-43	3,40E-41	No DEG
ENSG00000129946	SHC2	-3,30	-1,72	5,52E-11	6,55E-10	No DEG
ENSG00000141736	ERBB2	-3,30	-1,72	6,41E-42	9,54E-40	No DEG
ENSG00000112715	VEGFA	-3,30	-1,72	3,56E-11	4,35E-10	No DEG
ENSG00000108175	ZMIZ1	-3,30	-1,72	7,42E-31	5,58E-29	No DEG
ENSG00000111206	FOXM1	-3,30	-1,72	9,08E-04	3,40E-03	No DEG
ENSG00000237073	RP11-168K11.2	-3,30	-1,72	8,69E-04	3,26E-03	No DEG
ENSG00000267283	AC005306.3	-3,30	-1,72	2,13E-03	7,28E-03	No DEG
ENSG00000136944	LMX1B	-3,30	-1,72	1,22E-06	7,75E-06	No DEG
ENSG00000256538	RP11-847H18.3	3,30	1,72	1,40E-02	3,81E-02	No DEG
ENSG00000235034	C19orf81	3,30	1,72	5,07E-03	1,57E-02	No DEG
ENSG00000135636	DYSF	-3,30	-1,72	4,93E-36	5,18E-34	No DEG
ENSG00000166682	TMPRSS5	-3,30	-1,72	3,98E-06	2,33E-05	No DEG
ENSG00000256176	RP11-627K11.3	-3,29	-1,72	2,43E-03	8,20E-03	No DEG
ENSG00000166484	MAPK7	-3,29	-1,72	1,56E-16	3,37E-15	No DEG
ENSG00000136367	ZFHX2	-3,29	-1,72	3,14E-08	2,59E-07	No DEG
ENSG00000080031	PTPRH	-3,29	-1,72	2,96E-05	1,50E-04	DOWN
ENSG00000177191	B3GNT8	-3,29	-1,72	1,32E-06	8,37E-06	No DEG
ENSG00000100083	GGA1	-3,29	-1,72	9,64E-50	2,64E-47	No DEG
ENSG00000126062	TMEM115	-3,29	-1,72	2,35E-23	1,00E-21	No DEG
ENSG00000173402	DAG1	-3,29	-1,72	4,21E-44	7,26E-42	No DEG
ENSG00000171051	FPR1	-3,29	-1,72	6,69E-04	2,57E-03	No DEG
ENSG00000168758	SEMA4C	-3,29	-1,72	1,10E-17	2,71E-16	No DEG
ENSG00000160584	SIK3	-3,29	-1,72	1,41E-24	6,62E-23	No DEG
ENSG00000201574	RNU1-93P	3,29	1,72	1,29E-02	3,56E-02	No DEG
ENSG00000237438	CECR7	-3,29	-1,72	3,00E-04	1,24E-03	No DEG
ENSG00000103326	CAPN15	-3,29	-1,72	1,79E-18	4,74E-17	No DEG

ENSG00000178951	ZBTB7A	-3,29	-1,72	1,64E-28	1,05E-26	No DEG
ENSG00000174527	MYO1H	-3,29	-1,72	3,87E-05	1,92E-04	No DEG
ENSG00000142002	DPP9	-3,28	-1,72	7,45E-22	2,72E-20	No DEG
ENSG00000111540	RAB5B	-3,28	-1,72	1,70E-46	3,47E-44	No DEG
ENSG00000261198	RP11-26O3.1	-3,28	-1,71	4,65E-03	1,46E-02	No DEG
ENSG00000204444	APOM	-3,28	-1,71	3,39E-10	3,65E-09	No DEG
ENSG00000095397	DFNB31	-3,28	-1,71	3,23E-04	1,33E-03	No DEG
ENSG00000141012	GALNS	-3,28	-1,71	2,25E-17	5,39E-16	No DEG
ENSG00000008441	NFIX	-3,28	-1,71	2,36E-19	6,79E-18	No DEG
ENSG00000127419	TMEM175	-3,28	-1,71	2,19E-20	6,99E-19	No DEG
ENSG00000163132	MSX1	-3,28	-1,71	6,78E-07	4,52E-06	DOWN
ENSG00000101460	MAP1LC3A	-3,28	-1,71	3,38E-19	9,65E-18	No DEG
ENSG00000134817	APLNR	-3,28	-1,71	2,94E-04	1,22E-03	No DEG
ENSG00000113249	HAVCR1	-3,27	-1,71	1,02E-02	2,91E-02	No DEG
ENSG00000144891	AGTR1	3,27	1,71	4,36E-07	3,00E-06	No DEG
ENSG00000235763	SNRPGP5	-3,27	-1,71	9,17E-03	2,64E-02	No DEG
ENSG00000107816	LZTS2	-3,27	-1,71	2,09E-24	9,64E-23	No DEG
ENSG00000187800	PEAR1	-3,27	-1,71	5,76E-24	2,58E-22	No DEG
ENSG00000167600	CYP2S1	-3,27	-1,71	9,65E-04	3,59E-03	No DEG
ENSG00000068971	PPP2R5B	-3,27	-1,71	8,57E-23	3,43E-21	No DEG
ENSG00000184232	OAF	-3,27	-1,71	2,33E-11	2,91E-10	No DEG
ENSG00000107862	GBF1	-3,26	-1,71	8,05E-31	6,04E-29	No DEG
ENSG00000242028	HYPK	-3,26	-1,71	2,36E-07	1,70E-06	No DEG
ENSG00000199030	MIRLET7C	3,26	1,71	2,49E-03	8,36E-03	No DEG
ENSG00000068120	COASY	-3,26	-1,71	1,43E-20	4,67E-19	No DEG
ENSG00000267257	RP11-1151B14.4	-3,26	-1,71	3,19E-03	1,04E-02	No DEG
ENSG00000240970	RPL23AP64	-3,26	-1,71	1,24E-05	6,74E-05	No DEG
ENSG00000255200	AP003068.18	-3,26	-1,70	8,39E-05	3,88E-04	No DEG
ENSG00000130827	PLXNA3	-3,26	-1,70	3,19E-09	3,01E-08	No DEG
ENSG00000104725	NEFL	3,26	1,70	1,48E-02	4,01E-02	No DEG
ENSG00000165282	PIGO	-3,26	-1,70	1,42E-22	5,56E-21	No DEG
ENSG00000213055	AC064852.5	-3,26	-1,70	2,69E-05	1,37E-04	No DEG

ENSG00000215104	CHMP1B2P	3,26	1,70	3,97E-04	1,61E-03	No DEG
ENSG00000198133	TMEM229B	-3,26	-1,70	5,25E-08	4,19E-07	No DEG
ENSG00000245532	NEAT1	-3,25	-1,70	6,73E-04	2,59E-03	No DEG
ENSG00000156136	DCK	3,25	1,70	3,38E-18	8,70E-17	No DEG
ENSG00000223459	FAM115B	-3,25	-1,70	9,78E-17	2,18E-15	No DEG
ENSG00000149972	CNTN5	3,25	1,70	1,08E-02	3,05E-02	No DEG
ENSG00000272654	RP11-422P24.11	3,25	1,70	5,39E-06	3,09E-05	No DEG
ENSG00000160325	CACFD1	-3,25	-1,70	2,63E-18	6,82E-17	No DEG
ENSG00000251322	SHANK3	-3,25	-1,70	4,54E-20	1,40E-18	No DEG
ENSG00000214391	RP11-121L10.3	-3,25	-1,70	6,34E-05	3,01E-04	No DEG
ENSG00000164818	HEATR2	-3,25	-1,70	9,13E-22	3,31E-20	No DEG
ENSG00000147119	CHST7	3,25	1,70	5,63E-10	5,91E-09	No DEG
ENSG00000166183	ASPG	-3,25	-1,70	6,24E-08	4,93E-07	No DEG
ENSG00000186446	ZNF501	3,25	1,70	4,48E-08	3,61E-07	No DEG
ENSG00000149532	CPSF7	-3,24	-1,70	4,31E-42	6,44E-40	No DEG
ENSG00000182500	ORAI1	-3,24	-1,70	3,23E-12	4,48E-11	No DEG
ENSG00000236867	RP3-522J7.5	-3,24	-1,70	2,93E-03	9,68E-03	No DEG
ENSG00000230649	AC024084.1	3,24	1,70	1,47E-02	3,98E-02	UP
ENSG00000266651	RP11-138I1.3	-3,24	-1,70	7,49E-03	2,21E-02	No DEG
ENSG00000261586	RP11-923I11.6	-3,24	-1,70	4,98E-04	1,97E-03	No DEG
ENSG00000185085	INTS5	-3,24	-1,70	3,88E-29	2,57E-27	No DEG
ENSG00000197093	GAL3ST4	-3,24	-1,70	1,27E-05	6,89E-05	No DEG
ENSG00000236208	C10orf71-AS1	3,24	1,69	8,68E-07	5,67E-06	No DEG
ENSG00000224460	RP11-439L18.2	-3,24	-1,69	2,61E-05	1,33E-04	No DEG
ENSG00000263723	SNORD39	-3,24	-1,69	6,65E-03	1,99E-02	No DEG
ENSG00000154358	OBSCN	-3,23	-1,69	9,79E-23	3,90E-21	No DEG
ENSG00000146701	MDH2	-3,23	-1,69	8,85E-24	3,92E-22	No DEG
ENSG00000142327	RNPEPL1	-3,23	-1,69	6,61E-23	2,67E-21	No DEG
ENSG00000267160	RP11-1072C15.4	3,23	1,69	7,38E-05	3,45E-04	No DEG
ENSG00000081320	STK17B	3,23	1,69	2,09E-13	3,35E-12	UP
ENSG00000212054	AL354764.1	3,23	1,69	1,36E-02	3,73E-02	No DEG
ENSG00000236478	AC012513.4	-3,23	-1,69	1,73E-05	9,14E-05	No DEG

ENSG00000157502	MUM1L1	3,23	1,69	2,95E-09	2,80E-08	No DEG
ENSG00000094963	FMO2	3,23	1,69	8,96E-09	7,98E-08	No DEG
ENSG00000187922	LCN10	-3,23	-1,69	8,07E-04	3,05E-03	No DEG
ENSG00000204618	RNF39	-3,23	-1,69	9,99E-05	4,55E-04	No DEG
ENSG00000230626	RP11-286H14.4	-3,22	-1,69	3,36E-04	1,38E-03	No DEG
ENSG00000229539	RP11-119B16.2	-3,22	-1,69	5,54E-04	2,17E-03	No DEG
ENSG00000105894	PTN	3,22	1,69	3,49E-09	3,29E-08	No DEG
ENSG00000223949	RP11-24J23.2	-3,22	-1,69	1,14E-03	4,18E-03	DOWN
ENSG00000102996	MMP15	-3,22	-1,69	1,69E-07	1,24E-06	No DEG
ENSG00000104825	NFKBIB	-3,22	-1,69	1,73E-17	4,20E-16	No DEG
ENSG00000127580	WDR24	-3,22	-1,69	4,74E-21	1,63E-19	No DEG
ENSG00000233292	RP11-341B24.3	-3,22	-1,69	1,64E-02	4,37E-02	No DEG
ENSG00000236801	RPL24P8	3,22	1,69	7,25E-09	6,53E-08	No DEG
ENSG00000026036	RTEL1-TNFRSF6B	-3,22	-1,69	6,58E-08	5,17E-07	No DEG
ENSG00000137337	MDC1	-3,22	-1,69	1,70E-18	4,49E-17	No DEG
ENSG00000114805	PLCH1	3,22	1,69	1,89E-04	8,15E-04	No DEG
ENSG00000013619	MAMLD1	-3,22	-1,69	5,84E-10	6,11E-09	No DEG
ENSG00000255776	RP11-436I9.3	3,22	1,69	1,35E-02	3,71E-02	UP
ENSG00000267801	RP11-552F3.9	-3,22	-1,69	1,34E-04	5,92E-04	No DEG
ENSG00000264304	RP11-20B24.7	-3,21	-1,68	1,45E-02	3,92E-02	No DEG
ENSG00000110848	CD69	3,21	1,68	1,73E-04	7,53E-04	UP
ENSG00000151364	KCTD14	-3,21	-1,68	1,62E-02	4,31E-02	No DEG
ENSG00000246575	AC093162.5	-3,21	-1,68	1,83E-05	9,65E-05	No DEG
ENSG00000116221	MRPL37	-3,21	-1,68	3,00E-37	3,38E-35	No DEG
ENSG00000204569	PPP1R10	-3,21	-1,68	2,39E-26	1,28E-24	No DEG
ENSG00000234004	RP11-543B16.1	3,21	1,68	1,30E-04	5,76E-04	No DEG
ENSG00000011009	LYPLA2	-3,21	-1,68	1,49E-21	5,30E-20	No DEG
ENSG00000236540	AC006547.13	-3,21	-1,68	3,46E-10	3,71E-09	No DEG
ENSG00000204428	LY6G5C	-3,21	-1,68	3,01E-06	1,80E-05	No DEG
ENSG00000249868	RP11-63E5.6	-3,21	-1,68	1,87E-08	1,60E-07	No DEG
ENSG00000164920	OSR2	-3,21	-1,68	2,03E-06	1,24E-05	No DEG
ENSG00000100429	HDAC10	-3,21	-1,68	1,40E-03	5,00E-03	No DEG

ENSG00000188596	C12orf55	3,21	1,68	6,05E-08	4,79E-07	UP
ENSG00000102030	NAA10	-3,21	-1,68	5,62E-16	1,15E-14	No DEG
ENSG00000239677	PDZRN3-AS1	3,21	1,68	8,26E-09	7,39E-08	No DEG
ENSG00000140691	ARMC5	-3,21	-1,68	6,25E-12	8,40E-11	No DEG
ENSG00000164932	CTHRC1	3,21	1,68	2,06E-11	2,59E-10	No DEG
ENSG00000165821	SALL2	-3,21	-1,68	3,05E-07	2,16E-06	No DEG
ENSG00000152931	PART1	3,21	1,68	2,23E-05	1,15E-04	No DEG
ENSG00000256705	CTD-2017C7.1	-3,20	-1,68	5,37E-05	2,58E-04	No DEG
ENSG00000171612	SLC25A33	3,20	1,68	7,57E-10	7,80E-09	No DEG
ENSG00000106178	CCL24	3,20	1,68	5,45E-03	1,67E-02	UP
ENSG00000179604	CDC42EP4	-3,20	-1,68	1,74E-11	2,21E-10	No DEG
ENSG00000186716	BCR	-3,20	-1,68	5,31E-15	9,98E-14	No DEG
ENSG00000244384	RN7SL359P	3,20	1,68	6,49E-03	1,95E-02	No DEG
ENSG00000134824	FADS2	-3,20	-1,68	6,71E-08	5,26E-07	No DEG
ENSG00000224097	RP11-472B18.1	3,20	1,68	3,65E-03	1,17E-02	No DEG
ENSG00000178662	CSRNP3	3,20	1,68	2,49E-06	1,51E-05	No DEG
ENSG00000160781	PAQR6	-3,20	-1,68	1,86E-06	1,15E-05	No DEG
ENSG00000235888	AF064858.8	-3,20	-1,68	2,08E-03	7,14E-03	No DEG
ENSG00000261114	RP11-325K4.2	3,20	1,68	3,02E-04	1,25E-03	No DEG
ENSG00000146476	C6orf211	3,20	1,68	1,63E-16	3,53E-15	No DEG
ENSG00000062485	CS	-3,20	-1,68	6,25E-38	7,43E-36	No DEG
ENSG00000127452	FBXL12	-3,20	-1,68	2,53E-21	8,82E-20	No DEG
ENSG00000149115	TNKS1BP1	-3,20	-1,68	1,43E-18	3,82E-17	No DEG
ENSG00000223414	LINC00473	-3,19	-1,68	9,13E-05	4,18E-04	DOWN
ENSG00000251399	RP11-234K19.1	3,19	1,68	6,61E-04	2,54E-03	No DEG
ENSG00000166323	C11orf65	3,19	1,67	6,16E-09	5,60E-08	No DEG
ENSG00000204758	CTC-308K20.1	-3,19	-1,67	4,65E-03	1,46E-02	No DEG
ENSG00000105366	SIGLEC8	3,19	1,67	1,25E-02	3,47E-02	UP
ENSG00000169188	APEX2	-3,19	-1,67	6,39E-23	2,60E-21	No DEG
ENSG00000145194	ECE2	-3,19	-1,67	4,53E-06	2,63E-05	No DEG
ENSG00000214222	TUBBP2	-3,19	-1,67	1,93E-03	6,66E-03	No DEG
ENSG00000126767	ELK1	-3,19	-1,67	2,59E-20	8,20E-19	No DEG

ENSG00000267848	AL118506.1	-3,19	-1,67	6,60E-03	1,98E-02	No DEG
ENSG00000229622	MTND5P2	-3,19	-1,67	1,39E-02	3,81E-02	No DEG
ENSG00000214655	ZSWIM8	-3,19	-1,67	9,45E-46	1,86E-43	No DEG
ENSG00000103043	VAC14	-3,19	-1,67	9,79E-31	7,26E-29	No DEG
ENSG00000259212	CTD-3065B20.2	-3,19	-1,67	1,29E-05	6,97E-05	No DEG
ENSG00000266124	MIR5587	-3,18	-1,67	1,07E-02	3,03E-02	No DEG
ENSG00000104967	NOVA2	-3,18	-1,67	1,41E-14	2,55E-13	No DEG
ENSG00000183426	NPIPA1	-3,18	-1,67	1,06E-07	8,03E-07	No DEG
ENSG00000228384	AC007040.6	-3,18	-1,67	1,36E-02	3,72E-02	No DEG
ENSG00000104879	CKM	-3,18	-1,67	5,74E-12	7,76E-11	No DEG
ENSG00000137207	YIPF3	-3,18	-1,67	5,36E-35	5,14E-33	No DEG
ENSG00000125731	SH2D3A	-3,18	-1,67	2,83E-06	1,70E-05	No DEG
ENSG00000115107	STEAP3	-3,18	-1,67	2,80E-11	3,46E-10	No DEG
ENSG00000243854	RN7SL67P	-3,18	-1,67	7,19E-05	3,38E-04	No DEG
ENSG00000114770	ABCC5	-3,18	-1,67	1,85E-10	2,05E-09	No DEG
ENSG00000235731	AC124997.1	-3,18	-1,67	3,50E-03	1,13E-02	No DEG
ENSG00000171388	APLN	-3,17	-1,67	9,37E-04	3,49E-03	No DEG
ENSG00000264932	Y_RNA	-3,17	-1,67	3,84E-03	1,23E-02	No DEG
ENSG00000273352	RP11-61L19.3	3,17	1,67	1,78E-03	6,19E-03	No DEG
ENSG00000237017	AC012314.8	-3,17	-1,67	1,39E-03	4,95E-03	No DEG
ENSG00000267612	CTD-3116E22.7	3,17	1,67	1,08E-02	3,04E-02	No DEG
ENSG00000127774	EMC6	3,17	1,67	5,60E-06	3,20E-05	No DEG
ENSG00000143862	ARL8A	-3,17	-1,67	5,07E-24	2,28E-22	No DEG
ENSG00000123374	CDK2	-3,17	-1,67	7,66E-07	5,04E-06	No DEG
ENSG00000182541	LIMK2	-3,17	-1,67	6,97E-16	1,43E-14	No DEG
ENSG00000150681	RGS18	3,17	1,66	1,82E-08	1,56E-07	UP
ENSG00000130985	UBA1	-3,17	-1,66	1,02E-34	9,75E-33	No DEG
ENSG00000215513	PI4KAP1	-3,17	-1,66	1,81E-04	7,86E-04	No DEG
ENSG00000228594	C1orf233	-3,17	-1,66	6,09E-05	2,90E-04	No DEG
ENSG00000259935	RP11-519C12.1	3,17	1,66	6,48E-04	2,50E-03	No DEG
ENSG00000261396	CTD-2012K14.2	-3,17	-1,66	1,08E-02	3,04E-02	No DEG
ENSG00000105655	ISYNA1	-3,17	-1,66	2,06E-10	2,27E-09	No DEG

ENSG00000164675	IQUB	3,17	1,66	4,06E-06	2,38E-05	No DEG
ENSG00000197153	HIST1H3J	3,17	1,66	5,26E-03	1,62E-02	UP
ENSG00000164142	FAM160A1	-3,16	-1,66	1,72E-22	6,65E-21	No DEG
ENSG00000134030	CTIF	-3,16	-1,66	3,31E-23	1,39E-21	No DEG
ENSG00000134343	ANO3	3,16	1,66	5,20E-03	1,61E-02	No DEG
ENSG00000132768	DPH2	-3,16	-1,66	2,45E-14	4,29E-13	No DEG
ENSG00000104964	AES	-3,16	-1,66	8,77E-28	5,28E-26	No DEG
ENSG00000181035	SLC25A42	-3,16	-1,66	9,21E-15	1,70E-13	No DEG
ENSG00000204967	PCDHA4	3,16	1,66	4,91E-03	1,53E-02	No DEG
ENSG00000256222	MTRNR2L3	-3,16	-1,66	1,04E-04	4,73E-04	No DEG
ENSG00000186594	MIR22HG	-3,16	-1,66	2,78E-06	1,67E-05	No DEG
ENSG00000262884	CTD-3060P21.1	-3,16	-1,66	6,04E-03	1,83E-02	No DEG
ENSG00000255320	RP11-755F10.1	-3,16	-1,66	1,79E-03	6,23E-03	No DEG
ENSG00000204740	MALRD1	3,16	1,66	1,77E-06	1,09E-05	No DEG
ENSG00000267701	RP11-28F1.2	3,16	1,66	2,43E-03	8,18E-03	No DEG
ENSG00000130821	SLC6A8	-3,16	-1,66	5,89E-25	2,84E-23	No DEG
ENSG00000172638	EFEMP2	-3,16	-1,66	1,49E-28	9,51E-27	No DEG
ENSG00000135083	CCNJL	-3,16	-1,66	4,35E-07	3,00E-06	No DEG
ENSG00000090857	PDPR	-3,16	-1,66	2,10E-11	2,64E-10	No DEG
ENSG00000218582	GAPDHP63	-3,16	-1,66	2,29E-07	1,65E-06	No DEG
ENSG00000185942	NKAIN3	3,16	1,66	5,91E-04	2,30E-03	No DEG
ENSG00000112658	SRF	-3,16	-1,66	1,75E-16	3,77E-15	No DEG
ENSG00000171552	BCL2L1	-3,15	-1,66	6,51E-23	2,64E-21	No DEG
ENSG00000186187	ZNRF1	-3,15	-1,66	2,26E-20	7,23E-19	No DEG
ENSG00000173208	ABCD2	3,15	1,66	6,64E-08	5,21E-07	No DEG
ENSG00000188820	FAM26F	3,15	1,66	3,52E-03	1,14E-02	UP
ENSG00000188112	C6orf132	-3,15	-1,66	3,71E-06	2,19E-05	No DEG
ENSG00000181856	SLC2A4	-3,15	-1,66	2,10E-17	5,05E-16	No DEG
ENSG00000248874	C5orf17	3,15	1,66	4,47E-03	1,40E-02	No DEG
ENSG00000079385	CEACAM1	-3,15	-1,66	1,81E-06	1,12E-05	No DEG
ENSG00000232118	BACH1-AS1	-3,15	-1,66	1,41E-03	5,02E-03	No DEG
ENSG00000129353	SLC44A2	-3,15	-1,66	4,88E-20	1,50E-18	No DEG

ENSG00000228097	RP11-39K24.12	-3,15	-1,66	2,09E-03	7,15E-03	No DEG
ENSG00000237141	DNAJC19P1	-3,15	-1,65	1,57E-02	4,20E-02	No DEG
ENSG00000179094	PER1	-3,15	-1,65	5,83E-07	3,92E-06	No DEG
ENSG00000237512	UNC5B-AS1	-3,15	-1,65	1,85E-06	1,14E-05	DOWN
ENSG00000272341	RP1-151F17.2	3,15	1,65	3,01E-08	2,49E-07	No DEG
ENSG00000179583	CIITA	-3,15	-1,65	9,75E-10	9,89E-09	No DEG
ENSG00000262209	PCDHGB3	-3,15	-1,65	3,58E-13	5,58E-12	No DEG
ENSG00000158555	GDPD5	-3,15	-1,65	1,03E-07	7,89E-07	No DEG
ENSG00000106524	ANKMY2	3,15	1,65	1,24E-14	2,26E-13	No DEG
ENSG00000186056	MATN1-AS1	-3,15	-1,65	3,66E-03	1,18E-02	No DEG
ENSG00000110079	MS4A4A	3,15	1,65	1,40E-08	1,21E-07	UP
ENSG00000185019	UBOX5	-3,15	-1,65	3,34E-16	7,05E-15	No DEG
ENSG00000071564	TCF3	-3,15	-1,65	4,60E-24	2,07E-22	No DEG
ENSG00000260005	AC027601.1	-3,14	-1,65	2,71E-03	9,01E-03	No DEG
ENSG00000150756	FAM173B	3,14	1,65	1,10E-14	2,01E-13	No DEG
ENSG00000232814	COL4A2-AS1	-3,14	-1,65	1,02E-03	3,78E-03	No DEG
ENSG00000128340	RAC2	-3,14	-1,65	9,62E-07	6,24E-06	UP
ENSG00000259969	RP11-999E24.3	3,14	1,65	2,06E-06	1,26E-05	No DEG
ENSG00000228888	RP4-764O22.2	3,14	1,65	4,75E-04	1,89E-03	No DEG
ENSG00000184640	sept-09	-3,14	-1,65	1,99E-25	9,94E-24	No DEG
ENSG00000133250	ZNF414	-3,14	-1,65	1,27E-06	8,06E-06	No DEG
ENSG00000185340	GAS2L1	-3,14	-1,65	7,14E-15	1,33E-13	No DEG
ENSG00000273445	RP11-1399P15.1	-3,14	-1,65	1,91E-03	6,61E-03	No DEG
ENSG00000187534	CTC-471F3.4	-3,14	-1,65	1,08E-04	4,89E-04	No DEG
ENSG00000241319	SETP6	-3,14	-1,65	1,30E-03	4,68E-03	DOWN
ENSG00000136546	SCN7A	3,14	1,65	5,43E-13	8,29E-12	No DEG
ENSG00000250137	RP11-380P13.1	-3,14	-1,65	2,01E-03	6,93E-03	DOWN
ENSG00000251785	RNA5SP20	3,14	1,65	2,38E-04	1,01E-03	No DEG
ENSG00000142748	FCN3	-3,14	-1,65	2,84E-05	1,44E-04	DOWN
ENSG00000075089	ACTR6	3,14	1,65	5,40E-20	1,65E-18	No DEG
ENSG00000135472	FAIM2	-3,14	-1,65	3,06E-05	1,55E-04	No DEG
ENSG00000174791	RIN1	-3,14	-1,65	1,31E-05	7,09E-05	No DEG

ENSG00000250350	RP11-731D1.3	-3,13	-1,65	1,55E-02	4,15E-02	No DEG
ENSG00000188818	ZDHHC11	-3,13	-1,65	5,46E-05	2,63E-04	No DEG
ENSG00000270673	YTHDF3-AS1	3,13	1,65	2,88E-04	1,20E-03	No DEG
ENSG00000239887	C1orf226	-3,13	-1,65	3,81E-06	2,24E-05	No DEG
ENSG00000117155	SSX2IP	3,13	1,65	1,12E-18	3,04E-17	No DEG
ENSG00000140105	WARS	-3,13	-1,65	9,41E-08	7,22E-07	No DEG
ENSG00000230006	ANKRD36BP2	3,13	1,65	2,56E-04	1,08E-03	UP
ENSG00000272980	RP11-517H2.6	3,13	1,65	2,38E-04	1,01E-03	No DEG
ENSG00000177169	ULK1	-3,13	-1,65	9,84E-18	2,44E-16	No DEG
ENSG00000254682	RP11-660L16.2	-3,13	-1,65	9,26E-05	4,23E-04	No DEG
ENSG00000180458	CTD-3064H18.4	3,13	1,64	1,12E-04	5,06E-04	No DEG
ENSG00000234737	KRT18P15	-3,13	-1,64	1,02E-03	3,78E-03	No DEG
ENSG00000126262	FFAR2	-3,13	-1,64	1,02E-02	2,91E-02	No DEG
ENSG00000121753	BAI2	-3,13	-1,64	3,96E-10	4,22E-09	No DEG
ENSG00000235090	RPL7L1P3	-3,12	-1,64	4,46E-03	1,40E-02	No DEG
ENSG00000167972	ABCA3	-3,12	-1,64	1,82E-20	5,87E-19	No DEG
ENSG00000248126	CTD-2012J19.2	-3,12	-1,64	9,69E-03	2,77E-02	No DEG
ENSG00000068885	IFT80	3,12	1,64	5,13E-15	9,68E-14	No DEG
ENSG00000269688	AC008982.2	-3,12	-1,64	1,06E-03	3,90E-03	No DEG
ENSG00000170145	SIK2	-3,12	-1,64	1,29E-16	2,83E-15	No DEG
ENSG00000166963	MAP1A	-3,12	-1,64	4,09E-06	2,39E-05	No DEG
ENSG00000213900	RPS17P1	3,12	1,64	1,44E-02	3,91E-02	No DEG
ENSG00000034152	MAP2K3	-3,12	-1,64	9,04E-11	1,04E-09	No DEG
ENSG00000119616	FCF1	3,12	1,64	8,32E-44	1,38E-41	No DEG
ENSG00000119820	YIPF4	3,12	1,64	8,27E-33	7,05E-31	No DEG
ENSG00000128524	ATP6V1F	-3,12	-1,64	5,22E-11	6,20E-10	No DEG
ENSG00000188277	C15orf62	-3,12	-1,64	7,46E-07	4,93E-06	No DEG
ENSG00000206384	COL6A6	3,12	1,64	4,38E-04	1,75E-03	No DEG
ENSG00000142186	SCYL1	-3,12	-1,64	3,96E-35	3,82E-33	No DEG
ENSG00000141858	SAMD1	-3,12	-1,64	1,09E-14	2,00E-13	No DEG
ENSG00000260672	RP11-1006G14.1	-3,11	-1,64	1,14E-02	3,20E-02	No DEG
ENSG00000249453	RP13-497K6.1	-3,11	-1,64	1,03E-03	3,81E-03	No DEG

ENSG00000184634	MED12	-3,11	-1,64	1,59E-27	9,44E-26	No DEG
ENSG00000212456	RNVU1-13	3,11	1,64	3,24E-03	1,06E-02	UP
ENSG00000165832	TRUB1	3,11	1,64	6,56E-08	5,16E-07	No DEG
ENSG00000227329	RP11-258C19.4	3,11	1,64	2,05E-05	1,07E-04	No DEG
ENSG00000267352	SH3GL1P3	-3,11	-1,64	1,28E-10	1,45E-09	No DEG
ENSG00000178075	GRAMD1C	3,11	1,64	1,11E-05	6,06E-05	UP
ENSG00000185122	HSF1	-3,11	-1,64	1,07E-19	3,15E-18	No DEG
ENSG00000235821	IFITM4P	-3,11	-1,64	1,81E-03	6,30E-03	No DEG
ENSG00000125648	SLC25A23	-3,11	-1,64	5,17E-23	2,13E-21	No DEG
ENSG00000099937	SERPIND1	-3,11	-1,64	2,72E-04	1,14E-03	No DEG
ENSG00000147586	MRPS28	3,11	1,64	1,41E-28	9,01E-27	No DEG
ENSG00000167778	SPRYD3	-3,11	-1,64	1,53E-32	1,28E-30	No DEG
ENSG00000156515	HK1	-3,11	-1,64	1,75E-19	5,10E-18	No DEG
ENSG00000114650	SCAP	-3,11	-1,64	6,38E-33	5,56E-31	No DEG
ENSG00000267519	MIR24-2	-3,11	-1,64	8,75E-05	4,02E-04	No DEG
ENSG00000249855	EEF1A1P19	3,11	1,64	1,67E-08	1,44E-07	No DEG
ENSG00000228816	AK3P5	-3,11	-1,64	7,20E-04	2,75E-03	No DEG
ENSG00000168610	STAT3	-3,11	-1,64	2,57E-08	2,15E-07	No DEG
ENSG00000159251	ACTC1	-3,11	-1,64	3,84E-11	4,66E-10	No DEG
ENSG00000135632	SMYD5	-3,11	-1,63	5,17E-19	1,45E-17	No DEG
ENSG00000108961	RANGRF	-3,11	-1,63	9,68E-04	3,60E-03	No DEG
ENSG00000079462	PAFAH1B3	-3,10	-1,63	1,36E-05	7,32E-05	No DEG
ENSG00000109118	PHF12	-3,10	-1,63	2,50E-20	7,94E-19	No DEG
ENSG00000155282	RP11-195B21.3	3,10	1,63	3,27E-03	1,07E-02	No DEG
ENSG00000185615	PDIA2	3,10	1,63	1,41E-04	6,22E-04	No DEG
ENSG00000144451	SPAG16	3,10	1,63	3,48E-17	8,14E-16	No DEG
ENSG00000124067	SLC12A4	-3,10	-1,63	2,07E-34	1,93E-32	No DEG
ENSG00000133111	RFXAP	3,10	1,63	3,94E-09	3,68E-08	No DEG
ENSG00000188811	NHLRC3	3,10	1,63	1,18E-21	4,24E-20	No DEG
ENSG00000260084	RP11-615I2.1	-3,10	-1,63	1,60E-02	4,27E-02	No DEG
ENSG00000213304	CTC-398G3.1	-3,10	-1,63	9,46E-03	2,71E-02	No DEG
ENSG00000123901	GPR83	3,10	1,63	7,93E-03	2,32E-02	UP

ENSG00000231130	HLA-T	-3,10	-1,63	7,64E-03	2,25E-02	No DEG
ENSG00000249031	SUMO2P6	-3,10	-1,63	1,49E-03	5,28E-03	No DEG
ENSG00000129151	BBOX1	3,10	1,63	8,26E-03	2,41E-02	No DEG
ENSG00000205581	HMGN1	3,10	1,63	6,64E-26	3,45E-24	No DEG
ENSG00000213197	AC012066.1	-3,10	-1,63	7,14E-04	2,73E-03	No DEG
ENSG00000148384	INPP5E	-3,10	-1,63	7,25E-26	3,74E-24	No DEG
ENSG00000188991	SLC15A5	3,10	1,63	1,60E-02	4,28E-02	No DEG
ENSG00000123064	DDX54	-3,10	-1,63	4,86E-27	2,78E-25	No DEG
ENSG00000245468	RP11-367J11.3	3,10	1,63	3,10E-05	1,57E-04	No DEG
ENSG00000223561	AC003090.1	3,09	1,63	3,92E-04	1,59E-03	No DEG
ENSG00000259125	RP11-545N8.3	-3,09	-1,63	9,20E-03	2,64E-02	No DEG
ENSG00000149503	INCENP	-3,09	-1,63	7,92E-18	1,98E-16	No DEG
ENSG00000141503	MINK1	-3,09	-1,63	7,32E-29	4,79E-27	No DEG
ENSG00000204305	AGER	-3,09	-1,63	4,97E-06	2,87E-05	No DEG
ENSG00000142513	ACPT	-3,09	-1,63	3,55E-03	1,15E-02	No DEG
ENSG00000255946	RP11-173P15.7	-3,09	-1,63	1,33E-02	3,66E-02	No DEG
ENSG00000238178	RP11-431J24.2	-3,09	-1,63	3,17E-03	1,04E-02	No DEG
ENSG00000130518	KIAA1683	-3,09	-1,63	2,38E-04	1,01E-03	No DEG
ENSG00000164294	GPX8	3,09	1,63	1,10E-07	8,36E-07	No DEG
ENSG00000177731	FLII	-3,09	-1,63	9,72E-37	1,06E-34	No DEG
ENSG00000079435	LIPE	-3,09	-1,63	9,80E-19	2,68E-17	No DEG
ENSG00000108819	PPP1R9B	-3,09	-1,63	9,57E-16	1,93E-14	No DEG
ENSG00000089225	TBX5	-3,09	-1,63	2,85E-10	3,10E-09	No DEG
ENSG00000100918	REC8	-3,09	-1,63	2,63E-07	1,88E-06	No DEG
ENSG00000242899	RPL7P16	3,09	1,63	4,30E-06	2,51E-05	No DEG
ENSG00000150540	HNMT	3,09	1,63	2,75E-23	1,17E-21	No DEG
ENSG00000260628	RP11-1166P10.1	-3,09	-1,63	5,90E-03	1,79E-02	No DEG
ENSG00000112232	KHDRBS2	3,09	1,63	3,82E-03	1,22E-02	UP
ENSG00000222108	RNA5SP317	-3,09	-1,63	2,85E-03	9,45E-03	No DEG
ENSG00000230225	MTND5P14	-3,09	-1,63	6,15E-03	1,86E-02	No DEG
ENSG00000115902	SLC1A4	-3,09	-1,63	5,77E-08	4,58E-07	No DEG
ENSG00000092068	SLC7A8	-3,09	-1,63	5,63E-07	3,79E-06	No DEG

ENSG00000271108	KATNBL1P5	3,08	1,63	1,35E-02	3,69E-02	No DEG
ENSG00000142684	ZNF593	-3,08	-1,62	5,59E-03	1,71E-02	No DEG
ENSG00000128951	DUT	3,08	1,62	2,15E-21	7,56E-20	No DEG
ENSG00000008838	MED24	-3,08	-1,62	1,50E-46	3,10E-44	No DEG
ENSG00000115207	GTF3C2	-3,08	-1,62	2,12E-23	9,06E-22	No DEG
ENSG00000243667	WDR92	3,08	1,62	1,63E-13	2,64E-12	No DEG
ENSG00000254858	MPV17L2	-3,08	-1,62	2,42E-12	3,42E-11	No DEG
ENSG00000087111	PIGS	-3,08	-1,62	2,47E-32	2,05E-30	No DEG
ENSG00000184313	MROH7	-3,08	-1,62	1,27E-04	5,65E-04	No DEG
ENSG00000238273	AC012360.6	-3,08	-1,62	1,70E-06	1,06E-05	No DEG
ENSG00000076928	ARHGEF1	-3,08	-1,62	2,37E-17	5,63E-16	No DEG
ENSG00000120784	ZFP30	3,08	1,62	3,04E-15	5,85E-14	No DEG
ENSG00000255786	RP11-809N8.6	3,08	1,62	1,41E-02	3,84E-02	No DEG
ENSG00000237207	RP11-24B13.1	-3,08	-1,62	1,08E-02	3,06E-02	No DEG
ENSG00000266970	RP11-806H10.4	-3,08	-1,62	1,66E-02	4,41E-02	No DEG
ENSG00000130522	JUND	-3,08	-1,62	3,35E-08	2,75E-07	No DEG
ENSG00000261728	RP11-307O13.1	3,08	1,62	1,54E-03	5,44E-03	No DEG
ENSG00000168993	CPLX1	-3,08	-1,62	6,95E-06	3,92E-05	No DEG
ENSG00000242716	RNA5-8S5	3,08	1,62	1,59E-03	5,60E-03	No DEG
ENSG00000163348	PYGO2	-3,08	-1,62	3,30E-26	1,75E-24	No DEG
ENSG00000266066	POLRMTP1	-3,08	-1,62	5,69E-03	1,74E-02	No DEG
ENSG00000174238	PITPNA	-3,08	-1,62	1,10E-30	8,06E-29	No DEG
ENSG00000241493	RP11-274B21.5	-3,08	-1,62	6,99E-03	2,08E-02	No DEG
ENSG00000139410	SDSL	3,08	1,62	2,42E-08	2,04E-07	No DEG
ENSG00000205790	AC005594.3	-3,08	-1,62	9,87E-03	2,82E-02	No DEG
ENSG00000247228	RP11-296I10.3	-3,07	-1,62	1,40E-07	1,05E-06	No DEG
ENSG00000156030	ELMSAN1	-3,07	-1,62	1,77E-18	4,69E-17	No DEG
ENSG00000261664	RP11-275F13.1	-3,07	-1,62	2,00E-03	6,88E-03	No DEG
ENSG00000273173	SNURF	3,07	1,62	2,78E-09	2,65E-08	No DEG
ENSG00000107147	KCNT1	-3,07	-1,62	6,38E-07	4,27E-06	No DEG
ENSG00000006047	YBX2	-3,07	-1,62	4,41E-10	4,68E-09	No DEG
ENSG00000225511	LINC00475	3,07	1,62	4,27E-03	1,35E-02	No DEG

ENSG00000227359	AC017074.2	-3,07	-1,62	1,45E-03	5,14E-03	No DEG
ENSG00000183675	PTPN20B	3,07	1,62	3,67E-08	2,99E-07	No DEG
ENSG00000112414	GPR126	3,07	1,62	8,57E-22	3,12E-20	UP
ENSG00000135476	ESPL1	-3,07	-1,62	6,61E-04	2,54E-03	No DEG
ENSG00000265666	CTD-2267D19.2	-3,07	-1,62	1,12E-05	6,13E-05	No DEG
ENSG00000223117	RN7SKP296	-3,07	-1,62	4,44E-03	1,40E-02	No DEG
ENSG00000188636	LDOC1L	-3,07	-1,62	6,46E-42	9,58E-40	No DEG
ENSG00000259877	RP11-46C24.7	3,07	1,62	1,53E-06	9,57E-06	No DEG
ENSG00000159753	RLTPR	-3,07	-1,62	8,72E-05	4,01E-04	UP
ENSG00000120049	KCNIP2	-3,07	-1,62	4,27E-03	1,35E-02	No DEG
ENSG00000112139	MDGA1	-3,07	-1,62	8,88E-09	7,92E-08	No DEG
ENSG00000231845	HMGB3P14	-3,06	-1,62	4,65E-06	2,69E-05	No DEG
ENSG00000125912	NCLN	-3,06	-1,62	1,46E-22	5,71E-21	No DEG
ENSG00000158488	CD1E	3,06	1,62	9,40E-03	2,70E-02	UP
ENSG00000172771	EFCAB12	-3,06	-1,62	3,94E-03	1,26E-02	No DEG
ENSG00000125834	STK35	-3,06	-1,62	1,34E-20	4,36E-19	No DEG
ENSG00000234663	AC104820.2	3,06	1,61	7,40E-03	2,19E-02	UP
ENSG00000229020	AKR7A2P1	-3,06	-1,61	2,30E-05	1,19E-04	No DEG
ENSG00000170382	LRRN2	-3,06	-1,61	1,26E-05	6,81E-05	No DEG
ENSG00000251867	Y_RNA	3,06	1,61	3,56E-05	1,78E-04	No DEG
ENSG00000227649	RP11-522L3.9	-3,06	-1,61	1,64E-02	4,36E-02	No DEG
ENSG00000221983	UBA52	-3,06	-1,61	1,19E-51	3,76E-49	No DEG
ENSG00000196388	INCA1	-3,06	-1,61	5,52E-07	3,73E-06	No DEG
ENSG00000185924	RTN4RL1	-3,06	-1,61	7,27E-04	2,77E-03	No DEG
ENSG00000065000	AP3D1	-3,06	-1,61	9,34E-30	6,45E-28	No DEG
ENSG00000225415	RP3-509I19.1	3,06	1,61	1,05E-03	3,87E-03	No DEG
ENSG00000158856	DMTN	-3,06	-1,61	4,19E-21	1,44E-19	No DEG
ENSG00000135439	AGAP2	-3,06	-1,61	8,00E-08	6,20E-07	No DEG
ENSG00000198576	ARC	-3,06	-1,61	7,72E-03	2,27E-02	No DEG
ENSG00000105290	APLP1	-3,06	-1,61	5,04E-09	4,64E-08	No DEG
ENSG00000256006	AC084117.3	-3,06	-1,61	1,24E-03	4,47E-03	No DEG
ENSG00000119927	GPAM	3,06	1,61	5,92E-08	4,69E-07	No DEG

ENSG00000106701	FSD1L	3,06	1,61	6,59E-14	1,11E-12	No DEG
ENSG00000100316	RPL3	3,05	1,61	2,39E-14	4,19E-13	No DEG
ENSG00000227032	RP11-34E5.4	-3,05	-1,61	7,67E-03	2,26E-02	No DEG
ENSG00000106804	C5	3,05	1,61	9,63E-10	9,78E-09	No DEG
ENSG00000186743	TPI1P3	-3,05	-1,61	2,68E-04	1,12E-03	No DEG
ENSG00000178038	ALS2CL	-3,05	-1,61	9,65E-18	2,40E-16	No DEG
ENSG00000249456	RP11-298J20.4	3,05	1,61	1,44E-06	9,03E-06	No DEG
ENSG00000112312	GMNN	3,05	1,61	2,31E-13	3,66E-12	No DEG
ENSG00000240405	RP11-460N16.1	3,05	1,61	1,50E-02	4,04E-02	No DEG
ENSG00000008710	PKD1	-3,05	-1,61	3,33E-17	7,83E-16	No DEG
ENSG00000266139	MIR4435-2	-3,05	-1,61	1,74E-02	4,61E-02	No DEG
ENSG00000203813	HIST1H3H	3,05	1,61	2,95E-07	2,09E-06	UP
ENSG00000165238	WNK2	-3,05	-1,61	1,09E-12	1,60E-11	No DEG
ENSG00000179165	PXT1	-3,05	-1,61	1,01E-02	2,88E-02	No DEG
ENSG00000207654	MIR128-1	3,05	1,61	1,55E-02	4,16E-02	No DEG
ENSG00000137817	PARP6	-3,04	-1,61	3,95E-26	2,07E-24	No DEG
ENSG00000234592	RP11-41L14.1	-3,04	-1,61	1,52E-02	4,10E-02	No DEG
ENSG00000221947	XKR9	3,04	1,61	1,43E-04	6,31E-04	No DEG
ENSG00000116194	ANGPTL1	3,04	1,61	2,19E-11	2,74E-10	No DEG
ENSG00000235917	RP11-39K24.14	-3,04	-1,60	3,87E-03	1,24E-02	No DEG
ENSG00000139914	FITM1	-3,04	-1,60	1,28E-16	2,81E-15	No DEG
ENSG00000181013	C17orf47	-3,04	-1,60	1,62E-02	4,32E-02	No DEG
ENSG00000223956	RP4-710M16.2	-3,04	-1,60	5,34E-03	1,65E-02	No DEG
ENSG00000039523	FAM65A	-3,04	-1,60	6,04E-49	1,49E-46	No DEG
ENSG00000198933	TBKBP1	-3,04	-1,60	1,08E-19	3,18E-18	No DEG
ENSG00000186106	ANKRD46	3,04	1,60	1,74E-17	4,23E-16	No DEG
ENSG00000112874	NUDT12	3,04	1,60	6,94E-12	9,26E-11	No DEG
ENSG00000014216	CAPN1	-3,04	-1,60	1,32E-26	7,19E-25	No DEG
ENSG00000225285	RP4-758J18.10	-3,03	-1,60	2,35E-03	7,95E-03	No DEG
ENSG00000174417	TRHR	-3,03	-1,60	3,50E-04	1,43E-03	No DEG
ENSG00000143196	DPT	3,03	1,60	5,41E-10	5,68E-09	UP
ENSG00000178026	FAM211B	-3,03	-1,60	2,18E-10	2,40E-09	No DEG

ENSG00000182087	TMEM259	-3,03	-1,60	1,79E-29	1,21E-27	No DEG
ENSG00000159496	RGL4	-3,03	-1,60	5,86E-07	3,94E-06	No DEG
ENSG00000237738	RNF216-IT1	-3,03	-1,60	7,27E-03	2,15E-02	No DEG
ENSG00000166558	SLC38A8	-3,03	-1,60	1,33E-02	3,65E-02	No DEG
ENSG00000235454	RP11-678B3.2	3,03	1,60	8,95E-03	2,58E-02	No DEG
ENSG00000142227	EMP3	-3,03	-1,60	6,77E-08	5,30E-07	No DEG
ENSG00000145088	EAF2	3,03	1,60	5,64E-10	5,91E-09	UP
ENSG00000235777	DPYD-AS2	3,03	1,60	1,06E-07	8,06E-07	UP
ENSG00000160685	ZBTB7B	-3,03	-1,60	4,87E-23	2,01E-21	No DEG
ENSG00000270523	RP11-145P16.3	3,03	1,60	4,84E-03	1,51E-02	No DEG
ENSG00000010818	HIVEP2	-3,03	-1,60	1,23E-12	1,81E-11	No DEG
ENSG00000184922	FMNL1	-3,02	-1,60	8,04E-11	9,35E-10	No DEG
ENSG00000011478	QPCTL	-3,02	-1,60	9,58E-08	7,34E-07	No DEG
ENSG00000269001	ZNF818P	3,02	1,60	9,30E-08	7,14E-07	No DEG
ENSG00000243824	RP11-434O22.1	-3,02	-1,60	7,09E-04	2,71E-03	No DEG
ENSG00000184661	CDCA2	-3,02	-1,60	8,18E-05	3,79E-04	DOWN
ENSG00000256452	RP11-667M19.10	-3,02	-1,60	1,93E-03	6,67E-03	No DEG
ENSG00000257953	RP11-620J15.1	-3,02	-1,59	8,54E-04	3,21E-03	No DEG
ENSG00000105229	PIAS4	-3,02	-1,59	4,53E-14	7,72E-13	No DEG
ENSG00000113369	ARRDC3	3,02	1,59	6,74E-12	9,02E-11	No DEG
ENSG00000157600	TMEM164	-3,02	-1,59	8,46E-11	9,79E-10	No DEG
ENSG00000203791	METTL10	3,02	1,59	9,31E-28	5,60E-26	No DEG
ENSG00000155959	VBP1	3,02	1,59	1,29E-27	7,72E-26	No DEG
ENSG00000264070	DND1P1	-3,02	-1,59	5,19E-05	2,50E-04	No DEG
ENSG00000257613	RP11-320P7.1	3,02	1,59	2,21E-04	9,42E-04	No DEG
ENSG00000266171	RP11-769O8.1	-3,02	-1,59	4,01E-04	1,62E-03	No DEG
ENSG00000147113	CXorf36	-3,01	-1,59	3,76E-18	9,65E-17	No DEG
ENSG00000255182	CTD-2517M22.14	-3,01	-1,59	3,03E-03	9,98E-03	No DEG
ENSG00000251451	GS1-124K5.6	3,01	1,59	1,94E-03	6,68E-03	No DEG
ENSG00000226862	RP11-569A11.1	-3,01	-1,59	7,82E-03	2,30E-02	No DEG
ENSG00000196498	NCOR2	-3,01	-1,59	4,57E-20	1,41E-18	No DEG
ENSG00000163683	SMIM14	3,01	1,59	4,81E-27	2,76E-25	No DEG

ENSG00000213639	PPP1CB	3,01	1,59	7,97E-33	6,85E-31	No DEG
ENSG00000165548	TMEM63C	-3,01	-1,59	5,92E-03	1,80E-02	No DEG
ENSG00000010803	SCMH1	-3,01	-1,59	8,33E-43	1,31E-40	No DEG
ENSG00000100979	PLTP	-3,01	-1,59	2,93E-07	2,08E-06	No DEG
ENSG00000268324	LUZPP1	-3,01	-1,59	3,78E-06	2,23E-05	No DEG
ENSG00000250510	GPR162	-3,01	-1,59	9,66E-07	6,26E-06	No DEG
ENSG00000168685	IL7R	3,01	1,59	1,44E-03	5,12E-03	UP
ENSG00000272327	RP11-1002K11.1	3,01	1,59	5,47E-03	1,68E-02	No DEG
ENSG00000250986	AC141928.1	-3,01	-1,59	5,95E-08	4,72E-07	No DEG
ENSG00000179532	DNHD1	-3,01	-1,59	7,72E-12	1,02E-10	No DEG
ENSG00000071894	CPSF1	-3,00	-1,59	6,06E-21	2,06E-19	No DEG
ENSG00000163666	HESX1	3,00	1,59	1,22E-03	4,41E-03	No DEG
ENSG00000078098	FAP	3,00	1,59	1,48E-04	6,51E-04	No DEG
ENSG00000149499	EML3	-3,00	-1,59	4,96E-10	5,23E-09	No DEG
ENSG00000166667	SPDYE6	-3,00	-1,59	2,10E-03	7,17E-03	No DEG
ENSG00000161395	PGAP3	-3,00	-1,59	4,04E-12	5,56E-11	No DEG
ENSG00000110108	TMEM109	-3,00	-1,59	1,09E-40	1,52E-38	No DEG
ENSG00000255308	RP11-428C19.4	-3,00	-1,59	6,95E-04	2,66E-03	DOWN
ENSG00000137571	SLCO5A1	-3,00	-1,59	2,98E-07	2,11E-06	DOWN
ENSG00000155011	DKK2	3,00	1,59	1,85E-05	9,71E-05	No DEG
ENSG00000115541	HSPE1	3,00	1,59	1,15E-14	2,10E-13	No DEG
ENSG00000239224	RN7SL546P	3,00	1,59	6,45E-03	1,94E-02	No DEG
ENSG00000170043	TRAPPC1	-3,00	-1,59	7,38E-26	3,80E-24	No DEG
ENSG00000271771	RP11-1250I15.3	3,00	1,59	2,39E-03	8,08E-03	No DEG
ENSG00000198373	WWP2	-3,00	-1,59	1,91E-24	8,83E-23	No DEG
ENSG00000265150	RN7SL2	3,00	1,59	7,01E-19	1,94E-17	No DEG
ENSG00000053438	NNAT	-3,00	-1,59	5,23E-07	3,55E-06	No DEG
ENSG00000137474	MYO7A	-3,00	-1,58	2,96E-12	4,13E-11	No DEG
ENSG00000136653	RASSF5	-3,00	-1,58	2,73E-05	1,39E-04	No DEG
ENSG00000073849	ST6GAL1	-3,00	-1,58	1,00E-22	3,98E-21	No DEG
ENSG00000171471	MAP1LC3B2	3,00	1,58	1,91E-04	8,23E-04	No DEG
ENSG00000117480	FAAH	-3,00	-1,58	1,86E-05	9,75E-05	No DEG

ENSG00000135956	TMEM127	-3,00	-1,58	1,24E-26	6,80E-25	No DEG
ENSG00000233966	UBE2SP1	-3,00	-1,58	1,50E-03	5,32E-03	No DEG
ENSG00000137343	ATAT1	-3,00	-1,58	4,49E-11	5,40E-10	No DEG
ENSG00000037280	FLT4	-2,99	-1,58	3,86E-29	2,56E-27	No DEG
ENSG00000139044	B4GALNT3	-2,99	-1,58	3,04E-11	3,74E-10	No DEG
ENSG00000272108	AC005754.8	2,99	1,58	3,37E-05	1,69E-04	No DEG
ENSG00000073792	IGF2BP2	-2,99	-1,58	8,36E-19	2,30E-17	No DEG
ENSG00000235904	RBMS3-AS3	2,99	1,58	6,26E-07	4,19E-06	No DEG
ENSG00000272003	RP11-23P13.7	-2,99	-1,58	1,63E-12	2,36E-11	No DEG
ENSG00000257497	RP11-585P4.5	-2,99	-1,58	1,01E-03	3,74E-03	No DEG
ENSG00000105967	TFEC	2,99	1,58	6,31E-09	5,72E-08	UP
ENSG00000178209	PLEC	-2,99	-1,58	2,16E-29	1,46E-27	No DEG
ENSG00000230453	ANKRD18B	2,99	1,58	8,18E-03	2,39E-02	No DEG
ENSG00000258086	RP11-753H16.5	-2,99	-1,58	5,40E-03	1,66E-02	No DEG
ENSG00000100968	NFATC4	-2,99	-1,58	5,50E-11	6,53E-10	No DEG
ENSG00000182195	LDOC1	-2,99	-1,58	1,17E-05	6,36E-05	No DEG
ENSG00000113594	LIFR	2,99	1,58	1,07E-11	1,40E-10	No DEG
ENSG00000205085	FAM71F2	-2,99	-1,58	9,36E-06	5,18E-05	No DEG
ENSG00000250116	RP11-417F21.1	-2,99	-1,58	2,78E-06	1,67E-05	No DEG
ENSG00000099337	KCNK6	-2,99	-1,58	5,96E-05	2,84E-04	No DEG
ENSG00000232098	CTD-2619J13.14	-2,99	-1,58	4,53E-07	3,11E-06	No DEG
ENSG00000152582	SPEF2	2,99	1,58	3,42E-05	1,71E-04	UP
ENSG00000171806	METTL18	2,99	1,58	7,29E-11	8,51E-10	No DEG
ENSG00000228219	NPM1P30	-2,99	-1,58	8,70E-03	2,52E-02	No DEG
ENSG00000227409	ZMYM4-AS1	2,99	1,58	9,43E-04	3,51E-03	No DEG
ENSG00000178966	RMI1	2,99	1,58	2,85E-09	2,71E-08	No DEG
ENSG00000134901	KDELC1	2,99	1,58	5,48E-13	8,35E-12	No DEG
ENSG00000203615	AC069200.1	-2,99	-1,58	1,69E-03	5,92E-03	No DEG
ENSG00000173210	ABLIM3	-2,99	-1,58	3,72E-15	7,08E-14	No DEG
ENSG00000165443	PHYHIPL	2,99	1,58	1,46E-03	5,19E-03	No DEG
ENSG00000100084	HIRA	-2,99	-1,58	1,21E-12	1,78E-11	No DEG
ENSG00000213859	KCTD11	-2,99	-1,58	8,57E-15	1,58E-13	No DEG

ENSG00000174740	PABPC5	2,99	1,58	6,45E-07	4,31E-06	No DEG
ENSG00000175573	C11orf68	-2,99	-1,58	6,46E-29	4,24E-27	No DEG
ENSG00000184702	sept-05	-2,98	-1,58	7,76E-12	1,03E-10	No DEG
ENSG00000117318	ID3	-2,98	-1,58	9,17E-12	1,20E-10	No DEG
ENSG00000229729	RP11-159G9.5	2,98	1,58	5,30E-20	1,62E-18	No DEG
ENSG00000168772	CXXC4	2,98	1,58	4,94E-06	2,85E-05	No DEG
ENSG00000196811	CHRNG	-2,98	-1,58	5,39E-04	2,12E-03	No DEG
ENSG00000184985	SORCS2	-2,98	-1,58	8,43E-07	5,52E-06	No DEG
ENSG00000241211	IQCJ-SCHIP1-AS1	-2,98	-1,58	9,42E-04	3,51E-03	DOWN
ENSG00000103740	ACSBG1	-2,98	-1,58	1,77E-03	6,18E-03	No DEG
ENSG00000263826	RP11-573D15.9	-2,98	-1,58	1,27E-04	5,68E-04	No DEG
ENSG00000115806	GORASP2	-2,98	-1,58	4,54E-36	4,80E-34	No DEG
ENSG00000172469	MANEA	2,98	1,58	1,04E-23	4,58E-22	No DEG
ENSG00000159387	IRX6	-2,98	-1,58	1,93E-05	1,01E-04	No DEG
ENSG00000163629	PTPN13	2,98	1,58	6,78E-12	9,05E-11	No DEG
ENSG00000179172	HNRNPCL1	2,98	1,58	2,97E-03	9,79E-03	No DEG
ENSG00000095383	TBC1D2	-2,98	-1,58	9,99E-09	8,84E-08	No DEG
ENSG00000000971	CFH	2,98	1,57	3,60E-10	3,85E-09	No DEG
ENSG00000162148	PPP1R32	-2,98	-1,57	4,29E-05	2,10E-04	No DEG
ENSG00000105374	NKG7	-2,98	-1,57	1,17E-03	4,27E-03	UP
ENSG00000198646	NCOA6	-2,98	-1,57	5,01E-30	3,51E-28	No DEG
ENSG00000225643	RP11-70P17.1	-2,98	-1,57	4,39E-03	1,38E-02	No DEG
ENSG00000135063	FAM189A2	-2,98	-1,57	2,08E-14	3,68E-13	No DEG
ENSG00000253837	RP11-177H13.2	-2,98	-1,57	3,30E-05	1,66E-04	No DEG
ENSG00000188013	MEIS3P2	-2,97	-1,57	3,29E-06	1,96E-05	No DEG
ENSG00000091128	LAMB4	2,97	1,57	1,18E-04	5,30E-04	No DEG
ENSG00000156858	PRR14	-2,97	-1,57	8,32E-18	2,08E-16	No DEG
ENSG00000182511	FES	-2,97	-1,57	6,51E-08	5,13E-07	No DEG
ENSG00000115758	ODC1	2,97	1,57	9,46E-12	1,24E-10	No DEG
ENSG00000227586	RP11-162A23.5	2,97	1,57	1,86E-02	4,86E-02	No DEG
ENSG00000176476	CCDC101	-2,97	-1,57	1,24E-16	2,73E-15	No DEG
ENSG00000162522	KIAA1522	-2,97	-1,57	3,47E-12	4,80E-11	No DEG

ENSG00000213361	RP4-778K6.1	-2,97	-1,57	1,25E-02	3,45E-02	No DEG
ENSG00000272148	RP11-195B17.1	-2,97	-1,57	6,43E-04	2,48E-03	No DEG
ENSG00000133739	LRRCC1	2,97	1,57	1,28E-11	1,65E-10	No DEG
ENSG00000239405	TMED10P2	-2,97	-1,57	3,15E-03	1,03E-02	No DEG
ENSG00000262246	CORO7	-2,97	-1,57	4,95E-08	3,97E-07	No DEG
ENSG00000261934	PCDHGA9	-2,97	-1,57	2,24E-14	3,95E-13	No DEG
ENSG00000138395	CDK15	2,97	1,57	8,92E-05	4,09E-04	No DEG
ENSG00000165804	ZNF219	-2,97	-1,57	1,75E-22	6,76E-21	No DEG
ENSG00000131951	LRRC9	2,97	1,57	8,88E-05	4,08E-04	No DEG
ENSG00000196678	ERI2	2,97	1,57	1,08E-10	1,23E-09	No DEG
ENSG00000124802	EEF1E1	2,97	1,57	4,23E-12	5,81E-11	No DEG
ENSG00000103241	FOXF1	-2,97	-1,57	2,12E-03	7,24E-03	No DEG
ENSG00000158169	FANCC	-2,97	-1,57	1,87E-08	1,60E-07	No DEG
ENSG00000232259	RP11-4C20.3	-2,96	-1,57	5,51E-05	2,65E-04	No DEG
ENSG00000252396	RN7SKP195	-2,96	-1,57	9,43E-05	4,31E-04	No DEG
ENSG00000160789	LMNA	-2,96	-1,57	1,56E-09	1,54E-08	No DEG
ENSG00000107263	RAPGEF1	-2,96	-1,57	1,58E-47	3,53E-45	No DEG
ENSG00000227544	AC018647.3	2,96	1,57	1,89E-09	1,85E-08	No DEG
ENSG00000185215	TNFAIP2	-2,96	-1,57	2,74E-07	1,95E-06	No DEG
ENSG00000167207	NOD2	-2,96	-1,57	8,14E-05	3,77E-04	No DEG
ENSG00000099991	CABIN1	-2,96	-1,57	1,34E-24	6,27E-23	No DEG
ENSG00000144476	ACKR3	-2,96	-1,57	3,97E-08	3,22E-07	No DEG
ENSG00000152782	PANK1	2,96	1,56	1,54E-06	9,63E-06	No DEG
ENSG00000169057	MECP2	-2,96	-1,56	2,64E-51	8,03E-49	No DEG
ENSG00000126746	ZNF384	-2,96	-1,56	2,48E-25	1,23E-23	No DEG
ENSG00000253733	LZTS1-AS1	-2,96	-1,56	8,84E-03	2,55E-02	No DEG
ENSG00000185236	RAB11B	-2,96	-1,56	4,40E-20	1,36E-18	No DEG
ENSG00000100280	AP1B1	-2,96	-1,56	7,03E-25	3,36E-23	No DEG
ENSG00000130758	MAP3K10	-2,96	-1,56	1,23E-15	2,45E-14	No DEG
ENSG00000186815	TPCN1	-2,95	-1,56	9,02E-10	9,19E-09	No DEG
ENSG00000200556	RNU6-103P	-2,95	-1,56	1,92E-03	6,63E-03	No DEG
ENSG00000197191	C9orf169	2,95	1,56	1,06E-02	2,99E-02	No DEG

ENSG00000236853	OR2R1P	-2,95	-1,56	1,79E-02	4,71E-02	No DEG
ENSG00000148985	PGAP2	-2,95	-1,56	1,48E-13	2,42E-12	No DEG
ENSG00000200312	RN7SKP255	-2,95	-1,56	2,13E-26	1,15E-24	No DEG
ENSG00000166341	DCHS1	-2,95	-1,56	1,75E-19	5,10E-18	No DEG
ENSG00000240338	RP11-331F4.4	-2,95	-1,56	3,07E-03	1,01E-02	No DEG
ENSG00000124615	MOCS1	-2,95	-1,56	1,19E-12	1,76E-11	No DEG
ENSG00000175105	ZNF654	2,95	1,56	2,01E-20	6,45E-19	No DEG
ENSG00000167608	TMC4	-2,95	-1,56	8,57E-06	4,76E-05	No DEG
ENSG00000214243	AC004980.10	-2,95	-1,56	1,10E-02	3,11E-02	No DEG
ENSG00000197744	PTMAP2	-2,95	-1,56	3,99E-05	1,97E-04	No DEG
ENSG00000116786	PLEKHM2	-2,95	-1,56	7,95E-29	5,17E-27	No DEG
ENSG00000008853	RHOBTB2	-2,95	-1,56	3,43E-12	4,75E-11	No DEG
ENSG00000214300	SPDYE3	-2,95	-1,56	3,96E-08	3,21E-07	No DEG
ENSG00000182264	IZUMO1	-2,95	-1,56	2,77E-03	9,21E-03	No DEG
ENSG00000168591	TMUB2	-2,94	-1,56	1,21E-31	9,59E-30	No DEG
ENSG00000116871	MAP7D1	-2,94	-1,56	4,25E-22	1,58E-20	No DEG
ENSG00000143761	ARF1	-2,94	-1,56	6,68E-44	1,13E-41	No DEG
ENSG00000182010	RTKN2	2,94	1,56	7,44E-04	2,83E-03	UP
ENSG00000230189	GS1-124K5.2	2,94	1,56	6,62E-05	3,13E-04	No DEG
ENSG00000230280	HNRNPA1P59	-2,94	-1,56	3,40E-04	1,39E-03	No DEG
ENSG00000130300	PLVAP	-2,94	-1,56	1,39E-03	4,97E-03	No DEG
ENSG00000202415	RN7SKP269	-2,94	-1,56	3,05E-03	1,00E-02	No DEG
ENSG00000108785	HSD17B1P1	-2,94	-1,56	4,99E-06	2,87E-05	No DEG
ENSG00000092051	JPH4	-2,94	-1,56	6,55E-04	2,53E-03	No DEG
ENSG00000103227	LMF1	-2,94	-1,56	7,38E-10	7,62E-09	No DEG
ENSG00000166949	SMAD3	-2,94	-1,56	1,99E-10	2,19E-09	No DEG
ENSG00000135631	RAB11FIP5	-2,94	-1,55	2,60E-13	4,11E-12	No DEG
ENSG00000133884	DPF2	-2,94	-1,55	4,44E-28	2,75E-26	No DEG
ENSG00000197614	MFAP5	2,94	1,55	1,38E-09	1,38E-08	No DEG
ENSG00000100075	SLC25A1	-2,94	-1,55	4,80E-19	1,35E-17	No DEG
ENSG00000184497	TMEM255B	-2,94	-1,55	1,59E-06	9,90E-06	No DEG
ENSG00000103653	CSK	-2,94	-1,55	3,21E-21	1,11E-19	No DEG

ENSG00000233901	RP11-65J3.1	-2,94	-1,55	3,42E-03	1,11E-02	No DEG
ENSG00000255408	PCDHA3	2,94	1,55	2,32E-03	7,85E-03	No DEG
ENSG00000260774	CTD-2083E4.4	-2,93	-1,55	4,05E-04	1,64E-03	No DEG
ENSG00000172508	CARNS1	-2,93	-1,55	1,72E-07	1,26E-06	No DEG
ENSG00000230393	AC092667.2	2,93	1,55	1,74E-02	4,60E-02	No DEG
ENSG00000104899	AMH	-2,93	-1,55	1,41E-02	3,84E-02	No DEG
ENSG00000106018	VIPR2	-2,93	-1,55	2,33E-17	5,56E-16	No DEG
ENSG00000270028	RP11-380L11.4	-2,93	-1,55	3,37E-03	1,09E-02	No DEG
ENSG00000269858	EGLN2	-2,93	-1,55	8,04E-12	1,06E-10	No DEG
ENSG00000254685	FPGT	2,93	1,55	4,48E-17	1,04E-15	No DEG
ENSG00000266975	CTC-425F1.2	-2,93	-1,55	1,82E-02	4,77E-02	No DEG
ENSG00000228205	RP11-778D9.4	2,93	1,55	6,34E-04	2,45E-03	No DEG
ENSG00000198624	CCDC69	-2,93	-1,55	6,36E-09	5,76E-08	No DEG
ENSG00000200351	Y_RNA	2,93	1,55	3,27E-03	1,07E-02	No DEG
ENSG00000269473	CTD-2619J13.19	-2,93	-1,55	1,94E-04	8,38E-04	No DEG
ENSG00000236762	RP11-159H3.1	2,93	1,55	2,21E-03	7,53E-03	No DEG
ENSG00000023608	SNAPC1	2,93	1,55	1,77E-11	2,24E-10	No DEG
ENSG00000105438	KDELR1	-2,93	-1,55	3,91E-32	3,20E-30	No DEG
ENSG00000224821	COL4A2-AS2	-2,93	-1,55	7,97E-05	3,70E-04	No DEG
ENSG00000102100	SLC35A2	-2,93	-1,55	2,07E-20	6,63E-19	No DEG
ENSG00000171303	KCNK3	-2,93	-1,55	2,30E-04	9,76E-04	No DEG
ENSG00000159720	ATP6V0D1	-2,93	-1,55	2,66E-24	1,22E-22	No DEG
ENSG00000231066	NPM1P9	-2,92	-1,55	1,74E-02	4,60E-02	No DEG
ENSG00000271009	RP11-346C20.3	2,92	1,55	1,56E-05	8,29E-05	No DEG
ENSG00000165915	SLC39A13	-2,92	-1,55	7,74E-33	6,67E-31	No DEG
ENSG00000163399	ATP1A1	-2,92	-1,55	5,84E-13	8,90E-12	No DEG
ENSG00000173456	RNF26	-2,92	-1,55	6,21E-12	8,35E-11	No DEG
ENSG00000258584	FAM181A-AS1	-2,92	-1,55	1,79E-02	4,70E-02	No DEG
ENSG00000233791	LINC01136	-2,92	-1,55	1,57E-02	4,20E-02	No DEG
ENSG00000135424	ITGA7	-2,92	-1,55	1,84E-11	2,33E-10	No DEG
ENSG00000099624	ATP5D	-2,92	-1,55	5,71E-19	1,59E-17	No DEG
ENSG00000186198	SLC51B	-2,92	-1,55	1,05E-05	5,74E-05	No DEG

ENSG00000089692	LAG3	-2,92	-1,55	2,78E-03	9,23E-03	UP
ENSG00000259081	RP11-488C13.6	-2,92	-1,55	3,51E-05	1,75E-04	No DEG
ENSG00000140905	GCSH	2,92	1,55	8,85E-11	1,02E-09	No DEG
ENSG00000186281	GPAT2	-2,92	-1,55	6,60E-07	4,40E-06	No DEG
ENSG00000103202	NME4	-2,92	-1,55	2,58E-35	2,52E-33	No DEG
ENSG00000255737	AGAP2-AS1	-2,92	-1,55	1,33E-02	3,65E-02	No DEG
ENSG00000224555	RP11-438N5.2	2,92	1,55	1,49E-02	4,03E-02	No DEG
ENSG00000113163	COL4A3BP	2,92	1,54	2,06E-13	3,30E-12	No DEG
ENSG00000255201	RP11-350N15.4	-2,92	-1,54	3,12E-03	1,02E-02	No DEG
ENSG00000095627	TDRD1	2,92	1,54	3,78E-08	3,08E-07	No DEG
ENSG00000142733	MAP3K6	-2,92	-1,54	7,44E-08	5,81E-07	No DEG
ENSG00000099954	CECR2	-2,92	-1,54	3,56E-07	2,49E-06	No DEG
ENSG00000167701	GPT	-2,92	-1,54	9,12E-12	1,20E-10	No DEG
ENSG00000258733	CTD-2341M24.1	2,91	1,54	4,51E-04	1,80E-03	UP
ENSG00000067225	PKM	-2,91	-1,54	1,15E-16	2,54E-15	No DEG
ENSG00000005513	SOX8	-2,91	-1,54	3,35E-05	1,68E-04	No DEG
ENSG00000085644	ZNF213	-2,91	-1,54	3,44E-20	1,07E-18	No DEG
ENSG00000225921	NOL7	2,91	1,54	5,41E-13	8,26E-12	No DEG
ENSG00000200842	Y_RNA	-2,91	-1,54	5,68E-03	1,74E-02	No DEG
ENSG00000234513	AC073072.7	-2,91	-1,54	4,12E-03	1,31E-02	No DEG
ENSG00000136840	ST6GALNAC4	-2,91	-1,54	6,77E-12	9,05E-11	No DEG
ENSG00000249234	RP11-452J21.2	2,91	1,54	2,81E-04	1,17E-03	No DEG
ENSG00000230724	LINC01001	-2,91	-1,54	3,90E-06	2,29E-05	No DEG
ENSG00000268093	AC022154.7	-2,91	-1,54	9,18E-03	2,64E-02	No DEG
ENSG00000151704	KCNJ1	-2,91	-1,54	1,01E-03	3,73E-03	No DEG
ENSG00000198157	HMGN5	2,91	1,54	3,95E-04	1,60E-03	No DEG
ENSG00000174851	YIF1A	-2,91	-1,54	2,79E-13	4,40E-12	No DEG
ENSG00000162065	TBC1D24	-2,91	-1,54	2,10E-15	4,10E-14	No DEG
ENSG00000224163	RP11-309L24.6	-2,91	-1,54	1,31E-05	7,09E-05	No DEG
ENSG00000240342	RPS2P5	2,91	1,54	1,01E-19	3,01E-18	No DEG
ENSG00000264727	RP11-680C21.1	2,91	1,54	5,30E-03	1,63E-02	No DEG
ENSG00000213362	FTH1P12	-2,91	-1,54	3,89E-03	1,24E-02	No DEG

ENSG00000223547	ZNF844	2,91	1,54	2,52E-08	2,12E-07	No DEG
ENSG00000101162	TUBB1	-2,91	-1,54	2,49E-04	1,05E-03	No DEG
ENSG00000099860	GADD45B	-2,91	-1,54	7,76E-04	2,94E-03	DOWN
ENSG00000237872	POU5F1P4	-2,90	-1,54	3,62E-03	1,17E-02	No DEG
ENSG00000160216	AGPAT3	-2,90	-1,54	7,18E-21	2,41E-19	No DEG
ENSG00000189350	FAM179A	-2,90	-1,54	7,34E-03	2,17E-02	DOWN
ENSG00000181004	BBS12	2,90	1,54	5,27E-07	3,57E-06	No DEG
ENSG00000248641	HMGA1P2	-2,90	-1,54	5,36E-03	1,65E-02	No DEG
ENSG00000108379	WNT3	-2,90	-1,54	1,89E-05	9,93E-05	No DEG
ENSG00000141140	MYO19	-2,90	-1,54	3,61E-15	6,87E-14	No DEG
ENSG00000198618	PPIAP22	2,90	1,54	2,12E-18	5,55E-17	No DEG
ENSG00000229953	RP11-284F21.7	-2,90	-1,54	1,89E-02	4,93E-02	No DEG
ENSG00000225140	RP11-809C18.3	-2,90	-1,54	2,01E-04	8,64E-04	No DEG
ENSG00000263470	RP11-160O5.1	2,90	1,54	5,05E-04	1,99E-03	No DEG
ENSG00000116473	RAP1A	2,90	1,54	2,13E-42	3,26E-40	No DEG
ENSG00000167360	OR51Q1	-2,90	-1,54	1,40E-02	3,83E-02	No DEG
ENSG00000199846	RNU1-72P	-2,90	-1,54	1,83E-02	4,80E-02	No DEG
ENSG00000167685	ZNF444	-2,90	-1,54	2,13E-19	6,16E-18	No DEG
ENSG00000129450	SIGLEC9	-2,90	-1,54	1,17E-04	5,23E-04	No DEG
ENSG00000103042	SLC38A7	-2,90	-1,54	3,69E-16	7,74E-15	No DEG
ENSG00000256630	RP11-428I12.1	2,90	1,54	1,51E-02	4,06E-02	No DEG
ENSG00000136197	C7orf25	2,90	1,54	7,76E-06	4,34E-05	No DEG
ENSG00000100106	TRIOBP	-2,90	-1,53	1,96E-18	5,14E-17	No DEG
ENSG00000099817	POLR2E	-2,90	-1,53	2,91E-22	1,10E-20	No DEG
ENSG00000253731	PCDHGA6	-2,90	-1,53	2,51E-14	4,39E-13	No DEG
ENSG00000201672	SNORD113-4	2,90	1,53	1,32E-03	4,73E-03	No DEG
ENSG00000260526	RP11-73K9.2	2,90	1,53	8,97E-06	4,97E-05	No DEG
ENSG00000168890	TMEM150A	-2,90	-1,53	1,87E-16	4,03E-15	No DEG
ENSG00000103111	MON1B	-2,90	-1,53	4,70E-20	1,45E-18	No DEG
ENSG00000145936	KCNMB1	-2,89	-1,53	2,54E-08	2,13E-07	No DEG
ENSG00000186842	LINC00846	-2,89	-1,53	9,68E-05	4,42E-04	No DEG
ENSG00000201512	SNORA71C	-2,89	-1,53	1,53E-02	4,11E-02	No DEG

ENSG00000133313	CNDP2	-2,89	-1,53	2,62E-26	1,40E-24	No DEG
ENSG00000186350	RXRA	-2,89	-1,53	2,04E-20	6,55E-19	No DEG
ENSG00000071794	HLTF	2,89	1,53	3,18E-13	4,98E-12	No DEG
ENSG00000165704	HPRT1	2,89	1,53	6,92E-21	2,33E-19	No DEG
ENSG00000229110	AC006355.3	-2,89	-1,53	2,91E-03	9,62E-03	No DEG
ENSG00000250902	SMAD1-AS1	-2,89	-1,53	6,59E-03	1,98E-02	No DEG
ENSG00000106290	TAF6	-2,89	-1,53	1,88E-24	8,71E-23	No DEG
ENSG00000241544	RP11-6F2.5	-2,89	-1,53	6,88E-03	2,05E-02	No DEG
ENSG00000130816	DNMT1	-2,89	-1,53	4,03E-26	2,11E-24	No DEG
ENSG00000227077	AC107983.4	-2,89	-1,53	1,43E-07	1,06E-06	No DEG
ENSG00000264271	RN7SL488P	2,89	1,53	1,83E-02	4,79E-02	No DEG
ENSG00000260853	RP11-264B17.2	-2,89	-1,53	5,15E-03	1,59E-02	No DEG
ENSG00000100401	RANGAP1	-2,89	-1,53	1,10E-18	3,01E-17	No DEG
ENSG00000168310	IRF2	-2,89	-1,53	4,21E-13	6,50E-12	No DEG
ENSG00000215105	TTC3P1	2,89	1,53	3,86E-10	4,12E-09	No DEG
ENSG00000165891	E2F7	-2,89	-1,53	1,46E-03	5,18E-03	No DEG
ENSG00000155090	KLF10	-2,89	-1,53	5,16E-05	2,49E-04	No DEG
ENSG00000242267	SKINTL	2,89	1,53	1,28E-03	4,62E-03	No DEG
ENSG00000105737	GRIK5	-2,89	-1,53	1,18E-06	7,52E-06	No DEG
ENSG00000102468	HTR2A	2,89	1,53	6,25E-03	1,89E-02	No DEG
ENSG00000149609	C20orf144	-2,89	-1,53	1,88E-04	8,14E-04	No DEG
ENSG00000033100	CHPF2	-2,89	-1,53	2,66E-15	5,14E-14	No DEG
ENSG00000145703	IQGAP2	2,89	1,53	1,16E-15	2,31E-14	UP
ENSG00000184672	RALYL	2,89	1,53	4,52E-05	2,20E-04	No DEG
ENSG00000122035	RASL11A	-2,89	-1,53	3,51E-05	1,75E-04	No DEG
ENSG00000271945	RP11-354K4.2	2,89	1,53	3,60E-03	1,16E-02	No DEG
ENSG00000171056	SOX7	-2,88	-1,53	1,23E-11	1,59E-10	No DEG
ENSG00000106089	STX1A	-2,88	-1,53	3,72E-05	1,85E-04	No DEG
ENSG00000179294	C17orf96	-2,88	-1,53	1,66E-03	5,82E-03	No DEG
ENSG00000230912	RP3-508I15.10	-2,88	-1,53	8,38E-03	2,44E-02	No DEG
ENSG00000151650	VENTX	-2,88	-1,53	7,14E-05	3,35E-04	No DEG
ENSG00000272579	RP11-101E13.5	2,88	1,53	2,44E-20	7,77E-19	No DEG

ENSG00000116514	RNF19B	-2,88	-1,53	6,17E-15	1,15E-13	No DEG
ENSG00000223238	RNA5SP294	2,88	1,53	1,64E-02	4,37E-02	No DEG
ENSG00000239111	snoU13	2,88	1,53	1,63E-02	4,33E-02	No DEG
ENSG00000063761	ADCK1	-2,88	-1,53	8,68E-10	8,85E-09	No DEG
ENSG00000250770	RP5-1063M23.1	-2,88	-1,53	4,23E-04	1,70E-03	No DEG
ENSG00000254112	KB-1205A7.1	-2,88	-1,53	1,07E-03	3,94E-03	No DEG
ENSG00000270945	HSPE1P7	-2,88	-1,53	7,68E-03	2,26E-02	No DEG
ENSG00000136859	ANGPTL2	-2,88	-1,53	6,18E-07	4,14E-06	No DEG
ENSG00000267490	CTD-3116E22.6	2,88	1,53	1,05E-02	2,98E-02	No DEG
ENSG00000243437	RN7SL370P	-2,88	-1,52	3,39E-03	1,10E-02	No DEG
ENSG00000087074	PPP1R15A	-2,88	-1,52	4,57E-22	1,70E-20	No DEG
ENSG00000249623	RP11-610J23.1	-2,88	-1,52	2,47E-03	8,30E-03	No DEG
ENSG00000099364	FBXL19	-2,88	-1,52	7,59E-17	1,71E-15	No DEG
ENSG00000112640	PPP2R5D	-2,88	-1,52	1,40E-19	4,08E-18	No DEG
ENSG00000254035	RP11-281O15.4	-2,87	-1,52	3,19E-03	1,04E-02	No DEG
ENSG00000203855	HSD3BP4	-2,87	-1,52	5,08E-03	1,57E-02	No DEG
ENSG00000105245	NUMBL	-2,87	-1,52	4,27E-12	5,86E-11	No DEG
ENSG00000183397	C19orf71	-2,87	-1,52	2,26E-06	1,38E-05	No DEG
ENSG00000251196	RP11-54F2.1	-2,87	-1,52	4,31E-04	1,73E-03	No DEG
ENSG00000109736	MFSD10	-2,87	-1,52	1,34E-15	2,66E-14	No DEG
ENSG00000272631	RP11-359E3.4	-2,87	-1,52	4,22E-04	1,70E-03	No DEG
ENSG00000204173	LRRC37A5P	2,87	1,52	1,23E-04	5,49E-04	No DEG
ENSG00000172322	CLEC12A	2,87	1,52	3,61E-03	1,16E-02	UP
ENSG00000176714	CCDC121	2,87	1,52	1,02E-10	1,17E-09	No DEG
ENSG00000095319	NUP188	-2,87	-1,52	6,65E-19	1,85E-17	No DEG
ENSG00000229841	RP11-361K17.2	2,87	1,52	4,85E-10	5,12E-09	No DEG
ENSG00000261253	AC137932.6	-2,87	-1,52	1,87E-04	8,08E-04	No DEG
ENSG00000266798	AC078816.1	2,87	1,52	1,78E-02	4,68E-02	No DEG
ENSG00000105793	GTPBP10	2,87	1,52	1,94E-15	3,79E-14	No DEG
ENSG00000268927	FLJ00418	-2,87	-1,52	1,44E-02	3,91E-02	No DEG
ENSG00000225880	LINC00115	-2,87	-1,52	6,29E-06	3,56E-05	No DEG
ENSG00000204351	SKIV2L	-2,86	-1,52	1,14E-25	5,82E-24	No DEG

ENSG00000229891	Z83851.1	2,86	1,52	4,26E-04	1,71E-03	No DEG
ENSG00000150281	CTF1	-2,86	-1,52	3,13E-07	2,21E-06	No DEG
ENSG00000174944	P2RY14	2,86	1,52	1,10E-08	9,66E-08	No DEG
ENSG00000142065	ZFP14	2,86	1,52	6,61E-13	1,00E-11	No DEG
ENSG00000230701	FBXW4P1	-2,86	-1,52	3,39E-03	1,10E-02	No DEG
ENSG00000103249	CLCN7	-2,86	-1,52	7,66E-20	2,32E-18	No DEG
ENSG00000155034	FBXL18	-2,86	-1,52	5,34E-14	9,02E-13	No DEG
ENSG00000142156	COL6A1	-2,86	-1,52	3,70E-15	7,05E-14	No DEG
ENSG00000270081	RP5-935K16.1	2,86	1,52	6,24E-19	1,74E-17	No DEG
ENSG00000154269	ENPP3	2,86	1,52	2,34E-03	7,94E-03	No DEG
ENSG00000158292	GPR153	-2,86	-1,52	1,66E-08	1,43E-07	No DEG
ENSG00000108219	TSPAN14	-2,86	-1,52	9,90E-27	5,50E-25	No DEG
ENSG00000258768	CTD-2292M16.8	2,86	1,52	1,07E-06	6,87E-06	No DEG
ENSG00000186130	ZBTB6	2,86	1,52	5,91E-22	2,18E-20	No DEG
ENSG00000180221	TPT1P10	-2,86	-1,52	1,66E-03	5,84E-03	No DEG
ENSG00000119048	UBE2B	2,86	1,52	6,79E-32	5,44E-30	No DEG
ENSG00000177426	TGIF1	-2,86	-1,52	8,61E-07	5,63E-06	No DEG
ENSG00000245928	RP11-630D6.5	-2,86	-1,52	9,80E-03	2,80E-02	No DEG
ENSG00000143252	SDHC	2,86	1,52	7,96E-15	1,48E-13	No DEG
ENSG00000259431	THTPA	-2,86	-1,52	1,23E-09	1,23E-08	No DEG
ENSG00000197063	MAFG	-2,86	-1,52	9,31E-17	2,08E-15	No DEG
ENSG00000248714	RP11-1079K10.3	-2,86	-1,52	8,44E-03	2,45E-02	No DEG
ENSG00000089199	CHGB	-2,86	-1,52	3,40E-04	1,39E-03	No DEG
ENSG00000162600	OMA1	2,86	1,51	2,74E-11	3,39E-10	No DEG
ENSG00000198189	HSD17B11	2,86	1,51	1,90E-20	6,12E-19	No DEG
ENSG00000249077	RP11-478C1.8	2,86	1,51	1,45E-02	3,94E-02	No DEG
ENSG00000183513	COA5	2,86	1,51	4,01E-16	8,36E-15	No DEG
ENSG00000182021	RP11-381O7.3	-2,86	-1,51	1,20E-03	4,35E-03	No DEG
ENSG00000073050	XRCC1	-2,86	-1,51	6,05E-17	1,38E-15	No DEG
ENSG00000151176	PLBD2	-2,86	-1,51	1,89E-22	7,25E-21	No DEG
ENSG00000166845	C18orf54	2,86	1,51	2,41E-10	2,64E-09	No DEG
ENSG00000259308	RP11-382A20.1	-2,85	-1,51	4,27E-03	1,35E-02	No DEG

ENSG00000262766	RP11-196G11.4	-2,85	-1,51	9,07E-05	4,16E-04	No DEG
ENSG00000169894	MUC3A	-2,85	-1,51	5,90E-03	1,79E-02	No DEG
ENSG00000185847	RP1-46F2.2	-2,85	-1,51	4,81E-04	1,91E-03	No DEG
ENSG00000244144	RP11-757F18.3	-2,85	-1,51	6,48E-03	1,95E-02	No DEG
ENSG00000207370	Y_RNA	2,85	1,51	2,13E-04	9,11E-04	No DEG
ENSG00000232573	RPL3P4	2,85	1,51	2,33E-05	1,20E-04	No DEG
ENSG00000266208	CTD-2267D19.3	-2,85	-1,51	1,89E-05	9,93E-05	No DEG
ENSG00000273240	RP11-455J20.3	2,85	1,51	1,32E-03	4,72E-03	No DEG
ENSG00000146094	DOK3	-2,85	-1,51	3,16E-06	1,88E-05	No DEG
ENSG00000161692	DBF4B	-2,85	-1,51	8,10E-10	8,32E-09	No DEG
ENSG00000232184	RP11-370K11.1	2,85	1,51	1,14E-05	6,20E-05	No DEG
ENSG00000141564	RPTOR	-2,85	-1,51	3,82E-22	1,43E-20	No DEG
ENSG00000226443	GAPDHP32	-2,85	-1,51	1,85E-02	4,85E-02	No DEG
ENSG00000227110	LMCD1-AS1	2,85	1,51	1,28E-05	6,91E-05	No DEG
ENSG00000066735	KIF26A	-2,84	-1,51	8,01E-11	9,31E-10	No DEG
ENSG00000105281	SLC1A5	-2,84	-1,51	4,51E-06	2,62E-05	No DEG
ENSG00000184574	LPAR5	-2,84	-1,51	1,35E-06	8,53E-06	No DEG
ENSG00000259807	RP11-426C22.4	-2,84	-1,51	7,22E-03	2,14E-02	No DEG
ENSG00000213777	CTD-2224J9.8	-2,84	-1,51	1,11E-03	4,05E-03	No DEG
ENSG00000206754	SNORD101	-2,84	-1,51	8,62E-03	2,50E-02	No DEG
ENSG00000104497	SNX16	2,84	1,51	3,02E-21	1,05E-19	No DEG
ENSG00000105698	USF2	-2,84	-1,51	1,19E-23	5,18E-22	No DEG
ENSG00000177628	GBA	-2,84	-1,51	1,28E-20	4,18E-19	No DEG
ENSG00000166455	C16orf46	-2,84	-1,51	1,26E-04	5,64E-04	No DEG
ENSG00000115282	TTC31	-2,84	-1,51	2,34E-16	4,99E-15	No DEG
ENSG00000104327	CALB1	2,84	1,51	1,16E-05	6,29E-05	No DEG
ENSG00000184258	CDR1	-2,84	-1,51	2,97E-13	4,67E-12	No DEG
ENSG00000224557	HLA-DPB2	2,84	1,50	1,90E-02	4,97E-02	UP
ENSG00000079974	RABL2B	-2,84	-1,50	1,69E-10	1,88E-09	No DEG
ENSG00000177000	MTHFR	-2,84	-1,50	2,58E-09	2,47E-08	No DEG
ENSG00000133477	FAM83F	-2,84	-1,50	7,57E-07	4,99E-06	No DEG
ENSG00000182378	PLCXD1	-2,83	-1,50	5,63E-07	3,80E-06	No DEG

ENSG00000174516	PELI3	-2,83	-1,50	2,28E-14	4,01E-13	No DEG
ENSG00000114956	DGUOK	-2,83	-1,50	5,53E-22	2,05E-20	No DEG
ENSG00000235043	TECRP1	-2,83	-1,50	5,61E-03	1,72E-02	No DEG
ENSG00000204231	RXRB	-2,83	-1,50	1,65E-22	6,42E-21	No DEG
ENSG00000110318	KIAA1377	2,83	1,50	1,22E-05	6,63E-05	No DEG
ENSG00000167614	TTYH1	-2,83	-1,50	4,92E-06	2,84E-05	No DEG
ENSG00000196187	TMEM63A	-2,83	-1,50	2,51E-17	5,96E-16	No DEG
ENSG00000172426	RSPH9	-2,83	-1,50	1,01E-06	6,50E-06	No DEG
