# Supplementary material for: Epigenetic regulation of transcription factor binding motifs promotes Th1 response in Chagas disease cardiomyopathy
Source: Front Immunol. 2022 Aug 22;13:958200. doi: 10.3389/fimmu.2022.958200 (PMC9441916; doi:10.3389/fimmu.2022.958200)
Supplement: Supplementary Table 1 — Biological samples included in this study. [file DataSheet_1.zip › Supplementary Material/Supplementary Table 6.pdf]

Supplementary table 6. Gene ontology analysis based on diffrentially expressed genes between control and severe CCC and/or between control and DCM.

ID	Term	Corrected pvalue	Levels	Nb. Genes	% Associated Genes	%Genes associated to CCC	%Genes associated to DCM	Genes associated to CCC	Genes associated to DCM
GO:0009612	response to mechanical stimulus	1,65E-02	[3]	57,00	24,46	32,03	67,97	[ANGPT2, BTG2, CASP8, CXCL10, CXCR4, DPEP2, FOSB, FOSL1, MPO, PTK2B, STAT1, THBS1, TIFAB, TLR8, TMC2, TNFSF14]	[ACTA1, AGT, ANO3, ARHGDIA, ASIC3, ATP1A1, ATP1A3, BAG3, BTG2, CHI3L1, CITED2, CLCN6, COL1A1, DAG1, ENDOG, ENG, FADD, FASN, FOSL1, GATA4, GPI, HTR2A, IL1B, JUND, JUP, KCNK2, LTBR, MAP3K14, MAPK3, NPPA, P2RX3, PIEZO1, PKD1, POSTN, PPL, PTN, RELA, SHANK3, SLC9A1, SRC, THBS1, TNFRSF1A, TRPV4, WHRN]
GO:0061302	smooth muscle cell-matrix adhesion	1,38E-02	[5]	6,00	100,00	17,76	82,24	[PLAU]	[APOD, DDR1, EFEMP2, PLAU, SERPINE1, VTN]
GO:0070647	protein modification by small protein conjugation or removal	1,62E-02	[6, 7]	115,00	9,50	27,63	72,37	[ABCB11, ANGPT1, AREG, ARRDC5, CDC20B, CDKN2A, CISH, DEPDC1B, FBXL16, FBXL19, FBXO41, H2BC14, H2BC9, KLHL41, NLRP3, NUP210, OTULINL, PTPN22, RNF122, RNF182, SOCS1, SOCS3, TNFAIP3, TNFSF13B, TNIP3, TRAF1, TRIM14, UBD]	[AAAS, ABCB11, ABL1, ACTB, ANGPT1, AREG, ASB16, ASB6, BCOR, CDCA3, CDK1, CISH, DAXX, DCAF15, DTX1, DTX2, DTX3, EPOP, FBXL12, FBXL18, FBXL19, FEM1A, GSK3A, HCFC1, HECTD3, HGS, HLTF, IRF2BPL, KCTD11, LIMK1, MARCHF11, MBD6, MED12, MED24, MGRN1, MYC, NHLRC3, NOD2, NUP188, PAF1, PCGF2, PEF1, PELI3, PEX14, PHC1, PIAS4, PML, PPP1R11, RAD23A, RANGAP1, RASD2, RASSF5, RELA, RNF122, RNF126, RNF157, RNF167, RNF182, RNF19B, RNF208, RNF26, RNF39, RNU1-93P, RUSC1, SEPTIN4, SHARPIN, SMAD3, SOCS3, SPATA2, SPHK1, STX1A, SYVN1, TICAM1, TNFAIP1, TNIP1, TRIB1, TRIB3, TRIM47, TRIM62, TRIM8, UBA1, UBA52, UBE2B, UBE2L6, UBE2QL1, UBOX5, USP2, WDTC1, WFS1, WWP2, ZC3H12A, ZMIZ1, ZNF598, ZNRF1. ZSWIM8]
GO:0097006	regulation of plasma lipoprotein particle levels	1,60E-02	[2, 3]	31,00	30,69	28,50	71,50	[CEL, LCP1, LDLR, LPAL2, MPO, PLA2G7, SOAT2, TREM2]	[ABCA7, ABCC8, ABCD1, ADIPOQ, AGT, AGTR1, AP2A1, APOA1, APOM, ARF1, CEL, CES3, CETP, CSK, EHD1, LCAT, LDLR, LDLRAP1, LIPG, MFSD2A, PLA2G2A, PLA2G7, PLAGL2, PLTP, PRKACA, ZDHHC8]
GO:0097028	dendritic cell differentiation	4,36E-02	[6, 7, 8, 9, 10]	19,00	37,25	61,29	38,71	[BATF, BLK, CAMK4, CCL19, FLT3, IRF4, IRF8, LILRB1, TREM2, TRPM2, UBD]	[AGER, BATF2, CCR7, DHRS2, FLT3, LGALS9, LTBR, RELB, SPI1]
GO:0010469	regulation of signaling receptor activity	4,00E-02	[3, 4, 5, 6]	50,00	24,75	40,61	59,39	[AREG, CALCR, CCL2, CCL5, CCR2, DAPK1, DLGAP2, EREG, GPRC5A, IFNG, LILRB4, NCF1, NPTX2, PLAU, PSCA, PTK2B, RASGRF1, RGS9, SHISA8]	[ARC, AREG, CALCR, CAMK2B, CAPN1, CCL2, CGA, CHGA, DKK2, DLGAP4, GPRC5A, GPRIN1, HBEGF, HFE, IL18BP, IL1RN, LY6E, LYNX1, MINK1, MTRNR2L1, MTRNR2L10, MTRNR2L12, MTRNR2L2, MTRNR2L3, MTRNR2L6, MTRNR2L8, MTRNR2L9, NEFL, NLGN2, PLAU, PRRT1, SERPINE1, SHANK3, SLC6A9, SRC, TNF]
GO:1902563	regulation of neutrophil activation	2,82E-02	[4, 5, 6, 7]	8,00	72,73	61,83	38,17	[CD177, ITGB2, PRAM1, PTAFR, SYK]	[CD177, GRN, PLA2G2A, SPI1]
GO:0002577	regulation of antigen processing and presentation	4,12E-04	[3, 4]	14,00	60,87	53,53	46,47	[CCL19, CD74, FGL2, HLA-DOA, PYCARD, THBS1, TREM2, WAS]	[CCL21, CCR7, CD74, GNAO1, HFE, HLA-DOB, NOD2, THBS1, WAS]
GO:0003013	circulatory system process	2,46E-02	[3]	128,00	19,72	29,96	70,04	[ABCC3, ADORA3, ANGPT1, ANPEP, AREG, ATP2A3, CNR1, CXCL10, CXCR4, CYP11B1, DRD1, EDN2, EPX, GPR4, HOPX, IRX3, KCNK15, KNG1, LVRN, MYH6, NAMPT, NLRP3, NPPB, NPPC, P2RY2, PIK3CG, PTAFR, PTGER2, SCNN1B, SLC8A3, SMTNL2, STAT1, SUCNR1, TBXAS1, XCL2]	[ABCC5, ABCC8, ACTC1, ADIPOQ, AGER, AGT, AGTR1, ANGPT1, APLN, AREG, ATP1A1, ATP1A3, ATP1B2, CACNA1H, CEACAM1, CHGA, CNR1, CRP, CYB5R3, CYP21A2, DHX34, E2F4, EDN2, FOXC2, FXYD6, GATA4, GJD3, GNAI2, GNAO1, GNB3, GPER1, GPR37L1, GPR4, GRIP2, GSK3A, HBB, HBEGF, HCN4, HOPX, HRH2, HSPB7, HTR1B, HTR2A, ICAM1, IRX3, ITPR3, JUP, KCNH2, KCNIP2, KCNJ5, KCNK15, KCNK3, KCNK6, KCNMB2, KCTD11, KNG1, MAP2K3, MECP2, MFSD2A, MME, MYH6, NOS3, NPPA, NPPB, NPR3, NPRL3, P2RY2, PCDHA4, PDGFB, POSTN, PPP1R13L, PRKACA, PTP4A3, PTPRU, RAMP2, RANGRF, RASL10B, RNU1-93P, S100A1, SCN5A, SERPING1, SLC1A4, SLC1A5, SLC29A1, SLC29A2, SLC29A4, SLC2A4, SLC38A5, SLC4A3, SLC5A6, SLC6A17, SLC6A9, SLC7A1, SLC7A5, SLC7A8, SLC9A1, SLC9A3R1, SMAD3, SMTNL2, SRC, SREBF1, TBX5, TIMP1, TRPV4, VEGFA, ZC3H12A]
GO:0006968	cellular defense response	8,65E-04	[4]	24,00	40,00	80,40	19,60	[CCR2, CCR3, CCR5, CXCL9, FCMR, FOSL1, GNLY, ITK, KIR3DL2, KLRC2, KLRC3, KLRG1, NCF1, NCR1, PRF1, PTK2B, SH2D1A, SPN, TRAT1]	[C5AR1, FOSL1, LGALS3BP, RELA, TCIRG1, ZNF395]
GO:0031295	T cell costimulation	6,87E-03	[3, 6, 7, 8, 9]	23,00	37,10	79,54	20,46	[BTLA, CARD11, CCL19, CD3E, CD40LG, CD5, CD80, CD86, CTLA4, GRAP2, ICOS, KLRK1, LCK, LILRB4, PTPN6, TNFSF13B, TNFSF14, VAV1]	[BTLA, CCL21, CCR7, CSK, PDCD1, SRC]

GO:0007267	cell-cell signaling	5,31E-03	[2, 3]	317,00	17,33	37,46	62,54	[APBA2, AREG, BHLHA15, BLK, BTK, C1QA, C1QTNF1, CACNA1E, CAMK4, CCDC88C, CCL17, CCL2, CCL22, CCL24, CCL3, CCL4, CCL5, CCR2, CCR5, CD200R1, CD33, CDH1, CEL, CNR1, CNR2, CXCL10, CXCL11, CXCL5, CXCL6, CXCL9, DCC, DEPDC1B, DLGAP2, DPEP2, DRD1, EGR3, ELAVL4, EREG, FASLG, FCRL2, FFAR4, FOLR2, FOXP3, FRZB, GABBR2, GRAP2, HLA-DRB1, IFNG, IL18, IL26, ITGB2, KCNJ10, KLF5, KLRG1, KMO, LEF1, LILRB1, LTA, LTB, LYPD6B, MIDN, MUSK, MYB, MYCBPAP, NAMPT, NAPSB, NLRP3, NPPC, NPTX2, NR4A2, NSG1, P2RY12, PDE6G, PLCB2, PNOC, PRKCB, PTK2B, RAB3B, RASGRF1, RGS10, RIMBP3B, SALL1, SDC1, SH2D1A, SHISA8, SIRPG, SLC24A4, SLC8A3, SMPD3, STXBP2, SUCNR1, SYK, THY1, TNFAIP3, TNFSF8, TNR, TRABD2A, TRPM2, UBD, UCP2, WNT1, WNT10A, WNT10B, XCL1]	[ABCC8, ABL1, ABR, ACP4, ADCY1, ADGRE5, ADGRG1, ADIPOQ, AGER, AGT, AGTR1, AMH, AP2A1, APLN, ARC, AREG, ARF1, BCL7B, BCL9L, BCR, BMP8A, BSN, C1QTNF1, C2CD2L, CACNA1E, CACNB4, CALB1, CAMK2B, CCL2, CCL21, CCL24, CEL, CELSR2, CGA, CHGA, CHRNG, CNR1, COL1A1, CPLX1, CPNE6, CRHR2, CSNK1E, CTF1, CTHRC1, CX3CL1, CXXC4, DAB2IP, DAG1, DAGLA, DGKZ, DHX34, DKK2, DLGAP4, DOC2B, DVL3, ECE2, EFNA3, FFAR2, FOXM1, FRMD8, GABBR2, GABRR2, GAL3ST4, GATA4, GIT1, GJA4, GJD3, GLRA1, GNAI2, GNAO1, GPER1, GPI, GPRIN1, GRIK5, GRM2, GSK3A, HCN4, HCRTR1, HESX1, HFE, HRH2, HTR1B, HTR2A, IFT80, IL1B, IL1RN, ILK, INHA, ITGA3, ITPR3, JPH4, JUP, KCNIP2, KCNJ5, KCNK3, KCNMB1, KCTD11, KLF10, KLF5, KPNA2, LGR6, LRRC4, LRRC8A, LY6E, LYNX1, LZTS1, LZTS2, MAP1A, MARK2, MARK4, MECP2, MED12, MIDN, MINK1, MME, MOV10, NAPA, NDP, NFATC4, NGF, NKD2, NLGN2, NNAT, NODAL, NOTCH1, NPPA, NR1D1, NR4A2, NRARP, NRGN, NSG1, NSMF, OTOF, P2RX3, P2RX5, PAF1, PCDH1, PCDHA4, PCDHA6, PDGFB, PFN1, PIAS4, PIP5K1C, PKD1, PORCN, PPARD, PPP1R9B, PPP2R1A, PRKACA, PRKAR2B, PRRT1, PRRT2, PTGES, PTGIR, PTN, PTPN23, PTPRN, PTPRU, PYGO2, RAB11B, RAB11FIP5, RAB3A, RANGRF, RAP1A, RARA, RASL10B, RELA, RNU1-93P, RPH3A, S1PR2, SCN5A, SEPTIN5, SEPTIN9, SH2D3C, SH3GL1, SHANK3, SIDT2, SLC12A4, SLC1A4, SLC25A23, SLC29A1, SLC30A1, SLC4A10, SLC9A3R1, SMAD3, SMARCA4, SORCS2, SOX10, SOX13, SOX7, SRC, SREBF1, SRF, STAT3, STX1A, STX1B, SV2A, SYN1, SYNGAP1, SYT2, SYT7, TBC1D24, TBX5, TCF7L1, TCIRG1, TGFB1I1, TLE2, TLE3, TLE5, TMEM198, TMEM88, TNF, TRPV4, TSC2, TSKU, UBA52, UBE2B, VANGL2, VAX2, VIPR2, WNK2, WNT3, WNT9B, ZDHHC12, ZYX]
GO:0002260	lymphocyte homeostasis	4,68E-03	[3, 6]	24,00	36,92	66,03	33,97	[CD74, CORO1A, DOCK10, FOXP3, GAPT, GPR174, LMO1, NCKAP1L, SIT1, TNFAIP3, TNFRSF13B, TNFRSF17, TNFSF13B, TNFSF14, TSC22D3]	[ABL1, CD74, FADD, FASN, GPAM, JAK3, LGALS9, SH2B2, SPNS2, TCIRG1]
GO:0007600	sensory perception	3,28E-03	[4]	92,00	8,87	35,13	64,87	[BFSP2, C1QTNF1, CCL2, CCL3, CCR2, CNR1, CNR2, CRB2, CXCR4, FFAR4, GRXCR2, GUCY2D, KCNJ10, LEF1, MYO3B, OPN5, OR2I1P, PDE6G, PLCB2, PNOC, PTAFR, RGS9, SCNN1B, SLC24A4, TACSTD2, TMC2, TMPRSS3, WNT10B]	[ADCY3, AOC2, ASIC3, ATP6V1B1, ATPSCKMT, BBS10, C1QTNF1, C5AR1, CCL2, CNNM4, CNR1, CNTN5, COL1A1, GABRR2, GJD3, GLRA1, GNAI2, GRIK5, HPS1, HTR2A, ICAM1, ITPR3, KCNQ4, KERA, LUM, MAPK3, MECP2, MED12, MME, MYH7B, MYO3A, MYO7A, MYO7B, NDP, NLGN2, OR2H2, OR4C3, OR51Q1, OR52H1, OR52N1, OR52N4, OR52N5, OR56B2P, OTOF, P2RX3, PCDHA4, PCDHA6, PDE4A, PITPNA, PTGES, RAB3A, RABGGTA, RCVRN, RDH5, RGS16, RP1L1, RTP3, SLC4A10, SLC52A3, SLC9A1, SLC9A3R1, TMEM100, TRIOBP, VAX2, WFS1, WHRN, ZFHX2]
GO:0046643	regulation of gamma-delta T cell activation	2,82E-02	[6, 7, 8]	8,00	72,73	79,54	20,46	[EGR3, LEF1, LILRB1, NCKAP1L, PTPRC, SYK]	[NOD2, SOX13]
GO:0046718	viral entry into host cell	4,94E-04	[3, 6]	46,00	29,49	48,41	51,59	[ANPEP, CCR5, CD4, CD74, CD80, CD86, CR2, CXCR4, FCN1, FCN3, HLA-DRB1, ITGB7, LAMP3, LDLR, MOG, MRC1, NLRP3, PTX3, SELPLG, SIGLEC1, TRIM14]	[AGTR1, CD74, CDK1, CIITA, CLDN9, DAG1, EPHA2, FCN3, HAVCR1, HTR2A, HYAL1, ICAM1, IFITM1, IFITM2, IFITM3, ITGA5, LDLR, LGALS9, LY6E, MRC1, NECTIN1, NECTIN2, PVR, SLC1A5, SLC52A2, SMPD1, TRIM62, TRIM8, WWP2]
GO:0051090	regulation of DNA-binding transcription factor activity	2,75E-03	[3, 6, 7, 8, 9, 10, 11, 12, 13]	98,00	21,92	40,69	59,31	[ABRA, ADAM8, ADORA3, AIM2, AREG, BHLHE40, BMP7, BTK, CARD11, CD40LG, CDKN2A, EOMES, FOSL1, FOXP3, GFI1, HCLS1, IKBKE, IL18, IL18RAP, ITGB2, NFAM1, NLRC3, NLRP2, NLRP3, PIM1, PRKCB, PRKCQ, PYCARD, RTKN2, TLR6, TNFAIP3, TRAF1, TRIM14, WNT1, WNT10B, XCL1]	[ABRA, ADCY1, AGER, AGT, AMH, AREG, ARHGEF5, BHLHE40, CRAT, CRTC2, CX3CL1, DAB2IP, FLOT2, FOSL1, FOXS1, GLIS2, HDAC5, HEYL, HSF1, ICAM1, ID3, IL1B, IRAK2, JUP, LGALS9, LPAR5, LTF, MAP3K10, MAPK3, MAVS, MEN1, NFKB2, NFKBIB, NLRC5, NOD2, NODAL, PEX14, PIAS4, PIDD1, PIM1, PKD1, PPRC1, RELA, RGCC, RTKN2, SIK1, SMAD3, SMARCA4, SMARCB1, SPHK1, SPI1, SRF, STAT3, STING1, TCF3, TCF7L1, TICAM1, TNF, TRIB1, TRIM62, TRIM8, UBA52, VEGFA, VSIG2, WFS1, WWP2, ZBTB7A, ZC3H12A]
GO:0051091	positive regulation of DNA-binding transcription factor activity	3,93E-03	[4, 7, 8, 9, 10, 11, 12, 13, 14]	68,00	24,11	39,31	60,69	[ABRA, ADAM8, AIM2, AREG, BTK, CARD11, CD40LG, FOSL1, HCLS1, IKBKE, IL18, IL18RAP, ITGB2, NFAM1, NLRP3, PRKCB, PRKCQ, PYCARD, RTKN2, TLR6, TRAF1, TRIM14, WNT1, WNT10B]	[ABRA, ADCY1, AGER, AGT, AMH, AREG, ARHGEF5, CRAT, CRTC2, CX3CL1, FLOT2, FOSL1, HDAC5, HSF1, ICAM1, IL1B, IRAK2, JUP, LGALS9, LPAR5, LTF, MAVS, NFKB2, NFKBIB, NOD2, NODAL, PIDD1, PPRC1, RELA, RGCC, RTKN2, SMAD3, SMARCA4, SMARCB1, SPHK1, SRF, STAT3, STING1, TCF3, TCF7L1, TICAM1, TNF, TRIM62, TRIM8, UBA52, VEGFA, VSIG2, ZBTB7A]
GO:0043491	protein kinase B signaling	2,07E-03	[4, 5, 6]	66,00	24,72	45,50	54,50	[ADAM8, ANGPT1, AREG, BANK1, C1QTNF1, CALCR, CASS4, CCL19, CCL2, CCL3, CCL5, CD19, CD80, CD86, EREG, FLT3, HCLS1, ICOS, IL18, IL26, LCK, P2RY12, PIK3CG, PIK3R5, RAC2, SMPD3, THBS1, TRAT1, VAV1]	[ANGPT1, AREG, C1QTNF1, CALCR, CCL2, CCL21, CCR7, CHI3L1, CX3CL1, DAG1, ENG, EPHA2, ERBB2, FAM110A, FGFR4, FLT3, GAB2, GATA4, GPER1, GRM2, HBEGF, IL1B, ILK, LOX, MAZ, MEIS3P1, MSTN, OSM, PDGFB, PDGFRB, PEAR1, PLK3, PPP2R5B, RAC2, RASD2, RHOG, SH2B3, SLC9A3R1, SRC, THBS1, THPO, TMEM100, TNF, TRIB3, TSC2]
GO:0051896	regulation of protein kinase B signaling	3,78E-02	[5, 6, 7]	58,00	23,87	46,35	53,65	[ADAM8, ANGPT1, AREG, BANK1, C1QTNF1, CALCR, CASS4, CCL19, CCL3, CD19, CD80, CD86, EREG, FLT3, HCLS1, ICOS, IL18, IL26, LCK, P2RY12, PIK3CG, PIK3R5, RAC2, THBS1, TRAT1, VAV1]	[ANGPT1, AREG, C1QTNF1, CALCR, CCL21, CCR7, CHI3L1, CX3CL1, DAG1, ENG, EPHA2, ERBB2, FAM110A, FGFR4, FLT3, GAB2, GATA4, GPER1, GRM2, HBEGF, ILK, MAZ, MEIS3P1, MSTN, OSM, PDGFB, PDGFRB, PPP2R5B, RAC2, RASD2, RHOG, SH2B3, SLC9A3R1, SRC, THBS1, THPO, TNF, TRIB3, TSC2]

GO:0034612	response to tumor necrosis factor	3,48E-03	[5]	78,00	23,35	49,11	50,89	[AIM2, CASP8, CCL17, CCL19, CCL2, CCL22, CCL24, CCL3, CCL3L1, CCL4, CCL4L1, CCL5, CD27, CD40LG, LTA, LTB, PTK2B, PYCARD, SELE, SMPD3, STAT1, SYK, THBS1, TNFAIP3, TNFRSF11B, TNFRSF13B, TNFRSF17, TNFRSF18, TNFSF13B, TNFSF14, TNFSF8, TRAF1, UBD, XCL1, XCL2]	[ACTN4, ADAMTS7, ADIPOQ, APOA1, CCL11, CCL2, CCL21, CCL24, CD14, CHI3L1, CLIP3, COL1A1, CX3CL1, DAB2IP, ENDOG, FABP4, FADD, FASN, GBA, GPER1, HYAL1, ICAM1, IL18BP, ILK, LTBR, MAP3K14, MAPK3, NR1D1, PIAS4, PLVAP, POSTN, RELA, RNU1-93P, SELE, SHARPIN, SLC2A4, SMPD1, SMPD4, SPATA2, SPHK1, SPPL2B, THBS1, TNF, TNFRSF1A, TNFSF12, ZC3H12A, ZFP36]
GO:0071356	cellular response to tumor necrosis factor	2,96E-03	[6]	73,00	23,78	49,86	50,14	[AIM2, CASP8, CCL17, CCL19, CCL2, CCL22, CCL24, CCL3, CCL3L1, CCL4, CCL4L1, CCL5, CD27, CD40LG, LTA, LTB, PTK2B, PYCARD, SMPD3, STAT1, SYK, THBS1, TNFAIP3, TNFRSF11B, TNFRSF13B, TNFRSF17, TNFRSF18, TNFSF13B, TNFSF14, TNFSF8, TRAF1, XCL1, XCL2]	[ACTN4, ADAMTS7, ADIPOQ, APOA1, CCL11, CCL2, CCL21, CCL24, CHI3L1, CLIP3, COL1A1, CX3CL1, DAB2IP, FABP4, FADD, FASN, GBA, GPER1, HYAL1, ICAM1, IL18BP, ILK, LTBR, MAP3K14, MAPK3, NR1D1, PIAS4, PLVAP, POSTN, RELA, RNU1-93P, SHARPIN, SLC2A4, SMPD4, SPHK1, SPPL2B, THBS1, TNF, TNFRSF1A, TNFSF12, ZC3H12A, ZFP36]
GO:0001771	immunological synapse formation	2,88E-03	[4, 5]	10,00	71,43	66,03	33,97	[CCL19, CD6, CORO1A, DOCK2, DOCK8, PRF1]	[CCL21, CCR7, MSN, ZNF395]
GO:0001768	establishment of T cell polarity	1,47E-02	[5, 6]	9,00	69,23	50,90	49,10	[CCL19, CRTAM, DOCK2, DOCK8]	[CCL21, CCR7, CYP26B1, FLOT2, MYH9]
GO:0007167	enzyme linked receptor protein signaling pathway	2,53E-03	[4, 5, 6]	209,00	18,66	36,91	63,09	[ANGPT1, ANGPT2, AREG, BLK, BLNK, BMP7, BMPR1B, CASS4, CD3E, CD4, CD7, CD8A, CD8B, CHRDL1, CLNK, CRB2, CSF1R, CYBB, DOK2, EPHA6, EREG, ERFE, FAM83B, FASLG, FGR, FLT3, FOLR2, FUT7, GALNT3, GDF6, GFRA2, GPRC5A, GUCY2D, LCK, LCP2, LEF1, LIF, MATK, MMP9, MSX1, MUSK, MZB1, NAMPT, NCF1, NCKAP1L, NELL2, NPPB, NPPC, NR4A3, PDE6G, PRKCB, PRKCQ, PTK2B, RBPMS2, SH2D2A, SH2D6, SLA, SMPD3, SOCS1, SOCS3, STAP1, STYK1, SYK, THBS1, TNMD, TRAT1, VAV1, VWC2L, WNT1, ZAP70]	[ABL1, ACP4, ACTB, ADIPOQ, AGT, AKT1S1, AMH, ANGPT1, ANGPTL1, AP2A1, APLN, APOA1, APOD, AREG, ASPN, ATP6V0D1, ATP6V0E2, ATP6V1B1, ATP6V1F, BCAR1, BCL9L, BCR, BMP8A, C20orf27, CEACAM1, CITED2, CLNK, CNKSR1, COL1A1, CREB3L1, CSF1, CSPG4, CTF1, DAB2IP, DDR1, DGKD, DOK5, EFNA3, ENG, EPHA2, EPN1, ERBB2, ERFE, ESM1, FAM83G, FES, FGFR4, FGFRL1, FLT3, FLT4, FOXC2, FURIN, GAB2, GATA4, GDF6, GFRA3, GIT1, GNAI2, GPER1, GPI, GPRC5A, GPRIN1, GRB7, GSK3A, HBEGF, HESX1, HFE, HGS, HSPB1, IL1B, ILK, INHA, ITGA3, ITGA5, JAK3, KCP, LIF, LIFR, LOX, LRG1, LRRC32, MAPK3, MED12, MEGF8, MEN1, MSTN, MSX1, MUC20, NDST1, NFATC4, NGF, NGFR, NODAL, NOTCH1, NPPA, NPPB, NR4A3, PDGFB, PDGFRB, PDK2, PLAUR, PML, POLR2E, POLR2L, PRDM16, PTGIR, PTN, PTP4A3, PTPRU, PXN, RAPGEF1, RBM14, RBPMS2, RELA, RGMA, RHBDF1, RHBDF2, RNF126, SH2B1, SH2B2, SH2B3, SH2D6, SHC2, SHC4, SHKBP1, SIK2, SLC2A4, SLC39A5, SLC9A3R1, SMAD3, SOCS3, SPI1, SRC, SREBF1, STAT3, SYNGAP1, TCIRG1, TGFB1I1, THBS1, TMEM100, TNK1, TNK2, TNMD, TNS2, TSC2, UBA52, VASN, VEGFA, VTN, ZBTB7A, ZBTB7B, ZC3H3, ZMIZ1, ZMIZ2, ZYX]
GO:0007169	transmembrane receptor protein tyrosine kinase signaling pathway	5,46E-03	[5, 6, 7]	150,00	19,66	40,86	59,14	[ANGPT1, ANGPT2, AREG, BLK, BLNK, CASS4, CD3E, CD4, CD7, CD8A, CD8B, CLNK, CSF1R, CYBB, DOK2, EPHA6, EREG, ERFE, FAM83B, FASLG, FGR, FLT3, FUT7, GALNT3, GFRA2, GPRC5A, LCK, LCP2, MATK, MMP9, MUSK, MZB1, NAMPT, NCF1, NCKAP1L, NELL2, NR4A3, PDE6G, PRKCB, PRKCQ, PTK2B, SH2D2A, SH2D6, SLA, SMPD3, SOCS1, SOCS3, STAP1, STYK1, SYK, THBS1, TNMD, TRAT1, VAV1, WNT1, ZAP70]	[ABL1, ACP4, ACTB, ADIPOQ, AGT, AKT1S1, ANGPT1, ANGPTL1, AP2A1, APLN, APOD, AREG, ATP6V0D1, ATP6V0E2, ATP6V1B1, ATP6V1F, BCAR1, BCR, CEACAM1, CLNK, CNKSR1, COL1A1, CREB3L1, CSF1, CSPG4, DAB2IP, DDR1, DGKD, DOK5, EFNA3, EPHA2, EPN1, ERBB2, ERFE, ESM1, FES, FGFR4, FGFRL1, FLT3, FLT4, FOXC2, GAB2, GFRA3, GIT1, GNAI2, GPER1, GPRC5A, GPRIN1, GRB7, GSK3A, HBEGF, HGS, HSPB1, IL1B, ITGA5, JAK3, LOX, MAPK3, MED12, MSTN, MUC20, NDST1, NFATC4, NGF, NGFR, NR4A3, PDGFB, PDGFRB, PDK2, PLAUR, POLR2E, POLR2L, PTGIR, PTP4A3, PXN, RAPGEF1, RELA, RHBDF1, RHBDF2, RNF126, SH2B1, SH2B2, SH2B3, SH2D6, SHC2, SHC4, SHKBP1, SIK2, SLC2A4, SLC9A3R1, SOCS3, SRC, SREBF1, STAT3, SYNGAP1, TCIRG1, THBS1, TNK1, TNK2, TNMD, TNS2, TSC2, VEGFA, VTN, ZBTB7B]
GO:0034113	heterotypic cell-cell adhesion	6,49E-05	[4]	27,00	41,54	50,90	49,10	[ALOX15, BMP7, CD2, CD200R1, ITGA4, ITGAX, ITGB2, ITGB7, LCK, PTPRC, SKAP1, THY1]	[ADIPOQ, AGER, APOA1, FLOT2, IL1B, IL1RN, ITGA5, ITGA7, ITGAD, IZUMO1, JUP, MAPK7, MYADM, NINJ1, TNF]
GO:0034114	regulation of heterotypic cell-cell adhesion	9,88E-03	[5, 6]	14,00	50,00	41,85	58,15	[ALOX15, BMP7, LCK, SKAP1, THY1]	[ADIPOQ, AGER, APOA1, FLOT2, IL1B, IL1RN, MAPK7, MYADM, TNF]
GO:0007249	I-kappaB kinase/NF-kappaB signaling	1,39E-04	[4, 5, 6]	74,00	25,43	42,54	57,46	[ANGPT1, BCL3, BTK, CARD11, CASP8, CCL19, CD4, CD74, CLEC6A, FASLG, HLA-DRB1, IKBKE, LILRB4, NLRC3, NLRP6, PIM2, PRKCB, PYCARD, RHOH, STAT1, TIFAB, TLR8, TNFAIP3, TNIP3, TRAF1, TREM2, TRIM14, UBD]	[ABL1, ADIPOQ, ANGPT1, BCL3, BRD4, CC2D1A, CCL21, CCR7, CD14, CD74, CX3CL1, DAB2IP, ECM1, FADD, HSPB1, IL1B, IRAK2, JMJD8, LGALS9, LTBR, LTF, MAP3K14, MAVS, NKIRAS2, NOD2, NR1D1, PER1, PIDD1, RELA, RELB, RNU1-93P, SECTM1, SHARPIN, SHISA5, SLC44A2, SPHK2, TBKBP1, TGM2, TIAF1, TICAM1, TNF, TNFRSF1A, TNIP1, TRAF4, TRIM62, TRIM8, UBA52, VSIG2, ZC3H12A]
GO:0043122	regulation of I-kappaB kinase/NF-kappaB signaling	2,28E-03	[5, 6, 7]	63,00	25,10	43,13	56,87	[ANGPT1, CARD11, CASP8, CCL19, CD4, CD74, CLEC6A, FASLG, HLA-DRB1, IKBKE, LILRB4, NLRC3, NLRP6, PIM2, PRKCB, PYCARD, RHOH, STAT1, TNFAIP3, TNIP3, TRAF1, TREM2, TRIM14, UBD]	[ABL1, ADIPOQ, ANGPT1, BRD4, CC2D1A, CCL21, CCR7, CD74, CX3CL1, DAB2IP, ECM1, FADD, HSPB1, IL1B, JMJD8, LGALS9, LTBR, LTF, MAP3K14, MAVS, NOD2, NR1D1, PER1, PIDD1, RELA, RNU1-93P, SECTM1, SHARPIN, SHISA5, SLC44A2, SPHK2, TGM2, TICAM1, TNF, TNFRSF1A, TNIP1, TRAF4, TRIM62, TRIM8, VSIG2, ZC3H12A]
GO:0045058	T cell selection	5,04E-05	[2, 6, 7, 8, 9, 10, 11, 12]	24,00	45,28	87,18	12,82	[BATF, BCL11B, CARD11, CD3D, CD3E, CD3G, CD4, CD74, DOCK2, FOXP3, IL12RB1, IRF4, LY9, PTPRC, SLAMF6, SPN, SYK, TBX21, THEMIS, TOX, ZAP70]	[CCR7, CD74, SRF, STAT3]
GO:0043368	positive T cell selection	9,37E-05	[3, 7, 8, 9, 10, 11, 12, 13]	20,00	50,00	88,60	11,40	[BATF, BCL11B, CD3D, CD3E, CD3G, CD74, DOCK2, FOXP3, IL12RB1, IRF4, LY9, PTPRC, SLAMF6, SPN, TBX21, THEMIS, TOX, ZAP70]	[CD74, SRF, STAT3]
GO:0002361	CD4-positive, CD25-positive, alpha-beta regulatory T cell differentiation	2,70E-04	[7, 8, 9, 10, 11, 12, 13, 14]	8,00	100,00	90,07	9,93	[FOXP3, FUT7, HLA-DRA, HLA-DRB1, IFNG, PLA2G2D, TOX]	[LGALS9]
GO:0045066	regulatory T cell differentiation	1,43E-02	[6, 7, 8, 9, 10, 11, 12]	16,00	44,44	75,67	24,33	[CTLA4, FOXP3, FUT7, HLA-DRA, HLA-DRB1, IFNG, LAG3, LILRB4, PLA2G2D, SOCS1, TNFRSF18, TOX]	[BCL6, LAG3, LGALS9, SOX12, VSIR]

GO:0060627	regulation of vesicle-mediated transport	6,31E-05	[3, 4, 5]	124,00	22,18	40,13	59,87	[ALOX15, ANGPT1, BLK, C2, CACNA1I, CBARP, CCL19, CCL2, CCR2, CD177, CD22, CD300LF, CD84, CNR1, CORO1A, DOCK2, FGR, IFNG, IL2RB, IL2RG, ITGB2, KLRC2, LILRB1, LPAL2, NCKAP1L, PRAM1, PRKCB, PTAFR, PTPRC, PTX3, PYCARD, RAB15, RAB27B, RAB3B, RAB3C, RAC2, SDC1, SELE, SEPTIN1, SIRPG, SMPD3, STAP1, STXBP2, SYK, TREM2]	[ABCA7, ABL1, ADCY1, ADIPOQ, ANGPT1, ANKRD13B, AP2A1, APLN, APOA1, ARC, ARF1, ARHGAP1, ATP13A2, BSN, CACNA1H, CBARP, CCL2, CCL21, CD14, CD177, CEACAM1, CLIP3, CNR1, CPLX1, CSK, DGKD, DOC2B, DYSF, EHD1, EHD2, FES, FOXF1, GAB2, GAS1, GATA2, GIT1, GNAI2, GRIK5, HFE, HGS, HTR1B, HTR2A, IL15RA, IL1B, IL4R, LDLRAP1, LGALS9, MAPK3, MSN, MYO18A, NOD2, NOTCH1, NPPA, PACSIN1, PCDHA4, PRKCSH, PROM2, PRRT2, PTPN23, RAB11B, RAB15, RAB3A, RAB5B, RAC2, RAP1A, RUBCN, SELE, SEPTIN4, SEPTIN5, SERPINE1, SH3GL1, SMPD1, SPHK1, SPHK2, SPI1, SRC, STX1A, STX1B, SYN1, SYT2, SYT7, TGM2, TNF, TNK2, TSC2, VEGFA, VTN]
GO:0050764	regulation of phagocytosis	2,23E-03	[4, 5, 6]	33,00	33,00	59,31	40,69	[ALOX15, C2, CCL2, CD300LF, DOCK2, FGR, IFNG, IL2RB, IL2RG, LPAL2, NCKAP1L, PTPRC, PTX3, PYCARD, SIRPG, STAP1, SYK, TREM2]	[ABCA7, ADIPOQ, APOA1, CCL2, CSK, DYSF, GATA2, IL15RA, IL1B, MYO18A, NOD2, RAP1A, SPHK1, SYT7, TGM2, TNF]
GO:0050766	positive regulation of phagocytosis	1,53E-02	[3, 4, 5, 6, 7]	24,00	34,78	66,03	33,97	[C2, CCL2, CD300LF, DOCK2, IFNG, IL2RB, IL2RG, LPAL2, NCKAP1L, PTPRC, PTX3, PYCARD, SIRPG, STAP1, TREM2]	[ABCA7, APOA1, CCL2, GATA2, IL15RA, IL1B, MYO18A, NOD2, RAP1A, TNF]
GO:0048518	positive regulation of biological process	5,77E-18	[1, 2, 3]	1107,00	16,89	40,46	59,54	[ABCB11, ABRA, ADAM8, ADCY7, AIF1, AIM2, AKNA, ALOX15, ALOX15B, ANGPT1, ANGPT2, APBB1IP, AREG, ASCL2, ATF3, ATP1B4, ATP2A3, BANK1, BATF, BCL11B, BCL3, BHLHA15, BLK, BLNK, BMF, BMP7, BMPR1B, BRIP1, BTK, BTLA, BTN3A1, BTN3A2, C1QA, C1QB, C1QC, C1QTNF1, C2, C8G, CACNA1I, CALCR, CAMK4, CARD11, CARMIL2, CASP8, CASS4, CCDC88B, CCL17, CCL19, CCL2, CCL22, CCL24, CCL3, CCL3L1, CCL4, CCL4L1, CCL5, CCR2, CCR3, CCR4, CD177, CD180, CD19, CD1B, CD1C, CD1E, CD2, CD22, CD226, CD244, CD247, CD27, CD300LF, CD33, CD3D, CD3E, CD3G, CD4, CD40LG, CD5, CD53, CD6, CD74, CD79A, CD80, CD84, CD86, CDC20B, CDCA2, CDH1, CDH4, CDKN2A, CEL, CFP, CLCF1, CLEC10A, CLEC4D, CLEC4E, CLEC6A, CLNK, CNR1, CORO1A, CR2, CRABP2, CRB2, CREM, CRTAM, CSF1R, CST7, CTLA4, CTSS, CXCL10, CXCL11, CXCL5, CXCL9, CXCR4, CXorf21, CYBB, CYP11B1, DAPK1, DEPDC1B, DOCK2, DOCK8, DPEP2, DRD1, E2F8, EAF2, EBI3, EDN2, EGR3, ELAVL4, ELF3, EOMES, EPHA6, EPX, EREG, ERFE, EVI2B, FASLG, FCN1, FCN3, FCRL3, FFAR4, FGD2, FGD3, FGR, FLT3, FOLR2, FOSB, FOSL1, FOXP3, FUT7, FYB1, GABBR2, GADD45B, GBP5, GDF6, GFI1, GLIS1, GMFG, GPM6A, GPR174, GPR4, GPR55, GPRC5A, GPSM3, GRAP2, GZMA, GZMB, H1-3, HCLS1, HCST, HLA-DMB, HLA-DPA1, HLA-DPB1, HLA-DQA1, HLA-DRA, HLA-DRB1, HLA-DRB5, HOPX, ICOS, IFNG, IGLL5, IKBKE, IKZF3, IL10RA, IL12RB1, IL12RB2, IL16, IL18, IL18RAP, IL26, IL2RB, IL2RG, IL5RA, IL7R, INPP5D, IQCJ-SCHIP1, IQGAP2, IRF4, IRF5, IRF8, IRX3, ITGA4, ITGAX, ITGB2, ITK, JAML, JCHAIN, KCNJ10, KCNN4, KIAA1324, KLF5, KLRC2, KLRD1, KLRG1, KLRK1, KMO, KNG1, LAG3, LAMP3, LAX1, LCK, LCP1, LCP2, LDLR, LEF1, LIF, LILRB1, LILRB4, LMCD1, LMO1, LPAL2, LRRC38, LTA, LTB, LY86, LY9, MACC1, MAFF, MAP4K1, MAPK13, MATK, MCOLN2, MEDAG, MID1IP1, MIXL1, MLC1, MMP9, MOG, MS4A1, MSX1, MUC16, MUC19, MUC6, MUSK, MYB, MYBL2, MYO1G, MZB1, NAMPT, NCF1, NCKAP1L, NCR3, NEK5, NELL2, NFAM1	[ABCA3, ABCA7, ABCB11, ABCC8, ABCD1, ABCD2, ABI3, ABL1, ABLIM3, ABR, ABRA, ACACB, ACKR3, ACTA1, ACTB, ACTC1, ACTN1, ACTN4, ADAM19, ADAMTSL4, ADCK1, ADCY1, ADCY3, ADCY4, ADCY9, ADGRG1, ADGRL1, ADIPOQ, AGAP2, AGER, AGPAT1, AGT, AGTR1, ALPK3, AMH, ANGPT1, ANGPTL4, AP2A1, AP3D1, APLN, APLNR, APOA1, AQP3, ARAF, ARC, AREG, ARF1, ARHGDIA, ARHGEF1, ARHGEF15, ARHGEF16, ARHGEF5, ARID1A, ARTN, ATAT1, ATF5, ATP13A2, ATP1A1, ATP1A3, ATP1B2, ATP1B4, ATPSCKMT, BAG3, BAG6, BCAR1, BCL2L1, BCL3, BCL6, BCL9L, BEND6, BMP8A, BRD4, BRPF3, BTLA, C15orf62, C1QTNF1, C20orf27, C2CD2L, C5, C5AR1, C8G, CACNA1H, CALB1, CALCR, CAMK2B, CAMTA2, CAPN1, CAPNS1, CARM1, CARMIL2, CASZ1, CC2D1A, CCDC87, CCL11, CCL2, CCL21, CCL24, CCR7, CD14, CD177, CD1C, CD1E, CD276, CD74, CDC25B, CDC42EP1, CDC42EP2, CDC42EP4, CDCA2, CDH4, CDK1, CDK2, CDKN1A, CEACAM1, CEL, CETP, CFH, CFL1, CGA, CHCHD10, CHERP, CHGA, CHI3L1, CIITA, CITED2, CITED4, CIZ1, CLCF1, CLEC4E, CLIP3, CLNK, CNOT3, CNR1, CNTN6, COL1A1, CPN2, CPNE5, CPNE6, CRAT, CREB3L1, CRHR2, CRIP2, CRP, CRTC2, CSF1, CSK, CSNK1E, CSPG4, CSRNP3, CST7, CTC1, CTF1, CTHRC1, CTIF, CTSD, CX3CL1, CYP26B1, CYP27B1, DAB2IP, DAG1, DAXX, DBF4B, DDR1, DDX39B, DENND2B, DGKD, DGKZ, DHX34, DIRAS1, DISP3, DKK2, DLG5, DMTN, DNMT1, DOC2B, DOK5, DPAGT1, DPF2, DTX1, DUSP5, DVL3, DXO, DYRK1B, E2F4, E2F7, E2F8, EAF2, ECM1, ECM2, EDC4, EDN2, EEF1E1, EFEMP2, EFNA3, EGLN2, EHD1, EHD2, EIF4EBP1, EIF4G1, ELF3, ELF4, ELK1, ENDOG, ENG, ENPP3, EPHA2, ERBB2, ERFE, ESM1, ESPL1, ETS2, ETV6, FABP4, FADD, FAM110A, FAP, FASN, FCN3, FEM1A, FES, FFAR2, FGFR4, FHL5, FHOD1, FITM1, FLOT2, FLT3, FLT4, FOSL1, FOSL2, FOXC2, FOXF1, FOXM1, FOXS1, FPR1, FRMD8, FURIN, GAB2, GABBR2, GADD45B, GATA2, GATA4, GBA, GCSAM, GDF6, GDI1, GGA1, GGA3, GIT1, GLIS2, GLMP, GLRA1, GNAI2, GNAO1, GPAM

GO:0050794	regulation of cellular process	3,02E-10	[2, 3]	1748,00	15,03	38,78	61,22	[ABCB11, ABRA, ACP5, ADAM8, ADAMDEC1, ADAMTS9, ADCY7, ADGRE3, ADGRE4P, ADGRG5, ADGRG6, ADORA3, AIF1, AIM2, AKNA, ALOX15, ALOX15B, ANGPT1, ANGPT2, ANO9, ANPEP, APBB1IP, APOBEC3A, APOBEC3D, APOBEC3G, APOBEC3H, ARAP2, AREG, ARHGAP15, ARHGAP30, ARHGAP45, ASCL2, ASGR2, ATF3, ATP1B4, ATP2A3, BANK1, BATF, BCL11B, BCL2L14, BCL2L15, BCL3, BHLHA15, BHLHE22, BHLHE40, BLK, BLNK, BMF, BMP7, BMPR1B, BRIP1, BTG2, BTK, BTLA, BTN3A1, BTN3A2, C1QA, C1QC, C1QTNF1, C2, CACNA1E, CACNA1I, CALCR, CAMK4, CARD11, CARD17, CARMIL2, CASKIN1, CASP8, CASS4, CBARP, CCDC88B, CCDC88C, CCL17, CCL19, CCL2, CCL22, CCL24, CCL3, CCL3L1, CCL4, CCL4L1, CCL4L2, CCL5, CCR2, CCR3, CCR4, CCR5, CCR8, CD177, CD180, CD19, CD1B, CD1C, CD1E, CD2, CD200R1, CD22, CD226, CD244, CD247, CD27, CD300LF, CD33, CD3D, CD3E, CD3G, CD4, CD40LG, CD5, CD53, CD6, CD7, CD74, CD79A, CD80, CD84, CD86, CD8A, CD8B, CDC20B, CDCA2, CDH1, CDH4, CDKN2A, CEL, CHRDL1, CISH, CLCF1, CLCNKA, CLEC10A, CLEC1B, CLEC4D, CLEC4E, CLEC6A, CLNK, CMTM5, CNN1, CNR1, CNR2, CORO1A, CORO2A, COTL1, CR2, CRABP2, CRB2, CREM, CRTAM, CSF1R, CSF2RA, CST7, CTLA4, CTSS, CXCL10, CXCL11, CXCL5, CXCL6, CXCL9, CXCR3, CXCR4, CXCR6, CXorf21, CYBB, CYSLTR1, CYTIP, DAPK1, DAPP1, DCC, DEPDC1B, DERL3, DLGAP2, DOCK10, DOCK2, DOCK8, DOK2, DPEP1, DPEP2, DPT, DRC1, DRD1, DTHD1, DUSP2, DUSP8, E2F8, EAF2, EBI3, EDN2, EGR3, ELAVL4, ELF3, EOMES, EPHA6, EPX, EREG, ERFE, ERMN, EVI2B, FAM122C, FAM83B, FASLG, FATE1, FBXL19, FCGR2C, FCMR, FCN1, FCN3, FCRL1, FCRL2, FCRL3, FCRL5, FCRL6, FCRLA, FFAR4, FGD2, FGD3, FGL2, FGR, FIBIN, FLT3, FOLR2, FOSB, FOSL1, FOXP3, FRZB, FUT7, FYB1, GABBR2, GADD45B, GALNT3, GBP5, GDF6, GFI1, GFRA2, GLIS1, GMFG, GMIP, GNMT, GPM6A, GPR132, GPR141, GPR150, GPR171, GPR174, GPR19, GPR34, GPR4, GPR55, GPR83, GPRC5A, GPSM3, GRAP2	[AAAS, ABCA3, ABCA7, ABCB11, ABCC8, ABCD1, ABCD2, ABI3, ABL1, ABLIM3, ABR, ABRA, ACACB, ACKR1, ACKR3, ACP4, ACTB, ACTN1, ACTN4, ADAM19, ADAMTS7, ADAMTS8, ADAMTS9, ADAMTSL4, ADCK1, ADCY1, ADCY3, ADCY4, ADCY9, ADGRB2, ADGRE5, ADGRG1, ADGRG6, ADGRL1, ADIPOQ, AGAP2, AGER, AGPAT1, AGT, AGTR1, AKT1S1, ALPK3, AMH, ANGPT1, ANGPTL1, ANGPTL4, ANGPTL8, ANKK1, ANKRD13B, ANKRD52, AP2A1, AP3D1, APLN, APLNR, APLP1, APOA1, APOBEC3C, APOD, APOM, AQP3, ARAF, ARAP3, ARC, AREG, ARF1, ARGFX, ARHGAP1, ARHGAP23, ARHGAP39, ARHGDIA, ARHGEF1, ARHGEF15, ARHGEF16, ARHGEF5, ARID1A, ARID5A, ARMT1, ARRDC3, ARTN, ASB16, ASB6, ASIC3, ASPN, ATAT1, ATF5, ATN1, ATP13A2, ATP1A1, ATP1A3, ATP1B2, ATP1B4, ATP6V0D1, ATP6V0E2, ATP6V1B1, ATP6V1F, ATPSCKMT, ATXN2L, BAG3, BAG6, BAHD1, BATF2, BAZ2A, BBS12, BCAM, BCAR1, BCKDK, BCL2L1, BCL3, BCL6, BCL7B, BCL9L, BCOR, BCR, BEND6, BHLHE40, BMP8A, BRD4, BRPF3, BSN, BTG2, BTLA, C11orf65, C15orf62, C18orf54, C1QTNF1, C20orf27, C2CD2L, C5, C5AR1, CABIN1, CABLES2, CACNA1E, CACNA1H, CACNB4, CALB1, CALCR, CAMK2B, CAMSAP3, CAMTA2, CAPN1, CAPNS1, CARM1, CARMIL2, CASKIN1, CASZ1, CBARP, CC2D1A, CCDC106, CCL11, CCL2, CCL21, CCL24, CCNG2, CCNJL, CCR7, CD14, CD177, CD1C, CD1E, CD276, CD74, CDC25B, CDC42EP1, CDC42EP2, CDC42EP4, CDCA2, CDH4, CDK1, CDK15, CDK18, CDK2, CDK2AP2, CDKL2, CDKN1A, CEACAM1, CEL, CELSR2, CERT1, CETP, CFAP298-TCP10L, CFH, CFL1, CGA, CHCHD10, CHERP, CHGA, CHGB, CHI3L1, CHRNG, CIC, CIITA, CISH, CITED2, CITED4, CIZ1, CLC, CLCF1, CLCN6, CLCNKA, CLEC1B, CLEC4E, CLIP3, CLNK, CLPTM1, CMTM1, CMTM5, CNKSR1, CNN1, CNOT3, CNR1, CNTN6, COL1A1, CPLX1, CPNE5, CPNE6, CPSF7, CPTP, CRAT, CREB3L1, CRHR2, CRIP2, CRP, CRTC2, CRYAB, CSDC2, CSF1, CSK, CSNK1E, CSPG4, CSRNP3, CST7, CTC1, CTDSP1, CTF1, CTHRC1
GO:0007165	signal transduction	1,01E-19	[2, 3, 4]	1126,00	17,01	42,72	57,28	[ABRA, ADAM8, ADCY7, ADGRE3, ADGRE4P, ADGRG5, ADGRG6, ADORA3, AIF1, AIM2, ALOX15, ALOX15B, ANGPT1, ANGPT2, ANPEP, APBB1IP, ARAP2, AREG, ARHGAP15, ARHGAP30, ARHGAP45, ASGR2, ATF3, ATP2A3, BANK1, BATF, BCL11B, BCL2L14, BCL3, BHLHA15, BLK, BLNK, BMF, BMP7, BMPR1B, BRIP1, BTG2, BTK, BTLA, BTN3A1, BTN3A2, C1QTNF1, CACNA1I, CALCR, CAMK4, CARD11, CASKIN1, CASP8, CASS4, CCDC88C, CCL17, CCL19, CCL2, CCL22, CCL24, CCL3, CCL3L1, CCL4, CCL4L1, CCL4L2, CCL5, CCR2, CCR3, CCR4, CCR5, CCR8, CD177, CD180, CD19, CD2, CD200R1, CD22, CD226, CD244, CD247, CD27, CD300LF, CD33, CD3D, CD3E, CD3G, CD4, CD40LG, CD5, CD53, CD6, CD7, CD74, CD79A, CD80, CD86, CD8A, CD8B, CDKN2A, CHRDL1, CISH, CLCF1, CLEC10A, CLEC1B, CLEC4D, CLEC4E, CLEC6A, CLNK, CMTM5, CNR1, CNR2, CORO1A, CORO2A, CR2, CRABP2, CRB2, CREM, CSF1R, CSF2RA, CTLA4, CTSS, CXCL10, CXCL11, CXCL5, CXCL6, CXCL9, CXCR3, CXCR4, CXCR6, CXorf21, CYBB, CYSLTR1, DAPK1, DAPP1, DCC, DEPDC1B, DERL3, DLGAP2, DOCK10, DOCK2, DOCK8, DOK2, DPEP2, DRD1, DTHD1, DUSP2, DUSP8, E2F8, EBI3, EDN2, ELF3, EPHA6, EPX, EREG, ERFE, FAM83B, FASLG, FCGR2C, FCN1, FCRL1, FCRL2, FCRL3, FCRL5, FCRL6, FCRLA, FFAR4, FGD2, FGD3, FGR, FIBIN, FLT3, FOLR2, FOXP3, FRZB, FUT7, FYB1, GABBR2, GADD45B, GALNT3, GDF6, GFI1, GFRA2, GMFG, GMIP, GPR132, GPR141, GPR150, GPR171, GPR174, GPR19, GPR34, GPR4, GPR55, GPR83, GPRC5A, GRAP2, GUCY2D, GZMA, GZMB, H3C10, H3C11, H3C12, H3C7, HASPIN, HCLS1, HCST, HLA-DPA1, HLA-DPB1, HLA-DQA1, HLA-DRA, HLA-DRB1, HLA-DRB5, HORMAD1, HSH2D, ICOS, IER3, IFNG, IGLL5, IKBKE, IL10RA, IL12RB1, IL12RB2, IL16, IL18, IL18RAP, IL21R, IL22, IL26, IL2RB, IL2RG, IL5RA, IL7R, IL9R, INPP5D, IQCJ-SCHIP1, IQGAP2, IRF4, IRF5, IRF8, ITGA4, ITGAL, ITGAX, ITGB2, ITGB7, ITK, JPH1, JSRP1, KCNN4, KLF5, KLRB1, KLRC1, KLRC2, KLRD1, KLRG1, KLRK1, KNG1, LAG3, LAX1, LCK,	[ABCA7, ABL1, ABR, ABRA, ACKR1, ACKR3, ACP4, ACTB, ACTN4, ADCY1, ADCY3, ADCY4, ADCY9, ADGRB2, ADGRE5, ADGRG1, ADGRG6, ADGRL1, ADIPOQ, AGAP2, AGER, AGPAT1, AGT, AGTR1, AKT1S1, ALPK3, AMH, ANGPT1, ANGPTL1, ANGPTL8, AP2A1, APLN, APLNR, APLP1, APOA1, APOD, ARAF, ARAP3, ARC, AREG, ARF1, ARHGAP1, ARHGAP23, ARHGAP39, ARHGDIA, ARHGEF1, ARHGEF16, ARHGEF5, ARID1A, ARRDC3, ARTN, ASB16, ASB6, ASIC3, ASPN, ATP6V0D1, ATP6V0E2, ATP6V1B1, ATP6V1F, BAG3, BAG6, BCAM, BCAR1, BCL2L1, BCL3, BCL6, BCL7B, BCL9L, BCR, BEND6, BMP8A, BRD4, BSN, BTG2, BTLA, C15orf62, C1QTNF1, C20orf27, C5, C5AR1, CABIN1, CALCR, CAMK2B, CAPN1, CARM1, CASKIN1, CC2D1A, CCL11, CCL2, CCL21, CCL24, CCR7, CD14, CD177, CD276, CD74, CDC42EP1, CDC42EP2, CDC42EP4, CDK1, CDK2, CDKL2, CDKN1A, CEACAM1, CELSR2, CERT1, CETP, CFL1, CGA, CHCHD10, CHERP, CHGA, CHGB, CHI3L1, CHRNG, CIITA, CISH, CITED2, CLCF1, CLCN6, CLEC1B, CLEC4E, CLIP3, CLNK, CMTM1, CMTM5, CNKSR1, CNOT3, CNR1, CNTN6, COL1A1, CRAT, CREB3L1, CRHR2, CRYAB, CSF1, CSK, CSNK1E, CSPG4, CTF1, CTHRC1, CX3CL1, CXXC4, CYP26B1, CYP27B1, DAB2IP, DAG1, DAGLA, DAXX, DCHS1, DDR1, DDX39B, DDX54, DEDD, DENND2B, DENND4B, DGKD, DGKZ, DHX34, DIRAS1, DISP2, DISP3, DKK2, DLG5, DLGAP4, DMTN, DNMT1, DOCK6, DOK3, DOK5, DOT1L, DPF2, DTX1, DTX2, DTX3, DUSP5, DUSP8, DVL3, E2F4, E2F7, E2F8, ECE2, ECM1, EDN2, EEF1E1, EFNA3, EGLN2, EIF4EBP1, ELF3, ENG, EPHA2, EPN1, ERBB2, ERFE, ESM1, EXTL1, FADD, FAIM2, FAM110A, FAM83F, FAM83G, FASN, FES, FFAR2, FGFR4, FGFRL1, FIBIN, FLOT2, FLT3, FLT4, FOXC2, FOXF1, FOXM1, FPR1, FRMD8, FURIN, GAB2, GABBR2, GABRR2, GADD45B, GAS1, GATA4, GBA, GBF1, GCSAM, GDF6, GDI1, GFRA3, GIT1, GLIS2, GLRA1, GNAI2, GNAO1, GNB3, GNRHR, GPAM, GPBAR1, GPER1, GPI, GPR137, GPR153, GPR162, GPR17, GPR20, GPR3, GPR34, GPR37L1, GPR4, GPR75, GPR83,

GO:0009611	response to wounding	1,04E-03	[3]	145,00	20,37	38,42	61,58	[ADORA3, AIF1, ALOX15, BLK, C1QTNF1, CARMIL2, CCR2, CD177, CD40LG, CEL, CLEC1B, CXCR4, EPX, EREG, FOLR2, GPR4, H3C10, H3C11, H3C12, H3C7, HGFAC, JAML, KNG1, LCK, LCP1, LCP2, MAFF, NLRP6, NR4A3, P2RY12, PIK3CG, PIK3R5, PLAU, PLEK, PRKCB, PRKCQ, PTPN6, SCUBE1, SDC1, SERPINA1, SIGLEC10, SYK, TFPI2, THBS1, TNFAIP3, TNR, TREM2, TRPM2, TSPAN8, VAV1, WAS, WNT1]	[ABCC8, ACTB, ACTN1, AGER, APOA1, APOD, C1QTNF1, CARMIL2, CD177, CDK1, CDKN1A, CEACAM1, CEL, CLEC1B, COL1A1, CX3CL1, DAG1, DDR1, DGKD, DGKZ, DMTN, DOCK6, DYSF, EHD1, EHD2, EPPK1, ERBB2, FAM110A, FAP, FOXC2, FOXF1, GATA2, GATA4, GIT1, GNAI2, GPR4, GRN, H3C10, H3C12, HBB, HBD, HBEGF, HBG1, HBG2, HGFAC, HSPB1, ID3, ILK, IRF2, ITGA5, ITPR3, KCNK2, KNG1, LOX, MAFG, MAPK3, MAPK8IP3, MYC, MYH9, MYL9, NEFL, NFE2, NOS3, NOTCH4, NR4A3, P2RX3, P2RX5, PDGFB, PDGFRB, PEAR1, PLAU, PLAUR, PLEC, POSTN, POU5F1P4, PPARD, PPL, PRKACA, PRKAR2B, PTN, RAB3A, RANGAP1, RGMA, RHBDF2, RIPOR1, RTN4RL1, RTN4RL2, SELENOP, SERPIND1, SERPINE1, SERPING1, SH2B1, SH2B2, SH2B3, SMAD3, SMPD1, SRC, SRF, SYT7, THBS1, TIMP1, TNF, TSKU, TSPAN8, VEGFA, VTN, WAS, ZFP36]
GO:0050878	regulation of body fluid levels	4,55E-04	[3]	122,00	21,52	36,18	63,82	[ADCY7, AQP5, BLK, C1QTNF1, CD177, CD40LG, CEL, CLEC1B, H3C10, H3C11, H3C12, H3C7, HGFAC, KCNN4, KNG1, LCK, LCP2, MAFF, NLRP3, NLRP6, NPPB, NR4A3, P2RY12, P2RY2, PIK3CG, PIK3R5, PLAU, PLEK, PRKCB, PRKCQ, PTPN6, SCNN1B, SCUBE1, SERPINA1, SYK, TFPI2, THBS1, TIFAB, TRPM2, TSPAN8, VAV1, WAS]	[ACTB, ACTN1, ADCY1, ADCY3, ADCY4, ADCY9, APLN, APLNR, AQP3, AQP5, ATP6V1B1, C1QTNF1, CD177, CEACAM1, CEL, CELSR2, CLEC1B, COASY, COL1A1, CYP26B1, CYP27B1, DDR1, DGKD, DGKZ, DMTN, DOCK6, EHD1, EHD2, ELOVL1, FA2H, FAM110A, FAP, FLG, GATA2, GATA4, GBA, GNAI2, GPI, H3C10, H3C12, HBB, HBD, HBG1, HBG2, HGFAC, HSPB1, ILK, IRF2, ITPR3, KNG1, LSR, MAFG, MAPK3, MLLT6, MTCO2P12, MYH9, MYL9, NCOR2, NFE2, NOS3, NPPB, NPR3, NPRL3, NR4A3, P2RX3, P2RX5, P2RY2, PDGFB, PEAR1, PLAU, PLAUR, PRKACA, PRKAR2B, PTPRU, RHBDF2, RMRP, SELENOP, SERPIND1, SERPINE1, SERPING1, SH2B1, SH2B2, SH2B3, SLC29A1, SRC, SRF, THBS1, TRPV4, TSPAN8, USF2, VDR, VEGFA, VTN, WAS, WFS1, ZBTB7B]
GO:0042060	wound healing	2,26E-02	[4]	116,00	20,17	38,77	61,23	[ALOX15, BLK, C1QTNF1, CARMIL2, CD177, CD40LG, CEL, CLEC1B, CXCR4, EREG, GPR4, H3C10, H3C11, H3C12, H3C7, HGFAC, JAML, KNG1, LCK, LCP1, LCP2, MAFF, NLRP6, NR4A3, P2RY12, PIK3CG, PIK3R5, PLAU, PLEK, PRKCB, PRKCQ, PTPN6, SCUBE1, SDC1, SERPINA1, SYK, TFPI2, THBS1, TNFAIP3, TRPM2, TSPAN8, VAV1, WAS]	[ABCC8, ACTB, ACTN1, C1QTNF1, CARMIL2, CD177, CDKN1A, CEACAM1, CEL, CLEC1B, COL1A1, CX3CL1, DAG1, DDR1, DGKD, DGKZ, DMTN, DOCK6, DYSF, EHD1, EHD2, EPPK1, ERBB2, FAM110A, FAP, FOXC2, GATA2, GATA4, GNAI2, GPR4, H3C10, H3C12, HBB, HBD, HBEGF, HBG1, HBG2, HGFAC, HSPB1, ILK, IRF2, ITGA5, ITPR3, KNG1, LOX, MAFG, MAPK3, MYH9, MYL9, NFE2, NOS3, NOTCH4, NR4A3, P2RX3, P2RX5, PDGFB, PDGFRB, PEAR1, PLAU, PLAUR, PLEC, POSTN, PPARD, PPL, PRKACA, PRKAR2B, RAB3A, RHBDF2, SELENOP, SERPIND1, SERPINE1, SERPING1, SH2B1, SH2B2, SH2B3, SMAD3, SMPD1, SRC, SRF, SYT7, THBS1, TIMP1, TNF, TSKU, TSPAN8, VEGFA, VTN, WAS]
GO:0002532	production of molecular mediator involved in inflammatory response	3,22E-03	[2, 5]	28,00	34,57	56,44	43,56	[ADCY7, BTK, CD6, GBP5, GPSM3, LILRB4, NLRC3, NLRP7, PLD4, PYCARD, SLAMF8, SYK, TLR6, TREM2]	[ABCD1, ABCD2, APOD, GRN, IL4R, NOD2, NPPA, PER1, PLD3, SERPINE1, STAT3, TICAM1, TNF, ZC3H12A]
GO:0002534	cytokine production involved in inflammatory response	1,07E-02	[3, 6]	21,00	38,18	58,77	41,23	[ADCY7, CD6, GBP5, GPSM3, LILRB4, NLRC3, NLRP7, PLD4, PYCARD, TLR6, TREM2]	[ABCD1, ABCD2, APOD, NOD2, PER1, PLD3, STAT3, TICAM1, TNF, ZC3H12A]
GO:1900015	regulation of cytokine production involved in inflammatory response	1,07E-02	[4, 5, 6, 7]	21,00	38,18	58,77	41,23	[ADCY7, CD6, GBP5, GPSM3, LILRB4, NLRC3, NLRP7, PLD4, PYCARD, TLR6, TREM2]	[ABCD1, ABCD2, APOD, NOD2, PER1, PLD3, STAT3, TICAM1, TNF, ZC3H12A]
GO:0048018	receptor ligand activity	4,73E-03	[3, 4, 5]	111,00	21,10	53,62	46,38	[AREG, BMP7, C1QTNF1, CCL17, CCL19, CCL2, CCL22, CCL24, CCL3, CCL3L1, CCL4, CCL4L1, CCL4L2, CCL5, CD40LG, CLCF1, CMTM5, CXCL10, CXCL11, CXCL5, CXCL6, CXCL9, EBI3, EDN2, EPX, EREG, ERFE, FASLG, GDF6, GMFG, IFNG, IL16, IL18, IL22, IL26, KLF5, LIF, LTA, LTB, MACC1, NAMPT, NLRP3, NPPB, NPPC, PNOC, PROK1, RLN2, SEMA4A, SEMA4D, TNFRSF11B, TNFSF13B, TNFSF14, TNFSF8, WNT1, WNT10A, WNT10B, XCL1, XCL2]	[ADIPOQ, AGT, AMH, ANGPTL8, APLN, APOA1, AREG, ARTN, BMP8A, C1QTNF1, C5, CCL11, CCL2, CCL21, CCL24, CDC42EP2, CGA, CHGA, CHGB, CLCF1, CMTM1, CMTM5, CSF1, CTF1, CX3CL1, EDN2, ERFE, GDF6, GNAI2, GPI, GRN, HBEGF, IL1B, IL1RN, IL32, INHA, KLF5, LIF, MEN1, MSTN, NDP, NGF, NODAL, NPPA, NPPB, OGN, OSGIN1, OSM, PDGFB, PTN, SECTM1, SEMA3F, SEMA4B, SEMA4C, SEMA6B, SEMA6C, THPO, TIMP1, TNF, TNFSF12, TOR2A, UCN2, VEGFA, WNT3, WNT9B]
GO:0005125	cytokine activity	6,61E-07	[4, 5, 6]	73,00	28,63	60,64	39,36	[AREG, BMP7, CCL17, CCL19, CCL2, CCL22, CCL24, CCL3, CCL3L1, CCL4, CCL4L1, CCL4L2, CCL5, CD40LG, CLCF1, CMTM5, CXCL10, CXCL11, CXCL5, CXCL6, CXCL9, EBI3, EPX, FASLG, GDF6, IFNG, IL16, IL18, IL22, IL26, KLF5, LIF, LTA, LTB, NAMPT, TNFRSF11B, TNFSF13B, TNFSF14, TNFSF8, WNT1, WNT10A, WNT10B, XCL1, XCL2]	[ADIPOQ, AMH, AREG, BMP8A, C5, CCL11, CCL2, CCL21, CCL24, CLCF1, CMTM1, CMTM5, CSF1, CTF1, CX3CL1, GDF6, GPI, GRN, IL1B, IL1RN, IL32, INHA, KLF5, LIF, MEN1, MSTN, NDP, NODAL, OSM, SECTM1, THPO, TIMP1, TNF, TNFSF12, VEGFA, WNT3, WNT9B]
GO:0008009	chemokine activity	1,68E-04	[5, 6, 7]	23,00	44,23	77,86	22,14	[CCL17, CCL19, CCL2, CCL22, CCL24, CCL3, CCL3L1, CCL4, CCL4L1, CCL4L2, CCL5, CXCL10, CXCL11, CXCL5, CXCL6, CXCL9, KLF5, XCL1, XCL2]	[C5, CCL11, CCL2, CCL21, CCL24, CX3CL1, KLF5]

GO:0008610	lipid biosynthetic process	4,03E-03	[4]	165,00	19,37	32,07	67,93	[ABCB11, AKR1D1, ALOX15, ALOX15B, BCO1, C1QTNF1, CCL19, CD19, CD74, CERKL, CERS3, CISH, CPNE7, CYP11B1, CYP4F22, DEGS2, DHRS9, EDN2, ETNPPL, FGR, FLT3, GAL3ST2, GFI1, IFNG, INPP5D, LCP1, LDLR, LPAL2, MID1IP1, MUSK, NLRP3, P2RY12, PIK3C2G, PIK3CG, PIK3IP1, PIK3R5, PLA2G2D, PTK2B, SH2D2A, SLA2, SLC27A2, SMPD3, SOAT2, SOCS1, SOCS3, TBXAS1, UGT8]	[A4GALT, ABCA3, ABCB11, ABCD1, ABCD2, ABHD4, ACACB, ACSBG1, ADIPOQ, AGAP2, AGPAT1, AGPAT3, AMH, APOA1, ARF1, ARMC5, ATP1A1, ATP1A3, B4GALT3, C1QTNF1, CACNA1H, CCL21, CCR7, CD74, CEACAM1, CERT1, CGA, CHGA, CISH, CPNE6, CYB5R3, CYP21A2, CYP27B1, CYP2D6, DAB2IP, DGKD, DGKZ, DHRS9, DPAGT1, DPM2, EDN2, ELOVL1, FA2H, FADS2, FADS3, FASN, FGFR4, FITM1, FLT3, GAL3ST3, GAL3ST4, GBA, GNAI2, GPAM, GPAT2, GPER1, HSD17B11, HTR2A, IL1B, INPP5E, INPP5J, INPPL1, ISYNA1, LCAT, LDLR, LIPG, LPCAT4, MALRD1, MBOAT7, MFSD2A, MID1IP1, NOD2, NR1D1, PDGFB, PDGFRB, PGAP2, PGAP3, PI4KA, PI4KAP1, PI4KAP2, PIGO, PIGQ, PIGS, PIGT, PIGZ, PIK3C2B, PIK3C2G, PIP5K1C, PISD, PITPNM1, PITPNM2, PLA2G2A, PNPLA2, POR, PPARD, PRKCSH, PRXL2B, PTGDS, PTGES, PTPN13, RNU1-93P, RUBCN, SBF1, SCAP, SH2D3C, SIK1, SLC44A2, SMPD1, SMPD4, SNAI1, SOCS3, SPHK1, SPHK2, SPNS2, SRC, SREBF1, ST6GALNAC4, TECR, TMEM150A, TMEM189, TMEM256-PLSCR3, TNF, TNFRSF1A, TP53INP2, TRIB3, TTC7B, VAC14, VDR, WDTC1]
GO:0019216	regulation of lipid metabolic process	1,08E-02	[4, 5]	89,00	21,87	28,44	71,56	[ABCB11, BCL11B, C1QTNF1, CCL19, CD19, CD74, CNR1, ERFE, FGR, FLT3, GFI1, IFNG, LDLR, LPAL2, MID1IP1, NLRP3, NR4A3, P2RY12, PIK3CG, PIK3IP1, PTK2B, SH2D2A, SMPD3]	[ABCA3, ABCA7, ABCB11, ABCD1, ABCD2, ACACB, ADIPOQ, AGAP2, AGT, AGTR1, ANGPTL8, APOA1, APOD, ARF1, ARMC5, ATP1A1, ATP1A3, C1QTNF1, CARM1, CCL21, CCR7, CD74, CEACAM1, CGA, CHGA, CNR1, CYP27B1, DAB2IP, DGKZ, DISP3, ERFE, FASN, FGFR4, FLT3, GNAI2, GNB3, GPAM, GPER1, HTR2A, IL1B, LDLR, LDLRAP1, LSR, MALRD1, MBOAT7, MFSD2A, MID1IP1, NCOA6, NCOR2, NOD2, NR1D1, NR4A3, PCDHA4, PDGFB, PDGFRB, PDK2, PDPR, PNPLA2, POR, PPARD, RNU1-93P, RUBCN, RXRA, SCAP, SERPINA3, SIK1, SNAI1, SPHK1, SPHK2, SRC, SREBF1, TNF, TNFRSF1A, TRIB3, WDTC1]
GO:0045834	positive regulation of lipid metabolic process	2,22E-03	[3, 4, 5, 6]	46,00	28,05	35,06	64,94	[CCL19, CD19, CD74, FGR, FLT3, IFNG, LDLR, LPAL2, MID1IP1, NLRP3, NR4A3, P2RY12, PTK2B, SH2D2A, SMPD3]	[ABCD1, ABCD2, ADIPOQ, AGAP2, AGT, AGTR1, APOA1, CCL21, CCR7, CD74, CGA, CHGA, DGKZ, DISP3, FLT3, HTR2A, IL1B, LDLR, LDLRAP1, MFSD2A, MID1IP1, NOD2, NR1D1, NR4A3, PDGFB, PDGFRB, PNPLA2, POR, PPARD, RNU1-93P, SCAP, SPHK2, SRC, SREBF1, TNF, TNFRSF1A]
GO:0045087	innate immune response	6,57E-06	[4, 5, 6]	215,00	20,02	67,24	32,76	[ADAM8, AIF1, AIM2, APOBEC3A, APOBEC3D, APOBEC3G, APOBEC3H, BLK, BTK, C1QA, C1QB, C1QC, C2, C8G, CARD11, CASP8, CCL17, CCL19, CCL2, CCL22, CCL24, CCL3, CCL3L1, CCL4, CCL4L1, CCL5, CD177, CD180, CD226, CD244, CD6, CD74, CD84, CD96, CFP, CLEC10A, CLEC4D, CLEC4E, CLEC6A, CLNK, CORO1A, CR2, CRTAM, CSF1R, CXCL10, CXorf21, CYBB, DAPK1, EREG, FASLG, FCN1, FCN3, FGR, GBP5, GBP6, GZMB, GZMM, HLA-DPA1, HLA-DPB1, HLA-DQA1, HLA-DRA, HLA-DRB1, HLA-DRB5, IFNG, IGLL5, IKBKE, IL12RB1, IL18, IL18RAP, IRF4, IRF5, IRF8, JCHAIN, KIR2DL3, KLRC1, KLRC2, KLRD1, KLRG1, KLRK1, LAG3, LCK, LILRB1, LY86, LY9, MCOLN2, MPEG1, MRC1, MUC16, MUC19, MUC6, NCF1, NCR1, NCR3, NLRC3, NLRP2, NLRP3, NLRP6, NLRP9, OASL, PIK3CG, PLD4, PRDM1, PSTPIP1, PTAFR, PTK2B, PTPN22, PTPN6, PTX3, PYCARD, PYHIN1, RNASE6, RNASET2, SH2D1A, SIGLEC10, SIGLEC16, SLA, SLAMF6, SLAMF7, SLAMF8, SMPDL3B, SOCS1, SOCS3, STAT1, STYK1, SYK, TLR10, TLR6, TLR8, TNFAIP3, TNFAIP8L2, TREM2, TRIM14, UBD, VAV1, VNN1, WAS, XCL1, XCL2, ZAP70, ZBP1, ZNF683]	[AGER, AMH, APOBEC3C, ARID5A, C5, C8G, CAMK2B, CCL11, CCL2, CCL21, CCL24, CD14, CD177, CD300E, CD74, CDC42EP2, CDC42EP4, CEACAM1, CFH, CHGA, CIITA, CLEC4E, CLNK, CRP, CSF1, CX3CL1, CYP27B1, DAB2IP, ELF4, FADD, FCN3, FES, FFAR2, GNAO1, GPER1, GRN, HFE, HK1, ICAM1, IFITM1, IFITM2, IFITM3, IRF2, IRF9, JAK3, LAG3, LGALS9, LILRA5, LTF, MAVS, MRC1, MUC19, MUC20, MUC3A, MYC, NECTIN2, NLRC5, NLRP9, NOD2, PLEKHM2, PML, PQBP1, PRKACA, PVR, RBM14, RELA, RELB, RNASE2, RNF19B, RNF39, RNU1-93P, SERPING1, SMPD1, SOCS3, SPI1, SRC, STING1, TBKBP1, TICAM1, TNK1, TNK2, TRIM29, TRIM62, TRIM8, VNN1, WAS, ZCCHC3, ZDHHC11, ZYX]
GO:0034341	response to interferon-gamma	2,42E-05	[5, 6, 7]	60,00	28,71	62,31	37,69	[AIF1, CCL17, CCL19, CCL2, CCL22, CCL24, CCL3, CCL3L1, CCL4, CCL4L1, CCL5, CD74, DAPK1, FASLG, GBP5, GBP6, HLA-DPA1, HLA-DPB1, HLA-DQA1, HLA-DRA, HLA-DRB1, HLA-DRB5, IFNG, IL12RB1, IRF4, IRF5, IRF8, MRC1, OASL, PTAFR, SOCS1, SOCS3, STAT1, UBD, WAS, XCL1, XCL2]	[CAMK2B, CCL11, CCL2, CCL21, CCL24, CD74, CDC42EP2, CDC42EP4, CIITA, CX3CL1, CYP27B1, GNAO1, HFE, ICAM1, IFITM1, IFITM2, IFITM3, IRF2, IRF9, LGALS9, MRC1, MYC, NLRC5, PML, SOCS3, TRIM62, TRIM8, WAS, ZYX]
GO:0071346	cellular response to interferon-gamma	1,10E-04	[6, 7, 8]	54,00	28,72	65,39	34,61	[AIF1, CCL17, CCL19, CCL2, CCL22, CCL24, CCL3, CCL3L1, CCL4, CCL4L1, CCL5, DAPK1, FASLG, GBP5, GBP6, HLA-DPA1, HLA-DPB1, HLA-DQA1, HLA-DRA, HLA-DRB1, HLA-DRB5, IFNG, IL12RB1, IRF4, IRF5, IRF8, MRC1, OASL, PTAFR, SOCS1, SOCS3, STAT1, WAS, XCL1, XCL2]	[CAMK2B, CCL11, CCL2, CCL21, CCL24, CDC42EP2, CDC42EP4, CIITA, CX3CL1, GNAO1, HFE, ICAM1, IRF2, IRF9, LGALS9, MRC1, MYC, NLRC5, PML, SOCS3, TRIM62, TRIM8, WAS, ZYX]

GO:0042127	regulation of cell population proliferation	3,58E-15	[3, 4]	364,00	20,90	41,57	58,43	[ADORA3, AIF1, ALOX15B, ANGPT1, AREG, ASCL2, ATF3, BCL11B, BLK, BMP7, BMPR1B, BRIP1, BTG2, BTK, CARD11, CCDC88B, CCL19, CCL2, CCL24, CCL3L1, CCL5, CCR2, CCR3, CD22, CD33, CD3E, CD40LG, CD6, CD74, CD80, CD86, CDKN2A, CEL, CLCF1, CNN1, CORO1A, CRTAM, CSF1R, CTLA4, CXCL10, CXCL11, CXCL5, CXCL9, DPEP2, DPT, EAF2, EBI3, EDN2, EGR3, EPX, EREG, FASLG, FCRL3, FLT3, FOLR2, FOSL1, FOXP3, FRZB, HCLS1, HLA-DMB, HLA-DPA1, HLA-DPB1, HLA-DRB1, IFNG, IKZF3, IL12RB1, IL12RB2, IL18, IL26, IL5RA, IL7R, IL9R, INPP5D, ITGA4, ITGAX, JAML, KLF5, KLHL41, LEF1, LIF, LILRB1, LILRB4, LMO1, LST1, LTA, MATK, MMP9, MSX1, MYB, MZB1, NAMPT, NCKAP1L, NELL2, NLRC3, NLRP3, NPPC, NR4A1, NR4A3, NTN1, PIM1, PIM2, PLA2G2D, PLAAT4, PLAC8, PLAU, PRDM1, PRKCQ, PROK1, PTAFR, PTGFR, PTK2B, PTPN22, PTPN6, PTPRC, PYCARD, RAC2, RARRES1, RASAL3, RBPMS2, RTKN2, RUNX3, SASH3, SIRPG, SMPD3, SPN, STAT1, SULT2B1, SYK, THBS1, TNFAIP3, TNFRSF13B, TNFSF13B, TNFSF8, TNMD, TOX, TREM2, WNT1, WNT10B, XCL1, ZAP70]	[ABCC8, ABL1, ACKR3, ADAMTS8, ADGRG1, ADIPOQ, AGAP2, AGER, AGT, AGTR1, AMH, ANGPT1, APLN, APLNR, APOD, AREG, ATF5, BAG6, BCL2L1, BCL6, BTG2, C18orf54, C5AR1, CAPN1, CAPNS1, CARM1, CCL11, CCL2, CCL24, CD276, CD74, CDC25B, CDK1, CDK2, CDKN1A, CEACAM1, CEL, CGA, CHGA, CITED2, CLC, CLCF1, CNN1, CRIP2, CRP, CSF1, CSK, CTC1, CTF1, CTHRC1, CX3CL1, CYP27B1, DAB2IP, DBF4B, DDR1, DDX39B, DHRS2, DISP3, DLG5, DNMT1, DPT, E2F4, E2F7, EAF2, ECM1, EDN2, EEF1E1, EFEMP2, ENG, ENPP3, EPPK1, ERBB2, ESM1, FA2H, FADD, FAP, FES, FGFR4, FGFRL1, FLT3, FLT4, FOSL1, FOSL2, FOXF1, FOXM1, GAB2, GATA2, GNAI2, GPAM, GPBAR1, GPER1, GPR37L1, GRN, HBEGF, HGS, HMGA1, HMGN1, HMGN5, HSF1, HTR1B, HTR2A, HYAL1, IDO1, IFITM1, IFT80, IL1B, IL4R, IL5RA, IL7R, ILK, INCA1, INHA, INPPL1, JAK3, JUNB, JUND, JUP, KCNK2, KLF10, KLF5, KMT2D, KSR1, LAMA5, LDLRAP1, LDOC1, LGALS9, LIF, LIFR, LIMS2, LMNA, LRG1, LRRC32, LTF, LZTS2, MAFG, MAPK3, MARCKSL1, MARK4, MAZ, MECP2, MED25, MEF2D, MEIS3P1, MEIS3P2, MEN1, MNT, MSTN, MSX1, MYC, NACC1, NF2, NGF, NGFR, NOD2, NODAL, NOP2, NOS3, NOTCH1, NPR3, NPRL3, NR1D1, NR4A1, NR4A3, NRARP, NTN1, OGN, OSGIN1, OSM, OSR2, P2RY6, PDF, PDGFB, PDGFRB, PIM1, PLAU, PLCD3, PLXNB1, PML, POR, PPARD, PPP1R9B, PTGES, PTGFR, PTGIR, PTN, PTPRN, PTPRU, PYGO2, RAC2, RARA, RARRES1, RASSF5, RBPMS2, RELA, RGCC, RHOG, RMRP, RNF126, RNU1-93P, RPTOR, RTKN2, S100A11, S1PR2, SCN5A, SELENON, SH2B3, SHC4, SIX5, SLC25A33, SLC35F6, SLC7A1, SLC9A3R1, SMAD3, SMARCB1, SMIM22, SOX10, SOX7, SOX8, SPEG, SPHK1, SPHK2, SRF, SSBP3, SSTR2, STAT3, TBX5, TCIRG1, TGFB1I1, TGM2, THBS1, THPO, TICAM1, TIMP1, TMEM115, TMEM127, TNF, TNFSF12, TNMD, TNS2, TP53I11, TRIB1, TSC2, VDR, VEGFA, VIPR2, VSIR, WARS1, WNK2, WNT3, ZBTB7B, ZFP36, ZMIZ1, ZNF335, ZNF580, ZNF609]
GO:0008284	positive regulation of cell population proliferation	9,33E-09	[3, 4, 5]	211,00	21,53	44,36	55,64	[AIF1, AREG, ATF3, CARD11, CCDC88B, CCL19, CCL24, CCL5, CCR2, CCR3, CD3E, CD40LG, CD6, CD74, CD80, CD86, CLCF1, CORO1A, CSF1R, CXCL10, CXCL5, EBI3, EDN2, EGR3, EPX, EREG, FASLG, FCRL3, FLT3, FOLR2, FOSL1, FOXP3, HCLS1, HLA-DMB, HLA-DPA1, HLA-DPB1, IFNG, IL12RB1, IL12RB2, IL18, IL5RA, IL7R, ITGA4, ITGAX, JAML, KLF5, LEF1, LIF, LTA, MATK, MMP9, MYB, MZB1, NAMPT, NCKAP1L, NELL2, NLRP3, NR4A1, NR4A3, NTN1, PIM1, PLAC8, PRKCQ, PROK1, PTAFR, PTGFR, PTK2B, PTPN22, PTPN6, PTPRC, PYCARD, RAC2, RASAL3, RBPMS2, RTKN2, SASH3, SIRPG, SMPD3, STAT1, SYK, THBS1, TNFAIP3, TNFSF13B, TOX, WNT1, WNT10B, XCL1, ZAP70]	[ABL1, ADGRG1, AGAP2, AGER, AGT, AGTR1, AMH, APLN, APLNR, AREG, BCL2L1, BCL6, C5AR1, CAPN1, CAPNS1, CARM1, CCL11, CCL24, CD276, CD74, CDC25B, CDK1, CDK2, CDKN1A, CGA, CHGA, CLCF1, CRIP2, CSF1, CTC1, CTF1, CTHRC1, CX3CL1, DBF4B, DDX39B, DISP3, DNMT1, ECM1, EDN2, ERBB2, ESM1, FADD, FGFR4, FLT3, FLT4, FOSL1, FOSL2, FOXF1, FOXM1, GAB2, GATA2, GNAI2, GPAM, GPBAR1, GPER1, GPR37L1, GRN, HBEGF, HMGN5, HSF1, HTR1B, HTR2A, HYAL1, IL1B, IL5RA, IL7R, ILK, JAK3, KLF5, KMT2D, LDLRAP1, LGALS9, LIF, LIFR, LRG1, LTF, MAPK3, MARCKSL1, MARK4, MAZ, MECP2, MEF2D, MEIS3P1, MEIS3P2, MEN1, MYC, NACC1, NGFR, NOD2, NODAL, NOP2, NOTCH1, NR4A1, NR4A3, NRARP, NTN1, OSM, OSR2, P2RY6, PDF, PDGFB, PDGFRB, PIM1, PML, PPARD, PTGFR, PTN, PTPRN, RAC2, RARA, RBPMS2, RELA, RHOG, RMRP, RNU1-93P, RPTOR, RTKN2, S1PR2, SCN5A, SELENON, SHC4, SLC25A33, SLC35F6, SLC7A1, SOX10, SOX8, SPHK1, SPHK2, SSBP3, STAT3, TBX5, TCIRG1, TGM2, THBS1, THPO, TICAM1, TIMP1, TNF, TNFSF12, VEGFA, ZMIZ1, ZNF335, ZNF580]
GO:0008285	negative regulation of cell population proliferation	3,74E-05	[3, 4, 5]	157,00	21,05	40,15	59,85	[ADORA3, AIF1, ALOX15B, AREG, ASCL2, BCL11B, BLK, BMP7, BMPR1B, BRIP1, BTG2, BTK, CCL2, CCL3L1, CD33, CD80, CD86, CDKN2A, CEL, CNN1, CRTAM, CTLA4, DPT, EAF2, EREG, FOSL1, FOXP3, FRZB, HLA-DRB1, IFNG, IL26, INPP5D, LIF, LILRB1, LILRB4, LST1, LTA, MSX1, NLRC3, NPPC, PIM2, PLA2G2D, PLAAT4, PTK2B, PTPN6, RARRES1, RUNX3, SIRPG, SPN, STAT1, SULT2B1, THBS1, TNFAIP3, TNFRSF13B, TNMD, TREM2, WNT10B, XCL1]	[ABCC8, ACKR3, ADAMTS8, ADGRG1, ADIPOQ, APLN, APOD, AREG, ATF5, BCL6, BTG2, C18orf54, CCL2, CDKN1A, CEACAM1, CEL, CITED2, CNN1, CRP, CSK, CYP27B1, DAB2IP, DDR1, DHRS2, DLG5, DPT, E2F7, EAF2, EEF1E1, EFEMP2, ENPP3, EPPK1, ERBB2, FAP, FGFRL1, FOSL1, GATA2, GPER1, HGS, HMGA1, IFITM1, IFT80, IL1B, ILK, INCA1, INPPL1, KCNK2, KLF10, LDOC1, LGALS9, LIF, LIMS2, LMNA, LRRC32, LZTS2, MED25, MEN1, MNT, MSTN, MSX1, MYC, NF2, NGF, NGFR, NODAL, NOS3, NOTCH1, NPR3, NPRL3, OGN, OSM, PLXNB1, PML, PPARD, PTGES, PTGIR, PTN, PTPRU, RARA, RARRES1, RASSF5, RGCC, RMRP, S100A11, SH2B3, SIX5, SLC9A3R1, SMAD3, SMARCB1, SOX10, SOX7, SPEG, SRF, SSTR2, STAT3, TBX5, TGFB1I1, THBS1, TMEM115, TMEM127, TNF, TNMD, TNS2, TP53I11, TRIB1, TSC2, VDR, VIPR2, VSIR, WARS1, WNK2, ZBTB7B]
GO:0048017	inositol lipid-mediated signaling	1,94E-06	[4, 5, 6]	63,00	29,86	38,78	61,22	[ANGPT1, CCL5, CSF1R, EPX, FGR, FLT3, HCLS1, HCST, IER3, IL18, NCF1, NLRC3, PIK3C2G, PIK3CG, PIK3IP1, PIK3R5, PLCB2, PLCH2, PTAFR, PTPN6, SEMA4D, TREM2]	[AGAP2, AGT, ANGPT1, CEACAM1, CRAT, DAB2IP, ERBB2, FLT3, GAB2, GPER1, HTR2A, IER3, INPP5E, MAPK3, MAZ, NGF, NPR3, NPRL3, OSM, PDGFB, PDGFRB, PEAR1, PI4KA, PI4KAP1, PI4KAP2, PIK3C2B, PIK3C2G, PIP5K1C, PITPNM2, PLCD3, PLCH1, PLCZ1, PLXNB1, PPARD, PPP2R5B, PRKCSH, PTPN13, RNU1-93P, SELENOP, SLC9A3R1, SRC, TIMP1, TNF, TSC2, UNC5B]
GO:0014065	phosphatidylinositol 3-kinase signaling	1,32E-03	[6, 7, 8]	47,00	28,31	41,41	58,59	[ANGPT1, CCL5, EPX, FGR, FLT3, HCLS1, HCST, IER3, IL18, NCF1, NLRC3, PIK3C2G, PIK3CG, PIK3IP1, PIK3R5, PTPN6, SEMA4D, TREM2]	[AGAP2, AGT, ANGPT1, CEACAM1, CRAT, DAB2IP, ERBB2, FLT3, GPER1, HTR2A, IER3, INPP5E, MAPK3, MAZ, OSM, PDGFB, PDGFRB, PEAR1, PIK3C2B, PIK3C2G, PIP5K1C, PLXNB1, PPARD, PPP2R5B, PTPN13, RNU1-93P, SELENOP, SLC9A3R1, SRC, TIMP1, TNF, TSC2, UNC5B]

GO:0014066	regulation of phosphatidylinositol 3-kinase signaling	2,36E-03	[5, 6, 7, 8, 9]	41,00	29,71	44,93	55,07	[ANGPT1, CCL5, EPX, FGR, FLT3, HCLS1, HCST, IER3, IL18, NCF1, NLRC3, PIK3CG, PIK3IP1, PIK3R5, PTPN6, SEMA4D, TREM2]	[AGAP2, AGT, ANGPT1, CEACAM1, CRAT, DAB2IP, FLT3, GPER1, IER3, INPP5E, MAPK3, MAZ, OSM, PDGFB, PDGFRB, PIP5K1C, PLXNB1, PPARD, PPP2R5B, PTPN13, SELENOP, SLC9A3R1, SRC, TIMP1, TNF, TSC2, UNC5B]
GO:0033002	muscle cell proliferation	5,83E-05	[3]	58,00	28,43	35,36	64,64	[AIF1, ANGPT1, CCL5, CNN1, EREG, IFNG, IL18, MMP9, MYB, NAMPT, NPPC, NR4A3, PIM1, PTAFR, RBPMS2, SMPD3, STAT1, THBS1, TNFAIP3]	[ADIPOQ, AGT, ANGPT1, APLN, APOD, CDK1, CDKN1A, CITED2, CNN1, CX3CL1, DDX39B, DNMT1, EFEMP2, FOXC2, GNAI2, GPER1, HBEGF, HTR1B, ILK, KCNK2, LDLRAP1, MEF2D, MSTN, MYC, NOTCH1, NPR3, NPRL3, NR4A3, OGN, P2RY6, PDGFB, PDGFRB, PIM1, PPARD, PTGIR, RBPMS2, SELENON, SIX5, STAT3, TBX5, TGM2, THBS1, TNF, TRIB1, VIPR2]
GO:0048659	smooth muscle cell proliferation	9,19E-05	[4]	46,00	31,08	40,03	59,97	[AIF1, CCL5, CNN1, EREG, IFNG, IL18, MMP9, MYB, NAMPT, NPPC, NR4A3, PTAFR, RBPMS2, SMPD3, STAT1, THBS1, TNFAIP3]	[ADIPOQ, AGT, APLN, APOD, CDKN1A, CNN1, CX3CL1, DDX39B, DNMT1, EFEMP2, GNAI2, GPER1, HBEGF, HTR1B, ILK, LDLRAP1, MEF2D, MYC, NPR3, NPRL3, NR4A3, OGN, P2RY6, PDGFB, PDGFRB, PPARD, PTGIR, RBPMS2, TGM2, THBS1, TNF, TRIB1, VIPR2]
GO:0048660	regulation of smooth muscle cell proliferation	3,74E-05	[4, 5]	46,00	31,72	40,03	59,97	[AIF1, CCL5, CNN1, EREG, IFNG, IL18, MMP9, MYB, NAMPT, NPPC, NR4A3, PTAFR, RBPMS2, SMPD3, STAT1, THBS1, TNFAIP3]	[ADIPOQ, AGT, APLN, APOD, CDKN1A, CNN1, CX3CL1, DDX39B, DNMT1, EFEMP2, GNAI2, GPER1, HBEGF, HTR1B, ILK, LDLRAP1, MEF2D, MYC, NPR3, NPRL3, NR4A3, OGN, P2RY6, PDGFB, PDGFRB, PPARD, PTGIR, RBPMS2, TGM2, THBS1, TNF, TRIB1, VIPR2]
GO:0048661	positive regulation of smooth muscle cell proliferation	4,19E-02	[4, 5, 6]	28,00	30,77	48,34	51,66	[AIF1, CCL5, EREG, IL18, MMP9, MYB, NAMPT, NR4A3, PTAFR, RBPMS2, SMPD3, STAT1, THBS1]	[AGT, CX3CL1, DDX39B, DNMT1, GNAI2, HBEGF, HTR1B, LDLRAP1, MEF2D, MYC, NR4A3, P2RY6, PDGFB, PDGFRB, RBPMS2, TGM2, THBS1, TNF]
GO:0042116	macrophage activation	2,53E-02	[4, 5]	32,00	29,91	52,41	47,59	[AIF1, C1QA, CCL3, CD74, CST7, EDN2, IFNG, ITGB2, LDLR, NAMPT, PTPRC, STAP1, SUCNR1, SYK, THBS1, TLR6, TREM2]	[AGER, AMH, C5AR1, CD74, CST7, CX3CL1, DYSF, EDN2, GRN, IL4R, JUND, LDLR, MYO18A, NR1D1, SBNO2, SPHK1, THBS1, TICAM1, TNF, ZC3H12A]
GO:0150076	neuroinflammatory response	1,24E-03	[5]	18,00	47,37	56,44	43,56	[CCL3, CD200R1, CST7, IFNG, IL18, LDLR, MMP9, PTPRC, STAP1, TREM2]	[ADCY1, CST7, CX3CL1, DAGLA, GRN, IL1B, LDLR, NR1D1, SPHK1, TNF]
GO:0001774	microglial cell activation	7,67E-03	[5, 6]	19,00	41,30	63,34	36,66	[AIF1, C1QA, CCL3, CST7, IFNG, ITGB2, LDLR, NAMPT, PTPRC, STAP1, TLR6, TREM2]	[AGER, C5AR1, CST7, CX3CL1, GRN, LDLR, NR1D1, SPHK1, TNF]
GO:0150077	regulation of neuroinflammatory response	5,75E-03	[5, 6, 7]	16,00	47,06	56,44	43,56	[CCL3, CD200R1, CST7, IL18, LDLR, MMP9, PTPRC, STAP1, TREM2]	[CST7, CX3CL1, DAGLA, GRN, IL1B, LDLR, NR1D1, SPHK1, TNF]
GO:1903038	negative regulation of leukocyte cell-cell adhesion	9,60E-03	[5, 6, 7]	39,00	28,47	64,65	35,35	[CD74, CD80, CD86, CDKN2A, CRTAM, CTLA4, FGL2, FOXP3, HLA-DRB1, LAG3, LAX1, LILRB1, LILRB4, NCKAP1L, PLA2G2D, PTPN22, PTPN6, RUNX3, SOCS1, SPN, TBX21, TIGIT, TNFAIP8L2, XCL1]	[BCL6, CCL21, CD74, CEACAM1, DLG5, DTX1, ERBB2, HFE, IL4R, JAK3, LAG3, LGALS9, LRRC32, NRARP, VSIR, ZBTB7B, ZC3H12A]
GO:0036037	CD8-positive, alpha-beta T cell activation	1,46E-03	[6, 7]	15,00	53,57	83,83	16,17	[CRTAM, EOMES, LILRB1, LILRB4, NCKAP1L, PTPN22, RUNX3, SOCS1, TNFSF8, TOX, WDFY4, XCL1]	[HFE, VSIR, ZBTB7B]
GO:0002710	negative regulation of T cell mediated immunity	3,56E-02	[6, 7, 8]	13,00	48,15	76,41	23,59	[FOXP3, IL7R, KLRC1, KLRD1, LILRB1, LILRB4, NCKAP1L, PTPRC, TBX21, XCL1]	[CEACAM1, HFE, IL7R, NOD2]
GO:2001185	regulation of CD8-positive, alpha-beta T cell activation	1,67E-02	[7, 8, 9]	11,00	57,89	77,55	22,45	[CRTAM, LILRB1, LILRB4, NCKAP1L, PTPN22, RUNX3, SOCS1, XCL1]	[HFE, VSIR, ZBTB7B]
GO:0004896	cytokine receptor activity	1,19E-04	[5, 6, 7]	35,00	35,00	69,82	30,18	[CCR2, CCR3, CCR4, CCR5, CCR8, CD4, CD74, CSF2RA, CXCR3, CXCR4, CXCR6, EBI3, FLT3, GFRA2, IL10RA, IL12RB1, IL12RB2, IL18RAP, IL21R, IL2RB, IL2RG, IL5RA, IL7R, IL9R, XCR1]	[ACKR3, CCR7, CD74, FLT3, GFRA3, GPR17, GPR75, IL15RA, IL22RA1, IL3RA, IL4R, IL5RA, IL7R, LIFR]
GO:0070098	chemokine-mediated signaling pathway	5,99E-08	[5, 6, 7]	38,00	42,22	75,14	24,86	[CCL17, CCL19, CCL2, CCL22, CCL24, CCL3, CCL3L1, CCL4, CCL4L1, CCL5, CCR2, CCR3, CCR4, CCR5, CCR8, CXCL10, CXCL11, CXCL5, CXCL6, CXCL9, CXCR3, CXCR4, CXCR6, PTK2B, TREM2, XCL1, XCL2, XCR1]	[ACKR1, ACKR3, CCL11, CCL2, CCL21, CCL24, CCR7, CX3CL1, GPR17, GPR75, SH2B3, THPO]
GO:1990869	cellular response to chemokine	3,10E-08	[6]	41,00	41,00	72,85	27,15	[CCL17, CCL19, CCL2, CCL22, CCL24, CCL3, CCL3L1, CCL4, CCL4L1, CCL5, CCR2, CCR3, CCR4, CCR5, CCR8, CXCL10, CXCL11, CXCL5, CXCL6, CXCL9, CXCR3, CXCR4, CXCR6, DOCK8, PTK2B, TREM2, XCL1, XCL2, XCR1]	[ACKR1, ACKR3, CCL11, CCL2, CCL21, CCL24, CCR7, CX3CL1, GPR17, GPR75, LOX, SH2B3, THPO, ZC3H12A]
GO:0004950	chemokine receptor activity	3,56E-02	[6, 7, 8, 9]	13,00	48,15	74,46	25,54	[CCR2, CCR3, CCR4, CCR5, CCR8, CXCR3, CXCR4, CXCR6, XCR1]	[ACKR3, CCR7, GPR17, GPR75]
GO:0000902	cell morphogenesis	2,31E-02	[3, 4]	201,00	18,21	33,55	66,45	[ADAM8, AREG, BCL11B, BMP7, BMPR1B, CASS4, CCDC88C, CCL2, CCL24, CCL3, CDH4, CEL, COL22A1, CORO1A, CRABP2, CSF1R, CXCR4, DCC, DOCK10, DOK2, ELAVL4, EMB, EPHA6, ERMN, FGD2, FGD3, FGR, GFRA2, GPM6A, GRXCR2, IL7R, ITGA4, ITGB2, ITGB7, LEF1, LST1, MSX1, NCKAP1L, NELL2, NPPC, NR4A2, NTN1, PARVG, PEAK3, PLPPR4, PLXNC1, PRKCQ, PTK2B, PTPN6, RAC2, RHOH, SEMA4A, SEMA4D, ST14, TACSTD2, THY1, TNMD, TNR, UGT8, VSIG1]	[ABI3, ABL1, ACTB, ACTN1, ACTN4, ADCY1, ALDOA, APOA1, ARC, AREG, ARHGDIA, ARTN, BCL6, BCL9L, C15orf62, CAMK2B, CCL11, CCL2, CCL24, CDC42EP1, CDC42EP2, CDC42EP4, CDH4, CEL, CELSR2, CERT1, CFL1, CNTN6, COL22A1, CPNE5, CPNE6, CYP27B1, DAB2IP, DAG1, DCHS1, DDR1, DMTN, DOK5, DPYSL4, DSCAML1, DVL3, EFNA3, EPHA2, ERBB2, FBLIM1, FES, FMNL1, GAB2, GDI1, GFRA3, GPM6A, GRB7, HPRT1, ICAM1, IL7R, ILK, ITGA7, LAMA5, LAMB4, LGR6, LIMK1, LIMS2, LINGO1, LMX1A, LYPLA2, LZTS1, LZTS3, MAP1A, MAP1S, MAPK3, MAPK7, MARK2, MED12, MEGF8, MFSD2A, MICALL2, MINK1, MOV10, MSN, MSX1, MYADM, MYH7B, MYH9, MYO7A, NBEAL2, NECTIN1, NEFL, NFATC4, NGF, NGFR, NODAL, NOTCH1, NOTCH3, NOTCH4, NR4A2, NSMF, NTN1, NUMBL, OMA1, PACSIN1, PALM, PALM2AKAP2, PARP6, PARVB, PDLIM7, PHACTR1, PLEKHO1, PLXNA1, PLXNA3, PLXNB1, POSTN, PQBP1, PRAG1, PTN, PTPRU, PXN, RAB3A, RAC2, RGMA, RHOBTB2, RHOG, RILPL1, RNF157, ROBO4, SEMA3F, SEMA4B, SEMA4C, SEMA6B, SEMA6C, SHANK3, SIDT2, SIPA1L3, SLC9A3R1, SPI1, SPTB, SRC, SRF, SYNGAP1, SYT2, SZT2, TESK1, TNMD, TRIOBP, TSKU, ULK1, UNC5B, VAX2, VDR, VEGFA, WHRN, WNT3, ZNF335, ZSWIM4, ZSWIM8]

GO:0022603	regulation of anatomical structure morphogenesis	1,53E-04	[3, 4]	198,00	19,58	30,83	69,17	[ADAMTS9, ANGPT2, AREG, BMP7, CASS4, CCL2, CCL24, CCL3, CCR2, CCR3, CDH4, CORO1A, CRABP2, CRB2, CSF1R, CXCL10, CXCR4, CYBB, DCC, ERMN, FASLG, FGD2, FGD3, FGR, GPR4, ITGAX, ITGB2, LIF, LST1, MSX1, NPPB, NTN1, PEAK3, PLXNC1, PRKCB, PROK1, PTK2B, RAC2, RHOH, RLN2, SEMA4A, SEMA4D, SLC18A1, STAT1, TACSTD2, THBS1, THY1, TNFAIP3, TNFRSF11B, TNFSF13B, TNMD, TNR, WNT1, WNT10A]	[ABCC8, ABI3, ABL1, ACTN4, ADAMTS9, ADCK1, ADGRB2, AGT, ALDOA, ANGPTL4, AP2A1, APLNR, APOA1, ARC, AREG, ARHGDIA, BCL6, BCL9L, C11orf65, C15orf62, C5, C5AR1, CAMK2B, CCL11, CCL2, CCL24, CDC42EP1, CDC42EP2, CDC42EP4, CDH4, CEACAM1, CELSR2, CFL1, CHI3L1, CITED2, CPNE5, CPNE6, CREB3L1, CSF1, CTHRC1, DAB2IP, DAG1, DMTN, DVL3, ECM1, EFNA3, ENG, EPHA2, EPN1, ERBB2, FBLIM1, FES, FLT4, FMNL1, FOXC2, GATA2, GATA4, GDI1, GPR4, GRN, HGS, HSPB1, HSPG2, HYAL1, ICAM1, IL1B, ILK, ITGA5, ITGA7, JMJD8, JUP, LIF, LIMK1, LIMS2, LINGO1, LRG1, LZTS1, LZTS3, MAPK7, MARK2, MECP2, MED12, MEGF8, MFSD2A, MOV10, MSN, MSX1, MYADM, MYH7B, MYH9, MYO19, NEFL, NFATC4, NGF, NGFR, NINJ1, NODAL, NOS3, NOTCH1, NPPB, NSMF, NTN1, OMA1, PAK4, PALM, PALM2AKAP2, PARP6, PARVB, PFN1, PHLDB1, PKM, PLEKHO1, PLXNA1, PLXNA3, PLXNB1, PML, POSTN, PQBP1, PRAG1, PTN, RAC2, RAMP2, RGCC, RGMA, RHOBTB2, RHOG, RNF157, RNU1-93P, S100A1, SEMA3F, SEMA4B, SEMA4C, SEMA6B, SEMA6C, SERPINE1, SHANK3, SLC9A3R1, SOX8, SPHK1, SRC, SRF, STAT3, SYNGAP1, SYT2, TESK1, THBS1, TNF, TNFSF12, TNMD, TRIOBP, ULK1, VANGL2, VEGFA, WARS1, WNT3, WNT9B, ZC3H12A]
GO:0022604	regulation of cell morphogenesis	4,32E-03	[4, 5]	76,00	23,24	31,93	68,07	[CASS4, CCL2, CCL24, CCL3, CORO1A, CSF1R, CXCR4, ERMN, FGD2, FGD3, FGR, ITGB2, LST1, PEAK3, PLXNC1, PTK2B, RAC2, RHOH, SEMA4A, SEMA4D, TACSTD2]	[ABL1, ACTN4, ALDOA, APOA1, ARC, ARHGDIA, BCL9L, C15orf62, CAMK2B, CCL11, CCL2, CCL24, CDC42EP1, CDC42EP2, CDC42EP4, CFL1, CPNE5, CPNE6, DAG1, DMTN, DVL3, FBLIM1, FES, FMNL1, ICAM1, ILK, ITGA7, LIMS2, MARK2, MED12, MFSD2A, MOV10, MSN, MYADM, MYH7B, MYH9, PALM, PALM2AKAP2, PARP6, PARVB, PLEKHO1, PLXNA1, PLXNA3, PLXNB1, POSTN, PRAG1, PTN, RAC2, RHOBTB2, RHOG, RNF157, SLC9A3R1, SRC, SYNGAP1, SYT2, TESK1, TRIOBP, VEGFA]
GO:0008360	regulation of cell shape	3,28E-05	[3, 5, 6]	50,00	30,67	39,99	60,01	[CCL2, CCL24, CCL3, CORO1A, CSF1R, ERMN, FGD2, FGD3, FGR, ITGB2, LST1, PEAK3, PLXNC1, PTK2B, RAC2, RHOH, SEMA4A, SEMA4D]	[ALDOA, ARHGDIA, C15orf62, CCL11, CCL2, CCL24, CDC42EP1, CDC42EP2, CDC42EP4, DAG1, DMTN, FBLIM1, FES, FMNL1, ICAM1, ITGA7, MARK2, MSN, MYH7B, MYH9, PALM, PALM2AKAP2, PARVB, PLEKHO1, PLXNA1, PLXNA3, PLXNB1, PRAG1, PTN, RAC2, RHOBTB2, RHOG, SLC9A3R1, SYNGAP1, VEGFA]
GO:0042100	B cell proliferation	1,60E-02	[5, 6]	31,00	30,69	80,81	19,19	[BLK, BTK, CARD11, CD180, CD19, CD22, CD40LG, CD74, CD79A, CDKN2A, CLCF1, CR2, CTLA4, FCRL3, GAPT, IKZF3, IL7R, INPP5D, LEF1, MS4A1, MZB1, NCKAP1L, PTPRC, SASH3, TNFRSF13B, TNFSF13B]	[ABL1, AMH, BCL6, CD74, CDKN1A, CLCF1, IL7R, TICAM1]
GO:0030098	lymphocyte differentiation	4,55E-19	[4, 5, 6, 7, 8, 9, 10]	129,00	32,66	72,38	27,62	[ADAM8, BATF, BCL11B, BCL3, BLNK, BTK, CAMK4, CARD11, CCL19, CCR2, CD19, CD2, CD27, CD3D, CD3E, CD3G, CD4, CD40LG, CD74, CD79A, CD80, CD86, CD8A, CDKN2A, CLCF1, CR2, CRTAM, CTLA4, DOCK10, DOCK2, EGR3, EOMES, FCRL3, FGL2, FLT3, FOXP3, FUT7, HLA-DOA, HLA-DRA, HLA-DRB1, IFNG, IKZF1, IKZF3, IL12RB1, IL18, IL7R, INPP5D, IRF4, IRF8, ITGA4, ITK, KLRC1, LAG3, LCK, LEF1, LILRB4, LY9, MS4A1, MYB, NCKAP1L, NFAM1, NLRP3, PLA2G2D, POU2AF1, POU2F2, PRDM1, PTK2B, PTPN22, PTPN6, PTPRC, RHOH, RUNX3, SASH3, SEMA4A, SLAMF6, SLAMF8, SOCS1, SPN, SYK, TBX21, TESPA1, THEMIS, TNFRSF18, TNFSF13B, TNFSF8, TOX, VAV1, VNN1, WNT1, ZAP70, ZNF683]	[ABL1, AP3D1, ARMC5, BCL3, BCL6, CCR7, CD74, CLCF1, CLPTM1, CYP26B1, DTX1, ERBB2, FADD, FLT3, GBA, GNAO1, HDAC5, IL15RA, IL1B, IL4R, IL7R, INHA, JAK3, KDELR1, LAG3, LGALS9, LRRC8A, NRARP, RARA, RELB, SOX12, SOX13, SPI1, SRF, STAT3, SYVN1, TCF3, TCF7L1, TCIRG1, VNN1, VSIR, ZBTB7A, ZBTB7B, ZC3H12A, ZMIZ1]
GO:1903131	mononuclear cell differentiation	6,89E-22	[5, 6, 7, 8, 9]	146,00	32,52	69,78	30,22	[ADAM8, BATF, BCL11B, BCL3, BLK, BLNK, BTK, CAMK4, CARD11, CCL19, CCR2, CD19, CD2, CD27, CD3D, CD3E, CD3G, CD4, CD40LG, CD74, CD79A, CD80, CD86, CD8A, CDKN2A, CLCF1, CR2, CRTAM, CSF1R, CTLA4, DOCK10, DOCK2, EGR3, EOMES, FCRL3, FGL2, FLT3, FOXP3, FUT7, HLA-DOA, HLA-DRA, HLA-DRB1, IFNG, IKZF1, IKZF3, IL12RB1, IL18, IL7R, INPP5D, IRF4, IRF8, ITGA4, ITK, KLRC1, LAG3, LCK, LEF1, LILRB1, LILRB4, LY9, MS4A1, MT1G, MYB, NCKAP1L, NFAM1, NLRP3, PLA2G2D, POU2AF1, POU2F2, PRDM1, PTK2B, PTPN22, PTPN6, PTPRC, RHOH, RUNX3, SASH3, SEMA4A, SLAMF6, SLAMF8, SOCS1, SPN, SYK, TBX21, TESPA1, THEMIS, TNFRSF18, TNFSF13B, TNFSF8, TOX, TREM2, TRPM2, UBD, VAV1, VNN1, WNT1, ZAP70, ZNF683]	[ABL1, AGER, AP3D1, ARMC5, BATF2, BCL3, BCL6, CCR7, CD74, CLCF1, CLPTM1, CSF1, CYP26B1, DHRS2, DTX1, ERBB2, FADD, FASN, FES, FLT3, GBA, GNAO1, HDAC5, IL15RA, IL1B, IL4R, IL7R, INHA, JAK3, KDELR1, LAG3, LGALS9, LRRC8A, LTBR, MYC, MYH9, NRARP, RARA, RELB, SOX12, SOX13, SPI1, SRF, STAT3, SYVN1, TCF3, TCF7L1, TCIRG1, VEGFA, VNN1, VSIR, ZBTB7A, ZBTB7B, ZC3H12A, ZMIZ1]
GO:0030183	B cell differentiation	1,40E-02	[5, 6, 7, 8, 9, 10, 11]	41,00	27,52	69,39	30,61	[BCL3, BLNK, BTK, CARD11, CD19, CD27, CD40LG, CD79A, CLCF1, CR2, DOCK10, FCRL3, FLT3, IKZF3, INPP5D, IRF8, ITGA4, MS4A1, NCKAP1L, NFAM1, POU2AF1, POU2F2, PTK2B, PTPN6, PTPRC, SLAMF8, SYK, TNFSF13B]	[ABL1, BCL3, BCL6, CLCF1, FLT3, GNAO1, HDAC5, INHA, JAK3, LRRC8A, SPI1, SYVN1, TCF3, TCF7L1, TCIRG1, ZBTB7A]
GO:0032609	interferon-gamma production	4,81E-07	[3, 6]	43,00	37,07	71,47	28,53	[BCL3, BTN3A1, BTN3A2, CCR2, CD2, CD226, CD244, CD3E, CD96, CRTAM, EBI3, FOXP3, HLA-DPA1, HLA-DPB1, HLA-DRB1, IL12RB1, IL12RB2, IL18, IRF8, LILRB1, LILRB4, LTA, NLRP6, PTPN22, PYCARD, SASH3, SLAMF6, TLR8, XCL1]	[ABL1, BCL3, CCR7, CD14, CD276, FADD, IL1B, INHA, LGALS9, NOD2, RARA, SLC7A5, TNF, VSIR, ZC3H12A]
GO:0032649	regulation of interferon-gamma production	4,81E-07	[4, 5, 6, 7]	43,00	37,07	71,47	28,53	[BCL3, BTN3A1, BTN3A2, CCR2, CD2, CD226, CD244, CD3E, CD96, CRTAM, EBI3, FOXP3, HLA-DPA1, HLA-DPB1, HLA-DRB1, IL12RB1, IL12RB2, IL18, IRF8, LILRB1, LILRB4, LTA, NLRP6, PTPN22, PYCARD, SASH3, SLAMF6, TLR8, XCL1]	[ABL1, BCL3, CCR7, CD14, CD276, FADD, IL1B, INHA, LGALS9, NOD2, RARA, SLC7A5, TNF, VSIR, ZC3H12A]

GO:0042098	T cell proliferation	9,29E-09	[5, 6]	68,00	32,23	73,87	26,13	[AIF1, BTN3A1, CARD11, CCDC88B, CCL19, CCL5, CCR2, CD3E, CD40LG, CD6, CD80, CD86, CDKN2A, CLCF1, CORO1A, CRTAM, CTLA4, DOCK2, EBI3, EPX, FOXP3, HLA-DMB, HLA-DPA1, HLA-DPB1, HLA-DRB1, IL12RB1, IL18, LILRB1, LILRB4, LMO1, NCKAP1L, PIK3CG, PLA2G2D, PRKCQ, PTPN22, PTPN6, PTPRC, PYCARD, RAC2, RASAL3, SASH3, SPN, SYK, TNFSF13B, TNFSF14, TNFSF8, XCL1, ZAP70]	[ABL1, AGER, ARMC5, CD276, CLC, CLCF1, DLG5, ELF4, ERBB2, FADD, GPAM, IDO1, IL1B, JAK3, LGALS9, LRRC32, MSN, RAC2, SLC7A1, TIMP1, VSIR, ZBTB7B]
GO:0032729	positive regulation of interferon-gamma production	1,56E-05	[4, 5, 6, 7, 8]	31,00	40,26	76,80	23,20	[BCL3, BTN3A1, BTN3A2, CCR2, CD2, CD226, CD244, CD3E, CRTAM, EBI3, HLA-DPA1, HLA-DPB1, IL12RB1, IL12RB2, IL18, IRF8, LILRB1, LTA, PTPN22, PYCARD, SASH3, SLAMF6, TLR8]	[ABL1, BCL3, CD14, CD276, FADD, IL1B, LGALS9, SLC7A5, TNF]
GO:0044093	positive regulation of molecular function	1,91E-07	[3]	325,00	18,99	40,88	59,12	[ABRA, ADAM8, ADCY7, AIM2, ANGPT1, ARAP2, AREG, ARHGAP45, ATP2A3, BTK, CARD11, CASP8, CASS4, CCL17, CCL19, CCL2, CCL22, CCL24, CCL3, CCL3L1, CCL4, CCL4L1, CCL5, CCR2, CD19, CD33, CD4, CD40LG, CD74, CD86, CDC20B, CDKN2A, CSF1R, CTSS, CXCR4, DAPK1, DOCK10, DOCK8, DPEP2, EDN2, EPHA6, EPX, EREG, FASLG, FCRL3, FGD2, FGR, FLT3, FOSL1, GADD45B, GBA3, GMIP, GPR55, GPRC5A, HCLS1, HLA-DRB1, IFNG, IKBKE, IL18, IL18RAP, IRF4, ITGB2, ITK, LCK, LCP2, LPAR2, LRRC38, MAP4K1, MID1IP1, MMP9, MUSK, NCF1, NCKAP1L, NEK5, NFAM1, NLRP2, NLRP3, NLRP6, NMUR1, NR4A2, P2RY12, PDE6G, PIK3CG, PIK3R5, PLAAT4, PLCB2, PLEK, PRKCB, PRKCQ, PROK1, PTAFR, PTK2B, PTPRC, PYCARD, PYHIN1, RASGRF1, RGS1, RGS10, RGS9, RTKN2, S1PR4, SELE, SEMA4D, SH2D1A, SOCS1, STAP1, SYK, TBC1D10C, THBS1, THY1, TLR6, TRAF1, TREM2, TRIM14, VAV1, WNT1, WNT10B, XCL1, XCL2]	[ABCC8, ABL1, ABR, ABRA, ACTN4, ADCY1, ADCY3, ADCY4, ADCY9, ADIPOQ, AGAP2, AGER, AGT, AGTR1, AMH, ANGPT1, APOA1, ARAF, ARAP3, ARC, AREG, ARHGEF15, ARHGEF16, ARHGEF5, ARTN, ATP1B2, ATPSCKMT, BCR, C5, C5AR1, CCL11, CCL2, CCL21, CCL24, CCR7, CD74, CDC25B, CDKN1A, CHI3L1, CRAT, CRTC2, CSF1, CSK, CSPG4, CTHRC1, CTSD, CX3CL1, CYP27B1, DAB2IP, DAG1, DAXX, DBF4B, DDR1, DGKZ, DIRAS1, DUSP5, DVL3, EDN2, EFNA3, EIF4G1, ELMOD1, EPHA2, ERBB2, FADD, FASN, FGFR4, FLOT2, FLT3, FLT4, FOSL1, FPR1, GADD45B, GMNN, GNAI2, GNAO1, GPER1, GPRC5A, GPRIN1, GRN, GSK3A, HBEGF, HDAC5, HFE, HMGN1, HSF1, HSPE1, HTR2A, ICAM1, IL1B, ILK, INCENP, IRAK2, JUP, KCNIP2, KSR1, LGALS9, LILRA5, LPAR5, LRRC52, LTF, MAP2K3, MAP3K10, MAP3K11, MAP3K14, MAP3K6, MAP3K9, MAPK3, MAPK8IP3, MARK2, MAVS, MED25, MEN1, MID1IP1, MINK1, MLST8, MTSS2, MUC20, MYC, NAPA, NEK5, NFKB2, NFKBIB, NGF, NGFR, NOD2, NODAL, NOS3, NPPA, NPR3, NPRL3, NR4A2, OMA1, ORAI1, P2RY6, PAK4, PCOLCE, PDGFB, PDGFRB, PFN1, PIDD1, PKD1, PLAUR, PLXNB1, PML, POR, PPP1R15A, PPRC1, PRKACA, PRKAR2B, PYGO2, RAMP2, RANGAP1, RAP1A, RAP1GAP2, RAPGEF1, RARA, RCVRN, RELA, RGCC, RGL3, RGMA, RGS16, RHOG, RMRP, RNU1-93P, RPLP1, RPTOR, RTKN2, RXRA, S100A1, S1PR2, SELE, SERINC2, SH3BP1, SHANK3, SHC2, SIPA1L3, SLC25A23, SLC6A9, SLC9A1, SMAD3, SMARCA4, SMARCB1, SOX7, SPATC1L, SPHK1, SRC, SRF, STAC2, STAT3, STING1, SYDE1, SYNGAP1, TBC1D10B, TBC1D13, TBC1D17, TBC1D2, TBC1D22B, TBC1D25, TCF3, TCF7L1, TGM2, THBS1, TICAM1, TIMP1, TNF, TNIP1, TRAF4, TRIB3, TRIM62, TRIM8, TSC2, UBA52, VAC14, VDR, VEGFA, VSIG2, VSIR, VTN, WNK2, WNT9B, XRCC1, ZBTB7A]
GO:0043085	positive regulation of catalytic activity	1,49E-05	[4]	253,00	19,15	42,90	57,10	[ADAM8, ADCY7, AIM2, ANGPT1, ARAP2, AREG, ARHGAP45, ATP2A3, CASP8, CASS4, CCL17, CCL19, CCL2, CCL22, CCL24, CCL3, CCL3L1, CCL4, CCL4L1, CCL5, CD19, CD33, CD4, CD40LG, CD74, CD86, CDC20B, CDKN2A, CSF1R, CXCR4, DAPK1, DOCK10, DOCK8, EDN2, EPHA6, EPX, EREG, FASLG, FCRL3, FGD2, FGR, FLT3, GADD45B, GBA3, GMIP, GPR55, GPRC5A, HLA-DRB1, IFNG, IKBKE, IL18, ITGB2, ITK, LCK, LCP2, LPAR2, MAP4K1, MID1IP1, MUSK, NCF1, NCKAP1L, NEK5, NLRP2, NLRP3, NLRP6, NMUR1, NR4A2, P2RY12, PDE6G, PIK3CG, PIK3R5, PLAAT4, PLCB2, PLEK, PRKCQ, PROK1, PTAFR, PTK2B, PTPRC, PYCARD, RASGRF1, RGS1, RGS10, S1PR4, SELE, SEMA4D, SH2D1A, SOCS1, STAP1, SYK, TBC1D10C, THBS1, THY1, TLR6, TREM2, VAV1, XCL1, XCL2]	[ABL1, ABR, ADCY1, ADCY3, ADCY4, ADCY9, ADIPOQ, AGAP2, AGER, AGT, AGTR1, ANGPT1, APOA1, ARAF, ARAP3, AREG, ARHGEF15, ARHGEF16, ARHGEF5, ARTN, ATPSCKMT, BCR, C5, C5AR1, CCL11, CCL2, CCL21, CCL24, CCR7, CD74, CDC25B, CDKN1A, CHI3L1, CSF1, CSK, CSPG4, CTSD, CX3CL1, CYP27B1, DAB2IP, DAG1, DAXX, DBF4B, DDR1, DGKZ, DIRAS1, DUSP5, DVL3, EDN2, EFNA3, ELMOD1, EPHA2, ERBB2, FADD, FASN, FGFR4, FLT3, FLT4, FPR1, GADD45B, GNAI2, GNAO1, GPER1, GPRC5A, GPRIN1, GRN, GSK3A, HBEGF, HMGN1, HSF1, HSPE1, HTR2A, ICAM1, IL1B, ILK, INCENP, IRAK2, KSR1, LGALS9, LILRA5, LTF, MAP2K3, MAP3K10, MAP3K11, MAP3K14, MAP3K6, MAP3K9, MAPK3, MAPK8IP3, MARK2, MID1IP1, MINK1, MLST8, MTSS2, MUC20, MYC, NEK5, NGF, NGFR, NOD2, NODAL, NOS3, NPR3, NPRL3, NR4A2, OMA1, ORAI1, P2RY6, PAK4, PCOLCE, PDGFB, PDGFRB, PIDD1, PKD1, PLXNB1, PML, POR, PPP1R15A, PRKACA, PRKAR2B, RANGAP1, RAP1A, RAP1GAP2, RAPGEF1, RCVRN, RGCC, RGL3, RGMA, RGS16, RHOG, RNU1-93P, RPLP1, RPTOR, S100A1, S1PR2, SELE, SERINC2, SH3BP1, SHC2, SIPA1L3, SLC25A23, SMAD3, SOX7, SPATC1L, SRC, STAT3, SYDE1, SYNGAP1, TBC1D10B, TBC1D13, TBC1D17, TBC1D2, TBC1D22B, TBC1D25, TGM2, THBS1, TIMP1, TNF, TNIP1, TRAF4, TRIB3, TSC2, UBA52, VAC14, VDR, VEGFA, VSIR, WNT9B, XRCC1]
GO:0043087	regulation of GTPase activity	3,79E-02	[5]	79,00	21,82	46,60	53,40	[ARAP2, ARHGAP15, ARHGAP45, CCL17, CCL19, CCL2, CCL22, CCL24, CCL3, CCL3L1, CCL4, CCL4L1, CCL5, DOCK10, DOCK8, FGD2, FGD3, GMIP, IQGAP2, KLRK1, PLXNC1, PTK2B, PYCARD, RASAL3, RASGRF1, RGS1, RGS10, SEMA4D, TBC1D10C, THY1, VAV1, XCL1, XCL2]	[ABR, ARAP3, ARHGEF15, ARHGEF16, ARHGEF5, BCL6, BCR, CCL11, CCL2, CCL21, CCL24, CCR7, CX3CL1, DAB2IP, DVL3, ELMOD1, EPHA2, ERBB2, GNAO1, ICAM1, IQGAP2, MLST8, MTSS2, PLXNA1, PLXNA3, PLXNB1, PROM2, RANGAP1, RAP1A, RAP1GAP2, RAPGEF1, RGL3, RGMA, RGS16, RHOG, SBF1, SH3BP1, SIPA1L3, SLC25A23, SYDE1, SYNGAP1, TBC1D10B, TBC1D13, TBC1D17, TBC1D2, TBC1D22B, TBC1D25, TGM2, TSC2]

GO:0051345	positive regulation of hydrolase activity	3,09E-03	[5]	126,00	20,62	47,93	52,07	[AIM2, ARAP2, ARHGAP45, ATP2A3, CASP8, CCL17, CCL19, CCL2, CCL22, CCL24, CCL3, CCL3L1, CCL4, CCL4L1, CCL5, CD33, CD86, CDKN2A, DAPK1, DOCK10, DOCK8, FASLG, FCRL3, GBA3, GMIP, GPR55, IFNG, ITK, LCK, LPAR2, NEK5, NLRP2, NLRP3, NLRP6, NMUR1, P2RY12, PLCB2, PLEK, PTAFR, PTK2B, PTPRC, PYCARD, RASGRF1, RGS1, RGS10, S1PR4, SELE, SEMA4D, SYK, TBC1D10C, THY1, VAV1, XCL1, XCL2]	[ABR, AGER, AGT, AGTR1, APOA1, ARAP3, ARHGEF15, ARHGEF16, ARHGEF5, BCR, C5AR1, CCL11, CCL2, CCL21, CCL24, CCR7, CTSD, CX3CL1, DVL3, EFNA3, ELMOD1, EPHA2, ERBB2, FADD, FASN, GNAO1, GPER1, GPRIN1, GRN, HSF1, HSPE1, HTR2A, ICAM1, LGALS9, MTSS2, MYC, NEK5, NGF, NGFR, NODAL, P2RY6, PCOLCE, PDGFRB, PIDD1, PLXNB1, PML, PPP1R15A, RANGAP1, RAP1A, RAP1GAP2, RAPGEF1, RGL3, RGMA, RGS16, RHOG, RNU1-93P, SELE, SH3BP1, SIPA1L3, SLC25A23, SMAD3, SOX7, STAT3, SYDE1, SYNGAP1, TBC1D10B, TBC1D13, TBC1D17, TBC1D2, TBC1D22B, TBC1D25, TGM2, TNF, TSC2, VEGFA, VSIR]
GO:0043547	positive regulation of GTPase activity	1,39E-02	[6]	64,00	23,70	44,14	55,86	[ARAP2, ARHGAP45, CCL17, CCL19, CCL2, CCL22, CCL24, CCL3, CCL3L1, CCL4, CCL4L1, CCL5, DOCK10, DOCK8, GMIP, PTK2B, RASGRF1, RGS1, RGS10, SEMA4D, TBC1D10C, THY1, VAV1, XCL1, XCL2]	[ABR, ARAP3, ARHGEF15, ARHGEF16, ARHGEF5, BCR, CCL11, CCL2, CCL21, CCL24, CCR7, CX3CL1, DVL3, ELMOD1, EPHA2, ERBB2, GNAO1, ICAM1, MTSS2, PLXNB1, RANGAP1, RAP1A, RAP1GAP2, RAPGEF1, RGL3, RGMA, RGS16, RHOG, SH3BP1, SIPA1L3, SLC25A23, SYDE1, SYNGAP1, TBC1D10B, TBC1D13, TBC1D17, TBC1D2, TBC1D22B, TBC1D25, TGM2, TSC2]
GO:0050778	positive regulation of immune response	1,07E-08	[3, 4, 5]	186,00	22,25	69,27	30,73	[ADAM8, AIM2, BLK, BLNK, BTK, BTN3A1, BTN3A2, C1QA, C1QB, C1QC, C2, C8G, CARD11, CCL19, CCL5, CCR2, CD177, CD19, CD1B, CD1C, CD1E, CD22, CD226, CD247, CD3D, CD3E, CD3G, CD4, CD74, CD79A, CD80, CD86, CFP, CLCF1, CLEC10A, CLEC4D, CLEC4E, CLEC6A, CLNK, CNR1, CR2, CRTAM, CTLA4, CXCL10, CXorf21, CYP11B1, EREG, FCN1, FCN3, FCRL3, FGR, FOXP3, FYB1, GBP5, GRAP2, HLA-DMB, HLA-DPA1, HLA-DPB1, HLA-DQA1, HLA-DRA, HLA-DRB1, HLA-DRB5, IFNG, IGLL5, IKBKE, IL12RB1, IL18, IL18RAP, INPP5D, ITGB2, ITK, KCNN4, KLRC2, KLRD1, KLRK1, LAG3, LAX1, LCK, LCP2, LILRB1, LILRB4, LTA, MOG, MS4A1, MUC16, MUC19, MUC6, MYB, MYO1G, NCKAP1L, NCR3, NFAM1, NLRP3, NR4A3, PAX5, PRAM1, PRKCB, PRKCQ, PTAFR, PTPN22, PTPN6, PTPRC, PYCARD, PYHIN1, SASH3, SH2D1A, SIGLEC16, SKAP1, SLA2, SLAMF6, STAP1, SYK, TBX21, TESPA1, THEMIS, THEMIS2, THY1, TLR8, TNFSF13B, TRAT1, TREM2, UBASH3A, VAV1, WAS, XCL1, ZAP70, ZBP1]	[ABL1, ACTB, BAG6, BCAR1, C5, C5AR1, C8G, CCR7, CD177, CD1C, CD1E, CD276, CD74, CEACAM1, CFH, CLCF1, CLEC4E, CLNK, CNR1, CPN2, CRP, CSK, DGKZ, FADD, FCN3, FFAR2, FPR1, GAB2, GATA2, GCSAM, GNAO1, GRN, IL1B, IL4R, LAG3, LGALS9, LIMK1, MAPK3, MAVS, MUC19, MUC20, MUC3A, NCOR2, NECTIN2, NLRC5, NOD2, NPPA, NR4A3, PCDHA4, PQBP1, PRKACA, PVR, RAP1A, RARA, RBM14, RELA, RELB, RGCC, RHBDF2, RNU1-93P, SERPING1, SH2B2, SLC7A8, SPHK2, SPI1, SRC, STING1, TNF, TRIM62, VTN, WAS, ZC3H12A, ZCCHC3]
GO:0002253	activation of immune response	5,32E-04	[2, 4, 5, 6]	132,00	20,99	69,52	30,48	[AIM2, BLK, BLNK, BTK, BTN3A1, BTN3A2, C1QA, C1QB, C1QC, C2, C8G, CARD11, CD19, CD22, CD226, CD247, CD3D, CD3E, CD3G, CD4, CD79A, CFP, CLEC10A, CLEC4D, CLEC4E, CLEC6A, CR2, CTLA4, CXCL10, CYP11B1, FCN1, FCN3, FCRL3, FGR, FOXP3, FYB1, GRAP2, HLA-DPA1, HLA-DPB1, HLA-DQA1, HLA-DRA, HLA-DRB1, HLA-DRB5, IGLL5, INPP5D, ITK, KCNN4, KLRC2, KLRD1, LAX1, LCK, LCP2, LILRB4, MOG, MS4A1, MUC16, MUC19, MUC6, MYO1G, NCKAP1L, NCR3, NFAM1, NR4A3, PAX5, PRAM1, PRKCB, PRKCQ, PTPN22, PTPN6, PTPRC, PYCARD, PYHIN1, SH2D1A, SKAP1, SLA2, STAP1, SYK, TESPA1, THEMIS, THEMIS2, THY1, TRAT1, TREM2, UBASH3A, VAV1, WAS, ZAP70, ZBP1]	[ABL1, ACTB, BAG6, BCAR1, C5, C5AR1, C8G, CCR7, CD276, CEACAM1, CFH, CLEC4E, CPN2, CRP, CSK, DGKZ, FCN3, FFAR2, FPR1, GCSAM, GNAO1, IL1B, LIMK1, MAPK3, MAVS, MUC19, MUC20, MUC3A, NCOR2, NECTIN2, NOD2, NR4A3, PQBP1, PRKACA, RAP1A, RBM14, RELA, RELB, RGCC, RHBDF2, RNU1-93P, SERPING1, SH2B2, SLC7A8, SRC, STING1, VTN, WAS, ZC3H12A, ZCCHC3]
GO:0050854	regulation of antigen receptor-mediated signaling pathway	1,39E-04	[4, 5, 6, 7, 8, 9, 10]	27,00	40,30	81,93	18,07	[BLK, CARD11, CD19, CD22, CD226, FCRL3, KCNN4, LCK, LILRB4, NFAM1, PAX5, PRKCB, PTPN22, PTPN6, PTPRC, SH2D1A, SLA2, STAP1, TESPA1, THY1, UBASH3A]	[CCR7, CEACAM1, DGKZ, GCSAM, NECTIN2, RELA]
GO:0050857	positive regulation of antigen receptor-mediated signaling pathway	4,64E-02	[4, 5, 6, 7, 8, 9, 10, 11]	12,00	50,00	79,54	20,46	[CARD11, CD226, KCNN4, LCK, NFAM1, PRKCB, PTPRC, STAP1, TESPA1]	[CCR7, NECTIN2, RELA]
GO:0050852	T cell receptor signaling pathway	1,90E-03	[6, 7, 8, 9, 10]	57,00	26,03	77,69	22,31	[BTN3A1, BTN3A2, CARD11, CD226, CD247, CD3D, CD3E, CD3G, CD4, CTLA4, FOXP3, FYB1, GRAP2, HLA-DPA1, HLA-DPB1, HLA-DQA1, HLA-DRA, HLA-DRB1, HLA-DRB5, INPP5D, ITK, KCNN4, LCK, LCP2, LILRB4, MOG, NR4A3, PRAM1, PRKCQ, PTPN22, PTPN6, PTPRC, SH2D1A, SKAP1, SLA2, TESPA1, THEMIS, THEMIS2, THY1, TRAT1, UBASH3A, WAS, ZAP70]	[ABL1, BCAR1, CCR7, CD276, CEACAM1, CSK, DGKZ, GNAO1, NCOR2, NECTIN2, NR4A3, RELA, RHBDF2, RNU1-93P, WAS, ZC3H12A]
GO:0050856	regulation of T cell receptor signaling pathway	2,29E-02	[5, 6, 7, 8, 9, 10, 11]	17,00	41,46	75,67	24,33	[CARD11, CD226, KCNN4, LCK, LILRB4, PTPN22, PTPN6, SH2D1A, SLA2, TESPA1, THY1, UBASH3A]	[CCR7, CEACAM1, DGKZ, NECTIN2, RELA]
GO:0030097	hemopoiesis	1,27E-16	[4, 5, 6, 7]	235,00	24,28	59,15	40,85	[ADAM8, ANGPT1, BATF, BCL11B, BCL3, BLK, BLNK, BTK, C1QC, CALCR, CAMK4, CARD11, CASP8, CCL19, CCL3, CCR2, CD19, CD2, CD27, CD3D, CD3E, CD3G, CD4, CD40LG, CD74, CD79A, CD80, CD86, CD8A, CDKN2A, CLCF1, CR2, CRTAM, CSF1R, CTLA4, DOCK10, DOCK2, EGR3, EOMES, EPX, EVI2B, FCRL3, FGL2, FLT3, FOXP3, FUT7, GFI1, GPR171, GPR55, H3C10, H3C11, H3C12, H3C7, HCLS1, HLA-DOA, HLA-DRA, HLA-DRB1, IFNG, IKZF1, IKZF3, IL12RB1, IL18, IL7R, INPP5D, IRF4, IRF8, ITGA4, ITK, KLRC1, LAG3, LCK, LEF1, LILRB1, LILRB4, LMO1, LRRC17, LY9, MIXL1, MMP9, MS4A1, MT1G, MYB, NCKAP1L, NFAM1, NLRP3, NR4A3, PIM1, PLA2G2D, PLD4, PLEK, POU2AF1, POU2F2, PRDM1, PRKCB, PRKCQ, PTK2B, PTPN22, PTPN6, PTPRC, RHEX, RHOH, RTKN2, RUNX3, SASH3, SELPLG, SEMA4A, SLAMF6, SLAMF8, SLC8A3, SMPD3, SNX10, SOCS1, SPN, STAT1, SYK, TBX21, TESPA1, THBS1, THEMIS, TNFRSF13B, TNFRSF18, TNFSF13B, TNFSF8, TOX, TREM2, TRPM2, UBD, VAV1, VNN1, WNT1, WNT10B, ZAP70, ZNF683]	[ABL1, ACTN1, ADIPOQ, AGER, ANGPT1, AP3D1, ARMC5, BATF2, BCL3, BCL6, CALCR, CCR7, CD74, CDK2, CDKN1A, CEACAM1, CITED2, CLCF1, CLPTM1, CRIP2, CSF1, CTC1, CYP26B1, DHRS2, DMTN, DPF2, DTX1, EPHA2, ERBB2, ETV6, FADD, FASN, FES, FLT3, FLT4, GAB2, GATA2, GBA, GNAO1, GPR137, H3C10, H3C12, H4C3, HDAC5, HSF1, IL15RA, IL1B, IL4R, IL7R, INHA, JAK3, JUNB, KCP, KDELR1, KLF10, KMT2D, LAG3, LGALS9, LOX, LRRC17, LRRC8A, LTBR, LTF, MFAP5, MKNK2, MOV10, MYC, MYH9, MYL9, NBEAL2, NCOA6, NFE2, NOTCH1, NOTCH4, NR4A3, NRARP, OSM, PAF1, PDGFB, PIM1, PML, PTN, RARA, RELB, RMRP, RNU1-93P, RTKN2, RXRA, SBNO2, SETD1A, SH2B3, SIPA1L3, SNX10, SOX12, SOX13, SPI1, SRC, SRF, SSBP3, STAT3, SYVN1, TCF3, TCF7L1, TCIRG1, TFE3, THBS1, THPO, TIMP1, TNF, TRIB1, VEGFA, VNN1, VSIR, ZBTB7A, ZBTB7B, ZC3H12A, ZFP36, ZMIZ1, ZNF385A]

GO:0002521	leukocyte differentiation	9,44E-24	[4, 5, 6, 7, 8]	177,00	30,73	63,92	36,08	[ADAM8, BATF, BCL11B, BCL3, BLK, BLNK, BTK, C1QC, CALCR, CAMK4, CARD11, CASP8, CCL19, CCL3, CCR2, CD19, CD2, CD27, CD3D, CD3E, CD3G, CD4, CD40LG, CD74, CD79A, CD80, CD86, CD8A, CDKN2A, CLCF1, CR2, CRTAM, CSF1R, CTLA4, DOCK10, DOCK2, EGR3, EOMES, EVI2B, FCRL3, FGL2, FLT3, FOXP3, FUT7, GPR55, HCLS1, HLA-DOA, HLA-DRA, HLA-DRB1, IFNG, IKZF1, IKZF3, IL12RB1, IL18, IL7R, INPP5D, IRF4, IRF8, ITGA4, ITK, KLRC1, LAG3, LCK, LEF1, LILRB1, LILRB4, LRRC17, LY9, MMP9, MS4A1, MT1G, MYB, NCKAP1L, NFAM1, NLRP3, PLA2G2D, POU2AF1, POU2F2, PRDM1, PTK2B, PTPN22, PTPN6, PTPRC, RHOH, RUNX3, SASH3, SEMA4A, SLAMF6, SLAMF8, SNX10, SOCS1, SPN, SYK, TBX21, TESPA1, THEMIS, TNFRSF18, TNFSF13B, TNFSF8, TOX, TREM2, TRPM2, UBD, VAV1, VNN1, WNT1, ZAP70, ZNF683]	[ABL1, ADIPOQ, AGER, AP3D1, ARMC5, BATF2, BCL3, BCL6, CALCR, CCR7, CD74, CDK2, CDKN1A, CEACAM1, CITED2, CLCF1, CLPTM1, CSF1, CYP26B1, DHRS2, DTX1, EPHA2, ERBB2, FADD, FASN, FES, FLT3, GAB2, GATA2, GBA, GNAO1, GPR137, HDAC5, HSF1, IL15RA, IL1B, IL4R, IL7R, INHA, JAK3, JUNB, KDELR1, KLF10, LAG3, LGALS9, LRRC17, LRRC8A, LTBR, LTF, MYC, MYH9, NRARP, PML, RARA, RELB, RMRP, RXRA, SBNO2, SNX10, SOX12, SOX13, SPI1, SRC, SRF, STAT3, SYVN1, TCF3, TCF7L1, TCIRG1, TFE3, TNF, TRIB1, VEGFA, VNN1, VSIR, ZBTB7A, ZBTB7B, ZC3H12A, ZMIZ1]
GO:0030099	myeloid cell differentiation	5,32E-05	[4, 5, 6, 7, 8]	104,00	23,37	44,67	55,33	[BATF, C1QC, CALCR, CAMK4, CASP8, CCL19, CCL3, CD4, CD74, CSF1R, EPX, EVI2B, GPR171, GPR55, H3C10, H3C11, H3C12, H3C7, HCLS1, HLA-DRB1, IFNG, IKZF1, INPP5D, IRF4, IRF8, LEF1, LILRB1, LILRB4, LRRC17, MMP9, MT1G, NCKAP1L, NR4A3, PRKCB, PRKCQ, PTK2B, PTPN6, RHEX, SNX10, STAT1, THBS1, TREM2, UBD]	[ACTN1, ADIPOQ, BATF2, BCL6, CALCR, CCR7, CD74, CDK2, CDKN1A, CEACAM1, CITED2, CSF1, DHRS2, DMTN, EPHA2, FADD, FASN, FES, GAB2, GATA2, GBA, GPR137, H3C10, H3C12, H4C3, HSF1, INHA, JAK3, JUNB, KLF10, KMT2D, LGALS9, LOX, LRRC17, LTBR, LTF, MOV10, MYC, MYH9, MYL9, NBEAL2, NCOA6, NFE2, NR4A3, PAF1, PML, RARA, RELB, RMRP, RXRA, SBNO2, SETD1A, SH2B3, SNX10, SPI1, SRC, SRF, STAT3, TCIRG1, TFE3, THBS1, THPO, TIMP1, TNF, TRIB1, VEGFA, ZBTB7A, ZFP36, ZNF385A]
GO:1902105	regulation of leukocyte differentiation	4,19E-15	[4, 5, 6, 7, 8, 9]	97,00	33,92	65,84	34,16	[ADAM8, BTK, C1QC, CAMK4, CARD11, CASP8, CCL19, CCL3, CCR2, CD2, CD27, CD4, CD74, CD80, CD86, CDKN2A, CRTAM, CTLA4, EGR3, EVI2B, FCRL3, FGL2, FOXP3, GPR55, HCLS1, HLA-DOA, HLA-DRA, HLA-DRB1, IFNG, IKZF3, IL12RB1, IL18, IL7R, INPP5D, IRF4, LAG3, LEF1, LILRB1, LILRB4, LRRC17, MYB, NCKAP1L, NFAM1, NLRP3, PRDM1, PTPN6, PTPRC, RHOH, RUNX3, SASH3, SLAMF8, SOCS1, SYK, TBX21, TESPA1, TNFRSF18, TOX, TREM2, VNN1, ZAP70, ZNF683]	[ABL1, ADIPOQ, AGER, AP3D1, BCL6, CD74, CEACAM1, CLPTM1, CSF1, CYP26B1, DTX1, ERBB2, FADD, FES, GATA2, GPR137, HSF1, IL15RA, IL4R, IL7R, INHA, JAK3, KLF10, LAG3, LGALS9, LRRC17, LTF, MYC, NRARP, RARA, RMRP, SOX12, SOX13, TFE3, TNF, TRIB1, VNN1, VSIR, ZBTB7B, ZC3H12A, ZMIZ1]
GO:0002573	myeloid leukocyte differentiation	4,84E-06	[5, 6, 7, 8, 9]	65,00	28,76	43,93	56,07	[BATF, C1QC, CALCR, CAMK4, CASP8, CCL19, CCL3, CD4, CD74, CSF1R, EVI2B, GPR55, HCLS1, HLA-DRB1, IFNG, INPP5D, IRF4, LEF1, LILRB1, LILRB4, LRRC17, MMP9, MT1G, SNX10, TREM2, UBD]	[ADIPOQ, BATF2, CALCR, CCR7, CD74, CDK2, CDKN1A, CEACAM1, CITED2, CSF1, DHRS2, EPHA2, FADD, FASN, FES, GAB2, GATA2, GBA, GPR137, HSF1, INHA, JUNB, KLF10, LGALS9, LRRC17, LTBR, LTF, MYC, MYH9, PML, RARA, RELB, RMRP, RXRA, SBNO2, SNX10, SPI1, SRC, TCIRG1, TFE3, TNF, TRIB1, VEGFA]
GO:0030851	granulocyte differentiation	4,78E-02	[6, 7, 8, 9, 10]	17,00	39,53	35,06	64,94	[C1QC, EVI2B, HCLS1, INPP5D, LEF1]	[ADIPOQ, CDK2, CDKN1A, CEACAM1, CITED2, FASN, GATA2, PML, RARA, RXRA, SPI1, TRIB1]
GO:0032879	regulation of localization	1,05E-09	[2, 3]	515,00	18,06	37,10	62,90	[ABCB11, ADAM8, ADAMTS9, ADORA3, AIF1, ALOX15, ALOX15B, ANGPT1, ANGPT2, ANO9, AREG, BLK, BMP7, C1QTNF1, C2, CACNA1E, CACNA1I, CALCR, CARMIL2, CASP8, CASS4, CBARP, CCL19, CCL2, CCL24, CCL3, CCL4, CCL5, CCR2, CCR5, CD177, CD19, CD200R1, CD22, CD247, CD300LF, CD33, CD4, CD74, CD84, CDH1, CLCNKA, CNR1, CORO1A, CSF1R, CTSS, CXCL10, CXCL11, CXCL9, CXCR3, CXCR4, CYBB, DAPK1, DERL3, DOCK10, DOCK2, DOCK8, DPEP1, DRC1, DRD1, EDN2, EPX, EREG, ERFE, FASLG, FCRL3, FFAR4, FGR, FUT7, GABBR2, GPSM3, GZMB, HCLS1, HCN1, HLA-DRB1, IFNG, IKBKE, IL16, IL2RB, IL2RG, ITGA4, ITGAX, ITGB2, JPH1, JSRP1, KCNA3, KCNA7, KCNJ10, KCNK15, KCNN4, KCNQ5, KLRC2, KLRK1, KMO, LCK, LCP1, LEF1, LIF, LILRB1, LILRB4, LPAL2, LRRC38, MCOLN2, MCOLN3, MIDN, MLC1, MMP9, MYB, NCKAP1L, NELL2, NLRP3, NLRP6, NPPB, NR4A3, NTN1, P2RY12, P2RY2, PIK3CG, PIM1, PLA2G7, PLAU, PLXNC1, PRAM1, PRKCB, PTAFR, PTK2B, PTPN22, PTPN6, PTPRC, PTX3, PYCARD, PYHIN1, RAB15, RAB27B, RAB3B, RAB3C, RAC2, RASGRF1, RGS9, RHOH, RRAD, SDC1, SELE, SEMA4A, SEMA4D, SEPTIN1, SHISA8, SIRPG, SLAMF8, SLC31A2, SMPD3, SPINK1, SPN, STAP1, STXBP2, SYK, TACSTD2, TCAF2, THBS1, THY1, TIFAB, TMC2, TNFRSF18, TREM2, TRIM14, TRPM2, UBD, UCP2, WAS, XCL1, XCL2, XCR1, XG]	[AAAS, ABCA3, ABCA7, ABCB11, ABCC8, ABI3, ABL1, ABLIM3, ACACB, ACKR3, ACTB, ACTN1, ACTN4, ADAMTS9, ADCY1, ADGRG1, ADIPOQ, AGER, AGT, AGTR1, AMH, ANGPT1, ANKRD13B, AP2A1, AP3D1, APLN, APLNR, APOA1, APOD, ARC, AREG, ARF1, ARHGAP1, ARHGDIA, ARHGEF16, ARHGEF5, ATP13A2, ATP1A1, ATP1A3, ATP1B2, ATPSCKMT, BAG3, BCAR1, BCL2L1, BCL6, BMP8A, BSN, C11orf65, C1QTNF1, C2CD2L, C5, C5AR1, CACNA1E, CACNA1H, CACNB4, CALCR, CAMK2B, CAMSAP3, CAPN1, CARMIL2, CBARP, CCL11, CCL2, CCL21, CCL24, CCR7, CD14, CD177, CD74, CDK1, CEACAM1, CELSR2, CETP, CFAP298-TCP10L, CFH, CGA, CHCHD10, CHERP, CHGA, CITED2, CLCNKA, CLIP3, CNR1, COL1A1, CPLX1, CRP, CRYAB, CSF1, CSK, CSNK1E, CTSD, CX3CL1, DAB2IP, DAG1, DGKD, DISP3, DLG5, DMTN, DOC2B, DRC1, DVL3, DYSF, ECM1, EDN2, EHD1, EHD2, ENG, EPHA2, EPPK1, ERBB2, ERFE, FADD, FAM110A, FES, FFAR2, FITM1, FLT4, FOXC2, FOXF1, FURIN, FXYD5, FXYD6, GAB2, GABBR2, GAS1, GATA2, GATA4, GBF1, GCSAM, GDI1, GIT1, GLIS2, GNAI2, GNAO1, GPER1, GPI, GPRIN1, GRB7, GRIK5, GRM2, GRN, GSK3A, HBEGF, HCN3, HCN4, HDAC5, HDAC7, HFE, HGS, HSPB1, HTR1B, HTR2A, HVCN1, HYAL1, ICAM1, IFITM1, IL15RA, IL1B, IL4R, ILK, INHA, INPP5E, ITGA3, ITPR3, JPH4, JUP, KCNA7, KCNC3, KCNH2, KCNIP2, KCNJ1, KCNJ5, KCNJ9, KCNK15, KCNK6, KCNMB1, KCNMB2, KCNQ4, KCTD11, LAMA5, LDLRAP1, LGALS9, LGR6, LIF, LILRA5, LIMK2, LIPG, LMNA, LRRC52, LRRC8A, LZTS1, LZTS2, MAP1A, MAP2K3, MAPK3, MARK4, MAVS, MAZ, MECP2, MICALL2, MIDN, MIEN1, MINK1, MLLT6, MSN, MSTN, MYADM, MYC, MYO18A, NDC80, NEFL, NF2, NGFR, NKAIN3, NKD2, NLGN2, NNAT, NOD2, NODAL, NOS3, NOTCH1, NPPA, NPPB, NR1D1, NR4A3, NSMF, NTN1, NUCB1, NUTF2, ORAI1, OSGIN1, P2RX3, P2RX5, P2RY2, P2RY6, PACSIN1, PCDHA4, PDGFB, PDGFRB, PER1, PFN1, PHACTR1, PIM1, PLA2G7, PLAU, PLEKHM2, PLK3, PLTP, PLVAP, PLXNA1

GO:0006810	transport	5,25E-04 [3]	844,00	15,69	35,92	64,08	[ABCB11, ABCC3, ABRA, ACAP1, ADAM8, ADAMTS9, ADGRE3, AIF1, ALOX15, ANGPT1, ANO9, ANPEP, APBA2, AQP5, AREG, ARHGAP45, ARRDC5, ASGR2, ATP10B, ATP1B4, ATP2A3, BHLHA15, BICDL1, BIN2, BLK, BMF, BTK, C1QTNF1, C2, CA12, CACNA1E, CACNA1I, CALCR, CALHM6, CASP8, CBARP, CCDC88B, CCDC88C, CCL19, CCL2, CCL3, CCL4, CCL5, CCR2, CCR5, CD177, CD19, CD22, CD247, CD300LF, CD33, CD3G, CD4, CD5, CD53, CD6, CD74, CD84, CDH1, CEACAM21, CEACAM4, CEL, CFP, CLCNKA, CLEC10A, CLEC12A, CLEC4D, CLNK, CNR1, CORO1A, COTL1, CP, CTSS, CTSW, CXCL10, CXCL11, CXCL9, CXCR4, CYBB, CYSLTR1, DAPK1, DAW1, DENND1C, DERL3, DOCK2, DRD1, EMB, EPX, ERFE, EXOC3L4, FASLG, FCHO1, FCN1, FCN3, FCRL3, FFAR4, FGL2, FGR, FOLR2, FRMPD3, GABBR2, GCNT3, GMFG, GPM6A, GRAMD1B, GRAMD1C, GZMB, HCLS1, HCN1, HLA-DRB1, HSPA7, IFNG, IGLL5, IL10RA, IL16, IL2RB, IL2RG, IPCEF1, IQGAP2, IRF8, ITGA4, ITGAL, ITGAX, ITGB2, JAKMIP1, JCHAIN, JPH1, JSRP1, KCNA3, KCNA7, KCNJ10, KCNK15, KCNN4, KCNQ5, KLF5, KLRC2, KLRG1, KMO, KNG1, LAIR1, LCK, LCP1, LDLR, LILRB1, LILRB4, LPAL2, LRMP, LRRC38, LY75, LYZ, MCOLN2, MCOLN3, MID1IP1, MIDN, MLC1, MMP9, MPO, MRC1, MREG, MS4A1, MYB, MYO1F, MYO1G, NAPSB, NCF1, NCKAP1L, NFAM1, NLRP3, NLRP6, NMUR1, NPPB, NPPC, NR4A3, NSG1, NTN1, NUP210, ORM2, P2RY12, P2RY2, PIK3CG, PIM1, PLA2G2D, PLAC8, PLAU, PLD4, PLEK, PRAM1, PRF1, PRKCB, PSTPIP1, PTAFR, PTGDR, PTK2B, PTPN22, PTPN6, PTPRC, PTX3, PYCARD, RAB15, RAB27B, RAB37, RAB39B, RAB3B, RAB3C, RAB44, RAC2, RASGRF1, RGS9, RHOH, RINL, RNASET2, RRAD, SCNN1B, SDC1, SELE, SELL, SEPTIN1, SERPINA1, SH2D2A, SHISA8, SIDT1, SIGLEC1, SIRPG, SLC12A3, SLC16A6, SLC16A9, SLC18A1, SLC22A31, SLC24A4, SLC25A48, SLC27A2, SLC31A2, SLC36A2, SLC5A2, SLC7A5P1, SLC7A7, SLC8A3, SLCO5A1, SMPD3, SNX10, SNX20, SOAT2, SPEF2, SPINK1, SPNS3, STAP1, STARD5, STXBP2, SUCNR1, SYK, SYTL1	[AAAS, ABCA3, ABCA7, ABCB11, ABCC5, ABCC8, ABCD1, ABCD2, ABCG4, ABL1, ABLIM3, ABRA, ACACB, ACKR3, ACTB, ACTN1, ACTN4, ADAMTS8, ADAMTS9, ADCY1, ADGRE5, ADIPOQ, AGAP2, AGER, AGT, AGTR1, ALDOA, ALPK3, AMH, ANGPT1, ANKRD13B, ANO3, ANO7, ANXA11, AP1B1, AP2A1, AP3D1, APLN, APLNR, APLP1, APOA1, APOD, APOM, AQP3, AQP5, ARAP3, ARC, AREG, ARF1, ARHGAP1, ARHGEF5, ARL8A, ARRDC3, ARSA, ASIC3, ATG9A, ATP13A1, ATP13A2, ATP1A1, ATP1A3, ATP1B2, ATP1B4, ATP5F1D, ATP5MC2, ATP6V0D1, ATP6V0E2, ATP6V1B1, ATP6V1F, ATPSCKMT, BAG3, BBS12, BCL2L1, BMP8A, BRPF3, BSN, C11orf65, C1QTNF1, C2CD2L, C5AR1, CACFD1, CACNA1E, CACNA1H, CACNB4, CALCR, CALHM6, CAMK2B, CAMSAP3, CAPN1, CBARP, CCL2, CCL21, CCR7, CD14, CD177, CD74, CDK1, CDKN1A, CEACAM1, CECR2, CEL, CELSR2, CERT1, CETN3, CETP, CFH, CGA, CHCHD10, CHERP, CHGA, CHI3L1, CHRNG, CLCN6, CLCN7, CLCNKA, CLDN15, CLEC12A, CLIP3, CLNK, CNNM4, CNR1, COASY, COL1A1, COMMD3, CORO7, COX6A2, COX8A, CPLX1, CPNE6, CPSF1, CPTP, CRAT, CREB3L1, CRP, CRTC2, CRYAB, CSK, CSNK1E, CTSA, CTSD, CX3CL1, CYB5R3, CYP27B1, DDR1, DDX39B, DENND1C, DGKD, DISP3, DMTN, DOC2B, DOK3, DSC1, DYNLRB1, DYSF, ECM1, EHD1, EHD2, ENG, ENPP3, EPN1, EPN3, ERBB2, ERFE, F8A1, FABP4, FAM110A, FCN3, FES, FFAR2, FHOD1, FLOT2, FOXF1, FPR1, FTH1P19, FURIN, FXYD5, FXYD6, G6PC3, GAB2, GABBR2, GABRR2, GALNS, GAS1, GATA2, GBF1, GCNT3, GDI1, GGA1, GGA3, GIT1, GJA4, GJD3, GLRA1, GNAI2, GNAO1, GPER1, GPI, GPM6A, GPR84, GPRIN1, GRAMD1A, GRAMD1B, GRAMD1C, GRIK5, GRIP2, GRM2, GRN, GSK3A, GTPBP2, HAVCR1, HBA1, HBA2, HBB, HBD, HBG1, HBG2, HCN3, HCN4, HFE, HGS, HMGA1, HPS1, HRH2, HSF1, HSPB1, HSPG2, HTR1B, HTR2A, HVCN1, ICAM1, IFT80, IGF2BP2, IL15RA, IL1B, IL1RN, IL4R, IMPDH1, INHA, INPPL1, IPO13, IQGAP2, ITPR3, JPH4, JUP, KATNB1, KCNA7, KCNC3, KCNH2, KCNIP2, KCNJ1, KCNJ5
GO:0055085	transmembrane transport	3,39E-03 [2, 4]	307,00	17,51	35,41	64,59	[ABCB11, ABCC3, ANO9, AQP5, ATP1B4, ATP2A3, BHLHA15, C1QTNF1, CACNA1E, CACNA1I, CALCR, CALHM6, CBARP, CCL19, CCL2, CCL3, CCR2, CCR5, CD19, CLCNKA, CNR1, CORO1A, CTSS, CXCL10, CXCL11, CXCL9, CYBB, DAPK1, DRD1, EMB, EPX, ERFE, FFAR4, FOLR2, GABBR2, GPM6A, HCN1, IFNG, JPH1, JSRP1, KCNA3, KCNA7, KCNJ10, KCNK15, KCNN4, KCNQ5, LCK, LRRC38, MCOLN2, MCOLN3, MID1IP1, MMP9, NR4A3, P2RY12, PIK3CG, PIM1, PRF1, PRKCB, PTAFR, PTK2B, PTPN22, PTPN6, PTPRC, RASGRF1, RGS9, RRAD, SCNN1B, SHISA8, SIDT1, SLC12A3, SLC16A6, SLC16A9, SLC18A1, SLC22A31, SLC24A4, SLC25A48, SLC27A2, SLC31A2, SLC36A2, SLC5A2, SLC7A5P1, SLC7A7, SLC8A3, SLCO5A1, SPNS3, TCAF2, THBS1, THY1, TMC2, TMC3, TMC8, TREM2, TRPM2, TSC22D3, UBD, UCP2, UNC80, XCL1, XCR1]	[ABCA3, ABCA7, ABCB11, ABCC5, ABCC8, ABCD1, ABCD2, ABCG4, ABL1, ACACB, ACTB, ACTN4, ADAMTS8, ADIPOQ, AGT, ANO3, ANO7, APLNR, AQP3, AQP5, ARC, ARF1, ASIC3, ATP13A1, ATP13A2, ATP1A1, ATP1A3, ATP1B2, ATP1B4, ATP5F1D, ATP5MC2, ATP6V0D1, ATP6V0E2, ATP6V1B1, ATP6V1F, ATPSCKMT, C1QTNF1, CACNA1E, CACNA1H, CACNB4, CALCR, CALHM6, CAMK2B, CAPN1, CBARP, CCL2, CCL21, CCR7, CEACAM1, CFH, CHERP, CHRNG, CLCN6, CLCN7, CLCNKA, CLIP3, CNNM4, CNR1, COX6A2, COX8A, CPLX1, CX3CL1, DMTN, ERFE, FXYD5, FXYD6, GABBR2, GABRR2, GJA4, GJD3, GLRA1, GNAI2, GPER1, GPM6A, GPRIN1, GRIK5, GRM2, GSK3A, HCN3, HCN4, HFE, HTR1B, HTR2A, HVCN1, IL1B, ITPR3, JPH4, KCNA7, KCNC3, KCNH2, KCNIP2, KCNJ1, KCNJ5, KCNJ9, KCNK15, KCNK2, KCNK3, KCNK6, KCNMB1, KCNMB2, KCNQ4, KCNT1, LRRC52, LRRC8A, LYNX1, MED12, MFSD10, MFSD2A, MICU3, MID1IP1, MINK1, MTCO2P12, MYC, NDUFA4L2, NEFL, NIPAL4, NLGN2, NPPA, NR4A3, ORAI1, P2RX3, P2RX5, P2RY6, PCDHA4, PEX14, PIEZO1, PIM1, PKD1, PLP2, PM20D1, PPARD, PRAF2, PRKACA, PRRT1, RANGRF, RAP1A, RNU1-93P, RRAD, RTN2, S100A1, SCN5A, SCN7A, SELENON, SHANK3, SIDT2, SLC10A3, SLC12A4, SLC15A5, SLC16A5, SLC16A9, SLC17A3, SLC1A4, SLC1A5, SLC22A7, SLC25A1, SLC25A22, SLC25A23, SLC25A33, SLC25A39, SLC25A42, SLC25A44, SLC29A1, SLC29A2, SLC29A4, SLC2A4, SLC2A6, SLC30A1, SLC30A3, SLC35A2, SLC35A4, SLC35C1, SLC35F6, SLC36A2, SLC38A11, SLC38A5, SLC38A7, SLC38A8, SLC39A13, SLC39A5, SLC41A1, SLC44A2, SLC48A1, SLC4A10, SLC4A11, SLC4A2, SLC4A3, SLC51B, SLC52A2, SLC52A3, SLC5A10, SLC5A6, SLC6A12, SLC6A17, SLC6A8, SLC6A9, SLC7A1, SLC7A5, SLC7A8, SLC9A1, SLC9A3R1, SLCO4A1, SLCO4C1, SLCO5A1, SLN, SPHK2, SPNS2, STAC2, STX1A, SV2A, SYNGAP1, TAPBP, TCIRG1, THBS1, TIMP1, TMC3, TMC4, TMEM109, TMEM175, TMEM63A, TMEM63B, TMEM63C, TNF, TPCN1, TRIB3, TRPV4, TTYH1, TTYH2, TTYH3,

GO:0051049	regulation of transport	6,48E-06	[3, 4]	339,00	18,29	39,41	60,59	[ABCB11, ADAM8, ALOX15, ANGPT1, ANO9, BLK, C1QTNF1, C2, CACNA1E, CACNA1I, CALCR, CASP8, CBARP, CCL19, CCL2, CCL3, CCL4, CCL5, CCR2, CD177, CD19, CD22, CD300LF, CD33, CD4, CD74, CD84, CDH1, CLCNKA, CNR1, CORO1A, CTSS, CXCL10, CXCL11, CXCL9, CXCR4, CYBB, DAPK1, DERL3, DOCK2, DRD1, EPX, ERFE, FASLG, FCRL3, FFAR4, FGR, GABBR2, GZMB, HCLS1, HCN1, HLA-DRB1, IFNG, IL16, IL2RB, IL2RG, ITGB2, JPH1, JSRP1, KCNA3, KCNA7, KCNJ10, KCNK15, KCNN4, KCNQ5, KLRC2, KMO, LCP1, LILRB1, LPAL2, LRRC38, MIDN, MLC1, MMP9, MYB, NCKAP1L, NLRP3, NLRP6, NPPB, NR4A3, P2RY12, P2RY2, PIK3CG, PIM1, PRAM1, PRKCB, PTAFR, PTK2B, PTPN22, PTPN6, PTPRC, PTX3, PYCARD, RAB15, RAB27B, RAB3B, RAB3C, RAC2, RASGRF1, RGS9, RRAD, SDC1, SELE, SEPTIN1, SHISA8, SIRPG, SLC31A2, SMPD3, SPINK1, STAP1, STXBP2, SYK, TCAF2, THBS1, THY1, TIFAB, TMC2, TREM2, TRPM2, UBD, UCP2, XCL1]	[AAAS, ABCA3, ABCA7, ABCB11, ABCC8, ABL1, ABLIM3, ACTB, ACTN4, ADCY1, ADIPOQ, AGT, AGTR1, ANGPT1, ANKRD13B, AP2A1, APLN, APLNR, APOA1, APOD, ARC, ARF1, ARHGAP1, ARHGEF5, ATP13A2, ATP1A1, ATP1A3, ATP1B2, ATPSCKMT, BAG3, BMP8A, BSN, C11orf65, C1QTNF1, C2CD2L, CACNA1E, CACNA1H, CACNB4, CALCR, CAMK2B, CAPN1, CBARP, CCL2, CCL21, CD14, CD177, CD74, CDK1, CEACAM1, CETP, CFH, CHCHD10, CHGA, CLCNKA, CLIP3, CNR1, CPLX1, CRYAB, CSK, CX3CL1, DGKD, DISP3, DMTN, DOC2B, DYSF, EHD1, EHD2, ERBB2, ERFE, FES, FFAR2, FOXF1, FURIN, FXYD5, FXYD6, GAB2, GABBR2, GAS1, GATA2, GDI1, GIT1, GNAI2, GNAO1, GPER1, GPRIN1, GRIK5, GRM2, GSK3A, HCN3, HCN4, HFE, HGS, HTR1B, HTR2A, HVCN1, ICAM1, IL15RA, IL1B, IL4R, INHA, ITPR3, JPH4, JUP, KCNA7, KCNC3, KCNH2, KCNIP2, KCNJ1, KCNJ5, KCNJ9, KCNK15, KCNK6, KCNMB1, KCNMB2, KCNQ4, KCTD11, LDLRAP1, LGALS9, LILRA5, LIPG, LRRC52, LRRC8A, LZTS1, MAPK3, MAVS, MICALL2, MIDN, MINK1, MLLT6, MSN, MYC, MYO18A, NEFL, NKAIN3, NLGN2, NNAT, NOD2, NOTCH1, NPPA, NPPB, NR1D1, NR4A3, NUCB1, NUTF2, ORAI1, P2RX3, P2RX5, P2RY2, P2RY6, PACSIN1, PCDHA4, PDGFB, PDGFRB, PER1, PIM1, PLK3, PLTP, PM20D1, PML, PPARD, PRKACA, PRKCSH, PROM2, PRRT1, PRRT2, PTGES, PTPN23, RAB11B, RAB11FIP5, RAB15, RAB3A, RAB5B, RAC2, RANGAP1, RANGRF, RAP1A, RASL10B, RCVRN, RGCC, RHBDF1, RHBDF2, RIPOR1, RRAD, RTN2, RUBCN, RXRA, S100A1, SCN5A, SCN7A, SEC16A, SELE, SELENON, SEPTIN4, SEPTIN5, SERPINE1, SH3GL1, SHANK3, SIDT2, SIK1, SLC30A1, SLC30A3, SLC35F6, SLC51B, SLC6A9, SLC9A1, SLC9A3R1, SLN, SMAD3, SMPD1, SPHK1, SPHK2, SPI1, SPINK1, SRC, SREBF1, STAC2, STX1A, STX1B, SV2A, SYN1, SYT2, SYT7, TCIRG1, TCTEX1D2, TGM2, THBS1, TIMP1, TM9SF4, TMEM109, TMEM14A, TNF, TNFRSF1A, TNK2, TPCN1, TRIB3, TSC2, VEGFA, VTN, WFS1, WNK2, WWP2, ZC3H12A, ZDHHC8]
GO:0006811	ion transport	1,68E-07	[4]	320,00	19,07	38,58	61,42	[ABCB11, ABCC3, ANO9, ATP1B4, ATP2A3, BHLHA15, BTK, CA12, CACNA1E, CACNA1I, CALCR, CALHM6, CBARP, CCL19, CCL2, CCL3, CCL4, CCL5, CCR2, CCR5, CD19, CD33, CD4, CD84, CLCNKA, CNR1, CORO1A, CP, CTSS, CXCL10, CXCL11, CXCL9, CXCR4, CYBB, CYSLTR1, DAPK1, DRD1, EMB, EPX, FCRL3, FOLR2, GABBR2, GPM6A, HCN1, IFNG, IL16, JPH1, JSRP1, KCNA3, KCNA7, KCNJ10, KCNK15, KCNN4, KCNQ5, KMO, LCK, LILRB1, LRRC38, MCOLN2, MCOLN3, MLC1, MMP9, MS4A1, NLRP3, NMUR1, P2RY12, PIK3CG, PLA2G2D, PRKCB, PTAFR, PTK2B, PTPN22, PTPN6, PTPRC, RAB3B, RASGRF1, RGS9, RRAD, SCNN1B, SHISA8, SLC12A3, SLC16A6, SLC16A9, SLC18A1, SLC22A31, SLC24A4, SLC25A48, SLC27A2, SLC31A2, SLC36A2, SLC5A2, SLC7A5P1, SLC7A7, SLC8A3, SLCO5A1, SPINK1, STARD5, SYK, TCAF2, THY1, TMC2, TMC3, TMC8, TREM2, TRPM2, TSC22D3, UBD, UCP2, UNC80, XCL1, XCR1]	[ABCB11, ABCC5, ABCC8, ABL1, ACTN4, ADAMTS8, AGT, ANO3, ANO7, APLNR, ARC, ARF1, ARHGAP1, ASIC3, ATP13A1, ATP13A2, ATP1A1, ATP1A3, ATP1B2, ATP1B4, ATP5F1D, ATP5MC2, ATP6V0D1, ATP6V0E2, ATP6V1B1, ATP6V1F, ATPSCKMT, CACNA1E, CACNA1H, CACNB4, CALCR, CALHM6, CAMK2B, CAPN1, CBARP, CCL2, CCL21, CCR7, CEACAM1, CERT1, CFH, CHERP, CHRNG, CLCN6, CLCN7, CLCNKA, CLDN15, CNNM4, CNR1, COMMD3, COX6A2, COX8A, CPTP, CX3CL1, CYP27B1, ENPP3, FTH1P19, FXYD5, FXYD6, GABBR2, GABRR2, GJA4, GJD3, GLRA1, GNAI2, GNAO1, GPER1, GPM6A, GPRIN1, GRIK5, GRM2, HBA1, HBB, HCN3, HCN4, HFE, HTR1B, HTR2A, HVCN1, ICAM1, IL1B, ITPR3, JPH4, KCNA7, KCNC3, KCNH2, KCNIP2, KCNJ1, KCNJ5, KCNJ9, KCNK15, KCNK2, KCNK3, KCNK6, KCNMB1, KCNMB2, KCNQ4, KCNT1, LILRA5, LRRC52, LRRC8A, LTF, LYNX1, MED12, MFSD10, MFSD2A, MICU3, MINK1, MLLT6, MTCO2P12, MYC, NDUFA4L2, NECTIN1, NEFL, NIPAL4, NKAIN3, NLGN2, NPPA, ORAI1, P2RX3, P2RX5, P2RY6, PCDHA4, PDGFB, PDGFRB, PER1, PIEZO1, PKD1, PLA2G2A, PLCZ1, PLP2, PM20D1, PML, PRAF2, PRKACA, PRRT1, PTGES, RAB11B, RAB3A, RAMP2, RANGRF, RCVRN, RNU1-93P, RRAD, RXRA, S100A1, SCN5A, SCN7A, SELENON, SHANK3, SIK1, SLC10A3, SLC12A4, SLC16A5, SLC16A9, SLC17A3, SLC1A4, SLC1A5, SLC22A7, SLC25A1, SLC25A22, SLC25A23, SLC25A42, SLC29A4, SLC2A6, SLC30A1, SLC30A3, SLC35A2, SLC36A2, SLC38A11, SLC38A5, SLC38A7, SLC38A8, SLC39A13, SLC39A5, SLC41A1, SLC44A2, SLC48A1, SLC4A10, SLC4A11, SLC4A2, SLC4A3, SLC51B, SLC52A2, SLC52A3, SLC5A10, SLC5A6, SLC6A12, SLC6A17, SLC6A8, SLC6A9, SLC7A1, SLC7A5, SLC7A8, SLC9A1, SLC9A3R1, SLCO4A1, SLCO4C1, SLCO5A1, SLN, SPHK2, SPINK1, STAC2, STEAP3, STX1A, SV2A, SYNGAP1, SYT2, SYT7, TCIRG1, TIMP1, TMC3, TMC4, TMEM109, TMEM175, TMEM63A, TMEM63B, TMEM63C, TPCN1, TRPV4, TTYH1, TTYH2, TTYH3, UNC80, VDR, VPS9D1, WFS1, WNK2,

GO:0046903	secretion	6,04E-05	[4]	292,00	18,39	42,91	57,09	[ABCB11, ABCC3, ADAM8, ADGRE3, ANPEP, AQP5, ARHGAP45, BIN2, BLK, BTK, C1QTNF1, CACNA1E, CACNA1I, CBARP, CCL3, CCL5, CCR2, CD177, CD33, CD53, CD74, CD84, CEACAM21, CEL, CFP, CLEC12A, CLEC4D, CLNK, CNR1, CORO1A, COTL1, CTSS, CTSW, CYBB, DOCK2, EPX, EXOC3L4, FCN1, FFAR4, FGL2, FGR, FRMPD3, GMFG, HLA-DRB1, IFNG, IQGAP2, ITGAL, ITGAX, ITGB2, KCNN4, KLF5, KLRC2, KLRG1, KMO, KNG1, LAIR1, LILRB1, LRMP, LYZ, MIDN, MMP9, MPO, MYB, MYO1G, NAPSB, NCKAP1L, NFAM1, NLRP3, NLRP6, NPPB, NR4A3, ORM2, P2RY12, P2RY2, PIK3CG, PLA2G2D, PLAC8, PLAU, PLEK, PRAM1, PRKCB, PTAFR, PTGDR, PTPN6, PTPRC, PTX3, PYCARD, RAB15, RAB27B, RAB37, RAB3B, RAB3C, RAB44, RAC2, RNASET2, SCNN1B, SDC1, SELL, SEPTIN1, SERPINA1, SLC18A1, SLC27A2, SMPD3, SNX10, STXBP2, SUCNR1, SYK, SYTL1, TBC1D10C, THBS1, TIFAB, TREM2, TRPM2, TXNDC5, UCP2, VNN1]	[ABCB11, ABCC8, ACTN1, ACTN4, ADCY1, ADGRE5, ADIPOQ, AGT, AGTR1, ALDOA, AMH, APLN, APOA1, AQP5, ARF1, ARL8A, ARSA, ATP13A2, BMP8A, BRPF3, C1QTNF1, C2CD2L, C5AR1, CACNA1E, CACNA1H, CAPN1, CBARP, CD14, CD177, CD74, CEACAM1, CEL, CELSR2, CGA, CHGA, CHI3L1, CLEC12A, CLNK, CNR1, COASY, COMMD3, CPLX1, CRAT, CREB3L1, CTSA, CTSD, CYB5R3, CYP27B1, DDR1, DMTN, DOC2B, DOK3, DSC1, ECM1, ENG, FES, FFAR2, FOXF1, FPR1, FURIN, GAB2, GALNS, GATA2, GIT1, GNAI2, GPER1, GPI, GPR84, GRIK5, GRM2, GRN, GTPBP2, HBB, HFE, HGS, HRH2, HTR1B, HTR2A, HVCN1, IL1B, IL1RN, IL4R, IMPDH1, INHA, IQGAP2, ITPR3, JUP, KCTD11, KLF5, KNG1, LGALS3BP, LGALS9, LRG1, LRRC8A, LTF, LY6E, MEN1, METTL7A, MIDN, MME, MTCO2P12, MYC, MYH9, MYO18A, NAPA, NBEAL2, NCOR2, NHLRC3, NKD2, NLGN2, NNAT, NOTCH1, NPPA, NPPB, NPR3, NPRL3, NR1D1, NR4A3, OTOF, P2RY2, PCDHA4, PDGFB, PIP5K1C, PKM, PLA2G2A, PLAU, PLAUR, PORCN, PPARD, PRKCSH, PRRT2, PTGDS, PTGES, PTPN23, PTPRN, RAB11B, RAB11FIP5, RAB15, RAB3A, RAB3IL1, RAB5B, RAC2, RAP1A, RASL10B, RGCC, RHBDF1, RHBDF2, RHOG, RMRP, RNASE2, RNU1-93P, RPH3A, S100A11, SELENOP, SEPTIN4, SEPTIN5, SEPTIN9, SERPINA3, SERPINE1, SERPING1, SIDT2, SIGLEC9, SLC1A5, SLC29A1, SLC30A1, SLC44A2, SLC51B, SLC7A8, SLC9A3R1, SLCO4C1, SNX10, SPHK2, SPI1, SREBF1, STEAP3, STING1, STK11IP, STX1A, STX1B, SV2A, SYN1, SYT2, SYT7, TCIRG1, THBS1, TIMP1, TMEM179B, TMEM63A, TNF, TNFAIP2, TNFRSF1A, TRAPPC1, TRPV4, TSPAN14, TUBA4A, USF2, VDR, VEGFA, VNN1, ZBTB7B]
GO:0070661	leukocyte proliferation	5,30E-14	[3]	106,00	31,45	69,17	30,83	[AIF1, BLK, BTK, BTN3A1, CARD11, CCDC88B, CCL19, CCL5, CCR2, CD180, CD19, CD22, CD3E, CD40LG, CD6, CD74, CD79A, CD80, CD86, CDKN2A, CLCF1, CORO1A, CR2, CRTAM, CSF1R, CTLA4, DOCK2, EBI3, EPX, FCRL3, FLT3, FOXP3, GAPT, HLA-DMB, HLA-DPA1, HLA-DPB1, HLA-DRB1, IKZF3, IL12RB1, IL18, IL5RA, IL7R, INPP5D, LEF1, LILRB1, LILRB4, LMO1, LST1, MS4A1, MZB1, NCKAP1L, PIK3CG, PLA2G2D, PRKCQ, PTPN22, PTPN6, PTPRC, PYCARD, RAC2, RASAL3, SASH3, SPN, SYK, TNFAIP3, TNFRSF13B, TNFSF13B, TNFSF14, TNFSF8, TREM2, XCL1, ZAP70]	[ABL1, AGER, AMH, ARMC5, BCL6, CD276, CD74, CDKN1A, CLC, CLCF1, CRP, CSF1, CX3CL1, DLG5, ELF4, ENPP3, ERBB2, FADD, FLT3, GBA, GPAM, HPRT1, IDO1, IL1B, IL5RA, IL7R, JAK3, LGALS9, LRRC32, MAPK3, MSN, NPR3, NPRL3, RAC2, SLC7A1, TCIRG1, TICAM1, TIMP1, VSIR, ZBTB7B, ZNF335]
GO:0032943	mononuclear cell proliferation	8,21E-12	[4]	95,00	30,84	72,46	27,54	[AIF1, BLK, BTK, BTN3A1, CARD11, CCDC88B, CCL19, CCL5, CCR2, CD180, CD19, CD22, CD3E, CD40LG, CD6, CD74, CD79A, CD80, CD86, CDKN2A, CLCF1, CORO1A, CR2, CRTAM, CTLA4, DOCK2, EBI3, EPX, FCRL3, FLT3, FOXP3, GAPT, HLA-DMB, HLA-DPA1, HLA-DPB1, HLA-DRB1, IKZF3, IL12RB1, IL18, IL7R, INPP5D, LEF1, LILRB1, LILRB4, LMO1, LST1, MS4A1, MZB1, NCKAP1L, PIK3CG, PLA2G2D, PRKCQ, PTPN22, PTPN6, PTPRC, PYCARD, RAC2, RASAL3, SASH3, SPN, SYK, TNFRSF13B, TNFSF13B, TNFSF14, TNFSF8, XCL1, ZAP70]	[ABL1, AGER, AMH, ARMC5, BCL6, CD276, CD74, CDKN1A, CLC, CLCF1, CRP, CSF1, DLG5, ELF4, ERBB2, FADD, FLT3, GPAM, HPRT1, IDO1, IL1B, IL7R, JAK3, LGALS9, LRRC32, MSN, RAC2, SLC7A1, TICAM1, TIMP1, VSIR, ZBTB7B, ZNF335]
GO:0046651	lymphocyte proliferation	2,19E-11	[4, 5]	93,00	30,69	73,69	26,31	[AIF1, BLK, BTK, BTN3A1, CARD11, CCDC88B, CCL19, CCL5, CCR2, CD180, CD19, CD22, CD3E, CD40LG, CD6, CD74, CD79A, CD80, CD86, CDKN2A, CLCF1, CORO1A, CR2, CRTAM, CTLA4, DOCK2, EBI3, EPX, FCRL3, FLT3, FOXP3, GAPT, HLA-DMB, HLA-DPA1, HLA-DPB1, HLA-DRB1, IKZF3, IL12RB1, IL18, IL7R, INPP5D, LEF1, LILRB1, LILRB4, LMO1, LST1, MS4A1, MZB1, NCKAP1L, PIK3CG, PLA2G2D, PRKCQ, PTPN22, PTPN6, PTPRC, PYCARD, RAC2, RASAL3, SASH3, SPN, SYK, TNFRSF13B, TNFSF13B, TNFSF14, TNFSF8, XCL1, ZAP70]	[ABL1, AGER, AMH, ARMC5, BCL6, CD276, CD74, CDKN1A, CLC, CLCF1, DLG5, ELF4, ERBB2, FADD, FLT3, GPAM, HPRT1, IDO1, IL1B, IL7R, JAK3, LGALS9, LRRC32, MSN, RAC2, SLC7A1, TICAM1, TIMP1, VSIR, ZBTB7B, ZNF335]
GO:0042100	B cell proliferation	1,60E-02	[5, 6]	31,00	30,69	80,81	19,19	[BLK, BTK, CARD11, CD180, CD19, CD22, CD40LG, CD74, CD79A, CDKN2A, CLCF1, CR2, CTLA4, FCRL3, GAPT, IKZF3, IL7R, INPP5D, LEF1, MS4A1, MZB1, NCKAP1L, PTPRC, SASH3, TNFRSF13B, TNFSF13B]	[ABL1, AMH, BCL6, CD74, CDKN1A, CLCF1, IL7R, TICAM1]
GO:0050670	regulation of lymphocyte proliferation	2,85E-09	[5, 6, 7]	75,00	31,51	74,46	25,54	[AIF1, BLK, BTK, CARD11, CCDC88B, CCL19, CCL5, CCR2, CD22, CD3E, CD40LG, CD6, CD74, CD80, CD86, CDKN2A, CLCF1, CORO1A, CRTAM, CTLA4, EBI3, EPX, FCRL3, FOXP3, HLA-DMB, HLA-DPA1, HLA-DPB1, HLA-DRB1, IKZF3, IL12RB1, IL18, INPP5D, LILRB1, LILRB4, LMO1, LST1, MZB1, NCKAP1L, PLA2G2D, PRKCQ, PTPN22, PTPN6, PTPRC, PYCARD, RAC2, RASAL3, SASH3, SPN, SYK, TNFRSF13B, TNFSF13B, TNFSF8, XCL1, ZAP70]	[AGER, AMH, BCL6, CD276, CD74, CDKN1A, CLC, CLCF1, DLG5, ERBB2, FADD, GPAM, IDO1, IL1B, JAK3, LGALS9, LRRC32, RAC2, SLC7A1, TICAM1, TIMP1, VSIR, ZBTB7B, ZNF335]
GO:0030183	B cell differentiation	1,40E-02	[5, 6, 7, 8, 9, 10, 11]	41,00	27,52	69,39	30,61	[BCL3, BLNK, BTK, CARD11, CD19, CD27, CD40LG, CD79A, CLCF1, CR2, DOCK10, FCRL3, FLT3, IKZF3, INPP5D, IRF8, ITGA4, MS4A1, NCKAP1L, NFAM1, POU2AF1, POU2F2, PTK2B, PTPN6, PTPRC, SLAMF8, SYK, TNFSF13B]	[ABL1, BCL3, BCL6, CLCF1, FLT3, GNAO1, HDAC5, INHA, JAK3, LRRC8A, SPI1, SYVN1, TCF3, TCF7L1, TCIRG1, ZBTB7A]
GO:0032613	interleukin-10 production	2,74E-02	[3, 6]	21,00	36,21	69,39	30,61	[BCL3, CD40LG, EPX, FOXP3, HLA-DRB1, IRF4, LILRB1, LILRB4, PYCARD, SASH3, SYK, TIGIT, TREM2, XCL1]	[AGER, BCL3, JAK3, LGALS9, LILRA5, NOD2, STAT3, VSIR]
GO:0032615	interleukin-12 production	2,50E-02	[3, 6]	22,00	35,48	66,84	33,16	[ACP5, CCL19, CD40LG, IFNG, IL16, IRF5, IRF8, LILRB1, LTB, SCIMP, SYK, THBS1, TIGIT, TLR8]	[AGER, CCR7, JAK3, LGALS9, LILRA5, MAST2, NOD2, RELA, THBS1]
GO:0032633	interleukin-4 production	1,24E-02	[3, 6]	15,00	46,88	83,83	16,17	[CD3E, CD40LG, CD86, EPX, FOXP3, HLA-DRB1, IRF4, LEF1, NLRP3, PRKCQ, SASH3, SYK]	[LGALS9, RARA, SLC7A5]

GO:0032653	regulation of interleukin-10 production	2,74E-02	[4, 5, 6, 7]	21,00	36,21	69,39	30,61	[BCL3, CD40LG, EPX, FOXP3, HLA-DRB1, IRF4, LILRB1, LILRB4, PYCARD, SASH3, SYK, TIGIT, TREM2, XCL1]	[AGER, BCL3, JAK3, LGALS9, LILRA5, NOD2, STAT3, VSIR]
GO:0032655	regulation of interleukin-12 production	2,50E-02	[4, 5, 6, 7]	22,00	35,48	66,84	33,16	[ACP5, CCL19, CD40LG, IFNG, IL16, IRF5, IRF8, LILRB1, LTB, SCIMP, SYK, THBS1, TIGIT, TLR8]	[AGER, CCR7, JAK3, LGALS9, LILRA5, MAST2, NOD2, RELA, THBS1]
GO:0032673	regulation of interleukin-4 production	1,24E-02	[4, 5, 6, 7]	15,00	46,88	83,83	16,17	[CD3E, CD40LG, CD86, EPX, FOXP3, HLA-DRB1, IRF4, LEF1, NLRP3, PRKCQ, SASH3, SYK]	[LGALS9, RARA, SLC7A5]
GO:0032753	positive regulation of interleukin-4 production	1,24E-02	[4, 5, 6, 7, 8]	13,00	52,00	81,20	18,80	[CD3E, CD40LG, CD86, EPX, FOXP3, IRF4, NLRP3, PRKCQ, SASH3, SYK]	[LGALS9, RARA, SLC7A5]
GO:0051239	regulation of multicellular organismal process	4,67E-22	[2, 3]	570,00	20,09	43,33	56,67	[ACP5, ADAM8, ADAMTS9, ADCY7, ADORA3, AIF1, AIM2, ALOX15B, ANGPT1, ANGPT2, AREG, ASCL2, ATP2A3, BANK1, BATF, BCL3, BHLHE40, BMP7, BMPR1B, BTK, BTN3A1, BTN3A2, C1QC, C1QTNF1, CALCR, CAMK4, CARD11, CARD17, CASP8, CCDC88B, CCL19, CCL24, CCL3, CCR2, CCR3, CD2, CD200R1, CD226, CD244, CD27, CD33, CD3E, CD4, CD40LG, CD6, CD74, CD80, CD84, CD86, CD96, CDH4, CDKN2A, CEL, CLCF1, CLEC6A, CLNK, CNN1, CNR1, CRABP2, CRB2, CRTAM, CSF1R, CST7, CTLA4, CXCL10, CXCL6, CXCR4, CYBB, DCC, DLGAP2, DPEP1, EAF2, EBI3, EDN2, EGR3, EPX, EREG, EVI2B, FASLG, FCN1, FCRL3, FFAR4, FGL2, FGR, FOXP3, FRZB, GBP5, GDF6, GPR171, GPR4, GPR55, GPSM3, H3C10, H3C11, H3C12, H3C7, HCLS1, HLA-DOA, HLA-DPA1, HLA-DPB1, HLA-DRA, HLA-DRB1, HOPX, IFNG, IKBKE, IKZF3, IL12RB1, IL12RB2, IL16, IL18, IL26, IL5RA, IL7R, INPP5D, IRF4, IRF5, IRF8, IRX3, ITGAX, ITGB2, ITK, KCNK15, KNG1, LAG3, LCP1, LDLR, LEF1, LIF, LILRB1, LILRB4, LMCD1, LPAL2, LRRC17, LTA, LTB, LY9, MAFF, MAPK13, MCOLN2, MMP9, MOG, MYB, MYCL, MYH6, NCKAP1L, NELL2, NFAM1, NLRC3, NLRP2, NLRP3, NLRP6, NLRP7, NLRP9, NPPB, NPPC, NPTX2, NR4A2, NR4A3, NTN1, ORM2, P2RY2, PIK3CG, PIM1, PLAAT4, PLAC8, PLAU, PLD4, PLXNC1, POU2AF1, POU2F2, PRDM1, PRKCB, PRKCQ, PROK1, PTAFR, PTGER2, PTK2B, PTPN22, PTPN6, PTPRC, PYCARD, PYHIN1, RHEX, RHOH, RLN2, RUNX3, SASH3, SCIMP, SEMA4A, SEMA4D, SIGLEC16, SLAMF6, SLAMF8, SLC8A3, SMPD3, SMTNL2, SOCS1, STAT1, SULT2B1, SYK, TACSTD2, TBX21, TBXAS1, TESPA1, THBS1, THY1, TIFAB, TIGIT, TLR10, TLR6, TLR8, TNFAIP3, TNFRSF11B, TNFRSF18, TNMD, TNR, TOX, TREM2, TSPAN8, UBASH3A, UCP2, VNN1, WNT1, WNT10B, XCL1, ZAP70, ZBED2, ZBP1, ZNF683]	[ABCA7, ABCC8, ABCD1, ABCD2, ABL1, ACACB, ACKR1, ADAMTS5, ADAMTS7, ADAMTS9, ADGRB2, ADIPOQ, AGAP2, AGER, AGPAT1, AGT, AGTR1, AMH, ANGPT1, ANGPTL4, AP3D1, APLN, APLNR, APOA1, APOD, APOM, AQP3, AREG, ARHGDIA, ARRDC3, ASPN, ATP1A1, ATP1A3, ATP1B2, ATPSCKMT, BAG6, BCAR1, BCL3, BCL6, BCL9L, BCOR, BHLHE40, C1QTNF1, C5, C5AR1, CACNA1H, CALCR, CAMK2B, CCL11, CCL24, CCR7, CD14, CD276, CD74, CDH4, CDK1, CEACAM1, CEL, CFH, CFL1, CGA, CHGA, CHI3L1, CITED2, CLC, CLCF1, CLNK, CLPTM1, CNN1, CNOT3, CNR1, COL1A1, CPTP, CREB3L1, CRP, CSF1, CSK, CST7, CTDSP1, CX3CL1, CYP26B1, CYP27B1, DAB2IP, DAG1, DDX39B, DHX34, DLG5, DLGAP4, DMTN, DTX1, DXO, EAF2, ECM1, EDN2, EFNA3, ENG, EPHA2, EPN1, EPPK1, ERBB2, FA2H, FABP4, FADD, FAM110A, FAP, FES, FFAR2, FLT4, FOXC2, FOXS1, FRMD8, FURIN, FXYD6, GATA2, GATA4, GBA, GDF6, GDI1, GIT1, GJD3, GLRA1, GNAI2, GNAO1, GPAM, GPER1, GPI, GPR137, GPR3, GPR37L1, GPR4, GPRIN1, GRN, GSK3A, GTF2IRD1, H3C10, H3C12, H4C3, HBEGF, HCN4, HDAC5, HDAC7, HESX1, HEYL, HFE, HGS, HK1, HOPX, HRH2, HSF1, HSPB1, HSPB7, HSPG2, HTR2A, HYAL1, ICAM1, IGF2BP2, IL15RA, IL1B, IL4R, IL5RA, IL7R, ILK, INHA, IRX3, ITGA3, ITGA5, ITPR3, JAK3, JMJD8, JPH4, JUP, KCNH2, KCNIP2, KCNJ5, KCNK15, KCNK2, KCNK3, KCNK6, KCNMB2, KCTD11, KDM6B, KLF10, KMT2D, KNG1, LAG3, LAMA5, LCAT, LDLR, LDLRAP1, LGALS9, LIF, LILRA5, LIMK1, LINGO1, LIPG, LMCD1, LMNA, LOX, LRCH4, LRG1, LRRC17, LRRC32, LTF, LUM, MAFG, MAP2K3, MAPK3, MAPK7, MAST2, MAVS, MECP2, MED12, MEGF8, MFAP2, MFSD2A, MME, MOV10, MSTN, MYBPH, MYC, MYH6, MYL9, MYRF, NEFL, NF2, NFATC4, NFE2, NFKB2, NGF, NGFR, NINJ1, NLGN2, NLRC5, NLRP9, NOD2, NODAL, NOS3, NOTCH1, NOTCH4, NOVA2, NPAS2, NPPA, NPPB, NPR3, NPRL3, NR1D1, NR4A2, NR4A3, NRARP, NTN1, NUMBL, NUTF2, OMA1, OMD, OSM, OSR2, P2RX3, P2RY2, PAF1, PAK4, PARP6, PCDHA4, PDGFB
GO:0051093	negative regulation of developmental process	5,70E-04	[2, 3, 4]	183,00	19,55	38,59	61,41	[ADAMTS9, ANGPT2, AREG, ASCL2, BHLHA15, BMP7, C1QC, CCL17, CCL3, CCR2, CD74, CDKN2A, CMTM5, CTLA4, CXCL10, DCC, DPEP1, EAF2, EREG, FASLG, FGL2, FOXP3, FRZB, GLIS1, GPR171, GPR4, GPR55, IFNG, IL18, INPP5D, IRX3, LAG3, LDLR, LEF1, LILRB1, LILRB4, LRRC17, MIXL1, MMP9, MSX1, MYB, NAMPT, NPPB, NPPC, NTN1, P2RY12, PLAC8, PTK2B, RBPMS2, RUNX3, SEMA4A, SEMA4D, SLC18A1, SOCS1, STAT1, TACSTD2, TBX21, THBS1, THY1, TNFRSF11B, TNMD, TNR, TREM2, WNT1, WNT10B]	[ABCC8, ABL1, ACTN4, ADAMTS7, ADAMTS9, ADCK1, ADGRB2, ADIPOQ, AGT, AMH, AREG, ARHGDIA, ARHGEF15, ASPN, BBS12, BCL6, BCOR, C11orf65, CARM1, CCL11, CD74, CDKN1A, CEACAM1, CETP, CGA, CHERP, CHGA, CITED2, CMTM5, CREB3L1, CRP, CTDSP1, DAB2IP, DISP3, DMTN, DNMT1, DPF2, DTX1, DXO, EAF2, ECM1, EFNA3, EPHA2, EPN1, ERBB2, FOXC2, FOXM1, GATA2, GDI1, GPER1, GPR137, GPR37L1, GPR4, GSK3A, H4C3, HDAC5, HDAC7, HGS, HSPG2, ID3, IL1B, IL4R, INHA, IRX3, JAK3, KCNK2, LAG3, LDLR, LINGO1, LMX1A, LRRC17, LTF, MECP2, MSTN, MSX1, MYC, NFATC4, NGFR, NODAL, NOS3, NOTCH1, NOTCH4, NPPB, NR1D1, NRARP, NTN1, OMA1, PAF1, PDGFB, PLXNA3, PML, POSTN, PPARD, PTN, RAI1, RARA, RBPMS2, RGCC, RGMA, RMRP, RTN4RL1, SCAF1, SEMA3F, SEMA4B, SEMA4C, SEMA6B, SEMA6C, SERPINE1, SMAD3, SNAI1, SOX10, SOX8, SPDEF, SPI1, STAT3, SYNGAP1, TBX5, TGFB1I1, THBS1, TNF, TNMD, TRIB1, TRIB3, TRIM62, TRPV4, TSKU, ULK1, VASN, VEGFA, WNT3, WNT9B, ZBTB7B, ZC3H12A, ZFP36]

GO:0051241	negative regulation of multicellular organismal process	1,56E-08 [2, 3, 4]	224,00	21,11	41,07	58,93	[ACP5, ADAMTS9, ADCY7, ANGPT1, ANGPT2, ASCL2, BANK1, BCL3, BMP7, BTK, C1QC, C1QTNF1, CALCR, CARD17, CCL3, CCR2, CD200R1, CD33, CD74, CD84, CD96, CDKN2A, CEL, CTLA4, CXCL10, DCC, DPEP1, EAF2, EPX, FASLG, FFAR4, FGL2, FOXP3, FRZB, GPR4, GPR55, HLA-DRB1, IFNG, IKBKE, INPP5D, KNG1, LAG3, LDLR, LEF1, LILRB1, LILRB4, LPAL2, LRRC17, NCKAP1L, NLRC3, NLRP3, NLRP6, NLRP7, NPPB, NTN1, PIK3CG, PLAC8, PLAU, PTK2B, PTPN22, PTPN6, PTPRC, PYCARD, RUNX3, SEMA4A, SEMA4D, SLAMF8, SOCS1, STAT1, TACSTD2, TBX21, THBS1, THY1, TIFAB, TIGIT, TLR6, TLR8, TNFAIP3, TNFRSF11B, TNMD, TNR, TREM2, TSPAN8, WNT10B, XCL1]	[ABCC8, ABCD1, ABCD2, ADAMTS5, ADAMTS7, ADAMTS9, ADGRB2, ADIPOQ, AGER, AGT, ANGPT1, APLN, APOA1, APOD, APOM, ARHGDIA, ARRDC3, ASPN, ATP1A1, ATP1A3, BCL3, BCL6, BCOR, C1QTNF1, CALCR, CCL11, CCR7, CD74, CEACAM1, CEL, CGA, CHGA, CITED2, CPTP, CREB3L1, CSK, CTDSP1, CX3CL1, DAB2IP, DTX1, DXO, EAF2, ECM1, EFNA3, EPHA2, EPN1, EPPK1, ERBB2, FAM110A, FAP, FOXC2, FURIN, GATA2, GBA, GDI1, GIT1, GJD3, GLRA1, GNAI2, GPR137, GPR37L1, GPR4, GRN, GSK3A, HDAC5, HDAC7, HFE, HGS, HSPG2, IL1B, IL4R, INHA, JAK3, JUP, KCNK2, KNG1, LAG3, LDLR, LGALS9, LILRA5, LINGO1, LMNA, LRRC17, LRRC32, LTF, MAVS, MECP2, MSTN, MYC, NFATC4, NGFR, NLRC5, NOD2, NODAL, NOS3, NOTCH1, NOVA2, NPPA, NPPB, NPR3, NPRL3, NR1D1, NRARP, NTN1, NUTF2, OMA1, PDGFB, PLAU, PLAUR, PLXNA3, PML, PPP1R11, PTN, PTPN23, PTPRU, RAB11FIP5, RAI1, RARA, RELB, RGCC, RGMA, RMRP, SEMA3F, SEMA4B, SEMA4C, SEMA6B, SEMA6C, SERPINE1, SERPING1, SH2B3, SMAD3, SOX10, SOX8, SPI1, SRF, SYNGAP1, TBX5, TFE3, THBS1, TIMP1, TLE3, TNF, TNFRSF1A, TNMD, TRIB1, TRIM62, TSKU, TSPAN8, UBE2L6, ULK1, VASN, VSIR, VTN, WNT3, WNT9B, ZBTB7B, ZC3H12A, ZFP36]
GO:0045595	regulation of cell differentiation	2,28E-11 [3, 4]	337,00	20,17	43,12	56,88	[ADAM8, ALOX15B, AREG, ASCL2, BCL11B, BHLHA15, BHLHE40, BMP7, BMPR1B, BTK, C1QC, CAMK4, CARD11, CASP8, CASS4, CCL17, CCL19, CCL3, CCR2, CD2, CD27, CD4, CD74, CD80, CD86, CDH4, CDKN2A, CLCF1, CMTM5, CRABP2, CRB2, CRTAM, CTLA4, CXCL10, CXCL9, CXCR4, DCC, DPEP1, EGR3, EPX, EREG, EVI2B, FCRL3, FFAR4, FGL2, FOXP3, FRZB, GDF6, GLIS1, GPR171, GPR55, H3C10, H3C11, H3C12, H3C7, HCLS1, HLA-DOA, HLA-DRA, HLA-DRB1, HOPX, IFNG, IKZF3, IL12RB1, IL18, IL7R, INPP5D, IRF4, IRX3, KLF5, KLHL41, LAG3, LCP1, LDLR, LEF1, LIF, LILRB1, LILRB4, LMO1, LRRC17, LTA, MAFF, MEDAG, MIXL1, MMP9, MSX1, MUSK, MYB, MYCL, NCKAP1L, NEK5, NFAM1, NLRP3, NPPC, NR4A3, NTN1, P2RY12, PIM1, PLAAT4, PLXNC1, PRDM1, PRKCB, PRKCQ, PTK2B, PTPN6, PTPRC, RBPMS2, RHEX, RHOH, RUNX3, SASH3, SEMA4A, SEMA4D, SLAMF8, SOCS1, SOCS3, STAT1, SULT2B1, SYK, TACSTD2, TBX21, TESPA1, THBS1, THY1, TNFRSF18, TNR, TOX, TREM2, VNN1, VWC2L, WNT1, WNT10B, ZAP70, ZBED2, ZNF683]	[ABCC8, ABL1, ACTN4, ADAMTS7, ADIPOQ, AGER, AGT, AGTR1, AP3D1, APOA1, AQP3, AREG, ARHGDIA, ATAT1, BBS12, BCL6, BCL9L, BEND6, BHLHE40, CAMK2B, CARM1, CASZ1, CCL11, CD74, CDH4, CDK1, CEACAM1, CETP, CHERP, CLCF1, CLPTM1, CMTM5, COL1A1, CRP, CSF1, CSNK1E, CTDSP1, CTHRC1, CX3CL1, CYP26B1, CYP27B1, DAB2IP, DAG1, DDX39B, DISP3, DMTN, DNMT1, DPF2, DTX1, EFEMP2, EIF4G1, ENG, ERBB2, FADD, FES, FLOT2, GATA2, GATA4, GDF6, GDI1, GNB3, GPER1, GPR137, GPR37L1, GRN, GSK3A, H3C10, H3C12, H4C3, HDAC5, HDAC7, HEYL, HOPX, HSF1, HTR2A, ID3, IFITM1, IL15RA, IL1B, IL4R, IL7R, ILK, INHA, IRX3, JAK3, JUNB, JUND, KCTD11, KLF10, KLF5, KMT2D, LAG3, LDLR, LGALS9, LIF, LIMK1, LIMS2, LINGO1, LMX1A, LOX, LRRC17, LRRC8A, LTF, LZTS1, MAFG, MAMSTR, MAPK8IP3, MCRIP1, MECP2, MED12, MEGF8, MME, MOV10, MSTN, MSX1, MYADM, MYC, MYL9, NAP1L2, NEFL, NEK5, NF2, NFATC4, NFE2, NGF, NGFR, NODAL, NOTCH1, NOTCH4, NR1D1, NR4A3, NRARP, NTN1, NUMBL, OMA1, OSM, PAF1, PARP6, PDGFB, PDLIM7, PHLDB1, PIEZO1, PIM1, PLA2G2A, PLEKHB1, PLXNA1, PLXNA3, PLXNB1, POR, POSTN, PPARD, PPP1R13L, PPP2R1A, PRKACA, PTN, RAMP2, RAP1A, RARA, RBM38, RBPMS2, RELA, RGCC, RGMA, RMRP, RNU1-93P, S1PR2, SCAF1, SEMA3F, SEMA4B, SEMA4C, SEMA6B, SEMA6C, SERPINE1, SETD1A, SHANK3, SIK1, SLC25A23, SLC4A11, SMAD3, SNAI1, SOCS3, SOX10, SOX12, SOX13, SOX8, SPDEF, SPI1, SRF, SRRT, STAT3, SYNGAP1, TBX5, TCF3, TCF7L1, TCIRG1, TEAD2, TESK1, TFE3, TGFB1I1, TGM2, THBS1, THPO, TIMP1, TMEM100, TNF, TNFRSF1A, TRIB1, TRIB3, TRIM62, TRIOBP, TRPV4, TSKU, ULK1, VASN, VDR, VEGFA, VNN1, VSIG2, VSIR, WNT3, WNT9B, ZBTB7B, ZC3H12A, ZFHX2, ZFP36, ZMIZ1, ZNF219, ZNF335, ZNF385A]
GO:2000026	regulation of multicellular organismal development	3,52E-12 [3, 4]	296,00	21,07	44,92	55,08	[ADAM8, ADAMTS9, ALOX15B, ANGPT2, ASCL2, BHLHE40, BMP7, BMPR1B, BTK, C1QC, CAMK4, CARD11, CASP8, CCL19, CCL24, CCL3, CCR2, CCR3, CD2, CD27, CD4, CD74, CD80, CD86, CDH4, CDKN2A, CLCF1, CRABP2, CRB2, CRTAM, CST7, CTLA4, CXCL10, CXCR4, CYBB, DCC, DPEP1, EGR3, EVI2B, FASLG, FCRL3, FGL2, FOXP3, FRZB, GDF6, GPR171, GPR4, GPR55, H3C10, H3C11, H3C12, H3C7, HCLS1, HLA-DOA, HLA-DRA, HLA-DRB1, HOPX, IFNG, IKZF3, IL12RB1, IL18, IL7R, INPP5D, IRF4, ITGAX, ITGB2, LAG3, LDLR, LEF1, LIF, LILRB1, LILRB4, LRRC17, LTA, MAFF, MMP9, MYB, MYCL, MYH6, NCKAP1L, NFAM1, NLRP3, NPPB, NR4A3, NTN1, PIM1, PLAAT4, PLXNC1, PRDM1, PRKCB, PRKCQ, PROK1, PTK2B, PTPN6, PTPRC, RHEX, RHOH, RLN2, RUNX3, SASH3, SEMA4A, SEMA4D, SLAMF8, SMPD3, SOCS1, STAT1, SULT2B1, SYK, TBX21, TESPA1, THBS1, THY1, TNFAIP3, TNFRSF18, TNMD, TNR, TOX, TREM2, VNN1, WNT1, WNT10B, ZAP70, ZBED2, ZNF683]	[ABCC8, ABL1, ACACB, ADAMTS7, ADAMTS9, ADGRB2, ADIPOQ, AGER, AGT, ANGPTL4, AP3D1, APLNR, AQP3, ARHGDIA, ASPN, BAG6, BCL6, BCOR, BHLHE40, C5, C5AR1, CAMK2B, CCL11, CCL24, CD74, CDH4, CDK1, CEACAM1, CFL1, CHI3L1, CITED2, CLCF1, CLPTM1, CREB3L1, CSF1, CST7, CTDSP1, CX3CL1, CYP26B1, CYP27B1, DAB2IP, DAG1, DDX39B, DLG5, DTX1, ECM1, EFNA3, ENG, EPHA2, EPN1, ERBB2, FADD, FES, FOXC2, GATA2, GATA4, GDF6, GDI1, GPER1, GPR137, GPR37L1, GPR4, GRN, GSK3A, H3C10, H3C12, H4C3, HESX1, HEYL, HGS, HOPX, HSF1, HSPB1, HSPG2, HYAL1, IL15RA, IL1B, IL4R, IL7R, ILK, INHA, ITGA5, JAK3, JMJD8, JUP, KCNK2, KLF10, KMT2D, LAG3, LAMA5, LDLR, LGALS9, LIF, LIMK1, LINGO1, LOX, LRCH4, LRG1, LRRC17, LTF, MAFG, MAPK7, MECP2, MED12, MEGF8, MME, MOV10, MYC, MYH6, MYL9, MYRF, NEFL, NF2, NFATC4, NFE2, NGF, NGFR, NINJ1, NLGN2, NODAL, NOS3, NOTCH1, NOTCH4, NPPB, NR1D1, NR4A3, NRARP, NTN1, NUMBL, OMA1, OMD, OSR2, PAF1, PAK4, PARP6, PHLDB1, PIM1, PKM, PLXNA1, PLXNA3, PLXNB1, PML, POR, PPARD, PTN, RAMP2, RARA, RELA, RGCC, RGMA, RMRP, S100A1, S1PR2, SEMA3F, SEMA4B, SEMA4C, SEMA6B, SEMA6C, SERPINE1, SETD1A, SHANK3, SMAD3, SOX10, SOX12, SOX13, SOX8, SPHK1, SPI1, SRF, SRRT, STAT3, SYNGAP1, TBX5, TFE3, TGM2, THBS1, THPO, TMEM100, TNF, TNFRSF1A, TNFSF12, TNMD, TRIB1, ULK1, VDR, VEGFA, VNN1, VSIR, WARS1, WNT3, WNT9B, ZBTB7B, ZC3H12A, ZFP36, ZMIZ1, ZNF219, ZNF335]

GO:0045596	negative regulation of cell differentiation	1,17E-02 [3, 4, 5]	136,00	19,77	41,02	58,98	[AREG, ASCL2, BHLHA15, BMP7, C1QC, CCL17, CCL3, CD74, CDKN2A, CMTM5, CTLA4, CXCL10, DCC, DPEP1, EREG, FGL2, FOXP3, FRZB, GLIS1, GPR171, GPR55, IFNG, IL18, INPP5D, IRX3, LAG3, LDLR, LILRB1, LILRB4, LRRC17, MIXL1, MMP9, MSX1, MYB, NPPC, NTN1, P2RY12, PTK2B, RBPMS2, RUNX3, SEMA4A, SEMA4D, SOCS1, STAT1, TACSTD2, TBX21, THY1, TNR, TREM2, WNT1, WNT10B]	[ABCC8, ACTN4, ADAMTS7, ADIPOQ, AREG, ARHGDIA, BBS12, BCL6, CCL11, CD74, CEACAM1, CETP, CHERP, CMTM5, CRP, CTDSP1, DAB2IP, DISP3, DMTN, DNMT1, DPF2, DTX1, ERBB2, GATA2, GDI1, GPER1, GPR137, GPR37L1, GSK3A, H4C3, HDAC5, HDAC7, ID3, IL1B, IL4R, INHA, IRX3, JAK3, LAG3, LDLR, LINGO1, LMX1A, LRRC17, LTF, MECP2, MSTN, MSX1, MYC, NFATC4, NGFR, NODAL, NOTCH1, NOTCH4, NR1D1, NRARP, NTN1, OMA1, PAF1, PDGFB, PLXNA3, POSTN, PPARD, PTN, RARA, RBPMS2, RGMA, RMRP, SCAF1, SEMA3F, SEMA4B, SEMA4C, SEMA6B, SEMA6C, SMAD3, SNAI1, SOX10, SOX8, SPDEF, STAT3, SYNGAP1, TBX5, TGFB1I1, TNF, TRIB1, TRIB3, TRIM62, TRPV4, TSKU, ULK1, VASN, VEGFA, WNT3, ZBTB7B, ZC3H12A, ZFP36]
GO:0001818	negative regulation of cytokine production	9,12E-06 [3, 4, 5, 6, 7]	79,00	26,42	54,65	45,35	[ACP5, ADCY7, ANGPT1, BANK1, BCL3, BTK, CARD17, CD200R1, CD33, CD84, CD96, EPX, FFAR4, FOXP3, HLA-DRB1, IFNG, IKBKE, INPP5D, LAG3, LEF1, LILRB1, LILRB4, LPAL2, NCKAP1L, NLRC3, NLRP3, NLRP6, NLRP7, PTPN22, PTPN6, PTPRC, PYCARD, TBX21, THBS1, TIGIT, TLR6, TLR8, TNFAIP3, TREM2, XCL1]	[ABCD1, ABCD2, ADIPOQ, AGER, ANGPT1, APOA1, APOD, BCL3, BCL6, CCR7, CEACAM1, CPTP, CSK, CX3CL1, EPHA2, FURIN, GBA, GIT1, HDAC7, HFE, INHA, JAK3, LAG3, LGALS9, LILRA5, LRRC32, LTF, MAVS, NLRC5, NOD2, NUTF2, PML, PPP1R11, RARA, RELB, RGCC, THBS1, TNF, TSKU, UBE2L6, VSIR, ZC3H12A, ZFP36]
GO:0010721	negative regulation of cell development	2,92E-02 [4, 5, 6]	48,00	25,53	35,70	64,30	[ASCL2, BMP7, DCC, DPEP1, FRZB, LDLR, LILRB1, NPPC, NTN1, SEMA4A, SEMA4D, TACSTD2, THY1, TNR, TREM2]	[ABCC8, ACTN4, ARHGDIA, CCL11, CTDSP1, DMTN, GDI1, GPR37L1, GSK3A, IL1B, LDLR, LINGO1, LTF, NFATC4, NGFR, NODAL, NOTCH1, NR1D1, NTN1, OMA1, PLXNA3, POSTN, PTN, RGMA, SEMA3F, SEMA4B, SEMA4C, SEMA6B, SEMA6C, SOX10, SYNGAP1, TNF, ULK1, VEGFA, WNT3]
GO:0050866	negative regulation of cell activation	2,21E-10 [3, 4, 5]	72,00	33,49	63,17	36,83	[BANK1, BLK, BTK, C1QTNF1, CCR2, CD300LF, CD74, CD80, CD84, CD86, CDKN2A, CLNK, CNR1, CNR2, CRTAM, CST7, CTLA4, FGL2, FGR, FOXP3, HLA-DRB1, INPP5D, LAG3, LAX1, LDLR, LILRB1, LILRB4, LST1, NCKAP1L, PLA2G2D, PTPN22, PTPN6, PTPRC, RUNX3, SAMSN1, SOCS1, SPN, TBC1D10C, TBX21, TIGIT, TNFAIP3, TNFAIP8L2, TNFRSF13B, TREM2, XCL1]	[BCL6, C1QTNF1, CD74, CEACAM1, CLNK, CNR1, CST7, CX3CL1, DLG5, DTX1, ENPP3, ERBB2, FOXF1, GPER1, GRN, HFE, IL4R, INHA, JAK3, LAG3, LDLR, LGALS9, LRRC32, NOS3, NR1D1, NRARP, PCDHA4, PCDHA6, PDGFB, SH2B3, SPI1, VSIR, ZBTB7B, ZC3H12A]
GO:0002695	negative regulation of leukocyte activation	1,42E-10 [3, 4, 5, 6]	67,00	34,90	65,00	35,00	[BANK1, BLK, BTK, CCR2, CD300LF, CD74, CD80, CD84, CD86, CDKN2A, CLNK, CNR1, CNR2, CRTAM, CST7, CTLA4, FGL2, FGR, FOXP3, HLA-DRB1, INPP5D, LAG3, LAX1, LDLR, LILRB1, LILRB4, LST1, NCKAP1L, PLA2G2D, PTPN22, PTPN6, PTPRC, RUNX3, SAMSN1, SOCS1, SPN, TBC1D10C, TBX21, TIGIT, TNFAIP3, TNFAIP8L2, TNFRSF13B, XCL1]	[BCL6, CD74, CEACAM1, CLNK, CNR1, CST7, CX3CL1, DLG5, DTX1, ENPP3, ERBB2, FOXF1, GPER1, GRN, HFE, IL4R, INHA, JAK3, LAG3, LDLR, LGALS9, LRRC32, NR1D1, NRARP, PCDHA4, PCDHA6, SPI1, VSIR, ZBTB7B, ZC3H12A]
GO:0022408	negative regulation of cell-cell adhesion	2,36E-06 [4, 5, 6]	59,00	30,57	53,24	46,76	[AKNA, C1QTNF1, CD74, CD80, CD86, CDH1, CDKN2A, CRTAM, CTLA4, FGL2, FOXP3, HLA-DRB1, LAG3, LAX1, LILRB1, LILRB4, NCKAP1L, PLA2G2D, PTPN22, PTPN6, RUNX3, SOCS1, SPN, TBX21, TIGIT, TNFAIP8L2, TNR, WNT1, XCL1]	[ABL1, ADIPOQ, APOA1, BCL6, C1QTNF1, CCL21, CD74, CEACAM1, DLG5, DTX1, ERBB2, FXYD5, HFE, IL1RN, IL4R, JAK3, LAG3, LGALS9, LRRC32, MAPK7, MYADM, NF2, NOTCH1, NOTCH4, NRARP, RGCC, SH2B3, SPI1, TRPV4, VEGFA, VSIR, ZBTB7B, ZC3H12A]
GO:0051250	negative regulation of lymphocyte activation	1,91E-05 [4, 5, 6, 7]	50,00	31,25	71,59	28,41	[BANK1, BLK, BTK, CD74, CD80, CD86, CDKN2A, CLNK, CRTAM, CTLA4, FGL2, FGR, FOXP3, HLA-DRB1, INPP5D, LAG3, LAX1, LILRB1, LILRB4, LST1, NCKAP1L, PLA2G2D, PTPN22, PTPN6, RUNX3, SAMSN1, SOCS1, SPN, TBC1D10C, TBX21, TIGIT, TNFAIP3, TNFAIP8L2, TNFRSF13B, XCL1]	[BCL6, CD74, CEACAM1, CLNK, DLG5, DTX1, ERBB2, HFE, IL4R, INHA, JAK3, LAG3, LGALS9, LRRC32, NRARP, VSIR, ZBTB7B, ZC3H12A]
GO:1903706	regulation of hemopoiesis	2,05E-13 [3, 4, 5, 6, 7, 8]	122,00	28,98	61,99	38,01	[ADAM8, BTK, C1QC, CAMK4, CARD11, CASP8, CCL19, CCL3, CCR2, CD2, CD27, CD4, CD74, CD80, CD86, CDKN2A, CRTAM, CTLA4, EGR3, EVI2B, FCRL3, FGL2, FOXP3, GPR171, GPR55, H3C10, H3C11, H3C12, H3C7, HCLS1, HLA-DOA, HLA-DRA, HLA-DRB1, IFNG, IKZF3, IL12RB1, IL18, IL7R, INPP5D, IRF4, LAG3, LEF1, LILRB1, LILRB4, LRRC17, MYB, NCKAP1L, NFAM1, NLRP3, NR4A3, PRDM1, PRKCB, PRKCQ, PTK2B, PTPN6, PTPRC, RHEX, RHOH, RUNX3, SASH3, SLAMF8, SOCS1, STAT1, SYK, TBX21, TESPA1, THBS1, TNFRSF18, TOX, TREM2, VNN1, ZAP70, ZNF683]	[ABL1, ADIPOQ, AGER, AP3D1, BCL6, CD74, CEACAM1, CLPTM1, CSF1, CYP26B1, DTX1, ERBB2, FADD, FES, GATA2, GPR137, H3C10, H3C12, H4C3, HSF1, IL15RA, IL4R, IL7R, INHA, JAK3, KLF10, KMT2D, LAG3, LGALS9, LOX, LRRC17, LTF, MOV10, MYC, MYL9, NFE2, NR4A3, NRARP, PAF1, PTN, RARA, RMRP, SETD1A, SOX12, SOX13, SPI1, STAT3, TFE3, THBS1, THPO, TNF, TRIB1, VNN1, VSIR, ZBTB7B, ZC3H12A, ZFP36, ZMIZ1]
GO:1903038	negative regulation of leukocyte cell-cell adhesion	9,60E-03 [5, 6, 7]	39,00	28,47	64,65	35,35	[CD74, CD80, CD86, CDKN2A, CRTAM, CTLA4, FGL2, FOXP3, HLA-DRB1, LAG3, LAX1, LILRB1, LILRB4, NCKAP1L, PLA2G2D, PTPN22, PTPN6, RUNX3, SOCS1, SPN, TBX21, TIGIT, TNFAIP8L2, XCL1]	[BCL6, CCL21, CD74, CEACAM1, DLG5, DTX1, ERBB2, HFE, IL4R, JAK3, LAG3, LGALS9, LRRC32, NRARP, VSIR, ZBTB7B, ZC3H12A]
GO:1902105	regulation of leukocyte differentiation	4,19E-15 [4, 5, 6, 7, 8, 9]	97,00	33,92	65,84	34,16	[ADAM8, BTK, C1QC, CAMK4, CARD11, CASP8, CCL19, CCL3, CCR2, CD2, CD27, CD4, CD74, CD80, CD86, CDKN2A, CRTAM, CTLA4, EGR3, EVI2B, FCRL3, FGL2, FOXP3, GPR55, HCLS1, HLA-DOA, HLA-DRA, HLA-DRB1, IFNG, IKZF3, IL12RB1, IL18, IL7R, INPP5D, IRF4, LAG3, LEF1, LILRB1, LILRB4, LRRC17, MYB, NCKAP1L, NFAM1, NLRP3, PRDM1, PTPN6, PTPRC, RHOH, RUNX3, SASH3, SLAMF8, SOCS1, SYK, TBX21, TESPA1, TNFRSF18, TOX, TREM2, VNN1, ZAP70, ZNF683]	[ABL1, ADIPOQ, AGER, AP3D1, BCL6, CD74, CEACAM1, CLPTM1, CSF1, CYP26B1, DTX1, ERBB2, FADD, FES, GATA2, GPR137, HSF1, IL15RA, IL4R, IL7R, INHA, JAK3, KLF10, LAG3, LGALS9, LRRC17, LTF, MYC, NRARP, RARA, RMRP, SOX12, SOX13, TFE3, TNF, TRIB1, VNN1, VSIR, ZBTB7B, ZC3H12A, ZMIZ1]
GO:1902106	negative regulation of leukocyte differentiation	1,66E-03 [4, 5, 6, 7, 8, 9, 10]	34,00	32,69	49,68	50,32	[C1QC, CCL3, CD74, CDKN2A, CTLA4, FGL2, FOXP3, GPR55, INPP5D, LAG3, LILRB1, LILRB4, LRRC17, RUNX3, SOCS1, TBX21]	[ADIPOQ, BCL6, CD74, CEACAM1, DTX1, ERBB2, GATA2, GPR137, IL4R, INHA, JAK3, LAG3, LRRC17, LTF, MYC, NRARP, RARA, RMRP, TRIB1, ZBTB7B, ZC3H12A]
GO:0022613	ribonucleoprotein complex biogenesis	1,36E-04 [4]	30,00	6,05	4,28	95,72	[CDKN2A]	[CPSF7, DDX39B, DDX54, ERI2, FCF1, GTPBP10, LTB4R2, METTL18, MPV17L2, MRPS2, MYBBP1A, NOC4L, NOL6, NOP2, PELP1, RPL26, RPL3, RPLP1, RPS16, RPUSD1, RRP12, SF3A2, SPPL2B, SRPK3, TBL3, TNIP1, UBA52, WBP11, XAB2]

GO:0006396	RNA processing	3,45E-11	[5, 6, 7]	67,00	6,36	11,14	88,86	[CDKN2A, ELAVL4, KCNN4, KHDRBS2, M1AP, NLRP6]	[CHERP, CLASRP, CPSF1, CPSF7, CSDC2, DDX39B, DDX54, ELAVL2, ERI2, FCF1, HNRNPUL1, HSF1, INTS1, INTS5, KHDRBS2, LTB4R2, METTL1, METTL18, NCOR2, NOC4L, NOL6, NOP2, NOVA2, PAF1, PELP1, POLR2E, POLR2L, PPP1R9B, PPP2R1A, PQBP1, PRCC, PRKACA, RAVER1, RBM14, RBM38, RPL26, RPL3, RPLP1, RPS16, RPUSD1, RRP12, SCAF1, SF3A2, SF3B4, SMAD3, SNRPA, SPPL2B, SRPK3, SRRM2, SRRT, STAT3, TBL3, TNF, TNIP1, TRUB1, UBA52, WBP11, XAB2, ZBTB7A, ZC3H12A, ZC3H3, ZC3H7B]
GO:0016071	mRNA metabolic process	1,59E-04	[6, 7]	72,00	7,96	18,45	81,55	[CALCR, ELAVL4, EREG, IKBKE, KCNN4, KHDRBS2, NLRP6, PATL2, SLFN14, TENT5C, ZC3H12D]	[C5AR1, CALCR, CHERP, CLASRP, CNOT3, CPSF1, CPSF7, CSDC2, CTIF, DDX39B, DHX34, DXO, EDC4, EIF4G1, ELAVL2, GTPBP1, HNRNPUL1, HSF1, HSPB1, IGF2BP2, KHDRBS2, MOV10, NOVA2, NUDT12, PAF1, PATL1, PCBP3, PLEKHN1, PNLDC1, POLR2E, POLR2L, PPP2R1A, PQBP1, PRCC, PRKACA, RAVER1, RBM14, RBM38, RNU1-93P, RPL26, RPL3, RPLP1, RPS16, SAMD4B, SCAF1, SF3A2, SF3B4, SKIV2L, SNRPA, SREBF1, SRPK3, SRRM2, SRRT, STAT3, TNKS1BP1, TRUB1, UBA52, WBP11, XAB2, ZBTB7A, ZC3H12A, ZC3H3, ZFP36]
GO:0034660	ncRNA metabolic process	1,89E-03	[6, 7]	39,00	7,00	6,55	93,45	[CDKN2A, SLFN14]	[CPSF1, DDX54, EEF1E1, ERI2, FCF1, GPAT2, INTS1, INTS5, LTB4R2, METTL1, METTL18, NOC4L, NOL6, NOP2, PELP1, PIWIL1, RELA, RPL26, RPL3, RPLP1, RPS16, RPUSD1, RRP12, SMAD3, SPPL2B, SRRT, STAT3, TBL3, TDRD1, TNIP1, TRUB1, UBA52, WARS1, WBP11, YARS1, ZC3H12A, ZSWIM8]
GO:0006397	mRNA processing	4,89E-05	[6, 7, 8]	34,00	6,20	14,32	85,68	[ELAVL4, KCNN4, KHDRBS2, NLRP6]	[CHERP, CLASRP, CPSF1, CPSF7, CSDC2, DDX39B, ELAVL2, HNRNPUL1, HSF1, KHDRBS2, NOVA2, PAF1, POLR2E, POLR2L, PQBP1, PRCC, PRKACA, RAVER1, RBM14, RBM38, SCAF1, SF3A2, SF3B4, SNRPA, SRPK3, SRRM2, SRRT, WBP11, XAB2, ZBTB7A, ZC3H3]
GO:0008380	RNA splicing	7,15E-04	[6, 7, 8]	30,00	6,30	12,19	87,81	[ELAVL4, KCNN4, KHDRBS2]	[CHERP, CLASRP, CPSF1, CPSF7, DDX39B, ELAVL2, HNRNPUL1, KHDRBS2, NOVA2, POLR2E, POLR2L, PPP1R9B, PPP2R1A, PQBP1, PRCC, RAVER1, RBM14, RBM38, SCAF1, SF3A2, SF3B4, SNRPA, SRPK3, SRRM2, SRRT, WBP11, XAB2, ZBTB7A]
GO:0034470	ncRNA processing	9,98E-04	[6, 7, 8]	29,00	6,26	4,42	95,58	[CDKN2A]	[CPSF1, DDX54, ERI2, FCF1, INTS1, INTS5, LTB4R2, METTL1, METTL18, NOC4L, NOL6, NOP2, PELP1, RPL26, RPL3, RPLP1, RPS16, RPUSD1, RRP12, SMAD3, SPPL2B, SRRT, STAT3, TBL3, TNIP1, TRUB1, UBA52, WBP11]
GO:0000398	mRNA splicing, via spliceosome	4,26E-02	[7, 8, 9, 10, 11]	24,00	6,56	10,13	89,87	[KCNN4, KHDRBS2]	[CHERP, CPSF1, CPSF7, DDX39B, ELAVL2, HNRNPUL1, KHDRBS2, NOVA2, POLR2E, POLR2L, PQBP1, PRCC, RAVER1, RBM14, SF3A2, SF3B4, SNRPA, SRPK3, SRRM2, SRRT, WBP11, XAB2, ZBTB7A]
GO:0023051	regulation of signaling	5,81E-15	[2, 3]	675,00	18,15	37,12	62,88	[ABRA, ADAM8, ALOX15, ALOX15B, ANGPT1, ARAP2, AREG, ARHGAP15, ARHGAP30, ARHGAP45, ATF3, ATP2A3, BANK1, BCL2L14, BCL3, BLK, BMF, BMP7, BMPR1B, BRIP1, C1QTNF1, CACNA1E, CALCR, CARD11, CASP8, CASS4, CCDC88C, CCL17, CCL19, CCL2, CCL22, CCL24, CCL3, CCL3L1, CCL4, CCL4L1, CCL5, CCR2, CD177, CD180, CD19, CD22, CD226, CD27, CD300LF, CD3E, CD4, CD40LG, CD74, CD80, CD86, CDH1, CDKN2A, CEL, CHRDL1, CISH, CLEC6A, CNR1, CNR2, CRB2, CSF1R, CXCR4, CXorf21, DAPK1, DCC, DEPDC1B, DLGAP2, DPEP2, DRD1, DUSP2, DUSP8, ELAVL4, ELF3, EPX, EREG, ERFE, FASLG, FCRL3, FFAR4, FGD2, FGD3, FGR, FLT3, FOLR2, FOXP3, FRZB, FUT7, GADD45B, GDF6, GFI1, GMIP, GPR174, GPR4, GPR55, GPRC5A, GUCY2D, H3C10, H3C11, H3C12, H3C7, HCLS1, HCST, HLA-DRB1, ICOS, IER3, IFNG, IKBKE, IL10RA, IL18, IL26, IL7R, INPP5D, IQCJ-SCHIP1, IRF4, KCNJ10, KCNN4, KMO, LAX1, LCK, LEF1, LIF, LILRB4, LMCD1, LY86, MAP4K1, MIDN, MMP9, MSX1, MYB, MZB1, NCF1, NCKAP1L, NFAM1, NLRC3, NLRP3, NLRP6, NPFFR2, NPPC, NPTX2, NR4A2, NSG1, NTN1, OASL, P2RY10, P2RY12, PAX5, PDE6G, PIK3CG, PIK3IP1, PIK3R5, PIM2, PLAU, PLEK, PRDM1, PRKCB, PRKCQ, PROK1, PSCA, PSD4, PTK2B, PTPN22, PTPN6, PTPRC, PYCARD, PYHIN1, RAC2, RAD9B, RASAL3, RASGRF1, RBPMS2, RGS1, RGS10, RGS18, RGS9, RHOH, RMI2, RNASE6, RRAD, RTKN2, SALL1, SCIMP, SCUBE1, SEMA4D, SH2D1A, SHISA8, SLA, SLA2, SLC24A4, SLC8A3, SMPDL3B, SOCS1, SOCS3, SPINK1, STAP1, STAT1, SYK, TAGAP, TBC1D10C, TESPA1, THBS1, THY1, TLR6, TMC8, TNFAIP3, TNFSF14, TNIP3, TNMD, TNR, TRABD2A, TRAF1, TRAT1, TREM2, TRIM14, TRPM2, UBASH3A, UBD, UCP2, VAV1, VNN1, VWC2L, WNT1, WNT10B, XCL1, XCL2, ZAP70, ZBP1]	[ABCA7, ABCC8, ABL1, ABR, ABRA, ACKR3, ACP4, ACTN4, ADCY1, ADGRG1, ADIPOQ, AGAP2, AGER, AGPAT1, AGT, AGTR1, AKT1S1, AMH, ANGPT1, APLN, APLNR, APLP1, APOA1, APOD, ARAF, ARAP3, ARC, AREG, ARF1, ARHGAP1, ARHGAP23, ARHGAP39, ARHGDIA, ARHGEF1, ARHGEF16, ARHGEF5, ARRDC3, ARTN, ASPN, ATP1A1, ATP1A3, ATP1B2, BCL2L1, BCL3, BCL6, BCL9L, BCR, BEND6, BMP8A, BRD4, C1QTNF1, C20orf27, C2CD2L, C5, C5AR1, CACNA1E, CALB1, CALCR, CAMK2B, CAPN1, CARM1, CC2D1A, CCL11, CCL2, CCL21, CCL24, CCR7, CD14, CD177, CD74, CDK1, CDK2, CEACAM1, CEL, CGA, CHERP, CHGA, CHI3L1, CISH, CITED2, CLIP3, CNR1, CNTN6, COL1A1, CPLX1, CRAT, CREB3L1, CRHR2, CSF1, CSK, CSNK1E, CSPG4, CTHRC1, CX3CL1, CXXC4, CYP26B1, CYP27B1, DAB2IP, DAG1, DAXX, DDX39B, DENND2B, DENND4B, DGKD, DGKZ, DHX34, DIRAS1, DKK2, DLG5, DLGAP4, DMTN, DOC2B, DOK5, DOT1L, DTX1, DUSP5, DUSP8, DVL3, ECM1, EEF1E1, ELF3, ENG, EPHA2, EPN1, ERBB2, ERFE, ESM1, FADD, FAIM2, FAM110A, FASN, FFAR2, FGFR4, FLOT2, FLT3, FLT4, FOXM1, FPR1, FRMD8, FURIN, FXYD6, GAB2, GADD45B, GAS1, GATA4, GBA, GBF1, GCSAM, GDF6, GIT1, GJD3, GLIS2, GLRA1, GNAI2, GPAM, GPBAR1, GPER1, GPI, GPR137, GPR17, GPR20, GPR37L1, GPR4, GPRC5A, GPRIN1, GRB7, GRIK5, GRINA, GRM2, GSK3A, H3C10, H3C12, H4C3, HBEGF, HCRTR1, HDAC7, HEYL, HFE, HGS, HIPK4, HSPB1, HTR1B, HTR2A, ICAM1, IER3, IFT80, IL18BP, IL1B, IL1RN, IL7R, ILK, INCA1, INHA, INPP5E, IRAK2, ITGA3, ITGA5, ITPR3, ITPRIP, JAK3, JMJD8, JPH4, JUP, KCP, KCTD11, KIF26A, KLK14, KMT2D, KSR1, LGALS9, LGR6, LIF, LIFR, LILRA5, LIMS2, LMCD1, LMNA, LOX, LRG1, LRRC4, LRRC8A, LTBR, LTF, LY6E, LYNX1, LZTS1, LZTS2, MAP1A, MAP2K3, MAP3K10, MAP3K11, MAP3K14, MAP3K6, MAP3K9, MAPK3, MAPK7, MAPK8IP3, MAVS, MAZ, MECP2, MED12, MEGF8, MEIS3P1, MEN1, MGRN1, MIDN, MINK1, MLST8, MME, MNT, MSTN, MSX1, MTRNR2L1, MTRNR2L10, MTRNR2L12, MTRNR2L2, MTRNR2L3

GO:0048583	regulation of response to stimulus	4,40E-27 [2, 3]	851,00	18,88	45,12	54,88	[ABRA, ACP5, ADAM8, ADCY7, AIF1, AIM2, AKNA, ALOX15, ALOX15B, ANGPT1, ANGPT2, AOAH, APOBEC3G, ARAP2, AREG, ARHGAP15, ARHGAP30, ARHGAP45, ATF3, BANK1, BCL2L14, BCL3, BLK, BLNK, BMF, BMP7, BMPR1B, BRIP1, BTK, BTLA, BTN3A1, BTN3A2, C1QA, C1QB, C1QC, C1QTNF1, C2, C8G, CALCR, CARD11, CASP8, CASS4, CCDC88C, CCL17, CCL19, CCL2, CCL22, CCL24, CCL3, CCL3L1, CCL4, CCL4L1, CCL5, CCR2, CCR4, CD177, CD180, CD19, CD1B, CD1C, CD1E, CD200R1, CD22, CD226, CD247, CD27, CD300LF, CD33, CD3D, CD3E, CD3G, CD4, CD40LG, CD48, CD74, CD79A, CD80, CD84, CD86, CD8A, CD8B, CD96, CDKN2A, CEL, CFP, CHRDL1, CISH, CLCF1, CLEC10A, CLEC1B, CLEC4D, CLEC4E, CLEC6A, CLNK, CNR1, CNR2, CR2, CRB2, CRTAM, CSF1R, CST7, CTLA4, CXCL10, CXCL6, CXCR4, CXorf21, CYP11B1, CYSLTR1, DAPK1, DCC, DEPDC1B, DLGAP2, DPEP1, DPEP2, DRD1, DUSP2, DUSP8, EDN2, ELF3, EPX, EREG, ERFE, FASLG, FCGR2C, FCN1, FCN3, FCRL3, FFAR4, FGD2, FGD3, FGL2, FGR, FLT3, FOLR2, FOXP3, FRZB, FUT7, FYB1, GADD45B, GBP5, GDF6, GFI1, GMIP, GPR150, GPR174, GPR4, GPR55, GPR83, GPRC5A, GPSM3, GRAP2, GUCY2D, H3C10, H3C11, H3C12, H3C7, HCLS1, HCST, HLA-DMB, HLA-DPA1, HLA-DPB1, HLA-DQA1, HLA-DRA, HLA-DRB1, HLA-DRB5, HOPX, ICOS, IER3, IFNG, IGLL5, IKBKE, IL10RA, IL12RB1, IL16, IL18, IL18RAP, IL26, IL7R, INPP5D, IQCJ-SCHIP1, IRF4, ITGA4, ITGAL, ITGB2, ITGB7, ITK, JAML, KCNN4, KIR2DL2, KIR2DL3, KIR3DL2, KLRB1, KLRC1, KLRC2, KLRD1, KLRK1, KNG1, LAG3, LAIR1, LAIR2, LAX1, LCK, LCP1, LCP2, LDLR, LEF1, LIF, LILRA1, LILRB1, LILRB4, LMCD1, LTA, LY86, MAP4K1, MAPK13, MLC1, MMP9, MOG, MS4A1, MSX1, MUC16, MUC19, MUC6, MYB, MYO1G, MZB1, NAMPT, NCF1, NCKAP1L, NCR1, NCR3, NFAM1, NLRC3, NLRP3, NLRP6, NPFFR2, NPPC, NPTX2, NR4A2, NR4A3, NTN1, NUP210, OASL, P2RY10, P2RY12, PAX5, PDE6G, PIK3CG, PIK3IP1, PIK3R5, PILRA, PIM2, PLA2G2D, PLA2G7, PLAU, PLEK, PRAM1, PRDM1, PRKCB, PRKCQ	[AAAS, ABCA7, ABCC8, ABCD1, ABI3, ABL1, ABR, ABRA, ACKR3, ACP4, ACTB, ACTN4, ADCY1, ADCY3, ADCY4, ADCY9, ADGRG1, ADIPOQ, AGAP2, AGER, AGPAT1, AGT, AGTR1, AKT1S1, AMH, ANGPT1, APLN, APLNR, APLP1, APOA1, APOD, ARAF, ARAP3, ARC, AREG, ARHGAP1, ARHGAP23, ARHGAP39, ARHGDIA, ARHGEF1, ARHGEF16, ARHGEF5, ARMT1, ARRDC3, ARTN, ASPN, BAG3, BAG6, BCAR1, BCL2L1, BCL3, BCL6, BCL9L, BCR, BEND6, BMP8A, BRD4, BTLA, C1QTNF1, C20orf27, C5, C5AR1, C8G, CALCR, CAMK2B, CAPN1, CARM1, CC2D1A, CCL11, CCL2, CCL21, CCL24, CCR7, CD14, CD177, CD1C, CD1E, CD276, CD300E, CD74, CDK1, CDK2, CEACAM1, CEL, CFH, CGA, CHERP, CHGA, CHI3L1, CISH, CITED2, CLC, CLCF1, CLEC1B, CLEC4E, CLIP3, CLNK, CNR1, CNTN6, COL1A1, CPN2, CRAT, CREB3L1, CRHR2, CRP, CRYAB, CSF1, CSK, CSNK1E, CSPG4, CST7, CTHRC1, CX3CL1, CXXC4, CYP26B1, CYP27B1, DAB2IP, DAG1, DAGLA, DAXX, DDX39B, DENND2B, DENND4B, DGKD, DGKZ, DIRAS1, DKK2, DLG5, DLGAP4, DMTN, DOK5, DOT1L, DTX1, DUSP5, DUSP8, DVL3, ECM1, EDN2, EEF1E1, EIF4G1, ELF3, ENDOG, ENG, ENPP3, EPHA2, EPN1, EPPK1, ERBB2, ERFE, ESM1, FABP4, FADD, FAIM2, FAM110A, FAP, FASN, FCN3, FEM1A, FES, FFAR2, FGFR4, FLOT2, FLT3, FLT4, FOXC2, FOXF1, FOXM1, FPR1, FRMD8, FURIN, GAB2, GADD45B, GAS1, GATA2, GATA4, GBA, GBF1, GCSAM, GDF6, GIT1, GLIS2, GNAI2, GNAO1, GPAM, GPBAR1, GPER1, GPI, GPR137, GPR17, GPR20, GPR37L1, GPR4, GPR83, GPR84, GPRC5A, GPRIN1, GRB7, GRIK5, GRINA, GRM2, GRN, GSK3A, GTF2IRD1, H3C10, H3C12, H4C3, HBEGF, HCRTR1, HDAC10, HDAC7, HEYL, HFE, HGS, HIPK4, HMGA1, HOPX, HRH2, HSF1, HSPB1, HSPB8, HTR1B, HTR2A, ICAM1, IER3, IFITM1, IFT80, IL18BP, IL1B, IL1RN, IL4R, IL7R, ILK, INCA1, INHA, INPP5E, IRAK2, ITGA3, ITGA5, ITPRIP, JAK3, JMJD8, JUP, KCNK2, KCP, KCTD11, KIF26A, KLK14, KMT2D, KNG1, KSR1, LAG3, LDLR, LGALS9, LGR6, LIF, LIFR, LILRA5, LIMK1, LIMS2, LMCD1, LMNA, LOX, LRG1, LTBR
GO:0050794	regulation of cellular process	3,02E-10 [2, 3]	1748,00	15,03	38,78	61,22	[ABCB11, ABRA, ACP5, ADAM8, ADAMDEC1, ADAMTS9, ADCY7, ADGRE3, ADGRE4P, ADGRG5, ADGRG6, ADORA3, AIF1, AIM2, AKNA, ALOX15, ALOX15B, ANGPT1, ANGPT2, ANO9, ANPEP, APBB1IP, APOBEC3A, APOBEC3D, APOBEC3G, APOBEC3H, ARAP2, AREG, ARHGAP15, ARHGAP30, ARHGAP45, ASCL2, ASGR2, ATF3, ATP1B4, ATP2A3, BANK1, BATF, BCL11B, BCL2L14, BCL2L15, BCL3, BHLHA15, BHLHE22, BHLHE40, BLK, BLNK, BMF, BMP7, BMPR1B, BRIP1, BTG2, BTK, BTLA, BTN3A1, BTN3A2, C1QA, C1QC, C1QTNF1, C2, CACNA1E, CACNA1I, CALCR, CAMK4, CARD11, CARD17, CARMIL2, CASKIN1, CASP8, CASS4, CBARP, CCDC88B, CCDC88C, CCL17, CCL19, CCL2, CCL22, CCL24, CCL3, CCL3L1, CCL4, CCL4L1, CCL4L2, CCL5, CCR2, CCR3, CCR4, CCR5, CCR8, CD177, CD180, CD19, CD1B, CD1C, CD1E, CD2, CD200R1, CD22, CD226, CD244, CD247, CD27, CD300LF, CD33, CD3D, CD3E, CD3G, CD4, CD40LG, CD5, CD53, CD6, CD7, CD74, CD79A, CD80, CD84, CD86, CD8A, CD8B, CDC20B, CDCA2, CDH1, CDH4, CDKN2A, CEL, CHRDL1, CISH, CLCF1, CLCNKA, CLEC10A, CLEC1B, CLEC4D, CLEC4E, CLEC6A, CLNK, CMTM5, CNN1, CNR1, CNR2, CORO1A, CORO2A, COTL1, CR2, CRABP2, CRB2, CREM, CRTAM, CSF1R, CSF2RA, CST7, CTLA4, CTSS, CXCL10, CXCL11, CXCL5, CXCL6, CXCL9, CXCR3, CXCR4, CXCR6, CXorf21, CYBB, CYSLTR1, CYTIP, DAPK1, DAPP1, DCC, DEPDC1B, DERL3, DLGAP2, DOCK10, DOCK2, DOCK8, DOK2, DPEP1, DPEP2, DPT, DRC1, DRD1, DTHD1, DUSP2, DUSP8, E2F8, EAF2, EBI3, EDN2, EGR3, ELAVL4, ELF3, EOMES, EPHA6, EPX, EREG, ERFE, ERMN, EVI2B, FAM122C, FAM83B, FASLG, FATE1, FBXL19, FCGR2C, FCMR, FCN1, FCN3, FCRL1, FCRL2, FCRL3, FCRL5, FCRL6, FCRLA, FFAR4, FGD2, FGD3, FGL2, FGR, FIBIN, FLT3, FOLR2, FOSB, FOSL1, FOXP3, FRZB, FUT7, FYB1, GABBR2, GADD45B, GALNT3, GBP5, GDF6, GFI1, GFRA2, GLIS1, GMFG, GMIP, GNMT, GPM6A, GPR132, GPR141, GPR150, GPR171, GPR174, GPR19, GPR34, GPR4, GPR55, GPR83, GPRC5A, GPSM3, GRAP2,	[AAAS, ABCA3, ABCA7, ABCB11, ABCC8, ABCD1, ABCD2, ABI3, ABL1, ABLIM3, ABR, ABRA, ACACB, ACKR1, ACKR3, ACP4, ACTB, ACTN1, ACTN4, ADAM19, ADAMTS7, ADAMTS8, ADAMTS9, ADAMTSL4, ADCK1, ADCY1, ADCY3, ADCY4, ADCY9, ADGRB2, ADGRE5, ADGRG1, ADGRG6, ADGRL1, ADIPOQ, AGAP2, AGER, AGPAT1, AGT, AGTR1, AKT1S1, ALPK3, AMH, ANGPT1, ANGPTL1, ANGPTL4, ANGPTL8, ANKK1, ANKRD13B, ANKRD52, AP2A1, AP3D1, APLN, APLNR, APLP1, APOA1, APOBEC3C, APOD, APOM, AQP3, ARAF, ARAP3, ARC, AREG, ARF1, ARGFX, ARHGAP1, ARHGAP23, ARHGAP39, ARHGDIA, ARHGEF1, ARHGEF15, ARHGEF16, ARHGEF5, ARID1A, ARID5A, ARMT1, ARRDC3, ARTN, ASB16, ASB6, ASIC3, ASPN, ATAT1, ATF5, ATN1, ATP13A2, ATP1A1, ATP1A3, ATP1B2, ATP1B4, ATP6V0D1, ATP6V0E2, ATP6V1B1, ATP6V1F, ATPSCKMT, ATXN2L, BAG3, BAG6, BAHD1, BATF2, BAZ2A, BBS12, BCAM, BCAR1, BCKDK, BCL2L1, BCL3, BCL6, BCL7B, BCL9L, BCOR, BCR, BEND6, BHLHE40, BMP8A, BRD4, BRPF3, BSN, BTG2, BTLA, C11orf65, C15orf62, C18orf54, C1QTNF1, C20orf27, C2CD2L, C5, C5AR1, CABIN1, CABLES2, CACNA1E, CACNA1H, CACNB4, CALB1, CALCR, CAMK2B, CAMSAP3, CAMTA2, CAPN1, CAPNS1, CARM1, CARMIL2, CASKIN1, CASZ1, CBARP, CC2D1A, CCDC106, CCL11, CCL2, CCL21, CCL24, CCNG2, CCNJL, CCR7, CD14, CD177, CD1C, CD1E, CD276, CD74, CDC25B, CDC42EP1, CDC42EP2, CDC42EP4, CDCA2, CDH4, CDK1, CDK15, CDK18, CDK2, CDK2AP2, CDKL2, CDKN1A, CEACAM1, CEL, CELSR2, CERT1, CETP, CFAP298-TCP10L, CFH, CFL1, CGA, CHCHD10, CHERP, CHGA, CHGB, CHI3L1, CHRNG, CIC, CIITA, CISH, CITED2, CITED4, CIZ1, CLC, CLCF1, CLCN6, CLCNKA, CLEC1B, CLEC4E, CLIP3, CLNK, CLPTM1, CMTM1, CMTM5, CNKSR1, CNN1, CNOT3, CNR1, CNTN6, COL1A1, CPLX1, CPNE5, CPNE6, CPSF7, CPTP, CRAT, CREB3L1, CRHR2, CRIP2, CRP, CRTC2, CRYAB, CSDC2, CSF1, CSK, CSNK1E, CSPG4, CSRNP3, CST7, CTC1, CTDSP1, CTF1, CTHRC1,

GO:0007165	signal transduction	1,01E-19	[2, 3, 4]	1126,00	17,01	42,72	57,28	[ABRA, ADAM8, ADCY7, ADGRE3, ADGRE4P, ADGRG5, ADGRG6, ADORA3, AIF1, AIM2, ALOX15, ALOX15B, ANGPT1, ANGPT2, ANPEP, APBB1IP, ARAP2, AREG, ARHGAP15, ARHGAP30, ARHGAP45, ASGR2, ATF3, ATP2A3, BANK1, BATF, BCL11B, BCL2L14, BCL3, BHLHA15, BLK, BLNK, BMF, BMP7, BMPR1B, BRIP1, BTG2, BTK, BTLA, BTN3A1, BTN3A2, C1QTNF1, CACNA1I, CALCR, CAMK4, CARD11, CASKIN1, CASP8, CASS4, CCDC88C, CCL17, CCL19, CCL2, CCL22, CCL24, CCL3, CCL3L1, CCL4, CCL4L1, CCL4L2, CCL5, CCR2, CCR3, CCR4, CCR5, CCR8, CD177, CD180, CD19, CD2, CD200R1, CD22, CD226, CD244, CD247, CD27, CD300LF, CD33, CD3D, CD3E, CD3G, CD4, CD40LG, CD5, CD53, CD6, CD7, CD74, CD79A, CD80, CD86, CD8A, CD8B, CDKN2A, CHRDL1, CISH, CLCF1, CLEC10A, CLEC1B, CLEC4D, CLEC4E, CLEC6A, CLNK, CMTM5, CNR1, CNR2, CORO1A, CORO2A, CR2, CRABP2, CRB2, CREM, CSF1R, CSF2RA, CTLA4, CTSS, CXCL10, CXCL11, CXCL5, CXCL6, CXCL9, CXCR3, CXCR4, CXCR6, CXorf21, CYBB, CYSLTR1, DAPK1, DAPP1, DCC, DEPDC1B, DERL3, DLGAP2, DOCK10, DOCK2, DOCK8, DOK2, DPEP2, DRD1, DTHD1, DUSP2, DUSP8, E2F8, EBI3, EDN2, ELF3, EPHA6, EPX, EREG, ERFE, FAM83B, FASLG, FCGR2C, FCN1, FCRL1, FCRL2, FCRL3, FCRL5, FCRL6, FCRLA, FFAR4, FGD2, FGD3, FGR, FIBIN, FLT3, FOLR2, FOXP3, FRZB, FUT7, FYB1, GABBR2, GADD45B, GALNT3, GDF6, GFI1, GFRA2, GMFG, GMIP, GPR132, GPR141, GPR150, GPR171, GPR174, GPR19, GPR34, GPR4, GPR55, GPR83, GPRC5A, GRAP2, GUCY2D, GZMA, GZMB, H3C10, H3C11, H3C12, H3C7, HASPIN, HCLS1, HCST, HLA-DPA1, HLA-DPB1, HLA-DQA1, HLA-DRA, HLA-DRB1, HLA-DRB5, HORMAD1, HSH2D, ICOS, IER3, IFNG, IGLL5, IKBKE, IL10RA, IL12RB1, IL12RB2, IL16, IL18, IL18RAP, IL21R, IL22, IL26, IL2RB, IL2RG, IL5RA, IL7R, IL9R, INPP5D, IQCJ-SCHIP1, IQGAP2, IRF4, IRF5, IRF8, ITGA4, ITGAL, ITGAX, ITGB2, ITGB7, ITK, JPH1, JSRP1, KCNN4, KLF5, KLRB1, KLRC1, KLRC2, KLRD1, KLRG1, KLRK1, KNG1, LAG3, LAX1, LCK	[ABCA7, ABL1, ABR, ABRA, ACKR1, ACKR3, ACP4, ACTB, ACTN4, ADCY1, ADCY3, ADCY4, ADCY9, ADGRB2, ADGRE5, ADGRG1, ADGRG6, ADGRL1, ADIPOQ, AGAP2, AGER, AGPAT1, AGT, AGTR1, AKT1S1, ALPK3, AMH, ANGPT1, ANGPTL1, ANGPTL8, AP2A1, APLN, APLNR, APLP1, APOA1, APOD, ARAF, ARAP3, ARC, AREG, ARF1, ARHGAP1, ARHGAP23, ARHGAP39, ARHGDIA, ARHGEF1, ARHGEF16, ARHGEF5, ARID1A, ARRDC3, ARTN, ASB16, ASB6, ASIC3, ASPN, ATP6V0D1, ATP6V0E2, ATP6V1B1, ATP6V1F, BAG3, BAG6, BCAM, BCAR1, BCL2L1, BCL3, BCL6, BCL7B, BCL9L, BCR, BEND6, BMP8A, BRD4, BSN, BTG2, BTLA, C15orf62, C1QTNF1, C20orf27, C5, C5AR1, CABIN1, CALCR, CAMK2B, CAPN1, CARM1, CASKIN1, CC2D1A, CCL11, CCL2, CCL21, CCL24, CCR7, CD14, CD177, CD276, CD74, CDC42EP1, CDC42EP2, CDC42EP4, CDK1, CDK2, CDKL2, CDKN1A, CEACAM1, CELSR2, CERT1, CETP, CFL1, CGA, CHCHD10, CHERP, CHGA, CHGB, CHI3L1, CHRNG, CIITA, CISH, CITED2, CLCF1, CLCN6, CLEC1B, CLEC4E, CLIP3, CLNK, CMTM1, CMTM5, CNKSR1, CNOT3, CNR1, CNTN6, COL1A1, CRAT, CREB3L1, CRHR2, CRYAB, CSF1, CSK, CSNK1E, CSPG4, CTF1, CTHRC1, CX3CL1, CXXC4, CYP26B1, CYP27B1, DAB2IP, DAG1, DAGLA, DAXX, DCHS1, DDR1, DDX39B, DDX54, DEDD, DENND2B, DENND4B, DGKD, DGKZ, DHX34, DIRAS1, DISP2, DISP3, DKK2, DLG5, DLGAP4, DMTN, DNMT1, DOCK6, DOK3, DOK5, DOT1L, DPF2, DTX1, DTX2, DTX3, DUSP5, DUSP8, DVL3, E2F4, E2F7, E2F8, ECE2, ECM1, EDN2, EEF1E1, EFNA3, EGLN2, EIF4EBP1, ELF3, ENG, EPHA2, EPN1, ERBB2, ERFE, ESM1, EXTL1, FADD, FAIM2, FAM110A, FAM83F, FAM83G, FASN, FES, FFAR2, FGFR4, FGFRL1, FIBIN, FLOT2, FLT3, FLT4, FOXC2, FOXF1, FOXM1, FPR1, FRMD8, FURIN, GAB2, GABBR2, GABRR2, GADD45B, GAS1, GATA4, GBA, GBF1, GCSAM, GDF6, GDI1, GFRA3, GIT1, GLIS2, GLRA1, GNAI2, GNAO1, GNB3, GNRHR, GPAM, GPBAR1, GPER1, GPI, GPR137, GPR153, GPR162, GPR17, GPR20, GPR3, GPR34, GPR37L1, GPR4, GPR75, GPR83
GO:0010646	regulation of cell communication	2,64E-15	[3, 4]	671,00	18,23	37,26	62,74	[ABRA, ADAM8, ALOX15, ALOX15B, ANGPT1, ARAP2, AREG, ARHGAP15, ARHGAP30, ARHGAP45, ATF3, BANK1, BCL2L14, BCL3, BLK, BMF, BMP7, BMPR1B, BRIP1, C1QTNF1, CACNA1E, CALCR, CARD11, CASP8, CASS4, CCDC88C, CCL17, CCL19, CCL2, CCL22, CCL24, CCL3, CCL3L1, CCL4, CCL4L1, CCL5, CCR2, CD177, CD180, CD19, CD22, CD226, CD27, CD300LF, CD3E, CD4, CD40LG, CD74, CD80, CD86, CDH1, CDKN2A, CEL, CHRDL1, CISH, CLEC6A, CNR1, CNR2, CRB2, CSF1R, CXCR4, CXorf21, DAPK1, DCC, DEPDC1B, DLGAP2, DPEP2, DRD1, DUSP2, DUSP8, ELAVL4, ELF3, EPX, EREG, ERFE, FASLG, FCRL3, FFAR4, FGD2, FGD3, FGR, FLT3, FOLR2, FOXP3, FRZB, FUT7, GADD45B, GDF6, GFI1, GMIP, GPR174, GPR4, GPR55, GPRC5A, GUCY2D, H3C10, H3C11, H3C12, H3C7, HCLS1, HCST, HLA-DRB1, ICOS, IER3, IFNG, IKBKE, IL10RA, IL18, IL26, IL7R, INPP5D, IQCJ-SCHIP1, IRF4, IRX3, KCNJ10, KCNN4, KMO, LAX1, LCK, LEF1, LIF, LILRB4, LMCD1, LY86, MAP4K1, MIDN, MMP9, MSX1, MYB, MZB1, NCF1, NCKAP1L, NFAM1, NLRC3, NLRP3, NLRP6, NPFFR2, NPPC, NPTX2, NR4A2, NSG1, NTN1, OASL, P2RY10, P2RY12, PAX5, PDE6G, PIK3CG, PIK3IP1, PIK3R5, PIM2, PLAU, PLEK, PRDM1, PRKCB, PRKCQ, PROK1, PSCA, PSD4, PTK2B, PTPN22, PTPN6, PTPRC, PYCARD, PYHIN1, RAC2, RAD9B, RASAL3, RASGRF1, RBPMS2, RGS1, RGS10, RGS18, RGS9, RHOH, RMI2, RNASE6, RRAD, RTKN2, SALL1, SCIMP, SCUBE1, SEMA4D, SH2D1A, SHISA8, SLA, SLA2, SLC24A4, SLC8A3, SMPDL3B, SOCS1, SOCS3, SPINK1, STAP1, STAT1, SYK, TAGAP, TBC1D10C, TESPA1, THBS1, THY1, TLR6, TMC8, TNFAIP3, TNFSF14, TNIP3, TNMD, TNR, TRABD2A, TRAF1, TRAT1, TREM2, TRIM14, TRPM2, UBASH3A, UBD, UCP2, VAV1, VNN1, VWC2L, WNT1, WNT10B, XCL1, XCL2, ZAP70, ZBP1]	[ABCA7, ABCC8, ABL1, ABR, ABRA, ACKR3, ACP4, ACTN4, ADCY1, ADGRG1, ADIPOQ, AGAP2, AGER, AGPAT1, AGT, AGTR1, AKT1S1, AMH, ANGPT1, APLN, APLNR, APLP1, APOA1, APOD, ARAF, ARAP3, ARC, AREG, ARF1, ARHGAP1, ARHGAP23, ARHGAP39, ARHGDIA, ARHGEF1, ARHGEF16, ARHGEF5, ARRDC3, ARTN, ASPN, BCL2L1, BCL3, BCL6, BCL9L, BCR, BEND6, BMP8A, BRD4, C1QTNF1, C20orf27, C2CD2L, C5, C5AR1, CACNA1E, CALB1, CALCR, CAMK2B, CAPN1, CARM1, CC2D1A, CCL11, CCL2, CCL21, CCL24, CCR7, CD14, CD177, CD74, CDK1, CDK2, CEACAM1, CEL, CGA, CHERP, CHGA, CHI3L1, CISH, CITED2, CLIP3, CNR1, CNTN6, COL1A1, CPLX1, CRAT, CREB3L1, CRHR2, CSF1, CSK, CSNK1E, CSPG4, CTHRC1, CX3CL1, CXXC4, CYP26B1, CYP27B1, DAB2IP, DAG1, DAXX, DDX39B, DENND2B, DENND4B, DGKD, DGKZ, DHX34, DIRAS1, DKK2, DLG5, DLGAP4, DMTN, DOC2B, DOK5, DOT1L, DTX1, DUSP5, DUSP8, DVL3, ECM1, EEF1E1, ELF3, ENG, EPHA2, EPN1, ERBB2, ERFE, ESM1, FADD, FAIM2, FAM110A, FASN, FFAR2, FGFR4, FLOT2, FLT3, FLT4, FOXM1, FPR1, FRMD8, FURIN, GAB2, GADD45B, GAS1, GATA4, GBA, GBF1, GCSAM, GDF6, GIT1, GJD3, GLIS2, GLRA1, GNAI2, GPAM, GPBAR1, GPER1, GPI, GPR137, GPR17, GPR20, GPR37L1, GPR4, GPRC5A, GPRIN1, GRB7, GRIK5, GRINA, GRM2, GSK3A, H3C10, H3C12, H4C3, HBEGF, HCRTR1, HDAC7, HEYL, HFE, HGS, HIPK4, HSPB1, HTR1B, HTR2A, ICAM1, IER3, IFT80, IL18BP, IL1B, IL1RN, IL7R, ILK, INCA1, INHA, INPP5E, IRAK2, IRX3, ITGA3, ITGA5, ITPR3, ITPRIP, JAK3, JMJD8, JPH4, JUP, KCP, KCTD11, KIF26A, KLK14, KMT2D, KSR1, LGALS9, LGR6, LIF, LIFR, LILRA5, LIMS2, LMCD1, LMNA, LOX, LRG1, LRRC4, LRRC8A, LTBR, LTF, LY6E, LYNX1, LZTS1, LZTS2, MAP1A, MAP2K3, MAP3K10, MAP3K11, MAP3K14, MAP3K6, MAP3K9, MAPK3, MAPK7, MAPK8IP3, MAVS, MAZ, MECP2, MED12, MEGF8, MEIS3P1, MEN1, MGRN1, MIDN, MINK1, MLST8, MME, MNT, MSTN, MSX1, MTRNR2L1, MTRNR2L10, MTRNR2L12, MTRNR2L2, MTRNR2L3, MTRNR2L6, MTRNR2L8, MTRNR2L9,

GO:0007166	cell surface receptor signaling pathway	1,32E-23	[3, 4, 5]	662,00	19,60	47,87	52,13	[ADGRE3, ADGRE4P, ADGRG5, ADGRG6, ADORA3, AIM2, ALOX15, ANGPT1, ANGPT2, AREG, ASGR2, ATF3, BATF, BCL2L14, BCL3, BLK, BLNK, BMP7, BMPR1B, BTK, BTLA, BTN3A1, BTN3A2, CALCR, CARD11, CASP8, CASS4, CCDC88C, CCL17, CCL19, CCL2, CCL22, CCL24, CCL3, CCL3L1, CCL4, CCL4L1, CCL5, CCR2, CCR3, CCR4, CCR5, CCR8, CD177, CD180, CD19, CD2, CD200R1, CD22, CD226, CD247, CD27, CD300LF, CD3D, CD3E, CD3G, CD4, CD40LG, CD6, CD7, CD74, CD79A, CD80, CD86, CD8A, CD8B, CHRDL1, CISH, CLCF1, CLEC10A, CLEC1B, CLEC4D, CLEC4E, CLEC6A, CLNK, CORO1A, CR2, CRB2, CSF1R, CSF2RA, CTLA4, CXCL10, CXCL11, CXCL5, CXCL6, CXCL9, CXCR3, CXCR4, CXCR6, CYBB, CYSLTR1, DAPK1, DCC, DEPDC1B, DLGAP2, DOK2, DPEP2, DRD1, EBI3, EDN2, ELF3, EPHA6, EPX, EREG, ERFE, FAM83B, FASLG, FCGR2C, FCN1, FCRL1, FCRL2, FCRL3, FCRL5, FCRL6, FCRLA, FGR, FLT3, FOLR2, FOXP3, FRZB, FUT7, FYB1, GALNT3, GDF6, GFI1, GFRA2, GPR171, GPR34, GPRC5A, GRAP2, GUCY2D, H3C10, H3C11, H3C12, H3C7, HCLS1, HLA-DPA1, HLA-DPB1, HLA-DQA1, HLA-DRA, HLA-DRB1, HLA-DRB5, IFNG, IGLL5, IKBKE, IL10RA, IL12RB1, IL12RB2, IL16, IL18, IL18RAP, IL21R, IL22, IL26, IL2RB, IL2RG, IL5RA, IL7R, IL9R, INPP5D, IRF4, IRF5, IRF8, ITGA4, ITGAL, ITGAX, ITGB2, ITGB7, ITK, KCNN4, KLRB1, KLRC1, KLRC2, KLRD1, KLRG1, LAG3, LAX1, LCK, LCP1, LCP2, LEF1, LIF, LILRA1, LILRB1, LILRB4, LTA, LTB, LY86, LYPD6B, MATK, MMP9, MOG, MS4A1, MSX1, MUC16, MUC19, MUC6, MUSK, MYO1G, MZB1, NAMPT, NCF1, NCKAP1L, NCR3, NELL2, NFAM1, NLRP3, NPPB, NPPC, NPTX2, NR4A2, NR4A3, NTN1, OASL, P2RY10, P2RY12, P2RY13, P2RY2, PAX5, PDE6G, PIM1, PLCB2, PLEK, PLXNC1, PRAM1, PRKCB, PRKCQ, PSTPIP1, PTAFR, PTK2B, PTPN22, PTPN6, PTPRC, PYCARD, RASGRF1, RBPMS2, RGS9, RHEX, SALL1, SCIMP, SCUBE1, SDC1, SEMA4A, SEMA4D, SH2D1A, SH2D2A, SH2D6, SHISA8, SKAP1, SLA, SLA2, SLC8A3, SMPD3, SOCS1, SOCS3, SPN, STAP1, STAT1, STYK1, SYK, TESPA1, THBS1	[ABCA7, ABL1, ACKR1, ACKR3, ACP4, ACTB, ACTN4, ADGRB2, ADGRE5, ADGRG1, ADGRG6, ADGRL1, ADIPOQ, AGAP2, AGER, AGPAT1, AGT, AKT1S1, AMH, ANGPT1, ANGPTL1, AP2A1, APLN, APOA1, APOD, ARC, AREG, ARF1, ARHGDIA, ASPN, ATP6V0D1, ATP6V0E2, ATP6V1B1, ATP6V1F, BAG3, BAG6, BCAR1, BCL2L1, BCL3, BCL6, BCL7B, BCL9L, BCR, BEND6, BMP8A, BTLA, C20orf27, C5, C5AR1, CABIN1, CALCR, CAMK2B, CAPN1, CCL11, CCL2, CCL21, CCL24, CCR7, CD14, CD177, CD276, CD74, CDKN1A, CEACAM1, CELSR2, CFL1, CHRNG, CIITA, CISH, CITED2, CLCF1, CLEC1B, CLEC4E, CLIP3, CLNK, CNKSR1, CNTN6, COL1A1, CREB3L1, CRHR2, CSF1, CSK, CSNK1E, CSPG4, CTF1, CTHRC1, CX3CL1, CXXC4, DAB2IP, DAGLA, DAXX, DDR1, DEDD, DGKD, DGKZ, DISP2, DISP3, DKK2, DLG5, DLGAP4, DMTN, DOK5, DOT1L, DTX1, DTX2, DTX3, DVL3, ECM1, EDN2, EFNA3, ELF3, ENG, EPHA2, EPN1, ERBB2, ERFE, ESM1, FADD, FAM83G, FASN, FES, FFAR2, FGFR4, FGFRL1, FLT3, FLT4, FOXC2, FOXF1, FOXM1, FPR1, FRMD8, FURIN, GAB2, GABRR2, GAS1, GATA4, GCSAM, GDF6, GFRA3, GIT1, GLIS2, GLRA1, GNAI2, GNAO1, GPBAR1, GPER1, GPI, GPR17, GPR34, GPR37L1, GPR75, GPRC5A, GPRIN1, GRB7, GRIK5, GRIP2, GRM2, GSK3A, H3C10, H3C12, HBEGF, HESX1, HEYL, HFE, HGS, HSF1, HSPB1, ICAM1, IFITM1, IFITM2, IFITM3, IFT80, IL15RA, IL18BP, IL1B, IL1RN, IL22RA1, IL32, IL3RA, IL4R, IL5RA, IL7R, ILK, INHA, INPPL1, IQUB, IRAK2, IRF2, IRF9, ITGA3, ITGA5, ITGA7, ITGAD, ITPRIP, JAK3, JUNB, JUP, KCP, LAG3, LAMA5, LGR6, LIF, LIFR, LILRB5, LIMK1, LIMS2, LMNA, LOX, LRG1, LRRC32, LTBR, LTF, LY6E, LYNX1, LZTS2, MAP3K10, MAP3K14, MAPK3, MAPK7, MARK2, MARK4, MAVS, MECP2, MED12, MEGF8, MEN1, MGRN1, MINK1, MKNK2, MOV10, MSN, MSTN, MSX1, MUC19, MUC20, MUC3A, MYC, MYH9, NCOR2, NDP, NDST1, NECTIN2, NEFL, NF2, NFATC4, NGF, NGFR, NKD2, NLGN2, NLRC5, NOD2, NODAL, NOS3, NOTCH1, NOTCH3, NOTCH4, NPPA, NPPB, NR4A2, NR4A3, NRARP, NTN1, NUMBL, OMA1, OSM
GO:0009966	regulation of signal transduction	1,31E-14	[3, 4, 5]	609,00	18,48	37,99	62,01	[ABRA, ADAM8, ALOX15, ALOX15B, ANGPT1, ARAP2, AREG, ARHGAP15, ARHGAP30, ARHGAP45, ATF3, BANK1, BCL2L14, BCL3, BLK, BMF, BMP7, BMPR1B, BRIP1, C1QTNF1, CALCR, CARD11, CASP8, CASS4, CCDC88C, CCL17, CCL19, CCL2, CCL22, CCL24, CCL3, CCL3L1, CCL4, CCL4L1, CCL5, CCR2, CD177, CD180, CD19, CD22, CD226, CD27, CD300LF, CD3E, CD4, CD40LG, CD74, CD80, CD86, CDKN2A, CHRDL1, CISH, CLEC6A, CRB2, CSF1R, CXCR4, CXorf21, DAPK1, DCC, DEPDC1B, DLGAP2, DPEP2, DUSP2, DUSP8, ELF3, EPX, EREG, ERFE, FASLG, FCRL3, FFAR4, FGD2, FGD3, FGR, FLT3, FOLR2, FOXP3, FRZB, FUT7, GADD45B, GDF6, GFI1, GMIP, GPR174, GPR4, GPR55, GPRC5A, GUCY2D, H3C10, H3C11, H3C12, H3C7, HCLS1, HCST, HLA-DRB1, ICOS, IER3, IFNG, IKBKE, IL10RA, IL18, IL26, IL7R, INPP5D, IQCJ-SCHIP1, IRF4, KCNN4, LAX1, LCK, LEF1, LIF, LILRB4, LMCD1, LY86, MAP4K1, MMP9, MSX1, MZB1, NCF1, NCKAP1L, NFAM1, NLRC3, NLRP3, NLRP6, NPFFR2, NPPC, NPTX2, NR4A2, NTN1, OASL, P2RY10, P2RY12, PAX5, PDE6G, PIK3CG, PIK3IP1, PIK3R5, PIM2, PLAU, PLEK, PRDM1, PRKCB, PRKCQ, PROK1, PSCA, PSD4, PTK2B, PTPN22, PTPN6, PTPRC, PYCARD, PYHIN1, RAC2, RAD9B, RASAL3, RASGRF1, RBPMS2, RGS1, RGS10, RGS18, RGS9, RHOH, RMI2, RNASE6, RRAD, RTKN2, SALL1, SCIMP, SCUBE1, SEMA4D, SH2D1A, SHISA8, SLA, SLA2, SLC8A3, SMPDL3B, SOCS1, SOCS3, SPINK1, STAP1, STAT1, SYK, TAGAP, TBC1D10C, TESPA1, THBS1, THY1, TLR6, TMC8, TNFAIP3, TNFSF14, TNIP3, TNMD, TRABD2A, TRAF1, TRAT1, TREM2, TRIM14, UBASH3A, UBD, VAV1, VNN1, VWC2L, WNT1, WNT10B, XCL1, XCL2, ZAP70, ZBP1]	[ABCA7, ABL1, ABR, ABRA, ACKR3, ACP4, ACTN4, ADGRG1, ADIPOQ, AGAP2, AGER, AGPAT1, AGT, AKT1S1, AMH, ANGPT1, APLN, APLNR, APLP1, APOA1, APOD, ARAF, ARAP3, ARC, AREG, ARHGAP1, ARHGAP23, ARHGAP39, ARHGDIA, ARHGEF1, ARHGEF16, ARHGEF5, ARRDC3, ARTN, ASPN, BCL2L1, BCL3, BCL6, BCL9L, BCR, BEND6, BMP8A, BRD4, C1QTNF1, C20orf27, C5, C5AR1, CALCR, CAMK2B, CAPN1, CARM1, CC2D1A, CCL11, CCL2, CCL21, CCL24, CCR7, CD14, CD177, CD74, CDK1, CDK2, CEACAM1, CGA, CHERP, CHGA, CHI3L1, CISH, CITED2, CLIP3, CNTN6, COL1A1, CRAT, CREB3L1, CSF1, CSK, CSNK1E, CSPG4, CTHRC1, CX3CL1, CXXC4, CYP26B1, CYP27B1, DAB2IP, DAG1, DAXX, DDX39B, DENND2B, DENND4B, DGKD, DGKZ, DIRAS1, DKK2, DLG5, DLGAP4, DMTN, DOK5, DOT1L, DTX1, DUSP5, DUSP8, DVL3, ECM1, EEF1E1, ELF3, ENG, EPHA2, EPN1, ERBB2, ERFE, ESM1, FADD, FAIM2, FAM110A, FASN, FGFR4, FLOT2, FLT3, FLT4, FOXM1, FPR1, FRMD8, FURIN, GAB2, GADD45B, GAS1, GATA4, GBA, GBF1, GCSAM, GDF6, GIT1, GLIS2, GNAI2, GPAM, GPBAR1, GPER1, GPI, GPR137, GPR17, GPR20, GPR37L1, GPR4, GPRC5A, GPRIN1, GRB7, GRIK5, GRINA, GRM2, GSK3A, H3C10, H3C12, H4C3, HBEGF, HCRTR1, HDAC7, HEYL, HFE, HGS, HIPK4, HSPB1, HTR1B, HTR2A, ICAM1, IER3, IFT80, IL18BP, IL1B, IL1RN, IL7R, ILK, INCA1, INHA, INPP5E, IRAK2, ITGA3, ITGA5, ITPRIP, JAK3, JMJD8, JUP, KCP, KCTD11, KIF26A, KLK14, KMT2D, KSR1, LGALS9, LGR6, LIF, LIFR, LILRA5, LIMS2, LMCD1, LMNA, LOX, LRG1, LTBR, LTF, LY6E, LYNX1, LZTS2, MAP2K3, MAP3K10, MAP3K11, MAP3K14, MAP3K6, MAP3K9, MAPK3, MAPK7, MAPK8IP3, MAVS, MAZ, MED12, MEGF8, MEIS3P1, MEN1, MGRN1, MINK1, MLST8, MNT, MSTN, MSX1, MTRNR2L1, MTRNR2L10, MTRNR2L12, MTRNR2L2, MTRNR2L3, MTRNR2L6, MTRNR2L8, MTRNR2L9, MUC20, MYADM, MYC, NCLN, NCOR2, NDC80, NDST1, NECTIN2, NEFL, NF2, NFATC4, NGF, NGFR, NINJ1, NKD2, NLGN2, NLRC5, NOD2, NODAL, NOP2, NOS3, NOTCH1, NOTCH3, NOTCH4,

GO:0035556	intracellular signal transduction	2,94E-19	[3, 4, 5]	582,00	19,50	41,01	58,99	[ABRA, ADAM8, ADCY7, ADGRG6, AIF1, ALOX15, ANGPT1, ARAP2, AREG, ARHGAP15, ARHGAP30, ARHGAP45, ATF3, ATP2A3, BANK1, BATF, BCL11B, BCL3, BHLHA15, BLK, BLNK, BMF, BMP7, BRIP1, BTG2, BTK, C1QTNF1, CALCR, CAMK4, CARD11, CASP8, CASS4, CCDC88C, CCL17, CCL19, CCL2, CCL22, CCL24, CCL3, CCL3L1, CCL4, CCL4L1, CCL5, CCR2, CCR3, CCR4, CCR5, CCR8, CD19, CD200R1, CD22, CD27, CD3E, CD4, CD40LG, CD74, CD80, CD86, CDKN2A, CISH, CLEC6A, CLNK, CORO2A, CSF1R, CSF2RA, CXCR3, CXCR4, CXCR6, DAPK1, DEPDC1B, DOCK10, DOCK2, DOCK8, DOK2, DUSP2, DUSP8, E2F8, EDN2, EPX, EREG, FASLG, FCRL3, FFAR4, FGD2, FGD3, FGR, FIBIN, FLT3, GADD45B, GDF6, GFRA2, GMIP, GPR174, GPR4, GPR55, GRAP2, GUCY2D, HASPIN, HCLS1, HCST, HLA-DRB1, HORMAD1, ICOS, IER3, IKBKE, IL18, IL26, IL2RB, IL2RG, IL5RA, INPP5D, IQCJ-SCHIP1, ITK, JPH1, JSRP1, KCNN4, KLRG1, LAX1, LCK, LCP1, LCP2, LIF, LILRB4, LMCD1, MAP4K1, MAPK13, MCOLN2, MCOLN3, MMP9, MSX1, MYZAP, NCF1, NCKAP1L, NFAM1, NLRC3, NLRP3, NLRP6, NMUR1, NPFFR2, NPPB, NPPC, NR4A3, NTN1, OASL, P2RY10, P2RY12, PDE6G, PIK3C2G, PIK3CG, PIK3IP1, PIK3R5, PIM2, PLCB2, PLCH2, PLEK, PRDM1, PRKCB, PRKCQ, PROK1, PSCA, PSD4, PTAFR, PTGFR, PTK2B, PTPN22, PTPN6, PTPN7, PTPRC, PYCARD, PYHIN1, RAB15, RAB39B, RAC2, RAD9B, RASAL3, RASGRF1, RGS9, RHOH, RMI2, RNASE6, RRAD, RTKN2, SCIMP, SELE, SEMA4D, SH2D6, SIRPG, SLA2, SMPD3, SOCS1, SOCS3, SPINK1, STAT1, STK17B, SYK, TAGAP, TBC1D10C, THBS1, TIFAB, TLR6, TLR8, TNFAIP3, TNFSF13B, TNFSF14, TNIP3, TRAF1, TRAT1, TREM2, TRIM14, TRPM2, UBD, VAV1, VNN1, WAS, WNT1, XCL1, XCL2, XCR1, ZAP70, ZNF385B]	[ABCA7, ABL1, ABR, ABRA, ACKR3, ACTN4, ADCY1, ADCY3, ADCY4, ADCY9, ADGRB2, ADGRG1, ADGRG6, ADGRL1, ADIPOQ, AGAP2, AGER, AGT, AGTR1, AKT1S1, ALPK3, AMH, ANGPT1, APLNR, APOA1, ARAF, ARAP3, AREG, ARHGAP1, ARHGAP23, ARHGAP39, ARHGDIA, ARHGEF1, ARHGEF16, ARHGEF5, ARTN, ASB16, ASB6, BAG6, BCL2L1, BCL3, BCL6, BCR, BRD4, BTG2, C15orf62, C1QTNF1, C20orf27, C5, C5AR1, CALCR, CAMK2B, CARM1, CC2D1A, CCL11, CCL2, CCL21, CCL24, CCR7, CD14, CD74, CDC42EP1, CDC42EP2, CDC42EP4, CDK1, CDK2, CDKN1A, CEACAM1, CFL1, CHERP, CHI3L1, CISH, CLNK, CNOT3, CRAT, CREB3L1, CRYAB, CSF1, CSK, CSPG4, CX3CL1, DAB2IP, DAG1, DAXX, DCHS1, DDX39B, DENND2B, DENND4B, DGKD, DGKZ, DHX34, DIRAS1, DLG5, DMTN, DNMT1, DOCK6, DOK3, DOK5, DOT1L, DUSP5, DUSP8, DVL3, E2F4, E2F7, E2F8, ECM1, EDN2, EEF1E1, EIF4EBP1, ENG, EPHA2, ERBB2, FADD, FAM110A, FASN, FGFR4, FIBIN, FLT3, FLT4, FOXM1, FPR1, GAB2, GADD45B, GATA4, GBA, GBF1, GDF6, GDI1, GFRA3, GNAI2, GPBAR1, GPER1, GPI, GPR137, GPR17, GPR20, GPR37L1, GPR4, GPRIN1, GRIK5, GRINA, GRM2, GSK3A, HBEGF, HCRTR1, HDAC7, HESX1, HGS, HIPK4, HSF1, HSPB1, HTR2A, HUNK, ICAM1, IER3, IL1B, IL3RA, IL5RA, ILK, INPP5E, IRAK2, ITGA3, ITPR3, JAK3, JMJD8, JPH4, KCTD11, KSR1, LGALS9, LIF, LILRA5, LIMK1, LMCD1, LOX, LTBR, LTF, MAP2K3, MAP3K10, MAP3K11, MAP3K14, MAP3K6, MAP3K9, MAPK3, MAPK4, MAPK7, MAPK8IP3, MARK2, MARK4, MAST2, MAVS, MAZ, MDC1, MED12, MEIS3P1, MEN1, MGRN1, MINK1, MKNK2, MLST8, MSTN, MSX1, MUC20, MYADM, MYBBP1A, MYC, MYZAP, NDC80, NDST1, NEFL, NF2, NFATC4, NFKB2, NGF, NGFR, NKIRAS2, NLRC5, NOD2, NODAL, NOP2, NOS3, NOTCH1, NPFFR2, NPPA, NPPB, NPR3, NPRL3, NR1D1, NR4A3, NTN1, NUCB1, OBSCN, OMA1, OSM, P2RX3, P2RX5, P2RY6, PAK4, PDE4A, PDGFB, PDGFRB, PDK2, PEAR1, PER1, PI4KA, PI4KAP1, PI4KAP2, PIAS4, PIDD1, PIK3C2B, PIK3C2G, PIP5K1C, PISD, PITPNM2
GO:0012501	programmed cell death	6,56E-06	[3]	393,00	17,85	39,40	60,60	[ADAM8, AIF1, AIM2, ALOX15B, ANGPT1, AREG, ATF3, ATP2A3, BCL11B, BCL2L14, BCL2L15, BCL3, BLK, BMF, BMP7, BMPR1B, BTG2, BTK, CARD11, CARD17, CASP8, CCL19, CCL2, CCL3, CCL5, CD2, CD27, CD3E, CD3G, CD40LG, CD5, CD74, CDKN2A, CEL, CERS3, CLCF1, CNR1, CORO1A, CTLA4, CXCL10, CXCR3, CXCR4, DAPK1, DCC, DOCK8, DPEP1, EAF2, EGR3, EPX, EVI2B, FASLG, FATE1, FCMR, FFAR4, FGD2, FGD3, FLT3, FOSL1, GADD45B, GDF6, GZMA, GZMB, GZMH, GZMM, HCLS1, IER3, IFNG, IKBKE, IKZF3, IL7R, INPP5D, IRF5, ITGA4, ITGB2, KNG1, KRT85, LAMP3, LCK, LEF1, LILRB1, LIPM, LTA, MMP9, MPO, MSX1, MYB, MYBL2, MZB1, NCF1, NCKAP1L, NLRP2, NLRP3, NLRP6, NLRP9, NR4A1, NR4A2, NSG1, NTN1, NUGGC, PIK3CG, PIM1, PIM2, PLAC8, PPP2R2B, PRF1, PRKCB, PRKCQ, PTGFR, PTK2B, PTPN6, PTPRC, PTPRH, PYCARD, RASSF6, RNF122, RTKN2, SEMA4D, SOCS3, ST14, STAT1, STK17B, SYK, TEX11, THBS1, TMC8, TNFAIP3, TNFAIP8L2, TNFRSF11B, TNFRSF18, TNFSF14, TRAF1, TREM2, TSC22D3, TXNDC5, UBD, UCP2, VAV1, VNN1, WNT1, WNT10B, ZBP1, ZNF385B]	[ABL1, ABR, ACKR3, ACTN1, ACTN4, ADAMTSL4, ADIPOQ, AGAP2, AGT, AKT1S1, AMH, ANGPT1, ANGPTL4, APLP1, ARAF, AREG, ARHGDIA, ARHGEF1, ARHGEF16, ARHGEF5, ARTN, ATF5, ATN1, BAG3, BAG6, BCAR1, BCL2L1, BCL3, BCL6, BCL7B, BTG2, C5AR1, CAPN1, CAPNS1, CCL2, CCL21, CCR7, CD14, CD74, CDIP1, CDK1, CDKN1A, CECR2, CEL, CFL1, CHCHD10, CHERP, CHI3L1, CITED2, CLC, CLCF1, CLIP3, CNR1, CRAT, CREB3L1, CRYAB, CSRNP3, CTSD, CX3CL1, CYP26B1, CYP27B1, DAB2IP, DAXX, DEDD, DHRS2, DLG5, DNMT1, DPF2, DSC1, EAF2, EEF1E1, EGLN2, ENDOG, ENG, EPHA2, ESPL1, ETV6, FADD, FAIM2, FAP, FASN, FLG, FLOT2, FLT3, FLT4, FOSL1, FOXC2, FURIN, GADD45B, GAS1, GATA2, GATA4, GBA, GDF6, GNAI2, GPAM, GPER1, GPI, GRIK5, GRINA, GRN, GSK3A, HMGN5, HSF1, HSPB1, HSPE1, HYPK, ICAM1, ID3, IDO1, IER3, IL1B, IL7R, ILK, INCA1, ITGA5, ITPRIP, JAK3, JUP, KDELR1, KLK14, KNG1, LGALS9, LIMS2, LMNA, LOX, LTBR, LTF, MAP1S, MAP3K10, MAP3K11, MAP3K9, MAPK3, MAPK7, MARK4, MAZ, MECP2, MED12, MEF2D, MEIS3P1, MEN1, MFSD10, MIEN1, MKNK2, MNT, MSX1, MTRNR2L1, MTRNR2L10, MTRNR2L12, MTRNR2L2, MTRNR2L3, MTRNR2L6, MTRNR2L8, MTRNR2L9, MYBBP1A, MYC, MYO18A, NEFL, NF2, NFATC4, NGF, NGFR, NINJ1, NLRP9, NOD2, NODAL, NOS3, NOTCH1, NR4A1, NR4A2, NSG1, NSMF, NTN1, OBSCN, OMA1, OSGIN1, PACS2, PAK4, PCDHA4, PCGF2, PDCD1, PDGFRB, PDK2, PIAS4, PIDD1, PIGT, PIM1, PKM, PLAGL2, PLAUR, PLEKHG2, PLEKHN1, PLK3, PML, POR, PPARD, PPL, PPP1R10, PPP1R13L, PPP1R15A, PPP2R1A, PTGFR, PTN, PTPRH, PXT1, RABGGTA, RAMP2, RARA, RASSF5, RELA, RELT, RGCC, RMRP, RNF122, RNF157, RNU1-93P, RPL26, RTKN2, SCAF1, SEPTIN4, SERPINE1, SHARPIN, SHC4, SHISA5, SIK1, SLC35F6, SLC9A1, SLC9A3R1, SMAD3, SMPD1, SNAI1, SOCS3, SOX10, SOX7, SOX8, SPATA2, SPDEF, SPHK1, SPHK2, SPI1, SRC, STEAP3, STK17B, STK40, SYNGAP1, SYVN1, TAF6, TCIRG1, TEX261, TGM2, THBS1, TIAF1, TICAM1, TIMP1,

GO:0010941	regulation of cell death	6,17E-09	[3, 4]	338,00	19,37	39,51	60,49	[ADAM8, AIF1, ANGPT1, AREG, ATF3, BCL11B, BCL2L14, BCL2L15, BCL3, BLK, BMF, BMP7, BMPR1B, BTG2, BTK, C1QA, CALCR, CARD11, CARD17, CASP8, CCL19, CCL2, CCL3, CCL5, CD200R1, CD27, CD3E, CD3G, CD40LG, CD74, CDKN2A, CEL, CLCF1, CLNK, CNR1, CORO1A, CTLA4, CXCL10, CXCR4, DAPK1, DCC, DOCK8, DPEP1, EGR3, EPX, EVI2B, FASLG, FATE1, FCMR, FFAR4, FGD2, FGD3, FLT3, FOSL1, GADD45B, GZMA, HCLS1, IER3, IFNG, IKZF3, IL7R, INPP5D, IRF5, ITGA4, ITGB2, KIR3DL2, KMO, KNG1, LAMP3, LCK, LEF1, LILRB1, LTA, MMP9, MPO, MSX1, MYB, MYBL2, NCKAP1L, NLRP3, NR4A1, NR4A2, NR4A3, NUGGC, PIK3CG, PIM1, PIM2, PLAC8, PRF1, PRKCQ, PTGFR, PTK2B, PTPN6, PTPRC, PYCARD, RASSF6, RNF122, RTKN2, SEMA4D, SOCS3, STAT1, STK17B, SYK, TEX11, THBS1, TLR6, TMC8, TNFAIP3, TNFAIP8L2, TNFRSF18, TNFSF14, TRAF1, TREM2, TRPM2, TSC22D3, TXNDC5, UBD, UCP2, VAV1, VNN1, WNT1, WNT10B, ZBP1]	[ABL1, ABR, ACKR3, ACTN1, ACTN4, ADAMTSL4, ADIPOQ, AGAP2, AGT, AKT1S1, AMH, ANGPT1, ANGPTL4, ARAF, AREG, ARHGDIA, ARHGEF1, ARHGEF16, ARHGEF5, ATF5, ATP13A2, BAG3, BAG6, BCAR1, BCL2L1, BCL3, BCL6, BTG2, C5AR1, CALCR, CCL2, CCL21, CCR7, CD74, CDK1, CDKN1A, CEL, CFL1, CHERP, CHGA, CITED2, CLCF1, CLIP3, CLNK, CNR1, CRAT, CREB3L1, CRYAB, CSF1, CSRNP3, CTSD, CX3CL1, CYP27B1, DAB2IP, DAXX, DEDD, DHRS2, DLG5, DNMT1, EEF1E1, EGLN2, EIF4G1, ELK1, ENDOG, ENG, FADD, FAIM2, FAP, FASN, FLOT2, FLT3, FLT4, FOSL1, FOXC2, GADD45B, GAS1, GATA2, GATA4, GBA, GNAI2, GPAM, GPER1, GPI, GPR37L1, GPR75, GRIK5, GRINA, GRN, GSK3A, HBA1, HBB, HMGN5, HSF1, HSPB1, HYPK, ICAM1, ID3, IDO1, IER3, IL1B, IL7R, ILK, INCA1, ITGA5, ITPRIP, JAK3, KATNB1, KCNK2, KNG1, LGALS9, LIMS2, LMNA, LOX, LTBR, LTF, MAP3K10, MAP3K11, MAP3K9, MAPK3, MAPK7, MARK4, MAZ, MECP2, MED12, MEIS3P1, MEN1, MIEN1, MNT, MSX1, MTCO2P12, MTRNR2L1, MTRNR2L10, MTRNR2L12, MTRNR2L2, MTRNR2L3, MTRNR2L6, MTRNR2L8, MTRNR2L9, MYBBP1A, MYC, MYO18A, NEFL, NF2, NFATC4, NGF, NGFR, NOD2, NOS3, NOTCH1, NPAS2, NR4A1, NR4A2, NR4A3, NSMF, OBSCN, OMA1, OSGIN1, PAK4, PCDHA4, PCGF2, PDCD1, PDGFRB, PIAS4, PIDD1, PIM1, PLAGL2, PLAUR, PLEKHG2, PLEKHN1, PLK3, PM20D1, PML, POR, PPARD, PPP1R10, PTGFR, PTN, PXT1, RABGGTA, RAMP2, RARA, RASSF5, RELA, RGCC, RILPL1, RMRP, RNF122, RNF157, RNU1-93P, RPL26, RTKN2, SCAF1, SEPTIN4, SERPINE1, SIK1, SLC35F6, SLC9A1, SLC9A3R1, SMAD3, SMPD1, SNAI1, SOCS3, SOX10, SOX8, SPATA2, SPDEF, SPHK1, SPHK2, SRC, STAT3, STEAP3, STK17B, STK40, SYNGAP1, SYVN1, TEAD2, TEX261, TGM2, THBS1, TIAF1, TIMP1, TLE5, TMEM109, TMEM14A, TMEM161A, TMEM256-PLSCR3, TMEM259, TNF, TNFRSF1A, TNFSF12, TRAF4, UBA52, UBE2B, UNC5B, VDR, VEGFA, VNN1, VSIG2, WFS1, ZBTB7A, ZC3H12A, ZFP36, ZNF385A, ZNF395]
GO:0006915	apoptotic process	9,62E-07	[4]	373,00	18,29	39,67	60,33	[ADAM8, AIF1, AIM2, ALOX15B, ANGPT1, AREG, ATF3, ATP2A3, BCL11B, BCL2L14, BCL2L15, BCL3, BLK, BMF, BMP7, BMPR1B, BTG2, BTK, CARD11, CARD17, CASP8, CCL19, CCL2, CCL3, CCL5, CD2, CD27, CD3E, CD3G, CD40LG, CD5, CD74, CDKN2A, CEL, CLCF1, CNR1, CORO1A, CTLA4, CXCL10, CXCR3, CXCR4, DAPK1, DCC, DOCK8, DPEP1, EAF2, EGR3, EPX, EVI2B, FASLG, FATE1, FCMR, FFAR4, FGD2, FGD3, FLT3, FOSL1, GADD45B, GDF6, GZMA, GZMB, GZMH, GZMM, HCLS1, IER3, IFNG, IKBKE, IKZF3, IL7R, INPP5D, IRF5, ITGA4, ITGB2, KNG1, LAMP3, LCK, LEF1, LILRB1, LTA, MMP9, MPO, MSX1, MYB, MYBL2, MZB1, NCF1, NCKAP1L, NLRP2, NLRP3, NR4A1, NR4A2, NSG1, NTN1, NUGGC, PIK3CG, PIM1, PIM2, PLAC8, PPP2R2B, PRF1, PRKCB, PRKCQ, PTGFR, PTK2B, PTPN6, PTPRC, PTPRH, PYCARD, RASSF6, RNF122, RTKN2, SEMA4D, SOCS3, STAT1, STK17B, SYK, TEX11, THBS1, TMC8, TNFAIP3, TNFAIP8L2, TNFRSF11B, TNFRSF18, TNFSF14, TRAF1, TREM2, TSC22D3, TXNDC5, UBD, UCP2, VAV1, VNN1, WNT1, WNT10B, ZBP1, ZNF385B]	[ABL1, ABR, ACKR3, ACTN1, ACTN4, ADAMTSL4, ADIPOQ, AGAP2, AGT, AKT1S1, AMH, ANGPT1, ANGPTL4, APLP1, ARAF, AREG, ARHGDIA, ARHGEF1, ARHGEF16, ARHGEF5, ARTN, ATF5, ATN1, BAG3, BAG6, BCAR1, BCL2L1, BCL3, BCL6, BCL7B, BTG2, C5AR1, CCL2, CCL21, CCR7, CD14, CD74, CDIP1, CDK1, CDKN1A, CECR2, CEL, CFL1, CHCHD10, CHERP, CHI3L1, CITED2, CLC, CLCF1, CLIP3, CNR1, CRAT, CREB3L1, CRYAB, CSRNP3, CTSD, CX3CL1, CYP27B1, DAB2IP, DAXX, DEDD, DHRS2, DLG5, DNMT1, DPF2, EAF2, EEF1E1, EGLN2, ENDOG, ENG, EPHA2, ESPL1, ETV6, FADD, FAIM2, FAP, FASN, FLT3, FLT4, FOSL1, FOXC2, GADD45B, GAS1, GATA2, GATA4, GBA, GDF6, GNAI2, GPAM, GPER1, GPI, GRIK5, GRINA, GRN, GSK3A, HMGN5, HSF1, HSPB1, HSPE1, HYPK, ICAM1, ID3, IDO1, IER3, IL1B, IL7R, ILK, INCA1, ITGA5, ITPRIP, JAK3, KDELR1, KNG1, LGALS9, LIMS2, LMNA, LOX, LTBR, LTF, MAP1S, MAP3K10, MAP3K11, MAP3K9, MAPK3, MAPK7, MAZ, MECP2, MED12, MEF2D, MEIS3P1, MEN1, MFSD10, MIEN1, MKNK2, MNT, MSX1, MTRNR2L1, MTRNR2L10, MTRNR2L12, MTRNR2L2, MTRNR2L3, MTRNR2L6, MTRNR2L8, MTRNR2L9, MYBBP1A, MYC, MYO18A, NEFL, NF2, NFATC4, NGF, NGFR, NOD2, NODAL, NOS3, NOTCH1, NR4A1, NR4A2, NSG1, NSMF, NTN1, OBSCN, OMA1, OSGIN1, PACS2, PAK4, PCDHA4, PCGF2, PDCD1, PDGFRB, PDK2, PIAS4, PIDD1, PIGT, PIM1, PLAGL2, PLAUR, PLEKHG2, PLEKHN1, PLK3, PML, POR, PPARD, PPP1R10, PPP1R13L, PPP1R15A, PPP2R1A, PTGFR, PTN, PTPRH, PXT1, RABGGTA, RAMP2, RARA, RASSF5, RELA, RELT, RGCC, RMRP, RNF122, RNF157, RNU1-93P, RPL26, RTKN2, SCAF1, SEPTIN4, SERPINE1, SHARPIN, SHC4, SHISA5, SIK1, SLC35F6, SLC9A1, SLC9A3R1, SMAD3, SMPD1, SNAI1, SOCS3, SOX10, SOX7, SOX8, SPDEF, SPHK1, SPHK2, SPI1, SRC, STEAP3, STK17B, STK40, SYNGAP1, SYVN1, TAF6, TCIRG1, TEX261, TGM2, THBS1, TIAF1, TICAM1, TIMP1, TLE5, TMEM109, TMEM14A, TMEM161A, TMEM214, TMEM256-PLSCR3, TNF, TNFAIP1, TNFRSF1A,
GO:0010942	positive regulation of cell death	3,10E-03	[3, 4, 5]	134,00	20,33	42,48	57,52	[ADAM8, ATF3, BLK, BMF, BMP7, BMPR1B, C1QA, CALCR, CASP8, CCL2, CCL3, CCL5, CD40LG, CDKN2A, CEL, CNR1, CTLA4, DAPK1, FASLG, FGD2, FGD3, FOSL1, GADD45B, GZMA, IFNG, INPP5D, IRF5, ITGA4, ITGB2, KMO, KNG1, LCK, LILRB1, LTA, MMP9, MSX1, MYB, MYBL2, NR4A1, PRF1, PTPRC, PYCARD, RNF122, STK17B, SYK, THBS1, TLR6, TRPM2, UBD, UCP2, VAV1, WNT10B, ZBP1]	[ABL1, ABR, ADAMTSL4, ADIPOQ, AGT, ARHGEF1, ARHGEF16, ARHGEF5, BCL2L1, BCL6, CALCR, CCL2, CDKN1A, CEL, CLIP3, CNR1, CSRNP3, CTSD, CYP27B1, DAB2IP, DAXX, EEF1E1, EIF4G1, ELK1, ENDOG, FADD, FAP, FASN, FOSL1, GADD45B, GPER1, GRIK5, GRN, GSK3A, HBA1, HBB, ID3, IDO1, INCA1, KATNB1, KCNK2, KNG1, LGALS9, LTBR, MAP3K10, MAP3K11, MAP3K9, MARK4, MSX1, MTCO2P12, MYBBP1A, NFATC4, NGF, NGFR, NOTCH1, NR4A1, OBSCN, OMA1, OSGIN1, PCDHA4, PDCD1, PDGFRB, PIAS4, PIDD1, PLAGL2, PLEKHG2, PLEKHN1, PML, PTN, PXT1, RGCC, RNF122, RPL26, SEPTIN4, SIK1, SLC9A1, SLC9A3R1, SMPD1, SPDEF, SPHK2, SRC, STK17B, TEX261, TGM2, THBS1, TLE5, TNF, TNFRSF1A, TNFSF12, UBA52, VDR, ZC3H12A, ZNF395]

GO:0043067	regulation of programmed cell death	1,39E-07	[4, 5]	308,00	19,25	39,53	60,47	[ADAM8, AIF1, ANGPT1, AREG, ATF3, BCL11B, BCL2L14, BCL2L15, BCL3, BLK, BMF, BMP7, BMPR1B, BTG2, BTK, CARD11, CARD17, CASP8, CCL19, CCL2, CCL3, CCL5, CD27, CD3E, CD3G, CD40LG, CD74, CDKN2A, CEL, CLCF1, CNR1, CORO1A, CTLA4, CXCL10, CXCR4, DAPK1, DOCK8, DPEP1, EGR3, EPX, EVI2B, FASLG, FATE1, FCMR, FFAR4, FGD2, FGD3, FLT3, FOSL1, GADD45B, GZMA, HCLS1, IER3, IFNG, IKZF3, IL7R, INPP5D, IRF5, ITGA4, KNG1, LAMP3, LCK, LEF1, LILRB1, LTA, MMP9, MPO, MSX1, MYB, MYBL2, NCKAP1L, NLRP3, NR4A1, NR4A2, NUGGC, PIK3CG, PIM1, PIM2, PLAC8, PRF1, PRKCQ, PTGFR, PTK2B, PTPN6, PTPRC, PYCARD, RASSF6, RNF122, RTKN2, SEMA4D, SOCS3, STAT1, STK17B, SYK, TEX11, THBS1, TMC8, TNFAIP3, TNFAIP8L2, TNFRSF18, TNFSF14, TRAF1, TREM2, TSC22D3, TXNDC5, UBD, UCP2, VAV1, VNN1, WNT1, WNT10B, ZBP1]	[ABL1, ABR, ACKR3, ACTN1, ACTN4, ADAMTSL4, ADIPOQ, AGAP2, AGT, AKT1S1, AMH, ANGPT1, ANGPTL4, ARAF, AREG, ARHGDIA, ARHGEF1, ARHGEF16, ARHGEF5, ATF5, BAG3, BAG6, BCAR1, BCL2L1, BCL3, BCL6, BTG2, C5AR1, CCL2, CCL21, CCR7, CD74, CDK1, CDKN1A, CEL, CFL1, CHERP, CITED2, CLCF1, CLIP3, CNR1, CRAT, CREB3L1, CRYAB, CSRNP3, CTSD, CX3CL1, CYP27B1, DAB2IP, DAXX, DEDD, DHRS2, DLG5, DNMT1, EEF1E1, EGLN2, ENDOG, ENG, FADD, FAIM2, FAP, FASN, FLOT2, FLT3, FLT4, FOSL1, FOXC2, GADD45B, GAS1, GATA2, GATA4, GBA, GNAI2, GPAM, GPER1, GPI, GRIK5, GRINA, GRN, GSK3A, HMGN5, HSF1, HSPB1, HYPK, ICAM1, ID3, IDO1, IER3, IL1B, IL7R, ILK, INCA1, ITGA5, ITPRIP, JAK3, KNG1, LGALS9, LIMS2, LMNA, LOX, LTBR, LTF, MAP3K10, MAP3K11, MAP3K9, MAPK3, MAPK7, MARK4, MAZ, MECP2, MED12, MEIS3P1, MEN1, MIEN1, MNT, MSX1, MTRNR2L1, MTRNR2L10, MTRNR2L12, MTRNR2L2, MTRNR2L3, MTRNR2L6, MTRNR2L8, MTRNR2L9, MYBBP1A, MYC, MYO18A, NEFL, NF2, NFATC4, NGF, NGFR, NOD2, NOS3, NOTCH1, NR4A1, NR4A2, NSMF, OBSCN, OMA1, OSGIN1, PAK4, PCDHA4, PCGF2, PDCD1, PDGFRB, PIAS4, PIDD1, PIM1, PLAGL2, PLAUR, PLEKHG2, PLEKHN1, PLK3, PML, POR, PPARD, PPP1R10, PTGFR, PTN, PXT1, RABGGTA, RAMP2, RARA, RASSF5, RELA, RGCC, RMRP, RNF122, RNF157, RNU1-93P, RPL26, RTKN2, SCAF1, SEPTIN4, SERPINE1, SIK1, SLC35F6, SLC9A1, SLC9A3R1, SMAD3, SMPD1, SNAI1, SOCS3, SOX10, SOX8, SPATA2, SPDEF, SPHK1, SPHK2, SRC, STEAP3, STK17B, STK40, SYNGAP1, SYVN1, TEX261, TGM2, THBS1, TIAF1, TIMP1, TLE5, TMEM14A, TMEM161A, TMEM256-PLSCR3, TNF, TNFRSF1A, TNFSF12, TRAF4, UBA52, UBE2B, UNC5B, VDR, VEGFA, VNN1, VSIG2, WFS1, ZBTB7A, ZC3H12A, ZFP36, ZNF385A, ZNF395]
GO:0043068	positive regulation of programmed cell death	2,29E-02	[4, 5, 6]	119,00	20,07	41,50	58,50	[ADAM8, ATF3, BLK, BMF, BMP7, BMPR1B, CASP8, CCL2, CCL3, CCL5, CD40LG, CDKN2A, CEL, CNR1, CTLA4, DAPK1, FASLG, FGD2, FGD3, FOSL1, GADD45B, GZMA, IFNG, INPP5D, IRF5, ITGA4, KNG1, LCK, LILRB1, LTA, MMP9, MSX1, MYB, MYBL2, NR4A1, PRF1, PTPRC, PYCARD, RNF122, STK17B, SYK, THBS1, UBD, VAV1, WNT10B, ZBP1]	[ABL1, ABR, ADAMTSL4, ADIPOQ, AGT, ARHGEF1, ARHGEF16, ARHGEF5, BCL2L1, BCL6, CCL2, CDKN1A, CEL, CLIP3, CNR1, CSRNP3, CTSD, CYP27B1, DAB2IP, EEF1E1, ENDOG, FADD, FAP, FASN, FOSL1, GADD45B, GPER1, GRIK5, GRN, GSK3A, ID3, IDO1, INCA1, KNG1, LGALS9, LTBR, MAP3K10, MAP3K11, MAP3K9, MARK4, MSX1, MYBBP1A, NFATC4, NGF, NGFR, NOTCH1, NR4A1, OBSCN, OMA1, OSGIN1, PCDHA4, PDCD1, PDGFRB, PIAS4, PIDD1, PLAGL2, PLEKHG2, PLEKHN1, PML, PTN, PXT1, RGCC, RNF122, RPL26, SEPTIN4, SIK1, SLC9A1, SLC9A3R1, SMPD1, SPDEF, SPHK2, SRC, STK17B, TEX261, TGM2, THBS1, TLE5, TNF, TNFRSF1A, TNFSF12, UBA52, VDR, ZC3H12A, ZNF395]
GO:0042981	regulation of apoptotic process	1,37E-07	[5, 6]	301,00	19,33	39,31	60,69	[ADAM8, AIF1, ANGPT1, AREG, ATF3, BCL11B, BCL2L14, BCL2L15, BCL3, BLK, BMF, BMP7, BMPR1B, BTG2, BTK, CARD11, CARD17, CASP8, CCL19, CCL2, CCL3, CCL5, CD27, CD3E, CD3G, CD40LG, CD74, CDKN2A, CEL, CLCF1, CNR1, CORO1A, CTLA4, CXCL10, DAPK1, DOCK8, DPEP1, EGR3, EPX, EVI2B, FASLG, FATE1, FCMR, FFAR4, FGD2, FGD3, FLT3, FOSL1, GADD45B, GZMA, HCLS1, IER3, IFNG, IKZF3, IL7R, INPP5D, IRF5, ITGA4, KNG1, LAMP3, LCK, LEF1, LILRB1, LTA, MMP9, MPO, MSX1, MYB, MYBL2, NCKAP1L, NLRP3, NR4A1, NR4A2, NUGGC, PIK3CG, PIM1, PIM2, PLAC8, PRKCQ, PTGFR, PTK2B, PTPN6, PTPRC, PYCARD, RASSF6, RNF122, RTKN2, SEMA4D, SOCS3, STAT1, STK17B, TEX11, THBS1, TMC8, TNFAIP3, TNFAIP8L2, TNFRSF18, TNFSF14, TRAF1, TREM2, TSC22D3, TXNDC5, UBD, UCP2, VAV1, VNN1, WNT1, WNT10B, ZBP1]	[ABL1, ABR, ACKR3, ACTN1, ACTN4, ADAMTSL4, ADIPOQ, AGAP2, AGT, AKT1S1, AMH, ANGPT1, ANGPTL4, ARAF, AREG, ARHGDIA, ARHGEF1, ARHGEF16, ARHGEF5, ATF5, BAG3, BAG6, BCAR1, BCL2L1, BCL3, BCL6, BTG2, C5AR1, CCL2, CCL21, CCR7, CD74, CDK1, CDKN1A, CEL, CFL1, CHERP, CITED2, CLCF1, CLIP3, CNR1, CRAT, CREB3L1, CRYAB, CSRNP3, CTSD, CX3CL1, CYP27B1, DAB2IP, DAXX, DEDD, DHRS2, DLG5, DNMT1, EEF1E1, EGLN2, ENDOG, ENG, FADD, FAIM2, FAP, FASN, FLT3, FLT4, FOSL1, FOXC2, GADD45B, GAS1, GATA2, GATA4, GBA, GNAI2, GPAM, GPER1, GPI, GRIK5, GRINA, GRN, GSK3A, HMGN5, HSF1, HSPB1, HYPK, ICAM1, ID3, IDO1, IER3, IL1B, IL7R, ILK, INCA1, ITGA5, ITPRIP, JAK3, KNG1, LGALS9, LIMS2, LMNA, LOX, LTBR, LTF, MAP3K10, MAP3K11, MAP3K9, MAPK3, MAPK7, MAZ, MECP2, MED12, MEIS3P1, MEN1, MIEN1, MNT, MSX1, MTRNR2L1, MTRNR2L10, MTRNR2L12, MTRNR2L2, MTRNR2L3, MTRNR2L6, MTRNR2L8, MTRNR2L9, MYBBP1A, MYC, MYO18A, NEFL, NF2, NFATC4, NGF, NGFR, NOD2, NOS3, NOTCH1, NR4A1, NR4A2, NSMF, OBSCN, OMA1, OSGIN1, PAK4, PCDHA4, PCGF2, PDCD1, PDGFRB, PIAS4, PIDD1, PIM1, PLAGL2, PLAUR, PLEKHG2, PLEKHN1, PLK3, PML, POR, PPARD, PPP1R10, PTGFR, PTN, PXT1, RABGGTA, RAMP2, RARA, RASSF5, RELA, RGCC, RMRP, RNF122, RNF157, RNU1-93P, RPL26, RTKN2, SCAF1, SEPTIN4, SERPINE1, SIK1, SLC35F6, SLC9A1, SLC9A3R1, SMAD3, SMPD1, SNAI1, SOCS3, SOX10, SOX8, SPDEF, SPHK1, SPHK2, SRC, STEAP3, STK17B, STK40, SYNGAP1, SYVN1, TEX261, TGM2, THBS1, TIAF1, TIMP1, TLE5, TMEM14A, TMEM161A, TMEM256-PLSCR3, TNF, TNFRSF1A, TNFSF12, TRAF4, UBA52, UBE2B, UNC5B, VDR, VEGFA, VNN1, VSIG2, WFS1, ZBTB7A, ZC3H12A, ZFP36, ZNF385A]

GO:0043066	negative regulation of apoptotic process	1,47E-02	[5, 6, 7]	177,00	18,77	37,10	62,90	[ADAM8, AIF1, ANGPT1, AREG, BCL11B, BCL3, BMP7, BTG2, CASP8, CCL19, CCL2, CCL5, CD27, CD40LG, CD74, CLCF1, CORO1A, DOCK8, DPEP1, EGR3, EPX, EVI2B, FASLG, FATE1, FCMR, FFAR4, HCLS1, IER3, IL7R, LAMP3, LEF1, LILRB1, MMP9, MPO, MSX1, NCKAP1L, NLRP3, NR4A1, NR4A2, NUGGC, PIK3CG, PIM1, PIM2, PLAC8, PRKCQ, PTGFR, PTK2B, RTKN2, SEMA4D, SOCS3, TEX11, THBS1, TNFAIP3, TNFRSF18, TNFSF14, TREM2, TSC22D3, TXNDC5, UCP2, VNN1, WNT1]	[ABL1, ACKR3, AGAP2, AMH, ANGPT1, ANGPTL4, ARAF, AREG, ARHGDIA, ATF5, BAG3, BAG6, BCL2L1, BCL3, BCL6, BTG2, C5AR1, CCL2, CCL21, CCR7, CD74, CDK1, CDKN1A, CFL1, CITED2, CLCF1, CRAT, CREB3L1, CRYAB, CX3CL1, DHRS2, DNMT1, FADD, FAIM2, FASN, FLT4, FOXC2, GATA2, GATA4, GBA, GNAI2, GPAM, GPI, GRIK5, GRINA, GRN, HMGN5, HSF1, HSPB1, HYPK, ICAM1, IER3, IL1B, IL7R, ILK, ITGA5, ITPRIP, JAK3, LIMS2, LMNA, LTF, MAPK3, MAPK7, MAZ, MECP2, MED12, MEIS3P1, MEN1, MIEN1, MNT, MSX1, MTRNR2L1, MTRNR2L10, MTRNR2L12, MTRNR2L2, MTRNR2L3, MTRNR2L6, MTRNR2L8, MTRNR2L9, MYC, MYO18A, NEFL, NFATC4, NGF, NGFR, NOD2, NOS3, NOTCH1, NR4A1, NR4A2, PAK4, PCGF2, PDGFRB, PIDD1, PIM1, PLAUR, PLK3, POR, PPARD, PPP1R10, PTGFR, RAMP2, RELA, RNF157, RNU1-93P, RTKN2, SERPINE1, SLC35F6, SLC9A1, SMAD3, SNAI1, SOCS3, SOX10, SOX8, SPHK1, SRC, STK40, SYNGAP1, SYVN1, THBS1, TIAF1, TIMP1, TMEM14A, TMEM161A, TNF, UBA52, UBE2B, UNC5B, VEGFA, VNN1, VSIG2, WFS1, ZC3H12A, ZNF385A]
GO:0050793	regulation of developmental process	1,56E-12	[2, 3]	488,00	18,90	38,56	61,44	[ADAM8, ADAMTS9, ALOX15B, ANGPT2, AREG, ASCL2, BCL11B, BHLHA15, BHLHE40, BMP7, BMPR1B, BTK, C1QC, CAMK4, CARD11, CASP8, CASS4, CCL17, CCL19, CCL2, CCL24, CCL3, CCR2, CCR3, CD2, CD27, CD4, CD53, CD74, CD80, CD86, CDH4, CDKN2A, CLCF1, CMTM5, CORO1A, CRABP2, CRB2, CRTAM, CSF1R, CST7, CTLA4, CXCL10, CXCL9, CXCR4, CYBB, DCC, DPEP1, EAF2, EGR3, ELAVL4, EPX, EREG, ERMN, EVI2B, FASLG, FCRL3, FFAR4, FGD2, FGD3, FGL2, FGR, FOXP3, FRZB, GDF6, GLIS1, GPR171, GPR4, GPR55, H3C10, H3C11, H3C12, H3C7, HCLS1, HLA-DOA, HLA-DRA, HLA-DRB1, HOPX, IFNG, IKZF3, IL12RB1, IL18, IL7R, INPP5D, IRF4, IRX3, ITGAX, ITGB2, KLF5, KLHL41, LAG3, LCP1, LDLR, LEF1, LIF, LILRB1, LILRB4, LMO1, LRRC17, LST1, LTA, MAFF, MEDAG, MIXL1, MMP9, MSX1, MUSK, MYB, MYCL, MYH6, NAMPT, NCKAP1L, NEK5, NFAM1, NLRP3, NPPB, NPPC, NR4A3, NTN1, P2RY12, PEAK3, PIM1, PLAAT4, PLAC8, PLXNC1, PRDM1, PRKCB, PRKCQ, PROK1, PTK2B, PTPN6, PTPRC, RAC2, RBPMS2, RHEX, RHOH, RLN2, RUNX3, SASH3, SEMA4A, SEMA4D, SLAMF8, SLC18A1, SMPD3, SOCS1, SOCS3, STAT1, SULT2B1, SYK, TACSTD2, TBX21, TESPA1, THBS1, THY1, TIFAB, TNFAIP3, TNFRSF11B, TNFRSF18, TNFSF13B, TNMD, TNR, TOX, TREM2, VNN1, VWC2L, WNT1, WNT10A, WNT10B, ZAP70, ZBED2, ZNF683]	[ABCC8, ABI3, ABL1, ACACB, ACTN4, ADAMTS7, ADAMTS9, ADCK1, ADGRB2, ADGRL1, ADIPOQ, AGAP2, AGER, AGT, AGTR1, ALDOA, AMH, ANGPTL4, AP2A1, AP3D1, APLNR, APOA1, AQP3, ARC, AREG, ARF1, ARHGDIA, ARHGEF15, ASPN, ATAT1, BAG6, BBS12, BCL6, BCL9L, BCOR, BEND6, BHLHE40, C11orf65, C15orf62, C5, C5AR1, CAMK2B, CARM1, CASZ1, CCL11, CCL2, CCL24, CD74, CDC42EP1, CDC42EP2, CDC42EP4, CDH4, CDK1, CDKN1A, CEACAM1, CELSR2, CETP, CFL1, CGA, CHERP, CHGA, CHI3L1, CITED2, CLCF1, CLPTM1, CMTM5, CNOT3, COL1A1, CPNE5, CPNE6, CREB3L1, CRP, CSF1, CSNK1E, CST7, CTDSP1, CTHRC1, CX3CL1, CYP26B1, CYP27B1, DAB2IP, DAG1, DDX39B, DISP3, DLG5, DMTN, DNMT1, DPF2, DTX1, DVL3, DXO, EAF2, ECM1, EEF1E1, EFEMP2, EFNA3, EHD1, EHD2, EIF4G1, ENG, EPHA2, EPN1, EPPK1, ERBB2, FADD, FBLIM1, FES, FLOT2, FLT4, FMNL1, FOXC2, FOXM1, FOXS1, GATA2, GATA4, GDF6, GDI1, GNB3, GPAM, GPER1, GPR137, GPR37L1, GPR4, GRN, GSK3A, H3C10, H3C12, H4C3, HDAC5, HDAC7, HESX1, HEYL, HGS, HMGA1, HMGN1, HOPX, HSF1, HSPB1, HSPG2, HTR2A, HYAL1, ICAM1, ID3, IFITM1, IL15RA, IL1B, IL4R, IL7R, ILK, INHA, IRX3, ITGA5, ITGA7, JAK3, JMJD8, JUNB, JUND, JUP, KCNK2, KCTD11, KLF10, KLF5, KMT2D, LAG3, LAMA5, LDLR, LGALS9, LIF, LIMK1, LIMS2, LINGO1, LMNA, LMX1A, LOX, LRCH4, LRG1, LRRC17, LRRC8A, LTF, LZTS1, LZTS3, MAFG, MAMSTR, MAPK7, MAPK8IP3, MARK2, MCRIP1, MECP2, MED12, MEGF8, MFSD2A, MME, MOV10, MSN, MSTN, MSX1, MYADM, MYC, MYH6, MYH7B, MYH9, MYL9, MYO19, MYRF, NAP1L2, NEFL, NEK5, NF2, NFATC4, NFE2, NGF, NGFR, NINJ1, NLGN2, NODAL, NOS3, NOTCH1, NOTCH3, NOTCH4, NPPB, NR1D1, NR4A3, NRARP, NSMF, NTN1, NUMBL, OMA1, OMD, OSM, OSR2, PACSIN1, PAF1, PAK4, PALM, PALM2AKAP2, PARP6, PARVB, PDGFB, PDLIM7, PFN1, PHLDB1, PIEZO1, PIM1, PKM, PLA2G2A, PLEKHB1, PLEKHO1, PLXNA1, PLXNA3, PLXNB1, PML, POR, POSTN, PPARD, PPP1R13L, PPP2R1A, PQBP1, PRAG1
GO:0051094	positive regulation of developmental process	3,34E-08	[2, 3, 4]	267,00	20,09	37,54	62,46	[ADAM8, ALOX15B, ANGPT2, AREG, BMP7, BMPR1B, BTK, CASP8, CASS4, CCL19, CCL24, CCR3, CD27, CD4, CD53, CD74, CD80, CD86, CDH4, CDKN2A, CLCF1, CRABP2, CRB2, CXCL9, CXCR4, CYBB, EGR3, ELAVL4, EPX, EVI2B, FFAR4, FOXP3, GDF6, HCLS1, HLA-DRA, HLA-DRB1, HOPX, IFNG, IL12RB1, IL18, IL7R, INPP5D, IRX3, ITGAX, ITGB2, KLF5, LCP1, LEF1, LIF, LILRB4, LTA, MEDAG, MIXL1, MYB, NCKAP1L, NEK5, NLRP3, NPPC, NTN1, PIM1, PLAAT4, PLXNC1, PRKCB, PTK2B, PTPRC, RHEX, RHOH, RLN2, RUNX3, SASH3, SEMA4A, SEMA4D, SOCS1, SOCS3, STAT1, SULT2B1, SYK, TACSTD2, TBX21, TESPA1, THBS1, TNFSF13B, TOX, TREM2, VNN1, VWC2L, WNT1, WNT10B, ZAP70, ZBED2]	[ABI3, ABL1, ACACB, ADGRL1, ADIPOQ, AGAP2, AGER, AGT, AGTR1, ANGPTL4, AP3D1, APLNR, APOA1, AREG, ARF1, ARHGDIA, BCL6, BCL9L, BEND6, C5, C5AR1, CAMK2B, CARM1, CCL11, CCL24, CD74, CDH4, CDK1, CEACAM1, CFL1, CHI3L1, CITED2, CLCF1, COL1A1, CPNE5, CPNE6, CSF1, CSNK1E, CTHRC1, CX3CL1, CYP26B1, CYP27B1, DAB2IP, DAG1, DDX39B, DLG5, DMTN, DVL3, ECM1, EEF1E1, EFEMP2, EHD1, EHD2, EIF4G1, ENG, FADD, FES, FOXC2, FOXS1, GATA2, GATA4, GDF6, GDI1, GPAM, GPER1, GRN, HEYL, HMGA1, HOPX, HSF1, HSPB1, HTR2A, HYAL1, IFITM1, IL15RA, IL1B, IL4R, IL7R, ILK, IRX3, ITGA5, JMJD8, JUNB, JUND, JUP, KCTD11, KLF10, KLF5, LGALS9, LIF, LIMK1, LIMS2, LMNA, LRCH4, LRG1, LRRC8A, LTF, LZTS1, MAMSTR, MAPK8IP3, MED12, MEGF8, MME, MYADM, MYC, NAP1L2, NEFL, NEK5, NF2, NGF, NGFR, NINJ1, NLGN2, NODAL, NOS3, NOTCH1, NTN1, NUMBL, OMA1, OSR2, PACSIN1, PAK4, PARP6, PDGFB, PDLIM7, PHLDB1, PIEZO1, PIM1, PKM, PLA2G2A, PLXNA1, PLXNA3, PLXNB1, POR, PPARD, PPP1R13L, PTN, RAMP2, RAP1A, RARA, RELA, RGCC, RNF157, S100A1, S1PR2, SERPINE1, SHANK3, SMAD3, SNAI1, SOCS3, SOX10, SOX12, SOX13, SOX8, SPDEF, SPHK1, SRC, SRF, SRRT, SSBP3, STAT3, SYDE1, SYT2, TBX5, TCF3, TCF7L1, TEAD4, TESK1, TFE3, TGFB1I1, TGM2, THBS1, THPO, TIMP1, TMEM100, TNF, TNFRSF1A, TNFSF12, TRIB1, TRIOBP, VDR, VEGFA, VNN1, VSIG2, VSIR, WNT3, YPEL3, ZBTB7B, ZC3H12A, ZFP36, ZMIZ1, ZNF219, ZNF335, ZNF385A]

GO:0051240	positive regulation of multicellular organismal process	8,22E-15	[2, 3, 4]	325,00	21,42	48,82	51,18	[ADAM8, AIF1, AIM2, ALOX15B, ANGPT2, AREG, BATF, BCL3, BMP7, BMPR1B, BTK, BTN3A1, BTN3A2, C1QTNF1, CAMK4, CARD11, CASP8, CCDC88B, CCL19, CCL24, CCL3, CCR2, CCR3, CD2, CD226, CD244, CD27, CD3E, CD4, CD40LG, CD6, CD74, CD80, CD86, CDH4, CLCF1, CLEC6A, CLNK, CNR1, CRABP2, CRB2, CRTAM, CSF1R, CST7, CXCR4, CYBB, EBI3, EDN2, EGR3, EPX, EREG, EVI2B, FCN1, FFAR4, FGR, FOXP3, GBP5, GDF6, GPSM3, HCLS1, HLA-DPA1, HLA-DPB1, HLA-DRA, HLA-DRB1, IFNG, IL12RB1, IL12RB2, IL16, IL18, IL26, IL7R, INPP5D, IRF4, IRF5, IRF8, ITGAX, ITGB2, ITK, LCP1, LDLR, LEF1, LIF, LILRB1, LILRB4, LPAL2, LTA, LTB, LY9, MAPK13, MCOLN2, MYB, NCKAP1L, NFAM1, NLRP2, NLRP3, NLRP9, NPPB, NR4A3, NTN1, ORM2, P2RY2, PIK3CG, PIM1, PLAAT4, PLAC8, PLXNC1, POU2AF1, POU2F2, PRKCB, PRKCQ, PTAFR, PTGER2, PTK2B, PTPN22, PTPRC, PYCARD, PYHIN1, RHOH, RLN2, RUNX3, SASH3, SCIMP, SEMA4A, SEMA4D, SIGLEC16, SLAMF6, SMTNL2, SOCS1, STAT1, SYK, TBX21, TBXAS1, TESPA1, THBS1, TIGIT, TLR6, TLR8, TNR, TOX, TREM2, UCP2, VNN1, WNT1, WNT10B, XCL1, ZAP70, ZBED2, ZBP1]	[ABCA7, ABCC8, ABL1, ACACB, ADIPOQ, AGAP2, AGER, AGPAT1, AGT, AMH, ANGPTL4, AP3D1, APLN, APLNR, AREG, ARHGDIA, ATP1A1, ATP1A3, ATPSCKMT, BCL3, BCL6, BCL9L, C1QTNF1, C5, C5AR1, CAMK2B, CCL11, CCL24, CCR7, CD14, CD276, CD74, CDH4, CDK1, CFH, CFL1, CHGA, CHI3L1, CITED2, CLCF1, CLNK, CNOT3, CNR1, COL1A1, CSF1, CST7, CX3CL1, CYP27B1, DAG1, DDX39B, DHX34, DLG5, DMTN, ECM1, EDN2, ENG, FABP4, FADD, FES, FFAR2, FLT4, FOXC2, FOXS1, FRMD8, FURIN, GATA2, GATA4, GBA, GDF6, GDI1, GNAI2, GPAM, GPER1, GPR3, GRN, GSK3A, HK1, HRH2, HSF1, HSPB1, HTR2A, HYAL1, ICAM1, IL15RA, IL1B, IL4R, IL7R, ILK, INHA, ITGA5, JMJD8, JUP, KDM6B, KLF10, LDLR, LDLRAP1, LGALS9, LIF, LILRA5, LIMK1, LIPG, LRCH4, LRG1, LTF, LUM, MAVS, MED12, MEGF8, MFAP2, MME, MYC, MYRF, NEFL, NFATC4, NFKB2, NGF, NGFR, NINJ1, NLGN2, NLRP9, NOD2, NODAL, NOS3, NOTCH1, NPAS2, NPPA, NPPB, NR4A3, NTN1, NUMBL, OMA1, OSM, OSR2, P2RX3, P2RY2, PAK4, PARP6, PCDHA4, PDGFB, PIM1, PKM, PLXNA1, PLXNA3, PLXNB1, POLR2E, POLR2L, POR, POSTN, PQBP1, PRDM16, PTN, RAMP2, RARA, RELA, RGCC, RNU1-93P, S100A1, SERPINE1, SHANK3, SLC7A5, SLC9A1, SLN, SMAD3, SMTNL2, SNAI1, SOX10, SOX12, SOX13, SOX8, SPHK1, SPHK2, SRC, SRF, SRRT, STAT3, STING1, SYDE1, TBX5, TEAD4, TGFB1I1, TGM2, THBS1, TICAM1, TNF, TNFSF12, TRIB1, TRPV4, TSKU, VDR, VEGFA, VNN1, VSIR, WNT3, YBX2, ZBTB7B, ZC3H12A, ZCCHC3, ZMIZ1, ZNF219, ZNF335, ZNF580]
GO:0045595	regulation of cell differentiation	2,28E-11	[3, 4]	337,00	20,17	43,12	56,88	[ADAM8, ALOX15B, AREG, ASCL2, BCL11B, BHLHA15, BHLHE40, BMP7, BMPR1B, BTK, C1QC, CAMK4, CARD11, CASP8, CASS4, CCL17, CCL19, CCL3, CCR2, CD2, CD27, CD4, CD74, CD80, CD86, CDH4, CDKN2A, CLCF1, CMTM5, CRABP2, CRB2, CRTAM, CTLA4, CXCL10, CXCL9, CXCR4, DCC, DPEP1, EGR3, EPX, EREG, EVI2B, FCRL3, FFAR4, FGL2, FOXP3, FRZB, GDF6, GLIS1, GPR171, GPR55, H3C10, H3C11, H3C12, H3C7, HCLS1, HLA-DOA, HLA-DRA, HLA-DRB1, HOPX, IFNG, IKZF3, IL12RB1, IL18, IL7R, INPP5D, IRF4, IRX3, KLF5, KLHL41, LAG3, LCP1, LDLR, LEF1, LIF, LILRB1, LILRB4, LMO1, LRRC17, LTA, MAFF, MEDAG, MIXL1, MMP9, MSX1, MUSK, MYB, MYCL, NCKAP1L, NEK5, NFAM1, NLRP3, NPPC, NR4A3, NTN1, P2RY12, PIM1, PLAAT4, PLXNC1, PRDM1, PRKCB, PRKCQ, PTK2B, PTPN6, PTPRC, RBPMS2, RHEX, RHOH, RUNX3, SASH3, SEMA4A, SEMA4D, SLAMF8, SOCS1, SOCS3, STAT1, SULT2B1, SYK, TACSTD2, TBX21, TESPA1, THBS1, THY1, TNFRSF18, TNR, TOX, TREM2, VNN1, VWC2L, WNT1, WNT10B, ZAP70, ZBED2, ZNF683]	[ABCC8, ABL1, ACTN4, ADAMTS7, ADIPOQ, AGER, AGT, AGTR1, AP3D1, APOA1, AQP3, AREG, ARHGDIA, ATAT1, BBS12, BCL6, BCL9L, BEND6, BHLHE40, CAMK2B, CARM1, CASZ1, CCL11, CD74, CDH4, CDK1, CEACAM1, CETP, CHERP, CLCF1, CLPTM1, CMTM5, COL1A1, CRP, CSF1, CSNK1E, CTDSP1, CTHRC1, CX3CL1, CYP26B1, CYP27B1, DAB2IP, DAG1, DDX39B, DISP3, DMTN, DNMT1, DPF2, DTX1, EFEMP2, EIF4G1, ENG, ERBB2, FADD, FES, FLOT2, GATA2, GATA4, GDF6, GDI1, GNB3, GPER1, GPR137, GPR37L1, GRN, GSK3A, H3C10, H3C12, H4C3, HDAC5, HDAC7, HEYL, HOPX, HSF1, HTR2A, ID3, IFITM1, IL15RA, IL1B, IL4R, IL7R, ILK, INHA, IRX3, JAK3, JUNB, JUND, KCTD11, KLF10, KLF5, KMT2D, LAG3, LDLR, LGALS9, LIF, LIMK1, LIMS2, LINGO1, LMX1A, LOX, LRRC17, LRRC8A, LTF, LZTS1, MAFG, MAMSTR, MAPK8IP3, MCRIP1, MECP2, MED12, MEGF8, MME, MOV10, MSTN, MSX1, MYADM, MYC, MYL9, NAP1L2, NEFL, NEK5, NF2, NFATC4, NFE2, NGF, NGFR, NODAL, NOTCH1, NOTCH4, NR1D1, NR4A3, NRARP, NTN1, NUMBL, OMA1, OSM, PAF1, PARP6, PDGFB, PDLIM7, PHLDB1, PIEZO1, PIM1, PLA2G2A, PLEKHB1, PLXNA1, PLXNA3, PLXNB1, POR, POSTN, PPARD, PPP1R13L, PPP2R1A, PRKACA, PTN, RAMP2, RAP1A, RARA, RBM38, RBPMS2, RELA, RGCC, RGMA, RMRP, RNU1-93P, S1PR2, SCAF1, SEMA3F, SEMA4B, SEMA4C, SEMA6B, SEMA6C, SERPINE1, SETD1A, SHANK3, SIK1, SLC25A23, SLC4A11, SMAD3, SNAI1, SOCS3, SOX10, SOX12, SOX13, SOX8, SPDEF, SPI1, SRF, SRRT, STAT3, SYNGAP1, TBX5, TCF3, TCF7L1, TCIRG1, TEAD2, TESK1, TFE3, TGFB1I1, TGM2, THBS1, THPO, TIMP1, TMEM100, TNF, TNFRSF1A, TRIB1, TRIB3, TRIM62, TRIOBP, TRPV4, TSKU, ULK1, VASN, VDR, VEGFA, VNN1, VSIG2, VSIR, WNT3, WNT9B, ZBTB7B, ZC3H12A, ZFHX2, ZFP36, ZMIZ1, ZNF219, ZNF335, ZNF385A]

GO:2000026	regulation of multicellular organismal development	3,52E-12	[3, 4]	296,00	21,07	44,92	55,08	[ADAM8, ADAMTS9, ALOX15B, ANGPT2, ASCL2, BHLHE40, BMP7, BMPR1B, BTK, C1QC, CAMK4, CARD11, CASP8, CCL19, CCL24, CCL3, CCR2, CCR3, CD2, CD27, CD4, CD74, CD80, CD86, CDH4, CDKN2A, CLCF1, CRABP2, CRB2, CRTAM, CST7, CTLA4, CXCL10, CXCR4, CYBB, DCC, DPEP1, EGR3, EVI2B, FASLG, FCRL3, FGL2, FOXP3, FRZB, GDF6, GPR171, GPR4, GPR55, H3C10, H3C11, H3C12, H3C7, HCLS1, HLA-DOA, HLA-DRA, HLA-DRB1, HOPX, IFNG, IKZF3, IL12RB1, IL18, IL7R, INPP5D, IRF4, ITGAX, ITGB2, LAG3, LDLR, LEF1, LIF, LILRB1, LILRB4, LRRC17, LTA, MAFF, MMP9, MYB, MYCL, MYH6, NCKAP1L, NFAM1, NLRP3, NPPB, NR4A3, NTN1, PIM1, PLAAT4, PLXNC1, PRDM1, PRKCB, PRKCQ, PROK1, PTK2B, PTPN6, PTPRC, RHEX, RHOH, RLN2, RUNX3, SASH3, SEMA4A, SEMA4D, SLAMF8, SMPD3, SOCS1, STAT1, SULT2B1, SYK, TBX21, TESPA1, THBS1, THY1, TNFAIP3, TNFRSF18, TNMD, TNR, TOX, TREM2, VNN1, WNT1, WNT10B, ZAP70, ZBED2, ZNF683]	[ABCC8, ABL1, ACACB, ADAMTS7, ADAMTS9, ADGRB2, ADIPOQ, AGER, AGT, ANGPTL4, AP3D1, APLNR, AQP3, ARHGDIA, ASPN, BAG6, BCL6, BCOR, BHLHE40, C5, C5AR1, CAMK2B, CCL11, CCL24, CD74, CDH4, CDK1, CEACAM1, CFL1, CHI3L1, CITED2, CLCF1, CLPTM1, CREB3L1, CSF1, CST7, CTDSP1, CX3CL1, CYP26B1, CYP27B1, DAB2IP, DAG1, DDX39B, DLG5, DTX1, ECM1, EFNA3, ENG, EPHA2, EPN1, ERBB2, FADD, FES, FOXC2, GATA2, GATA4, GDF6, GDI1, GPER1, GPR137, GPR37L1, GPR4, GRN, GSK3A, H3C10, H3C12, H4C3, HESX1, HEYL, HGS, HOPX, HSF1, HSPB1, HSPG2, HYAL1, IL15RA, IL1B, IL4R, IL7R, ILK, INHA, ITGA5, JAK3, JMJD8, JUP, KCNK2, KLF10, KMT2D, LAG3, LAMA5, LDLR, LGALS9, LIF, LIMK1, LINGO1, LOX, LRCH4, LRG1, LRRC17, LTF, MAFG, MAPK7, MECP2, MED12, MEGF8, MME, MOV10, MYC, MYH6, MYL9, MYRF, NEFL, NF2, NFATC4, NFE2, NGF, NGFR, NINJ1, NLGN2, NODAL, NOS3, NOTCH1, NOTCH4, NPPB, NR1D1, NR4A3, NRARP, NTN1, NUMBL, OMA1, OMD, OSR2, PAF1, PAK4, PARP6, PHLDB1, PIM1, PKM, PLXNA1, PLXNA3, PLXNB1, PML, POR, PPARD, PTN, RAMP2, RARA, RELA, RGCC, RGMA, RMRP, S100A1, S1PR2, SEMA3F, SEMA4B, SEMA4C, SEMA6B, SEMA6C, SERPINE1, SETD1A, SHANK3, SMAD3, SOX10, SOX12, SOX13, SOX8, SPHK1, SPI1, SRF, SRRT, STAT3, SYNGAP1, TBX5, TFE3, TGM2, THBS1, THPO, TMEM100, TNF, TNFRSF1A, TNFSF12, TNMD, TRIB1, ULK1, VDR, VEGFA, VNN1, VSIR, WARS1, WNT3, WNT9B, ZBTB7B, ZC3H12A, ZFP36, ZMIZ1, ZNF219, ZNF335]
GO:0045597	positive regulation of cell differentiation	2,37E-08	[3, 4, 5]	192,00	21,87	40,86	59,14	[ADAM8, ALOX15B, AREG, BMP7, BMPR1B, BTK, CASP8, CASS4, CCL19, CD27, CD4, CD74, CD80, CD86, CDH4, CLCF1, CRABP2, CRB2, CXCL9, CXCR4, EGR3, EPX, EVI2B, FFAR4, FOXP3, GDF6, HCLS1, HLA-DRA, HLA-DRB1, HOPX, IFNG, IL12RB1, IL18, IL7R, INPP5D, IRX3, KLF5, LCP1, LEF1, LIF, LILRB4, LTA, MEDAG, MYB, NCKAP1L, NEK5, NLRP3, NPPC, NTN1, PIM1, PLAAT4, PLXNC1, PTPRC, RHEX, RHOH, RUNX3, SASH3, SEMA4D, SOCS1, SOCS3, STAT1, SULT2B1, SYK, TACSTD2, TESPA1, TOX, TREM2, VNN1, VWC2L, WNT10B, ZAP70, ZBED2]	[ABL1, ADIPOQ, AGER, AGT, AGTR1, AP3D1, APOA1, AREG, ARHGDIA, BCL6, BCL9L, BEND6, CAMK2B, CARM1, CD74, CDH4, CEACAM1, CLCF1, COL1A1, CSF1, CSNK1E, CTHRC1, CX3CL1, CYP26B1, CYP27B1, DAG1, DDX39B, DMTN, EFEMP2, EIF4G1, ENG, FADD, FES, GATA2, GATA4, GDF6, GDI1, GPER1, HEYL, HOPX, HSF1, HTR2A, IFITM1, IL15RA, IL1B, IL4R, IL7R, ILK, IRX3, JUNB, JUND, KCTD11, KLF10, KLF5, LGALS9, LIF, LIMK1, LIMS2, LRRC8A, LTF, LZTS1, MAMSTR, MAPK8IP3, MED12, MEGF8, MME, MYADM, MYC, NAP1L2, NEFL, NEK5, NF2, NGF, NGFR, NOTCH1, NTN1, NUMBL, OMA1, PARP6, PDLIM7, PIEZO1, PIM1, PLA2G2A, PLXNA1, PLXNA3, PLXNB1, POR, PPARD, PPP1R13L, PTN, RAMP2, RAP1A, RARA, RELA, RGCC, S1PR2, SERPINE1, SHANK3, SMAD3, SNAI1, SOCS3, SOX10, SOX12, SOX13, SOX8, SPDEF, SRF, SRRT, STAT3, TBX5, TCF3, TCF7L1, TESK1, TFE3, TGFB1I1, TGM2, THPO, TIMP1, TMEM100, TNF, TRIB1, TRIOBP, VDR, VEGFA, VNN1, VSIG2, VSIR, WNT3, ZBTB7B, ZC3H12A, ZFP36, ZMIZ1, ZNF219, ZNF335, ZNF385A]
GO:1903706	regulation of hemopoiesis	2,05E-13	[3, 4, 5, 6, 7, 8]	122,00	28,98	61,99	38,01	[ADAM8, BTK, C1QC, CAMK4, CARD11, CASP8, CCL19, CCL3, CCR2, CD2, CD27, CD4, CD74, CD80, CD86, CDKN2A, CRTAM, CTLA4, EGR3, EVI2B, FCRL3, FGL2, FOXP3, GPR171, GPR55, H3C10, H3C11, H3C12, H3C7, HCLS1, HLA-DOA, HLA-DRA, HLA-DRB1, IFNG, IKZF3, IL12RB1, IL18, IL7R, INPP5D, IRF4, LAG3, LEF1, LILRB1, LILRB4, LRRC17, MYB, NCKAP1L, NFAM1, NLRP3, NR4A3, PRDM1, PRKCB, PRKCQ, PTK2B, PTPN6, PTPRC, RHEX, RHOH, RUNX3, SASH3, SLAMF8, SOCS1, STAT1, SYK, TBX21, TESPA1, THBS1, TNFRSF18, TOX, TREM2, VNN1, ZAP70, ZNF683]	[ABL1, ADIPOQ, AGER, AP3D1, BCL6, CD74, CEACAM1, CLPTM1, CSF1, CYP26B1, DTX1, ERBB2, FADD, FES, GATA2, GPR137, H3C10, H3C12, H4C3, HSF1, IL15RA, IL4R, IL7R, INHA, JAK3, KLF10, KMT2D, LAG3, LGALS9, LOX, LRRC17, LTF, MOV10, MYC, MYL9, NFE2, NR4A3, NRARP, PAF1, PTN, RARA, RMRP, SETD1A, SOX12, SOX13, SPI1, STAT3, TFE3, THBS1, THPO, TNF, TRIB1, VNN1, VSIR, ZBTB7B, ZC3H12A, ZFP36, ZMIZ1]
GO:1902105	regulation of leukocyte differentiation	4,19E-15	[4, 5, 6, 7, 8, 9]	97,00	33,92	65,84	34,16	[ADAM8, BTK, C1QC, CAMK4, CARD11, CASP8, CCL19, CCL3, CCR2, CD2, CD27, CD4, CD74, CD80, CD86, CDKN2A, CRTAM, CTLA4, EGR3, EVI2B, FCRL3, FGL2, FOXP3, GPR55, HCLS1, HLA-DOA, HLA-DRA, HLA-DRB1, IFNG, IKZF3, IL12RB1, IL18, IL7R, INPP5D, IRF4, LAG3, LEF1, LILRB1, LILRB4, LRRC17, MYB, NCKAP1L, NFAM1, NLRP3, PRDM1, PTPN6, PTPRC, RHOH, RUNX3, SASH3, SLAMF8, SOCS1, SYK, TBX21, TESPA1, TNFRSF18, TOX, TREM2, VNN1, ZAP70, ZNF683]	[ABL1, ADIPOQ, AGER, AP3D1, BCL6, CD74, CEACAM1, CLPTM1, CSF1, CYP26B1, DTX1, ERBB2, FADD, FES, GATA2, GPR137, HSF1, IL15RA, IL4R, IL7R, INHA, JAK3, KLF10, LAG3, LGALS9, LRRC17, LTF, MYC, NRARP, RARA, RMRP, SOX12, SOX13, TFE3, TNF, TRIB1, VNN1, VSIR, ZBTB7B, ZC3H12A, ZMIZ1]
GO:1902107	positive regulation of leukocyte differentiation	3,68E-08	[4, 5, 6, 7, 8, 9, 10]	55,00	34,59	67,95	32,05	[ADAM8, BTK, CASP8, CCL19, CD27, CD4, CD74, CD80, CD86, EGR3, EVI2B, FOXP3, HCLS1, HLA-DRA, HLA-DRB1, IFNG, IL12RB1, IL18, IL7R, INPP5D, LEF1, LILRB4, MYB, NCKAP1L, NLRP3, PTPRC, RHOH, RUNX3, SASH3, SOCS1, SYK, TESPA1, TOX, TREM2, VNN1, ZAP70]	[AGER, AP3D1, BCL6, CD74, CSF1, FADD, FES, HSF1, IL15RA, IL4R, IL7R, KLF10, LGALS9, RARA, SOX12, SOX13, TNF, TRIB1, VNN1, VSIR, ZBTB7B, ZMIZ1]

GO:0048518	positive regulation of biological process	5,77E-18	[1, 2, 3]	1107,00	16,89	40,46	59,54	[ABCB11, ABRA, ADAM8, ADCY7, AIF1, AIM2, AKNA, ALOX15, ALOX15B, ANGPT1, ANGPT2, APBB1IP, AREG, ASCL2, ATF3, ATP1B4, ATP2A3, BANK1, BATF, BCL11B, BCL3, BHLHA15, BLK, BLNK, BMF, BMP7, BMPR1B, BRIP1, BTK, BTLA, BTN3A1, BTN3A2, C1QA, C1QB, C1QC, C1QTNF1, C2, C8G, CACNA1I, CALCR, CAMK4, CARD11, CARMIL2, CASP8, CASS4, CCDC88B, CCL17, CCL19, CCL2, CCL22, CCL24, CCL3, CCL3L1, CCL4, CCL4L1, CCL5, CCR2, CCR3, CCR4, CD177, CD180, CD19, CD1B, CD1C, CD1E, CD2, CD22, CD226, CD244, CD247, CD27, CD300LF, CD33, CD3D, CD3E, CD3G, CD4, CD40LG, CD5, CD53, CD6, CD74, CD79A, CD80, CD84, CD86, CDC20B, CDCA2, CDH1, CDH4, CDKN2A, CEL, CFP, CLCF1, CLEC10A, CLEC4D, CLEC4E, CLEC6A, CLNK, CNR1, CORO1A, CR2, CRABP2, CRB2, CREM, CRTAM, CSF1R, CST7, CTLA4, CTSS, CXCL10, CXCL11, CXCL5, CXCL9, CXCR4, CXorf21, CYBB, CYP11B1, DAPK1, DEPDC1B, DOCK2, DOCK8, DPEP2, DRD1, E2F8, EAF2, EBI3, EDN2, EGR3, ELAVL4, ELF3, EOMES, EPHA6, EPX, EREG, ERFE, EVI2B, FASLG, FCN1, FCN3, FCRL3, FFAR4, FGD2, FGD3, FGR, FLT3, FOLR2, FOSB, FOSL1, FOXP3, FUT7, FYB1, GABBR2, GADD45B, GBP5, GDF6, GFI1, GLIS1, GMFG, GPM6A, GPR174, GPR4, GPR55, GPRC5A, GPSM3, GRAP2, GZMA, GZMB, H1-3, HCLS1, HCST, HLA-DMB, HLA-DPA1, HLA-DPB1, HLA-DQA1, HLA-DRA, HLA-DRB1, HLA-DRB5, HOPX, ICOS, IFNG, IGLL5, IKBKE, IKZF3, IL10RA, IL12RB1, IL12RB2, IL16, IL18, IL18RAP, IL26, IL2RB, IL2RG, IL5RA, IL7R, INPP5D, IQCJ-SCHIP1, IQGAP2, IRF4, IRF5, IRF8, IRX3, ITGA4, ITGAX, ITGB2, ITK, JAML, JCHAIN, KCNJ10, KCNN4, KIAA1324, KLF5, KLRC2, KLRD1, KLRG1, KLRK1, KMO, KNG1, LAG3, LAMP3, LAX1, LCK, LCP1, LCP2, LDLR, LEF1, LIF, LILRB1, LILRB4, LMCD1, LMO1, LPAL2, LRRC38, LTA, LTB, LY86, LY9, MACC1, MAFF, MAP4K1, MAPK13, MATK, MCOLN2, MEDAG, MID1IP1, MIXL1, MLC1, MMP9, MOG, MS4A1, MSX1, MUC16, MUC19, MUC6, MUSK, MYB, MYBL2, MYO1G, MZB1, NAMPT, NCF1, NCKAP1L, NCR3, NEK5, NELL2, NFAM1	[ABCA3, ABCA7, ABCB11, ABCC8, ABCD1, ABCD2, ABI3, ABL1, ABLIM3, ABR, ABRA, ACACB, ACKR3, ACTA1, ACTB, ACTC1, ACTN1, ACTN4, ADAM19, ADAMTSL4, ADCK1, ADCY1, ADCY3, ADCY4, ADCY9, ADGRG1, ADGRL1, ADIPOQ, AGAP2, AGER, AGPAT1, AGT, AGTR1, ALPK3, AMH, ANGPT1, ANGPTL4, AP2A1, AP3D1, APLN, APLNR, APOA1, AQP3, ARAF, ARC, AREG, ARF1, ARHGDIA, ARHGEF1, ARHGEF15, ARHGEF16, ARHGEF5, ARID1A, ARTN, ATAT1, ATF5, ATP13A2, ATP1A1, ATP1A3, ATP1B2, ATP1B4, ATPSCKMT, BAG3, BAG6, BCAR1, BCL2L1, BCL3, BCL6, BCL9L, BEND6, BMP8A, BRD4, BRPF3, BTLA, C15orf62, C1QTNF1, C20orf27, C2CD2L, C5, C5AR1, C8G, CACNA1H, CALB1, CALCR, CAMK2B, CAMTA2, CAPN1, CAPNS1, CARM1, CARMIL2, CASZ1, CC2D1A, CCDC87, CCL11, CCL2, CCL21, CCL24, CCR7, CD14, CD177, CD1C, CD1E, CD276, CD74, CDC25B, CDC42EP1, CDC42EP2, CDC42EP4, CDCA2, CDH4, CDK1, CDK2, CDKN1A, CEACAM1, CEL, CETP, CFH, CFL1, CGA, CHCHD10, CHERP, CHGA, CHI3L1, CIITA, CITED2, CITED4, CIZ1, CLCF1, CLEC4E, CLIP3, CLNK, CNOT3, CNR1, CNTN6, COL1A1, CPN2, CPNE5, CPNE6, CRAT, CREB3L1, CRHR2, CRIP2, CRP, CRTC2, CSF1, CSK, CSNK1E, CSPG4, CSRNP3, CST7, CTC1, CTF1, CTHRC1, CTIF, CTSD, CX3CL1, CYP26B1, CYP27B1, DAB2IP, DAG1, DAXX, DBF4B, DDR1, DDX39B, DENND2B, DGKD, DGKZ, DHX34, DIRAS1, DISP3, DKK2, DLG5, DMTN, DNMT1, DOC2B, DOK5, DPAGT1, DPF2, DTX1, DUSP5, DVL3, DXO, DYRK1B, E2F4, E2F7, E2F8, EAF2, ECM1, ECM2, EDC4, EDN2, EEF1E1, EFEMP2, EFNA3, EGLN2, EHD1, EHD2, EIF4EBP1, EIF4G1, ELF3, ELF4, ELK1, ENDOG, ENG, ENPP3, EPHA2, ERBB2, ERFE, ESM1, ESPL1, ETS2, ETV6, FABP4, FADD, FAM110A, FAP, FASN, FCN3, FEM1A, FES, FFAR2, FGFR4, FHL5, FHOD1, FITM1, FLOT2, FLT3, FLT4, FOSL1, FOSL2, FOXC2, FOXF1, FOXM1, FOXS1, FPR1, FRMD8, FURIN, GAB2, GABBR2, GADD45B, GATA2, GATA4, GBA, GCSAM, GDF6, GDI1, GGA1, GGA3, GIT1, GLIS2, GLMP, GLRA1, GNAI2, GNAO1, GPAM
GO:0023051	regulation of signaling	5,81E-15	[2, 3]	675,00	18,15	37,12	62,88	[ABRA, ADAM8, ALOX15, ALOX15B, ANGPT1, ARAP2, AREG, ARHGAP15, ARHGAP30, ARHGAP45, ATF3, ATP2A3, BANK1, BCL2L14, BCL3, BLK, BMF, BMP7, BMPR1B, BRIP1, C1QTNF1, CACNA1E, CALCR, CARD11, CASP8, CASS4, CCDC88C, CCL17, CCL19, CCL2, CCL22, CCL24, CCL3, CCL3L1, CCL4, CCL4L1, CCL5, CCR2, CD177, CD180, CD19, CD22, CD226, CD27, CD300LF, CD3E, CD4, CD40LG, CD74, CD80, CD86, CDH1, CDKN2A, CEL, CHRDL1, CISH, CLEC6A, CNR1, CNR2, CRB2, CSF1R, CXCR4, CXorf21, DAPK1, DCC, DEPDC1B, DLGAP2, DPEP2, DRD1, DUSP2, DUSP8, ELAVL4, ELF3, EPX, EREG, ERFE, FASLG, FCRL3, FFAR4, FGD2, FGD3, FGR, FLT3, FOLR2, FOXP3, FRZB, FUT7, GADD45B, GDF6, GFI1, GMIP, GPR174, GPR4, GPR55, GPRC5A, GUCY2D, H3C10, H3C11, H3C12, H3C7, HCLS1, HCST, HLA-DRB1, ICOS, IER3, IFNG, IKBKE, IL10RA, IL18, IL26, IL7R, INPP5D, IQCJ-SCHIP1, IRF4, KCNJ10, KCNN4, KMO, LAX1, LCK, LEF1, LIF, LILRB4, LMCD1, LY86, MAP4K1, MIDN, MMP9, MSX1, MYB, MZB1, NCF1, NCKAP1L, NFAM1, NLRC3, NLRP3, NLRP6, NPFFR2, NPPC, NPTX2, NR4A2, NSG1, NTN1, OASL, P2RY10, P2RY12, PAX5, PDE6G, PIK3CG, PIK3IP1, PIK3R5, PIM2, PLAU, PLEK, PRDM1, PRKCB, PRKCQ, PROK1, PSCA, PSD4, PTK2B, PTPN22, PTPN6, PTPRC, PYCARD, PYHIN1, RAC2, RAD9B, RASAL3, RASGRF1, RBPMS2, RGS1, RGS10, RGS18, RGS9, RHOH, RMI2, RNASE6, RRAD, RTKN2, SALL1, SCIMP, SCUBE1, SEMA4D, SH2D1A, SHISA8, SLA, SLA2, SLC24A4, SLC8A3, SMPDL3B, SOCS1, SOCS3, SPINK1, STAP1, STAT1, SYK, TAGAP, TBC1D10C, TESPA1, THBS1, THY1, TLR6, TMC8, TNFAIP3, TNFSF14, TNIP3, TNMD, TNR, TRABD2A, TRAF1, TRAT1, TREM2, TRIM14, TRPM2, UBASH3A, UBD, UCP2, VAV1, VNN1, VWC2L, WNT1, WNT10B, XCL1, XCL2, ZAP70, ZBP1]	[ABCA7, ABCC8, ABL1, ABR, ABRA, ACKR3, ACP4, ACTN4, ADCY1, ADGRG1, ADIPOQ, AGAP2, AGER, AGPAT1, AGT, AGTR1, AKT1S1, AMH, ANGPT1, APLN, APLNR, APLP1, APOA1, APOD, ARAF, ARAP3, ARC, AREG, ARF1, ARHGAP1, ARHGAP23, ARHGAP39, ARHGDIA, ARHGEF1, ARHGEF16, ARHGEF5, ARRDC3, ARTN, ASPN, ATP1A1, ATP1A3, ATP1B2, BCL2L1, BCL3, BCL6, BCL9L, BCR, BEND6, BMP8A, BRD4, C1QTNF1, C20orf27, C2CD2L, C5, C5AR1, CACNA1E, CALB1, CALCR, CAMK2B, CAPN1, CARM1, CC2D1A, CCL11, CCL2, CCL21, CCL24, CCR7, CD14, CD177, CD74, CDK1, CDK2, CEACAM1, CEL, CGA, CHERP, CHGA, CHI3L1, CISH, CITED2, CLIP3, CNR1, CNTN6, COL1A1, CPLX1, CRAT, CREB3L1, CRHR2, CSF1, CSK, CSNK1E, CSPG4, CTHRC1, CX3CL1, CXXC4, CYP26B1, CYP27B1, DAB2IP, DAG1, DAXX, DDX39B, DENND2B, DENND4B, DGKD, DGKZ, DHX34, DIRAS1, DKK2, DLG5, DLGAP4, DMTN, DOC2B, DOK5, DOT1L, DTX1, DUSP5, DUSP8, DVL3, ECM1, EEF1E1, ELF3, ENG, EPHA2, EPN1, ERBB2, ERFE, ESM1, FADD, FAIM2, FAM110A, FASN, FFAR2, FGFR4, FLOT2, FLT3, FLT4, FOXM1, FPR1, FRMD8, FURIN, FXYD6, GAB2, GADD45B, GAS1, GATA4, GBA, GBF1, GCSAM, GDF6, GIT1, GJD3, GLIS2, GLRA1, GNAI2, GPAM, GPBAR1, GPER1, GPI, GPR137, GPR17, GPR20, GPR37L1, GPR4, GPRC5A, GPRIN1, GRB7, GRIK5, GRINA, GRM2, GSK3A, H3C10, H3C12, H4C3, HBEGF, HCRTR1, HDAC7, HEYL, HFE, HGS, HIPK4, HSPB1, HTR1B, HTR2A, ICAM1, IER3, IFT80, IL18BP, IL1B, IL1RN, IL7R, ILK, INCA1, INHA, INPP5E, IRAK2, ITGA3, ITGA5, ITPR3, ITPRIP, JAK3, JMJD8, JPH4, JUP, KCP, KCTD11, KIF26A, KLK14, KMT2D, KSR1, LGALS9, LGR6, LIF, LIFR, LILRA5, LIMS2, LMCD1, LMNA, LOX, LRG1, LRRC4, LRRC8A, LTBR, LTF, LY6E, LYNX1, LZTS1, LZTS2, MAP1A, MAP2K3, MAP3K10, MAP3K11, MAP3K14, MAP3K6, MAP3K9, MAPK3, MAPK7, MAPK8IP3, MAVS, MAZ, MECP2, MED12, MEGF8, MEIS3P1, MEN1, MGRN1, MIDN, MINK1, MLST8, MME, MNT, MSTN, MSX1, MTRNR2L1, MTRNR2L10, MTRNR2L12, MTRNR2L2, MTRNR2L3,

GO:0048583	regulation of response to stimulus	4,40E-27	[2, 3]	851,00	18,88	45,12	54,88	[ABRA, ACP5, ADAM8, ADCY7, AIF1, AIM2, AKNA, ALOX15, ALOX15B, ANGPT1, ANGPT2, AOAH, APOBEC3G, ARAP2, AREG, ARHGAP15, ARHGAP30, ARHGAP45, ATF3, BANK1, BCL2L14, BCL3, BLK, BLNK, BMF, BMP7, BMPR1B, BRIP1, BTK, BTLA, BTN3A1, BTN3A2, C1QA, C1QB, C1QC, C1QTNF1, C2, C8G, CALCR, CARD11, CASP8, CASS4, CCDC88C, CCL17, CCL19, CCL2, CCL22, CCL24, CCL3, CCL3L1, CCL4, CCL4L1, CCL5, CCR2, CCR4, CD177, CD180, CD19, CD1B, CD1C, CD1E, CD200R1, CD22, CD226, CD247, CD27, CD300LF, CD33, CD3D, CD3E, CD3G, CD4, CD40LG, CD48, CD74, CD79A, CD80, CD84, CD86, CD8A, CD8B, CD96, CDKN2A, CEL, CFP, CHRDL1, CISH, CLCF1, CLEC10A, CLEC1B, CLEC4D, CLEC4E, CLEC6A, CLNK, CNR1, CNR2, CR2, CRB2, CRTAM, CSF1R, CST7, CTLA4, CXCL10, CXCL6, CXCR4, CXorf21, CYP11B1, CYSLTR1, DAPK1, DCC, DEPDC1B, DLGAP2, DPEP1, DPEP2, DRD1, DUSP2, DUSP8, EDN2, ELF3, EPX, EREG, ERFE, FASLG, FCGR2C, FCN1, FCN3, FCRL3, FFAR4, FGD2, FGD3, FGL2, FGR, FLT3, FOLR2, FOXP3, FRZB, FUT7, FYB1, GADD45B, GBP5, GDF6, GFI1, GMIP, GPR150, GPR174, GPR4, GPR55, GPR83, GPRC5A, GPSM3, GRAP2, GUCY2D, H3C10, H3C11, H3C12, H3C7, HCLS1, HCST, HLA-DMB, HLA-DPA1, HLA-DPB1, HLA-DQA1, HLA-DRA, HLA-DRB1, HLA-DRB5, HOPX, ICOS, IER3, IFNG, IGLL5, IKBKE, IL10RA, IL12RB1, IL16, IL18, IL18RAP, IL26, IL7R, INPP5D, IQCJ-SCHIP1, IRF4, ITGA4, ITGAL, ITGB2, ITGB7, ITK, JAML, KCNN4, KIR2DL2, KIR2DL3, KIR3DL2, KLRB1, KLRC1, KLRC2, KLRD1, KLRK1, KNG1, LAG3, LAIR1, LAIR2, LAX1, LCK, LCP1, LCP2, LDLR, LEF1, LIF, LILRA1, LILRB1, LILRB4, LMCD1, LTA, LY86, MAP4K1, MAPK13, MLC1, MMP9, MOG, MS4A1, MSX1, MUC16, MUC19, MUC6, MYB, MYO1G, MZB1, NAMPT, NCF1, NCKAP1L, NCR1, NCR3, NFAM1, NLRC3, NLRP3, NLRP6, NPFFR2, NPPC, NPTX2, NR4A2, NR4A3, NTN1, NUP210, OASL, P2RY10, P2RY12, PAX5, PDE6G, PIK3CG, PIK3IP1, PIK3R5, PILRA, PIM2, PLA2G2D, PLA2G7, PLAU, PLEK, PRAM1, PRDM1, PRKCB, PRKCQ	[AAAS, ABCA7, ABCC8, ABCD1, ABI3, ABL1, ABR, ABRA, ACKR3, ACP4, ACTB, ACTN4, ADCY1, ADCY3, ADCY4, ADCY9, ADGRG1, ADIPOQ, AGAP2, AGER, AGPAT1, AGT, AGTR1, AKT1S1, AMH, ANGPT1, APLN, APLNR, APLP1, APOA1, APOD, ARAF, ARAP3, ARC, AREG, ARHGAP1, ARHGAP23, ARHGAP39, ARHGDIA, ARHGEF1, ARHGEF16, ARHGEF5, ARMT1, ARRDC3, ARTN, ASPN, BAG3, BAG6, BCAR1, BCL2L1, BCL3, BCL6, BCL9L, BCR, BEND6, BMP8A, BRD4, BTLA, C1QTNF1, C20orf27, C5, C5AR1, C8G, CALCR, CAMK2B, CAPN1, CARM1, CC2D1A, CCL11, CCL2, CCL21, CCL24, CCR7, CD14, CD177, CD1C, CD1E, CD276, CD300E, CD74, CDK1, CDK2, CEACAM1, CEL, CFH, CGA, CHERP, CHGA, CHI3L1, CISH, CITED2, CLC, CLCF1, CLEC1B, CLEC4E, CLIP3, CLNK, CNR1, CNTN6, COL1A1, CPN2, CRAT, CREB3L1, CRHR2, CRP, CRYAB, CSF1, CSK, CSNK1E, CSPG4, CST7, CTHRC1, CX3CL1, CXXC4, CYP26B1, CYP27B1, DAB2IP, DAG1, DAGLA, DAXX, DDX39B, DENND2B, DENND4B, DGKD, DGKZ, DIRAS1, DKK2, DLG5, DLGAP4, DMTN, DOK5, DOT1L, DTX1, DUSP5, DUSP8, DVL3, ECM1, EDN2, EEF1E1, EIF4G1, ELF3, ENDOG, ENG, ENPP3, EPHA2, EPN1, EPPK1, ERBB2, ERFE, ESM1, FABP4, FADD, FAIM2, FAM110A, FAP, FASN, FCN3, FEM1A, FES, FFAR2, FGFR4, FLOT2, FLT3, FLT4, FOXC2, FOXF1, FOXM1, FPR1, FRMD8, FURIN, GAB2, GADD45B, GAS1, GATA2, GATA4, GBA, GBF1, GCSAM, GDF6, GIT1, GLIS2, GNAI2, GNAO1, GPAM, GPBAR1, GPER1, GPI, GPR137, GPR17, GPR20, GPR37L1, GPR4, GPR83, GPR84, GPRC5A, GPRIN1, GRB7, GRIK5, GRINA, GRM2, GRN, GSK3A, GTF2IRD1, H3C10, H3C12, H4C3, HBEGF, HCRTR1, HDAC10, HDAC7, HEYL, HFE, HGS, HIPK4, HMGA1, HOPX, HRH2, HSF1, HSPB1, HSPB8, HTR1B, HTR2A, ICAM1, IER3, IFITM1, IFT80, IL18BP, IL1B, IL1RN, IL4R, IL7R, ILK, INCA1, INHA, INPP5E, IRAK2, ITGA3, ITGA5, ITPRIP, JAK3, JMJD8, JUP, KCNK2, KCP, KCTD11, KIF26A, KLK14, KMT2D, KNG1, KSR1, LAG3, LDLR, LGALS9, LGR6, LIF, LIFR, LILRA5, LIMK1, LIMS2, LMCD1, LMNA, LOX, LRG1, LTBR
GO:0050794	regulation of cellular process	3,02E-10	[2, 3]	1748,00	15,03	38,78	61,22	[ABCB11, ABRA, ACP5, ADAM8, ADAMDEC1, ADAMTS9, ADCY7, ADGRE3, ADGRE4P, ADGRG5, ADGRG6, ADORA3, AIF1, AIM2, AKNA, ALOX15, ALOX15B, ANGPT1, ANGPT2, ANO9, ANPEP, APBB1IP, APOBEC3A, APOBEC3D, APOBEC3G, APOBEC3H, ARAP2, AREG, ARHGAP15, ARHGAP30, ARHGAP45, ASCL2, ASGR2, ATF3, ATP1B4, ATP2A3, BANK1, BATF, BCL11B, BCL2L14, BCL2L15, BCL3, BHLHA15, BHLHE22, BHLHE40, BLK, BLNK, BMF, BMP7, BMPR1B, BRIP1, BTG2, BTK, BTLA, BTN3A1, BTN3A2, C1QA, C1QC, C1QTNF1, C2, CACNA1E, CACNA1I, CALCR, CAMK4, CARD11, CARD17, CARMIL2, CASKIN1, CASP8, CASS4, CBARP, CCDC88B, CCDC88C, CCL17, CCL19, CCL2, CCL22, CCL24, CCL3, CCL3L1, CCL4, CCL4L1, CCL4L2, CCL5, CCR2, CCR3, CCR4, CCR5, CCR8, CD177, CD180, CD19, CD1B, CD1C, CD1E, CD2, CD200R1, CD22, CD226, CD244, CD247, CD27, CD300LF, CD33, CD3D, CD3E, CD3G, CD4, CD40LG, CD5, CD53, CD6, CD7, CD74, CD79A, CD80, CD84, CD86, CD8A, CD8B, CDC20B, CDCA2, CDH1, CDH4, CDKN2A, CEL, CHRDL1, CISH, CLCF1, CLCNKA, CLEC10A, CLEC1B, CLEC4D, CLEC4E, CLEC6A, CLNK, CMTM5, CNN1, CNR1, CNR2, CORO1A, CORO2A, COTL1, CR2, CRABP2, CRB2, CREM, CRTAM, CSF1R, CSF2RA, CST7, CTLA4, CTSS, CXCL10, CXCL11, CXCL5, CXCL6, CXCL9, CXCR3, CXCR4, CXCR6, CXorf21, CYBB, CYSLTR1, CYTIP, DAPK1, DAPP1, DCC, DEPDC1B, DERL3, DLGAP2, DOCK10, DOCK2, DOCK8, DOK2, DPEP1, DPEP2, DPT, DRC1, DRD1, DTHD1, DUSP2, DUSP8, E2F8, EAF2, EBI3, EDN2, EGR3, ELAVL4, ELF3, EOMES, EPHA6, EPX, EREG, ERFE, ERMN, EVI2B, FAM122C, FAM83B, FASLG, FATE1, FBXL19, FCGR2C, FCMR, FCN1, FCN3, FCRL1, FCRL2, FCRL3, FCRL5, FCRL6, FCRLA, FFAR4, FGD2, FGD3, FGL2, FGR, FIBIN, FLT3, FOLR2, FOSB, FOSL1, FOXP3, FRZB, FUT7, FYB1, GABBR2, GADD45B, GALNT3, GBP5, GDF6, GFI1, GFRA2, GLIS1, GMFG, GMIP, GNMT, GPM6A, GPR132, GPR141, GPR150, GPR171, GPR174, GPR19, GPR34, GPR4, GPR55, GPR83, GPRC5A, GPSM3, GRAP2,	[AAAS, ABCA3, ABCA7, ABCB11, ABCC8, ABCD1, ABCD2, ABI3, ABL1, ABLIM3, ABR, ABRA, ACACB, ACKR1, ACKR3, ACP4, ACTB, ACTN1, ACTN4, ADAM19, ADAMTS7, ADAMTS8, ADAMTS9, ADAMTSL4, ADCK1, ADCY1, ADCY3, ADCY4, ADCY9, ADGRB2, ADGRE5, ADGRG1, ADGRG6, ADGRL1, ADIPOQ, AGAP2, AGER, AGPAT1, AGT, AGTR1, AKT1S1, ALPK3, AMH, ANGPT1, ANGPTL1, ANGPTL4, ANGPTL8, ANKK1, ANKRD13B, ANKRD52, AP2A1, AP3D1, APLN, APLNR, APLP1, APOA1, APOBEC3C, APOD, APOM, AQP3, ARAF, ARAP3, ARC, AREG, ARF1, ARGFX, ARHGAP1, ARHGAP23, ARHGAP39, ARHGDIA, ARHGEF1, ARHGEF15, ARHGEF16, ARHGEF5, ARID1A, ARID5A, ARMT1, ARRDC3, ARTN, ASB16, ASB6, ASIC3, ASPN, ATAT1, ATF5, ATN1, ATP13A2, ATP1A1, ATP1A3, ATP1B2, ATP1B4, ATP6V0D1, ATP6V0E2, ATP6V1B1, ATP6V1F, ATPSCKMT, ATXN2L, BAG3, BAG6, BAHD1, BATF2, BAZ2A, BBS12, BCAM, BCAR1, BCKDK, BCL2L1, BCL3, BCL6, BCL7B, BCL9L, BCOR, BCR, BEND6, BHLHE40, BMP8A, BRD4, BRPF3, BSN, BTG2, BTLA, C11orf65, C15orf62, C18orf54, C1QTNF1, C20orf27, C2CD2L, C5, C5AR1, CABIN1, CABLES2, CACNA1E, CACNA1H, CACNB4, CALB1, CALCR, CAMK2B, CAMSAP3, CAMTA2, CAPN1, CAPNS1, CARM1, CARMIL2, CASKIN1, CASZ1, CBARP, CC2D1A, CCDC106, CCL11, CCL2, CCL21, CCL24, CCNG2, CCNJL, CCR7, CD14, CD177, CD1C, CD1E, CD276, CD74, CDC25B, CDC42EP1, CDC42EP2, CDC42EP4, CDCA2, CDH4, CDK1, CDK15, CDK18, CDK2, CDK2AP2, CDKL2, CDKN1A, CEACAM1, CEL, CELSR2, CERT1, CETP, CFAP298-TCP10L, CFH, CFL1, CGA, CHCHD10, CHERP, CHGA, CHGB, CHI3L1, CHRNG, CIC, CIITA, CISH, CITED2, CITED4, CIZ1, CLC, CLCF1, CLCN6, CLCNKA, CLEC1B, CLEC4E, CLIP3, CLNK, CLPTM1, CMTM1, CMTM5, CNKSR1, CNN1, CNOT3, CNR1, CNTN6, COL1A1, CPLX1, CPNE5, CPNE6, CPSF7, CPTP, CRAT, CREB3L1, CRHR2, CRIP2, CRP, CRTC2, CRYAB, CSDC2, CSF1, CSK, CSNK1E, CSPG4, CSRNP3, CST7, CTC1, CTDSP1, CTF1, CTHRC1,

GO:0009893	positive regulation of metabolic process	1,11E-07	[2, 3, 4]	669,00	16,88	38,30	61,70	[ABRA, ADAM8, ADCY7, AIF1, AIM2, AKNA, ALOX15B, ANGPT1, AREG, ASCL2, ATF3, ATP1B4, ATP2A3, BANK1, BATF, BCL11B, BCL3, BHLHA15, BLNK, BMP7, BMPR1B, BRIP1, BTN3A1, BTN3A2, C1QTNF1, CALCR, CAMK4, CARD11, CASP8, CASS4, CCDC88B, CCL19, CCL2, CCL3, CCL5, CCR2, CD177, CD19, CD2, CD226, CD244, CD33, CD3E, CD4, CD40LG, CD6, CD74, CD80, CD86, CDC20B, CDCA2, CDH1, CDKN2A, CLCF1, CLEC6A, CLNK, CREM, CRTAM, CSF1R, CXCL10, CXCR4, CYBB, DAPK1, DEPDC1B, DRD1, E2F8, EAF2, EBI3, EGR3, ELAVL4, ELF3, EOMES, EPHA6, EPX, EREG, FASLG, FCN1, FCRL3, FFAR4, FGD2, FGR, FLT3, FOSB, FOSL1, FOXP3, GADD45B, GBP5, GDF6, GLIS1, GPRC5A, GPSM3, H1-3, HCLS1, HLA-DPA1, HLA-DPB1, HLA-DRB1, IFNG, IKBKE, IKZF3, IL12RB1, IL12RB2, IL16, IL18, IL26, IL7R, IRF4, IRF5, IRF8, IRX3, ITGAX, ITGB2, ITK, JCHAIN, KIAA1324, KLF5, KLRG1, LAMP3, LCK, LCP1, LCP2, LDLR, LEF1, LIF, LILRB1, LMO1, LPAL2, LTA, LTB, LY9, MACC1, MAFF, MAP4K1, MAPK13, MCOLN2, MID1IP1, MIXL1, MMP9, MSX1, MUSK, MYB, MYBL2, MZB1, NAMPT, NCF1, NCKAP1L, NEK5, NFAM1, NLRP2, NLRP3, NLRP6, NLRP9, NR4A1, NR4A2, NR4A3, NSG1, ORM2, P2RY12, PATL2, PAX5, PBX4, PDE6G, PFKFB4, PIK3CG, PIK3R5, PIM1, PIM2, PLAC8, PLEK, POU2AF1, POU2F2, PRDM1, PRKCB, PRKCQ, PROK1, PTAFR, PTGFR, PTK2B, PTPN22, PTPRC, PTX3, PYCARD, PYHIN1, RLN2, RUFY4, RUNX3, SALL1, SASH3, SCIMP, SELE, SEMA4D, SH2D1A, SH2D2A, SIGLEC16, SKAP1, SLAMF6, SMPD3, SOCS1, SOCS3, SPIB, SPIC, STAP1, STAT1, STXBP2, SYK, TBX21, TENT5C, TFEC, THBS1, TIGIT, TLR6, TLR8, TNFAIP3, TNFRSF18, TNFSF13B, TNFSF8, TNIP3, TOX, TRABD2A, TREM2, TRERF1, TRIM14, UCP2, WAS, WNT1, WNT10A, WNT10B, XCL1, ZBP1, ZC3H12D, ZNF804A]	[ABCC8, ABCD1, ABCD2, ABL1, ABLIM3, ABRA, ACACB, ACTA1, ACTB, ACTC1, ACTN1, ACTN4, ADAM19, ADCY1, ADCY3, ADCY4, ADCY9, ADIPOQ, AGAP2, AGER, AGPAT1, AGT, AGTR1, ALPK3, AMH, ANGPT1, AP3D1, APLN, APLNR, APOA1, ARAF, AREG, ARHGEF5, ARID1A, ARTN, ATF5, ATP13A2, ATP1B4, BAG3, BAG6, BCL3, BCL6, BCL9L, BMP8A, BRD4, BRPF3, C1QTNF1, C5, C5AR1, CALCR, CAMTA2, CARM1, CASZ1, CCL2, CCL21, CCR7, CD14, CD177, CD276, CD74, CDC25B, CDCA2, CDK1, CDKN1A, CFH, CGA, CHCHD10, CHERP, CHGA, CHI3L1, CIITA, CITED2, CITED4, CIZ1, CLCF1, CLIP3, CLNK, CNOT3, COL1A1, CREB3L1, CRP, CRTC2, CSF1, CSK, CSNK1E, CSPG4, CSRNP3, CTC1, CTF1, CTIF, CTSD, CX3CL1, CYP26B1, CYP27B1, DAB2IP, DAG1, DAXX, DBF4B, DDR1, DDX39B, DGKZ, DHX34, DIRAS1, DISP3, DNMT1, DPAGT1, DPF2, DTX1, DUSP5, DVL3, DXO, DYRK1B, E2F4, E2F7, E2F8, EAF2, EDC4, EFNA3, EGLN2, EIF4G1, ELF3, ELF4, ELK1, ENDOG, ENG, EPHA2, ERBB2, ETS2, ETV6, FABP4, FADD, FASN, FFAR2, FGFR4, FHL5, FHOD1, FLT3, FLT4, FOSL1, FOSL2, FOXC2, FOXF1, FOXM1, FPR1, FRMD8, FURIN, GADD45B, GATA2, GATA4, GBA, GDF6, GGA1, GGA3, GLIS2, GLMP, GNAI2, GPER1, GPI, GPR3, GPRC5A, GPRIN1, GPT, GRN, GSK3A, GTPBP1, H1-9P, HBB, HBEGF, HCFC1, HDAC10, HDAC5, HEYL, HFE, HGS, HK1, HLTF, HMGA1, HMGN1, HMGN5, HPRT1, HSF1, HSPB1, HSPB8, HSPE1, HTR2A, HVCN1, ICAM1, ID3, IL1B, IL4R, IL7R, ILK, INCENP, INHA, IRAK2, IRF2, IRF2BPL, IRX3, IRX6, ITGA3, ITGA5, JUNB, JUND, JUP, KCNH2, KDM6B, KLF10, KLF5, KMT2D, KSR1, LDLR, LDLRAP1, LGALS9, LIF, LILRA5, LIMK2, LMNA, LMX1A, LMX1B, LRRC32, LTF, LUM, MAFG, MAMSTR, MAP2K3, MAP3K10, MAP3K11, MAP3K14, MAP3K6, MAP3K9, MAPK3, MAPK7, MAPK8IP3, MARK2, MAVS, MAZ, MECP2, MED12, MED16, MED24, MED25, MEF2D, MEIS3P1, MEIS3P2, MEN1, MFAP2, MFSD2A, MID1IP1, MINK1, MLLT6, MLST8, MOV10, MPV17L2, MSN, MSTN, MSX1, MTCO2P12, MUC20, MYBBP1A, MYC, MYH9, MYRF
GO:0048522	positive regulation of cellular process	9,17E-14	[2, 3, 4]	993,00	16,77	38,30	61,70	[ABRA, ADAM8, ADCY7, AIF1, AIM2, AKNA, ALOX15, ALOX15B, ANGPT1, APBB1IP, AREG, ASCL2, ATF3, ATP1B4, ATP2A3, BANK1, BATF, BCL11B, BCL3, BHLHA15, BLK, BMF, BMP7, BMPR1B, BRIP1, BTK, BTLA, C1QA, C1QTNF1, C2, CACNA1I, CALCR, CAMK4, CARD11, CARMIL2, CASP8, CASS4, CCDC88B, CCL17, CCL19, CCL2, CCL22, CCL24, CCL3, CCL3L1, CCL4, CCL4L1, CCL5, CCR2, CCR3, CD177, CD180, CD19, CD1B, CD1C, CD1E, CD2, CD226, CD244, CD247, CD27, CD300LF, CD33, CD3E, CD4, CD40LG, CD5, CD53, CD6, CD74, CD80, CD86, CDC20B, CDCA2, CDH1, CDH4, CDKN2A, CEL, CLCF1, CLEC4D, CLEC6A, CNR1, CORO1A, CRABP2, CRB2, CREM, CRTAM, CSF1R, CST7, CTLA4, CTSS, CXCL10, CXCL11, CXCL5, CXCL9, CXCR4, CXorf21, DAPK1, DEPDC1B, DOCK2, DOCK8, DPEP2, DRD1, E2F8, EAF2, EBI3, EDN2, EGR3, ELAVL4, ELF3, EOMES, EPHA6, EPX, EREG, ERFE, EVI2B, FASLG, FCRL3, FFAR4, FGD2, FGD3, FGR, FLT3, FOLR2, FOSB, FOSL1, FOXP3, FUT7, GABBR2, GADD45B, GBP5, GDF6, GFI1, GLIS1, GMFG, GPM6A, GPR174, GPR4, GPR55, GPRC5A, GPSM3, GRAP2, GZMA, GZMB, H1-3, HCLS1, HCST, HLA-DMB, HLA-DPA1, HLA-DPB1, HLA-DRA, HLA-DRB1, HOPX, ICOS, IFNG, IGLL5, IKBKE, IKZF3, IL10RA, IL12RB1, IL12RB2, IL18, IL18RAP, IL26, IL2RB, IL2RG, IL5RA, IL7R, INPP5D, IQCJ-SCHIP1, IQGAP2, IRF4, IRF5, IRF8, IRX3, ITGA4, ITGAX, ITGB2, JAML, KCNJ10, KCNN4, KIAA1324, KLF5, KLRC2, KLRD1, KLRG1, KLRK1, KMO, KNG1, LAG3, LCK, LCP1, LCP2, LDLR, LEF1, LIF, LILRB1, LILRB4, LMCD1, LMO1, LPAL2, LRRC38, LTA, LY86, MACC1, MAFF, MAP4K1, MATK, MEDAG, MID1IP1, MIXL1, MLC1, MMP9, MSX1, MUSK, MYB, MYBL2, MZB1, NAMPT, NCF1, NCKAP1L, NCR3, NEK5, NELL2, NFAM1, NLRP2, NLRP3, NLRP6, NPPC, NR4A1, NR4A2, NR4A3, NSG1, NTN1, OASL, P2RY10, P2RY12, PATL2, PAX5, PBX4, PDE6G, PFKFB4, PIK3CG, PIK3R5, PIM1, PIM2, PLA2G7, PLAAT4, PLAC8, PLEK, PLXNC1, POU2AF1, POU2F2, PRF1, PRKCB, PRKCQ, PROK1, PTAFR, PTGFR, PTK2B, PTPN22,	[ABCA3, ABCA7, ABCC8, ABCD1, ABCD2, ABI3, ABL1, ABLIM3, ABR, ABRA, ACACB, ACKR3, ACTN1, ACTN4, ADAM19, ADAMTSL4, ADCK1, ADCY1, ADCY3, ADCY4, ADCY9, ADGRG1, ADGRL1, ADIPOQ, AGAP2, AGER, AGPAT1, AGT, AGTR1, ALPK3, AMH, ANGPT1, AP2A1, AP3D1, APLN, APLNR, APOA1, ARAF, ARC, AREG, ARF1, ARHGDIA, ARHGEF1, ARHGEF15, ARHGEF16, ARHGEF5, ARID1A, ARTN, ATAT1, ATF5, ATP13A2, ATP1B2, ATP1B4, ATPSCKMT, BAG3, BAG6, BCAR1, BCL2L1, BCL3, BCL6, BCL9L, BEND6, BMP8A, BRD4, BRPF3, BTLA, C15orf62, C1QTNF1, C20orf27, C2CD2L, C5, C5AR1, CACNA1H, CALB1, CALCR, CAMK2B, CAMTA2, CAPN1, CAPNS1, CARM1, CARMIL2, CASZ1, CC2D1A, CCL11, CCL2, CCL21, CCL24, CCR7, CD14, CD177, CD1C, CD1E, CD276, CD74, CDC25B, CDC42EP1, CDC42EP2, CDC42EP4, CDCA2, CDH4, CDK1, CDK2, CDKN1A, CEACAM1, CEL, CGA, CHCHD10, CHERP, CHGA, CHI3L1, CIITA, CITED2, CITED4, CIZ1, CLCF1, CLIP3, CNOT3, CNR1, CNTN6, COL1A1, CPNE5, CPNE6, CRAT, CREB3L1, CRHR2, CRIP2, CRP, CRTC2, CSF1, CSK, CSNK1E, CSPG4, CSRNP3, CST7, CTC1, CTF1, CTHRC1, CTIF, CTSD, CX3CL1, CYP26B1, CYP27B1, DAB2IP, DAG1, DAXX, DBF4B, DDR1, DDX39B, DENND2B, DGKD, DGKZ, DHX34, DIRAS1, DISP3, DKK2, DLG5, DMTN, DNMT1, DOC2B, DOK5, DPAGT1, DPF2, DTX1, DUSP5, DVL3, DXO, DYRK1B, E2F4, E2F7, E2F8, EAF2, ECM1, ECM2, EDC4, EDN2, EEF1E1, EFEMP2, EFNA3, EHD1, EHD2, EIF4EBP1, EIF4G1, ELF3, ELF4, ELK1, ENDOG, ENG, EPHA2, ERBB2, ERFE, ESM1, ESPL1, ETS2, ETV6, FADD, FAM110A, FAP, FASN, FES, FGFR4, FHL5, FHOD1, FLOT2, FLT3, FLT4, FOSL1, FOSL2, FOXC2, FOXF1, FOXM1, FPR1, FURIN, GAB2, GABBR2, GADD45B, GATA2, GATA4, GBA, GDF6, GDI1, GIT1, GLIS2, GLMP, GNAI2, GNAO1, GPAM, GPBAR1, GPER1, GPI, GPM6A, GPR137, GPR17, GPR20, GPR37L1, GPR4, GPRC5A, GPRIN1, GPT, GRB7, GRIK5, GRN, GSK3A, GTPBP1, H1-9P, H4C3, HAVCR1, HBA1, HBB, HBEGF, HCFC1, HCRTR1, HDAC10, HDAC5, HDAC7, HEYL, HFE,

GO:0048584	positive regulation of response to stimulus	3,89E-17	[2, 3, 4]	491,00	19,87	48,73	51,27	[ABRA, ADAM8, AIF1, AIM2, ALOX15, ALOX15B, ANGPT1, AREG, ATF3, BANK1, BLK, BLNK, BMF, BMP7, BMPR1B, BTK, BTN3A1, BTN3A2, C1QA, C1QB, C1QC, C1QTNF1, C2, C8G, CALCR, CARD11, CASP8, CASS4, CCL17, CCL19, CCL2, CCL22, CCL24, CCL3, CCL3L1, CCL4, CCL4L1, CCL5, CCR2, CCR4, CD177, CD180, CD19, CD1B, CD1C, CD1E, CD22, CD226, CD247, CD27, CD300LF, CD3D, CD3E, CD3G, CD4, CD40LG, CD74, CD79A, CD80, CD86, CDKN2A, CFP, CLCF1, CLEC10A, CLEC4D, CLEC4E, CLEC6A, CLNK, CNR1, CR2, CRB2, CRTAM, CSF1R, CTLA4, CXCL10, CXCR4, CXorf21, CYP11B1, DEPDC1B, DPEP2, EDN2, ELF3, EPX, EREG, ERFE, FASLG, FCN1, FCN3, FCRL3, FFAR4, FGD2, FGR, FLT3, FOXP3, FYB1, GADD45B, GBP5, GDF6, GFI1, GPR174, GPR4, GPR55, GPSM3, GRAP2, HCLS1, HCST, HLA-DMB, HLA-DPA1, HLA-DPB1, HLA-DQA1, HLA-DRA, HLA-DRB1, HLA-DRB5, ICOS, IFNG, IGLL5, IKBKE, IL10RA, IL12RB1, IL16, IL18, IL18RAP, IL26, IL7R, INPP5D, IQCJ-SCHIP1, ITGB2, ITK, KCNN4, KLRC2, KLRD1, KLRK1, LAG3, LAX1, LCK, LCP1, LCP2, LDLR, LILRB1, LILRB4, LMCD1, LTA, LY86, MAP4K1, MAPK13, MMP9, MOG, MS4A1, MSX1, MUC16, MUC19, MUC6, MYB, MYO1G, NCF1, NCKAP1L, NCR3, NFAM1, NLRP3, NPPC, NR4A3, OASL, P2RY10, P2RY12, PAX5, PDE6G, PIK3CG, PIK3R5, PIM2, PLA2G7, PRAM1, PRKCB, PRKCQ, PROK1, PTAFR, PTK2B, PTPN22, PTPN6, PTPRC, PYCARD, PYHIN1, RAC2, RASGRF1, RTKN2, SALL1, SASH3, SCIMP, SCUBE1, SEMA4D, SH2D1A, SIGLEC16, SKAP1, SLA2, SLAMF6, SOCS1, STAP1, SUCNR1, SYK, TBX21, TESPA1, THBS1, THEMIS, THEMIS2, THY1, TLR10, TLR8, TNFAIP3, TNFSF13B, TNFSF14, TRAF1, TRAT1, TREM2, TRIM14, UBASH3A, UBD, VAV1, WAS, WNT1, WNT10B, XCL1, XCL2, ZAP70, ZBP1]	[ABCA7, ABI3, ABL1, ABRA, ACKR3, ACTB, ACTN4, ADGRG1, ADIPOQ, AGAP2, AGER, AGPAT1, AGT, AGTR1, AMH, ANGPT1, APOA1, ARAF, AREG, ARHGEF5, ARTN, BAG6, BCAR1, BCL2L1, BMP8A, BRD4, C1QTNF1, C20orf27, C5, C5AR1, C8G, CALCR, CC2D1A, CCL11, CCL2, CCL21, CCL24, CCR7, CD14, CD177, CD1C, CD1E, CD276, CD74, CEACAM1, CFH, CHERP, CHGA, CHI3L1, CITED2, CLCF1, CLEC4E, CLNK, CNR1, CNTN6, COL1A1, CPN2, CRAT, CRP, CSF1, CSK, CSNK1E, CSPG4, CX3CL1, CYP27B1, DAB2IP, DAXX, DENND2B, DGKD, DGKZ, DIRAS1, DKK2, DLG5, DMTN, DOK5, DUSP5, DVL3, ECM1, EDN2, EEF1E1, ELF3, ENDOG, ENG, ERBB2, ERFE, ESM1, FABP4, FADD, FAM110A, FASN, FCN3, FEM1A, FFAR2, FGFR4, FLT3, FLT4, FOXC2, FOXM1, FPR1, GAB2, GADD45B, GATA2, GATA4, GCSAM, GDF6, GNAI2, GNAO1, GPBAR1, GPER1, GPR137, GPR17, GPR20, GPR37L1, GPR4, GPRIN1, GRN, GSK3A, HBEGF, HCRTR1, HDAC10, HFE, HMGA1, HSPB1, HTR2A, ICAM1, IL1B, IL4R, IL7R, ILK, INCA1, INHA, IRAK2, ITGA5, JMJD8, JUP, KCNK2, KCP, KLK14, KMT2D, KSR1, LAG3, LDLR, LGALS9, LGR6, LILRA5, LIMK1, LIMS2, LMCD1, LRG1, LTBR, LTF, MAP2K3, MAP3K10, MAP3K11, MAP3K14, MAP3K6, MAP3K9, MAPK3, MAPK8IP3, MAVS, MAZ, MED12, MEGF8, MEIS3P1, MEN1, MINK1, MLST8, MSTN, MSX1, MUC19, MUC20, MUC3A, MYC, NCOR2, NDC80, NDST1, NECTIN2, NFATC4, NGF, NGFR, NINJ1, NLGN2, NLRC5, NOD2, NODAL, NOS3, NOTCH1, NOTCH4, NPAS2, NPPA, NR4A3, NRARP, OSM, P2RX3, P2RX5, P2RY6, PCDHA4, PDGFB, PDGFRB, PIAS4, PLA2G2A, PLA2G7, PLAGL2, PLAUR, PLXNB1, PML, POR, PPARD, PPP1R15A, PQBP1, PRAG1, PRKACA, PTN, PTP4A3, PVR, RAC2, RAD51AP1, RAP1A, RAPGEF1, RARA, RASD2, RBM14, RELA, RELB, RGCC, RHBDF2, RHOG, RNU1-93P, RPL26, RPTOR, RTKN2, S1PR2, SECTM1, SELENON, SELENOP, SEMA4C, SEPTIN4, SERPINE1, SERPING1, SGTA, SH2B2, SHANK3, SHARPIN, SHC2, SHISA5, SHKBP1, SIK3, SLC2A4, SLC44A2, SLC7A8, SLC9A1, SLC9A3R1, SMAD3, SMARCA4
GO:0010646	regulation of cell communication	2,64E-15	[3, 4]	671,00	18,23	37,26	62,74	[ABRA, ADAM8, ALOX15, ALOX15B, ANGPT1, ARAP2, AREG, ARHGAP15, ARHGAP30, ARHGAP45, ATF3, BANK1, BCL2L14, BCL3, BLK, BMF, BMP7, BMPR1B, BRIP1, C1QTNF1, CACNA1E, CALCR, CARD11, CASP8, CASS4, CCDC88C, CCL17, CCL19, CCL2, CCL22, CCL24, CCL3, CCL3L1, CCL4, CCL4L1, CCL5, CCR2, CD177, CD180, CD19, CD22, CD226, CD27, CD300LF, CD3E, CD4, CD40LG, CD74, CD80, CD86, CDH1, CDKN2A, CEL, CHRDL1, CISH, CLEC6A, CNR1, CNR2, CRB2, CSF1R, CXCR4, CXorf21, DAPK1, DCC, DEPDC1B, DLGAP2, DPEP2, DRD1, DUSP2, DUSP8, ELAVL4, ELF3, EPX, EREG, ERFE, FASLG, FCRL3, FFAR4, FGD2, FGD3, FGR, FLT3, FOLR2, FOXP3, FRZB, FUT7, GADD45B, GDF6, GFI1, GMIP, GPR174, GPR4, GPR55, GPRC5A, GUCY2D, H3C10, H3C11, H3C12, H3C7, HCLS1, HCST, HLA-DRB1, ICOS, IER3, IFNG, IKBKE, IL10RA, IL18, IL26, IL7R, INPP5D, IQCJ-SCHIP1, IRF4, IRX3, KCNJ10, KCNN4, KMO, LAX1, LCK, LEF1, LIF, LILRB4, LMCD1, LY86, MAP4K1, MIDN, MMP9, MSX1, MYB, MZB1, NCF1, NCKAP1L, NFAM1, NLRC3, NLRP3, NLRP6, NPFFR2, NPPC, NPTX2, NR4A2, NSG1, NTN1, OASL, P2RY10, P2RY12, PAX5, PDE6G, PIK3CG, PIK3IP1, PIK3R5, PIM2, PLAU, PLEK, PRDM1, PRKCB, PRKCQ, PROK1, PSCA, PSD4, PTK2B, PTPN22, PTPN6, PTPRC, PYCARD, PYHIN1, RAC2, RAD9B, RASAL3, RASGRF1, RBPMS2, RGS1, RGS10, RGS18, RGS9, RHOH, RMI2, RNASE6, RRAD, RTKN2, SALL1, SCIMP, SCUBE1, SEMA4D, SH2D1A, SHISA8, SLA, SLA2, SLC24A4, SLC8A3, SMPDL3B, SOCS1, SOCS3, SPINK1, STAP1, STAT1, SYK, TAGAP, TBC1D10C, TESPA1, THBS1, THY1, TLR6, TMC8, TNFAIP3, TNFSF14, TNIP3, TNMD, TNR, TRABD2A, TRAF1, TRAT1, TREM2, TRIM14, TRPM2, UBASH3A, UBD, UCP2, VAV1, VNN1, VWC2L, WNT1, WNT10B, XCL1, XCL2, ZAP70, ZBP1]	[ABCA7, ABCC8, ABL1, ABR, ABRA, ACKR3, ACP4, ACTN4, ADCY1, ADGRG1, ADIPOQ, AGAP2, AGER, AGPAT1, AGT, AGTR1, AKT1S1, AMH, ANGPT1, APLN, APLNR, APLP1, APOA1, APOD, ARAF, ARAP3, ARC, AREG, ARF1, ARHGAP1, ARHGAP23, ARHGAP39, ARHGDIA, ARHGEF1, ARHGEF16, ARHGEF5, ARRDC3, ARTN, ASPN, BCL2L1, BCL3, BCL6, BCL9L, BCR, BEND6, BMP8A, BRD4, C1QTNF1, C20orf27, C2CD2L, C5, C5AR1, CACNA1E, CALB1, CALCR, CAMK2B, CAPN1, CARM1, CC2D1A, CCL11, CCL2, CCL21, CCL24, CCR7, CD14, CD177, CD74, CDK1, CDK2, CEACAM1, CEL, CGA, CHERP, CHGA, CHI3L1, CISH, CITED2, CLIP3, CNR1, CNTN6, COL1A1, CPLX1, CRAT, CREB3L1, CRHR2, CSF1, CSK, CSNK1E, CSPG4, CTHRC1, CX3CL1, CXXC4, CYP26B1, CYP27B1, DAB2IP, DAG1, DAXX, DDX39B, DENND2B, DENND4B, DGKD, DGKZ, DHX34, DIRAS1, DKK2, DLG5, DLGAP4, DMTN, DOC2B, DOK5, DOT1L, DTX1, DUSP5, DUSP8, DVL3, ECM1, EEF1E1, ELF3, ENG, EPHA2, EPN1, ERBB2, ERFE, ESM1, FADD, FAIM2, FAM110A, FASN, FFAR2, FGFR4, FLOT2, FLT3, FLT4, FOXM1, FPR1, FRMD8, FURIN, GAB2, GADD45B, GAS1, GATA4, GBA, GBF1, GCSAM, GDF6, GIT1, GJD3, GLIS2, GLRA1, GNAI2, GPAM, GPBAR1, GPER1, GPI, GPR137, GPR17, GPR20, GPR37L1, GPR4, GPRC5A, GPRIN1, GRB7, GRIK5, GRINA, GRM2, GSK3A, H3C10, H3C12, H4C3, HBEGF, HCRTR1, HDAC7, HEYL, HFE, HGS, HIPK4, HSPB1, HTR1B, HTR2A, ICAM1, IER3, IFT80, IL18BP, IL1B, IL1RN, IL7R, ILK, INCA1, INHA, INPP5E, IRAK2, IRX3, ITGA3, ITGA5, ITPR3, ITPRIP, JAK3, JMJD8, JPH4, JUP, KCP, KCTD11, KIF26A, KLK14, KMT2D, KSR1, LGALS9, LGR6, LIF, LIFR, LILRA5, LIMS2, LMCD1, LMNA, LOX, LRG1, LRRC4, LRRC8A, LTBR, LTF, LY6E, LYNX1, LZTS1, LZTS2, MAP1A, MAP2K3, MAP3K10, MAP3K11, MAP3K14, MAP3K6, MAP3K9, MAPK3, MAPK7, MAPK8IP3, MAVS, MAZ, MECP2, MED12, MEGF8, MEIS3P1, MEN1, MGRN1, MIDN, MINK1, MLST8, MME, MNT, MSTN, MSX1, MTRNR2L1, MTRNR2L10, MTRNR2L12, MTRNR2L2, MTRNR2L3, MTRNR2L6, MTRNR2L8, MTRNR2L9,

GO:0010604	positive regulation of macromolecule metabolic process	1,05E-06	[3, 4, 5]	618,00	16,88	38,50	61,50	[ABRA, ADAM8, ADCY7, AIF1, AIM2, AKNA, ALOX15B, ANGPT1, AREG, ASCL2, ATF3, ATP1B4, ATP2A3, BANK1, BATF, BCL11B, BCL3, BHLHA15, BLNK, BMP7, BMPR1B, BRIP1, BTN3A1, BTN3A2, C1QTNF1, CALCR, CAMK4, CARD11, CASP8, CASS4, CCDC88B, CCL19, CCL2, CCL3, CCL5, CCR2, CD2, CD226, CD244, CD33, CD3E, CD4, CD40LG, CD6, CD74, CD80, CD86, CDC20B, CDCA2, CDH1, CDKN2A, CLCF1, CLEC6A, CLNK, CREM, CRTAM, CSF1R, CXCL10, CXCR4, CYBB, DAPK1, DEPDC1B, DRD1, E2F8, EAF2, EBI3, EGR3, ELAVL4, ELF3, EOMES, EPX, EREG, FASLG, FCN1, FCRL3, FGD2, FGR, FLT3, FOSB, FOSL1, FOXP3, GADD45B, GBP5, GDF6, GLIS1, GPRC5A, GPSM3, H1-3, HCLS1, HLA-DPA1, HLA-DPB1, HLA-DRB1, IFNG, IKBKE, IKZF3, IL12RB1, IL12RB2, IL16, IL18, IL26, IL7R, IRF4, IRF5, IRF8, IRX3, ITGAX, ITK, KLF5, KLRG1, LAMP3, LCK, LCP1, LCP2, LDLR, LEF1, LIF, LILRB1, LMO1, LPAL2, LTA, LTB, LY9, MACC1, MAFF, MAP4K1, MAPK13, MCOLN2, MIXL1, MMP9, MSX1, MUSK, MYB, MYBL2, MZB1, NAMPT, NCF1, NCKAP1L, NEK5, NFAM1, NLRP2, NLRP3, NLRP6, NLRP9, NR4A1, NR4A2, NR4A3, NSG1, ORM2, PATL2, PAX5, PBX4, PDE6G, PIK3CG, PIK3R5, PIM1, PIM2, PLAC8, POU2AF1, POU2F2, PRDM1, PRKCB, PRKCQ, PROK1, PTAFR, PTGFR, PTK2B, PTPN22, PTPRC, PYCARD, PYHIN1, RLN2, RUNX3, SALL1, SASH3, SCIMP, SELE, SEMA4D, SH2D1A, SIGLEC16, SKAP1, SLAMF6, SOCS1, SOCS3, SPIB, SPIC, STAP1, STAT1, STXBP2, SYK, TBX21, TENT5C, TFEC, THBS1, TIGIT, TLR6, TLR8, TNFAIP3, TNFRSF18, TNFSF13B, TNFSF8, TNIP3, TOX, TRABD2A, TREM2, TRERF1, TRIM14, WAS, WNT1, WNT10A, WNT10B, XCL1, ZBP1, ZC3H12D, ZNF804A]	[ABCC8, ABL1, ABLIM3, ABRA, ACTA1, ACTB, ACTC1, ACTN1, ACTN4, ADAM19, ADCY1, ADCY3, ADCY4, ADCY9, ADIPOQ, AGAP2, AGER, AGPAT1, AGT, AGTR1, ALPK3, AMH, ANGPT1, AP3D1, APLN, APLNR, ARAF, AREG, ARHGEF5, ARID1A, ARTN, ATF5, ATP13A2, ATP1B4, BAG6, BCL3, BCL6, BCL9L, BMP8A, BRD4, BRPF3, C1QTNF1, C5, C5AR1, CALCR, CAMTA2, CARM1, CASZ1, CCL2, CCL21, CCR7, CD14, CD276, CD74, CDC25B, CDCA2, CDK1, CDKN1A, CGA, CHCHD10, CHERP, CHGA, CHI3L1, CIITA, CITED2, CITED4, CIZ1, CLCF1, CLIP3, CLNK, CNOT3, COL1A1, CREB3L1, CRP, CRTC2, CSF1, CSK, CSNK1E, CSPG4, CSRNP3, CTC1, CTF1, CTIF, CTSD, CX3CL1, CYP26B1, CYP27B1, DAB2IP, DAG1, DAXX, DBF4B, DDX39B, DHX34, DIRAS1, DNMT1, DPF2, DTX1, DUSP5, DVL3, DXO, DYRK1B, E2F4, E2F7, E2F8, EAF2, EDC4, EFNA3, EGLN2, EIF4G1, ELF3, ELF4, ELK1, ENDOG, ENG, ERBB2, ETS2, ETV6, FADD, FASN, FFAR2, FGFR4, FHL5, FHOD1, FLT3, FLT4, FOSL1, FOSL2, FOXC2, FOXF1, FOXM1, FPR1, FRMD8, FURIN, GADD45B, GATA2, GATA4, GBA, GDF6, GGA1, GGA3, GLIS2, GLMP, GPER1, GPI, GPRC5A, GPRIN1, GRN, GSK3A, GTPBP1, H1-9P, HBEGF, HCFC1, HDAC10, HDAC5, HEYL, HFE, HGS, HK1, HLTF, HMGA1, HMGN1, HMGN5, HSF1, HSPB1, HSPE1, HTR2A, ICAM1, ID3, IL1B, IL4R, IL7R, ILK, INCENP, INHA, IRAK2, IRF2, IRF2BPL, IRX3, IRX6, ITGA3, ITGA5, JUNB, JUND, JUP, KCNH2, KDM6B, KLF10, KLF5, KMT2D, KSR1, LDLR, LGALS9, LIF, LILRA5, LIMK2, LMNA, LMX1A, LMX1B, LRRC32, LTF, LUM, MAFG, MAMSTR, MAP2K3, MAP3K10, MAP3K11, MAP3K14, MAP3K6, MAP3K9, MAPK3, MAPK7, MAPK8IP3, MARK2, MAVS, MAZ, MECP2, MED12, MED16, MED24, MED25, MEF2D, MEIS3P1, MEIS3P2, MEN1, MINK1, MLLT6, MLST8, MOV10, MPV17L2, MSN, MSTN, MSX1, MUC20, MYBBP1A, MYC, MYH9, MYRF, NAP1L2, NCOA6, NCOR2, NDP, NEK5, NFATC4, NFE2, NFIC, NFIX, NFKB2, NFKBIB, NGF, NGFR, NKD2, NLRC5, NLRP9, NOD2, NODAL, NOS3, NOTCH1, NOTCH3, NOTCH4, NPAS2, NR1D1, NR4A1, NR4A2
GO:0031325	positive regulation of cellular metabolic process	1,24E-03	[3, 4, 5]	560,00	16,33	33,95	66,05	[ABRA, ADAM8, ADCY7, AIF1, AIM2, AKNA, ANGPT1, AREG, ASCL2, ATF3, ATP1B4, ATP2A3, BANK1, BATF, BCL11B, BCL3, BHLHA15, BMP7, BMPR1B, BRIP1, CALCR, CAMK4, CASP8, CASS4, CCL19, CCL5, CD177, CD19, CD244, CD33, CD3E, CD4, CD40LG, CD74, CD80, CD86, CDC20B, CDCA2, CDH1, CDKN2A, CLCF1, CREM, CSF1R, CXCL10, CXCR4, DAPK1, DEPDC1B, E2F8, EAF2, EGR3, ELF3, EOMES, EPHA6, EPX, EREG, FASLG, FCRL3, FGD2, FGR, FLT3, FOSB, FOSL1, FOXP3, GADD45B, GDF6, GLIS1, GPRC5A, H1-3, HCLS1, HLA-DRB1, IFNG, IKBKE, IKZF3, IL18, IL26, IRF4, IRF5, IRF8, IRX3, ITGB2, KIAA1324, KLF5, KLRG1, LCK, LCP2, LDLR, LEF1, LIF, LILRB1, LMO1, LPAL2, MACC1, MAFF, MAP4K1, MID1IP1, MIXL1, MMP9, MSX1, MUSK, MYB, MYBL2, NAMPT, NCF1, NCKAP1L, NEK5, NLRP2, NLRP3, NLRP6, NR4A1, NR4A2, NR4A3, NSG1, P2RY12, PATL2, PAX5, PBX4, PDE6G, PFKFB4, PIK3CG, PIK3R5, PIM1, PIM2, PLAC8, PLEK, POU2AF1, POU2F2, PRKCB, PRKCQ, PROK1, PTAFR, PTK2B, PTPN22, PTPRC, PTX3, PYCARD, PYHIN1, RUFY4, RUNX3, SALL1, SELE, SEMA4D, SH2D1A, SKAP1, SMPD3, SOCS1, SOCS3, SPIB, SPIC, STAP1, STAT1, STXBP2, SYK, TBX21, TFEC, THBS1, TLR6, TNFAIP3, TNFRSF18, TNFSF8, TNIP3, TOX, TRABD2A, TREM2, TRERF1, TRIM14, WAS, WNT1, WNT10B, ZC3H12D]	[ABCD1, ABCD2, ABL1, ABLIM3, ABRA, ACACB, ACTN1, ACTN4, ADCY1, ADCY3, ADCY4, ADCY9, ADIPOQ, AGAP2, AGER, AGPAT1, AGT, AGTR1, ALPK3, AMH, ANGPT1, AP3D1, APLN, APLNR, APOA1, ARAF, AREG, ARHGEF5, ARID1A, ARTN, ATF5, ATP1B4, BAG3, BAG6, BCL3, BCL6, BCL9L, BMP8A, BRD4, BRPF3, C5, C5AR1, CALCR, CAMTA2, CARM1, CASZ1, CCL21, CCR7, CD177, CD74, CDC25B, CDCA2, CDK1, CDKN1A, CGA, CHCHD10, CHERP, CHGA, CHI3L1, CIITA, CITED2, CITED4, CIZ1, CLCF1, CLIP3, CNOT3, COL1A1, CREB3L1, CRP, CRTC2, CSF1, CSK, CSNK1E, CSPG4, CSRNP3, CTC1, CTF1, CTIF, CTSD, CX3CL1, CYP27B1, DAB2IP, DAG1, DAXX, DBF4B, DDR1, DDX39B, DGKZ, DHX34, DIRAS1, DNMT1, DPAGT1, DPF2, DTX1, DUSP5, DVL3, DXO, DYRK1B, E2F4, E2F7, E2F8, EAF2, EDC4, EFNA3, EIF4G1, ELF3, ELF4, ELK1, ENDOG, ENG, EPHA2, ERBB2, ETS2, ETV6, FADD, FASN, FGFR4, FHL5, FHOD1, FLT3, FLT4, FOSL1, FOSL2, FOXC2, FOXF1, FOXM1, FPR1, FURIN, GADD45B, GATA2, GATA4, GBA, GDF6, GLIS2, GLMP, GNAI2, GPER1, GPRC5A, GPRIN1, GPT, GRN, GSK3A, GTPBP1, H1-9P, HBB, HBEGF, HCFC1, HDAC10, HDAC5, HEYL, HFE, HLTF, HMGA1, HMGN1, HMGN5, HPRT1, HSF1, HSPB8, HSPE1, HTR2A, HVCN1, ICAM1, IL1B, ILK, INCENP, INHA, IRAK2, IRF2, IRF2BPL, IRX3, IRX6, ITGA5, JUNB, JUND, JUP, KCNH2, KDM6B, KLF10, KLF5, KMT2D, KSR1, LDLR, LGALS9, LIF, LILRA5, LIMK2, LMNA, LMX1A, LMX1B, LTF, LUM, MAFG, MAMSTR, MAP2K3, MAP3K10, MAP3K11, MAP3K14, MAP3K6, MAP3K9, MAPK3, MAPK7, MAPK8IP3, MARK2, MAVS, MAZ, MECP2, MED12, MED16, MED24, MED25, MEF2D, MEIS3P1, MEIS3P2, MEN1, MFSD2A, MID1IP1, MINK1, MLLT6, MLST8, MOV10, MPV17L2, MSN, MSTN, MSX1, MTCO2P12, MUC20, MYC, MYH9, MYRF, NAP1L2, NCOA6, NDP, NEK5, NFATC4, NFE2, NFIC, NFIX, NFKB2, NFKBIB, NGF, NGFR, NKD2, NLRC5, NOD2, NODAL, NOS3, NOTCH1, NOTCH3, NOTCH4, NPAS2, NR1D1, NR4A1, NR4A2, NR4A3, NSG1, NSMF, OMA1, OSM, OSR2, P2RY6, PAF1, PAK4, PATL1, PCOLCE,

GO:0051173	positive regulation of nitrogen compound metabolic process	1,29E-02	[3, 4, 5]	523,00	16,17	33,88	66,12	[ABRA, ADAM8, ADCY7, AIF1, AIM2, AKNA, ANGPT1, AREG, ASCL2, ATF3, ATP1B4, ATP2A3, BANK1, BATF, BCL11B, BCL3, BHLHA15, BMP7, BMPR1B, BRIP1, CALCR, CAMK4, CASP8, CASS4, CCL19, CCL5, CD33, CD3E, CD4, CD40LG, CD74, CD80, CD86, CDC20B, CDCA2, CDH1, CDKN2A, CLCF1, CREM, CSF1R, CXCL10, CXCR4, DAPK1, DEPDC1B, E2F8, EAF2, EGR3, ELF3, EOMES, EPX, EREG, FASLG, FCRL3, FGD2, FLT3, FOSB, FOSL1, FOXP3, GADD45B, GDF6, GLIS1, GPRC5A, H1-3, HCLS1, HLA-DRB1, IFNG, IKBKE, IKZF3, IL18, IL26, IRF4, IRF5, IRF8, IRX3, ITGB2, KLF5, KLRG1, LCK, LCP2, LDLR, LEF1, LIF, LILRB1, LMO1, MACC1, MAFF, MAP4K1, MIXL1, MMP9, MSX1, MUSK, MYB, MYBL2, NAMPT, NCF1, NCKAP1L, NEK5, NLRP2, NLRP3, NLRP6, NR4A1, NR4A2, NR4A3, PATL2, PAX5, PBX4, PDE6G, PFKFB4, PIK3CG, PIK3R5, PIM1, PIM2, PLAC8, POU2AF1, POU2F2, PRKCB, PRKCQ, PROK1, PTAFR, PTK2B, PTPN22, PTPRC, PTX3, PYCARD, PYHIN1, RUNX3, SALL1, SEMA4D, SH2D1A, SKAP1, SMPD3, SOCS1, SOCS3, SPIB, SPIC, STAP1, STAT1, STXBP2, SYK, TBX21, TFEC, THBS1, TLR6, TNFAIP3, TNFRSF18, TNFSF13B, TNFSF8, TNIP3, TOX, TRABD2A, TREM2, TRERF1, TRIM14, WAS, WNT1, WNT10B, ZC3H12D]	[ABL1, ABLIM3, ABRA, ACTN1, ACTN4, ADCY1, ADCY3, ADCY4, ADCY9, ADIPOQ, AGAP2, AGER, AGT, AGTR1, ALPK3, AMH, ANGPT1, AP3D1, APLN, APLNR, ARAF, AREG, ARHGEF5, ARID1A, ARTN, ATF5, ATP1B4, BAG6, BCL3, BCL6, BCL9L, BMP8A, BRD4, C5, C5AR1, CALCR, CAMTA2, CARM1, CASZ1, CCL21, CCR7, CD74, CDC25B, CDCA2, CDKN1A, CGA, CHCHD10, CHERP, CHGA, CHI3L1, CIITA, CITED2, CITED4, CIZ1, CLCF1, CLIP3, CNOT3, COL1A1, CREB3L1, CRTC2, CSF1, CSK, CSNK1E, CSPG4, CSRNP3, CTF1, CTIF, CTSD, CX3CL1, CYP27B1, DAB2IP, DAG1, DAXX, DBF4B, DDX39B, DIRAS1, DNMT1, DPF2, DTX1, DUSP5, DVL3, DXO, DYRK1B, E2F4, E2F7, E2F8, EAF2, EDC4, EFNA3, EGLN2, EIF4G1, ELF3, ELF4, ELK1, ENDOG, ENG, ERBB2, ETS2, ETV6, FADD, FASN, FGFR4, FHL5, FHOD1, FLT3, FLT4, FOSL1, FOSL2, FOXC2, FOXF1, FOXM1, FPR1, FURIN, GADD45B, GATA2, GATA4, GBA, GDF6, GGA1, GGA3, GLIS2, GLMP, GPER1, GPRC5A, GPRIN1, GRN, GSK3A, GTPBP1, H1-9P, HBB, HBEGF, HCFC1, HDAC10, HDAC5, HEYL, HFE, HLTF, HMGA1, HMGN1, HMGN5, HPRT1, HSF1, HSPE1, HTR2A, ICAM1, IL1B, ILK, INCENP, INHA, IRAK2, IRF2, IRF2BPL, IRX3, IRX6, ITGA5, JUNB, JUND, JUP, KCNH2, KDM6B, KLF10, KLF5, KMT2D, KSR1, LDLR, LGALS9, LIF, LILRA5, LIMK2, LMNA, LMX1A, LMX1B, LTF, LUM, MAFG, MAMSTR, MAP2K3, MAP3K10, MAP3K11, MAP3K14, MAP3K6, MAP3K9, MAPK3, MAPK7, MAPK8IP3, MARK2, MAVS, MAZ, MECP2, MED12, MED16, MED24, MED25, MEF2D, MEIS3P1, MEIS3P2, MEN1, MINK1, MLLT6, MLST8, MOV10, MPV17L2, MSN, MSTN, MSX1, MTCO2P12, MUC20, MYC, MYH9, MYRF, NAP1L2, NCOA6, NDP, NEK5, NFATC4, NFE2, NFIC, NFIX, NFKB2, NFKBIB, NGF, NGFR, NKD2, NLRC5, NOD2, NODAL, NOS3, NOTCH1, NOTCH3, NOTCH4, NPAS2, NR1D1, NR4A1, NR4A2, NR4A3, NSMF, OMA1, OSM, OSR2, PAF1, PAK4, PATL1, PCOLCE, PDGFB, PDGFRB, PEF1, PELP1, PER1, PHF1, PIAS4, PIDD1, PIM1, PKD1, PKM, PLAGL2, PLAUR, PLEKHN1, PLK3, PML, PNLDC1, PPARD, PPP1R10, PPP1R15A
GO:0045937	positive regulation of phosphate metabolic process	6,68E-03	[5, 6, 7]	198,00	18,63	38,47	61,53	[ADAM8, ADCY7, AIF1, ANGPT1, AREG, BANK1, BMP7, CALCR, CASS4, CCL19, CCL5, CD19, CD244, CD33, CD3E, CD4, CD40LG, CD74, CD80, CD86, CDCA2, CLCF1, CSF1R, CXCR4, EPHA6, EPX, EREG, FCRL3, FGD2, FGR, FLT3, GADD45B, GDF6, GPRC5A, HCLS1, HLA-DRB1, IFNG, IKBKE, IL18, LCP2, LIF, MAP4K1, MMP9, MUSK, NCF1, NCKAP1L, NLRP3, NR4A3, P2RY12, PDE6G, PFKFB4, PIK3CG, PIK3R5, PLEK, PROK1, PTAFR, PTK2B, PTPRC, SEMA4D, SH2D1A, SOCS1, SOCS3, STAP1, SYK, THBS1, TLR6, TNFRSF18, TREM2, WNT1]	[ABL1, ADCY1, ADCY3, ADCY4, ADCY9, ADIPOQ, AGAP2, AGER, AGT, AMH, ANGPT1, APLN, APLNR, ARAF, AREG, ARHGEF5, ARTN, BMP8A, C5, C5AR1, CALCR, CCL21, CCR7, CD74, CDC25B, CDCA2, CDKN1A, CHI3L1, CLCF1, CLIP3, CSF1, CSK, CSPG4, CTF1, CX3CL1, DAB2IP, DAG1, DAXX, DBF4B, DDR1, DGKZ, DHX34, DIRAS1, DUSP5, DVL3, EIF4G1, ENG, EPHA2, ERBB2, FASN, FGFR4, FLT3, FLT4, FPR1, GADD45B, GBA, GDF6, GPER1, GPRC5A, GSK3A, HBEGF, HFE, HSF1, HTR2A, ICAM1, IL1B, ILK, INCENP, INHA, IRAK2, ITGA5, KSR1, LIF, LILRA5, LIMK2, LTF, MAP2K3, MAP3K10, MAP3K11, MAP3K14, MAP3K6, MAP3K9, MAPK3, MAPK8IP3, MARK2, MAVS, MINK1, MLST8, MSTN, MTCO2P12, MUC20, MYC, NGF, NOD2, NODAL, NOS3, NR4A3, NSMF, OMA1, OSM, P2RY6, PAK4, PDGFB, PDGFRB, PKD1, PLAUR, PPP1R15A, PPP2R5D, PRKACA, PRKAR2B, PROM2, RAP1A, RAPGEF1, RCVRN, RGCC, RHBDF2, RNU1-93P, RPLP1, RPTOR, S1PR2, SH2D3A, SH2D3C, SHC2, SMAD3, SMPD1, SOCS3, SPATC1L, SPHK1, SRC, STAT3, TESK1, THBS1, THPO, TIMP1, TNF, TNFRSF1A, TNK2, TNKS1BP1, TRAF4, UBA52, VAC14, VEGFA, VTN]

GO:0006468	protein phosphorylation	3,68E-07	[6, 7]	326,00	18,89	38,39	61,61	[ADAM8, ADCY7, AIF1, ANGPT1, AREG, ATF3, BANK1, BLK, BMP7, BMPR1B, BTK, CALCR, CAMK4, CASS4, CCDC88C, CCL19, CCL2, CCL3, CCL5, CD3E, CD4, CD40LG, CD74, CD80, CD86, CDKN2A, CLCF1, CSF1R, CXCL10, CXCR4, DAPK1, DUSP2, DUSP8, EPHA6, EPX, EREG, FGD2, FGR, FLT3, GADD45B, GDF6, GFRA2, GMFG, GPRC5A, GUCY2D, HASPIN, HCLS1, HCST, HLA-DRB1, IFNG, IKBKE, IL12RB2, IL18, ITGB2, ITK, LAX1, LCK, LCP2, LIF, LILRB4, MAP4K1, MAPK13, MATK, MMP9, MUSK, MYO3B, NCF1, NCKAP1L, NEK5, NELL2, NLRP3, NLRP6, NPFFR2, NR4A3, PDE6G, PEAK3, PIK3CG, PIK3R5, PIM1, PIM2, PRKCB, PRKCQ, PROK1, PTK2B, PTPN22, PTPN6, PTPRC, PTPRH, PYCARD, RAC2, RUNX3, SAMSN1, SEMA4D, SH2D1A, SLA, SLC8A3, SMPD3, SOCS1, SOCS3, SPINK1, STAP1, STK17B, STYK1, SYK, THBS1, THY1, TLR6, TLR8, TNFAIP3, TNFRSF18, TREM2, WNT1, ZAP70]	[AATK, ABL1, ABR, ACP4, ACTB, ADCK1, ADCY1, ADCY3, ADCY4, ADCY9, ADIPOQ, AGAP2, AGER, AGT, AKT1S1, ALPK3, AMH, ANGPT1, ANKK1, APLNR, APOA1, ARAF, AREG, ARHGEF5, ARTN, ATP13A2, BCKDK, BCR, BMP8A, BRD4, C5, C5AR1, CALCR, CAMK2B, CCL11, CCL2, CCL21, CCNG2, CCNJL, CCR7, CD74, CDC25B, CDK1, CDK15, CDK18, CDK2, CDKL2, CDKN1A, CEACAM1, CHI3L1, CIITA, CLCF1, CLIP3, CSF1, CSK, CSNK1E, CSPG4, CTDSP1, CTF1, CX3CL1, DAB2IP, DAG1, DAXX, DBF4B, DBNDD1, DDR1, DGUOK, DIRAS1, DMTN, DUSP5, DUSP8, DVL3, DYRK1B, EFNA3, EIF4G1, ENG, EPHA2, ERBB2, FABP4, FASN, FES, FGFR4, FGFRL1, FLT3, FLT4, FPR1, GADD45B, GBA, GDF6, GPER1, GPI, GPRC5A, GSK3A, HBEGF, HFE, HGS, HIPK4, HSF1, HSPB1, HSPB8, HTR2A, HUNK, ICAM1, IL1B, ILK, INCA1, INCENP, INHA, INPP5J, IRAK2, ITGA5, ITPRIP, JAK3, KSR1, LIF, LILRA5, LIMK1, LIMK2, LIPE, LMTK3, LOX, LTF, MAP2K3, MAP3K10, MAP3K11, MAP3K14, MAP3K6, MAP3K9, MAPK3, MAPK4, MAPK7, MAPK8IP3, MARK2, MARK4, MAST2, MAVS, MEN1, MINK1, MKNK2, MLLT1, MLST8, MSTN, MUC20, MYADM, MYO3A, NEK5, NF2, NGF, NOD2, NODAL, NPFFR2, NPPA, NR4A3, OBSCN, OMA1, OSM, PAK4, PDGFB, PDGFRB, PDK2, PIM1, PKD1, PKN3, PLAUR, PLK3, PML, PPP1R9B, PPP2R1A, PPP2R5B, PPP2R5D, PRAG1, PRKACA, PRKAR2B, PROM2, PRRT1, PTPN13, PTPRH, PTPRU, RAC2, RAP1A, RAPGEF1, RARA, RGCC, RHBDF2, RNU1-93P, RPLP1, RPTOR, RSKR, S1PR2, SBK1, SCYL1, SH2B3, SH2D3A, SH2D3C, SHC2, SIK1, SIK2, SIK3, SLC9A3R1, SMPD1, SOCS3, SPATC1L, SPEG, SPHK1, SPINK1, SRC, SRPK3, STAT3, STK17B, STK35, STK40, TESK1, TEX14, THBS1, THPO, TIMP1, TNF, TNFRSF1A, TNK1, TNK2, TNKS1BP1, TRAF4, TRIB1, TRIB3, TSC2, TSSK3, UBA52, UBE2B, ULK1, VEGFA, VTN, WARS1, WNK2, WNT9B, ZC3H12A]
GO:0042325	regulation of phosphorylation	3,73E-03	[6, 7]	261,00	17,91	39,44	60,56	[ADAM8, ADCY7, AIF1, ANGPT1, AREG, ATF3, BANK1, BMP7, CALCR, CASS4, CCDC88C, CCL19, CCL5, CD19, CD3E, CD4, CD40LG, CD74, CD80, CD86, CDKN2A, CISH, CLCF1, CSF1R, CXCL10, CXCR4, DUSP2, DUSP8, EPHA6, EPX, EREG, FGD2, FGR, FLT3, GADD45B, GDF6, GFRA2, GMFG, GPRC5A, HCLS1, HLA-DRB1, IFNG, IKBKE, IL18, ITGB2, LAX1, LCP2, LIF, LILRB4, MAP4K1, MIDN, MMP9, MUSK, NCF1, NCKAP1L, NLRP3, NPFFR2, NR4A3, NUP210, P2RY12, PDE6G, PFKFB4, PIK3CG, PIK3IP1, PIK3R5, PROK1, PTK2B, PTPN22, PTPN6, PTPRC, PTPRH, PYCARD, RAC2, RHOH, SAMSN1, SEMA4D, SH2D1A, SLA2, SLAMF8, SLC8A3, SMPD3, SOCS1, SOCS3, SPINK1, STAP1, SYK, THBS1, THY1, TLR6, TLR8, TNFAIP3, TNFRSF18, TREM2, WNT1]	[AAAS, ABL1, ACP4, ACTB, ADCY1, ADCY3, ADCY4, ADCY9, ADIPOQ, AGAP2, AGER, AGT, AKT1S1, AMH, ANGPT1, APLN, APLNR, APOA1, ARAF, AREG, ARHGEF5, ARTN, BMP8A, BRD4, C5, C5AR1, CALCR, CCL21, CCNG2, CCNJL, CCR7, CD74, CDC25B, CDKN1A, CEACAM1, CHI3L1, CISH, CLCF1, CLIP3, CSF1, CSK, CSPG4, CTDSP1, CTF1, CX3CL1, DAB2IP, DAG1, DAXX, DBF4B, DBNDD1, DDR1, DGKZ, DIRAS1, DMTN, DUSP5, DUSP8, DVL3, EIF4G1, ENG, EPHA2, ERBB2, FABP4, FASN, FGFR4, FLT3, FLT4, FPR1, GADD45B, GBA, GDF6, GIT1, GPER1, GPRC5A, GSK3A, HBEGF, HFE, HGS, HSF1, HSPB1, HTR2A, ICAM1, IL1B, ILK, INCA1, INCENP, INHA, INPP5J, IRAK2, ITGA5, ITPRIP, JMJD8, KSR1, LIF, LILRA5, LIMK2, LOX, LTF, MAP2K3, MAP3K10, MAP3K11, MAP3K14, MAP3K6, MAP3K9, MAPK3, MAPK8IP3, MARK2, MAVS, MEN1, MIDN, MINK1, MLLT1, MLST8, MSTN, MUC20, MYADM, MYC, NF2, NGF, NLRC5, NOD2, NODAL, NPFFR2, NPPA, NR4A3, NUP188, OMA1, OSM, PAK4, PDGFB, PDGFRB, PKD1, PLAUR, PML, PPP1R9B, PPP2R1A, PPP2R5B, PPP2R5D, PRKACA, PRKAR2B, PROM2, PTPN13, PTPRH, PTPRU, RAC2, RAP1A, RAPGEF1, RGCC, RHBDF2, RNU1-93P, RPLP1, RPTOR, RUBCN, S1PR2, SH2B3, SH2D3A, SH2D3C, SHC2, SLC2A6, SLC9A3R1, SMPD1, SOCS3, SPATC1L, SPHK1, SPINK1, SRC, STAT3, TESK1, THBS1, THPO, TIMP1, TNF, TNFRSF1A, TNK2, TNKS1BP1, TRAF4, TRIB1, TRIB3, TSC2, UBA52, UBE2B, VAC14, VEGFA, VTN, WARS1, WNT9B, ZBTB7A, ZC3H12A]
GO:0001932	regulation of protein phosphorylation	1,95E-03	[6, 7, 8]	235,00	18,39	38,75	61,25	[ADAM8, ADCY7, AIF1, ANGPT1, AREG, ATF3, BANK1, BMP7, CALCR, CASS4, CCDC88C, CCL19, CCL5, CD3E, CD4, CD40LG, CD74, CD80, CD86, CDKN2A, CLCF1, CSF1R, CXCL10, CXCR4, DUSP2, DUSP8, EPX, EREG, FGD2, FGR, FLT3, GADD45B, GDF6, GFRA2, GMFG, GPRC5A, HCLS1, HLA-DRB1, IFNG, IKBKE, IL18, ITGB2, LAX1, LCP2, LIF, LILRB4, MAP4K1, MMP9, MUSK, NCF1, NCKAP1L, NLRP3, NPFFR2, NR4A3, PDE6G, PIK3CG, PIK3R5, PROK1, PTK2B, PTPN22, PTPN6, PTPRC, PTPRH, PYCARD, RAC2, SAMSN1, SEMA4D, SH2D1A, SLC8A3, SMPD3, SOCS1, SOCS3, SPINK1, STAP1, SYK, THBS1, THY1, TLR6, TLR8, TNFAIP3, TNFRSF18, TREM2, WNT1]	[ABL1, ACP4, ACTB, ADCY1, ADCY3, ADCY4, ADCY9, ADIPOQ, AGAP2, AGER, AGT, AKT1S1, AMH, ANGPT1, APLNR, APOA1, ARAF, AREG, ARHGEF5, ARTN, BMP8A, BRD4, C5, C5AR1, CALCR, CCL21, CCNG2, CCNJL, CCR7, CD74, CDC25B, CDKN1A, CEACAM1, CHI3L1, CLCF1, CLIP3, CSF1, CSK, CSPG4, CTDSP1, CTF1, CX3CL1, DAB2IP, DAG1, DAXX, DBF4B, DBNDD1, DIRAS1, DMTN, DUSP5, DUSP8, DVL3, EIF4G1, ENG, ERBB2, FABP4, FASN, FLT3, FLT4, FPR1, GADD45B, GBA, GDF6, GPER1, GPRC5A, GSK3A, HBEGF, HFE, HGS, HSF1, HSPB1, HTR2A, ICAM1, IL1B, ILK, INCA1, INCENP, INHA, INPP5J, IRAK2, ITGA5, ITPRIP, KSR1, LIF, LILRA5, LIMK2, LOX, LTF, MAP2K3, MAP3K10, MAP3K11, MAP3K14, MAP3K6, MAP3K9, MAPK3, MAPK8IP3, MARK2, MAVS, MEN1, MINK1, MLLT1, MLST8, MSTN, MUC20, MYADM, NF2, NGF, NOD2, NODAL, NPFFR2, NPPA, NR4A3, OMA1, OSM, PAK4, PDGFB, PDGFRB, PKD1, PLAUR, PML, PPP1R9B, PPP2R1A, PPP2R5B, PPP2R5D, PRKACA, PRKAR2B, PROM2, PTPN13, PTPRH, PTPRU, RAC2, RAP1A, RAPGEF1, RGCC, RHBDF2, RNU1-93P, RPLP1, RPTOR, S1PR2, SH2B3, SH2D3A, SH2D3C, SHC2, SLC9A3R1, SMPD1, SOCS3, SPATC1L, SPHK1, SPINK1, SRC, STAT3, TESK1, THBS1, THPO, TIMP1, TNF, TNFRSF1A, TNK2, TNKS1BP1, TRAF4, TRIB1, TRIB3, TSC2, UBA52, UBE2B, VEGFA, VTN, WARS1, WNT9B, ZC3H12A]

GO:0042327	positive regulation of phosphorylation	2,10E-02	[6, 7, 8]	181,00	18,55	38,39	61,61	[ADAM8, ADCY7, AIF1, ANGPT1, AREG, BANK1, BMP7, CALCR, CASS4, CCL19, CCL5, CD19, CD3E, CD4, CD40LG, CD74, CD80, CD86, CLCF1, CSF1R, CXCR4, EPHA6, EPX, EREG, FGD2, FGR, FLT3, GADD45B, GDF6, GPRC5A, HCLS1, HLA-DRB1, IFNG, IKBKE, IL18, LCP2, LIF, MAP4K1, MMP9, MUSK, NCF1, NCKAP1L, NLRP3, NR4A3, P2RY12, PDE6G, PFKFB4, PIK3CG, PIK3R5, PROK1, PTK2B, PTPRC, SEMA4D, SH2D1A, SOCS1, SOCS3, STAP1, SYK, THBS1, TLR6, TNFRSF18, TREM2, WNT1]	[ABL1, ADCY1, ADCY3, ADCY4, ADCY9, ADIPOQ, AGAP2, AGER, AGT, AMH, ANGPT1, APLN, APLNR, ARAF, AREG, ARHGEF5, ARTN, BMP8A, C5, C5AR1, CALCR, CCL21, CCR7, CD74, CDC25B, CDKN1A, CHI3L1, CLCF1, CLIP3, CSF1, CSK, CSPG4, CTF1, CX3CL1, DAB2IP, DAG1, DAXX, DBF4B, DDR1, DGKZ, DIRAS1, DUSP5, DVL3, EIF4G1, ENG, EPHA2, ERBB2, FASN, FGFR4, FLT3, FLT4, FPR1, GADD45B, GDF6, GPER1, GPRC5A, GSK3A, HBEGF, HFE, HSF1, HTR2A, ICAM1, IL1B, ILK, INCENP, INHA, IRAK2, ITGA5, KSR1, LIF, LILRA5, LIMK2, LTF, MAP2K3, MAP3K10, MAP3K11, MAP3K14, MAP3K6, MAP3K9, MAPK3, MAPK8IP3, MARK2, MAVS, MINK1, MLST8, MSTN, MUC20, MYC, NGF, NOD2, NODAL, NR4A3, OMA1, OSM, PAK4, PDGFB, PDGFRB, PKD1, PLAUR, PRKACA, PRKAR2B, PROM2, RAP1A, RAPGEF1, RGCC, RHBDF2, RNU1-93P, RPLP1, RPTOR, S1PR2, SH2D3A, SH2D3C, SHC2, SOCS3, SPATC1L, SPHK1, SRC, STAT3, TESK1, THBS1, THPO, TIMP1, TNF, TNFRSF1A, TNK2, TNKS1BP1, TRAF4, UBA52, VAC14, VEGFA, VTN]
GO:0001934	positive regulation of protein phosphorylation	1,34E-02	[6, 7, 8, 9]	169,00	18,95	37,74	62,26	[ADAM8, ADCY7, AIF1, ANGPT1, AREG, BANK1, BMP7, CALCR, CASS4, CCL19, CCL5, CD3E, CD4, CD40LG, CD74, CD80, CD86, CLCF1, CSF1R, CXCR4, EPX, EREG, FGD2, FLT3, GADD45B, GDF6, GPRC5A, HCLS1, HLA-DRB1, IFNG, IKBKE, IL18, LCP2, LIF, MAP4K1, MMP9, MUSK, NCF1, NCKAP1L, NLRP3, NR4A3, PDE6G, PIK3CG, PIK3R5, PROK1, PTK2B, PTPRC, SEMA4D, SH2D1A, SOCS1, SOCS3, STAP1, SYK, THBS1, TLR6, TNFRSF18, TREM2, WNT1]	[ABL1, ADCY1, ADCY3, ADCY4, ADCY9, ADIPOQ, AGAP2, AGER, AGT, AMH, ANGPT1, APLNR, ARAF, AREG, ARHGEF5, ARTN, BMP8A, C5, C5AR1, CALCR, CCL21, CCR7, CD74, CDC25B, CDKN1A, CHI3L1, CLCF1, CLIP3, CSF1, CSK, CSPG4, CTF1, CX3CL1, DAB2IP, DAG1, DAXX, DBF4B, DIRAS1, DUSP5, DVL3, EIF4G1, ENG, ERBB2, FASN, FLT3, FLT4, FPR1, GADD45B, GDF6, GPER1, GPRC5A, GSK3A, HBEGF, HFE, HSF1, HTR2A, ICAM1, IL1B, ILK, INCENP, INHA, IRAK2, ITGA5, KSR1, LIF, LILRA5, LIMK2, LTF, MAP2K3, MAP3K10, MAP3K11, MAP3K14, MAP3K6, MAP3K9, MAPK3, MAPK8IP3, MARK2, MAVS, MINK1, MLST8, MSTN, MUC20, NGF, NOD2, NODAL, NR4A3, OMA1, OSM, PAK4, PDGFB, PDGFRB, PKD1, PLAUR, PRKACA, PRKAR2B, PROM2, RAP1A, RAPGEF1, RGCC, RHBDF2, RNU1-93P, RPLP1, RPTOR, S1PR2, SH2D3A, SH2D3C, SHC2, SOCS3, SPATC1L, SPHK1, SRC, STAT3, TESK1, THBS1, THPO, TIMP1, TNF, TNFRSF1A, TNK2, TNKS1BP1, TRAF4, UBA52, VEGFA, VTN]
GO:0004672	protein kinase activity	4,72E-06	[7, 8]	251,00	19,43	38,38	61,62	[ADAM8, ADCY7, ANGPT1, AREG, BLK, BMP7, BMPR1B, BTK, CAMK4, CASS4, CCL19, CCL2, CCL3, CCL5, CD4, CD40LG, CD74, CD86, CDKN2A, CSF1R, CXCL10, CXCR4, DAPK1, DUSP2, DUSP8, EPHA6, EPX, EREG, FGD2, FGR, FLT3, GADD45B, GMFG, GPRC5A, GUCY2D, HASPIN, IFNG, IKBKE, IL18, ITK, LAX1, LCK, LCP2, LILRB4, MAP4K1, MAPK13, MATK, MUSK, MYO3B, NCF1, NCKAP1L, NEK5, NELL2, NLRP3, NLRP6, NPFFR2, NR4A3, PDE6G, PEAK3, PIK3CG, PIK3R5, PIM1, PIM2, PRKCB, PRKCQ, PROK1, PTK2B, PTPN22, PTPN6, PTPRC, PTPRH, PYCARD, RAC2, SH2D1A, SLA, SLC8A3, SOCS1, SOCS3, STAP1, STK17B, STYK1, SYK, THBS1, THY1, TLR6, TNFAIP3, ZAP70]	[AATK, ABL1, ABR, ACP4, ACTB, ADCK1, ADCY1, ADCY3, ADCY4, ADCY9, ADIPOQ, AGAP2, AGER, AGT, AKT1S1, ALPK3, ANGPT1, ANKK1, ARAF, AREG, ARHGEF5, BCKDK, BCR, C5, C5AR1, CAMK2B, CCL2, CCL21, CCNG2, CCNJL, CCR7, CD74, CDC25B, CDK1, CDK15, CDK18, CDK2, CDKL2, CDKN1A, CEACAM1, CHI3L1, CIITA, CSF1, CSK, CSNK1E, CSPG4, DAB2IP, DAG1, DAXX, DBF4B, DBNDD1, DDR1, DIRAS1, DUSP5, DUSP8, DVL3, DYRK1B, EFNA3, ENG, EPHA2, ERBB2, FABP4, FES, FGFR4, FGFRL1, FLT3, FLT4, FPR1, GADD45B, GBA, GPI, GPRC5A, GSK3A, HBEGF, HGS, HIPK4, HSPB1, HSPB8, HTR2A, HUNK, IL1B, ILK, INCA1, INCENP, IRAK2, ITPRIP, JAK3, KSR1, LILRA5, LIMK1, LIMK2, LMTK3, LTF, MAP2K3, MAP3K10, MAP3K11, MAP3K14, MAP3K6, MAP3K9, MAPK3, MAPK4, MAPK7, MAPK8IP3, MARK2, MARK4, MAST2, MEN1, MINK1, MKNK2, MLLT1, MLST8, MUC20, MYO3A, NEK5, NF2, NGF, NOD2, NPFFR2, NPPA, NR4A3, OBSCN, OMA1, PAK4, PDGFB, PDGFRB, PDK2, PIM1, PKD1, PKN3, PLK3, PPP1R9B, PPP2R1A, PRAG1, PRKACA, PRKAR2B, PTPRH, PTPRU, RAC2, RAP1A, RAPGEF1, RGCC, RHBDF2, RNU1-93P, RPLP1, RPTOR, RSKR, S1PR2, SBK1, SCYL1, SH2B3, SHC2, SIK1, SIK2, SIK3, SLC9A3R1, SMPD1, SOCS3, SPATC1L, SPEG, SRC, SRPK3, STK17B, STK35, STK40, TESK1, TEX14, THBS1, TIMP1, TNF, TNK1, TNK2, TRAF4, TRIB1, TRIB3, TSC2, TSSK3, UBA52, ULK1, VEGFA, WARS1, WNK2]
GO:0046777	protein autophosphorylation	2,25E-02	[7, 8]	60,00	23,90	35,36	64,64	[BTK, CAMK4, CSF1R, DAPK1, FGR, FLT3, GFRA2, ITK, MAP4K1, MUSK, NR4A3, PIM1, PIM2, PTK2B, PTPRC, STK17B, SYK, THY1, ZAP70]	[AATK, ABL1, ADIPOQ, ALPK3, ARTN, ATP13A2, BCR, CAMK2B, CSK, DDR1, DYRK1B, ENG, ERBB2, FES, FGFR4, FLT3, FLT4, GPI, HIPK4, MAP3K10, MAP3K11, MAP3K9, MAPK3, MARK2, MINK1, MKNK2, MYO3A, NR4A3, PDGFB, PDGFRB, PIM1, PPP2R5B, PPP2R5D, PRKACA, RHBDF2, SIK1, SIK2, SRC, STK17B, TESK1, TNK1, TNKS1BP1, ULK1, VEGFA, WNK2]
GO:0018108	peptidyl-tyrosine phosphorylation	2,35E-06	[7, 8, 9]	102,00	24,76	51,10	48,90	[ANGPT1, AREG, BANK1, BLK, BTK, CASS4, CCL5, CD3E, CD4, CD74, CD80, CLCF1, CSF1R, EPHA6, EPX, EREG, FGR, FLT3, GPRC5A, HCLS1, IFNG, IL12RB2, IL18, ITGB2, ITK, LCK, LIF, LILRB4, MATK, MUSK, NCF1, NELL2, NR4A3, PTK2B, PTPN22, PTPN6, PTPRC, SAMSN1, SEMA4D, SLA, SOCS1, SOCS3, SPINK1, STAP1, STYK1, SYK, THY1, TNFRSF18, TREM2, ZAP70]	[AATK, ABL1, ADIPOQ, AGT, AMH, ANGPT1, AREG, CD74, CLCF1, CSK, CSPG4, CTF1, DDR1, DMTN, DYRK1B, EFNA3, EPHA2, ERBB2, FES, FGFR4, FGFRL1, FLT3, FLT4, GPRC5A, HBEGF, HIPK4, HSF1, HTR2A, ICAM1, ITGA5, JAK3, LIF, LILRA5, LMTK3, MAP2K3, MAP3K10, MAP3K11, MAP3K9, MAPK3, MLST8, NF2, NOD2, NR4A3, OSM, PDGFB, PDGFRB, PPP2R1A, RHBDF2, SCYL1, SH2B3, SOCS3, SPINK1, SRC, STAT3, TESK1, TIMP1, TNF, TNFRSF1A, TNK1, TNK2, VEGFA, VTN]
GO:0050730	regulation of peptidyl-tyrosine phosphorylation	1,24E-02	[7, 8, 9, 10]	66,00	23,66	55,14	44,86	[ANGPT1, AREG, BANK1, CASS4, CCL5, CD3E, CD4, CD74, CD80, CLCF1, CSF1R, EPX, EREG, FLT3, GPRC5A, HCLS1, IFNG, IL18, ITGB2, LIF, LILRB4, NCF1, NR4A3, PTK2B, PTPN22, PTPN6, PTPRC, SAMSN1, SEMA4D, SOCS1, SOCS3, SPINK1, STAP1, SYK, THY1, TNFRSF18, TREM2]	[ABL1, ADIPOQ, AGT, AMH, ANGPT1, AREG, CD74, CLCF1, CSPG4, CTF1, DMTN, FLT3, GPRC5A, HBEGF, HSF1, HTR2A, ICAM1, ITGA5, LIF, LILRA5, MLST8, NF2, NOD2, NR4A3, OSM, PDGFB, PPP2R1A, RHBDF2, SH2B3, SOCS3, SPINK1, SRC, STAT3, TIMP1, TNF, TNFRSF1A, TNK2, VEGFA, VTN]

GO:0050731	positive regulation of peptidyl-tyrosine phosphorylation	2,46E-02	[7, 8, 9, 10, 11]	53,00	24,88	52,37	47,63	[ANGPT1, AREG, BANK1, CASS4, CCL5, CD3E, CD4, CD74, CD80, CLCF1, CSF1R, EPX, EREG, FLT3, HCLS1, IFNG, IL18, LIF, NCF1, NR4A3, PTK2B, PTPRC, SEMA4D, SOCS3, STAP1, SYK, TNFRSF18, TREM2]	[ABL1, ADIPOQ, AGT, AMH, ANGPT1, AREG, CD74, CLCF1, CSPG4, CTF1, FLT3, HBEGF, HSF1, HTR2A, ICAM1, ITGA5, LIF, LILRA5, MLST8, NOD2, NR4A3, OSM, PDGFB, RHBDF2, SOCS3, SRC, STAT3, TIMP1, TNF, TNFRSF1A, TNK2, VEGFA, VTN]
GO:0046638	positive regulation of alpha-beta T cell differentiation	2,60E-05	[7, 8, 9, 10, 11, 12, 13, 14]	23,00	47,92	82,35	17,65	[CCL19, CD80, CD86, FOXP3, HLA-DRA, HLA-DRB1, IFNG, IL12RB1, IL18, LILRB4, MYB, NCKAP1L, NLRP3, RUNX3, SASH3, SOCS1, SYK, ZAP70]	[AP3D1, IL4R, LGALS9, RARA, ZBTB7B]
GO:0043370	regulation of CD4-positive, alpha-beta T cell differentiation	1,98E-05	[8, 9, 10, 11, 12, 13, 14]	24,00	47,06	75,88	24,12	[CCL19, CD80, CD86, FOXP3, HLA-DRA, HLA-DRB1, IFNG, IL12RB1, IL18, IRF4, MYB, NCKAP1L, NLRP3, RUNX3, SASH3, SOCS1, TBX21]	[BCL6, IL4R, JAK3, LGALS9, RARA, ZBTB7B, ZC3H12A]
GO:0043372	positive regulation of CD4-positive, alpha-beta T cell differentiation	2,05E-05	[8, 9, 10, 11, 12, 13, 14, 15]	18,00	58,06	81,93	18,07	[CCL19, CD80, CD86, FOXP3, HLA-DRA, HLA-DRB1, IFNG, IL12RB1, IL18, MYB, NCKAP1L, NLRP3, SASH3, SOCS1]	[IL4R, LGALS9, RARA, ZBTB7B]
GO:0032613	interleukin-10 production	2,74E-02	[3, 6]	21,00	36,21	69,39	30,61	[BCL3, CD40LG, EPX, FOXP3, HLA-DRB1, IRF4, LILRB1, LILRB4, PYCARD, SASH3, SYK, TIGIT, TREM2, XCL1]	[AGER, BCL3, JAK3, LGALS9, LILRA5, NOD2, STAT3, VSIR]
GO:0032633	interleukin-4 production	1,24E-02	[3, 6]	15,00	46,88	83,83	16,17	[CD3E, CD40LG, CD86, EPX, FOXP3, HLA-DRB1, IRF4, LEF1, NLRP3, PRKCQ, SASH3, SYK]	[LGALS9, RARA, SLC7A5]
GO:0032653	regulation of interleukin-10 production	2,74E-02	[4, 5, 6, 7]	21,00	36,21	69,39	30,61	[BCL3, CD40LG, EPX, FOXP3, HLA-DRB1, IRF4, LILRB1, LILRB4, PYCARD, SASH3, SYK, TIGIT, TREM2, XCL1]	[AGER, BCL3, JAK3, LGALS9, LILRA5, NOD2, STAT3, VSIR]
GO:0032673	regulation of interleukin-4 production	1,24E-02	[4, 5, 6, 7]	15,00	46,88	83,83	16,17	[CD3E, CD40LG, CD86, EPX, FOXP3, HLA-DRB1, IRF4, LEF1, NLRP3, PRKCQ, SASH3, SYK]	[LGALS9, RARA, SLC7A5]
GO:0032753	positive regulation of interleukin-4 production	1,24E-02	[4, 5, 6, 7, 8]	13,00	52,00	81,20	18,80	[CD3E, CD40LG, CD86, EPX, FOXP3, IRF4, NLRP3, PRKCQ, SASH3, SYK]	[LGALS9, RARA, SLC7A5]
GO:0046635	positive regulation of alpha-beta T cell activation	6,90E-08	[6, 7, 8, 9]	32,00	47,06	84,88	15,12	[CCL19, CCR2, CD3E, CD80, CD86, EBI3, FOXP3, HLA-DRA, HLA-DRB1, IFNG, IL12RB1, IL18, LILRB4, MYB, NCKAP1L, NLRP3, PRKCQ, PTPN22, PTPRC, RASAL3, RUNX3, SASH3, SOCS1, SYK, XCL1, ZAP70]	[AP3D1, GNAO1, IL4R, LGALS9, RARA, ZBTB7B]
GO:2000514	regulation of CD4-positive, alpha-beta T cell activation	4,57E-05	[7, 8, 9]	28,00	41,18	73,23	26,77	[CCL19, CD80, CD86, FOXP3, HLA-DRA, HLA-DRB1, IFNG, IL12RB1, IL18, IRF4, MYB, NCKAP1L, NLRP3, PRKCQ, RUNX3, SASH3, SOCS1, TBX21, XCL1]	[AGER, BCL6, IL4R, JAK3, LGALS9, RARA, VSIR, ZBTB7B, ZC3H12A]
GO:2000516	positive regulation of CD4-positive, alpha-beta T cell activation	3,03E-05	[7, 8, 9, 10]	20,00	52,63	83,83	16,17	[CCL19, CD80, CD86, FOXP3, HLA-DRA, HLA-DRB1, IFNG, IL12RB1, IL18, MYB, NCKAP1L, NLRP3, PRKCQ, SASH3, SOCS1, XCL1]	[IL4R, LGALS9, RARA, ZBTB7B]
GO:0008610	lipid biosynthetic process	4,03E-03	[4]	165,00	19,37	32,07	67,93	[ABCB11, AKR1D1, ALOX15, ALOX15B, BCO1, C1QTNF1, CCL19, CD19, CD74, CERKL, CERS3, CISH, CPNE7, CYP11B1, CYP4F22, DEGS2, DHRS9, EDN2, ETNPPL, FGR, FLT3, GAL3ST2, GFI1, IFNG, INPP5D, LCP1, LDLR, LPAL2, MID1IP1, MUSK, NLRP3, P2RY12, PIK3C2G, PIK3CG, PIK3IP1, PIK3R5, PLA2G2D, PTK2B, SH2D2A, SLA2, SLC27A2, SMPD3, SOAT2, SOCS1, SOCS3, TBXAS1, UGT8]	[A4GALT, ABCA3, ABCB11, ABCD1, ABCD2, ABHD4, ACACB, ACSBG1, ADIPOQ, AGAP2, AGPAT1, AGPAT3, AMH, APOA1, ARF1, ARMC5, ATP1A1, ATP1A3, B4GALT3, C1QTNF1, CACNA1H, CCL21, CCR7, CD74, CEACAM1, CERT1, CGA, CHGA, CISH, CPNE6, CYB5R3, CYP21A2, CYP27B1, CYP2D6, DAB2IP, DGKD, DGKZ, DHRS9, DPAGT1, DPM2, EDN2, ELOVL1, FA2H, FADS2, FADS3, FASN, FGFR4, FITM1, FLT3, GAL3ST3, GAL3ST4, GBA, GNAI2, GPAM, GPAT2, GPER1, HSD17B11, HTR2A, IL1B, INPP5E, INPP5J, INPPL1, ISYNA1, LCAT, LDLR, LIPG, LPCAT4, MALRD1, MBOAT7, MFSD2A, MID1IP1, NOD2, NR1D1, PDGFB, PDGFRB, PGAP2, PGAP3, PI4KA, PI4KAP1, PI4KAP2, PIGO, PIGQ, PIGS, PIGT, PIGZ, PIK3C2B, PIK3C2G, PIP5K1C, PISD, PITPNM1, PITPNM2, PLA2G2A, PNPLA2, POR, PPARD, PRKCSH, PRXL2B, PTGDS, PTGES, PTPN13, RNU1-93P, RUBCN, SBF1, SCAP, SH2D3C, SIK1, SLC44A2, SMPD1, SMPD4, SNAI1, SOCS3, SPHK1, SPHK2, SPNS2, SRC, SREBF1, ST6GALNAC4, TECR, TMEM150A, TMEM189, TMEM256-PLSCR3, TNF, TNFRSF1A, TP53INP2, TRIB3, TTC7B, VAC14, VDR, WDTC1]
GO:0006644	phospholipid metabolic process	2,53E-03	[4, 5]	105,00	21,65	35,41	64,59	[ALOX15, ALOX15B, CCL19, CD19, CISH, CPNE7, CSF1R, ETNPPL, FGR, FLT3, INPP5D, LCP1, LDLR, LPAL2, MUSK, P2RY12, PIK3C2G, PIK3CG, PIK3IP1, PIK3R5, PLA2G2D, PLA2G7, PLAAT4, PLCB2, PLCH2, PLEK, PLPPR4, PTK2B, SLA2, SMPD3, SMPDL3B, SOCS1, SOCS3]	[ABCA3, ABHD4, AGAP2, AGPAT1, AGPAT3, APOA1, ARF1, CCL21, CCR7, CETP, CISH, CPNE6, DAB2IP, DGKD, DGKZ, DPAGT1, DPM2, FITM1, FLT3, GNB3, GPAM, GPAT2, HTR2A, INPP5E, INPP5J, INPPL1, ISYNA1, LCAT, LDLR, LIPG, LPCAT4, MBOAT7, MECP2, MFSD2A, NOD2, PDGFB, PDGFRB, PDXP, PGAP2, PGAP3, PI4KA, PI4KAP1, PI4KAP2, PIGO, PIGQ, PIGS, PIGT, PIGZ, PIK3C2B, PIK3C2G, PIP5K1C, PISD, PITPNM1, PITPNM2, PLA1A, PLA2G2A, PLA2G7, PLBD2, PPARD, PRKCSH, PTPN13, RNU1-93P, RUBCN, SBF1, SERINC2, SH2D3C, SLC44A2, SMPD1, SMPD4, SOCS3, SPHK2, SRC, TMEM150A, TMEM256-PLSCR3, TMEM86B, TP53INP2, TTC7B, VAC14]
GO:0019216	regulation of lipid metabolic process	1,08E-02	[4, 5]	89,00	21,87	28,44	71,56	[ABCB11, BCL11B, C1QTNF1, CCL19, CD19, CD74, CNR1, ERFE, FGR, FLT3, GFI1, IFNG, LDLR, LPAL2, MID1IP1, NLRP3, NR4A3, P2RY12, PIK3CG, PIK3IP1, PTK2B, SH2D2A, SMPD3]	[ABCA3, ABCA7, ABCB11, ABCD1, ABCD2, ACACB, ADIPOQ, AGAP2, AGT, AGTR1, ANGPTL8, APOA1, APOD, ARF1, ARMC5, ATP1A1, ATP1A3, C1QTNF1, CARM1, CCL21, CCR7, CD74, CEACAM1, CGA, CHGA, CNR1, CYP27B1, DAB2IP, DGKZ, DISP3, ERFE, FASN, FGFR4, FLT3, GNAI2, GNB3, GPAM, GPER1, HTR2A, IL1B, LDLR, LDLRAP1, LSR, MALRD1, MBOAT7, MFSD2A, MID1IP1, NCOA6, NCOR2, NOD2, NR1D1, NR4A3, PCDHA4, PDGFB, PDGFRB, PDK2, PDPR, PNPLA2, POR, PPARD, RNU1-93P, RUBCN, RXRA, SCAP, SERPINA3, SIK1, SNAI1, SPHK1, SPHK2, SRC, SREBF1, TNF, TNFRSF1A, TRIB3, WDTC1]

GO:0046486	glycerolipid metabolic process	1,37E-02	[4, 5]	105,00	20,92	30,42	69,58	[ALOX15, CCL19, CD19, CISH, CPNE7, CSF1R, ETNPPL, FGR, FLT3, INPP5D, LCP1, LDLR, LPAL2, P2RY12, PIK3C2G, PIK3CG, PIK3IP1, PIK3R5, PLA2G2D, PLA2G7, PLAAT4, PLCB2, PLCH2, PLEK, PTK2B, SLA2, SOCS1, SOCS3]	[ABCA3, ABHD4, AGAP2, AGPAT1, AGPAT3, APOA1, ARF1, CCL21, CCR7, CETP, CISH, CPNE6, CRAT, CYP21A2, DAB2IP, DAGLA, DGKD, DGKZ, DPM2, FA2H, FAAH, FABP4, FLT3, GNB3, GPAM, GPAT2, HTR2A, INPP5E, INPP5J, INPPL1, LCAT, LDLR, LIPE, LIPG, LMF1, LPCAT4, LYPLA2, MBOAT7, MECP2, MFSD2A, NOD2, PDGFB, PDGFRB, PDXP, PGAP2, PGAP3, PI4KA, PI4KAP1, PI4KAP2, PIGO, PIGQ, PIGS, PIGT, PIGZ, PIK3C2B, PIK3C2G, PIP5K1C, PISD, PITPNM1, PITPNM2, PLA1A, PLA2G2A, PLA2G7, PNPLA2, PRKCSH, PTPN13, RNU1-93P, RUBCN, SBF1, SERINC2, SH2D3C, SIK1, SLC44A2, SMPD4, SOCS3, SRC, SREBF1, TMEM150A, TMEM256-PLSCR3, TMEM86B, TP53INP2, TTC7B, VAC14]
GO:0090407	organophosphate biosynthetic process	3,53E-02	[4, 5]	138,00	19,35	28,78	71,22	[ADCY7, ALOX15, AMPD1, CARD11, CCL19, CD19, CD244, CISH, CPNE7, ETNPPL, FGR, FLT3, GUCY2D, INPP5D, KMO, LPAL2, MUSK, NAMPT, NPPB, NPPC, P2RY12, PIK3C2G, PIK3CG, PIK3IP1, PIK3R5, PLA2G2D, PLEK, PTAFR, PTK2B, RRM2, SLA2, SOCS1, SOCS3, TREM2]	[ABHD4, ACACB, ACSBG1, ADCY1, ADCY3, ADCY4, ADCY9, AGAP2, AGPAT1, AGPAT3, ALDOA, AMPD2, APOA1, ARF1, ATP5F1D, ATP5MC2, ATPSCKMT, CCL21, CCR7, CISH, CKM, COASY, CPNE6, CYP21A2, DAB2IP, DCK, DGKD, DGKZ, DGUOK, DHX34, DPAGT1, DPM2, DUT, ELOVL1, FASN, FITM1, FLT3, GPAM, GPAT2, GPER1, HPRT1, HTR2A, IDO1, IMPDH1, INPP5E, INPP5J, INPPL1, ISYNA1, LCAT, LPCAT4, MBOAT7, MFSD2A, MOCS1, MTCO2P12, MYC, NME4, NOD2, NOS3, NPPA, NPPB, NUDT12, P2RY6, PANK1, PDGFB, PDGFRB, PDK2, PDPR, PGAP2, PGAP3, PI4KA, PI4KAP1, PI4KAP2, PIGO, PIGQ, PIGS, PIGT, PIGZ, PIK3C2B, PIK3C2G, PIP5K1C, PISD, PITPNM1, PITPNM2, PLA2G2A, PPARD, PPT2, PPT2-EGFL8, PRKCSH, PTPN13, RCVRN, RMRP, RNU1-93P, RUBCN, SBF1, SH2D3C, SLC25A1, SLC44A2, SOCS3, SPHK2, SRC, STAT3, TECR, TK1, TMEM150A, TMEM256-PLSCR3, TP53INP2, TTC7B, VAC14, VPS9D1]
GO:0008654	phospholipid biosynthetic process	1,44E-02	[4, 5, 6]	81,00	22,19	29,83	70,17	[ALOX15, CCL19, CD19, CISH, CPNE7, ETNPPL, FGR, FLT3, INPP5D, LPAL2, MUSK, P2RY12, PIK3C2G, PIK3CG, PIK3IP1, PIK3R5, PLA2G2D, PTK2B, SLA2, SOCS1, SOCS3]	[ABHD4, AGAP2, AGPAT1, AGPAT3, APOA1, ARF1, CCL21, CCR7, CISH, CPNE6, DAB2IP, DGKD, DGKZ, DPAGT1, DPM2, FITM1, FLT3, GPAM, GPAT2, HTR2A, INPP5E, INPP5J, INPPL1, ISYNA1, LCAT, LPCAT4, MBOAT7, MFSD2A, NOD2, PDGFB, PDGFRB, PGAP2, PGAP3, PI4KA, PI4KAP1, PI4KAP2, PIGO, PIGQ, PIGS, PIGT, PIGZ, PIK3C2B, PIK3C2G, PIP5K1C, PISD, PITPNM1, PITPNM2, PLA2G2A, PPARD, PRKCSH, PTPN13, RNU1-93P, RUBCN, SBF1, SH2D3C, SLC44A2, SOCS3, SPHK2, SRC, TMEM150A, TMEM256-PLSCR3, TP53INP2, TTC7B, VAC14]
GO:0045017	glycerolipid biosynthetic process	1,82E-02	[4, 5, 6]	80,00	22,16	31,15	68,85	[ALOX15, CCL19, CD19, CISH, CPNE7, ETNPPL, FGR, FLT3, INPP5D, LCP1, LDLR, LPAL2, P2RY12, PIK3C2G, PIK3CG, PIK3IP1, PIK3R5, PLA2G2D, PTK2B, SLA2, SOCS1, SOCS3]	[ABHD4, AGAP2, AGPAT1, AGPAT3, APOA1, ARF1, CCL21, CCR7, CISH, CPNE6, DAB2IP, DGKD, DGKZ, DPM2, FLT3, GPAM, GPAT2, HTR2A, INPP5E, INPP5J, INPPL1, LCAT, LDLR, LPCAT4, MBOAT7, MFSD2A, NOD2, PDGFB, PDGFRB, PGAP2, PGAP3, PI4KA, PI4KAP1, PI4KAP2, PIGO, PIGQ, PIGS, PIGT, PIGZ, PIK3C2B, PIK3C2G, PIP5K1C, PISD, PITPNM1, PITPNM2, PLA2G2A, PNPLA2, PRKCSH, PTPN13, RNU1-93P, RUBCN, SBF1, SH2D3C, SIK1, SLC44A2, SOCS3, SRC, SREBF1, TMEM150A, TMEM256-PLSCR3, TP53INP2, TTC7B, VAC14]
GO:0006650	glycerophospholipid metabolic process	5,63E-03	[5, 6]	90,00	22,06	33,64	66,36	[ALOX15, CCL19, CD19, CISH, CPNE7, CSF1R, ETNPPL, FGR, FLT3, INPP5D, LDLR, LPAL2, P2RY12, PIK3C2G, PIK3CG, PIK3IP1, PIK3R5, PLA2G2D, PLA2G7, PLAAT4, PLCB2, PLCH2, PLEK, PTK2B, SLA2, SOCS1, SOCS3]	[ABCA3, ABHD4, AGAP2, AGPAT1, AGPAT3, APOA1, ARF1, CCL21, CCR7, CETP, CISH, CPNE6, DAB2IP, DGKD, DGKZ, DPM2, FLT3, GPAM, GPAT2, HTR2A, INPP5E, INPP5J, INPPL1, LCAT, LDLR, LPCAT4, MBOAT7, MECP2, MFSD2A, NOD2, PDGFB, PDGFRB, PDXP, PGAP2, PGAP3, PI4KA, PI4KAP1, PI4KAP2, PIGO, PIGQ, PIGS, PIGT, PIGZ, PIK3C2B, PIK3C2G, PIP5K1C, PISD, PITPNM1, PITPNM2, PLA1A, PLA2G2A, PLA2G7, PRKCSH, PTPN13, RNU1-93P, RUBCN, SBF1, SERINC2, SH2D3C, SLC44A2, SMPD4, SOCS3, SRC, TMEM150A, TMEM256-PLSCR3, TMEM86B, TP53INP2, TTC7B, VAC14]
GO:0046474	glycerophospholipid biosynthetic process	5,17E-03	[5, 6, 7]	75,00	23,29	30,52	69,48	[ALOX15, CCL19, CD19, CISH, CPNE7, ETNPPL, FGR, FLT3, INPP5D, LPAL2, P2RY12, PIK3C2G, PIK3CG, PIK3IP1, PIK3R5, PLA2G2D, PTK2B, SLA2, SOCS1, SOCS3]	[ABHD4, AGAP2, AGPAT1, AGPAT3, APOA1, ARF1, CCL21, CCR7, CISH, CPNE6, DAB2IP, DGKD, DGKZ, DPM2, FLT3, GPAM, GPAT2, HTR2A, INPP5E, INPP5J, INPPL1, LCAT, LPCAT4, MBOAT7, MFSD2A, NOD2, PDGFB, PDGFRB, PGAP2, PGAP3, PI4KA, PI4KAP1, PI4KAP2, PIGO, PIGQ, PIGS, PIGT, PIGZ, PIK3C2B, PIK3C2G, PIP5K1C, PISD, PITPNM1, PITPNM2, PLA2G2A, PRKCSH, PTPN13, RNU1-93P, RUBCN, SBF1, SH2D3C, SLC44A2, SOCS3, SRC, TMEM150A, TMEM256-PLSCR3, TP53INP2, TTC7B, VAC14]
GO:0046488	phosphatidylinositol metabolic process	7,12E-03	[6, 7]	59,00	25,00	37,60	62,40	[CCL19, CD19, CISH, CSF1R, FGR, FLT3, INPP5D, P2RY12, PIK3C2G, PIK3CG, PIK3IP1, PIK3R5, PLA2G2D, PLCB2, PLCH2, PLEK, PTK2B, SLA2, SOCS1, SOCS3]	[AGAP2, ARF1, CCL21, CCR7, CISH, DAB2IP, DGKZ, DPM2, FLT3, HTR2A, INPP5E, INPP5J, INPPL1, MBOAT7, NOD2, PDGFB, PDGFRB, PGAP2, PGAP3, PI4KA, PI4KAP1, PI4KAP2, PIGO, PIGQ, PIGS, PIGT, PIGZ, PIK3C2B, PIK3C2G, PIP5K1C, PITPNM1, PITPNM2, PLA2G2A, PTPN13, RNU1-93P, RUBCN, SBF1, SOCS3, SRC, TMEM150A, TP53INP2, TTC7B, VAC14]
GO:0006661	phosphatidylinositol biosynthetic process	2,45E-03	[6, 7, 8]	53,00	26,63	31,64	68,36	[CCL19, CD19, CISH, FGR, FLT3, INPP5D, P2RY12, PIK3C2G, PIK3CG, PIK3IP1, PIK3R5, PTK2B, SLA2, SOCS1, SOCS3]	[AGAP2, ARF1, CCL21, CCR7, CISH, DAB2IP, DGKZ, DPM2, FLT3, HTR2A, INPP5E, INPP5J, INPPL1, MBOAT7, NOD2, PDGFB, PDGFRB, PGAP2, PGAP3, PI4KA, PI4KAP1, PI4KAP2, PIGO, PIGQ, PIGS, PIGT, PIGZ, PIK3C2B, PIK3C2G, PIP5K1C, PITPNM1, PITPNM2, PTPN13, RNU1-93P, RUBCN, SBF1, SOCS3, SRC, TMEM150A, TP53INP2, TTC7B, VAC14]

GO:0035004	phosphatidylinositol 3-kinase activity	1,16E-02	[7, 8, 9, 10]	24,00	35,29	56,44	43,56	[CCL19, CD19, CISH, FGR, FLT3, P2RY12, PIK3C2G, PIK3CG, PIK3IP1, PIK3R5, PTK2B, SLA2, SOCS1, SOCS3]	[AGAP2, CCL21, CCR7, CISH, DAB2IP, FLT3, NOD2, PDGFB, PDGFRB, PIK3C2B, PIK3C2G, RUBCN, SOCS3, SRC]
GO:0034762	regulation of transmembrane transport	6,62E-03	[3, 4, 5]	126,00	20,32	44,86	55,14	[ANO9, C1QTNF1, CACNA1E, CACNA1I, CALCR, CBARP, CCL2, CCR2, CD19, CLCNKA, CORO1A, CTSS, CXCL10, CXCL11, CXCL9, CYBB, DAPK1, DRD1, EPX, ERFE, FFAR4, GABBR2, HCN1, IFNG, JPH1, JSRP1, KCNA3, KCNA7, KCNJ10, KCNK15, KCNN4, KCNQ5, LRRC38, MMP9, NR4A3, PIK3CG, PIM1, PRKCB, PTAFR, PTK2B, PTPN22, PTPN6, RASGRF1, RGS9, RRAD, SHISA8, SLC31A2, TCAF2, THBS1, THY1, TMC2, TREM2, UBD, XCL1]	[ABCC8, ABL1, ACTB, ACTN4, ADIPOQ, AGT, APLNR, ARC, ARF1, ATP1B2, ATPSCKMT, C1QTNF1, CACNA1E, CACNA1H, CACNB4, CALCR, CAMK2B, CAPN1, CBARP, CCL2, CFH, CLCNKA, CLIP3, CX3CL1, ERFE, FXYD5, FXYD6, GABBR2, GNAI2, GPER1, GPRIN1, GSK3A, HCN3, HCN4, HVCN1, IL1B, JPH4, KCNA7, KCNC3, KCNH2, KCNIP2, KCNJ1, KCNJ5, KCNJ9, KCNK15, KCNK6, KCNMB1, KCNMB2, KCNQ4, LRRC52, MINK1, MYC, NEFL, NLGN2, NPPA, NR4A3, P2RY6, PIM1, PM20D1, PRKACA, PRRT1, RANGRF, RAP1A, RRAD, RTN2, S100A1, SCN5A, SCN7A, SELENON, SHANK3, SLC30A1, SLC6A9, SLC9A1, SLC9A3R1, SLN, SPHK2, STAC2, TCIRG1, THBS1, TIMP1, TMEM109, TNF, TPCN1, TRIB3, WNK2, WWP2]
GO:0043269	regulation of ion transport	2,13E-05	[4, 5]	156,00	21,22	44,98	55,02	[ABCB11, ANO9, CACNA1E, CACNA1I, CALCR, CBARP, CCL2, CCL3, CCL4, CCL5, CCR2, CD19, CD33, CD4, CD84, CLCNKA, CNR1, CORO1A, CTSS, CXCL10, CXCL11, CXCL9, CXCR4, CYBB, DAPK1, DRD1, EPX, FCRL3, GABBR2, HCN1, IFNG, IL16, JPH1, JSRP1, KCNA3, KCNA7, KCNJ10, KCNK15, KCNN4, KCNQ5, KMO, LILRB1, LRRC38, MMP9, NLRP3, P2RY12, PIK3CG, PTAFR, PTK2B, PTPN22, PTPN6, RAB3B, RASGRF1, RGS9, RRAD, SHISA8, SLC31A2, SPINK1, SYK, TCAF2, THY1, TMC2, TREM2, UBD, XCL1]	[ABCB11, ABCC8, ABL1, ACTN4, AGT, APLNR, ARC, ARF1, ATP1A1, ATP1A3, ATP1B2, ATPSCKMT, CACNA1E, CACNA1H, CACNB4, CALCR, CAMK2B, CAPN1, CBARP, CCL2, CFH, CLCNKA, CNR1, CX3CL1, FXYD5, FXYD6, GABBR2, GNAI2, GNAO1, GPER1, GPRIN1, GRM2, HCN3, HCN4, HFE, HTR1B, HTR2A, HVCN1, ICAM1, IL1B, JPH4, KCNA7, KCNC3, KCNH2, KCNIP2, KCNJ1, KCNJ5, KCNJ9, KCNK15, KCNK6, KCNMB1, KCNMB2, KCNQ4, LILRA5, LRRC52, MINK1, MLLT6, NEFL, NKAIN3, NLGN2, NPPA, ORAI1, P2RX3, P2RX5, P2RY6, PCDHA4, PDGFB, PDGFRB, PER1, PM20D1, PML, PRKACA, PRRT1, PTGES, RAB11B, RAB3A, RANGRF, RCVRN, RRAD, S100A1, SCN5A, SCN7A, SELENON, SHANK3, SIK1, SLC30A1, SLC6A9, SLC9A1, SLC9A3R1, SLN, SPHK2, SPINK1, STAC2, SV2A, SYT2, SYT7, TCIRG1, TIMP1, TMEM109, TPCN1, WFS1, WNK2, WWP2]
GO:0034765	regulation of ion transmembrane transport	1,63E-02	[4, 5, 6]	108,00	20,61	46,52	53,48	[ANO9, CACNA1E, CACNA1I, CALCR, CBARP, CCL2, CCR2, CD19, CLCNKA, CORO1A, CTSS, CXCL10, CXCL11, CXCL9, CYBB, DAPK1, DRD1, EPX, GABBR2, HCN1, IFNG, JPH1, JSRP1, KCNA3, KCNA7, KCNJ10, KCNK15, KCNN4, KCNQ5, LRRC38, MMP9, PIK3CG, PTAFR, PTK2B, PTPN22, PTPN6, RASGRF1, RGS9, RRAD, SHISA8, SLC31A2, TCAF2, THY1, TMC2, TREM2, UBD, XCL1]	[ABCC8, ABL1, ACTN4, AGT, APLNR, ARC, ARF1, ATP1B2, ATPSCKMT, CACNA1E, CACNA1H, CACNB4, CALCR, CAMK2B, CAPN1, CBARP, CCL2, CFH, CLCNKA, CX3CL1, FXYD5, FXYD6, GABBR2, GPER1, GPRIN1, HCN3, HCN4, HVCN1, JPH4, KCNA7, KCNC3, KCNH2, KCNIP2, KCNJ1, KCNJ5, KCNJ9, KCNK15, KCNK6, KCNMB1, KCNMB2, KCNQ4, LRRC52, MINK1, NEFL, NLGN2, NPPA, P2RY6, PM20D1, PRKACA, PRRT1, RANGRF, RRAD, S100A1, SCN5A, SCN7A, SELENON, SHANK3, SLC30A1, SLC6A9, SLC9A1, SLC9A3R1, SLN, SPHK2, STAC2, TCIRG1, TIMP1, TMEM109, TPCN1, WNK2, WWP2]
GO:0010959	regulation of metal ion transport	7,95E-04	[5, 6, 7]	68,00	25,28	51,67	48,33	[CALCR, CBARP, CCL2, CCL3, CCL4, CCL5, CD19, CD33, CD4, CD84, CORO1A, CXCL10, CXCL11, CXCL9, CXCR4, DRD1, EPX, FCRL3, IL16, JPH1, JSRP1, LILRB1, P2RY12, PIK3CG, PTK2B, PTPN22, PTPN6, RGS9, RRAD, SPINK1, THY1, TMC2, XCL1]	[ABL1, AGT, APLNR, ATP1B2, CACNB4, CALCR, CAMK2B, CBARP, CCL2, CX3CL1, GNAI2, GNAO1, GPER1, GPRIN1, ICAM1, JPH4, KCNH2, KCNIP2, LILRA5, NPPA, ORAI1, P2RX3, P2RX5, P2RY6, PDGFB, PDGFRB, PML, PRKACA, RCVRN, RRAD, S100A1, SELENON, SLC30A1, SLC9A1, SLN, SPHK2, SPINK1, STAC2, TIMP1, WFS1]
GO:0030001	metal ion transport	2,89E-05	[6]	173,00	20,60	42,80	57,20	[ATP2A3, BHLHA15, CACNA1E, CACNA1I, CALCR, CBARP, CCL19, CCL2, CCL3, CCL4, CCL5, CCR5, CD19, CD33, CD4, CD84, CNR1, CORO1A, CXCL10, CXCL11, CXCL9, CXCR4, CYSLTR1, DRD1, EPX, FCRL3, GPM6A, HCN1, IL16, JPH1, JSRP1, KCNA3, KCNA7, KCNJ10, KCNK15, KCNN4, KCNQ5, LCK, LILRB1, LRRC38, MCOLN2, MCOLN3, MS4A1, NMUR1, P2RY12, PIK3CG, PRKCB, PTK2B, PTPN22, PTPN6, PTPRC, RGS9, RRAD, SCNN1B, SLC12A3, SLC18A1, SLC24A4, SLC31A2, SLC5A2, SLC8A3, SPINK1, THY1, TMC2, TREM2, TRPM2, XCL1, XCR1]	[ABCC8, ABL1, ACTN4, AGT, APLNR, ASIC3, ATP1A1, ATP1A3, ATP1B2, CACNA1E, CACNA1H, CACNB4, CALCR, CAMK2B, CBARP, CCL2, CCL21, CCR7, CFH, CHERP, CNNM4, CNR1, CX3CL1, CYP27B1, FXYD5, FXYD6, GJA4, GNAI2, GNAO1, GPER1, GPM6A, GPRIN1, GRIK5, GRM2, HCN3, HCN4, HTR1B, HTR2A, ICAM1, ITPR3, JPH4, KCNA7, KCNC3, KCNH2, KCNIP2, KCNJ1, KCNJ5, KCNJ9, KCNK15, KCNK2, KCNK3, KCNK6, KCNMB1, KCNMB2, KCNQ4, KCNT1, LILRA5, LRRC52, MED12, MFSD2A, MICU3, NIPAL4, NPPA, ORAI1, P2RX3, P2RX5, P2RY6, PCDHA4, PDGFB, PDGFRB, PKD1, PLCZ1, PML, PRKACA, RAMP2, RANGRF, RCVRN, RNU1-93P, RRAD, S100A1, SCN5A, SCN7A, SELENON, SLC10A3, SLC12A4, SLC17A3, SLC25A23, SLC30A1, SLC30A3, SLC39A13, SLC39A5, SLC41A1, SLC4A10, SLC4A11, SLC5A10, SLC5A6, SLC6A12, SLC6A8, SLC6A9, SLC9A1, SLC9A3R1, SLN, SPHK2, SPINK1, STAC2, STX1A, SYNGAP1, TIMP1, TMEM175, TPCN1, TRPV4, TTYH1, VDR, WFS1, WNK2, WWP2]
GO:1904062	regulation of cation transmembrane transport	4,99E-02	[5, 6, 7]	85,00	21,25	45,19	54,81	[CALCR, CBARP, CCL2, CCR2, CD19, CORO1A, CTSS, CXCL10, CXCL11, CXCL9, DAPK1, DRD1, EPX, GABBR2, IFNG, JPH1, JSRP1, KCNJ10, KCNN4, LRRC38, MMP9, PIK3CG, PTK2B, PTPN22, PTPN6, RASGRF1, RGS9, RRAD, SHISA8, SLC31A2, THY1, TMC2, TREM2, UBD, XCL1]	[ABCC8, ABL1, ACTN4, AGT, APLNR, ARC, ARF1, ATP1B2, ATPSCKMT, CACNB4, CALCR, CAMK2B, CAPN1, CBARP, CCL2, CFH, CX3CL1, FXYD5, FXYD6, GABBR2, GPER1, GPRIN1, JPH4, KCNH2, KCNIP2, KCNJ5, KCNJ9, KCNMB1, KCNMB2, LRRC52, MINK1, NEFL, NLGN2, NPPA, P2RY6, PM20D1, PRKACA, PRRT1, RANGRF, RRAD, S100A1, SCN5A, SELENON, SHANK3, SLC30A1, SLC6A9, SLC9A1, SLC9A3R1, SLN, SPHK2, STAC2, TCIRG1, TIMP1, WNK2, WWP2]
GO:0006816	calcium ion transport	5,98E-05	[7]	108,00	22,98	53,02	46,98	[ATP2A3, BHLHA15, CACNA1E, CACNA1I, CALCR, CBARP, CCL19, CCL2, CCL3, CCL4, CCL5, CCR5, CD19, CD33, CD4, CD84, CNR1, CORO1A, CXCL10, CXCL11, CXCL9, CXCR4, CYSLTR1, DRD1, EPX, FCRL3, GPM6A, IL16, JPH1, JSRP1, KCNN4, LCK, LILRB1, MCOLN2, MCOLN3, MS4A1, NMUR1, P2RY12, PIK3CG, PRKCB, PTK2B, PTPN22, PTPN6, PTPRC, RGS9, RRAD, SLC24A4, SLC8A3, SPINK1, THY1, TMC2, TRPM2, XCL1, XCR1]	[ABL1, AGT, APLNR, CACNA1E, CACNA1H, CACNB4, CALCR, CAMK2B, CBARP, CCL2, CCL21, CCR7, CHERP, CNR1, CX3CL1, CYP27B1, GJA4, GNAI2, GNAO1, GPER1, GPM6A, GPRIN1, GRM2, HTR1B, HTR2A, ICAM1, ITPR3, JPH4, LILRA5, MED12, MICU3, NPPA, ORAI1, P2RX3, P2RX5, P2RY6, PCDHA4, PDGFB, PDGFRB, PKD1, PLCZ1, PML, PRKACA, RAMP2, RCVRN, RNU1-93P, RRAD, S100A1, SELENON, SLC25A23, SLC30A1, SLC9A1, SLN, SPHK2, SPINK1, STAC2, STX1A, TIMP1, TPCN1, TRPV4, VDR, WFS1]

GO:0051924	regulation of calcium ion transport	2,15E-03	[6, 7, 8]	65,00	25,00	53,61	46,39	[CALCR, CBARP, CCL2, CCL3, CCL4, CCL5, CD19, CD33, CD4, CD84, CORO1A, CXCL10, CXCL11, CXCL9, CXCR4, DRD1, EPX, FCRL3, IL16, JPH1, JSRP1, LILRB1, P2RY12, PIK3CG, PTK2B, PTPN22, PTPN6, RGS9, RRAD, SPINK1, THY1, TMC2, XCL1]	[ABL1, AGT, APLNR, CACNB4, CALCR, CAMK2B, CBARP, CCL2, CX3CL1, GNAI2, GNAO1, GPER1, GPRIN1, ICAM1, JPH4, LILRA5, NPPA, ORAI1, P2RX3, P2RX5, P2RY6, PDGFB, PDGFRB, PML, PRKACA, RCVRN, RRAD, S100A1, SELENON, SLC30A1, SLC9A1, SLN, SPHK2, SPINK1, STAC2, TIMP1, WFS1]
GO:0060401	cytosolic calcium ion transport	1,60E-03	[8]	52,00	27,08	59,20	40,80	[ATP2A3, CALCR, CCL19, CCL3, CCR5, CD19, CORO1A, CXCL10, CXCL11, CXCL9, DRD1, EPX, JPH1, JSRP1, LCK, MCOLN2, MCOLN3, MS4A1, PTK2B, PTPN6, PTPRC, RGS9, SLC24A4, SLC8A3, THY1, TRPM2, XCL1, XCR1]	[ABL1, APLNR, CALCR, CCL21, CCR7, CHERP, CX3CL1, GPER1, GPRIN1, HTR2A, ITPR3, JPH4, MED12, MICU3, P2RX3, P2RX5, P2RY6, PML, PRKACA, RNU1-93P, SELENON, SLC25A23, TIMP1, TPCN1, TRPV4]
GO:0060402	calcium ion transport into cytosol	2,59E-03	[4, 9, 10, 12]	47,00	27,98	60,49	39,51	[CALCR, CCL19, CCL3, CCR5, CD19, CORO1A, CXCL10, CXCL11, CXCL9, DRD1, EPX, JPH1, JSRP1, LCK, MCOLN2, MCOLN3, MS4A1, PTK2B, PTPN6, PTPRC, SLC24A4, SLC8A3, THY1, TRPM2, XCL1, XCR1]	[ABL1, APLNR, CALCR, CCL21, CCR7, CHERP, CX3CL1, GPER1, GPRIN1, HTR2A, ITPR3, JPH4, P2RX3, P2RX5, P2RY6, PML, PRKACA, RNU1-93P, SELENON, TIMP1, TPCN1, TRPV4]
GO:0051209	release of sequestered calcium ion into cytosol	6,28E-03	[4, 5, 6, 7, 8, 10, 11, 12, 13, 14]	36,00	30,00	64,46	35,54	[CCL19, CCL3, CCR5, CD19, CORO1A, CXCL10, CXCL11, CXCL9, DRD1, JPH1, JSRP1, LCK, MCOLN2, MCOLN3, PTK2B, PTPN6, PTPRC, THY1, TRPM2, XCL1, XCR1]	[ABL1, APLNR, CCL21, CCR7, CHERP, CX3CL1, GPER1, HTR2A, ITPR3, JPH4, P2RY6, PRKACA, RNU1-93P, SELENON, TPCN1]
GO:0097553	calcium ion transmembrane import into cytosol	8,62E-03	[5, 7, 9, 10, 11, 13]	41,00	28,28	63,34	36,66	[CALCR, CCL19, CCL3, CCR5, CD19, CORO1A, CXCL10, CXCL11, CXCL9, DRD1, JPH1, JSRP1, LCK, MCOLN2, MCOLN3, PTK2B, PTPN6, PTPRC, SLC24A4, SLC8A3, THY1, TRPM2, XCL1, XCR1]	[ABL1, APLNR, CALCR, CCL21, CCR7, CHERP, CX3CL1, GPER1, GPRIN1, HTR2A, ITPR3, JPH4, P2RY6, PRKACA, RNU1-93P, SELENON, TPCN1, TRPV4]
GO:0030155	regulation of cell adhesion	5,14E-25	[3, 4]	218,00	28,57	53,48	46,52	[ADAM8, ADAMDEC1, AIF1, AKNA, ALOX15, ANGPT1, ANGPT2, APBB1IP, BLK, BMP7, BTLA, C1QTNF1, CARD11, CASS4, CCDC88B, CCL19, CCL2, CCL5, CCR2, CD27, CD3E, CD4, CD40LG, CD5, CD6, CD74, CD80, CD86, CDH1, CDKN2A, CORO1A, CRTAM, CTLA4, CXCR3, CXCR4, CYTIP, DOCK8, EBI3, EGR3, EPX, FGL2, FOXP3, FUT7, GPR4, GRAP2, HLA-DMB, HLA-DPA1, HLA-DPB1, HLA-DRA, HLA-DRB1, ICOS, IFNG, IL12RB1, IL18, IL7R, ITGA4, ITGB2, KLRK1, KNG1, LAG3, LAX1, LCK, LEF1, LIF, LILRB1, LILRB4, MYB, NCKAP1L, NLRP3, NR4A3, P2RY12, PIK3CG, PLA2G2D, PLAU, PLXNC1, PRKCQ, PTAFR, PTK2B, PTPN22, PTPN6, PTPRC, PYCARD, RAC2, RASAL3, RHOH, RUNX3, SASH3, SELE, SEMA4D, SIRPG, SKAP1, SOCS1, SPN, SYK, TACSTD2, TBX21, TESPA1, THBS1, THY1, TIGIT, TNFAIP8L2, TNFRSF18, TNFSF13B, TNFSF14, TNR, VAV1, VNN1, WNT1, XCL1, ZAP70]	[ABL1, ACTN4, ADAM19, ADGRG1, ADIPOQ, AGER, ANGPT1, AP3D1, APOA1, APOD, ARHGDIA, BCL6, BTLA, C1QTNF1, CAMSAP3, CCL2, CCL21, CCR7, CD276, CD74, CEACAM1, CELSR2, CITED2, COL1A1, CSF1, CSK, CX3CL1, DAG1, DDR1, DLG5, DMTN, DTX1, ECM2, EFEMP2, EPHA2, ERBB2, FADD, FES, FLOT2, FOXC2, FOXF1, FXYD5, GNAO1, GPAM, GPR4, HFE, HYAL1, ICAM1, IL1B, IL1RN, IL4R, IL7R, ILK, ITGA3, JAK3, JUP, KNG1, LAG3, LAMA5, LGALS9, LIF, LIMS2, LRFN3, LRRC32, MAPK7, MDGA1, MEN1, MINK1, MYADM, NF2, NOD2, NODAL, NOTCH1, NOTCH4, NR4A3, NRARP, OMA1, PDCD1, PDGFB, PIEZO1, PKD1, PLAU, PLAUR, PLXNA1, PLXNA3, PLXNB1, PML, POSTN, PPP1CB, PPP2R1A, PTN, PTPN23, PTPRU, RAC2, RARA, RELA, RGCC, SELE, SELENOP, SERPINE1, SH2B3, SLC7A1, SLC9A1, SMAD3, SOX12, SOX13, SPI1, SRC, SRF, SYNGAP1, TESK1, TFE3, TGM2, THBS1, TIMP1, TNF, TRIOBP, TRPV4, VEGFA, VNN1, VSIR, ZBTB7B, ZC3H12A, ZMIZ1]
GO:0007162	negative regulation of cell adhesion	8,18E-08	[3, 4, 5]	84,00	28,19	48,63	51,37	[ADAMDEC1, AKNA, ANGPT1, ANGPT2, C1QTNF1, CD74, CD80, CD86, CDH1, CDKN2A, CRTAM, CTLA4, FGL2, FOXP3, HLA-DRB1, KNG1, LAG3, LAX1, LILRB1, LILRB4, NCKAP1L, PLA2G2D, PLXNC1, PTPN22, PTPN6, PTPRC, RUNX3, SEMA4D, SOCS1, SPN, TACSTD2, TBX21, THBS1, TIGIT, TNFAIP8L2, TNR, WNT1, XCL1]	[ABL1, ACTN4, ADIPOQ, ANGPT1, APOA1, APOD, ARHGDIA, BCL6, C1QTNF1, CCL21, CD74, CEACAM1, COL1A1, CX3CL1, DLG5, DMTN, DTX1, ERBB2, FXYD5, HFE, IL1RN, IL4R, JAK3, KNG1, LAG3, LGALS9, LRRC32, MAPK7, MEN1, MYADM, NF2, NOTCH1, NOTCH4, NRARP, OMA1, PLXNA1, PLXNA3, PLXNB1, POSTN, PTPRU, RGCC, SERPINE1, SH2B3, SPI1, SRC, SYNGAP1, THBS1, TRPV4, VEGFA, VSIR, ZBTB7B, ZC3H12A]
GO:0050866	negative regulation of cell activation	2,21E-10	[3, 4, 5]	72,00	33,49	63,17	36,83	[BANK1, BLK, BTK, C1QTNF1, CCR2, CD300LF, CD74, CD80, CD84, CD86, CDKN2A, CLNK, CNR1, CNR2, CRTAM, CST7, CTLA4, FGL2, FGR, FOXP3, HLA-DRB1, INPP5D, LAG3, LAX1, LDLR, LILRB1, LILRB4, LST1, NCKAP1L, PLA2G2D, PTPN22, PTPN6, PTPRC, RUNX3, SAMSN1, SOCS1, SPN, TBC1D10C, TBX21, TIGIT, TNFAIP3, TNFAIP8L2, TNFRSF13B, TREM2, XCL1]	[BCL6, C1QTNF1, CD74, CEACAM1, CLNK, CNR1, CST7, CX3CL1, DLG5, DTX1, ENPP3, ERBB2, FOXF1, GPER1, GRN, HFE, IL4R, INHA, JAK3, LAG3, LDLR, LGALS9, LRRC32, NOS3, NR1D1, NRARP, PCDHA4, PCDHA6, PDGFB, SH2B3, SPI1, VSIR, ZBTB7B, ZC3H12A]
GO:0002695	negative regulation of leukocyte activation	1,42E-10	[3, 4, 5, 6]	67,00	34,90	65,00	35,00	[BANK1, BLK, BTK, CCR2, CD300LF, CD74, CD80, CD84, CD86, CDKN2A, CLNK, CNR1, CNR2, CRTAM, CST7, CTLA4, FGL2, FGR, FOXP3, HLA-DRB1, INPP5D, LAG3, LAX1, LDLR, LILRB1, LILRB4, LST1, NCKAP1L, PLA2G2D, PTPN22, PTPN6, PTPRC, RUNX3, SAMSN1, SOCS1, SPN, TBC1D10C, TBX21, TIGIT, TNFAIP3, TNFAIP8L2, TNFRSF13B, XCL1]	[BCL6, CD74, CEACAM1, CLNK, CNR1, CST7, CX3CL1, DLG5, DTX1, ENPP3, ERBB2, FOXF1, GPER1, GRN, HFE, IL4R, INHA, JAK3, LAG3, LDLR, LGALS9, LRRC32, NR1D1, NRARP, PCDHA4, PCDHA6, SPI1, VSIR, ZBTB7B, ZC3H12A]
GO:0022407	regulation of cell-cell adhesion	2,49E-25	[4, 5]	155,00	33,70	60,59	39,41	[ADAM8, AIF1, AKNA, ALOX15, BLK, BMP7, BTLA, C1QTNF1, CARD11, CCDC88B, CCL19, CCL2, CCL5, CCR2, CD27, CD3E, CD4, CD40LG, CD5, CD6, CD74, CD80, CD86, CDH1, CDKN2A, CORO1A, CRTAM, CTLA4, DOCK8, EBI3, EGR3, EPX, FGL2, FOXP3, FUT7, GRAP2, HLA-DMB, HLA-DPA1, HLA-DPB1, HLA-DRA, HLA-DRB1, ICOS, IFNG, IL12RB1, IL18, IL7R, ITGA4, ITGB2, KLRK1, LAG3, LAX1, LCK, LEF1, LILRB1, LILRB4, MYB, NCKAP1L, NLRP3, NR4A3, PLA2G2D, PRKCQ, PTAFR, PTPN22, PTPN6, PTPRC, PYCARD, RASAL3, RHOH, RUNX3, SASH3, SELE, SIRPG, SKAP1, SOCS1, SPN, SYK, TBX21, TESPA1, THY1, TIGIT, TNFAIP8L2, TNFSF13B, TNFSF14, TNR, VAV1, VNN1, WNT1, XCL1, ZAP70]	[ABL1, ADAM19, ADIPOQ, AGER, AP3D1, APOA1, BCL6, BTLA, C1QTNF1, CCL2, CCL21, CCR7, CD276, CD74, CEACAM1, CELSR2, CITED2, CSK, CX3CL1, DLG5, DMTN, DTX1, ERBB2, FADD, FLOT2, FXYD5, GNAO1, GPAM, HFE, ICAM1, IL1B, IL1RN, IL4R, IL7R, JAK3, LAG3, LGALS9, LRFN3, LRRC32, MAPK7, MDGA1, MINK1, MYADM, NF2, NOD2, NODAL, NOTCH1, NOTCH4, NR4A3, NRARP, PDCD1, PIEZO1, PLAUR, PTPN23, PTPRU, RARA, RELA, RGCC, SELE, SELENOP, SH2B3, SLC7A1, SOX12, SOX13, SPI1, SRC, TIMP1, TNF, TRPV4, VEGFA, VNN1, VSIR, ZBTB7B, ZC3H12A, ZMIZ1]
GO:0022408	negative regulation of cell-cell adhesion	2,36E-06	[4, 5, 6]	59,00	30,57	53,24	46,76	[AKNA, C1QTNF1, CD74, CD80, CD86, CDH1, CDKN2A, CRTAM, CTLA4, FGL2, FOXP3, HLA-DRB1, LAG3, LAX1, LILRB1, LILRB4, NCKAP1L, PLA2G2D, PTPN22, PTPN6, RUNX3, SOCS1, SPN, TBX21, TIGIT, TNFAIP8L2, TNR, WNT1, XCL1]	[ABL1, ADIPOQ, APOA1, BCL6, C1QTNF1, CCL21, CD74, CEACAM1, DLG5, DTX1, ERBB2, FXYD5, HFE, IL1RN, IL4R, JAK3, LAG3, LGALS9, LRRC32, MAPK7, MYADM, NF2, NOTCH1, NOTCH4, NRARP, RGCC, SH2B3, SPI1, TRPV4, VEGFA, VSIR, ZBTB7B, ZC3H12A]
GO:0051250	negative regulation of lymphocyte activation	1,91E-05	[4, 5, 6, 7]	50,00	31,25	71,59	28,41	[BANK1, BLK, BTK, CD74, CD80, CD86, CDKN2A, CLNK, CRTAM, CTLA4, FGL2, FGR, FOXP3, HLA-DRB1, INPP5D, LAG3, LAX1, LILRB1, LILRB4, LST1, NCKAP1L, PLA2G2D, PTPN22, PTPN6, RUNX3, SAMSN1, SOCS1, SPN, TBC1D10C, TBX21, TIGIT, TNFAIP3, TNFAIP8L2, TNFRSF13B, XCL1]	[BCL6, CD74, CEACAM1, CLNK, DLG5, DTX1, ERBB2, HFE, IL4R, INHA, JAK3, LAG3, LGALS9, LRRC32, NRARP, VSIR, ZBTB7B, ZC3H12A]

GO:1903037	regulation of leukocyte cell-cell adhesion	3,98E-20	[5, 6]	119,00	34,69	69,53	30,47	[ADAM8, AIF1, BTLA, CARD11, CCDC88B, CCL19, CCL2, CCL5, CCR2, CD27, CD3E, CD4, CD40LG, CD5, CD6, CD74, CD80, CD86, CDKN2A, CORO1A, CRTAM, CTLA4, DOCK8, EBI3, EGR3, EPX, FGL2, FOXP3, FUT7, GRAP2, HLA-DMB, HLA-DPA1, HLA-DPB1, HLA-DRA, HLA-DRB1, ICOS, IFNG, IL12RB1, IL18, IL7R, ITGA4, ITGB2, KLRK1, LAG3, LAX1, LCK, LEF1, LILRB1, LILRB4, MYB, NCKAP1L, NLRP3, NR4A3, PLA2G2D, PRKCQ, PTAFR, PTPN22, PTPN6, PTPRC, PYCARD, RASAL3, RHOH, RUNX3, SASH3, SELE, SIRPG, SKAP1, SOCS1, SPN, SYK, TBX21, TESPA1, THY1, TIGIT, TNFAIP8L2, TNFSF13B, TNFSF14, VAV1, VNN1, XCL1, ZAP70]	[AGER, AP3D1, BCL6, BTLA, CCL2, CCL21, CCR7, CD276, CD74, CEACAM1, CSK, DLG5, DTX1, ERBB2, FADD, FLOT2, GNAO1, GPAM, HFE, ICAM1, IL1B, IL4R, IL7R, JAK3, LAG3, LGALS9, LRRC32, NOD2, NR4A3, NRARP, PDCD1, RARA, RELA, SELE, SELENOP, SLC7A1, SOX12, SOX13, SRC, TIMP1, TNF, VNN1, VSIR, ZBTB7B, ZC3H12A, ZMIZ1]
GO:0050863	regulation of T cell activation	2,38E-22	[5, 6, 7]	126,00	35,39	70,57	29,43	[ADAM8, AIF1, BTLA, CAMK4, CARD11, CCDC88B, CCL19, CCL2, CCL5, CCR2, CD2, CD27, CD3E, CD4, CD40LG, CD5, CD6, CD74, CD80, CD86, CDKN2A, CLCF1, CORO1A, CRTAM, CTLA4, DOCK8, EBI3, EGR3, EPX, FGL2, FOXP3, GRAP2, HLA-DMB, HLA-DOA, HLA-DPA1, HLA-DPB1, HLA-DRA, HLA-DRB1, ICOS, IFNG, IL12RB1, IL18, IL7R, IRF4, KLRK1, LAG3, LAX1, LCK, LEF1, LILRB1, LILRB4, LMO1, MYB, NCKAP1L, NLRP3, PLA2G2D, PRDM1, PRKCQ, PTPN22, PTPN6, PTPRC, PYCARD, RAC2, RASAL3, RHOH, RUNX3, SASH3, SIRPG, SIT1, SOCS1, SPN, SYK, TBX21, TESPA1, THY1, TIGIT, TNFAIP8L2, TNFRSF18, TNFSF13B, TNFSF14, TNFSF8, TOX, VAV1, VNN1, XCL1, ZAP70, ZNF683]	[ABL1, AGER, AP3D1, BCL6, BTLA, CCL2, CCL21, CCR7, CD276, CD74, CEACAM1, CLC, CLCF1, CLPTM1, CSK, CYP26B1, DLG5, DTX1, ERBB2, FADD, FLOT2, GNAO1, GPAM, HFE, IDO1, IL1B, IL4R, IL7R, JAK3, LAG3, LGALS9, LRRC32, NOD2, NRARP, PDCD1, RAC2, RARA, SLC7A1, SOX12, SOX13, SRC, TIMP1, VNN1, VSIR, ZBTB7B, ZC3H12A, ZMIZ1]
GO:1903038	negative regulation of leukocyte cell-cell adhesion	9,60E-03	[5, 6, 7]	39,00	28,47	64,65	35,35	[CD74, CD80, CD86, CDKN2A, CRTAM, CTLA4, FGL2, FOXP3, HLA-DRB1, LAG3, LAX1, LILRB1, LILRB4, NCKAP1L, PLA2G2D, PTPN22, PTPN6, RUNX3, SOCS1, SPN, TBX21, TIGIT, TNFAIP8L2, XCL1]	[BCL6, CCL21, CD74, CEACAM1, DLG5, DTX1, ERBB2, HFE, IL4R, JAK3, LAG3, LGALS9, LRRC32, NRARP, VSIR, ZBTB7B, ZC3H12A]
GO:1902106	negative regulation of leukocyte differentiation	1,66E-03	[4, 5, 6, 7, 8, 9, 10]	34,00	32,69	49,68	50,32	[C1QC, CCL3, CD74, CDKN2A, CTLA4, FGL2, FOXP3, GPR55, INPP5D, LAG3, LILRB1, LILRB4, LRRC17, RUNX3, SOCS1, TBX21]	[ADIPOQ, BCL6, CD74, CEACAM1, DTX1, ERBB2, GATA2, GPR137, IL4R, INHA, JAK3, LAG3, LRRC17, LTF, MYC, NRARP, RARA, RMRP, TRIB1, ZBTB7B, ZC3H12A]
GO:0045580	regulation of T cell differentiation	2,55E-13	[6, 7, 8, 9, 10, 11, 12]	62,00	40,79	72,16	27,84	[ADAM8, CAMK4, CARD11, CCL19, CCR2, CD2, CD27, CD74, CD80, CD86, CDKN2A, CRTAM, CTLA4, EGR3, FGL2, FOXP3, HLA-DOA, HLA-DRA, HLA-DRB1, IFNG, IL12RB1, IL18, IL7R, IRF4, LAG3, LEF1, LILRB4, MYB, NCKAP1L, NLRP3, PRDM1, PTPRC, RHOH, RUNX3, SASH3, SOCS1, SYK, TBX21, TESPA1, TNFRSF18, TOX, VNN1, ZAP70, ZNF683]	[ABL1, AP3D1, BCL6, CD74, CLPTM1, CYP26B1, DTX1, ERBB2, IL4R, IL7R, JAK3, LAG3, LGALS9, NRARP, RARA, SOX12, SOX13, VNN1, VSIR, ZBTB7B, ZC3H12A, ZMIZ1]
GO:0010033	response to organic substance	5,79E-16	[3]	652,00	18,46	41,35	58,65	[AANAT, ACP5, ADCY7, AIF1, AIM2, ALOX15, AMPD1, ANGPT1, ANGPT2, AREG, ATF3, BATF, BHLHA15, BMP7, BMPR1B, BRIP1, BTG2, BTK, C1QTNF1, CALCR, CARD17, CASP8, CCL17, CCL19, CCL2, CCL22, CCL24, CCL3, CCL3L1, CCL4, CCL4L1, CCL5, CCR2, CCR3, CCR4, CCR5, CCR8, CD180, CD27, CD300LF, CD4, CD40LG, CD6, CD74, CD80, CD86, CD96, CDH1, CHRDL1, CISH, CLCF1, CNR1, CNR2, CORO1A, CRB2, CSF1R, CSF2RA, CTSS, CXCL10, CXCL11, CXCL5, CXCL6, CXCL9, CXCR3, CXCR4, CXCR6, CYBB, CYP11B1, DAPK1, DERL3, DNAJB5, DOCK8, DPEP1, DPEP2, DRD1, EBI3, EDN2, EGR3, ELAVL4, EPX, EREG, ERFE, ERP27, FASLG, FIBIN, FLT3, FOLR2, FOSB, FOSL1, FOXP3, FUT7, GALNT3, GBP5, GBP6, GDF6, GFI1, GFPT2, GFRA2, GLDC, GPR83, GRAMD1B, GRAMD1C, GRAP2, H3C10, H3C11, H3C12, H3C7, HCLS1, HCN1, HLA-DPA1, HLA-DPB1, HLA-DQA1, HLA-DRA, HLA-DRB1, HLA-DRB5, HSPA7, IFNG, IKBKE, IL10RA, IL12RB1, IL12RB2, IL16, IL18, IL18RAP, IL21R, IL22, IL26, IL2RB, IL2RG, IL5RA, IL7R, IL9R, INPP5D, IRF4, IRF5, IRF8, ITGA4, ITGAX, ITGB2, KLF5, KLRG1, KMO, LAMP3, LCK, LCP1, LDLR, LEF1, LIF, LILRB1, LILRB4, LPAL2, LTA, LTB, LY86, MAP4K1, MAPK13, MLC1, MMP9, MPO, MRC1, MSX1, MT1G, MUSK, MYB, MYBL2, MZB1, NAMPT, NELL2, NLRP3, NLRP7, NPPC, NR4A1, NR4A2, NR4A3, NSG1, NUGGC, OASL, P2RY12, P2RY13, P2RY2, PIK3CG, PIM1, PRKCB, PRKCQ, PTAFR, PTGER2, PTGFR, PTK2B, PTPN22, PTPN6, PTPN7, PTPRC, PYCARD, PYHIN1, RAB15, RBPMS2, RGS10, RGS9, RHEX, RUFY4, RUNX3, SCIMP, SCNN1B, SDC1, SELE, SH2D2A, SLA, SLA2, SLC8A3, SMPD3, SNX10, SOCS1, SOCS3, STAP1, STAT1, SYK, TBX21, TBXAS1, THBS1, TLR6, TNFAIP3, TNFRSF11B, TNFRSF13B, TNFRSF17, TNFRSF18, TNFSF13B, TNFSF14, TNFSF8, TNIP3, TNMD, TRAF1, TREM2, TRERF1, TRPM2, UBD, UCP2, VAV1, VWC2L, WAS, WNT1, WNT10A, WNT10B, XCL1, XCL2, XCR1, ZBP1]	[ABCA3, ABCA7, ABCC8, ABCG4, ABL1, ACACB, ACKR1, ACKR3, ACSBG1, ACTA1, ACTB, ACTC1, ACTN4, ADAMTS7, ADCY1, ADCY3, ADCY4, ADCY9, ADIPOQ, AGER, AGPAT1, AGT, AGTR1, AKT1S1, ALPL, AMH, ANGPT1, APLN, APLP1, APOA1, APOM, AQP3, AREG, ARF1, ARHGDIA, ARID1A, ARID5A, ARSA, ASPN, ATAT1, ATP1A1, ATP1A3, ATP6V0D1, ATP6V0E2, ATP6V1B1, ATP6V1F, BAG3, BAG6, BCAR1, BCL2L1, BCL6, BCL9L, BMP8A, BTG2, C1QTNF1, C20orf27, C2CD2L, C5AR1, CACNA1H, CALB1, CALCR, CAMK2B, CARM1, CCL11, CCL2, CCL21, CCL24, CCR7, CD14, CD74, CDC42EP2, CDC42EP4, CDK1, CDKN1A, CEACAM1, CFL1, CGA, CHERP, CHGA, CHI3L1, CIITA, CISH, CITED2, CITED4, CLCF1, CLIP3, CNR1, COASY, COL1A1, COL6A1, CRAT, CREB3L1, CRHR2, CRYAB, CSF1, CSK, CTF1, CX3CL1, CYP21A2, CYP26B1, CYP27B1, DAB2IP, DAG1, DAXX, DDX54, DGKD, DHX34, DMTN, DNAJB5, DNMT1, DPAGT1, DTX1, ECM1, EDN2, EGLN2, EHD1, EIF4EBP1, ELK1, ENDOG, ENG, EPHA2, ERBB2, ERFE, EXTL1, FABP4, FADD, FAM83G, FASN, FES, FFAR2, FGFR4, FGFRL1, FIBIN, FLT3, FLT4, FOSL1, FOXC2, FOXF1, FOXRED2, FPR1, FURIN, GAB2, GAS1, GAS2L1, GATA2, GATA4, GBA, GDF6, GET4, GFPT2, GFRA3, GIT1, GJD3, GLRA1, GNAI2, GNAO1, GNRHR, GPAM, GPBAR1, GPER1, GPI, GPR17, GPR75, GPR83, GPRIN1, GPT, GPX3, GRAMD1A, GRAMD1B, GRAMD1C, GRB7, GRIK5, GRM2, GSK3A, H3C10, H3C12, H4C3, HCFC1, HCN3, HCN4, HDAC5, HEYL, HFE, HGS, HNMT, HPRT1, HRH2, HSF1, HSPB1, HSPB7, HSPB8, HSPE1, HTR1B, HTR2A, HYAL1, ICAM1, ID3, IFITM1, IFITM2, IFITM3, IL15RA, IL18BP, IL1B, IL1RN, IL22RA1, IL32, IL3RA, IL4R, IL5RA, IL7R, ILK, INPPL1, IRAK2, IRF2, IRF9, ITGA3, ITGA5, JAK3, JUNB, JUND, JUP, KCNMB1, KCP, KCTD11, KLF10, KLF5, KMT2D, LAMA5, LDLR, LDLRAP1, LDOC1, LGALS9, LIF, LIFR, LMNA, LOX, LRG1, LRRC32, LTBR, LTF, LUM, LY6E, LZTS1, MAP1LC3A, MAP2K3, MAP3K14, MAPK3, MAPK7, MAVS, MED12, MEGF8, MEN1, MICALL1, MME, MMP15, MRC1, MSC, MSN, MSTN

GO:0051707	response to other organism	1,26E-11	[2, 4]	341,00	20,19	61,24	38,76	[ACP5, ADAM8, AIF1, AIM2, APOBEC3A, APOBEC3D, APOBEC3G, APOBEC3H, BANK1, BATF, BCL3, BLK, BTK, C1QA, C1QB, C1QC, C2, C8G, CARD11, CARD17, CASP8, CCDC88B, CCL17, CCL19, CCL2, CCL22, CCL24, CCL3, CCL3L1, CCL4, CCL4L1, CCL5, CCR4, CCR5, CD177, CD180, CD226, CD244, CD4, CD6, CD74, CD80, CD84, CD86, CD96, CFP, CLEC10A, CLEC4D, CLEC4E, CLEC6A, CLNK, CNR1, CNR2, CORO1A, COTL1, CR2, CRTAM, CSF1R, CXCL10, CXCL11, CXCL5, CXCL6, CXCL9, CXCR4, CXorf21, CYBB, DAPK1, EPX, EREG, FASLG, FCN1, FCN3, FER1L6, FGL2, FGR, FOSL1, FOXP3, GBP5, GBP6, GFI1, GNLY, GPM6A, GZMA, GZMB, GZMM, HLA-DPA1, HLA-DPB1, HLA-DQA1, HLA-DRA, HLA-DRB1, HLA-DRB5, IFNG, IGLL5, IKBKE, IKZF3, IL10RA, IL12RB1, IL12RB2, IL18, IL18RAP, IL7R, IRF4, IRF5, IRF8, ITGAX, JCHAIN, KIR2DL3, KLRC1, KLRC2, KLRD1, KLRG1, KLRK1, KMO, LAG3, LCK, LILRB1, LTA, LY86, LY9, LYZ, MCOLN2, MPEG1, MPO, MRC1, MS4A1, MUC16, MUC19, MUC6, NCF1, NCR1, NCR3, NLRC3, NLRP2, NLRP3, NLRP6, NLRP7, NLRP9, NPPC, NR4A1, NUGGC, OASL, PIK3CG, PIM2, PLAC8, PLD4, POU2AF1, POU2F2, PRDM1, PRF1, PSTPIP1, PTAFR, PTGFR, PTK2B, PTPN22, PTPN6, PTPRC, PTX3, PYCARD, PYHIN1, RGS1, RNASE6, RNASET2, SCIMP, SCNN1B, SELE, SH2D1A, SIGLEC10, SIGLEC16, SLA, SLAMF6, SLAMF7, SLAMF8, SMPDL3B, SOCS1, SOCS3, SPN, STAP1, STAT1, STYK1, SYK, TBX21, TLR10, TLR6, TLR8, TNFAIP3, TNFAIP8L2, TNFSF8, TNIP3, TREM2, TRIM14, UBD, VAV1, VNN1, WAS, WDFY4, XCL1, XCL2, ZAP70, ZBP1, ZNF683]	[ABCC8, ABL1, ADAMTS5, ADIPOQ, AGER, ALPL, AMH, APOBEC3C, ARF1, ARID5A, ARMC5, BATF2, BCL2L1, BCL3, C5, C5AR1, C8G, CAMK2B, CCL11, CCL2, CCL21, CCL24, CCR7, CD14, CD177, CD300E, CD74, CDC42EP2, CDC42EP4, CEACAM1, CFH, CFL1, CHGA, CIITA, CLEC4E, CLNK, CNR1, CRP, CSF1, CX3CL1, CYP21A2, CYP27B1, DAB2IP, ELF4, EPHA2, FABP4, FADD, FCN3, FES, FFAR2, FOSL1, GBF1, GIT1, GNAO1, GPAM, GPER1, GPM6A, GRN, HFE, HK1, HMGA1, HNRNPUL1, HSF1, HSPB1, HYAL1, ICAM1, IFITM1, IFITM2, IFITM3, IL18BP, IL1B, IL22RA1, IL4R, IL7R, IRAK2, IRF2, IRF9, JAK3, JUND, KCTD11, LAG3, LDOC1, LGALS9, LILRA5, LRG1, LTF, MAP3K14, MAPK3, MAVS, MECP2, MOV10, MRC1, MUC19, MUC20, MUC3A, MYC, NCOR2, NECTIN2, NFKB2, NFKBIB, NLRC5, NLRP9, NOD2, NOS3, NOTCH1, NR1D1, NR4A1, ODC1, PAF1, PCDHA4, PCDHA6, PLA2G2A, PLEKHM2, PML, PPARD, PPP1R11, PQBP1, PRKACA, PTGES, PTGFR, PTGIR, PVR, RARA, RBM14, RELA, RELB, RNASE2, RNF19B, RNF26, RNF39, RNU1-93P, SBNO2, SCN7A, SELE, SELENOP, SERPINE1, SERPING1, SMPD1, SOCS3, SPI1, SRC, STING1, TBKBP1, TICAM1, TIMP1, TMEM229B, TMEM256-PLSCR3, TNF, TNFRSF1A, TNK1, TNK2, TRIB1, TRIM29, TRIM62, TRIM8, UBA52, VNN1, WAS, ZC3H12A, ZCCHC3, ZDHHC11, ZFP36, ZNF395, ZYX]
GO:0071216	cellular response to biotic stimulus	2,89E-05	[3]	65,00	27,78	58,63	41,37	[BTK, CARD17, CCL2, CCL3, CCL5, CCR5, CD180, CD6, CD80, CD86, CXCL10, CXCL11, CXCL5, CXCL6, CXCL9, GFI1, IL18, IRF8, KMO, LILRB1, LY86, MRC1, NLRP3, NLRP7, NUGGC, PTAFR, PTPN22, PYCARD, SCIMP, STAP1, SYK, TLR6, TNFAIP3, TNIP3, TREM2]	[ABL1, CCL2, CD14, CX3CL1, DAB2IP, GIT1, HSF1, ICAM1, IL1B, IRAK2, LDOC1, LTF, MAPK3, MRC1, NFKBIB, NOD2, NR1D1, PAF1, PPARD, RARA, RELA, SBNO2, SERPINE1, SPI1, SRC, TICAM1, TMEM256-PLSCR3, TNF, TRIB1, WFS1, ZC3H12A, ZFP36]
GO:1901700	response to oxygen-containing compound	6,42E-09	[3]	351,00	19,19	40,36	59,64	[AANAT, ACP5, ADCY7, AIF1, ANGPT2, AREG, BMP7, BRIP1, BTG2, BTK, C1QTNF1, CALCR, CARD17, CASP8, CCL19, CCL2, CCL3, CCL5, CCR5, CD180, CD27, CD6, CD80, CD86, CD96, CDH1, CNR1, CNR2, CXCL10, CXCL11, CXCL5, CXCL6, CXCL9, CYBB, CYP11B1, DAPK1, DPEP1, DPEP2, DRD1, ELAVL4, EPX, EREG, ERFE, FASLG, FIBIN, FOLR2, FOSB, FOSL1, FOXP3, FUT7, GFI1, GLDC, GRAMD1B, GRAMD1C, HCN1, HLA-DRB1, IL10RA, IL12RB2, IL18, IL18RAP, IRF5, IRF8, ITGA4, KLF5, KLRG1, KMO, LCP1, LDLR, LILRB1, LPAL2, LTA, LY86, MAP4K1, MAPK13, MLC1, MMP9, MPO, MRC1, MUSK, MYB, MZB1, NAMPT, NCF1, NLRP3, NLRP7, NME8, NPPC, NR4A1, NR4A2, NR4A3, NSG1, NUGGC, P2RY12, P2RY2, PIK3CG, PIM1, PRKCB, PRKCQ, PTAFR, PTGER2, PTGFR, PTK2B, PTPN22, PYCARD, RAB15, RGS10, RGS9, SCIMP, SCNN1B, SDC1, SELE, SH2D2A, SLC8A3, SMPD3, SOCS1, SOCS3, STAP1, STAT1, TBXAS1, THBS1, TLR6, TNFAIP3, TNIP3, TREM2, TRERF1, TRPM2, UCP2, WNT1, WNT10B]	[ABCC8, ABL1, ACTB, ACTC1, ADCY1, ADCY3, ADCY4, ADCY9, ADIPOQ, AGER, AGT, AGTR1, ALPL, APLP1, APOD, APOM, AQP3, AREG, ARSA, ATP1A1, ATP1A3, ATP6V0D1, ATP6V0E2, ATP6V1B1, ATP6V1F, BCAR1, BCL2L1, BTG2, C1QTNF1, C2CD2L, C5AR1, CALCR, CARM1, CCL2, CCL21, CCR7, CD14, CDK1, CDK2, CDKN1A, CEACAM1, CNR1, COASY, COL1A1, COL6A1, CRAT, CRHR2, CRYAB, CSK, CX3CL1, CYP21A2, CYP26B1, CYP27B1, DAB2IP, DAG1, DHX34, DMTN, DNMT1, DPAGT1, EIF4EBP1, ELK1, ENDOG, ERFE, FADD, FANCC, FES, FFAR2, FGFR4, FIBIN, FOSL1, FOXC2, GATA4, GBA, GIT1, GJD3, GLRA1, GNAI2, GNAO1, GNRHR, GPAM, GPBAR1, GPER1, GPI, GPR37L1, GPRIN1, GPT, GPX3, GRAMD1A, GRAMD1B, GRAMD1C, GRB7, GRIK5, GRM2, GSK3A, HBA1, HBB, HCN3, HCN4, HDAC5, HNMT, HRH2, HSF1, HTR1B, HTR2A, HYAL1, ICAM1, ID3, IL18BP, IL1B, INPPL1, IRAK2, JAK3, JUND, JUP, KCNMB1, KCTD11, KDM6B, KLF10, KLF5, LDLR, LDOC1, LGALS9, LTF, LY6E, MAP1LC3A, MAPK3, MAPK7, MED12, MEN1, MMP15, MRC1, MSN, MSTN, MTHFR, MYC, NCOR2, NDOR1, NEFL, NFKB2, NFKBIB, NGFR, NOD2, NOS3, NOTCH1, NPPA, NR1D1, NR4A1, NR4A2, NR4A3, NSG1, NSMF, P2RX3, P2RX5, P2RY2, P2RY6, PAF1, PALM, PCDHA4, PCDHA6, PCGF2, PDGFB, PDGFRB, PDK2, PER1, PIM1, PKM, PLK3, POR, POSTN, PPARD, PPP1R9B, PRKACA, PRKAR2B, PTGES, PTGFR, PTGIR, PTN, PTPRN, PXN, QDPR, RAB11B, RAB11FIP5, RAB15, RAMP2, RANGAP1, RAP1A, RAPGEF1, RARA, RELA, RMI1, RMRP, RNU1-93P, RPTOR, RXRA, RXRB, SBNO2, SCAP, SELE, SELENOP, SERPINE1, SH2B2, SIDT2, SIK2, SLC10A3, SLC25A33, SLC29A1, SLC2A4, SLC9A1, SLC9A3R1, SMARCA4, SMARCB1, SMPD1, SOCS3, SOX10, SPHK1, SPHK2, SPI1, SRC, SREBF1, SRF, SSTR2, STAT3, SZT2, TCIRG1, TEAD2, TGM2, THBS1, TICAM1, TIMP1, TMEM161A, TMEM256-PLSCR3, TNF, TNS2, TRIB1, TRIB3, TRPV4, TSC2, VDR, WBP2, WDTC1, WNT3, WNT9B, XRCC1, ZBTB7B, ZC3H12A, ZFP36, ZNF580]

GO:0009725	response to hormone	7,90E-04	[3, 4]	196,00	19,23	31,47	68,53	[AANAT, ADCY7, AIF1, AREG, BMP7, BRIP1, BTG2, C1QTNF1, CALCR, CCL19, CTSS, CYBB, CYP11B1, EBI3, EPX, EREG, ERFE, FIBIN, FLT3, FOSB, FOSL1, FUT7, GPR83, H3C10, H3C11, H3C12, H3C7, HCLS1, IL22, LCP1, LPAL2, MUSK, MZB1, NAMPT, NR4A1, NR4A2, NR4A3, PRKCB, PRKCQ, PTAFR, PTGER2, PTGFR, PTK2B, RAB15, SCNN1B, SDC1, SH2D2A, SOCS1, SOCS3, STAT1, THBS1, TNFRSF11B, TRERF1, UCP2, WNT1, WNT10B]	[ABCA3, ABCC8, ACSBG1, ACTA1, ADCY1, ADCY3, ADCY4, ADCY9, ADIPOQ, AGT, AGTR1, ALPL, APOA1, AREG, ARID1A, ARID5A, ARSA, ATAT1, ATP1A1, ATP1A3, ATP6V0D1, ATP6V0E2, ATP6V1B1, ATP6V1F, BCAR1, BTG2, C1QTNF1, CACNA1H, CALCR, CARM1, CCL21, CCR7, CDKN1A, CEACAM1, CGA, CHERP, CHGA, CITED2, CITED4, COASY, COL1A1, CRAT, CRHR2, CSK, CYP21A2, CYP27B1, DAG1, DAXX, DDX54, DPAGT1, EGLN2, ENG, ERFE, FIBIN, FLT3, FOSL1, FOXC2, GAS2L1, GBA, GNAI2, GNRHR, GPAM, GPER1, GPI, GPR83, GPT, GRB7, GSK3A, H3C10, H3C12, H4C3, HDAC5, HEYL, HNMT, HSF1, HTR1B, ICAM1, IL1B, IL1RN, IL4R, INPPL1, ITGA3, JAK3, JUND, KCTD11, KMT2D, LOX, MED12, MEN1, MSTN, MYC, NCOA6, NCOR2, NEFL, NODAL, NOS3, NOTCH1, NPPA, NR1D1, NR4A1, NR4A2, NR4A3, NSMF, P2RY6, PDGFRB, PDK2, PELP1, PER1, PKM, POR, PPARD, PPP1R9B, PRKACA, PRKAR2B, PTGFR, PTN, PTPRN, PTPRU, PXN, QDPR, RAB15, RAMP2, RANGAP1, RARA, RBM14, RELA, RXRA, RXRB, SCAF1, SCAP, SH2B2, SIK2, SLC25A33, SLC2A4, SLC9A1, SMARCA4, SOCS3, SOX10, SRC, SREBF1, SRF, SSTR2, STAT3, TCIRG1, THBS1, TIMP1, TNF, TNS2, TRIB3, TRPV4, TSC2, UCN2, WBP2, WDTC1, ZBTB7A, ZBTB7B, ZFP36, ZMIZ1]
GO:0014070	response to organic cyclic compound	8,35E-04	[4]	203,00	19,08	35,12	64,88	[AANAT, AIF1, ANGPT2, AREG, BMP7, BTG2, C1QTNF1, CALCR, CASP8, CCL2, CCL3, CCL5, CCR5, CDH1, CNR1, CXCL10, CXCR4, CYBB, CYP11B1, DPEP1, DPEP2, DRD1, ELAVL4, EPX, FIBIN, FLT3, FOLR2, FOSB, FOSL1, FOXP3, GLDC, GPR83, GRAMD1B, GRAMD1C, H3C10, H3C11, H3C12, H3C7, HCN1, IL18, IL22, KLF5, LPAL2, MLC1, MUSK, NAMPT, NLRP3, NR4A3, NSG1, P2RY12, P2RY13, P2RY2, PIK3CG, PIM1, PTAFR, PTGFR, PTK2B, RGS9, SCNN1B, SDC1, SLC8A3, STAT1, THBS1, TRERF1, TRPM2, WNT10B]	[ABCA3, ABL1, ACACB, ACSBG1, ACTA1, ACTB, ADCY1, ADCY3, ADIPOQ, AGT, ALPL, AMH, APLP1, AQP3, AREG, ARHGDIA, ARID1A, ATAT1, ATP1A1, ATP1A3, BCL2L1, BTG2, C1QTNF1, CALCR, CARM1, CCL2, CDK1, CDKN1A, CIITA, CNR1, COL1A1, CRAT, CRYAB, CYP21A2, CYP27B1, DAG1, DAXX, DDX54, DHX34, DMTN, EGLN2, ELK1, ENDOG, ENG, FADD, FES, FGFR4, FIBIN, FLT3, FOSL1, FOXF1, GBA, GNAI2, GNAO1, GPAM, GPBAR1, GPER1, GPI, GPR83, GRAMD1A, GRAMD1B, GRAMD1C, GRM2, GSK3A, H3C10, H3C12, H4C3, HCFC1, HCN3, HCN4, HEYL, HNMT, HRH2, HSF1, HTR1B, HTR2A, ICAM1, ID3, IL1B, IL1RN, JUND, JUP, KCNMB1, KCTD11, KLF5, KMT2D, LOX, LUM, MAP1LC3A, MAPK3, MAVS, MED12, MMP15, MSN, MSTN, MTHFR, MYC, NCOR2, NEFL, NOD2, NODAL, NOTCH1, NR1D1, NR4A3, NSG1, P2RX3, P2RX5, P2RY2, P2RY6, PALM, PCDHA4, PDGFB, PDGFRB, PER1, PIM1, POSTN, PPARD, PPP1R9B, PQBP1, PRKACA, PRKAR2B, PTGES, PTGFR, PTN, PTPRN, PTPRU, RAMP2, RAP1A, RAPGEF1, RARA, RELA, RMRP, RNU1-93P, RXRA, RXRB, SELENON, SLC9A1, SLC9A3R1, SMARCA4, SMPD1, SOX10, SRC, SREBF1, SSTR2, STAT3, STING1, TGM2, THBS1, TICAM1, TIMP1, TNF, VDR, WBP2, ZBTB7A, ZC3H12A, ZCCHC3, ZFP36, ZMIZ1]
GO:0033993	response to lipid	5,91E-09	[4]	216,00	21,51	43,79	56,21	[AANAT, ACP5, AIF1, AREG, BMP7, C1QTNF1, CALCR, CARD17, CASP8, CCL2, CCL3, CCL5, CCR5, CD180, CD6, CD80, CD86, CD96, CNR1, CNR2, CXCL10, CXCL11, CXCL5, CXCL6, CXCL9, CYBB, CYP11B1, DPEP1, DPEP2, EPX, FASLG, FIBIN, FLT3, FOSB, FOSL1, FOXP3, GFI1, GLDC, GPR83, GRAMD1B, GRAMD1C, H3C10, H3C11, H3C12, H3C7, IL10RA, IL12RB2, IL18, IL22, IRF8, KMO, LCP1, LDLR, LILRB1, LPAL2, LTA, LY86, MAP4K1, MLC1, MPO, MRC1, MUSK, MYB, NLRP3, NLRP7, NPPC, NR4A1, NR4A3, NUGGC, PIM1, PTAFR, PTGFR, PTK2B, PTPN22, PYCARD, RGS9, SCIMP, SCNN1B, SDC1, SELE, STAP1, TBXAS1, THBS1, TNFAIP3, TNIP3, TREM2, TRERF1, UCP2, WNT10B]	[ABCA3, ABCC8, ABL1, ACSBG1, ACTA1, ADCY1, ADCY3, ADIPOQ, AGT, ALPL, AQP3, AREG, ARID1A, ATAT1, ATP1A1, ATP1A3, C1QTNF1, C5AR1, CALCR, CARM1, CCL2, CCR7, CD14, CDKN1A, CNR1, COL1A1, CRAT, CRYAB, CX3CL1, CYP21A2, CYP26B1, CYP27B1, DAB2IP, DAG1, DAXX, DDX54, EGLN2, ELK1, ENDOG, ENG, FES, FFAR2, FGFR4, FIBIN, FLT3, FOSL1, GATA2, GATA4, GBA, GIT1, GNAI2, GPAM, GPBAR1, GPER1, GPI, GPR83, GPX3, GRAMD1A, GRAMD1B, GRAMD1C, H3C10, H3C12, H4C3, HEYL, HNMT, HSF1, HTR1B, ICAM1, ID3, IL18BP, IL1B, IL1RN, IRAK2, JUND, KCNMB1, KCTD11, KMT2D, LDLR, LDOC1, LGALS9, LOX, LTF, MAPK3, MMP15, MRC1, MSN, MSTN, MYC, NCOR2, NEFL, NFKB2, NFKBIB, NOD2, NODAL, NOS3, NOTCH1, NR1D1, NR4A1, NR4A3, PAF1, PCDHA4, PCDHA6, PDGFRB, PER1, PIM1, POSTN, PPARD, PPP1R9B, PTGES, PTGFR, PTGIR, PTN, PTPRU, RAMP2, RARA, RELA, RMRP, RXRA, RXRB, SBNO2, SELE, SELENOP, SERPINE1, SLC10A3, SMARCA4, SOX10, SPHK2, SPI1, SRC, SREBF1, SSTR2, STAT3, TEAD2, THBS1, TICAM1, TIMP1, TMEM161A, TMEM256-PLSCR3, TNF, TRIB1, VDR, WBP2, WNT3, WNT9B, ZBTB7A, ZC3H12A, ZFP36, ZMIZ1]

GO:1901701	cellular response to oxygen-containing compound	5,30E-07	[4]	255,00	19,78	41,02	58,98	[AANAT, ADCY7, AIF1, AREG, BMP7, BRIP1, BTK, CARD17, CCL2, CCL3, CCL5, CCR5, CD180, CD6, CD80, CD86, CDH1, CXCL10, CXCL11, CXCL5, CXCL6, CXCL9, CYBB, CYP11B1, DAPK1, DPEP1, DRD1, ERFE, FOLR2, FUT7, GFI1, GRAMD1B, GRAMD1C, HCN1, HLA-DRB1, IL18, IL18RAP, IRF8, ITGA4, KLF5, KMO, LCP1, LDLR, LILRB1, LY86, MAP4K1, MAPK13, MLC1, MMP9, MPO, MRC1, MYB, MZB1, NAMPT, NCF1, NLRP3, NLRP7, NME8, NR4A1, NR4A2, NR4A3, NSG1, NUGGC, P2RY12, P2RY2, PIK3CG, PIM1, PRKCB, PRKCQ, PTAFR, PTGER2, PTGFR, PTK2B, PTPN22, PYCARD, RAB15, RGS10, RGS9, SCIMP, SCNN1B, SLC8A3, SMPD3, SOCS1, SOCS3, STAP1, STAT1, TLR6, TNFAIP3, TNIP3, TREM2, TRERF1, TRPM2, UCP2, WNT1, WNT10B]	[ABL1, ACTB, ADCY1, ADCY3, ADCY4, ADCY9, ADIPOQ, AGER, AGT, AGTR1, APLP1, AREG, ATP1A3, ATP6V0D1, ATP6V0E2, ATP6V1B1, ATP6V1F, BCAR1, BCL2L1, C2CD2L, CCL2, CD14, CDK1, CDK2, CEACAM1, COASY, COL1A1, COL6A1, CRHR2, CSK, CX3CL1, CYP21A2, CYP26B1, CYP27B1, DAB2IP, DAG1, DHX34, DMTN, DNMT1, DPAGT1, ELK1, ENDOG, ERFE, FANCC, FES, FFAR2, FOXC2, GATA4, GIT1, GLRA1, GNAI2, GNAO1, GNRHR, GPBAR1, GPER1, GPR37L1, GPRIN1, GPT, GRAMD1A, GRAMD1B, GRAMD1C, GRB7, GRIK5, GSK3A, HCN3, HCN4, HDAC5, HRH2, HSF1, HTR1B, HTR2A, ICAM1, ID3, IL18BP, IL1B, IRAK2, JAK3, JUP, KCNMB1, KDM6B, KLF10, KLF5, LDLR, LDOC1, LTF, LY6E, MAP1LC3A, MAPK3, MAPK7, MED12, MEN1, MRC1, MSN, MSTN, MYC, NDOR1, NFKBIB, NGFR, NOD2, NOS3, NR1D1, NR4A1, NR4A2, NR4A3, NSG1, NSMF, P2RX3, P2RY2, P2RY6, PAF1, PALM, PCGF2, PDGFB, PDK2, PIM1, PKM, POR, PPARD, PPP1R9B, PRKACA, PRKAR2B, PTGFR, PTN, PTPRN, PXN, RAB11B, RAB11FIP5, RAB15, RANGAP1, RAP1A, RAPGEF1, RARA, RELA, RMRP, RNU1-93P, RPTOR, SBNO2, SERPINE1, SH2B2, SIDT2, SIK2, SLC25A33, SLC29A1, SLC2A4, SLC9A1, SLC9A3R1, SMARCA4, SMARCB1, SOCS3, SOX10, SPHK1, SPHK2, SPI1, SRC, SREBF1, SRF, SSTR2, STAT3, SZT2, TCIRG1, TEAD2, TGM2, TICAM1, TMEM256-PLSCR3, TNF, TNS2, TRIB1, TRIB3, TSC2, VDR, WDTC1, WNT3, WNT9B, ZBTB7B, ZC3H12A, ZFP36, ZNF580]
GO:0002237	response to molecule of bacterial origin	4,09E-07	[4, 6]	98,00	25,86	54,54	45,46	[ACP5, CARD17, CASP8, CCL2, CCL3, CCL5, CCR5, CD180, CD6, CD80, CD86, CD96, CNR1, CNR2, CXCL10, CXCL11, CXCL5, CXCL6, CXCL9, EPX, FASLG, FOXP3, GFI1, IL10RA, IL12RB2, IL18, IRF5, IRF8, KMO, LILRB1, LTA, LY86, MPO, MRC1, NLRP3, NLRP7, NPPC, NR4A1, NUGGC, PTAFR, PTGFR, PTPN22, PYCARD, SCIMP, SELE, STAP1, TLR6, TNFAIP3, TNIP3, TREM2]	[ABCC8, ABL1, ALPL, C5AR1, CCL2, CCR7, CD14, CNR1, CX3CL1, CYP21A2, CYP27B1, DAB2IP, GIT1, HSF1, ICAM1, IL18BP, IL1B, IRAK2, JUND, KCTD11, LDOC1, LGALS9, LTF, MAPK3, MRC1, NFKB2, NFKBIB, NOD2, NOS3, NOTCH1, NR1D1, NR4A1, PAF1, PCDHA4, PCDHA6, PPARD, PTGES, PTGFR, PTGIR, RARA, RELA, SBNO2, SELE, SELENOP, SERPINE1, SPI1, SRC, TICAM1, TIMP1, TMEM256-PLSCR3, TNF, TRIB1, ZC3H12A, ZFP36]
GO:0071396	cellular response to lipid	3,22E-03	[5]	129,00	20,51	42,61	57,39	[AREG, BMP7, CARD17, CCL2, CCL3, CCL5, CCR5, CD180, CD6, CD80, CD86, CXCL10, CXCL11, CXCL5, CXCL6, CXCL9, FLT3, GFI1, GRAMD1B, GRAMD1C, H3C10, H3C11, H3C12, H3C7, IL18, IRF8, KMO, LCP1, LDLR, LILRB1, LY86, MAP4K1, MLC1, MRC1, MYB, NLRP3, NLRP7, NR4A3, NUGGC, PIM1, PTAFR, PTK2B, PTPN22, PYCARD, SCIMP, SCNN1B, STAP1, TNFAIP3, TNIP3, TRERF1, WNT10B]	[ABL1, ADCY1, ADCY3, AREG, ARID1A, ATP1A1, ATP1A3, CARM1, CCL2, CD14, COL1A1, CX3CL1, CYP21A2, CYP26B1, CYP27B1, DAB2IP, DAG1, DAXX, DDX54, EGLN2, ELK1, FES, FFAR2, FLT3, GIT1, GPAM, GPBAR1, GPER1, GRAMD1A, GRAMD1B, GRAMD1C, H3C10, H3C12, H4C3, HEYL, HSF1, ICAM1, ID3, IL1B, IRAK2, KCNMB1, KMT2D, LDLR, LDOC1, LTF, MAPK3, MRC1, MSN, MSTN, MYC, NCOR2, NFKBIB, NOD2, NODAL, NR1D1, NR4A3, PAF1, PER1, PIM1, PPARD, PPP1R9B, PTN, RARA, RELA, RMRP, RXRA, RXRB, SBNO2, SERPINE1, SMARCA4, SOX10, SPHK2, SPI1, SRC, SREBF1, SSTR2, TEAD2, TICAM1, TMEM256-PLSCR3, TNF, TRIB1, VDR, WBP2, WNT3, WNT9B, ZBTB7A, ZC3H12A, ZFP36, ZMIZ1]
GO:0032496	response to lipopolysaccharide	1,25E-07	[4, 5, 7]	96,00	26,67	53,53	46,47	[ACP5, CARD17, CASP8, CCL2, CCL3, CCL5, CCR5, CD180, CD6, CD80, CD86, CD96, CNR1, CNR2, CXCL10, CXCL11, CXCL5, CXCL6, CXCL9, EPX, FASLG, FOXP3, GFI1, IL10RA, IL12RB2, IL18, IRF8, KMO, LILRB1, LTA, LY86, MPO, MRC1, NLRP3, NLRP7, NPPC, NR4A1, NUGGC, PTAFR, PTGFR, PTPN22, PYCARD, SCIMP, SELE, STAP1, TNFAIP3, TNIP3, TREM2]	[ABCC8, ABL1, ALPL, C5AR1, CCL2, CCR7, CD14, CNR1, CX3CL1, CYP21A2, CYP27B1, DAB2IP, GIT1, HSF1, ICAM1, IL18BP, IL1B, IRAK2, JUND, KCTD11, LDOC1, LGALS9, LTF, MAPK3, MRC1, NFKB2, NFKBIB, NOD2, NOS3, NOTCH1, NR1D1, NR4A1, PAF1, PCDHA4, PCDHA6, PPARD, PTGES, PTGFR, PTGIR, RARA, RELA, SBNO2, SELE, SELENOP, SERPINE1, SPI1, SRC, TICAM1, TIMP1, TMEM256-PLSCR3, TNF, TRIB1, ZC3H12A, ZFP36]
GO:0071219	cellular response to molecule of bacterial origin	1,01E-05	[4, 5, 7]	62,00	28,97	57,97	42,03	[CARD17, CCL2, CCL3, CCL5, CCR5, CD180, CD6, CD80, CD86, CXCL10, CXCL11, CXCL5, CXCL6, CXCL9, GFI1, IL18, IRF8, KMO, LILRB1, LY86, MRC1, NLRP3, NLRP7, NUGGC, PTAFR, PTPN22, PYCARD, SCIMP, STAP1, TLR6, TNFAIP3, TNIP3, TREM2]	[ABL1, CCL2, CD14, CX3CL1, DAB2IP, GIT1, HSF1, ICAM1, IL1B, IRAK2, LDOC1, LTF, MAPK3, MRC1, NFKBIB, NOD2, NR1D1, PAF1, PPARD, RARA, RELA, SBNO2, SERPINE1, SPI1, SRC, TICAM1, TMEM256-PLSCR3, TNF, TRIB1, ZC3H12A, ZFP36]
GO:0071222	cellular response to lipopolysaccharide	8,17E-06	[5, 6, 8]	60,00	29,41	56,44	43,56	[CARD17, CCL2, CCL3, CCL5, CCR5, CD180, CD6, CD80, CD86, CXCL10, CXCL11, CXCL5, CXCL6, CXCL9, GFI1, IL18, IRF8, KMO, LILRB1, LY86, MRC1, NLRP3, NLRP7, NUGGC, PTAFR, PTPN22, PYCARD, SCIMP, STAP1, TNFAIP3, TNIP3]	[ABL1, CCL2, CD14, CX3CL1, DAB2IP, GIT1, HSF1, ICAM1, IL1B, IRAK2, LDOC1, LTF, MAPK3, MRC1, NFKBIB, NOD2, NR1D1, PAF1, PPARD, RARA, RELA, SBNO2, SERPINE1, SPI1, SRC, TICAM1, TMEM256-PLSCR3, TNF, TRIB1, ZC3H12A, ZFP36]

GO:0002682	regulation of immune system process	1,99E-30	[2, 3]	430,00	23,55	62,05	37,95	[ADAM8, ADCY7, AIF1, AIM2, ALOX15, ANGPT1, APOBEC3G, BANK1, BLK, BLNK, BTK, BTLA, BTN3A1, BTN3A2, C1QA, C1QB, C1QC, C1QTNF1, C2, C8G, CALCR, CAMK4, CARD11, CASP8, CCDC88B, CCL19, CCL2, CCL24, CCL3, CCL4, CCL5, CCR2, CD177, CD19, CD1B, CD1C, CD1E, CD2, CD200R1, CD22, CD226, CD244, CD247, CD27, CD300LF, CD33, CD3D, CD3E, CD3G, CD4, CD40LG, CD48, CD5, CD6, CD74, CD79A, CD80, CD84, CD86, CD8A, CD8B, CD96, CDKN2A, CFP, CLCF1, CLEC10A, CLEC1B, CLEC4D, CLEC4E, CLEC6A, CLNK, CNR1, CNR2, CORO1A, CR2, CRTAM, CSF1R, CST7, CTLA4, CXCL10, CXCL6, CXCR3, CXorf21, CYP11B1, CYSLTR1, DOCK8, DPEP1, DRD1, EBI3, EDN2, EGR3, EPX, EREG, EVI2B, FCGR2C, FCN1, FCN3, FCRL3, FGL2, FGR, FOXP3, FUT7, FYB1, GBP5, GPR150, GPR171, GPR55, GPR83, GPSM3, GRAP2, H3C10, H3C11, H3C12, H3C7, HCLS1, HCST, HLA-DMB, HLA-DOA, HLA-DPA1, HLA-DPB1, HLA-DQA1, HLA-DRA, HLA-DRB1, HLA-DRB5, ICOS, IFNG, IGLL5, IKBKE, IKZF3, IL12RB1, IL18, IL18RAP, IL7R, INPP5D, IRF4, ITGA4, ITGAL, ITGB2, ITGB7, ITK, JAML, KCNN4, KIR2DL2, KIR2DL3, KIR3DL2, KLRB1, KLRC1, KLRC2, KLRD1, KLRK1, LAG3, LAIR1, LAIR2, LAX1, LCK, LCP2, LDLR, LEF1, LILRA1, LILRB1, LILRB4, LMO1, LPAL2, LRRC17, LST1, LTA, MOG, MS4A1, MUC16, MUC19, MUC6, MYB, MYO1G, MZB1, NCKAP1L, NCR1, NCR3, NFAM1, NLRC3, NLRP3, NLRP6, NR4A3, ORM2, P2RY12, PAX5, PILRA, PLA2G2D, PLA2G7, PRAM1, PRDM1, PRKCB, PRKCQ, PTAFR, PTGDR, PTGER2, PTK2B, PTPN22, PTPN6, PTPRC, PYCARD, PYHIN1, RAC2, RASAL3, RHEX, RHOH, RLN2, RUNX3, SAMSN1, SASH3, SCIMP, SELE, SELL, SH2D1A, SIGLEC16, SIRPG, SIT1, SKAP1, SLA2, SLAMF6, SLAMF7, SLAMF8, SMPD3, SOCS1, SOCS3, SPN, STAP1, STAT1, STXBP2, SYK, TBC1D10C, TBX21, TESPA1, THBS1, THEMIS, THEMIS2, THY1, TIGIT, TLR6, TLR8, TNFAIP3, TNFAIP8L2, TNFRSF13B, TNFRSF18, TNFSF13B, TNFSF14, TNFSF8, TOX, TRAT1, TREM2, TSC22D3, UBASH3A, VAV1, VNN1, WAS, XCL1, XCL2, XG, ZAP70, ZBP1	[ABL1, ACTB, ADCY1, ADCY3, ADCY4, ADCY9, ADIPOQ, AGER, AMH, ANGPT1, AP3D1, APOA1, APOD, AQP3, BAG6, BCAR1, BCL6, BTLA, C1QTNF1, C5, C5AR1, C8G, CALCR, CCL2, CCL21, CCL24, CCR7, CD177, CD1C, CD1E, CD276, CD300E, CD74, CDKN1A, CEACAM1, CFH, CGA, CHGA, CLC, CLCF1, CLEC1B, CLEC4E, CLNK, CLPTM1, CNR1, COL1A1, CPN2, CRHR2, CRP, CSF1, CSK, CST7, CX3CL1, CYP26B1, DCAF15, DGKZ, DLG5, DTX1, ECM1, EDN2, ENPP3, ERBB2, FADD, FCN3, FES, FFAR2, FLOT2, FOXF1, FPR1, FURIN, GAB2, GATA2, GCSAM, GNAI2, GNAO1, GPAM, GPBAR1, GPER1, GPI, GPR137, GPR20, GPR83, GPR84, GRN, H3C10, H3C12, H4C3, HAVCR1, HFE, HK1, HLA-DOB, HRH2, HSF1, ICAM1, IDO1, IFITM1, IL15RA, IL1B, IL4R, IL7R, INHA, JAK3, JUND, KLF10, KMT2D, LAG3, LDLR, LGALS9, LIMK1, LOX, LRRC17, LRRC32, LTF, MAPK3, MAVS, MOV10, MSN, MSTN, MUC19, MUC20, MUC3A, MYC, MYH9, MYL9, MYO18A, NCOR2, NECTIN2, NFE2, NLRC5, NOD2, NPPA, NR1D1, NR4A3, NRARP, PAF1, PCDHA4, PCDHA6, PDCD1, PLA2G2A, PLA2G7, PLVAP, PQBP1, PRKACA, PRKAR2B, PTGIR, PTN, PVR, RAC2, RAMP2, RAP1A, RARA, RBM14, RELA, RELB, RGCC, RHBDF2, RHOG, RMRP, RNF26, RNU1-93P, SELE, SELENOP, SERPINE1, SERPING1, SETD1A, SH2B2, SIGLEC9, SLC7A1, SLC7A5, SLC7A8, SMAD3, SOCS3, SOX12, SOX13, SPHK1, SPHK2, SPI1, SPNS2, SPPL2B, SRC, STAT3, STING1, TFE3, THBS1, THPO, TICAM1, TIMP1, TNF, TRIB1, TRIM62, TRPV4, VEGFA, VIPR2, VNN1, VSIR, VTN, WAS, XG, ZBTB7B, ZC3H12A, ZCCHC3, ZDHHC11, ZFP36, ZMIZ1, ZNF335, ZNF580]
GO:0002683	negative regulation of immune system process	1,16E-10	[2, 3, 4]	156,00	24,68	57,59	42,41	[ADCY7, ALOX15, ANGPT1, BANK1, BLK, BTK, C1QC, C1QTNF1, CALCR, CCL2, CCL3, CCR2, CD200R1, CD22, CD247, CD300LF, CD3G, CD74, CD80, CD84, CD86, CD96, CDKN2A, CLNK, CNR1, CNR2, CR2, CRTAM, CST7, CTLA4, CYSLTR1, DPEP1, DRD1, FCRL3, FGL2, FGR, FOXP3, GPR150, GPR55, GPR83, HLA-DOA, HLA-DRB1, IL7R, INPP5D, KLRC1, KLRD1, KLRK1, LAG3, LAX1, LDLR, LILRB1, LILRB4, LPAL2, LRRC17, LST1, NCKAP1L, NLRC3, NLRP3, NLRP6, PLA2G2D, PTGDR, PTGER2, PTPN22, PTPN6, PTPRC, RLN2, RUNX3, SAMSN1, SH2D1A, SLA2, SLAMF8, SOCS1, SPN, STAP1, SYK, TBC1D10C, TBX21, THBS1, THY1, TIGIT, TNFAIP3, TNFAIP8L2, TNFRSF13B, TREM2, TSC22D3, UBASH3A, XCL1]	[ADCY1, ADCY3, ADCY4, ADCY9, ADIPOQ, AMH, ANGPT1, APOA1, APOD, BCL6, C1QTNF1, C5, CALCR, CCL2, CCL21, CD74, CEACAM1, CGA, CHGA, CLNK, CNR1, CRHR2, CST7, CX3CL1, DGKZ, DLG5, DTX1, ENPP3, ERBB2, FADD, FOXF1, FURIN, GATA2, GCSAM, GNAI2, GPAM, GPBAR1, GPER1, GPR137, GPR20, GPR83, GPR84, GRN, HFE, HLA-DOB, HRH2, IL4R, IL7R, INHA, JAK3, LAG3, LDLR, LGALS9, LRRC17, LRRC32, LTF, MYC, MYH9, NLRC5, NOD2, NR1D1, NRARP, PCDHA4, PCDHA6, PDCD1, PRKACA, PRKAR2B, PTGIR, RAMP2, RARA, RHBDF2, RMRP, RNF26, SERPING1, SPI1, SRC, THBS1, TNF, TRIB1, VIPR2, VSIR, ZBTB7B, ZC3H12A]
GO:0048585	negative regulation of response to stimulus	4,01E-07	[2, 3, 4]	356,00	18,56	39,96	60,04	[ACP5, ADCY7, AIF1, ALOX15, ANGPT2, AOAH, AREG, ATF3, BANK1, BCL3, BMP7, BMPR1B, C1QTNF1, CALCR, CASP8, CCDC88C, CCL2, CCL5, CCR2, CD200R1, CD22, CD247, CD300LF, CD3E, CD3G, CD74, CD84, CD96, CEL, CHRDL1, CISH, CNR2, CR2, CST7, CTLA4, CYSLTR1, DCC, DPEP1, DRD1, DUSP2, DUSP8, EPX, EREG, FASLG, FCRL3, FFAR4, FGL2, FGR, FOXP3, FRZB, GPR150, GPR83, GPRC5A, HLA-DRB1, IL7R, INPP5D, IRF4, KLRC1, KLRD1, KLRK1, KNG1, LAX1, LCP1, LDLR, LIF, LILRB1, LILRB4, MMP9, NAMPT, NCKAP1L, NLRC3, NLRP3, NLRP6, NR4A2, NR4A3, NTN1, PIK3IP1, PLAU, PLEK, PRKCB, PRKCQ, PSCA, PTGDR, PTGER2, PTPN22, PTPN6, PTPRC, PYCARD, RASAL3, RBPMS2, RGS1, RGS10, RGS18, RGS9, RHOH, RLN2, RMI2, RTKN2, SAMSN1, SEMA4A, SEMA4D, SH2D1A, SIGLEC10, SLA2, SLAMF8, SMPDL3B, SOCS1, SOCS3, SPINK1, STAP1, STAT1, SYK, TBC1D10C, TBX21, THBS1, THY1, TLR6, TNFAIP3, TNFAIP8L2, TNIP3, TNMD, TNR, TRABD2A, TREM2, TSPAN8, UBASH3A, UCP2, VNN1, VWC2L, WNT1, XCL1]	[ABCA7, ABCC8, ABL1, ACKR3, ACP4, ADCY1, ADCY3, ADCY4, ADCY9, ADIPOQ, AGAP2, AGT, AKT1S1, AMH, APLN, APLNR, APLP1, APOA1, APOD, AREG, ARRDC3, ASPN, BCL2L1, BCL3, BCL6, BCL9L, BEND6, C1QTNF1, C5, CALCR, CCL2, CD14, CD74, CEACAM1, CEL, CGA, CHGA, CISH, CREB3L1, CRHR2, CSK, CST7, CTHRC1, CX3CL1, CXXC4, CYP26B1, DAB2IP, DAG1, DDX39B, DGKD, DGKZ, DKK2, DLG5, DUSP5, DUSP8, DVL3, ECM1, ENPP3, EPHA2, EPN1, EPPK1, ERBB2, FADD, FAIM2, FAM110A, FAP, FASN, FEM1A, FOXF1, FOXM1, FRMD8, FURIN, GATA4, GBA, GIT1, GLIS2, GNAI2, GPBAR1, GPER1, GPI, GPR20, GPR37L1, GPR83, GPR84, GPRC5A, GRB7, GRINA, GRN, GSK3A, HBEGF, HDAC7, HEYL, HFE, HGS, HRH2, HSF1, HSPB1, HTR1B, ICAM1, IFT80, IL1B, IL1RN, IL4R, IL7R, INPP5E, ITGA3, ITPRIP, JAK3, KIF26A, KLK14, KNG1, LDLR, LGALS9, LIF, LMNA, LTF, LZTS2, MAP2K3, MAPK7, MAZ, MED12, MEGF8, MEIS3P1, MEN1, MGRN1, MNT, MSTN, MYADM, MYC, MYH9, NCOR2, NF2, NFATC4, NGFR, NKD2, NLRC5, NOD2, NODAL, NOS3, NOTCH1, NOTCH3, NPPA, NPRL3, NR1D1, NR4A2, NR4A3, NRARP, NTN1, OMA1, PALM, PCDHA6, PCGF2, PDCD1, PDGFB, PER1, PIAS4, PLAU, PLAUR, PLXNA3, PPARD, PPP1R10, PPP1R15A, PPP2R1A, PPP2R5B, PRDM16, PRKACA, PRKAR2B, PTGIR, PTPRU, RADX, RAMP2, RBPMS2, RELA, RGMA, RGS16, RGS18, RHBDF2, RMI2, RNF126, RNF157, RNF26, RNU1-93P, RTKN2, RTN4RL1, S1PR2, SEMA3F, SEMA4B, SEMA4C, SEMA6B, SEMA6C, SERPINE1, SERPING1, SGTA, SH2B3, SH3BP1, SHARPIN, SLC25A23, SLC35C1, SLC35F6, SLC9A3R1, SMAD3, SMARCA4, SMPD1, SNAI1, SOCS3, SOX10, SOX13, SPI1, SPINK1, SRC, SYNGAP1, SYVN1, SZT2, TGFB1I1, THBS1, TICAM1, TIMP1, TLE2, TLE3, TLE5, TMEM127, TMEM14A, TMEM161A, TMEM88, TNF, TNFAIP1, TNFRSF1A, TNIP1, TNMD, TNS2, TRIB1, TRIB3, TSC2, TSKU, TSPAN8, UBA52, UBE2B, UBQLN4, UNC5B, VASN, VIPR2, VNN1, VSIG2, VTN, WFS1, WNK2, WNT3, WWP2, XRCC1, ZBTB7A, ZBTB7B, ZC3H12A, ZFP36

GO:0032101	regulation of response to external stimulus	2,76E-10	[3, 4]	266,00	20,94	53,55	46,45	[ACP5, ADAM8, ADCY7, AIF1, AIM2, AKNA, ALOX15, ANGPT2, AOAH, APOBEC3G, BTK, C1QTNF1, CALCR, CARD11, CASP8, CCL19, CCL2, CCL24, CCL3, CCL4, CCL5, CCR2, CCR4, CD180, CD200R1, CD226, CD247, CD3G, CD74, CD96, CEL, CLEC10A, CLEC4D, CLEC4E, CLEC6A, CLNK, CNR1, CNR2, CRTAM, CSF1R, CST7, CXCL10, CXCL6, CXCR4, CXorf21, CYSLTR1, DPEP1, DRD1, EDN2, EREG, FCN1, FFAR4, FGL2, FGR, FOXP3, FUT7, GBP5, GPR150, GPR4, GPR83, GPSM3, GUCY2D, HLA-DRB1, HOPX, IFNG, IKBKE, IL12RB1, IL16, IL18, IL18RAP, KLRC1, KLRC2, KLRD1, KLRK1, KNG1, LAG3, LCP1, LDLR, LILRB1, LTA, LY86, MAPK13, MMP9, MUC16, MUC19, MUC6, NCKAP1L, NCR1, NCR3, NLRC3, NLRP3, NLRP6, OASL, P2RY12, PDE6G, PIK3CG, PLA2G2D, PLA2G7, PLAU, PTGDR, PTGER2, PTK2B, PTPN22, PTPN6, PTPRC, PYCARD, PYHIN1, RAC2, RLN2, SCIMP, SELE, SEMA4A, SEMA4D, SH2D1A, SIGLEC10, SIGLEC16, SLAMF6, SLAMF8, SMPDL3B, SOCS1, SOCS3, STAP1, STAT1, SUCNR1, SYK, THBS1, TLR10, TLR8, TNFAIP3, TNFAIP8L2, TNR, TREM2, TSPAN8, VAV1, XCL1, XCL2, ZBP1]	[ABCC8, ADCY1, ADCY3, ADCY4, ADCY9, ADIPOQ, AGER, AGT, AGTR1, AMH, APOA1, ARTN, BCL6, BRD4, C1QTNF1, C5, C5AR1, CALCR, CCL2, CCL21, CCL24, CCR7, CD74, CEACAM1, CEL, CGA, CHGA, CLEC4E, CLNK, CNR1, CRHR2, CSF1, CST7, CX3CL1, CYP27B1, DAGLA, DMTN, EDN2, ENPP3, EPPK1, FABP4, FADD, FAM110A, FAP, FEM1A, FFAR2, FOXF1, FURIN, GBA, GIT1, GNAI2, GPBAR1, GPER1, GPR20, GPR4, GPR83, GPR84, GRN, HOPX, HRH2, HSPB1, IL1B, KNG1, LAG3, LDLR, LGALS9, LILRA5, LTF, MAPK3, MAPK7, MAVS, MEGF8, MEN1, MSTN, MUC19, MUC20, MUC3A, MYH9, NECTIN2, NINJ1, NLRC5, NOD2, NOS3, NOTCH1, NPPA, NR1D1, OSM, PCDHA4, PCDHA6, PDGFB, PDGFRB, PLA2G2A, PLA2G7, PLAU, PLAUR, PLXNA3, PPARD, PQBP1, PRKACA, PRKAR2B, PTGES, PTGIR, PTN, PTPRU, PVR, RAC2, RAMP2, RBM14, RELA, RELB, RGMA, RHBDF2, RNF26, RNU1-93P, RTN4RL1, SBNO2, SELE, SEMA3F, SEMA4B, SEMA4C, SEMA6B, SEMA6C, SERPINE1, SERPING1, SH2B3, SHARPIN, SMAD3, SOCS3, SPATA2, SPHK1, SPI1, SRC, STING1, TGM2, THBS1, TNF, TNFRSF1A, TNIP1, TRIB1, TRIM62, TRPV4, TSPAN8, VEGFA, VIPR2, VTN, WNT3, ZC3H12A, ZCCHC3, ZDHHC11, ZFP36, ZNF580, ZSWIM4, ZSWIM8, ZYX]
GO:0050776	regulation of immune response	2,87E-17	[3, 4]	286,00	23,08	66,69	33,31	[ADAM8, ADCY7, AIM2, ALOX15, APOBEC3G, BLK, BLNK, BTK, BTLA, BTN3A1, BTN3A2, C1QA, C1QB, C1QC, C1QTNF1, C2, C8G, CALCR, CARD11, CASP8, CCL19, CCL5, CCR2, CD177, CD19, CD1B, CD1C, CD1E, CD200R1, CD22, CD226, CD247, CD300LF, CD33, CD3D, CD3E, CD3G, CD4, CD40LG, CD48, CD74, CD79A, CD80, CD84, CD86, CD8A, CD8B, CD96, CFP, CLCF1, CLEC10A, CLEC1B, CLEC4D, CLEC4E, CLEC6A, CLNK, CNR1, CR2, CRTAM, CTLA4, CXCL10, CXCL6, CXorf21, CYP11B1, CYSLTR1, DPEP1, DRD1, EREG, FCGR2C, FCN1, FCN3, FCRL3, FGL2, FGR, FOXP3, FUT7, FYB1, GBP5, GPR150, GPR83, GRAP2, HCST, HLA-DMB, HLA-DPA1, HLA-DPB1, HLA-DQA1, HLA-DRA, HLA-DRB1, HLA-DRB5, IFNG, IGLL5, IKBKE, IL12RB1, IL18, IL18RAP, IL7R, INPP5D, IRF4, ITGA4, ITGAL, ITGB2, ITGB7, ITK, JAML, KCNN4, KIR2DL2, KIR2DL3, KIR3DL2, KLRB1, KLRC1, KLRC2, KLRD1, KLRK1, LAG3, LAIR1, LAIR2, LAX1, LCK, LCP2, LILRA1, LILRB1, LILRB4, LTA, MOG, MS4A1, MUC16, MUC19, MUC6, MYB, MYO1G, NCKAP1L, NCR1, NCR3, NFAM1, NLRC3, NLRP3, NLRP6, NR4A3, PAX5, PILRA, PLA2G2D, PRAM1, PRKCB, PRKCQ, PTAFR, PTGDR, PTGER2, PTPN22, PTPN6, PTPRC, PYCARD, PYHIN1, RAC2, RLN2, SAMSN1, SASH3, SELL, SH2D1A, SIGLEC16, SKAP1, SLA2, SLAMF6, SLAMF7, SLAMF8, SOCS1, SOCS3, SPN, STAP1, STAT1, STXBP2, SYK, TBX21, TESPA1, THEMIS, THEMIS2, THY1, TLR8, TNFAIP3, TNFSF13B, TRAT1, TREM2, UBASH3A, VAV1, WAS, XCL1, ZAP70, ZBP1]	[ABL1, ACTB, ADCY1, ADCY3, ADCY4, ADCY9, AGER, BAG6, BCAR1, BCL6, BTLA, C1QTNF1, C5, C5AR1, C8G, CALCR, CCR7, CD177, CD1C, CD1E, CD276, CD300E, CD74, CEACAM1, CFH, CGA, CHGA, CLC, CLCF1, CLEC1B, CLEC4E, CLNK, CNR1, COL1A1, CPN2, CRHR2, CRP, CSK, DGKZ, ECM1, ENPP3, FADD, FCN3, FES, FFAR2, FOXF1, FPR1, FURIN, GAB2, GATA2, GCSAM, GNAI2, GNAO1, GPBAR1, GPR20, GPR83, GPR84, GRN, HFE, HRH2, ICAM1, IFITM1, IL1B, IL4R, IL7R, JAK3, LAG3, LGALS9, LIMK1, MAPK3, MAVS, MUC19, MUC20, MUC3A, MYH9, NCOR2, NECTIN2, NLRC5, NOD2, NPPA, NR4A3, PCDHA4, PDCD1, PQBP1, PRKACA, PRKAR2B, PTGIR, PVR, RAC2, RAMP2, RAP1A, RARA, RBM14, RELA, RELB, RGCC, RHBDF2, RNF26, RNU1-93P, SERPING1, SH2B2, SIGLEC9, SLC7A8, SMAD3, SOCS3, SPHK2, SPI1, SPNS2, SPPL2B, SRC, STING1, TNF, TRIM62, VIPR2, VSIR, VTN, WAS, ZBTB7B, ZC3H12A, ZCCHC3, ZDHHC11]
GO:0050865	regulation of cell activation	2,09E-24	[3, 4]	204,00	29,18	65,18	34,82	[ADAM8, AIF1, BANK1, BLK, BTK, BTLA, C1QTNF1, CAMK4, CARD11, CCDC88B, CCL19, CCL2, CCL3, CCL5, CCR2, CD177, CD19, CD2, CD22, CD226, CD27, CD300LF, CD33, CD3E, CD4, CD40LG, CD5, CD6, CD74, CD80, CD84, CD86, CDKN2A, CLCF1, CLEC4D, CLNK, CNR1, CNR2, CORO1A, CRTAM, CST7, CTLA4, DOCK8, EBI3, EGR3, EPX, FCRL3, FGL2, FGR, FOXP3, GRAP2, HLA-DMB, HLA-DOA, HLA-DPA1, HLA-DPB1, HLA-DRA, HLA-DRB1, ICOS, IFNG, IGLL5, IKZF3, IL12RB1, IL18, IL7R, INPP5D, IRF4, ITGB2, KLRC2, KLRK1, LAG3, LAX1, LCK, LDLR, LEF1, LILRB1, LILRB4, LMO1, LST1, MYB, MZB1, NCKAP1L, NFAM1, NLRP3, NR4A3, PLA2G2D, PLEK, PRAM1, PRDM1, PRKCQ, PTAFR, PTPN22, PTPN6, PTPRC, PYCARD, RAC2, RASAL3, RHOH, RUNX3, SAMSN1, SASH3, SIRPG, SIT1, SLAMF8, SOCS1, SPN, STAP1, STXBP2, SYK, TBC1D10C, TBX21, TESPA1, THBS1, THEMIS2, THY1, TIGIT, TLR6, TNFAIP3, TNFAIP8L2, TNFRSF13B, TNFRSF18, TNFSF13B, TNFSF14, TNFSF8, TOX, TREM2, VAV1, VNN1, XCL1, ZAP70, ZNF683]	[ABL1, AGER, AMH, AP3D1, BCL6, BTLA, C1QTNF1, CCL2, CCL21, CCR7, CD177, CD276, CD74, CDKN1A, CEACAM1, CLC, CLCF1, CLNK, CLPTM1, CNR1, CSK, CST7, CX3CL1, CYP26B1, DCAF15, DLG5, DMTN, DTX1, ENPP3, ERBB2, FADD, FES, FLOT2, FOXF1, GAB2, GATA2, GNAO1, GPAM, GPER1, GRN, HAVCR1, HFE, IDO1, IL15RA, IL1B, IL4R, IL7R, INHA, JAK3, JUND, LAG3, LDLR, LGALS9, LILRA5, LRRC32, MYO18A, NECTIN2, NOD2, NOS3, NPPA, NR1D1, NR4A3, NRARP, PCDHA4, PCDHA6, PDCD1, PDGFB, PDGFRB, PLA2G2A, RAC2, RARA, RHBDF2, SELENOP, SH2B3, SLC7A1, SOX12, SOX13, SPHK1, SPHK2, SPI1, SRC, THBS1, TICAM1, TIMP1, VNN1, VSIR, ZBTB7B, ZC3H12A, ZMIZ1, ZNF335]

GO:0002694	regulation of leukocyte activation	2,18E-24	[3, 4, 5]	194,00	29,85	67,19	32,81	[ADAM8, AIF1, BANK1, BLK, BTK, BTLA, CAMK4, CARD11, CCDC88B, CCL19, CCL2, CCL3, CCL5, CCR2, CD177, CD19, CD2, CD22, CD226, CD27, CD300LF, CD33, CD3E, CD4, CD40LG, CD5, CD6, CD74, CD80, CD84, CD86, CDKN2A, CLCF1, CLEC4D, CLNK, CNR1, CNR2, CORO1A, CRTAM, CST7, CTLA4, DOCK8, EBI3, EGR3, EPX, FCRL3, FGL2, FGR, FOXP3, GRAP2, HLA-DMB, HLA-DOA, HLA-DPA1, HLA-DPB1, HLA-DRA, HLA-DRB1, ICOS, IFNG, IGLL5, IKZF3, IL12RB1, IL18, IL7R, INPP5D, IRF4, ITGB2, KLRC2, KLRK1, LAG3, LAX1, LCK, LDLR, LEF1, LILRB1, LILRB4, LMO1, LST1, MYB, MZB1, NCKAP1L, NFAM1, NLRP3, NR4A3, PLA2G2D, PRAM1, PRDM1, PRKCQ, PTAFR, PTPN22, PTPN6, PTPRC, PYCARD, RAC2, RASAL3, RHOH, RUNX3, SAMSN1, SASH3, SIRPG, SIT1, SLAMF8, SOCS1, SPN, STAP1, STXBP2, SYK, TBC1D10C, TBX21, TESPA1, THBS1, THEMIS2, THY1, TIGIT, TLR6, TNFAIP3, TNFAIP8L2, TNFRSF13B, TNFRSF18, TNFSF13B, TNFSF14, TNFSF8, TOX, TREM2, VAV1, VNN1, XCL1, ZAP70, ZNF683]	[ABL1, AGER, AMH, AP3D1, BCL6, BTLA, CCL2, CCL21, CCR7, CD177, CD276, CD74, CDKN1A, CEACAM1, CLC, CLCF1, CLNK, CLPTM1, CNR1, CSK, CST7, CX3CL1, CYP26B1, DCAF15, DLG5, DTX1, ENPP3, ERBB2, FADD, FES, FLOT2, FOXF1, GAB2, GATA2, GNAO1, GPAM, GPER1, GRN, HAVCR1, HFE, IDO1, IL15RA, IL1B, IL4R, IL7R, INHA, JAK3, JUND, LAG3, LDLR, LGALS9, LRRC32, MYO18A, NECTIN2, NOD2, NPPA, NR1D1, NR4A3, NRARP, PCDHA4, PCDHA6, PDCD1, PLA2G2A, RAC2, RARA, SLC7A1, SOX12, SOX13, SPHK1, SPHK2, SPI1, SRC, THBS1, TICAM1, TIMP1, VNN1, VSIR, ZBTB7B, ZC3H12A, ZMIZ1, ZNF335]
GO:0032102	negative regulation of response to external stimulus	5,21E-03	[3, 4, 5]	124,00	20,46	45,28	54,72	[ACP5, ADCY7, AIF1, ANGPT2, AOAH, C1QTNF1, CALCR, CCL2, CD200R1, CD247, CD3G, CD96, CEL, CNR2, CST7, CYSLTR1, DPEP1, DRD1, FFAR4, FGL2, FGR, FOXP3, GPR150, GPR83, HLA-DRB1, KLRC1, KLRD1, KLRK1, KNG1, LDLR, LILRB1, NLRC3, NLRP3, NLRP6, PLAU, PTGDR, PTGER2, PTPRC, RLN2, SEMA4A, SEMA4D, SIGLEC10, SLAMF8, SMPDL3B, SOCS3, STAP1, SYK, THBS1, TNFAIP3, TNFAIP8L2, TNR, TREM2, TSPAN8]	[ABCC8, ADCY1, ADCY3, ADCY4, ADCY9, ADIPOQ, AMH, APOA1, C1QTNF1, C5, CALCR, CCL2, CEACAM1, CEL, CGA, CHGA, CRHR2, CST7, CX3CL1, ENPP3, EPPK1, FAM110A, FAP, FEM1A, FOXF1, FURIN, GBA, GIT1, GNAI2, GPBAR1, GPER1, GPR20, GPR83, GPR84, GRN, HRH2, KNG1, LDLR, LGALS9, LTF, MAPK7, MYH9, NLRC5, NOD2, NOS3, NOTCH1, NR1D1, PCDHA6, PDGFB, PLAU, PLAUR, PLXNA3, PPARD, PRKACA, PRKAR2B, PTGIR, PTPRU, RAMP2, RGMA, RHBDF2, RNF26, RTN4RL1, SEMA3F, SEMA4B, SEMA4C, SEMA6B, SEMA6C, SERPINE1, SERPING1, SH2B3, SHARPIN, SMAD3, SOCS3, SRC, THBS1, TNF, TNFRSF1A, TRIB1, TSPAN8, VIPR2, VTN, WNT3, ZFP36]
GO:0050866	negative regulation of cell activation	2,21E-10	[3, 4, 5]	72,00	33,49	63,17	36,83	[BANK1, BLK, BTK, C1QTNF1, CCR2, CD300LF, CD74, CD80, CD84, CD86, CDKN2A, CLNK, CNR1, CNR2, CRTAM, CST7, CTLA4, FGL2, FGR, FOXP3, HLA-DRB1, INPP5D, LAG3, LAX1, LDLR, LILRB1, LILRB4, LST1, NCKAP1L, PLA2G2D, PTPN22, PTPN6, PTPRC, RUNX3, SAMSN1, SOCS1, SPN, TBC1D10C, TBX21, TIGIT, TNFAIP3, TNFAIP8L2, TNFRSF13B, TREM2, XCL1]	[BCL6, C1QTNF1, CD74, CEACAM1, CLNK, CNR1, CST7, CX3CL1, DLG5, DTX1, ENPP3, ERBB2, FOXF1, GPER1, GRN, HFE, IL4R, INHA, JAK3, LAG3, LDLR, LGALS9, LRRC32, NOS3, NR1D1, NRARP, PCDHA4, PCDHA6, PDGFB, SH2B3, SPI1, VSIR, ZBTB7B, ZC3H12A]
GO:0002695	negative regulation of leukocyte activation	1,42E-10	[3, 4, 5, 6]	67,00	34,90	65,00	35,00	[BANK1, BLK, BTK, CCR2, CD300LF, CD74, CD80, CD84, CD86, CDKN2A, CLNK, CNR1, CNR2, CRTAM, CST7, CTLA4, FGL2, FGR, FOXP3, HLA-DRB1, INPP5D, LAG3, LAX1, LDLR, LILRB1, LILRB4, LST1, NCKAP1L, PLA2G2D, PTPN22, PTPN6, PTPRC, RUNX3, SAMSN1, SOCS1, SPN, TBC1D10C, TBX21, TIGIT, TNFAIP3, TNFAIP8L2, TNFRSF13B, XCL1]	[BCL6, CD74, CEACAM1, CLNK, CNR1, CST7, CX3CL1, DLG5, DTX1, ENPP3, ERBB2, FOXF1, GPER1, GRN, HFE, IL4R, INHA, JAK3, LAG3, LDLR, LGALS9, LRRC32, NR1D1, NRARP, PCDHA4, PCDHA6, SPI1, VSIR, ZBTB7B, ZC3H12A]
GO:0031347	regulation of defense response	9,84E-09	[4, 5]	197,00	21,91	56,44	43,56	[ACP5, ADAM8, ADCY7, AIM2, AKNA, ALOX15, AOAH, APOBEC3G, BTK, C1QTNF1, CALCR, CARD11, CASP8, CCL24, CCL3, CCL5, CCR2, CD200R1, CD226, CD247, CD3G, CD96, CLEC10A, CLEC4D, CLEC4E, CLEC6A, CLNK, CNR1, CNR2, CRTAM, CST7, CXCL6, CXorf21, CYSLTR1, DPEP1, DRD1, EREG, FCN1, FFAR4, FGL2, FGR, FOXP3, FUT7, GBP5, GPR150, GPR4, GPR83, GPSM3, HLA-DRB1, IFNG, IKBKE, IL12RB1, IL16, IL18, IL18RAP, KLRC1, KLRC2, KLRD1, KLRK1, LAG3, LCP1, LDLR, LILRB1, LTA, MAPK13, MMP9, MUC16, MUC19, MUC6, NCR1, NCR3, NLRC3, NLRP3, NLRP6, PIK3CG, PLA2G2D, PLA2G7, PTGDR, PTGER2, PTPN22, PTPN6, PTPRC, PYCARD, PYHIN1, RLN2, SELE, SH2D1A, SIGLEC10, SIGLEC16, SLAMF6, SLAMF8, SMPDL3B, SOCS1, SOCS3, STAP1, STAT1, SUCNR1, SYK, TLR10, TLR8, TNFAIP3, TNFAIP8L2, TREM2, VAV1, XCL1, ZBP1]	[ADCY1, ADCY3, ADCY4, ADCY9, ADIPOQ, AGER, AGT, AGTR1, APOA1, BCL6, BRD4, C1QTNF1, CALCR, CCL24, CCR7, CEACAM1, CGA, CHGA, CLEC4E, CLNK, CNR1, CRHR2, CST7, CX3CL1, DAGLA, ENPP3, FABP4, FADD, FEM1A, FFAR2, FOXF1, FURIN, GBA, GIT1, GNAI2, GPBAR1, GPER1, GPR20, GPR4, GPR83, GPR84, GRN, HRH2, IL1B, LAG3, LDLR, LGALS9, LILRA5, MAPK3, MAPK7, MAVS, MUC19, MUC20, MUC3A, MYH9, NECTIN2, NINJ1, NLRC5, NOD2, NPAS2, NPPA, NR1D1, OSM, PCDHA4, PCDHA6, PLA2G2A, PLA2G7, PPARD, PQBP1, PRKACA, PRKAR2B, PTGES, PTGIR, PVR, RAMP2, RBM14, RELA, RELB, RHBDF2, RNF26, RNU1-93P, SBNO2, SELE, SERPINE1, SERPING1, SHANK3, SHARPIN, SMAD3, SOCS3, SPATA2, SPHK1, SPI1, SRC, STING1, TGM2, TNF, TNFRSF1A, TNIP1, TRIM62, TRPV4, VIPR2, ZC3H12A, ZCCHC3, ZDHHC11, ZFP36, ZYX]
GO:0050727	regulation of inflammatory response	2,55E-07	[4, 5, 6]	132,00	23,61	51,21	48,79	[ACP5, ADAM8, ADCY7, AKNA, ALOX15, AOAH, BTK, C1QTNF1, CALCR, CCL24, CCL3, CCL5, CCR2, CD200R1, CD247, CD3G, CNR1, CNR2, CST7, CYSLTR1, DPEP1, DRD1, FFAR4, FGR, FOXP3, FUT7, GPR150, GPR4, GPR83, GPSM3, HLA-DRB1, IFNG, IL16, IL18, LCP1, LDLR, LTA, MAPK13, MMP9, NLRC3, NLRP3, NLRP6, PIK3CG, PLA2G2D, PLA2G7, PTGDR, PTGER2, PTPRC, PYCARD, RLN2, SELE, SIGLEC10, SLAMF8, SMPDL3B, SOCS3, STAP1, SUCNR1, SYK, TLR10, TNFAIP3, TNFAIP8L2, TREM2, XCL1, ZBP1]	[ADCY1, ADCY3, ADCY4, ADCY9, ADIPOQ, AGER, AGT, AGTR1, APOA1, BCL6, BRD4, C1QTNF1, CALCR, CCL24, CCR7, CGA, CHGA, CNR1, CRHR2, CST7, CX3CL1, DAGLA, ENPP3, FABP4, FEM1A, FFAR2, FOXF1, FURIN, GBA, GIT1, GNAI2, GPBAR1, GPER1, GPR20, GPR4, GPR83, GPR84, GRN, HRH2, IL1B, LDLR, LILRA5, MAPK7, MYH9, NINJ1, NOD2, NPPA, NR1D1, OSM, PCDHA4, PCDHA6, PLA2G2A, PLA2G7, PPARD, PRKACA, PRKAR2B, PTGES, PTGIR, RAMP2, RELA, RHBDF2, SBNO2, SELE, SERPINE1, SHARPIN, SMAD3, SOCS3, SPATA2, SPHK1, SRC, STING1, TGM2, TNF, TNFRSF1A, TNIP1, TRPV4, VIPR2, ZFP36, ZYX]

GO:0051249	regulation of lymphocyte activation	2,62E-15	[4, 5, 6]	154,00	27,60	71,79	28,21	[ADAM8, AIF1, BANK1, BLK, BTK, BTLA, CAMK4, CARD11, CCDC88B, CCL19, CCL2, CCL5, CCR2, CD19, CD2, CD22, CD27, CD3E, CD4, CD40LG, CD5, CD6, CD74, CD80, CD86, CDKN2A, CLCF1, CLNK, CORO1A, CRTAM, CTLA4, DOCK8, EBI3, EGR3, EPX, FCRL3, FGL2, FGR, FOXP3, GRAP2, HLA-DMB, HLA-DOA, HLA-DPA1, HLA-DPB1, HLA-DRA, HLA-DRB1, ICOS, IFNG, IGLL5, IKZF3, IL12RB1, IL18, IL7R, INPP5D, IRF4, KLRC2, KLRK1, LAG3, LAX1, LCK, LEF1, LILRB1, LILRB4, LMO1, LST1, MYB, MZB1, NCKAP1L, NFAM1, NLRP3, PLA2G2D, PRDM1, PRKCQ, PTPN22, PTPN6, PTPRC, PYCARD, RAC2, RASAL3, RHOH, RUNX3, SAMSN1, SASH3, SIRPG, SIT1, SLAMF8, SOCS1, SPN, SYK, TBC1D10C, TBX21, TESPA1, THEMIS2, THY1, TIGIT, TNFAIP3, TNFAIP8L2, TNFRSF13B, TNFRSF18, TNFSF13B, TNFSF14, TNFSF8, TOX, VAV1, VNN1, XCL1, ZAP70, ZNF683]	[ABL1, AGER, AMH, AP3D1, BCL6, BTLA, CCL2, CCL21, CCR7, CD276, CD74, CDKN1A, CEACAM1, CLC, CLCF1, CLNK, CLPTM1, CSK, CYP26B1, DCAF15, DLG5, DTX1, ERBB2, FADD, FLOT2, GNAO1, GPAM, HFE, IDO1, IL15RA, IL1B, IL4R, IL7R, INHA, JAK3, LAG3, LGALS9, LRRC32, NOD2, NRARP, PDCD1, RAC2, RARA, SLC7A1, SOX12, SOX13, SRC, TICAM1, TIMP1, VNN1, VSIR, ZBTB7B, ZC3H12A, ZMIZ1, ZNF335]
GO:0051250	negative regulation of lymphocyte activation	1,91E-05	[4, 5, 6, 7]	50,00	31,25	71,59	28,41	[BANK1, BLK, BTK, CD74, CD80, CD86, CDKN2A, CLNK, CRTAM, CTLA4, FGL2, FGR, FOXP3, HLA-DRB1, INPP5D, LAG3, LAX1, LILRB1, LILRB4, LST1, NCKAP1L, PLA2G2D, PTPN22, PTPN6, RUNX3, SAMSN1, SOCS1, SPN, TBC1D10C, TBX21, TIGIT, TNFAIP3, TNFAIP8L2, TNFRSF13B, XCL1]	[BCL6, CD74, CEACAM1, CLNK, DLG5, DTX1, ERBB2, HFE, IL4R, INHA, JAK3, LAG3, LGALS9, LRRC32, NRARP, VSIR, ZBTB7B, ZC3H12A]
GO:0048519	negative regulation of biological process	6,13E-07	[1, 2, 3]	928,00	16,02	39,03	60,97	[ACP5, ADAM8, ADAMDEC1, ADAMTS9, ADCY7, ADORA3, AIF1, AKNA, ALOX15, ALOX15B, ANGPT1, ANGPT2, ANO9, AOAH, APOBEC3A, APOBEC3D, APOBEC3G, APOBEC3H, AREG, ASCL2, ATF3, BANK1, BCL11B, BCL3, BHLHA15, BHLHE40, BLK, BMF, BMP7, BMPR1B, BRIP1, BTG2, BTK, C1QC, C1QTNF1, CALCR, CARD17, CARMIL2, CASP8, CBARP, CCDC88C, CCL17, CCL19, CCL2, CCL3, CCL3L1, CCL5, CCR2, CCR5, CD19, CD200R1, CD22, CD247, CD27, CD300LF, CD33, CD3E, CD3G, CD40LG, CD74, CD80, CD84, CD86, CD96, CDH1, CDKN2A, CEL, CHRDL1, CISH, CLCF1, CLNK, CMTM5, CNN1, CNR1, CNR2, CORO1A, CR2, CRB2, CREM, CRTAM, CST7, CTLA4, CXCL10, CXCL11, CXCL9, CYSLTR1, DAPK1, DCC, DEPDC1B, DERL3, DOCK8, DPEP1, DPEP2, DPT, DRD1, DUSP2, DUSP8, E2F8, EAF2, EGR3, ELAVL4, ELF3, EOMES, EPX, EREG, ERFE, EVI2B, FAM122C, FASLG, FATE1, FCMR, FCN1, FCN3, FCRL3, FFAR4, FGL2, FGR, FOSB, FOSL1, FOXP3, FRZB, GADD45B, GFI1, GFRA2, GLIS1, GMFG, GMIP, GPR132, GPR150, GPR171, GPR4, GPR55, GPR83, GPRC5A, GZMA, GZMB, H1-3, H2AC13, H2AC14, H2AC16, H3C10, H3C11, H3C12, H3C7, HCLS1, HLA-DOA, HLA-DRB1, HOPX, HORMAD1, IER3, IFNG, IKBKE, IKZF1, IL10RA, IL18, IL26, IL7R, INPP5D, IQCJ-SCHIP1, IRF4, IRF8, IRX3, ITGB2, JPH1, JSRP1, KCNN4, KIR3DL2, KLF5, KLRC1, KLRD1, KLRK1, KNG1, LAG3, LAMP3, LAX1, LCK, LCP1, LDLR, LEF1, LIF, LILRB1, LILRB4, LMCD1, LMO1, LPAL2, LRRC17, LST1, LTA, MCOLN2, MCOLN3, MID1IP1, MIDN, MIXL1, MMP9, MPO, MSX1, MT1F, MT1G, MYB, NAMPT, NCKAP1L, NLRC3, NLRP3, NLRP6, NLRP7, NPPB, NPPC, NR4A1, NR4A2, NR4A3, NTN1, NUGGC, NUP210, OASL, P2RY12, PAPLN, PARP15, PATL2, PAX5, PIK3CG, PIK3IP1, PIM1, PIM2, PLA2G2D, PLAAT4, PLAC8, PLAU, PLEK, PLXNC1, PRDM1, PRKCB, PRKCQ, PSCA, PTGDR, PTGER2, PTGFR, PTK2B, PTPN22, PTPN6, PTPRC, PTPRH, PTX3, PYCARD, RAD9B, RARRES1, RASAL3, RBPMS2, RGS1, RGS10, RGS18, RGS9, RHOH, RLN2, RMI2, RNASE6, RRAD, RRM2, RTKN2, RTN1	[AAAS, ABCA7, ABCC8, ABCD1, ABCD2, ABI3, ABL1, ACACB, ACKR3, ACP4, ACTN1, ACTN4, ADAMTS5, ADAMTS7, ADAMTS8, ADAMTS9, ADCK1, ADCY1, ADCY3, ADCY4, ADCY9, ADGRB2, ADGRG1, ADIPOQ, AGAP2, AGER, AGT, AKT1S1, ALPK3, AMH, ANGPT1, ANGPTL4, ANKRD13B, ANKRD52, AP2A1, APLN, APLNR, APLP1, APOA1, APOBEC3C, APOD, APOM, ARAF, ARC, AREG, ARF1, ARHGAP1, ARHGDIA, ARHGEF15, ARID5A, ARRDC3, ARTN, ASPN, ATF5, ATN1, ATP13A2, ATP1A1, ATP1A3, ATP1B2, BAG3, BAG6, BAHD1, BAZ2A, BBS12, BCKDK, BCL2L1, BCL3, BCL6, BCL9L, BCOR, BEND6, BHLHE40, BMP8A, BTG2, C11orf65, C18orf54, C1QTNF1, C5, C5AR1, CABIN1, CALCR, CAMSAP3, CARM1, CARMIL2, CBARP, CC2D1A, CCL11, CCL2, CCL21, CCR7, CD14, CD74, CDK1, CDK2, CDK2AP2, CDKN1A, CEACAM1, CEL, CETP, CFH, CFL1, CGA, CHERP, CHGA, CIC, CIITA, CISH, CITED2, CLCF1, CLIP3, CLNK, CMTM5, CNN1, CNOT3, CNR1, COL1A1, CPTP, CRAT, CREB3L1, CRHR2, CRP, CRYAB, CSDC2, CSF1, CSK, CST7, CTC1, CTDSP1, CTHRC1, CTIF, CTSA, CX3CL1, CXXC4, CYP26B1, CYP27B1, CYP2D6, DAB2IP, DAG1, DAXX, DBNDD1, DDR1, DDX39B, DDX54, DEDD, DGKD, DGKZ, DGUOK, DHRS2, DHX34, DISP3, DKK2, DLG5, DMTN, DNMT1, DOT1L, DPF2, DPT, DTX1, DUSP5, DUSP8, DVL3, DXO, DYSF, E2F4, E2F7, E2F8, EAF2, ECM1, EDC4, EEF1E1, EFEMP2, EFNA3, EIF4EBP1, EIF4G1, ELF3, ENG, ENPP3, EPHA2, EPN1, EPPK1, ERBB2, ERF, ERFE, ESPL1, ETS2, ETV6, F8A1, FABP4, FADD, FAIM2, FAM110A, FAM122C, FAP, FASN, FCN3, FEM1A, FGFRL1, FLII, FLOT2, FLT4, FOSL1, FOXC2, FOXF1, FOXM1, FOXP4, FOXS1, FRMD8, FURIN, FXYD5, GADD45B, GAS1, GATA2, GATA4, GATAD2A, GBA, GCSAM, GDI1, GGA3, GIT1, GJD3, GLIS2, GLRA1, GMNN, GNAI2, GNAO1, GPAM, GPBAR1, GPER1, GPI, GPR137, GPR20, GPR37L1, GPR4, GPR83, GPR84, GPRC5A, GRB7, GRIK5, GRINA, GRN, GSK3A, GTF2IRD1, GTPBP1, H1-9P, H3C10, H3C12, H4C3, HBEGF, HCFC1, HDAC10, HDAC5, HDAC7, HESX1, HEYL, HFE, HGS, HIRA, HLA-DOB

GO:0023051	regulation of signaling	5,81E-15	[2, 3]	675,00	18,15	37,12	62,88	[ABRA, ADAM8, ALOX15, ALOX15B, ANGPT1, ARAP2, AREG, ARHGAP15, ARHGAP30, ARHGAP45, ATF3, ATP2A3, BANK1, BCL2L14, BCL3, BLK, BMF, BMP7, BMPR1B, BRIP1, C1QTNF1, CACNA1E, CALCR, CARD11, CASP8, CASS4, CCDC88C, CCL17, CCL19, CCL2, CCL22, CCL24, CCL3, CCL3L1, CCL4, CCL4L1, CCL5, CCR2, CD177, CD180, CD19, CD22, CD226, CD27, CD300LF, CD3E, CD4, CD40LG, CD74, CD80, CD86, CDH1, CDKN2A, CEL, CHRDL1, CISH, CLEC6A, CNR1, CNR2, CRB2, CSF1R, CXCR4, CXorf21, DAPK1, DCC, DEPDC1B, DLGAP2, DPEP2, DRD1, DUSP2, DUSP8, ELAVL4, ELF3, EPX, EREG, ERFE, FASLG, FCRL3, FFAR4, FGD2, FGD3, FGR, FLT3, FOLR2, FOXP3, FRZB, FUT7, GADD45B, GDF6, GFI1, GMIP, GPR174, GPR4, GPR55, GPRC5A, GUCY2D, H3C10, H3C11, H3C12, H3C7, HCLS1, HCST, HLA-DRB1, ICOS, IER3, IFNG, IKBKE, IL10RA, IL18, IL26, IL7R, INPP5D, IQCJ-SCHIP1, IRF4, KCNJ10, KCNN4, KMO, LAX1, LCK, LEF1, LIF, LILRB4, LMCD1, LY86, MAP4K1, MIDN, MMP9, MSX1, MYB, MZB1, NCF1, NCKAP1L, NFAM1, NLRC3, NLRP3, NLRP6, NPFFR2, NPPC, NPTX2, NR4A2, NSG1, NTN1, OASL, P2RY10, P2RY12, PAX5, PDE6G, PIK3CG, PIK3IP1, PIK3R5, PIM2, PLAU, PLEK, PRDM1, PRKCB, PRKCQ, PROK1, PSCA, PSD4, PTK2B, PTPN22, PTPN6, PTPRC, PYCARD, PYHIN1, RAC2, RAD9B, RASAL3, RASGRF1, RBPMS2, RGS1, RGS10, RGS18, RGS9, RHOH, RMI2, RNASE6, RRAD, RTKN2, SALL1, SCIMP, SCUBE1, SEMA4D, SH2D1A, SHISA8, SLA, SLA2, SLC24A4, SLC8A3, SMPDL3B, SOCS1, SOCS3, SPINK1, STAP1, STAT1, SYK, TAGAP, TBC1D10C, TESPA1, THBS1, THY1, TLR6, TMC8, TNFAIP3, TNFSF14, TNIP3, TNMD, TNR, TRABD2A, TRAF1, TRAT1, TREM2, TRIM14, TRPM2, UBASH3A, UBD, UCP2, VAV1, VNN1, VWC2L, WNT1, WNT10B, XCL1, XCL2, ZAP70, ZBP1]	[ABCA7, ABCC8, ABL1, ABR, ABRA, ACKR3, ACP4, ACTN4, ADCY1, ADGRG1, ADIPOQ, AGAP2, AGER, AGPAT1, AGT, AGTR1, AKT1S1, AMH, ANGPT1, APLN, APLNR, APLP1, APOA1, APOD, ARAF, ARAP3, ARC, AREG, ARF1, ARHGAP1, ARHGAP23, ARHGAP39, ARHGDIA, ARHGEF1, ARHGEF16, ARHGEF5, ARRDC3, ARTN, ASPN, ATP1A1, ATP1A3, ATP1B2, BCL2L1, BCL3, BCL6, BCL9L, BCR, BEND6, BMP8A, BRD4, C1QTNF1, C20orf27, C2CD2L, C5, C5AR1, CACNA1E, CALB1, CALCR, CAMK2B, CAPN1, CARM1, CC2D1A, CCL11, CCL2, CCL21, CCL24, CCR7, CD14, CD177, CD74, CDK1, CDK2, CEACAM1, CEL, CGA, CHERP, CHGA, CHI3L1, CISH, CITED2, CLIP3, CNR1, CNTN6, COL1A1, CPLX1, CRAT, CREB3L1, CRHR2, CSF1, CSK, CSNK1E, CSPG4, CTHRC1, CX3CL1, CXXC4, CYP26B1, CYP27B1, DAB2IP, DAG1, DAXX, DDX39B, DENND2B, DENND4B, DGKD, DGKZ, DHX34, DIRAS1, DKK2, DLG5, DLGAP4, DMTN, DOC2B, DOK5, DOT1L, DTX1, DUSP5, DUSP8, DVL3, ECM1, EEF1E1, ELF3, ENG, EPHA2, EPN1, ERBB2, ERFE, ESM1, FADD, FAIM2, FAM110A, FASN, FFAR2, FGFR4, FLOT2, FLT3, FLT4, FOXM1, FPR1, FRMD8, FURIN, FXYD6, GAB2, GADD45B, GAS1, GATA4, GBA, GBF1, GCSAM, GDF6, GIT1, GJD3, GLIS2, GLRA1, GNAI2, GPAM, GPBAR1, GPER1, GPI, GPR137, GPR17, GPR20, GPR37L1, GPR4, GPRC5A, GPRIN1, GRB7, GRIK5, GRINA, GRM2, GSK3A, H3C10, H3C12, H4C3, HBEGF, HCRTR1, HDAC7, HEYL, HFE, HGS, HIPK4, HSPB1, HTR1B, HTR2A, ICAM1, IER3, IFT80, IL18BP, IL1B, IL1RN, IL7R, ILK, INCA1, INHA, INPP5E, IRAK2, ITGA3, ITGA5, ITPR3, ITPRIP, JAK3, JMJD8, JPH4, JUP, KCP, KCTD11, KIF26A, KLK14, KMT2D, KSR1, LGALS9, LGR6, LIF, LIFR, LILRA5, LIMS2, LMCD1, LMNA, LOX, LRG1, LRRC4, LRRC8A, LTBR, LTF, LY6E, LYNX1, LZTS1, LZTS2, MAP1A, MAP2K3, MAP3K10, MAP3K11, MAP3K14, MAP3K6, MAP3K9, MAPK3, MAPK7, MAPK8IP3, MAVS, MAZ, MECP2, MED12, MEGF8, MEIS3P1, MEN1, MGRN1, MIDN, MINK1, MLST8, MME, MNT, MSTN, MSX1, MTRNR2L1, MTRNR2L10, MTRNR2L12, MTRNR2L2, MTRNR2L3
GO:0048583	regulation of response to stimulus	4,40E-27	[2, 3]	851,00	18,88	45,12	54,88	[ABRA, ACP5, ADAM8, ADCY7, AIF1, AIM2, AKNA, ALOX15, ALOX15B, ANGPT1, ANGPT2, AOAH, APOBEC3G, ARAP2, AREG, ARHGAP15, ARHGAP30, ARHGAP45, ATF3, BANK1, BCL2L14, BCL3, BLK, BLNK, BMF, BMP7, BMPR1B, BRIP1, BTK, BTLA, BTN3A1, BTN3A2, C1QA, C1QB, C1QC, C1QTNF1, C2, C8G, CALCR, CARD11, CASP8, CASS4, CCDC88C, CCL17, CCL19, CCL2, CCL22, CCL24, CCL3, CCL3L1, CCL4, CCL4L1, CCL5, CCR2, CCR4, CD177, CD180, CD19, CD1B, CD1C, CD1E, CD200R1, CD22, CD226, CD247, CD27, CD300LF, CD33, CD3D, CD3E, CD3G, CD4, CD40LG, CD48, CD74, CD79A, CD80, CD84, CD86, CD8A, CD8B, CD96, CDKN2A, CEL, CFP, CHRDL1, CISH, CLCF1, CLEC10A, CLEC1B, CLEC4D, CLEC4E, CLEC6A, CLNK, CNR1, CNR2, CR2, CRB2, CRTAM, CSF1R, CST7, CTLA4, CXCL10, CXCL6, CXCR4, CXorf21, CYP11B1, CYSLTR1, DAPK1, DCC, DEPDC1B, DLGAP2, DPEP1, DPEP2, DRD1, DUSP2, DUSP8, EDN2, ELF3, EPX, EREG, ERFE, FASLG, FCGR2C, FCN1, FCN3, FCRL3, FFAR4, FGD2, FGD3, FGL2, FGR, FLT3, FOLR2, FOXP3, FRZB, FUT7, FYB1, GADD45B, GBP5, GDF6, GFI1, GMIP, GPR150, GPR174, GPR4, GPR55, GPR83, GPRC5A, GPSM3, GRAP2, GUCY2D, H3C10, H3C11, H3C12, H3C7, HCLS1, HCST, HLA-DMB, HLA-DPA1, HLA-DPB1, HLA-DQA1, HLA-DRA, HLA-DRB1, HLA-DRB5, HOPX, ICOS, IER3, IFNG, IGLL5, IKBKE, IL10RA, IL12RB1, IL16, IL18, IL18RAP, IL26, IL7R, INPP5D, IQCJ-SCHIP1, IRF4, ITGA4, ITGAL, ITGB2, ITGB7, ITK, JAML, KCNN4, KIR2DL2, KIR2DL3, KIR3DL2, KLRB1, KLRC1, KLRC2, KLRD1, KLRK1, KNG1, LAG3, LAIR1, LAIR2, LAX1, LCK, LCP1, LCP2, LDLR, LEF1, LIF, LILRA1, LILRB1, LILRB4, LMCD1, LTA, LY86, MAP4K1, MAPK13, MLC1, MMP9, MOG, MS4A1, MSX1, MUC16, MUC19, MUC6, MYB, MYO1G, MZB1, NAMPT, NCF1, NCKAP1L, NCR1, NCR3, NFAM1, NLRC3, NLRP3, NLRP6, NPFFR2, NPPC, NPTX2, NR4A2, NR4A3, NTN1, NUP210, OASL, P2RY10, P2RY12, PAX5, PDE6G, PIK3CG, PIK3IP1, PIK3R5, PILRA, PIM2, PLA2G2D, PLA2G7, PLAU, PLEK, PRAM1, PRDM1, PRKCB, PRKCQ,	[AAAS, ABCA7, ABCC8, ABCD1, ABI3, ABL1, ABR, ABRA, ACKR3, ACP4, ACTB, ACTN4, ADCY1, ADCY3, ADCY4, ADCY9, ADGRG1, ADIPOQ, AGAP2, AGER, AGPAT1, AGT, AGTR1, AKT1S1, AMH, ANGPT1, APLN, APLNR, APLP1, APOA1, APOD, ARAF, ARAP3, ARC, AREG, ARHGAP1, ARHGAP23, ARHGAP39, ARHGDIA, ARHGEF1, ARHGEF16, ARHGEF5, ARMT1, ARRDC3, ARTN, ASPN, BAG3, BAG6, BCAR1, BCL2L1, BCL3, BCL6, BCL9L, BCR, BEND6, BMP8A, BRD4, BTLA, C1QTNF1, C20orf27, C5, C5AR1, C8G, CALCR, CAMK2B, CAPN1, CARM1, CC2D1A, CCL11, CCL2, CCL21, CCL24, CCR7, CD14, CD177, CD1C, CD1E, CD276, CD300E, CD74, CDK1, CDK2, CEACAM1, CEL, CFH, CGA, CHERP, CHGA, CHI3L1, CISH, CITED2, CLC, CLCF1, CLEC1B, CLEC4E, CLIP3, CLNK, CNR1, CNTN6, COL1A1, CPN2, CRAT, CREB3L1, CRHR2, CRP, CRYAB, CSF1, CSK, CSNK1E, CSPG4, CST7, CTHRC1, CX3CL1, CXXC4, CYP26B1, CYP27B1, DAB2IP, DAG1, DAGLA, DAXX, DDX39B, DENND2B, DENND4B, DGKD, DGKZ, DIRAS1, DKK2, DLG5, DLGAP4, DMTN, DOK5, DOT1L, DTX1, DUSP5, DUSP8, DVL3, ECM1, EDN2, EEF1E1, EIF4G1, ELF3, ENDOG, ENG, ENPP3, EPHA2, EPN1, EPPK1, ERBB2, ERFE, ESM1, FABP4, FADD, FAIM2, FAM110A, FAP, FASN, FCN3, FEM1A, FES, FFAR2, FGFR4, FLOT2, FLT3, FLT4, FOXC2, FOXF1, FOXM1, FPR1, FRMD8, FURIN, GAB2, GADD45B, GAS1, GATA2, GATA4, GBA, GBF1, GCSAM, GDF6, GIT1, GLIS2, GNAI2, GNAO1, GPAM, GPBAR1, GPER1, GPI, GPR137, GPR17, GPR20, GPR37L1, GPR4, GPR83, GPR84, GPRC5A, GPRIN1, GRB7, GRIK5, GRINA, GRM2, GRN, GSK3A, GTF2IRD1, H3C10, H3C12, H4C3, HBEGF, HCRTR1, HDAC10, HDAC7, HEYL, HFE, HGS, HIPK4, HMGA1, HOPX, HRH2, HSF1, HSPB1, HSPB8, HTR1B, HTR2A, ICAM1, IER3, IFITM1, IFT80, IL18BP, IL1B, IL1RN, IL4R, IL7R, ILK, INCA1, INHA, INPP5E, IRAK2, ITGA3, ITGA5, ITPRIP, JAK3, JMJD8, JUP, KCNK2, KCP, KCTD11, KIF26A, KLK14, KMT2D, KNG1, KSR1, LAG3, LDLR, LGALS9, LGR6, LIF, LIFR, LILRA5, LIMK1, LIMS2, LMCD1, LMNA, LOX, LRG1, LTBR,
GO:0002683	negative regulation of immune system process	1,16E-10	[2, 3, 4]	156,00	24,68	57,59	42,41	[ADCY7, ALOX15, ANGPT1, BANK1, BLK, BTK, C1QC, C1QTNF1, CALCR, CCL2, CCL3, CCR2, CD200R1, CD22, CD247, CD300LF, CD3G, CD74, CD80, CD84, CD86, CD96, CDKN2A, CLNK, CNR1, CNR2, CR2, CRTAM, CST7, CTLA4, CYSLTR1, DPEP1, DRD1, FCRL3, FGL2, FGR, FOXP3, GPR150, GPR55, GPR83, HLA-DOA, HLA-DRB1, IL7R, INPP5D, KLRC1, KLRD1, KLRK1, LAG3, LAX1, LDLR, LILRB1, LILRB4, LPAL2, LRRC17, LST1, NCKAP1L, NLRC3, NLRP3, NLRP6, PLA2G2D, PTGDR, PTGER2, PTPN22, PTPN6, PTPRC, RLN2, RUNX3, SAMSN1, SH2D1A, SLA2, SLAMF8, SOCS1, SPN, STAP1, SYK, TBC1D10C, TBX21, THBS1, THY1, TIGIT, TNFAIP3, TNFAIP8L2, TNFRSF13B, TREM2, TSC22D3, UBASH3A, XCL1]	[ADCY1, ADCY3, ADCY4, ADCY9, ADIPOQ, AMH, ANGPT1, APOA1, APOD, BCL6, C1QTNF1, C5, CALCR, CCL2, CCL21, CD74, CEACAM1, CGA, CHGA, CLNK, CNR1, CRHR2, CST7, CX3CL1, DGKZ, DLG5, DTX1, ENPP3, ERBB2, FADD, FOXF1, FURIN, GATA2, GCSAM, GNAI2, GPAM, GPBAR1, GPER1, GPR137, GPR20, GPR83, GPR84, GRN, HFE, HLA-DOB, HRH2, IL4R, IL7R, INHA, JAK3, LAG3, LDLR, LGALS9, LRRC17, LRRC32, LTF, MYC, MYH9, NLRC5, NOD2, NR1D1, NRARP, PCDHA4, PCDHA6, PDCD1, PRKACA, PRKAR2B, PTGIR, RAMP2, RARA, RHBDF2, RMRP, RNF26, SERPING1, SPI1, SRC, THBS1, TNF, TRIB1, VIPR2, VSIR, ZBTB7B, ZC3H12A]

GO:0023057	negative regulation of signaling	5,59E-04	[2, 3, 4]	269,00	18,24	33,37	66,63	[AREG, ATF3, BANK1, BCL3, BMP7, BMPR1B, CASP8, CCDC88C, CCL5, CD22, CD300LF, CD3E, CD74, CHRDL1, CISH, CNR2, DCC, DUSP2, DUSP8, EPX, EREG, FASLG, FCRL3, FFAR4, FRZB, GPRC5A, INPP5D, IRF4, LAX1, LIF, LILRB4, MIDN, MMP9, NLRC3, NLRP3, NLRP6, NR4A2, NTN1, PIK3IP1, PLEK, PRKCB, PRKCQ, PSCA, PTK2B, PTPN22, PTPN6, PTPRC, PYCARD, RASAL3, RBPMS2, RGS1, RGS10, RGS18, RGS9, RHOH, RTKN2, SH2D1A, SLA2, SLC24A4, SMPDL3B, SOCS1, SOCS3, SPINK1, STAP1, STAT1, TBC1D10C, THBS1, THY1, TLR6, TNFAIP3, TNIP3, TNMD, TNR, TRABD2A, TREM2, UBASH3A, UCP2, VNN1, VWC2L, WNT1]	[ABCA7, ABCC8, ABL1, ACKR3, ACP4, ADIPOQ, AGAP2, AGER, AGT, AKT1S1, AMH, APLN, APLNR, APLP1, APOA1, APOD, ARC, AREG, ARF1, ARRDC3, ASPN, BCL2L1, BCL3, BCL6, BCL9L, BEND6, BMP8A, CD14, CD74, CEACAM1, CHGA, CISH, CREB3L1, CSK, CTHRC1, CX3CL1, CXXC4, CYP26B1, DAB2IP, DAG1, DDX39B, DGKD, DGKZ, DKK2, DLG5, DUSP5, DUSP8, DVL3, ECM1, EPHA2, EPN1, ERBB2, FADD, FAIM2, FASN, FOXM1, FRMD8, GATA4, GBA, GJD3, GLIS2, GLRA1, GNAI2, GPER1, GPI, GPR37L1, GPRC5A, GRB7, GRIK5, GRINA, GSK3A, HBEGF, HDAC7, HEYL, HGS, HSPB1, HTR1B, HTR2A, ICAM1, IFT80, IL1B, IL1RN, INHA, INPP5E, ITGA3, ITPRIP, KIF26A, KLK14, LIF, LMNA, LTF, LZTS2, MAP2K3, MAPK7, MAZ, MED12, MEGF8, MEIS3P1, MEN1, MGRN1, MIDN, MNT, MSTN, MYADM, MYC, NCOR2, NF2, NFATC4, NGFR, NKD2, NLRC5, NOD2, NODAL, NOS3, NOTCH1, NOTCH3, NPPA, NPRL3, NR1D1, NR4A2, NRARP, NTN1, OMA1, PALM, PCDHA6, PCGF2, PER1, PIAS4, PLAUR, PPP1R10, PPP1R15A, PPP2R1A, PPP2R5B, PRDM16, PRKACA, PRRT1, PTGIR, PTPRU, RAB11FIP5, RAP1A, RBPMS2, RELA, RGS16, RGS18, RNF126, RNF157, RNU1-93P, RTKN2, S1PR2, SERPINE1, SH2B3, SH3BP1, SHANK3, SLC25A23, SLC30A1, SLC35C1, SLC35F6, SLC9A3R1, SMAD3, SMARCA4, SMPD1, SNAI1, SOCS3, SORCS2, SOX10, SOX13, SPI1, SPINK1, SRC, SREBF1, SRF, SYNGAP1, SYVN1, SZT2, TGFB1I1, THBS1, TICAM1, TIMP1, TLE2, TLE3, TLE5, TMEM127, TMEM14A, TMEM161A, TMEM88, TNF, TNFAIP1, TNIP1, TNMD, TNS2, TRIB1, TRIB3, TSC2, TSKU, UBA52, UBE2B, UNC5B, VASN, VNN1, VSIG2, WFS1, WNK2, WWP2, ZBTB7A, ZC3H12A, ZNF385A, ZNF653]
GO:0048523	negative regulation of cellular process	5,39E-08	[2, 3, 4]	842,00	16,40	38,68	61,32	[ACP5, ADAM8, ADAMDEC1, ADAMTS9, ADORA3, AIF1, AKNA, ALOX15B, ANGPT1, ANGPT2, ANO9, APOBEC3A, APOBEC3D, APOBEC3G, APOBEC3H, AREG, ASCL2, ATF3, BANK1, BCL11B, BCL3, BHLHA15, BHLHE40, BLK, BMF, BMP7, BMPR1B, BRIP1, BTG2, BTK, C1QC, C1QTNF1, CARD17, CARMIL2, CASP8, CBARP, CCDC88C, CCL17, CCL19, CCL2, CCL3, CCL3L1, CCL5, CCR2, CCR5, CD19, CD200R1, CD22, CD27, CD300LF, CD33, CD3E, CD40LG, CD74, CD80, CD84, CD86, CDH1, CDKN2A, CEL, CHRDL1, CISH, CLCF1, CLNK, CMTM5, CNN1, CNR1, CNR2, CORO1A, CRB2, CREM, CRTAM, CST7, CTLA4, CXCL10, CXCL11, CXCL9, DAPK1, DCC, DEPDC1B, DERL3, DOCK8, DPEP1, DPEP2, DPT, DRD1, DUSP2, DUSP8, E2F8, EAF2, EGR3, ELAVL4, ELF3, EOMES, EPX, EREG, ERFE, EVI2B, FAM122C, FASLG, FATE1, FCMR, FCN3, FCRL3, FFAR4, FGL2, FGR, FOSB, FOSL1, FOXP3, FRZB, GADD45B, GFI1, GFRA2, GLIS1, GMFG, GPR132, GPR171, GPR55, GPRC5A, GZMA, GZMB, H1-3, H2AC13, H2AC14, H2AC16, H3C10, H3C11, H3C12, H3C7, HCLS1, HLA-DRB1, HOPX, HORMAD1, IER3, IFNG, IKBKE, IKZF1, IL10RA, IL18, IL26, IL7R, INPP5D, IQCJ-SCHIP1, IRF4, IRF8, IRX3, ITGB2, JPH1, JSRP1, KCNN4, KIR3DL2, KLF5, KLRC1, KLRD1, KLRK1, KNG1, LAG3, LAMP3, LAX1, LCK, LCP1, LDLR, LEF1, LIF, LILRB1, LILRB4, LMCD1, LMO1, LPAL2, LRRC17, LST1, LTA, MCOLN2, MCOLN3, MID1IP1, MIDN, MIXL1, MMP9, MPO, MSX1, MYB, NAMPT, NCKAP1L, NLRC3, NLRP3, NLRP6, NLRP7, NPPB, NPPC, NR4A1, NR4A2, NR4A3, NTN1, NUGGC, P2RY12, PAPLN, PARP15, PATL2, PAX5, PIK3CG, PIK3IP1, PIM1, PIM2, PLA2G2D, PLAAT4, PLAC8, PLEK, PLXNC1, PRDM1, PRKCB, PRKCQ, PSCA, PTGFR, PTK2B, PTPN22, PTPN6, PTPRC, PTPRH, PTX3, PYCARD, RAD9B, RARRES1, RASAL3, RBPMS2, RGS1, RGS10, RGS18, RGS9, RHOH, RMI2, RNASE6, RRAD, RRM2, RTKN2, RTN1, RUNX3, SALL1, SAMSN1, SCML4, SEMA4A, SEMA4D, SERPINA1, SH2D1A, SIRPG, SLA2, SLAMF8, SLC18A1, SLC24A4, SLC8A3, SMPDL3B, SOCS1, SOCS3, SPINK1	[ABCA7, ABCC8, ABCD1, ABCD2, ABI3, ABL1, ACACB, ACKR3, ACP4, ACTN1, ACTN4, ADAMTS7, ADAMTS8, ADAMTS9, ADCK1, ADGRG1, ADIPOQ, AGAP2, AGER, AGT, AKT1S1, ALPK3, AMH, ANGPT1, ANGPTL4, ANKRD13B, ANKRD52, APLN, APLNR, APLP1, APOA1, APOBEC3C, APOD, APOM, ARAF, ARC, AREG, ARF1, ARHGAP1, ARHGDIA, ARHGEF15, ARID5A, ARRDC3, ARTN, ASPN, ATF5, ATN1, ATP13A2, ATP1A1, ATP1A3, ATP1B2, BAG3, BAG6, BAHD1, BAZ2A, BBS12, BCKDK, BCL2L1, BCL3, BCL6, BCL9L, BCOR, BEND6, BHLHE40, BMP8A, BTG2, C11orf65, C18orf54, C1QTNF1, C5, C5AR1, CABIN1, CAMSAP3, CARM1, CARMIL2, CBARP, CC2D1A, CCL11, CCL2, CCL21, CCR7, CD14, CD74, CDK1, CDK2, CDK2AP2, CDKN1A, CEACAM1, CEL, CETP, CFH, CFL1, CHERP, CHGA, CIC, CIITA, CISH, CITED2, CLCF1, CLIP3, CLNK, CMTM5, CNN1, CNOT3, CNR1, COL1A1, CPTP, CRAT, CREB3L1, CRP, CRYAB, CSF1, CSK, CST7, CTC1, CTDSP1, CTHRC1, CTSA, CX3CL1, CXXC4, CYP26B1, CYP27B1, CYP2D6, DAB2IP, DAG1, DAXX, DBNDD1, DDR1, DDX39B, DDX54, DEDD, DGKD, DGKZ, DGUOK, DHRS2, DHX34, DISP3, DKK2, DLG5, DMTN, DNMT1, DOT1L, DPF2, DPT, DTX1, DUSP5, DUSP8, DVL3, DYSF, E2F4, E2F7, E2F8, EAF2, ECM1, EDC4, EEF1E1, EFEMP2, EIF4EBP1, EIF4G1, ELF3, ENG, ENPP3, EPHA2, EPN1, EPPK1, ERBB2, ERF, ERFE, ESPL1, ETS2, ETV6, F8A1, FABP4, FADD, FAIM2, FAM122C, FAP, FASN, FCN3, FGFRL1, FLII, FLT4, FOSL1, FOXC2, FOXF1, FOXM1, FOXP4, FOXS1, FRMD8, FURIN, FXYD5, GADD45B, GAS1, GATA2, GATA4, GATAD2A, GBA, GCSAM, GDI1, GGA3, GIT1, GJD3, GLIS2, GLRA1, GMNN, GNAI2, GPAM, GPER1, GPI, GPR137, GPR37L1, GPRC5A, GRB7, GRIK5, GRINA, GRN, GSK3A, GTF2IRD1, H1-9P, H3C10, H3C12, H4C3, HBEGF, HCFC1, HDAC10, HDAC5, HDAC7, HESX1, HEYL, HFE, HGS, HIRA, HMGA1, HMGN5, HOPX, HSF1, HSPB1, HTR1B, HTR2A, HYAL1, HYPK, ICAM1, ID3, IER3, IFITM1, IFT80, IGF2BP2, IL1B, IL1RN, IL4R, IL7R, ILK, INCA1, INHA, INPP5E, INPP5J, INPPL1, IRF2, IRF2BPL, IRX3, ITGA3

GO:0048585	negative regulation of response to stimulus	4,01E-07 [2, 3, 4]	356,00	18,56	39,96	60,04	[ACP5, ADCY7, AIF1, ALOX15, ANGPT2, AOAH, AREG, ATF3, BANK1, BCL3, BMP7, BMPR1B, C1QTNF1, CALCR, CASP8, CCDC88C, CCL2, CCL5, CCR2, CD200R1, CD22, CD247, CD300LF, CD3E, CD3G, CD74, CD84, CD96, CEL, CHRDL1, CISH, CNR2, CR2, CST7, CTLA4, CYSLTR1, DCC, DPEP1, DRD1, DUSP2, DUSP8, EPX, EREG, FASLG, FCRL3, FFAR4, FGL2, FGR, FOXP3, FRZB, GPR150, GPR83, GPRC5A, HLA-DRB1, IL7R, INPP5D, IRF4, KLRC1, KLRD1, KLRK1, KNG1, LAX1, LCP1, LDLR, LIF, LILRB1, LILRB4, MMP9, NAMPT, NCKAP1L, NLRC3, NLRP3, NLRP6, NR4A2, NR4A3, NTN1, PIK3IP1, PLAU, PLEK, PRKCB, PRKCQ, PSCA, PTGDR, PTGER2, PTPN22, PTPN6, PTPRC, PYCARD, RASAL3, RBPMS2, RGS1, RGS10, RGS18, RGS9, RHOH, RLN2, RMI2, RTKN2, SAMSN1, SEMA4A, SEMA4D, SH2D1A, SIGLEC10, SLA2, SLAMF8, SMPDL3B, SOCS1, SOCS3, SPINK1, STAP1, STAT1, SYK, TBC1D10C, TBX21, THBS1, THY1, TLR6, TNFAIP3, TNFAIP8L2, TNIP3, TNMD, TNR, TRABD2A, TREM2, TSPAN8, UBASH3A, UCP2, VNN1, VWC2L, WNT1, XCL1]	[ABCA7, ABCC8, ABL1, ACKR3, ACP4, ADCY1, ADCY3, ADCY4, ADCY9, ADIPOQ, AGAP2, AGT, AKT1S1, AMH, APLN, APLNR, APLP1, APOA1, APOD, AREG, ARRDC3, ASPN, BCL2L1, BCL3, BCL6, BCL9L, BEND6, C1QTNF1, C5, CALCR, CCL2, CD14, CD74, CEACAM1, CEL, CGA, CHGA, CISH, CREB3L1, CRHR2, CSK, CST7, CTHRC1, CX3CL1, CXXC4, CYP26B1, DAB2IP, DAG1, DDX39B, DGKD, DGKZ, DKK2, DLG5, DUSP5, DUSP8, DVL3, ECM1, ENPP3, EPHA2, EPN1, EPPK1, ERBB2, FADD, FAIM2, FAM110A, FAP, FASN, FEM1A, FOXF1, FOXM1, FRMD8, FURIN, GATA4, GBA, GIT1, GLIS2, GNAI2, GPBAR1, GPER1, GPI, GPR20, GPR37L1, GPR83, GPR84, GPRC5A, GRB7, GRINA, GRN, GSK3A, HBEGF, HDAC7, HEYL, HFE, HGS, HRH2, HSF1, HSPB1, HTR1B, ICAM1, IFT80, IL1B, IL1RN, IL4R, IL7R, INPP5E, ITGA3, ITPRIP, JAK3, KIF26A, KLK14, KNG1, LDLR, LGALS9, LIF, LMNA, LTF, LZTS2, MAP2K3, MAPK7, MAZ, MED12, MEGF8, MEIS3P1, MEN1, MGRN1, MNT, MSTN, MYADM, MYC, MYH9, NCOR2, NF2, NFATC4, NGFR, NKD2, NLRC5, NOD2, NODAL, NOS3, NOTCH1, NOTCH3, NPPA, NPRL3, NR1D1, NR4A2, NR4A3, NRARP, NTN1, OMA1, PALM, PCDHA6, PCGF2, PDCD1, PDGFB, PER1, PIAS4, PLAU, PLAUR, PLXNA3, PPARD, PPP1R10, PPP1R15A, PPP2R1A, PPP2R5B, PRDM16, PRKACA, PRKAR2B, PTGIR, PTPRU, RADX, RAMP2, RBPMS2, RELA, RGMA, RGS16, RGS18, RHBDF2, RMI2, RNF126, RNF157, RNF26, RNU1-93P, RTKN2, RTN4RL1, S1PR2, SEMA3F, SEMA4B, SEMA4C, SEMA6B, SEMA6C, SERPINE1, SERPING1, SGTA, SH2B3, SH3BP1, SHARPIN, SLC25A23, SLC35C1, SLC35F6, SLC9A3R1, SMAD3, SMARCA4, SMPD1, SNAI1, SOCS3, SOX10, SOX13, SPI1, SPINK1, SRC, SYNGAP1, SYVN1, SZT2, TGFB1I1, THBS1, TICAM1, TIMP1, TLE2, TLE3, TLE5, TMEM127, TMEM14A, TMEM161A, TMEM88, TNF, TNFAIP1, TNFRSF1A, TNIP1, TNMD, TNS2, TRIB1, TRIB3, TSC2, TSKU, TSPAN8, UBA52, UBE2B, UBQLN4, UNC5B, VASN, VIPR2, VNN1, VSIG2, VTN, WFS1, WNK2, WNT3, WWP2, XRCC1, ZBTB7A, ZBTB7B, ZC3H12A, ZFP36
GO:0010646	regulation of cell communication	2,64E-15 [3, 4]	671,00	18,23	37,26	62,74	[ABRA, ADAM8, ALOX15, ALOX15B, ANGPT1, ARAP2, AREG, ARHGAP15, ARHGAP30, ARHGAP45, ATF3, BANK1, BCL2L14, BCL3, BLK, BMF, BMP7, BMPR1B, BRIP1, C1QTNF1, CACNA1E, CALCR, CARD11, CASP8, CASS4, CCDC88C, CCL17, CCL19, CCL2, CCL22, CCL24, CCL3, CCL3L1, CCL4, CCL4L1, CCL5, CCR2, CD177, CD180, CD19, CD22, CD226, CD27, CD300LF, CD3E, CD4, CD40LG, CD74, CD80, CD86, CDH1, CDKN2A, CEL, CHRDL1, CISH, CLEC6A, CNR1, CNR2, CRB2, CSF1R, CXCR4, CXorf21, DAPK1, DCC, DEPDC1B, DLGAP2, DPEP2, DRD1, DUSP2, DUSP8, ELAVL4, ELF3, EPX, EREG, ERFE, FASLG, FCRL3, FFAR4, FGD2, FGD3, FGR, FLT3, FOLR2, FOXP3, FRZB, FUT7, GADD45B, GDF6, GFI1, GMIP, GPR174, GPR4, GPR55, GPRC5A, GUCY2D, H3C10, H3C11, H3C12, H3C7, HCLS1, HCST, HLA-DRB1, ICOS, IER3, IFNG, IKBKE, IL10RA, IL18, IL26, IL7R, INPP5D, IQCJ-SCHIP1, IRF4, IRX3, KCNJ10, KCNN4, KMO, LAX1, LCK, LEF1, LIF, LILRB4, LMCD1, LY86, MAP4K1, MIDN, MMP9, MSX1, MYB, MZB1, NCF1, NCKAP1L, NFAM1, NLRC3, NLRP3, NLRP6, NPFFR2, NPPC, NPTX2, NR4A2, NSG1, NTN1, OASL, P2RY10, P2RY12, PAX5, PDE6G, PIK3CG, PIK3IP1, PIK3R5, PIM2, PLAU, PLEK, PRDM1, PRKCB, PRKCQ, PROK1, PSCA, PSD4, PTK2B, PTPN22, PTPN6, PTPRC, PYCARD, PYHIN1, RAC2, RAD9B, RASAL3, RASGRF1, RBPMS2, RGS1, RGS10, RGS18, RGS9, RHOH, RMI2, RNASE6, RRAD, RTKN2, SALL1, SCIMP, SCUBE1, SEMA4D, SH2D1A, SHISA8, SLA, SLA2, SLC24A4, SLC8A3, SMPDL3B, SOCS1, SOCS3, SPINK1, STAP1, STAT1, SYK, TAGAP, TBC1D10C, TESPA1, THBS1, THY1, TLR6, TMC8, TNFAIP3, TNFSF14, TNIP3, TNMD, TNR, TRABD2A, TRAF1, TRAT1, TREM2, TRIM14, TRPM2, UBASH3A, UBD, UCP2, VAV1, VNN1, VWC2L, WNT1, WNT10B, XCL1, XCL2, ZAP70, ZBP1]	[ABCA7, ABCC8, ABL1, ABR, ABRA, ACKR3, ACP4, ACTN4, ADCY1, ADGRG1, ADIPOQ, AGAP2, AGER, AGPAT1, AGT, AGTR1, AKT1S1, AMH, ANGPT1, APLN, APLNR, APLP1, APOA1, APOD, ARAF, ARAP3, ARC, AREG, ARF1, ARHGAP1, ARHGAP23, ARHGAP39, ARHGDIA, ARHGEF1, ARHGEF16, ARHGEF5, ARRDC3, ARTN, ASPN, BCL2L1, BCL3, BCL6, BCL9L, BCR, BEND6, BMP8A, BRD4, C1QTNF1, C20orf27, C2CD2L, C5, C5AR1, CACNA1E, CALB1, CALCR, CAMK2B, CAPN1, CARM1, CC2D1A, CCL11, CCL2, CCL21, CCL24, CCR7, CD14, CD177, CD74, CDK1, CDK2, CEACAM1, CEL, CGA, CHERP, CHGA, CHI3L1, CISH, CITED2, CLIP3, CNR1, CNTN6, COL1A1, CPLX1, CRAT, CREB3L1, CRHR2, CSF1, CSK, CSNK1E, CSPG4, CTHRC1, CX3CL1, CXXC4, CYP26B1, CYP27B1, DAB2IP, DAG1, DAXX, DDX39B, DENND2B, DENND4B, DGKD, DGKZ, DHX34, DIRAS1, DKK2, DLG5, DLGAP4, DMTN, DOC2B, DOK5, DOT1L, DTX1, DUSP5, DUSP8, DVL3, ECM1, EEF1E1, ELF3, ENG, EPHA2, EPN1, ERBB2, ERFE, ESM1, FADD, FAIM2, FAM110A, FASN, FFAR2, FGFR4, FLOT2, FLT3, FLT4, FOXM1, FPR1, FRMD8, FURIN, GAB2, GADD45B, GAS1, GATA4, GBA, GBF1, GCSAM, GDF6, GIT1, GJD3, GLIS2, GLRA1, GNAI2, GPAM, GPBAR1, GPER1, GPI, GPR137, GPR17, GPR20, GPR37L1, GPR4, GPRC5A, GPRIN1, GRB7, GRIK5, GRINA, GRM2, GSK3A, H3C10, H3C12, H4C3, HBEGF, HCRTR1, HDAC7, HEYL, HFE, HGS, HIPK4, HSPB1, HTR1B, HTR2A, ICAM1, IER3, IFT80, IL18BP, IL1B, IL1RN, IL7R, ILK, INCA1, INHA, INPP5E, IRAK2, IRX3, ITGA3, ITGA5, ITPR3, ITPRIP, JAK3, JMJD8, JPH4, JUP, KCP, KCTD11, KIF26A, KLK14, KMT2D, KSR1, LGALS9, LGR6, LIF, LIFR, LILRA5, LIMS2, LMCD1, LMNA, LOX, LRG1, LRRC4, LRRC8A, LTBR, LTF, LY6E, LYNX1, LZTS1, LZTS2, MAP1A, MAP2K3, MAP3K10, MAP3K11, MAP3K14, MAP3K6, MAP3K9, MAPK3, MAPK7, MAPK8IP3, MAVS, MAZ, MECP2, MED12, MEGF8, MEIS3P1, MEN1, MGRN1, MIDN, MINK1, MLST8, MME, MNT, MSTN, MSX1, MTRNR2L1, MTRNR2L10, MTRNR2L12, MTRNR2L2, MTRNR2L3, MTRNR2L6, MTRNR2L8, MTRNR2L9,

GO:0032101	regulation of response to external stimulus	2,76E-10	[3, 4]	266,00	20,94	53,55	46,45	[ACP5, ADAM8, ADCY7, AIF1, AIM2, AKNA, ALOX15, ANGPT2, AOAH, APOBEC3G, BTK, C1QTNF1, CALCR, CARD11, CASP8, CCL19, CCL2, CCL24, CCL3, CCL4, CCL5, CCR2, CCR4, CD180, CD200R1, CD226, CD247, CD3G, CD74, CD96, CEL, CLEC10A, CLEC4D, CLEC4E, CLEC6A, CLNK, CNR1, CNR2, CRTAM, CSF1R, CST7, CXCL10, CXCL6, CXCR4, CXorf21, CYSLTR1, DPEP1, DRD1, EDN2, EREG, FCN1, FFAR4, FGL2, FGR, FOXP3, FUT7, GBP5, GPR150, GPR4, GPR83, GPSM3, GUCY2D, HLA-DRB1, HOPX, IFNG, IKBKE, IL12RB1, IL16, IL18, IL18RAP, KLRC1, KLRC2, KLRD1, KLRK1, KNG1, LAG3, LCP1, LDLR, LILRB1, LTA, LY86, MAPK13, MMP9, MUC16, MUC19, MUC6, NCKAP1L, NCR1, NCR3, NLRC3, NLRP3, NLRP6, OASL, P2RY12, PDE6G, PIK3CG, PLA2G2D, PLA2G7, PLAU, PTGDR, PTGER2, PTK2B, PTPN22, PTPN6, PTPRC, PYCARD, PYHIN1, RAC2, RLN2, SCIMP, SELE, SEMA4A, SEMA4D, SH2D1A, SIGLEC10, SIGLEC16, SLAMF6, SLAMF8, SMPDL3B, SOCS1, SOCS3, STAP1, STAT1, SUCNR1, SYK, THBS1, TLR10, TLR8, TNFAIP3, TNFAIP8L2, TNR, TREM2, TSPAN8, VAV1, XCL1, XCL2, ZBP1]	[ABCC8, ADCY1, ADCY3, ADCY4, ADCY9, ADIPOQ, AGER, AGT, AGTR1, AMH, APOA1, ARTN, BCL6, BRD4, C1QTNF1, C5, C5AR1, CALCR, CCL2, CCL21, CCL24, CCR7, CD74, CEACAM1, CEL, CGA, CHGA, CLEC4E, CLNK, CNR1, CRHR2, CSF1, CST7, CX3CL1, CYP27B1, DAGLA, DMTN, EDN2, ENPP3, EPPK1, FABP4, FADD, FAM110A, FAP, FEM1A, FFAR2, FOXF1, FURIN, GBA, GIT1, GNAI2, GPBAR1, GPER1, GPR20, GPR4, GPR83, GPR84, GRN, HOPX, HRH2, HSPB1, IL1B, KNG1, LAG3, LDLR, LGALS9, LILRA5, LTF, MAPK3, MAPK7, MAVS, MEGF8, MEN1, MSTN, MUC19, MUC20, MUC3A, MYH9, NECTIN2, NINJ1, NLRC5, NOD2, NOS3, NOTCH1, NPPA, NR1D1, OSM, PCDHA4, PCDHA6, PDGFB, PDGFRB, PLA2G2A, PLA2G7, PLAU, PLAUR, PLXNA3, PPARD, PQBP1, PRKACA, PRKAR2B, PTGES, PTGIR, PTN, PTPRU, PVR, RAC2, RAMP2, RBM14, RELA, RELB, RGMA, RHBDF2, RNF26, RNU1-93P, RTN4RL1, SBNO2, SELE, SEMA3F, SEMA4B, SEMA4C, SEMA6B, SEMA6C, SERPINE1, SERPING1, SH2B3, SHARPIN, SMAD3, SOCS3, SPATA2, SPHK1, SPI1, SRC, STING1, TGM2, THBS1, TNF, TNFRSF1A, TNIP1, TRIB1, TRIM62, TRPV4, TSPAN8, VEGFA, VIPR2, VTN, WNT3, ZC3H12A, ZCCHC3, ZDHHC11, ZFP36, ZNF580, ZSWIM4, ZSWIM8, ZYX]
GO:0080134	regulation of response to stress	3,32E-08	[3, 4]	319,00	19,33	46,88	53,12	[ACP5, ADAM8, ADCY7, AIM2, AKNA, ALOX15, AOAH, APOBEC3G, ATF3, BMP7, BTK, C1QTNF1, CALCR, CARD11, CASP8, CCL19, CCL24, CCL3, CCL5, CCR2, CD200R1, CD226, CD247, CD27, CD3G, CD74, CD96, CDKN2A, CEL, CLEC10A, CLEC4D, CLEC4E, CLEC6A, CLNK, CNR1, CNR2, CRTAM, CST7, CXCL6, CXCR4, CXorf21, CYSLTR1, DEPDC1B, DPEP1, DRD1, EPX, EREG, FCN1, FFAR4, FGL2, FGR, FOXP3, FUT7, GADD45B, GBP5, GDF6, GPR150, GPR4, GPR83, GPSM3, HLA-DRB1, IER3, IFNG, IKBKE, IL12RB1, IL16, IL18, IL18RAP, IL26, KLRC1, KLRC2, KLRD1, KLRK1, KNG1, LAG3, LCP1, LDLR, LILRB1, LTA, MAPK13, MLC1, MMP9, MSX1, MUC16, MUC19, MUC6, NAMPT, NCF1, NCR1, NCR3, NLRC3, NLRP3, NLRP6, NR4A3, NUP210, PIK3CG, PLA2G2D, PLA2G7, PLAU, PTGDR, PTGER2, PTK2B, PTPN22, PTPN6, PTPRC, PYCARD, PYHIN1, RLN2, RMI2, SCIMP, SELE, SH2D1A, SIGLEC10, SIGLEC16, SLAMF6, SLAMF8, SMPDL3B, SOCS1, SOCS3, STAP1, STAT1, SUCNR1, SYK, THBS1, THY1, TLR10, TLR6, TLR8, TNFAIP3, TNFAIP8L2, TNR, TRAF1, TREM2, TRPM2, TSPAN8, VAV1, VNN1, WAS, WNT1, XCL1, ZBP1]	[AAAS, ABCA7, ABCC8, ABCD1, ABI3, ABL1, ACKR3, ADCY1, ADCY3, ADCY4, ADCY9, ADIPOQ, AGER, AGT, AGTR1, AKT1S1, AMH, APOA1, ARMT1, BAG3, BAG6, BCL2L1, BCL6, BRD4, C1QTNF1, CALCR, CAMK2B, CCL21, CCL24, CCR7, CD74, CEACAM1, CEL, CGA, CHGA, CLEC4E, CLNK, CNR1, CREB3L1, CRHR2, CRYAB, CST7, CX3CL1, DAB2IP, DAGLA, DDX39B, DMTN, EEF1E1, EIF4G1, ENDOG, ENPP3, EPPK1, FABP4, FADD, FAM110A, FAP, FASN, FEM1A, FFAR2, FLT4, FOXC2, FOXF1, FOXM1, FURIN, GADD45B, GATA4, GBA, GDF6, GIT1, GNAI2, GPBAR1, GPER1, GPR20, GPR37L1, GPR4, GPR83, GPR84, GRIK5, GRINA, GRN, HBEGF, HDAC10, HMGA1, HRH2, HSF1, HSPB1, HSPB8, IER3, IL1B, KCNK2, KNG1, LAG3, LDLR, LGALS9, LILRA5, LMNA, LTBR, MAP3K10, MAP3K11, MAPK3, MAPK7, MAPK8IP3, MAVS, MED12, MEN1, MINK1, MLST8, MSX1, MUC19, MUC20, MUC3A, MYC, MYH9, NECTIN2, NINJ1, NLRC5, NOD2, NOS3, NPAS2, NPPA, NR1D1, NR4A3, NUP188, OSM, PCDHA4, PCDHA6, PDGFB, PER1, PIAS4, PLA2G2A, PLA2G7, PLAU, PLAUR, PML, PPARD, PPP1R10, PPP1R15A, PQBP1, PRKACA, PRKAR2B, PTGES, PTGIR, PTN, PVR, RAD51AP1, RADX, RAMP2, RBM14, RELA, RELB, RGMA, RHBDF2, RMI2, RNF26, RNU1-93P, RPL26, RPTOR, RTEL1-TNFRSF6B, RTN4RL1, SBNO2, SELE, SELENON, SEMA4C, SERPINE1, SERPING1, SGTA, SH2B3, SHANK3, SHARPIN, SLC25A23, SMAD3, SNAI1, SOCS3, SPATA2, SPHK1, SPI1, SRC, STING1, SYVN1, TGM2, THBS1, TIMP1, TMEM161A, TMEM259, TNF, TNFRSF1A, TNIP1, TRAF4, TRIM62, TRPV4, TSPAN8, UBQLN4, VEGFA, VIPR2, VNN1, VTN, WAS, WFS1, XRCC1, YPEL3, ZBTB7B, ZC3H12A, ZCCHC3, ZDHHC11, ZFP36, ZNF385A, ZYX]

GO:0009966	regulation of signal transduction	1,31E-14	[3, 4, 5]	609,00	18,48	37,99	62,01	[ABRA, ADAM8, ALOX15, ALOX15B, ANGPT1, ARAP2, AREG, ARHGAP15, ARHGAP30, ARHGAP45, ATF3, BANK1, BCL2L14, BCL3, BLK, BMF, BMP7, BMPR1B, BRIP1, C1QTNF1, CALCR, CARD11, CASP8, CASS4, CCDC88C, CCL17, CCL19, CCL2, CCL22, CCL24, CCL3, CCL3L1, CCL4, CCL4L1, CCL5, CCR2, CD177, CD180, CD19, CD22, CD226, CD27, CD300LF, CD3E, CD4, CD40LG, CD74, CD80, CD86, CDKN2A, CHRDL1, CISH, CLEC6A, CRB2, CSF1R, CXCR4, CXorf21, DAPK1, DCC, DEPDC1B, DLGAP2, DPEP2, DUSP2, DUSP8, ELF3, EPX, EREG, ERFE, FASLG, FCRL3, FFAR4, FGD2, FGD3, FGR, FLT3, FOLR2, FOXP3, FRZB, FUT7, GADD45B, GDF6, GFI1, GMIP, GPR174, GPR4, GPR55, GPRC5A, GUCY2D, H3C10, H3C11, H3C12, H3C7, HCLS1, HCST, HLA-DRB1, ICOS, IER3, IFNG, IKBKE, IL10RA, IL18, IL26, IL7R, INPP5D, IQCJ-SCHIP1, IRF4, KCNN4, LAX1, LCK, LEF1, LIF, LILRB4, LMCD1, LY86, MAP4K1, MMP9, MSX1, MZB1, NCF1, NCKAP1L, NFAM1, NLRC3, NLRP3, NLRP6, NPFFR2, NPPC, NPTX2, NR4A2, NTN1, OASL, P2RY10, P2RY12, PAX5, PDE6G, PIK3CG, PIK3IP1, PIK3R5, PIM2, PLAU, PLEK, PRDM1, PRKCB, PRKCQ, PROK1, PSCA, PSD4, PTK2B, PTPN22, PTPN6, PTPRC, PYCARD, PYHIN1, RAC2, RAD9B, RASAL3, RASGRF1, RBPMS2, RGS1, RGS10, RGS18, RGS9, RHOH, RMI2, RNASE6, RRAD, RTKN2, SALL1, SCIMP, SCUBE1, SEMA4D, SH2D1A, SHISA8, SLA, SLA2, SLC8A3, SMPDL3B, SOCS1, SOCS3, SPINK1, STAP1, STAT1, SYK, TAGAP, TBC1D10C, TESPA1, THBS1, THY1, TLR6, TMC8, TNFAIP3, TNFSF14, TNIP3, TNMD, TRABD2A, TRAF1, TRAT1, TREM2, TRIM14, UBASH3A, UBD, VAV1, VNN1, VWC2L, WNT1, WNT10B, XCL1, XCL2, ZAP70, ZBP1]	[ABCA7, ABL1, ABR, ABRA, ACKR3, ACP4, ACTN4, ADGRG1, ADIPOQ, AGAP2, AGER, AGPAT1, AGT, AKT1S1, AMH, ANGPT1, APLN, APLNR, APLP1, APOA1, APOD, ARAF, ARAP3, ARC, AREG, ARHGAP1, ARHGAP23, ARHGAP39, ARHGDIA, ARHGEF1, ARHGEF16, ARHGEF5, ARRDC3, ARTN, ASPN, BCL2L1, BCL3, BCL6, BCL9L, BCR, BEND6, BMP8A, BRD4, C1QTNF1, C20orf27, C5, C5AR1, CALCR, CAMK2B, CAPN1, CARM1, CC2D1A, CCL11, CCL2, CCL21, CCL24, CCR7, CD14, CD177, CD74, CDK1, CDK2, CEACAM1, CGA, CHERP, CHGA, CHI3L1, CISH, CITED2, CLIP3, CNTN6, COL1A1, CRAT, CREB3L1, CSF1, CSK, CSNK1E, CSPG4, CTHRC1, CX3CL1, CXXC4, CYP26B1, CYP27B1, DAB2IP, DAG1, DAXX, DDX39B, DENND2B, DENND4B, DGKD, DGKZ, DIRAS1, DKK2, DLG5, DLGAP4, DMTN, DOK5, DOT1L, DTX1, DUSP5, DUSP8, DVL3, ECM1, EEF1E1, ELF3, ENG, EPHA2, EPN1, ERBB2, ERFE, ESM1, FADD, FAIM2, FAM110A, FASN, FGFR4, FLOT2, FLT3, FLT4, FOXM1, FPR1, FRMD8, FURIN, GAB2, GADD45B, GAS1, GATA4, GBA, GBF1, GCSAM, GDF6, GIT1, GLIS2, GNAI2, GPAM, GPBAR1, GPER1, GPI, GPR137, GPR17, GPR20, GPR37L1, GPR4, GPRC5A, GPRIN1, GRB7, GRIK5, GRINA, GRM2, GSK3A, H3C10, H3C12, H4C3, HBEGF, HCRTR1, HDAC7, HEYL, HFE, HGS, HIPK4, HSPB1, HTR1B, HTR2A, ICAM1, IER3, IFT80, IL18BP, IL1B, IL1RN, IL7R, ILK, INCA1, INHA, INPP5E, IRAK2, ITGA3, ITGA5, ITPRIP, JAK3, JMJD8, JUP, KCP, KCTD11, KIF26A, KLK14, KMT2D, KSR1, LGALS9, LGR6, LIF, LIFR, LILRA5, LIMS2, LMCD1, LMNA, LOX, LRG1, LTBR, LTF, LY6E, LYNX1, LZTS2, MAP2K3, MAP3K10, MAP3K11, MAP3K14, MAP3K6, MAP3K9, MAPK3, MAPK7, MAPK8IP3, MAVS, MAZ, MED12, MEGF8, MEIS3P1, MEN1, MGRN1, MINK1, MLST8, MNT, MSTN, MSX1, MTRNR2L1, MTRNR2L10, MTRNR2L12, MTRNR2L2, MTRNR2L3, MTRNR2L6, MTRNR2L8, MTRNR2L9, MUC20, MYADM, MYC, NCLN, NCOR2, NDC80, NDST1, NECTIN2, NEFL, NF2, NFATC4, NGF, NGFR, NINJ1, NKD2, NLGN2, NLRC5, NOD2, NODAL, NOP2, NOS3, NOTCH1, NOTCH3, NOTCH4
GO:0010648	negative regulation of cell communication	4,49E-04	[3, 4, 5]	269,00	18,26	33,37	66,63	[AREG, ATF3, BANK1, BCL3, BMP7, BMPR1B, CASP8, CCDC88C, CCL5, CD22, CD300LF, CD3E, CD74, CHRDL1, CISH, CNR2, DCC, DUSP2, DUSP8, EPX, EREG, FASLG, FCRL3, FFAR4, FRZB, GPRC5A, INPP5D, IRF4, LAX1, LIF, LILRB4, MIDN, MMP9, NLRC3, NLRP3, NLRP6, NR4A2, NTN1, PIK3IP1, PLEK, PRKCB, PRKCQ, PSCA, PTK2B, PTPN22, PTPN6, PTPRC, PYCARD, RASAL3, RBPMS2, RGS1, RGS10, RGS18, RGS9, RHOH, RTKN2, SH2D1A, SLA2, SLC24A4, SMPDL3B, SOCS1, SOCS3, SPINK1, STAP1, STAT1, TBC1D10C, THBS1, THY1, TLR6, TNFAIP3, TNIP3, TNMD, TNR, TRABD2A, TREM2, UBASH3A, UCP2, VNN1, VWC2L, WNT1]	[ABCA7, ABCC8, ABL1, ACKR3, ACP4, ADIPOQ, AGAP2, AGER, AGT, AKT1S1, AMH, APLN, APLNR, APLP1, APOA1, APOD, ARC, AREG, ARF1, ARRDC3, ASPN, BCL2L1, BCL3, BCL6, BCL9L, BEND6, BMP8A, CD14, CD74, CEACAM1, CHGA, CISH, CREB3L1, CSK, CTHRC1, CX3CL1, CXXC4, CYP26B1, DAB2IP, DAG1, DDX39B, DGKD, DGKZ, DKK2, DLG5, DUSP5, DUSP8, DVL3, ECM1, EPHA2, EPN1, ERBB2, FADD, FAIM2, FASN, FOXM1, FRMD8, GATA4, GBA, GJD3, GLIS2, GLRA1, GNAI2, GPER1, GPI, GPR37L1, GPRC5A, GRB7, GRIK5, GRINA, GSK3A, HBEGF, HDAC7, HEYL, HGS, HSPB1, HTR1B, HTR2A, ICAM1, IFT80, IL1B, IL1RN, INHA, INPP5E, ITGA3, ITPRIP, KIF26A, KLK14, LIF, LMNA, LTF, LZTS2, MAP2K3, MAPK7, MAZ, MED12, MEGF8, MEIS3P1, MEN1, MGRN1, MIDN, MNT, MSTN, MYADM, MYC, NCOR2, NF2, NFATC4, NGFR, NKD2, NLRC5, NOD2, NODAL, NOS3, NOTCH1, NOTCH3, NPPA, NPRL3, NR1D1, NR4A2, NRARP, NTN1, OMA1, PALM, PCDHA6, PCGF2, PER1, PIAS4, PLAUR, PPP1R10, PPP1R15A, PPP2R1A, PPP2R5B, PRDM16, PRKACA, PRRT1, PTGIR, PTPRU, RAB11FIP5, RAP1A, RBPMS2, RELA, RGS16, RGS18, RNF126, RNF157, RNU1-93P, RTKN2, S1PR2, SERPINE1, SH2B3, SH3BP1, SHANK3, SLC25A23, SLC30A1, SLC35C1, SLC35F6, SLC9A3R1, SMAD3, SMARCA4, SMPD1, SNAI1, SOCS3, SORCS2, SOX10, SOX13, SPI1, SPINK1, SRC, SREBF1, SRF, SYNGAP1, SYVN1, SZT2, TGFB1I1, THBS1, TICAM1, TIMP1, TLE2, TLE3, TLE5, TMEM127, TMEM14A, TMEM161A, TMEM88, TNF, TNFAIP1, TNIP1, TNMD, TNS2, TRIB1, TRIB3, TSC2, TSKU, UBA52, UBE2B, UNC5B, VASN, VNN1, VSIG2, WFS1, WNK2, WWP2, ZBTB7A, ZC3H12A, ZNF385A, ZNF653]
GO:0032102	negative regulation of response to external stimulus	5,21E-03	[3, 4, 5]	124,00	20,46	45,28	54,72	[ACP5, ADCY7, AIF1, ANGPT2, AOAH, C1QTNF1, CALCR, CCL2, CD200R1, CD247, CD3G, CD96, CEL, CNR2, CST7, CYSLTR1, DPEP1, DRD1, FFAR4, FGL2, FGR, FOXP3, GPR150, GPR83, HLA-DRB1, KLRC1, KLRD1, KLRK1, KNG1, LDLR, LILRB1, NLRC3, NLRP3, NLRP6, PLAU, PTGDR, PTGER2, PTPRC, RLN2, SEMA4A, SEMA4D, SIGLEC10, SLAMF8, SMPDL3B, SOCS3, STAP1, SYK, THBS1, TNFAIP3, TNFAIP8L2, TNR, TREM2, TSPAN8]	[ABCC8, ADCY1, ADCY3, ADCY4, ADCY9, ADIPOQ, AMH, APOA1, C1QTNF1, C5, CALCR, CCL2, CEACAM1, CEL, CGA, CHGA, CRHR2, CST7, CX3CL1, ENPP3, EPPK1, FAM110A, FAP, FEM1A, FOXF1, FURIN, GBA, GIT1, GNAI2, GPBAR1, GPER1, GPR20, GPR83, GPR84, GRN, HRH2, KNG1, LDLR, LGALS9, LTF, MAPK7, MYH9, NLRC5, NOD2, NOS3, NOTCH1, NR1D1, PCDHA6, PDGFB, PLAU, PLAUR, PLXNA3, PPARD, PRKACA, PRKAR2B, PTGIR, PTPRU, RAMP2, RGMA, RHBDF2, RNF26, RTN4RL1, SEMA3F, SEMA4B, SEMA4C, SEMA6B, SEMA6C, SERPINE1, SERPING1, SH2B3, SHARPIN, SMAD3, SOCS3, SRC, THBS1, TNF, TNFRSF1A, TRIB1, TSPAN8, VIPR2, VTN, WNT3, ZFP36]

GO:0009968	negative regulation of signal transduction	1,93E-02	[3, 4, 5, 6]	242,00	17,74	33,71	66,29	[AREG, ATF3, BANK1, BCL3, BMP7, BMPR1B, CASP8, CCDC88C, CCL5, CD22, CD300LF, CD3E, CD74, CHRDL1, CISH, DCC, DUSP2, DUSP8, EPX, EREG, FASLG, FCRL3, FRZB, GPRC5A, INPP5D, IRF4, LAX1, LIF, LILRB4, MMP9, NLRC3, NLRP3, NLRP6, NR4A2, NTN1, PIK3IP1, PLEK, PRKCB, PRKCQ, PSCA, PTPN22, PTPN6, PTPRC, PYCARD, RASAL3, RBPMS2, RGS1, RGS10, RGS18, RGS9, RHOH, RTKN2, SH2D1A, SLA2, SMPDL3B, SOCS1, SOCS3, SPINK1, STAP1, STAT1, TBC1D10C, THBS1, THY1, TLR6, TNFAIP3, TNIP3, TNMD, TRABD2A, TREM2, UBASH3A, VNN1, VWC2L, WNT1]
								[ABCA7, ABL1, ACKR3, ACP4, ADIPOQ, AGAP2, AGT, AKT1S1, AMH, APLN, APLNR, APLP1, APOA1, APOD, AREG, ARRDC3, ASPN, BCL2L1, BCL3, BCL6, BCL9L, BEND6, CD14, CD74, CEACAM1, CISH, CREB3L1, CSK, CTHRC1, CX3CL1, CXXC4, CYP26B1, DAB2IP, DAG1, DDX39B, DGKD, DGKZ, DKK2, DLG5, DUSP5, DUSP8, DVL3, ECM1, EPHA2, EPN1, ERBB2, FADD, FAIM2, FASN, FOXM1, FRMD8, GATA4, GBA, GLIS2, GNAI2, GPER1, GPI, GPR37L1, GPRC5A, GRB7, GRINA, GSK3A, HBEGF, HDAC7, HEYL, HGS, HSPB1, HTR1B, ICAM1, IFT80, IL1B, IL1RN, INPP5E, ITGA3, ITPRIP, KIF26A, KLK14, LIF, LMNA, LTF, LZTS2, MAP2K3, MAPK7, MAZ, MED12, MEGF8, MEIS3P1, MEN1, MGRN1, MNT, MSTN, MYADM, MYC, NCOR2, NF2, NFATC4, NGFR, NKD2, NLRC5, NOD2, NODAL, NOS3, NOTCH1, NOTCH3, NPPA, NPRL3, NR1D1, NR4A2, NRARP, NTN1, OMA1, PALM, PCGF2, PER1, PIAS4, PLAUR, PPP1R10, PPP1R15A, PPP2R1A, PPP2R5B, PRDM16, PRKACA, PTGIR, PTPRU, RBPMS2, RELA, RGS16, RGS18, RNF126, RNF157, RNU1-93P, RTKN2, S1PR2, SERPINE1, SH2B3, SH3BP1, SLC25A23, SLC35C1, SLC35F6, SLC9A3R1, SMAD3, SMARCA4, SMPD1, SNAI1, SOCS3, SOX10, SOX13, SPI1, SPINK1, SRC, SYNGAP1, SYVN1, SZT2, TGFB1I1, THBS1, TICAM1, TIMP1, TLE2, TLE3, TLE5, TMEM127, TMEM14A, TMEM161A, TMEM88, TNF, TNFAIP1, TNIP1, TNMD, TNS2, TRIB1, TRIB3, TSC2, TSKU, UBA52, UBE2B, UNC5B, VASN, VNN1, VSIG2, WFS1, WNK2, WWP2, ZBTB7A, ZC3H12A, ZNF385A, ZNF653]

GO:0051241	negative regulation of multicellular organismal process	1,56E-08	[2, 3, 4]	224,00	21,11	41,07	58,93	[ACP5, ADAMTS9, ADCY7, ANGPT1, ANGPT2, ASCL2, BANK1, BCL3, BMP7, BTK, C1QC, C1QTNF1, CALCR, CARD17, CCL3, CCR2, CD200R1, CD33, CD74, CD84, CD96, CDKN2A, CEL, CTLA4, CXCL10, DCC, DPEP1, EAF2, EPX, FASLG, FFAR4, FGL2, FOXP3, FRZB, GPR4, GPR55, HLA-DRB1, IFNG, IKBKE, INPP5D, KNG1, LAG3, LDLR, LEF1, LILRB1, LILRB4, LPAL2, LRRC17, NCKAP1L, NLRC3, NLRP3, NLRP6, NLRP7, NPPB, NTN1, PIK3CG, PLAC8, PLAU, PTK2B, PTPN22, PTPN6, PTPRC, PYCARD, RUNX3, SEMA4A, SEMA4D, SLAMF8, SOCS1, STAT1, TACSTD2, TBX21, THBS1, THY1, TIFAB, TIGIT, TLR6, TLR8, TNFAIP3, TNFRSF11B, TNMD, TNR, TREM2, TSPAN8, WNT10B, XCL1]	[ABCC8, ABCD1, ABCD2, ADAMTS5, ADAMTS7, ADAMTS9, ADGRB2, ADIPOQ, AGER, AGT, ANGPT1, APLN, APOA1, APOD, APOM, ARHGDIA, ARRDC3, ASPN, ATP1A1, ATP1A3, BCL3, BCL6, BCOR, C1QTNF1, CALCR, CCL11, CCR7, CD74, CEACAM1, CEL, CGA, CHGA, CITED2, CPTP, CREB3L1, CSK, CTDSP1, CX3CL1, DAB2IP, DTX1, DXO, EAF2, ECM1, EFNA3, EPHA2, EPN1, EPPK1, ERBB2, FAM110A, FAP, FOXC2, FURIN, GATA2, GBA, GDI1, GIT1, GJD3, GLRA1, GNAI2, GPR137, GPR37L1, GPR4, GRN, GSK3A, HDAC5, HDAC7, HFE, HGS, HSPG2, IL1B, IL4R, INHA, JAK3, JUP, KCNK2, KNG1, LAG3, LDLR, LGALS9, LILRA5, LINGO1, LMNA, LRRC17, LRRC32, LTF, MAVS, MECP2, MSTN, MYC, NFATC4, NGFR, NLRC5, NOD2, NODAL, NOS3, NOTCH1, NOVA2, NPPA, NPPB, NPR3, NPRL3, NR1D1, NRARP, NTN1, NUTF2, OMA1, PDGFB, PLAU, PLAUR, PLXNA3, PML, PPP1R11, PTN, PTPN23, PTPRU, RAB11FIP5, RAI1, RARA, RELB, RGCC, RGMA, RMRP, SEMA3F, SEMA4B, SEMA4C, SEMA6B, SEMA6C, SERPINE1, SERPING1, SH2B3, SMAD3, SOX10, SOX8, SPI1, SRF, SYNGAP1, TBX5, TFE3, THBS1, TIMP1, TLE3, TNF, TNFRSF1A, TNMD, TRIB1, TRIM62, TSKU, TSPAN8, UBE2L6, ULK1, VASN, VSIR, VTN, WNT3, WNT9B, ZBTB7B, ZC3H12A, ZFP36]
GO:0001816	cytokine production	6,35E-20	[2, 5]	213,00	26,66	59,72	40,28	[ACP5, ADAM8, ADCY7, AIF1, AIM2, ALOX15B, ANGPT1, BANK1, BATF, BCL3, BTK, BTN3A1, BTN3A2, CAMK4, CARD11, CARD17, CASP8, CCDC88B, CCL19, CCL3, CCR2, CD2, CD200R1, CD226, CD244, CD33, CD3E, CD4, CD40LG, CD6, CD74, CD80, CD84, CD86, CD96, CLCF1, CLEC6A, CLNK, CRTAM, CSF1R, CXCL6, CYBB, EBI3, EPX, EREG, FCN1, FFAR4, FGR, FOXP3, GBP5, GPSM3, HLA-DPA1, HLA-DPB1, HLA-DRB1, IFNG, IKBKE, IL12RB1, IL12RB2, IL16, IL18, IL26, IL5RA, INPP5D, IRF4, IRF5, IRF8, ITK, LAG3, LCP1, LEF1, LILRB1, LILRB4, LPAL2, LTA, LTB, LY9, MAPK13, MCOLN2, MOG, MYB, NCKAP1L, NFAM1, NLRC3, NLRP2, NLRP3, NLRP6, NLRP7, NLRP9, NR4A3, ORM2, PIK3CG, PLD4, POU2AF1, POU2F2, PRKCQ, PTAFR, PTPN22, PTPN6, PTPRC, PYCARD, PYHIN1, SASH3, SCIMP, SIGLEC16, SLAMF6, SOCS1, STAT1, SYK, TBX21, THBS1, TIGIT, TLR10, TLR6, TLR8, TNFAIP3, TREM2, UBASH3A, XCL1, ZBP1]	[ABCC8, ABCD1, ABCD2, ABL1, ACKR1, ADGRG1, ADIPOQ, AGER, AGPAT1, AGT, AMH, ANGPT1, APOA1, APOD, BCL3, BCL6, C5, C5AR1, CCR7, CD14, CD276, CD74, CEACAM1, CHI3L1, CLC, CLCF1, CLNK, CPTP, CRP, CSK, CX3CL1, EPHA2, FADD, FFAR2, FLT4, FRMD8, FURIN, GATA4, GBA, GIT1, GPAM, HDAC7, HFE, HK1, HSPB1, IGF2BP2, IL1B, IL4R, IL5RA, INHA, JAK3, JPH4, LAG3, LGALS9, LILRA5, LRRC32, LTF, LUM, MAP2K3, MAST2, MAVS, NFATC4, NFKB2, NLRC5, NLRP9, NOD2, NODAL, NR4A3, NUTF2, OSM, PER1, PLD3, PML, POLR2E, POLR2L, POSTN, PPP1R11, PQBP1, RARA, RELA, RELB, RGCC, RNF26, RNU1-93P, SERPINE1, SLC7A5, SMAD3, SPHK1, SPHK2, SRC, STAT3, STING1, THBS1, TICAM1, TNF, TRPV4, TSKU, UBE2L6, VSIR, ZBTB7B, ZC3H12A, ZCCHC3, ZFP36, ZNF580]
GO:0001817	regulation of cytokine production	4,02E-20	[3, 4, 5, 6]	212,00	26,77	59,95	40,05	[ACP5, ADAM8, ADCY7, AIF1, AIM2, ALOX15B, ANGPT1, BANK1, BATF, BCL3, BTK, BTN3A1, BTN3A2, CAMK4, CARD11, CARD17, CASP8, CCDC88B, CCL19, CCL3, CCR2, CD2, CD200R1, CD226, CD244, CD33, CD3E, CD4, CD40LG, CD6, CD74, CD80, CD84, CD86, CD96, CLCF1, CLEC6A, CLNK, CRTAM, CSF1R, CXCL6, CYBB, EBI3, EPX, EREG, FCN1, FFAR4, FGR, FOXP3, GBP5, GPSM3, HLA-DPA1, HLA-DPB1, HLA-DRB1, IFNG, IKBKE, IL12RB1, IL12RB2, IL16, IL18, IL26, IL5RA, INPP5D, IRF4, IRF5, IRF8, ITK, LAG3, LCP1, LEF1, LILRB1, LILRB4, LPAL2, LTA, LTB, LY9, MAPK13, MCOLN2, MOG, MYB, NCKAP1L, NFAM1, NLRC3, NLRP2, NLRP3, NLRP6, NLRP7, NLRP9, NR4A3, ORM2, PIK3CG, PLD4, POU2AF1, POU2F2, PRKCQ, PTAFR, PTPN22, PTPN6, PTPRC, PYCARD, PYHIN1, SASH3, SCIMP, SIGLEC16, SLAMF6, SOCS1, STAT1, SYK, TBX21, THBS1, TIGIT, TLR10, TLR6, TLR8, TNFAIP3, TREM2, UBASH3A, XCL1, ZBP1]	[ABCC8, ABCD1, ABCD2, ABL1, ACKR1, ADIPOQ, AGER, AGPAT1, AGT, AMH, ANGPT1, APOA1, APOD, BCL3, BCL6, C5, C5AR1, CCR7, CD14, CD276, CD74, CEACAM1, CHI3L1, CLC, CLCF1, CLNK, CPTP, CRP, CSK, CX3CL1, EPHA2, FADD, FFAR2, FLT4, FRMD8, FURIN, GATA4, GBA, GIT1, GPAM, HDAC7, HFE, HK1, HSPB1, IGF2BP2, IL1B, IL4R, IL5RA, INHA, JAK3, JPH4, LAG3, LGALS9, LILRA5, LRRC32, LTF, LUM, MAP2K3, MAST2, MAVS, NFATC4, NFKB2, NLRC5, NLRP9, NOD2, NODAL, NR4A3, NUTF2, OSM, PER1, PLD3, PML, POLR2E, POLR2L, POSTN, PPP1R11, PQBP1, RARA, RELA, RELB, RGCC, RNF26, RNU1-93P, SERPINE1, SLC7A5, SMAD3, SPHK1, SPHK2, SRC, STAT3, STING1, THBS1, TICAM1, TNF, TRPV4, TSKU, UBE2L6, VSIR, ZBTB7B, ZC3H12A, ZCCHC3, ZFP36, ZNF580]

GO:0032612	interleukin-1 production	2,00E-05	[3, 6]	41,00	34,17	62,34	37,66	[ACP5, AIM2, CARD17, CASP8, CCL19, CCL3, CD33, FFAR4, GBP5, IFNG, IL16, LCP1, LILRB4, NLRP2, NLRP3, NLRP7, ORM2, PYCARD, PYHIN1, TLR6, TLR8, TNFAIP3, TREM2]	[AGER, APOA1, CCR7, CEACAM1, CPTP, CX3CL1, GIT1, HK1, HSPB1, LGALS9, LILRA5, NOD2, PML, SMAD3, SPHK1, STAT3, TNF, ZC3H12A]
GO:0032635	interleukin-6 production	5,93E-03	[3, 6]	42,00	28,38	68,62	31,38	[AIF1, BANK1, CD200R1, CD74, EREG, FOXP3, IFNG, IL16, INPP5D, LCP1, LILRB4, MAPK13, NCKAP1L, NLRC3, POU2AF1, POU2F2, PTAFR, PTPN22, PTPN6, PYCARD, SCIMP, SIGLEC16, SYK, TLR6, TLR8, TNFAIP3, TREM2]	[AGER, CD74, CSK, CX3CL1, GBA, IL1B, LGALS9, LILRA5, MAVS, NOD2, SPHK2, STAT3, TICAM1, TNF, TRPV4, ZC3H12A]
GO:0071706	tumor necrosis factor superfamily cytokine production	1,34E-03	[3, 6]	49,00	28,00	58,32	41,68	[ACP5, ADAM8, ANGPT1, BCL3, CCL19, CCL3, CCR2, CD2, CD33, CD86, CYBB, FOXP3, IFNG, LCP1, LILRB1, NLRC3, ORM2, PTAFR, PTPN22, PTPN6, PTPRC, PYCARD, SASH3, SYK, THBS1, TNFAIP3, TREM2]	[ABCC8, ADIPOQ, AGER, AMH, ANGPT1, BCL3, CD14, CX3CL1, FADD, FRMD8, HSPB1, LGALS9, LILRA5, LTF, MAVS, NFATC4, NOD2, RARA, SPHK2, STAT3, THBS1, TICAM1, VSIR, ZC3H12A, ZFP36]
GO:0001818	negative regulation of cytokine production	9,12E-06	[3, 4, 5, 6, 7]	79,00	26,42	54,65	45,35	[ACP5, ADCY7, ANGPT1, BANK1, BCL3, BTK, CARD17, CD200R1, CD33, CD84, CD96, EPX, FFAR4, FOXP3, HLA-DRB1, IFNG, IKBKE, INPP5D, LAG3, LEF1, LILRB1, LILRB4, LPAL2, NCKAP1L, NLRC3, NLRP3, NLRP6, NLRP7, PTPN22, PTPN6, PTPRC, PYCARD, TBX21, THBS1, TIGIT, TLR6, TLR8, TNFAIP3, TREM2, XCL1]	[ABCD1, ABCD2, ADIPOQ, AGER, ANGPT1, APOA1, APOD, BCL3, BCL6, CCR7, CEACAM1, CPTP, CSK, CX3CL1, EPHA2, FURIN, GBA, GIT1, HDAC7, HFE, INHA, JAK3, LAG3, LGALS9, LILRA5, LRRC32, LTF, MAVS, NLRC5, NOD2, NUTF2, PML, PPP1R11, RARA, RELB, RGCC, THBS1, TNF, TSKU, UBE2L6, VSIR, ZC3H12A, ZFP36]
GO:0032611	interleukin-1 beta production	8,13E-07	[4, 7]	39,00	38,61	62,64	37,36	[ACP5, AIM2, CARD17, CASP8, CCL19, CCL3, CD33, FFAR4, GBP5, IFNG, LCP1, LILRB4, NLRP2, NLRP3, NLRP7, ORM2, PYCARD, PYHIN1, TLR6, TLR8, TNFAIP3, TREM2]	[AGER, APOA1, CCR7, CPTP, CX3CL1, GIT1, HK1, HSPB1, LGALS9, LILRA5, NOD2, PML, SMAD3, SPHK1, STAT3, TNF, ZC3H12A]
GO:0032640	tumor necrosis factor production	2,81E-03	[4, 7]	47,00	27,81	56,44	43,56	[ACP5, ANGPT1, BCL3, CCL19, CCL3, CCR2, CD2, CD33, CYBB, FOXP3, IFNG, LCP1, LILRB1, NLRC3, ORM2, PTAFR, PTPN22, PTPN6, PTPRC, PYCARD, SASH3, SYK, THBS1, TNFAIP3, TREM2]	[ABCC8, ADIPOQ, AGER, AMH, ANGPT1, BCL3, CD14, CX3CL1, FADD, FRMD8, HSPB1, LGALS9, LILRA5, LTF, MAVS, NFATC4, NOD2, RARA, SPHK2, STAT3, THBS1, TICAM1, VSIR, ZC3H12A, ZFP36]
GO:0032652	regulation of interleukin-1 production	2,00E-05	[4, 5, 6, 7]	41,00	34,17	62,34	37,66	[ACP5, AIM2, CARD17, CASP8, CCL19, CCL3, CD33, FFAR4, GBP5, IFNG, IL16, LCP1, LILRB4, NLRP2, NLRP3, NLRP7, ORM2, PYCARD, PYHIN1, TLR6, TLR8, TNFAIP3, TREM2]	[AGER, APOA1, CCR7, CEACAM1, CPTP, CX3CL1, GIT1, HK1, HSPB1, LGALS9, LILRA5, NOD2, PML, SMAD3, SPHK1, STAT3, TNF, ZC3H12A]
GO:0032675	regulation of interleukin-6 production	5,93E-03	[4, 5, 6, 7]	42,00	28,38	68,62	31,38	[AIF1, BANK1, CD200R1, CD74, EREG, FOXP3, IFNG, IL16, INPP5D, LCP1, LILRB4, MAPK13, NCKAP1L, NLRC3, POU2AF1, POU2F2, PTAFR, PTPN22, PTPN6, PYCARD, SCIMP, SIGLEC16, SYK, TLR6, TLR8, TNFAIP3, TREM2]	[AGER, CD74, CSK, CX3CL1, GBA, IL1B, LGALS9, LILRA5, MAVS, NOD2, SPHK2, STAT3, TICAM1, TNF, TRPV4, ZC3H12A]
GO:1903555	regulation of tumor necrosis factor superfamily cytokine production	1,34E-03	[4, 5, 6, 7]	49,00	28,00	58,32	41,68	[ACP5, ADAM8, ANGPT1, BCL3, CCL19, CCL3, CCR2, CD2, CD33, CD86, CYBB, FOXP3, IFNG, LCP1, LILRB1, NLRC3, ORM2, PTAFR, PTPN22, PTPN6, PTPRC, PYCARD, SASH3, SYK, THBS1, TNFAIP3, TREM2]	[ABCC8, ADIPOQ, AGER, AMH, ANGPT1, BCL3, CD14, CX3CL1, FADD, FRMD8, HSPB1, LGALS9, LILRA5, LTF, MAVS, NFATC4, NOD2, RARA, SPHK2, STAT3, THBS1, TICAM1, VSIR, ZC3H12A, ZFP36]
GO:0032692	negative regulation of interleukin-1 production	1,43E-02	[4, 5, 6, 7, 8]	16,00	44,44	62,49	37,51	[ACP5, CARD17, CD33, FFAR4, LILRB4, NLRP3, NLRP7, TNFAIP3, TREM2]	[APOA1, CEACAM1, CPTP, CX3CL1, GIT1, PML, ZC3H12A]
GO:0032651	regulation of interleukin-1 beta production	8,13E-07	[5, 6, 7, 8]	39,00	38,61	62,64	37,36	[ACP5, AIM2, CARD17, CASP8, CCL19, CCL3, CD33, FFAR4, GBP5, IFNG, LCP1, LILRB4, NLRP2, NLRP3, NLRP7, ORM2, PYCARD, PYHIN1, TLR6, TLR8, TNFAIP3, TREM2]	[AGER, APOA1, CCR7, CPTP, CX3CL1, GIT1, HK1, HSPB1, LGALS9, LILRA5, NOD2, PML, SMAD3, SPHK1, STAT3, TNF, ZC3H12A]
GO:0032680	regulation of tumor necrosis factor production	2,81E-03	[5, 6, 7, 8]	47,00	27,81	56,44	43,56	[ACP5, ANGPT1, BCL3, CCL19, CCL3, CCR2, CD2, CD33, CYBB, FOXP3, IFNG, LCP1, LILRB1, NLRC3, ORM2, PTAFR, PTPN22, PTPN6, PTPRC, PYCARD, SASH3, SYK, THBS1, TNFAIP3, TREM2]	[ABCC8, ADIPOQ, AGER, AMH, ANGPT1, BCL3, CD14, CX3CL1, FADD, FRMD8, HSPB1, LGALS9, LILRA5, LTF, MAVS, NFATC4, NOD2, RARA, SPHK2, STAT3, THBS1, TICAM1, VSIR, ZC3H12A, ZFP36]
GO:0050793	regulation of developmental process	1,56E-12	[2, 3]	488,00	18,90	38,56	61,44	[ADAM8, ADAMTS9, ALOX15B, ANGPT2, AREG, ASCL2, BCL11B, BHLHA15, BHLHE40, BMP7, BMPR1B, BTK, C1QC, CAMK4, CARD11, CASP8, CASS4, CCL17, CCL19, CCL2, CCL24, CCL3, CCR2, CCR3, CD2, CD27, CD4, CD53, CD74, CD80, CD86, CDH4, CDKN2A, CLCF1, CMTM5, CORO1A, CRABP2, CRB2, CRTAM, CSF1R, CST7, CTLA4, CXCL10, CXCL9, CXCR4, CYBB, DCC, DPEP1, EAF2, EGR3, ELAVL4, EPX, EREG, ERMN, EVI2B, FASLG, FCRL3, FFAR4, FGD2, FGD3, FGL2, FGR, FOXP3, FRZB, GDF6, GLIS1, GPR171, GPR4, GPR55, H3C10, H3C11, H3C12, H3C7, HCLS1, HLA-DOA, HLA-DRA, HLA-DRB1, HOPX, IFNG, IKZF3, IL12RB1, IL18, IL7R, INPP5D, IRF4, IRX3, ITGAX, ITGB2, KLF5, KLHL41, LAG3, LCP1, LDLR, LEF1, LIF, LILRB1, LILRB4, LMO1, LRRC17, LST1, LTA, MAFF, MEDAG, MIXL1, MMP9, MSX1, MUSK, MYB, MYCL, MYH6, NAMPT, NCKAP1L, NEK5, NFAM1, NLRP3, NPPB, NPPC, NR4A3, NTN1, P2RY12, PEAK3, PIM1, PLAAT4, PLAC8, PLXNC1, PRDM1, PRKCB, PRKCQ, PROK1, PTK2B, PTPN6, PTPRC, RAC2, RBPMS2, RHEX, RHOH, RLN2, RUNX3, SASH3, SEMA4A, SEMA4D, SLAMF8, SLC18A1, SMPD3, SOCS1, SOCS3, STAT1, SULT2B1, SYK, TACSTD2, TBX21, TESPA1, THBS1, THY1, TIFAB, TNFAIP3, TNFRSF11B, TNFRSF18, TNFSF13B, TNMD, TNR, TOX, TREM2, VNN1, VWC2L, WNT1, WNT10A, WNT10B, ZAP70, ZBED2, ZNF683]	[ABCC8, ABI3, ABL1, ACACB, ACTN4, ADAMTS7, ADAMTS9, ADCK1, ADGRB2, ADGRL1, ADIPOQ, AGAP2, AGER, AGT, AGTR1, ALDOA, AMH, ANGPTL4, AP2A1, AP3D1, APLNR, APOA1, AQP3, ARC, AREG, ARF1, ARHGDIA, ARHGEF15, ASPN, ATAT1, BAG6, BBS12, BCL6, BCL9L, BCOR, BEND6, BHLHE40, C11orf65, C15orf62, C5, C5AR1, CAMK2B, CARM1, CASZ1, CCL11, CCL2, CCL24, CD74, CDC42EP1, CDC42EP2, CDC42EP4, CDH4, CDK1, CDKN1A, CEACAM1, CELSR2, CETP, CFL1, CGA, CHERP, CHGA, CHI3L1, CITED2, CLCF1, CLPTM1, CMTM5, CNOT3, COL1A1, CPNE5, CPNE6, CREB3L1, CRP, CSF1, CSNK1E, CST7, CTDSP1, CTHRC1, CX3CL1, CYP26B1, CYP27B1, DAB2IP, DAG1, DDX39B, DISP3, DLG5, DMTN, DNMT1, DPF2, DTX1, DVL3, DXO, EAF2, ECM1, EEF1E1, EFEMP2, EFNA3, EHD1, EHD2, EIF4G1, ENG, EPHA2, EPN1, EPPK1, ERBB2, FADD, FBLIM1, FES, FLOT2, FLT4, FMNL1, FOXC2, FOXM1, FOXS1, GATA2, GATA4, GDF6, GDI1, GNB3, GPAM, GPER1, GPR137, GPR37L1, GPR4, GRN, GSK3A, H3C10, H3C12, H4C3, HDAC5, HDAC7, HESX1, HEYL, HGS, HMGA1, HMGN1, HOPX, HSF1, HSPB1, HSPG2, HTR2A, HYAL1, ICAM1, ID3, IFITM1, IL15RA, IL1B, IL4R, IL7R, ILK, INHA, IRX3, ITGA5, ITGA7, JAK3, JMJD8, JUNB, JUND, JUP, KCNK2, KCTD11, KLF10, KLF5, KMT2D, LAG3, LAMA5, LDLR, LGALS9, LIF, LIMK1, LIMS2, LINGO1, LMNA, LMX1A, LOX, LRCH4, LRG1, LRRC17, LRRC8A, LTF, LZTS1, LZTS3, MAFG, MAMSTR, MAPK7, MAPK8IP3, MARK2, MCRIP1, MECP2, MED12, MEGF8, MFSD2A, MME, MOV10, MSN, MSTN, MSX1, MYADM, MYC, MYH6, MYH7B, MYH9, MYL9, MYO19, MYRF, NAP1L2, NEFL, NEK5, NF2, NFATC4, NFE2, NGF, NGFR, NINJ1, NLGN2, NODAL, NOS3, NOTCH1, NOTCH3, NOTCH4, NPPB, NR1D1, NR4A3, NRARP, NSMF, NTN1, NUMBL, OMA1, OMD, OSM, OSR2, PACSIN1, PAF1, PAK4, PALM, PALM2AKAP2, PARP6, PARVB, PDGFB, PDLIM7, PFN1, PHLDB1, PIEZO1, PIM1, PKM, PLA2G2A, PLEKHB1, PLEKHO1, PLXNA1, PLXNA3, PLXNB1, PML, POR, POSTN, PPARD, PPP1R13L, PPP2R1A, PQBP1, PRAG1,

GO:0051239	regulation of multicellular organismal process	4,67E-22	[2, 3]	570,00	20,09	43,33	56,67	[ACP5, ADAM8, ADAMTS9, ADCY7, ADORA3, AIF1, AIM2, ALOX15B, ANGPT1, ANGPT2, AREG, ASCL2, ATP2A3, BANK1, BATF, BCL3, BHLHE40, BMP7, BMPR1B, BTK, BTN3A1, BTN3A2, C1QC, C1QTNF1, CALCR, CAMK4, CARD11, CARD17, CASP8, CCDC88B, CCL19, CCL24, CCL3, CCR2, CCR3, CD2, CD200R1, CD226, CD244, CD27, CD33, CD3E, CD4, CD40LG, CD6, CD74, CD80, CD84, CD86, CD96, CDH4, CDKN2A, CEL, CLCF1, CLEC6A, CLNK, CNN1, CNR1, CRABP2, CRB2, CRTAM, CSF1R, CST7, CTLA4, CXCL10, CXCL6, CXCR4, CYBB, DCC, DLGAP2, DPEP1, EAF2, EBI3, EDN2, EGR3, EPX, EREG, EVI2B, FASLG, FCN1, FCRL3, FFAR4, FGL2, FGR, FOXP3, FRZB, GBP5, GDF6, GPR171, GPR4, GPR55, GPSM3, H3C10, H3C11, H3C12, H3C7, HCLS1, HLA-DOA, HLA-DPA1, HLA-DPB1, HLA-DRA, HLA-DRB1, HOPX, IFNG, IKBKE, IKZF3, IL12RB1, IL12RB2, IL16, IL18, IL26, IL5RA, IL7R, INPP5D, IRF4, IRF5, IRF8, IRX3, ITGAX, ITGB2, ITK, KCNK15, KNG1, LAG3, LCP1, LDLR, LEF1, LIF, LILRB1, LILRB4, LMCD1, LPAL2, LRRC17, LTA, LTB, LY9, MAFF, MAPK13, MCOLN2, MMP9, MOG, MYB, MYCL, MYH6, NCKAP1L, NELL2, NFAM1, NLRC3, NLRP2, NLRP3, NLRP6, NLRP7, NLRP9, NPPB, NPPC, NPTX2, NR4A2, NR4A3, NTN1, ORM2, P2RY2, PIK3CG, PIM1, PLAAT4, PLAC8, PLAU, PLD4, PLXNC1, POU2AF1, POU2F2, PRDM1, PRKCB, PRKCQ, PROK1, PTAFR, PTGER2, PTK2B, PTPN22, PTPN6, PTPRC, PYCARD, PYHIN1, RHEX, RHOH, RLN2, RUNX3, SASH3, SCIMP, SEMA4A, SEMA4D, SIGLEC16, SLAMF6, SLAMF8, SLC8A3, SMPD3, SMTNL2, SOCS1, STAT1, SULT2B1, SYK, TACSTD2, TBX21, TBXAS1, TESPA1, THBS1, THY1, TIFAB, TIGIT, TLR10, TLR6, TLR8, TNFAIP3, TNFRSF11B, TNFRSF18, TNMD, TNR, TOX, TREM2, TSPAN8, UBASH3A, UCP2, VNN1, WNT1, WNT10B, XCL1, ZAP70, ZBED2, ZBP1, ZNF683]	[ABCA7, ABCC8, ABCD1, ABCD2, ABL1, ACACB, ACKR1, ADAMTS5, ADAMTS7, ADAMTS9, ADGRB2, ADIPOQ, AGAP2, AGER, AGPAT1, AGT, AGTR1, AMH, ANGPT1, ANGPTL4, AP3D1, APLN, APLNR, APOA1, APOD, APOM, AQP3, AREG, ARHGDIA, ARRDC3, ASPN, ATP1A1, ATP1A3, ATP1B2, ATPSCKMT, BAG6, BCAR1, BCL3, BCL6, BCL9L, BCOR, BHLHE40, C1QTNF1, C5, C5AR1, CACNA1H, CALCR, CAMK2B, CCL11, CCL24, CCR7, CD14, CD276, CD74, CDH4, CDK1, CEACAM1, CEL, CFH, CFL1, CGA, CHGA, CHI3L1, CITED2, CLC, CLCF1, CLNK, CLPTM1, CNN1, CNOT3, CNR1, COL1A1, CPTP, CREB3L1, CRP, CSF1, CSK, CST7, CTDSP1, CX3CL1, CYP26B1, CYP27B1, DAB2IP, DAG1, DDX39B, DHX34, DLG5, DLGAP4, DMTN, DTX1, DXO, EAF2, ECM1, EDN2, EFNA3, ENG, EPHA2, EPN1, EPPK1, ERBB2, FA2H, FABP4, FADD, FAM110A, FAP, FES, FFAR2, FLT4, FOXC2, FOXS1, FRMD8, FURIN, FXYD6, GATA2, GATA4, GBA, GDF6, GDI1, GIT1, GJD3, GLRA1, GNAI2, GNAO1, GPAM, GPER1, GPI, GPR137, GPR3, GPR37L1, GPR4, GPRIN1, GRN, GSK3A, GTF2IRD1, H3C10, H3C12, H4C3, HBEGF, HCN4, HDAC5, HDAC7, HESX1, HEYL, HFE, HGS, HK1, HOPX, HRH2, HSF1, HSPB1, HSPB7, HSPG2, HTR2A, HYAL1, ICAM1, IGF2BP2, IL15RA, IL1B, IL4R, IL5RA, IL7R, ILK, INHA, IRX3, ITGA3, ITGA5, ITPR3, JAK3, JMJD8, JPH4, JUP, KCNH2, KCNIP2, KCNJ5, KCNK15, KCNK2, KCNK3, KCNK6, KCNMB2, KCTD11, KDM6B, KLF10, KMT2D, KNG1, LAG3, LAMA5, LCAT, LDLR, LDLRAP1, LGALS9, LIF, LILRA5, LIMK1, LINGO1, LIPG, LMCD1, LMNA, LOX, LRCH4, LRG1, LRRC17, LRRC32, LTF, LUM, MAFG, MAP2K3, MAPK3, MAPK7, MAST2, MAVS, MECP2, MED12, MEGF8, MFAP2, MFSD2A, MME, MOV10, MSTN, MYBPH, MYC, MYH6, MYL9, MYRF, NEFL, NF2, NFATC4, NFE2, NFKB2, NGF, NGFR, NINJ1, NLGN2, NLRC5, NLRP9, NOD2, NODAL, NOS3, NOTCH1, NOTCH4, NOVA2, NPAS2, NPPA, NPPB, NPR3, NPRL3, NR1D1, NR4A2, NR4A3, NRARP, NTN1, NUMBL, NUTF2, OMA1, OMD, OSM, OSR2, P2RX3, P2RY2, PAF1, PAK4, PARP6, PCDHA4, PDGFB
GO:0002684	positive regulation of immune system process	4,58E-20	[2, 3, 4]	281,00	24,16	64,49	35,51	[ADAM8, AIF1, AIM2, BLK, BLNK, BTK, BTLA, BTN3A1, BTN3A2, C1QA, C1QB, C1QC, C2, C8G, CAMK4, CARD11, CASP8, CCDC88B, CCL19, CCL2, CCL24, CCL3, CCL4, CCL5, CCR2, CD177, CD19, CD1B, CD1C, CD1E, CD2, CD22, CD226, CD244, CD247, CD27, CD3D, CD3E, CD3G, CD4, CD40LG, CD5, CD6, CD74, CD79A, CD80, CD84, CD86, CFP, CLCF1, CLEC10A, CLEC4D, CLEC4E, CLEC6A, CLNK, CNR1, CORO1A, CR2, CRTAM, CSF1R, CTLA4, CXCL10, CXorf21, CYP11B1, DOCK8, EBI3, EDN2, EGR3, EPX, EREG, EVI2B, FCN1, FCN3, FCRL3, FGR, FOXP3, FYB1, GBP5, GPSM3, GRAP2, HCLS1, HLA-DMB, HLA-DPA1, HLA-DPB1, HLA-DQA1, HLA-DRA, HLA-DRB1, HLA-DRB5, ICOS, IFNG, IGLL5, IKBKE, IL12RB1, IL18, IL18RAP, IL7R, INPP5D, ITGA4, ITGB2, ITK, KCNN4, KLRC2, KLRD1, KLRK1, LAG3, LAX1, LCK, LCP2, LEF1, LILRB1, LILRB4, LTA, MOG, MS4A1, MUC16, MUC19, MUC6, MYB, MYO1G, MZB1, NCKAP1L, NCR3, NFAM1, NLRP3, NR4A3, P2RY12, PAX5, PLA2G7, PRAM1, PRKCB, PRKCQ, PTAFR, PTK2B, PTPN22, PTPN6, PTPRC, PYCARD, PYHIN1, RAC2, RASAL3, RHOH, RUNX3, SASH3, SCIMP, SH2D1A, SIGLEC16, SIRPG, SKAP1, SLA2, SLAMF6, SOCS1, SPN, STAP1, STXBP2, SYK, TBX21, TESPA1, THBS1, THEMIS, THEMIS2, THY1, TLR6, TLR8, TNFRSF18, TNFSF13B, TNFSF14, TOX, TRAT1, TREM2, UBASH3A, VAV1, VNN1, WAS, XCL1, XCL2, XG, ZAP70, ZBP1]	[ABL1, ACTB, AGER, AMH, AP3D1, AQP3, BAG6, BCAR1, BCL6, BTLA, C5, C5AR1, C8G, CCL2, CCL21, CCL24, CCR7, CD177, CD1C, CD1E, CD276, CD74, CDKN1A, CEACAM1, CFH, CLCF1, CLEC4E, CLNK, CNR1, CPN2, CRP, CSF1, CSK, CX3CL1, DGKZ, EDN2, ENPP3, FADD, FCN3, FES, FFAR2, FLOT2, FOXF1, FPR1, GAB2, GATA2, GCSAM, GNAO1, GPAM, GPI, GRN, HAVCR1, HK1, HSF1, ICAM1, IL15RA, IL1B, IL4R, IL7R, JAK3, JUND, KLF10, LAG3, LGALS9, LIMK1, MAPK3, MAVS, MSTN, MUC19, MUC20, MUC3A, MYO18A, NCOR2, NECTIN2, NLRC5, NOD2, NPPA, NR4A3, PCDHA4, PDCD1, PLA2G7, PLVAP, PQBP1, PRKACA, PTN, PVR, RAC2, RAP1A, RARA, RBM14, RELA, RELB, RGCC, RHBDF2, RNU1-93P, SELENOP, SERPINE1, SERPING1, SH2B2, SLC7A1, SLC7A5, SLC7A8, SOX12, SOX13, SPHK2, SPI1, SRC, STING1, THBS1, TICAM1, TIMP1, TNF, TRIB1, TRIM62, TRPV4, VEGFA, VNN1, VSIR, VTN, WAS, XG, ZBTB7B, ZC3H12A, ZCCHC3, ZMIZ1, ZNF335, ZNF580]

GO:0009893	positive regulation of metabolic process	1,11E-07	[2, 3, 4]	669,00	16,88	38,30	61,70	[ABRA, ADAM8, ADCY7, AIF1, AIM2, AKNA, ALOX15B, ANGPT1, AREG, ASCL2, ATF3, ATP1B4, ATP2A3, BANK1, BATF, BCL11B, BCL3, BHLHA15, BLNK, BMP7, BMPR1B, BRIP1, BTN3A1, BTN3A2, C1QTNF1, CALCR, CAMK4, CARD11, CASP8, CASS4, CCDC88B, CCL19, CCL2, CCL3, CCL5, CCR2, CD177, CD19, CD2, CD226, CD244, CD33, CD3E, CD4, CD40LG, CD6, CD74, CD80, CD86, CDC20B, CDCA2, CDH1, CDKN2A, CLCF1, CLEC6A, CLNK, CREM, CRTAM, CSF1R, CXCL10, CXCR4, CYBB, DAPK1, DEPDC1B, DRD1, E2F8, EAF2, EBI3, EGR3, ELAVL4, ELF3, EOMES, EPHA6, EPX, EREG, FASLG, FCN1, FCRL3, FFAR4, FGD2, FGR, FLT3, FOSB, FOSL1, FOXP3, GADD45B, GBP5, GDF6, GLIS1, GPRC5A, GPSM3, H1-3, HCLS1, HLA-DPA1, HLA-DPB1, HLA-DRB1, IFNG, IKBKE, IKZF3, IL12RB1, IL12RB2, IL16, IL18, IL26, IL7R, IRF4, IRF5, IRF8, IRX3, ITGAX, ITGB2, ITK, JCHAIN, KIAA1324, KLF5, KLRG1, LAMP3, LCK, LCP1, LCP2, LDLR, LEF1, LIF, LILRB1, LMO1, LPAL2, LTA, LTB, LY9, MACC1, MAFF, MAP4K1, MAPK13, MCOLN2, MID1IP1, MIXL1, MMP9, MSX1, MUSK, MYB, MYBL2, MZB1, NAMPT, NCF1, NCKAP1L, NEK5, NFAM1, NLRP2, NLRP3, NLRP6, NLRP9, NR4A1, NR4A2, NR4A3, NSG1, ORM2, P2RY12, PATL2, PAX5, PBX4, PDE6G, PFKFB4, PIK3CG, PIK3R5, PIM1, PIM2, PLAC8, PLEK, POU2AF1, POU2F2, PRDM1, PRKCB, PRKCQ, PROK1, PTAFR, PTGFR, PTK2B, PTPN22, PTPRC, PTX3, PYCARD, PYHIN1, RLN2, RUFY4, RUNX3, SALL1, SASH3, SCIMP, SELE, SEMA4D, SH2D1A, SH2D2A, SIGLEC16, SKAP1, SLAMF6, SMPD3, SOCS1, SOCS3, SPIB, SPIC, STAP1, STAT1, STXBP2, SYK, TBX21, TENT5C, TFEC, THBS1, TIGIT, TLR6, TLR8, TNFAIP3, TNFRSF18, TNFSF13B, TNFSF8, TNIP3, TOX, TRABD2A, TREM2, TRERF1, TRIM14, UCP2, WAS, WNT1, WNT10A, WNT10B, XCL1, ZBP1, ZC3H12D, ZNF804A]	[ABCC8, ABCD1, ABCD2, ABL1, ABLIM3, ABRA, ACACB, ACTA1, ACTB, ACTC1, ACTN1, ACTN4, ADAM19, ADCY1, ADCY3, ADCY4, ADCY9, ADIPOQ, AGAP2, AGER, AGPAT1, AGT, AGTR1, ALPK3, AMH, ANGPT1, AP3D1, APLN, APLNR, APOA1, ARAF, AREG, ARHGEF5, ARID1A, ARTN, ATF5, ATP13A2, ATP1B4, BAG3, BAG6, BCL3, BCL6, BCL9L, BMP8A, BRD4, BRPF3, C1QTNF1, C5, C5AR1, CALCR, CAMTA2, CARM1, CASZ1, CCL2, CCL21, CCR7, CD14, CD177, CD276, CD74, CDC25B, CDCA2, CDK1, CDKN1A, CFH, CGA, CHCHD10, CHERP, CHGA, CHI3L1, CIITA, CITED2, CITED4, CIZ1, CLCF1, CLIP3, CLNK, CNOT3, COL1A1, CREB3L1, CRP, CRTC2, CSF1, CSK, CSNK1E, CSPG4, CSRNP3, CTC1, CTF1, CTIF, CTSD, CX3CL1, CYP26B1, CYP27B1, DAB2IP, DAG1, DAXX, DBF4B, DDR1, DDX39B, DGKZ, DHX34, DIRAS1, DISP3, DNMT1, DPAGT1, DPF2, DTX1, DUSP5, DVL3, DXO, DYRK1B, E2F4, E2F7, E2F8, EAF2, EDC4, EFNA3, EGLN2, EIF4G1, ELF3, ELF4, ELK1, ENDOG, ENG, EPHA2, ERBB2, ETS2, ETV6, FABP4, FADD, FASN, FFAR2, FGFR4, FHL5, FHOD1, FLT3, FLT4, FOSL1, FOSL2, FOXC2, FOXF1, FOXM1, FPR1, FRMD8, FURIN, GADD45B, GATA2, GATA4, GBA, GDF6, GGA1, GGA3, GLIS2, GLMP, GNAI2, GPER1, GPI, GPR3, GPRC5A, GPRIN1, GPT, GRN, GSK3A, GTPBP1, H1-9P, HBB, HBEGF, HCFC1, HDAC10, HDAC5, HEYL, HFE, HGS, HK1, HLTF, HMGA1, HMGN1, HMGN5, HPRT1, HSF1, HSPB1, HSPB8, HSPE1, HTR2A, HVCN1, ICAM1, ID3, IL1B, IL4R, IL7R, ILK, INCENP, INHA, IRAK2, IRF2, IRF2BPL, IRX3, IRX6, ITGA3, ITGA5, JUNB, JUND, JUP, KCNH2, KDM6B, KLF10, KLF5, KMT2D, KSR1, LDLR, LDLRAP1, LGALS9, LIF, LILRA5, LIMK2, LMNA, LMX1A, LMX1B, LRRC32, LTF, LUM, MAFG, MAMSTR, MAP2K3, MAP3K10, MAP3K11, MAP3K14, MAP3K6, MAP3K9, MAPK3, MAPK7, MAPK8IP3, MARK2, MAVS, MAZ, MECP2, MED12, MED16, MED24, MED25, MEF2D, MEIS3P1, MEIS3P2, MEN1, MFAP2, MFSD2A, MID1IP1, MINK1, MLLT6, MLST8, MOV10, MPV17L2, MSN, MSTN, MSX1, MTCO2P12, MUC20, MYBBP1A, MYC, MYH9, MYRF
GO:0051094	positive regulation of developmental process	3,34E-08	[2, 3, 4]	267,00	20,09	37,54	62,46	[ADAM8, ALOX15B, ANGPT2, AREG, BMP7, BMPR1B, BTK, CASP8, CASS4, CCL19, CCL24, CCR3, CD27, CD4, CD53, CD74, CD80, CD86, CDH4, CDKN2A, CLCF1, CRABP2, CRB2, CXCL9, CXCR4, CYBB, EGR3, ELAVL4, EPX, EVI2B, FFAR4, FOXP3, GDF6, HCLS1, HLA-DRA, HLA-DRB1, HOPX, IFNG, IL12RB1, IL18, IL7R, INPP5D, IRX3, ITGAX, ITGB2, KLF5, LCP1, LEF1, LIF, LILRB4, LTA, MEDAG, MIXL1, MYB, NCKAP1L, NEK5, NLRP3, NPPC, NTN1, PIM1, PLAAT4, PLXNC1, PRKCB, PTK2B, PTPRC, RHEX, RHOH, RLN2, RUNX3, SASH3, SEMA4A, SEMA4D, SOCS1, SOCS3, STAT1, SULT2B1, SYK, TACSTD2, TBX21, TESPA1, THBS1, TNFSF13B, TOX, TREM2, VNN1, VWC2L, WNT1, WNT10B, ZAP70, ZBED2]	[ABI3, ABL1, ACACB, ADGRL1, ADIPOQ, AGAP2, AGER, AGT, AGTR1, ANGPTL4, AP3D1, APLNR, APOA1, AREG, ARF1, ARHGDIA, BCL6, BCL9L, BEND6, C5, C5AR1, CAMK2B, CARM1, CCL11, CCL24, CD74, CDH4, CDK1, CEACAM1, CFL1, CHI3L1, CITED2, CLCF1, COL1A1, CPNE5, CPNE6, CSF1, CSNK1E, CTHRC1, CX3CL1, CYP26B1, CYP27B1, DAB2IP, DAG1, DDX39B, DLG5, DMTN, DVL3, ECM1, EEF1E1, EFEMP2, EHD1, EHD2, EIF4G1, ENG, FADD, FES, FOXC2, FOXS1, GATA2, GATA4, GDF6, GDI1, GPAM, GPER1, GRN, HEYL, HMGA1, HOPX, HSF1, HSPB1, HTR2A, HYAL1, IFITM1, IL15RA, IL1B, IL4R, IL7R, ILK, IRX3, ITGA5, JMJD8, JUNB, JUND, JUP, KCTD11, KLF10, KLF5, LGALS9, LIF, LIMK1, LIMS2, LMNA, LRCH4, LRG1, LRRC8A, LTF, LZTS1, MAMSTR, MAPK8IP3, MED12, MEGF8, MME, MYADM, MYC, NAP1L2, NEFL, NEK5, NF2, NGF, NGFR, NINJ1, NLGN2, NODAL, NOS3, NOTCH1, NTN1, NUMBL, OMA1, OSR2, PACSIN1, PAK4, PARP6, PDGFB, PDLIM7, PHLDB1, PIEZO1, PIM1, PKM, PLA2G2A, PLXNA1, PLXNA3, PLXNB1, POR, PPARD, PPP1R13L, PTN, RAMP2, RAP1A, RARA, RELA, RGCC, RNF157, S100A1, S1PR2, SERPINE1, SHANK3, SMAD3, SNAI1, SOCS3, SOX10, SOX12, SOX13, SOX8, SPDEF, SPHK1, SRC, SRF, SRRT, SSBP3, STAT3, SYDE1, SYT2, TBX5, TCF3, TCF7L1, TEAD4, TESK1, TFE3, TGFB1I1, TGM2, THBS1, THPO, TIMP1, TMEM100, TNF, TNFRSF1A, TNFSF12, TRIB1, TRIOBP, VDR, VEGFA, VNN1, VSIG2, VSIR, WNT3, YPEL3, ZBTB7B, ZC3H12A, ZFP36, ZMIZ1, ZNF219, ZNF335, ZNF385A]

GO:0051240	positive regulation of multicellular organismal process	8,22E-15	[2, 3, 4]	325,00	21,42	48,82	51,18	[ADAM8, AIF1, AIM2, ALOX15B, ANGPT2, AREG, BATF, BCL3, BMP7, BMPR1B, BTK, BTN3A1, BTN3A2, C1QTNF1, CAMK4, CARD11, CASP8, CCDC88B, CCL19, CCL24, CCL3, CCR2, CCR3, CD2, CD226, CD244, CD27, CD3E, CD4, CD40LG, CD6, CD74, CD80, CD86, CDH4, CLCF1, CLEC6A, CLNK, CNR1, CRABP2, CRB2, CRTAM, CSF1R, CST7, CXCR4, CYBB, EBI3, EDN2, EGR3, EPX, EREG, EVI2B, FCN1, FFAR4, FGR, FOXP3, GBP5, GDF6, GPSM3, HCLS1, HLA-DPA1, HLA-DPB1, HLA-DRA, HLA-DRB1, IFNG, IL12RB1, IL12RB2, IL16, IL18, IL26, IL7R, INPP5D, IRF4, IRF5, IRF8, ITGAX, ITGB2, ITK, LCP1, LDLR, LEF1, LIF, LILRB1, LILRB4, LPAL2, LTA, LTB, LY9, MAPK13, MCOLN2, MYB, NCKAP1L, NFAM1, NLRP2, NLRP3, NLRP9, NPPB, NR4A3, NTN1, ORM2, P2RY2, PIK3CG, PIM1, PLAAT4, PLAC8, PLXNC1, POU2AF1, POU2F2, PRKCB, PRKCQ, PTAFR, PTGER2, PTK2B, PTPN22, PTPRC, PYCARD, PYHIN1, RHOH, RLN2, RUNX3, SASH3, SCIMP, SEMA4A, SEMA4D, SIGLEC16, SLAMF6, SMTNL2, SOCS1, STAT1, SYK, TBX21, TBXAS1, TESPA1, THBS1, TIGIT, TLR6, TLR8, TNR, TOX, TREM2, UCP2, VNN1, WNT1, WNT10B, XCL1, ZAP70, ZBED2, ZBP1]	[ABCA7, ABCC8, ABL1, ACACB, ADIPOQ, AGAP2, AGER, AGPAT1, AGT, AMH, ANGPTL4, AP3D1, APLN, APLNR, AREG, ARHGDIA, ATP1A1, ATP1A3, ATPSCKMT, BCL3, BCL6, BCL9L, C1QTNF1, C5, C5AR1, CAMK2B, CCL11, CCL24, CCR7, CD14, CD276, CD74, CDH4, CDK1, CFH, CFL1, CHGA, CHI3L1, CITED2, CLCF1, CLNK, CNOT3, CNR1, COL1A1, CSF1, CST7, CX3CL1, CYP27B1, DAG1, DDX39B, DHX34, DLG5, DMTN, ECM1, EDN2, ENG, FABP4, FADD, FES, FFAR2, FLT4, FOXC2, FOXS1, FRMD8, FURIN, GATA2, GATA4, GBA, GDF6, GDI1, GNAI2, GPAM, GPER1, GPR3, GRN, GSK3A, HK1, HRH2, HSF1, HSPB1, HTR2A, HYAL1, ICAM1, IL15RA, IL1B, IL4R, IL7R, ILK, INHA, ITGA5, JMJD8, JUP, KDM6B, KLF10, LDLR, LDLRAP1, LGALS9, LIF, LILRA5, LIMK1, LIPG, LRCH4, LRG1, LTF, LUM, MAVS, MED12, MEGF8, MFAP2, MME, MYC, MYRF, NEFL, NFATC4, NFKB2, NGF, NGFR, NINJ1, NLGN2, NLRP9, NOD2, NODAL, NOS3, NOTCH1, NPAS2, NPPA, NPPB, NR4A3, NTN1, NUMBL, OMA1, OSM, OSR2, P2RX3, P2RY2, PAK4, PARP6, PCDHA4, PDGFB, PIM1, PKM, PLXNA1, PLXNA3, PLXNB1, POLR2E, POLR2L, POR, POSTN, PQBP1, PRDM16, PTN, RAMP2, RARA, RELA, RGCC, RNU1-93P, S100A1, SERPINE1, SHANK3, SLC7A5, SLC9A1, SLN, SMAD3, SMTNL2, SNAI1, SOX10, SOX12, SOX13, SOX8, SPHK1, SPHK2, SRC, SRF, SRRT, STAT3, STING1, SYDE1, TBX5, TEAD4, TGFB1I1, TGM2, THBS1, TICAM1, TNF, TNFSF12, TRIB1, TRPV4, TSKU, VDR, VEGFA, VNN1, VSIR, WNT3, YBX2, ZBTB7B, ZC3H12A, ZCCHC3, ZMIZ1, ZNF219, ZNF335, ZNF580]
GO:0001816	cytokine production	6,35E-20	[2, 5]	213,00	26,66	59,72	40,28	[ACP5, ADAM8, ADCY7, AIF1, AIM2, ALOX15B, ANGPT1, BANK1, BATF, BCL3, BTK, BTN3A1, BTN3A2, CAMK4, CARD11, CARD17, CASP8, CCDC88B, CCL19, CCL3, CCR2, CD2, CD200R1, CD226, CD244, CD33, CD3E, CD4, CD40LG, CD6, CD74, CD80, CD84, CD86, CD96, CLCF1, CLEC6A, CLNK, CRTAM, CSF1R, CXCL6, CYBB, EBI3, EPX, EREG, FCN1, FFAR4, FGR, FOXP3, GBP5, GPSM3, HLA-DPA1, HLA-DPB1, HLA-DRB1, IFNG, IKBKE, IL12RB1, IL12RB2, IL16, IL18, IL26, IL5RA, INPP5D, IRF4, IRF5, IRF8, ITK, LAG3, LCP1, LEF1, LILRB1, LILRB4, LPAL2, LTA, LTB, LY9, MAPK13, MCOLN2, MOG, MYB, NCKAP1L, NFAM1, NLRC3, NLRP2, NLRP3, NLRP6, NLRP7, NLRP9, NR4A3, ORM2, PIK3CG, PLD4, POU2AF1, POU2F2, PRKCQ, PTAFR, PTPN22, PTPN6, PTPRC, PYCARD, PYHIN1, SASH3, SCIMP, SIGLEC16, SLAMF6, SOCS1, STAT1, SYK, TBX21, THBS1, TIGIT, TLR10, TLR6, TLR8, TNFAIP3, TREM2, UBASH3A, XCL1, ZBP1]	[ABCC8, ABCD1, ABCD2, ABL1, ACKR1, ADGRG1, ADIPOQ, AGER, AGPAT1, AGT, AMH, ANGPT1, APOA1, APOD, BCL3, BCL6, C5, C5AR1, CCR7, CD14, CD276, CD74, CEACAM1, CHI3L1, CLC, CLCF1, CLNK, CPTP, CRP, CSK, CX3CL1, EPHA2, FADD, FFAR2, FLT4, FRMD8, FURIN, GATA4, GBA, GIT1, GPAM, HDAC7, HFE, HK1, HSPB1, IGF2BP2, IL1B, IL4R, IL5RA, INHA, JAK3, JPH4, LAG3, LGALS9, LILRA5, LRRC32, LTF, LUM, MAP2K3, MAST2, MAVS, NFATC4, NFKB2, NLRC5, NLRP9, NOD2, NODAL, NR4A3, NUTF2, OSM, PER1, PLD3, PML, POLR2E, POLR2L, POSTN, PPP1R11, PQBP1, RARA, RELA, RELB, RGCC, RNF26, RNU1-93P, SERPINE1, SLC7A5, SMAD3, SPHK1, SPHK2, SRC, STAT3, STING1, THBS1, TICAM1, TNF, TRPV4, TSKU, UBE2L6, VSIR, ZBTB7B, ZC3H12A, ZCCHC3, ZFP36, ZNF580]
GO:0045595	regulation of cell differentiation	2,28E-11	[3, 4]	337,00	20,17	43,12	56,88	[ADAM8, ALOX15B, AREG, ASCL2, BCL11B, BHLHA15, BHLHE40, BMP7, BMPR1B, BTK, C1QC, CAMK4, CARD11, CASP8, CASS4, CCL17, CCL19, CCL3, CCR2, CD2, CD27, CD4, CD74, CD80, CD86, CDH4, CDKN2A, CLCF1, CMTM5, CRABP2, CRB2, CRTAM, CTLA4, CXCL10, CXCL9, CXCR4, DCC, DPEP1, EGR3, EPX, EREG, EVI2B, FCRL3, FFAR4, FGL2, FOXP3, FRZB, GDF6, GLIS1, GPR171, GPR55, H3C10, H3C11, H3C12, H3C7, HCLS1, HLA-DOA, HLA-DRA, HLA-DRB1, HOPX, IFNG, IKZF3, IL12RB1, IL18, IL7R, INPP5D, IRF4, IRX3, KLF5, KLHL41, LAG3, LCP1, LDLR, LEF1, LIF, LILRB1, LILRB4, LMO1, LRRC17, LTA, MAFF, MEDAG, MIXL1, MMP9, MSX1, MUSK, MYB, MYCL, NCKAP1L, NEK5, NFAM1, NLRP3, NPPC, NR4A3, NTN1, P2RY12, PIM1, PLAAT4, PLXNC1, PRDM1, PRKCB, PRKCQ, PTK2B, PTPN6, PTPRC, RBPMS2, RHEX, RHOH, RUNX3, SASH3, SEMA4A, SEMA4D, SLAMF8, SOCS1, SOCS3, STAT1, SULT2B1, SYK, TACSTD2, TBX21, TESPA1, THBS1, THY1, TNFRSF18, TNR, TOX, TREM2, VNN1, VWC2L, WNT1, WNT10B, ZAP70, ZBED2, ZNF683]	[ABCC8, ABL1, ACTN4, ADAMTS7, ADIPOQ, AGER, AGT, AGTR1, AP3D1, APOA1, AQP3, AREG, ARHGDIA, ATAT1, BBS12, BCL6, BCL9L, BEND6, BHLHE40, CAMK2B, CARM1, CASZ1, CCL11, CD74, CDH4, CDK1, CEACAM1, CETP, CHERP, CLCF1, CLPTM1, CMTM5, COL1A1, CRP, CSF1, CSNK1E, CTDSP1, CTHRC1, CX3CL1, CYP26B1, CYP27B1, DAB2IP, DAG1, DDX39B, DISP3, DMTN, DNMT1, DPF2, DTX1, EFEMP2, EIF4G1, ENG, ERBB2, FADD, FES, FLOT2, GATA2, GATA4, GDF6, GDI1, GNB3, GPER1, GPR137, GPR37L1, GRN, GSK3A, H3C10, H3C12, H4C3, HDAC5, HDAC7, HEYL, HOPX, HSF1, HTR2A, ID3, IFITM1, IL15RA, IL1B, IL4R, IL7R, ILK, INHA, IRX3, JAK3, JUNB, JUND, KCTD11, KLF10, KLF5, KMT2D, LAG3, LDLR, LGALS9, LIF, LIMK1, LIMS2, LINGO1, LMX1A, LOX, LRRC17, LRRC8A, LTF, LZTS1, MAFG, MAMSTR, MAPK8IP3, MCRIP1, MECP2, MED12, MEGF8, MME, MOV10, MSTN, MSX1, MYADM, MYC, MYL9, NAP1L2, NEFL, NEK5, NF2, NFATC4, NFE2, NGF, NGFR, NODAL, NOTCH1, NOTCH4, NR1D1, NR4A3, NRARP, NTN1, NUMBL, OMA1, OSM, PAF1, PARP6, PDGFB, PDLIM7, PHLDB1, PIEZO1, PIM1, PLA2G2A, PLEKHB1, PLXNA1, PLXNA3, PLXNB1, POR, POSTN, PPARD, PPP1R13L, PPP2R1A, PRKACA, PTN, RAMP2, RAP1A, RARA, RBM38, RBPMS2, RELA, RGCC, RGMA, RMRP, RNU1-93P, S1PR2, SCAF1, SEMA3F, SEMA4B, SEMA4C, SEMA6B, SEMA6C, SERPINE1, SETD1A, SHANK3, SIK1, SLC25A23, SLC4A11, SMAD3, SNAI1, SOCS3, SOX10, SOX12, SOX13, SOX8, SPDEF, SPI1, SRF, SRRT, STAT3, SYNGAP1, TBX5, TCF3, TCF7L1, TCIRG1, TEAD2, TESK1, TFE3, TGFB1I1, TGM2, THBS1, THPO, TIMP1, TMEM100, TNF, TNFRSF1A, TRIB1, TRIB3, TRIM62, TRIOBP, TRPV4, TSKU, ULK1, VASN, VDR, VEGFA, VNN1, VSIG2, VSIR, WNT3, WNT9B, ZBTB7B, ZC3H12A, ZFHX2, ZFP36, ZMIZ1, ZNF219, ZNF335, ZNF385A]

GO:2000026	regulation of multicellular organismal development	3,52E-12	[3, 4]	296,00	21,07	44,92	55,08	[ADAM8, ADAMTS9, ALOX15B, ANGPT2, ASCL2, BHLHE40, BMP7, BMPR1B, BTK, C1QC, CAMK4, CARD11, CASP8, CCL19, CCL24, CCL3, CCR2, CCR3, CD2, CD27, CD4, CD74, CD80, CD86, CDH4, CDKN2A, CLCF1, CRABP2, CRB2, CRTAM, CST7, CTLA4, CXCL10, CXCR4, CYBB, DCC, DPEP1, EGR3, EVI2B, FASLG, FCRL3, FGL2, FOXP3, FRZB, GDF6, GPR171, GPR4, GPR55, H3C10, H3C11, H3C12, H3C7, HCLS1, HLA-DOA, HLA-DRA, HLA-DRB1, HOPX, IFNG, IKZF3, IL12RB1, IL18, IL7R, INPP5D, IRF4, ITGAX, ITGB2, LAG3, LDLR, LEF1, LIF, LILRB1, LILRB4, LRRC17, LTA, MAFF, MMP9, MYB, MYCL, MYH6, NCKAP1L, NFAM1, NLRP3, NPPB, NR4A3, NTN1, PIM1, PLAAT4, PLXNC1, PRDM1, PRKCB, PRKCQ, PROK1, PTK2B, PTPN6, PTPRC, RHEX, RHOH, RLN2, RUNX3, SASH3, SEMA4A, SEMA4D, SLAMF8, SMPD3, SOCS1, STAT1, SULT2B1, SYK, TBX21, TESPA1, THBS1, THY1, TNFAIP3, TNFRSF18, TNMD, TNR, TOX, TREM2, VNN1, WNT1, WNT10B, ZAP70, ZBED2, ZNF683]	[ABCC8, ABL1, ACACB, ADAMTS7, ADAMTS9, ADGRB2, ADIPOQ, AGER, AGT, ANGPTL4, AP3D1, APLNR, AQP3, ARHGDIA, ASPN, BAG6, BCL6, BCOR, BHLHE40, C5, C5AR1, CAMK2B, CCL11, CCL24, CD74, CDH4, CDK1, CEACAM1, CFL1, CHI3L1, CITED2, CLCF1, CLPTM1, CREB3L1, CSF1, CST7, CTDSP1, CX3CL1, CYP26B1, CYP27B1, DAB2IP, DAG1, DDX39B, DLG5, DTX1, ECM1, EFNA3, ENG, EPHA2, EPN1, ERBB2, FADD, FES, FOXC2, GATA2, GATA4, GDF6, GDI1, GPER1, GPR137, GPR37L1, GPR4, GRN, GSK3A, H3C10, H3C12, H4C3, HESX1, HEYL, HGS, HOPX, HSF1, HSPB1, HSPG2, HYAL1, IL15RA, IL1B, IL4R, IL7R, ILK, INHA, ITGA5, JAK3, JMJD8, JUP, KCNK2, KLF10, KMT2D, LAG3, LAMA5, LDLR, LGALS9, LIF, LIMK1, LINGO1, LOX, LRCH4, LRG1, LRRC17, LTF, MAFG, MAPK7, MECP2, MED12, MEGF8, MME, MOV10, MYC, MYH6, MYL9, MYRF, NEFL, NF2, NFATC4, NFE2, NGF, NGFR, NINJ1, NLGN2, NODAL, NOS3, NOTCH1, NOTCH4, NPPB, NR1D1, NR4A3, NRARP, NTN1, NUMBL, OMA1, OMD, OSR2, PAF1, PAK4, PARP6, PHLDB1, PIM1, PKM, PLXNA1, PLXNA3, PLXNB1, PML, POR, PPARD, PTN, RAMP2, RARA, RELA, RGCC, RGMA, RMRP, S100A1, S1PR2, SEMA3F, SEMA4B, SEMA4C, SEMA6B, SEMA6C, SERPINE1, SETD1A, SHANK3, SMAD3, SOX10, SOX12, SOX13, SOX8, SPHK1, SPI1, SRF, SRRT, STAT3, SYNGAP1, TBX5, TFE3, TGM2, THBS1, THPO, TMEM100, TNF, TNFRSF1A, TNFSF12, TNMD, TRIB1, ULK1, VDR, VEGFA, VNN1, VSIR, WARS1, WNT3, WNT9B, ZBTB7B, ZC3H12A, ZFP36, ZMIZ1, ZNF219, ZNF335]
GO:0010604	positive regulation of macromolecule metabolic process	1,05E-06	[3, 4, 5]	618,00	16,88	38,50	61,50	[ABRA, ADAM8, ADCY7, AIF1, AIM2, AKNA, ALOX15B, ANGPT1, AREG, ASCL2, ATF3, ATP1B4, ATP2A3, BANK1, BATF, BCL11B, BCL3, BHLHA15, BLNK, BMP7, BMPR1B, BRIP1, BTN3A1, BTN3A2, C1QTNF1, CALCR, CAMK4, CARD11, CASP8, CASS4, CCDC88B, CCL19, CCL2, CCL3, CCL5, CCR2, CD2, CD226, CD244, CD33, CD3E, CD4, CD40LG, CD6, CD74, CD80, CD86, CDC20B, CDCA2, CDH1, CDKN2A, CLCF1, CLEC6A, CLNK, CREM, CRTAM, CSF1R, CXCL10, CXCR4, CYBB, DAPK1, DEPDC1B, DRD1, E2F8, EAF2, EBI3, EGR3, ELAVL4, ELF3, EOMES, EPX, EREG, FASLG, FCN1, FCRL3, FGD2, FGR, FLT3, FOSB, FOSL1, FOXP3, GADD45B, GBP5, GDF6, GLIS1, GPRC5A, GPSM3, H1-3, HCLS1, HLA-DPA1, HLA-DPB1, HLA-DRB1, IFNG, IKBKE, IKZF3, IL12RB1, IL12RB2, IL16, IL18, IL26, IL7R, IRF4, IRF5, IRF8, IRX3, ITGAX, ITK, KLF5, KLRG1, LAMP3, LCK, LCP1, LCP2, LDLR, LEF1, LIF, LILRB1, LMO1, LPAL2, LTA, LTB, LY9, MACC1, MAFF, MAP4K1, MAPK13, MCOLN2, MIXL1, MMP9, MSX1, MUSK, MYB, MYBL2, MZB1, NAMPT, NCF1, NCKAP1L, NEK5, NFAM1, NLRP2, NLRP3, NLRP6, NLRP9, NR4A1, NR4A2, NR4A3, NSG1, ORM2, PATL2, PAX5, PBX4, PDE6G, PIK3CG, PIK3R5, PIM1, PIM2, PLAC8, POU2AF1, POU2F2, PRDM1, PRKCB, PRKCQ, PROK1, PTAFR, PTGFR, PTK2B, PTPN22, PTPRC, PYCARD, PYHIN1, RLN2, RUNX3, SALL1, SASH3, SCIMP, SELE, SEMA4D, SH2D1A, SIGLEC16, SKAP1, SLAMF6, SOCS1, SOCS3, SPIB, SPIC, STAP1, STAT1, STXBP2, SYK, TBX21, TENT5C, TFEC, THBS1, TIGIT, TLR6, TLR8, TNFAIP3, TNFRSF18, TNFSF13B, TNFSF8, TNIP3, TOX, TRABD2A, TREM2, TRERF1, TRIM14, WAS, WNT1, WNT10A, WNT10B, XCL1, ZBP1, ZC3H12D, ZNF804A]	[ABCC8, ABL1, ABLIM3, ABRA, ACTA1, ACTB, ACTC1, ACTN1, ACTN4, ADAM19, ADCY1, ADCY3, ADCY4, ADCY9, ADIPOQ, AGAP2, AGER, AGPAT1, AGT, AGTR1, ALPK3, AMH, ANGPT1, AP3D1, APLN, APLNR, ARAF, AREG, ARHGEF5, ARID1A, ARTN, ATF5, ATP13A2, ATP1B4, BAG6, BCL3, BCL6, BCL9L, BMP8A, BRD4, BRPF3, C1QTNF1, C5, C5AR1, CALCR, CAMTA2, CARM1, CASZ1, CCL2, CCL21, CCR7, CD14, CD276, CD74, CDC25B, CDCA2, CDK1, CDKN1A, CGA, CHCHD10, CHERP, CHGA, CHI3L1, CIITA, CITED2, CITED4, CIZ1, CLCF1, CLIP3, CLNK, CNOT3, COL1A1, CREB3L1, CRP, CRTC2, CSF1, CSK, CSNK1E, CSPG4, CSRNP3, CTC1, CTF1, CTIF, CTSD, CX3CL1, CYP26B1, CYP27B1, DAB2IP, DAG1, DAXX, DBF4B, DDX39B, DHX34, DIRAS1, DNMT1, DPF2, DTX1, DUSP5, DVL3, DXO, DYRK1B, E2F4, E2F7, E2F8, EAF2, EDC4, EFNA3, EGLN2, EIF4G1, ELF3, ELF4, ELK1, ENDOG, ENG, ERBB2, ETS2, ETV6, FADD, FASN, FFAR2, FGFR4, FHL5, FHOD1, FLT3, FLT4, FOSL1, FOSL2, FOXC2, FOXF1, FOXM1, FPR1, FRMD8, FURIN, GADD45B, GATA2, GATA4, GBA, GDF6, GGA1, GGA3, GLIS2, GLMP, GPER1, GPI, GPRC5A, GPRIN1, GRN, GSK3A, GTPBP1, H1-9P, HBEGF, HCFC1, HDAC10, HDAC5, HEYL, HFE, HGS, HK1, HLTF, HMGA1, HMGN1, HMGN5, HSF1, HSPB1, HSPE1, HTR2A, ICAM1, ID3, IL1B, IL4R, IL7R, ILK, INCENP, INHA, IRAK2, IRF2, IRF2BPL, IRX3, IRX6, ITGA3, ITGA5, JUNB, JUND, JUP, KCNH2, KDM6B, KLF10, KLF5, KMT2D, KSR1, LDLR, LGALS9, LIF, LILRA5, LIMK2, LMNA, LMX1A, LMX1B, LRRC32, LTF, LUM, MAFG, MAMSTR, MAP2K3, MAP3K10, MAP3K11, MAP3K14, MAP3K6, MAP3K9, MAPK3, MAPK7, MAPK8IP3, MARK2, MAVS, MAZ, MECP2, MED12, MED16, MED24, MED25, MEF2D, MEIS3P1, MEIS3P2, MEN1, MINK1, MLLT6, MLST8, MOV10, MPV17L2, MSN, MSTN, MSX1, MUC20, MYBBP1A, MYC, MYH9, MYRF, NAP1L2, NCOA6, NCOR2, NDP, NEK5, NFATC4, NFE2, NFIC, NFIX, NFKB2, NFKBIB, NGF, NGFR, NKD2, NLRC5, NLRP9, NOD2, NODAL, NOS3, NOTCH1, NOTCH3, NOTCH4, NPAS2, NR1D1, NR4A1, NR4A2
GO:0032103	positive regulation of response to external stimulus	8,85E-08	[3, 4, 5]	131,00	24,08	57,80	42,20	[ADAM8, AIF1, AIM2, BTK, CARD11, CCL19, CCL24, CCL3, CCL4, CCL5, CCR2, CCR4, CD180, CD226, CD74, CLEC10A, CLEC4D, CLEC4E, CLEC6A, CLNK, CNR1, CRTAM, CSF1R, CXCL10, CXCR4, CXorf21, EDN2, EREG, FCN1, GBP5, GPR4, GPSM3, IFNG, IKBKE, IL16, IL18, IL18RAP, KLRC2, KLRD1, KLRK1, LAG3, LCP1, LDLR, LTA, LY86, MAPK13, MUC16, MUC19, MUC6, NCKAP1L, NCR3, OASL, P2RY12, PIK3CG, PLA2G7, PTK2B, PYCARD, PYHIN1, RAC2, SCIMP, SH2D1A, SIGLEC16, SLAMF6, STAP1, SUCNR1, SYK, THBS1, TLR10, TLR8, TREM2, VAV1, XCL1, XCL2, ZBP1]	[AGER, AGT, AGTR1, ARTN, C5AR1, CCL21, CCL24, CCR7, CD74, CLEC4E, CLNK, CNR1, CSF1, CX3CL1, EDN2, FABP4, FADD, FEM1A, FFAR2, GPR4, GRN, HSPB1, IL1B, LAG3, LDLR, LGALS9, LILRA5, MAPK3, MAVS, MEGF8, MEN1, MSTN, MUC19, MUC20, MUC3A, NECTIN2, NINJ1, NLRC5, NOD2, NPPA, OSM, PCDHA4, PDGFB, PDGFRB, PLA2G2A, PLA2G7, PQBP1, PRKACA, PTN, PVR, RAC2, RBM14, RELA, RELB, RNU1-93P, SERPINE1, SMAD3, SPI1, SRC, STING1, TGM2, THBS1, TNF, TNFRSF1A, TNIP1, TRIM62, TRPV4, VEGFA, ZCCHC3, ZNF580]

GO:0045597	positive regulation of cell differentiation	2,37E-08	[3, 4, 5]	192,00	21,87	40,86	59,14	[ADAM8, ALOX15B, AREG, BMP7, BMPR1B, BTK, CASP8, CASS4, CCL19, CD27, CD4, CD74, CD80, CD86, CDH4, CLCF1, CRABP2, CRB2, CXCL9, CXCR4, EGR3, EPX, EVI2B, FFAR4, FOXP3, GDF6, HCLS1, HLA-DRA, HLA-DRB1, HOPX, IFNG, IL12RB1, IL18, IL7R, INPP5D, IRX3, KLF5, LCP1, LEF1, LIF, LILRB4, LTA, MEDAG, MYB, NCKAP1L, NEK5, NLRP3, NPPC, NTN1, PIM1, PLAAT4, PLXNC1, PTPRC, RHEX, RHOH, RUNX3, SASH3, SEMA4D, SOCS1, SOCS3, STAT1, SULT2B1, SYK, TACSTD2, TESPA1, TOX, TREM2, VNN1, VWC2L, WNT10B, ZAP70, ZBED2]	[ABL1, ADIPOQ, AGER, AGT, AGTR1, AP3D1, APOA1, AREG, ARHGDIA, BCL6, BCL9L, BEND6, CAMK2B, CARM1, CD74, CDH4, CEACAM1, CLCF1, COL1A1, CSF1, CSNK1E, CTHRC1, CX3CL1, CYP26B1, CYP27B1, DAG1, DDX39B, DMTN, EFEMP2, EIF4G1, ENG, FADD, FES, GATA2, GATA4, GDF6, GDI1, GPER1, HEYL, HOPX, HSF1, HTR2A, IFITM1, IL15RA, IL1B, IL4R, IL7R, ILK, IRX3, JUNB, JUND, KCTD11, KLF10, KLF5, LGALS9, LIF, LIMK1, LIMS2, LRRC8A, LTF, LZTS1, MAMSTR, MAPK8IP3, MED12, MEGF8, MME, MYADM, MYC, NAP1L2, NEFL, NEK5, NF2, NGF, NGFR, NOTCH1, NTN1, NUMBL, OMA1, PARP6, PDLIM7, PIEZO1, PIM1, PLA2G2A, PLXNA1, PLXNA3, PLXNB1, POR, PPARD, PPP1R13L, PTN, RAMP2, RAP1A, RARA, RELA, RGCC, S1PR2, SERPINE1, SHANK3, SMAD3, SNAI1, SOCS3, SOX10, SOX12, SOX13, SOX8, SPDEF, SRF, SRRT, STAT3, TBX5, TCF3, TCF7L1, TESK1, TFE3, TGFB1I1, TGM2, THPO, TIMP1, TMEM100, TNF, TRIB1, TRIOBP, VDR, VEGFA, VNN1, VSIG2, VSIR, WNT3, ZBTB7B, ZC3H12A, ZFP36, ZMIZ1, ZNF219, ZNF335, ZNF385A]
GO:0001817	regulation of cytokine production	4,02E-20	[3, 4, 5, 6]	212,00	26,77	59,95	40,05	[ACP5, ADAM8, ADCY7, AIF1, AIM2, ALOX15B, ANGPT1, BANK1, BATF, BCL3, BTK, BTN3A1, BTN3A2, CAMK4, CARD11, CARD17, CASP8, CCDC88B, CCL19, CCL3, CCR2, CD2, CD200R1, CD226, CD244, CD33, CD3E, CD4, CD40LG, CD6, CD74, CD80, CD84, CD86, CD96, CLCF1, CLEC6A, CLNK, CRTAM, CSF1R, CXCL6, CYBB, EBI3, EPX, EREG, FCN1, FFAR4, FGR, FOXP3, GBP5, GPSM3, HLA-DPA1, HLA-DPB1, HLA-DRB1, IFNG, IKBKE, IL12RB1, IL12RB2, IL16, IL18, IL26, IL5RA, INPP5D, IRF4, IRF5, IRF8, ITK, LAG3, LCP1, LEF1, LILRB1, LILRB4, LPAL2, LTA, LTB, LY9, MAPK13, MCOLN2, MOG, MYB, NCKAP1L, NFAM1, NLRC3, NLRP2, NLRP3, NLRP6, NLRP7, NLRP9, NR4A3, ORM2, PIK3CG, PLD4, POU2AF1, POU2F2, PRKCQ, PTAFR, PTPN22, PTPN6, PTPRC, PYCARD, PYHIN1, SASH3, SCIMP, SIGLEC16, SLAMF6, SOCS1, STAT1, SYK, TBX21, THBS1, TIGIT, TLR10, TLR6, TLR8, TNFAIP3, TREM2, UBASH3A, XCL1, ZBP1]	[ABCC8, ABCD1, ABCD2, ABL1, ACKR1, ADIPOQ, AGER, AGPAT1, AGT, AMH, ANGPT1, APOA1, APOD, BCL3, BCL6, C5, C5AR1, CCR7, CD14, CD276, CD74, CEACAM1, CHI3L1, CLC, CLCF1, CLNK, CPTP, CRP, CSK, CX3CL1, EPHA2, FADD, FFAR2, FLT4, FRMD8, FURIN, GATA4, GBA, GIT1, GPAM, HDAC7, HFE, HK1, HSPB1, IGF2BP2, IL1B, IL4R, IL5RA, INHA, JAK3, JPH4, LAG3, LGALS9, LILRA5, LRRC32, LTF, LUM, MAP2K3, MAST2, MAVS, NFATC4, NFKB2, NLRC5, NLRP9, NOD2, NODAL, NR4A3, NUTF2, OSM, PER1, PLD3, PML, POLR2E, POLR2L, POSTN, PPP1R11, PQBP1, RARA, RELA, RELB, RGCC, RNF26, RNU1-93P, SERPINE1, SLC7A5, SMAD3, SPHK1, SPHK2, SRC, STAT3, STING1, THBS1, TICAM1, TNF, TRPV4, TSKU, UBE2L6, VSIR, ZBTB7B, ZC3H12A, ZCCHC3, ZFP36, ZNF580]
GO:0001818	negative regulation of cytokine production	9,12E-06	[3, 4, 5, 6, 7]	79,00	26,42	54,65	45,35	[ACP5, ADCY7, ANGPT1, BANK1, BCL3, BTK, CARD17, CD200R1, CD33, CD84, CD96, EPX, FFAR4, FOXP3, HLA-DRB1, IFNG, IKBKE, INPP5D, LAG3, LEF1, LILRB1, LILRB4, LPAL2, NCKAP1L, NLRC3, NLRP3, NLRP6, NLRP7, PTPN22, PTPN6, PTPRC, PYCARD, TBX21, THBS1, TIGIT, TLR6, TLR8, TNFAIP3, TREM2, XCL1]	[ABCD1, ABCD2, ADIPOQ, AGER, ANGPT1, APOA1, APOD, BCL3, BCL6, CCR7, CEACAM1, CPTP, CSK, CX3CL1, EPHA2, FURIN, GBA, GIT1, HDAC7, HFE, INHA, JAK3, LAG3, LGALS9, LILRA5, LRRC32, LTF, MAVS, NLRC5, NOD2, NUTF2, PML, PPP1R11, RARA, RELB, RGCC, THBS1, TNF, TSKU, UBE2L6, VSIR, ZC3H12A, ZFP36]
GO:0001819	positive regulation of cytokine production	2,05E-13	[3, 4, 5, 6, 7]	142,00	27,26	64,89	35,11	[ADAM8, AIF1, AIM2, ALOX15B, BATF, BCL3, BTN3A1, BTN3A2, CAMK4, CARD11, CASP8, CCDC88B, CCL19, CCL3, CCR2, CD2, CD226, CD244, CD3E, CD4, CD40LG, CD6, CD74, CD80, CD86, CLEC6A, CLNK, CRTAM, CSF1R, CYBB, EBI3, EPX, EREG, FCN1, FGR, FOXP3, GBP5, GPSM3, HLA-DPA1, HLA-DPB1, IFNG, IL12RB1, IL12RB2, IL16, IL18, IL26, IRF4, IRF5, IRF8, ITK, LCP1, LILRB1, LPAL2, LTA, LTB, LY9, MAPK13, MCOLN2, MYB, NFAM1, NLRP2, NLRP3, NLRP9, NR4A3, ORM2, PIK3CG, POU2AF1, POU2F2, PRKCQ, PTAFR, PTPN22, PTPRC, PYCARD, PYHIN1, SASH3, SCIMP, SIGLEC16, SLAMF6, STAT1, SYK, THBS1, TIGIT, TLR6, TLR8, TREM2, XCL1, ZBP1]	[ABCC8, ABL1, ADIPOQ, AGER, AGPAT1, AGT, AMH, BCL3, C5, C5AR1, CCR7, CD14, CD276, CD74, CHI3L1, CLNK, CX3CL1, FADD, FFAR2, FLT4, FRMD8, FURIN, GATA4, HK1, HSPB1, IL1B, IL4R, LGALS9, LILRA5, LUM, MAVS, NFATC4, NFKB2, NLRP9, NOD2, NODAL, NR4A3, OSM, POLR2E, POLR2L, POSTN, PQBP1, RARA, RELA, RGCC, RNU1-93P, SERPINE1, SLC7A5, SMAD3, SPHK1, SPHK2, SRC, STAT3, STING1, THBS1, TICAM1, TNF, TRPV4, ZBTB7B, ZCCHC3, ZNF580]
GO:0010628	positive regulation of gene expression	4,44E-08	[4, 5, 6]	249,00	20,34	51,15	48,85	[ADAM8, AIF1, AIM2, ALOX15B, ANGPT1, AREG, ATF3, BATF, BCL3, BLNK, BMP7, BTN3A1, BTN3A2, C1QTNF1, CALCR, CAMK4, CARD11, CASP8, CCDC88B, CCL19, CCL3, CCL5, CCR2, CD2, CD226, CD244, CD3E, CD4, CD40LG, CD6, CD74, CD80, CD86, CDKN2A, CLCF1, CLEC6A, CLNK, CRTAM, CSF1R, CYBB, DRD1, EBI3, ELAVL4, EPX, EREG, FCN1, FGR, FOXP3, GBP5, GPSM3, H1-3, HLA-DPA1, HLA-DPB1, IFNG, IKBKE, IL12RB1, IL12RB2, IL16, IL18, IL26, IL7R, IRF4, IRF5, IRF8, ITGAX, ITK, LAMP3, LCP1, LDLR, LEF1, LIF, LILRB1, LPAL2, LTA, LTB, LY9, MAPK13, MCOLN2, MUSK, MYB, MZB1, NFAM1, NLRP2, NLRP3, NLRP9, NR4A3, ORM2, PIK3CG, POU2AF1, POU2F2, PRDM1, PRKCQ, PTAFR, PTGFR, PTK2B, PTPN22, PTPRC, PYCARD, PYHIN1, RLN2, SASH3, SCIMP, SIGLEC16, SLAMF6, STAP1, STAT1, SYK, TBX21, TENT5C, THBS1, TIGIT, TLR6, TLR8, TREM2, WNT10A, XCL1, ZBP1, ZNF804A]	[ABCC8, ABL1, ACTA1, ACTB, ACTC1, ADAM19, ADIPOQ, AGER, AGPAT1, AGT, AMH, ANGPT1, AREG, ARID1A, ATP13A2, BCL3, C1QTNF1, C5, C5AR1, CALCR, CCR7, CD14, CD276, CD74, CDK1, CHI3L1, CITED2, CLCF1, CLNK, CRP, CSF1, CTIF, CX3CL1, CYP26B1, CYP27B1, DDX39B, DHX34, DNMT1, EIF4G1, ENG, ERBB2, FADD, FFAR2, FGFR4, FLT4, FRMD8, FURIN, GATA2, GATA4, GPER1, GPI, GSK3A, H1-9P, HCFC1, HFE, HGS, HK1, HMGA1, HMGN5, HSF1, HSPB1, ID3, IL1B, IL4R, IL7R, ITGA3, LDLR, LGALS9, LIF, LILRA5, LMNA, LRRC32, LUM, MAFG, MAPK3, MAVS, MAZ, MOV10, MPV17L2, MSN, MYBBP1A, MYC, MYH9, NCOR2, NFATC4, NFKB2, NGF, NKD2, NLRP9, NOD2, NODAL, NOS3, NOTCH1, NR4A3, OSM, OSR2, PDGFB, PKM, POLR2E, POLR2L, POSTN, POU5F1P4, PPARD, PPP1R15A, PQBP1, PRKCSH, PTGFR, RAMP2, RARA, RELA, RELB, RGCC, RNU1-93P, RPL26, SERPINE1, SF3B4, SLC35A4, SLC7A5, SMAD3, SOX10, SOX8, SPHK1, SPHK2, SPI1, SRC, STAT3, STING1, TAF1C, THBS1, TICAM1, TNF, TRPV4, VDR, VEGFA, VSIR, WARS1, WBP2, WHRN, WNT3, ZBTB7B, ZC3H12A, ZCCHC3, ZFP36, ZMIZ1, ZMIZ2, ZNF580]

GO:0051049	regulation of transport	6,48E-06	[3, 4]	339,00	18,29	39,41	60,59	[ABCB11, ADAM8, ALOX15, ANGPT1, ANO9, BLK, C1QTNF1, C2, CACNA1E, CACNA1I, CALCR, CASP8, CBARP, CCL19, CCL2, CCL3, CCL4, CCL5, CCR2, CD177, CD19, CD22, CD300LF, CD33, CD4, CD74, CD84, CDH1, CLCNKA, CNR1, CORO1A, CTSS, CXCL10, CXCL11, CXCL9, CXCR4, CYBB, DAPK1, DERL3, DOCK2, DRD1, EPX, ERFE, FASLG, FCRL3, FFAR4, FGR, GABBR2, GZMB, HCLS1, HCN1, HLA-DRB1, IFNG, IL16, IL2RB, IL2RG, ITGB2, JPH1, JSRP1, KCNA3, KCNA7, KCNJ10, KCNK15, KCNN4, KCNQ5, KLRC2, KMO, LCP1, LILRB1, LPAL2, LRRC38, MIDN, MLC1, MMP9, MYB, NCKAP1L, NLRP3, NLRP6, NPPB, NR4A3, P2RY12, P2RY2, PIK3CG, PIM1, PRAM1, PRKCB, PTAFR, PTK2B, PTPN22, PTPN6, PTPRC, PTX3, PYCARD, RAB15, RAB27B, RAB3B, RAB3C, RAC2, RASGRF1, RGS9, RRAD, SDC1, SELE, SEPTIN1, SHISA8, SIRPG, SLC31A2, SMPD3, SPINK1, STAP1, STXBP2, SYK, TCAF2, THBS1, THY1, TIFAB, TMC2, TREM2, TRPM2, UBD, UCP2, XCL1]	[AAAS, ABCA3, ABCA7, ABCB11, ABCC8, ABL1, ABLIM3, ACTB, ACTN4, ADCY1, ADIPOQ, AGT, AGTR1, ANGPT1, ANKRD13B, AP2A1, APLN, APLNR, APOA1, APOD, ARC, ARF1, ARHGAP1, ARHGEF5, ATP13A2, ATP1A1, ATP1A3, ATP1B2, ATPSCKMT, BAG3, BMP8A, BSN, C11orf65, C1QTNF1, C2CD2L, CACNA1E, CACNA1H, CACNB4, CALCR, CAMK2B, CAPN1, CBARP, CCL2, CCL21, CD14, CD177, CD74, CDK1, CEACAM1, CETP, CFH, CHCHD10, CHGA, CLCNKA, CLIP3, CNR1, CPLX1, CRYAB, CSK, CX3CL1, DGKD, DISP3, DMTN, DOC2B, DYSF, EHD1, EHD2, ERBB2, ERFE, FES, FFAR2, FOXF1, FURIN, FXYD5, FXYD6, GAB2, GABBR2, GAS1, GATA2, GDI1, GIT1, GNAI2, GNAO1, GPER1, GPRIN1, GRIK5, GRM2, GSK3A, HCN3, HCN4, HFE, HGS, HTR1B, HTR2A, HVCN1, ICAM1, IL15RA, IL1B, IL4R, INHA, ITPR3, JPH4, JUP, KCNA7, KCNC3, KCNH2, KCNIP2, KCNJ1, KCNJ5, KCNJ9, KCNK15, KCNK6, KCNMB1, KCNMB2, KCNQ4, KCTD11, LDLRAP1, LGALS9, LILRA5, LIPG, LRRC52, LRRC8A, LZTS1, MAPK3, MAVS, MICALL2, MIDN, MINK1, MLLT6, MSN, MYC, MYO18A, NEFL, NKAIN3, NLGN2, NNAT, NOD2, NOTCH1, NPPA, NPPB, NR1D1, NR4A3, NUCB1, NUTF2, ORAI1, P2RX3, P2RX5, P2RY2, P2RY6, PACSIN1, PCDHA4, PDGFB, PDGFRB, PER1, PIM1, PLK3, PLTP, PM20D1, PML, PPARD, PRKACA, PRKCSH, PROM2, PRRT1, PRRT2, PTGES, PTPN23, RAB11B, RAB11FIP5, RAB15, RAB3A, RAB5B, RAC2, RANGAP1, RANGRF, RAP1A, RASL10B, RCVRN, RGCC, RHBDF1, RHBDF2, RIPOR1, RRAD, RTN2, RUBCN, RXRA, S100A1, SCN5A, SCN7A, SEC16A, SELE, SELENON, SEPTIN4, SEPTIN5, SERPINE1, SH3GL1, SHANK3, SIDT2, SIK1, SLC30A1, SLC30A3, SLC35F6, SLC51B, SLC6A9, SLC9A1, SLC9A3R1, SLN, SMAD3, SMPD1, SPHK1, SPHK2, SPI1, SPINK1, SRC, SREBF1, STAC2, STX1A, STX1B, SV2A, SYN1, SYT2, SYT7, TCIRG1, TCTEX1D2, TGM2, THBS1, TIMP1, TM9SF4, TMEM109, TMEM14A, TNF, TNFRSF1A, TNK2, TPCN1, TRIB3, TSC2, VEGFA, VTN, WFS1, WNK2, WWP2, ZC3H12A, ZDHHC8]
GO:0051050	positive regulation of transport	1,01E-03	[2, 3, 4, 5]	192,00	19,22	42,40	57,60	[ABCB11, ADAM8, ANGPT1, BLK, C1QTNF1, C2, CACNA1I, CALCR, CASP8, CCL19, CCL2, CCL3, CCL4, CCL5, CCR2, CD177, CD19, CD300LF, CD33, CDH1, CNR1, CTSS, CXCL10, CXCL11, CXCL9, DOCK2, DRD1, ERFE, FASLG, FFAR4, FGR, GABBR2, GZMB, HCLS1, HLA-DRB1, IFNG, IL2RB, IL2RG, ITGB2, KCNJ10, KCNN4, KLRC2, KMO, LPAL2, LRRC38, MLC1, MYB, NCKAP1L, NLRP3, NPPB, NR4A3, P2RY12, P2RY2, PTAFR, PTPN22, PTPRC, PTX3, PYCARD, RAB15, RAB27B, RAB3B, RAC2, RGS9, SDC1, SELE, SIRPG, SMPD3, STAP1, SYK, TCAF2, THY1, TREM2, TRPM2, UBD, XCL1]	[ABCA3, ABCA7, ABCB11, ABCC8, ABL1, ABLIM3, ACTB, ACTN4, ADIPOQ, AGT, ANGPT1, AP2A1, APLN, APLNR, APOA1, ARC, ARF1, ARHGEF5, ATP13A2, ATP1B2, ATPSCKMT, BAG3, C1QTNF1, C2CD2L, CACNA1H, CALCR, CCL2, CCL21, CD14, CD177, CDK1, CETP, CFH, CHCHD10, CLIP3, CNR1, CX3CL1, DGKD, DOC2B, EHD1, EHD2, ERBB2, ERFE, GAB2, GABBR2, GATA2, GNAI2, GPER1, GPRIN1, GSK3A, HFE, HGS, IL15RA, IL1B, IL4R, INHA, JUP, KCNH2, KCNIP2, KCNJ5, KCNJ9, KCNMB1, KCNMB2, LDLRAP1, LILRA5, LIPG, LRRC52, LRRC8A, LZTS1, MAVS, MICALL2, MLLT6, MSN, MYO18A, NLGN2, NNAT, NOD2, NPPA, NPPB, NR4A3, NUTF2, ORAI1, P2RX3, P2RX5, P2RY2, P2RY6, PCDHA4, PDGFB, PDGFRB, PLK3, PLTP, PPARD, PRKACA, PRRT2, PTGES, PTPN23, RAB15, RAB3A, RAC2, RAP1A, RASL10B, RGCC, RIPOR1, RXRA, S100A1, SCN5A, SEC16A, SELE, SERPINE1, SH3GL1, SHANK3, SLC30A3, SLC51B, SLC6A9, SLC9A1, SLC9A3R1, SMAD3, SMPD1, SPHK2, SRC, STAC2, STX1A, STX1B, SYT7, TM9SF4, TNF, VEGFA, VTN, WFS1, WNK2, ZC3H12A, ZDHHC8]
GO:0002699	positive regulation of immune effector process	5,71E-10	[3, 4, 5]	79,00	31,60	67,30	32,70	[BLK, BTK, CAMK4, CCL19, CCR2, CD177, CD1B, CD1C, CD1E, CD226, CD244, CD74, CD80, CD84, CD86, CLCF1, CLNK, CRTAM, FGR, FOXP3, HLA-DMB, HLA-DRA, HLA-DRB1, IFNG, IL12RB1, IL18, IL18RAP, ITGB2, KLRC2, KLRD1, KLRK1, LAG3, LILRB1, LTA, MYB, MZB1, NCR3, NLRP3, NR4A3, PTAFR, PTPN22, PTPRC, RAC2, SASH3, SCIMP, SH2D1A, SLAMF6, STAP1, STXBP2, SYK, TBX21, TREM2, VAV1, XCL1]	[CD177, CD1C, CD1E, CD74, CLCF1, CLNK, ENPP3, FADD, FES, FFAR2, FOXF1, GAB2, GATA2, GPI, HK1, IL1B, IL4R, LAG3, LGALS9, MAVS, MYO18A, NECTIN2, NOD2, NPPA, NR4A3, PVR, RAC2, RAP1A, RARA, SLC7A5, SPHK2, SPI1, TICAM1, TNF]
GO:0032418	lysosome localization	2,87E-03	[4]	29,00	34,12	40,43	59,57	[BLK, BTK, CD84, CLNK, FGR, NR4A3, PIK3CG, PTGDR, RAC2, STXBP2, SYK]	[CHGA, CLNK, FES, FOXF1, GAB2, GATA2, IL4R, KLC2, KXD1, LGALS9, MYH9, NPPA, NR4A3, PLEKHM1, PLEKHM2, PTGDS, RAB3A, RAC2, SLC7A8, SPHK2, TFEB]
GO:0060627	regulation of vesicle-mediated transport	6,31E-05	[3, 4, 5]	124,00	22,18	40,13	59,87	[ALOX15, ANGPT1, BLK, C2, CACNA1I, CBARP, CCL19, CCL2, CCR2, CD177, CD22, CD300LF, CD84, CNR1, CORO1A, DOCK2, FGR, IFNG, IL2RB, IL2RG, ITGB2, KLRC2, LILRB1, LPAL2, NCKAP1L, PRAM1, PRKCB, PTAFR, PTPRC, PTX3, PYCARD, RAB15, RAB27B, RAB3B, RAB3C, RAC2, SDC1, SELE, SEPTIN1, SIRPG, SMPD3, STAP1, STXBP2, SYK, TREM2]	[ABCA7, ABL1, ADCY1, ADIPOQ, ANGPT1, ANKRD13B, AP2A1, APLN, APOA1, ARC, ARF1, ARHGAP1, ATP13A2, BSN, CACNA1H, CBARP, CCL2, CCL21, CD14, CD177, CEACAM1, CLIP3, CNR1, CPLX1, CSK, DGKD, DOC2B, DYSF, EHD1, EHD2, FES, FOXF1, GAB2, GAS1, GATA2, GIT1, GNAI2, GRIK5, HFE, HGS, HTR1B, HTR2A, IL15RA, IL1B, IL4R, LDLRAP1, LGALS9, MAPK3, MSN, MYO18A, NOD2, NOTCH1, NPPA, PACSIN1, PCDHA4, PRKCSH, PROM2, PRRT2, PTPN23, RAB11B, RAB15, RAB3A, RAB5B, RAC2, RAP1A, RUBCN, SELE, SEPTIN4, SEPTIN5, SERPINE1, SH3GL1, SMPD1, SPHK1, SPHK2, SPI1, SRC, STX1A, STX1B, SYN1, SYT2, SYT7, TGM2, TNF, TNK2, TSC2, VEGFA, VTN]

GO:0002703	regulation of leukocyte mediated immunity	3,88E-12	[4, 5]	79,00	34,05	68,35	31,65	[BLK, BTK, CAMK4, CCR2, CD177, CD1B, CD1C, CD1E, CD226, CD84, CD96, CLCF1, CLNK, CR2, CRTAM, CXCL6, FGR, FOXP3, FUT7, HLA-DRA, HLA-DRB1, IL12RB1, IL18, IL18RAP, IL7R, ITGB2, KLRC1, KLRC2, KLRD1, KLRK1, LAG3, LILRB1, LILRB4, LTA, NCKAP1L, NCR1, NCR3, NLRP3, PRAM1, PTAFR, PTPN6, PTPRC, RAC2, SASH3, SCIMP, SH2D1A, SLAMF6, STAP1, STXBP2, SYK, TBX21, TREM2, VAV1, WAS, XCL1]	[AGER, BCL6, CD177, CD1C, CD1E, CEACAM1, CLC, CLCF1, CLNK, FADD, FES, FOXF1, GAB2, GATA2, HFE, ICAM1, IL1B, IL4R, IL7R, JAK3, LAG3, LGALS9, MAVS, NECTIN2, NOD2, NPPA, PVR, RAC2, SPHK2, SPI1, TICAM1, TNF, WAS]
GO:0006887	exocytosis	1,79E-06	[3, 4, 5, 6]	202,00	20,57	47,31	52,69	[ADAM8, ADGRE3, ANPEP, ARHGAP45, BIN2, BLK, BTK, CACNA1I, CBARP, CCL3, CCL5, CCR2, CD177, CD33, CD53, CD84, CEACAM21, CFP, CLEC12A, CLEC4D, CLNK, CNR1, CORO1A, COTL1, CTSS, CTSW, CYBB, DOCK2, EPX, EXOC3L4, FCN1, FGL2, FGR, FRMPD3, GMFG, IFNG, IQGAP2, ITGAL, ITGAX, ITGB2, KLRC2, KNG1, LAIR1, LRMP, LYZ, MMP9, MPO, MYO1G, NAPSB, NCKAP1L, NFAM1, NR4A3, ORM2, PIK3CG, PLAC8, PLAU, PLEK, PRAM1, PRKCB, PTAFR, PTGDR, PTPN6, PTPRC, PTX3, PYCARD, RAB15, RAB27B, RAB37, RAB3B, RAB3C, RAB44, RAC2, RNASET2, SDC1, SELL, SEPTIN1, SERPINA1, SLC18A1, SLC27A2, SMPD3, STXBP2, SYK, SYTL1, TBC1D10C, THBS1, TRPM2, TXNDC5, VNN1]	[ACTN1, ACTN4, ADCY1, ADGRE5, ALDOA, AMH, APOA1, ARF1, ARL8A, ARSA, ATP13A2, BRPF3, C5AR1, CACNA1H, CAPN1, CBARP, CD14, CD177, CEACAM1, CHGA, CHI3L1, CLEC12A, CLNK, CNR1, COMMD3, CPLX1, CRAT, CTSA, CTSD, CYB5R3, DMTN, DOC2B, DOK3, DSC1, ECM1, FES, FOXF1, FPR1, GAB2, GALNS, GATA2, GIT1, GNAI2, GPI, GPR84, GRIK5, GRN, GTPBP2, HBB, HFE, HGS, HTR1B, HTR2A, HVCN1, IL4R, IMPDH1, IQGAP2, JUP, KNG1, LGALS3BP, LGALS9, LRG1, LTF, METTL7A, MME, MYH9, NAPA, NBEAL2, NHLRC3, NKD2, NOTCH1, NPPA, NR4A3, OTOF, PCDHA4, PDGFB, PIP5K1C, PKM, PLAU, PLAUR, PRKCSH, PRRT2, PTGDS, RAB11B, RAB11FIP5, RAB15, RAB3A, RAB3IL1, RAB5B, RAC2, RAP1A, RHOG, RNASE2, RNU1-93P, S100A11, SELENOP, SEPTIN4, SEPTIN5, SEPTIN9, SERPINA3, SERPINE1, SERPING1, SIGLEC9, SLC44A2, SLC7A8, SLCO4C1, SPHK2, SPI1, STING1, STK11IP, STX1A, STX1B, SV2A, SYN1, SYT2, SYT7, TCIRG1, THBS1, TIMP1, TMEM179B, TMEM63A, TNFAIP2, TRAPPC1, TSPAN14, TUBA4A, VEGFA, VNN1]
GO:0045576	mast cell activation	1,32E-07	[4, 5]	33,00	45,21	52,41	47,59	[BLK, BTK, CD226, CD300LF, CD84, CLNK, CNR1, CNR2, FGR, LCP2, NR4A3, PIK3CG, PTGDR, RAC2, RHOH, STXBP2, SYK]	[CHGA, CLNK, CNR1, ENPP3, FES, FOXF1, GAB2, GATA2, HAVCR1, IL4R, LGALS9, NECTIN2, NPPA, NR4A3, PCDHA4, PCDHA6, PTGDS, RAC2, SLC7A8, SPHK2]
GO:0033003	regulation of mast cell activation	7,64E-07	[4, 5, 6]	24,00	53,33	47,11	52,89	[BLK, CD226, CD300LF, CD84, CNR1, CNR2, FGR, NR4A3, RAC2, STXBP2, SYK]	[CNR1, ENPP3, FES, FOXF1, GAB2, GATA2, HAVCR1, IL4R, LGALS9, NECTIN2, NPPA, NR4A3, PCDHA4, PCDHA6, RAC2, SPHK2]
GO:0050764	regulation of phagocytosis	2,23E-03	[4, 5, 6]	33,00	33,00	59,31	40,69	[ALOX15, C2, CCL2, CD300LF, DOCK2, FGR, IFNG, IL2RB, IL2RG, LPAL2, NCKAP1L, PTPRC, PTX3, PYCARD, SIRPG, STAP1, SYK, TREM2]	[ABCA7, ADIPOQ, APOA1, CCL2, CSK, DYSF, GATA2, IL15RA, IL1B, MYO18A, NOD2, RAP1A, SPHK1, SYT7, TGM2, TNF]
GO:0002279	mast cell activation involved in immune response	8,58E-04	[5, 6]	23,00	41,07	48,72	51,28	[BLK, BTK, CD84, CLNK, FGR, NR4A3, PIK3CG, PTGDR, RAC2, STXBP2, SYK]	[CHGA, CLNK, ENPP3, FES, FOXF1, GAB2, GATA2, IL4R, LGALS9, NPPA, NR4A3, PTGDS, RAC2, SLC7A8, SPHK2]
GO:0002886	regulation of myeloid leukocyte mediated immunity	4,09E-05	[5, 6]	26,00	43,33	59,92	40,08	[BLK, BTK, CAMK4, CCR2, CD177, CD84, CXCL6, FGR, ITGB2, PRAM1, PTAFR, RAC2, STAP1, STXBP2, SYK]	[CD177, FES, FOXF1, GAB2, GATA2, IL4R, LGALS9, MAVS, NPPA, RAC2, SPHK2, SPI1, TICAM1]
GO:0017157	regulation of exocytosis	1,70E-03	[4, 5, 6, 7]	57,00	26,15	46,68	53,32	[BLK, CACNA1I, CBARP, CCR2, CD177, CD84, CNR1, FGR, IFNG, ITGB2, KLRC2, NCKAP1L, PRAM1, PRKCB, PTAFR, RAB15, RAB27B, RAB3B, RAB3C, RAC2, SDC1, SEPTIN1, SMPD3, STXBP2, SYK]	[ADCY1, ARF1, ATP13A2, CACNA1H, CBARP, CD177, CEACAM1, CNR1, CPLX1, DOC2B, FES, FOXF1, GAB2, GATA2, GIT1, GNAI2, HGS, HTR1B, HTR2A, IL4R, LGALS9, NOTCH1, NPPA, PCDHA4, RAB15, RAB3A, RAC2, RAP1A, SEPTIN4, SEPTIN5, SPHK2, SPI1, STX1A, STX1B, SYN1, SYT2, SYT7]
GO:0033005	positive regulation of mast cell activation	3,21E-02	[4, 5, 6, 7]	11,00	55,00	39,31	60,69	[CD226, FGR, NR4A3, SYK]	[GAB2, GATA2, HAVCR1, IL4R, NECTIN2, NPPA, NR4A3, SPHK2]
GO:0033006	regulation of mast cell activation involved in immune response	7,61E-03	[4, 5, 6, 7]	15,00	48,39	43,74	56,26	[BLK, CD84, FGR, RAC2, STXBP2, SYK]	[ENPP3, FES, FOXF1, GAB2, GATA2, IL4R, LGALS9, NPPA, RAC2, SPHK2]
GO:0043300	regulation of leukocyte degranulation	9,22E-06	[4, 5, 6, 7, 8, 9]	23,00	50,00	58,40	41,60	[BLK, CCR2, CD177, CD84, FGR, ITGB2, KLRC2, NCKAP1L, PRAM1, PTAFR, RAC2, STXBP2, SYK]	[CD177, CEACAM1, FES, FOXF1, GAB2, GATA2, IL4R, LGALS9, NPPA, RAC2, SPHK2, SPI1]
GO:1903305	regulation of regulated secretory pathway	5,55E-04	[5, 6, 7, 8]	45,00	29,80	42,16	57,84	[BLK, CACNA1I, CBARP, CCR2, CD177, CD84, CNR1, FGR, ITGB2, KLRC2, NCKAP1L, PRAM1, PRKCB, PTAFR, RAB15, RAC2, STXBP2, SYK]	[ADCY1, ARF1, CACNA1H, CBARP, CD177, CEACAM1, CNR1, DOC2B, FES, FOXF1, GAB2, GATA2, GIT1, GNAI2, HTR1B, HTR2A, IL4R, LGALS9, NOTCH1, NPPA, PCDHA4, RAB15, RAB3A, RAC2, RAP1A, SEPTIN5, SPHK2, SPI1, STX1A, SYN1, SYT2, SYT7]
GO:1903307	positive regulation of regulated secretory pathway	2,03E-02	[5, 6, 7, 8, 9]	21,00	36,84	43,74	56,26	[CACNA1I, CD177, CNR1, FGR, ITGB2, KLRC2, PTAFR, RAB15, SYK]	[ARF1, CACNA1H, CD177, CNR1, DOC2B, GAB2, GATA2, IL4R, NPPA, PCDHA4, RAB15, RAB3A, SPHK2, STX1A, SYT7]

GO:0048518	positive regulation of biological process	5,77E-18	[1, 2, 3]	1107,00	16,89	40,46	59,54	[ABCB11, ABRA, ADAM8, ADCY7, AIF1, AIM2, AKNA, ALOX15, ALOX15B, ANGPT1, ANGPT2, APBB1IP, AREG, ASCL2, ATF3, ATP1B4, ATP2A3, BANK1, BATF, BCL11B, BCL3, BHLHA15, BLK, BLNK, BMF, BMP7, BMPR1B, BRIP1, BTK, BTLA, BTN3A1, BTN3A2, C1QA, C1QB, C1QC, C1QTNF1, C2, C8G, CACNA1I, CALCR, CAMK4, CARD11, CARMIL2, CASP8, CASS4, CCDC88B, CCL17, CCL19, CCL2, CCL22, CCL24, CCL3, CCL3L1, CCL4, CCL4L1, CCL5, CCR2, CCR3, CCR4, CD177, CD180, CD19, CD1B, CD1C, CD1E, CD2, CD22, CD226, CD244, CD247, CD27, CD300LF, CD33, CD3D, CD3E, CD3G, CD4, CD40LG, CD5, CD53, CD6, CD74, CD79A, CD80, CD84, CD86, CDC20B, CDCA2, CDH1, CDH4, CDKN2A, CEL, CFP, CLCF1, CLEC10A, CLEC4D, CLEC4E, CLEC6A, CLNK, CNR1, CORO1A, CR2, CRABP2, CRB2, CREM, CRTAM, CSF1R, CST7, CTLA4, CTSS, CXCL10, CXCL11, CXCL5, CXCL9, CXCR4, CXorf21, CYBB, CYP11B1, DAPK1, DEPDC1B, DOCK2, DOCK8, DPEP2, DRD1, E2F8, EAF2, EBI3, EDN2, EGR3, ELAVL4, ELF3, EOMES, EPHA6, EPX, EREG, ERFE, EVI2B, FASLG, FCN1, FCN3, FCRL3, FFAR4, FGD2, FGD3, FGR, FLT3, FOLR2, FOSB, FOSL1, FOXP3, FUT7, FYB1, GABBR2, GADD45B, GBP5, GDF6, GFI1, GLIS1, GMFG, GPM6A, GPR174, GPR4, GPR55, GPRC5A, GPSM3, GRAP2, GZMA, GZMB, H1-3, HCLS1, HCST, HLA-DMB, HLA-DPA1, HLA-DPB1, HLA-DQA1, HLA-DRA, HLA-DRB1, HLA-DRB5, HOPX, ICOS, IFNG, IGLL5, IKBKE, IKZF3, IL10RA, IL12RB1, IL12RB2, IL16, IL18, IL18RAP, IL26, IL2RB, IL2RG, IL5RA, IL7R, INPP5D, IQCJ-SCHIP1, IQGAP2, IRF4, IRF5, IRF8, IRX3, ITGA4, ITGAX, ITGB2, ITK, JAML, JCHAIN, KCNJ10, KCNN4, KIAA1324, KLF5, KLRC2, KLRD1, KLRG1, KLRK1, KMO, KNG1, LAG3, LAMP3, LAX1, LCK, LCP1, LCP2, LDLR, LEF1, LIF, LILRB1, LILRB4, LMCD1, LMO1, LPAL2, LRRC38, LTA, LTB, LY86, LY9, MACC1, MAFF, MAP4K1, MAPK13, MATK, MCOLN2, MEDAG, MID1IP1, MIXL1, MLC1, MMP9, MOG, MS4A1, MSX1, MUC16, MUC19, MUC6, MUSK, MYB, MYBL2, MYO1G, MZB1, NAMPT, NCF1, NCKAP1L, NCR3, NEK5, NELL2, NFAM1	[ABCA3, ABCA7, ABCB11, ABCC8, ABCD1, ABCD2, ABI3, ABL1, ABLIM3, ABR, ABRA, ACACB, ACKR3, ACTA1, ACTB, ACTC1, ACTN1, ACTN4, ADAM19, ADAMTSL4, ADCK1, ADCY1, ADCY3, ADCY4, ADCY9, ADGRG1, ADGRL1, ADIPOQ, AGAP2, AGER, AGPAT1, AGT, AGTR1, ALPK3, AMH, ANGPT1, ANGPTL4, AP2A1, AP3D1, APLN, APLNR, APOA1, AQP3, ARAF, ARC, AREG, ARF1, ARHGDIA, ARHGEF1, ARHGEF15, ARHGEF16, ARHGEF5, ARID1A, ARTN, ATAT1, ATF5, ATP13A2, ATP1A1, ATP1A3, ATP1B2, ATP1B4, ATPSCKMT, BAG3, BAG6, BCAR1, BCL2L1, BCL3, BCL6, BCL9L, BEND6, BMP8A, BRD4, BRPF3, BTLA, C15orf62, C1QTNF1, C20orf27, C2CD2L, C5, C5AR1, C8G, CACNA1H, CALB1, CALCR, CAMK2B, CAMTA2, CAPN1, CAPNS1, CARM1, CARMIL2, CASZ1, CC2D1A, CCDC87, CCL11, CCL2, CCL21, CCL24, CCR7, CD14, CD177, CD1C, CD1E, CD276, CD74, CDC25B, CDC42EP1, CDC42EP2, CDC42EP4, CDCA2, CDH4, CDK1, CDK2, CDKN1A, CEACAM1, CEL, CETP, CFH, CFL1, CGA, CHCHD10, CHERP, CHGA, CHI3L1, CIITA, CITED2, CITED4, CIZ1, CLCF1, CLEC4E, CLIP3, CLNK, CNOT3, CNR1, CNTN6, COL1A1, CPN2, CPNE5, CPNE6, CRAT, CREB3L1, CRHR2, CRIP2, CRP, CRTC2, CSF1, CSK, CSNK1E, CSPG4, CSRNP3, CST7, CTC1, CTF1, CTHRC1, CTIF, CTSD, CX3CL1, CYP26B1, CYP27B1, DAB2IP, DAG1, DAXX, DBF4B, DDR1, DDX39B, DENND2B, DGKD, DGKZ, DHX34, DIRAS1, DISP3, DKK2, DLG5, DMTN, DNMT1, DOC2B, DOK5, DPAGT1, DPF2, DTX1, DUSP5, DVL3, DXO, DYRK1B, E2F4, E2F7, E2F8, EAF2, ECM1, ECM2, EDC4, EDN2, EEF1E1, EFEMP2, EFNA3, EGLN2, EHD1, EHD2, EIF4EBP1, EIF4G1, ELF3, ELF4, ELK1, ENDOG, ENG, ENPP3, EPHA2, ERBB2, ERFE, ESM1, ESPL1, ETS2, ETV6, FABP4, FADD, FAM110A, FAP, FASN, FCN3, FEM1A, FES, FFAR2, FGFR4, FHL5, FHOD1, FITM1, FLOT2, FLT3, FLT4, FOSL1, FOSL2, FOXC2, FOXF1, FOXM1, FOXS1, FPR1, FRMD8, FURIN, GAB2, GABBR2, GADD45B, GATA2, GATA4, GBA, GCSAM, GDF6, GDI1, GGA1, GGA3, GIT1, GLIS2, GLMP, GLRA1, GNAI2, GNAO1, GPAM
GO:0009893	positive regulation of metabolic process	1,11E-07	[2, 3, 4]	669,00	16,88	38,30	61,70	[ABRA, ADAM8, ADCY7, AIF1, AIM2, AKNA, ALOX15B, ANGPT1, AREG, ASCL2, ATF3, ATP1B4, ATP2A3, BANK1, BATF, BCL11B, BCL3, BHLHA15, BLNK, BMP7, BMPR1B, BRIP1, BTN3A1, BTN3A2, C1QTNF1, CALCR, CAMK4, CARD11, CASP8, CASS4, CCDC88B, CCL19, CCL2, CCL3, CCL5, CCR2, CD177, CD19, CD2, CD226, CD244, CD33, CD3E, CD4, CD40LG, CD6, CD74, CD80, CD86, CDC20B, CDCA2, CDH1, CDKN2A, CLCF1, CLEC6A, CLNK, CREM, CRTAM, CSF1R, CXCL10, CXCR4, CYBB, DAPK1, DEPDC1B, DRD1, E2F8, EAF2, EBI3, EGR3, ELAVL4, ELF3, EOMES, EPHA6, EPX, EREG, FASLG, FCN1, FCRL3, FFAR4, FGD2, FGR, FLT3, FOSB, FOSL1, FOXP3, GADD45B, GBP5, GDF6, GLIS1, GPRC5A, GPSM3, H1-3, HCLS1, HLA-DPA1, HLA-DPB1, HLA-DRB1, IFNG, IKBKE, IKZF3, IL12RB1, IL12RB2, IL16, IL18, IL26, IL7R, IRF4, IRF5, IRF8, IRX3, ITGAX, ITGB2, ITK, JCHAIN, KIAA1324, KLF5, KLRG1, LAMP3, LCK, LCP1, LCP2, LDLR, LEF1, LIF, LILRB1, LMO1, LPAL2, LTA, LTB, LY9, MACC1, MAFF, MAP4K1, MAPK13, MCOLN2, MID1IP1, MIXL1, MMP9, MSX1, MUSK, MYB, MYBL2, MZB1, NAMPT, NCF1, NCKAP1L, NEK5, NFAM1, NLRP2, NLRP3, NLRP6, NLRP9, NR4A1, NR4A2, NR4A3, NSG1, ORM2, P2RY12, PATL2, PAX5, PBX4, PDE6G, PFKFB4, PIK3CG, PIK3R5, PIM1, PIM2, PLAC8, PLEK, POU2AF1, POU2F2, PRDM1, PRKCB, PRKCQ, PROK1, PTAFR, PTGFR, PTK2B, PTPN22, PTPRC, PTX3, PYCARD, PYHIN1, RLN2, RUFY4, RUNX3, SALL1, SASH3, SCIMP, SELE, SEMA4D, SH2D1A, SH2D2A, SIGLEC16, SKAP1, SLAMF6, SMPD3, SOCS1, SOCS3, SPIB, SPIC, STAP1, STAT1, STXBP2, SYK, TBX21, TENT5C, TFEC, THBS1, TIGIT, TLR6, TLR8, TNFAIP3, TNFRSF18, TNFSF13B, TNFSF8, TNIP3, TOX, TRABD2A, TREM2, TRERF1, TRIM14, UCP2, WAS, WNT1, WNT10A, WNT10B, XCL1, ZBP1, ZC3H12D, ZNF804A]	[ABCC8, ABCD1, ABCD2, ABL1, ABLIM3, ABRA, ACACB, ACTA1, ACTB, ACTC1, ACTN1, ACTN4, ADAM19, ADCY1, ADCY3, ADCY4, ADCY9, ADIPOQ, AGAP2, AGER, AGPAT1, AGT, AGTR1, ALPK3, AMH, ANGPT1, AP3D1, APLN, APLNR, APOA1, ARAF, AREG, ARHGEF5, ARID1A, ARTN, ATF5, ATP13A2, ATP1B4, BAG3, BAG6, BCL3, BCL6, BCL9L, BMP8A, BRD4, BRPF3, C1QTNF1, C5, C5AR1, CALCR, CAMTA2, CARM1, CASZ1, CCL2, CCL21, CCR7, CD14, CD177, CD276, CD74, CDC25B, CDCA2, CDK1, CDKN1A, CFH, CGA, CHCHD10, CHERP, CHGA, CHI3L1, CIITA, CITED2, CITED4, CIZ1, CLCF1, CLIP3, CLNK, CNOT3, COL1A1, CREB3L1, CRP, CRTC2, CSF1, CSK, CSNK1E, CSPG4, CSRNP3, CTC1, CTF1, CTIF, CTSD, CX3CL1, CYP26B1, CYP27B1, DAB2IP, DAG1, DAXX, DBF4B, DDR1, DDX39B, DGKZ, DHX34, DIRAS1, DISP3, DNMT1, DPAGT1, DPF2, DTX1, DUSP5, DVL3, DXO, DYRK1B, E2F4, E2F7, E2F8, EAF2, EDC4, EFNA3, EGLN2, EIF4G1, ELF3, ELF4, ELK1, ENDOG, ENG, EPHA2, ERBB2, ETS2, ETV6, FABP4, FADD, FASN, FFAR2, FGFR4, FHL5, FHOD1, FLT3, FLT4, FOSL1, FOSL2, FOXC2, FOXF1, FOXM1, FPR1, FRMD8, FURIN, GADD45B, GATA2, GATA4, GBA, GDF6, GGA1, GGA3, GLIS2, GLMP, GNAI2, GPER1, GPI, GPR3, GPRC5A, GPRIN1, GPT, GRN, GSK3A, GTPBP1, H1-9P, HBB, HBEGF, HCFC1, HDAC10, HDAC5, HEYL, HFE, HGS, HK1, HLTF, HMGA1, HMGN1, HMGN5, HPRT1, HSF1, HSPB1, HSPB8, HSPE1, HTR2A, HVCN1, ICAM1, ID3, IL1B, IL4R, IL7R, ILK, INCENP, INHA, IRAK2, IRF2, IRF2BPL, IRX3, IRX6, ITGA3, ITGA5, JUNB, JUND, JUP, KCNH2, KDM6B, KLF10, KLF5, KMT2D, KSR1, LDLR, LDLRAP1, LGALS9, LIF, LILRA5, LIMK2, LMNA, LMX1A, LMX1B, LRRC32, LTF, LUM, MAFG, MAMSTR, MAP2K3, MAP3K10, MAP3K11, MAP3K14, MAP3K6, MAP3K9, MAPK3, MAPK7, MAPK8IP3, MARK2, MAVS, MAZ, MECP2, MED12, MED16, MED24, MED25, MEF2D, MEIS3P1, MEIS3P2, MEN1, MFAP2, MFSD2A, MID1IP1, MINK1, MLLT6, MLST8, MOV10, MPV17L2, MSN, MSTN, MSX1, MTCO2P12, MUC20, MYBBP1A, MYC, MYH9, MYRF,

GO:0044093	positive regulation of molecular function	1,91E-07 [3]	325,00	18,99	40,88	59,12	[ABRA, ADAM8, ADCY7, AIM2, ANGPT1, ARAP2, AREG, ARHGAP45, ATP2A3, BTK, CARD11, CASP8, CASS4, CCL17, CCL19, CCL2, CCL22, CCL24, CCL3, CCL3L1, CCL4, CCL4L1, CCL5, CCR2, CD19, CD33, CD4, CD40LG, CD74, CD86, CDC20B, CDKN2A, CSF1R, CTSS, CXCR4, DAPK1, DOCK10, DOCK8, DPEP2, EDN2, EPHA6, EPX, EREG, FASLG, FCRL3, FGD2, FGR, FLT3, FOSL1, GADD45B, GBA3, GMIP, GPR55, GPRC5A, HCLS1, HLA-DRB1, IFNG, IKBKE, IL18, IL18RAP, IRF4, ITGB2, ITK, LCK, LCP2, LPAR2, LRRC38, MAP4K1, MID1IP1, MMP9, MUSK, NCF1, NCKAP1L, NEK5, NFAM1, NLRP2, NLRP3, NLRP6, NMUR1, NR4A2, P2RY12, PDE6G, PIK3CG, PIK3R5, PLAAT4, PLCB2, PLEK, PRKCB, PRKCQ, PROK1, PTAFR, PTK2B, PTPRC, PYCARD, PYHIN1, RASGRF1, RGS1, RGS10, RGS9, RTKN2, S1PR4, SELE, SEMA4D, SH2D1A, SOCS1, STAP1, SYK, TBC1D10C, THBS1, THY1, TLR6, TRAF1, TREM2, TRIM14, VAV1, WNT1, WNT10B, XCL1, XCL2]	[ABCC8, ABL1, ABR, ABRA, ACTN4, ADCY1, ADCY3, ADCY4, ADCY9, ADIPOQ, AGAP2, AGER, AGT, AGTR1, AMH, ANGPT1, APOA1, ARAF, ARAP3, ARC, AREG, ARHGEF15, ARHGEF16, ARHGEF5, ARTN, ATP1B2, ATPSCKMT, BCR, C5, C5AR1, CCL11, CCL2, CCL21, CCL24, CCR7, CD74, CDC25B, CDKN1A, CHI3L1, CRAT, CRTC2, CSF1, CSK, CSPG4, CTHRC1, CTSD, CX3CL1, CYP27B1, DAB2IP, DAG1, DAXX, DBF4B, DDR1, DGKZ, DIRAS1, DUSP5, DVL3, EDN2, EFNA3, EIF4G1, ELMOD1, EPHA2, ERBB2, FADD, FASN, FGFR4, FLOT2, FLT3, FLT4, FOSL1, FPR1, GADD45B, GMNN, GNAI2, GNAO1, GPER1, GPRC5A, GPRIN1, GRN, GSK3A, HBEGF, HDAC5, HFE, HMGN1, HSF1, HSPE1, HTR2A, ICAM1, IL1B, ILK, INCENP, IRAK2, JUP, KCNIP2, KSR1, LGALS9, LILRA5, LPAR5, LRRC52, LTF, MAP2K3, MAP3K10, MAP3K11, MAP3K14, MAP3K6, MAP3K9, MAPK3, MAPK8IP3, MARK2, MAVS, MED25, MEN1, MID1IP1, MINK1, MLST8, MTSS2, MUC20, MYC, NAPA, NEK5, NFKB2, NFKBIB, NGF, NGFR, NOD2, NODAL, NOS3, NPPA, NPR3, NPRL3, NR4A2, OMA1, ORAI1, P2RY6, PAK4, PCOLCE, PDGFB, PDGFRB, PFN1, PIDD1, PKD1, PLAUR, PLXNB1, PML, POR, PPP1R15A, PPRC1, PRKACA, PRKAR2B, PYGO2, RAMP2, RANGAP1, RAP1A, RAP1GAP2, RAPGEF1, RARA, RCVRN, RELA, RGCC, RGL3, RGMA, RGS16, RHOG, RMRP, RNU1-93P, RPLP1, RPTOR, RTKN2, RXRA, S100A1, S1PR2, SELE, SERINC2, SH3BP1, SHANK3, SHC2, SIPA1L3, SLC25A23, SLC6A9, SLC9A1, SMAD3, SMARCA4, SMARCB1, SOX7, SPATC1L, SPHK1, SRC, SRF, STAC2, STAT3, STING1, SYDE1, SYNGAP1, TBC1D10B, TBC1D13, TBC1D17, TBC1D2, TBC1D22B, TBC1D25, TCF3, TCF7L1, TGM2, THBS1, TICAM1, TIMP1, TNF, TNIP1, TRAF4, TRIB3, TRIM62, TRIM8, TSC2, UBA52, VAC14, VDR, VEGFA, VSIG2, VSIR, VTN, WNK2, WNT9B, XRCC1, ZBTB7A]
GO:0048522	positive regulation of cellular process	9,17E-14 [2, 3, 4]	993,00	16,77	38,30	61,70	[ABRA, ADAM8, ADCY7, AIF1, AIM2, AKNA, ALOX15, ALOX15B, ANGPT1, APBB1IP, AREG, ASCL2, ATF3, ATP1B4, ATP2A3, BANK1, BATF, BCL11B, BCL3, BHLHA15, BLK, BMF, BMP7, BMPR1B, BRIP1, BTK, BTLA, C1QA, C1QTNF1, C2, CACNA1I, CALCR, CAMK4, CARD11, CARMIL2, CASP8, CASS4, CCDC88B, CCL17, CCL19, CCL2, CCL22, CCL24, CCL3, CCL3L1, CCL4, CCL4L1, CCL5, CCR2, CCR3, CD177, CD180, CD19, CD1B, CD1C, CD1E, CD2, CD226, CD244, CD247, CD27, CD300LF, CD33, CD3E, CD4, CD40LG, CD5, CD53, CD6, CD74, CD80, CD86, CDC20B, CDCA2, CDH1, CDH4, CDKN2A, CEL, CLCF1, CLEC4D, CLEC6A, CNR1, CORO1A, CRABP2, CRB2, CREM, CRTAM, CSF1R, CST7, CTLA4, CTSS, CXCL10, CXCL11, CXCL5, CXCL9, CXCR4, CXorf21, DAPK1, DEPDC1B, DOCK2, DOCK8, DPEP2, DRD1, E2F8, EAF2, EBI3, EDN2, EGR3, ELAVL4, ELF3, EOMES, EPHA6, EPX, EREG, ERFE, EVI2B, FASLG, FCRL3, FFAR4, FGD2, FGD3, FGR, FLT3, FOLR2, FOSB, FOSL1, FOXP3, FUT7, GABBR2, GADD45B, GBP5, GDF6, GFI1, GLIS1, GMFG, GPM6A, GPR174, GPR4, GPR55, GPRC5A, GPSM3, GRAP2, GZMA, GZMB, H1-3, HCLS1, HCST, HLA-DMB, HLA-DPA1, HLA-DPB1, HLA-DRA, HLA-DRB1, HOPX, ICOS, IFNG, IGLL5, IKBKE, IKZF3, IL10RA, IL12RB1, IL12RB2, IL18, IL18RAP, IL26, IL2RB, IL2RG, IL5RA, IL7R, INPP5D, IQCJ-SCHIP1, IQGAP2, IRF4, IRF5, IRF8, IRX3, ITGA4, ITGAX, ITGB2, JAML, KCNJ10, KCNN4, KIAA1324, KLF5, KLRC2, KLRD1, KLRG1, KLRK1, KMO, KNG1, LAG3, LCK, LCP1, LCP2, LDLR, LEF1, LIF, LILRB1, LILRB4, LMCD1, LMO1, LPAL2, LRRC38, LTA, LY86, MACC1, MAFF, MAP4K1, MATK, MEDAG, MID1IP1, MIXL1, MLC1, MMP9, MSX1, MUSK, MYB, MYBL2, MZB1, NAMPT, NCF1, NCKAP1L, NCR3, NEK5, NELL2, NFAM1, NLRP2, NLRP3, NLRP6, NPPC, NR4A1, NR4A2, NR4A3, NSG1, NTN1, OASL, P2RY10, P2RY12, PATL2, PAX5, PBX4, PDE6G, PFKFB4, PIK3CG, PIK3R5, PIM1, PIM2, PLA2G7, PLAAT4, PLAC8, PLEK, PLXNC1, POU2AF1, POU2F2, PRF1, PRKCB, PRKCQ, PROK1, PTAFR, PTGER[illegible], PTK2B, PTPN22	[ABCA3, ABCA7, ABCC8, ABCD1, ABCD2, ABI3, ABL1, ABLIM3, ABR, ABRA, ACACB, ACKR3, ACTN1, ACTN4, ADAM19, ADAMTSL4, ADCK1, ADCY1, ADCY3, ADCY4, ADCY9, ADGRG1, ADGRL1, ADIPOQ, AGAP2, AGER, AGPAT1, AGT, AGTR1, ALPK3, AMH, ANGPT1, AP2A1, AP3D1, APLN, APLNR, APOA1, ARAF, ARC, AREG, ARF1, ARHGDIA, ARHGEF1, ARHGEF15, ARHGEF16, ARHGEF5, ARID1A, ARTN, ATAT1, ATF5, ATP13A2, ATP1B2, ATP1B4, ATPSCKMT, BAG3, BAG6, BCAR1, BCL2L1, BCL3, BCL6, BCL9L, BEND6, BMP8A, BRD4, BRPF3, BTLA, C15orf62, C1QTNF1, C20orf27, C2CD2L, C5, C5AR1, CACNA1H, CALB1, CALCR, CAMK2B, CAMTA2, CAPN1, CAPNS1, CARM1, CARMIL2, CASZ1, CC2D1A, CCL11, CCL2, CCL21, CCL24, CCR7, CD14, CD177, CD1C, CD1E, CD276, CD74, CDC25B, CDC42EP1, CDC42EP2, CDC42EP4, CDCA2, CDH4, CDK1, CDK2, CDKN1A, CEACAM1, CEL, CGA, CHCHD10, CHERP, CHGA, CHI3L1, CIITA, CITED2, CITED4, CIZ1, CLCF1, CLIP3, CNOT3, CNR1, CNTN6, COL1A1, CPNE5, CPNE6, CRAT, CREB3L1, CRHR2, CRIP2, CRP, CRTC2, CSF1, CSK, CSNK1E, CSPG4, CSRNP3, CST7, CTC1, CTF1, CTHRC1, CTIF, CTSD, CX3CL1, CYP26B1, CYP27B1, DAB2IP, DAG1, DAXX, DBF4B, DDR1, DDX39B, DENND2B, DGKD, DGKZ, DHX34, DIRAS1, DISP3, DKK2, DLG5, DMTN, DNMT1, DOC2B, DOK5, DPAGT1, DPF2, DTX1, DUSP5, DVL3, DXO, DYRK1B, E2F4, E2F7, E2F8, EAF2, ECM1, ECM2, EDC4, EDN2, EEF1E1, EFEMP2, EFNA3, EHD1, EHD2, EIF4EBP1, EIF4G1, ELF3, ELF4, ELK1, ENDOG, ENG, EPHA2, ERBB2, ERFE, ESM1, ESPL1, ETS2, ETV6, FADD, FAM110A, FAP, FASN, FES, FGFR4, FHL5, FHOD1, FLOT2, FLT3, FLT4, FOSL1, FOSL2, FOXC2, FOXF1, FOXM1, FPR1, FURIN, GAB2, GABBR2, GADD45B, GATA2, GATA4, GBA, GDF6, GDI1, GIT1, GLIS2, GLMP, GNAI2, GNAO1, GPAM, GPBAR1, GPER1, GPI, GPM6A, GPR137, GPR17, GPR20, GPR37L1, GPR4, GPRC5A, GPRIN1, GPT, GRB7, GRIK5, GRN, GSK3A, GTPBP1, H1-9P, H4C3, HAVCR1, HBA1, HBB, HBEGF, HCFC1, HCRTR1, HDAC10, HDAC5, HDAC7, HEYL, HFE

GO:0009891	positive regulation of biosynthetic process	2,10E-02	[3, 4, 5]	350,00	16,84	33,59	66,41	[ABRA, AIF1, AKNA, AREG, ASCL2, ATF3, ATP1B4, BATF, BCL11B, BCL3, BHLHA15, BMP7, BMPR1B, BRIP1, CAMK4, CCL19, CCL2, CCL5, CD244, CD4, CD74, CD80, CD86, CDH1, CDKN2A, CREM, CXCL10, E2F8, EAF2, EGR3, ELF3, EOMES, EPX, EREG, FOSB, FOSL1, FOXP3, GDF6, GLIS1, H1-3, HCLS1, HLA-DRB1, IFNG, IKZF3, IL18, IL26, IRF4, IRF5, IRF8, IRX3, ITGB2, KLF5, KLRG1, LDLR, LEF1, LIF, LILRB1, LMO1, MACC1, MAFF, MID1IP1, MIXL1, MSX1, MYB, MYBL2, NAMPT, NCF1, NLRP3, NR4A1, NR4A2, NR4A3, PAX5, PBX4, PIM1, PIM2, PLAC8, POU2AF1, POU2F2, PRKCB, PRKCQ, PTAFR, PTK2B, PTX3, PYHIN1, RUNX3, SALL1, SH2D2A, SKAP1, SMPD3, SPIB, SPIC, STAT1, STXBP2, TBX21, TFEC, THBS1, TLR6, TNFSF8, TNIP3, TREM2, TRERF1, TRIM14, WAS, WNT1, WNT10B]	[ABCD1, ABCD2, ABL1, ABLIM3, ABRA, ACTN1, ACTN4, AGAP2, AGT, ALPK3, AP3D1, APLN, APOA1, AREG, ARID1A, ATF5, ATP1B4, BCL3, BCL9L, BRD4, BRPF3, CAMTA2, CARM1, CASZ1, CCL2, CCL21, CCR7, CD74, CDK1, CGA, CHCHD10, CHERP, CHGA, CIITA, CITED2, CITED4, CIZ1, COL1A1, CREB3L1, CRTC2, CSRNP3, CTC1, CTIF, CX3CL1, CYP27B1, DAB2IP, DAXX, DBF4B, DDX39B, DHX34, DPAGT1, DPF2, DTX1, DVL3, DXO, DYRK1B, E2F4, E2F7, E2F8, EAF2, EIF4G1, ELF3, ELF4, ELK1, ENG, ERBB2, ETS2, ETV6, FADD, FGFR4, FHL5, FHOD1, FOSL1, FOSL2, FOXC2, FOXF1, FOXM1, GATA2, GATA4, GBA, GDF6, GLIS2, GLMP, GPER1, GPRIN1, GPT, GSK3A, H1-9P, HBB, HCFC1, HDAC5, HEYL, HLTF, HMGA1, HMGN1, HMGN5, HSF1, HTR2A, ICAM1, IL1B, ILK, IRF2, IRF2BPL, IRX3, IRX6, JUNB, JUND, JUP, KCNH2, KDM6B, KLF10, KLF5, KMT2D, LDLR, LIF, LMX1A, LMX1B, LUM, MAFG, MAMSTR, MAP2K3, MAPK3, MAPK7, MAVS, MAZ, MECP2, MED12, MED16, MED24, MED25, MEF2D, MEIS3P1, MEIS3P2, MEN1, MFSD2A, MID1IP1, MLLT6, MPV17L2, MSTN, MSX1, MTCO2P12, MYC, MYRF, NCOA6, NDP, NFATC4, NFE2, NFIC, NFIX, NFKB2, NFKBIB, NGFR, NLRC5, NOD2, NODAL, NOS3, NOTCH1, NOTCH3, NOTCH4, NPAS2, NR1D1, NR4A1, NR4A2, NR4A3, OSR2, P2RY6, PDGFB, PDGFRB, PELP1, PER1, PHF1, PIM1, PKD1, PKM, PLAGL2, PML, POR, PPARD, PPP1R15A, PPRC1, PQBP1, PRDM16, PRKCSH, PTPRN, RAI1, RARA, RASL11A, RBM14, RCVRN, RELA, RFXAP, RGCC, RGMA, RNU1-93P, RPL26, RPTOR, RXRA, RXRB, SALL2, SBNO2, SCAF1, SCAP, SERPINE1, SIX5, SLC35A4, SLC51B, SLC6A9, SLC9A1, SMAD3, SMARCA4, SMARCB1, SNAI1, SOX10, SOX12, SOX7, SOX8, SPDEF, SPHK2, SPI1, SRC, SRCAP, SREBF1, SRF, SSBP3, STAT3, STING1, TAF6, TBX5, TCF3, TCF7L1, TEAD2, TEAD3, TEAD4, TFE3, TFEB, TFEC, TGFB1I1, THBS1, TICAM1, TIMP1, TNF, TNFAIP1, TNFRSF1A, TNIP1, TOX2, TP53INP2, TRIM62, TRIM8, UBA52, USF2, VDR, VEGFA, VENTX, WAS, WBP2, WIZ, ZBTB7B, ZC3H12A, ZMIZ1, ZMIZ2, ZNF219, ZNF335, ZNF384
GO:0010604	positive regulation of macromolecule metabolic process	1,05E-06	[3, 4, 5]	618,00	16,88	38,50	61,50	[ABRA, ADAM8, ADCY7, AIF1, AIM2, AKNA, ALOX15B, ANGPT1, AREG, ASCL2, ATF3, ATP1B4, ATP2A3, BANK1, BATF, BCL11B, BCL3, BHLHA15, BLNK, BMP7, BMPR1B, BRIP1, BTN3A1, BTN3A2, C1QTNF1, CALCR, CAMK4, CARD11, CASP8, CASS4, CCDC88B, CCL19, CCL2, CCL3, CCL5, CCR2, CD2, CD226, CD244, CD33, CD3E, CD4, CD40LG, CD6, CD74, CD80, CD86, CDC20B, CDCA2, CDH1, CDKN2A, CLCF1, CLEC6A, CLNK, CREM, CRTAM, CSF1R, CXCL10, CXCR4, CYBB, DAPK1, DEPDC1B, DRD1, E2F8, EAF2, EBI3, EGR3, ELAVL4, ELF3, EOMES, EPX, EREG, FASLG, FCN1, FCRL3, FGD2, FGR, FLT3, FOSB, FOSL1, FOXP3, GADD45B, GBP5, GDF6, GLIS1, GPRC5A, GPSM3, H1-3, HCLS1, HLA-DPA1, HLA-DPB1, HLA-DRB1, IFNG, IKBKE, IKZF3, IL12RB1, IL12RB2, IL16, IL18, IL26, IL7R, IRF4, IRF5, IRF8, IRX3, ITGAX, ITK, KLF5, KLRG1, LAMP3, LCK, LCP1, LCP2, LDLR, LEF1, LIF, LILRB1, LMO1, LPAL2, LTA, LTB, LY9, MACC1, MAFF, MAP4K1, MAPK13, MCOLN2, MIXL1, MMP9, MSX1, MUSK, MYB, MYBL2, MZB1, NAMPT, NCF1, NCKAP1L, NEK5, NFAM1, NLRP2, NLRP3, NLRP6, NLRP9, NR4A1, NR4A2, NR4A3, NSG1, ORM2, PATL2, PAX5, PBX4, PDE6G, PIK3CG, PIK3R5, PIM1, PIM2, PLAC8, POU2AF1, POU2F2, PRDM1, PRKCB, PRKCQ, PROK1, PTAFR, PTGFR, PTK2B, PTPN22, PTPRC, PYCARD, PYHIN1, RLN2, RUNX3, SALL1, SASH3, SCIMP, SELE, SEMA4D, SH2D1A, SIGLEC16, SKAP1, SLAMF6, SOCS1, SOCS3, SPIB, SPIC, STAP1, STAT1, STXBP2, SYK, TBX21, TENT5C, TFEC, THBS1, TIGIT, TLR6, TLR8, TNFAIP3, TNFRSF18, TNFSF13B, TNFSF8, TNIP3, TOX, TRABD2A, TREM2, TRERF1, TRIM14, WAS, WNT1, WNT10A, WNT10B, XCL1, ZBP1, ZC3H12D, ZNF804A]	[ABCC8, ABL1, ABLIM3, ABRA, ACTA1, ACTB, ACTC1, ACTN1, ACTN4, ADAM19, ADCY1, ADCY3, ADCY4, ADCY9, ADIPOQ, AGAP2, AGER, AGPAT1, AGT, AGTR1, ALPK3, AMH, ANGPT1, AP3D1, APLN, APLNR, ARAF, AREG, ARHGEF5, ARID1A, ARTN, ATF5, ATP13A2, ATP1B4, BAG6, BCL3, BCL6, BCL9L, BMP8A, BRD4, BRPF3, C1QTNF1, C5, C5AR1, CALCR, CAMTA2, CARM1, CASZ1, CCL2, CCL21, CCR7, CD14, CD276, CD74, CDC25B, CDCA2, CDK1, CDKN1A, CGA, CHCHD10, CHERP, CHGA, CHI3L1, CIITA, CITED2, CITED4, CIZ1, CLCF1, CLIP3, CLNK, CNOT3, COL1A1, CREB3L1, CRP, CRTC2, CSF1, CSK, CSNK1E, CSPG4, CSRNP3, CTC1, CTF1, CTIF, CTSD, CX3CL1, CYP26B1, CYP27B1, DAB2IP, DAG1, DAXX, DBF4B, DDX39B, DHX34, DIRAS1, DNMT1, DPF2, DTX1, DUSP5, DVL3, DXO, DYRK1B, E2F4, E2F7, E2F8, EAF2, EDC4, EFNA3, EGLN2, EIF4G1, ELF3, ELF4, ELK1, ENDOG, ENG, ERBB2, ETS2, ETV6, FADD, FASN, FFAR2, FGFR4, FHL5, FHOD1, FLT3, FLT4, FOSL1, FOSL2, FOXC2, FOXF1, FOXM1, FPR1, FRMD8, FURIN, GADD45B, GATA2, GATA4, GBA, GDF6, GGA1, GGA3, GLIS2, GLMP, GPER1, GPI, GPRC5A, GPRIN1, GRN, GSK3A, GTPBP1, H1-9P, HBEGF, HCFC1, HDAC10, HDAC5, HEYL, HFE, HGS, HK1, HLTF, HMGA1, HMGN1, HMGN5, HSF1, HSPB1, HSPE1, HTR2A, ICAM1, ID3, IL1B, IL4R, IL7R, ILK, INCENP, INHA, IRAK2, IRF2, IRF2BPL, IRX3, IRX6, ITGA3, ITGA5, JUNB, JUND, JUP, KCNH2, KDM6B, KLF10, KLF5, KMT2D, KSR1, LDLR, LGALS9, LIF, LILRA5, LIMK2, LMNA, LMX1A, LMX1B, LRRC32, LTF, LUM, MAFG, MAMSTR, MAP2K3, MAP3K10, MAP3K11, MAP3K14, MAP3K6, MAP3K9, MAPK3, MAPK7, MAPK8IP3, MARK2, MAVS, MAZ, MECP2, MED12, MED16, MED24, MED25, MEF2D, MEIS3P1, MEIS3P2, MEN1, MINK1, MLLT6, MLST8, MOV10, MPV17L2, MSN, MSTN, MSX1, MUC20, MYBBP1A, MYC, MYH9, MYRF, NAP1L2, NCOA6, NCOR2, NDP, NEK5, NFATC4, NFE2, NFIC, NFIX, NFKB2, NFKBIB, NGF, NGFR, NKD2, NLRC5, NLRP9, NOD2, NODAL, NOS3, NOTCH1, NOTCH3, NOTCH4, NPAS2, NR1D1, NR4A1, NR4A2,

GO:0031325	positive regulation of cellular metabolic process	1,24E-03	[3, 4, 5]	560,00	16,33	33,95	66,05	[ABRA, ADAM8, ADCY7, AIF1, AIM2, AKNA, ANGPT1, AREG, ASCL2, ATF3, ATP1B4, ATP2A3, BANK1, BATF, BCL11B, BCL3, BHLHA15, BMP7, BMPR1B, BRIP1, CALCR, CAMK4, CASP8, CASS4, CCL19, CCL5, CD177, CD19, CD244, CD33, CD3E, CD4, CD40LG, CD74, CD80, CD86, CDC20B, CDCA2, CDH1, CDKN2A, CLCF1, CREM, CSF1R, CXCL10, CXCR4, DAPK1, DEPDC1B, E2F8, EAF2, EGR3, ELF3, EOMES, EPHA6, EPX, EREG, FASLG, FCRL3, FGD2, FGR, FLT3, FOSB, FOSL1, FOXP3, GADD45B, GDF6, GLIS1, GPRC5A, H1-3, HCLS1, HLA-DRB1, IFNG, IKBKE, IKZF3, IL18, IL26, IRF4, IRF5, IRF8, IRX3, ITGB2, KIAA1324, KLF5, KLRG1, LCK, LCP2, LDLR, LEF1, LIF, LILRB1, LMO1, LPAL2, MACC1, MAFF, MAP4K1, MID1IP1, MIXL1, MMP9, MSX1, MUSK, MYB, MYBL2, NAMPT, NCF1, NCKAP1L, NEK5, NLRP2, NLRP3, NLRP6, NR4A1, NR4A2, NR4A3, NSG1, P2RY12, PATL2, PAX5, PBX4, PDE6G, PFKFB4, PIK3CG, PIK3R5, PIM1, PIM2, PLAC8, PLEK, POU2AF1, POU2F2, PRKCB, PRKCQ, PROK1, PTAFR, PTK2B, PTPN22, PTPRC, PTX3, PYCARD, PYHIN1, RUFY4, RUNX3, SALL1, SELE, SEMA4D, SH2D1A, SKAP1, SMPD3, SOCS1, SOCS3, SPIB, SPIC, STAP1, STAT1, STXBP2, SYK, TBX21, TFEC, THBS1, TLR6, TNFAIP3, TNFRSF18, TNFSF8, TNIP3, TOX, TRABD2A, TREM2, TRERF1, TRIM14, WAS, WNT1, WNT10B, ZC3H12D]	[ABCD1, ABCD2, ABL1, ABLIM3, ABRA, ACACB, ACTN1, ACTN4, ADCY1, ADCY3, ADCY4, ADCY9, ADIPOQ, AGAP2, AGER, AGPAT1, AGT, AGTR1, ALPK3, AMH, ANGPT1, AP3D1, APLN, APLNR, APOA1, ARAF, AREG, ARHGEF5, ARID1A, ARTN, ATF5, ATP1B4, BAG3, BAG6, BCL3, BCL6, BCL9L, BMP8A, BRD4, BRPF3, C5, C5AR1, CALCR, CAMTA2, CARM1, CASZ1, CCL21, CCR7, CD177, CD74, CDC25B, CDCA2, CDK1, CDKN1A, CGA, CHCHD10, CHERP, CHGA, CHI3L1, CIITA, CITED2, CITED4, CIZ1, CLCF1, CLIP3, CNOT3, COL1A1, CREB3L1, CRP, CRTC2, CSF1, CSK, CSNK1E, CSPG4, CSRNP3, CTC1, CTF1, CTIF, CTSD, CX3CL1, CYP27B1, DAB2IP, DAG1, DAXX, DBF4B, DDR1, DDX39B, DGKZ, DHX34, DIRAS1, DNMT1, DPAGT1, DPF2, DTX1, DUSP5, DVL3, DXO, DYRK1B, E2F4, E2F7, E2F8, EAF2, EDC4, EFNA3, EIF4G1, ELF3, ELF4, ELK1, ENDOG, ENG, EPHA2, ERBB2, ETS2, ETV6, FADD, FASN, FGFR4, FHL5, FHOD1, FLT3, FLT4, FOSL1, FOSL2, FOXC2, FOXF1, FOXM1, FPR1, FURIN, GADD45B, GATA2, GATA4, GBA, GDF6, GLIS2, GLMP, GNAI2, GPER1, GPRC5A, GPRIN1, GPT, GRN, GSK3A, GTPBP1, H1-9P, HBB, HBEGF, HCFC1, HDAC10, HDAC5, HEYL, HFE, HLTF, HMGA1, HMGN1, HMGN5, HPRT1, HSF1, HSPB8, HSPE1, HTR2A, HVCN1, ICAM1, IL1B, ILK, INCENP, INHA, IRAK2, IRF2, IRF2BPL, IRX3, IRX6, ITGA5, JUNB, JUND, JUP, KCNH2, KDM6B, KLF10, KLF5, KMT2D, KSR1, LDLR, LGALS9, LIF, LILRA5, LIMK2, LMNA, LMX1A, LMX1B, LTF, LUM, MAFG, MAMSTR, MAP2K3, MAP3K10, MAP3K11, MAP3K14, MAP3K6, MAP3K9, MAPK3, MAPK7, MAPK8IP3, MARK2, MAVS, MAZ, MECP2, MED12, MED16, MED24, MED25, MEF2D, MEIS3P1, MEIS3P2, MEN1, MFSD2A, MID1IP1, MINK1, MLLT6, MLST8, MOV10, MPV17L2, MSN, MSTN, MSX1, MTCO2P12, MUC20, MYC, MYH9, MYRF, NAP1L2, NCOA6, NDP, NEK5, NFATC4, NFE2, NFIC, NFIX, NFKB2, NFKBIB, NGF, NGFR, NKD2, NLRC5, NOD2, NODAL, NOS3, NOTCH1, NOTCH3, NOTCH4, NPAS2, NR1D1, NR4A1, NR4A2, NR4A3, NSG1, NSMF, OMA1, OSM, OSR2, P2RY6, PAF1, PAK4, PATL1, PCOLCE
GO:0051173	positive regulation of nitrogen compound metabolic process	1,29E-02	[3, 4, 5]	523,00	16,17	33,88	66,12	[ABRA, ADAM8, ADCY7, AIF1, AIM2, AKNA, ANGPT1, AREG, ASCL2, ATF3, ATP1B4, ATP2A3, BANK1, BATF, BCL11B, BCL3, BHLHA15, BMP7, BMPR1B, BRIP1, CALCR, CAMK4, CASP8, CASS4, CCL19, CCL5, CD33, CD3E, CD4, CD40LG, CD74, CD80, CD86, CDC20B, CDCA2, CDH1, CDKN2A, CLCF1, CREM, CSF1R, CXCL10, CXCR4, DAPK1, DEPDC1B, E2F8, EAF2, EGR3, ELF3, EOMES, EPX, EREG, FASLG, FCRL3, FGD2, FLT3, FOSB, FOSL1, FOXP3, GADD45B, GDF6, GLIS1, GPRC5A, H1-3, HCLS1, HLA-DRB1, IFNG, IKBKE, IKZF3, IL18, IL26, IRF4, IRF5, IRF8, IRX3, ITGB2, KLF5, KLRG1, LCK, LCP2, LDLR, LEF1, LIF, LILRB1, LMO1, MACC1, MAFF, MAP4K1, MIXL1, MMP9, MSX1, MUSK, MYB, MYBL2, NAMPT, NCF1, NCKAP1L, NEK5, NLRP2, NLRP3, NLRP6, NR4A1, NR4A2, NR4A3, PATL2, PAX5, PBX4, PDE6G, PFKFB4, PIK3CG, PIK3R5, PIM1, PIM2, PLAC8, POU2AF1, POU2F2, PRKCB, PRKCQ, PROK1, PTAFR, PTK2B, PTPN22, PTPRC, PTX3, PYCARD, PYHIN1, RUNX3, SALL1, SEMA4D, SH2D1A, SKAP1, SMPD3, SOCS1, SOCS3, SPIB, SPIC, STAP1, STAT1, STXBP2, SYK, TBX21, TFEC, THBS1, TLR6, TNFAIP3, TNFRSF18, TNFSF13B, TNFSF8, TNIP3, TOX, TRABD2A, TREM2, TRERF1, TRIM14, WAS, WNT1, WNT10B, ZC3H12D]	[ABL1, ABLIM3, ABRA, ACTN1, ACTN4, ADCY1, ADCY3, ADCY4, ADCY9, ADIPOQ, AGAP2, AGER, AGT, AGTR1, ALPK3, AMH, ANGPT1, AP3D1, APLN, APLNR, ARAF, AREG, ARHGEF5, ARID1A, ARTN, ATF5, ATP1B4, BAG6, BCL3, BCL6, BCL9L, BMP8A, BRD4, C5, C5AR1, CALCR, CAMTA2, CARM1, CASZ1, CCL21, CCR7, CD74, CDC25B, CDCA2, CDKN1A, CGA, CHCHD10, CHERP, CHGA, CHI3L1, CIITA, CITED2, CITED4, CIZ1, CLCF1, CLIP3, CNOT3, COL1A1, CREB3L1, CRTC2, CSF1, CSK, CSNK1E, CSPG4, CSRNP3, CTF1, CTIF, CTSD, CX3CL1, CYP27B1, DAB2IP, DAG1, DAXX, DBF4B, DDX39B, DIRAS1, DNMT1, DPF2, DTX1, DUSP5, DVL3, DXO, DYRK1B, E2F4, E2F7, E2F8, EAF2, EDC4, EFNA3, EGLN2, EIF4G1, ELF3, ELF4, ELK1, ENDOG, ENG, ERBB2, ETS2, ETV6, FADD, FASN, FGFR4, FHL5, FHOD1, FLT3, FLT4, FOSL1, FOSL2, FOXC2, FOXF1, FOXM1, FPR1, FURIN, GADD45B, GATA2, GATA4, GBA, GDF6, GGA1, GGA3, GLIS2, GLMP, GPER1, GPRC5A, GPRIN1, GRN, GSK3A, GTPBP1, H1-9P, HBB, HBEGF, HCFC1, HDAC10, HDAC5, HEYL, HFE, HLTF, HMGA1, HMGN1, HMGN5, HPRT1, HSF1, HSPE1, HTR2A, ICAM1, IL1B, ILK, INCENP, INHA, IRAK2, IRF2, IRF2BPL, IRX3, IRX6, ITGA5, JUNB, JUND, JUP, KCNH2, KDM6B, KLF10, KLF5, KMT2D, KSR1, LDLR, LGALS9, LIF, LILRA5, LIMK2, LMNA, LMX1A, LMX1B, LTF, LUM, MAFG, MAMSTR, MAP2K3, MAP3K10, MAP3K11, MAP3K14, MAP3K6, MAP3K9, MAPK3, MAPK7, MAPK8IP3, MARK2, MAVS, MAZ, MECP2, MED12, MED16, MED24, MED25, MEF2D, MEIS3P1, MEIS3P2, MEN1, MINK1, MLLT6, MLST8, MOV10, MPV17L2, MSN, MSTN, MSX1, MTCO2P12, MUC20, MYC, MYH9, MYRF, NAP1L2, NCOA6, NDP, NEK5, NFATC4, NFE2, NFIC, NFIX, NFKB2, NFKBIB, NGF, NGFR, NKD2, NLRC5, NOD2, NODAL, NOS3, NOTCH1, NOTCH3, NOTCH4, NPAS2, NR1D1, NR4A1, NR4A2, NR4A3, NSMF, OMA1, OSM, OSR2, PAF1, PAK4, PATL1, PCOLCE, PDGFB, PDGFRB, PEF1, PELP1, PER1, PHF1, PIAS4, PIDD1, PIM1, PKD1, PKM, PLAGL2, PLAUR, PLEKHN1, PLK3, PML, PNLDC1, PPARD, PPP1R10, PPP1R15A,

GO:0010628	positive regulation of gene expression	4,44E-08	[4, 5, 6]	249,00	20,34	51,15	48,85	[ADAM8, AIF1, AIM2, ALOX15B, ANGPT1, AREG, ATF3, BATF, BCL3, BLNK, BMP7, BTN3A1, BTN3A2, C1QTNF1, CALCR, CAMK4, CARD11, CASP8, CCDC88B, CCL19, CCL3, CCL5, CCR2, CD2, CD226, CD244, CD3E, CD4, CD40LG, CD6, CD74, CD80, CD86, CDKN2A, CLCF1, CLEC6A, CLNK, CRTAM, CSF1R, CYBB, DRD1, EBI3, ELAVL4, EPX, EREG, FCN1, FGR, FOXP3, GBP5, GPSM3, H1-3, HLA-DPA1, HLA-DPB1, IFNG, IKBKE, IL12RB1, IL12RB2, IL16, IL18, IL26, IL7R, IRF4, IRF5, IRF8, ITGAX, ITK, LAMP3, LCP1, LDLR, LEF1, LIF, LILRB1, LPAL2, LTA, LTB, LY9, MAPK13, MCOLN2, MUSK, MYB, MZB1, NFAM1, NLRP2, NLRP3, NLRP9, NR4A3, ORM2, PIK3CG, POU2AF1, POU2F2, PRDM1, PRKCQ, PTAFR, PTGFR, PTK2B, PTPN22, PTPRC, PYCARD, PYHIN1, RLN2, SASH3, SCIMP, SIGLEC16, SLAMF6, STAP1, STAT1, SYK, TBX21, TENT5C, THBS1, TIGIT, TLR6, TLR8, TREM2, WNT10A, XCL1, ZBP1, ZNF804A]	[ABCC8, ABL1, ACTA1, ACTB, ACTC1, ADAM19, ADIPOQ, AGER, AGPAT1, AGT, AMH, ANGPT1, AREG, ARID1A, ATP13A2, BCL3, C1QTNF1, C5, C5AR1, CALCR, CCR7, CD14, CD276, CD74, CDK1, CHI3L1, CITED2, CLCF1, CLNK, CRP, CSF1, CTIF, CX3CL1, CYP26B1, CYP27B1, DDX39B, DHX34, DNMT1, EIF4G1, ENG, ERBB2, FADD, FFAR2, FGFR4, FLT4, FRMD8, FURIN, GATA2, GATA4, GPER1, GPI, GSK3A, H1-9P, HCFC1, HFE, HGS, HK1, HMGA1, HMGN5, HSF1, HSPB1, ID3, IL1B, IL4R, IL7R, ITGA3, LDLR, LGALS9, LIF, LILRA5, LMNA, LRRC32, LUM, MAFG, MAPK3, MAVS, MAZ, MOV10, MPV17L2, MSN, MYBBP1A, MYC, MYH9, NCOR2, NFATC4, NFKB2, NGF, NKD2, NLRP9, NOD2, NODAL, NOS3, NOTCH1, NR4A3, OSM, OSR2, PDGFB, PKM, POLR2E, POLR2L, POSTN, POU5F1P4, PPARD, PPP1R15A, PQBP1, PRKCSH, PTGFR, RAMP2, RARA, RELA, RELB, RGCC, RNU1-93P, RPL26, SERPINE1, SF3B4, SLC35A4, SLC7A5, SMAD3, SOX10, SOX8, SPHK1, SPHK2, SPI1, SRC, STAT3, STING1, TAF1C, THBS1, TICAM1, TNF, TRPV4, VDR, VEGFA, VSIR, WARS1, WBP2, WHRN, WNT3, ZBTB7B, ZC3H12A, ZCCHC3, ZFP36, ZMIZ1, ZMIZ2, ZNF580]
GO:0031328	positive regulation of cellular biosynthetic process	1,33E-02	[4, 5, 6]	347,00	16,96	33,33	66,67	[ABRA, AIF1, AKNA, AREG, ASCL2, ATF3, ATP1B4, BATF, BCL11B, BCL3, BHLHA15, BMP7, BMPR1B, BRIP1, CAMK4, CCL19, CCL5, CD244, CD4, CD74, CD80, CD86, CDH1, CDKN2A, CREM, CXCL10, E2F8, EAF2, EGR3, ELF3, EOMES, EPX, EREG, FOSB, FOSL1, FOXP3, GDF6, GLIS1, H1-3, HCLS1, HLA-DRB1, IFNG, IKZF3, IL18, IL26, IRF4, IRF5, IRF8, IRX3, ITGB2, KLF5, KLRG1, LDLR, LEF1, LIF, LILRB1, LMO1, MACC1, MAFF, MID1IP1, MIXL1, MSX1, MYB, MYBL2, NAMPT, NCF1, NLRP3, NR4A1, NR4A2, NR4A3, PAX5, PBX4, PIM1, PIM2, PLAC8, POU2AF1, POU2F2, PRKCB, PRKCQ, PTAFR, PTK2B, PTX3, PYHIN1, RUNX3, SALL1, SKAP1, SMPD3, SPIB, SPIC, STAT1, STXBP2, TBX21, TFEC, THBS1, TLR6, TNFSF8, TNIP3, TREM2, TRERF1, TRIM14, WAS, WNT1, WNT10B]	[ABCD1, ABCD2, ABL1, ABLIM3, ABRA, ACTN1, ACTN4, AGAP2, AGT, ALPK3, AP3D1, APLN, APOA1, AREG, ARID1A, ATF5, ATP1B4, BCL3, BCL9L, BRD4, BRPF3, CAMTA2, CARM1, CASZ1, CCL21, CCR7, CD74, CDK1, CGA, CHCHD10, CHERP, CHGA, CIITA, CITED2, CITED4, CIZ1, COL1A1, CREB3L1, CRTC2, CSRNP3, CTC1, CTIF, CX3CL1, CYP27B1, DAB2IP, DAXX, DBF4B, DDX39B, DHX34, DPAGT1, DPF2, DTX1, DVL3, DXO, DYRK1B, E2F4, E2F7, E2F8, EAF2, EIF4G1, ELF3, ELF4, ELK1, ENG, ERBB2, ETS2, ETV6, FADD, FGFR4, FHL5, FHOD1, FOSL1, FOSL2, FOXC2, FOXF1, FOXM1, GATA2, GATA4, GBA, GDF6, GLIS2, GLMP, GPER1, GPRIN1, GPT, GSK3A, H1-9P, HBB, HCFC1, HDAC5, HEYL, HLTF, HMGA1, HMGN1, HMGN5, HSF1, HTR2A, ICAM1, IL1B, ILK, IRF2, IRF2BPL, IRX3, IRX6, JUNB, JUND, JUP, KCNH2, KDM6B, KLF10, KLF5, KMT2D, LDLR, LIF, LMX1A, LMX1B, LUM, MAFG, MAMSTR, MAP2K3, MAPK3, MAPK7, MAVS, MAZ, MECP2, MED12, MED16, MED24, MED25, MEF2D, MEIS3P1, MEIS3P2, MEN1, MFSD2A, MID1IP1, MLLT6, MPV17L2, MSTN, MSX1, MTCO2P12, MYC, MYRF, NCOA6, NDP, NFATC4, NFE2, NFIC, NFIX, NFKB2, NFKBIB, NGFR, NLRC5, NOD2, NODAL, NOS3, NOTCH1, NOTCH3, NOTCH4, NPAS2, NR1D1, NR4A1, NR4A2, NR4A3, OSR2, P2RY6, PDGFB, PDGFRB, PELP1, PER1, PHF1, PIM1, PKD1, PKM, PLAGL2, PML, POR, PPARD, PPP1R15A, PPRC1, PQBP1, PRDM16, PRKCSH, PTPRN, RAI1, RARA, RASL11A, RBM14, RCVRN, RELA, RFXAP, RGCC, RGMA, RNU1-93P, RPL26, RPTOR, RXRA, RXRB, SALL2, SBNO2, SCAF1, SERPINE1, SIX5, SLC35A4, SLC51B, SLC6A9, SLC9A1, SMAD3, SMARCA4, SMARCB1, SNAI1, SOX10, SOX12, SOX7, SOX8, SPDEF, SPHK2, SPI1, SRC, SRCAP, SREBF1, SRF, SSBP3, STAT3, STING1, TAF6, TBX5, TCF3, TCF7L1, TEAD2, TEAD3, TEAD4, TFE3, TFEB, TFEC, TGFB1I1, THBS1, TICAM1, TIMP1, TNF, TNFAIP1, TNFRSF1A, TNIP1, TOX2, TP53INP2, TRIM62, TRIM8, UBA52, USF2, VDR, VEGFA, VENTX, WAS, WBP2, WIZ, ZBTB7B, ZC3H12A, ZMIZ1, ZMIZ2, ZNF219, ZNF335, ZNF384

GO:0019220	regulation of phosphate metabolic process	1,01E-03	[5, 6]	295,00	17,84	38,64	61,36	[ADAM8, ADCY7, AIF1, ANGPT1, AREG, ATF3, BANK1, BMP7, CALCR, CASS4, CCDC88C, CCL19, CCL5, CD19, CD244, CD33, CD3E, CD4, CD40LG, CD74, CD80, CD86, CDCA2, CDKN2A, CISH, CLCF1, CSF1R, CXCL10, CXCR4, DUSP2, DUSP8, EPHA6, EPX, EREG, FAM122C, FCRL3, FGD2, FGR, FLT3, GADD45B, GDF6, GFRA2, GMFG, GPRC5A, HCLS1, HLA-DRB1, IFNG, IKBKE, IL18, ITGB2, LAX1, LCP2, LDLR, LIF, LILRB4, MAP4K1, MIDN, MMP9, MUSK, NCF1, NCKAP1L, NLRP3, NPFFR2, NR4A3, NUP210, P2RY12, PDE6G, PFKFB4, PIK3CG, PIK3IP1, PIK3R5, PLEK, PPP1R42, PPP2R2B, PROK1, PTAFR, PTK2B, PTPN22, PTPN6, PTPRC, PTPRH, PYCARD, RAC2, RHOH, SAMSN1, SEMA4D, SH2D1A, SLA2, SLAMF8, SLC8A3, SMPD3, SOCS1, SOCS3, SPINK1, STAP1, SYK, THBS1, THY1, TLR6, TLR8, TNFAIP3, TNFRSF18, TREM2, WNT1]	[AAAS, ABCA3, ABL1, ACP4, ACTB, ADCY1, ADCY3, ADCY4, ADCY9, ADIPOQ, AGAP2, AGER, AGT, AKT1S1, AMH, ANGPT1, APLN, APLNR, APOA1, ARAF, AREG, ARF1, ARHGEF5, ARTN, ATPSCKMT, BMP8A, BRD4, C5, C5AR1, CABIN1, CALCR, CCL21, CCNG2, CCNJL, CCR7, CD74, CDC25B, CDCA2, CDKN1A, CEACAM1, CHI3L1, CISH, CLCF1, CLIP3, CSF1, CSK, CSPG4, CTDSP1, CTF1, CX3CL1, DAB2IP, DAG1, DAXX, DBF4B, DBNDD1, DDR1, DGKZ, DHX34, DIRAS1, DMTN, DUSP5, DUSP8, DVL3, EIF4G1, ENG, EPHA2, ERBB2, FABP4, FAM122C, FASN, FGFR4, FLT3, FLT4, FPR1, GADD45B, GBA, GDF6, GIT1, GNAI2, GNB3, GPER1, GPRC5A, GSK3A, HBEGF, HFE, HGS, HSF1, HSPB1, HTR2A, ICAM1, IL1B, ILK, INCA1, INCENP, INHA, INPP5J, IRAK2, ITGA5, ITPRIP, JMJD8, KSR1, LDLR, LIF, LILRA5, LIMK2, LOX, LTF, MAP2K3, MAP3K10, MAP3K11, MAP3K14, MAP3K6, MAP3K9, MAPK3, MAPK8IP3, MARK2, MAVS, MEN1, MFSD2A, MIDN, MINK1, MLLT1, MLST8, MSTN, MTCO2P12, MUC20, MYADM, MYC, NF2, NGF, NLRC5, NOD2, NODAL, NOS3, NPFFR2, NPPA, NR4A3, NSMF, NUP188, OMA1, OSM, P2RY6, PAK4, PDGFB, PDGFRB, PDK2, PDPR, PHACTR1, PKD1, PLAUR, PML, PPP1R10, PPP1R11, PPP1R15A, PPP1R16A, PPP1R9B, PPP2R1A, PPP2R5B, PPP2R5D, PPP6R1, PRKACA, PRKAR2B, PROM2, PTN, PTPN13, PTPRH, PTPRU, RAC2, RAP1A, RAPGEF1, RCVRN, RGCC, RHBDF2, RNU1-93P, RPLP1, RPTOR, RUBCN, S1PR2, SH2B3, SH2D3A, SH2D3C, SHC2, SLC2A6, SLC9A3R1, SMAD3, SMPD1, SOCS3, SPATC1L, SPHK1, SPHK2, SPINK1, SRC, STAT3, TESK1, THBS1, THPO, TIMP1, TNF, TNFRSF1A, TNK2, TNKS1BP1, TRAF4, TRIB1, TRIB3, TSC2, UBA52, UBE2B, VAC14, VEGFA, VTN, WARS1, WNT9B, ZBTB7A, ZC3H12A]
GO:0045937	positive regulation of phosphate metabolic process	6,68E-03	[5, 6, 7]	198,00	18,63	38,47	61,53	[ADAM8, ADCY7, AIF1, ANGPT1, AREG, BANK1, BMP7, CALCR, CASS4, CCL19, CCL5, CD19, CD244, CD33, CD3E, CD4, CD40LG, CD74, CD80, CD86, CDCA2, CLCF1, CSF1R, CXCR4, EPHA6, EPX, EREG, FCRL3, FGD2, FGR, FLT3, GADD45B, GDF6, GPRC5A, HCLS1, HLA-DRB1, IFNG, IKBKE, IL18, LCP2, LIF, MAP4K1, MMP9, MUSK, NCF1, NCKAP1L, NLRP3, NR4A3, P2RY12, PDE6G, PFKFB4, PIK3CG, PIK3R5, PLEK, PROK1, PTAFR, PTK2B, PTPRC, SEMA4D, SH2D1A, SOCS1, SOCS3, STAP1, SYK, THBS1, TLR6, TNFRSF18, TREM2, WNT1]	[ABL1, ADCY1, ADCY3, ADCY4, ADCY9, ADIPOQ, AGAP2, AGER, AGT, AMH, ANGPT1, APLN, APLNR, ARAF, AREG, ARHGEF5, ARTN, BMP8A, C5, C5AR1, CALCR, CCL21, CCR7, CD74, CDC25B, CDCA2, CDKN1A, CHI3L1, CLCF1, CLIP3, CSF1, CSK, CSPG4, CTF1, CX3CL1, DAB2IP, DAG1, DAXX, DBF4B, DDR1, DGKZ, DHX34, DIRAS1, DUSP5, DVL3, EIF4G1, ENG, EPHA2, ERBB2, FASN, FGFR4, FLT3, FLT4, FPR1, GADD45B, GBA, GDF6, GPER1, GPRC5A, GSK3A, HBEGF, HFE, HSF1, HTR2A, ICAM1, IL1B, ILK, INCENP, INHA, IRAK2, ITGA5, KSR1, LIF, LILRA5, LIMK2, LTF, MAP2K3, MAP3K10, MAP3K11, MAP3K14, MAP3K6, MAP3K9, MAPK3, MAPK8IP3, MARK2, MAVS, MINK1, MLST8, MSTN, MTCO2P12, MUC20, MYC, NGF, NOD2, NODAL, NOS3, NR4A3, NSMF, OMA1, OSM, P2RY6, PAK4, PDGFB, PDGFRB, PKD1, PLAUR, PPP1R15A, PPP2R5D, PRKACA, PRKAR2B, PROM2, RAP1A, RAPGEF1, RCVRN, RGCC, RHBDF2, RNU1-93P, RPLP1, RPTOR, S1PR2, SH2D3A, SH2D3C, SHC2, SMAD3, SMPD1, SOCS3, SPATC1L, SPHK1, SRC, STAT3, TESK1, THBS1, THPO, TIMP1, TNF, TNFRSF1A, TNK2, TNKS1BP1, TRAF4, UBA52, VAC14, VEGFA, VTN]
GO:0031401	positive regulation of protein modification process	1,63E-02	[5, 6, 7, 8]	207,00	18,22	36,05	63,95	[ADAM8, ADCY7, AIF1, ANGPT1, AREG, BANK1, BMP7, CALCR, CASS4, CCL19, CCL5, CD33, CD3E, CD4, CD40LG, CD74, CD80, CD86, CDC20B, CDCA2, CDKN2A, CLCF1, CSF1R, CXCR4, EPX, EREG, FCRL3, FGD2, FLT3, FOXP3, GADD45B, GDF6, GPRC5A, HCLS1, HLA-DRB1, IFNG, IKBKE, IL18, LCP2, LIF, MAP4K1, MMP9, MUSK, MYB, NCF1, NCKAP1L, NLRP3, NR4A3, PDE6G, PIK3CG, PIK3R5, PROK1, PTK2B, PTPN22, PTPRC, SEMA4D, SH2D1A, SOCS1, SOCS3, STAP1, SYK, THBS1, TLR6, TNFRSF18, TRABD2A, TREM2, WNT1]	[ABL1, ADCY1, ADCY3, ADCY4, ADCY9, ADIPOQ, AGAP2, AGER, AGT, AMH, ANGPT1, APLNR, ARAF, AREG, ARHGEF5, ARTN, BCL6, BMP8A, BRD4, C5, C5AR1, CALCR, CCL21, CCR7, CD74, CDC25B, CDCA2, CDKN1A, CHI3L1, CLCF1, CLIP3, CSF1, CSK, CSPG4, CTF1, CX3CL1, DAB2IP, DAG1, DAXX, DBF4B, DIRAS1, DNMT1, DUSP5, DVL3, EIF4G1, ENG, ERBB2, FASN, FLT3, FLT4, FPR1, GADD45B, GBA, GDF6, GPER1, GPRC5A, GSK3A, HBEGF, HFE, HSF1, HTR2A, ICAM1, IL1B, ILK, INCENP, INHA, IRAK2, ITGA5, KSR1, LIF, LILRA5, LIMK2, LMNA, LTF, MAP2K3, MAP3K10, MAP3K11, MAP3K14, MAP3K6, MAP3K9, MAPK3, MAPK8IP3, MARK2, MAVS, MECP2, MINK1, MLST8, MSTN, MUC20, NAP1L2, NGF, NOD2, NODAL, NR4A3, NSMF, OMA1, OSM, PAF1, PAK4, PDGFB, PDGFRB, PEF1, PHF1, PIAS4, PKD1, PLAUR, PPP1R15A, PPP2R5D, PRKACA, PRKAR2B, PROM2, RAP1A, RAPGEF1, RASD2, RASSF5, RGCC, RHBDF2, RNU1-93P, RPLP1, RPTOR, S1PR2, SEPTIN4, SH2D3A, SH2D3C, SHC2, SLC51B, SMARCB1, SMPD1, SOCS3, SPATC1L, SPHK1, SPHK2, SRC, SREBF1, STAT3, TESK1, THBS1, THPO, TICAM1, TIMP1, TNF, TNFRSF1A, TNIP1, TNK2, TNKS1BP1, TRAF4, TRIB3, UBA52, VEGFA, VTN, WBP2, WFS1, ZBTB7B, ZC3H12A]

GO:0001932	regulation of protein phosphorylation	1,95E-03	[6, 7, 8]	235,00	18,39	38,75	61,25	[ADAM8, ADCY7, AIF1, ANGPT1, AREG, ATF3, BANK1, BMP7, CALCR, CASS4, CCDC88C, CCL19, CCL5, CD3E, CD4, CD40LG, CD74, CD80, CD86, CDKN2A, CLCF1, CSF1R, CXCL10, CXCR4, DUSP2, DUSP8, EPX, EREG, FGD2, FGR, FLT3, GADD45B, GDF6, GFRA2, GMFG, GPRC5A, HCLS1, HLA-DRB1, IFNG, IKBKE, IL18, ITGB2, LAX1, LCP2, LIF, LILRB4, MAP4K1, MMP9, MUSK, NCF1, NCKAP1L, NLRP3, NPFFR2, NR4A3, PDE6G, PIK3CG, PIK3R5, PROK1, PTK2B, PTPN22, PTPN6, PTPRC, PTPRH, PYCARD, RAC2, SAMSN1, SEMA4D, SH2D1A, SLC8A3, SMPD3, SOCS1, SOCS3, SPINK1, STAP1, SYK, THBS1, THY1, TLR6, TLR8, TNFAIP3, TNFRSF18, TREM2, WNT1]	[ABL1, ACP4, ACTB, ADCY1, ADCY3, ADCY4, ADCY9, ADIPOQ, AGAP2, AGER, AGT, AKT1S1, AMH, ANGPT1, APLNR, APOA1, ARAF, AREG, ARHGEF5, ARTN, BMP8A, BRD4, C5, C5AR1, CALCR, CCL21, CCNG2, CCNJL, CCR7, CD74, CDC25B, CDKN1A, CEACAM1, CHI3L1, CLCF1, CLIP3, CSF1, CSK, CSPG4, CTDSP1, CTF1, CX3CL1, DAB2IP, DAG1, DAXX, DBF4B, DBNDD1, DIRAS1, DMTN, DUSP5, DUSP8, DVL3, EIF4G1, ENG, ERBB2, FABP4, FASN, FLT3, FLT4, FPR1, GADD45B, GBA, GDF6, GPER1, GPRC5A, GSK3A, HBEGF, HFE, HGS, HSF1, HSPB1, HTR2A, ICAM1, IL1B, ILK, INCA1, INCENP, INHA, INPP5J, IRAK2, ITGA5, ITPRIP, KSR1, LIF, LILRA5, LIMK2, LOX, LTF, MAP2K3, MAP3K10, MAP3K11, MAP3K14, MAP3K6, MAP3K9, MAPK3, MAPK8IP3, MARK2, MAVS, MEN1, MINK1, MLLT1, MLST8, MSTN, MUC20, MYADM, NF2, NGF, NOD2, NODAL, NPFFR2, NPPA, NR4A3, OMA1, OSM, PAK4, PDGFB, PDGFRB, PKD1, PLAUR, PML, PPP1R9B, PPP2R1A, PPP2R5B, PPP2R5D, PRKACA, PRKAR2B, PROM2, PTPN13, PTPRH, PTPRU, RAC2, RAP1A, RAPGEF1, RGCC, RHBDF2, RNU1-93P, RPLP1, RPTOR, S1PR2, SH2B3, SH2D3A, SH2D3C, SHC2, SLC9A3R1, SMPD1, SOCS3, SPATC1L, SPHK1, SPINK1, SRC, STAT3, TESK1, THBS1, THPO, TIMP1, TNF, TNFRSF1A, TNK2, TNKS1BP1, TRAF4, TRIB1, TRIB3, TSC2, UBA52, UBE2B, VEGFA, VTN, WARS1, WNT9B, ZC3H12A]
GO:0042327	positive regulation of phosphorylation	2,10E-02	[6, 7, 8]	181,00	18,55	38,39	61,61	[ADAM8, ADCY7, AIF1, ANGPT1, AREG, BANK1, BMP7, CALCR, CASS4, CCL19, CCL5, CD19, CD3E, CD4, CD40LG, CD74, CD80, CD86, CLCF1, CSF1R, CXCR4, EPHA6, EPX, EREG, FGD2, FGR, FLT3, GADD45B, GDF6, GPRC5A, HCLS1, HLA-DRB1, IFNG, IKBKE, IL18, LCP2, LIF, MAP4K1, MMP9, MUSK, NCF1, NCKAP1L, NLRP3, NR4A3, P2RY12, PDE6G, PFKFB4, PIK3CG, PIK3R5, PROK1, PTK2B, PTPRC, SEMA4D, SH2D1A, SOCS1, SOCS3, STAP1, SYK, THBS1, TLR6, TNFRSF18, TREM2, WNT1]	[ABL1, ADCY1, ADCY3, ADCY4, ADCY9, ADIPOQ, AGAP2, AGER, AGT, AMH, ANGPT1, APLN, APLNR, ARAF, AREG, ARHGEF5, ARTN, BMP8A, C5, C5AR1, CALCR, CCL21, CCR7, CD74, CDC25B, CDKN1A, CHI3L1, CLCF1, CLIP3, CSF1, CSK, CSPG4, CTF1, CX3CL1, DAB2IP, DAG1, DAXX, DBF4B, DDR1, DGKZ, DIRAS1, DUSP5, DVL3, EIF4G1, ENG, EPHA2, ERBB2, FASN, FGFR4, FLT3, FLT4, FPR1, GADD45B, GDF6, GPER1, GPRC5A, GSK3A, HBEGF, HFE, HSF1, HTR2A, ICAM1, IL1B, ILK, INCENP, INHA, IRAK2, ITGA5, KSR1, LIF, LILRA5, LIMK2, LTF, MAP2K3, MAP3K10, MAP3K11, MAP3K14, MAP3K6, MAP3K9, MAPK3, MAPK8IP3, MARK2, MAVS, MINK1, MLST8, MSTN, MUC20, MYC, NGF, NOD2, NODAL, NR4A3, OMA1, OSM, PAK4, PDGFB, PDGFRB, PKD1, PLAUR, PRKACA, PRKAR2B, PROM2, RAP1A, RAPGEF1, RGCC, RHBDF2, RNU1-93P, RPLP1, RPTOR, S1PR2, SH2D3A, SH2D3C, SHC2, SOCS3, SPATC1L, SPHK1, SRC, STAT3, TESK1, THBS1, THPO, TIMP1, TNF, TNFRSF1A, TNK2, TNKS1BP1, TRAF4, UBA52, VAC14, VEGFA, VTN]
GO:0001934	positive regulation of protein phosphorylation	1,34E-02	[6, 7, 8, 9]	169,00	18,95	37,74	62,26	[ADAM8, ADCY7, AIF1, ANGPT1, AREG, BANK1, BMP7, CALCR, CASS4, CCL19, CCL5, CD3E, CD4, CD40LG, CD74, CD80, CD86, CLCF1, CSF1R, CXCR4, EPX, EREG, FGD2, FLT3, GADD45B, GDF6, GPRC5A, HCLS1, HLA-DRB1, IFNG, IKBKE, IL18, LCP2, LIF, MAP4K1, MMP9, MUSK, NCF1, NCKAP1L, NLRP3, NR4A3, PDE6G, PIK3CG, PIK3R5, PROK1, PTK2B, PTPRC, SEMA4D, SH2D1A, SOCS1, SOCS3, STAP1, SYK, THBS1, TLR6, TNFRSF18, TREM2, WNT1]	[ABL1, ADCY1, ADCY3, ADCY4, ADCY9, ADIPOQ, AGAP2, AGER, AGT, AMH, ANGPT1, APLNR, ARAF, AREG, ARHGEF5, ARTN, BMP8A, C5, C5AR1, CALCR, CCL21, CCR7, CD74, CDC25B, CDKN1A, CHI3L1, CLCF1, CLIP3, CSF1, CSK, CSPG4, CTF1, CX3CL1, DAB2IP, DAG1, DAXX, DBF4B, DIRAS1, DUSP5, DVL3, EIF4G1, ENG, ERBB2, FASN, FLT3, FLT4, FPR1, GADD45B, GDF6, GPER1, GPRC5A, GSK3A, HBEGF, HFE, HSF1, HTR2A, ICAM1, IL1B, ILK, INCENP, INHA, IRAK2, ITGA5, KSR1, LIF, LILRA5, LIMK2, LTF, MAP2K3, MAP3K10, MAP3K11, MAP3K14, MAP3K6, MAP3K9, MAPK3, MAPK8IP3, MARK2, MAVS, MINK1, MLST8, MSTN, MUC20, NGF, NOD2, NODAL, NR4A3, OMA1, OSM, PAK4, PDGFB, PDGFRB, PKD1, PLAUR, PRKACA, PRKAR2B, PROM2, RAP1A, RAPGEF1, RGCC, RHBDF2, RNU1-93P, RPLP1, RPTOR, S1PR2, SH2D3A, SH2D3C, SHC2, SOCS3, SPATC1L, SPHK1, SRC, STAT3, TESK1, THBS1, THPO, TIMP1, TNF, TNFRSF1A, TNK2, TNKS1BP1, TRAF4, UBA52, VEGFA, VTN]

GO:0045893	positive regulation of transcription, DNA-templated	2,65E-02	[6, 7, 8, 9, 10, 11]	290,00	17,22	33,22	66,78	[ABRA, AKNA, AREG, ASCL2, ATF3, ATP1B4, BATF, BCL11B, BCL3, BHLHA15, BMP7, BMPR1B, BRIP1, CAMK4, CD4, CD74, CD80, CD86, CDH1, CDKN2A, CREM, CXCL10, E2F8, EAF2, EGR3, ELF3, EOMES, EPX, FOSB, FOSL1, FOXP3, GDF6, GLIS1, H1-3, HCLS1, HLA-DRB1, IKZF3, IL18, IL26, IRF4, IRF5, IRF8, IRX3, KLF5, KLRG1, LEF1, LIF, LILRB1, LMO1, MACC1, MAFF, MIXL1, MSX1, MYB, MYBL2, NAMPT, NCF1, NLRP3, NR4A1, NR4A2, NR4A3, PAX5, PBX4, PIM1, PIM2, PLAC8, POU2AF1, POU2F2, PRKCB, PYHIN1, RUNX3, SALL1, SKAP1, SPIB, SPIC, STAT1, STXBP2, TBX21, TFEC, TNFSF8, TNIP3, TRERF1, TRIM14, WAS, WNT1, WNT10B]	[ABL1, ABLIM3, ABRA, ACTN1, ACTN4, AGAP2, AGT, ALPK3, AP3D1, APLN, AREG, ARID1A, ATF5, ATP1B4, BCL3, BCL9L, BRD4, CAMTA2, CARM1, CASZ1, CD74, CGA, CHCHD10, CHERP, CHGA, CIITA, CITED2, CITED4, COL1A1, CREB3L1, CRTC2, CSRNP3, CX3CL1, CYP27B1, DAB2IP, DAXX, DDX39B, DPF2, DTX1, DVL3, DXO, DYRK1B, E2F4, E2F7, E2F8, EAF2, ELF3, ELF4, ELK1, ENG, ERBB2, ETS2, ETV6, FADD, FHL5, FHOD1, FOSL1, FOSL2, FOXC2, FOXF1, FOXM1, GATA2, GATA4, GDF6, GLIS2, GLMP, GPER1, GPRIN1, GSK3A, H1-9P, HCFC1, HDAC5, HEYL, HLTF, HMGA1, HMGN1, HMGN5, HSF1, IL1B, ILK, IRF2, IRF2BPL, IRX3, IRX6, JUNB, JUND, JUP, KCNH2, KDM6B, KLF10, KLF5, KMT2D, LIF, LMX1A, LMX1B, LUM, MAFG, MAMSTR, MAP2K3, MAPK3, MAPK7, MAVS, MAZ, MECP2, MED12, MED16, MED24, MED25, MEF2D, MEIS3P1, MEIS3P2, MEN1, MLLT6, MSTN, MSX1, MYC, MYRF, NCOA6, NDP, NFATC4, NFE2, NFIC, NFIX, NFKB2, NFKBIB, NGFR, NLRC5, NOD2, NODAL, NOTCH1, NOTCH3, NOTCH4, NPAS2, NR1D1, NR4A1, NR4A2, NR4A3, OSR2, PDGFB, PELP1, PER1, PHF1, PIM1, PKD1, PLAGL2, PML, PPARD, PPRC1, PQBP1, PRDM16, PTPRN, RAI1, RARA, RASL11A, RBM14, RELA, RFXAP, RGCC, RGMA, RPTOR, RXRA, RXRB, SALL2, SBNO2, SCAF1, SERPINE1, SIX5, SLC9A1, SMAD3, SMARCA4, SMARCB1, SNAI1, SOX10, SOX12, SOX7, SOX8, SPDEF, SPI1, SRC, SRCAP, SREBF1, SRF, SSBP3, STAT3, STING1, TAF6, TBX5, TCF3, TCF7L1, TEAD2, TEAD3, TEAD4, TFE3, TFEB, TFEC, TGFB1I1, TIMP1, TNF, TNFRSF1A, TNIP1, TOX2, TP53INP2, TRIM62, TRIM8, UBA52, USF2, VDR, VEGFA, VENTX, WAS, WBP2, ZBTB7B, ZC3H12A, ZMIZ1, ZMIZ2, ZNF219, ZNF335, ZNF384, ZNF395, ZNF574, ZNF580, ZNF593, ZNF609, ZNF629]
GO:0045944	positive regulation of transcription by RNA polymerase II	1,18E-02	[7, 8, 9, 10, 11, 12]	225,00	18,07	32,70	67,30	[ABRA, AKNA, AREG, ASCL2, ATF3, ATP1B4, BATF, BCL11B, BCL3, BHLHA15, BMP7, BMPR1B, BRIP1, CDKN2A, CREM, CXCL10, E2F8, EAF2, EGR3, ELF3, FOSB, FOSL1, FOXP3, GLIS1, HCLS1, IKZF3, IL18, IL26, IRF4, IRF5, IRF8, IRX3, KLF5, KLRG1, LEF1, LIF, LILRB1, LMO1, MACC1, MAFF, MIXL1, MSX1, MYB, MYBL2, NAMPT, NLRP3, NR4A1, NR4A2, NR4A3, PAX5, PLAC8, POU2AF1, POU2F2, SALL1, SKAP1, SPIB, SPIC, STAT1, STXBP2, TFEC, TNFSF8, TNIP3, TRERF1, WAS, WNT1, WNT10B]	[ABL1, ABLIM3, ABRA, AGAP2, AP3D1, APLN, AREG, ATF5, ATP1B4, BCL3, BCL9L, BRD4, CAMTA2, CASZ1, CGA, CHGA, CIITA, CITED2, CITED4, CREB3L1, CRTC2, CSRNP3, CX3CL1, CYP27B1, DAB2IP, DPF2, DVL3, E2F4, E2F7, E2F8, EAF2, ELF3, ELF4, ELK1, ENG, ETS2, ETV6, FADD, FHL5, FHOD1, FOSL1, FOSL2, FOXC2, FOXF1, FOXM1, GATA2, GATA4, GLIS2, GLMP, GPER1, GPRIN1, GSK3A, HCFC1, HDAC5, HEYL, HLTF, HMGA1, HSF1, IRF2, IRF2BPL, IRX3, IRX6, JUNB, JUND, JUP, KDM6B, KLF10, KLF5, KMT2D, LIF, LMX1A, LMX1B, LUM, MAFG, MAMSTR, MAPK3, MAPK7, MAVS, MECP2, MED12, MED16, MED24, MED25, MEF2D, MEIS3P1, MEIS3P2, MEN1, MLLT6, MSX1, MYC, NCOA6, NFATC4, NFIC, NFIX, NFKB2, NGFR, NLRC5, NOD2, NODAL, NOTCH1, NOTCH3, NOTCH4, NR1D1, NR4A1, NR4A2, NR4A3, OSR2, PDGFB, PELP1, PER1, PKD1, PLAGL2, PPARD, PPRC1, PRDM16, PTPRN, RARA, RBM14, RELA, RFXAP, RGCC, RGMA, RXRA, RXRB, SALL2, SBNO2, SERPINE1, SIX5, SLC9A1, SMAD3, SMARCA4, SMARCB1, SOX10, SOX12, SOX8, SPDEF, SPI1, SREBF1, SRF, SSBP3, STAT3, STING1, TAF6, TBX5, TCF3, TCF7L1, TEAD2, TEAD3, TEAD4, TFE3, TFEB, TFEC, TNF, TNFRSF1A, TNIP1, TOX2, UBA52, USF2, VDR, VEGFA, VENTX, WAS, WBP2, ZBTB7B, ZC3H12A, ZMIZ1, ZMIZ2, ZNF219, ZNF335, ZNF384, ZNF395, ZNF574, ZNF580, ZNF593, ZNF609, ZNF629]

GO:0023051	regulation of signaling	5,81E-15	[2, 3]	675,00	18,15	37,12	62,88	[ABRA, ADAM8, ALOX15, ALOX15B, ANGPT1, ARAP2, AREG, ARHGAP15, ARHGAP30, ARHGAP45, ATF3, ATP2A3, BANK1, BCL2L14, BCL3, BLK, BMF, BMP7, BMPR1B, BRIP1, C1QTNF1, CACNA1E, CALCR, CARD11, CASP8, CASS4, CCDC88C, CCL17, CCL19, CCL2, CCL22, CCL24, CCL3, CCL3L1, CCL4, CCL4L1, CCL5, CCR2, CD177, CD180, CD19, CD22, CD226, CD27, CD300LF, CD3E, CD4, CD40LG, CD74, CD80, CD86, CDH1, CDKN2A, CEL, CHRDL1, CISH, CLEC6A, CNR1, CNR2, CRB2, CSF1R, CXCR4, CXorf21, DAPK1, DCC, DEPDC1B, DLGAP2, DPEP2, DRD1, DUSP2, DUSP8, ELAVL4, ELF3, EPX, EREG, ERFE, FASLG, FCRL3, FFAR4, FGD2, FGD3, FGR, FLT3, FOLR2, FOXP3, FRZB, FUT7, GADD45B, GDF6, GFI1, GMIP, GPR174, GPR4, GPR55, GPRC5A, GUCY2D, H3C10, H3C11, H3C12, H3C7, HCLS1, HCST, HLA-DRB1, ICOS, IER3, IFNG, IKBKE, IL10RA, IL18, IL26, IL7R, INPP5D, IQCJ-SCHIP1, IRF4, KCNJ10, KCNN4, KMO, LAX1, LCK, LEF1, LIF, LILRB4, LMCD1, LY86, MAP4K1, MIDN, MMP9, MSX1, MYB, MZB1, NCF1, NCKAP1L, NFAM1, NLRC3, NLRP3, NLRP6, NPFFR2, NPPC, NPTX2, NR4A2, NSG1, NTN1, OASL, P2RY10, P2RY12, PAX5, PDE6G, PIK3CG, PIK3IP1, PIK3R5, PIM2, PLAU, PLEK, PRDM1, PRKCB, PRKCQ, PROK1, PSCA, PSD4, PTK2B, PTPN22, PTPN6, PTPRC, PYCARD, PYHIN1, RAC2, RAD9B, RASAL3, RASGRF1, RBPMS2, RGS1, RGS10, RGS18, RGS9, RHOH, RMI2, RNASE6, RRAD, RTKN2, SALL1, SCIMP, SCUBE1, SEMA4D, SH2D1A, SHISA8, SLA, SLA2, SLC24A4, SLC8A3, SMPDL3B, SOCS1, SOCS3, SPINK1, STAP1, STAT1, SYK, TAGAP, TBC1D10C, TESPA1, THBS1, THY1, TLR6, TMC8, TNFAIP3, TNFSF14, TNIP3, TNMD, TNR, TRABD2A, TRAF1, TRAT1, TREM2, TRIM14, TRPM2, UBASH3A, UBD, UCP2, VAV1, VNN1, VWC2L, WNT1, WNT10B, XCL1, XCL2, ZAP70, ZBP1]	[ABCA7, ABCC8, ABL1, ABR, ABRA, ACKR3, ACP4, ACTN4, ADCY1, ADGRG1, ADIPOQ, AGAP2, AGER, AGPAT1, AGT, AGTR1, AKT1S1, AMH, ANGPT1, APLN, APLNR, APLP1, APOA1, APOD, ARAF, ARAP3, ARC, AREG, ARF1, ARHGAP1, ARHGAP23, ARHGAP39, ARHGDIA, ARHGEF1, ARHGEF16, ARHGEF5, ARRDC3, ARTN, ASPN, ATP1A1, ATP1A3, ATP1B2, BCL2L1, BCL3, BCL6, BCL9L, BCR, BEND6, BMP8A, BRD4, C1QTNF1, C20orf27, C2CD2L, C5, C5AR1, CACNA1E, CALB1, CALCR, CAMK2B, CAPN1, CARM1, CC2D1A, CCL11, CCL2, CCL21, CCL24, CCR7, CD14, CD177, CD74, CDK1, CDK2, CEACAM1, CEL, CGA, CHERP, CHGA, CHI3L1, CISH, CITED2, CLIP3, CNR1, CNTN6, COL1A1, CPLX1, CRAT, CREB3L1, CRHR2, CSF1, CSK, CSNK1E, CSPG4, CTHRC1, CX3CL1, CXXC4, CYP26B1, CYP27B1, DAB2IP, DAG1, DAXX, DDX39B, DENND2B, DENND4B, DGKD, DGKZ, DHX34, DIRAS1, DKK2, DLG5, DLGAP4, DMTN, DOC2B, DOK5, DOT1L, DTX1, DUSP5, DUSP8, DVL3, ECM1, EEF1E1, ELF3, ENG, EPHA2, EPN1, ERBB2, ERFE, ESM1, FADD, FAIM2, FAM110A, FASN, FFAR2, FGFR4, FLOT2, FLT3, FLT4, FOXM1, FPR1, FRMD8, FURIN, FXYD6, GAB2, GADD45B, GAS1, GATA4, GBA, GBF1, GCSAM, GDF6, GIT1, GJD3, GLIS2, GLRA1, GNAI2, GPAM, GPBAR1, GPER1, GPI, GPR137, GPR17, GPR20, GPR37L1, GPR4, GPRC5A, GPRIN1, GRB7, GRIK5, GRINA, GRM2, GSK3A, H3C10, H3C12, H4C3, HBEGF, HCRTR1, HDAC7, HEYL, HFE, HGS, HIPK4, HSPB1, HTR1B, HTR2A, ICAM1, IER3, IFT80, IL18BP, IL1B, IL1RN, IL7R, ILK, INCA1, INHA, INPP5E, IRAK2, ITGA3, ITGA5, ITPR3, ITPRIP, JAK3, JMJD8, JPH4, JUP, KCP, KCTD11, KIF26A, KLK14, KMT2D, KSR1, LGALS9, LGR6, LIF, LIFR, LILRA5, LIMS2, LMCD1, LMNA, LOX, LRG1, LRRC4, LRRC8A, LTBR, LTF, LY6E, LYNX1, LZTS1, LZTS2, MAP1A, MAP2K3, MAP3K10, MAP3K11, MAP3K14, MAP3K6, MAP3K9, MAPK3, MAPK7, MAPK8IP3, MAVS, MAZ, MECP2, MED12, MEGF8, MEIS3P1, MEN1, MGRN1, MIDN, MINK1, MLST8, MME, MNT, MSTN, MSX1, MTRNR2L1, MTRNR2L10, MTRNR2L12, MTRNR2L2, MTRNR2L3
GO:0048583	regulation of response to stimulus	4,40E-27	[2, 3]	851,00	18,88	45,12	54,88	[ABRA, ACP5, ADAM8, ADCY7, AIF1, AIM2, AKNA, ALOX15, ALOX15B, ANGPT1, ANGPT2, AOAH, APOBEC3G, ARAP2, AREG, ARHGAP15, ARHGAP30, ARHGAP45, ATF3, BANK1, BCL2L14, BCL3, BLK, BLNK, BMF, BMP7, BMPR1B, BRIP1, BTK, BTLA, BTN3A1, BTN3A2, C1QA, C1QB, C1QC, C1QTNF1, C2, C8G, CALCR, CARD11, CASP8, CASS4, CCDC88C, CCL17, CCL19, CCL2, CCL22, CCL24, CCL3, CCL3L1, CCL4, CCL4L1, CCL5, CCR2, CCR4, CD177, CD180, CD19, CD1B, CD1C, CD1E, CD200R1, CD22, CD226, CD247, CD27, CD300LF, CD33, CD3D, CD3E, CD3G, CD4, CD40LG, CD48, CD74, CD79A, CD80, CD84, CD86, CD8A, CD8B, CD96, CDKN2A, CEL, CFP, CHRDL1, CISH, CLCF1, CLEC10A, CLEC1B, CLEC4D, CLEC4E, CLEC6A, CLNK, CNR1, CNR2, CR2, CRB2, CRTAM, CSF1R, CST7, CTLA4, CXCL10, CXCL6, CXCR4, CXorf21, CYP11B1, CYSLTR1, DAPK1, DCC, DEPDC1B, DLGAP2, DPEP1, DPEP2, DRD1, DUSP2, DUSP8, EDN2, ELF3, EPX, EREG, ERFE, FASLG, FCGR2C, FCN1, FCN3, FCRL3, FFAR4, FGD2, FGD3, FGL2, FGR, FLT3, FOLR2, FOXP3, FRZB, FUT7, FYB1, GADD45B, GBP5, GDF6, GFI1, GMIP, GPR150, GPR174, GPR4, GPR55, GPR83, GPRC5A, GPSM3, GRAP2, GUCY2D, H3C10, H3C11, H3C12, H3C7, HCLS1, HCST, HLA-DMB, HLA-DPA1, HLA-DPB1, HLA-DQA1, HLA-DRA, HLA-DRB1, HLA-DRB5, HOPX, ICOS, IER3, IFNG, IGLL5, IKBKE, IL10RA, IL12RB1, IL16, IL18, IL18RAP, IL26, IL7R, INPP5D, IQCJ-SCHIP1, IRF4, ITGA4, ITGAL, ITGB2, ITGB7, ITK, JAML, KCNN4, KIR2DL2, KIR2DL3, KIR3DL2, KLRB1, KLRC1, KLRC2, KLRD1, KLRK1, KNG1, LAG3, LAIR1, LAIR2, LAX1, LCK, LCP1, LCP2, LDLR, LEF1, LIF, LILRA1, LILRB1, LILRB4, LMCD1, LTA, LY86, MAP4K1, MAPK13, MLC1, MMP9, MOG, MS4A1, MSX1, MUC16, MUC19, MUC6, MYB, MYO1G, MZB1, NAMPT, NCF1, NCKAP1L, NCR1, NCR3, NFAM1, NLRC3, NLRP3, NLRP6, NPFFR2, NPPC, NPTX2, NR4A2, NR4A3, NTN1, NUP210, OASL, P2RY10, P2RY12, PAX5, PDE6G, PIK3CG, PIK3IP1, PIK3R5, PILRA, PIM2, PLA2G2D, PLA2G7, PLAU, PLEK, PRAM1, PRDM1, PRKCB, PRKCQ,	[AAAS, ABCA7, ABCC8, ABCD1, ABI3, ABL1, ABR, ABRA, ACKR3, ACP4, ACTB, ACTN4, ADCY1, ADCY3, ADCY4, ADCY9, ADGRG1, ADIPOQ, AGAP2, AGER, AGPAT1, AGT, AGTR1, AKT1S1, AMH, ANGPT1, APLN, APLNR, APLP1, APOA1, APOD, ARAF, ARAP3, ARC, AREG, ARHGAP1, ARHGAP23, ARHGAP39, ARHGDIA, ARHGEF1, ARHGEF16, ARHGEF5, ARMT1, ARRDC3, ARTN, ASPN, BAG3, BAG6, BCAR1, BCL2L1, BCL3, BCL6, BCL9L, BCR, BEND6, BMP8A, BRD4, BTLA, C1QTNF1, C20orf27, C5, C5AR1, C8G, CALCR, CAMK2B, CAPN1, CARM1, CC2D1A, CCL11, CCL2, CCL21, CCL24, CCR7, CD14, CD177, CD1C, CD1E, CD276, CD300E, CD74, CDK1, CDK2, CEACAM1, CEL, CFH, CGA, CHERP, CHGA, CHI3L1, CISH, CITED2, CLC, CLCF1, CLEC1B, CLEC4E, CLIP3, CLNK, CNR1, CNTN6, COL1A1, CPN2, CRAT, CREB3L1, CRHR2, CRP, CRYAB, CSF1, CSK, CSNK1E, CSPG4, CST7, CTHRC1, CX3CL1, CXXC4, CYP26B1, CYP27B1, DAB2IP, DAG1, DAGLA, DAXX, DDX39B, DENND2B, DENND4B, DGKD, DGKZ, DIRAS1, DKK2, DLG5, DLGAP4, DMTN, DOK5, DOT1L, DTX1, DUSP5, DUSP8, DVL3, ECM1, EDN2, EEF1E1, EIF4G1, ELF3, ENDOG, ENG, ENPP3, EPHA2, EPN1, EPPK1, ERBB2, ERFE, ESM1, FABP4, FADD, FAIM2, FAM110A, FAP, FASN, FCN3, FEM1A, FES, FFAR2, FGFR4, FLOT2, FLT3, FLT4, FOXC2, FOXF1, FOXM1, FPR1, FRMD8, FURIN, GAB2, GADD45B, GAS1, GATA2, GATA4, GBA, GBF1, GCSAM, GDF6, GIT1, GLIS2, GNAI2, GNAO1, GPAM, GPBAR1, GPER1, GPI, GPR137, GPR17, GPR20, GPR37L1, GPR4, GPR83, GPR84, GPRC5A, GPRIN1, GRB7, GRIK5, GRINA, GRM2, GRN, GSK3A, GTF2IRD1, H3C10, H3C12, H4C3, HBEGF, HCRTR1, HDAC10, HDAC7, HEYL, HFE, HGS, HIPK4, HMGA1, HOPX, HRH2, HSF1, HSPB1, HSPB8, HTR1B, HTR2A, ICAM1, IER3, IFITM1, IFT80, IL18BP, IL1B, IL1RN, IL4R, IL7R, ILK, INCA1, INHA, INPP5E, IRAK2, ITGA3, ITGA5, ITPRIP, JAK3, JMJD8, JUP, KCNK2, KCP, KCTD11, KIF26A, KLK14, KMT2D, KNG1, KSR1, LAG3, LDLR, LGALS9, LGR6, LIF, LIFR, LILRA5, LIMK1, LIMS2, LMCD1, LMNA, LOX, LRG1, LTBR,

GO:0007165	signal transduction	1,01E-19	[2, 3, 4]	1126,00	17,01	42,72	57,28	[ABRA, ADAM8, ADCY7, ADGRE3, ADGRE4P, ADGRG5, ADGRG6, ADORA3, AIF1, AIM2, ALOX15, ALOX15B, ANGPT1, ANGPT2, ANPEP, APBB1IP, ARAP2, AREG, ARHGAP15, ARHGAP30, ARHGAP45, ASGR2, ATF3, ATP2A3, BANK1, BATF, BCL11B, BCL2L14, BCL3, BHLHA15, BLK, BLNK, BMF, BMP7, BMPR1B, BRIP1, BTG2, BTK, BTLA, BTN3A1, BTN3A2, C1QTNF1, CACNA1I, CALCR, CAMK4, CARD11, CASKIN1, CASP8, CASS4, CCDC88C, CCL17, CCL19, CCL2, CCL22, CCL24, CCL3, CCL3L1, CCL4, CCL4L1, CCL4L2, CCL5, CCR2, CCR3, CCR4, CCR5, CCR8, CD177, CD180, CD19, CD2, CD200R1, CD22, CD226, CD244, CD247, CD27, CD300LF, CD33, CD3D, CD3E, CD3G, CD4, CD40LG, CD5, CD53, CD6, CD7, CD74, CD79A, CD80, CD86, CD8A, CD8B, CDKN2A, CHRDL1, CISH, CLCF1, CLEC10A, CLEC1B, CLEC4D, CLEC4E, CLEC6A, CLNK, CMTM5, CNR1, CNR2, CORO1A, CORO2A, CR2, CRABP2, CRB2, CREM, CSF1R, CSF2RA, CTLA4, CTSS, CXCL10, CXCL11, CXCL5, CXCL6, CXCL9, CXCR3, CXCR4, CXCR6, CXorf21, CYBB, CYSLTR1, DAPK1, DAPP1, DCC, DEPDC1B, DERL3, DLGAP2, DOCK10, DOCK2, DOCK8, DOK2, DPEP2, DRD1, DTHD1, DUSP2, DUSP8, E2F8, EBI3, EDN2, ELF3, EPHA6, EPX, EREG, ERFE, FAM83B, FASLG, FCGR2C, FCN1, FCRL1, FCRL2, FCRL3, FCRL5, FCRL6, FCRLA, FFAR4, FGD2, FGD3, FGR, FIBIN, FLT3, FOLR2, FOXP3, FRZB, FUT7, FYB1, GABBR2, GADD45B, GALNT3, GDF6, GFI1, GFRA2, GMFG, GMIP, GPR132, GPR141, GPR150, GPR171, GPR174, GPR19, GPR34, GPR4, GPR55, GPR83, GPRC5A, GRAP2, GUCY2D, GZMA, GZMB, H3C10, H3C11, H3C12, H3C7, HASPIN, HCLS1, HCST, HLA-DPA1, HLA-DPB1, HLA-DQA1, HLA-DRA, HLA-DRB1, HLA-DRB5, HORMAD1, HSH2D, ICOS, IER3, IFNG, IGLL5, IKBKE, IL10RA, IL12RB1, IL12RB2, IL16, IL18, IL18RAP, IL21R, IL22, IL26, IL2RB, IL2RG, IL5RA, IL7R, IL9R, INPP5D, IQCJ-SCHIP1, IQGAP2, IRF4, IRF5, IRF8, ITGA4, ITGAL, ITGAX, ITGB2, ITGB7, ITK, JPH1, JSRP1, KCNN4, KLF5, KLRB1, KLRC1, KLRC2, KLRD1, KLRG1, KLRK1, KNG1, LAG3, LAX1, LCK	[ABCA7, ABL1, ABR, ABRA, ACKR1, ACKR3, ACP4, ACTB, ACTN4, ADCY1, ADCY3, ADCY4, ADCY9, ADGRB2, ADGRE5, ADGRG1, ADGRG6, ADGRL1, ADIPOQ, AGAP2, AGER, AGPAT1, AGT, AGTR1, AKT1S1, ALPK3, AMH, ANGPT1, ANGPTL1, ANGPTL8, AP2A1, APLN, APLNR, APLP1, APOA1, APOD, ARAF, ARAP3, ARC, AREG, ARF1, ARHGAP1, ARHGAP23, ARHGAP39, ARHGDIA, ARHGEF1, ARHGEF16, ARHGEF5, ARID1A, ARRDC3, ARTN, ASB16, ASB6, ASIC3, ASPN, ATP6V0D1, ATP6V0E2, ATP6V1B1, ATP6V1F, BAG3, BAG6, BCAM, BCAR1, BCL2L1, BCL3, BCL6, BCL7B, BCL9L, BCR, BEND6, BMP8A, BRD4, BSN, BTG2, BTLA, C15orf62, C1QTNF1, C20orf27, C5, C5AR1, CABIN1, CALCR, CAMK2B, CAPN1, CARM1, CASKIN1, CC2D1A, CCL11, CCL2, CCL21, CCL24, CCR7, CD14, CD177, CD276, CD74, CDC42EP1, CDC42EP2, CDC42EP4, CDK1, CDK2, CDKL2, CDKN1A, CEACAM1, CELSR2, CERT1, CETP, CFL1, CGA, CHCHD10, CHERP, CHGA, CHGB, CHI3L1, CHRNG, CIITA, CISH, CITED2, CLCF1, CLCN6, CLEC1B, CLEC4E, CLIP3, CLNK, CMTM1, CMTM5, CNKSR1, CNOT3, CNR1, CNTN6, COL1A1, CRAT, CREB3L1, CRHR2, CRYAB, CSF1, CSK, CSNK1E, CSPG4, CTF1, CTHRC1, CX3CL1, CXXC4, CYP26B1, CYP27B1, DAB2IP, DAG1, DAGLA, DAXX, DCHS1, DDR1, DDX39B, DDX54, DEDD, DENND2B, DENND4B, DGKD, DGKZ, DHX34, DIRAS1, DISP2, DISP3, DKK2, DLG5, DLGAP4, DMTN, DNMT1, DOCK6, DOK3, DOK5, DOT1L, DPF2, DTX1, DTX2, DTX3, DUSP5, DUSP8, DVL3, E2F4, E2F7, E2F8, ECE2, ECM1, EDN2, EEF1E1, EFNA3, EGLN2, EIF4EBP1, ELF3, ENG, EPHA2, EPN1, ERBB2, ERFE, ESM1, EXTL1, FADD, FAIM2, FAM110A, FAM83F, FAM83G, FASN, FES, FFAR2, FGFR4, FGFRL1, FIBIN, FLOT2, FLT3, FLT4, FOXC2, FOXF1, FOXM1, FPR1, FRMD8, FURIN, GAB2, GABBR2, GABRR2, GADD45B, GAS1, GATA4, GBA, GBF1, GCSAM, GDF6, GDI1, GFRA3, GIT1, GLIS2, GLRA1, GNAI2, GNAO1, GNB3, GNRHR, GPAM, GPBAR1, GPER1, GPI, GPR137, GPR153, GPR162, GPR17, GPR20, GPR3, GPR34, GPR37L1, GPR4, GPR75, GPR83
GO:0010033	response to organic substance	5,79E-16	[3]	652,00	18,46	41,35	58,65	[AANAT, ACP5, ADCY7, AIF1, AIM2, ALOX15, AMPD1, ANGPT1, ANGPT2, AREG, ATF3, BATF, BHLHA15, BMP7, BMPR1B, BRIP1, BTG2, BTK, C1QTNF1, CALCR, CARD17, CASP8, CCL17, CCL19, CCL2, CCL22, CCL24, CCL3, CCL3L1, CCL4, CCL4L1, CCL5, CCR2, CCR3, CCR4, CCR5, CCR8, CD180, CD27, CD300LF, CD4, CD40LG, CD6, CD74, CD80, CD86, CD96, CDH1, CHRDL1, CISH, CLCF1, CNR1, CNR2, CORO1A, CRB2, CSF1R, CSF2RA, CTSS, CXCL10, CXCL11, CXCL5, CXCL6, CXCL9, CXCR3, CXCR4, CXCR6, CYBB, CYP11B1, DAPK1, DERL3, DNAJB5, DOCK8, DPEP1, DPEP2, DRD1, EBI3, EDN2, EGR3, ELAVL4, EPX, EREG, ERFE, ERP27, FASLG, FIBIN, FLT3, FOLR2, FOSB, FOSL1, FOXP3, FUT7, GALNT3, GBP5, GBP6, GDF6, GFI1, GFPT2, GFRA2, GLDC, GPR83, GRAMD1B, GRAMD1C, GRAP2, H3C10, H3C11, H3C12, H3C7, HCLS1, HCN1, HLA-DPA1, HLA-DPB1, HLA-DQA1, HLA-DRA, HLA-DRB1, HLA-DRB5, HSPA7, IFNG, IKBKE, IL10RA, IL12RB1, IL12RB2, IL16, IL18, IL18RAP, IL21R, IL22, IL26, IL2RB, IL2RG, IL5RA, IL7R, IL9R, INPP5D, IRF4, IRF5, IRF8, ITGA4, ITGAX, ITGB2, KLF5, KLRG1, KMO, LAMP3, LCK, LCP1, LDLR, LEF1, LIF, LILRB1, LILRB4, LPAL2, LTA, LTB, LY86, MAP4K1, MAPK13, MLC1, MMP9, MPO, MRC1, MSX1, MT1G, MUSK, MYB, MYBL2, MZB1, NAMPT, NELL2, NLRP3, NLRP7, NPPC, NR4A1, NR4A2, NR4A3, NSG1, NUGGC, OASL, P2RY12, P2RY13, P2RY2, PIK3CG, PIM1, PRKCB, PRKCQ, PTAFR, PTGER2, PTGFR, PTK2B, PTPN22, PTPN6, PTPN7, PTPRC, PYCARD, PYHIN1, RAB15, RBPMS2, RGS10, RGS9, RHEX, RUFY4, RUNX3, SCIMP, SCNN1B, SDC1, SELE, SH2D2A, SLA, SLA2, SLC8A3, SMPD3, SNX10, SOCS1, SOCS3, STAP1, STAT1, SYK, TBX21, TBXAS1, THBS1, TLR6, TNFAIP3, TNFRSF11B, TNFRSF13B, TNFRSF17, TNFRSF18, TNFSF13B, TNFSF14, TNFSF8, TNIP3, TNMD, TRAF1, TREM2, TRERF1, TRPM2, UBD, UCP2, VAV1, VWC2L, WAS, WNT1, WNT10A, WNT10B, XCL1, XCL2, XCR1, ZBP1]	[ABCA3, ABCA7, ABCC8, ABCG4, ABL1, ACACB, ACKR1, ACKR3, ACSBG1, ACTA1, ACTB, ACTC1, ACTN4, ADAMTS7, ADCY1, ADCY3, ADCY4, ADCY9, ADIPOQ, AGER, AGPAT1, AGT, AGTR1, AKT1S1, ALPL, AMH, ANGPT1, APLN, APLP1, APOA1, APOM, AQP3, AREG, ARF1, ARHGDIA, ARID1A, ARID5A, ARSA, ASPN, ATAT1, ATP1A1, ATP1A3, ATP6V0D1, ATP6V0E2, ATP6V1B1, ATP6V1F, BAG3, BAG6, BCAR1, BCL2L1, BCL6, BCL9L, BMP8A, BTG2, C1QTNF1, C20orf27, C2CD2L, C5AR1, CACNA1H, CALB1, CALCR, CAMK2B, CARM1, CCL11, CCL2, CCL21, CCL24, CCR7, CD14, CD74, CDC42EP2, CDC42EP4, CDK1, CDKN1A, CEACAM1, CFL1, CGA, CHERP, CHGA, CHI3L1, CIITA, CISH, CITED2, CITED4, CLCF1, CLIP3, CNR1, COASY, COL1A1, COL6A1, CRAT, CREB3L1, CRHR2, CRYAB, CSF1, CSK, CTF1, CX3CL1, CYP21A2, CYP26B1, CYP27B1, DAB2IP, DAG1, DAXX, DDX54, DGKD, DHX34, DMTN, DNAJB5, DNMT1, DPAGT1, DTX1, ECM1, EDN2, EGLN2, EHD1, EIF4EBP1, ELK1, ENDOG, ENG, EPHA2, ERBB2, ERFE, EXTL1, FABP4, FADD, FAM83G, FASN, FES, FFAR2, FGFR4, FGFRL1, FIBIN, FLT3, FLT4, FOSL1, FOXC2, FOXF1, FOXRED2, FPR1, FURIN, GAB2, GAS1, GAS2L1, GATA2, GATA4, GBA, GDF6, GET4, GFPT2, GFRA3, GIT1, GJD3, GLRA1, GNAI2, GNAO1, GNRHR, GPAM, GPBAR1, GPER1, GPI, GPR17, GPR75, GPR83, GPRIN1, GPT, GPX3, GRAMD1A, GRAMD1B, GRAMD1C, GRB7, GRIK5, GRM2, GSK3A, H3C10, H3C12, H4C3, HCFC1, HCN3, HCN4, HDAC5, HEYL, HFE, HGS, HNMT, HPRT1, HRH2, HSF1, HSPB1, HSPB7, HSPB8, HSPE1, HTR1B, HTR2A, HYAL1, ICAM1, ID3, IFITM1, IFITM2, IFITM3, IL15RA, IL18BP, IL1B, IL1RN, IL22RA1, IL32, IL3RA, IL4R, IL5RA, IL7R, ILK, INPPL1, IRAK2, IRF2, IRF9, ITGA3, ITGA5, JAK3, JUNB, JUND, JUP, KCNMB1, KCP, KCTD11, KLF10, KLF5, KMT2D, LAMA5, LDLR, LDLRAP1, LDOC1, LGALS9, LIF, LIFR, LMNA, LOX, LRG1, LRRC32, LTBR, LTF, LUM, LY6E, LZTS1, MAP1LC3A, MAP2K3, MAP3K14, MAPK3, MAPK7, MAVS, MED12, MEGF8, MEN1, MICALL1, MME, MMP15, MRC1, MSC, MSN, MSTN,

GO:0048584	positive regulation of response to stimulus	3,89E-17	[2, 3, 4]	491,00	19,87	48,73	51,27	[ABRA, ADAM8, AIF1, AIM2, ALOX15, ALOX15B, ANGPT1, AREG, ATF3, BANK1, BLK, BLNK, BMF, BMP7, BMPR1B, BTK, BTN3A1, BTN3A2, C1QA, C1QB, C1QC, C1QTNF1, C2, C8G, CALCR, CARD11, CASP8, CASS4, CCL17, CCL19, CCL2, CCL22, CCL24, CCL3, CCL3L1, CCL4, CCL4L1, CCL5, CCR2, CCR4, CD177, CD180, CD19, CD1B, CD1C, CD1E, CD22, CD226, CD247, CD27, CD300LF, CD3D, CD3E, CD3G, CD4, CD40LG, CD74, CD79A, CD80, CD86, CDKN2A, CFP, CLCF1, CLEC10A, CLEC4D, CLEC4E, CLEC6A, CLNK, CNR1, CR2, CRB2, CRTAM, CSF1R, CTLA4, CXCL10, CXCR4, CXorf21, CYP11B1, DEPDC1B, DPEP2, EDN2, ELF3, EPX, EREG, ERFE, FASLG, FCN1, FCN3, FCRL3, FFAR4, FGD2, FGR, FLT3, FOXP3, FYB1, GADD45B, GBP5, GDF6, GFI1, GPR174, GPR4, GPR55, GPSM3, GRAP2, HCLS1, HCST, HLA-DMB, HLA-DPA1, HLA-DPB1, HLA-DQA1, HLA-DRA, HLA-DRB1, HLA-DRB5, ICOS, IFNG, IGLL5, IKBKE, IL10RA, IL12RB1, IL16, IL18, IL18RAP, IL26, IL7R, INPP5D, IQCJ-SCHIP1, ITGB2, ITK, KCNN4, KLRC2, KLRD1, KLRK1, LAG3, LAX1, LCK, LCP1, LCP2, LDLR, LILRB1, LILRB4, LMCD1, LTA, LY86, MAP4K1, MAPK13, MMP9, MOG, MS4A1, MSX1, MUC16, MUC19, MUC6, MYB, MYO1G, NCF1, NCKAP1L, NCR3, NFAM1, NLRP3, NPPC, NR4A3, OASL, P2RY10, P2RY12, PAX5, PDE6G, PIK3CG, PIK3R5, PIM2, PLA2G7, PRAM1, PRKCB, PRKCQ, PROK1, PTAFR, PTK2B, PTPN22, PTPN6, PTPRC, PYCARD, PYHIN1, RAC2, RASGRF1, RTKN2, SALL1, SASH3, SCIMP, SCUBE1, SEMA4D, SH2D1A, SIGLEC16, SKAP1, SLA2, SLAMF6, SOCS1, STAP1, SUCNR1, SYK, TBX21, TESPA1, THBS1, THEMIS, THEMIS2, THY1, TLR10, TLR8, TNFAIP3, TNFSF13B, TNFSF14, TRAF1, TRAT1, TREM2, TRIM14, UBASH3A, UBD, VAV1, WAS, WNT1, WNT10B, XCL1, XCL2, ZAP70, ZBP1]	[ABCA7, ABI3, ABL1, ABRA, ACKR3, ACTB, ACTN4, ADGRG1, ADIPOQ, AGAP2, AGER, AGPAT1, AGT, AGTR1, AMH, ANGPT1, APOA1, ARAF, AREG, ARHGEF5, ARTN, BAG6, BCAR1, BCL2L1, BMP8A, BRD4, C1QTNF1, C20orf27, C5, C5AR1, C8G, CALCR, CC2D1A, CCL11, CCL2, CCL21, CCL24, CCR7, CD14, CD177, CD1C, CD1E, CD276, CD74, CEACAM1, CFH, CHERP, CHGA, CHI3L1, CITED2, CLCF1, CLEC4E, CLNK, CNR1, CNTN6, COL1A1, CPN2, CRAT, CRP, CSF1, CSK, CSNK1E, CSPG4, CX3CL1, CYP27B1, DAB2IP, DAXX, DENND2B, DGKD, DGKZ, DIRAS1, DKK2, DLG5, DMTN, DOK5, DUSP5, DVL3, ECM1, EDN2, EEF1E1, ELF3, ENDOG, ENG, ERBB2, ERFE, ESM1, FABP4, FADD, FAM110A, FASN, FCN3, FEM1A, FFAR2, FGFR4, FLT3, FLT4, FOXC2, FOXM1, FPR1, GAB2, GADD45B, GATA2, GATA4, GCSAM, GDF6, GNAI2, GNAO1, GPBAR1, GPER1, GPR137, GPR17, GPR20, GPR37L1, GPR4, GPRIN1, GRN, GSK3A, HBEGF, HCRTR1, HDAC10, HFE, HMGA1, HSPB1, HTR2A, ICAM1, IL1B, IL4R, IL7R, ILK, INCA1, INHA, IRAK2, ITGA5, JMJD8, JUP, KCNK2, KCP, KLK14, KMT2D, KSR1, LAG3, LDLR, LGALS9, LGR6, LILRA5, LIMK1, LIMS2, LMCD1, LRG1, LTBR, LTF, MAP2K3, MAP3K10, MAP3K11, MAP3K14, MAP3K6, MAP3K9, MAPK3, MAPK8IP3, MAVS, MAZ, MED12, MEGF8, MEIS3P1, MEN1, MINK1, MLST8, MSTN, MSX1, MUC19, MUC20, MUC3A, MYC, NCOR2, NDC80, NDST1, NECTIN2, NFATC4, NGF, NGFR, NINJ1, NLGN2, NLRC5, NOD2, NODAL, NOS3, NOTCH1, NOTCH4, NPAS2, NPPA, NR4A3, NRARP, OSM, P2RX3, P2RX5, P2RY6, PCDHA4, PDGFB, PDGFRB, PIAS4, PLA2G2A, PLA2G7, PLAGL2, PLAUR, PLXNB1, PML, POR, PPARD, PPP1R15A, PQBP1, PRAG1, PRKACA, PTN, PTP4A3, PVR, RAC2, RAD51AP1, RAP1A, RAPGEF1, RARA, RASD2, RBM14, RELA, RELB, RGCC, RHBDF2, RHOG, RNU1-93P, RPL26, RPTOR, RTKN2, S1PR2, SECTM1, SELENON, SELENOP, SEMA4C, SEPTIN4, SERPINE1, SERPING1, SGTA, SH2B2, SHANK3, SHARPIN, SHC2, SHISA5, SHKBP1, SIK3, SLC2A4, SLC44A2, SLC7A8, SLC9A1, SLC9A3R1, SMAD3, SMARCA4
GO:0070887	cellular response to chemical stimulus	3,49E-19	[3]	666,00	18,89	40,52	59,48	[AANAT, ADAM8, ADCY7, AIF1, AIM2, ALOX15, ANGPT1, ANGPT2, APOBEC3A, AQP5, AREG, ATF3, B3GAT1, BATF, BHLHA15, BIN2, BMP7, BMPR1B, BRIP1, BTK, CARD17, CASP8, CCL17, CCL19, CCL2, CCL22, CCL24, CCL3, CCL3L1, CCL4, CCL4L1, CCL4L2, CCL5, CCR2, CCR3, CCR4, CCR5, CCR8, CD180, CD27, CD300LF, CD4, CD40LG, CD6, CD74, CD80, CD86, CDH1, CHRDL1, CISH, CLCF1, CNR2, CORO1A, CPNE7, CRB2, CSF1R, CSF2RA, CTSS, CXCL10, CXCL11, CXCL5, CXCL6, CXCL9, CXCR3, CXCR4, CXCR6, CYBB, CYP11B1, DAPK1, DERL3, DOCK8, DPEP1, DPEP2, DRD1, EBI3, EDN2, EGR3, ELAVL4, EPX, EREG, ERFE, FASLG, FLT3, FOLR2, FOSB, FUT7, GALNT3, GBP5, GBP6, GDF6, GFI1, GFPT2, GFRA2, GPSM3, GRAMD1B, GRAMD1C, GRAP2, H3C10, H3C11, H3C12, H3C7, HCLS1, HCN1, HLA-DPA1, HLA-DPB1, HLA-DQA1, HLA-DRA, HLA-DRB1, HLA-DRB5, HSPA7, IFNG, IKBKE, IL10RA, IL12RB1, IL12RB2, IL16, IL18, IL18RAP, IL21R, IL22, IL26, IL2RB, IL2RG, IL5RA, IL7R, IL9R, INPP5D, IPCEF1, IRF4, IRF5, IRF8, ITGA4, ITGAX, ITGB2, JAML, KLF5, KLRK1, KMO, LCK, LCP1, LDLR, LEF1, LIF, LILRB1, LILRB4, LTA, LTB, LY86, MAP4K1, MAPK13, MLC1, MMP9, MPO, MRC1, MSX1, MT1F, MT1G, MT1HL1, MYB, MYBL2, MZB1, NAMPT, NCF1, NCKAP1L, NELL2, NLRP3, NLRP7, NME8, NR4A1, NR4A2, NR4A3, NSG1, NTN1, NUGGC, OASL, P2RY12, P2RY13, P2RY2, PIK3CG, PIM1, PLA2G7, PRKCB, PRKCQ, PTAFR, PTGER2, PTGFR, PTK2B, PTPN22, PTPN6, PTPN7, PTPRC, PYCARD, PYHIN1, RAB15, RAC2, RBPMS2, RGS10, RGS9, RHEX, RUFY4, SCIMP, SCNN1B, SDC1, SLA, SLA2, SLAMF8, SLC8A3, SLFN14, SMPD3, SNX10, SOCS1, SOCS3, STAP1, STAT1, SYK, TBX21, THBS1, TLR6, TNFAIP3, TNFRSF11B, TNFRSF13B, TNFRSF17, TNFRSF18, TNFSF13B, TNFSF14, TNFSF8, TNIP3, TNMD, TRAF1, TREM2, TRERF1, TRPM2, UCP2, VAV1, VNN1, VWC2L, WAS, WNT1, WNT10A, WNT10B, XCL1, XCL2, XCR1, ZBP1]	[ABCA7, ABCC8, ABCD1, ABCG4, ABL1, ACKR1, ACKR3, ACTA1, ACTB, ACTN4, ADAMTS7, ADCY1, ADCY3, ADCY4, ADCY9, ADIPOQ, AGER, AGPAT1, AGT, AGTR1, AKT1S1, ALPL, AMH, ANGPT1, AOC2, APLN, APLP1, APOA1, APOM, AQP3, AQP5, AREG, ARF1, ARHGDIA, ARHGEF16, ARID1A, ARID5A, ASPN, ATP13A2, ATP1A1, ATP1A3, ATP6V0D1, ATP6V0E2, ATP6V1B1, ATP6V1F, BAG3, BAG6, BCAR1, BCL2L1, BCL6, BCL9L, BMP8A, C20orf27, C2CD2L, C5, C5AR1, CACNA1H, CALB1, CAMK2B, CARM1, CCL11, CCL2, CCL21, CCL24, CCR7, CD14, CD74, CDC42EP2, CDC42EP4, CDK1, CDK2, CDKN1A, CEACAM1, CES2, CES3, CFL1, CGA, CHERP, CHGA, CHI3L1, CIITA, CISH, CITED2, CLCF1, CLIP3, COASY, COL1A1, COL6A1, CPNE5, CPNE6, CRAT, CREB3L1, CRHR2, CSF1, CSK, CTF1, CX3CL1, CYB5R3, CYP21A2, CYP26B1, CYP27B1, CYP2A6, CYP2A7, CYP2D6, CYP2G1P, CYP2S1, DAB2IP, DAG1, DAXX, DDX54, DHRS2, DHX34, DMTN, DNMT1, DPAGT1, DTX1, ECM1, EDN2, EGLN2, EHD1, EIF4EBP1, ELK1, ENDOG, ENG, EPHA2, ERBB2, ERFE, EXTL1, FABP4, FADD, FAM83G, FANCC, FASN, FES, FFAR2, FGFR4, FGFRL1, FLT3, FLT4, FMO2, FMO3, FOXC2, FOXF1, FPR1, FURIN, GAB2, GAS1, GAS2L1, GATA4, GBA, GBF1, GDF6, GFPT2, GFRA3, GIT1, GLRA1, GNAI2, GNAO1, GNRHR, GPAM, GPBAR1, GPER1, GPI, GPR17, GPR37L1, GPR75, GPRIN1, GPT, GPX3, GPX8, GRAMD1A, GRAMD1B, GRAMD1C, GRB7, GRIK5, GSK3A, H3C10, H3C12, H4C3, HBA1, HBB, HBD, HBEGF, HBG1, HBG2, HCFC1, HCN3, HCN4, HDAC5, HESX1, HEYL, HFE, HGS, HRH2, HSF1, HSPB1, HSPB8, HTR1B, HTR2A, HVCN1, HYAL1, ICAM1, ID3, IFITM1, IFITM2, IFITM3, IL15RA, IL18BP, IL1B, IL1RN, IL22RA1, IL32, IL3RA, IL4R, IL5RA, IL7R, ILK, INPPL1, IRAK2, IRF2, IRF9, ITGA3, ITGA5, JAK3, JUNB, JUND, JUP, KCNH2, KCNK2, KCNK3, KCNMB1, KCP, KCTD11, KDM6B, KLF10, KLF5, KMT2D, LAMA5, LDLR, LDLRAP1, LDOC1, LGALS9, LIF, LIFR, LMNA, LOX, LRG1, LRRC32, LTBR, LTF, LY6E, LZTS1, MAP1LC3A, MAP2K3, MAP3K14, MAPK3, MAPK7,

GO:1901698	response to nitrogen compound	3,25E-02 [3]	219,00	17,89	35,80	64,20	[AANAT, ADCY7, AIF1, AREG, BMP7, BRIP1, BTG2, C1QTNF1, CALCR, CCL19, CDH1, CNR1, CNR2, CXCR4, CYBB, CYP11B1, DERL3, DPEP1, DRD1, ELAVL4, EREG, ERFE, FLT3, FOLR2, FOSB, FOSL1, FOXP3, FUT7, GLDC, HCN1, IRF5, ITGA4, ITGB2, KLF5, LCP1, LDLR, MAPK13, MMP9, MUSK, MZB1, NAMPT, NR4A1, NR4A2, NR4A3, NSG1, P2RY12, P2RY2, PIK3CG, PRKCB, PRKCQ, PTAFR, PTK2B, PTPN22, RAB15, RGS10, RGS9, SDC1, SH2D2A, SLC8A3, SMPD3, SOCS1, SOCS3, STAT1, SYK, TLR6, TNFAIP3, TREM2, TRPM2, UCP2, WNT1, WNT10B]	[ABCC8, ABL1, ACTA1, ACTB, ADCY1, ADCY3, ADCY4, ADCY9, ADIPOQ, AGER, AGT, AGTR1, APLP1, AREG, ATP1A3, ATP6V0D1, ATP6V0E2, ATP6V1B1, ATP6V1F, BAG6, BCAR1, BCL2L1, BTG2, C1QTNF1, C5AR1, CALCR, CARM1, CCR7, CDK1, CDK2, CDKN1A, CEACAM1, CIITA, CNR1, COASY, COL1A1, COL6A1, CRAT, CRHR2, CSK, CYP21A2, DAG1, DAXX, DHX34, DMTN, DNMT1, DPAGT1, ERFE, FADD, FLT3, FOSL1, FOXC2, FOXRED2, GET4, GLRA1, GNAI2, GNAO1, GNRHR, GPER1, GPI, GPRIN1, GPT, GRB7, GRM2, GSK3A, HCN3, HCN4, HDAC5, HNMT, HPRT1, HRH2, HSF1, HTR1B, HTR2A, ICAM1, IL1B, INPPL1, JAK3, JUND, JUP, KCTD11, KLF10, KLF5, LDLR, LDOC1, LY6E, MAP1LC3A, MAPK3, MAVS, MED12, MEN1, MSTN, MTHFR, MYC, NCOR2, NEFL, NGFR, NOD2, NOTCH1, NPPA, NR4A1, NR4A2, NR4A3, NSG1, NSMF, P2RX3, P2RX5, P2RY2, P2RY6, PALM, PCDHA4, PCDHA6, PDK2, PER1, PKM, POR, PPP1R9B, PQBP1, PRKACA, PRKAR2B, PTN, PTPRN, PXN, QDPR, RAB15, RANGAP1, RAP1A, RAPGEF1, RELA, RHBDD2, RMRP, RNU1-93P, RPTOR, SCAP, SELENON, SGTA, SH2B2, SIK2, SLC25A33, SLC2A4, SLC9A1, SLC9A3R1, SMPD1, SOCS3, SRC, SREBF1, STAT3, STING1, SYVN1, TCIRG1, TGM2, TICAM1, TIMP1, TMEM259, TMUB1, TMUB2, TNF, TNS2, TRIB3, TRPV4, TSC2, WDTC1, WFS1, ZBTB7B, ZCCHC3]
GO:1901700	response to oxygen-containing compound	6,42E-09 [3]	351,00	19,19	40,36	59,64	[AANAT, ACP5, ADCY7, AIF1, ANGPT2, AREG, BMP7, BRIP1, BTG2, BTK, C1QTNF1, CALCR, CARD17, CASP8, CCL19, CCL2, CCL3, CCL5, CCR5, CD180, CD27, CD6, CD80, CD86, CD96, CDH1, CNR1, CNR2, CXCL10, CXCL11, CXCL5, CXCL6, CXCL9, CYBB, CYP11B1, DAPK1, DPEP1, DPEP2, DRD1, ELAVL4, EPX, EREG, ERFE, FASLG, FIBIN, FOLR2, FOSB, FOSL1, FOXP3, FUT7, GFI1, GLDC, GRAMD1B, GRAMD1C, HCN1, HLA-DRB1, IL10RA, IL12RB2, IL18, IL18RAP, IRF5, IRF8, ITGA4, KLF5, KLRG1, KMO, LCP1, LDLR, LILRB1, LPAL2, LTA, LY86, MAP4K1, MAPK13, MLC1, MMP9, MPO, MRC1, MUSK, MYB, MZB1, NAMPT, NCF1, NLRP3, NLRP7, NME8, NPPC, NR4A1, NR4A2, NR4A3, NSG1, NUGGC, P2RY12, P2RY2, PIK3CG, PIM1, PRKCB, PRKCQ, PTAFR, PTGER2, PTGFR, PTK2B, PTPN22, PYCARD, RAB15, RGS10, RGS9, SCIMP, SCNN1B, SDC1, SELE, SH2D2A, SLC8A3, SMPD3, SOCS1, SOCS3, STAP1, STAT1, TBXAS1, THBS1, TLR6, TNFAIP3, TNIP3, TREM2, TRERF1, TRPM2, UCP2, WNT1, WNT10B]	[ABCC8, ABL1, ACTB, ACTC1, ADCY1, ADCY3, ADCY4, ADCY9, ADIPOQ, AGER, AGT, AGTR1, ALPL, APLP1, APOD, APOM, AQP3, AREG, ARSA, ATP1A1, ATP1A3, ATP6V0D1, ATP6V0E2, ATP6V1B1, ATP6V1F, BCAR1, BCL2L1, BTG2, C1QTNF1, C2CD2L, C5AR1, CALCR, CARM1, CCL2, CCL21, CCR7, CD14, CDK1, CDK2, CDKN1A, CEACAM1, CNR1, COASY, COL1A1, COL6A1, CRAT, CRHR2, CRYAB, CSK, CX3CL1, CYP21A2, CYP26B1, CYP27B1, DAB2IP, DAG1, DHX34, DMTN, DNMT1, DPAGT1, EIF4EBP1, ELK1, ENDOG, ERFE, FADD, FANCC, FES, FFAR2, FGFR4, FIBIN, FOSL1, FOXC2, GATA4, GBA, GIT1, GJD3, GLRA1, GNAI2, GNAO1, GNRHR, GPAM, GPBAR1, GPER1, GPI, GPR37L1, GPRIN1, GPT, GPX3, GRAMD1A, GRAMD1B, GRAMD1C, GRB7, GRIK5, GRM2, GSK3A, HBA1, HBB, HCN3, HCN4, HDAC5, HNMT, HRH2, HSF1, HTR1B, HTR2A, HYAL1, ICAM1, ID3, IL18BP, IL1B, INPPL1, IRAK2, JAK3, JUND, JUP, KCNMB1, KCTD11, KDM6B, KLF10, KLF5, LDLR, LDOC1, LGALS9, LTF, LY6E, MAP1LC3A, MAPK3, MAPK7, MED12, MEN1, MMP15, MRC1, MSN, MSTN, MTHFR, MYC, NCOR2, NDOR1, NEFL, NFKB2, NFKBIB, NGFR, NOD2, NOS3, NOTCH1, NPPA, NR1D1, NR4A1, NR4A2, NR4A3, NSG1, NSMF, P2RX3, P2RX5, P2RY2, P2RY6, PAF1, PALM, PCDHA4, PCDHA6, PCGF2, PDGFB, PDGFRB, PDK2, PER1, PIM1, PKM, PLK3, POR, POSTN, PPARD, PPP1R9B, PRKACA, PRKAR2B, PTGES, PTGFR, PTGIR, PTN, PTPRN, PXN, QDPR, RAB11B, RAB11FIP5, RAB15, RAMP2, RANGAP1, RAP1A, RAPGEF1, RARA, RELA, RMI1, RMRP, RNU1-93P, RPTOR, RXRA, RXRB, SBNO2, SCAP, SELE, SELENOP, SERPINE1, SH2B2, SIDT2, SIK2, SLC10A3, SLC25A33, SLC29A1, SLC2A4, SLC9A1, SLC9A3R1, SMARCA4, SMARCB1, SMPD1, SOCS3, SOX10, SPHK1, SPHK2, SPI1, SRC, SREBF1, SRF, SSTR2, STAT3, SZT2, TCIRG1, TEAD2, TGM2, THBS1, TICAM1, TIMP1, TMEM161A, TMEM256-PLSCR3, TNF, TNS2, TRIB1, TRIB3, TRPV4, TSC2, VDR, WBP2, WDTC1, WNT3, WNT9B, XRCC1, ZBTB7B, ZC3H12A, ZFP36, ZNF580]
GO:0009725	response to hormone	7,90E-04 [3, 4]	196,00	19,23	31,47	68,53	[AANAT, ADCY7, AIF1, AREG, BMP7, BRIP1, BTG2, C1QTNF1, CALCR, CCL19, CTSS, CYBB, CYP11B1, EBI3, EPX, EREG, ERFE, FIBIN, FLT3, FOSB, FOSL1, FUT7, GPR83, H3C10, H3C11, H3C12, H3C7, HCLS1, IL22, LCP1, LPAL2, MUSK, MZB1, NAMPT, NR4A1, NR4A2, NR4A3, PRKCB, PRKCQ, PTAFR, PTGER2, PTGFR, PTK2B, RAB15, SCNN1B, SDC1, SH2D2A, SOCS1, SOCS3, STAT1, THBS1, TNFRSF11B, TRERF1, UCP2, WNT1, WNT10B]	[ABCA3, ABCC8, ACSBG1, ACTA1, ADCY1, ADCY3, ADCY4, ADCY9, ADIPOQ, AGT, AGTR1, ALPL, APOA1, AREG, ARID1A, ARID5A, ARSA, ATAT1, ATP1A1, ATP1A3, ATP6V0D1, ATP6V0E2, ATP6V1B1, ATP6V1F, BCAR1, BTG2, C1QTNF1, CACNA1H, CALCR, CARM1, CCL21, CCR7, CDKN1A, CEACAM1, CGA, CHERP, CHGA, CITED2, CITED4, COASY, COL1A1, CRAT, CRHR2, CSK, CYP21A2, CYP27B1, DAG1, DAXX, DDX54, DPAGT1, EGLN2, ENG, ERFE, FIBIN, FLT3, FOSL1, FOXC2, GAS2L1, GBA, GNAI2, GNRHR, GPAM, GPER1, GPI, GPR83, GPT, GRB7, GSK3A, H3C10, H3C12, H4C3, HDAC5, HEYL, HNMT, HSF1, HTR1B, ICAM1, IL1B, IL1RN, IL4R, INPPL1, ITGA3, JAK3, JUND, KCTD11, KMT2D, LOX, MED12, MEN1, MSTN, MYC, NCOA6, NCOR2, NEFL, NODAL, NOS3, NOTCH1, NPPA, NR1D1, NR4A1, NR4A2, NR4A3, NSMF, P2RY6, PDGFRB, PDK2, PELP1, PER1, PKM, POR, PPARD, PPP1R9B, PRKACA, PRKAR2B, PTGFR, PTN, PTPRN, PTPRU, PXN, QDPR, RAB15, RAMP2, RANGAP1, RARA, RBM14, RELA, RXRA, RXRB, SCAF1, SCAP, SH2B2, SIK2, SLC25A33, SLC2A4, SLC9A1, SMARCA4, SOCS3, SOX10, SRC, SREBF1, SRF, SSTR2, STAT3, TCIRG1, THBS1, TIMP1, TNF, TNS2, TRIB3, TRPV4, TSC2, UCN2, WBP2, WDTC1, ZBTB7A, ZBTB7B, ZFP36, ZMIZ1]

GO:0010646	regulation of cell communication	2,64E-15	[3, 4]	671,00	18,23	37,26	62,74	[ABRA, ADAM8, ALOX15, ALOX15B, ANGPT1, ARAP2, AREG, ARHGAP15, ARHGAP30, ARHGAP45, ATF3, BANK1, BCL2L14, BCL3, BLK, BMF, BMP7, BMPR1B, BRIP1, C1QTNF1, CACNA1E, CALCR, CARD11, CASP8, CASS4, CCDC88C, CCL17, CCL19, CCL2, CCL22, CCL24, CCL3, CCL3L1, CCL4, CCL4L1, CCL5, CCR2, CD177, CD180, CD19, CD22, CD226, CD27, CD300LF, CD3E, CD4, CD40LG, CD74, CD80, CD86, CDH1, CDKN2A, CEL, CHRDL1, CISH, CLEC6A, CNR1, CNR2, CRB2, CSF1R, CXCR4, CXorf21, DAPK1, DCC, DEPDC1B, DLGAP2, DPEP2, DRD1, DUSP2, DUSP8, ELAVL4, ELF3, EPX, EREG, ERFE, FASLG, FCRL3, FFAR4, FGD2, FGD3, FGR, FLT3, FOLR2, FOXP3, FRZB, FUT7, GADD45B, GDF6, GFI1, GMIP, GPR174, GPR4, GPR55, GPRC5A, GUCY2D, H3C10, H3C11, H3C12, H3C7, HCLS1, HCST, HLA-DRB1, ICOS, IER3, IFNG, IKBKE, IL10RA, IL18, IL26, IL7R, INPP5D, IQCJ-SCHIP1, IRF4, IRX3, KCNJ10, KCNN4, KMO, LAX1, LCK, LEF1, LIF, LILRB4, LMCD1, LY86, MAP4K1, MIDN, MMP9, MSX1, MYB, MZB1, NCF1, NCKAP1L, NFAM1, NLRC3, NLRP3, NLRP6, NPFFR2, NPPC, NPTX2, NR4A2, NSG1, NTN1, OASL, P2RY10, P2RY12, PAX5, PDE6G, PIK3CG, PIK3IP1, PIK3R5, PIM2, PLAU, PLEK, PRDM1, PRKCB, PRKCQ, PROK1, PSCA, PSD4, PTK2B, PTPN22, PTPN6, PTPRC, PYCARD, PYHIN1, RAC2, RAD9B, RASAL3, RASGRF1, RBPMS2, RGS1, RGS10, RGS18, RGS9, RHOH, RMI2, RNASE6, RRAD, RTKN2, SALL1, SCIMP, SCUBE1, SEMA4D, SH2D1A, SHISA8, SLA, SLA2, SLC24A4, SLC8A3, SMPDL3B, SOCS1, SOCS3, SPINK1, STAP1, STAT1, SYK, TAGAP, TBC1D10C, TESPA1, THBS1, THY1, TLR6, TMC8, TNFAIP3, TNFSF14, TNIP3, TNMD, TNR, TRABD2A, TRAF1, TRAT1, TREM2, TRIM14, TRPM2, UBASH3A, UBD, UCP2, VAV1, VNN1, VWC2L, WNT1, WNT10B, XCL1, XCL2, ZAP70, ZBP1]	[ABCA7, ABCC8, ABL1, ABR, ABRA, ACKR3, ACP4, ACTN4, ADCY1, ADGRG1, ADIPOQ, AGAP2, AGER, AGPAT1, AGT, AGTR1, AKT1S1, AMH, ANGPT1, APLN, APLNR, APLP1, APOA1, APOD, ARAF, ARAP3, ARC, AREG, ARF1, ARHGAP1, ARHGAP23, ARHGAP39, ARHGDIA, ARHGEF1, ARHGEF16, ARHGEF5, ARRDC3, ARTN, ASPN, BCL2L1, BCL3, BCL6, BCL9L, BCR, BEND6, BMP8A, BRD4, C1QTNF1, C20orf27, C2CD2L, C5, C5AR1, CACNA1E, CALB1, CALCR, CAMK2B, CAPN1, CARM1, CC2D1A, CCL11, CCL2, CCL21, CCL24, CCR7, CD14, CD177, CD74, CDK1, CDK2, CEACAM1, CEL, CGA, CHERP, CHGA, CHI3L1, CISH, CITED2, CLIP3, CNR1, CNTN6, COL1A1, CPLX1, CRAT, CREB3L1, CRHR2, CSF1, CSK, CSNK1E, CSPG4, CTHRC1, CX3CL1, CXXC4, CYP26B1, CYP27B1, DAB2IP, DAG1, DAXX, DDX39B, DENND2B, DENND4B, DGKD, DGKZ, DHX34, DIRAS1, DKK2, DLG5, DLGAP4, DMTN, DOC2B, DOK5, DOT1L, DTX1, DUSP5, DUSP8, DVL3, ECM1, EEF1E1, ELF3, ENG, EPHA2, EPN1, ERBB2, ERFE, ESM1, FADD, FAIM2, FAM110A, FASN, FFAR2, FGFR4, FLOT2, FLT3, FLT4, FOXM1, FPR1, FRMD8, FURIN, GAB2, GADD45B, GAS1, GATA4, GBA, GBF1, GCSAM, GDF6, GIT1, GJD3, GLIS2, GLRA1, GNAI2, GPAM, GPBAR1, GPER1, GPI, GPR137, GPR17, GPR20, GPR37L1, GPR4, GPRC5A, GPRIN1, GRB7, GRIK5, GRINA, GRM2, GSK3A, H3C10, H3C12, H4C3, HBEGF, HCRTR1, HDAC7, HEYL, HFE, HGS, HIPK4, HSPB1, HTR1B, HTR2A, ICAM1, IER3, IFT80, IL18BP, IL1B, IL1RN, IL7R, ILK, INCA1, INHA, INPP5E, IRAK2, IRX3, ITGA3, ITGA5, ITPR3, ITPRIP, JAK3, JMJD8, JPH4, JUP, KCP, KCTD11, KIF26A, KLK14, KMT2D, KSR1, LGALS9, LGR6, LIF, LIFR, LILRA5, LIMS2, LMCD1, LMNA, LOX, LRG1, LRRC4, LRRC8A, LTBR, LTF, LY6E, LYNX1, LZTS1, LZTS2, MAP1A, MAP2K3, MAP3K10, MAP3K11, MAP3K14, MAP3K6, MAP3K9, MAPK3, MAPK7, MAPK8IP3, MAVS, MAZ, MECP2, MED12, MEGF8, MEIS3P1, MEN1, MGRN1, MIDN, MINK1, MLST8, MME, MNT, MSTN, MSX1, MTRNR2L1, MTRNR2L10, MTRNR2L12, MTRNR2L2, MTRNR2L3, MTRNR2L6, MTRNR2L8, MTRNR2L9
GO:0007166	cell surface receptor signaling pathway	1,32E-23	[3, 4, 5]	662,00	19,60	47,87	52,13	[ADGRE3, ADGRE4P, ADGRG5, ADGRG6, ADORA3, AIM2, ALOX15, ANGPT1, ANGPT2, AREG, ASGR2, ATF3, BATF, BCL2L14, BCL3, BLK, BLNK, BMP7, BMPR1B, BTK, BTLA, BTN3A1, BTN3A2, CALCR, CARD11, CASP8, CASS4, CCDC88C, CCL17, CCL19, CCL2, CCL22, CCL24, CCL3, CCL3L1, CCL4, CCL4L1, CCL5, CCR2, CCR3, CCR4, CCR5, CCR8, CD177, CD180, CD19, CD2, CD200R1, CD22, CD226, CD247, CD27, CD300LF, CD3D, CD3E, CD3G, CD4, CD40LG, CD6, CD7, CD74, CD79A, CD80, CD86, CD8A, CD8B, CHRDL1, CISH, CLCF1, CLEC10A, CLEC1B, CLEC4D, CLEC4E, CLEC6A, CLNK, CORO1A, CR2, CRB2, CSF1R, CSF2RA, CTLA4, CXCL10, CXCL11, CXCL5, CXCL6, CXCL9, CXCR3, CXCR4, CXCR6, CYBB, CYSLTR1, DAPK1, DCC, DEPDC1B, DLGAP2, DOK2, DPEP2, DRD1, EBI3, EDN2, ELF3, EPHA6, EPX, EREG, ERFE, FAM83B, FASLG, FCGR2C, FCN1, FCRL1, FCRL2, FCRL3, FCRL5, FCRL6, FCRLA, FGR, FLT3, FOLR2, FOXP3, FRZB, FUT7, FYB1, GALNT3, GDF6, GFI1, GFRA2, GPR171, GPR34, GPRC5A, GRAP2, GUCY2D, H3C10, H3C11, H3C12, H3C7, HCLS1, HLA-DPA1, HLA-DPB1, HLA-DQA1, HLA-DRA, HLA-DRB1, HLA-DRB5, IFNG, IGLL5, IKBKE, IL10RA, IL12RB1, IL12RB2, IL16, IL18, IL18RAP, IL21R, IL22, IL26, IL2RB, IL2RG, IL5RA, IL7R, IL9R, INPP5D, IRF4, IRF5, IRF8, ITGA4, ITGAL, ITGAX, ITGB2, ITGB7, ITK, KCNN4, KLRB1, KLRC1, KLRC2, KLRD1, KLRG1, LAG3, LAX1, LCK, LCP1, LCP2, LEF1, LIF, LILRA1, LILRB1, LILRB4, LTA, LTB, LY86, LYPD6B, MATK, MMP9, MOG, MS4A1, MSX1, MUC16, MUC19, MUC6, MUSK, MYO1G, MZB1, NAMPT, NCF1, NCKAP1L, NCR3, NELL2, NFAM1, NLRP3, NPPB, NPPC, NPTX2, NR4A2, NR4A3, NTN1, OASL, P2RY10, P2RY12, P2RY13, P2RY2, PAX5, PDE6G, PIM1, PLCB2, PLEK, PLXNC1, PRAM1, PRKCB, PRKCQ, PSTPIP1, PTAFR, PTK2B, PTPN22, PTPN6, PTPRC, PYCARD, RASGRF1, RBPMS2, RGS9, RHEX, SALL1, SCIMP, SCUBE1, SDC1, SEMA4A, SEMA4D, SH2D1A, SH2D2A, SH2D6, SHISA8, SKAP1, SLA, SLA2, SLC8A3, SMPD3, SOCS1, SOCS3, SPN, STAP1, STAT1, STYK1, SYK, TESPA1, THBS1,	[ABCA7, ABL1, ACKR1, ACKR3, ACP4, ACTB, ACTN4, ADGRB2, ADGRE5, ADGRG1, ADGRG6, ADGRL1, ADIPOQ, AGAP2, AGER, AGPAT1, AGT, AKT1S1, AMH, ANGPT1, ANGPTL1, AP2A1, APLN, APOA1, APOD, ARC, AREG, ARF1, ARHGDIA, ASPN, ATP6V0D1, ATP6V0E2, ATP6V1B1, ATP6V1F, BAG3, BAG6, BCAR1, BCL2L1, BCL3, BCL6, BCL7B, BCL9L, BCR, BEND6, BMP8A, BTLA, C20orf27, C5, C5AR1, CABIN1, CALCR, CAMK2B, CAPN1, CCL11, CCL2, CCL21, CCL24, CCR7, CD14, CD177, CD276, CD74, CDKN1A, CEACAM1, CELSR2, CFL1, CHRNG, CIITA, CISH, CITED2, CLCF1, CLEC1B, CLEC4E, CLIP3, CLNK, CNKSR1, CNTN6, COL1A1, CREB3L1, CRHR2, CSF1, CSK, CSNK1E, CSPG4, CTF1, CTHRC1, CX3CL1, CXXC4, DAB2IP, DAGLA, DAXX, DDR1, DEDD, DGKD, DGKZ, DISP2, DISP3, DKK2, DLG5, DLGAP4, DMTN, DOK5, DOT1L, DTX1, DTX2, DTX3, DVL3, ECM1, EDN2, EFNA3, ELF3, ENG, EPHA2, EPN1, ERBB2, ERFE, ESM1, FADD, FAM83G, FASN, FES, FFAR2, FGFR4, FGFRL1, FLT3, FLT4, FOXC2, FOXF1, FOXM1, FPR1, FRMD8, FURIN, GAB2, GABRR2, GAS1, GATA4, GCSAM, GDF6, GFRA3, GIT1, GLIS2, GLRA1, GNAI2, GNAO1, GPBAR1, GPER1, GPI, GPR17, GPR34, GPR37L1, GPR75, GPRC5A, GPRIN1, GRB7, GRIK5, GRIP2, GRM2, GSK3A, H3C10, H3C12, HBEGF, HESX1, HEYL, HFE, HGS, HSF1, HSPB1, ICAM1, IFITM1, IFITM2, IFITM3, IFT80, IL15RA, IL18BP, IL1B, IL1RN, IL22RA1, IL32, IL3RA, IL4R, IL5RA, IL7R, ILK, INHA, INPPL1, IQUB, IRAK2, IRF2, IRF9, ITGA3, ITGA5, ITGA7, ITGAD, ITPRIP, JAK3, JUNB, JUP, KCP, LAG3, LAMA5, LGR6, LIF, LIFR, LILRB5, LIMK1, LIMS2, LMNA, LOX, LRG1, LRRC32, LTBR, LTF, LY6E, LYNX1, LZTS2, MAP3K10, MAP3K14, MAPK3, MAPK7, MARK2, MARK4, MAVS, MECP2, MED12, MEGF8, MEN1, MGRN1, MINK1, MKNK2, MOV10, MSN, MSTN, MSX1, MUC19, MUC20, MUC3A, MYC, MYH9, NCOR2, NDP, NDST1, NECTIN2, NEFL, NF2, NFATC4, NGF, NGFR, NKD2, NLGN2, NLRC5, NOD2, NODAL, NOS3, NOTCH1, NOTCH3, NOTCH4, NPPA, NPPB, NR4A2, NR4A3, NRARP, NTN1, NUMBL, OMA1, OSM,

GO:0009966	regulation of signal transduction	1,31E-14	[3, 4, 5]	609,00	18,48	37,99	62,01	[ABRA, ADAM8, ALOX15, ALOX15B, ANGPT1, ARAP2, AREG, ARHGAP15, ARHGAP30, ARHGAP45, ATF3, BANK1, BCL2L14, BCL3, BLK, BMF, BMP7, BMPR1B, BRIP1, C1QTNF1, CALCR, CARD11, CASP8, CASS4, CCDC88C, CCL17, CCL19, CCL2, CCL22, CCL24, CCL3, CCL3L1, CCL4, CCL4L1, CCL5, CCR2, CD177, CD180, CD19, CD22, CD226, CD27, CD300LF, CD3E, CD4, CD40LG, CD74, CD80, CD86, CDKN2A, CHRDL1, CISH, CLEC6A, CRB2, CSF1R, CXCR4, CXorf21, DAPK1, DCC, DEPDC1B, DLGAP2, DPEP2, DUSP2, DUSP8, ELF3, EPX, EREG, ERFE, FASLG, FCRL3, FFAR4, FGD2, FGD3, FGR, FLT3, FOLR2, FOXP3, FRZB, FUT7, GADD45B, GDF6, GFI1, GMIP, GPR174, GPR4, GPR55, GPRC5A, GUCY2D, H3C10, H3C11, H3C12, H3C7, HCLS1, HCST, HLA-DRB1, ICOS, IER3, IFNG, IKBKE, IL10RA, IL18, IL26, IL7R, INPP5D, IQCJ-SCHIP1, IRF4, KCNN4, LAX1, LCK, LEF1, LIF, LILRB4, LMCD1, LY86, MAP4K1, MMP9, MSX1, MZB1, NCF1, NCKAP1L, NFAM1, NLRC3, NLRP3, NLRP6, NPFFR2, NPPC, NPTX2, NR4A2, NTN1, OASL, P2RY10, P2RY12, PAX5, PDE6G, PIK3CG, PIK3IP1, PIK3R5, PIM2, PLAU, PLEK, PRDM1, PRKCB, PRKCQ, PROK1, PSCA, PSD4, PTK2B, PTPN22, PTPN6, PTPRC, PYCARD, PYHIN1, RAC2, RAD9B, RASAL3, RASGRF1, RBPMS2, RGS1, RGS10, RGS18, RGS9, RHOH, RMI2, RNASE6, RRAD, RTKN2, SALL1, SCIMP, SCUBE1, SEMA4D, SH2D1A, SHISA8, SLA, SLA2, SLC8A3, SMPDL3B, SOCS1, SOCS3, SPINK1, STAP1, STAT1, SYK, TAGAP, TBC1D10C, TESPA1, THBS1, THY1, TLR6, TMC8, TNFAIP3, TNFSF14, TNIP3, TNMD, TRABD2A, TRAF1, TRAT1, TREM2, TRIM14, UBASH3A, UBD, VAV1, VNN1, VWC2L, WNT1, WNT10B, XCL1, XCL2, ZAP70, ZBP1]	[ABCA7, ABL1, ABR, ABRA, ACKR3, ACP4, ACTN4, ADGRG1, ADIPOQ, AGAP2, AGER, AGPAT1, AGT, AKT1S1, AMH, ANGPT1, APLN, APLNR, APLP1, APOA1, APOD, ARAF, ARAP3, ARC, AREG, ARHGAP1, ARHGAP23, ARHGAP39, ARHGDIA, ARHGEF1, ARHGEF16, ARHGEF5, ARRDC3, ARTN, ASPN, BCL2L1, BCL3, BCL6, BCL9L, BCR, BEND6, BMP8A, BRD4, C1QTNF1, C20orf27, C5, C5AR1, CALCR, CAMK2B, CAPN1, CARM1, CC2D1A, CCL11, CCL2, CCL21, CCL24, CCR7, CD14, CD177, CD74, CDK1, CDK2, CEACAM1, CGA, CHERP, CHGA, CHI3L1, CISH, CITED2, CLIP3, CNTN6, COL1A1, CRAT, CREB3L1, CSF1, CSK, CSNK1E, CSPG4, CTHRC1, CX3CL1, CXXC4, CYP26B1, CYP27B1, DAB2IP, DAG1, DAXX, DDX39B, DENND2B, DENND4B, DGKD, DGKZ, DIRAS1, DKK2, DLG5, DLGAP4, DMTN, DOK5, DOT1L, DTX1, DUSP5, DUSP8, DVL3, ECM1, EEF1E1, ELF3, ENG, EPHA2, EPN1, ERBB2, ERFE, ESM1, FADD, FAIM2, FAM110A, FASN, FGFR4, FLOT2, FLT3, FLT4, FOXM1, FPR1, FRMD8, FURIN, GAB2, GADD45B, GAS1, GATA4, GBA, GBF1, GCSAM, GDF6, GIT1, GLIS2, GNAI2, GPAM, GPBAR1, GPER1, GPI, GPR137, GPR17, GPR20, GPR37L1, GPR4, GPRC5A, GPRIN1, GRB7, GRIK5, GRINA, GRM2, GSK3A, H3C10, H3C12, H4C3, HBEGF, HCRTR1, HDAC7, HEYL, HFE, HGS, HIPK4, HSPB1, HTR1B, HTR2A, ICAM1, IER3, IFT80, IL18BP, IL1B, IL1RN, IL7R, ILK, INCA1, INHA, INPP5E, IRAK2, ITGA3, ITGA5, ITPRIP, JAK3, JMJD8, JUP, KCP, KCTD11, KIF26A, KLK14, KMT2D, KSR1, LGALS9, LGR6, LIF, LIFR, LILRA5, LIMS2, LMCD1, LMNA, LOX, LRG1, LTBR, LTF, LY6E, LYNX1, LZTS2, MAP2K3, MAP3K10, MAP3K11, MAP3K14, MAP3K6, MAP3K9, MAPK3, MAPK7, MAPK8IP3, MAVS, MAZ, MED12, MEGF8, MEIS3P1, MEN1, MGRN1, MINK1, MLST8, MNT, MSTN, MSX1, MTRNR2L1, MTRNR2L10, MTRNR2L12, MTRNR2L2, MTRNR2L3, MTRNR2L6, MTRNR2L8, MTRNR2L9, MUC20, MYADM, MYC, NCLN, NCOR2, NDC80, NDST1, NECTIN2, NEFL, NF2, NFATC4, NGF, NGFR, NINJ1, NKD2, NLGN2, NLRC5, NOD2, NODAL, NOP2, NOS3, NOTCH1, NOTCH3, NOTCH4
GO:0010243	response to organonitrogen compound	7,10E-03	[4]	209,00	18,40	36,76	63,24	[AANAT, ADCY7, AIF1, AREG, BMP7, BRIP1, BTG2, C1QTNF1, CALCR, CDH1, CNR1, CNR2, CXCR4, CYBB, CYP11B1, DERL3, DPEP1, DRD1, ELAVL4, EREG, ERFE, FLT3, FOLR2, FOSB, FOSL1, FOXP3, FUT7, GLDC, HCN1, IRF5, ITGA4, ITGB2, KLF5, LCP1, LDLR, MAPK13, MMP9, MUSK, MZB1, NAMPT, NR4A1, NR4A2, NR4A3, NSG1, P2RY12, P2RY2, PIK3CG, PRKCB, PRKCQ, PTAFR, PTK2B, PTPN22, RAB15, RGS10, RGS9, SDC1, SH2D2A, SLC8A3, SMPD3, SOCS1, SOCS3, STAT1, SYK, TLR6, TNFAIP3, TREM2, TRPM2, UCP2, WNT1, WNT10B]	[ABCC8, ABL1, ACTA1, ACTB, ADCY1, ADCY3, ADCY4, ADCY9, ADIPOQ, AGER, AGT, AGTR1, APLP1, AREG, ATP1A3, ATP6V0D1, ATP6V0E2, ATP6V1B1, ATP6V1F, BAG6, BCAR1, BCL2L1, BTG2, C1QTNF1, C5AR1, CALCR, CARM1, CDK1, CDKN1A, CEACAM1, CNR1, COASY, COL1A1, COL6A1, CRAT, CRHR2, CSK, CYP21A2, DAG1, DHX34, DMTN, DNMT1, DPAGT1, ERFE, FADD, FLT3, FOSL1, FOXC2, FOXRED2, GET4, GLRA1, GNAI2, GNAO1, GNRHR, GPER1, GPI, GPRIN1, GPT, GRB7, GRM2, GSK3A, HCN3, HCN4, HDAC5, HNMT, HPRT1, HRH2, HSF1, HTR1B, HTR2A, ICAM1, IL1B, INPPL1, JAK3, JUND, JUP, KCTD11, KLF10, KLF5, LDLR, LDOC1, MAP1LC3A, MAPK3, MED12, MEN1, MSTN, MTHFR, MYC, NCOR2, NEFL, NGFR, NOD2, NOTCH1, NPPA, NR4A1, NR4A2, NR4A3, NSG1, NSMF, P2RX3, P2RX5, P2RY2, P2RY6, PALM, PCDHA4, PCDHA6, PDK2, PER1, PKM, POR, PPP1R9B, PRKACA, PRKAR2B, PTN, PTPRN, PXN, QDPR, RAB15, RANGAP1, RAP1A, RAPGEF1, RELA, RHBDD2, RMRP, RNU1-93P, RPTOR, SCAP, SELENON, SGTA, SH2B2, SIK2, SLC25A33, SLC2A4, SLC9A1, SLC9A3R1, SMPD1, SOCS3, SRC, SREBF1, STAT3, SYVN1, TCIRG1, TGM2, TICAM1, TIMP1, TMEM259, TMUB1, TMUB2, TNF, TNS2, TRIB3, TRPV4, TSC2, WDTC1, WFS1, ZBTB7B]
GO:0014070	response to organic cyclic compound	8,35E-04	[4]	203,00	19,08	35,12	64,88	[AANAT, AIF1, ANGPT2, AREG, BMP7, BTG2, C1QTNF1, CALCR, CASP8, CCL2, CCL3, CCL5, CCR5, CDH1, CNR1, CXCL10, CXCR4, CYBB, CYP11B1, DPEP1, DPEP2, DRD1, ELAVL4, EPX, FIBIN, FLT3, FOLR2, FOSB, FOSL1, FOXP3, GLDC, GPR83, GRAMD1B, GRAMD1C, H3C10, H3C11, H3C12, H3C7, HCN1, IL18, IL22, KLF5, LPAL2, MLC1, MUSK, NAMPT, NLRP3, NR4A3, NSG1, P2RY12, P2RY13, P2RY2, PIK3CG, PIM1, PTAFR, PTGFR, PTK2B, RGS9, SCNN1B, SDC1, SLC8A3, STAT1, THBS1, TRERF1, TRPM2, WNT10B]	[ABCA3, ABL1, ACACB, ACSBG1, ACTA1, ACTB, ADCY1, ADCY3, ADIPOQ, AGT, ALPL, AMH, APLP1, AQP3, AREG, ARHGDIA, ARID1A, ATAT1, ATP1A1, ATP1A3, BCL2L1, BTG2, C1QTNF1, CALCR, CARM1, CCL2, CDK1, CDKN1A, CIITA, CNR1, COL1A1, CRAT, CRYAB, CYP21A2, CYP27B1, DAG1, DAXX, DDX54, DHX34, DMTN, EGLN2, ELK1, ENDOG, ENG, FADD, FES, FGFR4, FIBIN, FLT3, FOSL1, FOXF1, GBA, GNAI2, GNAO1, GPAM, GPBAR1, GPER1, GPI, GPR83, GRAMD1A, GRAMD1B, GRAMD1C, GRM2, GSK3A, H3C10, H3C12, H4C3, HCFC1, HCN3, HCN4, HEYL, HNMT, HRH2, HSF1, HTR1B, HTR2A, ICAM1, ID3, IL1B, IL1RN, JUND, JUP, KCNMB1, KCTD11, KLF5, KMT2D, LOX, LUM, MAP1LC3A, MAPK3, MAVS, MED12, MMP15, MSN, MSTN, MTHFR, MYC, NCOR2, NEFL, NOD2, NODAL, NOTCH1, NR1D1, NR4A3, NSG1, P2RX3, P2RX5, P2RY2, P2RY6, PALM, PCDHA4, PDGFB, PDGFRB, PER1, PIM1, POSTN, PPARD, PPP1R9B, PQBP1, PRKACA, PRKAR2B, PTGES, PTGFR, PTN, PTPRN, PTPRU, RAMP2, RAP1A, RAPGEF1, RARA, RELA, RMRP, RNU1-93P, RXRA, RXRB, SELENON, SLC9A1, SLC9A3R1, SMARCA4, SMPD1, SOX10, SRC, SREBF1, SSTR2, STAT3, STING1, TGM2, THBS1, TICAM1, TIMP1, TNF, VDR, WBP2, ZBTB7A, ZC3H12A, ZCCHC3, ZFP36, ZMIZ1]

GO:0033993	response to lipid	5,91E-09	[4]	216,00	21,51	43,79	56,21	[AANAT, ACP5, AIF1, AREG, BMP7, C1QTNF1, CALCR, CARD17, CASP8, CCL2, CCL3, CCL5, CCR5, CD180, CD6, CD80, CD86, CD96, CNR1, CNR2, CXCL10, CXCL11, CXCL5, CXCL6, CXCL9, CYBB, CYP11B1, DPEP1, DPEP2, EPX, FASLG, FIBIN, FLT3, FOSB, FOSL1, FOXP3, GFI1, GLDC, GPR83, GRAMD1B, GRAMD1C, H3C10, H3C11, H3C12, H3C7, IL10RA, IL12RB2, IL18, IL22, IRF8, KMO, LCP1, LDLR, LILRB1, LPAL2, LTA, LY86, MAP4K1, MLC1, MPO, MRC1, MUSK, MYB, NLRP3, NLRP7, NPPC, NR4A1, NR4A3, NUGGC, PIM1, PTAFR, PTGFR, PTK2B, PTPN22, PYCARD, RGS9, SCIMP, SCNN1B, SDC1, SELE, STAP1, TBXAS1, THBS1, TNFAIP3, TNIP3, TREM2, TRERF1, UCP2, WNT10B]	[ABCA3, ABCC8, ABL1, ACSBG1, ACTA1, ADCY1, ADCY3, ADIPOQ, AGT, ALPL, AQP3, AREG, ARID1A, ATAT1, ATP1A1, ATP1A3, C1QTNF1, C5AR1, CALCR, CARM1, CCL2, CCR7, CD14, CDKN1A, CNR1, COL1A1, CRAT, CRYAB, CX3CL1, CYP21A2, CYP26B1, CYP27B1, DAB2IP, DAG1, DAXX, DDX54, EGLN2, ELK1, ENDOG, ENG, FES, FFAR2, FGFR4, FIBIN, FLT3, FOSL1, GATA2, GATA4, GBA, GIT1, GNAI2, GPAM, GPBAR1, GPER1, GPI, GPR83, GPX3, GRAMD1A, GRAMD1B, GRAMD1C, H3C10, H3C12, H4C3, HEYL, HNMT, HSF1, HTR1B, ICAM1, ID3, IL18BP, IL1B, IL1RN, IRAK2, JUND, KCNMB1, KCTD11, KMT2D, LDLR, LDOC1, LGALS9, LOX, LTF, MAPK3, MMP15, MRC1, MSN, MSTN, MYC, NCOR2, NEFL, NFKB2, NFKBIB, NOD2, NODAL, NOS3, NOTCH1, NR1D1, NR4A1, NR4A3, PAF1, PCDHA4, PCDHA6, PDGFRB, PER1, PIM1, POSTN, PPARD, PPP1R9B, PTGES, PTGFR, PTGIR, PTN, PTPRU, RAMP2, RARA, RELA, RMRP, RXRA, RXRB, SBNO2, SELE, SELENOP, SERPINE1, SLC10A3, SMARCA4, SOX10, SPHK2, SPI1, SRC, SREBF1, SSTR2, STAT3, TEAD2, THBS1, TICAM1, TIMP1, TMEM161A, TMEM256-PLSCR3, TNF, TRIB1, VDR, WBP2, WNT3, WNT9B, ZBTB7A, ZC3H12A, ZFP36, ZMIZ1]
GO:0034097	response to cytokine	3,79E-20	[4]	302,00	23,59	52,48	47,52	[AANAT, ACP5, AIF1, AIM2, ALOX15, ANGPT1, BATF, BTK, CASP8, CCL17, CCL19, CCL2, CCL22, CCL24, CCL3, CCL3L1, CCL4, CCL4L1, CCL5, CCR2, CCR3, CCR4, CCR5, CCR8, CD27, CD300LF, CD4, CD40LG, CD74, CD80, CD86, CISH, CLCF1, CORO1A, CSF1R, CSF2RA, CXCL10, CXCL11, CXCL5, CXCL6, CXCL9, CXCR3, CXCR4, CXCR6, DAPK1, DOCK8, EBI3, EDN2, EPX, EREG, FASLG, FLT3, FOSL1, GBP5, GBP6, GFI1, GFPT2, GFRA2, GRAP2, H3C10, H3C11, H3C12, H3C7, HCLS1, HLA-DPA1, HLA-DPB1, HLA-DQA1, HLA-DRA, HLA-DRB1, HLA-DRB5, IFNG, IKBKE, IL10RA, IL12RB1, IL12RB2, IL16, IL18, IL18RAP, IL21R, IL22, IL26, IL2RB, IL2RG, IL5RA, IL7R, IL9R, INPP5D, IRF4, IRF5, IRF8, ITGA4, ITGAX, ITGB2, KLF5, KMO, LAMP3, LCK, LCP1, LEF1, LIF, LILRB4, LTA, LTB, MAPK13, MMP9, MRC1, MYBL2, NELL2, NLRP7, NR4A3, OASL, PIM1, PTAFR, PTK2B, PTPN6, PTPN7, PTPRC, PYCARD, PYHIN1, RHEX, RUFY4, SDC1, SELE, SLA, SLA2, SMPD3, SNX10, SOCS1, SOCS3, STAP1, STAT1, SYK, THBS1, TNFAIP3, TNFRSF11B, TNFRSF13B, TNFRSF17, TNFRSF18, TNFSF13B, TNFSF14, TNFSF8, TRAF1, TREM2, UBD, VAV1, WAS, XCL1, XCL2, XCR1, ZBP1]	[ABCG4, ACKR1, ACKR3, ACTN4, ADAMTS7, ADIPOQ, AGPAT1, AMH, ANGPT1, APOA1, ARF1, BCL2L1, BCL6, CAMK2B, CCL11, CCL2, CCL21, CCL24, CCR7, CD14, CD74, CDC42EP2, CDC42EP4, CDKN1A, CEACAM1, CFL1, CHI3L1, CIITA, CISH, CLCF1, CLIP3, COL1A1, CSF1, CSK, CTF1, CX3CL1, CYP27B1, DAB2IP, DTX1, ECM1, EDN2, ENDOG, FABP4, FADD, FASN, FLT3, FOSL1, FOXF1, FPR1, GAB2, GBA, GFPT2, GFRA3, GNAO1, GPER1, GPR17, GPR75, GSK3A, H3C10, H3C12, HFE, HNMT, HYAL1, ICAM1, IFITM1, IFITM2, IFITM3, IL15RA, IL18BP, IL1B, IL1RN, IL22RA1, IL32, IL3RA, IL4R, IL5RA, IL7R, ILK, INPPL1, IRAK2, IRF2, IRF9, JAK3, JUNB, KLF5, LAMA5, LDLRAP1, LGALS9, LIF, LIFR, LOX, LTBR, MAP3K14, MAPK3, MAPK7, MAVS, MME, MRC1, MSC, MSN, MTHFR, MYC, NFKB2, NLRC5, NOD2, NR1D1, NR4A3, NUMBL, OSM, PCOLCE2, PDGFB, PELI3, PIAS4, PIM1, PITPNA, PLP2, PLVAP, PML, POSTN, PRKACA, PTGES, PTP4A3, PTPN13, PTPN20, PTPN23, PTPRN, RAPGEF1, RARA, RELA, RELB, RHBDF2, RNU1-93P, RPL3, RPS16, SBNO2, SELE, SELENOP, SH2B2, SH2B3, SHARPIN, SLC2A4, SMAD3, SMARCA4, SMPD1, SMPD4, SNX10, SOCS3, SPATA2, SPHK1, SPI1, SPPL2B, SRC, SRF, SRM, STAT3, STING1, STX1A, TCIRG1, TEX14, THBS1, THPO, TIMP1, TLE5, TNF, TNFRSF1A, TNFSF12, TRIM62, TRIM8, TUBA1B, UBA52, VEGFA, VSIG2, WAS, ZC3H12A, ZFP36, ZYX]
GO:0071310	cellular response to organic substance	5,91E-19	[4]	565,00	19,58	42,38	57,62	[AANAT, ADCY7, AIF1, AIM2, ALOX15, ANGPT1, ANGPT2, AREG, ATF3, BATF, BHLHA15, BMP7, BMPR1B, BRIP1, BTK, CARD17, CASP8, CCL17, CCL19, CCL2, CCL22, CCL24, CCL3, CCL3L1, CCL4, CCL4L1, CCL5, CCR2, CCR3, CCR4, CCR5, CCR8, CD180, CD27, CD300LF, CD4, CD40LG, CD6, CD74, CD80, CD86, CDH1, CHRDL1, CISH, CLCF1, CORO1A, CRB2, CSF1R, CSF2RA, CTSS, CXCL10, CXCL11, CXCL5, CXCL6, CXCL9, CXCR3, CXCR4, CXCR6, CYBB, CYP11B1, DAPK1, DERL3, DOCK8, DPEP2, DRD1, EBI3, EDN2, EGR3, ELAVL4, EPX, EREG, ERFE, FASLG, FLT3, FOLR2, FOSB, FUT7, GALNT3, GBP5, GBP6, GDF6, GFI1, GFPT2, GFRA2, GRAMD1B, GRAMD1C, GRAP2, H3C10, H3C11, H3C12, H3C7, HCLS1, HCN1, HLA-DPA1, HLA-DPB1, HLA-DQA1, HLA-DRA, HLA-DRB1, HLA-DRB5, HSPA7, IFNG, IKBKE, IL10RA, IL12RB1, IL12RB2, IL16, IL18, IL18RAP, IL21R, IL22, IL26, IL2RB, IL2RG, IL5RA, IL7R, IL9R, INPP5D, IRF4, IRF5, IRF8, ITGA4, ITGAX, ITGB2, KLF5, KMO, LCK, LCP1, LDLR, LEF1, LIF, LILRB1, LILRB4, LTA, LTB, LY86, MAP4K1, MAPK13, MLC1, MMP9, MRC1, MSX1, MT1G, MYB, MYBL2, MZB1, NAMPT, NELL2, NLRP3, NLRP7, NR4A1, NR4A2, NR4A3, NSG1, NUGGC, OASL, P2RY12, P2RY13, P2RY2, PIK3CG, PIM1, PRKCB, PRKCQ, PTAFR, PTGER2, PTGFR, PTK2B, PTPN22, PTPN6, PTPN7, PTPRC, PYCARD, PYHIN1, RAB15, RBPMS2, RGS9, RHEX, RUFY4, SCIMP, SCNN1B, SDC1, SLA, SLA2, SLC8A3, SMPD3, SNX10, SOCS1, SOCS3, STAP1, STAT1, SYK, TBX21, THBS1, TLR6, TNFAIP3, TNFRSF11B, TNFRSF13B, TNFRSF17, TNFRSF18, TNFSF13B, TNFSF14, TNFSF8, TNIP3, TNMD, TRAF1, TREM2, TRERF1, TRPM2, UCP2, VAV1, VWC2L, WAS, WNT1, WNT10A, WNT10B, XCL1, XCL2, XCR1, ZBP1]	[ABCA7, ABCC8, ABCG4, ABL1, ACKR1, ACKR3, ACTA1, ACTB, ACTN4, ADAMTS7, ADCY1, ADCY3, ADCY4, ADCY9, ADIPOQ, AGER, AGPAT1, AGT, AGTR1, AKT1S1, ALPL, AMH, ANGPT1, APLN, APLP1, APOA1, AREG, ARF1, ARHGDIA, ARID1A, ARID5A, ASPN, ATP1A1, ATP1A3, ATP6V0D1, ATP6V0E2, ATP6V1B1, ATP6V1F, BAG3, BAG6, BCAR1, BCL2L1, BCL6, BCL9L, BMP8A, C20orf27, C2CD2L, CACNA1H, CALB1, CAMK2B, CARM1, CCL11, CCL2, CCL21, CCL24, CCR7, CD14, CD74, CDC42EP2, CDC42EP4, CDKN1A, CEACAM1, CFL1, CGA, CHERP, CHGA, CHI3L1, CIITA, CISH, CITED2, CLCF1, CLIP3, COASY, COL1A1, COL6A1, CRAT, CREB3L1, CRHR2, CSF1, CSK, CTF1, CX3CL1, CYP21A2, CYP26B1, CYP27B1, DAB2IP, DAG1, DAXX, DDX54, DHX34, DMTN, DNMT1, DPAGT1, DTX1, ECM1, EDN2, EGLN2, EHD1, ELK1, ENDOG, ENG, ERBB2, ERFE, EXTL1, FABP4, FADD, FAM83G, FASN, FES, FFAR2, FGFR4, FGFRL1, FLT3, FLT4, FOXC2, FOXF1, FPR1, FURIN, GAB2, GAS1, GAS2L1, GATA4, GBA, GDF6, GFPT2, GFRA3, GIT1, GLRA1, GNAI2, GNAO1, GNRHR, GPAM, GPBAR1, GPER1, GPI, GPR17, GPR75, GPRIN1, GPT, GRAMD1A, GRAMD1B, GRAMD1C, GRB7, GRIK5, GSK3A, H3C10, H3C12, H4C3, HCFC1, HCN3, HCN4, HDAC5, HEYL, HFE, HGS, HRH2, HSF1, HSPB1, HSPB8, HTR1B, HTR2A, HYAL1, ICAM1, ID3, IFITM1, IFITM2, IFITM3, IL15RA, IL18BP, IL1B, IL1RN, IL22RA1, IL32, IL3RA, IL4R, IL5RA, IL7R, ILK, INPPL1, IRAK2, IRF2, IRF9, ITGA3, ITGA5, JAK3, JUNB, JUP, KCNMB1, KCP, KCTD11, KLF10, KLF5, KMT2D, LAMA5, LDLR, LDLRAP1, LDOC1, LGALS9, LIF, LIFR, LMNA, LOX, LRG1, LRRC32, LTBR, LTF, LZTS1, MAP2K3, MAP3K14, MAPK3, MAPK7, MAVS, MED12, MEGF8, MEN1, MICALL1, MME, MRC1, MSC, MSN, MSTN, MSX1, MTSS2, MYC, NCOR2, NDOR1, NDST1, NFKBIB, NGF, NGFR, NLRC5, NOD2, NODAL, NOTCH1, NR1D1, NR4A1, NR4A2, NR4A3, NSG1, NSMF, NUMBL, OSM, P2RX3, P2RY2, P2RY6, PAF1, PALM, PCOLCE2, PDGFB, PDGFRB, PDK2, PELI3, PELP1, PER1, PIAS4, PIM1, PITPNA, PKM, PLP2, PLVAP, PML, POLR2[illegible], POLR2[illegible]

GO:1901699	cellular response to nitrogen compound	2,55E-02	[4]	147,00	19,22	33,73	66,27	[AANAT, ADCY7, AIF1, BRIP1, CDH1, CYBB, CYP11B1, DPEP1, DRD1, ERFE, FOLR2, FUT7, HCN1, ITGA4, KLF5, LCP1, MAPK13, MZB1, NAMPT, NR4A1, NR4A2, NR4A3, NSG1, P2RY12, P2RY2, PIK3CG, PRKCB, PRKCQ, PTAFR, PTPN22, RAB15, RGS10, RGS9, SLC8A3, SMPD3, SOCS1, SOCS3, STAT1, TLR6, TREM2, TRPM2, UCP2, WNT1, WNT10B]	[ABL1, ACTA1, ACTB, ADCY1, ADCY3, ADCY4, ADCY9, ADIPOQ, AGER, AGT, AGTR1, APLP1, ATP1A3, ATP6V0D1, ATP6V0E2, ATP6V1B1, ATP6V1F, BCAR1, BCL2L1, CDK2, CEACAM1, CIITA, COASY, COL1A1, COL6A1, CRHR2, CSK, CYP21A2, DAXX, DHX34, DMTN, DNMT1, DPAGT1, ERFE, FOXC2, GLRA1, GNAI2, GNAO1, GNRHR, GPER1, GPRIN1, GPT, GRB7, GSK3A, HCN3, HCN4, HDAC5, HRH2, HSF1, HTR1B, HTR2A, ICAM1, IL1B, JAK3, JUP, KLF10, KLF5, LDOC1, LY6E, MAPK3, MAVS, MED12, MEN1, MSTN, MYC, NGFR, NOD2, NR4A1, NR4A2, NR4A3, NSG1, NSMF, P2RX3, P2RY2, P2RY6, PALM, PDK2, PKM, POR, PPP1R9B, PQBP1, PRKACA, PRKAR2B, PXN, RAB15, RANGAP1, RAP1A, RAPGEF1, RELA, RNU1-93P, RPTOR, SELENON, SH2B2, SIK2, SLC25A33, SLC2A4, SLC9A1, SLC9A3R1, SOCS3, SRC, SREBF1, STAT3, STING1, TCIRG1, TGM2, TNF, TNS2, TRIB3, TSC2, WDTC1, ZBTB7B, ZCCHC3]
GO:1901701	cellular response to oxygen-containing compound	5,30E-07	[4]	255,00	19,78	41,02	58,98	[AANAT, ADCY7, AIF1, AREG, BMP7, BRIP1, BTK, CARD17, CCL2, CCL3, CCL5, CCR5, CD180, CD6, CD80, CD86, CDH1, CXCL10, CXCL11, CXCL5, CXCL6, CXCL9, CYBB, CYP11B1, DAPK1, DPEP1, DRD1, ERFE, FOLR2, FUT7, GFI1, GRAMD1B, GRAMD1C, HCN1, HLA-DRB1, IL18, IL18RAP, IRF8, ITGA4, KLF5, KMO, LCP1, LDLR, LILRB1, LY86, MAP4K1, MAPK13, MLC1, MMP9, MPO, MRC1, MYB, MZB1, NAMPT, NCF1, NLRP3, NLRP7, NME8, NR4A1, NR4A2, NR4A3, NSG1, NUGGC, P2RY12, P2RY2, PIK3CG, PIM1, PRKCB, PRKCQ, PTAFR, PTGER2, PTGFR, PTK2B, PTPN22, PYCARD, RAB15, RGS10, RGS9, SCIMP, SCNN1B, SLC8A3, SMPD3, SOCS1, SOCS3, STAP1, STAT1, TLR6, TNFAIP3, TNIP3, TREM2, TRERF1, TRPM2, UCP2, WNT1, WNT10B]	[ABL1, ACTB, ADCY1, ADCY3, ADCY4, ADCY9, ADIPOQ, AGER, AGT, AGTR1, APLP1, AREG, ATP1A3, ATP6V0D1, ATP6V0E2, ATP6V1B1, ATP6V1F, BCAR1, BCL2L1, C2CD2L, CCL2, CD14, CDK1, CDK2, CEACAM1, COASY, COL1A1, COL6A1, CRHR2, CSK, CX3CL1, CYP21A2, CYP26B1, CYP27B1, DAB2IP, DAG1, DHX34, DMTN, DNMT1, DPAGT1, ELK1, ENDOG, ERFE, FANCC, FES, FFAR2, FOXC2, GATA4, GIT1, GLRA1, GNAI2, GNAO1, GNRHR, GPBAR1, GPER1, GPR37L1, GPRIN1, GPT, GRAMD1A, GRAMD1B, GRAMD1C, GRB7, GRIK5, GSK3A, HCN3, HCN4, HDAC5, HRH2, HSF1, HTR1B, HTR2A, ICAM1, ID3, IL18BP, IL1B, IRAK2, JAK3, JUP, KCNMB1, KDM6B, KLF10, KLF5, LDLR, LDOC1, LTF, LY6E, MAP1LC3A, MAPK3, MAPK7, MED12, MEN1, MRC1, MSN, MSTN, MYC, NDOR1, NFKBIB, NGFR, NOD2, NOS3, NR1D1, NR4A1, NR4A2, NR4A3, NSG1, NSMF, P2RX3, P2RY2, P2RY6, PAF1, PALM, PCGF2, PDGFB, PDK2, PIM1, PKM, POR, PPARD, PPP1R9B, PRKACA, PRKAR2B, PTGFR, PTN, PTPRN, PXN, RAB11B, RAB11FIP5, RAB15, RANGAP1, RAP1A, RAPGEF1, RARA, RELA, RMRP, RNU1-93P, RPTOR, SBNO2, SERPINE1, SH2B2, SIDT2, SIK2, SLC25A33, SLC29A1, SLC2A4, SLC9A1, SLC9A3R1, SMARCA4, SMARCB1, SOCS3, SOX10, SPHK1, SPHK2, SPI1, SRC, SREBF1, SRF, SSTR2, STAT3, SZT2, TCIRG1, TEAD2, TGM2, TICAM1, TMEM256-PLSCR3, TNF, TNS2, TRIB1, TRIB3, TSC2, VDR, WDTC1, WNT3, WNT9B, ZBTB7B, ZC3H12A, ZFP36, ZNF580]
GO:0071417	cellular response to organonitrogen compound	2,41E-02	[4, 5]	137,00	19,49	34,35	65,65	[AANAT, ADCY7, AIF1, BRIP1, CDH1, CYBB, CYP11B1, DRD1, ERFE, FOLR2, FUT7, HCN1, ITGA4, KLF5, LCP1, MAPK13, MZB1, NAMPT, NR4A1, NR4A2, NR4A3, NSG1, P2RY12, P2RY2, PIK3CG, PRKCB, PRKCQ, PTAFR, PTPN22, RAB15, RGS9, SLC8A3, SMPD3, SOCS1, SOCS3, STAT1, TLR6, TREM2, TRPM2, UCP2, WNT1, WNT10B]	[ABL1, ACTA1, ACTB, ADCY1, ADCY3, ADCY4, ADCY9, ADIPOQ, AGER, AGT, AGTR1, APLP1, ATP1A3, ATP6V0D1, ATP6V0E2, ATP6V1B1, ATP6V1F, BCAR1, BCL2L1, CEACAM1, COASY, COL1A1, COL6A1, CRHR2, CSK, CYP21A2, DHX34, DMTN, DNMT1, DPAGT1, ERFE, FOXC2, GLRA1, GNAI2, GNAO1, GNRHR, GPER1, GPRIN1, GPT, GRB7, GSK3A, HCN3, HCN4, HDAC5, HRH2, HSF1, HTR1B, HTR2A, ICAM1, IL1B, JAK3, JUP, KLF10, KLF5, LDOC1, MAPK3, MED12, MEN1, MSTN, MYC, NGFR, NOD2, NR4A1, NR4A2, NR4A3, NSG1, NSMF, P2RX3, P2RY2, P2RY6, PALM, PDK2, PKM, POR, PPP1R9B, PRKACA, PRKAR2B, PXN, RAB15, RANGAP1, RAP1A, RAPGEF1, RELA, RNU1-93P, RPTOR, SELENON, SH2B2, SIK2, SLC25A33, SLC2A4, SLC9A1, SLC9A3R1, SOCS3, SRC, SREBF1, STAT3, TCIRG1, TGM2, TNF, TNS2, TRIB3, TSC2, WDTC1, ZBTB7B]
GO:1901652	response to peptide	3,04E-02	[4, 5]	116,00	20,10	35,62	64,38	[AANAT, ADCY7, AREG, BMP7, BRIP1, BTG2, C1QTNF1, CALCR, CYBB, CYP11B1, EREG, ERFE, FUT7, IRF5, ITGA4, KLF5, LCP1, MMP9, MUSK, MZB1, NAMPT, NR4A1, NR4A2, NR4A3, PRKCB, PRKCQ, PTPN22, RAB15, SH2D2A, SMPD3, SOCS1, SOCS3, STAT1, TLR6, TNFAIP3, TREM2, UCP2, WNT1]	[ABCC8, ADCY1, ADCY3, ADCY4, ADCY9, ADIPOQ, AGER, AGT, AGTR1, AREG, ATP1A3, ATP6V0D1, ATP6V0E2, ATP6V1B1, ATP6V1F, BCAR1, BTG2, C1QTNF1, CALCR, CEACAM1, COASY, COL1A1, CRAT, CRHR2, CSK, CYP21A2, DAG1, DPAGT1, ERFE, FOXC2, GNAI2, GNRHR, GPER1, GPRIN1, GPT, GRB7, GSK3A, HDAC5, HSF1, ICAM1, IL1B, INPPL1, JAK3, JUND, KLF10, KLF5, LDOC1, MED12, MEN1, MSTN, MYC, NEFL, NGFR, NOD2, NOTCH1, NPPA, NR4A1, NR4A2, NR4A3, PDK2, PKM, POR, PPP1R9B, PRKACA, PRKAR2B, PTPRN, PXN, QDPR, RAB15, RANGAP1, RELA, SCAP, SH2B2, SIK2, SLC25A33, SLC2A4, SLC9A1, SOCS3, SRC, SREBF1, STAT3, TCIRG1, TIMP1, TNS2, TRIB3, TRPV4, TSC2, WDTC1, ZBTB7B]

GO:0019221	cytokine-mediated signaling pathway	2,14E-17	[4, 5, 6]	216,00	25,35	57,71	42,29	[AIM2, ALOX15, ANGPT1, BATF, CASP8, CCL17, CCL19, CCL2, CCL22, CCL24, CCL3, CCL3L1, CCL4, CCL4L1, CCL5, CCR2, CCR3, CCR4, CCR5, CCR8, CD27, CD300LF, CD4, CD40LG, CD74, CD80, CD86, CISH, CLCF1, CSF1R, CSF2RA, CXCL10, CXCL11, CXCL5, CXCL6, CXCL9, CXCR3, CXCR4, CXCR6, EBI3, EDN2, EPX, EREG, FASLG, FLT3, GFI1, GFRA2, GRAP2, H3C10, H3C11, H3C12, H3C7, HLA-DPA1, HLA-DPB1, HLA-DQA1, HLA-DRA, HLA-DRB1, HLA-DRB5, IFNG, IKBKE, IL10RA, IL12RB1, IL12RB2, IL16, IL18, IL18RAP, IL21R, IL22, IL26, IL2RB, IL2RG, IL5RA, IL7R, IL9R, INPP5D, IRF4, IRF5, IRF8, ITGAX, ITGB2, LCK, LCP1, LIF, LILRB4, LTA, LTB, MMP9, NR4A3, OASL, PIM1, PTAFR, PTK2B, PTPN6, PTPRC, PYCARD, RHEX, SDC1, SLA, SLA2, SOCS1, SOCS3, STAP1, STAT1, SYK, TNFAIP3, TNFRSF11B, TNFRSF13B, TNFRSF17, TNFRSF18, TNFSF13B, TNFSF14, TNFSF8, TRAF1, TREM2, VAV1, XCL1, XCL2, XCR1, ZBP1]	[ACKR1, ACKR3, ACTN4, ADIPOQ, AGPAT1, AMH, ANGPT1, APOA1, ARF1, BCL2L1, BCL6, CAMK2B, CCL11, CCL2, CCL21, CCL24, CCR7, CD74, CDKN1A, CEACAM1, CFL1, CIITA, CISH, CLCF1, CLIP3, CSF1, CSK, CTF1, CX3CL1, ECM1, EDN2, FADD, FASN, FLT3, FPR1, GAB2, GFRA3, GNAO1, GPR17, GPR75, H3C10, H3C12, HFE, ICAM1, IFITM1, IFITM2, IFITM3, IL15RA, IL18BP, IL1B, IL1RN, IL22RA1, IL32, IL3RA, IL4R, IL5RA, IL7R, ILK, INPPL1, IRAK2, IRF2, IRF9, JAK3, JUNB, LAMA5, LIF, LIFR, LTBR, MAP3K14, MAPK3, MAVS, MSN, MYC, NLRC5, NOD2, NR4A3, NUMBL, OSM, PDGFB, PELI3, PIAS4, PIM1, PITPNA, PLP2, PLVAP, PML, PRKACA, PTPRN, RAPGEF1, RELA, RHBDF2, RNU1-93P, SH2B2, SH2B3, SHARPIN, SMARCA4, SOCS3, SPATA2, SPHK1, SPI1, SPPL2B, SRC, STAT3, STX1A, THPO, TIMP1, TNF, TNFRSF1A, TNFSF12, TRIM62, TRIM8, UBA52, VEGFA]
GO:0071345	cellular response to cytokine stimulus	4,97E-19	[5]	282,00	23,76	53,83	46,17	[AIF1, AIM2, ALOX15, ANGPT1, BATF, BTK, CASP8, CCL17, CCL19, CCL2, CCL22, CCL24, CCL3, CCL3L1, CCL4, CCL4L1, CCL5, CCR2, CCR3, CCR4, CCR5, CCR8, CD27, CD300LF, CD4, CD40LG, CD74, CD80, CD86, CISH, CLCF1, CORO1A, CSF1R, CSF2RA, CXCL10, CXCL11, CXCL5, CXCL6, CXCL9, CXCR3, CXCR4, CXCR6, DAPK1, DOCK8, EBI3, EDN2, EPX, EREG, FASLG, FLT3, GBP5, GBP6, GFI1, GFPT2, GFRA2, GRAP2, H3C10, H3C11, H3C12, H3C7, HCLS1, HLA-DPA1, HLA-DPB1, HLA-DQA1, HLA-DRA, HLA-DRB1, HLA-DRB5, IFNG, IKBKE, IL10RA, IL12RB1, IL12RB2, IL16, IL18, IL18RAP, IL21R, IL22, IL26, IL2RB, IL2RG, IL5RA, IL7R, IL9R, INPP5D, IRF4, IRF5, IRF8, ITGA4, ITGAX, ITGB2, KLF5, KMO, LCK, LCP1, LEF1, LIF, LILRB4, LTA, LTB, MAPK13, MMP9, MRC1, MYBL2, NELL2, NLRP7, NR4A3, OASL, PIM1, PTAFR, PTK2B, PTPN6, PTPN7, PTPRC, PYCARD, PYHIN1, RHEX, RUFY4, SDC1, SLA, SLA2, SMPD3, SNX10, SOCS1, SOCS3, STAP1, STAT1, SYK, THBS1, TNFAIP3, TNFRSF11B, TNFRSF13B, TNFRSF17, TNFRSF18, TNFSF13B, TNFSF14, TNFSF8, TRAF1, TREM2, VAV1, WAS, XCL1, XCL2, XCR1, ZBP1]	[ABCG4, ACKR1, ACKR3, ACTN4, ADAMTS7, ADIPOQ, AGPAT1, AMH, ANGPT1, APOA1, ARF1, BCL2L1, BCL6, CAMK2B, CCL11, CCL2, CCL21, CCL24, CCR7, CD74, CDC42EP2, CDC42EP4, CDKN1A, CEACAM1, CFL1, CHI3L1, CIITA, CISH, CLCF1, CLIP3, COL1A1, CSF1, CSK, CTF1, CX3CL1, DAB2IP, DTX1, ECM1, EDN2, FABP4, FADD, FASN, FLT3, FOXF1, FPR1, GAB2, GBA, GFPT2, GFRA3, GNAO1, GPER1, GPR17, GPR75, GSK3A, H3C10, H3C12, HFE, HYAL1, ICAM1, IFITM1, IFITM2, IFITM3, IL15RA, IL18BP, IL1B, IL1RN, IL22RA1, IL32, IL3RA, IL4R, IL5RA, IL7R, ILK, INPPL1, IRAK2, IRF2, IRF9, JAK3, JUNB, KLF5, LAMA5, LDLRAP1, LGALS9, LIF, LIFR, LOX, LTBR, MAP3K14, MAPK3, MAVS, MME, MRC1, MSC, MSN, MYC, NLRC5, NOD2, NR1D1, NR4A3, NUMBL, OSM, PCOLCE2, PDGFB, PELI3, PIAS4, PIM1, PITPNA, PLP2, PLVAP, PML, POSTN, PRKACA, PTP4A3, PTPN13, PTPN20, PTPN23, PTPRN, RAPGEF1, RELA, RHBDF2, RNU1-93P, RPL3, RPS16, SBNO2, SH2B2, SH2B3, SHARPIN, SLC2A4, SMAD3, SMARCA4, SMPD4, SNX10, SOCS3, SPATA2, SPHK1, SPI1, SPPL2B, SRC, SRM, STAT3, STING1, STX1A, TCIRG1, TEX14, THBS1, THPO, TIMP1, TNF, TNFRSF1A, TNFSF12, TRIM62, TRIM8, TUBA1B, UBA52, VEGFA, VSIG2, WAS, ZC3H12A, ZFP36, ZYX]
GO:0071396	cellular response to lipid	3,22E-03	[5]	129,00	20,51	42,61	57,39	[AREG, BMP7, CARD17, CCL2, CCL3, CCL5, CCR5, CD180, CD6, CD80, CD86, CXCL10, CXCL11, CXCL5, CXCL6, CXCL9, FLT3, GFI1, GRAMD1B, GRAMD1C, H3C10, H3C11, H3C12, H3C7, IL18, IRF8, KMO, LCP1, LDLR, LILRB1, LY86, MAP4K1, MLC1, MRC1, MYB, NLRP3, NLRP7, NR4A3, NUGGC, PIM1, PTAFR, PTK2B, PTPN22, PYCARD, SCIMP, SCNN1B, STAP1, TNFAIP3, TNIP3, TRERF1, WNT10B]	[ABL1, ADCY1, ADCY3, AREG, ARID1A, ATP1A1, ATP1A3, CARM1, CCL2, CD14, COL1A1, CX3CL1, CYP21A2, CYP26B1, CYP27B1, DAB2IP, DAG1, DAXX, DDX54, EGLN2, ELK1, FES, FFAR2, FLT3, GIT1, GPAM, GPBAR1, GPER1, GRAMD1A, GRAMD1B, GRAMD1C, H3C10, H3C12, H4C3, HEYL, HSF1, ICAM1, ID3, IL1B, IRAK2, KCNMB1, KMT2D, LDLR, LDOC1, LTF, MAPK3, MRC1, MSN, MSTN, MYC, NCOR2, NFKBIB, NOD2, NODAL, NR1D1, NR4A3, PAF1, PER1, PIM1, PPARD, PPP1R9B, PTN, RARA, RELA, RMRP, RXRA, RXRB, SBNO2, SERPINE1, SMARCA4, SOX10, SPHK2, SPI1, SRC, SREBF1, SSTR2, TEAD2, TICAM1, TMEM256-PLSCR3, TNF, TRIB1, VDR, WBP2, WNT3, WNT9B, ZBTB7A, ZC3H12A, ZFP36, ZMIZ1]

GO:0045321	leukocyte activation	1,83E-35	[2, 3]	374,00	25,97	63,37	36,63	[ADAM8, ADGRE3, AIF1, ANPEP, APBB1IP, ARHGAP45, BANK1, BATF, BCL11B, BCL3, BIN2, BLK, BLNK, BTK, BTLA, BTN3A1, C1QA, CAMK4, CARD11, CASP8, CCDC88B, CCL19, CCL2, CCL3, CCL5, CCR2, CD177, CD180, CD19, CD1C, CD2, CD22, CD226, CD244, CD27, CD300LF, CD33, CD3D, CD3E, CD3G, CD4, CD40LG, CD5, CD53, CD6, CD7, CD74, CD79A, CD80, CD84, CD86, CD8A, CD8B, CDKN2A, CEACAM21, CFP, CLCF1, CLEC12A, CLEC4D, CLNK, CNR1, CNR2, CORO1A, COTL1, CR2, CRTAM, CST7, CTLA4, CTSS, CXCL6, CYBB, DOCK10, DOCK2, DOCK8, EBI3, EDN2, EGR3, EOMES, EPX, FCN1, FCRL1, FCRL3, FCRLA, FGL2, FGR, FLT3, FOXP3, FRMPD3, FUT7, GAPT, GMFG, GRAP2, HLA-DMB, HLA-DOA, HLA-DPA1, HLA-DPB1, HLA-DRA, HLA-DRB1, ICOS, IFNG, IGLL5, IKZF1, IKZF3, IL12RB1, IL18, IL18RAP, IL21R, IL7R, INPP5D, IQGAP2, IRF4, IRF8, ITGA4, ITGAL, ITGAX, ITGB2, ITK, JAML, KLRC1, KLRC2, KLRK1, LAG3, LAIR1, LAX1, LCK, LCP1, LCP2, LDLR, LEF1, LILRB1, LILRB4, LMO1, LRMP, LST1, LY9, LYZ, MMP9, MPO, MS4A1, MT1G, MYB, MZB1, NAMPT, NCKAP1L, NCR1, NCR3, NFAM1, NLRC3, NLRP3, NR4A3, ORM2, PIK3CG, PLA2G2D, PLAC8, PLAU, POU2AF1, POU2F2, PRAM1, PRDM1, PRF1, PRKCB, PRKCQ, PTAFR, PTGDR, PTK2B, PTPN22, PTPN6, PTPRC, PTX3, PYCARD, RAB37, RAB44, RAC2, RASAL3, RHOH, RNASET2, RUNX3, SAMSN1, SASH3, SCNN1B, SELL, SELPLG, SEMA4A, SERPINA1, SIRPG, SIT1, SLA2, SLAMF6, SLAMF7, SLAMF8, SLC18A1, SLC27A2, SOCS1, SPN, STAP1, STXBP2, SUCNR1, SYK, TBC1D10C, TBX21, TESPA1, THBS1, THEMIS, THEMIS2, THY1, TIGIT, TLR6, TNFAIP3, TNFAIP8L2, TNFRSF13B, TNFRSF18, TNFSF13B, TNFSF14, TNFSF8, TOX, TREM2, TRPM2, TXNDC5, UBD, VAV1, VNN1, WAS, WDFY4, WNT1, XCL1, ZAP70, ZNF683]	[ABL1, ADGRE5, AGER, ALDOA, AMH, AP3D1, ARL8A, ARMC5, ARSA, ARTN, BAG6, BATF2, BCL3, BCL6, BTLA, C5AR1, CAPN1, CCL2, CCL21, CCR7, CD14, CD177, CD1C, CD276, CD74, CDKN1A, CEACAM1, CHGA, CHI3L1, CLC, CLCF1, CLEC12A, CLNK, CLPTM1, CNR1, COMMD3, CRAT, CSF1, CSK, CST7, CTSA, CTSD, CX3CL1, CYB5R3, CYP26B1, DCAF15, DHRS2, DLG5, DOK3, DSC1, DTX1, DYSF, EDN2, ELF4, ENPP3, ERBB2, FADD, FES, FLOT2, FLT3, FOXF1, FPR1, GAB2, GALNS, GATA2, GBA, GNAO1, GPAM, GPER1, GPI, GPR84, GRN, HAVCR1, HBB, HDAC5, HFE, HPRT1, HVCN1, ICAM1, IDO1, IL15RA, IL1B, IL4R, IL7R, IMPDH1, INHA, IQGAP2, JAK3, JUND, JUP, KDELR1, LAG3, LDLR, LGALS9, LRG1, LRRC32, LRRC8A, LTBR, LTF, METTL7A, MME, MSN, MYH9, MYO18A, NBEAL2, NECTIN2, NHLRC3, NOD2, NPPA, NR1D1, NR4A3, NRARP, PCDHA4, PCDHA6, PDCD1, PKM, PLA2G2A, PLAU, PLAUR, PRKCSH, PTGDS, RAB3A, RAB5B, RAC2, RAP1A, RARA, RELB, RHOG, RNASE2, RNU1-93P, S100A11, SBNO2, SERPINA3, SIGLEC9, SLC44A2, SLC7A1, SLC7A8, SLCO4C1, SMAD3, SOX12, SOX13, SPHK1, SPHK2, SPI1, SRC, SRF, STAT3, STING1, STK11IP, SYVN1, TCF3, TCF7L1, TCIRG1, THBS1, TICAM1, TIMP1, TMEM179B, TMEM63A, TNF, TRAPPC1, TSPAN14, VNN1, VSIR, WAS, ZBTB7A, ZBTB7B, ZC3H12A, ZMIZ1, ZNF335, ZNF395]
GO:0002250	adaptive immune response	1,69E-05	[3]	167,00	20,93	74,84	25,16	[ADCY7, ALOX15, BATF, BCL3, BTK, BTLA, BTN3A1, BTN3A2, C1QA, C1QB, C1QC, C2, C8G, CAMK4, CCL19, CCR2, CD19, CD1B, CD1C, CD1E, CD226, CD244, CD247, CD27, CD3D, CD3E, CD3G, CD4, CD40LG, CD48, CD6, CD7, CD74, CD79A, CD80, CD84, CD86, CD8A, CD8B, CLCF1, CLEC10A, CLEC4D, CLEC6A, CR2, CRTAM, CTLA4, CTSS, CXCL10, EBI3, EOMES, FOXP3, FUT7, GZMM, HLA-DMA, HLA-DMB, HLA-DOA, HLA-DPA1, HLA-DPB1, HLA-DQA1, HLA-DRA, HLA-DRB1, HLA-DRB5, IFNG, IGLL5, IL12RB1, IL18, IL7R, INPP5D, IRF4, ITK, JCHAIN, KLRC1, KLRC2, KLRD1, KLRK1, LAG3, LAIR1, LAMP3, LAX1, LEF1, LILRA1, LILRB1, LILRB4, LTA, LY9, MCOLN2, MYO1G, NCKAP1L, NLRP3, NR4A3, PIK3CG, PRDM1, PRF1, PRKCB, PRKCQ, PTK2B, PTPN6, PTPRC, PYCARD, SAMSN1, SASH3, SEMA4A, SH2D1A, SIGLEC10, SIT1, SKAP1, SLA2, SLAMF6, SLAMF7, SPN, SYK, TBX21, THEMIS, TLR8, TNFAIP3, TNFRSF13B, TNFRSF17, TNFSF13B, TRAT1, TREM2, WAS, XCL1, ZAP70, ZNF683]	[AGER, ARTN, BCL3, BCL6, BTLA, C5, C8G, CD1C, CD1E, CD74, CEACAM1, CLC, CLCF1, CRP, CSK, FADD, GNAO1, HFE, HLA-DOB, HPRT1, ICAM1, IL18BP, IL1B, IL4R, IL7R, JAK3, LAG3, LILRB5, NCOR2, NECTIN2, NFKB2, NOD2, NR4A3, ORAI1, PDCD1, PPL, PRKACA, PVR, RAP1A, RAP1GAP2, RELB, RHBDF2, RNF19B, SERPING1, SLC7A8, STAT3, TCIRG1, TFE3, TFEB, TNF, WAS, ZBTB7B, ZC3H12A, ZNF395]
GO:0002263	cell activation involved in immune response	9,70E-12	[3]	189,00	23,74	60,07	39,93	[ADAM8, ADGRE3, ANPEP, APBB1IP, ARHGAP45, BATF, BCL3, BIN2, BLK, BTK, CCL19, CCL3, CCR2, CD177, CD180, CD19, CD1C, CD244, CD33, CD40LG, CD53, CD74, CD80, CD84, CD86, CEACAM21, CFP, CLCF1, CLEC12A, CLEC4D, CLNK, CORO1A, COTL1, CTSS, CYBB, DOCK10, DOCK2, EOMES, EPX, FCN1, FGL2, FGR, FOXP3, FRMPD3, GAPT, GMFG, HLA-DMB, HLA-DRA, HLA-DRB1, IFNG, IL12RB1, IL18, IQGAP2, IRF4, IRF8, ITGAL, ITGAX, ITGB2, KLRC2, LAIR1, LCP1, LEF1, LILRB1, LRMP, LY9, LYZ, MMP9, MPO, MYB, NCKAP1L, NFAM1, NLRP3, NR4A3, ORM2, PIK3CG, PLAC8, PLAU, POU2AF1, PRAM1, PTAFR, PTGDR, PTK2B, PTPN6, PTPRC, PTX3, PYCARD, RAB37, RAB44, RAC2, RNASET2, SCNN1B, SELL, SEMA4A, SERPINA1, SLAMF6, SLC18A1, SLC27A2, SPN, STXBP2, SUCNR1, SYK, TBC1D10C, TBX21, TREM2, TRPM2, TXNDC5, VNN1, ZNF683]	[ABL1, ADGRE5, ALDOA, AMH, ARL8A, ARSA, ARTN, BCL3, BCL6, C5AR1, CAPN1, CD14, CD177, CD1C, CD74, CEACAM1, CHGA, CHI3L1, CLCF1, CLEC12A, CLNK, COMMD3, CRAT, CTSA, CTSD, CYB5R3, DOK3, DSC1, DYSF, ENPP3, FES, FOXF1, FPR1, GAB2, GALNS, GATA2, GBF1, GPI, GPR84, GRN, HBB, HFE, HVCN1, ICAM1, IL4R, IMPDH1, IQGAP2, JAK3, JUP, LGALS9, LRG1, LTF, METTL7A, MME, NBEAL2, NHLRC3, NPPA, NR4A3, PKM, PLAU, PLAUR, PRKCSH, PTGDS, RAB3A, RAB5B, RAC2, RAP1A, RARA, RELB, RHOG, RNASE2, RNU1-93P, S100A11, SBNO2, SERPINA3, SIGLEC9, SLC44A2, SLC7A8, SLCO4C1, SPHK2, SPI1, STAT3, STING1, STK11IP, TCIRG1, TICAM1, TMEM179B, TMEM63A, TRAPPC1, TSPAN14, VNN1, ZBTB7B, ZC3H12A]

GO:0002443	leukocyte mediated immunity	5,71E-10 [3]	218,00	21,91	61,60	38,40	[ADAM8, ADGRE3, ANPEP, ARHGAP45, BATF, BCL3, BIN2, BLK, BTK, BTN3A2, C1QA, C1QB, C1QC, C2, C8G, CAMK4, CCL3, CCR2, CD177, CD19, CD1B, CD1C, CD1E, CD226, CD27, CD33, CD40LG, CD53, CD74, CD84, CD8A, CD96, CEACAM21, CFP, CLCF1, CLEC12A, CLEC4D, CLNK, CORO1A, COTL1, CR2, CRTAM, CTSS, CXCL10, CXCL6, CYBB, DOCK2, EPX, FCN1, FGL2, FGR, FOXP3, FRMPD3, FUT7, GMFG, GZMB, GZMM, HLA-DRA, HLA-DRB1, IGLL5, IL12RB1, IL18, IL18RAP, IL7R, INPP5D, IQGAP2, ITGAL, ITGAX, ITGB2, KLRC1, KLRC2, KLRD1, KLRK1, LAG3, LAIR1, LILRB1, LILRB4, LRMP, LTA, LYZ, MMP9, MPO, MYO1G, NCKAP1L, NCR1, NCR3, NFAM1, NLRP3, NLRP6, NR4A3, ORM2, PIK3CG, PLAC8, PLAU, PRAM1, PRF1, PTAFR, PTGDR, PTPN6, PTPRC, PTX3, PYCARD, RAB37, RAB44, RAC2, RNASET2, SASH3, SCIMP, SCNN1B, SELL, SERPINA1, SH2D1A, SLA2, SLAMF6, SLAMF7, SLC18A1, SLC27A2, STAP1, STXBP2, SYK, TBC1D10C, TBX21, TLR8, TREM2, TRPM2, TXNDC5, VAV1, VNN1, WAS, XCL1]	[ADGRE5, AGER, ALDOA, AMH, ARL8A, ARSA, ARTN, BCL3, BCL6, C5, C5AR1, C8G, CAPN1, CD14, CD177, CD1C, CD1E, CD74, CEACAM1, CHGA, CHI3L1, CLC, CLCF1, CLEC12A, CLNK, COMMD3, CRAT, CRP, CTSA, CTSD, CYB5R3, DOK3, DSC1, FADD, FES, FOXF1, FPR1, GAB2, GALNS, GATA2, GNAO1, GPI, GPR84, GRN, HBB, HFE, HPRT1, HVCN1, ICAM1, IL1B, IL4R, IL7R, IMPDH1, IQGAP2, JAK3, JUP, LAG3, LGALS9, LRG1, LTF, MAVS, METTL7A, MME, NBEAL2, NECTIN2, NHLRC3, NOD2, NPPA, NR4A3, PKM, PLAU, PLAUR, PLEKHM2, PRKCSH, PTGDS, PVR, RAB3A, RAB5B, RAC2, RAP1A, RHOG, RNASE2, RNF19B, RNU1-93P, S100A11, SERPINA3, SERPING1, SIGLEC9, SLC44A2, SLC7A8, SLCO4C1, SPHK2, SPI1, STING1, STK11IP, TCIRG1, TICAM1, TMEM179B, TMEM63A, TNF, TRAPPC1, TSPAN14, VNN1, WAS, ZNF395]
GO:0006810	transport	5,25E-04 [3]	844,00	15,69	35,92	64,08	[ABCB11, ABCC3, ABRA, ACAP1, ADAM8, ADAMTS9, ADGRE3, AIF1, ALOX15, ANGPT1, ANO9, ANPEP, APBA2, AQP5, AREG, ARHGAP45, ARRDC5, ASGR2, ATP10B, ATP1B4, ATP2A3, BHLHA15, BICDL1, BIN2, BLK, BMF, BTK, C1QTNF1, C2, CA12, CACNA1E, CACNA1I, CALCR, CALHM6, CASP8, CBARP, CCDC88B, CCDC88C, CCL19, CCL2, CCL3, CCL4, CCL5, CCR2, CCR5, CD177, CD19, CD22, CD247, CD300LF, CD33, CD3G, CD4, CD5, CD53, CD6, CD74, CD84, CDH1, CEACAM21, CEACAM4, CEL, CFP, CLCNKA, CLEC10A, CLEC12A, CLEC4D, CLNK, CNR1, CORO1A, COTL1, CP, CTSS, CTSW, CXCL10, CXCL11, CXCL9, CXCR4, CYBB, CYSLTR1, DAPK1, DAW1, DENND1C, DERL3, DOCK2, DRD1, EMB, EPX, ERFE, EXOC3L4, FASLG, FCHO1, FCN1, FCN3, FCRL3, FFAR4, FGL2, FGR, FOLR2, FRMPD3, GABBR2, GCNT3, GMFG, GPM6A, GRAMD1B, GRAMD1C, GZMB, HCLS1, HCN1, HLA-DRB1, HSPA7, IFNG, IGLL5, IL10RA, IL16, IL2RB, IL2RG, IPCEF1, IQGAP2, IRF8, ITGA4, ITGAL, ITGAX, ITGB2, JAKMIP1, JCHAIN, JPH1, JSRP1, KCNA3, KCNA7, KCNJ10, KCNK15, KCNN4, KCNQ5, KLF5, KLRC2, KLRG1, KMO, KNG1, LAIR1, LCK, LCP1, LDLR, LILRB1, LILRB4, LPAL2, LRMP, LRRC38, LY75, LYZ, MCOLN2, MCOLN3, MID1IP1, MIDN, MLC1, MMP9, MPO, MRC1, MREG, MS4A1, MYB, MYO1F, MYO1G, NAPSB, NCF1, NCKAP1L, NFAM1, NLRP3, NLRP6, NMUR1, NPPB, NPPC, NR4A3, NSG1, NTN1, NUP210, ORM2, P2RY12, P2RY2, PIK3CG, PIM1, PLA2G2D, PLAC8, PLAU, PLD4, PLEK, PRAM1, PRF1, PRKCB, PSTPIP1, PTAFR, PTGDR, PTK2B, PTPN22, PTPN6, PTPRC, PTX3, PYCARD, RAB15, RAB27B, RAB37, RAB39B, RAB3B, RAB3C, RAB44, RAC2, RASGRF1, RGS9, RHOH, RINL, RNASET2, RRAD, SCNN1B, SDC1, SELE, SELL, SEPTIN1, SERPINA1, SH2D2A, SHISA8, SIDT1, SIGLEC1, SIRPG, SLC12A3, SLC16A6, SLC16A9, SLC18A1, SLC22A31, SLC24A4, SLC25A48, SLC27A2, SLC31A2, SLC36A2, SLC5A2, SLC7A5P1, SLC7A7, SLC8A3, SLCO5A1, SMPD3, SNX10, SNX20, SOAT2, SPEF2, SPINK1, SPNS3, STAP1, STARD5, STXBP2, SUCNR1, SYK, SYTL1	[AAAS, ABCA3, ABCA7, ABCB11, ABCC5, ABCC8, ABCD1, ABCD2, ABCG4, ABL1, ABLIM3, ABRA, ACACB, ACKR3, ACTB, ACTN1, ACTN4, ADAMTS8, ADAMTS9, ADCY1, ADGRE5, ADIPOQ, AGAP2, AGER, AGT, AGTR1, ALDOA, ALPK3, AMH, ANGPT1, ANKRD13B, ANO3, ANO7, ANXA11, AP1B1, AP2A1, AP3D1, APLN, APLNR, APLP1, APOA1, APOD, APOM, AQP3, AQP5, ARAP3, ARC, AREG, ARF1, ARHGAP1, ARHGEF5, ARL8A, ARRDC3, ARSA, ASIC3, ATG9A, ATP13A1, ATP13A2, ATP1A1, ATP1A3, ATP1B2, ATP1B4, ATP5F1D, ATP5MC2, ATP6V0D1, ATP6V0E2, ATP6V1B1, ATP6V1F, ATPSCKMT, BAG3, BBS12, BCL2L1, BMP8A, BRPF3, BSN, C11orf65, C1QTNF1, C2CD2L, C5AR1, CACFD1, CACNA1E, CACNA1H, CACNB4, CALCR, CALHM6, CAMK2B, CAMSAP3, CAPN1, CBARP, CCL2, CCL21, CCR7, CD14, CD177, CD74, CDK1, CDKN1A, CEACAM1, CECR2, CEL, CELSR2, CERT1, CETN3, CETP, CFH, CGA, CHCHD10, CHERP, CHGA, CHI3L1, CHRNG, CLCN6, CLCN7, CLCNKA, CLDN15, CLEC12A, CLIP3, CLNK, CNNM4, CNR1, COASY, COL1A1, COMMD3, CORO7, COX6A2, COX8A, CPLX1, CPNE6, CPSF1, CPTP, CRAT, CREB3L1, CRP, CRTC2, CRYAB, CSK, CSNK1E, CTSA, CTSD, CX3CL1, CYB5R3, CYP27B1, DDR1, DDX39B, DENND1C, DGKD, DISP3, DMTN, DOC2B, DOK3, DSC1, DYNLRB1, DYSF, ECM1, EHD1, EHD2, ENG, ENPP3, EPN1, EPN3, ERBB2, ERFE, F8A1, FABP4, FAM110A, FCN3, FES, FFAR2, FHOD1, FLOT2, FOXF1, FPR1, FTH1P19, FURIN, FXYD5, FXYD6, G6PC3, GAB2, GABBR2, GABRR2, GALNS, GAS1, GATA2, GBF1, GCNT3, GDI1, GGA1, GGA3, GIT1, GJA4, GJD3, GLRA1, GNAI2, GNAO1, GPER1, GPI, GPM6A, GPR84, GPRIN1, GRAMD1A, GRAMD1B, GRAMD1C, GRIK5, GRIP2, GRM2, GRN, GSK3A, GTPBP2, HAVCR1, HBA1, HBA2, HBB, HBD, HBG1, HBG2, HCN3, HCN4, HFE, HGS, HMGA1, HPS1, HRH2, HSF1, HSPB1, HSPG2, HTR1B, HTR2A, HVCN1, ICAM1, IFT80, IGF2BP2, IL15RA, IL1B, IL1RN, IL4R, IMPDH1, INHA, INPPL1, IPO13, IQGAP2, ITPR3, JPH4, JUP, KATNB1, KCNA7, KCNC3, KCNH2, KCNIP2, KCNJ1, KCNJ5
GO:0140352	export from cell	2,72E-04 [2, 4]	273,00	18,32	42,74	57,26	[ADAM8, ADGRE3, ANPEP, ARHGAP45, BIN2, BLK, BTK, C1QTNF1, CACNA1E, CACNA1I, CBARP, CCL3, CCL5, CCR2, CD177, CD33, CD53, CD84, CEACAM21, CFP, CLEC12A, CLEC4D, CLNK, CNR1, CORO1A, COTL1, CTSS, CTSW, CYBB, DOCK2, EPX, EXOC3L4, FCN1, FFAR4, FGL2, FGR, FRMPD3, GMFG, HLA-DRB1, IFNG, IQGAP2, ITGAL, ITGAX, ITGB2, KCNN4, KLF5, KLRC2, KLRG1, KMO, KNG1, LAIR1, LILRB1, LRMP, LYZ, MIDN, MMP9, MPO, MYB, MYO1G, NAPSB, NCKAP1L, NFAM1, NLRP3, NR4A3, ORM2, P2RY12, PIK3CG, PLAC8, PLAU, PLEK, PRAM1, PRKCB, PTAFR, PTGDR, PTPN6, PTPRC, PTX3, PYCARD, RAB15, RAB27B, RAB37, RAB3B, RAB3C, RAB44, RAC2, RGS9, RNASET2, SDC1, SELL, SEPTIN1, SERPINA1, SLC18A1, SLC27A2, SLC8A3, SMPD3, STXBP2, SUCNR1, SYK, SYTL1, TBC1D10C, THBS1, TREM2, TRPM2, TXNDC5, UCP2, VNN1]	[ABCC5, ABCC8, ACTN1, ACTN4, ADCY1, ADGRE5, ADIPOQ, AGT, AGTR1, ALDOA, AMH, APLN, APOA1, ARF1, ARL8A, ARSA, ATP13A2, ATP1A1, ATP1A3, ATP1B2, BMP8A, BRPF3, C1QTNF1, C2CD2L, C5AR1, CACNA1E, CACNA1H, CAPN1, CBARP, CD14, CD177, CEACAM1, CGA, CHGA, CHI3L1, CLEC12A, CLNK, CNR1, COMMD3, CPLX1, CRAT, CREB3L1, CTSA, CTSD, CYB5R3, DMTN, DOC2B, DOK3, DSC1, ECM1, ENG, FES, FFAR2, FOXF1, FPR1, FURIN, GAB2, GALNS, GATA2, GIT1, GNAI2, GPER1, GPI, GPR84, GRIK5, GRM2, GRN, GTPBP2, HBB, HFE, HGS, HTR1B, HTR2A, HVCN1, IL1B, IL1RN, IL4R, IMPDH1, INHA, IQGAP2, ITPR3, JUP, KCNH2, KCNIP2, KCTD11, KLF5, KNG1, LGALS3BP, LGALS9, LRG1, LRRC8A, LTF, LY6E, MEN1, METTL7A, MIDN, MME, MYH9, MYO18A, NAPA, NBEAL2, NHLRC3, NKD2, NLGN2, NNAT, NOTCH1, NPPA, NR1D1, NR4A3, OTOF, PCDHA4, PDGFB, PIP5K1C, PKM, PLAU, PLAUR, PORCN, PPARD, PRKCSH, PRRT2, PTGDS, PTGES, PTPN23, PTPRN, RAB11B, RAB11FIP5, RAB15, RAB3A, RAB3IL1, RAB5B, RAC2, RAP1A, RASL10B, RGCC, RHBDF1, RHBDF2, RHOG, RNASE2, RNU1-93P, RPH3A, S100A11, SELENOP, SEPTIN4, SEPTIN5, SEPTIN9, SERPINA3, SERPINE1, SERPING1, SIDT2, SIGLEC9, SLC17A3, SLC1A5, SLC29A4, SLC30A1, SLC44A2, SLC7A8, SLC9A1, SLCO4C1, SPHK2, SPI1, SREBF1, STEAP3, STING1, STK11IP, STX1A, STX1B, SV2A, SYN1, SYT2, SYT7, TCIRG1, THBS1, TIMP1, TMEM179B, TMEM63A, TNF, TNFAIP2, TNFRSF1A, TRAPPC1, TRPV4, TSPAN14, TUBA4A, VEGFA, VNN1]

GO:0002274	myeloid leukocyte activation	2,33E-14	[3, 4]	184,00	25,34	54,94	45,06	[ADAM8, ADGRE3, AIF1, ANPEP, ARHGAP45, BATF, BIN2, BLK, BTK, C1QA, CAMK4, CCL3, CCL5, CCR2, CD177, CD2, CD226, CD300LF, CD33, CD53, CD74, CD84, CEACAM21, CFP, CLEC12A, CLEC4D, CLNK, CNR1, CNR2, COTL1, CST7, CTSS, CXCL6, CYBB, DOCK2, EDN2, EPX, FCN1, FGL2, FGR, FRMPD3, GMFG, IFNG, IL18, IL18RAP, IQGAP2, IRF4, ITGAL, ITGAX, ITGB2, LAIR1, LCP2, LDLR, LILRB4, LRMP, LYZ, MMP9, MPO, MT1G, NAMPT, NCKAP1L, NFAM1, NR4A3, ORM2, PIK3CG, PLAC8, PLAU, PRAM1, PTAFR, PTGDR, PTPN6, PTPRC, PTX3, PYCARD, RAB37, RAB44, RAC2, RHOH, RNASET2, SCNN1B, SELL, SERPINA1, SLC18A1, SLC27A2, STAP1, STXBP2, SUCNR1, SYK, TBC1D10C, THBS1, TLR6, TREM2, TRPM2, TXNDC5, UBD, VNN1]	[ADGRE5, AGER, ALDOA, AMH, ARL8A, ARSA, BATF2, C5AR1, CAPN1, CD14, CD177, CD74, CEACAM1, CHGA, CHI3L1, CLEC12A, CLNK, CNR1, COMMD3, CRAT, CSF1, CST7, CTSA, CTSD, CX3CL1, CYB5R3, DHRS2, DOK3, DSC1, DYSF, EDN2, ENPP3, FES, FOXF1, FPR1, GAB2, GALNS, GATA2, GPI, GPR84, GRN, HAVCR1, HBB, HFE, HVCN1, IL4R, IMPDH1, IQGAP2, JUND, JUP, LDLR, LGALS9, LRG1, LTBR, LTF, METTL7A, MME, MYO18A, NBEAL2, NECTIN2, NHLRC3, NPPA, NR1D1, NR4A3, PCDHA4, PCDHA6, PKM, PLA2G2A, PLAU, PLAUR, PRKCSH, PTGDS, RAB3A, RAB5B, RAC2, RAP1A, RELB, RHOG, RNASE2, RNU1-93P, S100A11, SBNO2, SERPINA3, SIGLEC9, SLC44A2, SLC7A8, SLCO4C1, SPHK1, SPHK2, SPI1, STING1, STK11IP, TCIRG1, THBS1, TICAM1, TMEM179B, TMEM63A, TNF, TRAPPC1, TSPAN14, VNN1, ZC3H12A]
GO:0002697	regulation of immune effector process	1,81E-11	[3, 4]	126,00	27,21	66,43	33,57	[ANGPT1, BLK, BTK, C1QA, C1QB, C1QC, C2, C8G, CAMK4, CCL19, CCR2, CD177, CD19, CD1B, CD1C, CD1E, CD22, CD226, CD244, CD40LG, CD74, CD80, CD84, CD86, CD96, CFP, CLCF1, CLNK, CR2, CRTAM, CXCL10, CXCL6, CYP11B1, FCRL3, FGL2, FGR, FOXP3, FUT7, HLA-DMB, HLA-DRA, HLA-DRB1, IFNG, IL12RB1, IL18, IL18RAP, IL7R, IRF4, ITGB2, KLRC1, KLRC2, KLRD1, KLRK1, LAG3, LILRB1, LILRB4, LPAL2, LTA, MYB, MZB1, NCKAP1L, NCR1, NCR3, NLRP3, NR4A3, PRAM1, PTAFR, PTPN22, PTPN6, PTPRC, RAC2, SASH3, SCIMP, SH2D1A, SLAMF6, SLAMF8, STAP1, STXBP2, SYK, TBX21, TREM2, VAV1, WAS, XCL1, ZNF683]	[AGER, ANGPT1, APOA1, BCL6, C5, C5AR1, C8G, CD177, CD1C, CD1E, CD74, CEACAM1, CFH, CLC, CLCF1, CLNK, CPN2, ENPP3, FADD, FES, FFAR2, FOXF1, GAB2, GATA2, GPI, GRN, HFE, HK1, ICAM1, IL1B, IL4R, IL7R, JAK3, LAG3, LGALS9, MAVS, MYO18A, NECTIN2, NOD2, NPPA, NR4A3, PVR, RAC2, RAP1A, RARA, SERPING1, SLC7A5, SPHK2, SPI1, TICAM1, TNF, VTN, WAS, ZBTB7B, ZC3H12A]
GO:0050776	regulation of immune response	2,87E-17	[3, 4]	286,00	23,08	66,69	33,31	[ADAM8, ADCY7, AIM2, ALOX15, APOBEC3G, BLK, BLNK, BTK, BTLA, BTN3A1, BTN3A2, C1QA, C1QB, C1QC, C1QTNF1, C2, C8G, CALCR, CARD11, CASP8, CCL19, CCL5, CCR2, CD177, CD19, CD1B, CD1C, CD1E, CD200R1, CD22, CD226, CD247, CD300LF, CD33, CD3D, CD3E, CD3G, CD4, CD40LG, CD48, CD74, CD79A, CD80, CD84, CD86, CD8A, CD8B, CD96, CFP, CLCF1, CLEC10A, CLEC1B, CLEC4D, CLEC4E, CLEC6A, CLNK, CNR1, CR2, CRTAM, CTLA4, CXCL10, CXCL6, CXorf21, CYP11B1, CYSLTR1, DPEP1, DRD1, EREG, FCGR2C, FCN1, FCN3, FCRL3, FGL2, FGR, FOXP3, FUT7, FYB1, GBP5, GPR150, GPR83, GRAP2, HCST, HLA-DMB, HLA-DPA1, HLA-DPB1, HLA-DQA1, HLA-DRA, HLA-DRB1, HLA-DRB5, IFNG, IGLL5, IKBKE, IL12RB1, IL18, IL18RAP, IL7R, INPP5D, IRF4, ITGA4, ITGAL, ITGB2, ITGB7, ITK, JAML, KCNN4, KIR2DL2, KIR2DL3, KIR3DL2, KLRB1, KLRC1, KLRC2, KLRD1, KLRK1, LAG3, LAIR1, LAIR2, LAX1, LCK, LCP2, LILRA1, LILRB1, LILRB4, LTA, MOG, MS4A1, MUC16, MUC19, MUC6, MYB, MYO1G, NCKAP1L, NCR1, NCR3, NFAM1, NLRC3, NLRP3, NLRP6, NR4A3, PAX5, PILRA, PLA2G2D, PRAM1, PRKCB, PRKCQ, PTAFR, PTGDR, PTGER2, PTPN22, PTPN6, PTPRC, PYCARD, PYHIN1, RAC2, RLN2, SAMSN1, SASH3, SELL, SH2D1A, SIGLEC16, SKAP1, SLA2, SLAMF6, SLAMF7, SLAMF8, SOCS1, SOCS3, SPN, STAP1, STAT1, STXBP2, SYK, TBX21, TESPA1, THEMIS, THEMIS2, THY1, TLR8, TNFAIP3, TNFSF13B, TRAT1, TREM2, UBASH3A, VAV1, WAS, XCL1, ZAP70, ZBP1]	[ABL1, ACTB, ADCY1, ADCY3, ADCY4, ADCY9, AGER, BAG6, BCAR1, BCL6, BTLA, C1QTNF1, C5, C5AR1, C8G, CALCR, CCR7, CD177, CD1C, CD1E, CD276, CD300E, CD74, CEACAM1, CFH, CGA, CHGA, CLC, CLCF1, CLEC1B, CLEC4E, CLNK, CNR1, COL1A1, CPN2, CRHR2, CRP, CSK, DGKZ, ECM1, ENPP3, FADD, FCN3, FES, FFAR2, FOXF1, FPR1, FURIN, GAB2, GATA2, GCSAM, GNAI2, GNAO1, GPBAR1, GPR20, GPR83, GPR84, GRN, HFE, HRH2, ICAM1, IFITM1, IL1B, IL4R, IL7R, JAK3, LAG3, LGALS9, LIMK1, MAPK3, MAVS, MUC19, MUC20, MUC3A, MYH9, NCOR2, NECTIN2, NLRC5, NOD2, NPPA, NR4A3, PCDHA4, PDCD1, PQBP1, PRKACA, PRKAR2B, PTGIR, PVR, RAC2, RAMP2, RAP1A, RARA, RBM14, RELA, RELB, RGCC, RHBDF2, RNF26, RNU1-93P, SERPING1, SH2B2, SIGLEC9, SLC7A8, SMAD3, SOCS3, SPHK2, SPI1, SPNS2, SPPL2B, SRC, STING1, TNF, TRIM62, VIPR2, VSIR, VTN, WAS, ZBTB7B, ZC3H12A, ZCCHC3, ZDHHC11]

GO:0051049	regulation of transport	6,48E-06	[3, 4]	339,00	18,29	39,41	60,59	[ABCB11, ADAM8, ALOX15, ANGPT1, ANO9, BLK, C1QTNF1, C2, CACNA1E, CACNA1I, CALCR, CASP8, CBARP, CCL19, CCL2, CCL3, CCL4, CCL5, CCR2, CD177, CD19, CD22, CD300LF, CD33, CD4, CD74, CD84, CDH1, CLCNKA, CNR1, CORO1A, CTSS, CXCL10, CXCL11, CXCL9, CXCR4, CYBB, DAPK1, DERL3, DOCK2, DRD1, EPX, ERFE, FASLG, FCRL3, FFAR4, FGR, GABBR2, GZMB, HCLS1, HCN1, HLA-DRB1, IFNG, IL16, IL2RB, IL2RG, ITGB2, JPH1, JSRP1, KCNA3, KCNA7, KCNJ10, KCNK15, KCNN4, KCNQ5, KLRC2, KMO, LCP1, LILRB1, LPAL2, LRRC38, MIDN, MLC1, MMP9, MYB, NCKAP1L, NLRP3, NLRP6, NPPB, NR4A3, P2RY12, P2RY2, PIK3CG, PIM1, PRAM1, PRKCB, PTAFR, PTK2B, PTPN22, PTPN6, PTPRC, PTX3, PYCARD, RAB15, RAB27B, RAB3B, RAB3C, RAC2, RASGRF1, RGS9, RRAD, SDC1, SELE, SEPTIN1, SHISA8, SIRPG, SLC31A2, SMPD3, SPINK1, STAP1, STXBP2, SYK, TCAF2, THBS1, THY1, TIFAB, TMC2, TREM2, TRPM2, UBD, UCP2, XCL1]	[AAAS, ABCA3, ABCA7, ABCB11, ABCC8, ABL1, ABLIM3, ACTB, ACTN4, ADCY1, ADIPOQ, AGT, AGTR1, ANGPT1, ANKRD13B, AP2A1, APLN, APLNR, APOA1, APOD, ARC, ARF1, ARHGAP1, ARHGEF5, ATP13A2, ATP1A1, ATP1A3, ATP1B2, ATPSCKMT, BAG3, BMP8A, BSN, C11orf65, C1QTNF1, C2CD2L, CACNA1E, CACNA1H, CACNB4, CALCR, CAMK2B, CAPN1, CBARP, CCL2, CCL21, CD14, CD177, CD74, CDK1, CEACAM1, CETP, CFH, CHCHD10, CHGA, CLCNKA, CLIP3, CNR1, CPLX1, CRYAB, CSK, CX3CL1, DGKD, DISP3, DMTN, DOC2B, DYSF, EHD1, EHD2, ERBB2, ERFE, FES, FFAR2, FOXF1, FURIN, FXYD5, FXYD6, GAB2, GABBR2, GAS1, GATA2, GDI1, GIT1, GNAI2, GNAO1, GPER1, GPRIN1, GRIK5, GRM2, GSK3A, HCN3, HCN4, HFE, HGS, HTR1B, HTR2A, HVCN1, ICAM1, IL15RA, IL1B, IL4R, INHA, ITPR3, JPH4, JUP, KCNA7, KCNC3, KCNH2, KCNIP2, KCNJ1, KCNJ5, KCNJ9, KCNK15, KCNK6, KCNMB1, KCNMB2, KCNQ4, KCTD11, LDLRAP1, LGALS9, LILRA5, LIPG, LRRC52, LRRC8A, LZTS1, MAPK3, MAVS, MICALL2, MIDN, MINK1, MLLT6, MSN, MYC, MYO18A, NEFL, NKAIN3, NLGN2, NNAT, NOD2, NOTCH1, NPPA, NPPB, NR1D1, NR4A3, NUCB1, NUTF2, ORAI1, P2RX3, P2RX5, P2RY2, P2RY6, PACSIN1, PCDHA4, PDGFB, PDGFRB, PER1, PIM1, PLK3, PLTP, PM20D1, PML, PPARD, PRKACA, PRKCSH, PROM2, PRRT1, PRRT2, PTGES, PTPN23, RAB11B, RAB11FIP5, RAB15, RAB3A, RAB5B, RAC2, RANGAP1, RANGRF, RAP1A, RASL10B, RCVRN, RGCC, RHBDF1, RHBDF2, RIPOR1, RRAD, RTN2, RUBCN, RXRA, S100A1, SCN5A, SCN7A, SEC16A, SELE, SELENON, SEPTIN4, SEPTIN5, SERPINE1, SH3GL1, SHANK3, SIDT2, SIK1, SLC30A1, SLC30A3, SLC35F6, SLC51B, SLC6A9, SLC9A1, SLC9A3R1, SLN, SMAD3, SMPD1, SPHK1, SPHK2, SPI1, SPINK1, SRC, SREBF1, STAC2, STX1A, STX1B, SV2A, SYN1, SYT2, SYT7, TCIRG1, TCTEX1D2, TGM2, THBS1, TIMP1, TM9SF4, TMEM109, TMEM14A, TNF, TNFRSF1A, TNK2, TPCN1, TRIB3, TSC2, VEGFA, VTN, WFS1, WNK2, WWP2, ZC3H12A, ZDHHC8]
GO:0002444	myeloid leukocyte mediated immunity	9,78E-06	[4]	138,00	22,19	54,41	45,59	[ADAM8, ADGRE3, ANPEP, ARHGAP45, BIN2, BLK, BTK, CAMK4, CCL3, CCR2, CD177, CD33, CD53, CD84, CEACAM21, CFP, CLEC12A, CLEC4D, CLNK, COTL1, CTSS, CXCL6, CYBB, DOCK2, EPX, FCN1, FGL2, FGR, FRMPD3, GMFG, IQGAP2, ITGAL, ITGAX, ITGB2, LAIR1, LRMP, LYZ, MMP9, MPO, NCKAP1L, NFAM1, NLRP6, NR4A3, ORM2, PIK3CG, PLAC8, PLAU, PRAM1, PTAFR, PTGDR, PTPN6, PTPRC, PTX3, PYCARD, RAB37, RAB44, RAC2, RNASET2, SCNN1B, SELL, SERPINA1, SLC18A1, SLC27A2, STAP1, STXBP2, SYK, TBC1D10C, TRPM2, TXNDC5, VNN1]	[ADGRE5, ALDOA, AMH, ARL8A, ARSA, C5AR1, CAPN1, CD14, CD177, CEACAM1, CHGA, CHI3L1, CLEC12A, CLNK, COMMD3, CRAT, CTSA, CTSD, CYB5R3, DOK3, DSC1, FES, FOXF1, FPR1, GAB2, GALNS, GATA2, GPI, GPR84, GRN, HBB, HFE, HVCN1, IL4R, IMPDH1, IQGAP2, JUP, LGALS9, LRG1, LTF, MAVS, METTL7A, MME, NBEAL2, NHLRC3, NPPA, NR4A3, PKM, PLAU, PLAUR, PRKCSH, PTGDS, RAB3A, RAB5B, RAC2, RAP1A, RHOG, RNASE2, RNU1-93P, S100A11, SERPINA3, SIGLEC9, SLC44A2, SLC7A8, SLCO4C1, SPHK2, SPI1, STING1, STK11IP, TCIRG1, TICAM1, TMEM179B, TMEM63A, TRAPPC1, TSPAN14, VNN1]
GO:0002449	lymphocyte mediated immunity	1,17E-02	[4]	89,00	21,76	71,24	28,76	[BATF, BCL3, BTK, BTN3A2, C1QA, C1QB, C1QC, C2, C8G, CCR2, CD19, CD1B, CD1C, CD1E, CD226, CD27, CD40LG, CD74, CD8A, CD96, CLCF1, CLNK, CORO1A, CR2, CRTAM, CXCL10, FOXP3, FUT7, GZMB, GZMM, HLA-DRA, HLA-DRB1, IGLL5, IL12RB1, IL18, IL18RAP, IL7R, INPP5D, KLRC1, KLRC2, KLRD1, KLRK1, LAG3, LILRB1, LILRB4, LTA, MYO1G, NCKAP1L, NCR1, NCR3, NLRP3, PRF1, PTPN6, PTPRC, SASH3, SH2D1A, SLA2, SLAMF6, SLAMF7, TBX21, TLR8, TREM2, VAV1, WAS, XCL1]	[AGER, ARTN, BCL3, BCL6, C5, C8G, CD1C, CD1E, CD74, CEACAM1, CLC, CLCF1, CLNK, CRP, FADD, GNAO1, HFE, HPRT1, ICAM1, IL1B, IL4R, IL7R, LAG3, LGALS9, NECTIN2, NOD2, PLEKHM2, PVR, RNF19B, SERPING1, TCIRG1, TNF, WAS, ZNF395]
GO:0002460	adaptive immune response based on somatic recombination of immune receptors built from immunoglobulin superfamily domains	2,41E-03	[4]	93,00	22,30	70,05	29,95	[BATF, BCL3, BTK, BTN3A2, C1QA, C1QB, C1QC, C2, C8G, CCL19, CCR2, CD19, CD1B, CD1C, CD1E, CD226, CD27, CD4, CD40LG, CD74, CD80, CD8A, CLCF1, CLEC6A, CR2, CRTAM, CXCL10, EBI3, FOXP3, FUT7, GZMM, HLA-DRA, HLA-DRB1, IGLL5, IL12RB1, IL18, IL7R, INPP5D, IRF4, KLRC1, KLRD1, LEF1, LILRB1, LILRB4, LTA, LY9, MYO1G, NCKAP1L, NLRP3, PRF1, PRKCQ, PTPN6, PTPRC, SASH3, SEMA4A, SLA2, SLAMF6, SPN, TBX21, TLR8, TNFAIP3, TNFSF13B, TREM2, WAS, XCL1]	[AGER, ARTN, BCL3, BCL6, C5, C8G, CD1C, CD1E, CD74, CEACAM1, CLC, CLCF1, CRP, FADD, GNAO1, HFE, HPRT1, ICAM1, IL18BP, IL1B, IL4R, IL7R, JAK3, NECTIN2, NFKB2, NOD2, PVR, RELB, SERPING1, STAT3, TCIRG1, TNF, WAS, ZBTB7B, ZC3H12A, ZNF395]
GO:0002699	positive regulation of immune effector process	5,71E-10	[3, 4, 5]	79,00	31,60	67,30	32,70	[BLK, BTK, CAMK4, CCL19, CCR2, CD177, CD1B, CD1C, CD1E, CD226, CD244, CD74, CD80, CD84, CD86, CLCF1, CLNK, CRTAM, FGR, FOXP3, HLA-DMB, HLA-DRA, HLA-DRB1, IFNG, IL12RB1, IL18, IL18RAP, ITGB2, KLRC2, KLRD1, KLRK1, LAG3, LILRB1, LTA, MYB, MZB1, NCR3, NLRP3, NR4A3, PTAFR, PTPN22, PTPRC, RAC2, SASH3, SCIMP, SH2D1A, SLAMF6, STAP1, STXBP2, SYK, TBX21, TREM2, VAV1, XCL1]	[CD177, CD1C, CD1E, CD74, CLCF1, CLNK, ENPP3, FADD, FES, FFAR2, FOXF1, GAB2, GATA2, GPI, HK1, IL1B, IL4R, LAG3, LGALS9, MAVS, MYO18A, NECTIN2, NOD2, NPPA, NR4A3, PVR, RAC2, RAP1A, RARA, SLC7A5, SPHK2, SPI1, TICAM1, TNF]

GO:0032940	secretion by cell	7,48E-04	[3, 5]	262,00	18,26	43,50	56,50	[ADAM8, ADGRE3, ANPEP, ARHGAP45, BIN2, BLK, BTK, C1QTNF1, CACNA1E, CACNA1I, CBARP, CCL3, CCL5, CCR2, CD177, CD33, CD53, CD84, CEACAM21, CFP, CLEC12A, CLEC4D, CLNK, CNR1, CORO1A, COTL1, CTSS, CTSW, CYBB, DOCK2, EPX, EXOC3L4, FCN1, FFAR4, FGL2, FGR, FRMPD3, GMFG, HLA-DRB1, IFNG, IQGAP2, ITGAL, ITGAX, ITGB2, KCNN4, KLF5, KLRC2, KLRG1, KMO, KNG1, LAIR1, LILRB1, LRMP, LYZ, MIDN, MMP9, MPO, MYB, MYO1G, NAPSB, NCKAP1L, NFAM1, NLRP3, NR4A3, ORM2, P2RY12, PIK3CG, PLAC8, PLAU, PLEK, PRAM1, PRKCB, PTAFR, PTGDR, PTPN6, PTPRC, PTX3, PYCARD, RAB15, RAB27B, RAB37, RAB3B, RAB3C, RAB44, RAC2, RNASET2, SDC1, SELL, SEPTIN1, SERPINA1, SLC18A1, SLC27A2, SMPD3, STXBP2, SUCNR1, SYK, SYTL1, TBC1D10C, THBS1, TREM2, TRPM2, TXNDC5, UCP2, VNN1]	[ABCC8, ACTN1, ACTN4, ADCY1, ADGRE5, ADIPOQ, AGT, AGTR1, ALDOA, AMH, APLN, APOA1, ARF1, ARL8A, ARSA, ATP13A2, BMP8A, BRPF3, C1QTNF1, C2CD2L, C5AR1, CACNA1E, CACNA1H, CAPN1, CBARP, CD14, CD177, CEACAM1, CGA, CHGA, CHI3L1, CLEC12A, CLNK, CNR1, COMMD3, CPLX1, CRAT, CREB3L1, CTSA, CTSD, CYB5R3, DMTN, DOC2B, DOK3, DSC1, ECM1, ENG, FES, FFAR2, FOXF1, FPR1, FURIN, GAB2, GALNS, GATA2, GIT1, GNAI2, GPER1, GPI, GPR84, GRIK5, GRM2, GRN, GTPBP2, HBB, HFE, HGS, HTR1B, HTR2A, HVCN1, IL1B, IL1RN, IL4R, IMPDH1, INHA, IQGAP2, ITPR3, JUP, KCTD11, KLF5, KNG1, LGALS3BP, LGALS9, LRG1, LRRC8A, LTF, LY6E, MEN1, METTL7A, MIDN, MME, MYH9, MYO18A, NAPA, NBEAL2, NHLRC3, NKD2, NLGN2, NNAT, NOTCH1, NPPA, NR1D1, NR4A3, OTOF, PCDHA4, PDGFB, PIP5K1C, PKM, PLAU, PLAUR, PORCN, PPARD, PRKCSH, PRRT2, PTGDS, PTGES, PTPN23, PTPRN, RAB11B, RAB11FIP5, RAB15, RAB3A, RAB3IL1, RAB5B, RAC2, RAP1A, RASL10B, RGCC, RHBDF1, RHBDF2, RHOG, RNASE2, RNU1-93P, RPH3A, S100A11, SELENOP, SEPTIN4, SEPTIN5, SEPTIN9, SERPINA3, SERPINE1, SERPING1, SIDT2, SIGLEC9, SLC1A5, SLC30A1, SLC44A2, SLC7A8, SLCO4C1, SPHK2, SPI1, SREBF1, STEAP3, STING1, STK11IP, STX1A, STX1B, SV2A, SYN1, SYT2, SYT7, TCIRG1, THBS1, TIMP1, TMEM179B, TMEM63A, TNF, TNFAIP2, TNFRSF1A, TRAPPC1, TRPV4, TSPAN14, TUBA4A, VEGFA, VNN1]
GO:0046903	secretion	6,04E-05	[4]	292,00	18,39	42,91	57,09	[ABCB11, ABCC3, ADAM8, ADGRE3, ANPEP, AQP5, ARHGAP45, BIN2, BLK, BTK, C1QTNF1, CACNA1E, CACNA1I, CBARP, CCL3, CCL5, CCR2, CD177, CD33, CD53, CD74, CD84, CEACAM21, CEL, CFP, CLEC12A, CLEC4D, CLNK, CNR1, CORO1A, COTL1, CTSS, CTSW, CYBB, DOCK2, EPX, EXOC3L4, FCN1, FFAR4, FGL2, FGR, FRMPD3, GMFG, HLA-DRB1, IFNG, IQGAP2, ITGAL, ITGAX, ITGB2, KCNN4, KLF5, KLRC2, KLRG1, KMO, KNG1, LAIR1, LILRB1, LRMP, LYZ, MIDN, MMP9, MPO, MYB, MYO1G, NAPSB, NCKAP1L, NFAM1, NLRP3, NLRP6, NPPB, NR4A3, ORM2, P2RY12, P2RY2, PIK3CG, PLA2G2D, PLAC8, PLAU, PLEK, PRAM1, PRKCB, PTAFR, PTGDR, PTPN6, PTPRC, PTX3, PYCARD, RAB15, RAB27B, RAB37, RAB3B, RAB3C, RAB44, RAC2, RNASET2, SCNN1B, SDC1, SELL, SEPTIN1, SERPINA1, SLC18A1, SLC27A2, SMPD3, SNX10, STXBP2, SUCNR1, SYK, SYTL1, TBC1D10C, THBS1, TIFAB, TREM2, TRPM2, TXNDC5, UCP2, VNN1]	[ABCB11, ABCC8, ACTN1, ACTN4, ADCY1, ADGRE5, ADIPOQ, AGT, AGTR1, ALDOA, AMH, APLN, APOA1, AQP5, ARF1, ARL8A, ARSA, ATP13A2, BMP8A, BRPF3, C1QTNF1, C2CD2L, C5AR1, CACNA1E, CACNA1H, CAPN1, CBARP, CD14, CD177, CD74, CEACAM1, CEL, CELSR2, CGA, CHGA, CHI3L1, CLEC12A, CLNK, CNR1, COASY, COMMD3, CPLX1, CRAT, CREB3L1, CTSA, CTSD, CYB5R3, CYP27B1, DDR1, DMTN, DOC2B, DOK3, DSC1, ECM1, ENG, FES, FFAR2, FOXF1, FPR1, FURIN, GAB2, GALNS, GATA2, GIT1, GNAI2, GPER1, GPI, GPR84, GRIK5, GRM2, GRN, GTPBP2, HBB, HFE, HGS, HRH2, HTR1B, HTR2A, HVCN1, IL1B, IL1RN, IL4R, IMPDH1, INHA, IQGAP2, ITPR3, JUP, KCTD11, KLF5, KNG1, LGALS3BP, LGALS9, LRG1, LRRC8A, LTF, LY6E, MEN1, METTL7A, MIDN, MME, MTCO2P12, MYC, MYH9, MYO18A, NAPA, NBEAL2, NCOR2, NHLRC3, NKD2, NLGN2, NNAT, NOTCH1, NPPA, NPPB, NPR3, NPRL3, NR1D1, NR4A3, OTOF, P2RY2, PCDHA4, PDGFB, PIP5K1C, PKM, PLA2G2A, PLAU, PLAUR, PORCN, PPARD, PRKCSH, PRRT2, PTGDS, PTGES, PTPN23, PTPRN, RAB11B, RAB11FIP5, RAB15, RAB3A, RAB3IL1, RAB5B, RAC2, RAP1A, RASL10B, RGCC, RHBDF1, RHBDF2, RHOG, RMRP, RNASE2, RNU1-93P, RPH3A, S100A11, SELENOP, SEPTIN4, SEPTIN5, SEPTIN9, SERPINA3, SERPINE1, SERPING1, SIDT2, SIGLEC9, SLC1A5, SLC29A1, SLC30A1, SLC44A2, SLC51B, SLC7A8, SLC9A3R1, SLCO4C1, SNX10, SPHK2, SPI1, SREBF1, STEAP3, STING1, STK11IP, STX1A, STX1B, SV2A, SYN1, SYT2, SYT7, TCIRG1, THBS1, TIMP1, TMEM179B, TMEM63A, TNF, TNFAIP2, TNFRSF1A, TRAPPC1, TRPV4, TSPAN14, TUBA4A, USF2, VDR, VEGFA, VNN1, ZBTB7B]
GO:0060627	regulation of vesicle-mediated transport	6,31E-05	[3, 4, 5]	124,00	22,18	40,13	59,87	[ALOX15, ANGPT1, BLK, C2, CACNA1I, CBARP, CCL19, CCL2, CCR2, CD177, CD22, CD300LF, CD84, CNR1, CORO1A, DOCK2, FGR, IFNG, IL2RB, IL2RG, ITGB2, KLRC2, LILRB1, LPAL2, NCKAP1L, PRAM1, PRKCB, PTAFR, PTPRC, PTX3, PYCARD, RAB15, RAB27B, RAB3B, RAB3C, RAC2, SDC1, SELE, SEPTIN1, SIRPG, SMPD3, STAP1, STXBP2, SYK, TREM2]	[ABCA7, ABL1, ADCY1, ADIPOQ, ANGPT1, ANKRD13B, AP2A1, APLN, APOA1, ARC, ARF1, ARHGAP1, ATP13A2, BSN, CACNA1H, CBARP, CCL2, CCL21, CD14, CD177, CEACAM1, CLIP3, CNR1, CPLX1, CSK, DGKD, DOC2B, DYSF, EHD1, EHD2, FES, FOXF1, GAB2, GAS1, GATA2, GIT1, GNAI2, GRIK5, HFE, HGS, HTR1B, HTR2A, IL15RA, IL1B, IL4R, LDLRAP1, LGALS9, MAPK3, MSN, MYO18A, NOD2, NOTCH1, NPPA, PACSIN1, PCDHA4, PRKCSH, PROM2, PRRT2, PTPN23, RAB11B, RAB15, RAB3A, RAB5B, RAC2, RAP1A, RUBCN, SELE, SEPTIN4, SEPTIN5, SERPINE1, SH3GL1, SMPD1, SPHK1, SPHK2, SPI1, SRC, STX1A, STX1B, SYN1, SYT2, SYT7, TGM2, TNF, TNK2, TSC2, VEGFA, VTN]
GO:0002275	myeloid cell activation involved in immune response	1,74E-06	[4, 5]	139,00	22,64	53,41	46,59	[ADAM8, ADGRE3, ANPEP, ARHGAP45, BIN2, BLK, BTK, CCL3, CCR2, CD177, CD33, CD53, CD84, CEACAM21, CFP, CLEC12A, CLEC4D, CLNK, COTL1, CTSS, CYBB, DOCK2, EPX, FCN1, FGL2, FGR, FRMPD3, GMFG, IFNG, IQGAP2, ITGAL, ITGAX, ITGB2, LAIR1, LRMP, LYZ, MMP9, MPO, NCKAP1L, NFAM1, NR4A3, ORM2, PIK3CG, PLAC8, PLAU, PRAM1, PTAFR, PTGDR, PTPN6, PTPRC, PTX3, PYCARD, RAB37, RAB44, RAC2, RNASET2, SCNN1B, SELL, SERPINA1, SLC18A1, SLC27A2, STXBP2, SUCNR1, SYK, TBC1D10C, TREM2, TRPM2, TXNDC5, VNN1]	[ADGRE5, ALDOA, AMH, ARL8A, ARSA, C5AR1, CAPN1, CD14, CD177, CEACAM1, CHGA, CHI3L1, CLEC12A, CLNK, COMMD3, CRAT, CTSA, CTSD, CYB5R3, DOK3, DSC1, DYSF, ENPP3, FES, FOXF1, FPR1, GAB2, GALNS, GATA2, GPI, GPR84, GRN, HBB, HFE, HVCN1, IL4R, IMPDH1, IQGAP2, JUP, LGALS9, LRG1, LTF, METTL7A, MME, NBEAL2, NHLRC3, NPPA, NR4A3, PKM, PLAU, PLAUR, PRKCSH, PTGDS, RAB3A, RAB5B, RAC2, RAP1A, RHOG, RNASE2, RNU1-93P, S100A11, SBNO2, SERPINA3, SIGLEC9, SLC44A2, SLC7A8, SLCO4C1, SPHK2, SPI1, STING1, STK11IP, TCIRG1, TICAM1, TMEM179B, TMEM63A, TRAPPC1, TSPAN14, VNN1]

GO:0002285	lymphocyte activation involved in immune response	2,42E-05	[4, 5]	58,00	29,00	76,00	24,00	[APBB1IP, BATF, BCL3, CCL19, CD180, CD19, CD1C, CD244, CD40LG, CD74, CD80, CD86, CLCF1, CORO1A, DOCK10, EOMES, FGL2, FOXP3, GAPT, HLA-DMB, HLA-DRA, HLA-DRB1, IFNG, IL12RB1, IL18, IRF4, IRF8, ITGAL, KLRC2, LCP1, LEF1, LILRB1, LY9, MYB, NCKAP1L, NLRP3, POU2AF1, PTK2B, PTPRC, SEMA4A, SLAMF6, SPN, TBX21, ZNF683]	[ABL1, ARTN, BCL3, BCL6, CD1C, CD74, CEACAM1, CLCF1, ICAM1, IL4R, JAK3, LGALS9, RARA, RELB, SPI1, STAT3, ZBTB7B, ZC3H12A]
GO:0002703	regulation of leukocyte mediated immunity	3,88E-12	[4, 5]	79,00	34,05	68,35	31,65	[BLK, BTK, CAMK4, CCR2, CD177, CD1B, CD1C, CD1E, CD226, CD84, CD96, CLCF1, CLNK, CR2, CRTAM, CXCL6, FGR, FOXP3, FUT7, HLA-DRA, HLA-DRB1, IL12RB1, IL18, IL18RAP, IL7R, ITGB2, KLRC1, KLRC2, KLRD1, KLRK1, LAG3, LILRB1, LILRB4, LTA, NCKAP1L, NCR1, NCR3, NLRP3, PRAM1, PTAFR, PTPN6, PTPRC, RAC2, SASH3, SCIMP, SH2D1A, SLAMF6, STAP1, STXBP2, SYK, TBX21, TREM2, VAV1, WAS, XCL1]	[AGER, BCL6, CD177, CD1C, CD1E, CEACAM1, CLC, CLCF1, CLNK, FADD, FES, FOXF1, GAB2, GATA2, HFE, ICAM1, IL1B, IL4R, IL7R, JAK3, LAG3, LGALS9, MAVS, NECTIN2, NOD2, NPPA, PVR, RAC2, SPHK2, SPI1, TICAM1, TNF, WAS]
GO:0006887	exocytosis	1,79E-06	[3, 4, 5, 6]	202,00	20,57	47,31	52,69	[ADAM8, ADGRE3, ANPEP, ARHGAP45, BIN2, BLK, BTK, CACNA1I, CBARP, CCL3, CCL5, CCR2, CD177, CD33, CD53, CD84, CEACAM21, CFP, CLEC12A, CLEC4D, CLNK, CNR1, CORO1A, COTL1, CTSS, CTSW, CYBB, DOCK2, EPX, EXOC3L4, FCN1, FGL2, FGR, FRMPD3, GMFG, IFNG, IQGAP2, ITGAL, ITGAX, ITGB2, KLRC2, KNG1, LAIR1, LRMP, LYZ, MMP9, MPO, MYO1G, NAPSB, NCKAP1L, NFAM1, NR4A3, ORM2, PIK3CG, PLAC8, PLAU, PLEK, PRAM1, PRKCB, PTAFR, PTGDR, PTPN6, PTPRC, PTX3, PYCARD, RAB15, RAB27B, RAB37, RAB3B, RAB3C, RAB44, RAC2, RNASET2, SDC1, SELL, SEPTIN1, SERPINA1, SLC18A1, SLC27A2, SMPD3, STXBP2, SYK, SYTL1, TBC1D10C, THBS1, TRPM2, TXNDC5, VNN1]	[ACTN1, ACTN4, ADCY1, ADGRE5, ALDOA, AMH, APOA1, ARF1, ARL8A, ARSA, ATP13A2, BRPF3, C5AR1, CACNA1H, CAPN1, CBARP, CD14, CD177, CEACAM1, CHGA, CHI3L1, CLEC12A, CLNK, CNR1, COMMD3, CPLX1, CRAT, CTSA, CTSD, CYB5R3, DMTN, DOC2B, DOK3, DSC1, ECM1, FES, FOXF1, FPR1, GAB2, GALNS, GATA2, GIT1, GNAI2, GPI, GPR84, GRIK5, GRN, GTPBP2, HBB, HFE, HGS, HTR1B, HTR2A, HVCN1, IL4R, IMPDH1, IQGAP2, JUP, KNG1, LGALS3BP, LGALS9, LRG1, LTF, METTL7A, MME, MYH9, NAPA, NBEAL2, NHLRC3, NKD2, NOTCH1, NPPA, NR4A3, OTOF, PCDHA4, PDGFB, PIP5K1C, PKM, PLAU, PLAUR, PRKCSH, PRRT2, PTGDS, RAB11B, RAB11FIP5, RAB15, RAB3A, RAB3IL1, RAB5B, RAC2, RAP1A, RHOG, RNASE2, RNU1-93P, S100A11, SELENOP, SEPTIN4, SEPTIN5, SEPTIN9, SERPINA3, SERPINE1, SERPING1, SIGLEC9, SLC44A2, SLC7A8, SLCO4C1, SPHK2, SPI1, STING1, STK11IP, STX1A, STX1B, SV2A, SYN1, SYT2, SYT7, TCIRG1, THBS1, TIMP1, TMEM179B, TMEM63A, TNFAIP2, TRAPPC1, TSPAN14, TUBA4A, VEGFA, VNN1]
GO:0036230	granulocyte activation	1,49E-03	[4, 5]	119,00	21,21	56,44	43,56	[ADAM8, ADGRE3, ANPEP, ARHGAP45, BIN2, CCL3, CCL5, CCR2, CD177, CD33, CD53, CEACAM21, CFP, CLEC12A, CLEC4D, COTL1, CTSS, CXCL6, CYBB, DOCK2, EPX, FCN1, FGL2, FGR, FRMPD3, GMFG, IL18, IL18RAP, IQGAP2, ITGAL, ITGAX, ITGB2, LAIR1, LRMP, LYZ, MMP9, MPO, NCKAP1L, NFAM1, ORM2, PLAC8, PLAU, PRAM1, PTAFR, PTPN6, PTPRC, PTX3, PYCARD, RAB37, RAB44, RNASET2, SCNN1B, SELL, SERPINA1, SLC18A1, SLC27A2, STXBP2, SYK, TBC1D10C, TRPM2, TXNDC5, VNN1]	[ADGRE5, ALDOA, AMH, ARL8A, ARSA, C5AR1, CAPN1, CD14, CD177, CEACAM1, CHI3L1, CLEC12A, COMMD3, CRAT, CTSA, CTSD, CYB5R3, DOK3, DSC1, ENPP3, FPR1, GALNS, GPI, GPR84, GRN, HBB, HFE, HVCN1, IMPDH1, IQGAP2, JUP, LRG1, LTF, METTL7A, MME, NBEAL2, NHLRC3, PKM, PLA2G2A, PLAU, PLAUR, PRKCSH, RAB3A, RAB5B, RAP1A, RHOG, RNASE2, RNU1-93P, S100A11, SERPINA3, SIGLEC9, SLC44A2, SLCO4C1, SPI1, STING1, STK11IP, TCIRG1, TMEM179B, TMEM63A, TRAPPC1, TSPAN14, VNN1]
GO:0002446	neutrophil mediated immunity	2,14E-02	[5]	113,00	20,32	55,60	44,40	[ADAM8, ADGRE3, ANPEP, ARHGAP45, BIN2, CD177, CD33, CD53, CEACAM21, CFP, CLEC12A, CLEC4D, COTL1, CTSS, CXCL6, CYBB, DOCK2, EPX, FCN1, FGL2, FGR, FRMPD3, GMFG, IQGAP2, ITGAL, ITGAX, ITGB2, LAIR1, LRMP, LYZ, MMP9, MPO, NCKAP1L, NFAM1, NLRP6, ORM2, PLAC8, PLAU, PRAM1, PTAFR, PTPN6, PTPRC, PTX3, PYCARD, RAB37, RAB44, RNASET2, SCNN1B, SELL, SERPINA1, SLC18A1, SLC27A2, STXBP2, SYK, TBC1D10C, TRPM2, TXNDC5, VNN1]	[ADGRE5, ALDOA, AMH, ARL8A, ARSA, C5AR1, CAPN1, CD14, CD177, CEACAM1, CHI3L1, CLEC12A, COMMD3, CRAT, CTSA, CTSD, CYB5R3, DOK3, DSC1, FPR1, GALNS, GPI, GPR84, GRN, HBB, HFE, HVCN1, IMPDH1, IQGAP2, JUP, LRG1, LTF, METTL7A, MME, NBEAL2, NHLRC3, PKM, PLAU, PLAUR, PRKCSH, RAB3A, RAB5B, RAP1A, RHOG, RNASE2, RNU1-93P, S100A11, SERPINA3, SIGLEC9, SLC44A2, SLCO4C1, SPI1, STING1, STK11IP, TCIRG1, TMEM179B, TMEM63A, TRAPPC1, TSPAN14, VNN1]
GO:0017157	regulation of exocytosis	1,70E-03	[4, 5, 6, 7]	57,00	26,15	46,68	53,32	[BLK, CACNA1I, CBARP, CCR2, CD177, CD84, CNR1, FGR, IFNG, ITGB2, KLRC2, NCKAP1L, PRAM1, PRKCB, PTAFR, RAB15, RAB27B, RAB3B, RAB3C, RAC2, SDC1, SEPTIN1, SMPD3, STXBP2, SYK]	[ADCY1, ARF1, ATP13A2, CACNA1H, CBARP, CD177, CEACAM1, CNR1, CPLX1, DOC2B, FES, FOXF1, GAB2, GATA2, GIT1, GNAI2, HGS, HTR1B, HTR2A, IL4R, LGALS9, NOTCH1, NPPA, PCDHA4, RAB15, RAB3A, RAC2, RAP1A, SEPTIN4, SEPTIN5, SPHK2, SPI1, STX1A, STX1B, SYN1, SYT2, SYT7]
GO:0042119	neutrophil activation	4,66E-03	[5, 6]	116,00	20,90	56,03	43,97	[ADAM8, ADGRE3, ANPEP, ARHGAP45, BIN2, CCL5, CD177, CD33, CD53, CEACAM21, CFP, CLEC12A, CLEC4D, COTL1, CTSS, CXCL6, CYBB, DOCK2, EPX, FCN1, FGL2, FGR, FRMPD3, GMFG, IL18, IL18RAP, IQGAP2, ITGAL, ITGAX, ITGB2, LAIR1, LRMP, LYZ, MMP9, MPO, NCKAP1L, NFAM1, ORM2, PLAC8, PLAU, PRAM1, PTAFR, PTPN6, PTPRC, PTX3, PYCARD, RAB37, RAB44, RNASET2, SCNN1B, SELL, SERPINA1, SLC18A1, SLC27A2, STXBP2, SYK, TBC1D10C, TRPM2, TXNDC5, VNN1]	[ADGRE5, ALDOA, AMH, ARL8A, ARSA, C5AR1, CAPN1, CD14, CD177, CEACAM1, CHI3L1, CLEC12A, COMMD3, CRAT, CTSA, CTSD, CYB5R3, DOK3, DSC1, FPR1, GALNS, GPI, GPR84, GRN, HBB, HFE, HVCN1, IMPDH1, IQGAP2, JUP, LRG1, LTF, METTL7A, MME, NBEAL2, NHLRC3, PKM, PLA2G2A, PLAU, PLAUR, PRKCSH, RAB3A, RAB5B, RAP1A, RHOG, RNASE2, RNU1-93P, S100A11, SERPINA3, SIGLEC9, SLC44A2, SLCO4C1, SPI1, STING1, STK11IP, TCIRG1, TMEM179B, TMEM63A, TRAPPC1, TSPAN14, VNN1]
GO:0043299	leukocyte degranulation	1,01E-05	[3, 4, 5, 6, 7, 8]	133,00	22,32	53,98	46,02	[ADAM8, ADGRE3, ANPEP, ARHGAP45, BIN2, BLK, BTK, CCL3, CCR2, CD177, CD33, CD53, CD84, CEACAM21, CFP, CLEC12A, CLEC4D, CLNK, CORO1A, COTL1, CTSS, CYBB, DOCK2, EPX, FCN1, FGL2, FGR, FRMPD3, GMFG, IQGAP2, ITGAL, ITGAX, ITGB2, KLRC2, LAIR1, LRMP, LYZ, MMP9, MPO, NCKAP1L, NFAM1, NR4A3, ORM2, PIK3CG, PLAC8, PLAU, PRAM1, PTAFR, PTGDR, PTPN6, PTPRC, PTX3, PYCARD, RAB37, RAB44, RAC2, RNASET2, SELL, SERPINA1, SLC18A1, SLC27A2, STXBP2, SYK, TBC1D10C, TRPM2, TXNDC5, VNN1]	[ADGRE5, ALDOA, AMH, ARL8A, ARSA, C5AR1, CAPN1, CD14, CD177, CEACAM1, CHGA, CHI3L1, CLEC12A, CLNK, COMMD3, CRAT, CTSA, CTSD, CYB5R3, DOK3, DSC1, FES, FOXF1, FPR1, GAB2, GALNS, GATA2, GPI, GPR84, GRN, HBB, HFE, HVCN1, IL4R, IMPDH1, IQGAP2, JUP, LGALS9, LRG1, LTF, METTL7A, MME, NBEAL2, NHLRC3, NPPA, NR4A3, PKM, PLAU, PLAUR, PRKCSH, PTGDS, RAB3A, RAB5B, RAC2, RAP1A, RHOG, RNASE2, RNU1-93P, S100A11, SERPINA3, SIGLEC9, SLC44A2, SLC7A8, SLCO4C1, SPHK2, SPI1, STING1, STK11IP, TCIRG1, TMEM179B, TMEM63A, TRAPPC1, TSPAN14, VNN1]

GO:0045055	regulated exocytosis	2,13E-07	[4, 5, 6, 7]	186,00	21,48	45,51	54,49	[ADAM8, ADGRE3, ANPEP, ARHGAP45, BIN2, BLK, BTK, CACNA1I, CBARP, CCL3, CCR2, CD177, CD33, CD53, CD84, CEACAM21, CFP, CLEC12A, CLEC4D, CLNK, CNR1, CORO1A, COTL1, CTSS, CTSW, CYBB, DOCK2, EPX, FCN1, FGL2, FGR, FRMPD3, GMFG, IQGAP2, ITGAL, ITGAX, ITGB2, KLRC2, KNG1, LAIR1, LRMP, LYZ, MMP9, MPO, NAPSB, NCKAP1L, NFAM1, NR4A3, ORM2, PIK3CG, PLAC8, PLAU, PLEK, PRAM1, PRKCB, PTAFR, PTGDR, PTPN6, PTPRC, PTX3, PYCARD, RAB15, RAB27B, RAB37, RAB44, RAC2, RNASET2, SELL, SERPINA1, SLC18A1, SLC27A2, STXBP2, SYK, TBC1D10C, THBS1, TRPM2, TXNDC5, VNN1]	[ACTN1, ACTN4, ADCY1, ADGRE5, ALDOA, AMH, APOA1, ARF1, ARL8A, ARSA, BRPF3, C5AR1, CACNA1H, CAPN1, CBARP, CD14, CD177, CEACAM1, CHGA, CHI3L1, CLEC12A, CLNK, CNR1, COMMD3, CPLX1, CRAT, CTSA, CTSD, CYB5R3, DMTN, DOC2B, DOK3, DSC1, ECM1, FES, FOXF1, FPR1, GAB2, GALNS, GATA2, GIT1, GNAI2, GPI, GPR84, GRIK5, GRN, GTPBP2, HBB, HFE, HTR1B, HTR2A, HVCN1, IL4R, IMPDH1, IQGAP2, JUP, KNG1, LGALS3BP, LGALS9, LRG1, LTF, METTL7A, MME, MYH9, NAPA, NBEAL2, NHLRC3, NOTCH1, NPPA, NR4A3, OTOF, PCDHA4, PDGFB, PIP5K1C, PKM, PLAU, PLAUR, PRKCSH, PRRT2, PTGDS, RAB11B, RAB11FIP5, RAB15, RAB3A, RAB5B, RAC2, RAP1A, RHOG, RNASE2, RNU1-93P, S100A11, SELENOP, SEPTIN5, SEPTIN9, SERPINA3, SERPINE1, SERPING1, SIGLEC9, SLC44A2, SLC7A8, SLCO4C1, SPHK2, SPI1, STING1, STK11IP, STX1A, STX1B, SV2A, SYN1, SYT2, SYT7, TCIRG1, THBS1, TIMP1, TMEM179B, TMEM63A, TRAPPC1, TSPAN14, TUBA4A, VEGFA, VNN1]
GO:0002283	neutrophil activation involved in immune response	2,25E-02	[5, 6, 7]	111,00	20,44	54,74	45,26	[ADAM8, ADGRE3, ANPEP, ARHGAP45, BIN2, CD177, CD33, CD53, CEACAM21, CFP, CLEC12A, CLEC4D, COTL1, CTSS, CYBB, DOCK2, EPX, FCN1, FGL2, FGR, FRMPD3, GMFG, IQGAP2, ITGAL, ITGAX, ITGB2, LAIR1, LRMP, LYZ, MMP9, MPO, NCKAP1L, NFAM1, ORM2, PLAC8, PLAU, PRAM1, PTAFR, PTPN6, PTPRC, PTX3, PYCARD, RAB37, RAB44, RNASET2, SCNN1B, SELL, SERPINA1, SLC18A1, SLC27A2, STXBP2, SYK, TBC1D10C, TRPM2, TXNDC5, VNN1]	[ADGRE5, ALDOA, AMH, ARL8A, ARSA, C5AR1, CAPN1, CD14, CD177, CEACAM1, CHI3L1, CLEC12A, COMMD3, CRAT, CTSA, CTSD, CYB5R3, DOK3, DSC1, FPR1, GALNS, GPI, GPR84, GRN, HBB, HFE, HVCN1, IMPDH1, IQGAP2, JUP, LRG1, LTF, METTL7A, MME, NBEAL2, NHLRC3, PKM, PLAU, PLAUR, PRKCSH, RAB3A, RAB5B, RAP1A, RHOG, RNASE2, RNU1-93P, S100A11, SERPINA3, SIGLEC9, SLC44A2, SLCO4C1, SPI1, STING1, STK11IP, TCIRG1, TMEM179B, TMEM63A, TRAPPC1, TSPAN14, VNN1]
GO:0043367	CD4-positive, alpha-beta T cell differentiation	3,67E-08	[7, 8, 9, 10, 11, 12, 13]	37,00	43,53	76,08	23,92	[BATF, BCL3, CCL19, CD80, CD86, FOXP3, FUT7, HLA-DRA, HLA-DRB1, IFNG, IL12RB1, IL18, IRF4, LEF1, LY9, MYB, NCKAP1L, NLRP3, PLA2G2D, RUNX3, SASH3, SEMA4A, SLAMF6, SOCS1, SPN, TBX21, TOX]	[ARMC5, BCL3, BCL6, IL4R, JAK3, LGALS9, RARA, RELB, STAT3, ZBTB7B, ZC3H12A]
GO:0046637	regulation of alpha-beta T cell differentiation	1,29E-06	[7, 8, 9, 10, 11, 12, 13]	30,00	44,78	78,09	21,91	[CCL19, CD80, CD86, FOXP3, HLA-DRA, HLA-DRB1, IFNG, IL12RB1, IL18, IRF4, LILRB4, MYB, NCKAP1L, NLRP3, PRDM1, RUNX3, SASH3, SOCS1, SYK, TBX21, ZAP70, ZNF683]	[AP3D1, BCL6, IL4R, JAK3, LGALS9, RARA, ZBTB7B, ZC3H12A]
GO:0043370	regulation of CD4-positive, alpha-beta T cell differentiation	1,98E-05	[8, 9, 10, 11, 12, 13, 14]	24,00	47,06	75,88	24,12	[CCL19, CD80, CD86, FOXP3, HLA-DRA, HLA-DRB1, IFNG, IL12RB1, IL18, IRF4, MYB, NCKAP1L, NLRP3, RUNX3, SASH3, SOCS1, TBX21]	[BCL6, IL4R, JAK3, LGALS9, RARA, ZBTB7B, ZC3H12A]
GO:0002683	negative regulation of immune system process	1,16E-10	[2, 3, 4]	156,00	24,68	57,59	42,41	[ADCY7, ALOX15, ANGPT1, BANK1, BLK, BTK, C1QC, C1QTNF1, CALCR, CCL2, CCL3, CCR2, CD200R1, CD22, CD247, CD300LF, CD3G, CD74, CD80, CD84, CD86, CD96, CDKN2A, CLNK, CNR1, CNR2, CR2, CRTAM, CST7, CTLA4, CYSLTR1, DPEP1, DRD1, FCRL3, FGL2, FGR, FOXP3, GPR150, GPR55, GPR83, HLA-DOA, HLA-DRB1, IL7R, INPP5D, KLRC1, KLRD1, KLRK1, LAG3, LAX1, LDLR, LILRB1, LILRB4, LPAL2, LRRC17, LST1, NCKAP1L, NLRC3, NLRP3, NLRP6, PLA2G2D, PTGDR, PTGER2, PTPN22, PTPN6, PTPRC, RLN2, RUNX3, SAMSN1, SH2D1A, SLA2, SLAMF8, SOCS1, SPN, STAP1, SYK, TBC1D10C, TBX21, THBS1, THY1, TIGIT, TNFAIP3, TNFAIP8L2, TNFRSF13B, TREM2, TSC22D3, UBASH3A, XCL1]	[ADCY1, ADCY3, ADCY4, ADCY9, ADIPOQ, AMH, ANGPT1, APOA1, APOD, BCL6, C1QTNF1, C5, CALCR, CCL2, CCL21, CD74, CEACAM1, CGA, CHGA, CLNK, CNR1, CRHR2, CST7, CX3CL1, DGKZ, DLG5, DTX1, ENPP3, ERBB2, FADD, FOXF1, FURIN, GATA2, GCSAM, GNAI2, GPAM, GPBAR1, GPER1, GPR137, GPR20, GPR83, GPR84, GRN, HFE, HLA-DOB, HRH2, IL4R, IL7R, INHA, JAK3, LAG3, LDLR, LGALS9, LRRC17, LRRC32, LTF, MYC, MYH9, NLRC5, NOD2, NR1D1, NRARP, PCDHA4, PCDHA6, PDCD1, PRKACA, PRKAR2B, PTGIR, RAMP2, RARA, RHBDF2, RMRP, RNF26, SERPING1, SPI1, SRC, THBS1, TNF, TRIB1, VIPR2, VSIR, ZBTB7B, ZC3H12A]
GO:0050865	regulation of cell activation	2,09E-24	[3, 4]	204,00	29,18	65,18	34,82	[ADAM8, AIF1, BANK1, BLK, BTK, BTLA, C1QTNF1, CAMK4, CARD11, CCDC88B, CCL19, CCL2, CCL3, CCL5, CCR2, CD177, CD19, CD2, CD22, CD226, CD27, CD300LF, CD33, CD3E, CD4, CD40LG, CD5, CD6, CD74, CD80, CD84, CD86, CDKN2A, CLCF1, CLEC4D, CLNK, CNR1, CNR2, CORO1A, CRTAM, CST7, CTLA4, DOCK8, EBI3, EGR3, EPX, FCRL3, FGL2, FGR, FOXP3, GRAP2, HLA-DMB, HLA-DOA, HLA-DPA1, HLA-DPB1, HLA-DRA, HLA-DRB1, ICOS, IFNG, IGLL5, IKZF3, IL12RB1, IL18, IL7R, INPP5D, IRF4, ITGB2, KLRC2, KLRK1, LAG3, LAX1, LCK, LDLR, LEF1, LILRB1, LILRB4, LMO1, LST1, MYB, MZB1, NCKAP1L, NFAM1, NLRP3, NR4A3, PLA2G2D, PLEK, PRAM1, PRDM1, PRKCQ, PTAFR, PTPN22, PTPN6, PTPRC, PYCARD, RAC2, RASAL3, RHOH, RUNX3, SAMSN1, SASH3, SIRPG, SIT1, SLAMF8, SOCS1, SPN, STAP1, STXBP2, SYK, TBC1D10C, TBX21, TESPA1, THBS1, THEMIS2, THY1, TIGIT, TLR6, TNFAIP3, TNFAIP8L2, TNFRSF13B, TNFRSF18, TNFSF13B, TNFSF14, TNFSF8, TOX, TREM2, VAV1, VNN1, XCL1, ZAP70, ZNF683]	[ABL1, AGER, AMH, AP3D1, BCL6, BTLA, C1QTNF1, CCL2, CCL21, CCR7, CD177, CD276, CD74, CDKN1A, CEACAM1, CLC, CLCF1, CLNK, CLPTM1, CNR1, CSK, CST7, CX3CL1, CYP26B1, DCAF15, DLG5, DMTN, DTX1, ENPP3, ERBB2, FADD, FES, FLOT2, FOXF1, GAB2, GATA2, GNAO1, GPAM, GPER1, GRN, HAVCR1, HFE, IDO1, IL15RA, IL1B, IL4R, IL7R, INHA, JAK3, JUND, LAG3, LDLR, LGALS9, LILRA5, LRRC32, MYO18A, NECTIN2, NOD2, NOS3, NPPA, NR1D1, NR4A3, NRARP, PCDHA4, PCDHA6, PDCD1, PDGFB, PDGFRB, PLA2G2A, RAC2, RARA, RHBDF2, SELENOP, SH2B3, SLC7A1, SOX12, SOX13, SPHK1, SPHK2, SPI1, SRC, THBS1, TICAM1, TIMP1, VNN1, VSIR, ZBTB7B, ZC3H12A, ZMIZ1, ZNF335]

GO:0002694	regulation of leukocyte activation	2,18E-24	[3, 4, 5]	194,00	29,85	67,19	32,81	[ADAM8, AIF1, BANK1, BLK, BTK, BTLA, CAMK4, CARD11, CCDC88B, CCL19, CCL2, CCL3, CCL5, CCR2, CD177, CD19, CD2, CD22, CD226, CD27, CD300LF, CD33, CD3E, CD4, CD40LG, CD5, CD6, CD74, CD80, CD84, CD86, CDKN2A, CLCF1, CLEC4D, CLNK, CNR1, CNR2, CORO1A, CRTAM, CST7, CTLA4, DOCK8, EBI3, EGR3, EPX, FCRL3, FGL2, FGR, FOXP3, GRAP2, HLA-DMB, HLA-DOA, HLA-DPA1, HLA-DPB1, HLA-DRA, HLA-DRB1, ICOS, IFNG, IGLL5, IKZF3, IL12RB1, IL18, IL7R, INPP5D, IRF4, ITGB2, KLRC2, KLRK1, LAG3, LAX1, LCK, LDLR, LEF1, LILRB1, LILRB4, LMO1, LST1, MYB, MZB1, NCKAP1L, NFAM1, NLRP3, NR4A3, PLA2G2D, PRAM1, PRDM1, PRKCQ, PTAFR, PTPN22, PTPN6, PTPRC, PYCARD, RAC2, RASAL3, RHOH, RUNX3, SAMSN1, SASH3, SIRPG, SIT1, SLAMF8, SOCS1, SPN, STAP1, STXBP2, SYK, TBC1D10C, TBX21, TESPA1, THBS1, THEMIS2, THY1, TIGIT, TLR6, TNFAIP3, TNFAIP8L2, TNFRSF13B, TNFRSF18, TNFSF13B, TNFSF14, TNFSF8, TOX, TREM2, VAV1, VNN1, XCL1, ZAP70, ZNF683]	[ABL1, AGER, AMH, AP3D1, BCL6, BTLA, CCL2, CCL21, CCR7, CD177, CD276, CD74, CDKN1A, CEACAM1, CLC, CLCF1, CLNK, CLPTM1, CNR1, CSK, CST7, CX3CL1, CYP26B1, DCAF15, DLG5, DTX1, ENPP3, ERBB2, FADD, FES, FLOT2, FOXF1, GAB2, GATA2, GNAO1, GPAM, GPER1, GRN, HAVCR1, HFE, IDO1, IL15RA, IL1B, IL4R, IL7R, INHA, JAK3, JUND, LAG3, LDLR, LGALS9, LRRC32, MYO18A, NECTIN2, NOD2, NPPA, NR1D1, NR4A3, NRARP, PCDHA4, PCDHA6, PDCD1, PLA2G2A, RAC2, RARA, SLC7A1, SOX12, SOX13, SPHK1, SPHK2, SPI1, SRC, THBS1, TICAM1, TIMP1, VNN1, VSIR, ZBTB7B, ZC3H12A, ZMIZ1, ZNF335]
GO:0002698	negative regulation of immune effector process	4,35E-04	[3, 4, 5]	37,00	33,04	60,19	39,81	[ANGPT1, CCR2, CD22, CD84, CD96, CR2, FCRL3, FGL2, FOXP3, IL7R, KLRC1, KLRD1, LILRB1, LILRB4, LPAL2, NCKAP1L, PTPN6, PTPRC, SLAMF8, TBX21, XCL1]	[ANGPT1, APOA1, BCL6, CEACAM1, ENPP3, FOXF1, GRN, HFE, IL4R, IL7R, JAK3, LGALS9, NOD2, SERPING1, SPI1, TNF, ZBTB7B, ZC3H12A]
GO:0007159	leukocyte cell-cell adhesion	4,87E-21	[4]	129,00	34,04	69,75	30,25	[ADAM8, AIF1, BMP7, BTLA, CARD11, CCDC88B, CCL19, CCL2, CCL5, CCR2, CD177, CD27, CD3E, CD4, CD40LG, CD5, CD6, CD74, CD80, CD86, CDKN2A, CORO1A, CRTAM, CTLA4, DOCK8, EBI3, EGR3, EPX, FGL2, FOXP3, FUT7, GRAP2, HLA-DMB, HLA-DPA1, HLA-DPB1, HLA-DRA, HLA-DRB1, ICOS, IFNG, IL12RB1, IL18, IL7R, ITGA4, ITGAL, ITGB2, ITGB7, KLRK1, LAG3, LAX1, LCK, LEF1, LILRB1, LILRB4, MYB, NCKAP1L, NLRP3, NR4A3, PLA2G2D, PRKCQ, PTAFR, PTPN22, PTPN6, PTPRC, PYCARD, RAC2, RASAL3, RHOH, RUNX3, SASH3, SELE, SELL, SELPLG, SEMA4D, SIRPG, SKAP1, SOCS1, SPN, SYK, TBX21, TESPA1, THY1, TIGIT, TNFAIP8L2, TNFSF13B, TNFSF14, VAV1, VNN1, XCL1, ZAP70]	[AGER, AP3D1, BCL6, BTLA, CCL2, CCL21, CCR7, CD177, CD276, CD74, CEACAM1, CSK, DLG5, DTX1, ERBB2, FADD, FLOT2, GNAO1, GPAM, HFE, ICAM1, IL1B, IL4R, IL7R, JAK3, LAG3, LGALS9, LRRC32, MSN, NOD2, NR4A3, NRARP, PDCD1, RAC2, RARA, RELA, SELE, SELENOP, SLC7A1, SOX12, SOX13, SRC, TIMP1, TNF, TNIP1, VNN1, VSIR, ZBTB7B, ZC3H12A, ZMIZ1]
GO:0007162	negative regulation of cell adhesion	8,18E-08	[3, 4, 5]	84,00	28,19	48,63	51,37	[ADAMDEC1, AKNA, ANGPT1, ANGPT2, C1QTNF1, CD74, CD80, CD86, CDH1, CDKN2A, CRTAM, CTLA4, FGL2, FOXP3, HLA-DRB1, KNG1, LAG3, LAX1, LILRB1, LILRB4, NCKAP1L, PLA2G2D, PLXNC1, PTPN22, PTPN6, PTPRC, RUNX3, SEMA4D, SOCS1, SPN, TACSTD2, TBX21, THBS1, TIGIT, TNFAIP8L2, TNR, WNT1, XCL1]	[ABL1, ACTN4, ADIPOQ, ANGPT1, APOA1, APOD, ARHGDIA, BCL6, C1QTNF1, CCL21, CD74, CEACAM1, COL1A1, CX3CL1, DLG5, DMTN, DTX1, ERBB2, FXYD5, HFE, IL1RN, IL4R, JAK3, KNG1, LAG3, LGALS9, LRRC32, MAPK7, MEN1, MYADM, NF2, NOTCH1, NOTCH4, NRARP, OMA1, PLXNA1, PLXNA3, PLXNB1, POSTN, PTPRU, RGCC, SERPINE1, SH2B3, SPI1, SRC, SYNGAP1, THBS1, TRPV4, VEGFA, VSIR, ZBTB7B, ZC3H12A]
GO:0050866	negative regulation of cell activation	2,21E-10	[3, 4, 5]	72,00	33,49	63,17	36,83	[BANK1, BLK, BTK, C1QTNF1, CCR2, CD300LF, CD74, CD80, CD84, CD86, CDKN2A, CLNK, CNR1, CNR2, CRTAM, CST7, CTLA4, FGL2, FGR, FOXP3, HLA-DRB1, INPP5D, LAG3, LAX1, LDLR, LILRB1, LILRB4, LST1, NCKAP1L, PLA2G2D, PTPN22, PTPN6, PTPRC, RUNX3, SAMSN1, SOCS1, SPN, TBC1D10C, TBX21, TIGIT, TNFAIP3, TNFAIP8L2, TNFRSF13B, TREM2, XCL1]	[BCL6, C1QTNF1, CD74, CEACAM1, CLNK, CNR1, CST7, CX3CL1, DLG5, DTX1, ENPP3, ERBB2, FOXF1, GPER1, GRN, HFE, IL4R, INHA, JAK3, LAG3, LDLR, LGALS9, LRRC32, NOS3, NR1D1, NRARP, PCDHA4, PCDHA6, PDGFB, SH2B3, SPI1, VSIR, ZBTB7B, ZC3H12A]
GO:0002695	negative regulation of leukocyte activation	1,42E-10	[3, 4, 5, 6]	67,00	34,90	65,00	35,00	[BANK1, BLK, BTK, CCR2, CD300LF, CD74, CD80, CD84, CD86, CDKN2A, CLNK, CNR1, CNR2, CRTAM, CST7, CTLA4, FGL2, FGR, FOXP3, HLA-DRB1, INPP5D, LAG3, LAX1, LDLR, LILRB1, LILRB4, LST1, NCKAP1L, PLA2G2D, PTPN22, PTPN6, PTPRC, RUNX3, SAMSN1, SOCS1, SPN, TBC1D10C, TBX21, TIGIT, TNFAIP3, TNFAIP8L2, TNFRSF13B, XCL1]	[BCL6, CD74, CEACAM1, CLNK, CNR1, CST7, CX3CL1, DLG5, DTX1, ENPP3, ERBB2, FOXF1, GPER1, GRN, HFE, IL4R, INHA, JAK3, LAG3, LDLR, LGALS9, LRRC32, NR1D1, NRARP, PCDHA4, PCDHA6, SPI1, VSIR, ZBTB7B, ZC3H12A]
GO:0070663	regulation of leukocyte proliferation	1,96E-10	[4, 5]	82,00	31,54	71,80	28,20	[AIF1, BLK, BTK, CARD11, CCDC88B, CCL19, CCL5, CCR2, CD22, CD3E, CD40LG, CD6, CD74, CD80, CD86, CDKN2A, CLCF1, CORO1A, CRTAM, CSF1R, CTLA4, EBI3, EPX, FCRL3, FOXP3, HLA-DMB, HLA-DPA1, HLA-DPB1, HLA-DRB1, IKZF3, IL12RB1, IL18, IL5RA, INPP5D, LILRB1, LILRB4, LMO1, LST1, MZB1, NCKAP1L, PLA2G2D, PRKCQ, PTPN22, PTPN6, PTPRC, PYCARD, RAC2, RASAL3, SASH3, SPN, SYK, TNFAIP3, TNFRSF13B, TNFSF13B, TNFSF8, XCL1, ZAP70]	[AGER, AMH, BCL6, CD276, CD74, CDKN1A, CLC, CLCF1, CRP, CSF1, DLG5, ENPP3, ERBB2, FADD, GPAM, IDO1, IL1B, IL5RA, JAK3, LGALS9, LRRC32, MAPK3, RAC2, SLC7A1, TICAM1, TIMP1, VSIR, ZBTB7B, ZNF335]
GO:0002820	negative regulation of adaptive immune response	1,38E-02	[4, 5, 6]	22,00	36,67	66,84	33,16	[ALOX15, CR2, FOXP3, IL7R, KLRC1, KLRD1, LILRB1, LILRB4, NCKAP1L, PTPN6, PTPRC, SAMSN1, TBX21, XCL1]	[BCL6, CEACAM1, HFE, IL4R, IL7R, JAK3, NOD2, ZBTB7B, ZC3H12A]
GO:0022408	negative regulation of cell-cell adhesion	2,36E-06	[4, 5, 6]	59,00	30,57	53,24	46,76	[AKNA, C1QTNF1, CD74, CD80, CD86, CDH1, CDKN2A, CRTAM, CTLA4, FGL2, FOXP3, HLA-DRB1, LAG3, LAX1, LILRB1, LILRB4, NCKAP1L, PLA2G2D, PTPN22, PTPN6, RUNX3, SOCS1, SPN, TBX21, TIGIT, TNFAIP8L2, TNR, WNT1, XCL1]	[ABL1, ADIPOQ, APOA1, BCL6, C1QTNF1, CCL21, CD74, CEACAM1, DLG5, DTX1, ERBB2, FXYD5, HFE, IL1RN, IL4R, JAK3, LAG3, LGALS9, LRRC32, MAPK7, MYADM, NF2, NOTCH1, NOTCH4, NRARP, RGCC, SH2B3, SPI1, TRPV4, VEGFA, VSIR, ZBTB7B, ZC3H12A]

GO:0051249	regulation of lymphocyte activation	2,62E-15	[4, 5, 6]	154,00	27,60	71,79	28,21	[ADAM8, AIF1, BANK1, BLK, BTK, BTLA, CAMK4, CARD11, CCDC88B, CCL19, CCL2, CCL5, CCR2, CD19, CD2, CD22, CD27, CD3E, CD4, CD40LG, CD5, CD6, CD74, CD80, CD86, CDKN2A, CLCF1, CLNK, CORO1A, CRTAM, CTLA4, DOCK8, EBI3, EGR3, EPX, FCRL3, FGL2, FGR, FOXP3, GRAP2, HLA-DMB, HLA-DOA, HLA-DPA1, HLA-DPB1, HLA-DRA, HLA-DRB1, ICOS, IFNG, IGLL5, IKZF3, IL12RB1, IL18, IL7R, INPP5D, IRF4, KLRC2, KLRK1, LAG3, LAX1, LCK, LEF1, LILRB1, LILRB4, LMO1, LST1, MYB, MZB1, NCKAP1L, NFAM1, NLRP3, PLA2G2D, PRDM1, PRKCQ, PTPN22, PTPN6, PTPRC, PYCARD, RAC2, RASAL3, RHOH, RUNX3, SAMSN1, SASH3, SIRPG, SIT1, SLAMF8, SOCS1, SPN, SYK, TBC1D10C, TBX21, TESPA1, THEMIS2, THY1, TIGIT, TNFAIP3, TNFAIP8L2, TNFRSF13B, TNFRSF18, TNFSF13B, TNFSF14, TNFSF8, TOX, VAV1, VNN1, XCL1, ZAP70, ZNF683]	[ABL1, AGER, AMH, AP3D1, BCL6, BTLA, CCL2, CCL21, CCR7, CD276, CD74, CDKN1A, CEACAM1, CLC, CLCF1, CLNK, CLPTM1, CSK, CYP26B1, DCAF15, DLG5, DTX1, ERBB2, FADD, FLOT2, GNAO1, GPAM, HFE, IDO1, IL15RA, IL1B, IL4R, IL7R, INHA, JAK3, LAG3, LGALS9, LRRC32, NOD2, NRARP, PDCD1, RAC2, RARA, SLC7A1, SOX12, SOX13, SRC, TICAM1, TIMP1, VNN1, VSIR, ZBTB7B, ZC3H12A, ZMIZ1, ZNF335]
GO:0002286	T cell activation involved in immune response	4,84E-06	[5, 6]	41,00	35,65	73,52	26,48	[APBB1IP, BATF, BCL3, CCL19, CD1C, CD74, CD80, CD86, EOMES, FGL2, FOXP3, HLA-DMB, HLA-DRA, HLA-DRB1, IFNG, IL12RB1, IL18, IRF4, ITGAL, LCP1, LEF1, LILRB1, LY9, MYB, NCKAP1L, NLRP3, SEMA4A, SLAMF6, SPN, TBX21]	[BCL3, BCL6, CD1C, CD74, CEACAM1, ICAM1, IL4R, JAK3, LGALS9, RARA, RELB, STAT3, ZBTB7B, ZC3H12A]
GO:0032944	regulation of mononuclear cell proliferation	5,50E-10	[5, 6]	77,00	31,82	72,91	27,09	[AIF1, BLK, BTK, CARD11, CCDC88B, CCL19, CCL5, CCR2, CD22, CD3E, CD40LG, CD6, CD74, CD80, CD86, CDKN2A, CLCF1, CORO1A, CRTAM, CTLA4, EBI3, EPX, FCRL3, FOXP3, HLA-DMB, HLA-DPA1, HLA-DPB1, HLA-DRB1, IKZF3, IL12RB1, IL18, INPP5D, LILRB1, LILRB4, LMO1, LST1, MZB1, NCKAP1L, PLA2G2D, PRKCQ, PTPN22, PTPN6, PTPRC, PYCARD, RAC2, RASAL3, SASH3, SPN, SYK, TNFRSF13B, TNFSF13B, TNFSF8, XCL1, ZAP70]	[AGER, AMH, BCL6, CD276, CD74, CDKN1A, CLC, CLCF1, CRP, CSF1, DLG5, ERBB2, FADD, GPAM, IDO1, IL1B, JAK3, LGALS9, LRRC32, RAC2, SLC7A1, TICAM1, TIMP1, VSIR, ZBTB7B, ZNF335]
GO:0046631	alpha-beta T cell activation	1,31E-14	[5, 6]	66,00	41,25	76,60	23,40	[BATF, BCL11B, BCL3, CCL19, CCR2, CD3E, CD80, CD86, CRTAM, DOCK2, EBI3, EOMES, FOXP3, FUT7, HLA-DRA, HLA-DRB1, IFNG, IL12RB1, IL18, IRF4, ITK, LEF1, LILRB1, LILRB4, LY9, MYB, NCKAP1L, NLRP3, PLA2G2D, PRDM1, PRKCQ, PTPN22, PTPRC, RASAL3, RUNX3, SASH3, SEMA4A, SLAMF6, SOCS1, SPN, SYK, TBX21, TNFSF8, TOX, WDFY4, XCL1, ZAP70, ZNF683]	[ABL1, AGER, AP3D1, ARMC5, BCL3, BCL6, ELF4, GNAO1, HFE, IL4R, JAK3, LGALS9, RARA, RELB, STAT3, TCIRG1, VSIR, ZBTB7B, ZC3H12A]
GO:0051250	negative regulation of lymphocyte activation	1,91E-05	[4, 5, 6, 7]	50,00	31,25	71,59	28,41	[BANK1, BLK, BTK, CD74, CD80, CD86, CDKN2A, CLNK, CRTAM, CTLA4, FGL2, FGR, FOXP3, HLA-DRB1, INPP5D, LAG3, LAX1, LILRB1, LILRB4, LST1, NCKAP1L, PLA2G2D, PTPN22, PTPN6, RUNX3, SAMSN1, SOCS1, SPN, TBC1D10C, TBX21, TIGIT, TNFAIP3, TNFAIP8L2, TNFRSF13B, XCL1]	[BCL6, CD74, CEACAM1, CLNK, DLG5, DTX1, ERBB2, HFE, IL4R, INHA, JAK3, LAG3, LGALS9, LRRC32, NRARP, VSIR, ZBTB7B, ZC3H12A]
GO:1903037	regulation of leukocyte cell-cell adhesion	3,98E-20	[5, 6]	119,00	34,69	69,53	30,47	[ADAM8, AIF1, BTLA, CARD11, CCDC88B, CCL19, CCL2, CCL5, CCR2, CD27, CD3E, CD4, CD40LG, CD5, CD6, CD74, CD80, CD86, CDKN2A, CORO1A, CRTAM, CTLA4, DOCK8, EBI3, EGR3, EPX, FGL2, FOXP3, FUT7, GRAP2, HLA-DMB, HLA-DPA1, HLA-DPB1, HLA-DRA, HLA-DRB1, ICOS, IFNG, IL12RB1, IL18, IL7R, ITGA4, ITGB2, KLRK1, LAG3, LAX1, LCK, LEF1, LILRB1, LILRB4, MYB, NCKAP1L, NLRP3, NR4A3, PLA2G2D, PRKCQ, PTAFR, PTPN22, PTPN6, PTPRC, PYCARD, RASAL3, RHOH, RUNX3, SASH3, SELE, SIRPG, SKAP1, SOCS1, SPN, SYK, TBX21, TESPA1, THY1, TIGIT, TNFAIP8L2, TNFSF13B, TNFSF14, VAV1, VNN1, XCL1, ZAP70]	[AGER, AP3D1, BCL6, BTLA, CCL2, CCL21, CCR7, CD276, CD74, CEACAM1, CSK, DLG5, DTX1, ERBB2, FADD, FLOT2, GNAO1, GPAM, HFE, ICAM1, IL1B, IL4R, IL7R, JAK3, LAG3, LGALS9, LRRC32, NOD2, NR4A3, NRARP, PDCD1, RARA, RELA, SELE, SELENOP, SLC7A1, SOX12, SOX13, SRC, TIMP1, TNF, VNN1, VSIR, ZBTB7B, ZC3H12A, ZMIZ1]
GO:0002823	negative regulation of adaptive immune response based on somatic recombination of immune receptors built from immunoglobulin superfamily domains	4,05E-02	[5, 6, 7]	20,00	36,36	63,34	36,66	[CR2, FOXP3, IL7R, KLRC1, KLRD1, LILRB1, LILRB4, NCKAP1L, PTPN6, PTPRC, TBX21, XCL1]	[BCL6, CEACAM1, HFE, IL4R, IL7R, JAK3, NOD2, ZBTB7B, ZC3H12A]
GO:0050670	regulation of lymphocyte proliferation	2,85E-09	[5, 6, 7]	75,00	31,51	74,46	25,54	[AIF1, BLK, BTK, CARD11, CCDC88B, CCL19, CCL5, CCR2, CD22, CD3E, CD40LG, CD6, CD74, CD80, CD86, CDKN2A, CLCF1, CORO1A, CRTAM, CTLA4, EBI3, EPX, FCRL3, FOXP3, HLA-DMB, HLA-DPA1, HLA-DPB1, HLA-DRB1, IKZF3, IL12RB1, IL18, INPP5D, LILRB1, LILRB4, LMO1, LST1, MZB1, NCKAP1L, PLA2G2D, PRKCQ, PTPN22, PTPN6, PTPRC, PYCARD, RAC2, RASAL3, SASH3, SPN, SYK, TNFRSF13B, TNFSF13B, TNFSF8, XCL1, ZAP70]	[AGER, AMH, BCL6, CD276, CD74, CDKN1A, CLC, CLCF1, DLG5, ERBB2, FADD, GPAM, IDO1, IL1B, JAK3, LGALS9, LRRC32, RAC2, SLC7A1, TICAM1, TIMP1, VSIR, ZBTB7B, ZNF335]
GO:0050863	regulation of T cell activation	2,38E-22	[5, 6, 7]	126,00	35,39	70,57	29,43	[ADAM8, AIF1, BTLA, CAMK4, CARD11, CCDC88B, CCL19, CCL2, CCL5, CCR2, CD2, CD27, CD3E, CD4, CD40LG, CD5, CD6, CD74, CD80, CD86, CDKN2A, CLCF1, CORO1A, CRTAM, CTLA4, DOCK8, EBI3, EGR3, EPX, FGL2, FOXP3, GRAP2, HLA-DMB, HLA-DOA, HLA-DPA1, HLA-DPB1, HLA-DRA, HLA-DRB1, ICOS, IFNG, IL12RB1, IL18, IL7R, IRF4, KLRK1, LAG3, LAX1, LCK, LEF1, LILRB1, LILRB4, LMO1, MYB, NCKAP1L, NLRP3, PLA2G2D, PRDM1, PRKCQ, PTPN22, PTPN6, PTPRC, PYCARD, RAC2, RASAL3, RHOH, RUNX3, SASH3, SIRPG, SIT1, SOCS1, SPN, SYK, TBX21, TESPA1, THY1, TIGIT, TNFAIP8L2, TNFRSF18, TNFSF13B, TNFSF14, TNFSF8, TOX, VAV1, VNN1, XCL1, ZAP70, ZNF683]	[ABL1, AGER, AP3D1, BCL6, BTLA, CCL2, CCL21, CCR7, CD276, CD74, CEACAM1, CLC, CLCF1, CLPTM1, CSK, CYP26B1, DLG5, DTX1, ERBB2, FADD, FLOT2, GNAO1, GPAM, HFE, IDO1, IL1B, IL4R, IL7R, JAK3, LAG3, LGALS9, LRRC32, NOD2, NRARP, PDCD1, RAC2, RARA, SLC7A1, SOX12, SOX13, SRC, TIMP1, VNN1, VSIR, ZBTB7B, ZC3H12A, ZMIZ1]
GO:1903038	negative regulation of leukocyte cell-cell adhesion	9,60E-03	[5, 6, 7]	39,00	28,47	64,65	35,35	[CD74, CD80, CD86, CDKN2A, CRTAM, CTLA4, FGL2, FOXP3, HLA-DRB1, LAG3, LAX1, LILRB1, LILRB4, NCKAP1L, PLA2G2D, PTPN22, PTPN6, RUNX3, SOCS1, SPN, TBX21, TIGIT, TNFAIP8L2, XCL1]	[BCL6, CCL21, CD74, CEACAM1, DLG5, DTX1, ERBB2, HFE, IL4R, JAK3, LAG3, LGALS9, LRRC32, NRARP, VSIR, ZBTB7B, ZC3H12A]

GO:0035710	CD4-positive, alpha-beta T cell activation	4,57E-08	[6, 7]	42,00	40,00	72,85	27,15	[BATF, BCL3, CCL19, CD80, CD86, FOXP3, FUT7, HLA-DRA, HLA-DRB1, IFNG, IL12RB1, IL18, IRF4, LEF1, LY9, MYB, NCKAP1L, NLRP3, PLA2G2D, PRKCQ, RUNX3, SASH3, SEMA4A, SLAMF6, SOCS1, SPN, TBX21, TOX, XCL1]	[AGER, ARMC5, BCL3, BCL6, IL4R, JAK3, LGALS9, RARA, RELB, STAT3, TCIRG1, VSIR, ZBTB7B, ZC3H12A]
GO:0036037	CD8-positive, alpha-beta T cell activation	1,46E-03	[6, 7]	15,00	53,57	83,83	16,17	[CRTAM, EOMES, LILRB1, LILRB4, NCKAP1L, PTPN22, RUNX3, SOCS1, TNFSF8, TOX, WDFY4, XCL1]	[HFE, VSIR, ZBTB7B]
GO:1902105	regulation of leukocyte differentiation	4,19E-15	[4, 5, 6, 7, 8, 9]	97,00	33,92	65,84	34,16	[ADAM8, BTK, C1QC, CAMK4, CARD11, CASP8, CCL19, CCL3, CCR2, CD2, CD27, CD4, CD74, CD80, CD86, CDKN2A, CRTAM, CTLA4, EGR3, EVI2B, FCRL3, FGL2, FOXP3, GPR55, HCLS1, HLA-DOA, HLA-DRA, HLA-DRB1, IFNG, IKZF3, IL12RB1, IL18, IL7R, INPP5D, IRF4, LAG3, LEF1, LILRB1, LILRB4, LRRC17, MYB, NCKAP1L, NFAM1, NLRP3, PRDM1, PTPN6, PTPRC, RHOH, RUNX3, SASH3, SLAMF8, SOCS1, SYK, TBX21, TESPA1, TNFRSF18, TOX, TREM2, VNN1, ZAP70, ZNF683]	[ABL1, ADIPOQ, AGER, AP3D1, BCL6, CD74, CEACAM1, CLPTM1, CSF1, CYP26B1, DTX1, ERBB2, FADD, FES, GATA2, GPR137, HSF1, IL15RA, IL4R, IL7R, INHA, JAK3, KLF10, LAG3, LGALS9, LRRC17, LTF, MYC, NRARP, RARA, RMRP, SOX12, SOX13, TFE3, TNF, TRIB1, VNN1, VSIR, ZBTB7B, ZC3H12A, ZMIZ1]
GO:0042129	regulation of T cell proliferation	4,97E-08	[6, 7, 8]	60,00	32,97	76,00	24,00	[AIF1, CARD11, CCDC88B, CCL19, CCL5, CCR2, CD3E, CD40LG, CD6, CD80, CD86, CDKN2A, CLCF1, CORO1A, CRTAM, CTLA4, EBI3, EPX, FOXP3, HLA-DMB, HLA-DPA1, HLA-DPB1, HLA-DRB1, IL12RB1, IL18, LILRB1, LILRB4, LMO1, NCKAP1L, PLA2G2D, PRKCQ, PTPN22, PTPN6, PTPRC, PYCARD, RAC2, RASAL3, SASH3, SPN, SYK, TNFSF13B, TNFSF8, XCL1, ZAP70]	[AGER, CD276, CLC, CLCF1, DLG5, ERBB2, FADD, GPAM, IDO1, IL1B, JAK3, LGALS9, LRRC32, RAC2, SLC7A1, TIMP1, VSIR, ZBTB7B]
GO:0046634	regulation of alpha-beta T cell activation	4,95E-09	[6, 7, 8]	44,00	41,12	77,55	22,45	[CCL19, CCR2, CD3E, CD80, CD86, CRTAM, EBI3, FOXP3, HLA-DRA, HLA-DRB1, IFNG, IL12RB1, IL18, IRF4, LILRB1, LILRB4, MYB, NCKAP1L, NLRP3, PRDM1, PRKCQ, PTPN22, PTPRC, RASAL3, RUNX3, SASH3, SOCS1, SYK, TBX21, XCL1, ZAP70, ZNF683]	[AGER, AP3D1, BCL6, GNAO1, HFE, IL4R, JAK3, LGALS9, RARA, VSIR, ZBTB7B, ZC3H12A]
GO:1902106	negative regulation of leukocyte differentiation	1,66E-03	[4, 5, 6, 7, 8, 9, 10]	34,00	32,69	49,68	50,32	[C1QC, CCL3, CD74, CDKN2A, CTLA4, FGL2, FOXP3, GPR55, INPP5D, LAG3, LILRB1, LILRB4, LRRC17, RUNX3, SOCS1, TBX21]	[ADIPOQ, BCL6, CD74, CEACAM1, DTX1, ERBB2, GATA2, GPR137, IL4R, INHA, JAK3, LAG3, LRRC17, LTF, MYC, NRARP, RARA, RMRP, TRIB1, ZBTB7B, ZC3H12A]
GO:0045619	regulation of lymphocyte differentiation	9,57E-15	[5, 6, 7, 8, 9, 10, 11]	71,00	39,66	73,36	26,64	[ADAM8, BTK, CAMK4, CARD11, CCL19, CCR2, CD2, CD27, CD74, CD80, CD86, CDKN2A, CRTAM, CTLA4, EGR3, FCRL3, FGL2, FOXP3, HLA-DOA, HLA-DRA, HLA-DRB1, IFNG, IKZF3, IL12RB1, IL18, IL7R, INPP5D, IRF4, LAG3, LEF1, LILRB4, MYB, NCKAP1L, NFAM1, NLRP3, PRDM1, PTPN6, PTPRC, RHOH, RUNX3, SASH3, SLAMF8, SOCS1, SYK, TBX21, TESPA1, TNFRSF18, TOX, VNN1, ZAP70, ZNF683]	[ABL1, AP3D1, BCL6, CD74, CLPTM1, CYP26B1, DTX1, ERBB2, IL15RA, IL4R, IL7R, INHA, JAK3, LAG3, LGALS9, NRARP, RARA, SOX12, SOX13, VNN1, VSIR, ZBTB7B, ZC3H12A, ZMIZ1]
GO:2000514	regulation of CD4-positive, alpha-beta T cell activation	4,57E-05	[7, 8, 9]	28,00	41,18	73,23	26,77	[CCL19, CD80, CD86, FOXP3, HLA-DRA, HLA-DRB1, IFNG, IL12RB1, IL18, IRF4, MYB, NCKAP1L, NLRP3, PRKCQ, RUNX3, SASH3, SOCS1, TBX21, XCL1]	[AGER, BCL6, IL4R, JAK3, LGALS9, RARA, VSIR, ZBTB7B, ZC3H12A]
GO:2001185	regulation of CD8-positive, alpha-beta T cell activation	1,67E-02	[7, 8, 9]	11,00	57,89	77,55	22,45	[CRTAM, LILRB1, LILRB4, NCKAP1L, PTPN22, RUNX3, SOCS1, XCL1]	[HFE, VSIR, ZBTB7B]
GO:0045580	regulation of T cell differentiation	2,55E-13	[6, 7, 8, 9, 10, 11, 12]	62,00	40,79	72,16	27,84	[ADAM8, CAMK4, CARD11, CCL19, CCR2, CD2, CD27, CD74, CD80, CD86, CDKN2A, CRTAM, CTLA4, EGR3, FGL2, FOXP3, HLA-DOA, HLA-DRA, HLA-DRB1, IFNG, IL12RB1, IL18, IL7R, IRF4, LAG3, LEF1, LILRB4, MYB, NCKAP1L, NLRP3, PRDM1, PTPRC, RHOH, RUNX3, SASH3, SOCS1, SYK, TBX21, TESPA1, TNFRSF18, TOX, VNN1, ZAP70, ZNF683]	[ABL1, AP3D1, BCL6, CD74, CLPTM1, CYP26B1, DTX1, ERBB2, IL4R, IL7R, JAK3, LAG3, LGALS9, NRARP, RARA, SOX12, SOX13, VNN1, VSIR, ZBTB7B, ZC3H12A, ZMIZ1]
GO:0007275	multicellular organism development	7,51E-10	[2, 3]	944,00	16,40	35,83	64,17	[ACP5, ADAM8, ADAMTS4, ADAMTS9, ADGRG6, AIF1, AKNA, ALOX15, ALOX15B, ANGPT1, ANGPT2, ANGPTL6, ANPEP, APBA2, AQP5, AREG, ASCL2, ASGR2, ATF3, BATF, BCAS1, BCL11B, BCL3, BFSP2, BHLHA15, BHLHE22, BHLHE40, BICDL1, BLK, BLNK, BMP7, BMPR1B, BRIP1, BTG2, BTK, C1QA, C1QB, C1QC, C1QTNF1, CALCR, CAMK4, CARD11, CASP8, CCL17, CCL19, CCL2, CCL24, CCL3, CCR2, CCR3, CCR4, CD19, CD2, CD27, CD3D, CD3E, CD3G, CD4, CD40LG, CD74, CD79A, CD80, CD86, CD8A, CDH1, CDH4, CDKN2A, CEL, CERS3, CHRDL1, CHRDL2, CLCF1, CNR1, COL22A1, COL9A2, CR2, CRABP2, CRB2, CREM, CRTAM, CSF1R, CST7, CTLA4, CXCL10, CXCR3, CXCR4, CYBB, DAW1, DCC, DOCK10, DOCK2, DOK2, DPEP1, DPEP2, DRC1, DRD1, DUSP2, E2F8, EAF2, EDN2, EGR3, ELAVL3, ELAVL4, ELF3, EMB, ENKUR, EOMES, EPHA6, EPX, EREG, EVI2B, FASLG, FCRL3, FGL2, FGR, FLT3, FOLR2, FOSL1, FOXP3, FRZB, FUT7, GADD45B, GCNT3, GDF6, GFI1, GFRA2, GPM6A, GPR171, GPR4, GPR55, GRXCR2, H3C10, H3C11, H3C12, H3C7, HCLS1, HCN1, HLA-DOA, HLA-DRA, HLA-DRB1, HOPX, HORMAD1, ICOS, IFNG, IKZF1, IKZF3, IL12RB1, IL18, IL7R, INPP5D, IRF4, IRF8, IRX3, ITGA4, ITGAX, ITGB2, ITK, JPH1, KCNJ10, KLF5, KLHL41, KLRC1, KRT85, LAG3, LCK, LCP1, LDLR, LEF1, LIF, LILRB1, LILRB4, LIPM, LMO1, LPAL2, LRFN1, LRRC17, LST1, LTA, LTB, LY9, MAFF, MCOLN3, MIXL1, MMP9, MOG, MS4A1, MSLN, MSX1, MT1G, MUSK, MYB, MYCBPAP, MYCL, MYH6, MYO3B, NAMPT, NCKAP1L, NELL2, NFAM1, NLRP14, NLRP3, NME8, NPPB, NPPC, NR4A1, NR4A2, NR4A3, NTN1, OPN5, P2RY12, PAX5, PBX4, PCDHA2, PIK3CG, PIM1, PLA2G2D, PLAAT4, PLD4, PLEK, PLPPR4, PLXNC1, POU2AF1, POU2F2, PRDM1, PRKCB, PRKCQ, PROK1, PTK2B, PTPN22, PTPN6, PTPRC, PTX3, RAC2, RASGRF1, RBPMS2, RGS9, RHEX, RHOH, RLN2, RNASE6, RRAD, RRM2, RTKN2, RUNX3, SALL1, SASH3, SCUBE1, SDC1, SELPLG, SEMA4A, SEMA4D, SH2D2A, SLAMF6, SLAMF8, SLC24A4, SLC8A3, SMPD3, SNX10, SOCS1, SOCS3, SPEF2, SPIC, SPN	[AATK, ABCA3, ABCC8, ABCD1, ABCD2, ABI3, ABL1, ACACB, ACKR3, ACP4, ACSBG1, ACTA1, ACTB, ACTC1, ACTN1, ADAM19, ADAMTS4, ADAMTS5, ADAMTS7, ADAMTS9, ADCY1, ADCY9, ADGRB2, ADGRG1, ADGRG6, ADGRL1, ADIPOQ, AGAP2, AGER, AGT, AGTR1, ALPK3, ALPL, AMH, ANGPT1, ANGPTL1, ANGPTL2, ANGPTL4, ANKRD52, AP1B1, AP2A1, AP3D1, APLN, APLNR, APLP1, APOA1, APOD, AQP3, AQP5, ARC, AREG, ARF1, ARHGDIA, ARHGEF15, ARID1A, ARMC5, ARRDC3, ARSA, ARTN, ASPN, ATAT1, ATF5, ATN1, ATP1B2, ATP6V0D1, ATP6V1B1, BAG3, BAG6, BATF2, BCL2L1, BCL3, BCL6, BCL9L, BCOR, BCR, BEND6, BGN, BHLHE40, BMP8A, BSN, BTBD2, BTG2, C1QTNF1, C5, C5AR1, CACNA1H, CALB1, CALCR, CAMK2B, CAMSAP3, CAPN1, CAPNS1, CARM1, CASZ1, CCDC120, CCDC85C, CCL11, CCL2, CCL24, CCR7, CD74, CDH4, CDK1, CDK2, CDKN1A, CEACAM1, CECR2, CEL, CELSR2, CERT1, CFH, CFL1, CGA, CHERP, CHGA, CHI3L1, CHRDL2, CIC, CITED2, CLC, CLCF1, CLPTM1, CMIP, CNNM4, CNOT3, CNR1, CNTN5, CNTN6, COASY, COL1A1, COL22A1, COL27A1, COL6A1, CPNE5, CPNE6, CRAT, CREB3L1, CRIP2, CRYAB, CSF1, CSK, CSNK1E, CSPG4, CST7, CTC1, CTDSP1, CTF1, CTHRC1, CX3CL1, CXXC4, CYP21A2, CYP26B1, CYP27B1, DAB2IP, DAG1, DAGLA, DCHS1, DDR1, DDX39B, DEDD, DGKD, DGUOK, DHRS2, DISP3, DKK2, DLG5, DMC1, DMRTA1, DMTN, DNMT1, DOK5, DPF2, DPYSL4, DRC1, DSC1, DSCAML1, DTX1, DUSP5, DVL3, DXO, DYRK1B, E2F4, E2F7, E2F8, EAF2, EBF4, ECM1, EDN2, EFEMP2, EFNA3, EHD1, EIF4EBP1, EIF4G1, ELF3, ELK1, ELOVL1, ENDOG, ENG, EPHA2, EPN1, EPOP, ERBB2, ESM1, ETS2, ETV6, EXTL1, FA2H, FADD, FAIM2, FANCC, FAP, FASN, FES, FGFR4, FGFRL1, FHL3, FLG, FLII, FLT3, FLT4, FOSL1, FOSL2, FOXC2, FOXF1, FOXS1, FURIN, GAB2, GADD45B, GATA2, GATA4, GBA, GCNT3, GDF6, GDI1, GDPD5, GFRA3, GIT1, GJA4, GJD3, GLIS2, GMNN, GNAI2, GNAO1, GNRHR, GPER1, GPI, GPM6A, GPR137, GPR37L1, GPR4, GPRIN1, GRB7, GRN, GSK3A

GO:0009653	anatomical structure morphogenesis	2,29E-06	[2, 3]	493,00	17,35	31,64	68,36	[ACP5, ADAM8, ADAMTS9, ADGRG6, ANGPT1, ANGPT2, ANGPTL6, ANPEP, AQP5, AREG, BCL11B, BCL3, BMP7, BMPR1B, CARMIL2, CASP8, CASS4, CCDC88C, CCL2, CCL24, CCL3, CCR2, CCR3, CD53, CDH4, CEL, COL22A1, CORO1A, CPM, CRABP2, CRB2, CSF1R, CXCL10, CXCL9, CXCR3, CXCR4, CYBB, DCC, DOCK10, DOK2, DUSP2, E2F8, EGR3, ELAVL4, ELF3, EMB, EOMES, EPHA6, EREG, ERMN, FASLG, FGD2, FGD3, FGR, FOLR2, FRZB, GCNT3, GFRA2, GPM6A, GPR4, GRXCR2, HCN1, HOPX, IER3, IL18, IL7R, IRX3, ITGA4, ITGAX, ITGB2, ITGB7, KLF5, KLHL41, LCP1, LDLR, LEF1, LIF, LST1, MIXL1, MMP9, MSX1, MYH6, MYO3B, NCKAP1L, NELL2, NPPB, NPPC, NR4A1, NR4A2, NR4A3, NTN1, OPN5, PARVG, PAX5, PBX4, PEAK3, PIK3CG, PIM1, PLPPR4, PLXNC1, PRDM1, PRKCB, PRKCQ, PROK1, PTK2B, PTPN6, RAC2, RBPMS2, RHOH, RLN2, SALL1, SDC1, SEMA4A, SEMA4D, SH2D2A, SLC18A1, SLC24A4, SMPD3, SNX10, SOCS3, SPEF2, ST14, STAT1, SYK, TACSTD2, TBX21, THBS1, THY1, TIFAB, TNFAIP3, TNFRSF11B, TNFSF13B, TNMD, TNR, TREM2, UGT8, VSIG1, WNT1, WNT10A, WNT10B]	[ABCC8, ABI3, ABL1, ACKR3, ACP4, ACRBP, ACTA1, ACTB, ACTC1, ACTN1, ACTN4, ADAMTS5, ADAMTS9, ADCK1, ADCY1, ADGRB2, ADGRG1, ADGRG6, ADIPOQ, AGT, AGTR1, ALDOA, ALPL, AMH, ANGPT1, ANGPTL1, ANGPTL2, ANGPTL4, AP2A1, APLN, APLNR, APLP1, APOA1, APOD, AQP3, AQP5, ARC, AREG, ARHGDIA, ARHGEF15, ARMC5, ARTN, ASPN, ATP6V1B1, BCL2L1, BCL3, BCL6, BCL9L, BCOR, C11orf65, C15orf62, C5, C5AR1, CACNA1H, CALB1, CAMK2B, CAMSAP3, CAPN1, CARMIL2, CCL11, CCL2, CCL24, CDC42EP1, CDC42EP2, CDC42EP4, CDH4, CEACAM1, CECR2, CEL, CELSR2, CERT1, CFH, CFL1, CHI3L1, CITED2, CNNM4, CNOT3, CNTN6, COL1A1, COL22A1, COL27A1, COL6A1, CPNE5, CPNE6, CREB3L1, CRISPLD1, CRYAB, CSF1, CSPG4, CTHRC1, CX3CL1, CYP26B1, CYP27B1, DAB2IP, DAG1, DCHS1, DDR1, DLG5, DMTN, DOK5, DPYSL4, DSCAML1, DUSP5, DVL3, DYRK1B, E2F4, E2F7, E2F8, ECM1, EFEMP2, EFNA3, EHD1, EHD2, ELF3, ENG, EPHA2, EPN1, ERBB2, ESM1, ETS2, FAIM2, FANCC, FAP, FBLIM1, FES, FGFRL1, FLII, FLT4, FMNL1, FOXC2, FOXF1, FOXS1, FURIN, GAB2, GATA2, GATA4, GBA, GCNT3, GDI1, GFRA3, GJD3, GMNN, GNAO1, GPI, GPM6A, GPR4, GRB7, GRN, GSK3A, HBEGF, HDAC5, HDAC7, HESX1, HEYL, HGS, HIRA, HMGN1, HOPX, HPRT1, HSPB1, HSPG2, HYAL1, ICAM1, ID3, IER3, IFT80, IGF2BP2, IL1B, IL1RN, IL4R, IL7R, ILK, INPPL1, IRX3, ITGA3, ITGA5, ITGA7, JMJD8, JUNB, JUP, KCNQ4, KDM6B, KLF5, KLK14, LAMA5, LAMB4, LDLR, LGR6, LIF, LIMK1, LIMS2, LINGO1, LMX1A, LOX, LRG1, LTF, LY6E, LYPLA2, LZTS1, LZTS2, LZTS3, MAP1A, MAP1S, MAPK3, MAPK7, MARK2, MBOAT7, MECP2, MED12, MEF2D, MEGF8, MEIS3P1, MEIS3P2, MEN1, MFAP2, MFAP5, MFSD2A, MICALL2, MINK1, MMP15, MOV10, MSN, MSX1, MTHFR, MYADM, MYC, MYH6, MYH7B, MYH9, MYO18B, MYO19, MYO3A, MYO7A, NBEAL2, NDST1, NECTIN1, NECTIN2, NEFL, NF2, NFATC4, NFKB2, NGF, NGFR, NINJ1, NODAL, NOL6, NOS3, NOTCH1, NOTCH3, NOTCH4, NPPA, NPPB, NPRL3, NR4A1, NR4A2, NR4A3, NRARP
GO:0050793	regulation of developmental process	1,56E-12	[2, 3]	488,00	18,90	38,56	61,44	[ADAM8, ADAMTS9, ALOX15B, ANGPT2, AREG, ASCL2, BCL11B, BHLHA15, BHLHE40, BMP7, BMPR1B, BTK, C1QC, CAMK4, CARD11, CASP8, CASS4, CCL17, CCL19, CCL2, CCL24, CCL3, CCR2, CCR3, CD2, CD27, CD4, CD53, CD74, CD80, CD86, CDH4, CDKN2A, CLCF1, CMTM5, CORO1A, CRABP2, CRB2, CRTAM, CSF1R, CST7, CTLA4, CXCL10, CXCL9, CXCR4, CYBB, DCC, DPEP1, EAF2, EGR3, ELAVL4, EPX, EREG, ERMN, EVI2B, FASLG, FCRL3, FFAR4, FGD2, FGD3, FGL2, FGR, FOXP3, FRZB, GDF6, GLIS1, GPR171, GPR4, GPR55, H3C10, H3C11, H3C12, H3C7, HCLS1, HLA-DOA, HLA-DRA, HLA-DRB1, HOPX, IFNG, IKZF3, IL12RB1, IL18, IL7R, INPP5D, IRF4, IRX3, ITGAX, ITGB2, KLF5, KLHL41, LAG3, LCP1, LDLR, LEF1, LIF, LILRB1, LILRB4, LMO1, LRRC17, LST1, LTA, MAFF, MEDAG, MIXL1, MMP9, MSX1, MUSK, MYB, MYCL, MYH6, NAMPT, NCKAP1L, NEK5, NFAM1, NLRP3, NPPB, NPPC, NR4A3, NTN1, P2RY12, PEAK3, PIM1, PLAAT4, PLAC8, PLXNC1, PRDM1, PRKCB, PRKCQ, PROK1, PTK2B, PTPN6, PTPRC, RAC2, RBPMS2, RHEX, RHOH, RLN2, RUNX3, SASH3, SEMA4A, SEMA4D, SLAMF8, SLC18A1, SMPD3, SOCS1, SOCS3, STAT1, SULT2B1, SYK, TACSTD2, TBX21, TESPA1, THBS1, THY1, TIFAB, TNFAIP3, TNFRSF11B, TNFRSF18, TNFSF13B, TNMD, TNR, TOX, TREM2, VNN1, VWC2L, WNT1, WNT10A, WNT10B, ZAP70, ZBED2, ZNF683]	[ABCC8, ABI3, ABL1, ACACB, ACTN4, ADAMTS7, ADAMTS9, ADCK1, ADGRB2, ADGRL1, ADIPOQ, AGAP2, AGER, AGT, AGTR1, ALDOA, AMH, ANGPTL4, AP2A1, AP3D1, APLNR, APOA1, AQP3, ARC, AREG, ARF1, ARHGDIA, ARHGEF15, ASPN, ATAT1, BAG6, BBS12, BCL6, BCL9L, BCOR, BEND6, BHLHE40, C11orf65, C15orf62, C5, C5AR1, CAMK2B, CARM1, CASZ1, CCL11, CCL2, CCL24, CD74, CDC42EP1, CDC42EP2, CDC42EP4, CDH4, CDK1, CDKN1A, CEACAM1, CELSR2, CETP, CFL1, CGA, CHERP, CHGA, CHI3L1, CITED2, CLCF1, CLPTM1, CMTM5, CNOT3, COL1A1, CPNE5, CPNE6, CREB3L1, CRP, CSF1, CSNK1E, CST7, CTDSP1, CTHRC1, CX3CL1, CYP26B1, CYP27B1, DAB2IP, DAG1, DDX39B, DISP3, DLG5, DMTN, DNMT1, DPF2, DTX1, DVL3, DXO, EAF2, ECM1, EEF1E1, EFEMP2, EFNA3, EHD1, EHD2, EIF4G1, ENG, EPHA2, EPN1, EPPK1, ERBB2, FADD, FBLIM1, FES, FLOT2, FLT4, FMNL1, FOXC2, FOXM1, FOXS1, GATA2, GATA4, GDF6, GDI1, GNB3, GPAM, GPER1, GPR137, GPR37L1, GPR4, GRN, GSK3A, H3C10, H3C12, H4C3, HDAC5, HDAC7, HESX1, HEYL, HGS, HMGA1, HMGN1, HOPX, HSF1, HSPB1, HSPG2, HTR2A, HYAL1, ICAM1, ID3, IFITM1, IL15RA, IL1B, IL4R, IL7R, ILK, INHA, IRX3, ITGA5, ITGA7, JAK3, JMJD8, JUNB, JUND, JUP, KCNK2, KCTD11, KLF10, KLF5, KMT2D, LAG3, LAMA5, LDLR, LGALS9, LIF, LIMK1, LIMS2, LINGO1, LMNA, LMX1A, LOX, LRCH4, LRG1, LRRC17, LRRC8A, LTF, LZTS1, LZTS3, MAFG, MAMSTR, MAPK7, MAPK8IP3, MARK2, MCRIP1, MECP2, MED12, MEGF8, MFSD2A, MME, MOV10, MSN, MSTN, MSX1, MYADM, MYC, MYH6, MYH7B, MYH9, MYL9, MYO19, MYRF, NAP1L2, NEFL, NEK5, NF2, NFATC4, NFE2, NGF, NGFR, NINJ1, NLGN2, NODAL, NOS3, NOTCH1, NOTCH3, NOTCH4, NPPB, NR1D1, NR4A3, NRARP, NSMF, NTN1, NUMBL, OMA1, OMD, OSM, OSR2, PACSIN1, PAF1, PAK4, PALM, PALM2AKAP2, PARP6, PARVB, PDGFB, PDLIM7, PFN1, PHLDB1, PIEZO1, PIM1, PKM, PLA2G2A, PLEKHB1, PLEKHO1, PLXNA1, PLXNA3, PLXNB1, PML, POR, POSTN, PPARD, PPP1R13L, PPP2R1A, PQBP1, PRAG1,

GO:0051239	regulation of multicellular organismal process	4,67E-22	[2, 3]	570,00	20,09	43,33	56,67	[ACP5, ADAM8, ADAMTS9, ADCY7, ADORA3, AIF1, AIM2, ALOX15B, ANGPT1, ANGPT2, AREG, ASCL2, ATP2A3, BANK1, BATF, BCL3, BHLHE40, BMP7, BMPR1B, BTK, BTN3A1, BTN3A2, C1QC, C1QTNF1, CALCR, CAMK4, CARD11, CARD17, CASP8, CCDC88B, CCL19, CCL24, CCL3, CCR2, CCR3, CD2, CD200R1, CD226, CD244, CD27, CD33, CD3E, CD4, CD40LG, CD6, CD74, CD80, CD84, CD86, CD96, CDH4, CDKN2A, CEL, CLCF1, CLEC6A, CLNK, CNN1, CNR1, CRABP2, CRB2, CRTAM, CSF1R, CST7, CTLA4, CXCL10, CXCL6, CXCR4, CYBB, DCC, DLGAP2, DPEP1, EAF2, EBI3, EDN2, EGR3, EPX, EREG, EVI2B, FASLG, FCN1, FCRL3, FFAR4, FGL2, FGR, FOXP3, FRZB, GBP5, GDF6, GPR171, GPR4, GPR55, GPSM3, H3C10, H3C11, H3C12, H3C7, HCLS1, HLA-DOA, HLA-DPA1, HLA-DPB1, HLA-DRA, HLA-DRB1, HOPX, IFNG, IKBKE, IKZF3, IL12RB1, IL12RB2, IL16, IL18, IL26, IL5RA, IL7R, INPP5D, IRF4, IRF5, IRF8, IRX3, ITGAX, ITGB2, ITK, KCNK15, KNG1, LAG3, LCP1, LDLR, LEF1, LIF, LILRB1, LILRB4, LMCD1, LPAL2, LRRC17, LTA, LTB, LY9, MAFF, MAPK13, MCOLN2, MMP9, MOG, MYB, MYCL, MYH6, NCKAP1L, NELL2, NFAM1, NLRC3, NLRP2, NLRP3, NLRP6, NLRP7, NLRP9, NPPB, NPPC, NPTX2, NR4A2, NR4A3, NTN1, ORM2, P2RY2, PIK3CG, PIM1, PLAAT4, PLAC8, PLAU, PLD4, PLXNC1, POU2AF1, POU2F2, PRDM1, PRKCB, PRKCQ, PROK1, PTAFR, PTGER2, PTK2B, PTPN22, PTPN6, PTPRC, PYCARD, PYHIN1, RHEX, RHOH, RLN2, RUNX3, SASH3, SCIMP, SEMA4A, SEMA4D, SIGLEC16, SLAMF6, SLAMF8, SLC8A3, SMPD3, SMTNL2, SOCS1, STAT1, SULT2B1, SYK, TACSTD2, TBX21, TBXAS1, TESPA1, THBS1, THY1, TIFAB, TIGIT, TLR10, TLR6, TLR8, TNFAIP3, TNFRSF11B, TNFRSF18, TNMD, TNR, TOX, TREM2, TSPAN8, UBASH3A, UCP2, VNN1, WNT1, WNT10B, XCL1, ZAP70, ZBED2, ZBP1, ZNF683]	[ABCA7, ABCC8, ABCD1, ABCD2, ABL1, ACACB, ACKR1, ADAMTS5, ADAMTS7, ADAMTS9, ADGRB2, ADIPOQ, AGAP2, AGER, AGPAT1, AGT, AGTR1, AMH, ANGPT1, ANGPTL4, AP3D1, APLN, APLNR, APOA1, APOD, APOM, AQP3, AREG, ARHGDIA, ARRDC3, ASPN, ATP1A1, ATP1A3, ATP1B2, ATPSCKMT, BAG6, BCAR1, BCL3, BCL6, BCL9L, BCOR, BHLHE40, C1QTNF1, C5, C5AR1, CACNA1H, CALCR, CAMK2B, CCL11, CCL24, CCR7, CD14, CD276, CD74, CDH4, CDK1, CEACAM1, CEL, CFH, CFL1, CGA, CHGA, CHI3L1, CITED2, CLC, CLCF1, CLNK, CLPTM1, CNN1, CNOT3, CNR1, COL1A1, CPTP, CREB3L1, CRP, CSF1, CSK, CST7, CTDSP1, CX3CL1, CYP26B1, CYP27B1, DAB2IP, DAG1, DDX39B, DHX34, DLG5, DLGAP4, DMTN, DTX1, DXO, EAF2, ECM1, EDN2, EFNA3, ENG, EPHA2, EPN1, EPPK1, ERBB2, FA2H, FABP4, FADD, FAM110A, FAP, FES, FFAR2, FLT4, FOXC2, FOXS1, FRMD8, FURIN, FXYD6, GATA2, GATA4, GBA, GDF6, GDI1, GIT1, GJD3, GLRA1, GNAI2, GNAO1, GPAM, GPER1, GPI, GPR137, GPR3, GPR37L1, GPR4, GPRIN1, GRN, GSK3A, GTF2IRD1, H3C10, H3C12, H4C3, HBEGF, HCN4, HDAC5, HDAC7, HESX1, HEYL, HFE, HGS, HK1, HOPX, HRH2, HSF1, HSPB1, HSPB7, HSPG2, HTR2A, HYAL1, ICAM1, IGF2BP2, IL15RA, IL1B, IL4R, IL5RA, IL7R, ILK, INHA, IRX3, ITGA3, ITGA5, ITPR3, JAK3, JMJD8, JPH4, JUP, KCNH2, KCNIP2, KCNJ5, KCNK15, KCNK2, KCNK3, KCNK6, KCNMB2, KCTD11, KDM6B, KLF10, KMT2D, KNG1, LAG3, LAMA5, LCAT, LDLR, LDLRAP1, LGALS9, LIF, LILRA5, LIMK1, LINGO1, LIPG, LMCD1, LMNA, LOX, LRCH4, LRG1, LRRC17, LRRC32, LTF, LUM, MAFG, MAP2K3, MAPK3, MAPK7, MAST2, MAVS, MECP2, MED12, MEGF8, MFAP2, MFSD2A, MME, MOV10, MSTN, MYBPH, MYC, MYH6, MYL9, MYRF, NEFL, NF2, NFATC4, NFE2, NFKB2, NGF, NGFR, NINJ1, NLGN2, NLRC5, NLRP9, NOD2, NODAL, NOS3, NOTCH1, NOTCH4, NOVA2, NPAS2, NPPA, NPPB, NPR3, NPRL3, NR1D1, NR4A2, NR4A3, NRARP, NTN1, NUMBL, NUTF2, OMA1, OMD, OSM, OSR2, P2RX3, P2RY2, PAF1, PAK4, PARP6, PCDHA4, PDGFB [illegible]
GO:0002684	positive regulation of immune system process	4,58E-20	[2, 3, 4]	281,00	24,16	64,49	35,51	[ADAM8, AIF1, AIM2, BLK, BLNK, BTK, BTLA, BTN3A1, BTN3A2, C1QA, C1QB, C1QC, C2, C8G, CAMK4, CARD11, CASP8, CCDC88B, CCL19, CCL2, CCL24, CCL3, CCL4, CCL5, CCR2, CD177, CD19, CD1B, CD1C, CD1E, CD2, CD22, CD226, CD244, CD247, CD27, CD3D, CD3E, CD3G, CD4, CD40LG, CD5, CD6, CD74, CD79A, CD80, CD84, CD86, CFP, CLCF1, CLEC10A, CLEC4D, CLEC4E, CLEC6A, CLNK, CNR1, CORO1A, CR2, CRTAM, CSF1R, CTLA4, CXCL10, CXorf21, CYP11B1, DOCK8, EBI3, EDN2, EGR3, EPX, EREG, EVI2B, FCN1, FCN3, FCRL3, FGR, FOXP3, FYB1, GBP5, GPSM3, GRAP2, HCLS1, HLA-DMB, HLA-DPA1, HLA-DPB1, HLA-DQA1, HLA-DRA, HLA-DRB1, HLA-DRB5, ICOS, IFNG, IGLL5, IKBKE, IL12RB1, IL18, IL18RAP, IL7R, INPP5D, ITGA4, ITGB2, ITK, KCNN4, KLRC2, KLRD1, KLRK1, LAG3, LAX1, LCK, LCP2, LEF1, LILRB1, LILRB4, LTA, MOG, MS4A1, MUC16, MUC19, MUC6, MYB, MYO1G, MZB1, NCKAP1L, NCR3, NFAM1, NLRP3, NR4A3, P2RY12, PAX5, PLA2G7, PRAM1, PRKCB, PRKCQ, PTAFR, PTK2B, PTPN22, PTPN6, PTPRC, PYCARD, PYHIN1, RAC2, RASAL3, RHOH, RUNX3, SASH3, SCIMP, SH2D1A, SIGLEC16, SIRPG, SKAP1, SLA2, SLAMF6, SOCS1, SPN, STAP1, STXBP2, SYK, TBX21, TESPA1, THBS1, THEMIS, THEMIS2, THY1, TLR6, TLR8, TNFRSF18, TNFSF13B, TNFSF14, TOX, TRAT1, TREM2, UBASH3A, VAV1, VNN1, WAS, XCL1, XCL2, XG, ZAP70, ZBP1]	[ABL1, ACTB, AGER, AMH, AP3D1, AQP3, BAG6, BCAR1, BCL6, BTLA, C5, C5AR1, C8G, CCL2, CCL21, CCL24, CCR7, CD177, CD1C, CD1E, CD276, CD74, CDKN1A, CEACAM1, CFH, CLCF1, CLEC4E, CLNK, CNR1, CPN2, CRP, CSF1, CSK, CX3CL1, DGKZ, EDN2, ENPP3, FADD, FCN3, FES, FFAR2, FLOT2, FOXF1, FPR1, GAB2, GATA2, GCSAM, GNAO1, GPAM, GPI, GRN, HAVCR1, HK1, HSF1, ICAM1, IL15RA, IL1B, IL4R, IL7R, JAK3, JUND, KLF10, LAG3, LGALS9, LIMK1, MAPK3, MAVS, MSTN, MUC19, MUC20, MUC3A, MYO18A, NCOR2, NECTIN2, NLRC5, NOD2, NPPA, NR4A3, PCDHA4, PDCD1, PLA2G7, PLVAP, PQBP1, PRKACA, PTN, PVR, RAC2, RAP1A, RARA, RBM14, RELA, RELB, RGCC, RHBDF2, RNU1-93P, SELENOP, SERPINE1, SERPING1, SH2B2, SLC7A1, SLC7A5, SLC7A8, SOX12, SOX13, SPHK2, SPI1, SRC, STING1, THBS1, TICAM1, TIMP1, TNF, TRIB1, TRIM62, TRPV4, VEGFA, VNN1, VSIR, VTN, WAS, XG, ZBTB7B, ZC3H12A, ZCCHC3, ZMIZ1, ZNF335, ZNF580]

GO:0030154	cell differentiation	4,96E-11 [3]	766,00	17,12	39,27	60,73	[ADAM8, ADGRG6, AKNA, ALOX15B, ANGPT1, ANGPT2, ANGPTL6, ANPEP, AREG, ASCL2, ATF3, BATF, BCL11B, BCL3, BFSP2, BHLHA15, BHLHE22, BHLHE40, BICDL1, BLK, BLNK, BMP7, BMPR1B, BRIP1, BTG2, BTK, C1QA, C1QC, CALCR, CAMK4, CARD11, CASP8, CASS4, CCDC88C, CCL17, CCL19, CCL2, CCL3, CCR2, CCR4, CD19, CD2, CD27, CD3D, CD3E, CD3G, CD4, CD40LG, CD53, CD74, CD79A, CD80, CD86, CD8A, CDH1, CDH4, CDKN2A, CERS3, CFAP54, CHRDL1, CHRDL2, CLCF1, CMTM5, CNR1, COL22A1, CR2, CRABP2, CRB2, CREM, CRTAM, CSF1R, CTLA4, CXCL10, CXCL9, CXCR4, DCC, DHRS9, DOCK10, DOCK2, DOK2, DPEP1, DRD1, E2F8, EGR3, ELAVL3, ELAVL4, ELF3, EMB, EOMES, EPHA6, EPX, EREG, EVI2B, FASLG, FCRL3, FCRLA, FFAR4, FGL2, FGR, FLT3, FOLR2, FOXP3, FRZB, FUT7, GADD45B, GDF6, GFI1, GFRA2, GLIS1, GPM6A, GPR171, GPR4, GPR55, GRXCR2, H3C10, H3C11, H3C12, H3C7, HCLS1, HCN1, HLA-DOA, HLA-DRA, HLA-DRB1, HOPX, HORMAD1, IFNG, IKZF1, IKZF3, IL12RB1, IL18, IL7R, INPP5D, IRF4, IRF8, IRX3, ITGA4, ITGB2, ITGB7, ITK, KCNJ10, KLF5, KLHL41, KLRC1, KLRK1, KRT85, LAG3, LCK, LCP1, LDLR, LEF1, LIF, LILRB1, LILRB4, LIPM, LMO1, LRRC17, LST1, LTA, LY9, M1AP, MAFF, MCOLN3, MEDAG, MIXL1, MMP9, MREG, MS4A1, MSX1, MT1G, MUSK, MYB, MYCBPAP, MYCL, MYH6, NCKAP1L, NEK5, NELL2, NFAM1, NLRP14, NLRP3, NME8, NPPC, NR4A1, NR4A2, NR4A3, NTN1, P2RY12, PARVG, PAX5, PBX4, PIM1, PLA2G2D, PLAAT4, PLAC8, PLD4, PLEK, PLPPR4, PLXNC1, POU2AF1, POU2F2, PRDM1, PRKCB, PRKCQ, PTK2B, PTPN22, PTPN6, PTPRC, PTX3, RAC2, RASGRF1, RBPMS2, RHEX, RHOH, RIMBP3B, RUNX3, SALL1, SASH3, SCUBE1, SDC1, SEMA4A, SEMA4D, SH2D2A, SLA, SLAMF6, SLAMF8, SLC8A3, SLFN14, SMPD3, SNX10, SOAT2, SOCS1, SOCS3, SPEF2, SPIB, SPIC, SPINK1, SPN, ST14, STAP1, STAT1, STYK1, SULT2B1, SYK, TACSTD2, TBX21, TESPA1, THBS1, THEMIS, THY1, TMEM132E, TNFRSF13B, TNFRSF18, TNFSF13B, TNFSF8, TNMD, TNR, TOX	[ABCC8, ABI3, ABL1, ACKR3, ACP4, ACRBP, ACTA1, ACTB, ACTC1, ACTN1, ACTN4, ADAMTS5, ADAMTS7, ADAMTSL4, ADCY1, ADGRG1, ADGRG6, ADIPOQ, AGER, AGT, AGTR1, ALPK3, ALPL, AMH, ANGPT1, ANGPTL8, ANKRD52, AP2A1, AP3D1, APLNR, APOA1, APOD, AQP3, ARC, AREG, ARF1, ARHGDIA, ARMC5, ARTN, ATAT1, ATF5, ATP1B2, BAG6, BATF2, BBS12, BCL2L1, BCL3, BCL6, BCL7B, BCL9L, BCR, BEND6, BHLHE40, BMP8A, BTBD2, BTG2, C5AR1, CACNA1H, CALCR, CAMK2B, CAMSAP3, CAPN1, CAPNS1, CARM1, CASZ1, CCDC87, CCL11, CCL2, CCL21, CCR7, CD74, CDC25B, CDH4, CDK1, CDK2, CDKN1A, CEACAM1, CECR2, CELSR2, CETP, CFAP157, CFAP54, CFH, CFL1, CHERP, CHRDL2, CITED2, CLCF1, CLIP3, CLPTM1, CMTM5, CNOT3, CNR1, CNTN6, COL1A1, COL22A1, COL27A1, COL6A1, CPNE5, CPNE6, CRAT, CREB3L1, CRP, CSF1, CSK, CSNK1E, CSPG4, CTDSP1, CTF1, CTHRC1, CX3CL1, CYP21A2, CYP26B1, CYP27B1, DAB2IP, DAG1, DAGLA, DCHS1, DDR1, DDX39B, DGUOK, DHRS2, DHRS9, DISP3, DLG5, DMC1, DMRTA1, DMTN, DNMT1, DOK5, DPF2, DPYSL4, DSC1, DSCAML1, DTX1, DVL3, DYRK1B, DYSF, E2F4, E2F7, E2F8, ECM1, EFEMP2, EFNA3, EHD1, EHD2, EIF4G1, ELF3, ELF4, ELK1, ENG, EPHA2, EPOP, ERBB2, ERF, ETS2, ETV6, FA2H, FABP4, FADD, FAIM2, FANCC, FASN, FES, FFAR2, FLG, FLII, FLOT2, FLT3, FLT4, FOSL2, FOXC2, FOXF1, FOXS1, FURIN, GAB2, GADD45B, GAS1, GATA2, GATA4, GBA, GDF6, GDI1, GFRA3, GIT1, GJD3, GLIS2, GNAO1, GNB3, GORASP2, GPER1, GPM6A, GPR137, GPR37L1, GPR4, GPRIN1, GRB7, GRN, GSK3A, H1-9P, H3C10, H3C12, H4C3, HDAC10, HDAC5, HDAC7, HESX1, HEYL, HID1, HIVEP3, HOPX, HPRT1, HSF1, HSPE1, HSPG2, HTR2A, ICAM1, ID3, IFITM1, IFT80, IL15RA, IL1B, IL4R, IL7R, ILK, INHA, INPP5J, IRX3, IRX6, ITGA3, ITGA5, ITGA7, JAK3, JUNB, JUND, JUP, KATNB1, KCNIP2, KCP, KCTD11, KDELR1, KDM6B, KIAA1522, KLF10, KLF5, KLK14, KMT2D, KRTAP5-1, LAG3, LAMA5, LAMB4, LDLR, LGALS9, LGR6, LIF, LIMK1, LIMS2, LINGO1, LMNA, LMX1A, LMX1B, LOX
GO:0048646	anatomical structure formation involved in morphogenesis	2,44E-07 [2, 3, 4]	237,00	20,26	33,83	66,17	[ADAM8, ADAMTS9, ADGRG6, ANGPT1, ANGPT2, ANGPTL6, ANPEP, AREG, BCL3, BMP7, CASP8, CCL2, CCL24, CCR2, CCR3, CD53, CEL, COL22A1, CRB2, CXCL10, CXCL9, CXCR3, CXCR4, CYBB, DUSP2, E2F8, EGR3, EOMES, EREG, FASLG, FOLR2, GPR4, HOPX, IL18, IRX3, ITGA4, ITGAX, ITGB2, KLF5, KLHL41, LEF1, MIXL1, MMP9, MSX1, MYH6, NELL2, NPPB, NR4A1, PIK3CG, PIM1, PRKCB, PROK1, PTK2B, PTPN6, RLN2, SALL1, SEMA4A, SH2D2A, SLC24A4, SMPD3, ST14, STAT1, SYK, THBS1, THY1, TIFAB, TNFAIP3, TNFSF13B, TNMD, TREM2, UGT8, WNT1, WNT10B]	[ABCC8, ABL1, ACKR3, ACRBP, ACTA1, ACTC1, ACTN1, ADAMTS5, ADAMTS9, ADGRB2, ADGRG1, ADGRG6, AGT, AGTR1, ANGPT1, ANGPTL1, ANGPTL2, ANGPTL4, APLN, APLNR, APOD, AREG, ARMC5, BCL3, BCL6, C5, C5AR1, CACNA1H, CALB1, CCL11, CCL2, CCL24, CEACAM1, CECR2, CEL, CHI3L1, CITED2, CNNM4, CNOT3, COL1A1, COL22A1, COL6A1, CREB3L1, CSPG4, CTHRC1, CX3CL1, DAB2IP, DAG1, DCHS1, DUSP5, DVL3, DYRK1B, E2F7, E2F8, ECM1, EFNA3, EHD1, EHD2, ENG, EPHA2, EPN1, ERBB2, ESM1, ETS2, FAIM2, FAP, FLII, FLT4, FOXC2, FOXF1, FURIN, GATA2, GATA4, GBA, GPI, GPR4, GRN, HDAC5, HDAC7, HESX1, HEYL, HGS, HOPX, HSPB1, HSPG2, HYAL1, IL1B, IL4R, ILK, IRX3, ITGA3, ITGA5, ITGA7, JMJD8, JUNB, JUP, KDM6B, KLF5, LRG1, MAPK3, MAPK7, MBOAT7, MECP2, MED12, MEN1, MMP15, MSX1, MTHFR, MYH6, MYH9, NBEAL2, NECTIN1, NECTIN2, NF2, NFATC4, NFKB2, NGFR, NINJ1, NODAL, NOL6, NOS3, NOTCH1, NOTCH4, NPPB, NR4A1, NRARP, OBSCN, PAF1, PAK4, PDGFRB, PEF1, PFN1, PHLDB1, PIM1, PKM, PLCD3, PLEKHO1, PML, PNLDC1, PRKACA, PTN, RAMP2, RARA, RELT, RFX2, RGCC, RGMA, ROBO4, S100A1, SBNO2, SDK2, SEMA4C, SERPINE1, SHANK3, SMAD3, SNAI1, SOX7, SOX8, SPHK1, SRF, SSBP3, STAT3, TBX5, TCIRG1, TCOF1, TEAD2, TEAD4, TGM2, THBS1, TMEM100, TNF, TNFAIP2, TNFSF12, TNMD, TSC2, UNC5B, VANGL2, VEGFA, VTN, WARS1, WHRN, WNT3, WNT9B, ZC3H12A, ZNF219]
GO:0051093	negative regulation of developmental process	5,70E-04 [2, 3, 4]	183,00	19,55	38,59	61,41	[ADAMTS9, ANGPT2, AREG, ASCL2, BHLHA15, BMP7, C1QC, CCL17, CCL3, CCR2, CD74, CDKN2A, CMTM5, CTLA4, CXCL10, DCC, DPEP1, EAF2, EREG, FASLG, FGL2, FOXP3, FRZB, GLIS1, GPR171, GPR4, GPR55, IFNG, IL18, INPP5D, IRX3, LAG3, LDLR, LEF1, LILRB1, LILRB4, LRRC17, MIXL1, MMP9, MSX1, MYB, NAMPT, NPPB, NPPC, NTN1, P2RY12, PLAC8, PTK2B, RBPMS2, RUNX3, SEMA4A, SEMA4D, SLC18A1, SOCS1, STAT1, TACSTD2, TBX21, THBS1, THY1, TNFRSF11B, TNMD, TNR, TREM2, WNT1, WNT10B]	[ABCC8, ABL1, ACTN4, ADAMTS7, ADAMTS9, ADCK1, ADGRB2, ADIPOQ, AGT, AMH, AREG, ARHGDIA, ARHGEF15, ASPN, BBS12, BCL6, BCOR, C11orf65, CARM1, CCL11, CD74, CDKN1A, CEACAM1, CETP, CGA, CHERP, CHGA, CITED2, CMTM5, CREB3L1, CRP, CTDSP1, DAB2IP, DISP3, DMTN, DNMT1, DPF2, DTX1, DXO, EAF2, ECM1, EFNA3, EPHA2, EPN1, ERBB2, FOXC2, FOXM1, GATA2, GDI1, GPER1, GPR137, GPR37L1, GPR4, GSK3A, H4C3, HDAC5, HDAC7, HGS, HSPG2, ID3, IL1B, IL4R, INHA, IRX3, JAK3, KCNK2, LAG3, LDLR, LINGO1, LMX1A, LRRC17, LTF, MECP2, MSTN, MSX1, MYC, NFATC4, NGFR, NODAL, NOS3, NOTCH1, NOTCH4, NPPB, NR1D1, NRARP, NTN1, OMA1, PAF1, PDGFB, PLXNA3, PML, POSTN, PPARD, PTN, RAI1, RARA, RBPMS2, RGCC, RGMA, RMRP, RTN4RL1, SCAF1, SEMA3F, SEMA4B, SEMA4C, SEMA6B, SEMA6C, SERPINE1, SMAD3, SNAI1, SOX10, SOX8, SPDEF, SPI1, STAT3, SYNGAP1, TBX5, TGFB1I1, THBS1, TNF, TNMD, TRIB1, TRIB3, TRIM62, TRPV4, TSKU, ULK1, VASN, VEGFA, WNT3, WNT9B, ZBTB7B, ZC3H12A, ZFP36]

GO:0051094	positive regulation of developmental process	3,34E-08	[2, 3, 4]	267,00	20,09	37,54	62,46	[ADAM8, ALOX15B, ANGPT2, AREG, BMP7, BMPR1B, BTK, CASP8, CASS4, CCL19, CCL24, CCR3, CD27, CD4, CD53, CD74, CD80, CD86, CDH4, CDKN2A, CLCF1, CRABP2, CRB2, CXCL9, CXCR4, CYBB, EGR3, ELAVL4, EPX, EVI2B, FFAR4, FOXP3, GDF6, HCLS1, HLA-DRA, HLA-DRB1, HOPX, IFNG, IL12RB1, IL18, IL7R, INPP5D, IRX3, ITGAX, ITGB2, KLF5, LCP1, LEF1, LIF, LILRB4, LTA, MEDAG, MIXL1, MYB, NCKAP1L, NEK5, NLRP3, NPPC, NTN1, PIM1, PLAAT4, PLXNC1, PRKCB, PTK2B, PTPRC, RHEX, RHOH, RLN2, RUNX3, SASH3, SEMA4A, SEMA4D, SOCS1, SOCS3, STAT1, SULT2B1, SYK, TACSTD2, TBX21, TESPA1, THBS1, TNFSF13B, TOX, TREM2, VNN1, VWC2L, WNT1, WNT10B, ZAP70, ZBED2]	[ABI3, ABL1, ACACB, ADGRL1, ADIPOQ, AGAP2, AGER, AGT, AGTR1, ANGPTL4, AP3D1, APLNR, APOA1, AREG, ARF1, ARHGDIA, BCL6, BCL9L, BEND6, C5, C5AR1, CAMK2B, CARM1, CCL11, CCL24, CD74, CDH4, CDK1, CEACAM1, CFL1, CHI3L1, CITED2, CLCF1, COL1A1, CPNE5, CPNE6, CSF1, CSNK1E, CTHRC1, CX3CL1, CYP26B1, CYP27B1, DAB2IP, DAG1, DDX39B, DLG5, DMTN, DVL3, ECM1, EEF1E1, EFEMP2, EHD1, EHD2, EIF4G1, ENG, FADD, FES, FOXC2, FOXS1, GATA2, GATA4, GDF6, GDI1, GPAM, GPER1, GRN, HEYL, HMGA1, HOPX, HSF1, HSPB1, HTR2A, HYAL1, IFITM1, IL15RA, IL1B, IL4R, IL7R, ILK, IRX3, ITGA5, JMJD8, JUNB, JUND, JUP, KCTD11, KLF10, KLF5, LGALS9, LIF, LIMK1, LIMS2, LMNA, LRCH4, LRG1, LRRC8A, LTF, LZTS1, MAMSTR, MAPK8IP3, MED12, MEGF8, MME, MYADM, MYC, NAP1L2, NEFL, NEK5, NF2, NGF, NGFR, NINJ1, NLGN2, NODAL, NOS3, NOTCH1, NTN1, NUMBL, OMA1, OSR2, PACSIN1, PAK4, PARP6, PDGFB, PDLIM7, PHLDB1, PIEZO1, PIM1, PKM, PLA2G2A, PLXNA1, PLXNA3, PLXNB1, POR, PPARD, PPP1R13L, PTN, RAMP2, RAP1A, RARA, RELA, RGCC, RNF157, S100A1, S1PR2, SERPINE1, SHANK3, SMAD3, SNAI1, SOCS3, SOX10, SOX12, SOX13, SOX8, SPDEF, SPHK1, SRC, SRF, SRRT, SSBP3, STAT3, SYDE1, SYT2, TBX5, TCF3, TCF7L1, TEAD4, TESK1, TFE3, TGFB1I1, TGM2, THBS1, THPO, TIMP1, TMEM100, TNF, TNFRSF1A, TNFSF12, TRIB1, TRIOBP, VDR, VEGFA, VNN1, VSIG2, VSIR, WNT3, YPEL3, ZBTB7B, ZC3H12A, ZFP36, ZMIZ1, ZNF219, ZNF335, ZNF385A]
GO:0051240	positive regulation of multicellular organismal process	8,22E-15	[2, 3, 4]	325,00	21,42	48,82	51,18	[ADAM8, AIF1, AIM2, ALOX15B, ANGPT2, AREG, BATF, BCL3, BMP7, BMPR1B, BTK, BTN3A1, BTN3A2, C1QTNF1, CAMK4, CARD11, CASP8, CCDC88B, CCL19, CCL24, CCL3, CCR2, CCR3, CD2, CD226, CD244, CD27, CD3E, CD4, CD40LG, CD6, CD74, CD80, CD86, CDH4, CLCF1, CLEC6A, CLNK, CNR1, CRABP2, CRB2, CRTAM, CSF1R, CST7, CXCR4, CYBB, EBI3, EDN2, EGR3, EPX, EREG, EVI2B, FCN1, FFAR4, FGR, FOXP3, GBP5, GDF6, GPSM3, HCLS1, HLA-DPA1, HLA-DPB1, HLA-DRA, HLA-DRB1, IFNG, IL12RB1, IL12RB2, IL16, IL18, IL26, IL7R, INPP5D, IRF4, IRF5, IRF8, ITGAX, ITGB2, ITK, LCP1, LDLR, LEF1, LIF, LILRB1, LILRB4, LPAL2, LTA, LTB, LY9, MAPK13, MCOLN2, MYB, NCKAP1L, NFAM1, NLRP2, NLRP3, NLRP9, NPPB, NR4A3, NTN1, ORM2, P2RY2, PIK3CG, PIM1, PLAAT4, PLAC8, PLXNC1, POU2AF1, POU2F2, PRKCB, PRKCQ, PTAFR, PTGER2, PTK2B, PTPN22, PTPRC, PYCARD, PYHIN1, RHOH, RLN2, RUNX3, SASH3, SCIMP, SEMA4A, SEMA4D, SIGLEC16, SLAMF6, SMTNL2, SOCS1, STAT1, SYK, TBX21, TBXAS1, TESPA1, THBS1, TIGIT, TLR6, TLR8, TNR, TOX, TREM2, UCP2, VNN1, WNT1, WNT10B, XCL1, ZAP70, ZBED2, ZBP1]	[ABCA7, ABCC8, ABL1, ACACB, ADIPOQ, AGAP2, AGER, AGPAT1, AGT, AMH, ANGPTL4, AP3D1, APLN, APLNR, AREG, ARHGDIA, ATP1A1, ATP1A3, ATPSCKMT, BCL3, BCL6, BCL9L, C1QTNF1, C5, C5AR1, CAMK2B, CCL11, CCL24, CCR7, CD14, CD276, CD74, CDH4, CDK1, CFH, CFL1, CHGA, CHI3L1, CITED2, CLCF1, CLNK, CNOT3, CNR1, COL1A1, CSF1, CST7, CX3CL1, CYP27B1, DAG1, DDX39B, DHX34, DLG5, DMTN, ECM1, EDN2, ENG, FABP4, FADD, FES, FFAR2, FLT4, FOXC2, FOXS1, FRMD8, FURIN, GATA2, GATA4, GBA, GDF6, GDI1, GNAI2, GPAM, GPER1, GPR3, GRN, GSK3A, HK1, HRH2, HSF1, HSPB1, HTR2A, HYAL1, ICAM1, IL15RA, IL1B, IL4R, IL7R, ILK, INHA, ITGA5, JMJD8, JUP, KDM6B, KLF10, LDLR, LDLRAP1, LGALS9, LIF, LILRA5, LIMK1, LIPG, LRCH4, LRG1, LTF, LUM, MAVS, MED12, MEGF8, MFAP2, MME, MYC, MYRF, NEFL, NFATC4, NFKB2, NGF, NGFR, NINJ1, NLGN2, NLRP9, NOD2, NODAL, NOS3, NOTCH1, NPAS2, NPPA, NPPB, NR4A3, NTN1, NUMBL, OMA1, OSM, OSR2, P2RX3, P2RY2, PAK4, PARP6, PCDHA4, PDGFB, PIM1, PKM, PLXNA1, PLXNA3, PLXNB1, POLR2E, POLR2L, POR, POSTN, PQBP1, PRDM16, PTN, RAMP2, RARA, RELA, RGCC, RNU1-93P, S100A1, SERPINE1, SHANK3, SLC7A5, SLC9A1, SLN, SMAD3, SMTNL2, SNAI1, SOX10, SOX12, SOX13, SOX8, SPHK1, SPHK2, SRC, SRF, SRRT, STAT3, STING1, SYDE1, TBX5, TEAD4, TGFB1I1, TGM2, THBS1, TICAM1, TNF, TNFSF12, TRIB1, TRPV4, TSKU, VDR, VEGFA, VNN1, VSIR, WNT3, YBX2, ZBTB7B, ZC3H12A, ZCCHC3, ZMIZ1, ZNF219, ZNF335, ZNF580]
GO:0051241	negative regulation of multicellular organismal process	1,56E-08	[2, 3, 4]	224,00	21,11	41,07	58,93	[ACP5, ADAMTS9, ADCY7, ANGPT1, ANGPT2, ASCL2, BANK1, BCL3, BMP7, BTK, C1QC, C1QTNF1, CALCR, CARD17, CCL3, CCR2, CD200R1, CD33, CD74, CD84, CD96, CDKN2A, CEL, CTLA4, CXCL10, DCC, DPEP1, EAF2, EPX, FASLG, FFAR4, FGL2, FOXP3, FRZB, GPR4, GPR55, HLA-DRB1, IFNG, IKBKE, INPP5D, KNG1, LAG3, LDLR, LEF1, LILRB1, LILRB4, LPAL2, LRRC17, NCKAP1L, NLRC3, NLRP3, NLRP6, NLRP7, NPPB, NTN1, PIK3CG, PLAC8, PLAU, PTK2B, PTPN22, PTPN6, PTPRC, PYCARD, RUNX3, SEMA4A, SEMA4D, SLAMF8, SOCS1, STAT1, TACSTD2, TBX21, THBS1, THY1, TIFAB, TIGIT, TLR6, TLR8, TNFAIP3, TNFRSF11B, TNMD, TNR, TREM2, TSPAN8, WNT10B, XCL1]	[ABCC8, ABCD1, ABCD2, ADAMTS5, ADAMTS7, ADAMTS9, ADGRB2, ADIPOQ, AGER, AGT, ANGPT1, APLN, APOA1, APOD, APOM, ARHGDIA, ARRDC3, ASPN, ATP1A1, ATP1A3, BCL3, BCL6, BCOR, C1QTNF1, CALCR, CCL11, CCR7, CD74, CEACAM1, CEL, CGA, CHGA, CITED2, CPTP, CREB3L1, CSK, CTDSP1, CX3CL1, DAB2IP, DTX1, DXO, EAF2, ECM1, EFNA3, EPHA2, EPN1, EPPK1, ERBB2, FAM110A, FAP, FOXC2, FURIN, GATA2, GBA, GDI1, GIT1, GJD3, GLRA1, GNAI2, GPR137, GPR37L1, GPR4, GRN, GSK3A, HDAC5, HDAC7, HFE, HGS, HSPG2, IL1B, IL4R, INHA, JAK3, JUP, KCNK2, KNG1, LAG3, LDLR, LGALS9, LILRA5, LINGO1, LMNA, LRRC17, LRRC32, LTF, MAVS, MECP2, MSTN, MYC, NFATC4, NGFR, NLRC5, NOD2, NODAL, NOS3, NOTCH1, NOVA2, NPPA, NPPB, NPR3, NPRL3, NR1D1, NRARP, NTN1, NUTF2, OMA1, PDGFB, PLAU, PLAUR, PLXNA3, PML, PPP1R11, PTN, PTPN23, PTPRU, RAB11FIP5, RAI1, RARA, RELB, RGCC, RGMA, RMRP, SEMA3F, SEMA4B, SEMA4C, SEMA6B, SEMA6C, SERPINE1, SERPING1, SH2B3, SMAD3, SOX10, SOX8, SPI1, SRF, SYNGAP1, TBX5, TFE3, THBS1, TIMP1, TLE3, TNF, TNFRSF1A, TNMD, TRIB1, TRIM62, TSKU, TSPAN8, UBE2L6, ULK1, VASN, VSIR, VTN, WNT3, WNT9B, ZBTB7B, ZC3H12A, ZFP36]

GO:0000902	cell morphogenesis	2,31E-02	[3, 4]	201,00	18,21	33,55	66,45	[ADAM8, AREG, BCL11B, BMP7, BMPR1B, CASS4, CCDC88C, CCL2, CCL24, CCL3, CDH4, CEL, COL22A1, CORO1A, CRABP2, CSF1R, CXCR4, DCC, DOCK10, DOK2, ELAVL4, EMB, EPHA6, ERMN, FGD2, FGD3, FGR, GFRA2, GPM6A, GRXCR2, IL7R, ITGA4, ITGB2, ITGB7, LEF1, LST1, MSX1, NCKAP1L, NELL2, NPPC, NR4A2, NTN1, PARVG, PEAK3, PLPPR4, PLXNC1, PRKCQ, PTK2B, PTPN6, RAC2, RHOH, SEMA4A, SEMA4D, ST14, TACSTD2, THY1, TNMD, TNR, UGT8, VSIG1]	[ABI3, ABL1, ACTB, ACTN1, ACTN4, ADCY1, ALDOA, APOA1, ARC, AREG, ARHGDIA, ARTN, BCL6, BCL9L, C15orf62, CAMK2B, CCL11, CCL2, CCL24, CDC42EP1, CDC42EP2, CDC42EP4, CDH4, CEL, CELSR2, CERT1, CFL1, CNTN6, COL22A1, CPNE5, CPNE6, CYP27B1, DAB2IP, DAG1, DCHS1, DDR1, DMTN, DOK5, DPYSL4, DSCAML1, DVL3, EFNA3, EPHA2, ERBB2, FBLIM1, FES, FMNL1, GAB2, GDI1, GFRA3, GPM6A, GRB7, HPRT1, ICAM1, IL7R, ILK, ITGA7, LAMA5, LAMB4, LGR6, LIMK1, LIMS2, LINGO1, LMX1A, LYPLA2, LZTS1, LZTS3, MAP1A, MAP1S, MAPK3, MAPK7, MARK2, MED12, MEGF8, MFSD2A, MICALL2, MINK1, MOV10, MSN, MSX1, MYADM, MYH7B, MYH9, MYO7A, NBEAL2, NECTIN1, NEFL, NFATC4, NGF, NGFR, NODAL, NOTCH1, NOTCH3, NOTCH4, NR4A2, NSMF, NTN1, NUMBL, OMA1, PACSIN1, PALM, PALM2AKAP2, PARP6, PARVB, PDLIM7, PHACTR1, PLEKHO1, PLXNA1, PLXNA3, PLXNB1, POSTN, PQBP1, PRAG1, PTN, PTPRU, PXN, RAB3A, RAC2, RGMA, RHOBTB2, RHOG, RILPL1, RNF157, ROBO4, SEMA3F, SEMA4B, SEMA4C, SEMA6B, SEMA6C, SHANK3, SIDT2, SIPA1L3, SLC9A3R1, SPI1, SPTB, SRC, SRF, SYNGAP1, SYT2, SZT2, TESK1, TNMD, TRIOBP, TSKU, ULK1, UNC5B, VAX2, VDR, VEGFA, WHRN, WNT3, ZNF335, ZSWIM4, ZSWIM8]
GO:0001816	cytokine production	6,35E-20	[2, 5]	213,00	26,66	59,72	40,28	[ACP5, ADAM8, ADCY7, AIF1, AIM2, ALOX15B, ANGPT1, BANK1, BATF, BCL3, BTK, BTN3A1, BTN3A2, CAMK4, CARD11, CARD17, CASP8, CCDC88B, CCL19, CCL3, CCR2, CD2, CD200R1, CD226, CD244, CD33, CD3E, CD4, CD40LG, CD6, CD74, CD80, CD84, CD86, CD96, CLCF1, CLEC6A, CLNK, CRTAM, CSF1R, CXCL6, CYBB, EBI3, EPX, EREG, FCN1, FFAR4, FGR, FOXP3, GBP5, GPSM3, HLA-DPA1, HLA-DPB1, HLA-DRB1, IFNG, IKBKE, IL12RB1, IL12RB2, IL16, IL18, IL26, IL5RA, INPP5D, IRF4, IRF5, IRF8, ITK, LAG3, LCP1, LEF1, LILRB1, LILRB4, LPAL2, LTA, LTB, LY9, MAPK13, MCOLN2, MOG, MYB, NCKAP1L, NFAM1, NLRC3, NLRP2, NLRP3, NLRP6, NLRP7, NLRP9, NR4A3, ORM2, PIK3CG, PLD4, POU2AF1, POU2F2, PRKCQ, PTAFR, PTPN22, PTPN6, PTPRC, PYCARD, PYHIN1, SASH3, SCIMP, SIGLEC16, SLAMF6, SOCS1, STAT1, SYK, TBX21, THBS1, TIGIT, TLR10, TLR6, TLR8, TNFAIP3, TREM2, UBASH3A, XCL1, ZBP1]	[ABCC8, ABCD1, ABCD2, ABL1, ACKR1, ADGRG1, ADIPOQ, AGER, AGPAT1, AGT, AMH, ANGPT1, APOA1, APOD, BCL3, BCL6, C5, C5AR1, CCR7, CD14, CD276, CD74, CEACAM1, CHI3L1, CLC, CLCF1, CLNK, CPTP, CRP, CSK, CX3CL1, EPHA2, FADD, FFAR2, FLT4, FRMD8, FURIN, GATA4, GBA, GIT1, GPAM, HDAC7, HFE, HK1, HSPB1, IGF2BP2, IL1B, IL4R, IL5RA, INHA, JAK3, JPH4, LAG3, LGALS9, LILRA5, LRRC32, LTF, LUM, MAP2K3, MAST2, MAVS, NFATC4, NFKB2, NLRC5, NLRP9, NOD2, NODAL, NR4A3, NUTF2, OSM, PER1, PLD3, PML, POLR2E, POLR2L, POSTN, PPP1R11, PQBP1, RARA, RELA, RELB, RGCC, RNF26, RNU1-93P, SERPINE1, SLC7A5, SMAD3, SPHK1, SPHK2, SRC, STAT3, STING1, THBS1, TICAM1, TNF, TRPV4, TSKU, UBE2L6, VSIR, ZBTB7B, ZC3H12A, ZCCHC3, ZFP36, ZNF580]
GO:0022603	regulation of anatomical structure morphogenesis	1,53E-04	[3, 4]	198,00	19,58	30,83	69,17	[ADAMTS9, ANGPT2, AREG, BMP7, CASS4, CCL2, CCL24, CCL3, CCR2, CCR3, CDH4, CORO1A, CRABP2, CRB2, CSF1R, CXCL10, CXCR4, CYBB, DCC, ERMN, FASLG, FGD2, FGD3, FGR, GPR4, ITGAX, ITGB2, LIF, LST1, MSX1, NPPB, NTN1, PEAK3, PLXNC1, PRKCB, PROK1, PTK2B, RAC2, RHOH, RLN2, SEMA4A, SEMA4D, SLC18A1, STAT1, TACSTD2, THBS1, THY1, TNFAIP3, TNFRSF11B, TNFSF13B, TNMD, TNR, WNT1, WNT10A]	[ABCC8, ABI3, ABL1, ACTN4, ADAMTS9, ADCK1, ADGRB2, AGT, ALDOA, ANGPTL4, AP2A1, APLNR, APOA1, ARC, AREG, ARHGDIA, BCL6, BCL9L, C11orf65, C15orf62, C5, C5AR1, CAMK2B, CCL11, CCL2, CCL24, CDC42EP1, CDC42EP2, CDC42EP4, CDH4, CEACAM1, CELSR2, CFL1, CHI3L1, CITED2, CPNE5, CPNE6, CREB3L1, CSF1, CTHRC1, DAB2IP, DAG1, DMTN, DVL3, ECM1, EFNA3, ENG, EPHA2, EPN1, ERBB2, FBLIM1, FES, FLT4, FMNL1, FOXC2, GATA2, GATA4, GDI1, GPR4, GRN, HGS, HSPB1, HSPG2, HYAL1, ICAM1, IL1B, ILK, ITGA5, ITGA7, JMJD8, JUP, LIF, LIMK1, LIMS2, LINGO1, LRG1, LZTS1, LZTS3, MAPK7, MARK2, MECP2, MED12, MEGF8, MFSD2A, MOV10, MSN, MSX1, MYADM, MYH7B, MYH9, MYO19, NEFL, NFATC4, NGF, NGFR, NINJ1, NODAL, NOS3, NOTCH1, NPPB, NSMF, NTN1, OMA1, PAK4, PALM, PALM2AKAP2, PARP6, PARVB, PFN1, PHLDB1, PKM, PLEKHO1, PLXNA1, PLXNA3, PLXNB1, PML, POSTN, PQBP1, PRAG1, PTN, RAC2, RAMP2, RGCC, RGMA, RHOBTB2, RHOG, RNF157, RNU1-93P, S100A1, SEMA3F, SEMA4B, SEMA4C, SEMA6B, SEMA6C, SERPINE1, SHANK3, SLC9A3R1, SOX8, SPHK1, SRC, SRF, STAT3, SYNGAP1, SYT2, TESK1, THBS1, TNF, TNFSF12, TNMD, TRIOBP, ULK1, VANGL2, VEGFA, WARS1, WNT3, WNT9B, ZC3H12A]

GO:0035295	tube development	2,14E-05	[3, 4]	219,00	19,64	32,95	67,05	[ADAM8, ADAMTS9, ANGPT1, ANGPT2, ANGPTL6, ANPEP, AREG, BMP7, BRIP1, CASP8, CCL2, CCL24, CCR2, CCR3, CEL, COL22A1, CSF1R, CXCL10, CXCR3, CXCR4, CYBB, E2F8, EDN2, EGR3, EOMES, EREG, FASLG, FOLR2, GPR4, HOPX, IL18, IRX3, ITGAX, ITGB2, KLF5, LDLR, LEF1, LIF, MIXL1, NELL2, NPPB, NR4A1, NTN1, PIK3CG, PRDM1, PRKCB, PROK1, PTK2B, PTPN6, RBPMS2, RLN2, SALL1, SDC1, SEMA4A, SH2D2A, SMPD3, ST14, STAT1, SYK, TACSTD2, TBX21, THBS1, THY1, TNFAIP3, TNMD, WNT1]	[ABCA3, ABCC8, ABL1, ACKR3, ADAMTS9, ADGRB2, ADGRG1, AGT, AGTR1, ANGPT1, ANGPTL1, ANGPTL2, ANGPTL4, APLN, APLNR, APOD, AREG, BAG6, C5, C5AR1, CALB1, CCL11, CCL2, CCL24, CDKN1A, CEACAM1, CECR2, CEL, CHI3L1, CITED2, COL22A1, CRAT, CREB3L1, CSF1, CSPG4, CTHRC1, CX3CL1, CYP21A2, CYP27B1, DAB2IP, DAG1, DCHS1, DDR1, DLG5, DVL3, E2F7, E2F8, ECM1, EDN2, EFEMP2, EFNA3, EIF4EBP1, ELK1, ENG, EPHA2, EPN1, ERBB2, ESM1, FAP, FLT4, FOXC2, FOXF1, GATA2, GATA4, GJD3, GPR4, GRN, HDAC5, HDAC7, HESX1, HEYL, HGS, HOPX, HSPB1, HSPG2, HYAL1, IL1B, ILK, IRX3, ITGA3, ITGA5, JMJD8, JUNB, JUP, KLF5, LAMA5, LDLR, LIF, LOX, LRG1, LZTS2, MAPK3, MAPK7, MECP2, MED12, MEGF8, MEN1, MME, MTHFR, MYC, MYH9, MYO18B, NFATC4, NGFR, NINJ1, NODAL, NOS3, NOTCH1, NOTCH4, NPPB, NPRL3, NR4A1, NRARP, NTN1, OMA1, PAK4, PDGFRB, PFN1, PKD1, PKM, PLCD3, PML, PRKACA, PTN, RAB3A, RAMP2, RAP1A, RARA, RBPMS2, REC8, RGCC, RGMA, ROBO4, S100A1, SALL2, SELENON, SEMA4C, SERPINE1, SHANK3, SLC4A2, SMAD3, SOX10, SOX8, SPATA2, SPDEF, SPHK1, SPI1, SRC, SREBF1, SRF, SSBP3, STAT3, STK40, SYNGAP1, TBX5, TEAD2, TGM2, THBS1, TMEM100, TNF, TNFAIP2, TNFSF12, TNMD, TRAF4, TSC2, UNC5B, VANGL2, VDR, VEGFA, WARS1, WNT9B, ZC3H12A, ZMIZ1, ZNF358]
GO:0045595	regulation of cell differentiation	2,28E-11	[3, 4]	337,00	20,17	43,12	56,88	[ADAM8, ALOX15B, AREG, ASCL2, BCL11B, BHLHA15, BHLHE40, BMP7, BMPR1B, BTK, C1QC, CAMK4, CARD11, CASP8, CASS4, CCL17, CCL19, CCL3, CCR2, CD2, CD27, CD4, CD74, CD80, CD86, CDH4, CDKN2A, CLCF1, CMTM5, CRABP2, CRB2, CRTAM, CTLA4, CXCL10, CXCL9, CXCR4, DCC, DPEP1, EGR3, EPX, EREG, EVI2B, FCRL3, FFAR4, FGL2, FOXP3, FRZB, GDF6, GLIS1, GPR171, GPR55, H3C10, H3C11, H3C12, H3C7, HCLS1, HLA-DOA, HLA-DRA, HLA-DRB1, HOPX, IFNG, IKZF3, IL12RB1, IL18, IL7R, INPP5D, IRF4, IRX3, KLF5, KLHL41, LAG3, LCP1, LDLR, LEF1, LIF, LILRB1, LILRB4, LMO1, LRRC17, LTA, MAFF, MEDAG, MIXL1, MMP9, MSX1, MUSK, MYB, MYCL, NCKAP1L, NEK5, NFAM1, NLRP3, NPPC, NR4A3, NTN1, P2RY12, PIM1, PLAAT4, PLXNC1, PRDM1, PRKCB, PRKCQ, PTK2B, PTPN6, PTPRC, RBPMS2, RHEX, RHOH, RUNX3, SASH3, SEMA4A, SEMA4D, SLAMF8, SOCS1, SOCS3, STAT1, SULT2B1, SYK, TACSTD2, TBX21, TESPA1, THBS1, THY1, TNFRSF18, TNR, TOX, TREM2, VNN1, VWC2L, WNT1, WNT10B, ZAP70, ZBED2, ZNF683]	[ABCC8, ABL1, ACTN4, ADAMTS7, ADIPOQ, AGER, AGT, AGTR1, AP3D1, APOA1, AQP3, AREG, ARHGDIA, ATAT1, BBS12, BCL6, BCL9L, BEND6, BHLHE40, CAMK2B, CARM1, CASZ1, CCL11, CD74, CDH4, CDK1, CEACAM1, CETP, CHERP, CLCF1, CLPTM1, CMTM5, COL1A1, CRP, CSF1, CSNK1E, CTDSP1, CTHRC1, CX3CL1, CYP26B1, CYP27B1, DAB2IP, DAG1, DDX39B, DISP3, DMTN, DNMT1, DPF2, DTX1, EFEMP2, EIF4G1, ENG, ERBB2, FADD, FES, FLOT2, GATA2, GATA4, GDF6, GDI1, GNB3, GPER1, GPR137, GPR37L1, GRN, GSK3A, H3C10, H3C12, H4C3, HDAC5, HDAC7, HEYL, HOPX, HSF1, HTR2A, ID3, IFITM1, IL15RA, IL1B, IL4R, IL7R, ILK, INHA, IRX3, JAK3, JUNB, JUND, KCTD11, KLF10, KLF5, KMT2D, LAG3, LDLR, LGALS9, LIF, LIMK1, LIMS2, LINGO1, LMX1A, LOX, LRRC17, LRRC8A, LTF, LZTS1, MAFG, MAMSTR, MAPK8IP3, MCRIP1, MECP2, MED12, MEGF8, MME, MOV10, MSTN, MSX1, MYADM, MYC, MYL9, NAP1L2, NEFL, NEK5, NF2, NFATC4, NFE2, NGF, NGFR, NODAL, NOTCH1, NOTCH4, NR1D1, NR4A3, NRARP, NTN1, NUMBL, OMA1, OSM, PAF1, PARP6, PDGFB, PDLIM7, PHLDB1, PIEZO1, PIM1, PLA2G2A, PLEKHB1, PLXNA1, PLXNA3, PLXNB1, POR, POSTN, PPARD, PPP1R13L, PPP2R1A, PRKACA, PTN, RAMP2, RAP1A, RARA, RBM38, RBPMS2, RELA, RGCC, RGMA, RMRP, RNU1-93P, S1PR2, SCAF1, SEMA3F, SEMA4B, SEMA4C, SEMA6B, SEMA6C, SERPINE1, SETD1A, SHANK3, SIK1, SLC25A23, SLC4A11, SMAD3, SNAI1, SOCS3, SOX10, SOX12, SOX13, SOX8, SPDEF, SPI1, SRF, SRRT, STAT3, SYNGAP1, TBX5, TCF3, TCF7L1, TCIRG1, TEAD2, TESK1, TFE3, TGFB1I1, TGM2, THBS1, THPO, TIMP1, TMEM100, TNF, TNFRSF1A, TRIB1, TRIB3, TRIM62, TRIOBP, TRPV4, TSKU, ULK1, VASN, VDR, VEGFA, VNN1, VSIG2, VSIR, WNT3, WNT9B, ZBTB7B, ZC3H12A, ZFHX2, ZFP36, ZMIZ1, ZNF219, ZNF335, ZNF385A]

GO:0048468	cell development	8,30E-04	[3, 4]	378,00	17,23	32,19	67,81	[ADGRG6, ANGPT2, AREG, ASCL2, BCL11B, BFSP2, BHLHA15, BHLHE40, BICDL1, BLK, BMP7, BMPR1B, BRIP1, BTG2, BTK, C1QA, CASS4, CCDC88C, CCL19, CD3E, CDH1, CDH4, CLCF1, CNR1, COL22A1, CRABP2, CRB2, CXCR4, DCC, DOCK10, DOK2, DPEP1, DRD1, ELAVL4, EMB, EPHA6, EPX, EREG, EVI2B, FASLG, FOLR2, FOXP3, FRZB, GFI1, GFRA2, GPM6A, GPR4, GRXCR2, HCN1, HORMAD1, IFNG, IRX3, ITGA4, ITGB7, KCNJ10, KLF5, KLHL41, LDLR, LEF1, LIF, LILRB1, LST1, LTA, MYB, MYH6, NCKAP1L, NELL2, NPPC, NR4A2, NTN1, PARVG, PBX4, PLPPR4, PLXNC1, POU2F2, PRDM1, PRKCQ, PTK2B, PTPN6, PTPRC, PTX3, RAC2, RASGRF1, RHEX, RHOH, RIMBP3B, RUNX3, SDC1, SEMA4A, SEMA4D, SLAMF8, SLFN14, SMPD3, SPEF2, SPINK1, ST14, TACSTD2, THY1, TMEM132E, TNMD, TNR, TOX, TREM2, UGT8, VSIG1, WNT10B, ZNF804A]	[ABCC8, ABI3, ABL1, ACP4, ACRBP, ACTA1, ACTB, ACTC1, ACTN1, ACTN4, ADAMTSL4, ADCY1, ADGRG6, ADIPOQ, AGER, AGT, ALPK3, AMH, ANGPTL8, AP2A1, APOA1, APOD, ARC, AREG, ARF1, ARHGDIA, ARTN, ATAT1, ATF5, ATP1B2, BCL2L1, BCL6, BHLHE40, BTG2, C5AR1, CAMK2B, CAMSAP3, CARM1, CCL11, CCL21, CDC25B, CDH4, CDK1, CDKN1A, CECR2, CELSR2, CFAP157, CFL1, CITED2, CLCF1, CNR1, CNTN6, COL22A1, COL27A1, CPNE5, CPNE6, CTDSP1, CTF1, CTHRC1, CX3CL1, DAB2IP, DAG1, DDR1, DDX39B, DGUOK, DLG5, DMC1, DMRTA1, DMTN, DOK5, DPYSL4, DSCAML1, DVL3, DYSF, E2F4, ECM1, EFEMP2, EFNA3, EHD1, ENG, EPHA2, ERBB2, FA2H, FANCC, FASN, FES, FLII, FOSL2, FOXC2, GAB2, GATA2, GATA4, GBA, GDI1, GFRA3, GIT1, GJD3, GNAO1, GPER1, GPM6A, GPR37L1, GPR4, GPRIN1, GRB7, GRN, GSK3A, H1-9P, HDAC10, HEYL, HID1, HPRT1, ICAM1, IL1B, ILK, INPP5J, IRX3, IRX6, ITGA3, JUND, KATNB1, KCNIP2, KCTD11, KLF5, KMT2D, LAMA5, LAMB4, LDLR, LGR6, LIF, LIMK1, LIMS2, LINGO1, LMNA, LMX1A, LSR, LTF, LYPLA2, LZTS1, LZTS3, MAP1A, MAP1S, MAPK3, MAPK7, MAPK8IP3, MARK2, MECP2, MED12, MEGF8, MFSD2A, MICALL1, MICALL2, MINK1, MME, MOV10, MSN, MYADM, MYC, MYH6, MYH9, MYO7A, MYRF, NBEAL2, NECTIN1, NECTIN2, NEFL, NF2, NFATC4, NGF, NGFR, NODAL, NOL6, NOTCH1, NOTCH3, NOTCH4, NPPA, NR1D1, NR4A2, NSMF, NTN1, NUMBL, OBSCN, OGDH, OMA1, PACSIN1, PARP6, PARVB, PCDHA4, PDGFB, PDGFRB, PDLIM7, PEF1, PHACTR1, PISD, PIWIL1, PLXNA1, PLXNA3, PLXNB1, POSTN, PPP1R9B, PQBP1, PRAG1, PRKACA, PTN, PTPRU, PXN, PYGO2, RAB1B, RAB3A, RAC2, RAMP2, RAP1A, RAPGEF1, RARA, REC8, RELA, RFX2, RGMA, RHOG, RILPL1, RMRP, RNF157, RNU1-93P, ROBO4, RP1L1, RTN4RL1, RTN4RL2, S1PR2, SAMD14, SBF1, SCYL1, SELENON, SEMA3F, SEMA4B, SEMA4C, SEMA6B, SEMA6C, SF3A2, SH2B3, SHANK3, SIDT2, SIPA1L3, SIX5, SLC4A10, SLC9A3R1, SMAD3, SOX10, SOX8, SPAG16, SPDEF, SPEF2, SPI1, SPINK1, SPTB, SRC
GO:0048731	system development	7,36E-13	[3, 4]	877,00	17,00	36,59	63,41	[ACP5, ADAM8, ADAMTS4, ADAMTS9, ADGRG6, AIF1, AKNA, ALOX15, ALOX15B, ANGPT1, ANGPT2, ANGPTL6, ANPEP, APBA2, AQP5, AREG, ASCL2, ASGR2, ATF3, BATF, BCAS1, BCL11B, BCL3, BFSP2, BHLHA15, BHLHE22, BHLHE40, BICDL1, BLK, BLNK, BMP7, BMPR1B, BRIP1, BTG2, BTK, C1QA, C1QB, C1QC, C1QTNF1, CALCR, CAMK4, CARD11, CASP8, CCL19, CCL2, CCL24, CCL3, CCR2, CCR3, CCR4, CD19, CD2, CD27, CD3D, CD3E, CD3G, CD4, CD40LG, CD74, CD79A, CD80, CD86, CD8A, CDH1, CDH4, CDKN2A, CEL, CERS3, CHRDL1, CHRDL2, CLCF1, CNR1, COL22A1, COL9A2, CR2, CRABP2, CRB2, CRTAM, CSF1R, CST7, CTLA4, CXCL10, CXCR3, CXCR4, CYBB, DAW1, DCC, DOCK10, DOCK2, DOK2, DPEP1, DPEP2, DRC1, DRD1, E2F8, EAF2, EDN2, EGR3, ELAVL3, ELAVL4, ELF3, EMB, EOMES, EPHA6, EPX, EREG, EVI2B, FASLG, FCRL3, FGL2, FGR, FLT3, FOLR2, FOSL1, FOXP3, FRZB, FUT7, GCNT3, GDF6, GFI1, GFRA2, GPM6A, GPR171, GPR4, GPR55, GRXCR2, H3C10, H3C11, H3C12, H3C7, HCLS1, HCN1, HLA-DOA, HLA-DRA, HLA-DRB1, HOPX, ICOS, IFNG, IKZF1, IKZF3, IL12RB1, IL18, IL7R, INPP5D, IRF4, IRF8, IRX3, ITGA4, ITGAX, ITGB2, ITK, JPH1, KCNJ10, KLF5, KLHL41, KLRC1, KRT85, LAG3, LCK, LCP1, LDLR, LEF1, LIF, LILRB1, LILRB4, LIPM, LMO1, LPAL2, LRFN1, LRRC17, LST1, LTA, LTB, LY9, MAFF, MCOLN3, MIXL1, MMP9, MOG, MS4A1, MSLN, MSX1, MT1G, MUSK, MYB, MYCL, MYH6, MYO3B, NAMPT, NCKAP1L, NELL2, NFAM1, NLRP3, NPPB, NPPC, NR4A1, NR4A2, NR4A3, NTN1, OPN5, P2RY12, PAX5, PBX4, PCDHA2, PIK3CG, PIM1, PLA2G2D, PLAAT4, PLD4, PLEK, PLPPR4, PLXNC1, POU2AF1, POU2F2, PRDM1, PRKCB, PRKCQ, PROK1, PTK2B, PTPN22, PTPN6, PTPRC, PTX3, RAC2, RASGRF1, RBPMS2, RGS9, RHEX, RHOH, RLN2, RNASE6, RRAD, RTKN2, RUNX3, SALL1, SASH3, SCUBE1, SDC1, SELPLG, SEMA4A, SEMA4D, SH2D2A, SLAMF6, SLAMF8, SLC24A4, SLC8A3, SMPD3, SNX10, SOCS1, SOCS3, SPEF2, SPN, ST14, STAP1, STAT1, SYK, TACSTD2, TBX21, TESPA1, TEX11, THBS1, THEMIS, THY1, TIFAB,	[AATK, ABCA3, ABCC8, ABCD1, ABCD2, ABI3, ABL1, ACACB, ACKR3, ACP4, ACSBG1, ACTA1, ACTB, ACTC1, ACTN1, ADAM19, ADAMTS4, ADAMTS5, ADAMTS7, ADAMTS9, ADCY1, ADGRB2, ADGRG1, ADGRG6, ADGRL1, ADIPOQ, AGAP2, AGER, AGT, AGTR1, ALPK3, ALPL, AMH, ANGPT1, ANGPTL1, ANGPTL2, ANGPTL4, ANKRD52, AP1B1, AP2A1, AP3D1, APLN, APLNR, APLP1, APOA1, APOD, AQP3, AQP5, ARC, AREG, ARF1, ARHGDIA, ARHGEF15, ARID1A, ARMC5, ARRDC3, ARSA, ARTN, ASPN, ATAT1, ATF5, ATN1, ATP1B2, ATP6V0D1, ATP6V1B1, BAG3, BAG6, BATF2, BCL2L1, BCL3, BCL6, BCL9L, BCOR, BCR, BEND6, BGN, BHLHE40, BMP8A, BSN, BTBD2, BTG2, C1QTNF1, C5, C5AR1, CACNA1H, CALB1, CALCR, CAMK2B, CAMSAP3, CAPN1, CAPNS1, CARM1, CASZ1, CCDC85C, CCL11, CCL2, CCL24, CCR7, CD74, CDH4, CDK1, CDK2, CDKN1A, CEACAM1, CECR2, CEL, CELSR2, CERT1, CFH, CFL1, CGA, CHERP, CHGA, CHI3L1, CHRDL2, CIC, CITED2, CLC, CLCF1, CLPTM1, CNNM4, CNR1, CNTN5, CNTN6, COASY, COL1A1, COL22A1, COL27A1, CPNE5, CPNE6, CRAT, CREB3L1, CRIP2, CRYAB, CSF1, CSK, CSNK1E, CSPG4, CST7, CTC1, CTDSP1, CTF1, CTHRC1, CX3CL1, CYP21A2, CYP26B1, CYP27B1, DAB2IP, DAG1, DAGLA, DCHS1, DDR1, DDX39B, DEDD, DGUOK, DHRS2, DISP3, DLG5, DMC1, DMRTA1, DMTN, DNMT1, DOK5, DPF2, DPYSL4, DRC1, DSC1, DSCAML1, DTX1, DVL3, DXO, DYRK1B, E2F4, E2F7, E2F8, EAF2, ECM1, EDN2, EFEMP2, EFNA3, EHD1, EIF4EBP1, EIF4G1, ELF3, ELK1, ELOVL1, ENG, EPHA2, EPN1, EPOP, ERBB2, ESM1, ETS2, ETV6, EXTL1, FA2H, FADD, FAIM2, FANCC, FAP, FASN, FES, FGFRL1, FHL3, FLG, FLT3, FLT4, FOSL1, FOSL2, FOXC2, FOXF1, FOXS1, FURIN, GAB2, GATA2, GATA4, GBA, GCNT3, GDF6, GDI1, GDPD5, GFRA3, GIT1, GJA4, GJD3, GLIS2, GMNN, GNAI2, GNAO1, GPER1, GPM6A, GPR137, GPR37L1, GPR4, GPRIN1, GRB7, GRN, GSK3A, GYS1, H3C10, H3C12, H4C3, HAPLN1, HAPLN3, HBEGF, HCN4, HDAC10, HDAC5, HDAC7, HESX1, HEYL, HFE, HGS, HID1, HIVEP3, HMGN1, HNMT,

GO:2000026	regulation of multicellular organismal development	3,52E-12	[3, 4]	296,00	21,07	44,92	55,08	[ADAM8, ADAMTS9, ALOX15B, ANGPT2, ASCL2, BHLHE40, BMP7, BMPR1B, BTK, C1QC, CAMK4, CARD11, CASP8, CCL19, CCL24, CCL3, CCR2, CCR3, CD2, CD27, CD4, CD74, CD80, CD86, CDH4, CDKN2A, CLCF1, CRABP2, CRB2, CRTAM, CST7, CTLA4, CXCL10, CXCR4, CYBB, DCC, DPEP1, EGR3, EVI2B, FASLG, FCRL3, FGL2, FOXP3, FRZB, GDF6, GPR171, GPR4, GPR55, H3C10, H3C11, H3C12, H3C7, HCLS1, HLA-DOA, HLA-DRA, HLA-DRB1, HOPX, IFNG, IKZF3, IL12RB1, IL18, IL7R, INPP5D, IRF4, ITGAX, ITGB2, LAG3, LDLR, LEF1, LIF, LILRB1, LILRB4, LRRC17, LTA, MAFF, MMP9, MYB, MYCL, MYH6, NCKAP1L, NFAM1, NLRP3, NPPB, NR4A3, NTN1, PIM1, PLAAT4, PLXNC1, PRDM1, PRKCB, PRKCQ, PROK1, PTK2B, PTPN6, PTPRC, RHEX, RHOH, RLN2, RUNX3, SASH3, SEMA4A, SEMA4D, SLAMF8, SMPD3, SOCS1, STAT1, SULT2B1, SYK, TBX21, TESPA1, THBS1, THY1, TNFAIP3, TNFRSF18, TNMD, TNR, TOX, TREM2, VNN1, WNT1, WNT10B, ZAP70, ZBED2, ZNF683]	[ABCC8, ABL1, ACACB, ADAMTS7, ADAMTS9, ADGRB2, ADIPOQ, AGER, AGT, ANGPTL4, AP3D1, APLNR, AQP3, ARHGDIA, ASPN, BAG6, BCL6, BCOR, BHLHE40, C5, C5AR1, CAMK2B, CCL11, CCL24, CD74, CDH4, CDK1, CEACAM1, CFL1, CHI3L1, CITED2, CLCF1, CLPTM1, CREB3L1, CSF1, CST7, CTDSP1, CX3CL1, CYP26B1, CYP27B1, DAB2IP, DAG1, DDX39B, DLG5, DTX1, ECM1, EFNA3, ENG, EPHA2, EPN1, ERBB2, FADD, FES, FOXC2, GATA2, GATA4, GDF6, GDI1, GPER1, GPR137, GPR37L1, GPR4, GRN, GSK3A, H3C10, H3C12, H4C3, HESX1, HEYL, HGS, HOPX, HSF1, HSPB1, HSPG2, HYAL1, IL15RA, IL1B, IL4R, IL7R, ILK, INHA, ITGA5, JAK3, JMJD8, JUP, KCNK2, KLF10, KMT2D, LAG3, LAMA5, LDLR, LGALS9, LIF, LIMK1, LINGO1, LOX, LRCH4, LRG1, LRRC17, LTF, MAFG, MAPK7, MECP2, MED12, MEGF8, MME, MOV10, MYC, MYH6, MYL9, MYRF, NEFL, NF2, NFATC4, NFE2, NGF, NGFR, NINJ1, NLGN2, NODAL, NOS3, NOTCH1, NOTCH4, NPPB, NR1D1, NR4A3, NRARP, NTN1, NUMBL, OMA1, OMD, OSR2, PAF1, PAK4, PARP6, PHLDB1, PIM1, PKM, PLXNA1, PLXNA3, PLXNB1, PML, POR, PPARD, PTN, RAMP2, RARA, RELA, RGCC, RGMA, RMRP, S100A1, S1PR2, SEMA3F, SEMA4B, SEMA4C, SEMA6B, SEMA6C, SERPINE1, SETD1A, SHANK3, SMAD3, SOX10, SOX12, SOX13, SOX8, SPHK1, SPI1, SRF, SRRT, STAT3, SYNGAP1, TBX5, TFE3, TGM2, THBS1, THPO, TMEM100, TNF, TNFRSF1A, TNFSF12, TNMD, TRIB1, ULK1, VDR, VEGFA, VNN1, VSIR, WARS1, WNT3, WNT9B, ZBTB7B, ZC3H12A, ZFP36, ZMIZ1, ZNF219, ZNF335]
GO:0002520	immune system development	6,27E-14	[2, 4, 5]	246,00	22,86	58,85	41,15	[ADAM8, ANGPT1, BATF, BCL11B, BCL3, BLK, BLNK, BTK, C1QC, CALCR, CAMK4, CARD11, CASP8, CCL19, CCL3, CCR2, CCR4, CD19, CD2, CD27, CD3D, CD3E, CD3G, CD4, CD40LG, CD74, CD79A, CD80, CD86, CD8A, CDKN2A, CLCF1, CR2, CRTAM, CSF1R, CTLA4, DOCK10, DOCK2, EGR3, EOMES, EPX, EVI2B, FCRL3, FGL2, FLT3, FOXP3, FUT7, GFI1, GPR171, GPR55, H3C10, H3C11, H3C12, H3C7, HCLS1, HLA-DOA, HLA-DRA, HLA-DRB1, ICOS, IFNG, IKZF1, IKZF3, IL12RB1, IL18, IL7R, INPP5D, IRF4, IRF8, ITGA4, ITK, KLRC1, LAG3, LCK, LEF1, LILRB1, LILRB4, LMO1, LRRC17, LTA, LTB, LY9, MIXL1, MMP9, MS4A1, MT1G, MYB, NCKAP1L, NFAM1, NLRP3, NR4A3, PIM1, PLA2G2D, PLD4, PLEK, POU2AF1, POU2F2, PRDM1, PRKCB, PRKCQ, PTK2B, PTPN22, PTPN6, PTPRC, RHEX, RHOH, RTKN2, RUNX3, SASH3, SELPLG, SEMA4A, SLAMF6, SLAMF8, SLC8A3, SMPD3, SNX10, SOCS1, SPN, STAT1, SYK, TBX21, TESPA1, THBS1, THEMIS, TNFAIP3, TNFRSF13B, TNFRSF18, TNFSF13B, TNFSF8, TOX, TREM2, TRPM2, UBD, VAV1, VNN1, WNT1, WNT10B, ZAP70, ZNF683]	[ABL1, ACTN1, ADIPOQ, AGER, ANGPT1, AP3D1, ARMC5, ARTN, BATF2, BCL3, BCL6, CALCR, CCR7, CD74, CDK2, CDKN1A, CEACAM1, CITED2, CLC, CLCF1, CLPTM1, CRIP2, CSF1, CTC1, CYP26B1, DHRS2, DMTN, DPF2, DTX1, EPHA2, ERBB2, ETV6, FADD, FASN, FES, FLT3, FLT4, GAB2, GATA2, GBA, GNAO1, GPR137, H3C10, H3C12, H4C3, HDAC5, HSF1, IL15RA, IL1B, IL4R, IL7R, INHA, JAK3, JUNB, KCP, KDELR1, KLF10, KMT2D, LAG3, LGALS9, LOX, LRRC17, LRRC8A, LTBR, LTF, MAPK3, MFAP5, MKNK2, MOV10, MYC, MYH9, MYL9, NBEAL2, NCOA6, NFE2, NFKB2, NOTCH1, NOTCH4, NR4A3, NRARP, OSM, PAF1, PDGFB, PIM1, PML, PTN, RARA, RELB, RMRP, RNU1-93P, RTKN2, RXRA, SBNO2, SETD1A, SH2B3, SIPA1L3, SMAD3, SNX10, SOX12, SOX13, SPI1, SPNS2, SRC, SRF, SSBP3, STAT3, SYVN1, TCF3, TCF7L1, TCIRG1, TFE3, THBS1, THPO, TIMP1, TNF, TRIB1, VEGFA, VNN1, VSIR, ZBTB7A, ZBTB7B, ZC3H12A, ZFP36, ZMIZ1, ZNF385A]
GO:0035239	tube morphogenesis	5,43E-06	[3, 4, 5]	186,00	20,74	33,98	66,02	[ADAM8, ADAMTS9, ANGPT1, ANGPT2, ANGPTL6, ANPEP, AREG, BMP7, CASP8, CCL2, CCL24, CCR2, CCR3, CEL, COL22A1, CSF1R, CXCL10, CXCR3, CXCR4, CYBB, E2F8, EGR3, EOMES, EREG, FASLG, FOLR2, GPR4, IL18, IRX3, ITGAX, ITGB2, KLF5, LDLR, LEF1, NELL2, NPPB, NR4A1, NTN1, PIK3CG, PRDM1, PRKCB, PROK1, PTK2B, RBPMS2, RLN2, SALL1, SEMA4A, SH2D2A, ST14, STAT1, SYK, TACSTD2, TBX21, THBS1, THY1, TNFAIP3, TNMD, WNT1]	[ABCC8, ABL1, ACKR3, ADAMTS9, ADGRB2, ADGRG1, AGT, AGTR1, ANGPT1, ANGPTL1, ANGPTL2, ANGPTL4, APLN, APLNR, APOD, AREG, C5, C5AR1, CCL11, CCL2, CCL24, CEACAM1, CECR2, CEL, CHI3L1, CITED2, COL22A1, CREB3L1, CSF1, CSPG4, CTHRC1, CX3CL1, CYP27B1, DAB2IP, DAG1, DCHS1, DDR1, DLG5, DVL3, E2F7, E2F8, ECM1, EFEMP2, EFNA3, ENG, EPHA2, EPN1, ERBB2, ESM1, FAP, FLT4, FOXC2, FOXF1, GATA2, GATA4, GJD3, GPR4, GRN, HDAC5, HDAC7, HESX1, HGS, HSPB1, HSPG2, HYAL1, IL1B, ILK, IRX3, ITGA5, JMJD8, JUNB, JUP, KLF5, LAMA5, LDLR, LOX, LRG1, LZTS2, MAPK7, MECP2, MED12, MEGF8, MEN1, MTHFR, MYC, MYH9, MYO18B, NFATC4, NGFR, NINJ1, NODAL, NOS3, NOTCH1, NOTCH4, NPPB, NPRL3, NR4A1, NRARP, NTN1, PAK4, PDGFRB, PFN1, PKD1, PKM, PLCD3, PML, PRKACA, PTN, RAMP2, RAP1A, RARA, RBPMS2, RGCC, RGMA, ROBO4, S100A1, SEMA4C, SERPINE1, SHANK3, SMAD3, SOX10, SOX8, SPHK1, SPI1, SRC, SRF, STAT3, SYNGAP1, TBX5, TEAD2, TGM2, THBS1, TMEM100, TNF, TNFAIP2, TNFSF12, TNMD, TSC2, UNC5B, VANGL2, VDR, VEGFA, WARS1, WNT9B, ZC3H12A, ZMIZ1]
GO:0045596	negative regulation of cell differentiation	1,17E-02	[3, 4, 5]	136,00	19,77	41,02	58,98	[AREG, ASCL2, BHLHA15, BMP7, C1QC, CCL17, CCL3, CD74, CDKN2A, CMTM5, CTLA4, CXCL10, DCC, DPEP1, EREG, FGL2, FOXP3, FRZB, GLIS1, GPR171, GPR55, IFNG, IL18, INPP5D, IRX3, LAG3, LDLR, LILRB1, LILRB4, LRRC17, MIXL1, MMP9, MSX1, MYB, NPPC, NTN1, P2RY12, PTK2B, RBPMS2, RUNX3, SEMA4A, SEMA4D, SOCS1, STAT1, TACSTD2, TBX21, THY1, TNR, TREM2, WNT1, WNT10B]	[ABCC8, ACTN4, ADAMTS7, ADIPOQ, AREG, ARHGDIA, BBS12, BCL6, CCL11, CD74, CEACAM1, CETP, CHERP, CMTM5, CRP, CTDSP1, DAB2IP, DISP3, DMTN, DNMT1, DPF2, DTX1, ERBB2, GATA2, GDI1, GPER1, GPR137, GPR37L1, GSK3A, H4C3, HDAC5, HDAC7, ID3, IL1B, IL4R, INHA, IRX3, JAK3, LAG3, LDLR, LINGO1, LMX1A, LRRC17, LTF, MECP2, MSTN, MSX1, MYC, NFATC4, NGFR, NODAL, NOTCH1, NOTCH4, NR1D1, NRARP, NTN1, OMA1, PAF1, PDGFB, PLXNA3, POSTN, PPARD, PTN, RARA, RBPMS2, RGMA, RMRP, SCAF1, SEMA3F, SEMA4B, SEMA4C, SEMA6B, SEMA6C, SMAD3, SNAI1, SOX10, SOX8, SPDEF, STAT3, SYNGAP1, TBX5, TGFB1I1, TNF, TRIB1, TRIB3, TRIM62, TRPV4, TSKU, ULK1, VASN, VEGFA, WNT3, ZBTB7B, ZC3H12A, ZFP36]

GO:0045597	positive regulation of cell differentiation	2,37E-08	[3, 4, 5]	192,00	21,87	40,86	59,14	[ADAM8, ALOX15B, AREG, BMP7, BMPR1B, BTK, CASP8, CASS4, CCL19, CD27, CD4, CD74, CD80, CD86, CDH4, CLCF1, CRABP2, CRB2, CXCL9, CXCR4, EGR3, EPX, EVI2B, FFAR4, FOXP3, GDF6, HCLS1, HLA-DRA, HLA-DRB1, HOPX, IFNG, IL12RB1, IL18, IL7R, INPP5D, IRX3, KLF5, LCP1, LEF1, LIF, LILRB4, LTA, MEDAG, MYB, NCKAP1L, NEK5, NLRP3, NPPC, NTN1, PIM1, PLAAT4, PLXNC1, PTPRC, RHEX, RHOH, RUNX3, SASH3, SEMA4D, SOCS1, SOCS3, STAT1, SULT2B1, SYK, TACSTD2, TESPA1, TOX, TREM2, VNN1, VWC2L, WNT10B, ZAP70, ZBED2]	[ABL1, ADIPOQ, AGER, AGT, AGTR1, AP3D1, APOA1, AREG, ARHGDIA, BCL6, BCL9L, BEND6, CAMK2B, CARM1, CD74, CDH4, CEACAM1, CLCF1, COL1A1, CSF1, CSNK1E, CTHRC1, CX3CL1, CYP26B1, CYP27B1, DAG1, DDX39B, DMTN, EFEMP2, EIF4G1, ENG, FADD, FES, GATA2, GATA4, GDF6, GDI1, GPER1, HEYL, HOPX, HSF1, HTR2A, IFITM1, IL15RA, IL1B, IL4R, IL7R, ILK, IRX3, JUNB, JUND, KCTD11, KLF10, KLF5, LGALS9, LIF, LIMK1, LIMS2, LRRC8A, LTF, LZTS1, MAMSTR, MAPK8IP3, MED12, MEGF8, MME, MYADM, MYC, NAP1L2, NEFL, NEK5, NF2, NGF, NGFR, NOTCH1, NTN1, NUMBL, OMA1, PARP6, PDLIM7, PIEZO1, PIM1, PLA2G2A, PLXNA1, PLXNA3, PLXNB1, POR, PPARD, PPP1R13L, PTN, RAMP2, RAP1A, RARA, RELA, RGCC, S1PR2, SERPINE1, SHANK3, SMAD3, SNAI1, SOCS3, SOX10, SOX12, SOX13, SOX8, SPDEF, SRF, SRRT, STAT3, TBX5, TCF3, TCF7L1, TESK1, TFE3, TGFB1I1, TGM2, THPO, TIMP1, TMEM100, TNF, TRIB1, TRIOBP, VDR, VEGFA, VNN1, VSIG2, VSIR, WNT3, ZBTB7B, ZC3H12A, ZFP36, ZMIZ1, ZNF219, ZNF335, ZNF385A]
GO:0048513	animal organ development	1,47E-10	[3, 4, 5]	665,00	17,44	39,71	60,29	[ACP5, ADAM8, ADAMTS9, ADGRG6, AIF1, AKNA, ALOX15, ALOX15B, ANGPT1, ANGPT2, AQP5, AREG, ASCL2, ASGR2, ATF3, BATF, BCL11B, BCL3, BFSP2, BLK, BLNK, BMP7, BMPR1B, BRIP1, BTG2, BTK, C1QB, C1QC, C1QTNF1, CALCR, CAMK4, CARD11, CASP8, CCL19, CCL2, CCL3, CCR2, CD19, CD2, CD27, CD3D, CD3E, CD3G, CD4, CD40LG, CD74, CD79A, CD80, CD86, CD8A, CDH1, CDKN2A, CEL, CERS3, CHRDL1, CHRDL2, CLCF1, CR2, CRB2, CRTAM, CSF1R, CTLA4, CXCL10, CXCR4, DAW1, DCC, DOCK10, DOCK2, DPEP1, DPEP2, DRC1, DRD1, E2F8, EAF2, EDN2, EGR3, ELAVL4, ELF3, EOMES, EPX, EREG, EVI2B, FASLG, FCRL3, FGL2, FGR, FLT3, FOLR2, FOSL1, FOXP3, FRZB, FUT7, GCNT3, GDF6, GFI1, GPM6A, GPR171, GPR4, GPR55, GRXCR2, H3C10, H3C11, H3C12, H3C7, HCLS1, HCN1, HLA-DOA, HLA-DRA, HLA-DRB1, HOPX, IFNG, IKZF1, IKZF3, IL12RB1, IL18, IL7R, INPP5D, IRF4, IRF8, IRX3, ITGA4, ITGAX, ITK, JPH1, KLF5, KLHL41, KLRC1, KRT85, LAG3, LCK, LCP1, LEF1, LIF, LILRB1, LILRB4, LIPM, LMO1, LPAL2, LRRC17, LTA, LTB, LY9, MAFF, MCOLN3, MIXL1, MMP9, MS4A1, MSLN, MSX1, MT1G, MYB, MYCL, MYH6, MYO3B, NAMPT, NCKAP1L, NELL2, NFAM1, NLRP3, NPPB, NPPC, NR4A1, NR4A2, NR4A3, NTN1, OPN5, P2RY12, PAX5, PBX4, PIM1, PLA2G2D, PLAAT4, PLD4, PLEK, PLPPR4, POU2AF1, POU2F2, PRDM1, PRKCB, PRKCQ, PTK2B, PTPN22, PTPN6, PTPRC, PTX3, RBPMS2, RHEX, RHOH, RNASE6, RRAD, RTKN2, RUNX3, SALL1, SASH3, SCUBE1, SDC1, SELPLG, SEMA4A, SEMA4D, SLAMF6, SLAMF8, SLC24A4, SLC8A3, SMPD3, SNX10, SOCS1, SOCS3, SPEF2, SPN, ST14, STAT1, SYK, TACSTD2, TBX21, TESPA1, TEX11, THBS1, THEMIS, THY1, TIFAB, TMEM132E, TNFAIP3, TNFRSF11B, TNFRSF13B, TNFRSF18, TNFSF13B, TNFSF8, TNR, TOX, TREM2, TRPM2, UBD, UCP2, VAV1, VNN1, WNT1, WNT10A, WNT10B, ZAP70, ZBED2, ZNF683]	[AATK, ABCA3, ABL1, ACACB, ACP4, ACTA1, ACTB, ACTC1, ACTN1, ADAM19, ADAMTS5, ADAMTS7, ADAMTS9, ADCY1, ADGRG1, ADGRG6, ADIPOQ, AGAP2, AGER, AGT, AGTR1, ALPK3, ALPL, AMH, ANGPT1, ANKRD52, AP1B1, AP2A1, AP3D1, APLN, APLNR, APLP1, APOA1, APOD, AQP3, AQP5, AREG, ARHGEF15, ARMC5, ARRDC3, ARTN, ASPN, ATAT1, ATF5, ATP1B2, ATP6V0D1, ATP6V1B1, BAG3, BAG6, BATF2, BCL2L1, BCL3, BCL6, BCL9L, BCOR, BCR, BGN, BMP8A, BTG2, C1QTNF1, C5AR1, CACNA1H, CALB1, CALCR, CAPN1, CAPNS1, CCDC85C, CCL11, CCL2, CCR7, CD74, CDK1, CDK2, CDKN1A, CEACAM1, CECR2, CEL, CELSR2, CERT1, CFH, CGA, CHGA, CHI3L1, CHRDL2, CIC, CITED2, CLCF1, CLPTM1, CNNM4, COASY, COL1A1, COL27A1, CRAT, CRIP2, CRYAB, CSF1, CSK, CSNK1E, CTC1, CTF1, CTHRC1, CYP21A2, CYP26B1, CYP27B1, DAB2IP, DAG1, DCHS1, DDR1, DDX39B, DEDD, DHRS2, DLG5, DMC1, DMRTA1, DMTN, DPF2, DRC1, DSC1, DSCAML1, DTX1, DVL3, DYRK1B, E2F4, E2F7, E2F8, EAF2, ECM1, EDN2, EFEMP2, EIF4EBP1, ELF3, ELK1, ELOVL1, ENG, EPHA2, EPN1, ERBB2, ETV6, FA2H, FADD, FAIM2, FANCC, FASN, FES, FGFRL1, FHL3, FLG, FLT3, FLT4, FOSL1, FOSL2, FOXC2, FOXF1, FURIN, GAB2, GATA2, GATA4, GBA, GCNT3, GDF6, GIT1, GJD3, GLIS2, GMNN, GNAI2, GNAO1, GPER1, GPM6A, GPR137, GPR37L1, GPR4, GPRIN1, GSK3A, GYS1, H3C10, H3C12, H4C3, HBEGF, HCN4, HDAC5, HESX1, HEYL, HFE, HID1, HIVEP3, HMGN1, HNMT, HOPX, HPRT1, HSF1, HSPB7, HSPG2, HYAL1, ICAM1, ID3, IFT80, IL15RA, IL1B, IL4R, IL7R, ILK, INHA, INPPL1, IRX3, ITGA3, ITGA7, JAK3, JUNB, JUP, KCNK2, KCNK3, KCNQ4, KCP, KCTD11, KDELR1, KDM6B, KERA, KLF10, KLF5, KLK14, KMT2D, KRTAP5-1, LAG3, LAMA5, LAMB4, LGALS9, LIF, LIMK2, LIMS2, LMNA, LMX1A, LOX, LRP10, LRRC17, LRRC8A, LSR, LTBR, LTF, LUM, LY6E, LZTS1, LZTS2, MAMLD1, MAP1S, MAPK3, MBOAT7, MCRIP1, MDGA1, MDGA2, MECP2, MED12, MEF2D, MEGF8, MEIS3P1, MEIS3P2, MEN1, MEAP2, MEAP5, MESD2A, MKNK2, MME
GO:0001817	regulation of cytokine production	4,02E-20	[3, 4, 5, 6]	212,00	26,77	59,95	40,05	[ACP5, ADAM8, ADCY7, AIF1, AIM2, ALOX15B, ANGPT1, BANK1, BATF, BCL3, BTK, BTN3A1, BTN3A2, CAMK4, CARD11, CARD17, CASP8, CCDC88B, CCL19, CCL3, CCR2, CD2, CD200R1, CD226, CD244, CD33, CD3E, CD4, CD40LG, CD6, CD74, CD80, CD84, CD86, CD96, CLCF1, CLEC6A, CLNK, CRTAM, CSF1R, CXCL6, CYBB, EBI3, EPX, EREG, FCN1, FFAR4, FGR, FOXP3, GBP5, GPSM3, HLA-DPA1, HLA-DPB1, HLA-DRB1, IFNG, IKBKE, IL12RB1, IL12RB2, IL16, IL18, IL26, IL5RA, INPP5D, IRF4, IRF5, IRF8, ITK, LAG3, LCP1, LEF1, LILRB1, LILRB4, LPAL2, LTA, LTB, LY9, MAPK13, MCOLN2, MOG, MYB, NCKAP1L, NFAM1, NLRC3, NLRP2, NLRP3, NLRP6, NLRP7, NLRP9, NR4A3, ORM2, PIK3CG, PLD4, POU2AF1, POU2F2, PRKCQ, PTAFR, PTPN22, PTPN6, PTPRC, PYCARD, PYHIN1, SASH3, SCIMP, SIGLEC16, SLAMF6, SOCS1, STAT1, SYK, TBX21, THBS1, TIGIT, TLR10, TLR6, TLR8, TNFAIP3, TREM2, UBASH3A, XCL1, ZBP1]	[ABCC8, ABCD1, ABCD2, ABL1, ACKR1, ADIPOQ, AGER, AGPAT1, AGT, AMH, ANGPT1, APOA1, APOD, BCL3, BCL6, C5, C5AR1, CCR7, CD14, CD276, CD74, CEACAM1, CHI3L1, CLC, CLCF1, CLNK, CPTP, CRP, CSK, CX3CL1, EPHA2, FADD, FFAR2, FLT4, FRMD8, FURIN, GATA4, GBA, GIT1, GPAM, HDAC7, HFE, HK1, HSPB1, IGF2BP2, IL1B, IL4R, IL5RA, INHA, JAK3, JPH4, LAG3, LGALS9, LILRA5, LRRC32, LTF, LUM, MAP2K3, MAST2, MAVS, NFATC4, NFKB2, NLRC5, NLRP9, NOD2, NODAL, NR4A3, NUTF2, OSM, PER1, PLD3, PML, POLR2E, POLR2L, POSTN, PPP1R11, PQBP1, RARA, RELA, RELB, RGCC, RNF26, RNU1-93P, SERPINE1, SLC7A5, SMAD3, SPHK1, SPHK2, SRC, STAT3, STING1, THBS1, TICAM1, TNF, TRPV4, TSKU, UBE2L6, VSIR, ZBTB7B, ZC3H12A, ZCCHC3, ZFP36, ZNF580]

GO:0007399	nervous system development	3,92E-03	[4, 5]	431,00	16,71	28,92	71,08	[ADGRG6, AIF1, AKNA, APBA2, AREG, ASCL2, BCAS1, BCL11B, BHLHA15, BHLHE22, BHLHE40, BICDL1, BLK, BMP7, BMPR1B, BTG2, C1QA, CCL2, CCL3, CCR2, CCR4, CD3E, CDH1, CDH4, CEL, CHRDL1, CLCF1, CNR1, CRABP2, CRB2, CST7, CXCR4, DCC, DOCK10, DOK2, DPEP1, DRD1, EGR3, ELAVL3, ELAVL4, EMB, EOMES, EPHA6, EPX, FOLR2, FOXP3, GFI1, GFRA2, GPM6A, GRXCR2, HCN1, IFNG, IRX3, ITGA4, ITGAX, KCNJ10, LDLR, LEF1, LIF, LRFN1, LST1, LTA, MCOLN3, MOG, MSX1, MUSK, MYB, MYCL, NCKAP1L, NELL2, NPPC, NR4A2, NTN1, P2RY12, PAX5, PBX4, PCDHA2, PLPPR4, PLXNC1, PRDM1, PRKCQ, PTK2B, RAC2, RASGRF1, RGS9, RHOH, RNASE6, RRAD, RUNX3, SALL1, SEMA4A, SEMA4D, SLC8A3, SPEF2, ST14, STAP1, THY1, TIFAB, TMEM132E, TNR, TOX, TREM2, UGT8, VWC2L, WNT1, WNT10A, WNT10B, ZNF804A]	[AATK, ABCC8, ABCD1, ABCD2, ABI3, ABL1, ACP4, ACSBG1, ACTB, ADCY1, ADGRB2, ADGRG1, ADGRG6, ADGRL1, AGER, AGT, AP2A1, APLP1, APOA1, APOD, ARC, AREG, ARF1, ARHGDIA, ARHGEF15, ARID1A, ARSA, ARTN, ATAT1, ATF5, ATN1, ATP1B2, ATP6V0D1, BAG3, BAG6, BCL6, BEND6, BHLHE40, BSN, BTBD2, BTG2, C5AR1, CAMK2B, CAMSAP3, CARM1, CASZ1, CCDC85C, CCL11, CCL2, CDH4, CDK1, CECR2, CEL, CELSR2, CFL1, CHERP, CIC, CITED2, CLCF1, CNR1, CNTN5, CNTN6, CPNE5, CPNE6, CSF1, CSK, CSNK1E, CSPG4, CST7, CTDSP1, CTF1, CTHRC1, CX3CL1, DAB2IP, DAG1, DAGLA, DCHS1, DDR1, DGUOK, DISP3, DLG5, DOK5, DPF2, DPYSL4, DSCAML1, DTX1, DVL3, EFNA3, EHD1, EIF4G1, ENG, EPHA2, EPOP, ERBB2, ETV6, FA2H, FAIM2, FANCC, FES, GAB2, GATA2, GBA, GDI1, GDPD5, GFRA3, GIT1, GLIS2, GNAO1, GPER1, GPM6A, GPR37L1, GPRIN1, GRB7, GRN, GSK3A, HAPLN1, HAPLN3, HDAC10, HESX1, HEYL, HNMT, HPRT1, HSPG2, ID3, IGF2BP2, IGSF9B, IL1B, ILK, INPP5J, IRF2BPL, IRX3, IRX6, ITGA3, JRKL, KATNB1, KCNIP2, KCNK3, KCTD11, KDM6B, KIF26A, LAMA5, LDLR, LGR6, LIF, LIMK1, LINGO1, LMX1A, LMX1B, LRCH4, LRFN3, LRRC4, LSR, LYPLA2, LZTS1, LZTS3, MALL, MAP1A, MAP1S, MAPK3, MAPK7, MAPK8IP3, MARCKSL1, MARK2, MARK4, MBOAT7, MDGA1, MDGA2, MECP2, MED12, MEF2D, MEGF8, MEIS3P1, MEIS3P2, MEN1, MFSD2A, MICALL1, MICALL2, MINK1, MME, MOV10, MSI1, MSX1, MTHFR, MYC, MYO7A, MYRF, NAP1L2, NAPA, NCOA6, NDP, NDST1, NECTIN1, NEFL, NF2, NFATC4, NGF, NGFR, NINJ1, NLGN2, NNAT, NODAL, NOTCH1, NOTCH3, NPAS2, NR1D1, NR4A2, NRGN, NRSN2, NSMF, NTN1, NUMBL, OGDH, OMA1, P2RX5, PACSIN1, PAFAH1B3, PALM, PARP6, PCDH1, PCDHA10, PCDHA11, PCDHA2, PCDHA3, PCDHA4, PCDHA6, PDLIM7, PFN1, PHACTR1, PIGT, PISD, PITPNM1, PKD1, PLXNA1, PLXNA3, PLXNB1, POSTN, POU6F1, PPARD, PPP1R9B, PPP2R5D, PQBP1, PRAG1, PRKACA, PTN, PTPRU, PYGO2, RAB3A, RAC2, RAP1A, RAPGEF1, RAPGEFL1, RARA, RELA
GO:0022604	regulation of cell morphogenesis	4,32E-03	[4, 5]	76,00	23,24	31,93	68,07	[CASS4, CCL2, CCL24, CCL3, CORO1A, CSF1R, CXCR4, ERMN, FGD2, FGD3, FGR, ITGB2, LST1, PEAK3, PLXNC1, PTK2B, RAC2, RHOH, SEMA4A, SEMA4D, TACSTD2]	[ABL1, ACTN4, ALDOA, APOA1, ARC, ARHGDIA, BCL9L, C15orf62, CAMK2B, CCL11, CCL2, CCL24, CDC42EP1, CDC42EP2, CDC42EP4, CFL1, CPNE5, CPNE6, DAG1, DMTN, DVL3, FBLIM1, FES, FMNL1, ICAM1, ILK, ITGA7, LIMS2, MARK2, MED12, MFSD2A, MOV10, MSN, MYADM, MYH7B, MYH9, PALM, PALM2AKAP2, PARP6, PARVB, PLEKHO1, PLXNA1, PLXNA3, PLXNB1, POSTN, PRAG1, PTN, RAC2, RHOBTB2, RHOG, RNF157, SLC9A3R1, SRC, SYNGAP1, SYT2, TESK1, TRIOBP, VEGFA]
GO:0048534	hematopoietic or lymphoid organ development	4,52E-16	[3, 4, 5, 6]	241,00	23,86	58,71	41,29	[ADAM8, ANGPT1, BATF, BCL11B, BCL3, BLK, BLNK, BTK, C1QC, CALCR, CAMK4, CARD11, CASP8, CCL19, CCL3, CCR2, CD19, CD2, CD27, CD3D, CD3E, CD3G, CD4, CD40LG, CD74, CD79A, CD80, CD86, CD8A, CDKN2A, CLCF1, CR2, CRTAM, CSF1R, CTLA4, DOCK10, DOCK2, EGR3, EOMES, EPX, EVI2B, FCRL3, FGL2, FLT3, FOXP3, FUT7, GFI1, GPR171, GPR55, H3C10, H3C11, H3C12, H3C7, HCLS1, HLA-DOA, HLA-DRA, HLA-DRB1, IFNG, IKZF1, IKZF3, IL12RB1, IL18, IL7R, INPP5D, IRF4, IRF8, ITGA4, ITK, KLRC1, LAG3, LCK, LEF1, LILRB1, LILRB4, LMO1, LRRC17, LTA, LTB, LY9, MIXL1, MMP9, MS4A1, MT1G, MYB, NCKAP1L, NFAM1, NLRP3, NR4A3, PIM1, PLA2G2D, PLD4, PLEK, POU2AF1, POU2F2, PRDM1, PRKCB, PRKCQ, PTK2B, PTPN22, PTPN6, PTPRC, RHEX, RHOH, RTKN2, RUNX3, SASH3, SELPLG, SEMA4A, SLAMF6, SLAMF8, SLC8A3, SMPD3, SNX10, SOCS1, SPN, STAT1, SYK, TBX21, TESPA1, THBS1, THEMIS, TNFRSF13B, TNFRSF18, TNFSF13B, TNFSF8, TOX, TREM2, TRPM2, UBD, VAV1, VNN1, WNT1, WNT10B, ZAP70, ZNF683]	[ABL1, ACTN1, ADIPOQ, AGER, ANGPT1, AP3D1, ARMC5, ARTN, BATF2, BCL3, BCL6, CALCR, CCR7, CD74, CDK2, CDKN1A, CEACAM1, CITED2, CLCF1, CLPTM1, CRIP2, CSF1, CTC1, CYP26B1, DHRS2, DMTN, DPF2, DTX1, EPHA2, ERBB2, ETV6, FADD, FASN, FES, FLT3, FLT4, GAB2, GATA2, GBA, GNAO1, GPR137, H3C10, H3C12, H4C3, HDAC5, HSF1, IL15RA, IL1B, IL4R, IL7R, INHA, JAK3, JUNB, KCP, KDELR1, KLF10, KMT2D, LAG3, LGALS9, LOX, LRRC17, LRRC8A, LTBR, LTF, MAPK3, MFAP5, MKNK2, MOV10, MYC, MYH9, MYL9, NBEAL2, NCOA6, NFE2, NFKB2, NOTCH1, NOTCH4, NR4A3, NRARP, OSM, PAF1, PDGFB, PIM1, PML, PTN, RARA, RELB, RMRP, RNU1-93P, RTKN2, RXRA, SBNO2, SETD1A, SH2B3, SIPA1L3, SNX10, SOX12, SOX13, SPI1, SPNS2, SRC, SRF, SSBP3, STAT3, SYVN1, TCF3, TCF7L1, TCIRG1, TFE3, THBS1, THPO, TIMP1, TNF, TRIB1, VEGFA, VNN1, VSIR, ZBTB7A, ZBTB7B, ZC3H12A, ZFP36, ZMIZ1, ZNF385A]

GO:0072359	circulatory system development	3,68E-04	[4, 5]	220,00	18,98	32,45	67,55	[ADAM8, ADAMTS9, ADGRG6, ANGPT1, ANGPT2, ANGPTL6, ANPEP, BMP7, CASP8, CCL2, CCL24, CCR2, CCR3, CEL, COL22A1, CRB2, CXCL10, CXCR3, CXCR4, CYBB, DAW1, DPEP1, DPEP2, DRC1, E2F8, EGR3, EOMES, EREG, FASLG, FOLR2, FOSL1, GPR4, HOPX, IL18, IRX3, ITGAX, ITGB2, KLF5, LDLR, LEF1, LIF, MIXL1, MSX1, MYH6, NELL2, NPPB, NR4A1, PIK3CG, PIM1, PRDM1, PRKCB, PROK1, PTK2B, RLN2, SALL1, SCUBE1, SEMA4A, SH2D2A, SOCS3, STAT1, SYK, TBX21, THBS1, THY1, TNFAIP3, TNMD]	[ABCC8, ABL1, ACACB, ACKR3, ACTC1, ADAMTS9, ADGRB2, ADGRG1, ADGRG6, AGT, AGTR1, ALPK3, ANGPT1, ANGPTL1, ANGPTL2, ANGPTL4, AP1B1, APLN, APLNR, APOD, ARHGEF15, BCOR, C5, C5AR1, CCL11, CCL2, CCL24, CDK1, CDKN1A, CEACAM1, CEL, CERT1, CHI3L1, CITED2, COL1A1, COL22A1, CREB3L1, CSPG4, CX3CL1, DAB2IP, DAG1, DCHS1, DDX39B, DNMT1, DRC1, E2F7, E2F8, ECM1, EFEMP2, EFNA3, ENG, EPHA2, EPN1, ERBB2, ESM1, FAP, FGFRL1, FLT4, FOSL1, FOXC2, FOXF1, FOXS1, GATA2, GATA4, GJA4, GJD3, GPER1, GPR4, GRN, GSK3A, GYS1, HCN4, HDAC5, HDAC7, HEYL, HGS, HOPX, HSPB1, HSPB7, HSPG2, HYAL1, ID3, IL1B, ILK, IRX3, ITGA3, ITGA5, JMJD8, JUNB, JUP, KCNK2, KDM6B, KLF5, LDLR, LIF, LMNA, LOX, LRG1, LY6E, MAPK3, MAPK7, MECP2, MED12, MEF2D, MEGF8, MEN1, MOSPD3, MSC, MSX1, MYH6, MYH9, MYO18B, NCOA6, NDST1, NFATC4, NGFR, NINJ1, NODAL, NOL6, NOS3, NOTCH1, NOTCH4, NPPA, NPPB, NPRL3, NR4A1, NRARP, PAK4, PDGFB, PDGFRB, PDLIM7, PIM1, PKD1, PKM, PLCD3, PML, POU6F1, PPARD, PPP1R13L, PTN, RAMP2, RAP1A, RARA, RGCC, RNU1-93P, ROBO4, S100A1, SCN5A, SERPINE1, SIK1, SLC9A1, SMAD3, SNAI1, SOCS3, SPHK1, SPHK2, SPI1, SRF, STAT3, SYNGAP1, SYPL2, TBX5, TEAD2, THBS1, TMEM100, TNF, TNFAIP2, TNFRSF1A, TNFSF12, TNMD, TSC2, UNC5B, VANGL2, VEGFA, VSIG2, WARS1, ZC3H12A, ZMIZ1]
GO:0001819	positive regulation of cytokine production	2,05E-13	[3, 4, 5, 6, 7]	142,00	27,26	64,89	35,11	[ADAM8, AIF1, AIM2, ALOX15B, BATF, BCL3, BTN3A1, BTN3A2, CAMK4, CARD11, CASP8, CCDC88B, CCL19, CCL3, CCR2, CD2, CD226, CD244, CD3E, CD4, CD40LG, CD6, CD74, CD80, CD86, CLEC6A, CLNK, CRTAM, CSF1R, CYBB, EBI3, EPX, EREG, FCN1, FGR, FOXP3, GBP5, GPSM3, HLA-DPA1, HLA-DPB1, IFNG, IL12RB1, IL12RB2, IL16, IL18, IL26, IRF4, IRF5, IRF8, ITK, LCP1, LILRB1, LPAL2, LTA, LTB, LY9, MAPK13, MCOLN2, MYB, NFAM1, NLRP2, NLRP3, NLRP9, NR4A3, ORM2, PIK3CG, POU2AF1, POU2F2, PRKCQ, PTAFR, PTPN22, PTPRC, PYCARD, PYHIN1, SASH3, SCIMP, SIGLEC16, SLAMF6, STAT1, SYK, THBS1, TIGIT, TLR6, TLR8, TREM2, XCL1, ZBP1]	[ABCC8, ABL1, ADIPOQ, AGER, AGPAT1, AGT, AMH, BCL3, C5, C5AR1, CCR7, CD14, CD276, CD74, CHI3L1, CLNK, CX3CL1, FADD, FFAR2, FLT4, FRMD8, FURIN, GATA4, HK1, HSPB1, IL1B, IL4R, LGALS9, LILRA5, LUM, MAVS, NFATC4, NFKB2, NLRP9, NOD2, NODAL, NR4A3, OSM, POLR2E, POLR2L, POSTN, PQBP1, RARA, RELA, RGCC, RNU1-93P, SERPINE1, SLC7A5, SMAD3, SPHK1, SPHK2, SRC, STAT3, STING1, THBS1, TICAM1, TNF, TRPV4, ZBTB7B, ZCCHC3, ZNF580]
GO:0001944	vasculature development	6,73E-05	[4, 5, 6]	158,00	20,82	34,14	65,86	[ADAM8, ADAMTS9, ANGPT1, ANGPT2, ANGPTL6, ANPEP, BMP7, CASP8, CCL2, CCL24, CCR2, CCR3, CEL, COL22A1, CXCL10, CXCR3, CXCR4, CYBB, E2F8, EGR3, EREG, FASLG, FOLR2, FOSL1, GPR4, IL18, ITGAX, ITGB2, KLF5, LDLR, LEF1, LIF, NELL2, NPPB, NR4A1, PIK3CG, PRDM1, PRKCB, PROK1, PTK2B, RLN2, SEMA4A, SH2D2A, SOCS3, STAT1, SYK, THBS1, THY1, TNFAIP3, TNMD]	[ABCC8, ABL1, ACKR3, ADAMTS9, ADGRB2, ADGRG1, AGT, AGTR1, ANGPT1, ANGPTL1, ANGPTL2, ANGPTL4, APLN, APLNR, APOD, ARHGEF15, C5, C5AR1, CCL11, CCL2, CCL24, CEACAM1, CEL, CHI3L1, CITED2, COL1A1, COL22A1, CREB3L1, CSPG4, CX3CL1, DAB2IP, DAG1, DNMT1, E2F7, E2F8, ECM1, EFEMP2, EFNA3, ENG, EPHA2, EPN1, ERBB2, ESM1, FAP, FLT4, FOSL1, FOXC2, FOXF1, FOXS1, GATA2, GATA4, GJA4, GJD3, GPER1, GPR4, GRN, HDAC5, HDAC7, HGS, HSPB1, HSPG2, HYAL1, IL1B, ITGA5, JMJD8, JUNB, JUP, KLF5, LDLR, LIF, LOX, LRG1, MAPK7, MECP2, MEGF8, MEN1, MYH9, MYO18B, NDST1, NFATC4, NGFR, NINJ1, NODAL, NOS3, NOTCH1, NOTCH4, NPPB, NPRL3, NR4A1, NRARP, PAK4, PDGFB, PDGFRB, PKD1, PKM, PLCD3, PML, PTN, RAMP2, RAP1A, RGCC, ROBO4, S100A1, SERPINE1, SOCS3, SPHK1, SPHK2, SPI1, SRF, STAT3, SYNGAP1, TBX5, TEAD2, THBS1, TMEM100, TNF, TNFAIP2, TNFSF12, TNMD, UNC5B, VEGFA, VSIG2, WARS1, ZC3H12A, ZMIZ1]
GO:0010628	positive regulation of gene expression	4,44E-08	[4, 5, 6]	249,00	20,34	51,15	48,85	[ADAM8, AIF1, AIM2, ALOX15B, ANGPT1, AREG, ATF3, BATF, BCL3, BLNK, BMP7, BTN3A1, BTN3A2, C1QTNF1, CALCR, CAMK4, CARD11, CASP8, CCDC88B, CCL19, CCL3, CCL5, CCR2, CD2, CD226, CD244, CD3E, CD4, CD40LG, CD6, CD74, CD80, CD86, CDKN2A, CLCF1, CLEC6A, CLNK, CRTAM, CSF1R, CYBB, DRD1, EBI3, ELAVL4, EPX, EREG, FCN1, FGR, FOXP3, GBP5, GPSM3, H1-3, HLA-DPA1, HLA-DPB1, IFNG, IKBKE, IL12RB1, IL12RB2, IL16, IL18, IL26, IL7R, IRF4, IRF5, IRF8, ITGAX, ITK, LAMP3, LCP1, LDLR, LEF1, LIF, LILRB1, LPAL2, LTA, LTB, LY9, MAPK13, MCOLN2, MUSK, MYB, MZB1, NFAM1, NLRP2, NLRP3, NLRP9, NR4A3, ORM2, PIK3CG, POU2AF1, POU2F2, PRDM1, PRKCQ, PTAFR, PTGFR, PTK2B, PTPN22, PTPRC, PYCARD, PYHIN1, RLN2, SASH3, SCIMP, SIGLEC16, SLAMF6, STAP1, STAT1, SYK, TBX21, TENT5C, THBS1, TIGIT, TLR6, TLR8, TREM2, WNT10A, XCL1, ZBP1, ZNF804A]	[ABCC8, ABL1, ACTA1, ACTB, ACTC1, ADAM19, ADIPOQ, AGER, AGPAT1, AGT, AMH, ANGPT1, AREG, ARID1A, ATP13A2, BCL3, C1QTNF1, C5, C5AR1, CALCR, CCR7, CD14, CD276, CD74, CDK1, CHI3L1, CITED2, CLCF1, CLNK, CRP, CSF1, CTIF, CX3CL1, CYP26B1, CYP27B1, DDX39B, DHX34, DNMT1, EIF4G1, ENG, ERBB2, FADD, FFAR2, FGFR4, FLT4, FRMD8, FURIN, GATA2, GATA4, GPER1, GPI, GSK3A, H1-9P, HCFC1, HFE, HGS, HK1, HMGA1, HMGN5, HSF1, HSPB1, ID3, IL1B, IL4R, IL7R, ITGA3, LDLR, LGALS9, LIF, LILRA5, LMNA, LRRC32, LUM, MAFG, MAPK3, MAVS, MAZ, MOV10, MPV17L2, MSN, MYBBP1A, MYC, MYH9, NCOR2, NFATC4, NFKB2, NGF, NKD2, NLRP9, NOD2, NODAL, NOS3, NOTCH1, NR4A3, OSM, OSR2, PDGFB, PKM, POLR2E, POLR2L, POSTN, POU5F1P4, PPARD, PPP1R15A, PQBP1, PRKCSH, PTGFR, RAMP2, RARA, RELA, RELB, RGCC, RNU1-93P, RPL26, SERPINE1, SF3B4, SLC35A4, SLC7A5, SMAD3, SOX10, SOX8, SPHK1, SPHK2, SPI1, SRC, STAT3, STING1, TAF1C, THBS1, TICAM1, TNF, TRPV4, VDR, VEGFA, VSIR, WARS1, WBP2, WHRN, WNT3, ZBTB7B, ZC3H12A, ZCCHC3, ZFP36, ZMIZ1, ZMIZ2, ZNF580]

GO:0022008	neurogenesis	1,94E-02	[4, 5, 6]	301,00	17,19	32,28	67,72	[ADGRG6, AKNA, AREG, ASCL2, BCL11B, BHLHA15, BHLHE22, BHLHE40, BICDL1, BLK, BMP7, BMPR1B, BTG2, C1QA, CCL2, CCL3, CCR2, CCR4, CD3E, CDH1, CDH4, CLCF1, CNR1, CRABP2, CRB2, CXCR4, DCC, DOCK10, DOK2, DPEP1, DRD1, ELAVL4, EMB, EPHA6, EPX, FOLR2, GFI1, GFRA2, GPM6A, GRXCR2, HCN1, IFNG, IRX3, ITGA4, KCNJ10, LDLR, LEF1, LIF, LST1, LTA, MCOLN3, MYB, MYCL, NCKAP1L, NELL2, NPPC, NR4A2, NTN1, P2RY12, PBX4, PLPPR4, PLXNC1, PRDM1, PRKCQ, PTK2B, RAC2, RASGRF1, RHOH, RUNX3, SALL1, SEMA4A, SEMA4D, SLC8A3, STAP1, THY1, TMEM132E, TNR, TOX, TREM2, UGT8, VWC2L, WNT1, WNT10A, WNT10B, ZNF804A]	[ABCC8, ABI3, ABL1, ACP4, ACTB, ADCY1, ADGRG1, ADGRG6, AGER, AGT, AP2A1, APOA1, APOD, ARC, AREG, ARF1, ARHGDIA, ARTN, ATAT1, ATF5, ATP1B2, BCL6, BEND6, BHLHE40, BTBD2, BTG2, C5AR1, CAMK2B, CAMSAP3, CARM1, CASZ1, CCL11, CCL2, CDH4, CDK1, CECR2, CELSR2, CFL1, CLCF1, CNR1, CNTN6, CPNE5, CPNE6, CSF1, CSK, CSNK1E, CSPG4, CTDSP1, CTF1, CTHRC1, CX3CL1, DAB2IP, DAG1, DAGLA, DCHS1, DDR1, DGUOK, DISP3, DLG5, DOK5, DPYSL4, DSCAML1, DTX1, DVL3, EFNA3, EHD1, EIF4G1, EPHA2, EPOP, ERBB2, ETV6, FA2H, FAIM2, FES, GAB2, GATA2, GBA, GDI1, GFRA3, GIT1, GNAO1, GPER1, GPM6A, GPR37L1, GPRIN1, GRB7, GRN, GSK3A, HDAC10, HEYL, HPRT1, ID3, IL1B, ILK, INPP5J, IRX3, IRX6, ITGA3, KATNB1, KCNIP2, KCTD11, LAMA5, LDLR, LGR6, LIF, LIMK1, LINGO1, LMX1A, LMX1B, LYPLA2, LZTS1, LZTS3, MAP1A, MAP1S, MAPK3, MAPK7, MAPK8IP3, MARK2, MBOAT7, MDGA1, MDGA2, MECP2, MED12, MEGF8, MFSD2A, MICALL1, MICALL2, MINK1, MME, MOV10, MYC, MYO7A, MYRF, NAP1L2, NAPA, NECTIN1, NEFL, NF2, NFATC4, NGF, NGFR, NNAT, NOTCH1, NOTCH3, NR1D1, NR4A2, NSMF, NTN1, NUMBL, OGDH, OMA1, PACSIN1, PARP6, PCDHA4, PDLIM7, PHACTR1, PIGT, PISD, PLXNA1, PLXNA3, PLXNB1, POSTN, PPP1R9B, PQBP1, PRAG1, PTN, PTPRU, RAB3A, RAC2, RAP1A, RAPGEF1, RARA, RELA, RGMA, RHOG, RMRP, RNF157, ROBO4, RP1L1, RTN4RL1, RTN4RL2, SAMD14, SCYL1, SDK2, SEMA3F, SEMA4B, SEMA4C, SEMA6B, SEMA6C, SF3A2, SHANK3, SLC4A10, SLC9A3R1, SOX10, SOX13, SOX8, SPTB, SRC, SRF, SRRT, STAT3, STMND1, STX1B, SYN1, SYNGAP1, SYT2, SZT2, TBC1D24, TCF3, TCF7L1, TEAD3, TGM2, TIMP1, TNF, TRIOBP, TRPV4, TSKU, ULK1, UNC5B, VAX2, VEGFA, WDR62, WHRN, WNT3, WNT9B, ZFHX2, ZMIZ1, ZNF335, ZNF609, ZSWIM4, ZSWIM8]
GO:0120036	plasma membrane bounded cell projection organization	3,31E-02	[5]	291,00	17,17	28,13	71,87	[AIF1, AREG, BCL11B, BICDL1, BLK, BMP7, BMPR1B, BTG2, CARMIL2, CCL19, CD3E, CDH1, CDH4, CFAP54, CNR1, CORO1A, CRABP2, CSF1R, CXCR4, DAW1, DCC, DNAH8, DOCK10, DOK2, DRC1, ELAVL4, EMB, EPHA6, EPX, FGD2, FGD3, FOLR2, GFI1, GFRA2, GPM6A, GRXCR2, ITGA4, KLF5, KLHL41, LST1, NCKAP1L, NELL2, NME8, NPPC, NR4A2, NTN1, P2RY12, PLEK, PLPPR4, PLXNC1, PRKCQ, PTK2B, RAC2, RASGRF1, RHOH, SEMA4A, SEMA4D, SNX10, SPEF2, STAP1, TACSTD2, TBC1D10C, THY1, TNR, TOX, TREM2, TRPM2, UGT8, WAS, WNT1, ZNF804A]	[ABCD1, ABCD2, ABI3, ABL1, ABLIM3, ACP4, ACTB, ADCY1, AGER, AGT, ALPK3, AP2A1, APOA1, APOD, ARC, AREG, ARF1, ARHGDIA, ARTN, ATAT1, ATP1A3, ATP1B2, ATP6V0D1, BBS10, BBS12, BTG2, C11orf88, C15orf62, CAMK2B, CAMSAP3, CARM1, CARMIL2, CCL21, CCR7, CDC42EP1, CDC42EP2, CDC42EP4, CDH4, CDK1, CECR2, CELSR2, CEP126, CFAP157, CFAP54, CFL1, CNR1, CNTN6, CPNE5, CPNE6, CSNK1E, CSPG4, CTHRC1, CX3CL1, DAB2IP, DAG1, DDR1, DGUOK, DLG5, DMTN, DNAAF5, DNAH17, DOK5, DPYSL4, DRC1, DSCAML1, DVL3, DYNLRB1, E2F4, EFNA3, EHD1, EHD2, EMP3, EPHA2, ERBB2, FES, FXYD5, GAB2, GBA, GBF1, GDI1, GFRA3, GNAO1, GPM6A, GPRIN1, GRB7, GRN, GSK3A, HPRT1, ICAM1, IFT80, ILK, INPP5E, INPP5J, INPPL1, IQUB, ITGA3, KATNB1, KIF19, KLF5, LAMA5, LGR6, LIMK1, LIMK2, LINGO1, LMX1A, LYPLA2, LZTS1, LZTS3, MAP1A, MAP1S, MAPK3, MAPK7, MAPK8IP3, MARK2, MARK4, MECP2, MED12, MEGF8, MFSD2A, MICALL1, MICALL2, MIEN1, MINK1, MOV10, MSTN, MTSS2, MYH9, MYO7A, NECTIN1, NECTIN2, NEFL, NFATC4, NGF, NGFR, NOTCH1, NOTCH3, NR4A2, NSMF, NTN1, NUMBL, OMA1, PACSIN1, PALM, PARP6, PARVB, PCDHA4, PDLIM7, PFN1, PHACTR1, PISD, PKD1, PLEKHM1, PLEKHO1, PLXNA1, PLXNA3, PLXNB1, POSTN, PPP1R9B, PPP2R1A, PQBP1, PRAG1, PRKACA, PRKAR2B, PRKCSH, PTN, PTPN23, PTPRU, RAB3A, RABL2B, RAC2, RAP1A, RAPGEF1, RFX2, RGMA, RHOG, RILPL1, RMRP, RNF157, ROBO4, RP1L1, RSPH9, RTN4RL1, RTN4RL2, S1PR2, SAMD14, SEMA3F, SEMA4B, SEMA4C, SEMA6B, SEMA6C, SEPTIN9, SF3A2, SHANK3, SLC9A3R1, SNX10, SPAG16, SPEF2, SPTB, SRC, SRF, SSX2IP, STMND1, STX1B, SYNGAP1, SYT2, SZT2, TBC1D10B, TBC1D13, TBC1D17, TBC1D2, TBC1D22B, TBC1D24, TCIRG1, TCTEX1D2, TESK1, TIMP1, TMEM17, TRIOBP, TRPV4, TSKU, TUBA4A, TUBG1, UBE2B, ULK1, UNC5B, VANGL2, VAX2, VEGFA, WAS, WDR92, WHRN, WNT3, ZNF335, ZSWIM4, ZSWIM8]

GO:0001568	blood vessel development	2,47E-05	[3, 5, 6, 7]	154,00	21,21	34,41	65,59	[ADAM8, ADAMTS9, ANGPT1, ANGPT2, ANGPTL6, ANPEP, BMP7, CASP8, CCL2, CCL24, CCR2, CCR3, CEL, COL22A1, CXCL10, CXCR3, CXCR4, CYBB, E2F8, EGR3, EREG, FASLG, FOLR2, FOSL1, GPR4, IL18, ITGAX, ITGB2, KLF5, LDLR, LEF1, NELL2, NPPB, NR4A1, PIK3CG, PRDM1, PRKCB, PROK1, PTK2B, RLN2, SEMA4A, SH2D2A, SOCS3, STAT1, SYK, THBS1, THY1, TNFAIP3, TNMD]	[ABCC8, ABL1, ACKR3, ADAMTS9, ADGRB2, ADGRG1, AGT, AGTR1, ANGPT1, ANGPTL1, ANGPTL2, ANGPTL4, APLN, APLNR, APOD, C5, C5AR1, CCL11, CCL2, CCL24, CEACAM1, CEL, CHI3L1, CITED2, COL1A1, COL22A1, CREB3L1, CSPG4, CX3CL1, DAB2IP, DAG1, E2F7, E2F8, ECM1, EFEMP2, EFNA3, ENG, EPHA2, EPN1, ERBB2, ESM1, FAP, FLT4, FOSL1, FOXC2, FOXF1, FOXS1, GATA2, GATA4, GJA4, GJD3, GPER1, GPR4, GRN, HDAC5, HDAC7, HGS, HSPB1, HSPG2, HYAL1, IL1B, ITGA5, JMJD8, JUNB, JUP, KLF5, LDLR, LOX, LRG1, MAPK7, MECP2, MEGF8, MEN1, MYH9, MYO18B, NDST1, NFATC4, NGFR, NINJ1, NODAL, NOS3, NOTCH1, NOTCH4, NPPB, NPRL3, NR4A1, NRARP, PAK4, PDGFB, PDGFRB, PKD1, PKM, PLCD3, PML, PTN, RAMP2, RAP1A, RGCC, ROBO4, S100A1, SERPINE1, SOCS3, SPHK1, SPHK2, SPI1, SRF, STAT3, SYNGAP1, TBX5, TEAD2, THBS1, TMEM100, TNF, TNFAIP2, TNFSF12, TNMD, UNC5B, VEGFA, WARS1, ZC3H12A, ZMIZ1]
GO:0030097	hemopoiesis	1,27E-16	[4, 5, 6, 7]	235,00	24,28	59,15	40,85	[ADAM8, ANGPT1, BATF, BCL11B, BCL3, BLK, BLNK, BTK, C1QC, CALCR, CAMK4, CARD11, CASP8, CCL19, CCL3, CCR2, CD19, CD2, CD27, CD3D, CD3E, CD3G, CD4, CD40LG, CD74, CD79A, CD80, CD86, CD8A, CDKN2A, CLCF1, CR2, CRTAM, CSF1R, CTLA4, DOCK10, DOCK2, EGR3, EOMES, EPX, EVI2B, FCRL3, FGL2, FLT3, FOXP3, FUT7, GFI1, GPR171, GPR55, H3C10, H3C11, H3C12, H3C7, HCLS1, HLA-DOA, HLA-DRA, HLA-DRB1, IFNG, IKZF1, IKZF3, IL12RB1, IL18, IL7R, INPP5D, IRF4, IRF8, ITGA4, ITK, KLRC1, LAG3, LCK, LEF1, LILRB1, LILRB4, LMO1, LRRC17, LY9, MIXL1, MMP9, MS4A1, MT1G, MYB, NCKAP1L, NFAM1, NLRP3, NR4A3, PIM1, PLA2G2D, PLD4, PLEK, POU2AF1, POU2F2, PRDM1, PRKCB, PRKCQ, PTK2B, PTPN22, PTPN6, PTPRC, RHEX, RHOH, RTKN2, RUNX3, SASH3, SELPLG, SEMA4A, SLAMF6, SLAMF8, SLC8A3, SMPD3, SNX10, SOCS1, SPN, STAT1, SYK, TBX21, TESPA1, THBS1, THEMIS, TNFRSF13B, TNFRSF18, TNFSF13B, TNFSF8, TOX, TREM2, TRPM2, UBD, VAV1, VNN1, WNT1, WNT10B, ZAP70, ZNF683]	[ABL1, ACTN1, ADIPOQ, AGER, ANGPT1, AP3D1, ARMC5, BATF2, BCL3, BCL6, CALCR, CCR7, CD74, CDK2, CDKN1A, CEACAM1, CITED2, CLCF1, CLPTM1, CRIP2, CSF1, CTC1, CYP26B1, DHRS2, DMTN, DPF2, DTX1, EPHA2, ERBB2, ETV6, FADD, FASN, FES, FLT3, FLT4, GAB2, GATA2, GBA, GNAO1, GPR137, H3C10, H3C12, H4C3, HDAC5, HSF1, IL15RA, IL1B, IL4R, IL7R, INHA, JAK3, JUNB, KCP, KDELR1, KLF10, KMT2D, LAG3, LGALS9, LOX, LRRC17, LRRC8A, LTBR, LTF, MFAP5, MKNK2, MOV10, MYC, MYH9, MYL9, NBEAL2, NCOA6, NFE2, NOTCH1, NOTCH4, NR4A3, NRARP, OSM, PAF1, PDGFB, PIM1, PML, PTN, RARA, RELB, RMRP, RNU1-93P, RTKN2, RXRA, SBNO2, SETD1A, SH2B3, SIPA1L3, SNX10, SOX12, SOX13, SPI1, SRC, SRF, SSBP3, STAT3, SYVN1, TCF3, TCF7L1, TCIRG1, TFE3, THBS1, THPO, TIMP1, TNF, TRIB1, VEGFA, VNN1, VSIR, ZBTB7A, ZBTB7B, ZC3H12A, ZFP36, ZMIZ1, ZNF385A]
GO:1903706	regulation of hemopoiesis	2,05E-13	[3, 4, 5, 6, 7, 8]	122,00	28,98	61,99	38,01	[ADAM8, BTK, C1QC, CAMK4, CARD11, CASP8, CCL19, CCL3, CCR2, CD2, CD27, CD4, CD74, CD80, CD86, CDKN2A, CRTAM, CTLA4, EGR3, EVI2B, FCRL3, FGL2, FOXP3, GPR171, GPR55, H3C10, H3C11, H3C12, H3C7, HCLS1, HLA-DOA, HLA-DRA, HLA-DRB1, IFNG, IKZF3, IL12RB1, IL18, IL7R, INPP5D, IRF4, LAG3, LEF1, LILRB1, LILRB4, LRRC17, MYB, NCKAP1L, NFAM1, NLRP3, NR4A3, PRDM1, PRKCB, PRKCQ, PTK2B, PTPN6, PTPRC, RHEX, RHOH, RUNX3, SASH3, SLAMF8, SOCS1, STAT1, SYK, TBX21, TESPA1, THBS1, TNFRSF18, TOX, TREM2, VNN1, ZAP70, ZNF683]	[ABL1, ADIPOQ, AGER, AP3D1, BCL6, CD74, CEACAM1, CLPTM1, CSF1, CYP26B1, DTX1, ERBB2, FADD, FES, GATA2, GPR137, H3C10, H3C12, H4C3, HSF1, IL15RA, IL4R, IL7R, INHA, JAK3, KLF10, KMT2D, LAG3, LGALS9, LOX, LRRC17, LTF, MOV10, MYC, MYL9, NFE2, NR4A3, NRARP, PAF1, PTN, RARA, RMRP, SETD1A, SOX12, SOX13, SPI1, STAT3, TFE3, THBS1, THPO, TNF, TRIB1, VNN1, VSIR, ZBTB7B, ZC3H12A, ZFP36, ZMIZ1]
GO:0001525	angiogenesis	6,80E-06	[3, 4, 5, 6, 7, 8, 9]	125,00	22,85	36,97	63,03	[ADAM8, ADAMTS9, ANGPT1, ANGPT2, ANGPTL6, ANPEP, CASP8, CCL2, CCL24, CCR2, CCR3, CEL, COL22A1, CXCL10, CXCR3, CXCR4, CYBB, E2F8, EGR3, EREG, FASLG, GPR4, IL18, ITGAX, ITGB2, KLF5, LEF1, NELL2, NPPB, NR4A1, PIK3CG, PRKCB, PROK1, PTK2B, RLN2, SEMA4A, SH2D2A, STAT1, SYK, THBS1, THY1, TNFAIP3, TNMD]	[ABCC8, ABL1, ACKR3, ADAMTS9, ADGRB2, ADGRG1, AGT, AGTR1, ANGPT1, ANGPTL1, ANGPTL2, ANGPTL4, APLN, APLNR, APOD, C5, C5AR1, CCL11, CCL2, CCL24, CEACAM1, CEL, CHI3L1, COL22A1, CREB3L1, CSPG4, CX3CL1, DAB2IP, DAG1, E2F7, E2F8, ECM1, EFNA3, ENG, EPHA2, EPN1, ERBB2, ESM1, FAP, FLT4, FOXC2, GATA2, GATA4, GPR4, GRN, HDAC5, HDAC7, HGS, HSPB1, HSPG2, HYAL1, IL1B, ITGA5, JMJD8, JUP, KLF5, LRG1, MAPK7, MECP2, MEN1, MYH9, NFATC4, NGFR, NINJ1, NODAL, NOS3, NOTCH1, NOTCH4, NPPB, NR4A1, NRARP, PAK4, PDGFRB, PKM, PLCD3, PML, PTN, RAMP2, RGCC, ROBO4, S100A1, SERPINE1, SPHK1, SRF, STAT3, THBS1, TMEM100, TNF, TNFAIP2, TNFSF12, TNMD, UNC5B, VEGFA, WARS1, ZC3H12A]
GO:0002521	leukocyte differentiation	9,44E-24	[4, 5, 6, 7, 8]	177,00	30,73	63,92	36,08	[ADAM8, BATF, BCL11B, BCL3, BLK, BLNK, BTK, C1QC, CALCR, CAMK4, CARD11, CASP8, CCL19, CCL3, CCR2, CD19, CD2, CD27, CD3D, CD3E, CD3G, CD4, CD40LG, CD74, CD79A, CD80, CD86, CD8A, CDKN2A, CLCF1, CR2, CRTAM, CSF1R, CTLA4, DOCK10, DOCK2, EGR3, EOMES, EVI2B, FCRL3, FGL2, FLT3, FOXP3, FUT7, GPR55, HCLS1, HLA-DOA, HLA-DRA, HLA-DRB1, IFNG, IKZF1, IKZF3, IL12RB1, IL18, IL7R, INPP5D, IRF4, IRF8, ITGA4, ITK, KLRC1, LAG3, LCK, LEF1, LILRB1, LILRB4, LRRC17, LY9, MMP9, MS4A1, MT1G, MYB, NCKAP1L, NFAM1, NLRP3, PLA2G2D, POU2AF1, POU2F2, PRDM1, PTK2B, PTPN22, PTPN6, PTPRC, RHOH, RUNX3, SASH3, SEMA4A, SLAMF6, SLAMF8, SNX10, SOCS1, SPN, SYK, TBX21, TESPA1, THEMIS, TNFRSF18, TNFSF13B, TNFSF8, TOX, TREM2, TRPM2, UBD, VAV1, VNN1, WNT1, ZAP70, ZNF683]	[ABL1, ADIPOQ, AGER, AP3D1, ARMC5, BATF2, BCL3, BCL6, CALCR, CCR7, CD74, CDK2, CDKN1A, CEACAM1, CITED2, CLCF1, CLPTM1, CSF1, CYP26B1, DHRS2, DTX1, EPHA2, ERBB2, FADD, FASN, FES, FLT3, GAB2, GATA2, GBA, GNAO1, GPR137, HDAC5, HSF1, IL15RA, IL1B, IL4R, IL7R, INHA, JAK3, JUNB, KDELR1, KLF10, LAG3, LGALS9, LRRC17, LRRC8A, LTBR, LTF, MYC, MYH9, NRARP, PML, RARA, RELB, RMRP, RXRA, SBNO2, SNX10, SOX12, SOX13, SPI1, SRC, SRF, STAT3, SYVN1, TCF3, TCF7L1, TCIRG1, TFE3, TNF, TRIB1, VEGFA, VNN1, VSIR, ZBTB7A, ZBTB7B, ZC3H12A, ZMIZ1]

GO:0048514	blood vessel morphogenesis	8,73E-06	[4, 5, 6, 7, 8]	142,00	22,05	35,14	64,86	[ADAM8, ADAMTS9, ANGPT1, ANGPT2, ANGPTL6, ANPEP, CASP8, CCL2, CCL24, CCR2, CCR3, CEL, COL22A1, CXCL10, CXCR3, CXCR4, CYBB, E2F8, EGR3, EREG, FASLG, FOLR2, GPR4, IL18, ITGAX, ITGB2, KLF5, LDLR, LEF1, NELL2, NPPB, NR4A1, PIK3CG, PRDM1, PRKCB, PROK1, PTK2B, RLN2, SEMA4A, SH2D2A, STAT1, SYK, THBS1, THY1, TNFAIP3, TNMD]	[ABCC8, ABL1, ACKR3, ADAMTS9, ADGRB2, ADGRG1, AGT, AGTR1, ANGPT1, ANGPTL1, ANGPTL2, ANGPTL4, APLN, APLNR, APOD, C5, C5AR1, CCL11, CCL2, CCL24, CEACAM1, CEL, CHI3L1, CITED2, COL22A1, CREB3L1, CSPG4, CX3CL1, DAB2IP, DAG1, E2F7, E2F8, ECM1, EFEMP2, EFNA3, ENG, EPHA2, EPN1, ERBB2, ESM1, FAP, FLT4, FOXC2, FOXF1, GATA2, GATA4, GJD3, GPR4, GRN, HDAC5, HDAC7, HGS, HSPB1, HSPG2, HYAL1, IL1B, ITGA5, JMJD8, JUNB, JUP, KLF5, LDLR, LOX, LRG1, MAPK7, MECP2, MEN1, MYH9, MYO18B, NFATC4, NGFR, NINJ1, NODAL, NOS3, NOTCH1, NOTCH4, NPPB, NPRL3, NR4A1, NRARP, PAK4, PDGFRB, PKM, PLCD3, PML, PTN, RAMP2, RAP1A, RGCC, ROBO4, S100A1, SERPINE1, SPHK1, SPI1, SRF, STAT3, SYNGAP1, TBX5, TEAD2, THBS1, TMEM100, TNF, TNFAIP2, TNFSF12, TNMD, UNC5B, VEGFA, WARS1, ZC3H12A, ZMIZ1]
GO:1902105	regulation of leukocyte differentiation	4,19E-15	[4, 5, 6, 7, 8, 9]	97,00	33,92	65,84	34,16	[ADAM8, BTK, C1QC, CAMK4, CARD11, CASP8, CCL19, CCL3, CCR2, CD2, CD27, CD4, CD74, CD80, CD86, CDKN2A, CRTAM, CTLA4, EGR3, EVI2B, FCRL3, FGL2, FOXP3, GPR55, HCLS1, HLA-DOA, HLA-DRA, HLA-DRB1, IFNG, IKZF3, IL12RB1, IL18, IL7R, INPP5D, IRF4, LAG3, LEF1, LILRB1, LILRB4, LRRC17, MYB, NCKAP1L, NFAM1, NLRP3, PRDM1, PTPN6, PTPRC, RHOH, RUNX3, SASH3, SLAMF8, SOCS1, SYK, TBX21, TESPA1, TNFRSF18, TOX, TREM2, VNN1, ZAP70, ZNF683]	[ABL1, ADIPOQ, AGER, AP3D1, BCL6, CD74, CEACAM1, CLPTM1, CSF1, CYP26B1, DTX1, ERBB2, FADD, FES, GATA2, GPR137, HSF1, IL15RA, IL4R, IL7R, INHA, JAK3, KLF10, LAG3, LGALS9, LRRC17, LTF, MYC, NRARP, RARA, RMRP, SOX12, SOX13, TFE3, TNF, TRIB1, VNN1, VSIR, ZBTB7B, ZC3H12A, ZMIZ1]
GO:0045765	regulation of angiogenesis	2,95E-04	[4, 5, 6, 7, 8, 9, 10]	74,00	25,00	32,95	67,05	[ADAMTS9, ANGPT2, CCL24, CCR2, CCR3, CXCL10, CXCR4, CYBB, FASLG, GPR4, ITGAX, ITGB2, NPPB, PRKCB, PROK1, PTK2B, RLN2, SEMA4A, STAT1, THBS1, TNFAIP3, TNMD]	[ABCC8, ABL1, ADAMTS9, ADGRB2, AGT, ANGPTL4, APLNR, C5, C5AR1, CCL11, CCL24, CEACAM1, CHI3L1, CREB3L1, DAB2IP, ECM1, EFNA3, ENG, EPHA2, EPN1, ERBB2, FOXC2, GATA2, GATA4, GPR4, GRN, HGS, HSPB1, HSPG2, HYAL1, IL1B, ITGA5, JMJD8, JUP, LRG1, MAPK7, MECP2, NINJ1, NODAL, NOS3, NPPB, PAK4, PKM, PML, PTN, RAMP2, RGCC, S100A1, SERPINE1, SPHK1, STAT3, THBS1, TNF, TNFSF12, TNMD, VEGFA, WARS1, ZC3H12A]
GO:0032879	regulation of localization	1,05E-09	[2, 3]	515,00	18,06	37,10	62,90	[ABCB11, ADAM8, ADAMTS9, ADORA3, AIF1, ALOX15, ALOX15B, ANGPT1, ANGPT2, ANO9, AREG, BLK, BMP7, C1QTNF1, C2, CACNA1E, CACNA1I, CALCR, CARMIL2, CASP8, CASS4, CBARP, CCL19, CCL2, CCL24, CCL3, CCL4, CCL5, CCR2, CCR5, CD177, CD19, CD200R1, CD22, CD247, CD300LF, CD33, CD4, CD74, CD84, CDH1, CLCNKA, CNR1, CORO1A, CSF1R, CTSS, CXCL10, CXCL11, CXCL9, CXCR3, CXCR4, CYBB, DAPK1, DERL3, DOCK10, DOCK2, DOCK8, DPEP1, DRC1, DRD1, EDN2, EPX, EREG, ERFE, FASLG, FCRL3, FFAR4, FGR, FUT7, GABBR2, GPSM3, GZMB, HCLS1, HCN1, HLA-DRB1, IFNG, IKBKE, IL16, IL2RB, IL2RG, ITGA4, ITGAX, ITGB2, JPH1, JSRP1, KCNA3, KCNA7, KCNJ10, KCNK15, KCNN4, KCNQ5, KLRC2, KLRK1, KMO, LCK, LCP1, LEF1, LIF, LILRB1, LILRB4, LPAL2, LRRC38, MCOLN2, MCOLN3, MIDN, MLC1, MMP9, MYB, NCKAP1L, NELL2, NLRP3, NLRP6, NPPB, NR4A3, NTN1, P2RY12, P2RY2, PIK3CG, PIM1, PLA2G7, PLAU, PLXNC1, PRAM1, PRKCB, PTAFR, PTK2B, PTPN22, PTPN6, PTPRC, PTX3, PYCARD, PYHIN1, RAB15, RAB27B, RAB3B, RAB3C, RAC2, RASGRF1, RGS9, RHOH, RRAD, SDC1, SELE, SEMA4A, SEMA4D, SEPTIN1, SHISA8, SIRPG, SLAMF8, SLC31A2, SMPD3, SPINK1, SPN, STAP1, STXBP2, SYK, TACSTD2, TCAF2, THBS1, THY1, TIFAB, TMC2, TNFRSF18, TREM2, TRIM14, TRPM2, UBD, UCP2, WAS, XCL1, XCL2, XCR1, XG]	[AAAS, ABCA3, ABCA7, ABCB11, ABCC8, ABI3, ABL1, ABLIM3, ACACB, ACKR3, ACTB, ACTN1, ACTN4, ADAMTS9, ADCY1, ADGRG1, ADIPOQ, AGER, AGT, AGTR1, AMH, ANGPT1, ANKRD13B, AP2A1, AP3D1, APLN, APLNR, APOA1, APOD, ARC, AREG, ARF1, ARHGAP1, ARHGDIA, ARHGEF16, ARHGEF5, ATP13A2, ATP1A1, ATP1A3, ATP1B2, ATPSCKMT, BAG3, BCAR1, BCL2L1, BCL6, BMP8A, BSN, C11orf65, C1QTNF1, C2CD2L, C5, C5AR1, CACNA1E, CACNA1H, CACNB4, CALCR, CAMK2B, CAMSAP3, CAPN1, CARMIL2, CBARP, CCL11, CCL2, CCL21, CCL24, CCR7, CD14, CD177, CD74, CDK1, CEACAM1, CELSR2, CETP, CFAP298-TCP10L, CFH, CGA, CHCHD10, CHERP, CHGA, CITED2, CLCNKA, CLIP3, CNR1, COL1A1, CPLX1, CRP, CRYAB, CSF1, CSK, CSNK1E, CTSD, CX3CL1, DAB2IP, DAG1, DGKD, DISP3, DLG5, DMTN, DOC2B, DRC1, DVL3, DYSF, ECM1, EDN2, EHD1, EHD2, ENG, EPHA2, EPPK1, ERBB2, ERFE, FADD, FAM110A, FES, FFAR2, FITM1, FLT4, FOXC2, FOXF1, FURIN, FXYD5, FXYD6, GAB2, GABBR2, GAS1, GATA2, GATA4, GBF1, GCSAM, GDI1, GIT1, GLIS2, GNAI2, GNAO1, GPER1, GPI, GPRIN1, GRB7, GRIK5, GRM2, GRN, GSK3A, HBEGF, HCN3, HCN4, HDAC5, HDAC7, HFE, HGS, HSPB1, HTR1B, HTR2A, HVCN1, HYAL1, ICAM1, IFITM1, IL15RA, IL1B, IL4R, ILK, INHA, INPP5E, ITGA3, ITPR3, JPH4, JUP, KCNA7, KCNC3, KCNH2, KCNIP2, KCNJ1, KCNJ5, KCNJ9, KCNK15, KCNK6, KCNMB1, KCNMB2, KCNQ4, KCTD11, LAMA5, LDLRAP1, LGALS9, LGR6, LIF, LILRA5, LIMK2, LIPG, LMNA, LRRC52, LRRC8A, LZTS1, LZTS2, MAP1A, MAP2K3, MAPK3, MARK4, MAVS, MAZ, MECP2, MICALL2, MIDN, MIEN1, MINK1, MLLT6, MSN, MSTN, MYADM, MYC, MYO18A, NDC80, NEFL, NF2, NGFR, NKAIN3, NKD2, NLGN2, NNAT, NOD2, NODAL, NOS3, NOTCH1, NPPA, NPPB, NR1D1, NR4A3, NSMF, NTN1, NUCB1, NUTF2, ORAI1, OSGIN1, P2RX3, P2RX5, P2RY2, P2RY6, PACSIN1, PCDHA4, PDGFB, PDGFRB, PER1, PFN1, PHACTR1, PIM1, PLA2G7, PLAU, PLEKHM2, PLK3, PLTP, PLVAP, PLXNA1

GO:0006810	transport	5,25E-04	[3]	844,00	15,69	35,92	64,08	[ABCB11, ABCC3, ABRA, ACAP1, ADAM8, ADAMTS9, ADGRE3, AIF1, ALOX15, ANGPT1, ANO9, ANPEP, APBA2, AQP5, AREG, ARHGAP45, ARRDC5, ASGR2, ATP10B, ATP1B4, ATP2A3, BHLHA15, BICDL1, BIN2, BLK, BMF, BTK, C1QTNF1, C2, CA12, CACNA1E, CACNA1I, CALCR, CALHM6, CASP8, CBARP, CCDC88B, CCDC88C, CCL19, CCL2, CCL3, CCL4, CCL5, CCR2, CCR5, CD177, CD19, CD22, CD247, CD300LF, CD33, CD3G, CD4, CD5, CD53, CD6, CD74, CD84, CDH1, CEACAM21, CEACAM4, CEL, CFP, CLCNKA, CLEC10A, CLEC12A, CLEC4D, CLNK, CNR1, CORO1A, COTL1, CP, CTSS, CTSW, CXCL10, CXCL11, CXCL9, CXCR4, CYBB, CYSLTR1, DAPK1, DAW1, DENND1C, DERL3, DOCK2, DRD1, EMB, EPX, ERFE, EXOC3L4, FASLG, FCHO1, FCN1, FCN3, FCRL3, FFAR4, FGL2, FGR, FOLR2, FRMPD3, GABBR2, GCNT3, GMFG, GPM6A, GRAMD1B, GRAMD1C, GZMB, HCLS1, HCN1, HLA-DRB1, HSPA7, IFNG, IGLL5, IL10RA, IL16, IL2RB, IL2RG, IPCEF1, IQGAP2, IRF8, ITGA4, ITGAL, ITGAX, ITGB2, JAKMIP1, JCHAIN, JPH1, JSRP1, KCNA3, KCNA7, KCNJ10, KCNK15, KCNN4, KCNQ5, KLF5, KLRC2, KLRG1, KMO, KNG1, LAIR1, LCK, LCP1, LDLR, LILRB1, LILRB4, LPAL2, LRMP, LRRC38, LY75, LYZ, MCOLN2, MCOLN3, MID1IP1, MIDN, MLC1, MMP9, MPO, MRC1, MREG, MS4A1, MYB, MYO1F, MYO1G, NAPSB, NCF1, NCKAP1L, NFAM1, NLRP3, NLRP6, NMUR1, NPPB, NPPC, NR4A3, NSG1, NTN1, NUP210, ORM2, P2RY12, P2RY2, PIK3CG, PIM1, PLA2G2D, PLAC8, PLAU, PLD4, PLEK, PRAM1, PRF1, PRKCB, PSTPIP1, PTAFR, PTGDR, PTK2B, PTPN22, PTPN6, PTPRC, PTX3, PYCARD, RAB15, RAB27B, RAB37, RAB39B, RAB3B, RAB3C, RAB44, RAC2, RASGRF1, RGS9, RHOH, RINL, RNASET2, RRAD, SCNN1B, SDC1, SELE, SELL, SEPTIN1, SERPINA1, SH2D2A, SHISA8, SIDT1, SIGLEC1, SIRPG, SLC12A3, SLC16A6, SLC16A9, SLC18A1, SLC22A31, SLC24A4, SLC25A48, SLC27A2, SLC31A2, SLC36A2, SLC5A2, SLC7A5P1, SLC7A7, SLC8A3, SLCO5A1, SMPD3, SNX10, SNX20, SOAT2, SPEF2, SPINK1, SPNS3, STAP1, STARD5, STXBP2, SUCNR1, SYK, SYTL1	[AAAS, ABCA3, ABCA7, ABCB11, ABCC5, ABCC8, ABCD1, ABCD2, ABCG4, ABL1, ABLIM3, ABRA, ACACB, ACKR3, ACTB, ACTN1, ACTN4, ADAMTS8, ADAMTS9, ADCY1, ADGRE5, ADIPOQ, AGAP2, AGER, AGT, AGTR1, ALDOA, ALPK3, AMH, ANGPT1, ANKRD13B, ANO3, ANO7, ANXA11, AP1B1, AP2A1, AP3D1, APLN, APLNR, APLP1, APOA1, APOD, APOM, AQP3, AQP5, ARAP3, ARC, AREG, ARF1, ARHGAP1, ARHGEF5, ARL8A, ARRDC3, ARSA, ASIC3, ATG9A, ATP13A1, ATP13A2, ATP1A1, ATP1A3, ATP1B2, ATP1B4, ATP5F1D, ATP5MC2, ATP6V0D1, ATP6V0E2, ATP6V1B1, ATP6V1F, ATPSCKMT, BAG3, BBS12, BCL2L1, BMP8A, BRPF3, BSN, C11orf65, C1QTNF1, C2CD2L, C5AR1, CACFD1, CACNA1E, CACNA1H, CACNB4, CALCR, CALHM6, CAMK2B, CAMSAP3, CAPN1, CBARP, CCL2, CCL21, CCR7, CD14, CD177, CD74, CDK1, CDKN1A, CEACAM1, CECR2, CEL, CELSR2, CERT1, CETN3, CETP, CFH, CGA, CHCHD10, CHERP, CHGA, CHI3L1, CHRNG, CLCN6, CLCN7, CLCNKA, CLDN15, CLEC12A, CLIP3, CLNK, CNNM4, CNR1, COASY, COL1A1, COMMD3, CORO7, COX6A2, COX8A, CPLX1, CPNE6, CPSF1, CPTP, CRAT, CREB3L1, CRP, CRTC2, CRYAB, CSK, CSNK1E, CTSA, CTSD, CX3CL1, CYB5R3, CYP27B1, DDR1, DDX39B, DENND1C, DGKD, DISP3, DMTN, DOC2B, DOK3, DSC1, DYNLRB1, DYSF, ECM1, EHD1, EHD2, ENG, ENPP3, EPN1, EPN3, ERBB2, ERFE, F8A1, FABP4, FAM110A, FCN3, FES, FFAR2, FHOD1, FLOT2, FOXF1, FPR1, FTH1P19, FURIN, FXYD5, FXYD6, G6PC3, GAB2, GABBR2, GABRR2, GALNS, GAS1, GATA2, GBF1, GCNT3, GDI1, GGA1, GGA3, GIT1, GJA4, GJD3, GLRA1, GNAI2, GNAO1, GPER1, GPI, GPM6A, GPR84, GPRIN1, GRAMD1A, GRAMD1B, GRAMD1C, GRIK5, GRIP2, GRM2, GRN, GSK3A, GTPBP2, HAVCR1, HBA1, HBA2, HBB, HBD, HBG1, HBG2, HCN3, HCN4, HFE, HGS, HMGA1, HPS1, HRH2, HSF1, HSPB1, HSPG2, HTR1B, HTR2A, HVCN1, ICAM1, IFT80, IGF2BP2, IL15RA, IL1B, IL1RN, IL4R, IMPDH1, INHA, INPPL1, IPO13, IQGAP2, ITPR3, JPH4, JUP, KATNB1, KCNA7, KCNC3, KCNH2, KCNIP2, KCNJ1, KCNJ5
GO:0019725	cellular homeostasis	7,14E-05	[2, 4]	203,00	19,67	43,62	56,38	[ATP2A3, C1QTNF1, CALCR, CCL19, CCL2, CCL3, CCL5, CCR2, CCR3, CCR4, CCR5, CCR8, CD19, CD200R1, CD52, CNR1, CORO1A, CP, CXCL10, CXCL11, CXCL9, CXCR3, CXCR4, CXCR6, CXorf21, CYBB, CYSLTR1, DRD1, EDN2, EPX, ERFE, FASLG, FATE1, FFAR4, GPR174, GPR4, GPR55, HKDC1, HLA-DRB1, IFNG, JPH1, JSRP1, KCNN4, KNG1, LCK, LPAR2, MCOLN2, MCOLN3, MPO, MS4A1, MT1F, MT1G, MT1HL1, NCF1, NELL2, NLRP3, P2RY10, P2RY2, PIK3CG, PRKCB, PTGDR, PTGER2, PTGFR, PTK2B, PTPN6, PTPRC, RAC2, S1PR4, SLAMF8, SLC12A3, SLC24A4, SLC31A2, SLC8A3, TBXAS1, THY1, TREM2, TRPM2, UCP2, XCL1, XCR1]	[ABCC8, ABL1, ACKR3, AGER, AGT, AGTR1, ALDOA, AP3D1, APLNR, ARF1, ATP13A1, ATP13A2, ATP1A1, ATP1A3, ATP1B2, ATP6V0D1, ATP6V0E2, ATP6V1B1, ATP6V1F, BAG3, C1QTNF1, C2CD2L, C5AR1, CALB1, CALCR, CCL11, CCL2, CCL21, CCR7, CHERP, CLCN6, CNR1, CTC1, CX3CL1, CYP27B1, E2F4, EDN2, EGLN2, ENDOG, ERFE, FPR1, FTH1P19, GATA2, GATA4, GIT1, GNB3, GPER1, GPR17, GPR20, GPR4, GPRIN1, GRIK5, GRINA, GRN, HCRTR1, HFE, HK1, HTR1B, HTR2A, HVCN1, ICAM1, ITPR3, JPH4, KCNK3, KNG1, LOX, LRRC8A, LTF, MAFG, MAP1A, MAPK3, MED12, MEN1, MICU3, MSTN, MT1E, MYC, NGFR, NOS3, NR1D1, P2RX3, P2RX5, P2RY2, P2RY6, PACS2, PCDHA4, PKD1, PLCZ1, PML, PPARD, PRKACA, PTGFR, PTGIR, PTPRN, RAB11B, RAB11FIP5, RAC2, RMRP, RNU1-93P, SELENON, SHANK3, SIDT2, SLC12A4, SLC25A23, SLC29A1, SLC30A1, SLC30A3, SLC39A13, SLC39A5, SLC41A1, SLC4A10, SLC4A11, SLC4A2, SLC4A3, SLC6A9, SLC9A1, SLC9A3R1, SMAD3, SMARCA4, SMARCB1, SRF, SV2A, SYPL2, TCIRG1, TGM2, TIMP1, TM9SF4, TMEM175, TNS2, TPCN1, TRPV4, VDR, WDTC1, WFS1]

GO:0042592	homeostatic process	1,59E-10	[3]	399,00	19,17	39,06	60,94	[ABCB11, ACP5, ADAM8, ADCY7, ANGPT1, ASGR2, ATP2A3, BHLHA15, C1QTNF1, CA12, CALCR, CARD11, CCL19, CCL2, CCL3, CCL5, CCR2, CCR3, CCR4, CCR5, CCR8, CD19, CD200R1, CD52, CD74, CEL, CNR1, CORO1A, CP, CRB2, CSF1R, CXCL10, CXCL11, CXCL6, CXCL9, CXCR3, CXCR4, CXCR6, CXorf21, CYBB, CYP11B1, CYSLTR1, DOCK10, DRD1, EDN2, EPX, ERFE, FASLG, FATE1, FFAR4, FLT3, FOXP3, GAPT, GPR174, GPR4, GPR55, GRAMD1B, HCLS1, HKDC1, HLA-DRB1, IFNG, IKZF1, IL18, IL7R, INPP5D, IRF4, IRX3, JCHAIN, JPH1, JSRP1, KCNJ10, KCNN4, KNG1, LCK, LCP1, LDLR, LMO1, LPAL2, LPAR2, LYZ, MCOLN2, MCOLN3, MPO, MS4A1, MT1F, MT1G, MT1HL1, MUC6, NCF1, NCKAP1L, NELL2, NLRP3, NPPB, NR4A3, P2RY10, P2RY2, PIK3CG, PLAC8, PRKCB, PRKCQ, PTGDR, PTGER2, PTGFR, PTK2B, PTPN6, PTPRC, RAC2, RHEX, S1PR4, SASH3, SCNN1B, SIT1, SLAMF8, SLC12A3, SLC24A4, SLC31A2, SLC8A3, SNX10, SOAT2, STAT1, SUCNR1, SYK, TBXAS1, THY1, TMC8, TNFAIP3, TNFRSF11B, TNFRSF13B, TNFRSF17, TNFSF13B, TNFSF14, TREM2, TRPM2, TSC22D3, UCP2, UNC80, VSIG1, WNT10B, XCL1, XCR1]	[ABCA3, ABCB11, ABCC8, ABCD1, ABCG4, ABHD4, ABL1, ACACB, ACKR3, ACTB, ADAMTS5, ADCK1, ADCY1, ADCY3, ADCY4, ADCY9, ADGRG1, ADIPOQ, AGER, AGT, AGTR1, ALDOA, ALPK3, ANGPT1, ANGPTL4, ANGPTL8, AP3D1, APLN, APLNR, APOA1, APOM, AQP3, ARF1, ARRDC3, ARTN, ATP13A1, ATP13A2, ATP1A1, ATP1A3, ATP1B2, ATP6V0D1, ATP6V0E2, ATP6V1B1, ATP6V1F, BAG3, BBS10, BBS12, BCL6, BMP8A, C1QTNF1, C2CD2L, C5AR1, CALB1, CALCR, CCL11, CCL2, CCL21, CCR7, CD74, CDK2, CEL, CERT1, CETP, CFH, CHERP, CITED2, CLCN6, CNNM4, CNOT3, CNR1, CRTC2, CSF1, CSK, CTC1, CX3CL1, CYP21A2, CYP26B1, CYP27B1, DISP3, DMTN, E2F4, EDN2, EGLN2, EHD1, EIF4G1, ELOVL1, ENDOG, ERFE, ESAM, FA2H, FABP4, FADD, FANCC, FASN, FFAR2, FGFR4, FLG, FLT3, FOXC2, FPR1, FTH1P19, GATA2, GATA4, GBA, GIT1, GLIS2, GNAI2, GNB3, GPAM, GPER1, GPI, GPR137, GPR17, GPR20, GPR3, GPR4, GPRIN1, GRAMD1B, GRIK5, GRINA, GRM2, GRN, H4C3, HCN4, HCRTR1, HFE, HK1, HSF1, HSPB1, HTR1B, HTR2A, HVCN1, ICAM1, IL1B, IL4R, IL7R, INHA, IRX3, ITPR3, JAK3, JPH4, KCNH2, KCNK3, KDM6B, KNG1, LCAT, LDLR, LDLRAP1, LGALS9, LIPG, LOX, LRRC8A, LSR, LTF, MAFG, MALL, MALRD1, MAP1A, MAPK3, MED12, MEN1, MFAP2, MFSD2A, MICU3, MLLT6, MSTN, MT1E, MYC, NGFR, NOD2, NOS3, NOTCH1, NOVA2, NPPB, NPR3, NPRL3, NR1D1, NR4A3, OMA1, P2RX3, P2RX5, P2RY2, P2RY6, PACS2, PCDHA4, PDK2, PKD1, PLA2G2A, PLCZ1, PLEKHM1, PM20D1, PML, PNPLA2, PPARD, PPP1R10, PPP1R13L, PRDM16, PRKACA, PRKAR2B, PTGES, PTGFR, PTGIR, PTPRN, RAB11B, RAB11FIP5, RAC2, RANGRF, RMI1, RMRP, RNU1-93P, RP1L1, RTEL1-TNFRSF6B, SCN5A, SCN7A, SELENON, SERPINA3, SH2B2, SH2B3, SHANK3, SIDT2, SLC12A4, SLC25A23, SLC25A44, SLC29A1, SLC2A4, SLC30A1, SLC30A3, SLC39A13, SLC39A5, SLC41A1, SLC4A10, SLC4A11, SLC4A2, SLC4A3, SLC6A9, SLC7A8, SLC9A1, SLC9A3R1, SLN, SLX4, SMAD3, SMARCA4, SMARCB1, SNX10, SPI1, SPNS2, SRC,
GO:0048871	multicellular organismal homeostasis	7,94E-03	[2, 4]	119,00	20,48	34,50	65,50	[ACP5, ADAM8, ADCY7, ANGPT1, CCR2, CEL, CNR1, CORO1A, CRB2, CSF1R, CXCR4, EDN2, FFAR4, GPR55, IL18, INPP5D, IRF4, IRX3, JCHAIN, LDLR, LYZ, MUC6, NLRP3, NR4A3, PLAC8, PTK2B, RAC2, SASH3, SCNN1B, SNX10, SYK, TNFAIP3, TNFRSF11B, TRPM2, UCP2, VSIG1, WNT10B]	[ABCA3, ABCC8, ACACB, ACTB, ADAMTS5, ADCY1, ADCY3, ADCY4, ADCY9, ADIPOQ, ALPK3, ANGPT1, APLN, AQP3, ARRDC3, ATP1B2, ATP6V1B1, BBS10, BBS12, BMP8A, CEL, CFH, CNOT3, CNR1, CSF1, CSK, CYP26B1, EDN2, EIF4G1, ELOVL1, ESAM, FA2H, FABP4, FLG, FOXC2, GATA2, GBA, GPR137, GPR3, HFE, HSF1, HSPB1, HTR2A, IL1B, IL4R, IRX3, KDM6B, LDLR, LSR, LTF, MFAP2, MFSD2A, NOD2, NOS3, NOTCH1, NOVA2, NPR3, NPRL3, NR1D1, NR4A3, OMA1, PCDHA4, PLEKHM1, PM20D1, PPP1R13L, PRDM16, PRKACA, PRKAR2B, PTGES, RAC2, RP1L1, SERPINA3, SLC25A44, SLN, SNX10, SRC, SRF, STAT3, TCIRG1, TFE3, TLE3, TNF, TNS2, TP53INP2, TRPV4, TSKU, VEGFA, WFS1, WHRN, YBX2, ZBTB7B]
GO:0055085	transmembrane transport	3,39E-03	[2, 4]	307,00	17,51	35,41	64,59	[ABCB11, ABCC3, ANO9, AQP5, ATP1B4, ATP2A3, BHLHA15, C1QTNF1, CACNA1E, CACNA1I, CALCR, CALHM6, CBARP, CCL19, CCL2, CCL3, CCR2, CCR5, CD19, CLCNKA, CNR1, CORO1A, CTSS, CXCL10, CXCL11, CXCL9, CYBB, DAPK1, DRD1, EMB, EPX, ERFE, FFAR4, FOLR2, GABBR2, GPM6A, HCN1, IFNG, JPH1, JSRP1, KCNA3, KCNA7, KCNJ10, KCNK15, KCNN4, KCNQ5, LCK, LRRC38, MCOLN2, MCOLN3, MID1IP1, MMP9, NR4A3, P2RY12, PIK3CG, PIM1, PRF1, PRKCB, PTAFR, PTK2B, PTPN22, PTPN6, PTPRC, RASGRF1, RGS9, RRAD, SCNN1B, SHISA8, SIDT1, SLC12A3, SLC16A6, SLC16A9, SLC18A1, SLC22A31, SLC24A4, SLC25A48, SLC27A2, SLC31A2, SLC36A2, SLC5A2, SLC7A5P1, SLC7A7, SLC8A3, SLCO5A1, SPNS3, TCAF2, THBS1, THY1, TMC2, TMC3, TMC8, TREM2, TRPM2, TSC22D3, UBD, UCP2, UNC80, XCL1, XCR1]	[ABCA3, ABCA7, ABCB11, ABCC5, ABCC8, ABCD1, ABCD2, ABCG4, ABL1, ACACB, ACTB, ACTN4, ADAMTS8, ADIPOQ, AGT, ANO3, ANO7, APLNR, AQP3, AQP5, ARC, ARF1, ASIC3, ATP13A1, ATP13A2, ATP1A1, ATP1A3, ATP1B2, ATP1B4, ATP5F1D, ATP5MC2, ATP6V0D1, ATP6V0E2, ATP6V1B1, ATP6V1F, ATPSCKMT, C1QTNF1, CACNA1E, CACNA1H, CACNB4, CALCR, CALHM6, CAMK2B, CAPN1, CBARP, CCL2, CCL21, CCR7, CEACAM1, CFH, CHERP, CHRNG, CLCN6, CLCN7, CLCNKA, CLIP3, CNNM4, CNR1, COX6A2, COX8A, CPLX1, CX3CL1, DMTN, ERFE, FXYD5, FXYD6, GABBR2, GABRR2, GJA4, GJD3, GLRA1, GNAI2, GPER1, GPM6A, GPRIN1, GRIK5, GRM2, GSK3A, HCN3, HCN4, HFE, HTR1B, HTR2A, HVCN1, IL1B, ITPR3, JPH4, KCNA7, KCNC3, KCNH2, KCNIP2, KCNJ1, KCNJ5, KCNJ9, KCNK15, KCNK2, KCNK3, KCNK6, KCNMB1, KCNMB2, KCNQ4, KCNT1, LRRC52, LRRC8A, LYNX1, MED12, MFSD10, MFSD2A, MICU3, MID1IP1, MINK1, MTCO2P12, MYC, NDUFA4L2, NEFL, NIPAL4, NLGN2, NPPA, NR4A3, ORAI1, P2RX3, P2RX5, P2RY6, PCDHA4, PEX14, PIEZO1, PIM1, PKD1, PLP2, PM20D1, PPARD, PRAF2, PRKACA, PRRT1, RANGRF, RAP1A, RNU1-93P, RRAD, RTN2, S100A1, SCN5A, SCN7A, SELENON, SHANK3, SIDT2, SLC10A3, SLC12A4, SLC15A5, SLC16A5, SLC16A9, SLC17A3, SLC1A4, SLC1A5, SLC22A7, SLC25A1, SLC25A22, SLC25A23, SLC25A33, SLC25A39, SLC25A42, SLC25A44, SLC29A1, SLC29A2, SLC29A4, SLC2A4, SLC2A6, SLC30A1, SLC30A3, SLC35A2, SLC35A4, SLC35C1, SLC35F6, SLC36A2, SLC38A11, SLC38A5, SLC38A7, SLC38A8, SLC39A13, SLC39A5, SLC41A1, SLC44A2, SLC48A1, SLC4A10, SLC4A11, SLC4A2, SLC4A3, SLC51B, SLC52A2, SLC52A3, SLC5A10, SLC5A6, SLC6A12, SLC6A17, SLC6A8, SLC6A9, SLC7A1, SLC7A5, SLC7A8, SLC9A1, SLC9A3R1, SLCO4A1, SLCO4C1, SLCO5A1, SLN, SPHK2, SPNS2, STAC2, STX1A, SV2A, SYNGAP1, TAPBP, TCIRG1, THBS1, TIMP1, TMC3, TMC4, TMEM109, TMEM175, TMEM63A, TMEM63B, TMEM63C, TNF, TPCN1, TRIB3, TRPV4, TTYH1, TTYH2, TTYH3,

GO:0140352	export from cell	2,72E-04	[2, 4]	273,00	18,32	42,74	57,26	[ADAM8, ADGRE3, ANPEP, ARHGAP45, BIN2, BLK, BTK, C1QTNF1, CACNA1E, CACNA1I, CBARP, CCL3, CCL5, CCR2, CD177, CD33, CD53, CD84, CEACAM21, CFP, CLEC12A, CLEC4D, CLNK, CNR1, CORO1A, COTL1, CTSS, CTSW, CYBB, DOCK2, EPX, EXOC3L4, FCN1, FFAR4, FGL2, FGR, FRMPD3, GMFG, HLA-DRB1, IFNG, IQGAP2, ITGAL, ITGAX, ITGB2, KCNN4, KLF5, KLRC2, KLRG1, KMO, KNG1, LAIR1, LILRB1, LRMP, LYZ, MIDN, MMP9, MPO, MYB, MYO1G, NAPSB, NCKAP1L, NFAM1, NLRP3, NR4A3, ORM2, P2RY12, PIK3CG, PLAC8, PLAU, PLEK, PRAM1, PRKCB, PTAFR, PTGDR, PTPN6, PTPRC, PTX3, PYCARD, RAB15, RAB27B, RAB37, RAB3B, RAB3C, RAB44, RAC2, RGS9, RNASET2, SDC1, SELL, SEPTIN1, SERPINA1, SLC18A1, SLC27A2, SLC8A3, SMPD3, STXBP2, SUCNR1, SYK, SYTL1, TBC1D10C, THBS1, TREM2, TRPM2, TXNDC5, UCP2, VNN1]	[ABCC5, ABCC8, ACTN1, ACTN4, ADCY1, ADGRE5, ADIPOQ, AGT, AGTR1, ALDOA, AMH, APLN, APOA1, ARF1, ARL8A, ARSA, ATP13A2, ATP1A1, ATP1A3, ATP1B2, BMP8A, BRPF3, C1QTNF1, C2CD2L, C5AR1, CACNA1E, CACNA1H, CAPN1, CBARP, CD14, CD177, CEACAM1, CGA, CHGA, CHI3L1, CLEC12A, CLNK, CNR1, COMMD3, CPLX1, CRAT, CREB3L1, CTSA, CTSD, CYB5R3, DMTN, DOC2B, DOK3, DSC1, ECM1, ENG, FES, FFAR2, FOXF1, FPR1, FURIN, GAB2, GALNS, GATA2, GIT1, GNAI2, GPER1, GPI, GPR84, GRIK5, GRM2, GRN, GTPBP2, HBB, HFE, HGS, HTR1B, HTR2A, HVCN1, IL1B, IL1RN, IL4R, IMPDH1, INHA, IQGAP2, ITPR3, JUP, KCNH2, KCNIP2, KCTD11, KLF5, KNG1, LGALS3BP, LGALS9, LRG1, LRRC8A, LTF, LY6E, MEN1, METTL7A, MIDN, MME, MYH9, MYO18A, NAPA, NBEAL2, NHLRC3, NKD2, NLGN2, NNAT, NOTCH1, NPPA, NR1D1, NR4A3, OTOF, PCDHA4, PDGFB, PIP5K1C, PKM, PLAU, PLAUR, PORCN, PPARD, PRKCSH, PRRT2, PTGDS, PTGES, PTPN23, PTPRN, RAB11B, RAB11FIP5, RAB15, RAB3A, RAB3IL1, RAB5B, RAC2, RAP1A, RASL10B, RGCC, RHBDF1, RHBDF2, RHOG, RNASE2, RNU1-93P, RPH3A, S100A11, SELENOP, SEPTIN4, SEPTIN5, SEPTIN9, SERPINA3, SERPINE1, SERPING1, SIDT2, SIGLEC9, SLC17A3, SLC1A5, SLC29A4, SLC30A1, SLC44A2, SLC7A8, SLC9A1, SLCO4C1, SPHK2, SPI1, SREBF1, STEAP3, STING1, STK11IP, STX1A, STX1B, SV2A, SYN1, SYT2, SYT7, TCIRG1, THBS1, TIMP1, TMEM179B, TMEM63A, TNF, TNFAIP2, TNFRSF1A, TRAPPC1, TRPV4, TSPAN14, TUBA4A, VEGFA, VNN1]
GO:0051049	regulation of transport	6,48E-06	[3, 4]	339,00	18,29	39,41	60,59	[ABCB11, ADAM8, ALOX15, ANGPT1, ANO9, BLK, C1QTNF1, C2, CACNA1E, CACNA1I, CALCR, CASP8, CBARP, CCL19, CCL2, CCL3, CCL4, CCL5, CCR2, CD177, CD19, CD22, CD300LF, CD33, CD4, CD74, CD84, CDH1, CLCNKA, CNR1, CORO1A, CTSS, CXCL10, CXCL11, CXCL9, CXCR4, CYBB, DAPK1, DERL3, DOCK2, DRD1, EPX, ERFE, FASLG, FCRL3, FFAR4, FGR, GABBR2, GZMB, HCLS1, HCN1, HLA-DRB1, IFNG, IL16, IL2RB, IL2RG, ITGB2, JPH1, JSRP1, KCNA3, KCNA7, KCNJ10, KCNK15, KCNN4, KCNQ5, KLRC2, KMO, LCP1, LILRB1, LPAL2, LRRC38, MIDN, MLC1, MMP9, MYB, NCKAP1L, NLRP3, NLRP6, NPPB, NR4A3, P2RY12, P2RY2, PIK3CG, PIM1, PRAM1, PRKCB, PTAFR, PTK2B, PTPN22, PTPN6, PTPRC, PTX3, PYCARD, RAB15, RAB27B, RAB3B, RAB3C, RAC2, RASGRF1, RGS9, RRAD, SDC1, SELE, SEPTIN1, SHISA8, SIRPG, SLC31A2, SMPD3, SPINK1, STAP1, STXBP2, SYK, TCAF2, THBS1, THY1, TIFAB, TMC2, TREM2, TRPM2, UBD, UCP2, XCL1]	[AAAS, ABCA3, ABCA7, ABCB11, ABCC8, ABL1, ABLIM3, ACTB, ACTN4, ADCY1, ADIPOQ, AGT, AGTR1, ANGPT1, ANKRD13B, AP2A1, APLN, APLNR, APOA1, APOD, ARC, ARF1, ARHGAP1, ARHGEF5, ATP13A2, ATP1A1, ATP1A3, ATP1B2, ATPSCKMT, BAG3, BMP8A, BSN, C11orf65, C1QTNF1, C2CD2L, CACNA1E, CACNA1H, CACNB4, CALCR, CAMK2B, CAPN1, CBARP, CCL2, CCL21, CD14, CD177, CD74, CDK1, CEACAM1, CETP, CFH, CHCHD10, CHGA, CLCNKA, CLIP3, CNR1, CPLX1, CRYAB, CSK, CX3CL1, DGKD, DISP3, DMTN, DOC2B, DYSF, EHD1, EHD2, ERBB2, ERFE, FES, FFAR2, FOXF1, FURIN, FXYD5, FXYD6, GAB2, GABBR2, GAS1, GATA2, GDI1, GIT1, GNAI2, GNAO1, GPER1, GPRIN1, GRIK5, GRM2, GSK3A, HCN3, HCN4, HFE, HGS, HTR1B, HTR2A, HVCN1, ICAM1, IL15RA, IL1B, IL4R, INHA, ITPR3, JPH4, JUP, KCNA7, KCNC3, KCNH2, KCNIP2, KCNJ1, KCNJ5, KCNJ9, KCNK15, KCNK6, KCNMB1, KCNMB2, KCNQ4, KCTD11, LDLRAP1, LGALS9, LILRA5, LIPG, LRRC52, LRRC8A, LZTS1, MAPK3, MAVS, MICALL2, MIDN, MINK1, MLLT6, MSN, MYC, MYO18A, NEFL, NKAIN3, NLGN2, NNAT, NOD2, NOTCH1, NPPA, NPPB, NR1D1, NR4A3, NUCB1, NUTF2, ORAI1, P2RX3, P2RX5, P2RY2, P2RY6, PACSIN1, PCDHA4, PDGFB, PDGFRB, PER1, PIM1, PLK3, PLTP, PM20D1, PML, PPARD, PRKACA, PRKCSH, PROM2, PRRT1, PRRT2, PTGES, PTPN23, RAB11B, RAB11FIP5, RAB15, RAB3A, RAB5B, RAC2, RANGAP1, RANGRF, RAP1A, RASL10B, RCVRN, RGCC, RHBDF1, RHBDF2, RIPOR1, RRAD, RTN2, RUBCN, RXRA, S100A1, SCN5A, SCN7A, SEC16A, SELE, SELENON, SEPTIN4, SEPTIN5, SERPINE1, SH3GL1, SHANK3, SIDT2, SIK1, SLC30A1, SLC30A3, SLC35F6, SLC51B, SLC6A9, SLC9A1, SLC9A3R1, SLN, SMAD3, SMPD1, SPHK1, SPHK2, SPI1, SPINK1, SRC, SREBF1, STAC2, STX1A, STX1B, SV2A, SYN1, SYT2, SYT7, TCIRG1, TCTEX1D2, TGM2, THBS1, TIMP1, TM9SF4, TMEM109, TMEM14A, TNF, TNFRSF1A, TNK2, TPCN1, TRIB3, TSC2, VEGFA, VTN, WFS1, WNK2, WWP2, ZC3H12A, ZDHHC8]
GO:0051050	positive regulation of transport	1,01E-03	[2, 3, 4, 5]	192,00	19,22	42,40	57,60	[ABCB11, ADAM8, ANGPT1, BLK, C1QTNF1, C2, CACNA1I, CALCR, CASP8, CCL19, CCL2, CCL3, CCL4, CCL5, CCR2, CD177, CD19, CD300LF, CD33, CDH1, CNR1, CTSS, CXCL10, CXCL11, CXCL9, DOCK2, DRD1, ERFE, FASLG, FFAR4, FGR, GABBR2, GZMB, HCLS1, HLA-DRB1, IFNG, IL2RB, IL2RG, ITGB2, KCNJ10, KCNN4, KLRC2, KMO, LPAL2, LRRC38, MLC1, MYB, NCKAP1L, NLRP3, NPPB, NR4A3, P2RY12, P2RY2, PTAFR, PTPN22, PTPRC, PTX3, PYCARD, RAB15, RAB27B, RAB3B, RAC2, RGS9, SDC1, SELE, SIRPG, SMPD3, STAP1, SYK, TCAF2, THY1, TREM2, TRPM2, UBD, XCL1]	[ABCA3, ABCA7, ABCB11, ABCC8, ABL1, ABLIM3, ACTB, ACTN4, ADIPOQ, AGT, ANGPT1, AP2A1, APLN, APLNR, APOA1, ARC, ARF1, ARHGEF5, ATP13A2, ATP1B2, ATPSCKMT, BAG3, C1QTNF1, C2CD2L, CACNA1H, CALCR, CCL2, CCL21, CD14, CD177, CDK1, CETP, CFH, CHCHD10, CLIP3, CNR1, CX3CL1, DGKD, DOC2B, EHD1, EHD2, ERBB2, ERFE, GAB2, GABBR2, GATA2, GNAI2, GPER1, GPRIN1, GSK3A, HFE, HGS, IL15RA, IL1B, IL4R, INHA, JUP, KCNH2, KCNIP2, KCNJ5, KCNJ9, KCNMB1, KCNMB2, LDLRAP1, LILRA5, LIPG, LRRC52, LRRC8A, LZTS1, MAVS, MICALL2, MLLT6, MSN, MYO18A, NLGN2, NNAT, NOD2, NPPA, NPPB, NR4A3, NUTF2, ORAI1, P2RX3, P2RX5, P2RY2, P2RY6, PCDHA4, PDGFB, PDGFRB, PLK3, PLTP, PPARD, PRKACA, PRRT2, PTGES, PTPN23, RAB15, RAB3A, RAC2, RAP1A, RASL10B, RGCC, RIPOR1, RXRA, S100A1, SCN5A, SEC16A, SELE, SERPINE1, SH3GL1, SHANK3, SLC30A3, SLC51B, SLC6A9, SLC9A1, SLC9A3R1, SMAD3, SMPD1, SPHK2, SRC, STAC2, STX1A, STX1B, SYT7, TM9SF4, TNF, VEGFA, VTN, WFS1, WNK2, ZC3H12A, ZDHHC8]

GO:0006811	ion transport	1,68E-07 [4]	320,00	19,07	38,58	61,42	[ABCB11, ABCC3, ANO9, ATP1B4, ATP2A3, BHLHA15, BTK, CA12, CACNA1E, CACNA1I, CALCR, CALHM6, CBARP, CCL19, CCL2, CCL3, CCL4, CCL5, CCR2, CCR5, CD19, CD33, CD4, CD84, CLCNKA, CNR1, CORO1A, CP, CTSS, CXCL10, CXCL11, CXCL9, CXCR4, CYBB, CYSLTR1, DAPK1, DRD1, EMB, EPX, FCRL3, FOLR2, GABBR2, GPM6A, HCN1, IFNG, IL16, JPH1, JSRP1, KCNA3, KCNA7, KCNJ10, KCNK15, KCNN4, KCNQ5, KMO, LCK, LILRB1, LRRC38, MCOLN2, MCOLN3, MLC1, MMP9, MS4A1, NLRP3, NMUR1, P2RY12, PIK3CG, PLA2G2D, PRKCB, PTAFR, PTK2B, PTPN22, PTPN6, PTPRC, RAB3B, RASGRF1, RGS9, RRAD, SCNN1B, SHISA8, SLC12A3, SLC16A6, SLC16A9, SLC18A1, SLC22A31, SLC24A4, SLC25A48, SLC27A2, SLC31A2, SLC36A2, SLC5A2, SLC7A5P1, SLC7A7, SLC8A3, SLCO5A1, SPINK1, STARD5, SYK, TCAF2, THY1, TMC2, TMC3, TMC8, TREM2, TRPM2, TSC22D3, UBD, UCP2, UNC80, XCL1, XCR1]	[ABCB11, ABCC5, ABCC8, ABL1, ACTN4, ADAMTS8, AGT, ANO3, ANO7, APLNR, ARC, ARF1, ARHGAP1, ASIC3, ATP13A1, ATP13A2, ATP1A1, ATP1A3, ATP1B2, ATP1B4, ATP5F1D, ATP5MC2, ATP6V0D1, ATP6V0E2, ATP6V1B1, ATP6V1F, ATPSCKMT, CACNA1E, CACNA1H, CACNB4, CALCR, CALHM6, CAMK2B, CAPN1, CBARP, CCL2, CCL21, CCR7, CEACAM1, CERT1, CFH, CHERP, CHRNG, CLCN6, CLCN7, CLCNKA, CLDN15, CNNM4, CNR1, COMMD3, COX6A2, COX8A, CPTP, CX3CL1, CYP27B1, ENPP3, FTH1P19, FXYD5, FXYD6, GABBR2, GABRR2, GJA4, GJD3, GLRA1, GNAI2, GNAO1, GPER1, GPM6A, GPRIN1, GRIK5, GRM2, HBA1, HBB, HCN3, HCN4, HFE, HTR1B, HTR2A, HVCN1, ICAM1, IL1B, ITPR3, JPH4, KCNA7, KCNC3, KCNH2, KCNIP2, KCNJ1, KCNJ5, KCNJ9, KCNK15, KCNK2, KCNK3, KCNK6, KCNMB1, KCNMB2, KCNQ4, KCNT1, LILRA5, LRRC52, LRRC8A, LTF, LYNX1, MED12, MFSD10, MFSD2A, MICU3, MINK1, MLLT6, MTCO2P12, MYC, NDUFA4L2, NECTIN1, NEFL, NIPAL4, NKAIN3, NLGN2, NPPA, ORAI1, P2RX3, P2RX5, P2RY6, PCDHA4, PDGFB, PDGFRB, PER1, PIEZO1, PKD1, PLA2G2A, PLCZ1, PLP2, PM20D1, PML, PRAF2, PRKACA, PRRT1, PTGES, RAB11B, RAB3A, RAMP2, RANGRF, RCVRN, RNU1-93P, RRAD, RXRA, S100A1, SCN5A, SCN7A, SELENON, SHANK3, SIK1, SLC10A3, SLC12A4, SLC16A5, SLC16A9, SLC17A3, SLC1A4, SLC1A5, SLC22A7, SLC25A1, SLC25A22, SLC25A23, SLC25A42, SLC29A4, SLC2A6, SLC30A1, SLC30A3, SLC35A2, SLC36A2, SLC38A11, SLC38A5, SLC38A7, SLC38A8, SLC39A13, SLC39A5, SLC41A1, SLC44A2, SLC48A1, SLC4A10, SLC4A11, SLC4A2, SLC4A3, SLC51B, SLC52A2, SLC52A3, SLC5A10, SLC5A6, SLC6A12, SLC6A17, SLC6A8, SLC6A9, SLC7A1, SLC7A5, SLC7A8, SLC9A1, SLC9A3R1, SLCO4A1, SLCO4C1, SLCO5A1, SLN, SPHK2, SPINK1, STAC2, STEAP3, STX1A, SV2A, SYNGAP1, SYT2, SYT7, TCIRG1, TIMP1, TMC3, TMC4, TMEM109, TMEM175, TMEM63A, TMEM63B, TMEM63C, TPCN1, TRPV4, TTYH1, TTYH2, TTYH3, UNC80, VDR, VPS9D1, WFS1, WNK2
GO:0022857	transmembrane transporter activity	2,67E-02 [3, 5]	244,00	17,63	31,12	68,88	[ABCB11, ABCC3, ANO9, AQP5, ATP1B4, ATP2A3, CACNA1E, CACNA1I, CALHM6, CBARP, CCL2, CCR2, CLCNKA, CNR1, CTSS, CYBB, DAPK1, EPX, GPM6A, HCN1, IFNG, JPH1, JSRP1, KCNA3, KCNA7, KCNJ10, KCNK15, KCNN4, KCNQ5, LRRC38, MCOLN2, MCOLN3, MMP9, PIM1, PRF1, PRKCB, PTAFR, PTK2B, RASGRF1, RGS9, RRAD, SCNN1B, SHISA8, SIDT1, SLC12A3, SLC16A6, SLC16A9, SLC18A1, SLC22A31, SLC24A4, SLC25A48, SLC27A2, SLC31A2, SLC36A2, SLC5A2, SLC7A5P1, SLC7A7, SLC8A3, SLCO5A1, SPNS3, TCAF2, TMC2, TMC3, TMC8, TREM2, TRPM2, UCP2, UNC80]	[ABCA3, ABCA7, ABCB11, ABCC5, ABCC8, ABCD1, ABCD2, ABCG4, ACTB, ACTN4, ADAMTS8, ANO3, ANO7, AQP3, AQP5, ARC, ASIC3, ATP13A1, ATP13A2, ATP1A1, ATP1A3, ATP1B2, ATP1B4, ATP5F1D, ATP5MC2, ATP6V0D1, ATP6V0E2, ATP6V1B1, ATP6V1F, ATPSCKMT, CACNA1E, CACNA1H, CACNB4, CALHM6, CAMK2B, CAPN1, CBARP, CCL2, CEACAM1, CFH, CHRNG, CLCN6, CLCN7, CLCNKA, CNNM4, CNR1, COX6A2, COX8A, CPLX1, FXYD5, FXYD6, GABRR2, GJA4, GJD3, GLRA1, GPM6A, GPRIN1, GRIK5, GRM2, HCN3, HCN4, HTR1B, HVCN1, ITPR3, JPH4, KCNA7, KCNC3, KCNH2, KCNIP2, KCNJ1, KCNJ5, KCNJ9, KCNK15, KCNK2, KCNK3, KCNK6, KCNMB1, KCNMB2, KCNQ4, KCNT1, LRRC52, LRRC8A, LYNX1, MFSD10, MFSD2A, MINK1, MTCO2P12, NDUFA4L2, NEFL, NIPAL4, NLGN2, NPPA, ORAI1, P2RX3, P2RX5, P2RY6, PCDHA4, PIEZO1, PIM1, PKD1, PLP2, PM20D1, PRKACA, PRRT1, RANGRF, RRAD, S100A1, SCN5A, SCN7A, SELENON, SHANK3, SIDT2, SLC10A3, SLC12A4, SLC15A5, SLC16A5, SLC16A9, SLC17A3, SLC1A4, SLC1A5, SLC22A7, SLC25A1, SLC25A22, SLC25A23, SLC25A33, SLC25A42, SLC25A44, SLC29A1, SLC29A2, SLC29A4, SLC2A4, SLC2A6, SLC30A1, SLC30A3, SLC35A2, SLC35A4, SLC35C1, SLC35F6, SLC36A2, SLC38A11, SLC38A5, SLC38A7, SLC38A8, SLC39A13, SLC39A5, SLC41A1, SLC44A2, SLC48A1, SLC4A10, SLC4A11, SLC4A2, SLC4A3, SLC51B, SLC52A2, SLC52A3, SLC5A10, SLC5A6, SLC6A12, SLC6A17, SLC6A8, SLC6A9, SLC7A1, SLC7A5, SLC7A8, SLC9A1, SLC9A3R1, SLCO4A1, SLCO4C1, SLCO5A1, SLN, SPHK2, SPNS2, STAC2, STX1A, SV2A, SYNGAP1, TAPBP, TCIRG1, TIMP1, TMC3, TMC4, TMEM109, TMEM175, TMEM63A, TMEM63B, TMEM63C, TPCN1, TRPV4, TTYH1, TTYH2, TTYH3, UNC80, WNK2, WWP2, ZNF395]

GO:0034220	ion transmembrane transport	2,16E-04	[3, 5]	244,00	18,73	36,41	63,59	[ANO9, ATP1B4, ATP2A3, BHLHA15, CACNA1E, CACNA1I, CALCR, CALHM6, CBARP, CCL19, CCL2, CCL3, CCR2, CCR5, CD19, CLCNKA, CNR1, CORO1A, CTSS, CXCL10, CXCL11, CXCL9, CYBB, DAPK1, DRD1, EMB, EPX, FOLR2, GABBR2, GPM6A, HCN1, IFNG, JPH1, JSRP1, KCNA3, KCNA7, KCNJ10, KCNK15, KCNN4, KCNQ5, LCK, LRRC38, MCOLN2, MCOLN3, MMP9, P2RY12, PIK3CG, PRKCB, PTAFR, PTK2B, PTPN22, PTPN6, PTPRC, RASGRF1, RGS9, RRAD, SCNN1B, SHISA8, SLC12A3, SLC18A1, SLC24A4, SLC25A48, SLC31A2, SLC36A2, SLC5A2, SLC8A3, SLCO5A1, TCAF2, THY1, TMC2, TMC3, TMC8, TREM2, TRPM2, TSC22D3, UBD, UCP2, UNC80, XCL1, XCR1]	[ABCC5, ABCC8, ABL1, ACTN4, ADAMTS8, AGT, ANO3, ANO7, APLNR, ARC, ARF1, ASIC3, ATP13A1, ATP13A2, ATP1A1, ATP1A3, ATP1B2, ATP1B4, ATP5F1D, ATP5MC2, ATP6V0D1, ATP6V0E2, ATP6V1B1, ATP6V1F, ATPSCKMT, CACNA1E, CACNA1H, CACNB4, CALCR, CALHM6, CAMK2B, CAPN1, CBARP, CCL2, CCL21, CCR7, CFH, CHERP, CHRNG, CLCN6, CLCN7, CLCNKA, CNNM4, CNR1, COX6A2, COX8A, CX3CL1, FXYD5, FXYD6, GABBR2, GABRR2, GJD3, GLRA1, GPER1, GPM6A, GPRIN1, GRIK5, GRM2, HCN3, HCN4, HFE, HTR1B, HTR2A, HVCN1, ITPR3, JPH4, KCNA7, KCNC3, KCNH2, KCNIP2, KCNJ1, KCNJ5, KCNJ9, KCNK15, KCNK2, KCNK3, KCNK6, KCNMB1, KCNMB2, KCNQ4, KCNT1, LRRC52, LRRC8A, LYNX1, MED12, MFSD10, MFSD2A, MICU3, MINK1, MTCO2P12, MYC, NDUFA4L2, NEFL, NIPAL4, NLGN2, NPPA, ORAI1, P2RX3, P2RX5, P2RY6, PCDHA4, PIEZO1, PKD1, PLP2, PM20D1, PRAF2, PRKACA, PRRT1, RANGRF, RNU1-93P, RRAD, S100A1, SCN5A, SCN7A, SELENON, SHANK3, SLC10A3, SLC12A4, SLC17A3, SLC1A4, SLC1A5, SLC22A7, SLC25A1, SLC25A22, SLC25A23, SLC25A42, SLC29A4, SLC2A6, SLC30A1, SLC30A3, SLC35A2, SLC36A2, SLC38A5, SLC38A7, SLC39A13, SLC39A5, SLC41A1, SLC44A2, SLC4A10, SLC4A11, SLC4A2, SLC4A3, SLC52A2, SLC52A3, SLC5A10, SLC5A6, SLC6A12, SLC6A17, SLC6A8, SLC6A9, SLC7A1, SLC7A5, SLC7A8, SLC9A1, SLC9A3R1, SLCO4A1, SLCO4C1, SLCO5A1, SLN, SPHK2, STAC2, STX1A, SYNGAP1, TCIRG1, TIMP1, TMC3, TMC4, TMEM109, TMEM175, TMEM63A, TMEM63B, TMEM63C, TPCN1, TRPV4, TTYH1, TTYH2, TTYH3, UNC80, VPS9D1, WNK2, WWP2]
GO:0034762	regulation of transmembrane transport	6,62E-03	[3, 4, 5]	126,00	20,32	44,86	55,14	[ANO9, C1QTNF1, CACNA1E, CACNA1I, CALCR, CBARP, CCL2, CCR2, CD19, CLCNKA, CORO1A, CTSS, CXCL10, CXCL11, CXCL9, CYBB, DAPK1, DRD1, EPX, ERFE, FFAR4, GABBR2, HCN1, IFNG, JPH1, JSRP1, KCNA3, KCNA7, KCNJ10, KCNK15, KCNN4, KCNQ5, LRRC38, MMP9, NR4A3, PIK3CG, PIM1, PRKCB, PTAFR, PTK2B, PTPN22, PTPN6, RASGRF1, RGS9, RRAD, SHISA8, SLC31A2, TCAF2, THBS1, THY1, TMC2, TREM2, UBD, XCL1]	[ABCC8, ABL1, ACTB, ACTN4, ADIPOQ, AGT, APLNR, ARC, ARF1, ATP1B2, ATPSCKMT, C1QTNF1, CACNA1E, CACNA1H, CACNB4, CALCR, CAMK2B, CAPN1, CBARP, CCL2, CFH, CLCNKA, CLIP3, CX3CL1, ERFE, FXYD5, FXYD6, GABBR2, GNAI2, GPER1, GPRIN1, GSK3A, HCN3, HCN4, HVCN1, IL1B, JPH4, KCNA7, KCNC3, KCNH2, KCNIP2, KCNJ1, KCNJ5, KCNJ9, KCNK15, KCNK6, KCNMB1, KCNMB2, KCNQ4, LRRC52, MINK1, MYC, NEFL, NLGN2, NPPA, NR4A3, P2RY6, PIM1, PM20D1, PRKACA, PRRT1, RANGRF, RAP1A, RRAD, RTN2, S100A1, SCN5A, SCN7A, SELENON, SHANK3, SLC30A1, SLC6A9, SLC9A1, SLC9A3R1, SLN, SPHK2, STAC2, TCIRG1, THBS1, TIMP1, TMEM109, TNF, TPCN1, TRIB3, WNK2, WWP2]
GO:0048878	chemical homeostasis	1,59E-09	[4]	264,00	20,67	39,31	60,69	[ABCB11, ADCY7, ASGR2, ATP2A3, BHLHA15, C1QTNF1, CA12, CALCR, CCL19, CCL3, CCL5, CCR2, CCR3, CCR4, CCR5, CCR8, CD19, CD200R1, CD52, CNR1, CORO1A, CP, CXCL10, CXCL11, CXCL9, CXCR3, CXCR4, CXCR6, CXorf21, CYP11B1, CYSLTR1, DRD1, EDN2, EPX, ERFE, FASLG, FATE1, FFAR4, GPR174, GPR4, GPR55, GRAMD1B, HKDC1, HLA-DRB1, IFNG, IL18, JPH1, JSRP1, KCNJ10, KNG1, LCK, LCP1, LDLR, LPAL2, LPAR2, MCOLN2, MCOLN3, MS4A1, MT1F, MT1G, MT1HL1, NLRP3, NPPB, P2RY10, P2RY2, PIK3CG, PRKCB, PTGDR, PTGER2, PTGFR, PTK2B, PTPN6, PTPRC, S1PR4, SCNN1B, SLAMF8, SLC12A3, SLC24A4, SLC31A2, SLC8A3, SNX10, SOAT2, STAT1, SUCNR1, TBXAS1, THY1, TMC8, TREM2, TRPM2, UCP2, UNC80, XCL1, XCR1]	[ABCA3, ABCB11, ABCC8, ABCD1, ABCG4, ABHD4, ABL1, ACKR3, ADCK1, ADCY1, ADCY3, ADCY4, ADCY9, ADIPOQ, AGER, AGT, AGTR1, ANGPTL4, ANGPTL8, AP3D1, APLNR, APOA1, APOM, AQP3, ARF1, ATP13A1, ATP13A2, ATP1A1, ATP1A3, ATP1B2, ATP6V0D1, ATP6V0E2, ATP6V1B1, ATP6V1F, C1QTNF1, C2CD2L, C5AR1, CALB1, CALCR, CCL11, CCL21, CCR7, CERT1, CETP, CFH, CHERP, CNNM4, CNR1, CRTC2, CX3CL1, CYP21A2, CYP26B1, CYP27B1, DISP3, EDN2, EHD1, ELOVL1, ENDOG, ERFE, FA2H, FABP4, FFAR2, FGFR4, FLG, FPR1, FTH1P19, GATA2, GATA4, GBA, GNAI2, GPAM, GPER1, GPI, GPR17, GPR20, GPR4, GPRIN1, GRAMD1B, GRIK5, GRINA, GRM2, GRN, HCN4, HCRTR1, HFE, HK1, HTR1B, HTR2A, HVCN1, ICAM1, ITPR3, JPH4, KCNH2, KCNK3, KNG1, LCAT, LDLR, LDLRAP1, LIPG, LRRC8A, LSR, LTF, MAFG, MALL, MALRD1, MAPK3, MED12, MEN1, MICU3, MLLT6, MT1E, MYC, NGFR, NPPB, NR1D1, P2RX3, P2RX5, P2RY2, P2RY6, PACS2, PCDHA4, PDK2, PKD1, PLCZ1, PML, PNPLA2, PPARD, PRKACA, PRKAR2B, PTGFR, PTGIR, PTPRN, RAB11B, RAB11FIP5, RANGRF, RMI1, RMRP, RNU1-93P, SCN5A, SCN7A, SELENON, SIDT2, SLC12A4, SLC25A23, SLC29A1, SLC2A4, SLC30A1, SLC30A3, SLC39A13, SLC39A5, SLC41A1, SLC4A10, SLC4A11, SLC4A2, SLC4A3, SLC6A9, SLC7A8, SLC9A1, SLC9A3R1, SMAD3, SMARCA4, SMARCB1, SNX10, SRC, SRF, STAT3, STEAP3, SV2A, SYPL2, TBX5, TCIRG1, TGM2, TIMP1, TM9SF4, TMEM175, TMEM256-PLSCR3, TPCN1, TRPV4, TSKU, UNC80, USF2, VDR, VEGFA, WDTC1, WFS1, WNK2]

GO:0055082	cellular chemical homeostasis	4,98E-05	[3, 5]	180,00	20,29	44,36	55,64	[ATP2A3, C1QTNF1, CALCR, CCL19, CCL3, CCL5, CCR2, CCR3, CCR4, CCR5, CCR8, CD19, CD200R1, CD52, CNR1, CORO1A, CP, CXCL10, CXCL11, CXCL9, CXCR3, CXCR4, CXCR6, CXorf21, CYSLTR1, DRD1, EDN2, EPX, ERFE, FASLG, FATE1, FFAR4, GPR174, GPR4, GPR55, HKDC1, HLA-DRB1, IFNG, JPH1, JSRP1, KNG1, LCK, LPAR2, MCOLN2, MCOLN3, MS4A1, MT1F, MT1G, MT1HL1, NLRP3, P2RY10, P2RY2, PIK3CG, PRKCB, PTGDR, PTGER2, PTGFR, PTK2B, PTPN6, PTPRC, S1PR4, SLAMF8, SLC24A4, SLC31A2, SLC8A3, TBXAS1, THY1, TREM2, TRPM2, UCP2, XCL1, XCR1]	[ABCC8, ABL1, ACKR3, AGER, AGT, AGTR1, AP3D1, APLNR, ARF1, ATP13A1, ATP13A2, ATP1A1, ATP1A3, ATP1B2, ATP6V0D1, ATP6V0E2, ATP6V1B1, ATP6V1F, C1QTNF1, C2CD2L, C5AR1, CALB1, CALCR, CCL11, CCL21, CCR7, CHERP, CNR1, CX3CL1, CYP27B1, EDN2, ENDOG, ERFE, FPR1, FTH1P19, GATA2, GATA4, GPER1, GPR17, GPR20, GPR4, GPRIN1, GRIK5, GRINA, GRN, HCRTR1, HFE, HK1, HTR1B, HTR2A, HVCN1, ICAM1, ITPR3, JPH4, KCNK3, KNG1, LRRC8A, LTF, MAFG, MAPK3, MED12, MEN1, MICU3, MT1E, MYC, NGFR, NR1D1, P2RX3, P2RX5, P2RY2, P2RY6, PACS2, PCDHA4, PKD1, PLCZ1, PML, PPARD, PRKACA, PTGFR, PTGIR, PTPRN, RAB11B, RAB11FIP5, RMRP, RNU1-93P, SELENON, SIDT2, SLC25A23, SLC29A1, SLC30A1, SLC30A3, SLC39A13, SLC39A5, SLC41A1, SLC4A10, SLC4A11, SLC4A2, SLC4A3, SLC6A9, SLC9A1, SLC9A3R1, SMAD3, SMARCA4, SMARCB1, SRF, SV2A, SYPL2, TCIRG1, TGM2, TIMP1, TM9SF4, TMEM175, TPCN1, TRPV4, VDR, WDTC1, WFS1]
GO:0060627	regulation of vesicle-mediated transport	6,31E-05	[3, 4, 5]	124,00	22,18	40,13	59,87	[ALOX15, ANGPT1, BLK, C2, CACNA1I, CBARP, CCL19, CCL2, CCR2, CD177, CD22, CD300LF, CD84, CNR1, CORO1A, DOCK2, FGR, IFNG, IL2RB, IL2RG, ITGB2, KLRC2, LILRB1, LPAL2, NCKAP1L, PRAM1, PRKCB, PTAFR, PTPRC, PTX3, PYCARD, RAB15, RAB27B, RAB3B, RAB3C, RAC2, SDC1, SELE, SEPTIN1, SIRPG, SMPD3, STAP1, STXBP2, SYK, TREM2]	[ABCA7, ABL1, ADCY1, ADIPOQ, ANGPT1, ANKRD13B, AP2A1, APLN, APOA1, ARC, ARF1, ARHGAP1, ATP13A2, BSN, CACNA1H, CBARP, CCL2, CCL21, CD14, CD177, CEACAM1, CLIP3, CNR1, CPLX1, CSK, DGKD, DOC2B, DYSF, EHD1, EHD2, FES, FOXF1, GAB2, GAS1, GATA2, GIT1, GNAI2, GRIK5, HFE, HGS, HTR1B, HTR2A, IL15RA, IL1B, IL4R, LDLRAP1, LGALS9, MAPK3, MSN, MYO18A, NOD2, NOTCH1, NPPA, PACSIN1, PCDHA4, PRKCSH, PROM2, PRRT2, PTPN23, RAB11B, RAB15, RAB3A, RAB5B, RAC2, RAP1A, RUBCN, SELE, SEPTIN4, SEPTIN5, SERPINE1, SH3GL1, SMPD1, SPHK1, SPHK2, SPI1, SRC, STX1A, STX1B, SYN1, SYT2, SYT7, TGM2, TNF, TNK2, TSC2, VEGFA, VTN]
GO:0043269	regulation of ion transport	2,13E-05	[4, 5]	156,00	21,22	44,98	55,02	[ABCB11, ANO9, CACNA1E, CACNA1I, CALCR, CBARP, CCL2, CCL3, CCL4, CCL5, CCR2, CD19, CD33, CD4, CD84, CLCNKA, CNR1, CORO1A, CTSS, CXCL10, CXCL11, CXCL9, CXCR4, CYBB, DAPK1, DRD1, EPX, FCRL3, GABBR2, HCN1, IFNG, IL16, JPH1, JSRP1, KCNA3, KCNA7, KCNJ10, KCNK15, KCNN4, KCNQ5, KMO, LILRB1, LRRC38, MMP9, NLRP3, P2RY12, PIK3CG, PTAFR, PTK2B, PTPN22, PTPN6, RAB3B, RASGRF1, RGS9, RRAD, SHISA8, SLC31A2, SPINK1, SYK, TCAF2, THY1, TMC2, TREM2, UBD, XCL1]	[ABCB11, ABCC8, ABL1, ACTN4, AGT, APLNR, ARC, ARF1, ATP1A1, ATP1A3, ATP1B2, ATPSCKMT, CACNA1E, CACNA1H, CACNB4, CALCR, CAMK2B, CAPN1, CBARP, CCL2, CFH, CLCNKA, CNR1, CX3CL1, FXYD5, FXYD6, GABBR2, GNAI2, GNAO1, GPER1, GPRIN1, GRM2, HCN3, HCN4, HFE, HTR1B, HTR2A, HVCN1, ICAM1, IL1B, JPH4, KCNA7, KCNC3, KCNH2, KCNIP2, KCNJ1, KCNJ5, KCNJ9, KCNK15, KCNK6, KCNMB1, KCNMB2, KCNQ4, LILRA5, LRRC52, MINK1, MLLT6, NEFL, NKAIN3, NLGN2, NPPA, ORAI1, P2RX3, P2RX5, P2RY6, PCDHA4, PDGFB, PDGFRB, PER1, PM20D1, PML, PRKACA, PRRT1, PTGES, RAB11B, RAB3A, RANGRF, RCVRN, RRAD, S100A1, SCN5A, SCN7A, SELENON, SHANK3, SIK1, SLC30A1, SLC6A9, SLC9A1, SLC9A3R1, SLN, SPHK2, SPINK1, STAC2, SV2A, SYT2, SYT7, TCIRG1, TIMP1, TMEM109, TPCN1, WFS1, WNK2, WWP2]
GO:0006812	cation transport	1,41E-06	[5]	252,00	19,66	38,10	61,90	[ATP1B4, ATP2A3, BHLHA15, BTK, CACNA1E, CACNA1I, CALCR, CALHM6, CBARP, CCL19, CCL2, CCL3, CCL4, CCL5, CCR2, CCR5, CD19, CD33, CD4, CD84, CNR1, CORO1A, CP, CTSS, CXCL10, CXCL11, CXCL9, CXCR4, CYSLTR1, DAPK1, DRD1, EPX, FCRL3, GABBR2, GPM6A, HCN1, IFNG, IL16, JPH1, JSRP1, KCNA3, KCNA7, KCNJ10, KCNK15, KCNN4, KCNQ5, LCK, LILRB1, LRRC38, MCOLN2, MCOLN3, MMP9, MS4A1, NMUR1, P2RY12, PIK3CG, PRKCB, PTK2B, PTPN22, PTPN6, PTPRC, RAB3B, RASGRF1, RGS9, RRAD, SCNN1B, SHISA8, SLC12A3, SLC18A1, SLC24A4, SLC25A48, SLC31A2, SLC36A2, SLC5A2, SLC8A3, SPINK1, SYK, THY1, TMC2, TREM2, TRPM2, UBD, UCP2, UNC80, XCL1, XCR1]	[ABCC5, ABCC8, ABL1, ACTN4, AGT, APLNR, ARC, ARF1, ARHGAP1, ASIC3, ATP13A1, ATP13A2, ATP1A1, ATP1A3, ATP1B2, ATP1B4, ATP5F1D, ATP5MC2, ATP6V0D1, ATP6V0E2, ATP6V1B1, ATP6V1F, ATPSCKMT, CACNA1E, CACNA1H, CACNB4, CALCR, CALHM6, CAMK2B, CAPN1, CBARP, CCL2, CCL21, CCR7, CFH, CHERP, CHRNG, CNNM4, CNR1, COMMD3, COX6A2, COX8A, CX3CL1, CYP27B1, FTH1P19, FXYD5, FXYD6, GABBR2, GJA4, GNAI2, GNAO1, GPER1, GPM6A, GPRIN1, GRIK5, GRM2, HCN3, HCN4, HFE, HTR1B, HTR2A, HVCN1, ICAM1, ITPR3, JPH4, KCNA7, KCNC3, KCNH2, KCNIP2, KCNJ1, KCNJ5, KCNJ9, KCNK15, KCNK2, KCNK3, KCNK6, KCNMB1, KCNMB2, KCNQ4, KCNT1, LILRA5, LRRC52, LTF, MED12, MFSD2A, MICU3, MINK1, MLLT6, MTCO2P12, NDUFA4L2, NECTIN1, NEFL, NIPAL4, NKAIN3, NLGN2, NPPA, ORAI1, P2RX3, P2RX5, P2RY6, PCDHA4, PDGFB, PDGFRB, PER1, PIEZO1, PKD1, PLCZ1, PM20D1, PML, PRKACA, PRRT1, RAB11B, RAB3A, RAMP2, RANGRF, RCVRN, RNU1-93P, RRAD, S100A1, SCN5A, SCN7A, SELENON, SHANK3, SIK1, SLC10A3, SLC12A4, SLC17A3, SLC1A4, SLC1A5, SLC25A23, SLC25A42, SLC29A4, SLC30A1, SLC30A3, SLC36A2, SLC38A11, SLC38A5, SLC38A7, SLC38A8, SLC39A13, SLC39A5, SLC41A1, SLC44A2, SLC48A1, SLC4A10, SLC4A11, SLC5A10, SLC5A6, SLC6A12, SLC6A17, SLC6A8, SLC6A9, SLC7A1, SLC7A5, SLC7A8, SLC9A1, SLC9A3R1, SLN, SPHK2, SPINK1, STAC2, STEAP3, STX1A, SYNGAP1, SYT2, SYT7, TCIRG1, TIMP1, TMEM175, TMEM63A, TMEM63B, TMEM63C, TPCN1, TRPV4, TTYH1, UNC80, VDR, VPS9D1, WFS1, WNK2, WWP2]

GO:0006873	cellular ion homeostasis	1,87E-05	[4, 6]	153,00	21,40	48,38	51,62	[ATP2A3, C1QTNF1, CALCR, CCL19, CCL3, CCL5, CCR2, CCR3, CCR4, CCR5, CCR8, CD19, CD200R1, CD52, CNR1, CORO1A, CP, CXCL10, CXCL11, CXCL9, CXCR3, CXCR4, CXCR6, CXorf21, CYSLTR1, DRD1, EDN2, EPX, ERFE, FASLG, FATE1, FFAR4, GPR174, GPR4, GPR55, IFNG, JPH1, JSRP1, KNG1, LCK, LPAR2, MCOLN2, MCOLN3, MS4A1, MT1F, MT1G, MT1HL1, NLRP3, P2RY10, P2RY2, PIK3CG, PRKCB, PTGDR, PTGER2, PTGFR, PTK2B, PTPN6, PTPRC, S1PR4, SLAMF8, SLC24A4, SLC31A2, SLC8A3, TBXAS1, THY1, TRPM2, XCL1, XCR1]	[ABL1, ACKR3, AGT, AGTR1, AP3D1, APLNR, ARF1, ATP13A1, ATP13A2, ATP1A1, ATP1A3, ATP1B2, ATP6V0D1, ATP6V0E2, ATP6V1B1, ATP6V1F, C1QTNF1, C5AR1, CALB1, CALCR, CCL11, CCL21, CCR7, CHERP, CNR1, CX3CL1, CYP27B1, EDN2, ERFE, FPR1, FTH1P19, GATA2, GPER1, GPR17, GPR20, GPR4, GPRIN1, GRIK5, GRINA, GRN, HCRTR1, HFE, HTR1B, HTR2A, HVCN1, ITPR3, JPH4, KCNK3, KNG1, LTF, MAFG, MAPK3, MICU3, MT1E, MYC, P2RX3, P2RX5, P2RY2, P2RY6, PACS2, PCDHA4, PKD1, PLCZ1, PML, PRKACA, PTGFR, PTGIR, RNU1-93P, SELENON, SLC25A23, SLC30A1, SLC30A3, SLC39A13, SLC39A5, SLC41A1, SLC4A10, SLC4A11, SLC4A2, SLC4A3, SLC6A9, SLC9A1, SLC9A3R1, SMAD3, SV2A, SYPL2, TCIRG1, TGM2, TIMP1, TM9SF4, TMEM175, TPCN1, TRPV4, VDR, WFS1]
GO:0019932	second-messenger-mediated signaling	1,06E-04	[4, 5, 6]	88,00	24,18	50,78	49,22	[ADGRG6, BHLHA15, BTK, C1QTNF1, CCL3, CCL4, CCR2, CCR3, CCR4, CCR5, CCR8, CD22, CD3E, CD4, CXCR3, CXCR4, CXCR6, EDN2, FFAR4, JPH1, JSRP1, KLRG1, LMCD1, MCOLN2, MCOLN3, NMUR1, NPPB, NPPC, P2RY12, PLEK, PTGFR, PTK2B, PTPRC, SELE, SLA2, SPINK1, SYK, TBC1D10C, THBS1, TREM2, TRPM2, XCR1, ZAP70]	[ACKR3, ADCY1, ADGRB2, ADGRG6, ADGRL1, AGT, AGTR1, APLNR, C1QTNF1, CCR7, CHERP, DGKD, DHX34, DMTN, EDN2, FPR1, GNAI2, GPRIN1, ITPR3, JPH4, KSR1, LMCD1, MAPK7, MGRN1, NFATC4, NOS3, NPPA, NPPB, P2RX3, P2RX5, P2RY6, PDE4A, PDK2, PPP1R9B, PPP2R1A, PRKACA, PRKAR2B, PTGFR, RASD2, RNF157, RNU1-93P, SAMD14, SELE, SELENON, SELENOP, SLC7A8, SLC9A1, SLC9A3R1, SPHK1, SPINK1, THBS1, TMEM100, TPCN1, UBE2B]
GO:0034765	regulation of ion transmembrane transport	1,63E-02	[4, 5, 6]	108,00	20,61	46,52	53,48	[ANO9, CACNA1E, CACNA1I, CALCR, CBARP, CCL2, CCR2, CD19, CLCNKA, CORO1A, CTSS, CXCL10, CXCL11, CXCL9, CYBB, DAPK1, DRD1, EPX, GABBR2, HCN1, IFNG, JPH1, JSRP1, KCNA3, KCNA7, KCNJ10, KCNK15, KCNN4, KCNQ5, LRRC38, MMP9, PIK3CG, PTAFR, PTK2B, PTPN22, PTPN6, RASGRF1, RGS9, RRAD, SHISA8, SLC31A2, TCAF2, THY1, TMC2, TREM2, UBD, XCL1]	[ABCC8, ABL1, ACTN4, AGT, APLNR, ARC, ARF1, ATP1B2, ATPSCKMT, CACNA1E, CACNA1H, CACNB4, CALCR, CAMK2B, CAPN1, CBARP, CCL2, CFH, CLCNKA, CX3CL1, FXYD5, FXYD6, GABBR2, GPER1, GPRIN1, HCN3, HCN4, HVCN1, JPH4, KCNA7, KCNC3, KCNH2, KCNIP2, KCNJ1, KCNJ5, KCNJ9, KCNK15, KCNK6, KCNMB1, KCNMB2, KCNQ4, LRRC52, MINK1, NEFL, NLGN2, NPPA, P2RY6, PM20D1, PRKACA, PRRT1, RANGRF, RRAD, S100A1, SCN5A, SCN7A, SELENON, SHANK3, SLC30A1, SLC6A9, SLC9A1, SLC9A3R1, SLN, SPHK2, STAC2, TCIRG1, TIMP1, TMEM109, TPCN1, WNK2, WWP2]
GO:0050801	ion homeostasis	3,09E-07	[5]	181,00	21,55	45,70	54,30	[ATP2A3, C1QTNF1, CA12, CALCR, CCL19, CCL3, CCL5, CCR2, CCR3, CCR4, CCR5, CCR8, CD19, CD200R1, CD52, CNR1, CORO1A, CP, CXCL10, CXCL11, CXCL9, CXCR3, CXCR4, CXCR6, CXorf21, CYSLTR1, DRD1, EDN2, EPX, ERFE, FASLG, FATE1, FFAR4, GPR174, GPR4, GPR55, IFNG, JPH1, JSRP1, KCNJ10, KNG1, LCK, LPAR2, MCOLN2, MCOLN3, MS4A1, MT1F, MT1G, MT1HL1, NLRP3, NPPB, P2RY10, P2RY2, PIK3CG, PRKCB, PTGDR, PTGER2, PTGFR, PTK2B, PTPN6, PTPRC, S1PR4, SCNN1B, SLAMF8, SLC12A3, SLC24A4, SLC31A2, SLC8A3, SNX10, TBXAS1, THY1, TMC8, TRPM2, UNC80, XCL1, XCR1]	[ABCD1, ABL1, ACKR3, AGT, AGTR1, AP3D1, APLNR, ARF1, ATP13A1, ATP13A2, ATP1A1, ATP1A3, ATP1B2, ATP6V0D1, ATP6V0E2, ATP6V1B1, ATP6V1F, C1QTNF1, C5AR1, CALB1, CALCR, CCL11, CCL21, CCR7, CFH, CHERP, CNNM4, CNR1, CX3CL1, CYP21A2, CYP27B1, EDN2, ERFE, FGFR4, FPR1, FTH1P19, GATA2, GNAI2, GPER1, GPR17, GPR20, GPR4, GPRIN1, GRIK5, GRINA, GRM2, GRN, HCN4, HCRTR1, HFE, HTR1B, HTR2A, HVCN1, ITPR3, JPH4, KCNH2, KCNK3, KNG1, LTF, MAFG, MAPK3, MICU3, MLLT6, MT1E, MYC, NPPB, P2RX3, P2RX5, P2RY2, P2RY6, PACS2, PCDHA4, PDK2, PKD1, PLCZ1, PML, PRKACA, PTGFR, PTGIR, RANGRF, RNU1-93P, SCN5A, SCN7A, SELENON, SLC12A4, SLC25A23, SLC30A1, SLC30A3, SLC39A13, SLC39A5, SLC41A1, SLC4A10, SLC4A11, SLC4A2, SLC4A3, SLC6A9, SLC7A8, SLC9A1, SLC9A3R1, SMAD3, SNX10, SRC, STEAP3, SV2A, SYPL2, TBX5, TCIRG1, TGM2, TIMP1, TM9SF4, TMEM175, TPCN1, TRPV4, UNC80, VDR, WFS1, WNK2]
GO:0098655	cation transmembrane transport	1,56E-04	[4, 6]	204,00	19,45	37,50	62,50	[ATP1B4, ATP2A3, BHLHA15, CACNA1E, CACNA1I, CALCR, CALHM6, CBARP, CCL19, CCL2, CCL3, CCR2, CCR5, CD19, CNR1, CORO1A, CTSS, CXCL10, CXCL11, CXCL9, DAPK1, DRD1, EPX, GABBR2, GPM6A, HCN1, IFNG, JPH1, JSRP1, KCNA3, KCNA7, KCNJ10, KCNK15, KCNN4, KCNQ5, LCK, LRRC38, MCOLN2, MCOLN3, MMP9, P2RY12, PIK3CG, PRKCB, PTK2B, PTPN22, PTPN6, PTPRC, RASGRF1, RGS9, RRAD, SCNN1B, SHISA8, SLC12A3, SLC18A1, SLC24A4, SLC25A48, SLC31A2, SLC36A2, SLC5A2, SLC8A3, THY1, TMC2, TREM2, TRPM2, UBD, UCP2, UNC80, XCL1, XCR1]	[ABCC5, ABCC8, ABL1, ACTN4, AGT, APLNR, ARC, ARF1, ASIC3, ATP13A1, ATP13A2, ATP1A1, ATP1A3, ATP1B2, ATP1B4, ATP5F1D, ATP5MC2, ATP6V0D1, ATP6V0E2, ATP6V1B1, ATP6V1F, ATPSCKMT, CACNA1E, CACNA1H, CACNB4, CALCR, CALHM6, CAMK2B, CAPN1, CBARP, CCL2, CCL21, CCR7, CFH, CHERP, CHRNG, CNNM4, CNR1, COX6A2, COX8A, CX3CL1, FXYD5, FXYD6, GABBR2, GPER1, GPM6A, GPRIN1, GRIK5, GRM2, HCN3, HCN4, HFE, HTR1B, HTR2A, HVCN1, ITPR3, JPH4, KCNA7, KCNC3, KCNH2, KCNIP2, KCNJ1, KCNJ5, KCNJ9, KCNK15, KCNK2, KCNK3, KCNK6, KCNMB1, KCNMB2, KCNQ4, KCNT1, LRRC52, MED12, MFSD2A, MICU3, MINK1, MTCO2P12, NDUFA4L2, NEFL, NIPAL4, NLGN2, NPPA, ORAI1, P2RX3, P2RX5, P2RY6, PCDHA4, PIEZO1, PKD1, PM20D1, PRKACA, PRRT1, RANGRF, RNU1-93P, RRAD, S100A1, SCN5A, SCN7A, SELENON, SHANK3, SLC10A3, SLC12A4, SLC17A3, SLC1A4, SLC1A5, SLC25A23, SLC25A42, SLC29A4, SLC30A1, SLC30A3, SLC36A2, SLC38A5, SLC38A7, SLC39A13, SLC39A5, SLC41A1, SLC44A2, SLC4A10, SLC4A11, SLC5A10, SLC5A6, SLC6A12, SLC6A17, SLC6A8, SLC6A9, SLC7A1, SLC7A5, SLC7A8, SLC9A1, SLC9A3R1, SLN, SPHK2, STAC2, STX1A, SYNGAP1, TCIRG1, TIMP1, TMEM175, TMEM63A, TMEM63B, TMEM63C, TPCN1, TRPV4, TTYH1, UNC80, VPS9D1, WNK2, WWP2]
GO:0010959	regulation of metal ion transport	7,95E-04	[5, 6, 7]	68,00	25,28	51,67	48,33	[CALCR, CBARP, CCL2, CCL3, CCL4, CCL5, CD19, CD33, CD4, CD84, CORO1A, CXCL10, CXCL11, CXCL9, CXCR4, DRD1, EPX, FCRL3, IL16, JPH1, JSRP1, LILRB1, P2RY12, PIK3CG, PTK2B, PTPN22, PTPN6, RGS9, RRAD, SPINK1, THY1, TMC2, XCL1]	[ABL1, AGT, APLNR, ATP1B2, CACNB4, CALCR, CAMK2B, CBARP, CCL2, CX3CL1, GNAI2, GNAO1, GPER1, GPRIN1, ICAM1, JPH4, KCNH2, KCNIP2, LILRA5, NPPA, ORAI1, P2RX3, P2RX5, P2RY6, PDGFB, PDGFRB, PML, PRKACA, RCVRN, RRAD, S100A1, SELENON, SLC30A1, SLC9A1, SLN, SPHK2, SPINK1, STAC2, TIMP1, WFS1]

GO:0019722	calcium-mediated signaling	6,74E-04	[5, 6, 7]	62,00	26,05	59,40	40,60	[BHLHA15, BTK, CCL3, CCL4, CCR2, CCR3, CCR4, CCR5, CCR8, CD22, CD3E, CD4, CXCR3, CXCR4, CXCR6, EDN2, JPH1, JSRP1, LMCD1, MCOLN2, MCOLN3, NMUR1, P2RY12, PLEK, PTGFR, PTK2B, PTPRC, SELE, SLA2, SYK, TBC1D10C, TREM2, TRPM2, XCR1, ZAP70]	[ACKR3, ADGRB2, ADGRL1, AGTR1, CCR7, CHERP, DMTN, EDN2, GPRIN1, ITPR3, JPH4, LMCD1, MAPK7, NFATC4, P2RX3, P2RX5, P2RY6, PDK2, PPP1R9B, PRKACA, PTGFR, RNU1-93P, SAMD14, SELE, SELENON, SELENOP, SLC7A8, SLC9A1, SPHK1, TMEM100, TPCN1]
GO:0030001	metal ion transport	2,89E-05	[6]	173,00	20,60	42,80	57,20	[ATP2A3, BHLHA15, CACNA1E, CACNA1I, CALCR, CBARP, CCL19, CCL2, CCL3, CCL4, CCL5, CCR5, CD19, CD33, CD4, CD84, CNR1, CORO1A, CXCL10, CXCL11, CXCL9, CXCR4, CYSLTR1, DRD1, EPX, FCRL3, GPM6A, HCN1, IL16, JPH1, JSRP1, KCNA3, KCNA7, KCNJ10, KCNK15, KCNN4, KCNQ5, LCK, LILRB1, LRRC38, MCOLN2, MCOLN3, MS4A1, NMUR1, P2RY12, PIK3CG, PRKCB, PTK2B, PTPN22, PTPN6, PTPRC, RGS9, RRAD, SCNN1B, SLC12A3, SLC18A1, SLC24A4, SLC31A2, SLC5A2, SLC8A3, SPINK1, THY1, TMC2, TREM2, TRPM2, XCL1, XCR1]	[ABCC8, ABL1, ACTN4, AGT, APLNR, ASIC3, ATP1A1, ATP1A3, ATP1B2, CACNA1E, CACNA1H, CACNB4, CALCR, CAMK2B, CBARP, CCL2, CCL21, CCR7, CFH, CHERP, CNNM4, CNR1, CX3CL1, CYP27B1, FXYD5, FXYD6, GJA4, GNAI2, GNAO1, GPER1, GPM6A, GPRIN1, GRIK5, GRM2, HCN3, HCN4, HTR1B, HTR2A, ICAM1, ITPR3, JPH4, KCNA7, KCNC3, KCNH2, KCNIP2, KCNJ1, KCNJ5, KCNJ9, KCNK15, KCNK2, KCNK3, KCNK6, KCNMB1, KCNMB2, KCNQ4, KCNT1, LILRA5, LRRC52, MED12, MFSD2A, MICU3, NIPAL4, NPPA, ORAI1, P2RX3, P2RX5, P2RY6, PCDHA4, PDGFB, PDGFRB, PKD1, PLCZ1, PML, PRKACA, RAMP2, RANGRF, RCVRN, RNU1-93P, RRAD, S100A1, SCN5A, SCN7A, SELENON, SLC10A3, SLC12A4, SLC17A3, SLC25A23, SLC30A1, SLC30A3, SLC39A13, SLC39A5, SLC41A1, SLC4A10, SLC4A11, SLC5A10, SLC5A6, SLC6A12, SLC6A8, SLC6A9, SLC9A1, SLC9A3R1, SLN, SPHK2, SPINK1, STAC2, STX1A, SYNGAP1, TIMP1, TMEM175, TPCN1, TRPV4, TTYH1, VDR, WFS1, WNK2, WWP2]
GO:0030003	cellular cation homeostasis	1,82E-05	[5, 7]	150,00	21,46	48,17	51,83	[ATP2A3, C1QTNF1, CALCR, CCL19, CCL3, CCL5, CCR2, CCR3, CCR4, CCR5, CCR8, CD19, CD200R1, CD52, CNR1, CORO1A, CP, CXCL10, CXCL11, CXCL9, CXCR3, CXCR4, CXCR6, CXorf21, CYSLTR1, DRD1, EDN2, EPX, ERFE, FASLG, FATE1, FFAR4, GPR174, GPR4, GPR55, IFNG, JPH1, JSRP1, KNG1, LCK, LPAR2, MCOLN2, MCOLN3, MS4A1, MT1F, MT1G, MT1HL1, NLRP3, P2RY10, PIK3CG, PRKCB, PTGDR, PTGER2, PTGFR, PTK2B, PTPN6, PTPRC, S1PR4, SLAMF8, SLC24A4, SLC31A2, SLC8A3, THY1, TRPM2, XCL1, XCR1]	[ABL1, ACKR3, AGT, AGTR1, AP3D1, APLNR, ARF1, ATP13A1, ATP13A2, ATP1A1, ATP1A3, ATP1B2, ATP6V0D1, ATP6V0E2, ATP6V1B1, ATP6V1F, C1QTNF1, C5AR1, CALB1, CALCR, CCL11, CCL21, CCR7, CHERP, CNR1, CX3CL1, CYP27B1, EDN2, ERFE, FPR1, FTH1P19, GATA2, GPER1, GPR17, GPR20, GPR4, GPRIN1, GRIK5, GRINA, GRN, HCRTR1, HFE, HTR1B, HTR2A, HVCN1, ITPR3, JPH4, KCNK3, KNG1, LTF, MAFG, MAPK3, MICU3, MT1E, MYC, P2RX3, P2RX5, P2RY6, PACS2, PCDHA4, PKD1, PLCZ1, PML, PRKACA, PTGFR, PTGIR, RNU1-93P, SELENON, SLC25A23, SLC30A1, SLC30A3, SLC39A13, SLC39A5, SLC41A1, SLC4A10, SLC4A11, SLC4A2, SLC4A3, SLC6A9, SLC9A1, SMAD3, SV2A, SYPL2, TCIRG1, TGM2, TIMP1, TM9SF4, TMEM175, TPCN1, TRPV4, VDR, WFS1]
GO:0055080	cation homeostasis	3,97E-06	[6]	166,00	21,36	47,63	52,37	[ATP2A3, C1QTNF1, CALCR, CCL19, CCL3, CCL5, CCR2, CCR3, CCR4, CCR5, CCR8, CD19, CD200R1, CD52, CNR1, CORO1A, CP, CXCL10, CXCL11, CXCL9, CXCR3, CXCR4, CXCR6, CXorf21, CYSLTR1, DRD1, EDN2, EPX, ERFE, FASLG, FATE1, FFAR4, GPR174, GPR4, GPR55, IFNG, JPH1, JSRP1, KCNJ10, KNG1, LCK, LPAR2, MCOLN2, MCOLN3, MS4A1, MT1F, MT1G, MT1HL1, NLRP3, NPPB, P2RY10, PIK3CG, PRKCB, PTGDR, PTGER2, PTGFR, PTK2B, PTPN6, PTPRC, S1PR4, SCNN1B, SLAMF8, SLC12A3, SLC24A4, SLC31A2, SLC8A3, SNX10, THY1, TMC8, TRPM2, UNC80, XCL1, XCR1]	[ABL1, ACKR3, AGT, AGTR1, AP3D1, APLNR, ARF1, ATP13A1, ATP13A2, ATP1A1, ATP1A3, ATP1B2, ATP6V0D1, ATP6V0E2, ATP6V1B1, ATP6V1F, C1QTNF1, C5AR1, CALB1, CALCR, CCL11, CCL21, CCR7, CHERP, CNNM4, CNR1, CX3CL1, CYP27B1, EDN2, ERFE, FPR1, FTH1P19, GATA2, GNAI2, GPER1, GPR17, GPR20, GPR4, GPRIN1, GRIK5, GRINA, GRN, HCRTR1, HFE, HTR1B, HTR2A, HVCN1, ITPR3, JPH4, KCNH2, KCNK3, KNG1, LTF, MAFG, MAPK3, MICU3, MLLT6, MT1E, MYC, NPPB, P2RX3, P2RX5, P2RY6, PACS2, PCDHA4, PDK2, PKD1, PLCZ1, PML, PRKACA, PTGFR, PTGIR, RNU1-93P, SCN7A, SELENON, SLC12A4, SLC25A23, SLC30A1, SLC30A3, SLC39A13, SLC39A5, SLC41A1, SLC4A10, SLC4A11, SLC4A2, SLC4A3, SLC6A9, SLC7A8, SLC9A1, SMAD3, SNX10, STEAP3, SV2A, SYPL2, TCIRG1, TGM2, TIMP1, TM9SF4, TMEM175, TPCN1, TRPV4, UNC80, VDR, WFS1]
GO:0098771	inorganic ion homeostasis	4,00E-06	[6]	168,00	21,27	47,97	52,03	[ATP2A3, C1QTNF1, CA12, CALCR, CCL19, CCL3, CCL5, CCR2, CCR3, CCR4, CCR5, CCR8, CD19, CD200R1, CD52, CNR1, CORO1A, CP, CXCL10, CXCL11, CXCL9, CXCR3, CXCR4, CXCR6, CXorf21, CYSLTR1, DRD1, EDN2, EPX, ERFE, FASLG, FATE1, FFAR4, GPR174, GPR4, GPR55, IFNG, JPH1, JSRP1, KCNJ10, KNG1, LCK, LPAR2, MCOLN2, MCOLN3, MS4A1, MT1F, MT1G, MT1HL1, NLRP3, NPPB, P2RY10, PIK3CG, PRKCB, PTGDR, PTGER2, PTGFR, PTK2B, PTPN6, PTPRC, S1PR4, SCNN1B, SLAMF8, SLC12A3, SLC24A4, SLC31A2, SLC8A3, SNX10, TBXAS1, THY1, TMC8, TRPM2, XCL1, XCR1]	[ABL1, ACKR3, AGT, AGTR1, AP3D1, APLNR, ARF1, ATP13A1, ATP13A2, ATP1A1, ATP1A3, ATP1B2, ATP6V0D1, ATP6V0E2, ATP6V1B1, ATP6V1F, C1QTNF1, C5AR1, CALB1, CALCR, CCL11, CCL21, CCR7, CHERP, CNNM4, CNR1, CX3CL1, CYP27B1, EDN2, ERFE, FGFR4, FPR1, FTH1P19, GATA2, GNAI2, GPER1, GPR17, GPR20, GPR4, GPRIN1, GRIK5, GRINA, GRN, HCRTR1, HFE, HTR1B, HTR2A, HVCN1, ITPR3, JPH4, KCNH2, KCNK3, KNG1, LTF, MAFG, MAPK3, MICU3, MLLT6, MT1E, MYC, NPPB, P2RX3, P2RX5, P2RY6, PACS2, PCDHA4, PDK2, PKD1, PLCZ1, PML, PRKACA, PTGFR, PTGIR, RNU1-93P, SCN7A, SELENON, SLC12A4, SLC25A23, SLC30A1, SLC30A3, SLC39A13, SLC39A5, SLC41A1, SLC4A10, SLC4A2, SLC4A3, SLC6A9, SLC7A8, SLC9A1, SLC9A3R1, SMAD3, SNX10, STEAP3, SV2A, SYPL2, TCIRG1, TGM2, TIMP1, TM9SF4, TMEM175, TPCN1, TRPV4, VDR, WFS1]
GO:1904062	regulation of cation transmembrane transport	4,99E-02	[5, 6, 7]	85,00	21,25	45,19	54,81	[CALCR, CBARP, CCL2, CCR2, CD19, CORO1A, CTSS, CXCL10, CXCL11, CXCL9, DAPK1, DRD1, EPX, GABBR2, IFNG, JPH1, JSRP1, KCNJ10, KCNN4, LRRC38, MMP9, PIK3CG, PTK2B, PTPN22, PTPN6, RASGRF1, RGS9, RRAD, SHISA8, SLC31A2, THY1, TMC2, TREM2, UBD, XCL1]	[ABCC8, ABL1, ACTN4, AGT, APLNR, ARC, ARF1, ATP1B2, ATPSCKMT, CACNB4, CALCR, CAMK2B, CAPN1, CBARP, CCL2, CFH, CX3CL1, FXYD5, FXYD6, GABBR2, GPER1, GPRIN1, JPH4, KCNH2, KCNIP2, KCNJ5, KCNJ9, KCNMB1, KCNMB2, LRRC52, MINK1, NEFL, NLGN2, NPPA, P2RY6, PM20D1, PRKACA, PRRT1, RANGRF, RRAD, S100A1, SCN5A, SELENON, SHANK3, SLC30A1, SLC6A9, SLC9A1, SLC9A3R1, SLN, SPHK2, STAC2, TCIRG1, TIMP1, WNK2, WWP2]
GO:0051208	sequestering of calcium ion	1,20E-02	[3, 4, 8, 10]	37,00	29,13	62,97	37,03	[CCL19, CCL3, CCR5, CD19, CORO1A, CXCL10, CXCL11, CXCL9, DRD1, JPH1, JSRP1, LCK, MCOLN2, MCOLN3, PTK2B, PTPN6, PTPRC, THY1, TRPM2, XCL1, XCR1]	[ABL1, APLNR, CCL21, CCR7, CHERP, CX3CL1, GPER1, HTR2A, ITPR3, JPH4, P2RY6, PRKACA, RNU1-93P, SELENON, SLC25A23, TPCN1]

GO:0051282	regulation of sequestering of calcium ion	4,27E-03	[3, 4, 5, 9, 11]	37,00	30,08	62,97	37,03	[CCL19, CCL3, CCR5, CD19, CORO1A, CXCL10, CXCL11, CXCL9, DRD1, JPH1, JSRP1, LCK, MCOLN2, MCOLN3, PTK2B, PTPN6, PTPRC, THY1, TRPM2, XCL1, XCR1]	[ABL1, APLNR, CCL21, CCR7, CHERP, CX3CL1, GPER1, HTR2A, ITPR3, JPH4, P2RY6, PRKACA, RNU1-93P, SELENON, SLC25A23, TPCN1]
GO:0006816	calcium ion transport	5,98E-05	[7]	108,00	22,98	53,02	46,98	[ATP2A3, BHLHA15, CACNA1E, CACNA1I, CALCR, CBARP, CCL19, CCL2, CCL3, CCL4, CCL5, CCR5, CD19, CD33, CD4, CD84, CNR1, CORO1A, CXCL10, CXCL11, CXCL9, CXCR4, CYSLTR1, DRD1, EPX, FCRL3, GPM6A, IL16, JPH1, JSRP1, KCNN4, LCK, LILRB1, MCOLN2, MCOLN3, MS4A1, NMUR1, P2RY12, PIK3CG, PRKCB, PTK2B, PTPN22, PTPN6, PTPRC, RGS9, RRAD, SLC24A4, SLC8A3, SPINK1, THY1, TMC2, TRPM2, XCL1, XCR1]	[ABL1, AGT, APLNR, CACNA1E, CACNA1H, CACNB4, CALCR, CAMK2B, CBARP, CCL2, CCL21, CCR7, CHERP, CNR1, CX3CL1, CYP27B1, GJA4, GNAI2, GNAO1, GPER1, GPM6A, GPRIN1, GRM2, HTR1B, HTR2A, ICAM1, ITPR3, JPH4, LILRA5, MED12, MICU3, NPPA, ORAI1, P2RX3, P2RX5, P2RY6, PCDHA4, PDGFB, PDGFRB, PKD1, PLCZ1, PML, PRKACA, RAMP2, RCVRN, RNU1-93P, RRAD, S100A1, SELENON, SLC25A23, SLC30A1, SLC9A1, SLN, SPHK2, SPINK1, STAC2, STX1A, TIMP1, TPCN1, TRPV4, VDR, WFS1]
GO:0006875	cellular metal ion homeostasis	5,04E-05	[6, 8]	134,00	21,75	50,82	49,18	[ATP2A3, C1QTNF1, CALCR, CCL19, CCL3, CCL5, CCR2, CCR3, CCR4, CCR5, CCR8, CD19, CD200R1, CD52, CNR1, CORO1A, CP, CXCL10, CXCL11, CXCL9, CXCR3, CXCR4, CXCR6, CYSLTR1, DRD1, EDN2, EPX, ERFE, FATE1, FFAR4, GPR174, GPR4, GPR55, IFNG, JPH1, JSRP1, KNG1, LCK, LPAR2, MCOLN2, MCOLN3, MS4A1, MT1F, MT1G, MT1HL1, NLRP3, P2RY10, PIK3CG, PRKCB, PTGDR, PTGER2, PTGFR, PTK2B, PTPN6, PTPRC, S1PR4, SLC24A4, SLC31A2, SLC8A3, THY1, TRPM2, XCL1, XCR1]	[ABL1, ACKR3, AGT, AGTR1, AP3D1, APLNR, ARF1, ATP13A1, ATP13A2, ATP1A1, ATP1A3, ATP1B2, ATP6V0D1, C1QTNF1, C5AR1, CALB1, CALCR, CCL11, CCL21, CCR7, CHERP, CNR1, CX3CL1, CYP27B1, EDN2, ERFE, FPR1, FTH1P19, GATA2, GPER1, GPR17, GPR20, GPR4, GPRIN1, GRIK5, GRINA, HCRTR1, HFE, HTR1B, HTR2A, ITPR3, JPH4, KCNK3, KNG1, LTF, MICU3, MT1E, MYC, P2RX3, P2RX5, P2RY6, PACS2, PCDHA4, PKD1, PLCZ1, PML, PRKACA, PTGFR, PTGIR, RNU1-93P, SELENON, SLC25A23, SLC30A1, SLC30A3, SLC39A13, SLC39A5, SLC41A1, SLC6A9, SLC9A1, SMAD3, SV2A, SYPL2, TCIRG1, TGM2, TIMP1, TPCN1, TRPV4, VDR, WFS1]
GO:0022836	gated channel activity	2,26E-02	[6, 8]	100,00	20,83	38,00	62,00	[ANO9, CACNA1E, CACNA1I, CBARP, CCL2, CCR2, CLCNKA, CNR1, CYBB, DAPK1, HCN1, IFNG, JPH1, JSRP1, KCNA3, KCNA7, KCNJ10, KCNK15, KCNN4, KCNQ5, LRRC38, MCOLN2, MCOLN3, PTAFR, PTK2B, RASGRF1, RGS9, RRAD, SCNN1B, SHISA8, TMC2, TMC3, TMC8, TREM2, TRPM2]	[ABCC8, ANO3, ANO7, ARC, ASIC3, CACNA1E, CACNA1H, CACNB4, CAMK2B, CAPN1, CBARP, CCL2, CHRNG, CLCN6, CLCN7, CLCNKA, CNR1, GABRR2, GLRA1, GPRIN1, GRIK5, HCN3, HCN4, HTR1B, HVCN1, ITPR3, JPH4, KCNA7, KCNC3, KCNH2, KCNIP2, KCNJ1, KCNJ5, KCNJ9, KCNK15, KCNK2, KCNK3, KCNK6, KCNMB1, KCNMB2, KCNQ4, KCNT1, LRRC52, MINK1, NEFL, NLGN2, NPPA, P2RX3, P2RX5, P2RY6, PCDHA4, PIEZO1, PRKACA, PRRT1, RRAD, S100A1, SCN5A, SCN7A, SELENON, SHANK3, SLC17A3, SLC6A9, STAC2, TMC3, TMC4, TMEM109, TMEM63A, TMEM63B, TMEM63C, TPCN1, TRPV4, TTYH1, TTYH2, TTYH3]
GO:0051924	regulation of calcium ion transport	2,15E-03	[6, 7, 8]	65,00	25,00	53,61	46,39	[CALCR, CBARP, CCL2, CCL3, CCL4, CCL5, CD19, CD33, CD4, CD84, CORO1A, CXCL10, CXCL11, CXCL9, CXCR4, DRD1, EPX, FCRL3, IL16, JPH1, JSRP1, LILRB1, P2RY12, PIK3CG, PTK2B, PTPN22, PTPN6, RGS9, RRAD, SPINK1, THY1, TMC2, XCL1]	[ABL1, AGT, APLNR, CACNB4, CALCR, CAMK2B, CBARP, CCL2, CX3CL1, GNAI2, GNAO1, GPER1, GPRIN1, ICAM1, JPH4, LILRA5, NPPA, ORAI1, P2RX3, P2RX5, P2RY6, PDGFB, PDGFRB, PML, PRKACA, RCVRN, RRAD, S100A1, SELENON, SLC30A1, SLC9A1, SLN, SPHK2, SPINK1, STAC2, TIMP1, WFS1]
GO:0055065	metal ion homeostasis	1,49E-05	[7]	149,00	21,56	49,83	50,17	[ATP2A3, C1QTNF1, CALCR, CCL19, CCL3, CCL5, CCR2, CCR3, CCR4, CCR5, CCR8, CD19, CD200R1, CD52, CNR1, CORO1A, CP, CXCL10, CXCL11, CXCL9, CXCR3, CXCR4, CXCR6, CYSLTR1, DRD1, EDN2, EPX, ERFE, FATE1, FFAR4, GPR174, GPR4, GPR55, IFNG, JPH1, JSRP1, KCNJ10, KNG1, LCK, LPAR2, MCOLN2, MCOLN3, MS4A1, MT1F, MT1G, MT1HL1, NLRP3, NPPB, P2RY10, PIK3CG, PRKCB, PTGDR, PTGER2, PTGFR, PTK2B, PTPN6, PTPRC, S1PR4, SCNN1B, SLC12A3, SLC24A4, SLC31A2, SLC8A3, SNX10, THY1, TMC8, TRPM2, XCL1, XCR1]	[ABL1, ACKR3, AGT, AGTR1, AP3D1, APLNR, ARF1, ATP13A1, ATP13A2, ATP1A1, ATP1A3, ATP1B2, ATP6V0D1, ATP6V1B1, C1QTNF1, C5AR1, CALB1, CALCR, CCL11, CCL21, CCR7, CHERP, CNNM4, CNR1, CX3CL1, CYP27B1, EDN2, ERFE, FPR1, FTH1P19, GATA2, GNAI2, GPER1, GPR17, GPR20, GPR4, GPRIN1, GRIK5, GRINA, HCRTR1, HFE, HTR1B, HTR2A, ITPR3, JPH4, KCNH2, KCNK3, KNG1, LTF, MICU3, MLLT6, MT1E, MYC, NPPB, P2RX3, P2RX5, P2RY6, PACS2, PCDHA4, PKD1, PLCZ1, PML, PRKACA, PTGFR, PTGIR, RNU1-93P, SCN7A, SELENON, SLC12A4, SLC25A23, SLC30A1, SLC30A3, SLC39A13, SLC39A5, SLC41A1, SLC6A9, SLC7A8, SLC9A1, SMAD3, SNX10, STEAP3, SV2A, SYPL2, TCIRG1, TGM2, TIMP1, TPCN1, TRPV4, VDR, WFS1]
GO:0072503	cellular divalent inorganic cation homeostasis	6,33E-06	[6, 8]	119,00	23,29	53,29	46,71	[ATP2A3, C1QTNF1, CALCR, CCL19, CCL3, CCL5, CCR2, CCR3, CCR4, CCR5, CCR8, CD19, CD200R1, CD52, CNR1, CORO1A, CXCL10, CXCL11, CXCL9, CXCR3, CXCR4, CXCR6, CYSLTR1, DRD1, EDN2, EPX, FATE1, FFAR4, GPR174, GPR4, GPR55, JPH1, JSRP1, KNG1, LCK, LPAR2, MCOLN2, MCOLN3, MS4A1, MT1F, MT1G, MT1HL1, NLRP3, P2RY10, PIK3CG, PRKCB, PTGDR, PTGER2, PTGFR, PTK2B, PTPN6, PTPRC, S1PR4, SLC24A4, SLC8A3, THY1, TRPM2, XCL1, XCR1]	[ABL1, ACKR3, AGT, AGTR1, AP3D1, APLNR, ATP13A1, ATP13A2, C1QTNF1, C5AR1, CALB1, CALCR, CCL11, CCL21, CCR7, CHERP, CNR1, CX3CL1, CYP27B1, EDN2, FPR1, GATA2, GPER1, GPR17, GPR20, GPR4, GPRIN1, GRIK5, GRINA, HCRTR1, HTR1B, HTR2A, ITPR3, JPH4, KCNK3, KNG1, MICU3, MT1E, P2RX3, P2RX5, P2RY6, PACS2, PCDHA4, PKD1, PLCZ1, PML, PRKACA, PTGFR, PTGIR, RNU1-93P, SELENON, SLC25A23, SLC30A1, SLC30A3, SLC39A13, SLC39A5, SLC41A1, SMAD3, SV2A, SYPL2, TCIRG1, TGM2, TIMP1, TPCN1, TRPV4, VDR, WFS1]
GO:0072507	divalent inorganic cation homeostasis	3,69E-06	[7]	123,00	23,21	53,03	46,97	[ATP2A3, C1QTNF1, CALCR, CCL19, CCL3, CCL5, CCR2, CCR3, CCR4, CCR5, CCR8, CD19, CD200R1, CD52, CNR1, CORO1A, CXCL10, CXCL11, CXCL9, CXCR3, CXCR4, CXCR6, CYSLTR1, DRD1, EDN2, EPX, FATE1, FFAR4, GPR174, GPR4, GPR55, JPH1, JSRP1, KNG1, LCK, LPAR2, MCOLN2, MCOLN3, MS4A1, MT1F, MT1G, MT1HL1, NLRP3, P2RY10, PIK3CG, PRKCB, PTGDR, PTGER2, PTGFR, PTK2B, PTPN6, PTPRC, S1PR4, SLC24A4, SLC8A3, SNX10, THY1, TMC8, TRPM2, XCL1, XCR1]	[ABL1, ACKR3, AGT, AGTR1, AP3D1, APLNR, ATP13A1, ATP13A2, ATP6V1B1, C1QTNF1, C5AR1, CALB1, CALCR, CCL11, CCL21, CCR7, CHERP, CNNM4, CNR1, CX3CL1, CYP27B1, EDN2, FPR1, GATA2, GPER1, GPR17, GPR20, GPR4, GPRIN1, GRIK5, GRINA, HCRTR1, HTR1B, HTR2A, ITPR3, JPH4, KCNK3, KNG1, MICU3, MT1E, P2RX3, P2RX5, P2RY6, PACS2, PCDHA4, PKD1, PLCZ1, PML, PRKACA, PTGFR, PTGIR, RNU1-93P, SELENON, SLC25A23, SLC30A1, SLC30A3, SLC39A13, SLC39A5, SLC41A1, SMAD3, SNX10, SV2A, SYPL2, TCIRG1, TGM2, TIMP1, TPCN1, TRPV4, VDR, WFS1]

GO:0006874	cellular calcium ion homeostasis	1,92E-05	[7, 9]	110,00	23,31	54,33	45,67	[ATP2A3, C1QTNF1, CALCR, CCL19, CCL3, CCL5, CCR2, CCR3, CCR4, CCR5, CCR8, CD19, CD200R1, CD52, CNR1, CORO1A, CXCL10, CXCL11, CXCL9, CXCR3, CXCR4, CXCR6, CYSLTR1, DRD1, EDN2, EPX, FATE1, FFAR4, GPR174, GPR4, GPR55, JPH1, JSRP1, KNG1, LCK, LPAR2, MCOLN2, MCOLN3, MS4A1, NLRP3, P2RY10, PIK3CG, PRKCB, PTGDR, PTGER2, PTGFR, PTK2B, PTPN6, PTPRC, S1PR4, SLC24A4, SLC8A3, THY1, TRPM2, XCL1, XCR1]	[ABL1, ACKR3, AGT, AGTR1, APLNR, ATP13A1, ATP13A2, C1QTNF1, C5AR1, CALB1, CALCR, CCL11, CCL21, CCR7, CHERP, CNR1, CX3CL1, CYP27B1, EDN2, FPR1, GATA2, GPER1, GPR17, GPR20, GPR4, GPRIN1, GRIK5, GRINA, HCRTR1, HTR1B, HTR2A, ITPR3, JPH4, KCNK3, KNG1, MICU3, P2RX3, P2RX5, P2RY6, PACS2, PCDHA4, PKD1, PLCZ1, PML, PRKACA, PTGFR, PTGIR, RNU1-93P, SELENON, SLC25A23, SLC30A1, SMAD3, SV2A, SYPL2, TCIRG1, TGM2, TIMP1, TPCN1, TRPV4, VDR, WFS1]
GO:0055074	calcium ion homeostasis	2,79E-05	[8]	112,00	23,14	53,97	46,03	[ATP2A3, C1QTNF1, CALCR, CCL19, CCL3, CCL5, CCR2, CCR3, CCR4, CCR5, CCR8, CD19, CD200R1, CD52, CNR1, CORO1A, CXCL10, CXCL11, CXCL9, CXCR3, CXCR4, CXCR6, CYSLTR1, DRD1, EDN2, EPX, FATE1, FFAR4, GPR174, GPR4, GPR55, JPH1, JSRP1, KNG1, LCK, LPAR2, MCOLN2, MCOLN3, MS4A1, NLRP3, P2RY10, PIK3CG, PRKCB, PTGDR, PTGER2, PTGFR, PTK2B, PTPN6, PTPRC, S1PR4, SLC24A4, SLC8A3, SNX10, THY1, TRPM2, XCL1, XCR1]	[ABL1, ACKR3, AGT, AGTR1, APLNR, ATP13A1, ATP13A2, ATP6V1B1, C1QTNF1, C5AR1, CALB1, CALCR, CCL11, CCL21, CCR7, CHERP, CNR1, CX3CL1, CYP27B1, EDN2, FPR1, GATA2, GPER1, GPR17, GPR20, GPR4, GPRIN1, GRIK5, GRINA, HCRTR1, HTR1B, HTR2A, ITPR3, JPH4, KCNK3, KNG1, MICU3, P2RX3, P2RX5, P2RY6, PACS2, PCDHA4, PKD1, PLCZ1, PML, PRKACA, PTGFR, PTGIR, RNU1-93P, SELENON, SLC25A23, SLC30A1, SMAD3, SNX10, SV2A, SYPL2, TCIRG1, TGM2, TIMP1, TPCN1, TRPV4, VDR, WFS1]
GO:0060401	cytosolic calcium ion transport	1,60E-03	[8]	52,00	27,08	59,20	40,80	[ATP2A3, CALCR, CCL19, CCL3, CCR5, CD19, CORO1A, CXCL10, CXCL11, CXCL9, DRD1, EPX, JPH1, JSRP1, LCK, MCOLN2, MCOLN3, MS4A1, PTK2B, PTPN6, PTPRC, RGS9, SLC24A4, SLC8A3, THY1, TRPM2, XCL1, XCR1]	[ABL1, APLNR, CALCR, CCL21, CCR7, CHERP, CX3CL1, GPER1, GPRIN1, HTR2A, ITPR3, JPH4, MED12, MICU3, P2RX3, P2RX5, P2RY6, PML, PRKACA, RNU1-93P, SELENON, SLC25A23, TIMP1, TPCN1, TRPV4]
GO:0060402	calcium ion transport into cytosol	2,59E-03	[4, 9, 10, 12]	47,00	27,98	60,49	39,51	[CALCR, CCL19, CCL3, CCR5, CD19, CORO1A, CXCL10, CXCL11, CXCL9, DRD1, EPX, JPH1, JSRP1, LCK, MCOLN2, MCOLN3, MS4A1, PTK2B, PTPN6, PTPRC, SLC24A4, SLC8A3, THY1, TRPM2, XCL1, XCR1]	[ABL1, APLNR, CALCR, CCL21, CCR7, CHERP, CX3CL1, GPER1, GPRIN1, HTR2A, ITPR3, JPH4, P2RX3, P2RX5, P2RY6, PML, PRKACA, RNU1-93P, SELENON, TIMP1, TPCN1, TRPV4]
GO:0051209	release of sequestered calcium ion into cytosol	6,28E-03	[4, 5, 6, 7, 8, 10, 11, 12, 13, 14]	36,00	30,00	64,46	35,54	[CCL19, CCL3, CCR5, CD19, CORO1A, CXCL10, CXCL11, CXCL9, DRD1, JPH1, JSRP1, LCK, MCOLN2, MCOLN3, PTK2B, PTPN6, PTPRC, THY1, TRPM2, XCL1, XCR1]	[ABL1, APLNR, CCL21, CCR7, CHERP, CX3CL1, GPER1, HTR2A, ITPR3, JPH4, P2RY6, PRKACA, RNU1-93P, SELENON, TPCN1]
GO:0051480	regulation of cytosolic calcium ion concentration	2,17E-05	[8, 10]	91,00	24,66	59,43	40,57	[C1QTNF1, CALCR, CCL19, CCL3, CCR2, CCR3, CCR4, CCR5, CCR8, CD19, CD200R1, CD52, CNR1, CORO1A, CXCL10, CXCL11, CXCL9, CXCR3, CXCR4, CXCR6, CYSLTR1, DRD1, EDN2, EPX, FFAR4, GPR174, GPR4, GPR55, JPH1, JSRP1, KNG1, LCK, LPAR2, MCOLN2, MCOLN3, MS4A1, NLRP3, P2RY10, PIK3CG, PTGDR, PTGER2, PTGFR, PTK2B, PTPN6, PTPRC, S1PR4, SLC24A4, SLC8A3, THY1, TRPM2, XCL1, XCR1]	[ABL1, ACKR3, AGT, AGTR1, APLNR, C1QTNF1, C5AR1, CALB1, CALCR, CCL21, CCR7, CHERP, CNR1, CX3CL1, EDN2, FPR1, GATA2, GPER1, GPR17, GPR20, GPR4, GPRIN1, HCRTR1, HTR1B, HTR2A, ITPR3, JPH4, KCNK3, KNG1, P2RX3, P2RX5, P2RY6, PCDHA4, PKD1, PLCZ1, PML, PRKACA, PTGFR, PTGIR, RNU1-93P, SELENON, SMAD3, TGM2, TIMP1, TPCN1, TRPV4]
GO:0097553	calcium ion transmembrane import into cytosol	8,62E-03	[5, 7, 9, 10, 11, 13]	41,00	28,28	63,34	36,66	[CALCR, CCL19, CCL3, CCR5, CD19, CORO1A, CXCL10, CXCL11, CXCL9, DRD1, JPH1, JSRP1, LCK, MCOLN2, MCOLN3, PTK2B, PTPN6, PTPRC, SLC24A4, SLC8A3, THY1, TRPM2, XCL1, XCR1]	[ABL1, APLNR, CALCR, CCL21, CCR7, CHERP, CX3CL1, GPER1, GPRIN1, HTR2A, ITPR3, JPH4, P2RY6, PRKACA, RNU1-93P, SELENON, TPCN1, TRPV4]
GO:0032879	regulation of localization	1,05E-09	[2, 3]	515,00	18,06	37,10	62,90	[ABCB11, ADAM8, ADAMTS9, ADORA3, AIF1, ALOX15, ALOX15B, ANGPT1, ANGPT2, ANO9, AREG, BLK, BMP7, C1QTNF1, C2, CACNA1E, CACNA1I, CALCR, CARMIL2, CASP8, CASS4, CBARP, CCL19, CCL2, CCL24, CCL3, CCL4, CCL5, CCR2, CCR5, CD177, CD19, CD200R1, CD22, CD247, CD300LF, CD33, CD4, CD74, CD84, CDH1, CLCNKA, CNR1, CORO1A, CSF1R, CTSS, CXCL10, CXCL11, CXCL9, CXCR3, CXCR4, CYBB, DAPK1, DERL3, DOCK10, DOCK2, DOCK8, DPEP1, DRC1, DRD1, EDN2, EPX, EREG, ERFE, FASLG, FCRL3, FFAR4, FGR, FUT7, GABBR2, GPSM3, GZMB, HCLS1, HCN1, HLA-DRB1, IFNG, IKBKE, IL16, IL2RB, IL2RG, ITGA4, ITGAX, ITGB2, JPH1, JSRP1, KCNA3, KCNA7, KCNJ10, KCNK15, KCNN4, KCNQ5, KLRC2, KLRK1, KMO, LCK, LCP1, LEF1, LIF, LILRB1, LILRB4, LPAL2, LRRC38, MCOLN2, MCOLN3, MIDN, MLC1, MMP9, MYB, NCKAP1L, NELL2, NLRP3, NLRP6, NPPB, NR4A3, NTN1, P2RY12, P2RY2, PIK3CG, PIM1, PLA2G7, PLAU, PLXNC1, PRAM1, PRKCB, PTAFR, PTK2B, PTPN22, PTPN6, PTPRC, PTX3, PYCARD, PYHIN1, RAB15, RAB27B, RAB3B, RAB3C, RAC2, RASGRF1, RGS9, RHOH, RRAD, SDC1, SELE, SEMA4A, SEMA4D, SEPTIN1, SHISA8, SIRPG, SLAMF8, SLC31A2, SMPD3, SPINK1, SPN, STAP1, STXBP2, SYK, TACSTD2, TCAF2, THBS1, THY1, TIFAB, TMC2, TNFRSF18, TREM2, TRIM14, TRPM2, UBD, UCP2, WAS, XCL1, XCL2, XCR1, XG]	[AAAS, ABCA3, ABCA7, ABCB11, ABCC8, ABI3, ABL1, ABLIM3, ACACB, ACKR3, ACTB, ACTN1, ACTN4, ADAMTS9, ADCY1, ADGRG1, ADIPOQ, AGER, AGT, AGTR1, AMH, ANGPT1, ANKRD13B, AP2A1, AP3D1, APLN, APLNR, APOA1, APOD, ARC, AREG, ARF1, ARHGAP1, ARHGDIA, ARHGEF16, ARHGEF5, ATP13A2, ATP1A1, ATP1A3, ATP1B2, ATPSCKMT, BAG3, BCAR1, BCL2L1, BCL6, BMP8A, BSN, C11orf65, C1QTNF1, C2CD2L, C5, C5AR1, CACNA1E, CACNA1H, CACNB4, CALCR, CAMK2B, CAMSAP3, CAPN1, CARMIL2, CBARP, CCL11, CCL2, CCL21, CCL24, CCR7, CD14, CD177, CD74, CDK1, CEACAM1, CELSR2, CETP, CFAP298-TCP10L, CFH, CGA, CHCHD10, CHERP, CHGA, CITED2, CLCNKA, CLIP3, CNR1, COL1A1, CPLX1, CRP, CRYAB, CSF1, CSK, CSNK1E, CTSD, CX3CL1, DAB2IP, DAG1, DGKD, DISP3, DLG5, DMTN, DOC2B, DRC1, DVL3, DYSF, ECM1, EDN2, EHD1, EHD2, ENG, EPHA2, EPPK1, ERBB2, ERFE, FADD, FAM110A, FES, FFAR2, FITM1, FLT4, FOXC2, FOXF1, FURIN, FXYD5, FXYD6, GAB2, GABBR2, GAS1, GATA2, GATA4, GBF1, GCSAM, GDI1, GIT1, GLIS2, GNAI2, GNAO1, GPER1, GPI, GPRIN1, GRB7, GRIK5, GRM2, GRN, GSK3A, HBEGF, HCN3, HCN4, HDAC5, HDAC7, HFE, HGS, HSPB1, HTR1B, HTR2A, HVCN1, HYAL1, ICAM1, IFITM1, IL15RA, IL1B, IL4R, ILK, INHA, INPP5E, ITGA3, ITPR3, JPH4, JUP, KCNA7, KCNC3, KCNH2, KCNIP2, KCNJ1, KCNJ5, KCNJ9, KCNK15, KCNK6, KCNMB1, KCNMB2, KCNQ4, KCTD11, LAMA5, LDLRAP1, LGALS9, LGR6, LIF, LILRA5, LIMK2, LIPG, LMNA, LRRC52, LRRC8A, LZTS1, LZTS2, MAP1A, MAP2K3, MAPK3, MARK4, MAVS, MAZ, MECP2, MICALL2, MIDN, MIEN1, MINK1, MLLT6, MSN, MSTN, MYADM, MYC, MYO18A, NDC80, NEFL, NF2, NGFR, NKAIN3, NKD2, NLGN2, NNAT, NOD2, NODAL, NOS3, NOTCH1, NPPA, NPPB, NR1D1, NR4A3, NSMF, NTN1, NUCB1, NUTF2, ORAI1, OSGIN1, P2RX3, P2RX5, P2RY2, P2RY6, PACSIN1, PCDHA4, PDGFB, PDGFRB, PER1, PFN1, PHACTR1, PIM1, PLA2G7, PLAU, PLEKHM2, PLK3, PLTP, PLVAP, PLXNA1

GO:0040012	regulation of locomotion	6,83E-08	[2, 3]	217,00	20,93	38,54	61,46	[ADAM8, ADAMTS9, ADORA3, AIF1, ALOX15B, ANGPT1, ANGPT2, BMP7, CARMIL2, CASS4, CCL19, CCL2, CCL24, CCL3, CCL4, CCL5, CCR2, CCR4, CD200R1, CD74, CDH1, CORO1A, CSF1R, CXCL10, CXCR3, CXCR4, DOCK10, DOCK8, DPEP1, DRD1, EDN2, EREG, FGR, FUT7, GPSM3, IFNG, IL16, ITGA4, ITGAX, KLRK1, LEF1, MMP9, NCKAP1L, NELL2, NR4A3, NTN1, P2RY12, PIK3CG, PLA2G7, PLAU, PLXNC1, PTAFR, PTK2B, PTPN22, PTPRC, PYCARD, RAC2, RHOH, SELE, SEMA4A, SEMA4D, SLAMF8, SMPD3, SPN, STAP1, SUCNR1, TACSTD2, TCAF2, THBS1, THY1, TNFRSF18, TREM2, WAS, XCL1, XCL2, XG]	[ABCC8, ABI3, ABL1, ACKR3, ACTN4, ADAMTS9, ADGRG1, ADIPOQ, AGER, AGT, AMH, ANGPT1, APOD, ARHGDIA, ARRDC3, ARTN, ATP1B2, BCAR1, C5, C5AR1, CAMK2B, CAMSAP3, CARMIL2, CCL11, CCL2, CCL21, CCL24, CCR7, CD74, CEACAM1, CGA, CHGA, CITED2, COL1A1, CSF1, CX3CL1, DAB2IP, DAG1, DLG5, DMTN, ECM1, EDN2, ENG, EPHA2, EPPK1, ERBB2, FADD, FAM110A, FES, FLT4, FOXC2, FOXF1, GATA2, GCSAM, GNAI2, GNB3, GPER1, GPI, GRB7, GRN, HBEGF, HDAC5, HDAC7, HSPB1, HYAL1, ICAM1, IFITM1, IL1B, ILK, ITGA3, JUP, LAMA5, LGALS9, LGR6, LMNA, MAP2K3, MAPK3, MAZ, MECP2, MEGF8, MEN1, MIEN1, MINK1, MSN, MSTN, MYADM, MYC, NF2, NGFR, NOD2, NODAL, NOS3, NOTCH1, NR4A3, NSMF, NTN1, OSGIN1, P2RY6, PDGFB, PDGFRB, PFN1, PHACTR1, PLA2G7, PLAU, PLVAP, PLXNA1, PLXNA3, PLXNB1, POSTN, PPARD, PRAG1, PTN, PTPN23, PTPRU, RAC2, RGCC, RHOG, RIPOR1, ROBO4, S100A11, SELE, SELENOP, SEMA3F, SEMA4B, SEMA4C, SEMA6B, SEMA6C, SERPINE1, SH3BP1, SLC9A3R1, SMAD3, SMIM22, SNAI1, SPHK1, SPI1, SRC, SRF, SSX2IP, STAT3, SYDE1, TBX5, THBS1, TIMP1, TNF, TRIB1, TRPV4, VEGFA, VSIR, VTN, WAS, WNT3, XG, ZC3H12A, ZNF580, ZNF609, ZSWIM4, ZSWIM8]
GO:0048870	cell motility	6,24E-10	[2, 3]	354,00	19,50	44,98	55,02	[ADAM8, ADAMTS9, ADORA3, AIF1, ALOX15B, ANGPT1, ANGPT2, BCL11B, BIN2, BMP7, CACNA1I, CARMIL2, CASS4, CCL17, CCL19, CCL2, CCL22, CCL24, CCL3, CCL3L1, CCL4, CCL4L1, CCL4L2, CCL5, CCR2, CCR3, CCR4, CCR5, CCR8, CD177, CD2, CD200R1, CD244, CD48, CD74, CD84, CDH1, CEACAM21, CEL, CFAP54, CNR2, CORO1A, CRB2, CRTAM, CSF1R, CXCL10, CXCL11, CXCL5, CXCL6, CXCL9, CXCR3, CXCR4, CXCR6, DCC, DEPDC1B, DNAH8, DOCK10, DOCK8, DOK2, DPEP1, DRC1, DRD1, EDN2, EGR3, ENKUR, EPX, EREG, FGR, FOLR2, FUT7, GPM6A, GPSM3, IFNG, IL16, INPP5D, ITGA4, ITGAL, ITGAX, ITGB2, ITGB7, JAML, JCHAIN, KLF5, KLRK1, LCK, LCP1, LEF1, MCOLN2, MIXL1, MMP9, MYO1G, NCKAP1L, NELL2, NLRP3, NME8, NR4A1, NR4A2, NR4A3, NTN1, P2RY12, PIK3C2G, PIK3CG, PLA2G7, PLAU, PLXNC1, PRKCQ, PSTPIP2, PTAFR, PTK2B, PTPN22, PTPN6, PTPRC, PYCARD, RAC2, RHOH, SDC1, SELE, SELL, SELPLG, SEMA4A, SEMA4D, SIRPG, SLAMF8, SLC7A7, SMPD3, SPEF2, SPN, STAP1, STAT1, SYK, TACSTD2, TBX21, TCAF2, THBS1, THY1, TNFAIP3, TNFRSF18, TNR, TREM2, TRPM2, VAV1, WAS, XCL1, XCL2, XCR1, XG, ZAP70]	[ABCC8, ABI3, ABL1, ACKR3, ACTB, ACTN4, ADAMTS9, ADCY3, ADGRG1, ADIPOQ, AGER, AGT, AGTR1, AMH, ANGPT1, APOA1, APOD, ARC, ARHGDIA, ARHGEF16, ARTN, ATP1B2, BCAR1, C11orf88, C5, C5AR1, CAMK2B, CAMSAP3, CARMIL2, CCL11, CCL2, CCL21, CCL24, CCR7, CD177, CD74, CDK1, CEACAM1, CEL, CELSR2, CFAP157, CFAP54, CFL1, CGA, CHGA, CITED2, COL1A1, CSF1, CSPG4, CTHRC1, CX3CL1, DAB2IP, DAG1, DCHS1, DDR1, DGKZ, DHX34, DLG5, DMTN, DNAH17, DRC1, ECM1, EDN2, ENG, EPHA2, EPPK1, ERBB2, ESAM, FADD, FAM110A, FAP, FES, FFAR2, FGFR4, FLT4, FMNL1, FOXC2, FOXF1, GATA2, GBA, GBF1, GCSAM, GFRA3, GNAI2, GPER1, GPI, GPM6A, GRB7, GRN, GSK3A, HBEGF, HDAC5, HDAC7, HSPB1, HYAL1, ICAM1, IFITM1, IL1B, ILK, ITGA3, ITGA5, JUP, KLF5, LAMA5, LAMB4, LGALS9, LGR6, LMNA, LOX, LTB4R2, MAP2K3, MAPK3, MARK2, MAZ, MBOAT7, MDGA1, MECP2, MEGF8, MEN1, MIEN1, MINK1, MSN, MSTN, MYADM, MYC, MYH9, MYO18A, NF2, NGFR, NINJ1, NOD2, NODAL, NOS3, NOTCH1, NR4A1, NR4A2, NR4A3, NSMF, NTN1, OGDH, OMA1, OSGIN1, P2RY6, PAK4, PCDHA6, PDGFB, PDGFRB, PFN1, PHACTR1, PIK3C2B, PIK3C2G, PIP5K1C, PKN3, PLA2G7, PLAU, PLEKHO1, PLTP, PLVAP, PLXNA1, PLXNA3, PLXNB1, PML, POSTN, PPARD, PPP1R9B, PRAG1, PRKCSH, PTN, PTP4A3, PTPN23, PTPRU, PXN, RAC2, RGCC, RHBDF1, RHOG, RIPOR1, RNU1-93P, ROBO4, RSPH9, S100A11, SELE, SELENOP, SEMA3F, SEMA4B, SEMA4C, SEMA6B, SEMA6C, SEPTIN4, SERPINE1, SH3BP1, SLC25A23, SLC7A5, SLC7A8, SLC9A1, SLC9A3R1, SMAD3, SMIM22, SNAI1, SOX10, SOX8, SPAG16, SPEF2, SPHK1, SPI1, SPNS2, SRC, SRF, SSX2IP, STAT3, SYDE1, TBX5, TESK1, THBS1, TIMP1, TMEM201, TNF, TNFAIP1, TNFSF12, TRIB1, TRPV4, TSPAN11, UBE2B, VANGL2, VEGFA, VSIR, VTN, WAS, XG, ZC3H12A, ZMIZ1, ZNF580, ZNF609]

GO:0006810	transport	5,25E-04 [3]	844,00	15,69	35,92	64,08	[ABCB11, ABCC3, ABRA, ACAP1, ADAM8, ADAMTS9, ADGRE3, AIF1, ALOX15, ANGPT1, ANO9, ANPEP, APBA2, AQP5, AREG, ARHGAP45, ARRDC5, ASGR2, ATP10B, ATP1B4, ATP2A3, BHLHA15, BICDL1, BIN2, BLK, BMF, BTK, C1QTNF1, C2, CA12, CACNA1E, CACNA1I, CALCR, CALHM6, CASP8, CBARP, CCDC88B, CCDC88C, CCL19, CCL2, CCL3, CCL4, CCL5, CCR2, CCR5, CD177, CD19, CD22, CD247, CD300LF, CD33, CD3G, CD4, CD5, CD53, CD6, CD74, CD84, CDH1, CEACAM21, CEACAM4, CEL, CFP, CLCNKA, CLEC10A, CLEC12A, CLEC4D, CLNK, CNR1, CORO1A, COTL1, CP, CTSS, CTSW, CXCL10, CXCL11, CXCL9, CXCR4, CYBB, CYSLTR1, DAPK1, DAW1, DENND1C, DERL3, DOCK2, DRD1, EMB, EPX, ERFE, EXOC3L4, FASLG, FCHO1, FCN1, FCN3, FCRL3, FFAR4, FGL2, FGR, FOLR2, FRMPD3, GABBR2, GCNT3, GMFG, GPM6A, GRAMD1B, GRAMD1C, GZMB, HCLS1, HCN1, HLA-DRB1, HSPA7, IFNG, IGLL5, IL10RA, IL16, IL2RB, IL2RG, IPCEF1, IQGAP2, IRF8, ITGA4, ITGAL, ITGAX, ITGB2, JAKMIP1, JCHAIN, JPH1, JSRP1, KCNA3, KCNA7, KCNJ10, KCNK15, KCNN4, KCNQ5, KLF5, KLRC2, KLRG1, KMO, KNG1, LAIR1, LCK, LCP1, LDLR, LILRB1, LILRB4, LPAL2, LRMP, LRRC38, LY75, LYZ, MCOLN2, MCOLN3, MID1IP1, MIDN, MLC1, MMP9, MPO, MRC1, MREG, MS4A1, MYB, MYO1F, MYO1G, NAPSB, NCF1, NCKAP1L, NFAM1, NLRP3, NLRP6, NMUR1, NPPB, NPPC, NR4A3, NSG1, NTN1, NUP210, ORM2, P2RY12, P2RY2, PIK3CG, PIM1, PLA2G2D, PLAC8, PLAU, PLD4, PLEK, PRAM1, PRF1, PRKCB, PSTPIP1, PTAFR, PTGDR, PTK2B, PTPN22, PTPN6, PTPRC, PTX3, PYCARD, RAB15, RAB27B, RAB37, RAB39B, RAB3B, RAB3C, RAB44, RAC2, RASGRF1, RGS9, RHOH, RINL, RNASET2, RRAD, SCNN1B, SDC1, SELE, SELL, SEPTIN1, SERPINA1, SH2D2A, SHISA8, SIDT1, SIGLEC1, SIRPG, SLC12A3, SLC16A6, SLC16A9, SLC18A1, SLC22A31, SLC24A4, SLC25A48, SLC27A2, SLC31A2, SLC36A2, SLC5A2, SLC7A5P1, SLC7A7, SLC8A3, SLCO5A1, SMPD3, SNX10, SNX20, SOAT2, SPEF2, SPINK1, SPNS3, STAP1, STARD5, STXBP2, SUCNR1, SYK, SYTL1	[AAAS, ABCA3, ABCA7, ABCB11, ABCC5, ABCC8, ABCD1, ABCD2, ABCG4, ABL1, ABLIM3, ABRA, ACACB, ACKR3, ACTB, ACTN1, ACTN4, ADAMTS8, ADAMTS9, ADCY1, ADGRE5, ADIPOQ, AGAP2, AGER, AGT, AGTR1, ALDOA, ALPK3, AMH, ANGPT1, ANKRD13B, ANO3, ANO7, ANXA11, AP1B1, AP2A1, AP3D1, APLN, APLNR, APLP1, APOA1, APOD, APOM, AQP3, AQP5, ARAP3, ARC, AREG, ARF1, ARHGAP1, ARHGEF5, ARL8A, ARRDC3, ARSA, ASIC3, ATG9A, ATP13A1, ATP13A2, ATP1A1, ATP1A3, ATP1B2, ATP1B4, ATP5F1D, ATP5MC2, ATP6V0D1, ATP6V0E2, ATP6V1B1, ATP6V1F, ATPSCKMT, BAG3, BBS12, BCL2L1, BMP8A, BRPF3, BSN, C11orf65, C1QTNF1, C2CD2L, C5AR1, CACFD1, CACNA1E, CACNA1H, CACNB4, CALCR, CALHM6, CAMK2B, CAMSAP3, CAPN1, CBARP, CCL2, CCL21, CCR7, CD14, CD177, CD74, CDK1, CDKN1A, CEACAM1, CECR2, CEL, CELSR2, CERT1, CETN3, CETP, CFH, CGA, CHCHD10, CHERP, CHGA, CHI3L1, CHRNG, CLCN6, CLCN7, CLCNKA, CLDN15, CLEC12A, CLIP3, CLNK, CNNM4, CNR1, COASY, COL1A1, COMMD3, CORO7, COX6A2, COX8A, CPLX1, CPNE6, CPSF1, CPTP, CRAT, CREB3L1, CRP, CRTC2, CRYAB, CSK, CSNK1E, CTSA, CTSD, CX3CL1, CYB5R3, CYP27B1, DDR1, DDX39B, DENND1C, DGKD, DISP3, DMTN, DOC2B, DOK3, DSC1, DYNLRB1, DYSF, ECM1, EHD1, EHD2, ENG, ENPP3, EPN1, EPN3, ERBB2, ERFE, F8A1, FABP4, FAM110A, FCN3, FES, FFAR2, FHOD1, FLOT2, FOXF1, FPR1, FTH1P19, FURIN, FXYD5, FXYD6, G6PC3, GAB2, GABBR2, GABRR2, GALNS, GAS1, GATA2, GBF1, GCNT3, GDI1, GGA1, GGA3, GIT1, GJA4, GJD3, GLRA1, GNAI2, GNAO1, GPER1, GPI, GPM6A, GPR84, GPRIN1, GRAMD1A, GRAMD1B, GRAMD1C, GRIK5, GRIP2, GRM2, GRN, GSK3A, GTPBP2, HAVCR1, HBA1, HBA2, HBB, HBD, HBG1, HBG2, HCN3, HCN4, HFE, HGS, HMGA1, HPS1, HRH2, HSF1, HSPB1, HSPG2, HTR1B, HTR2A, HVCN1, ICAM1, IFT80, IGF2BP2, IL15RA, IL1B, IL1RN, IL4R, IMPDH1, INHA, INPPL1, IPO13, IQGAP2, ITPR3, JPH4, JUP, KATNB1, KCNA7, KCNC3, KCNH2, KCNIP2, KCNJ1, KCNJ5
GO:0040017	positive regulation of locomotion	7,24E-07 [2, 3, 4]	136,00	23,13	42,21	57,79	[ADAM8, AIF1, ANGPT1, BMP7, CARMIL2, CASS4, CCL19, CCL24, CCL3, CCL4, CCL5, CCR2, CCR4, CD74, CORO1A, CSF1R, CXCL10, CXCR4, DOCK8, DRD1, EDN2, FGR, GPSM3, IFNG, IL16, ITGA4, ITGAX, LEF1, MMP9, NCKAP1L, NELL2, NR4A3, NTN1, P2RY12, PIK3CG, PLA2G7, PTAFR, PTK2B, PTPRC, PYCARD, RAC2, SEMA4A, SEMA4D, SPN, SUCNR1, TCAF2, THBS1, THY1, TNFRSF18, TREM2, XCL1, XCL2, XG]	[ABL1, ACKR3, ACTN4, AGER, AGT, ANGPT1, ARTN, BCAR1, C5AR1, CARMIL2, CCL11, CCL21, CCL24, CCR7, CD74, CGA, CHGA, COL1A1, CSF1, CX3CL1, DAB2IP, DMTN, EDN2, FADD, FAM110A, FLT4, FOXC2, FOXF1, GATA2, GNAI2, GPER1, GPI, GRB7, GRN, HBEGF, HDAC7, HSPB1, HYAL1, ICAM1, IL1B, ILK, ITGA3, LGALS9, LGR6, MAP2K3, MAPK3, MAZ, MEGF8, MEN1, MIEN1, MSTN, MYADM, MYC, NOS3, NOTCH1, NR4A3, NSMF, NTN1, P2RY6, PDGFB, PDGFRB, PFN1, PLA2G7, PLVAP, POSTN, PTN, RAC2, RIPOR1, S100A11, SELENOP, SEMA3F, SEMA4B, SEMA4C, SEMA6B, SEMA6C, SERPINE1, SMAD3, SMIM22, SNAI1, SPHK1, SPI1, SRC, STAT3, SYDE1, THBS1, TNF, TRPV4, VEGFA, VSIR, VTN, XG, ZC3H12A, ZNF580, ZNF609]
GO:0006935	chemotaxis	8,74E-14 [3, 4]	178,00	25,32	51,30	48,70	[ADAM8, AIF1, ANGPT1, ANGPT2, BCL11B, BIN2, BMP7, BMPR1B, CCL17, CCL19, CCL2, CCL22, CCL24, CCL3, CCL3L1, CCL4, CCL4L1, CCL4L2, CCL5, CCR2, CCR3, CCR4, CCR5, CCR8, CD74, CDH4, CMTM5, CNR2, CORO1A, CSF1R, CXCL10, CXCL11, CXCL5, CXCL6, CXCL9, CXCR3, CXCR4, CXCR6, CYSLTR1, DCC, DOCK2, DOK2, DPEP1, EDN2, EGR3, EMB, EPHA6, FOLR2, FOSL1, GFRA2, GPSM3, IL16, ITGB2, JAML, KLF5, KLRK1, LEF1, NCKAP1L, NELL2, NR4A1, NTN1, P2RY12, PIK3C2G, PIK3CG, PLA2G7, PLAU, PLXNC1, PRKCQ, PTAFR, PTK2B, RAC2, RHOH, SEMA4A, SEMA4D, SLAMF8, SPN, STAP1, SUCNR1, SYK, THBS1, TNR, TREM2, TRPM2, VAV1, XCL1, XCL2, XCR1]	[ACKR3, AGER, AGTR1, AMH, ANGPT1, APOA1, ARHGEF16, ARTN, BCAR1, C5, C5AR1, CCL11, CCL2, CCL21, CCL24, CCR7, CD74, CDH4, CHGA, CMTM1, CMTM5, CNTN6, CSF1, CX3CL1, DAG1, DHX34, DOK5, DPYSL4, DSCAML1, EDN2, EFNA3, EPHA2, ERBB2, FES, FFAR2, FOSL1, FPR1, GAB2, GBF1, GFRA3, GRB7, HBEGF, HSPB1, KLF5, LAMA5, LGALS9, LGR6, LMX1A, LOX, LTB4R2, LYPLA2, LZTS1, MAPK3, MAPK7, MEGF8, MEN1, MSTN, NECTIN1, NGFR, NINJ1, NOD2, NOTCH1, NOTCH3, NR4A1, NTN1, OMA1, PCDHA6, PDGFB, PDGFRB, PDLIM7, PIK3C2G, PIP5K1C, PLA2G7, PLAU, PLAUR, PLP2, PLXNA1, PLXNA3, PLXNB1, PRKCSH, PTN, PTPRU, RAC2, RHOG, RNASE2, ROBO4, SEMA3F, SEMA4B, SEMA4C, SEMA6B, SEMA6C, SERPIND1, SERPINE1, SHANK3, SMAD3, SPI1, SPTB, SRC, THBS1, TRPV4, TSC2, UNC5B, VEGFA, WNT3, ZNF580, ZSWIM4, ZSWIM8]

GO:0016477	cell migration	1,00E-11	[3, 4]	330,00	20,36	45,52	54,48	[ADAM8, ADAMTS9, ADORA3, AIF1, ALOX15B, ANGPT1, ANGPT2, BCL11B, BIN2, BMP7, CARMIL2, CASS4, CCL17, CCL19, CCL2, CCL22, CCL24, CCL3, CCL3L1, CCL4, CCL4L1, CCL4L2, CCL5, CCR2, CCR3, CCR4, CCR5, CCR8, CD177, CD2, CD200R1, CD244, CD48, CD74, CD84, CDH1, CEACAM21, CEL, CNR2, CORO1A, CRB2, CRTAM, CSF1R, CXCL10, CXCL11, CXCL5, CXCL6, CXCL9, CXCR3, CXCR4, CXCR6, DCC, DEPDC1B, DOCK10, DOCK8, DOK2, DPEP1, DRD1, EDN2, EGR3, EPX, FGR, FOLR2, FUT7, GPM6A, GPSM3, IFNG, IL16, INPP5D, ITGA4, ITGAL, ITGAX, ITGB2, ITGB7, JAML, JCHAIN, KLF5, KLRK1, LCK, LCP1, LEF1, MCOLN2, MIXL1, MMP9, MYO1G, NCKAP1L, NELL2, NLRP3, NR4A1, NR4A2, NR4A3, NTN1, P2RY12, PIK3C2G, PIK3CG, PLA2G7, PLAU, PLXNC1, PRKCQ, PSTPIP2, PTAFR, PTK2B, PTPN22, PTPN6, PTPRC, PYCARD, RAC2, RHOH, SDC1, SELE, SELL, SELPLG, SEMA4A, SEMA4D, SIRPG, SLAMF8, SLC7A7, SMPD3, SPN, STAP1, STAT1, SYK, TACSTD2, TBX21, TCAF2, THBS1, THY1, TNFAIP3, TNFRSF18, TNR, TREM2, TRPM2, VAV1, XCL1, XCL2, XCR1, XG, ZAP70]	[ABCC8, ABI3, ABL1, ACKR3, ACTN4, ADAMTS9, ADGRG1, ADIPOQ, AGER, AGT, AGTR1, AMH, ANGPT1, APOA1, APOD, ARC, ARHGDIA, ARHGEF16, ARTN, ATP1B2, BCAR1, C5, C5AR1, CAMK2B, CAMSAP3, CARMIL2, CCL11, CCL2, CCL21, CCL24, CCR7, CD177, CD74, CDK1, CEACAM1, CEL, CELSR2, CGA, CHGA, CITED2, COL1A1, CSF1, CSPG4, CTHRC1, CX3CL1, DAB2IP, DAG1, DCHS1, DDR1, DGKZ, DHX34, DLG5, DMTN, ECM1, EDN2, ENG, EPHA2, EPPK1, ESAM, FADD, FAM110A, FAP, FFAR2, FGFR4, FLT4, FMNL1, FOXC2, FOXF1, GATA2, GBA, GBF1, GCSAM, GFRA3, GNAI2, GPER1, GPI, GPM6A, GRB7, GRN, GSK3A, HBEGF, HDAC5, HDAC7, HSPB1, HYAL1, ICAM1, IFITM1, IL1B, ILK, ITGA3, ITGA5, JUP, KLF5, LAMA5, LAMB4, LGALS9, LGR6, LMNA, LOX, LTB4R2, MAP2K3, MAPK3, MARK2, MAZ, MBOAT7, MDGA1, MECP2, MEGF8, MEN1, MIEN1, MINK1, MSN, MSTN, MYADM, MYC, MYH9, MYO18A, NF2, NGFR, NINJ1, NOD2, NODAL, NOS3, NOTCH1, NR4A1, NR4A2, NR4A3, NSMF, NTN1, OGDH, OMA1, OSGIN1, P2RY6, PAK4, PCDHA6, PDGFB, PDGFRB, PFN1, PHACTR1, PIK3C2B, PIK3C2G, PIP5K1C, PKN3, PLA2G7, PLAU, PLEKHO1, PLVAP, PLXNA1, PLXNA3, PLXNB1, PML, POSTN, PPARD, PPP1R9B, PRAG1, PTN, PTP4A3, PTPN23, PTPRU, PXN, RAC2, RGCC, RHBDF1, RHOG, RIPOR1, RNU1-93P, ROBO4, S100A11, SELE, SELENOP, SEMA3F, SEMA4B, SEMA4C, SEMA6B, SEMA6C, SERPINE1, SH3BP1, SLC25A23, SLC7A5, SLC7A8, SLC9A1, SLC9A3R1, SMAD3, SMIM22, SNAI1, SOX10, SOX8, SPHK1, SPI1, SPNS2, SRC, SRF, STAT3, SYDE1, TBX5, TESK1, THBS1, TIMP1, TMEM201, TNF, TNFAIP1, TNFSF12, TRIB1, TRPV4, TSPAN11, VANGL2, VEGFA, VSIR, VTN, XG, ZC3H12A, ZMIZ1, ZNF580, ZNF609]
GO:0030155	regulation of cell adhesion	5,14E-25	[3, 4]	218,00	28,57	53,48	46,52	[ADAM8, ADAMDEC1, AIF1, AKNA, ALOX15, ANGPT1, ANGPT2, APBB1IP, BLK, BMP7, BTLA, C1QTNF1, CARD11, CASS4, CCDC88B, CCL19, CCL2, CCL5, CCR2, CD27, CD3E, CD4, CD40LG, CD5, CD6, CD74, CD80, CD86, CDH1, CDKN2A, CORO1A, CRTAM, CTLA4, CXCR3, CXCR4, CYTIP, DOCK8, EBI3, EGR3, EPX, FGL2, FOXP3, FUT7, GPR4, GRAP2, HLA-DMB, HLA-DPA1, HLA-DPB1, HLA-DRA, HLA-DRB1, ICOS, IFNG, IL12RB1, IL18, IL7R, ITGA4, ITGB2, KLRK1, KNG1, LAG3, LAX1, LCK, LEF1, LIF, LILRB1, LILRB4, MYB, NCKAP1L, NLRP3, NR4A3, P2RY12, PIK3CG, PLA2G2D, PLAU, PLXNC1, PRKCQ, PTAFR, PTK2B, PTPN22, PTPN6, PTPRC, PYCARD, RAC2, RASAL3, RHOH, RUNX3, SASH3, SELE, SEMA4D, SIRPG, SKAP1, SOCS1, SPN, SYK, TACSTD2, TBX21, TESPA1, THBS1, THY1, TIGIT, TNFAIP8L2, TNFRSF18, TNFSF13B, TNFSF14, TNR, VAV1, VNN1, WNT1, XCL1, ZAP70]	[ABL1, ACTN4, ADAM19, ADGRG1, ADIPOQ, AGER, ANGPT1, AP3D1, APOA1, APOD, ARHGDIA, BCL6, BTLA, C1QTNF1, CAMSAP3, CCL2, CCL21, CCR7, CD276, CD74, CEACAM1, CELSR2, CITED2, COL1A1, CSF1, CSK, CX3CL1, DAG1, DDR1, DLG5, DMTN, DTX1, ECM2, EFEMP2, EPHA2, ERBB2, FADD, FES, FLOT2, FOXC2, FOXF1, FXYD5, GNAO1, GPAM, GPR4, HFE, HYAL1, ICAM1, IL1B, IL1RN, IL4R, IL7R, ILK, ITGA3, JAK3, JUP, KNG1, LAG3, LAMA5, LGALS9, LIF, LIMS2, LRFN3, LRRC32, MAPK7, MDGA1, MEN1, MINK1, MYADM, NF2, NOD2, NODAL, NOTCH1, NOTCH4, NR4A3, NRARP, OMA1, PDCD1, PDGFB, PIEZO1, PKD1, PLAU, PLAUR, PLXNA1, PLXNA3, PLXNB1, PML, POSTN, PPP1CB, PPP2R1A, PTN, PTPN23, PTPRU, RAC2, RARA, RELA, RGCC, SELE, SELENOP, SERPINE1, SH2B3, SLC7A1, SLC9A1, SMAD3, SOX12, SOX13, SPI1, SRC, SRF, SYNGAP1, TESK1, TFE3, TGM2, THBS1, TIMP1, TNF, TRIOBP, TRPV4, VEGFA, VNN1, VSIR, ZBTB7B, ZC3H12A, ZMIZ1]
GO:0051049	regulation of transport	6,48E-06	[3, 4]	339,00	18,29	39,41	60,59	[ABCB11, ADAM8, ALOX15, ANGPT1, ANO9, BLK, C1QTNF1, C2, CACNA1E, CACNA1I, CALCR, CASP8, CBARP, CCL19, CCL2, CCL3, CCL4, CCL5, CCR2, CD177, CD19, CD22, CD300LF, CD33, CD4, CD74, CD84, CDH1, CLCNKA, CNR1, CORO1A, CTSS, CXCL10, CXCL11, CXCL9, CXCR4, CYBB, DAPK1, DERL3, DOCK2, DRD1, EPX, ERFE, FASLG, FCRL3, FFAR4, FGR, GABBR2, GZMB, HCLS1, HCN1, HLA-DRB1, IFNG, IL16, IL2RB, IL2RG, ITGB2, JPH1, JSRP1, KCNA3, KCNA7, KCNJ10, KCNK15, KCNN4, KCNQ5, KLRC2, KMO, LCP1, LILRB1, LPAL2, LRRC38, MIDN, MLC1, MMP9, MYB, NCKAP1L, NLRP3, NLRP6, NPPB, NR4A3, P2RY12, P2RY2, PIK3CG, PIM1, PRAM1, PRKCB, PTAFR, PTK2B, PTPN22, PTPN6, PTPRC, PTX3, PYCARD, RAB15, RAB27B, RAB3B, RAB3C, RAC2, RASGRF1, RGS9, RRAD, SDC1, SELE, SEPTIN1, SHISA8, SIRPG, SLC31A2, SMPD3, SPINK1, STAP1, STXBP2, SYK, TCAF2, THBS1, THY1, TIFAB, TMC2, TREM2, TRPM2, UBD, UCP2, XCL1]	[AAAS, ABCA3, ABCA7, ABCB11, ABCC8, ABL1, ABLIM3, ACTB, ACTN4, ADCY1, ADIPOQ, AGT, AGTR1, ANGPT1, ANKRD13B, AP2A1, APLN, APLNR, APOA1, APOD, ARC, ARF1, ARHGAP1, ARHGEF5, ATP13A2, ATP1A1, ATP1A3, ATP1B2, ATPSCKMT, BAG3, BMP8A, BSN, C11orf65, C1QTNF1, C2CD2L, CACNA1E, CACNA1H, CACNB4, CALCR, CAMK2B, CAPN1, CBARP, CCL2, CCL21, CD14, CD177, CD74, CDK1, CEACAM1, CETP, CFH, CHCHD10, CHGA, CLCNKA, CLIP3, CNR1, CPLX1, CRYAB, CSK, CX3CL1, DGKD, DISP3, DMTN, DOC2B, DYSF, EHD1, EHD2, ERBB2, ERFE, FES, FFAR2, FOXF1, FURIN, FXYD5, FXYD6, GAB2, GABBR2, GAS1, GATA2, GDI1, GIT1, GNAI2, GNAO1, GPER1, GPRIN1, GRIK5, GRM2, GSK3A, HCN3, HCN4, HFE, HGS, HTR1B, HTR2A, HVCN1, ICAM1, IL15RA, IL1B, IL4R, INHA, ITPR3, JPH4, JUP, KCNA7, KCNC3, KCNH2, KCNIP2, KCNJ1, KCNJ5, KCNJ9, KCNK15, KCNK6, KCNMB1, KCNMB2, KCNQ4, KCTD11, LDLRAP1, LGALS9, LILRA5, LIPG, LRRC52, LRRC8A, LZTS1, MAPK3, MAVS, MICALL2, MIDN, MINK1, MLLT6, MSN, MYC, MYO18A, NEFL, NKAIN3, NLGN2, NNAT, NOD2, NOTCH1, NPPA, NPPB, NR1D1, NR4A3, NUCB1, NUTF2, ORAI1, P2RX3, P2RX5, P2RY2, P2RY6, PACSIN1, PCDHA4, PDGFB, PDGFRB, PER1, PIM1, PLK3, PLTP, PM20D1, PML, PPARD, PRKACA, PRKCSH, PROM2, PRRT1, PRRT2, PTGES, PTPN23, RAB11B, RAB11FIP5, RAB15, RAB3A, RAB5B, RAC2, RANGAP1, RANGRF, RAP1A, RASL10B, RCVRN, RGCC, RHBDF1, RHBDF2, RIPOR1, RRAD, RTN2, RUBCN, RXRA, S100A1, SCN5A, SCN7A, SEC16A, SELE, SELENON, SEPTIN4, SEPTIN5, SERPINE1, SH3GL1, SHANK3, SIDT2, SIK1, SLC30A1, SLC30A3, SLC35F6, SLC51B, SLC6A9, SLC9A1, SLC9A3R1, SLN, SMAD3, SMPD1, SPHK1, SPHK2, SPI1, SPINK1, SRC, SREBF1, STAC2, STX1A, STX1B, SV2A, SYN1, SYT2, SYT7, TCIRG1, TCTEX1D2, TGM2, THBS1, TIMP1, TM9SF4, TMEM109, TMEM14A, TNF, TNFRSF1A, TNK2, TPCN1, TRIB3, TSC2, VEGFA, VTN, WFS1, WNK2, WWP2, ZC3H12A, ZDHHC8]

GO:0051050	positive regulation of transport	1,01E-03	[2, 3, 4, 5]	192,00	19,22	42,40	57,60	[ABCB11, ADAM8, ANGPT1, BLK, C1QTNF1, C2, CACNA1I, CALCR, CASP8, CCL19, CCL2, CCL3, CCL4, CCL5, CCR2, CD177, CD19, CD300LF, CD33, CDH1, CNR1, CTSS, CXCL10, CXCL11, CXCL9, DOCK2, DRD1, ERFE, FASLG, FFAR4, FGR, GABBR2, GZMB, HCLS1, HLA-DRB1, IFNG, IL2RB, IL2RG, ITGB2, KCNJ10, KCNN4, KLRC2, KMO, LPAL2, LRRC38, MLC1, MYB, NCKAP1L, NLRP3, NPPB, NR4A3, P2RY12, P2RY2, PTAFR, PTPN22, PTPRC, PTX3, PYCARD, RAB15, RAB27B, RAB3B, RAC2, RGS9, SDC1, SELE, SIRPG, SMPD3, STAP1, SYK, TCAF2, THY1, TREM2, TRPM2, UBD, XCL1]	[ABCA3, ABCA7, ABCB11, ABCC8, ABL1, ABLIM3, ACTB, ACTN4, ADIPOQ, AGT, ANGPT1, AP2A1, APLN, APLNR, APOA1, ARC, ARF1, ARHGEF5, ATP13A2, ATP1B2, ATPSCKMT, BAG3, C1QTNF1, C2CD2L, CACNA1H, CALCR, CCL2, CCL21, CD14, CD177, CDK1, CETP, CFH, CHCHD10, CLIP3, CNR1, CX3CL1, DGKD, DOC2B, EHD1, EHD2, ERBB2, ERFE, GAB2, GABBR2, GATA2, GNAI2, GPER1, GPRIN1, GSK3A, HFE, HGS, IL15RA, IL1B, IL4R, INHA, JUP, KCNH2, KCNIP2, KCNJ5, KCNJ9, KCNMB1, KCNMB2, LDLRAP1, LILRA5, LIPG, LRRC52, LRRC8A, LZTS1, MAVS, MICALL2, MLLT6, MSN, MYO18A, NLGN2, NNAT, NOD2, NPPA, NPPB, NR4A3, NUTF2, ORAI1, P2RX3, P2RX5, P2RY2, P2RY6, PCDHA4, PDGFB, PDGFRB, PLK3, PLTP, PPARD, PRKACA, PRRT2, PTGES, PTPN23, RAB15, RAB3A, RAC2, RAP1A, RASL10B, RGCC, RIPOR1, RXRA, S100A1, SCN5A, SEC16A, SELE, SERPINE1, SH3GL1, SHANK3, SLC30A3, SLC51B, SLC6A9, SLC9A1, SLC9A3R1, SMAD3, SMPD1, SPHK2, SRC, STAC2, STX1A, STX1B, SYT7, TM9SF4, TNF, VEGFA, VTN, WFS1, WNK2, ZC3H12A, ZDHHC8]
GO:0051270	regulation of cellular component movement	6,89E-07	[3, 4]	220,00	20,41	37,04	62,96	[ADAM8, ADAMTS9, ADORA3, AIF1, ALOX15B, ANGPT1, ANGPT2, BMP7, CARMIL2, CASS4, CCL19, CCL2, CCL24, CCL3, CCL4, CCL5, CCR2, CD200R1, CD74, CDH1, CORO1A, CSF1R, CXCL10, CXCR3, CXCR4, DOCK10, DOCK8, DPEP1, DRC1, DRD1, EDN2, EREG, FGR, FUT7, GPSM3, IFNG, ITGA4, ITGAX, KLRK1, LEF1, MMP9, NCKAP1L, NELL2, NR4A3, NTN1, P2RY12, PIK3CG, PLA2G7, PLAU, PLXNC1, PTAFR, PTK2B, PTPN22, PTPRC, PYCARD, RAC2, RHOH, SELE, SEMA4A, SEMA4D, SLAMF8, SMPD3, SPN, STAP1, TACSTD2, TCAF2, THBS1, THY1, TNFRSF18, TREM2, WAS, XCL1, XCL2, XG]	[ABCC8, ABI3, ABL1, ACKR3, ACTN1, ACTN4, ADAMTS9, ADGRG1, ADIPOQ, AGER, AGT, AMH, ANGPT1, APOD, ARHGDIA, ATP1A1, ATP1A3, ATP1B2, BCAR1, BCL6, C5, C5AR1, CAMK2B, CAMSAP3, CARMIL2, CCL11, CCL2, CCL21, CCL24, CCR7, CD74, CEACAM1, CFAP298-TCP10L, CGA, CHGA, CITED2, COL1A1, CSF1, CX3CL1, DAB2IP, DAG1, DLG5, DMTN, DRC1, ECM1, EDN2, ENG, EPHA2, EPPK1, ERBB2, FADD, FAM110A, FES, FLT4, FOXC2, FOXF1, GATA2, GATA4, GCSAM, GNAI2, GPER1, GPI, GRB7, GRN, HBEGF, HCN4, HDAC5, HDAC7, HSPB1, HYAL1, ICAM1, IFITM1, IL1B, ILK, ITGA3, JUP, LAMA5, LGALS9, LGR6, LMNA, LZTS1, MAP2K3, MAPK3, MAZ, MECP2, MIEN1, MINK1, MSN, MSTN, MYADM, MYC, NF2, NGFR, NOD2, NODAL, NOS3, NOTCH1, NR4A3, NSMF, NTN1, OSGIN1, P2RY6, PDGFB, PDGFRB, PFN1, PHACTR1, PLA2G7, PLAU, PLVAP, PLXNA1, PLXNA3, PLXNB1, POSTN, PPARD, PRAG1, PTN, PTPN23, PTPRU, RAC2, RANGRF, RGCC, RHOG, RIPOR1, ROBO4, S100A11, SCN5A, SELE, SELENOP, SEMA3F, SEMA4B, SEMA4C, SEMA6B, SEMA6C, SERPINE1, SH3BP1, SLC9A3R1, SMAD3, SMIM22, SNAI1, SPHK1, SPI1, SRC, SRF, SSX2IP, STAT3, SYDE1, TBX5, TCTEX1D2, THBS1, TIMP1, TNF, TRIB1, TRPV4, VEGFA, VSIR, VTN, WAS, XG, ZC3H12A, ZNF580, ZNF609, ZSWIM4, ZSWIM8]
GO:0050900	leukocyte migration	2,35E-12	[2, 4, 5]	145,00	26,36	64,71	35,29	[ADAM8, AIF1, ANGPT1, ANGPT2, CCL17, CCL19, CCL2, CCL22, CCL24, CCL3, CCL3L1, CCL4, CCL4L1, CCL5, CCR2, CCR5, CD177, CD2, CD200R1, CD244, CD48, CD74, CD84, CEACAM21, CNR2, CORO1A, CRTAM, CSF1R, CXCL10, CXCL11, CXCL5, CXCL6, CXCL9, CXCR3, CXCR4, DOCK8, DOK2, DPEP1, EDN2, EPX, FOLR2, FUT7, GPSM3, IL16, INPP5D, ITGA4, ITGAL, ITGAX, ITGB2, ITGB7, JAML, JCHAIN, KLF5, KLRK1, LCK, MCOLN2, MMP9, MYO1G, NCKAP1L, NLRP3, P2RY12, PIK3CG, PLA2G7, PTAFR, PTK2B, PTPN22, PTPN6, PYCARD, RAC2, RHOH, SDC1, SELE, SELL, SELPLG, SIRPG, SLAMF8, SLC7A7, SMPD3, SPN, STAP1, SYK, TBX21, THBS1, THY1, TNFRSF18, TREM2, TRPM2, VAV1, XCL1. XCL2. XG. ZAP70]	[AGER, AMH, ANGPT1, APOD, ARTN, ATP1B2, C5, C5AR1, CCL11, CCL2, CCL21, CCL24, CCR7, CD177, CD74, CEACAM1, CHGA, COL1A1, CSF1, CX3CL1, DHX34, ECM1, EDN2, ESAM, FADD, FFAR2, GBA, GBF1, GCSAM, GRB7, ICAM1, ITGA3, ITGA5, KLF5, LGALS9, MAPK3, MEN1, MSN, MSTN, MYH9, NINJ1, NOD2, PCDHA6, PDGFB, PIP5K1C, PLA2G7, PLVAP, PTN, PTPRU, RAC2, RHOG, SELE, SELENOP, SERPINE1, SLC7A5, SLC7A8, SPI1, SPNS2, SRC, THBS1, TNF, TRPV4, VEGFA, XG, ZNF580]
GO:0032103	positive regulation of response to external stimulus	8,85E-08	[3, 4, 5]	131,00	24,08	57,80	42,20	[ADAM8, AIF1, AIM2, BTK, CARD11, CCL19, CCL24, CCL3, CCL4, CCL5, CCR2, CCR4, CD180, CD226, CD74, CLEC10A, CLEC4D, CLEC4E, CLEC6A, CLNK, CNR1, CRTAM, CSF1R, CXCL10, CXCR4, CXorf21, EDN2, EREG, FCN1, GBP5, GPR4, GPSM3, IFNG, IKBKE, IL16, IL18, IL18RAP, KLRC2, KLRD1, KLRK1, LAG3, LCP1, LDLR, LTA, LY86, MAPK13, MUC16, MUC19, MUC6, NCKAP1L, NCR3, OASL, P2RY12, PIK3CG, PLA2G7, PTK2B, PYCARD, PYHIN1, RAC2, SCIMP, SH2D1A, SIGLEC16, SLAMF6, STAP1, SUCNR1, SYK, THBS1, TLR10, TLR8, TREM2, VAV1, XCL1, XCL2, ZBP1]	[AGER, AGT, AGTR1, ARTN, C5AR1, CCL21, CCL24, CCR7, CD74, CLEC4E, CLNK, CNR1, CSF1, CX3CL1, EDN2, FABP4, FADD, FEM1A, FFAR2, GPR4, GRN, HSPB1, IL1B, LAG3, LDLR, LGALS9, LILRA5, MAPK3, MAVS, MEGF8, MEN1, MSTN, MUC19, MUC20, MUC3A, NECTIN2, NINJ1, NLRC5, NOD2, NPPA, OSM, PCDHA4, PDGFB, PDGFRB, PLA2G2A, PLA2G7, PQBP1, PRKACA, PTN, PVR, RAC2, RBM14, RELA, RELB, RNU1-93P, SERPINE1, SMAD3, SPI1, SRC, STING1, TGM2, THBS1, TNF, TNFRSF1A, TNIP1, TRIM62, TRPV4, VEGFA, ZCCHC3, ZNF580]
GO:0050920	regulation of chemotaxis	6,17E-05	[3, 4, 5]	66,00	27,05	49,49	50,51	[AIF1, ANGPT2, CCL19, CCL2, CCL3, CCL4, CCL5, CCR2, CCR4, CD74, CSF1R, CXCL10, CXCR4, EDN2, GPSM3, IL16, KLRK1, NCKAP1L, P2RY12, PLA2G7, PTK2B, RAC2, SEMA4A, SEMA4D, SLAMF8, STAP1, SUCNR1, THBS1, TREM2, XCL1, XCL2]	[AGER, AMH, ARTN, C5, C5AR1, CCL2, CCL21, CCR7, CD74, CSF1, EDN2, HSPB1, LGALS9, MAPK3, MEGF8, MEN1, MSTN, NOD2, NOTCH1, PDGFB, PDGFRB, PLA2G7, PLXNA3, PTN, PTPRU, RAC2, SEMA3F, SEMA4B, SEMA4C, SEMA6B, SEMA6C, SERPINE1, SMAD3, SPI1, THBS1, TRPV4, VEGFA, WNT3, ZNF580, ZSWIM4, ZSWIM8]

GO:0051272	positive regulation of cellular component movement	7,49E-06	[3, 4, 5]	132,00	22,49	41,06	58,94	[ADAM8, AIF1, ANGPT1, BMP7, CARMIL2, CASS4, CCL19, CCL24, CCL3, CCL4, CCL5, CCR2, CD74, CORO1A, CSF1R, CXCL10, CXCR4, DOCK8, DRD1, EDN2, FGR, GPSM3, IFNG, ITGA4, ITGAX, LEF1, MMP9, NCKAP1L, NELL2, NR4A3, NTN1, P2RY12, PIK3CG, PLA2G7, PTAFR, PTK2B, PTPRC, PYCARD, RAC2, SEMA4A, SEMA4D, SPN, TCAF2, THBS1, THY1, TNFRSF18, TREM2, XCL1, XCL2, XG]	[ABL1, ACKR3, ACTN4, AGER, AGT, ANGPT1, BCAR1, BCL6, C5AR1, CARMIL2, CCL11, CCL21, CCL24, CCR7, CD74, CGA, CHGA, COL1A1, CSF1, CX3CL1, DAB2IP, DMTN, EDN2, FADD, FAM110A, FLT4, FOXC2, FOXF1, GATA2, GNAI2, GPER1, GPI, GRB7, GRN, HBEGF, HDAC7, HSPB1, HYAL1, ICAM1, IL1B, ILK, ITGA3, LGALS9, LGR6, LZTS1, MAP2K3, MAPK3, MAZ, MIEN1, MSTN, MYADM, MYC, NOS3, NOTCH1, NR4A3, NSMF, NTN1, P2RY6, PDGFB, PDGFRB, PFN1, PLA2G7, PLVAP, POSTN, PTN, RAC2, RIPOR1, S100A11, SELENOP, SEMA3F, SEMA4B, SEMA4C, SEMA6B, SEMA6C, SERPINE1, SMAD3, SMIM22, SNAI1, SPHK1, SPI1, SRC, STAT3, SYDE1, THBS1, TNF, TRPV4, VEGFA, VSIR, VTN, XG, ZC3H12A, ZNF580, ZNF609]
GO:2000145	regulation of cell motility	4,54E-07	[3, 4, 5]	206,00	20,79	38,83	61,17	[ADAM8, ADAMTS9, ADORA3, AIF1, ALOX15B, ANGPT1, ANGPT2, BMP7, CARMIL2, CASS4, CCL19, CCL2, CCL24, CCL3, CCL4, CCL5, CCR2, CD200R1, CD74, CDH1, CORO1A, CSF1R, CXCL10, CXCR3, CXCR4, DOCK10, DOCK8, DPEP1, DRD1, EDN2, EREG, FGR, FUT7, GPSM3, IFNG, ITGA4, ITGAX, KLRK1, LEF1, MMP9, NCKAP1L, NELL2, NR4A3, NTN1, P2RY12, PIK3CG, PLA2G7, PLAU, PLXNC1, PTAFR, PTK2B, PTPN22, PTPRC, PYCARD, RAC2, RHOH, SELE, SEMA4A, SEMA4D, SLAMF8, SMPD3, SPN, STAP1, TACSTD2, TCAF2, THBS1, THY1, TNFRSF18, TREM2, WAS, XCL1, XCL2, XG]	[ABCC8, ABI3, ABL1, ACKR3, ACTN4, ADAMTS9, ADGRG1, ADIPOQ, AGER, AGT, AMH, ANGPT1, APOD, ARHGDIA, ATP1B2, BCAR1, C5, C5AR1, CAMK2B, CAMSAP3, CARMIL2, CCL11, CCL2, CCL21, CCL24, CCR7, CD74, CEACAM1, CGA, CHGA, CITED2, COL1A1, CSF1, CX3CL1, DAB2IP, DAG1, DLG5, DMTN, ECM1, EDN2, ENG, EPHA2, EPPK1, ERBB2, FADD, FAM110A, FES, FLT4, FOXC2, FOXF1, GATA2, GCSAM, GNAI2, GPER1, GPI, GRB7, GRN, HBEGF, HDAC5, HDAC7, HSPB1, HYAL1, ICAM1, IFITM1, IL1B, ILK, ITGA3, JUP, LAMA5, LGALS9, LGR6, LMNA, MAP2K3, MAPK3, MAZ, MECP2, MIEN1, MINK1, MSN, MSTN, MYADM, MYC, NF2, NGFR, NOD2, NODAL, NOS3, NOTCH1, NR4A3, NSMF, NTN1, OSGIN1, P2RY6, PDGFB, PDGFRB, PFN1, PHACTR1, PLA2G7, PLAU, PLVAP, PLXNA1, PLXNA3, PLXNB1, POSTN, PPARD, PRAG1, PTN, PTPN23, PTPRU, RAC2, RGCC, RHOG, RIPOR1, ROBO4, S100A11, SELE, SELENOP, SEMA3F, SEMA4B, SEMA4C, SEMA6B, SEMA6C, SERPINE1, SH3BP1, SLC9A3R1, SMAD3, SMIM22, SNAI1, SPHK1, SPI1, SRC, SRF, SSX2IP, STAT3, SYDE1, TBX5, THBS1, TIMP1, TNF, TRIB1, TRPV4, VEGFA, VSIR, VTN, WAS, XG, ZC3H12A, ZNF580, ZNF609]
GO:0043269	regulation of ion transport	2,13E-05	[4, 5]	156,00	21,22	44,98	55,02	[ABCB11, ANO9, CACNA1E, CACNA1I, CALCR, CBARP, CCL2, CCL3, CCL4, CCL5, CCR2, CD19, CD33, CD4, CD84, CLCNKA, CNR1, CORO1A, CTSS, CXCL10, CXCL11, CXCL9, CXCR4, CYBB, DAPK1, DRD1, EPX, FCRL3, GABBR2, HCN1, IFNG, IL16, JPH1, JSRP1, KCNA3, KCNA7, KCNJ10, KCNK15, KCNN4, KCNQ5, KMO, LILRB1, LRRC38, MMP9, NLRP3, P2RY12, PIK3CG, PTAFR, PTK2B, PTPN22, PTPN6, RAB3B, RASGRF1, RGS9, RRAD, SHISA8, SLC31A2, SPINK1, SYK, TCAF2, THY1, TMC2, TREM2, UBD, XCL1]	[ABCB11, ABCC8, ABL1, ACTN4, AGT, APLNR, ARC, ARF1, ATP1A1, ATP1A3, ATP1B2, ATPSCKMT, CACNA1E, CACNA1H, CACNB4, CALCR, CAMK2B, CAPN1, CBARP, CCL2, CFH, CLCNKA, CNR1, CX3CL1, FXYD5, FXYD6, GABBR2, GNAI2, GNAO1, GPER1, GPRIN1, GRM2, HCN3, HCN4, HFE, HTR1B, HTR2A, HVCN1, ICAM1, IL1B, JPH4, KCNA7, KCNC3, KCNH2, KCNIP2, KCNJ1, KCNJ5, KCNJ9, KCNK15, KCNK6, KCNMB1, KCNMB2, KCNQ4, LILRA5, LRRC52, MINK1, MLLT6, NEFL, NKAIN3, NLGN2, NPPA, ORAI1, P2RX3, P2RX5, P2RY6, PCDHA4, PDGFB, PDGFRB, PER1, PM20D1, PML, PRKACA, PRRT1, PTGES, RAB11B, RAB3A, RANGRF, RCVRN, RRAD, S100A1, SCN5A, SCN7A, SELENON, SHANK3, SIK1, SLC30A1, SLC6A9, SLC9A1, SLC9A3R1, SLN, SPHK2, SPINK1, STAC2, SV2A, SYT2, SYT7, TCIRG1, TIMP1, TMEM109, TPCN1, WFS1, WNK2, WWP2]
GO:0050921	positive regulation of chemotaxis	8,46E-04	[3, 4, 5, 6]	45,00	29,22	54,46	45,54	[AIF1, CCL19, CCL3, CCL4, CCL5, CCR2, CCR4, CD74, CSF1R, CXCL10, CXCR4, EDN2, GPSM3, IL16, NCKAP1L, P2RY12, PLA2G7, PTK2B, RAC2, SUCNR1, THBS1, TREM2, XCL1, XCL2]	[AGER, ARTN, C5AR1, CCL21, CCR7, CD74, CSF1, EDN2, HSPB1, LGALS9, MAPK3, MEGF8, MEN1, MSTN, PDGFB, PDGFRB, PLA2G7, PTN, RAC2, SERPINE1, SMAD3, SPI1, THBS1, TRPV4, VEGFA, ZNF580]
GO:0060326	cell chemotaxis	4,95E-10	[4, 5]	97,00	28,96	61,02	38,98	[ADAM8, AIF1, BIN2, CCL17, CCL19, CCL2, CCL22, CCL24, CCL3, CCL3L1, CCL4, CCL4L1, CCL4L2, CCL5, CCR2, CCR3, CCR4, CCR5, CCR8, CD74, CNR2, CORO1A, CSF1R, CXCL10, CXCL11, CXCL5, CXCL6, CXCL9, CXCR3, CXCR4, CXCR6, DPEP1, EDN2, EGR3, FOLR2, GPSM3, IL16, ITGB2, JAML, KLF5, KLRK1, LEF1, NCKAP1L, NR4A1, PIK3CG, PLA2G7, PRKCQ, PTK2B, RAC2, SLAMF8, STAP1, SYK, THBS1, TRPM2, VAV1, XCL1, XCL2, XCR1]	[ACKR3, AGTR1, AMH, ARHGEF16, BCAR1, C5, C5AR1, CCL11, CCL2, CCL21, CCL24, CCR7, CD74, CHGA, CSF1, CX3CL1, DHX34, EDN2, EPHA2, FFAR2, GBF1, HBEGF, HSPB1, KLF5, LGALS9, LOX, MAPK3, MEN1, MSTN, NINJ1, NOD2, NOTCH1, NR4A1, PCDHA6, PDGFB, PDGFRB, PIP5K1C, PLA2G7, PTN, PTPRU, RAC2, RHOG, SERPINE1, SPI1, THBS1, TRPV4, VEGFA, ZNF580]
GO:2000147	positive regulation of cell motility	4,74E-06	[3, 4, 5, 6]	130,00	22,73	41,59	58,41	[ADAM8, AIF1, ANGPT1, BMP7, CARMIL2, CASS4, CCL19, CCL24, CCL3, CCL4, CCL5, CCR2, CD74, CORO1A, CSF1R, CXCL10, CXCR4, DOCK8, DRD1, EDN2, FGR, GPSM3, IFNG, ITGA4, ITGAX, LEF1, MMP9, NCKAP1L, NELL2, NR4A3, NTN1, P2RY12, PIK3CG, PLA2G7, PTAFR, PTK2B, PTPRC, PYCARD, RAC2, SEMA4A, SEMA4D, SPN, TCAF2, THBS1, THY1, TNFRSF18, TREM2, XCL1, XCL2, XG]	[ABL1, ACKR3, ACTN4, AGER, AGT, ANGPT1, BCAR1, C5AR1, CARMIL2, CCL11, CCL21, CCL24, CCR7, CD74, CGA, CHGA, COL1A1, CSF1, CX3CL1, DAB2IP, DMTN, EDN2, FADD, FAM110A, FLT4, FOXC2, FOXF1, GATA2, GNAI2, GPER1, GPI, GRB7, GRN, HBEGF, HDAC7, HSPB1, HYAL1, ICAM1, IL1B, ILK, ITGA3, LGALS9, LGR6, MAP2K3, MAPK3, MAZ, MIEN1, MSTN, MYADM, MYC, NOS3, NOTCH1, NR4A3, NSMF, NTN1, P2RY6, PDGFB, PDGFRB, PFN1, PLA2G7, PLVAP, POSTN, PTN, RAC2, RIPOR1, S100A11, SELENOP, SEMA3F, SEMA4B, SEMA4C, SEMA6B, SEMA6C, SERPINE1, SMAD3, SMIM22, SNAI1, SPHK1, SPI1, SRC, STAT3, SYDE1, THBS1, TNF, TRPV4, VEGFA, VSIR, VTN, XG, ZC3H12A, ZNF580, ZNF609]
GO:0030595	leukocyte chemotaxis	6,15E-08	[3, 5, 6]	75,00	29,76	62,85	37,15	[ADAM8, AIF1, CCL17, CCL19, CCL2, CCL22, CCL24, CCL3, CCL3L1, CCL4, CCL4L1, CCL5, CCR2, CCR5, CD74, CNR2, CORO1A, CSF1R, CXCL10, CXCL11, CXCL5, CXCL6, CXCL9, CXCR3, CXCR4, DPEP1, EDN2, FOLR2, GPSM3, IL16, ITGB2, JAML, KLF5, KLRK1, NCKAP1L, PIK3CG, PLA2G7, PTK2B, RAC2, SLAMF8, STAP1, SYK, THBS1, TRPM2, VAV1, XCL1, XCL2]	[AMH, C5, C5AR1, CCL11, CCL2, CCL21, CCL24, CCR7, CD74, CHGA, CSF1, CX3CL1, DHX34, EDN2, FFAR2, GBF1, KLF5, LGALS9, MAPK3, MEN1, MSTN, NINJ1, NOD2, PCDHA6, PDGFB, PIP5K1C, PLA2G7, PTN, PTPRU, RAC2, SERPINE1, SPI1, THBS1, TRPV4, VEGFA, ZNF580]

GO:0045123	cellular extravasation	7,03E-03	[3, 5, 6]	26,00	34,67	66,03	33,97	[ADAM8, CCL2, CCR2, CD177, FUT7, ITGA4, ITGAL, ITGB2, ITGB7, JAML, PIK3CG, PTAFR, SELE, SELL, SELPLG, SPN, THY1, XG]	[AGER, CCL2, CCL21, CD177, CX3CL1, FADD, ICAM1, PLVAP, SELE, SELENOP, TNF, XG]
GO:0071674	mononuclear cell migration	8,97E-10	[3, 5, 6]	69,00	33,33	64,78	35,22	[ADAM8, AIF1, CCL17, CCL19, CCL2, CCL22, CCL24, CCL3, CCL3L1, CCL4, CCL4L1, CCL5, CCR2, CCR5, CD200R1, CRTAM, CSF1R, CXCL10, CXCL11, CXCR3, CXCR4, DOCK8, FOLR2, FUT7, ITGA4, ITGAL, ITGB7, JAML, KLF5, KLRK1, MYO1G, PIK3CG, PLA2G7, PTK2B, PYCARD, SLAMF8, SPN, TBX21, THBS1, TRPM2, XCL1, XCL2, XG, ZAP70]	[AGER, APOD, ARTN, C5AR1, CCL11, CCL2, CCL21, CCL24, CCR7, CSF1, CX3CL1, ECM1, FADD, GBA, GCSAM, ICAM1, KLF5, LGALS9, MAPK3, MSN, MSTN, PDGFB, PLA2G7, PTPRU, SERPINE1, SPI1, SPNS2, THBS1, TNF, TRPV4, XG]
GO:0097529	myeloid leukocyte migration	3,66E-07	[3, 5, 6]	71,00	29,34	64,17	35,83	[ADAM8, AIF1, CCL17, CCL19, CCL2, CCL22, CCL24, CCL3, CCL3L1, CCL4, CCL4L1, CCL5, CCR2, CD177, CD200R1, CD74, CSF1R, CXCL10, CXCL11, CXCL5, CXCL6, CXCL9, DPEP1, EDN2, EPX, FOLR2, FUT7, ITGB2, JAML, KLF5, MCOLN2, NCKAP1L, P2RY12, PIK3CG, PLA2G7, PTK2B, RAC2, RHOH, SLAMF8, STAP1, SYK, THBS1, TREM2, VAV1, XCL1, XCL2, XG]	[AGER, AMH, C5, C5AR1, CCL11, CCL2, CCL21, CCL24, CCR7, CD177, CD74, CHGA, CSF1, CX3CL1, DHX34, EDN2, GBF1, KLF5, MAPK3, MEN1, MSTN, NOD2, PDGFB, PIP5K1C, PLA2G7, PTPRU, RAC2, RHOG, SERPINE1, SPI1, THBS1, TRPV4, VEGFA, XG]
GO:0002685	regulation of leukocyte migration	6,64E-08	[3, 4, 5, 6, 7]	69,00	30,67	58,94	41,06	[ADAM8, AIF1, CCL19, CCL2, CCL24, CCL3, CCL4, CCL5, CCR2, CD200R1, CD74, CSF1R, CXCL10, CXCR3, DOCK8, EDN2, FUT7, GPSM3, ITGA4, KLRK1, NCKAP1L, P2RY12, PLA2G7, PTAFR, PTK2B, PTPN22, PYCARD, RAC2, RHOH, SELE, SLAMF8, SMPD3, SPN, STAP1, THBS1, THY1, TNFRSF18, TREM2, XCL1, XCL2, XG]	[AGER, AMH, APOD, C5, C5AR1, CCL2, CCL21, CCL24, CCR7, CD74, CSF1, CX3CL1, ECM1, EDN2, FADD, GCSAM, ICAM1, LGALS9, MAPK3, MSN, MSTN, NOD2, PLA2G7, PLVAP, PTN, RAC2, RHOG, SELE, SELENOP, SERPINE1, SPI1, THBS1, TNF, TRPV4, VEGFA, XG, ZNF580]
GO:0005125	cytokine activity	6,61E-07	[4, 5, 6]	73,00	28,63	60,64	39,36	[AREG, BMP7, CCL17, CCL19, CCL2, CCL22, CCL24, CCL3, CCL3L1, CCL4, CCL4L1, CCL4L2, CCL5, CD40LG, CLCF1, CMTM5, CXCL10, CXCL11, CXCL5, CXCL6, CXCL9, EBI3, EPX, FASLG, GDF6, IFNG, IL16, IL18, IL22, IL26, KLF5, LIF, LTA, LTB, NAMPT, TNFRSF11B, TNFSF13B, TNFSF14, TNFSF8, WNT1, WNT10A, WNT10B, XCL1, XCL2]	[ADIPOQ, AMH, AREG, BMP8A, C5, CCL11, CCL2, CCL21, CCL24, CLCF1, CMTM1, CMTM5, CSF1, CTF1, CX3CL1, GDF6, GPI, GRN, IL1B, IL1RN, IL32, INHA, KLF5, LIF, MEN1, MSTN, NDP, NODAL, OSM, SECTM1, THPO, TIMP1, TNF, TNFSF12, VEGFA, WNT3, WNT9B]
GO:0030334	regulation of cell migration	2,89E-08	[4, 5, 6]	200,00	21,57	38,98	61,02	[ADAM8, ADAMTS9, ADORA3, AIF1, ALOX15B, ANGPT1, ANGPT2, BMP7, CARMIL2, CASS4, CCL19, CCL2, CCL24, CCL3, CCL4, CCL5, CCR2, CD200R1, CD74, CDH1, CORO1A, CSF1R, CXCL10, CXCR3, CXCR4, DOCK10, DOCK8, DPEP1, DRD1, EDN2, FGR, FUT7, GPSM3, IFNG, ITGA4, ITGAX, KLRK1, LEF1, MMP9, NCKAP1L, NELL2, NR4A3, NTN1, P2RY12, PIK3CG, PLA2G7, PLAU, PLXNC1, PTAFR, PTK2B, PTPN22, PTPRC, PYCARD, RAC2, RHOH, SELE, SEMA4A, SEMA4D, SLAMF8, SMPD3, SPN, STAP1, TACSTD2, TCAF2, THBS1, THY1, TNFRSF18, TREM2, XCL1, XCL2, XG]	[ABCC8, ABI3, ABL1, ACKR3, ACTN4, ADAMTS9, ADGRG1, ADIPOQ, AGER, AGT, AMH, ANGPT1, APOD, ARHGDIA, ATP1B2, BCAR1, C5, C5AR1, CAMK2B, CAMSAP3, CARMIL2, CCL11, CCL2, CCL21, CCL24, CCR7, CD74, CEACAM1, CGA, CHGA, CITED2, COL1A1, CSF1, CX3CL1, DAB2IP, DAG1, DLG5, DMTN, ECM1, EDN2, ENG, EPHA2, EPPK1, FADD, FAM110A, FLT4, FOXC2, FOXF1, GATA2, GCSAM, GNAI2, GPER1, GPI, GRB7, GRN, HBEGF, HDAC5, HDAC7, HSPB1, HYAL1, ICAM1, IFITM1, IL1B, ILK, ITGA3, JUP, LAMA5, LGALS9, LGR6, LMNA, MAP2K3, MAPK3, MAZ, MECP2, MIEN1, MINK1, MSN, MSTN, MYADM, MYC, NF2, NGFR, NOD2, NODAL, NOS3, NOTCH1, NR4A3, NSMF, NTN1, OSGIN1, P2RY6, PDGFB, PDGFRB, PFN1, PHACTR1, PLA2G7, PLAU, PLVAP, PLXNA1, PLXNA3, PLXNB1, POSTN, PPARD, PTN, PTPN23, PTPRU, RAC2, RGCC, RHOG, RIPOR1, ROBO4, S100A11, SELE, SELENOP, SEMA3F, SEMA4B, SEMA4C, SEMA6B, SEMA6C, SERPINE1, SH3BP1, SLC9A3R1, SMAD3, SMIM22, SNAI1, SPHK1, SPI1, SRC, SRF, STAT3, SYDE1, TBX5, THBS1, TIMP1, TNF, TRIB1, TRPV4, VEGFA, VSIR, VTN, XG, ZC3H12A, ZNF580, ZNF609]
GO:0002687	positive regulation of leukocyte migration	2,59E-07	[3, 4, 5, 6, 7, 8]	50,00	34,72	59,01	40,99	[ADAM8, AIF1, CCL19, CCL24, CCL3, CCL4, CCL5, CCR2, CD74, CSF1R, CXCL10, DOCK8, EDN2, GPSM3, ITGA4, NCKAP1L, P2RY12, PLA2G7, PTAFR, PTK2B, PYCARD, RAC2, SPN, THBS1, THY1, TNFRSF18, TREM2, XCL1, XCL2, XG]	[AGER, C5AR1, CCL21, CCL24, CCR7, CD74, CSF1, CX3CL1, EDN2, FADD, ICAM1, LGALS9, MAPK3, MSTN, PLA2G7, PLVAP, PTN, RAC2, SELENOP, SERPINE1, SPI1, THBS1, TNF, TRPV4, VEGFA, XG, ZNF580]
GO:0072676	lymphocyte migration	2,45E-09	[4, 6, 7]	48,00	39,67	72,73	27,27	[ADAM8, AIF1, CCL17, CCL19, CCL2, CCL22, CCL24, CCL3, CCL3L1, CCL4, CCL4L1, CCL5, CCR2, CD200R1, CRTAM, CXCL10, CXCL11, CXCR3, DOCK8, FUT7, ITGA4, ITGAL, ITGB7, KLF5, KLRK1, MYO1G, PIK3CG, PTK2B, PYCARD, SPN, TBX21, XCL1, XCL2, XG, ZAP70]	[APOD, ARTN, CCL11, CCL2, CCL21, CCL24, CCR7, CX3CL1, ECM1, FADD, GBA, GCSAM, ICAM1, KLF5, MSN, SPNS2, XG]
GO:0097530	granulocyte migration	9,87E-07	[4, 6, 7]	53,00	32,72	67,23	32,77	[ADAM8, CCL17, CCL19, CCL2, CCL22, CCL24, CCL3, CCL3L1, CCL4, CCL4L1, CCL5, CD177, CD74, CSF1R, CXCL10, CXCL11, CXCL5, CXCL6, CXCL9, DPEP1, EDN2, EPX, FUT7, ITGB2, JAML, KLF5, MCOLN2, NCKAP1L, PIK3CG, RAC2, RHOH, SLAMF8, SYK, THBS1, VAV1, XCL1, XCL2, XG]	[C5AR1, CCL11, CCL2, CCL21, CCL24, CCR7, CD177, CD74, CSF1, CX3CL1, DHX34, EDN2, GBF1, KLF5, MAPK3, MEN1, MSTN, NOD2, PIP5K1C, RAC2, RHOG, THBS1, TRPV4, XG]
GO:1905517	macrophage migration	1,70E-02	[4, 6, 7]	23,00	35,38	61,83	38,17	[CCL2, CCL3, CCL5, CCR2, CD200R1, CSF1R, EDN2, KLF5, MCOLN2, P2RY12, PTK2B, SLAMF8, STAP1, THBS1, TREM2]	[AMH, C5, C5AR1, CCL2, CSF1, CX3CL1, EDN2, KLF5, MAPK3, MSTN, THBS1, TRPV4]
GO:0002688	regulation of leukocyte chemotaxis	2,87E-02	[4, 5, 6, 7, 8]	38,00	27,74	56,44	43,56	[AIF1, CCL19, CCL2, CCL3, CCL4, CCL5, CCR2, CD74, CSF1R, CXCL10, EDN2, GPSM3, KLRK1, NCKAP1L, PLA2G7, PTK2B, RAC2, SLAMF8, STAP1, THBS1, XCL1, XCL2]	[AMH, C5, C5AR1, CCL2, CCL21, CCR7, CD74, CSF1, EDN2, LGALS9, MAPK3, MSTN, NOD2, PLA2G7, PTN, RAC2, SERPINE1, SPI1, THBS1, TRPV4, VEGFA, ZNF580]
GO:0008009	chemokine activity	1,68E-04	[5, 6, 7]	23,00	44,23	77,86	22,14	[CCL17, CCL19, CCL2, CCL22, CCL24, CCL3, CCL3L1, CCL4, CCL4L1, CCL4L2, CCL5, CXCL10, CXCL11, CXCL5, CXCL6, CXCL9, KLF5, XCL1, XCL2]	[C5, CCL11, CCL2, CCL21, CCL24, CX3CL1, KLF5]
GO:0048247	lymphocyte chemotaxis	6,38E-03	[4, 5, 6, 7, 8]	24,00	36,36	81,93	18,07	[ADAM8, CCL17, CCL19, CCL2, CCL22, CCL24, CCL3, CCL3L1, CCL4, CCL4L1, CCL5, CCR2, CXCL10, CXCL11, CXCR3, KLF5, KLRK1, PIK3CG, PTK2B, XCL1, XCL2]	[CCL11, CCL2, CCL21, CCL24, CX3CL1, KLF5]
GO:0070098	chemokine-mediated signaling pathway	5,99E-08	[5, 6, 7]	38,00	42,22	75,14	24,86	[CCL17, CCL19, CCL2, CCL22, CCL24, CCL3, CCL3L1, CCL4, CCL4L1, CCL5, CCR2, CCR3, CCR4, CCR5, CCR8, CXCL10, CXCL11, CXCL5, CXCL6, CXCL9, CXCR3, CXCR4, CXCR6, PTK2B, TREM2, XCL1, XCL2, XCR1]	[ACKR1, ACKR3, CCL11, CCL2, CCL21, CCL24, CCR7, CX3CL1, GPR17, GPR75, SH2B3, THPO]

GO:0071621	granulocyte chemotaxis	5,27E-05	[4, 5, 6, 7, 8]	44,00	32,12	64,92	35,08	[CCL17, CCL19, CCL2, CCL22, CCL24, CCL3, CCL3L1, CCL4, CCL4L1, CCL5, CD74, CSF1R, CXCL10, CXCL11, CXCL5, CXCL6, CXCL9, DPEP1, EDN2, ITGB2, JAML, KLF5, NCKAP1L, PIK3CG, RAC2, SYK, THBS1, VAV1, XCL1, XCL2]	[C5AR1, CCL11, CCL2, CCL21, CCL24, CCR7, CD74, CSF1, CX3CL1, DHX34, EDN2, GBF1, KLF5, MAPK3, MEN1, MSTN, NOD2, PIP5K1C, RAC2, THBS1, TRPV4]
GO:0071675	regulation of mononuclear cell migration	5,63E-04	[4, 5, 6, 7, 8]	38,00	31,93	57,64	42,36	[ADAM8, AIF1, CCL2, CCL3, CCL4, CCL5, CCR2, CD200R1, CSF1R, CXCL10, DOCK8, ITGA4, KLRK1, PLA2G7, PTK2B, PYCARD, SLAMF8, SPN, THBS1, XCL1, XCL2]	[AGER, APOD, C5AR1, CCL2, CCL21, CCR7, CSF1, ECM1, FADD, GCSAM, LGALS9, MAPK3, MSN, MSTN, PLA2G7, SERPINE1, SPI1, THBS1, TNF, TRPV4]
GO:1990869	cellular response to chemokine	3,10E-08	[6]	41,00	41,00	72,85	27,15	[CCL17, CCL19, CCL2, CCL22, CCL24, CCL3, CCL3L1, CCL4, CCL4L1, CCL5, CCR2, CCR3, CCR4, CCR5, CCR8, CXCL10, CXCL11, CXCL5, CXCL6, CXCL9, CXCR3, CXCR4, CXCR6, DOCK8, PTK2B, TREM2, XCL1, XCL2, XCR1]	[ACKR1, ACKR3, CCL11, CCL2, CCL21, CCL24, CCR7, CX3CL1, GPR17, GPR75, LOX, SH2B3, THPO, ZC3H12A]
GO:0002690	positive regulation of leukocyte chemotaxis	1,60E-02	[4, 5, 6, 7, 8, 9]	31,00	30,69	56,44	43,56	[AIF1, CCL19, CCL3, CCL4, CCL5, CCR2, CD74, CSF1R, CXCL10, EDN2, GPSM3, NCKAP1L, PLA2G7, PTK2B, RAC2, THBS1, XCL1, XCL2]	[C5AR1, CCL21, CCR7, CD74, CSF1, EDN2, LGALS9, MAPK3, MSTN, PLA2G7, PTN, RAC2, SERPINE1, SPI1, THBS1, TRPV4, VEGFA, ZNF580]
GO:0071677	positive regulation of mononuclear cell migration	3,91E-02	[4, 5, 6, 7, 8, 9]	23,00	33,82	68,35	31,65	[ADAM8, AIF1, CCL3, CCL4, CCL5, CCR2, CXCL10, DOCK8, ITGA4, PLA2G7, PTK2B, PYCARD, SPN, XCL1, XCL2]	[AGER, CCL21, CCR7, FADD, LGALS9, PLA2G7, SERPINE1, SPI1, TNF]
GO:0072677	eosinophil migration	2,14E-02	[5, 7, 8]	13,00	50,00	59,69	40,31	[ADAM8, CCL2, CCL24, CCL3, CCL4, CCL4L1, CCL5, EPX]	[CCL11, CCL2, CCL21, CCL24, CX3CL1, DHX34, MEN1]
GO:0072678	T cell migration	3,10E-05	[5, 7, 8]	28,00	41,79	78,09	21,91	[ADAM8, AIF1, CCL2, CCL3, CCL5, CCR2, CD200R1, CXCL10, CXCL11, CXCR3, DOCK8, ITGA4, ITGAL, ITGB7, MYO1G, PIK3CG, PYCARD, SPN, XCL1, XCL2, XG, ZAP70]	[APOD, CCL2, CCL21, ECM1, FADD, ICAM1, MSN, XG]
GO:1990266	neutrophil migration	2,22E-05	[5, 7, 8]	44,00	33,33	72,73	27,27	[ADAM8, CCL17, CCL19, CCL2, CCL22, CCL24, CCL3, CCL3L1, CCL4, CCL4L1, CCL5, CD177, CD74, CXCL10, CXCL11, CXCL5, CXCL6, CXCL9, DPEP1, EDN2, FUT7, ITGB2, JAML, KLF5, MCOLN2, NCKAP1L, PIK3CG, RAC2, RHOH, SLAMF8, SYK, VAV1, XCL1, XCL2, XG]	[C5AR1, CCL11, CCL2, CCL21, CCL24, CCR7, CD177, CD74, CX3CL1, EDN2, GBF1, KLF5, NOD2, PIP5K1C, RAC2, RHOG, XG]
GO:0030593	neutrophil chemotaxis	8,59E-04	[5, 6, 7, 8, 9]	36,00	32,43	72,16	27,84	[CCL17, CCL19, CCL2, CCL22, CCL24, CCL3, CCL3L1, CCL4, CCL4L1, CCL5, CD74, CXCL10, CXCL11, CXCL5, CXCL6, CXCL9, DPEP1, EDN2, ITGB2, JAML, KLF5, NCKAP1L, PIK3CG, RAC2, SYK, VAV1, XCL1, XCL2]	[C5AR1, CCL11, CCL2, CCL21, CCL24, CCR7, CD74, CX3CL1, EDN2, GBF1, KLF5, NOD2, PIP5K1C, RAC2]
GO:2000401	regulation of lymphocyte migration	3,58E-03	[5, 6, 7, 8, 9]	23,00	38,33	75,88	24,12	[ADAM8, AIF1, CCL2, CCL3, CCL4, CCL5, CCR2, CD200R1, CXCL10, DOCK8, ITGA4, KLRK1, PTK2B, PYCARD, SPN, XCL1, XCL2]	[APOD, CCL2, CCL21, ECM1, FADD, GCSAM, MSN]
GO:0004950	chemokine receptor activity	3,56E-02	[6, 7, 8, 9]	13,00	48,15	74,46	25,54	[CCR2, CCR3, CCR4, CCR5, CCR8, CXCR3, CXCR4, CXCR6, XCR1]	[ACKR3, CCR7, GPR17, GPR75]
GO:2000403	positive regulation of lymphocyte migration	3,04E-02	[5, 6, 7, 8, 9, 10]	15,00	44,12	89,39	10,61	[ADAM8, AIF1, CCL3, CCL4, CCL5, CCR2, DOCK8, ITGA4, PTK2B, PYCARD, SPN, XCL1, XCL2]	[CCL21, FADD]
GO:0048518	positive regulation of biological process	5,77E-18	[1, 2, 3]	1107,00	16,89	40,46	59,54	[ABCB11, ABRA, ADAM8, ADCY7, AIF1, AIM2, AKNA, ALOX15, ALOX15B, ANGPT1, ANGPT2, APBB1IP, AREG, ASCL2, ATF3, ATP1B4, ATP2A3, BANK1, BATF, BCL11B, BCL3, BHLHA15, BLK, BLNK, BMF, BMP7, BMPR1B, BRIP1, BTK, BTLA, BTN3A1, BTN3A2, C1QA, C1QB, C1QC, C1QTNF1, C2, C8G, CACNA1I, CALCR, CAMK4, CARD11, CARMIL2, CASP8, CASS4, CCDC88B, CCL17, CCL19, CCL2, CCL22, CCL24, CCL3, CCL3L1, CCL4, CCL4L1, CCL5, CCR2, CCR3, CCR4, CD177, CD180, CD19, CD1B, CD1C, CD1E, CD2, CD22, CD226, CD244, CD247, CD27, CD300LF, CD33, CD3D, CD3E, CD3G, CD4, CD40LG, CD5, CD53, CD6, CD74, CD79A, CD80, CD84, CD86, CDC20B, CDCA2, CDH1, CDH4, CDKN2A, CEL, CFP, CLCF1, CLEC10A, CLEC4D, CLEC4E, CLEC6A, CLNK, CNR1, CORO1A, CR2, CRABP2, CRB2, CREM, CRTAM, CSF1R, CST7, CTLA4, CTSS, CXCL10, CXCL11, CXCL5, CXCL9, CXCR4, CXorf21, CYBB, CYP11B1, DAPK1, DEPDC1B, DOCK2, DOCK8, DPEP2, DRD1, E2F8, EAF2, EBI3, EDN2, EGR3, ELAVL4, ELF3, EOMES, EPHA6, EPX, EREG, ERFE, EVI2B, FASLG, FCN1, FCN3, FCRL3, FFAR4, FGD2, FGD3, FGR, FLT3, FOLR2, FOSB, FOSL1, FOXP3, FUT7, FYB1, GABBR2, GADD45B, GBP5, GDF6, GFI1, GLIS1, GMFG, GPM6A, GPR174, GPR4, GPR55, GPRC5A, GPSM3, GRAP2, GZMA, GZMB, H1-3, HCLS1, HCST, HLA-DMB, HLA-DPA1, HLA-DPB1, HLA-DQA1, HLA-DRA, HLA-DRB1, HLA-DRB5, HOPX, ICOS, IFNG, IGLL5, IKBKE, IKZF3, IL10RA, IL12RB1, IL12RB2, IL16, IL18, IL18RAP, IL26, IL2RB, IL2RG, IL5RA, IL7R, INPP5D, IQCJ-SCHIP1, IQGAP2, IRF4, IRF5, IRF8, IRX3, ITGA4, ITGAX, ITGB2, ITK, JAML, JCHAIN, KCNJ10, KCNN4, KIAA1324, KLF5, KLRC2, KLRD1, KLRG1, KLRK1, KMO, KNG1, LAG3, LAMP3, LAX1, LCK, LCP1, LCP2, LDLR, LEF1, LIF, LILRB1, LILRB4, LMCD1, LMO1, LPAL2, LRRC38, LTA, LTB, LY86, LY9, MACC1, MAFF, MAP4K1, MAPK13, MATK, MCOLN2, MEDAG, MID1IP1, MIXL1, MLC1, MMP9, MOG, MS4A1, MSX1, MUC16, MUC19, MUC6, MUSK, MYB, MYBL2, MYO1G, MZB1, NAMPT, NCF1, NCKAP1L, NCR3, NEK5, NELL2, NFAM1 [illegible]	[ABCA3, ABCA7, ABCB11, ABCC8, ABCD1, ABCD2, ABI3, ABL1, ABLIM3, ABR, ABRA, ACACB, ACKR3, ACTA1, ACTB, ACTC1, ACTN1, ACTN4, ADAM19, ADAMTSL4, ADCK1, ADCY1, ADCY3, ADCY4, ADCY9, ADGRG1, ADGRL1, ADIPOQ, AGAP2, AGER, AGPAT1, AGT, AGTR1, ALPK3, AMH, ANGPT1, ANGPTL4, AP2A1, AP3D1, APLN, APLNR, APOA1, AQP3, ARAF, ARC, AREG, ARF1, ARHGDIA, ARHGEF1, ARHGEF15, ARHGEF16, ARHGEF5, ARID1A, ARTN, ATAT1, ATF5, ATP13A2, ATP1A1, ATP1A3, ATP1B2, ATP1B4, ATPSCKMT, BAG3, BAG6, BCAR1, BCL2L1, BCL3, BCL6, BCL9L, BEND6, BMP8A, BRD4, BRPF3, BTLA, C15orf62, C1QTNF1, C20orf27, C2CD2L, C5, C5AR1, C8G, CACNA1H, CALB1, CALCR, CAMK2B, CAMTA2, CAPN1, CAPNS1, CARM1, CARMIL2, CASZ1, CC2D1A, CCDC87, CCL11, CCL2, CCL21, CCL24, CCR7, CD14, CD177, CD1C, CD1E, CD276, CD74, CDC25B, CDC42EP1, CDC42EP2, CDC42EP4, CDCA2, CDH4, CDK1, CDK2, CDKN1A, CEACAM1, CEL, CETP, CFH, CFL1, CGA, CHCHD10, CHERP, CHGA, CHI3L1, CIITA, CITED2, CITED4, CIZ1, CLCF1, CLEC4E, CLIP3, CLNK, CNOT3, CNR1, CNTN6, COL1A1, CPN2, CPNE5, CPNE6, CRAT, CREB3L1, CRHR2, CRIP2, CRP, CRTC2, CSF1, CSK, CSNK1E, CSPG4, CSRNP3, CST7, CTC1, CTF1, CTHRC1, CTIF, CTSD, CX3CL1, CYP26B1, CYP27B1, DAB2IP, DAG1, DAXX, DBF4B, DDR1, DDX39B, DENND2B, DGKD, DGKZ, DHX34, DIRAS1, DISP3, DKK2, DLG5, DMTN, DNMT1, DOC2B, DOK5, DPAGT1, DPF2, DTX1, DUSP5, DVL3, DXO, DYRK1B, E2F4, E2F7, E2F8, EAF2, ECM1, ECM2, EDC4, EDN2, EEF1E1, EFEMP2, EFNA3, EGLN2, EHD1, EHD2, EIF4EBP1, EIF4G1, ELF3, ELF4, ELK1, ENDOG, ENG, ENPP3, EPHA2, ERBB2, ERFE, ESM1, ESPL1, ETS2, ETV6, FABP4, FADD, FAM110A, FAP, FASN, FCN3, FEM1A, FES, FFAR2, FGFR4, FHL5, FHOD1, FITM1, FLOT2, FLT3, FLT4, FOSL1, FOSL2, FOXC2, FOXF1, FOXM1, FOXS1, FPR1, FRMD8, FURIN, GAB2, GABBR2, GADD45B, GATA2, GATA4, GBA, GCSAM, GDF6, GDI1, GGA1, GGA3, GIT1, GLIS2, GLMP, GLRA1, GNAI2, GNAO1, GPAM [illegible]

GO:0048519	negative regulation of biological process	6,13E-07	[1, 2, 3]	928,00	16,02	39,03	60,97	[ACP5, ADAM8, ADAMDEC1, ADAMTS9, ADCY7, ADORA3, AIF1, AKNA, ALOX15, ALOX15B, ANGPT1, ANGPT2, ANO9, AOAH, APOBEC3A, APOBEC3D, APOBEC3G, APOBEC3H, AREG, ASCL2, ATF3, BANK1, BCL11B, BCL3, BHLHA15, BHLHE40, BLK, BMF, BMP7, BMPR1B, BRIP1, BTG2, BTK, C1QC, C1QTNF1, CALCR, CARD17, CARMIL2, CASP8, CBARP, CCDC88C, CCL17, CCL19, CCL2, CCL3, CCL3L1, CCL5, CCR2, CCR5, CD19, CD200R1, CD22, CD247, CD27, CD300LF, CD33, CD3E, CD3G, CD40LG, CD74, CD80, CD84, CD86, CD96, CDH1, CDKN2A, CEL, CHRDL1, CISH, CLCF1, CLNK, CMTM5, CNN1, CNR1, CNR2, CORO1A, CR2, CRB2, CREM, CRTAM, CST7, CTLA4, CXCL10, CXCL11, CXCL9, CYSLTR1, DAPK1, DCC, DEPDC1B, DERL3, DOCK8, DPEP1, DPEP2, DPT, DRD1, DUSP2, DUSP8, E2F8, EAF2, EGR3, ELAVL4, ELF3, EOMES, EPX, EREG, ERFE, EVI2B, FAM122C, FASLG, FATE1, FCMR, FCN1, FCN3, FCRL3, FFAR4, FGL2, FGR, FOSB, FOSL1, FOXP3, FRZB, GADD45B, GFI1, GFRA2, GLIS1, GMFG, GMIP, GPR132, GPR150, GPR171, GPR4, GPR55, GPR83, GPRC5A, GZMA, GZMB, H1-3, H2AC13, H2AC14, H2AC16, H3C10, H3C11, H3C12, H3C7, HCLS1, HLA-DOA, HLA-DRB1, HOPX, HORMAD1, IER3, IFNG, IKBKE, IKZF1, IL10RA, IL18, IL26, IL7R, INPP5D, IQCJ-SCHIP1, IRF4, IRF8, IRX3, ITGB2, JPH1, JSRP1, KCNN4, KIR3DL2, KLF5, KLRC1, KLRD1, KLRK1, KNG1, LAG3, LAMP3, LAX1, LCK, LCP1, LDLR, LEF1, LIF, LILRB1, LILRB4, LMCD1, LMO1, LPAL2, LRRC17, LST1, LTA, MCOLN2, MCOLN3, MID1IP1, MIDN, MIXL1, MMP9, MPO, MSX1, MT1F, MT1G, MYB, NAMPT, NCKAP1L, NLRC3, NLRP3, NLRP6, NLRP7, NPPB, NPPC, NR4A1, NR4A2, NR4A3, NTN1, NUGGC, NUP210, OASL, P2RY12, PAPLN, PARP15, PATL2, PAX5, PIK3CG, PIK3IP1, PIM1, PIM2, PLA2G2D, PLAAT4, PLAC8, PLAU, PLEK, PLXNC1, PRDM1, PRKCB, PRKCQ, PSCA, PTGDR, PTGER2, PTGFR, PTK2B, PTPN22, PTPN6, PTPRC, PTPRH, PTX3, PYCARD, RAD9B, RARRES1, RASAL3, RBPMS2, RGS1, RGS10, RGS18, RGS9, RHOH, RLN2, RMI2, RNASE6, RRAD, RRM2, RTKN2, RTN1	[AAAS, ABCA7, ABCC8, ABCD1, ABCD2, ABI3, ABL1, ACACB, ACKR3, ACP4, ACTN1, ACTN4, ADAMTS5, ADAMTS7, ADAMTS8, ADAMTS9, ADCK1, ADCY1, ADCY3, ADCY4, ADCY9, ADGRB2, ADGRG1, ADIPOQ, AGAP2, AGER, AGT, AKT1S1, ALPK3, AMH, ANGPT1, ANGPTL4, ANKRD13B, ANKRD52, AP2A1, APLN, APLNR, APLP1, APOA1, APOBEC3C, APOD, APOM, ARAF, ARC, AREG, ARF1, ARHGAP1, ARHGDIA, ARHGEF15, ARID5A, ARRDC3, ARTN, ASPN, ATF5, ATN1, ATP13A2, ATP1A1, ATP1A3, ATP1B2, BAG3, BAG6, BAHD1, BAZ2A, BBS12, BCKDK, BCL2L1, BCL3, BCL6, BCL9L, BCOR, BEND6, BHLHE40, BMP8A, BTG2, C11orf65, C18orf54, C1QTNF1, C5, C5AR1, CABIN1, CALCR, CAMSAP3, CARM1, CARMIL2, CBARP, CC2D1A, CCL11, CCL2, CCL21, CCR7, CD14, CD74, CDK1, CDK2, CDK2AP2, CDKN1A, CEACAM1, CEL, CETP, CFH, CFL1, CGA, CHERP, CHGA, CIC, CIITA, CISH, CITED2, CLCF1, CLIP3, CLNK, CMTM5, CNN1, CNOT3, CNR1, COL1A1, CPTP, CRAT, CREB3L1, CRHR2, CRP, CRYAB, CSDC2, CSF1, CSK, CST7, CTC1, CTDSP1, CTHRC1, CTIF, CTSA, CX3CL1, CXXC4, CYP26B1, CYP27B1, CYP2D6, DAB2IP, DAG1, DAXX, DBNDD1, DDR1, DDX39B, DDX54, DEDD, DGKD, DGKZ, DGUOK, DHRS2, DHX34, DISP3, DKK2, DLG5, DMTN, DNMT1, DOT1L, DPF2, DPT, DTX1, DUSP5, DUSP8, DVL3, DXO, DYSF, E2F4, E2F7, E2F8, EAF2, ECM1, EDC4, EEF1E1, EFEMP2, EFNA3, EIF4EBP1, EIF4G1, ELF3, ENG, ENPP3, EPHA2, EPN1, EPPK1, ERBB2, ERF, ERFE, ESPL1, ETS2, ETV6, F8A1, FABP4, FADD, FAIM2, FAM110A, FAM122C, FAP, FASN, FCN3, FEM1A, FGFRL1, FLII, FLOT2, FLT4, FOSL1, FOXC2, FOXF1, FOXM1, FOXP4, FOXS1, FRMD8, FURIN, FXYD5, GADD45B, GAS1, GATA2, GATA4, GATAD2A, GBA, GCSAM, GDI1, GGA3, GIT1, GJD3, GLIS2, GLRA1, GMNN, GNAI2, GNAO1, GPAM, GPBAR1, GPER1, GPI, GPR137, GPR20, GPR37L1, GPR4, GPR83, GPR84, GPRC5A, GRB7, GRIK5, GRINA, GRN, GSK3A, GTF2IRD1, GTPBP1, H1-9P, H3C10, H3C12, H4C3, HBEGF, HCFC1, HDAC10, HDAC5, HDAC7, HESX1, HEYL, HFE, HGS, HIRA, HLA-DOB
GO:0002682	regulation of immune system process	1,99E-30	[2, 3]	430,00	23,55	62,05	37,95	[ADAM8, ADCY7, AIF1, AIM2, ALOX15, ANGPT1, APOBEC3G, BANK1, BLK, BLNK, BTK, BTLA, BTN3A1, BTN3A2, C1QA, C1QB, C1QC, C1QTNF1, C2, C8G, CALCR, CAMK4, CARD11, CASP8, CCDC88B, CCL19, CCL2, CCL24, CCL3, CCL4, CCL5, CCR2, CD177, CD19, CD1B, CD1C, CD1E, CD2, CD200R1, CD22, CD226, CD244, CD247, CD27, CD300LF, CD33, CD3D, CD3E, CD3G, CD4, CD40LG, CD48, CD5, CD6, CD74, CD79A, CD80, CD84, CD86, CD8A, CD8B, CD96, CDKN2A, CFP, CLCF1, CLEC10A, CLEC1B, CLEC4D, CLEC4E, CLEC6A, CLNK, CNR1, CNR2, CORO1A, CR2, CRTAM, CSF1R, CST7, CTLA4, CXCL10, CXCL6, CXCR3, CXorf21, CYP11B1, CYSLTR1, DOCK8, DPEP1, DRD1, EBI3, EDN2, EGR3, EPX, EREG, EVI2B, FCGR2C, FCN1, FCN3, FCRL3, FGL2, FGR, FOXP3, FUT7, FYB1, GBP5, GPR150, GPR171, GPR55, GPR83, GPSM3, GRAP2, H3C10, H3C11, H3C12, H3C7, HCLS1, HCST, HLA-DMB, HLA-DOA, HLA-DPA1, HLA-DPB1, HLA-DQA1, HLA-DRA, HLA-DRB1, HLA-DRB5, ICOS, IFNG, IGLL5, IKBKE, IKZF3, IL12RB1, IL18, IL18RAP, IL7R, INPP5D, IRF4, ITGA4, ITGAL, ITGB2, ITGB7, ITK, JAML, KCNN4, KIR2DL2, KIR2DL3, KIR3DL2, KLRB1, KLRC1, KLRC2, KLRD1, KLRK1, LAG3, LAIR1, LAIR2, LAX1, LCK, LCP2, LDLR, LEF1, LILRA1, LILRB1, LILRB4, LMO1, LPAL2, LRRC17, LST1, LTA, MOG, MS4A1, MUC16, MUC19, MUC6, MYB, MYO1G, MZB1, NCKAP1L, NCR1, NCR3, NFAM1, NLRC3, NLRP3, NLRP6, NR4A3, ORM2, P2RY12, PAX5, PILRA, PLA2G2D, PLA2G7, PRAM1, PRDM1, PRKCB, PRKCQ, PTAFR, PTGDR, PTGER2, PTK2B, PTPN22, PTPN6, PTPRC, PYCARD, PYHIN1, RAC2, RASAL3, RHEX, RHOH, RLN2, RUNX3, SAMSN1, SASH3, SCIMP, SELE, SELL, SH2D1A, SIGLEC16, SIRPG, SIT1, SKAP1, SLA2, SLAMF6, SLAMF7, SLAMF8, SMPD3, SOCS1, SOCS3, SPN, STAP1, STAT1, STXBP2, SYK, TBC1D10C, TBX21, TESPA1, THBS1, THEMIS, THEMIS2, THY1, TIGIT, TLR6, TLR8, TNFAIP3, TNFAIP8L2, TNFRSF13B, TNFRSF18, TNFSF13B, TNFSF14, TNFSF8, TOX, TRAT1, TREM2, TSC22D3, UBASH3A, VAV1, VNN1, WAS, XCL1, XCL2, XG, ZAP70, ZBP1,	[ABL1, ACTB, ADCY1, ADCY3, ADCY4, ADCY9, ADIPOQ, AGER, AMH, ANGPT1, AP3D1, APOA1, APOD, AQP3, BAG6, BCAR1, BCL6, BTLA, C1QTNF1, C5, C5AR1, C8G, CALCR, CCL2, CCL21, CCL24, CCR7, CD177, CD1C, CD1E, CD276, CD300E, CD74, CDKN1A, CEACAM1, CFH, CGA, CHGA, CLC, CLCF1, CLEC1B, CLEC4E, CLNK, CLPTM1, CNR1, COL1A1, CPN2, CRHR2, CRP, CSF1, CSK, CST7, CX3CL1, CYP26B1, DCAF15, DGKZ, DLG5, DTX1, ECM1, EDN2, ENPP3, ERBB2, FADD, FCN3, FES, FFAR2, FLOT2, FOXF1, FPR1, FURIN, GAB2, GATA2, GCSAM, GNAI2, GNAO1, GPAM, GPBAR1, GPER1, GPI, GPR137, GPR20, GPR83, GPR84, GRN, H3C10, H3C12, H4C3, HAVCR1, HFE, HK1, HLA-DOB, HRH2, HSF1, ICAM1, IDO1, IFITM1, IL15RA, IL1B, IL4R, IL7R, INHA, JAK3, JUND, KLF10, KMT2D, LAG3, LDLR, LGALS9, LIMK1, LOX, LRRC17, LRRC32, LTF, MAPK3, MAVS, MOV10, MSN, MSTN, MUC19, MUC20, MUC3A, MYC, MYH9, MYL9, MYO18A, NCOR2, NECTIN2, NFE2, NLRC5, NOD2, NPPA, NR1D1, NR4A3, NRARP, PAF1, PCDHA4, PCDHA6, PDCD1, PLA2G2A, PLA2G7, PLVAP, PQBP1, PRKACA, PRKAR2B, PTGIR, PTN, PVR, RAC2, RAMP2, RAP1A, RARA, RBM14, RELA, RELB, RGCC, RHBDF2, RHOG, RMRP, RNF26, RNU1-93P, SELE, SELENOP, SERPINE1, SERPING1, SETD1A, SH2B2, SIGLEC9, SLC7A1, SLC7A5, SLC7A8, SMAD3, SOCS3, SOX12, SOX13, SPHK1, SPHK2, SPI1, SPNS2, SPPL2B, SRC, STAT3, STING1, TFE3, THBS1, THPO, TICAM1, TIMP1, TNF, TRIB1, TRIM62, TRPV4, VEGFA, VIPR2, VNN1, VSIR, VTN, WAS, XG, ZBTB7B, ZC3H12A, ZCCHC3, ZDHHC11, ZFP36, ZMIZ1, ZNF335, ZNF580]

GO:0023051	regulation of signaling	5,81E-15	[2, 3]	675,00	18,15	37,12	62,88	[ABRA, ADAM8, ALOX15, ALOX15B, ANGPT1, ARAP2, AREG, ARHGAP15, ARHGAP30, ARHGAP45, ATF3, ATP2A3, BANK1, BCL2L14, BCL3, BLK, BMF, BMP7, BMPR1B, BRIP1, C1QTNF1, CACNA1E, CALCR, CARD11, CASP8, CASS4, CCDC88C, CCL17, CCL19, CCL2, CCL22, CCL24, CCL3, CCL3L1, CCL4, CCL4L1, CCL5, CCR2, CD177, CD180, CD19, CD22, CD226, CD27, CD300LF, CD3E, CD4, CD40LG, CD74, CD80, CD86, CDH1, CDKN2A, CEL, CHRDL1, CISH, CLEC6A, CNR1, CNR2, CRB2, CSF1R, CXCR4, CXorf21, DAPK1, DCC, DEPDC1B, DLGAP2, DPEP2, DRD1, DUSP2, DUSP8, ELAVL4, ELF3, EPX, EREG, ERFE, FASLG, FCRL3, FFAR4, FGD2, FGD3, FGR, FLT3, FOLR2, FOXP3, FRZB, FUT7, GADD45B, GDF6, GFI1, GMIP, GPR174, GPR4, GPR55, GPRC5A, GUCY2D, H3C10, H3C11, H3C12, H3C7, HCLS1, HCST, HLA-DRB1, ICOS, IER3, IFNG, IKBKE, IL10RA, IL18, IL26, IL7R, INPP5D, IQCJ-SCHIP1, IRF4, KCNJ10, KCNN4, KMO, LAX1, LCK, LEF1, LIF, LILRB4, LMCD1, LY86, MAP4K1, MIDN, MMP9, MSX1, MYB, MZB1, NCF1, NCKAP1L, NFAM1, NLRC3, NLRP3, NLRP6, NPFFR2, NPPC, NPTX2, NR4A2, NSG1, NTN1, OASL, P2RY10, P2RY12, PAX5, PDE6G, PIK3CG, PIK3IP1, PIK3R5, PIM2, PLAU, PLEK, PRDM1, PRKCB, PRKCQ, PROK1, PSCA, PSD4, PTK2B, PTPN22, PTPN6, PTPRC, PYCARD, PYHIN1, RAC2, RAD9B, RASAL3, RASGRF1, RBPMS2, RGS1, RGS10, RGS18, RGS9, RHOH, RMI2, RNASE6, RRAD, RTKN2, SALL1, SCIMP, SCUBE1, SEMA4D, SH2D1A, SHISA8, SLA, SLA2, SLC24A4, SLC8A3, SMPDL3B, SOCS1, SOCS3, SPINK1, STAP1, STAT1, SYK, TAGAP, TBC1D10C, TESPA1, THBS1, THY1, TLR6, TMC8, TNFAIP3, TNFSF14, TNIP3, TNMD, TNR, TRABD2A, TRAF1, TRAT1, TREM2, TRIM14, TRPM2, UBASH3A, UBD, UCP2, VAV1, VNN1, VWC2L, WNT1, WNT10B, XCL1, XCL2, ZAP70, ZBP1]	[ABCA7, ABCC8, ABL1, ABR, ABRA, ACKR3, ACP4, ACTN4, ADCY1, ADGRG1, ADIPOQ, AGAP2, AGER, AGPAT1, AGT, AGTR1, AKT1S1, AMH, ANGPT1, APLN, APLNR, APLP1, APOA1, APOD, ARAF, ARAP3, ARC, AREG, ARF1, ARHGAP1, ARHGAP23, ARHGAP39, ARHGDIA, ARHGEF1, ARHGEF16, ARHGEF5, ARRDC3, ARTN, ASPN, ATP1A1, ATP1A3, ATP1B2, BCL2L1, BCL3, BCL6, BCL9L, BCR, BEND6, BMP8A, BRD4, C1QTNF1, C20orf27, C2CD2L, C5, C5AR1, CACNA1E, CALB1, CALCR, CAMK2B, CAPN1, CARM1, CC2D1A, CCL11, CCL2, CCL21, CCL24, CCR7, CD14, CD177, CD74, CDK1, CDK2, CEACAM1, CEL, CGA, CHERP, CHGA, CHI3L1, CISH, CITED2, CLIP3, CNR1, CNTN6, COL1A1, CPLX1, CRAT, CREB3L1, CRHR2, CSF1, CSK, CSNK1E, CSPG4, CTHRC1, CX3CL1, CXXC4, CYP26B1, CYP27B1, DAB2IP, DAG1, DAXX, DDX39B, DENND2B, DENND4B, DGKD, DGKZ, DHX34, DIRAS1, DKK2, DLG5, DLGAP4, DMTN, DOC2B, DOK5, DOT1L, DTX1, DUSP5, DUSP8, DVL3, ECM1, EEF1E1, ELF3, ENG, EPHA2, EPN1, ERBB2, ERFE, ESM1, FADD, FAIM2, FAM110A, FASN, FFAR2, FGFR4, FLOT2, FLT3, FLT4, FOXM1, FPR1, FRMD8, FURIN, FXYD6, GAB2, GADD45B, GAS1, GATA4, GBA, GBF1, GCSAM, GDF6, GIT1, GJD3, GLIS2, GLRA1, GNAI2, GPAM, GPBAR1, GPER1, GPI, GPR137, GPR17, GPR20, GPR37L1, GPR4, GPRC5A, GPRIN1, GRB7, GRIK5, GRINA, GRM2, GSK3A, H3C10, H3C12, H4C3, HBEGF, HCRTR1, HDAC7, HEYL, HFE, HGS, HIPK4, HSPB1, HTR1B, HTR2A, ICAM1, IER3, IFT80, IL18BP, IL1B, IL1RN, IL7R, ILK, INCA1, INHA, INPP5E, IRAK2, ITGA3, ITGA5, ITPR3, ITPRIP, JAK3, JMJD8, JPH4, JUP, KCP, KCTD11, KIF26A, KLK14, KMT2D, KSR1, LGALS9, LGR6, LIF, LIFR, LILRA5, LIMS2, LMCD1, LMNA, LOX, LRG1, LRRC4, LRRC8A, LTBR, LTF, LY6E, LYNX1, LZTS1, LZTS2, MAP1A, MAP2K3, MAP3K10, MAP3K11, MAP3K14, MAP3K6, MAP3K9, MAPK3, MAPK7, MAPK8IP3, MAVS, MAZ, MECP2, MED12, MEGF8, MEIS3P1, MEN1, MGRN1, MIDN, MINK1, MLST8, MME, MNT, MSTN, MSX1, MTRNR2L1, MTRNR2L10, MTRNR2L12, MTRNR2L2, MTRNR2L3
GO:0048583	regulation of response to stimulus	4,40E-27	[2, 3]	851,00	18,88	45,12	54,88	[ABRA, ACP5, ADAM8, ADCY7, AIF1, AIM2, AKNA, ALOX15, ALOX15B, ANGPT1, ANGPT2, AOAH, APOBEC3G, ARAP2, AREG, ARHGAP15, ARHGAP30, ARHGAP45, ATF3, BANK1, BCL2L14, BCL3, BLK, BLNK, BMF, BMP7, BMPR1B, BRIP1, BTK, BTLA, BTN3A1, BTN3A2, C1QA, C1QB, C1QC, C1QTNF1, C2, C8G, CALCR, CARD11, CASP8, CASS4, CCDC88C, CCL17, CCL19, CCL2, CCL22, CCL24, CCL3, CCL3L1, CCL4, CCL4L1, CCL5, CCR2, CCR4, CD177, CD180, CD19, CD1B, CD1C, CD1E, CD200R1, CD22, CD226, CD247, CD27, CD300LF, CD33, CD3D, CD3E, CD3G, CD4, CD40LG, CD48, CD74, CD79A, CD80, CD84, CD86, CD8A, CD8B, CD96, CDKN2A, CEL, CFP, CHRDL1, CISH, CLCF1, CLEC10A, CLEC1B, CLEC4D, CLEC4E, CLEC6A, CLNK, CNR1, CNR2, CR2, CRB2, CRTAM, CSF1R, CST7, CTLA4, CXCL10, CXCL6, CXCR4, CXorf21, CYP11B1, CYSLTR1, DAPK1, DCC, DEPDC1B, DLGAP2, DPEP1, DPEP2, DRD1, DUSP2, DUSP8, EDN2, ELF3, EPX, EREG, ERFE, FASLG, FCGR2C, FCN1, FCN3, FCRL3, FFAR4, FGD2, FGD3, FGL2, FGR, FLT3, FOLR2, FOXP3, FRZB, FUT7, FYB1, GADD45B, GBP5, GDF6, GFI1, GMIP, GPR150, GPR174, GPR4, GPR55, GPR83, GPRC5A, GPSM3, GRAP2, GUCY2D, H3C10, H3C11, H3C12, H3C7, HCLS1, HCST, HLA-DMB, HLA-DPA1, HLA-DPB1, HLA-DQA1, HLA-DRA, HLA-DRB1, HLA-DRB5, HOPX, ICOS, IER3, IFNG, IGLL5, IKBKE, IL10RA, IL12RB1, IL16, IL18, IL18RAP, IL26, IL7R, INPP5D, IQCJ-SCHIP1, IRF4, ITGA4, ITGAL, ITGB2, ITGB7, ITK, JAML, KCNN4, KIR2DL2, KIR2DL3, KIR3DL2, KLRB1, KLRC1, KLRC2, KLRD1, KLRK1, KNG1, LAG3, LAIR1, LAIR2, LAX1, LCK, LCP1, LCP2, LDLR, LEF1, LIF, LILRA1, LILRB1, LILRB4, LMCD1, LTA, LY86, MAP4K1, MAPK13, MLC1, MMP9, MOG, MS4A1, MSX1, MUC16, MUC19, MUC6, MYB, MYO1G, MZB1, NAMPT, NCF1, NCKAP1L, NCR1, NCR3, NFAM1, NLRC3, NLRP3, NLRP6, NPFFR2, NPPC, NPTX2, NR4A2, NR4A3, NTN1, NUP210, OASL, P2RY10, P2RY12, PAX5, PDE6G, PIK3CG, PIK3IP1, PIK3R5, PILRA, PIM2, PLA2G2D, PLA2G7, PLAU, PLEK, PRAM1, PRDM1, PRKCB, PRKCQ,	[AAAS, ABCA7, ABCC8, ABCD1, ABI3, ABL1, ABR, ABRA, ACKR3, ACP4, ACTB, ACTN4, ADCY1, ADCY3, ADCY4, ADCY9, ADGRG1, ADIPOQ, AGAP2, AGER, AGPAT1, AGT, AGTR1, AKT1S1, AMH, ANGPT1, APLN, APLNR, APLP1, APOA1, APOD, ARAF, ARAP3, ARC, AREG, ARHGAP1, ARHGAP23, ARHGAP39, ARHGDIA, ARHGEF1, ARHGEF16, ARHGEF5, ARMT1, ARRDC3, ARTN, ASPN, BAG3, BAG6, BCAR1, BCL2L1, BCL3, BCL6, BCL9L, BCR, BEND6, BMP8A, BRD4, BTLA, C1QTNF1, C20orf27, C5, C5AR1, C8G, CALCR, CAMK2B, CAPN1, CARM1, CC2D1A, CCL11, CCL2, CCL21, CCL24, CCR7, CD14, CD177, CD1C, CD1E, CD276, CD300E, CD74, CDK1, CDK2, CEACAM1, CEL, CFH, CGA, CHERP, CHGA, CHI3L1, CISH, CITED2, CLC, CLCF1, CLEC1B, CLEC4E, CLIP3, CLNK, CNR1, CNTN6, COL1A1, CPN2, CRAT, CREB3L1, CRHR2, CRP, CRYAB, CSF1, CSK, CSNK1E, CSPG4, CST7, CTHRC1, CX3CL1, CXXC4, CYP26B1, CYP27B1, DAB2IP, DAG1, DAGLA, DAXX, DDX39B, DENND2B, DENND4B, DGKD, DGKZ, DIRAS1, DKK2, DLG5, DLGAP4, DMTN, DOK5, DOT1L, DTX1, DUSP5, DUSP8, DVL3, ECM1, EDN2, EEF1E1, EIF4G1, ELF3, ENDOG, ENG, ENPP3, EPHA2, EPN1, EPPK1, ERBB2, ERFE, ESM1, FABP4, FADD, FAIM2, FAM110A, FAP, FASN, FCN3, FEM1A, FES, FFAR2, FGFR4, FLOT2, FLT3, FLT4, FOXC2, FOXF1, FOXM1, FPR1, FRMD8, FURIN, GAB2, GADD45B, GAS1, GATA2, GATA4, GBA, GBF1, GCSAM, GDF6, GIT1, GLIS2, GNAI2, GNAO1, GPAM, GPBAR1, GPER1, GPI, GPR137, GPR17, GPR20, GPR37L1, GPR4, GPR83, GPR84, GPRC5A, GPRIN1, GRB7, GRIK5, GRINA, GRM2, GRN, GSK3A, GTF2IRD1, H3C10, H3C12, H4C3, HBEGF, HCRTR1, HDAC10, HDAC7, HEYL, HFE, HGS, HIPK4, HMGA1, HOPX, HRH2, HSF1, HSPB1, HSPB8, HTR1B, HTR2A, ICAM1, IER3, IFITM1, IFT80, IL18BP, IL1B, IL1RN, IL4R, IL7R, ILK, INCA1, INHA, INPP5E, IRAK2, ITGA3, ITGA5, ITPRIP, JAK3, JMJD8, JUP, KCNK2, KCP, KCTD11, KIF26A, KLK14, KMT2D, KNG1, KSR1, LAG3, LDLR, LGALS9, LGR6, LIF, LIFR, LILRA5, LIMK1, LIMS2, LMCD1, LMNA, LOX, LRG1, LTBR,

GO:0002684	positive regulation of immune system process	4,58E-20	[2, 3, 4]	281,00	24,16	64,49	35,51	[ADAM8, AIF1, AIM2, BLK, BLNK, BTK, BTLA, BTN3A1, BTN3A2, C1QA, C1QB, C1QC, C2, C8G, CAMK4, CARD11, CASP8, CCDC88B, CCL19, CCL2, CCL24, CCL3, CCL4, CCL5, CCR2, CD177, CD19, CD1B, CD1C, CD1E, CD2, CD22, CD226, CD244, CD247, CD27, CD3D, CD3E, CD3G, CD4, CD40LG, CD5, CD6, CD74, CD79A, CD80, CD84, CD86, CFP, CLCF1, CLEC10A, CLEC4D, CLEC4E, CLEC6A, CLNK, CNR1, CORO1A, CR2, CRTAM, CSF1R, CTLA4, CXCL10, CXorf21, CYP11B1, DOCK8, EBI3, EDN2, EGR3, EPX, EREG, EVI2B, FCN1, FCN3, FCRL3, FGR, FOXP3, FYB1, GBP5, GPSM3, GRAP2, HCLS1, HLA-DMB, HLA-DPA1, HLA-DPB1, HLA-DQA1, HLA-DRA, HLA-DRB1, HLA-DRB5, ICOS, IFNG, IGLL5, IKBKE, IL12RB1, IL18, IL18RAP, IL7R, INPP5D, ITGA4, ITGB2, ITK, KCNN4, KLRC2, KLRD1, KLRK1, LAG3, LAX1, LCK, LCP2, LEF1, LILRB1, LILRB4, LTA, MOG, MS4A1, MUC16, MUC19, MUC6, MYB, MYO1G, MZB1, NCKAP1L, NCR3, NFAM1, NLRP3, NR4A3, P2RY12, PAX5, PLA2G7, PRAM1, PRKCB, PRKCQ, PTAFR, PTK2B, PTPN22, PTPN6, PTPRC, PYCARD, PYHIN1, RAC2, RASAL3, RHOH, RUNX3, SASH3, SCIMP, SH2D1A, SIGLEC16, SIRPG, SKAP1, SLA2, SLAMF6, SOCS1, SPN, STAP1, STXBP2, SYK, TBX21, TESPA1, THBS1, THEMIS, THEMIS2, THY1, TLR6, TLR8, TNFRSF18, TNFSF13B, TNFSF14, TOX, TRAT1, TREM2, UBASH3A, VAV1, VNN1, WAS, XCL1, XCL2, XG, ZAP70, ZBP1]	[ABL1, ACTB, AGER, AMH, AP3D1, AQP3, BAG6, BCAR1, BCL6, BTLA, C5, C5AR1, C8G, CCL2, CCL21, CCL24, CCR7, CD177, CD1C, CD1E, CD276, CD74, CDKN1A, CEACAM1, CFH, CLCF1, CLEC4E, CLNK, CNR1, CPN2, CRP, CSF1, CSK, CX3CL1, DGKZ, EDN2, ENPP3, FADD, FCN3, FES, FFAR2, FLOT2, FOXF1, FPR1, GAB2, GATA2, GCSAM, GNAO1, GPAM, GPI, GRN, HAVCR1, HK1, HSF1, ICAM1, IL15RA, IL1B, IL4R, IL7R, JAK3, JUND, KLF10, LAG3, LGALS9, LIMK1, MAPK3, MAVS, MSTN, MUC19, MUC20, MUC3A, MYO18A, NCOR2, NECTIN2, NLRC5, NOD2, NPPA, NR4A3, PCDHA4, PDCD1, PLA2G7, PLVAP, PQBP1, PRKACA, PTN, PVR, RAC2, RAP1A, RARA, RBM14, RELA, RELB, RGCC, RHBDF2, RNU1-93P, SELENOP, SERPINE1, SERPING1, SH2B2, SLC7A1, SLC7A5, SLC7A8, SOX12, SOX13, SPHK2, SPI1, SRC, STING1, THBS1, TICAM1, TIMP1, TNF, TRIB1, TRIM62, TRPV4, VEGFA, VNN1, VSIR, VTN, WAS, XG, ZBTB7B, ZC3H12A, ZCCHC3, ZMIZ1, ZNF335, ZNF580]
GO:0007165	signal transduction	1,01E-19	[2, 3, 4]	1126,00	17,01	42,72	57,28	[ABRA, ADAM8, ADCY7, ADGRE3, ADGRE4P, ADGRG5, ADGRG6, ADORA3, AIF1, AIM2, ALOX15, ALOX15B, ANGPT1, ANGPT2, ANPEP, APBB1IP, ARAP2, AREG, ARHGAP15, ARHGAP30, ARHGAP45, ASGR2, ATF3, ATP2A3, BANK1, BATF, BCL11B, BCL2L14, BCL3, BHLHA15, BLK, BLNK, BMF, BMP7, BMPR1B, BRIP1, BTG2, BTK, BTLA, BTN3A1, BTN3A2, C1QTNF1, CACNA1I, CALCR, CAMK4, CARD11, CASKIN1, CASP8, CASS4, CCDC88C, CCL17, CCL19, CCL2, CCL22, CCL24, CCL3, CCL3L1, CCL4, CCL4L1, CCL4L2, CCL5, CCR2, CCR3, CCR4, CCR5, CCR8, CD177, CD180, CD19, CD2, CD200R1, CD22, CD226, CD244, CD247, CD27, CD300LF, CD33, CD3D, CD3E, CD3G, CD4, CD40LG, CD5, CD53, CD6, CD7, CD74, CD79A, CD80, CD86, CD8A, CD8B, CDKN2A, CHRDL1, CISH, CLCF1, CLEC10A, CLEC1B, CLEC4D, CLEC4E, CLEC6A, CLNK, CMTM5, CNR1, CNR2, CORO1A, CORO2A, CR2, CRABP2, CRB2, CREM, CSF1R, CSF2RA, CTLA4, CTSS, CXCL10, CXCL11, CXCL5, CXCL6, CXCL9, CXCR3, CXCR4, CXCR6, CXorf21, CYBB, CYSLTR1, DAPK1, DAPP1, DCC, DEPDC1B, DERL3, DLGAP2, DOCK10, DOCK2, DOCK8, DOK2, DPEP2, DRD1, DTHD1, DUSP2, DUSP8, E2F8, EBI3, EDN2, ELF3, EPHA6, EPX, EREG, ERFE, FAM83B, FASLG, FCGR2C, FCN1, FCRL1, FCRL2, FCRL3, FCRL5, FCRL6, FCRLA, FFAR4, FGD2, FGD3, FGR, FIBIN, FLT3, FOLR2, FOXP3, FRZB, FUT7, FYB1, GABBR2, GADD45B, GALNT3, GDF6, GFI1, GFRA2, GMFG, GMIP, GPR132, GPR141, GPR150, GPR171, GPR174, GPR19, GPR34, GPR4, GPR55, GPR83, GPRC5A, GRAP2, GUCY2D, GZMA, GZMB, H3C10, H3C11, H3C12, H3C7, HASPIN, HCLS1, HCST, HLA-DPA1, HLA-DPB1, HLA-DQA1, HLA-DRA, HLA-DRB1, HLA-DRB5, HORMAD1, HSH2D, ICOS, IER3, IFNG, IGLL5, IKBKE, IL10RA, IL12RB1, IL12RB2, IL16, IL18, IL18RAP, IL21R, IL22, IL26, IL2RB, IL2RG, IL5RA, IL7R, IL9R, INPP5D, IQCJ-SCHIP1, IQGAP2, IRF4, IRF5, IRF8, ITGA4, ITGAL, ITGAX, ITGB2, ITGB7, ITK, JPH1, JSRP1, KCNN4, KLF5, KLRB1, KLRC1, KLRC2, KLRD1, KLRG1, KLRK1, KNG1, LAG3, LAX1, LCK	[ABCA7, ABL1, ABR, ABRA, ACKR1, ACKR3, ACP4, ACTB, ACTN4, ADCY1, ADCY3, ADCY4, ADCY9, ADGRB2, ADGRE5, ADGRG1, ADGRG6, ADGRL1, ADIPOQ, AGAP2, AGER, AGPAT1, AGT, AGTR1, AKT1S1, ALPK3, AMH, ANGPT1, ANGPTL1, ANGPTL8, AP2A1, APLN, APLNR, APLP1, APOA1, APOD, ARAF, ARAP3, ARC, AREG, ARF1, ARHGAP1, ARHGAP23, ARHGAP39, ARHGDIA, ARHGEF1, ARHGEF16, ARHGEF5, ARID1A, ARRDC3, ARTN, ASB16, ASB6, ASIC3, ASPN, ATP6V0D1, ATP6V0E2, ATP6V1B1, ATP6V1F, BAG3, BAG6, BCAM, BCAR1, BCL2L1, BCL3, BCL6, BCL7B, BCL9L, BCR, BEND6, BMP8A, BRD4, BSN, BTG2, BTLA, C15orf62, C1QTNF1, C20orf27, C5, C5AR1, CABIN1, CALCR, CAMK2B, CAPN1, CARM1, CASKIN1, CC2D1A, CCL11, CCL2, CCL21, CCL24, CCR7, CD14, CD177, CD276, CD74, CDC42EP1, CDC42EP2, CDC42EP4, CDK1, CDK2, CDKL2, CDKN1A, CEACAM1, CELSR2, CERT1, CETP, CFL1, CGA, CHCHD10, CHERP, CHGA, CHGB, CHI3L1, CHRNG, CIITA, CISH, CITED2, CLCF1, CLCN6, CLEC1B, CLEC4E, CLIP3, CLNK, CMTM1, CMTM5, CNKSR1, CNOT3, CNR1, CNTN6, COL1A1, CRAT, CREB3L1, CRHR2, CRYAB, CSF1, CSK, CSNK1E, CSPG4, CTF1, CTHRC1, CX3CL1, CXXC4, CYP26B1, CYP27B1, DAB2IP, DAG1, DAGLA, DAXX, DCHS1, DDR1, DDX39B, DDX54, DEDD, DENND2B, DENND4B, DGKD, DGKZ, DHX34, DIRAS1, DISP2, DISP3, DKK2, DLG5, DLGAP4, DMTN, DNMT1, DOCK6, DOK3, DOK5, DOT1L, DPF2, DTX1, DTX2, DTX3, DUSP5, DUSP8, DVL3, E2F4, E2F7, E2F8, ECE2, ECM1, EDN2, EEF1E1, EFNA3, EGLN2, EIF4EBP1, ELF3, ENG, EPHA2, EPN1, ERBB2, ERFE, ESM1, EXTL1, FADD, FAIM2, FAM110A, FAM83F, FAM83G, FASN, FES, FFAR2, FGFR4, FGFRL1, FIBIN, FLOT2, FLT3, FLT4, FOXC2, FOXF1, FOXM1, FPR1, FRMD8, FURIN, GAB2, GABBR2, GABRR2, GADD45B, GAS1, GATA4, GBA, GBF1, GCSAM, GDF6, GDI1, GFRA3, GIT1, GLIS2, GLRA1, GNAI2, GNAO1, GNB3, GNRHR, GPAM, GPBAR1, GPER1, GPI, GPR137, GPR153, GPR162, GPR17, GPR20, GPR3, GPR34, GPR37L1, GPR4, GPR75, GPR83

GO:0023056	positive regulation of signaling	3,12E-10	[2, 3, 4]	366,00	19,44	40,33	59,67	[ABRA, ADAM8, ALOX15, ALOX15B, ANGPT1, AREG, ATF3, BANK1, BLK, BMF, BMP7, BMPR1B, C1QTNF1, CALCR, CARD11, CASP8, CASS4, CCL17, CCL19, CCL2, CCL22, CCL24, CCL3, CCL3L1, CCL4, CCL4L1, CCL5, CCR2, CD180, CD19, CD226, CD27, CD300LF, CD3E, CD4, CD40LG, CD74, CD80, CD86, CLEC6A, CNR1, CRB2, CSF1R, CXCR4, CXorf21, DEPDC1B, DPEP2, DRD1, ELF3, EPX, EREG, ERFE, FASLG, FCRL3, FFAR4, FGD2, FGR, FLT3, GADD45B, GDF6, GFI1, GPR174, GPR4, GPR55, HCLS1, HCST, HLA-DRB1, ICOS, IKBKE, IL10RA, IL18, IL26, IL7R, IQCJ-SCHIP1, KCNN4, KMO, LCK, LMCD1, LY86, MAP4K1, MMP9, MSX1, MYB, NCF1, NCKAP1L, NFAM1, NPPC, NSG1, OASL, P2RY10, P2RY12, PDE6G, PIK3CG, PIK3R5, PIM2, PRKCB, PROK1, PTK2B, PTPN22, PTPN6, PTPRC, PYCARD, PYHIN1, RAC2, RASGRF1, RTKN2, SALL1, SCIMP, SCUBE1, SEMA4D, SLC24A4, SLC8A3, SOCS1, STAP1, SYK, TESPA1, THBS1, TNFAIP3, TNFSF14, TNR, TRAF1, TRAT1, TREM2, TRIM14, TRPM2, UBD, VAV1, WNT1, WNT10B, XCL1, XCL2, ZAP70, ZBP1]	[ABCA7, ABL1, ABRA, ACKR3, ACTN4, ADCY1, ADGRG1, ADIPOQ, AGAP2, AGER, AGPAT1, AGT, AMH, ANGPT1, APLN, APOA1, ARAF, ARC, AREG, ARHGEF5, BCL2L1, BMP8A, BRD4, C1QTNF1, C20orf27, C2CD2L, C5, C5AR1, CALB1, CALCR, CC2D1A, CCL11, CCL2, CCL21, CCL24, CCR7, CD14, CD74, CHERP, CHGA, CHI3L1, CITED2, CNR1, CNTN6, COL1A1, CRAT, CRHR2, CSF1, CSK, CSNK1E, CSPG4, CX3CL1, CYP27B1, DAB2IP, DAXX, DENND2B, DGKD, DIRAS1, DKK2, DLG5, DMTN, DOC2B, DOK5, DUSP5, DVL3, ECM1, EEF1E1, ELF3, ENG, ERBB2, ERFE, ESM1, FADD, FAM110A, FASN, FGFR4, FLT3, FLT4, FOXM1, FPR1, GAB2, GADD45B, GATA4, GBA, GDF6, GNAI2, GPBAR1, GPER1, GPR137, GPR17, GPR20, GPR37L1, GPR4, GPRIN1, GRIK5, GSK3A, HBEGF, HCRTR1, HFE, HTR2A, ICAM1, IL1B, IL7R, ILK, INCA1, INHA, IRAK2, ITGA5, ITPR3, JMJD8, JUP, KCP, KLK14, KMT2D, KSR1, LGALS9, LGR6, LILRA5, LIMS2, LMCD1, LRG1, LRRC8A, LTBR, LTF, MAP2K3, MAP3K10, MAP3K11, MAP3K14, MAP3K6, MAP3K9, MAPK3, MAPK8IP3, MAVS, MAZ, MECP2, MED12, MEIS3P1, MEN1, MINK1, MLST8, MME, MSTN, MSX1, MUC20, NDC80, NDST1, NECTIN2, NFATC4, NGF, NGFR, NINJ1, NLGN2, NLRC5, NNAT, NOD2, NODAL, NOS3, NOTCH1, NOTCH4, NPPA, NRARP, NRGN, NSG1, OSM, P2RX3, P2RX5, P2RY6, PCDHA4, PDGFB, PDGFRB, PIAS4, PLA2G2A, PLAGL2, PLAUR, PLXNB1, PML, POR, PPARD, PRAG1, PRRT1, PTN, PTP4A3, PTPN23, RAC2, RAP1A, RAPGEF1, RASD2, RASL10B, RELA, RHOG, RNU1-93P, RPL26, RPTOR, RTKN2, S1PR2, SECTM1, SELENOP, SEMA4C, SEPTIN4, SHANK3, SHARPIN, SHC2, SHISA5, SHKBP1, SIK3, SLC2A4, SLC44A2, SLC9A1, SLC9A3R1, SMAD3, SMARCA4, SMARCB1, SORBS3, SPATC1L, SPHK1, SPHK2, SPI1, SRC, STAT3, STX1A, STX1B, TBX5, TGFB1I1, TGM2, THBS1, THPO, TICAM1, TIMP1, TMEM198, TNF, TNFRSF1A, TNFSF12, TRAF4, TRIB3, TRIM62, TRIM8, TRPV4, TSPAN14, UBA52, UBE2B, UNC5B, VEGFA, VSIG2, VTN, WBP2, WDR24, WNK2, WNT3, ZBTB7B, ZC3H12A, ZC3H3, ZCCHC3
GO:0023057	negative regulation of signaling	5,59E-04	[2, 3, 4]	269,00	18,24	33,37	66,63	[AREG, ATF3, BANK1, BCL3, BMP7, BMPR1B, CASP8, CCDC88C, CCL5, CD22, CD300LF, CD3E, CD74, CHRDL1, CISH, CNR2, DCC, DUSP2, DUSP8, EPX, EREG, FASLG, FCRL3, FFAR4, FRZB, GPRC5A, INPP5D, IRF4, LAX1, LIF, LILRB4, MIDN, MMP9, NLRC3, NLRP3, NLRP6, NR4A2, NTN1, PIK3IP1, PLEK, PRKCB, PRKCQ, PSCA, PTK2B, PTPN22, PTPN6, PTPRC, PYCARD, RASAL3, RBPMS2, RGS1, RGS10, RGS18, RGS9, RHOH, RTKN2, SH2D1A, SLA2, SLC24A4, SMPDL3B, SOCS1, SOCS3, SPINK1, STAP1, STAT1, TBC1D10C, THBS1, THY1, TLR6, TNFAIP3, TNIP3, TNMD, TNR, TRABD2A, TREM2, UBASH3A, UCP2, VNN1, VWC2L, WNT1]	[ABCA7, ABCC8, ABL1, ACKR3, ACP4, ADIPOQ, AGAP2, AGER, AGT, AKT1S1, AMH, APLN, APLNR, APLP1, APOA1, APOD, ARC, AREG, ARF1, ARRDC3, ASPN, BCL2L1, BCL3, BCL6, BCL9L, BEND6, BMP8A, CD14, CD74, CEACAM1, CHGA, CISH, CREB3L1, CSK, CTHRC1, CX3CL1, CXXC4, CYP26B1, DAB2IP, DAG1, DDX39B, DGKD, DGKZ, DKK2, DLG5, DUSP5, DUSP8, DVL3, ECM1, EPHA2, EPN1, ERBB2, FADD, FAIM2, FASN, FOXM1, FRMD8, GATA4, GBA, GJD3, GLIS2, GLRA1, GNAI2, GPER1, GPI, GPR37L1, GPRC5A, GRB7, GRIK5, GRINA, GSK3A, HBEGF, HDAC7, HEYL, HGS, HSPB1, HTR1B, HTR2A, ICAM1, IFT80, IL1B, IL1RN, INHA, INPP5E, ITGA3, ITPRIP, KIF26A, KLK14, LIF, LMNA, LTF, LZTS2, MAP2K3, MAPK7, MAZ, MED12, MEGF8, MEIS3P1, MEN1, MGRN1, MIDN, MNT, MSTN, MYADM, MYC, NCOR2, NF2, NFATC4, NGFR, NKD2, NLRC5, NOD2, NODAL, NOS3, NOTCH1, NOTCH3, NPPA, NPRL3, NR1D1, NR4A2, NRARP, NTN1, OMA1, PALM, PCDHA6, PCGF2, PER1, PIAS4, PLAUR, PPP1R10, PPP1R15A, PPP2R1A, PPP2R5B, PRDM16, PRKACA, PRRT1, PTGIR, PTPRU, RAB11FIP5, RAP1A, RBPMS2, RELA, RGS16, RGS18, RNF126, RNF157, RNU1-93P, RTKN2, S1PR2, SERPINE1, SH2B3, SH3BP1, SHANK3, SLC25A23, SLC30A1, SLC35C1, SLC35F6, SLC9A3R1, SMAD3, SMARCA4, SMPD1, SNAI1, SOCS3, SORCS2, SOX10, SOX13, SPI1, SPINK1, SRC, SREBF1, SRF, SYNGAP1, SYVN1, SZT2, TGFB1I1, THBS1, TICAM1, TIMP1, TLE2, TLE3, TLE5, TMEM127, TMEM14A, TMEM161A, TMEM88, TNF, TNFAIP1, TNIP1, TNMD, TNS2, TRIB1, TRIB3, TSC2, TSKU, UBA52, UBE2B, UNC5B, VASN, VNN1, VSIG2, WFS1, WNK2, WWP2, ZBTB7A, ZC3H12A, ZNF385A, ZNF653]

GO:0044093	positive regulation of molecular function	1,91E-07	[3]	325,00	18,99	40,88	59,12	[ABRA, ADAM8, ADCY7, AIM2, ANGPT1, ARAP2, AREG, ARHGAP45, ATP2A3, BTK, CARD11, CASP8, CASS4, CCL17, CCL19, CCL2, CCL22, CCL24, CCL3, CCL3L1, CCL4, CCL4L1, CCL5, CCR2, CD19, CD33, CD4, CD40LG, CD74, CD86, CDC20B, CDKN2A, CSF1R, CTSS, CXCR4, DAPK1, DOCK10, DOCK8, DPEP2, EDN2, EPHA6, EPX, EREG, FASLG, FCRL3, FGD2, FGR, FLT3, FOSL1, GADD45B, GBA3, GMIP, GPR55, GPRC5A, HCLS1, HLA-DRB1, IFNG, IKBKE, IL18, IL18RAP, IRF4, ITGB2, ITK, LCK, LCP2, LPAR2, LRRC38, MAP4K1, MID1IP1, MMP9, MUSK, NCF1, NCKAP1L, NEK5, NFAM1, NLRP2, NLRP3, NLRP6, NMUR1, NR4A2, P2RY12, PDE6G, PIK3CG, PIK3R5, PLAAT4, PLCB2, PLEK, PRKCB, PRKCQ, PROK1, PTAFR, PTK2B, PTPRC, PYCARD, PYHIN1, RASGRF1, RGS1, RGS10, RGS9, RTKN2, S1PR4, SELE, SEMA4D, SH2D1A, SOCS1, STAP1, SYK, TBC1D10C, THBS1, THY1, TLR6, TRAF1, TREM2, TRIM14, VAV1, WNT1, WNT10B, XCL1, XCL2]	[ABCC8, ABL1, ABR, ABRA, ACTN4, ADCY1, ADCY3, ADCY4, ADCY9, ADIPOQ, AGAP2, AGER, AGT, AGTR1, AMH, ANGPT1, APOA1, ARAF, ARAP3, ARC, AREG, ARHGEF15, ARHGEF16, ARHGEF5, ARTN, ATP1B2, ATPSCKMT, BCR, C5, C5AR1, CCL11, CCL2, CCL21, CCL24, CCR7, CD74, CDC25B, CDKN1A, CHI3L1, CRAT, CRTC2, CSF1, CSK, CSPG4, CTHRC1, CTSD, CX3CL1, CYP27B1, DAB2IP, DAG1, DAXX, DBF4B, DDR1, DGKZ, DIRAS1, DUSP5, DVL3, EDN2, EFNA3, EIF4G1, ELMOD1, EPHA2, ERBB2, FADD, FASN, FGFR4, FLOT2, FLT3, FLT4, FOSL1, FPR1, GADD45B, GMNN, GNAI2, GNAO1, GPER1, GPRC5A, GPRIN1, GRN, GSK3A, HBEGF, HDAC5, HFE, HMGN1, HSF1, HSPE1, HTR2A, ICAM1, IL1B, ILK, INCENP, IRAK2, JUP, KCNIP2, KSR1, LGALS9, LILRA5, LPAR5, LRRC52, LTF, MAP2K3, MAP3K10, MAP3K11, MAP3K14, MAP3K6, MAP3K9, MAPK3, MAPK8IP3, MARK2, MAVS, MED25, MEN1, MID1IP1, MINK1, MLST8, MTSS2, MUC20, MYC, NAPA, NEK5, NFKB2, NFKBIB, NGF, NGFR, NOD2, NODAL, NOS3, NPPA, NPR3, NPRL3, NR4A2, OMA1, ORAI1, P2RY6, PAK4, PCOLCE, PDGFB, PDGFRB, PFN1, PIDD1, PKD1, PLAUR, PLXNB1, PML, POR, PPP1R15A, PPRC1, PRKACA, PRKAR2B, PYGO2, RAMP2, RANGAP1, RAP1A, RAP1GAP2, RAPGEF1, RARA, RCVRN, RELA, RGCC, RGL3, RGMA, RGS16, RHOG, RMRP, RNU1-93P, RPLP1, RPTOR, RTKN2, RXRA, S100A1, S1PR2, SELE, SERINC2, SH3BP1, SHANK3, SHC2, SIPA1L3, SLC25A23, SLC6A9, SLC9A1, SMAD3, SMARCA4, SMARCB1, SOX7, SPATC1L, SPHK1, SRC, SRF, STAC2, STAT3, STING1, SYDE1, SYNGAP1, TBC1D10B, TBC1D13, TBC1D17, TBC1D2, TBC1D22B, TBC1D25, TCF3, TCF7L1, TGM2, THBS1, TICAM1, TIMP1, TNF, TNIP1, TRAF4, TRIB3, TRIM62, TRIM8, TSC2, UBA52, VAC14, VDR, VEGFA, VSIG2, VSIR, VTN, WNK2, WNT9B, XRCC1, ZBTB7A]
GO:0048522	positive regulation of cellular process	9,17E-14	[2, 3, 4]	993,00	16,77	38,30	61,70	[ABRA, ADAM8, ADCY7, AIF1, AIM2, AKNA, ALOX15, ALOX15B, ANGPT1, APBB1IP, AREG, ASCL2, ATF3, ATP1B4, ATP2A3, BANK1, BATF, BCL11B, BCL3, BHLHA15, BLK, BMF, BMP7, BMPR1B, BRIP1, BTK, BTLA, C1QA, C1QTNF1, C2, CACNA1I, CALCR, CAMK4, CARD11, CARMIL2, CASP8, CASS4, CCDC88B, CCL17, CCL19, CCL2, CCL22, CCL24, CCL3, CCL3L1, CCL4, CCL4L1, CCL5, CCR2, CCR3, CD177, CD180, CD19, CD1B, CD1C, CD1E, CD2, CD226, CD244, CD247, CD27, CD300LF, CD33, CD3E, CD4, CD40LG, CD5, CD53, CD6, CD74, CD80, CD86, CDC20B, CDCA2, CDH1, CDH4, CDKN2A, CEL, CLCF1, CLEC4D, CLEC6A, CNR1, CORO1A, CRABP2, CRB2, CREM, CRTAM, CSF1R, CST7, CTLA4, CTSS, CXCL10, CXCL11, CXCL5, CXCL9, CXCR4, CXorf21, DAPK1, DEPDC1B, DOCK2, DOCK8, DPEP2, DRD1, E2F8, EAF2, EBI3, EDN2, EGR3, ELAVL4, ELF3, EOMES, EPHA6, EPX, EREG, ERFE, EVI2B, FASLG, FCRL3, FFAR4, FGD2, FGD3, FGR, FLT3, FOLR2, FOSB, FOSL1, FOXP3, FUT7, GABBR2, GADD45B, GBP5, GDF6, GFI1, GLIS1, GMFG, GPM6A, GPR174, GPR4, GPR55, GPRC5A, GPSM3, GRAP2, GZMA, GZMB, H1-3, HCLS1, HCST, HLA-DMB, HLA-DPA1, HLA-DPB1, HLA-DRA, HLA-DRB1, HOPX, ICOS, IFNG, IGLL5, IKBKE, IKZF3, IL10RA, IL12RB1, IL12RB2, IL18, IL18RAP, IL26, IL2RB, IL2RG, IL5RA, IL7R, INPP5D, IQCJ-SCHIP1, IQGAP2, IRF4, IRF5, IRF8, IRX3, ITGA4, ITGAX, ITGB2, JAML, KCNJ10, KCNN4, KIAA1324, KLF5, KLRC2, KLRD1, KLRG1, KLRK1, KMO, KNG1, LAG3, LCK, LCP1, LCP2, LDLR, LEF1, LIF, LILRB1, LILRB4, LMCD1, LMO1, LPAL2, LRRC38, LTA, LY86, MACC1, MAFF, MAP4K1, MATK, MEDAG, MID1IP1, MIXL1, MLC1, MMP9, MSX1, MUSK, MYB, MYBL2, MZB1, NAMPT, NCF1, NCKAP1L, NCR3, NEK5, NELL2, NFAM1, NLRP2, NLRP3, NLRP6, NPPC, NR4A1, NR4A2, NR4A3, NSG1, NTN1, OASL, P2RY10, P2RY12, PATL2, PAX5, PBX4, PDE6G, PFKFB4, PIK3CG, PIK3R5, PIM1, PIM2, PLA2G7, PLAAT4, PLAC8, PLEK, PLXNC1, POU2AF1, POU2F2, PRF1, PRKCB, PRKCQ, PROK1, PTAFR, PTGER, PTK2B, PTPN22	[ABCA3, ABCA7, ABCC8, ABCD1, ABCD2, ABI3, ABL1, ABLIM3, ABR, ABRA, ACACB, ACKR3, ACTN1, ACTN4, ADAM19, ADAMTSL4, ADCK1, ADCY1, ADCY3, ADCY4, ADCY9, ADGRG1, ADGRL1, ADIPOQ, AGAP2, AGER, AGPAT1, AGT, AGTR1, ALPK3, AMH, ANGPT1, AP2A1, AP3D1, APLN, APLNR, APOA1, ARAF, ARC, AREG, ARF1, ARHGDIA, ARHGEF1, ARHGEF15, ARHGEF16, ARHGEF5, ARID1A, ARTN, ATAT1, ATF5, ATP13A2, ATP1B2, ATP1B4, ATPSCKMT, BAG3, BAG6, BCAR1, BCL2L1, BCL3, BCL6, BCL9L, BEND6, BMP8A, BRD4, BRPF3, BTLA, C15orf62, C1QTNF1, C20orf27, C2CD2L, C5, C5AR1, CACNA1H, CALB1, CALCR, CAMK2B, CAMTA2, CAPN1, CAPNS1, CARM1, CARMIL2, CASZ1, CC2D1A, CCL11, CCL2, CCL21, CCL24, CCR7, CD14, CD177, CD1C, CD1E, CD276, CD74, CDC25B, CDC42EP1, CDC42EP2, CDC42EP4, CDCA2, CDH4, CDK1, CDK2, CDKN1A, CEACAM1, CEL, CGA, CHCHD10, CHERP, CHGA, CHI3L1, CIITA, CITED2, CITED4, CIZ1, CLCF1, CLIP3, CNOT3, CNR1, CNTN6, COL1A1, CPNE5, CPNE6, CRAT, CREB3L1, CRHR2, CRIP2, CRP, CRTC2, CSF1, CSK, CSNK1E, CSPG4, CSRNP3, CST7, CTC1, CTF1, CTHRC1, CTIF, CTSD, CX3CL1, CYP26B1, CYP27B1, DAB2IP, DAG1, DAXX, DBF4B, DDR1, DDX39B, DENND2B, DGKD, DGKZ, DHX34, DIRAS1, DISP3, DKK2, DLG5, DMTN, DNMT1, DOC2B, DOK5, DPAGT1, DPF2, DTX1, DUSP5, DVL3, DXO, DYRK1B, E2F4, E2F7, E2F8, EAF2, ECM1, ECM2, EDC4, EDN2, EEF1E1, EFEMP2, EFNA3, EHD1, EHD2, EIF4EBP1, EIF4G1, ELF3, ELF4, ELK1, ENDOG, ENG, EPHA2, ERBB2, ERFE, ESM1, ESPL1, ETS2, ETV6, FADD, FAM110A, FAP, FASN, FES, FGFR4, FHL5, FHOD1, FLOT2, FLT3, FLT4, FOSL1, FOSL2, FOXC2, FOXF1, FOXM1, FPR1, FURIN, GAB2, GABBR2, GADD45B, GATA2, GATA4, GBA, GDF6, GDI1, GIT1, GLIS2, GLMP, GNAI2, GNAO1, GPAM, GPBAR1, GPER1, GPI, GPM6A, GPR137, GPR17, GPR20, GPR37L1, GPR4, GPRC5A, GPRIN1, GPT, GRB7, GRIK5, GRN, GSK3A, GTPBP1, H1-9P, H4C3, HAVCR1, HBA1, HBB, HBEGF, HCFC1, HCRTR1, HDAC10, HDAC5, HDAC7, HEYL, HFE

GO:0048523	negative regulation of cellular process	5,39E-08	[2, 3, 4]	842,00	16,40	38,68	61,32	[ACP5, ADAM8, ADAMDEC1, ADAMTS9, ADORA3, AIF1, AKNA, ALOX15B, ANGPT1, ANGPT2, ANO9, APOBEC3A, APOBEC3D, APOBEC3G, APOBEC3H, AREG, ASCL2, ATF3, BANK1, BCL11B, BCL3, BHLHA15, BHLHE40, BLK, BMF, BMP7, BMPR1B, BRIP1, BTG2, BTK, C1QC, C1QTNF1, CARD17, CARMIL2, CASP8, CBARP, CCDC88C, CCL17, CCL19, CCL2, CCL3, CCL3L1, CCL5, CCR2, CCR5, CD19, CD200R1, CD22, CD27, CD300LF, CD33, CD3E, CD40LG, CD74, CD80, CD84, CD86, CDH1, CDKN2A, CEL, CHRDL1, CISH, CLCF1, CLNK, CMTM5, CNN1, CNR1, CNR2, CORO1A, CRB2, CREM, CRTAM, CST7, CTLA4, CXCL10, CXCL11, CXCL9, DAPK1, DCC, DEPDC1B, DERL3, DOCK8, DPEP1, DPEP2, DPT, DRD1, DUSP2, DUSP8, E2F8, EAF2, EGR3, ELAVL4, ELF3, EOMES, EPX, EREG, ERFE, EVI2B, FAM122C, FASLG, FATE1, FCMR, FCN3, FCRL3, FFAR4, FGL2, FGR, FOSB, FOSL1, FOXP3, FRZB, GADD45B, GFI1, GFRA2, GLIS1, GMFG, GPR132, GPR171, GPR55, GPRC5A, GZMA, GZMB, H1-3, H2AC13, H2AC14, H2AC16, H3C10, H3C11, H3C12, H3C7, HCLS1, HLA-DRB1, HOPX, HORMAD1, IER3, IFNG, IKBKE, IKZF1, IL10RA, IL18, IL26, IL7R, INPP5D, IQCJ-SCHIP1, IRF4, IRF8, IRX3, ITGB2, JPH1, JSRP1, KCNN4, KIR3DL2, KLF5, KLRC1, KLRD1, KLRK1, KNG1, LAG3, LAMP3, LAX1, LCK, LCP1, LDLR, LEF1, LIF, LILRB1, LILRB4, LMCD1, LMO1, LPAL2, LRRC17, LST1, LTA, MCOLN2, MCOLN3, MID1IP1, MIDN, MIXL1, MMP9, MPO, MSX1, MYB, NAMPT, NCKAP1L, NLRC3, NLRP3, NLRP6, NLRP7, NPPB, NPPC, NR4A1, NR4A2, NR4A3, NTN1, NUGGC, P2RY12, PAPLN, PARP15, PATL2, PAX5, PIK3CG, PIK3IP1, PIM1, PIM2, PLA2G2D, PLAAT4, PLAC8, PLEK, PLXNC1, PRDM1, PRKCB, PRKCQ, PSCA, PTGFR, PTK2B, PTPN22, PTPN6, PTPRC, PTPRH, PTX3, PYCARD, RAD9B, RARRES1, RASAL3, RBPMS2, RGS1, RGS10, RGS18, RGS9, RHOH, RMI2, RNASE6, RRAD, RRM2, RTKN2, RTN1, RUNX3, SALL1, SAMSN1, SCML4, SEMA4A, SEMA4D, SERPINA1, SH2D1A, SIRPG, SLA2, SLAMF8, SLC18A1, SLC24A4, SLC8A3, SMPDL3B, SOCS1, SOCS3, SPINK1	[ABCA7, ABCC8, ABCD1, ABCD2, ABI3, ABL1, ACACB, ACKR3, ACP4, ACTN1, ACTN4, ADAMTS7, ADAMTS8, ADAMTS9, ADCK1, ADGRG1, ADIPOQ, AGAP2, AGER, AGT, AKT1S1, ALPK3, AMH, ANGPT1, ANGPTL4, ANKRD13B, ANKRD52, APLN, APLNR, APLP1, APOA1, APOBEC3C, APOD, APOM, ARAF, ARC, AREG, ARF1, ARHGAP1, ARHGDIA, ARHGEF15, ARID5A, ARRDC3, ARTN, ASPN, ATF5, ATN1, ATP13A2, ATP1A1, ATP1A3, ATP1B2, BAG3, BAG6, BAHD1, BAZ2A, BBS12, BCKDK, BCL2L1, BCL3, BCL6, BCL9L, BCOR, BEND6, BHLHE40, BMP8A, BTG2, C11orf65, C18orf54, C1QTNF1, C5, C5AR1, CABIN1, CAMSAP3, CARM1, CARMIL2, CBARP, CC2D1A, CCL11, CCL2, CCL21, CCR7, CD14, CD74, CDK1, CDK2, CDK2AP2, CDKN1A, CEACAM1, CEL, CETP, CFH, CFL1, CHERP, CHGA, CIC, CIITA, CISH, CITED2, CLCF1, CLIP3, CLNK, CMTM5, CNN1, CNOT3, CNR1, COL1A1, CPTP, CRAT, CREB3L1, CRP, CRYAB, CSF1, CSK, CST7, CTC1, CTDSP1, CTHRC1, CTSA, CX3CL1, CXXC4, CYP26B1, CYP27B1, CYP2D6, DAB2IP, DAG1, DAXX, DBNDD1, DDR1, DDX39B, DDX54, DEDD, DGKD, DGKZ, DGUOK, DHRS2, DHX34, DISP3, DKK2, DLG5, DMTN, DNMT1, DOT1L, DPF2, DPT, DTX1, DUSP5, DUSP8, DVL3, DYSF, E2F4, E2F7, E2F8, EAF2, ECM1, EDC4, EEF1E1, EFEMP2, EIF4EBP1, EIF4G1, ELF3, ENG, ENPP3, EPHA2, EPN1, EPPK1, ERBB2, ERF, ERFE, ESPL1, ETS2, ETV6, F8A1, FABP4, FADD, FAIM2, FAM122C, FAP, FASN, FCN3, FGFRL1, FLII, FLT4, FOSL1, FOXC2, FOXF1, FOXM1, FOXP4, FOXS1, FRMD8, FURIN, FXYD5, GADD45B, GAS1, GATA2, GATA4, GATAD2A, GBA, GCSAM, GDI1, GGA3, GIT1, GJD3, GLIS2, GLRA1, GMNN, GNAI2, GPAM, GPER1, GPI, GPR137, GPR37L1, GPRC5A, GRB7, GRIK5, GRINA, GRN, GSK3A, GTF2IRD1, H1-9P, H3C10, H3C12, H4C3, HBEGF, HCFC1, HDAC10, HDAC5, HDAC7, HESX1, HEYL, HFE, HGS, HIRA, HMGA1, HMGN5, HOPX, HSF1, HSPB1, HTR1B, HTR2A, HYAL1, HYPK, ICAM1, ID3, IER3, IFITM1, IFT80, IGF2BP2, IL1B, IL1RN, IL4R, IL7R, ILK, INCA1, INHA, INPP5E, INPP5J, INPPL1, IRF2, IRF2BPL, IRX3, ITGA3
GO:0048584	positive regulation of response to stimulus	3,89E-17	[2, 3, 4]	491,00	19,87	48,73	51,27	[ABRA, ADAM8, AIF1, AIM2, ALOX15, ALOX15B, ANGPT1, AREG, ATF3, BANK1, BLK, BLNK, BMF, BMP7, BMPR1B, BTK, BTN3A1, BTN3A2, C1QA, C1QB, C1QC, C1QTNF1, C2, C8G, CALCR, CARD11, CASP8, CASS4, CCL17, CCL19, CCL2, CCL22, CCL24, CCL3, CCL3L1, CCL4, CCL4L1, CCL5, CCR2, CCR4, CD177, CD180, CD19, CD1B, CD1C, CD1E, CD22, CD226, CD247, CD27, CD300LF, CD3D, CD3E, CD3G, CD4, CD40LG, CD74, CD79A, CD80, CD86, CDKN2A, CFP, CLCF1, CLEC10A, CLEC4D, CLEC4E, CLEC6A, CLNK, CNR1, CR2, CRB2, CRTAM, CSF1R, CTLA4, CXCL10, CXCR4, CXorf21, CYP11B1, DEPDC1B, DPEP2, EDN2, ELF3, EPX, EREG, ERFE, FASLG, FCN1, FCN3, FCRL3, FFAR4, FGD2, FGR, FLT3, FOXP3, FYB1, GADD45B, GBP5, GDF6, GFI1, GPR174, GPR4, GPR55, GPSM3, GRAP2, HCLS1, HCST, HLA-DMB, HLA-DPA1, HLA-DPB1, HLA-DQA1, HLA-DRA, HLA-DRB1, HLA-DRB5, ICOS, IFNG, IGLL5, IKBKE, IL10RA, IL12RB1, IL16, IL18, IL18RAP, IL26, IL7R, INPP5D, IQCJ-SCHIP1, ITGB2, ITK, KCNN4, KLRC2, KLRD1, KLRK1, LAG3, LAX1, LCK, LCP1, LCP2, LDLR, LILRB1, LILRB4, LMCD1, LTA, LY86, MAP4K1, MAPK13, MMP9, MOG, MS4A1, MSX1, MUC16, MUC19, MUC6, MYB, MYO1G, NCF1, NCKAP1L, NCR3, NFAM1, NLRP3, NPPC, NR4A3, OASL, P2RY10, P2RY12, PAX5, PDE6G, PIK3CG, PIK3R5, PIM2, PLA2G7, PRAM1, PRKCB, PRKCQ, PROK1, PTAFR, PTK2B, PTPN22, PTPN6, PTPRC, PYCARD, PYHIN1, RAC2, RASGRF1, RTKN2, SALL1, SASH3, SCIMP, SCUBE1, SEMA4D, SH2D1A, SIGLEC16, SKAP1, SLA2, SLAMF6, SOCS1, STAP1, SUCNR1, SYK, TBX21, TESPA1, THBS1, THEMIS, THEMIS2, THY1, TLR10, TLR8, TNFAIP3, TNFSF13B, TNFSF14, TRAF1, TRAT1, TREM2, TRIM14, UBASH3A, UBD, VAV1, WAS, WNT1, WNT10B, XCL1, XCL2, ZAP70, ZBP1]	[ABCA7, ABI3, ABL1, ABRA, ACKR3, ACTB, ACTN4, ADGRG1, ADIPOQ, AGAP2, AGER, AGPAT1, AGT, AGTR1, AMH, ANGPT1, APOA1, ARAF, AREG, ARHGEF5, ARTN, BAG6, BCAR1, BCL2L1, BMP8A, BRD4, C1QTNF1, C20orf27, C5, C5AR1, C8G, CALCR, CC2D1A, CCL11, CCL2, CCL21, CCL24, CCR7, CD14, CD177, CD1C, CD1E, CD276, CD74, CEACAM1, CFH, CHERP, CHGA, CHI3L1, CITED2, CLCF1, CLEC4E, CLNK, CNR1, CNTN6, COL1A1, CPN2, CRAT, CRP, CSF1, CSK, CSNK1E, CSPG4, CX3CL1, CYP27B1, DAB2IP, DAXX, DENND2B, DGKD, DGKZ, DIRAS1, DKK2, DLG5, DMTN, DOK5, DUSP5, DVL3, ECM1, EDN2, EEF1E1, ELF3, ENDOG, ENG, ERBB2, ERFE, ESM1, FABP4, FADD, FAM110A, FASN, FCN3, FEM1A, FFAR2, FGFR4, FLT3, FLT4, FOXC2, FOXM1, FPR1, GAB2, GADD45B, GATA2, GATA4, GCSAM, GDF6, GNAI2, GNAO1, GPBAR1, GPER1, GPR137, GPR17, GPR20, GPR37L1, GPR4, GPRIN1, GRN, GSK3A, HBEGF, HCRTR1, HDAC10, HFE, HMGA1, HSPB1, HTR2A, ICAM1, IL1B, IL4R, IL7R, ILK, INCA1, INHA, IRAK2, ITGA5, JMJD8, JUP, KCNK2, KCP, KLK14, KMT2D, KSR1, LAG3, LDLR, LGALS9, LGR6, LILRA5, LIMK1, LIMS2, LMCD1, LRG1, LTBR, LTF, MAP2K3, MAP3K10, MAP3K11, MAP3K14, MAP3K6, MAP3K9, MAPK3, MAPK8IP3, MAVS, MAZ, MED12, MEGF8, MEIS3P1, MEN1, MINK1, MLST8, MSTN, MSX1, MUC19, MUC20, MUC3A, MYC, NCOR2, NDC80, NDST1, NECTIN2, NFATC4, NGF, NGFR, NINJ1, NLGN2, NLRC5, NOD2, NODAL, NOS3, NOTCH1, NOTCH4, NPAS2, NPPA, NR4A3, NRARP, OSM, P2RX3, P2RX5, P2RY6, PCDHA4, PDGFB, PDGFRB, PIAS4, PLA2G2A, PLA2G7, PLAGL2, PLAUR, PLXNB1, PML, POR, PPARD, PPP1R15A, PQBP1, PRAG1, PRKACA, PTN, PTP4A3, PVR, RAC2, RAD51AP1, RAP1A, RAPGEF1, RARA, RASD2, RBM14, RELA, RELB, RGCC, RHBDF2, RHOG, RNU1-93P, RPL26, RPTOR, RTKN2, S1PR2, SECTM1, SELENON, SELENOP, SEMA4C, SEPTIN4, SERPINE1, SERPING1, SGTA, SH2B2, SHANK3, SHARPIN, SHC2, SHISA5, SHKBP1, SIK3, SLC2A4, SLC44A2, SLC7A8, SLC9A1, SLC9A3R1, SMAD3, SMARCA4,

GO:0048585	negative regulation of response to stimulus	4,01E-07	[2, 3, 4]	356,00	18,56	39,96	60,04	[ACP5, ADCY7, AIF1, ALOX15, ANGPT2, AOAH, AREG, ATF3, BANK1, BCL3, BMP7, BMPR1B, C1QTNF1, CALCR, CASP8, CCDC88C, CCL2, CCL5, CCR2, CD200R1, CD22, CD247, CD300LF, CD3E, CD3G, CD74, CD84, CD96, CEL, CHRDL1, CISH, CNR2, CR2, CST7, CTLA4, CYSLTR1, DCC, DPEP1, DRD1, DUSP2, DUSP8, EPX, EREG, FASLG, FCRL3, FFAR4, FGL2, FGR, FOXP3, FRZB, GPR150, GPR83, GPRC5A, HLA-DRB1, IL7R, INPP5D, IRF4, KLRC1, KLRD1, KLRK1, KNG1, LAX1, LCP1, LDLR, LIF, LILRB1, LILRB4, MMP9, NAMPT, NCKAP1L, NLRC3, NLRP3, NLRP6, NR4A2, NR4A3, NTN1, PIK3IP1, PLAU, PLEK, PRKCB, PRKCQ, PSCA, PTGDR, PTGER2, PTPN22, PTPN6, PTPRC, PYCARD, RASAL3, RBPMS2, RGS1, RGS10, RGS18, RGS9, RHOH, RLN2, RMI2, RTKN2, SAMSN1, SEMA4A, SEMA4D, SH2D1A, SIGLEC10, SLA2, SLAMF8, SMPDL3B, SOCS1, SOCS3, SPINK1, STAP1, STAT1, SYK, TBC1D10C, TBX21, THBS1, THY1, TLR6, TNFAIP3, TNFAIP8L2, TNIP3, TNMD, TNR, TRABD2A, TREM2, TSPAN8, UBASH3A, UCP2, VNN1, VWC2L, WNT1, XCL1]	[ABCA7, ABCC8, ABL1, ACKR3, ACP4, ADCY1, ADCY3, ADCY4, ADCY9, ADIPOQ, AGAP2, AGT, AKT1S1, AMH, APLN, APLNR, APLP1, APOA1, APOD, AREG, ARRDC3, ASPN, BCL2L1, BCL3, BCL6, BCL9L, BEND6, C1QTNF1, C5, CALCR, CCL2, CD14, CD74, CEACAM1, CEL, CGA, CHGA, CISH, CREB3L1, CRHR2, CSK, CST7, CTHRC1, CX3CL1, CXXC4, CYP26B1, DAB2IP, DAG1, DDX39B, DGKD, DGKZ, DKK2, DLG5, DUSP5, DUSP8, DVL3, ECM1, ENPP3, EPHA2, EPN1, EPPK1, ERBB2, FADD, FAIM2, FAM110A, FAP, FASN, FEM1A, FOXF1, FOXM1, FRMD8, FURIN, GATA4, GBA, GIT1, GLIS2, GNAI2, GPBAR1, GPER1, GPI, GPR20, GPR37L1, GPR83, GPR84, GPRC5A, GRB7, GRINA, GRN, GSK3A, HBEGF, HDAC7, HEYL, HFE, HGS, HRH2, HSF1, HSPB1, HTR1B, ICAM1, IFT80, IL1B, IL1RN, IL4R, IL7R, INPP5E, ITGA3, ITPRIP, JAK3, KIF26A, KLK14, KNG1, LDLR, LGALS9, LIF, LMNA, LTF, LZTS2, MAP2K3, MAPK7, MAZ, MED12, MEGF8, MEIS3P1, MEN1, MGRN1, MNT, MSTN, MYADM, MYC, MYH9, NCOR2, NF2, NFATC4, NGFR, NKD2, NLRC5, NOD2, NODAL, NOS3, NOTCH1, NOTCH3, NPPA, NPRL3, NR1D1, NR4A2, NR4A3, NRARP, NTN1, OMA1, PALM, PCDHA6, PCGF2, PDCD1, PDGFB, PER1, PIAS4, PLAU, PLAUR, PLXNA3, PPARD, PPP1R10, PPP1R15A, PPP2R1A, PPP2R5B, PRDM16, PRKACA, PRKAR2B, PTGIR, PTPRU, RADX, RAMP2, RBPMS2, RELA, RGMA, RGS16, RGS18, RHBDF2, RMI2, RNF126, RNF157, RNF26, RNU1-93P, RTKN2, RTN4RL1, S1PR2, SEMA3F, SEMA4B, SEMA4C, SEMA6B, SEMA6C, SERPINE1, SERPING1, SGTA, SH2B3, SH3BP1, SHARPIN, SLC25A23, SLC35C1, SLC35F6, SLC9A3R1, SMAD3, SMARCA4, SMPD1, SNAI1, SOCS3, SOX10, SOX13, SPI1, SPINK1, SRC, SYNGAP1, SYVN1, SZT2, TGFB1I1, THBS1, TICAM1, TIMP1, TLE2, TLE3, TLE5, TMEM127, TMEM14A, TMEM161A, TMEM88, TNF, TNFAIP1, TNFRSF1A, TNIP1, TNMD, TNS2, TRIB1, TRIB3, TSC2, TSKU, TSPAN8, UBA52, UBE2B, UBQLN4, UNC5B, VASN, VIPR2, VNN1, VSIG2, VTN, WFS1, WNK2, WNT3, WWP2, XRCC1, ZBTB7A, ZBTB7B, ZC3H12A, ZFP36
GO:0050790	regulation of catalytic activity	1,71E-05	[3]	446,00	17,37	42,64	57,36	[ACAP1, ADAM8, ADCY7, ADORA3, AIM2, ANGPT1, ARAP2, AREG, ARHGAP15, ARHGAP30, ARHGAP45, ATP2A3, BMP7, CALCR, CARD17, CASP8, CASS4, CCDC88C, CCL17, CCL19, CCL2, CCL22, CCL24, CCL3, CCL3L1, CCL4, CCL4L1, CCL5, CCR2, CD19, CD27, CD33, CD4, CD40LG, CD74, CD86, CDC20B, CDKN2A, CISH, CNR1, CNR2, CRB2, CSF1R, CST7, CXCL10, CXCR4, DAPK1, DENND1C, DENND2D, DEPDC1B, DOCK10, DOCK2, DOCK8, DPEP1, DRD1, DUSP2, DUSP8, EDN2, EPHA6, EPX, EREG, FAM122C, FASLG, FCRL3, FGD2, FGD3, FGR, FLT3, GABBR2, GADD45B, GBA3, GFI1, GMFG, GMIP, GPR55, GPRC5A, GPSM3, GZMA, HLA-DRB1, IFNG, IKBKE, IL18, IQGAP2, ITGB2, ITK, KLRK1, KNG1, LAMP3, LAX1, LCK, LCP1, LCP2, LILRB4, LPAL2, LPAR2, MAP4K1, MCF2L2, MID1IP1, MIDN, MMP9, MUSK, NCF1, NCKAP1L, NEK5, NLRP2, NLRP3, NLRP6, NLRP7, NMUR1, NPFFR2, NR4A1, NR4A2, OASL, P2RY12, P2RY13, PAPLN, PDE6G, PIK3CG, PIK3IP1, PIK3R5, PLAAT4, PLCB2, PLEK, PLXNC1, PPP1R42, PPP2R2B, PRKCQ, PROK1, PSD4, PTAFR, PTK2B, PTPN22, PTPN6, PTPRC, PTPRH, PTX3, PYCARD, RAC2, RARRES1, RASAL3, RASGRF1, RGS1, RGS10, RGS18, RGS9, RHOH, RINL, RLN2, S1PR4, SELE, SEMA4D, SERPINA1, SH2D1A, SLA2, SLAMF8, SLC8A3, SMAP2, SOCS1, SOCS3, SPINK1, STAP1, SYK, TAGAP, TBC1D10C, TFPI2, THBS1, THY1, TLR6, TNFAIP3, TNFSF14, TREM2, VAV1, WAS, XCL1, XCL2]	[ABL1, ABR, ACACB, ACP4, ACTB, ADCY1, ADCY3, ADCY4, ADCY9, ADIPOQ, AGAP2, AGER, AGFG2, AGT, AGTR1, AKT1S1, ALS2CL, ANGPT1, ANGPTL4, ANGPTL8, APOA1, ARAF, ARAP3, AREG, ARHGAP1, ARHGAP23, ARHGAP39, ARHGDIA, ARHGEF1, ARHGEF15, ARHGEF16, ARHGEF5, ARTN, ATP13A2, ATP1B2, ATPSCKMT, BAG3, BCL6, BCR, C5, C5AR1, CABIN1, CALCR, CAPN1, CCL11, CCL2, CCL21, CCL24, CCNG2, CCNJL, CCR7, CD74, CDC25B, CDC42EP2, CDKN1A, CEACAM1, CHI3L1, CISH, CNR1, COX6A2, CPN2, CRYAB, CSF1, CSK, CSPG4, CST7, CTSD, CX3CL1, CYP27B1, DAB2IP, DAG1, DAXX, DBF4B, DBNDD1, DDR1, DENND1C, DENND2B, DENND4B, DGKZ, DIRAS1, DOCK6, DPM2, DUSP5, DUSP8, DVL3, ECM1, EDN2, EFNA3, ELMOD1, EPHA2, ERBB2, FABP4, FADD, FAM122C, FASN, FEM1A, FGFR4, FLT3, FLT4, FPR1, FURIN, GABBR2, GADD45B, GBA, GBF1, GDI1, GIT1, GNAI2, GNAO1, GPER1, GPI, GPRC5A, GPRIN1, GRM2, GRN, GSK3A, HBEGF, HGS, HMGN1, HPS1, HSF1, HSPB1, HSPE1, HTR2A, ICAM1, IL1B, ILK, INCA1, INCENP, IQGAP2, IRAK2, ITPRIP, JMJD8, KATNB1, KNG1, KSR1, LGALS9, LILRA5, LIMK1, LIMK2, LMF1, LTB4R2, LTF, MAP2K3, MAP3K10, MAP3K11, MAP3K14, MAP3K6, MAP3K9, MAPK3, MAPK7, MAPK8IP3, MARK2, MEN1, MID1IP1, MIDN, MINK1, MLLT1, MLST8, MSTN, MTSS2, MUC20, MYC, NEK5, NF2, NGF, NGFR, NLRC5, NOD2, NODAL, NOS3, NOTCH1, NPFFR2, NPPA, NPR3, NPRL3, NR4A1, NR4A2, NUCB1, OBSCN, OMA1, ORAI1, P2RY6, PAK4, PALM, PCDHA4, PCDHA6, PCOLCE, PDGFB, PDGFRB, PFN1, PHACTR1, PIDD1, PISD, PKD1, PLAUR, PLEKHG2, PLXNA1, PLXNA3, PLXNB1, PML, POR, PPP1R10, PPP1R11, PPP1R15A, PPP1R16A, PPP1R9B, PPP2R1A, PPP2R5B, PPP2R5D, PPP6R1, PRKACA, PRKAR2B, PROM2, PTN, PTPRH, PTPRU, PYGO2, RAB3A, RAB3IL1, RAC2, RALGDS, RANGAP1, RANGRF, RAP1A, RAP1GAP2, RAPGEF1, RAPGEFL1, RARRES1, RASGEF1C, RCVRN, RGCC, RGL3, RGL4, RGMA, RGS16, RGS18, RHOG, RIN1, RNU1-93P, RPLP1, RPTOR, RUBCN,

GO:0010646	regulation of cell communication	2,64E-15	[3, 4]	671,00	18,23	37,26	62,74	[ABRA, ADAM8, ALOX15, ALOX15B, ANGPT1, ARAP2, AREG, ARHGAP15, ARHGAP30, ARHGAP45, ATF3, BANK1, BCL2L14, BCL3, BLK, BMF, BMP7, BMPR1B, BRIP1, C1QTNF1, CACNA1E, CALCR, CARD11, CASP8, CASS4, CCDC88C, CCL17, CCL19, CCL2, CCL22, CCL24, CCL3, CCL3L1, CCL4, CCL4L1, CCL5, CCR2, CD177, CD180, CD19, CD22, CD226, CD27, CD300LF, CD3E, CD4, CD40LG, CD74, CD80, CD86, CDH1, CDKN2A, CEL, CHRDL1, CISH, CLEC6A, CNR1, CNR2, CRB2, CSF1R, CXCR4, CXorf21, DAPK1, DCC, DEPDC1B, DLGAP2, DPEP2, DRD1, DUSP2, DUSP8, ELAVL4, ELF3, EPX, EREG, ERFE, FASLG, FCRL3, FFAR4, FGD2, FGD3, FGR, FLT3, FOLR2, FOXP3, FRZB, FUT7, GADD45B, GDF6, GFI1, GMIP, GPR174, GPR4, GPR55, GPRC5A, GUCY2D, H3C10, H3C11, H3C12, H3C7, HCLS1, HCST, HLA-DRB1, ICOS, IER3, IFNG, IKBKE, IL10RA, IL18, IL26, IL7R, INPP5D, IQCJ-SCHIP1, IRF4, IRX3, KCNJ10, KCNN4, KMO, LAX1, LCK, LEF1, LIF, LILRB4, LMCD1, LY86, MAP4K1, MIDN, MMP9, MSX1, MYB, MZB1, NCF1, NCKAP1L, NFAM1, NLRC3, NLRP3, NLRP6, NPFFR2, NPPC, NPTX2, NR4A2, NSG1, NTN1, OASL, P2RY10, P2RY12, PAX5, PDE6G, PIK3CG, PIK3IP1, PIK3R5, PIM2, PLAU, PLEK, PRDM1, PRKCB, PRKCQ, PROK1, PSCA, PSD4, PTK2B, PTPN22, PTPN6, PTPRC, PYCARD, PYHIN1, RAC2, RAD9B, RASAL3, RASGRF1, RBPMS2, RGS1, RGS10, RGS18, RGS9, RHOH, RMI2, RNASE6, RRAD, RTKN2, SALL1, SCIMP, SCUBE1, SEMA4D, SH2D1A, SHISA8, SLA, SLA2, SLC24A4, SLC8A3, SMPDL3B, SOCS1, SOCS3, SPINK1, STAP1, STAT1, SYK, TAGAP, TBC1D10C, TESPA1, THBS1, THY1, TLR6, TMC8, TNFAIP3, TNFSF14, TNIP3, TNMD, TNR, TRABD2A, TRAF1, TRAT1, TREM2, TRIM14, TRPM2, UBASH3A, UBD, UCP2, VAV1, VNN1, VWC2L, WNT1, WNT10B, XCL1, XCL2, ZAP70, ZBP1]	[ABCA7, ABCC8, ABL1, ABR, ABRA, ACKR3, ACP4, ACTN4, ADCY1, ADGRG1, ADIPOQ, AGAP2, AGER, AGPAT1, AGT, AGTR1, AKT1S1, AMH, ANGPT1, APLN, APLNR, APLP1, APOA1, APOD, ARAF, ARAP3, ARC, AREG, ARF1, ARHGAP1, ARHGAP23, ARHGAP39, ARHGDIA, ARHGEF1, ARHGEF16, ARHGEF5, ARRDC3, ARTN, ASPN, BCL2L1, BCL3, BCL6, BCL9L, BCR, BEND6, BMP8A, BRD4, C1QTNF1, C20orf27, C2CD2L, C5, C5AR1, CACNA1E, CALB1, CALCR, CAMK2B, CAPN1, CARM1, CC2D1A, CCL11, CCL2, CCL21, CCL24, CCR7, CD14, CD177, CD74, CDK1, CDK2, CEACAM1, CEL, CGA, CHERP, CHGA, CHI3L1, CISH, CITED2, CLIP3, CNR1, CNTN6, COL1A1, CPLX1, CRAT, CREB3L1, CRHR2, CSF1, CSK, CSNK1E, CSPG4, CTHRC1, CX3CL1, CXXC4, CYP26B1, CYP27B1, DAB2IP, DAG1, DAXX, DDX39B, DENND2B, DENND4B, DGKD, DGKZ, DHX34, DIRAS1, DKK2, DLG5, DLGAP4, DMTN, DOC2B, DOK5, DOT1L, DTX1, DUSP5, DUSP8, DVL3, ECM1, EEF1E1, ELF3, ENG, EPHA2, EPN1, ERBB2, ERFE, ESM1, FADD, FAIM2, FAM110A, FASN, FFAR2, FGFR4, FLOT2, FLT3, FLT4, FOXM1, FPR1, FRMD8, FURIN, GAB2, GADD45B, GAS1, GATA4, GBA, GBF1, GCSAM, GDF6, GIT1, GJD3, GLIS2, GLRA1, GNAI2, GPAM, GPBAR1, GPER1, GPI, GPR137, GPR17, GPR20, GPR37L1, GPR4, GPRC5A, GPRIN1, GRB7, GRIK5, GRINA, GRM2, GSK3A, H3C10, H3C12, H4C3, HBEGF, HCRTR1, HDAC7, HEYL, HFE, HGS, HIPK4, HSPB1, HTR1B, HTR2A, ICAM1, IER3, IFT80, IL18BP, IL1B, IL1RN, IL7R, ILK, INCA1, INHA, INPP5E, IRAK2, IRX3, ITGA3, ITGA5, ITPR3, ITPRIP, JAK3, JMJD8, JPH4, JUP, KCP, KCTD11, KIF26A, KLK14, KMT2D, KSR1, LGALS9, LGR6, LIF, LIFR, LILRA5, LIMS2, LMCD1, LMNA, LOX, LRG1, LRRC4, LRRC8A, LTBR, LTF, LY6E, LYNX1, LZTS1, LZTS2, MAP1A, MAP2K3, MAP3K10, MAP3K11, MAP3K14, MAP3K6, MAP3K9, MAPK3, MAPK7, MAPK8IP3, MAVS, MAZ, MECP2, MED12, MEGF8, MEIS3P1, MEN1, MGRN1, MIDN, MINK1, MLST8, MME, MNT, MSTN, MSX1, MTRNR2L1, MTRNR2L10, MTRNR2L12, MTRNR2L2, MTRNR2L3, MTRNR2L6, MTRNR2L8, MTRNR2L9
GO:0050776	regulation of immune response	2,87E-17	[3, 4]	286,00	23,08	66,69	33,31	[ADAM8, ADCY7, AIM2, ALOX15, APOBEC3G, BLK, BLNK, BTK, BTLA, BTN3A1, BTN3A2, C1QA, C1QB, C1QC, C1QTNF1, C2, C8G, CALCR, CARD11, CASP8, CCL19, CCL5, CCR2, CD177, CD19, CD1B, CD1C, CD1E, CD200R1, CD22, CD226, CD247, CD300LF, CD33, CD3D, CD3E, CD3G, CD4, CD40LG, CD48, CD74, CD79A, CD80, CD84, CD86, CD8A, CD8B, CD96, CFP, CLCF1, CLEC10A, CLEC1B, CLEC4D, CLEC4E, CLEC6A, CLNK, CNR1, CR2, CRTAM, CTLA4, CXCL10, CXCL6, CXorf21, CYP11B1, CYSLTR1, DPEP1, DRD1, EREG, FCGR2C, FCN1, FCN3, FCRL3, FGL2, FGR, FOXP3, FUT7, FYB1, GBP5, GPR150, GPR83, GRAP2, HCST, HLA-DMB, HLA-DPA1, HLA-DPB1, HLA-DQA1, HLA-DRA, HLA-DRB1, HLA-DRB5, IFNG, IGLL5, IKBKE, IL12RB1, IL18, IL18RAP, IL7R, INPP5D, IRF4, ITGA4, ITGAL, ITGB2, ITGB7, ITK, JAML, KCNN4, KIR2DL2, KIR2DL3, KIR3DL2, KLRB1, KLRC1, KLRC2, KLRD1, KLRK1, LAG3, LAIR1, LAIR2, LAX1, LCK, LCP2, LILRA1, LILRB1, LILRB4, LTA, MOG, MS4A1, MUC16, MUC19, MUC6, MYB, MYO1G, NCKAP1L, NCR1, NCR3, NFAM1, NLRC3, NLRP3, NLRP6, NR4A3, PAX5, PILRA, PLA2G2D, PRAM1, PRKCB, PRKCQ, PTAFR, PTGDR, PTGER2, PTPN22, PTPN6, PTPRC, PYCARD, PYHIN1, RAC2, RLN2, SAMSN1, SASH3, SELL, SH2D1A, SIGLEC16, SKAP1, SLA2, SLAMF6, SLAMF7, SLAMF8, SOCS1, SOCS3, SPN, STAP1, STAT1, STXBP2, SYK, TBX21, TESPA1, THEMIS, THEMIS2, THY1, TLR8, TNFAIP3, TNFSF13B, TRAT1, TREM2, UBASH3A, VAV1, WAS, XCL1, ZAP70, ZBP1]	[ABL1, ACTB, ADCY1, ADCY3, ADCY4, ADCY9, AGER, BAG6, BCAR1, BCL6, BTLA, C1QTNF1, C5, C5AR1, C8G, CALCR, CCR7, CD177, CD1C, CD1E, CD276, CD300E, CD74, CEACAM1, CFH, CGA, CHGA, CLC, CLCF1, CLEC1B, CLEC4E, CLNK, CNR1, COL1A1, CPN2, CRHR2, CRP, CSK, DGKZ, ECM1, ENPP3, FADD, FCN3, FES, FFAR2, FOXF1, FPR1, FURIN, GAB2, GATA2, GCSAM, GNAI2, GNAO1, GPBAR1, GPR20, GPR83, GPR84, GRN, HFE, HRH2, ICAM1, IFITM1, IL1B, IL4R, IL7R, JAK3, LAG3, LGALS9, LIMK1, MAPK3, MAVS, MUC19, MUC20, MUC3A, MYH9, NCOR2, NECTIN2, NLRC5, NOD2, NPPA, NR4A3, PCDHA4, PDCD1, PQBP1, PRKACA, PRKAR2B, PTGIR, PVR, RAC2, RAMP2, RAP1A, RARA, RBM14, RELA, RELB, RGCC, RHBDF2, RNF26, RNU1-93P, SERPING1, SH2B2, SIGLEC9, SLC7A8, SMAD3, SOCS3, SPHK2, SPI1, SPNS2, SPPL2B, SRC, STING1, TNF, TRIM62, VIPR2, VSIR, VTN, WAS, ZBTB7B, ZC3H12A, ZCCHC3, ZDHHC11]

GO:0080134	regulation of response to stress	3,32E-08	[3, 4]	319,00	19,33	46,88	53,12	[ACP5, ADAM8, ADCY7, AIM2, AKNA, ALOX15, AOAH, APOBEC3G, ATF3, BMP7, BTK, C1QTNF1, CALCR, CARD11, CASP8, CCL19, CCL24, CCL3, CCL5, CCR2, CD200R1, CD226, CD247, CD27, CD3G, CD74, CD96, CDKN2A, CEL, CLEC10A, CLEC4D, CLEC4E, CLEC6A, CLNK, CNR1, CNR2, CRTAM, CST7, CXCL6, CXCR4, CXorf21, CYSLTR1, DEPDC1B, DPEP1, DRD1, EPX, EREG, FCN1, FFAR4, FGL2, FGR, FOXP3, FUT7, GADD45B, GBP5, GDF6, GPR150, GPR4, GPR83, GPSM3, HLA-DRB1, IER3, IFNG, IKBKE, IL12RB1, IL16, IL18, IL18RAP, IL26, KLRC1, KLRC2, KLRD1, KLRK1, KNG1, LAG3, LCP1, LDLR, LILRB1, LTA, MAPK13, MLC1, MMP9, MSX1, MUC16, MUC19, MUC6, NAMPT, NCF1, NCR1, NCR3, NLRC3, NLRP3, NLRP6, NR4A3, NUP210, PIK3CG, PLA2G2D, PLA2G7, PLAU, PTGDR, PTGER2, PTK2B, PTPN22, PTPN6, PTPRC, PYCARD, PYHIN1, RLN2, RMI2, SCIMP, SELE, SH2D1A, SIGLEC10, SIGLEC16, SLAMF6, SLAMF8, SMPDL3B, SOCS1, SOCS3, STAP1, STAT1, SUCNR1, SYK, THBS1, THY1, TLR10, TLR6, TLR8, TNFAIP3, TNFAIP8L2, TNR, TRAF1, TREM2, TRPM2, TSPAN8, VAV1, VNN1, WAS, WNT1, XCL1, ZBP1]	[AAAS, ABCA7, ABCC8, ABCD1, ABI3, ABL1, ACKR3, ADCY1, ADCY3, ADCY4, ADCY9, ADIPOQ, AGER, AGT, AGTR1, AKT1S1, AMH, APOA1, ARMT1, BAG3, BAG6, BCL2L1, BCL6, BRD4, C1QTNF1, CALCR, CAMK2B, CCL21, CCL24, CCR7, CD74, CEACAM1, CEL, CGA, CHGA, CLEC4E, CLNK, CNR1, CREB3L1, CRHR2, CRYAB, CST7, CX3CL1, DAB2IP, DAGLA, DDX39B, DMTN, EEF1E1, EIF4G1, ENDOG, ENPP3, EPPK1, FABP4, FADD, FAM110A, FAP, FASN, FEM1A, FFAR2, FLT4, FOXC2, FOXF1, FOXM1, FURIN, GADD45B, GATA4, GBA, GDF6, GIT1, GNAI2, GPBAR1, GPER1, GPR20, GPR37L1, GPR4, GPR83, GPR84, GRIK5, GRINA, GRN, HBEGF, HDAC10, HMGA1, HRH2, HSF1, HSPB1, HSPB8, IER3, IL1B, KCNK2, KNG1, LAG3, LDLR, LGALS9, LILRA5, LMNA, LTBR, MAP3K10, MAP3K11, MAPK3, MAPK7, MAPK8IP3, MAVS, MED12, MEN1, MINK1, MLST8, MSX1, MUC19, MUC20, MUC3A, MYC, MYH9, NECTIN2, NINJ1, NLRC5, NOD2, NOS3, NPAS2, NPPA, NR1D1, NR4A3, NUP188, OSM, PCDHA4, PCDHA6, PDGFB, PER1, PIAS4, PLA2G2A, PLA2G7, PLAU, PLAUR, PML, PPARD, PPP1R10, PPP1R15A, PQBP1, PRKACA, PRKAR2B, PTGES, PTGIR, PTN, PVR, RAD51AP1, RADX, RAMP2, RBM14, RELA, RELB, RGMA, RHBDF2, RMI2, RNF26, RNU1-93P, RPL26, RPTOR, RTEL1-TNFRSF6B, RTN4RL1, SBNO2, SELE, SELENON, SEMA4C, SERPINE1, SERPING1, SGTA, SH2B3, SHANK3, SHARPIN, SLC25A23, SMAD3, SNAI1, SOCS3, SPATA2, SPHK1, SPI1, SRC, STING1, SYVN1, TGM2, THBS1, TIMP1, TMEM161A, TMEM259, TNF, TNFRSF1A, TNIP1, TRAF4, TRIM62, TRPV4, TSPAN8, UBQLN4, VEGFA, VIPR2, VNN1, VTN, WAS, WFS1, XRCC1, YPEL3, ZBTB7B, ZC3H12A, ZCCHC3, ZDHHC11, ZFP36, ZNF385A, ZYX]
GO:0006796	phosphate-containing compound metabolic process	6,96E-05	[4]	513,00	16,86	35,66	64,34	[ACP5, ADAM8, ADCY7, AIF1, ALOX15, ALOX15B, AMPD1, ANGPT1, AREG, ATF3, BANK1, BLK, BMP7, BMPR1B, BTK, CALCR, CAMK4, CARD11, CASS4, CCDC88C, CCL19, CCL2, CCL3, CCL5, CD19, CD244, CD33, CD3E, CD4, CD40LG, CD74, CD80, CD86, CDCA2, CDKN2A, CERKL, CISH, CLCF1, CPNE7, CSF1R, CXCL10, CXCR4, DAPK1, DAPP1, DUSP2, DUSP8, EPHA6, EPX, EREG, ETNPPL, FAM122C, FCRL3, FGD2, FGR, FLT3, GADD45B, GDF6, GFPT2, GFRA2, GMFG, GPRC5A, GUCY2D, HASPIN, HCLS1, HCST, HKDC1, HLA-DRB1, IFNG, IKBKE, IL12RB2, IL18, INPP5D, ITGB2, ITK, KMO, LAX1, LCK, LCP1, LCP2, LDLR, LIF, LILRB4, LPAL2, MAP4K1, MAPK13, MATK, MIDN, MMP9, MUSK, MYO3B, NAMPT, NCF1, NCKAP1L, NEK5, NELL2, NLRP3, NLRP6, NPFFR2, NPPB, NPPC, NR4A3, NUP210, P2RY12, PDE6G, PEAK3, PFKFB4, PIK3C2G, PIK3CG, PIK3IP1, PIK3R5, PIM1, PIM2, PLA2G2D, PLA2G7, PLAAT4, PLCB2, PLCH2, PLD4, PLEK, PLPPR4, PPP1R42, PPP2R2B, PRKCB, PRKCQ, PROK1, PTAFR, PTK2B, PTPN22, PTPN6, PTPN7, PTPRC, PTPRH, PYCARD, RAC2, RHOH, RRM2, RUNX3, SAMSN1, SEMA4D, SH2D1A, SLA, SLA2, SLAMF8, SLC8A3, SMPD3, SMPDL3B, SOCS1, SOCS3, SPINK1, STAP1, STK17B, STYK1, SULT2B1, SYK, THBS1, THY1, TLR6, TLR8, TNFAIP3, TNFRSF18, TREM2, WNT1, ZAP70]	[AAAS, AATK, ABCA3, ABCD1, ABHD4, ABL1, ABR, ACACB, ACP2, ACP4, ACSBG1, ACTB, ADCK1, ADCY1, ADCY3, ADCY4, ADCY9, ADIPOQ, AGAP2, AGER, AGPAT1, AGPAT3, AGT, AKT1S1, ALDOA, ALPK3, ALPL, AMH, AMPD2, ANGPT1, ANKK1, APLN, APLNR, APOA1, ARAF, AREG, ARF1, ARHGEF5, ARMT1, ARTN, ATP13A2, ATP1A1, ATP1A3, ATP5F1D, ATP5MC2, ATPSCKMT, BCKDK, BCR, BMP8A, BRD4, C5, C5AR1, CABIN1, CALCR, CAMK2B, CCL11, CCL2, CCL21, CCNG2, CCNJL, CCR7, CD74, CDC25B, CDCA2, CDK1, CDK15, CDK18, CDK2, CDKL2, CDKN1A, CEACAM1, CERT1, CETP, CHI3L1, CIITA, CISH, CKM, CLCF1, CLIP3, CNKSR1, COASY, CPNE6, CSF1, CSK, CSNK1E, CSPG4, CTDSP1, CTF1, CX3CL1, CYP21A2, DAB2IP, DAG1, DAXX, DBF4B, DBNDD1, DCK, DDR1, DGKD, DGKZ, DGUOK, DHX34, DIRAS1, DMTN, DPAGT1, DPM2, DUSP5, DUSP8, DUT, DVL3, DYRK1B, EFNA3, EIF4G1, ELOVL1, ENG, ENPP3, EPHA2, ERBB2, FABP4, FAM122C, FASN, FES, FGFR4, FGFRL1, FITM1, FLT3, FLT4, FPR1, G6PC3, GADD45B, GBA, GDF6, GFPT2, GIT1, GNAI2, GNB3, GPAM, GPAT2, GPER1, GPI, GPRC5A, GSK3A, GTPBP1, HBEGF, HFE, HGS, HIPK4, HK1, HPRT1, HSF1, HSPB1, HSPB8, HTR2A, HUNK, ICAM1, IDO1, IL1B, ILK, IMPDH1, INCA1, INCENP, INHA, INPP5E, INPP5J, INPPL1, IRAK2, ISYNA1, ITGA5, ITPRIP, JAK3, JMJD8, KSR1, LCAT, LDLR, LIF, LILRA5, LIMK1, LIMK2, LIPE, LIPG, LMTK3, LOX, LPCAT4, LTF, MAP2K3, MAP3K10, MAP3K11, MAP3K14, MAP3K6, MAP3K9, MAPK3, MAPK4, MAPK7, MAPK8IP3, MARK2, MARK4, MAST2, MAVS, MBOAT7, MECP2, MED12, MEN1, MFSD2A, MIDN, MINK1, MKNK2, MLLT1, MLST8, MOCS1, MSTN, MTCO2P12, MUC20, MYADM, MYC, MYO3A, NEK5, NF2, NGF, NLRC5, NME4, NOD2, NODAL, NOS3, NPFFR2, NPPA, NPPB, NR4A3, NSMF, NT5DC2, NUDT12, NUP188, OBSCN, OGDH, OMA1, OSM, P2RY6, PAK4, PANK1, PDE4A, PDGFB, PDGFRB, PDK2, PDPR, PDXP, PGAP2, PGAP3, PHACTR1, PI4KA, PI4KAP1, PI4KAP2, PIGO, PIGQ, PIGS, PIGT, PIGZ, PIK3C2B, PIK3C2G, PIM1, PIP5K1C, PISD, PITPNM1

GO:0007166	cell surface receptor signaling pathway	1,32E-23	[3, 4, 5]	662,00	19,60	47,87	52,13	[ADGRE3, ADGRE4P, ADGRG5, ADGRG6, ADORA3, AIM2, ALOX15, ANGPT1, ANGPT2, AREG, ASGR2, ATF3, BATF, BCL2L14, BCL3, BLK, BLNK, BMP7, BMPR1B, BTK, BTLA, BTN3A1, BTN3A2, CALCR, CARD11, CASP8, CASS4, CCDC88C, CCL17, CCL19, CCL2, CCL22, CCL24, CCL3, CCL3L1, CCL4, CCL4L1, CCL5, CCR2, CCR3, CCR4, CCR5, CCR8, CD177, CD180, CD19, CD2, CD200R1, CD22, CD226, CD247, CD27, CD300LF, CD3D, CD3E, CD3G, CD4, CD40LG, CD6, CD7, CD74, CD79A, CD80, CD86, CD8A, CD8B, CHRDL1, CISH, CLCF1, CLEC10A, CLEC1B, CLEC4D, CLEC4E, CLEC6A, CLNK, CORO1A, CR2, CRB2, CSF1R, CSF2RA, CTLA4, CXCL10, CXCL11, CXCL5, CXCL6, CXCL9, CXCR3, CXCR4, CXCR6, CYBB, CYSLTR1, DAPK1, DCC, DEPDC1B, DLGAP2, DOK2, DPEP2, DRD1, EBI3, EDN2, ELF3, EPHA6, EPX, EREG, ERFE, FAM83B, FASLG, FCGR2C, FCN1, FCRL1, FCRL2, FCRL3, FCRL5, FCRL6, FCRLA, FGR, FLT3, FOLR2, FOXP3, FRZB, FUT7, FYB1, GALNT3, GDF6, GFI1, GFRA2, GPR171, GPR34, GPRC5A, GRAP2, GUCY2D, H3C10, H3C11, H3C12, H3C7, HCLS1, HLA-DPA1, HLA-DPB1, HLA-DQA1, HLA-DRA, HLA-DRB1, HLA-DRB5, IFNG, IGLL5, IKBKE, IL10RA, IL12RB1, IL12RB2, IL16, IL18, IL18RAP, IL21R, IL22, IL26, IL2RB, IL2RG, IL5RA, IL7R, IL9R, INPP5D, IRF4, IRF5, IRF8, ITGA4, ITGAL, ITGAX, ITGB2, ITGB7, ITK, KCNN4, KLRB1, KLRC1, KLRC2, KLRD1, KLRG1, LAG3, LAX1, LCK, LCP1, LCP2, LEF1, LIF, LILRA1, LILRB1, LILRB4, LTA, LTB, LY86, LYPD6B, MATK, MMP9, MOG, MS4A1, MSX1, MUC16, MUC19, MUC6, MUSK, MYO1G, MZB1, NAMPT, NCF1, NCKAP1L, NCR3, NELL2, NFAM1, NLRP3, NPPB, NPPC, NPTX2, NR4A2, NR4A3, NTN1, OASL, P2RY10, P2RY12, P2RY13, P2RY2, PAX5, PDE6G, PIM1, PLCB2, PLEK, PLXNC1, PRAM1, PRKCB, PRKCQ, PSTPIP1, PTAFR, PTK2B, PTPN22, PTPN6, PTPRC, PYCARD, RASGRF1, RBPMS2, RGS9, RHEX, SALL1, SCIMP, SCUBE1, SDC1, SEMA4A, SEMA4D, SH2D1A, SH2D2A, SH2D6, SHISA8, SKAP1, SLA, SLA2, SLC8A3, SMPD3, SOCS1, SOCS3, SPN, STAP1, STAT1, STYK1, SYK, TESPA1, THBS1	[ABCA7, ABL1, ACKR1, ACKR3, ACP4, ACTB, ACTN4, ADGRB2, ADGRE5, ADGRG1, ADGRG6, ADGRL1, ADIPOQ, AGAP2, AGER, AGPAT1, AGT, AKT1S1, AMH, ANGPT1, ANGPTL1, AP2A1, APLN, APOA1, APOD, ARC, AREG, ARF1, ARHGDIA, ASPN, ATP6V0D1, ATP6V0E2, ATP6V1B1, ATP6V1F, BAG3, BAG6, BCAR1, BCL2L1, BCL3, BCL6, BCL7B, BCL9L, BCR, BEND6, BMP8A, BTLA, C20orf27, C5, C5AR1, CABIN1, CALCR, CAMK2B, CAPN1, CCL11, CCL2, CCL21, CCL24, CCR7, CD14, CD177, CD276, CD74, CDKN1A, CEACAM1, CELSR2, CFL1, CHRNG, CIITA, CISH, CITED2, CLCF1, CLEC1B, CLEC4E, CLIP3, CLNK, CNKSR1, CNTN6, COL1A1, CREB3L1, CRHR2, CSF1, CSK, CSNK1E, CSPG4, CTF1, CTHRC1, CX3CL1, CXXC4, DAB2IP, DAGLA, DAXX, DDR1, DEDD, DGKD, DGKZ, DISP2, DISP3, DKK2, DLG5, DLGAP4, DMTN, DOK5, DOT1L, DTX1, DTX2, DTX3, DVL3, ECM1, EDN2, EFNA3, ELF3, ENG, EPHA2, EPN1, ERBB2, ERFE, ESM1, FADD, FAM83G, FASN, FES, FFAR2, FGFR4, FGFRL1, FLT3, FLT4, FOXC2, FOXF1, FOXM1, FPR1, FRMD8, FURIN, GAB2, GABRR2, GAS1, GATA4, GCSAM, GDF6, GFRA3, GIT1, GLIS2, GLRA1, GNAI2, GNAO1, GPBAR1, GPER1, GPI, GPR17, GPR34, GPR37L1, GPR75, GPRC5A, GPRIN1, GRB7, GRIK5, GRIP2, GRM2, GSK3A, H3C10, H3C12, HBEGF, HESX1, HEYL, HFE, HGS, HSF1, HSPB1, ICAM1, IFITM1, IFITM2, IFITM3, IFT80, IL15RA, IL18BP, IL1B, IL1RN, IL22RA1, IL32, IL3RA, IL4R, IL5RA, IL7R, ILK, INHA, INPPL1, IQUB, IRAK2, IRF2, IRF9, ITGA3, ITGA5, ITGA7, ITGAD, ITPRIP, JAK3, JUNB, JUP, KCP, LAG3, LAMA5, LGR6, LIF, LIFR, LILRB5, LIMK1, LIMS2, LMNA, LOX, LRG1, LRRC32, LTBR, LTF, LY6E, LYNX1, LZTS2, MAP3K10, MAP3K14, MAPK3, MAPK7, MARK2, MARK4, MAVS, MECP2, MED12, MEGF8, MEN1, MGRN1, MINK1, MKNK2, MOV10, MSN, MSTN, MSX1, MUC19, MUC20, MUC3A, MYC, MYH9, NCOR2, NDP, NDST1, NECTIN2, NEFL, NF2, NFATC4, NGF, NGFR, NKD2, NLGN2, NLRC5, NOD2, NODAL, NOS3, NOTCH1, NOTCH3, NOTCH4, NPPA, NPPB, NR4A2, NR4A3, NRARP, NTN1, NUMBL, OMA1, OSM
GO:0009966	regulation of signal transduction	1,31E-14	[3, 4, 5]	609,00	18,48	37,99	62,01	[ABRA, ADAM8, ALOX15, ALOX15B, ANGPT1, ARAP2, AREG, ARHGAP15, ARHGAP30, ARHGAP45, ATF3, BANK1, BCL2L14, BCL3, BLK, BMF, BMP7, BMPR1B, BRIP1, C1QTNF1, CALCR, CARD11, CASP8, CASS4, CCDC88C, CCL17, CCL19, CCL2, CCL22, CCL24, CCL3, CCL3L1, CCL4, CCL4L1, CCL5, CCR2, CD177, CD180, CD19, CD22, CD226, CD27, CD300LF, CD3E, CD4, CD40LG, CD74, CD80, CD86, CDKN2A, CHRDL1, CISH, CLEC6A, CRB2, CSF1R, CXCR4, CXorf21, DAPK1, DCC, DEPDC1B, DLGAP2, DPEP2, DUSP2, DUSP8, ELF3, EPX, EREG, ERFE, FASLG, FCRL3, FFAR4, FGD2, FGD3, FGR, FLT3, FOLR2, FOXP3, FRZB, FUT7, GADD45B, GDF6, GFI1, GMIP, GPR174, GPR4, GPR55, GPRC5A, GUCY2D, H3C10, H3C11, H3C12, H3C7, HCLS1, HCST, HLA-DRB1, ICOS, IER3, IFNG, IKBKE, IL10RA, IL18, IL26, IL7R, INPP5D, IQCJ-SCHIP1, IRF4, KCNN4, LAX1, LCK, LEF1, LIF, LILRB4, LMCD1, LY86, MAP4K1, MMP9, MSX1, MZB1, NCF1, NCKAP1L, NFAM1, NLRC3, NLRP3, NLRP6, NPFFR2, NPPC, NPTX2, NR4A2, NTN1, OASL, P2RY10, P2RY12, PAX5, PDE6G, PIK3CG, PIK3IP1, PIK3R5, PIM2, PLAU, PLEK, PRDM1, PRKCB, PRKCQ, PROK1, PSCA, PSD4, PTK2B, PTPN22, PTPN6, PTPRC, PYCARD, PYHIN1, RAC2, RAD9B, RASAL3, RASGRF1, RBPMS2, RGS1, RGS10, RGS18, RGS9, RHOH, RMI2, RNASE6, RRAD, RTKN2, SALL1, SCIMP, SCUBE1, SEMA4D, SH2D1A, SHISA8, SLA, SLA2, SLC8A3, SMPDL3B, SOCS1, SOCS3, SPINK1, STAP1, STAT1, SYK, TAGAP, TBC1D10C, TESPA1, THBS1, THY1, TLR6, TMC8, TNFAIP3, TNFSF14, TNIP3, TNMD, TRABD2A, TRAF1, TRAT1, TREM2, TRIM14, UBASH3A, UBD, VAV1, VNN1, VWC2L, WNT1, WNT10B, XCL1, XCL2, ZAP70, ZBP1]	[ABCA7, ABL1, ABR, ABRA, ACKR3, ACP4, ACTN4, ADGRG1, ADIPOQ, AGAP2, AGER, AGPAT1, AGT, AKT1S1, AMH, ANGPT1, APLN, APLNR, APLP1, APOA1, APOD, ARAF, ARAP3, ARC, AREG, ARHGAP1, ARHGAP23, ARHGAP39, ARHGDIA, ARHGEF1, ARHGEF16, ARHGEF5, ARRDC3, ARTN, ASPN, BCL2L1, BCL3, BCL6, BCL9L, BCR, BEND6, BMP8A, BRD4, C1QTNF1, C20orf27, C5, C5AR1, CALCR, CAMK2B, CAPN1, CARM1, CC2D1A, CCL11, CCL2, CCL21, CCL24, CCR7, CD14, CD177, CD74, CDK1, CDK2, CEACAM1, CGA, CHERP, CHGA, CHI3L1, CISH, CITED2, CLIP3, CNTN6, COL1A1, CRAT, CREB3L1, CSF1, CSK, CSNK1E, CSPG4, CTHRC1, CX3CL1, CXXC4, CYP26B1, CYP27B1, DAB2IP, DAG1, DAXX, DDX39B, DENND2B, DENND4B, DGKD, DGKZ, DIRAS1, DKK2, DLG5, DLGAP4, DMTN, DOK5, DOT1L, DTX1, DUSP5, DUSP8, DVL3, ECM1, EEF1E1, ELF3, ENG, EPHA2, EPN1, ERBB2, ERFE, ESM1, FADD, FAIM2, FAM110A, FASN, FGFR4, FLOT2, FLT3, FLT4, FOXM1, FPR1, FRMD8, FURIN, GAB2, GADD45B, GAS1, GATA4, GBA, GBF1, GCSAM, GDF6, GIT1, GLIS2, GNAI2, GPAM, GPBAR1, GPER1, GPI, GPR137, GPR17, GPR20, GPR37L1, GPR4, GPRC5A, GPRIN1, GRB7, GRIK5, GRINA, GRM2, GSK3A, H3C10, H3C12, H4C3, HBEGF, HCRTR1, HDAC7, HEYL, HFE, HGS, HIPK4, HSPB1, HTR1B, HTR2A, ICAM1, IER3, IFT80, IL18BP, IL1B, IL1RN, IL7R, ILK, INCA1, INHA, INPP5E, IRAK2, ITGA3, ITGA5, ITPRIP, JAK3, JMJD8, JUP, KCP, KCTD11, KIF26A, KLK14, KMT2D, KSR1, LGALS9, LGR6, LIF, LIFR, LILRA5, LIMS2, LMCD1, LMNA, LOX, LRG1, LTBR, LTF, LY6E, LYNX1, LZTS2, MAP2K3, MAP3K10, MAP3K11, MAP3K14, MAP3K6, MAP3K9, MAPK3, MAPK7, MAPK8IP3, MAVS, MAZ, MED12, MEGF8, MEIS3P1, MEN1, MGRN1, MINK1, MLST8, MNT, MSTN, MSX1, MTRNR2L1, MTRNR2L10, MTRNR2L12, MTRNR2L2, MTRNR2L3, MTRNR2L6, MTRNR2L8, MTRNR2L9, MUC20, MYADM, MYC, NCLN, NCOR2, NDC80, NDST1, NECTIN2, NEFL, NF2, NFATC4, NGF, NGFR, NINJ1, NKD2, NLGN2, NLRC5, NOD2, NODAL, NOP2, NOS3, NOTCH1, NOTCH3, NOTCH4,

GO:0010604	positive regulation of macromolecule metabolic process	1,05E-06	[3, 4, 5]	618,00	16,88	38,50	61,50	[ABRA, ADAM8, ADCY7, AIF1, AIM2, AKNA, ALOX15B, ANGPT1, AREG, ASCL2, ATF3, ATP1B4, ATP2A3, BANK1, BATF, BCL11B, BCL3, BHLHA15, BLNK, BMP7, BMPR1B, BRIP1, BTN3A1, BTN3A2, C1QTNF1, CALCR, CAMK4, CARD11, CASP8, CASS4, CCDC88B, CCL19, CCL2, CCL3, CCL5, CCR2, CD2, CD226, CD244, CD33, CD3E, CD4, CD40LG, CD6, CD74, CD80, CD86, CDC20B, CDCA2, CDH1, CDKN2A, CLCF1, CLEC6A, CLNK, CREM, CRTAM, CSF1R, CXCL10, CXCR4, CYBB, DAPK1, DEPDC1B, DRD1, E2F8, EAF2, EBI3, EGR3, ELAVL4, ELF3, EOMES, EPX, EREG, FASLG, FCN1, FCRL3, FGD2, FGR, FLT3, FOSB, FOSL1, FOXP3, GADD45B, GBP5, GDF6, GLIS1, GPRC5A, GPSM3, H1-3, HCLS1, HLA-DPA1, HLA-DPB1, HLA-DRB1, IFNG, IKBKE, IKZF3, IL12RB1, IL12RB2, IL16, IL18, IL26, IL7R, IRF4, IRF5, IRF8, IRX3, ITGAX, ITK, KLF5, KLRG1, LAMP3, LCK, LCP1, LCP2, LDLR, LEF1, LIF, LILRB1, LMO1, LPAL2, LTA, LTB, LY9, MACC1, MAFF, MAP4K1, MAPK13, MCOLN2, MIXL1, MMP9, MSX1, MUSK, MYB, MYBL2, MZB1, NAMPT, NCF1, NCKAP1L, NEK5, NFAM1, NLRP2, NLRP3, NLRP6, NLRP9, NR4A1, NR4A2, NR4A3, NSG1, ORM2, PATL2, PAX5, PBX4, PDE6G, PIK3CG, PIK3R5, PIM1, PIM2, PLAC8, POU2AF1, POU2F2, PRDM1, PRKCB, PRKCQ, PROK1, PTAFR, PTGFR, PTK2B, PTPN22, PTPRC, PYCARD, PYHIN1, RLN2, RUNX3, SALL1, SASH3, SCIMP, SELE, SEMA4D, SH2D1A, SIGLEC16, SKAP1, SLAMF6, SOCS1, SOCS3, SPIB, SPIC, STAP1, STAT1, STXBP2, SYK, TBX21, TENT5C, TFEC, THBS1, TIGIT, TLR6, TLR8, TNFAIP3, TNFRSF18, TNFSF13B, TNFSF8, TNIP3, TOX, TRABD2A, TREM2, TRERF1, TRIM14, WAS, WNT1, WNT10A, WNT10B, XCL1, ZBP1, ZC3H12D, ZNF804A]	[ABCC8, ABL1, ABLIM3, ABRA, ACTA1, ACTB, ACTC1, ACTN1, ACTN4, ADAM19, ADCY1, ADCY3, ADCY4, ADCY9, ADIPOQ, AGAP2, AGER, AGPAT1, AGT, AGTR1, ALPK3, AMH, ANGPT1, AP3D1, APLN, APLNR, ARAF, AREG, ARHGEF5, ARID1A, ARTN, ATF5, ATP13A2, ATP1B4, BAG6, BCL3, BCL6, BCL9L, BMP8A, BRD4, BRPF3, C1QTNF1, C5, C5AR1, CALCR, CAMTA2, CARM1, CASZ1, CCL2, CCL21, CCR7, CD14, CD276, CD74, CDC25B, CDCA2, CDK1, CDKN1A, CGA, CHCHD10, CHERP, CHGA, CHI3L1, CIITA, CITED2, CITED4, CIZ1, CLCF1, CLIP3, CLNK, CNOT3, COL1A1, CREB3L1, CRP, CRTC2, CSF1, CSK, CSNK1E, CSPG4, CSRNP3, CTC1, CTF1, CTIF, CTSD, CX3CL1, CYP26B1, CYP27B1, DAB2IP, DAG1, DAXX, DBF4B, DDX39B, DHX34, DIRAS1, DNMT1, DPF2, DTX1, DUSP5, DVL3, DXO, DYRK1B, E2F4, E2F7, E2F8, EAF2, EDC4, EFNA3, EGLN2, EIF4G1, ELF3, ELF4, ELK1, ENDOG, ENG, ERBB2, ETS2, ETV6, FADD, FASN, FFAR2, FGFR4, FHL5, FHOD1, FLT3, FLT4, FOSL1, FOSL2, FOXC2, FOXF1, FOXM1, FPR1, FRMD8, FURIN, GADD45B, GATA2, GATA4, GBA, GDF6, GGA1, GGA3, GLIS2, GLMP, GPER1, GPI, GPRC5A, GPRIN1, GRN, GSK3A, GTPBP1, H1-9P, HBEGF, HCFC1, HDAC10, HDAC5, HEYL, HFE, HGS, HK1, HLTF, HMGA1, HMGN1, HMGN5, HSF1, HSPB1, HSPE1, HTR2A, ICAM1, ID3, IL1B, IL4R, IL7R, ILK, INCENP, INHA, IRAK2, IRF2, IRF2BPL, IRX3, IRX6, ITGA3, ITGA5, JUNB, JUND, JUP, KCNH2, KDM6B, KLF10, KLF5, KMT2D, KSR1, LDLR, LGALS9, LIF, LILRA5, LIMK2, LMNA, LMX1A, LMX1B, LRRC32, LTF, LUM, MAFG, MAMSTR, MAP2K3, MAP3K10, MAP3K11, MAP3K14, MAP3K6, MAP3K9, MAPK3, MAPK7, MAPK8IP3, MARK2, MAVS, MAZ, MECP2, MED12, MED16, MED24, MED25, MEF2D, MEIS3P1, MEIS3P2, MEN1, MINK1, MLLT6, MLST8, MOV10, MPV17L2, MSN, MSTN, MSX1, MUC20, MYBBP1A, MYC, MYH9, MYRF, NAP1L2, NCOA6, NCOR2, NDP, NEK5, NFATC4, NFE2, NFIC, NFIX, NFKB2, NFKBIB, NGF, NGFR, NKD2, NLRC5, NLRP9, NOD2, NODAL, NOS3, NOTCH1, NOTCH3, NOTCH4, NPAS2, NR1D1, NR4A1, NR4A2
GO:0010647	positive regulation of cell communication	2,16E-10	[3, 4, 5]	366,00	19,50	40,33	59,67	[ABRA, ADAM8, ALOX15, ALOX15B, ANGPT1, AREG, ATF3, BANK1, BLK, BMF, BMP7, BMPR1B, C1QTNF1, CALCR, CARD11, CASP8, CASS4, CCL17, CCL19, CCL2, CCL22, CCL24, CCL3, CCL3L1, CCL4, CCL4L1, CCL5, CCR2, CD180, CD19, CD226, CD27, CD300LF, CD3E, CD4, CD40LG, CD74, CD80, CD86, CLEC6A, CNR1, CRB2, CSF1R, CXCR4, CXorf21, DEPDC1B, DPEP2, DRD1, ELF3, EPX, EREG, ERFE, FASLG, FCRL3, FFAR4, FGD2, FGR, FLT3, GADD45B, GDF6, GFI1, GPR174, GPR4, GPR55, HCLS1, HCST, HLA-DRB1, ICOS, IKBKE, IL10RA, IL18, IL26, IL7R, IQCJ-SCHIP1, KCNN4, KMO, LCK, LMCD1, LY86, MAP4K1, MMP9, MSX1, MYB, NCF1, NCKAP1L, NFAM1, NPPC, NSG1, OASL, P2RY10, P2RY12, PDE6G, PIK3CG, PIK3R5, PIM2, PRKCB, PROK1, PTK2B, PTPN22, PTPN6, PTPRC, PYCARD, PYHIN1, RAC2, RASGRF1, RTKN2, SALL1, SCIMP, SCUBE1, SEMA4D, SLC24A4, SLC8A3, SOCS1, STAP1, SYK, TESPA1, THBS1, TNFAIP3, TNFSF14, TNR, TRAF1, TRAT1, TREM2, TRIM14, TRPM2, UBD, VAV1, WNT1, WNT10B, XCL1, XCL2, ZAP70, ZBP1]	[ABCA7, ABL1, ABRA, ACKR3, ACTN4, ADCY1, ADGRG1, ADIPOQ, AGAP2, AGER, AGPAT1, AGT, AMH, ANGPT1, APLN, APOA1, ARAF, ARC, AREG, ARHGEF5, BCL2L1, BMP8A, BRD4, C1QTNF1, C20orf27, C2CD2L, C5, C5AR1, CALB1, CALCR, CC2D1A, CCL11, CCL2, CCL21, CCL24, CCR7, CD14, CD74, CHERP, CHGA, CHI3L1, CITED2, CNR1, CNTN6, COL1A1, CRAT, CRHR2, CSF1, CSK, CSNK1E, CSPG4, CX3CL1, CYP27B1, DAB2IP, DAXX, DENND2B, DGKD, DIRAS1, DKK2, DLG5, DMTN, DOC2B, DOK5, DUSP5, DVL3, ECM1, EEF1E1, ELF3, ENG, ERBB2, ERFE, ESM1, FADD, FAM110A, FASN, FGFR4, FLT3, FLT4, FOXM1, FPR1, GAB2, GADD45B, GATA4, GBA, GDF6, GNAI2, GPBAR1, GPER1, GPR137, GPR17, GPR20, GPR37L1, GPR4, GPRIN1, GRIK5, GSK3A, HBEGF, HCRTR1, HFE, HTR2A, ICAM1, IL1B, IL7R, ILK, INCA1, INHA, IRAK2, ITGA5, ITPR3, JMJD8, JUP, KCP, KLK14, KMT2D, KSR1, LGALS9, LGR6, LILRA5, LIMS2, LMCD1, LRG1, LRRC8A, LTBR, LTF, MAP2K3, MAP3K10, MAP3K11, MAP3K14, MAP3K6, MAP3K9, MAPK3, MAPK8IP3, MAVS, MAZ, MECP2, MED12, MEIS3P1, MEN1, MINK1, MLST8, MME, MSTN, MSX1, MUC20, NDC80, NDST1, NECTIN2, NFATC4, NGF, NGFR, NINJ1, NLGN2, NLRC5, NNAT, NOD2, NODAL, NOS3, NOTCH1, NOTCH4, NPPA, NRARP, NRGN, NSG1, OSM, P2RX3, P2RX5, P2RY6, PCDHA4, PDGFB, PDGFRB, PIAS4, PLA2G2A, PLAGL2, PLAUR, PLXNB1, PML, POR, PPARD, PRAG1, PRRT1, PTN, PTP4A3, PTPN23, RAC2, RAP1A, RAPGEF1, RASD2, RASL10B, RELA, RHOG, RNU1-93P, RPL26, RPTOR, RTKN2, S1PR2, SECTM1, SELENOP, SEMA4C, SEPTIN4, SHANK3, SHARPIN, SHC2, SHISA5, SHKBP1, SIK3, SLC2A4, SLC44A2, SLC9A1, SLC9A3R1, SMAD3, SMARCA4, SMARCB1, SORBS3, SPATC1L, SPHK1, SPHK2, SPI1, SRC, STAT3, STX1A, STX1B, TBX5, TGFB1I1, TGM2, THBS1, THPO, TICAM1, TIMP1, TMEM198, TNF, TNFRSF1A, TNFSF12, TRAF4, TRIB3, TRIM62, TRIM8, TRPV4, TSPAN14, UBA52, UBE2B, UNC5B, VEGFA, VSIG2, VTN, WBP2, WDR24, WNK2, WNT3, ZBTB7B, ZC3H12A, ZC3H3, ZCCHC3,

GO:0010648	negative regulation of cell communication	4,49E-04	[3, 4, 5]	269,00	18,26	33,37	66,63	[AREG, ATF3, BANK1, BCL3, BMP7, BMPR1B, CASP8, CCDC88C, CCL5, CD22, CD300LF, CD3E, CD74, CHRDL1, CISH, CNR2, DCC, DUSP2, DUSP8, EPX, EREG, FASLG, FCRL3, FFAR4, FRZB, GPRC5A, INPP5D, IRF4, LAX1, LIF, LILRB4, MIDN, MMP9, NLRC3, NLRP3, NLRP6, NR4A2, NTN1, PIK3IP1, PLEK, PRKCB, PRKCQ, PSCA, PTK2B, PTPN22, PTPN6, PTPRC, PYCARD, RASAL3, RBPMS2, RGS1, RGS10, RGS18, RGS9, RHOH, RTKN2, SH2D1A, SLA2, SLC24A4, SMPDL3B, SOCS1, SOCS3, SPINK1, STAP1, STAT1, TBC1D10C, THBS1, THY1, TLR6, TNFAIP3, TNIP3, TNMD, TNR, TRABD2A, TREM2, UBASH3A, UCP2, VNN1, VWC2L, WNT1]	[ABCA7, ABCC8, ABL1, ACKR3, ACP4, ADIPOQ, AGAP2, AGER, AGT, AKT1S1, AMH, APLN, APLNR, APLP1, APOA1, APOD, ARC, AREG, ARF1, ARRDC3, ASPN, BCL2L1, BCL3, BCL6, BCL9L, BEND6, BMP8A, CD14, CD74, CEACAM1, CHGA, CISH, CREB3L1, CSK, CTHRC1, CX3CL1, CXXC4, CYP26B1, DAB2IP, DAG1, DDX39B, DGKD, DGKZ, DKK2, DLG5, DUSP5, DUSP8, DVL3, ECM1, EPHA2, EPN1, ERBB2, FADD, FAIM2, FASN, FOXM1, FRMD8, GATA4, GBA, GJD3, GLIS2, GLRA1, GNAI2, GPER1, GPI, GPR37L1, GPRC5A, GRB7, GRIK5, GRINA, GSK3A, HBEGF, HDAC7, HEYL, HGS, HSPB1, HTR1B, HTR2A, ICAM1, IFT80, IL1B, IL1RN, INHA, INPP5E, ITGA3, ITPRIP, KIF26A, KLK14, LIF, LMNA, LTF, LZTS2, MAP2K3, MAPK7, MAZ, MED12, MEGF8, MEIS3P1, MEN1, MGRN1, MIDN, MNT, MSTN, MYADM, MYC, NCOR2, NF2, NFATC4, NGFR, NKD2, NLRC5, NOD2, NODAL, NOS3, NOTCH1, NOTCH3, NPPA, NPRL3, NR1D1, NR4A2, NRARP, NTN1, OMA1, PALM, PCDHA6, PCGF2, PER1, PIAS4, PLAUR, PPP1R10, PPP1R15A, PPP2R1A, PPP2R5B, PRDM16, PRKACA, PRRT1, PTGIR, PTPRU, RAB11FIP5, RAP1A, RBPMS2, RELA, RGS16, RGS18, RNF126, RNF157, RNU1-93P, RTKN2, S1PR2, SERPINE1, SH2B3, SH3BP1, SHANK3, SLC25A23, SLC30A1, SLC35C1, SLC35F6, SLC9A3R1, SMAD3, SMARCA4, SMPD1, SNAI1, SOCS3, SORCS2, SOX10, SOX13, SPI1, SPINK1, SRC, SREBF1, SRF, SYNGAP1, SYVN1, SZT2, TGFB1I1, THBS1, TICAM1, TIMP1, TLE2, TLE3, TLE5, TMEM127, TMEM14A, TMEM161A, TMEM88, TNF, TNFAIP1, TNIP1, TNMD, TNS2, TRIB1, TRIB3, TSC2, TSKU, UBA52, UBE2B, UNC5B, VASN, VNN1, VSIG2, WFS1, WNK2, WWP2, ZBTB7A, ZC3H12A, ZNF385A, ZNF653]
GO:0031325	positive regulation of cellular metabolic process	1,24E-03	[3, 4, 5]	560,00	16,33	33,95	66,05	[ABRA, ADAM8, ADCY7, AIF1, AIM2, AKNA, ANGPT1, AREG, ASCL2, ATF3, ATP1B4, ATP2A3, BANK1, BATF, BCL11B, BCL3, BHLHA15, BMP7, BMPR1B, BRIP1, CALCR, CAMK4, CASP8, CASS4, CCL19, CCL5, CD177, CD19, CD244, CD33, CD3E, CD4, CD40LG, CD74, CD80, CD86, CDC20B, CDCA2, CDH1, CDKN2A, CLCF1, CREM, CSF1R, CXCL10, CXCR4, DAPK1, DEPDC1B, E2F8, EAF2, EGR3, ELF3, EOMES, EPHA6, EPX, EREG, FASLG, FCRL3, FGD2, FGR, FLT3, FOSB, FOSL1, FOXP3, GADD45B, GDF6, GLIS1, GPRC5A, H1-3, HCLS1, HLA-DRB1, IFNG, IKBKE, IKZF3, IL18, IL26, IRF4, IRF5, IRF8, IRX3, ITGB2, KIAA1324, KLF5, KLRG1, LCK, LCP2, LDLR, LEF1, LIF, LILRB1, LMO1, LPAL2, MACC1, MAFF, MAP4K1, MID1IP1, MIXL1, MMP9, MSX1, MUSK, MYB, MYBL2, NAMPT, NCF1, NCKAP1L, NEK5, NLRP2, NLRP3, NLRP6, NR4A1, NR4A2, NR4A3, NSG1, P2RY12, PATL2, PAX5, PBX4, PDE6G, PFKFB4, PIK3CG, PIK3R5, PIM1, PIM2, PLAC8, PLEK, POU2AF1, POU2F2, PRKCB, PRKCQ, PROK1, PTAFR, PTK2B, PTPN22, PTPRC, PTX3, PYCARD, PYHIN1, RUFY4, RUNX3, SALL1, SELE, SEMA4D, SH2D1A, SKAP1, SMPD3, SOCS1, SOCS3, SPIB, SPIC, STAP1, STAT1, STXBP2, SYK, TBX21, TFEC, THBS1, TLR6, TNFAIP3, TNFRSF18, TNFSF8, TNIP3, TOX, TRABD2A, TREM2, TRERF1, TRIM14, WAS, WNT1, WNT10B, ZC3H12D]	[ABCD1, ABCD2, ABL1, ABLIM3, ABRA, ACACB, ACTN1, ACTN4, ADCY1, ADCY3, ADCY4, ADCY9, ADIPOQ, AGAP2, AGER, AGPAT1, AGT, AGTR1, ALPK3, AMH, ANGPT1, AP3D1, APLN, APLNR, APOA1, ARAF, AREG, ARHGEF5, ARID1A, ARTN, ATF5, ATP1B4, BAG3, BAG6, BCL3, BCL6, BCL9L, BMP8A, BRD4, BRPF3, C5, C5AR1, CALCR, CAMTA2, CARM1, CASZ1, CCL21, CCR7, CD177, CD74, CDC25B, CDCA2, CDK1, CDKN1A, CGA, CHCHD10, CHERP, CHGA, CHI3L1, CIITA, CITED2, CITED4, CIZ1, CLCF1, CLIP3, CNOT3, COL1A1, CREB3L1, CRP, CRTC2, CSF1, CSK, CSNK1E, CSPG4, CSRNP3, CTC1, CTF1, CTIF, CTSD, CX3CL1, CYP27B1, DAB2IP, DAG1, DAXX, DBF4B, DDR1, DDX39B, DGKZ, DHX34, DIRAS1, DNMT1, DPAGT1, DPF2, DTX1, DUSP5, DVL3, DXO, DYRK1B, E2F4, E2F7, E2F8, EAF2, EDC4, EFNA3, EIF4G1, ELF3, ELF4, ELK1, ENDOG, ENG, EPHA2, ERBB2, ETS2, ETV6, FADD, FASN, FGFR4, FHL5, FHOD1, FLT3, FLT4, FOSL1, FOSL2, FOXC2, FOXF1, FOXM1, FPR1, FURIN, GADD45B, GATA2, GATA4, GBA, GDF6, GLIS2, GLMP, GNAI2, GPER1, GPRC5A, GPRIN1, GPT, GRN, GSK3A, GTPBP1, H1-9P, HBB, HBEGF, HCFC1, HDAC10, HDAC5, HEYL, HFE, HLTF, HMGA1, HMGN1, HMGN5, HPRT1, HSF1, HSPB8, HSPE1, HTR2A, HVCN1, ICAM1, IL1B, ILK, INCENP, INHA, IRAK2, IRF2, IRF2BPL, IRX3, IRX6, ITGA5, JUNB, JUND, JUP, KCNH2, KDM6B, KLF10, KLF5, KMT2D, KSR1, LDLR, LGALS9, LIF, LILRA5, LIMK2, LMNA, LMX1A, LMX1B, LTF, LUM, MAFG, MAMSTR, MAP2K3, MAP3K10, MAP3K11, MAP3K14, MAP3K6, MAP3K9, MAPK3, MAPK7, MAPK8IP3, MARK2, MAVS, MAZ, MECP2, MED12, MED16, MED24, MED25, MEF2D, MEIS3P1, MEIS3P2, MEN1, MFSD2A, MID1IP1, MINK1, MLLT6, MLST8, MOV10, MPV17L2, MSN, MSTN, MSX1, MTCO2P12, MUC20, MYC, MYH9, MYRF, NAP1L2, NCOA6, NDP, NEK5, NFATC4, NFE2, NFIC, NFIX, NFKB2, NFKBIB, NGF, NGFR, NKD2, NLRC5, NOD2, NODAL, NOS3, NOTCH1, NOTCH3, NOTCH4, NPAS2, NR1D1, NR4A1, NR4A2, NR4A3, NSG1, NSMF, OMA1, OSM, OSR2, P2RY6, PAF1, PAK4, PATL1, PCOLCE

GO:0035556	intracellular signal transduction	2,94E-19	[3, 4, 5]	582,00	19,50	41,01	58,99	[ABRA, ADAM8, ADCY7, ADGRG6, AIF1, ALOX15, ANGPT1, ARAP2, AREG, ARHGAP15, ARHGAP30, ARHGAP45, ATF3, ATP2A3, BANK1, BATF, BCL11B, BCL3, BHLHA15, BLK, BLNK, BMF, BMP7, BRIP1, BTG2, BTK, C1QTNF1, CALCR, CAMK4, CARD11, CASP8, CASS4, CCDC88C, CCL17, CCL19, CCL2, CCL22, CCL24, CCL3, CCL3L1, CCL4, CCL4L1, CCL5, CCR2, CCR3, CCR4, CCR5, CCR8, CD19, CD200R1, CD22, CD27, CD3E, CD4, CD40LG, CD74, CD80, CD86, CDKN2A, CISH, CLEC6A, CLNK, CORO2A, CSF1R, CSF2RA, CXCR3, CXCR4, CXCR6, DAPK1, DEPDC1B, DOCK10, DOCK2, DOCK8, DOK2, DUSP2, DUSP8, E2F8, EDN2, EPX, EREG, FASLG, FCRL3, FFAR4, FGD2, FGD3, FGR, FIBIN, FLT3, GADD45B, GDF6, GFRA2, GMIP, GPR174, GPR4, GPR55, GRAP2, GUCY2D, HASPIN, HCLS1, HCST, HLA-DRB1, HORMAD1, ICOS, IER3, IKBKE, IL18, IL26, IL2RB, IL2RG, IL5RA, INPP5D, IQCJ-SCHIP1, ITK, JPH1, JSRP1, KCNN4, KLRG1, LAX1, LCK, LCP1, LCP2, LIF, LILRB4, LMCD1, MAP4K1, MAPK13, MCOLN2, MCOLN3, MMP9, MSX1, MYZAP, NCF1, NCKAP1L, NFAM1, NLRC3, NLRP3, NLRP6, NMUR1, NPFFR2, NPPB, NPPC, NR4A3, NTN1, OASL, P2RY10, P2RY12, PDE6G, PIK3C2G, PIK3CG, PIK3IP1, PIK3R5, PIM2, PLCB2, PLCH2, PLEK, PRDM1, PRKCB, PRKCQ, PROK1, PSCA, PSD4, PTAFR, PTGFR, PTK2B, PTPN22, PTPN6, PTPN7, PTPRC, PYCARD, PYHIN1, RAB15, RAB39B, RAC2, RAD9B, RASAL3, RASGRF1, RGS9, RHOH, RMI2, RNASE6, RRAD, RTKN2, SCIMP, SELE, SEMA4D, SH2D6, SIRPG, SLA2, SMPD3, SOCS1, SOCS3, SPINK1, STAT1, STK17B, SYK, TAGAP, TBC1D10C, THBS1, TIFAB, TLR6, TLR8, TNFAIP3, TNFSF13B, TNFSF14, TNIP3, TRAF1, TRAT1, TREM2, TRIM14, TRPM2, UBD, VAV1, VNN1, WAS, WNT1, XCL1, XCL2, XCR1, ZAP70, ZNF385B]	[ABCA7, ABL1, ABR, ABRA, ACKR3, ACTN4, ADCY1, ADCY3, ADCY4, ADCY9, ADGRB2, ADGRG1, ADGRG6, ADGRL1, ADIPOQ, AGAP2, AGER, AGT, AGTR1, AKT1S1, ALPK3, AMH, ANGPT1, APLNR, APOA1, ARAF, ARAP3, AREG, ARHGAP1, ARHGAP23, ARHGAP39, ARHGDIA, ARHGEF1, ARHGEF16, ARHGEF5, ARTN, ASB16, ASB6, BAG6, BCL2L1, BCL3, BCL6, BCR, BRD4, BTG2, C15orf62, C1QTNF1, C20orf27, C5, C5AR1, CALCR, CAMK2B, CARM1, CC2D1A, CCL11, CCL2, CCL21, CCL24, CCR7, CD14, CD74, CDC42EP1, CDC42EP2, CDC42EP4, CDK1, CDK2, CDKN1A, CEACAM1, CFL1, CHERP, CHI3L1, CISH, CLNK, CNOT3, CRAT, CREB3L1, CRYAB, CSF1, CSK, CSPG4, CX3CL1, DAB2IP, DAG1, DAXX, DCHS1, DDX39B, DENND2B, DENND4B, DGKD, DGKZ, DHX34, DIRAS1, DLG5, DMTN, DNMT1, DOCK6, DOK3, DOK5, DOT1L, DUSP5, DUSP8, DVL3, E2F4, E2F7, E2F8, ECM1, EDN2, EEF1E1, EIF4EBP1, ENG, EPHA2, ERBB2, FADD, FAM110A, FASN, FGFR4, FIBIN, FLT3, FLT4, FOXM1, FPR1, GAB2, GADD45B, GATA4, GBA, GBF1, GDF6, GDI1, GFRA3, GNAI2, GPBAR1, GPER1, GPI, GPR137, GPR17, GPR20, GPR37L1, GPR4, GPRIN1, GRIK5, GRINA, GRM2, GSK3A, HBEGF, HCRTR1, HDAC7, HESX1, HGS, HIPK4, HSF1, HSPB1, HTR2A, HUNK, ICAM1, IER3, IL1B, IL3RA, IL5RA, ILK, INPP5E, IRAK2, ITGA3, ITPR3, JAK3, JMJD8, JPH4, KCTD11, KSR1, LGALS9, LIF, LILRA5, LIMK1, LMCD1, LOX, LTBR, LTF, MAP2K3, MAP3K10, MAP3K11, MAP3K14, MAP3K6, MAP3K9, MAPK3, MAPK4, MAPK7, MAPK8IP3, MARK2, MARK4, MAST2, MAVS, MAZ, MDC1, MED12, MEIS3P1, MEN1, MGRN1, MINK1, MKNK2, MLST8, MSTN, MSX1, MUC20, MYADM, MYBBP1A, MYC, MYZAP, NDC80, NDST1, NEFL, NF2, NFATC4, NFKB2, NGF, NGFR, NKIRAS2, NLRC5, NOD2, NODAL, NOP2, NOS3, NOTCH1, NPFFR2, NPPA, NPPB, NPR3, NPRL3, NR1D1, NR4A3, NTN1, NUCB1, OBSCN, OMA1, OSM, P2RX3, P2RX5, P2RY6, PAK4, PDE4A, PDGFB, PDGFRB, PDK2, PEAR1, PER1, PI4KA, PI4KAP1, PI4KAP2, PIAS4, PIDD1, PIK3C2B, PIK3C2G, PIP5K1C, PISD, PITPNM2
GO:0043085	positive regulation of catalytic activity	1,49E-05	[4]	253,00	19,15	42,90	57,10	[ADAM8, ADCY7, AIM2, ANGPT1, ARAP2, AREG, ARHGAP45, ATP2A3, CASP8, CASS4, CCL17, CCL19, CCL2, CCL22, CCL24, CCL3, CCL3L1, CCL4, CCL4L1, CCL5, CD19, CD33, CD4, CD40LG, CD74, CD86, CDC20B, CDKN2A, CSF1R, CXCR4, DAPK1, DOCK10, DOCK8, EDN2, EPHA6, EPX, EREG, FASLG, FCRL3, FGD2, FGR, FLT3, GADD45B, GBA3, GMIP, GPR55, GPRC5A, HLA-DRB1, IFNG, IKBKE, IL18, ITGB2, ITK, LCK, LCP2, LPAR2, MAP4K1, MID1IP1, MUSK, NCF1, NCKAP1L, NEK5, NLRP2, NLRP3, NLRP6, NMUR1, NR4A2, P2RY12, PDE6G, PIK3CG, PIK3R5, PLAAT4, PLCB2, PLEK, PRKCQ, PROK1, PTAFR, PTK2B, PTPRC, PYCARD, RASGRF1, RGS1, RGS10, S1PR4, SELE, SEMA4D, SH2D1A, SOCS1, STAP1, SYK, TBC1D10C, THBS1, THY1, TLR6, TREM2, VAV1, XCL1, XCL2]	[ABL1, ABR, ADCY1, ADCY3, ADCY4, ADCY9, ADIPOQ, AGAP2, AGER, AGT, AGTR1, ANGPT1, APOA1, ARAF, ARAP3, AREG, ARHGEF15, ARHGEF16, ARHGEF5, ARTN, ATPSCKMT, BCR, C5, C5AR1, CCL11, CCL2, CCL21, CCL24, CCR7, CD74, CDC25B, CDKN1A, CHI3L1, CSF1, CSK, CSPG4, CTSD, CX3CL1, CYP27B1, DAB2IP, DAG1, DAXX, DBF4B, DDR1, DGKZ, DIRAS1, DUSP5, DVL3, EDN2, EFNA3, ELMOD1, EPHA2, ERBB2, FADD, FASN, FGFR4, FLT3, FLT4, FPR1, GADD45B, GNAI2, GNAO1, GPER1, GPRC5A, GPRIN1, GRN, GSK3A, HBEGF, HMGN1, HSF1, HSPE1, HTR2A, ICAM1, IL1B, ILK, INCENP, IRAK2, KSR1, LGALS9, LILRA5, LTF, MAP2K3, MAP3K10, MAP3K11, MAP3K14, MAP3K6, MAP3K9, MAPK3, MAPK8IP3, MARK2, MID1IP1, MINK1, MLST8, MTSS2, MUC20, MYC, NEK5, NGF, NGFR, NOD2, NODAL, NOS3, NPR3, NPRL3, NR4A2, OMA1, ORAI1, P2RY6, PAK4, PCOLCE, PDGFB, PDGFRB, PIDD1, PKD1, PLXNB1, PML, POR, PPP1R15A, PRKACA, PRKAR2B, RANGAP1, RAP1A, RAP1GAP2, RAPGEF1, RCVRN, RGCC, RGL3, RGMA, RGS16, RHOG, RNU1-93P, RPLP1, RPTOR, S100A1, S1PR2, SELE, SERINC2, SH3BP1, SHC2, SIPA1L3, SLC25A23, SMAD3, SOX7, SPATC1L, SRC, STAT3, SYDE1, SYNGAP1, TBC1D10B, TBC1D13, TBC1D17, TBC1D2, TBC1D22B, TBC1D25, TGM2, THBS1, TIMP1, TNF, TNIP1, TRAF4, TRIB3, TSC2, UBA52, VAC14, VDR, VEGFA, VSIR, WNT9B, XRCC1]
GO:0050778	positive regulation of immune response	1,07E-08	[3, 4, 5]	186,00	22,25	69,27	30,73	[ADAM8, AIM2, BLK, BLNK, BTK, BTN3A1, BTN3A2, C1QA, C1QB, C1QC, C2, C8G, CARD11, CCL19, CCL5, CCR2, CD177, CD19, CD1B, CD1C, CD1E, CD22, CD226, CD247, CD3D, CD3E, CD3G, CD4, CD74, CD79A, CD80, CD86, CFP, CLCF1, CLEC10A, CLEC4D, CLEC4E, CLEC6A, CLNK, CNR1, CR2, CRTAM, CTLA4, CXCL10, CXorf21, CYP11B1, EREG, FCN1, FCN3, FCRL3, FGR, FOXP3, FYB1, GBP5, GRAP2, HLA-DMB, HLA-DPA1, HLA-DPB1, HLA-DQA1, HLA-DRA, HLA-DRB1, HLA-DRB5, IFNG, IGLL5, IKBKE, IL12RB1, IL18, IL18RAP, INPP5D, ITGB2, ITK, KCNN4, KLRC2, KLRD1, KLRK1, LAG3, LAX1, LCK, LCP2, LILRB1, LILRB4, LTA, MOG, MS4A1, MUC16, MUC19, MUC6, MYB, MYO1G, NCKAP1L, NCR3, NFAM1, NLRP3, NR4A3, PAX5, PRAM1, PRKCB, PRKCQ, PTAFR, PTPN22, PTPN6, PTPRC, PYCARD, PYHIN1, SASH3, SH2D1A, SIGLEC16, SKAP1, SLA2, SLAMF6, STAP1, SYK, TBX21, TESPA1, THEMIS, THEMIS2, THY1, TLR8, TNFSF13B, TRAT1, TREM2, UBASH3A, VAV1, WAS, XCL1, ZAP70, ZBP1]	[ABL1, ACTB, BAG6, BCAR1, C5, C5AR1, C8G, CCR7, CD177, CD1C, CD1E, CD276, CD74, CEACAM1, CFH, CLCF1, CLEC4E, CLNK, CNR1, CPN2, CRP, CSK, DGKZ, FADD, FCN3, FFAR2, FPR1, GAB2, GATA2, GCSAM, GNAO1, GRN, IL1B, IL4R, LAG3, LGALS9, LIMK1, MAPK3, MAVS, MUC19, MUC20, MUC3A, NCOR2, NECTIN2, NLRC5, NOD2, NPPA, NR4A3, PCDHA4, PQBP1, PRKACA, PVR, RAP1A, RARA, RBM14, RELA, RELB, RGCC, RHBDF2, RNU1-93P, SERPING1, SH2B2, SLC7A8, SPHK2, SPI1, SRC, STING1, TNF, TRIM62, VTN, WAS, ZC3H12A, ZCCHC3]

GO:0051173	positive regulation of nitrogen compound metabolic process	1,29E-02	[3, 4, 5]	523,00	16,17	33,88	66,12	[ABRA, ADAM8, ADCY7, AIF1, AIM2, AKNA, ANGPT1, AREG, ASCL2, ATF3, ATP1B4, ATP2A3, BANK1, BATF, BCL11B, BCL3, BHLHA15, BMP7, BMPR1B, BRIP1, CALCR, CAMK4, CASP8, CASS4, CCL19, CCL5, CD33, CD3E, CD4, CD40LG, CD74, CD80, CD86, CDC20B, CDCA2, CDH1, CDKN2A, CLCF1, CREM, CSF1R, CXCL10, CXCR4, DAPK1, DEPDC1B, E2F8, EAF2, EGR3, ELF3, EOMES, EPX, EREG, FASLG, FCRL3, FGD2, FLT3, FOSB, FOSL1, FOXP3, GADD45B, GDF6, GLIS1, GPRC5A, H1-3, HCLS1, HLA-DRB1, IFNG, IKBKE, IKZF3, IL18, IL26, IRF4, IRF5, IRF8, IRX3, ITGB2, KLF5, KLRG1, LCK, LCP2, LDLR, LEF1, LIF, LILRB1, LMO1, MACC1, MAFF, MAP4K1, MIXL1, MMP9, MSX1, MUSK, MYB, MYBL2, NAMPT, NCF1, NCKAP1L, NEK5, NLRP2, NLRP3, NLRP6, NR4A1, NR4A2, NR4A3, PATL2, PAX5, PBX4, PDE6G, PFKFB4, PIK3CG, PIK3R5, PIM1, PIM2, PLAC8, POU2AF1, POU2F2, PRKCB, PRKCQ, PROK1, PTAFR, PTK2B, PTPN22, PTPRC, PTX3, PYCARD, PYHIN1, RUNX3, SALL1, SEMA4D, SH2D1A, SKAP1, SMPD3, SOCS1, SOCS3, SPIB, SPIC, STAP1, STAT1, STXBP2, SYK, TBX21, TFEC, THBS1, TLR6, TNFAIP3, TNFRSF18, TNFSF13B, TNFSF8, TNIP3, TOX, TRABD2A, TREM2, TRERF1, TRIM14, WAS, WNT1, WNT10B, ZC3H12D]	[ABL1, ABLIM3, ABRA, ACTN1, ACTN4, ADCY1, ADCY3, ADCY4, ADCY9, ADIPOQ, AGAP2, AGER, AGT, AGTR1, ALPK3, AMH, ANGPT1, AP3D1, APLN, APLNR, ARAF, AREG, ARHGEF5, ARID1A, ARTN, ATF5, ATP1B4, BAG6, BCL3, BCL6, BCL9L, BMP8A, BRD4, C5, C5AR1, CALCR, CAMTA2, CARM1, CASZ1, CCL21, CCR7, CD74, CDC25B, CDCA2, CDKN1A, CGA, CHCHD10, CHERP, CHGA, CHI3L1, CIITA, CITED2, CITED4, CIZ1, CLCF1, CLIP3, CNOT3, COL1A1, CREB3L1, CRTC2, CSF1, CSK, CSNK1E, CSPG4, CSRNP3, CTF1, CTIF, CTSD, CX3CL1, CYP27B1, DAB2IP, DAG1, DAXX, DBF4B, DDX39B, DIRAS1, DNMT1, DPF2, DTX1, DUSP5, DVL3, DXO, DYRK1B, E2F4, E2F7, E2F8, EAF2, EDC4, EFNA3, EGLN2, EIF4G1, ELF3, ELF4, ELK1, ENDOG, ENG, ERBB2, ETS2, ETV6, FADD, FASN, FGFR4, FHL5, FHOD1, FLT3, FLT4, FOSL1, FOSL2, FOXC2, FOXF1, FOXM1, FPR1, FURIN, GADD45B, GATA2, GATA4, GBA, GDF6, GGA1, GGA3, GLIS2, GLMP, GPER1, GPRC5A, GPRIN1, GRN, GSK3A, GTPBP1, H1-9P, HBB, HBEGF, HCFC1, HDAC10, HDAC5, HEYL, HFE, HLTF, HMGA1, HMGN1, HMGN5, HPRT1, HSF1, HSPE1, HTR2A, ICAM1, IL1B, ILK, INCENP, INHA, IRAK2, IRF2, IRF2BPL, IRX3, IRX6, ITGA5, JUNB, JUND, JUP, KCNH2, KDM6B, KLF10, KLF5, KMT2D, KSR1, LDLR, LGALS9, LIF, LILRA5, LIMK2, LMNA, LMX1A, LMX1B, LTF, LUM, MAFG, MAMSTR, MAP2K3, MAP3K10, MAP3K11, MAP3K14, MAP3K6, MAP3K9, MAPK3, MAPK7, MAPK8IP3, MARK2, MAVS, MAZ, MECP2, MED12, MED16, MED24, MED25, MEF2D, MEIS3P1, MEIS3P2, MEN1, MINK1, MLLT6, MLST8, MOV10, MPV17L2, MSN, MSTN, MSX1, MTCO2P12, MUC20, MYC, MYH9, MYRF, NAP1L2, NCOA6, NDP, NEK5, NFATC4, NFE2, NFIC, NFIX, NFKB2, NFKBIB, NGF, NGFR, NKD2, NLRC5, NOD2, NODAL, NOS3, NOTCH1, NOTCH3, NOTCH4, NPAS2, NR1D1, NR4A1, NR4A2, NR4A3, NSMF, OMA1, OSM, OSR2, PAF1, PAK4, PATL1, PCOLCE, PDGFB, PDGFRB, PEF1, PELP1, PER1, PHF1, PIAS4, PIDD1, PIM1, PKD1, PKM, PLAGL2, PLAUR, PLEKHN1, PLK3, PML, PNLDC1, PPARD, PPP1R10, PPP1R15A
GO:0009967	positive regulation of signal transduction	8,98E-10	[3, 4, 5, 6]	332,00	19,71	40,73	59,27	[ABRA, ADAM8, ALOX15, ALOX15B, ANGPT1, AREG, ATF3, BANK1, BMF, BMP7, BMPR1B, C1QTNF1, CALCR, CARD11, CASP8, CASS4, CCL17, CCL19, CCL2, CCL22, CCL24, CCL3, CCL3L1, CCL4, CCL4L1, CCL5, CD180, CD19, CD226, CD27, CD300LF, CD3E, CD4, CD40LG, CD74, CD80, CD86, CLEC6A, CRB2, CSF1R, CXCR4, CXorf21, DEPDC1B, DPEP2, ELF3, EPX, EREG, ERFE, FASLG, FCRL3, FFAR4, FGD2, FGR, FLT3, GADD45B, GDF6, GFI1, GPR174, GPR4, GPR55, HCLS1, HCST, HLA-DRB1, ICOS, IKBKE, IL10RA, IL18, IL26, IL7R, IQCJ-SCHIP1, KCNN4, LCK, LMCD1, LY86, MAP4K1, MMP9, MSX1, NCF1, NCKAP1L, NFAM1, NPPC, OASL, P2RY10, P2RY12, PDE6G, PIK3CG, PIK3R5, PIM2, PRKCB, PROK1, PTK2B, PTPN22, PTPN6, PTPRC, PYCARD, PYHIN1, RAC2, RASGRF1, RTKN2, SALL1, SCIMP, SCUBE1, SEMA4D, SOCS1, STAP1, SYK, TESPA1, THBS1, TNFAIP3, TNFSF14, TRAF1, TRAT1, TREM2, TRIM14, UBD, VAV1, WNT1, WNT10B, XCL1, XCL2, ZAP70, ZBP1]	[ABCA7, ABL1, ABRA, ACKR3, ACTN4, ADGRG1, ADIPOQ, AGAP2, AGER, AGPAT1, AGT, AMH, ANGPT1, APOA1, ARAF, AREG, ARHGEF5, BCL2L1, BMP8A, BRD4, C1QTNF1, C20orf27, C5, C5AR1, CALCR, CC2D1A, CCL11, CCL2, CCL21, CCL24, CCR7, CD14, CD74, CHERP, CHGA, CHI3L1, CITED2, CNTN6, COL1A1, CRAT, CSF1, CSK, CSNK1E, CSPG4, CX3CL1, CYP27B1, DAB2IP, DAXX, DENND2B, DGKD, DIRAS1, DKK2, DLG5, DMTN, DOK5, DUSP5, DVL3, ECM1, EEF1E1, ELF3, ENG, ERBB2, ERFE, ESM1, FADD, FAM110A, FASN, FGFR4, FLT3, FLT4, FPR1, GAB2, GADD45B, GATA4, GDF6, GNAI2, GPBAR1, GPER1, GPR137, GPR17, GPR20, GPR37L1, GPR4, GPRIN1, GSK3A, HBEGF, HCRTR1, HFE, HTR2A, ICAM1, IL1B, IL7R, ILK, INCA1, INHA, IRAK2, ITGA5, JMJD8, JUP, KCP, KLK14, KMT2D, KSR1, LGALS9, LGR6, LILRA5, LIMS2, LMCD1, LRG1, LTBR, LTF, MAP2K3, MAP3K10, MAP3K11, MAP3K14, MAP3K6, MAP3K9, MAPK3, MAPK8IP3, MAVS, MAZ, MED12, MEIS3P1, MEN1, MINK1, MLST8, MSTN, MSX1, MUC20, NDC80, NDST1, NECTIN2, NFATC4, NGF, NGFR, NINJ1, NLGN2, NLRC5, NOD2, NODAL, NOS3, NOTCH1, NOTCH4, NPPA, NRARP, OSM, P2RX3, P2RX5, P2RY6, PDGFB, PDGFRB, PIAS4, PLA2G2A, PLAGL2, PLAUR, PLXNB1, PML, POR, PPARD, PRAG1, PTP4A3, RAC2, RAP1A, RAPGEF1, RASD2, RELA, RHOG, RNU1-93P, RPL26, RPTOR, RTKN2, S1PR2, SECTM1, SELENOP, SEMA4C, SEPTIN4, SHANK3, SHARPIN, SHC2, SHISA5, SHKBP1, SIK3, SLC2A4, SLC44A2, SLC9A1, SLC9A3R1, SMAD3, SMARCA4, SMARCB1, SORBS3, SPATC1L, SPHK1, SPHK2, SPI1, SRC, STAT3, STX1A, STX1B, TGFB1I1, TGM2, THBS1, THPO, TICAM1, TIMP1, TMEM198, TNF, TNFRSF1A, TNFSF12, TRAF4, TRIB3, TRIM62, TRIM8, TRPV4, TSPAN14, UBA52, UBE2B, UNC5B, VEGFA, VSIG2, VTN, WBP2, WDR24, WNK2, WNT3, ZBTB7B, ZC3H12A, ZC3H3, ZCCHC3, ZMIZ1, ZNF385A]

GO:0009968	negative regulation of signal transduction	1,93E-02	[3, 4, 5, 6]	242,00	17,74	33,71	66,29	[AREG, ATF3, BANK1, BCL3, BMP7, BMPR1B, CASP8, CCDC88C, CCL5, CD22, CD300LF, CD3E, CD74, CHRDL1, CISH, DCC, DUSP2, DUSP8, EPX, EREG, FASLG, FCRL3, FRZB, GPRC5A, INPP5D, IRF4, LAX1, LIF, LILRB4, MMP9, NLRC3, NLRP3, NLRP6, NR4A2, NTN1, PIK3IP1, PLEK, PRKCB, PRKCQ, PSCA, PTPN22, PTPN6, PTPRC, PYCARD, RASAL3, RBPMS2, RGS1, RGS10, RGS18, RGS9, RHOH, RTKN2, SH2D1A, SLA2, SMPDL3B, SOCS1, SOCS3, SPINK1, STAP1, STAT1, TBC1D10C, THBS1, THY1, TLR6, TNFAIP3, TNIP3, TNMD, TRABD2A, TREM2, UBASH3A, VNN1, VWC2L, WNT1]	[ABCA7, ABL1, ACKR3, ACP4, ADIPOQ, AGAP2, AGT, AKT1S1, AMH, APLN, APLNR, APLP1, APOA1, APOD, AREG, ARRDC3, ASPN, BCL2L1, BCL3, BCL6, BCL9L, BEND6, CD14, CD74, CEACAM1, CISH, CREB3L1, CSK, CTHRC1, CX3CL1, CXXC4, CYP26B1, DAB2IP, DAG1, DDX39B, DGKD, DGKZ, DKK2, DLG5, DUSP5, DUSP8, DVL3, ECM1, EPHA2, EPN1, ERBB2, FADD, FAIM2, FASN, FOXM1, FRMD8, GATA4, GBA, GLIS2, GNAI2, GPER1, GPI, GPR37L1, GPRC5A, GRB7, GRINA, GSK3A, HBEGF, HDAC7, HEYL, HGS, HSPB1, HTR1B, ICAM1, IFT80, IL1B, IL1RN, INPP5E, ITGA3, ITPRIP, KIF26A, KLK14, LIF, LMNA, LTF, LZTS2, MAP2K3, MAPK7, MAZ, MED12, MEGF8, MEIS3P1, MEN1, MGRN1, MNT, MSTN, MYADM, MYC, NCOR2, NF2, NFATC4, NGFR, NKD2, NLRC5, NOD2, NODAL, NOS3, NOTCH1, NOTCH3, NPPA, NPRL3, NR1D1, NR4A2, NRARP, NTN1, OMA1, PALM, PCGF2, PER1, PIAS4, PLAUR, PPP1R10, PPP1R15A, PPP2R1A, PPP2R5B, PRDM16, PRKACA, PTGIR, PTPRU, RBPMS2, RELA, RGS16, RGS18, RNF126, RNF157, RNU1-93P, RTKN2, S1PR2, SERPINE1, SH2B3, SH3BP1, SLC25A23, SLC35C1, SLC35F6, SLC9A3R1, SMAD3, SMARCA4, SMPD1, SNAI1, SOCS3, SOX10, SOX13, SPI1, SPINK1, SRC, SYNGAP1, SYVN1, SZT2, TGFB1I1, THBS1, TICAM1, TIMP1, TLE2, TLE3, TLE5, TMEM127, TMEM14A, TMEM161A, TMEM88, TNF, TNFAIP1, TNIP1, TNMD, TNS2, TRIB1, TRIB3, TSC2, TSKU, UBA52, UBE2B, UNC5B, VASN, VNN1, VSIG2, WFS1, WNK2, WWP2, ZBTB7A, ZC3H12A, ZNF385A, ZNF653]
GO:0000165	MAPK cascade	2,47E-07	[4, 5, 6]	210,00	20,83	40,27	59,73	[ADAM8, ALOX15, ANGPT1, AREG, ATF3, BANK1, BMP7, C1QTNF1, CALCR, CCL17, CCL19, CCL2, CCL22, CCL24, CCL3, CCL3L1, CCL4, CCL4L1, CCL5, CCR5, CD27, CD4, CD40LG, CD74, CSF1R, CSF2RA, CXCR4, DUSP2, DUSP8, EPX, EREG, FCRL3, FFAR4, FGD2, FLT3, GADD45B, GDF6, GFRA2, GPR55, HLA-DRB1, IKBKE, IL18, IL26, IL2RB, IL2RG, IL5RA, LAX1, LIF, LILRB4, MAP4K1, MAPK13, NCF1, NLRP3, NLRP6, NPFFR2, PDE6G, PIK3CG, PIK3R5, PRKCQ, PROK1, PSCA, PTK2B, PTPN22, PTPN6, PTPN7, PTPRC, PYCARD, RASAL3, RASGRF1, SCIMP, SYK, TBC1D10C, THBS1, TRAF1, TREM2, XCL1, XCL2]	[ABCA7, ABL1, ACKR3, ADIPOQ, AGER, AGT, AMH, ANGPT1, APOA1, ARAF, AREG, ARHGEF5, ARTN, BCL2L1, C1QTNF1, C5, C5AR1, CALCR, CAMK2B, CCL11, CCL2, CCL21, CCL24, CCR7, CD74, CDK1, CEACAM1, CHI3L1, CRYAB, CSK, CSPG4, CX3CL1, DAB2IP, DAG1, DAXX, DENND2B, DIRAS1, DOK5, DUSP5, DUSP8, DVL3, EPHA2, ERBB2, FASN, FGFR4, FLT3, FLT4, FOXM1, FPR1, GADD45B, GATA4, GBA, GDF6, GFRA3, GNAI2, GPBAR1, GPER1, GPI, GPR37L1, GPRIN1, GRIK5, HBEGF, HCRTR1, HESX1, HGS, HSF1, HTR2A, ICAM1, IL1B, IL3RA, IL5RA, ILK, IRAK2, JAK3, KCTD11, KSR1, LGALS9, LIF, LILRA5, LTBR, MAP2K3, MAP3K10, MAP3K11, MAP3K14, MAP3K6, MAP3K9, MAPK3, MAPK4, MAPK7, MAPK8IP3, MEN1, MINK1, MUC20, MYC, NDST1, NEFL, NF2, NGF, NOD2, NODAL, NOTCH1, NPFFR2, NPPA, P2RY6, PAK4, PDGFB, PDGFRB, PER1, PLA2G2A, PLVAP, PPP1CB, PPP2R1A, RALGDS, RAP1A, RAPGEF1, RGL3, RNU1-93P, S1PR2, SEMA4C, SH2B3, SH2D3A, SH2D3C, SHANK3, SHC2, SLC9A3R1, SMPD1, SORBS3, SPHK1, SPI1, SPTB, SRC, STK40, SYNGAP1, THBS1, THPO, TIMP1, TNF, TNIP1, TRAF4, TRIB1, TRIB3, TRPV4, UBA52, VEGFA, WNK2, ZC3H12A, ZDHHC9, ZFP36]
GO:0016310	phosphorylation	5,20E-06	[5]	372,00	18,05	37,87	62,13	[ADAM8, ADCY7, AIF1, ANGPT1, AREG, ATF3, BANK1, BLK, BMP7, BMPR1B, BTK, CALCR, CAMK4, CARD11, CASS4, CCDC88C, CCL19, CCL2, CCL3, CCL5, CD19, CD3E, CD4, CD40LG, CD74, CD80, CD86, CDKN2A, CERKL, CISH, CLCF1, CSF1R, CXCL10, CXCR4, DAPK1, DUSP2, DUSP8, EPHA6, EPX, EREG, FGD2, FGR, FLT3, GADD45B, GDF6, GFRA2, GMFG, GPRC5A, GUCY2D, HASPIN, HCLS1, HCST, HKDC1, HLA-DRB1, IFNG, IKBKE, IL12RB2, IL18, ITGB2, ITK, LAX1, LCK, LCP2, LIF, LILRB4, MAP4K1, MAPK13, MATK, MIDN, MMP9, MUSK, MYO3B, NCF1, NCKAP1L, NEK5, NELL2, NLRP3, NLRP6, NPFFR2, NR4A3, NUP210, P2RY12, PDE6G, PEAK3, PFKFB4, PIK3C2G, PIK3CG, PIK3IP1, PIK3R5, PIM1, PIM2, PRKCB, PRKCQ, PROK1, PTK2B, PTPN22, PTPN6, PTPRC, PTPRH, PYCARD, RAC2, RHOH, RUNX3, SAMSN1, SEMA4D, SH2D1A, SLA, SLA2, SLAMF8, SLC8A3, SMPD3, SOCS1, SOCS3, SPINK1, STAP1, STK17B, STYK1, SYK, THBS1, THY1, TLR6, TLR8, TNFAIP3, TNFRSF18, TREM2, WNT1, ZAP70]	[AAAS, AATK, ABL1, ABR, ACP4, ACTB, ADCK1, ADCY1, ADCY3, ADCY4, ADCY9, ADIPOQ, AGAP2, AGER, AGT, AKT1S1, ALDOA, ALPK3, AMH, ANGPT1, ANKK1, APLN, APLNR, APOA1, ARAF, AREG, ARHGEF5, ARTN, ATP13A2, BCKDK, BCR, BMP8A, BRD4, C5, C5AR1, CALCR, CAMK2B, CCL11, CCL2, CCL21, CCNG2, CCNJL, CCR7, CD74, CDC25B, CDK1, CDK15, CDK18, CDK2, CDKL2, CDKN1A, CEACAM1, CERT1, CHI3L1, CIITA, CISH, CKM, CLCF1, CLIP3, CNKSR1, COASY, CSF1, CSK, CSNK1E, CSPG4, CTDSP1, CTF1, CX3CL1, DAB2IP, DAG1, DAXX, DBF4B, DBNDD1, DCK, DDR1, DGKD, DGKZ, DGUOK, DIRAS1, DMTN, DUSP5, DUSP8, DVL3, DYRK1B, EFNA3, EIF4G1, ENG, EPHA2, ERBB2, FABP4, FASN, FES, FGFR4, FGFRL1, FLT3, FLT4, FPR1, GADD45B, GBA, GDF6, GIT1, GPER1, GPI, GPRC5A, GSK3A, HBEGF, HFE, HGS, HIPK4, HK1, HSF1, HSPB1, HSPB8, HTR2A, HUNK, ICAM1, IL1B, ILK, INCA1, INCENP, INHA, INPP5J, IRAK2, ITGA5, ITPRIP, JAK3, JMJD8, KSR1, LIF, LILRA5, LIMK1, LIMK2, LIPE, LMTK3, LOX, LTF, MAP2K3, MAP3K10, MAP3K11, MAP3K14, MAP3K6, MAP3K9, MAPK3, MAPK4, MAPK7, MAPK8IP3, MARK2, MARK4, MAST2, MAVS, MEN1, MIDN, MINK1, MKNK2, MLLT1, MLST8, MSTN, MUC20, MYADM, MYC, MYO3A, NEK5, NF2, NGF, NLRC5, NME4, NOD2, NODAL, NPFFR2, NPPA, NR4A3, NUP188, OBSCN, OGDH, OMA1, OSM, PAK4, PANK1, PDGFB, PDGFRB, PDK2, PI4KA, PI4KAP1, PI4KAP2, PIK3C2B, PIK3C2G, PIM1, PIP5K1C, PKD1, PKM, PKN3, PLAUR, PLK3, PML, PPP1R9B, PPP2R1A, PPP2R5B, PPP2R5D, PRAG1, PRKACA, PRKAR2B, PROM2, PRRT1, PTPN13, PTPRH, PTPRU, RAC2, RAP1A, RAPGEF1, RARA, RGCC, RHBDF2, RMRP, RNU1-93P, RPLP1, RPTOR, RSKR, RUBCN, S1PR2, SBK1, SCYL1, SH2B3, SH2D3A, SH2D3C, SHC2, SIK1, SIK2, SIK3, SLC2A6, SLC9A3R1, SMPD1, SOCS3, SPATC1L, SPEG, SPHK1, SPHK2, SPINK1, SRC, SRPK3, STAT3, STK17B, STK35, STK40, TESK1, TEX14, THBS1, THPO, TIMP1, TK1, TNF, TNFRSF1A, TNK1, TNK2, TNKS1BP1, TRAF4, TRIB1, TRIB3, TSC2, TSSK3, UBA52,

GO:0051345	positive regulation of hydrolase activity	3,09E-03	[5]	126,00	20,62	47,93	52,07	[AIM2, ARAP2, ARHGAP45, ATP2A3, CASP8, CCL17, CCL19, CCL2, CCL22, CCL24, CCL3, CCL3L1, CCL4, CCL4L1, CCL5, CD33, CD86, CDKN2A, DAPK1, DOCK10, DOCK8, FASLG, FCRL3, GBA3, GMIP, GPR55, IFNG, ITK, LCK, LPAR2, NEK5, NLRP2, NLRP3, NLRP6, NMUR1, P2RY12, PLCB2, PLEK, PTAFR, PTK2B, PTPRC, PYCARD, RASGRF1, RGS1, RGS10, S1PR4, SELE, SEMA4D, SYK, TBC1D10C, THY1, VAV1, XCL1, XCL2]	[ABR, AGER, AGT, AGTR1, APOA1, ARAP3, ARHGEF15, ARHGEF16, ARHGEF5, BCR, C5AR1, CCL11, CCL2, CCL21, CCL24, CCR7, CTSD, CX3CL1, DVL3, EFNA3, ELMOD1, EPHA2, ERBB2, FADD, FASN, GNAO1, GPER1, GPRIN1, GRN, HSF1, HSPE1, HTR2A, ICAM1, LGALS9, MTSS2, MYC, NEK5, NGF, NGFR, NODAL, P2RY6, PCOLCE, PDGFRB, PIDD1, PLXNB1, PML, PPP1R15A, RANGAP1, RAP1A, RAP1GAP2, RAPGEF1, RGL3, RGMA, RGS16, RHOG, RNU1-93P, SELE, SH3BP1, SIPA1L3, SLC25A23, SMAD3, SOX7, STAT3, SYDE1, SYNGAP1, TBC1D10B, TBC1D13, TBC1D17, TBC1D2, TBC1D22B, TBC1D25, TGM2, TNF, TSC2, VEGFA, VSIR]
GO:1902531	regulation of intracellular signal transduction	1,09E-10	[4, 5, 6]	375,00	19,47	39,85	60,15	[ABRA, ADAM8, ALOX15, ANGPT1, ARAP2, AREG, ARHGAP15, ARHGAP30, ARHGAP45, ATF3, BANK1, BCL3, BMF, BMP7, BRIP1, C1QTNF1, CALCR, CARD11, CASP8, CASS4, CCL17, CCL19, CCL2, CCL22, CCL24, CCL3, CCL3L1, CCL4, CCL4L1, CCL5, CD19, CD22, CD27, CD3E, CD4, CD40LG, CD74, CD80, CD86, CDKN2A, CLEC6A, CSF1R, CXCR4, DEPDC1B, DUSP2, DUSP8, EPX, EREG, FASLG, FCRL3, FFAR4, FGD2, FGD3, FGR, FLT3, GADD45B, GDF6, GMIP, GPR174, GPR4, GPR55, HCLS1, HCST, HLA-DRB1, ICOS, IER3, IKBKE, IL18, IL26, IQCJ-SCHIP1, LAX1, LCK, LIF, LILRB4, LMCD1, MAP4K1, MMP9, MSX1, NCF1, NCKAP1L, NLRC3, NLRP3, NLRP6, NPFFR2, NPPC, OASL, P2RY10, P2RY12, PDE6G, PIK3CG, PIK3IP1, PIK3R5, PIM2, PLEK, PRDM1, PRKCB, PROK1, PSCA, PSD4, PTK2B, PTPN22, PTPN6, PTPRC, PYCARD, PYHIN1, RAC2, RAD9B, RASAL3, RASGRF1, RHOH, RMI2, RNASE6, RRAD, RTKN2, SCIMP, SEMA4D, SLA2, SPINK1, STAT1, SYK, TAGAP, TBC1D10C, THBS1, TNFAIP3, TNFSF14, TNIP3, TRAF1, TRAT1, TREM2, TRIM14, UBD, VAV1, VNN1, XCL1, XCL2, ZAP70]	[ABCA7, ABL1, ABR, ABRA, ACKR3, ACTN4, ADGRG1, ADIPOQ, AGAP2, AGER, AGT, AKT1S1, AMH, ANGPT1, APLNR, APOA1, ARAF, ARAP3, AREG, ARHGAP1, ARHGAP23, ARHGAP39, ARHGDIA, ARHGEF1, ARHGEF16, ARHGEF5, ARTN, BCL2L1, BCL3, BCL6, BCR, BRD4, C1QTNF1, C20orf27, C5, C5AR1, CALCR, CC2D1A, CCL11, CCL2, CCL21, CCL24, CCR7, CD14, CD74, CDK1, CDK2, CEACAM1, CHERP, CHI3L1, CRAT, CREB3L1, CSF1, CSK, CSPG4, CX3CL1, DAB2IP, DAG1, DAXX, DDX39B, DENND2B, DENND4B, DGKD, DIRAS1, DLG5, DOK5, DUSP5, DUSP8, DVL3, ECM1, EEF1E1, ENG, EPHA2, ERBB2, FADD, FAM110A, FASN, FGFR4, FLT3, FLT4, FOXM1, FPR1, GAB2, GADD45B, GATA4, GBA, GBF1, GDF6, GNAI2, GPBAR1, GPER1, GPR137, GPR17, GPR20, GPR37L1, GPR4, GRIK5, GRINA, GRM2, GSK3A, HBEGF, HCRTR1, HDAC7, HIPK4, HSPB1, HTR2A, ICAM1, IER3, IL1B, ILK, INPP5E, IRAK2, ITGA3, JMJD8, KCTD11, KSR1, LGALS9, LIF, LILRA5, LMCD1, LTBR, LTF, MAP2K3, MAP3K10, MAP3K11, MAP3K14, MAP3K6, MAP3K9, MAPK3, MAPK7, MAPK8IP3, MAVS, MAZ, MED12, MEIS3P1, MEN1, MGRN1, MINK1, MLST8, MSTN, MSX1, MUC20, MYADM, MYC, NDC80, NDST1, NF2, NGF, NOD2, NODAL, NOP2, NOTCH1, NPFFR2, NPPA, NPRL3, NR1D1, OBSCN, OSM, P2RX3, P2RX5, P2RY6, PAK4, PDE4A, PDGFB, PDGFRB, PDK2, PER1, PIAS4, PIDD1, PIP5K1C, PISD, PLA2G2A, PLAGL2, PLAUR, PLEKHG2, PLK3, PLXNB1, PML, PPARD, PPP1R10, PPP1R13L, PPP2R1A, PPP2R5B, PRAG1, PRKAR2B, PTP4A3, PTPN13, RAC2, RAD9B, RAP1A, RAP1GAP2, RAPGEF1, RASD2, RELA, RHOG, RMI1, RMI2, RNF157, RNU1-93P, RPL26, RPTOR, RRAD, RTKN2, S1PR2, SECTM1, SELENOP, SEMA4C, SEPTIN4, SH2B2, SH2B3, SH3BP1, SHARPIN, SHC2, SHISA5, SIK3, SIPA1L3, SLC44A2, SLC9A1, SLC9A3R1, SMPD1, SNAI1, SORBS3, SPATC1L, SPHK1, SPHK2, SPI1, SPINK1, SRC, SSX2IP, STK40, SYDE1, SYNGAP1, SYVN1, SZT2, TAF6, TGM2, THBS1, THPO, TICAM1, TIMP1, TMEM100, TMEM127, TMEM161A, TNF, TNFAIP1, TNFRSF1A,
GO:0019220	regulation of phosphate metabolic process	1,01E-03	[5, 6]	295,00	17,84	38,64	61,36	[ADAM8, ADCY7, AIF1, ANGPT1, AREG, ATF3, BANK1, BMP7, CALCR, CASS4, CCDC88C, CCL19, CCL5, CD19, CD244, CD33, CD3E, CD4, CD40LG, CD74, CD80, CD86, CDCA2, CDKN2A, CISH, CLCF1, CSF1R, CXCL10, CXCR4, DUSP2, DUSP8, EPHA6, EPX, EREG, FAM122C, FCRL3, FGD2, FGR, FLT3, GADD45B, GDF6, GFRA2, GMFG, GPRC5A, HCLS1, HLA-DRB1, IFNG, IKBKE, IL18, ITGB2, LAX1, LCP2, LDLR, LIF, LILRB4, MAP4K1, MIDN, MMP9, MUSK, NCF1, NCKAP1L, NLRP3, NPFFR2, NR4A3, NUP210, P2RY12, PDE6G, PFKFB4, PIK3CG, PIK3IP1, PIK3R5, PLEK, PPP1R42, PPP2R2B, PROK1, PTAFR, PTK2B, PTPN22, PTPN6, PTPRC, PTPRH, PYCARD, RAC2, RHOH, SAMSN1, SEMA4D, SH2D1A, SLA2, SLAMF8, SLC8A3, SMPD3, SOCS1, SOCS3, SPINK1, STAP1, SYK, THBS1, THY1, TLR6, TLR8, TNFAIP3, TNFRSF18, TREM2, WNT1]	[AAAS, ABCA3, ABL1, ACP4, ACTB, ADCY1, ADCY3, ADCY4, ADCY9, ADIPOQ, AGAP2, AGER, AGT, AKT1S1, AMH, ANGPT1, APLN, APLNR, APOA1, ARAF, AREG, ARF1, ARHGEF5, ARTN, ATPSCKMT, BMP8A, BRD4, C5, C5AR1, CABIN1, CALCR, CCL21, CCNG2, CCNJL, CCR7, CD74, CDC25B, CDCA2, CDKN1A, CEACAM1, CHI3L1, CISH, CLCF1, CLIP3, CSF1, CSK, CSPG4, CTDSP1, CTF1, CX3CL1, DAB2IP, DAG1, DAXX, DBF4B, DBNDD1, DDR1, DGKZ, DHX34, DIRAS1, DMTN, DUSP5, DUSP8, DVL3, EIF4G1, ENG, EPHA2, ERBB2, FABP4, FAM122C, FASN, FGFR4, FLT3, FLT4, FPR1, GADD45B, GBA, GDF6, GIT1, GNAI2, GNB3, GPER1, GPRC5A, GSK3A, HBEGF, HFE, HGS, HSF1, HSPB1, HTR2A, ICAM1, IL1B, ILK, INCA1, INCENP, INHA, INPP5J, IRAK2, ITGA5, ITPRIP, JMJD8, KSR1, LDLR, LIF, LILRA5, LIMK2, LOX, LTF, MAP2K3, MAP3K10, MAP3K11, MAP3K14, MAP3K6, MAP3K9, MAPK3, MAPK8IP3, MARK2, MAVS, MEN1, MFSD2A, MIDN, MINK1, MLLT1, MLST8, MSTN, MTCO2P12, MUC20, MYADM, MYC, NF2, NGF, NLRC5, NOD2, NODAL, NOS3, NPFFR2, NPPA, NR4A3, NSMF, NUP188, OMA1, OSM, P2RY6, PAK4, PDGFB, PDGFRB, PDK2, PDPR, PHACTR1, PKD1, PLAUR, PML, PPP1R10, PPP1R11, PPP1R15A, PPP1R16A, PPP1R9B, PPP2R1A, PPP2R5B, PPP2R5D, PPP6R1, PRKACA, PRKAR2B, PROM2, PTN, PTPN13, PTPRH, PTPRU, RAC2, RAP1A, RAPGEF1, RCVRN, RGCC, RHBDF2, RNU1-93P, RPLP1, RPTOR, RUBCN, S1PR2, SH2B3, SH2D3A, SH2D3C, SHC2, SLC2A6, SLC9A3R1, SMAD3, SMPD1, SOCS3, SPATC1L, SPHK1, SPHK2, SPINK1, SRC, STAT3, TESK1, THBS1, THPO, TIMP1, TNF, TNFRSF1A, TNK2, TNKS1BP1, TRAF4, TRIB1, TRIB3, TSC2, UBA52, UBE2B, VAC14, VEGFA, VTN, WARS1, WNT9B, ZBTB7A, ZC3H12A]

GO:1902533	positive regulation of intracellular signal transduction	5,04E-11	[4, 5, 6, 7]	241,00	21,77	42,21	57,79	[ABRA, ADAM8, ALOX15, ANGPT1, AREG, BANK1, BMF, C1QTNF1, CALCR, CARD11, CASP8, CASS4, CCL17, CCL19, CCL2, CCL22, CCL24, CCL3, CCL3L1, CCL4, CCL4L1, CCL5, CD19, CD27, CD3E, CD4, CD40LG, CD74, CD80, CD86, CLEC6A, CSF1R, CXCR4, EPX, EREG, FASLG, FCRL3, FFAR4, FGD2, FGR, FLT3, GADD45B, GDF6, GPR174, GPR4, GPR55, HCLS1, HCST, HLA-DRB1, ICOS, IKBKE, IL18, IL26, IQCJ-SCHIP1, LCK, LMCD1, MAP4K1, MSX1, NCF1, NCKAP1L, NPPC, OASL, P2RY10, P2RY12, PDE6G, PIK3CG, PIK3R5, PIM2, PRKCB, PROK1, PTK2B, PTPN22, PTPN6, PTPRC, PYCARD, PYHIN1, RAC2, RASGRF1, RTKN2, SCIMP, SEMA4D, SYK, THBS1, TNFSF14, TRAF1, TRAT1, TREM2, TRIM14, UBD, VAV1, XCL1, XCL2, ZAP70]	[ABCA7, ABL1, ABRA, ACKR3, ACTN4, ADGRG1, ADIPOQ, AGAP2, AGER, AGT, AMH, ANGPT1, APOA1, ARAF, AREG, ARHGEF5, BCL2L1, BRD4, C1QTNF1, C20orf27, C5, C5AR1, CALCR, CC2D1A, CCL11, CCL2, CCL21, CCL24, CCR7, CD14, CD74, CHERP, CHI3L1, CRAT, CSF1, CSK, CSPG4, CX3CL1, DAB2IP, DAXX, DENND2B, DIRAS1, DLG5, DOK5, DUSP5, DVL3, ECM1, EEF1E1, ENG, ERBB2, FADD, FAM110A, FGFR4, FLT3, FLT4, FPR1, GAB2, GADD45B, GATA4, GDF6, GNAI2, GPBAR1, GPER1, GPR137, GPR17, GPR20, GPR37L1, GPR4, HBEGF, HCRTR1, HTR2A, ICAM1, IL1B, ILK, IRAK2, JMJD8, KSR1, LGALS9, LILRA5, LMCD1, LTBR, LTF, MAP2K3, MAP3K10, MAP3K11, MAP3K14, MAP3K6, MAP3K9, MAPK3, MAPK8IP3, MAVS, MAZ, MEIS3P1, MINK1, MLST8, MSX1, MUC20, NDC80, NDST1, NGF, NOD2, NODAL, NOTCH1, NPPA, OSM, P2RX3, P2RX5, P2RY6, PDGFB, PDGFRB, PIAS4, PLA2G2A, PLAGL2, PLXNB1, PPARD, PRAG1, PTP4A3, RAC2, RAP1A, RAPGEF1, RASD2, RELA, RHOG, RNU1-93P, RPL26, RPTOR, RTKN2, S1PR2, SECTM1, SELENOP, SEMA4C, SEPTIN4, SHARPIN, SHC2, SHISA5, SIK3, SLC44A2, SLC9A1, SLC9A3R1, SORBS3, SPATC1L, SPHK1, SPHK2, SPI1, SRC, TGM2, THBS1, THPO, TICAM1, TIMP1, TNF, TNFRSF1A, TRAF4, TRIB3, TRIM62, TRIM8, TRPV4, UBA52, UNC5B, VEGFA, VSIG2, WDR24, ZC3H12A, ZCCHC3, ZNF385A]
GO:0016301	kinase activity	4,14E-06	[6]	292,00	18,86	38,63	61,38	[ADAM8, ADCY7, ANGPT1, AREG, BLK, BMP7, BMPR1B, BTK, CAMK4, CARD11, CASS4, CCL19, CCL2, CCL3, CCL5, CD19, CD4, CD40LG, CD74, CD86, CDKN2A, CERKL, CISH, CSF1R, CXCL10, CXCR4, DAPK1, DUSP2, DUSP8, EPHA6, EPX, EREG, FGD2, FGR, FLT3, GADD45B, GMFG, GPRC5A, GUCY2D, HASPIN, HKDC1, HLA-DRB1, IFNG, IKBKE, IL18, ITK, LAX1, LCK, LCP2, LILRB4, MAP4K1, MAPK13, MATK, MIDN, MUSK, MYO3B, NCF1, NCKAP1L, NEK5, NELL2, NLRP3, NLRP6, NPFFR2, NR4A3, P2RY12, PDE6G, PEAK3, PFKFB4, PIK3C2G, PIK3CG, PIK3IP1, PIK3R5, PIM1, PIM2, PRKCB, PRKCQ, PROK1, PTK2B, PTPN22, PTPN6, PTPRC, PTPRH, PYCARD, RAC2, RHOH, SH2D1A, SLA, SLA2, SLAMF8, SLC8A3, SOCS1, SOCS3, STAP1, STK17B, STYK1, SYK, THBS1, THY1, TLR6, TNFAIP3, TREM2, ZAP70]	[AATK, ABL1, ABR, ACP4, ACTB, ADCK1, ADCY1, ADCY3, ADCY4, ADCY9, ADIPOQ, AGAP2, AGER, AGT, AKT1S1, ALPK3, ANGPT1, ANKK1, ARAF, AREG, ARHGEF5, ARTN, BCKDK, BCR, C5, C5AR1, CAMK2B, CCL2, CCL21, CCNG2, CCNJL, CCR7, CD74, CDC25B, CDK1, CDK15, CDK18, CDK2, CDKL2, CDKN1A, CEACAM1, CERT1, CHI3L1, CIITA, CISH, CKM, CNKSR1, COASY, CSF1, CSK, CSNK1E, CSPG4, DAB2IP, DAG1, DAXX, DBF4B, DBNDD1, DCK, DDR1, DGKD, DGKZ, DGUOK, DIRAS1, DUSP5, DUSP8, DVL3, DYRK1B, EFNA3, ENG, EPHA2, ERBB2, FABP4, FES, FGFR4, FGFRL1, FLT3, FLT4, FPR1, GADD45B, GBA, GPI, GPRC5A, GSK3A, HBEGF, HGS, HIPK4, HK1, HSPB1, HSPB8, HTR2A, HUNK, IL1B, ILK, INCA1, INCENP, IRAK2, ITPRIP, JAK3, KSR1, LILRA5, LIMK1, LIMK2, LMTK3, LTF, MAP2K3, MAP3K10, MAP3K11, MAP3K14, MAP3K6, MAP3K9, MAPK3, MAPK4, MAPK7, MAPK8IP3, MARK2, MARK4, MAST2, MEN1, MIDN, MINK1, MKNK2, MLLT1, MLST8, MSTN, MUC20, MYO3A, NEK5, NF2, NGF, NLRC5, NME4, NOD2, NPFFR2, NPPA, NR4A3, OBSCN, OMA1, PAK4, PANK1, PDGFB, PDGFRB, PDK2, PI4KA, PI4KAP1, PI4KAP2, PIK3C2B, PIK3C2G, PIM1, PIP5K1C, PKD1, PKM, PKN3, PLK3, PPP1R9B, PPP2R1A, PRAG1, PRKACA, PRKAR2B, PTPRH, PTPRU, RAC2, RAP1A, RAPGEF1, RGCC, RHBDF2, RMRP, RNU1-93P, RPLP1, RPTOR, RSKR, RUBCN, S1PR2, SBK1, SCYL1, SH2B3, SHC2, SIK1, SIK2, SIK3, SLC9A3R1, SMPD1, SOCS3, SPATC1L, SPEG, SPHK1, SPHK2, SRC, SRPK3, STK17B, STK35, STK40, TESK1, TEX14, THBS1, TIMP1, TK1, TNF, TNK1, TNK2, TRAF4, TRIB1, TRIB3, TSC2, TSSK3, UBA52, ULK1, VAC14, VEGFA, WARS1, WNK2]
GO:0043408	regulation of MAPK cascade	9,96E-07	[5, 6, 7]	166,00	21,76	41,10	58,90	[ADAM8, ALOX15, ANGPT1, AREG, ATF3, BANK1, BMP7, C1QTNF1, CALCR, CCL17, CCL19, CCL2, CCL22, CCL24, CCL3, CCL3L1, CCL4, CCL4L1, CCL5, CD27, CD4, CD40LG, CD74, CSF1R, CXCR4, DUSP2, DUSP8, EPX, FCRL3, FFAR4, FGD2, FLT3, GADD45B, GDF6, GPR55, HLA-DRB1, IKBKE, IL26, LAX1, LIF, LILRB4, MAP4K1, NCF1, NLRP6, NPFFR2, PDE6G, PIK3CG, PIK3R5, PROK1, PSCA, PTK2B, PTPN22, PTPN6, PTPRC, PYCARD, SCIMP, SYK, TBC1D10C, THBS1, TRAF1, TREM2, XCL1, XCL2]	[ABCA7, ABL1, ACKR3, ADIPOQ, AGER, AGT, AMH, ANGPT1, ARAF, AREG, ARHGEF5, C1QTNF1, C5, C5AR1, CALCR, CCL11, CCL2, CCL21, CCL24, CCR7, CD74, CEACAM1, CHI3L1, CSK, CSPG4, CX3CL1, DAB2IP, DAG1, DAXX, DENND2B, DIRAS1, DOK5, DUSP5, DUSP8, DVL3, EPHA2, ERBB2, FASN, FGFR4, FLT3, FLT4, FOXM1, FPR1, GADD45B, GATA4, GBA, GDF6, GNAI2, GPBAR1, GPER1, GPR37L1, GRIK5, HCRTR1, HTR2A, ICAM1, IL1B, ILK, IRAK2, KCTD11, KSR1, LGALS9, LIF, LILRA5, LTBR, MAP2K3, MAP3K10, MAP3K11, MAP3K14, MAP3K6, MAP3K9, MAPK3, MAPK8IP3, MEN1, MINK1, MUC20, MYC, NDST1, NF2, NGF, NOD2, NODAL, NOTCH1, NPFFR2, NPPA, P2RY6, PAK4, PDGFB, PDGFRB, PER1, PLA2G2A, PPP2R1A, RAP1A, RAPGEF1, RNU1-93P, S1PR2, SEMA4C, SH2B3, SHC2, SLC9A3R1, SMPD1, SORBS3, SPHK1, SPI1, SRC, STK40, SYNGAP1, THBS1, THPO, TIMP1, TNF, TNIP1, TRAF4, TRPV4, UBA52, VEGFA, WNK2, ZC3H12A]

GO:0045937	positive regulation of phosphate metabolic process	6,68E-03	[5, 6, 7]	198,00	18,63	38,47	61,53	[ADAM8, ADCY7, AIF1, ANGPT1, AREG, BANK1, BMP7, CALCR, CASS4, CCL19, CCL5, CD19, CD244, CD33, CD3E, CD4, CD40LG, CD74, CD80, CD86, CDCA2, CLCF1, CSF1R, CXCR4, EPHA6, EPX, EREG, FCRL3, FGD2, FGR, FLT3, GADD45B, GDF6, GPRC5A, HCLS1, HLA-DRB1, IFNG, IKBKE, IL18, LCP2, LIF, MAP4K1, MMP9, MUSK, NCF1, NCKAP1L, NLRP3, NR4A3, P2RY12, PDE6G, PFKFB4, PIK3CG, PIK3R5, PLEK, PROK1, PTAFR, PTK2B, PTPRC, SEMA4D, SH2D1A, SOCS1, SOCS3, STAP1, SYK, THBS1, TLR6, TNFRSF18, TREM2, WNT1]	[ABL1, ADCY1, ADCY3, ADCY4, ADCY9, ADIPOQ, AGAP2, AGER, AGT, AMH, ANGPT1, APLN, APLNR, ARAF, AREG, ARHGEF5, ARTN, BMP8A, C5, C5AR1, CALCR, CCL21, CCR7, CD74, CDC25B, CDCA2, CDKN1A, CHI3L1, CLCF1, CLIP3, CSF1, CSK, CSPG4, CTF1, CX3CL1, DAB2IP, DAG1, DAXX, DBF4B, DDR1, DGKZ, DHX34, DIRAS1, DUSP5, DVL3, EIF4G1, ENG, EPHA2, ERBB2, FASN, FGFR4, FLT3, FLT4, FPR1, GADD45B, GBA, GDF6, GPER1, GPRC5A, GSK3A, HBEGF, HFE, HSF1, HTR2A, ICAM1, IL1B, ILK, INCENP, INHA, IRAK2, ITGA5, KSR1, LIF, LILRA5, LIMK2, LTF, MAP2K3, MAP3K10, MAP3K11, MAP3K14, MAP3K6, MAP3K9, MAPK3, MAPK8IP3, MARK2, MAVS, MINK1, MLST8, MSTN, MTCO2P12, MUC20, MYC, NGF, NOD2, NODAL, NOS3, NR4A3, NSMF, OMA1, OSM, P2RY6, PAK4, PDGFB, PDGFRB, PKD1, PLAUR, PPP1R15A, PPP2R5D, PRKACA, PRKAR2B, PROM2, RAP1A, RAPGEF1, RCVRN, RGCC, RHBDF2, RNU1-93P, RPLP1, RPTOR, S1PR2, SH2D3A, SH2D3C, SHC2, SMAD3, SMPD1, SOCS3, SPATC1L, SPHK1, SRC, STAT3, TESK1, THBS1, THPO, TIMP1, TNF, TNFRSF1A, TNK2, TNKS1BP1, TRAF4, UBA52, VAC14, VEGFA, VTN]
GO:0070371	ERK1 and ERK2 cascade	6,42E-07	[5, 6, 7]	90,00	26,39	44,83	55,17	[ALOX15, ANGPT1, ATF3, CALCR, CCL17, CCL19, CCL2, CCL22, CCL24, CCL3, CCL3L1, CCL4, CCL4L1, CCL5, CD4, CD74, CSF1R, EPX, FFAR4, GPR55, HLA-DRB1, IL26, LIF, NLRP3, NLRP6, PSCA, PTK2B, PTPN22, PTPN6, PTPRC, PYCARD, SCIMP, SYK, TBC1D10C, TREM2, XCL1, XCL2]	[ABCA7, ABL1, ACKR3, ADIPOQ, AGER, AGT, AMH, ANGPT1, APOA1, ARHGEF5, C5AR1, CALCR, CCL11, CCL2, CCL21, CCL24, CCR7, CD74, CDK1, CEACAM1, CHI3L1, CSK, CX3CL1, DAB2IP, DENND2B, EPHA2, ERBB2, FGFR4, FLT4, GATA4, GNAI2, GPBAR1, GPER1, HCRTR1, HESX1, HTR2A, ICAM1, IL1B, LGALS9, LIF, MAPK3, MYC, NOD2, NODAL, NOTCH1, P2RY6, PDGFB, PDGFRB, PLA2G2A, RAP1A, RNU1-93P, SLC9A3R1, SRC, THPO, TIMP1, TNF, TNIP1, TRPV4, WNK2]
GO:0006468	protein phosphorylation	3,68E-07	[6, 7]	326,00	18,89	38,39	61,61	[ADAM8, ADCY7, AIF1, ANGPT1, AREG, ATF3, BANK1, BLK, BMP7, BMPR1B, BTK, CALCR, CAMK4, CASS4, CCDC88C, CCL19, CCL2, CCL3, CCL5, CD3E, CD4, CD40LG, CD74, CD80, CD86, CDKN2A, CLCF1, CSF1R, CXCL10, CXCR4, DAPK1, DUSP2, DUSP8, EPHA6, EPX, EREG, FGD2, FGR, FLT3, GADD45B, GDF6, GFRA2, GMFG, GPRC5A, GUCY2D, HASPIN, HCLS1, HCST, HLA-DRB1, IFNG, IKBKE, IL12RB2, IL18, ITGB2, ITK, LAX1, LCK, LCP2, LIF, LILRB4, MAP4K1, MAPK13, MATK, MMP9, MUSK, MYO3B, NCF1, NCKAP1L, NEK5, NELL2, NLRP3, NLRP6, NPFFR2, NR4A3, PDE6G, PEAK3, PIK3CG, PIK3R5, PIM1, PIM2, PRKCB, PRKCQ, PROK1, PTK2B, PTPN22, PTPN6, PTPRC, PTPRH, PYCARD, RAC2, RUNX3, SAMSN1, SEMA4D, SH2D1A, SLA, SLC8A3, SMPD3, SOCS1, SOCS3, SPINK1, STAP1, STK17B, STYK1, SYK, THBS1, THY1, TLR6, TLR8, TNFAIP3, TNFRSF18, TREM2, WNT1, ZAP70]	[AATK, ABL1, ABR, ACP4, ACTB, ADCK1, ADCY1, ADCY3, ADCY4, ADCY9, ADIPOQ, AGAP2, AGER, AGT, AKT1S1, ALPK3, AMH, ANGPT1, ANKK1, APLNR, APOA1, ARAF, AREG, ARHGEF5, ARTN, ATP13A2, BCKDK, BCR, BMP8A, BRD4, C5, C5AR1, CALCR, CAMK2B, CCL11, CCL2, CCL21, CCNG2, CCNJL, CCR7, CD74, CDC25B, CDK1, CDK15, CDK18, CDK2, CDKL2, CDKN1A, CEACAM1, CHI3L1, CIITA, CLCF1, CLIP3, CSF1, CSK, CSNK1E, CSPG4, CTDSP1, CTF1, CX3CL1, DAB2IP, DAG1, DAXX, DBF4B, DBNDD1, DDR1, DGUOK, DIRAS1, DMTN, DUSP5, DUSP8, DVL3, DYRK1B, EFNA3, EIF4G1, ENG, EPHA2, ERBB2, FABP4, FASN, FES, FGFR4, FGFRL1, FLT3, FLT4, FPR1, GADD45B, GBA, GDF6, GPER1, GPI, GPRC5A, GSK3A, HBEGF, HFE, HGS, HIPK4, HSF1, HSPB1, HSPB8, HTR2A, HUNK, ICAM1, IL1B, ILK, INCA1, INCENP, INHA, INPP5J, IRAK2, ITGA5, ITPRIP, JAK3, KSR1, LIF, LILRA5, LIMK1, LIMK2, LIPE, LMTK3, LOX, LTF, MAP2K3, MAP3K10, MAP3K11, MAP3K14, MAP3K6, MAP3K9, MAPK3, MAPK4, MAPK7, MAPK8IP3, MARK2, MARK4, MAST2, MAVS, MEN1, MINK1, MKNK2, MLLT1, MLST8, MSTN, MUC20, MYADM, MYO3A, NEK5, NF2, NGF, NOD2, NODAL, NPFFR2, NPPA, NR4A3, OBSCN, OMA1, OSM, PAK4, PDGFB, PDGFRB, PDK2, PIM1, PKD1, PKN3, PLAUR, PLK3, PML, PPP1R9B, PPP2R1A, PPP2R5B, PPP2R5D, PRAG1, PRKACA, PRKAR2B, PROM2, PRRT1, PTPN13, PTPRH, PTPRU, RAC2, RAP1A, RAPGEF1, RARA, RGCC, RHBDF2, RNU1-93P, RPLP1, RPTOR, RSKR, S1PR2, SBK1, SCYL1, SH2B3, SH2D3A, SH2D3C, SHC2, SIK1, SIK2, SIK3, SLC9A3R1, SMPD1, SOCS3, SPATC1L, SPEG, SPHK1, SPINK1, SRC, SRPK3, STAT3, STK17B, STK35, STK40, TESK1, TEX14, THBS1, THPO, TIMP1, TNF, TNFRSF1A, TNK1, TNK2, TNKS1BP1, TRAF4, TRIB1, TRIB3, TSC2, TSSK3, UBA52, UBE2B, ULK1, VEGFA, VTN, WARS1, WNK2, WNT9B, ZC3H12A]
GO:0031401	positive regulation of protein modification process	1,63E-02	[5, 6, 7, 8]	207,00	18,22	36,05	63,95	[ADAM8, ADCY7, AIF1, ANGPT1, AREG, BANK1, BMP7, CALCR, CASS4, CCL19, CCL5, CD33, CD3E, CD4, CD40LG, CD74, CD80, CD86, CDC20B, CDCA2, CDKN2A, CLCF1, CSF1R, CXCR4, EPX, EREG, FCRL3, FGD2, FLT3, FOXP3, GADD45B, GDF6, GPRC5A, HCLS1, HLA-DRB1, IFNG, IKBKE, IL18, LCP2, LIF, MAP4K1, MMP9, MUSK, MYB, NCF1, NCKAP1L, NLRP3, NR4A3, PDE6G, PIK3CG, PIK3R5, PROK1, PTK2B, PTPN22, PTPRC, SEMA4D, SH2D1A, SOCS1, SOCS3, STAP1, SYK, THBS1, TLR6, TNFRSF18, TRABD2A, TREM2, WNT1]	[ABL1, ADCY1, ADCY3, ADCY4, ADCY9, ADIPOQ, AGAP2, AGER, AGT, AMH, ANGPT1, APLNR, ARAF, AREG, ARHGEF5, ARTN, BCL6, BMP8A, BRD4, C5, C5AR1, CALCR, CCL21, CCR7, CD74, CDC25B, CDCA2, CDKN1A, CHI3L1, CLCF1, CLIP3, CSF1, CSK, CSPG4, CTF1, CX3CL1, DAB2IP, DAG1, DAXX, DBF4B, DIRAS1, DNMT1, DUSP5, DVL3, EIF4G1, ENG, ERBB2, FASN, FLT3, FLT4, FPR1, GADD45B, GBA, GDF6, GPER1, GPRC5A, GSK3A, HBEGF, HFE, HSF1, HTR2A, ICAM1, IL1B, ILK, INCENP, INHA, IRAK2, ITGA5, KSR1, LIF, LILRA5, LIMK2, LMNA, LTF, MAP2K3, MAP3K10, MAP3K11, MAP3K14, MAP3K6, MAP3K9, MAPK3, MAPK8IP3, MARK2, MAVS, MECP2, MINK1, MLST8, MSTN, MUC20, NAP1L2, NGF, NOD2, NODAL, NR4A3, NSMF, OMA1, OSM, PAF1, PAK4, PDGFB, PDGFRB, PEF1, PHF1, PIAS4, PKD1, PLAUR, PPP1R15A, PPP2R5D, PRKACA, PRKAR2B, PROM2, RAP1A, RAPGEF1, RASD2, RASSF5, RGCC, RHBDF2, RNU1-93P, RPLP1, RPTOR, S1PR2, SEPTIN4, SH2D3A, SH2D3C, SHC2, SLC51B, SMARCB1, SMPD1, SOCS3, SPATC1L, SPHK1, SPHK2, SRC, SREBF1, STAT3, TESK1, THBS1, THPO, TICAM1, TIMP1, TNF, TNFRSF1A, TNIP1, TNK2, TNKS1BP1, TRAF4, TRIB3, UBA52, VEGFA, VTN, WBP2, WFS1, ZBTB7B, ZC3H12A]

GO:0042325	regulation of phosphorylation	3,73E-03	[6, 7]	261,00	17,91	39,44	60,56	[ADAM8, ADCY7, AIF1, ANGPT1, AREG, ATF3, BANK1, BMP7, CALCR, CASS4, CCDC88C, CCL19, CCL5, CD19, CD3E, CD4, CD40LG, CD74, CD80, CD86, CDKN2A, CISH, CLCF1, CSF1R, CXCL10, CXCR4, DUSP2, DUSP8, EPHA6, EPX, EREG, FGD2, FGR, FLT3, GADD45B, GDF6, GFRA2, GMFG, GPRC5A, HCLS1, HLA-DRB1, IFNG, IKBKE, IL18, ITGB2, LAX1, LCP2, LIF, LILRB4, MAP4K1, MIDN, MMP9, MUSK, NCF1, NCKAP1L, NLRP3, NPFFR2, NR4A3, NUP210, P2RY12, PDE6G, PFKFB4, PIK3CG, PIK3IP1, PIK3R5, PROK1, PTK2B, PTPN22, PTPN6, PTPRC, PTPRH, PYCARD, RAC2, RHOH, SAMSN1, SEMA4D, SH2D1A, SLA2, SLAMF8, SLC8A3, SMPD3, SOCS1, SOCS3, SPINK1, STAP1, SYK, THBS1, THY1, TLR6, TLR8, TNFAIP3, TNFRSF18, TREM2, WNT1]	[AAAS, ABL1, ACP4, ACTB, ADCY1, ADCY3, ADCY4, ADCY9, ADIPOQ, AGAP2, AGER, AGT, AKT1S1, AMH, ANGPT1, APLN, APLNR, APOA1, ARAF, AREG, ARHGEF5, ARTN, BMP8A, BRD4, C5, C5AR1, CALCR, CCL21, CCNG2, CCNJL, CCR7, CD74, CDC25B, CDKN1A, CEACAM1, CHI3L1, CISH, CLCF1, CLIP3, CSF1, CSK, CSPG4, CTDSP1, CTF1, CX3CL1, DAB2IP, DAG1, DAXX, DBF4B, DBNDD1, DDR1, DGKZ, DIRAS1, DMTN, DUSP5, DUSP8, DVL3, EIF4G1, ENG, EPHA2, ERBB2, FABP4, FASN, FGFR4, FLT3, FLT4, FPR1, GADD45B, GBA, GDF6, GIT1, GPER1, GPRC5A, GSK3A, HBEGF, HFE, HGS, HSF1, HSPB1, HTR2A, ICAM1, IL1B, ILK, INCA1, INCENP, INHA, INPP5J, IRAK2, ITGA5, ITPRIP, JMJD8, KSR1, LIF, LILRA5, LIMK2, LOX, LTF, MAP2K3, MAP3K10, MAP3K11, MAP3K14, MAP3K6, MAP3K9, MAPK3, MAPK8IP3, MARK2, MAVS, MEN1, MIDN, MINK1, MLLT1, MLST8, MSTN, MUC20, MYADM, MYC, NF2, NGF, NLRC5, NOD2, NODAL, NPFFR2, NPPA, NR4A3, NUP188, OMA1, OSM, PAK4, PDGFB, PDGFRB, PKD1, PLAUR, PML, PPP1R9B, PPP2R1A, PPP2R5B, PPP2R5D, PRKACA, PRKAR2B, PROM2, PTPN13, PTPRH, PTPRU, RAC2, RAP1A, RAPGEF1, RGCC, RHBDF2, RNU1-93P, RPLP1, RPTOR, RUBCN, S1PR2, SH2B3, SH2D3A, SH2D3C, SHC2, SLC2A6, SLC9A3R1, SMPD1, SOCS3, SPATC1L, SPHK1, SPINK1, SRC, STAT3, TESK1, THBS1, THPO, TIMP1, TNF, TNFRSF1A, TNK2, TNKS1BP1, TRAF4, TRIB1, TRIB3, TSC2, UBA52, UBE2B, VAC14, VEGFA, VTN, WARS1, WNT9B, ZBTB7A, ZC3H12A]
GO:0043410	positive regulation of MAPK cascade	1,82E-06	[5, 6, 7, 8]	131,00	23,06	42,07	57,93	[ADAM8, ALOX15, ANGPT1, AREG, BANK1, C1QTNF1, CALCR, CCL17, CCL19, CCL2, CCL22, CCL24, CCL3, CCL3L1, CCL4, CCL4L1, CCL5, CD27, CD4, CD40LG, CD74, CSF1R, CXCR4, EPX, FCRL3, FFAR4, FGD2, FLT3, GADD45B, GDF6, GPR55, HLA-DRB1, IKBKE, IL26, MAP4K1, NCF1, PDE6G, PIK3CG, PIK3R5, PROK1, PTK2B, PTPN22, PTPRC, PYCARD, SCIMP, SYK, THBS1, TRAF1, TREM2, XCL1, XCL2]	[ABCA7, ABL1, ACKR3, AGER, AGT, AMH, ANGPT1, ARAF, AREG, ARHGEF5, C1QTNF1, C5, C5AR1, CALCR, CCL11, CCL2, CCL21, CCL24, CCR7, CD74, CHI3L1, CSK, CSPG4, CX3CL1, DAB2IP, DAXX, DENND2B, DIRAS1, DOK5, DUSP5, DVL3, ERBB2, FGFR4, FLT3, FLT4, FPR1, GADD45B, GATA4, GDF6, GNAI2, GPBAR1, GPER1, GPR37L1, HCRTR1, HTR2A, ICAM1, IL1B, ILK, IRAK2, KSR1, LGALS9, LILRA5, LTBR, MAP2K3, MAP3K10, MAP3K11, MAP3K14, MAP3K6, MAP3K9, MAPK3, MAPK8IP3, MINK1, MUC20, NDST1, NGF, NOD2, NODAL, NOTCH1, P2RY6, PDGFB, PDGFRB, PLA2G2A, RAP1A, RAPGEF1, RNU1-93P, S1PR2, SEMA4C, SHC2, SORBS3, SPHK1, SPI1, SRC, THBS1, THPO, TIMP1, TNF, TRAF4, TRPV4, UBA52, VEGFA, ZC3H12A]
GO:0043549	regulation of kinase activity	1,18E-02	[5, 7, 8]	181,00	18,76	41,50	58,50	[ADAM8, ADCY7, ANGPT1, AREG, BMP7, CASS4, CCL19, CCL5, CD19, CD4, CD40LG, CD74, CD86, CDKN2A, CISH, CSF1R, CXCL10, CXCR4, DUSP2, DUSP8, EPHA6, EPX, EREG, FGD2, FGR, FLT3, GADD45B, GMFG, GPRC5A, HLA-DRB1, IFNG, IKBKE, IL18, LAX1, LCP2, LILRB4, MAP4K1, MIDN, MUSK, NCF1, NCKAP1L, NPFFR2, P2RY12, PDE6G, PIK3CG, PIK3IP1, PIK3R5, PROK1, PTK2B, PTPN22, PTPN6, PTPRC, PTPRH, PYCARD, RAC2, RHOH, SH2D1A, SLA2, SLAMF8, SLC8A3, SOCS1, SOCS3, STAP1, SYK, THBS1, THY1, TLR6, TNFAIP3, TREM2]	[ABL1, ACP4, ACTB, ADCY1, ADCY3, ADCY4, ADCY9, ADIPOQ, AGAP2, AGER, AGT, AKT1S1, ANGPT1, ARAF, AREG, ARHGEF5, ARTN, C5, C5AR1, CCL21, CCNG2, CCNJL, CCR7, CD74, CDC25B, CDKN1A, CEACAM1, CHI3L1, CISH, CSF1, CSK, CSPG4, DAB2IP, DAG1, DAXX, DBF4B, DBNDD1, DDR1, DGKZ, DIRAS1, DUSP5, DUSP8, DVL3, EPHA2, ERBB2, FABP4, FGFR4, FLT3, FLT4, FPR1, GADD45B, GBA, GPRC5A, HBEGF, HGS, HSPB1, HTR2A, IL1B, ILK, INCA1, INCENP, IRAK2, ITPRIP, KSR1, LILRA5, LTF, MAP2K3, MAP3K10, MAP3K11, MAP3K14, MAP3K6, MAP3K9, MAPK3, MAPK8IP3, MARK2, MEN1, MIDN, MINK1, MLLT1, MLST8, MSTN, MUC20, NF2, NGF, NLRC5, NOD2, NPFFR2, NPPA, OMA1, PAK4, PDGFB, PDGFRB, PKD1, PPP2R1A, PRKACA, PRKAR2B, PTPRH, PTPRU, RAC2, RAP1A, RAPGEF1, RGCC, RNU1-93P, RPLP1, RPTOR, RUBCN, S1PR2, SH2B3, SHC2, SLC9A3R1, SMPD1, SOCS3, SPATC1L, SRC, TESK1, THBS1, TIMP1, TNF, TRAF4, TRIB1, TRIB3, TSC2, UBA52, VAC14, VEGFA, WARS1]
GO:0001932	regulation of protein phosphorylation	1,95E-03	[6, 7, 8]	235,00	18,39	38,75	61,25	[ADAM8, ADCY7, AIF1, ANGPT1, AREG, ATF3, BANK1, BMP7, CALCR, CASS4, CCDC88C, CCL19, CCL5, CD3E, CD4, CD40LG, CD74, CD80, CD86, CDKN2A, CLCF1, CSF1R, CXCL10, CXCR4, DUSP2, DUSP8, EPX, EREG, FGD2, FGR, FLT3, GADD45B, GDF6, GFRA2, GMFG, GPRC5A, HCLS1, HLA-DRB1, IFNG, IKBKE, IL18, ITGB2, LAX1, LCP2, LIF, LILRB4, MAP4K1, MMP9, MUSK, NCF1, NCKAP1L, NLRP3, NPFFR2, NR4A3, PDE6G, PIK3CG, PIK3R5, PROK1, PTK2B, PTPN22, PTPN6, PTPRC, PTPRH, PYCARD, RAC2, SAMSN1, SEMA4D, SH2D1A, SLC8A3, SMPD3, SOCS1, SOCS3, SPINK1, STAP1, SYK, THBS1, THY1, TLR6, TLR8, TNFAIP3, TNFRSF18, TREM2, WNT1]	[ABL1, ACP4, ACTB, ADCY1, ADCY3, ADCY4, ADCY9, ADIPOQ, AGAP2, AGER, AGT, AKT1S1, AMH, ANGPT1, APLNR, APOA1, ARAF, AREG, ARHGEF5, ARTN, BMP8A, BRD4, C5, C5AR1, CALCR, CCL21, CCNG2, CCNJL, CCR7, CD74, CDC25B, CDKN1A, CEACAM1, CHI3L1, CLCF1, CLIP3, CSF1, CSK, CSPG4, CTDSP1, CTF1, CX3CL1, DAB2IP, DAG1, DAXX, DBF4B, DBNDD1, DIRAS1, DMTN, DUSP5, DUSP8, DVL3, EIF4G1, ENG, ERBB2, FABP4, FASN, FLT3, FLT4, FPR1, GADD45B, GBA, GDF6, GPER1, GPRC5A, GSK3A, HBEGF, HFE, HGS, HSF1, HSPB1, HTR2A, ICAM1, IL1B, ILK, INCA1, INCENP, INHA, INPP5J, IRAK2, ITGA5, ITPRIP, KSR1, LIF, LILRA5, LIMK2, LOX, LTF, MAP2K3, MAP3K10, MAP3K11, MAP3K14, MAP3K6, MAP3K9, MAPK3, MAPK8IP3, MARK2, MAVS, MEN1, MINK1, MLLT1, MLST8, MSTN, MUC20, MYADM, NF2, NGF, NOD2, NODAL, NPFFR2, NPPA, NR4A3, OMA1, OSM, PAK4, PDGFB, PDGFRB, PKD1, PLAUR, PML, PPP1R9B, PPP2R1A, PPP2R5B, PPP2R5D, PRKACA, PRKAR2B, PROM2, PTPN13, PTPRH, PTPRU, RAC2, RAP1A, RAPGEF1, RGCC, RHBDF2, RNU1-93P, RPLP1, RPTOR, S1PR2, SH2B3, SH2D3A, SH2D3C, SHC2, SLC9A3R1, SMPD1, SOCS3, SPATC1L, SPHK1, SPINK1, SRC, STAT3, TESK1, THBS1, THPO, TIMP1, TNF, TNFRSF1A, TNK2, TNKS1BP1, TRAF4, TRIB1, TRIB3, TSC2, UBA52, UBE2B, VEGFA, VTN, WARS1, WNT9B, ZC3H12A]

GO:0042327	positive regulation of phosphorylation	2,10E-02	[6, 7, 8]	181,00	18,55	38,39	61,61	[ADAM8, ADCY7, AIF1, ANGPT1, AREG, BANK1, BMP7, CALCR, CASS4, CCL19, CCL5, CD19, CD3E, CD4, CD40LG, CD74, CD80, CD86, CLCF1, CSF1R, CXCR4, EPHA6, EPX, EREG, FGD2, FGR, FLT3, GADD45B, GDF6, GPRC5A, HCLS1, HLA-DRB1, IFNG, IKBKE, IL18, LCP2, LIF, MAP4K1, MMP9, MUSK, NCF1, NCKAP1L, NLRP3, NR4A3, P2RY12, PDE6G, PFKFB4, PIK3CG, PIK3R5, PROK1, PTK2B, PTPRC, SEMA4D, SH2D1A, SOCS1, SOCS3, STAP1, SYK, THBS1, TLR6, TNFRSF18, TREM2, WNT1]	[ABL1, ADCY1, ADCY3, ADCY4, ADCY9, ADIPOQ, AGAP2, AGER, AGT, AMH, ANGPT1, APLN, APLNR, ARAF, AREG, ARHGEF5, ARTN, BMP8A, C5, C5AR1, CALCR, CCL21, CCR7, CD74, CDC25B, CDKN1A, CHI3L1, CLCF1, CLIP3, CSF1, CSK, CSPG4, CTF1, CX3CL1, DAB2IP, DAG1, DAXX, DBF4B, DDR1, DGKZ, DIRAS1, DUSP5, DVL3, EIF4G1, ENG, EPHA2, ERBB2, FASN, FGFR4, FLT3, FLT4, FPR1, GADD45B, GDF6, GPER1, GPRC5A, GSK3A, HBEGF, HFE, HSF1, HTR2A, ICAM1, IL1B, ILK, INCENP, INHA, IRAK2, ITGA5, KSR1, LIF, LILRA5, LIMK2, LTF, MAP2K3, MAP3K10, MAP3K11, MAP3K14, MAP3K6, MAP3K9, MAPK3, MAPK8IP3, MARK2, MAVS, MINK1, MLST8, MSTN, MUC20, MYC, NGF, NOD2, NODAL, NR4A3, OMA1, OSM, PAK4, PDGFB, PDGFRB, PKD1, PLAUR, PRKACA, PRKAR2B, PROM2, RAP1A, RAPGEF1, RGCC, RHBDF2, RNU1-93P, RPLP1, RPTOR, S1PR2, SH2D3A, SH2D3C, SHC2, SOCS3, SPATC1L, SPHK1, SRC, STAT3, TESK1, THBS1, THPO, TIMP1, TNF, TNFRSF1A, TNK2, TNKS1BP1, TRAF4, UBA52, VAC14, VEGFA, VTN]
GO:0070372	regulation of ERK1 and ERK2 cascade	1,54E-06	[6, 7, 8]	84,00	26,75	46,35	53,65	[ALOX15, ANGPT1, ATF3, CALCR, CCL17, CCL19, CCL2, CCL22, CCL24, CCL3, CCL3L1, CCL4, CCL4L1, CCL5, CD4, CD74, CSF1R, EPX, FFAR4, GPR55, HLA-DRB1, IL26, LIF, NLRP6, PSCA, PTK2B, PTPN22, PTPN6, PTPRC, PYCARD, SCIMP, SYK, TBC1D10C, TREM2, XCL1, XCL2]	[ABCA7, ABL1, ACKR3, ADIPOQ, AGER, AMH, ANGPT1, ARHGEF5, C5AR1, CALCR, CCL11, CCL2, CCL21, CCL24, CCR7, CD74, CEACAM1, CHI3L1, CSK, CX3CL1, DAB2IP, DENND2B, EPHA2, ERBB2, FGFR4, FLT4, GATA4, GNAI2, GPBAR1, GPER1, HCRTR1, HTR2A, ICAM1, IL1B, LGALS9, LIF, MAPK3, NOD2, NODAL, NOTCH1, P2RY6, PDGFB, PDGFRB, PLA2G2A, RAP1A, RNU1-93P, SLC9A3R1, SRC, THPO, TIMP1, TNF, TNIP1, TRPV4, WNK2]
GO:0001934	positive regulation of protein phosphorylation	1,34E-02	[6, 7, 8, 9]	169,00	18,95	37,74	62,26	[ADAM8, ADCY7, AIF1, ANGPT1, AREG, BANK1, BMP7, CALCR, CASS4, CCL19, CCL5, CD3E, CD4, CD40LG, CD74, CD80, CD86, CLCF1, CSF1R, CXCR4, EPX, EREG, FGD2, FLT3, GADD45B, GDF6, GPRC5A, HCLS1, HLA-DRB1, IFNG, IKBKE, IL18, LCP2, LIF, MAP4K1, MMP9, MUSK, NCF1, NCKAP1L, NLRP3, NR4A3, PDE6G, PIK3CG, PIK3R5, PROK1, PTK2B, PTPRC, SEMA4D, SH2D1A, SOCS1, SOCS3, STAP1, SYK, THBS1, TLR6, TNFRSF18, TREM2, WNT1]	[ABL1, ADCY1, ADCY3, ADCY4, ADCY9, ADIPOQ, AGAP2, AGER, AGT, AMH, ANGPT1, APLNR, ARAF, AREG, ARHGEF5, ARTN, BMP8A, C5, C5AR1, CALCR, CCL21, CCR7, CD74, CDC25B, CDKN1A, CHI3L1, CLCF1, CLIP3, CSF1, CSK, CSPG4, CTF1, CX3CL1, DAB2IP, DAG1, DAXX, DBF4B, DIRAS1, DUSP5, DVL3, EIF4G1, ENG, ERBB2, FASN, FLT3, FLT4, FPR1, GADD45B, GDF6, GPER1, GPRC5A, GSK3A, HBEGF, HFE, HSF1, HTR2A, ICAM1, IL1B, ILK, INCENP, INHA, IRAK2, ITGA5, KSR1, LIF, LILRA5, LIMK2, LTF, MAP2K3, MAP3K10, MAP3K11, MAP3K14, MAP3K6, MAP3K9, MAPK3, MAPK8IP3, MARK2, MAVS, MINK1, MLST8, MSTN, MUC20, NGF, NOD2, NODAL, NR4A3, OMA1, OSM, PAK4, PDGFB, PDGFRB, PKD1, PLAUR, PRKACA, PRKAR2B, PROM2, RAP1A, RAPGEF1, RGCC, RHBDF2, RNU1-93P, RPLP1, RPTOR, S1PR2, SH2D3A, SH2D3C, SHC2, SOCS3, SPATC1L, SPHK1, SRC, STAT3, TESK1, THBS1, THPO, TIMP1, TNF, TNFRSF1A, TNK2, TNKS1BP1, TRAF4, UBA52, VEGFA, VTN]
GO:0004672	protein kinase activity	4,72E-06	[7, 8]	251,00	19,43	38,38	61,62	[ADAM8, ADCY7, ANGPT1, AREG, BLK, BMP7, BMPR1B, BTK, CAMK4, CASS4, CCL19, CCL2, CCL3, CCL5, CD4, CD40LG, CD74, CD86, CDKN2A, CSF1R, CXCL10, CXCR4, DAPK1, DUSP2, DUSP8, EPHA6, EPX, EREG, FGD2, FGR, FLT3, GADD45B, GMFG, GPRC5A, GUCY2D, HASPIN, IFNG, IKBKE, IL18, ITK, LAX1, LCK, LCP2, LILRB4, MAP4K1, MAPK13, MATK, MUSK, MYO3B, NCF1, NCKAP1L, NEK5, NELL2, NLRP3, NLRP6, NPFFR2, NR4A3, PDE6G, PEAK3, PIK3CG, PIK3R5, PIM1, PIM2, PRKCB, PRKCQ, PROK1, PTK2B, PTPN22, PTPN6, PTPRC, PTPRH, PYCARD, RAC2, SH2D1A, SLA, SLC8A3, SOCS1, SOCS3, STAP1, STK17B, STYK1, SYK, THBS1, THY1, TLR6, TNFAIP3, ZAP70]	[AATK, ABL1, ABR, ACP4, ACTB, ADCK1, ADCY1, ADCY3, ADCY4, ADCY9, ADIPOQ, AGAP2, AGER, AGT, AKT1S1, ALPK3, ANGPT1, ANKK1, ARAF, AREG, ARHGEF5, BCKDK, BCR, C5, C5AR1, CAMK2B, CCL2, CCL21, CCNG2, CCNJL, CCR7, CD74, CDC25B, CDK1, CDK15, CDK18, CDK2, CDKL2, CDKN1A, CEACAM1, CHI3L1, CIITA, CSF1, CSK, CSNK1E, CSPG4, DAB2IP, DAG1, DAXX, DBF4B, DBNDD1, DDR1, DIRAS1, DUSP5, DUSP8, DVL3, DYRK1B, EFNA3, ENG, EPHA2, ERBB2, FABP4, FES, FGFR4, FGFRL1, FLT3, FLT4, FPR1, GADD45B, GBA, GPI, GPRC5A, GSK3A, HBEGF, HGS, HIPK4, HSPB1, HSPB8, HTR2A, HUNK, IL1B, ILK, INCA1, INCENP, IRAK2, ITPRIP, JAK3, KSR1, LILRA5, LIMK1, LIMK2, LMTK3, LTF, MAP2K3, MAP3K10, MAP3K11, MAP3K14, MAP3K6, MAP3K9, MAPK3, MAPK4, MAPK7, MAPK8IP3, MARK2, MARK4, MAST2, MEN1, MINK1, MKNK2, MLLT1, MLST8, MUC20, MYO3A, NEK5, NF2, NGF, NOD2, NPFFR2, NPPA, NR4A3, OBSCN, OMA1, PAK4, PDGFB, PDGFRB, PDK2, PIM1, PKD1, PKN3, PLK3, PPP1R9B, PPP2R1A, PRAG1, PRKACA, PRKAR2B, PTPRH, PTPRU, RAC2, RAP1A, RAPGEF1, RGCC, RHBDF2, RNU1-93P, RPLP1, RPTOR, RSKR, S1PR2, SBK1, SCYL1, SH2B3, SHC2, SIK1, SIK2, SIK3, SLC9A3R1, SMPD1, SOCS3, SPATC1L, SPEG, SRC, SRPK3, STK17B, STK35, STK40, TESK1, TEX14, THBS1, TIMP1, TNF, TNK1, TNK2, TRAF4, TRIB1, TRIB3, TSC2, TSSK3, UBA52, ULK1, VEGFA, WARS1, WNK2]
GO:0070374	positive regulation of ERK1 and ERK2 cascade	6,07E-07	[6, 7, 8, 9]	66,00	30,00	47,22	52,78	[ALOX15, ANGPT1, CALCR, CCL17, CCL19, CCL2, CCL22, CCL24, CCL3, CCL3L1, CCL4, CCL4L1, CCL5, CD4, CD74, CSF1R, EPX, FFAR4, GPR55, HLA-DRB1, IL26, PTK2B, PTPN22, PTPRC, PYCARD, SCIMP, TREM2, XCL1, XCL2]	[ABCA7, ABL1, ACKR3, AGER, AMH, ANGPT1, C5AR1, CALCR, CCL11, CCL2, CCL21, CCL24, CCR7, CD74, CHI3L1, CX3CL1, DENND2B, FGFR4, FLT4, GATA4, GNAI2, GPBAR1, GPER1, HCRTR1, HTR2A, ICAM1, LGALS9, MAPK3, NOD2, NODAL, NOTCH1, P2RY6, PDGFB, PDGFRB, PLA2G2A, RAP1A, RNU1-93P, SRC, THPO, TIMP1, TNF, TRPV4]

GO:0018108	peptidyl-tyrosine phosphorylation	2,35E-06	[7, 8, 9]	102,00	24,76	51,10	48,90	[ANGPT1, AREG, BANK1, BLK, BTK, CASS4, CCL5, CD3E, CD4, CD74, CD80, CLCF1, CSF1R, EPHA6, EPX, EREG, FGR, FLT3, GPRC5A, HCLS1, IFNG, IL12RB2, IL18, ITGB2, ITK, LCK, LIF, LILRB4, MATK, MUSK, NCF1, NELL2, NR4A3, PTK2B, PTPN22, PTPN6, PTPRC, SAMSN1, SEMA4D, SLA, SOCS1, SOCS3, SPINK1, STAP1, STYK1, SYK, THY1, TNFRSF18, TREM2, ZAP70]	[AATK, ABL1, ADIPOQ, AGT, AMH, ANGPT1, AREG, CD74, CLCF1, CSK, CSPG4, CTF1, DDR1, DMTN, DYRK1B, EFNA3, EPHA2, ERBB2, FES, FGFR4, FGFRL1, FLT3, FLT4, GPRC5A, HBEGF, HIPK4, HSF1, HTR2A, ICAM1, ITGA5, JAK3, LIF, LILRA5, LMTK3, MAP2K3, MAP3K10, MAP3K11, MAP3K9, MAPK3, MLST8, NF2, NOD2, NR4A3, OSM, PDGFB, PDGFRB, PPP2R1A, RHBDF2, SCYL1, SH2B3, SOCS3, SPINK1, SRC, STAT3, TESK1, TIMP1, TNF, TNFRSF1A, TNK1, TNK2, VEGFA, VTN]
GO:0050730	regulation of peptidyl-tyrosine phosphorylation	1,24E-02	[7, 8, 9, 10]	66,00	23,66	55,14	44,86	[ANGPT1, AREG, BANK1, CASS4, CCL5, CD3E, CD4, CD74, CD80, CLCF1, CSF1R, EPX, EREG, FLT3, GPRC5A, HCLS1, IFNG, IL18, ITGB2, LIF, LILRB4, NCF1, NR4A3, PTK2B, PTPN22, PTPN6, PTPRC, SAMSN1, SEMA4D, SOCS1, SOCS3, SPINK1, STAP1, SYK, THY1, TNFRSF18, TREM2]	[ABL1, ADIPOQ, AGT, AMH, ANGPT1, AREG, CD74, CLCF1, CSPG4, CTF1, DMTN, FLT3, GPRC5A, HBEGF, HSF1, HTR2A, ICAM1, ITGA5, LIF, LILRA5, MLST8, NF2, NOD2, NR4A3, OSM, PDGFB, PPP2R1A, RHBDF2, SH2B3, SOCS3, SPINK1, SRC, STAT3, TIMP1, TNF, TNFRSF1A, TNK2, VEGFA, VTN]
GO:0050731	positive regulation of peptidyl-tyrosine phosphorylation	2,46E-02	[7, 8, 9, 10, 11]	53,00	24,88	52,37	47,63	[ANGPT1, AREG, BANK1, CASS4, CCL5, CD3E, CD4, CD74, CD80, CLCF1, CSF1R, EPX, EREG, FLT3, HCLS1, IFNG, IL18, LIF, NCF1, NR4A3, PTK2B, PTPRC, SEMA4D, SOCS3, STAP1, SYK, TNFRSF18, TREM2]	[ABL1, ADIPOQ, AGT, AMH, ANGPT1, AREG, CD74, CLCF1, CSPG4, CTF1, FLT3, HBEGF, HSF1, HTR2A, ICAM1, ITGA5, LIF, LILRA5, MLST8, NOD2, NR4A3, OSM, PDGFB, RHBDF2, SOCS3, SRC, STAT3, TIMP1, TNF, TNFRSF1A, TNK2, VEGFA, VTN]
GO:0043367	CD4-positive, alpha-beta T cell differentiation	3,67E-08	[7, 8, 9, 10, 11, 12, 13]	37,00	43,53	76,08	23,92	[BATF, BCL3, CCL19, CD80, CD86, FOXP3, FUT7, HLA-DRA, HLA-DRB1, IFNG, IL12RB1, IL18, IRF4, LEF1, LY9, MYB, NCKAP1L, NLRP3, PLA2G2D, RUNX3, SASH3, SEMA4A, SLAMF6, SOCS1, SPN, TBX21, TOX]	[ARMC5, BCL3, BCL6, IL4R, JAK3, LGALS9, RARA, RELB, STAT3, ZBTB7B, ZC3H12A]
GO:0046637	regulation of alpha-beta T cell differentiation	1,29E-06	[7, 8, 9, 10, 11, 12, 13]	30,00	44,78	78,09	21,91	[CCL19, CD80, CD86, FOXP3, HLA-DRA, HLA-DRB1, IFNG, IL12RB1, IL18, IRF4, LILRB4, MYB, NCKAP1L, NLRP3, PRDM1, RUNX3, SASH3, SOCS1, SYK, TBX21, ZAP70, ZNF683]	[AP3D1, BCL6, IL4R, JAK3, LGALS9, RARA, ZBTB7B, ZC3H12A]
GO:0043370	regulation of CD4-positive, alpha-beta T cell differentiation	1,98E-05	[8, 9, 10, 11, 12, 13, 14]	24,00	47,06	75,88	24,12	[CCL19, CD80, CD86, FOXP3, HLA-DRA, HLA-DRB1, IFNG, IL12RB1, IL18, IRF4, MYB, NCKAP1L, NLRP3, RUNX3, SASH3, SOCS1, TBX21]	[BCL6, IL4R, JAK3, LGALS9, RARA, ZBTB7B, ZC3H12A]
GO:0002682	regulation of immune system process	1,99E-30	[2, 3]	430,00	23,55	62,05	37,95	[ADAM8, ADCY7, AIF1, AIM2, ALOX15, ANGPT1, APOBEC3G, BANK1, BLK, BLNK, BTK, BTLA, BTN3A1, BTN3A2, C1QA, C1QB, C1QC, C1QTNF1, C2, C8G, CALCR, CAMK4, CARD11, CASP8, CCDC88B, CCL19, CCL2, CCL24, CCL3, CCL4, CCL5, CCR2, CD177, CD19, CD1B, CD1C, CD1E, CD2, CD200R1, CD22, CD226, CD244, CD247, CD27, CD300LF, CD33, CD3D, CD3E, CD3G, CD4, CD40LG, CD48, CD5, CD6, CD74, CD79A, CD80, CD84, CD86, CD8A, CD8B, CD96, CDKN2A, CFP, CLCF1, CLEC10A, CLEC1B, CLEC4D, CLEC4E, CLEC6A, CLNK, CNR1, CNR2, CORO1A, CR2, CRTAM, CSF1R, CST7, CTLA4, CXCL10, CXCL6, CXCR3, CXorf21, CYP11B1, CYSLTR1, DOCK8, DPEP1, DRD1, EBI3, EDN2, EGR3, EPX, EREG, EVI2B, FCGR2C, FCN1, FCN3, FCRL3, FGL2, FGR, FOXP3, FUT7, FYB1, GBP5, GPR150, GPR171, GPR55, GPR83, GPSM3, GRAP2, H3C10, H3C11, H3C12, H3C7, HCLS1, HCST, HLA-DMB, HLA-DOA, HLA-DPA1, HLA-DPB1, HLA-DQA1, HLA-DRA, HLA-DRB1, HLA-DRB5, ICOS, IFNG, IGLL5, IKBKE, IKZF3, IL12RB1, IL18, IL18RAP, IL7R, INPP5D, IRF4, ITGA4, ITGAL, ITGB2, ITGB7, ITK, JAML, KCNN4, KIR2DL2, KIR2DL3, KIR3DL2, KLRB1, KLRC1, KLRC2, KLRD1, KLRK1, LAG3, LAIR1, LAIR2, LAX1, LCK, LCP2, LDLR, LEF1, LILRA1, LILRB1, LILRB4, LMO1, LPAL2, LRRC17, LST1, LTA, MOG, MS4A1, MUC16, MUC19, MUC6, MYB, MYO1G, MZB1, NCKAP1L, NCR1, NCR3, NFAM1, NLRC3, NLRP3, NLRP6, NR4A3, ORM2, P2RY12, PAX5, PILRA, PLA2G2D, PLA2G7, PRAM1, PRDM1, PRKCB, PRKCQ, PTAFR, PTGDR, PTGER2, PTK2B, PTPN22, PTPN6, PTPRC, PYCARD, PYHIN1, RAC2, RASAL3, RHEX, RHOH, RLN2, RUNX3, SAMSN1, SASH3, SCIMP, SELE, SELL, SH2D1A, SIGLEC16, SIRPG, SIT1, SKAP1, SLA2, SLAMF6, SLAMF7, SLAMF8, SMPD3, SOCS1, SOCS3, SPN, STAP1, STAT1, STXBP2, SYK, TBC1D10C, TBX21, TESPA1, THBS1, THEMIS, THEMIS2, THY1, TIGIT, TLR6, TLR8, TNFAIP3, TNFAIP8L2, TNFRSF13B, TNFRSF18, TNFSF13B, TNFSF14, TNFSF8, TOX, TRAT1, TREM2, TSC22D3, UBASH3A, VAV1, VNN1, WAS, XCL1, XCL2, XG, ZAP70, ZBP1	[ABL1, ACTB, ADCY1, ADCY3, ADCY4, ADCY9, ADIPOQ, AGER, AMH, ANGPT1, AP3D1, APOA1, APOD, AQP3, BAG6, BCAR1, BCL6, BTLA, C1QTNF1, C5, C5AR1, C8G, CALCR, CCL2, CCL21, CCL24, CCR7, CD177, CD1C, CD1E, CD276, CD300E, CD74, CDKN1A, CEACAM1, CFH, CGA, CHGA, CLC, CLCF1, CLEC1B, CLEC4E, CLNK, CLPTM1, CNR1, COL1A1, CPN2, CRHR2, CRP, CSF1, CSK, CST7, CX3CL1, CYP26B1, DCAF15, DGKZ, DLG5, DTX1, ECM1, EDN2, ENPP3, ERBB2, FADD, FCN3, FES, FFAR2, FLOT2, FOXF1, FPR1, FURIN, GAB2, GATA2, GCSAM, GNAI2, GNAO1, GPAM, GPBAR1, GPER1, GPI, GPR137, GPR20, GPR83, GPR84, GRN, H3C10, H3C12, H4C3, HAVCR1, HFE, HK1, HLA-DOB, HRH2, HSF1, ICAM1, IDO1, IFITM1, IL15RA, IL1B, IL4R, IL7R, INHA, JAK3, JUND, KLF10, KMT2D, LAG3, LDLR, LGALS9, LIMK1, LOX, LRRC17, LRRC32, LTF, MAPK3, MAVS, MOV10, MSN, MSTN, MUC19, MUC20, MUC3A, MYC, MYH9, MYL9, MYO18A, NCOR2, NECTIN2, NFE2, NLRC5, NOD2, NPPA, NR1D1, NR4A3, NRARP, PAF1, PCDHA4, PCDHA6, PDCD1, PLA2G2A, PLA2G7, PLVAP, PQBP1, PRKACA, PRKAR2B, PTGIR, PTN, PVR, RAC2, RAMP2, RAP1A, RARA, RBM14, RELA, RELB, RGCC, RHBDF2, RHOG, RMRP, RNF26, RNU1-93P, SELE, SELENOP, SERPINE1, SERPING1, SETD1A, SH2B2, SIGLEC9, SLC7A1, SLC7A5, SLC7A8, SMAD3, SOCS3, SOX12, SOX13, SPHK1, SPHK2, SPI1, SPNS2, SPPL2B, SRC, STAT3, STING1, TFE3, THBS1, THPO, TICAM1, TIMP1, TNF, TRIB1, TRIM62, TRPV4, VEGFA, VIPR2, VNN1, VSIR, VTN, WAS, XG, ZBTB7B, ZC3H12A, ZCCHC3, ZDHHC11, ZFP36, ZMIZ1, ZNF335, ZNF580]
GO:0002250	adaptive immune response	1,69E-05	[3]	167,00	20,93	74,84	25,16	[ADCY7, ALOX15, BATF, BCL3, BTK, BTLA, BTN3A1, BTN3A2, C1QA, C1QB, C1QC, C2, C8G, CAMK4, CCL19, CCR2, CD19, CD1B, CD1C, CD1E, CD226, CD244, CD247, CD27, CD3D, CD3E, CD3G, CD4, CD40LG, CD48, CD6, CD7, CD74, CD79A, CD80, CD84, CD86, CD8A, CD8B, CLCF1, CLEC10A, CLEC4D, CLEC6A, CR2, CRTAM, CTLA4, CTSS, CXCL10, EBI3, EOMES, FOXP3, FUT7, GZMM, HLA-DMA, HLA-DMB, HLA-DOA, HLA-DPA1, HLA-DPB1, HLA-DQA1, HLA-DRA, HLA-DRB1, HLA-DRB5, IFNG, IGLL5, IL12RB1, IL18, IL7R, INPP5D, IRF4, ITK, JCHAIN, KLRC1, KLRC2, KLRD1, KLRK1, LAG3, LAIR1, LAMP3, LAX1, LEF1, LILRA1, LILRB1, LILRB4, LTA, LY9, MCOLN2, MYO1G, NCKAP1L, NLRP3, NR4A3, PIK3CG, PRDM1, PRF1, PRKCB, PRKCQ, PTK2B, PTPN6, PTPRC, PYCARD, SAMSN1, SASH3, SEMA4A, SH2D1A, SIGLEC10, SIT1, SKAP1, SLA2, SLAMF6, SLAMF7, SPN, SYK, TBX21, THEMIS, TLR8, TNFAIP3, TNFRSF13B, TNFRSF17, TNFSF13B, TRAT1, TREM2, WAS, XCL1, ZAP70, ZNF683]	[AGER, ARTN, BCL3, BCL6, BTLA, C5, C8G, CD1C, CD1E, CD74, CEACAM1, CLC, CLCF1, CRP, CSK, FADD, GNAO1, HFE, HLA-DOB, HPRT1, ICAM1, IL18BP, IL1B, IL4R, IL7R, JAK3, LAG3, LILRB5, NCOR2, NECTIN2, NFKB2, NOD2, NR4A3, ORAI1, PDCD1, PPL, PRKACA, PVR, RAP1A, RAP1GAP2, RELB, RHBDF2, RNF19B, SERPING1, SLC7A8, STAT3, TCIRG1, TFE3, TFEB, TNF, WAS, ZBTB7B, ZC3H12A, ZNF395]

GO:0002443	leukocyte mediated immunity	5,71E-10	[3]	218,00	21,91	61,60	38,40	[ADAM8, ADGRE3, ANPEP, ARHGAP45, BATF, BCL3, BIN2, BLK, BTK, BTN3A2, C1QA, C1QB, C1QC, C2, C8G, CAMK4, CCL3, CCR2, CD177, CD19, CD1B, CD1C, CD1E, CD226, CD27, CD33, CD40LG, CD53, CD74, CD84, CD8A, CD96, CEACAM21, CFP, CLCF1, CLEC12A, CLEC4D, CLNK, CORO1A, COTL1, CR2, CRTAM, CTSS, CXCL10, CXCL6, CYBB, DOCK2, EPX, FCN1, FGL2, FGR, FOXP3, FRMPD3, FUT7, GMFG, GZMB, GZMM, HLA-DRA, HLA-DRB1, IGLL5, IL12RB1, IL18, IL18RAP, IL7R, INPP5D, IQGAP2, ITGAL, ITGAX, ITGB2, KLRC1, KLRC2, KLRD1, KLRK1, LAG3, LAIR1, LILRB1, LILRB4, LRMP, LTA, LYZ, MMP9, MPO, MYO1G, NCKAP1L, NCR1, NCR3, NFAM1, NLRP3, NLRP6, NR4A3, ORM2, PIK3CG, PLAC8, PLAU, PRAM1, PRF1, PTAFR, PTGDR, PTPN6, PTPRC, PTX3, PYCARD, RAB37, RAB44, RAC2, RNASET2, SASH3, SCIMP, SCNN1B, SELL, SERPINA1, SH2D1A, SLA2, SLAMF6, SLAMF7, SLC18A1, SLC27A2, STAP1, STXBP2, SYK, TBC1D10C, TBX21, TLR8, TREM2, TRPM2, TXNDC5, VAV1, VNN1, WAS, XCL1]	[ADGRE5, AGER, ALDOA, AMH, ARL8A, ARSA, ARTN, BCL3, BCL6, C5, C5AR1, C8G, CAPN1, CD14, CD177, CD1C, CD1E, CD74, CEACAM1, CHGA, CHI3L1, CLC, CLCF1, CLEC12A, CLNK, COMMD3, CRAT, CRP, CTSA, CTSD, CYB5R3, DOK3, DSC1, FADD, FES, FOXF1, FPR1, GAB2, GALNS, GATA2, GNAO1, GPI, GPR84, GRN, HBB, HFE, HPRT1, HVCN1, ICAM1, IL1B, IL4R, IL7R, IMPDH1, IQGAP2, JAK3, JUP, LAG3, LGALS9, LRG1, LTF, MAVS, METTL7A, MME, NBEAL2, NECTIN2, NHLRC3, NOD2, NPPA, NR4A3, PKM, PLAU, PLAUR, PLEKHM2, PRKCSH, PTGDS, PVR, RAB3A, RAB5B, RAC2, RAP1A, RHOG, RNASE2, RNF19B, RNU1-93P, S100A11, SERPINA3, SERPING1, SIGLEC9, SLC44A2, SLC7A8, SLCO4C1, SPHK2, SPI1, STING1, STK11IP, TCIRG1, TICAM1, TMEM179B, TMEM63A, TNF, TRAPPC1, TSPAN14, VNN1, WAS, ZNF395]
GO:0002684	positive regulation of immune system process	4,58E-20	[2, 3, 4]	281,00	24,16	64,49	35,51	[ADAM8, AIF1, AIM2, BLK, BLNK, BTK, BTLA, BTN3A1, BTN3A2, C1QA, C1QB, C1QC, C2, C8G, CAMK4, CARD11, CASP8, CCDC88B, CCL19, CCL2, CCL24, CCL3, CCL4, CCL5, CCR2, CD177, CD19, CD1B, CD1C, CD1E, CD2, CD22, CD226, CD244, CD247, CD27, CD3D, CD3E, CD3G, CD4, CD40LG, CD5, CD6, CD74, CD79A, CD80, CD84, CD86, CFP, CLCF1, CLEC10A, CLEC4D, CLEC4E, CLEC6A, CLNK, CNR1, CORO1A, CR2, CRTAM, CSF1R, CTLA4, CXCL10, CXorf21, CYP11B1, DOCK8, EBI3, EDN2, EGR3, EPX, EREG, EVI2B, FCN1, FCN3, FCRL3, FGR, FOXP3, FYB1, GBP5, GPSM3, GRAP2, HCLS1, HLA-DMB, HLA-DPA1, HLA-DPB1, HLA-DQA1, HLA-DRA, HLA-DRB1, HLA-DRB5, ICOS, IFNG, IGLL5, IKBKE, IL12RB1, IL18, IL18RAP, IL7R, INPP5D, ITGA4, ITGB2, ITK, KCNN4, KLRC2, KLRD1, KLRK1, LAG3, LAX1, LCK, LCP2, LEF1, LILRB1, LILRB4, LTA, MOG, MS4A1, MUC16, MUC19, MUC6, MYB, MYO1G, MZB1, NCKAP1L, NCR3, NFAM1, NLRP3, NR4A3, P2RY12, PAX5, PLA2G7, PRAM1, PRKCB, PRKCQ, PTAFR, PTK2B, PTPN22, PTPN6, PTPRC, PYCARD, PYHIN1, RAC2, RASAL3, RHOH, RUNX3, SASH3, SCIMP, SH2D1A, SIGLEC16, SIRPG, SKAP1, SLA2, SLAMF6, SOCS1, SPN, STAP1, STXBP2, SYK, TBX21, TESPA1, THBS1, THEMIS, THEMIS2, THY1, TLR6, TLR8, TNFRSF18, TNFSF13B, TNFSF14, TOX, TRAT1, TREM2, UBASH3A, VAV1, VNN1, WAS, XCL1, XCL2, XG, ZAP70, ZBP1]	[ABL1, ACTB, AGER, AMH, AP3D1, AQP3, BAG6, BCAR1, BCL6, BTLA, C5, C5AR1, C8G, CCL2, CCL21, CCL24, CCR7, CD177, CD1C, CD1E, CD276, CD74, CDKN1A, CEACAM1, CFH, CLCF1, CLEC4E, CLNK, CNR1, CPN2, CRP, CSF1, CSK, CX3CL1, DGKZ, EDN2, ENPP3, FADD, FCN3, FES, FFAR2, FLOT2, FOXF1, FPR1, GAB2, GATA2, GCSAM, GNAO1, GPAM, GPI, GRN, HAVCR1, HK1, HSF1, ICAM1, IL15RA, IL1B, IL4R, IL7R, JAK3, JUND, KLF10, LAG3, LGALS9, LIMK1, MAPK3, MAVS, MSTN, MUC19, MUC20, MUC3A, MYO18A, NCOR2, NECTIN2, NLRC5, NOD2, NPPA, NR4A3, PCDHA4, PDCD1, PLA2G7, PLVAP, PQBP1, PRKACA, PTN, PVR, RAC2, RAP1A, RARA, RBM14, RELA, RELB, RGCC, RHBDF2, RNU1-93P, SELENOP, SERPINE1, SERPING1, SH2B2, SLC7A1, SLC7A5, SLC7A8, SOX12, SOX13, SPHK2, SPI1, SRC, STING1, THBS1, TICAM1, TIMP1, TNF, TRIB1, TRIM62, TRPV4, VEGFA, VNN1, VSIR, VTN, WAS, XG, ZBTB7B, ZC3H12A, ZCCHC3, ZMIZ1, ZNF335, ZNF580]
GO:0001909	leukocyte mediated cytotoxicity	3,75E-08	[3, 4]	48,00	37,21	74,46	25,54	[CD1B, CD1C, CD1E, CD226, CORO1A, CRTAM, CXCL6, GZMB, GZMM, HLA-DRA, HLA-DRB1, IL12RB1, IL18, IL18RAP, IL7R, KLRC1, KLRC2, KLRD1, KLRK1, LAG3, LILRB1, NCKAP1L, NCR1, NCR3, NLRP6, PRF1, PTPN6, PTPRC, SCNN1B, SH2D1A, SLAMF6, SLAMF7, STAP1, STXBP2, VAV1, XCL1]	[AGER, CD1C, CD1E, CEACAM1, FADD, HPRT1, ICAM1, IL7R, LAG3, LGALS9, NECTIN2, PLEKHM2, PVR, RNF19B, SPI1, ZNF395]
GO:0002697	regulation of immune effector process	1,81E-11	[3, 4]	126,00	27,21	66,43	33,57	[ANGPT1, BLK, BTK, C1QA, C1QB, C1QC, C2, C8G, CAMK4, CCL19, CCR2, CD177, CD19, CD1B, CD1C, CD1E, CD22, CD226, CD244, CD40LG, CD74, CD80, CD84, CD86, CD96, CFP, CLCF1, CLNK, CR2, CRTAM, CXCL10, CXCL6, CYP11B1, FCRL3, FGL2, FGR, FOXP3, FUT7, HLA-DMB, HLA-DRA, HLA-DRB1, IFNG, IL12RB1, IL18, IL18RAP, IL7R, IRF4, ITGB2, KLRC1, KLRC2, KLRD1, KLRK1, LAG3, LILRB1, LILRB4, LPAL2, LTA, MYB, MZB1, NCKAP1L, NCR1, NCR3, NLRP3, NR4A3, PRAM1, PTAFR, PTPN22, PTPN6, PTPRC, RAC2, SASH3, SCIMP, SH2D1A, SLAMF6, SLAMF8, STAP1, STXBP2, SYK, TBX21, TREM2, VAV1, WAS, XCL1, ZNF683]	[AGER, ANGPT1, APOA1, BCL6, C5, C5AR1, C8G, CD177, CD1C, CD1E, CD74, CEACAM1, CFH, CLC, CLCF1, CLNK, CPN2, ENPP3, FADD, FES, FFAR2, FOXF1, GAB2, GATA2, GPI, GRN, HFE, HK1, ICAM1, IL1B, IL4R, IL7R, JAK3, LAG3, LGALS9, MAVS, MYO18A, NECTIN2, NOD2, NPPA, NR4A3, PVR, RAC2, RAP1A, RARA, SERPING1, SLC7A5, SPHK2, SPI1, TICAM1, TNF, VTN, WAS, ZBTB7B, ZC3H12A]
GO:0031341	regulation of cell killing	3,88E-05	[3, 4]	40,00	33,90	73,52	26,48	[CASP8, CD1B, CD1C, CD1E, CD226, CRTAM, CXCL6, HLA-DRA, HLA-DRB1, IFNG, IL12RB1, IL18RAP, IL7R, KLRC1, KLRC2, KLRD1, KLRK1, LAG3, LILRB1, NCKAP1L, NCR1, NCR3, PRF1, PTPRC, SH2D1A, SLAMF6, STAP1, SYK, VAV1, XCL1]	[AGER, CD1C, CD1E, CEACAM1, FADD, ICAM1, IL7R, LAG3, LGALS9, MAPK3, NECTIN2, PVR, SPI1, ZNF395]

GO:0046649	lymphocyte activation	4,01E-25	[3, 4]	232,00	27,92	71,66	28,34	[ADAM8, AIF1, APBB1IP, BANK1, BATF, BCL11B, BCL3, BLK, BLNK, BTK, BTLA, BTN3A1, CAMK4, CARD11, CASP8, CCDC88B, CCL19, CCL2, CCL5, CCR2, CD180, CD19, CD1C, CD2, CD22, CD244, CD27, CD3D, CD3E, CD3G, CD4, CD40LG, CD5, CD6, CD7, CD74, CD79A, CD80, CD86, CD8A, CD8B, CDKN2A, CLCF1, CLNK, CORO1A, CR2, CRTAM, CTLA4, DOCK10, DOCK2, DOCK8, EBI3, EGR3, EOMES, EPX, FCRL1, FCRL3, FCRLA, FGL2, FGR, FLT3, FOXP3, FUT7, GAPT, GRAP2, HLA-DMB, HLA-DOA, HLA-DPA1, HLA-DPB1, HLA-DRA, HLA-DRB1, ICOS, IFNG, IGLL5, IKZF1, IKZF3, IL12RB1, IL18, IL21R, IL7R, INPP5D, IRF4, IRF8, ITGA4, ITGAL, ITGB2, ITK, JAML, KLRC1, KLRC2, KLRK1, LAG3, LAX1, LCK, LCP1, LEF1, LILRB1, LILRB4, LMO1, LST1, LY9, MS4A1, MYB, MZB1, NCKAP1L, NCR1, NCR3, NFAM1, NLRC3, NLRP3, PIK3CG, PLA2G2D, POU2AF1, POU2F2, PRDM1, PRF1, PRKCB, PRKCQ, PTK2B, PTPN22, PTPN6, PTPRC, PYCARD, RAC2, RASAL3, RHOH, RUNX3, SAMSN1, SASH3, SEMA4A, SIRPG, SIT1, SLA2, SLAMF6, SLAMF7, SLAMF8, SOCS1, SPN, SYK, TBC1D10C, TBX21, TESPA1, THEMIS, THEMIS2, THY1, TIGIT, TNFAIP3, TNFAIP8L2, TNFRSF13B, TNFRSF18, TNFSF13B, TNFSF14, TNFSF8, TOX, VAV1, VNN1, WAS, WDFY4, WNT1, XCL1, ZAP70, ZNF683]	[ABL1, AGER, AMH, AP3D1, ARMC5, ARTN, BAG6, BCL3, BCL6, BTLA, CCL2, CCL21, CCR7, CD1C, CD276, CD74, CDKN1A, CEACAM1, CLC, CLCF1, CLNK, CLPTM1, CSK, CYP26B1, DCAF15, DLG5, DTX1, ELF4, ERBB2, FADD, FLOT2, FLT3, GBA, GNAO1, GPAM, HDAC5, HFE, HPRT1, ICAM1, IDO1, IL15RA, IL1B, IL4R, IL7R, INHA, JAK3, KDELR1, LAG3, LGALS9, LRRC32, LRRC8A, MSN, MYH9, NOD2, NRARP, PDCD1, RAC2, RARA, RELB, SLC7A1, SLC7A8, SMAD3, SOX12, SOX13, SPI1, SRC, SRF, STAT3, SYVN1, TCF3, TCF7L1, TCIRG1, TICAM1, TIMP1, VNN1, VSIR, WAS, ZBTB7A, ZBTB7B, ZC3H12A, ZMIZ1, ZNF335, ZNF395]
GO:0050776	regulation of immune response	2,87E-17	[3, 4]	286,00	23,08	66,69	33,31	[ADAM8, ADCY7, AIM2, ALOX15, APOBEC3G, BLK, BLNK, BTK, BTLA, BTN3A1, BTN3A2, C1QA, C1QB, C1QC, C1QTNF1, C2, C8G, CALCR, CARD11, CASP8, CCL19, CCL5, CCR2, CD177, CD19, CD1B, CD1C, CD1E, CD200R1, CD22, CD226, CD247, CD300LF, CD33, CD3D, CD3E, CD3G, CD4, CD40LG, CD48, CD74, CD79A, CD80, CD84, CD86, CD8A, CD8B, CD96, CFP, CLCF1, CLEC10A, CLEC1B, CLEC4D, CLEC4E, CLEC6A, CLNK, CNR1, CR2, CRTAM, CTLA4, CXCL10, CXCL6, CXorf21, CYP11B1, CYSLTR1, DPEP1, DRD1, EREG, FCGR2C, FCN1, FCN3, FCRL3, FGL2, FGR, FOXP3, FUT7, FYB1, GBP5, GPR150, GPR83, GRAP2, HCST, HLA-DMB, HLA-DPA1, HLA-DPB1, HLA-DQA1, HLA-DRA, HLA-DRB1, HLA-DRB5, IFNG, IGLL5, IKBKE, IL12RB1, IL18, IL18RAP, IL7R, INPP5D, IRF4, ITGA4, ITGAL, ITGB2, ITGB7, ITK, JAML, KCNN4, KIR2DL2, KIR2DL3, KIR3DL2, KLRB1, KLRC1, KLRC2, KLRD1, KLRK1, LAG3, LAIR1, LAIR2, LAX1, LCK, LCP2, LILRA1, LILRB1, LILRB4, LTA, MOG, MS4A1, MUC16, MUC19, MUC6, MYB, MYO1G, NCKAP1L, NCR1, NCR3, NFAM1, NLRC3, NLRP3, NLRP6, NR4A3, PAX5, PILRA, PLA2G2D, PRAM1, PRKCB, PRKCQ, PTAFR, PTGDR, PTGER2, PTPN22, PTPN6, PTPRC, PYCARD, PYHIN1, RAC2, RLN2, SAMSN1, SASH3, SELL, SH2D1A, SIGLEC16, SKAP1, SLA2, SLAMF6, SLAMF7, SLAMF8, SOCS1, SOCS3, SPN, STAP1, STAT1, STXBP2, SYK, TBX21, TESPA1, THEMIS, THEMIS2, THY1, TLR8, TNFAIP3, TNFSF13B, TRAT1, TREM2, UBASH3A, VAV1, WAS, XCL1, ZAP70, ZBP1]	[ABL1, ACTB, ADCY1, ADCY3, ADCY4, ADCY9, AGER, BAG6, BCAR1, BCL6, BTLA, C1QTNF1, C5, C5AR1, C8G, CALCR, CCR7, CD177, CD1C, CD1E, CD276, CD300E, CD74, CEACAM1, CFH, CGA, CHGA, CLC, CLCF1, CLEC1B, CLEC4E, CLNK, CNR1, COL1A1, CPN2, CRHR2, CRP, CSK, DGKZ, ECM1, ENPP3, FADD, FCN3, FES, FFAR2, FOXF1, FPR1, FURIN, GAB2, GATA2, GCSAM, GNAI2, GNAO1, GPBAR1, GPR20, GPR83, GPR84, GRN, HFE, HRH2, ICAM1, IFITM1, IL1B, IL4R, IL7R, JAK3, LAG3, LGALS9, LIMK1, MAPK3, MAVS, MUC19, MUC20, MUC3A, MYH9, NCOR2, NECTIN2, NLRC5, NOD2, NPPA, NR4A3, PCDHA4, PDCD1, PQBP1, PRKACA, PRKAR2B, PTGIR, PVR, RAC2, RAMP2, RAP1A, RARA, RBM14, RELA, RELB, RGCC, RHBDF2, RNF26, RNU1-93P, SERPING1, SH2B2, SIGLEC9, SLC7A8, SMAD3, SOCS3, SPHK2, SPI1, SPNS2, SPPL2B, SRC, STING1, TNF, TRIM62, VIPR2, VSIR, VTN, WAS, ZBTB7B, ZC3H12A, ZCCHC3, ZDHHC11]
GO:0050865	regulation of cell activation	2,09E-24	[3, 4]	204,00	29,18	65,18	34,82	[ADAM8, AIF1, BANK1, BLK, BTK, BTLA, C1QTNF1, CAMK4, CARD11, CCDC88B, CCL19, CCL2, CCL3, CCL5, CCR2, CD177, CD19, CD2, CD22, CD226, CD27, CD300LF, CD33, CD3E, CD4, CD40LG, CD5, CD6, CD74, CD80, CD84, CD86, CDKN2A, CLCF1, CLEC4D, CLNK, CNR1, CNR2, CORO1A, CRTAM, CST7, CTLA4, DOCK8, EBI3, EGR3, EPX, FCRL3, FGL2, FGR, FOXP3, GRAP2, HLA-DMB, HLA-DOA, HLA-DPA1, HLA-DPB1, HLA-DRA, HLA-DRB1, ICOS, IFNG, IGLL5, IKZF3, IL12RB1, IL18, IL7R, INPP5D, IRF4, ITGB2, KLRC2, KLRK1, LAG3, LAX1, LCK, LDLR, LEF1, LILRB1, LILRB4, LMO1, LST1, MYB, MZB1, NCKAP1L, NFAM1, NLRP3, NR4A3, PLA2G2D, PLEK, PRAM1, PRDM1, PRKCQ, PTAFR, PTPN22, PTPN6, PTPRC, PYCARD, RAC2, RASAL3, RHOH, RUNX3, SAMSN1, SASH3, SIRPG, SIT1, SLAMF8, SOCS1, SPN, STAP1, STXBP2, SYK, TBC1D10C, TBX21, TESPA1, THBS1, THEMIS2, THY1, TIGIT, TLR6, TNFAIP3, TNFAIP8L2, TNFRSF13B, TNFRSF18, TNFSF13B, TNFSF14, TNFSF8, TOX, TREM2, VAV1, VNN1, XCL1, ZAP70, ZNF683]	[ABL1, AGER, AMH, AP3D1, BCL6, BTLA, C1QTNF1, CCL2, CCL21, CCR7, CD177, CD276, CD74, CDKN1A, CEACAM1, CLC, CLCF1, CLNK, CLPTM1, CNR1, CSK, CST7, CX3CL1, CYP26B1, DCAF15, DLG5, DMTN, DTX1, ENPP3, ERBB2, FADD, FES, FLOT2, FOXF1, GAB2, GATA2, GNAO1, GPAM, GPER1, GRN, HAVCR1, HFE, IDO1, IL15RA, IL1B, IL4R, IL7R, INHA, JAK3, JUND, LAG3, LDLR, LGALS9, LILRA5, LRRC32, MYO18A, NECTIN2, NOD2, NOS3, NPPA, NR1D1, NR4A3, NRARP, PCDHA4, PCDHA6, PDCD1, PDGFB, PDGFRB, PLA2G2A, RAC2, RARA, RHBDF2, SELENOP, SH2B3, SLC7A1, SOX12, SOX13, SPHK1, SPHK2, SPI1, SRC, THBS1, TICAM1, TIMP1, VNN1, VSIR, ZBTB7B, ZC3H12A, ZMIZ1, ZNF335]
GO:0002449	lymphocyte mediated immunity	1,17E-02	[4]	89,00	21,76	71,24	28,76	[BATF, BCL3, BTK, BTN3A2, C1QA, C1QB, C1QC, C2, C8G, CCR2, CD19, CD1B, CD1C, CD1E, CD226, CD27, CD40LG, CD74, CD8A, CD96, CLCF1, CLNK, CORO1A, CR2, CRTAM, CXCL10, FOXP3, FUT7, GZMB, GZMM, HLA-DRA, HLA-DRB1, IGLL5, IL12RB1, IL18, IL18RAP, IL7R, INPP5D, KLRC1, KLRC2, KLRD1, KLRK1, LAG3, LILRB1, LILRB4, LTA, MYO1G, NCKAP1L, NCR1, NCR3, NLRP3, PRF1, PTPN6, PTPRC, SASH3, SH2D1A, SLA2, SLAMF6, SLAMF7, TBX21, TLR8, TREM2, VAV1, WAS, XCL1]	[AGER, ARTN, BCL3, BCL6, C5, C8G, CD1C, CD1E, CD74, CEACAM1, CLC, CLCF1, CLNK, CRP, FADD, GNAO1, HFE, HPRT1, ICAM1, IL1B, IL4R, IL7R, LAG3, LGALS9, NECTIN2, NOD2, PLEKHM2, PVR, RNF19B, SERPING1, TCIRG1, TNF, WAS, ZNF395]

GO:0002460	adaptive immune response based on somatic recombination of immune receptors built from immunoglobulin superfamily domains	2,41E-03	[4]	93,00	22,30	70,05	29,95	[BATF, BCL3, BTK, BTN3A2, C1QA, C1QB, C1QC, C2, C8G, CCL19, CCR2, CD19, CD1B, CD1C, CD1E, CD226, CD27, CD4, CD40LG, CD74, CD80, CD8A, CLCF1, CLEC6A, CR2, CRTAM, CXCL10, EBI3, FOXP3, FUT7, GZMM, HLA-DRA, HLA-DRB1, IGLL5, IL12RB1, IL18, IL7R, INPP5D, IRF4, KLRC1, KLRD1, LEF1, LILRB1, LILRB4, LTA, LY9, MYO1G, NCKAP1L, NLRP3, PRF1, PRKCQ, PTPN6, PTPRC, SASH3, SEMA4A, SLA2, SLAMF6, SPN, TBX21, TLR8, TNFAIP3, TNFSF13B, TREM2, WAS, XCL1]	[AGER, ARTN, BCL3, BCL6, C5, C8G, CD1C, CD1E, CD74, CEACAM1, CLC, CLCF1, CRP, FADD, GNAO1, HFE, HPRT1, ICAM1, IL18BP, IL1B, IL4R, IL7R, JAK3, NECTIN2, NFKB2, NOD2, PVR, RELB, SERPING1, STAT3, TCIRG1, TNF, WAS, ZBTB7B, ZC3H12A, ZNF395]
GO:0002694	regulation of leukocyte activation	2,18E-24	[3, 4, 5]	194,00	29,85	67,19	32,81	[ADAM8, AIF1, BANK1, BLK, BTK, BTLA, CAMK4, CARD11, CCDC88B, CCL19, CCL2, CCL3, CCL5, CCR2, CD177, CD19, CD2, CD22, CD226, CD27, CD300LF, CD33, CD3E, CD4, CD40LG, CD5, CD6, CD74, CD80, CD84, CD86, CDKN2A, CLCF1, CLEC4D, CLNK, CNR1, CNR2, CORO1A, CRTAM, CST7, CTLA4, DOCK8, EBI3, EGR3, EPX, FCRL3, FGL2, FGR, FOXP3, GRAP2, HLA-DMB, HLA-DOA, HLA-DPA1, HLA-DPB1, HLA-DRA, HLA-DRB1, ICOS, IFNG, IGLL5, IKZF3, IL12RB1, IL18, IL7R, INPP5D, IRF4, ITGB2, KLRC2, KLRK1, LAG3, LAX1, LCK, LDLR, LEF1, LILRB1, LILRB4, LMO1, LST1, MYB, MZB1, NCKAP1L, NFAM1, NLRP3, NR4A3, PLA2G2D, PRAM1, PRDM1, PRKCQ, PTAFR, PTPN22, PTPN6, PTPRC, PYCARD, RAC2, RASAL3, RHOH, RUNX3, SAMSN1, SASH3, SIRPG, SIT1, SLAMF8, SOCS1, SPN, STAP1, STXBP2, SYK, TBC1D10C, TBX21, TESPA1, THBS1, THEMIS2, THY1, TIGIT, TLR6, TNFAIP3, TNFAIP8L2, TNFRSF13B, TNFRSF18, TNFSF13B, TNFSF14, TNFSF8, TOX, TREM2, VAV1, VNN1, XCL1, ZAP70, ZNF683]	[ABL1, AGER, AMH, AP3D1, BCL6, BTLA, CCL2, CCL21, CCR7, CD177, CD276, CD74, CDKN1A, CEACAM1, CLC, CLCF1, CLNK, CLPTM1, CNR1, CSK, CST7, CX3CL1, CYP26B1, DCAF15, DLG5, DTX1, ENPP3, ERBB2, FADD, FES, FLOT2, FOXF1, GAB2, GATA2, GNAO1, GPAM, GPER1, GRN, HAVCR1, HFE, IDO1, IL15RA, IL1B, IL4R, IL7R, INHA, JAK3, JUND, LAG3, LDLR, LGALS9, LRRC32, MYO18A, NECTIN2, NOD2, NPPA, NR1D1, NR4A3, NRARP, PCDHA4, PCDHA6, PDCD1, PLA2G2A, RAC2, RARA, SLC7A1, SOX12, SOX13, SPHK1, SPHK2, SPI1, SRC, THBS1, TICAM1, TIMP1, VNN1, VSIR, ZBTB7B, ZC3H12A, ZMIZ1, ZNF335]
GO:0002698	negative regulation of immune effector process	4,35E-04	[3, 4, 5]	37,00	33,04	60,19	39,81	[ANGPT1, CCR2, CD22, CD84, CD96, CR2, FCRL3, FGL2, FOXP3, IL7R, KLRC1, KLRD1, LILRB1, LILRB4, LPAL2, NCKAP1L, PTPN6, PTPRC, SLAMF8, TBX21, XCL1]	[ANGPT1, APOA1, BCL6, CEACAM1, ENPP3, FOXF1, GRN, HFE, IL4R, IL7R, JAK3, LGALS9, NOD2, SERPING1, SPI1, TNF, ZBTB7B, ZC3H12A]
GO:0002699	positive regulation of immune effector process	5,71E-10	[3, 4, 5]	79,00	31,60	67,30	32,70	[BLK, BTK, CAMK4, CCL19, CCR2, CD177, CD1B, CD1C, CD1E, CD226, CD244, CD74, CD80, CD84, CD86, CLCF1, CLNK, CRTAM, FGR, FOXP3, HLA-DMB, HLA-DRA, HLA-DRB1, IFNG, IL12RB1, IL18, IL18RAP, ITGB2, KLRC2, KLRD1, KLRK1, LAG3, LILRB1, LTA, MYB, MZB1, NCR3, NLRP3, NR4A3, PTAFR, PTPN22, PTPRC, RAC2, SASH3, SCIMP, SH2D1A, SLAMF6, STAP1, STXBP2, SYK, TBX21, TREM2, VAV1, XCL1]	[CD177, CD1C, CD1E, CD74, CLCF1, CLNK, ENPP3, FADD, FES, FFAR2, FOXF1, GAB2, GATA2, GPI, HK1, IL1B, IL4R, LAG3, LGALS9, MAVS, MYO18A, NECTIN2, NOD2, NPPA, NR4A3, PVR, RAC2, RAP1A, RARA, SLC7A5, SPHK2, SPI1, TICAM1, TNF]
GO:0031343	positive regulation of cell killing	5,36E-04	[3, 4, 5]	28,00	37,33	78,84	21,16	[CD1B, CD1C, CD1E, CD226, CRTAM, HLA-DRA, HLA-DRB1, IFNG, IL12RB1, IL18RAP, KLRC2, KLRD1, KLRK1, LAG3, NCR3, PRF1, PTPRC, SH2D1A, SLAMF6, STAP1, SYK, VAV1, XCL1]	[CD1C, CD1E, FADD, LAG3, NECTIN2, PVR, SPI1, ZNF395]
GO:0050778	positive regulation of immune response	1,07E-08	[3, 4, 5]	186,00	22,25	69,27	30,73	[ADAM8, AIM2, BLK, BLNK, BTK, BTN3A1, BTN3A2, C1QA, C1QB, C1QC, C2, C8G, CARD11, CCL19, CCL5, CCR2, CD177, CD19, CD1B, CD1C, CD1E, CD22, CD226, CD247, CD3D, CD3E, CD3G, CD4, CD74, CD79A, CD80, CD86, CFP, CLCF1, CLEC10A, CLEC4D, CLEC4E, CLEC6A, CLNK, CNR1, CR2, CRTAM, CTLA4, CXCL10, CXorf21, CYP11B1, EREG, FCN1, FCN3, FCRL3, FGR, FOXP3, FYB1, GBP5, GRAP2, HLA-DMB, HLA-DPA1, HLA-DPB1, HLA-DQA1, HLA-DRA, HLA-DRB1, HLA-DRB5, IFNG, IGLL5, IKBKE, IL12RB1, IL18, IL18RAP, INPP5D, ITGB2, ITK, KCNN4, KLRC2, KLRD1, KLRK1, LAG3, LAX1, LCK, LCP2, LILRB1, LILRB4, LTA, MOG, MS4A1, MUC16, MUC19, MUC6, MYB, MYO1G, NCKAP1L, NCR3, NFAM1, NLRP3, NR4A3, PAX5, PRAM1, PRKCB, PRKCQ, PTAFR, PTPN22, PTPN6, PTPRC, PYCARD, PYHIN1, SASH3, SH2D1A, SIGLEC16, SKAP1, SLA2, SLAMF6, STAP1, SYK, TBX21, TESPA1, THEMIS, THEMIS2, THY1, TLR8, TNFSF13B, TRAT1, TREM2, UBASH3A, VAV1, WAS, XCL1, ZAP70, ZBP1]	[ABL1, ACTB, BAG6, BCAR1, C5, C5AR1, C8G, CCR7, CD177, CD1C, CD1E, CD276, CD74, CEACAM1, CFH, CLCF1, CLEC4E, CLNK, CNR1, CPN2, CRP, CSK, DGKZ, FADD, FCN3, FFAR2, FPR1, GAB2, GATA2, GCSAM, GNAO1, GRN, IL1B, IL4R, LAG3, LGALS9, LIMK1, MAPK3, MAVS, MUC19, MUC20, MUC3A, NCOR2, NECTIN2, NLRC5, NOD2, NPPA, NR4A3, PCDHA4, PQBP1, PRKACA, PVR, RAP1A, RARA, RBM14, RELA, RELB, RGCC, RHBDF2, RNU1-93P, SERPING1, SH2B2, SLC7A8, SPHK2, SPI1, SRC, STING1, TNF, TRIM62, VTN, WAS, ZC3H12A, ZCCHC3]
GO:0050866	negative regulation of cell activation	2,21E-10	[3, 4, 5]	72,00	33,49	63,17	36,83	[BANK1, BLK, BTK, C1QTNF1, CCR2, CD300LF, CD74, CD80, CD84, CD86, CDKN2A, CLNK, CNR1, CNR2, CRTAM, CST7, CTLA4, FGL2, FGR, FOXP3, HLA-DRB1, INPP5D, LAG3, LAX1, LDLR, LILRB1, LILRB4, LST1, NCKAP1L, PLA2G2D, PTPN22, PTPN6, PTPRC, RUNX3, SAMSN1, SOCS1, SPN, TBC1D10C, TBX21, TIGIT, TNFAIP3, TNFAIP8L2, TNFRSF13B, TREM2, XCL1]	[BCL6, C1QTNF1, CD74, CEACAM1, CLNK, CNR1, CST7, CX3CL1, DLG5, DTX1, ENPP3, ERBB2, FOXF1, GPER1, GRN, HFE, IL4R, INHA, JAK3, LAG3, LDLR, LGALS9, LRRC32, NOS3, NR1D1, NRARP, PCDHA4, PCDHA6, PDGFB, SH2B3, SPI1, VSIR, ZBTB7B, ZC3H12A]
GO:0050867	positive regulation of cell activation	2,02E-12	[3, 4, 5]	127,00	27,79	69,03	30,97	[ADAM8, AIF1, BTK, BTLA, CARD11, CCDC88B, CCL19, CCL2, CCL3, CCL5, CCR2, CD177, CD2, CD226, CD27, CD3E, CD4, CD40LG, CD5, CD6, CD74, CD80, CD86, CLCF1, CLEC4D, CORO1A, CTLA4, DOCK8, EBI3, EGR3, EPX, FCRL3, FGR, FOXP3, GRAP2, HLA-DMB, HLA-DPA1, HLA-DPB1, HLA-DRA, HLA-DRB1, ICOS, IFNG, IGLL5, IL12RB1, IL18, IL7R, INPP5D, ITGB2, KLRC2, KLRK1, LCK, LEF1, LILRB1, LILRB4, MYB, NCKAP1L, NLRP3, NR4A3, PLEK, PRKCQ, PTAFR, PTPN22, PTPN6, PTPRC, PYCARD, RASAL3, RHOH, RUNX3, SASH3, SIRPG, SOCS1, STAP1, SYK, TBX21, TESPA1, THBS1, THY1, TLR6, TNFSF13B, TNFSF14, TOX, TREM2, VAV1, VNN1, XCL1, ZAP70]	[AGER, AMH, AP3D1, BCL6, BTLA, CCL2, CCL21, CCR7, CD177, CD276, CD74, CDKN1A, CLCF1, CSK, FADD, FLOT2, GAB2, GATA2, GNAO1, GPAM, HAVCR1, IL15RA, IL1B, IL4R, IL7R, JAK3, JUND, LGALS9, LILRA5, NECTIN2, NOD2, NPPA, NR4A3, PDCD1, PDGFRB, RARA, SELENOP, SLC7A1, SOX12, SOX13, SPHK2, SRC, THBS1, TICAM1, TIMP1, VNN1, VSIR, ZBTB7B, ZMIZ1, ZNF335]

GO:0002253	activation of immune response	5,32E-04	[2, 4, 5, 6]	132,00	20,99	69,52	30,48	[AIM2, BLK, BLNK, BTK, BTN3A1, BTN3A2, C1QA, C1QB, C1QC, C2, C8G, CARD11, CD19, CD22, CD226, CD247, CD3D, CD3E, CD3G, CD4, CD79A, CFP, CLEC10A, CLEC4D, CLEC4E, CLEC6A, CR2, CTLA4, CXCL10, CYP11B1, FCN1, FCN3, FCRL3, FGR, FOXP3, FYB1, GRAP2, HLA-DPA1, HLA-DPB1, HLA-DQA1, HLA-DRA, HLA-DRB1, HLA-DRB5, IGLL5, INPP5D, ITK, KCNN4, KLRC2, KLRD1, LAX1, LCK, LCP2, LILRB4, MOG, MS4A1, MUC16, MUC19, MUC6, MYO1G, NCKAP1L, NCR3, NFAM1, NR4A3, PAX5, PRAM1, PRKCB, PRKCQ, PTPN22, PTPN6, PTPRC, PYCARD, PYHIN1, SH2D1A, SKAP1, SLA2, STAP1, SYK, TESPA1, THEMIS, THEMIS2, THY1, TRAT1, TREM2, UBASH3A, VAV1, WAS, ZAP70, ZBP1]	[ABL1, ACTB, BAG6, BCAR1, C5, C5AR1, C8G, CCR7, CD276, CEACAM1, CFH, CLEC4E, CPN2, CRP, CSK, DGKZ, FCN3, FFAR2, FPR1, GCSAM, GNAO1, IL1B, LIMK1, MAPK3, MAVS, MUC19, MUC20, MUC3A, NCOR2, NECTIN2, NOD2, NR4A3, PQBP1, PRKACA, RAP1A, RBM14, RELA, RELB, RGCC, RHBDF2, RNU1-93P, SERPING1, SH2B2, SLC7A8, SRC, STING1, VTN, WAS, ZC3H12A, ZCCHC3]
GO:0002285	lymphocyte activation involved in immune response	2,42E-05	[4, 5]	58,00	29,00	76,00	24,00	[APBB1IP, BATF, BCL3, CCL19, CD180, CD19, CD1C, CD244, CD40LG, CD74, CD80, CD86, CLCF1, CORO1A, DOCK10, EOMES, FGL2, FOXP3, GAPT, HLA-DMB, HLA-DRA, HLA-DRB1, IFNG, IL12RB1, IL18, IRF4, IRF8, ITGAL, KLRC2, LCP1, LEF1, LILRB1, LY9, MYB, NCKAP1L, NLRP3, POU2AF1, PTK2B, PTPRC, SEMA4A, SLAMF6, SPN, TBX21, ZNF683]	[ABL1, ARTN, BCL3, BCL6, CD1C, CD74, CEACAM1, CLCF1, ICAM1, IL4R, JAK3, LGALS9, RARA, RELB, SPI1, STAT3, ZBTB7B, ZC3H12A]
GO:0002367	cytokine production involved in immune response	2,27E-03	[3, 6]	34,00	32,08	57,70	42,30	[ANGPT1, BTK, CAMK4, CCR2, CD226, CD74, CD96, CLCF1, CLNK, FOXP3, IL18, LILRB1, LILRB4, LPAL2, NLRP3, NR4A3, SASH3, SCIMP, TBX21, XCL1]	[ANGPT1, APOA1, BCL6, CD74, CLC, CLCF1, CLNK, FFAR2, HFE, HK1, IL1B, JAK3, MAVS, NOD2, NR4A3, SLC7A5, SPHK2, TICAM1, TNF]
GO:0002695	negative regulation of leukocyte activation	1,42E-10	[3, 4, 5, 6]	67,00	34,90	65,00	35,00	[BANK1, BLK, BTK, CCR2, CD300LF, CD74, CD80, CD84, CD86, CDKN2A, CLNK, CNR1, CNR2, CRTAM, CST7, CTLA4, FGL2, FGR, FOXP3, HLA-DRB1, INPP5D, LAG3, LAX1, LDLR, LILRB1, LILRB4, LST1, NCKAP1L, PLA2G2D, PTPN22, PTPN6, PTPRC, RUNX3, SAMSN1, SOCS1, SPN, TBC1D10C, TBX21, TIGIT, TNFAIP3, TNFAIP8L2, TNFRSF13B, XCL1]	[BCL6, CD74, CEACAM1, CLNK, CNR1, CST7, CX3CL1, DLG5, DTX1, ENPP3, ERBB2, FOXF1, GPER1, GRN, HFE, IL4R, INHA, JAK3, LAG3, LDLR, LGALS9, LRRC32, NR1D1, NRARP, PCDHA4, PCDHA6, SPI1, VSIR, ZBTB7B, ZC3H12A]
GO:0002696	positive regulation of leukocyte activation	9,04E-12	[3, 4, 5, 6]	123,00	27,64	70,09	29,91	[ADAM8, AIF1, BTK, BTLA, CARD11, CCDC88B, CCL19, CCL2, CCL3, CCL5, CCR2, CD177, CD2, CD226, CD27, CD3E, CD4, CD40LG, CD5, CD6, CD74, CD80, CD86, CLCF1, CLEC4D, CORO1A, CTLA4, DOCK8, EBI3, EGR3, EPX, FCRL3, FGR, FOXP3, GRAP2, HLA-DMB, HLA-DPA1, HLA-DPB1, HLA-DRA, HLA-DRB1, ICOS, IFNG, IGLL5, IL12RB1, IL18, IL7R, INPP5D, ITGB2, KLRC2, KLRK1, LCK, LEF1, LILRB1, LILRB4, MYB, NCKAP1L, NLRP3, NR4A3, PRKCQ, PTAFR, PTPN22, PTPN6, PTPRC, PYCARD, RASAL3, RHOH, RUNX3, SASH3, SIRPG, SOCS1, STAP1, SYK, TBX21, TESPA1, THBS1, THY1, TLR6, TNFSF13B, TNFSF14, TOX, TREM2, VAV1, VNN1, XCL1, ZAP70]	[AGER, AMH, AP3D1, BCL6, BTLA, CCL2, CCL21, CCR7, CD177, CD276, CD74, CDKN1A, CLCF1, CSK, FADD, FLOT2, GAB2, GATA2, GNAO1, GPAM, HAVCR1, IL15RA, IL1B, IL4R, IL7R, JAK3, JUND, LGALS9, NECTIN2, NOD2, NPPA, NR4A3, PDCD1, RARA, SLC7A1, SOX12, SOX13, SPHK2, SRC, THBS1, TICAM1, TIMP1, VNN1, VSIR, ZBTB7B, ZMIZ1, ZNF335]
GO:0002703	regulation of leukocyte mediated immunity	3,88E-12	[4, 5]	79,00	34,05	68,35	31,65	[BLK, BTK, CAMK4, CCR2, CD177, CD1B, CD1C, CD1E, CD226, CD84, CD96, CLCF1, CLNK, CR2, CRTAM, CXCL6, FGR, FOXP3, FUT7, HLA-DRA, HLA-DRB1, IL12RB1, IL18, IL18RAP, IL7R, ITGB2, KLRC1, KLRC2, KLRD1, KLRK1, LAG3, LILRB1, LILRB4, LTA, NCKAP1L, NCR1, NCR3, NLRP3, PRAM1, PTAFR, PTPN6, PTPRC, RAC2, SASH3, SCIMP, SH2D1A, SLAMF6, STAP1, STXBP2, SYK, TBX21, TREM2, VAV1, WAS, XCL1]	[AGER, BCL6, CD177, CD1C, CD1E, CEACAM1, CLC, CLCF1, CLNK, FADD, FES, FOXF1, GAB2, GATA2, HFE, ICAM1, IL1B, IL4R, IL7R, JAK3, LAG3, LGALS9, MAVS, NECTIN2, NOD2, NPPA, PVR, RAC2, SPHK2, SPI1, TICAM1, TNF, WAS]
GO:0002819	regulation of adaptive immune response	3,50E-06	[4, 5]	58,00	30,53	72,16	27,84	[ADCY7, ALOX15, BTK, CCL19, CCR2, CD1B, CD1C, CD1E, CD226, CD4, CD48, CD80, CLCF1, CLEC6A, CR2, FOXP3, FUT7, HLA-DRA, HLA-DRB1, IL12RB1, IL18, IL7R, KLRC1, KLRD1, LILRB1, LILRB4, LTA, NCKAP1L, NLRP3, PRKCQ, PTPN6, PTPRC, PYCARD, SAMSN1, SASH3, SKAP1, TBX21, TNFAIP3, TNFSF13B, TREM2, WAS, XCL1]	[AGER, BCL6, CD1C, CD1E, CEACAM1, CLC, CLCF1, FADD, GNAO1, HFE, IL1B, IL4R, IL7R, JAK3, NECTIN2, NOD2, PVR, TNF, WAS, ZBTB7B, ZC3H12A]
GO:0042110	T cell activation	1,53E-25	[4, 5]	169,00	32,50	70,64	29,36	[ADAM8, AIF1, APBB1IP, BATF, BCL11B, BCL3, BTLA, BTN3A1, CAMK4, CARD11, CASP8, CCDC88B, CCL19, CCL2, CCL5, CCR2, CD1C, CD2, CD27, CD3D, CD3E, CD3G, CD4, CD40LG, CD5, CD6, CD7, CD74, CD80, CD86, CD8A, CD8B, CDKN2A, CLCF1, CORO1A, CRTAM, CTLA4, DOCK2, DOCK8, EBI3, EGR3, EOMES, EPX, FGL2, FOXP3, FUT7, GRAP2, HLA-DMB, HLA-DOA, HLA-DPA1, HLA-DPB1, HLA-DRA, HLA-DRB1, ICOS, IFNG, IL12RB1, IL18, IL7R, IRF4, ITGAL, ITK, JAML, KLRC1, KLRK1, LAG3, LAX1, LCK, LCP1, LEF1, LILRB1, LILRB4, LMO1, LY9, MYB, NCKAP1L, NLRC3, NLRP3, PIK3CG, PLA2G2D, PRDM1, PRKCQ, PTPN22, PTPN6, PTPRC, PYCARD, RAC2, RASAL3, RHOH, RUNX3, SASH3, SEMA4A, SIRPG, SIT1, SLA2, SLAMF6, SOCS1, SPN, SYK, TBX21, TESPA1, THEMIS, THY1, TIGIT, TNFAIP8L2, TNFRSF18, TNFSF13B, TNFSF14, TNFSF8, TOX, VAV1, VNN1, WAS, WDFY4, WNT1, XCL1, ZAP70, ZNF683]	[ABL1, AGER, AP3D1, ARMC5, BCL3, BCL6, BTLA, CCL2, CCL21, CCR7, CD1C, CD276, CD74, CEACAM1, CLC, CLCF1, CLPTM1, CSK, CYP26B1, DLG5, DTX1, ELF4, ERBB2, FADD, FLOT2, GBA, GNAO1, GPAM, HFE, ICAM1, IDO1, IL1B, IL4R, IL7R, JAK3, KDELR1, LAG3, LGALS9, LRRC32, MSN, MYH9, NOD2, NRARP, PDCD1, RAC2, RARA, RELB, SLC7A1, SMAD3, SOX12, SOX13, SPI1, SRC, SRF, STAT3, TCIRG1, TIMP1, VNN1, VSIR, WAS, ZBTB7B, ZC3H12A, ZMIZ1]
GO:0001910	regulation of leukocyte mediated cytotoxicity	1,72E-06	[4, 5, 6]	34,00	40,96	73,73	26,27	[CD1B, CD1C, CD1E, CD226, CRTAM, CXCL6, HLA-DRA, HLA-DRB1, IL12RB1, IL18RAP, IL7R, KLRC1, KLRC2, KLRD1, KLRK1, LAG3, LILRB1, NCKAP1L, NCR1, NCR3, PTPRC, SH2D1A, SLAMF6, STAP1, VAV1, XCL1]	[AGER, CD1C, CD1E, CEACAM1, FADD, ICAM1, IL7R, LAG3, LGALS9, NECTIN2, PVR, SPI1]
GO:0001913	T cell mediated cytotoxicity	2,58E-04	[4, 5, 6]	23,00	43,40	67,46	32,54	[CD1B, CD1C, CD1E, CRTAM, GZMM, HLA-DRA, HLA-DRB1, IL12RB1, IL7R, KLRC1, KLRD1, LILRB1, NCKAP1L, PRF1, PTPRC, XCL1]	[AGER, CD1C, CD1E, CEACAM1, FADD, HPRT1, IL7R, NECTIN2, PVR, ZNF395]
GO:0002456	T cell mediated immunity	8,65E-06	[5]	41,00	35,04	68,85	31,15	[BTN3A2, CCR2, CD1B, CD1C, CD1E, CD8A, CLCF1, CRTAM, FOXP3, FUT7, GZMM, HLA-DRA, HLA-DRB1, IL12RB1, IL18, IL7R, KLRC1, KLRD1, LILRB1, LILRB4, MYO1G, NCKAP1L, NLRP3, PRF1, PTPRC, SASH3, TBX21, WAS, XCL1]	[AGER, CD1C, CD1E, CEACAM1, CLC, CLCF1, FADD, HFE, HPRT1, ICAM1, IL1B, IL7R, NECTIN2, NOD2, PVR, WAS, ZNF395]

GO:0002704	negative regulation of leukocyte mediated immunity	1,27E-02	[4, 5, 6]	23,00	35,94	68,35	31,65	[CCR2, CD84, CD96, CR2, FOXP3, IL7R, KLRC1, KLRD1, LILRB1, LILRB4, NCKAP1L, PTPN6, PTPRC, TBX21, XCL1]	[BCL6, CEACAM1, FOXF1, HFE, IL7R, JAK3, LGALS9, NOD2, SPI1]
GO:0002705	positive regulation of leukocyte mediated immunity	1,59E-04	[4, 5, 6]	44,00	31,43	75,14	24,86	[BTK, CAMK4, CD177, CD1B, CD1C, CD1E, CD226, CLCF1, CLNK, CRTAM, FOXP3, HLA-DRA, HLA-DRB1, IL12RB1, IL18, IL18RAP, ITGB2, KLRC2, KLRD1, KLRK1, LAG3, LTA, NCR3, NLRP3, PTAFR, PTPRC, SASH3, SCIMP, SH2D1A, SLAMF6, STAP1, TBX21, TREM2, VAV1, XCL1]	[CD177, CD1C, CD1E, CLCF1, CLNK, FADD, IL1B, LAG3, MAVS, NECTIN2, NOD2, PVR, SPI1, TICAM1, TNF]
GO:0002820	negative regulation of adaptive immune response	1,38E-02	[4, 5, 6]	22,00	36,67	66,84	33,16	[ALOX15, CR2, FOXP3, IL7R, KLRC1, KLRD1, LILRB1, LILRB4, NCKAP1L, PTPN6, PTPRC, SAMSN1, TBX21, XCL1]	[BCL6, CEACAM1, HFE, IL4R, IL7R, JAK3, NOD2, ZBTB7B, ZC3H12A]
GO:0051249	regulation of lymphocyte activation	2,62E-15	[4, 5, 6]	154,00	27,60	71,79	28,21	[ADAM8, AIF1, BANK1, BLK, BTK, BTLA, CAMK4, CARD11, CCDC88B, CCL19, CCL2, CCL5, CCR2, CD19, CD2, CD22, CD27, CD3E, CD4, CD40LG, CD5, CD6, CD74, CD80, CD86, CDKN2A, CLCF1, CLNK, CORO1A, CRTAM, CTLA4, DOCK8, EBI3, EGR3, EPX, FCRL3, FGL2, FGR, FOXP3, GRAP2, HLA-DMB, HLA-DOA, HLA-DPA1, HLA-DPB1, HLA-DRA, HLA-DRB1, ICOS, IFNG, IGLL5, IKZF3, IL12RB1, IL18, IL7R, INPP5D, IRF4, KLRC2, KLRK1, LAG3, LAX1, LCK, LEF1, LILRB1, LILRB4, LMO1, LST1, MYB, MZB1, NCKAP1L, NFAM1, NLRP3, PLA2G2D, PRDM1, PRKCQ, PTPN22, PTPN6, PTPRC, PYCARD, RAC2, RASAL3, RHOH, RUNX3, SAMSN1, SASH3, SIRPG, SIT1, SLAMF8, SOCS1, SPN, SYK, TBC1D10C, TBX21, TESPA1, THEMIS2, THY1, TIGIT, TNFAIP3, TNFAIP8L2, TNFRSF13B, TNFRSF18, TNFSF13B, TNFSF14, TNFSF8, TOX, VAV1, VNN1, XCL1, ZAP70, ZNF683]	[ABL1, AGER, AMH, AP3D1, BCL6, BTLA, CCL2, CCL21, CCR7, CD276, CD74, CDKN1A, CEACAM1, CLC, CLCF1, CLNK, CLPTM1, CSK, CYP26B1, DCAF15, DLG5, DTX1, ERBB2, FADD, FLOT2, GNAO1, GPAM, HFE, IDO1, IL15RA, IL1B, IL4R, IL7R, INHA, JAK3, LAG3, LGALS9, LRRC32, NOD2, NRARP, PDCD1, RAC2, RARA, SLC7A1, SOX12, SOX13, SRC, TICAM1, TIMP1, VNN1, VSIR, ZBTB7B, ZC3H12A, ZMIZ1, ZNF335]
GO:0001912	positive regulation of leukocyte mediated cytotoxicity	1,84E-04	[4, 5, 6, 7]	24,00	42,86	78,73	21,27	[CD1B, CD1C, CD1E, CD226, CRTAM, HLA-DRA, HLA-DRB1, IL12RB1, IL18RAP, KLRC2, KLRD1, KLRK1, LAG3, NCR3, PTPRC, SH2D1A, SLAMF6, STAP1, VAV1, XCL1]	[CD1C, CD1E, FADD, LAG3, NECTIN2, PVR, SPI1]
GO:0002286	T cell activation involved in immune response	4,84E-06	[5, 6]	41,00	35,65	73,52	26,48	[APBB1IP, BATF, BCL3, CCL19, CD1C, CD74, CD80, CD86, EOMES, FGL2, FOXP3, HLA-DMB, HLA-DRA, HLA-DRB1, IFNG, IL12RB1, IL18, IRF4, ITGAL, LCP1, LEF1, LILRB1, LY9, MYB, NCKAP1L, NLRP3, SEMA4A, SLAMF6, SPN, TBX21]	[BCL3, BCL6, CD1C, CD74, CEACAM1, ICAM1, IL4R, JAK3, LGALS9, RARA, RELB, STAT3, ZBTB7B, ZC3H12A]
GO:0002706	regulation of lymphocyte mediated immunity	8,41E-06	[5, 6]	53,00	30,99	73,66	26,34	[BTK, CCR2, CD1B, CD1C, CD1E, CD226, CD96, CLCF1, CLNK, CR2, CRTAM, FOXP3, FUT7, HLA-DRA, HLA-DRB1, IL12RB1, IL18, IL18RAP, IL7R, KLRC1, KLRC2, KLRD1, KLRK1, LAG3, LILRB1, LILRB4, LTA, NCKAP1L, NCR1, NCR3, NLRP3, PTPN6, PTPRC, SASH3, SH2D1A, SLAMF6, TBX21, TREM2, VAV1, WAS, XCL1]	[AGER, BCL6, CD1C, CD1E, CEACAM1, CLC, CLCF1, CLNK, FADD, HFE, IL1B, IL7R, LAG3, LGALS9, NECTIN2, NOD2, PVR, TNF, WAS]
GO:0002718	regulation of cytokine production involved in immune response	2,27E-03	[4, 5, 6, 7]	34,00	32,08	57,70	42,30	[ANGPT1, BTK, CAMK4, CCR2, CD226, CD74, CD96, CLCF1, CLNK, FOXP3, IL18, LILRB1, LILRB4, LPAL2, NLRP3, NR4A3, SASH3, SCIMP, TBX21, XCL1]	[ANGPT1, APOA1, BCL6, CD74, CLC, CLCF1, CLNK, FFAR2, HFE, HK1, IL1B, JAK3, MAVS, NOD2, NR4A3, SLC7A5, SPHK2, TICAM1, TNF]
GO:0002822	regulation of adaptive immune response based on somatic recombination of immune receptors built from immunoglobulin superfamily domains	5,77E-05	[5, 6]	52,00	29,71	68,96	31,04	[BTK, CCL19, CCR2, CD1B, CD1C, CD1E, CD226, CD4, CD80, CLCF1, CLEC6A, CR2, FOXP3, FUT7, HLA-DRA, HLA-DRB1, IL12RB1, IL18, IL7R, KLRC1, KLRD1, LILRB1, LILRB4, LTA, NCKAP1L, NLRP3, PRKCQ, PTPN6, PTPRC, SASH3, TBX21, TNFAIP3, TNFSF13B, TREM2, WAS, XCL1]	[AGER, BCL6, CD1C, CD1E, CEACAM1, CLC, CLCF1, FADD, GNAO1, HFE, IL1B, IL4R, IL7R, JAK3, NECTIN2, NOD2, PVR, TNF, WAS, ZBTB7B, ZC3H12A]
GO:0002886	regulation of myeloid leukocyte mediated immunity	4,09E-05	[5, 6]	26,00	43,33	59,92	40,08	[BLK, BTK, CAMK4, CCR2, CD177, CD84, CXCL6, FGR, ITGB2, PRAM1, PTAFR, RAC2, STAP1, STXBP2, SYK]	[CD177, FES, FOXF1, GAB2, GATA2, IL4R, LGALS9, MAVS, NPPA, RAC2, SPHK2, SPI1, TICAM1]
GO:0046631	alpha-beta T cell activation	1,31E-14	[5, 6]	66,00	41,25	76,60	23,40	[BATF, BCL11B, BCL3, CCL19, CCR2, CD3E, CD80, CD86, CRTAM, DOCK2, EBI3, EOMES, FOXP3, FUT7, HLA-DRA, HLA-DRB1, IFNG, IL12RB1, IL18, IRF4, ITK, LEF1, LILRB1, LILRB4, LY9, MYB, NCKAP1L, NLRP3, PLA2G2D, PRDM1, PRKCQ, PTPN22, PTPRC, RASAL3, RUNX3, SASH3, SEMA4A, SLAMF6, SOCS1, SPN, SYK, TBX21, TNFSF8, TOX, WDFY4, XCL1, ZAP70, ZNF683]	[ABL1, AGER, AP3D1, ARMC5, BCL3, BCL6, ELF4, GNAO1, HFE, IL4R, JAK3, LGALS9, RARA, RELB, STAT3, TCIRG1, VSIR, ZBTB7B, ZC3H12A]
GO:0051250	negative regulation of lymphocyte activation	1,91E-05	[4, 5, 6, 7]	50,00	31,25	71,59	28,41	[BANK1, BLK, BTK, CD74, CD80, CD86, CDKN2A, CLNK, CRTAM, CTLA4, FGL2, FGR, FOXP3, HLA-DRB1, INPP5D, LAG3, LAX1, LILRB1, LILRB4, LST1, NCKAP1L, PLA2G2D, PTPN22, PTPN6, RUNX3, SAMSN1, SOCS1, SPN, TBC1D10C, TBX21, TIGIT, TNFAIP3, TNFAIP8L2, TNFRSF13B, XCL1]	[BCL6, CD74, CEACAM1, CLNK, DLG5, DTX1, ERBB2, HFE, IL4R, INHA, JAK3, LAG3, LGALS9, LRRC32, NRARP, VSIR, ZBTB7B, ZC3H12A]
GO:0002228	natural killer cell mediated immunity	7,86E-04	[5, 6, 7]	27,00	37,50	77,28	22,72	[CD226, CD96, CLNK, CORO1A, CRTAM, GZMB, IL18, IL18RAP, KLRC1, KLRC2, KLRD1, KLRK1, LAG3, LILRB1, NCR1, NCR3, PTPN6, SH2D1A, SLAMF6, SLAMF7, VAV1]	[CEACAM1, CLNK, LAG3, LGALS9, NECTIN2, PLEKHM2, PVR, RNF19B]
GO:0002708	positive regulation of lymphocyte mediated immunity	6,96E-03	[5, 6, 7]	35,00	30,43	77,35	22,65	[BTK, CD1B, CD1C, CD1E, CD226, CLCF1, CLNK, CRTAM, FOXP3, HLA-DRA, HLA-DRB1, IL12RB1, IL18, IL18RAP, KLRC2, KLRD1, KLRK1, LAG3, LTA, NCR3, NLRP3, PTPRC, SASH3, SH2D1A, SLAMF6, TBX21, TREM2, VAV1, XCL1]	[CD1C, CD1E, CLCF1, CLNK, FADD, IL1B, LAG3, NECTIN2, NOD2, PVR, TNF]
GO:0002823	negative regulation of adaptive immune response based on somatic recombination of immune receptors built from immunoglobulin superfamily domains	4,05E-02	[5, 6, 7]	20,00	36,36	63,34	36,66	[CR2, FOXP3, IL7R, KLRC1, KLRD1, LILRB1, LILRB4, NCKAP1L, PTPN6, PTPRC, TBX21, XCL1]	[BCL6, CEACAM1, HFE, IL4R, IL7R, JAK3, NOD2, ZBTB7B, ZC3H12A]
GO:0042267	natural killer cell mediated cytotoxicity	4,33E-03	[4, 5, 6, 7, 8]	25,00	36,23	77,86	22,14	[CD226, CORO1A, CRTAM, GZMB, IL18, IL18RAP, KLRC1, KLRC2, KLRD1, KLRK1, LAG3, LILRB1, NCR1, NCR3, PTPN6, SH2D1A, SLAMF6, SLAMF7, VAV1]	[CEACAM1, LAG3, LGALS9, NECTIN2, PLEKHM2, PVR, RNF19B]

GO:0050863	regulation of T cell activation	2,38E-22	[5, 6, 7]	126,00	35,39	70,57	29,43	[ADAM8, AIF1, BTLA, CAMK4, CARD11, CCDC88B, CCL19, CCL2, CCL5, CCR2, CD2, CD27, CD3E, CD4, CD40LG, CD5, CD6, CD74, CD80, CD86, CDKN2A, CLCF1, CORO1A, CRTAM, CTLA4, DOCK8, EBI3, EGR3, EPX, FGL2, FOXP3, GRAP2, HLA-DMB, HLA-DOA, HLA-DPA1, HLA-DPB1, HLA-DRA, HLA-DRB1, ICOS, IFNG, IL12RB1, IL18, IL7R, IRF4, KLRK1, LAG3, LAX1, LCK, LEF1, LILRB1, LILRB4, LMO1, MYB, NCKAP1L, NLRP3, PLA2G2D, PRDM1, PRKCQ, PTPN22, PTPN6, PTPRC, PYCARD, RAC2, RASAL3, RHOH, RUNX3, SASH3, SIRPG, SIT1, SOCS1, SPN, SYK, TBX21, TESPA1, THY1, TIGIT, TNFAIP8L2, TNFRSF18, TNFSF13B, TNFSF14, TNFSF8, TOX, VAV1, VNN1, XCL1, ZAP70, ZNF683]	[ABL1, AGER, AP3D1, BCL6, BTLA, CCL2, CCL21, CCR7, CD276, CD74, CEACAM1, CLC, CLCF1, CLPTM1, CSK, CYP26B1, DLG5, DTX1, ERBB2, FADD, FLOT2, GNAO1, GPAM, HFE, IDO1, IL1B, IL4R, IL7R, JAK3, LAG3, LGALS9, LRRC32, NOD2, NRARP, PDCD1, RAC2, RARA, SLC7A1, SOX12, SOX13, SRC, TIMP1, VNN1, VSIR, ZBTB7B, ZC3H12A, ZMIZ1]
GO:1903038	negative regulation of leukocyte cell-cell adhesion	9,60E-03	[5, 6, 7]	39,00	28,47	64,65	35,35	[CD74, CD80, CD86, CDKN2A, CRTAM, CTLA4, FGL2, FOXP3, HLA-DRB1, LAG3, LAX1, LILRB1, LILRB4, NCKAP1L, PLA2G2D, PTPN22, PTPN6, RUNX3, SOCS1, SPN, TBX21, TIGIT, TNFAIP8L2, XCL1]	[BCL6, CCL21, CD74, CEACAM1, DLG5, DTX1, ERBB2, HFE, IL4R, JAK3, LAG3, LGALS9, LRRC32, NRARP, VSIR, ZBTB7B, ZC3H12A]
GO:0001914	regulation of T cell mediated cytotoxicity	1,24E-03	[5, 6, 7, 8]	18,00	47,37	67,80	32,20	[CD1B, CD1C, CD1E, HLA-DRA, HLA-DRB1, IL12RB1, IL7R, KLRC1, KLRD1, LILRB1, NCKAP1L, PTPRC, XCL1]	[AGER, CD1C, CD1E, CEACAM1, FADD, IL7R, NECTIN2, PVR]
GO:0002709	regulation of T cell mediated immunity	1,57E-04	[6, 7]	32,00	36,36	68,04	31,96	[CCR2, CD1B, CD1C, CD1E, CLCF1, FOXP3, FUT7, HLA-DRA, HLA-DRB1, IL12RB1, IL18, IL7R, KLRC1, KLRD1, LILRB1, LILRB4, NCKAP1L, NLRP3, PTPRC, SASH3, TBX21, WAS, XCL1]	[AGER, CD1C, CD1E, CEACAM1, CLC, CLCF1, FADD, HFE, IL1B, IL7R, NECTIN2, NOD2, PVR, WAS]
GO:0002715	regulation of natural killer cell mediated immunity	2,46E-03	[5, 6, 7, 8]	20,00	42,55	77,55	22,45	[CD226, CD96, CLNK, CRTAM, IL18RAP, KLRC1, KLRC2, KLRD1, KLRK1, LAG3, LILRB1, NCR1, NCR3, SH2D1A, SLAMF6, VAV1]	[CEACAM1, CLNK, LAG3, LGALS9, NECTIN2, PVR]
GO:0035710	CD4-positive, alpha-beta T cell activation	4,57E-08	[6, 7]	42,00	40,00	72,85	27,15	[BATF, BCL3, CCL19, CD80, CD86, FOXP3, FUT7, HLA-DRA, HLA-DRB1, IFNG, IL12RB1, IL18, IRF4, LEF1, LY9, MYB, NCKAP1L, NLRP3, PLA2G2D, PRKCQ, RUNX3, SASH3, SEMA4A, SLAMF6, SOCS1, SPN, TBX21, TOX, XCL1]	[AGER, ARMC5, BCL3, BCL6, IL4R, JAK3, LGALS9, RARA, RELB, STAT3, TCIRG1, VSIR, ZBTB7B, ZC3H12A]
GO:0043300	regulation of leukocyte degranulation	9,22E-06	[4, 5, 6, 7, 8, 9]	23,00	50,00	58,40	41,60	[BLK, CCR2, CD177, CD84, FGR, ITGB2, KLRC2, NCKAP1L, PRAM1, PTAFR, RAC2, STXBP2, SYK]	[CD177, CEACAM1, FES, FOXF1, GAB2, GATA2, IL4R, LGALS9, NPPA, RAC2, SPHK2, SPI1]
GO:0002710	negative regulation of T cell mediated immunity	3,56E-02	[6, 7, 8]	13,00	48,15	76,41	23,59	[FOXP3, IL7R, KLRC1, KLRD1, LILRB1, LILRB4, NCKAP1L, PTPRC, TBX21, XCL1]	[CEACAM1, HFE, IL7R, NOD2]
GO:0002717	positive regulation of natural killer cell mediated immunity	2,67E-02	[5, 6, 7, 8, 9]	14,00	46,67	79,54	20,46	[CD226, CLNK, CRTAM, IL18RAP, KLRC2, KLRD1, KLRK1, LAG3, NCR3, SH2D1A, SLAMF6, VAV1]	[CLNK, LAG3, NECTIN2, PVR]
GO:0030098	lymphocyte differentiation	4,55E-19	[4, 5, 6, 7, 8, 9, 10]	129,00	32,66	72,38	27,62	[ADAM8, BATF, BCL11B, BCL3, BLNK, BTK, CAMK4, CARD11, CCL19, CCR2, CD19, CD2, CD27, CD3D, CD3E, CD3G, CD4, CD40LG, CD74, CD79A, CD80, CD86, CD8A, CDKN2A, CLCF1, CR2, CRTAM, CTLA4, DOCK10, DOCK2, EGR3, EOMES, FCRL3, FGL2, FLT3, FOXP3, FUT7, HLA-DOA, HLA-DRA, HLA-DRB1, IFNG, IKZF1, IKZF3, IL12RB1, IL18, IL7R, INPP5D, IRF4, IRF8, ITGA4, ITK, KLRC1, LAG3, LCK, LEF1, LILRB4, LY9, MS4A1, MYB, NCKAP1L, NFAM1, NLRP3, PLA2G2D, POU2AF1, POU2F2, PRDM1, PTK2B, PTPN22, PTPN6, PTPRC, RHOH, RUNX3, SASH3, SEMA4A, SLAMF6, SLAMF8, SOCS1, SPN, SYK, TBX21, TESPA1, THEMIS, TNFRSF18, TNFSF13B, TNFSF8, TOX, VAV1, VNN1, WNT1, ZAP70, ZNF683]	[ABL1, AP3D1, ARMC5, BCL3, BCL6, CCR7, CD74, CLCF1, CLPTM1, CSF1, CYP26B1, DTX1, ERBB2, FADD, FLT3, GBA, GNAO1, HDAC5, IL15RA, IL1B, IL4R, IL7R, INHA, JAK3, KDELR1, LAG3, LGALS9, LRRC8A, NRARP, RARA, RELB, SOX12, SOX13, SPI1, SRF, STAT3, SYVN1, TCF3, TCF7L1, TCIRG1, VNN1, VSIR, ZBTB7A, ZBTB7B, ZC3H12A, ZMIZ1]
GO:0042269	regulation of natural killer cell mediated cytotoxicity	1,10E-02	[5, 6, 7, 8, 9]	18,00	41,86	78,39	21,61	[CD226, CRTAM, IL18RAP, KLRC1, KLRC2, KLRD1, KLRK1, LAG3, LILRB1, NCR1, NCR3, SH2D1A, SLAMF6, VAV1]	[CEACAM1, LAG3, LGALS9, NECTIN2, PVR]
GO:0046634	regulation of alpha-beta T cell activation	4,95E-09	[6, 7, 8]	44,00	41,12	77,55	22,45	[CCL19, CCR2, CD3E, CD80, CD86, CRTAM, EBI3, FOXP3, HLA-DRA, HLA-DRB1, IFNG, IL12RB1, IL18, IRF4, LILRB1, LILRB4, MYB, NCKAP1L, NLRP3, PRDM1, PRKCQ, PTPN22, PTPRC, RASAL3, RUNX3, SASH3, SOCS1, SYK, TBX21, XCL1, ZAP70, ZNF683]	[AGER, AP3D1, BCL6, GNAO1, HFE, IL4R, JAK3, LGALS9, RARA, VSIR, ZBTB7B, ZC3H12A]
GO:1903131	mononuclear cell differentiation	6,89E-22	[5, 6, 7, 8, 9]	146,00	32,52	69,78	30,22	[ADAM8, BATF, BCL11B, BCL3, BLK, BLNK, BTK, CAMK4, CARD11, CCL19, CCR2, CD19, CD2, CD27, CD3D, CD3E, CD3G, CD4, CD40LG, CD74, CD79A, CD80, CD86, CD8A, CDKN2A, CLCF1, CR2, CRTAM, CSF1R, CTLA4, DOCK10, DOCK2, EGR3, EOMES, FCRL3, FGL2, FLT3, FOXP3, FUT7, HLA-DOA, HLA-DRA, HLA-DRB1, IFNG, IKZF1, IKZF3, IL12RB1, IL18, IL7R, INPP5D, IRF4, IRF8, ITGA4, ITK, KLRC1, LAG3, LCK, LEF1, LILRB1, LILRB4, LY9, MS4A1, MT1G, MYB, NCKAP1L, NFAM1, NLRP3, PLA2G2D, POU2AF1, POU2F2, PRDM1, PTK2B, PTPN22, PTPN6, PTPRC, RHOH, RUNX3, SASH3, SEMA4A, SLAMF6, SLAMF8, SOCS1, SPN, SYK, TBX21, TESPA1, THEMIS, TNFRSF18, TNFSF13B, TNFSF8, TOX, TREM2, TRPM2, UBD, VAV1, VNN1, WNT1, ZAP70, ZNF683]	[ABL1, AGER, AP3D1, ARMC5, BATF2, BCL3, BCL6, CCR7, CD74, CLCF1, CLPTM1, CSF1, CYP26B1, DHRS2, DTX1, ERBB2, FADD, FASN, FES, FLT3, GBA, GNAO1, HDAC5, IL15RA, IL1B, IL4R, IL7R, INHA, JAK3, KDELR1, LAG3, LGALS9, LRRC8A, LTBR, MYC, MYH9, NRARP, RARA, RELB, SOX12, SOX13, SPI1, SRF, STAT3, SYVN1, TCF3, TCF7L1, TCIRG1, VEGFA, VNN1, VSIR, ZBTB7A, ZBTB7B, ZC3H12A, ZMIZ1]
GO:0046635	positive regulation of alpha-beta T cell activation	6,90E-08	[6, 7, 8, 9]	32,00	47,06	84,88	15,12	[CCL19, CCR2, CD3E, CD80, CD86, EBI3, FOXP3, HLA-DRA, HLA-DRB1, IFNG, IL12RB1, IL18, LILRB4, MYB, NCKAP1L, NLRP3, PRKCQ, PTPN22, PTPRC, RASAL3, RUNX3, SASH3, SOCS1, SYK, XCL1, ZAP70]	[AP3D1, GNAO1, IL4R, LGALS9, RARA, ZBTB7B]
GO:2000514	regulation of CD4-positive, alpha-beta T cell activation	4,57E-05	[7, 8, 9]	28,00	41,18	73,23	26,77	[CCL19, CD80, CD86, FOXP3, HLA-DRA, HLA-DRB1, IFNG, IL12RB1, IL18, IRF4, MYB, NCKAP1L, NLRP3, PRKCQ, RUNX3, SASH3, SOCS1, TBX21, XCL1]	[AGER, BCL6, IL4R, JAK3, LGALS9, RARA, VSIR, ZBTB7B, ZC3H12A]
GO:2001185	regulation of CD8-positive, alpha-beta T cell activation	1,67E-02	[7, 8, 9]	11,00	57,89	77,55	22,45	[CRTAM, LILRB1, LILRB4, NCKAP1L, PTPN22, RUNX3, SOCS1, XCL1]	[HFE, VSIR, ZBTB7B]

GO:0043367	CD4-positive, alpha-beta T cell differentiation	3,67E-08	[7, 8, 9, 10, 11, 12, 13]	37,00	43,53	76,08	23,92	[BATF, BCL3, CCL19, CD80, CD86, FOXP3, FUT7, HLA-DRA, HLA-DRB1, IFNG, IL12RB1, IL18, IRF4, LEF1, LY9, MYB, NCKAP1L, NLRP3, PLA2G2D, RUNX3, SASH3, SEMA4A, SLAMF6, SOCS1, SPN, TBX21, TOX]	[ARMC5, BCL3, BCL6, IL4R, JAK3, LGALS9, RARA, RELB, STAT3, ZBTB7B, ZC3H12A]
GO:0046637	regulation of alpha-beta T cell differentiation	1,29E-06	[7, 8, 9, 10, 11, 12, 13]	30,00	44,78	78,09	21,91	[CCL19, CD80, CD86, FOXP3, HLA-DRA, HLA-DRB1, IFNG, IL12RB1, IL18, IRF4, LILRB4, MYB, NCKAP1L, NLRP3, PRDM1, RUNX3, SASH3, SOCS1, SYK, TBX21, ZAP70, ZNF683]	[AP3D1, BCL6, IL4R, JAK3, LGALS9, RARA, ZBTB7B, ZC3H12A]
GO:0046638	positive regulation of alpha-beta T cell differentiation	2,60E-05	[7, 8, 9, 10, 11, 12, 13, 14]	23,00	47,92	82,35	17,65	[CCL19, CD80, CD86, FOXP3, HLA-DRA, HLA-DRB1, IFNG, IL12RB1, IL18, LILRB4, MYB, NCKAP1L, NLRP3, RUNX3, SASH3, SOCS1, SYK, ZAP70]	[AP3D1, IL4R, LGALS9, RARA, ZBTB7B]
GO:0045622	regulation of T-helper cell differentiation	1,99E-03	[4, 5, 9, 10, 11, 12, 13, 14, 15, 16]	18,00	46,15	72,16	27,84	[CCL19, CD80, CD86, FOXP3, HLA-DRA, HLA-DRB1, IL12RB1, IL18, IRF4, MYB, NLRP3, TBX21]	[BCL6, IL4R, JAK3, RARA, ZBTB7B, ZC3H12A]
GO:0043370	regulation of CD4-positive, alpha-beta T cell differentiation	1,98E-05	[8, 9, 10, 11, 12, 13, 14]	24,00	47,06	75,88	24,12	[CCL19, CD80, CD86, FOXP3, HLA-DRA, HLA-DRB1, IFNG, IL12RB1, IL18, IRF4, MYB, NCKAP1L, NLRP3, RUNX3, SASH3, SOCS1, TBX21]	[BCL6, IL4R, JAK3, LGALS9, RARA, ZBTB7B, ZC3H12A]
GO:0043372	positive regulation of CD4-positive, alpha-beta T cell differentiation	2,05E-05	[8, 9, 10, 11, 12, 13, 14, 15]	18,00	58,06	81,93	18,07	[CCL19, CD80, CD86, FOXP3, HLA-DRA, HLA-DRB1, IFNG, IL12RB1, IL18, MYB, NCKAP1L, NLRP3, SASH3, SOCS1]	[IL4R, LGALS9, RARA, ZBTB7B]
GO:0002682	regulation of immune system process	1,99E-30	[2, 3]	430,00	23,55	62,05	37,95	[ADAM8, ADCY7, AIF1, AIM2, ALOX15, ANGPT1, APOBEC3G, BANK1, BLK, BLNK, BTK, BTLA, BTN3A1, BTN3A2, C1QA, C1QB, C1QC, C1QTNF1, C2, C8G, CALCR, CAMK4, CARD11, CASP8, CCDC88B, CCL19, CCL2, CCL24, CCL3, CCL4, CCL5, CCR2, CD177, CD19, CD1B, CD1C, CD1E, CD2, CD200R1, CD22, CD226, CD244, CD247, CD27, CD300LF, CD33, CD3D, CD3E, CD3G, CD4, CD40LG, CD48, CD5, CD6, CD74, CD79A, CD80, CD84, CD86, CD8A, CD8B, CD96, CDKN2A, CFP, CLCF1, CLEC10A, CLEC1B, CLEC4D, CLEC4E, CLEC6A, CLNK, CNR1, CNR2, CORO1A, CR2, CRTAM, CSF1R, CST7, CTLA4, CXCL10, CXCL6, CXCR3, CXorf21, CYP11B1, CYSLTR1, DOCK8, DPEP1, DRD1, EBI3, EDN2, EGR3, EPX, EREG, EVI2B, FCGR2C, FCN1, FCN3, FCRL3, FGL2, FGR, FOXP3, FUT7, FYB1, GBP5, GPR150, GPR171, GPR55, GPR83, GPSM3, GRAP2, H3C10, H3C11, H3C12, H3C7, HCLS1, HCST, HLA-DMB, HLA-DOA, HLA-DPA1, HLA-DPB1, HLA-DQA1, HLA-DRA, HLA-DRB1, HLA-DRB5, ICOS, IFNG, IGLL5, IKBKE, IKZF3, IL12RB1, IL18, IL18RAP, IL7R, INPP5D, IRF4, ITGA4, ITGAL, ITGB2, ITGB7, ITK, JAML, KCNN4, KIR2DL2, KIR2DL3, KIR3DL2, KLRB1, KLRC1, KLRC2, KLRD1, KLRK1, LAG3, LAIR1, LAIR2, LAX1, LCK, LCP2, LDLR, LEF1, LILRA1, LILRB1, LILRB4, LMO1, LPAL2, LRRC17, LST1, LTA, MOG, MS4A1, MUC16, MUC19, MUC6, MYB, MYO1G, MZB1, NCKAP1L, NCR1, NCR3, NFAM1, NLRC3, NLRP3, NLRP6, NR4A3, ORM2, P2RY12, PAX5, PILRA, PLA2G2D, PLA2G7, PRAM1, PRDM1, PRKCB, PRKCQ, PTAFR, PTGDR, PTGER2, PTK2B, PTPN22, PTPN6, PTPRC, PYCARD, PYHIN1, RAC2, RASAL3, RHEX, RHOH, RLN2, RUNX3, SAMSN1, SASH3, SCIMP, SELE, SELL, SH2D1A, SIGLEC16, SIRPG, SIT1, SKAP1, SLA2, SLAMF6, SLAMF7, SLAMF8, SMPD3, SOCS1, SOCS3, SPN, STAP1, STAT1, STXBP2, SYK, TBC1D10C, TBX21, TESPA1, THBS1, THEMIS, THEMIS2, THY1, TIGIT, TLR6, TLR8, TNFAIP3, TNFAIP8L2, TNFRSF13B, TNFRSF18, TNFSF13B, TNFSF14, TNFSF8, TOX, TRAT1, TREM2, TSC22D3, UBASH3A, VAV1, VNN1, WAS, XCL1, XCL2, XG, ZAP70, ZBP1,	[ABL1, ACTB, ADCY1, ADCY3, ADCY4, ADCY9, ADIPOQ, AGER, AMH, ANGPT1, AP3D1, APOA1, APOD, AQP3, BAG6, BCAR1, BCL6, BTLA, C1QTNF1, C5, C5AR1, C8G, CALCR, CCL2, CCL21, CCL24, CCR7, CD177, CD1C, CD1E, CD276, CD300E, CD74, CDKN1A, CEACAM1, CFH, CGA, CHGA, CLC, CLCF1, CLEC1B, CLEC4E, CLNK, CLPTM1, CNR1, COL1A1, CPN2, CRHR2, CRP, CSF1, CSK, CST7, CX3CL1, CYP26B1, DCAF15, DGKZ, DLG5, DTX1, ECM1, EDN2, ENPP3, ERBB2, FADD, FCN3, FES, FFAR2, FLOT2, FOXF1, FPR1, FURIN, GAB2, GATA2, GCSAM, GNAI2, GNAO1, GPAM, GPBAR1, GPER1, GPI, GPR137, GPR20, GPR83, GPR84, GRN, H3C10, H3C12, H4C3, HAVCR1, HFE, HK1, HLA-DOB, HRH2, HSF1, ICAM1, IDO1, IFITM1, IL15RA, IL1B, IL4R, IL7R, INHA, JAK3, JUND, KLF10, KMT2D, LAG3, LDLR, LGALS9, LIMK1, LOX, LRRC17, LRRC32, LTF, MAPK3, MAVS, MOV10, MSN, MSTN, MUC19, MUC20, MUC3A, MYC, MYH9, MYL9, MYO18A, NCOR2, NECTIN2, NFE2, NLRC5, NOD2, NPPA, NR1D1, NR4A3, NRARP, PAF1, PCDHA4, PCDHA6, PDCD1, PLA2G2A, PLA2G7, PLVAP, PQBP1, PRKACA, PRKAR2B, PTGIR, PTN, PVR, RAC2, RAMP2, RAP1A, RARA, RBM14, RELA, RELB, RGCC, RHBDF2, RHOG, RMRP, RNF26, RNU1-93P, SELE, SELENOP, SERPINE1, SERPING1, SETD1A, SH2B2, SIGLEC9, SLC7A1, SLC7A5, SLC7A8, SMAD3, SOCS3, SOX12, SOX13, SPHK1, SPHK2, SPI1, SPNS2, SPPL2B, SRC, STAT3, STING1, TFE3, THBS1, THPO, TICAM1, TIMP1, TNF, TRIB1, TRIM62, TRPV4, VEGFA, VIPR2, VNN1, VSIR, VTN, WAS, XG, ZBTB7B, ZC3H12A, ZCCHC3, ZDHHC11, ZFP36, ZMIZ1, ZNF335, ZNF580]
GO:0040012	regulation of locomotion	6,83E-08	[2, 3]	217,00	20,93	38,54	61,46	[ADAM8, ADAMTS9, ADORA3, AIF1, ALOX15B, ANGPT1, ANGPT2, BMP7, CARMIL2, CASS4, CCL19, CCL2, CCL24, CCL3, CCL4, CCL5, CCR2, CCR4, CD200R1, CD74, CDH1, CORO1A, CSF1R, CXCL10, CXCR3, CXCR4, DOCK10, DOCK8, DPEP1, DRD1, EDN2, EREG, FGR, FUT7, GPSM3, IFNG, IL16, ITGA4, ITGAX, KLRK1, LEF1, MMP9, NCKAP1L, NELL2, NR4A3, NTN1, P2RY12, PIK3CG, PLA2G7, PLAU, PLXNC1, PTAFR, PTK2B, PTPN22, PTPRC, PYCARD, RAC2, RHOH, SELE, SEMA4A, SEMA4D, SLAMF8, SMPD3, SPN, STAP1, SUCNR1, TACSTD2, TCAF2, THBS1, THY1, TNFRSF18, TREM2, WAS, XCL1, XCL2, XG]	[ABCC8, ABI3, ABL1, ACKR3, ACTN4, ADAMTS9, ADGRG1, ADIPOQ, AGER, AGT, AMH, ANGPT1, APOD, ARHGDIA, ARRDC3, ARTN, ATP1B2, BCAR1, C5, C5AR1, CAMK2B, CAMSAP3, CARMIL2, CCL11, CCL2, CCL21, CCL24, CCR7, CD74, CEACAM1, CGA, CHGA, CITED2, COL1A1, CSF1, CX3CL1, DAB2IP, DAG1, DLG5, DMTN, ECM1, EDN2, ENG, EPHA2, EPPK1, ERBB2, FADD, FAM110A, FES, FLT4, FOXC2, FOXF1, GATA2, GCSAM, GNAI2, GNB3, GPER1, GPI, GRB7, GRN, HBEGF, HDAC5, HDAC7, HSPB1, HYAL1, ICAM1, IFITM1, IL1B, ILK, ITGA3, JUP, LAMA5, LGALS9, LGR6, LMNA, MAP2K3, MAPK3, MAZ, MECP2, MEGF8, MEN1, MIEN1, MINK1, MSN, MSTN, MYADM, MYC, NF2, NGFR, NOD2, NODAL, NOS3, NOTCH1, NR4A3, NSMF, NTN1, OSGIN1, P2RY6, PDGFB, PDGFRB, PFN1, PHACTR1, PLA2G7, PLAU, PLVAP, PLXNA1, PLXNA3, PLXNB1, POSTN, PPARD, PRAG1, PTN, PTPN23, PTPRU, RAC2, RGCC, RHOG, RIPOR1, ROBO4, S100A11, SELE, SELENOP, SEMA3F, SEMA4B, SEMA4C, SEMA6B, SEMA6C, SERPINE1, SH3BP1, SLC9A3R1, SMAD3, SMIM22, SNAI1, SPHK1, SPI1, SRC, SRF, SSX2IP, STAT3, SYDE1, TBX5, THBS1, TIMP1, TNF, TRIB1, TRPV4, VEGFA, VSIR, VTN, WAS, WNT3, XG, ZC3H12A, ZNF580, ZNF609, ZSWIM4, ZSWIM8]

GO:0045321	leukocyte activation	1,83E-35	[2, 3]	374,00	25,97	63,37	36,63	[ADAM8, ADGRE3, AIF1, ANPEP, APBB1IP, ARHGAP45, BANK1, BATF, BCL11B, BCL3, BIN2, BLK, BLNK, BTK, BTLA, BTN3A1, C1QA, CAMK4, CARD11, CASP8, CCDC88B, CCL19, CCL2, CCL3, CCL5, CCR2, CD177, CD180, CD19, CD1C, CD2, CD22, CD226, CD244, CD27, CD300LF, CD33, CD3D, CD3E, CD3G, CD4, CD40LG, CD5, CD53, CD6, CD7, CD74, CD79A, CD80, CD84, CD86, CD8A, CD8B, CDKN2A, CEACAM21, CFP, CLCF1, CLEC12A, CLEC4D, CLNK, CNR1, CNR2, CORO1A, COTL1, CR2, CRTAM, CST7, CTLA4, CTSS, CXCL6, CYBB, DOCK10, DOCK2, DOCK8, EBI3, EDN2, EGR3, EOMES, EPX, FCN1, FCRL1, FCRL3, FCRLA, FGL2, FGR, FLT3, FOXP3, FRMPD3, FUT7, GAPT, GMFG, GRAP2, HLA-DMB, HLA-DOA, HLA-DPA1, HLA-DPB1, HLA-DRA, HLA-DRB1, ICOS, IFNG, IGLL5, IKZF1, IKZF3, IL12RB1, IL18, IL18RAP, IL21R, IL7R, INPP5D, IQGAP2, IRF4, IRF8, ITGA4, ITGAL, ITGAX, ITGB2, ITK, JAML, KLRC1, KLRC2, KLRK1, LAG3, LAIR1, LAX1, LCK, LCP1, LCP2, LDLR, LEF1, LILRB1, LILRB4, LMO1, LRMP, LST1, LY9, LYZ, MMP9, MPO, MS4A1, MT1G, MYB, MZB1, NAMPT, NCKAP1L, NCR1, NCR3, NFAM1, NLRC3, NLRP3, NR4A3, ORM2, PIK3CG, PLA2G2D, PLAC8, PLAU, POU2AF1, POU2F2, PRAM1, PRDM1, PRF1, PRKCB, PRKCQ, PTAFR, PTGDR, PTK2B, PTPN22, PTPN6, PTPRC, PTX3, PYCARD, RAB37, RAB44, RAC2, RASAL3, RHOH, RNASET2, RUNX3, SAMSN1, SASH3, SCNN1B, SELL, SELPLG, SEMA4A, SERPINA1, SIRPG, SIT1, SLA2, SLAMF6, SLAMF7, SLAMF8, SLC18A1, SLC27A2, SOCS1, SPN, STAP1, STXBP2, SUCNR1, SYK, TBC1D10C, TBX21, TESPA1, THBS1, THEMIS, THEMIS2, THY1, TIGIT, TLR6, TNFAIP3, TNFAIP8L2, TNFRSF13B, TNFRSF18, TNFSF13B, TNFSF14, TNFSF8, TOX, TREM2, TRPM2, TXNDC5, UBD, VAV1, VNN1, WAS, WDFY4, WNT1, XCL1, ZAP70, ZNF683]	[ABL1, ADGRE5, AGER, ALDOA, AMH, AP3D1, ARL8A, ARMC5, ARSA, ARTN, BAG6, BATF2, BCL3, BCL6, BTLA, C5AR1, CAPN1, CCL2, CCL21, CCR7, CD14, CD177, CD1C, CD276, CD74, CDKN1A, CEACAM1, CHGA, CHI3L1, CLC, CLCF1, CLEC12A, CLNK, CLPTM1, CNR1, COMMD3, CRAT, CSF1, CSK, CST7, CTSA, CTSD, CX3CL1, CYB5R3, CYP26B1, DCAF15, DHRS2, DLG5, DOK3, DSC1, DTX1, DYSF, EDN2, ELF4, ENPP3, ERBB2, FADD, FES, FLOT2, FLT3, FOXF1, FPR1, GAB2, GALNS, GATA2, GBA, GNAO1, GPAM, GPER1, GPI, GPR84, GRN, HAVCR1, HBB, HDAC5, HFE, HPRT1, HVCN1, ICAM1, IDO1, IL15RA, IL1B, IL4R, IL7R, IMPDH1, INHA, IQGAP2, JAK3, JUND, JUP, KDELR1, LAG3, LDLR, LGALS9, LRG1, LRRC32, LRRC8A, LTBR, LTF, METTL7A, MME, MSN, MYH9, MYO18A, NBEAL2, NECTIN2, NHLRC3, NOD2, NPPA, NR1D1, NR4A3, NRARP, PCDHA4, PCDHA6, PDCD1, PKM, PLA2G2A, PLAU, PLAUR, PRKCSH, PTGDS, RAB3A, RAB5B, RAC2, RAP1A, RARA, RELB, RHOG, RNASE2, RNU1-93P, S100A11, SBNO2, SERPINA3, SIGLEC9, SLC44A2, SLC7A1, SLC7A8, SLCO4C1, SMAD3, SOX12, SOX13, SPHK1, SPHK2, SPI1, SRC, SRF, STAT3, STING1, STK11IP, SYVN1, TCF3, TCF7L1, TCIRG1, THBS1, TICAM1, TIMP1, TMEM179B, TMEM63A, TNF, TRAPPC1, TSPAN14, VNN1, VSIR, WAS, ZBTB7A, ZBTB7B, ZC3H12A, ZMIZ1, ZNF335, ZNF395]
GO:0048583	regulation of response to stimulus	4,40E-27	[2, 3]	851,00	18,88	45,12	54,88	[ABRA, ACP5, ADAM8, ADCY7, AIF1, AIM2, AKNA, ALOX15, ALOX15B, ANGPT1, ANGPT2, AOAH, APOBEC3G, ARAP2, AREG, ARHGAP15, ARHGAP30, ARHGAP45, ATF3, BANK1, BCL2L14, BCL3, BLK, BLNK, BMF, BMP7, BMPR1B, BRIP1, BTK, BTLA, BTN3A1, BTN3A2, C1QA, C1QB, C1QC, C1QTNF1, C2, C8G, CALCR, CARD11, CASP8, CASS4, CCDC88C, CCL17, CCL19, CCL2, CCL22, CCL24, CCL3, CCL3L1, CCL4, CCL4L1, CCL5, CCR2, CCR4, CD177, CD180, CD19, CD1B, CD1C, CD1E, CD200R1, CD22, CD226, CD247, CD27, CD300LF, CD33, CD3D, CD3E, CD3G, CD4, CD40LG, CD48, CD74, CD79A, CD80, CD84, CD86, CD8A, CD8B, CD96, CDKN2A, CEL, CFP, CHRDL1, CISH, CLCF1, CLEC10A, CLEC1B, CLEC4D, CLEC4E, CLEC6A, CLNK, CNR1, CNR2, CR2, CRB2, CRTAM, CSF1R, CST7, CTLA4, CXCL10, CXCL6, CXCR4, CXorf21, CYP11B1, CYSLTR1, DAPK1, DCC, DEPDC1B, DLGAP2, DPEP1, DPEP2, DRD1, DUSP2, DUSP8, EDN2, ELF3, EPX, EREG, ERFE, FASLG, FCGR2C, FCN1, FCN3, FCRL3, FFAR4, FGD2, FGD3, FGL2, FGR, FLT3, FOLR2, FOXP3, FRZB, FUT7, FYB1, GADD45B, GBP5, GDF6, GFI1, GMIP, GPR150, GPR174, GPR4, GPR55, GPR83, GPRC5A, GPSM3, GRAP2, GUCY2D, H3C10, H3C11, H3C12, H3C7, HCLS1, HCST, HLA-DMB, HLA-DPA1, HLA-DPB1, HLA-DQA1, HLA-DRA, HLA-DRB1, HLA-DRB5, HOPX, ICOS, IER3, IFNG, IGLL5, IKBKE, IL10RA, IL12RB1, IL16, IL18, IL18RAP, IL26, IL7R, INPP5D, IQCJ-SCHIP1, IRF4, ITGA4, ITGAL, ITGB2, ITGB7, ITK, JAML, KCNN4, KIR2DL2, KIR2DL3, KIR3DL2, KLRB1, KLRC1, KLRC2, KLRD1, KLRK1, KNG1, LAG3, LAIR1, LAIR2, LAX1, LCK, LCP1, LCP2, LDLR, LEF1, LIF, LILRA1, LILRB1, LILRB4, LMCD1, LTA, LY86, MAP4K1, MAPK13, MLC1, MMP9, MOG, MS4A1, MSX1, MUC16, MUC19, MUC6, MYB, MYO1G, MZB1, NAMPT, NCF1, NCKAP1L, NCR1, NCR3, NFAM1, NLRC3, NLRP3, NLRP6, NPFFR2, NPPC, NPTX2, NR4A2, NR4A3, NTN1, NUP210, OASL, P2RY10, P2RY12, PAX5, PDE6G, PIK3CG, PIK3IP1, PIK3R5, PILRA, PIM2, PLA2G2D, PLA2G7, PLAU, PLEK, PRAM1, PRDM1, PRKCB, PRKCQ	[AAAS, ABCA7, ABCC8, ABCD1, ABI3, ABL1, ABR, ABRA, ACKR3, ACP4, ACTB, ACTN4, ADCY1, ADCY3, ADCY4, ADCY9, ADGRG1, ADIPOQ, AGAP2, AGER, AGPAT1, AGT, AGTR1, AKT1S1, AMH, ANGPT1, APLN, APLNR, APLP1, APOA1, APOD, ARAF, ARAP3, ARC, AREG, ARHGAP1, ARHGAP23, ARHGAP39, ARHGDIA, ARHGEF1, ARHGEF16, ARHGEF5, ARMT1, ARRDC3, ARTN, ASPN, BAG3, BAG6, BCAR1, BCL2L1, BCL3, BCL6, BCL9L, BCR, BEND6, BMP8A, BRD4, BTLA, C1QTNF1, C20orf27, C5, C5AR1, C8G, CALCR, CAMK2B, CAPN1, CARM1, CC2D1A, CCL11, CCL2, CCL21, CCL24, CCR7, CD14, CD177, CD1C, CD1E, CD276, CD300E, CD74, CDK1, CDK2, CEACAM1, CEL, CFH, CGA, CHERP, CHGA, CHI3L1, CISH, CITED2, CLC, CLCF1, CLEC1B, CLEC4E, CLIP3, CLNK, CNR1, CNTN6, COL1A1, CPN2, CRAT, CREB3L1, CRHR2, CRP, CRYAB, CSF1, CSK, CSNK1E, CSPG4, CST7, CTHRC1, CX3CL1, CXXC4, CYP26B1, CYP27B1, DAB2IP, DAG1, DAGLA, DAXX, DDX39B, DENND2B, DENND4B, DGKD, DGKZ, DIRAS1, DKK2, DLG5, DLGAP4, DMTN, DOK5, DOT1L, DTX1, DUSP5, DUSP8, DVL3, ECM1, EDN2, EEF1E1, EIF4G1, ELF3, ENDOG, ENG, ENPP3, EPHA2, EPN1, EPPK1, ERBB2, ERFE, ESM1, FABP4, FADD, FAIM2, FAM110A, FAP, FASN, FCN3, FEM1A, FES, FFAR2, FGFR4, FLOT2, FLT3, FLT4, FOXC2, FOXF1, FOXM1, FPR1, FRMD8, FURIN, GAB2, GADD45B, GAS1, GATA2, GATA4, GBA, GBF1, GCSAM, GDF6, GIT1, GLIS2, GNAI2, GNAO1, GPAM, GPBAR1, GPER1, GPI, GPR137, GPR17, GPR20, GPR37L1, GPR4, GPR83, GPR84, GPRC5A, GPRIN1, GRB7, GRIK5, GRINA, GRM2, GRN, GSK3A, GTF2IRD1, H3C10, H3C12, H4C3, HBEGF, HCRTR1, HDAC10, HDAC7, HEYL, HFE, HGS, HIPK4, HMGA1, HOPX, HRH2, HSF1, HSPB1, HSPB8, HTR1B, HTR2A, ICAM1, IER3, IFITM1, IFT80, IL18BP, IL1B, IL1RN, IL4R, IL7R, ILK, INCA1, INHA, INPP5E, IRAK2, ITGA3, ITGA5, ITPRIP, JAK3, JMJD8, JUP, KCNK2, KCP, KCTD11, KIF26A, KLK14, KMT2D, KNG1, KSR1, LAG3, LDLR, LGALS9, LGR6, LIF, LIFR, LILRA5, LIMK1, LIMS2, LMCD1, LMNA, LOX, LRG1, LTBR

GO:0002250	adaptive immune response	1,69E-05	[3]	167,00	20,93	74,84	25,16	[ADCY7, ALOX15, BATF, BCL3, BTK, BTLA, BTN3A1, BTN3A2, C1QA, C1QB, C1QC, C2, C8G, CAMK4, CCL19, CCR2, CD19, CD1B, CD1C, CD1E, CD226, CD244, CD247, CD27, CD3D, CD3E, CD3G, CD4, CD40LG, CD48, CD6, CD7, CD74, CD79A, CD80, CD84, CD86, CD8A, CD8B, CLCF1, CLEC10A, CLEC4D, CLEC6A, CR2, CRTAM, CTLA4, CTSS, CXCL10, EBI3, EOMES, FOXP3, FUT7, GZMM, HLA-DMA, HLA-DMB, HLA-DOA, HLA-DPA1, HLA-DPB1, HLA-DQA1, HLA-DRA, HLA-DRB1, HLA-DRB5, IFNG, IGLL5, IL12RB1, IL18, IL7R, INPP5D, IRF4, ITK, JCHAIN, KLRC1, KLRC2, KLRD1, KLRK1, LAG3, LAIR1, LAMP3, LAX1, LEF1, LILRA1, LILRB1, LILRB4, LTA, LY9, MCOLN2, MYO1G, NCKAP1L, NLRP3, NR4A3, PIK3CG, PRDM1, PRF1, PRKCB, PRKCQ, PTK2B, PTPN6, PTPRC, PYCARD, SAMSN1, SASH3, SEMA4A, SH2D1A, SIGLEC10, SIT1, SKAP1, SLA2, SLAMF6, SLAMF7, SPN, SYK, TBX21, THEMIS, TLR8, TNFAIP3, TNFRSF13B, TNFRSF17, TNFSF13B, TRAT1, TREM2, WAS, XCL1, ZAP70, ZNF683]	[AGER, ARTN, BCL3, BCL6, BTLA, C5, C8G, CD1C, CD1E, CD74, CEACAM1, CLC, CLCF1, CRP, CSK, FADD, GNAO1, HFE, HLA-DOB, HPRT1, ICAM1, IL18BP, IL1B, IL4R, IL7R, JAK3, LAG3, LILRB5, NCOR2, NECTIN2, NFKB2, NOD2, NR4A3, ORAI1, PDCD1, PPL, PRKACA, PVR, RAP1A, RAP1GAP2, RELB, RHBDF2, RNF19B, SERPING1, SLC7A8, STAT3, TCIRG1, TFE3, TFEB, TNF, WAS, ZBTB7B, ZC3H12A, ZNF395]
GO:0002263	cell activation involved in immune response	9,70E-12	[3]	189,00	23,74	60,07	39,93	[ADAM8, ADGRE3, ANPEP, APBB1IP, ARHGAP45, BATF, BCL3, BIN2, BLK, BTK, CCL19, CCL3, CCR2, CD177, CD180, CD19, CD1C, CD244, CD33, CD40LG, CD53, CD74, CD80, CD84, CD86, CEACAM21, CFP, CLCF1, CLEC12A, CLEC4D, CLNK, CORO1A, COTL1, CTSS, CYBB, DOCK10, DOCK2, EOMES, EPX, FCN1, FGL2, FGR, FOXP3, FRMPD3, GAPT, GMFG, HLA-DMB, HLA-DRA, HLA-DRB1, IFNG, IL12RB1, IL18, IQGAP2, IRF4, IRF8, ITGAL, ITGAX, ITGB2, KLRC2, LAIR1, LCP1, LEF1, LILRB1, LRMP, LY9, LYZ, MMP9, MPO, MYB, NCKAP1L, NFAM1, NLRP3, NR4A3, ORM2, PIK3CG, PLAC8, PLAU, POU2AF1, PRAM1, PTAFR, PTGDR, PTK2B, PTPN6, PTPRC, PTX3, PYCARD, RAB37, RAB44, RAC2, RNASET2, SCNN1B, SELL, SEMA4A, SERPINA1, SLAMF6, SLC18A1, SLC27A2, SPN, STXBP2, SUCNR1, SYK, TBC1D10C, TBX21, TREM2, TRPM2, TXNDC5, VNN1, ZNF683]	[ABL1, ADGRE5, ALDOA, AMH, ARL8A, ARSA, ARTN, BCL3, BCL6, C5AR1, CAPN1, CD14, CD177, CD1C, CD74, CEACAM1, CHGA, CHI3L1, CLCF1, CLEC12A, CLNK, COMMD3, CRAT, CTSA, CTSD, CYB5R3, DOK3, DSC1, DYSF, ENPP3, FES, FOXF1, FPR1, GAB2, GALNS, GATA2, GBF1, GPI, GPR84, GRN, HBB, HFE, HVCN1, ICAM1, IL4R, IMPDH1, IQGAP2, JAK3, JUP, LGALS9, LRG1, LTF, METTL7A, MME, NBEAL2, NHLRC3, NPPA, NR4A3, PKM, PLAU, PLAUR, PRKCSH, PTGDS, RAB3A, RAB5B, RAC2, RAP1A, RARA, RELB, RHOG, RNASE2, RNU1-93P, S100A11, SBNO2, SERPINA3, SIGLEC9, SLC44A2, SLC7A8, SLCO4C1, SPHK2, SPI1, STAT3, STING1, STK11IP, TCIRG1, TICAM1, TMEM179B, TMEM63A, TRAPPC1, TSPAN14, VNN1, ZBTB7B, ZC3H12A]
GO:0002443	leukocyte mediated immunity	5,71E-10	[3]	218,00	21,91	61,60	38,40	[ADAM8, ADGRE3, ANPEP, ARHGAP45, BATF, BCL3, BIN2, BLK, BTK, BTN3A2, C1QA, C1QB, C1QC, C2, C8G, CAMK4, CCL3, CCR2, CD177, CD19, CD1B, CD1C, CD1E, CD226, CD27, CD33, CD40LG, CD53, CD74, CD84, CD8A, CD96, CEACAM21, CFP, CLCF1, CLEC12A, CLEC4D, CLNK, CORO1A, COTL1, CR2, CRTAM, CTSS, CXCL10, CXCL6, CYBB, DOCK2, EPX, FCN1, FGL2, FGR, FOXP3, FRMPD3, FUT7, GMFG, GZMB, GZMM, HLA-DRA, HLA-DRB1, IGLL5, IL12RB1, IL18, IL18RAP, IL7R, INPP5D, IQGAP2, ITGAL, ITGAX, ITGB2, KLRC1, KLRC2, KLRD1, KLRK1, LAG3, LAIR1, LILRB1, LILRB4, LRMP, LTA, LYZ, MMP9, MPO, MYO1G, NCKAP1L, NCR1, NCR3, NFAM1, NLRP3, NLRP6, NR4A3, ORM2, PIK3CG, PLAC8, PLAU, PRAM1, PRF1, PTAFR, PTGDR, PTPN6, PTPRC, PTX3, PYCARD, RAB37, RAB44, RAC2, RNASET2, SASH3, SCIMP, SCNN1B, SELL, SERPINA1, SH2D1A, SLA2, SLAMF6, SLAMF7, SLC18A1, SLC27A2, STAP1, STXBP2, SYK, TBC1D10C, TBX21, TLR8, TREM2, TRPM2, TXNDC5, VAV1, VNN1, WAS, XCL1]	[ADGRE5, AGER, ALDOA, AMH, ARL8A, ARSA, ARTN, BCL3, BCL6, C5, C5AR1, C8G, CAPN1, CD14, CD177, CD1C, CD1E, CD74, CEACAM1, CHGA, CHI3L1, CLC, CLCF1, CLEC12A, CLNK, COMMD3, CRAT, CRP, CTSA, CTSD, CYB5R3, DOK3, DSC1, FADD, FES, FOXF1, FPR1, GAB2, GALNS, GATA2, GNAO1, GPI, GPR84, GRN, HBB, HFE, HPRT1, HVCN1, ICAM1, IL1B, IL4R, IL7R, IMPDH1, IQGAP2, JAK3, JUP, LAG3, LGALS9, LRG1, LTF, MAVS, METTL7A, MME, NBEAL2, NECTIN2, NHLRC3, NOD2, NPPA, NR4A3, PKM, PLAU, PLAUR, PLEKHM2, PRKCSH, PTGDS, PVR, RAB3A, RAB5B, RAC2, RAP1A, RHOG, RNASE2, RNF19B, RNU1-93P, S100A11, SERPINA3, SERPING1, SIGLEC9, SLC44A2, SLC7A8, SLCO4C1, SPHK2, SPI1, STING1, STK11IP, TCIRG1, TICAM1, TMEM179B, TMEM63A, TNF, TRAPPC1, TSPAN14, VNN1, WAS, ZNF395]
GO:0002683	negative regulation of immune system process	1,16E-10	[2, 3, 4]	156,00	24,68	57,59	42,41	[ADCY7, ALOX15, ANGPT1, BANK1, BLK, BTK, C1QC, C1QTNF1, CALCR, CCL2, CCL3, CCR2, CD200R1, CD22, CD247, CD300LF, CD3G, CD74, CD80, CD84, CD86, CD96, CDKN2A, CLNK, CNR1, CNR2, CR2, CRTAM, CST7, CTLA4, CYSLTR1, DPEP1, DRD1, FCRL3, FGL2, FGR, FOXP3, GPR150, GPR55, GPR83, HLA-DOA, HLA-DRB1, IL7R, INPP5D, KLRC1, KLRD1, KLRK1, LAG3, LAX1, LDLR, LILRB1, LILRB4, LPAL2, LRRC17, LST1, NCKAP1L, NLRC3, NLRP3, NLRP6, PLA2G2D, PTGDR, PTGER2, PTPN22, PTPN6, PTPRC, RLN2, RUNX3, SAMSN1, SH2D1A, SLA2, SLAMF8, SOCS1, SPN, STAP1, SYK, TBC1D10C, TBX21, THBS1, THY1, TIGIT, TNFAIP3, TNFAIP8L2, TNFRSF13B, TREM2, TSC22D3, UBASH3A, XCL1]	[ADCY1, ADCY3, ADCY4, ADCY9, ADIPOQ, AMH, ANGPT1, APOA1, APOD, BCL6, C1QTNF1, C5, CALCR, CCL2, CCL21, CD74, CEACAM1, CGA, CHGA, CLNK, CNR1, CRHR2, CST7, CX3CL1, DGKZ, DLG5, DTX1, ENPP3, ERBB2, FADD, FOXF1, FURIN, GATA2, GCSAM, GNAI2, GPAM, GPBAR1, GPER1, GPR137, GPR20, GPR83, GPR84, GRN, HFE, HLA-DOB, HRH2, IL4R, IL7R, INHA, JAK3, LAG3, LDLR, LGALS9, LRRC17, LRRC32, LTF, MYC, MYH9, NLRC5, NOD2, NR1D1, NRARP, PCDHA4, PCDHA6, PDCD1, PRKACA, PRKAR2B, PTGIR, RAMP2, RARA, RHBDF2, RMRP, RNF26, SERPING1, SPI1, SRC, THBS1, TNF, TRIB1, VIPR2, VSIR, ZBTB7B, ZC3H12A]

GO:0002684	positive regulation of immune system process	4,58E-20	[2, 3, 4]	281,00	24,16	64,49	35,51	[ADAM8, AIF1, AIM2, BLK, BLNK, BTK, BTLA, BTN3A1, BTN3A2, C1QA, C1QB, C1QC, C2, C8G, CAMK4, CARD11, CASP8, CCDC88B, CCL19, CCL2, CCL24, CCL3, CCL4, CCL5, CCR2, CD177, CD19, CD1B, CD1C, CD1E, CD2, CD22, CD226, CD244, CD247, CD27, CD3D, CD3E, CD3G, CD4, CD40LG, CD5, CD6, CD74, CD79A, CD80, CD84, CD86, CFP, CLCF1, CLEC10A, CLEC4D, CLEC4E, CLEC6A, CLNK, CNR1, CORO1A, CR2, CRTAM, CSF1R, CTLA4, CXCL10, CXorf21, CYP11B1, DOCK8, EBI3, EDN2, EGR3, EPX, EREG, EVI2B, FCN1, FCN3, FCRL3, FGR, FOXP3, FYB1, GBP5, GPSM3, GRAP2, HCLS1, HLA-DMB, HLA-DPA1, HLA-DPB1, HLA-DQA1, HLA-DRA, HLA-DRB1, HLA-DRB5, ICOS, IFNG, IGLL5, IKBKE, IL12RB1, IL18, IL18RAP, IL7R, INPP5D, ITGA4, ITGB2, ITK, KCNN4, KLRC2, KLRD1, KLRK1, LAG3, LAX1, LCK, LCP2, LEF1, LILRB1, LILRB4, LTA, MOG, MS4A1, MUC16, MUC19, MUC6, MYB, MYO1G, MZB1, NCKAP1L, NCR3, NFAM1, NLRP3, NR4A3, P2RY12, PAX5, PLA2G7, PRAM1, PRKCB, PRKCQ, PTAFR, PTK2B, PTPN22, PTPN6, PTPRC, PYCARD, PYHIN1, RAC2, RASAL3, RHOH, RUNX3, SASH3, SCIMP, SH2D1A, SIGLEC16, SIRPG, SKAP1, SLA2, SLAMF6, SOCS1, SPN, STAP1, STXBP2, SYK, TBX21, TESPA1, THBS1, THEMIS, THEMIS2, THY1, TLR6, TLR8, TNFRSF18, TNFSF13B, TNFSF14, TOX, TRAT1, TREM2, UBASH3A, VAV1, VNN1, WAS, XCL1, XCL2, XG, ZAP70, ZBP1]	[ABL1, ACTB, AGER, AMH, AP3D1, AQP3, BAG6, BCAR1, BCL6, BTLA, C5, C5AR1, C8G, CCL2, CCL21, CCL24, CCR7, CD177, CD1C, CD1E, CD276, CD74, CDKN1A, CEACAM1, CFH, CLCF1, CLEC4E, CLNK, CNR1, CPN2, CRP, CSF1, CSK, CX3CL1, DGKZ, EDN2, ENPP3, FADD, FCN3, FES, FFAR2, FLOT2, FOXF1, FPR1, GAB2, GATA2, GCSAM, GNAO1, GPAM, GPI, GRN, HAVCR1, HK1, HSF1, ICAM1, IL15RA, IL1B, IL4R, IL7R, JAK3, JUND, KLF10, LAG3, LGALS9, LIMK1, MAPK3, MAVS, MSTN, MUC19, MUC20, MUC3A, MYO18A, NCOR2, NECTIN2, NLRC5, NOD2, NPPA, NR4A3, PCDHA4, PDCD1, PLA2G7, PLVAP, PQBP1, PRKACA, PTN, PVR, RAC2, RAP1A, RARA, RBM14, RELA, RELB, RGCC, RHBDF2, RNU1-93P, SELENOP, SERPINE1, SERPING1, SH2B2, SLC7A1, SLC7A5, SLC7A8, SOX12, SOX13, SPHK2, SPI1, SRC, STING1, THBS1, TICAM1, TIMP1, TNF, TRIB1, TRIM62, TRPV4, VEGFA, VNN1, VSIR, VTN, WAS, XG, ZBTB7B, ZC3H12A, ZCCHC3, ZMIZ1, ZNF335, ZNF580]
GO:0006952	defense response	1,24E-22	[3]	438,00	21,72	59,13	40,87	[ACP5, ADAM8, ADCY7, ADORA3, AIF1, AIM2, AKNA, ALOX15, AOAH, APOBEC3A, APOBEC3D, APOBEC3G, APOBEC3H, BATF, BCL3, BLK, BLNK, BMPR1B, BTK, C1QA, C1QB, C1QC, C1QTNF1, C2, C8G, CALCR, CAMK4, CARD11, CASP8, CCDC88B, CCL17, CCL19, CCL2, CCL22, CCL24, CCL3, CCL3L1, CCL4, CCL4L1, CCL5, CCR2, CCR3, CCR4, CCR5, CD177, CD180, CD200R1, CD226, CD244, CD247, CD3G, CD4, CD40LG, CD48, CD6, CD74, CD84, CD96, CEL, CFP, CLEC10A, CLEC1B, CLEC4D, CLEC4E, CLEC6A, CLNK, CNR1, CNR2, CORO1A, COTL1, CR2, CRTAM, CSF1R, CST7, CXCL10, CXCL11, CXCL5, CXCL6, CXCL9, CXCR3, CXCR4, CXCR6, CXorf21, CYBB, CYSLTR1, DAPK1, DPEP1, DRD1, ELF3, EPX, EREG, FASLG, FCMR, FCN1, FCN3, FFAR4, FGL2, FGR, FOLR2, FOSL1, FOXP3, FUT7, GBP5, GBP6, GNLY, GPR150, GPR4, GPR83, GPSM3, GZMB, GZMM, HLA-DPA1, HLA-DPB1, HLA-DQA1, HLA-DRA, HLA-DRB1, HLA-DRB5, IFNG, IGLL5, IKBKE, IL12RB1, IL16, IL18, IL18RAP, IL22, IL5RA, IL7R, IRF4, IRF5, IRF8, ITGAL, ITGAX, ITGB2, ITK, JCHAIN, KCNN4, KIR2DL3, KIR3DL2, KLRC1, KLRC2, KLRC3, KLRD1, KLRG1, KLRK1, KNG1, LAG3, LCK, LCP1, LDLR, LILRA1, LILRB1, LILRB4, LPAL2, LTA, LY75, LY86, LY9, LYZ, MAPK13, MCOLN2, MMP9, MPEG1, MPO, MRC1, MS4A1, MUC16, MUC19, MUC6, NCF1, NCR1, NCR3, NFAM1, NLRC3, NLRP2, NLRP3, NLRP6, NLRP7, NLRP9, OASL, ORM2, PIK3CG, PLA2G2D, PLA2G7, PLAC8, PLD4, PRDM1, PRF1, PRKCQ, PSTPIP1, PTAFR, PTGDR, PTGER2, PTGFR, PTK2B, PTPN22, PTPN6, PTPRC, PTPRCAP, PTX3, PYCARD, PYHIN1, RLN2, RNASE6, RNASET2, SCNN1B, SCUBE1, SDC1, SELE, SERPINA1, SH2D1A, SIGLEC1, SIGLEC10, SIGLEC16, SLA, SLAMF6, SLAMF7, SLAMF8, SMPDL3B, SOCS1, SOCS3, SP140, SPN, STAP1, STAT1, STYK1, SUCNR1, SYK, THBS1, THEMIS2, TLR10, TLR6, TLR8, TNFAIP3, TNFAIP8L2, TNFSF8, TNIP3, TRAT1, TREM2, TRIM14, UBD, VAV1, VNN1, WAS, XCL1, XCL2, XCR1, ZAP70, ZBP1, ZNF683]	[ABCD1, ABCD2, ABI3, ACKR1, ADAMTS5, ADCY1, ADCY3, ADCY4, ADCY9, ADGRE5, ADIPOQ, AGER, AGT, AGTR1, AMH, APOA1, APOBEC3C, APOD, ARID5A, ARMC5, BATF2, BCL2L1, BCL3, BCL6, BRD4, C1QTNF1, C5, C5AR1, C8G, CALCR, CAMK2B, CCL11, CCL2, CCL21, CCL24, CCR7, CD14, CD177, CD300E, CD74, CDC42EP2, CDC42EP4, CEACAM1, CEL, CFH, CGA, CHGA, CHI3L1, CHST1, CIITA, CLEC1B, CLEC4E, CLNK, CNR1, CRHR2, CRP, CSF1, CST7, CX3CL1, CYP26B1, CYP27B1, DAB2IP, DAGLA, DHX34, ECM1, EIF4G1, ELF3, ELF4, ENPP3, EPHA2, FABP4, FADD, FCN3, FEM1A, FES, FFAR2, FOSL1, FOXF1, FPR1, FURIN, GBA, GIT1, GNAI2, GNAO1, GPAM, GPBAR1, GPER1, GPR20, GPR4, GPR83, GPR84, GRIK5, GRN, HDAC5, HFE, HK1, HRH2, HSF1, HSPG2, HYAL1, ICAM1, IFITM1, IFITM2, IFITM3, IL1B, IL1RN, IL22RA1, IL32, IL4R, IL5RA, IL7R, IRAK2, IRF2, IRF9, JAK3, KDM6B, KNG1, LAG3, LDLR, LGALS3BP, LGALS9, LILRA5, LILRB5, LTB4R2, LTF, MAP2K3, MAP3K14, MAPK3, MAPK7, MAVS, MECP2, MEN1, MOV10, MRC1, MUC19, MUC20, MUC3A, MYC, MYH9, NDST1, NECTIN2, NFATC4, NINJ1, NLRC5, NLRP9, NOD2, NOTCH1, NPAS2, NPPA, NR1D1, OR2H2, OSM, PCDHA4, PCDHA6, PER1, PLA2G2A, PLA2G7, PLD3, PLEKHM2, PML, PPARD, PPP1R11, PQBP1, PRKACA, PRKAR2B, PTGES, PTGFR, PTGIR, PTN, PVR, RAMP2, RBM14, RELA, RELB, RHBDF2, RNASE2, RNF19B, RNF26, RNF39, RNU1-93P, SBNO2, SCYL1, SELE, SELENOP, SERPINA3, SERPINE1, SERPING1, SHANK3, SHARPIN, SMAD3, SMPD1, SOCS3, SPATA2, SPHK1, SPI1, SRC, STAT3, STING1, TBKBP1, TCIRG1, TGM2, THBS1, TICAM1, TIMP1, TNF, TNFRSF1A, TNIP1, TNK1, TNK2, TRIM29, TRIM62, TRIM8, TRPV4, VIPR2, VNN1, WAS, ZC3H12A, ZCCHC3, ZDHHC11, ZFP36, ZNF395, ZNF580, ZYX]

GO:0048584	positive regulation of response to stimulus	3,89E-17	[2, 3, 4]	491,00	19,87	48,73	51,27	[ABRA, ADAM8, AIF1, AIM2, ALOX15, ALOX15B, ANGPT1, AREG, ATF3, BANK1, BLK, BLNK, BMF, BMP7, BMPR1B, BTK, BTN3A1, BTN3A2, C1QA, C1QB, C1QC, C1QTNF1, C2, C8G, CALCR, CARD11, CASP8, CASS4, CCL17, CCL19, CCL2, CCL22, CCL24, CCL3, CCL3L1, CCL4, CCL4L1, CCL5, CCR2, CCR4, CD177, CD180, CD19, CD1B, CD1C, CD1E, CD22, CD226, CD247, CD27, CD300LF, CD3D, CD3E, CD3G, CD4, CD40LG, CD74, CD79A, CD80, CD86, CDKN2A, CFP, CLCF1, CLEC10A, CLEC4D, CLEC4E, CLEC6A, CLNK, CNR1, CR2, CRB2, CRTAM, CSF1R, CTLA4, CXCL10, CXCR4, CXorf21, CYP11B1, DEPDC1B, DPEP2, EDN2, ELF3, EPX, EREG, ERFE, FASLG, FCN1, FCN3, FCRL3, FFAR4, FGD2, FGR, FLT3, FOXP3, FYB1, GADD45B, GBP5, GDF6, GFI1, GPR174, GPR4, GPR55, GPSM3, GRAP2, HCLS1, HCST, HLA-DMB, HLA-DPA1, HLA-DPB1, HLA-DQA1, HLA-DRA, HLA-DRB1, HLA-DRB5, ICOS, IFNG, IGLL5, IKBKE, IL10RA, IL12RB1, IL16, IL18, IL18RAP, IL26, IL7R, INPP5D, IQCJ-SCHIP1, ITGB2, ITK, KCNN4, KLRC2, KLRD1, KLRK1, LAG3, LAX1, LCK, LCP1, LCP2, LDLR, LILRB1, LILRB4, LMCD1, LTA, LY86, MAP4K1, MAPK13, MMP9, MOG, MS4A1, MSX1, MUC16, MUC19, MUC6, MYB, MYO1G, NCF1, NCKAP1L, NCR3, NFAM1, NLRP3, NPPC, NR4A3, OASL, P2RY10, P2RY12, PAX5, PDE6G, PIK3CG, PIK3R5, PIM2, PLA2G7, PRAM1, PRKCB, PRKCQ, PROK1, PTAFR, PTK2B, PTPN22, PTPN6, PTPRC, PYCARD, PYHIN1, RAC2, RASGRF1, RTKN2, SALL1, SASH3, SCIMP, SCUBE1, SEMA4D, SH2D1A, SIGLEC16, SKAP1, SLA2, SLAMF6, SOCS1, STAP1, SUCNR1, SYK, TBX21, TESPA1, THBS1, THEMIS, THEMIS2, THY1, TLR10, TLR8, TNFAIP3, TNFSF13B, TNFSF14, TRAF1, TRAT1, TREM2, TRIM14, UBASH3A, UBD, VAV1, WAS, WNT1, WNT10B, XCL1, XCL2, ZAP70, ZBP1]	[ABCA7, ABI3, ABL1, ABRA, ACKR3, ACTB, ACTN4, ADGRG1, ADIPOQ, AGAP2, AGER, AGPAT1, AGT, AGTR1, AMH, ANGPT1, APOA1, ARAF, AREG, ARHGEF5, ARTN, BAG6, BCAR1, BCL2L1, BMP8A, BRD4, C1QTNF1, C20orf27, C5, C5AR1, C8G, CALCR, CC2D1A, CCL11, CCL2, CCL21, CCL24, CCR7, CD14, CD177, CD1C, CD1E, CD276, CD74, CEACAM1, CFH, CHERP, CHGA, CHI3L1, CITED2, CLCF1, CLEC4E, CLNK, CNR1, CNTN6, COL1A1, CPN2, CRAT, CRP, CSF1, CSK, CSNK1E, CSPG4, CX3CL1, CYP27B1, DAB2IP, DAXX, DENND2B, DGKD, DGKZ, DIRAS1, DKK2, DLG5, DMTN, DOK5, DUSP5, DVL3, ECM1, EDN2, EEF1E1, ELF3, ENDOG, ENG, ERBB2, ERFE, ESM1, FABP4, FADD, FAM110A, FASN, FCN3, FEM1A, FFAR2, FGFR4, FLT3, FLT4, FOXC2, FOXM1, FPR1, GAB2, GADD45B, GATA2, GATA4, GCSAM, GDF6, GNAI2, GNAO1, GPBAR1, GPER1, GPR137, GPR17, GPR20, GPR37L1, GPR4, GPRIN1, GRN, GSK3A, HBEGF, HCRTR1, HDAC10, HFE, HMGA1, HSPB1, HTR2A, ICAM1, IL1B, IL4R, IL7R, ILK, INCA1, INHA, IRAK2, ITGA5, JMJD8, JUP, KCNK2, KCP, KLK14, KMT2D, KSR1, LAG3, LDLR, LGALS9, LGR6, LILRA5, LIMK1, LIMS2, LMCD1, LRG1, LTBR, LTF, MAP2K3, MAP3K10, MAP3K11, MAP3K14, MAP3K6, MAP3K9, MAPK3, MAPK8IP3, MAVS, MAZ, MED12, MEGF8, MEIS3P1, MEN1, MINK1, MLST8, MSTN, MSX1, MUC19, MUC20, MUC3A, MYC, NCOR2, NDC80, NDST1, NECTIN2, NFATC4, NGF, NGFR, NINJ1, NLGN2, NLRC5, NOD2, NODAL, NOS3, NOTCH1, NOTCH4, NPAS2, NPPA, NR4A3, NRARP, OSM, P2RX3, P2RX5, P2RY6, PCDHA4, PDGFB, PDGFRB, PIAS4, PLA2G2A, PLA2G7, PLAGL2, PLAUR, PLXNB1, PML, POR, PPARD, PPP1R15A, PQBP1, PRAG1, PRKACA, PTN, PTP4A3, PVR, RAC2, RAD51AP1, RAP1A, RAPGEF1, RARA, RASD2, RBM14, RELA, RELB, RGCC, RHBDF2, RHOG, RNU1-93P, RPL26, RPTOR, RTKN2, S1PR2, SECTM1, SELENON, SELENOP, SEMA4C, SEPTIN4, SERPINE1, SERPING1, SGTA, SH2B2, SHANK3, SHARPIN, SHC2, SHISA5, SHKBP1, SIK3, SLC2A4, SLC44A2, SLC7A8, SLC9A1, SLC9A3R1, SMAD3, SMARCA4
GO:0048585	negative regulation of response to stimulus	4,01E-07	[2, 3, 4]	356,00	18,56	39,96	60,04	[ACP5, ADCY7, AIF1, ALOX15, ANGPT2, AOAH, AREG, ATF3, BANK1, BCL3, BMP7, BMPR1B, C1QTNF1, CALCR, CASP8, CCDC88C, CCL2, CCL5, CCR2, CD200R1, CD22, CD247, CD300LF, CD3E, CD3G, CD74, CD84, CD96, CEL, CHRDL1, CISH, CNR2, CR2, CST7, CTLA4, CYSLTR1, DCC, DPEP1, DRD1, DUSP2, DUSP8, EPX, EREG, FASLG, FCRL3, FFAR4, FGL2, FGR, FOXP3, FRZB, GPR150, GPR83, GPRC5A, HLA-DRB1, IL7R, INPP5D, IRF4, KLRC1, KLRD1, KLRK1, KNG1, LAX1, LCP1, LDLR, LIF, LILRB1, LILRB4, MMP9, NAMPT, NCKAP1L, NLRC3, NLRP3, NLRP6, NR4A2, NR4A3, NTN1, PIK3IP1, PLAU, PLEK, PRKCB, PRKCQ, PSCA, PTGDR, PTGER2, PTPN22, PTPN6, PTPRC, PYCARD, RASAL3, RBPMS2, RGS1, RGS10, RGS18, RGS9, RHOH, RLN2, RMI2, RTKN2, SAMSN1, SEMA4A, SEMA4D, SH2D1A, SIGLEC10, SLA2, SLAMF8, SMPDL3B, SOCS1, SOCS3, SPINK1, STAP1, STAT1, SYK, TBC1D10C, TBX21, THBS1, THY1, TLR6, TNFAIP3, TNFAIP8L2, TNIP3, TNMD, TNR, TRABD2A, TREM2, TSPAN8, UBASH3A, UCP2, VNN1, VWC2L, WNT1, XCL1]	[ABCA7, ABCC8, ABL1, ACKR3, ACP4, ADCY1, ADCY3, ADCY4, ADCY9, ADIPOQ, AGAP2, AGT, AKT1S1, AMH, APLN, APLNR, APLP1, APOA1, APOD, AREG, ARRDC3, ASPN, BCL2L1, BCL3, BCL6, BCL9L, BEND6, C1QTNF1, C5, CALCR, CCL2, CD14, CD74, CEACAM1, CEL, CGA, CHGA, CISH, CREB3L1, CRHR2, CSK, CST7, CTHRC1, CX3CL1, CXXC4, CYP26B1, DAB2IP, DAG1, DDX39B, DGKD, DGKZ, DKK2, DLG5, DUSP5, DUSP8, DVL3, ECM1, ENPP3, EPHA2, EPN1, EPPK1, ERBB2, FADD, FAIM2, FAM110A, FAP, FASN, FEM1A, FOXF1, FOXM1, FRMD8, FURIN, GATA4, GBA, GIT1, GLIS2, GNAI2, GPBAR1, GPER1, GPI, GPR20, GPR37L1, GPR83, GPR84, GPRC5A, GRB7, GRINA, GRN, GSK3A, HBEGF, HDAC7, HEYL, HFE, HGS, HRH2, HSF1, HSPB1, HTR1B, ICAM1, IFT80, IL1B, IL1RN, IL4R, IL7R, INPP5E, ITGA3, ITPRIP, JAK3, KIF26A, KLK14, KNG1, LDLR, LGALS9, LIF, LMNA, LTF, LZTS2, MAP2K3, MAPK7, MAZ, MED12, MEGF8, MEIS3P1, MEN1, MGRN1, MNT, MSTN, MYADM, MYC, MYH9, NCOR2, NF2, NFATC4, NGFR, NKD2, NLRC5, NOD2, NODAL, NOS3, NOTCH1, NOTCH3, NPPA, NPRL3, NR1D1, NR4A2, NR4A3, NRARP, NTN1, OMA1, PALM, PCDHA6, PCGF2, PDCD1, PDGFB, PER1, PIAS4, PLAU, PLAUR, PLXNA3, PPARD, PPP1R10, PPP1R15A, PPP2R1A, PPP2R5B, PRDM16, PRKACA, PRKAR2B, PTGIR, PTPRU, RADX, RAMP2, RBPMS2, RELA, RGMA, RGS16, RGS18, RHBDF2, RMI2, RNF126, RNF157, RNF26, RNU1-93P, RTKN2, RTN4RL1, S1PR2, SEMA3F, SEMA4B, SEMA4C, SEMA6B, SEMA6C, SERPINE1, SERPING1, SGTA, SH2B3, SH3BP1, SHARPIN, SLC25A23, SLC35C1, SLC35F6, SLC9A3R1, SMAD3, SMARCA4, SMPD1, SNAI1, SOCS3, SOX10, SOX13, SPI1, SPINK1, SRC, SYNGAP1, SYVN1, SZT2, TGFB1I1, THBS1, TICAM1, TIMP1, TLE2, TLE3, TLE5, TMEM127, TMEM14A, TMEM161A, TMEM88, TNF, TNFAIP1, TNFRSF1A, TNIP1, TNMD, TNS2, TRIB1, TRIB3, TSC2, TSKU, TSPAN8, UBA52, UBE2B, UBQLN4, UNC5B, VASN, VIPR2, VNN1, VSIG2, VTN, WFS1, WNK2, WNT3, WWP2, XRCC1, ZBTB7A, ZBTB7B, ZC3H12A, ZFP36,

GO:0051240	positive regulation of multicellular organismal process	8,22E-15	[2, 3, 4]	325,00	21,42	48,82	51,18	[ADAM8, AIF1, AIM2, ALOX15B, ANGPT2, AREG, BATF, BCL3, BMP7, BMPR1B, BTK, BTN3A1, BTN3A2, C1QTNF1, CAMK4, CARD11, CASP8, CCDC88B, CCL19, CCL24, CCL3, CCR2, CCR3, CD2, CD226, CD244, CD27, CD3E, CD4, CD40LG, CD6, CD74, CD80, CD86, CDH4, CLCF1, CLEC6A, CLNK, CNR1, CRABP2, CRB2, CRTAM, CSF1R, CST7, CXCR4, CYBB, EBI3, EDN2, EGR3, EPX, EREG, EVI2B, FCN1, FFAR4, FGR, FOXP3, GBP5, GDF6, GPSM3, HCLS1, HLA-DPA1, HLA-DPB1, HLA-DRA, HLA-DRB1, IFNG, IL12RB1, IL12RB2, IL16, IL18, IL26, IL7R, INPP5D, IRF4, IRF5, IRF8, ITGAX, ITGB2, ITK, LCP1, LDLR, LEF1, LIF, LILRB1, LILRB4, LPAL2, LTA, LTB, LY9, MAPK13, MCOLN2, MYB, NCKAP1L, NFAM1, NLRP2, NLRP3, NLRP9, NPPB, NR4A3, NTN1, ORM2, P2RY2, PIK3CG, PIM1, PLAAT4, PLAC8, PLXNC1, POU2AF1, POU2F2, PRKCB, PRKCQ, PTAFR, PTGER2, PTK2B, PTPN22, PTPRC, PYCARD, PYHIN1, RHOH, RLN2, RUNX3, SASH3, SCIMP, SEMA4A, SEMA4D, SIGLEC16, SLAMF6, SMTNL2, SOCS1, STAT1, SYK, TBX21, TBXAS1, TESPA1, THBS1, TIGIT, TLR6, TLR8, TNR, TOX, TREM2, UCP2, VNN1, WNT1, WNT10B, XCL1, ZAP70, ZBED2, ZBP1]	[ABCA7, ABCC8, ABL1, ACACB, ADIPOQ, AGAP2, AGER, AGPAT1, AGT, AMH, ANGPTL4, AP3D1, APLN, APLNR, AREG, ARHGDIA, ATP1A1, ATP1A3, ATPSCKMT, BCL3, BCL6, BCL9L, C1QTNF1, C5, C5AR1, CAMK2B, CCL11, CCL24, CCR7, CD14, CD276, CD74, CDH4, CDK1, CFH, CFL1, CHGA, CHI3L1, CITED2, CLCF1, CLNK, CNOT3, CNR1, COL1A1, CSF1, CST7, CX3CL1, CYP27B1, DAG1, DDX39B, DHX34, DLG5, DMTN, ECM1, EDN2, ENG, FABP4, FADD, FES, FFAR2, FLT4, FOXC2, FOXS1, FRMD8, FURIN, GATA2, GATA4, GBA, GDF6, GDI1, GNAI2, GPAM, GPER1, GPR3, GRN, GSK3A, HK1, HRH2, HSF1, HSPB1, HTR2A, HYAL1, ICAM1, IL15RA, IL1B, IL4R, IL7R, ILK, INHA, ITGA5, JMJD8, JUP, KDM6B, KLF10, LDLR, LDLRAP1, LGALS9, LIF, LILRA5, LIMK1, LIPG, LRCH4, LRG1, LTF, LUM, MAVS, MED12, MEGF8, MFAP2, MME, MYC, MYRF, NEFL, NFATC4, NFKB2, NGF, NGFR, NINJ1, NLGN2, NLRP9, NOD2, NODAL, NOS3, NOTCH1, NPAS2, NPPA, NPPB, NR4A3, NTN1, NUMBL, OMA1, OSM, OSR2, P2RX3, P2RY2, PAK4, PARP6, PCDHA4, PDGFB, PIM1, PKM, PLXNA1, PLXNA3, PLXNB1, POLR2E, POLR2L, POR, POSTN, PQBP1, PRDM16, PTN, RAMP2, RARA, RELA, RGCC, RNU1-93P, S100A1, SERPINE1, SHANK3, SLC7A5, SLC9A1, SLN, SMAD3, SMTNL2, SNAI1, SOX10, SOX12, SOX13, SOX8, SPHK1, SPHK2, SRC, SRF, SRRT, STAT3, STING1, SYDE1, TBX5, TEAD4, TGFB1I1, TGM2, THBS1, TICAM1, TNF, TNFSF12, TRIB1, TRPV4, TSKU, VDR, VEGFA, VNN1, VSIR, WNT3, YBX2, ZBTB7B, ZC3H12A, ZCCHC3, ZMIZ1, ZNF219, ZNF335, ZNF580]
GO:0051707	response to other organism	1,26E-11	[2, 4]	341,00	20,19	61,24	38,76	[ACP5, ADAM8, AIF1, AIM2, APOBEC3A, APOBEC3D, APOBEC3G, APOBEC3H, BANK1, BATF, BCL3, BLK, BTK, C1QA, C1QB, C1QC, C2, C8G, CARD11, CARD17, CASP8, CCDC88B, CCL17, CCL19, CCL2, CCL22, CCL24, CCL3, CCL3L1, CCL4, CCL4L1, CCL5, CCR4, CCR5, CD177, CD180, CD226, CD244, CD4, CD6, CD74, CD80, CD84, CD86, CD96, CFP, CLEC10A, CLEC4D, CLEC4E, CLEC6A, CLNK, CNR1, CNR2, CORO1A, COTL1, CR2, CRTAM, CSF1R, CXCL10, CXCL11, CXCL5, CXCL6, CXCL9, CXCR4, CXorf21, CYBB, DAPK1, EPX, EREG, FASLG, FCN1, FCN3, FER1L6, FGL2, FGR, FOSL1, FOXP3, GBP5, GBP6, GFI1, GNLY, GPM6A, GZMA, GZMB, GZMM, HLA-DPA1, HLA-DPB1, HLA-DQA1, HLA-DRA, HLA-DRB1, HLA-DRB5, IFNG, IGLL5, IKBKE, IKZF3, IL10RA, IL12RB1, IL12RB2, IL18, IL18RAP, IL7R, IRF4, IRF5, IRF8, ITGAX, JCHAIN, KIR2DL3, KLRC1, KLRC2, KLRD1, KLRG1, KLRK1, KMO, LAG3, LCK, LILRB1, LTA, LY86, LY9, LYZ, MCOLN2, MPEG1, MPO, MRC1, MS4A1, MUC16, MUC19, MUC6, NCF1, NCR1, NCR3, NLRC3, NLRP2, NLRP3, NLRP6, NLRP7, NLRP9, NPPC, NR4A1, NUGGC, OASL, PIK3CG, PIM2, PLAC8, PLD4, POU2AF1, POU2F2, PRDM1, PRF1, PSTPIP1, PTAFR, PTGFR, PTK2B, PTPN22, PTPN6, PTPRC, PTX3, PYCARD, PYHIN1, RGS1, RNASE6, RNASET2, SCIMP, SCNN1B, SELE, SH2D1A, SIGLEC10, SIGLEC16, SLA, SLAMF6, SLAMF7, SLAMF8, SMPDL3B, SOCS1, SOCS3, SPN, STAP1, STAT1, STYK1, SYK, TBX21, TLR10, TLR6, TLR8, TNFAIP3, TNFAIP8L2, TNFSF8, TNIP3, TREM2, TRIM14, UBD, VAV1, VNN1, WAS, WDFY4, XCL1, XCL2, ZAP70, ZBP1, ZNF683]	[ABCC8, ABL1, ADAMTS5, ADIPOQ, AGER, ALPL, AMH, APOBEC3C, ARF1, ARID5A, ARMC5, BATF2, BCL2L1, BCL3, C5, C5AR1, C8G, CAMK2B, CCL11, CCL2, CCL21, CCL24, CCR7, CD14, CD177, CD300E, CD74, CDC42EP2, CDC42EP4, CEACAM1, CFH, CFL1, CHGA, CIITA, CLEC4E, CLNK, CNR1, CRP, CSF1, CX3CL1, CYP21A2, CYP27B1, DAB2IP, ELF4, EPHA2, FABP4, FADD, FCN3, FES, FFAR2, FOSL1, GBF1, GIT1, GNAO1, GPAM, GPER1, GPM6A, GRN, HFE, HK1, HMGA1, HNRNPUL1, HSF1, HSPB1, HYAL1, ICAM1, IFITM1, IFITM2, IFITM3, IL18BP, IL1B, IL22RA1, IL4R, IL7R, IRAK2, IRF2, IRF9, JAK3, JUND, KCTD11, LAG3, LDOC1, LGALS9, LILRA5, LRG1, LTF, MAP3K14, MAPK3, MAVS, MECP2, MOV10, MRC1, MUC19, MUC20, MUC3A, MYC, NCOR2, NECTIN2, NFKB2, NFKBIB, NLRC5, NLRP9, NOD2, NOS3, NOTCH1, NR1D1, NR4A1, ODC1, PAF1, PCDHA4, PCDHA6, PLA2G2A, PLEKHM2, PML, PPARD, PPP1R11, PQBP1, PRKACA, PTGES, PTGFR, PTGIR, PVR, RARA, RBM14, RELA, RELB, RNASE2, RNF19B, RNF26, RNF39, RNU1-93P, SBNO2, SCN7A, SELE, SELENOP, SERPINE1, SERPING1, SMPD1, SOCS3, SPI1, SRC, STING1, TBKBP1, TICAM1, TIMP1, TMEM229B, TMEM256-PLSCR3, TNF, TNFRSF1A, TNK1, TNK2, TRIB1, TRIM29, TRIM62, TRIM8, UBA52, VNN1, WAS, ZC3H12A, ZCCHC3, ZDHHC11, ZFP36, ZNF395, ZYX]
GO:0070661	leukocyte proliferation	5,30E-14	[3]	106,00	31,45	69,17	30,83	[AIF1, BLK, BTK, BTN3A1, CARD11, CCDC88B, CCL19, CCL5, CCR2, CD180, CD19, CD22, CD3E, CD40LG, CD6, CD74, CD79A, CD80, CD86, CDKN2A, CLCF1, CORO1A, CR2, CRTAM, CSF1R, CTLA4, DOCK2, EBI3, EPX, FCRL3, FLT3, FOXP3, GAPT, HLA-DMB, HLA-DPA1, HLA-DPB1, HLA-DRB1, IKZF3, IL12RB1, IL18, IL5RA, IL7R, INPP5D, LEF1, LILRB1, LILRB4, LMO1, LST1, MS4A1, MZB1, NCKAP1L, PIK3CG, PLA2G2D, PRKCQ, PTPN22, PTPN6, PTPRC, PYCARD, RAC2, RASAL3, SASH3, SPN, SYK, TNFAIP3, TNFRSF13B, TNFSF13B, TNFSF14, TNFSF8, TREM2, XCL1, ZAP70]	[ABL1, AGER, AMH, ARMC5, BCL6, CD276, CD74, CDKN1A, CLC, CLCF1, CRP, CSF1, CX3CL1, DLG5, ELF4, ENPP3, ERBB2, FADD, FLT3, GBA, GPAM, HPRT1, IDO1, IL1B, IL5RA, IL7R, JAK3, LGALS9, LRRC32, MAPK3, MSN, NPR3, NPRL3, RAC2, SLC7A1, TCIRG1, TICAM1, TIMP1, VSIR, ZBTB7B, ZNF335]

GO:0098609	cell-cell adhesion	2,19E-23	[3]	245,00	26,63	50,34	49,66	[ADAM8, AIF1, AKNA, ALOX15, BLK, BMP7, BTLA, C1QTNF1, CARD11, CCDC88B, CCL19, CCL2, CCL5, CCR2, CD177, CD2, CD200R1, CD27, CD33, CD3E, CD4, CD40LG, CD5, CD6, CD74, CD80, CD84, CD86, CDH1, CDH16, CDH4, CDKN2A, CEL, CORO1A, CRB2, CRTAM, CTLA4, DOCK8, EBI3, EGR3, EMB, EPX, FGL2, FOXP3, FUT7, GRAP2, HLA-DMB, HLA-DPA1, HLA-DPB1, HLA-DRA, HLA-DRB1, ICOS, IFNG, IL12RB1, IL18, IL7R, ITGA4, ITGAL, ITGAX, ITGB2, ITGB7, JAML, KLRK1, LAG3, LAX1, LCK, LEF1, LILRB1, LILRB4, MYB, NCKAP1L, NLRP3, NR4A3, NTN1, P2RY12, PCDH20, PCDHA2, PIK3CG, PLA2G2D, PLEK, PRKCQ, PTAFR, PTPN22, PTPN6, PTPRC, PYCARD, RAC2, RASAL3, RHOH, RUNX3, SASH3, SELE, SELL, SELPLG, SEMA4D, SIGLEC1, SIRPG, SKAP1, SOCS1, SPN, SYK, TBX21, TESPA1, THY1, TIGIT, TNFAIP8L2, TNFSF13B, TNFSF14, TNR, VAV1, VNN1, WNT1, XCL1, XG, ZAP70]	[ABL1, ACTB, ACTN1, ADAM19, ADGRL1, ADIPOQ, AGER, AP3D1, APOA1, ATP1B2, BCL6, BTLA, C1QTNF1, CAMSAP3, CCL2, CCL21, CCR7, CD177, CD276, CD74, CDC42EP1, CDH16, CDH4, CEACAM1, CEL, CELSR2, CITED2, CLDN15, CLDN23, CLDN9, CNTN6, CSK, CX3CL1, DCHS1, DLG5, DMTN, DSC1, DSCAML1, DTX1, ERBB2, ESAM, FADD, FBLIM1, FGFRL1, FLOT2, FOXF1, FXYD5, GNAO1, GPAM, HBB, HFE, HSPB1, ICAM1, IL1B, IL1RN, IL4R, IL7R, ILK, ITGA5, ITGA7, ITGAD, IZUMO1, JAK3, JUP, LAG3, LAMA5, LGALS9, LIMS2, LRFN3, LRRC32, LRRC4, MAPK7, MDGA1, MINK1, MSN, MYADM, MYH9, MYL9, NECTIN1, NECTIN2, NF2, NINJ1, NLGN2, NOD2, NODAL, NOTCH1, NOTCH4, NR4A3, NRARP, NTN1, OBSCN, OMA1, PAK4, PCDH1, PCDHA10, PCDHA11, PCDHA2, PCDHA3, PCDHA4, PCDHA6, PCDHGA12, PCDHGA4, PCDHGA5, PCDHGA6, PCDHGA9, PCDHGB1, PCDHGB3, PCDHGC4, PCDHGC5, PDCD1, PEAR1, PIEZO1, PIP5K1C, PKD1, PLAUR, PLEKHA7, PTPN23, PTPRU, PVR, RAC2, RARA, RELA, RGCC, ROBO4, S100A11, SDK2, SELE, SELENOP, SH2B3, SLC7A1, SOX12, SOX13, SPI1, SRC, SRF, TIMP1, TNF, TNIP1, TRIM29, TRPV4, VEGFA, VNN1, VSIR, XG, ZBTB7B, ZC3H12A, ZMIZ1]
GO:0001816	cytokine production	6,35E-20	[2, 5]	213,00	26,66	59,72	40,28	[ACP5, ADAM8, ADCY7, AIF1, AIM2, ALOX15B, ANGPT1, BANK1, BATF, BCL3, BTK, BTN3A1, BTN3A2, CAMK4, CARD11, CARD17, CASP8, CCDC88B, CCL19, CCL3, CCR2, CD2, CD200R1, CD226, CD244, CD33, CD3E, CD4, CD40LG, CD6, CD74, CD80, CD84, CD86, CD96, CLCF1, CLEC6A, CLNK, CRTAM, CSF1R, CXCL6, CYBB, EBI3, EPX, EREG, FCN1, FFAR4, FGR, FOXP3, GBP5, GPSM3, HLA-DPA1, HLA-DPB1, HLA-DRB1, IFNG, IKBKE, IL12RB1, IL12RB2, IL16, IL18, IL26, IL5RA, INPP5D, IRF4, IRF5, IRF8, ITK, LAG3, LCP1, LEF1, LILRB1, LILRB4, LPAL2, LTA, LTB, LY9, MAPK13, MCOLN2, MOG, MYB, NCKAP1L, NFAM1, NLRC3, NLRP2, NLRP3, NLRP6, NLRP7, NLRP9, NR4A3, ORM2, PIK3CG, PLD4, POU2AF1, POU2F2, PRKCQ, PTAFR, PTPN22, PTPN6, PTPRC, PYCARD, PYHIN1, SASH3, SCIMP, SIGLEC16, SLAMF6, SOCS1, STAT1, SYK, TBX21, THBS1, TIGIT, TLR10, TLR6, TLR8, TNFAIP3, TREM2, UBASH3A, XCL1, ZBP1]	[ABCC8, ABCD1, ABCD2, ABL1, ACKR1, ADGRG1, ADIPOQ, AGER, AGPAT1, AGT, AMH, ANGPT1, APOA1, APOD, BCL3, BCL6, C5, C5AR1, CCR7, CD14, CD276, CD74, CEACAM1, CHI3L1, CLC, CLCF1, CLNK, CPTP, CRP, CSK, CX3CL1, EPHA2, FADD, FFAR2, FLT4, FRMD8, FURIN, GATA4, GBA, GIT1, GPAM, HDAC7, HFE, HK1, HSPB1, IGF2BP2, IL1B, IL4R, IL5RA, INHA, JAK3, JPH4, LAG3, LGALS9, LILRA5, LRRC32, LTF, LUM, MAP2K3, MAST2, MAVS, NFATC4, NFKB2, NLRC5, NLRP9, NOD2, NODAL, NR4A3, NUTF2, OSM, PER1, PLD3, PML, POLR2E, POLR2L, POSTN, PPP1R11, PQBP1, RARA, RELA, RELB, RGCC, RNF26, RNU1-93P, SERPINE1, SLC7A5, SMAD3, SPHK1, SPHK2, SRC, STAT3, STING1, THBS1, TICAM1, TNF, TRPV4, TSKU, UBE2L6, VSIR, ZBTB7B, ZC3H12A, ZCCHC3, ZFP36, ZNF580]
GO:0001909	leukocyte mediated cytotoxicity	3,75E-08	[3, 4]	48,00	37,21	74,46	25,54	[CD1B, CD1C, CD1E, CD226, CORO1A, CRTAM, CXCL6, GZMB, GZMM, HLA-DRA, HLA-DRB1, IL12RB1, IL18, IL18RAP, IL7R, KLRC1, KLRC2, KLRD1, KLRK1, LAG3, LILRB1, NCKAP1L, NCR1, NCR3, NLRP6, PRF1, PTPN6, PTPRC, SCNN1B, SH2D1A, SLAMF6, SLAMF7, STAP1, STXBP2, VAV1, XCL1]	[AGER, CD1C, CD1E, CEACAM1, FADD, HPRT1, ICAM1, IL7R, LAG3, LGALS9, NECTIN2, PLEKHM2, PVR, RNF19B, SPI1, ZNF395]
GO:0002274	myeloid leukocyte activation	2,33E-14	[3, 4]	184,00	25,34	54,94	45,06	[ADAM8, ADGRE3, AIF1, ANPEP, ARHGAP45, BATF, BIN2, BLK, BTK, C1QA, CAMK4, CCL3, CCL5, CCR2, CD177, CD2, CD226, CD300LF, CD33, CD53, CD74, CD84, CEACAM21, CFP, CLEC12A, CLEC4D, CLNK, CNR1, CNR2, COTL1, CST7, CTSS, CXCL6, CYBB, DOCK2, EDN2, EPX, FCN1, FGL2, FGR, FRMPD3, GMFG, IFNG, IL18, IL18RAP, IQGAP2, IRF4, ITGAL, ITGAX, ITGB2, LAIR1, LCP2, LDLR, LILRB4, LRMP, LYZ, MMP9, MPO, MT1G, NAMPT, NCKAP1L, NFAM1, NR4A3, ORM2, PIK3CG, PLAC8, PLAU, PRAM1, PTAFR, PTGDR, PTPN6, PTPRC, PTX3, PYCARD, RAB37, RAB44, RAC2, RHOH, RNASET2, SCNN1B, SELL, SERPINA1, SLC18A1, SLC27A2, STAP1, STXBP2, SUCNR1, SYK, TBC1D10C, THBS1, TLR6, TREM2, TRPM2, TXNDC5, UBD, VNN1]	[ADGRE5, AGER, ALDOA, AMH, ARL8A, ARSA, BATF2, C5AR1, CAPN1, CD14, CD177, CD74, CEACAM1, CHGA, CHI3L1, CLEC12A, CLNK, CNR1, COMMD3, CRAT, CSF1, CST7, CTSA, CTSD, CX3CL1, CYB5R3, DHRS2, DOK3, DSC1, DYSF, EDN2, ENPP3, FES, FOXF1, FPR1, GAB2, GALNS, GATA2, GPI, GPR84, GRN, HAVCR1, HBB, HFE, HVCN1, IL4R, IMPDH1, IQGAP2, JUND, JUP, LDLR, LGALS9, LRG1, LTBR, LTF, METTL7A, MME, MYO18A, NBEAL2, NECTIN2, NHLRC3, NPPA, NR1D1, NR4A3, PCDHA4, PCDHA6, PKM, PLA2G2A, PLAU, PLAUR, PRKCSH, PTGDS, RAB3A, RAB5B, RAC2, RAP1A, RELB, RHOG, RNASE2, RNU1-93P, S100A11, SBNO2, SERPINA3, SIGLEC9, SLC44A2, SLC7A8, SLCO4C1, SPHK1, SPHK2, SPI1, STING1, STK11IP, TCIRG1, THBS1, TICAM1, TMEM179B, TMEM63A, TNF, TRAPPC1, TSPAN14, VNN1, ZC3H12A]
GO:0002697	regulation of immune effector process	1,81E-11	[3, 4]	126,00	27,21	66,43	33,57	[ANGPT1, BLK, BTK, C1QA, C1QB, C1QC, C2, C8G, CAMK4, CCL19, CCR2, CD177, CD19, CD1B, CD1C, CD1E, CD22, CD226, CD244, CD40LG, CD74, CD80, CD84, CD86, CD96, CFP, CLCF1, CLNK, CR2, CRTAM, CXCL10, CXCL6, CYP11B1, FCRL3, FGL2, FGR, FOXP3, FUT7, HLA-DMB, HLA-DRA, HLA-DRB1, IFNG, IL12RB1, IL18, IL18RAP, IL7R, IRF4, ITGB2, KLRC1, KLRC2, KLRD1, KLRK1, LAG3, LILRB1, LILRB4, LPAL2, LTA, MYB, MZB1, NCKAP1L, NCR1, NCR3, NLRP3, NR4A3, PRAM1, PTAFR, PTPN22, PTPN6, PTPRC, RAC2, SASH3, SCIMP, SH2D1A, SLAMF6, SLAMF8, STAP1, STXBP2, SYK, TBX21, TREM2, VAV1, WAS, XCL1, ZNF683]	[AGER, ANGPT1, APOA1, BCL6, C5, C5AR1, C8G, CD177, CD1C, CD1E, CD74, CEACAM1, CFH, CLC, CLCF1, CLNK, CPN2, ENPP3, FADD, FES, FFAR2, FOXF1, GAB2, GATA2, GPI, GRN, HFE, HK1, ICAM1, IL1B, IL4R, IL7R, JAK3, LAG3, LGALS9, MAVS, MYO18A, NECTIN2, NOD2, NPPA, NR4A3, PVR, RAC2, RAP1A, RARA, SERPING1, SLC7A5, SPHK2, SPI1, TICAM1, TNF, VTN, WAS, ZBTB7B, ZC3H12A]

GO:0030155	regulation of cell adhesion	5,14E-25	[3, 4]	218,00	28,57	53,48	46,52	[ADAM8, ADAMDEC1, AIF1, AKNA, ALOX15, ANGPT1, ANGPT2, APBB1IP, BLK, BMP7, BTLA, C1QTNF1, CARD11, CASS4, CCDC88B, CCL19, CCL2, CCL5, CCR2, CD27, CD3E, CD4, CD40LG, CD5, CD6, CD74, CD80, CD86, CDH1, CDKN2A, CORO1A, CRTAM, CTLA4, CXCR3, CXCR4, CYTIP, DOCK8, EBI3, EGR3, EPX, FGL2, FOXP3, FUT7, GPR4, GRAP2, HLA-DMB, HLA-DPA1, HLA-DPB1, HLA-DRA, HLA-DRB1, ICOS, IFNG, IL12RB1, IL18, IL7R, ITGA4, ITGB2, KLRK1, KNG1, LAG3, LAX1, LCK, LEF1, LIF, LILRB1, LILRB4, MYB, NCKAP1L, NLRP3, NR4A3, P2RY12, PIK3CG, PLA2G2D, PLAU, PLXNC1, PRKCQ, PTAFR, PTK2B, PTPN22, PTPN6, PTPRC, PYCARD, RAC2, RASAL3, RHOH, RUNX3, SASH3, SELE, SEMA4D, SIRPG, SKAP1, SOCS1, SPN, SYK, TACSTD2, TBX21, TESPA1, THBS1, THY1, TIGIT, TNFAIP8L2, TNFRSF18, TNFSF13B, TNFSF14, TNR, VAV1, VNN1, WNT1, XCL1, ZAP70]	[ABL1, ACTN4, ADAM19, ADGRG1, ADIPOQ, AGER, ANGPT1, AP3D1, APOA1, APOD, ARHGDIA, BCL6, BTLA, C1QTNF1, CAMSAP3, CCL2, CCL21, CCR7, CD276, CD74, CEACAM1, CELSR2, CITED2, COL1A1, CSF1, CSK, CX3CL1, DAG1, DDR1, DLG5, DMTN, DTX1, ECM2, EFEMP2, EPHA2, ERBB2, FADD, FES, FLOT2, FOXC2, FOXF1, FXYD5, GNAO1, GPAM, GPR4, HFE, HYAL1, ICAM1, IL1B, IL1RN, IL4R, IL7R, ILK, ITGA3, JAK3, JUP, KNG1, LAG3, LAMA5, LGALS9, LIF, LIMS2, LRFN3, LRRC32, MAPK7, MDGA1, MEN1, MINK1, MYADM, NF2, NOD2, NODAL, NOTCH1, NOTCH4, NR4A3, NRARP, OMA1, PDCD1, PDGFB, PIEZO1, PKD1, PLAU, PLAUR, PLXNA1, PLXNA3, PLXNB1, PML, POSTN, PPP1CB, PPP2R1A, PTN, PTPN23, PTPRU, RAC2, RARA, RELA, RGCC, SELE, SELENOP, SERPINE1, SH2B3, SLC7A1, SLC9A1, SMAD3, SOX12, SOX13, SPI1, SRC, SRF, SYNGAP1, TESK1, TFE3, TGM2, THBS1, TIMP1, TNF, TRIOBP, TRPV4, VEGFA, VNN1, VSIR, ZBTB7B, ZC3H12A, ZMIZ1]
GO:0031341	regulation of cell killing	3,88E-05	[3, 4]	40,00	33,90	73,52	26,48	[CASP8, CD1B, CD1C, CD1E, CD226, CRTAM, CXCL6, HLA-DRA, HLA-DRB1, IFNG, IL12RB1, IL18RAP, IL7R, KLRC1, KLRC2, KLRD1, KLRK1, LAG3, LILRB1, NCKAP1L, NCR1, NCR3, PRF1, PTPRC, SH2D1A, SLAMF6, STAP1, SYK, VAV1, XCL1]	[AGER, CD1C, CD1E, CEACAM1, FADD, ICAM1, IL7R, LAG3, LGALS9, MAPK3, NECTIN2, PVR, SPI1, ZNF395]
GO:0032101	regulation of response to external stimulus	2,76E-10	[3, 4]	266,00	20,94	53,55	46,45	[ACP5, ADAM8, ADCY7, AIF1, AIM2, AKNA, ALOX15, ANGPT2, AOAH, APOBEC3G, BTK, C1QTNF1, CALCR, CARD11, CASP8, CCL19, CCL2, CCL24, CCL3, CCL4, CCL5, CCR2, CCR4, CD180, CD200R1, CD226, CD247, CD3G, CD74, CD96, CEL, CLEC10A, CLEC4D, CLEC4E, CLEC6A, CLNK, CNR1, CNR2, CRTAM, CSF1R, CST7, CXCL10, CXCL6, CXCR4, CXorf21, CYSLTR1, DPEP1, DRD1, EDN2, EREG, FCN1, FFAR4, FGL2, FGR, FOXP3, FUT7, GBP5, GPR150, GPR4, GPR83, GPSM3, GUCY2D, HLA-DRB1, HOPX, IFNG, IKBKE, IL12RB1, IL16, IL18, IL18RAP, KLRC1, KLRC2, KLRD1, KLRK1, KNG1, LAG3, LCP1, LDLR, LILRB1, LTA, LY86, MAPK13, MMP9, MUC16, MUC19, MUC6, NCKAP1L, NCR1, NCR3, NLRC3, NLRP3, NLRP6, OASL, P2RY12, PDE6G, PIK3CG, PLA2G2D, PLA2G7, PLAU, PTGDR, PTGER2, PTK2B, PTPN22, PTPN6, PTPRC, PYCARD, PYHIN1, RAC2, RLN2, SCIMP, SELE, SEMA4A, SEMA4D, SH2D1A, SIGLEC10, SIGLEC16, SLAMF6, SLAMF8, SMPDL3B, SOCS1, SOCS3, STAP1, STAT1, SUCNR1, SYK, THBS1, TLR10, TLR8, TNFAIP3, TNFAIP8L2, TNR, TREM2, TSPAN8, VAV1, XCL1, XCL2, ZBP1]	[ABCC8, ADCY1, ADCY3, ADCY4, ADCY9, ADIPOQ, AGER, AGT, AGTR1, AMH, APOA1, ARTN, BCL6, BRD4, C1QTNF1, C5, C5AR1, CALCR, CCL2, CCL21, CCL24, CCR7, CD74, CEACAM1, CEL, CGA, CHGA, CLEC4E, CLNK, CNR1, CRHR2, CSF1, CST7, CX3CL1, CYP27B1, DAGLA, DMTN, EDN2, ENPP3, EPPK1, FABP4, FADD, FAM110A, FAP, FEM1A, FFAR2, FOXF1, FURIN, GBA, GIT1, GNAI2, GPBAR1, GPER1, GPR20, GPR4, GPR83, GPR84, GRN, HOPX, HRH2, HSPB1, IL1B, KNG1, LAG3, LDLR, LGALS9, LILRA5, LTF, MAPK3, MAPK7, MAVS, MEGF8, MEN1, MSTN, MUC19, MUC20, MUC3A, MYH9, NECTIN2, NINJ1, NLRC5, NOD2, NOS3, NOTCH1, NPPA, NR1D1, OSM, PCDHA4, PCDHA6, PDGFB, PDGFRB, PLA2G2A, PLA2G7, PLAU, PLAUR, PLXNA3, PPARD, PQBP1, PRKACA, PRKAR2B, PTGES, PTGIR, PTN, PTPRU, PVR, RAC2, RAMP2, RBM14, RELA, RELB, RGMA, RHBDF2, RNF26, RNU1-93P, RTN4RL1, SBNO2, SELE, SEMA3F, SEMA4B, SEMA4C, SEMA6B, SEMA6C, SERPINE1, SERPING1, SH2B3, SHARPIN, SMAD3, SOCS3, SPATA2, SPHK1, SPI1, SRC, STING1, TGM2, THBS1, TNF, TNFRSF1A, TNIP1, TRIB1, TRIM62, TRPV4, TSPAN8, VEGFA, VIPR2, VTN, WNT3, ZC3H12A, ZCCHC3, ZDHHC11, ZFP36, ZNF580, ZSWIM4, ZSWIM8, ZYX]
GO:0045595	regulation of cell differentiation	2,28E-11	[3, 4]	337,00	20,17	43,12	56,88	[ADAM8, ALOX15B, AREG, ASCL2, BCL11B, BHLHA15, BHLHE40, BMP7, BMPR1B, BTK, C1QC, CAMK4, CARD11, CASP8, CASS4, CCL17, CCL19, CCL3, CCR2, CD2, CD27, CD4, CD74, CD80, CD86, CDH4, CDKN2A, CLCF1, CMTM5, CRABP2, CRB2, CRTAM, CTLA4, CXCL10, CXCL9, CXCR4, DCC, DPEP1, EGR3, EPX, EREG, EVI2B, FCRL3, FFAR4, FGL2, FOXP3, FRZB, GDF6, GLIS1, GPR171, GPR55, H3C10, H3C11, H3C12, H3C7, HCLS1, HLA-DOA, HLA-DRA, HLA-DRB1, HOPX, IFNG, IKZF3, IL12RB1, IL18, IL7R, INPP5D, IRF4, IRX3, KLF5, KLHL41, LAG3, LCP1, LDLR, LEF1, LIF, LILRB1, LILRB4, LMO1, LRRC17, LTA, MAFF, MEDAG, MIXL1, MMP9, MSX1, MUSK, MYB, MYCL, NCKAP1L, NEK5, NFAM1, NLRP3, NPPC, NR4A3, NTN1, P2RY12, PIM1, PLAAT4, PLXNC1, PRDM1, PRKCB, PRKCQ, PTK2B, PTPN6, PTPRC, RBPMS2, RHEX, RHOH, RUNX3, SASH3, SEMA4A, SEMA4D, SLAMF8, SOCS1, SOCS3, STAT1, SULT2B1, SYK, TACSTD2, TBX21, TESPA1, THBS1, THY1, TNFRSF18, TNR, TOX, TREM2, VNN1, VWC2L, WNT1, WNT10B, ZAP70, ZBED2, ZNF683]	[ABCC8, ABL1, ACTN4, ADAMTS7, ADIPOQ, AGER, AGT, AGTR1, AP3D1, APOA1, AQP3, AREG, ARHGDIA, ATAT1, BBS12, BCL6, BCL9L, BEND6, BHLHE40, CAMK2B, CARM1, CASZ1, CCL11, CD74, CDH4, CDK1, CEACAM1, CETP, CHERP, CLCF1, CLPTM1, CMTM5, COL1A1, CRP, CSF1, CSNK1E, CTDSP1, CTHRC1, CX3CL1, CYP26B1, CYP27B1, DAB2IP, DAG1, DDX39B, DISP3, DMTN, DNMT1, DPF2, DTX1, EFEMP2, EIF4G1, ENG, ERBB2, FADD, FES, FLOT2, GATA2, GATA4, GDF6, GDI1, GNB3, GPER1, GPR137, GPR37L1, GRN, GSK3A, H3C10, H3C12, H4C3, HDAC5, HDAC7, HEYL, HOPX, HSF1, HTR2A, ID3, IFITM1, IL15RA, IL1B, IL4R, IL7R, ILK, INHA, IRX3, JAK3, JUNB, JUND, KCTD11, KLF10, KLF5, KMT2D, LAG3, LDLR, LGALS9, LIF, LIMK1, LIMS2, LINGO1, LMX1A, LOX, LRRC17, LRRC8A, LTF, LZTS1, MAFG, MAMSTR, MAPK8IP3, MCRIP1, MECP2, MED12, MEGF8, MME, MOV10, MSTN, MSX1, MYADM, MYC, MYL9, NAP1L2, NEFL, NEK5, NF2, NFATC4, NFE2, NGF, NGFR, NODAL, NOTCH1, NOTCH4, NR1D1, NR4A3, NRARP, NTN1, NUMBL, OMA1, OSM, PAF1, PARP6, PDGFB, PDLIM7, PHLDB1, PIEZO1, PIM1, PLA2G2A, PLEKHB1, PLXNA1, PLXNA3, PLXNB1, POR, POSTN, PPARD, PPP1R13L, PPP2R1A, PRKACA, PTN, RAMP2, RAP1A, RARA, RBM38, RBPMS2, RELA, RGCC, RGMA, RMRP, RNU1-93P, S1PR2, SCAF1, SEMA3F, SEMA4B, SEMA4C, SEMA6B, SEMA6C, SERPINE1, SETD1A, SHANK3, SIK1, SLC25A23, SLC4A11, SMAD3, SNAI1, SOCS3, SOX10, SOX12, SOX13, SOX8, SPDEF, SPI1, SRF, SRRT, STAT3, SYNGAP1, TBX5, TCF3, TCF7L1, TCIRG1, TEAD2, TESK1, TFE3, TGFB1I1, TGM2, THBS1, THPO, TIMP1, TMEM100, TNF, TNFRSF1A, TRIB1, TRIB3, TRIM62, TRIOBP, TRPV4, TSKU, ULK1, VASN, VDR, VEGFA, VNN1, VSIG2, VSIR, WNT3, WNT9B, ZBTB7B, ZC3H12A, ZFHX2, ZFP36, ZMIZ1, ZNF219, ZNF335, ZNF385A]

GO:0046649	lymphocyte activation	4,01E-25	[3, 4]	232,00	27,92	71,66	28,34	[ADAM8, AIF1, APBB1IP, BANK1, BATF, BCL11B, BCL3, BLK, BLNK, BTK, BTLA, BTN3A1, CAMK4, CARD11, CASP8, CCDC88B, CCL19, CCL2, CCL5, CCR2, CD180, CD19, CD1C, CD2, CD22, CD244, CD27, CD3D, CD3E, CD3G, CD4, CD40LG, CD5, CD6, CD7, CD74, CD79A, CD80, CD86, CD8A, CD8B, CDKN2A, CLCF1, CLNK, CORO1A, CR2, CRTAM, CTLA4, DOCK10, DOCK2, DOCK8, EBI3, EGR3, EOMES, EPX, FCRL1, FCRL3, FCRLA, FGL2, FGR, FLT3, FOXP3, FUT7, GAPT, GRAP2, HLA-DMB, HLA-DOA, HLA-DPA1, HLA-DPB1, HLA-DRA, HLA-DRB1, ICOS, IFNG, IGLL5, IKZF1, IKZF3, IL12RB1, IL18, IL21R, IL7R, INPP5D, IRF4, IRF8, ITGA4, ITGAL, ITGB2, ITK, JAML, KLRC1, KLRC2, KLRK1, LAG3, LAX1, LCK, LCP1, LEF1, LILRB1, LILRB4, LMO1, LST1, LY9, MS4A1, MYB, MZB1, NCKAP1L, NCR1, NCR3, NFAM1, NLRC3, NLRP3, PIK3CG, PLA2G2D, POU2AF1, POU2F2, PRDM1, PRF1, PRKCB, PRKCQ, PTK2B, PTPN22, PTPN6, PTPRC, PYCARD, RAC2, RASAL3, RHOH, RUNX3, SAMSN1, SASH3, SEMA4A, SIRPG, SIT1, SLA2, SLAMF6, SLAMF7, SLAMF8, SOCS1, SPN, SYK, TBC1D10C, TBX21, TESPA1, THEMIS, THEMIS2, THY1, TIGIT, TNFAIP3, TNFAIP8L2, TNFRSF13B, TNFRSF18, TNFSF13B, TNFSF14, TNFSF8, TOX, VAV1, VNN1, WAS, WDFY4, WNT1, XCL1, ZAP70, ZNF683]	[ABL1, AGER, AMH, AP3D1, ARMC5, ARTN, BAG6, BCL3, BCL6, BTLA, CCL2, CCL21, CCR7, CD1C, CD276, CD74, CDKN1A, CEACAM1, CLC, CLCF1, CLNK, CLPTM1, CSK, CYP26B1, DCAF15, DLG5, DTX1, ELF4, ERBB2, FADD, FLOT2, FLT3, GBA, GNAO1, GPAM, HDAC5, HFE, HPRT1, ICAM1, IDO1, IL15RA, IL1B, IL4R, IL7R, INHA, JAK3, KDELR1, LAG3, LGALS9, LRRC32, LRRC8A, MSN, MYH9, NOD2, NRARP, PDCD1, RAC2, RARA, RELB, SLC7A1, SLC7A8, SMAD3, SOX12, SOX13, SPI1, SRC, SRF, STAT3, SYVN1, TCF3, TCF7L1, TCIRG1, TICAM1, TIMP1, VNN1, VSIR, WAS, ZBTB7A, ZBTB7B, ZC3H12A, ZMIZ1, ZNF335, ZNF395]
GO:0050776	regulation of immune response	2,87E-17	[3, 4]	286,00	23,08	66,69	33,31	[ADAM8, ADCY7, AIM2, ALOX15, APOBEC3G, BLK, BLNK, BTK, BTLA, BTN3A1, BTN3A2, C1QA, C1QB, C1QC, C1QTNF1, C2, C8G, CALCR, CARD11, CASP8, CCL19, CCL5, CCR2, CD177, CD19, CD1B, CD1C, CD1E, CD200R1, CD22, CD226, CD247, CD300LF, CD33, CD3D, CD3E, CD3G, CD4, CD40LG, CD48, CD74, CD79A, CD80, CD84, CD86, CD8A, CD8B, CD96, CFP, CLCF1, CLEC10A, CLEC1B, CLEC4D, CLEC4E, CLEC6A, CLNK, CNR1, CR2, CRTAM, CTLA4, CXCL10, CXCL6, CXorf21, CYP11B1, CYSLTR1, DPEP1, DRD1, EREG, FCGR2C, FCN1, FCN3, FCRL3, FGL2, FGR, FOXP3, FUT7, FYB1, GBP5, GPR150, GPR83, GRAP2, HCST, HLA-DMB, HLA-DPA1, HLA-DPB1, HLA-DQA1, HLA-DRA, HLA-DRB1, HLA-DRB5, IFNG, IGLL5, IKBKE, IL12RB1, IL18, IL18RAP, IL7R, INPP5D, IRF4, ITGA4, ITGAL, ITGB2, ITGB7, ITK, JAML, KCNN4, KIR2DL2, KIR2DL3, KIR3DL2, KLRB1, KLRC1, KLRC2, KLRD1, KLRK1, LAG3, LAIR1, LAIR2, LAX1, LCK, LCP2, LILRA1, LILRB1, LILRB4, LTA, MOG, MS4A1, MUC16, MUC19, MUC6, MYB, MYO1G, NCKAP1L, NCR1, NCR3, NFAM1, NLRC3, NLRP3, NLRP6, NR4A3, PAX5, PILRA, PLA2G2D, PRAM1, PRKCB, PRKCQ, PTAFR, PTGDR, PTGER2, PTPN22, PTPN6, PTPRC, PYCARD, PYHIN1, RAC2, RLN2, SAMSN1, SASH3, SELL, SH2D1A, SIGLEC16, SKAP1, SLA2, SLAMF6, SLAMF7, SLAMF8, SOCS1, SOCS3, SPN, STAP1, STAT1, STXBP2, SYK, TBX21, TESPA1, THEMIS, THEMIS2, THY1, TLR8, TNFAIP3, TNFSF13B, TRAT1, TREM2, UBASH3A, VAV1, WAS, XCL1, ZAP70, ZBP1]	[ABL1, ACTB, ADCY1, ADCY3, ADCY4, ADCY9, AGER, BAG6, BCAR1, BCL6, BTLA, C1QTNF1, C5, C5AR1, C8G, CALCR, CCR7, CD177, CD1C, CD1E, CD276, CD300E, CD74, CEACAM1, CFH, CGA, CHGA, CLC, CLCF1, CLEC1B, CLEC4E, CLNK, CNR1, COL1A1, CPN2, CRHR2, CRP, CSK, DGKZ, ECM1, ENPP3, FADD, FCN3, FES, FFAR2, FOXF1, FPR1, FURIN, GAB2, GATA2, GCSAM, GNAI2, GNAO1, GPBAR1, GPR20, GPR83, GPR84, GRN, HFE, HRH2, ICAM1, IFITM1, IL1B, IL4R, IL7R, JAK3, LAG3, LGALS9, LIMK1, MAPK3, MAVS, MUC19, MUC20, MUC3A, MYH9, NCOR2, NECTIN2, NLRC5, NOD2, NPPA, NR4A3, PCDHA4, PDCD1, PQBP1, PRKACA, PRKAR2B, PTGIR, PVR, RAC2, RAMP2, RAP1A, RARA, RBM14, RELA, RELB, RGCC, RHBDF2, RNF26, RNU1-93P, SERPING1, SH2B2, SIGLEC9, SLC7A8, SMAD3, SOCS3, SPHK2, SPI1, SPNS2, SPPL2B, SRC, STING1, TNF, TRIM62, VIPR2, VSIR, VTN, WAS, ZBTB7B, ZC3H12A, ZCCHC3, ZDHHC11]
GO:0050865	regulation of cell activation	2,09E-24	[3, 4]	204,00	29,18	65,18	34,82	[ADAM8, AIF1, BANK1, BLK, BTK, BTLA, C1QTNF1, CAMK4, CARD11, CCDC88B, CCL19, CCL2, CCL3, CCL5, CCR2, CD177, CD19, CD2, CD22, CD226, CD27, CD300LF, CD33, CD3E, CD4, CD40LG, CD5, CD6, CD74, CD80, CD84, CD86, CDKN2A, CLCF1, CLEC4D, CLNK, CNR1, CNR2, CORO1A, CRTAM, CST7, CTLA4, DOCK8, EBI3, EGR3, EPX, FCRL3, FGL2, FGR, FOXP3, GRAP2, HLA-DMB, HLA-DOA, HLA-DPA1, HLA-DPB1, HLA-DRA, HLA-DRB1, ICOS, IFNG, IGLL5, IKZF3, IL12RB1, IL18, IL7R, INPP5D, IRF4, ITGB2, KLRC2, KLRK1, LAG3, LAX1, LCK, LDLR, LEF1, LILRB1, LILRB4, LMO1, LST1, MYB, MZB1, NCKAP1L, NFAM1, NLRP3, NR4A3, PLA2G2D, PLEK, PRAM1, PRDM1, PRKCQ, PTAFR, PTPN22, PTPN6, PTPRC, PYCARD, RAC2, RASAL3, RHOH, RUNX3, SAMSN1, SASH3, SIRPG, SIT1, SLAMF8, SOCS1, SPN, STAP1, STXBP2, SYK, TBC1D10C, TBX21, TESPA1, THBS1, THEMIS2, THY1, TIGIT, TLR6, TNFAIP3, TNFAIP8L2, TNFRSF13B, TNFRSF18, TNFSF13B, TNFSF14, TNFSF8, TOX, TREM2, VAV1, VNN1, XCL1, ZAP70, ZNF683]	[ABL1, AGER, AMH, AP3D1, BCL6, BTLA, C1QTNF1, CCL2, CCL21, CCR7, CD177, CD276, CD74, CDKN1A, CEACAM1, CLC, CLCF1, CLNK, CLPTM1, CNR1, CSK, CST7, CX3CL1, CYP26B1, DCAF15, DLG5, DMTN, DTX1, ENPP3, ERBB2, FADD, FES, FLOT2, FOXF1, GAB2, GATA2, GNAO1, GPAM, GPER1, GRN, HAVCR1, HFE, IDO1, IL15RA, IL1B, IL4R, IL7R, INHA, JAK3, JUND, LAG3, LDLR, LGALS9, LILRA5, LRRC32, MYO18A, NECTIN2, NOD2, NOS3, NPPA, NR1D1, NR4A3, NRARP, PCDHA4, PCDHA6, PDCD1, PDGFB, PDGFRB, PLA2G2A, RAC2, RARA, RHBDF2, SELENOP, SH2B3, SLC7A1, SOX12, SOX13, SPHK1, SPHK2, SPI1, SRC, THBS1, TICAM1, TIMP1, VNN1, VSIR, ZBTB7B, ZC3H12A, ZMIZ1, ZNF335]

GO:0051270	regulation of cellular component movement	6,89E-07	[3, 4]	220,00	20,41	37,04	62,96	[ADAM8, ADAMTS9, ADORA3, AIF1, ALOX15B, ANGPT1, ANGPT2, BMP7, CARMIL2, CASS4, CCL19, CCL2, CCL24, CCL3, CCL4, CCL5, CCR2, CD200R1, CD74, CDH1, CORO1A, CSF1R, CXCL10, CXCR3, CXCR4, DOCK10, DOCK8, DPEP1, DRC1, DRD1, EDN2, EREG, FGR, FUT7, GPSM3, IFNG, ITGA4, ITGAX, KLRK1, LEF1, MMP9, NCKAP1L, NELL2, NR4A3, NTN1, P2RY12, PIK3CG, PLA2G7, PLAU, PLXNC1, PTAFR, PTK2B, PTPN22, PTPRC, PYCARD, RAC2, RHOH, SELE, SEMA4A, SEMA4D, SLAMF8, SMPD3, SPN, STAP1, TACSTD2, TCAF2, THBS1, THY1, TNFRSF18, TREM2, WAS, XCL1, XCL2, XG]	[ABCC8, ABI3, ABL1, ACKR3, ACTN1, ACTN4, ADAMTS9, ADGRG1, ADIPOQ, AGER, AGT, AMH, ANGPT1, APOD, ARHGDIA, ATP1A1, ATP1A3, ATP1B2, BCAR1, BCL6, C5, C5AR1, CAMK2B, CAMSAP3, CARMIL2, CCL11, CCL2, CCL21, CCL24, CCR7, CD74, CEACAM1, CFAP298-TCP10L, CGA, CHGA, CITED2, COL1A1, CSF1, CX3CL1, DAB2IP, DAG1, DLG5, DMTN, DRC1, ECM1, EDN2, ENG, EPHA2, EPPK1, ERBB2, FADD, FAM110A, FES, FLT4, FOXC2, FOXF1, GATA2, GATA4, GCSAM, GNAI2, GPER1, GPI, GRB7, GRN, HBEGF, HCN4, HDAC5, HDAC7, HSPB1, HYAL1, ICAM1, IFITM1, IL1B, ILK, ITGA3, JUP, LAMA5, LGALS9, LGR6, LMNA, LZTS1, MAP2K3, MAPK3, MAZ, MECP2, MIEN1, MINK1, MSN, MSTN, MYADM, MYC, NF2, NGFR, NOD2, NODAL, NOS3, NOTCH1, NR4A3, NSMF, NTN1, OSGIN1, P2RY6, PDGFB, PDGFRB, PFN1, PHACTR1, PLA2G7, PLAU, PLVAP, PLXNA1, PLXNA3, PLXNB1, POSTN, PPARD, PRAG1, PTN, PTPN23, PTPRU, RAC2, RANGRF, RGCC, RHOG, RIPOR1, ROBO4, S100A11, SCN5A, SELE, SELENOP, SEMA3F, SEMA4B, SEMA4C, SEMA6B, SEMA6C, SERPINE1, SH3BP1, SLC9A3R1, SMAD3, SMIM22, SNAI1, SPHK1, SPI1, SRC, SRF, SSX2IP, STAT3, SYDE1, TBX5, TCTEX1D2, THBS1, TIMP1, TNF, TRIB1, TRPV4, VEGFA, VSIR, VTN, WAS, XG, ZC3H12A, ZNF580, ZNF609, ZSWIM4, ZSWIM8]
GO:0080134	regulation of response to stress	3,32E-08	[3, 4]	319,00	19,33	46,88	53,12	[ACP5, ADAM8, ADCY7, AIM2, AKNA, ALOX15, AOAH, APOBEC3G, ATF3, BMP7, BTK, C1QTNF1, CALCR, CARD11, CASP8, CCL19, CCL24, CCL3, CCL5, CCR2, CD200R1, CD226, CD247, CD27, CD3G, CD74, CD96, CDKN2A, CEL, CLEC10A, CLEC4D, CLEC4E, CLEC6A, CLNK, CNR1, CNR2, CRTAM, CST7, CXCL6, CXCR4, CXorf21, CYSLTR1, DEPDC1B, DPEP1, DRD1, EPX, EREG, FCN1, FFAR4, FGL2, FGR, FOXP3, FUT7, GADD45B, GBP5, GDF6, GPR150, GPR4, GPR83, GPSM3, HLA-DRB1, IER3, IFNG, IKBKE, IL12RB1, IL16, IL18, IL18RAP, IL26, KLRC1, KLRC2, KLRD1, KLRK1, KNG1, LAG3, LCP1, LDLR, LILRB1, LTA, MAPK13, MLC1, MMP9, MSX1, MUC16, MUC19, MUC6, NAMPT, NCF1, NCR1, NCR3, NLRC3, NLRP3, NLRP6, NR4A3, NUP210, PIK3CG, PLA2G2D, PLA2G7, PLAU, PTGDR, PTGER2, PTK2B, PTPN22, PTPN6, PTPRC, PYCARD, PYHIN1, RLN2, RMI2, SCIMP, SELE, SH2D1A, SIGLEC10, SIGLEC16, SLAMF6, SLAMF8, SMPDL3B, SOCS1, SOCS3, STAP1, STAT1, SUCNR1, SYK, THBS1, THY1, TLR10, TLR6, TLR8, TNFAIP3, TNFAIP8L2, TNR, TRAF1, TREM2, TRPM2, TSPAN8, VAV1, VNN1, WAS, WNT1, XCL1, ZBP1]	[AAAS, ABCA7, ABCC8, ABCD1, ABI3, ABL1, ACKR3, ADCY1, ADCY3, ADCY4, ADCY9, ADIPOQ, AGER, AGT, AGTR1, AKT1S1, AMH, APOA1, ARMT1, BAG3, BAG6, BCL2L1, BCL6, BRD4, C1QTNF1, CALCR, CAMK2B, CCL21, CCL24, CCR7, CD74, CEACAM1, CEL, CGA, CHGA, CLEC4E, CLNK, CNR1, CREB3L1, CRHR2, CRYAB, CST7, CX3CL1, DAB2IP, DAGLA, DDX39B, DMTN, EEF1E1, EIF4G1, ENDOG, ENPP3, EPPK1, FABP4, FADD, FAM110A, FAP, FASN, FEM1A, FFAR2, FLT4, FOXC2, FOXF1, FOXM1, FURIN, GADD45B, GATA4, GBA, GDF6, GIT1, GNAI2, GPBAR1, GPER1, GPR20, GPR37L1, GPR4, GPR83, GPR84, GRIK5, GRINA, GRN, HBEGF, HDAC10, HMGA1, HRH2, HSF1, HSPB1, HSPB8, IER3, IL1B, KCNK2, KNG1, LAG3, LDLR, LGALS9, LILRA5, LMNA, LTBR, MAP3K10, MAP3K11, MAPK3, MAPK7, MAPK8IP3, MAVS, MED12, MEN1, MINK1, MLST8, MSX1, MUC19, MUC20, MUC3A, MYC, MYH9, NECTIN2, NINJ1, NLRC5, NOD2, NOS3, NPAS2, NPPA, NR1D1, NR4A3, NUP188, OSM, PCDHA4, PCDHA6, PDGFB, PER1, PIAS4, PLA2G2A, PLA2G7, PLAU, PLAUR, PML, PPARD, PPP1R10, PPP1R15A, PQBP1, PRKACA, PRKAR2B, PTGES, PTGIR, PTN, PVR, RAD51AP1, RADX, RAMP2, RBM14, RELA, RELB, RGMA, RHBDF2, RMI2, RNF26, RNU1-93P, RPL26, RPTOR, RTEL1-TNFRSF6B, RTN4RL1, SBNO2, SELE, SELENON, SEMA4C, SERPINE1, SERPING1, SGTA, SH2B3, SHANK3, SHARPIN, SLC25A23, SMAD3, SNAI1, SOCS3, SPATA2, SPHK1, SPI1, SRC, STING1, SYVN1, TGM2, THBS1, TIMP1, TMEM161A, TMEM259, TNF, TNFRSF1A, TNIP1, TRAF4, TRIM62, TRPV4, TSPAN8, UBQLN4, VEGFA, VIPR2, VNN1, VTN, WAS, WFS1, XRCC1, YPEL3, ZBTB7B, ZC3H12A, ZCCHC3, ZDHHC11, ZFP36, ZNF385A, ZYX]
GO:2000026	regulation of multicellular organismal development	3,52E-12	[3, 4]	296,00	21,07	44,92	55,08	[ADAM8, ADAMTS9, ALOX15B, ANGPT2, ASCL2, BHLHE40, BMP7, BMPR1B, BTK, C1QC, CAMK4, CARD11, CASP8, CCL19, CCL24, CCL3, CCR2, CCR3, CD2, CD27, CD4, CD74, CD80, CD86, CDH4, CDKN2A, CLCF1, CRABP2, CRB2, CRTAM, CST7, CTLA4, CXCL10, CXCR4, CYBB, DCC, DPEP1, EGR3, EVI2B, FASLG, FCRL3, FGL2, FOXP3, FRZB, GDF6, GPR171, GPR4, GPR55, H3C10, H3C11, H3C12, H3C7, HCLS1, HLA-DOA, HLA-DRA, HLA-DRB1, HOPX, IFNG, IKZF3, IL12RB1, IL18, IL7R, INPP5D, IRF4, ITGAX, ITGB2, LAG3, LDLR, LEF1, LIF, LILRB1, LILRB4, LRRC17, LTA, MAFF, MMP9, MYB, MYCL, MYH6, NCKAP1L, NFAM1, NLRP3, NPPB, NR4A3, NTN1, PIM1, PLAAT4, PLXNC1, PRDM1, PRKCB, PRKCQ, PROK1, PTK2B, PTPN6, PTPRC, RHEX, RHOH, RLN2, RUNX3, SASH3, SEMA4A, SEMA4D, SLAMF8, SMPD3, SOCS1, STAT1, SULT2B1, SYK, TBX21, TESPA1, THBS1, THY1, TNFAIP3, TNFRSF18, TNMD, TNR, TOX, TREM2, VNN1, WNT1, WNT10B, ZAP70, ZBED2, ZNF683]	[ABCC8, ABL1, ACACB, ADAMTS7, ADAMTS9, ADGRB2, ADIPOQ, AGER, AGT, ANGPTL4, AP3D1, APLNR, AQP3, ARHGDIA, ASPN, BAG6, BCL6, BCOR, BHLHE40, C5, C5AR1, CAMK2B, CCL11, CCL24, CD74, CDH4, CDK1, CEACAM1, CFL1, CHI3L1, CITED2, CLCF1, CLPTM1, CREB3L1, CSF1, CST7, CTDSP1, CX3CL1, CYP26B1, CYP27B1, DAB2IP, DAG1, DDX39B, DLG5, DTX1, ECM1, EFNA3, ENG, EPHA2, EPN1, ERBB2, FADD, FES, FOXC2, GATA2, GATA4, GDF6, GDI1, GPER1, GPR137, GPR37L1, GPR4, GRN, GSK3A, H3C10, H3C12, H4C3, HESX1, HEYL, HGS, HOPX, HSF1, HSPB1, HSPG2, HYAL1, IL15RA, IL1B, IL4R, IL7R, ILK, INHA, ITGA5, JAK3, JMJD8, JUP, KCNK2, KLF10, KMT2D, LAG3, LAMA5, LDLR, LGALS9, LIF, LIMK1, LINGO1, LOX, LRCH4, LRG1, LRRC17, LTF, MAFG, MAPK7, MECP2, MED12, MEGF8, MME, MOV10, MYC, MYH6, MYL9, MYRF, NEFL, NF2, NFATC4, NFE2, NGF, NGFR, NINJ1, NLGN2, NODAL, NOS3, NOTCH1, NOTCH4, NPPB, NR1D1, NR4A3, NRARP, NTN1, NUMBL, OMA1, OMD, OSR2, PAF1, PAK4, PARP6, PHLDB1, PIM1, PKM, PLXNA1, PLXNA3, PLXNB1, PML, POR, PPARD, PTN, RAMP2, RARA, RELA, RGCC, RGMA, RMRP, S100A1, S1PR2, SEMA3F, SEMA4B, SEMA4C, SEMA6B, SEMA6C, SERPINE1, SETD1A, SHANK3, SMAD3, SOX10, SOX12, SOX13, SOX8, SPHK1, SPI1, SRF, SRRT, STAT3, SYNGAP1, TBX5, TFE3, TGM2, THBS1, THPO, TMEM100, TNF, TNFRSF1A, TNFSF12, TNMD, TRIB1, ULK1, VDR, VEGFA, VNN1, VSIR, WARS1, WNT3, WNT9B, ZBTB7B, ZC3H12A, ZFP36, ZMIZ1, ZNF219, ZNF335]

GO:0002520	immune system development	6,27E-14	[2, 4, 5]	246,00	22,86	58,85	41,15	[ADAM8, ANGPT1, BATF, BCL11B, BCL3, BLK, BLNK, BTK, C1QC, CALCR, CAMK4, CARD11, CASP8, CCL19, CCL3, CCR2, CCR4, CD19, CD2, CD27, CD3D, CD3E, CD3G, CD4, CD40LG, CD74, CD79A, CD80, CD86, CD8A, CDKN2A, CLCF1, CR2, CRTAM, CSF1R, CTLA4, DOCK10, DOCK2, EGR3, EOMES, EPX, EVI2B, FCRL3, FGL2, FLT3, FOXP3, FUT7, GFI1, GPR171, GPR55, H3C10, H3C11, H3C12, H3C7, HCLS1, HLA-DOA, HLA-DRA, HLA-DRB1, ICOS, IFNG, IKZF1, IKZF3, IL12RB1, IL18, IL7R, INPP5D, IRF4, IRF8, ITGA4, ITK, KLRC1, LAG3, LCK, LEF1, LILRB1, LILRB4, LMO1, LRRC17, LTA, LTB, LY9, MIXL1, MMP9, MS4A1, MT1G, MYB, NCKAP1L, NFAM1, NLRP3, NR4A3, PIM1, PLA2G2D, PLD4, PLEK, POU2AF1, POU2F2, PRDM1, PRKCB, PRKCQ, PTK2B, PTPN22, PTPN6, PTPRC, RHEX, RHOH, RTKN2, RUNX3, SASH3, SELPLG, SEMA4A, SLAMF6, SLAMF8, SLC8A3, SMPD3, SNX10, SOCS1, SPN, STAT1, SYK, TBX21, TESPA1, THBS1, THEMIS, TNFAIP3, TNFRSF13B, TNFRSF18, TNFSF13B, TNFSF8, TOX, TREM2, TRPM2, UBD, VAV1, VNN1, WNT1, WNT10B, ZAP70, ZNF683]	[ABL1, ACTN1, ADIPOQ, AGER, ANGPT1, AP3D1, ARMC5, ARTN, BATF2, BCL3, BCL6, CALCR, CCR7, CD74, CDK2, CDKN1A, CEACAM1, CITED2, CLC, CLCF1, CLPTM1, CRIP2, CSF1, CTC1, CYP26B1, DHRS2, DMTN, DPF2, DTX1, EPHA2, ERBB2, ETV6, FADD, FASN, FES, FLT3, FLT4, GAB2, GATA2, GBA, GNAO1, GPR137, H3C10, H3C12, H4C3, HDAC5, HSF1, IL15RA, IL1B, IL4R, IL7R, INHA, JAK3, JUNB, KCP, KDELR1, KLF10, KMT2D, LAG3, LGALS9, LOX, LRRC17, LRRC8A, LTBR, LTF, MAPK3, MFAP5, MKNK2, MOV10, MYC, MYH9, MYL9, NBEAL2, NCOA6, NFE2, NFKB2, NOTCH1, NOTCH4, NR4A3, NRARP, OSM, PAF1, PDGFB, PIM1, PML, PTN, RARA, RELB, RMRP, RNU1-93P, RTKN2, RXRA, SBNO2, SETD1A, SH2B3, SIPA1L3, SMAD3, SNX10, SOX12, SOX13, SPI1, SPNS2, SRC, SRF, SSBP3, STAT3, SYVN1, TCF3, TCF7L1, TCIRG1, TFE3, THBS1, THPO, TIMP1, TNF, TRIB1, VEGFA, VNN1, VSIR, ZBTB7A, ZBTB7B, ZC3H12A, ZFP36, ZMIZ1, ZNF385A]
GO:0002444	myeloid leukocyte mediated immunity	9,78E-06	[4]	138,00	22,19	54,41	45,59	[ADAM8, ADGRE3, ANPEP, ARHGAP45, BIN2, BLK, BTK, CAMK4, CCL3, CCR2, CD177, CD33, CD53, CD84, CEACAM21, CFP, CLEC12A, CLEC4D, CLNK, COTL1, CTSS, CXCL6, CYBB, DOCK2, EPX, FCN1, FGL2, FGR, FRMPD3, GMFG, IQGAP2, ITGAL, ITGAX, ITGB2, LAIR1, LRMP, LYZ, MMP9, MPO, NCKAP1L, NFAM1, NLRP6, NR4A3, ORM2, PIK3CG, PLAC8, PLAU, PRAM1, PTAFR, PTGDR, PTPN6, PTPRC, PTX3, PYCARD, RAB37, RAB44, RAC2, RNASET2, SCNN1B, SELL, SERPINA1, SLC18A1, SLC27A2, STAP1, STXBP2, SYK, TBC1D10C, TRPM2, TXNDC5, VNN1]	[ADGRE5, ALDOA, AMH, ARL8A, ARSA, C5AR1, CAPN1, CD14, CD177, CEACAM1, CHGA, CHI3L1, CLEC12A, CLNK, COMMD3, CRAT, CTSA, CTSD, CYB5R3, DOK3, DSC1, FES, FOXF1, FPR1, GAB2, GALNS, GATA2, GPI, GPR84, GRN, HBB, HFE, HVCN1, IL4R, IMPDH1, IQGAP2, JUP, LGALS9, LRG1, LTF, MAVS, METTL7A, MME, NBEAL2, NHLRC3, NPPA, NR4A3, PKM, PLAU, PLAUR, PRKCSH, PTGDS, RAB3A, RAB5B, RAC2, RAP1A, RHOG, RNASE2, RNU1-93P, S100A11, SERPINA3, SIGLEC9, SLC44A2, SLC7A8, SLCO4C1, SPHK2, SPI1, STING1, STK11IP, TCIRG1, TICAM1, TMEM179B, TMEM63A, TRAPPC1, TSPAN14, VNN1]
GO:0002449	lymphocyte mediated immunity	1,17E-02	[4]	89,00	21,76	71,24	28,76	[BATF, BCL3, BTK, BTN3A2, C1QA, C1QB, C1QC, C2, C8G, CCR2, CD19, CD1B, CD1C, CD1E, CD226, CD27, CD40LG, CD74, CD8A, CD96, CLCF1, CLNK, CORO1A, CR2, CRTAM, CXCL10, FOXP3, FUT7, GZMB, GZMM, HLA-DRA, HLA-DRB1, IGLL5, IL12RB1, IL18, IL18RAP, IL7R, INPP5D, KLRC1, KLRC2, KLRD1, KLRK1, LAG3, LILRB1, LILRB4, LTA, MYO1G, NCKAP1L, NCR1, NCR3, NLRP3, PRF1, PTPN6, PTPRC, SASH3, SH2D1A, SLA2, SLAMF6, SLAMF7, TBX21, TLR8, TREM2, VAV1, WAS, XCL1]	[AGER, ARTN, BCL3, BCL6, C5, C8G, CD1C, CD1E, CD74, CEACAM1, CLC, CLCF1, CLNK, CRP, FADD, GNAO1, HFE, HPRT1, ICAM1, IL1B, IL4R, IL7R, LAG3, LGALS9, NECTIN2, NOD2, PLEKHM2, PVR, RNF19B, SERPING1, TCIRG1, TNF, WAS, ZNF395]
GO:0002460	adaptive immune response based on somatic recombination of immune receptors built from immunoglobulin superfamily domains	2,41E-03	[4]	93,00	22,30	70,05	29,95	[BATF, BCL3, BTK, BTN3A2, C1QA, C1QB, C1QC, C2, C8G, CCL19, CCR2, CD19, CD1B, CD1C, CD1E, CD226, CD27, CD4, CD40LG, CD74, CD80, CD8A, CLCF1, CLEC6A, CR2, CRTAM, CXCL10, EBI3, FOXP3, FUT7, GZMM, HLA-DRA, HLA-DRB1, IGLL5, IL12RB1, IL18, IL7R, INPP5D, IRF4, KLRC1, KLRD1, LEF1, LILRB1, LILRB4, LTA, LY9, MYO1G, NCKAP1L, NLRP3, PRF1, PRKCQ, PTPN6, PTPRC, SASH3, SEMA4A, SLA2, SLAMF6, SPN, TBX21, TLR8, TNFAIP3, TNFSF13B, TREM2, WAS, XCL1]	[AGER, ARTN, BCL3, BCL6, C5, C8G, CD1C, CD1E, CD74, CEACAM1, CLC, CLCF1, CRP, FADD, GNAO1, HFE, HPRT1, ICAM1, IL18BP, IL1B, IL4R, IL7R, JAK3, NECTIN2, NFKB2, NOD2, PVR, RELB, SERPING1, STAT3, TCIRG1, TNF, WAS, ZBTB7B, ZC3H12A, ZNF395]
GO:0002694	regulation of leukocyte activation	2,18E-24	[3, 4, 5]	194,00	29,85	67,19	32,81	[ADAM8, AIF1, BANK1, BLK, BTK, BTLA, CAMK4, CARD11, CCDC88B, CCL19, CCL2, CCL3, CCL5, CCR2, CD177, CD19, CD2, CD22, CD226, CD27, CD300LF, CD33, CD3E, CD4, CD40LG, CD5, CD6, CD74, CD80, CD84, CD86, CDKN2A, CLCF1, CLEC4D, CLNK, CNR1, CNR2, CORO1A, CRTAM, CST7, CTLA4, DOCK8, EBI3, EGR3, EPX, FCRL3, FGL2, FGR, FOXP3, GRAP2, HLA-DMB, HLA-DOA, HLA-DPA1, HLA-DPB1, HLA-DRA, HLA-DRB1, ICOS, IFNG, IGLL5, IKZF3, IL12RB1, IL18, IL7R, INPP5D, IRF4, ITGB2, KLRC2, KLRK1, LAG3, LAX1, LCK, LDLR, LEF1, LILRB1, LILRB4, LMO1, LST1, MYB, MZB1, NCKAP1L, NFAM1, NLRP3, NR4A3, PLA2G2D, PRAM1, PRDM1, PRKCQ, PTAFR, PTPN22, PTPN6, PTPRC, PYCARD, RAC2, RASAL3, RHOH, RUNX3, SAMSN1, SASH3, SIRPG, SIT1, SLAMF8, SOCS1, SPN, STAP1, STXBP2, SYK, TBC1D10C, TBX21, TESPA1, THBS1, THEMIS2, THY1, TIGIT, TLR6, TNFAIP3, TNFAIP8L2, TNFRSF13B, TNFRSF18, TNFSF13B, TNFSF14, TNFSF8, TOX, TREM2, VAV1, VNN1, XCL1, ZAP70, ZNF683]	[ABL1, AGER, AMH, AP3D1, BCL6, BTLA, CCL2, CCL21, CCR7, CD177, CD276, CD74, CDKN1A, CEACAM1, CLC, CLCF1, CLNK, CLPTM1, CNR1, CSK, CST7, CX3CL1, CYP26B1, DCAF15, DLG5, DTX1, ENPP3, ERBB2, FADD, FES, FLOT2, FOXF1, GAB2, GATA2, GNAO1, GPAM, GPER1, GRN, HAVCR1, HFE, IDO1, IL15RA, IL1B, IL4R, IL7R, INHA, JAK3, JUND, LAG3, LDLR, LGALS9, LRRC32, MYO18A, NECTIN2, NOD2, NPPA, NR1D1, NR4A3, NRARP, PCDHA4, PCDHA6, PDCD1, PLA2G2A, RAC2, RARA, SLC7A1, SOX12, SOX13, SPHK1, SPHK2, SPI1, SRC, THBS1, TICAM1, TIMP1, VNN1, VSIR, ZBTB7B, ZC3H12A, ZMIZ1, ZNF335]
GO:0002698	negative regulation of immune effector process	4,35E-04	[3, 4, 5]	37,00	33,04	60,19	39,81	[ANGPT1, CCR2, CD22, CD84, CD96, CR2, FCRL3, FGL2, FOXP3, IL7R, KLRC1, KLRD1, LILRB1, LILRB4, LPAL2, NCKAP1L, PTPN6, PTPRC, SLAMF8, TBX21, XCL1]	[ANGPT1, APOA1, BCL6, CEACAM1, ENPP3, FOXF1, GRN, HFE, IL4R, IL7R, JAK3, LGALS9, NOD2, SERPING1, SPI1, TNF, ZBTB7B, ZC3H12A]
GO:0002699	positive regulation of immune effector process	5,71E-10	[3, 4, 5]	79,00	31,60	67,30	32,70	[BLK, BTK, CAMK4, CCL19, CCR2, CD177, CD1B, CD1C, CD1E, CD226, CD244, CD74, CD80, CD84, CD86, CLCF1, CLNK, CRTAM, FGR, FOXP3, HLA-DMB, HLA-DRA, HLA-DRB1, IFNG, IL12RB1, IL18, IL18RAP, ITGB2, KLRC2, KLRD1, KLRK1, LAG3, LILRB1, LTA, MYB, MZB1, NCR3, NLRP3, NR4A3, PTAFR, PTPN22, PTPRC, RAC2, SASH3, SCIMP, SH2D1A, SLAMF6, STAP1, STXBP2, SYK, TBX21, TREM2, VAV1, XCL1]	[CD177, CD1C, CD1E, CD74, CLCF1, CLNK, ENPP3, FADD, FES, FFAR2, FOXF1, GAB2, GATA2, GPI, HK1, IL1B, IL4R, LAG3, LGALS9, MAVS, MYO18A, NECTIN2, NOD2, NPPA, NR4A3, PVR, RAC2, RAP1A, RARA, SLC7A5, SPHK2, SPI1, TICAM1, TNF]

GO:0006954	inflammatory response	7,89E-24	[4]	258,00	26,25	56,09	43,91	[ACP5, ADAM8, ADCY7, ADORA3, AIF1, AIM2, AKNA, ALOX15, AOAH, BLNK, BMPR1B, BTK, C1QTNF1, CALCR, CAMK4, CCL17, CCL19, CCL2, CCL22, CCL24, CCL3, CCL3L1, CCL4, CCL4L1, CCL5, CCR2, CCR3, CCR4, CCR5, CD180, CD200R1, CD247, CD3G, CD40LG, CD6, CD96, CEL, CNR1, CNR2, CSF1R, CST7, CXCL10, CXCL11, CXCL5, CXCL6, CXCL9, CXCR3, CXCR4, CXCR6, CYBB, CYSLTR1, DPEP1, DRD1, ELF3, EPX, FFAR4, FGR, FOLR2, FOXP3, FUT7, GBP5, GPR150, GPR4, GPR83, GPSM3, HLA-DRB1, IFNG, IL16, IL18, IL18RAP, IL22, IL5RA, IRF5, ITGAL, ITGB2, KLRG1, KNG1, LCP1, LDLR, LILRB4, LPAL2, LTA, LY75, LY86, LYZ, MAPK13, MMP9, MS4A1, NCR3, NFAM1, NLRC3, NLRP2, NLRP3, NLRP6, NLRP7, NLRP9, ORM2, PIK3CG, PLA2G2D, PLA2G7, PLD4, PRKCQ, PSTPIP1, PTAFR, PTGDR, PTGER2, PTGFR, PTPRC, PTX3, PYCARD, RLN2, SCNN1B, SCUBE1, SDC1, SELE, SERPINA1, SIGLEC1, SIGLEC10, SLAMF8, SMPDL3B, SOCS3, STAP1, SUCNR1, SYK, THBS1, THEMIS2, TLR10, TLR6, TLR8, TNFAIP3, TNFAIP8L2, TNIP3, TREM2, VNN1, XCL1, XCL2, XCR1, ZBP1]	[ABCD1, ABCD2, ACKR1, ADCY1, ADCY3, ADCY4, ADCY9, ADGRE5, ADIPOQ, AGER, AGT, AGTR1, AMH, APOA1, APOD, BCL6, BRD4, C1QTNF1, C5, C5AR1, CALCR, CCL11, CCL2, CCL21, CCL24, CCR7, CD14, CEL, CGA, CHGA, CHI3L1, CHST1, CIITA, CNR1, CRHR2, CRP, CSF1, CST7, CX3CL1, CYP26B1, DAB2IP, DAGLA, DHX34, ECM1, ELF3, ENPP3, EPHA2, FABP4, FEM1A, FFAR2, FOXF1, FPR1, FURIN, GBA, GIT1, GNAI2, GPBAR1, GPER1, GPR20, GPR4, GPR83, GPR84, GRN, HDAC5, HFE, HK1, HRH2, HSPG2, HYAL1, ICAM1, IL1B, IL1RN, IL4R, IL5RA, IRAK2, KDM6B, KNG1, LDLR, LGALS9, LILRA5, LTB4R2, MAP2K3, MAPK7, MEN1, MYH9, NDST1, NFATC4, NINJ1, NLRP9, NOD2, NOTCH1, NPPA, NR1D1, OSM, PCDHA4, PCDHA6, PER1, PLA2G2A, PLA2G7, PLD3, PPARD, PRKACA, PRKAR2B, PTGES, PTGFR, PTGIR, PTN, RAMP2, RELA, RELB, RHBDF2, SBNO2, SCYL1, SELE, SELENOP, SERPINA3, SERPINE1, SHARPIN, SMAD3, SOCS3, SPATA2, SPHK1, SRC, STAT3, STING1, TCIRG1, TGM2, THBS1, TICAM1, TIMP1, TNF, TNFRSF1A, TNIP1, TRPV4, VIPR2, VNN1, ZC3H12A, ZFP36, ZNF580, ZYX]
GO:0007159	leukocyte cell-cell adhesion	4,87E-21	[4]	129,00	34,04	69,75	30,25	[ADAM8, AIF1, BMP7, BTLA, CARD11, CCDC88B, CCL19, CCL2, CCL5, CCR2, CD177, CD27, CD3E, CD4, CD40LG, CD5, CD6, CD74, CD80, CD86, CDKN2A, CORO1A, CRTAM, CTLA4, DOCK8, EBI3, EGR3, EPX, FGL2, FOXP3, FUT7, GRAP2, HLA-DMB, HLA-DPA1, HLA-DPB1, HLA-DRA, HLA-DRB1, ICOS, IFNG, IL12RB1, IL18, IL7R, ITGA4, ITGAL, ITGB2, ITGB7, KLRK1, LAG3, LAX1, LCK, LEF1, LILRB1, LILRB4, MYB, NCKAP1L, NLRP3, NR4A3, PLA2G2D, PRKCQ, PTAFR, PTPN22, PTPN6, PTPRC, PYCARD, RAC2, RASAL3, RHOH, RUNX3, SASH3, SELE, SELL, SELPLG, SEMA4D, SIRPG, SKAP1, SOCS1, SPN, SYK, TBX21, TESPA1, THY1, TIGIT, TNFAIP8L2, TNFSF13B, TNFSF14, VAV1, VNN1, XCL1, ZAP70]	[AGER, AP3D1, BCL6, BTLA, CCL2, CCL21, CCR7, CD177, CD276, CD74, CEACAM1, CSK, DLG5, DTX1, ERBB2, FADD, FLOT2, GNAO1, GPAM, HFE, ICAM1, IL1B, IL4R, IL7R, JAK3, LAG3, LGALS9, LRRC32, MSN, NOD2, NR4A3, NRARP, PDCD1, RAC2, RARA, RELA, SELE, SELENOP, SLC7A1, SOX12, SOX13, SRC, TIMP1, TNF, TNIP1, VNN1, VSIR, ZBTB7B, ZC3H12A, ZMIZ1]
GO:0007162	negative regulation of cell adhesion	8,18E-08	[3, 4, 5]	84,00	28,19	48,63	51,37	[ADAMDEC1, AKNA, ANGPT1, ANGPT2, C1QTNF1, CD74, CD80, CD86, CDH1, CDKN2A, CRTAM, CTLA4, FGL2, FOXP3, HLA-DRB1, KNG1, LAG3, LAX1, LILRB1, LILRB4, NCKAP1L, PLA2G2D, PLXNC1, PTPN22, PTPN6, PTPRC, RUNX3, SEMA4D, SOCS1, SPN, TACSTD2, TBX21, THBS1, TIGIT, TNFAIP8L2, TNR, WNT1, XCL1]	[ABL1, ACTN4, ADIPOQ, ANGPT1, APOA1, APOD, ARHGDIA, BCL6, C1QTNF1, CCL21, CD74, CEACAM1, COL1A1, CX3CL1, DLG5, DMTN, DTX1, ERBB2, FXYD5, HFE, IL1RN, IL4R, JAK3, KNG1, LAG3, LGALS9, LRRC32, MAPK7, MEN1, MYADM, NF2, NOTCH1, NOTCH4, NRARP, OMA1, PLXNA1, PLXNA3, PLXNB1, POSTN, PTPRU, RGCC, SERPINE1, SH2B3, SPI1, SRC, SYNGAP1, THBS1, TRPV4, VEGFA, VSIR, ZBTB7B, ZC3H12A]
GO:0031343	positive regulation of cell killing	5,36E-04	[3, 4, 5]	28,00	37,33	78,84	21,16	[CD1B, CD1C, CD1E, CD226, CRTAM, HLA-DRA, HLA-DRB1, IFNG, IL12RB1, IL18RAP, KLRC2, KLRD1, KLRK1, LAG3, NCR3, PRF1, PTPRC, SH2D1A, SLAMF6, STAP1, SYK, VAV1, XCL1]	[CD1C, CD1E, FADD, LAG3, NECTIN2, PVR, SPI1, ZNF395]
GO:0032102	negative regulation of response to external stimulus	5,21E-03	[3, 4, 5]	124,00	20,46	45,28	54,72	[ACP5, ADCY7, AIF1, ANGPT2, AOAH, C1QTNF1, CALCR, CCL2, CD200R1, CD247, CD3G, CD96, CEL, CNR2, CST7, CYSLTR1, DPEP1, DRD1, FFAR4, FGL2, FGR, FOXP3, GPR150, GPR83, HLA-DRB1, KLRC1, KLRD1, KLRK1, KNG1, LDLR, LILRB1, NLRC3, NLRP3, NLRP6, PLAU, PTGDR, PTGER2, PTPRC, RLN2, SEMA4A, SEMA4D, SIGLEC10, SLAMF8, SMPDL3B, SOCS3, STAP1, SYK, THBS1, TNFAIP3, TNFAIP8L2, TNR, TREM2, TSPAN8]	[ABCC8, ADCY1, ADCY3, ADCY4, ADCY9, ADIPOQ, AMH, APOA1, C1QTNF1, C5, CALCR, CCL2, CEACAM1, CEL, CGA, CHGA, CRHR2, CST7, CX3CL1, ENPP3, EPPK1, FAM110A, FAP, FEM1A, FOXF1, FURIN, GBA, GIT1, GNAI2, GPBAR1, GPER1, GPR20, GPR83, GPR84, GRN, HRH2, KNG1, LDLR, LGALS9, LTF, MAPK7, MYH9, NLRC5, NOD2, NOS3, NOTCH1, NR1D1, PCDHA6, PDGFB, PLAU, PLAUR, PLXNA3, PPARD, PRKACA, PRKAR2B, PTGIR, PTPRU, RAMP2, RGMA, RHBDF2, RNF26, RTN4RL1, SEMA3F, SEMA4B, SEMA4C, SEMA6B, SEMA6C, SERPINE1, SERPING1, SH2B3, SHARPIN, SMAD3, SOCS3, SRC, THBS1, TNF, TNFRSF1A, TRIB1, TSPAN8, VIPR2, VTN, WNT3, ZFP36]
GO:0032103	positive regulation of response to external stimulus	8,85E-08	[3, 4, 5]	131,00	24,08	57,80	42,20	[ADAM8, AIF1, AIM2, BTK, CARD11, CCL19, CCL24, CCL3, CCL4, CCL5, CCR2, CCR4, CD180, CD226, CD74, CLEC10A, CLEC4D, CLEC4E, CLEC6A, CLNK, CNR1, CRTAM, CSF1R, CXCL10, CXCR4, CXorf21, EDN2, EREG, FCN1, GBP5, GPR4, GPSM3, IFNG, IKBKE, IL16, IL18, IL18RAP, KLRC2, KLRD1, KLRK1, LAG3, LCP1, LDLR, LTA, LY86, MAPK13, MUC16, MUC19, MUC6, NCKAP1L, NCR3, OASL, P2RY12, PIK3CG, PLA2G7, PTK2B, PYCARD, PYHIN1, RAC2, SCIMP, SH2D1A, SIGLEC16, SLAMF6, STAP1, SUCNR1, SYK, THBS1, TLR10, TLR8, TREM2, VAV1, XCL1, XCL2, ZBP1]	[AGER, AGT, AGTR1, ARTN, C5AR1, CCL21, CCL24, CCR7, CD74, CLEC4E, CLNK, CNR1, CSF1, CX3CL1, EDN2, FABP4, FADD, FEM1A, FFAR2, GPR4, GRN, HSPB1, IL1B, LAG3, LDLR, LGALS9, LILRA5, MAPK3, MAVS, MEGF8, MEN1, MSTN, MUC19, MUC20, MUC3A, NECTIN2, NINJ1, NLRC5, NOD2, NPPA, OSM, PCDHA4, PDGFB, PDGFRB, PLA2G2A, PLA2G7, PQBP1, PRKACA, PTN, PVR, RAC2, RBM14, RELA, RELB, RNU1-93P, SERPINE1, SMAD3, SPI1, SRC, STING1, TGM2, THBS1, TNF, TNFRSF1A, TNIP1, TRIM62, TRPV4, VEGFA, ZCCHC3, ZNF580]

GO:0032940	secretion by cell	7,48E-04	[3, 5]	262,00	18,26	43,50	56,50	[ADAM8, ADGRE3, ANPEP, ARHGAP45, BIN2, BLK, BTK, C1QTNF1, CACNA1E, CACNA1I, CBARP, CCL3, CCL5, CCR2, CD177, CD33, CD53, CD84, CEACAM21, CFP, CLEC12A, CLEC4D, CLNK, CNR1, CORO1A, COTL1, CTSS, CTSW, CYBB, DOCK2, EPX, EXOC3L4, FCN1, FFAR4, FGL2, FGR, FRMPD3, GMFG, HLA-DRB1, IFNG, IQGAP2, ITGAL, ITGAX, ITGB2, KCNN4, KLF5, KLRC2, KLRG1, KMO, KNG1, LAIR1, LILRB1, LRMP, LYZ, MIDN, MMP9, MPO, MYB, MYO1G, NAPSB, NCKAP1L, NFAM1, NLRP3, NR4A3, ORM2, P2RY12, PIK3CG, PLAC8, PLAU, PLEK, PRAM1, PRKCB, PTAFR, PTGDR, PTPN6, PTPRC, PTX3, PYCARD, RAB15, RAB27B, RAB37, RAB3B, RAB3C, RAB44, RAC2, RNASET2, SDC1, SELL, SEPTIN1, SERPINA1, SLC18A1, SLC27A2, SMPD3, STXBP2, SUCNR1, SYK, SYTL1, TBC1D10C, THBS1, TREM2, TRPM2, TXNDC5, UCP2, VNN1]	[ABCC8, ACTN1, ACTN4, ADCY1, ADGRE5, ADIPOQ, AGT, AGTR1, ALDOA, AMH, APLN, APOA1, ARF1, ARL8A, ARSA, ATP13A2, BMP8A, BRPF3, C1QTNF1, C2CD2L, C5AR1, CACNA1E, CACNA1H, CAPN1, CBARP, CD14, CD177, CEACAM1, CGA, CHGA, CHI3L1, CLEC12A, CLNK, CNR1, COMMD3, CPLX1, CRAT, CREB3L1, CTSA, CTSD, CYB5R3, DMTN, DOC2B, DOK3, DSC1, ECM1, ENG, FES, FFAR2, FOXF1, FPR1, FURIN, GAB2, GALNS, GATA2, GIT1, GNAI2, GPER1, GPI, GPR84, GRIK5, GRM2, GRN, GTPBP2, HBB, HFE, HGS, HTR1B, HTR2A, HVCN1, IL1B, IL1RN, IL4R, IMPDH1, INHA, IQGAP2, ITPR3, JUP, KCTD11, KLF5, KNG1, LGALS3BP, LGALS9, LRG1, LRRC8A, LTF, LY6E, MEN1, METTL7A, MIDN, MME, MYH9, MYO18A, NAPA, NBEAL2, NHLRC3, NKD2, NLGN2, NNAT, NOTCH1, NPPA, NR1D1, NR4A3, OTOF, PCDHA4, PDGFB, PIP5K1C, PKM, PLAU, PLAUR, PORCN, PPARD, PRKCSH, PRRT2, PTGDS, PTGES, PTPN23, PTPRN, RAB11B, RAB11FIP5, RAB15, RAB3A, RAB3IL1, RAB5B, RAC2, RAP1A, RASL10B, RGCC, RHBDF1, RHBDF2, RHOG, RNASE2, RNU1-93P, RPH3A, S100A11, SELENOP, SEPTIN4, SEPTIN5, SEPTIN9, SERPINA3, SERPINE1, SERPING1, SIDT2, SIGLEC9, SLC1A5, SLC30A1, SLC44A2, SLC7A8, SLCO4C1, SPHK2, SPI1, SREBF1, STEAP3, STING1, STK11IP, STX1A, STX1B, SV2A, SYN1, SYT2, SYT7, TCIRG1, THBS1, TIMP1, TMEM179B, TMEM63A, TNF, TNFAIP2, TNFRSF1A, TRAPPC1, TRPV4, TSPAN14, TUBA4A, VEGFA, VNN1]
GO:0032943	mononuclear cell proliferation	8,21E-12	[4]	95,00	30,84	72,46	27,54	[AIF1, BLK, BTK, BTN3A1, CARD11, CCDC88B, CCL19, CCL5, CCR2, CD180, CD19, CD22, CD3E, CD40LG, CD6, CD74, CD79A, CD80, CD86, CDKN2A, CLCF1, CORO1A, CR2, CRTAM, CTLA4, DOCK2, EBI3, EPX, FCRL3, FLT3, FOXP3, GAPT, HLA-DMB, HLA-DPA1, HLA-DPB1, HLA-DRB1, IKZF3, IL12RB1, IL18, IL7R, INPP5D, LEF1, LILRB1, LILRB4, LMO1, LST1, MS4A1, MZB1, NCKAP1L, PIK3CG, PLA2G2D, PRKCQ, PTPN22, PTPN6, PTPRC, PYCARD, RAC2, RASAL3, SASH3, SPN, SYK, TNFRSF13B, TNFSF13B, TNFSF14, TNFSF8, XCL1, ZAP70]	[ABL1, AGER, AMH, ARMC5, BCL6, CD276, CD74, CDKN1A, CLC, CLCF1, CRP, CSF1, DLG5, ELF4, ERBB2, FADD, FLT3, GPAM, HPRT1, IDO1, IL1B, IL7R, JAK3, LGALS9, LRRC32, MSN, RAC2, SLC7A1, TICAM1, TIMP1, VSIR, ZBTB7B, ZNF335]
GO:0045597	positive regulation of cell differentiation	2,37E-08	[3, 4, 5]	192,00	21,87	40,86	59,14	[ADAM8, ALOX15B, AREG, BMP7, BMPR1B, BTK, CASP8, CASS4, CCL19, CD27, CD4, CD74, CD80, CD86, CDH4, CLCF1, CRABP2, CRB2, CXCL9, CXCR4, EGR3, EPX, EVI2B, FFAR4, FOXP3, GDF6, HCLS1, HLA-DRA, HLA-DRB1, HOPX, IFNG, IL12RB1, IL18, IL7R, INPP5D, IRX3, KLF5, LCP1, LEF1, LIF, LILRB4, LTA, MEDAG, MYB, NCKAP1L, NEK5, NLRP3, NPPC, NTN1, PIM1, PLAAT4, PLXNC1, PTPRC, RHEX, RHOH, RUNX3, SASH3, SEMA4D, SOCS1, SOCS3, STAT1, SULT2B1, SYK, TACSTD2, TESPA1, TOX, TREM2, VNN1, VWC2L, WNT10B, ZAP70, ZBED2]	[ABL1, ADIPOQ, AGER, AGT, AGTR1, AP3D1, APOA1, AREG, ARHGDIA, BCL6, BCL9L, BEND6, CAMK2B, CARM1, CD74, CDH4, CEACAM1, CLCF1, COL1A1, CSF1, CSNK1E, CTHRC1, CX3CL1, CYP26B1, CYP27B1, DAG1, DDX39B, DMTN, EFEMP2, EIF4G1, ENG, FADD, FES, GATA2, GATA4, GDF6, GDI1, GPER1, HEYL, HOPX, HSF1, HTR2A, IFITM1, IL15RA, IL1B, IL4R, IL7R, ILK, IRX3, JUNB, JUND, KCTD11, KLF10, KLF5, LGALS9, LIF, LIMK1, LIMS2, LRRC8A, LTF, LZTS1, MAMSTR, MAPK8IP3, MED12, MEGF8, MME, MYADM, MYC, NAP1L2, NEFL, NEK5, NF2, NGF, NGFR, NOTCH1, NTN1, NUMBL, OMA1, PARP6, PDLIM7, PIEZO1, PIM1, PLA2G2A, PLXNA1, PLXNA3, PLXNB1, POR, PPARD, PPP1R13L, PTN, RAMP2, RAP1A, RARA, RELA, RGCC, S1PR2, SERPINE1, SHANK3, SMAD3, SNAI1, SOCS3, SOX10, SOX12, SOX13, SOX8, SPDEF, SRF, SRRT, STAT3, TBX5, TCF3, TCF7L1, TESK1, TFE3, TGFB1I1, TGM2, THPO, TIMP1, TMEM100, TNF, TRIB1, TRIOBP, VDR, VEGFA, VNN1, VSIG2, VSIR, WNT3, ZBTB7B, ZC3H12A, ZFP36, ZMIZ1, ZNF219, ZNF335, ZNF385A]
GO:0045785	positive regulation of cell adhesion	3,64E-19	[3, 4, 5]	143,00	31,09	59,01	40,99	[ADAM8, AIF1, ALOX15, ANGPT1, APBB1IP, BMP7, BTLA, CARD11, CASS4, CCDC88B, CCL19, CCL2, CCL5, CCR2, CD27, CD3E, CD4, CD40LG, CD5, CD6, CD74, CD80, CD86, CORO1A, CTLA4, DOCK8, EBI3, EGR3, EPX, FOXP3, FUT7, GRAP2, HLA-DMB, HLA-DPA1, HLA-DPB1, HLA-DRA, HLA-DRB1, ICOS, IFNG, IL12RB1, IL18, IL7R, ITGA4, ITGB2, KLRK1, LCK, LEF1, LIF, LILRB1, LILRB4, MYB, NCKAP1L, NLRP3, NR4A3, P2RY12, PRKCQ, PTAFR, PTK2B, PTPN22, PTPN6, PTPRC, PYCARD, RASAL3, RHOH, RUNX3, SASH3, SELE, SIRPG, SKAP1, SOCS1, SYK, TESPA1, THY1, TNFRSF18, TNFSF13B, TNFSF14, VAV1, VNN1, XCL1, ZAP70]	[ABL1, ADAM19, ADGRG1, AGER, ANGPT1, AP3D1, APOA1, BCL6, BTLA, CCL2, CCL21, CCR7, CD276, CD74, CITED2, CSF1, CSK, CX3CL1, DAG1, DMTN, ECM2, EFEMP2, ERBB2, FADD, FLOT2, FOXC2, FOXF1, GNAO1, GPAM, HYAL1, ICAM1, IL1B, IL4R, IL7R, ILK, ITGA3, JAK3, JUP, LGALS9, LIF, LIMS2, MYADM, NOD2, NODAL, NR4A3, PDCD1, PDGFB, PIEZO1, PLAUR, PTN, PTPN23, PTPRU, RARA, RELA, SELE, SELENOP, SLC7A1, SMAD3, SOX12, SOX13, SRC, TESK1, TFE3, TGM2, TIMP1, TNF, TRIOBP, VEGFA, VNN1, VSIR, ZBTB7B, ZMIZ1]

GO:0048513	animal organ development	1,47E-10	[3, 4, 5]	665,00	17,44	39,71	60,29	[ACP5, ADAM8, ADAMTS9, ADGRG6, AIF1, AKNA, ALOX15, ALOX15B, ANGPT1, ANGPT2, AQP5, AREG, ASCL2, ASGR2, ATF3, BATF, BCL11B, BCL3, BFSP2, BLK, BLNK, BMP7, BMPR1B, BRIP1, BTG2, BTK, C1QB, C1QC, C1QTNF1, CALCR, CAMK4, CARD11, CASP8, CCL19, CCL2, CCL3, CCR2, CD19, CD2, CD27, CD3D, CD3E, CD3G, CD4, CD40LG, CD74, CD79A, CD80, CD86, CD8A, CDH1, CDKN2A, CEL, CERS3, CHRDL1, CHRDL2, CLCF1, CR2, CRB2, CRTAM, CSF1R, CTLA4, CXCL10, CXCR4, DAW1, DCC, DOCK10, DOCK2, DPEP1, DPEP2, DRC1, DRD1, E2F8, EAF2, EDN2, EGR3, ELAVL4, ELF3, EOMES, EPX, EREG, EVI2B, FASLG, FCRL3, FGL2, FGR, FLT3, FOLR2, FOSL1, FOXP3, FRZB, FUT7, GCNT3, GDF6, GFI1, GPM6A, GPR171, GPR4, GPR55, GRXCR2, H3C10, H3C11, H3C12, H3C7, HCLS1, HCN1, HLA-DOA, HLA-DRA, HLA-DRB1, HOPX, IFNG, IKZF1, IKZF3, IL12RB1, IL18, IL7R, INPP5D, IRF4, IRF8, IRX3, ITGA4, ITGAX, ITK, JPH1, KLF5, KLHL41, KLRC1, KRT85, LAG3, LCK, LCP1, LEF1, LIF, LILRB1, LILRB4, LIPM, LMO1, LPAL2, LRRC17, LTA, LTB, LY9, MAFF, MCOLN3, MIXL1, MMP9, MS4A1, MSLN, MSX1, MT1G, MYB, MYCL, MYH6, MYO3B, NAMPT, NCKAP1L, NELL2, NFAM1, NLRP3, NPPB, NPPC, NR4A1, NR4A2, NR4A3, NTN1, OPN5, P2RY12, PAX5, PBX4, PIM1, PLA2G2D, PLAAT4, PLD4, PLEK, PLPPR4, POU2AF1, POU2F2, PRDM1, PRKCB, PRKCQ, PTK2B, PTPN22, PTPN6, PTPRC, PTX3, RBPMS2, RHEX, RHOH, RNASE6, RRAD, RTKN2, RUNX3, SALL1, SASH3, SCUBE1, SDC1, SELPLG, SEMA4A, SEMA4D, SLAMF6, SLAMF8, SLC24A4, SLC8A3, SMPD3, SNX10, SOCS1, SOCS3, SPEF2, SPN, ST14, STAT1, SYK, TACSTD2, TBX21, TESPA1, TEX11, THBS1, THEMIS, THY1, TIFAB, TMEM132E, TNFAIP3, TNFRSF11B, TNFRSF13B, TNFRSF18, TNFSF13B, TNFSF8, TNR, TOX, TREM2, TRPM2, UBD, UCP2, VAV1, VNN1, WNT1, WNT10A, WNT10B, ZAP70, ZBED2, ZNF683]	[AATK, ABCA3, ABL1, ACACB, ACP4, ACTA1, ACTB, ACTC1, ACTN1, ADAM19, ADAMTS5, ADAMTS7, ADAMTS9, ADCY1, ADGRG1, ADGRG6, ADIPOQ, AGAP2, AGER, AGT, AGTR1, ALPK3, ALPL, AMH, ANGPT1, ANKRD52, AP1B1, AP2A1, AP3D1, APLN, APLNR, APLP1, APOA1, APOD, AQP3, AQP5, AREG, ARHGEF15, ARMC5, ARRDC3, ARTN, ASPN, ATAT1, ATF5, ATP1B2, ATP6V0D1, ATP6V1B1, BAG3, BAG6, BATF2, BCL2L1, BCL3, BCL6, BCL9L, BCOR, BCR, BGN, BMP8A, BTG2, C1QTNF1, C5AR1, CACNA1H, CALB1, CALCR, CAPN1, CAPNS1, CCDC85C, CCL11, CCL2, CCR7, CD74, CDK1, CDK2, CDKN1A, CEACAM1, CECR2, CEL, CELSR2, CERT1, CFH, CGA, CHGA, CHI3L1, CHRDL2, CIC, CITED2, CLCF1, CLPTM1, CNNM4, COASY, COL1A1, COL27A1, CRAT, CRIP2, CRYAB, CSF1, CSK, CSNK1E, CTC1, CTF1, CTHRC1, CYP21A2, CYP26B1, CYP27B1, DAB2IP, DAG1, DCHS1, DDR1, DDX39B, DEDD, DHRS2, DLG5, DMC1, DMRTA1, DMTN, DPF2, DRC1, DSC1, DSCAML1, DTX1, DVL3, DYRK1B, E2F4, E2F7, E2F8, EAF2, ECM1, EDN2, EFEMP2, EIF4EBP1, ELF3, ELK1, ELOVL1, ENG, EPHA2, EPN1, ERBB2, ETV6, FA2H, FADD, FAIM2, FANCC, FASN, FES, FGFRL1, FHL3, FLG, FLT3, FLT4, FOSL1, FOSL2, FOXC2, FOXF1, FURIN, GAB2, GATA2, GATA4, GBA, GCNT3, GDF6, GIT1, GJD3, GLIS2, GMNN, GNAI2, GNAO1, GPER1, GPM6A, GPR137, GPR37L1, GPR4, GPRIN1, GSK3A, GYS1, H3C10, H3C12, H4C3, HBEGF, HCN4, HDAC5, HESX1, HEYL, HFE, HID1, HIVEP3, HMGN1, HNMT, HOPX, HPRT1, HSF1, HSPB7, HSPG2, HYAL1, ICAM1, ID3, IFT80, IL15RA, IL1B, IL4R, IL7R, ILK, INHA, INPPL1, IRX3, ITGA3, ITGA7, JAK3, JUNB, JUP, KCNK2, KCNK3, KCNQ4, KCP, KCTD11, KDELR1, KDM6B, KERA, KLF10, KLF5, KLK14, KMT2D, KRTAP5-1, LAG3, LAMA5, LAMB4, LGALS9, LIF, LIMK2, LIMS2, LMNA, LMX1A, LOX, LRP10, LRRC17, LRRC8A, LSR, LTBR, LTF, LUM, LY6E, LZTS1, LZTS2, MAMLD1, MAP1S, MAPK3, MBOAT7, MCRIP1, MDGA1, MDGA2, MECP2, MED12, MEF2D, MEGF8, MEIS3P1, MEIS3P2, MEN1, MFAP2, MFAP5, MFSD2A, MKNK2, MME
GO:0050778	positive regulation of immune response	1,07E-08	[3, 4, 5]	186,00	22,25	69,27	30,73	[ADAM8, AIM2, BLK, BLNK, BTK, BTN3A1, BTN3A2, C1QA, C1QB, C1QC, C2, C8G, CARD11, CCL19, CCL5, CCR2, CD177, CD19, CD1B, CD1C, CD1E, CD22, CD226, CD247, CD3D, CD3E, CD3G, CD4, CD74, CD79A, CD80, CD86, CFP, CLCF1, CLEC10A, CLEC4D, CLEC4E, CLEC6A, CLNK, CNR1, CR2, CRTAM, CTLA4, CXCL10, CXorf21, CYP11B1, EREG, FCN1, FCN3, FCRL3, FGR, FOXP3, FYB1, GBP5, GRAP2, HLA-DMB, HLA-DPA1, HLA-DPB1, HLA-DQA1, HLA-DRA, HLA-DRB1, HLA-DRB5, IFNG, IGLL5, IKBKE, IL12RB1, IL18, IL18RAP, INPP5D, ITGB2, ITK, KCNN4, KLRC2, KLRD1, KLRK1, LAG3, LAX1, LCK, LCP2, LILRB1, LILRB4, LTA, MOG, MS4A1, MUC16, MUC19, MUC6, MYB, MYO1G, NCKAP1L, NCR3, NFAM1, NLRP3, NR4A3, PAX5, PRAM1, PRKCB, PRKCQ, PTAFR, PTPN22, PTPN6, PTPRC, PYCARD, PYHIN1, SASH3, SH2D1A, SIGLEC16, SKAP1, SLA2, SLAMF6, STAP1, SYK, TBX21, TESPA1, THEMIS, THEMIS2, THY1, TLR8, TNFSF13B, TRAT1, TREM2, UBASH3A, VAV1, WAS, XCL1, ZAP70, ZBP1]	[ABL1, ACTB, BAG6, BCAR1, C5, C5AR1, C8G, CCR7, CD177, CD1C, CD1E, CD276, CD74, CEACAM1, CFH, CLCF1, CLEC4E, CLNK, CNR1, CPN2, CRP, CSK, DGKZ, FADD, FCN3, FFAR2, FPR1, GAB2, GATA2, GCSAM, GNAO1, GRN, IL1B, IL4R, LAG3, LGALS9, LIMK1, MAPK3, MAVS, MUC19, MUC20, MUC3A, NCOR2, NECTIN2, NLRC5, NOD2, NPPA, NR4A3, PCDHA4, PQBP1, PRKACA, PVR, RAP1A, RARA, RBM14, RELA, RELB, RGCC, RHBDF2, RNU1-93P, SERPING1, SH2B2, SLC7A8, SPHK2, SPI1, SRC, STING1, TNF, TRIM62, VTN, WAS, ZC3H12A, ZCCHC3]
GO:0050866	negative regulation of cell activation	2,21E-10	[3, 4, 5]	72,00	33,49	63,17	36,83	[BANK1, BLK, BTK, C1QTNF1, CCR2, CD300LF, CD74, CD80, CD84, CD86, CDKN2A, CLNK, CNR1, CNR2, CRTAM, CST7, CTLA4, FGL2, FGR, FOXP3, HLA-DRB1, INPP5D, LAG3, LAX1, LDLR, LILRB1, LILRB4, LST1, NCKAP1L, PLA2G2D, PTPN22, PTPN6, PTPRC, RUNX3, SAMSN1, SOCS1, SPN, TBC1D10C, TBX21, TIGIT, TNFAIP3, TNFAIP8L2, TNFRSF13B, TREM2, XCL1]	[BCL6, C1QTNF1, CD74, CEACAM1, CLNK, CNR1, CST7, CX3CL1, DLG5, DTX1, ENPP3, ERBB2, FOXF1, GPER1, GRN, HFE, IL4R, INHA, JAK3, LAG3, LDLR, LGALS9, LRRC32, NOS3, NR1D1, NRARP, PCDHA4, PCDHA6, PDGFB, SH2B3, SPI1, VSIR, ZBTB7B, ZC3H12A]
GO:0050867	positive regulation of cell activation	2,02E-12	[3, 4, 5]	127,00	27,79	69,03	30,97	[ADAM8, AIF1, BTK, BTLA, CARD11, CCDC88B, CCL19, CCL2, CCL3, CCL5, CCR2, CD177, CD2, CD226, CD27, CD3E, CD4, CD40LG, CD5, CD6, CD74, CD80, CD86, CLCF1, CLEC4D, CORO1A, CTLA4, DOCK8, EBI3, EGR3, EPX, FCRL3, FGR, FOXP3, GRAP2, HLA-DMB, HLA-DPA1, HLA-DPB1, HLA-DRA, HLA-DRB1, ICOS, IFNG, IGLL5, IL12RB1, IL18, IL7R, INPP5D, ITGB2, KLRC2, KLRK1, LCK, LEF1, LILRB1, LILRB4, MYB, NCKAP1L, NLRP3, NR4A3, PLEK, PRKCQ, PTAFR, PTPN22, PTPN6, PTPRC, PYCARD, RASAL3, RHOH, RUNX3, SASH3, SIRPG, SOCS1, STAP1, SYK, TBX21, TESPA1, THBS1, THY1, TLR6, TNFSF13B, TNFSF14, TOX, TREM2, VAV1, VNN1, XCL1, ZAP70]	[AGER, AMH, AP3D1, BCL6, BTLA, CCL2, CCL21, CCR7, CD177, CD276, CD74, CDKN1A, CLCF1, CSK, FADD, FLOT2, GAB2, GATA2, GNAO1, GPAM, HAVCR1, IL15RA, IL1B, IL4R, IL7R, JAK3, JUND, LGALS9, LILRA5, NECTIN2, NOD2, NPPA, NR4A3, PDCD1, PDGFRB, RARA, SELENOP, SLC7A1, SOX12, SOX13, SPHK2, SRC, THBS1, TICAM1, TIMP1, VNN1, VSIR, ZBTB7B, ZMIZ1, ZNF335]
GO:0050920	regulation of chemotaxis	6,17E-05	[3, 4, 5]	66,00	27,05	49,49	50,51	[AIF1, ANGPT2, CCL19, CCL2, CCL3, CCL4, CCL5, CCR2, CCR4, CD74, CSF1R, CXCL10, CXCR4, EDN2, GPSM3, IL16, KLRK1, NCKAP1L, P2RY12, PLA2G7, PTK2B, RAC2, SEMA4A, SEMA4D, SLAMF8, STAP1, SUCNR1, THBS1, TREM2, XCL1, XCL2]	[AGER, AMH, ARTN, C5, C5AR1, CCL2, CCL21, CCR7, CD74, CSF1, EDN2, HSPB1, LGALS9, MAPK3, MEGF8, MEN1, MSTN, NOD2, NOTCH1, PDGFB, PDGFRB, PLA2G7, PLXNA3, PTN, PTPRU, RAC2, SEMA3F, SEMA4B, SEMA4C, SEMA6B, SEMA6C, SERPINE1, SMAD3, SPI1, THBS1, TRPV4, VEGFA, WNT3, ZNF580, ZSWIM4, ZSWIM8]

GO:0098542	defense response to other organism	1,13E-07	[3, 4, 5]	259,00	19,98	65,48	34,52	[ACP5, ADAM8, AIF1, AIM2, APOBEC3A, APOBEC3D, APOBEC3G, APOBEC3H, BATF, BCL3, BLK, BTK, C1QA, C1QB, C1QC, C2, C8G, CARD11, CASP8, CCDC88B, CCL17, CCL19, CCL2, CCL22, CCL24, CCL3, CCL3L1, CCL4, CCL4L1, CCL5, CD177, CD180, CD226, CD244, CD4, CD6, CD74, CD84, CD96, CFP, CLEC10A, CLEC4D, CLEC4E, CLEC6A, CLNK, CORO1A, COTL1, CR2, CRTAM, CSF1R, CXCL10, CXCL6, CXCL9, CXorf21, CYBB, DAPK1, EPX, EREG, FASLG, FCN1, FCN3, FGL2, FGR, FOXP3, GBP5, GBP6, GNLY, GZMB, GZMM, HLA-DPA1, HLA-DPB1, HLA-DQA1, HLA-DRA, HLA-DRB1, HLA-DRB5, IFNG, IGLL5, IKBKE, IL12RB1, IL18, IL18RAP, IL7R, IRF4, IRF5, IRF8, ITGAX, JCHAIN, KIR2DL3, KLRC1, KLRC2, KLRD1, KLRG1, KLRK1, LAG3, LCK, LILRB1, LTA, LY86, LY9, LYZ, MCOLN2, MPEG1, MPO, MRC1, MUC16, MUC19, MUC6, NCF1, NCR1, NCR3, NLRC3, NLRP2, NLRP3, NLRP6, NLRP9, OASL, PIK3CG, PLAC8, PLD4, PRDM1, PRF1, PSTPIP1, PTAFR, PTK2B, PTPN22, PTPN6, PTPRC, PTX3, PYCARD, PYHIN1, RNASE6, RNASET2, SCNN1B, SH2D1A, SIGLEC10, SIGLEC16, SLA, SLAMF6, SLAMF7, SLAMF8, SMPDL3B, SOCS1, SOCS3, SPN, STAT1, STYK1, SYK, TLR10, TLR6, TLR8, TNFAIP3, TNFAIP8L2, TNFSF8, TREM2, TRIM14, UBD, VAV1, VNN1, WAS, XCL1, XCL2, ZAP70, ZBP1, ZNF683]	[ADAMTS5, AGER, AMH, APOBEC3C, ARID5A, ARMC5, BATF2, BCL2L1, BCL3, C5, C5AR1, C8G, CAMK2B, CCL11, CCL2, CCL21, CCL24, CD14, CD177, CD300E, CD74, CDC42EP2, CDC42EP4, CEACAM1, CFH, CHGA, CIITA, CLEC4E, CLNK, CRP, CSF1, CX3CL1, CYP27B1, DAB2IP, ELF4, EPHA2, FADD, FCN3, FES, FFAR2, GNAO1, GPAM, GPER1, GRN, HFE, HK1, ICAM1, IFITM1, IFITM2, IFITM3, IL22RA1, IL4R, IL7R, IRF2, IRF9, JAK3, LAG3, LGALS9, LILRA5, LTF, MAP3K14, MAPK3, MAVS, MOV10, MRC1, MUC19, MUC20, MUC3A, MYC, NECTIN2, NLRC5, NLRP9, NOD2, PLA2G2A, PLEKHM2, PML, PPP1R11, PQBP1, PRKACA, PVR, RBM14, RELA, RELB, RNASE2, RNF19B, RNF26, RNF39, RNU1-93P, SELENOP, SERPINE1, SERPING1, SMPD1, SOCS3, SPI1, SRC, STING1, TBKBP1, TICAM1, TNF, TNFRSF1A, TNK1, TNK2, TRIM29, TRIM62, TRIM8, VNN1, WAS, ZC3H12A, ZCCHC3, ZDHHC11, ZNF395, ZYX]
GO:2000145	regulation of cell motility	4,54E-07	[3, 4, 5]	206,00	20,79	38,83	61,17	[ADAM8, ADAMTS9, ADORA3, AIF1, ALOX15B, ANGPT1, ANGPT2, BMP7, CARMIL2, CASS4, CCL19, CCL2, CCL24, CCL3, CCL4, CCL5, CCR2, CD200R1, CD74, CDH1, CORO1A, CSF1R, CXCL10, CXCR3, CXCR4, DOCK10, DOCK8, DPEP1, DRD1, EDN2, EREG, FGR, FUT7, GPSM3, IFNG, ITGA4, ITGAX, KLRK1, LEF1, MMP9, NCKAP1L, NELL2, NR4A3, NTN1, P2RY12, PIK3CG, PLA2G7, PLAU, PLXNC1, PTAFR, PTK2B, PTPN22, PTPRC, PYCARD, RAC2, RHOH, SELE, SEMA4A, SEMA4D, SLAMF8, SMPD3, SPN, STAP1, TACSTD2, TCAF2, THBS1, THY1, TNFRSF18, TREM2, WAS, XCL1, XCL2, XG]	[ABCC8, ABI3, ABL1, ACKR3, ACTN4, ADAMTS9, ADGRG1, ADIPOQ, AGER, AGT, AMH, ANGPT1, APOD, ARHGDIA, ATP1B2, BCAR1, C5, C5AR1, CAMK2B, CAMSAP3, CARMIL2, CCL11, CCL2, CCL21, CCL24, CCR7, CD74, CEACAM1, CGA, CHGA, CITED2, COL1A1, CSF1, CX3CL1, DAB2IP, DAG1, DLG5, DMTN, ECM1, EDN2, ENG, EPHA2, EPPK1, ERBB2, FADD, FAM110A, FES, FLT4, FOXC2, FOXF1, GATA2, GCSAM, GNAI2, GPER1, GPI, GRB7, GRN, HBEGF, HDAC5, HDAC7, HSPB1, HYAL1, ICAM1, IFITM1, IL1B, ILK, ITGA3, JUP, LAMA5, LGALS9, LGR6, LMNA, MAP2K3, MAPK3, MAZ, MECP2, MIEN1, MINK1, MSN, MSTN, MYADM, MYC, NF2, NGFR, NOD2, NODAL, NOS3, NOTCH1, NR4A3, NSMF, NTN1, OSGIN1, P2RY6, PDGFB, PDGFRB, PFN1, PHACTR1, PLA2G7, PLAU, PLVAP, PLXNA1, PLXNA3, PLXNB1, POSTN, PPARD, PRAG1, PTN, PTPN23, PTPRU, RAC2, RGCC, RHOG, RIPOR1, ROBO4, S100A11, SELE, SELENOP, SEMA3F, SEMA4B, SEMA4C, SEMA6B, SEMA6C, SERPINE1, SH3BP1, SLC9A3R1, SMAD3, SMIM22, SNAI1, SPHK1, SPI1, SRC, SRF, SSX2IP, STAT3, SYDE1, TBX5, THBS1, TIMP1, TNF, TRIB1, TRPV4, VEGFA, VSIR, VTN, WAS, XG, ZC3H12A, ZNF580, ZNF609]
GO:0002253	activation of immune response	5,32E-04	[2, 4, 5, 6]	132,00	20,99	69,52	30,48	[AIM2, BLK, BLNK, BTK, BTN3A1, BTN3A2, C1QA, C1QB, C1QC, C2, C8G, CARD11, CD19, CD22, CD226, CD247, CD3D, CD3E, CD3G, CD4, CD79A, CFP, CLEC10A, CLEC4D, CLEC4E, CLEC6A, CR2, CTLA4, CXCL10, CYP11B1, FCN1, FCN3, FCRL3, FGR, FOXP3, FYB1, GRAP2, HLA-DPA1, HLA-DPB1, HLA-DQA1, HLA-DRA, HLA-DRB1, HLA-DRB5, IGLL5, INPP5D, ITK, KCNN4, KLRC2, KLRD1, LAX1, LCK, LCP2, LILRB4, MOG, MS4A1, MUC16, MUC19, MUC6, MYO1G, NCKAP1L, NCR3, NFAM1, NR4A3, PAX5, PRAM1, PRKCB, PRKCQ, PTPN22, PTPN6, PTPRC, PYCARD, PYHIN1, SH2D1A, SKAP1, SLA2, STAP1, SYK, TESPA1, THEMIS, THEMIS2, THY1, TRAT1, TREM2, UBASH3A, VAV1, WAS, ZAP70, ZBP1]	[ABL1, ACTB, BAG6, BCAR1, C5, C5AR1, C8G, CCR7, CD276, CEACAM1, CFH, CLEC4E, CPN2, CRP, CSK, DGKZ, FCN3, FFAR2, FPR1, GCSAM, GNAO1, IL1B, LIMK1, MAPK3, MAVS, MUC19, MUC20, MUC3A, NCOR2, NECTIN2, NOD2, NR4A3, PQBP1, PRKACA, RAP1A, RBM14, RELA, RELB, RGCC, RHBDF2, RNU1-93P, SERPING1, SH2B2, SLC7A8, SRC, STING1, VTN, WAS, ZC3H12A, ZCCHC3]
GO:0001817	regulation of cytokine production	4,02E-20	[3, 4, 5, 6]	212,00	26,77	59,95	40,05	[ACP5, ADAM8, ADCY7, AIF1, AIM2, ALOX15B, ANGPT1, BANK1, BATF, BCL3, BTK, BTN3A1, BTN3A2, CAMK4, CARD11, CARD17, CASP8, CCDC88B, CCL19, CCL3, CCR2, CD2, CD200R1, CD226, CD244, CD33, CD3E, CD4, CD40LG, CD6, CD74, CD80, CD84, CD86, CD96, CLCF1, CLEC6A, CLNK, CRTAM, CSF1R, CXCL6, CYBB, EBI3, EPX, EREG, FCN1, FFAR4, FGR, FOXP3, GBP5, GPSM3, HLA-DPA1, HLA-DPB1, HLA-DRB1, IFNG, IKBKE, IL12RB1, IL12RB2, IL16, IL18, IL26, IL5RA, INPP5D, IRF4, IRF5, IRF8, ITK, LAG3, LCP1, LEF1, LILRB1, LILRB4, LPAL2, LTA, LTB, LY9, MAPK13, MCOLN2, MOG, MYB, NCKAP1L, NFAM1, NLRC3, NLRP2, NLRP3, NLRP6, NLRP7, NLRP9, NR4A3, ORM2, PIK3CG, PLD4, POU2AF1, POU2F2, PRKCQ, PTAFR, PTPN22, PTPN6, PTPRC, PYCARD, PYHIN1, SASH3, SCIMP, SIGLEC16, SLAMF6, SOCS1, STAT1, SYK, TBX21, THBS1, TIGIT, TLR10, TLR6, TLR8, TNFAIP3, TREM2, UBASH3A, XCL1, ZBP1]	[ABCC8, ABCD1, ABCD2, ABL1, ACKR1, ADIPOQ, AGER, AGPAT1, AGT, AMH, ANGPT1, APOA1, APOD, BCL3, BCL6, C5, C5AR1, CCR7, CD14, CD276, CD74, CEACAM1, CHI3L1, CLC, CLCF1, CLNK, CPTP, CRP, CSK, CX3CL1, EPHA2, FADD, FFAR2, FLT4, FRMD8, FURIN, GATA4, GBA, GIT1, GPAM, HDAC7, HFE, HK1, HSPB1, IGF2BP2, IL1B, IL4R, IL5RA, INHA, JAK3, JPH4, LAG3, LGALS9, LILRA5, LRRC32, LTF, LUM, MAP2K3, MAST2, MAVS, NFATC4, NFKB2, NLRC5, NLRP9, NOD2, NODAL, NR4A3, NUTF2, OSM, PER1, PLD3, PML, POLR2E, POLR2L, POSTN, PPP1R11, PQBP1, RARA, RELA, RELB, RGCC, RNF26, RNU1-93P, SERPINE1, SLC7A5, SMAD3, SPHK1, SPHK2, SRC, STAT3, STING1, THBS1, TICAM1, TNF, TRPV4, TSKU, UBE2L6, VSIR, ZBTB7B, ZC3H12A, ZCCHC3, ZFP36, ZNF580]

GO:0002275	myeloid cell activation involved in immune response	1,74E-06	[4, 5]	139,00	22,64	53,41	46,59	[ADAM8, ADGRE3, ANPEP, ARHGAP45, BIN2, BLK, BTK, CCL3, CCR2, CD177, CD33, CD53, CD84, CEACAM21, CFP, CLEC12A, CLEC4D, CLNK, COTL1, CTSS, CYBB, DOCK2, EPX, FCN1, FGL2, FGR, FRMPD3, GMFG, IFNG, IQGAP2, ITGAL, ITGAX, ITGB2, LAIR1, LRMP, LYZ, MMP9, MPO, NCKAP1L, NFAM1, NR4A3, ORM2, PIK3CG, PLAC8, PLAU, PRAM1, PTAFR, PTGDR, PTPN6, PTPRC, PTX3, PYCARD, RAB37, RAB44, RAC2, RNASET2, SCNN1B, SELL, SERPINA1, SLC18A1, SLC27A2, STXBP2, SUCNR1, SYK, TBC1D10C, TREM2, TRPM2, TXNDC5, VNN1]	[ADGRE5, ALDOA, AMH, ARL8A, ARSA, C5AR1, CAPN1, CD14, CD177, CEACAM1, CHGA, CHI3L1, CLEC12A, CLNK, COMMD3, CRAT, CTSA, CTSD, CYB5R3, DOK3, DSC1, DYSF, ENPP3, FES, FOXF1, FPR1, GAB2, GALNS, GATA2, GPI, GPR84, GRN, HBB, HFE, HVCN1, IL4R, IMPDH1, IQGAP2, JUP, LGALS9, LRG1, LTF, METTL7A, MME, NBEAL2, NHLRC3, NPPA, NR4A3, PKM, PLAU, PLAUR, PRKCSH, PTGDS, RAB3A, RAB5B, RAC2, RAP1A, RHOG, RNASE2, RNU1-93P, S100A11, SBNO2, SERPINA3, SIGLEC9, SLC44A2, SLC7A8, SLCO4C1, SPHK2, SPI1, STING1, STK11IP, TCIRG1, TICAM1, TMEM179B, TMEM63A, TRAPPC1, TSPAN14, VNN1]
GO:0002285	lymphocyte activation involved in immune response	2,42E-05	[4, 5]	58,00	29,00	76,00	24,00	[APBB1IP, BATF, BCL3, CCL19, CD180, CD19, CD1C, CD244, CD40LG, CD74, CD80, CD86, CLCF1, CORO1A, DOCK10, EOMES, FGL2, FOXP3, GAPT, HLA-DMB, HLA-DRA, HLA-DRB1, IFNG, IL12RB1, IL18, IRF4, IRF8, ITGAL, KLRC2, LCP1, LEF1, LILRB1, LY9, MYB, NCKAP1L, NLRP3, POU2AF1, PTK2B, PTPRC, SEMA4A, SLAMF6, SPN, TBX21, ZNF683]	[ABL1, ARTN, BCL3, BCL6, CD1C, CD74, CEACAM1, CLCF1, ICAM1, IL4R, JAK3, LGALS9, RARA, RELB, SPI1, STAT3, ZBTB7B, ZC3H12A]
GO:0002367	cytokine production involved in immune response	2,27E-03	[3, 6]	34,00	32,08	57,70	42,30	[ANGPT1, BTK, CAMK4, CCR2, CD226, CD74, CD96, CLCF1, CLNK, FOXP3, IL18, LILRB1, LILRB4, LPAL2, NLRP3, NR4A3, SASH3, SCIMP, TBX21, XCL1]	[ANGPT1, APOA1, BCL6, CD74, CLC, CLCF1, CLNK, FFAR2, HFE, HK1, IL1B, JAK3, MAVS, NOD2, NR4A3, SLC7A5, SPHK2, TICAM1, TNF]
GO:0002695	negative regulation of leukocyte activation	1,42E-10	[3, 4, 5, 6]	67,00	34,90	65,00	35,00	[BANK1, BLK, BTK, CCR2, CD300LF, CD74, CD80, CD84, CD86, CDKN2A, CLNK, CNR1, CNR2, CRTAM, CST7, CTLA4, FGL2, FGR, FOXP3, HLA-DRB1, INPP5D, LAG3, LAX1, LDLR, LILRB1, LILRB4, LST1, NCKAP1L, PLA2G2D, PTPN22, PTPN6, PTPRC, RUNX3, SAMSN1, SOCS1, SPN, TBC1D10C, TBX21, TIGIT, TNFAIP3, TNFAIP8L2, TNFRSF13B, XCL1]	[BCL6, CD74, CEACAM1, CLNK, CNR1, CST7, CX3CL1, DLG5, DTX1, ENPP3, ERBB2, FOXF1, GPER1, GRN, HFE, IL4R, INHA, JAK3, LAG3, LDLR, LGALS9, LRRC32, NR1D1, NRARP, PCDHA4, PCDHA6, SPI1, VSIR, ZBTB7B, ZC3H12A]
GO:0002696	positive regulation of leukocyte activation	9,04E-12	[3, 4, 5, 6]	123,00	27,64	70,09	29,91	[ADAM8, AIF1, BTK, BTLA, CARD11, CCDC88B, CCL19, CCL2, CCL3, CCL5, CCR2, CD177, CD2, CD226, CD27, CD3E, CD4, CD40LG, CD5, CD6, CD74, CD80, CD86, CLCF1, CLEC4D, CORO1A, CTLA4, DOCK8, EBI3, EGR3, EPX, FCRL3, FGR, FOXP3, GRAP2, HLA-DMB, HLA-DPA1, HLA-DPB1, HLA-DRA, HLA-DRB1, ICOS, IFNG, IGLL5, IL12RB1, IL18, IL7R, INPP5D, ITGB2, KLRC2, KLRK1, LCK, LEF1, LILRB1, LILRB4, MYB, NCKAP1L, NLRP3, NR4A3, PRKCQ, PTAFR, PTPN22, PTPN6, PTPRC, PYCARD, RASAL3, RHOH, RUNX3, SASH3, SIRPG, SOCS1, STAP1, SYK, TBX21, TESPA1, THBS1, THY1, TLR6, TNFSF13B, TNFSF14, TOX, TREM2, VAV1, VNN1, XCL1, ZAP70]	[AGER, AMH, AP3D1, BCL6, BTLA, CCL2, CCL21, CCR7, CD177, CD276, CD74, CDKN1A, CLCF1, CSK, FADD, FLOT2, GAB2, GATA2, GNAO1, GPAM, HAVCR1, IL15RA, IL1B, IL4R, IL7R, JAK3, JUND, LGALS9, NECTIN2, NOD2, NPPA, NR4A3, PDCD1, RARA, SLC7A1, SOX12, SOX13, SPHK2, SRC, THBS1, TICAM1, TIMP1, VNN1, VSIR, ZBTB7B, ZMIZ1, ZNF335]
GO:0002703	regulation of leukocyte mediated immunity	3,88E-12	[4, 5]	79,00	34,05	68,35	31,65	[BLK, BTK, CAMK4, CCR2, CD177, CD1B, CD1C, CD1E, CD226, CD84, CD96, CLCF1, CLNK, CR2, CRTAM, CXCL6, FGR, FOXP3, FUT7, HLA-DRA, HLA-DRB1, IL12RB1, IL18, IL18RAP, IL7R, ITGB2, KLRC1, KLRC2, KLRD1, KLRK1, LAG3, LILRB1, LILRB4, LTA, NCKAP1L, NCR1, NCR3, NLRP3, PRAM1, PTAFR, PTPN6, PTPRC, RAC2, SASH3, SCIMP, SH2D1A, SLAMF6, STAP1, STXBP2, SYK, TBX21, TREM2, VAV1, WAS, XCL1]	[AGER, BCL6, CD177, CD1C, CD1E, CEACAM1, CLC, CLCF1, CLNK, FADD, FES, FOXF1, GAB2, GATA2, HFE, ICAM1, IL1B, IL4R, IL7R, JAK3, LAG3, LGALS9, MAVS, NECTIN2, NOD2, NPPA, PVR, RAC2, SPHK2, SPI1, TICAM1, TNF, WAS]
GO:0002819	regulation of adaptive immune response	3,50E-06	[4, 5]	58,00	30,53	72,16	27,84	[ADCY7, ALOX15, BTK, CCL19, CCR2, CD1B, CD1C, CD1E, CD226, CD4, CD48, CD80, CLCF1, CLEC6A, CR2, FOXP3, FUT7, HLA-DRA, HLA-DRB1, IL12RB1, IL18, IL7R, KLRC1, KLRD1, LILRB1, LILRB4, LTA, NCKAP1L, NLRP3, PRKCQ, PTPN6, PTPRC, PYCARD, SAMSN1, SASH3, SKAP1, TBX21, TNFAIP3, TNFSF13B, TREM2, WAS, XCL1]	[AGER, BCL6, CD1C, CD1E, CEACAM1, CLC, CLCF1, FADD, GNAO1, HFE, IL1B, IL4R, IL7R, JAK3, NECTIN2, NOD2, PVR, TNF, WAS, ZBTB7B, ZC3H12A]
GO:0006887	exocytosis	1,79E-06	[3, 4, 5, 6]	202,00	20,57	47,31	52,69	[ADAM8, ADGRE3, ANPEP, ARHGAP45, BIN2, BLK, BTK, CACNA1I, CBARP, CCL3, CCL5, CCR2, CD177, CD33, CD53, CD84, CEACAM21, CFP, CLEC12A, CLEC4D, CLNK, CNR1, CORO1A, COTL1, CTSS, CTSW, CYBB, DOCK2, EPX, EXOC3L4, FCN1, FGL2, FGR, FRMPD3, GMFG, IFNG, IQGAP2, ITGAL, ITGAX, ITGB2, KLRC2, KNG1, LAIR1, LRMP, LYZ, MMP9, MPO, MYO1G, NAPSB, NCKAP1L, NFAM1, NR4A3, ORM2, PIK3CG, PLAC8, PLAU, PLEK, PRAM1, PRKCB, PTAFR, PTGDR, PTPN6, PTPRC, PTX3, PYCARD, RAB15, RAB27B, RAB37, RAB3B, RAB3C, RAB44, RAC2, RNASET2, SDC1, SELL, SEPTIN1, SERPINA1, SLC18A1, SLC27A2, SMPD3, STXBP2, SYK, SYTL1, TBC1D10C, THBS1, TRPM2, TXNDC5, VNN1]	[ACTN1, ACTN4, ADCY1, ADGRE5, ALDOA, AMH, APOA1, ARF1, ARL8A, ARSA, ATP13A2, BRPF3, C5AR1, CACNA1H, CAPN1, CBARP, CD14, CD177, CEACAM1, CHGA, CHI3L1, CLEC12A, CLNK, CNR1, COMMD3, CPLX1, CRAT, CTSA, CTSD, CYB5R3, DMTN, DOC2B, DOK3, DSC1, ECM1, FES, FOXF1, FPR1, GAB2, GALNS, GATA2, GIT1, GNAI2, GPI, GPR84, GRIK5, GRN, GTPBP2, HBB, HFE, HGS, HTR1B, HTR2A, HVCN1, IL4R, IMPDH1, IQGAP2, JUP, KNG1, LGALS3BP, LGALS9, LRG1, LTF, METTL7A, MME, MYH9, NAPA, NBEAL2, NHLRC3, NKD2, NOTCH1, NPPA, NR4A3, OTOF, PCDHA4, PDGFB, PIP5K1C, PKM, PLAU, PLAUR, PRKCSH, PRRT2, PTGDS, RAB11B, RAB11FIP5, RAB15, RAB3A, RAB3IL1, RAB5B, RAC2, RAP1A, RHOG, RNASE2, RNU1-93P, S100A11, SELENOP, SEPTIN4, SEPTIN5, SEPTIN9, SERPINA3, SERPINE1, SERPING1, SIGLEC9, SLC44A2, SLC7A8, SLCO4C1, SPHK2, SPI1, STING1, STK11IP, STX1A, STX1B, SV2A, SYN1, SYT2, SYT7, TCIRG1, THBS1, TIMP1, TMEM179B, TMEM63A, TNFAIP2, TRAPPC1, TSPAN14, TUBA4A, VEGFA, VNN1]

GO:0022407	regulation of cell-cell adhesion	2,49E-25	[4, 5]	155,00	33,70	60,59	39,41	[ADAM8, AIF1, AKNA, ALOX15, BLK, BMP7, BTLA, C1QTNF1, CARD11, CCDC88B, CCL19, CCL2, CCL5, CCR2, CD27, CD3E, CD4, CD40LG, CD5, CD6, CD74, CD80, CD86, CDH1, CDKN2A, CORO1A, CRTAM, CTLA4, DOCK8, EBI3, EGR3, EPX, FGL2, FOXP3, FUT7, GRAP2, HLA-DMB, HLA-DPA1, HLA-DPB1, HLA-DRA, HLA-DRB1, ICOS, IFNG, IL12RB1, IL18, IL7R, ITGA4, ITGB2, KLRK1, LAG3, LAX1, LCK, LEF1, LILRB1, LILRB4, MYB, NCKAP1L, NLRP3, NR4A3, PLA2G2D, PRKCQ, PTAFR, PTPN22, PTPN6, PTPRC, PYCARD, RASAL3, RHOH, RUNX3, SASH3, SELE, SIRPG, SKAP1, SOCS1, SPN, SYK, TBX21, TESPA1, THY1, TIGIT, TNFAIP8L2, TNFSF13B, TNFSF14, TNR, VAV1, VNN1, WNT1, XCL1, ZAP70]	[ABL1, ADAM19, ADIPOQ, AGER, AP3D1, APOA1, BCL6, BTLA, C1QTNF1, CCL2, CCL21, CCR7, CD276, CD74, CEACAM1, CELSR2, CITED2, CSK, CX3CL1, DLG5, DMTN, DTX1, ERBB2, FADD, FLOT2, FXYD5, GNAO1, GPAM, HFE, ICAM1, IL1B, IL1RN, IL4R, IL7R, JAK3, LAG3, LGALS9, LRFN3, LRRC32, MAPK7, MDGA1, MINK1, MYADM, NF2, NOD2, NODAL, NOTCH1, NOTCH4, NR4A3, NRARP, PDCD1, PIEZO1, PLAUR, PTPN23, PTPRU, RARA, RELA, RGCC, SELE, SELENOP, SH2B3, SLC7A1, SOX12, SOX13, SPI1, SRC, TIMP1, TNF, TRPV4, VEGFA, VNN1, VSIR, ZBTB7B, ZC3H12A, ZMIZ1]
GO:0031347	regulation of defense response	9,84E-09	[4, 5]	197,00	21,91	56,44	43,56	[ACP5, ADAM8, ADCY7, AIM2, AKNA, ALOX15, AOAH, APOBEC3G, BTK, C1QTNF1, CALCR, CARD11, CASP8, CCL24, CCL3, CCL5, CCR2, CD200R1, CD226, CD247, CD3G, CD96, CLEC10A, CLEC4D, CLEC4E, CLEC6A, CLNK, CNR1, CNR2, CRTAM, CST7, CXCL6, CXorf21, CYSLTR1, DPEP1, DRD1, EREG, FCN1, FFAR4, FGL2, FGR, FOXP3, FUT7, GBP5, GPR150, GPR4, GPR83, GPSM3, HLA-DRB1, IFNG, IKBKE, IL12RB1, IL16, IL18, IL18RAP, KLRC1, KLRC2, KLRD1, KLRK1, LAG3, LCP1, LDLR, LILRB1, LTA, MAPK13, MMP9, MUC16, MUC19, MUC6, NCR1, NCR3, NLRC3, NLRP3, NLRP6, PIK3CG, PLA2G2D, PLA2G7, PTGDR, PTGER2, PTPN22, PTPN6, PTPRC, PYCARD, PYHIN1, RLN2, SELE, SH2D1A, SIGLEC10, SIGLEC16, SLAMF6, SLAMF8, SMPDL3B, SOCS1, SOCS3, STAP1, STAT1, SUCNR1, SYK, TLR10, TLR8, TNFAIP3, TNFAIP8L2, TREM2, VAV1, XCL1, ZBP1]	[ADCY1, ADCY3, ADCY4, ADCY9, ADIPOQ, AGER, AGT, AGTR1, APOA1, BCL6, BRD4, C1QTNF1, CALCR, CCL24, CCR7, CEACAM1, CGA, CHGA, CLEC4E, CLNK, CNR1, CRHR2, CST7, CX3CL1, DAGLA, ENPP3, FABP4, FADD, FEM1A, FFAR2, FOXF1, FURIN, GBA, GIT1, GNAI2, GPBAR1, GPER1, GPR20, GPR4, GPR83, GPR84, GRN, HRH2, IL1B, LAG3, LDLR, LGALS9, LILRA5, MAPK3, MAPK7, MAVS, MUC19, MUC20, MUC3A, MYH9, NECTIN2, NINJ1, NLRC5, NOD2, NPAS2, NPPA, NR1D1, OSM, PCDHA4, PCDHA6, PLA2G2A, PLA2G7, PPARD, PQBP1, PRKACA, PRKAR2B, PTGES, PTGIR, PVR, RAMP2, RBM14, RELA, RELB, RHBDF2, RNF26, RNU1-93P, SBNO2, SELE, SERPINE1, SERPING1, SHANK3, SHARPIN, SMAD3, SOCS3, SPATA2, SPHK1, SPI1, SRC, STING1, TGM2, TNF, TNFRSF1A, TNIP1, TRIM62, TRPV4, VIPR2, ZC3H12A, ZCCHC3, ZDHHC11, ZFP36, ZYX]
GO:0031349	positive regulation of defense response	6,95E-06	[3, 4, 5, 6]	96,00	24,87	57,84	42,16	[ADAM8, AIM2, BTK, CARD11, CCL24, CCL3, CCL5, CCR2, CD226, CLEC10A, CLEC4D, CLEC4E, CLEC6A, CLNK, CNR1, CRTAM, CXorf21, EREG, FCN1, GBP5, GPR4, GPSM3, IFNG, IKBKE, IL16, IL18, IL18RAP, KLRC2, KLRD1, KLRK1, LAG3, LCP1, LDLR, LTA, MAPK13, MUC16, MUC19, MUC6, NCR3, PIK3CG, PLA2G7, PYCARD, PYHIN1, SH2D1A, SIGLEC16, SLAMF6, STAP1, SUCNR1, SYK, TLR10, TLR8, TREM2, VAV1, ZBP1]	[AGT, AGTR1, CCL24, CCR7, CLEC4E, CLNK, CNR1, CX3CL1, FABP4, FADD, FEM1A, FFAR2, GPR4, GRN, IL1B, LAG3, LDLR, LILRA5, MAPK3, MAVS, MUC19, MUC20, MUC3A, NECTIN2, NINJ1, NLRC5, NOD2, NPAS2, NPPA, OSM, PCDHA4, PLA2G2A, PLA2G7, PQBP1, PRKACA, PVR, RBM14, RELA, RELB, RNU1-93P, SERPINE1, SPI1, SRC, STING1, TGM2, TNF, TNFRSF1A, TNIP1, TRIM62, TRPV4, ZCCHC3]
GO:0032609	interferon-gamma production	4,81E-07	[3, 6]	43,00	37,07	71,47	28,53	[BCL3, BTN3A1, BTN3A2, CCR2, CD2, CD226, CD244, CD3E, CD96, CRTAM, EBI3, FOXP3, HLA-DPA1, HLA-DPB1, HLA-DRB1, IL12RB1, IL12RB2, IL18, IRF8, LILRB1, LILRB4, LTA, NLRP6, PTPN22, PYCARD, SASH3, SLAMF6, TLR8, XCL1]	[ABL1, BCL3, CCR7, CD14, CD276, FADD, IL1B, INHA, LGALS9, NOD2, RARA, SLC7A5, TNF, VSIR, ZC3H12A]
GO:0032633	interleukin-4 production	1,24E-02	[3, 6]	15,00	46,88	83,83	16,17	[CD3E, CD40LG, CD86, EPX, FOXP3, HLA-DRB1, IRF4, LEF1, NLRP3, PRKCQ, SASH3, SYK]	[LGALS9, RARA, SLC7A5]
GO:0036230	granulocyte activation	1,49E-03	[4, 5]	119,00	21,21	56,44	43,56	[ADAM8, ADGRE3, ANPEP, ARHGAP45, BIN2, CCL3, CCL5, CCR2, CD177, CD33, CD53, CEACAM21, CFP, CLEC12A, CLEC4D, COTL1, CTSS, CXCL6, CYBB, DOCK2, EPX, FCN1, FGL2, FGR, FRMPD3, GMFG, IL18, IL18RAP, IQGAP2, ITGAL, ITGAX, ITGB2, LAIR1, LRMP, LYZ, MMP9, MPO, NCKAP1L, NFAM1, ORM2, PLAC8, PLAU, PRAM1, PTAFR, PTPN6, PTPRC, PTX3, PYCARD, RAB37, RAB44, RNASET2, SCNN1B, SELL, SERPINA1, SLC18A1, SLC27A2, STXBP2, SYK, TBC1D10C, TRPM2, TXNDC5, VNN1]	[ADGRE5, ALDOA, AMH, ARL8A, ARSA, C5AR1, CAPN1, CD14, CD177, CEACAM1, CHI3L1, CLEC12A, COMMD3, CRAT, CTSA, CTSD, CYB5R3, DOK3, DSC1, ENPP3, FPR1, GALNS, GPI, GPR84, GRN, HBB, HFE, HVCN1, IMPDH1, IQGAP2, JUP, LRG1, LTF, METTL7A, MME, NBEAL2, NHLRC3, PKM, PLA2G2A, PLAU, PLAUR, PRKCSH, RAB3A, RAB5B, RAP1A, RHOG, RNASE2, RNU1-93P, S100A11, SERPINA3, SIGLEC9, SLC44A2, SLCO4C1, SPI1, STING1, STK11IP, TCIRG1, TMEM179B, TMEM63A, TRAPPC1, TSPAN14, VNN1]
GO:0042110	T cell activation	1,53E-25	[4, 5]	169,00	32,50	70,64	29,36	[ADAM8, AIF1, APBB1IP, BATF, BCL11B, BCL3, BTLA, BTN3A1, CAMK4, CARD11, CASP8, CCDC88B, CCL19, CCL2, CCL5, CCR2, CD1C, CD2, CD27, CD3D, CD3E, CD3G, CD4, CD40LG, CD5, CD6, CD7, CD74, CD80, CD86, CD8A, CD8B, CDKN2A, CLCF1, CORO1A, CRTAM, CTLA4, DOCK2, DOCK8, EBI3, EGR3, EOMES, EPX, FGL2, FOXP3, FUT7, GRAP2, HLA-DMB, HLA-DOA, HLA-DPA1, HLA-DPB1, HLA-DRA, HLA-DRB1, ICOS, IFNG, IL12RB1, IL18, IL7R, IRF4, ITGAL, ITK, JAML, KLRC1, KLRK1, LAG3, LAX1, LCK, LCP1, LEF1, LILRB1, LILRB4, LMO1, LY9, MYB, NCKAP1L, NLRC3, NLRP3, PIK3CG, PLA2G2D, PRDM1, PRKCQ, PTPN22, PTPN6, PTPRC, PYCARD, RAC2, RASAL3, RHOH, RUNX3, SASH3, SEMA4A, SIRPG, SIT1, SLA2, SLAMF6, SOCS1, SPN, SYK, TBX21, TESPA1, THEMIS, THY1, TIGIT, TNFAIP8L2, TNFRSF18, TNFSF13B, TNFSF14, TNFSF8, TOX, VAV1, VNN1, WAS, WDFY4, WNT1, XCL1, ZAP70, ZNF683]	[ABL1, AGER, AP3D1, ARMC5, BCL3, BCL6, BTLA, CCL2, CCL21, CCR7, CD1C, CD276, CD74, CEACAM1, CLC, CLCF1, CLPTM1, CSK, CYP26B1, DLG5, DTX1, ELF4, ERBB2, FADD, FLOT2, GBA, GNAO1, GPAM, HFE, ICAM1, IDO1, IL1B, IL4R, IL7R, JAK3, KDELR1, LAG3, LGALS9, LRRC32, MSN, MYH9, NOD2, NRARP, PDCD1, RAC2, RARA, RELB, SLC7A1, SMAD3, SOX12, SOX13, SPI1, SRC, SRF, STAT3, TCIRG1, TIMP1, VNN1, VSIR, WAS, ZBTB7B, ZC3H12A, ZMIZ1]
GO:0046651	lymphocyte proliferation	2,19E-11	[4, 5]	93,00	30,69	73,69	26,31	[AIF1, BLK, BTK, BTN3A1, CARD11, CCDC88B, CCL19, CCL5, CCR2, CD180, CD19, CD22, CD3E, CD40LG, CD6, CD74, CD79A, CD80, CD86, CDKN2A, CLCF1, CORO1A, CR2, CRTAM, CTLA4, DOCK2, EBI3, EPX, FCRL3, FLT3, FOXP3, GAPT, HLA-DMB, HLA-DPA1, HLA-DPB1, HLA-DRB1, IKZF3, IL12RB1, IL18, IL7R, INPP5D, LEF1, LILRB1, LILRB4, LMO1, LST1, MS4A1, MZB1, NCKAP1L, PIK3CG, PLA2G2D, PRKCQ, PTPN22, PTPN6, PTPRC, PYCARD, RAC2, RASAL3, SASH3, SPN, SYK, TNFRSF13B, TNFSF13B, TNFSF14, TNFSF8, XCL1, ZAP70]	[ABL1, AGER, AMH, ARMC5, BCL6, CD276, CD74, CDKN1A, CLC, CLCF1, DLG5, ELF4, ERBB2, FADD, FLT3, GPAM, HPRT1, IDO1, IL1B, IL7R, JAK3, LGALS9, LRRC32, MSN, RAC2, SLC7A1, TICAM1, TIMP1, VSIR, ZBTB7B, ZNF335]

GO:0048534	hematopoietic or lymphoid organ development	4,52E-16	[3, 4, 5, 6]	241,00	23,86	58,71	41,29	[ADAM8, ANGPT1, BATF, BCL11B, BCL3, BLK, BLNK, BTK, C1QC, CALCR, CAMK4, CARD11, CASP8, CCL19, CCL3, CCR2, CD19, CD2, CD27, CD3D, CD3E, CD3G, CD4, CD40LG, CD74, CD79A, CD80, CD86, CD8A, CDKN2A, CLCF1, CR2, CRTAM, CSF1R, CTLA4, DOCK10, DOCK2, EGR3, EOMES, EPX, EVI2B, FCRL3, FGL2, FLT3, FOXP3, FUT7, GFI1, GPR171, GPR55, H3C10, H3C11, H3C12, H3C7, HCLS1, HLA-DOA, HLA-DRA, HLA-DRB1, IFNG, IKZF1, IKZF3, IL12RB1, IL18, IL7R, INPP5D, IRF4, IRF8, ITGA4, ITK, KLRC1, LAG3, LCK, LEF1, LILRB1, LILRB4, LMO1, LRRC17, LTA, LTB, LY9, MIXL1, MMP9, MS4A1, MT1G, MYB, NCKAP1L, NFAM1, NLRP3, NR4A3, PIM1, PLA2G2D, PLD4, PLEK, POU2AF1, POU2F2, PRDM1, PRKCB, PRKCQ, PTK2B, PTPN22, PTPN6, PTPRC, RHEX, RHOH, RTKN2, RUNX3, SASH3, SELPLG, SEMA4A, SLAMF6, SLAMF8, SLC8A3, SMPD3, SNX10, SOCS1, SPN, STAT1, SYK, TBX21, TESPA1, THBS1, THEMIS, TNFRSF13B, TNFRSF18, TNFSF13B, TNFSF8, TOX, TREM2, TRPM2, UBD, VAV1, VNN1, WNT1, WNT10B, ZAP70, ZNF683]	[ABL1, ACTN1, ADIPOQ, AGER, ANGPT1, AP3D1, ARMC5, ARTN, BATF2, BCL3, BCL6, CALCR, CCR7, CD74, CDK2, CDKN1A, CEACAM1, CITED2, CLCF1, CLPTM1, CRIP2, CSF1, CTC1, CYP26B1, DHRS2, DMTN, DPF2, DTX1, EPHA2, ERBB2, ETV6, FADD, FASN, FES, FLT3, FLT4, GAB2, GATA2, GBA, GNAO1, GPR137, H3C10, H3C12, H4C3, HDAC5, HSF1, IL15RA, IL1B, IL4R, IL7R, INHA, JAK3, JUNB, KCP, KDELR1, KLF10, KMT2D, LAG3, LGALS9, LOX, LRRC17, LRRC8A, LTBR, LTF, MAPK3, MFAP5, MKNK2, MOV10, MYC, MYH9, MYL9, NBEAL2, NCOA6, NFE2, NFKB2, NOTCH1, NOTCH4, NR4A3, NRARP, OSM, PAF1, PDGFB, PIM1, PML, PTN, RARA, RELB, RMRP, RNU1-93P, RTKN2, RXRA, SBNO2, SETD1A, SH2B3, SIPA1L3, SNX10, SOX12, SOX13, SPI1, SPNS2, SRC, SRF, SSBP3, STAT3, SYVN1, TCF3, TCF7L1, TCIRG1, TFE3, THBS1, THPO, TIMP1, TNF, TRIB1, VEGFA, VNN1, VSIR, ZBTB7A, ZBTB7B, ZC3H12A, ZFP36, ZMIZ1, ZNF385A]
GO:0050921	positive regulation of chemotaxis	8,46E-04	[3, 4, 5, 6]	45,00	29,22	54,46	45,54	[AIF1, CCL19, CCL3, CCL4, CCL5, CCR2, CCR4, CD74, CSF1R, CXCL10, CXCR4, EDN2, GPSM3, IL16, NCKAP1L, P2RY12, PLA2G7, PTK2B, RAC2, SUCNR1, THBS1, TREM2, XCL1, XCL2]	[AGER, ARTN, C5AR1, CCL21, CCR7, CD74, CSF1, EDN2, HSPB1, LGALS9, MAPK3, MEGF8, MEN1, MSTN, PDGFB, PDGFRB, PLA2G7, PTN, RAC2, SERPINE1, SMAD3, SPI1, THBS1, TRPV4, VEGFA, ZNF580]
GO:0060326	cell chemotaxis	4,95E-10	[4, 5]	97,00	28,96	61,02	38,98	[ADAM8, AIF1, BIN2, CCL17, CCL19, CCL2, CCL22, CCL24, CCL3, CCL3L1, CCL4, CCL4L1, CCL4L2, CCL5, CCR2, CCR3, CCR4, CCR5, CCR8, CD74, CNR2, CORO1A, CSF1R, CXCL10, CXCL11, CXCL5, CXCL6, CXCL9, CXCR3, CXCR4, CXCR6, DPEP1, EDN2, EGR3, FOLR2, GPSM3, IL16, ITGB2, JAML, KLF5, KLRK1, LEF1, NCKAP1L, NR4A1, PIK3CG, PLA2G7, PRKCQ, PTK2B, RAC2, SLAMF8, STAP1, SYK, THBS1, TRPM2, VAV1, XCL1, XCL2, XCR1]	[ACKR3, AGTR1, AMH, ARHGEF16, BCAR1, C5, C5AR1, CCL11, CCL2, CCL21, CCL24, CCR7, CD74, CHGA, CSF1, CX3CL1, DHX34, EDN2, EPHA2, FFAR2, GBF1, HBEGF, HSPB1, KLF5, LGALS9, LOX, MAPK3, MEN1, MSTN, NINJ1, NOD2, NOTCH1, NR4A1, PCDHA6, PDGFB, PDGFRB, PIP5K1C, PLA2G7, PTN, PTPRU, RAC2, RHOG, SERPINE1, SPI1, THBS1, TRPV4, VEGFA, ZNF580]
GO:0070663	regulation of leukocyte proliferation	1,96E-10	[4, 5]	82,00	31,54	71,80	28,20	[AIF1, BLK, BTK, CARD11, CCDC88B, CCL19, CCL5, CCR2, CD22, CD3E, CD40LG, CD6, CD74, CD80, CD86, CDKN2A, CLCF1, CORO1A, CRTAM, CSF1R, CTLA4, EBI3, EPX, FCRL3, FOXP3, HLA-DMB, HLA-DPA1, HLA-DPB1, HLA-DRB1, IKZF3, IL12RB1, IL18, IL5RA, INPP5D, LILRB1, LILRB4, LMO1, LST1, MZB1, NCKAP1L, PLA2G2D, PRKCQ, PTPN22, PTPN6, PTPRC, PYCARD, RAC2, RASAL3, SASH3, SPN, SYK, TNFAIP3, TNFRSF13B, TNFSF13B, TNFSF8, XCL1, ZAP70]	[AGER, AMH, BCL6, CD276, CD74, CDKN1A, CLC, CLCF1, CRP, CSF1, DLG5, ENPP3, ERBB2, FADD, GPAM, IDO1, IL1B, IL5RA, JAK3, LGALS9, LRRC32, MAPK3, RAC2, SLC7A1, TICAM1, TIMP1, VSIR, ZBTB7B, ZNF335]
GO:0030595	leukocyte chemotaxis	6,15E-08	[3, 5, 6]	75,00	29,76	62,85	37,15	[ADAM8, AIF1, CCL17, CCL19, CCL2, CCL22, CCL24, CCL3, CCL3L1, CCL4, CCL4L1, CCL5, CCR2, CCR5, CD74, CNR2, CORO1A, CSF1R, CXCL10, CXCL11, CXCL5, CXCL6, CXCL9, CXCR3, CXCR4, DPEP1, EDN2, FOLR2, GPSM3, IL16, ITGB2, JAML, KLF5, KLRK1, NCKAP1L, PIK3CG, PLA2G7, PTK2B, RAC2, SLAMF8, STAP1, SYK, THBS1, TRPM2, VAV1, XCL1, XCL2]	[AMH, C5, C5AR1, CCL11, CCL2, CCL21, CCL24, CCR7, CD74, CHGA, CSF1, CX3CL1, DHX34, EDN2, FFAR2, GBF1, KLF5, LGALS9, MAPK3, MEN1, MSTN, NINJ1, NOD2, PCDHA6, PDGFB, PIP5K1C, PLA2G7, PTN, PTPRU, RAC2, SERPINE1, SPI1, THBS1, TRPV4, VEGFA, ZNF580]
GO:0001819	positive regulation of cytokine production	2,05E-13	[3, 4, 5, 6, 7]	142,00	27,26	64,89	35,11	[ADAM8, AIF1, AIM2, ALOX15B, BATF, BCL3, BTN3A1, BTN3A2, CAMK4, CARD11, CASP8, CCDC88B, CCL19, CCL3, CCR2, CD2, CD226, CD244, CD3E, CD4, CD40LG, CD6, CD74, CD80, CD86, CLEC6A, CLNK, CRTAM, CSF1R, CYBB, EBI3, EPX, EREG, FCN1, FGR, FOXP3, GBP5, GPSM3, HLA-DPA1, HLA-DPB1, IFNG, IL12RB1, IL12RB2, IL16, IL18, IL26, IRF4, IRF5, IRF8, ITK, LCP1, LILRB1, LPAL2, LTA, LTB, LY9, MAPK13, MCOLN2, MYB, NFAM1, NLRP2, NLRP3, NLRP9, NR4A3, ORM2, PIK3CG, POU2AF1, POU2F2, PRKCQ, PTAFR, PTPN22, PTPRC, PYCARD, PYHIN1, SASH3, SCIMP, SIGLEC16, SLAMF6, STAT1, SYK, THBS1, TIGIT, TLR6, TLR8, TREM2, XCL1, ZBP1]	[ABCC8, ABL1, ADIPOQ, AGER, AGPAT1, AGT, AMH, BCL3, C5, C5AR1, CCR7, CD14, CD276, CD74, CHI3L1, CLNK, CX3CL1, FADD, FFAR2, FLT4, FRMD8, FURIN, GATA4, HK1, HSPB1, IL1B, IL4R, LGALS9, LILRA5, LUM, MAVS, NFATC4, NFKB2, NLRP9, NOD2, NODAL, NR4A3, OSM, POLR2E, POLR2L, POSTN, PQBP1, RARA, RELA, RGCC, RNU1-93P, SERPINE1, SLC7A5, SMAD3, SPHK1, SPHK2, SRC, STAT3, STING1, THBS1, TICAM1, TNF, TRPV4, ZBTB7B, ZCCHC3, ZNF580]
GO:0001910	regulation of leukocyte mediated cytotoxicity	1,72E-06	[4, 5, 6]	34,00	40,96	73,73	26,27	[CD1B, CD1C, CD1E, CD226, CRTAM, CXCL6, HLA-DRA, HLA-DRB1, IL12RB1, IL18RAP, IL7R, KLRC1, KLRC2, KLRD1, KLRK1, LAG3, LILRB1, NCKAP1L, NCR1, NCR3, PTPRC, SH2D1A, SLAMF6, STAP1, VAV1, XCL1]	[AGER, CD1C, CD1E, CEACAM1, FADD, ICAM1, IL7R, LAG3, LGALS9, NECTIN2, PVR, SPI1]
GO:0001913	T cell mediated cytotoxicity	2,58E-04	[4, 5, 6]	23,00	43,40	67,46	32,54	[CD1B, CD1C, CD1E, CRTAM, GZMM, HLA-DRA, HLA-DRB1, IL12RB1, IL7R, KLRC1, KLRD1, LILRB1, NCKAP1L, PRF1, PTPRC, XCL1]	[AGER, CD1C, CD1E, CEACAM1, FADD, HPRT1, IL7R, NECTIN2, PVR, ZNF395]
GO:0002237	response to molecule of bacterial origin	4,09E-07	[4, 6]	98,00	25,86	54,54	45,46	[ACP5, CARD17, CASP8, CCL2, CCL3, CCL5, CCR5, CD180, CD6, CD80, CD86, CD96, CNR1, CNR2, CXCL10, CXCL11, CXCL5, CXCL6, CXCL9, EPX, FASLG, FOXP3, GFI1, IL10RA, IL12RB2, IL18, IRF5, IRF8, KMO, LILRB1, LTA, LY86, MPO, MRC1, NLRP3, NLRP7, NPPC, NR4A1, NUGGC, PTAFR, PTGFR, PTPN22, PYCARD, SCIMP, SELE, STAP1, TLR6, TNFAIP3, TNIP3, TREM2]	[ABCC8, ABL1, ALPL, C5AR1, CCL2, CCR7, CD14, CNR1, CX3CL1, CYP21A2, CYP27B1, DAB2IP, GIT1, HSF1, ICAM1, IL18BP, IL1B, IRAK2, JUND, KCTD11, LDOC1, LGALS9, LTF, MAPK3, MRC1, NFKB2, NFKBIB, NOD2, NOS3, NOTCH1, NR1D1, NR4A1, PAF1, PCDHA4, PCDHA6, PPARD, PTGES, PTGFR, PTGIR, RARA, RELA, SBNO2, SELE, SELENOP, SERPINE1, SPI1, SRC, TICAM1, TIMP1, TMEM256-PLSCR3, TNF, TRIB1, ZC3H12A, ZFP36]

GO:0002446	neutrophil mediated immunity	2,14E-02	[5]	113,00	20,32	55,60	44,40	[ADAM8, ADGRE3, ANPEP, ARHGAP45, BIN2, CD177, CD33, CD53, CEACAM21, CFP, CLEC12A, CLEC4D, COTL1, CTSS, CXCL6, CYBB, DOCK2, EPX, FCN1, FGL2, FGR, FRMPD3, GMFG, IQGAP2, ITGAL, ITGAX, ITGB2, LAIR1, LRMP, LYZ, MMP9, MPO, NCKAP1L, NFAM1, NLRP6, ORM2, PLAC8, PLAU, PRAM1, PTAFR, PTPN6, PTPRC, PTX3, PYCARD, RAB37, RAB44, RNASET2, SCNN1B, SELL, SERPINA1, SLC18A1, SLC27A2, STXBP2, SYK, TBC1D10C, TRPM2, TXNDC5, VNN1]	[ADGRE5, ALDOA, AMH, ARL8A, ARSA, C5AR1, CAPN1, CD14, CD177, CEACAM1, CHI3L1, CLEC12A, COMMD3, CRAT, CTSA, CTSD, CYB5R3, DOK3, DSC1, FPR1, GALNS, GPI, GPR84, GRN, HBB, HFE, HVCN1, IMPDH1, IQGAP2, JUP, LRG1, LTF, METTL7A, MME, NBEAL2, NHLRC3, PKM, PLAU, PLAUR, PRKCSH, RAB3A, RAB5B, RAP1A, RHOG, RNASE2, RNU1-93P, S100A11, SERPINA3, SIGLEC9, SLC44A2, SLCO4C1, SPI1, STING1, STK11IP, TCIRG1, TMEM179B, TMEM63A, TRAPPC1, TSPAN14, VNN1]
GO:0002456	T cell mediated immunity	8,65E-06	[5]	41,00	35,04	68,85	31,15	[BTN3A2, CCR2, CD1B, CD1C, CD1E, CD8A, CLCF1, CRTAM, FOXP3, FUT7, GZMM, HLA-DRA, HLA-DRB1, IL12RB1, IL18, IL7R, KLRC1, KLRD1, LILRB1, LILRB4, MYO1G, NCKAP1L, NLRP3, PRF1, PTPRC, SASH3, TBX21, WAS, XCL1]	[AGER, CD1C, CD1E, CEACAM1, CLC, CLCF1, FADD, HFE, HPRT1, ICAM1, IL1B, IL7R, NECTIN2, NOD2, PVR, WAS, ZNF395]
GO:0002685	regulation of leukocyte migration	6,64E-08	[3, 4, 5, 6, 7]	69,00	30,67	58,94	41,06	[ADAM8, AIF1, CCL19, CCL2, CCL24, CCL3, CCL4, CCL5, CCR2, CD200R1, CD74, CSF1R, CXCL10, CXCR3, DOCK8, EDN2, FUT7, GPSM3, ITGA4, KLRK1, NCKAP1L, P2RY12, PLA2G7, PTAFR, PTK2B, PTPN22, PYCARD, RAC2, RHOH, SELE, SLAMF8, SMPD3, SPN, STAP1, THBS1, THY1, TNFRSF18, TREM2, XCL1, XCL2, XG]	[AGER, AMH, APOD, C5, C5AR1, CCL2, CCL21, CCL24, CCR7, CD74, CSF1, CX3CL1, ECM1, EDN2, FADD, GCSAM, ICAM1, LGALS9, MAPK3, MSN, MSTN, NOD2, PLA2G7, PLVAP, PTN, RAC2, RHOG, SELE, SELENOP, SERPINE1, SPI1, THBS1, TNF, TRPV4, VEGFA, XG, ZNF580]
GO:0002704	negative regulation of leukocyte mediated immunity	1,27E-02	[4, 5, 6]	23,00	35,94	68,35	31,65	[CCR2, CD84, CD96, CR2, FOXP3, IL7R, KLRC1, KLRD1, LILRB1, LILRB4, NCKAP1L, PTPN6, PTPRC, TBX21, XCL1]	[BCL6, CEACAM1, FOXF1, HFE, IL7R, JAK3, LGALS9, NOD2, SPI1]
GO:0002705	positive regulation of leukocyte mediated immunity	1,59E-04	[4, 5, 6]	44,00	31,43	75,14	24,86	[BTK, CAMK4, CD177, CD1B, CD1C, CD1E, CD226, CLCF1, CLNK, CRTAM, FOXP3, HLA-DRA, HLA-DRB1, IL12RB1, IL18, IL18RAP, ITGB2, KLRC2, KLRD1, KLRK1, LAG3, LTA, NCR3, NLRP3, PTAFR, PTPRC, SASH3, SCIMP, SH2D1A, SLAMF6, STAP1, TBX21, TREM2, VAV1, XCL1]	[CD177, CD1C, CD1E, CLCF1, CLNK, FADD, IL1B, LAG3, MAVS, NECTIN2, NOD2, PVR, SPI1, TICAM1, TNF]
GO:0002820	negative regulation of adaptive immune response	1,38E-02	[4, 5, 6]	22,00	36,67	66,84	33,16	[ALOX15, CR2, FOXP3, IL7R, KLRC1, KLRD1, LILRB1, LILRB4, NCKAP1L, PTPN6, PTPRC, SAMSN1, TBX21, XCL1]	[BCL6, CEACAM1, HFE, IL4R, IL7R, JAK3, NOD2, ZBTB7B, ZC3H12A]
GO:0022408	negative regulation of cell-cell adhesion	2,36E-06	[4, 5, 6]	59,00	30,57	53,24	46,76	[AKNA, C1QTNF1, CD74, CD80, CD86, CDH1, CDKN2A, CRTAM, CTLA4, FGL2, FOXP3, HLA-DRB1, LAG3, LAX1, LILRB1, LILRB4, NCKAP1L, PLA2G2D, PTPN22, PTPN6, RUNX3, SOCS1, SPN, TBX21, TIGIT, TNFAIP8L2, TNR, WNT1, XCL1]	[ABL1, ADIPOQ, APOA1, BCL6, C1QTNF1, CCL21, CD74, CEACAM1, DLG5, DTX1, ERBB2, FXYD5, HFE, IL1RN, IL4R, JAK3, LAG3, LGALS9, LRRC32, MAPK7, MYADM, NF2, NOTCH1, NOTCH4, NRARP, RGCC, SH2B3, SPI1, TRPV4, VEGFA, VSIR, ZBTB7B, ZC3H12A]
GO:0022409	positive regulation of cell-cell adhesion	4,07E-22	[4, 5, 6]	112,00	37,58	67,28	32,72	[ADAM8, AIF1, ALOX15, BMP7, BTLA, CARD11, CCDC88B, CCL19, CCL2, CCL5, CCR2, CD27, CD3E, CD4, CD40LG, CD5, CD6, CD74, CD80, CD86, CORO1A, CTLA4, DOCK8, EBI3, EGR3, EPX, FOXP3, FUT7, GRAP2, HLA-DMB, HLA-DPA1, HLA-DPB1, HLA-DRA, HLA-DRB1, ICOS, IFNG, IL12RB1, IL18, IL7R, ITGA4, ITGB2, KLRK1, LCK, LEF1, LILRB1, LILRB4, MYB, NCKAP1L, NLRP3, NR4A3, PRKCQ, PTAFR, PTPN22, PTPN6, PTPRC, PYCARD, RASAL3, RHOH, RUNX3, SASH3, SELE, SIRPG, SKAP1, SOCS1, SYK, TESPA1, THY1, TNFSF13B, TNFSF14, VAV1, VNN1, XCL1, ZAP70]	[ADAM19, AGER, AP3D1, BCL6, BTLA, CCL2, CCL21, CCR7, CD276, CD74, CITED2, CSK, CX3CL1, DMTN, FADD, FLOT2, GNAO1, GPAM, ICAM1, IL1B, IL4R, IL7R, JAK3, LGALS9, NOD2, NODAL, NR4A3, PDCD1, PIEZO1, PLAUR, PTPN23, PTPRU, RARA, RELA, SELE, SELENOP, SLC7A1, SOX12, SOX13, SRC, TIMP1, TNF, VNN1, VSIR, ZBTB7B, ZMIZ1]
GO:0030334	regulation of cell migration	2,89E-08	[4, 5, 6]	200,00	21,57	38,98	61,02	[ADAM8, ADAMTS9, ADORA3, AIF1, ALOX15B, ANGPT1, ANGPT2, BMP7, CARMIL2, CASS4, CCL19, CCL2, CCL24, CCL3, CCL4, CCL5, CCR2, CD200R1, CD74, CDH1, CORO1A, CSF1R, CXCL10, CXCR3, CXCR4, DOCK10, DOCK8, DPEP1, DRD1, EDN2, FGR, FUT7, GPSM3, IFNG, ITGA4, ITGAX, KLRK1, LEF1, MMP9, NCKAP1L, NELL2, NR4A3, NTN1, P2RY12, PIK3CG, PLA2G7, PLAU, PLXNC1, PTAFR, PTK2B, PTPN22, PTPRC, PYCARD, RAC2, RHOH, SELE, SEMA4A, SEMA4D, SLAMF8, SMPD3, SPN, STAP1, TACSTD2, TCAF2, THBS1, THY1, TNFRSF18, TREM2, XCL1, XCL2, XG]	[ABCC8, ABI3, ABL1, ACKR3, ACTN4, ADAMTS9, ADGRG1, ADIPOQ, AGER, AGT, AMH, ANGPT1, APOD, ARHGDIA, ATP1B2, BCAR1, C5, C5AR1, CAMK2B, CAMSAP3, CARMIL2, CCL11, CCL2, CCL21, CCL24, CCR7, CD74, CEACAM1, CGA, CHGA, CITED2, COL1A1, CSF1, CX3CL1, DAB2IP, DAG1, DLG5, DMTN, ECM1, EDN2, ENG, EPHA2, EPPK1, FADD, FAM110A, FLT4, FOXC2, FOXF1, GATA2, GCSAM, GNAI2, GPER1, GPI, GRB7, GRN, HBEGF, HDAC5, HDAC7, HSPB1, HYAL1, ICAM1, IFITM1, IL1B, ILK, ITGA3, JUP, LAMA5, LGALS9, LGR6, LMNA, MAP2K3, MAPK3, MAZ, MECP2, MIEN1, MINK1, MSN, MSTN, MYADM, MYC, NF2, NGFR, NOD2, NODAL, NOS3, NOTCH1, NR4A3, NSMF, NTN1, OSGIN1, P2RY6, PDGFB, PDGFRB, PFN1, PHACTR1, PLA2G7, PLAU, PLVAP, PLXNA1, PLXNA3, PLXNB1, POSTN, PPARD, PTN, PTPN23, PTPRU, RAC2, RGCC, RHOG, RIPOR1, ROBO4, S100A11, SELE, SELENOP, SEMA3F, SEMA4B, SEMA4C, SEMA6B, SEMA6C, SERPINE1, SH3BP1, SLC9A3R1, SMAD3, SMIM22, SNAI1, SPHK1, SPI1, SRC, SRF, STAT3, SYDE1, TBX5, THBS1, TIMP1, TNF, TRIB1, TRPV4, VEGFA, VSIR, VTN, XG, ZC3H12A, ZNF580, ZNF609]
GO:0042088	T-helper 1 type immune response	4,62E-02	[5]	18,00	38,30	68,96	31,04	[BCL3, CCL19, CCR2, CD80, EBI3, HLA-DRB1, IL12RB1, IL18, LEF1, SEMA4A, SPN, XCL1]	[BCL3, GNAO1, IL18BP, IL1B, IL4R, JAK3, RELB]

GO:0045087	innate immune response	6,57E-06	[4, 5, 6]	215,00	20,02	67,24	32,76	[ADAM8, AIF1, AIM2, APOBEC3A, APOBEC3D, APOBEC3G, APOBEC3H, BLK, BTK, C1QA, C1QB, C1QC, C2, C8G, CARD11, CASP8, CCL17, CCL19, CCL2, CCL22, CCL24, CCL3, CCL3L1, CCL4, CCL4L1, CCL5, CD177, CD180, CD226, CD244, CD6, CD74, CD84, CD96, CFP, CLEC10A, CLEC4D, CLEC4E, CLEC6A, CLNK, CORO1A, CR2, CRTAM, CSF1R, CXCL10, CXorf21, CYBB, DAPK1, EREG, FASLG, FCN1, FCN3, FGR, GBP5, GBP6, GZMB, GZMM, HLA-DPA1, HLA-DPB1, HLA-DQA1, HLA-DRA, HLA-DRB1, HLA-DRB5, IFNG, IGLL5, IKBKE, IL12RB1, IL18, IL18RAP, IRF4, IRF5, IRF8, JCHAIN, KIR2DL3, KLRC1, KLRC2, KLRD1, KLRG1, KLRK1, LAG3, LCK, LILRB1, LY86, LY9, MCOLN2, MPEG1, MRC1, MUC16, MUC19, MUC6, NCF1, NCR1, NCR3, NLRC3, NLRP2, NLRP3, NLRP6, NLRP9, OASL, PIK3CG, PLD4, PRDM1, PSTPIP1, PTAFR, PTK2B, PTPN22, PTPN6, PTX3, PYCARD, PYHIN1, RNASE6, RNASET2, SH2D1A, SIGLEC10, SIGLEC16, SLA, SLAMF6, SLAMF7, SLAMF8, SMPDL3B, SOCS1, SOCS3, STAT1, STYK1, SYK, TLR10, TLR6, TLR8, TNFAIP3, TNFAIP8L2, TREM2, TRIM14, UBD, VAV1, VNN1, WAS, XCL1, XCL2, ZAP70, ZBP1, ZNF683]	[AGER, AMH, APOBEC3C, ARID5A, C5, C8G, CAMK2B, CCL11, CCL2, CCL21, CCL24, CD14, CD177, CD300E, CD74, CDC42EP2, CDC42EP4, CEACAM1, CFH, CHGA, CIITA, CLEC4E, CLNK, CRP, CSF1, CX3CL1, CYP27B1, DAB2IP, ELF4, FADD, FCN3, FES, FFAR2, GNAO1, GPER1, GRN, HFE, HK1, ICAM1, IFITM1, IFITM2, IFITM3, IRF2, IRF9, JAK3, LAG3, LGALS9, LILRA5, LTF, MAVS, MRC1, MUC19, MUC20, MUC3A, MYC, NECTIN2, NLRC5, NLRP9, NOD2, PLEKHM2, PML, PQBP1, PRKACA, PVR, RBM14, RELA, RELB, RNASE2, RNF19B, RNF39, RNU1-93P, SERPING1, SMPD1, SOCS3, SPI1, SRC, STING1, TBKBP1, TICAM1, TNK1, TNK2, TRIM29, TRIM62, TRIM8, VNN1, WAS, ZCCHC3, ZDHHC11, ZYX]
GO:0050727	regulation of inflammatory response	2,55E-07	[4, 5, 6]	132,00	23,61	51,21	48,79	[ACP5, ADAM8, ADCY7, AKNA, ALOX15, AOAH, BTK, C1QTNF1, CALCR, CCL24, CCL3, CCL5, CCR2, CD200R1, CD247, CD3G, CNR1, CNR2, CST7, CYSLTR1, DPEP1, DRD1, FFAR4, FGR, FOXP3, FUT7, GPR150, GPR4, GPR83, GPSM3, HLA-DRB1, IFNG, IL16, IL18, LCP1, LDLR, LTA, MAPK13, MMP9, NLRC3, NLRP3, NLRP6, PIK3CG, PLA2G2D, PLA2G7, PTGDR, PTGER2, PTPRC, PYCARD, RLN2, SELE, SIGLEC10, SLAMF8, SMPDL3B, SOCS3, STAP1, SUCNR1, SYK, TLR10, TNFAIP3, TNFAIP8L2, TREM2, XCL1, ZBP1]	[ADCY1, ADCY3, ADCY4, ADCY9, ADIPOQ, AGER, AGT, AGTR1, APOA1, BCL6, BRD4, C1QTNF1, CALCR, CCL24, CCR7, CGA, CHGA, CNR1, CRHR2, CST7, CX3CL1, DAGLA, ENPP3, FABP4, FEM1A, FFAR2, FOXF1, FURIN, GBA, GIT1, GNAI2, GPBAR1, GPER1, GPR20, GPR4, GPR83, GPR84, GRN, HRH2, IL1B, LDLR, LILRA5, MAPK7, MYH9, NINJ1, NOD2, NPPA, NR1D1, OSM, PCDHA4, PCDHA6, PLA2G2A, PLA2G7, PPARD, PRKACA, PRKAR2B, PTGES, PTGIR, RAMP2, RELA, RHBDF2, SBNO2, SELE, SERPINE1, SHARPIN, SMAD3, SOCS3, SPATA2, SPHK1, SRC, STING1, TGM2, TNF, TNFRSF1A, TNIP1, TRPV4, VIPR2, ZFP36, ZYX]
GO:0051249	regulation of lymphocyte activation	2,62E-15	[4, 5, 6]	154,00	27,60	71,79	28,21	[ADAM8, AIF1, BANK1, BLK, BTK, BTLA, CAMK4, CARD11, CCDC88B, CCL19, CCL2, CCL5, CCR2, CD19, CD2, CD22, CD27, CD3E, CD4, CD40LG, CD5, CD6, CD74, CD80, CD86, CDKN2A, CLCF1, CLNK, CORO1A, CRTAM, CTLA4, DOCK8, EBI3, EGR3, EPX, FCRL3, FGL2, FGR, FOXP3, GRAP2, HLA-DMB, HLA-DOA, HLA-DPA1, HLA-DPB1, HLA-DRA, HLA-DRB1, ICOS, IFNG, IGLL5, IKZF3, IL12RB1, IL18, IL7R, INPP5D, IRF4, KLRC2, KLRK1, LAG3, LAX1, LCK, LEF1, LILRB1, LILRB4, LMO1, LST1, MYB, MZB1, NCKAP1L, NFAM1, NLRP3, PLA2G2D, PRDM1, PRKCQ, PTPN22, PTPN6, PTPRC, PYCARD, RAC2, RASAL3, RHOH, RUNX3, SAMSN1, SASH3, SIRPG, SIT1, SLAMF8, SOCS1, SPN, SYK, TBC1D10C, TBX21, TESPA1, THEMIS2, THY1, TIGIT, TNFAIP3, TNFAIP8L2, TNFRSF13B, TNFRSF18, TNFSF13B, TNFSF14, TNFSF8, TOX, VAV1, VNN1, XCL1, ZAP70, ZNF683]	[ABL1, AGER, AMH, AP3D1, BCL6, BTLA, CCL2, CCL21, CCR7, CD276, CD74, CDKN1A, CEACAM1, CLC, CLCF1, CLNK, CLPTM1, CSK, CYP26B1, DCAF15, DLG5, DTX1, ERBB2, FADD, FLOT2, GNAO1, GPAM, HFE, IDO1, IL15RA, IL1B, IL4R, IL7R, INHA, JAK3, LAG3, LGALS9, LRRC32, NOD2, NRARP, PDCD1, RAC2, RARA, SLC7A1, SOX12, SOX13, SRC, TICAM1, TIMP1, VNN1, VSIR, ZBTB7B, ZC3H12A, ZMIZ1, ZNF335]
GO:0070665	positive regulation of leukocyte proliferation	9,99E-07	[4, 5, 6]	52,00	33,12	71,06	28,94	[AIF1, CARD11, CCDC88B, CCL19, CCL5, CCR2, CD3E, CD40LG, CD6, CD74, CD80, CD86, CLCF1, CORO1A, CSF1R, EBI3, EPX, FCRL3, FOXP3, HLA-DMB, HLA-DPA1, HLA-DPB1, IL12RB1, IL18, IL5RA, NCKAP1L, PRKCQ, PTPN22, PTPRC, PYCARD, RASAL3, SASH3, SYK, TNFSF13B, XCL1, ZAP70]	[AGER, AMH, BCL6, CD276, CD74, CDKN1A, CLCF1, CSF1, FADD, GPAM, IL1B, IL5RA, JAK3, LGALS9, MAPK3, SLC7A1, TICAM1, TIMP1, ZNF335]
GO:0032496	response to lipopolysaccharide	1,25E-07	[4, 5, 7]	96,00	26,67	53,53	46,47	[ACP5, CARD17, CASP8, CCL2, CCL3, CCL5, CCR5, CD180, CD6, CD80, CD86, CD96, CNR1, CNR2, CXCL10, CXCL11, CXCL5, CXCL6, CXCL9, EPX, FASLG, FOXP3, GFI1, IL10RA, IL12RB2, IL18, IRF8, KMO, LILRB1, LTA, LY86, MPO, MRC1, NLRP3, NLRP7, NPPC, NR4A1, NUGGC, PTAFR, PTGFR, PTPN22, PYCARD, SCIMP, SELE, STAP1, TNFAIP3, TNIP3, TREM2]	[ABCC8, ABL1, ALPL, C5AR1, CCL2, CCR7, CD14, CNR1, CX3CL1, CYP21A2, CYP27B1, DAB2IP, GIT1, HSF1, ICAM1, IL18BP, IL1B, IRAK2, JUND, KCTD11, LDOC1, LGALS9, LTF, MAPK3, MRC1, NFKB2, NFKBIB, NOD2, NOS3, NOTCH1, NR1D1, NR4A1, PAF1, PCDHA4, PCDHA6, PPARD, PTGES, PTGFR, PTGIR, RARA, RELA, SBNO2, SELE, SELENOP, SERPINE1, SPI1, SRC, TICAM1, TIMP1, TMEM256-PLSCR3, TNF, TRIB1, ZC3H12A, ZFP36]
GO:0001912	positive regulation of leukocyte mediated cytotoxicity	1,84E-04	[4, 5, 6, 7]	24,00	42,86	78,73	21,27	[CD1B, CD1C, CD1E, CD226, CRTAM, HLA-DRA, HLA-DRB1, IL12RB1, IL18RAP, KLRC2, KLRD1, KLRK1, LAG3, NCR3, PTPRC, SH2D1A, SLAMF6, STAP1, VAV1, XCL1]	[CD1C, CD1E, FADD, LAG3, NECTIN2, PVR, SPI1]
GO:0002286	T cell activation involved in immune response	4,84E-06	[5, 6]	41,00	35,65	73,52	26,48	[APBB1IP, BATF, BCL3, CCL19, CD1C, CD74, CD80, CD86, EOMES, FGL2, FOXP3, HLA-DMB, HLA-DRA, HLA-DRB1, IFNG, IL12RB1, IL18, IRF4, ITGAL, LCP1, LEF1, LILRB1, LY9, MYB, NCKAP1L, NLRP3, SEMA4A, SLAMF6, SPN, TBX21]	[BCL3, BCL6, CD1C, CD74, CEACAM1, ICAM1, IL4R, JAK3, LGALS9, RARA, RELB, STAT3, ZBTB7B, ZC3H12A]
GO:0002687	positive regulation of leukocyte migration	2,59E-07	[3, 4, 5, 6, 7, 8]	50,00	34,72	59,01	40,99	[ADAM8, AIF1, CCL19, CCL24, CCL3, CCL4, CCL5, CCR2, CD74, CSF1R, CXCL10, DOCK8, EDN2, GPSM3, ITGA4, NCKAP1L, P2RY12, PLA2G7, PTAFR, PTK2B, PYCARD, RAC2, SPN, THBS1, THY1, TNFRSF18, TREM2, XCL1, XCL2, XG]	[AGER, C5AR1, CCL21, CCL24, CCR7, CD74, CSF1, CX3CL1, EDN2, FADD, ICAM1, LGALS9, MAPK3, MSTN, PLA2G7, PLVAP, PTN, RAC2, SELENOP, SERPINE1, SPI1, THBS1, TNF, TRPV4, VEGFA, XG, ZNF580]
GO:0002706	regulation of lymphocyte mediated immunity	8,41E-06	[5, 6]	53,00	30,99	73,66	26,34	[BTK, CCR2, CD1B, CD1C, CD1E, CD226, CD96, CLCF1, CLNK, CR2, CRTAM, FOXP3, FUT7, HLA-DRA, HLA-DRB1, IL12RB1, IL18, IL18RAP, IL7R, KLRC1, KLRC2, KLRD1, KLRK1, LAG3, LILRB1, LILRB4, LTA, NCKAP1L, NCR1, NCR3, NLRP3, PTPN6, PTPRC, SASH3, SH2D1A, SLAMF6, TBX21, TREM2, VAV1, WAS, XCL1]	[AGER, BCL6, CD1C, CD1E, CEACAM1, CLC, CLCF1, CLNK, FADD, HFE, IL1B, IL7R, LAG3, LGALS9, NECTIN2, NOD2, PVR, TNF, WAS]
GO:0002718	regulation of cytokine production involved in immune response	2,27E-03	[4, 5, 6, 7]	34,00	32,08	57,70	42,30	[ANGPT1, BTK, CAMK4, CCR2, CD226, CD74, CD96, CLCF1, CLNK, FOXP3, IL18, LILRB1, LILRB4, LPAL2, NLRP3, NR4A3, SASH3, SCIMP, TBX21, XCL1]	[ANGPT1, APOA1, BCL6, CD74, CLC, CLCF1, CLNK, FFAR2, HFE, HK1, IL1B, JAK3, MAVS, NOD2, NR4A3, SLC7A5, SPHK2, TICAM1, TNF]

GO:0002822	regulation of adaptive immune response based on somatic recombination of immune receptors built from immunoglobulin superfamily domains	5,77E-05	[5, 6]	52,00	29,71	68,96	31,04	[BTK, CCL19, CCR2, CD1B, CD1C, CD1E, CD226, CD4, CD80, CLCF1, CLEC6A, CR2, FOXP3, FUT7, HLA-DRA, HLA-DRB1, IL12RB1, IL18, IL7R, KLRC1, KLRD1, LILRB1, LILRB4, LTA, NCKAP1L, NLRP3, PRKCQ, PTPN6, PTPRC, SASH3, TBX21, TNFAIP3, TNFSF13B, TREM2, WAS, XCL1]	[AGER, BCL6, CD1C, CD1E, CEACAM1, CLC, CLCF1, FADD, GNAO1, HFE, IL1B, IL4R, IL7R, JAK3, NECTIN2, NOD2, PVR, TNF, WAS, ZBTB7B, ZC3H12A]
GO:0002886	regulation of myeloid leukocyte mediated immunity	4,09E-05	[5, 6]	26,00	43,33	59,92	40,08	[BLK, BTK, CAMK4, CCR2, CD177, CD84, CXCL6, FGR, ITGB2, PRAM1, PTAFR, RAC2, STAP1, STXBP2, SYK]	[CD177, FES, FOXF1, GAB2, GATA2, IL4R, LGALS9, MAVS, NPPA, RAC2, SPHK2, SPI1, TICAM1]
GO:0030097	hemopoiesis	1,27E-16	[4, 5, 6, 7]	235,00	24,28	59,15	40,85	[ADAM8, ANGPT1, BATF, BCL11B, BCL3, BLK, BLNK, BTK, C1QC, CALCR, CAMK4, CARD11, CASP8, CCL19, CCL3, CCR2, CD19, CD2, CD27, CD3D, CD3E, CD3G, CD4, CD40LG, CD74, CD79A, CD80, CD86, CD8A, CDKN2A, CLCF1, CR2, CRTAM, CSF1R, CTLA4, DOCK10, DOCK2, EGR3, EOMES, EPX, EVI2B, FCRL3, FGL2, FLT3, FOXP3, FUT7, GFI1, GPR171, GPR55, H3C10, H3C11, H3C12, H3C7, HCLS1, HLA-DOA, HLA-DRA, HLA-DRB1, IFNG, IKZF1, IKZF3, IL12RB1, IL18, IL7R, INPP5D, IRF4, IRF8, ITGA4, ITK, KLRC1, LAG3, LCK, LEF1, LILRB1, LILRB4, LMO1, LRRC17, LY9, MIXL1, MMP9, MS4A1, MT1G, MYB, NCKAP1L, NFAM1, NLRP3, NR4A3, PIM1, PLA2G2D, PLD4, PLEK, POU2AF1, POU2F2, PRDM1, PRKCB, PRKCQ, PTK2B, PTPN22, PTPN6, PTPRC, RHEX, RHOH, RTKN2, RUNX3, SASH3, SELPLG, SEMA4A, SLAMF6, SLAMF8, SLC8A3, SMPD3, SNX10, SOCS1, SPN, STAT1, SYK, TBX21, TESPA1, THBS1, THEMIS, TNFRSF13B, TNFRSF18, TNFSF13B, TNFSF8, TOX, TREM2, TRPM2, UBD, VAV1, VNN1, WNT1, WNT10B, ZAP70, ZNF683]	[ABL1, ACTN1, ADIPOQ, AGER, ANGPT1, AP3D1, ARMC5, BATF2, BCL3, BCL6, CALCR, CCR7, CD74, CDK2, CDKN1A, CEACAM1, CITED2, CLCF1, CLPTM1, CRIP2, CSF1, CTC1, CYP26B1, DHRS2, DMTN, DPF2, DTX1, EPHA2, ERBB2, ETV6, FADD, FASN, FES, FLT3, FLT4, GAB2, GATA2, GBA, GNAO1, GPR137, H3C10, H3C12, H4C3, HDAC5, HSF1, IL15RA, IL1B, IL4R, IL7R, INHA, JAK3, JUNB, KCP, KDELR1, KLF10, KMT2D, LAG3, LGALS9, LOX, LRRC17, LRRC8A, LTBR, LTF, MFAP5, MKNK2, MOV10, MYC, MYH9, MYL9, NBEAL2, NCOA6, NFE2, NOTCH1, NOTCH4, NR4A3, NRARP, OSM, PAF1, PDGFB, PIM1, PML, PTN, RARA, RELB, RMRP, RNU1-93P, RTKN2, RXRA, SBNO2, SETD1A, SH2B3, SIPA1L3, SNX10, SOX12, SOX13, SPI1, SRC, SRF, SSBP3, STAT3, SYVN1, TCF3, TCF7L1, TCIRG1, TFE3, THBS1, THPO, TIMP1, TNF, TRIB1, VEGFA, VNN1, VSIR, ZBTB7A, ZBTB7B, ZC3H12A, ZFP36, ZMIZ1, ZNF385A]
GO:0032649	regulation of interferon-gamma production	4,81E-07	[4, 5, 6, 7]	43,00	37,07	71,47	28,53	[BCL3, BTN3A1, BTN3A2, CCR2, CD2, CD226, CD244, CD3E, CD96, CRTAM, EBI3, FOXP3, HLA-DPA1, HLA-DPB1, HLA-DRB1, IL12RB1, IL12RB2, IL18, IRF8, LILRB1, LILRB4, LTA, NLRP6, PTPN22, PYCARD, SASH3, SLAMF6, TLR8, XCL1]	[ABL1, BCL3, CCR7, CD14, CD276, FADD, IL1B, INHA, LGALS9, NOD2, RARA, SLC7A5, TNF, VSIR, ZC3H12A]
GO:0032673	regulation of interleukin-4 production	1,24E-02	[4, 5, 6, 7]	15,00	46,88	83,83	16,17	[CD3E, CD40LG, CD86, EPX, FOXP3, HLA-DRB1, IRF4, LEF1, NLRP3, PRKCQ, SASH3, SYK]	[LGALS9, RARA, SLC7A5]
GO:0032944	regulation of mononuclear cell proliferation	5,50E-10	[5, 6]	77,00	31,82	72,91	27,09	[AIF1, BLK, BTK, CARD11, CCDC88B, CCL19, CCL5, CCR2, CD22, CD3E, CD40LG, CD6, CD74, CD80, CD86, CDKN2A, CLCF1, CORO1A, CRTAM, CTLA4, EBI3, EPX, FCRL3, FOXP3, HLA-DMB, HLA-DPA1, HLA-DPB1, HLA-DRB1, IKZF3, IL12RB1, IL18, INPP5D, LILRB1, LILRB4, LMO1, LST1, MZB1, NCKAP1L, PLA2G2D, PRKCQ, PTPN22, PTPN6, PTPRC, PYCARD, RAC2, RASAL3, SASH3, SPN, SYK, TNFRSF13B, TNFSF13B, TNFSF8, XCL1, ZAP70]	[AGER, AMH, BCL6, CD276, CD74, CDKN1A, CLC, CLCF1, CRP, CSF1, DLG5, ERBB2, FADD, GPAM, IDO1, IL1B, JAK3, LGALS9, LRRC32, RAC2, SLC7A1, TICAM1, TIMP1, VSIR, ZBTB7B, ZNF335]
GO:0042098	T cell proliferation	9,29E-09	[5, 6]	68,00	32,23	73,87	26,13	[AIF1, BTN3A1, CARD11, CCDC88B, CCL19, CCL5, CCR2, CD3E, CD40LG, CD6, CD80, CD86, CDKN2A, CLCF1, CORO1A, CRTAM, CTLA4, DOCK2, EBI3, EPX, FOXP3, HLA-DMB, HLA-DPA1, HLA-DPB1, HLA-DRB1, IL12RB1, IL18, LILRB1, LILRB4, LMO1, NCKAP1L, PIK3CG, PLA2G2D, PRKCQ, PTPN22, PTPN6, PTPRC, PYCARD, RAC2, RASAL3, SASH3, SPN, SYK, TNFSF13B, TNFSF14, TNFSF8, XCL1, ZAP70]	[ABL1, AGER, ARMC5, CD276, CLC, CLCF1, DLG5, ELF4, ERBB2, FADD, GPAM, IDO1, IL1B, JAK3, LGALS9, LRRC32, MSN, RAC2, SLC7A1, TIMP1, VSIR, ZBTB7B]
GO:0042100	B cell proliferation	1,60E-02	[5, 6]	31,00	30,69	80,81	19,19	[BLK, BTK, CARD11, CD180, CD19, CD22, CD40LG, CD74, CD79A, CDKN2A, CLCF1, CR2, CTLA4, FCRL3, GAPT, IKZF3, IL7R, INPP5D, LEF1, MS4A1, MZB1, NCKAP1L, PTPRC, SASH3, TNFRSF13B, TNFSF13B]	[ABL1, AMH, BCL6, CD74, CDKN1A, CLCF1, IL7R, TICAM1]
GO:0042119	neutrophil activation	4,66E-03	[5, 6]	116,00	20,90	56,03	43,97	[ADAM8, ADGRE3, ANPEP, ARHGAP45, BIN2, CCL5, CD177, CD33, CD53, CEACAM21, CFP, CLEC12A, CLEC4D, COTL1, CTSS, CXCL6, CYBB, DOCK2, EPX, FCN1, FGL2, FGR, FRMPD3, GMFG, IL18, IL18RAP, IQGAP2, ITGAL, ITGAX, ITGB2, LAIR1, LRMP, LYZ, MMP9, MPO, NCKAP1L, NFAM1, ORM2, PLAC8, PLAU, PRAM1, PTAFR, PTPN6, PTPRC, PTX3, PYCARD, RAB37, RAB44, RNASET2, SCNN1B, SELL, SERPINA1, SLC18A1, SLC27A2, STXBP2, SYK, TBC1D10C, TRPM2, TXNDC5, VNN1]	[ADGRE5, ALDOA, AMH, ARL8A, ARSA, C5AR1, CAPN1, CD14, CD177, CEACAM1, CHI3L1, CLEC12A, COMMD3, CRAT, CTSA, CTSD, CYB5R3, DOK3, DSC1, FPR1, GALNS, GPI, GPR84, GRN, HBB, HFE, HVCN1, IMPDH1, IQGAP2, JUP, LRG1, LTF, METTL7A, MME, NBEAL2, NHLRC3, PKM, PLA2G2A, PLAU, PLAUR, PRKCSH, RAB3A, RAB5B, RAP1A, RHOG, RNASE2, RNU1-93P, S100A11, SERPINA3, SIGLEC9, SLC44A2, SLCO4C1, SPI1, STING1, STK11IP, TCIRG1, TMEM179B, TMEM63A, TRAPPC1, TSPAN14, VNN1]
GO:0043299	leukocyte degranulation	1,01E-05	[3, 4, 5, 6, 7, 8]	133,00	22,32	53,98	46,02	[ADAM8, ADGRE3, ANPEP, ARHGAP45, BIN2, BLK, BTK, CCL3, CCR2, CD177, CD33, CD53, CD84, CEACAM21, CFP, CLEC12A, CLEC4D, CLNK, CORO1A, COTL1, CTSS, CYBB, DOCK2, EPX, FCN1, FGL2, FGR, FRMPD3, GMFG, IQGAP2, ITGAL, ITGAX, ITGB2, KLRC2, LAIR1, LRMP, LYZ, MMP9, MPO, NCKAP1L, NFAM1, NR4A3, ORM2, PIK3CG, PLAC8, PLAU, PRAM1, PTAFR, PTGDR, PTPN6, PTPRC, PTX3, PYCARD, RAB37, RAB44, RAC2, RNASET2, SELL, SERPINA1, SLC18A1, SLC27A2, STXBP2, SYK, TBC1D10C, TRPM2, TXNDC5, VNN1]	[ADGRE5, ALDOA, AMH, ARL8A, ARSA, C5AR1, CAPN1, CD14, CD177, CEACAM1, CHGA, CHI3L1, CLEC12A, CLNK, COMMD3, CRAT, CTSA, CTSD, CYB5R3, DOK3, DSC1, FES, FOXF1, FPR1, GAB2, GALNS, GATA2, GPI, GPR84, GRN, HBB, HFE, HVCN1, IL4R, IMPDH1, IQGAP2, JUP, LGALS9, LRG1, LTF, METTL7A, MME, NBEAL2, NHLRC3, NPPA, NR4A3, PKM, PLAU, PLAUR, PRKCSH, PTGDS, RAB3A, RAB5B, RAC2, RAP1A, RHOG, RNASE2, RNU1-93P, S100A11, SERPINA3, SIGLEC9, SLC44A2, SLC7A8, SLCO4C1, SPHK2, SPI1, STING1, STK11IP, TCIRG1, TMEM179B, TMEM63A, TRAPPC1, TSPAN14, VNN1]

GO:0045055	regulated exocytosis	2,13E-07	[4, 5, 6, 7]	186,00	21,48	45,51	54,49	[ADAM8, ADGRE3, ANPEP, ARHGAP45, BIN2, BLK, BTK, CACNA1I, CBARP, CCL3, CCR2, CD177, CD33, CD53, CD84, CEACAM21, CFP, CLEC12A, CLEC4D, CLNK, CNR1, CORO1A, COTL1, CTSS, CTSW, CYBB, DOCK2, EPX, FCN1, FGL2, FGR, FRMPD3, GMFG, IQGAP2, ITGAL, ITGAX, ITGB2, KLRC2, KNG1, LAIR1, LRMP, LYZ, MMP9, MPO, NAPSB, NCKAP1L, NFAM1, NR4A3, ORM2, PIK3CG, PLAC8, PLAU, PLEK, PRAM1, PRKCB, PTAFR, PTGDR, PTPN6, PTPRC, PTX3, PYCARD, RAB15, RAB27B, RAB37, RAB44, RAC2, RNASET2, SELL, SERPINA1, SLC18A1, SLC27A2, STXBP2, SYK, TBC1D10C, THBS1, TRPM2, TXNDC5, VNN1]	[ACTN1, ACTN4, ADCY1, ADGRE5, ALDOA, AMH, APOA1, ARF1, ARL8A, ARSA, BRPF3, C5AR1, CACNA1H, CAPN1, CBARP, CD14, CD177, CEACAM1, CHGA, CHI3L1, CLEC12A, CLNK, CNR1, COMMD3, CPLX1, CRAT, CTSA, CTSD, CYB5R3, DMTN, DOC2B, DOK3, DSC1, ECM1, FES, FOXF1, FPR1, GAB2, GALNS, GATA2, GIT1, GNAI2, GPI, GPR84, GRIK5, GRN, GTPBP2, HBB, HFE, HTR1B, HTR2A, HVCN1, IL4R, IMPDH1, IQGAP2, JUP, KNG1, LGALS3BP, LGALS9, LRG1, LTF, METTL7A, MME, MYH9, NAPA, NBEAL2, NHLRC3, NOTCH1, NPPA, NR4A3, OTOF, PCDHA4, PDGFB, PIP5K1C, PKM, PLAU, PLAUR, PRKCSH, PRRT2, PTGDS, RAB11B, RAB11FIP5, RAB15, RAB3A, RAB5B, RAC2, RAP1A, RHOG, RNASE2, RNU1-93P, S100A11, SELENOP, SEPTIN5, SEPTIN9, SERPINA3, SERPINE1, SERPING1, SIGLEC9, SLC44A2, SLC7A8, SLCO4C1, SPHK2, SPI1, STING1, STK11IP, STX1A, STX1B, SV2A, SYN1, SYT2, SYT7, TCIRG1, THBS1, TIMP1, TMEM179B, TMEM63A, TRAPPC1, TSPAN14, TUBA4A, VEGFA, VNN1]
GO:0046631	alpha-beta T cell activation	1,31E-14	[5, 6]	66,00	41,25	76,60	23,40	[BATF, BCL11B, BCL3, CCL19, CCR2, CD3E, CD80, CD86, CRTAM, DOCK2, EBI3, EOMES, FOXP3, FUT7, HLA-DRA, HLA-DRB1, IFNG, IL12RB1, IL18, IRF4, ITK, LEF1, LILRB1, LILRB4, LY9, MYB, NCKAP1L, NLRP3, PLA2G2D, PRDM1, PRKCQ, PTPN22, PTPRC, RASAL3, RUNX3, SASH3, SEMA4A, SLAMF6, SOCS1, SPN, SYK, TBX21, TNFSF8, TOX, WDFY4, XCL1, ZAP70, ZNF683]	[ABL1, AGER, AP3D1, ARMC5, BCL3, BCL6, ELF4, GNAO1, HFE, IL4R, JAK3, LGALS9, RARA, RELB, STAT3, TCIRG1, VSIR, ZBTB7B, ZC3H12A]
GO:0050729	positive regulation of inflammatory response	1,20E-03	[4, 5, 6, 7]	43,00	29,66	52,30	47,70	[ADAM8, BTK, CCL24, CCL3, CCR2, CNR1, GPR4, GPSM3, IFNG, IL16, IL18, LCP1, LDLR, LTA, MAPK13, PIK3CG, PLA2G7, STAP1, SUCNR1, TLR10, TREM2, ZBP1]	[AGT, AGTR1, CCL24, CCR7, CNR1, CX3CL1, FABP4, FEM1A, FFAR2, GPR4, GRN, IL1B, LDLR, LILRA5, NINJ1, NPPA, OSM, PCDHA4, PLA2G2A, PLA2G7, SERPINE1, TGM2, TNF, TNFRSF1A, TNIP1, TRPV4]
GO:0051250	negative regulation of lymphocyte activation	1,91E-05	[4, 5, 6, 7]	50,00	31,25	71,59	28,41	[BANK1, BLK, BTK, CD74, CD80, CD86, CDKN2A, CLNK, CRTAM, CTLA4, FGL2, FGR, FOXP3, HLA-DRB1, INPP5D, LAG3, LAX1, LILRB1, LILRB4, LST1, NCKAP1L, PLA2G2D, PTPN22, PTPN6, RUNX3, SAMSN1, SOCS1, SPN, TBC1D10C, TBX21, TIGIT, TNFAIP3, TNFAIP8L2, TNFRSF13B, XCL1]	[BCL6, CD74, CEACAM1, CLNK, DLG5, DTX1, ERBB2, HFE, IL4R, INHA, JAK3, LAG3, LGALS9, LRRC32, NRARP, VSIR, ZBTB7B, ZC3H12A]
GO:0051251	positive regulation of lymphocyte activation	1,50E-07	[4, 5, 6, 7]	103,00	25,88	71,60	28,40	[ADAM8, AIF1, BTK, BTLA, CARD11, CCDC88B, CCL19, CCL2, CCL5, CCR2, CD27, CD3E, CD4, CD40LG, CD5, CD6, CD74, CD80, CD86, CLCF1, CORO1A, CTLA4, DOCK8, EBI3, EGR3, EPX, FCRL3, FOXP3, GRAP2, HLA-DMB, HLA-DPA1, HLA-DPB1, HLA-DRA, HLA-DRB1, ICOS, IFNG, IGLL5, IL12RB1, IL18, IL7R, INPP5D, KLRC2, KLRK1, LCK, LEF1, LILRB1, LILRB4, MYB, NCKAP1L, NLRP3, PRKCQ, PTPN22, PTPN6, PTPRC, PYCARD, RASAL3, RHOH, RUNX3, SASH3, SIRPG, SOCS1, SYK, TBX21, TESPA1, THY1, TNFSF13B, TNFSF14, TOX, VAV1, VNN1, XCL1, ZAP70]	[AGER, AMH, AP3D1, BCL6, BTLA, CCL2, CCL21, CCR7, CD276, CD74, CDKN1A, CLCF1, CSK, FADD, FLOT2, GNAO1, GPAM, IL15RA, IL1B, IL4R, IL7R, JAK3, LGALS9, NOD2, PDCD1, RARA, SLC7A1, SOX12, SOX13, SRC, TICAM1, TIMP1, VNN1, VSIR, ZBTB7B, ZMIZ1, ZNF335]
GO:1903037	regulation of leukocyte cell-cell adhesion	3,98E-20	[5, 6]	119,00	34,69	69,53	30,47	[ADAM8, AIF1, BTLA, CARD11, CCDC88B, CCL19, CCL2, CCL5, CCR2, CD27, CD3E, CD4, CD40LG, CD5, CD6, CD74, CD80, CD86, CDKN2A, CORO1A, CRTAM, CTLA4, DOCK8, EBI3, EGR3, EPX, FGL2, FOXP3, FUT7, GRAP2, HLA-DMB, HLA-DPA1, HLA-DPB1, HLA-DRA, HLA-DRB1, ICOS, IFNG, IL12RB1, IL18, IL7R, ITGA4, ITGB2, KLRK1, LAG3, LAX1, LCK, LEF1, LILRB1, LILRB4, MYB, NCKAP1L, NLRP3, NR4A3, PLA2G2D, PRKCQ, PTAFR, PTPN22, PTPN6, PTPRC, PYCARD, RASAL3, RHOH, RUNX3, SASH3, SELE, SIRPG, SKAP1, SOCS1, SPN, SYK, TBX21, TESPA1, THY1, TIGIT, TNFAIP8L2, TNFSF13B, TNFSF14, VAV1, VNN1, XCL1, ZAP70]	[AGER, AP3D1, BCL6, BTLA, CCL2, CCL21, CCR7, CD276, CD74, CEACAM1, CSK, DLG5, DTX1, ERBB2, FADD, FLOT2, GNAO1, GPAM, HFE, ICAM1, IL1B, IL4R, IL7R, JAK3, LAG3, LGALS9, LRRC32, NOD2, NR4A3, NRARP, PDCD1, RARA, RELA, SELE, SELENOP, SLC7A1, SOX12, SOX13, SRC, TIMP1, TNF, VNN1, VSIR, ZBTB7B, ZC3H12A, ZMIZ1]
GO:1903706	regulation of hemopoiesis	2,05E-13	[3, 4, 5, 6, 7, 8]	122,00	28,98	61,99	38,01	[ADAM8, BTK, C1QC, CAMK4, CARD11, CASP8, CCL19, CCL3, CCR2, CD2, CD27, CD4, CD74, CD80, CD86, CDKN2A, CRTAM, CTLA4, EGR3, EVI2B, FCRL3, FGL2, FOXP3, GPR171, GPR55, H3C10, H3C11, H3C12, H3C7, HCLS1, HLA-DOA, HLA-DRA, HLA-DRB1, IFNG, IKZF3, IL12RB1, IL18, IL7R, INPP5D, IRF4, LAG3, LEF1, LILRB1, LILRB4, LRRC17, MYB, NCKAP1L, NFAM1, NLRP3, NR4A3, PRDM1, PRKCB, PRKCQ, PTK2B, PTPN6, PTPRC, RHEX, RHOH, RUNX3, SASH3, SLAMF8, SOCS1, STAT1, SYK, TBX21, TESPA1, THBS1, TNFRSF18, TOX, TREM2, VNN1, ZAP70, ZNF683]	[ABL1, ADIPOQ, AGER, AP3D1, BCL6, CD74, CEACAM1, CLPTM1, CSF1, CYP26B1, DTX1, ERBB2, FADD, FES, GATA2, GPR137, H3C10, H3C12, H4C3, HSF1, IL15RA, IL4R, IL7R, INHA, JAK3, KLF10, KMT2D, LAG3, LGALS9, LOX, LRRC17, LTF, MOV10, MYC, MYL9, NFE2, NR4A3, NRARP, PAF1, PTN, RARA, RMRP, SETD1A, SOX12, SOX13, SPI1, STAT3, TFE3, THBS1, THPO, TNF, TRIB1, VNN1, VSIR, ZBTB7B, ZC3H12A, ZFP36, ZMIZ1]
GO:0002228	natural killer cell mediated immunity	7,86E-04	[5, 6, 7]	27,00	37,50	77,28	22,72	[CD226, CD96, CLNK, CORO1A, CRTAM, GZMB, IL18, IL18RAP, KLRC1, KLRC2, KLRD1, KLRK1, LAG3, LILRB1, NCR1, NCR3, PTPN6, SH2D1A, SLAMF6, SLAMF7, VAV1]	[CEACAM1, CLNK, LAG3, LGALS9, NECTIN2, PLEKHM2, PVR, RNF19B]
GO:0002283	neutrophil activation involved in immune response	2,25E-02	[5, 6, 7]	111,00	20,44	54,74	45,26	[ADAM8, ADGRE3, ANPEP, ARHGAP45, BIN2, CD177, CD33, CD53, CEACAM21, CFP, CLEC12A, CLEC4D, COTL1, CTSS, CYBB, DOCK2, EPX, FCN1, FGL2, FGR, FRMPD3, GMFG, IQGAP2, ITGAL, ITGAX, ITGB2, LAIR1, LRMP, LYZ, MMP9, MPO, NCKAP1L, NFAM1, ORM2, PLAC8, PLAU, PRAM1, PTAFR, PTPN6, PTPRC, PTX3, PYCARD, RAB37, RAB44, RNASET2, SCNN1B, SELL, SERPINA1, SLC18A1, SLC27A2, STXBP2, SYK, TBC1D10C, TRPM2, TXNDC5, VNN1]	[ADGRE5, ALDOA, AMH, ARL8A, ARSA, C5AR1, CAPN1, CD14, CD177, CEACAM1, CHI3L1, CLEC12A, COMMD3, CRAT, CTSA, CTSD, CYB5R3, DOK3, DSC1, FPR1, GALNS, GPI, GPR84, GRN, HBB, HFE, HVCN1, IMPDH1, IQGAP2, JUP, LRG1, LTF, METTL7A, MME, NBEAL2, NHLRC3, PKM, PLAU, PLAUR, PRKCSH, RAB3A, RAB5B, RAP1A, RHOG, RNASE2, RNU1-93P, S100A11, SERPINA3, SIGLEC9, SLC44A2, SLCO4C1, SPI1, STING1, STK11IP, TCIRG1, TMEM179B, TMEM63A, TRAPPC1, TSPAN14, VNN1]

GO:0002521	leukocyte differentiation	9,44E-24	[4, 5, 6, 7, 8]	177,00	30,73	63,92	36,08	[ADAM8, BATF, BCL11B, BCL3, BLK, BLNK, BTK, C1QC, CALCR, CAMK4, CARD11, CASP8, CCL19, CCL3, CCR2, CD19, CD2, CD27, CD3D, CD3E, CD3G, CD4, CD40LG, CD74, CD79A, CD80, CD86, CD8A, CDKN2A, CLCF1, CR2, CRTAM, CSF1R, CTLA4, DOCK10, DOCK2, EGR3, EOMES, EVI2B, FCRL3, FGL2, FLT3, FOXP3, FUT7, GPR55, HCLS1, HLA-DOA, HLA-DRA, HLA-DRB1, IFNG, IKZF1, IKZF3, IL12RB1, IL18, IL7R, INPP5D, IRF4, IRF8, ITGA4, ITK, KLRC1, LAG3, LCK, LEF1, LILRB1, LILRB4, LRRC17, LY9, MMP9, MS4A1, MT1G, MYB, NCKAP1L, NFAM1, NLRP3, PLA2G2D, POU2AF1, POU2F2, PRDM1, PTK2B, PTPN22, PTPN6, PTPRC, RHOH, RUNX3, SASH3, SEMA4A, SLAMF6, SLAMF8, SNX10, SOCS1, SPN, SYK, TBX21, TESPA1, THEMIS, TNFRSF18, TNFSF13B, TNFSF8, TOX, TREM2, TRPM2, UBD, VAV1, VNN1, WNT1, ZAP70, ZNF683]	[ABL1, ADIPOQ, AGER, AP3D1, ARMC5, BATF2, BCL3, BCL6, CALCR, CCR7, CD74, CDK2, CDKN1A, CEACAM1, CITED2, CLCF1, CLPTM1, CSF1, CYP26B1, DHRS2, DTX1, EPHA2, ERBB2, FADD, FASN, FES, FLT3, GAB2, GATA2, GBA, GNAO1, GPR137, HDAC5, HSF1, IL15RA, IL1B, IL4R, IL7R, INHA, JAK3, JUNB, KDELR1, KLF10, LAG3, LGALS9, LRRC17, LRRC8A, LTBR, LTF, MYC, MYH9, NRARP, PML, RARA, RELB, RMRP, RXRA, SBNO2, SNX10, SOX12, SOX13, SPI1, SRC, SRF, STAT3, SYVN1, TCF3, TCF7L1, TCIRG1, TFE3, TNF, TRIB1, VEGFA, VNN1, VSIR, ZBTB7A, ZBTB7B, ZC3H12A, ZMIZ1]
GO:0002708	positive regulation of lymphocyte mediated immunity	6,96E-03	[5, 6, 7]	35,00	30,43	77,35	22,65	[BTK, CD1B, CD1C, CD1E, CD226, CLCF1, CLNK, CRTAM, FOXP3, HLA-DRA, HLA-DRB1, IL12RB1, IL18, IL18RAP, KLRC2, KLRD1, KLRK1, LAG3, LTA, NCR3, NLRP3, PTPRC, SASH3, SH2D1A, SLAMF6, TBX21, TREM2, VAV1, XCL1]	[CD1C, CD1E, CLCF1, CLNK, FADD, IL1B, LAG3, NECTIN2, NOD2, PVR, TNF]
GO:0002823	negative regulation of adaptive immune response based on somatic recombination of immune receptors built from immunoglobulin superfamily domains	4,05E-02	[5, 6, 7]	20,00	36,36	63,34	36,66	[CR2, FOXP3, IL7R, KLRC1, KLRD1, LILRB1, LILRB4, NCKAP1L, PTPN6, PTPRC, TBX21, XCL1]	[BCL6, CEACAM1, HFE, IL4R, IL7R, JAK3, NOD2, ZBTB7B, ZC3H12A]
GO:0030099	myeloid cell differentiation	5,32E-05	[4, 5, 6, 7, 8]	104,00	23,37	44,67	55,33	[BATF, C1QC, CALCR, CAMK4, CASP8, CCL19, CCL3, CD4, CD74, CSF1R, EPX, EVI2B, GPR171, GPR55, H3C10, H3C11, H3C12, H3C7, HCLS1, HLA-DRB1, IFNG, IKZF1, INPP5D, IRF4, IRF8, LEF1, LILRB1, LILRB4, LRRC17, MMP9, MT1G, NCKAP1L, NR4A3, PRKCB, PRKCQ, PTK2B, PTPN6, RHEX, SNX10, STAT1, THBS1, TREM2, UBD]	[ACTN1, ADIPOQ, BATF2, BCL6, CALCR, CCR7, CD74, CDK2, CDKN1A, CEACAM1, CITED2, CSF1, DHRS2, DMTN, EPHA2, FADD, FASN, FES, GAB2, GATA2, GBA, GPR137, H3C10, H3C12, H4C3, HSF1, INHA, JAK3, JUNB, KLF10, KMT2D, LGALS9, LOX, LRRC17, LTBR, LTF, MOV10, MYC, MYH9, MYL9, NBEAL2, NCOA6, NFE2, NR4A3, PAF1, PML, RARA, RELB, RMRP, RXRA, SBNO2, SETD1A, SH2B3, SNX10, SPI1, SRC, SRF, STAT3, TCIRG1, TFE3, THBS1, THPO, TIMP1, TNF, TRIB1, VEGFA, ZBTB7A, ZFP36, ZNF385A]
GO:0032753	positive regulation of interleukin-4 production	1,24E-02	[4, 5, 6, 7, 8]	13,00	52,00	81,20	18,80	[CD3E, CD40LG, CD86, EPX, FOXP3, IRF4, NLRP3, PRKCQ, SASH3, SYK]	[LGALS9, RARA, SLC7A5]
GO:0032946	positive regulation of mononuclear cell proliferation	1,15E-06	[5, 6, 7]	49,00	33,79	72,16	27,84	[AIF1, CARD11, CCDC88B, CCL19, CCL5, CCR2, CD3E, CD40LG, CD6, CD74, CD80, CD86, CLCF1, CORO1A, EBI3, EPX, FCRL3, FOXP3, HLA-DMB, HLA-DPA1, HLA-DPB1, IL12RB1, IL18, NCKAP1L, PRKCQ, PTPN22, PTPRC, PYCARD, RASAL3, SASH3, SYK, TNFSF13B, XCL1, ZAP70]	[AGER, AMH, BCL6, CD276, CD74, CDKN1A, CLCF1, CSF1, FADD, GPAM, IL1B, JAK3, LGALS9, SLC7A1, TICAM1, TIMP1, ZNF335]
GO:0034341	response to interferon-gamma	2,42E-05	[5, 6, 7]	60,00	28,71	62,31	37,69	[AIF1, CCL17, CCL19, CCL2, CCL22, CCL24, CCL3, CCL3L1, CCL4, CCL4L1, CCL5, CD74, DAPK1, FASLG, GBP5, GBP6, HLA-DPA1, HLA-DPB1, HLA-DQA1, HLA-DRA, HLA-DRB1, HLA-DRB5, IFNG, IL12RB1, IRF4, IRF5, IRF8, MRC1, OASL, PTAFR, SOCS1, SOCS3, STAT1, UBD, WAS, XCL1, XCL2]	[CAMK2B, CCL11, CCL2, CCL21, CCL24, CD74, CDC42EP2, CDC42EP4, CIITA, CX3CL1, CYP27B1, GNAO1, HFE, ICAM1, IFITM1, IFITM2, IFITM3, IRF2, IRF9, LGALS9, MRC1, MYC, NLRC5, PML, SOCS3, TRIM62, TRIM8, WAS, ZYX]
GO:0042267	natural killer cell mediated cytotoxicity	4,33E-03	[4, 5, 6, 7, 8]	25,00	36,23	77,86	22,14	[CD226, CORO1A, CRTAM, GZMB, IL18, IL18RAP, KLRC1, KLRC2, KLRD1, KLRK1, LAG3, LILRB1, NCR1, NCR3, PTPN6, SH2D1A, SLAMF6, SLAMF7, VAV1]	[CEACAM1, LAG3, LGALS9, NECTIN2, PLEKHM2, PVR, RNF19B]
GO:0050670	regulation of lymphocyte proliferation	2,85E-09	[5, 6, 7]	75,00	31,51	74,46	25,54	[AIF1, BLK, BTK, CARD11, CCDC88B, CCL19, CCL5, CCR2, CD22, CD3E, CD40LG, CD6, CD74, CD80, CD86, CDKN2A, CLCF1, CORO1A, CRTAM, CTLA4, EBI3, EPX, FCRL3, FOXP3, HLA-DMB, HLA-DPA1, HLA-DPB1, HLA-DRB1, IKZF3, IL12RB1, IL18, INPP5D, LILRB1, LILRB4, LMO1, LST1, MZB1, NCKAP1L, PLA2G2D, PRKCQ, PTPN22, PTPN6, PTPRC, PYCARD, RAC2, RASAL3, SASH3, SPN, SYK, TNFRSF13B, TNFSF13B, TNFSF8, XCL1, ZAP70]	[AGER, AMH, BCL6, CD276, CD74, CDKN1A, CLC, CLCF1, DLG5, ERBB2, FADD, GPAM, IDO1, IL1B, JAK3, LGALS9, LRRC32, RAC2, SLC7A1, TICAM1, TIMP1, VSIR, ZBTB7B, ZNF335]
GO:0050863	regulation of T cell activation	2,38E-22	[5, 6, 7]	126,00	35,39	70,57	29,43	[ADAM8, AIF1, BTLA, CAMK4, CARD11, CCDC88B, CCL19, CCL2, CCL5, CCR2, CD2, CD27, CD3E, CD4, CD40LG, CD5, CD6, CD74, CD80, CD86, CDKN2A, CLCF1, CORO1A, CRTAM, CTLA4, DOCK8, EBI3, EGR3, EPX, FGL2, FOXP3, GRAP2, HLA-DMB, HLA-DOA, HLA-DPA1, HLA-DPB1, HLA-DRA, HLA-DRB1, ICOS, IFNG, IL12RB1, IL18, IL7R, IRF4, KLRK1, LAG3, LAX1, LCK, LEF1, LILRB1, LILRB4, LMO1, MYB, NCKAP1L, NLRP3, PLA2G2D, PRDM1, PRKCQ, PTPN22, PTPN6, PTPRC, PYCARD, RAC2, RASAL3, RHOH, RUNX3, SASH3, SIRPG, SIT1, SOCS1, SPN, SYK, TBX21, TESPA1, THY1, TIGIT, TNFAIP8L2, TNFRSF18, TNFSF13B, TNFSF14, TNFSF8, TOX, VAV1, VNN1, XCL1, ZAP70, ZNF683]	[ABL1, AGER, AP3D1, BCL6, BTLA, CCL2, CCL21, CCR7, CD276, CD74, CEACAM1, CLC, CLCF1, CLPTM1, CSK, CYP26B1, DLG5, DTX1, ERBB2, FADD, FLOT2, GNAO1, GPAM, HFE, IDO1, IL1B, IL4R, IL7R, JAK3, LAG3, LGALS9, LRRC32, NOD2, NRARP, PDCD1, RAC2, RARA, SLC7A1, SOX12, SOX13, SRC, TIMP1, VNN1, VSIR, ZBTB7B, ZC3H12A, ZMIZ1]
GO:1903038	negative regulation of leukocyte cell-cell adhesion	9,60E-03	[5, 6, 7]	39,00	28,47	64,65	35,35	[CD74, CD80, CD86, CDKN2A, CRTAM, CTLA4, FGL2, FOXP3, HLA-DRB1, LAG3, LAX1, LILRB1, LILRB4, NCKAP1L, PLA2G2D, PTPN22, PTPN6, RUNX3, SOCS1, SPN, TBX21, TIGIT, TNFAIP8L2, XCL1]	[BCL6, CCL21, CD74, CEACAM1, DLG5, DTX1, ERBB2, HFE, IL4R, JAK3, LAG3, LGALS9, LRRC32, NRARP, VSIR, ZBTB7B, ZC3H12A]

GO:1903039	positive regulation of leukocyte cell-cell adhesion	2,87E-22	[5, 6, 7]	101,00	40,08	71,32	28,68	[ADAM8, AIF1, BTLA, CARD11, CCDC88B, CCL19, CCL2, CCL5, CCR2, CD27, CD3E, CD4, CD40LG, CD5, CD6, CD74, CD80, CD86, CORO1A, CTLA4, DOCK8, EBI3, EGR3, EPX, FOXP3, FUT7, GRAP2, HLA-DMB, HLA-DPA1, HLA-DPB1, HLA-DRA, HLA-DRB1, ICOS, IFNG, IL12RB1, IL18, IL7R, ITGA4, ITGB2, KLRK1, LCK, LEF1, LILRB1, LILRB4, MYB, NCKAP1L, NLRP3, NR4A3, PRKCQ, PTAFR, PTPN22, PTPN6, PTPRC, PYCARD, RASAL3, RHOH, RUNX3, SASH3, SELE, SIRPG, SKAP1, SOCS1, SYK, TESPA1, THY1, TNFSF13B, TNFSF14, VAV1, VNN1, XCL1, ZAP70]	[AGER, AP3D1, BCL6, BTLA, CCL2, CCL21, CCR7, CD276, CD74, CSK, FADD, FLOT2, GNAO1, GPAM, ICAM1, IL1B, IL4R, IL7R, JAK3, LGALS9, NOD2, NR4A3, PDCD1, RARA, RELA, SELE, SELENOP, SLC7A1, SOX12, SOX13, SRC, TIMP1, TNF, VNN1, VSIR, ZBTB7B, ZMIZ1]
GO:0001914	regulation of T cell mediated cytotoxicity	1,24E-03	[5, 6, 7, 8]	18,00	47,37	67,80	32,20	[CD1B, CD1C, CD1E, HLA-DRA, HLA-DRB1, IL12RB1, IL7R, KLRC1, KLRD1, LILRB1, NCKAP1L, PTPRC, XCL1]	[AGER, CD1C, CD1E, CEACAM1, FADD, IL7R, NECTIN2, PVR]
GO:0002709	regulation of T cell mediated immunity	1,57E-04	[6, 7]	32,00	36,36	68,04	31,96	[CCR2, CD1B, CD1C, CD1E, CLCF1, FOXP3, FUT7, HLA-DRA, HLA-DRB1, IL12RB1, IL18, IL7R, KLRC1, KLRD1, LILRB1, LILRB4, NCKAP1L, NLRP3, PTPRC, SASH3, TBX21, WAS, XCL1]	[AGER, CD1C, CD1E, CEACAM1, CLC, CLCF1, FADD, HFE, IL1B, IL7R, NECTIN2, NOD2, PVR, WAS]
GO:0002715	regulation of natural killer cell mediated immunity	2,46E-03	[5, 6, 7, 8]	20,00	42,55	77,55	22,45	[CD226, CD96, CLNK, CRTAM, IL18RAP, KLRC1, KLRC2, KLRD1, KLRK1, LAG3, LILRB1, NCR1, NCR3, SH2D1A, SLAMF6, VAV1]	[CEACAM1, CLNK, LAG3, LGALS9, NECTIN2, PVR]
GO:0035710	CD4-positive, alpha-beta T cell activation	4,57E-08	[6, 7]	42,00	40,00	72,85	27,15	[BATF, BCL3, CCL19, CD80, CD86, FOXP3, FUT7, HLA-DRA, HLA-DRB1, IFNG, IL12RB1, IL18, IRF4, LEF1, LY9, MYB, NCKAP1L, NLRP3, PLA2G2D, PRKCQ, RUNX3, SASH3, SEMA4A, SLAMF6, SOCS1, SPN, TBX21, TOX, XCL1]	[AGER, ARMC5, BCL3, BCL6, IL4R, JAK3, LGALS9, RARA, RELB, STAT3, TCIRG1, VSIR, ZBTB7B, ZC3H12A]
GO:0043300	regulation of leukocyte degranulation	9,22E-06	[4, 5, 6, 7, 8, 9]	23,00	50,00	58,40	41,60	[BLK, CCR2, CD177, CD84, FGR, ITGB2, KLRC2, NCKAP1L, PRAM1, PTAFR, RAC2, STXBP2, SYK]	[CD177, CEACAM1, FES, FOXF1, GAB2, GATA2, IL4R, LGALS9, NPPA, RAC2, SPHK2, SPI1]
GO:0050870	positive regulation of T cell activation	1,33E-18	[5, 6, 7, 8]	90,00	38,96	72,79	27,21	[ADAM8, AIF1, BTLA, CARD11, CCDC88B, CCL19, CCL2, CCL5, CCR2, CD27, CD3E, CD4, CD40LG, CD5, CD6, CD74, CD80, CD86, CORO1A, CTLA4, DOCK8, EBI3, EGR3, EPX, FOXP3, GRAP2, HLA-DMB, HLA-DPA1, HLA-DPB1, HLA-DRA, HLA-DRB1, ICOS, IFNG, IL12RB1, IL18, IL7R, KLRK1, LCK, LEF1, LILRB1, LILRB4, MYB, NCKAP1L, NLRP3, PRKCQ, PTPN22, PTPN6, PTPRC, PYCARD, RASAL3, RHOH, RUNX3, SASH3, SIRPG, SOCS1, SYK, TESPA1, THY1, TNFSF13B, TNFSF14, VAV1, VNN1, XCL1, ZAP70]	[AGER, AP3D1, BCL6, BTLA, CCL2, CCL21, CCR7, CD276, CD74, CSK, FADD, FLOT2, GNAO1, GPAM, IL1B, IL4R, IL7R, JAK3, LGALS9, NOD2, PDCD1, RARA, SLC7A1, SOX12, SOX13, SRC, TIMP1, VNN1, VSIR, ZBTB7B, ZMIZ1]
GO:1902105	regulation of leukocyte differentiation	4,19E-15	[4, 5, 6, 7, 8, 9]	97,00	33,92	65,84	34,16	[ADAM8, BTK, C1QC, CAMK4, CARD11, CASP8, CCL19, CCL3, CCR2, CD2, CD27, CD4, CD74, CD80, CD86, CDKN2A, CRTAM, CTLA4, EGR3, EVI2B, FCRL3, FGL2, FOXP3, GPR55, HCLS1, HLA-DOA, HLA-DRA, HLA-DRB1, IFNG, IKZF3, IL12RB1, IL18, IL7R, INPP5D, IRF4, LAG3, LEF1, LILRB1, LILRB4, LRRC17, MYB, NCKAP1L, NFAM1, NLRP3, PRDM1, PTPN6, PTPRC, RHOH, RUNX3, SASH3, SLAMF8, SOCS1, SYK, TBX21, TESPA1, TNFRSF18, TOX, TREM2, VNN1, ZAP70, ZNF683]	[ABL1, ADIPOQ, AGER, AP3D1, BCL6, CD74, CEACAM1, CLPTM1, CSF1, CYP26B1, DTX1, ERBB2, FADD, FES, GATA2, GPR137, HSF1, IL15RA, IL4R, IL7R, INHA, JAK3, KLF10, LAG3, LGALS9, LRRC17, LTF, MYC, NRARP, RARA, RMRP, SOX12, SOX13, TFE3, TNF, TRIB1, VNN1, VSIR, ZBTB7B, ZC3H12A, ZMIZ1]
GO:0002573	myeloid leukocyte differentiation	4,84E-06	[5, 6, 7, 8, 9]	65,00	28,76	43,93	56,07	[BATF, C1QC, CALCR, CAMK4, CASP8, CCL19, CCL3, CD4, CD74, CSF1R, EVI2B, GPR55, HCLS1, HLA-DRB1, IFNG, INPP5D, IRF4, LEF1, LILRB1, LILRB4, LRRC17, MMP9, MT1G, SNX10, TREM2, UBD]	[ADIPOQ, BATF2, CALCR, CCR7, CD74, CDK2, CDKN1A, CEACAM1, CITED2, CSF1, DHRS2, EPHA2, FADD, FASN, FES, GAB2, GATA2, GBA, GPR137, HSF1, INHA, JUNB, KLF10, LGALS9, LRRC17, LTBR, LTF, MYC, MYH9, PML, RARA, RELB, RMRP, RXRA, SBNO2, SNX10, SPI1, SRC, TCIRG1, TFE3, TNF, TRIB1, VEGFA]
GO:0002717	positive regulation of natural killer cell mediated immunity	2,67E-02	[5, 6, 7, 8, 9]	14,00	46,67	79,54	20,46	[CD226, CLNK, CRTAM, IL18RAP, KLRC2, KLRD1, KLRK1, LAG3, NCR3, SH2D1A, SLAMF6, VAV1]	[CLNK, LAG3, NECTIN2, PVR]
GO:0030098	lymphocyte differentiation	4,55E-19	[4, 5, 6, 7, 8, 9, 10]	129,00	32,66	72,38	27,62	[ADAM8, BATF, BCL11B, BCL3, BLNK, BTK, CAMK4, CARD11, CCL19, CCR2, CD19, CD2, CD27, CD3D, CD3E, CD3G, CD4, CD40LG, CD74, CD79A, CD80, CD86, CD8A, CDKN2A, CLCF1, CR2, CRTAM, CTLA4, DOCK10, DOCK2, EGR3, EOMES, FCRL3, FGL2, FLT3, FOXP3, FUT7, HLA-DOA, HLA-DRA, HLA-DRB1, IFNG, IKZF1, IKZF3, IL12RB1, IL18, IL7R, INPP5D, IRF4, IRF8, ITGA4, ITK, KLRC1, LAG3, LCK, LEF1, LILRB4, LY9, MS4A1, MYB, NCKAP1L, NFAM1, NLRP3, PLA2G2D, POU2AF1, POU2F2, PRDM1, PTK2B, PTPN22, PTPN6, PTPRC, RHOH, RUNX3, SASH3, SEMA4A, SLAMF6, SLAMF8, SOCS1, SPN, SYK, TBX21, TESPA1, THEMIS, TNFRSF18, TNFSF13B, TNFSF8, TOX, VAV1, VNN1, WNT1, ZAP70, ZNF683]	[ABL1, AP3D1, ARMC5, BCL3, BCL6, CCR7, CD74, CLCF1, CLPTM1, CYP26B1, DTX1, ERBB2, FADD, FLT3, GBA, GNAO1, HDAC5, IL15RA, IL1B, IL4R, IL7R, INHA, JAK3, KDELR1, LAG3, LGALS9, LRRC8A, NRARP, RARA, RELB, SOX12, SOX13, SPI1, SRF, STAT3, SYVN1, TCF3, TCF7L1, TCIRG1, VNN1, VSIR, ZBTB7A, ZBTB7B, ZC3H12A, ZMIZ1]
GO:0042129	regulation of T cell proliferation	4,97E-08	[6, 7, 8]	60,00	32,97	76,00	24,00	[AIF1, CARD11, CCDC88B, CCL19, CCL5, CCR2, CD3E, CD40LG, CD6, CD80, CD86, CDKN2A, CLCF1, CORO1A, CRTAM, CTLA4, EBI3, EPX, FOXP3, HLA-DMB, HLA-DPA1, HLA-DPB1, HLA-DRB1, IL12RB1, IL18, LILRB1, LILRB4, LMO1, NCKAP1L, PLA2G2D, PRKCQ, PTPN22, PTPN6, PTPRC, PYCARD, RAC2, RASAL3, SASH3, SPN, SYK, TNFSF13B, TNFSF8, XCL1, ZAP70]	[AGER, CD276, CLC, CLCF1, DLG5, ERBB2, FADD, GPAM, IDO1, IL1B, JAK3, LGALS9, LRRC32, RAC2, SLC7A1, TIMP1, VSIR, ZBTB7B]
GO:0042269	regulation of natural killer cell mediated cytotoxicity	1,10E-02	[5, 6, 7, 8, 9]	18,00	41,86	78,39	21,61	[CD226, CRTAM, IL18RAP, KLRC1, KLRC2, KLRD1, KLRK1, LAG3, LILRB1, NCR1, NCR3, SH2D1A, SLAMF6, VAV1]	[CEACAM1, LAG3, LGALS9, NECTIN2, PVR]
GO:0046634	regulation of alpha-beta T cell activation	4,95E-09	[6, 7, 8]	44,00	41,12	77,55	22,45	[CCL19, CCR2, CD3E, CD80, CD86, CRTAM, EBI3, FOXP3, HLA-DRA, HLA-DRB1, IFNG, IL12RB1, IL18, IRF4, LILRB1, LILRB4, MYB, NCKAP1L, NLRP3, PRDM1, PRKCQ, PTPN22, PTPRC, RASAL3, RUNX3, SASH3, SOCS1, SYK, TBX21, XCL1, ZAP70, ZNF683]	[AGER, AP3D1, BCL6, GNAO1, HFE, IL4R, JAK3, LGALS9, RARA, VSIR, ZBTB7B, ZC3H12A]
GO:1902106	negative regulation of leukocyte differentiation	1,66E-03	[4, 5, 6, 7, 8, 9, 10]	34,00	32,69	49,68	50,32	[C1QC, CCL3, CD74, CDKN2A, CTLA4, FGL2, FOXP3, GPR55, INPP5D, LAG3, LILRB1, LILRB4, LRRC17, RUNX3, SOCS1, TBX21]	[ADIPOQ, BCL6, CD74, CEACAM1, DTX1, ERBB2, GATA2, GPR137, IL4R, INHA, JAK3, LAG3, LRRC17, LTF, MYC, NRARP, RARA, RMRP, TRIB1, ZBTB7B, ZC3H12A]

GO:1902107	positive regulation of leukocyte differentiation	3,68E-08	[4, 5, 6, 7, 8, 9, 10]	55,00	34,59	67,95	32,05	[ADAM8, BTK, CASP8, CCL19, CD27, CD4, CD74, CD80, CD86, EGR3, EVI2B, FOXP3, HCLS1, HLA-DRA, HLA-DRB1, IFNG, IL12RB1, IL18, IL7R, INPP5D, LEF1, LILRB4, MYB, NCKAP1L, NLRP3, PTPRC, RHOH, RUNX3, SASH3, SOCS1, SYK, TESPA1, TOX, TREM2, VNN1, ZAP70]	[AGER, AP3D1, BCL6, CD74, CSF1, FADD, FES, HSF1, IL15RA, IL4R, IL7R, KLF10, LGALS9, RARA, SOX12, SOX13, TNF, TRIB1, VNN1, VSIR, ZBTB7B, ZMIZ1]
GO:1903131	mononuclear cell differentiation	6,89E-22	[5, 6, 7, 8, 9]	146,00	32,52	69,78	30,22	[ADAM8, BATF, BCL11B, BCL3, BLK, BLNK, BTK, CAMK4, CARD11, CCL19, CCR2, CD19, CD2, CD27, CD3D, CD3E, CD3G, CD4, CD40LG, CD74, CD79A, CD80, CD86, CD8A, CDKN2A, CLCF1, CR2, CRTAM, CSF1R, CTLA4, DOCK10, DOCK2, EGR3, EOMES, FCRL3, FGL2, FLT3, FOXP3, FUT7, HLA-DOA, HLA-DRA, HLA-DRB1, IFNG, IKZF1, IKZF3, IL12RB1, IL18, IL7R, INPP5D, IRF4, IRF8, ITGA4, ITK, KLRC1, LAG3, LCK, LEF1, LILRB1, LILRB4, LY9, MS4A1, MT1G, MYB, NCKAP1L, NFAM1, NLRP3, PLA2G2D, POU2AF1, POU2F2, PRDM1, PTK2B, PTPN22, PTPN6, PTPRC, RHOH, RUNX3, SASH3, SEMA4A, SLAMF6, SLAMF8, SOCS1, SPN, SYK, TBX21, TESPA1, THEMIS, TNFRSF18, TNFSF13B, TNFSF8, TOX, TREM2, TRPM2, UBD, VAV1, VNN1, WNT1, ZAP70, ZNF683]	[ABL1, AGER, AP3D1, ARMC5, BATF2, BCL3, BCL6, CCR7, CD74, CLCF1, CLPTM1, CSF1, CYP26B1, DHRS2, DTX1, ERBB2, FADD, FASN, FES, FLT3, GBA, GNAO1, HDAC5, IL15RA, IL1B, IL4R, IL7R, INHA, JAK3, KDELR1, LAG3, LGALS9, LRRC8A, LTBR, MYC, MYH9, NRARP, RARA, RELB, SOX12, SOX13, SPI1, SRF, STAT3, SYVN1, TCF3, TCF7L1, TCIRG1, VEGFA, VNN1, VSIR, ZBTB7A, ZBTB7B, ZC3H12A, ZMIZ1]
GO:0042102	positive regulation of T cell proliferation	7,83E-07	[6, 7, 8, 9]	40,00	38,10	81,69	18,31	[AIF1, CARD11, CCDC88B, CCL19, CCL5, CCR2, CD3E, CD40LG, CD6, CD80, CD86, CORO1A, EBI3, EPX, FOXP3, HLA-DMB, HLA-DPA1, HLA-DPB1, IL12RB1, IL18, NCKAP1L, PRKCQ, PTPN22, PTPRC, PYCARD, RASAL3, SASH3, SYK, TNFSF13B, XCL1, ZAP70]	[AGER, CD276, FADD, GPAM, IL1B, JAK3, LGALS9, SLC7A1, TIMP1]
GO:0046635	positive regulation of alpha-beta T cell activation	6,90E-08	[6, 7, 8, 9]	32,00	47,06	84,88	15,12	[CCL19, CCR2, CD3E, CD80, CD86, EBI3, FOXP3, HLA-DRA, HLA-DRB1, IFNG, IL12RB1, IL18, LILRB4, MYB, NCKAP1L, NLRP3, PRKCQ, PTPN22, PTPRC, RASAL3, RUNX3, SASH3, SOCS1, SYK, XCL1, ZAP70]	[AP3D1, GNAO1, IL4R, LGALS9, RARA, ZBTB7B]
GO:0030183	B cell differentiation	1,40E-02	[5, 6, 7, 8, 9, 10, 11]	41,00	27,52	69,39	30,61	[BCL3, BLNK, BTK, CARD11, CD19, CD27, CD40LG, CD79A, CLCF1, CR2, DOCK10, FCRL3, FLT3, IKZF3, INPP5D, IRF8, ITGA4, MS4A1, NCKAP1L, NFAM1, POU2AF1, POU2F2, PTK2B, PTPN6, PTPRC, SLAMF8, SYK, TNFSF13B]	[ABL1, BCL3, BCL6, CLCF1, FLT3, GNAO1, HDAC5, INHA, JAK3, LRRC8A, SPI1, SYVN1, TCF3, TCF7L1, TCIRG1, ZBTB7A]
GO:0030217	T cell differentiation	5,74E-17	[5, 6, 7, 8, 9, 10, 11]	97,00	35,66	72,16	27,84	[ADAM8, BATF, BCL11B, BCL3, CAMK4, CARD11, CCL19, CCR2, CD2, CD27, CD3D, CD3E, CD3G, CD4, CD74, CD80, CD86, CD8A, CDKN2A, CRTAM, CTLA4, DOCK2, EGR3, EOMES, FGL2, FOXP3, FUT7, HLA-DOA, HLA-DRA, HLA-DRB1, IFNG, IL12RB1, IL18, IL7R, IRF4, ITK, KLRC1, LAG3, LCK, LEF1, LILRB4, LY9, MYB, NCKAP1L, NLRP3, PLA2G2D, PRDM1, PTPN22, PTPRC, RHOH, RUNX3, SASH3, SEMA4A, SLAMF6, SOCS1, SPN, SYK, TBX21, TESPA1, THEMIS, TNFRSF18, TNFSF8, TOX, VAV1, VNN1, WNT1, ZAP70, ZNF683]	[ABL1, AP3D1, ARMC5, BCL3, BCL6, CCR7, CD74, CLPTM1, CYP26B1, DTX1, ERBB2, FADD, GBA, IL1B, IL4R, IL7R, JAK3, KDELR1, LAG3, LGALS9, NRARP, RARA, RELB, SOX12, SOX13, SPI1, SRF, STAT3, TCIRG1, VNN1, VSIR, ZBTB7B, ZC3H12A, ZMIZ1]
GO:0045619	regulation of lymphocyte differentiation	9,57E-15	[5, 6, 7, 8, 9, 10, 11]	71,00	39,66	73,36	26,64	[ADAM8, BTK, CAMK4, CARD11, CCL19, CCR2, CD2, CD27, CD74, CD80, CD86, CDKN2A, CRTAM, CTLA4, EGR3, FCRL3, FGL2, FOXP3, HLA-DOA, HLA-DRA, HLA-DRB1, IFNG, IKZF3, IL12RB1, IL18, IL7R, INPP5D, IRF4, LAG3, LEF1, LILRB4, MYB, NCKAP1L, NFAM1, NLRP3, PRDM1, PTPN6, PTPRC, RHOH, RUNX3, SASH3, SLAMF8, SOCS1, SYK, TBX21, TESPA1, TNFRSF18, TOX, VNN1, ZAP70, ZNF683]	[ABL1, AP3D1, BCL6, CD74, CLPTM1, CYP26B1, DTX1, ERBB2, IL15RA, IL4R, IL7R, INHA, JAK3, LAG3, LGALS9, NRARP, RARA, SOX12, SOX13, VNN1, VSIR, ZBTB7B, ZC3H12A, ZMIZ1]
GO:0050852	T cell receptor signaling pathway	1,90E-03	[6, 7, 8, 9, 10]	57,00	26,03	77,69	22,31	[BTN3A1, BTN3A2, CARD11, CD226, CD247, CD3D, CD3E, CD3G, CD4, CTLA4, FOXP3, FYB1, GRAP2, HLA-DPA1, HLA-DPB1, HLA-DQA1, HLA-DRA, HLA-DRB1, HLA-DRB5, INPP5D, ITK, KCNN4, LCK, LCP2, LILRB4, MOG, NR4A3, PRAM1, PRKCQ, PTPN22, PTPN6, PTPRC, SH2D1A, SKAP1, SLA2, TESPA1, THEMIS, THEMIS2, THY1, TRAT1, UBASH3A, WAS, ZAP70]	[ABL1, BCAR1, CCR7, CD276, CEACAM1, CSK, DGKZ, GNAO1, NCOR2, NECTIN2, NR4A3, RELA, RHBDF2, RNU1-93P, WAS, ZC3H12A]
GO:2000514	regulation of CD4-positive, alpha-beta T cell activation	4,57E-05	[7, 8, 9]	28,00	41,18	73,23	26,77	[CCL19, CD80, CD86, FOXP3, HLA-DRA, HLA-DRB1, IFNG, IL12RB1, IL18, IRF4, MYB, NCKAP1L, NLRP3, PRKCQ, RUNX3, SASH3, SOCS1, TBX21, XCL1]	[AGER, BCL6, IL4R, JAK3, LGALS9, RARA, VSIR, ZBTB7B, ZC3H12A]
GO:0045621	positive regulation of lymphocyte differentiation	4,57E-08	[5, 6, 7, 8, 9, 10, 11, 12]	42,00	40,00	74,15	25,85	[ADAM8, BTK, CCL19, CD27, CD74, CD80, CD86, EGR3, FOXP3, HLA-DRA, HLA-DRB1, IFNG, IL12RB1, IL18, IL7R, INPP5D, LEF1, LILRB4, MYB, NCKAP1L, NLRP3, PTPRC, RHOH, RUNX3, SASH3, SOCS1, SYK, TESPA1, TOX, VNN1, ZAP70]	[AP3D1, BCL6, CD74, IL15RA, IL4R, IL7R, LGALS9, RARA, SOX12, SOX13, VNN1, VSIR, ZBTB7B, ZMIZ1]
GO:2000516	positive regulation of CD4-positive, alpha-beta T cell activation	3,03E-05	[7, 8, 9, 10]	20,00	52,63	83,83	16,17	[CCL19, CD80, CD86, FOXP3, HLA-DRA, HLA-DRB1, IFNG, IL12RB1, IL18, MYB, NCKAP1L, NLRP3, PRKCQ, SASH3, SOCS1, XCL1]	[IL4R, LGALS9, RARA, ZBTB7B]
GO:0002292	T cell differentiation involved in immune response	7,33E-06	[6, 7, 8, 9, 10, 11, 12]	31,00	41,33	74,03	25,97	[BATF, BCL3, CCL19, CD80, CD86, EOMES, FGL2, FOXP3, HLA-DRA, HLA-DRB1, IFNG, IL12RB1, IL18, IRF4, LEF1, LY9, MYB, NLRP3, SEMA4A, SLAMF6, SPN, TBX21]	[BCL3, BCL6, IL4R, JAK3, LGALS9, RARA, RELB, STAT3, ZBTB7B, ZC3H12A]
GO:0045580	regulation of T cell differentiation	2,55E-13	[6, 7, 8, 9, 10, 11, 12]	62,00	40,79	72,16	27,84	[ADAM8, CAMK4, CARD11, CCL19, CCR2, CD2, CD27, CD74, CD80, CD86, CDKN2A, CRTAM, CTLA4, EGR3, FGL2, FOXP3, HLA-DOA, HLA-DRA, HLA-DRB1, IFNG, IL12RB1, IL18, IL7R, IRF4, LAG3, LEF1, LILRB4, MYB, NCKAP1L, NLRP3, PRDM1, PTPRC, RHOH, RUNX3, SASH3, SOCS1, SYK, TBX21, TESPA1, TNFRSF18, TOX, VNN1, ZAP70, ZNF683]	[ABL1, AP3D1, BCL6, CD74, CLPTM1, CYP26B1, DTX1, ERBB2, IL4R, IL7R, JAK3, LAG3, LGALS9, NRARP, RARA, SOX12, SOX13, VNN1, VSIR, ZBTB7B, ZC3H12A, ZMIZ1]
GO:0046632	alpha-beta T cell differentiation	7,53E-11	[6, 7, 8, 9, 10, 11, 12]	48,00	42,86	78,20	21,80	[BATF, BCL11B, BCL3, CCL19, CD80, CD86, EOMES, FOXP3, FUT7, HLA-DRA, HLA-DRB1, IFNG, IL12RB1, IL18, IRF4, ITK, LEF1, LILRB4, LY9, MYB, NCKAP1L, NLRP3, PLA2G2D, PRDM1, RUNX3, SASH3, SEMA4A, SLAMF6, SOCS1, SPN, SYK, TBX21, TNFSF8, TOX, ZAP70, ZNF683]	[ABL1, AP3D1, ARMC5, BCL3, BCL6, IL4R, JAK3, LGALS9, RARA, RELB, STAT3, ZBTB7B, ZC3H12A]

GO:0045582	positive regulation of T cell differentiation	1,32E-07	[6, 7, 8, 9, 10, 11, 12, 13]	38,00	41,30	73,62	26,38	[ADAM8, CCL19, CD27, CD74, CD80, CD86, EGR3, FOXP3, HLA-DRA, HLA-DRB1, IFNG, IL12RB1, IL18, IL7R, LEF1, LILRB4, MYB, NCKAP1L, NLRP3, PTPRC, RHOH, RUNX3, SASH3, SOCS1, SYK, TESPA1, VNN1, ZAP70]	[AP3D1, BCL6, CD74, IL4R, IL7R, LGALS9, RARA, SOX12, SOX13, VNN1, VSIR, ZBTB7B, ZMIZ1]
