# Supplementary material for: Epigenetic regulation of transcription factor binding motifs promotes Th1 response in Chagas disease cardiomyopathy
Source: Front Immunol. 2022 Aug 22;13:958200. doi: 10.3389/fimmu.2022.958200 (PMC9441916; doi:10.3389/fimmu.2022.958200)
Supplement: Supplementary Table 1 — Biological samples included in this study. [file DataSheet_1.zip › Supplementary Material/Supplementary Table 7.pdf]

Supplementary table 7. List of differentially expressed ncRNAs between control and severe CCC, or between control and dilated cardiomyopathies.

Gene ID	Gene name	Gene type	2FoldChange - CTRL vs CCC	pvalue - CTRL vs CCC	ected pvalue - CTRL vs CCC	DE - CTRL vs CCC	FoldChange - CTRL vs DCM	pvalue - CTRL vs DCM	cted pvalue - CTRL vs DCM	DE - CTRL vs DCM
ENSG00000271725	RP11-761I4.4	3prime_overlapping_ncrna	2,60E+00	1,90E-04	2,85E-03	Yes	1,57E-01	5,78E-01	7,12E-01	No
ENSG00000236423	LINC01134	lincRNA	-2,08E+00	1,73E-04	2,66E-03	Yes	-3,35E+00	7,46E-07	4,93E-06	Yes
ENSG00000176320	RP11-404O13.5	lincRNA	4,71E+00	3,45E-07	1,43E-05	Yes	2,66E+00	1,94E-03	6,68E-03	Yes
ENSG00000272275	RP11-791G15.2	lincRNA	-1,92E+00	9,86E-04	1,01E-02	Yes	-2,12E+00	1,02E-03	3,78E-03	Yes
ENSG00000234663	AC104820.2	lincRNA	5,42E+00	2,10E-18	7,66E-16	Yes	1,61E+00	7,40E-03	2,19E-02	Yes
ENSG00000270659	RP11-105N14.1	lincRNA	1,60E+00	1,66E-03	1,50E-02	Yes	3,24E+00	2,20E-09	2,13E-08	Yes
ENSG00000248986	RP11-774O3.1	lincRNA	-1,51E+00	1,15E-04	1,92E-03	Yes	-2,45E+00	2,30E-06	1,40E-05	Yes
ENSG00000246375	RP11-10L7.1	lincRNA	1,88E+00	2,22E-06	7,27E-05	Yes	1,75E+00	3,29E-05	1,66E-04	Yes
ENSG00000271538	RP11-326I11.4	lincRNA	1,60E+00	5,18E-04	6,26E-03	Yes	2,24E+00	4,84E-06	2,80E-05	Yes
ENSG00000250421	RP11-83M16.6	lincRNA	3,30E+00	1,82E-05	4,22E-04	Yes	3,61E+00	2,08E-05	1,08E-04	Yes
ENSG00000250994	AC005355.1	lincRNA	-1,62E+00	1,07E-03	1,08E-02	Yes	-2,22E+00	3,83E-04	1,55E-03	Yes
ENSG00000272279	RP11-157J24.2	lincRNA	3,78E+00	4,53E-04	5,64E-03	Yes	3,01E+00	1,81E-03	6,28E-03	Yes
ENSG00000223414	LINC00473	lincRNA	-2,65E+00	7,21E-09	4,66E-07	Yes	-1,68E+00	9,13E-05	4,18E-04	Yes
ENSG00000235994	RP3-470B24.5	lincRNA	-3,12E+00	5,12E-06	1,47E-04	Yes	-2,62E+00	5,21E-04	2,05E-03	Yes
ENSG00000236039	AC019117.2	lincRNA	-1,55E+00	5,29E-03	3,55E-02	Yes	-2,06E+00	3,68E-03	1,18E-02	Yes
ENSG00000272899	RP11-309L24.9	lincRNA	-2,55E+00	3,82E-05	7,75E-04	Yes	-2,98E+00	4,77E-04	1,90E-03	Yes
ENSG00000230649	AC024084.1	lincRNA	2,12E+00	4,57E-03	3,20E-02	Yes	1,70E+00	1,47E-02	3,98E-02	Yes
ENSG00000229807	XIST	lincRNA	8,18E+00	4,31E-12	5,40E-10	Yes	6,04E+00	2,41E-06	1,46E-05	Yes
ENSG00000253702	RP11-567J20.1	lincRNA	3,04E+00	1,84E-04	2,79E-03	Yes	2,18E+00	4,93E-03	1,53E-02	Yes
ENSG00000253669	KB-1732A1.1	lincRNA	-2,33E+00	3,51E-05	7,23E-04	Yes	-2,48E+00	1,10E-05	6,01E-05	Yes
ENSG00000226237	RP11-276H19.1	lincRNA	1,57E+00	9,82E-04	1,00E-02	Yes	1,89E+00	8,25E-05	3,82E-04	Yes
ENSG00000256443	RP11-794G24.1	lincRNA	-2,19E+00	7,83E-04	8,46E-03	Yes	-4,24E+00	7,49E-08	5,84E-07	Yes
ENSG00000255414	LINC01059	lincRNA	-2,01E+00	5,83E-05	1,11E-03	Yes	-2,75E+00	2,30E-06	1,40E-05	Yes
ENSG00000256540	RP11-598F7.6	lincRNA	-1,87E+00	3,32E-05	6,90E-04	Yes	-3,86E+00	4,09E-14	7,01E-13	Yes
ENSG00000235532	LINC00402	lincRNA	1,58E+00	2,41E-03	1,99E-02	Yes	1,73E+00	1,45E-03	5,15E-03	Yes
ENSG00000258733	CTD-2341M24.1	lincRNA	2,32E+00	3,51E-08	1,92E-06	Yes	1,54E+00	4,51E-04	1,80E-03	Yes
ENSG00000260953	RP11-426C22.6	lincRNA	-1,77E+00	4,03E-04	5,17E-03	Yes	-3,34E+00	2,34E-06	1,42E-05	Yes
ENSG00000261008	AC004158.2	lincRNA	2,81E+00	1,03E-04	1,75E-03	Yes	1,74E+00	6,39E-03	1,92E-02	Yes
ENSG00000272799	RP11-474N24.6	lincRNA	-2,10E+00	4,32E-03	3,07E-02	Yes	-3,43E+00	3,58E-03	1,15E-02	Yes
ENSG00000225978	HAR1A	lincRNA	2,33E+00	5,28E-04	6,35E-03	Yes	2,40E+00	2,75E-04	1,15E-03	Yes
ENSG00000223695	RP4-633O19__A.1	lincRNA	-3,03E+00	1,49E-05	3,57E-04	Yes	-3,47E+00	8,06E-06	4,50E-05	Yes
ENSG00000259834	RP11-284N8.3	lincRNA	3,49E+00	4,14E-21	2,48E-18	Yes	1,27E+00	4,25E-05	2,08E-04	No
ENSG00000223804	RP6-206I17.1	lincRNA	2,17E+00	5,59E-09	3,77E-07	Yes	1,04E+00	1,62E-03	5,70E-03	No
ENSG00000231734	RP6-206I17.2	lincRNA	2,03E+00	3,41E-03	2,59E-02	Yes	8,73E-01	5,25E-02	1,16E-01	No
ENSG00000215863	LINC01138	lincRNA	2,57E+00	2,64E-03	2,14E-02	Yes	-1,88E-02	9,35E-01	NA	No
ENSG00000235999	RP11-403I13.8	lincRNA	1,56E+00	1,23E-05	3,05E-04	Yes	-3,82E-01	2,61E-01	4,08E-01	No
ENSG00000234996	RP11-480I12.7	lincRNA	1,79E+00	7,21E-03	4,46E-02	Yes	4,22E-02	8,06E-01	NA	No
ENSG00000240219	RP11-430C7.5	lincRNA	3,29E+00	9,83E-07	3,57E-05	Yes	2,74E-01	4,44E-01	5,98E-01	No
ENSG00000232812	RP11-459K23.2	lincRNA	4,07E+00	2,59E-03	2,11E-02	Yes	1,10E-01	6,85E-01	NA	No
ENSG00000228058	RP11-552D4.1	lincRNA	3,73E+00	4,10E-03	2,95E-02	Yes	8,55E-02	8,64E-01	NA	No
ENSG00000227630	LINC01132	lincRNA	-1,50E+00	5,26E-04	6,34E-03	Yes	-9,87E-01	2,73E-02	6,73E-02	No
ENSG00000238005	RP11-443B7.1	lincRNA	1,59E+00	4,93E-03	3,39E-02	Yes	2,81E-01	4,22E-01	5,78E-01	No
ENSG00000235576	AC092580.4	lincRNA	5,01E+00	7,04E-13	1,02E-10	Yes	9,23E-02	7,10E-01	8,15E-01	No
ENSG00000236790	LINC00299	lincRNA	3,33E+00	1,48E-07	6,86E-06	Yes	6,52E-01	1,14E-01	2,17E-01	No
ENSG00000228784	LINC00954	lincRNA	1,77E+00	5,06E-06	1,46E-04	Yes	-5,55E-02	8,68E-01	9,20E-01	No
ENSG00000237638	AC007386.2	lincRNA	1,85E+00	6,80E-03	4,27E-02	Yes	-1,30E-01	7,12E-01	NA	No
ENSG00000230606	AC159540.1	lincRNA	2,08E+00	2,85E-06	9,01E-05	Yes	1,98E-01	5,68E-01	7,04E-01	No
ENSG00000227403	AC009299.3	lincRNA	2,55E+00	5,46E-04	6,48E-03	Yes	2,27E-01	4,82E-01	6,33E-01	No
ENSG00000235192	AC009495.2	lincRNA	2,73E+00	3,77E-04	4,92E-03	Yes	-6,86E-02	7,59E-01	NA	No
ENSG00000271151	RP11-394I13.2	lincRNA	1,55E+00	1,87E-03	1,65E-02	Yes	6,41E-01	1,20E-01	2,27E-01	No
ENSG00000226681	AC020595.1	lincRNA	3,76E+00	4,12E-03	2,96E-02	Yes	1,17E-01	5,77E-01	NA	No
ENSG00000229647	AC007879.7	lincRNA	2,01E+00	5,73E-03	3,76E-02	Yes	5,81E-01	7,72E-02	1,60E-01	No
ENSG00000237940	AC093642.3	lincRNA	2,65E+00	1,23E-06	4,33E-05	Yes	2,58E-01	4,73E-01	6,24E-01	No
ENSG00000272282	RP11-222K16.2	lincRNA	2,85E+00	1,66E-04	2,56E-03	Yes	3,24E-01	3,28E-01	4,83E-01	No
ENSG00000261786	RP4-555D20.2	lincRNA	5,11E+00	9,57E-04	9,87E-03	Yes	8,55E-02	8,64E-01	NA	No
ENSG00000241163	LINC00877	lincRNA	1,63E+00	3,08E-03	2,40E-02	Yes	-6,28E-02	8,23E-01	8,92E-01	No
ENSG00000271856	RP11-861A13.4	lincRNA	2,69E+00	8,79E-05	1,54E-03	Yes	-7,43E-02	7,86E-01	8,67E-01	No
ENSG00000239482	RP11-90K6.1	lincRNA	-1,56E+00	2,50E-04	3,56E-03	Yes	-1,22E+00	4,44E-03	1,40E-02	No
ENSG00000260217	RP11-809F4.3	lincRNA	2,90E+00	1,43E-03	1,34E-02	Yes	6,72E-01	4,37E-02	1,00E-01	No

ENSG00000251408	RP11-586D19.2	lincRNA	7,15E+00	4,26E-08	2,28E-06	Yes	2,24E-01	2,71E-01	NA	No
ENSG00000246526	RP11-539L10.2	lincRNA	2,34E+00	3,24E-06	1,01E-04	Yes	-2,82E-01	4,22E-01	5,78E-01	No
ENSG00000249988	RP11-669M16.1	lincRNA	2,14E+00	4,63E-03	3,23E-02	Yes	5,57E-01	9,69E-02	1,91E-01	No
ENSG00000250829	RP11-11N5.1	lincRNA	3,29E+00	7,18E-05	1,30E-03	Yes	6,53E-01	6,02E-02	1,30E-01	No
ENSG00000251628	RP11-371M22.1	lincRNA	-1,87E+00	2,28E-07	9,90E-06	Yes	-9,96E-01	3,69E-04	1,50E-03	No
ENSG00000250603	CTC-228N24.2	lincRNA	-2,23E+00	6,01E-04	6,95E-03	Yes	-5,08E-01	1,92E-01	3,24E-01	No
ENSG00000272023	CTC-350I8.1	lincRNA	1,93E+00	5,70E-03	3,75E-02	Yes	5,56E-01	9,46E-02	1,87E-01	No
ENSG00000253522	MIR146A	lincRNA	3,33E+00	2,24E-07	9,76E-06	Yes	1,36E+00	1,75E-02	4,62E-02	No
ENSG00000253686	CTB-43E15.3	lincRNA	3,25E+00	4,09E-04	5,23E-03	Yes	-9,52E-02	6,78E-01	NA	No
ENSG00000272053	RP11-367G6.3	lincRNA	3,37E+00	1,52E-05	3,62E-04	Yes	1,17E-01	6,29E-01	7,54E-01	No
ENSG00000204110	RP1-153P14.8	lincRNA	6,77E+00	7,58E-07	2,86E-05	Yes	1,00E-01	7,75E-01	NA	No
ENSG00000227920	RP1-153P14.5	lincRNA	5,97E+00	3,94E-05	7,92E-04	Yes	8,51E-02	8,64E-01	NA	No
ENSG00000223946	RP11-533O20.2	lincRNA	1,58E+00	1,54E-03	1,42E-02	Yes	-1,80E-01	6,08E-01	7,37E-01	No
ENSG00000237567	RP3-359N14.2	lincRNA	2,71E+00	2,77E-03	2,22E-02	Yes	-9,51E-02	8,30E-01	NA	No
ENSG00000230943	RP11-367G18.1	lincRNA	5,98E+00	1,09E-05	2,79E-04	Yes	7,30E-01	1,16E-02	3,25E-02	No
ENSG00000227681	RP11-307P5.1	lincRNA	2,30E+00	2,77E-03	2,21E-02	Yes	5,94E-01	1,16E-01	2,20E-01	No
ENSG00000270933	CTD-2227E11.1	lincRNA	3,16E+00	2,54E-04	3,61E-03	Yes	4,70E-01	9,44E-02	1,87E-01	No
ENSG00000272908	RP11-121A8.1	lincRNA	2,97E+00	2,31E-07	1,00E-05	Yes	2,16E-01	5,17E-01	6,62E-01	No
ENSG00000273142	RP11-458F8.4	lincRNA	2,19E+00	1,07E-04	1,80E-03	Yes	-2,59E-01	4,27E-01	5,82E-01	No
ENSG00000242258	LINC00996	lincRNA	3,62E+00	1,96E-07	8,69E-06	Yes	8,82E-03	9,77E-01	9,86E-01	No
ENSG00000223511	RP13-297E16.4	lincRNA	5,00E+00	6,30E-07	2,43E-05	Yes	1,53E-01	5,25E-01	NA	No
ENSG00000205663	RP11-706O15.5	lincRNA	1,78E+00	2,18E-03	1,85E-02	Yes	8,38E-02	7,83E-01	8,65E-01	No
ENSG00000270641	TSIX	lincRNA	1,16E+01	1,37E-08	8,26E-07	Yes	1,52E-01	3,14E-14	5,45E-13	No
ENSG00000233858	AC026904.1	lincRNA	1,99E+00	8,86E-05	1,55E-03	Yes	7,98E-01	6,61E-02	1,41E-01	No
ENSG00000261618	RP11-79H23.3	lincRNA	2,29E+00	1,12E-03	1,11E-02	Yes	1,70E-01	6,12E-01	7,41E-01	No
ENSG00000272264	RP11-92K15.3	lincRNA	3,37E+00	1,21E-04	1,99E-03	Yes	5,34E-02	7,57E-01	NA	No
ENSG00000248599	RP11-302J23.1	lincRNA	2,72E+00	3,59E-07	1,48E-05	Yes	1,87E+00	5,50E-01	6,90E-01	No
ENSG00000255080	RP11-1082L8.3	lincRNA	2,46E+00	4,01E-03	2,91E-02	Yes	1,22E+00	2,45E-02	6,15E-02	No
ENSG00000249816	LINC00964	lincRNA	-1,70E+00	3,21E-05	6,69E-04	Yes	-3,89E-01	2,88E-01	4,38E-01	No
ENSG00000245164	LINC00861	lincRNA	4,08E+00	2,82E-09	2,04E-07	Yes	9,62E-01	3,22E-02	7,73E-02	No
ENSG00000238113	RP11-262H14.1	lincRNA	2,19E+00	1,69E-09	1,27E-07	Yes	4,37E-01	1,53E-01	2,72E-01	No
ENSG00000226337	RP11-274B18.4	lincRNA	-1,90E+00	4,47E-03	3,15E-02	Yes	-7,63E-01	8,15E-02	1,67E-01	No
ENSG00000230537	RP11-305L7.1	lincRNA	1,69E+00	3,80E-04	4,94E-03	Yes	-2,30E-01	5,13E-01	6,59E-01	No
ENSG00000227531	RP11-202G18.1	lincRNA	1,92E+00	1,21E-03	1,18E-02	Yes	-3,50E-01	2,99E-01	4,51E-01	No
ENSG00000231528	FAM225A	lincRNA	1,85E+00	4,56E-03	3,19E-02	Yes	2,68E-01	3,64E-01	5,21E-01	No
ENSG00000237943	PRKCQ-AS1	lincRNA	2,75E+00	2,86E-08	1,61E-06	Yes	3,47E-01	3,39E-01	4,95E-01	No
ENSG00000238266	LINC00707	lincRNA	1,89E+00	2,07E-03	1,78E-02	Yes	-6,53E-01	9,81E-02	1,93E-01	No
ENSG00000233261	LINC00264	lincRNA	4,47E+00	1,93E-03	1,69E-02	Yes	1,09E-01	6,90E-01	NA	No
ENSG00000226667	RP11-292F22.5	lincRNA	4,83E+00	4,12E-04	5,25E-03	Yes	6,34E-02	9,56E-01	NA	No
ENSG00000254872	RP13-870H17.3	lincRNA	3,07E+00	8,19E-04	8,74E-03	Yes	9,94E-02	6,78E-01	NA	No
ENSG00000255299	RP11-655C2.3	lincRNA	1,68E+00	8,16E-03	4,88E-02	Yes	2,16E-01	4,45E-01	5,99E-01	No
ENSG00000255183	RP11-720D4.3	lincRNA	7,12E+00	1,62E-07	7,43E-06	Yes	2,40E-01	3,32E-01	NA	No
ENSG00000226091	LINC00937	lincRNA	1,89E+00	2,29E-04	3,32E-03	Yes	1,63E-01	6,38E-01	7,61E-01	No
ENSG00000256582	RP11-75L1.1	lincRNA	3,27E+00	2,15E-04	3,14E-03	Yes	-2,64E-02	9,20E-01	NA	No
ENSG00000256155	RP11-277P12.9	lincRNA	6,09E+00	6,78E-07	2,61E-05	Yes	1,87E-01	3,76E-01	NA	No
ENSG00000256039	RP11-291B21.2	lincRNA	7,00E+00	4,07E-32	1,11E-28	Yes	1,90E-01	5,39E-01	6,82E-01	No
ENSG00000258181	RP11-493L12.4	lincRNA	2,08E+00	1,69E-03	1,51E-02	Yes	3,86E-01	2,66E-01	4,14E-01	No
ENSG00000251301	RP11-81H14.2	lincRNA	3,33E+00	1,52E-07	7,00E-06	Yes	7,58E-02	8,25E-01	8,93E-01	No
ENSG00000189238	LINC00943	lincRNA	4,34E+00	1,17E-13	1,96E-11	Yes	2,12E-01	5,31E-01	6,74E-01	No
ENSG00000256128	LINC00944	lincRNA	4,02E+00	3,49E-11	3,65E-09	Yes	1,84E-02	9,51E-01	9,71E-01	No
ENSG00000238121	LINC00426	lincRNA	5,57E+00	3,65E-27	4,97E-24	Yes	1,41E+00	4,07E-03	1,29E-02	No
ENSG00000225039	LINC01058	lincRNA	2,66E+00	1,56E-03	1,43E-02	Yes	-7,79E-02	7,24E-01	NA	No
ENSG00000258929	RP11-58E21.3	lincRNA	2,18E+00	5,68E-06	1,61E-04	Yes	5,16E-01	1,73E-01	3,00E-01	No
ENSG00000258912	RP11-1079H9.1	lincRNA	2,13E+00	6,12E-04	7,04E-03	Yes	2,90E-01	3,73E-01	5,30E-01	No
ENSG00000246084	CTD-2506J14.1	lincRNA	8,10E+00	5,67E-11	5,72E-09	Yes	2,95E-01	2,11E-01	NA	No
ENSG00000258379	RP11-204N11.2	lincRNA	6,57E+00	6,11E-07	2,37E-05	Yes	1,74E-01	4,42E-01	NA	No
ENSG00000253701	AL928768.3	lincRNA	4,46E+00	3,72E-06	1,13E-04	Yes	-1,33E-01	7,12E-01	NA	No
ENSG00000244620	AL122127.25	lincRNA	2,26E+00	4,19E-03	3,00E-02	Yes	-1,30E-01	7,12E-01	NA	No
ENSG00000259772	RP11-16E12.2	lincRNA	3,20E+00	1,36E-09	1,05E-07	Yes	-6,92E-02	8,21E-01	8,91E-01	No
ENSG00000259731	RP11-326N17.1	lincRNA	6,95E+00	9,03E-08	4,48E-06	Yes	1,19E-01	5,59E-01	NA	No
ENSG00000259278	RP11-62C7.2	lincRNA	4,69E+00	1,20E-07	5,73E-06	Yes	7,45E-02	6,59E-01	NA	No
ENSG00000259727	RP11-1069G10.2	lincRNA	-1,93E+00	7,24E-04	7,98E-03	Yes	-1,08E+00	2,36E-02	5,96E-02	No

ENSG00000267539	RP11-138H8.7	lincRNA	2,37E+00	3,72E-03	2,75E-02	Yes	1,05E+00	2,90E-02	7,08E-02	No
ENSG00000258676	RP11-386M24.3	lincRNA	-1,70E+00	3,34E-03	2,55E-02	Yes	4,80E-01	1,95E-01	3,27E-01	No
ENSG00000270127	RP11-526I2.5	lincRNA	1,79E+00	2,92E-04	4,01E-03	Yes	4,89E-01	2,05E-01	3,41E-01	No
ENSG00000261218	RP11-960L18.1	lincRNA	2,73E+00	1,21E-03	1,18E-02	Yes	4,66E-02	7,93E-01	NA	No
ENSG00000266389	CTB-41I6.1	lincRNA	1,56E+00	3,68E-03	2,73E-02	Yes	-1,39E-01	6,28E-01	7,52E-01	No
ENSG00000266378	RP11-214O1.3	lincRNA	1,63E+00	1,26E-03	1,22E-02	Yes	3,36E-01	3,56E-01	5,13E-01	No
ENSG00000266999	AC015849.16	lincRNA	3,18E+00	2,89E-04	3,98E-03	Yes	1,16E-01	5,83E-01	7,16E-01	No
ENSG00000264198	RP11-94L15.2	lincRNA	4,63E+00	6,09E-23	4,68E-20	Yes	5,05E-01	1,55E-01	2,76E-01	No
ENSG00000266088	RP5-1028K7.2	lincRNA	6,14E+00	3,52E-05	7,24E-04	Yes	1,82E-01	4,08E-01	NA	No
ENSG00000244649	CTD-2377D24.6	lincRNA	5,42E+00	3,28E-05	6,84E-04	Yes	8,77E-02	8,64E-01	NA	No
ENSG00000265055	AC145343.2	lincRNA	-2,15E+00	4,01E-03	2,91E-02	Yes	-6,89E-01	1,05E-01	2,04E-01	No
ENSG00000265787	CYP4F35P	lincRNA	3,60E+00	6,17E-05	1,15E-03	Yes	7,40E-02	6,40E-01	NA	No
ENSG00000267311	RP11-99A1.2	lincRNA	4,26E+00	1,31E-03	1,26E-02	Yes	1,94E-01	3,62E-01	NA	No
ENSG00000235621	LINC00494	lincRNA	7,14E+00	7,54E-09	4,86E-07	Yes	9,02E-02	8,64E-01	NA	No
ENSG00000268027	AC006129.2	lincRNA	2,87E+00	5,30E-10	4,50E-08	Yes	5,40E-01	1,38E-01	2,52E-01	No
ENSG00000269220	LINC00528	lincRNA	2,33E+00	4,05E-06	1,21E-04	Yes	-3,11E-01	3,85E-01	5,43E-01	No
ENSG00000236499	LINC00896	lincRNA	1,99E+00	1,04E-04	1,77E-03	Yes	1,65E-01	6,40E-01	7,63E-01	No
ENSG00000225783	MIAT	lincRNA	3,21E+00	4,72E-19	1,97E-16	Yes	-1,05E+00	1,48E-04	6,53E-04	No
ENSG00000226751	AF127936.5	lincRNA	2,48E+00	7,05E-06	1,94E-04	Yes	1,08E+00	2,32E-02	5,86E-02	No
ENSG00000237484	AP000476.1	lincRNA	2,16E+00	3,50E-06	1,07E-04	Yes	9,62E-01	2,23E-02	5,69E-02	No
ENSG00000234883	MIR155HG	lincRNA	1,75E+00	1,19E-04	1,97E-03	Yes	8,94E-01	1,87E-02	4,88E-02	No
ENSG00000240754	RP11-38J22.6	lincRNA	6,47E+00	6,85E-07	2,63E-05	Yes	NA	NA	NA	NA
ENSG00000122043	LINC00544	lincRNA	5,42E+00	6,61E-05	1,22E-03	Yes	NA	NA	NA	NA
ENSG00000253364	RP11-731F5.2	lincRNA	4,40E+00	9,07E-04	9,49E-03	Yes	NA	NA	NA	NA
ENSG00000236481	AC002331.1	lincRNA	4,39E+00	1,38E-03	1,30E-02	Yes	NA	NA	NA	NA
ENSG00000272763	RP11-357H14.17	lincRNA	4,63E+00	8,74E-04	9,21E-03	Yes	NA	NA	NA	NA
ENSG00000199157	MIR208A	miRNA	-1,81E+00	5,58E-03	3,70E-02	Yes	-1,99E+00	7,95E-03	2,33E-02	Yes
ENSG00000265768	MIR4506	miRNA	-1,84E+00	2,26E-03	1,90E-02	Yes	-4,02E+00	1,42E-06	8,95E-06	Yes
ENSG00000264925	Z98949.1	miRNA	-2,28E+00	2,96E-05	6,28E-04	Yes	-4,17E+00	3,83E-08	3,11E-07	Yes
ENSG00000264773	MIR4420	miRNA	1,83E+00	6,95E-03	4,34E-02	Yes	-1,15E-01	6,53E-01	NA	No
ENSG00000216054	AC019201.1	miRNA	4,44E+00	9,08E-06	2,40E-04	Yes	6,48E-01	8,10E-02	1,66E-01	No
ENSG00000266705	MIR4437	miRNA	2,63E+00	2,12E-03	1,82E-02	Yes	-2,14E-02	9,21E-01	NA	No
ENSG00000263642	MIR4802	miRNA	2,25E+00	4,33E-03	3,07E-02	Yes	1,07E+00	3,10E-02	7,49E-02	No
ENSG00000216009	MIR874	miRNA	2,27E+00	2,12E-03	1,81E-02	Yes	3,24E-01	2,61E-01	4,08E-01	No
ENSG00000222086	AC010609.1	miRNA	3,47E+00	4,19E-04	5,31E-03	Yes	-2,34E-02	8,90E-01	NA	No
ENSG00000221743	Z95152.1	miRNA	1,76E+00	3,83E-03	2,81E-02	Yes	1,59E-01	5,70E-01	7,06E-01	No
ENSG00000207939	MIR223	miRNA	3,41E+00	1,23E-05	3,05E-04	Yes	1,05E+00	4,21E-02	9,71E-02	No
ENSG00000221299	Z83826.1	miRNA	-2,53E+00	2,20E-04	3,20E-03	Yes	-3,90E-01	2,96E-01	4,47E-01	No
ENSG00000266017	MIR4477A	miRNA	1,88E+00	1,06E-05	2,74E-04	Yes	3,38E-01	3,55E-01	5,11E-01	No
ENSG00000265154	MIR151B	miRNA	3,14E+00	1,18E-04	1,96E-03	Yes	-3,03E-02	8,94E-01	NA	No
ENSG00000264386	MIR4513	miRNA	2,50E+00	3,54E-03	2,66E-02	Yes	1,36E-02	9,38E-01	NA	No
ENSG00000221476	MIR1827	miRNA	2,53E+00	2,16E-03	1,84E-02	Yes	2,41E-01	3,58E-01	NA	No
ENSG00000263806	AL592188.3	miRNA	2,26E+00	7,62E-04	8,31E-03	Yes	3,58E-01	2,84E-01	4,34E-01	No
ENSG00000264781	hsa-mir-4537	miRNA	5,63E+00	1,56E-05	3,72E-04	Yes	NA	NA	NA	NA
ENSG00000265714	AL122127.3	miRNA	5,86E+00	1,58E-05	3,74E-04	Yes	NA	NA	NA	NA
ENSG00000238342	snoU13	snoRNA	3,08E+00	8,34E-04	8,88E-03	Yes	2,84E+00	2,20E-03	7,49E-03	Yes
ENSG00000201957	SNORA25	snoRNA	1,61E+00	3,73E-04	4,87E-03	Yes	-7,72E-01	7,88E-02	1,62E-01	No
ENSG00000238322	snoU13	snoRNA	1,97E+00	5,10E-03	3,46E-02	Yes	1,25E+00	2,63E-02	6,51E-02	No
ENSG00000238450	snoU13	snoRNA	2,72E+00	7,74E-04	8,39E-03	Yes	1,49E+00	1,86E-02	4,86E-02	No
ENSG00000252985	SNORD116	snoRNA	2,48E+00	8,50E-05	1,50E-03	Yes	1,68E-02	9,55E-01	9,74E-01	No
ENSG00000238528	snoU13	snoRNA	1,55E+00	5,41E-04	6,46E-03	Yes	1,33E-01	7,06E-01	8,11E-01	No
ENSG00000238685	ACA64	snoRNA	4,13E+00	1,71E-03	1,53E-02	Yes	1,14E-01	6,86E-01	NA	No
ENSG00000212456	RNVU1-13	snRNA	2,47E+00	1,05E-04	1,78E-03	Yes	1,64E+00	3,24E-03	1,06E-02	Yes
ENSG00000251889	RNU4-49P	snRNA	2,15E+00	5,77E-03	3,78E-02	Yes	3,13E+00	3,23E-04	1,33E-03	Yes
ENSG00000199377	RNU5F-1	snRNA	3,32E+00	2,71E-04	3,79E-03	Yes	1,70E-01	4,73E-01	NA	No
ENSG00000252105	RNU1-143P	snRNA	3,11E+00	1,80E-04	2,74E-03	Yes	5,51E-03	9,81E-01	NA	No
ENSG00000206965	RNU6-5P	snRNA	1,61E+00	5,50E-03	3,67E-02	Yes	2,78E-01	3,76E-01	5,33E-01	No
ENSG00000222389	RNU2-28P	snRNA	-2,14E+00	1,06E-04	1,79E-03	Yes	-7,21E-01	5,57E-02	1,22E-01	No
ENSG00000222057	RNU4-62P	snRNA	2,29E+00	2,67E-05	5,76E-04	Yes	-1,04E-01	7,61E-01	8,50E-01	No
ENSG00000206875	RNU6-761P	snRNA	2,16E+00	3,54E-03	2,66E-02	Yes	4,05E-01	2,41E-01	3,84E-01	No
ENSG00000251891	RNU7-79P	snRNA	-2,28E+00	6,92E-04	7,76E-03	Yes	-4,10E-01	2,76E-01	4,25E-01	No
ENSG00000242540	AC010729.1	3prime_overlapping_ncrna	1,63E-01	1,92E-01	NA	No	3,05E+00	1,73E-03	6,04E-03	Yes

ENSG00000233631	RP11-457M11.2	3prime_overlapping_ncrna	6,68E-01	3,90E-02	1,47E-01	No	2,21E+00	3,08E-06	1,84E-05	Yes
ENSG00000272084	RP5-1126H10.2	3prime_overlapping_ncrna	2,16E-02	9,23E-01	9,68E-01	No	-8,52E-01	5,77E-03	1,76E-02	No
ENSG00000243389	AC012442.5	3prime_overlapping_ncrna	2,82E-03	9,88E-01	9,96E-01	No	-3,97E-02	9,05E-01	9,43E-01	No
ENSG00000241772	AC092620.2	3prime_overlapping_ncrna	3,97E-01	1,24E-01	3,22E-01	No	-1,63E-02	9,60E-01	9,76E-01	No
ENSG00000241409	AC064852.4	3prime_overlapping_ncrna	2,11E-01	2,89E-01	5,35E-01	No	9,81E-01	3,79E-02	8,88E-02	No
ENSG00000250686	RP1-240B8.3	3prime_overlapping_ncrna	7,66E-02	3,80E-01	NA	No	1,21E-01	5,25E-01	NA	No
ENSG00000240143	RP4-753P9.3	3prime_overlapping_ncrna	4,37E-01	7,71E-02	2,34E-01	No	-1,05E+00	3,19E-02	NA	No
ENSG00000255343	RP11-299M14.2	3prime_overlapping_ncrna	1,88E-02	8,64E-01	NA	No	1,44E-01	4,88E-01	NA	No
ENSG00000257499	RP11-571M6.8	3prime_overlapping_ncrna	5,65E-01	2,36E-02	1,04E-01	No	-2,68E-01	3,62E-01	5,18E-01	No
ENSG00000267174	CTC-510F12.4	3prime_overlapping_ncrna	1,56E-01	2,24E-01	NA	No	1,66E-02	9,35E-01	NA	No
ENSG00000225880	LINC00115	lincRNA	2,89E-01	1,92E-01	4,21E-01	No	-1,52E+00	6,29E-06	3,56E-05	Yes
ENSG00000225285	RP4-758J18.10	lincRNA	-7,80E-01	2,11E-02	9,55E-02	No	-1,60E+00	2,35E-03	7,95E-03	Yes
ENSG00000272235	RP11-22L13.1	lincRNA	-8,34E-01	1,58E-03	1,44E-02	No	-2,02E+00	3,05E-08	2,52E-07	Yes
ENSG00000225643	RP11-70P17.1	lincRNA	-2,16E-02	9,18E-01	9,65E-01	No	-1,57E+00	4,39E-03	1,38E-02	Yes
ENSG00000234810	RP11-466L17.1	lincRNA	3,34E-01	1,08E-01	2,93E-01	No	2,54E+00	1,79E-04	7,79E-04	Yes
ENSG00000223956	RP4-710M16.2	lincRNA	-1,49E+00	5,12E-03	3,47E-02	No	-1,60E+00	5,34E-03	1,65E-02	Yes
ENSG00000270457	RP11-467C18.1	lincRNA	-1,24E+00	9,00E-03	5,24E-02	No	-3,32E+00	1,92E-04	8,27E-04	Yes
ENSG00000231252	RP11-436K8.1	lincRNA	1,46E+00	4,31E-03	3,06E-02	No	2,42E+00	2,19E-04	9,32E-04	Yes
ENSG00000231485	RP4-535B20.1	lincRNA	4,45E-01	9,59E-02	2,71E-01	No	2,35E+00	1,41E-05	7,57E-05	Yes
ENSG00000272864	RP11-17E13.2	lincRNA	6,47E-02	6,66E-01	8,36E-01	No	1,78E+00	5,08E-03	1,57E-02	Yes
ENSG00000273487	RP4-621B10.8	lincRNA	2,24E-01	1,52E-01	3,66E-01	No	3,14E+00	5,54E-06	3,17E-05	Yes
ENSG00000270066	SCARNA2	lincRNA	3,72E-01	1,02E-02	5,72E-02	No	2,53E+00	7,91E-52	2,58E-49	Yes
ENSG00000237291	RP11-782C8.4	lincRNA	5,83E-02	7,78E-01	8,99E-01	No	-3,96E+00	5,31E-06	3,05E-05	Yes
ENSG00000272654	RP11-422P24.11	lincRNA	8,95E-01	5,79E-03	3,79E-02	No	1,70E+00	5,39E-06	3,09E-05	Yes
ENSG00000260460	RP11-284F21.8	lincRNA	-2,09E-01	3,48E-01	5,97E-01	No	-1,94E+00	5,58E-04	2,18E-03	Yes
ENSG00000272068	RP11-284F21.9	lincRNA	-3,82E-01	8,36E-02	2,47E-01	No	-2,50E+00	2,99E-03	9,85E-03	Yes
ENSG00000224259	LINC01133	lincRNA	4,48E-01	3,11E-02	1,25E-01	No	1,81E+00	7,09E-03	2,11E-02	Yes
ENSG00000271811	RP1-79C4.4	lincRNA	-9,56E-02	6,66E-01	8,36E-01	No	-1,72E+00	1,79E-06	1,11E-05	Yes
ENSG00000233791	LINC01136	lincRNA	-4,72E-01	6,76E-02	2,14E-01	No	-1,55E+00	1,57E-02	4,20E-02	Yes
ENSG00000259865	RP11-488L18.10	lincRNA	6,61E-01	1,99E-02	9,15E-02	No	2,32E+00	3,32E-14	5,76E-13	Yes
ENSG00000233005	AC067959.1	lincRNA	1,38E-01	1,18E-01	NA	No	4,62E+00	1,80E-03	6,26E-03	Yes
ENSG00000270210	RP11-373D23.3	lincRNA	-2,45E-01	2,61E-01	5,05E-01	No	-3,58E+00	2,62E-04	1,10E-03	Yes
ENSG00000231327	AC016700.5	lincRNA	-1,85E-02	9,31E-01	9,72E-01	No	-2,02E+00	2,16E-03	7,37E-03	Yes
ENSG00000261600	RP11-575H3.1	lincRNA	3,54E-01	7,02E-02	2,20E-01	No	2,25E+00	3,46E-03	1,12E-02	Yes
ENSG00000270190	RP11-803D5.4	lincRNA	-1,11E-01	5,87E-01	7,88E-01	No	-4,65E+00	4,43E-07	3,05E-06	Yes
ENSG00000240350	AC017002.1	lincRNA	-4,07E-01	1,19E-01	3,12E-01	No	-2,83E+00	3,05E-06	1,83E-05	Yes
ENSG00000224959	AC017002.2	lincRNA	-6,64E-01	3,09E-02	1,25E-01	No	-2,26E+00	2,72E-06	1,64E-05	Yes
ENSG00000227359	AC017074.2	lincRNA	-7,16E-01	2,65E-02	1,12E-01	No	-1,62E+00	1,45E-03	5,14E-03	Yes
ENSG00000270081	RP5-935K16.1	lincRNA	2,14E-01	1,59E-01	3,76E-01	No	1,52E+00	6,24E-19	1,74E-17	Yes
ENSG00000226383	AC093375.1	lincRNA	4,60E-01	4,45E-02	1,61E-01	No	1,99E+00	9,11E-04	3,41E-03	Yes
ENSG00000225873	LINC00694	lincRNA	4,94E-02	8,24E-01	9,23E-01	No	-2,57E+00	3,41E-05	1,71E-04	Yes
ENSG00000240405	RP11-460N16.1	lincRNA	2,13E-01	1,17E-01	NA	No	1,61E+00	1,50E-02	4,04E-02	Yes
ENSG00000272597	RP11-446H18.6	lincRNA	4,64E-01	4,22E-02	1,55E-01	No	2,43E+00	1,52E-03	5,39E-03	Yes
ENSG00000241732	RP11-38P22.2	lincRNA	2,01E-01	3,07E-01	5,55E-01	No	2,13E+00	9,78E-12	1,28E-10	Yes
ENSG00000241544	RP11-6F2.5	lincRNA	-5,21E-01	5,44E-02	1,84E-01	No	-1,53E+00	6,88E-03	2,05E-02	Yes
ENSG00000273193	RP11-523G9.3	lincRNA	3,14E-01	1,49E-01	3,61E-01	No	2,04E+00	8,68E-05	4,00E-04	Yes
ENSG00000241231	RP11-275H4.1	lincRNA	5,14E-01	4,17E-02	1,54E-01	No	1,94E+00	8,90E-03	2,57E-02	Yes
ENSG00000223401	RP11-211G3.2	lincRNA	-1,26E+00	2,38E-03	1,98E-02	No	-1,93E+00	4,07E-05	2,00E-04	Yes
ENSG00000250986	AC141928.1	lincRNA	-6,96E-01	9,20E-04	9,58E-03	No	-1,59E+00	5,95E-08	4,72E-07	Yes
ENSG00000245468	RP11-367J11.3	lincRNA	7,49E-01	2,26E-02	1,00E-01	No	1,63E+00	3,10E-05	1,57E-04	Yes
ENSG00000250863	RP11-663P9.1	lincRNA	5,98E-02	4,04E-01	NA	No	4,77E+00	2,64E-03	8,82E-03	Yes
ENSG00000251399	RP11-234K19.1	lincRNA	1,26E+00	2,88E-03	2,28E-02	No	1,68E+00	6,61E-04	2,54E-03	Yes
ENSG00000273156	RP11-127B20.2	lincRNA	8,61E-02	6,91E-01	8,52E-01	No	2,00E+00	5,06E-06	2,91E-05	Yes
ENSG00000248161	RP11-499E18.1	lincRNA	2,03E-01	6,80E-02	NA	No	2,26E+00	5,42E-03	1,67E-02	Yes
ENSG00000260091	RP11-33B1.4	lincRNA	3,12E-01	2,06E-01	4,39E-01	No	-2,28E+00	1,43E-03	5,08E-03	Yes
ENSG00000250777	RP13-884E18.4	lincRNA	1,84E-01	1,88E-01	4,16E-01	No	2,06E+00	4,92E-03	1,53E-02	Yes
ENSG00000272784	RP11-335L23.5	lincRNA	7,55E-02	5,49E-01	NA	No	2,22E+00	3,25E-03	1,06E-02	Yes
ENSG00000249096	RP11-290F5.1	lincRNA	-2,44E-01	2,90E-01	5,37E-01	No	-2,01E+00	4,68E-07	3,21E-06	Yes
ENSG00000271771	RP11-1250I15.3	lincRNA	9,23E-01	1,81E-02	8,55E-02	No	1,59E+00	2,39E-03	8,08E-03	Yes
ENSG00000152931	PART1	lincRNA	4,43E-01	9,34E-02	2,66E-01	No	1,68E+00	2,23E-05	1,15E-04	Yes
ENSG00000272308	RP11-231G3.1	lincRNA	6,97E-01	2,83E-02	1,17E-01	No	1,73E+00	4,82E-03	1,50E-02	Yes
ENSG00000249364	RP11-434D9.1	lincRNA	1,14E+00	1,05E-02	5,82E-02	No	1,84E+00	1,32E-03	4,73E-03	Yes

ENSG00000247877	CTD-2001C12.1	lincRNA	1,05E+00	1,46E-02	7,39E-02	No	1,76E+00	3,60E-03	1,16E-02	Yes
ENSG00000253807	LINC01170	lincRNA	4,24E-01	4,69E-02	1,66E-01	No	2,69E+00	5,75E-04	2,24E-03	Yes
ENSG00000248107	CTC-339D2.1	lincRNA	7,84E-01	1,72E-02	8,24E-02	No	2,43E+00	3,45E-04	1,41E-03	Yes
ENSG00000250564	RP11-215P8.4	lincRNA	-3,01E-01	3,09E-02	1,25E-01	No	-2,13E+00	1,14E-02	3,19E-02	Yes
ENSG00000250635	CTD-3224K15.2	lincRNA	-2,05E-01	3,64E-01	6,11E-01	No	-2,00E+00	6,22E-03	1,88E-02	Yes
ENSG00000272108	AC005754.8	lincRNA	-2,87E-01	2,27E-01	4,66E-01	No	1,58E+00	3,37E-05	1,69E-04	Yes
ENSG00000249669	MIR143HG	lincRNA	-8,22E-01	1,27E-03	1,23E-02	No	-2,38E+00	5,03E-17	1,15E-15	Yes
ENSG00000253864	AC131025.8	lincRNA	-4,46E-01	7,09E-02	2,21E-01	No	-2,14E+00	8,80E-11	1,02E-09	Yes
ENSG00000269936	MIR145	lincRNA	-8,54E-01	1,02E-03	1,03E-02	No	-2,35E+00	1,22E-12	1,80E-11	Yes
ENSG00000253298	AC008703.1	lincRNA	-8,68E-01	1,78E-02	8,44E-02	No	-1,94E+00	5,72E-03	1,75E-02	Yes
ENSG00000250274	CTB-114C7.4	lincRNA	-6,56E-01	1,43E-02	7,27E-02	No	-3,42E+00	1,28E-03	4,61E-03	Yes
ENSG00000272142	RP11-428J1.5	lincRNA	7,95E-02	7,21E-01	8,70E-01	No	-1,77E+00	1,01E-03	3,74E-03	Yes
ENSG00000272341	RP1-151F17.2	lincRNA	3,45E-01	1,29E-01	3,30E-01	No	1,65E+00	3,01E-08	2,49E-07	Yes
ENSG00000224843	LINC00240	lincRNA	5,90E-01	4,27E-02	1,56E-01	No	1,91E+00	2,00E-05	1,04E-04	Yes
ENSG00000228789	HCG22	lincRNA	-3,07E-01	1,71E-01	3,95E-01	No	-2,63E+00	1,12E-03	4,10E-03	Yes
ENSG00000271761	XXbac-BPGBPG55C20.3	lincRNA	6,68E-03	9,52E-01	NA	No	2,03E+00	4,53E-03	1,42E-02	Yes
ENSG00000223633	RP11-143A22.1	lincRNA	2,16E-01	2,83E-01	5,30E-01	No	1,92E+00	2,85E-04	1,19E-03	Yes
ENSG00000271945	RP11-354K4.2	lincRNA	1,71E-01	4,38E-01	6,77E-01	No	1,53E+00	3,60E-03	1,16E-02	Yes
ENSG00000236013	RP3-332B22.1	lincRNA	3,83E-01	8,81E-02	2,56E-01	No	1,77E+00	3,53E-09	3,32E-08	Yes
ENSG00000234147	RP3-460G2.2	lincRNA	2,64E-02	8,66E-01	9,43E-01	No	2,17E+00	5,26E-05	2,54E-04	Yes
ENSG00000237851	RP1-67K17.4	lincRNA	-1,09E+00	1,18E-02	6,32E-02	No	-2,90E+00	3,12E-05	1,57E-04	Yes
ENSG00000224460	RP11-439L18.2	lincRNA	-1,44E+00	3,22E-04	4,33E-03	No	-1,69E+00	2,61E-05	1,33E-04	Yes
ENSG00000270419	CAHM	lincRNA	5,32E-01	5,31E-02	1,81E-01	No	1,98E+00	4,41E-09	4,10E-08	Yes
ENSG00000273100	RP11-302L19.3	lincRNA	-4,77E-02	8,03E-01	9,13E-01	No	-1,83E+00	3,64E-03	1,17E-02	Yes
ENSG00000272953	RP11-1275H24.2	lincRNA	2,38E-01	9,42E-02	2,68E-01	No	2,30E+00	4,63E-03	1,45E-02	Yes
ENSG00000223561	AC003090.1	lincRNA	6,38E-01	3,98E-02	1,49E-01	No	1,63E+00	3,92E-04	1,59E-03	Yes
ENSG00000227544	AC018647.3	lincRNA	1,25E-01	5,50E-01	7,62E-01	No	1,57E+00	1,89E-09	1,85E-08	Yes
ENSG00000229180	GS1-124K5.11	lincRNA	5,90E-02	7,62E-01	8,91E-01	No	-2,27E+00	9,39E-13	1,40E-11	Yes
ENSG00000237513	RP11-325F22.2	lincRNA	3,53E-01	9,89E-02	2,76E-01	No	-2,00E+00	3,99E-03	1,27E-02	Yes
ENSG00000239815	RP11-309L24.4	lincRNA	-1,18E+00	9,59E-03	5,48E-02	No	-3,18E+00	3,88E-05	1,92E-04	Yes
ENSG00000234449	RP11-706O15.3	lincRNA	-6,28E-01	2,48E-04	3,54E-03	No	-2,30E+00	1,07E-04	4,84E-04	Yes
ENSG00000270012	LL0XNC01-7P3.1	lincRNA	1,05E-01	5,64E-01	7,72E-01	No	-2,26E+00	5,37E-12	7,30E-11	Yes
ENSG00000253270	RP11-1105O14.1	lincRNA	-9,63E-01	2,35E-03	1,96E-02	No	-2,13E+00	4,42E-08	3,57E-07	Yes
ENSG00000253733	LZTS1-AS1	lincRNA	-3,99E-01	1,26E-01	3,25E-01	No	-1,56E+00	8,84E-03	2,55E-02	Yes
ENSG00000272327	RP11-1002K11.1	lincRNA	1,30E+00	5,29E-01	7,50E-01	No	1,59E+00	5,47E-03	1,68E-02	Yes
ENSG00000270673	YTHDF3-AS1	lincRNA	7,44E-02	6,95E-01	8,55E-01	No	1,65E+00	2,88E-04	1,20E-03	Yes
ENSG00000272010	CTD-3025N20.3	lincRNA	4,29E-01	1,00E-01	2,79E-01	No	2,83E+00	4,19E-10	4,46E-09	Yes
ENSG00000253339	RP11-434I12.3	lincRNA	-3,26E-01	4,02E-02	NA	No	-4,19E+00	1,55E-02	4,15E-02	Yes
ENSG00000253824	KB-173C10.2	lincRNA	2,47E-01	2,19E-01	4,56E-01	No	2,00E+00	1,55E-03	5,46E-03	Yes
ENSG00000271882	KB-1410C5.5	lincRNA	1,20E-01	3,80E-01	NA	No	2,87E+00	1,31E-04	5,84E-04	Yes
ENSG00000245330	KB-1471A8.1	lincRNA	7,12E-01	1,96E-02	9,04E-02	No	2,86E+00	1,15E-03	4,21E-03	Yes
ENSG00000231052	RP11-91N2.3	lincRNA	-5,17E-01	4,67E-03	3,25E-02	No	-3,53E+00	2,25E-03	7,65E-03	Yes
ENSG00000205636	LINC00583	lincRNA	4,56E-02	6,85E-01	NA	No	2,26E+00	3,99E-03	1,27E-02	Yes
ENSG00000269900	RMRP	lincRNA	2,38E-01	2,51E-01	4,93E-01	No	1,91E+00	4,57E-24	2,06E-22	Yes
ENSG00000182021	RP11-381O7.3	lincRNA	-8,83E-02	6,87E-01	8,50E-01	No	-1,51E+00	1,20E-03	4,35E-03	Yes
ENSG00000234506	RP11-274B18.2	lincRNA	-1,82E-01	2,93E-01	5,41E-01	No	-2,29E+00	3,67E-03	1,18E-02	Yes
ENSG00000270102	RP11-498E2.8	lincRNA	-4,64E-01	7,50E-02	2,30E-01	No	-1,79E+00	4,73E-03	1,48E-02	Yes
ENSG00000233901	RP11-65J3.1	lincRNA	3,82E-02	8,63E-01	9,41E-01	No	-1,55E+00	3,42E-03	1,11E-02	Yes
ENSG00000226676	RP11-589B3.6	lincRNA	-8,46E-02	6,35E-01	8,19E-01	No	-2,96E+00	1,85E-03	6,41E-03	Yes
ENSG00000226159	RP11-478K7.2	lincRNA	1,46E+00	2,90E-03	2,30E-02	No	1,85E+00	1,28E-03	4,61E-03	Yes
ENSG00000228261	RP11-127L20.3	lincRNA	-6,13E-01	4,34E-02	1,58E-01	No	-2,83E+00	1,77E-08	1,52E-07	Yes
ENSG00000232259	RP11-4C20.3	lincRNA	-1,50E+00	5,11E-04	6,19E-03	No	-1,57E+00	5,51E-05	2,65E-04	Yes
ENSG00000230724	LINC01001	lincRNA	2,39E-01	2,60E-01	5,04E-01	No	-1,54E+00	3,90E-06	2,29E-05	Yes
ENSG00000254401	RP11-179A10.1	lincRNA	-3,83E-01	6,81E-02	2,15E-01	No	-2,99E+00	6,83E-04	2,62E-03	Yes
ENSG00000261340	RP11-215H22.1	lincRNA	3,36E-01	1,77E-01	4,01E-01	No	2,17E+00	5,62E-07	3,79E-06	Yes
ENSG00000255375	RP1-65P5.5	lincRNA	-6,87E-01	1,47E-02	7,43E-02	No	-2,26E+00	3,91E-03	1,25E-02	Yes
ENSG00000244953	RP11-613D13.8	lincRNA	-8,40E-01	2,22E-02	9,91E-02	No	-2,22E+00	4,09E-05	2,01E-04	Yes
ENSG00000250230	RP11-855O10.2	lincRNA	-5,21E-02	8,02E-01	9,12E-01	No	-3,66E+00	1,11E-03	4,07E-03	Yes
ENSG00000245532	NEAT1	lincRNA	-9,75E-01	1,56E-02	7,71E-02	No	-1,70E+00	6,73E-04	2,59E-03	Yes
ENSG00000254810	RP11-672A2.4	lincRNA	-6,20E-01	3,67E-02	1,41E-01	No	-3,58E+00	8,89E-07	5,80E-06	Yes
ENSG00000270160	RP11-264E20.2	lincRNA	-2,00E-02	9,10E-01	9,63E-01	No	-5,88E+00	1,59E-03	5,59E-03	Yes
ENSG00000250770	RP5-1063M23.1	lincRNA	-3,27E-02	8,78E-01	9,49E-01	No	-1,53E+00	4,23E-04	1,70E-03	Yes

ENSG00000256995	RP11-114G22.1	lincRNA	-2,68E-01	9,20E-03	5,32E-02	No	-3,79E+00	2,15E-03	7,34E-03	Yes
ENSG00000257258	RP11-946L16.1	lincRNA	2,16E-01	5,17E-02	1,78E-01	No	1,79E+00	9,73E-03	2,78E-02	Yes
ENSG00000256538	RP11-847H18.3	lincRNA	5,73E-01	3,02E-02	1,23E-01	No	1,72E+00	1,40E-02	3,81E-02	Yes
ENSG00000261586	RP11-923I11.6	lincRNA	2,44E-02	9,13E-01	9,64E-01	No	-1,70E+00	4,98E-04	1,97E-03	Yes
ENSG00000256268	RP11-221N13.3	lincRNA	1,79E-01	2,98E-01	5,45E-01	No	1,74E+00	3,56E-03	1,15E-02	Yes
ENSG00000257613	RP11-320P7.1	lincRNA	9,31E-01	9,28E-03	5,35E-02	No	1,59E+00	2,21E-04	9,42E-04	Yes
ENSG00000258170	RP11-263K4.5	lincRNA	3,02E-01	4,33E-02	NA	No	2,99E+00	1,40E-02	3,81E-02	Yes
ENSG00000185847	RP1-46F2.2	lincRNA	-6,05E-01	4,93E-02	1,72E-01	No	-1,51E+00	4,81E-04	1,91E-03	Yes
ENSG00000271579	RP11-116D17.3	lincRNA	8,04E-01	2,00E-02	9,19E-02	No	2,29E+00	2,16E-03	7,37E-03	Yes
ENSG00000272849	RP11-347I19.8	lincRNA	1,46E-01	5,18E-01	7,42E-01	No	-2,45E+00	2,71E-05	1,38E-04	Yes
ENSG00000251497	RP11-197N18.7	lincRNA	-1,27E+00	2,46E-03	2,03E-02	No	-2,34E+00	1,50E-06	9,37E-06	Yes
ENSG00000255992	RP11-417L19.4	lincRNA	-3,66E-01	1,28E-01	3,28E-01	No	-2,51E+00	2,82E-03	9,35E-03	Yes
ENSG00000250790	RP11-46H11.3	lincRNA	3,56E-01	5,06E-02	1,75E-01	No	2,86E+00	5,58E-04	2,18E-03	Yes
ENSG00000261728	RP11-307O13.1	lincRNA	1,59E-01	4,13E-01	6,56E-01	No	1,62E+00	1,54E-03	5,44E-03	Yes
ENSG00000272349	RP11-397O8.7	lincRNA	6,43E-02	6,86E-01	8,49E-01	No	2,62E+00	2,95E-06	1,77E-05	Yes
ENSG00000258768	CTD-2292M16.8	lincRNA	1,23E+00	3,27E-05	6,83E-04	No	1,52E+00	1,07E-06	6,87E-06	Yes
ENSG00000258751	RP11-2G1.1	lincRNA	3,61E-01	1,07E-02	NA	No	3,45E+00	1,02E-02	2,89E-02	Yes
ENSG00000258753	RP11-794A8.1	lincRNA	-5,16E-01	7,14E-02	2,22E-01	No	-2,20E+00	5,11E-04	2,02E-03	Yes
ENSG00000250548	RP11-47I22.2	lincRNA	-1,69E-01	4,53E-01	6,89E-01	No	-1,84E+00	1,21E-04	5,41E-04	Yes
ENSG00000258943	RP11-696D21.2	lincRNA	-5,75E-01	5,53E-02	1,86E-01	No	-2,88E+00	3,82E-04	1,55E-03	Yes
ENSG00000258675	RP11-299L17.3	lincRNA	1,76E-01	1,83E-01	NA	No	2,88E+00	1,04E-03	3,85E-03	Yes
ENSG00000269963	RP11-73M18.9	lincRNA	6,55E-02	7,61E-01	8,90E-01	No	-2,17E+00	2,64E-03	8,82E-03	Yes
ENSG00000235731	AC124997.1	lincRNA	-9,15E-01	1,53E-02	7,62E-02	No	-1,67E+00	3,50E-03	1,13E-02	Yes
ENSG00000259395	RP11-475A13.2	lincRNA	-6,88E-01	3,57E-02	1,38E-01	No	-2,13E+00	2,74E-03	9,11E-03	Yes
ENSG00000272003	RP11-23P13.7	lincRNA	-6,77E-01	6,98E-04	7,80E-03	No	-1,58E+00	1,63E-12	2,36E-11	Yes
ENSG00000259438	CTD-2650P22.1	lincRNA	2,32E-01	8,18E-02	NA	No	2,08E+00	8,94E-03	2,58E-02	Yes
ENSG00000260672	RP11-1006G14.1	lincRNA	-1,61E-01	4,05E-01	6,49E-01	No	-1,64E+00	1,14E-02	3,20E-02	Yes
ENSG00000258551	RP11-661P17.1	lincRNA	1,83E-01	2,56E-01	4,99E-01	No	2,30E+00	3,27E-04	1,35E-03	Yes
ENSG00000259582	RP11-461F11.3	lincRNA	2,42E-01	8,94E-02	2,59E-01	No	1,80E+00	9,78E-03	2,79E-02	Yes
ENSG00000263105	RP11-95P2.3	lincRNA	-1,35E+00	6,36E-05	1,18E-03	No	-2,57E+00	4,01E-08	3,25E-07	Yes
ENSG00000259807	RP11-426C22.4	lincRNA	3,41E-01	1,61E-01	3,79E-01	No	-1,51E+00	7,22E-03	2,14E-02	Yes
ENSG00000261474	RP11-452L6.1	lincRNA	1,74E-01	4,01E-01	6,46E-01	No	-2,11E+00	1,55E-07	1,15E-06	Yes
ENSG00000261512	RP11-46D6.1	lincRNA	2,13E-01	3,17E-01	5,66E-01	No	-1,93E+00	1,19E-07	9,01E-07	Yes
ENSG00000263126	CTC-479C5.10	lincRNA	1,82E-02	9,35E-01	9,73E-01	No	-2,46E+00	1,75E-04	7,61E-04	Yes
ENSG00000271009	RP11-346C20.3	lincRNA	-6,32E-02	7,74E-01	8,97E-01	No	1,55E+00	1,56E-05	8,29E-05	Yes
ENSG00000260884	AC009120.5	lincRNA	-1,39E-02	9,48E-01	9,79E-01	No	-2,16E+00	4,38E-07	3,02E-06	Yes
ENSG00000270124	RP11-118F19.1	lincRNA	4,47E-02	8,38E-01	9,30E-01	No	-2,27E+00	2,86E-04	1,19E-03	Yes
ENSG00000261193	RP11-863P13.5	lincRNA	-7,10E-02	5,88E-01	7,89E-01	No	-4,07E+00	1,75E-02	4,62E-02	Yes
ENSG00000180422	LINC00304	lincRNA	5,01E-02	8,19E-01	9,21E-01	No	-2,22E+00	4,74E-03	1,48E-02	Yes
ENSG00000186594	MIR22HG	lincRNA	-1,07E+00	8,39E-04	8,91E-03	No	-1,66E+00	2,78E-06	1,67E-05	Yes
ENSG00000264727	RP11-680C21.1	lincRNA	1,67E-01	4,00E-01	6,45E-01	No	1,54E+00	5,30E-03	1,63E-02	Yes
ENSG00000266651	RP11-138I1.3	lincRNA	-3,48E-01	1,54E-01	3,69E-01	No	-1,70E+00	7,49E-03	2,21E-02	Yes
ENSG00000264940	SNORD3C	lincRNA	5,30E-01	4,37E-02	1,59E-01	No	1,80E+00	1,02E-03	3,78E-03	Yes
ENSG00000263603	CTD-2349P21.5	lincRNA	-1,66E-01	3,29E-01	5,77E-01	No	-2,42E+00	7,17E-03	2,13E-02	Yes
ENSG00000265139	RP11-227G15.2	lincRNA	7,91E-03	9,66E-01	9,86E-01	No	3,47E+00	2,40E-15	4,67E-14	Yes
ENSG00000248714	RP11-1079K10.3	lincRNA	3,43E-01	1,66E-01	3,87E-01	No	-1,52E+00	8,44E-03	2,45E-02	Yes
ENSG00000262006	RP11-700H6.4	lincRNA	-5,93E-02	7,69E-01	8,94E-01	No	-2,04E+00	5,58E-03	1,71E-02	Yes
ENSG00000263931	RP11-180P8.1	lincRNA	1,31E-02	9,51E-01	9,80E-01	No	2,32E+00	3,39E-08	2,77E-07	Yes
ENSG00000263470	RP11-160O5.1	lincRNA	6,80E-01	3,28E-02	1,30E-01	No	1,54E+00	5,05E-04	1,99E-03	Yes
ENSG00000267461	RP11-120M18.5	lincRNA	-6,13E-01	4,62E-02	1,65E-01	No	-2,07E+00	1,01E-03	3,75E-03	Yes
ENSG00000267194	RP1-193H18.2	lincRNA	8,97E-01	7,92E-03	4,78E-02	No	2,30E+00	1,36E-07	1,01E-06	Yes
ENSG00000260248	RP11-143K11.1	lincRNA	-4,09E-01	1,07E-01	2,91E-01	No	-1,80E+00	2,38E-03	8,05E-03	Yes
ENSG00000204277	RP11-219G17.4	lincRNA	-2,75E-01	1,36E-01	3,41E-01	No	-2,37E+00	8,06E-03	2,36E-02	Yes
ENSG00000266970	RP11-806H10.4	lincRNA	-9,95E-01	1,28E-02	6,71E-02	No	-1,62E+00	1,66E-02	4,41E-02	Yes
ENSG00000264345	RP11-958F21.1	lincRNA	3,68E-01	4,66E-02	1,66E-01	No	2,70E+00	6,19E-04	2,40E-03	Yes
ENSG00000267686	RP11-795H16.3	lincRNA	-1,13E+00	1,27E-02	6,66E-02	No	-2,01E+00	2,60E-03	8,69E-03	Yes
ENSG00000267279	RP11-879F14.2	lincRNA	5,78E-01	4,40E-02	1,59E-01	No	1,78E+00	4,36E-07	3,00E-06	Yes
ENSG00000228888	RP4-764O22.2	lincRNA	3,62E-01	1,53E-01	3,68E-01	No	1,65E+00	4,75E-04	1,89E-03	Yes
ENSG00000228539	RP11-526K17.2	lincRNA	-1,26E+00	4,85E-03	3,35E-02	No	-3,34E+00	1,95E-09	1,90E-08	Yes
ENSG00000231081	RP4-760C5.3	lincRNA	1,13E-01	6,14E-01	8,06E-01	No	-1,89E+00	6,69E-04	2,57E-03	Yes
ENSG00000267161	AC005943.5	lincRNA	-3,49E-01	1,67E-01	3,89E-01	No	-2,73E+00	1,51E-07	1,12E-06	Yes
ENSG00000267427	CTC-503J8.6	lincRNA	-5,29E-01	3,10E-04	4,21E-03	No	-2,88E+00	1,83E-45	3,55E-43	Yes

ENSG00000267612	CTD-3116E22.7	lincRNA	1,04E+00	1,45E-02	7,33E-02	No	1,67E+00	1,08E-02	3,04E-02	Yes
ENSG00000175898	CTD-2369P2.2	lincRNA	-7,57E-01	7,68E-03	4,67E-02	No	-2,83E+00	5,44E-13	8,30E-12	Yes
ENSG00000267519	MIR24-2	lincRNA	-8,64E-01	4,25E-03	3,04E-02	No	-1,64E+00	8,75E-05	4,02E-04	Yes
ENSG00000268751	SCGB1B2P	lincRNA	-2,76E-01	2,50E-01	4,92E-01	No	-1,87E+00	3,80E-03	1,22E-02	Yes
ENSG00000267381	CTD-2086O20.3	lincRNA	2,19E-01	2,89E-01	5,35E-01	No	1,85E+00	3,36E-17	7,89E-16	Yes
ENSG00000267473	AC005789.11	lincRNA	-5,08E-01	3,28E-02	1,30E-01	No	-2,31E+00	3,52E-03	1,14E-02	Yes
ENSG00000267242	AC069278.4	lincRNA	1,10E+00	1,00E-02	5,65E-02	No	2,03E+00	3,69E-05	1,84E-04	Yes
ENSG00000268816	CTD-3093M3.1	lincRNA	4,24E-01	1,12E-01	3,01E-01	No	2,13E+00	1,88E-05	9,86E-05	Yes
ENSG00000232098	CTD-2619J13.14	lincRNA	2,59E-01	1,65E-01	3,85E-01	No	-1,58E+00	4,53E-07	3,11E-06	Yes
ENSG00000269473	CTD-2619J13.19	lincRNA	-4,61E-01	8,44E-02	2,49E-01	No	-1,55E+00	1,94E-04	8,38E-04	Yes
ENSG00000237438	CECR7	lincRNA	-1,81E-01	4,28E-01	6,68E-01	No	-1,72E+00	3,00E-04	1,24E-03	Yes
ENSG00000272216	LL22NC03-2H8.5	lincRNA	5,58E-01	5,01E-02	1,74E-01	No	1,82E+00	5,04E-07	3,43E-06	Yes
ENSG00000229891	Z83851.1	lincRNA	3,81E-01	1,27E-01	3,26E-01	No	1,52E+00	4,26E-04	1,71E-03	Yes
ENSG00000270093	AP000473.8	lincRNA	1,71E-01	4,29E-01	6,69E-01	No	1,91E+00	1,12E-03	4,11E-03	Yes
ENSG00000232118	BACH1-AS1	lincRNA	-7,89E-01	2,39E-02	1,04E-01	No	-1,66E+00	1,41E-03	5,02E-03	Yes
ENSG00000186842	LINC00846	lincRNA	-6,50E-02	7,66E-01	8,93E-01	No	-1,53E+00	9,68E-05	4,42E-04	Yes
ENSG00000235888	AF064858.8	lincRNA	-1,55E-01	4,75E-01	7,07E-01	No	-1,68E+00	2,08E-03	7,14E-03	Yes
ENSG00000237721	AF064858.11	lincRNA	-4,73E-02	8,21E-01	9,22E-01	No	-1,91E+00	1,19E-02	3,32E-02	Yes
ENSG00000237232	ZNF295-AS1	lincRNA	-3,22E-01	1,93E-01	4,23E-01	No	-2,18E+00	7,98E-04	3,02E-03	Yes
ENSG00000237613	FAM138A	lincRNA	-4,23E-02	7,60E-01	NA	No	-9,82E-02	8,30E-01	NA	No
ENSG00000238009	RP11-34P13.7	lincRNA	-3,24E-02	8,50E-01	9,35E-01	No	-3,81E-01	2,40E-01	3,83E-01	No
ENSG00000241599	RP11-34P13.9	lincRNA	1,27E-03	9,95E-01	9,98E-01	No	8,55E-02	7,83E-01	8,65E-01	No
ENSG00000228463	AP006222.2	lincRNA	7,72E-01	1,04E-02	5,79E-02	No	3,02E-02	9,33E-01	9,60E-01	No
ENSG00000237094	RP4-669L17.10	lincRNA	-6,31E-01	4,13E-02	1,53E-01	No	-1,38E+00	6,14E-03	1,86E-02	No
ENSG00000236601	RP4-669L17.2	lincRNA	-5,51E-02	5,71E-01	NA	No	-1,48E-01	6,05E-01	NA	No
ENSG00000236743	RP5-857K21.15	lincRNA	1,16E-02	9,24E-01	NA	No	-1,48E-01	5,91E-01	NA	No
ENSG00000231709	RP5-857K21.1	lincRNA	-1,60E-02	9,01E-01	NA	No	-3,60E-01	2,02E-01	NA	No
ENSG00000235146	RP5-857K21.2	lincRNA	-8,43E-02	3,88E-01	NA	No	-1,97E-01	4,41E-01	NA	No
ENSG00000239664	RP5-857K21.3	lincRNA	1,27E-02	9,65E-01	NA	No	-2,61E-02	9,21E-01	NA	No
ENSG00000230021	RP5-857K21.4	lincRNA	6,54E-02	7,34E-01	8,76E-01	No	4,04E-01	2,82E-01	4,31E-01	No
ENSG00000223659	RP5-857K21.5	lincRNA	2,40E-02	8,05E-01	NA	No	-5,64E-02	8,61E-01	NA	No
ENSG00000235373	RP11-206L10.3	lincRNA	1,37E-02	9,53E-01	9,80E-01	No	-6,28E-01	7,48E-02	1,55E-01	No
ENSG00000240618	RP11-206L10.5	lincRNA	3,22E-02	8,51E-01	9,35E-01	No	4,45E-01	2,43E-01	3,86E-01	No
ENSG00000228327	RP11-206L10.2	lincRNA	6,48E-01	3,17E-03	2,45E-02	No	-2,78E-01	2,55E-01	4,01E-01	No
ENSG00000237491	RP11-206L10.9	lincRNA	5,54E-01	8,95E-03	5,22E-02	No	5,36E-02	8,05E-01	8,80E-01	No
ENSG00000177757	FAM87B	lincRNA	7,64E-01	9,70E-03	5,52E-02	No	-9,40E-02	7,69E-01	8,56E-01	No
ENSG00000230368	FAM41C	lincRNA	1,16E-01	2,47E-01	NA	No	2,82E-01	2,36E-01	3,79E-01	No
ENSG00000272438	RP11-54O7.16	lincRNA	-3,71E-02	6,51E-01	NA	No	-7,33E-02	6,81E-01	NA	No
ENSG00000230699	RP11-54O7.1	lincRNA	-6,46E-02	5,91E-01	NA	No	-7,64E-03	9,77E-01	9,86E-01	No
ENSG00000223764	RP11-54O7.3	lincRNA	1,40E+00	3,64E-03	2,71E-02	No	1,28E+00	8,23E-03	2,40E-02	No
ENSG00000272512	RP11-54O7.17	lincRNA	-7,54E-01	4,55E-03	3,19E-02	No	-7,11E-01	1,46E-02	3,97E-02	No
ENSG00000272141	RP11-465B22.8	lincRNA	3,74E-02	8,45E-01	NA	No	8,51E-02	8,64E-01	NA	No
ENSG00000260179	RP5-902P8.12	lincRNA	1,94E-01	2,68E-01	5,12E-01	No	3,45E-01	3,24E-01	4,78E-01	No
ENSG00000230415	RP5-902P8.10	lincRNA	2,88E-01	2,36E-01	4,75E-01	No	-7,71E-01	6,24E-02	1,34E-01	No
ENSG00000272455	RP4-758J18.13	lincRNA	7,59E-01	1,11E-02	6,04E-02	No	3,59E-01	2,86E-01	4,36E-01	No
ENSG00000234396	RP11-181G12.4	lincRNA	-1,23E-01	2,21E-01	NA	No	-2,56E-01	2,89E-01	NA	No
ENSG00000272449	RP3-395M20.12	lincRNA	1,09E-01	6,25E-01	8,13E-01	No	-1,08E+00	4,28E-03	1,35E-02	No
ENSG00000272088	RP11-168F9.2	lincRNA	-1,26E-02	9,02E-01	NA	No	-1,38E-01	5,04E-01	NA	No
ENSG00000228750	RP11-242F24.1	lincRNA	-5,73E-02	6,46E-01	NA	No	-1,36E-01	7,12E-01	NA	No
ENSG00000270171	RP11-338N10.1	lincRNA	8,47E-02	4,55E-01	NA	No	-1,52E-01	5,59E-01	NA	No
ENSG00000270035	RP11-338N10.2	lincRNA	2,40E-01	1,04E-01	NA	No	3,12E-01	2,13E-01	3,50E-01	No
ENSG00000269978	RP11-338N10.3	lincRNA	3,67E-01	8,03E-02	2,41E-01	No	1,86E-01	5,21E-01	6,66E-01	No
ENSG00000238290	RP11-431K24.1	lincRNA	2,92E-01	1,28E-01	3,28E-01	No	9,33E-01	5,67E-02	1,24E-01	No
ENSG00000228423	RP4-633I8.4	lincRNA	4,92E-02	8,23E-01	9,23E-01	No	-8,64E-01	4,25E-02	9,79E-02	No
ENSG00000228526	RP3-510D11.1	lincRNA	2,37E-01	2,06E-01	4,39E-01	No	-5,67E-02	8,60E-01	9,15E-01	No
ENSG00000234546	RP3-510D11.2	lincRNA	8,29E-02	6,42E-01	8,23E-01	No	-5,92E-02	8,49E-01	9,08E-01	No
ENSG00000235263	RP13-392I16.1	lincRNA	4,64E-03	9,92E-01	NA	No	2,73E-01	2,65E-01	4,12E-01	No
ENSG00000229484	RP5-888M10.2	lincRNA	2,48E-01	1,01E-01	2,79E-01	No	2,62E-01	3,55E-01	NA	No
ENSG00000272482	RP11-474O21.5	lincRNA	1,04E-01	6,40E-01	8,22E-01	No	-2,65E-02	9,29E-01	9,58E-01	No
ENSG00000231606	RP11-344F13.1	lincRNA	2,97E-02	8,30E-01	9,25E-01	No	9,41E-01	5,12E-02	1,14E-01	No
ENSG00000234593	RP4-704D23.1	lincRNA	-2,85E-03	9,78E-01	NA	No	4,24E-01	2,04E-01	3,39E-01	No
ENSG00000228140	RP3-467K16.4	lincRNA	4,87E-03	9,65E-01	NA	No	-1,03E-01	6,13E-01	NA	No

ENSG00000271742	RP4-680D5.9	lincRNA	-4,82E-02	6,30E-01	NA	No	-1,66E-01	4,09E-01	NA	No
ENSG00000226029	RP4-798A10.2	lincRNA	4,04E-01	9,07E-02	2,61E-01	No	-3,74E-01	2,08E-01	3,44E-01	No
ENSG00000261135	RP4-798A10.7	lincRNA	2,85E-02	7,37E-01	NA	No	3,60E-01	1,62E-01	NA	No
ENSG00000224174	RP4-798A10.4	lincRNA	-7,73E-02	3,22E-01	NA	No	-1,78E-01	3,89E-01	NA	No
ENSG00000233421	U1	lincRNA	-4,26E-01	8,31E-04	8,85E-03	No	-5,19E-01	1,86E-01	3,17E-01	No
ENSG00000271732	RP5-1182A14.5	lincRNA	1,98E-01	3,26E-01	5,76E-01	No	-2,14E-01	5,14E-01	6,60E-01	No
ENSG00000215908	CROCCP2	lincRNA	6,98E-01	1,63E-04	2,53E-03	No	1,45E-01	4,42E-01	5,96E-01	No
ENSG00000230239	RP11-108M9.2	lincRNA	-8,39E-02	6,48E-01	8,26E-01	No	-6,31E-02	8,46E-01	9,06E-01	No
ENSG00000228549	U1	lincRNA	1,23E-01	2,62E-01	5,06E-01	No	-3,80E-01	3,08E-01	4,61E-01	No
ENSG00000238142	RP11-108M9.4	lincRNA	-1,09E-01	5,74E-01	7,79E-01	No	7,25E-01	5,71E-02	1,25E-01	No
ENSG00000272426	RP11-108M9.6	lincRNA	5,27E-02	7,28E-01	8,73E-01	No	-3,40E-02	9,13E-01	9,48E-01	No
ENSG00000236648	RP11-473A10.2	lincRNA	2,35E-02	9,72E-01	NA	No	9,88E-02	7,75E-01	NA	No
ENSG00000227066	RP3-340N1.2	lincRNA	4,29E-02	7,70E-01	8,95E-01	No	-3,15E-01	2,79E-01	NA	No
ENSG00000236963	LINC01141	lincRNA	5,61E-02	5,78E-01	NA	No	-1,46E-01	5,99E-01	NA	No
ENSG00000228397	RP1-224A6.3	lincRNA	3,26E-02	8,15E-01	NA	No	-5,75E-02	7,91E-01	8,71E-01	No
ENSG00000218510	LINC00339	lincRNA	-8,76E-03	9,65E-01	9,86E-01	No	7,37E-01	2,35E-02	5,92E-02	No
ENSG00000271840	RP1-224A6.9	lincRNA	2,54E-01	2,85E-01	5,31E-01	No	4,76E-01	1,35E-01	2,48E-01	No
ENSG00000272796	RP1-74M1.3	lincRNA	4,08E-01	6,75E-03	4,25E-02	No	1,09E-01	6,85E-01	NA	No
ENSG00000261326	RP5-1057J7.6	lincRNA	2,49E-01	2,51E-01	4,93E-01	No	8,05E-01	1,85E-03	6,41E-03	No
ENSG00000271420	RP5-1057J7.7	lincRNA	-3,93E-01	1,24E-01	3,21E-01	No	-4,37E-01	2,49E-01	3,94E-01	No
ENSG00000235052	RP1-150O5.3	lincRNA	-6,59E-03	9,47E-01	NA	No	-6,63E-02	7,71E-01	NA	No
ENSG00000230023	RP11-10N16.2	lincRNA	-4,15E-02	7,60E-01	NA	No	-9,51E-02	8,30E-01	NA	No
ENSG00000272432	RP3-465N24.6	lincRNA	-3,96E-02	8,50E-01	9,35E-01	No	4,59E-01	2,13E-01	3,51E-01	No
ENSG00000241169	RP4-752I6.1	lincRNA	4,80E-01	7,96E-02	2,39E-01	No	4,20E-03	9,91E-01	9,95E-01	No
ENSG00000233975	RP11-288L9.1	lincRNA	-8,55E-02	3,51E-01	NA	No	-1,75E-01	3,13E-01	NA	No
ENSG00000271398	RP5-1092A3.5	lincRNA	-8,92E-02	4,52E-01	NA	No	6,00E-02	8,32E-01	8,98E-01	No
ENSG00000229388	RP11-442N24__B.1	lincRNA	-2,63E-01	2,39E-01	4,80E-01	No	-3,48E-01	2,24E-01	3,64E-01	No
ENSG00000270103	RNU11	lincRNA	2,31E-01	3,17E-01	5,66E-01	No	4,94E-01	1,32E-01	2,43E-01	No
ENSG00000225750	RP11-242O24.3	lincRNA	3,74E-02	8,11E-01	9,16E-01	No	-6,47E-02	8,30E-01	8,96E-01	No
ENSG00000230523	RP3-437I16.1	lincRNA	-6,17E-02	5,92E-01	NA	No	-1,83E-01	3,67E-01	NA	No
ENSG00000223382	RP1-65J11.1	lincRNA	-1,09E-01	5,16E-01	7,40E-01	No	-2,13E-02	9,48E-01	9,69E-01	No
ENSG00000223907	RP11-439L8.4	lincRNA	5,53E-02	6,17E-01	NA	No	1,13E-01	6,49E-01	NA	No
ENSG00000269967	RP11-84A19.4	lincRNA	-1,39E-01	5,06E-01	7,32E-01	No	-4,59E-01	2,01E-01	3,36E-01	No
ENSG00000228634	RP4-534N18.2	lincRNA	-1,74E-01	4,45E-01	6,82E-01	No	-6,65E-01	8,18E-02	1,67E-01	No
ENSG00000233775	RP4-811H24.9	lincRNA	6,78E-02	5,16E-01	NA	No	-1,28E-01	7,12E-01	NA	No
ENSG00000270115	RP11-415J8.7	lincRNA	1,09E+00	6,60E-03	4,18E-02	No	-9,41E-02	7,83E-01	8,65E-01	No
ENSG00000271914	RP4-789D17.5	lincRNA	-2,85E-02	8,38E-01	9,30E-01	No	-1,03E-01	7,17E-01	8,20E-01	No
ENSG00000224592	RP5-884C9.2	lincRNA	-3,08E-03	8,98E-01	NA	No	-1,28E-01	7,12E-01	NA	No
ENSG00000237899	RP4-739H11.3	lincRNA	2,33E-01	1,86E-01	4,13E-01	No	3,36E-01	3,22E-01	4,76E-01	No
ENSG00000235358	RP11-399E6.1	lincRNA	-5,97E-01	3,24E-02	1,29E-01	No	-1,82E-01	5,95E-01	7,26E-01	No
ENSG00000234917	RP5-994D16.3	lincRNA	7,52E-02	7,15E-01	8,67E-01	No	2,54E-01	4,77E-01	6,28E-01	No
ENSG00000227533	SLC2A1-AS1	lincRNA	3,64E-01	1,40E-01	3,47E-01	No	1,07E-02	9,78E-01	9,86E-01	No
ENSG00000230615	RP5-1198O20.4	lincRNA	4,31E-02	7,54E-01	NA	No	1,41E-01	6,10E-01	7,39E-01	No
ENSG00000225506	CYP4A22-AS1	lincRNA	-2,81E-02	7,60E-01	NA	No	-1,30E-01	4,53E-01	NA	No
ENSG00000225028	RP11-330M19.1	lincRNA	-5,97E-02	7,62E-01	8,91E-01	No	-5,44E-01	1,70E-01	2,95E-01	No
ENSG00000226133	RP4-683M8.2	lincRNA	-1,95E-02	9,17E-01	9,65E-01	No	-7,08E-01	8,72E-02	1,76E-01	No
ENSG00000231413	RP11-193P11.3	lincRNA	2,02E-02	8,45E-01	NA	No	-1,26E-01	7,12E-01	NA	No
ENSG00000223720	RP5-1024N4.2	lincRNA	-5,25E-02	6,74E-01	NA	No	-1,83E-01	4,84E-01	NA	No
ENSG00000271782	RP5-850O15.4	lincRNA	-3,79E-02	8,50E-01	9,35E-01	No	2,63E-01	4,60E-01	6,13E-01	No
ENSG00000203356	RP11-296A18.3	lincRNA	9,84E-02	5,94E-01	7,92E-01	No	4,41E-01	2,43E-01	3,87E-01	No
ENSG00000272371	RP11-25O10.2	lincRNA	2,17E-01	1,60E-01	NA	No	-8,71E-02	6,98E-01	NA	No
ENSG00000270172	RP5-1024G6.8	lincRNA	1,72E-01	4,52E-01	6,88E-01	No	5,37E-01	1,56E-01	2,77E-01	No
ENSG00000225675	RP4-784A16.5	lincRNA	2,20E-01	4,02E-02	NA	No	9,02E-02	8,64E-01	NA	No
ENSG00000226938	RP11-117D22.1	lincRNA	-5,47E-02	6,46E-01	NA	No	-1,30E-01	7,12E-01	NA	No
ENSG00000230138	RP11-117D22.2	lincRNA	4,26E-02	6,91E-01	NA	No	5,93E-02	8,08E-01	NA	No
ENSG00000272091	RP4-758J24.5	lincRNA	-5,14E-01	4,88E-02	1,71E-01	No	-5,84E-01	1,77E-02	4,67E-02	No
ENSG00000231090	RP11-101C11.1	lincRNA	4,04E-02	7,80E-01	NA	No	9,20E-02	8,64E-01	NA	No
ENSG00000233147	RP11-90C4.1	lincRNA	8,53E-03	9,83E-01	NA	No	7,47E-02	7,69E-01	NA	No
ENSG00000235612	RP1-158P9.1	lincRNA	-8,87E-02	5,59E-01	7,69E-01	No	-2,68E-01	3,94E-01	5,51E-01	No
ENSG00000260971	RP11-504A18.1	lincRNA	-1,28E+00	5,89E-03	3,84E-02	No	-4,73E-01	1,92E-01	3,24E-01	No
ENSG00000227935	RP6-102O10.1	lincRNA	-2,48E-01	1,30E-01	3,31E-01	No	-2,54E-01	1,27E-01	2,36E-01	No
ENSG00000235038	RP11-393I23.4	lincRNA	3,03E-01	3,65E-02	1,40E-01	No	-5,40E-04	9,80E-01	NA	No

ENSG00000234807	LINC01135	lincRNA	-5,54E-02	7,89E-01	9,05E-01	No	-3,13E-01	3,71E-01	5,28E-01	No
ENSG00000272226	RP11-63G10.3	lincRNA	1,60E-01	2,59E-01	5,03E-01	No	2,13E-01	5,15E-01	6,61E-01	No
ENSG00000231740	RP11-63G10.2	lincRNA	1,82E-01	1,64E-01	NA	No	-9,31E-02	6,66E-01	NA	No
ENSG00000232453	RP4-794H19.1	lincRNA	-4,62E-02	8,32E-01	9,27E-01	No	-2,27E-01	5,02E-01	6,50E-01	No
ENSG00000230812	RP4-794H19.4	lincRNA	4,69E-01	7,88E-02	2,38E-01	No	5,54E-01	1,63E-01	2,85E-01	No
ENSG00000241042	RP11-145M4.1	lincRNA	3,65E-02	7,60E-01	NA	No	8,93E-02	7,09E-01	NA	No
ENSG00000235215	RP11-145M4.2	lincRNA	6,74E-01	1,17E-02	NA	No	8,96E-02	6,82E-01	NA	No
ENSG00000237352	RP11-145M4.3	lincRNA	3,40E-01	2,25E-02	1,00E-01	No	9,25E-01	2,49E-02	6,24E-02	No
ENSG00000231816	RP4-782L23.1	lincRNA	8,85E-02	5,38E-01	7,55E-01	No	7,63E-02	7,93E-01	8,72E-01	No
ENSG00000226476	RP11-776H12.1	lincRNA	2,91E-01	7,82E-02	2,37E-01	No	1,22E+00	2,82E-02	6,91E-02	No
ENSG00000237853	RP5-833A20.1	lincRNA	1,56E-01	4,84E-01	7,14E-01	No	5,18E-01	5,45E-02	1,20E-01	No
ENSG00000271200	RP11-430G17.3	lincRNA	8,73E-02	6,34E-01	8,19E-01	No	2,77E-01	4,44E-01	5,98E-01	No
ENSG00000234318	RP4-771M4.3	lincRNA	2,12E-02	7,39E-01	NA	No	6,75E-02	7,01E-01	NA	No
ENSG00000271593	RP11-335E6.4	lincRNA	-4,23E-02	7,60E-01	NA	No	-9,82E-02	8,30E-01	NA	No
ENSG00000224209	LINC00466	lincRNA	4,41E-03	9,28E-01	NA	No	1,95E-02	8,90E-01	NA	No
ENSG00000226891	RP11-182I10.3	lincRNA	4,13E-01	9,19E-02	2,64E-01	No	3,18E-01	2,33E-01	3,75E-01	No
ENSG00000272506	RP4-535B20.4	lincRNA	1,00E-01	6,30E-01	8,16E-01	No	-1,23E-01	7,18E-01	8,20E-01	No
ENSG00000271992	RP11-42O15.3	lincRNA	-3,41E-02	8,06E-01	NA	No	4,95E-02	8,74E-01	9,25E-01	No
ENSG00000237726	RP11-386O9.2	lincRNA	1,95E-02	8,95E-01	9,56E-01	No	5,10E-01	1,61E-01	2,84E-01	No
ENSG00000225087	RP4-660H19.1	lincRNA	1,20E-01	2,12E-01	NA	No	2,01E-01	3,42E-01	NA	No
ENSG00000227016	RP11-262K1.1	lincRNA	3,75E-02	8,45E-01	NA	No	8,51E-02	8,64E-01	NA	No
ENSG00000233973	RP4-598G3.1	lincRNA	-6,89E-02	3,34E-01	NA	No	-1,70E-01	3,75E-01	NA	No
ENSG00000272855	RP5-1102E8.3	lincRNA	-2,27E-01	1,91E-01	4,21E-01	No	3,00E-02	9,31E-01	9,59E-01	No
ENSG00000230817	RP4-601K24.1	lincRNA	4,32E-01	3,09E-02	1,25E-01	No	3,75E-01	2,07E-01	NA	No
ENSG00000236268	RP11-170N11.1	lincRNA	-5,69E-02	6,46E-01	NA	No	-1,35E-01	7,12E-01	NA	No
ENSG00000231364	RP11-413G15.1	lincRNA	-1,22E-01	2,59E-01	NA	No	-1,18E-01	6,50E-01	7,70E-01	No
ENSG00000233008	RP11-475O6.1	lincRNA	7,30E-01	2,73E-02	1,14E-01	No	6,73E-01	8,77E-02	1,76E-01	No
ENSG00000237076	RP5-836J3.1	lincRNA	7,24E-02	4,69E-01	NA	No	-3,10E-02	8,70E-01	NA	No
ENSG00000271576	RP11-486G15.2	lincRNA	7,19E-01	2,85E-02	1,18E-01	No	9,29E-01	2,42E-02	6,08E-02	No
ENSG00000273264	RP11-131L23.2	lincRNA	-1,06E-02	9,37E-01	9,74E-01	No	-5,25E-02	8,58E-01	9,14E-01	No
ENSG00000272691	RP11-290M5.4	lincRNA	-1,83E-02	8,95E-01	9,56E-01	No	-1,33E-01	6,20E-01	7,46E-01	No
ENSG00000230285	RP11-290M5.2	lincRNA	6,95E-02	4,53E-01	NA	No	3,32E-01	1,44E-01	NA	No
ENSG00000267734	RP4-604K5.3	lincRNA	3,24E-01	1,85E-01	4,12E-01	No	-1,51E-02	9,61E-01	9,77E-01	No
ENSG00000267272	LINC01140	lincRNA	3,75E-03	9,88E-01	9,96E-01	No	1,55E-01	5,51E-01	6,91E-01	No
ENSG00000227290	RP4-544H6.2	lincRNA	1,89E-01	3,35E-01	5,84E-01	No	6,66E-01	1,10E-01	2,11E-01	No
ENSG00000224235	RP5-1027O11.1	lincRNA	-1,49E-01	1,42E-01	NA	No	1,08E-01	7,00E-01	8,08E-01	No
ENSG00000272931	RP5-943J3.2	lincRNA	-3,22E-02	7,83E-01	NA	No	-1,70E-02	9,42E-01	9,66E-01	No
ENSG00000272672	RP11-302M6.5	lincRNA	4,40E-01	8,08E-02	2,42E-01	No	-7,19E-02	8,28E-01	8,95E-01	No
ENSG00000229201	RP5-827O9.1	lincRNA	4,03E-02	7,82E-01	NA	No	6,13E-02	9,56E-01	NA	No
ENSG00000233593	RP4-665J23.1	lincRNA	-1,21E-01	5,56E-01	7,67E-01	No	2,02E-01	4,43E-01	5,97E-01	No
ENSG00000225446	RP4-665J23.2	lincRNA	-7,60E-02	3,78E-01	NA	No	-1,01E-01	6,13E-01	NA	No
ENSG00000272094	RP4-665J23.4	lincRNA	-2,46E-03	9,81E-01	NA	No	-1,45E-01	5,28E-01	NA	No
ENSG00000260464	RP4-561L24.3	lincRNA	2,32E-02	9,18E-01	9,65E-01	No	8,28E-01	1,99E-02	5,15E-02	No
ENSG00000231363	RP11-148B18.1	lincRNA	-5,45E-02	6,46E-01	NA	No	-1,28E-01	7,12E-01	NA	No
ENSG00000236098	RP11-148B18.4	lincRNA	-6,46E-02	6,93E-01	8,53E-01	No	-1,38E-01	6,44E-01	7,65E-01	No
ENSG00000226835	RP11-148B18.3	lincRNA	-2,00E-02	9,09E-01	9,62E-01	No	-6,39E-01	9,63E-02	1,90E-01	No
ENSG00000223675	RP11-86H7.6	lincRNA	-5,69E-02	4,65E-01	NA	No	-8,96E-02	7,16E-01	8,20E-01	No
ENSG00000235565	RP11-86H7.7	lincRNA	-1,17E+00	1,48E-03	1,38E-02	No	-1,06E+00	4,68E-03	1,47E-02	No
ENSG00000224081	LINC01057	lincRNA	-2,96E-01	1,87E-01	4,15E-01	No	-2,24E-01	4,11E-01	5,68E-01	No
ENSG00000228504	RP4-586O15.1	lincRNA	3,17E-02	9,36E-01	NA	No	9,18E-02	8,64E-01	NA	No
ENSG00000233907	RP4-617C6.1	lincRNA	-2,07E-01	4,48E-02	NA	No	-6,16E-01	4,37E-02	1,00E-01	No
ENSG00000228971	RP11-286B14.1	lincRNA	-1,07E-01	2,28E-01	NA	No	1,01E-01	7,22E-01	8,23E-01	No
ENSG00000231987	RP5-898J17.1	lincRNA	4,61E-02	6,23E-01	NA	No	8,55E-02	8,64E-01	NA	No
ENSG00000224445	RP11-413P11.1	lincRNA	-9,68E-02	5,10E-01	7,36E-01	No	-1,94E-01	5,53E-01	6,93E-01	No
ENSG00000223906	RP5-837M10.2	lincRNA	-4,23E-02	7,60E-01	NA	No	-2,42E-02	9,21E-01	NA	No
ENSG00000215869	RP11-364B6.1	lincRNA	-6,33E-02	4,04E-01	NA	No	-1,17E-01	5,60E-01	NA	No
ENSG00000230768	RP11-251P6.1	lincRNA	3,46E-02	9,36E-01	NA	No	8,58E-02	8,64E-01	NA	No
ENSG00000226822	RP11-356N1.2	lincRNA	3,06E-01	1,27E-01	3,25E-01	No	-1,94E-02	9,52E-01	9,72E-01	No
ENSG00000228420	RP4-735C1.6	lincRNA	-7,60E-03	9,00E-01	NA	No	-1,30E-01	7,12E-01	NA	No
ENSG00000261055	RP11-195M16.3	lincRNA	-3,18E-02	7,22E-01	NA	No	-1,04E-01	6,31E-01	NA	No
ENSG00000231346	LINC01160	lincRNA	-6,75E-02	7,40E-01	8,79E-01	No	-5,24E-01	1,75E-01	3,02E-01	No
ENSG00000231437	RP11-88H9.2	lincRNA	-2,64E-01	2,56E-01	4,99E-01	No	1,92E-02	9,51E-01	9,71E-01	No

ENSG00000231246	RP5-965F6.2	lincRNA	9,69E-01	1,32E-02	6,84E-02	No	1,01E+00	2,34E-02	5,92E-02	No
ENSG00000215866	RP11-426L16.8	lincRNA	3,87E-02	8,45E-01	NA	No	6,13E-02	9,56E-01	NA	No
ENSG00000224167	RP3-522D1.1	lincRNA	-8,74E-03	9,21E-01	NA	No	-1,59E-01	4,92E-01	NA	No
ENSG00000236066	RP11-389O22.1	lincRNA	-2,26E-01	3,17E-01	5,66E-01	No	-1,12E+00	2,13E-02	5,47E-02	No
ENSG00000238198	RP11-31F15.2	lincRNA	-2,20E-01	3,43E-01	5,91E-01	No	-2,41E-01	4,20E-01	5,76E-01	No
ENSG00000233730	RP4-666F24.3	lincRNA	-6,61E-02	5,64E-01	7,72E-01	No	1,69E-01	5,87E-01	7,19E-01	No
ENSG00000228127	RP11-12L8.1	lincRNA	4,98E-02	7,27E-01	8,73E-01	No	3,38E-01	3,17E-01	4,72E-01	No
ENSG00000235933	RP5-1185H19.2	lincRNA	5,08E-01	4,30E-02	1,57E-01	No	1,73E-01	5,74E-01	7,10E-01	No
ENSG00000233154	RP4-655J12.4	lincRNA	-4,32E-01	1,05E-01	2,88E-01	No	-1,07E+00	1,57E-02	4,20E-02	No
ENSG00000230381	RP4-655J12.5	lincRNA	-1,28E-01	3,48E-01	5,96E-01	No	-3,69E-01	2,72E-01	4,20E-01	No
ENSG00000224950	RP5-1086K13.1	lincRNA	-1,91E-01	3,90E-01	6,36E-01	No	-1,34E+00	4,54E-04	1,81E-03	No
ENSG00000272715	RP4-753F5.1	lincRNA	-1,50E-01	3,04E-01	5,52E-01	No	-4,23E-01	1,90E-01	3,23E-01	No
ENSG00000271427	RP11-188D8.1	lincRNA	3,18E-01	4,26E-02	1,56E-01	No	5,89E-01	8,37E-02	1,70E-01	No
ENSG00000226172	RP4-712E4.1	lincRNA	-6,07E-02	5,43E-01	NA	No	-1,62E-01	4,58E-01	NA	No
ENSG00000239216	RP4-712E4.2	lincRNA	-5,47E-02	6,46E-01	NA	No	-1,30E-01	7,12E-01	NA	No
ENSG00000227712	RP11-418J17.3	lincRNA	-3,68E-02	7,56E-01	NA	No	-2,31E-01	2,45E-01	NA	No
ENSG00000273406	RP11-114O18.1	lincRNA	9,29E-02	3,83E-01	NA	No	1,03E-01	7,75E-01	NA	No
ENSG00000227193	RP11-439A17.4	lincRNA	5,61E-03	9,55E-01	NA	No	7,04E-02	7,83E-01	NA	No
ENSG00000230806	RP11-343N15.1	lincRNA	6,08E-01	4,39E-02	1,59E-01	No	3,40E-01	3,36E-01	4,91E-01	No
ENSG00000269996	RP11-343N15.5	lincRNA	1,66E-01	2,80E-01	5,26E-01	No	6,25E-01	5,09E-05	2,46E-04	No
ENSG00000227082	AL592494.5	lincRNA	-6,07E-01	3,83E-02	1,45E-01	No	1,35E-01	6,75E-01	7,89E-01	No
ENSG00000231429	RP11-343N15.2	lincRNA	-1,69E-01	3,26E-01	5,75E-01	No	-4,46E-01	2,34E-01	3,76E-01	No
ENSG00000228826	RP11-344P13.4	lincRNA	1,37E-01	2,42E-01	NA	No	-1,87E-02	8,90E-01	NA	No
ENSG00000272583	RP11-344P13.6	lincRNA	3,67E-01	1,15E-02	NA	No	1,73E-01	4,32E-01	NA	No
ENSG00000224857	RP11-344P13.1	lincRNA	-1,39E-02	9,10E-01	NA	No	2,16E-01	3,16E-01	NA	No
ENSG00000227552	RP11-417J8.1	lincRNA	3,91E-02	8,45E-01	NA	No	8,64E-02	8,64E-01	NA	No
ENSG00000230880	RP11-417J8.3	lincRNA	1,02E-01	6,46E-01	8,25E-01	No	-1,36E-01	6,51E-01	7,71E-01	No
ENSG00000203849	RP11-417J8.6	lincRNA	-2,31E-02	9,13E-01	9,64E-01	No	3,82E-01	1,88E-01	3,20E-01	No
ENSG00000234978	RP11-423O2.5	lincRNA	5,81E-01	4,46E-02	1,61E-01	No	9,15E-01	1,27E-02	3,50E-02	No
ENSG00000232274	RP11-782C8.2	lincRNA	-4,00E-02	8,50E-01	9,35E-01	No	-5,71E-02	8,39E-01	9,02E-01	No
ENSG00000230850	RP11-782C8.1	lincRNA	1,78E-01	4,34E-01	6,74E-01	No	-1,42E-01	5,96E-01	7,27E-01	No
ENSG00000232336	RP11-782C8.3	lincRNA	-5,46E-02	7,52E-01	8,84E-01	No	-9,74E-03	9,76E-01	9,85E-01	No
ENSG00000225278	RP11-782C8.5	lincRNA	-2,24E-02	9,16E-01	9,65E-01	No	8,14E-02	7,45E-01	8,39E-01	No
ENSG00000242569	RP11-435B5.3	lincRNA	-9,21E-02	6,76E-01	8,43E-01	No	6,83E-01	5,45E-02	1,20E-01	No
ENSG00000185044	RP11-435B5.4	lincRNA	1,91E-01	4,05E-01	6,49E-01	No	2,71E-01	3,31E-01	4,86E-01	No
ENSG00000238261	RP11-435B5.5	lincRNA	-1,27E-01	5,69E-01	7,75E-01	No	5,76E-01	5,36E-02	1,19E-01	No
ENSG00000235566	RP11-435B5.7	lincRNA	3,15E-02	9,36E-01	NA	No	6,19E-02	9,56E-01	NA	No
ENSG00000224363	RP11-289H16.1	lincRNA	7,61E-01	2,87E-02	1,18E-01	No	3,05E-01	3,83E-01	5,40E-01	No
ENSG00000235398	LINC00623	lincRNA	1,36E+00	9,34E-05	1,62E-03	No	1,12E+00	2,91E-03	9,63E-03	No
ENSG00000272709	RP11-458D21.6	lincRNA	4,03E-01	2,92E-02	1,20E-01	No	3,75E-02	9,10E-01	NA	No
ENSG00000233396	RP11-458D21.1	lincRNA	-1,04E-01	6,19E-01	8,10E-01	No	2,16E-01	4,22E-01	5,78E-01	No
ENSG00000234225	RP4-704D21.2	lincRNA	-1,50E-01	5,06E-01	7,32E-01	No	-2,14E-01	4,91E-01	6,40E-01	No
ENSG00000234610	LINC00624	lincRNA	-3,28E-02	7,76E-01	NA	No	5,47E-01	1,39E-01	2,54E-01	No
ENSG00000227139	RP11-533N14.3	lincRNA	6,09E-02	5,49E-01	NA	No	1,25E-02	9,42E-01	NA	No
ENSG00000234190	RP11-433J22.3	lincRNA	6,21E-03	9,00E-01	NA	No	8,04E-02	6,51E-01	NA	No
ENSG00000226335	XXyac-YX155B6.6	lincRNA	9,43E-03	9,24E-01	NA	No	2,80E-01	2,69E-01	NA	No
ENSG00000238107	RP11-495P10.5	lincRNA	-1,69E-02	8,49E-01	NA	No	-3,34E-02	9,21E-01	NA	No
ENSG00000224481	RP11-495P10.3	lincRNA	6,20E-02	5,90E-01	NA	No	1,20E-02	9,73E-01	NA	No
ENSG00000272824	RP6-74O6.6	lincRNA	3,24E-01	5,08E-02	1,76E-01	No	5,51E-02	8,11E-01	NA	No
ENSG00000231551	RP11-495P10.1	lincRNA	4,98E-01	6,72E-02	2,14E-01	No	-5,84E-02	8,62E-01	9,17E-01	No
ENSG00000236140	RP11-89F3.2	lincRNA	5,68E-01	6,79E-03	4,27E-02	No	8,94E-02	6,43E-01	NA	No
ENSG00000237253	RP11-666A1.5	lincRNA	4,52E-02	7,45E-01	NA	No	8,99E-02	8,64E-01	NA	No
ENSG00000237343	RP11-763B22.4	lincRNA	3,24E-02	9,36E-01	NA	No	9,20E-02	8,64E-01	NA	No
ENSG00000235887	RP11-763B22.6	lincRNA	-5,54E-02	6,46E-01	NA	No	-1,30E-01	7,12E-01	NA	No
ENSG00000232527	RP11-14N7.2	lincRNA	-4,63E-01	5,73E-02	1,90E-01	No	-8,04E-01	2,34E-03	7,93E-03	No
ENSG00000232721	RP11-403I13.5	lincRNA	1,89E-01	8,01E-02	NA	No	6,03E-01	4,99E-02	1,12E-01	No
ENSG00000223779	RP11-403I13.4	lincRNA	1,30E+00	6,27E-06	1,76E-04	No	-4,10E-01	1,77E-01	3,05E-01	No
ENSG00000226067	LINC00869	lincRNA	6,79E-01	2,96E-02	1,21E-01	No	-7,53E-01	6,07E-02	1,31E-01	No
ENSG00000233430	RP11-403I13.9	lincRNA	9,08E-01	1,90E-02	8,84E-02	No	-4,14E-02	8,91E-01	9,35E-01	No
ENSG00000234571	RP5-998N21.4	lincRNA	2,71E-01	2,24E-01	4,62E-01	No	3,56E-01	3,28E-01	4,83E-01	No
ENSG00000268544	RP11-277L2.4	lincRNA	-7,42E-03	9,00E-01	NA	No	-1,30E-01	7,12E-01	NA	No
ENSG00000233368	RP11-277L2.3	lincRNA	6,67E-02	5,93E-01	NA	No	-1,10E-03	9,97E-01	NA	No

ENSG00000269614	RP11-277L2.5	lincRNA	-5,98E-03	8,03E-02	NA	No	-4,31E-02	8,92E-01	9,36E-01	No
ENSG00000229801	RP11-353N4.1	lincRNA	-4,66E-01	8,95E-02	2,59E-01	No	-1,14E+00	5,76E-03	1,76E-02	No
ENSG00000234232	RP11-353N4.5	lincRNA	-2,33E-01	2,57E-01	5,01E-01	No	-8,07E-01	7,30E-02	1,52E-01	No
ENSG00000223759	RP11-353N4.4	lincRNA	2,44E-01	2,23E-01	4,60E-01	No	-3,42E-01	2,78E-01	4,27E-01	No
ENSG00000228126	LINC00568	lincRNA	-1,16E-02	9,52E-01	9,80E-01	No	-1,87E-01	5,95E-01	7,26E-01	No
ENSG00000273481	RP11-126K1.9	lincRNA	1,50E-01	3,29E-01	NA	No	2,46E-01	3,87E-01	5,44E-01	No
ENSG00000269489	RP11-98D18.17	lincRNA	-3,73E-02	7,31E-01	NA	No	2,01E-01	3,47E-01	NA	No
ENSG00000236427	RP11-107M16.2	lincRNA	6,76E-03	9,77E-01	9,91E-01	No	6,43E-01	7,13E-02	1,49E-01	No
ENSG00000270361	RP11-307C12.13	lincRNA	-6,40E-02	6,51E-01	NA	No	-2,80E-01	3,06E-01	NA	No
ENSG00000236947	RP11-98G7.1	lincRNA	-4,71E-03	9,53E-01	NA	No	3,19E-02	8,77E-01	NA	No
ENSG00000272971	RP11-284F21.11	lincRNA	1,06E-01	5,58E-01	7,68E-01	No	1,67E-01	6,16E-01	7,44E-01	No
ENSG00000231666	RP11-404O13.1	lincRNA	-1,02E-01	3,98E-01	NA	No	-6,81E-02	8,27E-01	8,94E-01	No
ENSG00000235477	RP11-122G18.5	lincRNA	-2,44E-01	1,13E-01	3,03E-01	No	7,34E-01	5,46E-08	4,35E-07	No
ENSG00000273075	RP11-122G18.8	lincRNA	8,11E-01	2,26E-02	1,00E-01	No	5,69E-01	1,18E-01	2,23E-01	No
ENSG00000237756	RP11-77M5.1	lincRNA	6,15E-01	2,67E-02	1,13E-01	No	3,36E-01	3,08E-01	4,61E-01	No
ENSG00000273365	RP11-466F5.10	lincRNA	-1,91E-02	8,75E-01	NA	No	-7,25E-02	7,73E-01	NA	No
ENSG00000229588	RP11-479J7.2	lincRNA	-3,25E-02	8,47E-01	9,33E-01	No	-3,45E-02	9,16E-01	9,50E-01	No
ENSG00000237096	RP4-782G3.1	lincRNA	-1,44E-01	3,28E-01	5,77E-01	No	-1,72E-01	5,78E-01	7,13E-01	No
ENSG00000273160	RP11-104L21.3	lincRNA	5,35E-01	6,28E-02	2,03E-01	No	5,38E-02	8,78E-01	9,28E-01	No
ENSG00000228697	RP5-968D22.1	lincRNA	3,62E-02	6,82E-01	NA	No	2,82E-02	8,91E-01	NA	No
ENSG00000237658	RP5-968D22.3	lincRNA	-8,41E-02	4,35E-01	NA	No	-3,03E-01	2,03E-01	NA	No
ENSG00000235736	RP1-10C16.1	lincRNA	-2,28E-02	8,00E-01	NA	No	-7,83E-02	7,95E-01	NA	No
ENSG00000225826	LINC00626	lincRNA	-5,47E-02	6,46E-01	NA	No	-6,72E-02	8,00E-01	NA	No
ENSG00000234437	RP1-206D15.3	lincRNA	5,41E-02	6,48E-01	NA	No	5,25E-01	1,59E-01	2,81E-01	No
ENSG00000233985	RP11-297H3.3	lincRNA	-3,51E-01	4,65E-02	1,65E-01	No	-5,47E-01	1,46E-01	2,64E-01	No
ENSG00000224286	LINC01142	lincRNA	-2,19E-01	1,86E-01	4,14E-01	No	-9,04E-01	5,83E-02	1,27E-01	No
ENSG00000225545	RP11-545I10.2	lincRNA	-5,45E-02	6,46E-01	NA	No	-1,28E-01	7,12E-01	NA	No
ENSG00000235303	RP1-79C4.1	lincRNA	2,86E-02	8,41E-01	NA	No	-9,82E-02	8,30E-01	NA	No
ENSG00000224228	RP1-15D23.2	lincRNA	1,20E-01	2,45E-01	NA	No	7,43E-01	3,35E-02	8,00E-02	No
ENSG00000224000	RP3-471M13.2	lincRNA	3,42E-03	9,83E-01	NA	No	5,95E-02	7,29E-01	NA	No
ENSG00000226375	RP3-395P12.2	lincRNA	1,86E-01	2,92E-01	5,39E-01	No	9,10E-03	9,78E-01	9,87E-01	No
ENSG00000224977	RP11-160H22.3	lincRNA	-3,92E-03	9,89E-01	NA	No	1,90E-02	8,68E-01	NA	No
ENSG00000227373	RP11-160H22.5	lincRNA	-2,68E-01	2,58E-01	5,02E-01	No	1,07E-01	7,46E-01	8,40E-01	No
ENSG00000224718	RP11-222A5.1	lincRNA	-1,30E-01	2,46E-01	4,88E-01	No	-3,56E-01	1,32E-01	2,43E-01	No
ENSG00000227740	RP11-318C24.2	lincRNA	-6,48E-02	7,08E-01	8,62E-01	No	-4,39E-01	1,92E-01	3,24E-01	No
ENSG00000224968	RP1-35C21.1	lincRNA	-3,62E-02	6,58E-01	NA	No	-8,38E-02	7,27E-01	NA	No
ENSG00000227579	RP1-35C21.2	lincRNA	3,00E-02	6,46E-01	NA	No	4,84E-01	8,20E-02	1,67E-01	No
ENSG00000224687	RASAL2-AS1	lincRNA	-3,44E-02	8,74E-01	9,47E-01	No	3,56E-01	2,71E-01	4,19E-01	No
ENSG00000273384	RP5-1098D14.1	lincRNA	2,15E-01	7,45E-02	2,29E-01	No	2,84E-01	2,59E-01	NA	No
ENSG00000243062	RP11-12M5.1	lincRNA	-5,47E-02	6,46E-01	NA	No	-1,30E-01	7,12E-01	NA	No
ENSG00000231966	RP11-12M5.4	lincRNA	-1,04E-01	5,29E-01	7,49E-01	No	-3,34E-01	3,19E-01	4,74E-01	No
ENSG00000272906	RP11-533E19.7	lincRNA	2,28E-01	1,58E-01	3,74E-01	No	8,48E-01	5,97E-02	1,29E-01	No
ENSG00000261831	RP11-533E19.2	lincRNA	3,23E-02	6,96E-01	NA	No	2,24E-01	3,33E-01	4,88E-01	No
ENSG00000260360	RP11-533E19.5	lincRNA	1,40E-01	4,13E-01	6,56E-01	No	9,64E-01	3,63E-02	8,57E-02	No
ENSG00000261817	RP11-502H18.2	lincRNA	2,59E-02	7,65E-01	NA	No	7,00E-02	6,79E-01	NA	No
ENSG00000236719	OVAAL	lincRNA	-3,40E-01	1,73E-01	3,97E-01	No	-1,03E-01	7,09E-01	8,14E-01	No
ENSG00000225857	RP11-46A10.2	lincRNA	2,77E-01	2,22E-01	4,60E-01	No	-6,32E-01	1,28E-01	2,37E-01	No
ENSG00000272198	RP11-309G3.3	lincRNA	2,57E-02	9,09E-01	9,62E-01	No	8,96E-01	2,63E-03	8,79E-03	No
ENSG00000237292	RP11-540K16.1	lincRNA	-2,48E-01	1,62E-02	7,91E-02	No	-4,29E-01	1,83E-01	3,13E-01	No
ENSG00000225359	RP11-540K16.2	lincRNA	-9,27E-02	2,11E-01	NA	No	-1,57E-01	3,94E-01	NA	No
ENSG00000179452	RP11-380B22.1	lincRNA	-1,54E-01	1,30E-01	NA	No	-7,00E-02	8,01E-01	8,77E-01	No
ENSG00000224810	RP11-538D16.2	lincRNA	-6,48E-02	4,53E-01	NA	No	5,87E-02	7,76E-01	NA	No
ENSG00000225982	RP11-538D16.3	lincRNA	-1,11E-01	2,32E-01	NA	No	-1,61E-01	5,22E-01	NA	No
ENSG00000228918	GS1-122H1.2	lincRNA	-1,00E-01	4,05E-01	NA	No	-2,61E-01	2,13E-01	NA	No
ENSG00000203729	LINC00272	lincRNA	3,34E-01	1,45E-02	NA	No	1,30E-01	6,48E-01	NA	No
ENSG00000261504	RP11-317P15.4	lincRNA	-7,43E-02	7,27E-01	8,73E-01	No	-8,37E-01	5,97E-02	1,29E-01	No
ENSG00000261185	RP11-317P15.5	lincRNA	-1,04E-01	1,94E-01	NA	No	-2,20E-01	3,47E-01	NA	No
ENSG00000270009	RP1-127C7.6	lincRNA	-2,96E-02	8,04E-01	NA	No	3,95E-01	2,64E-01	4,12E-01	No
ENSG00000271387	RP11-382D12.2	lincRNA	3,14E-01	1,15E-01	3,06E-01	No	9,87E-01	4,39E-02	1,00E-01	No
ENSG00000273004	GS1-279B7.2	lincRNA	-3,25E-01	1,44E-01	3,54E-01	No	-9,36E-01	5,45E-02	1,20E-01	No
ENSG00000261024	GS1-279B7.1	lincRNA	2,31E-02	8,94E-01	9,56E-01	No	-2,70E-01	4,21E-01	5,77E-01	No
ENSG00000261729	GS1-204I12.4	lincRNA	4,26E-02	7,41E-01	NA	No	1,14E-01	6,31E-01	NA	No

ENSG00000273198	GS1-304P7.3	lincRNA	7,48E-02	4,07E-01	NA	No	2,33E-01	2,88E-01	NA	No
ENSG00000273129	RP5-973M2.2	lincRNA	1,53E-01	3,75E-01	6,22E-01	No	6,80E-01	1,09E-01	2,09E-01	No
ENSG00000231599	LINC01037	lincRNA	3,32E-02	9,36E-01	NA	No	1,40E-01	5,82E-01	NA	No
ENSG00000232212	RP11-398M15.1	lincRNA	-4,81E-02	6,47E-01	NA	No	3,09E-02	9,00E-01	9,41E-01	No
ENSG00000237457	RP11-547I7.2	lincRNA	-9,12E-02	4,48E-01	NA	No	1,20E-01	6,94E-01	8,03E-01	No
ENSG00000228215	RP11-541F9.2	lincRNA	-1,88E-02	8,19E-01	NA	No	-2,94E-02	8,90E-01	NA	No
ENSG00000236069	RP5-1011O1.3	lincRNA	-2,95E-01	1,80E-01	4,06E-01	No	-6,49E-01	1,12E-01	2,14E-01	No
ENSG00000234871	LINC01032	lincRNA	-1,30E-01	5,22E-01	7,44E-01	No	-1,10E+00	3,66E-02	8,63E-02	No
ENSG00000232077	LINC01031	lincRNA	7,94E-02	6,58E-01	8,32E-01	No	4,72E-01	2,17E-01	3,55E-01	No
ENSG00000226640	RP11-21J7.1	lincRNA	1,23E-01	2,75E-01	NA	No	9,76E-02	6,51E-01	NA	No
ENSG00000237011	RP11-98G13.1	lincRNA	8,52E-02	4,74E-01	NA	No	1,06E-01	6,21E-01	7,47E-01	No
ENSG00000227240	RP11-563D10.1	lincRNA	6,56E-02	6,24E-01	8,13E-01	No	2,25E-01	4,45E-01	5,99E-01	No
ENSG00000227421	RP11-476B1.1	lincRNA	5,27E-02	6,42E-01	NA	No	8,55E-02	8,64E-01	NA	No
ENSG00000229989	MIR181A1HG	lincRNA	6,10E-01	4,81E-03	3,33E-02	No	-2,68E-01	1,35E-01	2,48E-01	No
ENSG00000235492	RP11-16L9.4	lincRNA	-3,00E-02	7,31E-01	NA	No	-1,28E-01	7,12E-01	NA	No
ENSG00000231718	RP11-382E9.1	lincRNA	2,03E-02	8,25E-01	NA	No	-6,39E-02	8,00E-01	NA	No
ENSG00000203721	LINC00862	lincRNA	-1,40E-01	3,90E-01	6,36E-01	No	-3,39E-01	3,41E-01	4,97E-01	No
ENSG00000230623	RP11-469A15.2	lincRNA	-9,75E-03	9,03E-01	NA	No	1,86E-01	4,77E-01	6,28E-01	No
ENSG00000260088	RP11-92G12.3	lincRNA	3,88E-02	6,76E-01	NA	No	6,64E-02	7,93E-01	NA	No
ENSG00000229191	RP11-168O16.1	lincRNA	-2,29E-02	8,19E-01	NA	No	-3,93E-02	8,90E-01	NA	No
ENSG00000224818	RP11-134G8.8	lincRNA	1,25E-02	9,55E-01	9,81E-01	No	5,79E-02	8,22E-01	8,92E-01	No
ENSG00000223396	RPS10P7	lincRNA	5,97E-02	7,85E-01	9,03E-01	No	8,63E-02	7,75E-01	8,60E-01	No
ENSG00000225620	RP11-569A11.2	lincRNA	-5,59E-02	4,64E-01	NA	No	-8,26E-02	7,08E-01	NA	No
ENSG00000231507	RP11-134P9.3	lincRNA	-4,77E-01	7,70E-02	2,34E-01	No	-1,34E+00	7,53E-03	2,22E-02	No
ENSG00000176754	LINC00303	lincRNA	-4,46E-02	7,60E-01	NA	No	2,13E-03	9,87E-01	NA	No
ENSG00000228153	RP11-23I7.1	lincRNA	-1,24E-02	9,00E-01	NA	No	-1,57E-01	5,08E-01	NA	No
ENSG00000243636	RP11-164O23.7	lincRNA	1,27E-01	3,53E-01	NA	No	-1,15E-01	6,49E-01	NA	No
ENSG00000237074	RP11-6J21.2	lincRNA	6,07E-01	4,27E-02	1,56E-01	No	1,32E+00	1,58E-02	4,23E-02	No
ENSG00000226202	RP11-328D5.1	lincRNA	-7,79E-02	6,90E-01	8,51E-01	No	-1,39E-01	6,88E-01	7,99E-01	No
ENSG00000261453	RP11-565N2.1	lincRNA	3,38E-01	1,12E-02	6,11E-02	No	1,00E-01	7,75E-01	NA	No
ENSG00000259815	RP11-565N2.2	lincRNA	1,20E-01	2,12E-01	NA	No	6,68E-02	9,56E-01	NA	No
ENSG00000236950	RP5-1051D14.1	lincRNA	2,74E-01	3,08E-02	NA	No	8,55E-02	8,64E-01	NA	No
ENSG00000261252	RP11-318L16.6	lincRNA	1,71E-02	8,99E-01	NA	No	1,10E+00	3,08E-02	7,46E-02	No
ENSG00000228792	RP11-354K1.2	lincRNA	3,17E-02	9,36E-01	NA	No	9,02E-02	8,64E-01	NA	No
ENSG00000226251	RP11-15I11.3	lincRNA	-1,27E+00	2,52E-03	2,06E-02	No	-1,02E+00	1,43E-02	3,89E-02	No
ENSG00000224535	RP11-61J19.2	lincRNA	4,26E-02	7,31E-01	NA	No	8,58E-02	8,64E-01	NA	No
ENSG00000228067	RP11-61J19.3	lincRNA	-3,13E-02	7,31E-01	NA	No	-1,19E-02	9,82E-01	NA	No
ENSG00000260805	RP11-61J19.4	lincRNA	6,35E-02	7,75E-01	8,97E-01	No	3,56E-01	2,86E-01	4,36E-01	No
ENSG00000237980	RP11-338C15.3	lincRNA	1,91E-01	4,05E-01	6,49E-01	No	-3,83E-01	2,89E-01	4,39E-01	No
ENSG00000198468	FLVCR1-AS1	lincRNA	-7,34E-02	7,30E-01	8,75E-01	No	1,72E-01	6,22E-01	7,48E-01	No
ENSG00000225233	RP11-554K11.2	lincRNA	-1,73E-02	8,19E-01	NA	No	-1,36E-01	7,12E-01	NA	No
ENSG00000228255	RP11-323I1.1	lincRNA	3,32E-02	9,36E-01	NA	No	8,51E-02	8,64E-01	NA	No
ENSG00000225561	RP11-412H9.2	lincRNA	1,31E-02	8,37E-01	NA	No	8,39E-02	6,41E-01	NA	No
ENSG00000223842	RP11-135J2.3	lincRNA	-5,91E-02	7,87E-01	9,04E-01	No	1,36E-01	6,78E-01	7,91E-01	No
ENSG00000228536	RP11-392O17.1	lincRNA	-1,26E-01	5,66E-01	7,73E-01	No	-2,36E-01	4,85E-01	6,35E-01	No
ENSG00000230024	RP11-95P13.1	lincRNA	-2,27E-01	3,31E-01	5,80E-01	No	-2,82E-01	4,13E-01	5,70E-01	No
ENSG00000238232	RP11-95P13.2	lincRNA	-4,21E-02	7,60E-01	NA	No	1,15E-01	4,78E-01	NA	No
ENSG00000221571	RNU6ATAC35P	lincRNA	2,36E-02	8,55E-01	9,37E-01	No	2,68E-01	3,55E-01	5,11E-01	No
ENSG00000272823	RP11-295M18.6	lincRNA	1,12E-01	4,79E-01	7,10E-01	No	1,05E-01	6,89E-01	8,00E-01	No
ENSG00000238078	RP11-295M18.2	lincRNA	4,99E-01	7,48E-02	2,29E-01	No	4,30E-01	2,54E-01	4,00E-01	No
ENSG00000234754	RP11-421L10.1	lincRNA	-7,78E-02	6,42E-01	8,23E-01	No	-2,64E-01	4,28E-01	5,84E-01	No
ENSG00000227925	RP11-191N8.2	lincRNA	-5,47E-02	6,46E-01	NA	No	-1,30E-01	7,12E-01	NA	No
ENSG00000238042	RP11-815M8.1	lincRNA	1,41E-01	2,12E-01	NA	No	1,37E-02	9,87E-01	NA	No
ENSG00000232679	RP11-400N13.3	lincRNA	-4,49E-03	9,18E-01	NA	No	-2,37E-02	8,87E-01	NA	No
ENSG00000236230	RP11-400N13.1	lincRNA	3,31E-02	9,36E-01	NA	No	6,17E-02	9,56E-01	NA	No
ENSG00000228106	RP11-452F19.3	lincRNA	4,74E-01	4,17E-02	1,54E-01	No	1,14E+00	9,19E-07	5,99E-06	No
ENSG00000236846	RP11-239E10.2	lincRNA	3,39E-02	9,36E-01	NA	No	8,55E-02	8,64E-01	NA	No
ENSG00000226601	RP11-239E10.3	lincRNA	-1,73E-02	8,19E-01	NA	No	-7,38E-02	8,00E-01	NA	No
ENSG00000272645	RP11-504P24.8	lincRNA	6,02E-01	1,33E-02	6,88E-02	No	-1,13E+00	5,28E-04	2,07E-03	No
ENSG00000185495	RP11-504P24.4	lincRNA	-3,18E-02	8,67E-01	9,44E-01	No	7,00E-01	9,49E-02	1,88E-01	No
ENSG00000263182	RP11-504P24.6	lincRNA	1,84E-03	9,41E-01	NA	No	1,73E-02	9,87E-01	NA	No
ENSG00000225518	RP11-396C23.2	lincRNA	-1,69E-01	4,39E-01	6,78E-01	No	-1,12E+00	3,32E-02	7,93E-02	No

ENSG00000234277	CTD-2090I13.1	lincRNA	-5,73E-02	6,46E-01	NA	No	-3,12E-02	8,90E-01	NA	No
ENSG00000227711	RP11-275O4.3	lincRNA	6,57E-02	6,12E-01	8,04E-01	No	-3,62E-02	9,01E-01	9,41E-01	No
ENSG00000270104	RP11-245P10.6	lincRNA	-8,01E-02	3,29E-01	NA	No	-1,06E-01	6,76E-01	NA	No
ENSG00000177788	RP5-1061H20.4	lincRNA	1,32E-02	9,52E-01	9,80E-01	No	5,61E-01	1,08E-01	2,07E-01	No
ENSG00000226920	RP5-1068B5.3	lincRNA	2,19E-01	3,35E-01	5,84E-01	No	4,36E-01	1,66E-01	2,90E-01	No
ENSG00000225656	RP5-858B6.1	lincRNA	6,67E-02	7,30E-01	8,74E-01	No	2,71E-02	9,38E-01	9,63E-01	No
ENSG00000231768	RP5-855F14.1	lincRNA	6,76E-01	1,90E-02	8,86E-02	No	1,02E+00	1,85E-03	6,42E-03	No
ENSG00000230404	RP5-855F14.2	lincRNA	-1,23E-01	4,56E-01	6,91E-01	No	-1,83E-01	5,99E-01	7,29E-01	No
ENSG00000224939	LINC00184	lincRNA	8,82E-03	9,30E-01	NA	No	-9,79E-02	6,54E-01	NA	No
ENSG00000272362	RP4-781K5.9	lincRNA	4,19E-02	7,48E-01	NA	No	9,18E-02	8,64E-01	NA	No
ENSG00000228044	RP4-781K5.4	lincRNA	7,00E-02	4,42E-01	NA	No	3,80E-01	1,05E-01	NA	No
ENSG00000224037	RP4-781K5.7	lincRNA	2,06E-02	7,95E-01	NA	No	4,90E-02	7,97E-01	NA	No
ENSG00000237520	RP11-443B7.2	lincRNA	3,17E-02	9,36E-01	NA	No	6,67E-02	9,56E-01	NA	No
ENSG00000258082	RP11-443B7.3	lincRNA	5,16E-01	2,43E-02	1,06E-01	No	8,29E-01	6,02E-02	1,30E-01	No
ENSG00000273416	RP4-597N16.4	lincRNA	-8,70E-02	6,47E-01	8,26E-01	No	3,90E-01	2,91E-01	4,41E-01	No
ENSG00000237845	RP5-940F7.2	lincRNA	-2,93E-02	7,31E-01	NA	No	1,22E-01	5,65E-01	NA	No
ENSG00000229291	RP4-580N22.2	lincRNA	-9,41E-03	9,49E-01	9,79E-01	No	-1,28E-01	6,88E-01	7,99E-01	No
ENSG00000215808	LINC01139	lincRNA	1,13E-01	4,31E-01	6,71E-01	No	4,56E-02	8,89E-01	9,34E-01	No
ENSG00000231877	RP11-177F15.1	lincRNA	4,71E-02	5,83E-01	NA	No	8,64E-02	8,64E-01	NA	No
ENSG00000214837	RP11-261C10.3	lincRNA	3,42E-01	1,73E-01	3,96E-01	No	3,16E-01	3,71E-01	5,28E-01	No
ENSG00000226828	RP11-278H7.1	lincRNA	4,06E-02	6,89E-01	NA	No	2,02E-01	4,08E-01	NA	No
ENSG00000273175	RP11-11N7.4	lincRNA	1,54E-01	1,25E-01	3,23E-01	No	4,18E-01	1,51E-01	2,70E-01	No
ENSG00000260698	RP11-439E19.9	lincRNA	-1,19E-01	5,90E-01	7,90E-01	No	-5,16E-01	1,82E-01	3,11E-01	No
ENSG00000235021	RP11-439E19.7	lincRNA	7,43E-02	3,99E-01	NA	No	1,28E-01	4,98E-01	NA	No
ENSG00000232347	RP11-488L18.8	lincRNA	-1,92E-03	8,99E-01	NA	No	-1,26E-01	7,12E-01	NA	No
ENSG00000233684	AC079779.6	lincRNA	7,42E-02	4,22E-01	NA	No	1,73E-02	9,87E-01	NA	No
ENSG00000233633	AC093326.3	lincRNA	1,03E-01	3,16E-01	NA	No	1,84E-01	4,01E-01	NA	No
ENSG00000237667	LINC01115	lincRNA	4,86E-02	5,27E-01	NA	No	9,20E-02	8,64E-01	NA	No
ENSG00000231482	AC141930.2	lincRNA	2,45E-02	7,98E-01	NA	No	2,67E-01	3,32E-01	4,87E-01	No
ENSG00000203635	AC144450.2	lincRNA	-2,11E-01	1,93E-01	4,22E-01	No	-1,04E+00	3,74E-02	8,78E-02	No
ENSG00000237720	AC011995.1	lincRNA	7,53E-02	5,24E-01	NA	No	8,58E-02	8,64E-01	NA	No
ENSG00000234423	AC019118.2	lincRNA	1,69E-01	1,16E-01	NA	No	1,07E-01	6,96E-01	NA	No
ENSG00000236760	AC019118.3	lincRNA	1,06E-01	3,68E-01	NA	No	-1,26E-01	7,12E-01	NA	No
ENSG00000226649	AC019118.4	lincRNA	-1,90E-02	8,79E-01	NA	No	1,61E-02	9,53E-01	NA	No
ENSG00000271868	RP11-1293J14.1	lincRNA	-3,44E-03	9,85E-01	9,94E-01	No	-2,80E-02	9,33E-01	9,60E-01	No
ENSG00000242282	AC108488.4	lincRNA	2,17E-01	3,42E-01	5,90E-01	No	2,63E-01	3,45E-01	5,00E-01	No
ENSG00000227363	AC019172.2	lincRNA	-1,30E-01	1,79E-01	NA	No	-3,15E-01	1,86E-01	NA	No
ENSG00000231532	AC022311.1	lincRNA	3,88E-02	8,45E-01	NA	No	6,23E-02	9,56E-01	NA	No
ENSG00000236106	AC010729.2	lincRNA	-3,11E-02	7,31E-01	NA	No	-1,26E-01	7,12E-01	NA	No
ENSG00000205837	LINC00487	lincRNA	-1,32E-01	5,03E-01	7,30E-01	No	-5,72E-01	1,56E-01	2,76E-01	No
ENSG00000271947	RP11-439M11.1	lincRNA	-1,83E-01	1,71E-01	NA	No	-1,21E-01	6,76E-01	7,90E-01	No
ENSG00000272002	RP11-557L19.1	lincRNA	1,38E-03	9,93E-01	9,97E-01	No	-1,44E-01	5,61E-01	6,99E-01	No
ENSG00000235665	LINC00298	lincRNA	3,68E-01	2,39E-02	1,04E-01	No	1,68E-01	4,12E-01	NA	No
ENSG00000229740	U91324.1	lincRNA	1,46E+00	1,03E-02	5,75E-02	No	2,15E-01	3,23E-01	NA	No
ENSG00000231435	AC011747.3	lincRNA	2,81E-01	6,39E-02	NA	No	-1,26E-01	7,12E-01	NA	No
ENSG00000236008	AC011747.4	lincRNA	4,95E-01	7,30E-02	2,26E-01	No	2,98E-01	4,11E-01	5,68E-01	No
ENSG00000260837	RP11-434B12.1	lincRNA	-1,99E-01	2,87E-01	5,34E-01	No	7,87E-01	6,57E-05	3,11E-04	No
ENSG00000271855	RP11-214N9.1	lincRNA	1,44E-01	4,19E-01	6,61E-01	No	2,81E-01	3,94E-01	5,51E-01	No
ENSG00000240687	RP11-521D12.1	lincRNA	5,25E-02	6,46E-01	NA	No	1,26E-01	6,29E-01	7,53E-01	No
ENSG00000244260	RP11-521D12.2	lincRNA	-7,50E-03	9,00E-01	NA	No	8,77E-02	6,33E-01	NA	No
ENSG00000243491	RP11-521D12.5	lincRNA	2,70E-03	9,80E-01	NA	No	2,82E-01	3,31E-01	4,86E-01	No
ENSG00000269973	RP11-95D17.1	lincRNA	-5,13E-01	3,11E-02	1,25E-01	No	-5,74E-01	4,55E-02	1,03E-01	No
ENSG00000260077	RP11-254F7.2	lincRNA	2,43E-01	2,03E-01	4,36E-01	No	2,34E-01	2,27E-01	3,68E-01	No
ENSG00000272524	RP11-254F7.4	lincRNA	5,20E-04	9,41E-01	NA	No	1,35E-02	9,87E-01	NA	No
ENSG00000257135	RP11-320M2.1	lincRNA	4,64E-01	7,36E-02	2,27E-01	No	1,54E-01	6,21E-01	7,47E-01	No
ENSG00000217258	AC007249.3	lincRNA	4,21E-02	7,78E-01	8,99E-01	No	-3,62E-01	1,36E-01	NA	No
ENSG00000243819	RN7SL832P	lincRNA	1,78E-01	3,78E-01	6,24E-01	No	-7,90E-03	9,78E-01	9,87E-01	No
ENSG00000271952	RP11-245G13.2	lincRNA	-2,57E-02	9,01E-01	9,59E-01	No	1,09E+00	8,42E-05	3,89E-04	No
ENSG00000145063	AC062028.1	lincRNA	2,34E-02	8,13E-01	NA	No	-1,42E-01	6,24E-01	NA	No
ENSG00000231403	AC099344.3	lincRNA	3,15E-02	9,36E-01	NA	No	6,23E-02	9,56E-01	NA	No
ENSG00000224177	LINC00570	lincRNA	6,40E-02	4,74E-01	NA	No	9,18E-02	6,64E-01	NA	No
ENSG00000224184	AC096559.1	lincRNA	4,49E-02	6,64E-01	NA	No	2,37E-01	4,22E-01	5,78E-01	No

ENSG00000223360	AC096559.2	lincRNA	-5,24E-02	5,51E-01	NA	No	-1,65E-01	4,31E-01	NA	No
ENSG00000261117	RP11-333O1.1	lincRNA	-1,55E-01	4,82E-01	7,13E-01	No	-4,37E-01	2,51E-01	3,96E-01	No
ENSG00000230448	LINC00276	lincRNA	6,85E-03	9,54E-01	NA	No	-2,46E-01	2,67E-01	NA	No
ENSG00000235127	AC068286.1	lincRNA	-8,15E-02	4,39E-01	NA	No	-2,20E-01	2,95E-01	NA	No
ENSG00000223850	MYCNUN	lincRNA	1,86E-02	8,69E-01	NA	No	-9,72E-02	8,30E-01	NA	No
ENSG00000260331	RP11-111J6.2	lincRNA	-2,56E-02	8,94E-01	9,56E-01	No	3,10E-01	3,90E-01	5,47E-01	No
ENSG00000224626	AC106053.1	lincRNA	-2,17E-01	2,64E-01	5,08E-01	No	-3,59E-01	3,25E-01	4,80E-01	No
ENSG00000236204	AC092594.1	lincRNA	-1,09E-01	6,13E-01	8,05E-01	No	1,10E-01	7,02E-01	8,09E-01	No
ENSG00000234597	AC010096.1	lincRNA	-1,70E-01	NA	NA	No	-7,37E-01	NA	NA	No
ENSG00000237992	AC010096.2	lincRNA	-2,05E-01	NA	NA	No	-4,51E-01	NA	NA	No
ENSG00000235911	AC019055.1	lincRNA	-1,45E-01	NA	NA	No	-1,95E-01	NA	NA	No
ENSG00000271991	RP11-79O8.1	lincRNA	-8,36E-02	5,97E-01	7,95E-01	No	-3,85E-01	2,89E-01	4,40E-01	No
ENSG00000228950	AC023137.2	lincRNA	8,97E-02	6,18E-01	8,09E-01	No	4,24E-01	2,60E-01	4,07E-01	No
ENSG00000269976	RP11-130L8.2	lincRNA	-1,42E-02	9,30E-01	9,71E-01	No	1,97E-01	5,63E-01	7,00E-01	No
ENSG00000260396	AC012065.7	lincRNA	4,34E-01	9,87E-02	2,76E-01	No	6,34E-01	5,47E-02	1,20E-01	No
ENSG00000236436	AC012361.1	lincRNA	4,33E-02	7,20E-01	NA	No	9,18E-02	8,64E-01	NA	No
ENSG00000261012	RP11-116D2.1	lincRNA	4,65E-02	6,04E-01	NA	No	1,19E-01	5,49E-01	NA	No
ENSG00000231204	AC011752.1	lincRNA	1,83E-01	1,85E-01	4,12E-01	No	9,87E-01	4,93E-02	1,11E-01	No
ENSG00000235537	AC009411.2	lincRNA	3,18E-03	9,92E-01	NA	No	9,18E-02	6,82E-01	NA	No
ENSG00000228538	AC009411.1	lincRNA	-3,28E-02	7,56E-01	NA	No	-1,53E-01	5,62E-01	NA	No
ENSG00000229621	AC018742.1	lincRNA	-1,94E-01	3,35E-01	5,84E-01	No	-6,36E-02	8,52E-01	9,10E-01	No
ENSG00000232451	AC016768.1	lincRNA	3,15E-02	9,36E-01	NA	No	8,51E-02	8,64E-01	NA	No
ENSG00000235497	AC012506.4	lincRNA	3,85E-02	8,45E-01	NA	No	1,01E-01	7,75E-01	NA	No
ENSG00000261452	RP11-509E16.1	lincRNA	-1,48E-01	4,86E-01	7,16E-01	No	-2,51E-01	2,77E-01	4,26E-01	No
ENSG00000272048	RP11-458N5.1	lincRNA	-1,04E-01	5,86E-01	7,87E-01	No	-5,47E-02	8,73E-01	9,24E-01	No
ENSG00000235072	AC012074.2	lincRNA	1,10E+00	1,45E-02	7,33E-02	No	-2,72E-02	9,22E-01	9,54E-01	No
ENSG00000273233	RP11-713D19.1	lincRNA	1,26E-01	2,91E-01	5,38E-01	No	1,50E-01	5,70E-01	7,06E-01	No
ENSG00000235997	AC109642.1	lincRNA	1,75E-01	4,36E-01	6,76E-01	No	9,53E-01	4,82E-04	1,91E-03	No
ENSG00000223647	AL133249.1	lincRNA	4,11E-02	7,67E-01	NA	No	9,85E-02	7,75E-01	NA	No
ENSG00000273165	RP11-1057B6.1	lincRNA	-1,49E-01	5,08E-01	7,34E-01	No	-5,46E-01	1,46E-01	2,63E-01	No
ENSG00000272716	RP11-563N4.1	lincRNA	-1,20E-01	5,64E-01	7,72E-01	No	3,22E-01	3,61E-01	5,17E-01	No
ENSG00000272754	AL133245.2	lincRNA	4,69E-01	8,43E-02	2,48E-01	No	-1,22E+00	6,17E-03	1,86E-02	No
ENSG00000230876	LINC00486	lincRNA	1,34E-01	4,94E-01	7,23E-01	No	2,91E-01	4,22E-01	5,78E-01	No
ENSG00000236854	AL121656.5	lincRNA	5,44E-02	6,02E-01	NA	No	-1,39E-02	8,90E-01	NA	No
ENSG00000203386	AC009499.1	lincRNA	-4,43E-02	8,23E-01	9,23E-01	No	2,42E-01	4,98E-01	6,46E-01	No
ENSG00000237790	AC009499.2	lincRNA	-3,37E-02	7,96E-01	9,09E-01	No	-2,24E-01	3,70E-01	5,26E-01	No
ENSG00000226785	AC073218.1	lincRNA	-7,57E-02	4,85E-01	NA	No	-2,18E-01	3,91E-01	NA	No
ENSG00000228262	AC073218.2	lincRNA	1,08E-01	4,00E-01	NA	No	2,01E-01	3,86E-01	5,43E-01	No
ENSG00000272027	RP11-529E15.1	lincRNA	4,56E-02	6,38E-01	NA	No	6,67E-02	9,56E-01	NA	No
ENSG00000226994	AC012593.1	lincRNA	3,46E-02	9,36E-01	NA	No	1,14E-01	6,21E-01	NA	No
ENSG00000260025	RP11-490M8.1	lincRNA	-2,46E-01	2,83E-01	5,29E-01	No	5,97E-01	2,89E-02	7,07E-02	No
ENSG00000232028	AC007391.2	lincRNA	-1,37E-02	8,91E-01	NA	No	-1,57E-01	5,07E-01	NA	No
ENSG00000236572	AC006369.3	lincRNA	-2,16E-02	8,34E-01	NA	No	2,40E-01	4,35E-01	5,90E-01	No
ENSG00000237803	LINC00211	lincRNA	-4,65E-02	8,13E-01	9,17E-01	No	1,09E-01	7,53E-01	8,44E-01	No
ENSG00000229160	AC009229.6	lincRNA	2,49E-01	2,92E-01	5,40E-01	No	-1,13E+00	2,01E-02	5,20E-02	No
ENSG00000273006	RP11-314C9.2	lincRNA	5,91E-01	3,39E-02	1,33E-01	No	1,46E-01	5,74E-01	7,09E-01	No
ENSG00000227292	AC009229.5	lincRNA	1,45E-01	2,07E-01	4,40E-01	No	1,97E-01	4,64E-01	6,16E-01	No
ENSG00000231367	AC016995.3	lincRNA	1,16E-01	4,93E-01	7,22E-01	No	4,61E-01	2,19E-01	3,57E-01	No
ENSG00000235009	AC093822.1	lincRNA	-8,92E-03	9,01E-01	NA	No	-1,30E-01	7,12E-01	NA	No
ENSG00000233128	AC007317.1	lincRNA	6,61E-02	4,79E-01	NA	No	2,19E-01	2,88E-01	NA	No
ENSG00000214691	AC104654.1	lincRNA	1,66E-01	3,85E-01	6,31E-01	No	-8,76E-01	6,08E-02	1,32E-01	No
ENSG00000234362	AC104654.2	lincRNA	8,65E-02	3,34E-01	NA	No	1,47E-01	4,81E-01	NA	No
ENSG00000224739	AC016735.1	lincRNA	4,68E-02	7,44E-01	8,81E-01	No	-3,20E-02	9,02E-01	9,42E-01	No
ENSG00000231826	AC016735.2	lincRNA	1,80E-01	3,43E-01	5,91E-01	No	1,45E-02	9,68E-01	9,81E-01	No
ENSG00000233978	AC016735.3	lincRNA	1,40E-01	4,37E-01	6,76E-01	No	-4,25E-01	8,94E-02	NA	No
ENSG00000230587	AC093609.1	lincRNA	1,35E+00	4,36E-04	5,47E-03	No	4,35E-01	1,89E-01	3,21E-01	No
ENSG00000231156	AC093702.1	lincRNA	-1,82E-02	8,19E-01	NA	No	-8,44E-03	9,79E-01	NA	No
ENSG00000228100	AC016912.3	lincRNA	6,47E-02	3,72E-01	NA	No	8,92E-02	6,92E-01	NA	No
ENSG00000260977	RP11-333I13.1	lincRNA	-1,13E+00	8,07E-06	2,17E-04	No	-1,41E+00	1,27E-05	6,88E-05	No
ENSG00000234690	AC073283.4	lincRNA	-7,92E-02	6,28E-01	8,15E-01	No	-3,20E-01	3,05E-01	4,58E-01	No
ENSG00000226087	AC106869.2	lincRNA	-3,87E-03	9,67E-01	NA	No	2,27E-01	4,15E-01	NA	No
ENSG00000236824	BCYRN1	lincRNA	1,10E-01	5,32E-01	7,51E-01	No	9,48E-01	6,79E-07	4,52E-06	No

ENSG00000230773	AC079807.4	lincRNA	1,14E-01	1,27E-01	NA	No	6,13E-02	9,56E-01	NA	No
ENSG00000272663	RP11-191L17.1	lincRNA	-3,75E-01	1,13E-01	3,03E-01	No	-6,47E-01	1,11E-01	2,12E-01	No
ENSG00000231918	AC007682.1	lincRNA	6,40E-02	5,13E-01	NA	No	9,81E-02	7,75E-01	NA	No
ENSG00000236837	AC139712.4	lincRNA	3,46E-02	9,36E-01	NA	No	6,17E-02	9,56E-01	NA	No
ENSG00000228033	AC010967.2	lincRNA	9,77E-02	3,34E-01	NA	No	3,31E-01	2,41E-01	NA	No
ENSG00000232604	AC010967.3	lincRNA	6,28E-02	5,28E-01	NA	No	1,18E-01	5,67E-01	NA	No
ENSG00000272156	RP11-477N3.1	lincRNA	3,67E-01	1,23E-01	3,20E-01	No	7,30E-01	1,30E-02	3,58E-02	No
ENSG00000272180	RP11-481J13.1	lincRNA	-4,06E-01	1,02E-01	2,81E-01	No	-5,71E-01	1,53E-01	2,73E-01	No
ENSG00000226702	AC011306.2	lincRNA	3,31E-02	9,36E-01	NA	No	8,55E-02	8,64E-01	NA	No
ENSG00000229805	AC011306.1	lincRNA	-5,37E-02	6,46E-01	NA	No	-1,26E-01	7,12E-01	NA	No
ENSG00000233723	LINC01122	lincRNA	5,70E-02	5,95E-01	NA	No	8,51E-02	8,64E-01	NA	No
ENSG00000222030	AC007131.1	lincRNA	-1,24E-01	2,15E-01	NA	No	-2,96E-01	2,21E-01	NA	No
ENSG00000271955	RP11-444A22.1	lincRNA	-6,79E-02	3,81E-01	NA	No	-1,54E-01	4,65E-01	NA	No
ENSG00000233891	AC007131.2	lincRNA	-1,26E-01	1,94E-01	NA	No	-4,19E-03	9,87E-01	9,92E-01	No
ENSG00000228590	AC007381.3	lincRNA	8,62E-02	6,67E-01	8,37E-01	No	-9,64E-01	4,81E-02	1,08E-01	No
ENSG00000223929	AC007381.2	lincRNA	-4,91E-02	8,18E-01	9,20E-01	No	-1,92E-01	5,88E-01	7,20E-01	No
ENSG00000271889	RP11-493E12.1	lincRNA	3,43E-01	1,70E-01	3,92E-01	No	1,86E-01	6,00E-01	7,31E-01	No
ENSG00000228541	AC093159.1	lincRNA	8,33E-02	3,15E-01	NA	No	9,02E-02	8,64E-01	NA	No
ENSG00000228079	AC012368.1	lincRNA	7,93E-02	5,87E-01	7,88E-01	No	2,75E-01	4,14E-01	5,70E-01	No
ENSG00000230923	LINC00309	lincRNA	7,07E-02	4,39E-01	NA	No	8,64E-02	8,64E-01	NA	No
ENSG00000238012	AC114752.1	lincRNA	-1,83E-02	8,19E-01	NA	No	-2,72E-02	8,90E-01	NA	No
ENSG00000238201	AC114752.3	lincRNA	-1,97E-02	8,32E-01	NA	No	-5,43E-02	7,99E-01	NA	No
ENSG00000223863	AC008074.4	lincRNA	-6,78E-02	4,56E-01	NA	No	-1,57E-01	5,14E-01	NA	No
ENSG00000260101	RP11-568N6.1	lincRNA	3,99E-01	1,24E-01	3,22E-01	No	1,83E-01	6,04E-01	7,34E-01	No
ENSG00000226756	AC007365.3	lincRNA	1,26E-01	5,76E-01	7,80E-01	No	3,71E-01	2,43E-01	3,87E-01	No
ENSG00000234572	AC007880.1	lincRNA	-5,29E-03	9,91E-01	NA	No	2,98E-02	8,69E-01	NA	No
ENSG00000204929	AC074391.1	lincRNA	1,83E-01	4,25E-01	6,66E-01	No	4,87E-01	1,96E-01	3,29E-01	No
ENSG00000234255	AC012370.3	lincRNA	2,33E-01	1,78E-01	4,03E-01	No	2,38E-01	4,85E-01	6,35E-01	No
ENSG00000235725	AC007389.3	lincRNA	1,26E-01	2,89E-01	NA	No	6,89E-02	7,48E-01	NA	No
ENSG00000232164	AC092669.3	lincRNA	6,13E-02	6,11E-01	NA	No	9,93E-02	6,57E-01	NA	No
ENSG00000232046	AC007392.3	lincRNA	-5,90E-01	5,22E-02	1,79E-01	No	2,35E-02	9,46E-01	9,68E-01	No
ENSG00000235885	AC023115.2	lincRNA	3,39E-02	9,36E-01	NA	No	1,56E-01	5,16E-01	NA	No
ENSG00000230906	AC023115.1	lincRNA	4,24E-02	7,54E-01	NA	No	6,19E-02	9,56E-01	NA	No
ENSG00000236780	AC078941.1	lincRNA	3,81E-02	8,45E-01	NA	No	6,17E-02	9,56E-01	NA	No
ENSG00000236605	AC023115.4	lincRNA	-2,70E-03	8,97E-01	NA	No	-1,30E-01	7,12E-01	NA	No
ENSG00000224173	AC007422.1	lincRNA	-8,77E-02	4,45E-01	NA	No	-1,14E-01	6,59E-01	NA	No
ENSG00000235495	AC010987.5	lincRNA	-4,42E-02	7,60E-01	NA	No	4,50E-03	9,87E-01	NA	No
ENSG00000188971	RP11-427H3.3	lincRNA	-3,09E-01	2,99E-02	1,22E-01	No	-1,61E-01	2,83E-01	4,32E-01	No
ENSG00000231024	AC092431.3	lincRNA	2,46E-01	8,44E-02	2,49E-01	No	4,12E-01	1,61E-01	2,83E-01	No
ENSG00000233060	AC016700.2	lincRNA	-1,32E-03	9,91E-01	NA	No	-3,42E-02	8,90E-01	NA	No
ENSG00000237751	LINC01143	lincRNA	-1,38E-01	1,28E-01	NA	No	-2,86E-01	3,02E-01	NA	No
ENSG00000272735	RP11-467P9.1	lincRNA	7,44E-02	7,14E-01	8,66E-01	No	5,39E-01	1,64E-01	2,87E-01	No
ENSG00000273245	RP11-434P11.2	lincRNA	2,85E-01	2,36E-01	4,75E-01	No	-2,81E-01	3,67E-01	5,23E-01	No
ENSG00000235499	AC073046.25	lincRNA	1,22E+00	7,78E-05	1,40E-03	No	-6,97E-01	3,74E-02	8,78E-02	No
ENSG00000255989	RP11-711M9.1	lincRNA	5,27E-01	6,22E-02	2,02E-01	No	-4,57E-01	1,99E-01	3,33E-01	No
ENSG00000272711	RP11-259N19.1	lincRNA	6,11E-02	7,27E-01	8,73E-01	No	2,30E-01	4,96E-01	6,44E-01	No
ENSG00000204792	AC104135.3	lincRNA	-2,75E-02	7,74E-01	8,97E-01	No	-2,60E-01	4,37E-01	5,91E-01	No
ENSG00000236209	AC104135.2	lincRNA	-3,78E-02	7,16E-01	NA	No	8,95E-02	7,43E-01	8,37E-01	No
ENSG00000230836	AC104135.4	lincRNA	-3,81E-03	9,76E-01	9,91E-01	No	-2,78E-01	4,26E-01	5,81E-01	No
ENSG00000270996	RP11-342K6.4	lincRNA	-9,32E-02	5,52E-01	7,64E-01	No	1,94E-01	5,74E-01	7,09E-01	No
ENSG00000227088	AC084149.2	lincRNA	-1,14E-02	8,19E-01	NA	No	-1,26E-01	7,12E-01	NA	No
ENSG00000229494	AC012494.1	lincRNA	-4,78E-02	5,61E-01	NA	No	-1,33E-01	5,70E-01	NA	No
ENSG00000260840	RP11-60H5.1	lincRNA	7,04E-02	4,37E-01	NA	No	6,11E-02	7,05E-01	NA	No
ENSG00000232504	AC105053.4	lincRNA	2,61E-01	2,65E-01	5,09E-01	No	-2,17E-01	5,37E-01	6,80E-01	No
ENSG00000272564	RP11-548P2.2	lincRNA	2,78E-02	8,26E-01	9,24E-01	No	-1,24E-01	6,38E-01	7,61E-01	No
ENSG00000222041	LINC00152	lincRNA	2,71E-02	8,96E-01	9,57E-01	No	-7,38E-01	7,89E-03	2,31E-02	No
ENSG00000233757	AC092835.2	lincRNA	2,30E-01	1,74E-01	3,99E-01	No	7,93E-01	1,45E-05	7,75E-05	No
ENSG00000272913	RP11-440D17.3	lincRNA	-1,54E-02	9,42E-01	9,76E-01	No	-5,79E-01	9,45E-02	1,87E-01	No
ENSG00000273305	RP11-440D17.4	lincRNA	-5,20E-01	7,14E-02	2,22E-01	No	5,16E-01	1,38E-01	2,52E-01	No
ENSG00000232931	LINC00342	lincRNA	1,02E+00	8,26E-05	1,47E-03	No	1,57E-01	5,47E-01	6,87E-01	No
ENSG00000228873	AC012307.2	lincRNA	4,56E-02	6,40E-01	NA	No	9,81E-02	7,75E-01	NA	No
ENSG00000273265	RP11-353K11.1	lincRNA	4,49E-01	9,60E-02	2,71E-01	No	2,88E-01	4,02E-01	5,59E-01	No

ENSG00000241481	AC159540.2	lincRNA	8,70E-02	4,16E-01	NA	No	6,42E-02	7,08E-01	NA	No
ENSG00000226791	AC109826.1	lincRNA	4,04E-01	9,47E-02	2,69E-01	No	-1,81E-01	5,99E-01	7,29E-01	No
ENSG00000272902	RP11-299H21.1	lincRNA	2,93E-01	1,86E-01	4,13E-01	No	1,97E-02	9,45E-01	9,68E-01	No
ENSG00000223826	AC092570.2	lincRNA	-3,01E-02	7,31E-01	NA	No	-1,28E-01	7,12E-01	NA	No
ENSG00000236757	AC007251.2	lincRNA	8,13E-02	5,53E-01	7,65E-01	No	1,21E-02	9,67E-01	9,80E-01	No
ENSG00000224095	AC108051.1	lincRNA	-1,29E-01	2,11E-01	NA	No	-7,45E-04	9,97E-01	9,98E-01	No
ENSG00000227680	AC108051.2	lincRNA	5,84E-02	4,18E-01	NA	No	9,02E-02	8,64E-01	NA	No
ENSG00000224850	AC108051.3	lincRNA	-4,09E-02	7,39E-01	NA	No	4,55E-02	8,56E-01	9,13E-01	No
ENSG00000230690	AC013402.5	lincRNA	1,99E-02	7,91E-01	NA	No	7,86E-02	6,66E-01	NA	No
ENSG00000234177	LINC01114	lincRNA	-1,48E-02	8,19E-01	NA	No	-2,79E-02	8,90E-01	NA	No
ENSG00000226508	AC104655.3	lincRNA	4,74E-02	5,77E-01	NA	No	1,09E-01	6,81E-01	NA	No
ENSG00000272861	RP11-332H14.1	lincRNA	9,60E-02	5,51E-01	7,63E-01	No	-1,95E-01	5,08E-01	NA	No
ENSG00000235319	AC012360.4	lincRNA	2,43E-01	2,40E-01	4,81E-01	No	-1,10E-01	7,36E-01	8,33E-01	No
ENSG00000272994	RP11-332H14.2	lincRNA	-1,04E-01	6,13E-01	8,05E-01	No	5,40E-01	4,49E-02	1,02E-01	No
ENSG00000231505	AC108868.5	lincRNA	5,18E-03	9,73E-01	NA	No	-1,28E-01	7,12E-01	NA	No
ENSG00000224568	AC096669.3	lincRNA	1,34E-01	1,50E-01	NA	No	2,08E-01	3,21E-01	NA	No
ENSG00000259863	SH3RF3-AS1	lincRNA	2,08E-01	3,55E-01	6,03E-01	No	1,76E-01	5,25E-01	6,70E-01	No
ENSG00000204588	LINC01123	lincRNA	-2,92E-01	2,30E-01	4,68E-01	No	-1,04E+00	1,31E-02	3,59E-02	No
ENSG00000175701	LINC00116	lincRNA	-4,20E-01	2,81E-02	1,17E-01	No	4,19E-01	2,75E-02	6,76E-02	No
ENSG00000261760	RP11-1223D19.1	lincRNA	-2,17E-01	3,27E-01	5,77E-01	No	-6,23E-01	1,27E-01	2,37E-01	No
ENSG00000175772	LINC01106	lincRNA	-9,45E-02	6,69E-01	8,38E-01	No	-9,41E-01	2,61E-02	6,47E-02	No
ENSG00000184115	RP13-1039J1.2	lincRNA	-4,42E-02	7,60E-01	NA	No	6,13E-03	9,87E-01	NA	No
ENSG00000230499	AC108463.1	lincRNA	-1,51E-01	4,99E-01	7,27E-01	No	-4,08E-01	2,76E-01	4,25E-01	No
ENSG00000172965	MIR4435-1HG	lincRNA	-3,50E-01	1,10E-01	2,96E-01	No	-4,59E-01	8,95E-02	1,79E-01	No
ENSG00000271590	RP11-181E10.3	lincRNA	-1,39E-01	5,32E-01	7,51E-01	No	-4,28E-01	2,09E-01	3,45E-01	No
ENSG00000229118	AC068491.2	lincRNA	-2,02E-01	2,52E-01	4,94E-01	No	-5,94E-01	1,11E-01	2,13E-01	No
ENSG00000228251	AC012442.6	lincRNA	1,04E-01	6,26E-01	8,14E-01	No	-9,00E-02	7,93E-01	8,72E-01	No
ENSG00000237753	AC079922.3	lincRNA	8,16E-01	5,34E-03	3,58E-02	No	9,55E-01	2,35E-03	7,96E-03	No
ENSG00000272563	RP11-480C16.1	lincRNA	7,88E-02	3,66E-01	NA	No	-3,01E-02	8,90E-01	NA	No
ENSG00000234148	RP11-395L14.3	lincRNA	-5,37E-02	6,46E-01	NA	No	-1,26E-01	7,12E-01	NA	No
ENSG00000231943	RP11-395L14.4	lincRNA	1,04E-01	6,03E-01	8,00E-01	No	-2,11E-01	5,32E-01	6,76E-01	No
ENSG00000226516	FAM138B	lincRNA	7,98E-02	3,47E-01	NA	No	8,77E-02	8,64E-01	NA	No
ENSG00000233479	AC017074.1	lincRNA	-7,65E-02	4,02E-01	NA	No	-1,50E-01	4,50E-01	NA	No
ENSG00000244063	AC024704.2	lincRNA	1,15E-01	3,35E-01	NA	No	8,64E-02	8,64E-01	NA	No
ENSG00000228857	AC104653.1	lincRNA	2,84E-01	1,09E-01	2,95E-01	No	7,62E-01	8,29E-02	1,69E-01	No
ENSG00000270019	RP11-141B14.1	lincRNA	3,20E-01	1,87E-01	4,15E-01	No	-1,12E-01	7,38E-01	8,34E-01	No
ENSG00000234199	AC010982.1	lincRNA	-1,99E-02	8,80E-01	NA	No	-4,82E-02	8,40E-01	NA	No
ENSG00000243179	AC110769.3	lincRNA	1,14E-01	5,63E-01	7,71E-01	No	-2,56E-01	4,42E-01	5,96E-01	No
ENSG00000236255	AC009404.2	lincRNA	2,12E-01	3,42E-01	5,90E-01	No	-2,41E-01	3,89E-01	5,47E-01	No
ENSG00000226856	AC093901.1	lincRNA	-8,46E-02	3,24E-01	NA	No	-1,30E-02	9,46E-01	9,69E-01	No
ENSG00000235840	AC012363.13	lincRNA	7,38E-02	4,04E-01	NA	No	6,54E-02	9,56E-01	NA	No
ENSG00000237614	AC073257.2	lincRNA	5,06E-02	6,89E-01	NA	No	1,15E-01	6,43E-01	7,64E-01	No
ENSG00000271709	RP11-297J22.1	lincRNA	-1,87E-01	3,12E-01	5,61E-01	No	-9,06E-01	3,16E-02	7,60E-02	No
ENSG00000224655	AC018737.3	lincRNA	-1,88E-02	8,19E-01	NA	No	2,20E-02	9,15E-01	NA	No
ENSG00000226813	AC114813.1	lincRNA	3,92E-02	8,45E-01	NA	No	1,40E-01	9,56E-01	NA	No
ENSG00000235774	AC023347.1	lincRNA	-1,60E-02	8,19E-01	NA	No	1,14E-01	7,12E-01	NA	No
ENSG00000260634	RP11-521O16.1	lincRNA	-5,73E-02	6,46E-01	NA	No	-1,36E-01	7,12E-01	NA	No
ENSG00000236682	AC068282.3	lincRNA	-2,69E-02	9,00E-01	9,59E-01	No	-1,25E-01	7,03E-01	8,09E-01	No
ENSG00000272667	RP11-395A13.2	lincRNA	-1,61E-01	3,86E-01	6,32E-01	No	3,81E-01	4,32E-02	9,92E-02	No
ENSG00000229536	AC079776.1	lincRNA	-5,75E-03	9,42E-01	NA	No	4,14E-02	8,55E-01	NA	No
ENSG00000273073	RP11-109E12.1	lincRNA	7,00E-02	7,49E-01	8,83E-01	No	9,99E-01	2,38E-03	8,05E-03	No
ENSG00000224559	LINC01087	lincRNA	-4,78E-02	6,03E-01	NA	No	3,71E-01	2,21E-01	3,60E-01	No
ENSG00000229203	AC103564.7	lincRNA	4,19E-02	7,49E-01	NA	No	8,64E-02	8,64E-01	NA	No
ENSG00000272769	RP11-725P16.2	lincRNA	-1,95E-01	3,86E-01	6,32E-01	No	1,63E-01	6,36E-01	7,59E-01	No
ENSG00000223430	AC011243.1	lincRNA	-4,26E-01	5,09E-02	1,76E-01	No	-1,11E+00	3,37E-02	8,03E-02	No
ENSG00000224231	AC069394.1	lincRNA	9,48E-02	5,53E-01	7,65E-01	No	2,97E-01	3,95E-01	5,52E-01	No
ENSG00000228043	AC097721.2	lincRNA	1,35E-01	5,09E-01	7,35E-01	No	2,27E-01	4,06E-01	5,63E-01	No
ENSG00000230569	AC114763.1	lincRNA	1,01E-01	6,36E-01	8,19E-01	No	4,80E-02	8,87E-01	9,33E-01	No
ENSG00000260059	RP11-231E19.1	lincRNA	5,02E-01	7,39E-02	2,28E-01	No	4,06E-01	2,48E-01	3,92E-01	No
ENSG00000237772	AC092620.3	lincRNA	1,87E-01	1,61E-01	3,79E-01	No	1,12E-01	6,59E-01	NA	No
ENSG00000232606	AC010090.1	lincRNA	3,41E-02	9,36E-01	NA	No	1,44E-01	5,65E-01	NA	No
ENSG00000226674	TEX41	lincRNA	5,58E-01	3,33E-02	1,31E-01	No	1,38E+00	2,75E-04	1,15E-03	No

ENSG00000234940	AC023128.1	lincRNA	3,15E-02	9,36E-01	NA	No	8,55E-02	8,64E-01	NA	No
ENSG00000268580	RP11-514A9.1	lincRNA	3,80E-02	8,45E-01	NA	No	1,53E-01	5,23E-01	NA	No
ENSG00000231040	AC103881.1	lincRNA	-2,31E-02	8,19E-01	NA	No	-1,35E-01	7,12E-01	NA	No
ENSG00000162947	AC007364.1	lincRNA	1,88E-01	3,04E-01	5,52E-01	No	6,77E-01	1,06E-01	2,05E-01	No
ENSG00000231420	AC113610.1	lincRNA	5,04E-03	9,91E-01	NA	No	6,24E-03	9,80E-01	NA	No
ENSG00000236049	AC104777.2	lincRNA	1,03E-02	9,12E-01	NA	No	-2,72E-02	8,90E-01	NA	No
ENSG00000232359	AC104777.1	lincRNA	5,38E-02	6,28E-01	NA	No	1,01E-01	7,75E-01	NA	No
ENSG00000224048	AC104777.4	lincRNA	3,39E-02	9,36E-01	NA	No	1,14E-01	6,83E-01	NA	No
ENSG00000234584	AC019186.1	lincRNA	1,21E-01	5,85E-01	7,87E-01	No	4,30E-01	2,10E-01	3,47E-01	No
ENSG00000270557	RP11-546J1.1	lincRNA	4,37E-01	7,80E-02	2,36E-01	No	1,10E+00	2,64E-02	6,53E-02	No
ENSG00000271320	RP11-1152H14.1	lincRNA	-1,06E-01	2,44E-01	NA	No	1,43E-02	9,62E-01	9,78E-01	No
ENSG00000228586	AC005042.5	lincRNA	6,47E-02	5,19E-01	NA	No	-2,82E-02	8,82E-01	NA	No
ENSG00000226266	AC009961.3	lincRNA	-3,09E-01	1,28E-01	3,28E-01	No	-6,42E-02	7,94E-01	8,73E-01	No
ENSG00000233397	AC008063.3	lincRNA	-5,73E-02	6,46E-01	NA	No	-1,36E-01	7,12E-01	NA	No
ENSG00000237844	AC092684.1	lincRNA	-7,45E-02	5,09E-01	NA	No	-9,57E-02	7,04E-01	NA	No
ENSG00000229195	AC009495.4	lincRNA	-2,41E-02	8,06E-01	NA	No	1,88E-01	4,92E-01	6,41E-01	No
ENSG00000232411	AC009495.3	lincRNA	2,02E-01	3,39E-01	5,88E-01	No	-1,27E-01	6,97E-01	8,06E-01	No
ENSG00000228222	AC074363.1	lincRNA	3,60E-02	7,04E-01	NA	No	3,62E-01	2,05E-01	3,40E-01	No
ENSG00000234350	AC007405.4	lincRNA	-1,31E-01	3,02E-01	5,50E-01	No	-1,83E-01	5,60E-01	6,98E-01	No
ENSG00000239467	AC007405.6	lincRNA	-2,07E-01	3,51E-01	5,99E-01	No	6,99E-02	8,38E-01	9,01E-01	No
ENSG00000236651	AC104801.1	lincRNA	-4,23E-02	7,60E-01	NA	No	-9,82E-02	8,30E-01	NA	No
ENSG00000232555	AC104088.1	lincRNA	-4,86E-02	4,90E-01	NA	No	1,19E-02	9,56E-01	NA	No
ENSG00000224638	AC106900.6	lincRNA	4,20E-01	5,39E-02	1,83E-01	No	-1,12E-02	9,61E-01	NA	No
ENSG00000273258	RP11-394I13.3	lincRNA	1,98E-01	2,00E-01	4,31E-01	No	7,21E-01	7,89E-02	1,62E-01	No
ENSG00000260868	RP11-394I13.1	lincRNA	6,89E-02	6,37E-01	8,20E-01	No	2,35E-01	4,48E-01	6,01E-01	No
ENSG00000231453	AC018470.4	lincRNA	5,02E-02	5,80E-01	NA	No	7,98E-02	7,17E-01	NA	No
ENSG00000226853	AC010894.3	lincRNA	3,11E-01	2,04E-01	4,37E-01	No	4,20E-01	2,04E-01	3,39E-01	No
ENSG00000229779	AC016751.2	lincRNA	3,46E-02	9,36E-01	NA	No	1,08E-01	6,85E-01	NA	No
ENSG00000226363	AC009336.24	lincRNA	-7,97E-02	4,97E-01	NA	No	-2,12E-02	9,33E-01	9,60E-01	No
ENSG00000229434	AC017048.1	lincRNA	9,02E-02	3,71E-01	NA	No	3,01E-02	8,32E-01	NA	No
ENSG00000236231	AC017048.2	lincRNA	1,59E-01	1,40E-01	NA	No	1,39E+00	2,07E-02	5,34E-02	No
ENSG00000272551	RP11-324L17.1	lincRNA	1,34E-01	2,59E-01	NA	No	2,48E-01	3,18E-01	NA	No
ENSG00000163364	LINC01116	lincRNA	2,09E-02	9,15E-01	9,64E-01	No	9,63E-01	3,29E-02	7,88E-02	No
ENSG00000224577	LINC01117	lincRNA	2,82E-01	4,70E-02	1,67E-01	No	2,00E-01	3,72E-01	NA	No
ENSG00000230552	AC092162.1	lincRNA	5,03E-01	7,12E-02	2,22E-01	No	5,39E-01	1,72E-01	2,99E-01	No
ENSG00000236501	AC079305.11	lincRNA	1,84E-02	8,97E-01	NA	No	4,06E-02	8,93E-01	NA	No
ENSG00000260931	RP11-65L3.1	lincRNA	1,04E+00	1,25E-02	6,60E-02	No	1,88E-01	5,96E-01	7,27E-01	No
ENSG00000271141	RP11-171I2.4	lincRNA	5,47E-02	6,99E-01	NA	No	-9,35E-02	7,01E-01	NA	No
ENSG00000271401	RP11-171I2.3	lincRNA	-1,04E+00	2,58E-04	3,65E-03	No	-5,89E-01	1,89E-02	4,94E-02	No
ENSG00000238171	AC068196.1	lincRNA	4,77E-02	7,00E-01	NA	No	8,53E-02	8,64E-01	NA	No
ENSG00000272800	RP11-438L19.1	lincRNA	-1,93E-01	3,77E-01	6,23E-01	No	-1,50E-02	9,64E-01	9,79E-01	No
ENSG00000237877	AC097500.2	lincRNA	3,01E-01	1,15E-01	3,06E-01	No	7,72E-01	1,34E-04	5,94E-04	No
ENSG00000259915	RP11-410E4.1	lincRNA	-7,23E-02	7,40E-01	8,79E-01	No	3,97E-01	1,31E-01	2,42E-01	No
ENSG00000231689	LINC01090	lincRNA	-1,81E-02	8,19E-01	NA	No	-6,53E-02	8,00E-01	NA	No
ENSG00000227542	AC092614.2	lincRNA	5,93E-03	9,80E-01	9,92E-01	No	-5,53E-01	9,02E-02	1,80E-01	No
ENSG00000260142	RP11-764E7.1	lincRNA	3,31E-02	9,36E-01	NA	No	1,07E-01	6,96E-01	NA	No
ENSG00000230173	AC006196.1	lincRNA	4,19E-02	7,49E-01	NA	No	6,13E-02	9,56E-01	NA	No
ENSG00000224099	AC064834.1	lincRNA	6,97E-02	4,40E-01	NA	No	6,23E-02	9,56E-01	NA	No
ENSG00000234919	AC064834.3	lincRNA	3,84E-02	8,45E-01	NA	No	7,04E-01	3,32E-02	7,94E-02	No
ENSG00000237035	AC109589.1	lincRNA	-1,98E-01	1,99E-01	NA	No	-7,12E-01	3,92E-02	NA	No
ENSG00000236653	AC005235.1	lincRNA	-1,53E-02	8,19E-01	NA	No	3,43E-02	8,95E-01	NA	No
ENSG00000238217	AC093590.1	lincRNA	-3,60E-02	6,77E-01	NA	No	-1,57E-01	5,12E-01	NA	No
ENSG00000237166	AC007163.3	lincRNA	-5,73E-02	6,46E-01	NA	No	8,17E-02	6,09E-01	NA	No
ENSG00000260006	RP11-469M7.1	lincRNA	-2,11E-01	3,38E-01	5,87E-01	No	-1,13E-01	6,77E-01	7,90E-01	No
ENSG00000232719	RP11-13J8.1	lincRNA	7,75E-02	5,94E-01	7,93E-01	No	-1,28E-02	9,63E-01	9,79E-01	No
ENSG00000273209	RP11-107N15.1	lincRNA	1,93E-01	3,00E-01	5,48E-01	No	3,71E-01	3,07E-01	4,60E-01	No
ENSG00000272966	RP11-686O6.1	lincRNA	-2,23E-02	8,38E-01	NA	No	-5,73E-03	9,79E-01	NA	No
ENSG00000273456	RP11-686O6.2	lincRNA	1,17E-01	5,95E-01	7,93E-01	No	1,96E-01	5,55E-01	6,94E-01	No
ENSG00000237271	AC009498.1	lincRNA	3,41E-02	9,36E-01	NA	No	8,55E-02	8,64E-01	NA	No
ENSG00000227946	AC007383.3	lincRNA	6,45E-02	7,71E-01	8,96E-01	No	7,70E-01	1,84E-02	4,82E-02	No
ENSG00000235118	AC010731.4	lincRNA	-8,10E-01	2,49E-02	1,07E-01	No	-2,04E-01	5,48E-01	6,88E-01	No
ENSG00000260171	RP11-95H11.1	lincRNA	-2,66E-02	8,94E-01	9,56E-01	No	4,64E-01	2,04E-01	3,39E-01	No

ENSG00000229321	AC008269.2	lincRNA	1,58E-02	9,43E-01	9,77E-01	No	-4,96E-02	8,79E-01	9,28E-01	No
ENSG00000224342	AC007879.1	lincRNA	3,08E-01	3,82E-02	NA	No	4,67E-01	1,04E-01	2,01E-01	No
ENSG00000234902	AC007879.2	lincRNA	3,80E-01	9,02E-02	2,60E-01	No	-3,33E-02	9,13E-01	9,48E-01	No
ENSG00000225916	AC007879.4	lincRNA	-3,07E-03	9,81E-01	9,93E-01	No	-2,56E-01	2,87E-01	4,37E-01	No
ENSG00000240440	AC007879.3	lincRNA	2,22E-01	5,88E-02	NA	No	8,55E-02	8,64E-01	NA	No
ENSG00000225064	AC007879.6	lincRNA	3,44E-02	9,36E-01	NA	No	1,38E-01	4,52E-01	NA	No
ENSG00000224137	AC079767.4	lincRNA	1,48E-01	1,23E-01	3,20E-01	No	-1,28E-01	7,12E-01	NA	No
ENSG00000244567	AC096772.6	lincRNA	-9,78E-02	5,12E-01	7,38E-01	No	2,90E-01	2,36E-02	5,96E-02	No
ENSG00000234308	AC093381.2	lincRNA	1,74E-02	8,75E-01	NA	No	-1,22E-01	5,52E-01	NA	No
ENSG00000223373	AC108066.1	lincRNA	2,27E-01	1,45E-01	3,55E-01	No	6,19E-01	1,23E-01	2,30E-01	No
ENSG00000237525	AC012668.2	lincRNA	7,58E-02	5,77E-01	NA	No	5,49E-01	1,33E-01	2,45E-01	No
ENSG00000227981	AC012668.3	lincRNA	-4,42E-02	7,60E-01	NA	No	4,50E-03	9,87E-01	NA	No
ENSG00000235770	LINC00607	lincRNA	5,09E-01	6,40E-02	2,06E-01	No	7,71E-01	5,43E-02	1,20E-01	No
ENSG00000230838	AC093850.2	lincRNA	-1,98E-02	8,97E-01	9,57E-01	No	-3,00E-01	3,90E-01	5,48E-01	No
ENSG00000226276	AC093382.1	lincRNA	2,67E-01	3,39E-02	NA	No	1,66E-01	3,28E-01	NA	No
ENSG00000260804	RP11-566E18.3	lincRNA	-2,81E-01	1,34E-01	3,38E-01	No	-4,77E-03	9,81E-01	9,88E-01	No
ENSG00000229352	AC007563.3	lincRNA	-2,17E-02	8,07E-01	NA	No	2,85E-01	3,57E-01	5,13E-01	No
ENSG00000227021	AC007557.3	lincRNA	5,05E-02	6,19E-01	NA	No	1,25E-02	9,51E-01	NA	No
ENSG00000259855	RP11-574O16.1	lincRNA	1,25E-01	3,24E-01	NA	No	2,98E-01	3,00E-01	4,52E-01	No
ENSG00000233143	AC009492.1	lincRNA	-4,66E-02	8,16E-01	9,19E-01	No	-3,38E-01	3,58E-01	5,14E-01	No
ENSG00000272555	RP11-459I19.1	lincRNA	1,98E-01	2,94E-01	5,42E-01	No	2,78E-01	4,35E-01	5,89E-01	No
ENSG00000272644	RP11-33O4.1	lincRNA	-4,86E-01	6,89E-02	2,17E-01	No	-1,44E+00	1,51E-03	5,34E-03	No
ENSG00000267919	RP11-256I23.3	lincRNA	-7,03E-01	2,61E-02	1,11E-01	No	-1,45E+00	4,30E-04	1,72E-03	No
ENSG00000227308	AC009502.4	lincRNA	-8,47E-02	3,21E-01	NA	No	-2,95E-01	2,07E-01	3,43E-01	No
ENSG00000228909	AC008281.1	lincRNA	-6,67E-02	5,73E-01	NA	No	-3,29E-01	1,71E-01	NA	No
ENSG00000239498	AC114765.1	lincRNA	-2,38E-01	2,70E-01	5,15E-01	No	-6,11E-01	1,35E-01	2,47E-01	No
ENSG00000235337	AC114765.2	lincRNA	-1,62E-01	3,02E-01	NA	No	-5,78E-02	8,58E-01	9,15E-01	No
ENSG00000236451	AC067956.1	lincRNA	8,84E-02	6,05E-01	8,00E-01	No	3,94E-01	2,86E-01	4,36E-01	No
ENSG00000224819	AC093843.1	lincRNA	3,56E-01	1,55E-01	3,71E-01	No	1,32E+00	1,13E-02	3,16E-02	No
ENSG00000267034	RP11-384O8.1	lincRNA	-1,13E-01	6,07E-01	8,01E-01	No	1,31E+00	2,69E-03	8,95E-03	No
ENSG00000232784	AC067961.1	lincRNA	8,97E-02	3,73E-01	NA	No	-2,53E-02	8,90E-01	NA	No
ENSG00000273301	RP11-314B1.2	lincRNA	3,36E-01	1,08E-01	2,93E-01	No	2,39E-01	4,40E-01	5,94E-01	No
ENSG00000235070	AC068138.1	lincRNA	-7,99E-02	2,58E-01	NA	No	-1,75E-01	3,13E-01	NA	No
ENSG00000272622	RP11-395N3.2	lincRNA	-3,94E-01	1,28E-01	3,28E-01	No	-9,18E-02	7,71E-01	8,57E-01	No
ENSG00000232023	AC009410.1	lincRNA	9,44E-02	4,70E-01	NA	No	8,19E-02	7,49E-01	NA	No
ENSG00000228226	AC074019.1	lincRNA	2,65E-01	5,55E-02	1,86E-01	No	4,75E-01	1,45E-01	2,62E-01	No
ENSG00000226125	AC098823.3	lincRNA	1,49E-01	3,30E-01	5,79E-01	No	-2,96E-02	9,07E-01	9,45E-01	No
ENSG00000238062	SPATA3-AS1	lincRNA	8,14E-02	3,38E-01	NA	No	9,81E-02	7,75E-01	NA	No
ENSG00000228162	AC097713.3	lincRNA	-5,58E-02	6,46E-01	NA	No	-1,33E-01	7,12E-01	NA	No
ENSG00000231682	AC097713.4	lincRNA	1,25E-01	3,33E-01	NA	No	-5,99E-02	8,00E-01	NA	No
ENSG00000226542	AC114814.4	lincRNA	-2,07E-03	9,41E-01	NA	No	-1,03E-01	8,30E-01	NA	No
ENSG00000235293	AC114814.3	lincRNA	3,15E-02	9,36E-01	NA	No	8,55E-02	8,64E-01	NA	No
ENSG00000232328	AC011286.1	lincRNA	-5,45E-02	6,46E-01	NA	No	5,92E-02	7,75E-01	NA	No
ENSG00000224844	AC107079.1	lincRNA	-3,15E-01	1,85E-01	4,13E-01	No	-8,99E-01	5,42E-02	1,20E-01	No
ENSG00000222032	AC112721.2	lincRNA	7,47E-02	4,13E-01	NA	No	-1,26E-01	7,12E-01	NA	No
ENSG00000227107	AC096574.5	lincRNA	4,16E-01	6,35E-02	2,05E-01	No	-9,11E-01	9,78E-03	2,79E-02	No
ENSG00000225493	LINC01107	lincRNA	4,99E-02	6,36E-01	NA	No	-7,85E-02	6,95E-01	NA	No
ENSG00000234279	AC113618.1	lincRNA	3,15E-02	9,36E-01	NA	No	6,67E-02	9,56E-01	NA	No
ENSG00000227744	AC114788.2	lincRNA	1,05E-01	3,20E-01	NA	No	1,15E-02	9,11E-01	NA	No
ENSG00000227479	AC124861.1	lincRNA	7,93E-02	5,32E-01	7,51E-01	No	5,66E-01	1,21E-01	2,28E-01	No
ENSG00000273113	RP11-367H1.1	lincRNA	2,46E-01	2,44E-01	4,85E-01	No	2,66E-01	4,43E-01	5,98E-01	No
ENSG00000232002	AC093642.6	lincRNA	4,70E-02	7,75E-01	8,97E-01	No	3,49E-01	3,21E-01	4,75E-01	No
ENSG00000224957	AC090044.1	lincRNA	1,06E-01	6,35E-01	8,19E-01	No	6,48E-01	6,94E-02	1,46E-01	No
ENSG00000224239	AC090044.2	lincRNA	1,07E-01	2,90E-01	NA	No	-3,71E-02	8,36E-01	NA	No
ENSG00000235158	AC087430.1	lincRNA	-8,99E-03	9,39E-01	NA	No	-1,70E-01	4,19E-01	NA	No
ENSG00000227588	CNTN4-AS2	lincRNA	5,12E-02	7,62E-01	8,90E-01	No	2,21E-01	5,09E-01	6,56E-01	No
ENSG00000223727	AC026188.1	lincRNA	1,62E-01	3,87E-01	6,32E-01	No	6,15E-02	8,44E-01	9,05E-01	No
ENSG00000235947	EGOT	lincRNA	2,84E-01	2,33E-01	4,72E-01	No	-7,06E-02	8,15E-01	8,87E-01	No
ENSG00000230944	AC026202.5	lincRNA	3,13E-02	6,76E-01	NA	No	-1,36E-01	7,12E-01	NA	No
ENSG00000229642	AC027119.1	lincRNA	3,15E-02	9,36E-01	NA	No	8,77E-02	8,64E-01	NA	No
ENSG00000189229	AC069277.2	lincRNA	7,70E-02	4,45E-01	NA	No	2,99E-01	2,71E-01	4,19E-01	No
ENSG00000226258	GRM7-AS3	lincRNA	-1,25E-01	1,66E-01	NA	No	-8,97E-02	7,45E-01	8,39E-01	No

ENSG00000228351	AC018832.1	lincRNA	1,42E-01	3,44E-01	5,92E-01	No	3,89E-01	2,42E-01	3,86E-01	No
ENSG00000231401	AC023481.1	lincRNA	9,23E-01	1,81E-02	8,55E-02	No	1,24E+00	1,43E-02	3,89E-02	No
ENSG00000237697	LINC00312	lincRNA	-8,58E-01	5,98E-03	3,88E-02	No	-3,93E-01	2,05E-01	3,40E-01	No
ENSG00000224884	AC034187.2	lincRNA	-4,27E-02	5,28E-01	NA	No	-9,42E-02	6,06E-01	NA	No
ENSG00000235830	SRGAP3-AS4	lincRNA	-4,84E-02	8,08E-01	9,15E-01	No	5,78E-01	1,43E-01	2,59E-01	No
ENSG00000269975	RP11-58B17.2	lincRNA	3,41E-01	5,51E-02	1,85E-01	No	-4,48E-02	7,98E-01	NA	No
ENSG00000206567	AC022007.5	lincRNA	1,77E-01	4,35E-01	6,75E-01	No	-5,05E-01	1,44E-01	2,61E-01	No
ENSG00000232746	RP11-767C1.1	lincRNA	5,10E-02	6,65E-01	NA	No	1,27E-01	6,52E-01	NA	No
ENSG00000251576	RP11-536I6.1	lincRNA	2,70E-01	1,11E-01	2,99E-01	No	-2,05E-02	9,36E-01	9,62E-01	No
ENSG00000260902	RP11-95M5.1	lincRNA	-1,08E-01	3,59E-01	NA	No	-3,55E-01	1,95E-01	NA	No
ENSG00000269391	RP11-194G10.3	lincRNA	-6,47E-02	4,56E-01	NA	No	-1,57E-01	5,07E-01	NA	No
ENSG00000233570	LINC00690	lincRNA	-7,91E-02	3,10E-01	NA	No	-1,54E-01	4,28E-01	NA	No
ENSG00000229271	AC091493.2	lincRNA	-5,57E-02	6,46E-01	NA	No	-1,32E-01	7,12E-01	NA	No
ENSG00000226238	AC132807.1	lincRNA	3,39E-02	9,36E-01	NA	No	8,55E-02	8,64E-01	NA	No
ENSG00000231304	AC107622.1	lincRNA	1,84E-01	1,22E-01	NA	No	7,22E-01	4,63E-02	1,05E-01	No
ENSG00000261734	RP11-669C19.1	lincRNA	-1,61E-02	9,29E-01	9,71E-01	No	-2,11E-01	5,43E-01	6,85E-01	No
ENSG00000272511	RP11-180N14.1	lincRNA	-1,82E-01	4,20E-01	6,63E-01	No	7,22E-01	5,99E-03	1,82E-02	No
ENSG00000223351	ZNF385D-AS2	lincRNA	-3,62E-01	8,65E-02	2,53E-01	No	-3,60E-01	2,71E-01	4,19E-01	No
ENSG00000224074	LINC00691	lincRNA	9,68E-02	6,21E-01	8,11E-01	No	-6,64E-02	8,46E-01	9,06E-01	No
ENSG00000237838	AC133680.1	lincRNA	-8,31E-02	3,91E-01	NA	No	-1,87E-01	4,73E-01	NA	No
ENSG00000225386	AC099754.1	lincRNA	4,00E-02	8,45E-01	NA	No	8,64E-02	8,64E-01	NA	No
ENSG00000234165	AC114877.3	lincRNA	-8,63E-04	9,94E-01	NA	No	3,22E-01	3,13E-01	4,67E-01	No
ENSG00000271943	RP11-222K16.1	lincRNA	2,33E-02	8,98E-01	9,57E-01	No	5,62E-03	9,88E-01	9,93E-01	No
ENSG00000225548	AC098973.2	lincRNA	3,41E-02	9,36E-01	NA	No	1,14E-01	6,21E-01	NA	No
ENSG00000229243	AC098973.1	lincRNA	4,17E-02	7,65E-01	NA	No	1,69E-01	4,52E-01	NA	No
ENSG00000235493	AC092415.1	lincRNA	3,35E-01	2,59E-02	1,10E-01	No	2,84E-01	2,30E-01	3,71E-01	No
ENSG00000227260	AC116035.1	lincRNA	1,01E-01	4,70E-01	7,04E-01	No	4,45E-01	2,01E-01	3,35E-01	No
ENSG00000271324	RP11-10C24.2	lincRNA	-2,54E-01	2,58E-01	5,01E-01	No	2,43E-01	4,74E-01	6,25E-01	No
ENSG00000271643	RP11-10C24.3	lincRNA	-4,93E-01	6,62E-02	2,11E-01	No	6,22E-01	2,27E-02	5,76E-02	No
ENSG00000271020	RP11-10C24.1	lincRNA	-2,96E-01	2,21E-01	4,58E-01	No	1,37E-01	6,41E-01	7,63E-01	No
ENSG00000226320	AC018359.1	lincRNA	4,08E-02	7,74E-01	NA	No	1,38E-01	5,92E-01	NA	No
ENSG00000236452	AC123023.1	lincRNA	4,73E-02	5,80E-01	NA	No	8,58E-02	8,64E-01	NA	No
ENSG00000272334	RP11-129K12.1	lincRNA	3,97E-02	8,08E-01	9,15E-01	No	-3,36E-01	2,69E-01	4,18E-01	No
ENSG00000271835	RP11-129K12.4	lincRNA	2,71E-01	2,59E-01	5,03E-01	No	-1,26E-01	7,15E-01	8,18E-01	No
ENSG00000271653	RP11-259K5.1	lincRNA	-2,69E-02	7,40E-01	NA	No	1,40E-01	5,62E-01	7,00E-01	No
ENSG00000272452	RP11-391M1.4	lincRNA	-7,34E-01	1,62E-04	2,52E-03	No	-1,15E-01	5,36E-01	6,79E-01	No
ENSG00000226302	RP11-528N21.1	lincRNA	1,83E-02	8,35E-01	NA	No	-1,12E-01	5,93E-01	NA	No
ENSG00000231873	RP11-761N21.1	lincRNA	7,32E-02	6,05E-01	8,00E-01	No	4,13E-02	8,76E-01	9,26E-01	No
ENSG00000233096	RP11-520A21.1	lincRNA	3,31E-02	9,36E-01	NA	No	1,86E-01	3,93E-01	NA	No
ENSG00000227245	RP11-136C24.2	lincRNA	4,48E-01	7,19E-02	2,23E-01	No	3,66E-02	9,00E-01	9,41E-01	No
ENSG00000235886	RP4-672N11.1	lincRNA	-8,78E-02	3,55E-01	NA	No	-2,00E-01	4,31E-01	NA	No
ENSG00000272121	RP4-555D20.4	lincRNA	-1,97E-02	8,19E-01	NA	No	-1,35E-01	7,12E-01	NA	No
ENSG00000272077	RP11-348P10.2	lincRNA	-4,96E-01	3,21E-02	1,28E-01	No	-5,82E-01	1,23E-02	3,40E-02	No
ENSG00000271973	RP11-572O6.1	lincRNA	7,78E-01	2,22E-02	9,89E-02	No	2,83E-01	4,17E-01	5,73E-01	No
ENSG00000226913	BSN-AS2	lincRNA	2,10E-01	3,15E-01	5,64E-01	No	4,51E-01	2,37E-01	3,80E-01	No
ENSG00000230454	U73166.2	lincRNA	3,81E-01	9,89E-02	2,76E-01	No	-6,97E-01	1,95E-02	5,07E-02	No
ENSG00000273356	RP11-804H8.6	lincRNA	-1,12E+00	1,31E-03	1,26E-02	No	-1,23E+00	1,41E-03	5,02E-03	No
ENSG00000240777	RP11-58O15.1	lincRNA	4,13E-04	9,41E-01	NA	No	8,65E-03	9,87E-01	NA	No
ENSG00000241933	RP11-755B10.3	lincRNA	-3,16E-02	8,40E-01	9,30E-01	No	-6,67E-02	8,41E-01	9,03E-01	No
ENSG00000273493	RP11-80H18.4	lincRNA	-1,56E-01	3,10E-01	NA	No	-2,33E-01	4,84E-01	6,34E-01	No
ENSG00000272360	RP11-359I18.5	lincRNA	6,69E-03	9,70E-01	9,88E-01	No	3,72E-01	3,16E-01	4,70E-01	No
ENSG00000244342	LINC00698	lincRNA	-5,37E-02	6,46E-01	NA	No	-1,26E-01	7,12E-01	NA	No
ENSG00000271843	RP11-245J9.5	lincRNA	2,71E-01	2,40E-01	4,80E-01	No	-1,64E-01	5,58E-01	6,97E-01	No
ENSG00000189196	LINC00994	lincRNA	-2,26E-02	8,61E-01	9,40E-01	No	-2,83E-01	2,86E-01	NA	No
ENSG00000241884	RP11-85I21.1	lincRNA	3,61E-01	1,01E-02	5,68E-02	No	2,36E-01	3,03E-01	NA	No
ENSG00000241316	RP11-81N13.1	lincRNA	2,75E-01	8,84E-02	2,57E-01	No	7,18E-01	9,54E-05	4,35E-04	No
ENSG00000270562	RP11-154H23.3	lincRNA	5,17E-01	6,49E-02	2,08E-01	No	5,09E-01	1,26E-01	2,35E-01	No
ENSG00000273461	RP11-398A8.4	lincRNA	1,54E-01	1,64E-01	NA	No	6,68E-02	9,56E-01	NA	No
ENSG00000243083	LINC00870	lincRNA	4,60E-02	6,23E-01	NA	No	1,46E-01	5,55E-01	NA	No
ENSG00000260670	RP11-447M4.1	lincRNA	3,39E-02	9,36E-01	NA	No	8,58E-02	8,64E-01	NA	No
ENSG00000244345	RP11-654C22.2	lincRNA	-4,19E-03	9,91E-01	NA	No	-6,80E-02	8,00E-01	NA	No
ENSG00000242741	RP11-20B7.1	lincRNA	1,21E-02	9,42E-01	9,76E-01	No	1,94E-01	5,69E-01	7,06E-01	No

ENSG00000272690	RP11-803B1.8	lincRNA	2,19E-01	3,39E-01	5,88E-01	No	1,67E-01	6,29E-01	7,53E-01	No
ENSG00000272710	CTD-2026G6.3	lincRNA	3,65E-02	7,42E-01	NA	No	2,93E-02	9,12E-01	NA	No
ENSG00000242516	LINC00960	lincRNA	8,20E-03	9,42E-01	NA	No	1,50E-02	9,50E-01	9,71E-01	No
ENSG00000244461	RP11-354H21.1	lincRNA	4,19E-02	7,67E-01	NA	No	8,77E-02	8,64E-01	NA	No
ENSG00000240573	RP11-354H21.2	lincRNA	4,23E-02	7,45E-01	NA	No	9,94E-02	7,75E-01	NA	No
ENSG00000242828	RP11-47P18.1	lincRNA	2,33E-02	8,20E-01	NA	No	9,91E-02	7,12E-01	8,16E-01	No
ENSG00000242781	RP11-47P18.2	lincRNA	4,75E-02	5,44E-01	NA	No	1,37E-01	4,70E-01	NA	No
ENSG00000243694	RP11-6B4.1	lincRNA	4,50E-02	6,60E-01	NA	No	2,77E-01	2,49E-01	NA	No
ENSG00000241593	RP11-520D19.2	lincRNA	-1,14E-01	5,41E-01	7,57E-01	No	-5,30E-02	8,77E-01	9,26E-01	No
ENSG00000242190	RP11-142L1.1	lincRNA	-4,91E-02	7,48E-01	8,82E-01	No	-3,67E-02	9,07E-01	9,45E-01	No
ENSG00000239440	RP11-260O18.1	lincRNA	3,43E-02	7,24E-01	NA	No	2,41E-01	3,17E-01	NA	No
ENSG00000242641	LINC00971	lincRNA	3,75E-02	8,45E-01	NA	No	8,55E-02	8,64E-01	NA	No
ENSG00000239572	RP11-451B8.1	lincRNA	3,93E-02	7,28E-01	NA	No	-1,20E-01	4,73E-01	NA	No
ENSG00000239589	LINC00879	lincRNA	-5,73E-02	6,46E-01	NA	No	-2,72E-02	8,90E-01	NA	No
ENSG00000251088	RP11-325B23.2	lincRNA	4,40E-02	6,48E-01	NA	No	-1,47E-01	6,01E-01	NA	No
ENSG00000240476	LINC00973	lincRNA	-1,96E-02	8,22E-01	NA	No	1,24E-02	9,28E-01	NA	No
ENSG00000243296	RP11-779P15.1	lincRNA	3,13E-02	7,09E-01	NA	No	2,21E-01	3,08E-01	NA	No
ENSG00000243089	RP11-779P15.2	lincRNA	7,27E-02	4,29E-01	NA	No	4,14E-01	1,45E-01	NA	No
ENSG00000244464	RP11-201E8.1	lincRNA	3,13E-01	1,57E-01	3,73E-01	No	4,78E-01	2,08E-01	3,44E-01	No
ENSG00000273374	RP11-383I23.2	lincRNA	3,08E-01	1,88E-01	4,16E-01	No	4,07E-01	2,59E-01	4,06E-01	No
ENSG00000273488	RP11-114I8.4	lincRNA	2,38E-01	2,70E-01	5,15E-01	No	5,14E-01	4,00E-02	9,29E-02	No
ENSG00000214407	RP11-221J22.1	lincRNA	6,84E-02	5,89E-01	NA	No	1,02E-01	7,75E-01	NA	No
ENSG00000241280	RP11-221J22.2	lincRNA	3,46E-02	9,36E-01	NA	No	1,01E-01	7,75E-01	NA	No
ENSG00000241754	RP11-280H21.1	lincRNA	5,60E-03	9,52E-01	NA	No	6,41E-01	1,19E-01	2,25E-01	No
ENSG00000242759	LINC00882	lincRNA	-2,98E-01	1,14E-01	3,04E-01	No	3,70E-01	5,30E-02	1,17E-01	No
ENSG00000243701	LINC00883	lincRNA	8,59E-02	6,73E-01	8,41E-01	No	4,48E-01	6,59E-02	1,40E-01	No
ENSG00000239828	RP11-446H18.5	lincRNA	4,75E-01	8,01E-02	2,40E-01	No	9,67E-01	1,04E-02	2,94E-02	No
ENSG00000273125	RP11-115H18.1	lincRNA	2,40E-01	1,21E-01	3,17E-01	No	1,48E+00	2,03E-02	5,25E-02	No
ENSG00000241469	LINC00635	lincRNA	5,60E-02	6,02E-01	NA	No	2,18E-01	2,71E-01	NA	No
ENSG00000240423	LINC00636	lincRNA	-1,58E-02	8,44E-01	NA	No	6,66E-02	7,89E-01	NA	No
ENSG00000242029	RP11-457K10.1	lincRNA	-4,38E-02	5,30E-01	NA	No	-1,61E-01	4,63E-01	NA	No
ENSG00000243081	RP11-231E6.1	lincRNA	-3,23E-02	7,31E-01	NA	No	-1,33E-01	7,12E-01	NA	No
ENSG00000272761	RP11-572C15.6	lincRNA	6,34E-01	3,24E-02	1,29E-01	No	1,26E+00	6,03E-04	2,34E-03	No
ENSG00000240893	RP11-572C15.5	lincRNA	-7,61E-02	4,62E-01	NA	No	-1,22E-01	5,58E-01	NA	No
ENSG00000241219	RP11-572M11.1	lincRNA	-6,66E-02	7,19E-01	8,69E-01	No	-1,16E-01	7,01E-01	8,08E-01	No
ENSG00000243795	RP11-572M11.3	lincRNA	-2,20E-01	2,46E-01	4,88E-01	No	-1,78E-01	6,02E-01	7,32E-01	No
ENSG00000242659	RP11-271C24.2	lincRNA	3,31E-02	8,75E-01	9,48E-01	No	-3,21E-01	3,78E-01	5,35E-01	No
ENSG00000259976	RP11-553L6.5	lincRNA	-1,19E-01	4,37E-01	6,77E-01	No	6,79E-01	4,62E-05	2,25E-04	No
ENSG00000242290	RP11-197K3.1	lincRNA	-3,84E-02	8,60E-01	9,40E-01	No	9,88E-02	7,43E-01	8,38E-01	No
ENSG00000241295	ZBTB20-AS2	lincRNA	-8,69E-02	6,91E-01	8,52E-01	No	-4,51E-02	8,92E-01	9,36E-01	No
ENSG00000239946	ZBTB20-AS3	lincRNA	-3,33E-02	8,74E-01	9,47E-01	No	-6,77E-02	8,09E-01	8,82E-01	No
ENSG00000242767	ZBTB20-AS4	lincRNA	6,54E-01	1,23E-02	6,51E-02	No	1,38E-01	5,85E-01	7,18E-01	No
ENSG00000242880	RP11-190P13.2	lincRNA	1,06E-01	4,12E-01	6,55E-01	No	4,12E-01	2,14E-01	3,52E-01	No
ENSG00000239268	RP11-384F7.2	lincRNA	-1,51E-01	3,18E-01	5,66E-01	No	-2,84E-02	9,28E-01	9,58E-01	No
ENSG00000241213	RP11-768G7.2	lincRNA	3,56E-02	7,30E-01	NA	No	3,32E-01	2,32E-01	NA	No
ENSG00000272662	RP11-190C22.8	lincRNA	-8,90E-02	4,10E-01	NA	No	-2,75E-01	1,56E-01	NA	No
ENSG00000242622	RP11-18H7.1	lincRNA	3,37E-01	1,24E-01	3,21E-01	No	9,03E-01	3,70E-03	1,19E-02	No
ENSG00000273033	RP11-67L2.2	lincRNA	-4,70E-01	1,79E-02	8,48E-02	No	-6,88E-01	1,40E-03	4,99E-03	No
ENSG00000273454	RP11-797D24.3	lincRNA	-1,59E-01	4,74E-01	7,06E-01	No	-4,23E-01	2,58E-01	4,04E-01	No
ENSG00000272947	RP11-71H17.9	lincRNA	3,77E-01	1,44E-01	3,53E-01	No	3,34E-01	3,62E-01	5,18E-01	No
ENSG00000272840	RP11-379B18.6	lincRNA	3,87E-02	6,88E-01	NA	No	-5,64E-03	9,79E-01	NA	No
ENSG00000241288	RP11-379B18.5	lincRNA	4,76E-01	8,09E-02	2,42E-01	No	3,46E-01	2,86E-01	4,36E-01	No
ENSG00000248787	RP11-666A20.4	lincRNA	2,25E-01	2,56E-01	4,99E-01	No	2,31E-01	5,17E-01	6,63E-01	No
ENSG00000250934	RP11-71E19.1	lincRNA	5,24E-01	6,72E-02	2,14E-01	No	5,75E-01	1,48E-01	2,66E-01	No
ENSG00000251448	RP11-71E19.2	lincRNA	5,00E-01	4,67E-02	1,66E-01	No	7,10E-01	9,50E-02	1,88E-01	No
ENSG00000239921	RP11-59J16.1	lincRNA	-5,69E-02	6,46E-01	NA	No	-1,35E-01	7,12E-01	NA	No
ENSG00000240562	RP11-59J16.2	lincRNA	-1,32E-02	8,49E-01	NA	No	-9,82E-02	8,30E-01	NA	No
ENSG00000261159	RP11-723O4.9	lincRNA	4,18E-01	1,31E-02	NA	No	1,07E-01	6,96E-01	NA	No
ENSG00000273437	RP11-434H6.7	lincRNA	1,95E-01	3,87E-01	6,32E-01	No	-4,69E-01	1,83E-01	3,13E-01	No
ENSG00000248243	RP11-93K22.13	lincRNA	-5,22E-02	5,71E-01	NA	No	-5,16E-02	7,88E-01	NA	No
ENSG00000249846	RP11-77P16.4	lincRNA	9,82E-02	6,37E-01	8,20E-01	No	3,39E-02	9,25E-01	9,55E-01	No
ENSG00000272832	RP11-91K8.5	lincRNA	3,99E-02	6,37E-01	NA	No	1,27E-02	9,22E-01	NA	No

ENSG00000240006	RP11-200A1.1	lincRNA	-5,18E-02	6,84E-01	NA	No	2,78E-02	9,20E-01	9,52E-01	No
ENSG00000260633	RP11-375I20.6	lincRNA	3,85E-02	8,62E-01	9,40E-01	No	-1,08E+00	2,04E-02	5,26E-02	No
ENSG00000261763	RP11-442N1.2	lincRNA	-5,54E-02	5,61E-01	NA	No	-1,20E-01	6,25E-01	NA	No
ENSG00000250543	RP11-442N1.1	lincRNA	-6,37E-02	4,85E-01	NA	No	-4,79E-02	8,37E-01	NA	No
ENSG00000261826	RP11-691G17.1	lincRNA	-4,23E-02	7,60E-01	NA	No	-9,82E-02	8,30E-01	NA	No
ENSG00000242104	RP11-340E6.1	lincRNA	4,32E-02	7,25E-01	NA	No	8,55E-02	8,64E-01	NA	No
ENSG00000272565	RP11-485G4.2	lincRNA	4,93E-01	7,44E-04	8,16E-03	No	1,28E+00	2,92E-20	9,17E-19	No
ENSG00000268129	RP11-91G21.1	lincRNA	3,91E-02	8,45E-01	NA	No	1,80E-01	5,32E-01	NA	No
ENSG00000241679	RP11-80H8.4	lincRNA	9,73E-02	6,35E-01	8,19E-01	No	7,80E-02	8,23E-01	8,92E-01	No
ENSG00000244358	RP11-88H10.2	lincRNA	-4,90E-02	5,44E-01	NA	No	-1,09E-01	6,02E-01	NA	No
ENSG00000239922	RP11-71N10.1	lincRNA	-4,77E-02	6,59E-01	8,33E-01	No	3,21E-02	9,11E-01	9,47E-01	No
ENSG00000243885	RP11-278L15.2	lincRNA	-4,50E-02	6,64E-01	NA	No	-1,57E-01	4,48E-01	NA	No
ENSG00000242791	RP11-651P23.5	lincRNA	1,13E-01	5,74E-01	7,79E-01	No	-3,09E-01	3,84E-01	5,42E-01	No
ENSG00000243321	snoU13	lincRNA	-2,26E-02	7,73E-01	NA	No	-8,44E-02	6,42E-01	NA	No
ENSG00000241048	RP11-167H9.5	lincRNA	3,57E-02	7,74E-01	NA	No	1,21E-02	9,87E-01	NA	No
ENSG00000244541	RP11-167H9.6	lincRNA	-1,33E-02	8,81E-01	NA	No	-7,88E-02	6,82E-01	NA	No
ENSG00000243550	RP11-483E7.1	lincRNA	2,17E-01	3,70E-02	NA	No	2,12E-01	2,90E-01	NA	No
ENSG00000241151	RP11-454C18.1	lincRNA	1,87E-01	1,83E-01	4,09E-01	No	1,91E-01	5,28E-01	6,72E-01	No
ENSG00000239941	RP11-246A10.1	lincRNA	1,91E-01	1,87E-01	NA	No	1,47E-01	5,76E-01	NA	No
ENSG00000244545	RP11-788A4.1	lincRNA	5,46E-02	5,37E-01	NA	No	4,75E-02	7,54E-01	NA	No
ENSG00000241220	RP11-292E2.1	lincRNA	3,89E-01	5,63E-02	1,88E-01	No	3,27E-01	3,38E-01	4,93E-01	No
ENSG00000241912	RP11-292E2.2	lincRNA	1,45E-01	4,01E-01	6,45E-01	No	-1,26E-02	9,65E-01	9,79E-01	No
ENSG00000243012	RP11-292E2.4	lincRNA	1,05E-01	4,88E-01	7,18E-01	No	-8,69E-02	7,35E-01	8,32E-01	No
ENSG00000240045	RP11-451G4.2	lincRNA	-1,38E-01	2,67E-01	5,11E-01	No	4,73E-02	8,74E-01	9,25E-01	No
ENSG00000242790	RP11-451G4.3	lincRNA	-3,04E-02	7,31E-01	NA	No	-1,63E-02	8,90E-01	NA	No
ENSG00000240875	LINC00886	lincRNA	2,75E-01	1,46E-01	3,57E-01	No	2,61E-01	2,58E-01	4,05E-01	No
ENSG00000243629	LINC00880	lincRNA	4,09E-02	8,40E-01	9,30E-01	No	-5,63E-01	1,62E-01	2,85E-01	No
ENSG00000241135	LINC00881	lincRNA	-6,64E-01	1,75E-02	8,35E-02	No	-8,77E-01	5,10E-03	1,58E-02	No
ENSG00000272087	RP11-379F4.7	lincRNA	-1,36E+00	2,05E-05	4,63E-04	No	-4,55E-01	1,17E-01	2,22E-01	No
ENSG00000272440	RP11-379F4.6	lincRNA	-1,18E+00	1,08E-02	5,92E-02	No	-8,77E-01	5,05E-02	1,13E-01	No
ENSG00000240567	RP11-3P17.4	lincRNA	-1,62E-01	2,46E-01	4,88E-01	No	-6,02E-02	8,41E-01	9,03E-01	No
ENSG00000241636	RP11-71H9.2	lincRNA	5,71E-02	6,70E-01	NA	No	6,28E-01	1,15E-01	2,19E-01	No
ENSG00000241767	RP11-71H9.1	lincRNA	2,63E-01	4,48E-02	NA	No	5,74E-01	4,31E-02	9,91E-02	No
ENSG00000242924	RP11-298O21.6	lincRNA	1,04E-02	9,65E-01	9,85E-01	No	-2,73E-01	3,99E-01	5,56E-01	No
ENSG00000244214	RP11-298O21.7	lincRNA	1,98E-01	3,75E-01	6,21E-01	No	6,32E-01	2,72E-02	6,70E-02	No
ENSG00000244706	RP11-298O21.3	lincRNA	-1,61E-01	4,73E-01	7,06E-01	No	5,96E-01	7,28E-02	1,52E-01	No
ENSG00000240292	RP11-298O21.2	lincRNA	-7,65E-02	7,24E-01	8,71E-01	No	9,64E-01	1,71E-04	7,48E-04	No
ENSG00000244227	RP11-298O21.5	lincRNA	1,51E-01	4,97E-01	7,25E-01	No	1,32E+00	1,78E-05	9,39E-05	No
ENSG00000242268	RP11-368I23.2	lincRNA	-5,38E-02	5,74E-01	NA	No	1,09E-01	7,10E-01	8,14E-01	No
ENSG00000272797	RP11-368I23.3	lincRNA	-1,36E-02	8,19E-01	NA	No	8,01E-02	6,42E-01	NA	No
ENSG00000270141	TERC	lincRNA	1,45E-02	9,33E-01	9,72E-01	No	1,26E+00	5,34E-13	8,17E-12	No
ENSG00000242578	RP11-469J4.3	lincRNA	-2,63E-02	8,73E-01	9,47E-01	No	-5,04E-01	1,14E-01	2,17E-01	No
ENSG00000239739	RP11-373E16.4	lincRNA	3,95E-02	8,45E-01	NA	No	1,14E-01	6,27E-01	NA	No
ENSG00000240497	RP11-185E8.1	lincRNA	-5,74E-02	5,05E-01	NA	No	-5,99E-02	7,92E-01	NA	No
ENSG00000223387	RP11-408H1.3	lincRNA	-6,87E-02	6,74E-01	8,42E-01	No	-1,05E+00	3,85E-02	9,00E-02	No
ENSG00000223715	RP11-71G7.1	lincRNA	3,16E-02	7,77E-01	NA	No	-2,73E-01	2,74E-01	NA	No
ENSG00000232461	RP11-644C3.1	lincRNA	6,99E-02	4,48E-01	NA	No	1,17E-01	6,75E-01	NA	No
ENSG00000228308	RP11-255G21.1	lincRNA	-1,98E-03	9,80E-01	NA	No	-9,06E-02	6,43E-01	NA	No
ENSG00000203645	LINC00501	lincRNA	4,71E-01	7,17E-02	2,23E-01	No	1,15E+00	2,97E-04	1,23E-03	No
ENSG00000226782	RP11-706D8.3	lincRNA	8,86E-02	3,99E-01	NA	No	1,13E-01	6,49E-01	NA	No
ENSG00000228221	LINC00578	lincRNA	3,38E-01	6,15E-02	2,00E-01	No	4,92E-01	1,53E-01	2,73E-01	No
ENSG00000228561	RP11-114M1.1	lincRNA	3,24E-01	1,71E-02	NA	No	8,64E-02	8,64E-01	NA	No
ENSG00000231574	RP11-91K9.1	lincRNA	1,52E-01	1,96E-01	NA	No	1,58E-01	4,57E-01	NA	No
ENSG00000225790	RP11-2L8.1	lincRNA	3,70E-02	8,45E-01	NA	No	8,64E-02	8,64E-01	NA	No
ENSG00000223930	RP11-33A14.1	lincRNA	8,84E-01	4,73E-03	3,28E-02	No	5,44E-01	1,11E-01	2,13E-01	No
ENSG00000229102	RP11-360P21.2	lincRNA	3,94E-02	7,37E-01	NA	No	-4,70E-02	8,24E-01	NA	No
ENSG00000273190	RP11-255C15.4	lincRNA	-3,90E-01	1,33E-01	3,36E-01	No	4,86E-01	1,44E-01	2,61E-01	No
ENSG00000260743	RP11-255C15.3	lincRNA	-4,13E-01	1,18E-01	3,11E-01	No	-5,21E-01	1,25E-01	2,33E-01	No
ENSG00000269728	RP11-145M9.4	lincRNA	1,13E-01	5,22E-01	7,44E-01	No	1,43E+00	2,87E-06	1,72E-05	No
ENSG00000270178	RP11-494H4.3	lincRNA	-8,73E-02	4,68E-01	7,01E-01	No	-9,73E-02	7,56E-01	8,47E-01	No
ENSG00000241696	RP11-420J11.2	lincRNA	-1,45E-05	1,00E+00	NA	No	-6,91E-02	7,24E-01	NA	No
ENSG00000239774	RP11-496B10.3	lincRNA	3,49E-01	8,04E-02	2,41E-01	No	5,63E-01	1,27E-01	2,37E-01	No

ENSG00000239381	RP11-4B14.3	lincRNA	4,70E-02	5,85E-01	NA	No	1,25E-01	6,65E-01	NA	No
ENSG00000260377	RP11-646E18.4	lincRNA	3,32E-02	9,36E-01	NA	No	6,19E-02	9,56E-01	NA	No
ENSG00000273181	RP11-778D9.13	lincRNA	6,91E-02	4,46E-01	NA	No	5,95E-01	5,33E-02	1,18E-01	No
ENSG00000272970	RP11-329B9.4	lincRNA	3,36E-01	1,54E-01	3,69E-01	No	4,67E-01	2,23E-01	3,63E-01	No
ENSG00000273403	RP11-329B9.3	lincRNA	2,09E-01	2,31E-01	4,69E-01	No	9,81E-02	7,08E-01	8,13E-01	No
ENSG00000272922	RP11-329B9.5	lincRNA	3,69E-02	8,45E-01	NA	No	1,09E-01	6,85E-01	NA	No
ENSG00000229433	RP11-329B9.1	lincRNA	2,34E-02	9,72E-01	NA	No	1,00E-01	7,75E-01	NA	No
ENSG00000232233	RP11-573D15.2	lincRNA	-6,52E-02	6,89E-01	8,51E-01	No	-3,98E-01	1,84E-01	3,14E-01	No
ENSG00000224049	RP11-30O15.1	lincRNA	-5,58E-02	6,46E-01	NA	No	-1,33E-01	7,12E-01	NA	No
ENSG00000224187	RP11-132N15.3	lincRNA	-4,88E-01	6,26E-02	2,03E-01	No	-3,51E-01	3,30E-01	4,86E-01	No
ENSG00000225058	RP11-132N15.1	lincRNA	7,03E-02	4,44E-01	NA	No	3,88E-02	8,90E-01	NA	No
ENSG00000236412	RP11-132N15.2	lincRNA	-4,46E-02	7,60E-01	NA	No	8,72E-03	9,87E-01	NA	No
ENSG00000234238	RP11-430L16.1	lincRNA	4,19E-02	7,48E-01	NA	No	6,23E-02	9,56E-01	NA	No
ENSG00000234076	TPRG1-AS1	lincRNA	9,26E-02	6,51E-01	8,29E-01	No	1,90E-01	5,91E-01	7,22E-01	No
ENSG00000223812	RP11-197K6.1	lincRNA	1,22E-01	3,45E-01	NA	No	6,04E-01	1,10E-01	2,11E-01	No
ENSG00000232353	RP11-655G22.1	lincRNA	3,15E-02	9,36E-01	NA	No	8,64E-02	8,64E-01	NA	No
ENSG00000232130	RP11-143P4.2	lincRNA	-6,63E-02	4,07E-01	NA	No	-1,61E-01	4,63E-01	NA	No
ENSG00000214146	RP11-699L21.1	lincRNA	1,12E-01	4,76E-01	7,08E-01	No	1,22E-01	6,88E-01	7,99E-01	No
ENSG00000238043	RP11-699L21.2	lincRNA	3,86E-02	8,45E-01	NA	No	8,58E-02	8,64E-01	NA	No
ENSG00000225742	RP11-513G11.4	lincRNA	-5,69E-02	6,46E-01	NA	No	-4,54E-02	8,81E-01	NA	No
ENSG00000214145	LINC00887	lincRNA	-1,27E-01	3,08E-01	NA	No	-3,86E-01	1,40E-01	NA	No
ENSG00000272707	RP11-534C12.1	lincRNA	-3,27E-01	1,15E-01	3,06E-01	No	-7,55E-01	8,73E-02	1,76E-01	No
ENSG00000237222	AC090505.6	lincRNA	-7,12E-03	9,44E-01	NA	No	1,20E-01	6,58E-01	7,77E-01	No
ENSG00000272792	RP11-141C7.4	lincRNA	-6,57E-03	9,57E-01	NA	No	-1,02E-01	6,43E-01	NA	No
ENSG00000229178	AC069513.4	lincRNA	5,76E-02	6,74E-01	NA	No	4,79E-02	8,62E-01	9,17E-01	No
ENSG00000242086	LINC00969	lincRNA	-1,48E-01	4,07E-01	6,51E-01	No	2,04E-02	9,23E-01	9,54E-01	No
ENSG00000260261	RP11-480A16.1	lincRNA	2,93E-01	1,94E-01	4,24E-01	No	-5,76E-01	5,87E-02	1,28E-01	No
ENSG00000224652	LINC00885	lincRNA	-1,50E-02	8,19E-01	NA	No	-1,28E-01	7,12E-01	NA	No
ENSG00000270170	NCBP2-AS2	lincRNA	-4,57E-01	3,11E-02	1,25E-01	No	5,62E-01	5,66E-03	1,73E-02	No
ENSG00000235126	AC128709.3	lincRNA	-6,34E-02	4,58E-01	NA	No	-1,08E-01	6,15E-01	NA	No
ENSG00000229912	AC128709.4	lincRNA	3,40E-02	7,25E-01	NA	No	-9,72E-02	8,30E-01	NA	No
ENSG00000273308	RP11-496H1.2	lincRNA	4,06E-02	6,45E-01	NA	No	-1,57E-01	5,07E-01	NA	No
ENSG00000236833	AC024560.2	lincRNA	4,18E-02	7,50E-01	NA	No	8,77E-02	8,64E-01	NA	No
ENSG00000273375	RP11-803P9.1	lincRNA	-3,55E-01	1,61E-01	3,79E-01	No	-2,36E-01	4,74E-01	6,25E-01	No
ENSG00000236438	FAM157A	lincRNA	2,73E-01	2,16E-01	4,53E-01	No	-1,31E-01	6,71E-01	7,86E-01	No
ENSG00000250312	ZNF718	lincRNA	9,72E-02	5,74E-01	7,79E-01	No	8,44E-01	1,87E-06	1,15E-05	No
ENSG00000272885	RP11-2H3.6	lincRNA	-1,93E-02	8,84E-01	9,52E-01	No	4,82E-01	2,10E-01	3,47E-01	No
ENSG00000260262	RP11-440L14.3	lincRNA	2,88E-02	8,69E-01	9,45E-01	No	-3,72E-01	2,41E-01	3,85E-01	No
ENSG00000250259	RP11-460I19.2	lincRNA	-6,74E-02	4,32E-01	NA	No	-3,38E-02	8,71E-01	NA	No
ENSG00000272783	RP13-1016M1.2	lincRNA	-3,23E-01	1,67E-01	3,88E-01	No	-6,26E-01	1,26E-01	2,34E-01	No
ENSG00000244459	RP11-1398P2.1	lincRNA	-2,61E-02	7,94E-01	NA	No	1,74E-01	5,01E-01	6,49E-01	No
ENSG00000270195	RP11-572O17.1	lincRNA	1,08E+00	5,48E-03	3,66E-02	No	-2,82E-01	4,38E-01	5,92E-01	No
ENSG00000206113	RP11-503N18.1	lincRNA	1,21E-01	2,44E-01	NA	No	-4,13E-02	8,90E-01	NA	No
ENSG00000270090	RP11-529E10.7	lincRNA	-5,58E-02	6,46E-01	NA	No	-1,33E-01	7,12E-01	NA	No
ENSG00000248516	RP11-265O12.1	lincRNA	2,12E-02	8,84E-01	NA	No	-3,66E-02	8,78E-01	NA	No
ENSG00000273396	RP11-326I19.3	lincRNA	1,31E-02	9,65E-01	NA	No	-9,72E-02	8,30E-01	NA	No
ENSG00000249896	RP11-586D19.1	lincRNA	3,19E-01	6,24E-02	2,02E-01	No	-2,51E-01	3,00E-01	NA	No
ENSG00000251580	RP11-539L10.3	lincRNA	2,01E-01	3,81E-01	6,27E-01	No	1,15E+00	6,92E-03	2,06E-02	No
ENSG00000170846	AC093323.3	lincRNA	2,34E-01	1,81E-01	4,07E-01	No	8,20E-01	9,94E-07	6,43E-06	No
ENSG00000249145	RP11-774O3.2	lincRNA	-7,38E-02	5,74E-01	NA	No	-3,71E-01	1,83E-01	NA	No
ENSG00000251615	RP11-774O3.3	lincRNA	-1,52E-01	4,51E-01	6,87E-01	No	-5,71E-01	3,43E-02	8,15E-02	No
ENSG00000205959	RP11-689P11.2	lincRNA	2,59E-01	2,76E-01	5,22E-01	No	-3,63E-01	2,57E-01	4,03E-01	No
ENSG00000251152	RP11-281P23.1	lincRNA	-1,74E-01	3,42E-01	5,91E-01	No	-6,89E-02	8,34E-01	8,99E-01	No
ENSG00000249631	RP11-281P23.2	lincRNA	7,72E-02	7,12E-01	8,65E-01	No	7,68E-01	5,44E-02	1,20E-01	No
ENSG00000248300	RP11-74M11.2	lincRNA	-6,53E-02	7,27E-01	8,73E-01	No	2,12E-01	5,42E-01	6,84E-01	No
ENSG00000248262	RP11-1J7.1	lincRNA	1,03E-01	3,09E-01	NA	No	9,90E-02	7,75E-01	NA	No
ENSG00000251210	RP11-168E17.1	lincRNA	2,27E-01	2,79E-01	5,25E-01	No	1,23E+00	1,23E-02	3,42E-02	No
ENSG00000250371	RP11-22A3.2	lincRNA	-5,30E-01	4,83E-02	1,70E-01	No	-1,17E-01	6,96E-01	8,05E-01	No
ENSG00000250098	RP11-22A3.1	lincRNA	-9,60E-02	6,18E-01	8,08E-01	No	-4,75E-02	8,89E-01	9,34E-01	No
ENSG00000246095	LINC01096	lincRNA	3,46E-02	9,36E-01	NA	No	9,18E-02	8,64E-01	NA	No
ENSG00000250634	RP11-341G5.1	lincRNA	3,54E-02	8,54E-01	9,37E-01	No	-8,68E-02	7,97E-01	8,75E-01	No
ENSG00000250497	AC007126.1	lincRNA	-3,37E-01	1,79E-01	4,03E-01	No	2,26E-01	5,08E-01	6,55E-01	No

ENSG00000248698	LINC01085	lincRNA	-1,85E-04	9,98E-01	NA	No	4,63E-01	1,76E-01	3,03E-01	No
ENSG00000251412	AC006296.1	lincRNA	-3,27E-02	7,31E-01	NA	No	-1,30E-01	7,12E-01	NA	No
ENSG00000248360	LINC00504	lincRNA	4,82E-01	7,91E-02	2,38E-01	No	6,51E-01	1,10E-01	2,12E-01	No
ENSG00000247624	CPEB2-AS1	lincRNA	-9,15E-03	9,65E-01	9,86E-01	No	1,68E-01	6,14E-01	7,42E-01	No
ENSG00000250819	RP11-576E20.1	lincRNA	4,19E-02	7,48E-01	NA	No	8,58E-02	8,64E-01	NA	No
ENSG00000248515	RP11-608O21.1	lincRNA	2,35E-02	9,72E-01	NA	No	8,55E-02	8,64E-01	NA	No
ENSG00000249547	RP11-453O5.1	lincRNA	4,72E-03	9,73E-01	NA	No	-2,83E-02	8,90E-01	NA	No
ENSG00000251292	RP11-380P13.2	lincRNA	-7,95E-02	3,65E-01	NA	No	-4,04E-02	8,31E-01	NA	No
ENSG00000261121	RP11-496D24.2	lincRNA	-5,69E-02	6,46E-01	NA	No	-2,92E-02	8,90E-01	NA	No
ENSG00000271172	RP11-660M5.1	lincRNA	3,65E-01	6,40E-02	2,06E-01	No	2,90E-01	3,20E-01	4,75E-01	No
ENSG00000251009	RP13-494C23.1	lincRNA	-3,61E-02	NA	NA	No	-2,60E-01	NA	NA	No
ENSG00000249699	RP11-415C15.2	lincRNA	6,03E-03	9,90E-01	NA	No	2,03E-02	9,38E-01	NA	No
ENSG00000250064	RP11-123O22.1	lincRNA	-3,60E-02	6,78E-01	NA	No	1,75E-01	4,60E-01	NA	No
ENSG00000249228	RP11-769N22.1	lincRNA	-5,45E-02	6,46E-01	NA	No	-1,28E-01	7,12E-01	NA	No
ENSG00000251182	RP11-617I14.1	lincRNA	2,85E-02	8,22E-01	NA	No	1,62E-01	4,92E-01	NA	No
ENSG00000251129	RP11-734I18.1	lincRNA	-4,58E-02	NA	NA	No	5,60E-01	6,76E-02	1,43E-01	No
ENSG00000250954	RP11-79E3.3	lincRNA	-1,81E-01	7,28E-02	NA	No	-4,22E-01	1,21E-01	2,29E-01	No
ENSG00000250723	RP11-79E3.2	lincRNA	3,32E-02	9,36E-01	NA	No	1,35E-01	6,05E-01	NA	No
ENSG00000248215	RP11-722M1.1	lincRNA	3,15E-02	9,36E-01	NA	No	8,64E-02	8,64E-01	NA	No
ENSG00000248227	RP11-83C7.1	lincRNA	-3,22E-02	7,23E-01	NA	No	-1,50E-01	5,73E-01	NA	No
ENSG00000249534	RP11-83C7.2	lincRNA	3,15E-02	9,36E-01	NA	No	1,11E-01	6,71E-01	NA	No
ENSG00000271958	RP11-617D20.2	lincRNA	3,37E-03	9,41E-01	NA	No	4,77E-02	8,89E-01	NA	No
ENSG00000249685	RP11-360F5.3	lincRNA	-2,36E-02	9,11E-01	9,63E-01	No	-4,82E-01	2,00E-01	3,34E-01	No
ENSG00000255458	RP11-539G18.2	lincRNA	1,96E-01	3,96E-01	6,41E-01	No	2,26E-01	4,93E-01	6,42E-01	No
ENSG00000272862	RP11-814H16.2	lincRNA	-9,29E-03	9,46E-01	NA	No	3,07E-01	3,24E-01	4,79E-01	No
ENSG00000251517	RP11-109E24.1	lincRNA	7,11E-02	2,88E-01	NA	No	8,55E-02	8,64E-01	NA	No
ENSG00000251350	RP11-328N19.1	lincRNA	3,67E-02	8,14E-01	9,18E-01	No	5,59E-01	1,56E-01	2,77E-01	No
ENSG00000248744	RP11-362I1.1	lincRNA	3,68E-02	8,45E-01	NA	No	6,34E-02	9,56E-01	NA	No
ENSG00000259959	RP11-121C2.2	lincRNA	1,11E-01	5,92E-01	7,91E-01	No	6,69E-02	7,61E-01	8,50E-01	No
ENSG00000272576	RP11-365H22.2	lincRNA	2,84E-02	8,92E-01	9,55E-01	No	1,03E+00	5,34E-03	1,64E-02	No
ENSG00000251040	RP11-535C7.1	lincRNA	3,88E-02	8,45E-01	NA	No	6,54E-02	9,56E-01	NA	No
ENSG00000248866	USP46-AS1	lincRNA	2,65E-01	2,24E-01	4,62E-01	No	9,24E-01	1,06E-03	3,92E-03	No
ENSG00000260120	RP11-177B4.2	lincRNA	1,12E-01	4,09E-01	6,53E-01	No	1,39E-02	9,54E-01	9,73E-01	No
ENSG00000250302	RP11-752D24.3	lincRNA	8,55E-03	9,18E-01	NA	No	-3,12E-02	8,90E-01	NA	No
ENSG00000272650	RP11-571I18.4	lincRNA	7,92E-03	9,73E-01	9,89E-01	No	8,58E-01	1,18E-02	3,28E-02	No
ENSG00000251256	RP11-273B19.1	lincRNA	-5,37E-02	6,46E-01	NA	No	3,63E-02	8,84E-01	NA	No
ENSG00000273257	RP11-177J6.1	lincRNA	2,84E-02	8,55E-01	9,37E-01	No	2,29E-01	4,53E-01	6,06E-01	No
ENSG00000269921	RP11-646I6.5	lincRNA	-2,96E-01	2,24E-01	4,62E-01	No	-2,38E-01	4,92E-01	6,41E-01	No
ENSG00000270147	RP11-646I6.6	lincRNA	3,47E-02	8,39E-01	9,30E-01	No	5,51E-01	1,64E-01	2,87E-01	No
ENSG00000270109	RP11-393M11.2	lincRNA	2,25E-01	3,28E-01	5,77E-01	No	-2,42E-02	9,43E-01	9,66E-01	No
ENSG00000269949	RP11-738E22.3	lincRNA	7,57E-02	6,78E-01	8,44E-01	No	1,73E-03	9,97E-01	9,98E-01	No
ENSG00000250192	RP11-300M6.1	lincRNA	1,10E-01	3,21E-01	NA	No	2,13E-03	9,87E-01	NA	No
ENSG00000248505	RP11-319E12.1	lincRNA	-1,05E-02	8,49E-01	NA	No	1,57E-01	5,06E-01	NA	No
ENSG00000251459	RP11-319E12.2	lincRNA	-4,07E-01	6,28E-02	2,03E-01	No	2,84E-01	4,29E-01	5,85E-01	No
ENSG00000205682	RP11-798L4.1	lincRNA	2,22E-01	3,42E-01	5,91E-01	No	-5,97E-01	1,24E-01	2,31E-01	No
ENSG00000250775	RP11-12K22.1	lincRNA	1,37E+00	2,26E-03	1,90E-02	No	7,62E-01	7,64E-02	1,58E-01	No
ENSG00000250125	RP11-707A18.1	lincRNA	1,23E-01	5,10E-01	7,35E-01	No	2,18E-01	4,84E-01	6,34E-01	No
ENSG00000250846	RP11-807H7.1	lincRNA	-6,09E-02	5,50E-01	NA	No	-6,59E-02	7,57E-01	NA	No
ENSG00000248479	RP11-807H7.2	lincRNA	-5,73E-02	6,46E-01	NA	No	-1,36E-01	7,12E-01	NA	No
ENSG00000250075	RP11-584P21.2	lincRNA	7,27E-01	2,38E-02	1,04E-01	No	4,62E-01	1,89E-01	3,21E-01	No
ENSG00000272859	RP11-352E8.2	lincRNA	6,17E-02	6,27E-01	NA	No	4,93E-01	1,44E-01	2,60E-01	No
ENSG00000228277	AC112518.3	lincRNA	-5,73E-02	6,46E-01	NA	No	-1,36E-01	7,12E-01	NA	No
ENSG00000251185	RP11-542G1.1	lincRNA	-5,58E-02	6,46E-01	NA	No	-1,33E-01	7,12E-01	NA	No
ENSG00000251454	RP11-542G1.2	lincRNA	-4,32E-03	9,10E-01	NA	No	1,58E-02	9,38E-01	NA	No
ENSG00000248646	RP11-567N4.2	lincRNA	4,31E-02	7,25E-01	NA	No	8,55E-02	8,64E-01	NA	No
ENSG00000249036	RP11-625I7.1	lincRNA	2,93E-01	8,28E-02	2,45E-01	No	8,23E-02	7,36E-01	NA	No
ENSG00000251442	LINC01094	lincRNA	2,36E-01	3,13E-01	5,62E-01	No	6,15E-01	1,02E-01	1,99E-01	No
ENSG00000250334	LINC00989	lincRNA	8,79E-02	6,93E-01	8,53E-01	No	1,15E+00	2,07E-03	7,09E-03	No
ENSG00000248408	RP11-452C8.1	lincRNA	-1,10E-02	9,36E-01	NA	No	-2,55E-01	3,30E-01	NA	No
ENSG00000251321	PCAT4	lincRNA	4,66E-02	6,02E-01	NA	No	3,25E-01	1,73E-01	NA	No
ENSG00000250057	RP11-576N17.5	lincRNA	2,87E-01	4,41E-02	1,59E-01	No	4,50E-01	1,48E-01	2,65E-01	No
ENSG00000272856	RP11-710E1.2	lincRNA	1,54E-01	4,64E-01	6,98E-01	No	4,39E-01	2,41E-01	3,84E-01	No

ENSG00000271359	RP11-84C13.1	lincRNA	2,25E-01	3,15E-01	5,63E-01	No	-2,03E-01	5,00E-01	6,48E-01	No
ENSG00000270720	RP11-84C13.2	lincRNA	4,70E-01	1,05E-02	5,83E-02	No	4,48E-01	1,29E-01	2,40E-01	No
ENSG00000246541	RP11-363G15.2	lincRNA	5,32E-03	9,60E-01	NA	No	-1,33E-01	7,12E-01	NA	No
ENSG00000249951	RP11-554D13.1	lincRNA	-2,19E-01	2,94E-01	5,42E-01	No	-6,97E-01	1,04E-01	2,02E-01	No
ENSG00000249599	RP11-168E14.1	lincRNA	-8,54E-02	3,38E-01	NA	No	-1,77E-01	4,21E-01	NA	No
ENSG00000248510	RP11-145G20.1	lincRNA	2,42E-02	9,72E-01	NA	No	6,67E-02	9,56E-01	NA	No
ENSG00000272777	RP11-571L19.8	lincRNA	5,49E-02	7,76E-01	8,98E-01	No	3,08E-01	1,73E-01	3,00E-01	No
ENSG00000248740	RP11-328K4.1	lincRNA	2,22E-02	9,72E-01	NA	No	6,17E-02	9,56E-01	NA	No
ENSG00000250670	AC004063.1	lincRNA	-9,43E-02	5,13E-01	NA	No	-9,36E-02	7,62E-01	8,51E-01	No
ENSG00000248242	RP11-556I14.2	lincRNA	2,65E-02	8,31E-01	NA	No	6,81E-02	7,72E-01	8,58E-01	No
ENSG00000248373	RP11-556I14.1	lincRNA	3,31E-02	9,36E-01	NA	No	3,41E-01	2,19E-01	NA	No
ENSG00000251259	AC004069.2	lincRNA	5,59E-01	5,69E-02	1,90E-01	No	1,25E-01	7,19E-01	8,21E-01	No
ENSG00000250298	GIMD1	lincRNA	2,49E-02	9,72E-01	NA	No	1,12E-01	6,53E-01	NA	No
ENSG00000234492	RPL34-AS1	lincRNA	2,70E-01	2,42E-01	4,83E-01	No	7,59E-01	1,76E-02	4,64E-02	No
ENSG00000246774	AC004051.2	lincRNA	1,07E-01	3,64E-01	NA	No	4,90E-01	1,40E-01	2,55E-01	No
ENSG00000272795	RP11-602N24.3	lincRNA	7,16E-02	5,36E-01	NA	No	-3,88E-02	8,50E-01	NA	No
ENSG00000249519	RP11-777N19.1	lincRNA	3,46E-02	9,36E-01	NA	No	1,90E-01	3,78E-01	NA	No
ENSG00000248432	RP11-269F21.2	lincRNA	3,46E-02	9,36E-01	NA	No	9,02E-02	8,64E-01	NA	No
ENSG00000249815	RP11-269F21.3	lincRNA	5,99E-02	5,60E-01	NA	No	3,75E-01	1,81E-01	NA	No
ENSG00000272567	RP11-73K9.3	lincRNA	-1,10E-02	9,48E-01	9,79E-01	No	3,68E-01	3,19E-01	4,74E-01	No
ENSG00000250046	RP11-148B6.2	lincRNA	-3,85E-01	1,32E-01	3,34E-01	No	-2,06E-01	5,02E-01	6,50E-01	No
ENSG00000269893	SNHG8	lincRNA	-5,66E-01	1,00E-02	5,66E-02	No	5,91E-01	1,50E-02	4,04E-02	No
ENSG00000260404	RP11-384K6.6	lincRNA	2,31E-01	2,11E-01	4,46E-01	No	-6,54E-01	4,64E-03	1,45E-02	No
ENSG00000249356	RP11-21I10.2	lincRNA	-3,01E-01	1,74E-01	3,99E-01	No	-8,96E-02	7,23E-01	8,24E-01	No
ENSG00000250950	RP11-33B1.3	lincRNA	1,33E-01	5,35E-01	7,53E-01	No	-2,09E-01	4,82E-01	6,33E-01	No
ENSG00000250392	RP11-700N1.1	lincRNA	1,97E-01	3,61E-01	6,08E-01	No	1,08E+00	4,01E-05	1,97E-04	No
ENSG00000249409	RP11-501E14.1	lincRNA	4,11E-04	1,00E+00	1,00E+00	No	-1,96E-02	9,53E-01	9,73E-01	No
ENSG00000250344	RP11-647P12.1	lincRNA	-4,19E-02	6,36E-01	NA	No	-1,27E-01	5,05E-01	NA	No
ENSG00000249125	RP11-381N20.2	lincRNA	4,71E-02	5,85E-01	NA	No	1,57E-01	5,02E-01	NA	No
ENSG00000251526	RP11-381N20.1	lincRNA	6,49E-02	6,65E-01	NA	No	2,39E-01	4,56E-01	6,09E-01	No
ENSG00000249464	LINC01091	lincRNA	-2,24E-01	3,38E-01	5,87E-01	No	2,40E-01	4,52E-01	6,05E-01	No
ENSG00000226655	AC096732.2	lincRNA	7,03E-02	5,92E-01	7,91E-01	No	3,63E-01	2,69E-01	4,18E-01	No
ENSG00000249837	CTD-2325B11.1	lincRNA	-3,56E-02	7,40E-01	NA	No	3,77E-01	2,71E-01	4,20E-01	No
ENSG00000261083	RP11-93I21.3	lincRNA	-1,45E-01	5,15E-01	7,39E-01	No	3,37E-01	3,33E-01	4,88E-01	No
ENSG00000250945	RP11-810P8.1	lincRNA	3,46E-02	9,36E-01	NA	No	1,08E-01	6,83E-01	NA	No
ENSG00000248491	RP11-125O18.1	lincRNA	8,30E-02	2,95E-01	NA	No	-4,43E-03	9,79E-01	NA	No
ENSG00000251432	RP11-420A23.1	lincRNA	4,49E-01	8,18E-02	2,43E-01	No	7,16E-01	2,00E-02	5,17E-02	No
ENSG00000273077	RP11-130C6.1	lincRNA	1,01E-01	6,41E-01	8,22E-01	No	-7,73E-01	7,89E-02	1,62E-01	No
ENSG00000248187	RP11-184M15.1	lincRNA	-9,69E-03	9,53E-01	9,80E-01	No	-2,91E-01	3,80E-01	5,37E-01	No
ENSG00000248802	RP11-184M15.2	lincRNA	7,31E-02	6,60E-01	8,33E-01	No	-8,40E-02	7,89E-01	8,69E-01	No
ENSG00000249618	RP11-422J15.1	lincRNA	4,18E-02	7,50E-01	NA	No	9,85E-02	7,75E-01	NA	No
ENSG00000251676	RP11-614F17.2	lincRNA	-3,20E-02	7,29E-01	NA	No	1,33E-01	6,33E-01	7,57E-01	No
ENSG00000251555	RP11-745L13.2	lincRNA	-6,17E-02	6,83E-01	8,48E-01	No	-8,11E-02	8,05E-01	8,79E-01	No
ENSG00000251598	RP11-789C2.1	lincRNA	-3,59E-02	6,60E-01	NA	No	-1,58E-01	4,56E-01	NA	No
ENSG00000249463	RP11-1E22.1	lincRNA	3,17E-02	9,36E-01	NA	No	1,13E-01	6,42E-01	NA	No
ENSG00000250241	RP11-9G1.3	lincRNA	1,26E-02	8,42E-01	NA	No	-2,52E-02	8,90E-01	NA	No
ENSG00000251388	RP11-427M20.1	lincRNA	-1,46E-01	5,15E-01	7,39E-01	No	9,67E-02	7,82E-01	8,64E-01	No
ENSG00000248869	RP11-138I17.1	lincRNA	-4,24E-02	8,23E-01	9,23E-01	No	8,26E-01	6,27E-02	1,35E-01	No
ENSG00000250341	RP11-785F11.1	lincRNA	-1,57E-02	9,04E-01	9,60E-01	No	1,24E-01	6,36E-01	7,59E-01	No
ENSG00000251632	RP11-714L20.1	lincRNA	-3,54E-03	9,77E-01	9,91E-01	No	1,88E-01	5,63E-01	7,00E-01	No
ENSG00000250126	RP13-884E18.2	lincRNA	2,49E-01	3,77E-02	NA	No	9,11E-01	2,95E-02	7,19E-02	No
ENSG00000248307	LINC00616	lincRNA	-5,79E-02	5,40E-01	NA	No	-1,10E-01	6,18E-01	7,45E-01	No
ENSG00000249091	RP11-733C7.1	lincRNA	-3,91E-03	9,77E-01	NA	No	-4,89E-02	8,69E-01	NA	No
ENSG00000248397	LINC00498	lincRNA	-4,42E-02	7,60E-01	NA	No	4,50E-03	9,87E-01	NA	No
ENSG00000251372	LINC00499	lincRNA	-3,62E-02	7,33E-01	NA	No	1,87E-02	9,42E-01	NA	No
ENSG00000249381	LINC00500	lincRNA	-5,59E-02	4,67E-01	NA	No	-9,49E-02	6,06E-01	NA	No
ENSG00000250977	RP11-173E2.2	lincRNA	-8,43E-02	3,79E-01	NA	No	-2,43E-01	2,14E-01	NA	No
ENSG00000250501	RP11-98O2.1	lincRNA	-1,65E-01	1,37E-01	NA	No	-2,74E-01	3,56E-01	5,13E-01	No
ENSG00000250577	RP11-577B7.1	lincRNA	-8,38E-02	2,75E-01	NA	No	-1,45E-01	5,20E-01	NA	No
ENSG00000261268	RP11-308D13.3	lincRNA	-3,12E-02	6,83E-01	NA	No	-4,79E-02	7,72E-01	NA	No
ENSG00000273472	RP11-102N12.3	lincRNA	-6,68E-02	7,35E-01	8,77E-01	No	-3,45E-01	1,93E-01	3,26E-01	No
ENSG00000248810	RP11-362F19.1	lincRNA	2,65E-01	2,52E-01	4,94E-01	No	3,37E-01	3,52E-01	5,08E-01	No

ENSG00000272632	RP11-362F19.3	lincRNA	3,34E-01	1,73E-01	3,96E-01	No	1,06E+00	1,52E-02	4,10E-02	No
ENSG00000250141	RP11-208N20.1	lincRNA	4,06E-02	6,30E-01	NA	No	1,17E-01	5,19E-01	NA	No
ENSG00000251248	RP11-223C24.2	lincRNA	1,26E-01	5,75E-01	7,80E-01	No	7,08E-02	8,37E-01	9,01E-01	No
ENSG00000249806	RP11-223C24.1	lincRNA	-9,43E-02	6,68E-01	8,38E-01	No	-7,12E-01	3,82E-02	8,93E-02	No
ENSG00000250969	RP11-364L4.3	lincRNA	-4,23E-02	7,60E-01	NA	No	1,31E-02	9,87E-01	NA	No
ENSG00000251600	RP11-673E1.1	lincRNA	6,52E-02	7,61E-01	8,90E-01	No	7,85E-01	5,82E-02	1,27E-01	No
ENSG00000261129	RP11-361D14.2	lincRNA	4,11E-02	7,67E-01	NA	No	2,08E-01	3,21E-01	NA	No
ENSG00000250406	RP13-539F13.3	lincRNA	-9,99E-02	4,54E-01	6,90E-01	No	-1,73E-01	5,99E-01	7,29E-01	No
ENSG00000272727	RP11-142A22.4	lincRNA	1,26E-01	1,99E-01	NA	No	5,23E-01	9,91E-02	1,94E-01	No
ENSG00000260303	RP11-203B7.2	lincRNA	3,99E-02	5,98E-01	NA	No	-1,36E-01	7,12E-01	NA	No
ENSG00000248346	RP11-292D4.1	lincRNA	7,79E-02	4,90E-01	NA	No	2,11E-02	9,27E-01	NA	No
ENSG00000260090	RP11-292D4.3	lincRNA	2,16E-02	8,03E-01	NA	No	1,57E-02	9,30E-01	NA	No
ENSG00000251688	RP11-752L20.5	lincRNA	-8,29E-02	4,41E-01	NA	No	-1,45E-01	5,56E-01	6,95E-01	No
ENSG00000250357	RP11-503L19.1	lincRNA	3,50E-01	1,42E-01	3,51E-01	No	5,70E-01	1,53E-01	2,73E-01	No
ENSG00000249752	RP11-563M4.1	lincRNA	-2,83E-01	1,51E-01	3,64E-01	No	6,66E-02	8,49E-01	9,08E-01	No
ENSG00000250252	RP11-342A1.1	lincRNA	2,14E-01	8,82E-02	NA	No	1,65E-01	4,69E-01	NA	No
ENSG00000248210	RP11-24I21.1	lincRNA	6,84E-02	6,49E-01	8,27E-01	No	5,74E-01	1,36E-01	2,49E-01	No
ENSG00000234828	RP11-526A4.1	lincRNA	-4,62E-03	9,59E-01	NA	No	-1,61E-01	4,63E-01	NA	No
ENSG00000270265	RP11-731D1.4	lincRNA	-1,91E-01	3,58E-01	6,05E-01	No	-8,76E-01	3,76E-02	8,83E-02	No
ENSG00000251611	RP11-610P16.1	lincRNA	7,29E-02	6,79E-01	8,45E-01	No	-1,14E-01	7,39E-01	8,35E-01	No
ENSG00000249184	RP11-424M21.1	lincRNA	-5,44E-01	5,93E-02	1,95E-01	No	-3,80E-01	3,06E-01	4,59E-01	No
ENSG00000249708	RP11-503L23.1	lincRNA	3,17E-02	9,36E-01	NA	No	8,77E-02	8,64E-01	NA	No
ENSG00000248991	RP11-73G16.2	lincRNA	-2,36E-02	7,90E-01	NA	No	-6,91E-02	6,78E-01	NA	No
ENSG00000245954	RP11-18H21.1	lincRNA	1,19E+00	1,03E-02	5,74E-02	No	-3,26E-01	3,42E-01	4,98E-01	No
ENSG00000251377	RP11-18H21.3	lincRNA	5,89E-01	1,34E-02	6,94E-02	No	1,09E-01	6,04E-01	NA	No
ENSG00000270302	RP11-461L13.4	lincRNA	-3,63E-02	8,10E-01	NA	No	-1,51E-01	5,02E-01	NA	No
ENSG00000270883	RP11-461L13.5	lincRNA	-1,62E-01	4,63E-01	6,98E-01	No	-8,82E-01	4,75E-02	1,07E-01	No
ENSG00000270750	RP11-461L13.3	lincRNA	-4,76E-01	7,30E-02	2,26E-01	No	-1,19E-01	7,11E-01	8,15E-01	No
ENSG00000268471	MIR4453	lincRNA	2,78E-01	1,75E-01	3,99E-01	No	1,85E-01	4,38E-01	5,92E-01	No
ENSG00000251244	RP13-487K5.1	lincRNA	9,85E-01	5,64E-03	3,73E-02	No	4,89E-01	1,91E-01	3,24E-01	No
ENSG00000234111	RP11-364P22.1	lincRNA	1,50E-01	9,80E-02	NA	No	1,07E-01	6,99E-01	NA	No
ENSG00000249275	RP11-364P22.2	lincRNA	3,15E-02	9,36E-01	NA	No	8,55E-02	8,64E-01	NA	No
ENSG00000250488	RP11-6C14.1	lincRNA	-5,45E-02	6,46E-01	NA	No	6,17E-02	7,63E-01	NA	No
ENSG00000249425	RP11-502M1.2	lincRNA	4,03E-02	7,82E-01	NA	No	8,64E-02	8,64E-01	NA	No
ENSG00000248431	AC005150.1	lincRNA	-3,24E-02	8,76E-01	9,48E-01	No	5,50E-01	1,54E-01	2,73E-01	No
ENSG00000273449	RP11-218F10.3	lincRNA	3,51E-01	1,44E-01	3,54E-01	No	4,46E-01	1,18E-01	2,23E-01	No
ENSG00000273097	RP11-219C20.3	lincRNA	-3,99E-02	6,10E-01	NA	No	3,19E-01	2,50E-01	3,94E-01	No
ENSG00000249500	RP11-340B18.1	lincRNA	-6,37E-02	4,85E-01	NA	No	-1,53E-01	5,47E-01	NA	No
ENSG00000261646	RP11-489G11.3	lincRNA	7,77E-01	2,22E-02	9,90E-02	No	7,45E-01	4,22E-02	9,73E-02	No
ENSG00000261672	RP11-475B2.1	lincRNA	-1,26E-01	5,71E-01	7,77E-01	No	5,64E-01	1,10E-01	2,11E-01	No
ENSG00000250043	RP11-161D15.2	lincRNA	-8,94E-02	5,33E-01	7,52E-01	No	-7,00E-02	8,11E-01	8,83E-01	No
ENSG00000250708	RP11-161D15.1	lincRNA	-1,11E-01	5,47E-01	7,61E-01	No	1,67E-01	6,27E-01	7,52E-01	No
ENSG00000251216	RP11-161D15.3	lincRNA	-9,66E-02	5,98E-01	7,95E-01	No	-3,47E-02	9,14E-01	9,49E-01	No
ENSG00000248174	RP11-148L24.1	lincRNA	1,77E-01	4,34E-01	6,74E-01	No	2,20E-01	5,24E-01	6,69E-01	No
ENSG00000250957	RP11-248N22.1	lincRNA	-7,07E-02	7,12E-01	8,65E-01	No	5,88E-01	1,43E-01	2,59E-01	No
ENSG00000251213	RP11-248N22.2	lincRNA	-6,38E-03	9,37E-01	NA	No	-5,49E-02	7,72E-01	NA	No
ENSG00000249875	RP11-51M24.1	lincRNA	-4,70E-02	5,82E-01	NA	No	4,96E-02	7,77E-01	NA	No
ENSG00000250596	RP11-440I14.2	lincRNA	-1,18E-02	8,19E-01	NA	No	3,75E-02	9,10E-01	NA	No
ENSG00000251416	RP11-140M13.1	lincRNA	2,35E-02	9,72E-01	NA	No	2,73E-01	2,63E-01	NA	No
ENSG00000251504	LINC01099	lincRNA	1,97E-02	9,14E-01	9,64E-01	No	8,15E-01	5,28E-02	1,17E-01	No
ENSG00000249460	RP11-665C14.2	lincRNA	-2,73E-01	9,65E-02	2,72E-01	No	7,51E-02	8,28E-01	8,95E-01	No
ENSG00000251336	RP11-540E16.2	lincRNA	3,80E-02	8,45E-01	NA	No	8,58E-02	8,64E-01	NA	No
ENSG00000272646	RP11-188P17.2	lincRNA	1,04E-02	9,44E-01	9,77E-01	No	8,39E-02	7,90E-01	8,70E-01	No
ENSG00000273180	RP11-335L23.4	lincRNA	3,32E-02	9,36E-01	NA	No	8,51E-02	8,64E-01	NA	No
ENSG00000251359	WWC2-AS2	lincRNA	6,83E-01	1,66E-02	8,04E-02	No	8,01E-01	1,52E-02	4,10E-02	No
ENSG00000272744	RP11-367N14.3	lincRNA	-1,16E-01	5,03E-01	7,30E-01	No	-1,41E-01	6,66E-01	7,82E-01	No
ENSG00000180712	RP11-290F5.2	lincRNA	-4,24E-01	1,00E-01	2,79E-01	No	-1,42E+00	4,11E-04	1,66E-03	No
ENSG00000271646	RP11-326I11.3	lincRNA	3,05E-01	1,80E-01	4,06E-01	No	-1,35E-01	6,29E-01	7,53E-01	No
ENSG00000254233	RP11-242J7.1	lincRNA	7,37E-03	9,58E-01	9,83E-01	No	-3,91E-01	2,13E-01	3,50E-01	No
ENSG00000251230	RP11-701P16.5	lincRNA	1,03E-01	5,11E-01	7,36E-01	No	-3,62E-01	1,94E-01	3,27E-01	No
ENSG00000249173	LINC01093	lincRNA	-5,27E-02	5,37E-01	NA	No	-1,59E-01	4,88E-01	NA	No
ENSG00000250754	RP11-386B13.3	lincRNA	-1,37E-02	8,49E-01	NA	No	-1,01E-01	8,30E-01	NA	No

ENSG00000235149	RP11-45I20.1	lincRNA	-5,21E-02	5,52E-01	NA	No	5,46E-02	8,08E-01	NA	No
ENSG00000272218	RP11-11N5.3	lincRNA	-2,78E-02	8,26E-01	NA	No	2,38E-01	4,48E-01	6,02E-01	No
ENSG00000249742	RP11-217E13.1	lincRNA	2,32E-01	1,79E-01	4,04E-01	No	1,82E-01	4,28E-01	5,84E-01	No
ENSG00000250971	RP11-696F12.1	lincRNA	1,92E-01	1,08E-01	NA	No	4,03E-01	1,57E-01	2,78E-01	No
ENSG00000250658	RP11-138B4.1	lincRNA	1,21E-01	2,07E-01	4,40E-01	No	1,02E-01	6,60E-01	7,78E-01	No
ENSG00000250042	RP11-91J3.2	lincRNA	8,12E-02	3,16E-01	NA	No	5,57E-02	7,28E-01	NA	No
ENSG00000250620	RP11-91J3.3	lincRNA	-3,93E-03	9,72E-01	9,89E-01	No	1,97E-01	5,19E-01	6,65E-01	No
ENSG00000249378	LINC01060	lincRNA	-4,35E-02	7,89E-01	9,05E-01	No	-2,31E-01	4,52E-01	6,05E-01	No
ENSG00000251310	RP11-706F1.1	lincRNA	-6,60E-02	1,78E-01	4,03E-01	No	-1,41E-01	3,09E-01	4,62E-01	No
ENSG00000249877	RP11-706F1.2	lincRNA	-2,72E-02	6,71E-01	8,40E-01	No	-9,44E-02	5,45E-01	6,86E-01	No
ENSG00000250739	RP11-462G22.1	lincRNA	3,82E-02	8,45E-01	NA	No	3,76E-01	1,79E-01	NA	No
ENSG00000272566	RP11-462G22.2	lincRNA	-3,02E-02	7,92E-01	NA	No	5,94E-01	1,16E-01	2,20E-01	No
ENSG00000245685	AF146191.4	lincRNA	1,08E-02	9,60E-01	9,84E-01	No	-3,74E-01	3,13E-01	4,67E-01	No
ENSG00000250666	RP11-463J17.1	lincRNA	-9,15E-02	3,93E-01	NA	No	-9,45E-02	7,23E-01	8,24E-01	No
ENSG00000248949	RP11-661C8.2	lincRNA	4,03E-02	7,82E-01	NA	No	1,06E-01	7,03E-01	NA	No
ENSG00000272347	RP11-43F13.4	lincRNA	-1,93E-01	4,01E-01	6,45E-01	No	-9,89E-01	2,81E-02	6,90E-02	No
ENSG00000250584	RP11-325I22.2	lincRNA	-1,48E-02	8,19E-01	NA	No	-1,28E-01	7,12E-01	NA	No
ENSG00000251532	CTD-2245E15.3	lincRNA	1,75E-01	1,12E-01	NA	No	1,20E-01	5,44E-01	NA	No
ENSG00000271119	CTD-2012J19.3	lincRNA	6,14E-01	2,16E-02	9,70E-02	No	4,11E-01	1,86E-01	3,17E-01	No
ENSG00000248994	RP11-259O2.1	lincRNA	-1,16E-04	9,98E-01	9,99E-01	No	-2,36E-01	4,64E-01	6,17E-01	No
ENSG00000248597	RP11-259O2.2	lincRNA	9,32E-02	5,49E-01	7,62E-01	No	-9,73E-02	7,09E-01	8,14E-01	No
ENSG00000249731	RP11-259O2.3	lincRNA	2,79E-02	8,24E-01	NA	No	1,40E-01	5,88E-01	7,20E-01	No
ENSG00000259757	RP11-129I19.2	lincRNA	-6,42E-02	4,70E-01	NA	No	-7,68E-02	6,84E-01	NA	No
ENSG00000249994	CTD-2383I20.1	lincRNA	-5,73E-02	6,46E-01	NA	No	-7,72E-02	8,00E-01	NA	No
ENSG00000261037	CTC-471C19.1	lincRNA	3,17E-02	9,36E-01	NA	No	9,20E-02	8,64E-01	NA	No
ENSG00000250490	CTD-2324F15.2	lincRNA	7,26E-02	7,40E-01	8,79E-01	No	-6,00E-01	1,35E-01	2,48E-01	No
ENSG00000250056	LINC01018	lincRNA	-2,75E-01	2,44E-01	4,85E-01	No	-6,05E-01	7,27E-02	1,52E-01	No
ENSG00000272520	CTD-2044J15.2	lincRNA	1,85E-01	3,96E-01	6,41E-01	No	8,08E-01	3,67E-04	1,49E-03	No
ENSG00000248677	CTD-2044J15.1	lincRNA	-5,59E-02	7,85E-01	9,03E-01	No	-6,11E-01	1,34E-01	2,46E-01	No
ENSG00000251365	RP11-332J15.3	lincRNA	4,65E-02	6,04E-01	NA	No	8,51E-02	8,64E-01	NA	No
ENSG00000272071	RP11-332J15.4	lincRNA	3,15E-02	9,36E-01	NA	No	8,58E-02	8,64E-01	NA	No
ENSG00000250974	RP11-122F24.1	lincRNA	1,50E-01	2,61E-01	NA	No	5,20E-01	1,67E-01	2,92E-01	No
ENSG00000245729	RP11-480D4.1	lincRNA	-3,90E-02	7,62E-01	NA	No	-6,03E-02	7,86E-01	8,67E-01	No
ENSG00000249159	RP11-480D4.2	lincRNA	-9,83E-02	5,55E-01	7,66E-01	No	2,01E-03	9,97E-01	9,98E-01	No
ENSG00000272049	RP11-480D4.6	lincRNA	-2,87E-01	9,89E-02	2,76E-01	No	-5,00E-01	1,92E-01	3,24E-01	No
ENSG00000247516	MIR4458HG	lincRNA	-3,45E-01	1,57E-01	3,74E-01	No	4,21E-01	1,45E-01	2,61E-01	No
ENSG00000249782	RP11-417J1.1	lincRNA	3,41E-02	9,36E-01	NA	No	6,54E-02	9,56E-01	NA	No
ENSG00000250198	RP11-143A12.3	lincRNA	5,36E-03	9,81E-01	NA	No	-1,30E-01	7,12E-01	NA	No
ENSG00000251370	CTD-2201E9.1	lincRNA	-3,25E-01	1,78E-01	4,03E-01	No	-4,51E-02	8,76E-01	9,26E-01	No
ENSG00000250786	SNHG18	lincRNA	-3,07E-01	1,66E-01	3,86E-01	No	-2,82E-02	8,98E-01	9,40E-01	No
ENSG00000249781	CTD-2143L24.1	lincRNA	-2,78E-01	8,64E-02	NA	No	-6,91E-01	8,76E-02	1,76E-01	No
ENSG00000249807	CTD-2199O4.1	lincRNA	-3,21E-03	9,00E-01	NA	No	-2,28E-02	8,90E-01	NA	No
ENSG00000260515	CTD-2199O4.3	lincRNA	-1,19E-02	8,49E-01	NA	No	-9,82E-02	8,30E-01	NA	No
ENSG00000272417	CTD-2199O4.6	lincRNA	-2,55E-02	8,04E-01	NA	No	3,65E-01	2,50E-01	3,95E-01	No
ENSG00000271715	CTD-2256P15.5	lincRNA	1,32E-01	3,56E-01	6,03E-01	No	2,66E-02	9,28E-01	9,57E-01	No
ENSG00000272016	RP11-215G15.5	lincRNA	6,01E-02	7,87E-01	9,04E-01	No	-3,07E-01	3,86E-01	5,43E-01	No
ENSG00000272057	CTB-40H15.4	lincRNA	-3,99E-02	6,30E-01	NA	No	-3,65E-02	8,82E-01	NA	No
ENSG00000249332	RP1-251I12.1	lincRNA	3,17E-02	9,36E-01	NA	No	1,15E-01	6,02E-01	NA	No
ENSG00000248486	RP1-137K24.1	lincRNA	4,73E-02	5,74E-01	NA	No	1,14E-01	6,19E-01	NA	No
ENSG00000248150	RP1-167G20.1	lincRNA	1,44E-01	5,01E-01	7,28E-01	No	2,52E-01	4,76E-01	6,27E-01	No
ENSG00000250415	CTC-461F20.1	lincRNA	6,09E-02	7,05E-01	8,60E-01	No	-2,28E-01	4,07E-01	5,64E-01	No
ENSG00000249662	RP11-321E2.4	lincRNA	3,39E-02	9,36E-01	NA	No	2,62E-01	2,94E-01	NA	No
ENSG00000248693	CTD-2023M8.1	lincRNA	1,05E+00	1,23E-02	6,51E-02	No	-2,63E-01	4,51E-01	6,05E-01	No
ENSG00000251629	RP11-774D14.1	lincRNA	-1,21E-02	8,19E-01	NA	No	-2,53E-02	8,90E-01	NA	No
ENSG00000249359	RP11-374A4.1	lincRNA	7,20E-02	5,66E-01	7,74E-01	No	4,84E-01	1,29E-01	2,39E-01	No
ENSG00000272130	RP11-360I2.1	lincRNA	1,01E-01	5,55E-01	7,66E-01	No	6,71E-01	1,04E-01	2,02E-01	No
ENSG00000251273	RP11-549K20.1	lincRNA	2,92E-02	8,42E-01	9,31E-01	No	3,75E-01	2,78E-01	4,27E-01	No
ENSG00000245662	RP11-184E9.1	lincRNA	-3,45E-02	7,68E-01	8,94E-01	No	1,18E-01	7,89E-01	8,70E-01	No
ENSG00000251654	RP11-192H6.2	lincRNA	-4,15E-02	7,60E-01	NA	No	1,78E-01	1,01E-01	1,97E-01	No
ENSG00000260761	RP11-184E9.2	lincRNA	-6,39E-02	1,99E-01	4,31E-01	No	1,28E-02	9,49E-01	9,70E-01	No
ENSG00000249099	RP11-351N6.1	lincRNA	3,32E-02	9,36E-01	NA	No	9,02E-02	8,64E-01	NA	No
ENSG00000250337	LINC01021	lincRNA	-2,60E-02	8,64E-01	9,41E-01	No	5,43E-01	1,43E-01	2,59E-01	No

ENSG00000259786	CTD-2118P12.1	lincRNA	-5,69E-02	6,46E-01	NA	No	-1,35E-01	7,12E-01	NA	No
ENSG00000248813	RP11-5N11.4	lincRNA	2,05E-01	3,29E-01	5,78E-01	No	1,65E-01	6,40E-01	7,62E-01	No
ENSG00000248378	RP11-5N11.5	lincRNA	-8,77E-02	6,18E-01	8,09E-01	No	4,56E-01	2,29E-01	3,70E-01	No
ENSG00000250164	RP11-5N11.6	lincRNA	8,53E-02	7,02E-01	8,59E-01	No	-9,80E-01	3,46E-02	8,22E-02	No
ENSG00000272086	CTD-2186M15.3	lincRNA	-6,08E-01	1,54E-02	7,63E-02	No	-1,08E+00	9,26E-04	3,46E-03	No
ENSG00000250697	CTD-2066L21.3	lincRNA	2,04E-01	1,90E-01	4,20E-01	No	5,05E-02	8,34E-01	8,99E-01	No
ENSG00000248279	CTD-2218G20.2	lincRNA	5,75E-02	7,11E-01	8,64E-01	No	1,19E-01	6,78E-01	7,92E-01	No
ENSG00000249102	CTD-2066L21.1	lincRNA	7,07E-02	4,39E-01	NA	No	1,08E-01	6,85E-01	NA	No
ENSG00000251281	CTD-2066L21.2	lincRNA	4,27E-02	6,43E-01	NA	No	-6,59E-02	8,00E-01	NA	No
ENSG00000251443	RP11-113I22.1	lincRNA	-1,18E-01	4,97E-01	7,25E-01	No	7,19E-01	9,07E-02	1,81E-01	No
ENSG00000271874	CTD-2024P10.2	lincRNA	-1,04E-01	2,57E-01	NA	No	-1,79E-01	3,93E-01	NA	No
ENSG00000272103	CTD-2653M23.3	lincRNA	8,26E-02	4,66E-01	NA	No	7,04E-01	8,35E-02	1,70E-01	No
ENSG00000248587	GDNF-AS1	lincRNA	6,21E-01	4,42E-02	1,60E-01	No	1,04E+00	2,32E-02	5,87E-02	No
ENSG00000251189	CTD-2196P11.2	lincRNA	-3,21E-02	7,86E-01	NA	No	-5,58E-02	8,39E-01	9,02E-01	No
ENSG00000249911	RP11-122C5.1	lincRNA	-2,77E-03	9,78E-01	NA	No	-1,21E-01	5,59E-01	NA	No
ENSG00000249740	CTD-2127H9.1	lincRNA	-6,28E-02	7,74E-01	8,97E-01	No	1,55E-01	6,31E-01	7,55E-01	No
ENSG00000271334	CTD-2078B5.2	lincRNA	1,63E-01	4,43E-01	6,81E-01	No	1,10E+00	1,90E-02	4,96E-02	No
ENSG00000260786	RP11-112L7.1	lincRNA	-5,73E-02	6,46E-01	NA	No	-1,36E-01	7,12E-01	NA	No
ENSG00000271788	CTD-2201E18.5	lincRNA	-1,99E-01	2,28E-01	NA	No	-2,89E-01	3,88E-01	5,46E-01	No
ENSG00000272382	CTD-2035E11.4	lincRNA	9,64E-01	1,28E-02	6,69E-02	No	-7,24E-02	8,34E-01	8,99E-01	No
ENSG00000272144	CTD-2035E11.5	lincRNA	8,60E-01	1,01E-02	5,67E-02	No	5,44E-01	1,49E-01	2,67E-01	No
ENSG00000249203	RP11-473L15.3	lincRNA	1,03E-02	9,21E-01	NA	No	-4,45E-02	8,34E-01	NA	No
ENSG00000250418	RP11-503D12.1	lincRNA	-4,15E-02	7,60E-01	NA	No	-2,07E-02	9,21E-01	NA	No
ENSG00000248779	RP11-53O19.2	lincRNA	6,72E-02	7,31E-01	8,75E-01	No	3,65E-01	3,24E-01	4,78E-01	No
ENSG00000272335	RP11-53O19.3	lincRNA	-1,36E-03	9,92E-01	9,97E-01	No	1,06E+00	3,37E-06	2,00E-05	No
ENSG00000249405	RP11-317O24.1	lincRNA	1,49E-01	2,08E-01	NA	No	-5,77E-02	8,00E-01	NA	No
ENSG00000251573	CTD-2089N3.2	lincRNA	-1,73E-02	8,19E-01	NA	No	-1,36E-01	7,12E-01	NA	No
ENSG00000250447	CTD-2081C10.1	lincRNA	-1,27E-02	9,18E-01	NA	No	-7,60E-02	7,48E-01	NA	No
ENSG00000272416	CTD-2081C10.7	lincRNA	-1,78E-01	4,29E-01	6,70E-01	No	3,60E-01	3,08E-01	4,61E-01	No
ENSG00000249069	LINC01033	lincRNA	-8,70E-02	5,08E-01	NA	No	-1,54E-01	5,62E-01	7,00E-01	No
ENSG00000228650	AC008940.1	lincRNA	5,01E-02	6,58E-01	NA	No	-1,57E-01	4,84E-01	NA	No
ENSG00000249584	RP11-478P10.1	lincRNA	-4,32E-02	6,16E-01	NA	No	1,29E-03	9,94E-01	NA	No
ENSG00000248132	CTD-2037L6.2	lincRNA	-1,22E-01	3,12E-01	5,61E-01	No	-1,02E-01	6,98E-01	8,06E-01	No
ENSG00000249279	CTC-436P18.3	lincRNA	4,21E-02	7,65E-01	NA	No	1,18E-01	5,65E-01	NA	No
ENSG00000272370	RP11-307L14.1	lincRNA	-3,71E-02	8,59E-01	9,39E-01	No	7,25E-01	8,10E-02	1,66E-01	No
ENSG00000272354	RP11-307L14.2	lincRNA	5,26E-02	6,44E-01	NA	No	1,01E+00	1,56E-02	4,17E-02	No
ENSG00000269961	CTD-2033C11.1	lincRNA	-1,69E-02	9,20E-01	9,67E-01	No	1,94E-01	5,80E-01	7,14E-01	No
ENSG00000248846	CTD-2016O11.1	lincRNA	3,99E-02	7,51E-01	NA	No	-9,86E-02	6,18E-01	NA	No
ENSG00000251391	RP11-305P14.1	lincRNA	1,13E-02	9,39E-01	9,75E-01	No	5,67E-01	1,52E-01	2,71E-01	No
ENSG00000250313	RP11-5P22.3	lincRNA	2,84E-02	7,46E-01	NA	No	1,66E-01	3,46E-01	NA	No
ENSG00000270107	CTD-2306M10.1	lincRNA	2,90E-01	1,96E-01	4,27E-01	No	-1,43E-01	6,61E-01	7,78E-01	No
ENSG00000251206	CTD-2187J20.1	lincRNA	1,02E-01	3,25E-01	NA	No	8,46E-01	4,03E-02	9,35E-02	No
ENSG00000249736	RP11-83M16.5	lincRNA	3,96E-02	7,04E-01	NA	No	1,73E-01	5,09E-01	6,56E-01	No
ENSG00000248884	CTC-537E7.3	lincRNA	9,39E-02	3,66E-01	NA	No	3,73E-02	8,94E-01	NA	No
ENSG00000249335	CTC-340D7.1	lincRNA	-5,73E-02	6,46E-01	NA	No	-4,22E-02	8,90E-01	NA	No
ENSG00000249352	7SK	lincRNA	3,41E-02	9,36E-01	NA	No	6,17E-02	9,56E-01	NA	No
ENSG00000269983	RP11-497H16.9	lincRNA	-2,93E-01	1,76E-01	4,00E-01	No	-8,36E-02	7,93E-01	8,72E-01	No
ENSG00000254353	RP11-195E2.4	lincRNA	2,22E-02	9,13E-01	9,64E-01	No	-7,52E-02	8,25E-01	8,93E-01	No
ENSG00000250387	RP11-136K7.2	lincRNA	-5,58E-02	6,46E-01	NA	No	2,21E-01	3,15E-01	NA	No
ENSG00000253985	RP11-136K7.3	lincRNA	7,85E-02	3,67E-01	NA	No	4,25E-01	6,99E-02	1,47E-01	No
ENSG00000249981	RP11-136K7.1	lincRNA	2,22E-02	9,72E-01	NA	No	6,54E-02	9,56E-01	NA	No
ENSG00000272093	CTC-365E16.1	lincRNA	2,52E-01	2,50E-01	4,93E-01	No	-3,39E-01	2,06E-01	3,42E-01	No
ENSG00000272365	RP11-389C8.3	lincRNA	3,57E-01	1,34E-01	3,37E-01	No	5,60E-01	5,22E-02	1,16E-01	No
ENSG00000248371	CTC-347C20.2	lincRNA	3,15E-02	9,36E-01	NA	No	1,01E-01	7,75E-01	NA	No
ENSG00000251613	CTC-347C20.1	lincRNA	3,31E-02	9,36E-01	NA	No	1,10E-01	6,85E-01	NA	No
ENSG00000271926	CTD-2376I4.1	lincRNA	8,41E-02	4,71E-01	NA	No	8,53E-03	9,67E-01	NA	No
ENSG00000272081	CTD-2376I4.2	lincRNA	9,83E-01	1,53E-02	7,60E-02	No	1,81E-01	5,93E-01	7,25E-01	No
ENSG00000249743	RP11-60A8.1	lincRNA	3,15E-02	9,36E-01	NA	No	1,22E-01	5,34E-01	NA	No
ENSG00000272525	RP11-79P5.9	lincRNA	7,88E-02	6,95E-01	8,54E-01	No	2,19E-01	5,38E-01	6,81E-01	No
ENSG00000259968	RP11-428C6.2	lincRNA	-4,15E-02	7,60E-01	NA	No	-9,51E-02	8,30E-01	NA	No
ENSG00000248474	CTD-2292M14.1	lincRNA	-4,90E-02	5,44E-01	NA	No	-1,06E-01	6,02E-01	NA	No
ENSG00000249856	CTD-2503O16.4	lincRNA	5,83E-02	6,00E-01	NA	No	-1,30E-01	5,34E-01	NA	No

ENSG00000250889	RP11-229C3.2	lincRNA	5,04E-01	6,76E-02	2,14E-01	No	3,92E-01	2,92E-01	4,43E-01	No
ENSG00000271815	CTD-2235C13.3	lincRNA	1,93E-01	2,22E-01	4,60E-01	No	1,35E-02	9,64E-01	9,79E-01	No
ENSG00000272040	CTC-366B18.4	lincRNA	2,80E-01	2,26E-01	4,64E-01	No	6,90E-01	2,27E-02	5,76E-02	No
ENSG00000248127	CTC-235G5.3	lincRNA	7,97E-02	3,52E-01	NA	No	-1,02E-01	8,30E-01	NA	No
ENSG00000266045	RP11-466P24.7	lincRNA	4,48E-02	7,28E-01	8,73E-01	No	-3,86E-02	8,84E-01	9,31E-01	No
ENSG00000253572	RP11-107N7.1	lincRNA	-5,73E-02	6,46E-01	NA	No	-1,36E-01	7,12E-01	NA	No
ENSG00000245556	CTD-2037K23.2	lincRNA	4,13E-01	8,04E-02	2,41E-01	No	3,56E-01	1,92E-01	3,24E-01	No
ENSG00000251221	CTC-325J23.3	lincRNA	2,49E-02	9,72E-01	NA	No	8,51E-02	8,64E-01	NA	No
ENSG00000249483	CTD-2249K22.1	lincRNA	3,32E-02	9,36E-01	NA	No	9,20E-02	8,64E-01	NA	No
ENSG00000248393	CTD-2015A6.1	lincRNA	4,41E-02	6,47E-01	NA	No	-1,03E-01	8,30E-01	NA	No
ENSG00000248870	CTD-2015A6.2	lincRNA	-1,08E-01	2,10E-01	NA	No	-2,03E-01	3,35E-01	NA	No
ENSG00000248112	RP11-78C3.1	lincRNA	3,41E-02	9,36E-01	NA	No	1,28E-01	5,02E-01	NA	No
ENSG00000249857	CTD-2227C6.3	lincRNA	-7,19E-03	9,28E-01	NA	No	-1,56E-01	5,23E-01	NA	No
ENSG00000249664	CTD-2227C6.2	lincRNA	1,26E-01	4,61E-01	6,96E-01	No	1,60E-01	5,54E-01	6,94E-01	No
ENSG00000271862	RP11-343L5.2	lincRNA	1,12E-02	9,58E-01	9,83E-01	No	-4,29E-01	9,50E-02	1,88E-01	No
ENSG00000249842	CTD-2331D11.4	lincRNA	-1,66E-02	8,19E-01	NA	No	-1,36E-01	7,12E-01	NA	No
ENSG00000248701	CTC-493L21.2	lincRNA	3,32E-02	9,36E-01	NA	No	6,13E-02	9,56E-01	NA	No
ENSG00000249061	RP11-72L22.1	lincRNA	5,90E-02	5,71E-01	NA	No	8,77E-02	8,64E-01	NA	No
ENSG00000250156	CTC-498M16.2	lincRNA	-6,63E-01	3,89E-02	1,47E-01	No	-2,56E-01	4,73E-01	6,25E-01	No
ENSG00000271904	CTC-498M16.4	lincRNA	6,08E-02	4,96E-01	NA	No	1,49E-01	4,29E-01	NA	No
ENSG00000245526	LINC00461	lincRNA	4,44E-01	9,34E-02	2,66E-01	No	1,26E+00	8,36E-03	2,43E-02	No
ENSG00000250831	CTD-2232E5.2	lincRNA	-1,21E-02	8,90E-01	NA	No	1,43E-01	5,68E-01	NA	No
ENSG00000214942	AC113167.1	lincRNA	2,23E-02	9,72E-01	NA	No	1,11E-01	6,54E-01	NA	No
ENSG00000249265	AC113167.2	lincRNA	-6,93E-02	4,93E-01	NA	No	3,04E-01	3,36E-01	4,91E-01	No
ENSG00000212930	RP11-414H23.2	lincRNA	-1,98E-02	8,00E-01	NA	No	7,71E-02	7,10E-01	NA	No
ENSG00000251093	RP11-414H23.3	lincRNA	1,16E-02	8,99E-01	NA	No	1,05E-02	9,64E-01	NA	No
ENSG00000234292	RP11-213H15.1	lincRNA	-3,26E-01	1,64E-01	3,83E-01	No	-7,20E-01	7,08E-02	1,49E-01	No
ENSG00000248323	LUCAT1	lincRNA	-2,14E-01	3,37E-01	5,86E-01	No	2,99E-01	3,92E-01	5,49E-01	No
ENSG00000250049	RP11-348J24.2	lincRNA	5,56E-02	7,33E-01	8,76E-01	No	7,46E-02	8,10E-01	8,83E-01	No
ENSG00000248864	RP11-348J24.1	lincRNA	-1,25E+00	7,78E-03	4,71E-02	No	5,07E-01	1,84E-01	3,14E-01	No
ENSG00000249776	RP11-133F8.2	lincRNA	4,22E-02	7,45E-01	NA	No	6,19E-02	9,56E-01	NA	No
ENSG00000249984	CTC-529L17.2	lincRNA	3,46E-02	9,36E-01	NA	No	9,18E-02	8,64E-01	NA	No
ENSG00000248528	CTC-458G6.2	lincRNA	1,32E-02	8,66E-01	NA	No	-2,44E-02	8,81E-01	NA	No
ENSG00000272021	AC008592.8	lincRNA	-4,33E-01	5,02E-03	3,43E-02	No	-5,76E-01	1,09E-01	2,09E-01	No
ENSG00000250955	AC008592.7	lincRNA	2,62E-01	2,43E-01	4,84E-01	No	1,35E-01	6,97E-01	8,06E-01	No
ENSG00000250551	RP11-254I22.1	lincRNA	-1,29E-02	9,42E-01	9,76E-01	No	1,52E-01	6,62E-01	7,79E-01	No
ENSG00000249746	RP11-254I22.3	lincRNA	-6,37E-02	6,24E-01	8,13E-01	No	-2,12E-02	9,46E-01	9,68E-01	No
ENSG00000250158	RP11-254I22.2	lincRNA	-2,29E-01	2,29E-01	4,68E-01	No	-7,91E-01	7,42E-02	1,54E-01	No
ENSG00000251054	CTD-2215E18.3	lincRNA	1,18E-01	4,42E-01	6,80E-01	No	-7,16E-02	7,86E-01	8,67E-01	No
ENSG00000250331	RP11-1E3.1	lincRNA	2,62E-02	8,35E-01	9,28E-01	No	3,74E-02	8,86E-01	9,32E-01	No
ENSG00000248202	RP11-455B3.1	lincRNA	-1,44E-01	2,04E-01	4,37E-01	No	-1,08E-01	7,27E-01	8,27E-01	No
ENSG00000248489	CTD-2007H13.3	lincRNA	2,79E-01	2,34E-01	4,73E-01	No	8,27E-02	7,94E-01	8,73E-01	No
ENSG00000249787	RP11-346J10.1	lincRNA	2,14E-01	2,38E-01	4,78E-01	No	1,44E-01	6,32E-01	7,56E-01	No
ENSG00000250806	CTC-293G12.1	lincRNA	-4,46E-02	7,60E-01	NA	No	2,97E-03	9,87E-01	NA	No
ENSG00000248261	CTD-2333K2.1	lincRNA	3,75E-02	8,45E-01	NA	No	1,07E-01	6,99E-01	NA	No
ENSG00000250958	LINC00492	lincRNA	3,43E-03	9,90E-01	NA	No	-6,53E-02	8,00E-01	NA	No
ENSG00000247402	CTD-2340E1.2	lincRNA	-5,73E-02	6,46E-01	NA	No	-7,72E-02	8,00E-01	NA	No
ENSG00000250682	LINC00491	lincRNA	-4,48E-02	5,74E-01	NA	No	-6,53E-02	7,74E-01	NA	No
ENSG00000248757	CTD-2193G5.1	lincRNA	3,71E-01	1,05E-01	2,86E-01	No	2,27E-01	5,02E-01	6,50E-01	No
ENSG00000251026	RP11-138J23.1	lincRNA	2,35E-02	9,72E-01	NA	No	6,67E-02	9,56E-01	NA	No
ENSG00000251574	RP11-6N13.1	lincRNA	8,51E-02	4,38E-01	NA	No	6,68E-02	9,56E-01	NA	No
ENSG00000253584	CTD-2374C24.1	lincRNA	-1,18E-02	8,19E-01	NA	No	-2,34E-02	8,90E-01	NA	No
ENSG00000251027	CTC-254B4.1	lincRNA	-2,93E-02	7,93E-01	NA	No	1,10E-01	6,97E-01	8,06E-01	No
ENSG00000272523	LINC01023	lincRNA	-2,38E-01	3,06E-01	5,55E-01	No	2,39E-01	4,33E-01	5,87E-01	No
ENSG00000249476	CTD-2587M2.1	lincRNA	2,99E-01	2,07E-01	4,41E-01	No	-2,73E-01	3,86E-01	5,43E-01	No
ENSG00000271849	CTC-332L22.1	lincRNA	2,92E-02	7,94E-01	NA	No	1,53E-01	5,53E-01	6,92E-01	No
ENSG00000224032	EPB41L4A-AS1	lincRNA	-2,85E-01	6,29E-02	2,04E-01	No	8,11E-02	6,38E-01	7,61E-01	No
ENSG00000248175	CTC-428G20.3	lincRNA	-1,73E-01	2,31E-01	4,69E-01	No	7,90E-01	1,88E-08	1,61E-07	No
ENSG00000249021	CTC-505O3.3	lincRNA	-8,92E-02	3,59E-01	NA	No	-2,00E-01	3,41E-01	NA	No
ENSG00000272265	CTD-2287O16.4	lincRNA	2,19E-01	1,87E-01	4,15E-01	No	-2,00E-01	3,66E-01	NA	No
ENSG00000250015	CTC-339F2.2	lincRNA	-1,60E-01	4,38E-01	6,77E-01	No	-3,83E-01	1,72E-01	2,99E-01	No
ENSG00000249167	CTB-118N6.2	lincRNA	3,29E-02	8,62E-01	9,40E-01	No	-2,98E-01	3,70E-01	5,27E-01	No

ENSG00000251311	RP11-249M12.2	lincRNA	-5,73E-02	6,46E-01	NA	No	-1,36E-01	7,12E-01	NA	No
ENSG00000249797	CTD-3179P9.1	lincRNA	-6,80E-01	2,25E-02	1,00E-01	No	5,06E-01	1,36E-01	2,50E-01	No
ENSG00000250427	CTD-3179P9.2	lincRNA	-1,30E-01	5,33E-01	7,52E-01	No	-7,29E-01	9,23E-02	1,84E-01	No
ENSG00000250891	CTD-2281M20.1	lincRNA	4,46E-02	8,43E-01	9,32E-01	No	7,57E-01	3,41E-02	8,13E-02	No
ENSG00000249551	RP11-2N5.2	lincRNA	-1,80E-01	4,79E-02	NA	No	-2,39E-01	3,95E-01	5,52E-01	No
ENSG00000249128	RP11-2N5.1	lincRNA	4,46E-02	7,44E-01	8,80E-01	No	6,84E-03	9,81E-01	9,88E-01	No
ENSG00000249426	CTC-448D22.1	lincRNA	-8,09E-02	6,48E-01	8,27E-01	No	2,76E-01	4,42E-01	5,96E-01	No
ENSG00000251293	CTC-552D5.1	lincRNA	4,15E-02	7,67E-01	NA	No	6,54E-02	9,56E-01	NA	No
ENSG00000261036	RP11-574H6.1	lincRNA	4,04E-02	7,80E-01	NA	No	1,01E-01	7,75E-01	NA	No
ENSG00000248927	CTD-2334D19.1	lincRNA	5,54E-02	7,78E-01	8,99E-01	No	7,55E-02	8,29E-01	8,96E-01	No
ENSG00000249621	CTD-2544H17.1	lincRNA	4,94E-01	7,01E-02	2,20E-01	No	5,68E-02	8,57E-01	9,14E-01	No
ENSG00000251538	RP11-166A12.1	lincRNA	-4,60E-02	5,34E-01	NA	No	4,04E-03	9,85E-01	NA	No
ENSG00000251421	CTC-369A16.2	lincRNA	-1,16E-02	8,19E-01	NA	No	-1,28E-01	7,12E-01	NA	No
ENSG00000248600	CTD-2308B18.4	lincRNA	-4,46E-02	7,60E-01	NA	No	5,58E-02	7,64E-01	NA	No
ENSG00000248296	CTD-2308B18.3	lincRNA	-1,25E-01	2,12E-01	NA	No	-3,31E-01	9,44E-02	1,87E-01	No
ENSG00000249112	RP11-43D2.2	lincRNA	8,25E-03	9,70E-01	9,88E-01	No	2,52E-01	4,80E-01	6,31E-01	No
ENSG00000249677	RP11-395P13.3	lincRNA	3,17E-02	9,36E-01	NA	No	2,00E-01	3,41E-01	NA	No
ENSG00000250269	RP11-395P13.5	lincRNA	3,41E-02	9,36E-01	NA	No	1,18E-01	5,76E-01	NA	No
ENSG00000260192	RP11-756H20.1	lincRNA	6,02E-02	5,49E-01	NA	No	8,10E-02	7,16E-01	8,19E-01	No
ENSG00000248752	RP11-114J13.1	lincRNA	1,54E-01	3,06E-01	5,54E-01	No	5,33E-01	1,36E-01	2,49E-01	No
ENSG00000230561	CTC-228N24.1	lincRNA	3,04E-01	2,13E-01	4,49E-01	No	1,38E+00	2,74E-04	1,15E-03	No
ENSG00000245937	CTC-228N24.3	lincRNA	2,31E-01	1,05E-01	2,88E-01	No	5,91E-01	2,46E-05	1,26E-04	No
ENSG00000224015	AC063976.3	lincRNA	5,25E-02	6,56E-01	NA	No	5,90E-03	9,80E-01	NA	No
ENSG00000272203	AC004775.5	lincRNA	-1,85E-02	8,19E-01	NA	No	4,52E-03	9,27E-01	NA	No
ENSG00000271737	CTB-113I20.2	lincRNA	4,26E-02	8,48E-01	9,34E-01	No	3,83E-02	9,09E-01	9,46E-01	No
ENSG00000270177	CTD-2410N18.3	lincRNA	1,08E-01	6,25E-01	8,13E-01	No	-2,86E-01	3,65E-01	5,21E-01	No
ENSG00000251169	AC005355.2	lincRNA	4,26E-02	7,94E-01	9,08E-01	No	7,82E-02	8,04E-01	8,79E-01	No
ENSG00000248753	CTC-276P9.2	lincRNA	-4,15E-02	7,60E-01	NA	No	-1,11E-02	9,21E-01	NA	No
ENSG00000270123	VTRNA2-1	lincRNA	-5,58E-02	6,46E-01	NA	No	2,50E-02	8,82E-01	NA	No
ENSG00000271824	AC009014.3	lincRNA	1,95E-02	8,81E-01	9,50E-01	No	2,38E-01	4,36E-01	5,91E-01	No
ENSG00000270237	RP11-381K20.5	lincRNA	-3,90E-01	1,35E-01	3,39E-01	No	-1,96E-01	5,52E-01	6,92E-01	No
ENSG00000270697	RP11-381K20.4	lincRNA	-2,56E-01	2,53E-01	4,96E-01	No	6,51E-02	8,53E-01	9,11E-01	No
ENSG00000250260	RP11-325L7.2	lincRNA	-9,23E-02	6,40E-01	8,22E-01	No	-1,07E-01	7,57E-01	8,47E-01	No
ENSG00000272255	CTD-3224K15.3	lincRNA	6,79E-02	6,85E-01	8,49E-01	No	4,01E-02	8,99E-01	9,40E-01	No
ENSG00000249526	CTB-35F21.1	lincRNA	-8,64E-02	6,14E-01	8,05E-01	No	-5,69E-01	1,23E-01	2,30E-01	No
ENSG00000249131	CTB-35F21.2	lincRNA	-4,42E-02	5,99E-01	NA	No	-1,20E-01	5,48E-01	NA	No
ENSG00000272070	AC005618.6	lincRNA	-4,17E-01	3,95E-02	1,48E-01	No	-1,54E-01	4,07E-01	5,64E-01	No
ENSG00000261757	AC005592.3	lincRNA	-4,23E-02	7,16E-01	NA	No	7,04E-02	8,04E-01	8,79E-01	No
ENSG00000236714	AC005592.1	lincRNA	-6,78E-02	7,56E-01	8,87E-01	No	1,12E+00	1,40E-02	3,83E-02	No
ENSG00000249429	CTD-2050E21.1	lincRNA	4,29E-02	6,42E-01	NA	No	1,61E-01	4,42E-01	5,96E-01	No
ENSG00000251205	CTD-2050E21.2	lincRNA	4,57E-02	NA	NA	No	1,57E-01	6,13E-01	7,41E-01	No
ENSG00000251031	CTC-367F4.1	lincRNA	1,22E-01	2,33E-01	NA	No	-1,36E-01	7,12E-01	NA	No
ENSG00000250842	CTC-806A22.1	lincRNA	-1,52E-01	4,37E-01	6,76E-01	No	4,47E-01	2,23E-01	3,63E-01	No
ENSG00000248125	CTB-73N10.1	lincRNA	-2,37E-01	3,02E-01	5,50E-01	No	3,07E-01	3,63E-01	5,20E-01	No
ENSG00000248109	CTC-295J13.3	lincRNA	1,97E-02	8,72E-01	NA	No	-4,47E-02	8,56E-01	NA	No
ENSG00000250072	CTC-529P8.1	lincRNA	3,41E-01	1,01E-01	2,80E-01	No	5,15E-01	1,77E-01	3,04E-01	No
ENSG00000254333	CTC-367J11.1	lincRNA	-6,31E-02	4,79E-01	NA	No	-1,93E-01	3,52E-01	NA	No
ENSG00000254298	CTB-17P3.4	lincRNA	-1,38E-01	4,65E-01	6,99E-01	No	-4,52E-01	2,09E-01	3,45E-01	No
ENSG00000272112	CTB-113P19.5	lincRNA	9,81E-02	5,89E-01	7,89E-01	No	2,66E-01	4,48E-01	6,01E-01	No
ENSG00000254226	CTB-12O2.1	lincRNA	1,48E-01	3,85E-01	6,32E-01	No	1,18E-01	7,13E-01	8,16E-01	No
ENSG00000249484	AC091969.1	lincRNA	-7,19E-01	2,84E-02	1,17E-01	No	2,27E-01	5,18E-01	6,64E-01	No
ENSG00000251183	AC091962.3	lincRNA	-9,67E-04	9,64E-01	NA	No	-6,36E-02	7,60E-01	NA	No
ENSG00000261382	RP11-461L18.1	lincRNA	-1,58E-01	1,04E-01	NA	No	-3,54E-01	1,50E-01	NA	No
ENSG00000254293	CTB-158E9.1	lincRNA	6,80E-01	2,14E-02	9,64E-02	No	5,21E-01	1,48E-01	2,66E-01	No
ENSG00000253449	CTC-436K13.6	lincRNA	5,41E-02	5,14E-01	NA	No	8,11E-02	6,36E-01	NA	No
ENSG00000253424	CTC-436K13.3	lincRNA	9,87E-02	3,27E-01	NA	No	1,78E-01	4,14E-01	NA	No
ENSG00000253134	CTC-436K13.2	lincRNA	1,32E-01	1,38E-01	NA	No	8,64E-02	8,64E-01	NA	No
ENSG00000254350	RP11-542A14.1	lincRNA	3,35E-02	7,63E-01	NA	No	-1,35E-01	6,08E-01	NA	No
ENSG00000254135	RP11-32D16.1	lincRNA	-5,41E-03	9,10E-01	NA	No	-7,31E-02	8,00E-01	NA	No
ENSG00000245812	RP11-175K6.1	lincRNA	6,28E-01	1,21E-02	6,44E-02	No	3,56E-01	2,12E-01	3,50E-01	No
ENSG00000253315	CTB-11I22.2	lincRNA	3,41E-02	9,36E-01	NA	No	8,55E-02	8,64E-01	NA	No
ENSG00000249738	AC008697.1	lincRNA	5,16E-02	6,91E-01	NA	No	8,20E-02	7,79E-01	8,63E-01	No

ENSG00000254199	AC008691.1	lincRNA	-2,37E-02	8,19E-01	NA	No	-4,06E-02	8,90E-01	NA	No
ENSG00000253311	AC011343.1	lincRNA	3,21E-01	1,32E-01	3,34E-01	No	1,40E+00	9,26E-03	2,66E-02	No
ENSG00000253417	RP11-109J4.1	lincRNA	5,06E-03	9,05E-01	NA	No	-3,12E-02	8,90E-01	NA	No
ENSG00000254186	RP11-167P20.1	lincRNA	-7,33E-02	4,07E-01	NA	No	-1,15E-01	6,01E-01	NA	No
ENSG00000253693	CTC-535M15.2	lincRNA	3,32E-02	9,36E-01	NA	No	1,95E-01	3,50E-01	NA	No
ENSG00000253713	CTC-264O10.2	lincRNA	-6,38E-02	4,82E-01	NA	No	-1,54E-01	5,41E-01	NA	No
ENSG00000249601	CTB-27N1.1	lincRNA	4,04E-02	7,81E-01	NA	No	2,40E-01	2,37E-01	NA	No
ENSG00000235172	CTB-114C7.3	lincRNA	-3,19E-02	8,41E-01	9,31E-01	No	2,80E-01	4,35E-01	5,89E-01	No
ENSG00000254299	CTB-54I1.1	lincRNA	6,17E-02	5,32E-01	NA	No	3,89E-01	1,01E-01	NA	No
ENSG00000254295	CTC-308K20.2	lincRNA	8,45E-03	9,67E-01	NA	No	-1,02E-01	8,30E-01	NA	No
ENSG00000253968	CTB-32H22.1	lincRNA	-3,64E-02	6,74E-01	NA	No	1,62E-01	5,32E-01	NA	No
ENSG00000253768	CTB-33O18.1	lincRNA	4,22E-02	7,03E-01	NA	No	5,96E-02	8,01E-01	NA	No
ENSG00000254164	CTB-33O18.2	lincRNA	1,59E-03	9,92E-01	9,97E-01	No	-2,17E-01	2,87E-01	NA	No
ENSG00000254211	CTB-43E15.4	lincRNA	2,13E-02	8,81E-01	NA	No	-2,03E-01	4,26E-01	NA	No
ENSG00000249306	RP11-267A15.1	lincRNA	-1,71E-01	1,79E-01	4,04E-01	No	-4,56E-01	1,10E-01	2,12E-01	No
ENSG00000248596	RP11-844P9.2	lincRNA	1,10E-01	4,02E-01	NA	No	-5,59E-02	7,75E-01	NA	No
ENSG00000272459	RP11-1277A3.3	lincRNA	2,04E-01	1,93E-01	4,23E-01	No	5,37E-01	1,53E-01	2,73E-01	No
ENSG00000272431	RP11-281O15.8	lincRNA	1,82E-01	1,62E-01	3,81E-01	No	3,00E-01	3,09E-01	4,62E-01	No
ENSG00000253163	RP11-443C10.1	lincRNA	3,84E-03	9,41E-01	NA	No	-9,82E-02	8,30E-01	NA	No
ENSG00000253652	RP11-798K23.4	lincRNA	-1,91E-02	8,49E-01	NA	No	-1,03E-01	8,30E-01	NA	No
ENSG00000245060	LINC00847	lincRNA	4,57E-01	6,71E-03	4,23E-02	No	-3,39E-02	8,48E-01	9,07E-01	No
ENSG00000248473	CTC-338M12.2	lincRNA	-1,75E-02	8,19E-01	NA	No	3,59E-01	1,74E-01	3,01E-01	No
ENSG00000248103	CTC-338M12.9	lincRNA	4,20E-01	7,68E-02	2,34E-01	No	2,26E-01	4,57E-01	6,10E-01	No
ENSG00000238035	AC138035.2	lincRNA	4,09E-01	1,18E-01	3,11E-01	No	-8,22E-01	4,30E-02	9,88E-02	No
ENSG00000250765	AC138035.1	lincRNA	-1,42E-02	8,49E-01	NA	No	-9,51E-02	8,30E-01	NA	No
ENSG00000272463	RP11-532F6.3	lincRNA	9,68E-01	3,18E-03	2,46E-02	No	4,46E-01	1,77E-01	3,05E-01	No
ENSG00000271727	RP11-532F6.4	lincRNA	-1,46E-01	2,21E-01	NA	No	-2,71E-01	3,25E-01	4,80E-01	No
ENSG00000271911	RP11-532F6.5	lincRNA	2,06E-02	8,97E-01	9,57E-01	No	-2,53E-01	3,37E-01	4,92E-01	No
ENSG00000272485	RP11-284J1.1	lincRNA	7,46E-02	5,54E-01	7,66E-01	No	-8,07E-02	7,11E-01	NA	No
ENSG00000250903	GMDS-AS1	lincRNA	5,55E-01	2,18E-03	1,85E-02	No	1,95E-01	3,00E-01	4,52E-01	No
ENSG00000272465	RP1-136B1.1	lincRNA	7,08E-02	7,43E-01	8,80E-01	No	8,32E-02	8,13E-01	8,85E-01	No
ENSG00000230438	RP11-420G6.4	lincRNA	1,58E-01	4,78E-01	7,10E-01	No	-1,04E+00	2,25E-02	5,71E-02	No
ENSG00000244041	LINC01011	lincRNA	7,49E-01	5,83E-03	3,81E-02	No	-2,27E-01	4,48E-01	6,01E-01	No
ENSG00000270346	RP1-90J20.12	lincRNA	7,19E-02	7,47E-01	8,82E-01	No	-1,79E-02	9,55E-01	9,74E-01	No
ENSG00000230269	RP1-40E16.9	lincRNA	4,31E-02	7,25E-01	NA	No	6,07E-01	4,83E-02	1,09E-01	No
ENSG00000228793	RP1-223B1.1	lincRNA	-5,82E-02	4,64E-01	NA	No	-6,29E-02	7,49E-01	NA	No
ENSG00000233068	RP1-140K8.1	lincRNA	-4,32E-03	9,62E-01	9,84E-01	No	1,66E-01	5,61E-01	6,99E-01	No
ENSG00000260604	RP1-140K8.5	lincRNA	1,70E-01	4,01E-01	6,45E-01	No	1,44E+00	7,56E-05	3,53E-04	No
ENSG00000272248	RP3-406P24.4	lincRNA	-1,29E-02	9,18E-01	NA	No	1,83E-01	5,47E-01	6,87E-01	No
ENSG00000234817	RP3-400B16.1	lincRNA	1,21E-01	5,90E-01	7,90E-01	No	3,35E-01	2,57E-01	4,03E-01	No
ENSG00000237716	RP3-400B16.3	lincRNA	-1,26E-01	5,28E-01	7,49E-01	No	-5,80E-01	1,52E-01	2,71E-01	No
ENSG00000231811	RP3-527G5.1	lincRNA	-1,54E-01	4,72E-01	7,05E-01	No	-6,29E-01	1,15E-02	3,22E-02	No
ENSG00000260239	RP11-274H24.1	lincRNA	-1,12E-01	1,73E-01	NA	No	-2,13E-01	2,82E-01	NA	No
ENSG00000271978	RP11-428J1.4	lincRNA	-5,33E-02	7,31E-01	8,75E-01	No	-1,61E-01	6,28E-01	7,53E-01	No
ENSG00000233064	RP3-380B8.4	lincRNA	-6,41E-02	4,19E-01	NA	No	-1,40E-01	5,02E-01	NA	No
ENSG00000261211	RP1-80N2.3	lincRNA	1,36E-01	5,48E-01	7,62E-01	No	8,86E-01	1,25E-02	3,46E-02	No
ENSG00000226281	RP1-80N2.2	lincRNA	5,26E-02	8,12E-01	9,17E-01	No	5,04E-01	1,58E-01	2,78E-01	No
ENSG00000223342	RP3-429O6.1	lincRNA	-5,37E-02	6,46E-01	NA	No	-1,87E-02	8,90E-01	NA	No
ENSG00000251164	HULC	lincRNA	1,35E-02	9,34E-01	9,73E-01	No	6,63E-01	1,06E-01	2,05E-01	No
ENSG00000230939	RP11-314C16.1	lincRNA	4,77E-02	7,69E-01	8,94E-01	No	2,57E-01	4,53E-01	6,07E-01	No
ENSG00000272097	RP11-421M1.8	lincRNA	9,44E-02	6,10E-01	8,03E-01	No	-2,67E-01	3,55E-01	5,11E-01	No
ENSG00000271897	RP11-679B17.2	lincRNA	-3,18E-02	6,21E-01	NA	No	-1,55E-01	5,35E-01	NA	No
ENSG00000234427	RP3-413H6.2	lincRNA	-7,89E-02	5,80E-01	NA	No	-3,77E-02	8,95E-01	9,38E-01	No
ENSG00000229896	RP11-456H18.2	lincRNA	1,51E-02	8,93E-01	NA	No	-1,38E-01	5,30E-01	NA	No
ENSG00000272379	RP1-257A7.5	lincRNA	-7,76E-01	1,55E-04	2,44E-03	No	-6,98E-01	9,62E-03	2,75E-02	No
ENSG00000272209	RP3-500L14.2	lincRNA	-8,65E-02	3,14E-01	NA	No	-9,76E-02	6,79E-01	NA	No
ENSG00000230631	RP11-359N11.1	lincRNA	2,87E-03	9,81E-01	NA	No	-1,85E-01	3,69E-01	NA	No
ENSG00000226673	LINC01108	lincRNA	6,33E-01	3,57E-02	1,38E-01	No	7,10E-01	1,01E-01	1,97E-01	No
ENSG00000237346	RP3-448I9.2	lincRNA	3,41E-02	9,36E-01	NA	No	1,07E-01	6,96E-01	NA	No
ENSG00000234540	RP3-448I9.1	lincRNA	3,85E-02	8,45E-01	NA	No	9,18E-02	8,64E-01	NA	No
ENSG00000229646	RP11-330A16.1	lincRNA	6,70E-02	4,69E-01	NA	No	1,25E-01	6,67E-01	NA	No
ENSG00000234261	RP11-146I2.1	lincRNA	-1,14E-02	8,19E-01	NA	No	-1,26E-01	7,12E-01	NA	No

ENSG00000271888	RP11-560J1.2	lincRNA	-3,45E-02	8,08E-01	NA	No	2,61E-01	4,27E-01	5,83E-01	No
ENSG00000237404	RP3-471C18.2	lincRNA	4,43E-02	6,83E-01	NA	No	1,18E-01	5,75E-01	NA	No
ENSG00000227116	RP3-471C18.1	lincRNA	5,30E-03	9,41E-01	NA	No	4,23E-02	8,91E-01	NA	No
ENSG00000228412	RP4-625H18.2	lincRNA	7,56E-03	9,57E-01	9,83E-01	No	-3,36E-01	2,03E-01	3,38E-01	No
ENSG00000231754	RP11-204E9.1	lincRNA	-5,69E-02	6,46E-01	NA	No	-6,80E-02	8,00E-01	NA	No
ENSG00000272168	CASC15	lincRNA	4,50E-02	8,29E-01	9,25E-01	No	5,03E-01	3,43E-02	8,15E-02	No
ENSG00000260455	CASC14	lincRNA	-9,62E-02	6,62E-01	8,34E-01	No	1,19E+00	7,91E-04	2,99E-03	No
ENSG00000261568	RP11-524C21.2	lincRNA	4,71E-02	6,78E-01	NA	No	9,64E-03	9,62E-01	NA	No
ENSG00000235743	RP11-439H9.1	lincRNA	3,75E-02	8,45E-01	NA	No	1,39E-01	4,45E-01	NA	No
ENSG00000224164	RP3-369A17.4	lincRNA	-6,51E-02	4,43E-01	NA	No	-1,59E-01	4,91E-01	NA	No
ENSG00000272065	U91328.20	lincRNA	5,35E-01	5,57E-02	1,87E-01	No	-4,92E-02	8,75E-01	9,25E-01	No
ENSG00000272462	U91328.19	lincRNA	4,40E-03	9,86E-01	9,95E-01	No	1,33E-01	6,35E-01	7,58E-01	No
ENSG00000272810	U91328.22	lincRNA	4,15E-02	7,12E-01	NA	No	2,12E-02	9,19E-01	NA	No
ENSG00000228223	HCG11	lincRNA	4,78E-01	8,45E-03	5,00E-02	No	1,47E+00	2,87E-17	6,78E-16	No
ENSG00000261353	CTA-14H9.5	lincRNA	4,10E-01	1,17E-01	3,09E-01	No	1,33E+00	8,76E-05	4,03E-04	No
ENSG00000261584	RP11-457M11.5	lincRNA	1,05E+00	7,22E-03	4,47E-02	No	1,51E-02	9,65E-01	9,79E-01	No
ENSG00000272312	RP11-239L20.6	lincRNA	9,12E-02	3,85E-01	NA	No	4,53E-01	1,70E-01	2,96E-01	No
ENSG00000272468	RP1-86C11.7	lincRNA	-1,55E-01	2,32E-01	4,71E-01	No	-1,33E-01	6,66E-01	7,82E-01	No
ENSG00000271755	RP1-153G14.4	lincRNA	-3,89E-01	1,05E-01	2,88E-01	No	-1,50E-01	6,39E-01	7,62E-01	No
ENSG00000261839	RP1-265C24.8	lincRNA	7,56E-03	9,71E-01	9,88E-01	No	9,04E-02	7,96E-01	8,74E-01	No
ENSG00000235570	LINC00533	lincRNA	3,50E-02	9,36E-01	NA	No	6,34E-02	9,56E-01	NA	No
ENSG00000229274	XXbac-BPG13B8.10	lincRNA	2,71E-01	5,16E-02	1,77E-01	No	1,68E-01	4,28E-01	NA	No
ENSG00000272236	XXbac-BPG170G13.32	lincRNA	4,62E-02	6,91E-01	8,52E-01	No	1,33E-01	6,62E-01	7,79E-01	No
ENSG00000204625	HCG9	lincRNA	4,63E-02	8,32E-01	9,27E-01	No	7,39E-01	6,84E-02	1,45E-01	No
ENSG00000270604	HCG17	lincRNA	-1,06E-01	4,70E-01	7,04E-01	No	-1,84E-02	9,55E-01	9,74E-01	No
ENSG00000228022	HCG20	lincRNA	1,68E-01	4,59E-01	6,94E-01	No	-2,03E-02	9,50E-01	9,71E-01	No
ENSG00000271821	XXbac-BPG299F13.14	lincRNA	1,38E-01	4,63E-01	6,97E-01	No	9,20E-02	7,90E-01	8,70E-01	No
ENSG00000272221	XXbac-BPG181B23.7	lincRNA	-3,88E-01	1,24E-01	3,20E-01	No	-9,49E-01	4,10E-03	1,30E-02	No
ENSG00000230174	LINC01149	lincRNA	1,76E-02	8,83E-01	9,51E-01	No	1,73E-03	9,96E-01	9,98E-01	No
ENSG00000204261	TAPSAR1	lincRNA	9,55E-01	9,26E-05	1,61E-03	No	-4,09E-01	9,90E-02	1,94E-01	No
ENSG00000249346	LINC01016	lincRNA	-4,22E-03	9,41E-01	NA	No	5,12E-03	9,87E-01	NA	No
ENSG00000272374	RP3-329A5.8	lincRNA	1,77E-01	4,12E-01	6,55E-01	No	-5,78E-01	8,39E-02	1,70E-01	No
ENSG00000228559	RP3-340B19.3	lincRNA	9,67E-02	3,54E-01	NA	No	-1,35E-01	7,12E-01	NA	No
ENSG00000227516	RP5-973N23.4	lincRNA	1,62E-01	1,63E-01	NA	No	-5,19E-03	9,82E-01	NA	No
ENSG00000261068	RP11-7K24.3	lincRNA	-1,42E-01	4,60E-01	6,94E-01	No	-9,14E-01	5,35E-02	1,18E-01	No
ENSG00000231881	RP5-1120P11.3	lincRNA	-1,07E-01	4,32E-01	6,72E-01	No	-5,32E-01	6,48E-02	1,38E-01	No
ENSG00000262179	RP1-302G2.5	lincRNA	9,97E-01	1,82E-02	8,58E-02	No	-1,76E-02	9,40E-01	NA	No
ENSG00000237530	RP3-449H6.1	lincRNA	-6,10E-02	5,36E-01	NA	No	-1,47E-01	5,95E-01	NA	No
ENSG00000261080	RP1-166H4.2	lincRNA	3,63E-01	3,06E-02	1,24E-01	No	-1,30E-01	7,12E-01	NA	No
ENSG00000270761	RP11-385F7.1	lincRNA	4,84E-02	6,68E-01	NA	No	9,50E-01	4,81E-02	1,08E-01	No
ENSG00000225613	MIR133BHG	lincRNA	-5,19E-02	6,16E-01	NA	No	-1,72E-01	4,38E-01	NA	No
ENSG00000225791	TRAM2-AS1	lincRNA	7,83E-02	6,92E-01	8,53E-01	No	8,89E-01	1,12E-04	5,06E-04	No
ENSG00000271367	RP3-483K16.4	lincRNA	4,59E-02	7,49E-01	8,83E-01	No	-1,28E-01	6,29E-01	7,53E-01	No
ENSG00000231683	RP1-27K12.2	lincRNA	3,46E-02	9,36E-01	NA	No	8,55E-02	8,64E-01	NA	No
ENSG00000227885	RP11-79N23.1	lincRNA	1,56E-02	9,26E-01	9,69E-01	No	-2,03E-01	5,19E-01	6,64E-01	No
ENSG00000236740	RP11-411K7.1	lincRNA	-2,54E-01	2,68E-01	5,12E-01	No	4,07E-01	2,32E-01	3,74E-01	No
ENSG00000266579	RP1-71H19.2	lincRNA	1,56E-01	3,62E-01	6,10E-01	No	8,83E-01	6,09E-02	1,32E-01	No
ENSG00000231273	RP11-343D24.2	lincRNA	-4,23E-02	7,60E-01	NA	No	9,91E-02	6,39E-01	NA	No
ENSG00000272541	XXbac-BPGBPG55C20.1	lincRNA	1,52E-01	1,26E-01	NA	No	5,50E-02	7,95E-01	NA	No
ENSG00000272316	XXbac-BPGBPG55C20.2	lincRNA	6,93E-01	3,16E-03	2,45E-02	No	1,15E+00	3,36E-06	2,00E-05	No
ENSG00000225096	XXbac-BPG55C20.7	lincRNA	3,94E-01	1,23E-01	3,20E-01	No	7,50E-01	8,01E-02	1,64E-01	No
ENSG00000223504	RP11-542F9.1	lincRNA	-6,75E-02	3,80E-01	NA	No	3,14E-02	8,85E-01	9,32E-01	No
ENSG00000237643	RP11-462G2.1	lincRNA	2,05E-02	8,53E-01	NA	No	-3,38E-02	8,51E-01	NA	No
ENSG00000271967	RP11-134K13.4	lincRNA	-8,59E-03	9,61E-01	9,84E-01	No	-4,78E-02	8,81E-01	9,29E-01	No
ENSG00000230597	RP3-331H24.4	lincRNA	-1,24E-01	5,80E-01	7,83E-01	No	-1,11E+00	1,40E-02	3,81E-02	No
ENSG00000233237	LINC00472	lincRNA	-3,97E-01	1,68E-02	8,12E-02	No	-4,31E-02	8,06E-01	8,80E-01	No
ENSG00000223786	RP11-554D15.1	lincRNA	-2,40E-01	1,85E-01	4,12E-01	No	3,98E-01	2,87E-01	4,38E-01	No
ENSG00000272243	RP11-554D15.3	lincRNA	3,70E-01	8,28E-02	2,45E-01	No	4,16E-01	1,81E-01	3,10E-01	No
ENSG00000224583	RP11-554D15.4	lincRNA	-1,03E-01	2,68E-01	NA	No	1,59E-01	5,62E-01	7,00E-01	No
ENSG00000225793	RP1-234P15.4	lincRNA	-5,45E-01	4,93E-02	1,72E-01	No	-2,25E-01	4,70E-01	6,22E-01	No
ENSG00000238156	RP11-415D17.4	lincRNA	-5,18E-01	6,84E-02	2,16E-01	No	-5,74E-01	1,11E-01	2,12E-01	No
ENSG00000229495	RP11-173D14.3	lincRNA	4,98E-02	5,23E-01	NA	No	5,24E-01	8,97E-02	1,80E-01	No

ENSG00000231533	RP1-232L24.3	lincRNA	4,35E-02	7,20E-01	NA	No	1,08E-01	6,92E-01	NA	No
ENSG00000272137	RP11-177G23.2	lincRNA	-1,43E-01	5,23E-01	7,45E-01	No	3,40E-02	9,17E-01	9,51E-01	No
ENSG00000272129	RP11-250B2.6	lincRNA	-6,66E-01	9,45E-03	5,42E-02	No	4,67E-01	8,37E-02	1,70E-01	No
ENSG00000233967	RP11-250B2.3	lincRNA	1,80E-01	4,33E-01	6,73E-01	No	3,81E-01	2,35E-01	3,77E-01	No
ENSG00000260645	RP11-250B2.5	lincRNA	-1,44E-01	5,14E-01	7,38E-01	No	2,86E-01	3,91E-01	5,48E-01	No
ENSG00000224995	RP5-991C6.3	lincRNA	4,69E-03	9,58E-01	NA	No	-5,15E-02	7,73E-01	NA	No
ENSG00000226453	RP11-379B8.1	lincRNA	-4,26E-01	1,11E-01	2,99E-01	No	-1,14E+00	8,13E-03	2,37E-02	No
ENSG00000260574	RP11-801I18.1	lincRNA	-5,47E-02	6,46E-01	NA	No	-1,30E-01	7,12E-01	NA	No
ENSG00000228679	RP4-676J13.2	lincRNA	7,78E-02	5,02E-01	NA	No	-3,88E-02	8,49E-01	NA	No
ENSG00000230852	RP11-51G5.1	lincRNA	2,34E-02	9,72E-01	NA	No	8,55E-02	8,64E-01	NA	No
ENSG00000231776	RP1-90L14.1	lincRNA	4,88E-02	6,92E-01	NA	No	1,75E-01	4,25E-01	NA	No
ENSG00000234155	RP11-30P6.6	lincRNA	4,57E-02	6,33E-01	NA	No	1,02E-01	7,75E-01	NA	No
ENSG00000260271	RP1-45N11.1	lincRNA	-6,05E-02	4,97E-01	NA	No	2,41E-02	9,17E-01	NA	No
ENSG00000224944	CASC6	lincRNA	2,35E-02	9,72E-01	NA	No	8,55E-02	8,64E-01	NA	No
ENSG00000261038	RP1-149C7.1	lincRNA	1,29E-02	8,88E-01	NA	No	1,48E-01	4,97E-01	6,46E-01	No
ENSG00000220908	RP11-127B16.1	lincRNA	8,13E-02	5,21E-01	7,43E-01	No	1,56E-01	4,97E-01	6,46E-01	No
ENSG00000226684	RP3-418C23.2	lincRNA	4,37E-02	6,78E-01	NA	No	3,71E-01	1,94E-01	NA	No
ENSG00000224849	RP1-104O17.1	lincRNA	4,03E-02	7,82E-01	NA	No	1,21E-01	5,31E-01	NA	No
ENSG00000226207	RP1-104O17.3	lincRNA	4,12E-02	7,65E-01	NA	No	2,09E-01	3,13E-01	NA	No
ENSG00000271860	RP11-436D23.1	lincRNA	4,55E-02	6,45E-01	NA	No	6,34E-02	9,56E-01	NA	No
ENSG00000203809	LINC00577	lincRNA	-4,42E-02	7,60E-01	NA	No	-1,02E-01	8,30E-01	NA	No
ENSG00000272102	RP3-355L5.5	lincRNA	-1,00E-01	5,94E-01	7,93E-01	No	-3,87E-01	2,80E-01	4,30E-01	No
ENSG00000229654	RP1-60O19.2	lincRNA	3,17E-02	9,36E-01	NA	No	6,68E-02	9,56E-01	NA	No
ENSG00000235142	RP1-60O19.1	lincRNA	-2,00E-01	2,47E-01	4,89E-01	No	-8,58E-01	1,42E-02	3,86E-02	No
ENSG00000271734	RP1-111B22.3	lincRNA	2,67E-02	8,90E-01	9,54E-01	No	8,12E-03	9,81E-01	9,88E-01	No
ENSG00000225174	OSTM1-AS1	lincRNA	-1,87E-02	8,49E-01	NA	No	-2,88E-02	9,21E-01	NA	No
ENSG00000203801	LINC00222	lincRNA	6,38E-01	3,24E-02	1,29E-01	No	1,03E+00	9,21E-03	2,65E-02	No
ENSG00000232311	RP1-249I4.2	lincRNA	5,97E-01	1,93E-02	8,95E-02	No	1,30E+00	1,83E-02	4,81E-02	No
ENSG00000271789	RP5-1112D6.7	lincRNA	2,32E-01	3,22E-01	5,71E-01	No	4,39E-01	1,56E-01	2,76E-01	No
ENSG00000270661	RP1-142L7.9	lincRNA	-2,02E-01	3,81E-01	6,27E-01	No	1,16E-01	7,22E-01	8,23E-01	No
ENSG00000271208	RP1-142L7.8	lincRNA	-2,34E-01	2,75E-01	5,21E-01	No	3,55E-01	3,30E-01	4,85E-01	No
ENSG00000232299	RP1-105O18.1	lincRNA	-5,73E-02	6,46E-01	NA	No	-1,36E-01	7,12E-01	NA	No
ENSG00000232316	RP1-124C6.1	lincRNA	4,21E-02	7,67E-01	NA	No	1,18E-01	5,75E-01	NA	No
ENSG00000227502	RP1-249H1.4	lincRNA	1,53E-01	4,62E-01	6,97E-01	No	3,50E-01	3,41E-01	4,96E-01	No
ENSG00000228777	RP11-282C5.1	lincRNA	3,92E-02	8,45E-01	NA	No	1,33E-01	4,74E-01	NA	No
ENSG00000237021	RP3-486I3.7	lincRNA	1,37E-01	5,19E-01	7,42E-01	No	9,63E-01	2,68E-02	6,61E-02	No
ENSG00000240050	RP1-93H18.1	lincRNA	3,57E-01	1,57E-01	3,73E-01	No	6,47E-01	5,68E-02	1,24E-01	No
ENSG00000272403	RP1-93H18.7	lincRNA	9,55E-02	6,06E-01	8,01E-01	No	8,21E-01	4,53E-02	1,03E-01	No
ENSG00000272472	RP11-95G17.2	lincRNA	-2,19E-02	7,83E-01	NA	No	-9,11E-02	5,80E-01	NA	No
ENSG00000228704	RP11-138M12.1	lincRNA	1,84E-01	1,38E-01	NA	No	4,88E-01	1,24E-01	2,32E-01	No
ENSG00000224506	RP1-293L8.2	lincRNA	6,51E-02	6,99E-01	8,58E-01	No	2,23E-01	4,91E-01	6,40E-01	No
ENSG00000231842	RP11-527F13.1	lincRNA	2,22E-01	3,40E-01	5,88E-01	No	2,32E-01	5,11E-01	6,58E-01	No
ENSG00000226149	RP1-69D17.4	lincRNA	4,49E-01	8,67E-02	2,53E-01	No	5,72E-01	6,44E-02	1,38E-01	No
ENSG00000236673	RP11-69I8.2	lincRNA	-7,62E-02	3,32E-01	NA	No	-1,78E-01	3,92E-01	NA	No
ENSG00000236166	RP3-523C21.2	lincRNA	-6,74E-02	6,60E-01	8,33E-01	No	1,98E-01	5,63E-01	7,01E-01	No
ENSG00000228495	LINC01013	lincRNA	-1,80E-01	3,06E-01	5,54E-01	No	-2,83E-01	3,91E-01	5,48E-01	No
ENSG00000231023	LINC00326	lincRNA	5,89E-02	5,73E-01	NA	No	8,77E-02	8,64E-01	NA	No
ENSG00000272428	RP11-704J17.5	lincRNA	1,18E-01	4,17E-01	NA	No	1,48E+00	1,65E-02	4,39E-02	No
ENSG00000223586	RP4-662A9.2	lincRNA	1,38E-01	4,20E-01	6,63E-01	No	2,78E-01	4,09E-01	5,66E-01	No
ENSG00000231971	RP11-557H15.3	lincRNA	3,51E-01	1,16E-01	3,07E-01	No	3,01E-01	3,57E-01	5,14E-01	No
ENSG00000236700	LINC01010	lincRNA	3,97E-01	8,93E-02	2,59E-01	No	3,89E-01	2,96E-01	4,48E-01	No
ENSG00000232310	RP11-557H15.4	lincRNA	8,24E-01	1,37E-02	7,04E-02	No	4,61E-01	2,08E-01	3,44E-01	No
ENSG00000229722	RP11-557H15.5	lincRNA	8,38E-02	NA	NA	No	-3,63E-01	2,92E-01	4,43E-01	No
ENSG00000227723	CTA-31J9.2	lincRNA	5,57E-03	9,75E-01	9,90E-01	No	-2,14E-02	NA	NA	No
ENSG00000236389	RP1-287H17.1	lincRNA	4,89E-03	9,90E-01	NA	No	-1,26E-01	7,12E-01	NA	No
ENSG00000231028	LINC00271	lincRNA	2,24E-01	3,27E-01	5,76E-01	No	-1,18E-01	7,11E-01	8,15E-01	No
ENSG00000233534	RP1-38C16.2	lincRNA	-1,77E-02	8,19E-01	NA	No	2,14E-02	8,82E-01	NA	No
ENSG00000236378	RP11-394G3.2	lincRNA	-5,73E-02	6,46E-01	NA	No	-7,59E-02	8,00E-01	NA	No
ENSG00000260418	RP3-406A7.7	lincRNA	-4,94E-02	5,81E-01	NA	No	-1,53E-01	5,47E-01	NA	No
ENSG00000230533	RP11-95M15.1	lincRNA	3,50E-02	9,36E-01	NA	No	1,07E-01	6,96E-01	NA	No
ENSG00000234956	RP11-356I2.1	lincRNA	2,07E-01	5,57E-02	NA	No	1,08E-01	6,87E-01	NA	No
ENSG00000238099	RP11-12A2.3	lincRNA	4,38E-01	7,34E-03	4,52E-02	No	1,03E-01	7,75E-01	NA	No

ENSG00000231426	RP5-899B16.1	lincRNA	-9,92E-02	2,13E-01	NA	No	-6,79E-02	8,02E-01	8,78E-01	No
ENSG00000225148	RP5-899B16.2	lincRNA	-4,60E-02	6,17E-01	NA	No	-8,82E-02	6,98E-01	NA	No
ENSG00000259828	RP11-63E9.1	lincRNA	1,22E-01	5,85E-01	7,87E-01	No	1,16E+00	3,78E-04	1,53E-03	No
ENSG00000232618	RP11-439L18.1	lincRNA	-3,47E-01	1,35E-01	3,40E-01	No	-6,45E-01	1,20E-01	2,26E-01	No
ENSG00000270638	RP3-466P17.1	lincRNA	-2,05E-01	3,00E-01	5,48E-01	No	1,76E-01	6,19E-01	7,45E-01	No
ENSG00000227748	RP11-497D6.3	lincRNA	2,33E-03	9,80E-01	NA	No	-7,51E-02	7,48E-01	NA	No
ENSG00000272397	RP11-497D6.5	lincRNA	1,62E-01	3,91E-01	6,36E-01	No	6,51E-01	1,16E-01	2,21E-01	No
ENSG00000196634	RP3-337D23.3	lincRNA	1,28E-02	9,31E-01	NA	No	-9,55E-02	6,89E-01	NA	No
ENSG00000225135	RP11-361F15.2	lincRNA	-3,12E-01	1,26E-01	3,24E-01	No	1,35E+00	1,53E-10	1,72E-09	No
ENSG00000226249	RP11-307P5.2	lincRNA	1,32E-01	1,60E-01	NA	No	2,60E-01	2,31E-01	3,72E-01	No
ENSG00000234675	RP11-242F11.2	lincRNA	6,56E-01	3,09E-02	1,25E-01	No	4,36E-01	2,44E-01	3,88E-01	No
ENSG00000230205	RP11-631F7.2	lincRNA	8,72E-02	3,74E-01	NA	No	-1,28E-01	7,12E-01	NA	No
ENSG00000228408	RP1-111D6.3	lincRNA	4,49E-03	9,20E-01	NA	No	-4,13E-02	8,90E-01	NA	No
ENSG00000226599	RP11-136K14.3	lincRNA	2,56E-01	2,39E-01	4,79E-01	No	1,34E+00	1,89E-03	6,54E-03	No
ENSG00000271265	RP11-230C9.4	lincRNA	1,17E-01	4,00E-01	NA	No	4,89E-01	1,89E-01	3,21E-01	No
ENSG00000271551	RP11-230C9.2	lincRNA	3,80E-01	1,41E-01	3,48E-01	No	6,37E-01	1,06E-01	2,05E-01	No
ENSG00000271040	RP5-933K21.3	lincRNA	-5,11E-01	7,06E-02	2,21E-01	No	-2,93E-02	9,28E-01	9,57E-01	No
ENSG00000236537	RP11-732M18.3	lincRNA	-6,85E-02	6,42E-01	8,23E-01	No	5,83E-01	1,07E-04	4,85E-04	No
ENSG00000224478	RP11-13P5.1	lincRNA	1,45E-01	1,97E-01	NA	No	8,77E-02	8,64E-01	NA	No
ENSG00000237927	RP3-393E18.2	lincRNA	-2,05E-01	3,73E-01	6,19E-01	No	-4,82E-01	1,83E-01	3,13E-01	No
ENSG00000270949	RP11-288H12.4	lincRNA	2,71E-02	8,94E-01	9,56E-01	No	2,73E-02	9,04E-01	9,43E-01	No
ENSG00000228692	RP5-826L7.1	lincRNA	-8,37E-02	7,05E-01	8,60E-01	No	-5,13E-02	8,62E-01	9,17E-01	No
ENSG00000235538	RP1-230L10.1	lincRNA	9,79E-02	5,24E-01	7,45E-01	No	-3,50E-02	9,09E-01	9,46E-01	No
ENSG00000260422	RP1-155D22.2	lincRNA	-8,75E-02	5,15E-01	7,40E-01	No	-2,97E-01	3,01E-01	4,53E-01	No
ENSG00000230627	RP1-155D22.1	lincRNA	-9,61E-01	8,47E-03	5,01E-02	No	-1,03E+00	6,84E-03	2,04E-02	No
ENSG00000226739	RP11-347L18.1	lincRNA	1,66E-02	8,45E-01	NA	No	3,77E-02	8,85E-01	NA	No
ENSG00000236627	RP11-252P19.1	lincRNA	1,92E-02	8,88E-01	NA	No	1,35E-01	5,45E-01	NA	No
ENSG00000223942	RP11-252P19.2	lincRNA	-6,28E-02	5,05E-01	NA	No	-1,16E-01	6,10E-01	NA	No
ENSG00000272549	RP11-351J23.2	lincRNA	1,24E-01	2,89E-01	5,36E-01	No	-2,04E-02	9,22E-01	NA	No
ENSG00000203688	RP11-351J23.1	lincRNA	3,17E-01	2,44E-02	1,06E-01	No	-4,25E-03	9,80E-01	NA	No
ENSG00000229921	KIF25-AS1	lincRNA	-9,97E-02	4,96E-01	7,24E-01	No	3,15E-01	3,76E-01	5,33E-01	No
ENSG00000237987	RP11-503C24.2	lincRNA	3,32E-02	9,36E-01	NA	No	1,07E-01	6,98E-01	NA	No
ENSG00000260771	RP1-39J2.1	lincRNA	4,11E-02	7,67E-01	NA	No	8,77E-02	8,64E-01	NA	No
ENSG00000235815	RP1-125N5.2	lincRNA	-1,77E-02	8,19E-01	NA	No	-1,35E-01	7,12E-01	NA	No
ENSG00000225879	RP3-495K2.3	lincRNA	3,15E-02	9,36E-01	NA	No	9,20E-02	8,64E-01	NA	No
ENSG00000223485	RP11-417E7.1	lincRNA	-5,94E-02	6,52E-01	NA	No	-1,09E-01	6,53E-01	7,72E-01	No
ENSG00000261039	RP11-417E7.2	lincRNA	-4,68E-02	7,18E-01	8,69E-01	No	-3,30E-01	3,33E-01	4,88E-01	No
ENSG00000233085	XXyac-YX65C7_A.3	lincRNA	-5,69E-02	6,46E-01	NA	No	-1,35E-01	7,12E-01	NA	No
ENSG00000226194	RP1-137D17.1	lincRNA	-1,45E-01	3,38E-01	5,87E-01	No	7,01E-01	9,96E-02	1,95E-01	No
ENSG00000272848	RP1-137D17.2	lincRNA	2,37E-01	1,39E-01	3,45E-01	No	3,98E-02	8,60E-01	NA	No
ENSG00000229214	LINC00242	lincRNA	-2,06E-02	9,19E-01	9,66E-01	No	-3,26E-01	3,67E-01	5,24E-01	No
ENSG00000231690	LINC00574	lincRNA	4,40E-02	7,69E-01	8,94E-01	No	8,08E-02	7,96E-01	8,74E-01	No
ENSG00000271820	RP5-894D12.5	lincRNA	-1,88E-01	1,46E-01	3,56E-01	No	-4,35E-01	1,55E-01	2,75E-01	No
ENSG00000227508	RP5-894D12.3	lincRNA	2,55E-01	2,29E-01	4,67E-01	No	3,92E-02	9,09E-01	9,46E-01	No
ENSG00000271234	RP5-894D12.4	lincRNA	1,19E-02	9,08E-01	NA	No	7,07E-03	9,82E-01	NA	No
ENSG00000242611	AC093627.8	lincRNA	6,82E-02	4,48E-01	NA	No	9,93E-02	5,94E-01	NA	No
ENSG00000242474	AC093627.9	lincRNA	2,63E-01	2,70E-01	5,15E-01	No	5,31E-01	1,21E-01	2,27E-01	No
ENSG00000240859	AC093627.10	lincRNA	2,14E-01	3,56E-01	6,04E-01	No	1,40E-01	6,45E-01	7,66E-01	No
ENSG00000261795	RP11-90P13.1	lincRNA	-3,90E-01	1,26E-02	6,60E-02	No	-5,14E-01	1,13E-01	2,16E-01	No
ENSG00000239715	AC093627.11	lincRNA	-5,40E-02	6,88E-01	8,50E-01	No	-2,20E-01	3,94E-01	5,51E-01	No
ENSG00000249574	AC226118.1	lincRNA	1,11E-01	4,39E-01	6,78E-01	No	-1,71E-01	4,52E-01	6,05E-01	No
ENSG00000234471	AC147651.1	lincRNA	9,92E-02	3,24E-01	NA	No	2,12E-02	9,27E-01	NA	No
ENSG00000223855	AC147651.3	lincRNA	-6,06E-01	1,94E-03	1,69E-02	No	7,05E-01	2,17E-04	9,26E-04	No
ENSG00000273230	RP11-1246C19.1	lincRNA	4,99E-02	8,23E-01	9,23E-01	No	-8,62E-01	2,58E-02	6,41E-02	No
ENSG00000230487	PSMG3-AS1	lincRNA	1,81E-01	3,25E-01	5,74E-01	No	-4,95E-01	2,98E-02	7,25E-02	No
ENSG00000234977	AC074389.7	lincRNA	-4,83E-02	6,04E-01	NA	No	-1,65E-01	4,69E-01	NA	No
ENSG00000230914	AC004840.8	lincRNA	5,80E-02	5,83E-01	NA	No	6,34E-02	9,56E-01	NA	No
ENSG00000175873	AC004840.9	lincRNA	5,97E-01	6,61E-03	4,18E-02	No	-9,75E-01	1,87E-04	8,09E-04	No
ENSG00000231892	AC073316.2	lincRNA	-2,89E-02	7,31E-01	NA	No	-1,26E-01	7,12E-01	NA	No
ENSG00000273313	RBAKDN	lincRNA	-1,59E-02	8,97E-01	NA	No	-1,36E-01	7,12E-01	NA	No
ENSG00000234432	RP11-1275H24.1	lincRNA	-1,14E-02	9,56E-01	9,82E-01	No	1,75E-01	5,60E-01	6,98E-01	No
ENSG00000273084	RP11-1275H24.3	lincRNA	1,17E-01	5,64E-01	7,72E-01	No	4,39E-01	2,35E-01	3,77E-01	No

ENSG00000230733	AC092171.4	lincRNA	-2,81E-02	8,55E-01	9,37E-01	No	-1,50E+00	2,44E-12	3,44E-11	No
ENSG00000231704	AC004895.4	lincRNA	7,52E-02	4,56E-01	NA	No	-2,61E-02	8,90E-01	NA	No
ENSG00000260054	RP11-611L7.1	lincRNA	-2,20E-01	1,57E-01	3,73E-01	No	-1,21E+00	1,24E-09	1,25E-08	No
ENSG00000272732	RP5-1159O4.2	lincRNA	-2,05E-01	3,14E-01	5,63E-01	No	4,68E-01	2,02E-01	3,37E-01	No
ENSG00000272894	RP5-1159O4.1	lincRNA	3,67E-01	1,49E-01	3,61E-01	No	-3,28E-02	9,20E-01	9,53E-01	No
ENSG00000272745	RP5-1007H16.1	lincRNA	6,47E-02	5,06E-01	NA	No	1,09E-01	6,78E-01	NA	No
ENSG00000234718	AC007161.5	lincRNA	8,50E-01	1,36E-02	6,99E-02	No	7,89E-01	2,43E-02	6,11E-02	No
ENSG00000272328	RP4-594A5.1	lincRNA	1,74E-02	8,03E-01	NA	No	-7,72E-02	8,00E-01	NA	No
ENSG00000234710	AC060834.3	lincRNA	3,85E-02	8,45E-01	NA	No	1,13E-01	6,40E-01	NA	No
ENSG00000235431	AC006373.1	lincRNA	1,15E-02	9,73E-01	NA	No	-1,26E-01	7,12E-01	NA	No
ENSG00000271185	RP5-855F16.1	lincRNA	-9,45E-02	4,45E-01	NA	No	5,67E-01	1,32E-01	2,43E-01	No
ENSG00000226690	AC005281.1	lincRNA	-4,15E-02	7,60E-01	NA	No	-9,51E-02	8,30E-01	NA	No
ENSG00000229233	AC011891.5	lincRNA	2,34E-02	8,48E-01	NA	No	1,73E-02	9,87E-01	NA	No
ENSG00000229618	AC011288.2	lincRNA	-9,85E-01	5,51E-04	6,52E-03	No	-1,10E+00	1,07E-03	3,94E-03	No
ENSG00000237713	AC006000.5	lincRNA	-9,45E-01	4,90E-03	3,37E-02	No	-1,20E+00	3,29E-03	1,07E-02	No
ENSG00000227489	AC006458.3	lincRNA	4,18E-01	6,51E-03	4,14E-02	No	2,25E-01	2,58E-01	NA	No
ENSG00000229108	AC005550.4	lincRNA	-1,53E-01	4,99E-01	7,26E-01	No	9,47E-01	8,75E-03	2,53E-02	No
ENSG00000225974	AC005550.5	lincRNA	1,27E-01	5,14E-01	7,39E-01	No	1,16E+00	1,34E-02	3,67E-02	No
ENSG00000229379	AC006041.1	lincRNA	-1,17E-01	3,71E-01	NA	No	-4,04E-02	9,00E-01	9,41E-01	No
ENSG00000273477	RP11-196O16.1	lincRNA	-8,19E-02	6,92E-01	8,53E-01	No	1,44E+00	1,29E-08	1,13E-07	No
ENSG00000272537	GS1-166A23.1	lincRNA	2,55E-03	9,94E-01	NA	No	8,08E-02	6,99E-01	NA	No
ENSG00000272361	GS1-166A23.2	lincRNA	-9,37E-02	6,41E-01	8,22E-01	No	3,96E-01	2,84E-01	4,34E-01	No
ENSG00000236318	AC019117.1	lincRNA	-1,91E-01	2,29E-01	4,68E-01	No	-3,03E-01	3,62E-01	5,18E-01	No
ENSG00000226598	AC017060.1	lincRNA	-1,01E-01	5,45E-01	7,60E-01	No	-4,16E-01	1,58E-01	2,79E-01	No
ENSG00000228675	AC006482.1	lincRNA	2,49E-02	9,72E-01	NA	No	8,55E-02	8,64E-01	NA	No
ENSG00000223838	AC007091.1	lincRNA	1,69E-01	1,13E-01	NA	No	1,32E-01	4,79E-01	NA	No
ENSG00000237921	AC004543.2	lincRNA	4,43E-02	6,83E-01	NA	No	1,17E-01	5,88E-01	NA	No
ENSG00000226097	AC099342.1	lincRNA	-5,73E-02	6,46E-01	NA	No	-7,38E-02	8,00E-01	NA	No
ENSG00000238033	AC002480.2	lincRNA	-1,49E-01	3,85E-01	6,31E-01	No	-4,57E-01	1,50E-01	2,69E-01	No
ENSG00000235664	AC005682.8	lincRNA	6,85E-02	7,39E-01	8,78E-01	No	-4,26E-02	9,00E-01	9,41E-01	No
ENSG00000230658	KLHL7-AS1	lincRNA	3,06E-01	2,01E-01	4,33E-01	No	4,01E-01	2,40E-01	3,84E-01	No
ENSG00000226816	AC005082.12	lincRNA	-4,67E-02	8,30E-01	9,26E-01	No	2,90E-01	4,19E-01	5,75E-01	No
ENSG00000214870	AC004540.5	lincRNA	7,79E-04	9,99E-01	9,99E-01	No	1,31E-01	7,02E-01	8,09E-01	No
ENSG00000233760	AC004947.2	lincRNA	-4,39E-02	8,14E-01	9,18E-01	No	4,72E-01	2,02E-01	3,37E-01	No
ENSG00000253508	RP1-170O19.14	lincRNA	-5,69E-02	6,46E-01	NA	No	-1,35E-01	7,12E-01	NA	No
ENSG00000228421	AC005013.5	lincRNA	3,64E-01	6,71E-02	2,13E-01	No	5,03E-01	1,00E-01	1,96E-01	No
ENSG00000227017	AC007036.6	lincRNA	3,78E-01	1,37E-01	3,44E-01	No	-1,59E-01	6,36E-01	7,59E-01	No
ENSG00000264520	RP4-777O23.2	lincRNA	4,17E-01	1,02E-01	2,82E-01	No	2,22E-01	5,29E-01	6,73E-01	No
ENSG00000273014	RP11-225B17.2	lincRNA	-8,22E-01	9,70E-04	9,97E-03	No	-3,16E-01	1,31E-01	2,42E-01	No
ENSG00000272905	RP11-265E18.1	lincRNA	6,07E-02	7,55E-01	8,86E-01	No	7,57E-01	6,52E-02	1,39E-01	No
ENSG00000236494	RP11-89N17.4	lincRNA	8,05E-03	9,71E-01	9,88E-01	No	1,07E-01	7,60E-01	8,50E-01	No
ENSG00000233219	RP11-89N17.3	lincRNA	9,94E-02	4,43E-01	6,80E-01	No	-5,62E-02	7,92E-01	NA	No
ENSG00000226063	AC009531.2	lincRNA	-6,35E-02	7,72E-01	8,96E-01	No	-4,14E-01	2,63E-01	4,10E-01	No
ENSG00000228878	AC007551.3	lincRNA	7,79E-01	3,85E-03	2,83E-02	No	1,43E+00	1,03E-06	6,62E-06	No
ENSG00000232930	AC083864.3	lincRNA	-5,37E-02	6,46E-01	NA	No	-1,26E-01	7,12E-01	NA	No
ENSG00000272984	RP11-85E16.1	lincRNA	-1,68E-02	8,55E-01	NA	No	-1,72E-01	4,14E-01	NA	No
ENSG00000229424	AC007349.4	lincRNA	3,60E-02	7,48E-01	NA	No	-8,65E-02	7,11E-01	NA	No
ENSG00000235728	AC007349.5	lincRNA	3,52E-01	7,58E-02	2,32E-01	No	5,68E-02	8,27E-01	8,95E-01	No
ENSG00000188185	LINC00265	lincRNA	7,87E-01	6,28E-03	4,03E-02	No	-9,22E-02	7,22E-01	8,23E-01	No
ENSG00000232458	AC005029.1	lincRNA	8,85E-02	5,43E-01	7,58E-01	No	7,24E-03	9,77E-01	9,86E-01	No
ENSG00000224017	AC005022.1	lincRNA	-8,00E-03	9,28E-01	NA	No	2,91E-02	8,86E-01	NA	No
ENSG00000261019	RP11-111K18.2	lincRNA	-6,00E-02	7,84E-01	9,02E-01	No	1,18E+00	2,57E-04	1,08E-03	No
ENSG00000228434	AC004951.6	lincRNA	6,28E-02	7,75E-01	8,97E-01	No	-1,15E+00	1,70E-02	4,50E-02	No
ENSG00000235314	LINC00957	lincRNA	1,57E-01	4,82E-01	7,13E-01	No	1,85E-01	5,22E-01	6,67E-01	No
ENSG00000232956	SNHG15	lincRNA	-5,99E-01	2,10E-02	9,52E-02	No	-8,52E-01	4,08E-03	1,30E-02	No
ENSG00000237471	AC073115.6	lincRNA	5,04E-01	2,80E-03	2,23E-02	No	1,19E-01	5,56E-01	NA	No
ENSG00000229628	AC073115.7	lincRNA	1,99E-01	6,67E-02	NA	No	1,60E-01	4,80E-01	NA	No
ENSG00000229459	AC023669.1	lincRNA	4,55E-02	6,39E-01	NA	No	3,86E-01	2,57E-01	4,03E-01	No
ENSG00000237760	AC092657.2	lincRNA	4,20E-02	7,47E-01	NA	No	1,07E-01	6,98E-01	NA	No
ENSG00000229192	AC004870.3	lincRNA	5,46E-02	6,12E-01	NA	No	9,87E-02	7,75E-01	NA	No
ENSG00000236078	AC095067.1	lincRNA	9,23E-02	4,25E-01	6,66E-01	No	8,85E-02	7,30E-01	8,29E-01	No
ENSG00000205628	GS1-179L18.1	lincRNA	8,62E-02	4,45E-01	6,82E-01	No	4,88E-02	4,12E-01	NA	No

ENSG00000233977	RP11-310H4.2	lincRNA	5,67E-02	5,13E-01	NA	No	-1,56E-01	5,23E-01	NA	No
ENSG00000231394	RP11-310H4.3	lincRNA	3,41E-02	9,36E-01	NA	No	9,20E-02	8,64E-01	NA	No
ENSG00000261275	RP11-760D2.11	lincRNA	8,46E-02	5,86E-01	7,87E-01	No	2,42E-01	4,65E-01	6,18E-01	No
ENSG00000233288	RP11-760D2.5	lincRNA	3,39E-02	9,36E-01	NA	No	8,64E-02	8,64E-01	NA	No
ENSG00000236299	RP11-340I6.7	lincRNA	3,97E-02	8,45E-01	NA	No	1,10E-01	6,87E-01	NA	No
ENSG00000226581	RP11-340I6.8	lincRNA	7,94E-02	5,24E-01	NA	No	1,05E-01	6,53E-01	7,73E-01	No
ENSG00000179406	LINC00174	lincRNA	1,05E+00	7,90E-04	8,52E-03	No	-5,05E-01	9,84E-02	1,93E-01	No
ENSG00000223473	GS1-124K5.3	lincRNA	1,02E+00	1,53E-03	1,41E-02	No	3,23E-01	3,23E-01	4,78E-01	No
ENSG00000237310	GS1-124K5.4	lincRNA	-2,08E-02	9,21E-01	9,67E-01	No	7,85E-01	1,40E-02	3,82E-02	No
ENSG00000273448	RP11-166O4.6	lincRNA	-8,93E-02	6,87E-01	8,50E-01	No	4,33E-01	1,58E-01	2,78E-01	No
ENSG00000235475	RP11-166O4.5	lincRNA	6,70E-04	9,96E-01	9,98E-01	No	-1,01E-01	7,06E-01	8,11E-01	No
ENSG00000234215	RP5-942I16.1	lincRNA	6,14E-02	4,79E-01	NA	No	1,04E-01	6,56E-01	NA	No
ENSG00000273069	RP5-1186P10.2	lincRNA	-6,37E-02	4,85E-01	NA	No	-1,53E-01	5,47E-01	NA	No
ENSG00000225703	AC005522.7	lincRNA	4,45E-01	6,63E-02	2,11E-01	No	1,42E-01	6,54E-01	7,73E-01	No
ENSG00000273341	RP5-899E9.1	lincRNA	3,37E-01	1,10E-01	2,96E-01	No	5,07E-02	8,78E-01	9,28E-01	No
ENSG00000214293	RSBN1L-AS1	lincRNA	-2,75E-01	1,86E-01	4,13E-01	No	2,88E-01	2,11E-01	3,48E-01	No
ENSG00000233491	AC010091.1	lincRNA	-2,31E-02	8,19E-01	NA	No	9,42E-02	6,09E-01	NA	No
ENSG00000232019	AC074183.4	lincRNA	4,26E-02	7,31E-01	NA	No	1,07E-01	6,94E-01	NA	No
ENSG00000261462	CTA-254O6.1	lincRNA	1,91E-01	7,46E-02	NA	No	6,19E-02	9,56E-01	NA	No
ENSG00000182165	TP53TG1	lincRNA	-1,16E-01	4,81E-01	7,12E-01	No	7,62E-01	1,49E-04	6,58E-04	No
ENSG00000264868	CTB-167B5.2	lincRNA	-3,42E-01	1,06E-01	2,90E-01	No	2,78E-01	2,39E-01	3,82E-01	No
ENSG00000233420	AC002127.4	lincRNA	-5,37E-02	6,46E-01	NA	No	-6,19E-02	8,00E-01	NA	No
ENSG00000227863	AC002383.2	lincRNA	1,09E-01	1,92E-01	NA	No	5,29E-02	8,87E-01	NA	No
ENSG00000238358	RP5-1121E10.2	lincRNA	-1,82E-01	3,69E-01	6,15E-01	No	-6,08E-01	1,32E-01	2,43E-01	No
ENSG00000225498	AC002064.5	lincRNA	6,64E-02	6,88E-01	8,50E-01	No	-1,94E-01	5,24E-01	6,69E-01	No
ENSG00000243144	RP11-115N4.1	lincRNA	-3,23E-01	1,34E-01	3,38E-01	No	-3,68E-02	9,14E-01	9,49E-01	No
ENSG00000235450	RP11-142A5.1	lincRNA	7,35E-03	9,52E-01	NA	No	2,67E-02	9,22E-01	NA	No
ENSG00000223665	CTB-111F10.1	lincRNA	2,36E-01	1,72E-01	3,95E-01	No	2,70E-01	3,78E-01	5,35E-01	No
ENSG00000236453	AC003092.1	lincRNA	-5,73E-02	6,46E-01	NA	No	3,96E-02	8,10E-01	NA	No
ENSG00000272377	RP11-682N22.1	lincRNA	-2,21E-01	3,43E-01	5,91E-01	No	2,19E-01	4,58E-01	6,11E-01	No
ENSG00000244219	GS1-259H13.2	lincRNA	-3,10E-01	1,46E-01	3,57E-01	No	-1,20E+00	3,04E-02	7,38E-02	No
ENSG00000225329	RP11-325F22.5	lincRNA	-9,22E-02	3,94E-01	NA	No	-2,65E-01	3,10E-01	NA	No
ENSG00000267052	CTB-30L5.1	lincRNA	1,54E-01	3,50E-01	NA	No	2,95E-01	2,98E-01	4,50E-01	No
ENSG00000228742	RP5-884M6.1	lincRNA	-5,69E-02	6,46E-01	NA	No	-1,35E-01	7,12E-01	NA	No
ENSG00000225647	AC005487.2	lincRNA	-4,09E-02	5,25E-01	NA	No	-1,61E-01	4,63E-01	NA	No
ENSG00000229603	AC004014.3	lincRNA	3,15E-02	9,36E-01	NA	No	6,17E-02	9,56E-01	NA	No
ENSG00000230941	AC002386.1	lincRNA	-5,37E-02	6,46E-01	NA	No	-1,26E-01	7,12E-01	NA	No
ENSG00000226965	AC003088.1	lincRNA	1,24E-01	1,70E-01	NA	No	2,28E-01	3,69E-01	NA	No
ENSG00000223646	AC002463.3	lincRNA	1,17E-01	3,99E-01	6,44E-01	No	2,14E-01	4,34E-01	5,88E-01	No
ENSG00000234520	AC018464.3	lincRNA	-8,40E-01	8,63E-04	9,11E-03	No	6,28E-01	1,52E-02	4,09E-02	No
ENSG00000233607	AC068610.5	lincRNA	3,32E-02	9,36E-01	NA	No	1,74E-01	4,30E-01	NA	No
ENSG00000225535	AC068610.3	lincRNA	1,11E-01	5,23E-01	7,45E-01	No	-2,64E-01	3,87E-01	5,45E-01	No
ENSG00000231210	AC006159.3	lincRNA	-5,26E-02	4,77E-01	NA	No	1,53E-02	9,45E-01	NA	No
ENSG00000225546	RP11-328J2.1	lincRNA	-1,86E-02	8,19E-01	NA	No	-2,53E-02	8,90E-01	NA	No
ENSG00000233969	RP5-1006K12.1	lincRNA	4,11E-02	7,67E-01	NA	No	8,64E-02	8,64E-01	NA	No
ENSG00000234418	RP11-560I19.1	lincRNA	1,57E-01	3,83E-01	6,29E-01	No	1,17E+00	2,64E-02	6,54E-02	No
ENSG00000241324	RP5-921G16.2	lincRNA	6,91E-02	3,08E-01	NA	No	8,58E-02	8,64E-01	NA	No
ENSG00000224899	RP11-3B12.5	lincRNA	-3,42E-02	8,29E-01	9,25E-01	No	3,93E-01	2,90E-01	4,40E-01	No
ENSG00000237764	RP11-3B12.2	lincRNA	5,55E-02	6,99E-01	8,58E-01	No	2,42E-01	4,64E-01	6,17E-01	No
ENSG00000219445	RP11-3B12.3	lincRNA	3,12E-02	7,07E-01	NA	No	-1,78E-02	8,90E-01	NA	No
ENSG00000272915	RP11-62J1.4	lincRNA	1,42E-02	9,65E-01	NA	No	-9,82E-02	8,30E-01	NA	No
ENSG00000273270	RP11-212P7.2	lincRNA	-3,00E-01	1,79E-01	4,04E-01	No	-1,27E-01	6,33E-01	7,56E-01	No
ENSG00000271553	RP11-274B21.10	lincRNA	1,39E-01	5,17E-01	7,41E-01	No	-1,46E-01	6,73E-01	7,88E-01	No
ENSG00000270810	RP11-274B21.8	lincRNA	2,00E-02	9,27E-01	9,70E-01	No	-8,80E-01	5,16E-02	1,15E-01	No
ENSG00000271344	RP11-274B21.9	lincRNA	-5,51E-02	7,89E-01	9,05E-01	No	-7,85E-02	8,13E-01	8,85E-01	No
ENSG00000273329	RP11-448A19.1	lincRNA	1,06E+00	8,24E-09	5,27E-07	No	1,14E-01	5,26E-01	6,70E-01	No
ENSG00000259920	RP11-2E11.5	lincRNA	-1,36E-02	8,19E-01	NA	No	-1,33E-01	7,12E-01	NA	No
ENSG00000271522	RP11-36B6.1	lincRNA	-2,99E-01	9,22E-02	2,64E-01	No	-5,51E-01	1,31E-01	2,42E-01	No
ENSG00000233559	AC016831.7	lincRNA	3,69E-01	1,26E-01	3,25E-01	No	-7,18E-01	1,52E-02	4,08E-02	No
ENSG00000226380	MIR29A	lincRNA	3,87E-01	1,10E-01	2,96E-01	No	3,05E-01	2,49E-01	3,94E-01	No
ENSG00000271204	RP11-138A9.1	lincRNA	4,20E-01	8,87E-02	2,57E-01	No	6,68E-01	1,64E-02	4,37E-02	No
ENSG00000273319	RP11-138A9.2	lincRNA	5,64E-01	2,87E-02	1,18E-01	No	5,69E-01	3,81E-02	8,92E-02	No

ENSG00000273297	RP11-38M8.1	lincRNA	2,96E-01	1,75E-01	3,99E-01	No	2,36E-01	4,98E-01	6,47E-01	No
ENSG00000224375	AC009276.4	lincRNA	-8,63E-02	4,01E-01	NA	No	9,31E-02	7,38E-01	NA	No
ENSG00000232053	AC009784.3	lincRNA	3,54E-02	7,28E-01	NA	No	-3,74E-02	8,57E-01	NA	No
ENSG00000231114	AC078842.4	lincRNA	-1,05E-01	5,25E-01	7,46E-01	No	3,63E-01	3,13E-01	4,67E-01	No
ENSG00000228031	AC078842.3	lincRNA	4,30E-02	6,66E-01	NA	No	6,87E-02	7,51E-01	NA	No
ENSG00000273081	RP4-813F11.4	lincRNA	1,41E-01	5,24E-01	7,46E-01	No	1,44E+00	1,41E-06	8,87E-06	No
ENSG00000204990	RP5-842K16.1	lincRNA	-1,42E-01	5,29E-01	7,49E-01	No	-4,82E-01	1,80E-01	3,09E-01	No
ENSG00000261797	RP11-744I24.3	lincRNA	-1,95E-02	8,33E-01	NA	No	-8,09E-02	7,07E-01	NA	No
ENSG00000241657	TRBV11-2	lincRNA	3,67E-01	8,39E-02	2,47E-01	No	-1,73E-01	4,52E-01	NA	No
ENSG00000243583	RP4-669B10.3	lincRNA	5,00E-02	5,31E-01	NA	No	1,00E-01	7,75E-01	NA	No
ENSG00000230746	AC006007.1	lincRNA	1,62E-01	3,81E-01	6,27E-01	No	9,60E-01	3,94E-02	9,17E-02	No
ENSG00000261842	RP11-143I21.1	lincRNA	1,70E-01	4,04E-01	6,48E-01	No	3,27E-02	9,16E-01	9,50E-01	No
ENSG00000273293	RP11-445N20.3	lincRNA	3,54E-01	1,11E-01	2,98E-01	No	-2,61E-01	3,28E-01	NA	No
ENSG00000273011	RP11-728K20.3	lincRNA	2,27E-02	8,87E-01	9,53E-01	No	-3,17E-01	3,36E-01	4,91E-01	No
ENSG00000260555	RP11-728K20.2	lincRNA	1,34E-01	3,14E-01	5,63E-01	No	9,84E-02	7,25E-01	8,25E-01	No
ENSG00000241449	RP11-545G3.1	lincRNA	-3,93E-02	8,11E-01	9,16E-01	No	-2,00E-01	5,48E-01	6,88E-01	No
ENSG00000261455	LINC01003	lincRNA	-8,50E-02	6,99E-01	8,58E-01	No	1,21E+00	1,40E-03	4,98E-03	No
ENSG00000234722	AC073236.3	lincRNA	5,48E-02	5,39E-01	NA	No	-2,94E-02	8,90E-01	NA	No
ENSG00000273344	PAXIP1-AS1	lincRNA	1,06E-01	5,85E-01	7,87E-01	No	1,34E+00	1,27E-07	9,55E-07	No
ENSG00000273117	AC144652.1	lincRNA	3,30E-01	1,83E-01	4,09E-01	No	4,24E-01	2,34E-01	3,76E-01	No
ENSG00000233878	AC073133.1	lincRNA	8,09E-02	3,74E-01	NA	No	-1,11E-02	9,21E-01	NA	No
ENSG00000229660	RP5-1142J19.1	lincRNA	8,08E-02	6,79E-01	8,45E-01	No	-5,14E-01	1,39E-01	2,53E-01	No
ENSG00000234210	AC006372.4	lincRNA	1,11E-02	9,14E-01	NA	No	-1,69E-01	4,19E-01	NA	No
ENSG00000223872	AC006372.5	lincRNA	-5,73E-02	6,46E-01	NA	No	-1,36E-01	7,12E-01	NA	No
ENSG00000236871	LINC00106	lincRNA	8,83E-01	1,03E-02	5,74E-02	No	-2,21E-01	5,27E-01	6,72E-01	No
ENSG00000230542	LINC00102	lincRNA	1,84E-01	4,19E-01	6,62E-01	No	-3,59E-01	3,25E-01	4,79E-01	No
ENSG00000236120	RP11-733O18.1	lincRNA	1,74E-01	3,58E-01	6,05E-01	No	3,15E-01	3,62E-01	5,19E-01	No
ENSG00000231217	RP11-1M18.1	lincRNA	9,05E-02	5,79E-01	NA	No	-1,68E-01	5,00E-01	NA	No
ENSG00000230394	RP11-692P14.1	lincRNA	-1,29E-01	5,60E-01	7,70E-01	No	-1,08E-01	7,52E-01	8,44E-01	No
ENSG00000228543	GS1-519E5.1	lincRNA	4,03E-02	7,82E-01	NA	No	1,14E-01	6,18E-01	NA	No
ENSG00000227042	RP6-1O2.1	lincRNA	-1,48E-01	2,52E-01	NA	No	-4,98E-02	8,60E-01	9,15E-01	No
ENSG00000234129	RP11-120D5.1	lincRNA	2,18E-01	3,17E-01	5,66E-01	No	6,92E-01	9,42E-02	1,87E-01	No
ENSG00000261030	RP11-791M20.1	lincRNA	-2,30E-02	8,02E-01	NA	No	-1,99E-01	3,17E-01	NA	No
ENSG00000235385	GS1-600G8.5	lincRNA	4,28E-02	7,43E-01	NA	No	8,77E-02	8,64E-01	NA	No
ENSG00000212663	RP1-122K4.3	lincRNA	8,87E-02	3,34E-01	NA	No	4,54E-02	8,90E-01	NA	No
ENSG00000233067	RP11-40F8.2	lincRNA	-1,57E-02	9,02E-01	NA	No	-1,02E-01	7,33E-01	8,31E-01	No
ENSG00000237019	GS1-433O24.1	lincRNA	-6,40E-02	4,76E-01	NA	No	-1,55E-01	5,35E-01	NA	No
ENSG00000242021	RP11-268G12.3	lincRNA	-3,61E-02	7,22E-01	NA	No	2,85E-01	8,59E-01	NA	No
ENSG00000228933	RP11-268G12.1	lincRNA	3,87E-02	8,45E-01	NA	No	3,00E-01	1,80E-01	NA	No
ENSG00000223742	RP11-149B9.2	lincRNA	2,06E-02	7,94E-01	NA	No	1,01E-01	6,16E-01	NA	No
ENSG00000233928	RP11-305F18.1	lincRNA	4,59E-01	4,74E-02	1,68E-01	No	4,61E-01	1,87E-01	3,18E-01	No
ENSG00000233571	RP11-545D19.1	lincRNA	4,26E-02	7,31E-01	NA	No	6,54E-02	9,56E-01	NA	No
ENSG00000259977	AL121578.2	lincRNA	-3,42E-02	7,63E-01	8,91E-01	No	1,07E-01	7,41E-01	8,36E-01	No
ENSG00000235806	RP4-646N3.1	lincRNA	4,15E-02	7,66E-01	NA	No	1,64E-01	5,76E-01	7,11E-01	No
ENSG00000235304	RP11-265P11.2	lincRNA	3,80E-02	6,57E-01	NA	No	-1,26E-01	7,12E-01	NA	No
ENSG00000261435	MIR1587	lincRNA	3,27E-01	2,73E-02	1,14E-01	No	-3,95E-02	8,90E-01	NA	No
ENSG00000223486	AC092198.1	lincRNA	-4,52E-02	6,65E-01	NA	No	-1,85E-01	3,61E-01	NA	No
ENSG00000236393	RP11-320G24.1	lincRNA	-3,27E-01	1,06E-01	2,89E-01	No	-6,97E-01	9,83E-02	1,93E-01	No
ENSG00000223714	RP5-1172N10.2	lincRNA	6,94E-02	4,53E-01	NA	No	1,42E-01	5,70E-01	NA	No
ENSG00000231772	RP1-154K9.2	lincRNA	-5,45E-02	6,46E-01	NA	No	-9,51E-04	9,80E-01	NA	No
ENSG00000229563	RP11-245M24.1	lincRNA	1,69E-02	8,08E-01	NA	No	6,97E-02	6,55E-01	NA	No
ENSG00000270069	RP6-99M1.2	lincRNA	1,21E-01	5,87E-01	7,88E-01	No	7,48E-01	2,34E-02	5,92E-02	No
ENSG00000269902	RP6-99M1.3	lincRNA	1,19E-01	5,62E-01	7,71E-01	No	2,63E-01	4,53E-01	6,06E-01	No
ENSG00000231566	RP5-1158E12.3	lincRNA	2,39E-01	5,03E-02	1,74E-01	No	1,09E-01	5,52E-01	NA	No
ENSG00000236751	RP1-30G7.2	lincRNA	-3,82E-01	1,39E-01	3,45E-01	No	1,11E+00	6,02E-03	1,83E-02	No
ENSG00000230844	ZNF674-AS1	lincRNA	-3,69E-01	1,12E-01	3,00E-01	No	-3,42E-01	2,08E-01	3,44E-01	No
ENSG00000234390	USP27X-AS1	lincRNA	1,92E-01	4,00E-01	6,45E-01	No	5,51E-01	9,24E-02	1,84E-01	No
ENSG00000230317	RP11-104D21.3	lincRNA	3,17E-02	9,36E-01	NA	No	9,02E-02	8,64E-01	NA	No
ENSG00000226530	RP11-348F1.2	lincRNA	6,44E-02	7,41E-01	8,79E-01	No	4,16E-01	2,69E-01	4,18E-01	No
ENSG00000234766	RP11-56H2.2	lincRNA	4,03E-02	7,82E-01	NA	No	8,77E-02	8,64E-01	NA	No
ENSG00000234019	RP11-236P24.3	lincRNA	-1,47E-02	9,29E-01	9,71E-01	No	-6,09E-01	9,29E-02	1,85E-01	No
ENSG00000232593	LINC01155	lincRNA	2,61E-01	2,55E-01	4,98E-01	No	5,93E-01	3,81E-02	8,91E-02	No

ENSG00000270189	RP11-258C19.7	lincRNA	1,06E-01	6,06E-01	8,01E-01	No	1,09E+00	3,36E-05	1,68E-04	No
ENSG00000227486	RP13-188A5.1	lincRNA	9,26E-01	7,86E-03	4,75E-02	No	1,24E+00	1,11E-03	4,05E-03	No
ENSG00000230105	RP11-431N15.2	lincRNA	2,72E-02	8,15E-01	9,18E-01	No	8,64E-02	7,41E-01	8,36E-01	No
ENSG00000260118	RP6-201G10.2	lincRNA	-5,00E-01	5,79E-02	1,92E-01	No	-3,87E-01	2,83E-01	4,32E-01	No
ENSG00000215162	LINC00269	lincRNA	3,32E-02	9,36E-01	NA	No	6,68E-02	9,56E-01	NA	No
ENSG00000228160	RP13-57D9.3	lincRNA	3,12E-02	8,16E-01	NA	No	-1,42E-01	5,85E-01	NA	No
ENSG00000225470	JPX	lincRNA	3,86E-01	1,19E-02	6,36E-02	No	5,00E-01	1,14E-03	4,17E-03	No
ENSG00000228906	RP13-216E22.4	lincRNA	1,72E-01	4,31E-01	6,71E-01	No	-6,67E-01	3,62E-02	8,54E-02	No
ENSG00000230590	FTX	lincRNA	4,16E-02	8,37E-01	9,30E-01	No	-2,46E-01	2,95E-01	4,47E-01	No
ENSG00000226854	RP11-212D3.4	lincRNA	1,61E-01	1,44E-01	NA	No	2,26E-01	2,67E-01	NA	No
ENSG00000235461	RP5-928E24.2	lincRNA	4,26E-02	7,31E-01	NA	No	8,55E-02	8,64E-01	NA	No
ENSG00000225012	RP13-348B13.2	lincRNA	3,41E-02	9,36E-01	NA	No	8,64E-02	8,64E-01	NA	No
ENSG00000223546	LINC00630	lincRNA	3,23E-01	1,51E-02	7,54E-02	No	3,40E-01	3,78E-02	8,87E-02	No
ENSG00000239407	LL0XNC01-237H1.2	lincRNA	3,64E-01	3,10E-02	1,25E-01	No	5,36E-01	7,20E-03	2,14E-02	No
ENSG00000260802	LINC00890	lincRNA	1,06E-01	2,99E-01	NA	No	2,47E-01	3,05E-01	NA	No
ENSG00000235244	RP11-761E20.1	lincRNA	-1,55E-01	4,63E-01	6,97E-01	No	-2,23E-02	9,30E-01	9,59E-01	No
ENSG00000230159	RP11-232D9.3	lincRNA	1,05E-01	2,95E-01	NA	No	2,91E-01	2,76E-01	NA	No
ENSG00000203650	RP4-562J12.2	lincRNA	2,88E-01	2,10E-01	4,45E-01	No	9,60E-01	2,16E-02	5,53E-02	No
ENSG00000228139	GS1-421I3.4	lincRNA	1,26E-01	1,71E-01	NA	No	1,60E-01	3,56E-01	NA	No
ENSG00000260585	RP13-192B19.2	lincRNA	-5,61E-01	5,52E-02	1,86E-01	No	7,94E-01	4,74E-02	1,07E-01	No
ENSG00000232412	RP1-315G1.3	lincRNA	-3,11E-01	1,89E-01	4,18E-01	No	1,18E-01	7,36E-01	8,33E-01	No
ENSG00000260683	CTD-2076M15.1	lincRNA	-1,37E-01	1,52E-01	NA	No	1,11E-01	7,25E-01	8,25E-01	No
ENSG00000225689	RP1-30E17.2	lincRNA	-2,39E-02	8,19E-01	NA	No	8,08E-03	9,14E-01	NA	No
ENSG00000213468	RP11-453F18__B.1	lincRNA	4,10E-01	1,15E-01	3,06E-01	No	3,96E-01	2,29E-01	3,70E-01	No
ENSG00000223749	MIR503HG	lincRNA	2,16E-01	3,52E-01	6,01E-01	No	-1,34E-01	6,77E-01	7,91E-01	No
ENSG00000227060	LINC00629	lincRNA	2,15E-01	2,43E-01	4,84E-01	No	7,27E-01	8,82E-02	1,77E-01	No
ENSG00000236091	RP3-473B4.3	lincRNA	-4,07E-01	5,74E-02	1,91E-01	No	-1,15E+00	3,29E-02	7,87E-02	No
ENSG00000228372	RP11-85L21.4	lincRNA	-1,51E-02	8,49E-01	NA	No	-9,72E-02	8,30E-01	NA	No
ENSG00000196972	LINC00087	lincRNA	-1,60E-01	4,36E-01	6,76E-01	No	-5,67E-01	5,11E-02	1,14E-01	No
ENSG00000238210	RP13-210D15.4	lincRNA	-8,28E-02	2,83E-01	NA	No	-2,06E-01	3,21E-01	NA	No
ENSG00000178947	LINC00086	lincRNA	1,48E-01	5,10E-01	7,35E-01	No	-1,54E-01	5,68E-01	7,04E-01	No
ENSG00000232611	RP11-1114A5.4	lincRNA	1,96E-01	3,53E-01	6,01E-01	No	5,91E-01	2,67E-02	6,60E-02	No
ENSG00000238039	AF011889.2	lincRNA	8,36E-01	2,13E-02	9,60E-02	No	-1,60E-01	6,39E-01	7,61E-01	No
ENSG00000270052	WI2-80269A6.1	lincRNA	-4,06E-02	7,83E-01	NA	No	-2,17E-01	4,52E-01	NA	No
ENSG00000259886	U82695.10	lincRNA	-6,75E-01	1,99E-02	9,15E-02	No	-1,15E+00	3,08E-03	1,01E-02	No
ENSG00000182366	FAM87A	lincRNA	2,89E-01	1,02E-01	2,81E-01	No	5,99E-02	8,31E-01	8,97E-01	No
ENSG00000272005	RP11-91J19.4	lincRNA	2,08E-01	2,16E-01	4,52E-01	No	6,09E-01	1,78E-03	6,19E-03	No
ENSG00000253640	CTD-2281E23.1	lincRNA	1,17E-01	2,85E-01	NA	No	1,06E-01	6,13E-01	NA	No
ENSG00000260721	AF067845.1	lincRNA	6,91E-02	5,06E-01	NA	No	4,22E-02	8,50E-01	NA	No
ENSG00000253696	KBTBD11-OT1	lincRNA	-1,58E-01	2,50E-01	NA	No	4,25E-02	8,95E-01	9,38E-01	No
ENSG00000253764	RP11-439C15.4	lincRNA	9,38E-02	5,65E-01	7,72E-01	No	8,10E-01	7,29E-02	1,52E-01	No
ENSG00000270988	RP11-439C15.5	lincRNA	-2,10E-01	1,59E-01	NA	No	-7,03E-01	5,22E-02	1,16E-01	No
ENSG00000253805	RP11-1049H7.2	lincRNA	-1,06E-01	6,00E-01	7,97E-01	No	-4,27E-01	2,59E-01	4,06E-01	No
ENSG00000253444	AC133633.2	lincRNA	3,39E-02	9,36E-01	NA	No	9,88E-02	7,75E-01	NA	No
ENSG00000253855	AC133633.1	lincRNA	3,39E-02	9,36E-01	NA	No	1,19E-01	5,56E-01	NA	No
ENSG00000254319	RP11-134O21.1	lincRNA	1,44E-01	3,68E-01	6,14E-01	No	-8,47E-02	7,44E-01	8,38E-01	No
ENSG00000253853	GS1-57L11.1	lincRNA	3,11E-01	1,18E-01	3,11E-01	No	-7,75E-02	8,05E-01	8,80E-01	No
ENSG00000271743	CTD-2541M15.3	lincRNA	1,01E-01	6,31E-01	8,17E-01	No	-2,50E-01	4,78E-01	6,29E-01	No
ENSG00000271927	CTD-2541M15.4	lincRNA	-8,08E-02	6,84E-01	8,49E-01	No	-1,22E-01	5,85E-01	7,18E-01	No
ENSG00000215374	FAM66B	lincRNA	2,17E-01	3,49E-01	5,97E-01	No	5,58E-01	1,10E-01	2,10E-01	No
ENSG00000225725	FAM66E	lincRNA	3,60E-01	7,42E-02	2,28E-01	No	3,38E-01	2,52E-01	3,97E-01	No
ENSG00000253505	CTA-398F10.1	lincRNA	2,42E-02	9,72E-01	NA	No	9,02E-02	8,64E-01	NA	No
ENSG00000254153	CTA-398F10.2	lincRNA	-6,95E-02	3,11E-01	NA	No	-1,71E-01	3,60E-01	NA	No
ENSG00000253343	CTD-3023L14.3	lincRNA	-5,45E-02	6,46E-01	NA	No	-1,28E-01	7,12E-01	NA	No
ENSG00000254367	RP11-211C9.1	lincRNA	7,07E-02	5,37E-01	NA	No	7,30E-03	9,68E-01	NA	No
ENSG00000253252	RP11-10A14.6	lincRNA	2,20E-01	7,21E-02	NA	No	9,88E-02	7,75E-01	NA	No
ENSG00000248538	RP11-10A14.5	lincRNA	-2,83E-01	2,03E-01	4,36E-01	No	-3,66E-01	3,22E-01	4,76E-01	No
ENSG00000254235	RP11-115J16.1	lincRNA	-1,13E-02	8,89E-01	NA	No	-1,50E-01	4,22E-01	NA	No
ENSG00000254237	RP11-115J16.2	lincRNA	-5,69E-02	6,46E-01	NA	No	-2,55E-02	8,90E-01	NA	No
ENSG00000253641	RP11-981G7.2	lincRNA	-1,49E-01	5,08E-01	7,34E-01	No	-4,41E-01	1,85E-01	3,16E-01	No
ENSG00000253678	RP11-981G7.3	lincRNA	-1,02E-01	5,65E-01	7,73E-01	No	-2,90E-01	4,08E-01	5,64E-01	No
ENSG00000272505	RP11-981G7.6	lincRNA	-2,33E-01	2,51E-01	4,93E-01	No	-1,85E-01	5,04E-01	6,52E-01	No

ENSG00000254839	AF131215.6	lincRNA	1,64E-01	2,98E-01	5,45E-01	No	-7,28E-02	7,79E-01	8,63E-01	No
ENSG00000270076	AF131215.8	lincRNA	-1,34E-01	3,03E-01	NA	No	4,41E-02	8,84E-01	9,31E-01	No
ENSG00000170983	LINC00208	lincRNA	-6,63E-02	4,07E-01	NA	No	-1,13E-01	5,30E-01	NA	No
ENSG00000270074	LINC00965	lincRNA	-5,04E-01	7,48E-02	2,29E-01	No	-2,17E-01	5,44E-01	6,85E-01	No
ENSG00000270154	RP11-419I17.1	lincRNA	6,88E-02	7,27E-01	8,73E-01	No	-4,76E-01	2,01E-01	3,35E-01	No
ENSG00000254813	RP11-252C15.1	lincRNA	-1,20E-01	2,07E-01	NA	No	-2,62E-01	2,73E-01	NA	No
ENSG00000253184	RP11-13N12.2	lincRNA	3,15E-02	9,36E-01	NA	No	6,17E-02	9,56E-01	NA	No
ENSG00000249258	RP11-468H14.2	lincRNA	6,14E-02	7,83E-01	9,01E-01	No	-4,11E-01	1,89E-01	3,21E-01	No
ENSG00000271948	RP11-242F4.2	lincRNA	1,33E-02	9,51E-01	9,80E-01	No	1,30E+00	1,05E-04	4,78E-04	No
ENSG00000254054	RP11-156K13.3	lincRNA	2,49E-02	9,72E-01	NA	No	6,13E-02	9,56E-01	NA	No
ENSG00000253557	RP11-1080G15.1	lincRNA	-3,02E-01	1,90E-01	4,18E-01	No	-6,03E-01	1,39E-01	2,54E-01	No
ENSG00000254242	RP11-1080G15.2	lincRNA	-3,56E-02	7,28E-01	NA	No	-1,62E-01	4,60E-01	NA	No
ENSG00000253164	RP11-563N12.2	lincRNA	2,22E-04	9,41E-01	NA	No	-2,64E-02	9,21E-01	NA	No
ENSG00000254260	RP11-369E15.1	lincRNA	3,68E-02	8,45E-01	NA	No	6,34E-02	9,56E-01	NA	No
ENSG00000254092	RP11-369E15.3	lincRNA	4,47E-02	6,70E-01	NA	No	1,08E-01	6,91E-01	NA	No
ENSG00000253199	RP11-421P23.1	lincRNA	4,69E-02	5,89E-01	NA	No	8,58E-02	8,64E-01	NA	No
ENSG00000254040	RP11-421P23.2	lincRNA	-2,07E-02	8,19E-01	NA	No	-1,36E-01	7,12E-01	NA	No
ENSG00000253390	CTC-756D1.2	lincRNA	3,69E-01	1,75E-02	8,35E-02	No	1,47E-01	4,57E-01	NA	No
ENSG00000253986	CTC-756D1.3	lincRNA	4,28E-02	7,43E-01	NA	No	9,18E-02	8,64E-01	NA	No
ENSG00000254002	RP11-213G6.2	lincRNA	-1,78E-01	4,20E-01	6,63E-01	No	2,84E-01	4,18E-01	5,74E-01	No
ENSG00000253891	RP11-203E8.1	lincRNA	1,13E-01	2,38E-01	NA	No	1,12E-01	6,64E-01	NA	No
ENSG00000253476	RP11-395I14.2	lincRNA	1,63E-01	4,41E-01	6,79E-01	No	2,56E-01	3,49E-01	5,04E-01	No
ENSG00000253100	RP11-219J21.1	lincRNA	6,82E-03	9,89E-01	NA	No	3,75E-02	9,10E-01	NA	No
ENSG00000253342	RP11-219J21.2	lincRNA	8,49E-03	9,61E-01	NA	No	5,74E-02	8,05E-01	NA	No
ENSG00000254178	RP11-299D14.2	lincRNA	-7,15E-02	4,59E-01	NA	No	5,16E-03	9,87E-01	9,93E-01	No
ENSG00000253632	RP11-486M23.1	lincRNA	4,87E-01	4,60E-03	3,21E-02	No	2,10E-01	3,13E-01	NA	No
ENSG00000251191	LINC00589	lincRNA	-1,25E-03	9,74E-01	NA	No	2,95E-02	9,11E-01	NA	No
ENSG00000253490	AC145110.1	lincRNA	8,97E-02	2,67E-01	NA	No	1,07E-01	6,95E-01	NA	No
ENSG00000271869	RP11-51J9.5	lincRNA	1,48E-02	9,46E-01	9,78E-01	No	9,58E-01	1,18E-02	3,30E-02	No
ENSG00000272375	RP11-51J9.6	lincRNA	-7,81E-01	2,18E-02	9,76E-02	No	2,17E-02	9,42E-01	9,66E-01	No
ENSG00000247134	RP11-11N9.4	lincRNA	3,68E-01	1,14E-01	3,04E-01	No	1,29E+00	3,78E-07	2,63E-06	No
ENSG00000272338	RP11-722E23.2	lincRNA	1,12E+00	6,68E-04	7,54E-03	No	1,23E+00	2,16E-03	7,38E-03	No
ENSG00000253123	RP11-527N22.1	lincRNA	-7,80E-02	6,73E-01	8,41E-01	No	-3,14E-01	3,90E-01	5,48E-01	No
ENSG00000253746	RP11-527N22.2	lincRNA	4,12E-02	7,65E-01	NA	No	1,82E-01	3,99E-01	NA	No
ENSG00000253161	RP11-150O12.1	lincRNA	3,01E-01	9,09E-02	2,62E-01	No	1,76E-01	5,31E-01	6,74E-01	No
ENSG00000254290	RP11-150O12.3	lincRNA	-1,13E-01	4,18E-01	6,61E-01	No	1,55E-02	9,63E-01	9,78E-01	No
ENSG00000254306	RP11-150O12.4	lincRNA	-1,83E-02	8,19E-01	NA	No	3,73E-02	8,95E-01	NA	No
ENSG00000272092	RP11-350N15.5	lincRNA	-2,95E-02	8,91E-01	9,54E-01	No	-4,58E-01	1,90E-01	3,22E-01	No
ENSG00000254100	RP11-675F6.4	lincRNA	-4,58E-02	6,16E-01	NA	No	-1,51E-01	5,67E-01	NA	No
ENSG00000254143	RP11-470M17.2	lincRNA	-1,34E-01	3,19E-01	NA	No	-1,19E-01	7,06E-01	8,12E-01	No
ENSG00000253354	CTA-392C11.2	lincRNA	-5,37E-02	6,46E-01	NA	No	2,94E-03	9,81E-01	NA	No
ENSG00000272479	RP11-301G7.1	lincRNA	-5,47E-02	6,46E-01	NA	No	-1,30E-01	7,12E-01	NA	No
ENSG00000260588	RP11-930P14.2	lincRNA	4,07E-01	9,70E-02	2,73E-01	No	-3,53E-01	3,29E-01	4,85E-01	No
ENSG00000271938	RP11-589C21.6	lincRNA	2,22E-02	9,72E-01	NA	No	8,64E-02	8,64E-01	NA	No
ENSG00000272233	RP11-503E24.3	lincRNA	-1,65E-01	2,90E-01	5,37E-01	No	-1,06E-01	7,40E-01	8,35E-01	No
ENSG00000253745	RP11-350F16.1	lincRNA	3,84E-03	9,91E-01	NA	No	-1,26E-01	7,12E-01	NA	No
ENSG00000255366	RP11-1134I14.8	lincRNA	7,21E-01	2,74E-02	1,15E-01	No	8,68E-01	3,93E-02	9,16E-02	No
ENSG00000253688	RP11-567J20.2	lincRNA	1,30E-01	4,37E-01	6,77E-01	No	7,42E-01	8,79E-02	1,77E-01	No
ENSG00000253455	RP11-770E5.3	lincRNA	1,88E-01	1,05E-01	NA	No	1,89E-01	4,05E-01	NA	No
ENSG00000253892	RP11-770E5.2	lincRNA	3,31E-02	9,36E-01	NA	No	6,17E-02	9,56E-01	NA	No
ENSG00000272076	RP11-11C20.3	lincRNA	-7,04E-02	7,41E-01	8,79E-01	No	5,34E-01	1,38E-01	2,52E-01	No
ENSG00000272024	RP11-546K22.3	lincRNA	-2,77E-01	2,21E-01	4,58E-01	No	6,84E-01	6,29E-02	1,35E-01	No
ENSG00000253844	RP11-546K22.1	lincRNA	4,42E-03	9,74E-01	NA	No	-2,75E-01	2,61E-01	NA	No
ENSG00000253924	RP11-1023P17.2	lincRNA	5,58E-03	9,08E-01	NA	No	-3,95E-02	8,90E-01	NA	No
ENSG00000260484	RP11-1081M5.2	lincRNA	-6,27E-01	3,56E-02	1,38E-01	No	-5,56E-01	1,53E-01	2,73E-01	No
ENSG00000253369	RP11-1081M5.1	lincRNA	-8,25E-01	1,49E-02	7,48E-02	No	-6,07E-02	8,45E-01	9,06E-01	No
ENSG00000237807	RP11-400K9.4	lincRNA	-5,24E-01	1,71E-02	8,23E-02	No	-8,32E-01	2,30E-03	7,82E-03	No
ENSG00000260955	RP11-30L15.6	lincRNA	-1,24E-01	5,03E-01	7,30E-01	No	-2,35E-01	5,05E-01	6,53E-01	No
ENSG00000254142	RP11-53M11.3	lincRNA	-1,63E-02	8,93E-01	NA	No	5,94E-02	7,96E-01	8,74E-01	No
ENSG00000271722	RP11-446E9.2	lincRNA	1,91E-01	2,68E-01	5,12E-01	No	-3,60E-01	2,70E-01	4,18E-01	No
ENSG00000272343	RP11-140I16.3	lincRNA	7,13E-04	9,99E-01	9,99E-01	No	9,50E-01	3,34E-02	7,97E-02	No
ENSG00000246430	LINC00968	lincRNA	9,02E-01	1,01E-02	5,68E-02	No	1,38E+00	1,10E-03	4,04E-03	No

ENSG00000253139	RP11-17A4.3	lincRNA	1,60E-01	3,13E-01	5,62E-01	No	3,05E-01	2,54E-01	3,99E-01	No
ENSG00000254139	CTD-2339F6.1	lincRNA	-7,42E-02	6,84E-01	8,49E-01	No	8,81E-01	5,41E-02	1,19E-01	No
ENSG00000253376	RP11-44D19.1	lincRNA	-1,12E-02	8,96E-01	NA	No	-3,46E-02	8,90E-01	NA	No
ENSG00000253523	RP11-1112C15.2	lincRNA	-1,01E+00	4,37E-03	3,10E-02	No	-5,16E-01	1,33E-01	2,44E-01	No
ENSG00000253116	RP11-648L3.2	lincRNA	-1,75E-01	4,34E-01	6,74E-01	No	-5,59E-01	7,63E-02	1,58E-01	No
ENSG00000251396	RP11-163N6.2	lincRNA	5,13E-02	8,18E-01	9,20E-01	No	6,39E-01	4,30E-02	9,89E-02	No
ENSG00000228862	RP11-91I20.3	lincRNA	-8,74E-04	9,41E-01	NA	No	-9,82E-02	8,30E-01	NA	No
ENSG00000254869	RP11-91I20.2	lincRNA	1,55E-03	9,96E-01	NA	No	-7,46E-02	7,35E-01	NA	No
ENSG00000255289	RP11-91I20.1	lincRNA	-3,10E-02	7,31E-01	NA	No	-1,33E-01	7,12E-01	NA	No
ENSG00000254777	AC022182.1	lincRNA	3,15E-02	8,27E-01	9,24E-01	No	-7,10E-01	5,05E-02	1,13E-01	No
ENSG00000254802	AC022182.3	lincRNA	1,24E-01	2,22E-01	NA	No	-5,50E-02	8,00E-01	NA	No
ENSG00000254119	RP11-705O24.1	lincRNA	4,03E-02	7,82E-01	NA	No	8,51E-02	8,64E-01	NA	No
ENSG00000261542	RP11-16E18.3	lincRNA	2,63E-01	2,69E-01	5,14E-01	No	8,10E-01	4,15E-03	1,31E-02	No
ENSG00000253205	CTD-3046C4.1	lincRNA	3,17E-02	9,36E-01	NA	No	1,09E-01	6,87E-01	NA	No
ENSG00000254006	RP11-1D12.2	lincRNA	6,01E-02	4,04E-01	NA	No	6,17E-02	9,56E-01	NA	No
ENSG00000254081	CTD-3025N20.2	lincRNA	3,32E-02	9,36E-01	NA	No	1,07E-01	6,99E-01	NA	No
ENSG00000272192	CTD-2532N20.1	lincRNA	5,82E-02	6,21E-01	NA	No	-1,13E-01	6,01E-01	NA	No
ENSG00000253190	AC084082.3	lincRNA	6,22E-02	6,32E-01	NA	No	8,60E-02	7,32E-01	8,30E-01	No
ENSG00000253138	LINC00967	lincRNA	5,32E-02	6,36E-01	NA	No	6,19E-02	9,56E-01	NA	No
ENSG00000246145	RP11-346I3.4	lincRNA	1,14E-01	5,78E-01	7,82E-01	No	3,62E-01	3,17E-01	4,71E-01	No
ENSG00000271966	RP11-7F18.2	lincRNA	2,26E-01	2,96E-01	5,44E-01	No	5,42E-01	1,66E-01	2,90E-01	No
ENSG00000254337	RP11-865I6.2	lincRNA	4,61E-02	6,21E-01	NA	No	6,54E-02	9,56E-01	NA	No
ENSG00000253658	RP11-600K15.1	lincRNA	5,49E-02	4,63E-01	NA	No	3,36E-01	1,61E-01	NA	No
ENSG00000253479	RP11-744J10.3	lincRNA	4,03E-02	7,82E-01	NA	No	1,55E-01	5,19E-01	NA	No
ENSG00000253143	Metazoa_SRP	lincRNA	3,32E-02	9,36E-01	NA	No	1,36E-01	5,97E-01	NA	No
ENSG00000253967	RP11-333A23.4	lincRNA	2,22E-01	9,19E-02	2,64E-01	No	3,55E-01	5,12E-02	1,14E-01	No
ENSG00000253317	RP11-142A23.1	lincRNA	1,75E-01	4,43E-01	6,80E-01	No	-6,25E-03	9,84E-01	9,90E-01	No
ENSG00000253302	STAU2-AS1	lincRNA	-4,29E-03	9,83E-01	9,93E-01	No	-8,29E-01	2,39E-02	6,01E-02	No
ENSG00000254538	RP11-463D19.1	lincRNA	-1,40E-01	4,70E-01	7,03E-01	No	-3,04E-01	1,77E-01	3,05E-01	No
ENSG00000254288	RP11-6I2.3	lincRNA	-4,75E-02	7,87E-01	9,04E-01	No	-3,34E-01	3,40E-01	4,95E-01	No
ENSG00000253115	RP11-6I2.4	lincRNA	-4,10E-02	7,50E-01	8,83E-01	No	-9,08E-02	7,44E-01	8,38E-01	No
ENSG00000249395	CASC9	lincRNA	2,35E-02	7,90E-01	NA	No	1,66E-01	1,42E-01	2,58E-01	No
ENSG00000253416	RP11-48D4.2	lincRNA	-4,15E-02	7,60E-01	NA	No	2,43E-01	2,66E-01	NA	No
ENSG00000254266	RP11-594N15.2	lincRNA	-4,35E-01	8,45E-02	2,49E-01	No	-6,39E-01	1,39E-02	3,80E-02	No
ENSG00000272138	RP11-27N21.3	lincRNA	-1,50E-01	4,36E-01	6,76E-01	No	-2,68E-01	4,59E-01	6,12E-01	No
ENSG00000249328	RP11-26J3.1	lincRNA	-4,38E-01	1,04E-01	2,85E-01	No	-5,35E-01	1,56E-01	2,76E-01	No
ENSG00000254162	RP11-48B3.3	lincRNA	-1,22E-01	5,62E-01	7,71E-01	No	2,51E-01	2,66E-01	4,13E-01	No
ENSG00000260317	RP11-48B3.4	lincRNA	1,02E-01	6,44E-01	8,24E-01	No	8,35E-01	1,85E-02	4,83E-02	No
ENSG00000254060	RP11-172E10.1	lincRNA	2,06E-01	3,66E-01	6,13E-01	No	-1,17E-01	7,29E-01	8,28E-01	No
ENSG00000254177	RP11-1149M10.1	lincRNA	3,37E-01	1,73E-01	3,96E-01	No	1,41E-01	6,60E-01	7,77E-01	No
ENSG00000253214	RP11-1149M10.2	lincRNA	-3,55E-02	7,88E-01	9,05E-01	No	1,28E-01	6,76E-01	7,90E-01	No
ENSG00000272425	RP11-363E6.4	lincRNA	-1,89E-01	3,17E-01	5,66E-01	No	-9,28E-01	5,34E-02	1,18E-01	No
ENSG00000253778	RP11-386D6.2	lincRNA	-1,60E-01	2,47E-01	4,89E-01	No	-2,82E-01	3,74E-01	5,31E-01	No
ENSG00000271156	RP11-642C5.1	lincRNA	-1,28E-01	3,52E-01	NA	No	1,87E-01	5,81E-01	7,15E-01	No
ENSG00000251136	RP11-37B2.1	lincRNA	-8,67E-02	6,22E-01	8,12E-01	No	-2,20E-01	1,71E-01	2,96E-01	No
ENSG00000253394	LINC00534	lincRNA	5,21E-02	6,79E-01	8,45E-01	No	2,61E-01	3,82E-01	5,39E-01	No
ENSG00000253799	LINC01030	lincRNA	1,94E-01	7,33E-02	NA	No	1,58E-01	5,07E-01	NA	No
ENSG00000253901	RP11-122C21.1	lincRNA	-9,94E-02	2,85E-01	NA	No	-1,22E-01	6,35E-01	NA	No
ENSG00000253177	RP11-100L22.1	lincRNA	-1,18E-01	5,94E-01	7,93E-01	No	1,21E+00	1,69E-03	5,92E-03	No
ENSG00000254020	RP11-100L22.4	lincRNA	7,21E-02	4,24E-01	NA	No	8,55E-02	8,64E-01	NA	No
ENSG00000253577	RP11-100L22.3	lincRNA	-5,73E-02	6,46E-01	NA	No	-1,36E-01	7,12E-01	NA	No
ENSG00000253960	RP11-388K12.1	lincRNA	-5,47E-02	6,46E-01	NA	No	6,24E-03	9,82E-01	NA	No
ENSG00000254089	RP11-388K12.2	lincRNA	-6,42E-02	4,70E-01	NA	No	-3,52E-02	8,46E-01	NA	No
ENSG00000254057	RP3-388N13.3	lincRNA	2,56E-01	4,14E-02	NA	No	1,35E-02	9,87E-01	NA	No
ENSG00000264448	MIR378D2	lincRNA	1,24E-02	9,46E-01	9,78E-01	No	-1,42E-02	9,65E-01	9,79E-01	No
ENSG00000253585	KB-1184D12.1	lincRNA	-1,14E-02	9,27E-01	9,70E-01	No	-3,94E-01	1,25E-01	2,34E-01	No
ENSG00000253704	RP11-267M23.4	lincRNA	2,01E-01	3,86E-01	6,32E-01	No	3,54E-01	2,81E-01	4,30E-01	No
ENSG00000253773	KB-1047C11.2	lincRNA	1,45E+00	8,04E-03	4,83E-02	No	2,91E-01	3,60E-01	5,17E-01	No
ENSG00000253872	RP11-90D11.1	lincRNA	3,78E-02	7,06E-01	NA	No	-1,60E-02	9,44E-01	NA	No
ENSG00000270131	KB-1043D8.8	lincRNA	4,23E-02	7,41E-01	NA	No	1,32E-01	4,83E-01	NA	No
ENSG00000253105	KB-1448A5.1	lincRNA	8,56E-02	4,42E-01	NA	No	-2,80E-02	9,01E-01	NA	No
ENSG00000260640	KB-1000E4.2	lincRNA	-2,54E-01	2,05E-01	4,38E-01	No	-1,50E-01	6,53E-01	7,72E-01	No

ENSG00000254267	KB-1458E12.1	lincRNA	-6,06E-02	7,04E-01	8,60E-01	No	-1,48E-01	6,41E-01	7,63E-01	No
ENSG00000271930	RP11-44N12.5	lincRNA	1,89E-03	9,41E-01	NA	No	6,89E-02	7,80E-01	NA	No
ENSG00000229625	RP11-44N12.2	lincRNA	-2,40E-02	8,06E-01	NA	No	4,48E-02	8,13E-01	NA	No
ENSG00000253948	RP11-410L14.2	lincRNA	2,67E-01	2,59E-01	5,03E-01	No	2,91E-02	9,29E-01	9,58E-01	No
ENSG00000253217	KB-1991G8.1	lincRNA	3,31E-03	9,76E-01	NA	No	2,21E-02	9,30E-01	NA	No
ENSG00000253842	KB-173C10.1	lincRNA	3,75E-01	1,28E-01	3,28E-01	No	4,90E-01	1,07E-01	2,07E-01	No
ENSG00000253395	KB-1460A1.1	lincRNA	-1,02E-01	6,46E-01	8,25E-01	No	-4,52E-01	2,05E-01	3,41E-01	No
ENSG00000253737	KB-1460A1.3	lincRNA	-5,58E-02	6,46E-01	NA	No	-3,53E-02	8,90E-01	NA	No
ENSG00000261087	KB-1460A1.5	lincRNA	-1,69E-01	4,00E-01	6,45E-01	No	-4,11E-01	7,15E-02	1,50E-01	No
ENSG00000253679	KB-1410C5.2	lincRNA	3,32E-02	9,36E-01	NA	No	1,07E-01	6,96E-01	NA	No
ENSG00000253385	KB-1254G8.1	lincRNA	-2,06E-01	3,55E-01	6,03E-01	No	-2,55E-01	3,57E-01	5,14E-01	No
ENSG00000254281	KB-1507C5.4	lincRNA	1,55E-01	4,57E-01	6,92E-01	No	2,59E-02	9,43E-01	9,66E-01	No
ENSG00000266289	RP11-1C8.6	lincRNA	-2,14E-01	3,51E-01	6,00E-01	No	7,46E-01	2,33E-02	5,90E-02	No
ENSG00000261670	RP11-1C8.5	lincRNA	1,56E-01	7,97E-02	NA	No	1,96E-01	3,56E-01	NA	No
ENSG00000253526	RP11-152P17.3	lincRNA	1,78E-01	1,14E-01	NA	No	3,65E-01	1,66E-01	NA	No
ENSG00000254021	RP11-778D12.2	lincRNA	-5,73E-02	6,46E-01	NA	No	-1,36E-01	7,12E-01	NA	No
ENSG00000254615	RP11-395G23.3	lincRNA	6,51E-02	7,02E-01	8,59E-01	No	8,91E-01	5,32E-02	1,18E-01	No
ENSG00000253956	RP11-211F2.1	lincRNA	6,66E-02	6,67E-01	8,37E-01	No	8,02E-01	7,20E-02	1,51E-01	No
ENSG00000253122	RP11-238I10.1	lincRNA	-2,30E-02	8,90E-01	9,54E-01	No	5,03E-01	1,94E-01	3,27E-01	No
ENSG00000253717	KB-1299A7.2	lincRNA	6,81E-03	9,83E-01	NA	No	3,71E-01	1,27E-01	2,37E-01	No
ENSG00000253877	RP11-946L20.2	lincRNA	3,32E-02	9,36E-01	NA	No	8,64E-02	8,64E-01	NA	No
ENSG00000253434	RP11-1101K5.1	lincRNA	5,64E-01	5,66E-02	1,89E-01	No	1,09E+00	2,41E-02	6,06E-02	No
ENSG00000254262	RP11-58O3.2	lincRNA	4,71E-02	5,82E-01	NA	No	9,88E-02	7,75E-01	NA	No
ENSG00000249917	LINC00536	lincRNA	-1,09E-02	8,19E-01	NA	No	5,89E-02	7,71E-01	NA	No
ENSG00000253972	RP11-4K16.2	lincRNA	-3,88E-02	6,37E-01	NA	No	-1,50E-01	5,87E-01	NA	No
ENSG00000254343	RP11-760H22.2	lincRNA	2,70E-01	2,04E-01	4,37E-01	No	1,24E+00	5,72E-07	3,85E-06	No
ENSG00000248478	RP11-3G20.2	lincRNA	-3,04E-02	7,31E-01	NA	No	-6,72E-02	8,00E-01	NA	No
ENSG00000254018	RP11-785H20.1	lincRNA	-3,74E-03	9,71E-01	NA	No	8,61E-03	9,66E-01	NA	No
ENSG00000254303	RP11-398G24.2	lincRNA	-2,88E-02	8,29E-01	NA	No	-1,34E-03	9,95E-01	9,97E-01	No
ENSG00000255364	RP11-94A24.1	lincRNA	-2,28E-01	2,85E-01	5,31E-01	No	5,08E-01	1,88E-01	3,20E-01	No
ENSG00000255325	RP11-96B2.1	lincRNA	-1,16E-01	5,15E-01	7,39E-01	No	-2,79E-01	4,26E-01	5,81E-01	No
ENSG00000253819	LINC01151	lincRNA	-2,44E-01	2,96E-01	5,43E-01	No	6,10E-01	1,12E-01	2,14E-01	No
ENSG00000263443	RP11-973F15.2	lincRNA	-1,18E-01	5,96E-01	7,94E-01	No	-6,37E-01	5,32E-02	1,18E-01	No
ENSG00000272384	RP11-44N11.2	lincRNA	1,65E-01	4,52E-01	6,88E-01	No	-2,33E-01	5,03E-01	6,51E-01	No
ENSG00000272043	RP11-44N11.3	lincRNA	-1,29E-01	3,52E-01	NA	No	-1,11E-02	9,71E-01	9,83E-01	No
ENSG00000259631	RP11-557C18.4	lincRNA	-1,17E-01	2,58E-01	NA	No	9,17E-02	7,55E-01	8,46E-01	No
ENSG00000254113	RP11-174I12.2	lincRNA	3,17E-02	9,36E-01	NA	No	1,09E-01	6,78E-01	NA	No
ENSG00000214803	RP11-37N22.1	lincRNA	-1,19E+00	6,20E-03	4,00E-02	No	-8,80E-01	5,09E-02	1,14E-01	No
ENSG00000253227	RP11-383J24.1	lincRNA	-1,05E+00	9,91E-03	5,61E-02	No	-6,16E-01	1,20E-01	2,27E-01	No
ENSG00000271975	RP11-383J24.6	lincRNA	-6,22E-01	5,90E-05	1,12E-03	No	2,54E-01	2,31E-01	3,72E-01	No
ENSG00000245149	RNF139-AS1	lincRNA	2,52E-01	2,31E-01	4,70E-01	No	-4,63E-01	8,62E-02	1,74E-01	No
ENSG00000255491	RP11-1082L8.4	lincRNA	9,71E-02	3,57E-01	NA	No	6,26E-01	6,55E-02	1,40E-01	No
ENSG00000254227	RP11-622O11.4	lincRNA	4,66E-02	6,02E-01	NA	No	9,18E-02	8,64E-01	NA	No
ENSG00000253220	RP11-103H7.2	lincRNA	-5,98E-02	5,58E-01	NA	No	2,46E-02	9,33E-01	NA	No
ENSG00000253573	RP11-351C8.1	lincRNA	7,29E-02	4,40E-01	NA	No	6,68E-02	7,57E-01	NA	No
ENSG00000253438	PCAT1	lincRNA	-2,37E-01	3,06E-01	5,55E-01	No	-2,28E-01	5,07E-01	6,54E-01	No
ENSG00000253264	PCAT2	lincRNA	3,17E-01	1,41E-01	3,49E-01	No	1,81E-01	5,92E-01	7,23E-01	No
ENSG00000254166	CASC19	lincRNA	-1,26E-01	3,64E-01	6,11E-01	No	1,04E-02	9,73E-01	9,84E-01	No
ENSG00000247844	CCAT1	lincRNA	3,03E-02	8,48E-01	9,34E-01	No	-2,34E-01	4,50E-01	6,04E-01	No
ENSG00000253929	RP11-382A18.2	lincRNA	4,24E-02	6,21E-01	NA	No	1,11E-01	5,46E-01	NA	No
ENSG00000249375	CASC11	lincRNA	1,12E-01	3,99E-01	6,44E-01	No	-5,16E-02	8,11E-01	NA	No
ENSG00000229140	CCDC26	lincRNA	1,41E-01	1,21E-01	NA	No	9,18E-02	8,64E-01	NA	No
ENSG00000253926	RP11-26E5.1	lincRNA	1,22E-01	1,83E-01	NA	No	8,55E-02	8,64E-01	NA	No
ENSG00000254263	RP11-473O4.4	lincRNA	-1,12E-01	6,05E-01	8,00E-01	No	-7,45E-01	8,09E-02	1,66E-01	No
ENSG00000253720	RP11-473O4.3	lincRNA	-1,26E-01	5,33E-01	7,52E-01	No	-6,20E-01	1,33E-01	2,44E-01	No
ENSG00000253656	KB-1568E2.1	lincRNA	-3,05E-01	1,03E-01	2,84E-01	No	-2,64E-01	4,50E-01	6,04E-01	No
ENSG00000253521	HPYR1	lincRNA	-5,47E-02	6,46E-01	NA	No	-3,01E-02	8,90E-01	NA	No
ENSG00000261220	RP11-629O1.2	lincRNA	2,95E-01	2,24E-01	4,62E-01	No	4,96E-01	1,67E-01	2,92E-01	No
ENSG00000253593	RP11-157E21.1	lincRNA	-7,93E-02	3,56E-01	NA	No	-1,85E-01	3,69E-01	NA	No
ENSG00000253627	RP11-513H8.1	lincRNA	6,89E-02	4,59E-01	NA	No	8,51E-02	8,64E-01	NA	No
ENSG00000272456	CTD-2342N23.3	lincRNA	-3,09E-02	8,11E-01	NA	No	-2,41E-01	3,42E-01	NA	No
ENSG00000259820	AC083843.1	lincRNA	-2,11E-01	2,49E-01	4,91E-01	No	1,96E-01	3,21E-01	4,76E-01	No

ENSG00000254028	AC083843.2	lincRNA	1,35E-01	3,18E-01	5,67E-01	No	4,29E-01	2,08E-01	3,44E-01	No
ENSG00000253433	AC083843.3	lincRNA	-5,19E-02	7,30E-01	8,74E-01	No	3,13E-02	9,06E-01	9,44E-01	No
ENSG00000251218	RP11-1057B8.2	lincRNA	4,11E-02	7,67E-01	NA	No	2,47E-01	2,11E-01	NA	No
ENSG00000254083	RP11-452N4.1	lincRNA	-1,85E-02	8,19E-01	NA	No	-1,36E-01	7,12E-01	NA	No
ENSG00000254101	RP11-30J20.1	lincRNA	1,29E-01	2,84E-01	5,30E-01	No	9,71E-01	3,59E-02	8,48E-02	No
ENSG00000253288	RP11-238K6.1	lincRNA	4,55E-02	6,45E-01	NA	No	8,55E-02	8,64E-01	NA	No
ENSG00000253988	RP11-489O18.1	lincRNA	5,92E-03	9,62E-01	NA	No	3,00E-02	8,97E-01	9,39E-01	No
ENSG00000254258	RP11-398H6.1	lincRNA	-5,73E-02	6,46E-01	NA	No	-7,72E-02	8,00E-01	NA	No
ENSG00000259758	CASC7	lincRNA	-4,78E-01	1,90E-03	1,67E-02	No	-5,97E-01	2,77E-04	1,16E-03	No
ENSG00000254197	RP11-10J21.5	lincRNA	-4,54E-02	8,11E-01	9,16E-01	No	-6,92E-01	1,06E-01	2,06E-01	No
ENSG00000247317	RP11-273G15.2	lincRNA	1,37E-01	5,29E-01	7,50E-01	No	8,22E-02	8,13E-01	8,85E-01	No
ENSG00000271912	RP11-661A12.14	lincRNA	-1,04E-01	6,25E-01	8,13E-01	No	-1,09E-01	6,96E-01	8,05E-01	No
ENSG00000203499	FAM83H-AS1	lincRNA	1,75E-01	3,83E-01	6,30E-01	No	9,79E-02	7,81E-01	8,64E-01	No
ENSG00000254973	RP11-429J17.7	lincRNA	-3,29E-02	7,31E-01	NA	No	2,89E-02	9,15E-01	NA	No
ENSG00000254533	AF186192.1	lincRNA	-2,65E-01	2,06E-01	4,39E-01	No	-1,46E-01	6,70E-01	7,86E-01	No
ENSG00000263612	AF186192.6	lincRNA	9,46E-02	5,46E-01	7,61E-01	No	-1,33E-01	6,21E-01	7,47E-01	No
ENSG00000227917	RP11-143M1.3	lincRNA	-1,44E-01	2,53E-01	4,95E-01	No	-8,09E-01	6,03E-02	1,31E-01	No
ENSG00000231808	RP11-143M1.4	lincRNA	-3,25E-01	1,43E-01	3,52E-01	No	-7,23E-02	8,06E-01	8,80E-01	No
ENSG00000236511	RP11-32F11.2	lincRNA	-3,78E-02	6,23E-01	NA	No	-1,56E-01	5,23E-01	NA	No
ENSG00000232104	RP11-509J21.1	lincRNA	4,15E-01	9,43E-02	2,68E-01	No	5,69E-01	4,25E-02	9,79E-02	No
ENSG00000237359	RP11-509J21.2	lincRNA	9,68E-02	6,64E-01	8,36E-01	No	-2,23E-01	4,84E-01	6,34E-01	No
ENSG00000235326	RP11-509J21.3	lincRNA	6,48E-01	3,33E-02	1,31E-01	No	1,54E-01	6,55E-01	7,74E-01	No
ENSG00000226669	RP11-509J21.4	lincRNA	7,95E-02	6,99E-01	8,58E-01	No	-4,93E-01	2,05E-01	3,40E-01	No
ENSG00000273061	RP11-6J24.6	lincRNA	5,06E-02	8,13E-01	9,17E-01	No	9,39E-01	9,31E-03	2,67E-02	No
ENSG00000272866	RP11-12D24.10	lincRNA	-1,02E-01	2,87E-01	NA	No	-3,03E-02	9,05E-01	NA	No
ENSG00000231509	RP11-574F11.3	lincRNA	2,34E-01	2,46E-01	4,88E-01	No	-1,37E-01	6,53E-01	7,73E-01	No
ENSG00000228739	RP11-218I7.2	lincRNA	-5,37E-02	6,46E-01	NA	No	-1,26E-01	7,12E-01	NA	No
ENSG00000236924	RP11-390F4.6	lincRNA	2,02E-02	8,10E-01	NA	No	-2,10E-02	9,30E-01	NA	No
ENSG00000225489	RP11-390F4.3	lincRNA	1,47E+00	1,03E-02	5,75E-02	No	-8,11E-03	9,70E-01	NA	No
ENSG00000273056	RP11-77E14.2	lincRNA	-7,38E-02	6,30E-01	8,16E-01	No	1,06E-01	7,40E-01	8,36E-01	No
ENSG00000226376	RP11-29B9.1	lincRNA	-8,33E-02	3,59E-01	NA	No	-2,24E-01	3,66E-01	NA	No
ENSG00000225706	RP11-75C9.1	lincRNA	-6,76E-01	3,87E-02	1,46E-01	No	3,43E-01	3,13E-01	4,67E-01	No
ENSG00000270547	RP11-536O18.2	lincRNA	-8,93E-02	3,49E-01	5,97E-01	No	-1,96E-01	5,06E-01	6,54E-01	No
ENSG00000226197	RP11-536O18.1	lincRNA	-9,73E-01	1,26E-02	6,62E-02	No	2,84E-01	4,30E-01	5,85E-01	No
ENSG00000235533	RP11-284P20.3	lincRNA	-9,40E-02	6,16E-01	8,07E-01	No	8,57E-02	8,08E-01	8,81E-01	No
ENSG00000273399	RP11-408A13.3	lincRNA	-6,39E-02	7,67E-01	8,93E-01	No	-1,76E-01	5,99E-01	7,29E-01	No
ENSG00000272871	RP11-408A13.4	lincRNA	3,23E-01	1,85E-01	4,12E-01	No	2,30E-01	4,65E-01	6,18E-01	No
ENSG00000227167	RP11-570H19.2	lincRNA	-3,01E-02	7,31E-01	NA	No	7,54E-02	7,04E-01	NA	No
ENSG00000261402	RP11-378I6.1	lincRNA	7,71E-03	9,81E-01	NA	No	4,45E-02	8,71E-01	NA	No
ENSG00000227071	RP11-4E23.2	lincRNA	-1,70E-01	4,51E-01	6,87E-01	No	5,31E-01	1,51E-01	2,69E-01	No
ENSG00000261696	RP11-354P17.15	lincRNA	4,91E-02	5,47E-01	NA	No	1,63E-01	4,83E-01	NA	No
ENSG00000234840	RP11-399D6.2	lincRNA	-4,72E-02	8,05E-01	9,13E-01	No	9,69E-03	9,79E-01	9,87E-01	No
ENSG00000224549	RP11-370B11.3	lincRNA	9,83E-02	3,30E-01	NA	No	2,95E-01	1,06E-01	2,05E-01	No
ENSG00000233906	RP11-315I14.2	lincRNA	-3,25E-02	7,31E-01	NA	No	-2,79E-02	8,90E-01	NA	No
ENSG00000254261	RP11-18A15.1	lincRNA	3,15E-02	9,36E-01	NA	No	1,00E-01	7,75E-01	NA	No
ENSG00000253400	RP11-337A23.6	lincRNA	-2,91E-02	7,66E-01	NA	No	-1,57E-01	5,07E-01	NA	No
ENSG00000254396	RP11-56F10.3	lincRNA	1,85E-01	1,51E-01	NA	No	1,10E-01	6,40E-01	NA	No
ENSG00000231459	LINC00032	lincRNA	-2,15E-02	9,07E-01	9,61E-01	No	2,33E-01	5,14E-01	6,60E-01	No
ENSG00000260390	RP11-575I8.1	lincRNA	4,02E-02	8,45E-01	NA	No	1,63E-01	4,82E-01	NA	No
ENSG00000231193	RP11-462B18.2	lincRNA	-3,52E-02	6,76E-01	NA	No	-6,83E-03	9,75E-01	NA	No
ENSG00000260947	RP11-384P7.7	lincRNA	5,03E-01	6,02E-02	1,97E-01	No	1,40E+00	2,05E-03	7,03E-03	No
ENSG00000227388	RP11-112J3.16	lincRNA	3,53E-01	1,60E-01	3,77E-01	No	2,25E-02	9,49E-01	9,70E-01	No
ENSG00000235387	LINC00961	lincRNA	-8,27E-02	6,67E-01	8,37E-01	No	2,07E-01	2,80E-01	4,30E-01	No
ENSG00000260100	RP11-220I1.5	lincRNA	-3,66E-02	7,31E-01	NA	No	6,47E-02	6,88E-01	NA	No
ENSG00000233137	RP11-220I1.1	lincRNA	2,07E-01	2,37E-02	1,04E-01	No	7,03E-01	8,74E-12	1,15E-10	No
ENSG00000224648	RP11-397D12.4	lincRNA	-7,86E-03	9,30E-01	NA	No	-4,42E-03	9,82E-01	NA	No
ENSG00000228534	RP11-95H8.5	lincRNA	2,40E-02	9,72E-01	NA	No	6,23E-02	9,56E-01	NA	No
ENSG00000236361	RP11-395E19.7	lincRNA	-2,37E-02	8,19E-01	NA	No	-1,36E-01	7,12E-01	NA	No
ENSG00000204837	RP11-204M4.2	lincRNA	9,54E-03	9,68E-01	9,87E-01	No	1,43E+00	2,96E-05	1,50E-04	No
ENSG00000233244	CYP4F59P	lincRNA	1,64E-02	8,86E-01	NA	No	-1,26E-01	7,12E-01	NA	No
ENSG00000236322	RP11-327I22.8	lincRNA	-2,49E-01	2,06E-01	4,39E-01	No	-1,37E-01	6,90E-01	8,01E-01	No
ENSG00000233424	RP11-175I6.5	lincRNA	-6,45E-03	9,02E-01	NA	No	3,73E-02	8,94E-01	NA	No

ENSG00000229311	RP11-475I24.8	lincRNA	4,03E-02	7,82E-01	NA	No	1,10E-01	6,81E-01	NA	No
ENSG00000237357	RP11-475I24.3	lincRNA	1,78E-01	2,83E-01	5,30E-01	No	3,86E-01	2,92E-01	4,43E-01	No
ENSG00000223379	RP11-374M1.4	lincRNA	-1,28E-02	8,19E-01	NA	No	-1,28E-01	7,12E-01	NA	No
ENSG00000231527	RP11-374M1.2	lincRNA	-8,91E-02	3,78E-01	NA	No	-1,49E-01	4,74E-01	NA	No
ENSG00000232116	RP11-187C18.2	lincRNA	-1,79E-01	4,27E-01	6,68E-01	No	1,21E-02	9,73E-01	9,84E-01	No
ENSG00000225353	RP11-292F9.1	lincRNA	-4,46E-02	7,60E-01	NA	No	-3,34E-02	9,21E-01	NA	No
ENSG00000231212	RP11-111F5.3	lincRNA	-1,38E-02	8,19E-01	NA	No	3,10E-02	9,04E-01	NA	No
ENSG00000204802	RP11-111F5.4	lincRNA	-7,41E-02	5,56E-01	NA	No	1,96E-02	9,45E-01	9,68E-01	No
ENSG00000233651	RP11-111F5.8	lincRNA	1,55E-01	2,70E-01	5,14E-01	No	4,23E-01	2,33E-01	3,75E-01	No
ENSG00000232827	RP11-211N8.7	lincRNA	3,09E-02	6,90E-01	NA	No	2,53E-02	9,01E-01	NA	No
ENSG00000226007	RP11-211N8.2	lincRNA	-3,94E-02	7,65E-01	8,92E-01	No	-1,67E-02	9,52E-01	9,72E-01	No
ENSG00000234665	RP11-262H14.3	lincRNA	6,18E-03	9,74E-01	9,90E-01	No	-7,73E-01	8,11E-02	1,66E-01	No
ENSG00000170161	RP11-262H14.4	lincRNA	6,74E-01	1,42E-03	1,33E-02	No	7,29E-01	1,05E-03	3,86E-03	No
ENSG00000230857	RP11-318K12.2	lincRNA	-4,60E-02	5,96E-01	NA	No	7,92E-02	7,36E-01	NA	No
ENSG00000225411	RP11-764K9.1	lincRNA	2,38E-01	2,57E-02	NA	No	1,41E-01	4,37E-01	NA	No
ENSG00000231242	RP11-87H9.3	lincRNA	-2,27E-01	3,30E-01	5,79E-01	No	2,52E-01	4,70E-01	6,22E-01	No
ENSG00000237543	RP11-58A12.3	lincRNA	-3,83E-01	1,30E-01	3,31E-01	No	-4,19E-01	2,62E-01	4,09E-01	No
ENSG00000240907	BX255923.3	lincRNA	-1,50E-02	8,19E-01	NA	No	-1,28E-01	7,12E-01	NA	No
ENSG00000234394	RP11-561O23.5	lincRNA	6,88E-03	9,65E-01	NA	No	-1,02E-01	8,30E-01	NA	No
ENSG00000261447	RP11-109D9.4	lincRNA	1,69E-01	1,26E-01	NA	No	1,60E-01	4,96E-01	NA	No
ENSG00000225434	RP11-63P12.6	lincRNA	1,33E+00	1,86E-03	1,64E-02	No	8,77E-01	3,85E-02	9,00E-02	No
ENSG00000236849	RP11-151D14.1	lincRNA	4,71E-02	8,29E-01	9,25E-01	No	5,61E-02	8,69E-01	9,21E-01	No
ENSG00000232590	RP11-128I7.1	lincRNA	1,03E-01	6,34E-01	8,19E-01	No	6,59E-01	7,21E-02	1,51E-01	No
ENSG00000260564	RP11-403N16.3	lincRNA	4,56E-02	6,38E-01	NA	No	9,02E-02	8,64E-01	NA	No
ENSG00000230945	RP11-394O9.1	lincRNA	6,11E-02	5,39E-01	NA	No	8,55E-02	8,64E-01	NA	No
ENSG00000233926	RP11-154D17.1	lincRNA	1,46E-01	2,93E-01	NA	No	5,10E-02	8,22E-01	NA	No
ENSG00000228430	RP11-15B24.5	lincRNA	-8,31E-02	5,39E-01	7,56E-01	No	2,65E-02	9,25E-01	9,56E-01	No
ENSG00000227463	RP11-158D2.2	lincRNA	-2,47E-02	8,30E-01	NA	No	3,14E-01	3,25E-01	4,80E-01	No
ENSG00000230303	RP11-213G2.2	lincRNA	-1,60E-01	4,67E-01	7,00E-01	No	6,54E-01	8,31E-02	1,69E-01	No
ENSG00000269994	RP11-276H19.2	lincRNA	4,92E-01	3,79E-02	1,44E-01	No	1,05E+00	3,89E-02	9,07E-02	No
ENSG00000228189	RP13-60M5.2	lincRNA	3,50E-02	9,36E-01	NA	No	8,77E-02	8,64E-01	NA	No
ENSG00000231632	RP11-82L18.4	lincRNA	2,06E-01	2,21E-01	4,59E-01	No	6,70E-01	1,14E-01	2,17E-01	No
ENSG00000237372	RP11-316P17.2	lincRNA	-2,91E-02	7,10E-01	NA	No	4,98E-03	9,92E-01	NA	No
ENSG00000226206	RP5-1050E16.2	lincRNA	3,32E-02	9,36E-01	NA	No	1,10E-01	6,87E-01	NA	No
ENSG00000228216	RP11-367F23.1	lincRNA	4,33E-02	7,20E-01	NA	No	1,66E-01	4,53E-01	NA	No
ENSG00000260454	RP11-367F23.2	lincRNA	-1,17E-03	9,90E-01	NA	No	-1,79E-01	3,75E-01	NA	No
ENSG00000229694	RP11-305L7.6	lincRNA	-4,36E-01	1,05E-01	2,88E-01	No	-9,82E-01	1,55E-02	4,15E-02	No
ENSG00000237422	RP11-305L7.3	lincRNA	9,50E-01	1,65E-02	8,01E-02	No	-4,64E-01	2,25E-01	3,65E-01	No
ENSG00000235641	LINC00484	lincRNA	6,73E-01	1,24E-02	6,55E-02	No	-1,39E+00	1,91E-04	8,23E-04	No
ENSG00000273381	RP11-305L7.7	lincRNA	-7,03E-01	1,21E-02	6,46E-02	No	-1,07E+00	1,26E-03	4,54E-03	No
ENSG00000233081	RP11-440G5.2	lincRNA	8,63E-02	4,20E-01	NA	No	1,40E-01	5,57E-01	NA	No
ENSG00000236717	RP11-100G15.10	lincRNA	1,14E-01	2,29E-01	NA	No	2,72E-01	1,53E-01	NA	No
ENSG00000203364	RP11-370F5.4	lincRNA	2,11E-02	8,16E-01	NA	No	-6,59E-02	8,00E-01	NA	No
ENSG00000269946	RP11-2B6.3	lincRNA	6,45E-02	7,71E-01	8,95E-01	No	7,99E-01	1,74E-02	4,61E-02	No
ENSG00000269929	RP11-2B6.2	lincRNA	-8,64E-02	6,92E-01	8,53E-01	No	3,99E-01	1,79E-01	3,08E-01	No
ENSG00000230262	MIRLET7DHG	lincRNA	1,80E-01	3,93E-01	6,39E-01	No	1,43E-01	6,86E-01	7,97E-01	No
ENSG00000232063	RP11-307E17.8	lincRNA	-7,51E-02	7,33E-01	8,76E-01	No	5,43E-03	9,89E-01	9,94E-01	No
ENSG00000271384	RP11-435O5.7	lincRNA	-3,65E-02	8,34E-01	9,28E-01	No	-4,80E-01	1,67E-01	2,91E-01	No
ENSG00000237857	RP11-435O5.2	lincRNA	3,05E-01	2,06E-01	4,39E-01	No	8,29E-01	1,55E-02	4,16E-02	No
ENSG00000225194	LINC00092	lincRNA	3,53E-01	1,57E-01	3,73E-01	No	-9,42E-01	2,12E-02	5,43E-02	No
ENSG00000237212	RP11-569G13.2	lincRNA	-2,77E-02	7,91E-01	NA	No	-1,94E-01	3,38E-01	NA	No
ENSG00000224848	RP11-535M15.1	lincRNA	-2,80E-01	2,39E-01	4,79E-01	No	-2,20E-01	5,27E-01	6,72E-01	No
ENSG00000242375	RP11-498P14.3	lincRNA	8,33E-03	9,37E-01	NA	No	3,51E-01	2,59E-01	4,06E-01	No
ENSG00000203279	RP11-498P14.5	lincRNA	-5,55E-03	9,65E-01	NA	No	-8,36E-02	7,27E-01	NA	No
ENSG00000235494	RP11-498P14.4	lincRNA	9,17E-02	3,78E-01	NA	No	-2,39E-02	9,21E-01	NA	No
ENSG00000227269	RP11-96L7.2	lincRNA	2,29E-02	8,74E-01	NA	No	-9,82E-02	8,30E-01	NA	No
ENSG00000271086	NAMA	lincRNA	-3,51E-01	1,65E-01	3,85E-01	No	-1,11E+00	1,42E-02	3,86E-02	No
ENSG00000234860	RP11-547C13.1	lincRNA	-1,38E-02	8,77E-01	NA	No	4,25E-02	8,52E-01	NA	No
ENSG00000237461	RP11-554F20.1	lincRNA	-1,86E-01	4,04E-01	6,49E-01	No	-2,99E-01	3,94E-01	5,51E-01	No
ENSG00000270332	RP11-82L2.1	lincRNA	3,15E-02	8,88E-01	9,53E-01	No	9,71E-01	5,65E-03	1,73E-02	No
ENSG00000230013	RP11-217B7.3	lincRNA	-2,81E-01	1,42E-01	3,50E-01	No	-1,29E+00	2,26E-02	5,75E-02	No
ENSG00000225002	RP11-6F6.1	lincRNA	-3,12E-02	8,44E-01	9,32E-01	No	5,67E-02	8,50E-01	9,09E-01	No

ENSG00000234323	RP11-308N19.1	lincRNA	-2,97E-02	8,89E-01	9,54E-01	No	7,36E-01	1,99E-02	5,15E-02	No
ENSG00000229297	RP11-540H22.2	lincRNA	-1,19E-01	NA	NA	No	-1,93E-01	5,77E-01	7,12E-01	No
ENSG00000237062	RP11-308N19.3	lincRNA	8,94E-02	6,20E-01	8,10E-01	No	3,36E-01	3,53E-01	5,09E-01	No
ENSG00000234229	RP11-308N19.4	lincRNA	-3,68E-03	9,83E-01	9,93E-01	No	-3,99E-02	8,98E-01	9,40E-01	No
ENSG00000230030	RP11-505C13.1	lincRNA	-5,58E-02	6,46E-01	NA	No	8,68E-02	6,56E-01	NA	No
ENSG00000231678	RP11-339N8.1	lincRNA	-2,61E-02	8,33E-01	NA	No	1,95E-01	5,13E-01	6,59E-01	No
ENSG00000232896	RP11-410K21.2	lincRNA	-4,17E-02	6,23E-01	NA	No	-2,21E-02	9,17E-01	NA	No
ENSG00000228623	ZNF883	lincRNA	5,92E-01	1,43E-02	7,29E-02	No	1,23E+00	4,04E-05	1,99E-04	No
ENSG00000225684	FAM225B	lincRNA	6,89E-01	3,30E-02	1,31E-01	No	1,35E+00	1,00E-02	2,85E-02	No
ENSG00000233817	RP11-168K11.3	lincRNA	-5,51E-02	6,15E-01	8,07E-01	No	-5,54E-02	8,33E-01	8,98E-01	No
ENSG00000227482	RP11-18B16.2	lincRNA	-3,77E-02	7,11E-01	NA	No	-6,86E-02	7,65E-01	NA	No
ENSG00000235119	RP11-9M16.2	lincRNA	1,04E-01	3,56E-01	NA	No	4,44E-02	8,44E-01	NA	No
ENSG00000230054	RP11-402G3.5	lincRNA	1,12E-01	4,17E-01	6,60E-01	No	5,33E-02	8,56E-01	9,13E-01	No
ENSG00000230601	RP11-402G3.3	lincRNA	4,17E-02	7,65E-01	NA	No	6,19E-02	9,56E-01	NA	No
ENSG00000236461	RP11-523L1.2	lincRNA	4,95E-02	5,41E-01	NA	No	1,08E-01	6,91E-01	NA	No
ENSG00000234692	RP11-445L6.3	lincRNA	4,61E-02	6,21E-01	NA	No	9,98E-02	7,75E-01	NA	No
ENSG00000204148	LINC00474	lincRNA	-5,45E-02	6,46E-01	NA	No	-1,28E-01	7,12E-01	NA	No
ENSG00000228512	RP11-281A20.2	lincRNA	4,85E-01	4,90E-02	1,71E-01	No	8,60E-01	5,82E-02	1,27E-01	No
ENSG00000231901	RP11-281A20.1	lincRNA	1,05E-01	3,04E-01	NA	No	5,41E-01	1,05E-01	2,03E-01	No
ENSG00000261432	RP11-360A18.2	lincRNA	1,13E-01	2,70E-01	NA	No	1,96E-01	3,38E-01	NA	No
ENSG00000225960	RP11-360A18.1	lincRNA	-4,46E-02	7,60E-01	NA	No	-1,03E-01	8,30E-01	NA	No
ENSG00000269979	RP11-498E2.7	lincRNA	-3,88E-01	7,39E-02	2,27E-01	No	-8,94E-02	7,10E-01	8,15E-01	No
ENSG00000231149	RP11-10J18.3	lincRNA	-8,62E-04	8,99E-01	NA	No	-1,26E-01	7,12E-01	NA	No
ENSG00000229582	RP11-423C15.3	lincRNA	7,78E-02	3,67E-01	NA	No	7,02E-01	3,43E-02	8,16E-02	No
ENSG00000272960	RP11-339B21.15	lincRNA	-6,14E-02	7,65E-01	8,92E-01	No	1,41E-02	9,53E-01	9,73E-01	No
ENSG00000273281	RP11-339B21.14	lincRNA	1,21E-01	5,81E-01	7,83E-01	No	6,25E-01	1,46E-02	3,95E-02	No
ENSG00000272593	RP11-339B21.11	lincRNA	-2,70E-01	2,47E-01	4,88E-01	No	-6,35E-01	7,11E-02	1,49E-01	No
ENSG00000207955	MIR219-2	lincRNA	1,78E-02	9,23E-01	9,68E-01	No	-2,10E-01	5,42E-01	6,84E-01	No
ENSG00000267834	RP11-167N5.5	lincRNA	2,18E-02	9,15E-01	9,64E-01	No	-7,84E-01	7,76E-02	1,60E-01	No
ENSG00000268707	RP11-247A12.7	lincRNA	8,95E-02	5,62E-01	NA	No	-2,51E-01	2,96E-01	NA	No
ENSG00000235007	RP11-344B5.4	lincRNA	4,19E-02	7,48E-01	NA	No	6,34E-02	9,56E-01	NA	No
ENSG00000224307	RP11-344B5.2	lincRNA	8,56E-01	1,40E-02	7,16E-02	No	-4,69E-02	8,91E-01	9,35E-01	No
ENSG00000268615	RP11-65J3.15	lincRNA	1,59E-01	1,34E-01	NA	No	1,97E-01	3,41E-01	NA	No
ENSG00000230676	RP11-65J3.3	lincRNA	9,30E-03	9,61E-01	NA	No	1,08E-01	6,42E-01	NA	No
ENSG00000226355	RP11-65J3.2	lincRNA	3,31E-02	9,36E-01	NA	No	8,51E-02	8,64E-01	NA	No
ENSG00000261334	RP11-65J3.14	lincRNA	6,27E-02	6,55E-01	8,30E-01	No	-1,16E-01	6,35E-01	7,59E-01	No
ENSG00000239353	RP11-492E3.51	lincRNA	5,97E-02	6,85E-01	8,49E-01	No	-3,72E-01	2,23E-01	NA	No
ENSG00000270755	RP11-138E2.1	lincRNA	1,41E-01	3,93E-01	6,39E-01	No	6,31E-01	1,20E-01	2,27E-01	No
ENSG00000273473	LL09NC01-139C3.1	lincRNA	4,31E-02	6,39E-01	NA	No	4,81E-02	8,13E-01	NA	No
ENSG00000223729	RP11-145E17.2	lincRNA	-5,69E-02	6,46E-01	NA	No	-1,35E-01	7,12E-01	NA	No
ENSG00000228877	RP11-473E2.4	lincRNA	1,26E-01	4,79E-01	7,10E-01	No	3,56E-01	3,07E-01	4,60E-01	No
ENSG00000227150	RP11-473E2.3	lincRNA	-1,02E-02	8,49E-01	NA	No	-2,07E-02	9,21E-01	NA	No
ENSG00000236403	RP11-447M12.2	lincRNA	4,46E-02	7,42E-01	NA	No	9,88E-02	7,75E-01	NA	No
ENSG00000260193	RP11-83N9.5	lincRNA	-6,58E-01	2,86E-02	1,18E-01	No	-4,27E-01	2,40E-01	3,84E-01	No
ENSG00000264527	WI2-1959D15.1	lincRNA	-6,34E-02	4,94E-01	NA	No	-1,52E-01	5,59E-01	NA	No
ENSG00000235117	RP11-229P13.20	lincRNA	5,35E-01	6,07E-02	1,98E-01	No	-4,80E-01	1,75E-01	3,02E-01	No
ENSG00000237419	RP11-885N19.6	lincRNA	1,61E-02	8,44E-01	NA	No	-3,02E-02	8,75E-01	NA	No
ENSG00000233013	FAM157B	lincRNA	-3,27E-02	8,23E-01	9,23E-01	No	1,57E-01	6,40E-01	7,62E-01	No
ENSG00000272992	RP11-809C18.4	lincRNA	-1,81E-01	4,14E-01	6,57E-01	No	-1,38E+00	6,99E-03	2,08E-02	No
ENSG00000229205	LINC00200	lincRNA	3,09E-02	7,10E-01	NA	No	1,26E-01	5,62E-01	NA	No
ENSG00000273001	RP11-118K6.3	lincRNA	-7,27E-01	2,60E-02	1,11E-01	No	-2,96E-01	3,74E-01	5,31E-01	No
ENSG00000226762	RP11-298E9.6	lincRNA	3,32E-02	9,36E-01	NA	No	6,67E-02	9,56E-01	NA	No
ENSG00000233321	RP11-482E14.1	lincRNA	1,62E-01	1,39E-01	NA	No	5,10E-01	7,75E-02	NA	No
ENSG00000236892	RP11-482E14.2	lincRNA	-1,34E-02	8,19E-01	NA	No	-1,26E-01	7,12E-01	NA	No
ENSG00000230573	RP11-464C19.2	lincRNA	3,32E-02	9,36E-01	NA	No	8,55E-02	8,64E-01	NA	No
ENSG00000226005	RP11-464C19.3	lincRNA	-5,96E-03	9,75E-01	9,90E-01	No	9,96E-01	1,21E-02	3,36E-02	No
ENSG00000227101	RP11-433J20.2	lincRNA	2,35E-02	9,72E-01	NA	No	8,55E-02	8,64E-01	NA	No
ENSG00000236990	RP11-433J20.1	lincRNA	2,47E-03	9,89E-01	9,96E-01	No	4,03E-02	9,02E-01	9,42E-01	No
ENSG00000233117	LINC00702	lincRNA	-1,79E-01	4,21E-01	6,63E-01	No	2,17E-02	9,47E-01	9,69E-01	No
ENSG00000224382	LINC00703	lincRNA	3,32E-02	9,36E-01	NA	No	8,64E-02	8,64E-01	NA	No
ENSG00000231298	LINC00704	lincRNA	2,20E-02	8,95E-01	9,56E-01	No	1,37E+00	6,80E-03	2,03E-02	No
ENSG00000225269	LINC00705	lincRNA	5,33E-03	9,75E-01	9,90E-01	No	4,92E-01	2,05E-01	3,40E-01	No

ENSG00000231039	RP11-445P17.3	lincRNA	1,14E-01	4,95E-01	7,23E-01	No	-3,47E-01	1,62E-01	NA	No
ENSG00000224034	RP11-445P17.8	lincRNA	2,84E-02	7,81E-01	NA	No	2,77E-01	3,11E-01	4,64E-01	No
ENSG00000231483	RP11-336A10.5	lincRNA	-3,12E-03	9,54E-01	NA	No	-5,83E-02	7,35E-01	NA	No
ENSG00000228951	RP11-336A10.4	lincRNA	3,66E-03	9,89E-01	NA	No	-1,26E-01	7,12E-01	NA	No
ENSG00000272764	RP11-318E3.9	lincRNA	-9,33E-02	5,61E-01	7,70E-01	No	-2,41E-01	4,60E-01	6,13E-01	No
ENSG00000215244	RP11-563J2.2	lincRNA	1,14E+00	3,26E-04	4,37E-03	No	-4,97E-01	1,63E-01	2,85E-01	No
ENSG00000225948	RP11-554I8.1	lincRNA	2,51E-01	8,42E-02	NA	No	-1,26E-01	7,12E-01	NA	No
ENSG00000223784	RP11-554I8.2	lincRNA	-3,21E-01	1,93E-01	4,23E-01	No	3,32E-01	3,37E-01	4,93E-01	No
ENSG00000232591	RP5-1031D4.2	lincRNA	1,19E-01	2,07E-01	NA	No	1,10E-01	6,87E-01	NA	No
ENSG00000197308	GATA3-AS1	lincRNA	-8,62E-03	9,24E-01	NA	No	2,66E-03	9,92E-01	9,96E-01	No
ENSG00000226990	RP11-543F8.2	lincRNA	-3,96E-02	7,26E-01	NA	No	-1,10E-01	6,38E-01	NA	No
ENSG00000270111	RP11-271F18.4	lincRNA	-5,37E-02	6,46E-01	NA	No	-5,07E-02	8,00E-01	NA	No
ENSG00000225383	SFTA1P	lincRNA	4,48E-01	5,94E-02	1,95E-01	No	8,91E-01	4,71E-02	1,06E-01	No
ENSG00000231496	RP1-251M9.2	lincRNA	-9,49E-02	6,36E-01	8,20E-01	No	2,29E-01	5,11E-01	6,57E-01	No
ENSG00000271046	RP11-138I18.2	lincRNA	4,21E-02	6,75E-01	NA	No	-1,26E-01	7,12E-01	NA	No
ENSG00000272853	RP11-398C13.6	lincRNA	2,19E-01	3,37E-01	5,86E-01	No	5,52E-01	1,39E-01	2,54E-01	No
ENSG00000273153	RP11-406H21.2	lincRNA	3,56E-01	1,34E-01	3,38E-01	No	-3,20E-01	3,09E-01	4,62E-01	No
ENSG00000152487	ARL5B-AS1	lincRNA	9,54E-01	1,83E-03	1,62E-02	No	6,91E-01	2,52E-02	6,28E-02	No
ENSG00000230109	RP11-275N1.1	lincRNA	3,81E-02	8,45E-01	NA	No	1,13E-01	6,49E-01	NA	No
ENSG00000260205	RP11-108B14.5	lincRNA	-4,96E-02	5,81E-01	NA	No	-1,53E-01	5,47E-01	NA	No
ENSG00000261671	RP11-573G6.6	lincRNA	1,40E-01	4,95E-01	7,24E-01	No	1,32E+00	6,60E-03	1,98E-02	No
ENSG00000272516	RP11-573G6.9	lincRNA	4,85E-03	9,97E-01	NA	No	4,08E-01	1,91E-01	NA	No
ENSG00000272366	RP11-573G6.10	lincRNA	-1,17E-02	8,99E-01	NA	No	2,08E-02	9,04E-01	NA	No
ENSG00000273107	RP11-165A20.3	lincRNA	8,63E-01	2,10E-02	9,53E-02	No	2,57E-01	4,74E-01	6,25E-01	No
ENSG00000231422	RP11-80K21.3	lincRNA	4,17E-02	7,65E-01	NA	No	8,77E-02	8,64E-01	NA	No
ENSG00000236894	RP11-128B16.3	lincRNA	3,43E-01	1,35E-02	NA	No	9,81E-02	7,75E-01	NA	No
ENSG00000231976	LINC00202-2	lincRNA	4,43E-02	6,85E-01	NA	No	1,42E-01	5,73E-01	NA	No
ENSG00000232224	LINC00202-1	lincRNA	-5,99E-01	5,06E-02	1,75E-01	No	-1,44E+00	1,85E-03	6,42E-03	No
ENSG00000236983	LINC00614	lincRNA	-2,93E-01	1,11E-01	2,99E-01	No	-7,15E-01	7,51E-02	1,56E-01	No
ENSG00000262412	RP11-85G18.6	lincRNA	-1,44E-01	2,95E-01	NA	No	-3,82E-01	1,20E-01	NA	No
ENSG00000260151	RP11-748L13.6	lincRNA	7,74E-02	3,58E-01	NA	No	3,05E-01	1,89E-01	NA	No
ENSG00000233472	RP11-218D6.4	lincRNA	1,06E-01	4,09E-01	NA	No	1,10E-01	6,57E-01	NA	No
ENSG00000237128	RP11-351M16.3	lincRNA	-6,86E-02	4,48E-01	NA	No	-1,39E-01	5,24E-01	NA	No
ENSG00000228800	RP11-253D19.1	lincRNA	5,05E-02	5,27E-01	NA	No	1,71E-01	4,49E-01	NA	No
ENSG00000230998	RP11-14C22.3	lincRNA	-6,40E-02	4,76E-01	NA	No	-1,55E-01	5,35E-01	NA	No
ENSG00000272381	RP11-192P3.4	lincRNA	2,17E-01	3,49E-01	5,97E-01	No	1,21E-02	9,73E-01	9,84E-01	No
ENSG00000223834	RP11-472N13.2	lincRNA	1,66E-01	8,67E-02	NA	No	1,13E-01	6,28E-01	NA	No
ENSG00000237797	RP11-472N13.3	lincRNA	2,07E-01	2,38E-01	4,78E-01	No	9,39E-01	5,23E-02	1,16E-01	No
ENSG00000273038	RP11-479G22.8	lincRNA	-5,31E-01	4,62E-02	1,65E-01	No	-2,85E-01	3,38E-01	4,93E-01	No
ENSG00000229656	RP11-462L8.1	lincRNA	-5,53E-02	7,78E-01	8,99E-01	No	-3,69E-01	3,15E-01	4,69E-01	No
ENSG00000233387	RP11-342D11.3	lincRNA	1,73E-01	3,00E-01	5,48E-01	No	-1,41E-01	6,19E-01	7,46E-01	No
ENSG00000273012	RP11-90B22.1	lincRNA	3,96E-02	6,33E-01	NA	No	6,73E-01	6,38E-02	1,37E-01	No
ENSG00000233200	RP11-324I22.2	lincRNA	-2,35E-01	4,95E-02	NA	No	-3,61E+00	2,39E-02	6,01E-02	No
ENSG00000227313	RP11-309N24.1	lincRNA	-5,45E-02	6,46E-01	NA	No	4,11E-02	8,54E-01	NA	No
ENSG00000227475	RP11-810B23.1	lincRNA	-1,53E-02	8,19E-01	NA	No	-2,53E-02	8,90E-01	NA	No
ENSG00000237002	SNORA40	lincRNA	-5,69E-02	6,46E-01	NA	No	-1,35E-01	7,12E-01	NA	No
ENSG00000226578	RP11-258F22.1	lincRNA	6,34E-02	7,71E-01	8,95E-01	No	1,69E-01	6,30E-01	7,54E-01	No
ENSG00000260137	RP11-258F22.2	lincRNA	-2,03E-01	2,13E-01	4,49E-01	No	-3,73E-01	3,02E-01	4,55E-01	No
ENSG00000272983	RP11-508N22.12	lincRNA	3,26E-01	1,81E-01	4,06E-01	No	3,63E-01	1,70E-01	2,95E-01	No
ENSG00000273019	RP11-508N22.13	lincRNA	3,71E-02	6,78E-01	NA	No	-7,68E-02	6,84E-01	NA	No
ENSG00000226113	RP11-672F9.1	lincRNA	3,68E-02	8,45E-01	NA	No	9,02E-02	8,64E-01	NA	No
ENSG00000185904	LINC00839	lincRNA	-1,40E-02	9,20E-01	9,67E-01	No	6,70E-01	1,02E-01	1,99E-01	No
ENSG00000259869	AL022344.7	lincRNA	-9,97E-02	2,87E-01	NA	No	-5,44E-02	8,44E-01	9,05E-01	No
ENSG00000273008	RP11-351D16.3	lincRNA	-2,38E-02	9,02E-01	9,59E-01	No	8,04E-02	8,19E-01	8,89E-01	No
ENSG00000230555	RP11-517P14.2	lincRNA	-2,45E-02	9,09E-01	9,62E-01	No	-4,28E-01	1,83E-01	3,13E-01	No
ENSG00000204187	LINC00619	lincRNA	-6,45E-02	7,29E-01	8,74E-01	No	3,01E-01	4,07E-01	5,63E-01	No
ENSG00000226808	LINC00840	lincRNA	-8,67E-01	1,54E-02	7,62E-02	No	-1,36E-01	6,65E-01	7,81E-01	No
ENSG00000236769	RP11-168P8.5	lincRNA	-5,95E-02	5,13E-01	NA	No	-1,24E-01	5,70E-01	NA	No
ENSG00000227029	RP11-168P8.3	lincRNA	-1,38E-01	1,53E-01	3,67E-01	No	-2,61E-01	2,68E-01	4,16E-01	No
ENSG00000233395	LINC00841	lincRNA	-2,74E-01	2,83E-02	1,17E-01	No	-5,48E-01	8,11E-02	1,66E-01	No
ENSG00000223462	RP11-285G1.9	lincRNA	9,46E-01	1,32E-02	6,85E-02	No	-1,39E-01	6,91E-01	8,01E-01	No
ENSG00000227683	RP11-445N18.5	lincRNA	6,00E-02	5,58E-01	NA	No	1,12E-01	6,44E-01	NA	No

ENSG00000230761	RP11-342C24.8	lincRNA	6,33E-03	9,92E-01	NA	No	-1,30E-01	7,12E-01	NA	No
ENSG00000272430	RP11-38L15.8	lincRNA	-1,11E-01	5,66E-01	7,73E-01	No	6,24E-01	1,22E-01	2,29E-01	No
ENSG00000229227	RP11-38L15.2	lincRNA	-3,95E-02	7,60E-01	NA	No	8,58E-03	9,72E-01	NA	No
ENSG00000223477	LINC00842	lincRNA	2,90E-01	1,72E-01	3,95E-01	No	1,90E-01	5,81E-01	7,14E-01	No
ENSG00000259942	RP11-292F22.7	lincRNA	7,71E-03	9,11E-01	NA	No	-3,60E-02	8,90E-01	NA	No
ENSG00000232426	RP11-508M1.7	lincRNA	-2,09E-02	7,72E-01	NA	No	-3,12E-02	8,03E-01	NA	No
ENSG00000272225	RP11-592B15.9	lincRNA	2,35E-02	9,72E-01	NA	No	9,20E-02	8,64E-01	NA	No
ENSG00000178440	LINC00843	lincRNA	-1,32E-01	4,84E-01	7,14E-01	No	7,39E-02	7,45E-01	8,39E-01	No
ENSG00000261368	RP11-96B5.4	lincRNA	-7,20E-02	4,55E-01	NA	No	-5,99E-02	7,81E-01	NA	No
ENSG00000231131	RP11-346D6.6	lincRNA	-5,71E-02	5,14E-01	NA	No	-3,38E-02	8,32E-01	NA	No
ENSG00000223800	RP11-598C10.2	lincRNA	-7,23E-02	3,10E-01	NA	No	-1,79E-01	3,80E-01	NA	No
ENSG00000228527	RP11-179B15.5	lincRNA	1,47E-02	8,99E-01	NA	No	7,55E-02	7,82E-01	8,65E-01	No
ENSG00000261076	RP11-179B15.6	lincRNA	1,19E-01	2,14E-01	NA	No	1,25E-01	5,21E-01	NA	No
ENSG00000237949	LINC00844	lincRNA	-7,40E-01	2,16E-02	9,69E-02	No	9,97E-01	8,54E-03	2,48E-02	No
ENSG00000235140	RP11-135D11.2	lincRNA	-9,04E-02	2,33E-01	NA	No	-2,12E-01	2,89E-01	NA	No
ENSG00000227877	LINC00948	lincRNA	-1,23E-01	8,83E-02	NA	No	-2,99E-01	1,22E-01	NA	No
ENSG00000233643	RP11-491H19.1	lincRNA	4,77E-03	9,27E-01	NA	No	8,74E-02	6,64E-01	NA	No
ENSG00000234756	RP11-120C12.3	lincRNA	5,90E-02	5,71E-01	NA	No	8,55E-02	8,64E-01	NA	No
ENSG00000228566	RP11-170M17.1	lincRNA	1,79E-01	1,41E-01	NA	No	2,64E-01	2,75E-01	NA	No
ENSG00000226426	RP11-174J11.1	lincRNA	-2,97E-04	9,89E-01	NA	No	1,50E-02	9,14E-01	NA	No
ENSG00000224714	RP11-179K3.2	lincRNA	3,46E-02	9,36E-01	NA	No	8,77E-02	8,64E-01	NA	No
ENSG00000228065	RP11-222A11.1	lincRNA	1,72E-01	4,44E-01	6,81E-01	No	4,90E-01	1,04E-01	2,02E-01	No
ENSG00000272892	RP11-57G10.8	lincRNA	2,03E-01	1,75E-01	3,99E-01	No	2,41E-01	4,20E-01	5,76E-01	No
ENSG00000233590	RP11-153K11.3	lincRNA	-6,76E-03	8,90E-01	NA	No	9,65E-02	6,93E-01	NA	No
ENSG00000224222	RP11-262I2.2	lincRNA	-1,02E-01	4,53E-01	6,89E-01	No	-2,35E-01	4,05E-01	5,62E-01	No
ENSG00000259267	RP11-432J9.6	lincRNA	-6,42E-02	4,70E-01	NA	No	-1,56E-01	5,23E-01	NA	No
ENSG00000272988	RP11-150D20.5	lincRNA	5,53E-02	5,93E-01	NA	No	-1,30E-01	7,12E-01	NA	No
ENSG00000272630	RP11-344N10.5	lincRNA	1,23E-01	5,85E-01	7,87E-01	No	-2,00E-01	4,83E-01	6,33E-01	No
ENSG00000272791	RP11-464F9.22	lincRNA	1,08E-01	5,72E-01	7,77E-01	No	1,19E-01	7,35E-01	8,32E-01	No
ENSG00000272140	RP11-574K11.29	lincRNA	4,73E-01	6,78E-02	2,15E-01	No	-2,35E-01	4,07E-01	5,64E-01	No
ENSG00000267957	RP11-178G16.4	lincRNA	2,60E-02	8,92E-01	9,55E-01	No	7,00E-01	1,41E-03	5,02E-03	No
ENSG00000269772	RP11-178G16.5	lincRNA	-1,67E-01	3,90E-01	6,35E-01	No	3,52E-01	1,03E-01	2,01E-01	No
ENSG00000226051	ZNF503-AS1	lincRNA	2,90E-01	2,15E-01	4,51E-01	No	1,34E-01	6,97E-01	8,05E-01	No
ENSG00000270087	RP11-399K21.11	lincRNA	6,96E-02	6,87E-01	8,50E-01	No	3,02E-01	3,85E-01	5,42E-01	No
ENSG00000273248	RP11-399K21.13	lincRNA	-1,42E-02	8,49E-01	NA	No	-1,75E-02	9,21E-01	NA	No
ENSG00000272692	RP11-399K21.14	lincRNA	1,78E-01	3,41E-01	5,90E-01	No	-1,59E-01	6,04E-01	7,33E-01	No
ENSG00000230417	LINC00856	lincRNA	-1,72E-01	1,83E-01	4,09E-01	No	-6,12E-01	8,46E-02	1,71E-01	No
ENSG00000227136	LINC00595	lincRNA	1,65E-02	8,89E-01	NA	No	6,96E-02	7,76E-01	NA	No
ENSG00000229543	RP11-90J7.2	lincRNA	-5,45E-02	6,46E-01	NA	No	-1,28E-01	7,12E-01	NA	No
ENSG00000228683	RP11-31E13.2	lincRNA	2,46E-02	7,34E-01	NA	No	-4,69E-02	8,79E-01	NA	No
ENSG00000229569	RP11-481G8.2	lincRNA	2,07E-01	4,91E-02	NA	No	2,16E-01	2,96E-01	NA	No
ENSG00000244733	RP11-506M13.3	lincRNA	-4,32E-01	9,41E-02	2,67E-01	No	6,79E-03	9,81E-01	9,88E-01	No
ENSG00000225484	RP11-773D16.1	lincRNA	-4,05E-01	6,88E-02	2,17E-01	No	-4,17E-01	6,32E-02	1,36E-01	No
ENSG00000272447	RP11-182L21.6	lincRNA	-5,65E-01	2,64E-02	1,12E-01	No	2,33E-02	9,36E-01	9,62E-01	No
ENSG00000273372	RP11-479O17.10	lincRNA	-5,94E-01	3,28E-02	1,30E-01	No	-3,28E-01	3,22E-01	4,76E-01	No
ENSG00000237523	LINC00857	lincRNA	2,36E-02	9,15E-01	9,64E-01	No	8,29E-02	8,08E-01	8,81E-01	No
ENSG00000271738	RP11-137H2.6	lincRNA	-1,16E+00	5,89E-09	3,93E-07	No	-4,31E-01	2,26E-02	5,74E-02	No
ENSG00000229404	LINC00858	lincRNA	3,39E-02	9,36E-01	NA	No	8,55E-02	8,64E-01	NA	No
ENSG00000224914	LINC00863	lincRNA	-3,49E-01	5,64E-02	1,88E-01	No	-4,53E-01	1,47E-02	3,99E-02	No
ENSG00000225913	RP11-57C13.6	lincRNA	-3,43E-02	6,58E-01	NA	No	-6,30E-02	6,91E-01	NA	No
ENSG00000225836	RP11-80H5.6	lincRNA	-1,36E-02	8,19E-01	NA	No	2,00E-01	4,62E-01	NA	No
ENSG00000235100	RP11-80H5.9	lincRNA	3,08E-01	1,73E-01	3,96E-01	No	1,14E+00	2,20E-02	5,62E-02	No
ENSG00000240996	RP11-80H5.7	lincRNA	3,63E-01	1,44E-01	3,53E-01	No	1,23E+00	9,90E-03	2,82E-02	No
ENSG00000232229	LINC00865	lincRNA	9,91E-02	6,35E-01	8,19E-01	No	4,46E-01	2,37E-01	3,79E-01	No
ENSG00000236373	RP11-15K3.1	lincRNA	1,31E-02	8,37E-01	NA	No	5,07E-02	7,51E-01	NA	No
ENSG00000224750	RP11-94M14.2	lincRNA	1,21E-01	2,93E-01	NA	No	8,51E-02	8,64E-01	NA	No
ENSG00000225519	RP11-236B18.2	lincRNA	-2,26E-01	2,16E-01	4,53E-01	No	-4,44E-01	2,27E-01	3,68E-01	No
ENSG00000273124	RP11-236B18.5	lincRNA	-2,00E-01	2,29E-01	4,68E-01	No	-2,05E-01	5,08E-01	6,55E-01	No
ENSG00000224851	LINC00502	lincRNA	-6,12E-02	4,94E-01	NA	No	-1,25E-01	5,67E-01	NA	No
ENSG00000231829	RP11-310E22.5	lincRNA	-2,80E-01	1,87E-01	4,14E-01	No	-3,42E-01	3,43E-01	4,99E-01	No
ENSG00000227356	LINC00866	lincRNA	5,04E-03	9,65E-01	NA	No	-3,34E-02	9,21E-01	NA	No
ENSG00000224934	RP11-441O15.3	lincRNA	1,51E-01	4,65E-01	6,99E-01	No	2,03E-01	4,22E-01	5,78E-01	No

ENSG00000273030	RP11-285F16.1	lincRNA	4,85E-02	6,23E-01	NA	No	-2,85E-02	9,01E-01	NA	No
ENSG00000273476	RP11-108L7.14	lincRNA	8,38E-02	6,04E-01	8,00E-01	No	1,59E-01	6,11E-01	7,40E-01	No
ENSG00000273162	RP11-108L7.15	lincRNA	1,57E-01	2,91E-01	NA	No	1,92E-01	4,94E-01	6,43E-01	No
ENSG00000225208	RP11-107I14.2	lincRNA	1,83E-01	1,04E-01	NA	No	1,64E-01	4,37E-01	NA	No
ENSG00000230967	RP11-107I14.5	lincRNA	7,37E-02	4,05E-01	NA	No	6,54E-02	9,56E-01	NA	No
ENSG00000224817	RP11-190J1.3	lincRNA	5,58E-04	9,96E-01	NA	No	-3,15E-02	8,98E-01	NA	No
ENSG00000272933	RP11-47A8.5	lincRNA	-7,10E-01	9,47E-03	5,43E-02	No	3,79E-01	1,17E-01	2,22E-01	No
ENSG00000272912	RP11-724N1.1	lincRNA	-6,86E-02	5,79E-01	7,82E-01	No	-2,34E-01	3,63E-01	5,19E-01	No
ENSG00000225768	RP11-127O4.3	lincRNA	3,41E-02	9,36E-01	NA	No	6,67E-02	9,56E-01	NA	No
ENSG00000229981	RP11-215N21.1	lincRNA	-2,77E-02	8,39E-01	9,30E-01	No	8,08E-02	8,02E-01	8,78E-01	No
ENSG00000203434	RP11-163F15.1	lincRNA	2,42E-02	9,72E-01	NA	No	1,09E-01	6,87E-01	NA	No
ENSG00000273143	RP11-525A16.4	lincRNA	2,68E-01	3,96E-02	NA	No	2,50E-01	3,04E-01	NA	No
ENSG00000232470	RP11-313D6.3	lincRNA	1,30E-01	3,57E-01	6,05E-01	No	-2,21E-01	3,09E-01	NA	No
ENSG00000228484	RP11-106M7.1	lincRNA	-8,80E-02	6,64E-01	8,35E-01	No	-6,13E-01	1,24E-01	2,33E-01	No
ENSG00000236799	RP11-383C6.2	lincRNA	1,34E-01	3,23E-01	NA	No	2,69E-02	9,06E-01	NA	No
ENSG00000258114	CTA-109P11.4	lincRNA	-4,15E-02	7,60E-01	NA	No	-9,51E-02	8,30E-01	NA	No
ENSG00000234952	RP11-328K15.1	lincRNA	3,85E-02	8,45E-01	NA	No	8,58E-02	8,64E-01	NA	No
ENSG00000232139	LINC00867	lincRNA	-1,83E-03	9,83E-01	NA	No	3,51E-02	8,25E-01	NA	No
ENSG00000227307	RP11-95I16.2	lincRNA	-3,43E-02	7,31E-01	NA	No	-1,35E-01	7,12E-01	NA	No
ENSG00000230131	RP11-282I1.1	lincRNA	8,91E-02	6,63E-01	8,35E-01	No	-3,62E-01	3,14E-01	4,68E-01	No
ENSG00000225152	RP11-338O1.2	lincRNA	-1,26E-02	9,22E-01	NA	No	-2,17E-01	3,09E-01	NA	No
ENSG00000228021	RP11-383C5.3	lincRNA	1,77E-02	8,64E-01	NA	No	2,44E-02	9,25E-01	9,56E-01	No
ENSG00000224023	RP11-383C5.4	lincRNA	1,87E-01	3,44E-01	5,92E-01	No	1,38E-01	6,90E-01	8,00E-01	No
ENSG00000224190	RP11-442O18.2	lincRNA	2,32E-01	9,65E-02	NA	No	3,14E-01	1,84E-01	3,14E-01	No
ENSG00000227374	RP11-109A6.3	lincRNA	6,14E-01	3,96E-02	1,49E-01	No	-5,16E-01	1,85E-01	3,16E-01	No
ENSG00000237489	LINC00959	lincRNA	6,59E-01	2,13E-02	9,62E-02	No	6,16E-01	5,81E-02	1,27E-01	No
ENSG00000231705	RP11-432J24.2	lincRNA	-1,87E-02	8,84E-01	9,52E-01	No	-2,93E-01	3,05E-01	NA	No
ENSG00000226900	RP11-432J24.5	lincRNA	-1,01E+00	4,16E-04	5,28E-03	No	1,45E-01	5,41E-01	6,83E-01	No
ENSG00000234531	RP11-288G11.3	lincRNA	4,41E-02	6,93E-01	NA	No	1,17E-01	5,88E-01	NA	No
ENSG00000232903	LINC01166	lincRNA	-5,65E-03	9,72E-01	9,89E-01	No	-7,55E-01	3,54E-02	8,38E-02	No
ENSG00000224758	LINC01167	lincRNA	1,02E-01	3,56E-01	NA	No	-2,69E-03	9,66E-01	NA	No
ENSG00000240707	LINC01168	lincRNA	1,28E-01	3,87E-01	6,33E-01	No	-3,27E-01	1,68E-01	NA	No
ENSG00000270030	RP11-326C3.13	lincRNA	-9,61E-02	6,44E-01	8,24E-01	No	-1,41E+00	1,30E-02	3,58E-02	No
ENSG00000255328	RP11-326C3.12	lincRNA	1,03E-01	5,63E-01	7,71E-01	No	-3,44E-01	2,82E-01	4,31E-01	No
ENSG00000270972	RP11-326C3.15	lincRNA	8,56E-02	5,73E-01	7,78E-01	No	-4,67E-01	1,65E-01	2,89E-01	No
ENSG00000247095	MIR210HG	lincRNA	-7,53E-03	9,70E-01	9,88E-01	No	1,68E-01	5,61E-01	6,99E-01	No
ENSG00000229671	LINC01150	lincRNA	1,32E-01	3,36E-01	NA	No	-1,13E-01	6,17E-01	NA	No
ENSG00000232987	AC051649.6	lincRNA	4,51E-02	6,57E-01	NA	No	-1,55E-01	5,35E-01	NA	No
ENSG00000254757	RP13-726E6.1	lincRNA	3,28E-02	8,17E-01	NA	No	-9,51E-02	8,30E-01	NA	No
ENSG00000254480	RP11-23F23.2	lincRNA	-6,72E-02	5,62E-01	NA	No	-6,53E-02	7,85E-01	8,67E-01	No
ENSG00000255257	AC025016.1	lincRNA	5,27E-02	4,97E-01	NA	No	6,34E-02	9,56E-01	NA	No
ENSG00000267940	RP11-290F24.6	lincRNA	7,25E-01	3,18E-02	1,27E-01	No	4,84E-01	2,01E-01	3,35E-01	No
ENSG00000254443	RP11-304C12.3	lincRNA	-1,83E-01	2,24E-01	4,62E-01	No	-4,83E-01	1,23E-01	2,30E-01	No
ENSG00000255179	RP11-324J3.1	lincRNA	2,35E-02	9,72E-01	NA	No	1,07E-01	6,99E-01	NA	No
ENSG00000246820	RP11-379P15.1	lincRNA	5,32E-02	7,01E-01	8,59E-01	No	-2,92E-01	2,22E-01	NA	No
ENSG00000245522	RP11-540A21.2	lincRNA	-2,71E-01	2,52E-01	4,95E-01	No	-5,27E-01	1,28E-01	2,38E-01	No
ENSG00000250041	CTD-2003C8.2	lincRNA	-1,34E+00	3,14E-03	2,44E-02	No	-1,06E+00	1,96E-02	5,09E-02	No
ENSG00000255260	CTD-3224I3.3	lincRNA	-1,89E-01	1,04E-01	NA	No	-2,05E-01	4,81E-01	6,32E-01	No
ENSG00000254486	RP13-631K18.2	lincRNA	7,93E-02	3,59E-01	NA	No	1,72E-01	2,97E-01	NA	No
ENSG00000254991	RP13-631K18.3	lincRNA	5,90E-02	5,71E-01	NA	No	1,09E-01	6,78E-01	NA	No
ENSG00000255400	RP13-631K18.5	lincRNA	-1,84E-02	8,42E-01	NA	No	1,04E-01	6,61E-01	7,78E-01	No
ENSG00000254847	RP11-51B23.3	lincRNA	-3,43E-01	1,68E-01	3,90E-01	No	-8,97E-01	2,87E-02	7,03E-02	No
ENSG00000251381	LINC00958	lincRNA	-6,47E-02	4,56E-01	NA	No	-1,00E-01	6,01E-01	NA	No
ENSG00000254946	RP11-531H8.1	lincRNA	3,32E-02	9,36E-01	NA	No	1,10E-01	6,90E-01	NA	No
ENSG00000254789	RP11-531H8.2	lincRNA	-5,37E-03	8,99E-01	NA	No	7,98E-02	7,17E-01	NA	No
ENSG00000254645	RP11-396O20.2	lincRNA	-2,05E-02	8,19E-01	NA	No	-7,78E-04	9,82E-01	NA	No
ENSG00000255160	RP11-428C19.5	lincRNA	2,08E-01	3,47E-01	5,95E-01	No	-1,34E-01	6,94E-01	8,03E-01	No
ENSG00000270607	RP11-359E10.1	lincRNA	-1,23E-01	5,80E-01	7,83E-01	No	-1,86E-01	4,66E-01	6,19E-01	No
ENSG00000255418	RP11-266A24.1	lincRNA	1,88E-01	4,12E-01	6,55E-01	No	6,12E-01	9,19E-02	1,83E-01	No
ENSG00000255193	RP11-945A11.1	lincRNA	-3,01E-02	7,31E-01	NA	No	1,23E-01	5,69E-01	NA	No
ENSG00000254861	RP11-945A11.2	lincRNA	9,89E-03	9,66E-01	NA	No	-9,82E-02	8,30E-01	NA	No
ENSG00000254456	RP11-405K6.1	lincRNA	3,50E-03	9,89E-01	NA	No	1,81E-01	4,62E-01	NA	No

ENSG00000254754	RP11-20J1.1	lincRNA	2,52E-02	8,04E-01	NA	No	8,43E-02	6,54E-01	NA	No
ENSG00000255243	CTD-2507G9.1	lincRNA	9,77E-02	3,36E-01	NA	No	1,63E-01	4,85E-01	NA	No
ENSG00000254934	LINC00678	lincRNA	6,32E-01	3,39E-02	1,33E-01	No	9,19E-02	7,71E-01	8,57E-01	No
ENSG00000255322	RP11-22P4.1	lincRNA	2,34E-03	9,93E-01	9,97E-01	No	6,62E-02	8,39E-01	9,02E-01	No
ENSG00000254606	RP11-22P4.2	lincRNA	-4,88E-02	7,68E-01	8,93E-01	No	3,81E-02	9,11E-01	9,47E-01	No
ENSG00000248990	RP11-960D24.1	lincRNA	-1,16E-02	8,19E-01	NA	No	2,12E-02	9,27E-01	NA	No
ENSG00000249867	RP11-115J23.1	lincRNA	2,40E-02	9,72E-01	NA	No	6,17E-02	9,56E-01	NA	No
ENSG00000254530	RP11-460B17.3	lincRNA	-2,06E-02	8,60E-01	NA	No	4,55E-02	8,78E-01	9,27E-01	No
ENSG00000255117	RP5-1027O15.1	lincRNA	5,78E-02	5,69E-01	NA	No	8,55E-02	8,64E-01	NA	No
ENSG00000254734	CTD-3138F19.1	lincRNA	-1,10E-01	3,56E-01	6,04E-01	No	-1,08E-01	6,86E-01	7,98E-01	No
ENSG00000254532	RP11-624D11.2	lincRNA	-1,15E-01	3,52E-01	NA	No	-9,73E-02	7,42E-01	8,37E-01	No
ENSG00000254619	RP11-646J21.4	lincRNA	-5,47E-02	6,46E-01	NA	No	9,63E-02	6,51E-01	NA	No
ENSG00000255133	RP11-646J21.2	lincRNA	-4,33E-02	7,60E-01	NA	No	-1,01E-01	8,30E-01	NA	No
ENSG00000254686	RP1-276E15.1	lincRNA	-5,58E-02	6,46E-01	NA	No	-1,33E-01	7,12E-01	NA	No
ENSG00000196559	LINC00610	lincRNA	3,74E-01	1,10E-01	2,97E-01	No	-4,53E-01	1,69E-01	2,94E-01	No
ENSG00000254566	RP11-514F3.4	lincRNA	4,18E-02	7,50E-01	NA	No	1,35E-01	4,66E-01	NA	No
ENSG00000254562	RP11-64I17.1	lincRNA	-2,52E-01	5,81E-02	NA	No	-1,19E-01	6,75E-01	7,89E-01	No
ENSG00000255109	CTD-2572N17.1	lincRNA	-9,64E-03	8,49E-01	NA	No	-9,51E-02	8,30E-01	NA	No
ENSG00000255079	RP11-45A12.1	lincRNA	-6,80E-02	3,64E-01	NA	No	4,84E-02	8,24E-01	8,93E-01	No
ENSG00000255032	RP11-45A12.2	lincRNA	-1,53E-01	4,87E-01	7,16E-01	No	-3,75E-01	3,11E-01	4,64E-01	No
ENSG00000254427	RP11-430H10.1	lincRNA	8,50E-02	5,97E-01	7,95E-01	No	4,07E-01	2,29E-01	3,69E-01	No
ENSG00000254653	RP11-702F3.1	lincRNA	3,21E-01	1,95E-01	4,25E-01	No	2,76E-01	4,37E-01	5,91E-01	No
ENSG00000254639	CTD-2589M5.5	lincRNA	-1,16E-01	4,76E-01	7,08E-01	No	-2,01E-01	5,10E-01	6,57E-01	No
ENSG00000255433	AP000479.1	lincRNA	3,41E-02	9,36E-01	NA	No	6,17E-02	9,56E-01	NA	No
ENSG00000255146	RP11-659P15.1	lincRNA	-6,18E-02	7,02E-01	8,59E-01	No	3,67E-01	3,18E-01	4,73E-01	No
ENSG00000245571	AP001258.4	lincRNA	9,91E-01	3,18E-04	4,30E-03	No	2,28E-01	4,33E-01	5,87E-01	No
ENSG00000214797	RP11-1036E20.9	lincRNA	7,04E-02	6,02E-01	NA	No	-6,67E-02	7,68E-01	NA	No
ENSG00000255008	AP000442.4	lincRNA	6,64E-02	6,05E-01	NA	No	-6,20E-02	8,04E-01	NA	No
ENSG00000254952	AP001257.1	lincRNA	1,53E+00	1,68E-01	3,90E-01	No	1,10E+00	3,55E-02	8,41E-02	No
ENSG00000256733	RP11-881M11.8	lincRNA	2,60E-01	2,51E-02	1,08E-01	No	-3,12E-02	8,90E-01	NA	No
ENSG00000124915	RP11-467L20.10	lincRNA	2,07E-02	8,45E-01	NA	No	-9,72E-02	8,30E-01	NA	No
ENSG00000188070	C11orf95	lincRNA	-4,56E-01	1,43E-03	1,34E-02	No	-3,01E-01	1,06E-02	2,99E-02	No
ENSG00000255651	RP11-466C23.4	lincRNA	-1,69E-01	3,37E-01	5,86E-01	No	-2,84E-01	3,35E-01	4,90E-01	No
ENSG00000257086	RP11-783K16.13	lincRNA	4,70E-02	7,13E-01	NA	No	-1,94E-02	9,37E-01	NA	No
ENSG00000231680	AP003774.6	lincRNA	1,57E-01	1,11E-01	NA	No	1,70E-02	9,87E-01	NA	No
ENSG00000269038	AP001462.6	lincRNA	-7,17E-03	9,69E-01	9,87E-01	No	3,00E-01	4,09E-01	5,66E-01	No
ENSG00000269290	RP11-869B15.1	lincRNA	1,21E+00	9,22E-03	5,33E-02	No	7,23E-01	9,38E-02	1,86E-01	No
ENSG00000229719	MIR194-2	lincRNA	2,33E-02	8,38E-01	NA	No	-2,21E-01	3,52E-01	NA	No
ENSG00000251562	MALAT1	lincRNA	-1,76E-01	3,58E-01	6,05E-01	No	4,03E-01	1,05E-01	2,03E-01	No
ENSG00000255557	RP11-770G2.2	lincRNA	4,66E-01	4,47E-02	1,61E-01	No	1,05E-01	6,89E-01	7,99E-01	No
ENSG00000245156	RP11-867G23.3	lincRNA	2,78E-01	2,46E-01	4,87E-01	No	-8,61E-01	2,96E-02	7,21E-02	No
ENSG00000269990	CTD-3074O7.12	lincRNA	6,22E-01	9,49E-04	9,81E-03	No	-6,08E-01	7,46E-03	2,20E-02	No
ENSG00000269913	CTC-1337H24.1	lincRNA	3,41E-01	1,05E-01	2,87E-01	No	-1,66E-02	9,37E-01	9,63E-01	No
ENSG00000270169	CTC-1337H24.2	lincRNA	6,34E-02	6,21E-01	NA	No	2,96E-02	8,96E-01	NA	No
ENSG00000255318	RP11-655M14.13	lincRNA	-7,70E-02	7,21E-01	8,70E-01	No	-4,38E-01	2,47E-01	3,92E-01	No
ENSG00000251637	RP11-119D9.1	lincRNA	-9,29E-03	9,61E-01	9,84E-01	No	-2,70E-01	4,53E-01	6,06E-01	No
ENSG00000254610	RP5-903G2.2	lincRNA	-8,17E-03	9,01E-01	NA	No	-1,28E-01	7,12E-01	NA	No
ENSG00000260808	CTD-2007L18.5	lincRNA	1,64E-01	4,71E-01	7,04E-01	No	-2,76E-01	3,94E-01	5,51E-01	No
ENSG00000250508	RP11-757G1.6	lincRNA	3,86E-01	1,34E-01	3,38E-01	No	1,10E+00	4,06E-03	1,29E-02	No
ENSG00000260895	RP11-554A11.7	lincRNA	4,64E-01	2,65E-02	1,12E-01	No	7,68E-02	7,42E-01	NA	No
ENSG00000261070	RP11-554A11.8	lincRNA	2,55E-03	9,86E-01	9,95E-01	No	4,00E-02	8,98E-01	9,39E-01	No
ENSG00000260877	RP11-211G23.2	lincRNA	-5,47E-02	6,46E-01	NA	No	-1,30E-01	7,12E-01	NA	No
ENSG00000255980	AP000439.1	lincRNA	6,87E-02	4,61E-01	NA	No	8,64E-02	8,64E-01	NA	No
ENSG00000255606	AP000439.2	lincRNA	-7,51E-02	4,77E-01	NA	No	-5,07E-02	8,29E-01	NA	No
ENSG00000255774	AP000439.3	lincRNA	-5,37E-02	6,46E-01	NA	No	-2,44E-02	8,90E-01	NA	No
ENSG00000255191	RP11-626H12.1	lincRNA	1,19E-01	4,76E-01	7,08E-01	No	7,98E-02	8,15E-01	8,86E-01	No
ENSG00000254605	RP11-626H12.2	lincRNA	-3,04E-01	1,75E-01	3,99E-01	No	-1,19E+00	2,55E-02	6,36E-02	No
ENSG00000248844	RP11-626H12.3	lincRNA	-1,26E-01	3,65E-01	NA	No	-2,29E-01	4,11E-01	5,67E-01	No
ENSG00000254972	RP11-167J8.3	lincRNA	7,69E-02	3,79E-01	NA	No	8,64E-02	8,64E-01	NA	No
ENSG00000227467	RP11-169D4.1	lincRNA	4,82E-02	8,25E-01	9,24E-01	No	-2,26E-01	5,07E-01	6,54E-01	No
ENSG00000256568	RP11-800A3.3	lincRNA	-5,01E-02	8,18E-01	9,20E-01	No	-6,68E-01	4,67E-02	1,06E-01	No
ENSG00000256360	RP11-707G14.7	lincRNA	-1,31E-02	9,41E-01	9,75E-01	No	7,13E-01	8,64E-02	1,74E-01	No

ENSG00000246211	RP11-632K5.3	lincRNA	-5,09E-02	5,38E-01	NA	No	-1,57E-01	5,12E-01	NA	No
ENSG00000254837	AP001372.2	lincRNA	1,63E-01	4,36E-01	6,76E-01	No	8,69E-01	1,16E-03	4,21E-03	No
ENSG00000255136	CTD-2562J17.4	lincRNA	1,14E+00	1,22E-02	6,47E-02	No	3,68E-01	3,02E-01	4,55E-01	No
ENSG00000255362	RP11-619A14.3	lincRNA	2,78E-01	2,45E-01	4,86E-01	No	4,40E-01	1,85E-01	3,15E-01	No
ENSG00000255135	RP11-111M22.3	lincRNA	-6,85E-02	7,11E-01	8,64E-01	No	1,09E+00	2,50E-07	1,79E-06	No
ENSG00000255479	RP11-672A2.6	lincRNA	2,18E-02	8,04E-01	NA	No	-1,51E-01	5,67E-01	NA	No
ENSG00000255363	RP11-672A2.5	lincRNA	-7,49E-03	9,52E-01	9,80E-01	No	1,79E-01	5,29E-01	6,73E-01	No
ENSG00000254975	RP11-672A2.3	lincRNA	-2,55E-03	9,41E-01	NA	No	-1,01E-01	8,30E-01	NA	No
ENSG00000254761	RP11-672A2.1	lincRNA	-4,33E-02	7,60E-01	NA	No	-1,01E-01	8,30E-01	NA	No
ENSG00000251323	RP11-452H21.4	lincRNA	9,12E-02	6,81E-01	8,47E-01	No	-3,00E-01	3,89E-01	5,46E-01	No
ENSG00000255345	CTD-2337I7.1	lincRNA	7,28E-02	6,98E-01	8,57E-01	No	-2,95E-01	3,94E-01	5,51E-01	No
ENSG00000254885	RP11-802F5.1	lincRNA	3,77E-02	8,45E-01	NA	No	1,17E-01	5,84E-01	NA	No
ENSG00000254434	CTD-2555I5.1	lincRNA	4,26E-02	7,41E-01	NA	No	1,07E-01	6,99E-01	NA	No
ENSG00000255178	RP11-686G23.2	lincRNA	-1,48E-02	9,08E-01	NA	No	-6,15E-02	7,97E-01	8,74E-01	No
ENSG00000245832	RP11-179A16.1	lincRNA	1,33E-01	1,31E-01	NA	No	1,63E-01	3,35E-01	NA	No
ENSG00000254511	RP11-876F14.1	lincRNA	3,88E-02	8,45E-01	NA	No	6,17E-02	9,56E-01	NA	No
ENSG00000255382	RP11-718B12.2	lincRNA	2,54E-02	8,53E-01	NA	No	-6,15E-02	8,21E-01	8,91E-01	No
ENSG00000246067	RAB30-AS1	lincRNA	2,44E-01	1,96E-01	4,26E-01	No	3,16E-01	1,18E-01	2,22E-01	No
ENSG00000255503	RP11-113K21.4	lincRNA	-6,63E-02	7,45E-01	8,81E-01	No	-1,04E+00	3,75E-02	8,81E-02	No
ENSG00000254731	CTD-2005H7.1	lincRNA	1,87E-01	4,10E-01	6,53E-01	No	4,29E-01	2,56E-01	4,03E-01	No
ENSG00000255250	CTD-2005H7.2	lincRNA	6,80E-02	7,35E-01	8,77E-01	No	2,25E-01	5,29E-01	6,73E-01	No
ENSG00000269895	AP000654.4	lincRNA	2,23E-01	2,70E-01	5,14E-01	No	7,18E-01	7,87E-02	1,62E-01	No
ENSG00000246523	RP11-736K20.6	lincRNA	4,61E-01	7,46E-02	2,29E-01	No	8,54E-01	1,15E-02	3,21E-02	No
ENSG00000255187	RP11-699F16.2	lincRNA	-5,69E-02	6,46E-01	NA	No	2,90E-02	8,68E-01	NA	No
ENSG00000255233	RP11-755E23.3	lincRNA	-3,06E-02	7,32E-01	NA	No	-1,08E-01	6,08E-01	NA	No
ENSG00000250519	RP11-680H20.2	lincRNA	1,32E-01	4,43E-01	6,80E-01	No	-5,40E-01	1,06E-01	2,04E-01	No
ENSG00000257057	LINC01171	lincRNA	3,95E-02	8,45E-01	NA	No	8,58E-02	8,64E-01	NA	No
ENSG00000233536	RP11-867G2.5	lincRNA	9,06E-02	6,06E-01	8,01E-01	No	-5,46E-02	8,66E-01	9,19E-01	No
ENSG00000255666	RP11-867G2.6	lincRNA	1,83E-01	3,66E-01	6,13E-01	No	-1,50E-01	6,41E-01	7,63E-01	No
ENSG00000250390	RP11-338H14.1	lincRNA	9,74E-03	8,96E-01	NA	No	-1,51E-01	5,67E-01	NA	No
ENSG00000255039	RP11-882G5.1	lincRNA	-6,63E-02	4,07E-01	NA	No	-2,18E-02	8,95E-01	NA	No
ENSG00000254987	RP11-563P16.1	lincRNA	-5,33E-01	6,71E-02	2,13E-01	No	-7,09E-01	6,02E-02	1,30E-01	No
ENSG00000256422	RP11-886D15.1	lincRNA	1,36E-01	4,08E-01	6,52E-01	No	-2,30E-02	9,33E-01	9,60E-01	No
ENSG00000254998	RP11-94P11.4	lincRNA	-1,76E-01	8,14E-02	NA	No	-2,52E-01	3,68E-01	5,24E-01	No
ENSG00000261098	RP11-819C21.1	lincRNA	5,02E-01	3,66E-02	1,40E-01	No	3,22E-01	2,33E-01	3,75E-01	No
ENSG00000255528	RP11-25I9.2	lincRNA	-5,37E-02	6,46E-01	NA	No	-1,26E-01	7,12E-01	NA	No
ENSG00000254416	RP11-347E10.1	lincRNA	3,31E-02	9,36E-01	NA	No	1,09E-01	6,81E-01	NA	No
ENSG00000271390	RP11-89C3.3	lincRNA	4,61E-02	6,21E-01	NA	No	8,77E-02	8,64E-01	NA	No
ENSG00000271584	RP11-89C3.4	lincRNA	1,75E-01	1,02E-01	NA	No	1,25E-01	5,19E-01	NA	No
ENSG00000254990	RP11-108O10.2	lincRNA	-2,16E-01	1,56E-01	3,72E-01	No	-5,46E-01	7,90E-02	1,62E-01	No
ENSG00000254968	RP11-65M17.3	lincRNA	2,04E-02	8,57E-01	NA	No	-7,55E-02	7,44E-01	NA	No
ENSG00000255484	RP11-65M17.1	lincRNA	8,73E-02	2,79E-01	NA	No	1,10E-01	6,71E-01	NA	No
ENSG00000256972	AP000462.3	lincRNA	-7,90E-01	2,70E-02	1,14E-01	No	-6,95E-02	8,37E-01	9,01E-01	No
ENSG00000255580	AP000462.2	lincRNA	-3,21E-01	1,95E-01	4,25E-01	No	2,86E-01	4,15E-01	5,71E-01	No
ENSG00000256717	AP000797.3	lincRNA	1,42E-01	2,07E-01	NA	No	9,70E-02	6,46E-01	NA	No
ENSG00000246100	LINC00900	lincRNA	7,30E-01	2,94E-02	1,20E-01	No	9,05E-01	4,30E-02	9,89E-02	No
ENSG00000257067	AP000797.4	lincRNA	3,52E-02	7,12E-01	NA	No	2,67E-01	2,50E-01	NA	No
ENSG00000231611	AP006216.11	lincRNA	2,49E-01	2,88E-01	5,35E-01	No	8,43E-03	9,82E-01	9,89E-01	No
ENSG00000270403	RP11-35P15.1	lincRNA	2,71E-02	8,63E-01	9,41E-01	No	-1,28E-01	6,37E-01	7,60E-01	No
ENSG00000255121	RP11-110I1.12	lincRNA	7,06E-01	1,82E-02	8,60E-02	No	-2,74E-01	4,00E-01	5,57E-01	No
ENSG00000271751	RP11-110I1.14	lincRNA	-2,36E-01	3,02E-01	5,50E-01	No	-4,21E-01	2,60E-01	4,07E-01	No
ENSG00000255247	CTD-2523D13.1	lincRNA	2,67E-01	4,91E-02	NA	No	4,24E-02	8,85E-01	NA	No
ENSG00000255015	RP11-716H6.1	lincRNA	2,43E-01	1,88E-01	4,16E-01	No	5,67E-01	1,58E-01	2,79E-01	No
ENSG00000260209	RP11-680F20.10	lincRNA	-3,49E-02	7,68E-01	8,94E-01	No	-2,99E-02	9,09E-01	9,46E-01	No
ENSG00000254833	RP11-50B3.2	lincRNA	-1,21E-01	4,46E-01	NA	No	-5,00E-01	1,12E-01	NA	No
ENSG00000254938	RP11-688I9.4	lincRNA	-7,47E-02	6,66E-01	8,36E-01	No	2,91E-02	9,34E-01	9,61E-01	No
ENSG00000273415	RP11-702B10.2	lincRNA	3,96E-02	6,42E-01	NA	No	3,35E-02	8,45E-01	NA	No
ENSG00000255465	RP11-264E20.1	lincRNA	-4,63E-01	9,18E-02	2,63E-01	No	-1,17E+00	1,39E-02	3,80E-02	No
ENSG00000233220	LINC00167	lincRNA	-8,86E-02	6,33E-01	8,18E-01	No	-8,29E-03	9,79E-01	9,87E-01	No
ENSG00000175773	RP11-121M22.1	lincRNA	-4,84E-02	7,70E-01	8,95E-01	No	-1,31E-01	6,83E-01	7,96E-01	No
ENSG00000254842	RP11-890B15.2	lincRNA	9,69E-01	1,05E-02	5,83E-02	No	1,54E-01	6,60E-01	7,78E-01	No
ENSG00000255455	RP11-890B15.3	lincRNA	7,35E-02	5,57E-01	7,68E-01	No	1,83E-01	1,39E-01	2,53E-01	No

ENSG00000224795	AP003039.3	lincRNA	4,47E-02	6,72E-01	NA	No	1,10E-01	6,81E-01	NA	No
ENSG00000255406	RP11-713P17.5	lincRNA	2,35E-02	9,72E-01	NA	No	8,58E-02	8,64E-01	NA	No
ENSG00000204241	RP11-713P17.3	lincRNA	3,06E-01	2,12E-01	4,47E-01	No	4,18E-01	2,59E-01	4,06E-01	No
ENSG00000254648	RP11-713P17.4	lincRNA	6,72E-03	9,70E-01	9,88E-01	No	1,43E-01	6,71E-01	7,87E-01	No
ENSG00000251226	RP11-469N6.1	lincRNA	1,36E-01	3,28E-01	5,77E-01	No	4,14E-01	2,12E-01	3,49E-01	No
ENSG00000256672	RP11-218M22.2	lincRNA	-1,01E+00	1,10E-02	6,04E-02	No	-1,41E+00	2,28E-03	7,73E-03	No
ENSG00000249628	LINC00942	lincRNA	1,46E-01	2,14E-01	NA	No	6,10E-02	8,02E-01	NA	No
ENSG00000235049	LINC00940	lincRNA	3,12E-02	7,84E-01	NA	No	3,82E-02	8,82E-01	9,30E-01	No
ENSG00000256150	RP11-885B4.1	lincRNA	1,64E-01	2,81E-01	NA	No	-1,13E-01	5,86E-01	NA	No
ENSG00000255669	RP11-885B4.2	lincRNA	-6,29E-02	7,67E-01	8,93E-01	No	-9,09E-01	5,50E-02	1,21E-01	No
ENSG00000236908	RP5-1063M23.2	lincRNA	1,80E-01	4,24E-01	6,65E-01	No	-5,10E-01	1,87E-01	3,18E-01	No
ENSG00000255474	RP11-234B24.2	lincRNA	-9,33E-02	6,54E-01	8,30E-01	No	-5,34E-01	1,58E-01	2,79E-01	No
ENSG00000256988	RP11-234B24.4	lincRNA	-7,35E-02	3,54E-01	NA	No	9,60E-03	9,68E-01	NA	No
ENSG00000256417	RP11-1038A11.3	lincRNA	-6,24E-02	5,15E-01	NA	No	4,44E-01	7,16E-02	1,50E-01	No
ENSG00000256218	RP11-1038A11.2	lincRNA	-5,69E-02	6,46E-01	NA	No	-1,35E-01	7,12E-01	NA	No
ENSG00000255983	RP11-1038A11.1	lincRNA	-6,51E-02	4,43E-01	NA	No	1,64E-01	4,35E-01	5,89E-01	No
ENSG00000255775	RP3-454B23.1	lincRNA	6,10E-02	7,08E-01	8,62E-01	No	8,58E-01	6,32E-02	1,36E-01	No
ENSG00000256433	RP1-102E24.8	lincRNA	-9,41E-02	6,54E-01	8,30E-01	No	-8,00E-01	6,43E-02	1,37E-01	No
ENSG00000269892	RP4-761J14.10	lincRNA	-1,14E-01	4,74E-01	7,06E-01	No	-1,20E-02	9,71E-01	9,83E-01	No
ENSG00000272201	U47924.30	lincRNA	-1,25E-02	9,50E-01	9,79E-01	No	1,20E-01	7,32E-01	8,31E-01	No
ENSG00000215241	RP11-266K4.9	lincRNA	3,12E-01	1,98E-01	4,30E-01	No	-2,19E-01	5,38E-01	6,80E-01	No
ENSG00000256101	RP11-90D4.3	lincRNA	3,64E-02	8,16E-01	9,19E-01	No	-7,34E-02	8,04E-01	8,79E-01	No
ENSG00000255801	RP11-561P12.5	lincRNA	3,84E-01	1,69E-03	1,52E-02	No	2,97E-01	2,34E-02	5,92E-02	No
ENSG00000249790	RP11-20D14.6	lincRNA	3,87E-01	1,24E-01	3,21E-01	No	-2,86E-01	4,27E-01	5,82E-01	No
ENSG00000237248	LINC00987	lincRNA	1,54E-01	4,93E-01	7,22E-01	No	-4,50E-01	2,09E-01	3,46E-01	No
ENSG00000256427	RP11-118B22.4	lincRNA	5,60E-01	3,72E-02	1,42E-01	No	2,20E-01	5,24E-01	6,68E-01	No
ENSG00000260423	RP13-735L24.1	lincRNA	6,26E-02	7,35E-01	8,76E-01	No	1,95E-01	5,79E-01	7,13E-01	No
ENSG00000231560	AC091814.3	lincRNA	2,23E-01	4,36E-02	NA	No	1,13E-01	6,49E-01	NA	No
ENSG00000256288	RP11-277P12.10	lincRNA	1,09E-01	1,50E-01	NA	No	1,39E-01	4,47E-01	NA	No
ENSG00000256888	RP13-81N3.2	lincRNA	6,86E-02	4,53E-01	NA	No	4,91E-01	1,32E-01	2,44E-01	No
ENSG00000256373	RP11-711K1.8	lincRNA	-1,32E-01	3,62E-01	6,09E-01	No	-1,81E-01	5,47E-01	6,87E-01	No
ENSG00000256237	RP11-434C1.2	lincRNA	-1,14E-01	1,80E-01	NA	No	-2,56E-01	1,89E-01	NA	No
ENSG00000247157	RP11-434C1.1	lincRNA	-3,73E-01	1,41E-01	3,49E-01	No	-1,30E+00	1,30E-02	3,59E-02	No
ENSG00000255670	RP11-253I19.3	lincRNA	-1,87E-01	4,08E-01	6,52E-01	No	-4,94E-01	1,65E-01	2,89E-01	No
ENSG00000255621	RP11-377D9.3	lincRNA	1,70E-01	1,38E-01	NA	No	3,94E-01	1,44E-01	NA	No
ENSG00000256084	RP11-134N1.2	lincRNA	3,92E-02	7,38E-01	NA	No	-8,37E-02	6,60E-01	NA	No
ENSG00000255727	RP11-508P1.2	lincRNA	-1,92E-02	9,07E-01	9,61E-01	No	7,76E-02	8,05E-01	8,79E-01	No
ENSG00000255660	RERG-AS1	lincRNA	1,05E-01	6,36E-01	8,19E-01	No	1,54E-01	6,27E-01	7,52E-01	No
ENSG00000255565	RP11-6B19.1	lincRNA	1,09E-01	6,23E-01	8,12E-01	No	-2,14E-01	5,06E-01	6,53E-01	No
ENSG00000256953	RP11-6B19.2	lincRNA	9,37E-02	4,11E-01	NA	No	3,40E-01	2,28E-01	NA	No
ENSG00000255871	RP11-69C13.1	lincRNA	-1,85E-02	8,19E-01	NA	No	-4,06E-02	8,90E-01	NA	No
ENSG00000255910	RP11-405A12.2	lincRNA	6,68E-02	7,58E-01	8,88E-01	No	-8,42E-02	8,07E-01	8,81E-01	No
ENSG00000256287	RP11-664H17.1	lincRNA	3,34E-01	1,13E-01	3,02E-01	No	3,28E-01	3,69E-01	5,25E-01	No
ENSG00000256499	CTC-465D4.1	lincRNA	-8,03E-02	5,05E-01	7,32E-01	No	1,27E-01	7,04E-01	8,10E-01	No
ENSG00000256714	RP11-73M14.1	lincRNA	2,80E-01	6,99E-03	4,35E-02	No	6,23E-02	9,56E-01	NA	No
ENSG00000256973	RP11-359J14.2	lincRNA	3,55E-01	1,51E-01	3,65E-01	No	3,57E-01	2,58E-01	4,04E-01	No
ENSG00000256923	RP11-449P1.1	lincRNA	3,15E-02	9,36E-01	NA	No	8,55E-02	8,64E-01	NA	No
ENSG00000256321	RP11-153K16.1	lincRNA	-1,29E-01	7,77E-02	NA	No	-3,20E-01	1,42E-01	2,57E-01	No
ENSG00000255864	RP11-444D3.1	lincRNA	6,52E-01	2,61E-02	1,11E-01	No	1,09E+00	1,04E-03	3,84E-03	No
ENSG00000256120	RP11-778H2.1	lincRNA	2,68E-01	2,50E-01	4,92E-01	No	-7,40E-02	8,30E-01	8,97E-01	No
ENSG00000197503	LINC00477	lincRNA	4,55E-02	6,45E-01	NA	No	1,13E-01	6,38E-01	NA	No
ENSG00000255858	RP11-612B6.1	lincRNA	-1,34E-01	2,96E-01	NA	No	-1,41E-01	5,87E-01	7,19E-01	No
ENSG00000256185	RP11-612B6.2	lincRNA	4,43E-01	6,52E-02	2,09E-01	No	1,43E+00	1,15E-06	7,33E-06	No
ENSG00000256226	RP11-582E3.2	lincRNA	-2,56E-02	7,92E-01	NA	No	1,40E-01	5,75E-01	NA	No
ENSG00000248100	RP11-709A23.1	lincRNA	-5,01E-01	4,65E-02	1,65E-01	No	-8,58E-01	1,25E-02	3,45E-02	No
ENSG00000256504	RP11-1060J15.7	lincRNA	-3,65E-03	9,00E-01	NA	No	-6,19E-02	8,00E-01	NA	No
ENSG00000246331	RP11-77I22.2	lincRNA	-2,01E-02	8,88E-01	9,54E-01	No	6,71E-01	9,47E-02	1,87E-01	No
ENSG00000235884	LINC00941	lincRNA	-1,96E-01	2,94E-01	5,41E-01	No	-8,19E-01	5,12E-02	1,14E-01	No
ENSG00000226472	RP11-551L14.4	lincRNA	-1,92E-01	2,30E-01	NA	No	-9,54E-01	3,86E-02	9,01E-02	No
ENSG00000256984	RP11-551L14.6	lincRNA	-1,46E-01	1,59E-01	NA	No	-4,00E-01	1,59E-01	NA	No
ENSG00000256232	RP11-771K4.1	lincRNA	-5,69E-02	6,46E-01	NA	No	2,20E-02	8,91E-01	NA	No
ENSG00000255760	RP11-428G5.5	lincRNA	1,04E+00	1,23E-02	6,52E-02	No	1,39E-01	6,63E-01	7,80E-01	No

ENSG00000255652	RP11-313F23.4	lincRNA	-1,05E-01	5,54E-01	7,65E-01	No	-4,22E-01	2,56E-01	4,02E-01	No
ENSG00000257784	RP11-630C16.1	lincRNA	1,23E-01	2,21E-01	NA	No	1,72E-01	4,49E-01	NA	No
ENSG00000257239	RP11-630C16.2	lincRNA	7,39E-02	6,44E-01	8,24E-01	No	2,02E-01	5,24E-01	6,69E-01	No
ENSG00000257510	RP11-609L23.1	lincRNA	-4,23E-02	7,60E-01	NA	No	-9,82E-02	8,30E-01	NA	No
ENSG00000257114	RP11-25I15.3	lincRNA	1,91E-01	1,08E-01	NA	No	3,46E-01	1,62E-01	2,85E-01	No
ENSG00000257849	RP11-547C5.1	lincRNA	6,85E-02	4,65E-01	NA	No	1,98E-01	3,38E-01	NA	No
ENSG00000257373	RP11-547C5.2	lincRNA	2,43E-02	8,17E-01	NA	No	2,11E-01	3,76E-01	NA	No
ENSG00000258331	RP11-118A3.1	lincRNA	-3,55E-02	6,73E-01	NA	No	-1,38E-01	5,20E-01	NA	No
ENSG00000257742	RP11-350F4.2	lincRNA	6,43E-01	2,62E-03	2,12E-02	No	1,38E+00	1,74E-09	1,72E-08	No
ENSG00000257657	RP11-352M15.1	lincRNA	-6,46E-01	3,69E-02	1,41E-01	No	-3,88E-01	2,97E-01	4,49E-01	No
ENSG00000273015	LINC00938	lincRNA	-2,78E-01	1,05E-01	2,88E-01	No	-3,89E-01	3,18E-02	7,66E-02	No
ENSG00000257261	RP11-96H19.1	lincRNA	2,67E-01	1,95E-01	4,25E-01	No	-2,75E-01	2,42E-01	3,86E-01	No
ENSG00000257496	RP11-474P2.4	lincRNA	3,77E-01	1,33E-01	3,35E-01	No	6,50E-01	1,14E-01	2,17E-01	No
ENSG00000272369	RP11-446N19.1	lincRNA	1,03E-01	6,35E-01	8,19E-01	No	3,07E-01	3,20E-01	4,75E-01	No
ENSG00000257924	RP11-493L12.5	lincRNA	4,16E-02	7,67E-01	NA	No	8,55E-02	8,64E-01	NA	No
ENSG00000257906	RP1-90J4.1	lincRNA	6,10E-02	5,46E-01	NA	No	1,07E-01	6,99E-01	NA	No
ENSG00000257433	RP1-197B17.3	lincRNA	7,82E-01	7,22E-03	4,47E-02	No	-5,24E-01	1,08E-01	2,08E-01	No
ENSG00000257488	RP5-1057I20.2	lincRNA	4,11E-02	7,67E-01	NA	No	6,17E-02	9,56E-01	NA	No
ENSG00000258203	RP1-228P16.3	lincRNA	-2,64E-02	7,31E-01	NA	No	-5,99E-02	8,00E-01	NA	No
ENSG00000258234	RP11-370I10.2	lincRNA	1,29E-01	2,31E-01	NA	No	-3,46E-02	8,90E-01	NA	No
ENSG00000257660	RP11-579D7.4	lincRNA	2,46E-02	9,72E-01	NA	No	8,77E-02	8,64E-01	NA	No
ENSG00000257771	RP11-70F11.8	lincRNA	-1,56E-02	8,49E-01	NA	No	1,17E-01	6,31E-01	NA	No
ENSG00000260473	RP11-923I11.4	lincRNA	1,43E+00	1,46E-03	1,36E-02	No	-3,18E-01	3,45E-01	5,00E-01	No
ENSG00000259887	RP11-923I11.5	lincRNA	-1,16E-02	9,32E-01	9,72E-01	No	-3,43E-01	1,63E-01	NA	No
ENSG00000259884	RP11-1100L3.8	lincRNA	-2,21E-01	1,39E-01	3,45E-01	No	-7,01E-01	1,01E-01	1,98E-01	No
ENSG00000258279	LINC00592	lincRNA	3,15E-02	9,36E-01	NA	No	1,11E-01	6,54E-01	NA	No
ENSG00000257808	RP11-1136G11.8	lincRNA	-4,21E-02	7,60E-01	NA	No	-9,72E-02	8,30E-01	NA	No
ENSG00000260492	RP11-686F15.3	lincRNA	-7,52E-02	7,31E-01	8,75E-01	No	-7,38E-01	5,66E-02	1,24E-01	No
ENSG00000260597	AC012531.25	lincRNA	5,94E-02	6,06E-01	8,01E-01	No	-1,18E-01	5,16E-01	NA	No
ENSG00000248265	RP11-834C11.3	lincRNA	-5,01E-02	5,81E-01	NA	No	-6,76E-02	7,77E-01	NA	No
ENSG00000250742	RP11-834C11.4	lincRNA	1,10E-01	6,16E-01	8,07E-01	No	-9,54E-01	8,48E-03	2,46E-02	No
ENSG00000260470	RP11-834C11.11	lincRNA	1,19E-02	9,44E-01	9,77E-01	No	-8,04E-01	5,04E-02	1,13E-01	No
ENSG00000257534	RP11-834C11.10	lincRNA	1,13E-01	2,41E-01	NA	No	3,03E-02	9,09E-01	NA	No
ENSG00000257477	LINC01154	lincRNA	-2,16E-01	1,54E-01	3,69E-01	No	-6,92E-01	4,28E-02	9,85E-02	No
ENSG00000258001	RP11-756H6.1	lincRNA	-1,38E-03	9,89E-01	NA	No	1,63E-01	4,59E-01	NA	No
ENSG00000270039	RP11-571M6.17	lincRNA	-6,42E-02	7,05E-01	8,60E-01	No	7,45E-01	3,85E-04	1,56E-03	No
ENSG00000269903	RP11-571M6.18	lincRNA	4,11E-01	1,17E-01	3,08E-01	No	-8,58E-01	3,95E-02	9,19E-02	No
ENSG00000245651	RP11-620J15.2	lincRNA	-2,46E-01	2,95E-01	5,42E-01	No	8,47E-02	8,02E-01	8,78E-01	No
ENSG00000257698	RP11-620J15.3	lincRNA	-1,10E-02	9,55E-01	9,81E-01	No	1,04E+00	1,81E-07	1,33E-06	No
ENSG00000257541	RP11-58A17.4	lincRNA	3,26E-02	7,74E-01	NA	No	-1,61E-01	4,69E-01	NA	No
ENSG00000257259	RP11-767I20.1	lincRNA	-3,99E-01	1,27E-01	3,26E-01	No	7,10E-01	9,07E-02	1,81E-01	No
ENSG00000258117	RP11-1022B3.1	lincRNA	3,32E-02	9,36E-01	NA	No	8,58E-02	8,64E-01	NA	No
ENSG00000256199	RP11-439H13.2	lincRNA	-9,53E-03	9,24E-01	NA	No	-6,00E-02	7,57E-01	NA	No
ENSG00000255693	RP11-766N7.3	lincRNA	1,07E-01	4,42E-01	6,80E-01	No	-1,45E-01	4,81E-01	NA	No
ENSG00000248995	RP11-766N7.4	lincRNA	3,17E-02	9,36E-01	NA	No	8,77E-02	8,64E-01	NA	No
ENSG00000250748	RP11-230G5.2	lincRNA	-8,28E-01	1,22E-02	6,50E-02	No	-4,73E-01	1,41E-01	2,56E-01	No
ENSG00000255866	RP11-221N13.2	lincRNA	-6,87E-02	4,35E-01	NA	No	-1,23E-01	4,48E-01	NA	No
ENSG00000256248	RP11-123O10.4	lincRNA	-2,47E-02	8,09E-01	NA	No	-5,13E-02	8,10E-01	NA	No
ENSG00000257083	RP11-123O10.3	lincRNA	-3,70E-02	6,75E-01	NA	No	9,40E-02	7,01E-01	8,08E-01	No
ENSG00000256172	RP11-473M14.3	lincRNA	4,57E-02	6,37E-01	NA	No	1,01E-01	7,75E-01	NA	No
ENSG00000203585	RP11-542B15.1	lincRNA	1,33E-01	3,07E-01	NA	No	3,97E-01	1,45E-01	2,61E-01	No
ENSG00000255970	RP11-43N5.1	lincRNA	4,95E-01	7,63E-02	2,33E-01	No	1,46E-01	6,76E-01	7,90E-01	No
ENSG00000255772	GS1-410F4.4	lincRNA	-6,70E-02	7,59E-01	8,89E-01	No	6,98E-01	4,02E-02	9,32E-02	No
ENSG00000247363	RP11-637A17.2	lincRNA	3,98E-01	1,26E-01	3,24E-01	No	-3,99E-01	2,81E-01	4,31E-01	No
ENSG00000257507	RP11-956E11.1	lincRNA	1,64E-02	9,12E-01	9,63E-01	No	-1,26E-01	6,91E-01	8,01E-01	No
ENSG00000257139	RP11-320P7.2	lincRNA	1,78E+00	8,83E-03	5,17E-02	No	1,21E+00	3,17E-02	7,64E-02	No
ENSG00000257515	RP11-498M15.1	lincRNA	1,81E-01	2,30E-01	4,68E-01	No	7,49E-01	7,28E-02	1,52E-01	No
ENSG00000257410	RP11-2H8.2	lincRNA	-2,82E-01	2,11E-01	4,45E-01	No	1,26E-01	6,22E-01	7,48E-01	No
ENSG00000258077	RP11-114H23.1	lincRNA	1,42E-01	1,15E-01	NA	No	5,87E-01	4,87E-02	1,10E-01	No
ENSG00000257219	RP11-54A9.1	lincRNA	-5,86E-02	6,78E-01	8,44E-01	No	-2,23E-01	4,68E-01	6,20E-01	No
ENSG00000257526	RP11-20E24.1	lincRNA	9,18E-03	9,61E-01	9,84E-01	No	3,99E-01	2,85E-01	4,35E-01	No
ENSG00000231121	RP1-34H18.1	lincRNA	-1,93E-01	4,01E-01	6,46E-01	No	1,06E+00	3,34E-03	1,09E-02	No

ENSG00000257835	RP1-97G4.1	lincRNA	2,75E-02	8,90E-01	9,54E-01	No	1,18E-03	9,99E-01	9,99E-01	No
ENSG00000258066	RP11-781A6.1	lincRNA	-3,06E-02	8,59E-01	9,39E-01	No	-6,61E-03	9,81E-01	9,88E-01	No
ENSG00000257894	RP1-78O14.1	lincRNA	7,62E-02	4,59E-01	NA	No	1,24E-01	6,01E-01	NA	No
ENSG00000257474	RP11-359M6.1	lincRNA	-2,38E-01	2,39E-01	4,79E-01	No	-4,36E-01	2,48E-01	3,93E-01	No
ENSG00000258048	RP11-530C5.1	lincRNA	1,42E-01	4,75E-01	7,07E-01	No	7,92E-01	6,73E-02	1,43E-01	No
ENSG00000257467	RP11-121G22.3	lincRNA	7,39E-02	6,87E-01	8,50E-01	No	1,98E-01	5,70E-01	7,06E-01	No
ENSG00000257747	RP11-362A1.1	lincRNA	1,50E-02	8,83E-01	NA	No	-6,53E-02	7,10E-01	NA	No
ENSG00000257431	RP11-263K4.3	lincRNA	-2,08E-02	8,19E-01	NA	No	-1,33E-01	7,12E-01	NA	No
ENSG00000258338	RP11-87P13.2	lincRNA	1,60E-02	8,55E-01	NA	No	-2,28E-02	8,90E-01	NA	No
ENSG00000258815	RP11-408B11.2	lincRNA	-6,38E-02	3,83E-01	NA	No	2,29E-02	3,60E-01	NA	No
ENSG00000258178	RP11-18J9.3	lincRNA	-5,09E-03	9,10E-01	NA	No	-6,33E-03	9,80E-01	NA	No
ENSG00000246363	RP11-13A1.1	lincRNA	-1,06E-01	NA	NA	No	-2,77E-01	NA	NA	No
ENSG00000257156	RP11-13A1.3	lincRNA	-1,14E-01	2,28E-01	NA	No	-1,05E-01	6,61E-01	7,78E-01	No
ENSG00000271327	RP11-1109F11.3	lincRNA	3,71E-01	1,42E-01	3,51E-01	No	5,18E-01	8,73E-02	1,76E-01	No
ENSG00000271259	RP11-1109F11.5	lincRNA	2,79E-01	2,01E-01	4,33E-01	No	7,46E-01	6,71E-02	1,42E-01	No
ENSG00000271614	LINC00936	lincRNA	2,80E-01	2,21E-01	4,59E-01	No	8,57E-01	8,22E-03	2,40E-02	No
ENSG00000258216	RP11-654D12.2	lincRNA	8,81E-02	4,09E-01	NA	No	1,07E-01	6,99E-01	NA	No
ENSG00000257725	RP11-654D12.3	lincRNA	5,84E-02	5,78E-01	NA	No	1,30E-01	4,89E-01	NA	No
ENSG00000257194	RP11-567C2.1	lincRNA	-3,81E-02	6,59E-01	NA	No	-1,10E-01	6,09E-01	NA	No
ENSG00000258183	RP11-753N8.1	lincRNA	-4,15E-02	7,60E-01	NA	No	-9,51E-02	8,30E-01	NA	No
ENSG00000258100	RP11-121E16.1	lincRNA	2,12E-01	1,16E-01	NA	No	4,76E-01	9,30E-02	1,85E-01	No
ENSG00000205056	RP11-693J15.5	lincRNA	8,52E-02	4,40E-01	6,79E-01	No	-1,21E-01	6,54E-01	7,73E-01	No
ENSG00000257345	RP11-511B23.1	lincRNA	-1,52E-01	4,02E-01	6,46E-01	No	-3,01E-01	3,73E-01	5,30E-01	No
ENSG00000257746	RP11-202G11.2	lincRNA	2,51E-01	2,02E-01	4,34E-01	No	5,87E-01	1,44E-01	2,61E-01	No
ENSG00000258171	RP11-511B23.3	lincRNA	-1,53E-01	4,73E-01	7,06E-01	No	1,89E-01	5,81E-01	7,14E-01	No
ENSG00000271382	RP11-1060G2.2	lincRNA	-1,23E-01	5,17E-01	7,41E-01	No	-1,29E-01	7,06E-01	8,12E-01	No
ENSG00000257400	RP11-778J16.3	lincRNA	-1,03E-01	2,81E-01	NA	No	-2,61E-01	2,86E-01	NA	No
ENSG00000258313	RP11-167N24.5	lincRNA	4,33E-02	7,20E-01	NA	No	8,51E-02	8,64E-01	NA	No
ENSG00000258272	RP11-510I5.4	lincRNA	-4,21E-02	7,60E-01	NA	No	-9,72E-02	8,30E-01	NA	No
ENSG00000258131	RP11-541G9.1	lincRNA	-5,37E-02	6,46E-01	NA	No	-1,26E-01	7,12E-01	NA	No
ENSG00000258312	RP11-690J15.1	lincRNA	8,61E-02	4,59E-01	NA	No	7,54E-03	9,80E-01	NA	No
ENSG00000257543	RP11-321F8.4	lincRNA	-3,89E-02	7,64E-01	8,92E-01	No	3,49E-01	3,30E-01	4,85E-01	No
ENSG00000258308	RP11-554E23.2	lincRNA	5,98E-02	7,59E-01	8,89E-01	No	-7,56E-01	7,23E-02	1,51E-01	No
ENSG00000258169	LINC00485	lincRNA	2,03E-01	9,06E-02	NA	No	5,21E-02	8,17E-01	NA	No
ENSG00000257762	RP11-626I20.3	lincRNA	-8,48E-02	4,35E-01	6,75E-01	No	-4,37E-01	9,39E-02	1,86E-01	No
ENSG00000257859	CASC18	lincRNA	2,53E-01	2,03E-01	4,36E-01	No	4,51E-01	2,23E-01	3,63E-01	No
ENSG00000257890	RP11-114F10.2	lincRNA	2,91E-01	2,16E-01	4,52E-01	No	-6,86E-02	8,41E-01	9,03E-01	No
ENSG00000257398	RP11-554D14.6	lincRNA	-7,04E-02	6,72E-01	8,40E-01	No	-6,27E-02	8,42E-01	9,04E-01	No
ENSG00000264043	SNORA40	lincRNA	-2,70E-01	2,61E-01	5,04E-01	No	-3,51E-01	2,80E-01	4,29E-01	No
ENSG00000260987	RP11-423G4.7	lincRNA	6,60E-02	7,23E-01	8,71E-01	No	-2,44E-01	4,91E-01	6,40E-01	No
ENSG00000258240	RP1-46F2.3	lincRNA	-1,26E-02	9,36E-01	9,74E-01	No	1,74E-02	9,59E-01	9,76E-01	No
ENSG00000257595	RP3-473L9.4	lincRNA	-4,17E-03	9,73E-01	NA	No	2,38E-02	9,30E-01	NA	No
ENSG00000257877	RP3-462E2.3	lincRNA	-4,18E-01	9,45E-02	2,68E-01	No	-4,17E-01	1,36E-01	2,50E-01	No
ENSG00000234608	MAPKAPK5-AS1	lincRNA	-1,17E-01	5,56E-01	7,67E-01	No	2,64E-01	1,81E-01	3,10E-01	No
ENSG00000270018	RP3-462E2.5	lincRNA	9,40E-02	6,71E-01	8,40E-01	No	5,25E-01	1,18E-01	2,23E-01	No
ENSG00000249550	RP11-438N16.1	lincRNA	-5,73E-02	6,46E-01	NA	No	-6,98E-02	8,00E-01	NA	No
ENSG00000257817	RP4-601P9.2	lincRNA	2,34E-02	8,40E-01	NA	No	4,13E-02	8,47E-01	NA	No
ENSG00000257958	RP11-25E2.1	lincRNA	-1,05E-01	2,09E-01	NA	No	-2,38E-01	2,18E-01	NA	No
ENSG00000258102	RP11-809C9.2	lincRNA	1,05E-01	4,00E-01	NA	No	7,67E-02	7,58E-01	NA	No
ENSG00000258249	RP11-497G19.3	lincRNA	9,63E-02	3,48E-01	NA	No	4,19E-01	1,40E-01	NA	No
ENSG00000257883	RP11-497G19.1	lincRNA	6,45E-02	4,69E-01	NA	No	-2,72E-02	8,90E-01	NA	No
ENSG00000258285	RP11-103B5.2	lincRNA	3,53E-01	1,63E-01	3,82E-01	No	-2,13E-01	5,46E-01	6,87E-01	No
ENSG00000270482	RP11-131L12.2	lincRNA	1,01E-01	3,76E-01	NA	No	1,29E-01	6,20E-01	NA	No
ENSG00000248636	RP11-768F21.1	lincRNA	2,58E-01	2,62E-01	5,06E-01	No	-7,23E-02	8,35E-01	8,99E-01	No
ENSG00000256742	RP13-941N14.1	lincRNA	1,90E-01	3,07E-01	5,55E-01	No	-1,03E-01	7,47E-01	8,40E-01	No
ENSG00000212694	AC084018.1	lincRNA	3,98E-01	6,14E-02	2,00E-01	No	-1,14E+00	5,01E-06	2,88E-05	No
ENSG00000255856	RP11-87C12.5	lincRNA	6,65E-02	5,90E-01	NA	No	3,51E-01	2,76E-01	4,25E-01	No
ENSG00000257097	RP11-450K4.1	lincRNA	-1,43E-01	4,48E-01	6,85E-01	No	-7,05E-01	1,00E-01	1,96E-01	No
ENSG00000256249	RP11-324E6.6	lincRNA	-3,01E-02	7,31E-01	NA	No	-1,28E-01	7,12E-01	NA	No
ENSG00000256092	hsa-mir-8072	lincRNA	1,07E-01	6,25E-01	8,13E-01	No	-5,33E-02	8,52E-01	9,10E-01	No
ENSG00000247373	RP11-486O12.2	lincRNA	8,15E-01	3,55E-03	2,67E-02	No	-5,61E-01	1,13E-01	2,16E-01	No
ENSG00000269923	RP11-214K3.24	lincRNA	3,64E-03	9,86E-01	9,95E-01	No	-2,50E-01	4,39E-01	5,93E-01	No

ENSG00000256596	RP11-522N14.2	lincRNA	-9,69E-02	5,09E-01	7,35E-01	No	-4,83E-01	6,04E-02	1,31E-01	No
ENSG00000214650	RP11-83B20.1	lincRNA	6,04E-01	3,21E-02	1,28E-01	No	1,77E-01	6,03E-01	7,33E-01	No
ENSG00000256276	RP5-916L7.2	lincRNA	-5,45E-02	6,46E-01	NA	No	-2,89E-02	8,90E-01	NA	No
ENSG00000255595	RP4-809F18.1	lincRNA	4,16E-02	7,65E-01	NA	No	1,63E-01	4,86E-01	NA	No
ENSG00000214043	RP5-944M2.3	lincRNA	-6,34E-02	4,94E-01	NA	No	-5,65E-02	7,21E-01	NA	No
ENSG00000256732	RP11-407A16.3	lincRNA	-1,79E-02	8,41E-01	NA	No	-2,18E-02	8,95E-01	NA	No
ENSG00000255998	RP11-407A16.7	lincRNA	1,03E-01	3,05E-01	NA	No	6,32E-02	7,77E-01	NA	No
ENSG00000249345	RP11-575F12.1	lincRNA	-6,10E-02	5,36E-01	NA	No	1,22E-01	5,97E-01	NA	No
ENSG00000256001	RP11-575F12.2	lincRNA	-1,04E-01	5,07E-01	7,34E-01	No	-3,58E-02	9,14E-01	9,49E-01	No
ENSG00000257025	RP11-553N19.1	lincRNA	-2,82E-02	8,07E-01	9,14E-01	No	-2,76E-01	3,12E-01	4,66E-01	No
ENSG00000256250	RP11-989F5.1	lincRNA	-1,59E-01	4,45E-01	6,82E-01	No	-8,05E-01	7,21E-02	1,51E-01	No
ENSG00000256299	RP11-989F5.3	lincRNA	1,24E-01	5,70E-01	7,76E-01	No	-2,85E-01	4,30E-01	5,85E-01	No
ENSG00000256204	RP11-243M5.1	lincRNA	-2,03E-01	3,80E-01	6,26E-01	No	-4,10E-01	2,45E-01	3,89E-01	No
ENSG00000256151	RP11-76C10.5	lincRNA	2,36E-01	3,09E-01	5,58E-01	No	-2,25E-01	5,09E-01	6,56E-01	No
ENSG00000256810	RP11-76C10.2	lincRNA	4,68E-02	8,19E-01	9,21E-01	No	-9,48E-01	5,03E-02	1,13E-01	No
ENSG00000204603	RP11-638F5.1	lincRNA	-2,17E-02	8,32E-01	NA	No	-2,17E-01	3,57E-01	NA	No
ENSG00000256258	RP11-495K9.9	lincRNA	-1,75E-02	8,19E-01	NA	No	-1,33E-01	7,12E-01	NA	No
ENSG00000256424	RP11-495K9.7	lincRNA	3,32E-02	9,36E-01	NA	No	1,11E-01	6,54E-01	NA	No
ENSG00000255933	RP11-495K9.5	lincRNA	3,16E-01	1,91E-01	4,20E-01	No	-1,30E+00	1,24E-03	4,48E-03	No
ENSG00000256576	RP13-977J11.2	lincRNA	1,41E+00	1,52E-03	1,40E-02	No	-2,52E-01	4,83E-01	6,33E-01	No
ENSG00000256312	RP13-977J11.8	lincRNA	6,05E-02	6,02E-01	NA	No	2,36E-02	9,87E-01	NA	No
ENSG00000256783	RP11-503G7.1	lincRNA	4,46E-02	6,75E-01	NA	No	9,20E-02	8,64E-01	NA	No
ENSG00000256875	RP11-503G7.2	lincRNA	5,16E-02	5,12E-01	NA	No	1,22E-01	5,02E-01	NA	No
ENSG00000204583	LRCOL1	lincRNA	2,58E-01	1,73E-01	3,97E-01	No	1,23E-01	7,06E-01	8,12E-01	No
ENSG00000225316	LINC00350	lincRNA	-6,60E-02	4,16E-01	NA	No	-1,60E-01	4,74E-01	NA	No
ENSG00000236076	LINC01072	lincRNA	1,12E-02	9,45E-01	NA	No	-1,26E-01	7,12E-01	NA	No
ENSG00000229137	RP11-101P17.11	lincRNA	1,33E-01	4,37E-01	6,77E-01	No	1,16E+00	2,63E-02	6,52E-02	No
ENSG00000224429	LINC00422	lincRNA	7,66E-01	1,77E-02	8,42E-02	No	-2,34E-01	4,51E-01	6,05E-01	No
ENSG00000229556	RP11-363G2.4	lincRNA	4,17E-02	7,65E-01	NA	No	1,09E-01	6,90E-01	NA	No
ENSG00000262619	LINC00621	lincRNA	3,31E-02	9,36E-01	NA	No	1,01E-01	7,75E-01	NA	No
ENSG00000229483	LINC00362	lincRNA	3,46E-02	9,36E-01	NA	No	8,53E-02	8,64E-01	NA	No
ENSG00000229558	SACS-AS1	lincRNA	-2,68E-01	1,89E-01	4,18E-01	No	-3,20E-01	3,77E-01	5,34E-01	No
ENSG00000232977	LINC00327	lincRNA	-1,44E-01	5,22E-01	7,44E-01	No	9,37E-01	7,11E-03	2,11E-02	No
ENSG00000227893	LINC00352	lincRNA	-5,47E-02	6,46E-01	NA	No	-5,70E-02	8,00E-01	NA	No
ENSG00000228741	RP11-309I15.1	lincRNA	-3,94E-02	8,47E-01	9,33E-01	No	6,24E-01	1,10E-01	2,10E-01	No
ENSG00000260509	RP11-271M24.2	lincRNA	7,79E-02	5,37E-01	NA	No	1,07E-01	6,75E-01	NA	No
ENSG00000231983	LINC00415	lincRNA	3,15E-02	9,36E-01	NA	No	1,18E-01	5,75E-01	NA	No
ENSG00000260704	LINC00543	lincRNA	5,94E-02	4,15E-01	NA	No	8,77E-02	8,64E-01	NA	No
ENSG00000224511	LINC00365	lincRNA	1,02E-01	4,03E-01	NA	No	1,50E-01	5,63E-01	7,00E-01	No
ENSG00000232117	LINC00384	lincRNA	3,17E-01	1,61E-01	3,79E-01	No	-4,66E-01	2,24E-01	3,64E-01	No
ENSG00000236463	LINC00427	lincRNA	1,15E+00	9,42E-03	5,41E-02	No	1,46E-01	6,78E-01	7,92E-01	No
ENSG00000237879	LINC00398	lincRNA	6,08E-01	3,82E-02	1,45E-01	No	-4,61E-01	1,27E-01	NA	No
ENSG00000235822	LINC01073	lincRNA	-3,09E-01	2,08E-01	4,42E-01	No	-1,38E+00	2,80E-03	9,30E-03	No
ENSG00000227911	RP11-141M1.1	lincRNA	1,69E-01	4,51E-01	6,87E-01	No	1,89E-01	4,95E-01	6,44E-01	No
ENSG00000230490	RP11-141M1.3	lincRNA	1,46E-01	4,89E-01	7,19E-01	No	7,21E-01	1,04E-02	2,94E-02	No
ENSG00000234535	RP11-37L2.1	lincRNA	1,63E-01	4,73E-01	7,06E-01	No	5,30E-01	9,71E-02	1,91E-01	No
ENSG00000232986	RP11-179A7.2	lincRNA	-1,07E-02	9,59E-01	9,83E-01	No	-3,03E-01	3,50E-01	5,05E-01	No
ENSG00000271850	RP11-16D22.2	lincRNA	3,37E-01	9,90E-02	2,76E-01	No	1,62E-01	4,89E-01	6,38E-01	No
ENSG00000225179	LINC00457	lincRNA	1,60E-01	1,48E-01	NA	No	8,55E-02	8,64E-01	NA	No
ENSG00000271901	RP11-266E6.3	lincRNA	9,55E-02	4,53E-01	NA	No	1,35E-01	6,05E-01	NA	No
ENSG00000230390	LINC01048	lincRNA	2,40E-02	9,72E-01	NA	No	3,09E-01	1,97E-01	NA	No
ENSG00000223685	LINC00571	lincRNA	-9,07E-02	6,73E-01	8,41E-01	No	2,12E-01	4,62E-01	6,15E-01	No
ENSG00000230710	LINC00332	lincRNA	-3,02E-01	2,50E-02	NA	No	-7,37E-01	5,46E-02	1,20E-01	No
ENSG00000230058	RP11-172E9.2	lincRNA	-1,56E-02	9,28E-01	9,70E-01	No	3,79E-01	3,10E-01	4,63E-01	No
ENSG00000215483	LINC00598	lincRNA	1,52E-01	4,99E-01	7,26E-01	No	-2,09E-01	5,56E-01	6,95E-01	No
ENSG00000271216	LINC01050	lincRNA	-9,85E-02	2,19E-01	NA	No	-2,64E-01	2,21E-01	NA	No
ENSG00000238189	ENOX1-AS2	lincRNA	-1,20E-01	4,92E-01	7,22E-01	No	-1,20E-01	7,28E-01	8,27E-01	No
ENSG00000233821	ENOX1-AS1	lincRNA	5,13E-01	6,73E-02	2,14E-01	No	1,45E-01	6,60E-01	7,78E-01	No
ENSG00000270522	RP11-168P13.1	lincRNA	-2,51E-02	8,07E-01	NA	No	-1,60E-01	4,81E-01	NA	No
ENSG00000233725	LINC00284	lincRNA	2,80E-01	2,37E-01	4,77E-01	No	9,25E-01	2,11E-02	5,41E-02	No
ENSG00000226519	LINC00390	lincRNA	-1,71E-01	4,04E-01	6,48E-01	No	3,25E-01	3,72E-01	5,28E-01	No
ENSG00000230731	RP11-478K15.6	lincRNA	3,83E-01	1,13E-01	3,02E-01	No	9,19E-02	7,79E-01	8,63E-01	No

ENSG00000237361	LINC01071	lincRNA	9,93E-02	2,38E-01	NA	No	-2,72E-02	8,90E-01	NA	No
ENSG00000237585	LINC00407	lincRNA	3,68E-02	8,45E-01	NA	No	8,77E-02	8,64E-01	NA	No
ENSG00000228886	RP11-290D2.3	lincRNA	2,27E-01	3,07E-01	5,56E-01	No	-4,22E-01	2,60E-01	4,07E-01	No
ENSG00000251015	SLC25A30-AS1	lincRNA	3,15E-02	8,77E-01	9,48E-01	No	1,43E-01	6,84E-01	7,96E-01	No
ENSG00000261097	LINC00563	lincRNA	2,20E-02	7,70E-01	NA	No	-7,78E-03	9,79E-01	NA	No
ENSG00000231817	RP11-189B4.6	lincRNA	-7,33E-02	4,43E-01	NA	No	-2,32E-01	2,71E-01	NA	No
ENSG00000228573	RP11-279N8.1	lincRNA	-1,15E-01	4,57E-01	6,92E-01	No	-3,12E-01	3,15E-01	4,69E-01	No
ENSG00000234689	LINC00444	lincRNA	-2,49E-01	1,79E-01	4,04E-01	No	-8,13E-01	7,24E-02	1,51E-01	No
ENSG00000260388	LINC00562	lincRNA	7,90E-02	7,19E-01	8,69E-01	No	-1,61E-02	9,60E-01	9,76E-01	No
ENSG00000231473	LINC00441	lincRNA	1,48E-01	5,10E-01	7,36E-01	No	1,62E-01	6,39E-01	7,61E-01	No
ENSG00000233610	LINC00462	lincRNA	3,43E-03	9,90E-01	NA	No	-2,32E-02	8,90E-01	NA	No
ENSG00000229323	RP11-175B12.2	lincRNA	5,81E-02	5,31E-01	NA	No	3,23E-01	2,46E-01	NA	No
ENSG00000226792	LINC00371	lincRNA	8,80E-03	9,21E-01	NA	No	-1,23E-01	5,53E-01	NA	No
ENSG00000235660	LINC00345	lincRNA	2,11E-01	1,46E-01	NA	No	2,95E-01	2,98E-01	4,50E-01	No
ENSG00000232954	LINC00374	lincRNA	-1,63E-03	9,96E-01	NA	No	-2,52E-02	8,90E-01	NA	No
ENSG00000230040	LINC00364	lincRNA	1,26E-04	9,41E-01	NA	No	-1,02E-01	8,30E-01	NA	No
ENSG00000226846	LINC00348	lincRNA	4,19E-02	7,48E-01	NA	No	8,55E-02	8,64E-01	NA	No
ENSG00000236678	LINC00347	lincRNA	2,76E-02	8,28E-01	NA	No	6,23E-02	8,22E-01	8,91E-01	No
ENSG00000261206	LINC00561	lincRNA	-3,12E-03	9,79E-01	NA	No	5,57E-01	1,09E-01	2,09E-01	No
ENSG00000224933	LINC01034	lincRNA	1,18E-01	1,94E-01	NA	No	1,04E-01	7,75E-01	NA	No
ENSG00000225579	EDNRB-AS1	lincRNA	-6,20E-02	4,42E-01	NA	No	1,50E-01	5,99E-01	7,30E-01	No
ENSG00000233379	RP11-318G21.4	lincRNA	3,46E-02	9,36E-01	NA	No	9,85E-02	7,75E-01	NA	No
ENSG00000236133	LINC01069	lincRNA	-1,67E-03	9,89E-01	NA	No	3,00E-01	1,99E-01	3,33E-01	No
ENSG00000229249	LINC00446	lincRNA	2,40E-02	9,72E-01	NA	No	6,13E-02	9,56E-01	NA	No
ENSG00000227676	LINC01068	lincRNA	1,74E-02	8,76E-01	NA	No	-1,30E-01	5,72E-01	NA	No
ENSG00000229011	LINC01038	lincRNA	2,19E-02	8,83E-01	9,51E-01	No	2,25E-01	4,95E-01	6,44E-01	No
ENSG00000229175	LINC00382	lincRNA	3,88E-02	7,57E-01	NA	No	3,99E-01	1,74E-01	3,00E-01	No
ENSG00000229246	RP11-521J24.1	lincRNA	9,65E-02	5,72E-01	7,77E-01	No	9,96E-01	3,77E-02	8,85E-02	No
ENSG00000226317	LINC00351	lincRNA	4,12E-02	7,65E-01	NA	No	9,18E-02	8,64E-01	NA	No
ENSG00000228824	MIR4500HG	lincRNA	-2,18E-02	7,97E-01	NA	No	4,31E-02	8,07E-01	NA	No
ENSG00000231019	RP11-545P6.2	lincRNA	3,32E-02	9,36E-01	NA	No	8,51E-02	8,64E-01	NA	No
ENSG00000261446	LINC00559	lincRNA	5,33E-02	4,89E-01	NA	No	9,20E-02	8,64E-01	NA	No
ENSG00000234384	LINC01049	lincRNA	-5,08E-03	9,41E-01	NA	No	5,44E-02	7,90E-01	NA	No
ENSG00000231674	LINC00410	lincRNA	-8,41E-02	2,73E-01	NA	No	-1,76E-01	3,92E-01	NA	No
ENSG00000238230	LINC00391	lincRNA	4,16E-02	7,67E-01	NA	No	8,77E-02	8,64E-01	NA	No
ENSG00000227640	SOX21-AS1	lincRNA	4,04E-02	7,80E-01	NA	No	1,07E-01	6,95E-01	NA	No
ENSG00000247400	DNAJC3-AS1	lincRNA	3,97E-01	8,83E-02	2,57E-01	No	1,17E+00	8,85E-05	4,06E-04	No
ENSG00000243300	LINC00359	lincRNA	2,23E-03	9,62E-01	NA	No	-1,35E-01	7,12E-01	NA	No
ENSG00000233124	LINC00456	lincRNA	7,80E-02	4,35E-01	NA	No	-8,05E-03	9,70E-01	NA	No
ENSG00000270725	RP11-383H17.4	lincRNA	2,23E-02	9,72E-01	NA	No	6,17E-02	9,56E-01	NA	No
ENSG00000228889	UBAC2-AS1	lincRNA	2,52E-01	2,86E-01	5,32E-01	No	3,58E-01	2,71E-01	4,20E-01	No
ENSG00000229599	LINC00411	lincRNA	-6,33E-02	4,47E-01	NA	No	-1,72E-01	3,51E-01	NA	No
ENSG00000243319	FGF14-IT1	lincRNA	3,39E-01	1,60E-01	3,78E-01	No	1,21E+00	6,03E-03	1,83E-02	No
ENSG00000272143	FGF14-AS2	lincRNA	2,17E-01	2,78E-01	5,24E-01	No	1,21E+00	4,37E-10	4,65E-09	No
ENSG00000269942	RP11-29B2.5	lincRNA	2,93E-01	2,01E-01	4,33E-01	No	1,29E-01	6,51E-01	7,70E-01	No
ENSG00000272542	RP11-255P5.2	lincRNA	2,08E-01	3,46E-01	5,94E-01	No	-1,57E-01	6,54E-01	7,73E-01	No
ENSG00000272274	LINC00551	lincRNA	-1,21E-02	8,19E-01	NA	No	9,34E-02	6,63E-01	NA	No
ENSG00000230156	LINC00443	lincRNA	-5,73E-02	6,46E-01	NA	No	-2,72E-02	8,90E-01	NA	No
ENSG00000223617	LINC00370	lincRNA	-2,42E-01	1,26E-01	3,24E-01	No	-4,36E-01	2,03E-01	3,38E-01	No
ENSG00000236053	LINC01067	lincRNA	3,39E-02	9,36E-01	NA	No	6,68E-02	9,56E-01	NA	No
ENSG00000231428	LINC00396	lincRNA	-3,01E-02	7,31E-01	NA	No	3,20E-02	9,00E-01	NA	No
ENSG00000259831	LINC00567	lincRNA	-1,25E-03	9,74E-01	NA	No	-2,53E-02	8,90E-01	NA	No
ENSG00000225760	LINC00431	lincRNA	-2,76E-02	7,73E-01	8,96E-01	No	-6,92E-02	7,32E-01	8,30E-01	No
ENSG00000225870	LINC00368	lincRNA	-4,51E-02	6,31E-01	NA	No	-1,35E-01	4,54E-01	NA	No
ENSG00000260385	RP11-450H6.3	lincRNA	3,31E-02	9,36E-01	NA	No	8,77E-02	8,64E-01	NA	No
ENSG00000223626	LINC01044	lincRNA	-2,05E-02	8,19E-01	NA	No	-1,36E-01	7,12E-01	NA	No
ENSG00000267868	RP11-120K24.3	lincRNA	-1,12E-01	5,33E-01	7,52E-01	No	-6,57E-01	1,15E-01	2,20E-01	No
ENSG00000272695	GAS6-AS2	lincRNA	-4,00E-02	8,49E-01	9,34E-01	No	4,72E-01	7,84E-02	1,62E-01	No
ENSG00000229373	LINC00452	lincRNA	1,43E-02	8,76E-01	NA	No	-3,60E-02	8,90E-01	NA	No
ENSG00000260910	LINC00565	lincRNA	-1,62E-01	4,04E-01	6,48E-01	No	-1,15E+00	3,25E-02	7,79E-02	No
ENSG00000225210	AL589743.1	lincRNA	4,84E-02	8,10E-01	9,16E-01	No	9,72E-01	2,55E-02	6,35E-02	No
ENSG00000228294	BMS1P17	lincRNA	6,04E-02	5,78E-01	NA	No	4,37E-01	9,39E-02	1,86E-01	No

ENSG00000258188	RP11-146E13.4	lincRNA	1,03E-02	9,67E-01	NA	No	1,59E-01	3,98E-01	NA	No
ENSG00000244306	CTD-2314B22.3	lincRNA	4,25E-01	9,86E-02	2,76E-01	No	1,02E+00	2,13E-02	5,46E-02	No
ENSG00000215394	BMS1P18	lincRNA	2,75E-02	8,46E-01	9,33E-01	No	6,36E-01	1,16E-01	2,21E-01	No
ENSG00000259130	RP11-219E7.3	lincRNA	6,88E-03	9,06E-01	NA	No	-6,54E-02	8,00E-01	NA	No
ENSG00000258918	RP11-219E7.4	lincRNA	-4,23E-02	7,60E-01	NA	No	-9,82E-02	8,30E-01	NA	No
ENSG00000136315	RP11-84C10.2	lincRNA	-2,12E-01	7,99E-02	2,40E-01	No	-9,08E-01	1,31E-02	3,60E-02	No
ENSG00000258604	AL161668.5	lincRNA	-2,57E-01	2,34E-01	4,73E-01	No	-9,08E-02	7,91E-01	8,70E-01	No
ENSG00000259334	LINC00596	lincRNA	-6,77E-02	5,33E-01	7,52E-01	No	-3,51E-01	2,56E-01	4,02E-01	No
ENSG00000259321	RP11-468E2.5	lincRNA	1,02E-01	3,85E-01	NA	No	3,38E-01	2,99E-01	NA	No
ENSG00000258098	RP11-89K22.1	lincRNA	1,14E-01	5,23E-01	7,44E-01	No	1,28E+00	1,73E-02	4,57E-02	No
ENSG00000257986	RP11-314P15.2	lincRNA	-1,55E-02	9,10E-01	9,63E-01	No	6,61E-02	8,20E-01	8,90E-01	No
ENSG00000257845	RP11-626P14.1	lincRNA	-2,91E-03	9,89E-01	NA	No	1,76E-02	9,02E-01	NA	No
ENSG00000258081	RP11-384J4.2	lincRNA	-1,25E-03	9,41E-01	NA	No	6,73E-02	6,92E-01	NA	No
ENSG00000257612	RP11-384J4.1	lincRNA	-4,52E-02	6,07E-01	NA	No	-1,13E-01	5,94E-01	NA	No
ENSG00000258548	LINC00645	lincRNA	2,27E-01	1,04E-01	2,85E-01	No	1,00E+00	2,89E-02	7,07E-02	No
ENSG00000258038	CTD-2384A14.1	lincRNA	3,39E-02	9,36E-01	NA	No	8,77E-02	8,64E-01	NA	No
ENSG00000258028	RP11-148E17.1	lincRNA	4,36E-02	7,20E-01	NA	No	6,34E-02	9,56E-01	NA	No
ENSG00000257472	RP11-159D23.2	lincRNA	-6,16E-02	7,38E-01	8,78E-01	No	-1,77E-01	5,68E-01	7,04E-01	No
ENSG00000258860	RP11-561B11.3	lincRNA	-6,57E-02	4,24E-01	NA	No	-1,60E-01	4,77E-01	NA	No
ENSG00000257272	RP11-317N8.3	lincRNA	2,09E-01	2,74E-01	5,20E-01	No	5,82E-01	1,50E-01	2,69E-01	No
ENSG00000257585	LINC00609	lincRNA	4,27E-02	7,29E-01	NA	No	2,69E-01	2,69E-01	NA	No
ENSG00000259104	PTCSC3	lincRNA	3,17E-02	9,36E-01	NA	No	8,55E-02	8,64E-01	NA	No
ENSG00000259087	RP11-356O9.2	lincRNA	2,26E-01	1,31E-01	3,33E-01	No	3,48E-01	2,38E-01	3,81E-01	No
ENSG00000258414	RP11-356O9.1	lincRNA	-9,54E-04	9,93E-01	NA	No	1,11E+00	3,54E-02	8,39E-02	No
ENSG00000258649	CTD-2142D14.1	lincRNA	3,71E-02	6,87E-01	NA	No	1,87E-02	9,26E-01	NA	No
ENSG00000259072	RP11-96D24.1	lincRNA	-8,80E-02	6,67E-01	8,37E-01	No	-1,63E-01	6,40E-01	7,62E-01	No
ENSG00000259070	LINC00639	lincRNA	-4,20E-01	8,91E-02	2,58E-01	No	-1,16E+00	2,68E-04	1,12E-03	No
ENSG00000259083	RP11-407N17.4	lincRNA	2,24E-02	9,20E-01	9,67E-01	No	-9,51E-01	2,99E-02	7,26E-02	No
ENSG00000258526	RP11-111A21.1	lincRNA	1,50E-01	4,91E-01	7,20E-01	No	2,08E-02	9,54E-01	9,73E-01	No
ENSG00000258480	RP11-662J14.2	lincRNA	-4,21E-02	7,60E-01	NA	No	-9,72E-02	8,30E-01	NA	No
ENSG00000251363	RP11-129M6.1	lincRNA	-2,52E-02	8,72E-01	9,46E-01	No	4,88E-01	1,88E-01	3,20E-01	No
ENSG00000258636	CTD-2298J14.2	lincRNA	4,29E-01	6,57E-02	2,10E-01	No	1,19E+00	2,44E-02	6,12E-02	No
ENSG00000258850	RP11-214N1.1	lincRNA	-4,21E-02	7,60E-01	NA	No	-9,72E-02	8,30E-01	NA	No
ENSG00000259126	RP11-99L13.2	lincRNA	2,38E-02	8,04E-01	NA	No	4,81E-02	8,88E-01	NA	No
ENSG00000258487	RP11-99L13.1	lincRNA	2,22E-02	9,72E-01	NA	No	1,08E-01	6,87E-01	NA	No
ENSG00000258998	RP11-398E10.1	lincRNA	-3,41E-01	1,06E-01	2,90E-01	No	-1,98E-01	5,56E-01	6,95E-01	No
ENSG00000260046	RP11-454K7.3	lincRNA	1,42E-01	2,76E-01	5,22E-01	No	9,33E-01	5,52E-02	1,21E-01	No
ENSG00000257900	RP11-454K7.1	lincRNA	2,74E-01	2,05E-02	9,36E-02	No	8,68E-02	6,30E-01	NA	No
ENSG00000258845	RP11-945F5.1	lincRNA	1,72E-01	1,38E-01	NA	No	2,87E-01	2,33E-01	3,75E-01	No
ENSG00000258616	RP11-369C8.1	lincRNA	6,75E-02	4,41E-01	NA	No	1,99E-01	3,31E-01	NA	No
ENSG00000259129	LINC00648	lincRNA	3,30E-02	7,49E-01	8,83E-01	No	2,71E-01	3,88E-01	5,45E-01	No
ENSG00000258868	RP11-816J8.1	lincRNA	-4,54E-02	5,56E-01	NA	No	-1,61E-01	4,63E-01	NA	No
ENSG00000258946	RP11-58E21.4	lincRNA	1,94E-02	9,23E-01	9,68E-01	No	-5,83E-04	9,97E-01	9,99E-01	No
ENSG00000273307	RP11-58E21.7	lincRNA	2,37E-01	9,92E-02	NA	No	-6,72E-02	8,00E-01	NA	No
ENSG00000259071	RP11-247L20.4	lincRNA	-3,83E-01	1,04E-01	2,85E-01	No	9,25E-01	2,14E-04	9,14E-04	No
ENSG00000258857	RP11-247L20.3	lincRNA	1,33E-01	2,47E-01	NA	No	5,44E-02	8,85E-01	NA	No
ENSG00000259113	RP11-406H23.2	lincRNA	1,16E-01	5,79E-01	7,82E-01	No	3,82E-01	2,67E-01	4,14E-01	No
ENSG00000258711	RP11-218E20.3	lincRNA	-1,50E-01	8,78E-02	NA	No	-1,16E-01	5,99E-01	7,29E-01	No
ENSG00000258955	LINC00519	lincRNA	-3,68E-02	7,52E-01	8,84E-01	No	2,18E-02	9,45E-01	9,68E-01	No
ENSG00000258479	LINC00640	lincRNA	2,26E-01	2,91E-01	5,38E-01	No	-2,23E-01	5,28E-01	6,72E-01	No
ENSG00000258942	RP11-255G12.2	lincRNA	5,41E-02	7,23E-01	8,71E-01	No	-3,28E-01	1,23E-01	2,31E-01	No
ENSG00000258808	RP11-255G12.3	lincRNA	4,52E-02	6,53E-01	NA	No	6,17E-02	9,56E-01	NA	No
ENSG00000258537	FRMD6-AS2	lincRNA	9,21E-02	3,90E-01	NA	No	1,77E-01	4,26E-01	NA	No
ENSG00000258694	RP11-1033H12.1	lincRNA	-1,50E-02	8,49E-01	NA	No	1,70E-02	9,87E-01	NA	No
ENSG00000258698	RP11-589M4.3	lincRNA	-5,13E-03	9,01E-01	NA	No	5,07E-02	8,34E-01	NA	No
ENSG00000237356	AL163953.3	lincRNA	6,52E-01	2,86E-02	1,18E-01	No	3,05E-01	3,80E-01	5,37E-01	No
ENSG00000225680	AL163953.2	lincRNA	9,20E-02	2,79E-01	5,25E-01	No	4,99E-02	8,41E-01	NA	No
ENSG00000235269	AL162759.1	lincRNA	-3,55E-02	6,91E-01	NA	No	-1,31E-01	5,43E-01	NA	No
ENSG00000258413	RP11-665C16.6	lincRNA	2,11E-01	3,58E-01	6,05E-01	No	2,38E-01	4,49E-01	6,03E-01	No
ENSG00000258784	RP11-813I20.2	lincRNA	-1,55E-01	2,06E-01	4,39E-01	No	-5,47E-01	6,74E-02	1,43E-01	No
ENSG00000259868	RP11-1012E15.1	lincRNA	-1,97E-01	4,81E-02	NA	No	-4,98E-01	4,91E-02	1,10E-01	No
ENSG00000259483	RP11-930O11.2	lincRNA	4,15E-01	1,16E-01	3,07E-01	No	1,20E-01	7,20E-01	8,22E-01	No

ENSG00000259719	RP11-930O11.1	lincRNA	1,17E-01	5,87E-01	7,88E-01	No	2,73E-02	9,35E-01	9,61E-01	No
ENSG00000258803	RP11-624J12.1	lincRNA	4,23E-02	7,43E-01	NA	No	1,10E-01	6,87E-01	NA	No
ENSG00000258592	RP11-108M12.3	lincRNA	5,95E-02	4,11E-01	NA	No	7,25E-01	2,72E-02	6,71E-02	No
ENSG00000259008	RP11-932A10.1	lincRNA	-1,70E-01	3,39E-01	5,88E-01	No	-3,67E-01	3,08E-01	4,61E-01	No
ENSG00000258583	RP11-112J1.2	lincRNA	2,13E-02	8,67E-01	NA	No	2,31E-01	3,54E-01	NA	No
ENSG00000258556	CTD-2568P8.1	lincRNA	1,68E-01	1,23E-01	NA	No	8,58E-02	8,64E-01	NA	No
ENSG00000258777	HIF1A-AS1	lincRNA	-7,58E-02	4,54E-01	NA	No	-1,44E-01	5,80E-01	NA	No
ENSG00000258667	HIF1A-AS2	lincRNA	-2,76E-01	2,01E-01	4,34E-01	No	3,89E-01	1,42E-01	2,57E-01	No
ENSG00000258882	CTD-2277K2.1	lincRNA	4,03E-02	7,82E-01	NA	No	9,95E-02	7,75E-01	NA	No
ENSG00000186369	LINC00643	lincRNA	4,04E-02	7,80E-01	NA	No	1,17E-01	5,90E-01	NA	No
ENSG00000259093	RP11-1112J20.2	lincRNA	-1,03E-01	3,91E-01	NA	No	-2,33E-01	3,43E-01	NA	No
ENSG00000258809	CTD-2555O16.1	lincRNA	1,97E-02	8,87E-01	9,53E-01	No	-3,00E-01	2,00E-01	NA	No
ENSG00000272828	CTD-2555O16.3	lincRNA	-1,40E-01	4,91E-01	7,20E-01	No	-7,40E-01	8,91E-02	1,79E-01	No
ENSG00000255002	CTD-2509G16.2	lincRNA	4,04E-02	7,81E-01	NA	No	8,64E-02	8,64E-01	NA	No
ENSG00000258760	CTD-2509G16.5	lincRNA	1,94E-01	7,21E-02	NA	No	9,82E-02	7,75E-01	NA	No
ENSG00000258502	RP11-783L4.1	lincRNA	3,01E-02	7,83E-01	NA	No	4,42E-01	1,51E-01	2,70E-01	No
ENSG00000258561	RP11-72M17.1	lincRNA	4,83E-01	6,53E-02	2,09E-01	No	1,08E+00	1,98E-02	5,13E-02	No
ENSG00000258957	RP11-363J20.1	lincRNA	1,31E+00	9,87E-03	5,60E-02	No	9,40E-01	5,20E-02	1,16E-01	No
ENSG00000258520	RP11-363J20.2	lincRNA	-6,25E-02	7,31E-01	8,75E-01	No	-1,74E-01	6,16E-01	7,43E-01	No
ENSG00000257759	RP11-486O13.4	lincRNA	3,30E-01	1,21E-01	3,17E-01	No	3,25E-01	3,65E-01	5,21E-01	No
ENSG00000259158	ADAM20P1	lincRNA	1,27E-01	5,66E-01	7,73E-01	No	-1,18E-01	7,29E-01	8,28E-01	No
ENSG00000245466	CTD-2540L5.6	lincRNA	-1,36E-02	8,19E-01	NA	No	-1,33E-01	7,12E-01	NA	No
ENSG00000259153	RP6-65G23.3	lincRNA	2,47E-01	2,70E-01	5,15E-01	No	1,04E-01	7,64E-01	8,52E-01	No
ENSG00000269927	RP6-91H8.3	lincRNA	2,00E-01	1,78E-01	4,03E-01	No	-2,97E-01	2,29E-01	NA	No
ENSG00000259907	RP6-91H8.2	lincRNA	4,28E-02	7,31E-01	NA	No	1,19E-01	5,56E-01	NA	No
ENSG00000258517	RP6-91H8.1	lincRNA	1,36E-02	8,78E-01	NA	No	-8,15E-02	7,36E-01	NA	No
ENSG00000269945	RP6-91H8.5	lincRNA	5,18E-02	6,88E-01	8,50E-01	No	1,23E-01	7,01E-01	8,08E-01	No
ENSG00000258871	RP3-514A23.2	lincRNA	-1,05E-01	6,31E-01	8,17E-01	No	-8,34E-01	5,85E-02	1,27E-01	No
ENSG00000258813	RP11-109N23.4	lincRNA	-2,07E-02	9,07E-01	9,61E-01	No	-4,32E-01	2,49E-01	3,93E-01	No
ENSG00000258586	RP5-1021I20.2	lincRNA	-4,57E-02	5,68E-01	NA	No	4,91E-01	1,37E-01	2,51E-01	No
ENSG00000270000	RP3-449M8.9	lincRNA	1,20E-01	4,40E-01	6,79E-01	No	1,40E-01	6,17E-01	7,44E-01	No
ENSG00000259005	RP3-449M8.6	lincRNA	5,87E-01	4,44E-02	1,60E-01	No	-7,33E-01	8,82E-02	1,77E-01	No
ENSG00000258425	CTD-2207P18.1	lincRNA	1,36E-01	3,60E-01	6,08E-01	No	-2,86E-02	9,28E-01	9,58E-01	No
ENSG00000258976	CTD-2207P18.2	lincRNA	7,06E-01	3,16E-02	1,27E-01	No	4,18E-01	2,28E-01	3,68E-01	No
ENSG00000259687	RP11-293M10.5	lincRNA	1,46E-01	4,84E-01	7,15E-01	No	1,04E+00	2,36E-02	5,95E-02	No
ENSG00000258876	RP11-270M14.5	lincRNA	-5,53E-02	7,73E-01	8,96E-01	No	-6,40E-01	1,18E-01	2,24E-01	No
ENSG00000258402	RP11-516J2.1	lincRNA	1,05E-04	9,82E-01	NA	No	2,72E-01	2,59E-01	NA	No
ENSG00000259124	RP11-187O7.3	lincRNA	8,05E-02	6,87E-01	8,50E-01	No	-3,01E-01	4,05E-01	5,62E-01	No
ENSG00000258569	RP11-99E15.2	lincRNA	2,41E-01	2,77E-01	5,23E-01	No	3,01E-01	4,05E-01	5,62E-01	No
ENSG00000258301	RP11-488C13.5	lincRNA	8,18E-03	9,68E-01	9,87E-01	No	-6,24E-01	2,75E-02	6,77E-02	No
ENSG00000258602	RP11-7F17.7	lincRNA	-2,39E-01	2,28E-01	4,66E-01	No	-1,54E-01	5,88E-01	7,20E-01	No
ENSG00000246548	RP11-7F17.5	lincRNA	1,96E-01	3,82E-01	6,28E-01	No	-4,76E-01	2,16E-01	3,54E-01	No
ENSG00000258473	RP11-7F17.4	lincRNA	-2,29E-02	8,19E-01	NA	No	-1,35E-01	7,12E-01	NA	No
ENSG00000258819	RP11-7F17.3	lincRNA	6,32E-01	2,92E-02	1,20E-01	No	6,53E-02	8,41E-01	9,03E-01	No
ENSG00000258416	RP11-526N18.1	lincRNA	-1,05E-04	9,41E-01	NA	No	-1,03E-01	8,30E-01	NA	No
ENSG00000258743	RP11-406A9.2	lincRNA	3,68E-03	9,91E-01	NA	No	4,00E-01	1,63E-01	2,86E-01	No
ENSG00000205562	RP11-497E19.1	lincRNA	2,78E-02	8,17E-01	9,19E-01	No	9,39E-02	7,18E-01	8,21E-01	No
ENSG00000258859	RP11-594C13.1	lincRNA	-8,57E-02	2,77E-01	NA	No	-1,69E-01	4,62E-01	NA	No
ENSG00000258770	CTD-2007A10.1	lincRNA	-6,20E-02	5,19E-01	NA	No	-8,05E-02	7,07E-01	NA	No
ENSG00000258807	RP11-1152H15.1	lincRNA	3,94E-03	9,68E-01	NA	No	5,75E-02	8,02E-01	NA	No
ENSG00000258826	RP11-300J18.1	lincRNA	1,72E-01	2,79E-01	5,25E-01	No	-1,70E-01	4,50E-01	6,03E-01	No
ENSG00000258867	LINC01146	lincRNA	8,10E-01	1,91E-02	8,90E-02	No	5,44E-02	8,65E-01	9,19E-01	No
ENSG00000258678	RP11-1078H9.1	lincRNA	-5,37E-02	6,46E-01	NA	No	-1,26E-01	7,12E-01	NA	No
ENSG00000233208	LINC00642	lincRNA	3,46E-02	9,36E-01	NA	No	1,13E-01	6,43E-01	NA	No
ENSG00000258935	RP11-1078H9.2	lincRNA	-4,04E-02	8,44E-01	9,32E-01	No	-1,02E+00	4,32E-02	9,91E-02	No
ENSG00000258884	CTD-3035D6.2	lincRNA	-4,64E-02	7,47E-01	8,82E-01	No	-3,28E-01	2,56E-01	4,02E-01	No
ENSG00000260810	CTD-2547L24.4	lincRNA	2,87E-01	1,50E-01	3,64E-01	No	-1,74E-01	5,84E-01	7,17E-01	No
ENSG00000258481	RP11-472N19.3	lincRNA	1,12E-02	9,64E-01	NA	No	-1,02E-01	8,30E-01	NA	No
ENSG00000258742	RP11-862G15.1	lincRNA	-7,06E-03	9,37E-01	NA	No	-1,10E-01	6,07E-01	NA	No
ENSG00000258499	RP11-862G15.2	lincRNA	4,12E-02	7,65E-01	NA	No	1,10E-01	6,71E-01	NA	No
ENSG00000229645	LINC00341	lincRNA	4,07E-01	4,38E-02	1,59E-01	No	-5,83E-01	6,37E-04	2,46E-03	No
ENSG00000258390	RP11-1070N10.4	lincRNA	-4,06E-02	6,14E-01	NA	No	5,46E-02	8,00E-01	8,77E-01	No

ENSG00000258927	RP11-1070N10.5	lincRNA	3,32E-02	9,36E-01	NA	No	8,58E-02	8,64E-01	NA	No
ENSG00000258702	RP11-433J8.1	lincRNA	2,33E-01	1,35E-01	NA	No	1,41E-01	4,95E-01	6,44E-01	No
ENSG00000225163	LINC00618	lincRNA	2,49E-01	2,25E-01	4,63E-01	No	-3,81E-01	2,15E-01	NA	No
ENSG00000197176	RP11-76E12.1	lincRNA	3,41E-02	9,36E-01	NA	No	8,55E-02	8,64E-01	NA	No
ENSG00000247970	RP11-543C4.1	lincRNA	3,74E-02	8,50E-01	9,35E-01	No	-3,18E-01	3,54E-01	5,10E-01	No
ENSG00000258693	RP11-436M15.3	lincRNA	1,99E-02	8,79E-01	NA	No	4,02E-01	2,43E-01	3,86E-01	No
ENSG00000259031	CTD-2062F14.3	lincRNA	6,07E-02	6,02E-01	NA	No	-8,27E-02	6,98E-01	NA	No
ENSG00000273087	RP11-566J3.4	lincRNA	3,29E-02	6,68E-01	NA	No	7,55E-02	6,94E-01	NA	No
ENSG00000214548	MEG3	lincRNA	5,01E-01	3,57E-02	1,38E-01	No	-3,93E-01	1,48E-01	2,66E-01	No
ENSG00000258399	MEG8	lincRNA	-1,38E-01	5,18E-01	7,41E-01	No	7,18E-01	7,17E-03	2,13E-02	No
ENSG00000225746	AL132709.5	lincRNA	-4,60E-02	8,11E-01	9,16E-01	No	1,10E+00	6,14E-05	2,92E-04	No
ENSG00000232018	AL132709.8	lincRNA	-4,18E-02	8,47E-01	9,33E-01	No	5,48E-01	1,02E-01	1,98E-01	No
ENSG00000271417	RP11-909M7.3	lincRNA	-2,42E-01	2,17E-01	4,53E-01	No	6,62E-01	1,30E-02	3,58E-02	No
ENSG00000258861	MIR381HG	lincRNA	2,58E-02	8,90E-01	9,54E-01	No	-5,26E-01	1,40E-01	2,55E-01	No
ENSG00000223403	MEG9	lincRNA	1,50E-02	9,47E-01	9,78E-01	No	-1,13E+00	2,60E-02	6,46E-02	No
ENSG00000259004	RP11-8L8.2	lincRNA	8,18E-02	6,65E-01	8,36E-01	No	-1,72E-01	6,14E-01	7,42E-01	No
ENSG00000258498	DIO3OS	lincRNA	6,00E-01	4,01E-02	1,50E-01	No	2,51E-01	4,83E-01	6,33E-01	No
ENSG00000258919	RP11-1029J19.4	lincRNA	8,90E-02	3,01E-01	NA	No	3,42E-02	8,89E-01	NA	No
ENSG00000258404	RP11-1029J19.5	lincRNA	2,24E-01	8,55E-02	NA	No	-1,32E-01	7,12E-01	NA	No
ENSG00000271780	RP11-1017G21.5	lincRNA	4,23E-01	9,29E-02	2,65E-01	No	5,56E-01	8,40E-02	1,71E-01	No
ENSG00000272444	RP11-1017G21.6	lincRNA	-4,61E-01	8,99E-02	2,59E-01	No	-1,57E-01	6,42E-01	7,64E-01	No
ENSG00000259230	CTD-2555C10.3	lincRNA	1,30E-01	1,72E-01	NA	No	9,18E-02	8,64E-01	NA	No
ENSG00000259508	RP11-661D19.3	lincRNA	5,70E-02	5,89E-01	NA	No	9,02E-02	8,64E-01	NA	No
ENSG00000259717	LINC00677	lincRNA	-8,29E-03	9,59E-01	9,83E-01	No	-2,20E-01	4,58E-01	6,11E-01	No
ENSG00000251533	LINC00605	lincRNA	-1,37E-01	1,32E-01	NA	No	-1,14E-01	6,08E-01	NA	No
ENSG00000258735	LINC00637	lincRNA	2,21E-02	8,38E-01	NA	No	1,77E-01	4,86E-01	NA	No
ENSG00000258534	CTD-2134A5.4	lincRNA	2,21E-01	1,86E-01	4,14E-01	No	1,26E+00	2,68E-02	6,63E-02	No
ENSG00000258914	CTD-2134A5.3	lincRNA	5,87E-02	5,63E-01	NA	No	-1,49E-01	6,00E-01	NA	No
ENSG00000258913	RP11-260M19.2	lincRNA	6,51E-02	4,38E-01	NA	No	1,18E-02	9,25E-01	NA	No
ENSG00000260792	RP11-982M15.8	lincRNA	4,26E-02	7,31E-01	NA	No	9,20E-02	8,64E-01	NA	No
ENSG00000256050	RP11-982M15.6	lincRNA	1,02E-01	2,97E-01	NA	No	1,03E-01	6,25E-01	NA	No
ENSG00000258701	LINC00638	lincRNA	-8,27E-02	6,81E-01	8,46E-01	No	-7,05E-01	8,62E-02	1,74E-01	No
ENSG00000258811	CTD-3051D23.1	lincRNA	6,21E-03	9,43E-01	NA	No	-6,41E-02	6,81E-01	NA	No
ENSG00000259067	CTD-3051D23.3	lincRNA	3,15E-02	9,36E-01	NA	No	1,17E-01	5,78E-01	NA	No
ENSG00000257556	RP11-44N21.1	lincRNA	1,06E+00	7,60E-03	4,64E-02	No	3,19E-01	3,77E-01	5,34E-01	No
ENSG00000187156	LINC00221	lincRNA	-5,58E-02	6,46E-01	NA	No	-3,60E-02	8,90E-01	NA	No
ENSG00000258410	RP11-173D3.1	lincRNA	-1,49E-01	4,85E-01	7,15E-01	No	-7,62E-01	8,57E-02	1,73E-01	No
ENSG00000258654	RP11-509A17.3	lincRNA	7,83E-02	6,16E-01	8,07E-01	No	-8,61E-01	1,96E-02	5,09E-02	No
ENSG00000260409	RP11-403B2.7	lincRNA	2,31E-01	3,14E-01	5,63E-01	No	-1,79E-02	9,56E-01	9,74E-01	No
ENSG00000258710	CT60	lincRNA	-4,40E-02	6,84E-01	NA	No	-3,99E-02	8,68E-01	9,21E-01	No
ENSG00000247765	RP11-32B5.7	lincRNA	4,94E-01	7,54E-02	2,31E-01	No	7,19E-02	8,38E-01	9,01E-01	No
ENSG00000261622	RP11-484P15.1	lincRNA	-3,01E-02	7,31E-01	NA	No	2,33E-01	3,00E-01	NA	No
ENSG00000260551	PWRN2	lincRNA	4,19E-02	7,48E-01	NA	No	1,21E-01	5,33E-01	NA	No
ENSG00000261621	RP11-580I1.2	lincRNA	-2,81E-02	7,31E-01	NA	No	-1,30E-01	7,12E-01	NA	No
ENSG00000261598	RP11-107D24.2	lincRNA	-4,46E-02	7,60E-01	NA	No	-1,03E-01	8,30E-01	NA	No
ENSG00000260760	PWRN3	lincRNA	2,92E-01	3,27E-02	1,30E-01	No	2,54E-03	9,65E-01	NA	No
ENSG00000259905	PWRN1	lincRNA	2,33E-01	8,99E-01	9,58E-01	No	-3,23E-01	3,33E-01	4,88E-01	No
ENSG00000257647	RP11-701H24.3	lincRNA	-3,79E-01	6,32E-02	2,04E-01	No	1,20E-01	5,09E-01	6,56E-01	No
ENSG00000257151	PWAR6	lincRNA	-3,60E-01	3,91E-02	1,47E-01	No	9,24E-01	8,60E-08	6,64E-07	No
ENSG00000261069	SNORD116-20	lincRNA	-1,67E-01	4,47E-01	6,84E-01	No	4,51E-02	9,02E-01	9,42E-01	No
ENSG00000235160	AC009878.2	lincRNA	-3,07E-01	5,04E-02	1,75E-01	No	-5,58E-01	1,56E-01	2,76E-01	No
ENSG00000235518	AC011196.3	lincRNA	-5,37E-02	6,46E-01	NA	No	3,63E-02	8,84E-01	NA	No
ENSG00000259647	RP11-143J24.1	lincRNA	-4,59E-02	7,49E-01	8,83E-01	No	-4,74E-01	1,17E-01	NA	No
ENSG00000225930	AC026150.5	lincRNA	7,02E-02	5,10E-01	NA	No	-1,26E-01	7,12E-01	NA	No
ENSG00000270173	RP11-382B18.4	lincRNA	-1,83E-02	8,19E-01	NA	No	2,16E-01	4,59E-01	6,12E-01	No
ENSG00000260693	AC026150.8	lincRNA	1,37E-01	5,33E-01	7,52E-01	No	6,91E-01	4,46E-02	1,02E-01	No
ENSG00000269930	RP11-932O9.9	lincRNA	5,85E-03	9,74E-01	9,90E-01	No	8,79E-01	4,91E-02	1,10E-01	No
ENSG00000269974	RP11-932O9.10	lincRNA	1,09E-01	5,06E-01	7,32E-01	No	1,91E-01	5,31E-01	6,74E-01	No
ENSG00000259448	RP11-16E12.1	lincRNA	5,42E-01	6,09E-02	1,99E-01	No	3,35E-01	3,46E-01	5,02E-01	No
ENSG00000261064	RP11-1000B6.3	lincRNA	-1,72E-03	9,92E-01	9,97E-01	No	3,46E-01	1,94E-01	3,27E-01	No
ENSG00000262728	AC123768.4	lincRNA	9,33E-02	6,50E-01	8,27E-01	No	1,27E+00	1,96E-07	1,43E-06	No
ENSG00000244952	RP11-1000B6.5	lincRNA	5,18E-01	6,87E-02	2,17E-01	No	1,72E-01	6,17E-01	7,44E-01	No

ENSG00000259721	RP11-758N13.1	lincRNA	-6,98E-02	3,85E-01	NA	No	-1,64E-01	4,43E-01	NA	No
ENSG00000261304	CTD-2125J1.1	lincRNA	3,77E-02	8,45E-01	NA	No	6,23E-02	9,56E-01	NA	No
ENSG00000259181	RP11-463I20.3	lincRNA	9,09E-02	5,67E-01	7,74E-01	No	6,71E-02	8,01E-01	8,77E-01	No
ENSG00000259587	RP11-463I20.2	lincRNA	2,77E-02	8,33E-01	9,27E-01	No	-1,33E-01	6,64E-01	7,81E-01	No
ENSG00000259336	RP11-323I15.5	lincRNA	-4,85E-01	6,99E-02	2,19E-01	No	-1,03E-01	7,19E-01	8,21E-01	No
ENSG00000248079	DPH6-AS1	lincRNA	-2,86E-01	2,35E-01	4,74E-01	No	5,23E-01	1,02E-01	1,98E-01	No
ENSG00000259639	RP11-184D12.1	lincRNA	-1,04E-01	5,35E-01	7,53E-01	No	-2,79E-01	4,34E-01	5,88E-01	No
ENSG00000259245	RP11-684B21.1	lincRNA	4,85E-02	6,60E-01	NA	No	-2,95E-02	9,05E-01	NA	No
ENSG00000259737	RP11-475A13.1	lincRNA	-2,94E-01	1,43E-01	3,51E-01	No	-4,61E-01	1,70E-01	2,96E-01	No
ENSG00000259434	RP11-720L8.1	lincRNA	-1,90E-01	9,76E-02	NA	No	-1,42E-02	9,59E-01	9,76E-01	No
ENSG00000259225	RP11-1008C21.1	lincRNA	3,92E-01	7,38E-02	2,27E-01	No	6,18E-01	1,25E-01	2,34E-01	No
ENSG00000236914	RP11-1008C21.2	lincRNA	7,41E-01	1,33E-02	6,89E-02	No	5,68E-01	9,57E-02	1,89E-01	No
ENSG00000259380	RP11-346D14.1	lincRNA	-4,10E-02	6,13E-01	NA	No	-1,09E-01	5,50E-01	NA	No
ENSG00000259423	RP11-265N7.2	lincRNA	2,98E-01	1,50E-01	3,62E-01	No	-4,10E-01	1,93E-01	3,26E-01	No
ENSG00000259450	RP11-265N7.1	lincRNA	-7,96E-02	6,64E-01	8,35E-01	No	-8,14E-01	7,14E-02	1,50E-01	No
ENSG00000259447	RP11-462P6.1	lincRNA	-2,81E-01	1,70E-01	3,92E-01	No	-3,13E-01	3,81E-01	5,38E-01	No
ENSG00000259269	RP11-624L4.2	lincRNA	-1,73E-02	9,22E-01	9,68E-01	No	-3,29E-01	3,62E-01	5,18E-01	No
ENSG00000259390	RP11-27M9.1	lincRNA	-1,33E-01	3,10E-01	NA	No	-2,63E-01	3,13E-01	NA	No
ENSG00000246863	RP11-325N19.3	lincRNA	-4,54E-03	9,89E-01	NA	No	1,12E-01	4,99E-01	NA	No
ENSG00000248508	SRP14-AS1	lincRNA	-6,20E-01	9,91E-03	5,61E-02	No	3,29E-01	1,97E-01	3,30E-01	No
ENSG00000259584	RP11-521C20.2	lincRNA	-5,30E-02	7,16E-01	8,67E-01	No	-1,57E-01	6,19E-01	7,46E-01	No
ENSG00000259714	LINC00594	lincRNA	4,32E-02	7,25E-01	NA	No	9,18E-02	8,64E-01	NA	No
ENSG00000251161	RP11-540O11.1	lincRNA	5,33E-02	7,19E-01	8,69E-01	No	-2,65E-01	3,10E-01	NA	No
ENSG00000260814	RP11-107F6.3	lincRNA	-5,00E-01	5,88E-02	1,94E-01	No	5,23E-01	4,62E-02	1,05E-01	No
ENSG00000259499	RP11-616K22.2	lincRNA	1,89E-01	3,58E-01	6,05E-01	No	-3,89E-01	2,97E-01	4,49E-01	No
ENSG00000179523	EIF3J-AS1	lincRNA	-1,23E-02	9,41E-01	9,76E-01	No	4,60E-01	1,25E-02	3,47E-02	No
ENSG00000259200	RP11-718O11.1	lincRNA	-5,47E-02	6,46E-01	NA	No	-1,30E-01	7,12E-01	NA	No
ENSG00000259235	RP11-605F22.2	lincRNA	-5,73E-02	6,46E-01	NA	No	-2,75E-02	8,90E-01	NA	No
ENSG00000259705	RP11-227D13.1	lincRNA	3,61E-01	1,38E-01	3,45E-01	No	1,03E-01	7,67E-01	8,54E-01	No
ENSG00000259469	RP11-227D13.4	lincRNA	6,75E-03	9,53E-01	NA	No	-1,61E-01	4,80E-01	NA	No
ENSG00000259656	RP11-325E5.1	lincRNA	1,74E-01	2,24E-01	NA	No	5,62E-01	9,80E-02	1,92E-01	No
ENSG00000259715	CTD-3110H11.1	lincRNA	-7,61E-02	7,28E-01	8,73E-01	No	-4,26E-01	2,45E-01	3,89E-01	No
ENSG00000259618	RP11-562A8.5	lincRNA	-1,49E-01	5,01E-01	7,28E-01	No	-4,71E-03	9,87E-01	9,93E-01	No
ENSG00000259240	RP11-108K3.1	lincRNA	1,36E-02	9,10E-01	NA	No	-1,60E-01	4,62E-01	NA	No
ENSG00000259306	RP11-108K3.2	lincRNA	3,76E-02	7,55E-01	NA	No	9,95E-02	6,37E-01	NA	No
ENSG00000259178	CTD-2184D3.3	lincRNA	-5,58E-02	6,46E-01	NA	No	-1,33E-01	7,12E-01	NA	No
ENSG00000259327	CTD-2184D3.6	lincRNA	3,04E-01	2,62E-02	NA	No	1,08E-01	6,89E-01	NA	No
ENSG00000259577	RP11-430B1.2	lincRNA	3,66E-01	1,17E-01	3,09E-01	No	6,08E-02	8,54E-01	9,11E-01	No
ENSG00000261823	RP11-48G14.1	lincRNA	4,53E-01	5,50E-02	1,85E-01	No	4,32E-01	1,77E-01	3,05E-01	No
ENSG00000247982	LINC00926	lincRNA	3,23E-01	1,86E-01	4,13E-01	No	-8,98E-01	3,02E-02	7,33E-02	No
ENSG00000260172	RP11-358M11.3	lincRNA	3,31E-02	9,36E-01	NA	No	6,23E-02	9,56E-01	NA	No
ENSG00000261489	CTD-2515H24.3	lincRNA	1,46E-01	4,45E-01	6,82E-01	No	3,11E-01	3,89E-01	5,46E-01	No
ENSG00000261265	CTD-2515H24.4	lincRNA	1,11E-01	5,92E-01	7,92E-01	No	-3,95E-01	2,26E-01	3,67E-01	No
ENSG00000261219	RP11-344A16.2	lincRNA	-2,37E-01	2,97E-01	5,44E-01	No	2,50E-02	9,43E-01	9,66E-01	No
ENSG00000245975	RP11-30K9.6	lincRNA	-3,60E-01	1,56E-01	3,72E-01	No	1,29E-01	6,79E-01	7,92E-01	No
ENSG00000259616	RP11-507B12.2	lincRNA	9,05E-02	5,28E-01	7,49E-01	No	2,82E-01	3,86E-01	5,44E-01	No
ENSG00000259530	RP11-259A24.1	lincRNA	4,01E-01	1,94E-02	NA	No	3,57E-01	1,63E-01	NA	No
ENSG00000259675	RP11-507B12.1	lincRNA	1,09E-01	3,89E-01	6,35E-01	No	7,61E-01	7,74E-02	1,60E-01	No
ENSG00000259284	RP11-162I7.1	lincRNA	2,46E-02	9,72E-01	NA	No	6,34E-02	9,56E-01	NA	No
ENSG00000261296	RP11-299H22.6	lincRNA	1,93E-02	8,66E-01	NA	No	2,30E-02	9,35E-01	9,61E-01	No
ENSG00000259672	RP11-69G7.1	lincRNA	-5,74E-02	4,67E-01	NA	No	-1,59E-01	4,73E-01	NA	No
ENSG00000259437	RP11-798K3.4	lincRNA	4,03E-02	6,68E-01	NA	No	4,66E-01	1,40E-01	NA	No
ENSG00000259289	RP11-798K3.3	lincRNA	6,84E-02	5,94E-01	7,93E-01	No	-1,36E-01	5,66E-01	NA	No
ENSG00000259347	RP11-798K3.2	lincRNA	-6,10E-02	6,66E-01	8,37E-01	No	-1,26E-01	6,52E-01	7,72E-01	No
ENSG00000259673	IQCH-AS1	lincRNA	-3,73E-01	3,03E-02	1,23E-01	No	4,19E-01	2,05E-02	5,30E-02	No
ENSG00000270964	RP11-502I4.3	lincRNA	2,16E-01	3,54E-01	6,02E-01	No	3,14E-01	3,37E-01	4,92E-01	No
ENSG00000245719	RP11-34F13.2	lincRNA	-5,01E-02	5,68E-01	NA	No	-1,30E-01	5,26E-01	NA	No
ENSG00000260657	RP11-315D16.4	lincRNA	1,81E-02	8,97E-01	9,57E-01	No	-1,50E-01	4,29E-01	5,85E-01	No
ENSG00000261425	RP11-709B3.2	lincRNA	-2,36E-01	2,46E-01	4,88E-01	No	-5,28E-01	1,49E-02	4,02E-02	No
ENSG00000212766	LINC00277	lincRNA	5,72E-03	9,70E-01	9,88E-01	No	-1,68E-01	4,62E-01	6,14E-01	No
ENSG00000259504	RP11-352D13.5	lincRNA	-7,39E-03	9,45E-01	NA	No	-1,52E-01	5,67E-01	NA	No
ENSG00000259215	RP11-253M7.4	lincRNA	1,10E-01	4,24E-01	NA	No	-1,53E-01	4,67E-01	NA	No

ENSG00000245750	RP11-279F6.1	lincRNA	5,55E-01	9,39E-03	5,40E-02	No	7,17E-01	4,86E-02	1,09E-01	No
ENSG00000259641	RP11-279F6.3	lincRNA	1,07E-01	3,54E-01	NA	No	-1,55E-01	5,35E-01	NA	No
ENSG00000259703	LINC00593	lincRNA	9,77E-02	3,50E-01	NA	No	8,64E-02	8,64E-01	NA	No
ENSG00000259503	RP11-543G18.1	lincRNA	-4,23E-02	7,60E-01	NA	No	-9,82E-02	8,30E-01	NA	No
ENSG00000259473	RP11-96C21.1	lincRNA	-3,52E-02	6,64E-01	NA	No	-1,52E-01	5,67E-01	NA	No
ENSG00000259532	RP11-138H8.2	lincRNA	9,54E-02	6,65E-01	8,36E-01	No	5,64E-02	8,55E-01	9,12E-01	No
ENSG00000261423	RP11-1007O24.3	lincRNA	-5,84E-02	7,87E-01	9,04E-01	No	-1,33E-01	6,93E-01	8,02E-01	No
ENSG00000261187	RP11-1007O24.2	lincRNA	3,16E-01	1,87E-01	4,15E-01	No	2,40E-01	5,02E-01	6,50E-01	No
ENSG00000259783	RP11-1006G14.2	lincRNA	8,31E-02	5,59E-01	NA	No	-4,34E-02	8,71E-01	NA	No
ENSG00000260469	RP11-8P11.3	lincRNA	-3,67E-02	6,37E-01	NA	No	4,13E-02	8,14E-01	NA	No
ENSG00000261775	RP11-10O17.3	lincRNA	2,30E-01	2,15E-01	4,51E-01	No	-1,10E-01	6,88E-01	7,99E-01	No
ENSG00000260591	RP11-817O13.6	lincRNA	3,67E-01	1,50E-01	3,62E-01	No	-9,81E-02	7,77E-01	8,61E-01	No
ENSG00000260274	RP11-817O13.8	lincRNA	3,90E-01	1,32E-01	3,35E-01	No	5,38E-01	1,32E-01	2,44E-01	No
ENSG00000261043	MIR4313	lincRNA	8,81E-02	4,08E-01	NA	No	9,18E-02	8,64E-01	NA	No
ENSG00000270036	RP11-685G9.4	lincRNA	4,18E-02	7,50E-01	NA	No	1,13E-01	6,49E-01	NA	No
ENSG00000260787	RP11-797A18.4	lincRNA	9,49E-01	4,02E-03	2,92E-02	No	2,73E-02	9,33E-01	9,60E-01	No
ENSG00000259652	RP11-797A18.3	lincRNA	4,74E-01	7,69E-02	2,34E-01	No	-1,99E-01	5,70E-01	7,06E-01	No
ENSG00000259362	RP11-307C19.1	lincRNA	1,18E-01	5,56E-01	7,67E-01	No	4,46E-01	2,38E-01	3,81E-01	No
ENSG00000259420	RP11-307C19.2	lincRNA	1,24E-01	5,27E-01	7,48E-01	No	6,74E-02	8,47E-01	9,07E-01	No
ENSG00000261244	RP11-114H24.7	lincRNA	-3,23E-02	7,42E-01	NA	No	-1,58E-01	5,03E-01	NA	No
ENSG00000260988	RP11-285A1.1	lincRNA	7,64E-01	2,09E-02	9,50E-02	No	9,70E-02	7,60E-01	8,49E-01	No
ENSG00000259234	RP11-17L5.4	lincRNA	-9,08E-02	4,73E-01	NA	No	-2,39E-01	3,26E-01	NA	No
ENSG00000261712	RP11-358L4.1	lincRNA	4,80E-02	7,52E-01	8,84E-01	No	-1,27E-01	6,96E-01	8,05E-01	No
ENSG00000259548	RP11-38G5.2	lincRNA	9,04E-02	6,39E-01	8,21E-01	No	-4,44E-01	2,09E-01	3,45E-01	No
ENSG00000261229	RP11-38G5.4	lincRNA	3,06E-01	1,95E-01	4,25E-01	No	8,69E-02	8,05E-01	8,79E-01	No
ENSG00000259967	RP11-2E17.2	lincRNA	1,30E+00	9,58E-03	5,47E-02	No	-3,10E-02	8,90E-01	NA	No
ENSG00000259417	RP11-2E17.1	lincRNA	-8,28E-01	9,94E-03	5,62E-02	No	-9,41E-01	1,27E-02	3,51E-02	No
ENSG00000271983	RP11-28H5.2	lincRNA	1,76E-02	8,94E-01	NA	No	-4,74E-02	8,04E-01	NA	No
ENSG00000260619	RP11-775C24.3	lincRNA	1,67E-02	9,14E-01	9,64E-01	No	-1,99E-01	5,16E-01	6,62E-01	No
ENSG00000259372	CTD-2240J17.1	lincRNA	1,49E+00	6,51E-03	4,14E-02	No	2,27E-01	4,61E-01	6,14E-01	No
ENSG00000259543	CTD-2034I4.1	lincRNA	4,38E-01	1,67E-02	8,09E-02	No	1,13E-01	5,67E-01	NA	No
ENSG00000259594	CTD-2034I4.2	lincRNA	1,11E-01	5,18E-01	7,41E-01	No	-2,06E-02	9,51E-01	9,71E-01	No
ENSG00000259610	RP11-499F3.1	lincRNA	1,71E-01	2,84E-01	5,30E-01	No	-9,63E-03	9,64E-01	9,79E-01	No
ENSG00000259692	RP11-499F3.2	lincRNA	-1,40E-01	4,65E-01	6,99E-01	No	-1,93E-01	5,84E-01	7,17E-01	No
ENSG00000259445	RP11-276M12.1	lincRNA	5,74E-02	7,88E-01	9,05E-01	No	2,33E-04	9,98E-01	9,99E-01	No
ENSG00000228141	AC105339.1	lincRNA	1,86E-02	8,04E-01	NA	No	-1,35E-01	7,12E-01	NA	No
ENSG00000259694	RP13-262C2.3	lincRNA	-6,04E-02	5,29E-01	NA	No	-1,44E-01	5,24E-01	NA	No
ENSG00000259774	RP11-182J1.13	lincRNA	6,77E-03	9,65E-01	9,85E-01	No	-7,43E-02	8,17E-01	8,87E-01	No
ENSG00000259551	RP11-182J1.10	lincRNA	7,26E-01	3,60E-02	1,39E-01	No	2,53E-02	9,37E-01	9,62E-01	No
ENSG00000259560	RP11-648K4.2	lincRNA	-2,46E-01	3,00E-01	5,48E-01	No	7,48E-01	3,45E-02	8,20E-02	No
ENSG00000259527	LINC00052	lincRNA	2,39E-02	8,35E-01	NA	No	-4,65E-02	7,97E-01	NA	No
ENSG00000260305	NTRK3-AS1	lincRNA	1,93E-01	8,19E-02	NA	No	1,59E-01	5,00E-01	NA	No
ENSG00000249487	RP11-97O12.2	lincRNA	2,75E-02	8,62E-01	9,40E-01	No	-1,69E-01	6,02E-01	7,32E-01	No
ENSG00000259676	RP11-343B18.2	lincRNA	-1,19E-02	8,95E-01	NA	No	-1,59E-01	4,92E-01	NA	No
ENSG00000260123	RP11-326A19.4	lincRNA	-8,58E-02	4,22E-01	NA	No	9,36E-02	7,50E-01	8,43E-01	No
ENSG00000255571	LINC00925	lincRNA	6,84E-01	1,11E-02	6,04E-02	No	1,19E-01	6,91E-01	8,01E-01	No
ENSG00000261478	RP11-429B14.4	lincRNA	-5,37E-02	6,46E-01	NA	No	-1,26E-01	7,12E-01	NA	No
ENSG00000259685	CTD-2315E11.1	lincRNA	-4,78E-01	8,17E-02	2,43E-01	No	-1,52E-01	6,61E-01	7,78E-01	No
ENSG00000259894	RP11-697E2.4	lincRNA	2,25E-01	1,76E-01	4,00E-01	No	-3,63E-02	8,78E-01	9,27E-01	No
ENSG00000258954	RP11-692C24.1	lincRNA	-4,14E-02	6,25E-01	NA	No	3,84E-01	2,46E-01	3,90E-01	No
ENSG00000258765	RP11-692C24.2	lincRNA	3,15E-02	9,36E-01	NA	No	6,23E-02	9,56E-01	NA	No
ENSG00000258647	LINC00930	lincRNA	-1,99E-01	2,70E-01	5,14E-01	No	-3,06E-01	3,72E-01	5,29E-01	No
ENSG00000260337	RP11-386M24.6	lincRNA	6,69E-02	6,85E-01	8,49E-01	No	8,95E-01	5,33E-02	1,18E-01	No
ENSG00000258888	RP11-326A13.1	lincRNA	-5,46E-02	5,63E-01	NA	No	-3,36E-02	8,97E-01	9,39E-01	No
ENSG00000257060	RP11-266O8.1	lincRNA	3,36E-01	1,86E-02	NA	No	6,56E-01	3,69E-02	8,69E-02	No
ENSG00000258909	RP11-164C12.2	lincRNA	-1,73E-02	8,19E-01	NA	No	1,29E-02	9,10E-01	NA	No
ENSG00000258631	RP11-739G5.1	lincRNA	-4,09E-02	5,25E-01	NA	No	-9,14E-02	6,43E-01	NA	No
ENSG00000258754	CTD-3049M7.1	lincRNA	1,17E-01	5,27E-01	7,48E-01	No	4,27E-01	2,51E-01	3,96E-01	No
ENSG00000259123	RP11-76E17.2	lincRNA	1,27E-02	9,30E-01	NA	No	-1,43E-01	6,16E-01	NA	No
ENSG00000258476	RP11-76E17.3	lincRNA	3,50E-02	8,71E-01	9,46E-01	No	6,15E-01	1,25E-01	2,33E-01	No
ENSG00000258831	RP11-76E17.4	lincRNA	-5,65E-02	7,16E-01	8,67E-01	No	6,57E-02	8,41E-01	9,03E-01	No
ENSG00000259724	CTD-2643K12.3	lincRNA	5,26E-02	4,68E-01	NA	No	1,17E-01	5,93E-01	NA	No

ENSG00000260359	RP11-4F5.2	lincRNA	1,28E+00	7,01E-04	7,83E-03	No	-6,32E-01	1,11E-01	2,13E-01	No
ENSG00000248441	CTD-2536I1.1	lincRNA	3,21E-01	1,13E-01	3,03E-01	No	7,66E-01	3,87E-04	1,57E-03	No
ENSG00000259134	LINC00924	lincRNA	3,28E-01	1,18E-01	3,11E-01	No	5,17E-01	3,36E-02	8,02E-02	No
ENSG00000258489	RP11-398J10.2	lincRNA	-8,28E-02	6,16E-01	8,07E-01	No	-1,52E-02	9,59E-01	9,76E-01	No
ENSG00000259348	RP11-4G2.1	lincRNA	9,76E-02	5,69E-01	7,75E-01	No	1,02E-01	7,33E-01	8,31E-01	No
ENSG00000259702	RP11-236L14.1	lincRNA	-1,97E-01	2,88E-01	5,35E-01	No	-2,85E-01	4,31E-01	5,86E-01	No
ENSG00000259359	RP11-327J17.2	lincRNA	1,78E-01	4,31E-01	6,71E-01	No	9,85E-01	7,95E-03	2,33E-02	No
ENSG00000259763	RP11-327J17.1	lincRNA	8,55E-02	7,00E-01	8,58E-01	No	-9,95E-02	7,74E-01	8,59E-01	No
ENSG00000259275	RP11-522B15.3	lincRNA	1,14E-01	5,78E-01	7,81E-01	No	2,33E-01	5,13E-01	6,60E-01	No
ENSG00000259542	RP11-522B15.4	lincRNA	7,91E-02	5,40E-01	7,56E-01	No	-1,02E-01	7,01E-01	8,08E-01	No
ENSG00000259534	RP11-522B15.7	lincRNA	2,50E-02	7,74E-01	NA	No	5,45E-02	7,52E-01	NA	No
ENSG00000259403	RP11-315L6.1	lincRNA	-3,76E-02	7,16E-01	NA	No	-9,75E-04	9,99E-01	NA	No
ENSG00000259485	CTD-2147F2.1	lincRNA	-3,31E-01	1,56E-01	3,72E-01	No	-7,21E-01	9,73E-02	1,91E-01	No
ENSG00000259664	CTD-2147F2.2	lincRNA	-2,14E-01	3,52E-01	6,00E-01	No	1,82E-01	5,87E-01	7,19E-01	No
ENSG00000259524	RP11-461F11.1	lincRNA	-2,13E-01	3,53E-01	6,01E-01	No	6,06E-01	8,39E-02	1,70E-01	No
ENSG00000259590	RP11-20G13.3	lincRNA	-1,98E-02	8,73E-01	9,47E-01	No	-1,93E-01	4,31E-01	5,86E-01	No
ENSG00000259341	RP11-20G13.1	lincRNA	1,78E-01	1,30E-01	NA	No	-6,59E-02	8,00E-01	NA	No
ENSG00000189419	SPATA41	lincRNA	2,57E-01	1,67E-01	3,89E-01	No	7,97E-01	6,71E-02	1,42E-01	No
ENSG00000232386	RP11-66B24.2	lincRNA	-1,24E-02	9,01E-01	NA	No	-2,69E-02	9,15E-01	NA	No
ENSG00000272808	RP11-66B24.7	lincRNA	5,84E-02	6,21E-01	8,11E-01	No	-1,10E-01	6,19E-01	NA	No
ENSG00000259365	RP11-424I19.1	lincRNA	-2,50E-03	9,90E-01	NA	No	-7,78E-02	8,00E-01	NA	No
ENSG00000259182	RP11-424I19.2	lincRNA	2,12E-02	9,23E-01	9,68E-01	No	-5,12E-01	1,78E-01	3,06E-01	No
ENSG00000259553	MIR1302-11	lincRNA	-5,58E-02	6,46E-01	NA	No	-3,42E-02	8,90E-01	NA	No
ENSG00000268836	LA16c-OS12.2	lincRNA	1,61E-01	1,86E-01	NA	No	-1,02E-01	8,30E-01	NA	No
ENSG00000259840	LA16c-380A1.1	lincRNA	-7,13E-02	5,95E-01	NA	No	-3,07E-01	2,06E-01	NA	No
ENSG00000260807	RP11-161M6.2	lincRNA	2,37E-02	8,90E-01	9,54E-01	No	-8,67E-01	1,44E-03	5,12E-03	No
ENSG00000260646	LA16c-385E7.1	lincRNA	2,13E-01	2,49E-01	4,91E-01	No	1,74E-01	5,39E-01	6,82E-01	No
ENSG00000255198	SNHG9	lincRNA	6,34E-03	9,78E-01	9,92E-01	No	3,53E-01	2,64E-01	4,11E-01	No
ENSG00000260260	RP11-304L19.5	lincRNA	-4,17E-01	8,19E-02	2,44E-01	No	6,56E-01	1,65E-02	4,39E-02	No
ENSG00000260778	MIR940	lincRNA	5,06E-01	7,06E-02	2,21E-01	No	-7,15E-01	8,74E-02	1,76E-01	No
ENSG00000260874	RP11-715J22.4	lincRNA	1,17E-01	5,31E-01	7,51E-01	No	1,34E-01	6,72E-01	7,87E-01	No
ENSG00000260176	CTD-3126B10.4	lincRNA	1,12E-03	9,95E-01	9,98E-01	No	-2,17E-01	4,46E-01	6,00E-01	No
ENSG00000263280	LA16c-325D7.2	lincRNA	-4,38E-03	9,66E-01	NA	No	1,90E-01	5,12E-01	6,58E-01	No
ENSG00000262152	LINC00514	lincRNA	1,41E-01	3,37E-01	5,86E-01	No	-1,47E-01	4,76E-01	NA	No
ENSG00000270168	LA16c-380H5.4	lincRNA	6,51E-02	4,90E-01	NA	No	6,68E-02	9,56E-01	NA	No
ENSG00000272079	LA16c-380H5.5	lincRNA	-7,82E-02	7,16E-01	8,67E-01	No	-2,94E-01	3,61E-01	5,18E-01	No
ENSG00000262370	RP11-473M20.9	lincRNA	8,39E-01	7,49E-04	8,20E-03	No	3,85E-01	1,33E-01	2,44E-01	No
ENSG00000261889	RP11-473M20.16	lincRNA	7,66E-02	5,79E-01	7,82E-01	No	-3,07E-02	8,97E-01	9,39E-01	No
ENSG00000262468	RP11-95P2.1	lincRNA	1,21E-02	9,58E-01	9,83E-01	No	-6,23E-01	9,85E-02	1,93E-01	No
ENSG00000262686	AC005356.1	lincRNA	-3,72E-01	1,31E-01	3,33E-01	No	-2,11E-01	4,91E-01	6,40E-01	No
ENSG00000267070	RP11-10K17.6	lincRNA	-3,20E-02	7,31E-01	NA	No	-1,35E-01	7,12E-01	NA	No
ENSG00000260289	CTD-2535I10.1	lincRNA	-3,13E-02	7,31E-01	NA	No	-3,69E-02	8,90E-01	NA	No
ENSG00000260003	RP11-279O17.2	lincRNA	-7,97E-02	3,05E-01	NA	No	-2,07E-01	3,46E-01	NA	No
ENSG00000261319	RP11-279O17.1	lincRNA	-9,26E-02	2,20E-01	NA	No	-1,44E-01	4,80E-01	NA	No
ENSG00000260979	RP11-77H9.8	lincRNA	4,20E-02	8,30E-01	9,26E-01	No	-1,25E-01	7,19E-01	8,21E-01	No
ENSG00000261617	RP11-243A14.1	lincRNA	-1,01E-01	6,28E-01	8,15E-01	No	4,16E-01	2,55E-01	4,00E-01	No
ENSG00000261075	RP11-243A14.3	lincRNA	3,41E-02	9,36E-01	NA	No	1,01E-01	7,75E-01	NA	No
ENSG00000260071	RP11-418I22.2	lincRNA	1,91E-01	1,55E-01	3,71E-01	No	4,57E-01	1,48E-01	2,66E-01	No
ENSG00000260432	RP11-297M9.2	lincRNA	-1,31E-01	5,57E-01	7,68E-01	No	1,44E+00	2,76E-03	9,19E-03	No
ENSG00000260468	RP11-27M24.3	lincRNA	-1,01E-01	6,48E-01	8,27E-01	No	8,26E-02	8,07E-01	8,81E-01	No
ENSG00000263033	RP11-396B14.2	lincRNA	1,54E-01	4,18E-01	6,61E-01	No	-5,00E-01	1,03E-01	2,00E-01	No
ENSG00000262117	BCAR4	lincRNA	8,31E-02	3,22E-01	NA	No	1,06E-01	7,03E-01	NA	No
ENSG00000262801	U91319.1	lincRNA	3,35E-02	6,87E-01	NA	No	1,65E-01	4,89E-01	NA	No
ENSG00000262097	CTD-2135D7.5	lincRNA	3,71E-02	8,68E-01	9,44E-01	No	7,84E-01	2,59E-02	6,44E-02	No
ENSG00000261856	CTD-2135D7.4	lincRNA	3,18E-02	7,65E-01	NA	No	-6,62E-02	7,52E-01	NA	No
ENSG00000262454	RP11-65J21.3	lincRNA	-5,24E-01	3,42E-02	1,34E-01	No	1,11E+00	2,68E-05	1,37E-04	No
ENSG00000263257	RP11-65J21.4	lincRNA	-8,46E-02	6,91E-01	8,52E-01	No	2,98E-01	4,10E-01	5,66E-01	No
ENSG00000257264	MIR3179-1	lincRNA	-4,30E-02	5,53E-01	NA	No	-1,61E-01	4,63E-01	NA	No
ENSG00000258354	MIR3180-1	lincRNA	-9,28E-02	4,26E-01	NA	No	-1,42E-01	5,29E-01	NA	No
ENSG00000257391	MIR3180-4	lincRNA	5,99E-02	5,60E-01	NA	No	8,51E-02	8,64E-01	NA	No
ENSG00000259929	CTA-481E9.4	lincRNA	-5,47E-02	6,46E-01	NA	No	-3,01E-02	8,90E-01	NA	No
ENSG00000261357	RP11-626G11.1	lincRNA	6,28E-02	5,24E-01	NA	No	3,69E-01	1,91E-01	NA	No

ENSG00000260430	RP11-626G11.4	lincRNA	1,13E-01	4,15E-01	NA	No	-1,09E-01	6,09E-01	NA	No
ENSG00000189149	CRYM-AS1	lincRNA	3,35E-01	1,76E-01	4,00E-01	No	9,00E-01	8,48E-03	2,46E-02	No
ENSG00000260306	RP11-645C24.5	lincRNA	2,38E-02	9,11E-01	9,63E-01	No	2,19E-01	5,31E-01	6,75E-01	No
ENSG00000260973	RP11-105C19.2	lincRNA	1,01E-01	1,90E-01	NA	No	1,64E-01	4,77E-01	NA	No
ENSG00000260905	RP11-105C19.1	lincRNA	3,50E-02	6,51E-01	NA	No	1,82E-01	3,48E-01	NA	No
ENSG00000260136	CTD-2270L9.4	lincRNA	-2,78E-01	2,36E-01	4,75E-01	No	-3,82E-01	2,98E-01	4,50E-01	No
ENSG00000260482	CTD-2196E14.9	lincRNA	1,17E-01	5,17E-01	7,41E-01	No	3,96E-01	5,23E-02	1,16E-01	No
ENSG00000259806	CTD-2196E14.4	lincRNA	1,53E-01	4,57E-01	6,92E-01	No	8,89E-01	4,63E-04	1,85E-03	No
ENSG00000259888	CTD-2540M10.1	lincRNA	1,06E-01	2,71E-01	NA	No	6,34E-02	9,56E-01	NA	No
ENSG00000224310	AC012317.1	lincRNA	9,29E-02	2,35E-01	NA	No	8,55E-02	8,64E-01	NA	No
ENSG00000262155	RP11-266L9.5	lincRNA	1,51E-01	4,76E-01	7,08E-01	No	-5,10E-01	9,64E-02	1,90E-01	No
ENSG00000260448	RP11-449H11.1	lincRNA	3,13E-01	1,71E-01	3,94E-01	No	-3,44E-01	2,27E-01	3,67E-01	No
ENSG00000246465	RP11-57A19.2	lincRNA	-1,47E-01	5,12E-01	7,38E-01	No	-4,90E-01	1,97E-01	3,31E-01	No
ENSG00000251417	RP11-1348G14.4	lincRNA	9,94E-02	6,56E-01	8,31E-01	No	-6,06E-02	8,57E-01	9,14E-01	No
ENSG00000260517	RP11-426C22.5	lincRNA	1,18E-01	5,92E-01	7,91E-01	No	-6,52E-01	9,02E-02	1,80E-01	No
ENSG00000198106	SNX29P2	lincRNA	2,09E-01	3,59E-01	6,07E-01	No	-5,24E-01	5,23E-02	1,16E-01	No
ENSG00000260219	RP11-347C12.10	lincRNA	-7,86E-02	7,15E-01	8,67E-01	No	-3,18E-01	3,79E-01	5,36E-01	No
ENSG00000260899	RP11-2C24.4	lincRNA	-4,46E-02	7,60E-01	NA	No	-1,03E-01	8,30E-01	NA	No
ENSG00000260911	RP11-196G11.2	lincRNA	8,16E-01	2,31E-02	1,02E-01	No	1,31E-01	6,87E-01	7,98E-01	No
ENSG00000261925	RP11-388M20.9	lincRNA	-5,91E-02	7,76E-01	8,98E-01	No	-1,76E-01	6,16E-01	7,43E-01	No
ENSG00000260158	RP11-67H24.2	lincRNA	-4,20E-02	7,52E-01	8,84E-01	No	-3,11E-01	1,73E-01	NA	No
ENSG00000214614	RP11-989E6.3	lincRNA	-7,92E-02	4,55E-01	NA	No	-7,98E-02	7,68E-01	NA	No
ENSG00000260141	RP11-19N8.4	lincRNA	-2,73E-02	8,11E-01	NA	No	-1,28E-01	5,48E-01	NA	No
ENSG00000256642	LINC00273	lincRNA	1,22E-03	9,73E-01	NA	No	-5,07E-02	8,00E-01	NA	No
ENSG00000261788	RP11-480G7.1	lincRNA	-3,57E-01	8,26E-02	2,45E-01	No	8,00E-01	1,68E-05	8,92E-05	No
ENSG00000259821	RP11-169E6.4	lincRNA	-1,60E-02	8,19E-01	NA	No	8,96E-02	6,49E-01	NA	No
ENSG00000261231	RP11-523L20.2	lincRNA	-3,68E-02	7,56E-01	NA	No	-3,33E-01	1,66E-01	NA	No
ENSG00000260086	RP11-42I10.1	lincRNA	8,00E-03	9,61E-01	9,84E-01	No	3,82E-01	2,98E-01	4,50E-01	No
ENSG00000260052	CTC-527H23.3	lincRNA	2,94E-02	7,91E-01	NA	No	1,24E-01	5,89E-01	NA	No
ENSG00000272545	CTC-527H23.4	lincRNA	-4,99E-02	5,76E-01	NA	No	2,79E-02	9,02E-01	NA	No
ENSG00000259957	RP11-491F9.8	lincRNA	2,72E-02	8,80E-01	9,49E-01	No	-6,23E-01	1,31E-01	2,43E-01	No
ENSG00000260769	RP11-491F9.5	lincRNA	-1,12E-01	4,16E-01	6,59E-01	No	-6,88E-01	5,26E-02	1,17E-01	No
ENSG00000262950	RP11-189E14.5	lincRNA	6,59E-02	5,21E-01	NA	No	-1,28E-01	7,12E-01	NA	No
ENSG00000263110	RP11-189E14.3	lincRNA	4,28E-02	7,26E-01	NA	No	8,55E-02	8,64E-01	NA	No
ENSG00000261623	RP11-189E14.4	lincRNA	9,81E-03	9,12E-01	NA	No	-1,30E-01	7,12E-01	NA	No
ENSG00000261685	RP11-401P9.4	lincRNA	5,69E-01	5,31E-02	1,81E-01	No	-4,09E-02	9,02E-01	9,42E-01	No
ENSG00000261703	RP11-327F22.5	lincRNA	6,71E-02	5,54E-01	NA	No	3,30E-02	8,93E-01	NA	No
ENSG00000260042	CTD-2034I21.1	lincRNA	-6,63E-02	4,07E-01	NA	No	-1,61E-01	4,63E-01	NA	No
ENSG00000262714	RP11-44F14.8	lincRNA	2,19E-02	8,26E-01	NA	No	3,75E-02	9,10E-01	NA	No
ENSG00000259759	RP11-324D17.2	lincRNA	8,82E-02	2,80E-01	NA	No	2,84E-01	2,35E-01	NA	No
ENSG00000245694	CRNDE	lincRNA	-4,30E-01	6,07E-02	1,99E-01	No	1,10E+00	2,19E-06	1,34E-05	No
ENSG00000259711	CTD-3032H12.2	lincRNA	-2,51E-01	2,22E-01	4,60E-01	No	-4,27E-01	2,45E-01	3,89E-01	No
ENSG00000259725	CTD-3032H12.1	lincRNA	-4,34E-02	7,39E-01	8,78E-01	No	-1,45E-01	5,58E-01	6,96E-01	No
ENSG00000263207	RP11-26L20.4	lincRNA	1,51E-01	4,16E-01	6,59E-01	No	-2,03E-01	5,22E-01	6,67E-01	No
ENSG00000260135	RP11-212I21.2	lincRNA	2,91E-01	9,22E-02	2,64E-01	No	8,96E-02	7,63E-01	8,52E-01	No
ENSG00000261997	RP11-212I21.4	lincRNA	7,43E-01	4,01E-03	2,91E-02	No	4,33E-01	1,37E-01	2,50E-01	No
ENSG00000246379	RP11-461O7.1	lincRNA	-5,43E-01	2,04E-02	9,32E-02	No	-8,58E-02	7,49E-01	8,41E-01	No
ENSG00000260041	RP11-355E10.1	lincRNA	-1,05E-02	9,52E-01	9,80E-01	No	-7,03E-02	7,92E-01	8,71E-01	No
ENSG00000261013	CTD-2050B12.1	lincRNA	-2,55E-03	9,83E-01	9,93E-01	No	2,10E-01	4,85E-01	6,35E-01	No
ENSG00000260823	RP11-249C24.10	lincRNA	1,53E-01	3,76E-01	6,22E-01	No	-1,22E-01	6,87E-01	7,98E-01	No
ENSG00000260186	RP11-481J2.2	lincRNA	9,97E-02	6,52E-01	8,29E-01	No	3,17E-03	9,95E-01	9,97E-01	No
ENSG00000245768	RP11-410D17.2	lincRNA	-2,55E-01	2,66E-01	5,11E-01	No	-4,67E-02	8,91E-01	9,35E-01	No
ENSG00000261638	RP11-700H13.1	lincRNA	-8,75E-02	4,99E-01	7,26E-01	No	-4,99E-03	9,85E-01	9,91E-01	No
ENSG00000261807	RP11-430C1.1	lincRNA	-5,47E-02	6,46E-01	NA	No	-3,10E-02	8,90E-01	NA	No
ENSG00000260658	RP11-368L12.1	lincRNA	-1,22E-01	5,34E-01	7,53E-01	No	-6,00E-01	7,91E-03	2,32E-02	No
ENSG00000261743	RP11-2K6.2	lincRNA	-1,39E-01	5,28E-01	7,49E-01	No	-1,04E+00	1,29E-02	3,55E-02	No
ENSG00000261014	RP11-370P15.2	lincRNA	2,23E-02	9,72E-01	NA	No	1,08E-01	6,91E-01	NA	No
ENSG00000261653	RP11-21L1.1	lincRNA	6,99E-02	4,51E-01	NA	No	4,44E-01	9,40E-02	1,86E-01	No
ENSG00000260715	RP11-744D14.2	lincRNA	5,33E-01	4,65E-02	1,65E-01	No	1,47E+00	1,29E-02	3,55E-02	No
ENSG00000261028	AC012322.1	lincRNA	3,41E-01	8,73E-02	2,54E-01	No	5,29E-01	1,43E-01	2,59E-01	No
ENSG00000259846	RP11-467L24.1	lincRNA	-1,33E-01	2,32E-01	NA	No	-2,82E-01	2,90E-01	NA	No
ENSG00000261742	LINC00922	lincRNA	3,97E-02	8,45E-01	NA	No	1,09E-01	6,85E-01	NA	No

ENSG00000260695	RP11-513N24.1	lincRNA	-5,47E-02	6,46E-01	NA	No	-1,30E-01	7,12E-01	NA	No
ENSG00000246898	LINC00920	lincRNA	1,06E-01	6,32E-01	8,17E-01	No	4,16E-01	2,14E-01	3,51E-01	No
ENSG00000261519	Y_RNA	lincRNA	4,84E-02	5,51E-01	NA	No	8,51E-02	8,64E-01	NA	No
ENSG00000260558	RP11-63M22.1	lincRNA	2,78E-02	8,96E-01	9,57E-01	No	-1,45E-02	9,64E-01	9,79E-01	No
ENSG00000246777	RP11-61A14.4	lincRNA	-5,65E-02	6,43E-01	8,23E-01	No	-1,23E-01	6,72E-01	7,87E-01	No
ENSG00000261705	RP11-61A14.2	lincRNA	-9,76E-02	6,03E-01	7,99E-01	No	-6,39E-02	8,52E-01	9,10E-01	No
ENSG00000261088	RP11-61A14.3	lincRNA	-6,12E-03	9,75E-01	9,90E-01	No	7,67E-01	1,28E-03	4,61E-03	No
ENSG00000261386	CTD-2012K14.6	lincRNA	2,41E-01	3,04E-01	5,52E-01	No	-4,46E-01	2,19E-01	3,57E-01	No
ENSG00000260999	RP11-521L9.1	lincRNA	6,81E-03	9,54E-01	NA	No	-7,98E-02	7,17E-01	NA	No
ENSG00000260023	RP11-49C24.1	lincRNA	-8,07E-02	6,63E-01	8,35E-01	No	-1,17E+00	8,89E-05	4,08E-04	No
ENSG00000260612	RP11-432I5.4	lincRNA	-1,62E-03	9,91E-01	NA	No	5,51E-01	6,00E-02	1,30E-01	No
ENSG00000261673	RP11-328J14.1	lincRNA	1,38E-02	9,63E-01	NA	No	1,35E-02	9,87E-01	NA	No
ENSG00000260664	AC004158.3	lincRNA	1,43E-01	2,04E-01	NA	No	1,54E-01	5,24E-01	NA	No
ENSG00000272250	RP11-346C20.4	lincRNA	4,76E-01	8,68E-02	2,53E-01	No	5,06E-02	8,83E-01	9,30E-01	No
ENSG00000260880	HCCAT5	lincRNA	-8,52E-02	6,82E-01	8,47E-01	No	-4,03E-01	2,80E-01	4,30E-01	No
ENSG00000261227	AC140912.1	lincRNA	-2,60E-01	1,61E-01	3,79E-01	No	1,34E-01	7,02E-01	8,09E-01	No
ENSG00000258779	RP11-140I24.1	lincRNA	-6,55E-02	7,08E-01	8,62E-01	No	1,78E-01	6,02E-01	7,32E-01	No
ENSG00000258582	RP11-44L9.1	lincRNA	-1,60E-02	8,98E-01	NA	No	-2,55E-02	8,90E-01	NA	No
ENSG00000260687	RP11-44L9.2	lincRNA	-3,48E-02	6,55E-01	NA	No	-1,60E-01	4,74E-01	NA	No
ENSG00000259971	RP11-44L9.3	lincRNA	-2,78E-02	7,59E-01	NA	No	-9,64E-02	6,29E-01	NA	No
ENSG00000260848	CTD-2009A10.1	lincRNA	1,30E-01	2,98E-01	5,46E-01	No	-4,95E-02	8,21E-01	NA	No
ENSG00000259817	RP11-53L24.1	lincRNA	-5,83E-02	7,33E-01	8,76E-01	No	3,34E-01	3,64E-01	5,21E-01	No
ENSG00000261404	AC009120.4	lincRNA	-2,08E-01	2,94E-01	5,42E-01	No	-2,48E-01	4,86E-01	6,36E-01	No
ENSG00000261248	AC009120.10	lincRNA	6,36E-02	5,69E-01	NA	No	-8,81E-02	7,12E-01	NA	No
ENSG00000261079	RP11-252A24.3	lincRNA	8,29E-02	7,10E-01	8,63E-01	No	-5,44E-01	1,32E-01	2,43E-01	No
ENSG00000260539	RP11-252A24.7	lincRNA	-1,05E-02	9,53E-01	9,80E-01	No	-8,65E-01	3,51E-05	1,75E-04	No
ENSG00000261058	RP11-252E2.2	lincRNA	-5,69E-02	6,46E-01	NA	No	-7,41E-02	8,00E-01	NA	No
ENSG00000261313	RP11-2C15.1	lincRNA	-6,20E-02	5,30E-01	NA	No	-1,49E-01	5,91E-01	NA	No
ENSG00000250514	RP11-96P7.1	lincRNA	4,09E-02	5,96E-01	NA	No	4,45E-02	8,83E-01	NA	No
ENSG00000259995	CTD-2336H13.2	lincRNA	-6,08E-02	5,50E-01	NA	No	-8,07E-02	7,20E-01	NA	No
ENSG00000261722	RP11-679B19.2	lincRNA	-6,24E-02	5,15E-01	NA	No	-9,82E-02	6,63E-01	NA	No
ENSG00000261472	RP11-467I17.1	lincRNA	-2,19E-02	8,62E-01	9,40E-01	No	1,21E-01	7,09E-01	8,14E-01	No
ENSG00000260876	RP11-345M22.1	lincRNA	2,05E-01	2,49E-01	4,91E-01	No	-5,49E-02	8,27E-01	NA	No
ENSG00000261390	RP11-345M22.2	lincRNA	2,27E-01	2,67E-01	5,12E-01	No	5,17E-02	8,73E-01	9,24E-01	No
ENSG00000260896	RP11-314O13.1	lincRNA	1,22E-01	5,50E-01	7,63E-01	No	-7,53E-02	8,23E-01	8,92E-01	No
ENSG00000245059	RP11-303E16.7	lincRNA	7,70E-02	6,14E-01	8,06E-01	No	-2,94E-02	9,24E-01	9,55E-01	No
ENSG00000260682	7SK	lincRNA	1,47E-03	9,95E-01	9,98E-01	No	-1,02E+00	2,22E-08	1,88E-07	No
ENSG00000261029	CTD-2588J6.2	lincRNA	-5,37E-02	6,46E-01	NA	No	2,11E-02	9,35E-01	NA	No
ENSG00000260523	RP11-483P21.3	lincRNA	-4,81E-01	7,01E-02	2,20E-01	No	1,79E-01	5,22E-01	6,67E-01	No
ENSG00000260340	RP11-254F19.3	lincRNA	1,97E-02	8,40E-01	NA	No	-1,57E-01	5,14E-01	NA	No
ENSG00000179219	LINC00311	lincRNA	1,44E-01	3,34E-01	5,84E-01	No	2,09E-01	4,52E-01	6,05E-01	No
ENSG00000260417	CTD-2542L18.1	lincRNA	7,72E-01	1,98E-02	9,12E-02	No	1,52E-01	6,64E-01	7,80E-01	No
ENSG00000268804	RP11-542M13.3	lincRNA	5,00E-02	5,31E-01	NA	No	6,34E-02	9,56E-01	NA	No
ENSG00000269667	RP11-542M13.2	lincRNA	6,27E-02	5,19E-01	NA	No	9,18E-02	8,64E-01	NA	No
ENSG00000261177	RP11-805I24.1	lincRNA	-6,56E-02	4,29E-01	NA	No	-1,18E-01	5,72E-01	NA	No
ENSG00000268505	RP11-805I24.3	lincRNA	-1,82E-02	8,19E-01	NA	No	1,81E-02	9,14E-01	NA	No
ENSG00000268388	FENDRR	lincRNA	-1,11E-01	3,71E-01	NA	No	-4,22E-01	9,15E-02	NA	No
ENSG00000270020	RP11-463O9.9	lincRNA	-2,40E-01	1,95E-01	4,25E-01	No	-4,05E-01	2,61E-01	4,09E-01	No
ENSG00000260387	RP11-463O9.6	lincRNA	-7,69E-02	5,23E-01	NA	No	-4,23E-03	9,87E-01	NA	No
ENSG00000261161	RP11-58A18.1	lincRNA	9,03E-02	6,35E-01	8,19E-01	No	-1,08E-01	7,07E-01	8,12E-01	No
ENSG00000260026	CTD-2015G9.1	lincRNA	8,30E-02	4,49E-01	NA	No	1,64E-01	4,77E-01	NA	No
ENSG00000261175	CTD-2015G9.2	lincRNA	-1,60E-01	4,27E-01	6,67E-01	No	3,48E-01	3,44E-01	4,99E-01	No
ENSG00000261095	RP11-899L11.1	lincRNA	-2,32E-02	7,66E-01	NA	No	-1,21E-01	5,65E-01	NA	No
ENSG00000260750	RP11-482M8.1	lincRNA	-1,68E-01	3,10E-01	5,60E-01	No	3,38E-01	3,35E-01	4,91E-01	No
ENSG00000260177	RP4-536B24.3	lincRNA	-3,00E-02	7,31E-01	NA	No	-2,73E-02	8,84E-01	NA	No
ENSG00000260166	RP11-863P13.6	lincRNA	9,01E-02	4,63E-01	NA	No	-3,53E-02	8,94E-01	NA	No
ENSG00000205037	RP11-863P13.4	lincRNA	-1,65E-01	4,41E-01	6,80E-01	No	-5,61E-01	1,63E-01	2,86E-01	No
ENSG00000261327	RP11-863P13.3	lincRNA	4,26E-02	7,43E-01	NA	No	1,23E-01	6,58E-01	NA	No
ENSG00000205015	RP11-46C24.3	lincRNA	-1,53E-02	8,19E-01	NA	No	-1,30E-01	7,12E-01	NA	No
ENSG00000260637	RP11-46C24.5	lincRNA	3,55E-02	8,22E-01	NA	No	-1,23E-01	6,57E-01	NA	No
ENSG00000260507	RP11-356C4.3	lincRNA	-3,70E-02	6,32E-01	NA	No	-6,60E-02	6,93E-01	NA	No
ENSG00000261172	RP11-356C4.5	lincRNA	8,37E-02	5,11E-01	NA	No	-1,63E-01	4,52E-01	NA	No

ENSG00000260923	AC137934.1	lincRNA	1,42E-01	5,01E-01	7,28E-01	No	-2,38E-01	5,04E-01	6,52E-01	No
ENSG00000273172	AC108004.2	lincRNA	2,04E-03	9,92E-01	9,97E-01	No	-3,10E-01	3,77E-01	5,34E-01	No
ENSG00000263050	RP11-667K14.3	lincRNA	-1,22E-01	5,50E-01	7,63E-01	No	-1,34E+00	9,95E-05	4,53E-04	No
ENSG00000272911	RP11-74E22.6	lincRNA	1,09E-01	6,25E-01	8,13E-01	No	-1,30E+00	1,04E-02	2,95E-02	No
ENSG00000261848	CTD-2309O5.3	lincRNA	-5,73E-02	6,46E-01	NA	No	-1,36E-01	7,12E-01	NA	No
ENSG00000262194	CTD-3195I5.5	lincRNA	1,38E-01	5,38E-01	7,55E-01	No	-7,39E-01	8,26E-02	1,68E-01	No
ENSG00000262758	CTD-3195I5.1	lincRNA	2,07E-01	2,03E-01	4,36E-01	No	-4,93E-01	9,72E-03	2,78E-02	No
ENSG00000263312	RP11-459C13.1	lincRNA	-5,25E-04	9,95E-01	NA	No	-8,23E-02	6,67E-01	NA	No
ENSG00000261863	RP11-141J13.5	lincRNA	-1,42E-02	8,88E-01	NA	No	2,37E-01	3,74E-01	5,31E-01	No
ENSG00000262165	RP11-81A22.5	lincRNA	6,71E-02	7,45E-01	8,81E-01	No	-1,96E-01	5,78E-01	7,12E-01	No
ENSG00000263164	RP11-333E1.2	lincRNA	-2,74E-02	7,91E-01	NA	No	-1,89E-01	3,57E-01	NA	No
ENSG00000262099	CTC-524C5.5	lincRNA	1,11E-01	2,50E-01	NA	No	1,65E-01	4,68E-01	NA	No
ENSG00000262089	RP11-589P10.5	lincRNA	3,11E-01	1,77E-01	4,02E-01	No	-1,99E-01	5,72E-01	7,08E-01	No
ENSG00000263427	RP11-599B13.3	lincRNA	4,75E-02	5,70E-01	NA	No	2,17E-01	4,02E-01	NA	No
ENSG00000266824	RP11-599B13.7	lincRNA	-1,01E-02	9,27E-01	NA	No	-7,28E-03	9,75E-01	9,85E-01	No
ENSG00000178977	LINC00324	lincRNA	2,60E-01	2,01E-01	4,33E-01	No	-3,27E-01	2,30E-01	3,71E-01	No
ENSG00000269928	RP11-849F2.8	lincRNA	3,94E-01	1,02E-01	2,81E-01	No	-6,38E-01	6,38E-02	1,37E-01	No
ENSG00000269947	RP11-849F2.9	lincRNA	-1,47E-02	9,30E-01	9,71E-01	No	-3,06E-01	3,92E-01	5,49E-01	No
ENSG00000265975	CTB-41I6.2	lincRNA	1,21E+00	1,07E-02	5,87E-02	No	-1,22E-02	9,61E-01	9,77E-01	No
ENSG00000261433	CTC-297N7.1	lincRNA	-1,26E-01	5,44E-01	7,59E-01	No	-8,63E-01	6,29E-02	1,35E-01	No
ENSG00000273290	CTC-297N7.8	lincRNA	-3,66E-01	1,35E-02	6,96E-02	No	-5,26E-01	1,04E-03	3,83E-03	No
ENSG00000264016	CTC-297N7.9	lincRNA	-1,48E-02	9,02E-01	NA	No	3,26E-02	9,04E-01	9,43E-01	No
ENSG00000266114	RP11-963H4.5	lincRNA	3,85E-02	8,45E-01	NA	No	6,54E-02	9,56E-01	NA	No
ENSG00000263429	LINC00675	lincRNA	4,89E-02	5,86E-01	NA	No	2,01E-02	9,13E-01	NA	No
ENSG00000263508	RP11-963H4.3	lincRNA	5,14E-02	5,10E-01	NA	No	2,17E-01	2,73E-01	NA	No
ENSG00000179136	LINC00670	lincRNA	2,65E-01	2,65E-01	5,09E-01	No	6,92E-01	5,59E-02	1,23E-01	No
ENSG00000265494	RP11-131K5.2	lincRNA	1,70E-01	2,82E-01	5,28E-01	No	-2,43E-01	2,14E-01	NA	No
ENSG00000233852	AC005304.1	lincRNA	9,00E-02	3,56E-01	NA	No	4,16E-03	9,79E-01	NA	No
ENSG00000266744	RP11-131K5.1	lincRNA	2,46E-01	1,22E-01	3,18E-01	No	-2,45E-02	9,24E-01	NA	No
ENSG00000262786	RP11-214O1.1	lincRNA	-4,47E-02	5,48E-01	NA	No	-1,60E-01	4,74E-01	NA	No
ENSG00000266709	RP11-214O1.2	lincRNA	9,36E-01	7,03E-03	4,38E-02	No	-8,92E-01	1,98E-02	5,14E-02	No
ENSG00000230647	AC022816.2	lincRNA	1,38E-02	9,08E-01	NA	No	-1,45E-01	6,02E-01	NA	No
ENSG00000265163	CDRT8	lincRNA	8,24E-03	9,65E-01	NA	No	2,26E-01	2,63E-01	NA	No
ENSG00000230971	AC005703.3	lincRNA	-1,53E-02	8,19E-01	NA	No	-1,30E-01	7,12E-01	NA	No
ENSG00000237377	AC005703.2	lincRNA	2,92E-03	9,90E-01	9,96E-01	No	3,85E-01	2,96E-01	4,47E-01	No
ENSG00000266667	RP11-849N15.4	lincRNA	-3,10E-03	9,76E-01	NA	No	-1,32E-01	5,68E-01	NA	No
ENSG00000266538	RP11-385D13.3	lincRNA	5,43E-01	5,90E-02	1,94E-01	No	2,65E-01	4,43E-01	5,97E-01	No
ENSG00000265519	CTD-3157E16.1	lincRNA	-4,83E-01	6,01E-03	3,90E-02	No	-6,35E-01	1,26E-03	4,55E-03	No
ENSG00000237057	AC015922.6	lincRNA	-9,33E-02	2,61E-01	NA	No	-1,68E-01	4,04E-01	NA	No
ENSG00000230709	AC104024.1	lincRNA	2,26E-01	3,76E-02	NA	No	9,02E-02	8,64E-01	NA	No
ENSG00000230969	AC104024.2	lincRNA	4,34E-02	7,14E-01	NA	No	9,20E-02	8,64E-01	NA	No
ENSG00000260328	RP11-416I2.1	lincRNA	4,19E-02	7,48E-01	NA	No	9,20E-02	8,64E-01	NA	No
ENSG00000266498	RP11-45M22.5	lincRNA	-7,92E-02	6,41E-01	8,22E-01	No	-5,78E-01	1,50E-01	2,68E-01	No
ENSG00000223979	SMCR2	lincRNA	4,35E-02	5,79E-01	NA	No	2,00E-02	9,31E-01	NA	No
ENSG00000267350	RP1-178F10.3	lincRNA	1,66E-01	3,61E-01	6,08E-01	No	-6,80E-01	1,75E-03	6,12E-03	No
ENSG00000220161	RP1-37N7.3	lincRNA	-1,19E-01	3,27E-01	NA	No	-2,76E-01	2,46E-01	NA	No
ENSG00000265478	CTD-2145A24.3	lincRNA	3,10E-01	1,65E-01	3,86E-01	No	3,02E-01	4,07E-01	5,63E-01	No
ENSG00000265185	SNORD3B-1	lincRNA	6,30E-01	4,04E-02	1,51E-01	No	1,19E+00	3,52E-03	1,14E-02	No
ENSG00000262074	SNORD3B-2	lincRNA	2,25E-01	2,53E-01	4,95E-01	No	5,19E-01	1,64E-01	2,88E-01	No
ENSG00000230197	RP11-160E2.17	lincRNA	2,42E-02	9,72E-01	NA	No	6,17E-02	9,56E-01	NA	No
ENSG00000263394	RP11-160E2.19	lincRNA	-5,58E-02	6,46E-01	NA	No	-1,33E-01	7,12E-01	NA	No
ENSG00000262202	SNORD3D	lincRNA	-1,13E-01	6,08E-01	8,02E-01	No	1,67E-01	6,13E-01	7,42E-01	No
ENSG00000263934	SNORD3A	lincRNA	1,87E-01	4,01E-01	6,45E-01	No	2,42E-01	3,33E-01	4,88E-01	No
ENSG00000236022	RP11-160E2.16	lincRNA	2,42E-02	9,72E-01	NA	No	6,17E-02	9,56E-01	NA	No
ENSG00000227078	AC004448.2	lincRNA	-1,75E-02	8,75E-01	NA	No	-1,19E-01	5,88E-01	NA	No
ENSG00000235979	AC004448.5	lincRNA	-5,69E-02	6,46E-01	NA	No	-7,49E-02	8,00E-01	NA	No
ENSG00000263726	CTB-187M2.3	lincRNA	1,02E-01	3,13E-01	NA	No	1,18E-01	5,65E-01	NA	No
ENSG00000262681	RP11-311F12.1	lincRNA	6,03E-02	6,99E-01	8,57E-01	No	-3,56E-01	1,61E-01	NA	No
ENSG00000270091	RP11-78O7.2	lincRNA	7,98E-02	7,19E-01	8,69E-01	No	-9,65E-01	2,43E-02	6,10E-02	No
ENSG00000263494	AC004702.2	lincRNA	3,67E-02	8,36E-01	9,29E-01	No	6,57E-02	8,50E-01	9,09E-01	No
ENSG00000260907	AC008088.4	lincRNA	-1,69E-02	8,19E-01	NA	No	-1,26E-01	7,12E-01	NA	No
ENSG00000264215	RP11-283C24.1	lincRNA	-2,08E-02	8,19E-01	NA	No	-3,60E-02	8,90E-01	NA	No

ENSG00000236819	AC087393.1	lincRNA	-2,21E-02	8,63E-01	NA	No	1,71E-01	5,77E-01	7,11E-01	No
ENSG00000265043	RP11-728E14.3	lincRNA	-1,88E-02	8,19E-01	NA	No	-1,30E-01	7,12E-01	NA	No
ENSG00000265265	RP11-822E23.7	lincRNA	-1,25E-03	9,74E-01	NA	No	-1,30E-01	7,12E-01	NA	No
ENSG00000263609	RP11-1109M24.16	lincRNA	-1,37E-01	1,64E-01	NA	No	-2,31E-01	2,76E-01	NA	No
ENSG00000264956	RP11-1109M24.5	lincRNA	-6,57E-02	5,98E-01	NA	No	-3,41E-01	2,02E-01	NA	No
ENSG00000266795	RP11-744K17.9	lincRNA	-1,02E-02	8,49E-01	NA	No	-9,72E-02	8,30E-01	NA	No
ENSG00000261020	RP11-744K17.1	lincRNA	-2,59E-02	7,31E-01	NA	No	-1,26E-01	7,12E-01	NA	No
ENSG00000266885	RP11-744K17.2	lincRNA	1,26E-04	9,41E-01	NA	No	-1,02E-01	8,30E-01	NA	No
ENSG00000264469	RP11-173M1.8	lincRNA	-6,34E-02	7,60E-01	8,89E-01	No	7,02E-02	7,48E-01	8,41E-01	No
ENSG00000266313	RP11-173M1.4	lincRNA	1,00E-01	4,79E-01	7,10E-01	No	2,32E-01	4,48E-01	6,01E-01	No
ENSG00000267644	CTD-2008P7.10	lincRNA	-5,73E-02	6,46E-01	NA	No	-7,38E-02	8,00E-01	NA	No
ENSG00000267259	CTD-2008P7.9	lincRNA	8,18E-02	3,34E-01	NA	No	1,10E-01	6,87E-01	NA	No
ENSG00000260777	CTD-2008P7.1	lincRNA	4,02E-02	8,45E-01	NA	No	8,77E-02	8,64E-01	NA	No
ENSG00000264066	MIR451B	lincRNA	-3,20E-02	7,21E-01	NA	No	-1,53E-01	5,47E-01	NA	No
ENSG00000266111	RP11-296K13.4	lincRNA	6,78E-02	7,59E-01	8,89E-01	No	-1,66E-01	6,33E-01	7,56E-01	No
ENSG00000263860	RP11-218M11.3	lincRNA	1,04E-01	4,08E-01	NA	No	-1,56E-01	5,24E-01	NA	No
ENSG00000259928	RP11-218M11.1	lincRNA	7,00E-02	5,34E-01	NA	No	2,21E-02	9,24E-01	NA	No
ENSG00000265443	CTD-2349P21.6	lincRNA	7,98E-02	5,96E-01	7,94E-01	No	-1,65E-01	4,17E-01	NA	No
ENSG00000266490	CTD-2349P21.9	lincRNA	-2,02E-01	3,80E-01	6,26E-01	No	-1,18E+00	8,61E-03	2,50E-02	No
ENSG00000265743	RP11-848P1.3	lincRNA	1,06E+00	2,41E-03	1,99E-02	No	4,09E-01	2,25E-01	3,65E-01	No
ENSG00000266877	RP1-41C23.1	lincRNA	6,31E-03	9,66E-01	NA	No	-1,91E-01	4,02E-01	NA	No
ENSG00000263990	CTC-542B22.2	lincRNA	3,54E-01	1,59E-01	3,76E-01	No	-2,44E-01	4,87E-01	6,36E-01	No
ENSG00000266385	RP11-227G15.8	lincRNA	8,99E-02	6,70E-01	8,39E-01	No	-3,91E-01	2,88E-01	4,38E-01	No
ENSG00000264083	RP11-227G15.9	lincRNA	1,06E-01	5,88E-01	7,88E-01	No	-2,49E-01	4,87E-01	6,37E-01	No
ENSG00000264174	RP11-212E8.1	lincRNA	4,26E-02	7,41E-01	NA	No	8,77E-02	8,64E-01	NA	No
ENSG00000264622	RP11-642M2.1	lincRNA	-7,19E-04	9,41E-01	NA	No	-9,72E-02	8,30E-01	NA	No
ENSG00000267457	RP5-837J1.4	lincRNA	4,93E-01	7,64E-02	2,33E-01	No	6,80E-01	1,01E-01	1,98E-01	No
ENSG00000267364	RP11-47L3.1	lincRNA	1,57E-01	4,88E-01	7,18E-01	No	-5,06E-01	1,48E-01	2,65E-01	No
ENSG00000267711	RP11-686D22.5	lincRNA	1,53E-01	4,08E-01	6,52E-01	No	3,69E-01	3,05E-01	4,58E-01	No
ENSG00000267547	RP11-686D22.4	lincRNA	5,42E-01	6,17E-02	2,01E-01	No	8,89E-01	1,48E-02	4,01E-02	No
ENSG00000267035	RP11-1094M14.10	lincRNA	3,48E-01	1,31E-02	NA	No	9,20E-02	8,64E-01	NA	No
ENSG00000267321	RP11-1094M14.11	lincRNA	-2,93E-01	1,07E-01	2,91E-01	No	7,67E-01	2,20E-04	9,38E-04	No
ENSG00000267330	AC131056.5	lincRNA	-3,04E-02	7,31E-01	NA	No	-1,30E-01	7,12E-01	NA	No
ENSG00000267067	CTB-75G16.3	lincRNA	1,42E-01	2,81E-01	5,27E-01	No	-2,58E-02	9,13E-01	9,48E-01	No
ENSG00000267785	CTD-3194G12.2	lincRNA	4,97E-02	5,91E-01	NA	No	-1,78E-02	8,90E-01	NA	No
ENSG00000261005	CTB-58E17.1	lincRNA	-2,02E-01	3,46E-01	5,95E-01	No	4,75E-01	5,41E-02	1,19E-01	No
ENSG00000263874	LINC00672	lincRNA	4,90E-01	7,81E-02	2,36E-01	No	1,50E+00	2,76E-04	1,15E-03	No
ENSG00000266588	RP1-56K13.5	lincRNA	-9,29E-02	5,69E-01	7,75E-01	No	-3,88E-01	2,99E-01	4,51E-01	No
ENSG00000266013	CTD-2206N4.2	lincRNA	-4,33E-02	7,60E-01	NA	No	-1,01E-01	8,30E-01	NA	No
ENSG00000266753	RP11-690G19.3	lincRNA	2,96E-01	5,53E-02	1,86E-01	No	-3,69E-01	3,24E-02	7,76E-02	No
ENSG00000264968	RP11-387H17.4	lincRNA	-3,32E-01	1,77E-01	4,01E-01	No	-2,13E-01	4,65E-01	6,18E-01	No
ENSG00000265799	RP11-387H17.6	lincRNA	-7,18E-02	3,97E-01	NA	No	-3,11E-02	8,97E-01	9,39E-01	No
ENSG00000270145	CTD-2267D19.6	lincRNA	1,52E-01	2,98E-01	NA	No	8,72E-01	6,31E-02	1,35E-01	No
ENSG00000264488	RP11-605F20.1	lincRNA	-5,73E-02	6,46E-01	NA	No	-1,36E-01	7,12E-01	NA	No
ENSG00000267658	RP11-358B23.1	lincRNA	-4,93E-02	7,89E-01	9,05E-01	No	-1,06E+00	3,53E-02	8,36E-02	No
ENSG00000197291	RAMP2-AS1	lincRNA	1,51E-01	4,98E-01	7,26E-01	No	4,24E-01	5,92E-02	1,29E-01	No
ENSG00000213373	LINC00671	lincRNA	1,24E-01	4,38E-01	6,77E-01	No	3,88E-01	2,66E-01	4,13E-01	No
ENSG00000267002	RP11-242D8.1	lincRNA	5,36E-01	2,88E-02	1,19E-01	No	8,09E-02	7,53E-01	8,44E-01	No
ENSG00000188825	LINC00910	lincRNA	5,10E-01	1,69E-02	8,17E-02	No	-5,13E-01	2,59E-02	6,44E-02	No
ENSG00000267440	CTC-501O10.1	lincRNA	1,36E-02	9,28E-01	9,71E-01	No	-1,71E-01	4,83E-01	6,33E-01	No
ENSG00000267420	RP11-527L4.6	lincRNA	2,66E-03	9,62E-01	NA	No	-6,70E-02	7,34E-01	NA	No
ENSG00000267505	CTC-296K1.3	lincRNA	-1,32E-01	5,55E-01	7,66E-01	No	4,88E-01	1,52E-01	2,71E-01	No
ENSG00000267405	CTC-296K1.4	lincRNA	-1,57E-01	4,82E-01	7,12E-01	No	3,74E-01	2,92E-01	4,43E-01	No
ENSG00000267334	CTD-2534I21.8	lincRNA	1,62E-02	8,72E-01	NA	No	1,55E-02	9,87E-01	NA	No
ENSG00000267446	CTB-39G8.2	lincRNA	-4,46E-02	7,60E-01	NA	No	1,67E-01	3,37E-01	NA	No
ENSG00000266918	RP11-798G7.8	lincRNA	-7,70E-02	7,21E-01	8,70E-01	No	1,57E-01	6,45E-01	7,66E-01	No
ENSG00000267198	RP11-798G7.6	lincRNA	4,24E-01	4,48E-02	1,61E-01	No	6,18E-01	1,26E-01	2,34E-01	No
ENSG00000261886	RP11-63A1.1	lincRNA	1,15E-01	2,57E-01	NA	No	-6,48E-02	8,00E-01	NA	No
ENSG00000264243	RP11-6N17.1	lincRNA	-5,73E-02	6,46E-01	NA	No	-4,06E-02	8,90E-01	NA	No
ENSG00000266821	RP11-6N17.2	lincRNA	3,68E-02	6,35E-01	NA	No	1,93E-02	9,07E-01	NA	No
ENSG00000262837	RP11-304F15.7	lincRNA	2,68E-01	2,60E-01	5,04E-01	No	-1,15E+00	7,62E-03	2,25E-02	No
ENSG00000248954	RP11-304F15.4	lincRNA	-4,00E-02	7,71E-01	NA	No	-2,00E-01	4,92E-01	NA	No

ENSG00000248172	RP11-1094H24.3	lincRNA	-7,19E-04	9,41E-01	NA	No	-9,72E-02	8,30E-01	NA	No
ENSG00000246640	RP11-1094H24.4	lincRNA	-1,13E-01	6,07E-01	8,01E-01	No	-3,27E-01	3,71E-01	5,28E-01	No
ENSG00000249406	RP11-893F2.5	lincRNA	-6,94E-02	4,18E-01	NA	No	-1,55E-01	4,93E-01	NA	No
ENSG00000249451	RP11-94C24.6	lincRNA	-1,73E-01	3,08E-01	NA	No	-6,85E-01	6,86E-02	NA	No
ENSG00000247011	RP11-700H6.1	lincRNA	5,60E-01	5,53E-02	1,86E-01	No	1,79E-01	5,87E-01	7,19E-01	No
ENSG00000251665	RP11-700H6.2	lincRNA	6,57E-02	6,89E-01	8,51E-01	No	4,03E-02	9,03E-01	9,43E-01	No
ENSG00000249383	RP11-1018N14.1	lincRNA	-5,47E-02	6,46E-01	NA	No	-1,30E-01	7,12E-01	NA	No
ENSG00000267452	RP11-1018N14.5	lincRNA	2,22E-02	8,56E-01	NA	No	1,37E-02	9,87E-01	NA	No
ENSG00000263317	RP11-429O1.1	lincRNA	1,11E-01	2,86E-01	NA	No	3,20E-03	9,42E-01	NA	No
ENSG00000261589	CTC-462L7.1	lincRNA	2,36E-01	2,73E-01	5,18E-01	No	4,72E-01	6,25E-02	1,34E-01	No
ENSG00000262079	RP11-515O17.3	lincRNA	1,24E-01	2,96E-01	5,44E-01	No	1,11E-01	6,68E-01	7,84E-01	No
ENSG00000262052	RP11-763E3.1	lincRNA	-1,44E-01	3,43E-01	5,91E-01	No	-2,21E-01	4,95E-01	6,44E-01	No
ENSG00000262951	RP11-670E13.2	lincRNA	9,46E-02	5,57E-01	7,68E-01	No	1,71E-02	9,60E-01	9,77E-01	No
ENSG00000263004	RP11-166P13.3	lincRNA	-3,20E-01	1,69E-01	3,91E-01	No	3,88E-01	1,43E-01	2,59E-01	No
ENSG00000265542	RP11-60A24.3	lincRNA	-1,17E+00	7,01E-04	7,83E-03	No	-1,29E+00	9,58E-04	3,57E-03	No
ENSG00000264112	RP11-159D12.2	lincRNA	3,88E-01	7,78E-02	2,36E-01	No	5,00E-01	4,49E-02	1,02E-01	No
ENSG00000265313	RP11-567L7.6	lincRNA	9,92E-03	9,31E-01	NA	No	-6,58E-02	7,58E-01	NA	No
ENSG00000267302	RP11-178C3.2	lincRNA	-5,95E-02	6,94E-01	8,54E-01	No	-2,89E-01	3,84E-01	5,41E-01	No
ENSG00000267416	CTD-2319I12.2	lincRNA	1,23E-01	4,60E-01	6,94E-01	No	5,12E-01	1,70E-01	2,95E-01	No
ENSG00000267095	CTD-2319I12.5	lincRNA	4,66E-02	6,00E-01	NA	No	3,46E-01	1,45E-01	NA	No
ENSG00000266411	RP11-180P8.3	lincRNA	4,16E-02	8,07E-01	9,14E-01	No	-4,02E-02	9,04E-01	9,43E-01	No
ENSG00000265282	RP11-269G24.4	lincRNA	1,08E-01	4,89E-01	7,18E-01	No	-1,77E-01	3,93E-01	NA	No
ENSG00000265971	RP11-269G24.6	lincRNA	-4,21E-02	6,25E-01	NA	No	-1,45E-01	6,08E-01	NA	No
ENSG00000263489	CTC-264K15.6	lincRNA	4,52E-02	6,56E-01	NA	No	1,10E-01	6,87E-01	NA	No
ENSG00000264954	RP11-214C8.2	lincRNA	1,29E-02	9,39E-01	9,75E-01	No	-3,42E-02	9,10E-01	9,46E-01	No
ENSG00000266402	SNORA76	lincRNA	-6,14E-02	7,79E-01	9,00E-01	No	4,12E-01	1,79E-01	3,07E-01	No
ENSG00000265912	RP11-583F2.2	lincRNA	5,40E-01	5,17E-02	1,78E-01	No	3,81E-01	2,88E-01	4,38E-01	No
ENSG00000263520	CTD-2535L24.3	lincRNA	-4,15E-02	7,60E-01	NA	No	-9,51E-02	8,30E-01	NA	No
ENSG00000266176	RP11-855A2.5	lincRNA	-1,09E-01	4,54E-01	6,89E-01	No	-6,03E-02	8,47E-01	9,07E-01	No
ENSG00000267731	RP11-147L13.8	lincRNA	1,04E+00	3,20E-03	2,47E-02	No	4,09E-01	1,98E-01	3,32E-01	No
ENSG00000267659	RP11-118B18.1	lincRNA	-3,75E-03	9,82E-01	9,93E-01	No	-3,81E-01	3,06E-01	4,59E-01	No
ENSG00000267250	RP11-118B18.2	lincRNA	2,90E-02	7,66E-01	NA	No	-1,30E-01	7,12E-01	NA	No
ENSG00000267653	RP1-193H18.3	lincRNA	6,66E-01	2,88E-02	1,19E-01	No	3,63E-01	2,55E-01	4,00E-01	No
ENSG00000227517	AC003051.1	lincRNA	7,71E-02	3,74E-01	NA	No	9,20E-02	8,64E-01	NA	No
ENSG00000237560	AC004562.1	lincRNA	-1,88E-02	8,11E-01	NA	No	1,19E-01	6,05E-01	7,34E-01	No
ENSG00000267109	CTD-2378E21.1	lincRNA	-4,21E-02	7,60E-01	NA	No	-9,72E-02	8,30E-01	NA	No
ENSG00000256124	LINC01152	lincRNA	-2,62E-02	8,27E-01	NA	No	-2,29E-01	3,22E-01	NA	No
ENSG00000226101	AC007461.2	lincRNA	1,51E-01	4,62E-01	6,97E-01	No	5,72E-01	1,42E-01	2,58E-01	No
ENSG00000227036	LINC00511	lincRNA	-3,31E-01	1,84E-01	4,10E-01	No	-3,54E-03	9,90E-01	9,94E-01	No
ENSG00000264026	RP11-1124B17.1	lincRNA	3,17E-02	9,36E-01	NA	No	8,58E-02	8,64E-01	NA	No
ENSG00000263680	RP11-57A1.1	lincRNA	3,91E-02	8,45E-01	NA	No	1,87E-01	3,79E-01	NA	No
ENSG00000263893	CTD-3010D24.3	lincRNA	5,25E-02	6,48E-01	NA	No	1,26E-01	5,91E-01	7,23E-01	No
ENSG00000263574	CTD-2532D12.4	lincRNA	-1,10E-01	2,35E-01	NA	No	-2,57E-01	2,94E-01	NA	No
ENSG00000177338	LINC00469	lincRNA	-3,13E-02	7,31E-01	NA	No	-1,33E-01	7,12E-01	NA	No
ENSG00000261222	CTD-2006K23.1	lincRNA	-1,99E-02	8,82E-01	9,51E-01	No	-5,07E-01	1,21E-01	2,28E-01	No
ENSG00000265242	RP11-649A18.7	lincRNA	2,99E-01	9,63E-02	2,72E-01	No	-8,39E-01	5,88E-03	1,79E-02	No
ENSG00000267065	CTD-2246P4.1	lincRNA	4,28E-02	7,81E-01	9,01E-01	No	-1,13E-01	6,45E-01	7,66E-01	No
ENSG00000263718	RP11-285E9.6	lincRNA	3,31E-02	9,36E-01	NA	No	6,54E-02	9,56E-01	NA	No
ENSG00000267665	RP11-13K12.2	lincRNA	3,32E-02	9,36E-01	NA	No	8,51E-02	8,64E-01	NA	No
ENSG00000267506	RP11-13K12.1	lincRNA	-6,51E-01	4,18E-02	1,54E-01	No	-7,16E-01	7,51E-02	1,56E-01	No
ENSG00000267466	RP11-13K12.5	lincRNA	-1,60E-01	4,66E-01	7,00E-01	No	-6,51E-01	1,18E-01	2,24E-01	No
ENSG00000267790	RP11-316M20.1	lincRNA	-5,69E-02	6,46E-01	NA	No	-2,52E-02	8,90E-01	NA	No
ENSG00000267737	AC061992.2	lincRNA	-1,25E-01	4,69E-01	7,02E-01	No	-2,75E-01	4,00E-01	5,57E-01	No
ENSG00000267123	CTD-2357A8.3	lincRNA	1,76E-01	2,55E-01	4,98E-01	No	-1,86E-01	4,11E-01	NA	No
ENSG00000214105	CTD-2116F7.1	lincRNA	1,71E-01	1,51E-01	NA	No	-1,28E-01	7,12E-01	NA	No
ENSG00000262772	RP11-353N14.2	lincRNA	-5,31E-02	7,75E-01	8,97E-01	No	-1,52E-01	6,52E-01	7,71E-01	No
ENSG00000262585	RP11-353N14.5	lincRNA	-3,06E-02	7,26E-01	NA	No	2,29E-02	9,19E-01	NA	No
ENSG00000260369	CTD-2526A2.2	lincRNA	-9,80E-02	6,09E-01	8,03E-01	No	-8,15E-01	7,18E-02	1,50E-01	No
ENSG00000262873	CTD-2561B21.11	lincRNA	5,21E-02	7,49E-01	8,83E-01	No	-3,49E-01	3,12E-01	4,66E-01	No
ENSG00000262098	CTD-2561B21.10	lincRNA	5,44E-02	5,56E-01	NA	No	-1,26E-01	7,12E-01	NA	No
ENSG00000226137	BAIAP2-AS1	lincRNA	9,76E-02	5,93E-01	7,92E-01	No	-8,22E-02	7,12E-01	8,16E-01	No
ENSG00000185168	LINC00482	lincRNA	9,82E-02	6,42E-01	8,23E-01	No	-3,18E-01	3,81E-01	5,38E-01	No

ENSG00000262223	RP11-1055B8.3	lincRNA	-1,14E-01	5,08E-01	7,34E-01	No	-4,49E-01	1,72E-01	2,99E-01	No
ENSG00000262877	RP11-1055B8.4	lincRNA	5,53E-01	1,06E-02	5,84E-02	No	-1,42E+00	1,47E-05	7,85E-05	No
ENSG00000263731	RP11-498C9.15	lincRNA	1,06E-01	6,08E-01	8,02E-01	No	-6,55E-01	2,48E-02	6,22E-02	No
ENSG00000265692	RP13-516M14.4	lincRNA	1,52E-01	2,81E-01	5,27E-01	No	4,28E-02	8,68E-01	9,21E-01	No
ENSG00000260563	RP13-516M14.1	lincRNA	3,96E-01	1,28E-01	3,28E-01	No	2,51E-01	4,65E-01	6,18E-01	No
ENSG00000260011	RP13-20L14.1	lincRNA	1,74E-02	8,76E-01	NA	No	3,31E-02	8,93E-01	NA	No
ENSG00000263063	RP11-388C12.1	lincRNA	1,90E-01	2,58E-01	5,01E-01	No	3,42E-01	2,80E-01	4,30E-01	No
ENSG00000263321	RP11-388C12.5	lincRNA	2,77E-02	8,13E-01	NA	No	-1,15E-01	5,80E-01	NA	No
ENSG00000262663	RP11-497H17.1	lincRNA	5,86E-01	4,84E-02	1,70E-01	No	-9,82E-01	3,03E-02	7,35E-02	No
ENSG00000261888	AC144831.1	lincRNA	-4,41E-01	6,89E-02	2,17E-01	No	-1,43E+00	2,21E-05	1,14E-04	No
ENSG00000262898	AC139099.4	lincRNA	9,98E-03	9,66E-01	NA	No	-9,82E-02	8,30E-01	NA	No
ENSG00000262952	AC139099.6	lincRNA	-2,59E-03	9,75E-01	NA	No	-1,30E-01	7,12E-01	NA	No
ENSG00000262094	AC139099.5	lincRNA	5,05E-03	9,62E-01	NA	No	2,44E-01	3,27E-01	NA	No
ENSG00000263884	RP11-705O1.8	lincRNA	2,36E-02	9,15E-01	9,64E-01	No	4,94E-01	1,65E-01	2,88E-01	No
ENSG00000264514	RP11-720L2.4	lincRNA	-1,66E-02	9,36E-01	9,74E-01	No	2,64E-01	4,33E-01	5,88E-01	No
ENSG00000264433	RP11-720L2.3	lincRNA	-1,75E-01	4,09E-01	6,52E-01	No	-1,93E-01	5,84E-01	7,17E-01	No
ENSG00000273355	RP11-672L10.6	lincRNA	-4,72E-03	9,79E-01	9,92E-01	No	7,67E-01	6,22E-02	1,34E-01	No
ENSG00000263551	RP11-78F17.1	lincRNA	-2,04E-01	2,64E-01	5,08E-01	No	-1,60E-02	9,58E-01	9,75E-01	No
ENSG00000132204	LINC00470	lincRNA	-3,76E-01	8,13E-02	2,43E-01	No	6,69E-02	8,42E-01	9,04E-01	No
ENSG00000266450	CTD-2015H3.2	lincRNA	3,88E-02	8,45E-01	NA	No	6,67E-02	9,56E-01	NA	No
ENSG00000264080	CTD-2015H3.1	lincRNA	-2,81E-02	8,37E-01	9,30E-01	No	1,20E+00	2,52E-02	6,30E-02	No
ENSG00000266578	RP11-838N2.5	lincRNA	-4,23E-02	7,60E-01	NA	No	7,53E-02	7,59E-01	NA	No
ENSG00000266835	RP11-838N2.4	lincRNA	5,45E-02	6,65E-01	8,36E-01	No	1,39E-01	6,64E-01	7,81E-01	No
ENSG00000265091	RP11-835E18.5	lincRNA	-6,40E-02	4,76E-01	NA	No	2,12E-01	4,22E-01	5,77E-01	No
ENSG00000263753	LINC00667	lincRNA	-2,50E-01	6,02E-02	1,97E-01	No	2,52E-01	8,65E-02	1,75E-01	No
ENSG00000264575	LINC00526	lincRNA	7,52E-01	2,06E-02	9,38E-02	No	1,35E+00	3,99E-04	1,61E-03	No
ENSG00000264254	CTD-3096M3.1	lincRNA	1,63E-02	8,01E-01	NA	No	-3,46E-02	8,90E-01	NA	No
ENSG00000266288	CTD-3096M3.2	lincRNA	7,20E-02	5,43E-01	7,58E-01	No	9,42E-02	7,42E-01	8,37E-01	No
ENSG00000264707	RP11-760N9.1	lincRNA	3,64E-03	9,88E-01	9,96E-01	No	-5,42E-01	1,59E-01	2,81E-01	No
ENSG00000265933	LINC00668	lincRNA	4,13E-02	5,40E-01	NA	No	-9,39E-03	8,90E-01	NA	No
ENSG00000264964	RP11-888D10.3	lincRNA	1,03E-01	2,91E-01	NA	No	1,80E-02	9,29E-01	NA	No
ENSG00000273335	RP11-61L19.2	lincRNA	-1,36E-02	8,19E-01	NA	No	-3,60E-02	8,90E-01	NA	No
ENSG00000263627	RP11-692N5.1	lincRNA	6,60E-03	9,53E-01	NA	No	2,00E-01	4,47E-01	6,00E-01	No
ENSG00000265554	RP11-419J16.1	lincRNA	-4,23E-02	7,60E-01	NA	No	-9,82E-02	8,30E-01	NA	No
ENSG00000265728	RP11-883A18.3	lincRNA	-1,36E-02	9,25E-01	9,68E-01	No	-2,87E-01	2,62E-01	4,09E-01	No
ENSG00000267051	RP11-128P17.2	lincRNA	-5,69E-02	6,46E-01	NA	No	-1,35E-01	7,12E-01	NA	No
ENSG00000267079	RP11-820I16.1	lincRNA	-1,65E-01	1,83E-01	NA	No	-1,32E-01	6,17E-01	7,44E-01	No
ENSG00000267247	RP11-64C12.6	lincRNA	-2,37E-02	8,19E-01	NA	No	-1,36E-01	7,12E-01	NA	No
ENSG00000267069	RP11-64C12.8	lincRNA	1,30E-01	5,66E-01	7,74E-01	No	-8,34E-02	8,10E-01	8,83E-01	No
ENSG00000267108	RP11-861E21.1	lincRNA	6,26E-01	1,60E-02	7,86E-02	No	6,00E-01	7,39E-02	1,54E-01	No
ENSG00000267654	RP11-973H7.4	lincRNA	1,00E-01	1,95E-01	NA	No	8,51E-02	8,64E-01	NA	No
ENSG00000260302	RP11-973H7.1	lincRNA	1,69E-01	7,19E-02	2,23E-01	No	4,40E-02	7,69E-01	NA	No
ENSG00000267286	RP11-794M8.2	lincRNA	-4,15E-02	7,60E-01	NA	No	1,45E-01	5,48E-01	NA	No
ENSG00000264222	RP11-757O6.1	lincRNA	4,26E-01	1,29E-02	6,72E-02	No	3,00E-03	9,77E-01	NA	No
ENSG00000265766	CXADRP3	lincRNA	-5,58E-02	6,46E-01	NA	No	-1,33E-01	7,12E-01	NA	No
ENSG00000265786	RP11-527H14.6	lincRNA	-1,48E-02	8,19E-01	NA	No	-1,28E-01	7,12E-01	NA	No
ENSG00000266554	RP11-527H14.2	lincRNA	-1,43E-02	8,47E-01	NA	No	-4,31E-02	7,69E-01	NA	No
ENSG00000263350	RP11-13N13.2	lincRNA	-4,79E-02	8,26E-01	9,24E-01	No	8,42E-01	8,29E-03	2,41E-02	No
ENSG00000264149	RP11-268I9.3	lincRNA	3,17E-02	9,36E-01	NA	No	2,69E-01	2,74E-01	NA	No
ENSG00000266010	GATA6-AS1	lincRNA	-2,86E-01	1,39E-01	3,45E-01	No	2,40E-01	2,62E-01	4,09E-01	No
ENSG00000264825	RP11-627G18.4	lincRNA	4,58E-02	7,81E-01	9,01E-01	No	3,15E-01	3,82E-01	5,39E-01	No
ENSG00000273348	RP11-535A5.1	lincRNA	6,56E-01	3,79E-02	1,44E-01	No	1,26E+00	1,16E-03	4,22E-03	No
ENSG00000265752	RP11-403A21.1	lincRNA	-4,96E-03	9,71E-01	NA	No	8,85E-02	7,56E-01	8,46E-01	No
ENSG00000265750	RP11-799B12.4	lincRNA	-1,38E-01	5,37E-01	7,55E-01	No	-6,99E-01	8,12E-02	1,66E-01	No
ENSG00000273321	RP11-621L6.3	lincRNA	-1,43E-02	8,88E-01	NA	No	1,32E-01	6,30E-01	NA	No
ENSG00000264695	RP11-178F10.2	lincRNA	-1,88E-02	8,08E-01	NA	No	5,93E-02	7,63E-01	NA	No
ENSG00000265485	RP11-449D8.1	lincRNA	6,77E-02	7,35E-01	8,77E-01	No	1,35E+00	2,83E-03	9,37E-03	No
ENSG00000263553	RP11-449D8.2	lincRNA	2,27E-01	1,23E-01	NA	No	1,79E-01	4,52E-01	NA	No
ENSG00000266573	RP11-449D8.5	lincRNA	7,14E-02	6,06E-01	8,01E-01	No	1,46E-01	5,96E-01	7,27E-01	No
ENSG00000265552	RP11-958F21.3	lincRNA	5,95E-02	6,33E-01	NA	No	-1,32E-01	6,16E-01	NA	No
ENSG00000265374	RP11-57J16.1	lincRNA	3,17E-02	9,36E-01	NA	No	8,58E-02	8,64E-01	NA	No
ENSG00000264151	RP11-739N10.1	lincRNA	6,48E-03	9,67E-01	NA	No	-5,68E-02	8,00E-01	NA	No

ENSG00000266196	RP11-675P14.1	lincRNA	2,46E-02	9,72E-01	NA	No	9,88E-02	7,75E-01	NA	No
ENSG00000261521	RP11-408H20.1	lincRNA	-1,58E-01	3,05E-01	5,54E-01	No	5,94E-01	1,42E-01	2,58E-01	No
ENSG00000266521	RP11-650P15.1	lincRNA	-1,05E-03	9,83E-01	9,94E-01	No	1,69E-01	5,70E-01	7,06E-01	No
ENSG00000262477	AC021224.1	lincRNA	3,16E-03	9,41E-01	NA	No	-9,72E-02	8,30E-01	NA	No
ENSG00000267642	RP11-258B16.1	lincRNA	-4,58E-02	6,01E-01	NA	No	4,60E-02	8,44E-01	NA	No
ENSG00000268573	RP11-158H5.7	lincRNA	-6,10E-01	2,36E-03	1,96E-02	No	-7,98E-01	1,32E-04	5,87E-04	No
ENSG00000267583	RP11-322E11.5	lincRNA	1,39E-01	4,93E-01	7,22E-01	No	-1,25E-01	7,15E-01	8,19E-01	No
ENSG00000267397	RP11-873E20.1	lincRNA	-7,27E-01	3,11E-02	1,25E-01	No	-7,89E-01	5,31E-02	1,18E-01	No
ENSG00000267627	RP11-905K4.1	lincRNA	-4,42E-02	7,60E-01	NA	No	3,67E-03	9,87E-01	NA	No
ENSG00000267651	RP11-95O2.1	lincRNA	7,67E-01	1,77E-02	8,43E-02	No	-1,83E-02	9,56E-01	9,74E-01	No
ENSG00000267374	LINC00669	lincRNA	7,30E-02	5,79E-01	7,82E-01	No	-1,35E-01	5,03E-01	NA	No
ENSG00000267313	RP11-142I20.1	lincRNA	7,07E-02	6,93E-01	8,54E-01	No	5,64E-01	1,48E-01	2,66E-01	No
ENSG00000267586	LINC00907	lincRNA	2,78E-01	2,47E-01	4,88E-01	No	6,72E-02	8,46E-01	9,06E-01	No
ENSG00000267414	RP11-456K23.1	lincRNA	1,21E+00	3,50E-03	2,64E-02	No	1,12E+00	7,79E-03	2,29E-02	No
ENSG00000267167	RP11-687F6.1	lincRNA	-2,63E-01	2,65E-01	5,09E-01	No	-6,36E-01	5,02E-02	1,12E-01	No
ENSG00000266905	RP11-1058N17.1	lincRNA	-1,60E-02	8,97E-01	NA	No	2,51E-02	8,52E-01	NA	No
ENSG00000227115	RP11-267C16.1	lincRNA	1,89E-01	3,09E-01	5,57E-01	No	3,68E-01	2,87E-01	4,38E-01	No
ENSG00000263438	RP11-202D1.3	lincRNA	-1,60E-01	2,09E-01	NA	No	-3,38E-01	2,72E-01	NA	No
ENSG00000260433	RP11-202D1.2	lincRNA	5,90E-02	5,64E-01	NA	No	2,24E-02	8,87E-01	NA	No
ENSG00000267013	CTD-2171N6.1	lincRNA	-1,20E-02	9,53E-01	9,80E-01	No	6,43E-01	1,01E-01	1,97E-01	No
ENSG00000267325	RP11-397A16.2	lincRNA	1,09E-02	9,59E-01	9,83E-01	No	1,31E+00	7,75E-07	5,10E-06	No
ENSG00000260930	RP11-214L13.1	lincRNA	6,33E-02	6,08E-01	NA	No	5,59E-01	1,36E-01	2,49E-01	No
ENSG00000267327	CTD-2008L17.1	lincRNA	6,19E-01	4,10E-02	1,52E-01	No	8,72E-01	5,63E-02	1,23E-01	No
ENSG00000206129	CTD-2008L17.2	lincRNA	8,44E-02	6,59E-01	8,33E-01	No	8,57E-02	8,01E-01	8,77E-01	No
ENSG00000267057	RP11-456O19.2	lincRNA	1,28E-01	1,85E-01	NA	No	5,79E-02	8,14E-01	NA	No
ENSG00000267399	RP11-456O19.5	lincRNA	7,16E-02	4,17E-01	NA	No	9,88E-02	7,75E-01	NA	No
ENSG00000267712	RP11-456O19.4	lincRNA	-6,52E-02	5,76E-01	NA	No	-1,13E-01	6,87E-01	7,98E-01	No
ENSG00000258609	LINC-ROR	lincRNA	1,02E-01	3,11E-01	5,60E-01	No	-1,55E-01	5,35E-01	NA	No
ENSG00000267675	RP11-1151B14.2	lincRNA	3,50E-02	9,36E-01	NA	No	8,64E-02	8,64E-01	NA	No
ENSG00000267010	RP11-108P20.1	lincRNA	2,06E-01	3,00E-01	5,48E-01	No	4,86E-01	3,04E-02	7,36E-02	No
ENSG00000267501	RP11-108P20.2	lincRNA	3,82E-02	8,27E-01	9,24E-01	No	-6,26E-01	1,30E-01	2,40E-01	No
ENSG00000267098	RP11-325K19.1	lincRNA	-7,03E-02	2,89E-01	NA	No	-1,91E-01	3,42E-01	NA	No
ENSG00000267316	RP11-879F14.3	lincRNA	4,66E-01	8,95E-02	2,59E-01	No	7,08E-01	7,97E-02	1,64E-01	No
ENSG00000267175	RP11-879F14.1	lincRNA	2,73E-02	8,45E-01	NA	No	1,78E-01	4,09E-01	NA	No
ENSG00000267038	RP11-1096D5.2	lincRNA	4,22E-02	7,43E-01	NA	No	9,84E-02	7,75E-01	NA	No
ENSG00000260440	RP11-1096D5.1	lincRNA	9,09E-01	2,04E-02	9,33E-02	No	-3,57E-01	2,31E-01	3,73E-01	No
ENSG00000267134	RP11-146N18.1	lincRNA	3,32E-02	9,36E-01	NA	No	8,77E-02	8,64E-01	NA	No
ENSG00000260578	CTD-2541J13.1	lincRNA	3,34E-01	1,68E-01	3,90E-01	No	1,02E+00	4,18E-03	1,32E-02	No
ENSG00000265533	RP11-638L3.1	lincRNA	5,12E-02	5,17E-01	NA	No	2,38E-01	2,30E-01	NA	No
ENSG00000263594	RP11-28O3.2	lincRNA	4,69E-02	5,91E-01	NA	No	1,08E-01	6,89E-01	NA	No
ENSG00000265555	RP11-638L3.3	lincRNA	4,46E-02	6,75E-01	NA	No	8,77E-02	8,64E-01	NA	No
ENSG00000264869	RP11-526H11.1	lincRNA	4,40E-02	6,96E-01	NA	No	8,59E-02	8,64E-01	NA	No
ENSG00000265425	RP11-128D14.1	lincRNA	2,35E-02	9,72E-01	NA	No	1,10E-01	6,87E-01	NA	No
ENSG00000264705	RP11-674P19.2	lincRNA	-1,49E-01	5,06E-01	7,32E-01	No	-2,05E-01	5,37E-01	6,80E-01	No
ENSG00000266304	RP11-484N16.1	lincRNA	-9,66E-01	4,26E-03	3,04E-02	No	-8,59E-01	2,13E-02	5,46E-02	No
ENSG00000266278	RP11-41O4.2	lincRNA	-1,30E-01	1,57E-01	NA	No	7,31E-02	7,81E-01	8,64E-01	No
ENSG00000265781	RP11-384E22.1	lincRNA	-6,58E-02	4,30E-01	NA	No	-4,72E-02	8,36E-01	NA	No
ENSG00000265484	RP11-47G4.2	lincRNA	-2,20E-01	4,85E-02	NA	No	-5,69E-01	9,16E-02	NA	No
ENSG00000260676	RP11-510D19.1	lincRNA	-6,69E-02	4,09E-01	NA	No	-1,61E-01	4,69E-01	NA	No
ENSG00000265380	RP11-126K15.1	lincRNA	3,31E-02	9,36E-01	NA	No	1,40E-01	5,82E-01	NA	No
ENSG00000263655	RP11-25L3.3	lincRNA	-6,34E-02	4,94E-01	NA	No	-1,03E-01	6,39E-01	NA	No
ENSG00000264247	LINC00909	lincRNA	2,12E-02	8,68E-01	9,44E-01	No	7,78E-01	3,85E-07	2,67E-06	No
ENSG00000266774	RP11-321M21.1	lincRNA	-1,18E-02	8,19E-01	NA	No	-1,26E-01	7,12E-01	NA	No
ENSG00000266743	RP11-94B19.6	lincRNA	2,48E-01	1,67E-01	3,88E-01	No	6,86E-02	7,95E-01	8,74E-01	No
ENSG00000264260	RP11-94B19.1	lincRNA	2,70E-02	8,79E-01	9,49E-01	No	3,40E-01	3,42E-01	4,98E-01	No
ENSG00000264595	RP11-94B19.5	lincRNA	1,84E-01	2,90E-01	5,37E-01	No	4,30E-02	8,90E-01	9,35E-01	No
ENSG00000265717	RP11-94B19.7	lincRNA	3,51E-02	7,01E-01	NA	No	-1,39E-01	4,60E-01	NA	No
ENSG00000264212	RP11-94B19.3	lincRNA	-5,45E-02	6,46E-01	NA	No	1,56E-02	9,79E-01	NA	No
ENSG00000266256	LINC00683	lincRNA	2,89E-02	7,89E-01	NA	No	4,90E-02	8,31E-01	NA	No
ENSG00000266312	RP11-111H3.3	lincRNA	-1,97E-02	8,19E-01	NA	No	-1,35E-01	7,12E-01	NA	No
ENSG00000264015	RP11-176N18.2	lincRNA	-4,83E-02	5,97E-01	NA	No	3,79E-02	8,80E-01	9,28E-01	No
ENSG00000263146	RP11-849I19.1	lincRNA	6,25E-02	5,28E-01	NA	No	5,46E-01	2,72E-02	6,71E-02	No

ENSG00000267409	RP11-567M16.2	lincRNA	-1,21E-02	8,19E-01	NA	No	1,24E-01	4,92E-01	NA	No
ENSG00000178412	RP11-567M16.3	lincRNA	5,50E-02	6,73E-01	NA	No	-1,57E-01	5,07E-01	NA	No
ENSG00000266924	RP11-154H12.2	lincRNA	1,45E-02	8,21E-01	NA	No	-1,35E-01	7,12E-01	NA	No
ENSG00000230839	RP5-968J1.1	lincRNA	4,74E-02	7,67E-01	8,93E-01	No	1,02E-01	7,52E-01	8,44E-01	No
ENSG00000226644	RP11-128M1.1	lincRNA	-3,23E-02	6,22E-01	NA	No	-7,23E-02	6,98E-01	NA	No
ENSG00000226995	LINC00658	lincRNA	-2,74E-01	1,70E-01	3,93E-01	No	-1,39E+00	1,31E-02	3,59E-02	No
ENSG00000230563	RP5-828H9.1	lincRNA	7,42E-01	2,82E-02	1,17E-01	No	1,15E-01	7,33E-01	8,31E-01	No
ENSG00000205181	LINC00654	lincRNA	1,65E-01	4,58E-01	6,93E-01	No	9,68E-02	7,41E-01	8,36E-01	No
ENSG00000266908	RP5-1022P6.7	lincRNA	-7,20E-02	5,32E-01	NA	No	-2,46E-01	3,02E-01	NA	No
ENSG00000225640	RP5-1022P6.4	lincRNA	1,23E-01	3,84E-01	NA	No	-5,32E-02	8,17E-01	NA	No
ENSG00000229876	CASC20	lincRNA	3,27E-03	9,41E-01	NA	No	7,03E-03	9,87E-01	NA	No
ENSG00000232271	RP4-764O22.1	lincRNA	-4,15E-02	7,60E-01	NA	No	2,71E-01	3,31E-01	NA	No
ENSG00000238102	RP11-19D2.1	lincRNA	-1,84E-01	4,09E-01	6,52E-01	No	1,01E+00	4,19E-04	1,69E-03	No
ENSG00000243961	RP5-839B4.8	lincRNA	-5,47E-02	6,46E-01	NA	No	2,98E-02	8,93E-01	NA	No
ENSG00000230506	RP11-416N4.4	lincRNA	3,17E-02	9,36E-01	NA	No	8,77E-02	8,64E-01	NA	No
ENSG00000224961	RP1-278O22.1	lincRNA	1,12E-01	2,73E-01	NA	No	1,93E-01	3,57E-01	NA	No
ENSG00000234900	RP1-278O22.2	lincRNA	3,47E-01	1,39E-01	3,45E-01	No	-1,68E-01	6,15E-01	7,43E-01	No
ENSG00000232900	RP4-697P8.3	lincRNA	1,16E-01	5,59E-01	7,70E-01	No	-9,72E-02	7,67E-01	8,54E-01	No
ENSG00000270777	RP11-103J8.2	lincRNA	5,56E-02	4,79E-01	NA	No	-7,78E-03	9,79E-01	NA	No
ENSG00000230990	RP4-734C18.1	lincRNA	3,69E-02	8,45E-01	NA	No	1,03E-01	7,75E-01	NA	No
ENSG00000235292	RP4-796I8.1	lincRNA	5,30E-02	6,57E-01	NA	No	-1,27E-01	7,12E-01	NA	No
ENSG00000230437	RP11-102J14.1	lincRNA	-2,38E-02	7,62E-01	NA	No	-1,62E-05	9,99E-01	NA	No
ENSG00000233048	RP5-1069C8.2	lincRNA	4,23E-02	6,53E-01	NA	No	-1,42E-02	8,90E-01	NA	No
ENSG00000233625	RP5-905G11.3	lincRNA	1,46E-01	2,06E-01	NA	No	1,28E-01	4,83E-01	NA	No
ENSG00000230010	RP4-568F9.6	lincRNA	1,39E-01	4,78E-01	7,10E-01	No	4,09E-01	2,70E-01	4,18E-01	No
ENSG00000232388	LINC00493	lincRNA	-4,39E-01	1,31E-02	6,80E-02	No	3,72E-01	3,12E-02	7,52E-02	No
ENSG00000179935	LINC00652	lincRNA	6,68E-02	7,51E-01	8,84E-01	No	8,88E-02	8,00E-01	8,77E-01	No
ENSG00000273148	RP5-1068E13.7	lincRNA	3,34E-02	8,80E-01	9,50E-01	No	6,46E-01	3,09E-02	7,47E-02	No
ENSG00000233895	RP1-122P22.2	lincRNA	-1,83E-01	2,53E-01	4,95E-01	No	-4,27E-01	2,82E-02	6,92E-02	No
ENSG00000230400	RP11-359G22.2	lincRNA	-1,75E-02	8,19E-01	NA	No	-3,60E-02	8,90E-01	NA	No
ENSG00000233746	LINC00656	lincRNA	1,44E-02	9,11E-01	NA	No	-1,09E-01	5,34E-01	NA	No
ENSG00000230387	RP4-737E23.2	lincRNA	-1,41E-01	1,66E-01	NA	No	-4,06E-01	1,07E-01	2,06E-01	No
ENSG00000270001	RP11-218C14.8	lincRNA	7,14E-01	1,42E-02	7,21E-02	No	-1,02E+00	2,91E-02	7,12E-02	No
ENSG00000225056	RP11-218C14.5	lincRNA	7,47E-02	4,83E-01	NA	No	1,67E-02	9,80E-01	NA	No
ENSG00000230133	RP5-1100I6.2	lincRNA	-5,37E-02	6,46E-01	NA	No	-1,26E-01	7,12E-01	NA	No
ENSG00000231015	RP5-860P4.2	lincRNA	-2,15E-01	6,78E-02	NA	No	-4,92E-01	1,08E-01	NA	No
ENSG00000238129	RP3-410C9.2	lincRNA	-5,69E-02	6,46E-01	NA	No	-2,52E-02	8,90E-01	NA	No
ENSG00000205611	RP4-610C12.4	lincRNA	-2,32E-02	9,03E-01	9,60E-01	No	-8,04E-01	7,51E-02	1,56E-01	No
ENSG00000225490	RP4-610C12.3	lincRNA	1,87E-02	9,13E-01	9,64E-01	No	-3,45E-01	3,29E-01	4,84E-01	No
ENSG00000233354	LINC00028	lincRNA	1,19E-01	2,80E-01	5,26E-01	No	1,26E-01	5,88E-01	7,20E-01	No
ENSG00000198547	C20orf203	lincRNA	2,37E-01	2,04E-01	4,38E-01	No	-5,80E-02	8,63E-01	9,18E-01	No
ENSG00000260257	RP5-1085F17.3	lincRNA	-6,65E-01	1,94E-03	1,70E-02	No	-1,13E+00	2,90E-06	1,74E-05	No
ENSG00000228265	RP5-1125A11.1	lincRNA	4,77E-01	8,07E-02	2,41E-01	No	-1,95E-01	5,79E-01	7,13E-01	No
ENSG00000228386	RP5-1125A11.4	lincRNA	-8,26E-03	9,68E-01	9,87E-01	No	-3,89E-01	2,80E-01	4,30E-01	No
ENSG00000269202	RP4-614O4.12	lincRNA	2,15E-01	3,48E-01	5,96E-01	No	6,32E-01	9,67E-02	1,90E-01	No
ENSG00000268340	RP3-477O4.16	lincRNA	7,42E-02	5,34E-01	NA	No	1,59E-01	4,81E-01	NA	No
ENSG00000260032	LINC00657	lincRNA	-8,13E-02	5,09E-01	7,35E-01	No	9,93E-01	1,13E-20	3,71E-19	No
ENSG00000234139	RP4-550H1.4	lincRNA	6,68E-02	5,24E-01	NA	No	-1,02E-01	8,30E-01	NA	No
ENSG00000237063	RP4-550H1.5	lincRNA	3,15E-02	9,36E-01	NA	No	1,09E-01	6,87E-01	NA	No
ENSG00000224635	RP4-564F22.5	lincRNA	2,14E-02	9,01E-01	9,59E-01	No	-6,03E-01	1,26E-01	2,34E-01	No
ENSG00000235408	SNORA71B	lincRNA	-7,56E-02	6,96E-01	8,55E-01	No	-4,06E-01	2,78E-01	4,28E-01	No
ENSG00000229976	RP5-1031J8.1	lincRNA	-1,85E-03	9,85E-01	NA	No	-8,33E-02	6,94E-01	NA	No
ENSG00000228959	RP5-1121H13.3	lincRNA	-5,69E-02	6,46E-01	NA	No	-1,35E-01	7,12E-01	NA	No
ENSG00000223891	OSER1-AS1	lincRNA	-9,22E-02	6,22E-01	8,12E-01	No	8,61E-01	1,61E-04	7,07E-04	No
ENSG00000227477	STK4-AS1	lincRNA	3,21E-01	7,04E-02	2,20E-01	No	8,45E-01	6,27E-02	1,35E-01	No
ENSG00000236028	RP11-323C15.2	lincRNA	-3,91E-02	7,83E-01	9,02E-01	No	2,41E-01	4,72E-01	6,23E-01	No
ENSG00000224565	RP1-148H17.1	lincRNA	7,35E-02	6,50E-01	8,27E-01	No	7,77E-02	8,05E-01	8,80E-01	No
ENSG00000237423	RP11-347D21.3	lincRNA	-5,45E-02	6,46E-01	NA	No	-2,61E-02	8,90E-01	NA	No
ENSG00000231265	TRERNA1	lincRNA	-5,45E-02	6,46E-01	NA	No	-1,28E-01	7,12E-01	NA	No
ENSG00000237595	RP11-112L6.3	lincRNA	-1,93E-01	4,00E-01	6,45E-01	No	5,25E-01	1,44E-01	2,60E-01	No
ENSG00000234698	RP11-112L6.2	lincRNA	-2,60E-01	3,44E-02	1,35E-01	No	-3,95E-01	2,40E-01	3,83E-01	No
ENSG00000231742	RP11-112L6.4	lincRNA	-1,12E+00	3,89E-03	2,85E-02	No	-1,86E-01	5,83E-01	7,17E-01	No

ENSG00000224397	RP11-290F20.3	lincRNA	3,49E-01	1,29E-01	3,30E-01	No	-1,39E+00	1,43E-02	3,88E-02	No
ENSG00000203999	RP11-290F20.1	lincRNA	-1,92E-03	9,91E-01	9,97E-01	No	1,91E-04	9,99E-01	9,99E-01	No
ENSG00000233077	RP11-290F20.2	lincRNA	3,39E-02	8,29E-01	9,25E-01	No	-1,71E-01	5,90E-01	7,21E-01	No
ENSG00000227964	RP5-1112F19.2	lincRNA	4,24E-02	6,67E-01	NA	No	-1,26E-01	7,12E-01	NA	No
ENSG00000234948	RP4-723E3.1	lincRNA	-2,34E-02	8,07E-01	NA	No	-1,33E-01	7,12E-01	NA	No
ENSG00000232286	RP11-80K6.2	lincRNA	-5,37E-02	6,46E-01	NA	No	-6,12E-02	8,00E-01	NA	No
ENSG00000225657	RP5-843L14.1	lincRNA	-5,69E-02	6,46E-01	NA	No	-2,92E-02	8,90E-01	NA	No
ENSG00000226308	RP4-813D12.3	lincRNA	-3,31E-02	7,31E-01	NA	No	-1,35E-01	7,12E-01	NA	No
ENSG00000270279	RP4-806M20.5	lincRNA	-6,15E-02	5,36E-01	NA	No	-1,48E-01	5,95E-01	NA	No
ENSG00000268649	MIR296	lincRNA	1,38E-01	2,27E-01	NA	No	6,58E-02	7,33E-01	NA	No
ENSG00000268333	RP4-806M20.3	lincRNA	-1,87E-01	4,09E-01	6,53E-01	No	-3,47E-01	3,42E-01	4,98E-01	No
ENSG00000228340	RP5-1043L13.1	lincRNA	2,74E-01	2,49E-01	4,91E-01	No	-1,87E-01	5,84E-01	7,17E-01	No
ENSG00000233017	RP5-908M14.5	lincRNA	-1,48E-02	8,49E-01	NA	No	-1,01E-01	8,30E-01	NA	No
ENSG00000264490	WI2-87327B8.1	lincRNA	-4,35E-01	1,05E-01	2,87E-01	No	-1,03E+00	2,09E-02	5,38E-02	No
ENSG00000125514	LINC00029	lincRNA	3,41E-02	9,36E-01	NA	No	6,13E-02	9,56E-01	NA	No
ENSG00000260416	RP5-963E22.5	lincRNA	-3,74E-02	6,41E-01	NA	No	-1,49E-01	5,91E-01	NA	No
ENSG00000196421	LINC00176	lincRNA	6,99E-01	1,01E-02	5,70E-02	No	-6,97E-01	6,55E-02	1,40E-01	No
ENSG00000267290	AC092192.1	lincRNA	-5,37E-02	6,46E-01	NA	No	2,71E-02	9,24E-01	NA	No
ENSG00000261204	AC004449.6	lincRNA	4,47E-02	6,72E-01	NA	No	9,98E-02	7,75E-01	NA	No
ENSG00000267666	AC004156.3	lincRNA	5,34E-02	7,30E-01	8,74E-01	No	-2,13E-01	4,73E-01	6,25E-01	No
ENSG00000272473	AC006273.4	lincRNA	-4,54E-04	9,95E-01	9,98E-01	No	1,86E-02	9,50E-01	9,71E-01	No
ENSG00000267530	AC006273.5	lincRNA	-2,83E-02	8,62E-01	9,40E-01	No	1,19E-01	7,24E-01	8,24E-01	No
ENSG00000266927	AC006273.7	lincRNA	-7,05E-02	4,32E-01	NA	No	-2,03E-01	3,29E-01	NA	No
ENSG00000267778	AC004221.2	lincRNA	4,65E-02	6,07E-01	NA	No	2,41E-01	2,27E-01	NA	No
ENSG00000267372	AC005330.2	lincRNA	1,60E-01	2,10E-01	NA	No	4,20E-02	8,71E-01	NA	No
ENSG00000261526	CTB-31O20.2	lincRNA	4,65E-02	8,34E-01	9,28E-01	No	7,33E-01	2,66E-02	6,57E-02	No
ENSG00000267201	AC005624.2	lincRNA	1,05E-01	2,85E-01	NA	No	8,64E-02	8,64E-01	NA	No
ENSG00000267011	CTB-50L17.16	lincRNA	1,33E-01	3,04E-01	NA	No	-1,45E-01	6,02E-01	NA	No
ENSG00000268536	AC005523.3	lincRNA	4,65E-02	5,20E-01	NA	No	4,48E-01	1,38E-01	2,51E-01	No
ENSG00000223573	TINCR	lincRNA	3,67E-01	6,26E-02	2,03E-01	No	4,72E-02	8,18E-01	NA	No
ENSG00000214347	CTB-180A7.8	lincRNA	2,90E-02	7,71E-01	NA	No	4,04E-02	8,56E-01	NA	No
ENSG00000267939	CTD-2325M2.1	lincRNA	3,25E-01	1,41E-01	3,49E-01	No	8,86E-03	9,79E-01	9,87E-01	No
ENSG00000266912	CTC-325H20.2	lincRNA	1,33E-01	5,28E-01	7,49E-01	No	3,06E-01	2,54E-01	4,00E-01	No
ENSG00000267273	CTC-543D15.3	lincRNA	-4,42E-02	7,60E-01	NA	No	8,75E-03	9,87E-01	NA	No
ENSG00000266950	CTD-2623N2.5	lincRNA	4,62E-02	5,93E-01	NA	No	1,34E-02	9,87E-01	NA	No
ENSG00000267289	CTD-2623N2.11	lincRNA	3,20E-01	1,42E-01	3,51E-01	No	-7,76E-02	7,79E-01	8,63E-01	No
ENSG00000267100	ILF3-AS1	lincRNA	-9,07E-02	6,49E-01	8,27E-01	No	3,38E-01	1,91E-01	3,23E-01	No
ENSG00000197332	ZNF833P	lincRNA	-6,08E-02	7,77E-01	8,98E-01	No	2,15E-01	5,16E-01	6,62E-01	No
ENSG00000267089	CTC-499B15.8	lincRNA	5,66E-02	5,14E-01	NA	No	-1,36E-01	7,12E-01	NA	No
ENSG00000234773	CTD-2666L21.1	lincRNA	4,39E-02	8,40E-01	9,30E-01	No	7,45E-02	7,90E-01	8,70E-01	No
ENSG00000261280	CTD-3105H18.13	lincRNA	1,91E-01	3,68E-01	6,15E-01	No	1,13E+00	4,66E-05	2,27E-04	No
ENSG00000230310	CTD-2192J16.11	lincRNA	-4,96E-03	9,73E-01	9,89E-01	No	2,31E-01	4,84E-01	6,34E-01	No
ENSG00000268945	CTD-2192J16.26	lincRNA	-1,66E-02	8,87E-01	NA	No	-2,51E-01	3,08E-01	NA	No
ENSG00000267004	CTD-2659N19.4	lincRNA	2,23E-02	9,72E-01	NA	No	8,51E-02	8,64E-01	NA	No
ENSG00000267212	CTD-2659N19.9	lincRNA	1,47E-01	4,35E-01	6,74E-01	No	-7,43E-01	4,14E-02	9,57E-02	No
ENSG00000266897	AC005546.2	lincRNA	-3,22E-02	8,50E-01	9,35E-01	No	-1,46E+00	1,81E-02	4,76E-02	No
ENSG00000266913	CTC-548K16.2	lincRNA	5,03E-01	4,30E-02	1,57E-01	No	-2,48E-02	8,78E-01	NA	No
ENSG00000267147	CTC-548K16.1	lincRNA	4,70E-02	5,89E-01	NA	No	8,51E-02	8,64E-01	NA	No
ENSG00000269635	AC004257.1	lincRNA	-6,57E-02	4,24E-01	NA	No	-1,60E-01	4,77E-01	NA	No
ENSG00000270143	AC004257.3	lincRNA	5,67E-01	4,83E-02	1,70E-01	No	-2,22E-01	5,07E-01	6,54E-01	No
ENSG00000273218	LLNLR-246C6.1	lincRNA	9,25E-02	6,69E-01	8,38E-01	No	-1,08E+00	2,12E-02	5,45E-02	No
ENSG00000268673	LLNLR-249E10.1	lincRNA	-9,83E-02	2,94E-01	NA	No	-1,67E-01	4,43E-01	NA	No
ENSG00000267373	CTD-2231E14.5	lincRNA	3,49E-02	7,53E-01	NA	No	-1,18E-01	5,72E-01	NA	No
ENSG00000269399	CTD-3222D19.7	lincRNA	3,10E-01	2,07E-01	4,41E-01	No	-2,53E-01	4,48E-01	6,02E-01	No
ENSG00000268087	CTC-429P9.2	lincRNA	3,53E-01	1,10E-01	2,97E-01	No	-5,52E-01	7,24E-02	1,51E-01	No
ENSG00000269720	CTD-2521M24.5	lincRNA	1,11E-01	5,43E-01	7,59E-01	No	-2,91E-02	9,26E-01	9,56E-01	No
ENSG00000269640	CTD-2521M24.9	lincRNA	9,46E-01	2,45E-03	2,02E-02	No	-3,96E-01	2,09E-01	3,46E-01	No
ENSG00000269736	CTD-2521M24.11	lincRNA	9,63E-02	2,18E-01	NA	No	1,00E-01	7,75E-01	NA	No
ENSG00000269439	CTD-3131K8.2	lincRNA	4,04E-01	9,49E-02	2,69E-01	No	-1,42E+00	5,15E-04	2,03E-03	No
ENSG00000266904	LINC00663	lincRNA	4,12E-02	8,05E-01	9,13E-01	No	1,04E-01	5,85E-01	7,18E-01	No
ENSG00000267581	CTC-559E9.4	lincRNA	-3,51E-01	1,60E-01	3,78E-01	No	1,31E-02	9,72E-01	9,83E-01	No
ENSG00000269110	CTC-513N18.7	lincRNA	-3,27E-02	7,31E-01	NA	No	-1,30E-01	7,12E-01	NA	No

ENSG00000268560	CTD-2332E11.2	lincRNA	8,31E-02	5,15E-01	NA	No	6,96E-01	8,88E-02	1,78E-01	No
ENSG00000268658	LINC00664	lincRNA	-2,69E-01	2,24E-01	4,62E-01	No	1,49E-02	9,67E-01	9,81E-01	No
ENSG00000268555	RP11-678G14.3	lincRNA	-7,70E-01	1,31E-03	1,25E-02	No	-8,31E-01	8,83E-04	3,31E-03	No
ENSG00000268240	RP11-678G14.4	lincRNA	1,65E-02	9,19E-01	9,66E-01	No	-2,92E-02	9,30E-01	9,59E-01	No
ENSG00000268184	RP11-420K14.8	lincRNA	-7,23E-02	4,26E-01	NA	No	-6,44E-02	7,63E-01	NA	No
ENSG00000269504	AC003973.4	lincRNA	-6,73E-02	5,25E-01	NA	No	-7,27E-02	7,17E-01	8,20E-01	No
ENSG00000270947	AC025811.3	lincRNA	-6,13E-02	5,10E-01	NA	No	5,58E-02	8,26E-01	8,94E-01	No
ENSG00000180081	CTC-451A6.4	lincRNA	6,84E-02	7,00E-01	8,58E-01	No	5,65E-01	1,58E-01	2,79E-01	No
ENSG00000261558	CTD-2291D10.2	lincRNA	3,32E-02	9,36E-01	NA	No	6,23E-02	9,56E-01	NA	No
ENSG00000261615	CTD-2291D10.1	lincRNA	3,23E-02	7,34E-01	NA	No	-5,70E-02	8,00E-01	NA	No
ENSG00000269543	CTD-2291D10.3	lincRNA	8,49E-03	9,61E-01	NA	No	-1,26E-01	7,12E-01	NA	No
ENSG00000267886	CTD-2291D10.4	lincRNA	2,52E-01	1,92E-01	4,21E-01	No	1,70E-01	6,12E-01	7,40E-01	No
ENSG00000269416	CTB-175P5.4	lincRNA	4,21E-02	8,39E-01	9,30E-01	No	-1,16E+00	3,31E-02	7,91E-02	No
ENSG00000267924	RP11-255H23.4	lincRNA	1,82E-01	4,27E-01	6,68E-01	No	2,74E-02	9,34E-01	9,61E-01	No
ENSG00000268362	CTD-2017D11.1	lincRNA	2,73E-01	2,25E-01	4,63E-01	No	-5,52E-01	5,07E-02	1,13E-01	No
ENSG00000261824	LINC00662	lincRNA	-4,59E-02	7,68E-01	8,94E-01	No	3,25E-01	4,92E-02	1,11E-01	No
ENSG00000261770	CTC-459F4.1	lincRNA	2,33E-02	9,14E-01	9,64E-01	No	-2,21E-01	4,26E-01	5,81E-01	No
ENSG00000267575	CTC-459F4.3	lincRNA	1,99E-01	1,99E-01	4,31E-01	No	-1,95E-01	2,70E-01	4,18E-01	No
ENSG00000266977	CTC-459F4.5	lincRNA	1,42E-01	1,87E-01	NA	No	-1,08E-02	9,79E-01	NA	No
ENSG00000266928	CTB-70G10.1	lincRNA	5,40E-02	4,76E-01	NA	No	6,67E-02	9,56E-01	NA	No
ENSG00000267243	AC005307.3	lincRNA	3,78E-02	6,36E-01	NA	No	-1,03E-01	8,30E-01	NA	No
ENSG00000266893	AC005616.1	lincRNA	-1,77E-02	8,19E-01	NA	No	-1,33E-01	7,12E-01	NA	No
ENSG00000267537	AC005394.1	lincRNA	3,31E-02	9,36E-01	NA	No	8,77E-02	8,64E-01	NA	No
ENSG00000266976	AC079466.1	lincRNA	3,14E-02	7,22E-01	NA	No	1,65E-01	4,61E-01	NA	No
ENSG00000267528	AC011524.1	lincRNA	1,30E-01	5,32E-01	7,52E-01	No	4,15E-01	2,61E-01	4,08E-01	No
ENSG00000267683	AC008991.1	lincRNA	5,62E-02	7,51E-01	8,84E-01	No	-1,33E-02	9,67E-01	9,81E-01	No
ENSG00000267027	AC011524.2	lincRNA	1,38E-01	1,26E-01	NA	No	6,67E-02	9,56E-01	NA	No
ENSG00000267240	AC011524.3	lincRNA	1,89E-01	1,58E-01	NA	No	4,58E-01	1,50E-01	2,69E-01	No
ENSG00000267339	LINC00906	lincRNA	4,42E-02	8,17E-01	9,20E-01	No	1,36E-01	6,99E-01	8,06E-01	No
ENSG00000267498	CTB-32O4.2	lincRNA	-2,00E-01	3,51E-01	5,99E-01	No	-1,72E-01	6,18E-01	7,45E-01	No
ENSG00000267006	CTC-448F2.4	lincRNA	5,30E-01	5,41E-02	1,83E-01	No	9,55E-01	1,55E-03	5,47E-03	No
ENSG00000267760	CTC-565M22.1	lincRNA	-2,98E-02	6,60E-01	NA	No	-1,51E-01	5,67E-01	NA	No
ENSG00000267777	CTC-439O9.3	lincRNA	6,62E-02	6,25E-01	NA	No	5,10E-03	9,83E-01	NA	No
ENSG00000267636	AC008992.1	lincRNA	-4,23E-02	7,60E-01	NA	No	6,23E-02	7,91E-01	NA	No
ENSG00000267475	CTD-2538C1.2	lincRNA	-3,10E-01	1,76E-01	4,00E-01	No	-1,13E-01	6,74E-01	7,88E-01	No
ENSG00000267567	CTD-2538C1.3	lincRNA	1,22E-01	2,77E-01	NA	No	2,97E-03	9,80E-01	NA	No
ENSG00000266930	CTD-2085J24.4	lincRNA	3,99E-02	8,56E-01	9,38E-01	No	-1,04E+00	7,64E-03	2,25E-02	No
ENSG00000267130	CTD-2540B15.9	lincRNA	4,28E-02	7,31E-01	NA	No	9,90E-02	7,75E-01	NA	No
ENSG00000267767	CTC-523E23.4	lincRNA	1,72E-01	4,52E-01	6,88E-01	No	-5,37E-01	1,57E-01	2,77E-01	No
ENSG00000261754	CTC-523E23.1	lincRNA	4,83E-01	8,13E-02	2,43E-01	No	1,22E+00	3,98E-03	1,27E-02	No
ENSG00000269086	CTC-523E23.5	lincRNA	3,49E-01	1,45E-01	3,55E-01	No	-6,17E-02	8,31E-01	8,97E-01	No
ENSG00000271109	CTC-523E23.11	lincRNA	1,02E-01	6,42E-01	8,23E-01	No	-5,58E-01	1,47E-01	2,65E-01	No
ENSG00000271649	LINC00904	lincRNA	-2,08E-02	8,19E-01	NA	No	-1,33E-01	7,12E-01	NA	No
ENSG00000270544	CTC-523E23.14	lincRNA	9,61E-03	9,01E-01	NA	No	-5,93E-02	7,23E-01	NA	No
ENSG00000270876	CTC-523E23.8	lincRNA	-3,15E-02	8,74E-01	9,47E-01	No	3,41E-01	3,45E-01	5,01E-01	No
ENSG00000205786	AC002511.1	lincRNA	-2,41E-01	1,95E-01	4,25E-01	No	-4,50E-01	2,38E-01	3,81E-01	No
ENSG00000233214	AC002511.2	lincRNA	-4,13E-02	6,97E-01	NA	No	-9,61E-02	6,98E-01	8,06E-01	No
ENSG00000232680	AC002511.3	lincRNA	-3,61E-01	9,40E-02	2,67E-01	No	-1,30E+00	2,47E-02	6,19E-02	No
ENSG00000225872	AC002398.5	lincRNA	-1,97E-02	8,19E-01	NA	No	-7,54E-02	8,00E-01	NA	No
ENSG00000267786	AF038458.3	lincRNA	-2,21E-02	8,72E-01	9,46E-01	No	-2,14E-01	2,69E-01	NA	No
ENSG00000267053	CTD-3162L10.1	lincRNA	3,05E-01	2,12E-01	4,47E-01	No	-3,80E-01	2,91E-01	4,42E-01	No
ENSG00000232677	LINC00665	lincRNA	-2,58E-01	1,26E-01	3,24E-01	No	-4,65E-01	3,78E-03	1,21E-02	No
ENSG00000266935	CTD-3234P18.2	lincRNA	-1,51E-01	4,79E-01	7,11E-01	No	6,78E-01	1,43E-02	3,89E-02	No
ENSG00000225975	AC074138.3	lincRNA	3,28E-01	1,43E-01	3,52E-01	No	3,62E-01	1,80E-01	3,09E-01	No
ENSG00000267353	CTD-2162K18.3	lincRNA	-6,40E-02	4,76E-01	NA	No	-1,55E-01	5,35E-01	NA	No
ENSG00000226686	AC012309.5	lincRNA	-2,14E-01	3,57E-01	6,04E-01	No	-3,24E-01	3,35E-01	4,90E-01	No
ENSG00000267605	CTD-3220F14.1	lincRNA	-4,68E-01	8,63E-02	2,53E-01	No	-9,76E-01	2,97E-02	7,23E-02	No
ENSG00000267115	CTD-3064H18.2	lincRNA	-9,34E-02	5,02E-01	7,29E-01	No	7,67E-02	8,06E-01	8,80E-01	No
ENSG00000267152	CTD-2528L19.6	lincRNA	3,54E-02	8,59E-01	9,39E-01	No	8,55E-01	5,01E-05	2,42E-04	No
ENSG00000267640	CTD-2554C21.2	lincRNA	-1,28E-01	5,41E-01	7,57E-01	No	9,72E-01	3,36E-04	1,38E-03	No
ENSG00000225868	AC016582.2	lincRNA	-4,93E-02	8,20E-01	9,21E-01	No	6,44E-01	7,54E-02	1,56E-01	No
ENSG00000229481	CTD-2554C21.3	lincRNA	-3,06E-01	2,02E-01	4,34E-01	No	-5,13E-02	8,53E-01	9,11E-01	No

ENSG00000268913	AC026806.2	lincRNA	-1,91E-01	4,07E-01	6,51E-01	No	-9,44E-01	2,04E-02	5,26E-02	No
ENSG00000269486	CTC-360G5.9	lincRNA	1,22E-01	5,78E-01	7,81E-01	No	7,36E-01	1,81E-02	4,76E-02	No
ENSG00000269246	CTC-246B18.10	lincRNA	-1,54E-01	3,87E-01	6,33E-01	No	-2,71E-01	4,23E-01	5,79E-01	No
ENSG00000268166	CTC-471F3.6	lincRNA	3,50E-01	1,62E-01	3,80E-01	No	1,37E+00	1,23E-03	4,44E-03	No
ENSG00000269843	CTC-490E21.10	lincRNA	-2,46E-01	2,81E-01	5,27E-01	No	-6,92E-01	9,14E-02	1,82E-01	No
ENSG00000267107	AC011526.1	lincRNA	-7,12E-01	2,77E-03	2,22E-02	No	-1,72E-01	4,90E-01	6,40E-01	No
ENSG00000268355	AC006129.1	lincRNA	1,04E+00	8,28E-03	4,93E-02	No	7,62E-01	6,25E-02	1,34E-01	No
ENSG00000270164	AC006129.4	lincRNA	1,14E+00	9,76E-04	1,00E-02	No	4,22E-01	2,18E-01	3,56E-01	No
ENSG00000267839	AC020956.3	lincRNA	4,04E-02	7,80E-01	NA	No	9,18E-02	8,64E-01	NA	No
ENSG00000225877	AC004603.4	lincRNA	-2,43E-02	8,37E-01	9,30E-01	No	-1,23E-01	5,45E-01	6,86E-01	No
ENSG00000231412	CTC-490G23.2	lincRNA	-8,59E-02	3,65E-01	NA	No	-8,98E-02	6,50E-01	NA	No
ENSG00000268601	AC115522.3	lincRNA	-4,91E-01	3,38E-02	1,33E-01	No	-9,24E-01	4,90E-02	1,10E-01	No
ENSG00000267058	RP11-15A1.3	lincRNA	9,50E-02	6,14E-01	8,06E-01	No	1,08E-01	6,29E-01	7,53E-01	No
ENSG00000267045	AC006126.4	lincRNA	-1,59E-01	4,83E-01	7,13E-01	No	-9,14E-01	3,23E-02	7,76E-02	No
ENSG00000268401	CTC-344H19.4	lincRNA	-2,05E-02	8,92E-01	9,55E-01	No	-4,96E-01	1,02E-01	NA	No
ENSG00000269619	CTD-3023J11.2	lincRNA	6,62E-02	3,24E-01	NA	No	1,17E-01	5,80E-01	NA	No
ENSG00000268746	CTD-2571L23.8	lincRNA	-3,77E-02	7,83E-01	NA	No	-2,68E-01	2,87E-01	NA	No
ENSG00000268186	CTC-241F20.4	lincRNA	5,48E-02	4,94E-01	NA	No	-3,75E-02	8,90E-01	NA	No
ENSG00000269423	CTC-273B12.6	lincRNA	3,90E-02	8,45E-01	NA	No	6,68E-02	9,56E-01	NA	No
ENSG00000269814	CTC-273B12.10	lincRNA	2,95E-02	6,87E-01	NA	No	2,00E-02	8,87E-01	NA	No
ENSG00000267898	CTD-2639E6.9	lincRNA	4,70E-05	9,98E-01	9,99E-01	No	-1,04E+00	3,96E-02	9,21E-02	No
ENSG00000268655	CTB-60B18.10	lincRNA	1,78E-02	8,96E-01	9,57E-01	No	-3,47E-01	2,42E-01	NA	No
ENSG00000268287	CTB-60B18.18	lincRNA	-8,14E-02	3,87E-01	NA	No	-2,15E-01	3,94E-01	NA	No
ENSG00000197813	CTC-301O7.4	lincRNA	8,54E-01	1,34E-02	6,95E-02	No	8,18E-02	8,13E-01	8,85E-01	No
ENSG00000267890	CTD-2126E3.4	lincRNA	-3,31E-02	7,31E-01	NA	No	-1,35E-01	7,12E-01	NA	No
ENSG00000268518	CTD-2545M3.8	lincRNA	2,75E-01	2,48E-01	4,90E-01	No	7,06E-02	8,35E-01	8,99E-01	No
ENSG00000261341	CTD-2568A17.1	lincRNA	-1,65E-01	1,23E-01	NA	No	-2,82E-01	3,05E-01	NA	No
ENSG00000180279	CTD-2568A17.5	lincRNA	2,30E-02	7,93E-01	NA	No	-1,33E-01	7,12E-01	NA	No
ENSG00000268889	CTD-2616J11.14	lincRNA	-5,47E-02	6,46E-01	NA	No	-1,30E-01	7,12E-01	NA	No
ENSG00000269959	SPACA6P-AS	lincRNA	-3,62E-01	1,47E-01	3,59E-01	No	-6,14E-01	1,33E-01	2,45E-01	No
ENSG00000268316	AC006272.2	lincRNA	-3,68E-02	8,40E-01	9,30E-01	No	-4,84E-01	1,89E-01	3,21E-01	No
ENSG00000269483	AC006272.1	lincRNA	5,64E-01	5,79E-02	1,92E-01	No	3,75E-02	9,15E-01	9,50E-01	No
ENSG00000268458	CTC-471J1.9	lincRNA	-7,09E-02	6,50E-01	8,27E-01	No	-1,81E-01	5,57E-01	6,96E-01	No
ENSG00000213793	ZNF888	lincRNA	3,70E-01	1,48E-01	3,59E-01	No	2,96E-01	3,87E-01	5,44E-01	No
ENSG00000269646	CTD-2331H12.7	lincRNA	8,53E-02	4,00E-01	NA	No	1,41E-01	4,50E-01	NA	No
ENSG00000267943	CTD-2620I22.3	lincRNA	1,74E-01	3,32E-01	5,82E-01	No	-9,19E-02	7,80E-01	8,63E-01	No
ENSG00000268613	CTD-2620I22.7	lincRNA	-2,98E-01	2,20E-01	4,58E-01	No	5,94E-01	1,10E-01	2,11E-01	No
ENSG00000269051	CTD-2245F17.3	lincRNA	4,95E-01	4,82E-02	1,70E-01	No	3,22E-01	2,71E-01	4,20E-01	No
ENSG00000232324	AC008440.10	lincRNA	1,95E-01	2,35E-01	4,74E-01	No	-2,71E-01	2,05E-01	NA	No
ENSG00000269524	CTB-83J4.1	lincRNA	-1,18E-01	2,87E-01	5,34E-01	No	-5,29E-01	6,08E-02	1,31E-01	No
ENSG00000268164	CTD-2337J16.1	lincRNA	-2,58E-01	2,62E-01	5,05E-01	No	-5,03E-01	1,88E-01	3,20E-01	No
ENSG00000268802	CTD-2587H19.1	lincRNA	1,21E+00	6,28E-03	4,03E-02	No	3,72E-01	3,11E-01	4,65E-01	No
ENSG00000269873	CTD-2587H19.3	lincRNA	-1,57E-02	9,24E-01	9,68E-01	No	-6,67E-01	5,37E-02	1,19E-01	No
ENSG00000267838	AC008746.12	lincRNA	5,42E-01	5,71E-02	1,90E-01	No	-1,65E-01	6,35E-01	7,58E-01	No
ENSG00000269271	CTB-83J4.2	lincRNA	2,54E-02	8,34E-01	NA	No	2,92E-02	8,87E-01	NA	No
ENSG00000268734	CTB-61M7.2	lincRNA	5,06E-01	3,37E-02	1,33E-01	No	-4,23E-02	8,89E-01	9,34E-01	No
ENSG00000267649	CTD-2587H24.10	lincRNA	-4,62E-02	8,17E-01	9,19E-01	No	-1,43E+00	1,27E-02	3,51E-02	No
ENSG00000267093	CTD-2105E13.13	lincRNA	-6,05E-02	6,44E-01	8,24E-01	No	-1,18E-01	6,59E-01	7,77E-01	No
ENSG00000267730	CTD-2537I9.5	lincRNA	6,14E-02	6,56E-01	NA	No	7,64E-02	7,96E-01	8,74E-01	No
ENSG00000267183	AC010525.6	lincRNA	2,21E-01	3,39E-01	5,88E-01	No	-6,38E-01	7,96E-02	1,63E-01	No
ENSG00000267018	AC010525.7	lincRNA	4,85E-01	8,10E-02	2,42E-01	No	-5,03E-01	2,02E-01	3,36E-01	No
ENSG00000267522	CTD-2621I17.6	lincRNA	1,13E-01	2,25E-01	NA	No	1,02E-01	7,75E-01	NA	No
ENSG00000267606	AC006116.21	lincRNA	3,09E-02	8,78E-01	9,49E-01	No	-1,12E-01	7,48E-01	8,41E-01	No
ENSG00000267454	ZNF582-AS1	lincRNA	1,39E-01	5,29E-01	7,49E-01	No	-5,65E-01	8,86E-02	1,78E-01	No
ENSG00000166770	ZNF667-AS1	lincRNA	-1,61E-01	3,27E-01	5,76E-01	No	3,13E-02	8,51E-01	9,09E-01	No
ENSG00000267224	AC005498.4	lincRNA	1,61E-01	1,43E-01	NA	No	8,76E-01	2,25E-02	5,72E-02	No
ENSG00000267421	AC005498.3	lincRNA	1,21E-01	5,88E-01	7,88E-01	No	5,80E-01	9,53E-02	1,88E-01	No
ENSG00000268352	AC007228.5	lincRNA	7,21E-02	4,37E-01	NA	No	1,90E-01	3,76E-01	NA	No
ENSG00000268568	AC007228.9	lincRNA	8,67E-02	6,84E-01	8,49E-01	No	-2,48E-01	4,90E-01	6,40E-01	No
ENSG00000268654	MIMT1	lincRNA	2,15E-01	4,55E-02	NA	No	1,07E-01	6,99E-01	NA	No
ENSG00000268713	CTC-444N24.8	lincRNA	-1,64E-01	3,96E-01	6,41E-01	No	1,11E-01	6,09E-01	7,38E-01	No
ENSG00000268205	CTC-444N24.11	lincRNA	-4,20E-01	8,54E-03	5,05E-02	No	-6,63E-02	6,82E-01	7,94E-01	No

ENSG00000268266	AC003005.2	lincRNA	2,91E-02	8,21E-01	9,22E-01	No	2,98E-01	3,51E-01	5,06E-01	No
ENSG00000268392	AC003682.16	lincRNA	2,52E-01	2,88E-01	5,34E-01	No	5,76E-01	1,31E-01	2,41E-01	No
ENSG00000268543	CTD-2619J13.16	lincRNA	-2,39E-01	2,80E-01	5,26E-01	No	-7,03E-01	3,07E-02	7,43E-02	No
ENSG00000268912	CTD-2619J13.17	lincRNA	-4,06E-02	8,06E-01	9,14E-01	No	1,29E-01	4,68E-01	6,21E-01	No
ENSG00000231535	LINC00278	lincRNA	3,99E-02	7,17E-01	8,68E-01	No	2,60E-02	9,41E-01	9,65E-01	No
ENSG00000229308	AC010084.1	lincRNA	-1,12E-01	3,00E-01	NA	No	1,47E-01	6,01E-01	7,31E-01	No
ENSG00000233864	TTTY15	lincRNA	-5,75E-02	5,58E-01	7,69E-01	No	-5,54E-02	8,39E-01	9,02E-01	No
ENSG00000230663	FAM224B	lincRNA	-7,45E-02	3,46E-01	NA	No	-1,37E-01	4,66E-01	NA	No
ENSG00000251510	AC022486.1	lincRNA	-5,58E-02	6,46E-01	NA	No	-1,33E-01	7,12E-01	NA	No
ENSG00000176728	TTTY14	lincRNA	-8,54E-02	4,07E-01	6,51E-01	No	-1,76E-01	5,74E-01	7,09E-01	No
ENSG00000260197	RP11-424G14.1	lincRNA	-4,92E-02	6,63E-01	8,35E-01	No	1,01E-01	7,48E-01	8,41E-01	No
ENSG00000229236	TTTY10	lincRNA	-8,03E-02	4,72E-01	7,05E-01	No	-1,94E-01	5,25E-01	6,70E-01	No
ENSG00000228786	LINC00266-4P	lincRNA	-1,47E-01	1,41E-01	NA	No	-8,50E-02	7,30E-01	8,29E-01	No
ENSG00000100181	TPTEP1	lincRNA	-2,75E-03	9,87E-01	9,96E-01	No	-9,89E-02	7,22E-01	8,23E-01	No
ENSG00000267338	KB-7G2.9	lincRNA	-2,00E-02	8,95E-01	9,56E-01	No	1,48E+00	6,00E-03	1,82E-02	No
ENSG00000237689	AC007064.24	lincRNA	4,00E-02	7,29E-01	NA	No	2,74E-01	3,29E-01	4,85E-01	No
ENSG00000273203	AC006946.16	lincRNA	2,32E-01	2,32E-01	4,71E-01	No	-2,36E-01	4,88E-01	6,37E-01	No
ENSG00000273442	AC006946.17	lincRNA	-4,78E-02	6,75E-01	NA	No	1,03E-01	6,87E-01	7,98E-01	No
ENSG00000235295	XXbac-B476C20.17	lincRNA	-1,24E-02	9,48E-01	9,79E-01	No	-1,17E+00	2,88E-02	7,05E-02	No
ENSG00000273032	DGCR9	lincRNA	2,33E-02	8,16E-01	NA	No	-3,72E-02	8,41E-01	NA	No
ENSG00000272682	AC004471.10	lincRNA	-5,58E-02	7,26E-01	8,72E-01	No	-6,15E-01	5,11E-02	1,14E-01	No
ENSG00000225007	AC000067.1	lincRNA	-8,53E-02	3,31E-01	NA	No	-2,35E-01	3,61E-01	NA	No
ENSG00000273139	XXbac-B444P24.14	lincRNA	2,47E-01	2,76E-01	5,22E-01	No	4,19E-01	2,45E-01	3,89E-01	No
ENSG00000235704	AC023490.2	lincRNA	3,46E-02	9,36E-01	NA	No	6,17E-02	9,56E-01	NA	No
ENSG00000206176	AC023490.1	lincRNA	8,53E-02	4,53E-01	6,88E-01	No	1,13E-01	7,02E-01	8,09E-01	No
ENSG00000237476	XXbac-B135H6.15	lincRNA	1,33E-01	4,65E-01	6,99E-01	No	-2,05E-01	5,49E-01	6,89E-01	No
ENSG00000272829	XXbac-B135H6.18	lincRNA	1,15E-01	4,52E-01	6,88E-01	No	2,21E-01	4,77E-01	6,29E-01	No
ENSG00000197210	KB-1592A4.15	lincRNA	2,49E-02	8,46E-01	NA	No	1,56E-02	9,87E-01	NA	No
ENSG00000206140	TMEM191C	lincRNA	-5,60E-02	7,72E-01	8,96E-01	No	-4,18E-01	2,70E-01	4,18E-01	No
ENSG00000273342	KB-1440D3.14	lincRNA	4,55E-02	6,24E-01	NA	No	3,25E-02	9,09E-01	NA	No
ENSG00000234630	LL22NC03-2H8.4	lincRNA	1,39E-01	2,87E-01	5,34E-01	No	2,90E-01	3,71E-01	5,28E-01	No
ENSG00000236611	AP000343.2	lincRNA	7,57E-02	4,52E-01	NA	No	-2,72E-02	8,90E-01	NA	No
ENSG00000234928	AP000344.3	lincRNA	-5,45E-02	6,46E-01	NA	No	-1,28E-01	7,12E-01	NA	No
ENSG00000225413	AP000345.4	lincRNA	-5,47E-02	6,46E-01	NA	No	-1,30E-01	7,12E-01	NA	No
ENSG00000272733	KB-208E9.1	lincRNA	-1,10E-01	4,98E-01	7,25E-01	No	-2,41E-01	4,31E-01	5,86E-01	No
ENSG00000270041	LL22NC03-N27C7.1	lincRNA	-2,29E-01	3,18E-01	5,67E-01	No	-2,00E-01	5,17E-01	6,62E-01	No
ENSG00000273295	AP000350.5	lincRNA	5,66E-01	3,43E-02	1,34E-01	No	-8,41E-01	1,03E-02	2,93E-02	No
ENSG00000272787	KB-226F1.2	lincRNA	7,09E-02	7,41E-01	8,79E-01	No	-3,51E-01	3,36E-01	4,92E-01	No
ENSG00000236641	CTA-221G9.7	lincRNA	4,07E-02	6,02E-01	NA	No	-1,30E-01	7,12E-01	NA	No
ENSG00000272942	CTA-246H3.12	lincRNA	1,13E-01	2,49E-01	NA	No	9,02E-02	8,64E-01	NA	No
ENSG00000236464	CTA-796E4.3	lincRNA	-4,42E-02	7,60E-01	NA	No	-1,02E-01	8,30E-01	NA	No
ENSG00000260065	CTA-445C9.15	lincRNA	-7,19E-01	2,69E-03	2,17E-02	No	-8,83E-01	1,85E-03	6,41E-03	No
ENSG00000206028	CTA-373H7.7	lincRNA	9,55E-02	3,60E-01	NA	No	8,51E-02	8,64E-01	NA	No
ENSG00000244625	CTA-211A9.5	lincRNA	7,92E-01	3,51E-04	4,63E-03	No	-1,52E-01	4,64E-01	6,17E-01	No
ENSG00000223704	RP1-90L6.2	lincRNA	1,21E-01	5,79E-01	7,83E-01	No	-4,23E-01	1,81E-01	3,10E-01	No
ENSG00000235271	RP1-90L6.3	lincRNA	4,88E-01	8,10E-02	2,42E-01	No	6,81E-02	8,43E-01	9,04E-01	No
ENSG00000236858	CTA-992D9.6	lincRNA	2,66E-02	8,30E-01	NA	No	-1,77E-01	4,52E-01	NA	No
ENSG00000231405	CTA-992D9.7	lincRNA	-5,37E-02	6,46E-01	NA	No	-1,26E-01	7,12E-01	NA	No
ENSG00000233521	RP5-1172A22.1	lincRNA	5,03E-01	5,08E-02	1,76E-01	No	-1,79E-02	9,45E-01	NA	No
ENSG00000226741	CTA-929C8.6	lincRNA	1,78E-02	8,46E-01	NA	No	-1,30E-01	7,12E-01	NA	No
ENSG00000227838	RP1-213J1P__B.1	lincRNA	4,56E-02	6,38E-01	NA	No	1,07E-01	7,01E-01	NA	No
ENSG00000224050	RP1-90G24.6	lincRNA	1,79E-01	1,81E-01	4,07E-01	No	3,41E-01	2,25E-01	3,66E-01	No
ENSG00000273325	RP1-90G24.11	lincRNA	1,32E-01	1,52E-01	NA	No	3,26E-01	1,72E-01	NA	No
ENSG00000230736	RP1-149A16.3	lincRNA	1,42E-01	3,23E-01	5,72E-01	No	-5,51E-02	8,41E-01	9,03E-01	No
ENSG00000241954	RP1-149A16.17	lincRNA	6,33E-01	3,08E-02	1,25E-01	No	-1,00E-01	6,85E-01	NA	No
ENSG00000228622	RP1-302D9.1	lincRNA	-3,46E-02	6,59E-01	NA	No	-1,19E-02	9,50E-01	NA	No
ENSG00000231253	RP1-302D9.2	lincRNA	-2,37E-01	1,84E-01	4,10E-01	No	-5,36E-01	1,50E-01	2,69E-01	No
ENSG00000228587	RP1-41P2.7	lincRNA	-4,33E-02	7,60E-01	NA	No	-1,01E-01	8,30E-01	NA	No
ENSG00000228719	RP5-1119A7.14	lincRNA	-3,00E-01	NA	NA	No	-5,93E-01	NA	NA	No
ENSG00000237862	RP1-63G5.7	lincRNA	-4,23E-02	7,60E-01	NA	No	-9,82E-02	8,30E-01	NA	No
ENSG00000273214	RP5-1039K5.18	lincRNA	-6,29E-02	3,88E-01	NA	No	-1,71E-01	3,60E-01	NA	No
ENSG00000272720	CTA-228A9.3	lincRNA	3,14E-01	1,95E-01	4,25E-01	No	1,84E-01	5,97E-01	7,27E-01	No

ENSG00000235209	CTA-150C2.13	lincRNA	4,76E-02	5,66E-01	NA	No	1,68E-01	4,60E-01	NA	No
ENSG00000273424	CTA-223H9.9	lincRNA	1,99E-01	3,85E-01	6,31E-01	No	-5,14E-01	1,87E-01	3,19E-01	No
ENSG00000260655	CTA-250D10.23	lincRNA	4,19E-01	2,99E-02	1,22E-01	No	-2,90E-01	1,57E-01	2,78E-01	No
ENSG00000182057	Z83851.3	lincRNA	5,02E-01	6,36E-02	2,05E-01	No	3,51E-01	3,37E-01	4,93E-01	No
ENSG00000233903	Z83851.4	lincRNA	2,67E-01	2,61E-01	5,05E-01	No	-5,08E-01	1,91E-01	3,23E-01	No
ENSG00000273366	CTA-989H11.1	lincRNA	8,33E-03	9,58E-01	9,83E-01	No	5,60E-02	8,64E-01	9,18E-01	No
ENSG00000270022	RNU12	lincRNA	-1,39E-02	9,48E-01	9,79E-01	No	-1,24E-02	9,63E-01	9,78E-01	No
ENSG00000232655	CTA-397C4.2	lincRNA	-4,15E-02	7,60E-01	NA	No	-1,95E-02	9,21E-01	NA	No
ENSG00000187012	LINC00207	lincRNA	3,39E-02	9,36E-01	NA	No	9,18E-02	8,64E-01	NA	No
ENSG00000226328	CTA-217C2.1	lincRNA	-1,81E-02	9,11E-01	9,63E-01	No	3,64E-01	8,90E-02	1,79E-01	No
ENSG00000273243	CTA-217C2.2	lincRNA	1,71E-01	4,50E-01	6,86E-01	No	-2,29E-01	4,95E-01	6,44E-01	No
ENSG00000273287	CTA-268H5.14	lincRNA	1,64E-01	1,52E-01	NA	No	-2,00E-02	8,90E-01	NA	No
ENSG00000235091	WI2-85898F10.1	lincRNA	-1,67E-01	3,14E-01	5,63E-01	No	-3,45E-01	3,22E-01	4,77E-01	No
ENSG00000273145	CITF22-92A6.1	lincRNA	5,06E-01	6,31E-02	2,04E-01	No	-1,34E-01	6,76E-01	7,90E-01	No
ENSG00000231010	RP6-109B7.2	lincRNA	4,48E-01	7,42E-02	2,28E-01	No	-2,16E-02	9,32E-01	9,60E-01	No
ENSG00000205632	WI2-81516E3.1	lincRNA	3,32E-02	9,36E-01	NA	No	8,55E-02	8,64E-01	NA	No
ENSG00000260613	RP3-522J7.6	lincRNA	2,42E-02	9,72E-01	NA	No	1,53E-01	3,84E-01	NA	No
ENSG00000273192	CITF22-1A6.3	lincRNA	-2,16E-02	7,60E-01	NA	No	-9,53E-02	6,58E-01	NA	No
ENSG00000272666	CTA-384D8.35	lincRNA	2,01E-01	1,25E-01	3,22E-01	No	5,42E-04	9,71E-01	NA	No
ENSG00000272940	CTA-384D8.33	lincRNA	3,78E-02	6,55E-01	NA	No	-6,07E-02	8,00E-01	NA	No
ENSG00000232884	AF127936.3	lincRNA	1,32E+00	9,44E-03	5,42E-02	No	6,35E-01	1,21E-01	2,28E-01	No
ENSG00000235609	AF127936.7	lincRNA	-1,18E-01	5,75E-01	7,79E-01	No	8,36E-01	2,51E-03	8,43E-03	No
ENSG00000229425	AJ006998.2	lincRNA	5,80E-02	5,83E-01	NA	No	2,56E-01	2,99E-01	NA	No
ENSG00000215386	LINC00478	lincRNA	2,73E-01	1,99E-01	4,30E-01	No	1,08E+00	4,79E-05	2,33E-04	No
ENSG00000270071	AP001172.2	lincRNA	9,58E-02	6,68E-01	8,38E-01	No	4,12E-01	2,18E-01	3,57E-01	No
ENSG00000270139	AP001172.3	lincRNA	2,44E-01	2,81E-01	5,28E-01	No	6,09E-01	1,06E-01	2,05E-01	No
ENSG00000269950	AP000962.2	lincRNA	2,10E-01	3,65E-01	6,12E-01	No	-3,58E-01	2,81E-01	4,30E-01	No
ENSG00000228798	AP000473.5	lincRNA	3,13E-01	2,02E-01	4,34E-01	No	2,15E-01	5,12E-01	6,59E-01	No
ENSG00000237735	AP000473.6	lincRNA	9,86E-02	2,55E-01	NA	No	2,02E-01	3,56E-01	NA	No
ENSG00000232886	AF212831.2	lincRNA	-9,74E-02	4,92E-01	7,21E-01	No	-3,34E-02	9,13E-01	9,48E-01	No
ENSG00000240770	C21orf91-OT1	lincRNA	9,76E-02	5,92E-01	7,91E-01	No	1,89E-01	5,70E-01	7,06E-01	No
ENSG00000231755	CHODL-AS1	lincRNA	1,00E-01	1,96E-01	NA	No	2,80E-01	2,54E-01	NA	No
ENSG00000227330	AP000998.2	lincRNA	2,10E-01	3,22E-01	5,71E-01	No	-7,25E-02	8,29E-01	8,96E-01	No
ENSG00000237527	AF241725.6	lincRNA	-6,40E-02	4,76E-01	NA	No	-1,55E-01	5,35E-01	NA	No
ENSG00000226983	AP000235.2	lincRNA	3,15E-02	9,36E-01	NA	No	8,58E-02	8,64E-01	NA	No
ENSG00000232512	7SK	lincRNA	5,24E-02	6,48E-01	NA	No	6,68E-02	9,56E-01	NA	No
ENSG00000222042	AP000233.4	lincRNA	-1,34E-02	8,49E-01	NA	No	-9,82E-02	8,30E-01	NA	No
ENSG00000185433	LINC00158	lincRNA	1,03E+00	1,70E-02	8,19E-02	No	2,37E-01	4,45E-01	5,99E-01	No
ENSG00000229962	AP000221.1	lincRNA	9,77E-02	3,40E-01	NA	No	2,77E-01	2,50E-01	NA	No
ENSG00000273492	AP000230.1	lincRNA	-2,51E-01	2,89E-01	5,36E-01	No	-2,35E-01	5,04E-01	6,52E-01	No
ENSG00000229025	AP001595.1	lincRNA	-1,81E-02	8,19E-01	NA	No	-1,28E-01	7,12E-01	NA	No
ENSG00000273115	KB-1466C5.1	lincRNA	-8,70E-02	4,55E-01	NA	No	-2,59E-01	2,90E-01	NA	No
ENSG00000231236	AP001604.3	lincRNA	-9,73E-02	6,20E-01	8,10E-01	No	-1,41E-01	6,72E-01	7,87E-01	No
ENSG00000236332	AP001605.4	lincRNA	-1,05E-01	5,20E-01	7,42E-01	No	1,86E-01	5,76E-01	7,11E-01	No
ENSG00000234052	AP001607.1	lincRNA	1,70E-01	3,47E-01	5,95E-01	No	3,88E-01	2,50E-01	3,95E-01	No
ENSG00000225298	LINC00113	lincRNA	-8,36E-01	2,34E-02	1,03E-01	No	-5,30E-01	1,80E-01	3,09E-01	No
ENSG00000234083	AJ006995.3	lincRNA	-3,15E-02	8,52E-01	9,35E-01	No	-1,44E-01	6,74E-01	7,89E-01	No
ENSG00000178457	LINC00314	lincRNA	-4,94E-01	6,94E-02	2,18E-01	No	-2,29E-01	5,14E-01	6,60E-01	No
ENSG00000236532	AL035610.2	lincRNA	-1,76E-01	4,41E-01	6,80E-01	No	-7,66E-02	8,07E-01	8,81E-01	No
ENSG00000232855	AF131217.1	lincRNA	-2,58E-01	2,18E-01	4,55E-01	No	-7,98E-02	7,78E-01	8,62E-01	No
ENSG00000226935	LINC00161	lincRNA	1,46E-01	5,05E-01	7,32E-01	No	-4,61E-01	2,31E-01	3,72E-01	No
ENSG00000228817	BACH1-IT2	lincRNA	-2,97E-01	2,22E-01	4,59E-01	No	-8,24E-01	3,85E-02	8,99E-02	No
ENSG00000234509	AP000253.1	lincRNA	1,17E-01	5,61E-01	7,70E-01	No	8,65E-02	8,05E-01	8,79E-01	No
ENSG00000273091	AP000255.6	lincRNA	4,91E-02	7,99E-01	9,10E-01	No	-1,07E-01	7,58E-01	8,47E-01	No
ENSG00000230323	LINC00159	lincRNA	-4,25E-02	7,14E-01	NA	No	3,73E-01	3,42E-01	NA	No
ENSG00000261610	AP000265.1	lincRNA	-5,23E-02	6,51E-01	8,28E-01	No	-2,17E-01	4,26E-01	5,81E-01	No
ENSG00000228961	AP000282.3	lincRNA	3,41E-02	9,36E-01	NA	No	1,11E-01	6,71E-01	NA	No
ENSG00000232539	LINC00945	lincRNA	3,32E-02	9,36E-01	NA	No	8,58E-02	8,64E-01	NA	No
ENSG00000272659	AP000295.10	lincRNA	-4,14E-02	6,59E-01	NA	No	-7,00E-02	7,57E-01	NA	No
ENSG00000273102	AP000569.9	lincRNA	1,16E-01	4,00E-01	6,45E-01	No	-1,21E-01	5,48E-01	NA	No
ENSG00000227456	LINC00310	lincRNA	8,39E-02	7,04E-01	8,60E-01	No	-2,27E-01	5,23E-01	6,67E-01	No
ENSG00000214955	AP000318.2	lincRNA	-9,33E-02	5,16E-01	7,40E-01	No	-4,30E-01	1,69E-01	2,94E-01	No

ENSG00000273104	CMP21-97G8.1	lincRNA	-2,13E-03	9,86E-01	9,95E-01	No	-1,22E-01	6,38E-01	7,61E-01	No
ENSG00000272958	CMP21-97G8.2	lincRNA	7,39E-03	8,85E-01	NA	No	1,03E-02	9,16E-01	NA	No
ENSG00000230794	AF015720.3	lincRNA	-4,75E-03	9,53E-01	NA	No	9,76E-03	9,61E-01	NA	No
ENSG00000231106	AP000688.8	lincRNA	-6,27E-02	5,96E-01	7,94E-01	No	-2,66E-01	2,53E-01	3,99E-01	No
ENSG00000236119	AP000688.15	lincRNA	3,34E-04	9,88E-01	NA	No	-6,98E-02	7,97E-01	NA	No
ENSG00000233393	AP000688.29	lincRNA	3,00E-01	2,81E-02	NA	No	1,37E-01	5,97E-01	NA	No
ENSG00000231324	AP000696.2	lincRNA	3,46E-02	9,36E-01	NA	No	8,58E-02	8,64E-01	NA	No
ENSG00000224790	AP000704.5	lincRNA	-3,45E-01	1,56E-01	3,72E-01	No	-1,16E+00	2,69E-02	6,65E-02	No
ENSG00000230366	DSCR9	lincRNA	-8,77E-02	6,89E-01	8,51E-01	No	-7,09E-02	8,33E-01	8,99E-01	No
ENSG00000242553	AP001432.14	lincRNA	-5,48E-02	8,01E-01	9,12E-01	No	-2,55E-01	4,00E-01	5,57E-01	No
ENSG00000226012	AP001434.2	lincRNA	-3,69E-02	7,24E-01	NA	No	-3,82E-03	9,85E-01	9,91E-01	No
ENSG00000231231	AP001422.3	lincRNA	-4,32E-02	7,50E-01	NA	No	-1,70E-01	5,48E-01	6,88E-01	No
ENSG00000223806	LINC00114	lincRNA	4,56E-02	6,38E-01	NA	No	1,48E-01	5,45E-01	NA	No
ENSG00000229986	AP001042.1	lincRNA	-8,70E-02	2,53E-01	NA	No	-2,02E-01	3,15E-01	NA	No
ENSG00000205622	AF064858.6	lincRNA	-2,24E-01	9,26E-02	2,65E-01	No	-6,55E-01	6,88E-02	1,45E-01	No
ENSG00000229925	AP001043.1	lincRNA	-1,54E-01	8,62E-02	NA	No	-3,97E-01	1,18E-01	NA	No
ENSG00000234035	AP001044.2	lincRNA	-1,55E-01	1,45E-01	NA	No	-3,61E-01	2,00E-01	NA	No
ENSG00000232837	AF064858.7	lincRNA	-7,01E-02	4,37E-01	NA	No	-1,86E-01	3,73E-01	NA	No
ENSG00000237609	AF064858.10	lincRNA	1,14E-01	4,69E-01	7,03E-01	No	-2,54E-01	2,70E-01	4,19E-01	No
ENSG00000231713	AF064860.7	lincRNA	6,12E-02	4,36E-01	NA	No	-3,66E-02	8,90E-01	NA	No
ENSG00000225431	AP001626.1	lincRNA	4,04E-02	7,80E-01	NA	No	9,18E-02	8,64E-01	NA	No
ENSG00000233754	AP001628.7	lincRNA	2,94E-02	7,45E-01	NA	No	-9,39E-03	8,90E-01	NA	No
ENSG00000225218	AP001628.6	lincRNA	-6,67E-02	7,17E-01	8,67E-01	No	-7,69E-01	8,51E-02	1,72E-01	No
ENSG00000233056	ERVH48-1	lincRNA	6,86E-02	7,58E-01	8,88E-01	No	-2,10E-01	5,00E-01	6,48E-01	No
ENSG00000237864	LINC00322	lincRNA	-2,60E-01	5,50E-02	1,85E-01	No	-6,56E-01	1,86E-02	4,86E-02	No
ENSG00000237989	AP001046.5	lincRNA	1,75E-01	4,31E-01	6,71E-01	No	-1,01E+00	1,59E-03	5,59E-03	No
ENSG00000225637	AP001046.6	lincRNA	5,08E-02	6,69E-01	NA	No	8,64E-02	8,64E-01	NA	No
ENSG00000188660	LINC00319	lincRNA	1,01E+00	2,73E-03	2,19E-02	No	-1,12E-01	7,20E-01	8,22E-01	No
ENSG00000185186	LINC00313	lincRNA	1,39E-01	5,35E-01	7,53E-01	No	4,77E-01	1,90E-01	3,23E-01	No
ENSG00000223975	AP001048.4	lincRNA	9,02E-02	2,56E-01	NA	No	2,16E-01	2,95E-01	NA	No
ENSG00000225331	AP001055.6	lincRNA	4,44E-01	7,02E-02	2,20E-01	No	-7,94E-01	4,25E-02	9,79E-02	No
ENSG00000237604	AP001056.1	lincRNA	6,86E-01	3,44E-02	1,35E-01	No	-4,34E-01	2,49E-01	3,94E-01	No
ENSG00000232698	AP001058.3	lincRNA	1,40E-01	3,52E-01	6,00E-01	No	-2,58E-01	3,14E-01	NA	No
ENSG00000228709	AP001065.15	lincRNA	2,89E-01	1,04E-01	2,85E-01	No	1,43E-01	6,17E-01	7,44E-01	No
ENSG00000234880	LINC00163	lincRNA	-4,06E-01	5,30E-02	1,81E-01	No	-6,30E-01	1,11E-01	2,13E-01	No
ENSG00000182586	LINC00334	lincRNA	3,07E-02	7,87E-01	NA	No	-1,59E-01	4,42E-01	NA	No
ENSG00000223768	LINC00205	lincRNA	5,78E-01	2,30E-02	1,02E-01	No	8,88E-01	1,74E-03	6,08E-03	No
ENSG00000184274	LINC00315	lincRNA	5,49E-02	7,98E-01	9,09E-01	No	-9,00E-01	3,48E-02	8,26E-02	No
ENSG00000229382	BX322557.13	lincRNA	-5,37E-02	6,46E-01	NA	No	-1,26E-01	7,12E-01	NA	No
ENSG00000233922	AL133493.2	lincRNA	3,31E-02	9,36E-01	NA	No	1,05E-01	7,75E-01	NA	No
ENSG00000224413	AP001476.2	lincRNA	-4,49E-02	7,23E-01	8,71E-01	No	-2,62E-01	3,50E-01	5,06E-01	No
ENSG00000228235	AP001476.4	lincRNA	-6,14E-02	5,40E-01	NA	No	-1,47E-01	6,01E-01	NA	No
ENSG00000226115	AP001476.3	lincRNA	-1,27E-01	1,90E-01	NA	No	-1,99E-01	3,29E-01	4,84E-01	No
ENSG00000223823	RP11-465B22.5	lincRNA	4,56E-02	6,38E-01	NA	No	NA	NA	NA	NA
ENSG00000226374	RP13-614K11.2	lincRNA	3,15E-02	9,36E-01	NA	No	NA	NA	NA	NA
ENSG00000226852	RP1-212P9.2	lincRNA	3,31E-02	9,36E-01	NA	No	NA	NA	NA	NA
ENSG00000228682	RP11-439L8.3	lincRNA	3,34E-02	9,36E-01	NA	No	NA	NA	NA	NA
ENSG00000224863	RP5-1109J22.2	lincRNA	5,65E-02	5,95E-01	NA	No	NA	NA	NA	NA
ENSG00000223479	RP4-788P17.1	lincRNA	3,69E-02	8,45E-01	NA	No	NA	NA	NA	NA
ENSG00000237552	RP11-415A20.1	lincRNA	3,41E-02	9,36E-01	NA	No	NA	NA	NA	NA
ENSG00000234184	RP5-887A10.1	lincRNA	8,25E-02	3,23E-01	NA	No	NA	NA	NA	NA
ENSG00000259946	RP11-490G2.2	lincRNA	3,32E-02	9,36E-01	NA	No	NA	NA	NA	NA
ENSG00000226053	RP5-1070A16.1	lincRNA	3,32E-02	9,36E-01	NA	No	NA	NA	NA	NA
ENSG00000232650	RP5-834N19.1	lincRNA	3,90E-02	8,45E-01	NA	No	NA	NA	NA	NA
ENSG00000215859	RP6-74O6.2	lincRNA	2,42E-02	9,72E-01	NA	No	NA	NA	NA	NA
ENSG00000231196	RP11-495P10.8	lincRNA	3,15E-02	9,36E-01	NA	No	NA	NA	NA	NA
ENSG00000237588	RP11-66D17.3	lincRNA	2,40E-02	9,72E-01	NA	No	NA	NA	NA	NA
ENSG00000229961	RP11-71G12.1	lincRNA	5,78E-02	5,86E-01	NA	No	NA	NA	NA	NA
ENSG00000236206	RP11-306I1.2	lincRNA	4,36E-02	7,20E-01	NA	No	NA	NA	NA	NA
ENSG00000234142	RP11-276E17.2	lincRNA	7,12E-02	4,33E-01	NA	No	NA	NA	NA	NA
ENSG00000236720	RP11-63B19.1	lincRNA	4,91E-02	5,10E-01	NA	No	NA	NA	NA	NA
ENSG00000260976	GS1-120K12.4	lincRNA	2,22E-02	9,72E-01	NA	No	NA	NA	NA	NA

ENSG00000236030	LINC01036	lincRNA	1,01E-01	3,24E-01	NA	No	NA	NA	NA	NA
ENSG00000261642	RP11-309H21.3	lincRNA	3,32E-02	9,36E-01	NA	No	NA	NA	NA	NA
ENSG00000231714	RP11-476H20.1	lincRNA	3,15E-02	9,36E-01	NA	No	NA	NA	NA	NA
ENSG00000223881	RP11-553K8.2	lincRNA	4,69E-02	5,91E-01	NA	No	NA	NA	NA	NA
ENSG00000225172	RP11-16L9.2	lincRNA	3,32E-02	9,36E-01	NA	No	NA	NA	NA	NA
ENSG00000224717	RP11-576D8.4	lincRNA	4,52E-02	7,37E-01	NA	No	NA	NA	NA	NA
ENSG00000232436	RP11-103C3.1	lincRNA	2,40E-02	9,72E-01	NA	No	NA	NA	NA	NA
ENSG00000228437	RP11-400N13.2	lincRNA	3,78E-02	8,45E-01	NA	No	NA	NA	NA	NA
ENSG00000226643	RP11-358H9.1	lincRNA	3,41E-02	9,36E-01	NA	No	NA	NA	NA	NA
ENSG00000236372	RP5-865N13.1	lincRNA	3,17E-02	9,36E-01	NA	No	NA	NA	NA	NA
ENSG00000230628	RP4-781K5.6	lincRNA	6,51E-02	4,91E-01	NA	No	NA	NA	NA	NA
ENSG00000227185	RP11-544D21.1	lincRNA	3,50E-02	9,36E-01	NA	No	NA	NA	NA	NA
ENSG00000234116	RP11-261C10.1	lincRNA	3,32E-02	9,36E-01	NA	No	NA	NA	NA	NA
ENSG00000229703	XX-CR54.1	lincRNA	6,06E-02	5,51E-01	NA	No	NA	NA	NA	NA
ENSG00000235779	AC079779.5	lincRNA	1,77E-01	1,04E-01	NA	No	NA	NA	NA	NA
ENSG00000226277	AC105393.2	lincRNA	1,47E-01	1,11E-01	NA	No	NA	NA	NA	NA
ENSG00000227713	AC116609.1	lincRNA	3,39E-02	9,36E-01	NA	No	NA	NA	NA	NA
ENSG00000234275	AC017053.1	lincRNA	3,17E-02	9,36E-01	NA	No	NA	NA	NA	NA
ENSG00000260476	RP11-254F7.1	lincRNA	3,46E-02	9,36E-01	NA	No	NA	NA	NA	NA
ENSG00000228876	AC010745.2	lincRNA	2,22E-02	9,72E-01	NA	No	NA	NA	NA	NA
ENSG00000224400	AC010880.1	lincRNA	3,15E-02	9,36E-01	NA	No	NA	NA	NA	NA
ENSG00000228999	AC068490.1	lincRNA	3,32E-02	9,36E-01	NA	No	NA	NA	NA	NA
ENSG00000234207	AC096570.2	lincRNA	2,35E-02	9,72E-01	NA	No	NA	NA	NA	NA
ENSG00000259439	RP11-89K21.1	lincRNA	5,70E-02	5,95E-01	NA	No	NA	NA	NA	NA
ENSG00000228414	AC010733.4	lincRNA	4,65E-02	6,04E-01	NA	No	NA	NA	NA	NA
ENSG00000233694	AC007365.1	lincRNA	4,71E-02	5,83E-01	NA	No	NA	NA	NA	NA
ENSG00000237179	AC007392.4	lincRNA	5,61E-02	5,99E-01	NA	No	NA	NA	NA	NA
ENSG00000231781	AC079896.1	lincRNA	2,22E-02	9,72E-01	NA	No	NA	NA	NA	NA
ENSG00000232084	LINC01104	lincRNA	6,95E-02	4,52E-01	NA	No	NA	NA	NA	NA
ENSG00000228488	AC092168.4	lincRNA	4,28E-02	7,26E-01	NA	No	NA	NA	NA	NA
ENSG00000234781	LINC01103	lincRNA	4,53E-02	6,46E-01	NA	No	NA	NA	NA	NA
ENSG00000231626	AC013402.4	lincRNA	3,17E-02	9,36E-01	NA	No	NA	NA	NA	NA
ENSG00000228528	AC068057.1	lincRNA	3,15E-02	9,36E-01	NA	No	NA	NA	NA	NA
ENSG00000234162	AC009505.4	lincRNA	9,87E-02	3,27E-01	NA	No	NA	NA	NA	NA
ENSG00000237880	AC096669.2	lincRNA	3,17E-02	9,36E-01	NA	No	NA	NA	NA	NA
ENSG00000225744	AC017002.4	lincRNA	3,41E-02	9,36E-01	NA	No	NA	NA	NA	NA
ENSG00000235242	AC010982.2	lincRNA	3,31E-02	9,36E-01	NA	No	NA	NA	NA	NA
ENSG00000232740	AC062020.1	lincRNA	3,50E-02	9,36E-01	NA	No	NA	NA	NA	NA
ENSG00000204460	AC079586.1	lincRNA	3,39E-02	9,36E-01	NA	No	NA	NA	NA	NA
ENSG00000237574	AC079776.3	lincRNA	4,26E-02	7,41E-01	NA	No	NA	NA	NA	NA
ENSG00000223631	LINC01120	lincRNA	2,40E-02	9,72E-01	NA	No	NA	NA	NA	NA
ENSG00000235615	AJ239322.1	lincRNA	3,88E-02	8,45E-01	NA	No	NA	NA	NA	NA
ENSG00000224028	AC073928.2	lincRNA	3,31E-02	9,36E-01	NA	No	NA	NA	NA	NA
ENSG00000237220	AC104777.3	lincRNA	3,15E-02	9,36E-01	NA	No	NA	NA	NA	NA
ENSG00000224612	AC011901.2	lincRNA	3,15E-02	9,36E-01	NA	No	NA	NA	NA	NA
ENSG00000227708	AC079150.3	lincRNA	2,46E-02	9,72E-01	NA	No	NA	NA	NA	NA
ENSG00000238004	AC009227.3	lincRNA	3,17E-02	9,36E-01	NA	No	NA	NA	NA	NA
ENSG00000237477	AC093911.1	lincRNA	2,23E-02	9,72E-01	NA	No	NA	NA	NA	NA
ENSG00000227418	PCGEM1	lincRNA	3,15E-02	9,36E-01	NA	No	NA	NA	NA	NA
ENSG00000225539	AC018799.1	lincRNA	3,88E-02	8,45E-01	NA	No	NA	NA	NA	NA
ENSG00000235056	AC010983.1	lincRNA	4,63E-02	6,16E-01	NA	No	NA	NA	NA	NA
ENSG00000271893	RP11-762E8.1	lincRNA	3,31E-02	9,36E-01	NA	No	NA	NA	NA	NA
ENSG00000223466	AC064834.2	lincRNA	4,49E-02	6,60E-01	NA	No	NA	NA	NA	NA
ENSG00000272519	RP11-105N14.2	lincRNA	3,96E-02	8,45E-01	NA	No	NA	NA	NA	NA
ENSG00000196096	AC079610.2	lincRNA	8,00E-02	3,44E-01	NA	No	NA	NA	NA	NA
ENSG00000225166	AC012462.2	lincRNA	4,16E-02	7,65E-01	NA	No	NA	NA	NA	NA
ENSG00000234938	AC012668.1	lincRNA	2,35E-02	9,72E-01	NA	No	NA	NA	NA	NA
ENSG00000227824	AC122136.2	lincRNA	3,41E-02	9,36E-01	NA	No	NA	NA	NA	NA
ENSG00000227498	AC018359.3	lincRNA	3,24E-02	9,36E-01	NA	No	NA	NA	NA	NA
ENSG00000271192	RP4-555D20.3	lincRNA	3,15E-02	9,36E-01	NA	No	NA	NA	NA	NA
ENSG00000243572	RP11-889D3.2	lincRNA	3,32E-02	9,36E-01	NA	No	NA	NA	NA	NA
ENSG00000240708	RP11-64C1.1	lincRNA	4,03E-02	7,82E-01	NA	No	NA	NA	NA	NA

ENSG00000242317	RP11-875H7.5	lincRNA		4,26E-02	7,41E-01	NA	No	NA	NA	NA	NA
ENSG00000243903	RP11-285B24.1	lincRNA		3,32E-02	9,36E-01	NA	No	NA	NA	NA	NA
ENSG00000243149	RP11-543A18.1	lincRNA		4,11E-02	7,67E-01	NA	No	NA	NA	NA	NA
ENSG00000240241	RP11-314M24.1	lincRNA		3,50E-02	9,36E-01	NA	No	NA	NA	NA	NA
ENSG00000261364	RP11-59E19.4	lincRNA		3,32E-02	9,36E-01	NA	No	NA	NA	NA	NA
ENSG00000214381	LINC00488	lincRNA		3,85E-02	8,45E-01	NA	No	NA	NA	NA	NA
ENSG00000243276	RP11-384F7.1	lincRNA		3,15E-02	9,36E-01	NA	No	NA	NA	NA	NA
ENSG00000243733	RP11-80H8.3	lincRNA		3,41E-02	9,36E-01	NA	No	NA	NA	NA	NA
ENSG00000240095	RP11-88H10.3	lincRNA		3,85E-02	8,45E-01	NA	No	NA	NA	NA	NA
ENSG00000241131	RP11-639B1.1	lincRNA		3,17E-02	9,36E-01	NA	No	NA	NA	NA	NA
ENSG00000242536	RP11-290K4.2	lincRNA		3,50E-02	9,36E-01	NA	No	NA	NA	NA	NA
ENSG00000241369	CT64	lincRNA		4,28E-02	7,26E-01	NA	No	NA	NA	NA	NA
ENSG00000244128	RP11-85M11.2	lincRNA		4,65E-02	6,07E-01	NA	No	NA	NA	NA	NA
ENSG00000241882	RP11-637O11.2	lincRNA		3,46E-02	9,36E-01	NA	No	NA	NA	NA	NA
ENSG00000234717	TMEM212-AS1	lincRNA		2,46E-02	9,72E-01	NA	No	NA	NA	NA	NA
ENSG00000270321	RP11-2L8.2	lincRNA		4,46E-02	6,73E-01	NA	No	NA	NA	NA	NA
ENSG00000242512	RP11-416O18.1	lincRNA		7,81E-02	3,64E-01	NA	No	NA	NA	NA	NA
ENSG00000241098	RP11-139K4.1	lincRNA		4,96E-02	4,83E-01	NA	No	NA	NA	NA	NA
ENSG00000228952	RP11-567G11.1	lincRNA		3,15E-02	9,36E-01	NA	No	NA	NA	NA	NA
ENSG00000236864	RP11-44H4.1	lincRNA		3,39E-02	9,36E-01	NA	No	NA	NA	NA	NA
ENSG00000273009	RP11-352G9.1	lincRNA		2,34E-02	9,72E-01	NA	No	NA	NA	NA	NA
ENSG00000269908	RP11-1406H17.1	lincRNA		3,41E-02	9,36E-01	NA	No	NA	NA	NA	NA
ENSG00000249334	RP11-61G19.1	lincRNA		4,43E-01	4,44E-03	3,13E-02	No	NA	NA	NA	NA
ENSG00000248425	AC006296.2	lincRNA		3,50E-02	9,36E-01	NA	No	NA	NA	NA	NA
ENSG00000248238	RP11-3J1.1	lincRNA		2,35E-02	9,72E-01	NA	No	NA	NA	NA	NA
ENSG00000249441	RP11-94H6.1	lincRNA		2,35E-02	9,72E-01	NA	No	NA	NA	NA	NA
ENSG00000248837	RP11-412P11.1	lincRNA		1,51E-01	9,48E-02	NA	No	NA	NA	NA	NA
ENSG00000249645	RP11-552M14.1	lincRNA		4,47E-02	6,74E-01	NA	No	NA	NA	NA	NA
ENSG00000251325	RP11-415C15.3	lincRNA		3,32E-02	9,36E-01	NA	No	NA	NA	NA	NA
ENSG00000248176	RP11-472K22.1	lincRNA		3,17E-02	9,36E-01	NA	No	NA	NA	NA	NA
ENSG00000251434	RP11-315A17.1	lincRNA		4,73E-02	5,80E-01	NA	No	NA	NA	NA	NA
ENSG00000261761	RP11-103J17.2	lincRNA		3,41E-02	9,36E-01	NA	No	NA	NA	NA	NA
ENSG00000249667	RP11-213G21.1	lincRNA		3,97E-02	8,45E-01	NA	No	NA	NA	NA	NA
ENSG00000250338	RP11-395I6.2	lincRNA		9,55E-02	3,60E-01	NA	No	NA	NA	NA	NA
ENSG00000249122	RP11-227F19.2	lincRNA		3,39E-02	9,36E-01	NA	No	NA	NA	NA	NA
ENSG00000251264	RP11-545H22.1	lincRNA		4,04E-02	7,80E-01	NA	No	NA	NA	NA	NA
ENSG00000248317	RP11-463H12.2	lincRNA		3,39E-02	9,36E-01	NA	No	NA	NA	NA	NA
ENSG00000251339	RP11-506N2.1	lincRNA		4,04E-02	7,80E-01	NA	No	NA	NA	NA	NA
ENSG00000251266	RP11-340A13.2	lincRNA		2,22E-02	9,72E-01	NA	No	NA	NA	NA	NA
ENSG00000260265	RP11-44F21.5	lincRNA		3,46E-02	9,36E-01	NA	No	NA	NA	NA	NA
ENSG00000251383	RP11-542G1.3	lincRNA		3,50E-02	9,36E-01	NA	No	NA	NA	NA	NA
ENSG00000250735	RP11-567N4.3	lincRNA		3,32E-02	9,36E-01	NA	No	NA	NA	NA	NA
ENSG00000231782	LINC00575	lincRNA		4,48E-02	6,71E-01	NA	No	NA	NA	NA	NA
ENSG00000249171	RP11-767N15.1	lincRNA		4,04E-02	7,80E-01	NA	No	NA	NA	NA	NA
ENSG00000250103	RP11-380D23.2	lincRNA		3,31E-02	9,36E-01	NA	No	NA	NA	NA	NA
ENSG00000250791	RP11-55L3.1	lincRNA		2,35E-02	9,72E-01	NA	No	NA	NA	NA	NA
ENSG00000236922	AC092661.1	lincRNA		3,41E-02	9,36E-01	NA	No	NA	NA	NA	NA
ENSG00000229565	AC108056.1	lincRNA		3,31E-02	9,36E-01	NA	No	NA	NA	NA	NA
ENSG00000250149	RP11-399F2.2	lincRNA		3,15E-02	9,36E-01	NA	No	NA	NA	NA	NA
ENSG00000251326	RP11-521E5.1	lincRNA		3,15E-02	9,36E-01	NA	No	NA	NA	NA	NA
ENSG00000249847	RP11-192C21.2	lincRNA		3,15E-02	9,36E-01	NA	No	NA	NA	NA	NA
ENSG00000248434	RP11-553P9.2	lincRNA		3,41E-02	9,36E-01	NA	No	NA	NA	NA	NA
ENSG00000248456	RP11-553P9.3	lincRNA		3,39E-02	9,36E-01	NA	No	NA	NA	NA	NA
ENSG00000250865	RP11-780O17.1	lincRNA		3,15E-02	9,36E-01	NA	No	NA	NA	NA	NA
ENSG00000248330	LINC00613	lincRNA		4,28E-02	7,31E-01	NA	No	NA	NA	NA	NA
ENSG00000261166	RP11-775H9.3	lincRNA		2,34E-02	9,72E-01	NA	No	NA	NA	NA	NA
ENSG00000250665	RP11-775H9.1	lincRNA		4,19E-02	7,48E-01	NA	No	NA	NA	NA	NA
ENSG00000251024	RP11-203B7.1	lincRNA		2,42E-02	9,72E-01	NA	No	NA	NA	NA	NA
ENSG00000248809	LINC01095	lincRNA		4,16E-02	7,67E-01	NA	No	NA	NA	NA	NA
ENSG00000250706	RP11-18H21.2	lincRNA		4,99E-02	5,33E-01	NA	No	NA	NA	NA	NA
ENSG00000251511	RP11-171N4.1	lincRNA		3,15E-02	9,36E-01	NA	No	NA	NA	NA	NA
ENSG00000250180	RP11-138A23.2	lincRNA		4,33E-02	7,20E-01	NA	No	NA	NA	NA	NA

ENSG00000250968	RP11-322J23.1	lincRNA	2,40E-02	9,72E-01	NA	No	NA	NA	NA	NA
ENSG00000251584	RP11-440I14.3	lincRNA	4,12E-02	7,65E-01	NA	No	NA	NA	NA	NA
ENSG00000250993	RP11-404J23.1	lincRNA	2,22E-02	9,72E-01	NA	No	NA	NA	NA	NA
ENSG00000248370	RP11-366H4.1	lincRNA	3,50E-02	9,36E-01	NA	No	NA	NA	NA	NA
ENSG00000249430	CTD-2231H16.1	lincRNA	3,17E-02	9,36E-01	NA	No	NA	NA	NA	NA
ENSG00000260066	CTD-2587M23.1	lincRNA	4,62E-02	6,16E-01	NA	No	NA	NA	NA	NA
ENSG00000248118	LINC01019	lincRNA	4,88E-02	5,14E-01	NA	No	NA	NA	NA	NA
ENSG00000251168	CTD-2072I24.1	lincRNA	7,44E-02	4,05E-01	NA	No	NA	NA	NA	NA
ENSG00000260981	RP11-315A16.1	lincRNA	3,31E-02	9,36E-01	NA	No	NA	NA	NA	NA
ENSG00000249396	RP11-1C1.4	lincRNA	4,33E-02	7,20E-01	NA	No	NA	NA	NA	NA
ENSG00000250822	CTD-2139B15.4	lincRNA	6,61E-02	4,92E-01	NA	No	NA	NA	NA	NA
ENSG00000248605	CTD-2306M5.1	lincRNA	3,32E-02	9,36E-01	NA	No	NA	NA	NA	NA
ENSG00000250583	CTD-2233C11.2	lincRNA	6,74E-02	4,77E-01	NA	No	NA	NA	NA	NA
ENSG00000248539	CTD-2194L12.2	lincRNA	3,17E-02	9,36E-01	NA	No	NA	NA	NA	NA
ENSG00000251518	CTD-2130F23.2	lincRNA	3,46E-02	9,36E-01	NA	No	NA	NA	NA	NA
ENSG00000250585	LINC00604	lincRNA	4,04E-02	7,80E-01	NA	No	NA	NA	NA	NA
ENSG00000249276	RP11-8L21.1	lincRNA	2,40E-02	9,72E-01	NA	No	NA	NA	NA	NA
ENSG00000250122	CTD-2013M15.1	lincRNA	3,15E-02	9,36E-01	NA	No	NA	NA	NA	NA
ENSG00000271752	RP11-269M20.3	lincRNA	5,70E-02	5,95E-01	NA	No	NA	NA	NA	NA
ENSG00000250360	CTD-2089N3.1	lincRNA	3,17E-02	9,36E-01	NA	No	NA	NA	NA	NA
ENSG00000251376	CTD-2313F11.2	lincRNA	1,00E-01	2,00E-01	NA	No	NA	NA	NA	NA
ENSG00000249236	CTD-2227I18.1	lincRNA	4,21E-02	7,67E-01	NA	No	NA	NA	NA	NA
ENSG00000249198	RP11-772C9.1	lincRNA	4,71E-02	5,81E-01	NA	No	NA	NA	NA	NA
ENSG00000251575	CTD-2170G1.2	lincRNA	4,83E-02	5,38E-01	NA	No	NA	NA	NA	NA
ENSG00000249894	RP11-434D9.2	lincRNA	4,12E-02	7,73E-01	NA	No	NA	NA	NA	NA
ENSG00000249588	CTC-537E7.1	lincRNA	3,32E-02	9,36E-01	NA	No	NA	NA	NA	NA
ENSG00000249343	CTD-2275D24.2	lincRNA	3,31E-02	9,36E-01	NA	No	NA	NA	NA	NA
ENSG00000249023	CTD-2331D11.3	lincRNA	3,39E-02	9,36E-01	NA	No	NA	NA	NA	NA
ENSG00000248363	CTC-261N6.1	lincRNA	2,22E-02	9,72E-01	NA	No	NA	NA	NA	NA
ENSG00000250544	CTC-493L21.1	lincRNA	6,60E-02	4,93E-01	NA	No	NA	NA	NA	NA
ENSG00000249362	CTD-2316B1.1	lincRNA	3,17E-02	9,36E-01	NA	No	NA	NA	NA	NA
ENSG00000248708	CTD-2316B1.2	lincRNA	2,22E-02	9,72E-01	NA	No	NA	NA	NA	NA
ENSG00000250377	CTC-467M3.3	lincRNA	2,34E-02	9,72E-01	NA	No	NA	NA	NA	NA
ENSG00000250437	RP11-116A1.1	lincRNA	3,15E-02	9,36E-01	NA	No	NA	NA	NA	NA
ENSG00000249169	CTC-529L17.1	lincRNA	2,35E-02	9,72E-01	NA	No	NA	NA	NA	NA
ENSG00000251361	CTD-2091N23.1	lincRNA	2,34E-02	9,72E-01	NA	No	NA	NA	NA	NA
ENSG00000249959	RP11-252I13.2	lincRNA	2,23E-02	9,72E-01	NA	No	NA	NA	NA	NA
ENSG00000248440	RP11-231G15.1	lincRNA	3,39E-02	9,36E-01	NA	No	NA	NA	NA	NA
ENSG00000248428	CTC-551A13.1	lincRNA	3,15E-02	9,36E-01	NA	No	NA	NA	NA	NA
ENSG00000251132	RP11-438C19.2	lincRNA	3,50E-02	9,36E-01	NA	No	NA	NA	NA	NA
ENSG00000229855	CTC-546K23.1	lincRNA	2,22E-02	9,72E-01	NA	No	NA	NA	NA	NA
ENSG00000251662	CTC-369A16.3	lincRNA	3,46E-02	9,36E-01	NA	No	NA	NA	NA	NA
ENSG00000251214	CTD-2308B18.1	lincRNA	4,21E-02	7,67E-01	NA	No	NA	NA	NA	NA
ENSG00000248147	RP11-395P13.1	lincRNA	3,17E-02	9,36E-01	NA	No	NA	NA	NA	NA
ENSG00000251554	RP11-395P13.6	lincRNA	3,32E-02	9,36E-01	NA	No	NA	NA	NA	NA
ENSG00000251045	CTC-321K16.4	lincRNA	4,28E-02	7,26E-01	NA	No	NA	NA	NA	NA
ENSG00000250378	RP11-119J18.1	lincRNA	3,15E-02	9,36E-01	NA	No	NA	NA	NA	NA
ENSG00000253792	CTC-436K13.5	lincRNA	3,98E-02	8,45E-01	NA	No	NA	NA	NA	NA
ENSG00000253959	CTB-43E15.1	lincRNA	4,91E-02	5,47E-01	NA	No	NA	NA	NA	NA
ENSG00000253428	CTB-43E15.2	lincRNA	1,03E-01	2,91E-01	NA	No	NA	NA	NA	NA
ENSG00000228718	RP11-145H9.3	lincRNA	3,92E-02	8,45E-01	NA	No	NA	NA	NA	NA
ENSG00000205444	RP4-529N6.1	lincRNA	6,05E-02	5,51E-01	NA	No	NA	NA	NA	NA
ENSG00000224532	RP3-470L22.1	lincRNA	5,55E-02	6,16E-01	NA	No	NA	NA	NA	NA
ENSG00000203498	RP11-556O15.1	lincRNA	3,32E-02	9,36E-01	NA	No	NA	NA	NA	NA
ENSG00000229401	RP1-290I10.7	lincRNA	2,46E-02	9,72E-01	NA	No	NA	NA	NA	NA
ENSG00000242753	RP11-716O23.2	lincRNA	3,46E-02	9,36E-01	NA	No	NA	NA	NA	NA
ENSG00000231662	RP11-686D16.1	lincRNA	3,88E-02	8,45E-01	NA	No	NA	NA	NA	NA
ENSG00000233183	RP3-468B3.2	lincRNA	2,40E-02	9,72E-01	NA	No	NA	NA	NA	NA
ENSG00000226917	RP11-328M4.3	lincRNA	3,17E-02	9,36E-01	NA	No	NA	NA	NA	NA
ENSG00000236347	RP11-346D19.1	lincRNA	3,15E-02	9,36E-01	NA	No	NA	NA	NA	NA
ENSG00000225391	RP11-55K22.5	lincRNA	3,80E-02	8,45E-01	NA	No	NA	NA	NA	NA
ENSG00000226004	RP11-10J5.1	lincRNA	2,97E-01	5,65E-03	3,73E-02	No	NA	NA	NA	NA

ENSG00000229922	RP11-240M16.1	lincRNA	7,17E-02	4,28E-01	NA	No	NA	NA	NA	NA
ENSG00000236366	RP11-440G9.1	lincRNA	4,66E-02	6,02E-01	NA	No	NA	NA	NA	NA
ENSG00000231883	RP1-297M16.2	lincRNA	3,32E-02	9,36E-01	NA	No	NA	NA	NA	NA
ENSG00000224893	RP1-200K18.1	lincRNA	3,17E-02	9,36E-01	NA	No	NA	NA	NA	NA
ENSG00000236823	RP1-249F5.3	lincRNA	3,69E-02	8,45E-01	NA	No	NA	NA	NA	NA
ENSG00000234986	XX-C2158C12.1	lincRNA	3,86E-02	8,45E-01	NA	No	NA	NA	NA	NA
ENSG00000230423	XX-C2158C6.1	lincRNA	2,35E-02	9,72E-01	NA	No	NA	NA	NA	NA
ENSG00000225532	XX-C2158C6.3	lincRNA	4,75E-02	5,70E-01	NA	No	NA	NA	NA	NA
ENSG00000231476	AC074389.5	lincRNA	4,04E-02	7,81E-01	NA	No	NA	NA	NA	NA
ENSG00000236081	AC074389.9	lincRNA	5,29E-01	4,24E-03	3,03E-02	No	NA	NA	NA	NA
ENSG00000228334	AC024028.1	lincRNA	8,08E-02	3,33E-01	NA	No	NA	NA	NA	NA
ENSG00000267055	RP11-486P11.1	lincRNA	3,17E-02	9,36E-01	NA	No	NA	NA	NA	NA
ENSG00000233824	AC003985.1	lincRNA	3,46E-02	9,36E-01	NA	No	NA	NA	NA	NA
ENSG00000253308	RP1-170O19.17	lincRNA	2,46E-02	9,72E-01	NA	No	NA	NA	NA	NA
ENSG00000272556	RP11-638I8.1	lincRNA	2,84E-01	8,74E-03	5,14E-02	No	NA	NA	NA	NA
ENSG00000235620	AC020743.4	lincRNA	3,41E-02	9,36E-01	NA	No	NA	NA	NA	NA
ENSG00000236046	AC004920.3	lincRNA	4,09E-02	7,75E-01	NA	No	NA	NA	NA	NA
ENSG00000229403	RP4-718N17.2	lincRNA	3,88E-02	8,45E-01	NA	No	NA	NA	NA	NA
ENSG00000238131	RP11-806J6.1	lincRNA	2,35E-02	9,72E-01	NA	No	NA	NA	NA	NA
ENSG00000229881	RP11-321E8.4	lincRNA	3,91E-02	8,45E-01	NA	No	NA	NA	NA	NA
ENSG00000261467	RP11-731K22.1	lincRNA	3,31E-02	9,36E-01	NA	No	NA	NA	NA	NA
ENSG00000234113	AC008163.4	lincRNA	2,34E-02	9,72E-01	NA	No	NA	NA	NA	NA
ENSG00000235139	AC003984.1	lincRNA	5,17E-02	6,56E-01	NA	No	NA	NA	NA	NA
ENSG00000233942	AC004012.1	lincRNA	4,32E-02	7,25E-01	NA	No	NA	NA	NA	NA
ENSG00000233123	LINC01007	lincRNA	2,23E-02	9,72E-01	NA	No	NA	NA	NA	NA
ENSG00000234273	AC073071.1	lincRNA	4,04E-02	7,80E-01	NA	No	NA	NA	NA	NA
ENSG00000243574	RP11-264K23.1	lincRNA	3,32E-02	9,36E-01	NA	No	NA	NA	NA	NA
ENSG00000197462	AC005276.1	lincRNA	3,32E-02	9,36E-01	NA	No	NA	NA	NA	NA
ENSG00000272619	RP11-563K23.1	lincRNA	2,46E-02	9,72E-01	NA	No	NA	NA	NA	NA
ENSG00000237531	RP11-309M23.1	lincRNA	6,44E-02	5,09E-01	NA	No	NA	NA	NA	NA
ENSG00000234622	RP13-297E16.5	lincRNA	7,45E-02	3,86E-01	NA	No	NA	NA	NA	NA
ENSG00000205662	RP11-706O15.7	lincRNA	8,97E-02	2,58E-01	NA	No	NA	NA	NA	NA
ENSG00000226985	RP11-142G7.2	lincRNA	4,64E-02	6,11E-01	NA	No	NA	NA	NA	NA
ENSG00000272294	RP3-326L13.3	lincRNA	3,15E-02	9,36E-01	NA	No	NA	NA	NA	NA
ENSG00000206062	RP13-212L9.1	lincRNA	4,28E-02	7,41E-01	NA	No	NA	NA	NA	NA
ENSG00000230392	RP5-1139I1.1	lincRNA	2,22E-02	9,72E-01	NA	No	NA	NA	NA	NA
ENSG00000234493	GS1-421I3.2	lincRNA	5,73E-02	5,92E-01	NA	No	NA	NA	NA	NA
ENSG00000229702	RP1-274L7.1	lincRNA	4,15E-02	7,67E-01	NA	No	NA	NA	NA	NA
ENSG00000229269	RP11-298A8.2	lincRNA	3,15E-02	9,36E-01	NA	No	NA	NA	NA	NA
ENSG00000214915	XX-FW80269A6.1	lincRNA	3,15E-02	9,36E-01	NA	No	NA	NA	NA	NA
ENSG00000254160	CTD-2281E23.3	lincRNA	3,41E-02	9,36E-01	NA	No	NA	NA	NA	NA
ENSG00000249694	RP11-378A12.1	lincRNA	8,91E-02	2,62E-01	NA	No	NA	NA	NA	NA
ENSG00000253130	CTD-3023L14.2	lincRNA	4,55E-02	6,42E-01	NA	No	NA	NA	NA	NA
ENSG00000253887	RP11-10A14.7	lincRNA	3,41E-02	9,36E-01	NA	No	NA	NA	NA	NA
ENSG00000253735	RP11-115J16.3	lincRNA	2,46E-02	9,72E-01	NA	No	NA	NA	NA	NA
ENSG00000253230	LINC00599	lincRNA	9,46E-02	2,19E-01	NA	No	NA	NA	NA	NA
ENSG00000269954	RP11-148O21.6	lincRNA	2,21E-01	4,08E-02	NA	No	NA	NA	NA	NA
ENSG00000253496	RP11-13N12.1	lincRNA	3,15E-02	9,36E-01	NA	No	NA	NA	NA	NA
ENSG00000253147	RP11-369E15.4	lincRNA	3,17E-02	9,36E-01	NA	No	NA	NA	NA	NA
ENSG00000253182	RP11-486M23.2	lincRNA	3,32E-02	9,36E-01	NA	No	NA	NA	NA	NA
ENSG00000248964	RP11-94H18.1	lincRNA	3,31E-02	9,36E-01	NA	No	NA	NA	NA	NA
ENSG00000253604	RP11-489E7.1	lincRNA	3,39E-02	9,36E-01	NA	No	NA	NA	NA	NA
ENSG00000253279	FAM183CP	lincRNA	3,41E-02	9,36E-01	NA	No	NA	NA	NA	NA
ENSG00000254302	RP11-431M3.1	lincRNA	3,32E-02	9,36E-01	NA	No	NA	NA	NA	NA
ENSG00000253108	RP1-84O15.2	lincRNA	2,22E-02	9,72E-01	NA	No	NA	NA	NA	NA
ENSG00000254038	RP11-419C23.1	lincRNA	3,46E-02	9,36E-01	NA	No	NA	NA	NA	NA
ENSG00000254111	RP11-150O12.5	lincRNA	3,41E-02	9,36E-01	NA	No	NA	NA	NA	NA
ENSG00000253361	RP11-675F6.3	lincRNA	3,17E-02	9,36E-01	NA	No	NA	NA	NA	NA
ENSG00000254067	RP11-122L4.1	lincRNA	3,17E-02	9,36E-01	NA	No	NA	NA	NA	NA
ENSG00000253802	CTA-392C11.1	lincRNA	3,50E-02	9,36E-01	NA	No	NA	NA	NA	NA
ENSG00000253782	RP11-350F16.2	lincRNA	2,42E-02	9,72E-01	NA	No	NA	NA	NA	NA
ENSG00000253140	RP11-567J20.3	lincRNA	2,35E-02	9,72E-01	NA	No	NA	NA	NA	NA

ENSG00000253474	RP11-10H3.1	lincRNA	3,46E-02	9,36E-01	NA	No	NA	NA	NA	NA
ENSG00000253301	RP11-513O17.2	lincRNA	3,31E-02	9,36E-01	NA	No	NA	NA	NA	NA
ENSG00000253614	RP11-513O17.3	lincRNA	2,40E-02	9,72E-01	NA	No	NA	NA	NA	NA
ENSG00000254775	RP11-27P7.1	lincRNA	3,90E-02	8,45E-01	NA	No	NA	NA	NA	NA
ENSG00000254120	RP11-705O24.2	lincRNA	3,15E-02	9,36E-01	NA	No	NA	NA	NA	NA
ENSG00000253734	RP11-579E24.1	lincRNA	3,17E-02	9,36E-01	NA	No	NA	NA	NA	NA
ENSG00000254277	RP11-1144P22.1	lincRNA	3,31E-02	9,36E-01	NA	No	NA	NA	NA	NA
ENSG00000254300	LINC01111	lincRNA	4,18E-02	7,50E-01	NA	No	NA	NA	NA	NA
ENSG00000254366	RP11-38H17.1	lincRNA	2,22E-02	9,72E-01	NA	No	NA	NA	NA	NA
ENSG00000254001	RP11-91P17.1	lincRNA	3,50E-02	9,36E-01	NA	No	NA	NA	NA	NA
ENSG00000253503	RP11-653B10.1	lincRNA	2,42E-02	9,72E-01	NA	No	NA	NA	NA	NA
ENSG00000253836	RP11-731N10.1	lincRNA	3,15E-02	9,36E-01	NA	No	NA	NA	NA	NA
ENSG00000254394	CTD-2272D18.1	lincRNA	3,32E-02	9,36E-01	NA	No	NA	NA	NA	NA
ENSG00000253154	CTA-392E5.1	lincRNA	3,46E-02	9,36E-01	NA	No	NA	NA	NA	NA
ENSG00000253371	RP11-587H10.1	lincRNA	2,22E-02	9,72E-01	NA	No	NA	NA	NA	NA
ENSG00000254000	RP11-245A18.1	lincRNA	3,31E-02	9,36E-01	NA	No	NA	NA	NA	NA
ENSG00000255313	RP11-1082L8.2	lincRNA	3,32E-02	9,36E-01	NA	No	NA	NA	NA	NA
ENSG00000253470	RP11-697B24.1	lincRNA	3,41E-02	9,36E-01	NA	No	NA	NA	NA	NA
ENSG00000244791	RP11-65D17.1	lincRNA	3,46E-02	9,36E-01	NA	No	NA	NA	NA	NA
ENSG00000250400	LINC00977	lincRNA	7,69E-02	3,83E-01	NA	No	NA	NA	NA	NA
ENSG00000253783	CTD-2534J5.1	lincRNA	3,15E-02	9,36E-01	NA	No	NA	NA	NA	NA
ENSG00000253266	RP11-128L5.1	lincRNA	3,17E-02	9,36E-01	NA	No	NA	NA	NA	NA
ENSG00000229611	RP11-390F4.10	lincRNA	4,71E-02	5,82E-01	NA	No	NA	NA	NA	NA
ENSG00000231902	RP11-29B9.2	lincRNA	5,09E-02	6,67E-01	NA	No	NA	NA	NA	NA
ENSG00000234021	RP11-75C9.2	lincRNA	4,61E-02	6,21E-01	NA	No	NA	NA	NA	NA
ENSG00000236921	RP11-408N14.1	lincRNA	3,32E-02	9,36E-01	NA	No	NA	NA	NA	NA
ENSG00000226562	CYP4F26P	lincRNA	5,98E-02	5,61E-01	NA	No	NA	NA	NA	NA
ENSG00000227301	RP11-384P7.5	lincRNA	4,04E-02	7,80E-01	NA	No	NA	NA	NA	NA
ENSG00000235659	RP11-374M1.5	lincRNA	3,15E-02	9,36E-01	NA	No	NA	NA	NA	NA
ENSG00000229697	RP11-374M1.3	lincRNA	2,42E-02	9,72E-01	NA	No	NA	NA	NA	NA
ENSG00000236376	RP11-470F18.1	lincRNA	3,15E-02	9,36E-01	NA	No	NA	NA	NA	NA
ENSG00000260995	RP11-165H23.1	lincRNA	4,16E-02	7,67E-01	NA	No	NA	NA	NA	NA
ENSG00000232494	RP11-403N16.2	lincRNA	2,35E-02	9,72E-01	NA	No	NA	NA	NA	NA
ENSG00000267559	RP11-383M4.6	lincRNA	3,39E-02	9,36E-01	NA	No	NA	NA	NA	NA
ENSG00000225085	RP11-15B24.1	lincRNA	4,18E-02	7,50E-01	NA	No	NA	NA	NA	NA
ENSG00000229454	RP11-202I11.2	lincRNA	2,35E-02	9,72E-01	NA	No	NA	NA	NA	NA
ENSG00000229613	RP11-555F9.2	lincRNA	3,32E-02	9,36E-01	NA	No	NA	NA	NA	NA
ENSG00000228142	RP11-180I4.2	lincRNA	2,23E-02	9,72E-01	NA	No	NA	NA	NA	NA
ENSG00000260677	RP11-546O6.4	lincRNA	4,77E-02	5,64E-01	NA	No	NA	NA	NA	NA
ENSG00000226047	RP11-196I18.4	lincRNA	2,23E-02	9,72E-01	NA	No	NA	NA	NA	NA
ENSG00000228707	RP11-787B4.2	lincRNA	4,46E-02	6,75E-01	NA	No	NA	NA	NA	NA
ENSG00000233721	RP11-205K6.1	lincRNA	3,69E-02	8,45E-01	NA	No	NA	NA	NA	NA
ENSG00000237339	RP11-98L5.2	lincRNA	7,85E-02	3,58E-01	NA	No	NA	NA	NA	NA
ENSG00000234556	LINC00701	lincRNA	2,22E-02	9,72E-01	NA	No	NA	NA	NA	NA
ENSG00000234170	RP11-446F3.2	lincRNA	3,15E-02	9,36E-01	NA	No	NA	NA	NA	NA
ENSG00000234248	RP11-799O21.2	lincRNA	4,90E-01	1,16E-03	1,14E-02	No	NA	NA	NA	NA
ENSG00000234752	RP11-428L9.1	lincRNA	2,46E-02	9,72E-01	NA	No	NA	NA	NA	NA
ENSG00000223808	RP11-428L9.2	lincRNA	4,57E-02	6,35E-01	NA	No	NA	NA	NA	NA
ENSG00000227932	RP13-16H11.2	lincRNA	5,80E-02	5,77E-01	NA	No	NA	NA	NA	NA
ENSG00000235824	LINC00837	lincRNA	3,17E-02	9,36E-01	NA	No	NA	NA	NA	NA
ENSG00000232624	RP11-478H13.3	lincRNA	4,03E-02	7,82E-01	NA	No	NA	NA	NA	NA
ENSG00000241125	RP11-508N22.9	lincRNA	4,38E-02	7,01E-01	NA	No	NA	NA	NA	NA
ENSG00000228048	RP11-598C10.1	lincRNA	4,82E-02	5,55E-01	NA	No	NA	NA	NA	NA
ENSG00000254271	RP11-131N11.4	lincRNA	2,42E-02	9,72E-01	NA	No	NA	NA	NA	NA
ENSG00000230526	RP11-472G21.2	lincRNA	3,15E-02	9,36E-01	NA	No	NA	NA	NA	NA
ENSG00000272748	RP11-222G7.2	lincRNA	4,24E-02	7,41E-01	NA	No	NA	NA	NA	NA
ENSG00000230229	RP11-90J7.4	lincRNA	3,50E-02	9,36E-01	NA	No	NA	NA	NA	NA
ENSG00000223993	RP11-475D12.1	lincRNA	4,26E-02	7,31E-01	NA	No	NA	NA	NA	NA
ENSG00000238291	RP11-94M14.3	lincRNA	4,26E-02	7,31E-01	NA	No	NA	NA	NA	NA
ENSG00000228778	RP11-129J12.1	lincRNA	4,36E-02	7,20E-01	NA	No	NA	NA	NA	NA
ENSG00000237579	RP11-324L3.1	lincRNA	3,46E-02	9,36E-01	NA	No	NA	NA	NA	NA
ENSG00000229775	RP11-298H24.1	lincRNA	4,11E-02	7,67E-01	NA	No	NA	NA	NA	NA

ENSG00000223381	RP11-655H13.2	lincRNA	3,39E-02	9,36E-01	NA	No	NA	NA	NA	NA
ENSG00000234542	RP11-282I1.2	lincRNA	2,42E-02	9,72E-01	NA	No	NA	NA	NA	NA
ENSG00000235180	LINC00601	lincRNA	4,22E-02	7,43E-01	NA	No	NA	NA	NA	NA
ENSG00000234640	RP11-264E18.1	lincRNA	3,46E-02	9,36E-01	NA	No	NA	NA	NA	NA
ENSG00000270105	RP11-326C3.14	lincRNA	2,42E-02	9,72E-01	NA	No	NA	NA	NA	NA
ENSG00000254586	RP11-358H18.3	lincRNA	2,35E-02	9,72E-01	NA	No	NA	NA	NA	NA
ENSG00000255094	RP11-406D1.2	lincRNA	5,30E-02	6,39E-01	NA	No	NA	NA	NA	NA
ENSG00000254669	RP5-945I17.2	lincRNA	3,32E-02	9,36E-01	NA	No	NA	NA	NA	NA
ENSG00000254654	AC068858.1	lincRNA	3,15E-02	9,36E-01	NA	No	NA	NA	NA	NA
ENSG00000255447	CTD-2210P24.6	lincRNA	5,67E-02	4,45E-01	NA	No	NA	NA	NA	NA
ENSG00000267804	RP11-665N17.4	lincRNA	3,15E-02	9,36E-01	NA	No	NA	NA	NA	NA
ENSG00000259854	RP11-211G23.1	lincRNA	4,24E-02	7,41E-01	NA	No	NA	NA	NA	NA
ENSG00000255209	RP11-258O13.1	lincRNA	4,21E-02	7,67E-01	NA	No	NA	NA	NA	NA
ENSG00000255246	RP11-179A16.2	lincRNA	2,46E-02	9,72E-01	NA	No	NA	NA	NA	NA
ENSG00000255516	RP11-164N3.1	lincRNA	3,40E-02	9,36E-01	NA	No	NA	NA	NA	NA
ENSG00000255102	RP11-164N3.2	lincRNA	3,17E-02	9,36E-01	NA	No	NA	NA	NA	NA
ENSG00000255679	JRKL-AS1	lincRNA	3,41E-02	9,36E-01	NA	No	NA	NA	NA	NA
ENSG00000254587	RP11-360K13.1	lincRNA	4,19E-02	7,67E-01	NA	No	NA	NA	NA	NA
ENSG00000254830	RP11-99C10.1	lincRNA	3,15E-02	9,36E-01	NA	No	NA	NA	NA	NA
ENSG00000256281	RP11-136I14.2	lincRNA	3,17E-02	9,36E-01	NA	No	NA	NA	NA	NA
ENSG00000272575	RP11-702B10.1	lincRNA	2,35E-02	9,72E-01	NA	No	NA	NA	NA	NA
ENSG00000255258	RP11-448P19.1	lincRNA	7,33E-02	4,06E-01	NA	No	NA	NA	NA	NA
ENSG00000249054	RP11-598F7.1	lincRNA	4,26E-02	7,31E-01	NA	No	NA	NA	NA	NA
ENSG00000256969	RP11-320N7.2	lincRNA	2,34E-02	9,72E-01	NA	No	NA	NA	NA	NA
ENSG00000256790	RP11-429A20.3	lincRNA	2,34E-02	9,72E-01	NA	No	NA	NA	NA	NA
ENSG00000257084	U47924.27	lincRNA	2,42E-02	9,72E-01	NA	No	NA	NA	NA	NA
ENSG00000225231	AC091814.2	lincRNA	5,76E-01	5,81E-03	NA	No	NA	NA	NA	NA
ENSG00000257004	RP11-180M15.3	lincRNA	4,23E-02	7,43E-01	NA	No	NA	NA	NA	NA
ENSG00000258879	RP11-713N11.5	lincRNA	4,52E-02	6,54E-01	NA	No	NA	NA	NA	NA
ENSG00000258119	RP11-804F13.1	lincRNA	3,15E-02	9,36E-01	NA	No	NA	NA	NA	NA
ENSG00000260943	RP11-476D10.1	lincRNA	1,94E-01	6,10E-02	NA	No	NA	NA	NA	NA
ENSG00000249388	RP11-834C11.6	lincRNA	2,35E-02	9,72E-01	NA	No	NA	NA	NA	NA
ENSG00000257870	RP11-616L12.1	lincRNA	3,50E-02	9,36E-01	NA	No	NA	NA	NA	NA
ENSG00000256915	RP11-221N13.4	lincRNA	3,15E-02	9,36E-01	NA	No	NA	NA	NA	NA
ENSG00000256077	RP11-335O4.1	lincRNA	3,31E-02	9,36E-01	NA	No	NA	NA	NA	NA
ENSG00000256273	RP11-71J4.2	lincRNA	4,03E-02	7,81E-01	NA	No	NA	NA	NA	NA
ENSG00000257265	CTD-2021H9.1	lincRNA	3,15E-02	9,36E-01	NA	No	NA	NA	NA	NA
ENSG00000257183	RP11-274M17.3	lincRNA	2,35E-02	9,72E-01	NA	No	NA	NA	NA	NA
ENSG00000257329	RP11-290L1.5	lincRNA	3,32E-02	9,36E-01	NA	No	NA	NA	NA	NA
ENSG00000257741	RP11-540D4.3	lincRNA	3,32E-02	9,36E-01	NA	No	NA	NA	NA	NA
ENSG00000257124	RP11-384P14.1	lincRNA	3,31E-02	9,36E-01	NA	No	NA	NA	NA	NA
ENSG00000257729	RP11-788H18.1	lincRNA	4,46E-02	6,75E-01	NA	No	NA	NA	NA	NA
ENSG00000269916	RP11-193M21.1	lincRNA	3,17E-02	9,36E-01	NA	No	NA	NA	NA	NA
ENSG00000257912	RP11-248E9.5	lincRNA	4,33E-02	7,20E-01	NA	No	NA	NA	NA	NA
ENSG00000258125	RP11-1041F24.1	lincRNA	3,15E-02	9,36E-01	NA	No	NA	NA	NA	NA
ENSG00000257863	RP11-587P21.3	lincRNA	3,15E-02	9,36E-01	NA	No	NA	NA	NA	NA
ENSG00000257193	RP11-818F20.4	lincRNA	4,64E-02	6,11E-01	NA	No	NA	NA	NA	NA
ENSG00000249094	RP1-7G5.6	lincRNA	4,23E-02	7,43E-01	NA	No	NA	NA	NA	NA
ENSG00000257519	RP11-116D17.2	lincRNA	2,40E-02	9,72E-01	NA	No	NA	NA	NA	NA
ENSG00000258346	RP11-148B3.2	lincRNA	2,22E-02	9,72E-01	NA	No	NA	NA	NA	NA
ENSG00000258018	RP11-148B3.1	lincRNA	3,32E-02	9,36E-01	NA	No	NA	NA	NA	NA
ENSG00000258435	RP11-711D18.2	lincRNA	3,50E-02	9,36E-01	NA	No	NA	NA	NA	NA
ENSG00000256814	RP11-158L12.5	lincRNA	2,42E-02	9,72E-01	NA	No	NA	NA	NA	NA
ENSG00000257061	RP4-809F18.2	lincRNA	3,39E-02	9,36E-01	NA	No	NA	NA	NA	NA
ENSG00000249873	RP11-983C2.2	lincRNA	4,19E-02	7,48E-01	NA	No	NA	NA	NA	NA
ENSG00000256292	RP11-955H22.1	lincRNA	3,32E-02	9,36E-01	NA	No	NA	NA	NA	NA
ENSG00000256193	LINC00507	lincRNA	4,75E-02	5,70E-01	NA	No	NA	NA	NA	NA
ENSG00000259862	RP11-143E21.6	lincRNA	3,17E-02	9,36E-01	NA	No	NA	NA	NA	NA
ENSG00000256343	RP11-662M24.1	lincRNA	3,17E-02	9,36E-01	NA	No	NA	NA	NA	NA
ENSG00000230294	RP13-507P19.2	lincRNA	2,35E-02	9,72E-01	NA	No	NA	NA	NA	NA
ENSG00000256209	RP11-897M7.1	lincRNA	2,42E-02	9,72E-01	NA	No	NA	NA	NA	NA
ENSG00000249926	RP11-495K9.6	lincRNA	4,12E-02	7,65E-01	NA	No	NA	NA	NA	NA

ENSG00000266505	RP11-629E24.2	lincRNA	8,46E-02	4,43E-01	NA	No	NA	NA	NA	NA
ENSG00000236094	LINC00545	lincRNA	5,18E-02	5,04E-01	NA	No	NA	NA	NA	NA
ENSG00000235097	LINC00330	lincRNA	2,34E-02	9,72E-01	NA	No	NA	NA	NA	NA
ENSG00000223732	RP11-321C24.1	lincRNA	3,80E-02	8,45E-01	NA	No	NA	NA	NA	NA
ENSG00000233456	LINC01077	lincRNA	3,39E-02	9,36E-01	NA	No	NA	NA	NA	NA
ENSG00000271564	RP13-444H2.1	lincRNA	4,04E-02	7,80E-01	NA	No	NA	NA	NA	NA
ENSG00000261517	LINC00558	lincRNA	3,32E-02	9,36E-01	NA	No	NA	NA	NA	NA
ENSG00000227510	RP11-196P2.1	lincRNA	3,32E-02	9,36E-01	NA	No	NA	NA	NA	NA
ENSG00000219926	RP11-394A14.2	lincRNA	2,22E-02	9,72E-01	NA	No	NA	NA	NA	NA
ENSG00000227674	LINC00355	lincRNA	3,15E-02	9,36E-01	NA	No	NA	NA	NA	NA
ENSG00000234767	LINC01052	lincRNA	3,32E-02	9,36E-01	NA	No	NA	NA	NA	NA
ENSG00000235221	LINC00401	lincRNA	3,32E-02	9,36E-01	NA	No	NA	NA	NA	NA
ENSG00000224853	LINC00393	lincRNA	3,15E-02	9,36E-01	NA	No	NA	NA	NA	NA
ENSG00000223880	LINC01078	lincRNA	4,16E-02	7,65E-01	NA	No	NA	NA	NA	NA
ENSG00000233528	LINC00430	lincRNA	3,17E-02	9,36E-01	NA	No	NA	NA	NA	NA
ENSG00000272046	RP11-471M2.3	lincRNA	3,46E-02	9,36E-01	NA	No	NA	NA	NA	NA
ENSG00000229557	LINC00379	lincRNA	3,15E-02	9,36E-01	NA	No	NA	NA	NA	NA
ENSG00000234551	RP11-123H22.1	lincRNA	4,19E-02	7,48E-01	NA	No	NA	NA	NA	NA
ENSG00000260102	LINC01070	lincRNA	3,31E-02	9,36E-01	NA	No	NA	NA	NA	NA
ENSG00000226921	LINC00454	lincRNA	6,23E-02	5,24E-01	NA	No	NA	NA	NA	NA
ENSG00000248358	AE000658.31	lincRNA	3,17E-02	9,36E-01	NA	No	NA	NA	NA	NA
ENSG00000257869	CTD-2591A6.2	lincRNA	3,17E-02	9,36E-01	NA	No	NA	NA	NA	NA
ENSG00000257523	CTD-2384A14.2	lincRNA	4,67E-02	5,98E-01	NA	No	NA	NA	NA	NA
ENSG00000248975	CTD-2251F13.1	lincRNA	4,61E-02	6,21E-01	NA	No	NA	NA	NA	NA
ENSG00000259017	RP11-561B11.6	lincRNA	3,81E-02	8,45E-01	NA	No	NA	NA	NA	NA
ENSG00000258844	RP11-259K15.2	lincRNA	3,15E-02	9,36E-01	NA	No	NA	NA	NA	NA
ENSG00000258418	RP11-662J14.1	lincRNA	4,18E-02	7,50E-01	NA	No	NA	NA	NA	NA
ENSG00000258969	RP11-305B6.3	lincRNA	3,32E-02	9,36E-01	NA	No	NA	NA	NA	NA
ENSG00000258952	RP11-1042B17.5	lincRNA	2,35E-02	9,72E-01	NA	No	NA	NA	NA	NA
ENSG00000258689	RP6-65G23.1	lincRNA	8,36E-02	4,52E-01	NA	No	NA	NA	NA	NA
ENSG00000258994	RP11-26L16.1	lincRNA	3,17E-02	9,36E-01	NA	No	NA	NA	NA	NA
ENSG00000259028	RP11-325D8.1	lincRNA	3,39E-02	9,36E-01	NA	No	NA	NA	NA	NA
ENSG00000258798	RP11-895M11.3	lincRNA	3,31E-02	9,36E-01	NA	No	NA	NA	NA	NA
ENSG00000258630	RP11-725G5.2	lincRNA	3,46E-02	9,36E-01	NA	No	NA	NA	NA	NA
ENSG00000259026	RP11-907D1.1	lincRNA	3,15E-02	9,36E-01	NA	No	NA	NA	NA	NA
ENSG00000259119	RP11-1127D7.1	lincRNA	4,28E-02	7,41E-01	NA	No	NA	NA	NA	NA
ENSG00000258672	RP11-543C4.3	lincRNA	4,11E-02	7,73E-01	NA	No	NA	NA	NA	NA
ENSG00000230805	AL132709.1	lincRNA	3,31E-02	9,36E-01	NA	No	NA	NA	NA	NA
ENSG00000227468	AL928742.12	lincRNA	3,15E-02	9,36E-01	NA	No	NA	NA	NA	NA
ENSG00000233988	ADAM6	lincRNA	1,27E-01	1,69E-01	NA	No	NA	NA	NA	NA
ENSG00000259383	RP11-403B2.6	lincRNA	5,04E-02	4,56E-01	NA	No	NA	NA	NA	NA
ENSG00000206187	RP11-1084I9.1	lincRNA	3,41E-02	9,36E-01	NA	No	NA	NA	NA	NA
ENSG00000259747	RP11-275I4.2	lincRNA	1,30E-01	1,57E-01	NA	No	NA	NA	NA	NA
ENSG00000259198	RP11-133K1.6	lincRNA	4,16E-02	7,67E-01	NA	No	NA	NA	NA	NA
ENSG00000259669	RP11-643A5.2	lincRNA	2,22E-02	9,72E-01	NA	No	NA	NA	NA	NA
ENSG00000260937	RP11-548M13.1	lincRNA	3,32E-02	9,36E-01	NA	No	NA	NA	NA	NA
ENSG00000259459	RP11-321G12.1	lincRNA	2,22E-02	9,72E-01	NA	No	NA	NA	NA	NA
ENSG00000259457	RP11-279F6.2	lincRNA	3,41E-02	9,36E-01	NA	No	NA	NA	NA	NA
ENSG00000259218	LINC00928	lincRNA	3,32E-02	9,36E-01	NA	No	NA	NA	NA	NA
ENSG00000258483	AC144835.1	lincRNA	3,31E-02	9,36E-01	NA	No	NA	NA	NA	NA
ENSG00000259611	CTD-2544M6.2	lincRNA	2,42E-02	9,72E-01	NA	No	NA	NA	NA	NA
ENSG00000260496	RP11-161M6.3	lincRNA	4,71E-02	5,82E-01	NA	No	NA	NA	NA	NA
ENSG00000260532	LA16c-381G6.1	lincRNA	3,90E-02	8,45E-01	NA	No	NA	NA	NA	NA
ENSG00000259947	XX-DJ76P10__A.2	lincRNA	3,90E-02	8,45E-01	NA	No	NA	NA	NA	NA
ENSG00000270097	LA16c-380H5.3	lincRNA	3,32E-02	9,36E-01	NA	No	NA	NA	NA	NA
ENSG00000259925	CTA-363E6.2	lincRNA	3,15E-02	9,36E-01	NA	No	NA	NA	NA	NA
ENSG00000262756	AC009133.21	lincRNA	2,22E-02	9,72E-01	NA	No	NA	NA	NA	NA
ENSG00000261475	CTD-2014E2.6	lincRNA	2,34E-02	9,72E-01	NA	No	NA	NA	NA	NA
ENSG00000261541	RP11-626K17.3	lincRNA	4,17E-02	7,65E-01	NA	No	NA	NA	NA	NA
ENSG00000260958	RP11-488I20.8	lincRNA	4,04E-02	7,80E-01	NA	No	NA	NA	NA	NA
ENSG00000261325	AC140542.2	lincRNA	4,04E-02	7,81E-01	NA	No	NA	NA	NA	NA
ENSG00000259839	RP11-491F9.6	lincRNA	4,52E-02	6,56E-01	NA	No	NA	NA	NA	NA

ENSG00000261751	RP11-305A4.3	lincRNA	2,34E-02	9,72E-01	NA	No	NA	NA	NA	NA
ENSG00000261241	RP11-883G14.1	lincRNA	2,22E-02	9,72E-01	NA	No	NA	NA	NA	NA
ENSG00000261803	RP11-434E6.2	lincRNA	4,12E-02	7,65E-01	NA	No	NA	NA	NA	NA
ENSG00000261436	RP11-354I13.2	lincRNA	3,32E-02	9,36E-01	NA	No	NA	NA	NA	NA
ENSG00000259847	RP11-95H3.1	lincRNA	2,35E-02	9,72E-01	NA	No	NA	NA	NA	NA
ENSG00000260364	RP11-256I9.3	lincRNA	3,31E-02	9,36E-01	NA	No	NA	NA	NA	NA
ENSG00000261176	RP11-2L4.1	lincRNA	3,32E-02	9,36E-01	NA	No	NA	NA	NA	NA
ENSG00000270058	RP11-514D23.3	lincRNA	2,35E-02	9,72E-01	NA	No	NA	NA	NA	NA
ENSG00000260498	RP4-536B24.4	lincRNA	4,68E-02	5,94E-01	NA	No	NA	NA	NA	NA
ENSG00000261816	RP11-863P13.2	lincRNA	4,28E-02	7,26E-01	NA	No	NA	NA	NA	NA
ENSG00000264729	RP11-219A15.2	lincRNA	4,56E-02	6,40E-01	NA	No	NA	NA	NA	NA
ENSG00000264734	RP11-260A9.6	lincRNA	3,15E-02	9,36E-01	NA	No	NA	NA	NA	NA
ENSG00000265246	RP11-663N22.1	lincRNA	3,41E-02	9,36E-01	NA	No	NA	NA	NA	NA
ENSG00000266830	CTD-2008P7.8	lincRNA	2,22E-02	9,72E-01	NA	No	NA	NA	NA	NA
ENSG00000265046	CTC-542B22.1	lincRNA	2,40E-02	9,72E-01	NA	No	NA	NA	NA	NA
ENSG00000236377	AC084809.3	lincRNA	4,11E-02	7,67E-01	NA	No	NA	NA	NA	NA
ENSG00000267499	CTD-3194G12.1	lincRNA	4,04E-02	7,80E-01	NA	No	NA	NA	NA	NA
ENSG00000214546	AC087491.2	lincRNA	1,14E-01	2,27E-01	NA	No	NA	NA	NA	NA
ENSG00000226629	LINC00974	lincRNA	3,41E-02	9,36E-01	NA	No	NA	NA	NA	NA
ENSG00000267211	RP5-1067M6.3	lincRNA	5,24E-02	5,00E-01	NA	No	NA	NA	NA	NA
ENSG00000266979	RP11-1072C15.2	lincRNA	3,32E-02	9,36E-01	NA	No	NA	NA	NA	NA
ENSG00000264451	RP11-433M22.2	lincRNA	4,54E-02	6,47E-01	NA	No	NA	NA	NA	NA
ENSG00000262745	CTD-2377D24.8	lincRNA	3,35E-01	1,81E-02	NA	No	NA	NA	NA	NA
ENSG00000242407	CTD-2377D24.4	lincRNA	2,22E-02	9,72E-01	NA	No	NA	NA	NA	NA
ENSG00000254039	RP11-304F15.6	lincRNA	2,42E-02	9,72E-01	NA	No	NA	NA	NA	NA
ENSG00000263176	RP11-893F2.15	lincRNA	4,54E-02	6,44E-01	NA	No	NA	NA	NA	NA
ENSG00000266290	RP11-159D12.10	lincRNA	4,31E-02	7,22E-01	NA	No	NA	NA	NA	NA
ENSG00000260785	CASC17	lincRNA	3,17E-02	9,36E-01	NA	No	NA	NA	NA	NA
ENSG00000228639	AC005152.3	lincRNA	3,41E-02	9,36E-01	NA	No	NA	NA	NA	NA
ENSG00000234721	AC125421.1	lincRNA	4,71E-02	5,81E-01	NA	No	NA	NA	NA	NA
ENSG00000267535	LINC00868	lincRNA	3,32E-02	9,36E-01	NA	No	NA	NA	NA	NA
ENSG00000267521	RP11-87G24.6	lincRNA	4,04E-02	7,80E-01	NA	No	NA	NA	NA	NA
ENSG00000265121	RP11-285E9.5	lincRNA	2,35E-02	9,72E-01	NA	No	NA	NA	NA	NA
ENSG00000263154	RP11-1055B8.2	lincRNA	3,32E-02	9,36E-01	NA	No	NA	NA	NA	NA
ENSG00000262352	AP005530.1	lincRNA	3,15E-02	9,36E-01	NA	No	NA	NA	NA	NA
ENSG00000264265	RP11-14P20.1	lincRNA	2,40E-02	9,72E-01	NA	No	NA	NA	NA	NA
ENSG00000261738	RP11-945C19.1	lincRNA	3,15E-02	9,36E-01	NA	No	NA	NA	NA	NA
ENSG00000266961	RP11-973H7.5	lincRNA	3,17E-02	9,36E-01	NA	No	NA	NA	NA	NA
ENSG00000263862	RP11-107K17.1	lincRNA	3,15E-02	9,36E-01	NA	No	NA	NA	NA	NA
ENSG00000263382	RP11-552O4.1	lincRNA	3,15E-02	9,36E-01	NA	No	NA	NA	NA	NA
ENSG00000263688	RP11-386P4.1	lincRNA	2,35E-02	9,72E-01	NA	No	NA	NA	NA	NA
ENSG00000267413	RP11-636O21.1	lincRNA	7,17E-02	4,28E-01	NA	No	NA	NA	NA	NA
ENSG00000261715	RP11-653G8.2	lincRNA	3,32E-02	9,36E-01	NA	No	NA	NA	NA	NA
ENSG00000267337	CTC-782O7.1	lincRNA	3,70E-02	8,45E-01	NA	No	NA	NA	NA	NA
ENSG00000261307	RP11-157P23.2	lincRNA	3,15E-02	9,36E-01	NA	No	NA	NA	NA	NA
ENSG00000267674	RP11-813F20.2	lincRNA	3,15E-02	9,36E-01	NA	No	NA	NA	NA	NA
ENSG00000267732	RP11-456O19.3	lincRNA	3,46E-02	9,36E-01	NA	No	NA	NA	NA	NA
ENSG00000267593	RP11-108P20.4	lincRNA	4,12E-02	7,65E-01	NA	No	NA	NA	NA	NA
ENSG00000267462	RP11-866E20.3	lincRNA	4,28E-02	7,26E-01	NA	No	NA	NA	NA	NA
ENSG00000179676	LINC00305	lincRNA	3,41E-02	9,36E-01	NA	No	NA	NA	NA	NA
ENSG00000264699	RP11-421N8.2	lincRNA	4,63E-02	6,13E-01	NA	No	NA	NA	NA	NA
ENSG00000265995	RP11-736G13.1	lincRNA	3,32E-02	9,36E-01	NA	No	NA	NA	NA	NA
ENSG00000266014	RP11-94B19.2	lincRNA	5,92E-02	5,68E-01	NA	No	NA	NA	NA	NA
ENSG00000229055	AC034110.1	lincRNA	4,93E-02	5,31E-01	NA	No	NA	NA	NA	NA
ENSG00000265844	RP11-751H17.1	lincRNA	3,32E-02	9,36E-01	NA	No	NA	NA	NA	NA
ENSG00000267780	RP11-154H12.3	lincRNA	5,99E-02	5,60E-01	NA	No	NA	NA	NA	NA
ENSG00000224711	RP5-988G17.1	lincRNA	3,32E-02	9,36E-01	NA	No	NA	NA	NA	NA
ENSG00000225127	LINC00237	lincRNA	3,15E-02	9,36E-01	NA	No	NA	NA	NA	NA
ENSG00000204684	RP5-1004I9.1	lincRNA	2,42E-02	9,72E-01	NA	No	NA	NA	NA	NA
ENSG00000230725	RP4-738P15.1	lincRNA	3,85E-02	8,45E-01	NA	No	NA	NA	NA	NA
ENSG00000230324	RP4-705O1.1	lincRNA	5,98E-02	5,53E-01	NA	No	NA	NA	NA	NA
ENSG00000229771	RP4-644L1.2	lincRNA	3,85E-02	8,45E-01	NA	No	NA	NA	NA	NA

ENSG00000229957	RP5-998H6.2	lincRNA	3,32E-02	9,36E-01	NA	No	NA	NA	NA	NA
ENSG00000235415	AC005808.3	lincRNA	1,21E-01	1,91E-01	NA	No	NA	NA	NA	NA
ENSG00000230843	RP11-380D15.3	lincRNA	2,46E-02	9,72E-01	NA	No	NA	NA	NA	NA
ENSG00000227297	RP4-718J7.4	lincRNA	3,90E-02	8,45E-01	NA	No	NA	NA	NA	NA
ENSG00000225708	RP13-379L11.1	lincRNA	3,50E-02	9,36E-01	NA	No	NA	NA	NA	NA
ENSG00000254620	RP5-907D15.3	lincRNA	5,25E-02	6,45E-01	NA	No	NA	NA	NA	NA
ENSG00000272050	RP13-379O24.3	lincRNA	3,46E-02	9,36E-01	NA	No	NA	NA	NA	NA
ENSG00000237687	LINC00686	lincRNA	3,41E-02	9,36E-01	NA	No	NA	NA	NA	NA
ENSG00000272259	RP11-305P22.9	lincRNA	1,60E-01	1,29E-01	NA	No	NA	NA	NA	NA
ENSG00000231133	HAR1B	lincRNA	4,19E-01	1,61E-02	NA	No	NA	NA	NA	NA
ENSG00000231977	RP5-963E22.4	lincRNA	7,21E-02	4,24E-01	NA	No	NA	NA	NA	NA
ENSG00000267231	AC005786.5	lincRNA	4,16E-02	7,65E-01	NA	No	NA	NA	NA	NA
ENSG00000176840	MIR7-3HG	lincRNA	3,74E-02	8,45E-01	NA	No	NA	NA	NA	NA
ENSG00000268480	CTD-2586B10.1	lincRNA	3,46E-02	9,36E-01	NA	No	NA	NA	NA	NA
ENSG00000267723	CTD-2189E23.1	lincRNA	3,88E-02	8,45E-01	NA	No	NA	NA	NA	NA
ENSG00000266951	AD000091.2	lincRNA	3,39E-02	9,36E-01	NA	No	NA	NA	NA	NA
ENSG00000269043	CTC-513N18.6	lincRNA	8,89E-02	2,82E-01	NA	No	NA	NA	NA	NA
ENSG00000269199	AC003973.5	lincRNA	4,28E-02	7,41E-01	NA	No	NA	NA	NA	NA
ENSG00000269796	AC011516.1	lincRNA	2,35E-02	9,72E-01	NA	No	NA	NA	NA	NA
ENSG00000267696	CTB-151G24.1	lincRNA	3,17E-02	9,36E-01	NA	No	NA	NA	NA	NA
ENSG00000267012	CTC-360P9.1	lincRNA	3,88E-02	8,45E-01	NA	No	NA	NA	NA	NA
ENSG00000266985	CTD-2329C7.2	lincRNA	2,34E-02	9,72E-01	NA	No	NA	NA	NA	NA
ENSG00000269037	CTC-523E23.6	lincRNA	1,70E-01	1,19E-01	NA	No	NA	NA	NA	NA
ENSG00000269729	AC006262.4	lincRNA	3,15E-02	9,36E-01	NA	No	NA	NA	NA	NA
ENSG00000268621	AC006262.5	lincRNA	3,17E-02	9,36E-01	NA	No	NA	NA	NA	NA
ENSG00000267808	AC018755.17	lincRNA	3,74E-02	8,45E-01	NA	No	NA	NA	NA	NA
ENSG00000269758	CTD-2245F17.9	lincRNA	3,31E-02	9,36E-01	NA	No	NA	NA	NA	NA
ENSG00000269877	MIR371B	lincRNA	2,42E-02	9,72E-01	NA	No	NA	NA	NA	NA
ENSG00000268307	CTD-2619J13.13	lincRNA	4,24E-02	7,41E-01	NA	No	NA	NA	NA	NA
ENSG00000225255	LA16c-83F12.6	lincRNA	3,17E-02	9,36E-01	NA	No	NA	NA	NA	NA
ENSG00000235578	XXbac-B33L19.4	lincRNA	3,15E-02	9,36E-01	NA	No	NA	NA	NA	NA
ENSG00000225070	LL22NC03-23C6.13	lincRNA	3,80E-02	8,45E-01	NA	No	NA	NA	NA	NA
ENSG00000233577	RP3-462D8.2	lincRNA	3,31E-02	9,36E-01	NA	No	NA	NA	NA	NA
ENSG00000230051	CTA-929C8.8	lincRNA	3,32E-02	9,36E-01	NA	No	NA	NA	NA	NA
ENSG00000227519	CTA-342B11.1	lincRNA	2,35E-02	9,72E-01	NA	No	NA	NA	NA	NA
ENSG00000239674	RP1-127L4.7	lincRNA	3,80E-02	8,45E-01	NA	No	NA	NA	NA	NA
ENSG00000233080	CTA-714B7.5	lincRNA	9,84E-02	3,30E-01	NA	No	NA	NA	NA	NA
ENSG00000230107	CTA-126B4.7	lincRNA	9,64E-02	2,18E-01	NA	No	NA	NA	NA	NA
ENSG00000224271	RP11-191L9.4	lincRNA	3,41E-02	9,36E-01	NA	No	NA	NA	NA	NA
ENSG00000230345	RP13-455A7.1	lincRNA	3,95E-02	8,45E-01	NA	No	NA	NA	NA	NA
ENSG00000235154	CTA-280A3__B.2	lincRNA	3,46E-02	9,36E-01	NA	No	NA	NA	NA	NA
ENSG00000226954	RP5-983L19.2	lincRNA	5,28E-02	4,91E-01	NA	No	NA	NA	NA	NA
ENSG00000271308	AP003900.6	lincRNA	3,85E-02	8,45E-01	NA	No	NA	NA	NA	NA
ENSG00000229306	AP001464.4	lincRNA	3,15E-02	9,36E-01	NA	No	NA	NA	NA	NA
ENSG00000236471	AF127577.12	lincRNA	4,95E-02	4,91E-01	NA	No	NA	NA	NA	NA
ENSG00000224247	AJ009632.3	lincRNA	3,46E-02	9,36E-01	NA	No	NA	NA	NA	NA
ENSG00000226956	AP000432.2	lincRNA	3,31E-02	9,36E-01	NA	No	NA	NA	NA	NA
ENSG00000224141	AL109763.2	lincRNA	2,22E-02	9,72E-01	NA	No	NA	NA	NA	NA
ENSG00000234730	AF241725.4	lincRNA	3,17E-02	9,36E-01	NA	No	NA	NA	NA	NA
ENSG00000184856	LINC00308	lincRNA	3,41E-02	9,36E-01	NA	No	NA	NA	NA	NA
ENSG00000226996	AP000477.3	lincRNA	4,69E-02	5,91E-01	NA	No	NA	NA	NA	NA
ENSG00000230379	AP000146.2	lincRNA	4,12E-02	7,65E-01	NA	No	NA	NA	NA	NA
ENSG00000223870	AP000233.3	lincRNA	5,49E-02	6,09E-01	NA	No	NA	NA	NA	NA
ENSG00000237664	LINC00316	lincRNA	6,20E-02	5,29E-01	NA	No	NA	NA	NA	NA
ENSG00000264553	MIR4257	miRNA	-9,53E-02	5,51E-01	7,64E-01	No	-2,98E+00	6,63E-03	1,99E-02	Yes
ENSG00000265744	MIR4427	miRNA	-5,45E-01	3,69E-02	1,41E-01	No	-2,08E+00	8,58E-03	2,49E-02	Yes
ENSG00000266097	MIR5192	miRNA	-3,56E-01	1,32E-01	3,34E-01	No	-2,86E+00	1,05E-03	3,87E-03	Yes
ENSG00000266139	MIR4435-2	miRNA	-1,52E-01	4,23E-01	6,64E-01	No	-1,61E+00	1,74E-02	4,61E-02	Yes
ENSG00000263813	MIR3679	miRNA	-9,39E-02	6,63E-01	8,35E-01	No	-1,87E+00	2,48E-03	8,34E-03	Yes
ENSG00000207654	MIR128-1	miRNA	7,34E-02	5,06E-01	NA	No	1,61E+00	1,55E-02	4,16E-02	Yes
ENSG00000265396	MIR3128	miRNA	1,75E-01	1,60E-01	NA	No	4,48E+00	8,23E-04	3,11E-03	Yes
ENSG00000199121	MIR26B	miRNA	-6,30E-03	9,74E-01	9,90E-01	No	-2,40E+00	3,45E-03	1,12E-02	Yes

ENSG00000272543	MIR4787	miRNA	4,23E-03	9,41E-01	NA	No	4,12E+00	4,62E-05	2,25E-04	Yes
ENSG00000221264	MIR1284	miRNA	2,63E-01	1,01E-01	2,81E-01	No	2,23E+00	4,98E-03	1,55E-02	Yes
ENSG00000266798	AC078816.1	miRNA	1,17E-01	2,52E-01	NA	No	1,52E+00	1,78E-02	4,68E-02	Yes
ENSG00000266447	AC046143.2	miRNA	4,97E-02	6,49E-01	NA	No	3,50E+00	2,50E-05	1,28E-04	Yes
ENSG00000252616	AC079140.1	miRNA	-1,11E-01	5,57E-01	7,68E-01	No	-1,79E+00	1,23E-02	3,42E-02	Yes
ENSG00000264585	MIR4449	miRNA	9,84E-02	3,27E-01	NA	No	3,27E+00	3,57E-04	1,46E-03	Yes
ENSG00000264274	MIR4799	miRNA	5,03E-02	7,35E-01	8,77E-01	No	2,42E+00	7,96E-05	3,70E-04	Yes
ENSG00000222121	AL512290.1	miRNA	1,27E-01	1,78E-01	NA	No	3,91E+00	3,05E-03	1,00E-02	Yes
ENSG00000221157	AC009196.1	miRNA	4,86E-01	3,76E-02	1,43E-01	No	1,94E+00	8,22E-03	2,40E-02	Yes
ENSG00000207725	MIR222	miRNA	7,79E-02	4,78E-01	NA	No	2,32E+00	7,20E-03	2,14E-02	Yes
ENSG00000264604	AL158069.1	miRNA	-1,70E-01	4,00E-01	6,45E-01	No	2,86E+00	4,56E-05	2,22E-04	Yes
ENSG00000264392	AC002477.1	miRNA	2,29E-01	1,52E-01	3,66E-01	No	2,58E+00	1,01E-03	3,73E-03	Yes
ENSG00000207574	MIR661	miRNA	-4,57E-02	8,13E-01	9,17E-01	No	-1,75E+00	9,97E-03	2,84E-02	Yes
ENSG00000207609	MIR491	miRNA	3,50E-01	1,52E-01	3,66E-01	No	1,92E+00	8,58E-05	3,95E-04	Yes
ENSG00000241781	AL161626.1	miRNA	-2,54E-02	7,95E-01	NA	No	2,61E+00	5,49E-04	2,15E-03	Yes
ENSG00000207612	MIR604	miRNA	-3,33E-01	1,83E-01	4,09E-01	No	-2,26E+00	2,02E-05	1,05E-04	Yes
ENSG00000266591	AC022537.1	miRNA	2,09E-01	3,02E-01	5,50E-01	No	2,13E+00	4,27E-06	2,49E-05	Yes
ENSG00000264572	MIR4296	miRNA	-5,19E-02	8,01E-01	9,12E-01	No	-3,56E+00	2,13E-04	9,09E-04	Yes
ENSG00000264201	MIR4701	miRNA	-2,25E-01	3,00E-01	5,48E-01	No	-5,60E+00	7,37E-04	2,81E-03	Yes
ENSG00000239776	AC079949.1	miRNA	-6,61E-01	1,58E-02	7,79E-02	No	3,04E+00	3,26E-23	1,37E-21	Yes
ENSG00000212054	AL354764.1	miRNA	4,94E-02	6,29E-01	NA	No	1,69E+00	1,36E-02	3,73E-02	Yes
ENSG00000252813	AC108861.1	miRNA	-3,07E-02	8,72E-01	9,46E-01	No	-2,16E+00	3,79E-03	1,21E-02	Yes
ENSG00000221247	AC136704.1	miRNA	-4,04E-02	8,25E-01	9,24E-01	No	-1,75E+00	1,56E-02	4,18E-02	Yes
ENSG00000266124	MIR5587	miRNA	2,33E-01	3,04E-01	5,52E-01	No	-1,67E+00	1,07E-02	3,03E-02	Yes
ENSG00000266141	MIR2909	miRNA	3,19E-01	1,48E-02	NA	No	4,65E+00	8,09E-04	3,06E-03	Yes
ENSG00000207764	MIR133A2	miRNA	-1,79E-01	4,05E-01	6,50E-01	No	-3,26E+00	3,45E-05	1,72E-04	Yes
ENSG00000215946	MIR941-1	miRNA	-7,65E-01	2,89E-02	1,19E-01	No	-2,02E+00	5,71E-03	1,74E-02	Yes
ENSG00000207554	MIR647	miRNA	-6,30E-01	4,54E-02	1,63E-01	No	-2,24E+00	1,86E-03	6,45E-03	Yes
ENSG00000207808	MIR27A	miRNA	-5,06E-01	5,06E-02	1,75E-01	No	-2,11E+00	5,85E-03	1,78E-02	Yes
ENSG00000207780	MIR648	miRNA	-1,83E-01	3,91E-01	6,36E-01	No	-3,55E+00	2,94E-04	1,22E-03	Yes
ENSG00000264462	MIR3648	miRNA	1,13E+00	3,31E-03	2,54E-02	No	5,38E+00	1,62E-32	1,35E-30	Yes
ENSG00000264063	MIR3687	miRNA	1,37E+00	2,37E-03	1,97E-02	No	7,04E+00	3,00E-99	8,36E-96	Yes
ENSG00000207638	MIR99A	miRNA	1,18E+00	1,49E-02	7,48E-02	No	3,24E+00	1,07E-05	5,87E-05	Yes
ENSG00000199030	MIRLET7C	miRNA	1,63E-01	3,33E-01	5,82E-01	No	1,71E+00	2,49E-03	8,36E-03	Yes
ENSG00000207863	MIR125B2	miRNA	1,86E-01	2,81E-01	5,27E-01	No	1,73E+00	9,42E-03	2,70E-02	Yes
ENSG00000216197	AP001623.1	miRNA	4,46E-02	8,40E-01	9,30E-01	No	-2,39E+00	2,36E-08	1,99E-07	Yes
ENSG00000244180	AL592188.2	miRNA	1,02E+00	8,81E-01	9,50E-01	No	1,98E+00	1,00E-03	3,72E-03	Yes
ENSG00000264827	AL592188.4	miRNA	4,68E-01	9,13E-02	2,62E-01	No	4,44E+00	2,70E-28	1,70E-26	Yes
ENSG00000266219	AL592188.8	miRNA	3,88E-01	5,77E-02	1,91E-01	No	6,40E+00	6,77E-21	2,28E-19	Yes
ENSG00000243151	AL592188.1	miRNA	3,30E-01	1,72E-01	3,96E-01	No	2,64E+00	1,44E-12	2,09E-11	Yes
ENSG00000265807	AL592188.6	miRNA	2,46E-02	8,82E-01	9,50E-01	No	3,84E+00	7,08E-08	5,54E-07	Yes
ENSG00000265525	AL592188.5	miRNA	5,94E-03	9,95E-01	NA	No	4,72E+00	7,14E-07	4,73E-06	Yes
ENSG00000265830	AL592188.7	miRNA	7,76E-01	1,55E-02	7,66E-02	No	6,43E+00	9,17E-83	1,41E-79	Yes
ENSG00000264101	MIR4689	miRNA	7,67E-02	5,09E-01	NA	No	1,65E-01	4,74E-01	NA	No
ENSG00000266687	AL356261.1	miRNA	-2,74E-02	7,57E-01	NA	No	-1,38E-01	5,30E-01	NA	No
ENSG00000207865	MIR34A	miRNA	2,14E-01	2,62E-01	5,06E-01	No	-1,11E-01	7,06E-01	8,12E-01	No
ENSG00000265521	MIR5697	miRNA	3,64E-02	8,10E-01	9,16E-01	No	-2,30E-01	3,75E-01	NA	No
ENSG00000264881	MIR1273D	miRNA	-3,54E-02	6,18E-01	NA	No	-7,33E-02	6,81E-01	NA	No
ENSG00000263676	MIR4632	miRNA	4,24E-03	9,72E-01	NA	No	-1,96E-01	4,28E-01	NA	No
ENSG00000266358	AL359771.1	miRNA	-4,46E-02	7,60E-01	NA	No	-3,02E-02	9,21E-01	NA	No
ENSG00000221662	MIR1290	miRNA	1,81E-02	8,71E-01	NA	No	-1,02E-01	8,30E-01	NA	No
ENSG00000266098	AL137127.1	miRNA	-1,88E-02	9,24E-01	9,68E-01	No	-9,35E-01	5,44E-02	1,20E-01	No
ENSG00000221808	MIR1256	miRNA	-2,08E-01	2,29E-01	4,67E-01	No	-3,37E-01	3,38E-01	4,94E-01	No
ENSG00000266867	AL031005.1	miRNA	-6,00E-02	5,54E-01	NA	No	-7,11E-02	7,29E-01	NA	No
ENSG00000265422	MIR4684	miRNA	-1,55E-02	8,19E-01	NA	No	-1,30E-01	7,12E-01	NA	No
ENSG00000263793	MIR3115	miRNA	-1,09E-01	4,37E-01	NA	No	-1,66E-01	5,43E-01	NA	No
ENSG00000266802	MIR4419A	miRNA	-2,72E-02	8,57E-01	9,38E-01	No	-1,86E-01	5,09E-01	6,56E-01	No
ENSG00000264371	MIR4425	miRNA	-4,42E-02	7,60E-01	NA	No	-1,02E-01	8,30E-01	NA	No
ENSG00000221550	AL645859.1	miRNA	-6,75E-02	3,80E-01	NA	No	-1,29E-01	5,01E-01	NA	No
ENSG00000266580	MIR4254	miRNA	-6,42E-02	4,70E-01	NA	No	-1,56E-01	5,23E-01	NA	No
ENSG00000266203	MIR5585	miRNA	6,77E-03	8,94E-01	NA	No	-1,36E-01	7,12E-01	NA	No
ENSG00000266239	MIR3605	miRNA	-1,60E-01	4,56E-01	6,90E-01	No	-1,14E+00	2,41E-02	6,06E-02	No

ENSG00000263675	MIR5581	miRNA	6,03E-01	3,58E-02	1,39E-01	No	7,80E-01	7,94E-02	1,63E-01	No
ENSG00000198974	MIR30E	miRNA	2,03E-01	3,36E-01	5,85E-01	No	-1,70E-01	5,95E-01	7,26E-01	No
ENSG00000207962	MIR30C1	miRNA	1,67E-01	4,10E-01	6,53E-01	No	4,96E-01	2,03E-01	3,38E-01	No
ENSG00000264896	AL451006.1	miRNA	2,34E-02	9,72E-01	NA	No	9,85E-02	7,75E-01	NA	No
ENSG00000252146	AL390776.1	miRNA	4,26E-02	7,66E-01	8,93E-01	No	-1,04E-01	6,72E-01	7,87E-01	No
ENSG00000266294	AL136380.1	miRNA	3,90E-02	8,45E-01	NA	No	2,56E-01	2,99E-01	NA	No
ENSG00000265538	MIR4421	miRNA	4,34E-02	7,20E-01	NA	No	8,77E-02	8,64E-01	NA	No
ENSG00000264834	MIR1273F	miRNA	3,76E-02	7,35E-01	NA	No	-9,39E-02	6,84E-01	NA	No
ENSG00000265815	MIR1273G	miRNA	-6,03E-03	9,54E-01	NA	No	-1,19E-01	5,44E-01	NA	No
ENSG00000264454	AL049745.1	miRNA	4,52E-02	6,56E-01	NA	No	1,08E-01	6,91E-01	NA	No
ENSG00000223307	AC119674.1	miRNA	-3,03E-03	9,94E-01	NA	No	1,01E-01	5,27E-01	NA	No
ENSG00000238773	AL137855.1	miRNA	3,17E-02	9,36E-01	NA	No	8,57E-02	8,64E-01	NA	No
ENSG00000266150	MIR4711	miRNA	-5,37E-02	6,46E-01	NA	No	-1,26E-01	7,12E-01	NA	No
ENSG00000263380	AC096534.1	miRNA	3,54E-02	7,04E-01	NA	No	5,69E-01	7,57E-02	1,57E-01	No
ENSG00000264470	MIR4794	miRNA	8,87E-02	4,03E-01	NA	No	2,35E-01	3,45E-01	NA	No
ENSG00000265996	MIR3671	miRNA	1,92E-01	4,05E-01	6,49E-01	No	8,01E-02	8,04E-01	8,79E-01	No
ENSG00000199135	MIR101-1	miRNA	1,11E-02	9,46E-01	9,78E-01	No	1,15E+00	2,53E-02	6,31E-02	No
ENSG00000264720	MIR3117	miRNA	-4,70E-01	8,94E-02	2,59E-01	No	-8,88E-01	4,14E-02	9,58E-02	No
ENSG00000263542	AL157407.1	miRNA	1,35E-01	2,34E-01	NA	No	2,47E-01	2,60E-01	NA	No
ENSG00000221203	MIR1262	miRNA	6,98E-02	6,46E-01	8,25E-01	No	2,14E-01	5,01E-01	6,49E-01	No
ENSG00000207721	MIR186	miRNA	1,44E-01	5,10E-01	7,36E-01	No	8,15E-01	1,15E-03	4,19E-03	No
ENSG00000264239	AL360297.1	miRNA	-5,37E-02	6,46E-01	NA	No	7,32E-02	7,75E-01	NA	No
ENSG00000266832	AL445464.1	miRNA	-4,21E-02	7,60E-01	NA	No	1,56E-01	4,94E-01	NA	No
ENSG00000223231	AL035706.1	miRNA	4,21E-02	7,67E-01	NA	No	1,02E-01	7,75E-01	NA	No
ENSG00000251899	AC104169.1	miRNA	-1,42E-01	1,77E-01	NA	No	-1,80E-01	5,36E-01	6,79E-01	No
ENSG00000266110	MIR4423	miRNA	2,30E-02	8,45E-01	NA	No	1,60E-02	9,87E-01	NA	No
ENSG00000264380	AL590113.1	miRNA	-1,68E-01	2,72E-01	5,17E-01	No	-1,00E+00	3,20E-02	7,69E-02	No
ENSG00000221222	AL139139.1	miRNA	-1,60E-01	2,51E-01	4,93E-01	No	-1,55E-01	6,09E-01	7,38E-01	No
ENSG00000211575	MIR760	miRNA	-1,60E-02	9,21E-01	NA	No	4,39E-01	2,41E-01	3,84E-01	No
ENSG00000263526	MIR378G	miRNA	-1,32E-01	2,51E-01	NA	No	-2,57E-01	3,34E-01	NA	No
ENSG00000207750	MIR553	miRNA	1,72E-01	2,89E-01	5,35E-01	No	6,80E-01	1,04E-01	2,02E-01	No
ENSG00000216067	AC104457.1	miRNA	7,46E-02	5,66E-01	7,73E-01	No	-1,54E-03	9,95E-01	9,97E-01	No
ENSG00000265536	AL591042.1	miRNA	5,65E-02	6,28E-01	NA	No	1,23E-01	6,13E-01	NA	No
ENSG00000207709	MIR197	miRNA	2,87E-01	2,34E-01	4,73E-01	No	4,28E-01	2,29E-01	3,69E-01	No
ENSG00000264419	MIR548AC	miRNA	1,21E-02	9,17E-01	NA	No	2,55E-01	3,87E-01	5,44E-01	No
ENSG00000215930	MIR942	miRNA	1,86E-02	8,97E-01	NA	No	-2,89E-02	8,90E-01	NA	No
ENSG00000266657	AL583842.3	miRNA	-8,25E-02	6,10E-01	8,03E-01	No	-2,49E-01	4,42E-01	5,96E-01	No
ENSG00000239012	AL583842.1	miRNA	-5,43E-03	9,75E-01	9,90E-01	No	-5,02E-01	1,83E-01	3,13E-01	No
ENSG00000265466	AL583842.2	miRNA	4,79E-02	7,50E-01	8,84E-01	No	-1,39E-02	9,62E-01	9,77E-01	No
ENSG00000265715	MIR3118-1	miRNA	8,75E-02	4,14E-01	NA	No	6,13E-02	9,56E-01	NA	No
ENSG00000265661	MIR3118-2	miRNA	2,00E-01	3,30E-01	5,80E-01	No	-3,24E-01	3,48E-01	5,03E-01	No
ENSG00000266811	MIR3118-3	miRNA	5,66E-02	6,16E-01	8,07E-01	No	-1,06E-01	5,68E-01	NA	No
ENSG00000272302	BX004987.1	miRNA	-2,37E-02	7,70E-01	NA	No	7,03E-02	7,22E-01	NA	No
ENSG00000266739	AL138796.1	miRNA	-9,08E-02	5,97E-01	7,95E-01	No	-1,52E-01	6,64E-01	7,80E-01	No
ENSG00000212044	CR812485.1	miRNA	-4,33E-02	7,60E-01	NA	No	-1,01E-01	8,30E-01	NA	No
ENSG00000263825	AL358813.2	miRNA	-4,33E-02	7,60E-01	NA	No	-1,01E-01	8,30E-01	NA	No
ENSG00000207606	MIR554	miRNA	-6,20E-02	4,86E-01	NA	No	-1,57E-01	4,52E-01	NA	No
ENSG00000252817	AL590431.1	miRNA	2,93E-01	2,25E-01	4,63E-01	No	-4,79E-02	8,85E-01	9,32E-01	No
ENSG00000264349	MIR4258	miRNA	-8,02E-02	4,07E-01	NA	No	-1,78E-01	3,92E-01	NA	No
ENSG00000271748	MIR92B	miRNA	1,20E-02	9,65E-01	NA	No	-2,64E-02	9,21E-01	NA	No
ENSG00000216109	AL713999.1	miRNA	7,78E-02	5,35E-01	NA	No	-1,81E-01	4,50E-01	NA	No
ENSG00000207720	MIR555	miRNA	-1,30E-02	8,48E-01	NA	No	-1,46E-01	5,99E-01	NA	No
ENSG00000265960	AL365181.1	miRNA	2,22E-02	9,72E-01	NA	No	6,13E-02	9,56E-01	NA	No
ENSG00000211581	MIR765	miRNA	1,15E-01	2,52E-01	NA	No	1,25E-01	5,35E-01	NA	No
ENSG00000223009	AL138930.1	miRNA	1,86E-01	1,27E-01	NA	No	-1,26E-01	7,12E-01	NA	No
ENSG00000263548	MIR5187	miRNA	-1,14E-01	3,47E-01	NA	No	-3,45E-01	2,27E-01	NA	No
ENSG00000266144	MIR4654	miRNA	-9,58E-02	4,18E-01	NA	No	-2,76E-01	3,54E-01	NA	No
ENSG00000215952	MIR921	miRNA	2,07E-03	9,91E-01	9,97E-01	No	-2,04E-01	5,02E-01	6,50E-01	No
ENSG00000221545	MIR1255B2	miRNA	1,79E-02	9,11E-01	9,63E-01	No	-3,37E-01	2,08E-01	3,44E-01	No
ENSG00000263390	MIR3119-1	miRNA	-5,37E-02	6,46E-01	NA	No	1,20E-01	6,00E-01	NA	No
ENSG00000215924	BX284613.1	miRNA	-4,46E-02	7,60E-01	NA	No	-1,03E-01	8,30E-01	NA	No
ENSG00000221390	MIR1295A	miRNA	2,33E-01	7,84E-02	NA	No	2,01E-01	3,62E-01	NA	No

ENSG00000207949	MIR214	miRNA	7,85E-02	7,24E-01	8,72E-01	No	1,00E+00	4,50E-03	1,41E-02	No
ENSG00000208024	MIR199A2	miRNA	1,49E-01	4,16E-01	6,59E-01	No	4,51E-01	2,24E-01	3,63E-01	No
ENSG00000266125	AL022400.1	miRNA	-3,11E-02	6,95E-01	NA	No	-1,08E-01	6,32E-01	NA	No
ENSG00000265435	MIR3121	miRNA	1,80E-01	1,56E-01	NA	No	4,15E-01	1,54E-01	NA	No
ENSG00000266825	AL590085.1	miRNA	-1,14E-01	6,07E-01	8,01E-01	No	-6,21E-01	1,17E-01	2,21E-01	No
ENSG00000265042	AL137800.1	miRNA	4,08E-02	7,73E-01	NA	No	3,67E-01	2,38E-01	NA	No
ENSG00000221145	AL136987.1	miRNA	4,80E-02	6,14E-01	NA	No	4,03E-02	8,85E-01	NA	No
ENSG00000221680	MIR1278	miRNA	-1,04E-01	5,51E-01	7,64E-01	No	-3,31E-01	2,27E-01	3,68E-01	No
ENSG00000265986	MIR4735	miRNA	3,14E-01	4,95E-02	NA	No	2,98E-01	2,55E-01	NA	No
ENSG00000207975	MIR181B1	miRNA	3,79E-02	7,33E-01	NA	No	5,38E-01	1,23E-01	2,31E-01	No
ENSG00000207759	MIR181A1	miRNA	-1,06E-02	8,86E-01	NA	No	4,97E-01	1,40E-01	NA	No
ENSG00000264802	MIR5191	miRNA	8,24E-02	6,25E-01	8,13E-01	No	-2,71E-01	2,89E-01	NA	No
ENSG00000253044	AL691452.1	miRNA	-5,58E-02	6,46E-01	NA	No	-6,51E-02	8,00E-01	NA	No
ENSG00000252240	AC096643.1	miRNA	4,62E-02	6,16E-01	NA	No	9,18E-02	8,64E-01	NA	No
ENSG00000207590	MIR215	miRNA	1,04E-02	9,59E-01	9,83E-01	No	3,79E-01	3,10E-01	4,63E-01	No
ENSG00000207624	MIR194-1	miRNA	-1,91E-02	8,88E-01	NA	No	1,33E+00	1,92E-02	5,01E-02	No
ENSG00000221406	MIR320B2	miRNA	7,39E-02	6,03E-01	NA	No	-1,42E-01	5,06E-01	NA	No
ENSG00000266618	MIR4742	miRNA	1,72E-01	3,23E-01	NA	No	3,80E-01	2,57E-01	4,03E-01	No
ENSG00000265216	AL592310.1	miRNA	4,46E-02	7,72E-01	8,96E-01	No	-1,51E-01	6,18E-01	7,45E-01	No
ENSG00000264483	MIR5008	miRNA	1,84E-01	3,66E-01	6,13E-01	No	-1,01E+00	3,67E-02	8,65E-02	No
ENSG00000252131	AL844165.1	miRNA	-4,11E-02	6,54E-01	NA	No	-2,09E-02	9,28E-01	NA	No
ENSG00000263439	MIR4753	miRNA	5,45E-02	5,41E-01	NA	No	1,73E-01	3,95E-01	NA	No
ENSG00000222831	MIR1537	miRNA	7,24E-01	3,12E-02	1,25E-01	No	7,71E-01	8,38E-02	1,70E-01	No
ENSG00000266262	MIR4428	miRNA	-3,40E-01	1,75E-01	4,00E-01	No	-2,27E-01	4,97E-01	6,46E-01	No
ENSG00000265201	MIR4677	miRNA	1,56E-01	4,16E-01	6,59E-01	No	5,35E-01	1,74E-01	3,01E-01	No
ENSG00000238952	AC099757.1	miRNA	5,40E-02	6,25E-01	NA	No	1,08E-01	6,92E-01	NA	No
ENSG00000265583	AC011994.1	miRNA	3,31E-02	9,36E-01	NA	No	1,02E-01	7,75E-01	NA	No
ENSG00000265056	MIR548S	miRNA	-2,23E-01	2,20E-01	4,58E-01	No	-1,25E-01	7,10E-01	8,15E-01	No
ENSG00000264370	MIR3125	miRNA	2,80E-01	2,29E-01	4,67E-01	No	1,53E-01	6,63E-01	7,80E-01	No
ENSG00000221445	MIR1301	miRNA	-5,50E-02	5,51E-01	NA	No	2,90E-02	8,97E-01	NA	No
ENSG00000265321	MIR4263	miRNA	4,87E-02	7,94E-01	9,08E-01	No	1,34E-01	6,97E-01	8,06E-01	No
ENSG00000221326	AL121652.1	miRNA	-3,66E-01	1,12E-01	3,01E-01	No	-6,15E-01	1,25E-01	2,33E-01	No
ENSG00000263802	AL121655.1	miRNA	-5,02E-02	5,98E-01	NA	No	-5,07E-02	8,34E-01	NA	No
ENSG00000207653	MIR558	miRNA	-1,02E-01	5,87E-01	7,88E-01	No	-5,28E-01	1,81E-01	3,10E-01	No
ENSG00000265057	MIR4765	miRNA	-2,53E-02	7,77E-01	NA	No	7,71E-02	7,37E-01	8,33E-01	No
ENSG00000264267	AC069303.1	miRNA	-4,46E-02	7,60E-01	NA	No	-1,03E-01	8,30E-01	NA	No
ENSG00000221372	AC010739.1	miRNA	3,69E-03	9,61E-01	NA	No	-1,37E-01	5,57E-01	NA	No
ENSG00000252896	AC067957.1	miRNA	-4,46E-02	6,32E-01	NA	No	-6,14E-02	7,59E-01	NA	No
ENSG00000215968	AC007560.2	miRNA	4,49E-02	6,23E-01	NA	No	5,29E-01	9,41E-02	1,86E-01	No
ENSG00000216011	AC007682.2	miRNA	-1,03E-01	3,89E-01	NA	No	3,43E-02	8,86E-01	9,33E-01	No
ENSG00000272385	AC007682.3	miRNA	-6,20E-02	5,30E-01	NA	No	6,86E-01	7,87E-02	1,62E-01	No
ENSG00000264975	MIR4431	miRNA	3,15E-02	9,36E-01	NA	No	1,05E-01	7,75E-01	NA	No
ENSG00000264740	AC008064.1	miRNA	2,22E-02	9,72E-01	NA	No	1,14E-01	6,26E-01	NA	No
ENSG00000265452	MIR3682	miRNA	-6,37E-02	7,58E-01	8,88E-01	No	-2,44E-01	4,87E-01	6,37E-01	No
ENSG00000251738	AC073215.1	miRNA	8,48E-02	6,90E-01	8,51E-01	No	5,46E-01	1,37E-01	2,50E-01	No
ENSG00000266078	MIR4432	miRNA	1,60E-02	8,89E-01	NA	No	-1,93E-02	8,84E-01	NA	No
ENSG00000221085	AC096664.3	miRNA	-3,65E-03	9,00E-01	NA	No	3,83E-02	9,05E-01	NA	No
ENSG00000216115	AC017083.1	miRNA	3,36E-01	9,33E-02	2,66E-01	No	6,55E-02	8,13E-01	8,85E-01	No
ENSG00000221443	AC017083.3	miRNA	5,94E-02	5,66E-01	NA	No	8,55E-02	8,64E-01	NA	No
ENSG00000266649	MIR3126	miRNA	-5,92E-01	1,30E-02	NA	No	-5,03E-01	1,75E-01	3,02E-01	No
ENSG00000264051	AC007881.1	miRNA	3,69E-02	7,88E-01	9,05E-01	No	-1,35E-01	6,07E-01	7,36E-01	No
ENSG00000221087	AC096546.1	miRNA	2,80E-02	7,01E-01	NA	No	2,65E-02	8,80E-01	NA	No
ENSG00000263909	MIR5000	miRNA	1,37E-01	2,38E-01	NA	No	4,63E-02	8,54E-01	NA	No
ENSG00000265420	MIR4779	miRNA	-3,46E-02	6,27E-01	NA	No	-2,58E-03	9,89E-01	NA	No
ENSG00000265507	MIR4435-1	miRNA	-1,32E-01	3,88E-01	6,34E-01	No	-1,06E+00	1,98E-02	5,14E-02	No
ENSG00000265003	MIR4780	miRNA	-1,07E-01	5,73E-01	7,78E-01	No	-8,10E-01	7,17E-02	1,50E-01	No
ENSG00000221686	AC096579.1	miRNA	2,66E-01	1,80E-02	8,53E-02	No	6,34E-02	9,56E-01	NA	No
ENSG00000264764	MIR4772	miRNA	1,67E-01	1,79E-01	NA	No	1,27E-01	5,49E-01	NA	No
ENSG00000263685	AC010884.2	miRNA	-5,45E-02	6,46E-01	NA	No	-1,28E-01	7,12E-01	NA	No
ENSG00000264934	MIR4265	miRNA	1,98E-01	3,92E-01	6,37E-01	No	-1,27E+00	1,59E-02	4,26E-02	No
ENSG00000265965	MIR4266	miRNA	-6,95E-02	6,46E-01	8,25E-01	No	-1,71E-01	6,02E-01	7,32E-01	No
ENSG00000264979	MIR4436B2	miRNA	3,86E-02	8,45E-01	NA	No	6,23E-02	9,56E-01	NA	No

ENSG00000266063	MIR4771-2	miRNA	-5,37E-02	6,46E-01	NA	No	6,85E-02	7,95E-01	NA	No
ENSG00000221055	MIR1302-3	miRNA	-9,92E-02	2,48E-01	NA	No	-2,27E-01	2,50E-01	NA	No
ENSG00000265429	MIR4782	miRNA	-4,35E-02	7,12E-01	NA	No	4,64E-01	1,61E-01	2,83E-01	No
ENSG00000215984	AC110769.1	miRNA	1,05E-01	2,97E-01	NA	No	8,77E-02	8,64E-01	NA	No
ENSG00000264075	MIR4783	miRNA	5,23E-02	6,48E-01	NA	No	6,19E-02	9,56E-01	NA	No
ENSG00000265280	AC018804.1	miRNA	-1,44E-01	5,14E-01	7,39E-01	No	-7,15E-01	8,05E-02	1,65E-01	No
ENSG00000263783	MIR5590	miRNA	-6,29E-02	5,05E-01	NA	No	-1,24E-01	6,14E-01	NA	No
ENSG00000221169	AC013444.2	miRNA	7,40E-02	5,77E-01	NA	No	-9,35E-02	6,18E-01	NA	No
ENSG00000265049	AC009480.1	miRNA	5,90E-02	6,66E-01	8,36E-01	No	4,39E-01	2,14E-01	3,51E-01	No
ENSG00000263848	AC108057.1	miRNA	4,27E-02	7,29E-01	NA	No	1,14E-01	6,19E-01	NA	No
ENSG00000215934	AC013731.1	miRNA	-5,73E-02	6,46E-01	NA	No	-1,27E-02	9,79E-01	NA	No
ENSG00000221590	AC009299.1	miRNA	-2,55E-01	6,24E-02	2,02E-01	No	-6,34E-02	8,48E-01	9,08E-01	No
ENSG00000221234	AC009495.1	miRNA	-5,47E-02	6,46E-01	NA	No	-1,30E-01	7,12E-01	NA	No
ENSG00000265694	MIR4774	miRNA	8,04E-01	2,52E-02	1,08E-01	No	8,01E-01	6,46E-02	1,38E-01	No
ENSG00000215973	MIR933	miRNA	3,65E-02	8,66E-01	9,43E-01	No	-8,63E-01	6,50E-02	1,39E-01	No
ENSG00000265129	AC064837.1	miRNA	-3,31E-01	1,69E-01	3,91E-01	No	-4,85E-01	2,11E-01	3,48E-01	No
ENSG00000207951	MIR561	miRNA	-7,71E-02	5,50E-01	NA	No	1,74E-01	5,44E-01	6,86E-01	No
ENSG00000221502	MIR1245A	miRNA	8,29E-02	6,59E-01	8,33E-01	No	9,65E-01	3,59E-02	8,48E-02	No
ENSG00000264725	MIR3129	miRNA	4,03E-02	8,45E-01	NA	No	5,73E-01	8,14E-02	NA	No
ENSG00000238641	AC008122.1	miRNA	8,83E-02	2,79E-01	NA	No	1,91E-01	3,73E-01	NA	No
ENSG00000264798	AC105381.1	miRNA	-2,53E-02	8,82E-01	9,51E-01	No	-3,46E-01	3,36E-01	4,92E-01	No
ENSG00000263468	MIR3130-1	miRNA	3,04E-03	9,41E-01	NA	No	2,30E-02	9,87E-01	NA	No
ENSG00000253008	MIR2355	miRNA	-8,61E-02	6,95E-01	8,55E-01	No	-3,81E-01	2,18E-01	3,56E-01	No
ENSG00000265454	AC012362.1	miRNA	3,17E-02	9,36E-01	NA	No	6,34E-02	9,56E-01	NA	No
ENSG00000221782	MIR548F2	miRNA	-8,48E-02	6,29E-01	8,16E-01	No	9,50E-01	3,84E-02	8,97E-02	No
ENSG00000265252	MIR3132	miRNA	4,78E-03	9,70E-01	NA	No	-3,09E-01	2,01E-01	NA	No
ENSG00000266518	MIR4268	miRNA	1,59E-02	8,55E-01	NA	No	-2,28E-02	8,90E-01	NA	No
ENSG00000221199	AC114765.3	miRNA	-1,44E-03	9,41E-01	NA	No	3,48E-02	8,87E-01	NA	No
ENSG00000222096	AC019051.1	miRNA	3,63E-02	6,44E-01	NA	No	1,36E-02	9,38E-01	NA	No
ENSG00000221432	AC079834.2	miRNA	-3,66E-02	8,24E-01	9,23E-01	No	-3,77E-01	2,91E-01	4,41E-01	No
ENSG00000265621	AC013476.1	miRNA	-5,10E-02	6,13E-01	NA	No	-8,13E-02	7,41E-01	NA	No
ENSG00000263828	MIR4439	miRNA	2,68E-01	1,24E-02	6,55E-02	No	1,63E-01	4,83E-01	NA	No
ENSG00000263363	MIR5702	miRNA	3,52E-02	6,67E-01	NA	No	4,09E-03	9,87E-01	NA	No
ENSG00000221608	AC079235.1	miRNA	1,73E-01	3,52E-01	6,01E-01	No	4,88E-01	1,97E-01	3,31E-01	No
ENSG00000263774	AC017104.1	miRNA	3,97E-02	8,45E-01	NA	No	9,18E-02	8,64E-01	NA	No
ENSG00000207626	MIR562	miRNA	-3,89E-02	7,59E-01	8,89E-01	No	3,51E-02	9,09E-01	9,46E-01	No
ENSG00000221704	AC012305.1	miRNA	-4,42E-02	7,60E-01	NA	No	-1,02E-01	8,30E-01	NA	No
ENSG00000266109	MIR4440	miRNA	-9,79E-03	9,32E-01	NA	No	-1,12E-01	6,55E-01	NA	No
ENSG00000264518	AC017028.4	miRNA	1,59E-02	9,60E-01	NA	No	-9,51E-02	8,30E-01	NA	No
ENSG00000265979	AC017028.9	miRNA	5,29E-02	6,73E-01	NA	No	-1,03E-01	8,30E-01	NA	No
ENSG00000264730	AC017028.5	miRNA	1,08E-01	4,19E-01	NA	No	-1,49E-01	6,00E-01	NA	No
ENSG00000265891	AC017028.8	miRNA	4,39E-02	7,82E-01	9,01E-01	No	1,24E-01	6,93E-01	8,03E-01	No
ENSG00000264810	MIR4441	miRNA	1,12E-01	3,36E-01	NA	No	-2,34E-02	8,90E-01	NA	No
ENSG00000265416	AC017028.7	miRNA	5,49E-02	7,21E-01	8,70E-01	No	-3,37E-02	9,12E-01	9,48E-01	No
ENSG00000265215	MIR4269	miRNA	1,69E-01	4,08E-01	6,52E-01	No	1,74E-01	6,21E-01	7,48E-01	No
ENSG00000264292	MIR2467	miRNA	5,55E-02	7,50E-01	8,84E-01	No	-3,30E-01	3,68E-01	5,24E-01	No
ENSG00000264279	MIR4786	miRNA	-2,31E-01	2,57E-01	5,00E-01	No	-9,18E-01	5,37E-02	1,19E-01	No
ENSG00000221412	AC124861.2	miRNA	-1,90E-02	8,19E-01	NA	No	8,08E-03	9,14E-01	NA	No
ENSG00000266621	AC104841.1	miRNA	9,06E-02	4,99E-01	NA	No	2,81E-01	3,94E-01	5,51E-01	No
ENSG00000223036	AC024158.1	miRNA	1,71E-02	8,87E-01	NA	No	3,82E-02	8,76E-01	NA	No
ENSG00000266778	hsa-mir-548ba	miRNA	-5,47E-02	6,46E-01	NA	No	-1,30E-01	7,12E-01	NA	No
ENSG00000266001	AC018506.1	miRNA	-3,58E-02	7,67E-01	NA	No	-5,96E-02	8,23E-01	NA	No
ENSG00000221424	AC090954.1	miRNA	-4,21E-02	7,60E-01	NA	No	-9,72E-02	8,30E-01	NA	No
ENSG00000264354	MIR3134	miRNA	1,38E-01	4,70E-01	7,04E-01	No	-2,46E-02	9,40E-01	9,64E-01	No
ENSG00000207815	MIR563	miRNA	8,78E-03	8,99E-01	NA	No	3,49E-01	2,05E-01	NA	No
ENSG00000266745	MIR3135A	miRNA	1,60E-01	3,70E-01	6,16E-01	No	-2,69E-01	3,94E-01	5,51E-01	No
ENSG00000252306	AC104183.1	miRNA	-6,20E-02	5,27E-01	NA	No	-7,66E-02	7,19E-01	NA	No
ENSG00000221384	AC023798.1	miRNA	-3,49E-02	8,03E-01	9,12E-01	No	-6,78E-01	6,35E-02	1,36E-01	No
ENSG00000221506	AC092038.1	miRNA	2,29E-01	2,79E-01	5,25E-01	No	6,89E-01	1,05E-01	2,04E-01	No
ENSG00000265645	AC092038.2	miRNA	1,10E-01	5,76E-01	7,81E-01	No	-2,39E-01	4,86E-01	6,35E-01	No
ENSG00000265028	MIR4792	miRNA	3,15E-02	9,36E-01	NA	No	8,51E-02	8,64E-01	NA	No
ENSG00000264219	MIR4442	miRNA	1,78E-01	4,04E-01	6,48E-01	No	1,34E+00	4,01E-03	1,28E-02	No

ENSG00000263385	AC092798.1	miRNA	2,55E-02	8,33E-01	NA	No	5,58E-02	7,64E-01	NA	No
ENSG00000221573	AC099535.1	miRNA	-2,49E-02	7,78E-01	NA	No	-1,51E-01	5,67E-01	NA	No
ENSG00000238470	AC097361.1	miRNA	3,41E-02	9,36E-01	NA	No	8,51E-02	8,64E-01	NA	No
ENSG00000216169	AC098650.1	miRNA	1,88E-01	3,98E-01	6,43E-01	No	-4,22E-01	2,02E-01	3,37E-01	No
ENSG00000199075	MIR26A1	miRNA	1,30E-01	2,33E-01	NA	No	-1,04E-01	6,43E-01	NA	No
ENSG00000264724	AC099332.1	miRNA	2,34E-02	8,43E-01	NA	No	4,17E-02	8,85E-01	NA	No
ENSG00000221337	AC099537.1	miRNA	-4,46E-02	7,60E-01	NA	No	2,97E-03	9,87E-01	NA	No
ENSG00000251927	AC099539.1	miRNA	9,20E-02	3,81E-01	NA	No	1,92E-01	3,62E-01	NA	No
ENSG00000264306	AC104304.2	miRNA	-7,47E-03	9,58E-01	9,83E-01	No	-4,67E-01	1,53E-01	2,72E-01	No
ENSG00000266457	AC026318.1	miRNA	-2,50E-02	8,49E-01	NA	No	3,68E-02	8,91E-01	NA	No
ENSG00000199032	MIR425	miRNA	-1,54E-02	8,75E-01	NA	No	-8,88E-03	9,74E-01	NA	No
ENSG00000207605	MIR191	miRNA	2,34E-02	9,72E-01	NA	No	6,13E-02	9,56E-01	NA	No
ENSG00000207922	MIR566	miRNA	1,88E-02	8,67E-01	NA	No	-9,72E-02	8,30E-01	NA	No
ENSG00000199150	MIRLET7G	miRNA	7,26E-02	4,85E-01	NA	No	3,58E-01	2,32E-01	3,74E-01	No
ENSG00000264716	AC106827.2	miRNA	-1,37E-01	4,58E-01	6,92E-01	No	-2,36E-02	9,44E-01	9,67E-01	No
ENSG00000266400	AC113171.1	miRNA	3,39E-02	9,36E-01	NA	No	8,58E-02	8,64E-01	NA	No
ENSG00000265355	MIR3136	miRNA	8,94E-02	6,00E-01	7,97E-01	No	-2,05E-01	4,65E-01	NA	No
ENSG00000265722	AC099326.1	miRNA	1,17E-01	5,99E-01	7,96E-01	No	-1,27E-01	7,15E-01	8,18E-01	No
ENSG00000263689	AC128653.1	miRNA	2,49E-02	9,72E-01	NA	No	6,67E-02	9,56E-01	NA	No
ENSG00000266780	MIR4444-1	miRNA	-4,21E-02	7,60E-01	NA	No	-1,45E-02	9,21E-01	NA	No
ENSG00000264084	MIR5688	miRNA	3,17E-02	9,36E-01	NA	No	6,67E-02	9,56E-01	NA	No
ENSG00000264897	MIR3921	miRNA	-4,15E-02	7,60E-01	NA	No	-1,11E-02	9,21E-01	NA	No
ENSG00000207940	MIR567	miRNA	-2,56E-01	2,56E-02	1,09E-01	No	-5,85E-01	3,83E-02	8,97E-02	No
ENSG00000265253	MIR4446	miRNA	8,03E-02	3,70E-01	NA	No	-1,36E-01	7,12E-01	NA	No
ENSG00000207770	MIR568	miRNA	-5,29E-02	8,02E-01	9,12E-01	No	4,43E-01	1,07E-01	2,07E-01	No
ENSG00000264623	MIR4796	miRNA	1,72E-01	3,96E-01	6,41E-01	No	1,03E+00	3,20E-02	7,69E-02	No
ENSG00000263678	AC072031.1	miRNA	2,75E-01	4,97E-02	NA	No	1,30E-01	6,50E-01	NA	No
ENSG00000221211	AC078794.1	miRNA	1,27E-02	9,23E-01	9,68E-01	No	-1,31E-01	5,88E-01	7,20E-01	No
ENSG00000238512	AC117401.1	miRNA	-6,23E-02	7,53E-01	8,85E-01	No	6,22E-01	1,09E-01	2,09E-01	No
ENSG00000266383	MIR5002	miRNA	-3,16E-01	1,96E-01	4,26E-01	No	-7,24E-01	7,32E-02	1,53E-01	No
ENSG00000263775	AC080008.1	miRNA	-5,73E-02	6,46E-01	NA	No	-1,36E-01	7,12E-01	NA	No
ENSG00000265981	MIR544B	miRNA	3,01E-02	7,75E-01	NA	No	-1,01E-01	6,69E-01	NA	No
ENSG00000264986	MIR5092	miRNA	-5,45E-02	6,46E-01	NA	No	-1,28E-01	7,12E-01	NA	No
ENSG00000221681	AC092902.1	miRNA	5,20E-03	9,54E-01	NA	No	2,28E-02	9,19E-01	NA	No
ENSG00000221413	AC023593.1	miRNA	-5,45E-02	6,46E-01	NA	No	-1,28E-01	7,12E-01	NA	No
ENSG00000221067	MIR1280	miRNA	-4,85E-02	7,55E-01	8,86E-01	No	-6,35E-01	1,17E-01	2,22E-01	No
ENSG00000212073	AC121332.1	miRNA	-6,34E-02	4,94E-01	NA	No	-3,76E-02	8,56E-01	NA	No
ENSG00000265235	AC083908.1	miRNA	4,26E-02	6,79E-01	NA	No	-3,03E-02	9,21E-01	NA	No
ENSG00000216184	AC048346.1	miRNA	-2,64E-02	7,31E-01	NA	No	-1,26E-01	7,12E-01	NA	No
ENSG00000265391	AC112504.1	miRNA	-6,05E-02	5,46E-01	NA	No	-1,45E-01	6,08E-01	NA	No
ENSG00000211483	AC018450.1	miRNA	-5,58E-02	6,46E-01	NA	No	-1,33E-01	7,12E-01	NA	No
ENSG00000264330	MIR5186	miRNA	6,29E-02	5,16E-01	NA	No	1,12E-01	6,90E-01	NA	No
ENSG00000264146	AC021654.1	miRNA	-4,53E-02	7,51E-01	8,84E-01	No	-5,00E-01	1,04E-01	NA	No
ENSG00000207779	MIR15B	miRNA	2,23E-02	9,72E-01	NA	No	1,37E-01	5,95E-01	NA	No
ENSG00000198987	MIR16-2	miRNA	2,23E-02	9,72E-01	NA	No	9,86E-02	7,75E-01	NA	No
ENSG00000207717	MIR551B	miRNA	2,34E-02	9,72E-01	NA	No	9,18E-02	8,64E-01	NA	No
ENSG00000207963	MIR569	miRNA	-3,95E-02	8,17E-01	9,19E-01	No	4,79E-02	8,89E-01	9,34E-01	No
ENSG00000264711	AC007919.1	miRNA	7,83E-02	4,86E-01	NA	No	4,21E-02	8,91E-01	NA	No
ENSG00000264974	MIR4789	miRNA	5,08E-02	6,04E-01	NA	No	1,95E-02	9,87E-01	NA	No
ENSG00000265470	MIR548AQ	miRNA	4,99E-02	7,04E-01	8,60E-01	No	-1,61E-01	4,82E-01	NA	No
ENSG00000264114	AC063932.1	miRNA	-1,91E-01	4,01E-01	6,46E-01	No	-6,74E-01	8,89E-02	1,78E-01	No
ENSG00000207651	MIR28	miRNA	1,67E-01	4,14E-01	6,58E-01	No	1,51E-02	9,66E-01	9,80E-01	No
ENSG00000216058	MIR944	miRNA	-5,37E-02	6,46E-01	NA	No	-2,34E-02	8,90E-01	NA	No
ENSG00000221718	AC046143.1	miRNA	-9,32E-02	3,43E-01	NA	No	-1,08E-01	6,28E-01	NA	No
ENSG00000265333	MIR3137	miRNA	-3,21E-03	9,00E-01	NA	No	-1,26E-01	7,12E-01	NA	No
ENSG00000207650	MIR570	miRNA	-7,77E-01	8,30E-03	4,94E-02	No	-2,31E-01	4,40E-01	5,94E-01	No
ENSG00000265850	MIR4797	miRNA	2,84E-03	9,72E-01	NA	No	1,15E-01	6,25E-01	7,50E-01	No
ENSG00000207643	Z95704.1	miRNA	-4,09E-01	9,50E-02	2,69E-01	No	-1,02E+00	1,35E-02	3,71E-02	No
ENSG00000211553	AC118278.1	miRNA	-1,89E-01	4,08E-01	6,51E-01	No	-2,38E-01	4,60E-01	6,13E-01	No
ENSG00000207642	MIR571	miRNA	-9,98E-02	5,76E-01	7,80E-01	No	-1,93E-01	5,77E-01	7,12E-01	No
ENSG00000211482	AC092574.1	miRNA	1,35E-02	9,65E-01	NA	No	2,13E-01	3,25E-01	NA	No
ENSG00000252150	AC092574.2	miRNA	1,05E-01	6,28E-01	8,15E-01	No	-7,24E-01	9,16E-02	1,83E-01	No

ENSG00000264106	AC116562.2	miRNA	6,08E-02	5,48E-01	NA	No	9,18E-02	8,64E-01	NA	No
ENSG00000221301	AC116562.1	miRNA	-6,02E-02	5,50E-01	NA	No	-1,44E-01	6,13E-01	NA	No
ENSG00000265953	AC092463.1	miRNA	-4,23E-02	7,60E-01	NA	No	-9,82E-02	8,30E-01	NA	No
ENSG00000265550	AC097382.1	miRNA	2,22E-02	9,72E-01	NA	No	1,03E-01	7,75E-01	NA	No
ENSG00000207807	MIR95	miRNA	3,41E-02	9,36E-01	NA	No	8,55E-02	8,64E-01	NA	No
ENSG00000266246	AC116655.1	miRNA	-2,73E-02	7,31E-01	NA	No	-1,26E-01	7,12E-01	NA	No
ENSG00000265901	AC097493.1	miRNA	1,68E-02	7,83E-01	NA	No	-7,67E-03	9,79E-01	NA	No
ENSG00000264931	MIR3138	miRNA	-1,49E-02	9,35E-01	9,73E-01	No	-7,12E-01	9,74E-02	1,92E-01	No
ENSG00000207716	MIR572	miRNA	4,16E-02	7,73E-01	NA	No	1,12E-01	6,53E-01	NA	No
ENSG00000266669	AC097452.1	miRNA	-5,58E-02	6,46E-01	NA	No	-1,33E-01	7,12E-01	NA	No
ENSG00000266240	MIR5091	miRNA	1,87E-01	1,57E-01	NA	No	2,96E-01	2,74E-01	NA	No
ENSG00000207732	MIR218-1	miRNA	2,10E-01	1,21E-01	NA	No	1,80E-01	4,39E-01	NA	No
ENSG00000265001	AC092846.2	miRNA	-4,23E-02	7,60E-01	NA	No	-9,82E-02	8,30E-01	NA	No
ENSG00000207697	MIR573	miRNA	3,97E-01	1,27E-01	3,25E-01	No	8,11E-01	3,96E-02	9,20E-02	No
ENSG00000264496	AC108218.1	miRNA	1,26E-01	5,65E-01	7,73E-01	No	9,56E-02	7,83E-01	8,65E-01	No
ENSG00000265627	AC093807.1	miRNA	4,04E-02	7,81E-01	NA	No	8,51E-02	8,64E-01	NA	No
ENSG00000221495	AC098680.1	miRNA	-5,45E-02	6,46E-01	NA	No	-1,28E-01	7,12E-01	NA	No
ENSG00000207944	MIR574	miRNA	9,52E-02	5,16E-01	7,40E-01	No	-1,23E-02	9,65E-01	9,79E-01	No
ENSG00000221122	AC098869.1	miRNA	4,13E-02	6,07E-01	NA	No	3,49E-02	8,87E-01	NA	No
ENSG00000266058	AC084010.1	miRNA	8,90E-02	6,04E-01	8,00E-01	No	-8,35E-02	7,77E-01	8,61E-01	No
ENSG00000221747	AC118282.2	miRNA	-5,37E-02	6,46E-01	NA	No	-1,26E-01	7,12E-01	NA	No
ENSG00000221415	AC118282.1	miRNA	9,48E-02	3,31E-01	NA	No	-6,19E-02	8,00E-01	NA	No
ENSG00000266337	AC118282.4	miRNA	8,07E-02	3,47E-01	NA	No	2,39E-01	3,44E-01	NA	No
ENSG00000221075	AC119751.1	miRNA	-3,24E-02	7,93E-01	NA	No	1,48E-01	6,16E-01	NA	No
ENSG00000221334	AC119751.3	miRNA	-2,08E-01	1,43E-01	NA	No	-3,10E-01	3,16E-01	NA	No
ENSG00000221082	AC119751.2	miRNA	4,19E-03	9,82E-01	NA	No	1,42E-03	9,81E-01	NA	No
ENSG00000266656	AC104066.1	miRNA	3,17E-02	9,36E-01	NA	No	1,83E-01	4,05E-01	NA	No
ENSG00000222954	AC110611.1	miRNA	-7,28E-02	3,63E-01	NA	No	5,15E-02	8,54E-01	9,11E-01	No
ENSG00000265085	AC110810.1	miRNA	9,81E-02	4,68E-01	NA	No	-5,41E-02	7,82E-01	NA	No
ENSG00000264696	AC108078.1	miRNA	-1,58E-01	1,18E-01	NA	No	-3,81E-02	8,90E-01	9,34E-01	No
ENSG00000265931	AC112719.1	miRNA	2,40E-02	9,72E-01	NA	No	8,64E-02	8,64E-01	NA	No
ENSG00000221118	AC112249.1	miRNA	4,04E-02	7,81E-01	NA	No	1,04E-01	7,75E-01	NA	No
ENSG00000263445	MIR4450	miRNA	5,09E-02	6,67E-01	NA	No	1,38E-01	5,92E-01	NA	No
ENSG00000265314	MIR548AH	miRNA	3,91E-02	8,45E-01	NA	No	8,27E-01	3,85E-02	NA	No
ENSG00000221183	AC093897.1	miRNA	-1,22E-01	1,42E-01	NA	No	-2,78E-01	1,92E-01	NA	No
ENSG00000266270	MIR5096	miRNA	-2,80E-02	8,27E-01	NA	No	3,71E-02	8,94E-01	9,37E-01	No
ENSG00000207746	MIR575	miRNA	7,19E-02	4,25E-01	NA	No	2,69E-01	2,73E-01	NA	No
ENSG00000266421	MIR4451	miRNA	1,24E-01	5,82E-01	7,85E-01	No	3,61E-01	2,63E-01	4,10E-01	No
ENSG00000265774	AC098870.1	miRNA	2,98E-02	8,67E-01	9,44E-01	No	6,83E-02	8,39E-01	9,02E-01	No
ENSG00000266515	MIR4452	miRNA	3,32E-02	9,36E-01	NA	No	6,67E-02	9,56E-01	NA	No
ENSG00000271995	AC034154.1	miRNA	-4,42E-02	7,60E-01	NA	No	1,67E-02	9,87E-01	NA	No
ENSG00000265213	MIR3684	miRNA	-2,45E-01	1,81E-01	4,07E-01	No	-4,88E-01	1,90E-01	3,22E-01	No
ENSG00000221265	MIR1255A	miRNA	-1,23E-01	5,29E-01	7,49E-01	No	6,77E-01	1,00E-01	1,96E-01	No
ENSG00000238553	AF213884.3	miRNA	1,25E-01	2,90E-01	5,37E-01	No	2,47E-01	4,14E-01	5,71E-01	No
ENSG00000266046	AC084209.1	miRNA	2,49E-02	8,46E-01	NA	No	-9,51E-02	8,30E-01	NA	No
ENSG00000207988	MIR576	miRNA	1,16E-01	5,95E-01	7,93E-01	No	4,40E-01	2,18E-01	3,56E-01	No
ENSG00000199169	MIR367	miRNA	3,85E-02	8,45E-01	NA	No	8,83E-02	8,64E-01	NA	No
ENSG00000199145	MIR302D	miRNA	1,49E-02	8,96E-01	NA	No	9,52E-01	5,48E-02	1,21E-01	No
ENSG00000207927	MIR302A	miRNA	3,49E-02	8,01E-01	9,12E-01	No	6,89E-01	1,09E-01	2,09E-01	No
ENSG00000199102	MIR302C	miRNA	1,20E-01	4,77E-01	7,09E-01	No	7,51E-01	8,89E-02	1,78E-01	No
ENSG00000264927	AC093816.1	miRNA	-9,58E-02	2,06E-01	NA	No	-1,08E-01	6,55E-01	7,74E-01	No
ENSG00000216154	AC109357.1	miRNA	-5,69E-02	6,46E-01	NA	No	-1,35E-01	7,12E-01	NA	No
ENSG00000266277	AC096763.1	miRNA	-5,45E-02	6,46E-01	NA	No	7,86E-02	7,34E-01	NA	No
ENSG00000222526	AC060835.1	miRNA	-6,45E-02	3,75E-01	NA	No	-1,63E-01	3,75E-01	NA	No
ENSG00000238971	AC093766.1	miRNA	-6,83E-02	3,47E-01	NA	No	-1,67E-01	3,99E-01	NA	No
ENSG00000264953	AC093602.1	miRNA	-7,24E-02	3,92E-01	NA	No	-1,73E-01	4,02E-01	NA	No
ENSG00000265623	MIR3139	miRNA	2,08E-01	3,53E-01	6,01E-01	No	-1,19E-01	7,33E-01	8,31E-01	No
ENSG00000221657	AC097455.1	miRNA	-1,81E-01	1,14E-01	NA	No	-2,25E-01	3,92E-01	NA	No
ENSG00000264678	MIR3140	miRNA	7,48E-01	1,27E-02	6,66E-02	No	-1,25E-01	6,85E-01	7,97E-01	No
ENSG00000252375	AC099339.1	miRNA	4,35E-02	7,20E-01	NA	No	2,73E-01	2,55E-01	NA	No
ENSG00000266377	AC095064.1	miRNA	-3,80E-02	7,74E-01	NA	No	2,19E-01	4,97E-01	6,46E-01	No
ENSG00000264105	MIR3688-1	miRNA	2,76E-02	8,10E-01	NA	No	2,99E-01	3,26E-01	NA	No

ENSG00000216089	AC105316.1	miRNA	-3,90E-02	8,48E-01	9,34E-01	No	-2,24E-01	5,21E-01	6,66E-01	No
ENSG00000264535	AC093788.1	miRNA	1,57E-01	3,61E-01	6,08E-01	No	6,60E-02	8,43E-01	9,05E-01	No
ENSG00000207559	MIR578	miRNA	-3,48E-02	6,62E-01	NA	No	-6,82E-02	7,29E-01	NA	No
ENSG00000221296	MIR548T	miRNA	4,58E-02	6,13E-01	NA	No	-1,15E-01	6,53E-01	NA	No
ENSG00000221227	MIR1305	miRNA	-5,08E-02	5,75E-01	NA	No	2,31E-02	9,08E-01	NA	No
ENSG00000266698	MIR3945	miRNA	-1,99E-02	8,19E-01	NA	No	-1,35E-01	7,12E-01	NA	No
ENSG00000252382	AC108865.1	miRNA	2,22E-02	9,72E-01	NA	No	6,13E-02	9,56E-01	NA	No
ENSG00000263834	MIR4635	miRNA	-9,86E-02	5,30E-01	7,50E-01	No	-5,79E-01	9,15E-02	1,83E-01	No
ENSG00000266415	MIR4636	miRNA	-4,23E-02	7,16E-01	NA	No	-1,29E-01	5,60E-01	NA	No
ENSG00000264792	MIR4637	miRNA	-5,44E-01	5,33E-02	1,82E-01	No	-2,24E-01	5,30E-01	6,73E-01	No
ENSG00000216077	MIR887	miRNA	1,96E-01	2,52E-01	4,94E-01	No	-1,69E-01	4,88E-01	NA	No
ENSG00000265561	AC025458.1	miRNA	1,75E-01	3,67E-01	6,14E-01	No	8,60E-02	7,96E-01	8,74E-01	No
ENSG00000266243	MIR4279	miRNA	-3,42E-02	7,31E-01	NA	No	-7,54E-02	8,00E-01	NA	No
ENSG00000207956	MIR579	miRNA	-9,81E-02	4,63E-01	NA	No	-6,48E-02	8,35E-01	9,00E-01	No
ENSG00000272174	AC026703.2	miRNA	-4,88E-02	6,98E-01	NA	No	-3,84E-01	1,77E-01	NA	No
ENSG00000207756	MIR580	miRNA	5,65E-02	7,06E-01	8,61E-01	No	5,89E-02	8,49E-01	9,08E-01	No
ENSG00000238508	AC024569.1	miRNA	6,21E-02	7,77E-01	8,98E-01	No	-1,49E-01	6,59E-01	7,77E-01	No
ENSG00000207627	MIR581	miRNA	-5,26E-02	8,02E-01	9,12E-01	No	1,49E-01	6,70E-01	7,85E-01	No
ENSG00000265421	MIR4459	miRNA	-9,26E-02	6,74E-01	8,42E-01	No	3,13E-01	3,27E-01	4,82E-01	No
ENSG00000265135	MIR5687	miRNA	-4,17E-02	8,43E-01	9,32E-01	No	-7,56E-01	6,08E-02	1,32E-01	No
ENSG00000265665	AC008391.1	miRNA	-4,20E-02	5,82E-01	NA	No	-1,51E-01	5,67E-01	NA	No
ENSG00000202601	MIR582	miRNA	-3,48E-02	6,27E-01	NA	No	-9,03E-02	6,48E-01	NA	No
ENSG00000265613	AC143336.1	miRNA	8,18E-02	5,16E-01	7,40E-01	No	-2,15E-01	3,67E-01	NA	No
ENSG00000264099	MIR4803	miRNA	-2,73E-01	1,72E-01	3,95E-01	No	-1,71E-01	6,24E-01	7,50E-01	No
ENSG00000263593	MIR4804	miRNA	4,56E-02	7,54E-01	8,86E-01	No	2,95E-02	9,25E-01	9,56E-01	No
ENSG00000222316	AC008581.1	miRNA	7,99E-02	4,74E-01	NA	No	6,16E-01	7,59E-02	NA	No
ENSG00000222085	AC024568.1	miRNA	1,30E-01	5,45E-01	7,60E-01	No	-4,85E-02	8,87E-01	9,33E-01	No
ENSG00000264142	AC114969.1	miRNA	6,43E-02	4,61E-01	NA	No	-1,30E-01	7,12E-01	NA	No
ENSG00000216125	AC109496.1	miRNA	-6,34E-02	4,94E-01	NA	No	-2,82E-02	8,71E-01	NA	No
ENSG00000211498	AC093311.1	miRNA	-3,20E-02	7,10E-01	NA	No	2,66E-03	9,77E-01	NA	No
ENSG00000207578	MIR583	miRNA	5,63E-02	6,34E-01	NA	No	1,55E-01	4,94E-01	NA	No
ENSG00000264129	AC020900.2	miRNA	6,07E-02	7,30E-01	8,74E-01	No	-4,25E-02	8,89E-01	9,34E-01	No
ENSG00000221263	MIR548P	miRNA	1,65E-03	9,81E-01	NA	No	4,00E-01	1,59E-01	2,80E-01	No
ENSG00000221436	MIR548F3	miRNA	-1,34E-02	8,19E-01	NA	No	1,42E-01	5,27E-01	NA	No
ENSG00000266499	AC008536.1	miRNA	-1,62E-01	1,56E-01	NA	No	-9,66E-02	7,44E-01	NA	No
ENSG00000221778	AC034236.2	miRNA	1,03E-02	9,66E-01	NA	No	-9,72E-02	8,30E-01	NA	No
ENSG00000264536	MIR5706	miRNA	-1,26E-01	5,49E-01	7,62E-01	No	-2,32E-01	5,12E-01	6,58E-01	No
ENSG00000266587	AC027320.1	miRNA	-5,45E-02	6,46E-01	NA	No	-1,28E-01	7,12E-01	NA	No
ENSG00000212114	AC093267.1	miRNA	2,77E-01	9,49E-02	2,69E-01	No	-4,69E-02	8,10E-01	NA	No
ENSG00000265637	AC010235.1	miRNA	-1,75E-02	8,19E-01	NA	No	-1,33E-01	7,12E-01	NA	No
ENSG00000264563	MIR4633	miRNA	2,22E-02	9,72E-01	NA	No	1,02E-01	7,75E-01	NA	No
ENSG00000265691	MIR4460	miRNA	-9,41E-03	9,39E-01	NA	No	-1,52E-01	5,67E-01	NA	No
ENSG00000263597	MIR3936	miRNA	-3,73E-02	8,62E-01	9,41E-01	No	3,51E-02	9,16E-01	9,50E-01	No
ENSG00000221612	AC106775.1	miRNA	3,41E-02	9,36E-01	NA	No	9,20E-02	8,64E-01	NA	No
ENSG00000266475	AC106753.1	miRNA	-1,02E-01	5,84E-01	7,86E-01	No	-3,92E-01	2,74E-01	4,23E-01	No
ENSG00000252831	AC005215.1	miRNA	-4,46E-02	7,60E-01	NA	No	-1,03E-01	8,30E-01	NA	No
ENSG00000252968	AC005370.1	miRNA	1,67E-03	9,66E-01	NA	No	-1,51E-01	5,73E-01	NA	No
ENSG00000266478	MIR5197	miRNA	-7,00E-02	6,57E-01	8,32E-01	No	-1,24E-01	5,18E-01	6,63E-01	No
ENSG00000221467	AC132803.1	miRNA	2,23E-02	9,72E-01	NA	No	9,02E-02	8,64E-01	NA	No
ENSG00000207714	MIR584	miRNA	3,80E-02	8,45E-01	NA	No	9,18E-02	8,64E-01	NA	No
ENSG00000208035	MIR143	miRNA	-1,17E+00	3,61E-03	2,69E-02	No	-8,39E-01	5,01E-02	1,12E-01	No
ENSG00000221135	AC021078.1	miRNA	-1,50E-02	8,73E-01	NA	No	-6,23E-02	7,34E-01	NA	No
ENSG00000199047	MIR378A	miRNA	-6,76E-01	2,90E-02	1,19E-01	No	-1,16E+00	3,10E-02	7,49E-02	No
ENSG00000251710	AC010295.1	miRNA	-1,02E-02	9,10E-01	NA	No	8,46E-03	9,13E-01	NA	No
ENSG00000221070	AC008625.1	miRNA	1,64E-01	1,98E-01	NA	No	-2,00E-02	8,90E-01	NA	No
ENSG00000221430	MIR1294	miRNA	1,70E-01	1,95E-01	NA	No	-8,13E-02	7,21E-01	NA	No
ENSG00000221552	MIR1303	miRNA	-1,94E-02	8,45E-01	NA	No	3,48E-02	8,80E-01	NA	No
ENSG00000263361	MIR378H	miRNA	2,45E-01	2,70E-01	5,15E-01	No	-1,68E-01	6,20E-01	7,47E-01	No
ENSG00000199035	MIR103A1	miRNA	-8,47E-02	5,21E-01	7,43E-01	No	1,90E-01	5,74E-01	7,09E-01	No
ENSG00000207739	MIR218-2	miRNA	-1,25E-01	3,14E-01	NA	No	-2,18E-01	3,81E-01	NA	No
ENSG00000207619	MIR585	miRNA	-6,34E-02	4,94E-01	NA	No	-1,52E-01	5,59E-01	NA	No
ENSG00000263831	MIR378E	miRNA	2,75E-01	1,49E-01	3,62E-01	No	-1,69E-01	4,64E-01	NA	No

ENSG00000266671	AC113342.1	miRNA	-2,65E-02	8,75E-01	9,48E-01	No	-4,69E-01	1,89E-01	3,20E-01	No
ENSG00000222732	AC008671.1	miRNA	5,61E-02	7,86E-01	9,04E-01	No	-1,95E-01	5,58E-01	6,97E-01	No
ENSG00000216127	AC011407.1	miRNA	-5,82E-02	7,47E-01	8,82E-01	No	-3,40E-01	3,18E-01	4,72E-01	No
ENSG00000265160	MIR5003	miRNA	5,33E-02	7,72E-01	8,96E-01	No	-4,63E-01	2,28E-01	3,68E-01	No
ENSG00000266083	AC138965.1	miRNA	2,59E-01	4,28E-02	NA	No	1,24E-01	5,21E-01	NA	No
ENSG00000221464	MIR1271	miRNA	8,50E-02	6,02E-01	7,99E-01	No	-3,59E-01	1,87E-01	NA	No
ENSG00000198995	MIR340	miRNA	2,89E-02	8,45E-01	9,33E-01	No	-1,44E-01	5,52E-01	NA	No
ENSG00000263759	AC122714.1	miRNA	-4,42E-02	7,60E-01	NA	No	-1,02E-01	8,30E-01	NA	No
ENSG00000266252	AL031768.1	miRNA	4,66E-03	9,78E-01	9,91E-01	No	-8,01E-01	6,00E-02	1,30E-01	No
ENSG00000266750	MIR4645	miRNA	2,73E-01	6,48E-02	NA	No	1,73E-02	9,87E-01	NA	No
ENSG00000265083	MIR3691	miRNA	1,59E-01	1,75E-01	NA	No	1,52E-01	4,70E-01	NA	No
ENSG00000264541	AL162381.1	miRNA	2,42E-02	9,72E-01	NA	No	9,02E-02	8,64E-01	NA	No
ENSG00000263572	MIR5683	miRNA	2,45E-01	2,44E-01	4,85E-01	No	9,18E-02	7,89E-01	8,70E-01	No
ENSG00000263712	MIR4639	miRNA	2,65E-02	8,52E-01	NA	No	-4,12E-02	8,64E-01	9,18E-01	No
ENSG00000265642	AL034375.1	miRNA	-1,47E-02	9,20E-01	9,67E-01	No	-2,53E-01	3,26E-01	NA	No
ENSG00000252949	AL136303.1	miRNA	-3,74E-02	8,46E-01	9,33E-01	No	-2,28E-01	5,18E-01	6,63E-01	No
ENSG00000263391	AL512428.1	miRNA	1,15E+00	1,38E-02	7,09E-02	No	3,51E-01	3,16E-01	4,70E-01	No
ENSG00000264238	AL590084.1	miRNA	1,18E-01	2,03E-01	NA	No	1,02E-01	7,75E-01	NA	No
ENSG00000253017	AL160037.1	miRNA	3,15E-01	3,26E-02	1,29E-01	No	3,06E-01	2,22E-01	3,62E-01	No
ENSG00000215979	AL021917.1	miRNA	5,59E-01	4,61E-02	1,65E-01	No	-3,49E-01	3,22E-01	4,77E-01	No
ENSG00000238621	TRNAI2	miRNA	-6,65E-02	4,29E-01	NA	No	-7,84E-02	7,32E-01	NA	No
ENSG00000265565	MIR3143	miRNA	-3,28E-02	6,65E-01	NA	No	-1,59E-01	4,91E-01	NA	No
ENSG00000222894	AL662800.1	miRNA	4,89E-02	8,24E-01	9,23E-01	No	-9,62E-01	1,05E-03	3,86E-03	No
ENSG00000266776	MIR4646	miRNA	9,67E-02	4,42E-01	NA	No	8,55E-02	8,64E-01	NA	No
ENSG00000199036	MIR219-1	miRNA	5,88E-02	5,77E-01	NA	No	-7,04E-02	7,21E-01	NA	No
ENSG00000265527	MIR5690	miRNA	1,37E+00	8,62E-04	9,11E-03	No	1,80E-01	6,07E-01	7,36E-01	No
ENSG00000264878	Z85986.1	miRNA	-1,82E-04	9,98E-01	9,99E-01	No	-6,27E-01	1,21E-01	2,28E-01	No
ENSG00000238716	AL031905.1	miRNA	5,44E-02	5,54E-01	NA	No	8,07E-02	7,17E-01	NA	No
ENSG00000266494	MIR4641	miRNA	1,58E-02	9,65E-01	NA	No	-9,51E-02	8,30E-01	NA	No
ENSG00000266619	MIR4642	miRNA	-9,39E-02	5,24E-01	NA	No	-1,73E-01	5,66E-01	7,03E-01	No
ENSG00000207769	MIR586	miRNA	4,97E-02	5,38E-01	NA	No	6,66E-01	3,09E-02	7,47E-02	No
ENSG00000264056	MIR5685	miRNA	1,81E-01	4,02E-01	6,46E-01	No	-2,99E-01	4,04E-01	5,60E-01	No
ENSG00000212017	MIR548U	miRNA	7,14E-02	6,35E-01	8,19E-01	No	-6,49E-04	9,98E-01	9,99E-01	No
ENSG00000265498	AL158051.1	miRNA	-3,27E-02	7,22E-01	NA	No	9,33E-03	9,76E-01	NA	No
ENSG00000199094	MIR30C2	miRNA	-3,42E-01	1,73E-01	3,97E-01	No	5,58E-02	8,60E-01	9,16E-01	No
ENSG00000207827	MIR30A	miRNA	-5,17E-02	8,06E-01	9,14E-01	No	4,75E-01	1,91E-01	3,23E-01	No
ENSG00000264447	AL445256.1	miRNA	2,49E-02	9,72E-01	NA	No	1,03E-01	7,75E-01	NA	No
ENSG00000264609	AL603910.1	miRNA	1,56E-02	8,75E-01	NA	No	-1,30E-01	7,12E-01	NA	No
ENSG00000264884	AL590684.1	miRNA	-5,47E-02	6,46E-01	NA	No	7,98E-02	7,17E-01	NA	No
ENSG00000263533	MIR4463	miRNA	-5,74E-01	5,56E-02	1,87E-01	No	-7,93E-01	6,28E-02	1,35E-01	No
ENSG00000251969	AL356776.1	miRNA	-2,44E-01	2,89E-01	5,35E-01	No	2,03E-01	4,60E-01	6,13E-01	No
ENSG00000252309	AL391416.1	miRNA	1,76E-02	8,63E-01	NA	No	2,27E-01	4,30E-01	5,85E-01	No
ENSG00000221544	AL359709.1	miRNA	-3,31E-02	7,31E-01	NA	No	-3,93E-02	8,90E-01	NA	No
ENSG00000252918	AL359709.2	miRNA	-4,84E-02	6,05E-01	NA	No	-7,65E-02	7,32E-01	NA	No
ENSG00000239160	AL109947.1	miRNA	2,46E-02	9,72E-01	NA	No	6,19E-02	9,56E-01	NA	No
ENSG00000253037	AL109947.2	miRNA	-1,04E-02	9,17E-01	NA	No	-1,55E-01	4,93E-01	NA	No
ENSG00000266032	AL357515.1	miRNA	-1,15E-01	5,97E-01	7,95E-01	No	3,87E-02	9,09E-01	9,46E-01	No
ENSG00000207982	MIR548B	miRNA	-5,45E-02	6,46E-01	NA	No	2,98E-02	8,99E-01	NA	No
ENSG00000265725	MIR3144	miRNA	-8,65E-03	8,97E-01	NA	No	-1,33E-01	7,12E-01	NA	No
ENSG00000216015	AL354936.1	miRNA	-1,09E-02	8,94E-01	NA	No	-1,36E-01	7,12E-01	NA	No
ENSG00000265669	MIR548AJ1	miRNA	-5,37E-02	6,46E-01	NA	No	-1,26E-01	7,12E-01	NA	No
ENSG00000238921	AL360178.1	miRNA	-3,29E-02	7,31E-01	NA	No	-1,30E-01	7,12E-01	NA	No
ENSG00000266555	MIR3145	miRNA	7,29E-02	7,26E-01	8,72E-01	No	-5,28E-02	8,74E-01	9,25E-01	No
ENSG00000223322	AL033378.1	miRNA	1,69E-01	1,96E-01	NA	No	8,52E-01	5,37E-02	1,19E-01	No
ENSG00000221469	AL133260.1	miRNA	-9,30E-02	3,80E-01	NA	No	-3,24E-01	2,19E-01	NA	No
ENSG00000264814	MIR1273C	miRNA	-1,69E-01	4,11E-01	6,55E-01	No	1,71E-01	6,23E-01	7,49E-01	No
ENSG00000223286	AL121952.1	miRNA	1,02E-01	5,20E-01	7,42E-01	No	-2,30E-01	4,86E-01	6,36E-01	No
ENSG00000221456	MIR1202	miRNA	1,45E-01	2,64E-01	NA	No	-5,27E-02	7,89E-01	NA	No
ENSG00000271899	MIR4466	miRNA	9,03E-02	3,96E-01	NA	No	5,20E-01	7,60E-02	NA	No
ENSG00000266617	MIR3692	miRNA	-1,39E-01	3,44E-01	5,92E-01	No	-5,12E-01	7,08E-02	NA	No
ENSG00000265803	AL590703.1	miRNA	-1,69E-02	8,19E-01	NA	No	-1,26E-01	7,12E-01	NA	No
ENSG00000221021	AL035697.1	miRNA	-2,31E-01	1,49E-01	NA	No	-9,03E-01	3,36E-02	8,01E-02	No

ENSG00000252196	AL590482.1	miRNA	-3,68E-02	6,54E-01	NA	No	-7,14E-02	7,30E-01	NA	No
ENSG00000222958	MIR1913	miRNA	-7,82E-02	6,72E-01	8,41E-01	No	-1,15E-01	7,39E-01	8,35E-01	No
ENSG00000265828	MIR3939	miRNA	8,61E-02	6,14E-01	8,06E-01	No	1,44E-01	6,49E-01	7,69E-01	No
ENSG00000265654	AL513547.1	miRNA	1,07E-01	5,91E-01	7,91E-01	No	-4,28E-01	2,37E-01	3,79E-01	No
ENSG00000266245	MIR4644	miRNA	3,61E-02	8,58E-01	9,39E-01	No	1,41E-01	6,87E-01	7,98E-01	No
ENSG00000199023	MIR339	miRNA	-1,07E-01	4,70E-01	NA	No	-5,08E-02	8,67E-01	9,20E-01	No
ENSG00000265089	MIR4655	miRNA	1,43E-01	2,01E-01	NA	No	-3,03E-02	9,21E-01	NA	No
ENSG00000264357	MIR4648	miRNA	-3,64E-02	7,75E-01	NA	No	-3,17E-01	2,32E-01	NA	No
ENSG00000211544	AC069286.1	miRNA	-5,13E-02	6,52E-01	NA	No	-3,18E-01	2,61E-01	NA	No
ENSG00000207973	MIR589	miRNA	-8,07E-02	6,85E-01	8,49E-01	No	-1,10E+00	3,69E-02	8,69E-02	No
ENSG00000221011	AC079804.1	miRNA	7,20E-02	5,38E-01	NA	No	-1,30E-01	7,12E-01	NA	No
ENSG00000266121	AC092104.4	miRNA	-5,37E-02	6,46E-01	NA	No	-2,34E-02	8,90E-01	NA	No
ENSG00000265212	AC007009.2	miRNA	-8,44E-01	2,38E-02	1,04E-01	No	-2,10E-01	5,51E-01	6,91E-01	No
ENSG00000221393	MIR1302-6	miRNA	-1,08E-01	2,47E-01	NA	No	-4,87E-02	8,49E-01	9,08E-01	No
ENSG00000223030	AC091697.1	miRNA	-2,88E-01	1,55E-01	3,70E-01	No	-2,07E-01	5,29E-01	6,73E-01	No
ENSG00000265932	MIR3146	miRNA	3,18E-02	8,14E-01	NA	No	5,98E-02	8,26E-01	NA	No
ENSG00000221783	MIR1183	miRNA	1,02E-02	9,47E-01	9,78E-01	No	-7,19E-01	7,04E-02	1,48E-01	No
ENSG00000238976	AC004016.1	miRNA	4,39E-02	7,49E-01	NA	No	1,21E-01	6,63E-01	NA	No
ENSG00000223052	AC007096.1	miRNA	8,38E-02	5,04E-01	NA	No	5,85E-02	8,17E-01	NA	No
ENSG00000212024	MIR550A3	miRNA	-3,04E-01	1,19E-01	3,12E-01	No	5,84E-02	8,61E-01	9,17E-01	No
ENSG00000207771	MIR550A1	miRNA	1,11E-01	4,69E-01	7,03E-01	No	1,85E-01	5,58E-01	6,96E-01	No
ENSG00000223070	AC006377.1	miRNA	-9,91E-01	4,97E-03	3,40E-02	No	-1,64E-01	6,27E-01	7,52E-01	No
ENSG00000207573	MIR550A2	miRNA	-4,99E-01	7,46E-02	2,29E-01	No	-2,29E-01	5,19E-01	6,64E-01	No
ENSG00000221669	MIR548N	miRNA	-1,14E-01	3,82E-01	NA	No	3,60E-02	9,06E-01	9,44E-01	No
ENSG00000221156	AC007551.1	miRNA	8,91E-02	6,43E-01	8,23E-01	No	-1,59E-01	6,42E-01	7,64E-01	No
ENSG00000221561	AC087069.1	miRNA	1,81E-01	9,79E-02	NA	No	1,10E-01	6,75E-01	NA	No
ENSG00000221325	MIR1200	miRNA	4,48E-01	8,42E-02	2,48E-01	No	6,99E-01	8,88E-02	1,78E-01	No
ENSG00000266168	MIR3147	miRNA	-2,19E-03	9,81E-01	NA	No	1,87E-01	4,91E-01	6,40E-01	No
ENSG00000265017	AC092634.2	miRNA	2,35E-02	9,72E-01	NA	No	6,13E-02	9,56E-01	NA	No
ENSG00000266525	MIR4650-1	miRNA	3,96E-02	7,21E-01	NA	No	2,14E-02	9,26E-01	NA	No
ENSG00000265024	AC073089.1	miRNA	1,03E-01	3,54E-01	NA	No	2,36E-01	3,47E-01	5,02E-01	No
ENSG00000265600	AC006480.1	miRNA	-2,04E-01	2,83E-01	5,30E-01	No	-6,51E-01	1,02E-01	1,99E-01	No
ENSG00000265878	MIR3914-1	miRNA	2,23E-02	9,72E-01	NA	No	1,07E-01	6,99E-01	NA	No
ENSG00000266688	AC079398.1	miRNA	-3,11E-02	7,31E-01	NA	No	4,90E-02	8,44E-01	NA	No
ENSG00000266772	AC091738.2	miRNA	-5,23E-02	5,89E-01	NA	No	-1,61E-01	4,80E-01	NA	No
ENSG00000264494	MIR4650-2	miRNA	2,40E-02	9,72E-01	NA	No	6,17E-02	9,56E-01	NA	No
ENSG00000265724	MIR4284	miRNA	-1,01E-02	9,02E-01	NA	No	-1,30E-01	7,12E-01	NA	No
ENSG00000207741	MIR590	miRNA	3,08E-02	8,90E-01	9,54E-01	No	-5,05E-01	1,77E-01	3,05E-01	No
ENSG00000252378	AC004851.1	miRNA	-2,63E-01	5,44E-02	1,84E-01	No	-6,31E-01	2,44E-02	6,12E-02	No
ENSG00000221249	AC004980.1	miRNA	-3,32E-02	7,20E-01	NA	No	-7,23E-02	7,98E-01	NA	No
ENSG00000221262	AC005159.1	miRNA	-2,88E-02	8,91E-01	9,55E-01	No	2,41E-01	4,91E-01	6,40E-01	No
ENSG00000223029	AC004969.1	miRNA	4,35E-02	7,20E-01	NA	No	1,02E-01	7,75E-01	NA	No
ENSG00000221520	MIR1285-1	miRNA	2,58E-01	2,30E-01	4,68E-01	No	1,75E-01	6,03E-01	7,33E-01	No
ENSG00000208025	MIR591	miRNA	3,60E-02	8,45E-01	9,33E-01	No	4,35E-01	2,49E-01	3,93E-01	No
ENSG00000220987	AC084368.1	miRNA	-6,52E-02	7,37E-01	8,78E-01	No	-1,50E-01	6,57E-01	7,75E-01	No
ENSG00000221048	AC079781.1	miRNA	9,60E-03	8,46E-01	NA	No	3,11E-03	9,35E-01	NA	No
ENSG00000266318	MIR5692A2	miRNA	-3,26E-01	1,37E-01	3,43E-01	No	-1,20E+00	3,16E-02	7,62E-02	No
ENSG00000266668	MIR5692C2	miRNA	3,46E-01	1,32E-01	3,34E-01	No	3,14E-01	3,81E-01	5,38E-01	No
ENSG00000266019	MIR3609	miRNA	3,72E-01	3,34E-02	1,32E-01	No	9,88E-01	2,65E-08	2,21E-07	No
ENSG00000265324	AC005020.1	miRNA	-3,41E-02	7,30E-01	NA	No	-1,09E-01	5,91E-01	NA	No
ENSG00000221487	AC069294.1	miRNA	-1,75E-02	8,61E-01	NA	No	1,14E-01	6,73E-01	7,87E-01	No
ENSG00000207547	MIR25	miRNA	1,60E-01	4,32E-01	6,72E-01	No	-2,90E-01	4,13E-01	5,69E-01	No
ENSG00000207757	MIR93	miRNA	-7,12E-02	6,59E-01	8,33E-01	No	-2,69E-01	4,23E-01	5,78E-01	No
ENSG00000208036	MIR106B	miRNA	-5,43E-02	7,54E-01	8,86E-01	No	-2,88E-01	3,65E-01	5,21E-01	No
ENSG00000264642	AC005088.1	miRNA	2,74E-02	8,35E-01	NA	No	-1,87E-02	8,90E-01	NA	No
ENSG00000264675	MIR4285	miRNA	-7,91E-02	3,10E-01	NA	No	-1,83E-01	3,74E-01	NA	No
ENSG00000221510	MIR548O	miRNA	2,85E-02	7,71E-01	NA	No	4,29E-01	1,79E-01	3,08E-01	No
ENSG00000238392	AC093668.1	miRNA	-4,33E-02	7,60E-01	NA	No	3,55E-02	8,89E-01	NA	No
ENSG00000238997	AC105052.1	miRNA	2,99E-02	8,15E-01	NA	No	-1,01E-01	8,30E-01	NA	No
ENSG00000264045	AC073127.1	miRNA	-4,21E-02	7,60E-01	NA	No	-9,72E-02	8,30E-01	NA	No
ENSG00000221279	AC005161.1	miRNA	-3,68E-02	7,10E-01	NA	No	1,86E-01	4,74E-01	6,25E-01	No
ENSG00000272230	MIR3666	miRNA	-1,47E-02	8,80E-01	NA	No	1,21E-01	6,55E-01	NA	No

ENSG00000216076	AC002066.2	miRNA	-7,60E-03	8,84E-01	NA	No	8,04E-02	7,46E-01	NA	No
ENSG00000207588	MIR593	miRNA	1,13E-02	9,34E-01	9,73E-01	No	-2,80E-01	1,41E-01	NA	No
ENSG00000221429	AC018635.1	miRNA	-6,25E-02	5,42E-01	NA	No	3,83E-02	8,74E-01	NA	No
ENSG00000221401	AC025594.1	miRNA	-5,23E-02	5,62E-01	NA	No	-1,49E-01	5,91E-01	NA	No
ENSG00000263557	AC073320.1	miRNA	-4,33E-02	7,60E-01	NA	No	-1,01E-01	8,30E-01	NA	No
ENSG00000238844	AC083875.1	miRNA	5,21E-02	6,49E-01	NA	No	-3,28E-02	8,93E-01	NA	No
ENSG00000222219	AC091736.1	miRNA	5,53E-02	6,17E-01	NA	No	8,79E-02	8,64E-01	NA	No
ENSG00000220982	AC009784.1	miRNA	-3,44E-02	6,89E-01	NA	No	-6,18E-02	7,68E-01	NA	No
ENSG00000221732	AC006452.1	miRNA	-2,05E-02	8,19E-01	NA	No	-1,36E-01	7,12E-01	NA	No
ENSG00000265523	AC073647.1	miRNA	-3,73E-02	6,62E-01	NA	No	-1,55E-01	5,35E-01	NA	No
ENSG00000264918	AC005229.1	miRNA	-2,18E-02	9,20E-01	9,66E-01	No	-6,49E-01	5,65E-02	1,24E-01	No
ENSG00000265810	MIR3907	miRNA	-2,05E-02	8,19E-01	NA	No	-1,36E-01	7,12E-01	NA	No
ENSG00000207960	MIR153-2	miRNA	2,49E-02	9,72E-01	NA	No	6,23E-02	9,56E-01	NA	No
ENSG00000264510	BX649553.3	miRNA	9,31E-02	3,69E-01	NA	No	-9,51E-02	8,30E-01	NA	No
ENSG00000264819	BX649553.4	miRNA	2,04E-01	8,14E-02	NA	No	1,49E-01	4,91E-01	NA	No
ENSG00000263980	BX649553.2	miRNA	1,87E-01	1,14E-01	NA	No	-1,69E-02	9,77E-01	NA	No
ENSG00000265658	MIR3690	miRNA	1,18E-01	2,48E-01	NA	No	-9,82E-02	8,30E-01	NA	No
ENSG00000264268	MIR4767	miRNA	5,14E-02	7,31E-01	8,75E-01	No	2,16E-01	5,22E-01	6,67E-01	No
ENSG00000207628	MIR651	miRNA	-1,74E-01	5,74E-02	NA	No	-4,29E-01	1,61E-01	2,83E-01	No
ENSG00000222133	AC003666.1	miRNA	-5,69E-02	6,46E-01	NA	No	-1,53E-02	9,82E-01	NA	No
ENSG00000263652	MIR548AX	miRNA	-1,29E-01	3,86E-01	6,32E-01	No	1,27E-01	7,12E-01	8,16E-01	No
ENSG00000252648	AC078993.1	miRNA	3,74E-02	8,45E-01	NA	No	9,10E-02	8,64E-01	NA	No
ENSG00000238623	AC073597.1	miRNA	-5,73E-02	6,46E-01	NA	No	-1,36E-01	7,12E-01	NA	No
ENSG00000265465	MIR4768	miRNA	8,23E-02	5,60E-01	7,70E-01	No	8,36E-02	7,75E-01	8,60E-01	No
ENSG00000266822	AL928874.1	miRNA	4,27E-02	7,63E-01	NA	No	1,10E-01	6,71E-01	NA	No
ENSG00000263425	AC131011.1	miRNA	-6,53E-02	4,92E-01	NA	No	8,80E-03	9,72E-01	NA	No
ENSG00000264184	AC096509.1	miRNA	-4,23E-02	7,60E-01	NA	No	-9,82E-02	8,30E-01	NA	No
ENSG00000265506	AC004656.1	miRNA	-4,15E-02	7,60E-01	NA	No	4,07E-02	8,92E-01	NA	No
ENSG00000264224	AC004655.1	miRNA	4,30E-02	7,18E-01	NA	No	3,03E-02	8,92E-01	NA	No
ENSG00000264090	MIR4666B	miRNA	1,68E-02	8,73E-01	NA	No	-1,02E-01	8,30E-01	NA	No
ENSG00000263600	MIR3915	miRNA	-1,13E-01	3,44E-01	5,92E-01	No	4,10E-02	8,98E-01	9,39E-01	No
ENSG00000221348	MIR548F5	miRNA	-8,76E-02	3,36E-01	NA	No	5,61E-01	1,31E-01	2,42E-01	No
ENSG00000207870	MIR221	miRNA	1,51E-02	9,33E-01	9,72E-01	No	4,04E-02	8,97E-01	9,39E-01	No
ENSG00000263858	MIR4769	miRNA	-1,16E-02	8,96E-01	NA	No	-1,35E-01	7,12E-01	NA	No
ENSG00000207758	MIR532	miRNA	-1,37E-02	8,49E-01	NA	No	1,92E-02	9,87E-01	NA	No
ENSG00000207768	MIR188	miRNA	1,22E-02	9,66E-01	NA	No	-9,51E-02	8,30E-01	NA	No
ENSG00000265152	AF222686.1	miRNA	2,33E-02	7,49E-01	NA	No	8,46E-03	9,13E-01	NA	No
ENSG00000207785	MIR500A	miRNA	2,98E-03	9,75E-01	NA	No	-1,31E-01	5,83E-01	NA	No
ENSG00000208015	MIR362	miRNA	6,91E-02	4,49E-01	NA	No	9,93E-02	7,75E-01	NA	No
ENSG00000211538	MIR501	miRNA	-1,25E-02	8,03E-01	NA	No	-1,28E-01	7,12E-01	NA	No
ENSG00000239057	MIR500B	miRNA	-4,46E-02	7,60E-01	NA	No	-1,03E-01	8,30E-01	NA	No
ENSG00000207970	MIR660	miRNA	3,90E-02	8,45E-01	NA	No	6,34E-02	9,56E-01	NA	No
ENSG00000272080	MIR502	miRNA	3,69E-02	8,45E-01	NA	No	8,55E-02	8,64E-01	NA	No
ENSG00000265211	AL121865.1	miRNA	-4,54E-03	8,99E-01	NA	No	-6,39E-02	8,00E-01	NA	No
ENSG00000271886	MIR98	miRNA	5,14E-02	7,44E-01	NA	No	2,55E-01	4,52E-01	6,05E-01	No
ENSG00000208012	MIRLET7F2	miRNA	4,19E-02	7,48E-01	NA	No	9,11E-02	8,64E-01	NA	No
ENSG00000222532	MIR1468	miRNA	-5,58E-02	6,46E-01	NA	No	-1,33E-01	7,12E-01	NA	No
ENSG00000222596	AL445523.1	miRNA	1,18E-01	3,79E-01	NA	No	2,51E-01	3,94E-01	5,51E-01	No
ENSG00000215933	BX119917.1	miRNA	3,82E-01	1,26E-01	3,24E-01	No	-3,79E-01	2,85E-01	4,35E-01	No
ENSG00000202566	MIR421	miRNA	2,72E-01	2,43E-01	4,84E-01	No	-2,97E-01	3,16E-01	4,70E-01	No
ENSG00000212027	MIR374B	miRNA	1,50E-01	5,08E-01	7,34E-01	No	1,80E-01	4,99E-01	6,48E-01	No
ENSG00000207820	MIR545	miRNA	4,04E-02	7,83E-01	NA	No	-6,27E-02	7,89E-01	NA	No
ENSG00000199168	MIR374A	miRNA	-6,46E-02	6,44E-01	NA	No	-8,01E-02	7,95E-01	8,73E-01	No
ENSG00000264374	AL357115.1	miRNA	2,15E-01	2,86E-01	5,32E-01	No	-3,25E-01	3,31E-01	4,86E-01	No
ENSG00000199051	MIR361	miRNA	1,89E-01	2,16E-01	4,52E-01	No	-3,87E-02	8,66E-01	NA	No
ENSG00000221665	AL591708.1	miRNA	3,85E-02	8,45E-01	NA	No	8,55E-02	8,64E-01	NA	No
ENSG00000221132	AL035422.1	miRNA	4,31E-01	5,53E-02	1,86E-01	No	4,12E-01	2,49E-01	3,94E-01	No
ENSG00000266568	AL049610.1	miRNA	1,17E-01	4,39E-01	6,78E-01	No	-1,29E-01	5,96E-01	NA	No
ENSG00000221392	Z73964.1	miRNA	1,75E-02	8,73E-01	NA	No	-1,65E-01	5,14E-01	NA	No
ENSG00000222161	Z97356.1	miRNA	1,18E-01	3,98E-01	NA	No	-6,27E-02	7,90E-01	NA	No
ENSG00000263515	MIR548AN	miRNA	2,34E-02	9,72E-01	NA	No	8,55E-02	8,64E-01	NA	No
ENSG00000208013	MIR652	miRNA	-1,36E-01	4,06E-01	6,50E-01	No	-1,15E-01	7,23E-01	8,24E-01	No

ENSG00000265584	MIR3978	miRNA	-6,20E-02	7,66E-01	8,93E-01	No	-5,13E-01	1,93E-01	3,25E-01	No
ENSG00000264567	AL391803.1	miRNA	8,41E-02	5,04E-01	NA	No	-7,45E-03	9,83E-01	NA	No
ENSG00000221463	MIR1277	miRNA	-2,77E-02	7,96E-01	NA	No	7,76E-02	7,46E-01	NA	No
ENSG00000264952	AC004000.1	miRNA	-2,28E-02	7,98E-01	NA	No	-3,93E-02	8,90E-01	NA	No
ENSG00000211578	MIR766	miRNA	6,51E-02	4,58E-01	NA	No	-3,30E-02	9,21E-01	NA	No
ENSG00000265456	MIR3672	miRNA	3,31E-02	9,36E-01	NA	No	1,04E-01	7,75E-01	NA	No
ENSG00000221317	AL513487.1	miRNA	5,35E-01	6,03E-02	1,98E-01	No	7,94E-02	8,21E-01	8,90E-01	No
ENSG00000265082	AL356213.1	miRNA	-4,23E-02	7,60E-01	NA	No	-9,82E-02	8,30E-01	NA	No
ENSG00000266440	AL359542.1	miRNA	2,35E-02	8,65E-01	NA	No	3,43E-01	1,65E-01	NA	No
ENSG00000239594	MIR18B	miRNA	-1,69E-02	8,19E-01	NA	No	-1,33E-02	8,90E-01	NA	No
ENSG00000251962	AL591668.1	miRNA	1,38E-01	3,32E-01	5,81E-01	No	9,59E-01	5,46E-02	1,20E-01	No
ENSG00000207784	MIR542	miRNA	-5,37E-02	6,46E-01	NA	No	-1,26E-01	7,12E-01	NA	No
ENSG00000208005	MIR503	miRNA	-2,59E-02	7,31E-01	NA	No	6,84E-02	7,96E-01	NA	No
ENSG00000264284	AL391380.1	miRNA	2,23E-01	6,72E-02	NA	No	1,14E-01	6,30E-01	NA	No
ENSG00000266272	Z97632.1	miRNA	-4,15E-02	7,60E-01	NA	No	-9,51E-02	8,30E-01	NA	No
ENSG00000263845	AL390879.1	miRNA	9,02E-02	4,55E-01	NA	No	2,06E-01	4,21E-01	NA	No
ENSG00000207633	MIR505	miRNA	1,06E-01	5,65E-01	7,73E-01	No	-5,57E-02	8,58E-01	9,14E-01	No
ENSG00000263713	AC109994.1	miRNA	1,32E-02	9,67E-01	NA	No	-9,51E-02	8,30E-01	NA	No
ENSG00000266038	MIR4659B	miRNA	3,21E-02	7,58E-01	NA	No	1,82E-02	9,39E-01	NA	No
ENSG00000216206	AF228730.1	miRNA	-5,47E-02	6,46E-01	NA	No	1,25E-02	9,42E-01	NA	No
ENSG00000221027	AC068020.1	miRNA	1,23E-01	4,59E-01	6,93E-01	No	5,59E-02	8,37E-01	9,01E-01	No
ENSG00000264445	AC087269.2	miRNA	-5,45E-02	6,46E-01	NA	No	-1,28E-01	7,12E-01	NA	No
ENSG00000207701	MIR597	miRNA	9,95E-02	5,39E-01	7,56E-01	No	-1,50E-01	6,05E-01	7,34E-01	No
ENSG00000221320	AC023385.1	miRNA	1,43E-02	9,15E-01	NA	No	-1,97E-01	3,31E-01	NA	No
ENSG00000207600	MIR598	miRNA	-6,85E-02	4,16E-01	NA	No	-1,59E-01	4,82E-01	NA	No
ENSG00000266637	AC145124.1	miRNA	-4,23E-02	7,60E-01	NA	No	-9,82E-02	8,30E-01	NA	No
ENSG00000221714	AC130352.1	miRNA	4,61E-02	6,23E-01	NA	No	1,21E-01	6,71E-01	NA	No
ENSG00000238460	AC068587.2	miRNA	5,59E-02	6,28E-01	8,15E-01	No	-1,12E-01	5,79E-01	NA	No
ENSG00000264512	MIR5692A1	miRNA	3,30E-03	9,91E-01	NA	No	-1,28E-01	7,12E-01	NA	No
ENSG00000266206	MIR3926-2	miRNA	-4,21E-02	7,60E-01	NA	No	3,54E-01	2,06E-01	NA	No
ENSG00000265520	MIR548V	miRNA	4,37E-01	1,02E-01	2,81E-01	No	6,21E-01	1,00E-01	1,96E-01	No
ENSG00000251963	AC090420.1	miRNA	-5,96E-01	4,79E-02	1,69E-01	No	2,66E-02	9,37E-01	9,62E-01	No
ENSG00000208037	MIR320A	miRNA	-1,52E-01	2,09E-01	NA	No	-1,65E-01	5,76E-01	7,11E-01	No
ENSG00000265847	MIR4287	miRNA	-4,89E-02	5,14E-01	NA	No	-1,59E-01	4,91E-01	NA	No
ENSG00000265251	MIR4288	miRNA	-4,23E-02	7,60E-01	NA	No	-9,82E-02	8,30E-01	NA	No
ENSG00000263392	AC084262.2	miRNA	-6,30E-02	5,05E-01	NA	No	-1,52E-01	5,67E-01	NA	No
ENSG00000263372	MIR548AO	miRNA	5,09E-02	6,38E-01	NA	No	2,86E-02	9,87E-01	NA	No
ENSG00000221035	MIR486	miRNA	1,45E-02	9,18E-01	9,65E-01	No	-5,30E-02	8,65E-01	9,19E-01	No
ENSG00000266044	MIR4469	miRNA	4,88E-03	9,60E-01	NA	No	1,68E-01	5,26E-01	NA	No
ENSG00000211562	AC103686.1	miRNA	-5,45E-02	6,46E-01	NA	No	-2,72E-02	8,90E-01	NA	No
ENSG00000216204	AC018607.1	miRNA	3,31E-02	9,36E-01	NA	No	8,51E-02	8,64E-01	NA	No
ENSG00000266491	AC109335.1	miRNA	-3,55E-02	7,93E-01	9,07E-01	No	-1,79E-01	5,62E-01	6,99E-01	No
ENSG00000266712	hsa-mir-3149	miRNA	9,46E-02	5,47E-01	7,61E-01	No	1,48E-01	6,50E-01	7,70E-01	No
ENSG00000221185	AC103816.1	miRNA	3,95E-02	8,25E-01	9,24E-01	No	-1,71E-01	5,93E-01	7,24E-01	No
ENSG00000265157	AF070718.1	miRNA	-6,07E-02	4,99E-01	NA	No	-1,53E-01	5,47E-01	NA	No
ENSG00000266194	MIR4661	miRNA	-5,45E-02	6,46E-01	NA	No	-1,28E-01	7,12E-01	NA	No
ENSG00000263855	AC105081.1	miRNA	-5,73E-02	6,46E-01	NA	No	2,61E-02	8,47E-01	NA	No
ENSG00000223297	AC116154.1	miRNA	-9,61E-03	9,10E-01	NA	No	-1,35E-01	7,12E-01	NA	No
ENSG00000263842	AC104986.1	miRNA	8,95E-02	6,04E-01	8,00E-01	No	5,70E-01	1,54E-01	2,74E-01	No
ENSG00000207804	MIR599	miRNA	1,79E-02	9,06E-01	9,61E-01	No	-1,43E-01	6,10E-01	7,39E-01	No
ENSG00000216069	MIR875	miRNA	1,85E-02	8,96E-01	9,56E-01	No	4,20E-02	8,87E-01	9,33E-01	No
ENSG00000264991	AP001205.1	miRNA	-8,52E-03	9,59E-01	9,83E-01	No	2,17E-01	5,28E-01	6,72E-01	No
ENSG00000265898	AP003354.1	miRNA	-6,09E-02	5,10E-01	NA	No	-1,77E-01	3,94E-01	NA	No
ENSG00000265657	MIR3151	miRNA	1,40E-02	8,98E-01	NA	No	-1,06E-01	5,80E-01	NA	No
ENSG00000208032	MIR548A3	miRNA	-5,82E-02	7,02E-01	8,59E-01	No	-4,87E-01	1,91E-01	3,23E-01	No
ENSG00000221542	AC016405.2	miRNA	9,10E-02	5,57E-01	7,68E-01	No	-1,74E-01	5,15E-01	NA	No
ENSG00000266324	MIR4663	miRNA	5,92E-03	9,55E-01	NA	No	1,44E-02	9,47E-01	NA	No
ENSG00000207704	MIR548D1	miRNA	-1,84E-02	9,04E-01	9,60E-01	No	1,52E-01	6,63E-01	7,80E-01	No
ENSG00000221771	MIR1205	miRNA	2,85E-02	8,24E-01	NA	No	-1,24E-01	5,53E-01	NA	No
ENSG00000221176	MIR1207	miRNA	1,95E-01	1,09E-01	NA	No	-2,99E-02	8,85E-01	NA	No
ENSG00000221261	MIR1208	miRNA	-4,21E-02	7,60E-01	NA	No	-9,72E-02	8,30E-01	NA	No
ENSG00000264653	MIR5194	miRNA	-6,73E-02	7,20E-01	8,69E-01	No	-1,07E+00	3,86E-02	9,01E-02	No

ENSG00000222570	AF216667.1	miRNA	6,57E-03	9,65E-01	NA	No	-2,83E-02	8,90E-01	NA	No
ENSG00000207582	MIR30B	miRNA	-2,49E-02	8,93E-01	9,56E-01	No	2,94E-01	4,14E-01	5,70E-01	No
ENSG00000199153	MIR30D	miRNA	-2,81E-02	8,21E-01	NA	No	1,19E-01	6,98E-01	8,06E-01	No
ENSG00000254324	MIR151A	miRNA	-1,57E-01	3,86E-01	6,32E-01	No	-2,91E-01	3,49E-01	5,04E-01	No
ENSG00000263660	AC087793.1	miRNA	-1,11E-01	5,17E-01	NA	No	-4,70E-01	1,77E-01	NA	No
ENSG00000265660	MIR4664	miRNA	2,53E-02	8,78E-01	9,49E-01	No	-4,83E-01	1,25E-01	2,33E-01	No
ENSG00000264144	AC105049.1	miRNA	3,32E-02	9,36E-01	NA	No	8,77E-02	8,64E-01	NA	No
ENSG00000265295	AC084125.3	miRNA	1,50E-02	9,67E-01	NA	No	6,59E-02	7,95E-01	NA	No
ENSG00000221730	AL162419.1	miRNA	-4,15E-02	7,60E-01	NA	No	-9,51E-02	8,30E-01	NA	No
ENSG00000238362	AL158147.1	miRNA	1,65E-03	9,41E-01	NA	No	5,68E-02	7,81E-01	NA	No
ENSG00000223211	AL158150.1	miRNA	6,59E-02	4,92E-01	NA	No	8,55E-02	8,64E-01	NA	No
ENSG00000263790	MIR4473	miRNA	2,05E-01	2,86E-01	5,32E-01	No	1,75E-01	5,85E-01	7,18E-01	No
ENSG00000264941	MIR4474	miRNA	-2,09E-02	8,42E-01	NA	No	-2,02E-01	3,65E-01	NA	No
ENSG00000252324	AL163193.1	miRNA	3,45E-01	1,46E-01	3,57E-01	No	1,13E-01	7,47E-01	8,40E-01	No
ENSG00000266732	AL359922.1	miRNA	1,44E-01	4,19E-01	6,62E-01	No	-1,21E-01	6,48E-01	7,68E-01	No
ENSG00000216070	AL158155.1	miRNA	1,36E-02	9,17E-01	NA	No	2,67E-02	9,25E-01	9,55E-01	No
ENSG00000238638	AL353626.1	miRNA	4,75E-01	8,04E-02	2,41E-01	No	3,66E-01	2,90E-01	4,40E-01	No
ENSG00000264898	AL353626.2	miRNA	5,97E-01	3,33E-02	1,31E-01	No	2,30E-01	4,59E-01	6,12E-01	No
ENSG00000265285	AL591379.1	miRNA	1,98E-02	7,72E-01	NA	No	9,79E-02	6,70E-01	7,85E-01	No
ENSG00000265601	AL845321.1	miRNA	-2,28E-01	2,37E-01	4,76E-01	No	-2,79E-01	4,11E-01	5,67E-01	No
ENSG00000266021	AL353763.2	miRNA	2,35E-01	3,19E-01	5,67E-01	No	1,70E-01	6,13E-01	7,41E-01	No
ENSG00000238919	AL353763.1	miRNA	2,18E-01	3,44E-01	5,92E-01	No	9,34E-02	7,89E-01	8,69E-01	No
ENSG00000239070	MIR1299	miRNA	2,39E-01	3,11E-01	5,60E-01	No	2,78E-01	3,90E-01	5,47E-01	No
ENSG00000264681	AL445665.2	miRNA	-3,21E-01	7,81E-02	2,36E-01	No	-3,76E-01	2,88E-01	4,38E-01	No
ENSG00000239028	AL359955.1	miRNA	2,15E-01	1,36E-01	3,41E-01	No	6,75E-02	7,88E-01	8,69E-01	No
ENSG00000238826	AL359253.1	miRNA	-5,30E-02	6,37E-01	NA	No	9,43E-03	9,70E-01	9,82E-01	No
ENSG00000265488	AL359314.1	miRNA	1,36E-03	9,60E-01	NA	No	6,54E-02	7,09E-01	NA	No
ENSG00000265112	MIR3153	miRNA	3,70E-02	8,45E-01	NA	No	1,31E-01	6,43E-01	NA	No
ENSG00000264158	MIR4670	miRNA	5,40E-02	6,18E-01	NA	No	3,18E-02	8,87E-01	NA	No
ENSG00000199165	MIRLET7A1	miRNA	-1,41E-01	4,75E-01	7,07E-01	No	1,33E+00	2,77E-03	9,21E-03	No
ENSG00000199072	MIRLET7F1	miRNA	4,01E-02	8,41E-01	9,31E-01	No	7,91E-01	4,87E-02	1,10E-01	No
ENSG00000199133	MIRLET7D	miRNA	8,69E-03	9,64E-01	9,85E-01	No	4,58E-02	8,97E-01	9,39E-01	No
ENSG00000252153	MIR2278	miRNA	1,58E-01	3,86E-01	6,32E-01	No	6,42E-02	8,21E-01	8,90E-01	No
ENSG00000207563	MIR23B	miRNA	-9,26E-02	6,67E-01	8,37E-01	No	-4,91E-01	1,88E-01	3,20E-01	No
ENSG00000207864	MIR27B	miRNA	-6,95E-01	3,06E-02	1,24E-01	No	-7,70E-01	3,91E-02	9,11E-02	No
ENSG00000265367	AL160269.1	miRNA	-3,21E-03	9,00E-01	NA	No	-1,26E-01	7,12E-01	NA	No
ENSG00000221269	MIR1302-8	miRNA	9,70E-02	4,34E-01	NA	No	-3,39E-02	8,82E-01	NA	No
ENSG00000263475	AL353805.1	miRNA	-9,12E-02	6,08E-01	8,02E-01	No	9,75E-03	9,79E-01	9,87E-01	No
ENSG00000265068	AL591377.1	miRNA	-1,16E-01	5,62E-01	7,71E-01	No	-5,93E-01	1,39E-01	2,53E-01	No
ENSG00000222512	AL137019.1	miRNA	-5,73E-02	6,46E-01	NA	No	-7,59E-02	8,00E-01	NA	No
ENSG00000207698	MIR32	miRNA	3,18E-01	1,63E-01	3,82E-01	No	6,48E-01	1,19E-01	2,25E-01	No
ENSG00000265647	AL358815.1	miRNA	1,79E-01	1,51E-01	NA	No	2,38E-02	9,87E-01	NA	No
ENSG00000266315	MIR4668	miRNA	-3,32E-02	7,61E-01	NA	No	-1,66E-01	4,73E-01	NA	No
ENSG00000207726	MIR455	miRNA	3,32E-02	7,34E-01	NA	No	-1,39E-02	8,90E-01	NA	No
ENSG00000221772	AL353141.1	miRNA	2,97E-01	6,06E-02	1,98E-01	No	2,60E-02	9,14E-01	NA	No
ENSG00000216182	AL137024.1	miRNA	-6,52E-02	5,56E-01	NA	No	-1,39E-01	5,03E-01	NA	No
ENSG00000252940	AL357936.1	miRNA	-3,43E-02	7,31E-01	NA	No	-2,55E-02	8,90E-01	NA	No
ENSG00000272317	AL162424.2	miRNA	-1,41E-02	9,16E-01	NA	No	-2,64E-01	3,01E-01	NA	No
ENSG00000207991	MIR601	miRNA	-7,96E-03	9,65E-01	9,86E-01	No	-1,00E+00	4,57E-02	1,04E-01	No
ENSG00000222722	AL161790.1	miRNA	4,69E-02	6,37E-01	NA	No	-1,02E-01	6,39E-01	NA	No
ENSG00000264237	AL137846.1	miRNA	1,05E-01	5,86E-01	7,87E-01	No	-4,17E-01	2,56E-01	4,01E-01	No
ENSG00000207595	MIR181A2	miRNA	-8,07E-02	6,94E-01	8,54E-01	No	-2,21E-01	5,36E-01	6,79E-01	No
ENSG00000207737	MIR181B2	miRNA	-2,77E-01	1,88E-01	4,16E-01	No	-3,58E-01	3,32E-01	4,87E-01	No
ENSG00000264641	AL354928.1	miRNA	1,94E-02	8,63E-01	NA	No	-1,59E-01	4,82E-01	NA	No
ENSG00000264329	MIR3911	miRNA	-1,36E-01	3,65E-01	NA	No	-5,48E-01	1,10E-01	NA	No
ENSG00000263979	MIR4672	miRNA	1,29E-01	1,90E-01	NA	No	-6,53E-02	8,00E-01	NA	No
ENSG00000207581	MIR199B	miRNA	2,42E-02	9,72E-01	NA	No	8,55E-02	8,64E-01	NA	No
ENSG00000221521	AL158151.2	miRNA	-3,21E-02	7,66E-01	NA	No	-1,61E-01	4,69E-01	NA	No
ENSG00000263897	MIR4669	miRNA	-1,91E-02	8,85E-01	NA	No	-2,23E-01	3,53E-01	NA	No
ENSG00000264744	MIR3689C	miRNA	-5,52E-02	6,46E-01	NA	No	-1,31E-01	7,12E-01	NA	No
ENSG00000266060	AL603650.4	miRNA	2,23E-02	9,72E-01	NA	No	1,09E-01	6,78E-01	NA	No
ENSG00000264163	MIR3689B	miRNA	-1,30E-02	8,49E-01	NA	No	-9,72E-02	8,30E-01	NA	No

ENSG00000264136	MIR3689D2	miRNA	-6,04E-02	5,47E-01	NA	No	-9,79E-02	6,70E-01	NA	No
ENSG00000266827	MIR3689E	miRNA	3,85E-02	8,45E-01	NA	No	8,58E-02	8,64E-01	NA	No
ENSG00000263403	MIR4673	miRNA	1,18E-02	9,64E-01	NA	No	-1,03E-01	8,30E-01	NA	No
ENSG00000199161	MIR126	miRNA	1,55E-01	4,48E-01	6,85E-01	No	-2,55E-02	9,39E-01	9,64E-01	No
ENSG00000265806	MIR4292	miRNA	-1,15E+00	7,09E-03	4,41E-02	No	-4,51E-01	2,39E-01	3,82E-01	No
ENSG00000266507	MIR4479	miRNA	3,41E-02	9,36E-01	NA	No	1,13E-01	6,43E-01	NA	No
ENSG00000263933	AL365502.1	miRNA	4,32E-02	7,11E-01	NA	No	-2,07E-02	9,21E-01	NA	No
ENSG00000207693	MIR602	miRNA	7,65E-02	3,79E-01	NA	No	8,58E-02	8,64E-01	NA	No
ENSG00000263511	MIR5699	miRNA	-1,22E-01	4,43E-01	6,81E-01	No	-1,39E+00	2,01E-02	5,19E-02	No
ENSG00000263628	MIR3155A	miRNA	1,43E-01	2,97E-01	NA	No	3,64E-02	8,88E-01	NA	No
ENSG00000265653	MIR548AK	miRNA	-1,51E-02	8,49E-01	NA	No	3,82E-02	8,91E-01	NA	No
ENSG00000263584	MIR4480	miRNA	1,40E-01	1,38E-01	NA	No	1,37E-01	4,47E-01	NA	No
ENSG00000221331	MIR548Q	miRNA	-2,27E-02	7,98E-01	NA	No	5,74E-02	7,46E-01	NA	No
ENSG00000222235	AL157392.1	miRNA	6,29E-02	5,68E-01	NA	No	-2,37E-02	9,10E-01	NA	No
ENSG00000207937	MIR511-1	miRNA	1,31E-01	4,10E-01	6,54E-01	No	1,26E-01	6,78E-01	7,91E-01	No
ENSG00000207938	MIR511-2	miRNA	1,54E-01	4,10E-01	6,53E-01	No	4,65E-01	2,14E-01	3,52E-01	No
ENSG00000212072	AL353147.1	miRNA	4,03E-02	7,82E-01	NA	No	1,14E-01	6,19E-01	NA	No
ENSG00000265372	MIR4675	miRNA	-2,87E-02	7,31E-01	NA	No	-1,26E-01	7,12E-01	NA	No
ENSG00000222071	MIR1915	miRNA	-5,69E-02	6,46E-01	NA	No	1,43E-01	5,02E-01	NA	No
ENSG00000207930	MIR603	miRNA	1,60E-01	3,06E-01	5,54E-01	No	-1,12E-01	7,30E-01	8,29E-01	No
ENSG00000263577	AL160291.1	miRNA	-5,11E-02	6,70E-01	NA	No	-1,25E-01	6,60E-01	7,78E-01	No
ENSG00000222374	AC022021.1	miRNA	4,49E-02	6,63E-01	NA	No	6,17E-02	9,56E-01	NA	No
ENSG00000216035	MIR938	miRNA	-1,68E-01	4,51E-01	6,88E-01	No	-6,91E-01	9,36E-02	1,86E-01	No
ENSG00000263576	AL121748.1	miRNA	2,46E-02	9,72E-01	NA	No	6,23E-02	9,56E-01	NA	No
ENSG00000266228	MIR3611	miRNA	5,64E-02	7,33E-01	8,76E-01	No	-3,36E-01	3,06E-01	4,58E-01	No
ENSG00000266684	AL117337.1	miRNA	4,58E-01	1,71E-02	NA	No	1,41E-01	5,50E-01	NA	No
ENSG00000264800	MIR4294	miRNA	-1,33E-02	8,81E-01	NA	No	-1,34E-01	7,12E-01	NA	No
ENSG00000207813	MIR605	miRNA	7,13E-03	9,75E-01	9,90E-01	No	-3,87E-01	2,97E-01	4,49E-01	No
ENSG00000216022	AC068062.1	miRNA	-1,52E-01	4,32E-01	6,72E-01	No	8,63E-01	4,61E-02	1,05E-01	No
ENSG00000221063	MIR1296	miRNA	3,22E-02	8,85E-01	9,52E-01	No	-9,91E-01	2,40E-02	6,03E-02	No
ENSG00000253012	AC022538.1	miRNA	-2,79E-02	7,90E-01	NA	No	-2,67E-01	2,66E-01	NA	No
ENSG00000221159	AL356741.1	miRNA	-1,71E-01	2,93E-01	5,41E-01	No	-2,84E-01	3,63E-01	5,19E-01	No
ENSG00000221184	MIR1254-1	miRNA	-9,40E-02	6,55E-01	8,30E-01	No	-1,43E+00	2,54E-03	8,52E-03	No
ENSG00000221774	AC016821.1	miRNA	-9,55E-02	6,12E-01	8,05E-01	No	-1,00E+00	4,51E-02	1,03E-01	No
ENSG00000222631	AC025426.1	miRNA	-5,47E-02	6,46E-01	NA	No	-1,30E-01	7,12E-01	NA	No
ENSG00000216004	AL359832.1	miRNA	-6,60E-02	5,73E-01	NA	No	-1,80E-01	4,33E-01	NA	No
ENSG00000238766	AL513185.1	miRNA	2,54E-01	4,60E-02	NA	No	2,85E-01	2,44E-01	NA	No
ENSG00000266719	MIR4676	miRNA	4,76E-02	7,18E-01	NA	No	5,82E-01	8,12E-02	NA	No
ENSG00000266344	AL353731.1	miRNA	-4,42E-02	7,60E-01	NA	No	1,67E-02	9,87E-01	NA	No
ENSG00000263626	AL392111.1	miRNA	-4,21E-02	7,60E-01	NA	No	3,82E-02	8,91E-01	NA	No
ENSG00000207583	MIR606	miRNA	9,25E-01	1,35E-02	6,95E-02	No	8,94E-01	3,93E-02	9,15E-02	No
ENSG00000215921	AL731568.1	miRNA	3,77E-02	8,30E-01	9,26E-01	No	-1,96E-01	5,37E-01	6,80E-01	No
ENSG00000221232	AC012047.1	miRNA	1,86E-01	1,66E-01	3,87E-01	No	1,31E-01	6,15E-01	7,43E-01	No
ENSG00000238803	AL732479.1	miRNA	-6,34E-02	4,94E-01	NA	No	-6,91E-02	7,24E-01	NA	No
ENSG00000266769	AL353751.1	miRNA	-3,53E-02	7,21E-01	NA	No	-1,59E-01	4,92E-01	NA	No
ENSG00000198997	MIR107	miRNA	1,37E-01	2,21E-01	NA	No	3,39E-01	2,34E-01	NA	No
ENSG00000264076	AL158040.1	miRNA	-1,85E-02	8,49E-01	NA	No	-1,02E-01	8,30E-01	NA	No
ENSG00000266407	MIR3157	miRNA	2,54E-02	8,81E-01	9,50E-01	No	-4,61E-02	8,85E-01	9,31E-01	No
ENSG00000221465	AL162502.1	miRNA	7,02E-02	4,92E-01	NA	No	-2,32E-02	8,90E-01	NA	No
ENSG00000264677	AL355490.2	miRNA	3,13E-02	7,09E-01	NA	No	-1,64E-02	9,82E-01	NA	No
ENSG00000264610	MIR4685	miRNA	-3,13E-01	1,13E-01	3,02E-01	No	-1,24E+00	2,42E-02	6,09E-02	No
ENSG00000251989	AL160011.1	miRNA	-4,88E-02	8,04E-01	9,13E-01	No	-5,69E-01	1,58E-01	2,79E-01	No
ENSG00000202569	MIR146B	miRNA	-3,38E-02	7,59E-01	NA	No	-2,39E-01	3,27E-01	NA	No
ENSG00000221767	MIR1307	miRNA	1,59E-02	9,65E-01	NA	No	-9,51E-02	8,30E-01	NA	No
ENSG00000216083	MIR936	miRNA	-5,73E-02	6,46E-01	NA	No	-1,36E-01	7,12E-01	NA	No
ENSG00000266852	MIR4482-1	miRNA	-1,64E-01	4,55E-01	6,90E-01	No	-6,09E-01	1,27E-01	2,35E-01	No
ENSG00000264544	AL355512.2	miRNA	-5,58E-02	6,46E-01	NA	No	-1,33E-01	7,12E-01	NA	No
ENSG00000221214	MIR548E	miRNA	7,54E-02	7,08E-01	8,62E-01	No	5,10E-01	1,79E-01	3,07E-01	No
ENSG00000264763	MIR4295	miRNA	-1,23E-01	4,11E-01	6,55E-01	No	-4,50E-01	2,12E-01	3,49E-01	No
ENSG00000222197	AL359836.1	miRNA	1,57E-01	2,10E-01	NA	No	4,21E-02	8,47E-01	NA	No
ENSG00000215925	AL157788.1	miRNA	-5,37E-02	6,46E-01	NA	No	-1,26E-01	7,12E-01	NA	No
ENSG00000265719	MIR4681	miRNA	-2,03E-02	8,88E-01	NA	No	-1,28E-01	5,94E-01	NA	No

ENSG00000265370	MIR4682	miRNA	8,38E-02	4,50E-01	NA	No	-1,03E-01	8,30E-01	NA	No
ENSG00000265442	MIR3941	miRNA	7,12E-02	6,50E-01	8,28E-01	No	3,54E-01	3,07E-01	4,60E-01	No
ENSG00000265463	AC012391.1	miRNA	7,02E-02	4,34E-01	NA	No	1,61E-01	4,88E-01	NA	No
ENSG00000265092	MIR4484	miRNA	9,19E-02	5,52E-01	7,64E-01	No	-2,07E-01	4,68E-01	6,21E-01	No
ENSG00000215972	AL360176.1	miRNA	-3,10E-02	7,83E-01	NA	No	-1,48E-01	5,03E-01	NA	No
ENSG00000266061	AL355531.1	miRNA	4,58E-02	6,86E-01	NA	No	-1,60E-02	9,34E-01	NA	No
ENSG00000266676	MIR4297	miRNA	-2,74E-02	8,49E-01	NA	No	-2,37E-01	3,73E-01	NA	No
ENSG00000266547	AL161645.1	miRNA	9,89E-02	2,85E-01	NA	No	7,86E-02	6,57E-01	NA	No
ENSG00000199038	MIR210	miRNA	1,32E-01	2,40E-01	NA	No	7,52E-02	6,90E-01	NA	No
ENSG00000264671	AP006477.1	miRNA	4,47E-02	6,74E-01	NA	No	1,00E-01	7,75E-01	NA	No
ENSG00000252187	AC051649.1	miRNA	-1,80E-02	8,96E-01	9,57E-01	No	-4,24E-01	9,29E-02	1,85E-01	No
ENSG00000207805	MIR483	miRNA	-1,56E-02	8,58E-01	NA	No	-1,60E-01	4,77E-01	NA	No
ENSG00000263365	AC123788.1	miRNA	1,72E-02	8,96E-01	NA	No	-9,72E-02	8,30E-01	NA	No
ENSG00000266645	MIR4299	miRNA	5,28E-02	6,79E-01	NA	No	7,63E-02	7,79E-01	NA	No
ENSG00000221556	AC103794.1	miRNA	1,26E-02	9,32E-01	9,72E-01	No	-3,72E-02	8,99E-01	9,40E-01	No
ENSG00000266493	AC116533.3	miRNA	2,34E-02	9,72E-01	NA	No	6,68E-02	9,56E-01	NA	No
ENSG00000221322	AC116533.1	miRNA	-4,53E-02	6,56E-01	NA	No	1,25E-02	9,64E-01	9,79E-01	No
ENSG00000265210	MIR4486	miRNA	-2,22E-02	8,23E-01	NA	No	-8,44E-02	6,82E-01	NA	No
ENSG00000264309	MIR4694	miRNA	3,05E-02	8,25E-01	NA	No	5,14E-02	8,46E-01	9,06E-01	No
ENSG00000264478	AC100767.1	miRNA	3,46E-02	9,36E-01	NA	No	8,55E-02	8,64E-01	NA	No
ENSG00000207874	MIR610	miRNA	-4,15E-02	7,60E-01	NA	No	-9,51E-02	8,30E-01	NA	No
ENSG00000221338	AC108456.1	miRNA	1,49E-01	3,26E-01	NA	No	1,09E+00	3,33E-02	7,96E-02	No
ENSG00000251862	MIR1343	miRNA	9,82E-02	5,90E-01	7,90E-01	No	7,96E-01	6,99E-02	1,47E-01	No
ENSG00000263389	MIR3973	miRNA	1,25E-01	3,02E-01	NA	No	1,49E-01	5,21E-01	NA	No
ENSG00000263539	AC024475.1	miRNA	-3,58E-02	8,53E-01	9,36E-01	No	1,35E-01	7,00E-01	8,07E-01	No
ENSG00000263540	MIR5582	miRNA	-4,33E-02	7,60E-01	NA	No	5,26E-02	8,82E-01	NA	No
ENSG00000264583	MIR4487	miRNA	-5,73E-02	6,46E-01	NA	No	-1,36E-01	7,12E-01	NA	No
ENSG00000263693	MIR3161	miRNA	1,02E-01	3,57E-01	NA	No	-3,25E-02	8,85E-01	NA	No
ENSG00000266018	AP000781.1	miRNA	-2,04E-02	7,24E-01	NA	No	-1,42E-01	6,24E-01	NA	No
ENSG00000208009	MIR130A	miRNA	1,70E-02	8,77E-01	NA	No	-1,55E-01	5,29E-01	NA	No
ENSG00000264559	MIR3162	miRNA	-8,77E-03	9,50E-01	NA	No	-7,75E-02	7,77E-01	NA	No
ENSG00000238861	AP003064.1	miRNA	2,34E-02	7,83E-01	NA	No	-1,35E-01	7,12E-01	NA	No
ENSG00000265874	MIR4489	miRNA	1,99E-01	2,30E-01	NA	No	-7,42E-02	7,62E-01	NA	No
ENSG00000221553	AC004924.1	miRNA	-5,47E-02	6,46E-01	NA	No	-1,30E-01	7,12E-01	NA	No
ENSG00000222339	AP000807.2	miRNA	-1,52E-01	4,61E-01	6,96E-01	No	2,89E-01	4,20E-01	5,76E-01	No
ENSG00000212093	AP000807.1	miRNA	2,77E-01	2,99E-02	NA	No	1,99E-01	3,42E-01	NA	No
ENSG00000266854	AP003096.1	miRNA	-4,55E-02	6,12E-01	NA	No	-1,34E-01	4,39E-01	NA	No
ENSG00000265539	MIR3164	miRNA	-6,34E-02	7,12E-01	8,65E-01	No	-2,42E-01	4,65E-01	6,17E-01	No
ENSG00000221333	MIR548K	miRNA	6,49E-02	5,50E-01	7,63E-01	No	-2,17E-02	9,27E-01	NA	No
ENSG00000264091	AP002495.1	miRNA	3,49E-02	7,45E-01	NA	No	7,98E-02	7,48E-01	NA	No
ENSG00000266859	AP000719.1	miRNA	3,13E-01	1,59E-01	3,76E-01	No	2,05E-01	4,95E-01	6,44E-01	No
ENSG00000265902	MIR4696	miRNA	-5,69E-02	6,46E-01	NA	No	-1,35E-01	7,12E-01	NA	No
ENSG00000264095	AP000560.1	miRNA	2,03E-01	1,76E-01	4,00E-01	No	4,87E-02	8,64E-01	NA	No
ENSG00000265344	AP001992.1	miRNA	-5,98E-02	5,58E-01	NA	No	-7,55E-02	7,08E-01	NA	No
ENSG00000199090	MIR326	miRNA	4,77E-02	6,50E-01	NA	No	1,89E-01	3,90E-01	NA	No
ENSG00000212030	AP002498.1	miRNA	1,52E-01	3,23E-01	5,72E-01	No	3,24E-01	3,40E-01	4,96E-01	No
ENSG00000271767	AP002789.1	miRNA	4,17E-02	7,65E-01	NA	No	3,16E-01	1,77E-01	NA	No
ENSG00000211997	MIR708	miRNA	-8,65E-02	4,55E-01	NA	No	-3,64E-01	1,26E-01	NA	No
ENSG00000266570	MIR5579	miRNA	7,94E-02	4,23E-01	NA	No	-3,30E-02	9,21E-01	NA	No
ENSG00000264838	AP003305.1	miRNA	-1,90E-02	8,82E-01	9,51E-01	No	-1,90E-01	4,56E-01	6,09E-01	No
ENSG00000221586	MIR1261	miRNA	3,41E-02	9,36E-01	NA	No	4,12E-01	1,31E-01	NA	No
ENSG00000263966	AP000765.1	miRNA	2,64E-01	2,34E-01	4,72E-01	No	1,17E+00	1,81E-02	4,76E-02	No
ENSG00000221230	MIR548L	miRNA	2,32E-01	1,77E-01	4,01E-01	No	-1,05E-01	6,84E-01	7,96E-01	No
ENSG00000265208	AC015600.1	miRNA	3,88E-02	8,45E-01	NA	No	3,06E-01	2,07E-01	NA	No
ENSG00000264200	MIR4693	miRNA	-6,60E-02	4,86E-01	NA	No	-1,35E-01	5,79E-01	NA	No
ENSG00000264542	AP001282.1	miRNA	-7,24E-02	3,74E-01	NA	No	-1,78E-01	3,89E-01	NA	No
ENSG00000215954	AP002884.1	miRNA	2,86E-02	6,97E-01	NA	No	-8,04E-02	6,98E-01	NA	No
ENSG00000221112	AP000908.1	miRNA	3,65E-01	1,47E-01	3,58E-01	No	-1,07E+00	1,87E-02	4,89E-02	No
ENSG00000264344	AP000936.2	miRNA	-1,57E-02	8,75E-01	NA	No	-2,16E-01	3,63E-01	NA	No
ENSG00000207971	MIR125B1	miRNA	-2,32E-01	3,22E-01	5,71E-01	No	6,60E-01	4,70E-02	1,06E-01	No
ENSG00000198975	MIRLET7A2	miRNA	2,70E-02	8,63E-01	9,41E-01	No	-1,50E-02	9,62E-01	9,78E-01	No
ENSG00000207994	MIR100	miRNA	-5,11E-02	6,19E-01	NA	No	-3,76E-02	8,67E-01	NA	No

ENSG00000265357	MIR4493	miRNA	5,12E-03	9,73E-01	9,89E-01	No	-1,02E-01	7,16E-01	8,19E-01	No
ENSG00000266215	MIR3167	miRNA	-8,83E-02	3,33E-01	NA	No	-1,50E-01	4,68E-01	NA	No
ENSG00000264919	MIR4697	miRNA	-1,02E-01	6,41E-01	8,22E-01	No	-5,56E-01	1,24E-01	2,31E-01	No
ENSG00000266026	AC092111.1	miRNA	-6,02E-02	5,50E-01	NA	No	-6,36E-02	7,60E-01	NA	No
ENSG00000221307	AC092745.1	miRNA	3,80E-02	8,45E-01	NA	No	8,77E-02	8,64E-01	NA	No
ENSG00000207983	MIR613	miRNA	-5,47E-02	6,46E-01	NA	No	-1,30E-01	7,12E-01	NA	No
ENSG00000215923	AC007528.1	miRNA	-7,24E-02	5,34E-01	NA	No	-2,16E-01	2,92E-01	NA	No
ENSG00000221679	AC087237.1	miRNA	-3,50E-02	7,31E-01	NA	No	-1,36E-01	7,12E-01	NA	No
ENSG00000216192	MIR920	miRNA	2,59E-01	2,19E-01	4,56E-01	No	-3,95E-02	9,07E-01	9,45E-01	No
ENSG00000265012	AC024896.1	miRNA	2,43E-01	1,22E-01	NA	No	1,14E-01	6,28E-01	NA	No
ENSG00000266482	AC023157.1	miRNA	7,44E-02	4,55E-01	NA	No	2,57E-01	2,82E-01	NA	No
ENSG00000223082	AC121336.1	miRNA	-2,18E-01	3,12E-01	5,61E-01	No	-1,26E+00	1,89E-02	4,92E-02	No
ENSG00000222884	AC023513.1	miRNA	1,34E-02	8,34E-01	NA	No	1,25E-02	9,11E-01	NA	No
ENSG00000263838	MIR4698	miRNA	4,10E-01	5,09E-02	1,76E-01	No	7,94E-02	7,30E-01	NA	No
ENSG00000221604	MIR1293	miRNA	-4,42E-02	7,60E-01	NA	No	4,50E-03	9,87E-01	NA	No
ENSG00000199122	MIR148B	miRNA	1,12E-02	9,24E-01	NA	No	-1,49E-01	5,69E-01	NA	No
ENSG00000208028	MIR616	miRNA	-4,55E-01	7,89E-02	2,38E-01	No	-9,39E-01	5,19E-02	1,15E-01	No
ENSG00000266465	AC025165.1	miRNA	5,83E-02	5,80E-01	NA	No	1,40E-01	5,82E-01	NA	No
ENSG00000207789	MIR26A2	miRNA	-2,08E-02	8,98E-01	9,57E-01	No	-4,57E-01	2,23E-01	3,63E-01	No
ENSG00000199179	MIRLET7I	miRNA	-1,57E-02	9,35E-01	9,73E-01	No	-5,79E-01	1,47E-01	2,65E-01	No
ENSG00000207546	MIR548C	miRNA	-3,88E-02	7,46E-01	NA	No	-9,15E-02	7,14E-01	NA	No
ENSG00000266539	AC078889.1	miRNA	1,19E-01	5,32E-01	7,51E-01	No	2,59E-01	4,68E-01	6,20E-01	No
ENSG00000263910	AC133749.1	miRNA	-5,73E-02	6,46E-01	NA	No	-1,36E-01	7,12E-01	NA	No
ENSG00000264405	MIR3913-1	miRNA	-2,06E-02	8,34E-01	NA	No	-1,50E-01	5,87E-01	NA	No
ENSG00000221788	MIR1252	miRNA	-6,48E-02	4,51E-01	NA	No	-1,28E-01	5,25E-01	NA	No
ENSG00000207763	MIR617	miRNA	2,94E-01	1,29E-01	3,29E-01	No	4,95E-01	2,08E-01	3,44E-01	No
ENSG00000208022	MIR618	miRNA	1,04E-02	9,41E-01	9,76E-01	No	1,45E+00	8,12E-03	2,37E-02	No
ENSG00000265227	MIR4699	miRNA	3,83E-02	8,12E-01	9,17E-01	No	-1,11E-01	7,21E-01	8,23E-01	No
ENSG00000221121	AC083811.1	miRNA	3,15E-02	9,36E-01	NA	No	6,17E-02	9,56E-01	NA	No
ENSG00000266347	AC068641.1	miRNA	3,00E-01	2,19E-01	4,56E-01	No	-1,57E-01	6,54E-01	7,73E-01	No
ENSG00000221617	AC138123.1	miRNA	3,31E-02	9,36E-01	NA	No	1,65E-01	4,59E-01	NA	No
ENSG00000199172	MIR331	miRNA	3,92E-02	7,74E-01	8,97E-01	No	-8,32E-02	7,83E-01	8,65E-01	No
ENSG00000265917	MIR3685	miRNA	-6,22E-02	7,37E-01	8,77E-01	No	5,33E-01	1,67E-01	2,91E-01	No
ENSG00000221479	MIR1251	miRNA	8,91E-02	4,30E-01	NA	No	3,72E-02	8,62E-01	NA	No
ENSG00000207586	MIR135A2	miRNA	-1,34E-02	8,19E-01	NA	No	7,14E-02	7,83E-01	NA	No
ENSG00000221625	AC013283.1	miRNA	-9,84E-02	6,53E-01	8,29E-01	No	1,84E-01	5,17E-01	6,62E-01	No
ENSG00000239036	AC010209.1	miRNA	-5,45E-02	6,46E-01	NA	No	-1,28E-01	7,12E-01	NA	No
ENSG00000215976	AC078819.1	miRNA	5,23E-02	6,49E-01	NA	No	2,35E-01	3,55E-01	NA	No
ENSG00000264295	MIR3922	miRNA	2,07E-02	8,16E-01	NA	No	-9,95E-02	6,39E-01	NA	No
ENSG00000265054	AC090051.1	miRNA	1,15E-02	9,66E-01	NA	No	-2,50E-02	9,21E-01	NA	No
ENSG00000207622	MIR619	miRNA	-2,73E-02	8,87E-01	9,53E-01	No	-2,26E-01	5,06E-01	6,53E-01	No
ENSG00000221246	AC144522.1	miRNA	2,67E-01	7,03E-02	NA	No	1,30E-01	5,93E-01	NA	No
ENSG00000221386	AC002979.1	miRNA	-5,73E-02	6,46E-01	NA	No	-1,36E-01	7,12E-01	NA	No
ENSG00000266269	AC002395.1	miRNA	8,19E-03	9,86E-01	NA	No	7,13E-02	7,42E-01	NA	No
ENSG00000266370	MIR3657	miRNA	-5,38E-01	3,94E-02	1,48E-01	No	-7,79E-01	7,11E-02	1,49E-01	No
ENSG00000207967	MIR620	miRNA	-7,94E-02	6,25E-01	8,13E-01	No	8,77E-01	5,78E-02	1,26E-01	No
ENSG00000263768	AC060226.1	miRNA	1,94E-01	3,40E-01	5,88E-01	No	7,58E-01	7,66E-02	1,58E-01	No
ENSG00000221280	AC026366.1	miRNA	2,44E-01	5,28E-02	NA	No	1,20E-01	5,35E-01	NA	No
ENSG00000266704	MIR4498	miRNA	3,44E-02	9,36E-01	NA	No	8,87E-02	8,64E-01	NA	No
ENSG00000263913	AC004812.1	miRNA	2,46E-01	8,71E-02	NA	No	-1,43E-02	9,80E-01	NA	No
ENSG00000265526	MIR4304	miRNA	-1,04E-01	4,66E-01	7,00E-01	No	-5,76E-01	6,72E-02	1,43E-01	No
ENSG00000265345	MIR5188	miRNA	6,71E-02	5,40E-01	NA	No	5,25E-01	1,33E-01	2,45E-01	No
ENSG00000265635	MIR3612	miRNA	1,96E-01	9,40E-02	NA	No	5,19E-01	1,08E-01	NA	No
ENSG00000271905	AC137590.1	miRNA	6,00E-01	4,35E-02	1,58E-01	No	4,65E-01	2,14E-01	3,51E-01	No
ENSG00000263978	MIR4499	miRNA	9,82E-02	3,17E-01	NA	No	2,95E-02	9,11E-01	NA	No
ENSG00000252695	MIR2276	miRNA	-1,05E-01	3,21E-01	NA	No	-2,34E-01	2,35E-01	NA	No
ENSG00000266216	AL359538.2	miRNA	-6,75E-02	3,80E-01	NA	No	-1,64E-01	4,37E-01	NA	No
ENSG00000221670	AL596092.1	miRNA	-1,61E-01	2,33E-01	4,71E-01	No	-6,12E-01	1,08E-01	2,08E-01	No
ENSG00000221677	AL161898.1	miRNA	-5,45E-02	6,46E-01	NA	No	-1,28E-01	7,12E-01	NA	No
ENSG00000264288	AL138999.1	miRNA	2,17E-01	2,55E-01	4,98E-01	No	5,89E-01	1,42E-01	2,57E-01	No
ENSG00000263663	AL161891.1	miRNA	-1,26E-01	3,55E-01	NA	No	-1,73E-01	5,15E-01	6,61E-01	No
ENSG00000221519	AL390071.1	miRNA	1,55E-02	9,23E-01	9,68E-01	No	1,03E-01	7,57E-01	8,47E-01	No

ENSG00000266047	AL139377.1	miRNA	-1,39E-01	2,71E-01	NA	No	-3,55E-01	2,79E-01	4,28E-01	No
ENSG00000221100	AL138706.1	miRNA	1,45E-01	4,77E-01	7,09E-01	No	2,43E-01	4,97E-01	6,46E-01	No
ENSG00000212049	AL590007.1	miRNA	-9,10E-03	8,86E-01	NA	No	-1,27E-01	7,12E-01	NA	No
ENSG00000264171	MIR4305	miRNA	1,34E-01	4,43E-01	6,81E-01	No	2,70E-01	4,45E-01	5,99E-01	No
ENSG00000211491	MIR320D1	miRNA	-4,93E-02	7,46E-01	8,82E-01	No	-8,70E-02	7,78E-01	8,62E-01	No
ENSG00000207652	MIR621	miRNA	1,78E-01	4,33E-01	6,73E-01	No	5,01E-01	8,90E-02	1,79E-01	No
ENSG00000263907	AL354833.1	miRNA	-5,58E-02	6,46E-01	NA	No	-1,33E-01	7,12E-01	NA	No
ENSG00000263928	AL354833.2	miRNA	3,15E-02	9,36E-01	NA	No	8,64E-02	8,64E-01	NA	No
ENSG00000264190	MIR5006	miRNA	2,67E-02	8,20E-01	NA	No	3,93E-01	2,54E-01	NA	No
ENSG00000265679	AL139328.1	miRNA	-5,53E-03	9,69E-01	9,87E-01	No	-3,74E-01	1,63E-01	NA	No
ENSG00000221163	AL138963.1	miRNA	-6,53E-02	5,33E-01	NA	No	-2,16E-01	2,77E-01	NA	No
ENSG00000222391	AL137118.1	miRNA	-9,48E-04	8,99E-01	NA	No	-1,26E-01	7,12E-01	NA	No
ENSG00000264526	AL136123.1	miRNA	-1,92E-03	8,99E-01	NA	No	3,99E-03	9,81E-01	NA	No
ENSG00000266072	MIR5693	miRNA	-4,43E-02	7,94E-01	9,08E-01	No	-4,82E-01	2,01E-01	3,36E-01	No
ENSG00000263581	MIR548X2	miRNA	-2,99E-01	1,73E-01	3,97E-01	No	-8,46E-01	6,33E-02	1,36E-01	No
ENSG00000263561	MIR4704	miRNA	-5,45E-02	6,46E-01	NA	No	-1,28E-01	7,12E-01	NA	No
ENSG00000220981	AL391384.1	miRNA	-2,48E-01	2,53E-01	4,96E-01	No	-4,49E-01	2,43E-01	3,86E-01	No
ENSG00000263399	MIR3170	miRNA	-1,81E-02	8,19E-01	NA	No	-2,32E-02	8,90E-01	NA	No
ENSG00000207719	MIR623	miRNA	9,52E-03	9,67E-01	9,86E-01	No	-1,41E+00	1,18E-02	3,29E-02	No
ENSG00000263615	MIR4306	miRNA	7,10E-02	6,05E-01	8,00E-01	No	-2,70E-01	2,62E-01	NA	No
ENSG00000265164	MIR2681	miRNA	1,08E-02	9,35E-01	NA	No	1,23E-01	6,08E-01	NA	No
ENSG00000264482	MIR4705	miRNA	9,34E-02	5,12E-01	7,38E-01	No	1,08E-01	6,58E-01	7,77E-01	No
ENSG00000221650	MIR1267	miRNA	1,31E-01	2,60E-01	NA	No	2,20E-01	3,18E-01	NA	No
ENSG00000221473	AL138710.1	miRNA	1,49E-01	9,27E-02	NA	No	3,30E-01	1,66E-01	NA	No
ENSG00000264726	AL162711.1	miRNA	4,21E-02	6,75E-01	NA	No	-1,00E-01	6,37E-01	NA	No
ENSG00000264539	MIR548AR	miRNA	3,40E-02	7,85E-01	NA	No	8,34E-02	7,51E-01	NA	No
ENSG00000265450	MIR4502	miRNA	1,56E-02	8,86E-01	NA	No	-6,68E-02	7,61E-01	NA	No
ENSG00000215991	MIR208B	miRNA	2,76E-01	2,29E-01	4,67E-01	No	7,17E-01	8,11E-02	1,66E-01	No
ENSG00000223164	AL445384.1	miRNA	7,21E-02	4,18E-01	NA	No	9,91E-02	7,75E-01	NA	No
ENSG00000264797	AL356756.1	miRNA	6,35E-02	5,73E-01	NA	No	2,90E-01	2,38E-01	NA	No
ENSG00000207952	MIR624	miRNA	7,21E-02	4,96E-01	NA	No	1,40E-01	5,47E-01	NA	No
ENSG00000263784	AL136418.1	miRNA	-1,97E-02	8,45E-01	NA	No	1,52E-01	5,20E-01	NA	No
ENSG00000265244	AL358340.1	miRNA	3,77E-02	8,45E-01	NA	No	9,86E-02	7,75E-01	NA	No
ENSG00000263691	AL355885.1	miRNA	1,20E-01	2,83E-01	NA	No	5,55E-02	8,21E-01	NA	No
ENSG00000265530	AL121594.1	miRNA	5,47E-02	5,59E-01	NA	No	-1,26E-01	7,12E-01	NA	No
ENSG00000264234	AL161751.1	miRNA	-8,07E-02	6,85E-01	8,49E-01	No	-6,19E-02	8,55E-01	9,12E-01	No
ENSG00000221645	AL157791.1	miRNA	2,82E-01	1,96E-01	4,27E-01	No	8,64E-02	7,99E-01	8,76E-01	No
ENSG00000221695	AL390335.1	miRNA	-1,16E-03	9,40E-01	NA	No	5,66E-02	7,47E-01	NA	No
ENSG00000251731	AL512359.1	miRNA	2,22E-02	9,72E-01	NA	No	9,02E-02	8,64E-01	NA	No
ENSG00000265432	MIR4308	miRNA	-3,28E-02	7,74E-01	NA	No	-2,01E-01	3,19E-01	NA	No
ENSG00000252869	AL159140.1	miRNA	-2,72E-03	9,76E-01	NA	No	-3,77E-02	8,53E-01	NA	No
ENSG00000263629	MIR5586	miRNA	1,70E-01	1,03E-01	NA	No	1,44E-01	5,64E-01	NA	No
ENSG00000265711	AL049871.1	miRNA	1,13E-03	9,96E-01	9,98E-01	No	-4,05E-01	2,37E-01	3,80E-01	No
ENSG00000221537	MIR548H1	miRNA	9,36E-03	9,59E-01	9,83E-01	No	1,24E-01	7,03E-01	8,10E-01	No
ENSG00000264620	AL139022.1	miRNA	6,86E-02	6,80E-01	8,46E-01	No	-4,41E-01	1,34E-01	NA	No
ENSG00000266531	MIR4706	miRNA	-6,21E-02	7,01E-01	8,59E-01	No	-3,98E-01	1,63E-01	NA	No
ENSG00000207781	MIR625	miRNA	5,08E-02	6,69E-01	NA	No	1,44E-01	5,63E-01	NA	No
ENSG00000264741	MIR4505	miRNA	2,78E-01	1,61E-01	3,79E-01	No	-5,62E-01	1,39E-01	2,53E-01	No
ENSG00000212061	AC016543.1	miRNA	-1,19E-01	3,68E-01	NA	No	-5,95E-02	8,31E-01	8,97E-01	No
ENSG00000238758	AL512791.1	miRNA	7,22E-03	9,48E-01	NA	No	-1,57E-01	5,14E-01	NA	No
ENSG00000252748	AL096869.1	miRNA	-2,10E-02	8,50E-01	NA	No	-1,04E-01	6,03E-01	NA	No
ENSG00000265856	AL133153.1	miRNA	2,71E-01	6,12E-02	NA	No	-5,50E-02	8,00E-01	NA	No
ENSG00000264607	MIR3173	miRNA	1,10E-01	5,88E-01	7,89E-01	No	-3,45E-01	3,33E-01	4,88E-01	No
ENSG00000199082	MIR342	miRNA	2,55E-01	2,34E-02	NA	No	1,73E-01	4,25E-01	NA	No
ENSG00000198984	MIR345	miRNA	5,48E-02	6,18E-01	NA	No	6,17E-02	9,56E-01	NA	No
ENSG00000211574	MIR770	miRNA	8,49E-02	3,01E-01	NA	No	9,20E-02	8,64E-01	NA	No
ENSG00000272291	MIR665	miRNA	2,35E-02	9,72E-01	NA	No	6,17E-02	9,56E-01	NA	No
ENSG00000221077	AL117190.1	miRNA	1,78E-02	8,58E-01	NA	No	-5,76E-02	7,83E-01	NA	No
ENSG00000199005	MIR370	miRNA	1,43E-01	1,36E-01	NA	No	3,59E-03	9,87E-01	NA	No
ENSG00000199088	MIR379	miRNA	-1,97E-02	8,19E-01	NA	No	-1,35E-01	7,12E-01	NA	No
ENSG00000199109	MIR411	miRNA	-1,34E-02	8,38E-01	NA	No	3,98E-03	1,00E+00	NA	No
ENSG00000207749	MIR299	miRNA	2,95E-02	7,96E-01	NA	No	4,37E-02	8,95E-01	NA	No

ENSG00000198982	MIR380	miRNA	3,78E-02	8,45E-01	NA	No	3,20E-01	1,71E-01	NA	No
ENSG00000221745	MIR1197	miRNA	1,49E-02	9,65E-01	NA	No	4,94E-02	8,87E-01	NA	No
ENSG00000199069	MIR323A	miRNA	3,60E-02	7,03E-01	NA	No	4,25E-02	8,88E-01	NA	No
ENSG00000211582	MIR758	miRNA	9,10E-02	3,83E-01	NA	No	1,13E-01	5,79E-01	NA	No
ENSG00000207761	MIR329-1	miRNA	-1,53E-02	8,63E-01	NA	No	9,29E-02	7,10E-01	NA	No
ENSG00000207762	MIR329-2	miRNA	3,15E-02	9,36E-01	NA	No	1,45E-01	5,57E-01	NA	No
ENSG00000194717	MIR494	miRNA	-6,68E-02	5,11E-01	NA	No	-5,08E-02	8,26E-01	NA	No
ENSG00000221036	MIR1193	miRNA	1,56E-01	2,25E-01	4,63E-01	No	-1,44E-01	5,09E-01	NA	No
ENSG00000265032	AL132709.2	miRNA	5,08E-02	6,62E-01	NA	No	9,02E-02	8,64E-01	NA	No
ENSG00000207934	MIR654	miRNA	-2,46E-02	8,71E-01	9,46E-01	No	-2,49E-01	4,48E-01	6,02E-01	No
ENSG00000264882	MIR376A1	miRNA	-1,78E-02	8,13E-01	NA	No	-2,44E-02	8,90E-01	NA	No
ENSG00000215957	MIR300	miRNA	-4,70E-02	6,05E-01	NA	No	-8,86E-02	6,57E-01	NA	No
ENSG00000221525	MIR1185-1	miRNA	-3,72E-02	6,25E-01	NA	No	-4,48E-03	9,66E-01	NA	No
ENSG00000221614	MIR1185-2	miRNA	1,44E-02	8,76E-01	NA	No	-9,06E-02	6,44E-01	NA	No
ENSG00000199020	MIR381	miRNA	2,43E-02	8,21E-01	NA	No	-8,32E-02	7,15E-01	NA	No
ENSG00000207754	MIR487B	miRNA	4,30E-02	7,12E-01	NA	No	-1,26E-01	7,12E-01	NA	No
ENSG00000216099	MIR889	miRNA	1,43E-01	2,09E-01	NA	No	8,77E-02	8,64E-01	NA	No
ENSG00000207587	MIR544A	miRNA	1,14E-01	4,19E-01	NA	No	-1,10E-01	6,26E-01	NA	No
ENSG00000207646	MIR655	miRNA	-6,07E-02	4,99E-01	NA	No	3,31E-02	8,94E-01	NA	No
ENSG00000265250	AL132709.3	miRNA	-1,38E-02	9,10E-01	NA	No	-2,11E-01	4,01E-01	NA	No
ENSG00000207558	MIR487A	miRNA	4,44E-02	6,01E-01	NA	No	-3,53E-02	8,90E-01	NA	No
ENSG00000207742	MIR382	miRNA	4,57E-02	6,92E-01	NA	No	-5,42E-02	7,78E-01	NA	No
ENSG00000207993	MIR134	miRNA	-4,97E-02	7,20E-01	8,69E-01	No	1,93E-02	9,53E-01	9,73E-01	No
ENSG00000208027	MIR485	miRNA	-4,16E-02	6,46E-01	NA	No	2,89E-02	8,96E-01	NA	No
ENSG00000208004	MIR323B	miRNA	-6,14E-02	6,09E-01	NA	No	2,99E-01	3,67E-01	5,23E-01	No
ENSG00000207978	MIR154	miRNA	2,66E-02	7,66E-01	NA	No	-9,64E-02	6,13E-01	NA	No
ENSG00000207961	MIR496	miRNA	-2,77E-02	7,29E-01	NA	No	-3,92E-02	8,36E-01	NA	No
ENSG00000199015	MIR377	miRNA	-7,81E-02	4,07E-01	NA	No	-2,34E-01	3,04E-01	NA	No
ENSG00000216179	MIR541	miRNA	-4,04E-02	7,42E-01	NA	No	-1,95E-01	4,12E-01	NA	No
ENSG00000199107	MIR409	miRNA	6,22E-02	5,91E-01	NA	No	6,03E-02	7,70E-01	NA	No
ENSG00000199012	MIR412	miRNA	2,47E-03	9,41E-01	NA	No	-2,07E-02	9,21E-01	NA	No
ENSG00000199025	MIR369	miRNA	-1,51E-02	8,38E-01	NA	No	1,63E-02	9,53E-01	NA	No
ENSG00000199092	MIR410	miRNA	-3,38E-02	7,30E-01	NA	No	1,06E-02	9,76E-01	NA	No
ENSG00000207959	MIR656	miRNA	2,22E-02	9,72E-01	NA	No	1,01E-01	7,75E-01	NA	No
ENSG00000265677	AL137229.1	miRNA	2,84E-02	8,51E-01	NA	No	5,82E-01	9,20E-02	1,83E-01	No
ENSG00000264904	AL139300.1	miRNA	2,08E-03	9,41E-01	NA	No	9,17E-02	7,46E-01	NA	No
ENSG00000207568	MIR203	miRNA	4,46E-02	6,75E-01	NA	No	6,23E-02	9,56E-01	NA	No
ENSG00000265929	hsa-mir-5195	miRNA	3,15E-03	1,00E+00	NA	No	-1,30E-01	7,12E-01	NA	No
ENSG00000265673	MIR4508	miRNA	-6,98E-02	3,03E-01	NA	No	-1,72E-01	3,51E-01	NA	No
ENSG00000266517	MIR4715	miRNA	2,25E-02	8,72E-01	NA	No	-9,82E-02	8,30E-01	NA	No
ENSG00000265102	MIR3942	miRNA	-2,04E-03	9,90E-01	9,97E-01	No	1,74E-01	6,21E-01	7,47E-01	No
ENSG00000265098	MIR4510	miRNA	2,34E-02	9,72E-01	NA	No	1,01E-01	7,75E-01	NA	No
ENSG00000207766	MIR626	miRNA	-1,25E-01	3,36E-01	NA	No	9,21E-02	7,39E-01	8,35E-01	No
ENSG00000207712	MIR627	miRNA	-1,34E-03	9,92E-01	NA	No	7,52E-02	7,80E-01	NA	No
ENSG00000222311	AC084882.1	miRNA	3,74E-02	8,45E-01	NA	No	6,68E-02	9,56E-01	NA	No
ENSG00000266143	AC066612.1	miRNA	-6,10E-02	6,33E-01	NA	No	1,71E-01	5,76E-01	7,11E-01	No
ENSG00000264210	MIR4716	miRNA	1,29E-02	9,31E-01	NA	No	3,36E-02	9,09E-01	NA	No
ENSG00000264109	MIR4712	miRNA	1,65E-01	4,53E-01	6,89E-01	No	-3,52E-01	3,42E-01	4,97E-01	No
ENSG00000263437	AC020892.1	miRNA	-4,96E-02	5,88E-01	NA	No	-5,69E-02	7,90E-01	NA	No
ENSG00000207964	MIR628	miRNA	9,25E-02	5,32E-01	NA	No	4,40E-01	2,28E-01	3,69E-01	No
ENSG00000222586	AC010999.1	miRNA	-3,43E-01	1,73E-01	3,96E-01	No	-9,45E-01	1,43E-02	3,89E-02	No
ENSG00000221096	AC092757.1	miRNA	5,86E-02	6,19E-01	NA	No	-9,82E-02	8,30E-01	NA	No
ENSG00000253030	MIR2116	miRNA	-2,47E-01	2,63E-01	5,07E-01	No	-1,05E+00	3,90E-02	9,10E-02	No
ENSG00000215983	AC092756.1	miRNA	-5,81E-02	5,99E-01	NA	No	-2,27E-01	3,49E-01	NA	No
ENSG00000265317	AC103740.1	miRNA	-6,74E-02	6,46E-01	NA	No	-2,89E-01	3,32E-01	NA	No
ENSG00000211137	MIR190A	miRNA	2,42E-02	8,49E-01	NA	No	1,18E-01	6,49E-01	7,69E-01	No
ENSG00000221033	MIR1272	miRNA	-2,73E-01	5,68E-02	NA	No	-3,93E-01	2,10E-01	3,46E-01	No
ENSG00000264737	MIR4511	miRNA	-7,43E-02	3,49E-01	NA	No	-1,50E-01	4,80E-01	NA	No
ENSG00000266589	MIR4512	miRNA	1,04E-01	5,26E-01	7,47E-01	No	-3,65E-01	2,10E-01	NA	No
ENSG00000265195	MIR4312	miRNA	3,02E-01	1,61E-01	3,79E-01	No	2,80E-01	4,17E-01	5,73E-01	No
ENSG00000265047	AC026992.1	miRNA	-1,00E-01	4,03E-01	NA	No	-6,18E-02	8,22E-01	8,91E-01	No
ENSG00000207965	MIR629	miRNA	5,50E-02	5,50E-01	NA	No	-1,02E-01	8,30E-01	NA	No

ENSG00000223253	AC022872.1	miRNA	-1,79E-02	9,05E-01	9,60E-01	No	-4,68E-01	1,06E-01	2,05E-01	No
ENSG00000264950	AC113208.1	miRNA	-1,11E-01	3,22E-01	NA	No	-3,01E-01	2,26E-01	NA	No
ENSG00000266449	MIR3713	miRNA	-5,71E-04	8,98E-01	NA	No	-1,79E-02	8,84E-01	NA	No
ENSG00000266543	AC027811.1	miRNA	-1,69E-02	8,19E-01	NA	No	-1,26E-01	7,12E-01	NA	No
ENSG00000265855	MIR4514	miRNA	5,92E-02	6,82E-01	NA	No	-1,67E-01	4,26E-01	NA	No
ENSG00000221484	AC027807.1	miRNA	-1,90E-01	2,60E-01	5,04E-01	No	-3,57E-01	2,63E-01	4,10E-01	No
ENSG00000221634	MIR1276	miRNA	7,19E-02	5,93E-01	7,92E-01	No	-2,73E-01	2,94E-01	NA	No
ENSG00000221391	AC013489.1	miRNA	1,06E-01	3,79E-01	NA	No	1,26E-01	6,62E-01	NA	No
ENSG00000221630	MIR1179	miRNA	-2,11E-02	8,02E-01	NA	No	-1,33E-01	7,12E-01	NA	No
ENSG00000264966	MIR5094	miRNA	8,07E-02	4,74E-01	NA	No	-1,21E-01	6,14E-01	NA	No
ENSG00000264796	MIR5009	miRNA	8,62E-02	6,11E-01	8,04E-01	No	-2,87E-03	9,92E-01	9,95E-01	No
ENSG00000265871	MIR3174	miRNA	1,88E-01	1,82E-01	4,08E-01	No	2,26E-01	3,99E-01	5,56E-01	No
ENSG00000264173	MIR3175	miRNA	1,28E-01	5,49E-01	7,62E-01	No	1,39E-01	6,91E-01	8,01E-01	No
ENSG00000264480	MIR4714	miRNA	-2,37E-01	2,65E-01	5,09E-01	No	3,73E-01	2,99E-01	4,50E-01	No
ENSG00000264771	AC022819.1	miRNA	-1,67E-01	2,07E-01	NA	No	-2,18E-01	4,64E-01	6,17E-01	No
ENSG00000266235	MIR3176	miRNA	3,12E-01	4,95E-02	1,72E-01	No	5,44E-02	8,11E-01	8,83E-01	No
ENSG00000265820	MIR3177	miRNA	-9,49E-02	5,79E-01	7,82E-01	No	-6,20E-01	8,06E-02	1,65E-01	No
ENSG00000265867	MIR4516	miRNA	-4,17E-02	7,08E-01	NA	No	-1,72E-01	4,03E-01	NA	No
ENSG00000266643	MIR3677	miRNA	1,83E-01	2,33E-01	NA	No	6,01E-02	8,08E-01	NA	No
ENSG00000266232	MIR3178	miRNA	1,52E-01	2,47E-01	4,88E-01	No	2,49E-01	3,76E-01	5,33E-01	No
ENSG00000222055	AC092117.1	miRNA	-5,73E-02	6,46E-01	NA	No	-1,36E-01	7,12E-01	NA	No
ENSG00000266492	AC092122.2	miRNA	-1,08E-02	8,49E-01	NA	No	-9,51E-02	8,30E-01	NA	No
ENSG00000263984	AC092380.1	miRNA	-4,46E-02	7,60E-01	NA	No	-3,22E-02	9,21E-01	NA	No
ENSG00000207639	MIR193B	miRNA	-3,23E-02	7,20E-01	NA	No	-4,38E-02	8,49E-01	NA	No
ENSG00000199130	MIR365A	miRNA	-1,86E-01	1,42E-01	3,51E-01	No	-2,75E-01	3,22E-01	4,77E-01	No
ENSG00000238728	MIR1972-1	miRNA	2,05E-01	2,83E-01	5,29E-01	No	3,70E-01	3,18E-01	4,72E-01	No
ENSG00000265515	AC092287.1	miRNA	-6,49E-02	4,47E-01	NA	No	-1,10E-01	6,09E-01	NA	No
ENSG00000265462	MIR3680-1	miRNA	1,28E-01	5,28E-01	7,49E-01	No	-4,41E-01	2,40E-01	3,84E-01	No
ENSG00000266758	MIR3680-2	miRNA	6,80E-02	6,21E-01	NA	No	-2,25E-01	3,64E-01	NA	No
ENSG00000220993	AC007611.1	miRNA	-1,55E-02	8,49E-01	NA	No	-9,82E-02	8,30E-01	NA	No
ENSG00000221134	AC007339.1	miRNA	3,67E-02	7,15E-01	NA	No	-1,47E-01	5,95E-01	NA	No
ENSG00000265708	AC027348.1	miRNA	-2,58E-02	7,99E-01	NA	No	-1,41E-01	4,45E-01	NA	No
ENSG00000264947	hsa-mir-3181	miRNA	5,64E-02	6,77E-01	NA	No	-2,52E-02	9,27E-01	NA	No
ENSG00000265281	MIR3935	miRNA	2,86E-01	2,76E-02	1,15E-01	No	1,13E-02	9,87E-01	NA	No
ENSG00000223123	AC010546.1	miRNA	-5,73E-02	6,46E-01	NA	No	-6,70E-03	9,79E-01	NA	No
ENSG00000221789	AC130462.1	miRNA	3,46E-02	9,36E-01	NA	No	9,18E-02	8,64E-01	NA	No
ENSG00000239118	MIR1972-2	miRNA	-1,85E-01	2,70E-01	5,15E-01	No	-5,80E-01	1,20E-01	2,26E-01	No
ENSG00000221799	AC132068.1	miRNA	4,80E-03	9,69E-01	NA	No	-1,56E-01	4,83E-01	NA	No
ENSG00000265341	AC092332.1	miRNA	3,31E-02	9,36E-01	NA	No	6,17E-02	9,56E-01	NA	No
ENSG00000222299	AC009142.1	miRNA	-1,04E-01	4,76E-01	7,08E-01	No	-5,14E-01	1,40E-01	2,55E-01	No
ENSG00000263785	MIR3182	miRNA	-3,29E-02	8,13E-01	9,17E-01	No	-3,51E-01	1,98E-01	3,32E-01	No
ENSG00000265249	AC025280.1	miRNA	-2,94E-02	8,38E-01	9,30E-01	No	-5,67E-01	1,24E-01	2,32E-01	No
ENSG00000266307	MIR5093	miRNA	1,21E-01	2,46E-01	NA	No	1,77E-02	8,97E-01	NA	No
ENSG00000264203	AC092377.1	miRNA	1,49E-02	9,11E-01	NA	No	-2,79E-02	8,90E-01	NA	No
ENSG00000263456	MIR5189	miRNA	1,18E-01	3,32E-01	NA	No	-1,31E-01	7,12E-01	NA	No
ENSG00000273288	AC108004.5	miRNA	4,33E-02	7,05E-01	NA	No	-1,30E-01	7,12E-01	NA	No
ENSG00000264429	MIR3183	miRNA	7,03E-02	6,63E-01	8,35E-01	No	-5,55E-01	7,69E-02	NA	No
ENSG00000267195	MIR212	miRNA	-5,79E-02	4,23E-01	NA	No	-1,78E-01	2,79E-01	NA	No
ENSG00000264468	MIR4520A	miRNA	4,31E-02	7,25E-01	NA	No	1,02E-01	7,75E-01	NA	No
ENSG00000199053	MIR324	miRNA	-1,08E-01	5,75E-01	7,80E-01	No	-6,49E-01	1,10E-01	2,11E-01	No
ENSG00000266638	AC129492.1	miRNA	-3,11E-02	8,42E-01	9,31E-01	No	2,09E-01	4,87E-01	6,37E-01	No
ENSG00000221486	AC000003.1	miRNA	5,22E-02	7,12E-01	NA	No	-3,34E-01	2,35E-01	NA	No
ENSG00000253002	AC005725.1	miRNA	-1,11E-01	3,53E-01	NA	No	-3,00E-01	2,18E-01	NA	No
ENSG00000266297	MIR744	miRNA	-3,02E-02	8,56E-01	9,38E-01	No	4,12E-01	2,73E-01	4,22E-01	No
ENSG00000265503	MIR1269B	miRNA	-6,09E-02	5,10E-01	NA	No	-1,94E-01	3,80E-01	NA	No
ENSG00000221698	MIR548H3	miRNA	5,47E-02	6,05E-01	NA	No	-1,03E-01	8,30E-01	NA	No
ENSG00000265110	MIR4731	miRNA	-8,75E-02	6,48E-01	8,27E-01	No	7,87E-02	8,22E-01	8,91E-01	No
ENSG00000221355	MIR1288	miRNA	-3,81E-02	7,43E-01	NA	No	-1,29E-01	5,59E-01	NA	No
ENSG00000207839	MIR33B	miRNA	6,98E-02	4,52E-01	NA	No	-1,32E-01	7,12E-01	NA	No
ENSG00000221540	MIR1180	miRNA	-3,79E-01	1,11E-01	2,99E-01	No	-1,35E+00	2,25E-02	5,72E-02	No
ENSG00000263583	MIR4522	miRNA	-4,20E-02	5,82E-01	NA	No	-5,27E-02	7,96E-01	NA	No
ENSG00000264715	AC104996.1	miRNA	-5,73E-02	6,46E-01	NA	No	-1,36E-01	7,12E-01	NA	No

ENSG00000207614	MIR193A	miRNA	-1,27E-01	2,50E-01	NA	No	-8,68E-02	7,55E-01	NA	No
ENSG00000265976	MIR4725	miRNA	-1,44E-02	8,67E-01	NA	No	-6,01E-02	7,34E-01	NA	No
ENSG00000265930	MIR4734	miRNA	-4,46E-02	7,60E-01	NA	No	4,78E-02	8,68E-01	NA	No
ENSG00000265904	AC005288.1	miRNA	2,07E-01	2,49E-01	4,91E-01	No	-2,46E-02	9,28E-01	9,58E-01	No
ENSG00000265178	MIR4728	miRNA	5,82E-03	9,52E-01	NA	No	-9,88E-02	6,55E-01	NA	No
ENSG00000221555	AC068669.1	miRNA	1,13E-01	3,44E-01	NA	No	1,78E-02	9,38E-01	NA	No
ENSG00000221020	AC107993.1	miRNA	-5,58E-02	6,46E-01	NA	No	-1,33E-01	7,12E-01	NA	No
ENSG00000264314	MIR548AT	miRNA	1,26E-01	4,53E-01	6,89E-01	No	1,39E-01	6,33E-01	7,56E-01	No
ENSG00000265611	MIR5010	miRNA	-3,76E-02	8,27E-01	9,24E-01	No	-5,24E-01	1,46E-01	2,63E-01	No
ENSG00000264038	AC091132.2	miRNA	3,28E-02	9,36E-01	NA	No	8,70E-02	8,64E-01	NA	No
ENSG00000264999	MIR5089	miRNA	-2,20E-02	7,10E-01	NA	No	-1,45E-01	6,08E-01	NA	No
ENSG00000207947	MIR152	miRNA	1,05E-01	3,76E-01	NA	No	2,12E-01	4,12E-01	NA	No
ENSG00000221739	MIR1203	miRNA	8,44E-02	3,30E-01	NA	No	-1,28E-01	7,12E-01	NA	No
ENSG00000216168	AC100832.1	miRNA	-8,55E-02	2,56E-01	NA	No	-1,99E-01	3,17E-01	NA	No
ENSG00000211514	MIR454	miRNA	1,05E-01	4,19E-01	6,61E-01	No	5,94E-02	8,19E-01	8,89E-01	No
ENSG00000207996	MIR301A	miRNA	4,97E-02	5,34E-01	NA	No	4,18E-01	1,12E-01	NA	No
ENSG00000263857	MIR4729	miRNA	1,47E-01	4,94E-01	7,23E-01	No	-2,59E-01	4,68E-01	6,21E-01	No
ENSG00000263422	AC005702.1	miRNA	2,72E-01	2,58E-01	5,01E-01	No	-7,70E-01	6,39E-02	1,37E-01	No
ENSG00000266701	AC005702.4	miRNA	8,15E-03	9,65E-01	9,85E-01	No	-2,50E-01	4,23E-01	5,79E-01	No
ENSG00000265638	AC005702.3	miRNA	1,46E-02	9,33E-01	9,72E-01	No	-4,16E-01	2,11E-01	3,48E-01	No
ENSG00000263820	AC005702.2	miRNA	1,34E-01	5,32E-01	7,51E-01	No	-7,94E-01	7,79E-02	1,61E-01	No
ENSG00000264049	MIR4737	miRNA	3,31E-01	1,64E-01	3,83E-01	No	-3,95E-01	2,91E-01	4,41E-01	No
ENSG00000266566	AC009994.2	miRNA	-7,47E-02	5,99E-01	NA	No	3,81E-01	2,89E-01	4,39E-01	No
ENSG00000207943	MIR634	miRNA	3,70E-02	8,45E-01	NA	No	8,58E-02	8,64E-01	NA	No
ENSG00000207688	MIR548AA2	miRNA	-1,08E-02	9,46E-01	9,78E-01	No	-2,19E-01	5,17E-01	6,63E-01	No
ENSG00000265086	AC006534.2	miRNA	-2,92E-03	9,89E-01	NA	No	-1,36E-01	7,12E-01	NA	No
ENSG00000207561	MIR635	miRNA	2,37E-01	3,06E-01	5,55E-01	No	3,95E-01	2,06E-01	3,41E-01	No
ENSG00000265331	MIR4524B	miRNA	2,28E-01	3,21E-01	5,70E-01	No	7,49E-01	2,95E-02	7,19E-02	No
ENSG00000222545	AC080037.1	miRNA	2,76E-02	7,70E-01	NA	No	-8,16E-02	7,52E-01	NA	No
ENSG00000264060	MIR4316	miRNA	9,19E-02	3,71E-01	NA	No	6,13E-02	9,56E-01	NA	No
ENSG00000264961	MIR4730	miRNA	2,85E-02	8,51E-01	9,35E-01	No	-5,24E-02	8,42E-01	9,04E-01	No
ENSG00000207736	MIR657	miRNA	-3,63E-02	7,77E-01	NA	No	-3,76E-01	1,87E-01	NA	No
ENSG00000211563	MIR338	miRNA	-1,08E-02	8,76E-01	NA	No	-1,59E-01	4,91E-01	NA	No
ENSG00000221025	MIR1250	miRNA	-2,87E-02	7,31E-01	NA	No	-1,26E-01	7,12E-01	NA	No
ENSG00000266392	MIR4740	miRNA	-4,17E-02	7,75E-01	8,97E-01	No	3,73E-02	9,07E-01	9,45E-01	No
ENSG00000266189	MIR3186	miRNA	-7,62E-02	4,56E-01	NA	No	-1,52E-01	4,62E-01	NA	No
ENSG00000263853	AC139530.1	miRNA	-7,20E-02	6,83E-01	8,48E-01	No	-2,64E-01	4,12E-01	5,69E-01	No
ENSG00000266107	MIR4525	miRNA	3,86E-02	8,45E-01	NA	No	6,17E-02	9,56E-01	NA	No
ENSG00000264383	AC068014.1	miRNA	-1,45E-01	2,37E-01	NA	No	-3,64E-01	1,32E-01	NA	No
ENSG00000221631	AP000897.1	miRNA	6,30E-02	7,67E-01	8,93E-01	No	-3,61E-01	3,28E-01	4,82E-01	No
ENSG00000263827	AP005118.1	miRNA	-4,54E-02	8,17E-01	9,20E-01	No	-5,51E-02	8,72E-01	9,24E-01	No
ENSG00000212067	AP005227.1	miRNA	-1,35E-02	9,35E-01	9,73E-01	No	2,78E-01	4,22E-01	5,78E-01	No
ENSG00000266146	MIR5190	miRNA	1,15E-03	9,97E-01	9,99E-01	No	-2,54E-01	4,65E-01	6,17E-01	No
ENSG00000263527	MIR4526	miRNA	1,18E-01	5,06E-01	7,33E-01	No	-4,75E-02	8,64E-01	9,18E-01	No
ENSG00000252234	AC006557.1	miRNA	8,09E-02	6,98E-01	8,57E-01	No	-3,47E-01	3,31E-01	4,86E-01	No
ENSG00000216205	AC036178.1	miRNA	7,93E-02	5,54E-01	NA	No	8,09E-01	7,76E-02	1,60E-01	No
ENSG00000221493	MIR320C1	miRNA	3,26E-02	7,81E-01	NA	No	2,62E-01	4,30E-01	5,85E-01	No
ENSG00000264183	AC091038.1	miRNA	4,48E-05	9,86E-01	NA	No	1,63E-01	5,28E-01	NA	No
ENSG00000212051	MIR320C2	miRNA	4,12E-02	7,65E-01	NA	No	8,55E-02	8,64E-01	NA	No
ENSG00000252846	AC090206.1	miRNA	-2,93E-02	7,43E-01	NA	No	-1,61E-01	4,72E-01	NA	No
ENSG00000265063	AC009831.1	miRNA	3,64E-02	7,68E-01	NA	No	8,57E-02	7,39E-01	NA	No
ENSG00000221409	AC068506.1	miRNA	2,13E-02	8,93E-01	9,56E-01	No	1,82E-01	5,77E-01	7,12E-01	No
ENSG00000265957	MIR4319	miRNA	3,30E-02	8,62E-01	9,40E-01	No	-2,40E-01	4,81E-01	6,32E-01	No
ENSG00000266276	MIR4743	miRNA	-4,23E-02	6,39E-01	NA	No	-1,67E-01	4,27E-01	NA	No
ENSG00000263849	MIR4744	miRNA	-2,89E-02	8,17E-01	9,19E-01	No	-1,68E-01	4,90E-01	6,40E-01	No
ENSG00000221058	AC090666.1	miRNA	5,30E-03	9,41E-01	NA	No	-9,51E-02	8,30E-01	NA	No
ENSG00000264571	MIR4529	miRNA	-3,46E-02	6,80E-01	NA	No	1,65E-01	5,30E-01	NA	No
ENSG00000221536	AC091643.1	miRNA	3,46E-02	9,36E-01	NA	No	9,90E-02	7,75E-01	NA	No
ENSG00000263750	MIR548AV	miRNA	2,34E-02	9,72E-01	NA	No	1,50E-01	3,98E-01	NA	No
ENSG00000266085	AL109658.1	miRNA	1,31E-01	3,89E-01	6,35E-01	No	3,90E-02	9,02E-01	9,42E-01	No
ENSG00000263452	AL121899.1	miRNA	-2,05E-02	8,19E-01	NA	No	-1,36E-01	7,12E-01	NA	No
ENSG00000266355	AL117334.1	miRNA	4,31E-02	7,73E-01	8,96E-01	No	-2,85E-01	2,72E-01	4,20E-01	No

ENSG00000264957	AL109805.1	miRNA	5,39E-02	5,61E-01	NA	No	3,38E-01	2,09E-01	NA	No
ENSG00000199024	MIR103A2	miRNA	2,56E-01	1,18E-01	3,12E-01	No	1,28E+00	2,71E-02	6,69E-02	No
ENSG00000221201	AL031655.1	miRNA	1,01E-01	2,93E-01	NA	No	1,16E-01	5,96E-01	NA	No
ENSG00000222298	AL031655.2	miRNA	5,85E-02	6,15E-01	NA	No	4,22E-02	8,98E-01	NA	No
ENSG00000212111	AL121584.1	miRNA	-1,01E-01	3,23E-01	NA	No	-3,80E-02	8,57E-01	NA	No
ENSG00000221448	AL118509.1	miRNA	-2,76E-02	7,31E-01	NA	No	7,54E-02	7,32E-01	NA	No
ENSG00000251713	AL050321.1	miRNA	7,61E-02	5,25E-01	7,46E-01	No	2,03E-01	4,74E-01	6,25E-01	No
ENSG00000265137	MIR3192	miRNA	6,77E-02	7,35E-01	8,77E-01	No	-1,05E+00	4,13E-02	9,55E-02	No
ENSG00000264134	AL121893.1	miRNA	5,79E-02	5,64E-01	NA	No	-5,72E-02	7,69E-01	NA	No
ENSG00000221748	AL049647.1	miRNA	5,10E-02	4,00E-01	NA	No	1,31E-01	5,96E-01	NA	No
ENSG00000216017	AL139429.1	miRNA	-2,96E-02	7,31E-01	NA	No	-6,38E-02	8,00E-01	NA	No
ENSG00000239120	AL121761.1	miRNA	-4,15E-02	7,60E-01	NA	No	2,84E-02	9,87E-01	NA	No
ENSG00000221319	AL441988.1	miRNA	2,87E-01	5,71E-02	NA	No	4,26E-01	1,32E-01	2,43E-01	No
ENSG00000264395	MIR3193	miRNA	4,77E-03	9,79E-01	9,92E-01	No	-5,29E-01	1,59E-01	2,81E-01	No
ENSG00000265736	AL049539.1	miRNA	-5,20E-02	6,57E-01	NA	No	1,15E-01	6,83E-01	7,95E-01	No
ENSG00000264308	AL121897.1	miRNA	-1,87E-02	8,25E-01	NA	No	-1,45E-01	6,08E-01	NA	No
ENSG00000264616	MIR4755	miRNA	7,25E-02	7,30E-01	8,75E-01	No	-4,56E-02	8,94E-01	9,37E-01	No
ENSG00000207997	MIR644A	miRNA	8,38E-03	9,66E-01	9,86E-01	No	2,00E-02	9,54E-01	9,73E-01	No
ENSG00000207635	MIR499A	miRNA	1,40E-01	4,29E-01	6,69E-01	No	-2,53E-01	3,86E-01	NA	No
ENSG00000264888	AL121753.1	miRNA	3,86E-02	8,45E-01	NA	No	1,14E-01	6,27E-01	NA	No
ENSG00000263473	MIR548O2	miRNA	-2,96E-02	8,31E-01	NA	No	-2,12E-02	9,41E-01	NA	No
ENSG00000211534	AL121588.1	miRNA	3,48E-02	7,19E-01	NA	No	2,54E-02	9,87E-01	NA	No
ENSG00000265893	AL132772.1	miRNA	-4,21E-02	7,60E-01	NA	No	-9,72E-02	8,30E-01	NA	No
ENSG00000264901	MIR3616	miRNA	3,39E-02	9,36E-01	NA	No	1,19E-01	5,48E-01	NA	No
ENSG00000263770	AL031666.1	miRNA	1,60E-01	3,28E-01	NA	No	-1,59E-01	4,92E-01	NA	No
ENSG00000221294	AL021394.1	miRNA	7,25E-02	4,15E-01	NA	No	8,58E-02	8,64E-01	NA	No
ENSG00000251876	AL035106.1	miRNA	3,82E-02	7,71E-01	NA	No	-2,57E-01	3,32E-01	NA	No
ENSG00000208018	MIR645	miRNA	5,93E-01	3,55E-02	1,38E-01	No	-1,41E+00	2,90E-03	9,59E-03	No
ENSG00000221091	MIR1302-5	miRNA	4,76E-02	5,84E-01	NA	No	-3,66E-02	8,90E-01	NA	No
ENSG00000265062	AL133228.1	miRNA	-5,73E-02	6,46E-01	NA	No	-1,36E-01	7,12E-01	NA	No
ENSG00000266761	MIR3194	miRNA	1,20E-01	2,99E-01	NA	No	1,44E-02	9,35E-01	NA	No
ENSG00000252629	AL050316.1	miRNA	3,46E-02	8,41E-01	9,31E-01	No	3,10E-01	3,88E-01	5,46E-01	No
ENSG00000212084	AL121914.1	miRNA	3,02E-02	7,54E-01	NA	No	3,09E-01	2,00E-01	NA	No
ENSG00000216031	MIR298	miRNA	5,99E-02	5,59E-01	NA	No	9,02E-02	8,64E-01	NA	No
ENSG00000207802	MIR646	miRNA	2,00E-02	8,73E-01	9,47E-01	No	-3,08E-01	3,16E-01	NA	No
ENSG00000265617	MIR548AG2	miRNA	-7,29E-03	9,26E-01	NA	No	-6,50E-02	7,25E-01	NA	No
ENSG00000199017	MIR1-1	miRNA	3,63E-02	8,44E-01	9,32E-01	No	-3,57E-01	3,19E-01	4,73E-01	No
ENSG00000266104	MIR4326	miRNA	-1,29E-02	9,20E-01	NA	No	8,13E-02	7,70E-01	8,57E-01	No
ENSG00000216141	MIR941-2	miRNA	9,95E-02	4,62E-01	NA	No	-6,96E-02	7,79E-01	NA	No
ENSG00000216195	MIR941-3	miRNA	3,82E-02	7,41E-01	NA	No	-1,43E-01	6,16E-01	NA	No
ENSG00000221535	AC027319.1	miRNA	-1,99E-02	8,27E-01	NA	No	-1,61E-01	4,67E-01	NA	No
ENSG00000266483	AC010311.1	miRNA	-1,07E-01	3,04E-01	NA	No	-5,63E-02	8,24E-01	NA	No
ENSG00000265390	MIR4999	miRNA	7,11E-02	4,91E-01	NA	No	-1,90E-03	9,82E-01	NA	No
ENSG00000265528	AC092316.2	miRNA	9,68E-02	3,30E-01	NA	No	6,67E-02	9,56E-01	NA	No
ENSG00000265379	AC008752.2	miRNA	2,17E-01	2,03E-01	4,36E-01	No	6,19E-02	8,38E-01	9,01E-01	No
ENSG00000266247	AC008752.3	miRNA	-6,38E-02	6,19E-01	NA	No	-3,00E-01	2,45E-01	NA	No
ENSG00000264266	MIR4322	miRNA	1,44E-01	2,87E-01	5,34E-01	No	-1,51E-01	4,76E-01	NA	No
ENSG00000265879	MIR4748	miRNA	2,04E-03	9,91E-01	9,97E-01	No	-4,83E-01	8,63E-02	NA	No
ENSG00000207752	MIR199A1	miRNA	7,89E-03	9,53E-01	9,81E-01	No	-2,98E-01	2,18E-01	NA	No
ENSG00000263800	MIR5684	miRNA	4,75E-02	6,44E-01	NA	No	-5,42E-02	7,30E-01	NA	No
ENSG00000266721	MIR5695	miRNA	1,00E-01	2,75E-01	NA	No	-2,92E-02	8,90E-01	NA	No
ENSG00000222492	AC092069.1	miRNA	-4,15E-02	7,60E-01	NA	No	-1,75E-02	9,21E-01	NA	No
ENSG00000207980	MIR23A	miRNA	-2,45E-01	2,68E-01	5,12E-01	No	-1,44E+00	1,65E-02	4,40E-02	No
ENSG00000207613	MIR181C	miRNA	-5,47E-02	6,46E-01	NA	No	-1,30E-01	7,12E-01	NA	No
ENSG00000207585	MIR181D	miRNA	-1,55E-02	8,19E-01	NA	No	-6,79E-02	8,00E-01	NA	No
ENSG00000264175	MIR3189	miRNA	-6,13E-02	5,30E-01	NA	No	-1,48E-01	5,91E-01	NA	No
ENSG00000207821	MIR640	miRNA	1,88E-01	2,86E-01	5,32E-01	No	-1,19E-01	6,96E-01	8,05E-01	No
ENSG00000265339	AC011477.1	miRNA	3,05E-01	9,70E-03	5,52E-02	No	1,39E-01	5,82E-01	NA	No
ENSG00000264441	AC006539.1	miRNA	-1,37E-01	3,15E-01	5,64E-01	No	-7,92E-02	8,04E-01	8,79E-01	No
ENSG00000266156	AC010329.1	miRNA	7,58E-02	5,78E-01	NA	No	-8,11E-02	7,32E-01	NA	No
ENSG00000265650	AC012627.1	miRNA	3,37E-02	6,85E-01	NA	No	1,01E-01	6,06E-01	NA	No
ENSG00000263960	AC007773.1	miRNA	1,83E-01	3,45E-01	5,93E-01	No	-4,19E-01	2,38E-01	3,81E-01	No

ENSG00000264355	AC008738.2	miRNA	-1,39E-01	2,11E-01	NA	No	-2,75E-01	2,61E-01	NA	No
ENSG00000221584	AC008747.1	miRNA	-4,21E-02	7,60E-01	NA	No	-2,35E-02	9,21E-01	NA	No
ENSG00000221258	AC011465.1	miRNA	2,35E-02	9,72E-01	NA	No	8,89E-02	8,64E-01	NA	No
ENSG00000266559	MIR4530	miRNA	2,29E-01	2,50E-01	4,92E-01	No	2,78E-01	4,23E-01	5,79E-01	No
ENSG00000221233	AC007842.1	miRNA	-3,11E-02	7,07E-01	NA	No	1,37E-01	5,56E-01	NA	No
ENSG00000221051	AC118344.1	miRNA	5,67E-02	5,83E-01	NA	No	-1,39E-02	8,90E-01	NA	No
ENSG00000207631	MIR641	miRNA	5,92E-02	6,09E-01	NA	No	-5,19E-02	8,34E-01	NA	No
ENSG00000264133	AC006953.1	miRNA	-1,26E-03	9,64E-01	NA	No	-1,45E-01	6,08E-01	NA	No
ENSG00000207773	MIR642A	miRNA	-6,60E-02	4,14E-01	NA	No	-1,60E-01	4,71E-01	NA	No
ENSG00000211580	MIR769	miRNA	3,32E-02	9,36E-01	NA	No	8,58E-02	8,64E-01	NA	No
ENSG00000211513	MIR320E	miRNA	1,92E-01	1,37E-01	NA	No	-2,83E-02	8,90E-01	NA	No
ENSG00000221595	AC010458.1	miRNA	-1,13E-01	3,68E-01	NA	No	-2,32E-01	3,45E-01	NA	No
ENSG00000265407	MIR4324	miRNA	-6,63E-02	4,07E-01	NA	No	-1,11E-01	5,45E-01	NA	No
ENSG00000207550	MIR99B	miRNA	-1,08E-01	2,65E-01	NA	No	-2,28E-01	3,34E-01	NA	No
ENSG00000208002	MIR643	miRNA	1,04E-01	3,19E-01	NA	No	1,99E-01	4,26E-01	NA	No
ENSG00000221017	MIR1323	miRNA	-5,45E-02	6,46E-01	NA	No	-1,28E-01	7,12E-01	NA	No
ENSG00000207738	MIR520C	miRNA	-5,45E-02	6,46E-01	NA	No	-1,28E-01	7,12E-01	NA	No
ENSG00000207734	MIR517A	miRNA	-4,21E-02	7,60E-01	NA	No	-9,72E-02	8,30E-01	NA	No
ENSG00000207981	MIR519D	miRNA	-4,21E-02	7,60E-01	NA	No	-9,72E-02	8,30E-01	NA	No
ENSG00000207735	MIR520D	miRNA	-6,40E-02	4,76E-01	NA	No	-1,55E-01	5,35E-01	NA	No
ENSG00000266462	AC010327.1	miRNA	1,59E-01	2,28E-01	NA	No	1,54E-01	4,86E-01	NA	No
ENSG00000211571	AC010525.2	miRNA	5,21E-02	7,32E-01	8,76E-01	No	-2,93E-01	2,10E-01	NA	No
ENSG00000266631	AC006116.1	miRNA	1,93E-01	1,57E-01	NA	No	5,38E-02	8,11E-01	NA	No
ENSG00000265070	AC006115.1	miRNA	4,04E-02	7,80E-01	NA	No	8,55E-02	8,64E-01	NA	No
ENSG00000264453	AC003006.1	miRNA	-5,86E-02	5,36E-01	NA	No	-8,17E-02	7,19E-01	NA	No
ENSG00000266640	MIR4754	miRNA	1,76E-03	9,91E-01	NA	No	3,52E-02	9,06E-01	9,44E-01	No
ENSG00000263502	AC134878.2	miRNA	7,61E-02	4,92E-01	NA	No	9,64E-02	7,03E-01	NA	No
ENSG00000252855	AC134878.1	miRNA	-5,78E-02	5,33E-01	NA	No	1,11E-01	6,44E-01	7,65E-01	No
ENSG00000252586	AC002992.1	miRNA	-4,15E-02	7,60E-01	NA	No	-9,51E-02	8,30E-01	NA	No
ENSG00000264757	MIR3198-1	miRNA	4,57E-01	4,50E-02	1,62E-01	No	2,07E-01	4,77E-01	6,28E-01	No
ENSG00000265087	MIR4761	miRNA	-3,34E-02	7,09E-01	NA	No	-5,89E-02	8,10E-01	NA	No
ENSG00000208023	MIR185	miRNA	6,00E-02	5,97E-01	NA	No	-1,63E-01	4,45E-01	NA	No
ENSG00000265978	AL008721.1	miRNA	3,90E-02	8,45E-01	NA	No	4,22E-01	7,74E-02	1,60E-01	No
ENSG00000221760	MIR548J	miRNA	2,08E-01	1,09E-01	NA	No	1,13E-01	6,42E-01	NA	No
ENSG00000264073	MIR3199-2	miRNA	-2,79E-03	9,41E-01	NA	No	-1,01E-01	8,30E-01	NA	No
ENSG00000221736	AC003681.1	miRNA	7,00E-02	4,89E-01	NA	No	-3,43E-02	9,00E-01	NA	No
ENSG00000266012	MIR4764	miRNA	-1,78E-01	1,48E-01	3,60E-01	No	-1,67E-01	5,65E-01	7,02E-01	No
ENSG00000266320	MIR3909	miRNA	1,14E-01	4,42E-01	6,80E-01	No	-2,67E-01	2,59E-01	NA	No
ENSG00000207696	MIR659	miRNA	-5,76E-02	6,01E-01	NA	No	-1,52E-01	4,93E-01	NA	No
ENSG00000264505	MIR4534	miRNA	-8,11E-02	4,46E-01	NA	No	1,46E-03	9,96E-01	9,98E-01	No
ENSG00000252124	AL031594.1	miRNA	3,91E-02	8,46E-01	9,33E-01	No	3,16E-01	3,80E-01	5,37E-01	No
ENSG00000266594	MIR4766	miRNA	1,14E-01	4,70E-01	7,04E-01	No	2,42E-01	4,65E-01	6,17E-01	No
ENSG00000266175	AL080243.1	miRNA	-4,46E-02	7,60E-01	NA	No	-1,03E-01	8,30E-01	NA	No
ENSG00000263463	MIR378I	miRNA	2,72E-01	2,06E-01	4,39E-01	No	-5,10E-01	8,43E-02	NA	No
ENSG00000265106	Z93241.1	miRNA	4,12E-02	7,65E-01	NA	No	6,34E-02	9,56E-01	NA	No
ENSG00000221598	MIR1249	miRNA	-4,51E-02	6,90E-01	NA	No	-9,86E-03	9,62E-01	NA	No
ENSG00000264160	MIR4762	miRNA	7,44E-02	7,01E-01	8,59E-01	No	-3,85E-02	9,07E-01	9,45E-01	No
ENSG00000266533	MIR3619	miRNA	-5,93E-02	6,26E-01	8,14E-01	No	-2,92E-01	2,27E-01	3,68E-01	No
ENSG00000221672	Z97351.1	miRNA	1,44E-01	2,04E-01	NA	No	2,09E-01	3,85E-01	NA	No
ENSG00000238411	CR381670.1	miRNA	5,21E-02	5,05E-01	NA	No	1,68E-01	4,43E-01	NA	No
ENSG00000239179	CR392039.2	miRNA	-1,01E-01	5,63E-01	7,71E-01	No	5,78E-02	8,68E-01	9,21E-01	No
ENSG00000239107	CR392039.1	miRNA	-3,20E-01	1,52E-01	3,66E-01	No	-4,75E-01	2,20E-01	3,58E-01	No
ENSG00000263839	CR381653.1	miRNA	2,83E-03	9,89E-01	9,96E-01	No	-6,68E-02	8,45E-01	9,06E-01	No
ENSG00000221210	AP001137.1	miRNA	2,56E-02	7,75E-01	NA	No	-6,44E-03	9,77E-01	NA	No
ENSG00000238660	AP001340.1	miRNA	4,18E-02	7,50E-01	NA	No	6,34E-02	9,56E-01	NA	No
ENSG00000266133	MIR4759	miRNA	3,83E-02	8,45E-01	NA	No	3,03E-01	2,12E-01	NA	No
ENSG00000266892	AP000301.1	miRNA	6,48E-02	5,04E-01	NA	No	1,14E+00	2,11E-02	5,43E-02	No
ENSG00000266007	AP000318.1	miRNA	-7,23E-04	9,90E-01	NA	No	-1,31E-01	7,12E-01	NA	No
ENSG00000263681	MIR3197	miRNA	-4,48E-02	6,56E-01	NA	No	-1,91E-02	9,35E-01	NA	No
ENSG00000264580	MIR5692B	miRNA	-2,15E-02	8,09E-01	NA	No	1,64E-03	9,93E-01	NA	No
ENSG00000264009	AP001631.1	miRNA	-1,87E-02	8,00E-01	NA	No	-1,32E-01	7,12E-01	NA	No
ENSG00000221485	AC145212.2	miRNA	-4,09E-02	8,43E-01	9,32E-01	No	-3,45E-01	3,10E-01	4,63E-01	No

ENSG00000223061	AC145212.3	miRNA	2,63E-02	9,05E-01	9,60E-01	No	-6,42E-01	8,08E-02	1,65E-01	No
ENSG00000266831	MIR4273	miRNA	7,33E-02	7,40E-01	8,79E-01	No	4,96E-01	1,34E-01	2,47E-01	No
ENSG00000263662	AL591856.4	miRNA	1,65E-01	1,26E-01	NA	No	1,58E-01	4,67E-01	NA	No
ENSG00000266603	AL591856.7	miRNA	-1,26E-01	3,91E-01	6,36E-01	No	-3,02E-02	9,15E-01	9,50E-01	No
ENSG00000238617	AL591856.1	miRNA	-6,11E-02	6,41E-01	NA	No	-1,23E-01	6,15E-01	7,43E-01	No
ENSG00000264787	AL591856.6	miRNA	5,53E-02	6,17E-01	NA	No	1,12E-01	6,45E-01	NA	No
ENSG00000239051	AL591856.2	miRNA	-1,55E-02	8,49E-01	NA	No	7,01E-02	7,72E-01	NA	No
ENSG00000263936	AL592183.7	miRNA	-2,27E-01	1,81E-01	4,07E-01	No	-6,07E-01	1,02E-01	1,99E-01	No
ENSG00000239150	AL592183.6	miRNA	-9,05E-02	4,61E-01	NA	No	-1,83E-01	4,63E-01	NA	No
ENSG00000238303	AL592183.2	miRNA	1,72E-01	1,27E-01	NA	No	8,35E-02	7,07E-01	NA	No
ENSG00000238600	AL592183.3	miRNA	1,56E-01	1,97E-01	NA	No	8,97E-02	6,53E-01	NA	No
ENSG00000239060	AL592183.5	miRNA	7,97E-01	2,03E-02	9,30E-02	No	5,19E-01	1,75E-01	3,02E-01	No
ENSG00000238664	AL592183.4	miRNA	1,83E-01	1,81E-01	4,07E-01	No	-8,54E-03	9,73E-01	9,84E-01	No
ENSG00000221666	AC011841.6	miRNA	1,47E-01	3,01E-01	5,49E-01	No	-6,44E-02	7,82E-01	NA	No
ENSG00000221397	AC011841.4	miRNA	-7,30E-02	5,21E-01	NA	No	-1,50E-01	5,38E-01	6,81E-01	No
ENSG00000266596	AC011841.10	miRNA	-1,25E-01	2,85E-01	5,32E-01	No	-1,38E-01	5,74E-01	7,09E-01	No
ENSG00000265703	AC011841.9	miRNA	-3,58E-02	6,87E-01	NA	No	-4,37E-02	8,32E-01	NA	No
ENSG00000220994	AC011841.3	miRNA	-6,21E-02	5,02E-01	NA	No	-1,21E-01	5,46E-01	NA	No
ENSG00000221692	AC011841.7	miRNA	7,15E-02	5,97E-01	7,95E-01	No	1,31E-02	9,60E-01	9,77E-01	No
ENSG00000221570	AC011841.5	miRNA	-2,89E-02	8,34E-01	9,28E-01	No	-1,34E-01	6,05E-01	7,35E-01	No
ENSG00000263455	AC011841.8	miRNA	-6,96E-02	5,36E-01	NA	No	-1,41E-03	9,94E-01	9,97E-01	No
ENSG00000239867	BX088702.2	miRNA	3,43E-02	7,87E-01	NA	No	-9,82E-02	8,30E-01	NA	No
ENSG00000241601	CT867976.2	miRNA	-5,47E-02	6,46E-01	NA	No	-1,30E-01	7,12E-01	NA	No
ENSG00000222670	CU459211.1	miRNA	1,68E-01	2,41E-01	4,81E-01	No	3,15E-01	3,03E-01	4,55E-01	No
ENSG00000244656	CU463998.3	miRNA	-4,21E-02	7,60E-01	NA	No	-9,72E-02	8,30E-01	NA	No
ENSG00000263760	CU463998.4	miRNA	4,04E-02	7,80E-01	NA	No	1,08E-01	6,91E-01	NA	No
ENSG00000264728	CU442762.3	miRNA	-1,34E-01	4,70E-01	7,04E-01	No	-1,09E+00	3,71E-02	8,72E-02	No
ENSG00000238667	CU442762.2	miRNA	-7,03E-02	6,71E-01	8,40E-01	No	-2,91E-01	3,82E-01	5,39E-01	No
ENSG00000238477	CU442762.1	miRNA	1,74E-02	8,98E-01	NA	No	-3,36E-02	8,84E-01	NA	No
ENSG00000265557	CT867977.1	miRNA	2,35E-02	9,72E-01	NA	No	6,54E-02	9,56E-01	NA	No
ENSG00000207607	MIR200A	miRNA	2,49E-02	9,72E-01	NA	No	NA	NA	NA	NA
ENSG00000264341	MIR4417	miRNA	2,22E-02	9,72E-01	NA	No	NA	NA	NA	NA
ENSG00000216162	AL121988.1	miRNA	3,32E-02	9,36E-01	NA	No	NA	NA	NA	NA
ENSG00000264650	AL359473.1	miRNA	3,88E-02	8,45E-01	NA	No	NA	NA	NA	NA
ENSG00000221746	AL645730.1	miRNA	3,80E-02	8,45E-01	NA	No	NA	NA	NA	NA
ENSG00000221492	AL162430.1	miRNA	2,35E-02	9,72E-01	NA	No	NA	NA	NA	NA
ENSG00000264081	AL136985.1	miRNA	5,55E-02	6,16E-01	NA	No	NA	NA	NA	NA
ENSG00000263908	MIR3116-2	miRNA	2,34E-02	9,72E-01	NA	No	NA	NA	NA	NA
ENSG00000221655	AL354978.1	miRNA	2,04E-01	5,07E-02	NA	No	NA	NA	NA	NA
ENSG00000266388	AC093430.1	miRNA	3,15E-02	9,36E-01	NA	No	NA	NA	NA	NA
ENSG00000238787	AC093577.1	miRNA	3,35E-02	9,36E-01	NA	No	NA	NA	NA	NA
ENSG00000238883	AC114491.1	miRNA	6,97E-02	4,50E-01	NA	No	NA	NA	NA	NA
ENSG00000221538	AL365361.1	miRNA	3,17E-02	9,36E-01	NA	No	NA	NA	NA	NA
ENSG00000238532	AL355794.1	miRNA	4,21E-02	7,67E-01	NA	No	NA	NA	NA	NA
ENSG00000207933	MIR9-1	miRNA	3,15E-02	9,36E-01	NA	No	NA	NA	NA	NA
ENSG00000265589	AL359753.1	miRNA	6,43E-01	3,67E-03	2,73E-02	No	NA	NA	NA	NA
ENSG00000207729	MIR556	miRNA	3,46E-02	9,36E-01	NA	No	NA	NA	NA	NA
ENSG00000207974	MIR557	miRNA	2,35E-02	9,72E-01	NA	No	NA	NA	NA	NA
ENSG00000266417	MIR4424	miRNA	3,17E-02	9,36E-01	NA	No	NA	NA	NA	NA
ENSG00000221403	AC096633.1	miRNA	2,49E-02	9,72E-01	NA	No	NA	NA	NA	NA
ENSG00000221028	MIR1231	miRNA	2,34E-02	9,72E-01	NA	No	NA	NA	NA	NA
ENSG00000263570	AC074264.1	miRNA	3,46E-02	9,36E-01	NA	No	NA	NA	NA	NA
ENSG00000264010	MIR4429	miRNA	4,03E-02	7,82E-01	NA	No	NA	NA	NA	NA
ENSG00000266738	MIR4757	miRNA	4,46E-02	6,75E-01	NA	No	NA	NA	NA	NA
ENSG00000263957	AC105398.1	miRNA	5,84E-02	5,72E-01	NA	No	NA	NA	NA	NA
ENSG00000216093	AC009305.2	miRNA	2,42E-02	9,72E-01	NA	No	NA	NA	NA	NA
ENSG00000266861	AC097506.1	miRNA	3,50E-02	9,36E-01	NA	No	NA	NA	NA	NA
ENSG00000265812	AC007040.1	miRNA	2,23E-02	9,72E-01	NA	No	NA	NA	NA	NA
ENSG00000221579	AC093162.1	miRNA	5,78E-02	7,07E-01	NA	No	NA	NA	NA	NA
ENSG00000265510	MIR4436A	miRNA	3,39E-02	9,36E-01	NA	No	NA	NA	NA	NA
ENSG00000264157	MIR3127	miRNA	8,95E-02	3,90E-01	NA	No	NA	NA	NA	NA
ENSG00000221541	AC079753.1	miRNA	2,34E-02	9,72E-01	NA	No	NA	NA	NA	NA

ENSG00000265553	AC013275.1	miRNA	2,42E-02	9,72E-01	NA	No	NA	NA	NA	NA
ENSG00000207744	MIR10B	miRNA	3,17E-02	9,36E-01	NA	No	NA	NA	NA	NA
ENSG00000264755	MIR3131	miRNA	3,32E-02	9,36E-01	NA	No	NA	NA	NA	NA
ENSG00000221344	AC068946.1	miRNA	2,34E-02	9,72E-01	NA	No	NA	NA	NA	NA
ENSG00000263743	AC017028.2	miRNA	3,29E-02	9,36E-01	NA	No	NA	NA	NA	NA
ENSG00000264103	AC017028.3	miRNA	1,44E-01	1,59E-01	NA	No	NA	NA	NA	NA
ENSG00000266293	AC069276.1	miRNA	3,15E-02	9,36E-01	NA	No	NA	NA	NA	NA
ENSG00000264534	MIR378B	miRNA	3,41E-02	9,36E-01	NA	No	NA	NA	NA	NA
ENSG00000263573	MIR4270	miRNA	4,18E-02	7,50E-01	NA	No	NA	NA	NA	NA
ENSG00000252976	AC090644.1	miRNA	5,94E-02	5,66E-01	NA	No	NA	NA	NA	NA
ENSG00000207954	MIR138-1	miRNA	2,22E-02	9,72E-01	NA	No	NA	NA	NA	NA
ENSG00000238958	AC098649.1	miRNA	2,40E-02	9,72E-01	NA	No	NA	NA	NA	NA
ENSG00000252504	AC097015.1	miRNA	2,49E-02	9,72E-01	NA	No	NA	NA	NA	NA
ENSG00000221795	MIR1324	miRNA	3,50E-02	9,36E-01	NA	No	NA	NA	NA	NA
ENSG00000266233	AC021660.1	miRNA	2,42E-02	9,72E-01	NA	No	NA	NA	NA	NA
ENSG00000266030	AC078828.1	miRNA	3,17E-02	9,36E-01	NA	No	NA	NA	NA	NA
ENSG00000238593	AC078785.1	miRNA	3,79E-02	8,45E-01	NA	No	NA	NA	NA	NA
ENSG00000221737	MIR548I1	miRNA	3,32E-02	9,36E-01	NA	No	NA	NA	NA	NA
ENSG00000266849	AC083906.2	miRNA	2,35E-02	9,72E-01	NA	No	NA	NA	NA	NA
ENSG00000263959	AC092988.1	miRNA	2,34E-02	9,72E-01	NA	No	NA	NA	NA	NA
ENSG00000266757	AC092962.1	miRNA	2,23E-02	9,72E-01	NA	No	NA	NA	NA	NA
ENSG00000264614	MIR5588	miRNA	3,39E-02	9,36E-01	NA	No	NA	NA	NA	NA
ENSG00000221275	MIR548I2	miRNA	4,19E-02	7,67E-01	NA	No	NA	NA	NA	NA
ENSG00000215961	MIR297	miRNA	2,35E-02	9,72E-01	NA	No	NA	NA	NA	NA
ENSG00000264362	AC093875.1	miRNA	3,74E-02	8,45E-01	NA	No	NA	NA	NA	NA
ENSG00000266024	AF250324.1	miRNA	2,49E-02	9,72E-01	NA	No	NA	NA	NA	NA
ENSG00000265615	AC108105.1	miRNA	1,20E-01	1,85E-01	NA	No	NA	NA	NA	NA
ENSG00000263710	AC025175.1	miRNA	2,42E-02	9,72E-01	NA	No	NA	NA	NA	NA
ENSG00000265699	MIR548AE2	miRNA	2,46E-02	9,72E-01	NA	No	NA	NA	NA	NA
ENSG00000265919	MIR4280	miRNA	3,88E-02	8,45E-01	NA	No	NA	NA	NA	NA
ENSG00000263954	AC004769.1	miRNA	3,32E-02	9,36E-01	NA	No	NA	NA	NA	NA
ENSG00000266751	MIR3661	miRNA	2,22E-02	9,72E-01	NA	No	NA	NA	NA	NA
ENSG00000265875	AC011357.1	miRNA	2,21E-01	1,04E-01	NA	No	NA	NA	NA	NA
ENSG00000263401	AC008674.1	miRNA	3,68E-02	8,45E-01	NA	No	NA	NA	NA	NA
ENSG00000221394	MIR1229	miRNA	2,40E-02	9,72E-01	NA	No	NA	NA	NA	NA
ENSG00000263926	MIR4462	miRNA	3,31E-02	9,36E-01	NA	No	NA	NA	NA	NA
ENSG00000207604	MIR206	miRNA	2,22E-02	9,72E-01	NA	No	NA	NA	NA	NA
ENSG00000265187	AL109612.1	miRNA	3,32E-02	9,36E-01	NA	No	NA	NA	NA	NA
ENSG00000265419	AL359987.1	miRNA	3,39E-02	9,36E-01	NA	No	NA	NA	NA	NA
ENSG00000207632	MIR588	miRNA	3,17E-02	9,36E-01	NA	No	NA	NA	NA	NA
ENSG00000216097	AL357060.1	miRNA	3,32E-02	9,36E-01	NA	No	NA	NA	NA	NA
ENSG00000263514	MIR3668	miRNA	4,36E-02	7,20E-01	NA	No	NA	NA	NA	NA
ENSG00000264390	MIR4465	miRNA	3,39E-02	9,36E-01	NA	No	NA	NA	NA	NA
ENSG00000264679	AC092104.2	miRNA	4,11E-02	7,67E-01	NA	No	NA	NA	NA	NA
ENSG00000266287	MIR3683	miRNA	4,45E-02	6,77E-01	NA	No	NA	NA	NA	NA
ENSG00000263504	AC092104.1	miRNA	3,17E-02	9,36E-01	NA	No	NA	NA	NA	NA
ENSG00000265155	AC092104.3	miRNA	4,27E-02	7,29E-01	NA	No	NA	NA	NA	NA
ENSG00000266409	AC092104.5	miRNA	3,50E-02	9,36E-01	NA	No	NA	NA	NA	NA
ENSG00000263416	AC006145.1	miRNA	4,55E-02	6,45E-01	NA	No	NA	NA	NA	NA
ENSG00000211518	AC006988.1	miRNA	3,31E-02	9,36E-01	NA	No	NA	NA	NA	NA
ENSG00000208014	MIR653	miRNA	3,15E-02	9,36E-01	NA	No	NA	NA	NA	NA
ENSG00000207656	MIR489	miRNA	2,22E-02	9,72E-01	NA	No	NA	NA	NA	NA
ENSG00000264425	MIR4653	miRNA	3,31E-02	9,36E-01	NA	No	NA	NA	NA	NA
ENSG00000221356	AC004492.1	miRNA	2,42E-02	9,72E-01	NA	No	NA	NA	NA	NA
ENSG00000221442	MIR548F4	miRNA	3,32E-02	9,36E-01	NA	No	NA	NA	NA	NA
ENSG00000263835	BX649553.1	miRNA	3,16E-02	9,36E-01	NA	No	NA	NA	NA	NA
ENSG00000264861	AC108683.1	miRNA	4,03E-02	7,82E-01	NA	No	NA	NA	NA	NA
ENSG00000265819	AC092832.1	miRNA	3,31E-02	9,36E-01	NA	No	NA	NA	NA	NA
ENSG00000211991	MIR676	miRNA	3,75E-02	8,45E-01	NA	No	NA	NA	NA	NA
ENSG00000265597	AL590764.1	miRNA	2,23E-02	9,72E-01	NA	No	NA	NA	NA	NA
ENSG00000264855	AL590763.1	miRNA	3,41E-02	9,36E-01	NA	No	NA	NA	NA	NA
ENSG00000221005	AL161723.1	miRNA	3,15E-02	9,36E-01	NA	No	NA	NA	NA	NA

ENSG00000265471	AL034400.1	miRNA	4,36E-02	7,20E-01	NA	No	NA	NA	NA	NA
ENSG00000216114	AL033403.1	miRNA	2,40E-02	9,72E-01	NA	No	NA	NA	NA	NA
ENSG00000252583	MIR514B	miRNA	3,15E-02	9,36E-01	NA	No	NA	NA	NA	NA
ENSG00000265176	MIR3202-1	miRNA	2,23E-02	9,72E-01	NA	No	NA	NA	NA	NA
ENSG00000207826	MIR596	miRNA	3,15E-02	9,36E-01	NA	No	NA	NA	NA	NA
ENSG00000216123	AC012119.1	miRNA	4,66E-02	6,03E-01	NA	No	NA	NA	NA	NA
ENSG00000265075	MIR3622B	miRNA	3,35E-02	9,36E-01	NA	No	NA	NA	NA	NA
ENSG00000264408	MIR4470	miRNA	4,28E-02	7,26E-01	NA	No	NA	NA	NA	NA
ENSG00000266799	AP003356.1	miRNA	2,42E-02	9,72E-01	NA	No	NA	NA	NA	NA
ENSG00000238399	MIR2053	miRNA	2,35E-02	9,72E-01	NA	No	NA	NA	NA	NA
ENSG00000264680	hsa-mir-3669	miRNA	3,17E-02	9,36E-01	NA	No	NA	NA	NA	NA
ENSG00000199065	MIR101-2	miRNA	4,15E-02	7,58E-01	NA	No	NA	NA	NA	NA
ENSG00000215966	MIR876	miRNA	2,42E-02	9,72E-01	NA	No	NA	NA	NA	NA
ENSG00000264922	MIR4540	miRNA	3,17E-02	9,36E-01	NA	No	NA	NA	NA	NA
ENSG00000211556	AL157702.1	miRNA	3,41E-02	9,36E-01	NA	No	NA	NA	NA	NA
ENSG00000272241	AL160275.1	miRNA	2,23E-02	9,72E-01	NA	No	NA	NA	NA	NA
ENSG00000221734	AL390240.1	miRNA	3,32E-02	9,36E-01	NA	No	NA	NA	NA	NA
ENSG00000265662	AL354981.1	miRNA	2,40E-02	9,72E-01	NA	No	NA	NA	NA	NA
ENSG00000266583	MIR4478	miRNA	3,15E-02	9,36E-01	NA	No	NA	NA	NA	NA
ENSG00000265872	MIR3689A	miRNA	2,22E-02	9,72E-01	NA	No	NA	NA	NA	NA
ENSG00000265848	MIR3689D1	miRNA	3,15E-02	9,36E-01	NA	No	NA	NA	NA	NA
ENSG00000263507	AL603650.1	miRNA	2,34E-02	9,72E-01	NA	No	NA	NA	NA	NA
ENSG00000266514	MIR3689F	miRNA	3,79E-02	8,45E-01	NA	No	NA	NA	NA	NA
ENSG00000221272	AC067742.1	miRNA	2,77E-01	1,66E-02	8,07E-02	No	NA	NA	NA	NA
ENSG00000221094	AL671972.1	miRNA	3,91E-02	8,45E-01	NA	No	NA	NA	NA	NA
ENSG00000265990	AL356154.1	miRNA	3,31E-02	9,36E-01	NA	No	NA	NA	NA	NA
ENSG00000265375	MIR4679-2	miRNA	4,32E-02	7,25E-01	NA	No	NA	NA	NA	NA
ENSG00000207551	MIR608	miRNA	3,15E-02	9,36E-01	NA	No	NA	NA	NA	NA
ENSG00000263436	MIR3158-2	miRNA	4,19E-02	7,48E-01	NA	No	NA	NA	NA	NA
ENSG00000221293	AC009987.1	miRNA	4,18E-02	7,50E-01	NA	No	NA	NA	NA	NA
ENSG00000265395	MIR3944	miRNA	3,17E-02	9,36E-01	NA	No	NA	NA	NA	NA
ENSG00000264493	MIR4298	miRNA	1,74E-01	1,16E-01	NA	No	NA	NA	NA	NA
ENSG00000265258	MIR4686	miRNA	3,32E-02	9,36E-01	NA	No	NA	NA	NA	NA
ENSG00000221030	AC010930.1	miRNA	4,12E-02	7,65E-01	NA	No	NA	NA	NA	NA
ENSG00000266625	AC084859.1	miRNA	2,35E-02	9,72E-01	NA	No	NA	NA	NA	NA
ENSG00000264603	MIR3159	miRNA	3,27E-02	9,36E-01	NA	No	NA	NA	NA	NA
ENSG00000265910	AC090559.2	miRNA	2,23E-02	9,72E-01	NA	No	NA	NA	NA	NA
ENSG00000265783	AP000445.3	miRNA	4,28E-02	7,26E-01	NA	No	NA	NA	NA	NA
ENSG00000266006	MIR4488	miRNA	3,31E-02	9,36E-01	NA	No	NA	NA	NA	NA
ENSG00000263742	MIR3165	miRNA	3,31E-02	9,36E-01	NA	No	NA	NA	NA	NA
ENSG00000212089	AP002490.1	miRNA	6,05E-02	5,51E-01	NA	No	NA	NA	NA	NA
ENSG00000272036	MIR139	miRNA	3,24E-02	9,36E-01	NA	No	NA	NA	NA	NA
ENSG00000265064	MIR4692	miRNA	3,31E-02	9,36E-01	NA	No	NA	NA	NA	NA
ENSG00000221551	AP000478.1	miRNA	3,32E-02	9,36E-01	NA	No	NA	NA	NA	NA
ENSG00000266703	MIR4490	miRNA	2,34E-02	9,72E-01	NA	No	NA	NA	NA	NA
ENSG00000263885	MIR3920	miRNA	2,22E-02	9,72E-01	NA	No	NA	NA	NA	NA
ENSG00000264032	MIR4491	miRNA	4,24E-02	7,54E-01	NA	No	NA	NA	NA	NA
ENSG00000266633	AC006432.1	miRNA	2,35E-02	9,72E-01	NA	No	NA	NA	NA	NA
ENSG00000252445	AC007535.1	miRNA	3,17E-02	9,36E-01	NA	No	NA	NA	NA	NA
ENSG00000264906	MIR4494	miRNA	5,19E-02	6,54E-01	NA	No	NA	NA	NA	NA
ENSG00000265804	AC078864.2	miRNA	2,40E-02	9,72E-01	NA	No	NA	NA	NA	NA
ENSG00000238909	AC068643.1	miRNA	2,23E-02	9,72E-01	NA	No	NA	NA	NA	NA
ENSG00000222647	AC002070.1	miRNA	5,99E-02	5,60E-01	NA	No	NA	NA	NA	NA
ENSG00000266655	MIR3908	miRNA	2,46E-02	9,72E-01	NA	No	NA	NA	NA	NA
ENSG00000221749	AC107020.1	miRNA	2,22E-02	9,72E-01	NA	No	NA	NA	NA	NA
ENSG00000266230	AL591925.1	miRNA	2,34E-02	9,72E-01	NA	No	NA	NA	NA	NA
ENSG00000264864	MIR3613	miRNA	3,75E-02	8,45E-01	NA	No	NA	NA	NA	NA
ENSG00000212057	AL139318.1	miRNA	3,39E-02	9,36E-01	NA	No	NA	NA	NA	NA
ENSG00000207989	MIR493	miRNA	4,61E-02	6,21E-01	NA	No	NA	NA	NA	NA
ENSG00000212040	MIR543	miRNA	4,19E-02	7,49E-01	NA	No	NA	NA	NA	NA
ENSG00000271946	MIR376C	miRNA	3,80E-02	8,45E-01	NA	No	NA	NA	NA	NA
ENSG00000211990	AL117209.1	miRNA	8,47E-02	4,32E-01	NA	No	NA	NA	NA	NA

ENSG00000265291	MIR4710	miRNA	3,46E-02	9,36E-01	NA	No	NA	NA	NA	NA
ENSG00000263413	AL928742.1	miRNA	6,15E-02	5,34E-01	NA	No	NA	NA	NA	NA
ENSG00000265612	hsa-mir-4539	miRNA	3,41E-02	9,36E-01	NA	No	NA	NA	NA	NA
ENSG00000266706	AL122127.5	miRNA	3,33E-02	9,36E-01	NA	No	NA	NA	NA	NA
ENSG00000265517	AL122127.2	miRNA	9,02E-02	3,83E-01	NA	No	NA	NA	NA	NA
ENSG00000264473	hsa-mir-4538	miRNA	5,57E-02	6,06E-01	NA	No	NA	NA	NA	NA
ENSG00000263751	AL122127.1	miRNA	1,67E-01	1,36E-01	NA	No	NA	NA	NA	NA
ENSG00000266408	AL122127.4	miRNA	1,43E-01	1,21E-01	NA	No	NA	NA	NA	NA
ENSG00000265002	AC026495.1	miRNA	6,02E-02	5,49E-01	NA	No	NA	NA	NA	NA
ENSG00000221202	AC012050.1	miRNA	3,39E-02	9,36E-01	NA	No	NA	NA	NA	NA
ENSG00000221052	MIR1266	miRNA	4,68E-02	5,95E-01	NA	No	NA	NA	NA	NA
ENSG00000238870	AC009677.1	miRNA	3,39E-02	9,36E-01	NA	No	NA	NA	NA	NA
ENSG00000266542	MIR5572	miRNA	2,35E-02	9,72E-01	NA	No	NA	NA	NA	NA
ENSG00000208003	MIR549	miRNA	3,32E-02	9,36E-01	NA	No	NA	NA	NA	NA
ENSG00000264406	MIR548AP	miRNA	3,32E-02	9,36E-01	NA	No	NA	NA	NA	NA
ENSG00000221691	AC090825.2	miRNA	2,35E-02	9,72E-01	NA	No	NA	NA	NA	NA
ENSG00000252958	AC009065.3	miRNA	2,35E-02	9,72E-01	NA	No	NA	NA	NA	NA
ENSG00000264004	MIR4717	miRNA	2,23E-02	9,72E-01	NA	No	NA	NA	NA	NA
ENSG00000264694	AC092122.1	miRNA	3,85E-02	8,45E-01	NA	No	NA	NA	NA	NA
ENSG00000264733	MIR4718	miRNA	5,01E-02	6,77E-01	NA	No	NA	NA	NA	NA
ENSG00000266305	MIR4518	miRNA	6,62E-02	4,79E-01	NA	No	NA	NA	NA	NA
ENSG00000265616	AC136932.2	miRNA	2,49E-02	9,72E-01	NA	No	NA	NA	NA	NA
ENSG00000263441	AC012181.1	miRNA	2,22E-02	9,72E-01	NA	No	NA	NA	NA	NA
ENSG00000208017	MIR140	miRNA	3,41E-02	9,36E-01	NA	No	NA	NA	NA	NA
ENSG00000222190	MIR1910	miRNA	4,20E-02	7,65E-01	NA	No	NA	NA	NA	NA
ENSG00000266115	AC005375.1	miRNA	4,75E-02	5,65E-01	NA	No	NA	NA	NA	NA
ENSG00000263985	AC116407.1	miRNA	4,12E-02	7,65E-01	NA	No	NA	NA	NA	NA
ENSG00000221699	AC024610.1	miRNA	3,31E-02	9,36E-01	NA	No	NA	NA	NA	NA
ENSG00000266632	MIR4726	miRNA	2,46E-02	9,72E-01	NA	No	NA	NA	NA	NA
ENSG00000263791	MIR4727	miRNA	3,15E-02	9,36E-01	NA	No	NA	NA	NA	NA
ENSG00000210741	MIR196A1	miRNA	4,75E-02	7,21E-01	NA	No	NA	NA	NA	NA
ENSG00000265809	AC007114.1	miRNA	9,77E-02	3,40E-01	NA	No	NA	NA	NA	NA
ENSG00000265636	AC100787.1	miRNA	4,61E-02	6,21E-01	NA	No	NA	NA	NA	NA
ENSG00000266665	MIR4739	miRNA	2,35E-02	9,72E-01	NA	No	NA	NA	NA	NA
ENSG00000265481	AP006564.1	miRNA	2,22E-02	9,72E-01	NA	No	NA	NA	NA	NA
ENSG00000266530	MIR4318	miRNA	4,28E-02	7,26E-01	NA	No	NA	NA	NA	NA
ENSG00000221291	AC090660.1	miRNA	2,35E-02	9,72E-01	NA	No	NA	NA	NA	NA
ENSG00000252330	AC105105.1	miRNA	3,32E-02	9,36E-01	NA	No	NA	NA	NA	NA
ENSG00000221763	MIR1289-1	miRNA	3,31E-02	9,36E-01	NA	No	NA	NA	NA	NA
ENSG00000263645	AL079339.1	miRNA	2,22E-02	9,72E-01	NA	No	NA	NA	NA	NA
ENSG00000266140	MIR4533	miRNA	4,36E-02	7,20E-01	NA	No	NA	NA	NA	NA
ENSG00000263997	AC006130.1	miRNA	5,72E-02	5,94E-01	NA	No	NA	NA	NA	NA
ENSG00000266209	AC005783.1	miRNA	3,75E-02	8,45E-01	NA	No	NA	NA	NA	NA
ENSG00000264899	AC027319.2	miRNA	2,22E-02	9,72E-01	NA	No	NA	NA	NA	NA
ENSG00000263995	AC011499.1	miRNA	6,93E-02	4,55E-01	NA	No	NA	NA	NA	NA
ENSG00000238517	MIR1270-1	miRNA	3,24E-02	9,36E-01	NA	No	NA	NA	NA	NA
ENSG00000221147	MIR1270-2	miRNA	3,24E-02	9,36E-01	NA	No	NA	NA	NA	NA
ENSG00000221115	AC020910.1	miRNA	3,28E-02	9,36E-01	NA	No	NA	NA	NA	NA
ENSG00000222730	AC092296.1	miRNA	2,23E-02	9,72E-01	NA	No	NA	NA	NA	NA
ENSG00000266226	MIR4323	miRNA	5,10E-02	5,19E-01	NA	No	NA	NA	NA	NA
ENSG00000265122	AC010247.1	miRNA	2,49E-02	9,72E-01	NA	No	NA	NA	NA	NA
ENSG00000264413	AC011495.1	miRNA	2,22E-02	9,72E-01	NA	No	NA	NA	NA	NA
ENSG00000199143	MIR373	miRNA	3,50E-02	9,36E-01	NA	No	NA	NA	NA	NA
ENSG00000264799	AC009892.1	miRNA	3,31E-02	9,36E-01	NA	No	NA	NA	NA	NA
ENSG00000221039	MIR1286	miRNA	3,15E-02	9,36E-01	NA	No	NA	NA	NA	NA
ENSG00000207575	MIR649	miRNA	3,39E-02	9,36E-01	NA	No	NA	NA	NA	NA
ENSG00000212102	MIR301B	miRNA	3,15E-02	9,36E-01	NA	No	NA	NA	NA	NA
ENSG00000207834	D86994.2	miRNA	3,31E-02	9,36E-01	NA	No	NA	NA	NA	NA
ENSG00000207832	D87024.1	miRNA	4,28E-02	7,31E-01	NA	No	NA	NA	NA	NA
ENSG00000264824	hsa-mir-5571	miRNA	3,80E-02	8,45E-01	NA	No	NA	NA	NA	NA
ENSG00000264661	MIR3200	miRNA	3,35E-02	9,36E-01	NA	No	NA	NA	NA	NA
ENSG00000252142	AL022314.1	miRNA	9,98E-02	1,96E-01	NA	No	NA	NA	NA	NA

ENSG00000207932	MIR33A	miRNA	5,46E-02	6,17E-01	NA	No	NA	NA	NA	NA
ENSG00000264139	MIR3667	miRNA	4,16E-02	7,67E-01	NA	No	NA	NA	NA	NA
ENSG00000264055	AP000261.1	miRNA	3,32E-02	9,36E-01	NA	No	NA	NA	NA	NA
ENSG00000211590	MIR802	miRNA	9,10E-02	2,58E-01	NA	No	NA	NA	NA	NA
ENSG00000239196	AL591856.3	miRNA	3,45E-02	9,36E-01	NA	No	NA	NA	NA	NA
ENSG00000264001	CT867976.3	miRNA	3,69E-02	8,45E-01	NA	No	NA	NA	NA	NA
ENSG00000200087	SNORA73B	snoRNA	3,08E-01	1,19E-01	3,12E-01	No	1,89E+00	6,33E-18	1,60E-16	Yes
ENSG00000239111	snoU13	snoRNA	8,69E-02	3,08E-01	NA	No	1,53E+00	1,63E-02	4,33E-02	Yes
ENSG00000252010	SCARNA5	snoRNA	7,90E-01	2,83E-04	3,91E-03	No	2,28E+00	1,77E-35	1,77E-33	Yes
ENSG00000251791	SCARNA6	snoRNA	6,85E-01	1,18E-03	1,15E-02	No	2,36E+00	6,10E-40	8,14E-38	Yes
ENSG00000263723	SNORD39	snoRNA	-4,69E-01	8,51E-02	2,50E-01	No	-1,69E+00	6,65E-03	1,99E-02	Yes
ENSG00000199815	SNORA43	snoRNA	2,50E-01	5,04E-02	NA	No	2,80E+00	3,15E-03	1,03E-02	Yes
ENSG00000238359	snoU13	snoRNA	8,39E-01	2,59E-02	1,10E-01	No	3,42E+00	3,88E-06	2,28E-05	Yes
ENSG00000238541	snoU13	snoRNA	1,83E-01	4,22E-01	6,64E-01	No	-2,25E+00	1,22E-06	7,75E-06	Yes
ENSG00000212579	SNORA40	snoRNA	1,10E+00	6,98E-03	4,35E-02	No	1,76E+00	4,72E-04	1,88E-03	Yes
ENSG00000206754	SNORD101	snoRNA	-3,16E-01	1,56E-01	3,72E-01	No	-1,51E+00	8,62E-03	2,50E-02	Yes
ENSG00000207062	SNORA15	snoRNA	2,44E-01	6,95E-02	NA	No	2,51E+00	5,36E-03	1,65E-02	Yes
ENSG00000231587	SNORD62B	snoRNA	2,51E-02	9,09E-01	9,63E-01	No	-4,00E+00	1,56E-05	8,30E-05	Yes
ENSG00000212464	SNORA12	snoRNA	-1,23E-01	5,27E-01	7,48E-01	No	1,84E+00	3,13E-23	1,32E-21	Yes
ENSG00000201998	SNORA23	snoRNA	-4,65E-02	8,31E-01	9,26E-01	No	4,22E+00	3,90E-50	1,12E-47	Yes
ENSG00000212443	SNORA53	snoRNA	4,77E-02	8,30E-01	9,26E-01	No	2,23E+00	1,53E-11	1,96E-10	Yes
ENSG00000238344	SNORD126	snoRNA	2,07E-01	1,90E-01	NA	No	1,89E+00	1,13E-02	3,18E-02	Yes
ENSG00000252481	SCARNA13	snoRNA	2,29E-01	3,07E-01	5,55E-01	No	3,09E+00	6,73E-32	5,41E-30	Yes
ENSG00000252873	SNORD112	snoRNA	2,72E-02	8,00E-01	NA	No	2,23E+00	4,54E-03	1,42E-02	Yes
ENSG00000201672	SNORD113-4	snoRNA	2,29E-01	3,02E-01	5,50E-01	No	1,53E+00	1,32E-03	4,73E-03	Yes
ENSG00000201839	SNORD114-3	snoRNA	2,08E-01	1,81E-01	4,06E-01	No	1,99E+00	7,25E-03	2,15E-02	Yes
ENSG00000252009	SNORD112	snoRNA	1,59E-02	8,78E-01	NA	No	1,77E+00	6,87E-03	2,05E-02	Yes
ENSG00000251860	snoU13	snoRNA	-2,80E-01	1,65E-01	3,86E-01	No	-1,86E+00	1,35E-02	3,70E-02	Yes
ENSG00000221044	U3	snoRNA	-2,96E-01	1,82E-01	4,07E-01	No	-2,05E+00	4,95E-03	1,54E-02	Yes
ENSG00000252284	SNORD28	snoRNA	-3,40E-01	1,40E-01	3,48E-01	No	-2,31E+00	5,03E-03	1,56E-02	Yes
ENSG00000225091	SNORA71A	snoRNA	-5,38E-02	8,02E-01	9,12E-01	No	-2,03E+00	5,36E-03	1,65E-02	Yes
ENSG00000201512	SNORA71C	snoRNA	-3,47E-02	8,61E-01	9,40E-01	No	-1,53E+00	1,53E-02	4,11E-02	Yes
ENSG00000239157	snoU13	snoRNA	-8,10E-02	7,03E-01	8,59E-01	No	-2,33E+00	6,23E-04	2,41E-03	Yes
ENSG00000253007	SNORA76	snoRNA	-9,09E-01	1,50E-02	7,50E-02	No	-2,01E+00	3,84E-05	1,90E-04	Yes
ENSG00000221083	SNORA77	snoRNA	-4,92E-02	5,54E-01	NA	No	-1,09E-01	5,15E-01	NA	No
ENSG00000271794	snoU13	snoRNA	-2,86E-02	8,06E-01	NA	No	1,13E-01	6,85E-01	7,97E-01	No
ENSG00000239149	SNORA59A	snoRNA	-9,55E-01	3,15E-03	2,44E-02	No	-1,19E+00	3,12E-04	1,29E-03	No
ENSG00000251866	SCARNA21	snoRNA	-9,51E-02	5,15E-01	7,39E-01	No	-2,49E-01	4,21E-01	5,77E-01	No
ENSG00000238818	snoU13	snoRNA	1,73E-01	1,69E-01	NA	No	9,35E-01	4,09E-02	9,47E-02	No
ENSG00000239027	snoU13	snoRNA	4,41E-02	6,93E-01	NA	No	8,55E-02	8,64E-01	NA	No
ENSG00000238889	snoU13	snoRNA	9,16E-03	9,48E-01	9,79E-01	No	-1,08E-01	6,83E-01	7,95E-01	No
ENSG00000252190	SCARNA17	snoRNA	-1,70E-02	8,51E-01	NA	No	-1,92E-01	3,47E-01	NA	No
ENSG00000252691	SCARNA18	snoRNA	4,79E-03	9,61E-01	NA	No	-1,15E-01	5,86E-01	NA	No
ENSG00000252947	SCARNA1	snoRNA	5,68E-01	4,54E-02	1,63E-01	No	4,01E-01	2,48E-01	3,93E-01	No
ENSG00000238821	snoU13	snoRNA	1,65E-02	9,27E-01	9,70E-01	No	-8,11E-01	3,60E-02	NA	No
ENSG00000221539	SNORD99	snoRNA	-1,96E-01	2,87E-01	5,34E-01	No	-7,78E-01	8,10E-02	1,66E-01	No
ENSG00000252777	SCARNA24	snoRNA	-5,47E-02	6,46E-01	NA	No	-3,01E-02	8,90E-01	NA	No
ENSG00000200154	SNORD103A	snoRNA	4,09E-02	8,34E-01	9,28E-01	No	-2,66E-01	4,41E-01	5,96E-01	No
ENSG00000202107	SNORD103B	snoRNA	-8,00E-02	7,02E-01	8,59E-01	No	-4,52E-01	2,27E-01	3,67E-01	No
ENSG00000200181	SNORD85	snoRNA	-1,22E-01	4,57E-01	6,92E-01	No	3,19E-01	3,77E-01	5,33E-01	No
ENSG00000252728	SNORD112	snoRNA	-5,47E-02	6,46E-01	NA	No	-1,30E-01	7,12E-01	NA	No
ENSG00000201542	SNORA62	snoRNA	-1,89E-02	8,33E-01	NA	No	1,21E-01	6,36E-01	7,59E-01	No
ENSG00000201448	SNORA63	snoRNA	1,27E-01	3,43E-01	NA	No	2,59E-01	3,79E-01	5,36E-01	No
ENSG00000252448	SNORA63	snoRNA	4,76E-02	8,02E-01	9,12E-01	No	-5,51E-02	8,67E-01	9,20E-01	No
ENSG00000201457	SNORA55	snoRNA	-3,19E-01	1,93E-01	4,23E-01	No	-9,18E-01	4,46E-02	1,02E-01	No
ENSG00000200913	SNORD46	snoRNA	4,11E-01	1,04E-01	2,86E-01	No	-2,32E-01	4,54E-01	6,07E-01	No
ENSG00000202031	SNORD38A	snoRNA	-5,57E-02	6,46E-01	NA	No	-1,32E-01	7,12E-01	NA	No
ENSG00000238945	snoU13	snoRNA	-4,23E-02	7,60E-01	NA	No	-9,82E-02	8,30E-01	NA	No
ENSG00000212624	SNORA26	snoRNA	3,48E-02	8,39E-01	9,30E-01	No	-3,62E-01	2,24E-01	3,64E-01	No
ENSG00000239063	snoU13	snoRNA	3,47E-01	1,02E-01	2,82E-01	No	6,47E-01	1,13E-01	2,16E-01	No
ENSG00000238931	snoU13	snoRNA	-1,79E-02	8,60E-01	NA	No	-1,55E-01	5,29E-01	NA	No
ENSG00000199934	SNORD81	snoRNA	4,32E-02	7,25E-01	NA	No	1,03E-01	7,75E-01	NA	No

ENSG00000251795	SNORA66	snoRNA	-2,11E-01	3,62E-01	6,10E-01	No	-4,15E-01	1,84E-01	3,15E-01	No
ENSG00000207523	SNORA66	snoRNA	1,44E-01	5,15E-01	7,39E-01	No	7,47E-02	8,31E-01	8,97E-01	No
ENSG00000207022	SNORA51	snoRNA	-1,18E-01	3,36E-01	NA	No	-3,12E-01	2,41E-01	NA	No
ENSG00000238296	snoU13	snoRNA	1,64E-01	2,91E-01	NA	No	3,14E-01	2,80E-01	4,29E-01	No
ENSG00000200536	SNORA25	snoRNA	4,31E-02	7,25E-01	NA	No	9,20E-02	8,64E-01	NA	No
ENSG00000238975	snoU13	snoRNA	1,40E-01	5,28E-01	7,49E-01	No	-3,25E-01	3,62E-01	5,19E-01	No
ENSG00000238679	snoU13	snoRNA	-1,47E-03	9,89E-01	NA	No	-1,61E-01	4,69E-01	NA	No
ENSG00000238526	snoU13	snoRNA	-2,18E-01	1,30E-01	NA	No	-5,99E-01	8,73E-02	NA	No
ENSG00000253047	SNORA40	snoRNA	1,94E-01	2,22E-01	NA	No	3,88E-01	2,28E-01	3,68E-01	No
ENSG00000238511	snoU13	snoRNA	9,26E-02	6,02E-01	7,98E-01	No	-2,19E-01	4,33E-01	5,87E-01	No
ENSG00000201129	SNORA58	snoRNA	-5,69E-02	6,46E-01	NA	No	-1,35E-01	7,12E-01	NA	No
ENSG00000238805	snoU13	snoRNA	-3,33E-02	8,22E-01	9,22E-01	No	-2,80E-01	3,16E-01	4,70E-01	No
ENSG00000207475	SNORA42	snoRNA	1,14E-01	5,19E-01	7,42E-01	No	2,26E-01	5,09E-01	6,56E-01	No
ENSG00000252808	SCARNA4	snoRNA	-1,91E-01	3,53E-01	6,01E-01	No	-7,08E-01	9,84E-02	1,93E-01	No
ENSG00000212161	SNORD64	snoRNA	4,57E-02	6,37E-01	NA	No	1,27E-01	5,08E-01	NA	No
ENSG00000238934	ACA64	snoRNA	2,35E-02	9,72E-01	NA	No	6,17E-02	9,56E-01	NA	No
ENSG00000253060	SCARNA20	snoRNA	1,83E-02	8,24E-01	NA	No	2,71E-02	9,24E-01	NA	No
ENSG00000252354	SNORD112	snoRNA	3,27E-01	1,60E-02	NA	No	1,25E-01	6,64E-01	NA	No
ENSG00000251817	snoU13	snoRNA	1,31E-02	9,08E-01	NA	No	1,11E-01	6,92E-01	8,02E-01	No
ENSG00000208317	SNORD78	snoRNA	-9,16E-02	6,41E-01	8,22E-01	No	-1,47E-02	9,65E-01	9,79E-01	No
ENSG00000238872	snoU13	snoRNA	2,57E-02	8,80E-01	9,50E-01	No	1,61E-01	6,32E-01	7,56E-01	No
ENSG00000252906	SCARNA3	snoRNA	2,29E-01	3,07E-01	5,55E-01	No	3,27E-01	3,68E-01	5,25E-01	No
ENSG00000201791	SNORA63	snoRNA	-1,39E-02	9,03E-01	NA	No	-9,39E-02	6,90E-01	NA	No
ENSG00000212338	SNORA67	snoRNA	9,80E-02	3,33E-01	NA	No	2,63E-01	2,71E-01	NA	No
ENSG00000201619	SNORA67	snoRNA	-2,42E-02	8,88E-01	9,53E-01	No	1,38E-01	6,75E-01	7,89E-01	No
ENSG00000238754	snoU109	snoRNA	2,30E-01	1,55E-01	3,70E-01	No	3,88E-01	2,53E-01	3,98E-01	No
ENSG00000252241	U3	snoRNA	7,25E-02	4,25E-01	NA	No	1,09E-01	6,81E-01	NA	No
ENSG00000253042	SNORA70	snoRNA	-1,85E-01	4,05E-01	6,49E-01	No	5,28E-02	8,79E-01	9,28E-01	No
ENSG00000221643	SNORA77	snoRNA	-8,14E-01	2,14E-02	9,64E-02	No	-1,05E+00	2,60E-02	6,45E-02	No
ENSG00000252946	SNORD112	snoRNA	1,21E-01	2,20E-01	NA	No	-9,72E-02	8,30E-01	NA	No
ENSG00000201944	SNORA72	snoRNA	9,61E-02	6,13E-01	8,05E-01	No	3,27E-01	3,68E-01	5,24E-01	No
ENSG00000252853	SNORD112	snoRNA	-1,83E-01	1,53E-01	NA	No	-1,71E-01	5,53E-01	6,93E-01	No
ENSG00000238401	snoU13	snoRNA	-6,06E-02	5,43E-01	NA	No	-2,22E-02	8,35E-01	NA	No
ENSG00000212187	SNORA26	snoRNA	-1,85E-02	8,14E-01	NA	No	-5,34E-02	7,44E-01	NA	No
ENSG00000201544	SNORA16B	snoRNA	-6,28E-02	7,66E-01	8,93E-01	No	-1,09E+00	2,44E-02	6,11E-02	No
ENSG00000202498	SNORD116	snoRNA	-4,21E-02	7,60E-01	NA	No	-9,72E-02	8,30E-01	NA	No
ENSG00000238798	snoU13	snoRNA	-4,78E-02	6,01E-01	NA	No	-7,15E-02	7,26E-01	NA	No
ENSG00000238576	snoU13	snoRNA	-8,50E-02	5,46E-01	7,61E-01	No	-2,72E-01	3,92E-01	5,49E-01	No
ENSG00000222370	SNORA36B	snoRNA	1,88E-01	3,25E-01	5,74E-01	No	2,63E-01	4,46E-01	6,00E-01	No
ENSG00000239054	snoU13	snoRNA	4,26E-02	8,03E-01	9,13E-01	No	9,63E-02	7,75E-01	8,60E-01	No
ENSG00000238545	snoU13	snoRNA	3,90E-02	8,45E-01	NA	No	1,07E-01	6,99E-01	NA	No
ENSG00000212144	U8	snoRNA	-5,47E-02	6,46E-01	NA	No	-1,30E-01	7,12E-01	NA	No
ENSG00000207181	SNORA14B	snoRNA	2,42E-02	8,93E-01	9,56E-01	No	-2,57E-01	4,59E-01	6,12E-01	No
ENSG00000252290	SNORA25	snoRNA	-2,32E-01	3,23E-01	5,71E-01	No	5,48E-01	9,68E-02	1,91E-01	No
ENSG00000252495	SNORD112	snoRNA	2,49E-02	9,72E-01	NA	No	6,34E-02	9,56E-01	NA	No
ENSG00000238888	snoU13	snoRNA	-3,12E-02	8,50E-01	9,35E-01	No	3,56E-02	9,15E-01	9,49E-01	No
ENSG00000238462	snoU13	snoRNA	-6,69E-02	6,10E-01	8,03E-01	No	-2,65E-01	3,79E-01	5,36E-01	No
ENSG00000206898	SNORA51	snoRNA	4,18E-02	7,50E-01	NA	No	6,17E-02	9,56E-01	NA	No
ENSG00000206633	SNORA80B	snoRNA	6,10E-02	4,72E-01	NA	No	2,37E-01	3,33E-01	NA	No
ENSG00000212455	SNORA40	snoRNA	-2,81E-02	7,31E-01	NA	No	4,43E-02	8,28E-01	NA	No
ENSG00000251805	SCARNA21	snoRNA	1,62E-03	9,95E-01	9,98E-01	No	2,01E-01	5,69E-01	7,06E-01	No
ENSG00000202479	SNORD14	snoRNA	-4,78E-02	5,61E-01	NA	No	3,72E-02	8,70E-01	NA	No
ENSG00000206731	SNORA36	snoRNA	2,83E-02	8,57E-01	9,38E-01	No	-6,82E-01	6,03E-02	1,31E-01	No
ENSG00000264994	SNORD92	snoRNA	4,01E-02	7,45E-01	8,81E-01	No	-7,04E-02	7,83E-01	8,65E-01	No
ENSG00000265145	SNORD53	snoRNA	1,78E-01	3,45E-01	5,93E-01	No	2,84E-01	4,25E-01	5,81E-01	No
ENSG00000265706	SNORD53_SNORD92	snoRNA	1,20E-01	1,99E-01	NA	No	1,19E+00	2,04E-02	5,28E-02	No
ENSG00000252502	SNORD112	snoRNA	6,27E-02	6,41E-01	NA	No	6,45E-01	1,06E-01	2,04E-01	No
ENSG00000252473	SNORA67	snoRNA	1,63E-01	4,66E-01	7,00E-01	No	4,01E-01	1,53E-01	2,73E-01	No
ENSG00000252281	snoZ247	snoRNA	3,63E-02	6,54E-01	NA	No	4,31E-02	8,45E-01	NA	No
ENSG00000239052	snoU13	snoRNA	1,31E-01	4,65E-01	6,99E-01	No	2,02E-01	5,42E-01	6,84E-01	No
ENSG00000238756	snoU13	snoRNA	3,98E-02	6,58E-01	NA	No	-7,89E-02	7,70E-01	NA	No
ENSG00000212175	SNORA12	snoRNA	3,19E-01	1,96E-01	4,27E-01	No	2,54E-01	4,47E-01	6,01E-01	No

ENSG00000212168	SNORD78	snoRNA	1,04E-01	2,90E-01	NA	No	1,42E-01	5,73E-01	NA	No
ENSG00000206937	SNORA70B	snoRNA	2,75E-01	1,69E-01	3,91E-01	No	-1,71E-01	5,62E-01	7,00E-01	No
ENSG00000251775	ACA59	snoRNA	-5,07E-01	7,21E-02	2,24E-01	No	-5,36E-01	1,27E-01	2,36E-01	No
ENSG00000207016	SNORA36C	snoRNA	-1,01E-01	3,91E-01	NA	No	-3,23E-01	2,28E-01	3,69E-01	No
ENSG00000238708	snoU13	snoRNA	-2,68E-01	1,95E-01	4,26E-01	No	2,05E-01	5,64E-01	7,01E-01	No
ENSG00000238465	snoU13	snoRNA	-2,82E-01	2,41E-01	4,81E-01	No	1,07E+00	6,95E-04	2,66E-03	No
ENSG00000239072	snoU13	snoRNA	5,54E-03	9,26E-01	NA	No	-1,23E-01	6,14E-01	NA	No
ENSG00000239064	snoU13	snoRNA	1,54E-02	9,64E-01	NA	No	-1,01E-01	8,30E-01	NA	No
ENSG00000212378	SNORD78	snoRNA	-4,23E-02	7,60E-01	NA	No	3,16E-01	1,96E-01	NA	No
ENSG00000208772	SNORD94	snoRNA	8,07E-01	1,08E-02	5,91E-02	No	1,72E-01	5,85E-01	7,18E-01	No
ENSG00000238328	snoU13	snoRNA	1,66E-02	8,85E-01	NA	No	2,87E-01	2,80E-01	NA	No
ENSG00000212283	SNORD89	snoRNA	1,16E-01	5,22E-01	7,44E-01	No	-1,58E-01	6,28E-01	7,53E-01	No
ENSG00000212182	U3	snoRNA	-6,34E-02	4,94E-01	NA	No	-1,52E-01	5,59E-01	NA	No
ENSG00000238368	snoU13	snoRNA	-2,43E-02	9,01E-01	9,59E-01	No	-1,06E+00	3,90E-02	9,10E-02	No
ENSG00000238341	snoU13	snoRNA	-4,23E-02	7,60E-01	NA	No	-9,82E-02	8,30E-01	NA	No
ENSG00000238337	snoU13	snoRNA	4,43E-02	8,18E-01	9,20E-01	No	4,40E-01	2,46E-01	3,90E-01	No
ENSG00000208308	SNORA40	snoRNA	-1,45E-01	4,72E-01	7,05E-01	No	-3,84E-01	2,88E-01	4,38E-01	No
ENSG00000212181	SNORA48	snoRNA	2,84E-02	8,92E-01	9,55E-01	No	-1,51E-01	6,66E-01	7,82E-01	No
ENSG00000238481	snoU13	snoRNA	-2,07E-02	8,19E-01	NA	No	-1,36E-01	7,12E-01	NA	No
ENSG00000251721	snoZ5	snoRNA	-4,19E-01	6,66E-02	2,12E-01	No	3,40E-01	3,49E-01	5,05E-01	No
ENSG00000206869	SNORA70F	snoRNA	-3,17E-01	1,92E-01	4,21E-01	No	8,15E-02	8,13E-01	8,85E-01	No
ENSG00000206961	SNORA51	snoRNA	-5,41E-02	6,46E-01	NA	No	1,32E-01	6,23E-01	NA	No
ENSG00000252981	U3	snoRNA	-9,72E-02	6,09E-01	8,03E-01	No	-5,07E-01	1,97E-01	3,30E-01	No
ENSG00000238567	snoU13	snoRNA	7,94E-02	4,97E-01	NA	No	9,42E-02	6,84E-01	NA	No
ENSG00000239041	snoU13	snoRNA	-3,23E-02	6,65E-01	NA	No	-1,45E-01	6,02E-01	NA	No
ENSG00000238295	snoU13	snoRNA	8,75E-02	4,14E-01	NA	No	2,10E-01	4,26E-01	NA	No
ENSG00000252000	ACA59	snoRNA	9,41E-04	9,97E-01	9,99E-01	No	1,83E-01	5,95E-01	7,26E-01	No
ENSG00000238339	snoU13	snoRNA	-2,57E-01	1,95E-01	4,26E-01	No	-7,31E-01	9,49E-02	1,88E-01	No
ENSG00000202216	SNORA43	snoRNA	2,84E-02	7,75E-01	NA	No	1,53E-01	5,51E-01	6,91E-01	No
ENSG00000212581	U8	snoRNA	1,06E-01	2,72E-01	NA	No	1,16E-01	6,77E-01	NA	No
ENSG00000239161	snoU13	snoRNA	5,12E-02	7,94E-01	9,08E-01	No	1,15E-02	9,75E-01	9,85E-01	No
ENSG00000252923	SCARNA16	snoRNA	-1,05E-04	9,41E-01	NA	No	-1,03E-01	8,30E-01	NA	No
ENSG00000202434	SNORA4	snoRNA	1,66E-02	9,30E-01	9,72E-01	No	1,93E-02	9,56E-01	9,74E-01	No
ENSG00000212534	SNORD70	snoRNA	2,52E-01	1,95E-01	4,26E-01	No	-8,10E-02	7,89E-01	8,69E-01	No
ENSG00000212309	SNORD70	snoRNA	-1,55E-02	8,49E-01	NA	No	-2,22E-02	9,21E-01	NA	No
ENSG00000271852	SNORD11B	snoRNA	-1,26E-02	9,25E-01	NA	No	-3,25E-01	2,10E-01	NA	No
ENSG00000238317	SNORD11	snoRNA	1,79E-01	4,07E-01	6,51E-01	No	-3,45E-01	3,46E-01	5,01E-01	No
ENSG00000202059	SNORA1	snoRNA	-6,35E-03	9,69E-01	9,87E-01	No	-9,20E-01	5,39E-02	1,19E-01	No
ENSG00000207047	SNORD51	snoRNA	1,66E-01	1,13E-01	NA	No	2,96E-01	2,97E-01	NA	No
ENSG00000207274	SNORA70	snoRNA	-1,05E-02	8,49E-01	NA	No	1,64E-02	9,87E-01	NA	No
ENSG00000238736	snoU13	snoRNA	1,27E-02	9,65E-01	NA	No	-9,82E-02	8,30E-01	NA	No
ENSG00000252805	U3	snoRNA	-4,42E-02	7,60E-01	NA	No	-1,02E-01	8,30E-01	NA	No
ENSG00000238852	snoU13	snoRNA	3,74E-04	9,41E-01	NA	No	4,73E-02	8,89E-01	NA	No
ENSG00000212391	SNORA48	snoRNA	1,19E-01	5,86E-01	7,87E-01	No	1,87E-01	5,96E-01	7,27E-01	No
ENSG00000251801	SNORD112	snoRNA	6,11E-04	9,41E-01	NA	No	-9,51E-02	8,30E-01	NA	No
ENSG00000206885	SNORA75	snoRNA	-8,40E-02	6,92E-01	8,53E-01	No	-3,43E-01	3,50E-01	5,05E-01	No
ENSG00000207280	SNORD20	snoRNA	2,65E-02	9,00E-01	9,58E-01	No	-1,33E-02	9,68E-01	9,81E-01	No
ENSG00000202400	SNORD82	snoRNA	-1,24E-02	8,83E-01	NA	No	-3,77E-02	8,62E-01	NA	No
ENSG00000239170	snoU13	snoRNA	-1,11E-03	9,41E-01	NA	No	-9,82E-02	8,30E-01	NA	No
ENSG00000238642	snoU13	snoRNA	-1,61E-02	8,28E-01	NA	No	-1,45E-01	6,05E-01	NA	No
ENSG00000239140	snoU13	snoRNA	-3,11E-01	7,20E-02	2,24E-01	No	-4,65E-01	1,70E-01	2,95E-01	No
ENSG00000238891	snoU13	snoRNA	1,89E-01	1,11E-01	NA	No	2,08E-01	4,10E-01	NA	No
ENSG00000272166	SNORD5	snoRNA	2,22E-03	9,85E-01	NA	No	-1,39E-01	5,43E-01	NA	No
ENSG00000199927	U3	snoRNA	-9,02E-02	4,57E-01	NA	No	-8,80E-02	7,36E-01	8,33E-01	No
ENSG00000238646	snoU13	snoRNA	6,14E-02	7,15E-01	8,67E-01	No	8,07E-02	7,89E-01	8,69E-01	No
ENSG00000238929	snoU13	snoRNA	-9,84E-03	8,95E-01	NA	No	-1,35E-01	7,12E-01	NA	No
ENSG00000202517	SNORA64	snoRNA	-5,90E-02	7,66E-01	8,93E-01	No	6,98E-02	8,40E-01	9,03E-01	No
ENSG00000212145	U8	snoRNA	1,18E-01	2,22E-01	NA	No	1,26E-01	6,58E-01	NA	No
ENSG00000251938	snoU13	snoRNA	4,54E-02	7,64E-01	NA	No	2,62E-01	4,45E-01	5,99E-01	No
ENSG00000238350	snoU13	snoRNA	5,78E-02	6,39E-01	NA	No	8,36E-02	7,32E-01	NA	No
ENSG00000252787	SNORD19B	snoRNA	-9,01E-02	4,49E-01	NA	No	-1,68E-01	4,26E-01	NA	No
ENSG00000212493	SNORD19	snoRNA	-3,51E-01	1,48E-01	3,60E-01	No	-2,28E-01	5,20E-01	6,65E-01	No

ENSG00000222345	SNORD19	snoRNA	-8,73E-02	6,65E-01	8,36E-01	No	1,81E-01	6,08E-01	7,37E-01	No
ENSG00000212452	SNORD69	snoRNA	-3,18E-01	1,45E-01	3,55E-01	No	-8,70E-01	6,19E-02	1,33E-01	No
ENSG00000238565	snoU13	snoRNA	1,37E-01	3,33E-01	NA	No	-2,80E-03	9,89E-01	NA	No
ENSG00000207109	SNORD38	snoRNA	1,00E-01	6,09E-01	8,03E-01	No	-3,60E-01	3,10E-01	4,64E-01	No
ENSG00000212211	U3	snoRNA	6,55E-02	4,83E-01	NA	No	4,90E-01	9,08E-02	1,81E-01	No
ENSG00000200222	U3	snoRNA	3,31E-02	9,36E-01	NA	No	1,10E-01	6,85E-01	NA	No
ENSG00000238568	snoU13	snoRNA	3,15E-02	9,36E-01	NA	No	8,64E-02	8,64E-01	NA	No
ENSG00000238525	snoU13	snoRNA	-9,19E-02	5,58E-01	7,69E-01	No	-2,01E-01	5,30E-01	6,74E-01	No
ENSG00000202379	SNORA70	snoRNA	2,03E-02	9,16E-01	9,65E-01	No	-1,07E-03	9,96E-01	9,98E-01	No
ENSG00000238670	snoU13	snoRNA	-2,50E-03	9,90E-01	NA	No	1,76E-01	4,38E-01	NA	No
ENSG00000238480	snoU13	snoRNA	1,79E-01	1,07E-01	NA	No	3,85E-01	1,24E-01	2,32E-01	No
ENSG00000207002	SNORA5	snoRNA	-5,37E-02	6,46E-01	NA	No	-1,26E-01	7,12E-01	NA	No
ENSG00000207088	SNORA7B	snoRNA	1,26E-01	5,40E-01	7,56E-01	No	-1,47E-01	6,66E-01	7,82E-01	No
ENSG00000249020	SNORA58	snoRNA	9,95E-03	9,57E-01	9,82E-01	No	3,07E-01	3,97E-01	5,54E-01	No
ENSG00000238741	SCARNA7	snoRNA	4,65E-02	7,40E-01	8,79E-01	No	6,07E-01	3,47E-06	2,05E-05	No
ENSG00000200355	SNORA72	snoRNA	-9,73E-02	2,56E-01	NA	No	-3,07E-02	9,05E-01	9,43E-01	No
ENSG00000271842	snoU13	snoRNA	-2,90E-02	7,31E-01	NA	No	-4,11E-03	9,82E-01	NA	No
ENSG00000200288	SNORA18	snoRNA	2,22E-02	9,72E-01	NA	No	6,19E-02	9,56E-01	NA	No
ENSG00000239096	snoU13	snoRNA	3,60E-02	8,22E-01	9,22E-01	No	1,22E-01	6,92E-01	8,02E-01	No
ENSG00000201810	U8	snoRNA	3,17E-02	9,36E-01	NA	No	1,03E-01	7,75E-01	NA	No
ENSG00000201229	SNORA63	snoRNA	2,85E-02	8,16E-01	NA	No	1,61E-01	5,34E-01	6,77E-01	No
ENSG00000253092	SNORA81	snoRNA	7,03E-03	9,66E-01	9,86E-01	No	1,29E-01	7,06E-01	8,12E-01	No
ENSG00000251730	SNORA4	snoRNA	-1,45E-01	3,50E-01	NA	No	-3,91E-01	1,80E-01	NA	No
ENSG00000212158	SNORD66	snoRNA	5,95E-03	9,41E-01	NA	No	-9,72E-02	8,30E-01	NA	No
ENSG00000239146	snoU13	snoRNA	7,10E-02	4,27E-01	NA	No	8,53E-02	8,64E-01	NA	No
ENSG00000238902	snoU13	snoRNA	-6,33E-01	3,88E-02	1,46E-01	No	1,15E-02	9,73E-01	9,83E-01	No
ENSG00000212458	SNORA48	snoRNA	3,25E-02	7,59E-01	NA	No	2,86E-01	2,71E-01	4,19E-01	No
ENSG00000249784	SCARNA22	snoRNA	1,02E-01	4,49E-01	NA	No	-9,61E-02	6,84E-01	NA	No
ENSG00000202449	SNORA63	snoRNA	-3,22E-02	6,78E-01	NA	No	-9,67E-02	6,80E-01	NA	No
ENSG00000238383	snoU13	snoRNA	-4,14E-02	6,53E-01	NA	No	-1,84E-01	4,06E-01	NA	No
ENSG00000200999	SNORD74	snoRNA	1,50E-01	3,90E-01	6,36E-01	No	3,52E-01	3,27E-01	4,82E-01	No
ENSG00000201863	SNORA51	snoRNA	5,80E-02	6,01E-01	NA	No	-1,84E-01	4,30E-01	NA	No
ENSG00000238351	snoU13	snoRNA	4,98E-02	7,23E-01	NA	No	1,58E-01	5,93E-01	7,25E-01	No
ENSG00000238301	snoU13	snoRNA	-2,87E-02	7,31E-01	NA	No	1,71E-02	9,82E-01	NA	No
ENSG00000239068	snoU13	snoRNA	1,94E-01	3,93E-01	6,38E-01	No	7,48E-01	2,04E-02	5,28E-02	No
ENSG00000212588	SNORA26	snoRNA	-1,11E-01	6,07E-01	8,01E-01	No	-1,46E-01	6,11E-01	7,40E-01	No
ENSG00000238579	snoU13	snoRNA	-6,82E-02	6,84E-01	8,49E-01	No	-1,70E-01	6,06E-01	7,35E-01	No
ENSG00000238925	snoU13	snoRNA	-3,11E-02	7,31E-01	NA	No	-2,34E-02	8,90E-01	NA	No
ENSG00000202374	SNORA62	snoRNA	1,03E-01	5,73E-01	7,78E-01	No	4,84E-01	2,02E-01	3,37E-01	No
ENSG00000238318	snoU13	snoRNA	3,13E-01	1,57E-01	3,73E-01	No	1,96E-01	5,72E-01	7,08E-01	No
ENSG00000221639	SNORA3	snoRNA	8,46E-01	2,12E-02	9,59E-02	No	1,19E+00	6,89E-03	2,05E-02	No
ENSG00000199857	SNORD50	snoRNA	-4,15E-02	7,60E-01	NA	No	-9,51E-02	8,30E-01	NA	No
ENSG00000252834	snoR442	snoRNA	-3,60E-01	3,86E-02	NA	No	-1,43E+00	2,62E-02	NA	No
ENSG00000238948	snoU13	snoRNA	7,11E-02	6,17E-01	NA	No	2,36E-01	4,48E-01	6,01E-01	No
ENSG00000221245	SNORA11	snoRNA	1,40E-01	4,71E-01	7,04E-01	No	1,63E-01	6,28E-01	7,53E-01	No
ENSG00000238802	snoU13	snoRNA	3,41E-02	9,36E-01	NA	No	1,09E-01	6,78E-01	NA	No
ENSG00000239005	ACA64	snoRNA	-8,30E-03	9,66E-01	9,86E-01	No	-2,36E-01	5,07E-01	6,54E-01	No
ENSG00000238695	snoU13	snoRNA	-1,41E-02	8,49E-01	NA	No	1,70E-02	9,87E-01	NA	No
ENSG00000201264	SNORD73	snoRNA	-1,09E-01	4,53E-01	6,88E-01	No	-1,96E-01	5,15E-01	6,61E-01	No
ENSG00000271817	U3	snoRNA	-3,55E-02	8,66E-01	9,43E-01	No	-1,32E+00	1,96E-02	5,09E-02	No
ENSG00000238744	snoU13	snoRNA	2,75E-01	1,26E-01	3,24E-01	No	-4,95E-02	8,29E-01	NA	No
ENSG00000207171	SNORA51	snoRNA	-8,27E-02	6,60E-01	8,33E-01	No	-5,50E-01	1,68E-01	2,93E-01	No
ENSG00000201516	SNORA51	snoRNA	-5,37E-02	6,46E-01	NA	No	3,30E-02	9,17E-01	NA	No
ENSG00000252388	snoU13	snoRNA	-1,33E-02	8,19E-01	NA	No	1,93E-01	3,57E-01	NA	No
ENSG00000252048	U3	snoRNA	-7,09E-02	6,74E-01	8,42E-01	No	1,87E-02	9,54E-01	9,73E-01	No
ENSG00000238319	snoU13	snoRNA	8,14E-02	6,60E-01	8,33E-01	No	6,84E-01	1,03E-01	2,01E-01	No
ENSG00000238596	snoU13	snoRNA	-6,38E-02	6,17E-01	NA	No	-5,96E-02	8,03E-01	8,78E-01	No
ENSG00000251878	SNORD79	snoRNA	1,09E-02	9,36E-01	NA	No	-1,74E-01	4,61E-01	NA	No
ENSG00000239112	SNORD123	snoRNA	1,76E-02	8,72E-01	NA	No	5,93E-01	9,79E-02	1,92E-01	No
ENSG00000238864	snoU13	snoRNA	-9,79E-02	5,84E-01	7,86E-01	No	8,63E-02	7,99E-01	8,76E-01	No
ENSG00000199552	SNORA63	snoRNA	1,80E-03	9,83E-01	NA	No	-1,55E-01	5,35E-01	NA	No
ENSG00000212567	SNORA57	snoRNA	7,73E-02	7,11E-01	8,64E-01	No	-4,92E-01	2,06E-01	3,42E-01	No

ENSG00000212296	SNORD72	snoRNA	-2,30E-01	1,50E-01	NA	No	-1,29E-01	6,82E-01	7,95E-01	No
ENSG00000238326	snoU13	snoRNA	2,23E-02	9,72E-01	NA	No	1,38E-01	5,90E-01	NA	No
ENSG00000238717	snoU13	snoRNA	-2,01E-01	3,49E-01	5,98E-01	No	-5,32E-02	8,74E-01	9,25E-01	No
ENSG00000252904	SNORA76	snoRNA	2,77E-01	2,46E-01	4,87E-01	No	1,87E-01	5,86E-01	7,18E-01	No
ENSG00000212249	U8	snoRNA	-5,45E-02	6,46E-01	NA	No	-1,28E-01	7,12E-01	NA	No
ENSG00000238961	SNORA47	snoRNA	1,27E-01	5,28E-01	7,49E-01	No	4,52E-02	8,97E-01	9,39E-01	No
ENSG00000206592	SNORA18	snoRNA	-5,73E-02	6,46E-01	NA	No	4,75E-02	7,54E-01	NA	No
ENSG00000239159	snoU13	snoRNA	1,33E-01	3,78E-01	NA	No	1,36E-01	6,12E-01	NA	No
ENSG00000238835	SCARNA18	snoRNA	-8,43E-02	6,88E-01	8,50E-01	No	3,25E-02	9,26E-01	9,56E-01	No
ENSG00000206958	SNORA70	snoRNA	4,25E-03	9,41E-01	NA	No	-2,27E-02	9,21E-01	NA	No
ENSG00000252337	SNORA31	snoRNA	-6,34E-02	4,94E-01	NA	No	-1,52E-01	5,59E-01	NA	No
ENSG00000207177	SNORA51	snoRNA	6,86E-02	4,55E-01	NA	No	6,54E-02	9,56E-01	NA	No
ENSG00000238363	SNORA13	snoRNA	-1,11E-01	6,02E-01	7,98E-01	No	-4,21E-02	9,02E-01	9,42E-01	No
ENSG00000239011	snoU13	snoRNA	5,48E-02	4,69E-01	NA	No	8,77E-02	8,64E-01	NA	No
ENSG00000239084	snoU13	snoRNA	1,00E-01	5,89E-01	7,90E-01	No	1,16E-01	7,40E-01	8,35E-01	No
ENSG00000252295	snoU13	snoRNA	4,73E-01	8,65E-02	2,53E-01	No	4,45E-01	2,43E-01	3,87E-01	No
ENSG00000239103	snoU13	snoRNA	-1,56E-02	9,18E-01	9,66E-01	No	3,27E-01	3,69E-01	5,25E-01	No
ENSG00000222937	SNORD63	snoRNA	-2,33E-02	9,10E-01	9,63E-01	No	-8,71E-01	5,21E-02	1,16E-01	No
ENSG00000206989	SNORD63	snoRNA	-2,31E-01	1,55E-01	3,71E-01	No	-4,27E-01	2,42E-01	3,86E-01	No
ENSG00000252213	SNORA74	snoRNA	6,27E-02	7,78E-01	8,99E-01	No	-2,24E-01	5,02E-01	6,50E-01	No
ENSG00000200959	SNORA74A	snoRNA	-3,20E-01	1,96E-01	4,26E-01	No	9,45E-01	6,08E-03	1,84E-02	No
ENSG00000200051	SNORD45	snoRNA	-1,57E-02	8,98E-01	NA	No	2,34E-01	4,68E-01	6,20E-01	No
ENSG00000221043	U3	snoRNA	-2,31E-01	2,00E-01	4,32E-01	No	-2,00E-01	5,59E-01	6,98E-01	No
ENSG00000238369	snoU13	snoRNA	-1,50E-01	5,02E-01	7,29E-01	No	-2,75E-01	4,02E-01	5,59E-01	No
ENSG00000239191	snoU13	snoRNA	-2,72E-01	1,20E-01	3,14E-01	No	-4,34E-01	2,10E-01	3,46E-01	No
ENSG00000253065	SNORA40	snoRNA	-1,90E-01	3,65E-01	6,12E-01	No	-6,24E-01	1,30E-01	2,40E-01	No
ENSG00000252387	snoU13	snoRNA	-5,45E-02	6,46E-01	NA	No	-1,28E-01	7,12E-01	NA	No
ENSG00000212529	SNORA57	snoRNA	-1,25E-01	3,63E-01	6,11E-01	No	3,71E-01	3,13E-01	4,67E-01	No
ENSG00000212402	SNORA74B	snoRNA	1,03E-01	6,36E-01	8,19E-01	No	9,64E-01	1,59E-02	4,25E-02	No
ENSG00000238801	snoU13	snoRNA	4,89E-03	9,90E-01	NA	No	5,67E-02	8,10E-01	NA	No
ENSG00000251793	U3	snoRNA	-7,64E-02	4,73E-01	NA	No	1,85E-01	4,97E-01	6,46E-01	No
ENSG00000201785	SNORD117	snoRNA	-3,20E-02	8,41E-01	9,31E-01	No	-2,62E-01	4,19E-01	5,75E-01	No
ENSG00000200816	SNORA38	snoRNA	-7,08E-03	9,64E-01	9,85E-01	No	-2,20E-01	5,08E-01	6,55E-01	No
ENSG00000201823	SNORD48	snoRNA	-4,86E-02	5,84E-01	NA	No	-1,46E-01	6,01E-01	NA	No
ENSG00000239059	snoU13	snoRNA	1,94E-02	8,45E-01	NA	No	-1,60E-01	4,77E-01	NA	No
ENSG00000238484	snoU13	snoRNA	-3,78E-02	7,84E-01	NA	No	-1,17E-01	6,27E-01	NA	No
ENSG00000252687	SNORD112	snoRNA	-5,69E-02	6,46E-01	NA	No	-1,35E-01	7,12E-01	NA	No
ENSG00000200706	SNORD45	snoRNA	-4,15E-02	7,36E-01	NA	No	-2,35E-01	2,24E-01	NA	No
ENSG00000206977	SNORA8	snoRNA	2,89E-02	7,47E-01	NA	No	6,13E-03	9,87E-01	NA	No
ENSG00000221252	U3	snoRNA	1,33E-01	1,65E-01	NA	No	-1,36E-01	7,12E-01	NA	No
ENSG00000252218	SCARNA15	snoRNA	1,37E-03	9,86E-01	NA	No	-1,34E-02	9,34E-01	NA	No
ENSG00000251930	U3	snoRNA	-4,46E-02	7,60E-01	NA	No	-1,03E-01	8,30E-01	NA	No
ENSG00000221332	U3	snoRNA	-3,72E-01	1,36E-01	3,42E-01	No	1,87E-01	5,83E-01	7,16E-01	No
ENSG00000239132	snoU13	snoRNA	4,56E-02	6,38E-01	NA	No	8,57E-02	8,64E-01	NA	No
ENSG00000206886	SNORA70	snoRNA	4,46E-02	6,75E-01	NA	No	8,51E-02	8,64E-01	NA	No
ENSG00000222145	SNORA73	snoRNA	-2,97E-02	8,44E-01	9,32E-01	No	1,01E-01	7,61E-01	8,50E-01	No
ENSG00000200492	U3	snoRNA	4,61E-02	6,22E-01	NA	No	2,51E-01	1,94E-01	NA	No
ENSG00000238974	snoU13	snoRNA	2,20E-01	2,46E-01	4,88E-01	No	1,09E-01	7,10E-01	8,15E-01	No
ENSG00000238775	snoU13	snoRNA	7,53E-03	9,45E-01	NA	No	-7,80E-02	7,26E-01	NA	No
ENSG00000221500	SNORD100	snoRNA	-8,39E-01	2,31E-02	1,02E-01	No	-4,82E-01	2,03E-01	3,38E-01	No
ENSG00000200534	SNORA33	snoRNA	-1,88E-01	4,10E-01	6,53E-01	No	-7,99E-02	8,01E-01	8,77E-01	No
ENSG00000201807	SNORA27	snoRNA	1,07E-01	4,93E-01	7,22E-01	No	-1,40E-01	5,92E-01	NA	No
ENSG00000202343	SNORA2	snoRNA	9,89E-02	6,59E-01	8,33E-01	No	1,04E+00	3,83E-03	1,23E-02	No
ENSG00000238594	snoU13	snoRNA	-2,01E-01	1,43E-01	3,52E-01	No	-4,33E-02	8,94E-01	9,37E-01	No
ENSG00000238939	snoU13	snoRNA	8,59E-02	6,04E-01	8,00E-01	No	2,25E-01	4,99E-01	6,48E-01	No
ENSG00000238963	U8	snoRNA	-1,29E-01	4,81E-01	7,12E-01	No	-3,91E-01	2,30E-01	3,71E-01	No
ENSG00000207392	SNORA20	snoRNA	6,88E-03	9,65E-01	NA	No	1,58E-01	5,63E-01	NA	No
ENSG00000239136	snoU13	snoRNA	-1,91E-02	8,70E-01	NA	No	-9,32E-02	7,55E-01	8,46E-01	No
ENSG00000238857	snoU13	snoRNA	2,10E-01	1,03E-01	NA	No	3,10E-02	8,78E-01	NA	No
ENSG00000238781	snoU13	snoRNA	4,68E-03	9,80E-01	9,92E-01	No	-3,95E-01	2,28E-01	3,69E-01	No
ENSG00000207217	SNORA42	snoRNA	-6,96E-02	7,46E-01	8,82E-01	No	-5,06E-01	1,95E-01	3,27E-01	No
ENSG00000212422	U3	snoRNA	-2,24E-01	3,29E-01	5,78E-01	No	-1,54E-01	6,48E-01	7,69E-01	No

ENSG00000199470	SNORA64	snoRNA	-9,86E-02	3,40E-01	NA	No	-2,16E-01	2,78E-01	NA	No
ENSG00000199473	SNORA63	snoRNA	4,04E-02	7,81E-01	NA	No	6,54E-02	9,56E-01	NA	No
ENSG00000200753	SNORD56	snoRNA	-1,28E-01	3,61E-01	NA	No	-2,55E-01	4,00E-01	5,57E-01	No
ENSG00000221740	SNORD93	snoRNA	-1,83E-01	2,90E-01	5,36E-01	No	-2,78E-02	9,33E-01	9,60E-01	No
ENSG00000238906	snoU13	snoRNA	1,12E-01	2,68E-01	NA	No	3,41E-01	2,38E-01	NA	No
ENSG00000251999	SNORA31	snoRNA	5,40E-03	9,91E-01	NA	No	6,19E-02	8,22E-01	NA	No
ENSG00000238772	snoU13	snoRNA	1,44E-01	3,39E-01	5,88E-01	No	5,41E-01	1,57E-01	2,78E-01	No
ENSG00000200113	SNORA51	snoRNA	2,45E-01	2,56E-01	4,99E-01	No	8,63E-01	5,07E-02	1,13E-01	No
ENSG00000206838	SNORA5A	snoRNA	3,80E-01	1,24E-01	3,21E-01	No	1,18E+00	1,23E-04	5,51E-04	No
ENSG00000201772	SNORA5C	snoRNA	-3,50E-02	8,55E-01	9,37E-01	No	1,08E+00	7,21E-07	4,77E-06	No
ENSG00000206603	SNORA22	snoRNA	-7,89E-03	9,65E-01	9,85E-01	No	-3,61E-01	3,05E-01	4,58E-01	No
ENSG00000207168	SNORA15	snoRNA	3,41E-02	9,36E-01	NA	No	9,11E-02	8,64E-01	NA	No
ENSG00000238673	snoU13	snoRNA	6,60E-02	7,55E-01	8,86E-01	No	-3,13E-01	3,86E-01	5,43E-01	No
ENSG00000207344	SNORA22	snoRNA	1,32E-01	3,83E-01	6,29E-01	No	3,39E-01	3,32E-01	4,87E-01	No
ENSG00000206634	SNORA22	snoRNA	3,94E-01	1,09E-01	2,95E-01	No	5,64E-01	1,56E-01	2,76E-01	No
ENSG00000206785	SNORA15	snoRNA	7,22E-02	4,65E-01	NA	No	2,31E-01	2,72E-01	4,20E-01	No
ENSG00000252265	snoU13	snoRNA	3,98E-02	7,35E-01	NA	No	6,25E-02	7,95E-01	NA	No
ENSG00000238841	snoU13	snoRNA	3,23E-02	8,75E-01	9,48E-01	No	-4,17E-01	2,66E-01	4,13E-01	No
ENSG00000201643	SNORA14A	snoRNA	3,34E-02	8,36E-01	9,29E-01	No	-2,35E-01	4,70E-01	6,22E-01	No
ENSG00000238587	snoU13	snoRNA	2,23E-02	9,72E-01	NA	No	9,82E-02	7,75E-01	NA	No
ENSG00000238739	snoU13	snoRNA	-1,34E-01	5,49E-01	7,62E-01	No	4,39E-01	1,90E-01	3,22E-01	No
ENSG00000238384	snoU13	snoRNA	8,12E-02	6,73E-01	8,41E-01	No	-8,71E-02	7,97E-01	8,74E-01	No
ENSG00000238459	snoU13	snoRNA	3,98E-01	7,94E-03	4,78E-02	No	2,34E-01	3,50E-01	NA	No
ENSG00000239133	snoU13	snoRNA	3,42E-02	7,25E-01	NA	No	1,77E-01	4,24E-01	NA	No
ENSG00000252824	SNORA48	snoRNA	-8,44E-02	3,51E-01	NA	No	6,50E-02	8,10E-01	8,83E-01	No
ENSG00000251911	SNORD112	snoRNA	1,04E-02	9,22E-01	NA	No	-8,61E-02	6,58E-01	NA	No
ENSG00000238832	snoU109	snoRNA	-2,79E-01	2,31E-01	4,69E-01	No	-1,06E+00	5,57E-03	1,71E-02	No
ENSG00000238297	U3	snoRNA	2,63E-01	2,67E-01	5,12E-01	No	5,91E-01	7,12E-02	1,49E-01	No
ENSG00000238922	snoU13	snoRNA	1,34E-01	5,01E-01	7,28E-01	No	5,61E-01	1,61E-01	2,83E-01	No
ENSG00000252672	snoZ185	snoRNA	2,13E-01	2,56E-01	4,98E-01	No	6,36E-01	1,24E-01	2,32E-01	No
ENSG00000202023	SNORD81	snoRNA	3,66E-03	9,91E-01	NA	No	1,51E-01	4,69E-01	NA	No
ENSG00000239123	snoU13	snoRNA	-3,75E-03	9,73E-01	NA	No	-4,70E-02	8,64E-01	NA	No
ENSG00000238868	snoU13	snoRNA	-2,22E-03	9,86E-01	9,95E-01	No	-3,66E-02	9,02E-01	9,42E-01	No
ENSG00000199370	U3	snoRNA	3,46E-02	9,36E-01	NA	No	1,06E-01	7,03E-01	NA	No
ENSG00000252557	SNORD112	snoRNA	3,86E-02	8,45E-01	NA	No	1,01E-01	7,75E-01	NA	No
ENSG00000239045	snoU13	snoRNA	2,64E-01	1,83E-01	4,09E-01	No	1,16E+00	1,53E-02	4,12E-02	No
ENSG00000251848	snoU13	snoRNA	3,46E-02	9,36E-01	NA	No	8,55E-02	8,64E-01	NA	No
ENSG00000200620	SNORA7	snoRNA	1,45E-01	2,07E-01	NA	No	3,95E-01	1,52E-01	2,71E-01	No
ENSG00000201467	SNORA16	snoRNA	-4,33E-02	7,60E-01	NA	No	3,76E-02	8,88E-01	NA	No
ENSG00000201882	snoU2-30	snoRNA	5,73E-02	7,67E-01	8,93E-01	No	1,32E-02	9,71E-01	9,82E-01	No
ENSG00000201592	snoU2_19	snoRNA	-4,09E-01	1,15E-01	3,06E-01	No	-1,40E+00	1,99E-03	6,86E-03	No
ENSG00000251869	SCARNA23	snoRNA	5,51E-02	6,11E-01	NA	No	1,25E-01	6,66E-01	NA	No
ENSG00000201666	SNORD74	snoRNA	7,18E-02	6,23E-01	8,12E-01	No	9,13E-01	5,02E-02	1,12E-01	No
ENSG00000238969	snoU13	snoRNA	-5,02E-01	5,41E-02	1,83E-01	No	-1,23E+00	2,61E-02	6,47E-02	No
ENSG00000252050	SNORA31	snoRNA	3,15E-02	8,71E-01	9,46E-01	No	7,52E-01	6,54E-02	1,39E-01	No
ENSG00000252175	U3	snoRNA	-2,08E-02	8,19E-01	NA	No	-3,60E-02	8,90E-01	NA	No
ENSG00000221716	SNORA11	snoRNA	1,65E-01	4,13E-01	6,56E-01	No	1,40E-01	6,88E-01	7,99E-01	No
ENSG00000212434	U3	snoRNA	-1,01E-01	4,93E-01	7,22E-01	No	1,55E-01	6,50E-01	7,70E-01	No
ENSG00000252525	snoU13	snoRNA	9,07E-02	4,40E-01	6,79E-01	No	-1,43E-01	5,47E-01	NA	No
ENSG00000208883	SNORD96B	snoRNA	-3,95E-01	9,40E-02	2,67E-01	No	3,46E-01	3,44E-01	5,00E-01	No
ENSG00000238811	snoU13	snoRNA	2,02E-02	8,75E-01	NA	No	-9,72E-02	8,30E-01	NA	No
ENSG00000271907	SNORA35	snoRNA	3,85E-02	8,45E-01	NA	No	1,03E-01	7,75E-01	NA	No
ENSG00000252441	SNORA64	snoRNA	-5,92E-02	6,34E-01	NA	No	-2,12E-01	2,92E-01	NA	No
ENSG00000239182	SNORA35	snoRNA	2,43E-01	8,20E-02	NA	No	1,76E-01	4,62E-01	NA	No
ENSG00000206622	SNORA69	snoRNA	2,22E-02	9,72E-01	NA	No	8,51E-02	8,64E-01	NA	No
ENSG00000272179	snoU13	snoRNA	-9,16E-02	5,15E-01	NA	No	-2,35E-01	3,89E-01	NA	No
ENSG00000238485	snoU13	snoRNA	-1,56E-02	8,49E-01	NA	No	-9,82E-02	8,30E-01	NA	No
ENSG00000252719	SNORA18	snoRNA	1,28E-01	4,42E-01	6,80E-01	No	8,05E-02	7,68E-01	8,55E-01	No
ENSG00000207244	SNORA70	snoRNA	-4,15E-02	7,60E-01	NA	No	2,33E-02	9,87E-01	NA	No
ENSG00000252543	U3	snoRNA	-7,19E-04	9,41E-01	NA	No	-9,72E-02	8,30E-01	NA	No
ENSG00000252565	SNORD112	snoRNA	-3,23E-02	7,83E-01	NA	No	-1,32E-01	6,16E-01	7,43E-01	No
ENSG00000201157	SNORA62	snoRNA	-9,17E-03	9,45E-01	9,78E-01	No	1,01E-01	7,43E-01	8,37E-01	No

ENSG00000207027	SNORA67	snoRNA	5,64E-02	5,94E-01	NA	No	1,35E-01	6,03E-01	NA	No
ENSG00000238624	snoU13	snoRNA	-1,02E-01	4,87E-01	7,16E-01	No	-2,95E-01	2,62E-01	4,09E-01	No
ENSG00000252505	SNORA70	snoRNA	-4,05E-03	9,68E-01	NA	No	-1,47E-01	5,21E-01	NA	No
ENSG00000207199	SNORD38	snoRNA	-3,54E-01	1,61E-01	3,79E-01	No	-3,45E-01	3,45E-01	5,00E-01	No
ENSG00000238966	SNORD112	snoRNA	9,53E-02	6,71E-01	8,40E-01	No	-7,94E-02	8,05E-01	8,79E-01	No
ENSG00000199405	SNORA1	snoRNA	3,11E-02	7,56E-01	NA	No	1,89E-01	4,62E-01	6,15E-01	No
ENSG00000238433	snoU13	snoRNA	1,50E-03	9,85E-01	NA	No	-7,07E-02	7,29E-01	NA	No
ENSG00000238566	snoU13	snoRNA	-1,33E-03	8,83E-01	NA	No	2,35E-01	3,32E-01	NA	No
ENSG00000238791	snoU13	snoRNA	-3,98E-02	6,52E-01	NA	No	-1,49E-01	6,00E-01	NA	No
ENSG00000238372	snoU13	snoRNA	-8,27E-02	6,55E-01	8,30E-01	No	-4,40E-01	2,49E-01	3,94E-01	No
ENSG00000238533	snoU13	snoRNA	-7,05E-02	7,46E-01	8,81E-01	No	-6,18E-02	8,49E-01	9,08E-01	No
ENSG00000252559	SNORD112	snoRNA	6,04E-02	5,81E-01	NA	No	4,94E-01	1,15E-01	2,19E-01	No
ENSG00000238422	snoU13	snoRNA	-4,93E-02	7,71E-01	8,96E-01	No	5,07E-01	1,91E-01	3,23E-01	No
ENSG00000200075	SNORA25	snoRNA	-7,36E-02	6,97E-01	8,56E-01	No	-1,49E-01	6,61E-01	7,78E-01	No
ENSG00000212342	SNORA12	snoRNA	-1,06E-01	6,22E-01	8,12E-01	No	-3,88E-03	9,89E-01	9,94E-01	No
ENSG00000238654	snoU13	snoRNA	-4,41E-02	7,52E-01	8,84E-01	No	-1,33E-01	6,51E-01	7,71E-01	No
ENSG00000251733	SCARNA8	snoRNA	2,85E-02	8,53E-01	9,36E-01	No	-1,27E-01	6,32E-01	7,56E-01	No
ENSG00000238348	snoU13	snoRNA	-1,30E-02	9,24E-01	9,68E-01	No	7,17E-01	9,08E-02	1,81E-01	No
ENSG00000202189	SNORA30	snoRNA	2,59E-01	8,20E-02	NA	No	3,13E-01	2,10E-01	3,47E-01	No
ENSG00000238300	SNORD121B	snoRNA	-4,23E-02	7,60E-01	NA	No	1,10E-02	9,87E-01	NA	No
ENSG00000238886	SNORD121A	snoRNA	-5,45E-02	5,50E-01	NA	No	-2,00E-01	3,78E-01	NA	No
ENSG00000252617	SNORA70	snoRNA	-2,72E-02	7,31E-01	NA	No	-1,28E-01	7,12E-01	NA	No
ENSG00000238608	snoU13	snoRNA	-3,18E-01	7,84E-02	2,37E-01	No	-5,32E-01	1,52E-01	2,71E-01	No
ENSG00000252256	SNORD112	snoRNA	-5,34E-02	7,21E-01	8,70E-01	No	7,71E-02	8,08E-01	8,81E-01	No
ENSG00000212421	SNORA26	snoRNA	-4,46E-02	7,60E-01	NA	No	-1,03E-01	8,30E-01	NA	No
ENSG00000238996	snoU13	snoRNA	-4,23E-02	7,60E-01	NA	No	-9,82E-02	8,30E-01	NA	No
ENSG00000239183	SNORA84	snoRNA	-3,39E-02	8,28E-01	9,25E-01	No	-4,62E-01	1,50E-01	2,69E-01	No
ENSG00000238746	snoU13	snoRNA	4,03E-02	6,36E-01	NA	No	5,87E-02	7,33E-01	NA	No
ENSG00000251847	snoZ13_snr52	snoRNA	2,78E-01	6,43E-02	2,07E-01	No	2,16E-01	3,75E-01	5,32E-01	No
ENSG00000212447	SNORD90	snoRNA	-9,57E-03	9,21E-01	NA	No	2,07E-01	4,19E-01	NA	No
ENSG00000201302	SNORA65	snoRNA	2,88E-02	8,92E-01	9,55E-01	No	-4,08E-01	2,77E-01	4,26E-01	No
ENSG00000239055	snoU13	snoRNA	-1,43E-03	9,90E-01	NA	No	-2,17E-03	9,93E-01	NA	No
ENSG00000238298	snoU13	snoRNA	-2,64E-02	8,51E-01	9,35E-01	No	-3,71E-01	2,06E-01	3,42E-01	No
ENSG00000252582	SNORA31	snoRNA	-4,23E-02	7,60E-01	NA	No	-9,82E-02	8,30E-01	NA	No
ENSG00000235284	SNORD62A	snoRNA	1,36E-01	3,70E-01	6,16E-01	No	-1,74E-01	4,80E-01	NA	No
ENSG00000238657	snoU13	snoRNA	-4,09E-03	8,99E-01	NA	No	4,39E-02	8,87E-01	NA	No
ENSG00000199411	SNORD62	snoRNA	4,45E-02	6,77E-01	NA	No	1,16E-01	5,90E-01	NA	No
ENSG00000238824	snoU13	snoRNA	1,05E-01	2,93E-01	NA	No	-1,28E-01	7,12E-01	NA	No
ENSG00000272272	snoU13	snoRNA	2,04E-01	1,46E-01	NA	No	2,91E-01	2,74E-01	NA	No
ENSG00000238840	U8	snoRNA	-9,03E-02	4,38E-01	NA	No	2,56E-01	4,40E-01	5,94E-01	No
ENSG00000239148	U8	snoRNA	2,50E-03	9,64E-01	NA	No	4,63E-02	7,92E-01	NA	No
ENSG00000251922	SNORA14	snoRNA	7,48E-02	4,61E-01	NA	No	-1,30E-01	7,12E-01	NA	No
ENSG00000238900	snoU13	snoRNA	-7,89E-02	5,26E-01	NA	No	-1,13E-01	6,81E-01	NA	No
ENSG00000252438	SNORD45	snoRNA	2,19E-01	1,32E-01	3,34E-01	No	-1,55E-01	5,35E-01	NA	No
ENSG00000251959	snoR442	snoRNA	-7,50E-02	7,07E-01	8,62E-01	No	-5,44E-01	1,50E-01	2,69E-01	No
ENSG00000223027	SNORA57	snoRNA	-5,78E-02	7,18E-01	8,68E-01	No	7,77E-03	9,83E-01	9,90E-01	No
ENSG00000212411	SNORD115	snoRNA	-3,98E-01	8,98E-02	2,59E-01	No	-1,49E-01	6,69E-01	7,85E-01	No
ENSG00000238732	snoU13	snoRNA	3,81E-02	8,36E-01	9,29E-01	No	-1,27E-01	7,07E-01	8,13E-01	No
ENSG00000238970	snoU13	snoRNA	-8,55E-02	2,56E-01	NA	No	-4,12E-02	8,60E-01	9,16E-01	No
ENSG00000252203	snoR442	snoRNA	-1,67E-02	9,27E-01	9,70E-01	No	-5,22E-02	8,75E-01	9,26E-01	No
ENSG00000239000	snoU13	snoRNA	2,90E-01	1,37E-01	3,42E-01	No	9,86E-01	4,97E-02	1,11E-01	No
ENSG00000221182	SNORD98	snoRNA	2,35E-02	9,72E-01	NA	No	6,34E-02	9,56E-01	NA	No
ENSG00000238918	snoU13	snoRNA	4,13E-02	6,66E-01	NA	No	7,23E-03	9,87E-01	NA	No
ENSG00000200294	SNORA36	snoRNA	-2,73E-02	8,22E-01	NA	No	-2,27E-01	3,66E-01	NA	No
ENSG00000221164	SNORA11	snoRNA	7,70E-01	1,71E-02	8,24E-02	No	1,94E-01	5,72E-01	7,08E-01	No
ENSG00000238983	snoU13	snoRNA	3,41E-02	9,36E-01	NA	No	6,23E-02	9,56E-01	NA	No
ENSG00000252189	U3	snoRNA	-1,35E-01	4,28E-01	6,69E-01	No	-4,32E-01	2,45E-01	3,89E-01	No
ENSG00000238991	snoU13	snoRNA	-5,45E-02	6,46E-01	NA	No	2,26E-02	9,22E-01	NA	No
ENSG00000252993	SNORA25	snoRNA	2,45E-02	8,53E-01	NA	No	-6,32E-02	8,02E-01	NA	No
ENSG00000239091	snoU13	snoRNA	-2,43E-02	8,97E-01	9,57E-01	No	-1,34E-01	7,01E-01	8,08E-01	No
ENSG00000238620	snoU13	snoRNA	-5,73E-02	6,46E-01	NA	No	-1,36E-01	7,12E-01	NA	No
ENSG00000239125	snoU13	snoRNA	1,46E-01	3,35E-01	5,84E-01	No	1,45E-01	6,39E-01	7,62E-01	No

ENSG00000238577	snoU13	snoRNA	-3,53E-01	1,62E-01	3,80E-01	No	-7,50E-01	6,01E-02	1,30E-01	No
ENSG00000251836	U3	snoRNA	-3,46E-02	6,57E-01	NA	No	-9,10E-02	6,20E-01	NA	No
ENSG00000207468	SNORA19	snoRNA	2,46E-01	2,61E-01	5,05E-01	No	9,55E-01	3,32E-02	7,94E-02	No
ENSG00000222588	SNORA19	snoRNA	-1,65E-01	3,86E-01	6,32E-01	No	3,83E-01	3,02E-01	4,55E-01	No
ENSG00000207008	SNORA54	snoRNA	4,01E-01	1,17E-01	3,10E-01	No	-2,11E-01	5,53E-01	6,92E-01	No
ENSG00000238686	snoU13	snoRNA	-2,37E-01	8,21E-02	NA	No	-4,89E-01	1,69E-01	2,94E-01	No
ENSG00000206976	SNORA7	snoRNA	1,03E-01	4,19E-01	6,62E-01	No	-2,98E-02	9,00E-01	9,41E-01	No
ENSG00000252778	SCARNA20	snoRNA	6,13E-02	6,55E-01	8,30E-01	No	-4,25E-02	8,71E-01	9,23E-01	No
ENSG00000212607	SNORA45	snoRNA	2,15E-01	2,56E-01	4,99E-01	No	3,03E-01	3,90E-01	5,47E-01	No
ENSG00000252329	SCARNA16	snoRNA	-1,57E-01	4,42E-01	6,80E-01	No	-3,05E-01	3,97E-01	5,54E-01	No
ENSG00000272034	SNORD14A	snoRNA	1,99E-01	3,73E-01	6,19E-01	No	3,52E-01	3,37E-01	4,92E-01	No
ENSG00000207407	SNORA1	snoRNA	1,74E-01	1,06E-01	NA	No	1,97E-01	3,46E-01	NA	No
ENSG00000252427	SNORD67	snoRNA	1,94E-02	8,76E-01	NA	No	-2,07E-01	4,09E-01	NA	No
ENSG00000212135	SNORD67	snoRNA	9,55E-02	5,60E-01	7,70E-01	No	1,20E-01	7,17E-01	8,20E-01	No
ENSG00000252447	snoU13	snoRNA	7,15E-03	9,76E-01	9,90E-01	No	-6,28E-01	4,54E-02	1,03E-01	No
ENSG00000238763	snoU13	snoRNA	-3,15E-03	9,84E-01	9,94E-01	No	-5,14E-01	1,88E-01	3,20E-01	No
ENSG00000238892	snoU13	snoRNA	1,27E-01	4,47E-01	6,84E-01	No	2,04E-01	5,53E-01	6,92E-01	No
ENSG00000238768	snoU13	snoRNA	3,15E-02	9,36E-01	NA	No	1,01E-01	7,75E-01	NA	No
ENSG00000206913	SNORA7	snoRNA	-5,32E-03	9,70E-01	9,88E-01	No	-3,34E-01	2,55E-01	NA	No
ENSG00000206941	SNORD15A	snoRNA	-1,15E-02	8,49E-01	NA	No	-2,35E-02	9,21E-01	NA	No
ENSG00000207445	SNORD15B	snoRNA	-1,01E-01	6,47E-01	8,26E-01	No	1,37E-01	6,73E-01	7,88E-01	No
ENSG00000238995	snoU13	snoRNA	4,43E-02	7,25E-01	8,72E-01	No	2,31E-01	4,86E-01	6,36E-01	No
ENSG00000207221	SNORA70E	snoRNA	1,88E-01	1,81E-01	NA	No	-1,05E-01	6,82E-01	NA	No
ENSG00000202314	SNORD6	snoRNA	-1,19E-01	5,92E-01	7,91E-01	No	7,30E-01	5,49E-02	1,21E-01	No
ENSG00000238437	snoU13	snoRNA	-4,11E-02	8,19E-01	9,21E-01	No	2,76E-01	4,44E-01	5,98E-01	No
ENSG00000238388	snoU13	snoRNA	2,62E-02	8,49E-01	9,34E-01	No	6,02E-03	9,84E-01	9,90E-01	No
ENSG00000238724	snoU13	snoRNA	-3,01E-02	6,77E-01	NA	No	-3,39E-02	8,81E-01	NA	No
ENSG00000252992	SCARNA11	snoRNA	2,42E-02	8,00E-01	NA	No	3,70E-01	2,25E-01	NA	No
ENSG00000239079	snoU13	snoRNA	9,51E-02	4,06E-01	NA	No	2,00E-03	9,72E-01	NA	No
ENSG00000200879	SNORD14E	snoRNA	8,54E-02	6,56E-01	8,31E-01	No	1,08E-01	7,56E-01	8,47E-01	No
ENSG00000238855	snoU13	snoRNA	-7,22E-03	8,97E-01	NA	No	-1,27E-02	9,80E-01	NA	No
ENSG00000251898	SCARNA11	snoRNA	2,42E-02	9,72E-01	NA	No	8,70E-02	8,64E-01	NA	No
ENSG00000238795	SCARNA12	snoRNA	2,34E-02	9,72E-01	NA	No	8,77E-02	8,64E-01	NA	No
ENSG00000212440	SNORA75	snoRNA	1,42E-01	1,58E-01	NA	No	1,15E-01	6,47E-01	NA	No
ENSG00000212432	SNORA75	snoRNA	4,66E-02	7,38E-01	NA	No	1,88E-01	5,00E-01	NA	No
ENSG00000221611	SNORD88	snoRNA	3,87E-02	7,60E-01	NA	No	2,06E-02	9,87E-01	NA	No
ENSG00000212533	SNORA75	snoRNA	-7,49E-02	6,24E-01	8,13E-01	No	-7,23E-01	3,80E-02	NA	No
ENSG00000239033	snoU13	snoRNA	6,98E-02	5,50E-01	NA	No	9,77E-02	7,00E-01	8,08E-01	No
ENSG00000199571	SNORA22	snoRNA	-2,11E-01	2,16E-01	4,52E-01	No	-2,01E-01	5,52E-01	6,92E-01	No
ENSG00000252917	SNORA74	snoRNA	9,45E-03	9,64E-01	9,85E-01	No	-1,80E-01	6,06E-01	7,35E-01	No
ENSG00000221491	SNORA34	snoRNA	3,34E-01	1,60E-01	3,77E-01	No	8,33E-01	6,00E-02	1,30E-01	No
ENSG00000206612	SNORA2A	snoRNA	-1,42E-01	3,92E-01	6,38E-01	No	4,62E-02	8,94E-01	9,37E-01	No
ENSG00000207313	SNORA2B	snoRNA	-2,52E-01	2,78E-01	5,24E-01	No	-7,88E-01	7,53E-02	1,56E-01	No
ENSG00000207031	SNORD59A	snoRNA	1,00E-01	6,53E-01	8,30E-01	No	-1,62E-02	9,60E-01	9,76E-01	No
ENSG00000252883	SNORD112	snoRNA	3,31E-02	9,36E-01	NA	No	1,62E-01	4,88E-01	NA	No
ENSG00000238475	snoU13	snoRNA	-3,79E-02	6,71E-01	NA	No	-8,75E-02	6,68E-01	NA	No
ENSG00000238592	snoU13	snoRNA	-1,69E-02	8,19E-01	NA	No	7,54E-03	9,80E-01	NA	No
ENSG00000206650	SNORA70G	snoRNA	-5,93E-02	7,79E-01	8,99E-01	No	-7,77E-01	5,44E-02	1,20E-01	No
ENSG00000212461	SNORA17	snoRNA	-4,46E-01	9,91E-02	2,77E-01	No	-7,63E-01	6,04E-02	1,31E-01	No
ENSG00000251893	SNORA70	snoRNA	-5,82E-03	9,74E-01	NA	No	-7,41E-02	8,00E-01	NA	No
ENSG00000238361	snoU13	snoRNA	-5,45E-02	6,46E-01	NA	No	8,52E-02	7,34E-01	NA	No
ENSG00000239073	snoU13	snoRNA	-5,00E-02	6,34E-01	NA	No	-1,65E-01	4,54E-01	NA	No
ENSG00000251844	snoMe28S-Am2634	snoRNA	6,41E-02	5,03E-01	NA	No	1,17E-01	5,77E-01	NA	No
ENSG00000238940	snoU13	snoRNA	4,31E-02	7,07E-01	NA	No	1,42E-01	5,72E-01	NA	No
ENSG00000200897	SNORD74	snoRNA	4,18E-02	7,50E-01	NA	No	9,02E-02	8,64E-01	NA	No
ENSG00000252192	SNORA9	snoRNA	3,31E-01	1,38E-01	3,44E-01	No	1,45E-01	6,76E-01	7,90E-01	No
ENSG00000206897	SNORA9	snoRNA	3,66E-02	7,16E-01	NA	No	2,75E-01	3,35E-01	4,90E-01	No
ENSG00000238822	snoU13	snoRNA	-1,46E-01	3,37E-01	5,86E-01	No	-5,38E-01	1,12E-01	2,14E-01	No
ENSG00000208892	SNORA49	snoRNA	7,03E-02	7,34E-01	8,76E-01	No	2,21E-01	5,33E-01	6,76E-01	No
ENSG00000238665	snoU13	snoRNA	3,50E-02	9,36E-01	NA	No	9,02E-02	8,64E-01	NA	No
ENSG00000238893	snoU13	snoRNA	3,18E-02	7,34E-01	NA	No	-1,01E-01	8,30E-01	NA	No
ENSG00000238878	snoU13	snoRNA	7,98E-02	4,75E-01	NA	No	9,45E-01	1,69E-02	4,48E-02	No

ENSG00000212293	SNORA16	snoRNA	6,27E-01	4,36E-02	1,58E-01	No	2,36E-02	9,47E-01	9,69E-01	No
ENSG00000238408	snoU13	snoRNA	8,28E-02	7,10E-01	8,63E-01	No	5,79E-01	7,58E-02	1,57E-01	No
ENSG00000238651	snoU13	snoRNA	1,00E-01	5,83E-01	7,85E-01	No	6,57E-01	1,13E-01	2,16E-01	No
ENSG00000238932	snoU13	snoRNA	-4,23E-02	7,60E-01	NA	No	-9,82E-02	8,30E-01	NA	No
ENSG00000253051	SNORA31	snoRNA	-1,64E-01	4,67E-01	7,00E-01	No	6,89E-01	1,65E-02	4,39E-02	No
ENSG00000199477	SNORA31	snoRNA	-1,50E-01	2,57E-01	NA	No	-5,94E-02	8,44E-01	9,05E-01	No
ENSG00000252365	SNORD22	snoRNA	7,12E-02	4,91E-01	NA	No	6,66E-02	7,41E-01	NA	No
ENSG00000238463	snoU13	snoRNA	7,44E-03	9,64E-01	NA	No	1,28E-01	5,55E-01	NA	No
ENSG00000251901	snR65	snoRNA	2,75E-02	8,29E-01	NA	No	4,55E-01	2,05E-01	3,41E-01	No
ENSG00000201245	SNORA25	snoRNA	5,09E-02	6,67E-01	NA	No	8,55E-02	8,64E-01	NA	No
ENSG00000238305	snoU13	snoRNA	2,92E-02	7,92E-01	NA	No	-1,87E-02	8,90E-01	NA	No
ENSG00000201847	SNORD31	snoRNA	2,34E-02	9,72E-01	NA	No	1,42E-01	5,72E-01	NA	No
ENSG00000238629	snoU13	snoRNA	-4,41E-01	8,05E-02	2,41E-01	No	-1,42E+00	1,31E-02	3,60E-02	No
ENSG00000222489	SNORA79	snoRNA	-7,42E-01	2,25E-02	1,00E-01	No	-6,30E-01	5,77E-02	1,26E-01	No
ENSG00000199436	SNORD9	snoRNA	2,05E-01	1,19E-01	NA	No	2,65E-01	3,36E-01	NA	No
ENSG00000200785	SNORD8	snoRNA	-2,89E-01	1,83E-01	4,09E-01	No	-2,95E-01	4,13E-01	5,70E-01	No
ENSG00000212302	SNORD41	snoRNA	3,39E-02	9,36E-01	NA	No	1,13E-01	6,87E-01	NA	No
ENSG00000212270	SNORD37	snoRNA	5,36E-02	6,31E-01	NA	No	6,54E-02	9,56E-01	NA	No
ENSG00000253059	SNORA31	snoRNA	-3,28E-01	1,39E-01	3,46E-01	No	1,63E-02	9,64E-01	9,79E-01	No
ENSG00000238718	snoU13	snoRNA	2,23E-01	2,95E-01	5,43E-01	No	-2,96E-01	3,91E-01	5,48E-01	No
ENSG00000251858	SNORA31	snoRNA	3,23E-02	9,36E-01	NA	No	8,77E-02	8,64E-01	NA	No
ENSG00000212615	SNORD58	snoRNA	1,22E-01	3,21E-01	5,70E-01	No	1,81E-01	4,99E-01	6,47E-01	No
ENSG00000239043	SNORD127	snoRNA	3,32E-02	7,30E-01	NA	No	1,56E-01	5,36E-01	NA	No
ENSG00000252945	snoU83B	snoRNA	2,42E-02	9,72E-01	NA	No	9,87E-02	7,75E-01	NA	No
ENSG00000201376	SNORA70	snoRNA	5,93E-02	7,79E-01	8,99E-01	No	-2,11E-01	5,46E-01	6,87E-01	No
ENSG00000252800	SCARNA20	snoRNA	1,57E-01	1,63E-01	NA	No	5,11E-01	8,55E-02	1,73E-01	No
ENSG00000200693	U3	snoRNA	-3,50E-01	1,61E-01	3,80E-01	No	8,24E-01	1,94E-02	5,04E-02	No
ENSG00000252792	U3	snoRNA	4,57E-02	6,52E-01	NA	No	-2,32E-02	9,18E-01	NA	No
ENSG00000221060	SNORA11	snoRNA	3,41E-02	9,36E-01	NA	No	8,58E-02	8,64E-01	NA	No
ENSG00000207444	SNORD56B	snoRNA	1,92E-01	9,95E-02	NA	No	4,14E-02	8,90E-01	NA	No
ENSG00000238330	snoU13	snoRNA	1,55E-02	8,96E-01	NA	No	1,65E-01	5,05E-01	NA	No
ENSG00000222604	SNORA7	snoRNA	1,11E-01	2,55E-01	NA	No	2,29E-01	3,76E-01	NA	No
ENSG00000212371	SNORA46	snoRNA	-1,35E-01	3,94E-01	6,40E-01	No	-7,60E-01	4,24E-02	9,78E-02	No
ENSG00000221303	SNORA79	snoRNA	2,17E-01	2,25E-01	4,63E-01	No	2,70E-01	4,08E-01	5,65E-01	No
ENSG00000221102	SNORA11B	snoRNA	4,22E-01	9,69E-02	2,73E-01	No	4,57E-02	8,87E-01	9,33E-01	No
ENSG00000238776	snoU13	snoRNA	1,38E-01	3,90E-01	6,36E-01	No	5,55E-02	8,46E-01	9,06E-01	No
ENSG00000251769	SNORD112	snoRNA	1,56E-01	2,15E-01	NA	No	7,29E-01	8,12E-02	1,66E-01	No
ENSG00000202191	SNORD113-1	snoRNA	-2,78E-02	8,81E-01	9,50E-01	No	4,66E-01	2,23E-01	3,62E-01	No
ENSG00000251949	SNORD112	snoRNA	1,19E-01	5,67E-01	7,74E-01	No	5,06E-01	1,92E-01	3,25E-01	No
ENSG00000212384	SNORD113-2	snoRNA	5,75E-02	7,15E-01	8,66E-01	No	7,01E-01	9,46E-02	1,87E-01	No
ENSG00000201700	SNORD113-3	snoRNA	-1,04E-02	9,56E-01	9,82E-01	No	6,16E-01	1,21E-01	2,28E-01	No
ENSG00000272474	SNORD113-5	snoRNA	4,98E-02	6,34E-01	NA	No	8,01E-01	4,50E-02	1,03E-01	No
ENSG00000252144	SNORD112	snoRNA	7,21E-02	5,22E-01	NA	No	2,53E-01	3,36E-01	4,92E-01	No
ENSG00000200215	SNORD113-6	snoRNA	2,86E-02	7,78E-01	NA	No	3,96E-01	2,39E-01	3,83E-01	No
ENSG00000251918	SNORD112	snoRNA	6,62E-02	4,78E-01	NA	No	2,91E-01	3,11E-01	NA	No
ENSG00000200632	SNORD113-7	snoRNA	2,49E-02	9,72E-01	NA	No	4,49E-01	1,50E-01	NA	No
ENSG00000200367	SNORD113-8	snoRNA	-5,45E-02	6,76E-01	NA	No	1,38E-01	6,39E-01	7,62E-01	No
ENSG00000201950	SNORD113-9	snoRNA	-1,56E-01	3,37E-01	NA	No	5,59E-01	1,56E-01	2,77E-01	No
ENSG00000199575	SNORD114-1	snoRNA	-5,94E-01	4,42E-02	1,60E-01	No	1,14E+00	9,53E-03	2,73E-02	No
ENSG00000200823	SNORD114-2	snoRNA	2,26E-01	1,55E-01	3,70E-01	No	1,36E+00	2,37E-02	5,97E-02	No
ENSG00000200832	SNORD114-4	snoRNA	2,20E-01	9,47E-02	NA	No	6,06E-01	5,57E-02	1,22E-01	No
ENSG00000199798	SNORD114-5	snoRNA	-2,15E-02	8,55E-01	NA	No	-4,40E-02	8,67E-01	NA	No
ENSG00000200150	SNORD113	snoRNA	4,04E-02	7,59E-01	NA	No	5,03E-01	1,43E-01	2,59E-01	No
ENSG00000201263	SNORD114-6	snoRNA	5,98E-02	6,89E-01	NA	No	8,76E-01	4,72E-02	1,07E-01	No
ENSG00000199390	SNORD114-7	snoRNA	-1,91E-01	3,26E-01	5,75E-01	No	1,84E-01	5,93E-01	7,24E-01	No
ENSG00000201240	SNORD114-9	snoRNA	-2,00E-01	8,90E-02	NA	No	-1,43E-02	9,64E-01	9,79E-01	No
ENSG00000200279	SNORD114-10	snoRNA	-1,31E-01	4,28E-01	6,69E-01	No	8,40E-02	7,98E-01	8,76E-01	No
ENSG00000200608	SNORD114-11	snoRNA	-4,91E-03	9,73E-01	9,89E-01	No	2,33E-01	4,94E-01	6,43E-01	No
ENSG00000202270	SNORD114-12	snoRNA	2,40E-02	9,72E-01	NA	No	3,49E-01	2,00E-01	NA	No
ENSG00000201247	SNORD114-13	snoRNA	2,99E-02	7,35E-01	NA	No	4,98E-01	1,31E-01	2,42E-01	No
ENSG00000199593	SNORD114-14	snoRNA	-1,14E-01	3,33E-01	NA	No	2,93E-01	3,89E-01	5,47E-01	No
ENSG00000201557	SNORD114-15	snoRNA	-1,34E-01	1,83E-01	NA	No	2,97E-01	3,77E-01	5,34E-01	No

ENSG00000199914	SNORD114-16	snoRNA	1,88E-02	8,91E-01	NA	No	3,68E-01	2,88E-01	4,39E-01	No
ENSG00000201569	SNORD114-17	snoRNA	3,34E-02	7,56E-01	NA	No	7,04E-01	8,23E-02	1,68E-01	No
ENSG00000202142	SNORD114-18	snoRNA	1,18E-01	3,52E-01	NA	No	1,39E+00	2,45E-02	6,13E-02	No
ENSG00000199942	SNORD114-19	snoRNA	-6,70E-02	4,97E-01	NA	No	1,46E-01	6,15E-01	7,43E-01	No
ENSG00000201500	SNORD113	snoRNA	1,12E-02	9,26E-01	NA	No	8,51E-02	7,51E-01	8,43E-01	No
ENSG00000201036	SNORD113	snoRNA	4,38E-02	7,79E-01	8,99E-01	No	1,89E-01	5,28E-01	6,72E-01	No
ENSG00000201710	SNORD113	snoRNA	-1,89E-01	1,99E-01	4,31E-01	No	2,82E-01	4,29E-01	5,84E-01	No
ENSG00000202048	SNORD114-20	snoRNA	1,11E-01	5,85E-01	7,87E-01	No	6,06E-01	1,28E-01	2,37E-01	No
ENSG00000272344	SNORD114-21	snoRNA	8,36E-02	5,69E-01	7,76E-01	No	9,09E-01	5,27E-02	1,17E-01	No
ENSG00000202293	SNORD114-22	snoRNA	1,33E-02	9,65E-01	NA	No	6,49E-02	7,80E-01	NA	No
ENSG00000200406	SNORD114-23	snoRNA	-7,33E-02	5,09E-01	NA	No	7,66E-01	7,65E-02	1,58E-01	No
ENSG00000201899	SNORD114-24	snoRNA	3,39E-02	6,87E-01	NA	No	1,91E-01	3,21E-01	4,76E-01	No
ENSG00000200612	SNORD114-25	snoRNA	-1,04E-02	8,94E-01	NA	No	6,60E-01	5,41E-02	1,19E-01	No
ENSG00000200413	SNORD114-26	snoRNA	5,09E-02	6,67E-01	NA	No	4,03E-01	1,52E-01	NA	No
ENSG00000200480	SNORD114-28	snoRNA	-6,45E-02	4,55E-01	NA	No	2,04E-01	4,33E-01	5,87E-01	No
ENSG00000201689	SNORD114-29	snoRNA	-1,56E-02	8,49E-01	NA	No	1,10E-01	6,59E-01	NA	No
ENSG00000201318	SNORD114-30	snoRNA	6,17E-02	5,32E-01	NA	No	5,53E-01	9,16E-02	NA	No
ENSG00000200089	SNORD114-31	snoRNA	5,96E-02	6,34E-01	NA	No	2,06E-01	3,89E-01	5,46E-01	No
ENSG00000222095	SNORD113	snoRNA	8,71E-03	9,67E-01	NA	No	1,91E-02	9,87E-01	NA	No
ENSG00000222185	SNORD113	snoRNA	-7,00E-02	4,57E-01	NA	No	5,99E-01	1,07E-01	2,06E-01	No
ENSG00000239061	snoU13	snoRNA	3,83E-02	6,61E-01	NA	No	-1,35E-01	7,12E-01	NA	No
ENSG00000238853	snoU13	snoRNA	3,15E-01	3,97E-02	1,49E-01	No	2,71E-01	2,75E-01	NA	No
ENSG00000272533	SNORA28	snoRNA	8,98E-02	6,62E-01	8,35E-01	No	5,10E-01	3,96E-02	9,21E-02	No
ENSG00000238615	snoU13	snoRNA	3,01E-02	8,09E-01	NA	No	-1,24E-01	6,32E-01	NA	No
ENSG00000239014	SNORD108	snoRNA	-1,25E-01	4,52E-01	6,88E-01	No	4,55E-01	3,04E-03	9,99E-03	No
ENSG00000207063	SNORD116-1	snoRNA	3,19E-02	8,86E-01	9,53E-01	No	-5,13E-01	1,51E-01	2,70E-01	No
ENSG00000207001	SNORD116-2	snoRNA	2,17E-02	9,23E-01	9,68E-01	No	-3,46E-01	3,18E-01	4,73E-01	No
ENSG00000207014	SNORD116-3	snoRNA	-9,01E-02	6,70E-01	8,39E-01	No	-1,10E+00	2,92E-02	7,12E-02	No
ENSG00000207191	SNORD116-5	snoRNA	5,24E-02	7,38E-01	8,78E-01	No	-2,24E-01	4,34E-01	5,88E-01	No
ENSG00000207442	SNORD116-6	snoRNA	-1,02E-01	6,42E-01	8,23E-01	No	-1,23E+00	9,84E-04	3,65E-03	No
ENSG00000207133	SNORD116-7	snoRNA	1,95E-02	9,30E-01	9,71E-01	No	-1,15E+00	1,77E-02	4,67E-02	No
ENSG00000207093	SNORD116-8	snoRNA	-9,73E-02	6,42E-01	8,23E-01	No	-1,20E+00	2,81E-02	6,90E-02	No
ENSG00000206727	SNORD116-9	snoRNA	1,54E-01	4,99E-01	7,26E-01	No	-2,32E-01	4,81E-01	6,31E-01	No
ENSG00000200661	SNORD116-10	snoRNA	-2,23E-01	1,46E-01	3,56E-01	No	-3,91E-01	2,16E-01	3,54E-01	No
ENSG00000206609	SNORD116-11	snoRNA	-9,48E-02	5,94E-01	7,93E-01	No	-2,51E-01	4,44E-01	5,98E-01	No
ENSG00000207197	SNORD116-12	snoRNA	-3,19E-01	1,95E-01	4,25E-01	No	-1,37E+00	3,11E-03	1,02E-02	No
ENSG00000207137	SNORD116-13	snoRNA	-3,06E-01	1,43E-01	3,53E-01	No	-8,22E-02	7,39E-01	8,35E-01	No
ENSG00000206621	SNORD116-14	snoRNA	-5,67E-02	7,84E-01	9,02E-01	No	-7,09E-01	1,67E-02	4,44E-02	No
ENSG00000207174	SNORD116-15	snoRNA	6,82E-02	7,55E-01	8,86E-01	No	-1,04E-01	7,64E-01	8,52E-01	No
ENSG00000207263	SNORD116-16	snoRNA	4,41E-03	9,85E-01	9,95E-01	No	-5,34E-01	1,50E-01	2,69E-01	No
ENSG00000206656	SNORD116-17	snoRNA	6,98E-02	7,35E-01	8,77E-01	No	-3,76E-01	2,90E-01	4,40E-01	No
ENSG00000206688	SNORD116-18	snoRNA	-6,74E-01	8,54E-03	5,05E-02	No	-9,07E-01	2,80E-03	9,28E-03	No
ENSG00000207460	SNORD116-19	snoRNA	-5,61E-01	3,51E-02	1,36E-01	No	-9,23E-01	2,78E-03	9,24E-03	No
ENSG00000207375	SNORD116-23	snoRNA	-8,55E-02	6,82E-01	8,47E-01	No	-7,68E-01	7,30E-02	1,52E-01	No
ENSG00000207279	SNORD116-24	snoRNA	-1,23E-01	5,81E-01	7,84E-01	No	2,43E-01	4,17E-01	5,73E-01	No
ENSG00000252326	SNORD116-25	snoRNA	-1,85E-01	3,86E-01	6,32E-01	No	-5,64E-02	8,70E-01	9,22E-01	No
ENSG00000251815	SNORD116-26	snoRNA	-2,42E-01	3,02E-01	5,50E-01	No	-7,45E-02	8,21E-01	8,90E-01	No
ENSG00000251896	SNORD116-27	snoRNA	-2,08E-02	9,22E-01	9,68E-01	No	4,86E-01	1,56E-01	2,76E-01	No
ENSG00000207245	SNORD116-29	snoRNA	-1,58E-02	9,40E-01	9,75E-01	No	1,82E-01	5,83E-01	7,17E-01	No
ENSG00000252277	SNORD116-30	snoRNA	-2,15E-01	3,05E-01	5,53E-01	No	1,48E-01	6,65E-01	7,82E-01	No
ENSG00000212428	SNORD115	snoRNA	-5,68E-03	8,96E-01	NA	No	1,06E-02	9,77E-01	NA	No
ENSG00000201831	SNORD115-1	snoRNA	3,17E-02	9,36E-01	NA	No	1,18E-01	5,67E-01	NA	No
ENSG00000199712	SNORD115-2	snoRNA	1,45E-03	9,41E-01	NA	No	-9,82E-02	8,30E-01	NA	No
ENSG00000200680	SNORD115-4	snoRNA	9,27E-02	3,40E-01	NA	No	-2,75E-02	8,90E-01	NA	No
ENSG00000200503	SNORD115-5	snoRNA	7,01E-02	4,35E-01	NA	No	2,73E-01	2,59E-01	NA	No
ENSG00000200812	SNORD115-6	snoRNA	1,94E-02	8,87E-01	NA	No	-6,12E-02	8,00E-01	NA	No
ENSG00000200726	SNORD115-8	snoRNA	3,69E-02	8,45E-01	NA	No	1,10E-01	6,90E-01	NA	No
ENSG00000199782	SNORD115-9	snoRNA	-8,74E-04	9,41E-01	NA	No	1,15E-02	9,87E-01	NA	No
ENSG00000200486	SNORD115-11	snoRNA	3,46E-02	9,36E-01	NA	No	6,34E-02	9,56E-01	NA	No
ENSG00000199453	SNORD115-12	snoRNA	-6,42E-02	4,29E-01	NA	No	-1,40E-01	4,97E-01	NA	No
ENSG00000199960	SNORD115-14	snoRNA	-1,28E-02	8,46E-01	NA	No	-9,09E-02	6,82E-01	NA	No
ENSG00000201969	SNORD115-20	snoRNA	-3,03E-02	6,79E-01	NA	No	-1,43E-01	6,16E-01	NA	No

ENSG00000199833	SNORD115-21	snoRNA	4,64E-03	9,88E-01	NA	No	-2,94E-02	8,64E-01	NA	No
ENSG00000201326	SNORD115-22	snoRNA	7,18E-02	4,35E-01	NA	No	-9,72E-02	8,30E-01	NA	No
ENSG00000199489	SNORD115-25	snoRNA	6,46E-02	5,07E-01	NA	No	1,01E-01	7,75E-01	NA	No
ENSG00000201300	SNORD115-27	snoRNA	3,74E-02	8,45E-01	NA	No	8,51E-02	8,64E-01	NA	No
ENSG00000199704	SNORD115-29	snoRNA	3,50E-02	9,36E-01	NA	No	9,20E-02	8,64E-01	NA	No
ENSG00000200949	SNORD115-32	snoRNA	-1,16E-02	8,19E-01	NA	No	-2,32E-02	8,90E-01	NA	No
ENSG00000202499	SNORD115-36	snoRNA	-8,24E-02	3,35E-01	NA	No	-1,73E-01	4,02E-01	NA	No
ENSG00000200564	SNORD115-39	snoRNA	-1,47E-02	8,49E-01	NA	No	2,13E-03	9,87E-01	NA	No
ENSG00000272460	SNORD115-40	snoRNA	-1,59E-01	1,52E-01	NA	No	-3,56E-01	2,07E-01	NA	No
ENSG00000200478	SNORD115-41	snoRNA	-5,09E-02	6,35E-01	NA	No	-1,31E-01	5,80E-01	NA	No
ENSG00000201143	SNORD115-42	snoRNA	1,99E-02	8,81E-01	NA	No	-1,87E-02	8,90E-01	NA	No
ENSG00000202373	SNORD115-43	snoRNA	-5,86E-02	6,37E-01	NA	No	-2,46E-01	3,06E-01	NA	No
ENSG00000202261	SNORD115-44	snoRNA	-1,02E-02	8,19E-01	NA	No	-2,34E-02	8,90E-01	NA	No
ENSG00000212380	SNORD115-45	snoRNA	1,06E-01	2,11E-01	NA	No	4,41E-01	1,23E-01	NA	No
ENSG00000212528	SNORD115-47	snoRNA	3,41E-02	9,36E-01	NA	No	9,87E-02	7,75E-01	NA	No
ENSG00000239169	SNORD109B	snoRNA	2,18E-02	8,66E-01	NA	No	-1,64E-01	5,35E-01	NA	No
ENSG00000207430	U8	snoRNA	3,32E-02	9,36E-01	NA	No	9,18E-02	8,64E-01	NA	No
ENSG00000252602	U8	snoRNA	1,37E-01	2,27E-01	NA	No	8,51E-02	8,64E-01	NA	No
ENSG00000206987	U8	snoRNA	3,32E-02	9,36E-01	NA	No	8,55E-02	8,64E-01	NA	No
ENSG00000212415	SNORD77	snoRNA	2,96E-01	3,31E-02	NA	No	1,49E-01	4,89E-01	NA	No
ENSG00000212511	U3	snoRNA	-2,97E-04	9,89E-01	NA	No	-6,51E-02	8,00E-01	NA	No
ENSG00000238564	snoU13	snoRNA	4,18E-02	6,63E-01	NA	No	1,45E-01	4,58E-01	NA	No
ENSG00000239025	snoU13	snoRNA	1,53E-01	1,41E-01	NA	No	2,10E-01	2,78E-01	4,28E-01	No
ENSG00000238583	snoU13	snoRNA	4,86E-02	6,95E-01	NA	No	6,13E-02	9,56E-01	NA	No
ENSG00000238513	snoU13	snoRNA	-5,49E-02	5,41E-01	NA	No	-8,74E-02	7,04E-01	NA	No
ENSG00000239035	snoU13	snoRNA	6,30E-02	6,29E-01	NA	No	-1,67E-01	4,53E-01	NA	No
ENSG00000239100	snoU13	snoRNA	-1,92E-01	3,74E-01	6,21E-01	No	-3,59E-01	3,25E-01	4,80E-01	No
ENSG00000200318	U3	snoRNA	-6,14E-02	5,28E-01	NA	No	-7,70E-02	7,23E-01	NA	No
ENSG00000206903	SNORA24	snoRNA	4,96E-02	5,68E-01	NA	No	-2,03E-02	9,80E-01	NA	No
ENSG00000238715	snoU13	snoRNA	-1,33E-02	9,18E-01	NA	No	4,27E-01	2,46E-01	3,91E-01	No
ENSG00000238311	snoU13	snoRNA	1,36E-01	5,39E-01	7,56E-01	No	-5,25E-01	1,73E-01	3,00E-01	No
ENSG00000207119	U3	snoRNA	-3,96E-01	5,26E-02	NA	No	-1,22E+00	2,65E-02	6,56E-02	No
ENSG00000252722	SCARNA20	snoRNA	-6,61E-02	5,76E-01	NA	No	-1,18E-01	6,26E-01	7,51E-01	No
ENSG00000251881	SNORD112	snoRNA	1,61E-01	1,05E-01	NA	No	1,40E-01	5,69E-01	NA	No
ENSG00000200991	SNORA25	snoRNA	-1,02E+00	1,51E-02	7,56E-02	No	-2,33E-01	5,13E-01	6,59E-01	No
ENSG00000200677	SNORD18	snoRNA	8,82E-02	4,07E-01	NA	No	9,20E-02	8,64E-01	NA	No
ENSG00000239197	snoU109	snoRNA	9,35E-02	4,59E-01	NA	No	7,45E-04	9,96E-01	NA	No
ENSG00000238502	snoU13	snoRNA	2,18E-01	2,65E-01	5,09E-01	No	2,40E-01	4,47E-01	6,01E-01	No
ENSG00000206630	SNORD60	snoRNA	-1,53E-01	3,05E-01	5,53E-01	No	-2,12E-01	4,61E-01	6,13E-01	No
ENSG00000252405	snoMe28S-Am2634	snoRNA	-2,39E-01	9,57E-02	NA	No	-1,98E-01	5,28E-01	6,72E-01	No
ENSG00000272310	snoU13	snoRNA	4,26E-02	7,31E-01	NA	No	9,88E-02	7,75E-01	NA	No
ENSG00000238329	snoU13	snoRNA	-9,79E-02	6,52E-01	8,29E-01	No	2,74E-01	4,29E-01	5,84E-01	No
ENSG00000238954	snoU13	snoRNA	-5,42E-02	6,50E-01	NA	No	-5,95E-02	7,85E-01	NA	No
ENSG00000238712	snoU13	snoRNA	-5,42E-02	6,50E-01	NA	No	-7,88E-02	7,66E-01	NA	No
ENSG00000239172	snoU13	snoRNA	2,35E-02	9,72E-01	NA	No	8,53E-02	8,64E-01	NA	No
ENSG00000212593	SNORA75	snoRNA	1,89E-03	9,41E-01	NA	No	9,91E-02	6,39E-01	NA	No
ENSG00000200652	SNORA25	snoRNA	6,00E-02	5,53E-01	NA	No	1,52E-01	3,89E-01	NA	No
ENSG00000252461	SNORA43	snoRNA	-4,15E-02	7,60E-01	NA	No	-9,51E-02	8,30E-01	NA	No
ENSG00000238639	snoU13	snoRNA	4,42E-02	7,27E-01	NA	No	4,20E-02	8,93E-01	NA	No
ENSG00000239193	snoU13	snoRNA	-3,11E-02	7,31E-01	NA	No	-1,26E-01	7,12E-01	NA	No
ENSG00000199787	SNORA42	snoRNA	1,79E-02	8,71E-01	NA	No	-3,41E-02	9,21E-01	NA	No
ENSG00000206755	SNORA30	snoRNA	1,24E-01	5,22E-01	7,44E-01	No	-4,83E-01	2,03E-01	3,38E-01	No
ENSG00000238834	snoU13	snoRNA	5,11E-02	7,22E-01	NA	No	-2,74E-01	2,57E-01	NA	No
ENSG00000238544	snoU13	snoRNA	2,15E-02	9,18E-01	9,65E-01	No	-3,74E-01	3,13E-01	4,67E-01	No
ENSG00000238645	snoU13	snoRNA	3,84E-02	8,45E-01	NA	No	1,04E-01	7,75E-01	NA	No
ENSG00000207493	SNORA46	snoRNA	1,04E-01	5,69E-01	7,75E-01	No	5,72E-01	1,48E-01	2,65E-01	No
ENSG00000206952	SNORA50	snoRNA	1,12E-01	6,17E-01	8,08E-01	No	7,76E-02	8,05E-01	8,80E-01	No
ENSG00000239121	snoU13	snoRNA	-7,81E-02	6,66E-01	8,36E-01	No	-9,63E-01	4,89E-02	1,10E-01	No
ENSG00000212445	SNORA48	snoRNA	-6,69E-02	6,00E-01	NA	No	-8,53E-03	9,75E-01	9,85E-01	No
ENSG00000238343	snoU13	snoRNA	-7,53E-02	6,28E-01	8,15E-01	No	-5,38E-01	8,60E-02	1,74E-01	No
ENSG00000238683	snoU13	snoRNA	-1,08E-01	4,04E-01	6,48E-01	No	-5,17E-01	7,34E-02	1,53E-01	No
ENSG00000252443	SNORA62	snoRNA	3,23E-03	9,75E-01	NA	No	-1,33E-01	7,12E-01	NA	No

ENSG00000238734	snoU13	snoRNA	4,66E-02	7,06E-01	NA	No	1,28E-01	6,41E-01	7,63E-01	No
ENSG00000221514	SNORD111B	snoRNA	-9,16E-02	5,54E-01	7,66E-01	No	-2,38E-01	4,27E-01	5,83E-01	No
ENSG00000221066	SNORD111	snoRNA	-1,75E-01	2,90E-01	5,37E-01	No	-5,32E-01	1,43E-01	2,59E-01	No
ENSG00000263666	SNORA70D	snoRNA	-5,45E-02	6,46E-01	NA	No	2,12E-02	9,27E-01	NA	No
ENSG00000223224	SNORD71	snoRNA	1,80E-02	9,35E-01	9,73E-01	No	5,39E-02	8,73E-01	9,24E-01	No
ENSG00000252122	SNORA76	snoRNA	3,66E-03	9,91E-01	NA	No	5,99E-02	7,72E-01	NA	No
ENSG00000239024	snoU13	snoRNA	7,09E-02	7,28E-01	8,74E-01	No	-8,33E-01	6,86E-02	1,45E-01	No
ENSG00000238807	snoU13	snoRNA	1,52E-01	3,61E-01	6,09E-01	No	1,13E-01	7,11E-01	8,16E-01	No
ENSG00000252835	SCARNA21	snoRNA	1,21E+00	3,51E-08	1,92E-06	No	2,43E-01	8,20E-02	1,67E-01	No
ENSG00000212206	SNORA69	snoRNA	3,32E-02	9,36E-01	NA	No	8,79E-02	8,64E-01	NA	No
ENSG00000252305	SNORA74	snoRNA	5,21E-01	4,90E-02	1,71E-01	No	6,19E-01	1,33E-01	2,44E-01	No
ENSG00000238806	snoU13	snoRNA	-1,82E-01	4,21E-01	6,63E-01	No	4,40E-01	1,92E-01	3,25E-01	No
ENSG00000238691	snoU13	snoRNA	6,58E-02	5,17E-01	NA	No	-1,28E-01	7,12E-01	NA	No
ENSG00000252349	SNORA31	snoRNA	-6,71E-02	3,96E-01	NA	No	2,49E-02	9,26E-01	NA	No
ENSG00000252657	SNORA70	snoRNA	-2,34E-01	1,35E-01	NA	No	-3,32E-01	2,98E-01	4,50E-01	No
ENSG00000239129	snoU13	snoRNA	9,32E-03	9,46E-01	9,78E-01	No	-2,03E-01	4,88E-01	6,38E-01	No
ENSG00000252112	SNORD63	snoRNA	3,79E-02	7,09E-01	NA	No	1,48E-01	5,50E-01	NA	No
ENSG00000238858	snoU13	snoRNA	-6,31E-02	6,86E-01	8,49E-01	No	-4,38E-01	1,22E-01	NA	No
ENSG00000207297	SNORD7	snoRNA	-3,18E-02	8,15E-01	NA	No	6,35E-02	8,35E-01	8,99E-01	No
ENSG00000238793	SNORD124	snoRNA	2,67E-01	7,23E-02	NA	No	-2,22E-02	9,21E-01	NA	No
ENSG00000252088	snoU13	snoRNA	4,64E-03	9,92E-01	NA	No	-6,12E-02	8,00E-01	NA	No
ENSG00000200538	U3	snoRNA	2,23E-01	2,79E-02	NA	No	8,90E-02	8,64E-01	NA	No
ENSG00000238815	snoU13	snoRNA	1,14E-02	9,49E-01	9,79E-01	No	-9,24E-02	7,82E-01	8,64E-01	No
ENSG00000212195	U3	snoRNA	7,06E-01	2,90E-02	1,19E-01	No	7,19E-01	7,45E-02	1,55E-01	No
ENSG00000252577	SCARNA20	snoRNA	-2,52E-02	7,99E-01	NA	No	1,23E-01	6,28E-01	NA	No
ENSG00000238799	snoU13	snoRNA	-7,36E-02	6,43E-01	8,23E-01	No	-4,17E-01	2,17E-01	3,55E-01	No
ENSG00000199753	SNORD104	snoRNA	-3,52E-01	1,63E-01	3,81E-01	No	-4,72E-01	1,72E-01	2,98E-01	No
ENSG00000238612	snoU13	snoRNA	-1,57E-01	3,42E-01	5,91E-01	No	-5,60E-01	1,27E-01	2,36E-01	No
ENSG00000200394	SNORA38B	snoRNA	-2,38E-02	8,38E-01	NA	No	-1,61E-01	5,48E-01	6,88E-01	No
ENSG00000252274	SCARNA24	snoRNA	1,98E-02	8,96E-01	9,57E-01	No	2,35E-01	4,93E-01	6,43E-01	No
ENSG00000238418	snoU13	snoRNA	-5,18E-02	6,05E-01	NA	No	-8,04E-02	7,37E-01	NA	No
ENSG00000199961	SNORD1B	snoRNA	-4,91E-02	5,80E-01	NA	No	-1,28E-01	5,68E-01	NA	No
ENSG00000200063	SNORA30	snoRNA	-2,05E-02	8,38E-01	NA	No	-1,58E-01	5,03E-01	NA	No
ENSG00000238947	snoU13	snoRNA	3,39E-02	9,36E-01	NA	No	1,08E-01	6,89E-01	NA	No
ENSG00000238403	snoU13	snoRNA	-1,47E-01	3,49E-01	5,97E-01	No	-2,15E-01	4,70E-01	6,22E-01	No
ENSG00000238575	snoU109	snoRNA	1,47E-02	8,95E-01	NA	No	5,07E-01	1,88E-01	3,19E-01	No
ENSG00000238425	snoU13	snoRNA	2,11E-01	3,33E-01	5,82E-01	No	1,25E-02	9,72E-01	9,83E-01	No
ENSG00000238863	snoU13	snoRNA	3,50E-02	9,36E-01	NA	No	1,03E-01	7,75E-01	NA	No
ENSG00000238790	snoU13	snoRNA	3,36E-02	7,25E-01	NA	No	-2,73E-02	8,85E-01	NA	No
ENSG00000252576	snR65	snoRNA	9,74E-03	9,67E-01	NA	No	1,55E-02	9,87E-01	NA	No
ENSG00000199856	U3	snoRNA	-7,79E-02	7,24E-01	8,71E-01	No	3,57E-01	2,49E-01	3,93E-01	No
ENSG00000239087	snoU13	snoRNA	7,54E-02	7,01E-01	8,58E-01	No	1,67E-02	9,62E-01	9,78E-01	No
ENSG00000238309	snoU13	snoRNA	-1,14E-01	3,44E-01	NA	No	-1,83E-01	5,49E-01	6,90E-01	No
ENSG00000221139	SNORD23	snoRNA	-2,24E-02	8,26E-01	NA	No	-5,71E-02	7,90E-01	NA	No
ENSG00000252677	SNORA81	snoRNA	3,97E-02	8,45E-01	NA	No	6,23E-02	9,56E-01	NA	No
ENSG00000199977	SNORA73	snoRNA	-4,38E-03	9,79E-01	9,92E-01	No	-3,60E-01	3,05E-01	4,58E-01	No
ENSG00000238907	snoU13	snoRNA	-1,87E-01	3,76E-01	6,23E-01	No	1,39E-01	6,93E-01	8,02E-01	No
ENSG00000238537	snoU13	snoRNA	2,43E-02	7,72E-01	NA	No	-1,30E-01	7,12E-01	NA	No
ENSG00000252921	U3	snoRNA	4,19E-02	7,49E-01	NA	No	1,14E-01	6,19E-01	NA	No
ENSG00000238376	snoU13	snoRNA	-5,82E-02	5,61E-01	NA	No	3,50E-02	8,87E-01	NA	No
ENSG00000238982	snoU13	snoRNA	2,52E-02	8,77E-01	9,48E-01	No	1,57E-02	9,61E-01	9,77E-01	No
ENSG00000252078	SNORD112	snoRNA	1,64E-02	8,85E-01	NA	No	-5,51E-03	9,83E-01	NA	No
ENSG00000251992	SCARNA17	snoRNA	3,50E-01	6,43E-02	2,07E-01	No	4,83E-02	8,22E-01	8,91E-01	No
ENSG00000252139	SCARNA18	snoRNA	2,80E-01	2,00E-01	4,31E-01	No	1,29E-01	6,29E-01	7,53E-01	No
ENSG00000207233	SNORA37	snoRNA	1,92E-01	3,44E-01	5,92E-01	No	2,95E-01	3,98E-01	5,55E-01	No
ENSG00000212539	U3	snoRNA	-6,30E-02	7,08E-01	8,62E-01	No	-1,82E-03	9,94E-01	9,97E-01	No
ENSG00000238988	snoU13	snoRNA	5,19E-01	3,85E-03	2,83E-02	No	2,63E-01	1,77E-01	NA	No
ENSG00000251806	SNORD119	snoRNA	-1,47E-01	3,08E-01	NA	No	-2,44E-01	4,27E-01	NA	No
ENSG00000201346	U3	snoRNA	1,06E-02	9,25E-01	NA	No	-4,57E-02	8,25E-01	NA	No
ENSG00000252096	SNORA31	snoRNA	-1,08E-01	4,87E-01	NA	No	-1,66E-01	5,85E-01	7,18E-01	No
ENSG00000252058	snoU13	snoRNA	3,46E-02	9,36E-01	NA	No	1,68E-01	3,12E-01	NA	No
ENSG00000201348	RNU105B	snoRNA	1,53E-01	3,77E-01	6,24E-01	No	-8,13E-02	7,82E-01	8,64E-01	No

ENSG00000212232	SNORD17	snoRNA	3,41E-01	1,19E-01	3,12E-01	No	3,28E-01	9,69E-02	1,91E-01	No
ENSG00000201151	SNORD56	snoRNA	3,26E-02	7,32E-01	NA	No	4,99E-02	8,01E-01	NA	No
ENSG00000238549	snoU13	snoRNA	1,46E-01	5,12E-01	7,37E-01	No	-4,48E-01	2,37E-01	3,79E-01	No
ENSG00000200354	SNORA71D	snoRNA	-1,50E-01	3,57E-01	6,05E-01	No	-5,00E-01	1,21E-01	2,28E-01	No
ENSG00000199266	SNORA60	snoRNA	-6,15E-02	7,34E-01	8,76E-01	No	3,20E-01	3,81E-01	5,38E-01	No
ENSG00000209042	SNORD12C	snoRNA	-7,34E-02	7,12E-01	8,65E-01	No	-2,51E-01	4,81E-01	6,31E-01	No
ENSG00000222365	SNORD12B	snoRNA	-3,70E-01	1,49E-01	3,62E-01	No	-1,35E+00	1,05E-02	2,97E-02	No
ENSG00000212304	SNORD12	snoRNA	4,33E-01	1,05E-01	2,87E-01	No	3,02E-01	4,03E-01	5,60E-01	No
ENSG00000238294	snoU13	snoRNA	-4,65E-02	7,06E-01	NA	No	-3,67E-02	8,88E-01	9,33E-01	No
ENSG00000252408	SNORD38	snoRNA	2,15E-02	8,48E-01	NA	No	-6,25E-02	7,64E-01	NA	No
ENSG00000206775	SNORD37	snoRNA	-1,51E-01	2,97E-01	NA	No	-6,67E-01	5,23E-02	NA	No
ENSG00000209645	SNORD105	snoRNA	4,37E-03	9,46E-01	NA	No	-1,51E-01	5,67E-01	NA	No
ENSG00000238531	SNORD105B	snoRNA	9,56E-03	9,64E-01	NA	No	1,70E-02	9,87E-01	NA	No
ENSG00000238349	snoU13	snoRNA	5,08E-04	9,98E-01	NA	No	-2,74E-01	2,57E-01	NA	No
ENSG00000209702	SNORD41	snoRNA	7,48E-02	4,68E-01	NA	No	-1,32E-01	7,12E-01	NA	No
ENSG00000201388	SNORA68	snoRNA	2,25E-01	2,13E-01	4,48E-01	No	-1,83E-01	4,62E-01	NA	No
ENSG00000252230	SNORD111	snoRNA	8,21E-03	9,58E-01	9,83E-01	No	-1,96E-01	4,30E-01	5,85E-01	No
ENSG00000253027	SNORA70	snoRNA	-7,95E-02	2,47E-01	NA	No	-2,05E-01	2,45E-01	NA	No
ENSG00000252071	snoU13	snoRNA	-4,97E-02	8,15E-01	9,19E-01	No	-7,93E-01	4,89E-02	1,10E-01	No
ENSG00000221241	SNORD88A	snoRNA	-3,42E-02	7,78E-01	NA	No	-4,22E-02	8,58E-01	NA	No
ENSG00000251737	snoU13	snoRNA	5,88E-02	7,65E-01	8,92E-01	No	-4,10E-01	2,59E-01	4,06E-01	No
ENSG00000201209	SNORD42	snoRNA	7,95E-02	5,41E-01	7,57E-01	No	-1,86E-01	5,15E-01	6,61E-01	No
ENSG00000239127	SNORD125	snoRNA	2,03E-01	3,46E-01	5,94E-01	No	-2,45E-01	4,75E-01	6,26E-01	No
ENSG00000238910	snoU13	snoRNA	-1,90E-01	2,83E-01	5,30E-01	No	-1,01E+00	4,40E-02	1,01E-01	No
ENSG00000209482	SNORD83A	snoRNA	-8,88E-02	6,02E-01	7,99E-01	No	-4,47E-01	2,04E-01	3,39E-01	No
ENSG00000263764	SNORD43	snoRNA	-4,21E-02	7,60E-01	NA	No	-9,72E-02	8,30E-01	NA	No
ENSG00000238887	snoU13	snoRNA	3,31E-02	9,36E-01	NA	No	1,01E-01	7,75E-01	NA	No
ENSG00000238498	snoU13	snoRNA	6,04E-02	5,53E-01	NA	No	8,55E-02	8,64E-01	NA	No
ENSG00000201025	SNORD74	snoRNA	1,58E-01	3,70E-01	6,16E-01	No	9,90E-01	3,67E-02	8,65E-02	No
ENSG00000212479	U3	snoRNA	3,87E-02	8,45E-01	NA	No	1,10E-01	6,87E-01	NA	No
ENSG00000239171	snoU13	snoRNA	2,28E-01	2,26E-01	4,63E-01	No	4,41E-01	2,33E-01	3,75E-01	No
ENSG00000238390	SNORA81	snoRNA	3,58E-01	1,47E-01	3,58E-01	No	-4,03E-01	2,81E-01	4,31E-01	No
ENSG00000200792	SNORA80	snoRNA	8,15E-03	9,53E-01	9,81E-01	No	-3,37E-01	2,79E-01	NA	No
ENSG00000252045	SNORA33	snoRNA	2,41E-02	8,44E-01	NA	No	-1,26E-01	7,12E-01	NA	No
ENSG00000207098	SNORA70	snoRNA	-5,37E-02	6,46E-01	NA	No	-2,46E-02	8,86E-01	NA	No
ENSG00000221398	SNORA11	snoRNA	7,19E-02	4,03E-01	NA	No	-9,31E-02	6,39E-01	NA	No
ENSG00000238851	snoU13	snoRNA	-5,69E-02	6,46E-01	NA	No	1,71E-01	4,24E-01	NA	No
ENSG00000272015	SNORA62	snoRNA	-4,21E-02	7,60E-01	NA	No	-9,72E-02	8,30E-01	NA	No
ENSG00000251778	SNORA3	snoRNA	2,22E-02	9,72E-01	NA	No	8,55E-02	8,64E-01	NA	No
ENSG00000253085	SCARNA11	snoRNA	2,34E-02	9,72E-01	NA	No	NA	NA	NA	NA
ENSG00000238538	snoU13	snoRNA	3,39E-02	9,36E-01	NA	No	NA	NA	NA	NA
ENSG00000238986	snoU13	snoRNA	2,35E-01	3,47E-02	NA	No	NA	NA	NA	NA
ENSG00000201003	SNORA58	snoRNA	1,04E-01	2,81E-01	NA	No	NA	NA	NA	NA
ENSG00000199959	SNORA2	snoRNA	3,41E-02	9,36E-01	NA	No	NA	NA	NA	NA
ENSG00000212266	SNORA40	snoRNA	2,23E-02	9,72E-01	NA	No	NA	NA	NA	NA
ENSG00000252669	U3	snoRNA	3,32E-02	9,36E-01	NA	No	NA	NA	NA	NA
ENSG00000252790	SNORD112	snoRNA	2,35E-02	9,72E-01	NA	No	NA	NA	NA	NA
ENSG00000238571	snoU13	snoRNA	3,69E-02	8,45E-01	NA	No	NA	NA	NA	NA
ENSG00000201898	SNORA72	snoRNA	2,42E-02	9,72E-01	NA	No	NA	NA	NA	NA
ENSG00000206878	SNORA51	snoRNA	2,34E-02	9,72E-01	NA	No	NA	NA	NA	NA
ENSG00000238985	snoU13	snoRNA	2,23E-02	9,72E-01	NA	No	NA	NA	NA	NA
ENSG00000252011	SNORA25	snoRNA	3,32E-02	9,36E-01	NA	No	NA	NA	NA	NA
ENSG00000207187	SNORA64	snoRNA	4,41E-02	7,47E-01	NA	No	NA	NA	NA	NA
ENSG00000238696	snoU13	snoRNA	2,34E-02	9,72E-01	NA	No	NA	NA	NA	NA
ENSG00000221638	U3	snoRNA	3,15E-02	9,36E-01	NA	No	NA	NA	NA	NA
ENSG00000239077	snoU13	snoRNA	3,41E-02	9,36E-01	NA	No	NA	NA	NA	NA
ENSG00000200377	SNORD56	snoRNA	3,68E-02	8,45E-01	NA	No	NA	NA	NA	NA
ENSG00000238543	snoU13	snoRNA	3,15E-02	9,36E-01	NA	No	NA	NA	NA	NA
ENSG00000238662	snoU13	snoRNA	2,46E-02	9,72E-01	NA	No	NA	NA	NA	NA
ENSG00000253049	SNORA43	snoRNA	4,12E-02	7,65E-01	NA	No	NA	NA	NA	NA
ENSG00000239126	snoU13	snoRNA	4,48E-02	6,71E-01	NA	No	NA	NA	NA	NA
ENSG00000252409	SCARNA21	snoRNA	7,83E-02	4,91E-01	NA	No	NA	NA	NA	NA

ENSG00000202269	U8	snoRNA	3,84E-02	8,45E-01	NA	No	NA	NA	NA	NA
ENSG00000220986	SNORA50	snoRNA	3,15E-02	9,36E-01	NA	No	NA	NA	NA	NA
ENSG00000212363	SNORA40	snoRNA	3,32E-02	9,36E-01	NA	No	NA	NA	NA	NA
ENSG00000200235	SNORA27	snoRNA	4,12E-02	7,65E-01	NA	No	NA	NA	NA	NA
ENSG00000238611	snoU13	snoRNA	2,46E-02	9,72E-01	NA	No	NA	NA	NA	NA
ENSG00000238464	snoU13	snoRNA	3,41E-02	9,36E-01	NA	No	NA	NA	NA	NA
ENSG00000238628	snoU13	snoRNA	2,42E-02	9,72E-01	NA	No	NA	NA	NA	NA
ENSG00000238747	snoU13	snoRNA	3,15E-02	9,36E-01	NA	No	NA	NA	NA	NA
ENSG00000239015	snoU13	snoRNA	2,49E-02	9,72E-01	NA	No	NA	NA	NA	NA
ENSG00000238631	snoU13	snoRNA	2,34E-02	9,72E-01	NA	No	NA	NA	NA	NA
ENSG00000238394	snoU13	snoRNA	5,30E-02	6,39E-01	NA	No	NA	NA	NA	NA
ENSG00000252706	snoU13	snoRNA	3,39E-02	9,36E-01	NA	No	NA	NA	NA	NA
ENSG00000238453	snoU13	snoRNA	2,35E-02	9,72E-01	NA	No	NA	NA	NA	NA
ENSG00000212214	SNORA48	snoRNA	4,33E-02	7,20E-01	NA	No	NA	NA	NA	NA
ENSG00000252291	SNORA31	snoRNA	3,50E-02	9,36E-01	NA	No	NA	NA	NA	NA
ENSG00000238327	snoU13	snoRNA	2,46E-02	9,72E-01	NA	No	NA	NA	NA	NA
ENSG00000202231	SNORA9	snoRNA	4,73E-02	5,78E-01	NA	No	NA	NA	NA	NA
ENSG00000238466	snoU13	snoRNA	3,17E-02	9,36E-01	NA	No	NA	NA	NA	NA
ENSG00000264379	SNORD39	snoRNA	3,50E-02	9,36E-01	NA	No	NA	NA	NA	NA
ENSG00000252580	SNORA31	snoRNA	2,35E-02	9,72E-01	NA	No	NA	NA	NA	NA
ENSG00000238402	snoU13	snoRNA	2,35E-02	9,72E-01	NA	No	NA	NA	NA	NA
ENSG00000238598	snoU13	snoRNA	3,41E-02	9,36E-01	NA	No	NA	NA	NA	NA
ENSG00000223182	SNORA74	snoRNA	4,55E-02	6,42E-01	NA	No	NA	NA	NA	NA
ENSG00000253068	SNORD112	snoRNA	6,19E-02	5,30E-01	NA	No	NA	NA	NA	NA
ENSG00000212551	U3	snoRNA	3,32E-02	9,36E-01	NA	No	NA	NA	NA	NA
ENSG00000201733	SNORA43	snoRNA	5,17E-02	6,56E-01	NA	No	NA	NA	NA	NA
ENSG00000238666	snoU13	snoRNA	2,42E-02	9,72E-01	NA	No	NA	NA	NA	NA
ENSG00000239086	snoU13	snoRNA	2,34E-02	9,72E-01	NA	No	NA	NA	NA	NA
ENSG00000252204	SNORA25	snoRNA	3,17E-02	9,36E-01	NA	No	NA	NA	NA	NA
ENSG00000238395	snoU13	snoRNA	2,42E-02	9,72E-01	NA	No	NA	NA	NA	NA
ENSG00000238440	snoU13	snoRNA	3,75E-02	8,45E-01	NA	No	NA	NA	NA	NA
ENSG00000252128	SNORD27	snoRNA	4,60E-02	6,24E-01	NA	No	NA	NA	NA	NA
ENSG00000238483	snoU13	snoRNA	5,19E-02	5,03E-01	NA	No	NA	NA	NA	NA
ENSG00000252114	SNORA73	snoRNA	2,22E-02	9,72E-01	NA	No	NA	NA	NA	NA
ENSG00000200042	U3	snoRNA	3,17E-02	9,36E-01	NA	No	NA	NA	NA	NA
ENSG00000238960	snoU13	snoRNA	2,42E-02	9,72E-01	NA	No	NA	NA	NA	NA
ENSG00000201943	SNORD115-10	snoRNA	2,46E-02	9,72E-01	NA	No	NA	NA	NA	NA
ENSG00000201679	SNORD115-15	snoRNA	3,15E-02	9,36E-01	NA	No	NA	NA	NA	NA
ENSG00000199968	SNORD115-19	snoRNA	4,19E-02	7,65E-01	NA	No	NA	NA	NA	NA
ENSG00000200593	SNORD115-33	snoRNA	2,22E-02	9,72E-01	NA	No	NA	NA	NA	NA
ENSG00000200638	SNORD115-37	snoRNA	3,31E-02	9,36E-01	NA	No	NA	NA	NA	NA
ENSG00000201907	SNORD115-38	snoRNA	2,23E-02	9,72E-01	NA	No	NA	NA	NA	NA
ENSG00000252774	SNORA48	snoRNA	2,35E-02	9,72E-01	NA	No	NA	NA	NA	NA
ENSG00000239186	snoU13	snoRNA	3,28E-02	9,36E-01	NA	No	NA	NA	NA	NA
ENSG00000238946	snoU13	snoRNA	9,27E-02	3,84E-01	NA	No	NA	NA	NA	NA
ENSG00000238676	snoU13	snoRNA	7,30E-02	4,13E-01	NA	No	NA	NA	NA	NA
ENSG00000212565	SNORA68	snoRNA	3,50E-02	9,36E-01	NA	No	NA	NA	NA	NA
ENSG00000239173	snoU13	snoRNA	4,04E-02	7,81E-01	NA	No	NA	NA	NA	NA
ENSG00000199392	SNORA25	snoRNA	3,39E-02	9,36E-01	NA	No	NA	NA	NA	NA
ENSG00000271798	SNORA51	snoRNA	2,22E-02	9,72E-01	NA	No	NA	NA	NA	NA
ENSG00000212517	SNORA26	snoRNA	2,35E-02	9,72E-01	NA	No	NA	NA	NA	NA
ENSG00000238514	snoU13	snoRNA	2,35E-02	9,72E-01	NA	No	NA	NA	NA	NA
ENSG00000238838	snoU13	snoRNA	2,42E-02	9,72E-01	NA	No	NA	NA	NA	NA
ENSG00000252356	SNORD112	snoRNA	3,80E-02	8,45E-01	NA	No	NA	NA	NA	NA
ENSG00000238630	snoU13	snoRNA	4,61E-02	6,22E-01	NA	No	NA	NA	NA	NA
ENSG00000239137	snoU13	snoRNA	3,31E-02	9,36E-01	NA	No	NA	NA	NA	NA
ENSG00000251940	SNORA15	snoRNA	5,14E-02	5,11E-01	NA	No	NA	NA	NA	NA
ENSG00000252571	SCARNA17	snoRNA	2,40E-02	9,72E-01	NA	No	NA	NA	NA	NA
ENSG00000252143	SCARNA17	snoRNA	3,32E-02	9,36E-01	NA	No	NA	NA	NA	NA
ENSG00000252392	SNORD112	snoRNA	2,34E-02	9,72E-01	NA	No	NA	NA	NA	NA
ENSG00000207087	RNU6-242P	snRNA	3,23E-01	8,52E-02	2,50E-01	No	2,52E+00	7,45E-04	2,84E-03	Yes
ENSG00000201574	RNU1-93P	snRNA	1,35E-01	1,62E-01	NA	No	1,72E+00	1,29E-02	3,56E-02	Yes

ENSG00000222872	RNU4-78P	snRNA	-1,44E-01	4,88E-01	7,18E-01	No	-2,40E+00	2,98E-03	9,83E-03	Yes
ENSG00000212340	RNU6-739P	snRNA	-1,92E-01	2,15E-01	4,51E-01	No	-4,57E+00	1,09E-02	3,06E-02	Yes
ENSG00000212282	RNU6-578P	snRNA	1,82E-01	2,23E-01	4,61E-01	No	2,04E+00	6,16E-03	1,86E-02	Yes
ENSG00000252343	RNU2-34P	snRNA	-1,41E-01	3,89E-01	6,35E-01	No	-2,74E+00	9,74E-03	2,78E-02	Yes
ENSG00000201433	RNU6-335P	snRNA	5,20E-01	1,71E-02	8,24E-02	No	2,61E+00	2,24E-03	7,64E-03	Yes
ENSG00000201747	RNU6-534P	snRNA	3,31E-02	9,36E-01	NA	No	3,36E+00	7,50E-03	2,22E-02	Yes
ENSG00000199846	RNU1-72P	snRNA	-1,34E+00	1,10E-02	6,02E-02	No	-1,54E+00	1,83E-02	4,80E-02	Yes
ENSG00000222973	RNU2-25P	snRNA	1,42E+00	2,90E-03	2,30E-02	No	2,06E+00	8,71E-05	4,00E-04	Yes
ENSG00000212254	RNU6-124P	snRNA	1,06E-01	2,75E-01	NA	No	3,88E+00	3,78E-03	1,21E-02	Yes
ENSG00000221676	RNU6ATAC	snRNA	5,47E-02	5,18E-01	NA	No	2,59E+00	2,41E-03	8,14E-03	Yes
ENSG00000201390	RNU6-1141P	snRNA	1,86E-01	2,32E-01	4,71E-01	No	1,88E+00	3,52E-03	1,14E-02	Yes
ENSG00000200253	RNU6-529P	snRNA	-5,55E-02	7,85E-01	9,03E-01	No	1,88E+00	1,46E-05	7,84E-05	Yes
ENSG00000199237	RNU6-834P	snRNA	9,21E-02	2,94E-01	NA	No	2,46E+00	6,45E-03	1,94E-02	Yes
ENSG00000202538	RNU4-2	snRNA	4,23E-01	1,00E-01	2,79E-01	No	2,34E+00	1,43E-06	8,97E-06	Yes
ENSG00000223336	RNU2-6P	snRNA	-3,93E-02	8,42E-01	9,31E-01	No	2,97E+00	2,36E-15	4,59E-14	Yes
ENSG00000252469	RNU7-160P	snRNA	-8,25E-01	2,04E-02	9,32E-02	No	-2,37E+00	1,78E-03	6,19E-03	Yes
ENSG00000239151	RNU7-195P	snRNA	-3,35E-01	1,40E-01	3,48E-01	No	-1,84E+00	7,35E-03	2,18E-02	Yes
ENSG00000200556	RNU6-103P	snRNA	7,50E-02	7,32E-01	8,75E-01	No	-1,56E+00	1,92E-03	6,63E-03	Yes
ENSG00000202159	RNU6-742P	snRNA	7,61E-02	3,76E-01	NA	No	4,08E+00	2,82E-03	9,36E-03	Yes
ENSG00000223181	RNU6-1199P	snRNA	6,55E-02	4,86E-01	NA	No	1,38E-01	5,91E-01	NA	No
ENSG00000253022	RNU6-731P	snRNA	4,66E-01	6,99E-03	4,35E-02	No	-1,02E-01	8,30E-01	NA	No
ENSG00000200975	RNU1-7P	snRNA	-6,75E-02	3,80E-01	NA	No	-1,64E-01	4,37E-01	NA	No
ENSG00000201746	RNU6-828P	snRNA	-6,62E-02	6,63E-01	8,35E-01	No	-4,53E-01	1,43E-01	2,58E-01	No
ENSG00000199562	RNU6-37P	snRNA	1,66E-01	3,69E-01	6,15E-01	No	2,52E-01	4,69E-01	6,21E-01	No
ENSG00000253086	RNU6-537P	snRNA	2,40E-01	9,49E-02	2,69E-01	No	-1,45E-01	5,89E-01	NA	No
ENSG00000207451	RNU6-291P	snRNA	-1,93E-02	8,71E-01	NA	No	-6,75E-02	7,72E-01	NA	No
ENSG00000199347	RNU5E-1	snRNA	2,47E-02	9,00E-01	9,59E-01	No	4,02E-01	2,62E-01	4,09E-01	No
ENSG00000201801	RNU5E-4P	snRNA	-9,62E-03	9,43E-01	9,76E-01	No	-1,88E-01	5,63E-01	7,00E-01	No
ENSG00000221340	RNU6ATAC18P	snRNA	-1,06E-01	5,34E-01	7,53E-01	No	-3,11E-01	3,50E-01	5,05E-01	No
ENSG00000252151	RNU6-1265P	snRNA	3,41E-02	9,36E-01	NA	No	9,18E-02	8,64E-01	NA	No
ENSG00000206652	RNU1-1	snRNA	1,01E-01	6,45E-01	8,25E-01	No	4,45E-01	1,93E-01	3,26E-01	No
ENSG00000207513	RNU1-3	snRNA	3,99E-03	9,76E-01	NA	No	-7,06E-02	7,90E-01	NA	No
ENSG00000207389	RNU1-4	snRNA	1,08E-01	2,90E-01	NA	No	4,26E-01	1,39E-01	2,53E-01	No
ENSG00000207005	RNU1-2	snRNA	1,57E-01	3,55E-01	6,03E-01	No	3,33E-01	3,53E-01	5,09E-01	No
ENSG00000200403	RNU6-1099P	snRNA	6,36E-02	5,08E-01	NA	No	1,17E-01	5,78E-01	NA	No
ENSG00000251914	RNU7-200P	snRNA	2,22E-02	9,72E-01	NA	No	5,02E-01	1,43E-01	NA	No
ENSG00000206935	RNU6-514P	snRNA	5,14E-02	7,32E-01	8,75E-01	No	-2,86E-01	2,84E-01	NA	No
ENSG00000252578	RNU6-135P	snRNA	-1,01E-01	4,06E-01	6,50E-01	No	-1,25E-01	6,58E-01	7,76E-01	No
ENSG00000238482	RNU6-1208P	snRNA	-3,42E-01	1,31E-01	3,34E-01	No	-3,06E-01	3,95E-01	5,52E-01	No
ENSG00000252515	RNU6-1171P	snRNA	-1,51E-01	2,28E-01	NA	No	-4,26E-01	9,03E-02	1,81E-01	No
ENSG00000207237	RNU6-110P	snRNA	-4,54E-03	8,99E-01	NA	No	-1,28E-01	7,12E-01	NA	No
ENSG00000206888	RNU6-48P	snRNA	-1,80E-02	9,08E-01	NA	No	-2,81E-01	3,03E-01	NA	No
ENSG00000223062	RNU6-1245P	snRNA	-6,46E-02	4,59E-01	NA	No	-7,82E-02	7,50E-01	NA	No
ENSG00000221216	RNU6ATAC27P	snRNA	8,27E-02	5,62E-01	NA	No	-2,55E-01	3,80E-01	NA	No
ENSG00000222282	RNU6-584P	snRNA	-2,99E-03	9,76E-01	NA	No	-1,25E-02	9,77E-01	NA	No
ENSG00000212541	RNU6-510P	snRNA	-1,99E-01	3,10E-01	5,59E-01	No	-1,49E-01	6,70E-01	7,86E-01	No
ENSG00000199963	RNU6-605P	snRNA	-5,45E-02	6,46E-01	NA	No	-5,78E-02	8,00E-01	NA	No
ENSG00000206654	RNU6-608P	snRNA	-8,68E-02	3,49E-01	NA	No	6,26E-01	1,08E-01	2,09E-01	No
ENSG00000207508	RNU6-1237P	snRNA	-4,20E-02	7,39E-01	NA	No	1,40E-01	6,54E-01	7,73E-01	No
ENSG00000200254	RNU6-536P	snRNA	3,87E-02	6,81E-01	NA	No	5,44E-02	8,87E-01	NA	No
ENSG00000252201	RNU6-1058P	snRNA	4,10E-02	7,11E-01	NA	No	3,62E-01	2,52E-01	3,98E-01	No
ENSG00000200169	RNU5D-1	snRNA	6,48E-01	3,82E-02	1,45E-01	No	-3,29E-02	9,22E-01	9,54E-01	No
ENSG00000206700	RNU6-723P	snRNA	3,38E-01	1,24E-01	3,21E-01	No	7,10E-01	1,00E-01	1,96E-01	No
ENSG00000223175	RNU4-61P	snRNA	8,54E-02	6,04E-01	8,00E-01	No	-3,14E-02	9,18E-01	9,51E-01	No
ENSG00000207194	RNU6-1026P	snRNA	1,49E-01	4,43E-01	6,80E-01	No	-1,34E-01	6,95E-01	8,04E-01	No
ENSG00000252825	RNU6-1253P	snRNA	-4,61E-02	5,39E-01	NA	No	5,31E-02	8,44E-01	9,05E-01	No
ENSG00000206595	RNU6-877P	snRNA	5,63E-02	7,15E-01	8,66E-01	No	4,68E-02	8,87E-01	9,33E-01	No
ENSG00000252032	RNU6-1281P	snRNA	7,18E-02	4,33E-01	NA	No	6,17E-02	9,56E-01	NA	No
ENSG00000252018	RNU2-30P	snRNA	5,57E-02	5,91E-01	NA	No	-1,28E-01	7,12E-01	NA	No
ENSG00000206627	RNU6-969P	snRNA	1,97E-01	3,33E-01	5,82E-01	No	-1,83E-01	5,77E-01	7,12E-01	No
ENSG00000239007	RNU7-95P	snRNA	-5,58E-02	6,46E-01	NA	No	-7,31E-02	8,00E-01	NA	No
ENSG00000200575	RNU6-414P	snRNA	1,02E-01	3,83E-01	NA	No	7,98E-02	7,67E-01	NA	No

ENSG00000212360	RNU6-1177P	snRNA	3,15E-02	9,36E-01	NA	No	1,10E-01	6,81E-01	NA	No
ENSG00000251720	RNU7-123P	snRNA	1,12E-02	9,20E-01	NA	No	6,02E-01	1,22E-01	2,29E-01	No
ENSG00000207190	RNU6-809P	snRNA	-2,61E-01	1,07E-01	2,90E-01	No	-3,56E-01	2,88E-01	4,38E-01	No
ENSG00000212257	RNU6-1176P	snRNA	-7,53E-02	7,23E-01	8,71E-01	No	-1,54E-01	6,60E-01	7,78E-01	No
ENSG00000222624	RNU2-15P	snRNA	-6,43E-02	4,67E-01	NA	No	-1,56E-01	5,24E-01	NA	No
ENSG00000223152	RNU4-88P	snRNA	4,62E-02	6,17E-01	NA	No	2,03E-01	3,94E-01	NA	No
ENSG00000223263	RNU6-387P	snRNA	1,27E-01	5,18E-01	7,41E-01	No	-1,36E-01	6,86E-01	7,97E-01	No
ENSG00000238778	RNU7-80P	snRNA	-2,80E-01	1,72E-01	3,95E-01	No	-3,06E-01	3,84E-01	5,41E-01	No
ENSG00000252338	RNU6-503P	snRNA	-5,36E-02	5,14E-01	NA	No	-4,21E-02	8,58E-01	9,15E-01	No
ENSG00000251958	RNU6-1102P	snRNA	6,38E-02	5,16E-01	NA	No	2,81E-01	2,28E-01	NA	No
ENSG00000207234	RNU6-125P	snRNA	6,68E-01	3,78E-02	1,44E-01	No	-3,27E-01	3,69E-01	5,25E-01	No
ENSG00000201317	RNU4-59P	snRNA	-1,61E-01	3,48E-01	5,96E-01	No	2,90E-01	4,13E-01	5,69E-01	No
ENSG00000212248	RNU6-750P	snRNA	1,05E-01	3,02E-01	NA	No	1,09E-01	6,52E-01	NA	No
ENSG00000202259	RNU6-1318P	snRNA	-1,32E-01	4,69E-01	7,02E-01	No	-8,36E-03	9,80E-01	9,88E-01	No
ENSG00000206832	RNU6V	snRNA	-2,16E-02	9,16E-01	9,65E-01	No	-1,97E-01	5,79E-01	7,13E-01	No
ENSG00000200360	RNU6-792P	snRNA	-1,56E-02	8,19E-01	NA	No	-1,33E-01	7,12E-01	NA	No
ENSG00000201028	RNU6-151P	snRNA	-1,57E-03	9,92E-01	9,97E-01	No	-4,94E-01	2,02E-01	3,36E-01	No
ENSG00000252750	RNU7-70P	snRNA	1,25E-01	4,98E-01	7,26E-01	No	2,63E-01	4,60E-01	6,13E-01	No
ENSG00000212385	RNU6-817P	snRNA	2,03E-01	2,15E-01	4,51E-01	No	1,95E-01	4,70E-01	6,22E-01	No
ENSG00000201699	RNU1-59P	snRNA	-5,83E-01	4,32E-02	1,57E-01	No	-1,07E+00	3,80E-02	8,90E-02	No
ENSG00000222788	RNU2-38P	snRNA	-1,08E+00	4,30E-03	3,06E-02	No	1,38E-01	6,61E-01	7,78E-01	No
ENSG00000201558	RNVU1-6	snRNA	-1,73E-02	8,99E-01	9,58E-01	No	-9,79E-02	7,63E-01	8,52E-01	No
ENSG00000207418	RNVU1-7	snRNA	2,46E-02	9,72E-01	NA	No	6,67E-02	9,56E-01	NA	No
ENSG00000201142	RNVU1-8	snRNA	-4,23E-02	7,60E-01	NA	No	-9,82E-02	8,30E-01	NA	No
ENSG00000206585	RNVU1-9	snRNA	9,99E-03	9,64E-01	NA	No	-3,34E-02	9,21E-01	NA	No
ENSG00000199879	RNU1-120P	snRNA	4,62E-02	6,18E-01	NA	No	2,16E-01	2,93E-01	NA	No
ENSG00000202408	RNU1-122P	snRNA	-1,31E-02	9,26E-01	NA	No	1,13E-01	7,12E-01	8,16E-01	No
ENSG00000202064	RNVU1-11	snRNA	3,88E-02	8,45E-01	NA	No	6,17E-02	9,56E-01	NA	No
ENSG00000207501	RNVU1-14	snRNA	6,75E-01	3,11E-02	1,25E-01	No	5,40E-01	1,50E-01	2,69E-01	No
ENSG00000207205	RNVU1-15	snRNA	1,68E-01	3,58E-01	6,05E-01	No	-3,56E-01	3,16E-01	4,70E-01	No
ENSG00000206968	RNVU1-16	snRNA	6,37E-02	6,22E-01	NA	No	-1,01E-01	6,81E-01	NA	No
ENSG00000206737	RNVU1-18	snRNA	2,70E-01	2,37E-01	4,76E-01	No	5,12E-01	1,17E-01	2,22E-01	No
ENSG00000212544	RNVU1-19	snRNA	4,92E-01	2,20E-02	9,84E-02	No	1,48E-01	5,94E-01	7,25E-01	No
ENSG00000202496	RNVU1-20	snRNA	2,48E-01	1,76E-01	4,00E-01	No	1,42E-03	9,98E-01	9,99E-01	No
ENSG00000252925	RNU1-68P	snRNA	6,32E-01	2,38E-02	1,04E-01	No	-7,42E-02	7,30E-01	NA	No
ENSG00000222222	RNU2-17P	snRNA	1,37E-02	8,92E-01	NA	No	-1,55E-01	5,35E-01	NA	No
ENSG00000206931	RNU6-1042P	snRNA	5,29E-02	6,40E-01	NA	No	6,19E-02	9,56E-01	NA	No
ENSG00000200175	RNU6-1309P	snRNA	-5,68E-02	6,23E-01	NA	No	-7,04E-02	7,65E-01	NA	No
ENSG00000200759	RNU6-884P	snRNA	-8,52E-02	3,12E-01	NA	No	-1,76E-01	3,92E-01	NA	No
ENSG00000206635	RNU6-1062P	snRNA	1,81E-01	2,82E-01	5,29E-01	No	-2,37E-02	9,34E-01	9,61E-01	No
ENSG00000238365	RNU7-57P	snRNA	3,89E-02	8,25E-01	9,24E-01	No	-6,30E-01	6,34E-02	NA	No
ENSG00000207134	RNU6-106P	snRNA	-4,11E-02	7,15E-01	NA	No	-3,72E-01	1,82E-01	NA	No
ENSG00000207144	RNU6-1297P	snRNA	1,97E-01	1,26E-01	NA	No	6,69E-02	7,80E-01	NA	No
ENSG00000201614	RNU4-19P	snRNA	-1,92E-01	1,70E-01	NA	No	-2,60E-01	3,98E-01	NA	No
ENSG00000206921	RNU6-481P	snRNA	-4,19E-02	7,68E-01	8,94E-01	No	-5,18E-02	8,56E-01	9,13E-01	No
ENSG00000207082	RNU6-171P	snRNA	-1,61E-01	4,77E-01	7,09E-01	No	-7,54E-01	7,04E-02	1,48E-01	No
ENSG00000201270	RNU6-755P	snRNA	-2,08E-02	8,19E-01	NA	No	4,41E-02	7,98E-01	NA	No
ENSG00000206880	RNU6-1310P	snRNA	4,38E-02	7,56E-01	NA	No	7,04E-02	8,03E-01	8,78E-01	No
ENSG00000201126	RNU6-773P	snRNA	-1,20E-01	4,31E-01	NA	No	-3,98E-01	2,13E-01	3,50E-01	No
ENSG00000206684	RNU6-157P	snRNA	6,15E-01	1,29E-02	NA	No	1,41E+00	2,14E-02	5,48E-02	No
ENSG00000252552	RNU6-307P	snRNA	-5,37E-02	6,46E-01	NA	No	4,00E-01	1,90E-01	NA	No
ENSG00000272292	U6	snRNA	-8,14E-02	7,07E-01	8,62E-01	No	-1,46E+00	7,08E-03	2,11E-02	No
ENSG00000252222	RNU7-13P	snRNA	-1,04E-01	6,35E-01	8,19E-01	No	-2,14E-01	5,30E-01	6,74E-01	No
ENSG00000252860	RNU6-570P	snRNA	-1,38E-01	2,21E-01	NA	No	-1,35E-01	6,41E-01	7,63E-01	No
ENSG00000200942	RNU6-501P	snRNA	-3,21E-02	7,31E-01	NA	No	-6,98E-03	9,79E-01	NA	No
ENSG00000272262	U6	snRNA	-1,54E-01	2,09E-01	NA	No	-1,20E-01	7,02E-01	8,09E-01	No
ENSG00000206762	RNU6-418P	snRNA	1,79E-01	1,15E-01	NA	No	-9,51E-02	8,30E-01	NA	No
ENSG00000200972	RNU5A-8P	snRNA	-1,18E-02	8,49E-01	NA	No	1,70E-02	9,87E-01	NA	No
ENSG00000207491	RNU6-423P	snRNA	-4,69E-02	6,82E-01	NA	No	-2,14E-01	3,25E-01	NA	No
ENSG00000212157	RNU6-1319P	snRNA	3,31E-02	9,36E-01	NA	No	8,77E-02	8,64E-01	NA	No
ENSG00000206887	RNU6-1008P	snRNA	-3,53E-02	7,46E-01	NA	No	-2,14E-01	3,88E-01	NA	No
ENSG00000199672	RNU4-21P	snRNA	2,92E-01	9,41E-02	2,68E-01	No	2,06E-01	4,02E-01	5,59E-01	No

ENSG00000222986	RNU5A-5P	snRNA	7,50E-02	4,67E-01	NA	No	-7,00E-02	6,97E-01	NA	No
ENSG00000206835	RNU1-74P	snRNA	-2,77E-02	7,31E-01	NA	No	-1,28E-01	7,12E-01	NA	No
ENSG00000252056	RNU5E-2P	snRNA	6,42E-02	5,53E-01	NA	No	1,93E-01	4,34E-01	NA	No
ENSG00000222650	RNU2-70P	snRNA	-7,40E-02	6,37E-01	8,20E-01	No	1,36E-01	6,86E-01	7,97E-01	No
ENSG00000252282	RNU6-1089P	snRNA	1,93E-02	9,01E-01	9,59E-01	No	-4,00E-01	1,56E-01	2,76E-01	No
ENSG00000251754	RNU6-999P	snRNA	3,86E-02	5,94E-01	NA	No	1,12E-01	6,75E-01	NA	No
ENSG00000201170	RNU1-132P	snRNA	-1,34E-02	8,19E-01	NA	No	-2,00E-02	8,90E-01	NA	No
ENSG00000202184	RNU6-1283P	snRNA	-3,78E-02	8,17E-01	9,20E-01	No	-4,92E-01	1,26E-01	2,34E-01	No
ENSG00000200495	RNU6-1205P	snRNA	-5,17E-02	4,76E-01	NA	No	-1,60E-01	4,74E-01	NA	No
ENSG00000200034	RNU4-73P	snRNA	-1,73E-02	7,81E-01	NA	No	-1,42E-01	6,24E-01	NA	No
ENSG00000207267	RNU6-1081P	snRNA	-1,07E-01	4,55E-01	NA	No	4,54E-01	2,29E-01	3,70E-01	No
ENSG00000251718	RNU2-13P	snRNA	7,45E-02	4,05E-01	NA	No	6,67E-02	9,56E-01	NA	No
ENSG00000207170	RNU6-1215P	snRNA	4,18E-02	7,50E-01	NA	No	1,00E-01	7,75E-01	NA	No
ENSG00000238735	RNU7-113P	snRNA	1,04E-01	2,87E-01	NA	No	1,53E-01	5,21E-01	NA	No
ENSG00000222940	RNU6-370P	snRNA	-4,33E-02	7,60E-01	NA	No	-1,01E-01	8,30E-01	NA	No
ENSG00000206732	RNU6-936P	snRNA	-1,98E-01	3,70E-01	6,16E-01	No	-7,75E-01	7,98E-02	1,64E-01	No
ENSG00000199872	RNU6-942P	snRNA	8,46E-01	2,74E-02	1,15E-01	No	7,87E-02	7,73E-01	8,58E-01	No
ENSG00000200003	RNU6-986P	snRNA	4,19E-02	7,67E-01	NA	No	6,17E-02	9,56E-01	NA	No
ENSG00000201113	RNU6-647P	snRNA	7,36E-02	5,21E-01	NA	No	2,16E-01	4,17E-01	5,73E-01	No
ENSG00000252756	RNU6-577P	snRNA	-4,26E-01	1,10E-01	2,97E-01	No	-3,74E-01	3,15E-01	4,69E-01	No
ENSG00000252518	RNU6-846P	snRNA	-7,17E-01	2,66E-02	1,12E-01	No	-1,45E+00	9,05E-03	2,61E-02	No
ENSG00000199603	RNU6-951P	snRNA	4,54E-01	1,92E-02	8,94E-02	No	5,12E-02	8,18E-01	NA	No
ENSG00000207295	RNU6-851P	snRNA	-5,37E-02	6,46E-01	NA	No	-1,26E-01	7,12E-01	NA	No
ENSG00000206985	RNU6-198P	snRNA	5,93E-02	7,79E-01	8,99E-01	No	9,96E-02	7,76E-01	8,61E-01	No
ENSG00000252239	RNU6-1185P	snRNA	-2,86E-01	1,98E-01	4,29E-01	No	-1,14E-01	7,43E-01	8,37E-01	No
ENSG00000223245	RNU4-63P	snRNA	1,56E-01	2,24E-01	NA	No	-1,04E-01	6,61E-01	NA	No
ENSG00000200550	RNU6-137P	snRNA	5,18E-02	7,24E-01	8,71E-01	No	3,89E-01	2,78E-01	4,27E-01	No
ENSG00000252804	RNU6-958P	snRNA	-1,22E-01	4,56E-01	6,91E-01	No	-2,55E-01	4,62E-01	6,15E-01	No
ENSG00000200924	RNU6-1048P	snRNA	-5,00E-02	8,07E-01	9,14E-01	No	-8,40E-02	8,05E-01	8,80E-01	No
ENSG00000202227	RNU6-282P	snRNA	1,78E-01	2,95E-01	5,42E-01	No	4,25E-01	2,03E-01	3,38E-01	No
ENSG00000207456	RNU6-997P	snRNA	-4,15E-02	7,60E-01	NA	No	-9,51E-02	8,30E-01	NA	No
ENSG00000252934	RNU7-172P	snRNA	-1,65E-02	9,16E-01	9,65E-01	No	-3,07E-01	3,32E-01	4,87E-01	No
ENSG00000200086	RNU6-433P	snRNA	-2,13E-01	3,16E-01	5,65E-01	No	2,99E-02	9,33E-01	9,60E-01	No
ENSG00000239189	RNU6-634P	snRNA	2,22E-02	9,72E-01	NA	No	6,17E-02	9,56E-01	NA	No
ENSG00000200807	RNU1-32P	snRNA	-4,34E-02	5,82E-01	NA	No	-7,94E-02	7,32E-01	NA	No
ENSG00000201076	RNU4-51P	snRNA	2,02E-01	1,46E-01	3,57E-01	No	2,83E-01	2,98E-01	4,50E-01	No
ENSG00000252414	RNU6-100P	snRNA	-6,96E-02	7,38E-01	8,78E-01	No	2,66E-02	9,40E-01	9,65E-01	No
ENSG00000252892	RNU6-548P	snRNA	1,71E-01	2,90E-01	5,37E-01	No	-1,31E-02	9,59E-01	9,76E-01	No
ENSG00000199460	RNU6-1216P	snRNA	-6,68E-02	4,70E-01	NA	No	-1,62E-01	5,08E-01	NA	No
ENSG00000222536	RNU2-39P	snRNA	-1,54E-02	8,94E-01	NA	No	5,68E-01	1,49E-01	2,68E-01	No
ENSG00000252214	RNU6-542P	snRNA	2,25E-02	7,54E-01	NA	No	4,43E-02	7,90E-01	NA	No
ENSG00000200701	RNU6-674P	snRNA	-2,01E-02	8,58E-01	NA	No	1,92E-01	4,87E-01	6,37E-01	No
ENSG00000252478	RNU7-162P	snRNA	4,29E-02	7,29E-01	NA	No	1,85E-01	3,98E-01	NA	No
ENSG00000252293	RNU7-64P	snRNA	4,23E-03	9,41E-01	NA	No	-9,72E-02	8,30E-01	NA	No
ENSG00000199687	RNU1-38P	snRNA	3,15E-02	9,36E-01	NA	No	1,62E-01	4,88E-01	NA	No
ENSG00000200563	RNU6-640P	snRNA	-1,14E-01	2,92E-01	NA	No	3,65E-01	2,93E-01	4,44E-01	No
ENSG00000222724	RNU2-63P	snRNA	7,20E-01	1,79E-02	8,47E-02	No	2,83E-02	9,27E-01	9,57E-01	No
ENSG00000201806	RNU4-8P	snRNA	8,90E-03	9,59E-01	9,83E-01	No	-8,73E-02	7,88E-01	8,68E-01	No
ENSG00000238719	RNU7-96P	snRNA	3,33E-02	8,54E-01	9,37E-01	No	3,29E-01	3,67E-01	5,23E-01	No
ENSG00000202427	RNU6-744P	snRNA	-5,37E-02	6,46E-01	NA	No	-1,26E-01	7,12E-01	NA	No
ENSG00000264229	RNU4ATAC	snRNA	-4,53E-01	9,60E-02	2,71E-01	No	-1,55E-01	6,43E-01	7,64E-01	No
ENSG00000206963	RNU6-675P	snRNA	3,69E-02	7,43E-01	NA	No	3,16E-01	1,75E-01	NA	No
ENSG00000200250	RNU6-1147P	snRNA	-1,51E-01	4,02E-01	6,46E-01	No	-2,65E-01	4,18E-01	5,74E-01	No
ENSG00000202429	RNU4-48P	snRNA	3,18E-01	7,96E-02	2,39E-01	No	8,52E-01	7,06E-02	1,48E-01	No
ENSG00000252688	RNU6-579P	snRNA	-1,92E-01	1,42E-01	3,50E-01	No	-1,21E-01	6,91E-01	8,01E-01	No
ENSG00000201308	RNU6-512P	snRNA	5,51E-02	5,35E-01	NA	No	3,00E-02	8,88E-01	NA	No
ENSG00000212389	RNU6-1275P	snRNA	4,07E-02	8,26E-01	9,24E-01	No	5,79E-01	1,46E-01	2,63E-01	No
ENSG00000207270	RNU6-601P	snRNA	-3,43E-02	7,09E-01	NA	No	7,84E-02	7,74E-01	8,59E-01	No
ENSG00000251980	RNU6-436P	snRNA	5,30E-03	9,41E-01	NA	No	-9,51E-02	8,30E-01	NA	No
ENSG00000202029	RNU6-580P	snRNA	-9,06E-02	6,29E-01	8,16E-01	No	-8,74E-01	5,86E-02	1,28E-01	No
ENSG00000199348	RNU6-766P	snRNA	-4,99E-02	8,02E-01	9,12E-01	No	-3,46E-01	3,21E-01	4,76E-01	No
ENSG00000252779	RNU6-182P	snRNA	3,85E-02	8,16E-01	9,19E-01	No	-1,98E-01	5,11E-01	6,57E-01	No

ENSG00000199827	RNU6-1290P	snRNA	3,41E-02	9,36E-01	NA	No	9,93E-02	7,75E-01	NA	No
ENSG00000206788	RNU6-629P	snRNA	-5,45E-02	6,46E-01	NA	No	-1,28E-01	7,12E-01	NA	No
ENSG00000199626	RNU6-989P	snRNA	7,52E-02	3,96E-01	NA	No	2,48E-01	2,00E-01	NA	No
ENSG00000207402	RNU6-959P	snRNA	2,80E-01	7,84E-02	2,37E-01	No	2,25E-02	9,22E-01	NA	No
ENSG00000252130	RNU6-1045P	snRNA	-1,03E-01	6,01E-01	7,97E-01	No	-1,64E-01	6,37E-01	7,60E-01	No
ENSG00000201813	RNU6-915P	snRNA	3,46E-02	7,78E-01	NA	No	1,99E-01	5,18E-01	6,64E-01	No
ENSG00000201499	RNU6-312P	snRNA	1,46E-01	3,27E-01	5,76E-01	No	-3,79E-02	8,74E-01	NA	No
ENSG00000252916	RNU6-762P	snRNA	1,38E-01	3,58E-01	6,05E-01	No	3,66E-01	2,73E-01	4,22E-01	No
ENSG00000252148	RNU6-1206P	snRNA	-8,44E-02	4,15E-01	NA	No	4,99E-02	8,43E-01	NA	No
ENSG00000238829	RNU7-45P	snRNA	9,93E-02	6,56E-01	8,31E-01	No	-4,33E-01	1,95E-01	3,28E-01	No
ENSG00000222972	RNU6-651P	snRNA	6,87E-02	4,61E-01	NA	No	3,10E-01	1,89E-01	NA	No
ENSG00000212246	RNU6-360P	snRNA	1,21E-01	1,98E-01	NA	No	1,25E-01	6,64E-01	NA	No
ENSG00000207393	RNU6-136P	snRNA	-4,51E-02	6,06E-01	NA	No	1,29E-02	9,63E-01	NA	No
ENSG00000202016	RNU6-619P	snRNA	-5,37E-02	6,46E-01	NA	No	-1,26E-01	7,12E-01	NA	No
ENSG00000200281	RNU6-624P	snRNA	6,22E-02	7,61E-01	8,90E-01	No	1,32E+00	4,67E-03	1,46E-02	No
ENSG00000222344	RNU6-613P	snRNA	3,07E-01	9,62E-02	2,72E-01	No	7,42E-01	8,28E-02	1,69E-01	No
ENSG00000252333	RNU6-964P	snRNA	6,64E-02	4,85E-01	NA	No	8,55E-02	8,64E-01	NA	No
ENSG00000201044	RNU6-268P	snRNA	2,19E-01	2,07E-01	4,40E-01	No	-2,39E-01	3,54E-01	5,10E-01	No
ENSG00000223198	RNU2-22P	snRNA	1,17E-01	2,11E-01	NA	No	4,85E-01	9,43E-02	NA	No
ENSG00000252452	RNU6-107P	snRNA	2,49E-01	2,66E-01	5,10E-01	No	6,98E-02	8,41E-01	9,03E-01	No
ENSG00000238812	RNU7-127P	snRNA	-9,87E-03	9,13E-01	NA	No	-1,57E-01	5,07E-01	NA	No
ENSG00000200745	RNU1-31P	snRNA	1,23E-02	9,48E-01	9,79E-01	No	-4,68E-01	2,11E-01	3,48E-01	No
ENSG00000202341	RNU6-1051P	snRNA	3,39E-02	9,36E-01	NA	No	6,23E-02	9,56E-01	NA	No
ENSG00000251971	RNU6-1333P	snRNA	-3,69E-02	8,21E-01	9,22E-01	No	1,61E-02	9,56E-01	9,74E-01	No
ENSG00000202099	RNU6-234P	snRNA	-5,37E-02	6,46E-01	NA	No	-9,71E-03	8,90E-01	NA	No
ENSG00000252017	RNU6-1194P	snRNA	-5,69E-02	6,46E-01	NA	No	6,43E-02	6,89E-01	NA	No
ENSG00000253081	RNU4ATAC17P	snRNA	6,54E-02	5,89E-01	NA	No	6,30E-02	7,60E-01	NA	No
ENSG00000212327	RNU6-882P	snRNA	1,94E-01	6,74E-02	NA	No	1,08E-01	6,94E-01	NA	No
ENSG00000222348	RNU6-814P	snRNA	-1,27E-01	2,11E-01	NA	No	-2,63E-01	2,81E-01	NA	No
ENSG00000251777	RNU6-404P	snRNA	4,58E-02	6,50E-01	NA	No	2,35E-02	9,16E-01	NA	No
ENSG00000251774	RNU6-377P	snRNA	-2,31E-01	1,29E-01	NA	No	-2,50E-01	4,38E-01	5,92E-01	No
ENSG00000207163	RNU6-905P	snRNA	-1,55E-01	2,71E-01	5,15E-01	No	-1,02E-01	7,07E-01	8,12E-01	No
ENSG00000206926	RNU6-1024P	snRNA	1,90E-01	1,43E-01	NA	No	3,65E-01	2,19E-01	NA	No
ENSG00000271841	U7	snRNA	2,55E-01	1,41E-01	3,49E-01	No	2,22E-01	4,80E-01	6,31E-01	No
ENSG00000201545	RNU4-85P	snRNA	6,38E-03	9,41E-01	NA	No	5,89E-02	8,85E-01	NA	No
ENSG00000199577	RNU6-822P	snRNA	-4,23E-02	7,60E-01	NA	No	-2,50E-02	9,21E-01	NA	No
ENSG00000206807	RNU6-815P	snRNA	2,46E-02	9,72E-01	NA	No	9,18E-02	8,64E-01	NA	No
ENSG00000207251	RNU6-342P	snRNA	8,23E-02	5,74E-01	7,79E-01	No	-2,36E-02	9,39E-01	9,64E-01	No
ENSG00000252700	RNU7-110P	snRNA	1,68E-01	2,17E-01	NA	No	-5,82E-02	7,69E-01	NA	No
ENSG00000199594	RNU6-1301P	snRNA	1,61E-01	3,01E-01	5,49E-01	No	2,10E-01	4,97E-01	6,46E-01	No
ENSG00000239105	RNU7-73P	snRNA	-8,89E-03	9,63E-01	9,85E-01	No	-3,40E-02	9,21E-01	9,53E-01	No
ENSG00000206708	RNU6-1227P	snRNA	4,04E-02	7,81E-01	NA	No	1,00E-01	7,75E-01	NA	No
ENSG00000201570	RNU4-56P	snRNA	1,04E-01	2,98E-01	NA	No	1,18E-01	5,70E-01	NA	No
ENSG00000199906	RNU5B-2P	snRNA	1,79E-01	1,55E-01	NA	No	2,98E-01	2,25E-01	3,66E-01	No
ENSG00000252410	RNU5B-3P	snRNA	2,43E-01	1,84E-01	4,11E-01	No	-1,21E-01	6,22E-01	NA	No
ENSG00000221015	RNU6ATAC29P	snRNA	-7,58E-02	3,35E-01	NA	No	-1,76E-01	3,98E-01	NA	No
ENSG00000252768	RNU6-856P	snRNA	1,31E-01	4,79E-01	7,11E-01	No	-1,16E-01	7,28E-01	8,28E-01	No
ENSG00000221518	RNU6ATAC16P	snRNA	1,68E-01	4,26E-01	6,67E-01	No	3,96E-01	2,75E-01	4,24E-01	No
ENSG00000206918	RNU6-1181P	snRNA	2,35E-02	9,72E-01	NA	No	6,19E-02	9,56E-01	NA	No
ENSG00000202428	RNU6-108P	snRNA	7,69E-03	9,94E-01	NA	No	1,92E-02	9,42E-01	NA	No
ENSG00000206815	RNU6-483P	snRNA	1,51E-02	8,76E-01	NA	No	-1,30E-01	7,12E-01	NA	No
ENSG00000210841	RNU6ATAC26P	snRNA	5,65E-02	4,78E-01	NA	No	2,54E-02	9,87E-01	NA	No
ENSG00000252184	RNU2-10P	snRNA	-5,59E-02	7,81E-01	9,01E-01	No	-5,76E-01	1,54E-01	2,74E-01	No
ENSG00000252449	RNU6-139P	snRNA	-3,95E-02	6,27E-01	NA	No	1,79E-01	5,21E-01	NA	No
ENSG00000206759	RNU6-787P	snRNA	-2,89E-02	8,25E-01	9,24E-01	No	-1,02E-01	7,25E-01	8,25E-01	No
ENSG00000238959	RNU7-19P	snRNA	-1,17E-02	9,26E-01	9,69E-01	No	7,55E-02	7,94E-01	8,73E-01	No
ENSG00000223247	RNU2-64P	snRNA	4,28E-02	7,31E-01	NA	No	8,77E-02	8,64E-01	NA	No
ENSG00000239119	RNU7-119P	snRNA	-1,52E-01	4,84E-01	7,14E-01	No	2,15E-01	5,18E-01	6,64E-01	No
ENSG00000221059	RNU6ATAC6P	snRNA	8,15E-02	3,48E-01	NA	No	-1,36E-01	7,12E-01	NA	No
ENSG00000253062	RNU7-108P	snRNA	2,97E-01	1,54E-01	3,70E-01	No	-1,35E-01	6,43E-01	7,64E-01	No
ENSG00000206712	RNU6-26P	snRNA	-3,74E-01	1,33E-01	3,35E-01	No	-6,16E-01	1,34E-01	2,46E-01	No
ENSG00000207331	RNU6-1263P	snRNA	-5,98E-02	5,58E-01	NA	No	1,17E-02	9,81E-01	NA	No

ENSG00000201642	RNU6-865P	snRNA	-4,61E-02	6,21E-01	NA	No	1,53E-01	5,95E-01	7,26E-01	No
ENSG00000252348	RNU6-1256P	snRNA	-4,48E-02	5,48E-01	NA	No	-1,60E-01	4,74E-01	NA	No
ENSG00000252626	RNU6-1308P	snRNA	8,30E-03	9,46E-01	NA	No	-5,42E-02	7,78E-01	NA	No
ENSG00000206889	RNU6-1200P	snRNA	-5,45E-02	6,46E-01	NA	No	-5,78E-02	8,00E-01	NA	No
ENSG00000207310	RNU6-1127P	snRNA	7,48E-02	6,93E-01	8,53E-01	No	-1,88E-01	5,87E-01	7,19E-01	No
ENSG00000252751	RNU6-143P	snRNA	-7,22E-02	4,08E-01	NA	No	8,73E-02	7,34E-01	8,32E-01	No
ENSG00000272020	U1	snRNA	-1,15E-01	5,20E-01	7,43E-01	No	-1,52E-01	6,40E-01	7,62E-01	No
ENSG00000222627	RNU2-37P	snRNA	9,74E-02	4,78E-01	NA	No	-1,84E-01	4,31E-01	NA	No
ENSG00000252786	RNU6-1142P	snRNA	-4,42E-03	9,82E-01	9,93E-01	No	-1,21E-01	7,26E-01	8,26E-01	No
ENSG00000201550	RNU6-726P	snRNA	2,84E-02	6,74E-01	NA	No	6,56E-02	7,66E-01	NA	No
ENSG00000252641	RNU6-678P	snRNA	3,40E-01	5,08E-02	NA	No	5,17E-02	7,92E-01	NA	No
ENSG00000200571	RNU6-1284P	snRNA	2,17E-02	9,07E-01	9,61E-01	No	1,66E-03	9,97E-01	9,99E-01	No
ENSG00000251708	RNU7-198P	snRNA	-3,00E-02	7,61E-01	NA	No	-1,58E-01	5,03E-01	NA	No
ENSG00000252914	RNU6-789P	snRNA	-3,28E-02	7,76E-01	NA	No	-5,34E-02	8,23E-01	NA	No
ENSG00000252245	RNU6-736P	snRNA	-1,87E-01	3,30E-01	5,79E-01	No	-6,89E-01	1,01E-01	1,97E-01	No
ENSG00000200389	RNU6-509P	snRNA	-9,87E-02	5,21E-01	7,43E-01	No	-2,11E-01	5,40E-01	6,83E-01	No
ENSG00000206604	RNU6-425P	snRNA	2,05E-01	1,92E-01	4,22E-01	No	1,82E-01	5,28E-01	6,72E-01	No
ENSG00000251804	RNU6-1294P	snRNA	-4,97E-02	7,75E-01	8,97E-01	No	-5,17E-01	1,79E-01	3,08E-01	No
ENSG00000202125	RNU1-100P	snRNA	-6,42E-02	7,37E-01	8,77E-01	No	-6,62E-02	8,38E-01	9,01E-01	No
ENSG00000251787	RNU7-47P	snRNA	1,04E-01	4,44E-01	6,81E-01	No	1,54E-01	6,15E-01	7,43E-01	No
ENSG00000252581	RNU6-1098P	snRNA	3,92E-02	8,53E-01	9,36E-01	No	-4,05E-01	2,83E-01	4,33E-01	No
ENSG00000252172	RNU6-720P	snRNA	5,62E-01	4,92E-02	1,72E-01	No	1,25E+00	8,11E-03	2,37E-02	No
ENSG00000207323	RNU6-901P	snRNA	-1,59E-03	9,85E-01	NA	No	3,47E-01	1,73E-01	3,00E-01	No
ENSG00000252763	RNU2-31P	snRNA	-7,93E-01	2,64E-02	1,12E-01	No	-1,43E+00	1,32E-02	3,62E-02	No
ENSG00000201342	RNU4-38P	snRNA	4,43E-02	6,83E-01	NA	No	1,18E-01	5,68E-01	NA	No
ENSG00000199536	RNU6-315P	snRNA	-1,48E-01	4,95E-01	7,23E-01	No	-5,66E-01	1,59E-01	2,80E-01	No
ENSG00000252082	RNU6-547P	snRNA	3,89E-02	6,32E-01	NA	No	9,73E-02	6,55E-01	NA	No
ENSG00000201458	RNU4-4P	snRNA	-1,18E-01	2,37E-01	NA	No	-2,04E-01	3,82E-01	NA	No
ENSG00000199792	RNU6-1233P	snRNA	7,89E-03	9,54E-01	NA	No	1,58E-02	9,50E-01	9,71E-01	No
ENSG00000201648	RNU4-91P	snRNA	3,31E-02	9,36E-01	NA	No	3,21E-01	1,76E-01	NA	No
ENSG00000200882	RNU6-681P	snRNA	2,87E-01	1,95E-01	4,26E-01	No	1,20E+00	8,02E-03	2,35E-02	No
ENSG00000199986	RNU6-486P	snRNA	-3,04E-02	7,31E-01	NA	No	-1,30E-01	7,12E-01	NA	No
ENSG00000206932	RNU6-4P	snRNA	1,42E-01	1,99E-01	NA	No	1,85E-01	3,87E-01	NA	No
ENSG00000206600	RNU6-25P	snRNA	3,50E-02	9,36E-01	NA	No	6,34E-02	9,56E-01	NA	No
ENSG00000252620	RNU6ATAC24P	snRNA	3,37E-01	1,70E-01	3,92E-01	No	4,15E-01	2,62E-01	4,09E-01	No
ENSG00000239122	RNU2-11P	snRNA	-4,33E-02	6,38E-01	NA	No	-5,36E-02	7,59E-01	NA	No
ENSG00000252174	RNU7-18P	snRNA	-1,75E-01	2,12E-01	NA	No	-4,59E-01	1,85E-01	3,15E-01	No
ENSG00000206644	RNU6-1279P	snRNA	-5,47E-02	6,46E-01	NA	No	2,61E-01	2,69E-01	NA	No
ENSG00000201441	RNU6-646P	snRNA	7,85E-02	4,91E-01	NA	No	-5,41E-02	7,75E-01	NA	No
ENSG00000272359	U4	snRNA	-1,90E-01	2,96E-01	5,44E-01	No	-1,37E-01	6,88E-01	7,99E-01	No
ENSG00000206892	RNU6-42P	snRNA	-1,99E-01	1,89E-01	4,17E-01	No	-1,29E-01	6,81E-01	7,94E-01	No
ENSG00000199306	RNU6-858P	snRNA	2,46E-02	9,72E-01	NA	No	2,95E-01	2,17E-01	NA	No
ENSG00000201622	RNU6-621P	snRNA	-1,93E-02	8,49E-01	NA	No	1,44E-01	4,94E-01	NA	No
ENSG00000199335	RNU6-204P	snRNA	-1,41E-02	8,97E-01	NA	No	-1,77E-02	9,80E-01	NA	No
ENSG00000252235	RNU6-962P	snRNA	-1,31E-01	1,15E-01	NA	No	-2,69E-01	1,71E-01	NA	No
ENSG00000251758	RNU6-350P	snRNA	5,40E-02	6,25E-01	NA	No	1,21E-01	5,25E-01	NA	No
ENSG00000206675	RNU6-32P	snRNA	2,39E-01	4,25E-02	1,56E-01	No	3,41E-01	1,69E-01	2,94E-01	No
ENSG00000252796	RNU7-11P	snRNA	1,55E-01	2,48E-01	NA	No	2,92E-01	3,42E-01	NA	No
ENSG00000200455	RNU6-1112P	snRNA	-7,53E-03	9,01E-01	NA	No	1,29E-01	6,20E-01	NA	No
ENSG00000199790	RNU6-836P	snRNA	-6,61E-02	6,46E-01	NA	No	-2,05E-01	4,16E-01	NA	No
ENSG00000207198	RNU6-1195P	snRNA	1,00E-02	9,33E-01	NA	No	1,12E+00	3,72E-02	8,75E-02	No
ENSG00000200269	RNU6-838P	snRNA	-2,06E-02	8,19E-01	NA	No	1,49E-01	5,17E-01	NA	No
ENSG00000253050	RNU6-868P	snRNA	1,53E-01	1,33E-01	NA	No	8,66E-02	7,59E-01	NA	No
ENSG00000251722	RNU5E-3P	snRNA	5,31E-03	9,72E-01	9,89E-01	No	-1,92E-02	9,47E-01	9,69E-01	No
ENSG00000199924	RNU6-1252P	snRNA	3,36E-02	8,38E-01	9,30E-01	No	4,36E-01	2,41E-01	3,84E-01	No
ENSG00000207385	RNU6-310P	snRNA	3,06E-02	8,11E-01	NA	No	1,51E-01	5,81E-01	7,14E-01	No
ENSG00000202358	RNU6-652P	snRNA	2,67E-01	2,20E-01	4,58E-01	No	6,58E-01	1,08E-01	2,07E-01	No
ENSG00000251703	RNU6-998P	snRNA	6,87E-03	9,72E-01	9,89E-01	No	-7,25E-01	9,25E-02	1,84E-01	No
ENSG00000252104	RNU6-191P	snRNA	-3,78E-01	1,42E-01	3,51E-01	No	-5,17E-01	1,47E-01	2,65E-01	No
ENSG00000206629	RNU1-63P	snRNA	-9,03E-04	9,94E-01	9,98E-01	No	-3,06E-01	3,33E-01	4,88E-01	No
ENSG00000207428	RNU6-95P	snRNA	4,32E-02	7,25E-01	NA	No	1,35E-01	6,03E-01	NA	No
ENSG00000207448	RNU6-520P	snRNA	-1,43E-02	8,96E-01	NA	No	-6,72E-02	8,00E-01	NA	No

ENSG00000207058	RNU6-784P	snRNA	3,17E-02	9,36E-01	NA	No	1,07E-01	7,00E-01	NA	No
ENSG00000207007	RNU6-891P	snRNA	3,68E-02	8,45E-01	NA	No	8,51E-02	8,64E-01	NA	No
ENSG00000222644	RNU2-16P	snRNA	-4,21E-02	7,60E-01	NA	No	-9,72E-02	8,30E-01	NA	No
ENSG00000252062	RNU6-469P	snRNA	3,08E-02	7,84E-01	NA	No	1,70E-01	4,70E-01	6,22E-01	No
ENSG00000221440	RNU6ATAC31P	snRNA	3,57E-03	9,30E-01	NA	No	-7,72E-02	8,00E-01	NA	No
ENSG00000207524	RNU6-33P	snRNA	1,99E-01	2,58E-01	5,01E-01	No	4,75E-01	2,08E-01	3,44E-01	No
ENSG00000200622	RNU6-1059P	snRNA	-3,01E-02	7,31E-01	NA	No	4,88E-02	8,22E-01	NA	No
ENSG00000252548	RNU7-149P	snRNA	-3,46E-02	6,27E-01	NA	No	-1,06E-01	6,14E-01	NA	No
ENSG00000252739	RNU7-151P	snRNA	-9,93E-02	4,82E-01	NA	No	5,96E-02	8,54E-01	9,12E-01	No
ENSG00000200917	RNU6-553P	snRNA	-1,38E-01	5,19E-01	7,42E-01	No	-3,58E-01	3,21E-01	4,76E-01	No
ENSG00000222691	RNU6-733P	snRNA	-2,43E-01	9,46E-02	2,69E-01	No	-2,39E-01	3,94E-01	5,51E-01	No
ENSG00000206601	RNU6-431P	snRNA	1,07E-01	3,62E-01	NA	No	2,96E-01	2,88E-01	4,38E-01	No
ENSG00000207260	RNU6-35P	snRNA	-1,07E-01	4,47E-01	NA	No	-4,04E-01	2,12E-01	NA	No
ENSG00000206820	RNU1-138P	snRNA	-3,06E-01	1,36E-01	3,42E-01	No	-8,57E-01	6,38E-02	1,37E-01	No
ENSG00000223225	RNU6-1054P	snRNA	8,04E-03	9,41E-01	NA	No	-1,09E-01	6,37E-01	NA	No
ENSG00000201186	RNU4-33P	snRNA	-6,38E-02	4,76E-01	NA	No	7,16E-02	7,81E-01	8,64E-01	No
ENSG00000212359	RNU6-550P	snRNA	-1,28E-01	2,35E-01	NA	No	-2,82E-01	3,41E-01	4,96E-01	No
ENSG00000251821	RNU6-583P	snRNA	-3,47E-02	6,74E-01	NA	No	-9,54E-02	6,72E-01	NA	No
ENSG00000252503	RNU6-531P	snRNA	4,86E-01	6,17E-02	2,01E-01	No	-1,04E-01	7,56E-01	8,46E-01	No
ENSG00000200520	RNU6-1214P	snRNA	3,28E-02	6,96E-01	NA	No	-1,56E-02	9,82E-01	NA	No
ENSG00000239168	RNU7-197P	snRNA	4,63E-02	6,63E-01	NA	No	-2,89E-02	8,90E-01	NA	No
ENSG00000238721	RNU7-194P	snRNA	-1,43E-02	8,84E-01	NA	No	-2,95E-02	9,04E-01	NA	No
ENSG00000252544	RNU6-1282P	snRNA	-7,04E-02	6,49E-01	8,27E-01	No	-1,29E-01	6,86E-01	7,98E-01	No
ENSG00000252030	RNU6-1196P	snRNA	-1,31E-01	1,24E-01	NA	No	-2,59E-01	2,24E-01	NA	No
ENSG00000200350	RNU6-1285P	snRNA	-6,34E-02	7,08E-01	8,62E-01	No	4,54E-02	8,93E-01	9,37E-01	No
ENSG00000206703	RNU6-128P	snRNA	-4,74E-01	7,48E-02	2,29E-01	No	-9,85E-01	4,01E-02	9,30E-02	No
ENSG00000200974	RNU4-87P	snRNA	-3,28E-02	8,59E-01	9,39E-01	No	3,49E-02	9,20E-01	9,53E-01	No
ENSG00000206613	RNU6-1336P	snRNA	-1,66E-01	2,82E-01	5,29E-01	No	-5,91E-01	1,24E-01	2,31E-01	No
ENSG00000201176	RNU6-853P	snRNA	-7,48E-01	2,12E-02	9,56E-02	No	-8,03E-01	3,56E-02	8,42E-02	No
ENSG00000252898	RNU6-1096P	snRNA	8,19E-02	5,43E-01	7,58E-01	No	1,42E-01	6,32E-01	7,56E-01	No
ENSG00000252702	RNU7-158P	snRNA	-4,26E-02	7,70E-01	8,95E-01	No	5,94E-02	8,47E-01	9,07E-01	No
ENSG00000252157	RNU6-479P	snRNA	1,19E-02	9,34E-01	9,73E-01	No	-2,37E-01	4,21E-01	5,77E-01	No
ENSG00000251739	RNU6-1053P	snRNA	8,61E-02	6,24E-01	8,13E-01	No	-4,76E-01	1,14E-01	2,17E-01	No
ENSG00000202215	RNU1-51P	snRNA	6,42E-03	9,50E-01	NA	No	-1,68E-01	4,57E-01	NA	No
ENSG00000207312	RNU6-429P	snRNA	-8,71E-02	3,85E-01	NA	No	-1,72E-01	4,87E-01	NA	No
ENSG00000252908	RNU6-1003P	snRNA	9,10E-03	9,66E-01	NA	No	1,02E-01	6,33E-01	NA	No
ENSG00000252867	RNU6-909P	snRNA	-3,68E-02	6,31E-01	NA	No	-5,11E-02	7,81E-01	NA	No
ENSG00000252373	RNU6-358P	snRNA	-2,48E-02	8,24E-01	NA	No	9,99E-01	4,51E-02	1,03E-01	No
ENSG00000251887	RNU6-760P	snRNA	-1,95E-02	9,10E-01	9,63E-01	No	-1,40E+00	2,05E-02	5,29E-02	No
ENSG00000199731	RNU6-1079P	snRNA	-2,72E-03	9,86E-01	9,95E-01	No	1,25E-01	7,09E-01	8,14E-01	No
ENSG00000207052	RNU6-378P	snRNA	2,05E-01	2,81E-01	5,28E-01	No	2,39E-01	4,48E-01	6,01E-01	No
ENSG00000201623	RNU6-923P	snRNA	-6,63E-02	4,07E-01	NA	No	-1,61E-01	4,63E-01	NA	No
ENSG00000201223	RNU6-1305P	snRNA	-1,23E-01	3,59E-01	NA	No	-1,64E-01	5,48E-01	6,88E-01	No
ENSG00000251880	RNU7-75P	snRNA	9,68E-02	6,13E-01	8,05E-01	No	-2,93E-01	3,97E-01	5,54E-01	No
ENSG00000222743	RNU6-1190P	snRNA	2,03E-01	3,17E-01	5,66E-01	No	7,33E-01	8,83E-02	1,77E-01	No
ENSG00000206743	RNU6-484P	snRNA	3,27E-02	7,97E-01	NA	No	1,30E-01	6,63E-01	7,80E-01	No
ENSG00000253098	RNU7-161P	snRNA	-6,86E-02	6,26E-01	8,14E-01	No	1,22E-01	7,02E-01	8,09E-01	No
ENSG00000222960	RNU6-272P	snRNA	-1,93E-01	3,76E-01	6,22E-01	No	7,01E-01	5,97E-02	1,30E-01	No
ENSG00000253032	RNU6-299P	snRNA	3,50E-02	9,36E-01	NA	No	6,23E-02	9,56E-01	NA	No
ENSG00000202017	RNU6-806P	snRNA	4,66E-02	6,04E-01	NA	No	9,02E-02	8,64E-01	NA	No
ENSG00000199279	RNU6-661P	snRNA	5,43E-03	9,88E-01	NA	No	-1,27E-01	7,12E-01	NA	No
ENSG00000207352	RNU6-540P	snRNA	-2,12E-01	2,42E-01	4,82E-01	No	-8,40E-02	7,99E-01	8,76E-01	No
ENSG00000212260	RNU6-724P	snRNA	-4,21E-02	7,60E-01	NA	No	-9,72E-02	8,30E-01	NA	No
ENSG00000238913	RNU7-196P	snRNA	-1,02E-02	8,98E-01	NA	No	2,38E-04	9,79E-01	NA	No
ENSG00000207336	RNU6-658P	snRNA	-1,62E-01	3,94E-01	6,40E-01	No	4,99E-02	8,86E-01	9,33E-01	No
ENSG00000251724	RNU7-175P	snRNA	3,20E-02	7,49E-01	NA	No	1,44E-01	5,94E-01	7,26E-01	No
ENSG00000207333	RNU6-680P	snRNA	-7,79E-03	9,68E-01	NA	No	3,39E-01	2,12E-01	3,50E-01	No
ENSG00000252684	RNU6ATAC36P	snRNA	2,67E-03	9,91E-01	NA	No	-1,30E-01	7,12E-01	NA	No
ENSG00000206744	RNU6-620P	snRNA	3,17E-02	9,36E-01	NA	No	1,13E-01	6,88E-01	NA	No
ENSG00000207452	RNU6-606P	snRNA	-9,17E-04	9,95E-01	9,98E-01	No	-8,28E-01	5,01E-02	1,12E-01	No
ENSG00000200462	RNU6-727P	snRNA	-1,62E-02	8,11E-01	NA	No	-2,34E-02	8,90E-01	NA	No
ENSG00000199643	RNU4-90P	snRNA	-1,14E-02	8,19E-01	NA	No	-1,30E-01	7,12E-01	NA	No

ENSG00000212259	RNU6-308P	snRNA	-6,27E-02	7,51E-01	8,84E-01	No	5,43E-01	1,57E-01	2,78E-01	No
ENSG00000206997	RNU6-524P	snRNA	3,43E-01	1,27E-01	3,26E-01	No	9,78E-02	7,39E-01	8,35E-01	No
ENSG00000206698	RNU1-73P	snRNA	8,46E-03	9,23E-01	NA	No	4,24E-01	1,50E-01	2,69E-01	No
ENSG00000222610	RNU6-402P	snRNA	8,30E-02	6,68E-01	8,38E-01	No	7,44E-01	7,84E-02	1,61E-01	No
ENSG00000206593	RNU6-47P	snRNA	6,31E-02	5,11E-01	NA	No	5,40E-01	7,81E-02	1,61E-01	No
ENSG00000212370	RNU6-482P	snRNA	1,67E-01	2,68E-01	5,12E-01	No	1,36E+00	1,57E-02	4,20E-02	No
ENSG00000212457	RNU6-644P	snRNA	-7,02E-02	7,39E-01	8,78E-01	No	-1,53E-01	6,57E-01	7,76E-01	No
ENSG00000238904	RNU7-34P	snRNA	-4,15E-02	7,60E-01	NA	No	9,21E-02	6,92E-01	NA	No
ENSG00000206786	RNU6-701P	snRNA	2,21E-01	1,68E-01	3,89E-01	No	1,12E+00	3,23E-02	7,76E-02	No
ENSG00000222609	RNU4-69P	snRNA	-1,63E-01	1,87E-01	NA	No	6,75E-02	8,23E-01	8,92E-01	No
ENSG00000221562	RNU6ATAC10P	snRNA	-9,60E-02	5,95E-01	7,94E-01	No	3,62E-01	3,24E-01	4,79E-01	No
ENSG00000207222	RNU6-456P	snRNA	-5,69E-02	6,46E-01	NA	No	-1,35E-01	7,12E-01	NA	No
ENSG00000201298	RNU6-1164P	snRNA	1,09E-02	9,23E-01	NA	No	-1,49E-02	9,42E-01	NA	No
ENSG00000222266	RNU6-757P	snRNA	-1,71E-01	3,44E-01	5,92E-01	No	-8,91E-01	5,44E-02	1,20E-01	No
ENSG00000212460	RNU6-460P	snRNA	4,38E-02	7,02E-01	NA	No	8,59E-02	8,64E-01	NA	No
ENSG00000200378	RNU5B-4P	snRNA	-5,04E-02	8,01E-01	9,12E-01	No	-4,16E-01	2,62E-01	4,10E-01	No
ENSG00000253023	RNU7-156P	snRNA	2,46E-02	9,72E-01	NA	No	1,17E-01	5,78E-01	NA	No
ENSG00000238374	RNU7-180P	snRNA	6,00E-02	6,78E-01	NA	No	2,01E-01	4,90E-01	6,40E-01	No
ENSG00000251842	RNU6-732P	snRNA	3,46E-02	9,36E-01	NA	No	8,77E-02	8,64E-01	NA	No
ENSG00000199468	RNU6-556P	snRNA	4,51E-02	6,57E-01	NA	No	6,54E-02	9,56E-01	NA	No
ENSG00000252068	RNU6-390P	snRNA	-1,20E-02	8,49E-01	NA	No	-9,51E-02	8,30E-01	NA	No
ENSG00000206819	RNU6-260P	snRNA	2,61E-03	9,85E-01	9,94E-01	No	1,10E+00	2,73E-02	6,73E-02	No
ENSG00000200648	RNU6-226P	snRNA	-1,55E-01	3,90E-01	6,36E-01	No	-1,08E-01	7,54E-01	8,45E-01	No
ENSG00000206686	RNU6-1036P	snRNA	2,40E-02	9,72E-01	NA	No	9,02E-02	8,64E-01	NA	No
ENSG00000222533	RNU6-705P	snRNA	-3,54E-02	6,18E-01	NA	No	-1,56E-01	5,23E-01	NA	No
ENSG00000206702	RNU1-11P	snRNA	6,07E-02	7,53E-01	8,85E-01	No	-6,91E-02	8,41E-01	9,03E-01	No
ENSG00000252966	RNU7-91P	snRNA	-5,91E-02	7,63E-01	8,91E-01	No	2,81E-03	9,95E-01	9,97E-01	No
ENSG00000238987	RNU7-133P	snRNA	-5,45E-02	6,46E-01	NA	No	1,46E-03	9,80E-01	NA	No
ENSG00000201367	RNU6-522P	snRNA	1,18E-01	5,46E-01	7,61E-01	No	9,76E-01	3,04E-02	7,37E-02	No
ENSG00000201519	RNU6-645P	snRNA	1,79E-02	8,48E-01	9,34E-01	No	-1,06E-01	5,62E-01	NA	No
ENSG00000206881	RNU6-190P	snRNA	2,30E-01	1,46E-01	3,56E-01	No	8,56E-02	7,65E-01	8,53E-01	No
ENSG00000200681	RNU6-263P	snRNA	-5,37E-02	6,46E-01	NA	No	-5,09E-02	8,00E-01	NA	No
ENSG00000222431	RNU6-141P	snRNA	-1,16E-01	4,98E-01	7,26E-01	No	-2,30E-01	4,80E-01	6,31E-01	No
ENSG00000252046	RNU6-150P	snRNA	3,05E-01	3,95E-02	NA	No	2,00E-01	3,29E-01	NA	No
ENSG00000207490	RNU6-987P	snRNA	2,06E-01	1,89E-01	4,18E-01	No	2,80E-01	3,85E-01	5,43E-01	No
ENSG00000199289	RNU6-502P	snRNA	3,90E-02	8,45E-01	NA	No	8,51E-02	8,64E-01	NA	No
ENSG00000222800	RNU2-62P	snRNA	3,46E-02	9,36E-01	NA	No	9,18E-02	8,64E-01	NA	No
ENSG00000212240	RNU6-930P	snRNA	1,63E-01	3,87E-01	6,32E-01	No	2,83E-01	3,93E-01	5,49E-01	No
ENSG00000252743	RNU6-850P	snRNA	8,57E-02	4,62E-01	NA	No	-1,40E-01	5,50E-01	NA	No
ENSG00000223335	RNU6-603P	snRNA	-1,02E-02	8,19E-01	NA	No	3,09E-01	2,10E-01	3,46E-01	No
ENSG00000200597	RNU1-87P	snRNA	-1,53E-02	9,36E-01	9,74E-01	No	2,10E-01	5,46E-01	6,87E-01	No
ENSG00000206848	RNU6-890P	snRNA	-2,12E-02	9,15E-01	9,64E-01	No	-1,77E-01	6,14E-01	7,42E-01	No
ENSG00000200340	RNU1-105P	snRNA	-4,33E-01	2,88E-03	2,28E-02	No	-1,34E+00	1,79E-02	4,71E-02	No
ENSG00000206908	RNU1-136P	snRNA	3,17E-02	9,36E-01	NA	No	1,89E-01	4,93E-01	NA	No
ENSG00000238386	RNU7-48P	snRNA	3,40E-01	1,28E-01	3,28E-01	No	1,20E+00	2,51E-02	6,26E-02	No
ENSG00000207180	RNU6-411P	snRNA	3,46E-02	9,36E-01	NA	No	8,77E-02	8,64E-01	NA	No
ENSG00000207023	RNU6-975P	snRNA	-4,93E-02	6,62E-01	NA	No	-5,27E-02	8,18E-01	NA	No
ENSG00000201291	RNU1-34P	snRNA	-6,42E-01	4,31E-02	1,57E-01	No	1,41E-01	6,86E-01	7,97E-01	No
ENSG00000252498	RNU6-1016P	snRNA	7,90E-02	7,23E-01	8,71E-01	No	-1,77E-01	5,63E-01	7,00E-01	No
ENSG00000252156	RNU6-155P	snRNA	9,65E-02	3,35E-01	NA	No	1,44E-01	5,46E-01	NA	No
ENSG00000223044	RNU6-130P	snRNA	7,81E-02	7,19E-01	8,69E-01	No	-5,93E-01	1,31E-01	2,41E-01	No
ENSG00000222686	RNU4-72P	snRNA	2,43E-02	7,98E-01	NA	No	-1,26E-01	7,12E-01	NA	No
ENSG00000206715	RNU6-444P	snRNA	1,02E-02	9,45E-01	9,78E-01	No	-1,45E-01	6,48E-01	7,69E-01	No
ENSG00000252944	RNU6-897P	snRNA	-3,14E-02	8,51E-01	9,35E-01	No	5,17E-02	8,79E-01	9,28E-01	No
ENSG00000252444	RNU6-344P	snRNA	1,33E-03	9,94E-01	9,97E-01	No	1,81E-01	5,85E-01	7,18E-01	No
ENSG00000200295	RNU6-527P	snRNA	7,61E-02	4,16E-01	NA	No	9,27E-01	3,79E-02	8,87E-02	No
ENSG00000238420	RNU6-437P	snRNA	4,31E-02	6,71E-01	NA	No	-8,44E-02	6,58E-01	NA	No
ENSG00000200799	RNU6-770P	snRNA	-1,31E-02	8,82E-01	NA	No	4,01E-01	2,21E-01	3,61E-01	No
ENSG00000199944	RNU6-653P	snRNA	2,55E-02	8,96E-01	9,57E-01	No	5,07E-01	1,86E-01	3,16E-01	No
ENSG00000200522	RNU6-957P	snRNA	3,05E-02	8,01E-01	NA	No	7,09E-02	7,56E-01	NA	No
ENSG00000199360	RNU6-1115P	snRNA	-1,89E-03	8,99E-01	NA	No	-1,26E-01	7,12E-01	NA	No
ENSG00000207431	RNU6-906P	snRNA	-9,71E-03	9,04E-01	NA	No	9,49E-02	6,92E-01	NA	No

ENSG00000200926	RNU6-960P	snRNA	-1,35E-01	3,31E-01	NA	No	-9,13E-02	7,81E-01	8,64E-01	No
ENSG00000207044	RNU6-1226P	snRNA	-2,98E-01	1,76E-01	4,00E-01	No	-6,53E-01	1,18E-01	2,24E-01	No
ENSG00000200732	RNU6-194P	snRNA	1,11E+00	1,15E-02	6,23E-02	No	5,10E-01	1,92E-01	3,24E-01	No
ENSG00000199458	RNU4-35P	snRNA	2,79E-02	8,12E-01	NA	No	4,89E-02	8,81E-01	NA	No
ENSG00000222659	RNU2-8P	snRNA	3,50E-02	9,36E-01	NA	No	8,55E-02	8,64E-01	NA	No
ENSG00000252554	RNU6-861P	snRNA	2,98E-01	2,21E-01	4,59E-01	No	1,11E+00	2,44E-03	8,22E-03	No
ENSG00000207345	RNU6-1222P	snRNA	-1,01E-01	2,75E-01	NA	No	1,06E-01	7,00E-01	8,07E-01	No
ENSG00000252244	RNU7-3P	snRNA	1,47E-01	3,79E-01	6,25E-01	No	1,05E+00	3,57E-02	8,44E-02	No
ENSG00000202119	RNU6-302P	snRNA	5,69E-02	5,90E-01	NA	No	1,07E-01	6,98E-01	NA	No
ENSG00000252431	RNU6-1247P	snRNA	-5,66E-02	7,26E-01	8,72E-01	No	2,46E-03	9,95E-01	9,97E-01	No
ENSG00000207422	RNU6-813P	snRNA	-5,56E-02	5,54E-01	NA	No	-9,66E-02	6,64E-01	NA	No
ENSG00000200594	RNU6-824P	snRNA	-1,51E-01	4,73E-01	7,06E-01	No	-4,04E-01	2,79E-01	4,28E-01	No
ENSG00000252658	RNU6-786P	snRNA	6,29E-02	6,52E-01	8,29E-01	No	1,80E-01	5,71E-01	7,07E-01	No
ENSG00000251988	RNU4ATAC18P	snRNA	7,06E-01	3,06E-02	1,24E-01	No	-3,08E-02	9,18E-01	9,51E-01	No
ENSG00000252929	RNU6-218P	snRNA	4,04E-02	7,80E-01	NA	No	6,23E-02	9,56E-01	NA	No
ENSG00000206623	RNU6-979P	snRNA	6,33E-02	7,36E-01	8,77E-01	No	-6,10E-02	8,60E-01	9,16E-01	No
ENSG00000252821	RNU6-388P	snRNA	4,07E-02	7,73E-01	8,96E-01	No	4,94E-02	8,69E-01	9,21E-01	No
ENSG00000202431	RNU6-438P	snRNA	-1,82E-02	8,19E-01	NA	No	-1,35E-01	7,12E-01	NA	No
ENSG00000222869	RNU6-565P	snRNA	1,42E-01	2,96E-01	NA	No	1,49E-01	5,40E-01	NA	No
ENSG00000212450	RNU6-241P	snRNA	-5,37E-02	6,46E-01	NA	No	-1,26E-01	7,12E-01	NA	No
ENSG00000200815	RNU6-1091P	snRNA	2,92E-02	7,99E-01	NA	No	-1,31E-01	5,18E-01	NA	No
ENSG00000200670	RNU6-912P	snRNA	-2,90E-02	7,31E-01	NA	No	-1,30E-01	7,12E-01	NA	No
ENSG00000252126	RNU6-313P	snRNA	-4,46E-02	7,60E-01	NA	No	-3,30E-02	9,21E-01	NA	No
ENSG00000222364	RNU6-96P	snRNA	-4,72E-02	7,15E-01	NA	No	-1,91E-01	3,64E-01	NA	No
ENSG00000212568	RNU6-1254P	snRNA	-5,58E-02	6,46E-01	NA	No	-3,53E-02	8,90E-01	NA	No
ENSG00000252713	RNU6-1198P	snRNA	-7,38E-02	4,50E-01	NA	No	-1,82E-01	4,38E-01	NA	No
ENSG00000212545	RNU6-337P	snRNA	9,52E-02	4,05E-01	NA	No	-1,46E-03	9,80E-01	NA	No
ENSG00000212482	RNU6-530P	snRNA	-1,26E-01	5,00E-01	7,28E-01	No	-2,68E-01	4,58E-01	6,11E-01	No
ENSG00000206763	RNU6-10P	snRNA	-5,58E-02	6,88E-01	8,50E-01	No	-4,32E-01	1,82E-01	3,11E-01	No
ENSG00000201607	RNU4-16P	snRNA	-1,18E-02	8,49E-01	NA	No	5,47E-02	8,87E-01	NA	No
ENSG00000252643	RNU6-1136P	snRNA	-4,54E-02	7,39E-01	NA	No	-1,97E-01	4,21E-01	NA	No
ENSG00000201179	RNU6-1322P	snRNA	4,22E-01	4,78E-02	1,69E-01	No	1,01E-02	9,65E-01	NA	No
ENSG00000207090	RNU6-517P	snRNA	7,21E-02	6,65E-01	8,36E-01	No	3,31E-01	3,25E-01	4,79E-01	No
ENSG00000252003	RNU7-154P	snRNA	1,41E-01	2,98E-01	NA	No	9,00E-02	7,47E-01	NA	No
ENSG00000201104	RNU6-11P	snRNA	3,17E-02	9,36E-01	NA	No	1,07E-01	6,94E-01	NA	No
ENSG00000223189	RNU6-177P	snRNA	-6,99E-02	4,56E-01	NA	No	-2,10E-01	4,36E-01	5,90E-01	No
ENSG00000252280	RNU7-16P	snRNA	7,02E-02	4,33E-01	NA	No	1,18E-01	5,69E-01	NA	No
ENSG00000199627	RNU6-1010P	snRNA	1,26E+00	1,04E-02	5,80E-02	No	1,39E-01	6,58E-01	7,76E-01	No
ENSG00000272393	U6	snRNA	3,50E-02	9,36E-01	NA	No	6,13E-02	9,56E-01	NA	No
ENSG00000212303	RNU6-1154P	snRNA	4,03E-03	9,75E-01	NA	No	-1,39E-02	9,57E-01	NA	No
ENSG00000199700	RNU6-223P	snRNA	-7,33E-02	6,78E-01	8,44E-01	No	3,30E-01	3,60E-01	5,17E-01	No
ENSG00000199646	RNU6-1272P	snRNA	3,93E-02	8,45E-01	NA	No	9,92E-02	7,75E-01	NA	No
ENSG00000271932	U6	snRNA	-1,08E-01	3,98E-01	NA	No	1,18E-01	7,06E-01	8,11E-01	No
ENSG00000252523	RNU6-267P	snRNA	2,17E-01	4,45E-02	NA	No	1,40E-01	5,85E-01	NA	No
ENSG00000221507	RNU6ATAC40P	snRNA	1,89E-02	8,53E-01	NA	No	9,34E-02	6,27E-01	NA	No
ENSG00000206938	RNU4-31P	snRNA	-3,16E-02	8,58E-01	9,39E-01	No	8,60E-02	7,91E-01	8,70E-01	No
ENSG00000207435	RNU6-114P	snRNA	-5,73E-02	6,46E-01	NA	No	-1,36E-01	7,12E-01	NA	No
ENSG00000207202	RNU6-800P	snRNA	7,83E-02	3,90E-01	NA	No	3,58E-01	1,47E-01	NA	No
ENSG00000200566	RNU5F-7P	snRNA	-4,21E-02	7,60E-01	NA	No	-9,72E-02	8,30E-01	NA	No
ENSG00000252903	RNU6-894P	snRNA	-7,49E-02	5,31E-01	7,51E-01	No	6,69E-02	8,34E-01	8,99E-01	No
ENSG00000238606	RNU7-7P	snRNA	-3,71E-02	7,17E-01	NA	No	1,99E-01	4,79E-01	6,30E-01	No
ENSG00000212207	RNU6-1321P	snRNA	1,38E-01	2,02E-01	NA	No	5,19E-01	9,13E-02	NA	No
ENSG00000251807	RNU6-202P	snRNA	4,08E-01	5,47E-02	1,84E-01	No	1,02E+00	4,34E-02	9,96E-02	No
ENSG00000252113	RNU6-523P	snRNA	9,13E-02	6,40E-01	8,22E-01	No	-3,20E-01	3,46E-01	5,02E-01	No
ENSG00000199226	RNU6-50P	snRNA	1,54E-02	8,57E-01	NA	No	6,99E-01	8,71E-02	1,76E-01	No
ENSG00000206685	RNU6-1189P	snRNA	1,12E-01	4,67E-01	7,01E-01	No	6,00E-01	1,27E-01	2,36E-01	No
ENSG00000201659	RNU12-2P	snRNA	-4,62E-02	6,85E-01	NA	No	-2,05E-01	3,72E-01	NA	No
ENSG00000206723	RNU6-1056P	snRNA	2,49E-02	9,72E-01	NA	No	1,02E-01	7,75E-01	NA	No
ENSG00000223309	RNU6-722P	snRNA	1,15E-01	2,92E-01	NA	No	9,24E-02	6,35E-01	NA	No
ENSG00000206936	RNU4-52P	snRNA	-3,71E-02	7,31E-01	NA	No	2,59E-02	8,48E-01	NA	No
ENSG00000201341	RNU6-421P	snRNA	-5,45E-02	6,46E-01	NA	No	6,07E-02	7,68E-01	NA	No
ENSG00000222303	RNU6-935P	snRNA	-8,77E-03	9,30E-01	NA	No	-2,78E-02	9,02E-01	NA	No

ENSG00000252377	RNU6-504P	snRNA	9,58E-02	4,63E-01	6,97E-01	No	1,08E+00	3,72E-02	8,75E-02	No
ENSG00000251707	RNU7-37P	snRNA	2,26E-01	1,04E-01	NA	No	1,52E-01	5,29E-01	NA	No
ENSG00000207104	RNU6-434P	snRNA	-5,73E-02	6,46E-01	NA	No	-1,36E-01	7,12E-01	NA	No
ENSG00000252145	RNU6-1225P	snRNA	-5,58E-03	9,77E-01	NA	No	5,07E-01	1,38E-01	2,52E-01	No
ENSG00000206747	RNU6-245P	snRNA	2,04E-02	7,97E-01	NA	No	6,87E-02	6,61E-01	NA	No
ENSG00000212605	RNU1-56P	snRNA	-5,45E-02	6,46E-01	NA	No	-6,48E-02	8,00E-01	NA	No
ENSG00000201392	RNU6-1078P	snRNA	4,26E-02	7,43E-01	NA	No	1,01E-01	7,75E-01	NA	No
ENSG00000222810	RNU2-68P	snRNA	6,55E-02	5,55E-01	NA	No	2,87E-01	3,40E-01	4,95E-01	No
ENSG00000200906	RNU6-854P	snRNA	-1,14E-01	4,21E-01	6,63E-01	No	-4,66E-01	1,40E-01	2,55E-01	No
ENSG00000202024	RNU6-934P	snRNA	-4,23E-02	5,99E-01	NA	No	-4,99E-02	7,43E-01	NA	No
ENSG00000207291	RNU6-30P	snRNA	1,00E-02	9,63E-01	9,85E-01	No	9,75E-01	1,88E-02	4,90E-02	No
ENSG00000252810	RNU6-345P	snRNA	8,35E-02	5,31E-01	NA	No	1,26E-02	9,59E-01	NA	No
ENSG00000252294	RNU6-589P	snRNA	3,85E-02	7,79E-01	9,00E-01	No	1,53E-01	6,39E-01	7,62E-01	No
ENSG00000207175	RNU1-67P	snRNA	-4,18E-02	6,84E-01	NA	No	-1,44E-01	4,45E-01	NA	No
ENSG00000252627	RNU6-122P	snRNA	-2,02E-01	2,89E-01	5,36E-01	No	-1,88E-01	5,67E-01	7,03E-01	No
ENSG00000207081	RNU6-616P	snRNA	2,22E-02	9,72E-01	NA	No	6,67E-02	9,56E-01	NA	No
ENSG00000212510	RNU6-972P	snRNA	5,80E-02	5,98E-01	NA	No	-1,33E-02	8,90E-01	NA	No
ENSG00000221375	RNU6ATAC23P	snRNA	6,86E-02	6,31E-01	8,16E-01	No	-3,25E-02	8,99E-01	9,40E-01	No
ENSG00000239078	RNU7-55P	snRNA	-6,34E-02	4,94E-01	NA	No	-1,52E-01	5,59E-01	NA	No
ENSG00000207128	RNU6-729P	snRNA	-1,25E-01	3,84E-01	NA	No	-3,70E-03	9,90E-01	9,94E-01	No
ENSG00000222267	RNU6-892P	snRNA	-1,84E-02	8,80E-01	NA	No	1,98E-01	4,83E-01	6,33E-01	No
ENSG00000252067	RNU4-71P	snRNA	-6,29E-02	5,05E-01	NA	No	-1,51E-01	5,67E-01	NA	No
ENSG00000254172	RNU5A-3P	snRNA	3,45E-02	7,31E-01	NA	No	4,19E-02	8,55E-01	NA	No
ENSG00000253437	RP11-90P5.4	snRNA	6,62E-02	6,15E-01	NA	No	1,18E-01	5,72E-01	NA	No
ENSG00000253739	RP11-90P5.7	snRNA	6,72E-03	9,73E-01	9,89E-01	No	-7,89E-01	7,79E-02	1,61E-01	No
ENSG00000206852	RNU6-895P	snRNA	-1,78E-02	8,12E-01	NA	No	-4,93E-02	7,96E-01	NA	No
ENSG00000200731	RNU1-124P	snRNA	2,95E-01	1,68E-01	3,90E-01	No	2,38E-01	4,79E-01	6,30E-01	No
ENSG00000207369	RNU6-665P	snRNA	-7,26E-02	5,80E-01	NA	No	3,65E-02	8,89E-01	9,34E-01	No
ENSG00000251835	RNU6ATAC32P	snRNA	-7,06E-02	4,73E-01	NA	No	-7,67E-02	7,73E-01	NA	No
ENSG00000206975	RNU6-13P	snRNA	-5,69E-02	6,46E-01	NA	No	-1,35E-01	7,12E-01	NA	No
ENSG00000222488	RNU6-285P	snRNA	6,22E-02	4,86E-01	NA	No	6,07E-02	6,93E-01	NA	No
ENSG00000212348	RNU6-1300P	snRNA	3,26E-02	6,65E-01	NA	No	-7,59E-02	8,00E-01	NA	No
ENSG00000212485	RNU6-1197P	snRNA	5,61E-02	6,01E-01	NA	No	8,55E-02	8,64E-01	NA	No
ENSG00000212429	RNU11-6P	snRNA	2,11E-01	2,84E-01	5,31E-01	No	5,44E-02	8,73E-01	9,24E-01	No
ENSG00000206701	RNU6-1040P	snRNA	4,63E-02	6,10E-01	NA	No	5,21E-01	1,28E-01	2,37E-01	No
ENSG00000207359	RNU6-925P	snRNA	2,18E-01	1,40E-01	NA	No	4,07E-01	1,57E-01	2,78E-01	No
ENSG00000200525	RNU6-1209P	snRNA	-5,76E-02	6,85E-01	NA	No	2,49E-02	9,30E-01	9,59E-01	No
ENSG00000252910	RNU7-177P	snRNA	-6,77E-03	9,62E-01	9,85E-01	No	-6,49E-02	8,31E-01	8,97E-01	No
ENSG00000252755	RNU6-703P	snRNA	5,64E-02	4,51E-01	NA	No	2,49E-01	3,07E-01	NA	No
ENSG00000207378	RNU6-748P	snRNA	-1,29E-03	9,91E-01	9,97E-01	No	4,38E-01	2,27E-01	3,68E-01	No
ENSG00000252764	RNU6-1092P	snRNA	-4,42E-02	7,60E-01	NA	No	-1,02E-01	8,30E-01	NA	No
ENSG00000207399	RNU6-1011P	snRNA	-3,75E-02	8,32E-01	9,26E-01	No	-9,66E-02	7,79E-01	8,62E-01	No
ENSG00000251892	RNU7-84P	snRNA	5,01E-01	6,03E-02	1,98E-01	No	8,87E-01	5,66E-02	1,24E-01	No
ENSG00000207334	RNU6-12P	snRNA	-6,22E-02	5,30E-01	NA	No	-1,49E-01	5,91E-01	NA	No
ENSG00000222607	RNU6-628P	snRNA	-1,31E-01	2,81E-01	NA	No	-1,39E-01	6,16E-01	7,43E-01	No
ENSG00000252297	RNU6-875P	snRNA	2,67E-01	1,53E-01	NA	No	2,96E-01	3,43E-01	4,99E-01	No
ENSG00000206724	RNU6-756P	snRNA	-3,75E-02	7,28E-01	NA	No	-6,72E-02	7,66E-01	NA	No
ENSG00000207110	RNU1-106P	snRNA	4,01E-02	6,47E-01	NA	No	8,53E-02	6,47E-01	NA	No
ENSG00000222501	RNU4-25P	snRNA	2,86E-01	8,07E-02	2,42E-01	No	2,07E-01	4,30E-01	5,85E-01	No
ENSG00000253043	RNU7-181P	snRNA	5,85E-03	9,69E-01	9,87E-01	No	-5,59E-01	6,60E-02	NA	No
ENSG00000200304	RNU6-1255P	snRNA	6,36E-02	5,44E-01	NA	No	1,07E-01	6,19E-01	NA	No
ENSG00000252075	RNU7-109P	snRNA	-7,45E-02	5,20E-01	NA	No	-2,56E-01	2,90E-01	NA	No
ENSG00000202172	RNU6-1327P	snRNA	-7,32E-02	7,34E-01	8,76E-01	No	5,40E-01	1,26E-01	2,35E-01	No
ENSG00000239102	RNU7-185P	snRNA	-5,98E-02	4,94E-01	NA	No	-1,10E-01	6,21E-01	NA	No
ENSG00000199814	RNU6-1260P	snRNA	-3,65E-03	9,00E-01	NA	No	8,89E-03	9,80E-01	NA	No
ENSG00000251834	RNU6-319P	snRNA	-4,07E-02	8,01E-01	9,12E-01	No	-1,99E-01	5,36E-01	6,79E-01	No
ENSG00000207360	RNU6-14P	snRNA	2,35E-02	9,72E-01	NA	No	2,08E-01	4,34E-01	NA	No
ENSG00000252943	RNU6-264P	snRNA	4,30E-02	6,99E-01	NA	No	-1,37E-01	5,22E-01	NA	No
ENSG00000222202	RNU4-26P	snRNA	1,61E-01	2,62E-01	NA	No	1,30E-01	6,33E-01	NA	No
ENSG00000251748	RNU4ATAC11P	snRNA	-3,66E-02	8,61E-01	9,40E-01	No	-8,04E-01	7,01E-02	1,47E-01	No
ENSG00000252406	RNU7-36P	snRNA	9,76E-04	9,90E-01	NA	No	2,63E-02	8,91E-01	NA	No
ENSG00000251745	RNU7-124P	snRNA	-3,12E-02	8,27E-01	9,25E-01	No	2,78E-02	9,31E-01	9,59E-01	No

ENSG00000238529	RNU6-599P	snRNA	-4,15E-02	7,60E-01	NA	No	1,80E-02	9,87E-01	NA	No
ENSG00000238551	RNU6-1193P	snRNA	-4,23E-02	7,60E-01	NA	No	-9,82E-02	8,30E-01	NA	No
ENSG00000207000	RNU6-820P	snRNA	-3,20E-02	7,25E-01	NA	No	9,60E-02	6,44E-01	7,65E-01	No
ENSG00000222465	RNU2-5P	snRNA	3,00E-01	1,11E-01	2,98E-01	No	1,29E+00	1,39E-02	3,80E-02	No
ENSG00000212316	RNU6-1228P	snRNA	5,30E-03	9,41E-01	NA	No	-9,51E-02	8,30E-01	NA	No
ENSG00000201711	RNU6-1303P	snRNA	-2,32E-03	9,80E-01	NA	No	1,20E-02	9,51E-01	NA	No
ENSG00000207206	RNU6-1035P	snRNA	4,26E-02	7,41E-01	NA	No	8,51E-02	8,64E-01	NA	No
ENSG00000251752	RNU4-29P	snRNA	-1,21E-01	4,32E-01	6,72E-01	No	1,12E-01	7,33E-01	8,31E-01	No
ENSG00000199483	RNU4-15P	snRNA	3,86E-02	8,45E-01	NA	No	9,18E-02	8,64E-01	NA	No
ENSG00000222293	RNU2-36P	snRNA	4,63E-02	6,24E-01	NA	No	1,36E-01	5,32E-01	NA	No
ENSG00000252772	RNU6-714P	snRNA	2,46E-02	9,72E-01	NA	No	6,13E-02	9,56E-01	NA	No
ENSG00000252847	RNU2-46P	snRNA	1,01E-01	5,68E-01	7,74E-01	No	-6,76E-02	8,43E-01	9,05E-01	No
ENSG00000207276	RNU6-1160P	snRNA	2,80E-02	8,08E-01	NA	No	-1,47E-01	6,03E-01	NA	No
ENSG00000200106	RNU6-984P	snRNA	4,91E-02	5,06E-01	NA	No	1,13E-01	6,87E-01	NA	No
ENSG00000206658	RNU6-1039P	snRNA	1,33E-02	8,95E-01	NA	No	1,23E-01	5,90E-01	NA	No
ENSG00000206923	RNU6-432P	snRNA	-1,14E-02	9,42E-01	9,76E-01	No	4,69E-02	8,91E-01	9,35E-01	No
ENSG00000206907	RNU6-1013P	snRNA	-1,82E-02	8,19E-01	NA	No	9,34E-02	5,71E-01	NA	No
ENSG00000222327	RNU6-855P	snRNA	-8,27E-02	3,95E-01	NA	No	-1,76E-01	4,67E-01	NA	No
ENSG00000201444	RNU6-1082P	snRNA	1,18E-01	4,70E-01	7,03E-01	No	-1,08E-02	9,72E-01	9,83E-01	No
ENSG00000199313	RNU4-82P	snRNA	-1,00E-01	3,87E-01	6,33E-01	No	-4,54E-01	5,48E-02	1,21E-01	No
ENSG00000200554	RNU6-1020P	snRNA	4,94E-02	6,16E-01	NA	No	1,78E-01	4,47E-01	6,00E-01	No
ENSG00000238406	RNU7-171P	snRNA	-6,57E-02	4,24E-01	NA	No	1,78E-01	4,83E-01	6,33E-01	No
ENSG00000252622	RNU6-881P	snRNA	-6,05E-02	5,46E-01	NA	No	-4,45E-02	7,78E-01	NA	No
ENSG00000238924	RNU7-163P	snRNA	1,45E-01	4,42E-01	6,80E-01	No	3,08E-01	3,92E-01	5,49E-01	No
ENSG00000207124	RNU6-163P	snRNA	6,35E-02	5,19E-01	NA	No	9,18E-02	8,64E-01	NA	No
ENSG00000251957	RNU6-1095P	snRNA	6,80E-02	5,42E-01	NA	No	-1,72E-02	9,41E-01	NA	No
ENSG00000272507	U6	snRNA	1,80E-01	2,30E-01	4,68E-01	No	-8,72E-02	7,18E-01	NA	No
ENSG00000252118	RNU6ATAC39P	snRNA	-3,24E-02	8,15E-01	9,19E-01	No	-2,10E-01	3,64E-01	5,20E-01	No
ENSG00000272055	RNU6-6P	snRNA	-4,47E-02	5,48E-01	NA	No	-9,05E-02	6,20E-01	NA	No
ENSG00000252832	RNU6-1212P	snRNA	6,82E-02	4,79E-01	NA	No	3,62E-01	1,76E-01	3,03E-01	No
ENSG00000207347	RNU6-306P	snRNA	1,95E-02	8,96E-01	NA	No	-9,19E-03	9,73E-01	9,83E-01	No
ENSG00000206605	RNU6-946P	snRNA	-2,54E-02	8,97E-01	9,57E-01	No	-3,63E-01	3,20E-01	4,75E-01	No
ENSG00000199855	RNU6-490P	snRNA	3,59E-02	8,39E-01	9,30E-01	No	3,97E-01	2,89E-01	4,39E-01	No
ENSG00000252639	RNU2-24P	snRNA	5,34E-02	5,79E-01	NA	No	-2,19E-02	8,90E-01	NA	No
ENSG00000251839	RNU7-12P	snRNA	4,06E-02	6,59E-01	NA	No	-7,57E-02	7,31E-01	NA	No
ENSG00000200369	RNU6-666P	snRNA	3,37E-03	9,41E-01	NA	No	1,37E-01	4,98E-01	NA	No
ENSG00000207135	RNU6-452P	snRNA	-4,46E-02	7,60E-01	NA	No	1,52E-02	9,87E-01	NA	No
ENSG00000222092	RNU6-908P	snRNA	7,08E-03	9,46E-01	NA	No	-1,18E-01	4,67E-01	NA	No
ENSG00000253054	RNU7-77P	snRNA	3,83E-02	8,03E-01	9,13E-01	No	1,61E-01	6,15E-01	7,43E-01	No
ENSG00000200097	RNU6-1167P	snRNA	-4,46E-02	7,60E-01	NA	No	-1,03E-01	8,30E-01	NA	No
ENSG00000252132	RNU6-795P	snRNA	1,67E-01	3,05E-01	5,53E-01	No	2,18E-02	9,46E-01	9,68E-01	No
ENSG00000251783	RNU6-1170P	snRNA	5,57E-01	6,08E-03	NA	No	2,41E-01	3,27E-01	NA	No
ENSG00000252532	RNU7-193P	snRNA	-6,43E-02	4,67E-01	NA	No	-1,56E-01	5,24E-01	NA	No
ENSG00000238523	RNU7-107P	snRNA	1,08E-01	2,70E-01	NA	No	9,86E-02	7,75E-01	NA	No
ENSG00000223107	RNU2-72P	snRNA	1,03E-01	5,13E-01	7,38E-01	No	-1,99E-01	3,86E-01	5,43E-01	No
ENSG00000212520	RNU6-1250P	snRNA	-4,23E-02	7,60E-01	NA	No	-9,82E-02	8,30E-01	NA	No
ENSG00000206855	RNU6-571P	snRNA	-1,64E-01	3,78E-01	6,24E-01	No	-5,87E-01	1,24E-01	2,32E-01	No
ENSG00000238446	RNU7-38P	snRNA	8,14E-02	6,01E-01	NA	No	-2,61E-01	2,86E-01	NA	No
ENSG00000202513	RNU6-805P	snRNA	-4,32E-02	7,08E-01	NA	No	-1,11E-01	6,68E-01	7,84E-01	No
ENSG00000200356	RNU6-833P	snRNA	1,13E-01	2,40E-01	NA	No	8,77E-02	8,64E-01	NA	No
ENSG00000207327	RNU6-883P	snRNA	-5,08E-02	7,57E-01	8,88E-01	No	-3,54E-01	3,11E-01	4,65E-01	No
ENSG00000201954	RNU6-673P	snRNA	6,20E-02	5,83E-01	NA	No	-1,32E-02	9,49E-01	NA	No
ENSG00000199664	RNU6-1266P	snRNA	1,67E-02	8,09E-01	NA	No	-1,36E-01	7,12E-01	NA	No
ENSG00000212332	RNU6-780P	snRNA	2,96E-01	2,03E-01	4,36E-01	No	1,40E-01	6,90E-01	8,00E-01	No
ENSG00000200176	RNU1-19P	snRNA	-1,72E-01	1,92E-01	4,22E-01	No	-3,40E-01	3,10E-01	4,63E-01	No
ENSG00000201604	RNU6-740P	snRNA	1,98E-02	7,54E-01	NA	No	-1,36E-01	7,12E-01	NA	No
ENSG00000206631	RNU6-657P	snRNA	1,40E-01	2,24E-01	NA	No	-9,51E-02	8,30E-01	NA	No
ENSG00000207362	RNU6-422P	snRNA	-1,07E-01	5,18E-01	7,41E-01	No	-3,97E-01	2,75E-01	4,24E-01	No
ENSG00000222414	RNU2-59P	snRNA	-9,64E-02	6,58E-01	8,32E-01	No	6,08E-01	9,20E-02	1,83E-01	No
ENSG00000222051	RNU6-1165P	snRNA	2,83E-01	1,50E-01	3,62E-01	No	-1,29E-01	5,92E-01	NA	No
ENSG00000207029	RNU6-43P	snRNA	1,08E-01	3,26E-01	NA	No	3,91E-02	8,11E-01	NA	No
ENSG00000252994	RNU6-1231P	snRNA	-3,92E-02	7,20E-01	8,69E-01	No	-1,19E-01	7,01E-01	8,08E-01	No

ENSG00000201080	RNU6-372P	snRNA	7,49E-03	9,58E-01	NA	No	1,24E-02	9,92E-01	NA	No
ENSG00000212413	RNU11-3P	snRNA	-5,47E-02	6,62E-01	NA	No	-2,82E-01	2,40E-01	NA	No
ENSG00000272160	U4	snRNA	2,14E-02	9,04E-01	9,60E-01	No	-9,45E-02	7,74E-01	8,59E-01	No
ENSG00000253066	RNU6-709P	snRNA	3,55E-01	1,13E-01	3,03E-01	No	7,34E-01	8,25E-02	1,68E-01	No
ENSG00000252611	RNU6-1121P	snRNA	4,39E-02	6,97E-01	NA	No	1,50E-01	5,30E-01	NA	No
ENSG00000222629	RNU2-42P	snRNA	-4,56E-02	6,66E-01	NA	No	5,73E-01	1,46E-01	2,63E-01	No
ENSG00000222561	RNU6-1025P	snRNA	2,28E-02	8,72E-01	NA	No	4,10E-01	1,04E-01	2,02E-01	No
ENSG00000207308	RNU6-878P	snRNA	6,73E-03	9,53E-01	NA	No	-1,45E-01	4,55E-01	NA	No
ENSG00000201616	RNU1-91P	snRNA	1,15E-01	4,09E-01	NA	No	-4,63E-02	8,37E-01	NA	No
ENSG00000251934	RNU6-1143P	snRNA	-3,62E-01	8,60E-02	2,52E-01	No	-7,54E-01	8,20E-02	1,67E-01	No
ENSG00000238304	RNU7-50P	snRNA	-3,32E-02	7,95E-01	NA	No	-1,76E-01	5,57E-01	6,96E-01	No
ENSG00000252568	RNU7-28P	snRNA	2,75E-02	8,34E-01	NA	No	6,65E-01	1,02E-01	1,99E-01	No
ENSG00000251991	RNU7-49P	snRNA	1,14E-02	9,49E-01	9,79E-01	No	2,37E-01	4,99E-01	6,48E-01	No
ENSG00000251928	RNU6-585P	snRNA	3,84E-03	9,91E-01	NA	No	-1,33E-02	8,90E-01	NA	No
ENSG00000201586	RNU6-593P	snRNA	-1,28E-01	5,12E-01	7,37E-01	No	-2,42E-01	4,85E-01	6,35E-01	No
ENSG00000252327	RNU6-1302P	snRNA	-3,73E-02	7,31E-01	NA	No	-3,61E-02	8,76E-01	NA	No
ENSG00000200376	RNU5E-10P	snRNA	3,62E-02	6,79E-01	NA	No	1,73E-02	9,10E-01	NA	No
ENSG00000207153	RNU6-933P	snRNA	6,16E-02	4,40E-01	NA	No	-9,51E-02	8,30E-01	NA	No
ENSG00000252361	RNU6-118P	snRNA	2,04E-03	9,92E-01	9,97E-01	No	-1,91E-01	5,59E-01	6,97E-01	No
ENSG00000202089	RNU6-1306P	snRNA	-7,56E-03	9,60E-01	9,84E-01	No	-2,91E-01	3,46E-01	5,01E-01	No
ENSG00000222477	RNU2-23P	snRNA	-3,01E-02	7,31E-01	NA	No	-3,42E-02	8,79E-01	NA	No
ENSG00000199325	RNU4-39P	snRNA	4,61E-02	5,40E-01	NA	No	-4,10E-02	8,77E-01	NA	No
ENSG00000253024	RNU6-1238P	snRNA	8,43E-03	8,88E-01	NA	No	-3,86E-02	8,90E-01	NA	No
ENSG00000206783	RNU6-292P	snRNA	3,32E-02	9,36E-01	NA	No	6,17E-02	9,56E-01	NA	No
ENSG00000251919	RNU7-105P	snRNA	5,29E-03	9,65E-01	NA	No	-1,66E-01	4,67E-01	NA	No
ENSG00000206638	RNU6-672P	snRNA	7,99E-05	9,98E-01	9,99E-01	No	-9,81E-01	2,26E-02	5,74E-02	No
ENSG00000200152	RNU6-216P	snRNA	-2,02E-02	9,13E-01	9,64E-01	No	-2,66E-01	4,57E-01	6,10E-01	No
ENSG00000238880	RNU7-59P	snRNA	3,50E-02	9,36E-01	NA	No	8,89E-02	8,64E-01	NA	No
ENSG00000252494	RNU6-126P	snRNA	-2,31E-02	8,69E-01	NA	No	2,05E-01	5,28E-01	6,72E-01	No
ENSG00000206583	RNU6-1292P	snRNA	-8,40E-02	5,94E-01	7,93E-01	No	-2,73E-01	4,41E-01	5,95E-01	No
ENSG00000223015	RNU6-1135P	snRNA	8,24E-02	3,16E-01	NA	No	8,77E-02	8,64E-01	NA	No
ENSG00000199235	RNU6-1063P	snRNA	9,32E-02	3,59E-01	NA	No	8,64E-02	8,64E-01	NA	No
ENSG00000252081	RNU6-277P	snRNA	-4,79E-02	6,45E-01	NA	No	-3,83E-02	8,79E-01	NA	No
ENSG00000238444	RNU6-893P	snRNA	-1,16E-01	5,72E-01	7,78E-01	No	-4,12E-01	2,70E-01	4,18E-01	No
ENSG00000206772	RNU6-44P	snRNA	-4,99E-02	5,76E-01	NA	No	-4,19E-02	8,42E-01	NA	No
ENSG00000238998	RNU7-187P	snRNA	-1,65E-01	1,93E-01	NA	No	1,41E+00	9,86E-03	2,81E-02	No
ENSG00000201687	RNU6-1107P	snRNA	-8,30E-02	2,81E-01	NA	No	-1,73E-01	4,06E-01	NA	No
ENSG00000207185	RNU6-1157P	snRNA	-6,30E-02	7,33E-01	8,76E-01	No	-3,24E-01	3,42E-01	4,98E-01	No
ENSG00000207462	RNU6-376P	snRNA	-4,23E-02	7,60E-01	NA	No	-9,82E-02	8,30E-01	NA	No
ENSG00000222249	RNU6-262P	snRNA	-5,70E-02	5,79E-01	NA	No	9,83E-02	6,78E-01	7,91E-01	No
ENSG00000199709	RNU4-23P	snRNA	2,84E-02	8,42E-01	NA	No	1,00E-01	6,40E-01	NA	No
ENSG00000222844	RNU6-321P	snRNA	-4,62E-02	5,99E-01	NA	No	-1,03E-01	6,38E-01	NA	No
ENSG00000222297	RNU6-1156P	snRNA	1,09E-01	2,65E-01	NA	No	8,54E-02	6,97E-01	NA	No
ENSG00000222067	RNU4-86P	snRNA	-1,69E-02	8,49E-01	NA	No	-1,03E-01	8,30E-01	NA	No
ENSG00000212597	RNU6-876P	snRNA	-5,08E-02	4,54E-01	NA	No	-1,19E-01	5,89E-01	NA	No
ENSG00000199260	RNU6-874P	snRNA	-1,30E-01	3,53E-01	NA	No	2,31E-02	9,43E-01	9,66E-01	No
ENSG00000238370	RNU7-103P	snRNA	5,83E-02	5,80E-01	NA	No	1,20E-01	6,76E-01	NA	No
ENSG00000221439	RNU4ATAC16P	snRNA	-6,85E-02	7,45E-01	8,81E-01	No	-6,25E-02	8,52E-01	9,10E-01	No
ENSG00000238689	RNU7-166P	snRNA	4,21E-02	7,67E-01	NA	No	1,00E-01	7,75E-01	NA	No
ENSG00000252996	RNU6-1315P	snRNA	-4,42E-02	7,60E-01	NA	No	-1,02E-01	8,30E-01	NA	No
ENSG00000200345	RNU6-485P	snRNA	3,24E-02	7,60E-01	NA	No	-3,41E-02	9,21E-01	NA	No
ENSG00000238831	RNU7-189P	snRNA	2,97E-01	5,02E-02	1,74E-01	No	6,11E-02	7,59E-01	NA	No
ENSG00000212345	RNU6-700P	snRNA	4,04E-02	7,81E-01	NA	No	8,55E-02	8,64E-01	NA	No
ENSG00000253095	RNU6-676P	snRNA	6,34E-02	7,71E-01	8,96E-01	No	1,08E+00	8,72E-03	2,52E-02	No
ENSG00000251747	RNU7-60P	snRNA	-6,30E-02	5,05E-01	NA	No	-1,52E-01	5,67E-01	NA	No
ENSG00000199551	RNU6-545P	snRNA	-1,50E-02	8,99E-01	NA	No	2,91E-02	9,22E-01	9,54E-01	No
ENSG00000200105	RNU6-251P	snRNA	-4,69E-02	8,26E-01	9,24E-01	No	1,05E+00	6,72E-03	2,01E-02	No
ENSG00000200247	RNU6-254P	snRNA	1,65E-01	2,73E-01	NA	No	1,56E-01	5,62E-01	6,99E-01	No
ENSG00000200885	RNU1-146P	snRNA	-8,59E-02	3,41E-01	NA	No	-1,94E-01	3,38E-01	NA	No
ENSG00000252237	RNU4-54P	snRNA	1,89E-01	4,04E-01	6,48E-01	No	1,37E-01	6,92E-01	8,02E-01	No
ENSG00000200388	RNU6-618P	snRNA	-1,45E-03	9,86E-01	NA	No	4,21E-02	8,35E-01	NA	No
ENSG00000253063	RNU6-494P	snRNA	1,52E-01	4,05E-01	6,49E-01	No	-1,17E-01	6,99E-01	8,07E-01	No

ENSG00000199886	RNU6-249P	snRNA	1,87E-01	3,24E-01	5,73E-01	No	-6,00E-02	8,39E-01	9,02E-01	No
ENSG00000222635	RNU6-1203P	snRNA	-1,16E-02	8,19E-01	NA	No	3,23E-01	2,31E-01	NA	No
ENSG00000212496	RNU6-1093P	snRNA	-4,15E-02	7,60E-01	NA	No	1,26E-01	6,17E-01	NA	No
ENSG00000207136	RNU6-769P	snRNA	2,21E-02	8,14E-01	NA	No	3,04E-02	8,79E-01	NA	No
ENSG00000200183	RNU6-238P	snRNA	-6,76E-02	5,97E-01	NA	No	-5,78E-01	1,04E-01	NA	No
ENSG00000272028	U6	snRNA	1,54E-02	9,03E-01	NA	No	7,35E-02	7,61E-01	NA	No
ENSG00000199824	RNU6-199P	snRNA	8,84E-02	6,46E-01	8,25E-01	No	-1,52E-01	6,58E-01	7,76E-01	No
ENSG00000206992	RNU6-574P	snRNA	9,22E-02	5,07E-01	7,34E-01	No	-6,44E-02	7,95E-01	NA	No
ENSG00000252206	RNU7-40P	snRNA	2,13E-01	2,65E-01	5,09E-01	No	-3,15E-01	2,35E-01	3,77E-01	No
ENSG00000201579	RNU6-343P	snRNA	-1,86E-01	3,75E-01	6,21E-01	No	-4,22E-01	2,53E-01	3,99E-01	No
ENSG00000201198	RNU6-879P	snRNA	2,13E-02	9,14E-01	9,64E-01	No	1,61E-01	6,48E-01	7,68E-01	No
ENSG00000252865	RNU6-594P	snRNA	2,41E-02	8,05E-01	NA	No	5,12E-03	9,87E-01	NA	No
ENSG00000200814	RNU6-595P	snRNA	1,22E-01	5,45E-01	7,60E-01	No	5,94E-01	9,49E-02	1,88E-01	No
ENSG00000202034	RNU6-399P	snRNA	-3,47E-02	7,69E-01	NA	No	1,41E-01	6,41E-01	7,63E-01	No
ENSG00000251788	RNU5A-7P	snRNA	8,36E-02	4,43E-01	NA	No	2,71E-01	2,64E-01	NA	No
ENSG00000221564	RNU6ATAC42P	snRNA	-2,05E-02	8,19E-01	NA	No	-1,36E-01	7,12E-01	NA	No
ENSG00000252770	RNU7-4P	snRNA	6,07E-02	7,11E-01	8,64E-01	No	2,12E-01	4,95E-01	6,44E-01	No
ENSG00000222405	RNU4-65P	snRNA	4,04E-02	7,80E-01	NA	No	2,13E-01	4,16E-01	NA	No
ENSG00000252220	RNU6-977P	snRNA	1,44E-02	8,47E-01	NA	No	-2,75E-02	8,90E-01	NA	No
ENSG00000202534	RNU6-1329P	snRNA	-5,69E-02	6,46E-01	NA	No	-1,35E-01	7,12E-01	NA	No
ENSG00000212535	RNU6-808P	snRNA	1,38E-01	3,90E-01	NA	No	2,11E-01	4,79E-01	6,30E-01	No
ENSG00000201435	RNU4-24P	snRNA	-2,18E-01	3,47E-01	5,95E-01	No	-4,73E-02	8,85E-01	9,32E-01	No
ENSG00000222890	RNU6-1068P	snRNA	3,15E-02	9,36E-01	NA	No	9,83E-02	7,75E-01	NA	No
ENSG00000222255	RNU6-101P	snRNA	8,53E-02	6,60E-01	8,33E-01	No	2,71E-02	9,38E-01	9,64E-01	No
ENSG00000222932	RNU6-172P	snRNA	1,13E-01	2,96E-01	NA	No	6,59E-01	6,01E-02	1,30E-01	No
ENSG00000238457	RNU7-169P	snRNA	-1,37E-01	3,99E-01	6,44E-01	No	3,85E-01	2,95E-01	4,47E-01	No
ENSG00000200274	RNU4-32P	snRNA	-7,22E-01	2,44E-02	1,06E-01	No	-3,57E-01	3,06E-01	4,59E-01	No
ENSG00000272215	U7	snRNA	1,61E-01	3,92E-01	6,37E-01	No	1,70E-01	6,22E-01	7,48E-01	No
ENSG00000200665	RNU6-1188P	snRNA	1,16E-01	5,63E-01	7,71E-01	No	-9,98E-02	7,72E-01	8,58E-01	No
ENSG00000200795	RNU4-1	snRNA	1,13E-01	4,49E-01	6,85E-01	No	1,39E-01	6,28E-01	7,53E-01	No
ENSG00000239082	RNU7-170P	snRNA	1,24E-01	2,43E-01	NA	No	-3,20E-02	9,21E-01	NA	No
ENSG00000252079	RNU6-327P	snRNA	-3,68E-02	7,24E-01	NA	No	-1,49E-01	4,79E-01	NA	No
ENSG00000252269	RNU4ATAC12P	snRNA	2,92E-02	7,93E-01	NA	No	-1,26E-01	7,12E-01	NA	No
ENSG00000222726	RNU2-7P	snRNA	-1,44E-02	9,01E-01	9,59E-01	No	-1,58E-01	5,61E-01	6,99E-01	No
ENSG00000251902	RNU6-51P	snRNA	7,93E-02	6,12E-01	8,05E-01	No	-1,29E-01	6,64E-01	7,80E-01	No
ENSG00000201821	RNU4-9P	snRNA	-5,61E-02	7,24E-01	8,72E-01	No	-7,96E-01	7,05E-02	1,48E-01	No
ENSG00000207518	RNU6-59P	snRNA	-1,59E-02	9,02E-01	9,59E-01	No	-6,60E-02	8,30E-01	8,96E-01	No
ENSG00000252952	RNU6-58P	snRNA	-2,61E-01	2,19E-01	4,56E-01	No	-8,29E-02	8,10E-01	8,83E-01	No
ENSG00000200840	RNU6-82P	snRNA	3,23E-01	1,77E-01	4,01E-01	No	1,36E+00	3,82E-03	1,22E-02	No
ENSG00000202237	RNU6-53P	snRNA	-3,51E-02	7,62E-01	NA	No	-1,59E-01	4,87E-01	NA	No
ENSG00000252928	RNU6-64P	snRNA	4,46E-02	6,73E-01	NA	No	3,75E-01	1,10E-01	NA	No
ENSG00000252397	RNU5A-4P	snRNA	8,20E-02	4,32E-01	NA	No	4,52E-02	8,90E-01	NA	No
ENSG00000223280	RNU6-57P	snRNA	-3,05E-01	1,71E-01	3,94E-01	No	1,05E-02	9,77E-01	9,86E-01	No
ENSG00000206962	RNU6-74P	snRNA	3,42E-02	8,45E-01	9,33E-01	No	8,40E-01	5,88E-02	1,28E-01	No
ENSG00000252055	RNU6-69P	snRNA	-1,20E-01	3,98E-01	6,44E-01	No	-2,80E-01	3,92E-01	5,49E-01	No
ENSG00000201662	RNU6-60P	snRNA	-4,71E-01	7,78E-02	2,36E-01	No	-1,15E+00	1,82E-02	4,77E-02	No
ENSG00000239003	RNU7-88P	snRNA	-4,21E-02	7,60E-01	NA	No	1,31E-02	9,87E-01	NA	No
ENSG00000238500	RNU7-87P	snRNA	-5,39E-03	9,72E-01	9,89E-01	No	1,34E-01	6,79E-01	7,92E-01	No
ENSG00000206922	RNU6-80P	snRNA	2,26E-01	7,62E-02	NA	No	8,12E-01	4,10E-02	NA	No
ENSG00000199381	RNU6-79P	snRNA	7,63E-02	5,95E-01	7,94E-01	No	1,32E-02	9,68E-01	9,81E-01	No
ENSG00000252335	RNU6-62P	snRNA	3,79E-04	9,93E-01	NA	No	3,75E-01	2,22E-01	3,61E-01	No
ENSG00000207298	RNU6-83P	snRNA	1,22E-02	9,40E-01	9,75E-01	No	1,83E-01	6,02E-01	7,32E-01	No
ENSG00000201155	RNU1-24P	snRNA	3,63E-01	3,48E-02	1,36E-01	No	3,35E-01	1,70E-01	2,95E-01	No
ENSG00000202347	RNU1-16P	snRNA	-1,68E-01	4,44E-01	6,81E-01	No	-8,43E-02	8,06E-01	8,80E-01	No
ENSG00000202229	RNU6-1138P	snRNA	7,61E-02	3,85E-01	NA	No	1,24E-01	5,22E-01	NA	No
ENSG00000252347	RNU6-1046P	snRNA	5,37E-02	5,55E-01	NA	No	-1,02E-01	8,30E-01	NA	No
ENSG00000252686	RNU6-1234P	snRNA	-1,41E-03	9,89E-01	NA	No	4,05E-01	1,91E-01	3,23E-01	No
ENSG00000207440	RNU6-541P	snRNA	5,90E-02	5,69E-01	NA	No	2,81E-01	2,94E-01	4,45E-01	No
ENSG00000223087	RNU6-602P	snRNA	3,31E-02	9,36E-01	NA	No	9,81E-02	7,75E-01	NA	No
ENSG00000207412	RNU6-455P	snRNA	9,77E-02	4,86E-01	7,16E-01	No	-1,20E-02	9,67E-01	9,80E-01	No
ENSG00000201654	RNU6-7	snRNA	-1,33E-01	2,13E-01	NA	No	-3,36E-02	9,03E-01	9,43E-01	No
ENSG00000202337	RNU6-8	snRNA	-2,79E-02	8,07E-01	NA	No	1,49E+00	1,49E-02	4,03E-02	No

ENSG00000206596	RNU1-27P	snRNA	-1,89E-02	8,92E-01	NA	No	-2,16E-02	9,40E-01	NA	No
ENSG00000206588	RNU1-28P	snRNA	5,24E-02	6,28E-01	NA	No	-1,01E-01	7,01E-01	NA	No
ENSG00000251726	RNU7-41P	snRNA	-1,03E-01	5,03E-01	7,30E-01	No	-4,77E-01	1,54E-01	2,73E-01	No
ENSG00000207046	RNU6-886P	snRNA	-3,64E-02	7,31E-01	NA	No	5,00E-02	7,52E-01	NA	No
ENSG00000199739	RNU6-552P	snRNA	-5,47E-02	6,46E-01	NA	No	-1,30E-01	7,12E-01	NA	No
ENSG00000207366	RNU6-297P	snRNA	4,44E-02	6,79E-01	NA	No	1,57E-01	5,11E-01	NA	No
ENSG00000252474	RNU6-539P	snRNA	1,26E-01	5,15E-01	7,39E-01	No	1,74E-01	6,14E-01	7,42E-01	No
ENSG00000207004	RNU6-301P	snRNA	7,90E-02	4,78E-01	NA	No	4,98E-01	8,43E-02	NA	No
ENSG00000252019	RNU6ATAC9P	snRNA	1,83E-01	1,57E-01	3,73E-01	No	5,66E-02	7,96E-01	NA	No
ENSG00000252782	RNU6-341P	snRNA	-1,43E-01	5,15E-01	7,39E-01	No	-2,24E-01	5,23E-01	6,68E-01	No
ENSG00000252463	RNU6-852P	snRNA	4,34E-02	7,06E-01	NA	No	2,84E-01	3,25E-01	4,80E-01	No
ENSG00000202490	RNU6-597P	snRNA	-5,57E-03	9,69E-01	NA	No	-2,04E-01	4,82E-01	NA	No
ENSG00000207172	RNU6-1162P	snRNA	-1,93E-02	8,49E-01	NA	No	-1,03E-01	8,30E-01	NA	No
ENSG00000222640	RNU2-51P	snRNA	-5,38E-02	7,39E-01	8,78E-01	No	-4,89E-01	1,08E-01	NA	No
ENSG00000252263	RNU6-659P	snRNA	3,12E-03	9,76E-01	NA	No	1,85E-02	9,43E-01	NA	No
ENSG00000252839	RNU6-419P	snRNA	4,44E-02	7,38E-01	NA	No	8,55E-02	8,64E-01	NA	No
ENSG00000206924	RNU6-689P	snRNA	2,57E-02	7,23E-01	NA	No	3,61E-01	1,48E-01	2,66E-01	No
ENSG00000222990	RNU4-22P	snRNA	-1,51E-02	9,34E-01	9,73E-01	No	-1,97E-03	9,94E-01	9,97E-01	No
ENSG00000252720	RNU6-1258P	snRNA	3,17E-02	9,36E-01	NA	No	6,13E-02	9,56E-01	NA	No
ENSG00000222276	RNU2-33P	snRNA	7,56E-02	5,39E-01	NA	No	-7,61E-02	7,41E-01	NA	No
ENSG00000272439	U6	snRNA	-5,63E-02	7,72E-01	8,96E-01	No	-1,97E-01	5,75E-01	7,10E-01	No
ENSG00000199836	RNU1-47P	snRNA	-5,45E-02	6,46E-01	NA	No	-1,28E-01	7,12E-01	NA	No
ENSG00000207208	RNU6-790P	snRNA	1,99E-01	2,22E-01	4,59E-01	No	-1,39E-01	5,31E-01	NA	No
ENSG00000206969	RNU6-1316P	snRNA	7,39E-02	4,10E-01	NA	No	6,13E-02	9,56E-01	NA	No
ENSG00000201184	RNU4-68P	snRNA	1,28E-02	9,64E-01	NA	No	7,73E-02	7,08E-01	NA	No
ENSG00000212526	RNU6-466P	snRNA	7,37E-02	7,15E-01	8,66E-01	No	-3,94E-01	2,36E-01	3,79E-01	No
ENSG00000223313	RNU6-516P	snRNA	1,13E-02	9,52E-01	9,80E-01	No	-1,84E-01	5,69E-01	7,06E-01	No
ENSG00000200407	RNU6-1169P	snRNA	-3,35E-02	7,31E-01	NA	No	1,41E-01	4,87E-01	NA	No
ENSG00000201077	RNU6-188P	snRNA	5,47E-02	7,48E-01	8,82E-01	No	8,68E-01	5,71E-02	1,25E-01	No
ENSG00000206991	RNU6-610P	snRNA	-9,50E-02	3,49E-01	NA	No	-1,31E-01	6,11E-01	7,40E-01	No
ENSG00000206589	RNU6-354P	snRNA	3,90E-02	8,45E-01	NA	No	9,18E-02	8,64E-01	NA	No
ENSG00000238941	RNU6-953P	snRNA	-8,53E-03	9,10E-01	NA	No	-1,30E-01	7,12E-01	NA	No
ENSG00000238456	RNU6-1014P	snRNA	3,17E-02	9,36E-01	NA	No	9,94E-02	7,75E-01	NA	No
ENSG00000271819	U6	snRNA	5,32E-01	4,82E-02	1,70E-01	No	2,00E-01	5,34E-01	6,77E-01	No
ENSG00000272337	U6	snRNA	3,45E-02	8,52E-01	9,36E-01	No	-3,28E-01	3,48E-01	5,04E-01	No
ENSG00000199784	RNU6-1287P	snRNA	3,15E-02	9,36E-01	NA	No	8,77E-02	8,64E-01	NA	No
ENSG00000252984	RNU6-844P	snRNA	3,21E-02	7,23E-01	NA	No	-5,93E-02	7,77E-01	NA	No
ENSG00000200070	RNU4-80P	snRNA	-4,46E-02	7,08E-01	NA	No	-1,25E-01	6,30E-01	7,54E-01	No
ENSG00000199512	RNU6-212P	snRNA	1,70E-01	3,16E-01	5,65E-01	No	2,52E-01	4,40E-01	5,94E-01	No
ENSG00000207162	RNU6-549P	snRNA	1,61E-02	8,77E-01	NA	No	-2,91E-02	8,78E-01	NA	No
ENSG00000199568	RNU5A-1	snRNA	1,30E-01	5,03E-01	7,30E-01	No	2,39E-01	5,00E-01	6,48E-01	No
ENSG00000200156	RNU5B-1	snRNA	1,86E-02	8,76E-01	NA	No	1,02E+00	4,50E-02	1,03E-01	No
ENSG00000207449	RNU6-19P	snRNA	9,87E-02	3,27E-01	NA	No	8,51E-02	8,64E-01	NA	No
ENSG00000206625	RNU6-1	snRNA	2,67E-02	7,97E-01	NA	No	-1,39E-02	8,90E-01	NA	No
ENSG00000222094	RNU2-65P	snRNA	-2,85E-02	8,12E-01	NA	No	1,85E-01	4,96E-01	6,45E-01	No
ENSG00000252061	RNU6-415P	snRNA	-1,93E-02	9,22E-01	9,68E-01	No	-5,25E-01	1,79E-01	3,07E-01	No
ENSG00000212170	RNU1-77P	snRNA	1,71E-01	1,82E-01	NA	No	2,91E-01	2,85E-01	NA	No
ENSG00000212374	RNU6-401P	snRNA	3,07E-02	7,82E-01	NA	No	2,00E-01	4,44E-01	NA	No
ENSG00000200444	RNU6-1339P	snRNA	3,15E-02	9,36E-01	NA	No	1,15E-01	6,09E-01	NA	No
ENSG00000207037	RNU6-339P	snRNA	-2,25E-02	7,04E-01	NA	No	-1,46E-01	5,99E-01	NA	No
ENSG00000202081	RNU6-1280P	snRNA	-1,81E-01	4,28E-01	6,68E-01	No	-7,01E-01	4,41E-02	1,01E-01	No
ENSG00000207150	RNU6-185P	snRNA	3,32E-02	9,36E-01	NA	No	8,58E-02	8,64E-01	NA	No
ENSG00000212420	RNU6-1111P	snRNA	9,01E-02	5,25E-01	NA	No	1,50E-01	5,51E-01	6,91E-01	No
ENSG00000252645	RNU7-111P	snRNA	1,03E-01	6,24E-01	8,13E-01	No	2,17E-01	4,51E-01	6,05E-01	No
ENSG00000222076	RNU2-3P	snRNA	-2,12E-03	9,91E-01	NA	No	-3,66E-02	8,90E-01	NA	No
ENSG00000251819	RNU6-322P	snRNA	-6,35E-02	6,22E-01	8,12E-01	No	-2,59E-01	3,54E-01	5,11E-01	No
ENSG00000200095	RNU6-181P	snRNA	2,41E-01	6,04E-02	NA	No	1,11E-01	6,73E-01	NA	No
ENSG00000252614	RNU6-807P	snRNA	-1,44E-01	4,69E-01	7,02E-01	No	-1,09E+00	3,71E-02	8,73E-02	No
ENSG00000200204	RNU1-22P	snRNA	1,04E-01	3,42E-01	5,90E-01	No	-1,70E-01	4,23E-01	NA	No
ENSG00000238417	RNU7-63P	snRNA	4,56E-02	6,39E-01	NA	No	1,13E-01	6,32E-01	NA	No
ENSG00000199482	RNU6-633P	snRNA	-1,90E-02	8,70E-01	NA	No	5,89E-02	8,42E-01	9,03E-01	No
ENSG00000238964	RNU7-125P	snRNA	1,93E-01	6,97E-02	NA	No	6,19E-02	9,56E-01	NA	No

ENSG00000206778	RNU6-213P	snRNA	1,38E-01	1,51E-01	NA	No	3,17E-02	8,88E-01	NA	No
ENSG00000207167	RNU6-1340P	snRNA	-5,73E-02	6,46E-01	NA	No	-1,36E-01	7,12E-01	NA	No
ENSG00000200893	RNU6-944P	snRNA	2,19E-01	8,16E-02	NA	No	3,92E-01	1,56E-01	NA	No
ENSG00000207248	RNU6-1005P	snRNA	1,11E-02	9,45E-01	9,78E-01	No	5,72E-01	1,55E-01	2,75E-01	No
ENSG00000207042	RNU6-196P	snRNA	2,10E-01	1,30E-01	3,31E-01	No	8,18E-01	6,79E-02	1,44E-01	No
ENSG00000251706	RNU7-61P	snRNA	9,40E-02	3,34E-01	NA	No	1,52E-02	9,87E-01	NA	No
ENSG00000222287	RNU6-1043P	snRNA	2,72E-02	8,47E-01	NA	No	-9,22E-02	7,12E-01	NA	No
ENSG00000252569	RNU6-1153P	snRNA	-3,46E-03	9,83E-01	9,93E-01	No	1,36E-01	6,77E-01	7,90E-01	No
ENSG00000212379	RNU6-269P	snRNA	3,33E-02	6,66E-01	NA	No	-1,35E-01	7,12E-01	NA	No
ENSG00000252271	RNU6-1110P	snRNA	-5,92E-02	4,57E-01	NA	No	-1,79E-01	3,87E-01	NA	No
ENSG00000252026	RNU6-1262P	snRNA	9,34E-02	6,68E-01	8,38E-01	No	3,51E-02	9,18E-01	9,52E-01	No
ENSG00000222177	RNU4-30P	snRNA	1,42E-02	8,56E-01	NA	No	-3,54E-02	8,80E-01	NA	No
ENSG00000201164	RNU4-36P	snRNA	4,38E-02	7,01E-01	NA	No	6,67E-02	9,56E-01	NA	No
ENSG00000202497	RNU6-898P	snRNA	-1,06E-01	2,81E-01	NA	No	-2,56E-01	3,12E-01	NA	No
ENSG00000207083	RNU6-22P	snRNA	-6,63E-02	4,07E-01	NA	No	-6,63E-02	7,37E-01	NA	No
ENSG00000199301	RNU6-208P	snRNA	3,60E-04	9,41E-01	NA	No	4,55E-02	8,21E-01	NA	No
ENSG00000251868	RNU7-71P	snRNA	-2,82E-01	2,06E-01	4,39E-01	No	1,43E-01	6,83E-01	7,95E-01	No
ENSG00000251794	RNU6-237P	snRNA	1,41E-01	1,91E-01	NA	No	-7,59E-02	8,00E-01	NA	No
ENSG00000252101	RNU6-758P	snRNA	-6,69E-02	4,09E-01	NA	No	-5,17E-02	8,19E-01	NA	No
ENSG00000252311	RNU1-103P	snRNA	-4,53E-02	7,57E-01	NA	No	-2,57E-01	3,47E-01	NA	No
ENSG00000252887	RNU6-430P	snRNA	-2,60E-02	8,46E-01	NA	No	-2,10E-01	4,09E-01	NA	No
ENSG00000252579	RNU7-117P	snRNA	-5,04E-02	8,04E-01	9,13E-01	No	-1,56E-01	6,54E-01	7,73E-01	No
ENSG00000252363	RNU7-43P	snRNA	1,17E-01	2,38E-01	NA	No	3,90E-02	8,95E-01	NA	No
ENSG00000252707	RNU11-2P	snRNA	3,31E-01	1,85E-01	4,12E-01	No	6,13E-01	9,98E-02	1,95E-01	No
ENSG00000200437	RNU6-799P	snRNA	4,38E-02	7,50E-01	NA	No	2,29E-01	3,76E-01	NA	No
ENSG00000252383	RNU6-314P	snRNA	1,39E-01	3,78E-01	6,24E-01	No	-1,13E-01	6,88E-01	7,99E-01	No
ENSG00000199674	RNU6-862P	snRNA	4,43E-02	6,91E-01	NA	No	3,22E-01	2,83E-01	4,32E-01	No
ENSG00000206859	RNU6-767P	snRNA	-1,06E-01	6,11E-01	8,04E-01	No	-4,75E-01	2,20E-01	3,59E-01	No
ENSG00000212186	RNU6-258P	snRNA	3,94E-03	9,80E-01	9,92E-01	No	-2,42E-01	4,22E-01	5,78E-01	No
ENSG00000252971	RNU6-1057P	snRNA	-5,19E-02	5,42E-01	NA	No	-1,03E-01	6,38E-01	NA	No
ENSG00000253064	RNU6-711P	snRNA	-9,40E-02	4,84E-01	7,15E-01	No	-3,09E-01	3,41E-01	4,97E-01	No
ENSG00000202205	RNU6-1034P	snRNA	5,33E-02	7,43E-01	8,80E-01	No	2,37E-01	4,87E-01	6,37E-01	No
ENSG00000222858	RNU6-920P	snRNA	-1,95E-01	3,33E-01	5,82E-01	No	-1,76E-01	6,13E-01	7,41E-01	No
ENSG00000222679	RNU6-1267P	snRNA	9,46E-02	4,58E-01	NA	No	5,78E-02	8,13E-01	NA	No
ENSG00000212190	RNU6-298P	snRNA	1,15E-01	3,61E-01	NA	No	-1,01E-01	6,19E-01	NA	No
ENSG00000221038	RNU6ATAC7P	snRNA	-7,70E-02	4,70E-01	NA	No	-1,29E-01	6,23E-01	7,49E-01	No
ENSG00000253084	RNU6-840P	snRNA	4,16E-02	6,70E-01	NA	No	2,08E-01	3,06E-01	NA	No
ENSG00000252278	RNU6-866P	snRNA	7,39E-02	4,65E-01	NA	No	-6,67E-02	8,00E-01	NA	No
ENSG00000223091	RNU6-981P	snRNA	3,30E-02	7,83E-01	NA	No	-1,13E-01	5,94E-01	NA	No
ENSG00000222777	RNU6-233P	snRNA	7,94E-02	4,41E-01	NA	No	-1,28E-01	7,12E-01	NA	No
ENSG00000252698	RNU7-101P	snRNA	2,81E-01	9,80E-02	2,75E-01	No	5,34E-01	1,48E-01	2,66E-01	No
ENSG00000238419	RNU7-186P	snRNA	-1,27E-01	7,18E-02	NA	No	-2,40E-01	2,46E-01	3,90E-01	No
ENSG00000206954	RNU6-1201P	snRNA	-5,42E-03	9,64E-01	NA	No	-1,83E-01	4,86E-01	NA	No
ENSG00000207306	RNU6-1152P	snRNA	1,91E-01	1,42E-01	3,51E-01	No	-8,74E-02	6,58E-01	NA	No
ENSG00000251964	RNU7-135P	snRNA	3,37E-01	1,36E-02	7,00E-02	No	1,60E-01	4,46E-01	NA	No
ENSG00000238447	RNU7-134P	snRNA	8,16E-02	4,19E-01	NA	No	-2,00E-02	8,90E-01	NA	No
ENSG00000202077	RNU1-60P	snRNA	2,39E-01	1,64E-01	3,83E-01	No	5,89E-01	1,37E-01	2,51E-01	No
ENSG00000200997	RNU1-85P	snRNA	1,30E-01	3,63E-01	NA	No	2,08E-01	4,60E-01	6,13E-01	No
ENSG00000252212	RNU2-58P	snRNA	-6,95E-02	6,13E-01	NA	No	-2,34E-01	2,39E-01	NA	No
ENSG00000201524	RNU6-450P	snRNA	2,14E-02	8,91E-01	NA	No	-2,16E-01	3,18E-01	NA	No
ENSG00000200013	RNU6-623P	snRNA	6,61E-02	6,16E-01	NA	No	1,41E-01	5,75E-01	NA	No
ENSG00000199697	RNU6-446P	snRNA	-1,31E-01	2,22E-01	NA	No	6,62E-03	9,81E-01	NA	No
ENSG00000200560	RNU6-288P	snRNA	-3,72E-02	8,11E-01	NA	No	-1,01E-01	6,96E-01	8,05E-01	No
ENSG00000252242	RNU7-115P	snRNA	8,13E-03	9,44E-01	NA	No	-1,33E-01	5,77E-01	NA	No
ENSG00000252685	RNU6-928P	snRNA	2,27E-02	8,74E-01	NA	No	2,86E-02	9,87E-01	NA	No
ENSG00000200257	RNU6-97P	snRNA	6,02E-02	7,17E-01	8,68E-01	No	-1,63E-02	9,58E-01	9,76E-01	No
ENSG00000222808	RNU4-47P	snRNA	1,50E-01	4,79E-01	7,10E-01	No	-4,28E-02	8,98E-01	9,40E-01	No
ENSG00000238658	RNU6-625P	snRNA	-4,88E-02	6,47E-01	NA	No	-1,04E-01	7,07E-01	8,13E-01	No
ENSG00000252391	RNU6-638P	snRNA	2,42E-01	3,03E-01	5,51E-01	No	-4,43E-01	2,24E-01	3,64E-01	No
ENSG00000206687	RNU1-109P	snRNA	-1,74E-01	3,63E-01	6,10E-01	No	8,89E-02	7,97E-01	8,74E-01	No
ENSG00000252353	RNU7-25P	snRNA	-2,96E-01	1,55E-01	3,70E-01	No	-5,86E-01	1,44E-01	2,60E-01	No
ENSG00000200637	RNU5F-3P	snRNA	-6,99E-02	4,70E-01	NA	No	-1,78E-01	3,89E-01	NA	No

ENSG00000251994	RNU2-27P	snRNA	2,61E-01	2,46E-01	4,87E-01	No	-4,06E-01	2,76E-01	4,25E-01	No
ENSG00000212329	RNU6-316P	snRNA	3,70E-02	8,45E-01	NA	No	8,85E-02	8,64E-01	NA	No
ENSG00000221363	RNU6ATAC20P	snRNA	-3,32E-02	7,20E-01	NA	No	-1,32E-01	7,12E-01	NA	No
ENSG00000201852	RNU6-702P	snRNA	-2,63E-01	2,70E-01	5,15E-01	No	5,80E-01	1,07E-01	2,06E-01	No
ENSG00000206863	RNU5A-6P	snRNA	-2,14E-03	9,10E-01	NA	No	6,84E-02	7,70E-01	NA	No
ENSG00000206766	RNU6-435P	snRNA	4,63E-02	8,21E-01	9,22E-01	No	-6,98E-02	8,39E-01	9,02E-01	No
ENSG00000207160	RNU6-1289P	snRNA	5,66E-02	6,01E-01	NA	No	8,55E-02	8,64E-01	NA	No
ENSG00000252379	RNU6-1002P	snRNA	4,35E-02	7,20E-01	NA	No	1,07E-01	6,99E-01	NA	No
ENSG00000222320	RNU6-1050P	snRNA	1,26E-01	2,77E-01	5,23E-01	No	-1,05E-01	6,26E-01	NA	No
ENSG00000238333	RNU7-145P	snRNA	-6,83E-02	3,47E-01	NA	No	-1,67E-01	3,99E-01	NA	No
ENSG00000251939	RNU6-1278P	snRNA	-2,64E-02	8,30E-01	NA	No	-1,58E-01	4,89E-01	NA	No
ENSG00000206944	RNU6-708P	snRNA	-1,85E-02	8,44E-01	NA	No	-1,20E-02	9,38E-01	NA	No
ENSG00000253033	RNU7-191P	snRNA	-1,10E-02	8,49E-01	NA	No	4,79E-02	8,86E-01	NA	No
ENSG00000207154	RNU1-46P	snRNA	-5,45E-02	6,46E-01	NA	No	-1,28E-01	7,12E-01	NA	No
ENSG00000251870	RNU2-69P	snRNA	-4,05E-02	8,08E-01	9,15E-01	No	2,62E-02	9,35E-01	9,61E-01	No
ENSG00000206769	RNU6-116P	snRNA	4,33E-02	7,20E-01	NA	No	1,65E-01	4,75E-01	NA	No
ENSG00000206746	RNU6-142P	snRNA	3,00E-03	9,83E-01	NA	No	1,08E-01	7,34E-01	8,32E-01	No
ENSG00000207072	RNU6-39P	snRNA	1,75E-02	8,45E-01	NA	No	-1,02E-01	6,30E-01	NA	No
ENSG00000252097	RNU6-346P	snRNA	3,50E-02	9,36E-01	NA	No	6,34E-02	9,56E-01	NA	No
ENSG00000252103	RNU6-917P	snRNA	3,41E-02	9,36E-01	NA	No	1,14E-01	6,86E-01	NA	No
ENSG00000201294	RNU6-1019P	snRNA	3,00E-02	8,59E-01	9,39E-01	No	1,90E-01	5,74E-01	7,09E-01	No
ENSG00000199570	RNU6-228P	snRNA	3,09E-02	8,37E-01	NA	No	3,40E-01	2,12E-01	NA	No
ENSG00000201180	RNU6-115P	snRNA	-5,98E-02	5,58E-01	NA	No	5,53E-02	8,13E-01	NA	No
ENSG00000207355	RNU6-1257P	snRNA	7,06E-02	5,62E-01	NA	No	8,26E-02	7,42E-01	NA	No
ENSG00000251883	RNU6ATAC17P	snRNA	-5,69E-02	6,46E-01	NA	No	1,01E-02	9,14E-01	NA	No
ENSG00000201770	RNU6-384P	snRNA	1,07E-02	9,32E-01	NA	No	-1,28E-01	7,12E-01	NA	No
ENSG00000202150	RNU6-407P	snRNA	1,53E-01	2,60E-01	5,03E-01	No	5,28E-01	1,58E-01	2,79E-01	No
ENSG00000252549	RNU6-759P	snRNA	9,71E-02	6,58E-01	8,32E-01	No	-7,60E-01	7,15E-02	1,50E-01	No
ENSG00000201221	RNU4-40P	snRNA	1,54E-01	3,28E-01	5,77E-01	No	-5,01E-02	8,30E-01	8,97E-01	No
ENSG00000207053	RNU6-937P	snRNA	-3,39E-02	8,05E-01	9,13E-01	No	-3,93E-01	1,42E-01	2,58E-01	No
ENSG00000201372	RNU6-1251P	snRNA	-7,19E-04	9,41E-01	NA	No	-9,72E-02	8,30E-01	NA	No
ENSG00000252123	RNU7-72P	snRNA	1,47E-02	8,38E-01	NA	No	6,16E-02	7,49E-01	NA	No
ENSG00000206710	RNU6-147P	snRNA	4,23E-03	9,41E-01	NA	No	-9,72E-02	8,30E-01	NA	No
ENSG00000238777	RNU7-141P	snRNA	4,50E-03	9,59E-01	NA	No	-9,06E-02	6,43E-01	NA	No
ENSG00000199805	RNU1-134P	snRNA	-1,31E-01	4,52E-01	6,88E-01	No	-5,51E-01	1,28E-01	2,38E-01	No
ENSG00000207507	RNU6-9	snRNA	-3,13E-01	9,47E-02	NA	No	-2,30E-01	4,80E-01	6,31E-01	No
ENSG00000207357	RNU6-2	snRNA	-2,30E-02	8,46E-01	9,33E-01	No	5,96E-02	8,11E-01	8,83E-01	No
ENSG00000252933	RNU6-1223P	snRNA	1,18E-01	2,38E-01	NA	No	1,51E-01	4,86E-01	NA	No
ENSG00000238364	RNU7-140P	snRNA	-2,89E-01	4,64E-02	1,65E-01	No	-6,41E-01	2,50E-02	6,25E-02	No
ENSG00000206674	RNU6-945P	snRNA	2,11E-02	8,74E-01	NA	No	9,17E-02	7,46E-01	NA	No
ENSG00000223284	RNU6-195P	snRNA	-1,73E-02	9,27E-01	9,70E-01	No	-5,37E-01	1,60E-01	2,82E-01	No
ENSG00000253048	RNU4-60P	snRNA	-2,05E-02	8,19E-01	NA	No	-1,36E-01	7,12E-01	NA	No
ENSG00000253021	RNU6-902P	snRNA	-4,23E-02	7,60E-01	NA	No	1,31E-02	9,87E-01	NA	No
ENSG00000207003	RNU6-611P	snRNA	1,57E-01	3,80E-01	6,26E-01	No	-4,21E-01	1,37E-01	NA	No
ENSG00000199796	RNU6-924P	snRNA	5,32E-02	7,39E-01	8,78E-01	No	-2,24E-01	4,47E-01	6,01E-01	No
ENSG00000251843	RNU6-803P	snRNA	-6,05E-02	5,46E-01	NA	No	-1,45E-01	6,08E-01	NA	No
ENSG00000252334	RNU6-1337P	snRNA	4,97E-02	6,82E-01	NA	No	8,51E-02	8,64E-01	NA	No
ENSG00000252900	RNU6-303P	snRNA	-4,15E-02	7,60E-01	NA	No	-9,51E-02	8,30E-01	NA	No
ENSG00000252472	RNU6-521P	snRNA	-1,21E-01	1,81E-01	NA	No	-2,80E-01	2,42E-01	NA	No
ENSG00000252209	RNU1-48P	snRNA	-4,23E-02	7,60E-01	NA	No	-9,82E-02	8,30E-01	NA	No
ENSG00000252766	RNU6-255P	snRNA	-1,12E-01	3,21E-01	5,70E-01	No	5,84E-03	9,86E-01	9,92E-01	No
ENSG00000251952	RNU6-1219P	snRNA	-4,46E-02	7,60E-01	NA	No	-1,03E-01	8,30E-01	NA	No
ENSG00000207455	RNU6-331P	snRNA	-2,90E-02	8,89E-01	9,54E-01	No	-2,81E-01	4,37E-01	5,91E-01	No
ENSG00000222915	RNU6-564P	snRNA	5,96E-02	7,06E-01	NA	No	2,53E-01	4,44E-01	5,98E-01	No
ENSG00000199695	RNU6-1128P	snRNA	-6,38E-02	5,95E-01	NA	No	-2,36E-01	2,32E-01	NA	No
ENSG00000199248	RNU6-28P	snRNA	-8,90E-02	4,66E-01	7,00E-01	No	-1,96E-01	4,97E-01	6,45E-01	No
ENSG00000207227	RNU6-900P	snRNA	-7,94E-03	9,43E-01	NA	No	-9,97E-02	5,85E-01	NA	No
ENSG00000200683	RNU6-379P	snRNA	4,65E-02	6,06E-01	NA	No	6,34E-02	9,56E-01	NA	No
ENSG00000252859	RNU6-375P	snRNA	-1,93E-02	8,76E-01	NA	No	-1,20E-01	6,18E-01	NA	No
ENSG00000199865	RNU6-495P	snRNA	9,08E-02	4,00E-01	NA	No	-5,99E-02	7,92E-01	NA	No
ENSG00000207457	RNU6-476P	snRNA	-5,83E-02	7,34E-01	8,76E-01	No	-1,02E+00	3,98E-02	9,26E-02	No
ENSG00000206841	RNU6-409P	snRNA	5,97E-02	6,99E-01	8,58E-01	No	7,68E-01	8,20E-02	1,67E-01	No

ENSG00000239023	RNU1-98P	snRNA	3,31E-02	7,34E-01	NA	No	-9,82E-02	8,30E-01	NA	No
ENSG00000251972	RNU6-123P	snRNA	-2,04E-01	3,15E-01	5,63E-01	No	-2,60E-01	4,44E-01	5,98E-01	No
ENSG00000251894	RNU6-872P	snRNA	1,89E-01	2,38E-01	4,77E-01	No	-1,97E-01	4,90E-01	6,40E-01	No
ENSG00000252619	RNU6-1149P	snRNA	-3,21E-03	9,00E-01	NA	No	-1,26E-01	7,12E-01	NA	No
ENSG00000199598	RNU6-859P	snRNA	-9,53E-03	9,46E-01	9,78E-01	No	-4,06E-01	2,07E-01	3,43E-01	No
ENSG00000252606	RNU6-1150P	snRNA	-6,63E-02	4,07E-01	NA	No	-1,18E-01	5,34E-01	NA	No
ENSG00000202239	RNU6-396P	snRNA	-5,39E-03	9,66E-01	NA	No	2,31E-01	4,60E-01	6,13E-01	No
ENSG00000238421	U1	snRNA	4,85E-02	5,43E-01	NA	No	1,98E-01	3,04E-01	4,57E-01	No
ENSG00000252596	U6	snRNA	-2,34E-02	8,08E-01	NA	No	7,31E-02	7,17E-01	NA	No
ENSG00000222623	RNU6-1100P	snRNA	3,50E-02	9,36E-01	NA	No	NA	NA	NA	NA
ENSG00000251977	RNU6-991P	snRNA	3,15E-02	9,36E-01	NA	No	NA	NA	NA	NA
ENSG00000252429	RNU7-29P	snRNA	2,34E-02	9,72E-01	NA	No	NA	NA	NA	NA
ENSG00000222821	RNU4-27P	snRNA	3,88E-02	8,45E-01	NA	No	NA	NA	NA	NA
ENSG00000238653	RNU7-62P	snRNA	3,15E-02	9,36E-01	NA	No	NA	NA	NA	NA
ENSG00000212366	RNU6-1246P	snRNA	3,50E-02	9,36E-01	NA	No	NA	NA	NA	NA
ENSG00000252121	RNU6-970P	snRNA	2,49E-02	9,72E-01	NA	No	NA	NA	NA	NA
ENSG00000238310	RNU7-122P	snRNA	2,46E-02	9,72E-01	NA	No	NA	NA	NA	NA
ENSG00000194297	RNU1-75P	snRNA	4,11E-02	7,67E-01	NA	No	NA	NA	NA	NA
ENSG00000252135	RNVU1-2	snRNA	3,32E-02	9,36E-01	NA	No	NA	NA	NA	NA
ENSG00000207340	RNVU1-10	snRNA	2,40E-02	9,72E-01	NA	No	NA	NA	NA	NA
ENSG00000207349	RNVU1-17	snRNA	2,22E-02	9,72E-01	NA	No	NA	NA	NA	NA
ENSG00000252826	RNU1-92P	snRNA	3,41E-02	9,36E-01	NA	No	NA	NA	NA	NA
ENSG00000221255	RNU6ATAC37P	snRNA	4,18E-02	7,50E-01	NA	No	NA	NA	NA	NA
ENSG00000206915	RNU6-439P	snRNA	3,32E-02	9,36E-01	NA	No	NA	NA	NA	NA
ENSG00000238619	RNU6-775P	snRNA	2,42E-02	9,72E-01	NA	No	NA	NA	NA	NA
ENSG00000212410	RNU6-932P	snRNA	3,17E-02	9,36E-01	NA	No	NA	NA	NA	NA
ENSG00000252027	RNU6ATAC14P	snRNA	3,32E-02	9,36E-01	NA	No	NA	NA	NA	NA
ENSG00000207178	RNU6-1122P	snRNA	3,41E-02	9,36E-01	NA	No	NA	NA	NA	NA
ENSG00000206725	RNU6-1027P	snRNA	9,59E-02	2,21E-01	NA	No	NA	NA	NA	NA
ENSG00000252562	RNU6-922P	snRNA	8,88E-02	3,97E-01	NA	No	NA	NA	NA	NA
ENSG00000210181	RNU6ATAC4P	snRNA	3,15E-02	9,36E-01	NA	No	NA	NA	NA	NA
ENSG00000201065	RNU6-461P	snRNA	3,17E-02	9,36E-01	NA	No	NA	NA	NA	NA
ENSG00000212344	RNU6-823P	snRNA	4,33E-02	7,20E-01	NA	No	NA	NA	NA	NA
ENSG00000222357	RNU2-20P	snRNA	5,63E-02	5,96E-01	NA	No	NA	NA	NA	NA
ENSG00000199488	RNU1-70P	snRNA	4,26E-02	7,43E-01	NA	No	NA	NA	NA	NA
ENSG00000207505	RNU6-1105P	snRNA	3,32E-02	9,36E-01	NA	No	NA	NA	NA	NA
ENSG00000212146	RNU6-910P	snRNA	2,42E-02	9,72E-01	NA	No	NA	NA	NA	NA
ENSG00000212297	RNU6-821P	snRNA	4,38E-02	7,01E-01	NA	No	NA	NA	NA	NA
ENSG00000252913	RNU6-158P	snRNA	3,39E-02	9,36E-01	NA	No	NA	NA	NA	NA
ENSG00000252815	RNU6-410P	snRNA	3,70E-02	8,45E-01	NA	No	NA	NA	NA	NA
ENSG00000252880	RNU6-459P	snRNA	3,24E-02	9,36E-01	NA	No	NA	NA	NA	NA
ENSG00000252955	RNU4ATAC9P	snRNA	3,15E-02	9,36E-01	NA	No	NA	NA	NA	NA
ENSG00000251874	RNU6-615P	snRNA	3,69E-02	8,45E-01	NA	No	NA	NA	NA	NA
ENSG00000202000	RNU1-36P	snRNA	5,64E-02	4,44E-01	NA	No	NA	NA	NA	NA
ENSG00000201707	RNU6-818P	snRNA	4,48E-02	6,65E-01	NA	No	NA	NA	NA	NA
ENSG00000199326	RNU6-1298P	snRNA	2,49E-02	9,72E-01	NA	No	NA	NA	NA	NA
ENSG00000201752	RNU6-119P	snRNA	3,41E-02	9,36E-01	NA	No	NA	NA	NA	NA
ENSG00000223208	RNU6-1217P	snRNA	4,24E-02	7,41E-01	NA	No	NA	NA	NA	NA
ENSG00000252183	RNU6-948P	snRNA	8,46E-02	4,42E-01	NA	No	NA	NA	NA	NA
ENSG00000206782	RNU6-224P	snRNA	3,32E-02	9,36E-01	NA	No	NA	NA	NA	NA
ENSG00000207188	RNU6-569P	snRNA	2,40E-02	9,72E-01	NA	No	NA	NA	NA	NA
ENSG00000202157	RNU4-11P	snRNA	3,46E-02	9,36E-01	NA	No	NA	NA	NA	NA
ENSG00000251741	RNU4ATAC13P	snRNA	2,42E-02	9,72E-01	NA	No	NA	NA	NA	NA
ENSG00000223179	RNU6-373P	snRNA	2,46E-02	9,72E-01	NA	No	NA	NA	NA	NA
ENSG00000200347	RNU6-963P	snRNA	3,39E-02	9,36E-01	NA	No	NA	NA	NA	NA
ENSG00000207459	RNU6-1311P	snRNA	3,17E-02	9,36E-01	NA	No	NA	NA	NA	NA
ENSG00000199880	RNU6-888P	snRNA	2,22E-02	9,72E-01	NA	No	NA	NA	NA	NA
ENSG00000222790	RNU4-14P	snRNA	3,91E-02	8,45E-01	NA	No	NA	NA	NA	NA
ENSG00000222213	RNU6-588P	snRNA	4,72E-02	5,79E-01	NA	No	NA	NA	NA	NA
ENSG00000238610	RNU7-26P	snRNA	2,35E-02	9,72E-01	NA	No	NA	NA	NA	NA
ENSG00000201658	RNU6-283P	snRNA	2,35E-02	9,72E-01	NA	No	NA	NA	NA	NA
ENSG00000251916	RNU1-61P	snRNA	4,21E-02	7,47E-01	NA	No	NA	NA	NA	NA

ENSG00000252738	RNU6-515P	snRNA	3,17E-02	9,36E-01	NA	No	NA	NA	NA	NA
ENSG00000200224	RNU6-626P	snRNA	8,59E-02	2,90E-01	NA	No	NA	NA	NA	NA
ENSG00000252137	RNU6-1338P	snRNA	2,23E-02	9,72E-01	NA	No	NA	NA	NA	NA
ENSG00000223001	RNU2-61P	snRNA	3,17E-02	9,36E-01	NA	No	NA	NA	NA	NA
ENSG00000201386	RNU6-1163P	snRNA	3,15E-02	9,36E-01	NA	No	NA	NA	NA	NA
ENSG00000206857	RNU6-214P	snRNA	3,32E-02	9,36E-01	NA	No	NA	NA	NA	NA
ENSG00000223191	RNU6-293P	snRNA	2,34E-02	9,72E-01	NA	No	NA	NA	NA	NA
ENSG00000201292	RNU6-153P	snRNA	4,26E-02	7,31E-01	NA	No	NA	NA	NA	NA
ENSG00000252480	RNU6-719P	snRNA	3,17E-02	9,36E-01	NA	No	NA	NA	NA	NA
ENSG00000252023	RNU6-581P	snRNA	2,23E-02	9,72E-01	NA	No	NA	NA	NA	NA
ENSG00000199971	RNU6-797P	snRNA	3,32E-02	9,36E-01	NA	No	NA	NA	NA	NA
ENSG00000199283	RNU1-58P	snRNA	4,21E-02	7,67E-01	NA	No	NA	NA	NA	NA
ENSG00000206983	RNU6-49P	snRNA	3,84E-02	8,45E-01	NA	No	NA	NA	NA	NA
ENSG00000252930	RNU6-1015P	snRNA	3,46E-02	9,36E-01	NA	No	NA	NA	NA	NA
ENSG00000199368	RNU6-1084P	snRNA	3,32E-02	9,36E-01	NA	No	NA	NA	NA	NA
ENSG00000212407	RNU6-663P	snRNA	3,17E-02	9,36E-01	NA	No	NA	NA	NA	NA
ENSG00000222522	RNU6-519P	snRNA	3,46E-02	9,36E-01	NA	No	NA	NA	NA	NA
ENSG00000201231	RNU4-50P	snRNA	3,93E-02	8,45E-01	NA	No	NA	NA	NA	NA
ENSG00000206949	RNU6-1324P	snRNA	2,40E-02	9,72E-01	NA	No	NA	NA	NA	NA
ENSG00000223327	RNU2-71P	snRNA	3,32E-02	9,36E-01	NA	No	NA	NA	NA	NA
ENSG00000221387	RNU6ATAC8P	snRNA	3,50E-02	9,36E-01	NA	No	NA	NA	NA	NA
ENSG00000206695	RNU6-442P	snRNA	5,55E-02	6,08E-01	NA	No	NA	NA	NA	NA
ENSG00000207138	RNU6-869P	snRNA	2,22E-02	9,72E-01	NA	No	NA	NA	NA	NA
ENSG00000200941	RNU6-694P	snRNA	2,40E-02	9,72E-01	NA	No	NA	NA	NA	NA
ENSG00000222760	RNU4-53P	snRNA	4,24E-02	7,41E-01	NA	No	NA	NA	NA	NA
ENSG00000238415	RNU6-538P	snRNA	3,32E-02	9,36E-01	NA	No	NA	NA	NA	NA
ENSG00000202308	RNU6-996P	snRNA	3,94E-02	8,45E-01	NA	No	NA	NA	NA	NA
ENSG00000252521	RNU5D-2P	snRNA	4,36E-02	7,20E-01	NA	No	NA	NA	NA	NA
ENSG00000223156	RNU2-18P	snRNA	1,05E-01	2,97E-01	NA	No	NA	NA	NA	NA
ENSG00000253070	RNU6-794P	snRNA	2,42E-02	9,72E-01	NA	No	NA	NA	NA	NA
ENSG00000222314	RNU6-1118P	snRNA	2,23E-02	9,72E-01	NA	No	NA	NA	NA	NA
ENSG00000200817	RNU6-899P	snRNA	3,39E-02	9,36E-01	NA	No	NA	NA	NA	NA
ENSG00000207200	RNU6-45P	snRNA	6,08E-02	5,43E-01	NA	No	NA	NA	NA	NA
ENSG00000239099	RNU7-23P	snRNA	7,93E-02	3,52E-01	NA	No	NA	NA	NA	NA
ENSG00000206587	RNU6-46P	snRNA	2,22E-02	9,72E-01	NA	No	NA	NA	NA	NA
ENSG00000222663	RNU4-55P	snRNA	4,31E-02	7,29E-01	NA	No	NA	NA	NA	NA
ENSG00000201243	RNU6-654P	snRNA	2,46E-02	9,72E-01	NA	No	NA	NA	NA	NA
ENSG00000252255	RNU2-35P	snRNA	3,32E-02	9,36E-01	NA	No	NA	NA	NA	NA
ENSG00000201780	RNU6-275P	snRNA	4,40E-02	6,95E-01	NA	No	NA	NA	NA	NA
ENSG00000252421	RNU6-1069P	snRNA	4,57E-02	6,37E-01	NA	No	NA	NA	NA	NA
ENSG00000201296	RNU4-41P	snRNA	3,41E-02	9,36E-01	NA	No	NA	NA	NA	NA
ENSG00000252393	RNU6-1004P	snRNA	2,35E-02	9,72E-01	NA	No	NA	NA	NA	NA
ENSG00000252008	RNU6-927P	snRNA	3,15E-02	9,36E-01	NA	No	NA	NA	NA	NA
ENSG00000206812	RNU6-38P	snRNA	3,31E-02	9,36E-01	NA	No	NA	NA	NA	NA
ENSG00000251771	RNU6-1033P	snRNA	2,23E-02	9,72E-01	NA	No	NA	NA	NA	NA
ENSG00000251907	RNU6-240P	snRNA	4,19E-02	7,48E-01	NA	No	NA	NA	NA	NA
ENSG00000201136	RNU6-353P	snRNA	2,35E-02	9,72E-01	NA	No	NA	NA	NA	NA
ENSG00000252995	RNU6-667P	snRNA	3,97E-02	8,45E-01	NA	No	NA	NA	NA	NA
ENSG00000200424	RNU6-1155P	snRNA	5,39E-02	4,76E-01	NA	No	NA	NA	NA	NA
ENSG00000252339	RNU6-1061P	snRNA	3,32E-02	9,36E-01	NA	No	NA	NA	NA	NA
ENSG00000238731	RNU7-90P	snRNA	4,19E-02	7,48E-01	NA	No	NA	NA	NA	NA
ENSG00000252608	RNU6-1191P	snRNA	3,32E-02	9,36E-01	NA	No	NA	NA	NA	NA
ENSG00000222359	RNU6-355P	snRNA	2,35E-02	9,72E-01	NA	No	NA	NA	NA	NA
ENSG00000238704	RNU7-97P	snRNA	3,46E-02	9,36E-01	NA	No	NA	NA	NA	NA
ENSG00000212469	RNU6-1158P	snRNA	5,03E-02	6,74E-01	NA	No	NA	NA	NA	NA
ENSG00000207169	RNU6-24P	snRNA	4,64E-02	6,11E-01	NA	No	NA	NA	NA	NA
ENSG00000206827	RNU6-1032P	snRNA	3,88E-02	8,45E-01	NA	No	NA	NA	NA	NA
ENSG00000221806	RNU6ATAC34P	snRNA	4,63E-02	6,13E-01	NA	No	NA	NA	NA	NA
ENSG00000199497	RNU1-94P	snRNA	4,04E-02	7,80E-01	NA	No	NA	NA	NA	NA
ENSG00000222612	RNU2-52P	snRNA	2,34E-02	9,72E-01	NA	No	NA	NA	NA	NA
ENSG00000221046	RNU6ATAC38P	snRNA	2,35E-02	9,72E-01	NA	No	NA	NA	NA	NA
ENSG00000252654	RNU7-6P	snRNA	3,41E-02	9,36E-01	NA	No	NA	NA	NA	NA

ENSG00000200871	RNU6-810P	snRNA	3,87E-02	8,45E-01	NA	No	NA	NA	NA	NA
ENSG00000251985	RNU6-1161P	snRNA	2,35E-02	9,72E-01	NA	No	NA	NA	NA	NA
ENSG00000223287	RNU6-954P	snRNA	3,24E-02	9,36E-01	NA	No	NA	NA	NA	NA
ENSG00000235054	RP5-1166F10.1	lincRNA	NA	NA	NA	NA	1,10E-01	6,85E-01	NA	No
ENSG00000260972	RP1-58B11.1	lincRNA	NA	NA	NA	NA	8,58E-02	8,64E-01	NA	No
ENSG00000223643	RP11-108M9.1	lincRNA	NA	NA	NA	NA	6,68E-02	9,56E-01	NA	No
ENSG00000261025	RP11-84D1.2	lincRNA	NA	NA	NA	NA	8,79E-02	8,64E-01	NA	No
ENSG00000203620	RP11-84A19.2	lincRNA	NA	NA	NA	NA	1,44E-01	5,60E-01	NA	No
ENSG00000223944	RP5-1180C18.1	lincRNA	NA	NA	NA	NA	8,55E-02	8,64E-01	NA	No
ENSG00000225667	LINC00505	lincRNA	NA	NA	NA	NA	8,64E-02	8,64E-01	NA	No
ENSG00000229639	RP3-380B4.1	lincRNA	NA	NA	NA	NA	6,17E-02	9,56E-01	NA	No
ENSG00000229051	RP5-952N6.1	lincRNA	NA	NA	NA	NA	6,17E-02	9,56E-01	NA	No
ENSG00000225605	RP11-550H2.1	lincRNA	NA	NA	NA	NA	1,21E-01	6,68E-01	NA	No
ENSG00000260322	RP11-339A11.2	lincRNA	NA	NA	NA	NA	8,77E-02	8,64E-01	NA	No
ENSG00000233290	RP11-147G16.1	lincRNA	NA	NA	NA	NA	8,77E-02	8,64E-01	NA	No
ENSG00000230434	RP11-14O19.1	lincRNA	NA	NA	NA	NA	9,18E-02	8,64E-01	NA	No
ENSG00000237954	RP11-14O19.2	lincRNA	NA	NA	NA	NA	1,07E-01	6,95E-01	NA	No
ENSG00000230402	RP11-84O12.4	lincRNA	NA	NA	NA	NA	1,07E-01	6,95E-01	NA	No
ENSG00000226715	RP11-411H5.1	lincRNA	NA	NA	NA	NA	9,18E-02	8,64E-01	NA	No
ENSG00000233359	RP11-202K23.1	lincRNA	NA	NA	NA	NA	1,18E-01	5,67E-01	NA	No
ENSG00000232265	XXyac-YX155B6.5	lincRNA	NA	NA	NA	NA	8,77E-02	8,64E-01	NA	No
ENSG00000230687	RP5-1114G22.2	lincRNA	NA	NA	NA	NA	8,64E-02	8,64E-01	NA	No
ENSG00000232309	GS1-122H1.1	lincRNA	NA	NA	NA	NA	6,68E-02	9,56E-01	NA	No
ENSG00000272827	RP11-445P19.3	lincRNA	NA	NA	NA	NA	9,20E-02	8,64E-01	NA	No
ENSG00000230426	ERVMER61-1	lincRNA	NA	NA	NA	NA	9,90E-02	7,75E-01	NA	No
ENSG00000273093	RP11-532L16.3	lincRNA	NA	NA	NA	NA	1,07E-01	6,96E-01	NA	No
ENSG00000224260	RP1-272L16.1	lincRNA	NA	NA	NA	NA	1,24E-01	5,21E-01	NA	No
ENSG00000229983	RP11-15I11.2	lincRNA	NA	NA	NA	NA	6,19E-02	9,56E-01	NA	No
ENSG00000224359	RP11-467I20.2	lincRNA	NA	NA	NA	NA	6,19E-02	9,56E-01	NA	No
ENSG00000232694	XX-CR54.3	lincRNA	NA	NA	NA	NA	8,77E-02	8,64E-01	NA	No
ENSG00000228391	AC011995.3	lincRNA	NA	NA	NA	NA	1,08E-01	6,91E-01	NA	No
ENSG00000237401	AC107070.1	lincRNA	NA	NA	NA	NA	6,34E-02	9,56E-01	NA	No
ENSG00000236172	hsa-mir-7515	lincRNA	NA	NA	NA	NA	9,20E-02	8,64E-01	NA	No
ENSG00000236989	AC142119.1	lincRNA	NA	NA	NA	NA	6,23E-02	9,56E-01	NA	No
ENSG00000227047	AC012065.4	lincRNA	NA	NA	NA	NA	6,13E-02	9,56E-01	NA	No
ENSG00000225226	AC007250.4	lincRNA	NA	NA	NA	NA	6,19E-02	9,56E-01	NA	No
ENSG00000231815	AC007179.1	lincRNA	NA	NA	NA	NA	8,64E-02	8,64E-01	NA	No
ENSG00000227293	AC118345.1	lincRNA	NA	NA	NA	NA	8,51E-02	8,64E-01	NA	No
ENSG00000237013	AC010987.6	lincRNA	NA	NA	NA	NA	9,02E-02	8,64E-01	NA	No
ENSG00000230968	AC084149.1	lincRNA	NA	NA	NA	NA	8,58E-02	8,64E-01	NA	No
ENSG00000229209	AC073987.1	lincRNA	NA	NA	NA	NA	8,58E-02	8,64E-01	NA	No
ENSG00000225765	AC068535.3	lincRNA	NA	NA	NA	NA	8,58E-02	8,64E-01	NA	No
ENSG00000229457	AC006227.1	lincRNA	NA	NA	NA	NA	9,02E-02	8,64E-01	NA	No
ENSG00000231221	AC023672.2	lincRNA	NA	NA	NA	NA	8,51E-02	8,64E-01	NA	No
ENSG00000236485	RP1-156L9.1	lincRNA	NA	NA	NA	NA	8,77E-02	8,64E-01	NA	No
ENSG00000227902	AC062032.1	lincRNA	NA	NA	NA	NA	1,09E-01	6,78E-01	NA	No
ENSG00000236885	AC018731.3	lincRNA	NA	NA	NA	NA	6,23E-02	9,56E-01	NA	No
ENSG00000225258	AC009478.1	lincRNA	NA	NA	NA	NA	6,34E-02	9,56E-01	NA	No
ENSG00000236445	LINC00608	lincRNA	NA	NA	NA	NA	6,19E-02	9,56E-01	NA	No
ENSG00000226423	AC093642.4	lincRNA	NA	NA	NA	NA	8,55E-02	8,64E-01	NA	No
ENSG00000240895	RP11-119D18.1	lincRNA	NA	NA	NA	NA	9,94E-02	7,75E-01	NA	No
ENSG00000242816	RP11-768G7.3	lincRNA	NA	NA	NA	NA	6,67E-02	9,56E-01	NA	No
ENSG00000250271	RP11-64D22.5	lincRNA	NA	NA	NA	NA	1,10E-01	6,87E-01	NA	No
ENSG00000243953	RP11-439C8.2	lincRNA	NA	NA	NA	NA	9,20E-02	8,64E-01	NA	No
ENSG00000241383	RP11-152C17.1	lincRNA	NA	NA	NA	NA	8,77E-02	8,64E-01	NA	No
ENSG00000236385	RP11-114M1.2	lincRNA	NA	NA	NA	NA	1,18E-01	5,72E-01	NA	No
ENSG00000224406	RP11-48F14.1	lincRNA	NA	NA	NA	NA	1,11E-01	6,71E-01	NA	No
ENSG00000251679	RP11-669M16.2	lincRNA	NA	NA	NA	NA	1,05E-01	7,75E-01	NA	No
ENSG00000251049	RP11-685F15.1	lincRNA	NA	NA	NA	NA	8,77E-02	8,64E-01	NA	No
ENSG00000240152	RP11-16N2.1	lincRNA	NA	NA	NA	NA	6,23E-02	9,56E-01	NA	No
ENSG00000251331	RP11-689K5.3	lincRNA	NA	NA	NA	NA	1,02E-01	7,75E-01	NA	No
ENSG00000250920	RP11-297P16.4	lincRNA	NA	NA	NA	NA	1,04E-01	7,75E-01	NA	No

ENSG00000251170	RP11-729M20.1	lincRNA	NA	NA	NA	NA	6,23E-02	9,56E-01	NA	No
ENSG00000249613	RP13-612N21.1	lincRNA	NA	NA	NA	NA	6,19E-02	9,56E-01	NA	No
ENSG00000248656	RP11-255I10.2	lincRNA	NA	NA	NA	NA	8,77E-02	8,64E-01	NA	No
ENSG00000250855	RP11-269F21.1	lincRNA	NA	NA	NA	NA	1,08E-01	6,92E-01	NA	No
ENSG00000248173	RP11-659O3.1	lincRNA	NA	NA	NA	NA	8,77E-02	8,64E-01	NA	No
ENSG00000253825	RP11-96A1.5	lincRNA	NA	NA	NA	NA	8,64E-02	8,64E-01	NA	No
ENSG00000250102	RP11-314N14.1	lincRNA	NA	NA	NA	NA	9,88E-02	7,75E-01	NA	No
ENSG00000249000	CTD-2012I17.1	lincRNA	NA	NA	NA	NA	6,34E-02	9,56E-01	NA	No
ENSG00000248747	RP11-586L23.1	lincRNA	NA	NA	NA	NA	8,53E-02	8,64E-01	NA	No
ENSG00000251061	RP11-789C1.2	lincRNA	NA	NA	NA	NA	8,64E-02	8,64E-01	NA	No
ENSG00000248339	RP11-717H13.1	lincRNA	NA	NA	NA	NA	1,01E-01	7,75E-01	NA	No
ENSG00000251619	RP11-756P10.5	lincRNA	NA	NA	NA	NA	6,17E-02	9,56E-01	NA	No
ENSG00000250921	CTD-2008N3.1	lincRNA	NA	NA	NA	NA	9,94E-02	7,75E-01	NA	No
ENSG00000248131	CT49	lincRNA	NA	NA	NA	NA	9,18E-02	8,64E-01	NA	No
ENSG00000250453	CTD-2134P3.1	lincRNA	NA	NA	NA	NA	9,88E-02	7,75E-01	NA	No
ENSG00000248533	CTD-2130F23.1	lincRNA	NA	NA	NA	NA	8,58E-02	8,64E-01	NA	No
ENSG00000248461	CTD-2207A17.1	lincRNA	NA	NA	NA	NA	1,04E-01	7,75E-01	NA	No
ENSG00000249697	RP11-155L15.1	lincRNA	NA	NA	NA	NA	8,77E-02	8,64E-01	NA	No
ENSG00000248294	CTD-2023N9.2	lincRNA	NA	NA	NA	NA	1,17E-01	5,75E-01	NA	No
ENSG00000249436	CTD-2023N9.3	lincRNA	NA	NA	NA	NA	6,23E-02	9,56E-01	NA	No
ENSG00000250237	CTC-498J12.1	lincRNA	NA	NA	NA	NA	1,00E-01	7,75E-01	NA	No
ENSG00000250874	CTC-480C2.1	lincRNA	NA	NA	NA	NA	8,58E-02	8,64E-01	NA	No
ENSG00000250567	CTD-2154H6.1	lincRNA	NA	NA	NA	NA	6,19E-02	9,56E-01	NA	No
ENSG00000250358	RP11-159K7.2	lincRNA	NA	NA	NA	NA	8,51E-02	8,64E-01	NA	No
ENSG00000250438	CTD-2308B18.2	lincRNA	NA	NA	NA	NA	9,02E-02	8,64E-01	NA	No
ENSG00000248631	RP11-425E13.1	lincRNA	NA	NA	NA	NA	8,77E-02	8,64E-01	NA	No
ENSG00000250402	RP11-395P13.4	lincRNA	NA	NA	NA	NA	9,18E-02	8,64E-01	NA	No
ENSG00000251072	RP11-434D11.4	lincRNA	NA	NA	NA	NA	6,54E-02	9,56E-01	NA	No
ENSG00000249647	CTC-349C3.2	lincRNA	NA	NA	NA	NA	8,77E-02	8,64E-01	NA	No
ENSG00000253673	CTC-436K13.1	lincRNA	NA	NA	NA	NA	1,37E-01	5,90E-01	NA	No
ENSG00000253331	CTC-207P7.1	lincRNA	NA	NA	NA	NA	8,51E-02	8,64E-01	NA	No
ENSG00000253236	CTD-2313P7.1	lincRNA	NA	NA	NA	NA	8,77E-02	8,64E-01	NA	No
ENSG00000254066	CTB-181F24.1	lincRNA	NA	NA	NA	NA	8,77E-02	8,64E-01	NA	No
ENSG00000253141	CTB-164N12.1	lincRNA	NA	NA	NA	NA	1,71E-01	4,50E-01	NA	No
ENSG00000248943	RP11-1026M7.3	lincRNA	NA	NA	NA	NA	9,20E-02	8,64E-01	NA	No
ENSG00000229282	RP1-40E16.2	lincRNA	NA	NA	NA	NA	9,20E-02	8,64E-01	NA	No
ENSG00000233656	RP11-716O23.1	lincRNA	NA	NA	NA	NA	6,23E-02	9,56E-01	NA	No
ENSG00000228772	RP1-309H15.2	lincRNA	NA	NA	NA	NA	9,02E-02	8,64E-01	NA	No
ENSG00000233358	RP1-209A6.1	lincRNA	NA	NA	NA	NA	9,20E-02	8,64E-01	NA	No
ENSG00000225173	XXbac-BPG308K3.5	lincRNA	NA	NA	NA	NA	6,17E-02	9,56E-01	NA	No
ENSG00000225595	XXbac-BPG308K3.6	lincRNA	NA	NA	NA	NA	1,07E-01	6,99E-01	NA	No
ENSG00000256166	XXbac-BPG248L24.13	lincRNA	NA	NA	NA	NA	6,67E-02	9,56E-01	NA	No
ENSG00000236355	RP3-503A6.2	lincRNA	NA	NA	NA	NA	8,77E-02	8,64E-01	NA	No
ENSG00000236635	RP11-35J1.1	lincRNA	NA	NA	NA	NA	6,19E-02	9,56E-01	NA	No
ENSG00000235357	RP1-159G19.1	lincRNA	NA	NA	NA	NA	6,13E-02	9,56E-01	NA	No
ENSG00000234426	RP11-459O1.2	lincRNA	NA	NA	NA	NA	8,58E-02	8,64E-01	NA	No
ENSG00000224371	RP11-235G24.1	lincRNA	NA	NA	NA	NA	1,13E-01	6,49E-01	NA	No
ENSG00000236173	RP1-182D15.2	lincRNA	NA	NA	NA	NA	1,08E-01	6,89E-01	NA	No
ENSG00000227386	AC091705.1	lincRNA	NA	NA	NA	NA	6,19E-02	9,56E-01	NA	No
ENSG00000224322	AC004009.3	lincRNA	NA	NA	NA	NA	8,55E-02	8,64E-01	NA	No
ENSG00000234223	AC003988.1	lincRNA	NA	NA	NA	NA	8,64E-02	8,64E-01	NA	No
ENSG00000236938	AC003092.2	lincRNA	NA	NA	NA	NA	9,02E-02	8,64E-01	NA	No
ENSG00000234826	AC003084.2	lincRNA	NA	NA	NA	NA	1,21E-01	5,31E-01	NA	No
ENSG00000227869	RP11-807H17.1	lincRNA	NA	NA	NA	NA	1,17E-01	5,86E-01	NA	No
ENSG00000242078	RP11-738B7.1	lincRNA	NA	NA	NA	NA	6,19E-02	9,56E-01	NA	No
ENSG00000228569	AC073133.2	lincRNA	NA	NA	NA	NA	1,07E-01	6,99E-01	NA	No
ENSG00000236102	RP4-639J15.1	lincRNA	NA	NA	NA	NA	8,77E-02	8,64E-01	NA	No
ENSG00000225882	RP3-410B11.1	lincRNA	NA	NA	NA	NA	9,02E-02	8,64E-01	NA	No
ENSG00000234191	RP11-157D23.2	lincRNA	NA	NA	NA	NA	1,01E-01	7,75E-01	NA	No
ENSG00000224294	RP3-326I13.1	lincRNA	NA	NA	NA	NA	9,20E-02	8,64E-01	NA	No
ENSG00000215113	CXorf49B	lincRNA	NA	NA	NA	NA	6,13E-02	9,56E-01	NA	No
ENSG00000253452	RP11-473J6.1	lincRNA	NA	NA	NA	NA	9,18E-02	8,64E-01	NA	No

ENSG00000253388	RP11-150O12.2	lincRNA	NA	NA	NA	NA	6,67E-02	9,56E-01	NA	No
ENSG00000254321	RP11-495O10.1	lincRNA	NA	NA	NA	NA	1,21E-01	5,25E-01	NA	No
ENSG00000254082	CTD-2024D23.1	lincRNA	NA	NA	NA	NA	8,51E-02	8,64E-01	NA	No
ENSG00000254136	RP11-546K22.2	lincRNA	NA	NA	NA	NA	1,07E-01	6,94E-01	NA	No
ENSG00000215117	LINC00588	lincRNA	NA	NA	NA	NA	9,18E-02	8,64E-01	NA	No
ENSG00000253322	RP11-388G22.1	lincRNA	NA	NA	NA	NA	9,02E-02	8,64E-01	NA	No
ENSG00000253373	RP11-21C17.1	lincRNA	NA	NA	NA	NA	1,17E-01	6,81E-01	NA	No
ENSG00000260838	RP11-531A24.3	lincRNA	NA	NA	NA	NA	1,11E-01	6,54E-01	NA	No
ENSG00000253712	RP11-697M17.2	lincRNA	NA	NA	NA	NA	1,52E-01	5,16E-01	NA	No
ENSG00000254689	RP11-354A14.1	lincRNA	NA	NA	NA	NA	1,19E-01	5,56E-01	NA	No
ENSG00000254202	RP11-120I21.2	lincRNA	NA	NA	NA	NA	1,17E-01	5,88E-01	NA	No
ENSG00000254380	RP11-317J10.4	lincRNA	NA	NA	NA	NA	9,86E-02	7,75E-01	NA	No
ENSG00000253682	RP11-700E23.2	lincRNA	NA	NA	NA	NA	1,07E-01	6,98E-01	NA	No
ENSG00000254209	RP11-3G20.1	lincRNA	NA	NA	NA	NA	1,20E-01	5,44E-01	NA	No
ENSG00000253111	RP11-136O12.2	lincRNA	NA	NA	NA	NA	1,63E-01	4,83E-01	NA	No
ENSG00000218839	FAM138C	lincRNA	NA	NA	NA	NA	6,17E-02	9,56E-01	NA	No
ENSG00000204844	FAM74A3	lincRNA	NA	NA	NA	NA	6,68E-02	9,56E-01	NA	No
ENSG00000233074	RP11-327I22.6	lincRNA	NA	NA	NA	NA	8,55E-02	8,64E-01	NA	No
ENSG00000224303	RP11-327I22.5	lincRNA	NA	NA	NA	NA	8,55E-02	8,64E-01	NA	No
ENSG00000235523	RP11-63P12.7	lincRNA	NA	NA	NA	NA	9,18E-02	8,64E-01	NA	No
ENSG00000227809	RP11-171A24.2	lincRNA	NA	NA	NA	NA	1,22E-01	5,24E-01	NA	No
ENSG00000226798	RP11-289F5.1	lincRNA	NA	NA	NA	NA	8,55E-02	8,64E-01	NA	No
ENSG00000235819	RP11-359J6.1	lincRNA	NA	NA	NA	NA	1,13E-01	6,42E-01	NA	No
ENSG00000235601	RP11-231K24.2	lincRNA	NA	NA	NA	NA	9,18E-02	8,64E-01	NA	No
ENSG00000236130	RP11-23B15.1	lincRNA	NA	NA	NA	NA	8,55E-02	8,64E-01	NA	No
ENSG00000225564	RP11-341A22.2	lincRNA	NA	NA	NA	NA	9,86E-02	7,75E-01	NA	No
ENSG00000228714	RP11-284G10.1	lincRNA	NA	NA	NA	NA	6,67E-02	9,56E-01	NA	No
ENSG00000233569	RP11-500B12.1	lincRNA	NA	NA	NA	NA	1,08E-01	6,92E-01	NA	No
ENSG00000223795	RP11-473E2.2	lincRNA	NA	NA	NA	NA	1,27E-01	6,53E-01	NA	No
ENSG00000242147	RP13-463N16.6	lincRNA	NA	NA	NA	NA	6,23E-02	9,56E-01	NA	No
ENSG00000234306	RP11-271F18.1	lincRNA	NA	NA	NA	NA	1,03E-01	7,75E-01	NA	No
ENSG00000226447	RP11-393J16.4	lincRNA	NA	NA	NA	NA	9,02E-02	8,64E-01	NA	No
ENSG00000229466	RP11-56I23.1	lincRNA	NA	NA	NA	NA	8,51E-02	8,64E-01	NA	No
ENSG00000234474	RP11-501J20.2	lincRNA	NA	NA	NA	NA	6,23E-02	9,56E-01	NA	No
ENSG00000254670	CTC-497E21.3	lincRNA	NA	NA	NA	NA	1,27E-01	5,09E-01	NA	No
ENSG00000254695	RP11-396O20.1	lincRNA	NA	NA	NA	NA	1,08E-01	6,92E-01	NA	No
ENSG00000254819	RP11-430L3.1	lincRNA	NA	NA	NA	NA	1,12E-01	6,53E-01	NA	No
ENSG00000254526	RP11-466I1.1	lincRNA	NA	NA	NA	NA	6,17E-02	9,56E-01	NA	No
ENSG00000255227	RP11-460B17.2	lincRNA	NA	NA	NA	NA	1,07E-01	6,99E-01	NA	No
ENSG00000255477	RP11-63D14.1	lincRNA	NA	NA	NA	NA	1,07E-01	6,99E-01	NA	No
ENSG00000255175	RP11-277K23.1	lincRNA	NA	NA	NA	NA	1,25E-01	5,17E-01	NA	No
ENSG00000254651	RP11-430H10.3	lincRNA	NA	NA	NA	NA	6,17E-02	9,56E-01	NA	No
ENSG00000255426	CTD-2210P24.2	lincRNA	NA	NA	NA	NA	6,17E-02	9,56E-01	NA	No
ENSG00000255269	RP11-702F3.4	lincRNA	NA	NA	NA	NA	8,77E-02	8,64E-01	NA	No
ENSG00000255931	RP11-286N22.10	lincRNA	NA	NA	NA	NA	9,88E-02	7,75E-01	NA	No
ENSG00000255947	RP11-855O10.3	lincRNA	NA	NA	NA	NA	8,51E-02	8,64E-01	NA	No
ENSG00000232500	AP005273.1	lincRNA	NA	NA	NA	NA	8,55E-02	8,64E-01	NA	No
ENSG00000250105	CTD-3074O7.2	lincRNA	NA	NA	NA	NA	6,17E-02	9,56E-01	NA	No
ENSG00000261347	AP000439.5	lincRNA	NA	NA	NA	NA	1,50E-01	5,34E-01	NA	No
ENSG00000254699	RP11-12D16.2	lincRNA	NA	NA	NA	NA	8,51E-02	8,64E-01	NA	No
ENSG00000256684	RP11-49K4.2	lincRNA	NA	NA	NA	NA	6,23E-02	9,56E-01	NA	No
ENSG00000255502	RP11-379J13.2	lincRNA	NA	NA	NA	NA	1,07E-01	6,99E-01	NA	No
ENSG00000255689	RP11-136I14.5	lincRNA	NA	NA	NA	NA	8,51E-02	8,64E-01	NA	No
ENSG00000255124	RP11-680F20.5	lincRNA	NA	NA	NA	NA	1,11E-01	6,71E-01	NA	No
ENSG00000254790	RP11-680F20.4	lincRNA	NA	NA	NA	NA	6,34E-02	9,56E-01	NA	No
ENSG00000255087	RP11-168K9.2	lincRNA	NA	NA	NA	NA	1,08E-01	6,92E-01	NA	No
ENSG00000273409	RP11-480C22.1	lincRNA	NA	NA	NA	NA	1,18E-01	5,64E-01	NA	No
ENSG00000255512	AP005135.2	lincRNA	NA	NA	NA	NA	6,23E-02	9,56E-01	NA	No
ENSG00000257048	RP11-476M19.2	lincRNA	NA	NA	NA	NA	8,64E-02	8,64E-01	NA	No
ENSG00000256115	RP11-319E16.1	lincRNA	NA	NA	NA	NA	1,27E-01	5,09E-01	NA	No
ENSG00000256146	RP11-319E16.2	lincRNA	NA	NA	NA	NA	1,42E-01	1,45E-01	2,62E-01	No
ENSG00000257262	RP11-776A13.1	lincRNA	NA	NA	NA	NA	6,34E-02	9,56E-01	NA	No

ENSG00000223914	AC079630.2	lincRNA		NA	NA	NA	NA	8,55E-02	8,64E-01	NA	No
ENSG00000257587	RP11-711C17.1	lincRNA		NA	NA	NA	NA	8,64E-02	8,64E-01	NA	No
ENSG00000251138	RP11-81H3.2	lincRNA		NA	NA	NA	NA	1,10E-01	6,81E-01	NA	No
ENSG00000257995	RP11-632B21.1	lincRNA		NA	NA	NA	NA	4,04E-01	8,80E-02	1,77E-01	No
ENSG00000196243	LINC00615	lincRNA		NA	NA	NA	NA	6,34E-02	9,56E-01	NA	No
ENSG00000257893	RP11-587P21.2	lincRNA		NA	NA	NA	NA	9,18E-02	8,64E-01	NA	No
ENSG00000257470	RP11-397H6.1	lincRNA		NA	NA	NA	NA	8,55E-02	8,64E-01	NA	No
ENSG00000257141	RP11-554D14.7	lincRNA		NA	NA	NA	NA	3,23E-01	1,67E-01	NA	No
ENSG00000257407	RP11-1028N23.4	lincRNA		NA	NA	NA	NA	1,00E-01	7,75E-01	NA	No
ENSG00000255778	RP4-765H13.1	lincRNA		NA	NA	NA	NA	1,13E-01	6,49E-01	NA	No
ENSG00000256906	RP11-474D1.2	lincRNA		NA	NA	NA	NA	9,20E-02	8,64E-01	NA	No
ENSG00000227332	RP11-38M15.11	lincRNA		NA	NA	NA	NA	1,01E-01	7,75E-01	NA	No
ENSG00000233405	LINC01046	lincRNA		NA	NA	NA	NA	9,18E-02	8,64E-01	NA	No
ENSG00000226968	LINC00423	lincRNA		NA	NA	NA	NA	8,55E-02	8,64E-01	NA	No
ENSG00000236036	LINC00445	lincRNA		NA	NA	NA	NA	6,54E-02	9,56E-01	NA	No
ENSG00000236354	LINC00437	lincRNA		NA	NA	NA	NA	1,75E-01	4,25E-01	NA	No
ENSG00000225249	LINC00378	lincRNA		NA	NA	NA	NA	8,77E-02	8,64E-01	NA	No
ENSG00000237534	LINC00383	lincRNA		NA	NA	NA	NA	8,51E-02	8,64E-01	NA	No
ENSG00000226370	LINC00375	lincRNA		NA	NA	NA	NA	6,34E-02	9,56E-01	NA	No
ENSG00000261666	LINC00560	lincRNA		NA	NA	NA	NA	8,58E-02	8,64E-01	NA	No
ENSG00000229520	LINC00404	lincRNA		NA	NA	NA	NA	6,17E-02	9,56E-01	NA	No
ENSG00000224243	LINC00403	lincRNA		NA	NA	NA	NA	8,77E-02	8,64E-01	NA	No
ENSG00000260343	LINC01043	lincRNA		NA	NA	NA	NA	1,08E-01	6,92E-01	NA	No
ENSG00000257826	RP11-116N8.4	lincRNA		NA	NA	NA	NA	1,42E-01	5,71E-01	NA	No
ENSG00000258050	RP11-698F20.3	lincRNA		NA	NA	NA	NA	8,77E-02	8,64E-01	NA	No
ENSG00000258902	CTD-2128A3.2	lincRNA		NA	NA	NA	NA	1,25E-01	5,20E-01	NA	No
ENSG00000258814	CTD-2128A3.3	lincRNA		NA	NA	NA	NA	6,67E-02	9,56E-01	NA	No
ENSG00000259107	LINC00911	lincRNA		NA	NA	NA	NA	9,18E-02	8,64E-01	NA	No
ENSG00000259077	RP11-804L24.2	lincRNA		NA	NA	NA	NA	6,34E-02	9,56E-01	NA	No
ENSG00000259998	RP11-753D20.4	lincRNA		NA	NA	NA	NA	8,58E-02	8,64E-01	NA	No
ENSG00000259789	RP11-1078H9.6	lincRNA		NA	NA	NA	NA	6,13E-02	9,56E-01	NA	No
ENSG00000259443	RP11-403B2.5	lincRNA		NA	NA	NA	NA	8,55E-02	8,64E-01	NA	No
ENSG00000260232	PWRN4	lincRNA		NA	NA	NA	NA	8,77E-02	8,64E-01	NA	No
ENSG00000260780	RP11-580I1.1	lincRNA		NA	NA	NA	NA	9,02E-02	8,64E-01	NA	No
ENSG00000259011	RP11-2C7.1	lincRNA		NA	NA	NA	NA	8,55E-02	8,64E-01	NA	No
ENSG00000261480	RP11-578F21.6	lincRNA		NA	NA	NA	NA	4,87E-01	8,95E-02	NA	No
ENSG00000261709	SNORA11	lincRNA		NA	NA	NA	NA	1,18E-01	5,62E-01	NA	No
ENSG00000259681	RP11-96O20.2	lincRNA		NA	NA	NA	NA	1,02E-01	7,75E-01	NA	No
ENSG00000259255	RP11-627D16.1	lincRNA		NA	NA	NA	NA	8,59E-02	8,64E-01	NA	No
ENSG00000259572	RP11-198M11.2	lincRNA		NA	NA	NA	NA	8,55E-02	8,64E-01	NA	No
ENSG00000259941	RP11-48G14.2	lincRNA		NA	NA	NA	NA	6,23E-02	9,56E-01	NA	No
ENSG00000260957	RP11-299H22.7	lincRNA		NA	NA	NA	NA	6,17E-02	9,56E-01	NA	No
ENSG00000258433	RP11-255M2.1	lincRNA		NA	NA	NA	NA	8,77E-02	8,64E-01	NA	No
ENSG00000259312	RP11-522B15.5	lincRNA		NA	NA	NA	NA	8,77E-02	8,64E-01	NA	No
ENSG00000259604	RP11-66B24.1	lincRNA		NA	NA	NA	NA	1,07E-01	6,99E-01	NA	No
ENSG00000260009	RP11-65J21.1	lincRNA		NA	NA	NA	NA	6,67E-02	9,56E-01	NA	No
ENSG00000234186	C16orf82	lincRNA		NA	NA	NA	NA	9,18E-02	8,64E-01	NA	No
ENSG00000260626	RP11-23E10.3	lincRNA		NA	NA	NA	NA	1,04E-01	7,75E-01	NA	No
ENSG00000259912	CTC-527H23.2	lincRNA		NA	NA	NA	NA	6,67E-02	9,56E-01	NA	No
ENSG00000263082	CTD-2034I21.2	lincRNA		NA	NA	NA	NA	6,54E-02	9,56E-01	NA	No
ENSG00000261804	RP11-44F14.2	lincRNA		NA	NA	NA	NA	9,20E-02	8,64E-01	NA	No
ENSG00000260706	RP11-525K10.1	lincRNA		NA	NA	NA	NA	6,19E-02	9,56E-01	NA	No
ENSG00000268754	LINC01081	lincRNA		NA	NA	NA	NA	6,13E-02	9,56E-01	NA	No
ENSG00000261273	LA16c-444G7.1	lincRNA		NA	NA	NA	NA	8,51E-02	8,64E-01	NA	No
ENSG00000256982	CTD-2555A7.2	lincRNA		NA	NA	NA	NA	1,07E-01	7,01E-01	NA	No
ENSG00000273098	RP11-488L1.1	lincRNA		NA	NA	NA	NA	8,58E-02	8,64E-01	NA	No
ENSG00000265556	RP11-434D2.2	lincRNA		NA	NA	NA	NA	6,13E-02	9,56E-01	NA	No
ENSG00000267075	RP11-434D2.3	lincRNA		NA	NA	NA	NA	6,17E-02	9,56E-01	NA	No
ENSG00000264262	RP11-285M22.3	lincRNA		NA	NA	NA	NA	6,19E-02	9,56E-01	NA	No
ENSG00000225582	AC011193.1	lincRNA		NA	NA	NA	NA	8,55E-02	8,64E-01	NA	No
ENSG00000267578	AC015849.12	lincRNA		NA	NA	NA	NA	8,55E-02	8,64E-01	NA	No
ENSG00000267151	MIR2117	lincRNA		NA	NA	NA	NA	1,15E-01	6,02E-01	NA	No

ENSG00000262973	RP11-708H21.4	lincRNA	NA	NA	NA	NA	1,60E-01	4,96E-01	NA	No
ENSG00000265000	RP11-453A12.1	lincRNA	NA	NA	NA	NA	6,23E-02	9,56E-01	NA	No
ENSG00000267603	LINC01028	lincRNA	NA	NA	NA	NA	6,17E-02	9,56E-01	NA	No
ENSG00000262188	RP11-353N14.4	lincRNA	NA	NA	NA	NA	9,85E-02	7,75E-01	NA	No
ENSG00000272461	RP11-689C9.1	lincRNA	NA	NA	NA	NA	1,18E-01	5,62E-01	NA	No
ENSG00000263745	RP11-161I6.2	lincRNA	NA	NA	NA	NA	8,51E-02	8,64E-01	NA	No
ENSG00000259256	RP11-838N2.3	lincRNA	NA	NA	NA	NA	6,19E-02	9,56E-01	NA	No
ENSG00000266153	RP11-190I17.2	lincRNA	NA	NA	NA	NA	8,64E-02	8,64E-01	NA	No
ENSG00000264876	RP11-138E9.2	lincRNA	NA	NA	NA	NA	9,02E-02	8,64E-01	NA	No
ENSG00000260913	RP11-243E13.1	lincRNA	NA	NA	NA	NA	9,02E-02	8,64E-01	NA	No
ENSG00000264301	RP11-527H14.3	lincRNA	NA	NA	NA	NA	8,64E-02	8,64E-01	NA	No
ENSG00000265984	RP11-699A5.2	lincRNA	NA	NA	NA	NA	8,42E-01	4,26E-02	NA	No
ENSG00000265758	RP11-595B24.1	lincRNA	NA	NA	NA	NA	8,55E-02	8,64E-01	NA	No
ENSG00000265369	U3	lincRNA	NA	NA	NA	NA	8,64E-02	8,64E-01	NA	No
ENSG00000268566	RP11-687D19.1	lincRNA	NA	NA	NA	NA	6,19E-02	9,56E-01	NA	No
ENSG00000267284	RP11-397A16.1	lincRNA	NA	NA	NA	NA	9,85E-02	7,75E-01	NA	No
ENSG00000267172	RP11-397A16.3	lincRNA	NA	NA	NA	NA	8,51E-02	8,64E-01	NA	No
ENSG00000266952	RP11-909B2.1	lincRNA	NA	NA	NA	NA	8,55E-02	8,64E-01	NA	No
ENSG00000264634	RP11-659F24.1	lincRNA	NA	NA	NA	NA	1,00E-01	7,75E-01	NA	No
ENSG00000263958	RP11-676J15.1	lincRNA	NA	NA	NA	NA	8,55E-02	8,64E-01	NA	No
ENSG00000259779	RP11-231E4.2	lincRNA	NA	NA	NA	NA	8,55E-02	8,64E-01	NA	No
ENSG00000236387	RP11-307O10.1	lincRNA	NA	NA	NA	NA	9,18E-02	8,64E-01	NA	No
ENSG00000228482	RP5-859D4.3	lincRNA	NA	NA	NA	NA	8,55E-02	8,64E-01	NA	No
ENSG00000232448	RP11-416N4.1	lincRNA	NA	NA	NA	NA	1,10E-01	6,85E-01	NA	No
ENSG00000236985	RP5-1195D24.1	lincRNA	NA	NA	NA	NA	2,71E-01	2,65E-01	NA	No
ENSG00000232200	RP4-564O4.1	lincRNA	NA	NA	NA	NA	8,58E-02	8,64E-01	NA	No
ENSG00000271784	RP1-28H20.3	lincRNA	NA	NA	NA	NA	3,29E-01	1,44E-01	NA	No
ENSG00000267616	AC016626.2	lincRNA	NA	NA	NA	NA	8,55E-02	8,64E-01	NA	No
ENSG00000267406	CTD-2189E23.2	lincRNA	NA	NA	NA	NA	1,12E-01	6,53E-01	NA	No
ENSG00000267509	CTD-2043I16.1	lincRNA	NA	NA	NA	NA	9,02E-02	8,64E-01	NA	No
ENSG00000267599	CTD-2050I18.2	lincRNA	NA	NA	NA	NA	9,18E-02	8,64E-01	NA	No
ENSG00000267094	CTC-360P9.5	lincRNA	NA	NA	NA	NA	8,77E-02	8,64E-01	NA	No
ENSG00000267626	AC002115.9	lincRNA	NA	NA	NA	NA	1,37E-01	4,57E-01	NA	No
ENSG00000269842	MIR519A2	lincRNA	NA	NA	NA	NA	6,17E-02	9,56E-01	NA	No
ENSG00000178248	AP000345.1	lincRNA	NA	NA	NA	NA	8,51E-02	8,64E-01	NA	No
ENSG00000238195	CTA-503F6.2	lincRNA	NA	NA	NA	NA	1,98E-01	3,39E-01	NA	No
ENSG00000231495	RP1-213J1P__B.2	lincRNA	NA	NA	NA	NA	6,68E-02	9,56E-01	NA	No
ENSG00000273044	RP4-569D19.8	lincRNA	NA	NA	NA	NA	8,55E-02	8,64E-01	NA	No
ENSG00000235965	AP000431.1	lincRNA	NA	NA	NA	NA	8,64E-02	8,64E-01	NA	No
ENSG00000233236	AP001171.1	lincRNA	NA	NA	NA	NA	8,55E-02	8,64E-01	NA	No
ENSG00000233215	AP000472.2	lincRNA	NA	NA	NA	NA	6,34E-02	9,56E-01	NA	No
ENSG00000252841	Z98044.1	miRNA	NA	NA	NA	NA	1,17E-01	5,83E-01	NA	No
ENSG00000263381	MIR5584	miRNA	NA	NA	NA	NA	6,67E-02	9,56E-01	NA	No
ENSG00000221707	AL592294.1	miRNA	NA	NA	NA	NA	6,13E-02	9,56E-01	NA	No
ENSG00000212076	MIR761	miRNA	NA	NA	NA	NA	6,67E-02	9,56E-01	NA	No
ENSG00000264551	AC099791.1	miRNA	NA	NA	NA	NA	6,19E-02	9,56E-01	NA	No
ENSG00000221720	AC096951.1	miRNA	NA	NA	NA	NA	8,90E-02	8,64E-01	NA	No
ENSG00000221702	AL356270.1	miRNA	NA	NA	NA	NA	6,54E-02	9,56E-01	NA	No
ENSG00000221798	AC092812.1	miRNA	NA	NA	NA	NA	9,18E-02	8,64E-01	NA	No
ENSG00000252731	AL161793.1	miRNA	NA	NA	NA	NA	1,03E-01	7,75E-01	NA	No
ENSG00000264972	AL445675.1	miRNA	NA	NA	NA	NA	6,54E-02	9,56E-01	NA	No
ENSG00000221165	AL359983.1	miRNA	NA	NA	NA	NA	8,53E-02	8,64E-01	NA	No
ENSG00000221383	AC016907.1	miRNA	NA	NA	NA	NA	8,58E-02	8,64E-01	NA	No
ENSG00000238448	AL121652.2	miRNA	NA	NA	NA	NA	8,87E-02	8,64E-01	NA	No
ENSG00000212025	AC011748.1	miRNA	NA	NA	NA	NA	9,18E-02	8,64E-01	NA	No
ENSG00000216191	AC009234.2	miRNA	NA	NA	NA	NA	9,02E-02	8,64E-01	NA	No
ENSG00000211984	AC079117.2	miRNA	NA	NA	NA	NA	1,13E-01	6,49E-01	NA	No
ENSG00000263447	AC079117.3	miRNA	NA	NA	NA	NA	9,02E-02	8,64E-01	NA	No
ENSG00000263656	AC105402.1	miRNA	NA	NA	NA	NA	6,54E-02	9,56E-01	NA	No
ENSG00000221374	AC007242.1	miRNA	NA	NA	NA	NA	8,77E-02	8,64E-01	NA	No
ENSG00000221628	MIR1302-4	miRNA	NA	NA	NA	NA	6,34E-02	9,56E-01	NA	No
ENSG00000251849	AC010887.1	miRNA	NA	NA	NA	NA	1,13E-01	6,88E-01	NA	No

ENSG00000207611	MIR149	miRNA	NA	NA	NA	NA	6,68E-02	9,56E-01	NA	No
ENSG00000263752	MIR3133	miRNA	NA	NA	NA	NA	6,23E-02	9,56E-01	NA	No
ENSG00000221659	AC104441.1	miRNA	NA	NA	NA	NA	6,67E-02	9,56E-01	NA	No
ENSG00000238672	AC020626.1	miRNA	NA	NA	NA	NA	8,55E-02	8,64E-01	NA	No
ENSG00000266546	AC137674.2	miRNA	NA	NA	NA	NA	9,02E-02	8,64E-01	NA	No
ENSG00000264135	AC104306.2	miRNA	NA	NA	NA	NA	8,89E-02	8,64E-01	NA	No
ENSG00000264209	AC104448.1	miRNA	NA	NA	NA	NA	9,20E-02	8,64E-01	NA	No
ENSG00000222888	AC012467.1	miRNA	NA	NA	NA	NA	6,23E-02	9,56E-01	NA	No
ENSG00000265166	AF186996.1	miRNA	NA	NA	NA	NA	1,07E-01	6,96E-01	NA	No
ENSG00000221270	AC130888.1	miRNA	NA	NA	NA	NA	8,55E-02	8,64E-01	NA	No
ENSG00000222833	AC069067.1	miRNA	NA	NA	NA	NA	6,17E-02	9,56E-01	NA	No
ENSG00000263634	MIR3919	miRNA	NA	NA	NA	NA	9,94E-02	7,75E-01	NA	No
ENSG00000265090	AC104637.1	miRNA	NA	NA	NA	NA	6,68E-02	9,56E-01	NA	No
ENSG00000221120	MIR1224	miRNA	NA	NA	NA	NA	6,23E-02	9,56E-01	NA	No
ENSG00000266690	MIR4274	miRNA	NA	NA	NA	NA	1,06E-01	7,03E-01	NA	No
ENSG00000222262	AC092846.1	miRNA	NA	NA	NA	NA	8,58E-02	8,64E-01	NA	No
ENSG00000265035	AC079772.1	miRNA	NA	NA	NA	NA	8,51E-02	8,64E-01	NA	No
ENSG00000222437	AC118282.3	miRNA	NA	NA	NA	NA	6,19E-02	9,56E-01	NA	No
ENSG00000265720	AC097470.1	miRNA	NA	NA	NA	NA	9,18E-02	8,64E-01	NA	No
ENSG00000221499	AC078881.1	miRNA	NA	NA	NA	NA	1,07E-01	6,95E-01	NA	No
ENSG00000221244	AC116351.2	miRNA	NA	NA	NA	NA	8,64E-02	8,64E-01	NA	No
ENSG00000223200	AC008836.1	miRNA	NA	NA	NA	NA	9,18E-02	8,64E-01	NA	No
ENSG00000223149	AC010376.1	miRNA	NA	NA	NA	NA	8,51E-02	8,64E-01	NA	No
ENSG00000238518	AC020900.1	miRNA	NA	NA	NA	NA	1,29E-01	4,98E-01	NA	No
ENSG00000264039	AC114296.1	miRNA	NA	NA	NA	NA	9,18E-02	8,64E-01	NA	No
ENSG00000264890	AC011379.1	miRNA	NA	NA	NA	NA	1,00E-01	7,75E-01	NA	No
ENSG00000264241	AL138721.1	miRNA	NA	NA	NA	NA	6,13E-02	9,56E-01	NA	No
ENSG00000266675	AL606923.1	miRNA	NA	NA	NA	NA	8,64E-02	8,64E-01	NA	No
ENSG00000266346	AC019205.2	miRNA	NA	NA	NA	NA	2,06E-01	2,98E-01	NA	No
ENSG00000221599	AL136446.1	miRNA	NA	NA	NA	NA	6,67E-02	9,56E-01	NA	No
ENSG00000221434	AL132671.1	miRNA	NA	NA	NA	NA	6,13E-02	9,56E-01	NA	No
ENSG00000221336	AL356137.1	miRNA	NA	NA	NA	NA	8,64E-02	8,64E-01	NA	No
ENSG00000212101	AL080276.1	miRNA	NA	NA	NA	NA	1,11E-01	6,54E-01	NA	No
ENSG00000252986	AC147651.2	miRNA	NA	NA	NA	NA	1,04E-01	7,75E-01	NA	No
ENSG00000222101	AC091705.2	miRNA	NA	NA	NA	NA	1,14E-01	6,27E-01	NA	No
ENSG00000222994	AC006322.1	miRNA	NA	NA	NA	NA	1,09E-01	6,78E-01	NA	No
ENSG00000222482	AC005071.1	miRNA	NA	NA	NA	NA	6,54E-02	9,56E-01	NA	No
ENSG00000252111	AC002981.1	miRNA	NA	NA	NA	NA	6,19E-02	9,56E-01	NA	No
ENSG00000207995	MIR325	miRNA	NA	NA	NA	NA	1,07E-01	6,99E-01	NA	No
ENSG00000212032	AC007486.1	miRNA	NA	NA	NA	NA	2,14E-01	4,11E-01	NA	No
ENSG00000207755	MIR450A2	miRNA	NA	NA	NA	NA	3,27E-01	2,38E-01	NA	No
ENSG00000207818	MIR105-2	miRNA	NA	NA	NA	NA	6,13E-02	9,56E-01	NA	No
ENSG00000221567	AF228730.2	miRNA	NA	NA	NA	NA	6,68E-02	9,56E-01	NA	No
ENSG00000263407	MIR4660	miRNA	NA	NA	NA	NA	8,58E-02	8,64E-01	NA	No
ENSG00000265826	AC087203.1	miRNA	NA	NA	NA	NA	6,54E-02	9,56E-01	NA	No
ENSG00000266476	AC084262.3	miRNA	NA	NA	NA	NA	6,23E-02	9,56E-01	NA	No
ENSG00000221753	MIR1273A	miRNA	NA	NA	NA	NA	9,18E-02	8,64E-01	NA	No
ENSG00000211995	AP000428.1	miRNA	NA	NA	NA	NA	6,13E-02	9,56E-01	NA	No
ENSG00000199177	MIR31	miRNA	NA	NA	NA	NA	6,34E-02	9,56E-01	NA	No
ENSG00000266881	AL353662.4	miRNA	NA	NA	NA	NA	8,51E-02	8,64E-01	NA	No
ENSG00000266353	AL591471.1	miRNA	NA	NA	NA	NA	1,90E-01	3,69E-01	NA	No
ENSG00000263754	CR786580.2	miRNA	NA	NA	NA	NA	8,77E-02	8,64E-01	NA	No
ENSG00000264891	AL359091.1	miRNA	NA	NA	NA	NA	8,51E-02	8,64E-01	NA	No
ENSG00000263921	AC013737.1	miRNA	NA	NA	NA	NA	8,58E-02	8,64E-01	NA	No
ENSG00000221378	AL096864.1	miRNA	NA	NA	NA	NA	8,51E-02	8,64E-01	NA	No
ENSG00000207895	AL161652.1	miRNA	NA	NA	NA	NA	8,90E-02	8,64E-01	NA	No
ENSG00000264984	MIR5691	miRNA	NA	NA	NA	NA	9,02E-02	8,64E-01	NA	No
ENSG00000265467	AC090559.1	miRNA	NA	NA	NA	NA	8,64E-02	8,64E-01	NA	No
ENSG00000266550	AP002958.1	miRNA	NA	NA	NA	NA	8,79E-02	8,64E-01	NA	No
ENSG00000223101	AP002364.1	miRNA	NA	NA	NA	NA	8,80E-02	8,64E-01	NA	No
ENSG00000221565	AP004242.1	miRNA	NA	NA	NA	NA	1,13E-01	6,49E-01	NA	No
ENSG00000266043	MIR3649	miRNA	NA	NA	NA	NA	1,02E-01	7,75E-01	NA	No

ENSG00000221104	AC092865.1	miRNA	NA	NA	NA	NA	1,05E-01	7,75E-01	NA	No
ENSG00000265029	AC023158.1	miRNA	NA	NA	NA	NA	6,23E-02	9,56E-01	NA	No
ENSG00000266016	AC009779.1	miRNA	NA	NA	NA	NA	1,22E-01	5,04E-01	NA	No
ENSG00000266099	MIR5700	miRNA	NA	NA	NA	NA	8,77E-02	8,64E-01	NA	No
ENSG00000263364	AC021052.1	miRNA	NA	NA	NA	NA	6,19E-02	9,56E-01	NA	No
ENSG00000265710	AL161772.1	miRNA	NA	NA	NA	NA	6,19E-02	9,56E-01	NA	No
ENSG00000264226	MIR3168	miRNA	NA	NA	NA	NA	6,67E-02	9,56E-01	NA	No
ENSG00000264042	AL354833.3	miRNA	NA	NA	NA	NA	8,58E-02	8,64E-01	NA	No
ENSG00000266699	AL512655.1	miRNA	NA	NA	NA	NA	1,08E-01	6,92E-01	NA	No
ENSG00000238980	AL354806.1	miRNA	NA	NA	NA	NA	1,13E-01	6,49E-01	NA	No
ENSG00000263741	MIR548AS	miRNA	NA	NA	NA	NA	1,07E-01	6,99E-01	NA	No
ENSG00000266500	AL138498.1	miRNA	NA	NA	NA	NA	6,54E-02	9,56E-01	NA	No
ENSG00000221427	AL121819.1	miRNA	NA	NA	NA	NA	6,13E-02	9,56E-01	NA	No
ENSG00000199151	MIR337	miRNA	NA	NA	NA	NA	1,26E-01	6,57E-01	NA	No
ENSG00000202560	MIR539	miRNA	NA	NA	NA	NA	1,00E-01	7,75E-01	NA	No
ENSG00000263643	MIR4515	miRNA	NA	NA	NA	NA	6,13E-02	9,56E-01	NA	No
ENSG00000212063	AC112693.1	miRNA	NA	NA	NA	NA	6,19E-02	9,56E-01	NA	No
ENSG00000222409	AC103996.1	miRNA	NA	NA	NA	NA	6,19E-02	9,56E-01	NA	No
ENSG00000265529	AL031721.1	miRNA	NA	NA	NA	NA	1,04E-01	7,75E-01	NA	No
ENSG00000264115	hsa-mir-3180-4	miRNA	NA	NA	NA	NA	9,18E-02	8,64E-01	NA	No
ENSG00000221286	AC092289.1	miRNA	NA	NA	NA	NA	8,51E-02	8,64E-01	NA	No
ENSG00000263636	AC138304.1	miRNA	NA	NA	NA	NA	6,17E-02	9,56E-01	NA	No
ENSG00000265403	AC015884.1	miRNA	NA	NA	NA	NA	8,77E-02	8,64E-01	NA	No
ENSG00000267200	MIR132	miRNA	NA	NA	NA	NA	8,58E-02	8,64E-01	NA	No
ENSG00000222770	AC138761.1	miRNA	NA	NA	NA	NA	6,34E-02	9,56E-01	NA	No
ENSG00000265444	MIR4733	miRNA	NA	NA	NA	NA	8,51E-02	8,64E-01	NA	No
ENSG00000252789	AC006487.1	miRNA	NA	NA	NA	NA	8,64E-02	8,64E-01	NA	No
ENSG00000207552	MIR633	miRNA	NA	NA	NA	NA	8,51E-02	8,64E-01	NA	No
ENSG00000252785	AC103809.1	miRNA	NA	NA	NA	NA	8,77E-02	8,64E-01	NA	No
ENSG00000221621	AC009835.1	miRNA	NA	NA	NA	NA	6,13E-02	9,56E-01	NA	No
ENSG00000207778	MIR122	miRNA	NA	NA	NA	NA	8,64E-02	8,64E-01	NA	No
ENSG00000264059	AC013558.1	miRNA	NA	NA	NA	NA	6,13E-02	9,56E-01	NA	No
ENSG00000221220	AL035045.1	miRNA	NA	NA	NA	NA	6,17E-02	9,56E-01	NA	No
ENSG00000207972	MIR638	miRNA	NA	NA	NA	NA	6,23E-02	9,56E-01	NA	No
ENSG00000267959	MIR3188	miRNA	NA	NA	NA	NA	6,19E-02	9,56E-01	NA	No
ENSG00000223148	AC011478.1	miRNA	NA	NA	NA	NA	1,11E-01	6,54E-01	NA	No
ENSG00000264141	MIR3928	miRNA	NA	NA	NA	NA	6,34E-02	9,56E-01	NA	No
ENSG00000263854	AL591856.5	miRNA	NA	NA	NA	NA	8,58E-02	8,64E-01	NA	No
ENSG00000221597	BX088702.1	miRNA	NA	NA	NA	NA	8,64E-02	8,64E-01	NA	No
ENSG00000239144	snoU13	snoRNA	NA	NA	NA	NA	8,55E-02	8,64E-01	NA	No
ENSG00000238618	snoU13	snoRNA	NA	NA	NA	NA	8,55E-02	8,64E-01	NA	No
ENSG00000221040	U3	snoRNA	NA	NA	NA	NA	1,03E-01	7,75E-01	NA	No
ENSG00000252236	SNORA26	snoRNA	NA	NA	NA	NA	8,68E-02	8,64E-01	NA	No
ENSG00000238430	snoU13	snoRNA	NA	NA	NA	NA	8,55E-02	8,64E-01	NA	No
ENSG00000238722	snoU13	snoRNA	NA	NA	NA	NA	6,34E-02	9,56E-01	NA	No
ENSG00000206647	SNORA2	snoRNA	NA	NA	NA	NA	1,07E-01	6,99E-01	NA	No
ENSG00000202537	U8	snoRNA	NA	NA	NA	NA	9,18E-02	8,64E-01	NA	No
ENSG00000238546	snoU13	snoRNA	NA	NA	NA	NA	8,77E-02	8,64E-01	NA	No
ENSG00000238377	SNORD61	snoRNA	NA	NA	NA	NA	6,17E-02	9,56E-01	NA	No
ENSG00000253076	SNORD112	snoRNA	NA	NA	NA	NA	8,58E-02	8,64E-01	NA	No
ENSG00000238992	snoU13	snoRNA	NA	NA	NA	NA	1,21E-01	6,63E-01	NA	No
ENSG00000238701	snoU13	snoRNA	NA	NA	NA	NA	6,13E-02	9,56E-01	NA	No
ENSG00000201827	SNORA33	snoRNA	NA	NA	NA	NA	1,00E-01	7,75E-01	NA	No
ENSG00000238400	snoU13	snoRNA	NA	NA	NA	NA	8,58E-02	8,64E-01	NA	No
ENSG00000238334	snoU13	snoRNA	NA	NA	NA	NA	1,22E-01	5,25E-01	NA	No
ENSG00000238458	snoU13	snoRNA	NA	NA	NA	NA	6,17E-02	9,56E-01	NA	No
ENSG00000238375	snoU13	snoRNA	NA	NA	NA	NA	6,17E-02	9,56E-01	NA	No
ENSG00000212587	SNORA40	snoRNA	NA	NA	NA	NA	6,17E-02	9,56E-01	NA	No
ENSG00000238938	snoU13	snoRNA	NA	NA	NA	NA	6,54E-02	9,56E-01	NA	No
ENSG00000201407	SNORA68	snoRNA	NA	NA	NA	NA	8,55E-02	8,64E-01	NA	No
ENSG00000207215	U3	snoRNA	NA	NA	NA	NA	1,11E-01	6,54E-01	NA	No
ENSG00000206776	SNORA32	snoRNA	NA	NA	NA	NA	9,18E-02	8,64E-01	NA	No

ENSG00000238901	snoU13	snoRNA	NA	NA	NA	NA	6,54E-02	9,56E-01	NA	No
ENSG00000252724	SNORA70	snoRNA	NA	NA	NA	NA	8,58E-02	8,64E-01	NA	No
ENSG00000251909	U8	snoRNA	NA	NA	NA	NA	6,19E-02	9,56E-01	NA	No
ENSG00000252537	SNORA31	snoRNA	NA	NA	NA	NA	6,68E-02	9,56E-01	NA	No
ENSG00000200545	U3	snoRNA	NA	NA	NA	NA	1,05E-01	7,75E-01	NA	No
ENSG00000238355	snoU13	snoRNA	NA	NA	NA	NA	6,67E-02	9,56E-01	NA	No
ENSG00000252679	snoU13	snoRNA	NA	NA	NA	NA	6,13E-02	9,56E-01	NA	No
ENSG00000223294	SNORD83	snoRNA	NA	NA	NA	NA	1,10E-01	6,81E-01	NA	No
ENSG00000201502	SNORD74	snoRNA	NA	NA	NA	NA	1,09E-01	6,78E-01	NA	No
ENSG00000238800	snoU13	snoRNA	NA	NA	NA	NA	1,00E-01	7,75E-01	NA	No
ENSG00000272464	snoU13	snoRNA	NA	NA	NA	NA	9,18E-02	8,64E-01	NA	No
ENSG00000238895	snoU13	snoRNA	NA	NA	NA	NA	8,55E-02	8,64E-01	NA	No
ENSG00000200636	SNORD114-27	snoRNA	NA	NA	NA	NA	1,18E-01	5,61E-01	NA	No
ENSG00000202275	SNORD51	snoRNA	NA	NA	NA	NA	6,19E-02	9,56E-01	NA	No
ENSG00000239083	snoU13	snoRNA	NA	NA	NA	NA	8,55E-02	8,64E-01	NA	No
ENSG00000199970	SNORD115-3	snoRNA	NA	NA	NA	NA	8,55E-02	8,64E-01	NA	No
ENSG00000200757	SNORD115-16	snoRNA	NA	NA	NA	NA	6,34E-02	9,56E-01	NA	No
ENSG00000202188	SNORD115-31	snoRNA	NA	NA	NA	NA	8,58E-02	8,64E-01	NA	No
ENSG00000199311	SNORD115-34	snoRNA	NA	NA	NA	NA	6,19E-02	9,56E-01	NA	No
ENSG00000201992	SNORD115-35	snoRNA	NA	NA	NA	NA	8,64E-02	8,64E-01	NA	No
ENSG00000201634	SNORD115-48	snoRNA	NA	NA	NA	NA	6,23E-02	9,56E-01	NA	No
ENSG00000252425	SNORA18	snoRNA	NA	NA	NA	NA	6,17E-02	9,56E-01	NA	No
ENSG00000238767	snoU13	snoRNA	NA	NA	NA	NA	8,66E-02	8,64E-01	NA	No
ENSG00000238981	snoU13	snoRNA	NA	NA	NA	NA	6,34E-02	9,56E-01	NA	No
ENSG00000252040	snoU109	snoRNA	NA	NA	NA	NA	8,64E-02	8,64E-01	NA	No
ENSG00000252572	SNORD112	snoRNA	NA	NA	NA	NA	1,71E-01	4,49E-01	NA	No
ENSG00000212626	SNORA48	snoRNA	NA	NA	NA	NA	6,17E-02	9,56E-01	NA	No
ENSG00000239089	snoU13	snoRNA	NA	NA	NA	NA	6,54E-02	9,56E-01	NA	No
ENSG00000200237	SNORA70	snoRNA	NA	NA	NA	NA	9,84E-02	7,75E-01	NA	No
ENSG00000199783	SNORD56	snoRNA	NA	NA	NA	NA	9,18E-02	8,64E-01	NA	No
ENSG00000238314	snoU13	snoRNA	NA	NA	NA	NA	6,23E-02	9,56E-01	NA	No
ENSG00000264452	snoZ6	snoRNA	NA	NA	NA	NA	6,17E-02	9,56E-01	NA	No
ENSG00000207106	RNVU1-4	snRNA	NA	NA	NA	NA	6,23E-02	9,56E-01	NA	No
ENSG00000206828	RNVU1-5	snRNA	NA	NA	NA	NA	8,59E-02	8,64E-01	NA	No
ENSG00000201105	RNU1-137P	snRNA	NA	NA	NA	NA	6,19E-02	9,56E-01	NA	No
ENSG00000206764	RNU6-152P	snRNA	NA	NA	NA	NA	8,64E-02	8,64E-01	NA	No
ENSG00000253097	RNU2-19P	snRNA	NA	NA	NA	NA	8,58E-02	8,64E-01	NA	No
ENSG00000252073	RNU6-947P	snRNA	NA	NA	NA	NA	6,17E-02	9,56E-01	NA	No
ENSG00000207364	RNU6-939P	snRNA	NA	NA	NA	NA	6,17E-02	9,56E-01	NA	No
ENSG00000238830	RNU7-46P	snRNA	NA	NA	NA	NA	8,55E-02	8,64E-01	NA	No
ENSG00000206718	RNU6-546P	snRNA	NA	NA	NA	NA	6,23E-02	9,56E-01	NA	No
ENSG00000212167	RNU6-763P	snRNA	NA	NA	NA	NA	1,07E-01	6,98E-01	NA	No
ENSG00000223096	RNU5E-9P	snRNA	NA	NA	NA	NA	9,18E-02	8,64E-01	NA	No
ENSG00000212269	RNU6-788P	snRNA	NA	NA	NA	NA	9,20E-02	8,64E-01	NA	No
ENSG00000201033	RNU1-96P	snRNA	NA	NA	NA	NA	1,19E-01	5,54E-01	NA	No
ENSG00000201452	RNU6-1317P	snRNA	NA	NA	NA	NA	1,04E-01	7,75E-01	NA	No
ENSG00000212489	RNU6-1109P	snRNA	NA	NA	NA	NA	9,81E-02	7,75E-01	NA	No
ENSG00000200184	RNU1-20P	snRNA	NA	NA	NA	NA	8,55E-02	8,64E-01	NA	No
ENSG00000212368	RNU6-1000P	snRNA	NA	NA	NA	NA	8,77E-02	8,64E-01	NA	No
ENSG00000207307	RNU6-145P	snRNA	NA	NA	NA	NA	1,54E-01	5,24E-01	NA	No
ENSG00000202485	RNU6-499P	snRNA	NA	NA	NA	NA	8,77E-02	8,64E-01	NA	No
ENSG00000199529	RNU6-462P	snRNA	NA	NA	NA	NA	6,13E-02	9,56E-01	NA	No
ENSG00000238441	RNU7-130P	snRNA	NA	NA	NA	NA	1,14E-01	6,29E-01	NA	No
ENSG00000212561	RNU6-381P	snRNA	NA	NA	NA	NA	6,17E-02	9,56E-01	NA	No
ENSG00000210678	RNU6ATAC2P	snRNA	NA	NA	NA	NA	8,51E-02	8,64E-01	NA	No
ENSG00000212215	RNU6-913P	snRNA	NA	NA	NA	NA	1,13E-01	6,35E-01	NA	No
ENSG00000201869	RNU6-294P	snRNA	NA	NA	NA	NA	1,61E-01	4,93E-01	NA	No
ENSG00000252861	RNU6-448P	snRNA	NA	NA	NA	NA	8,70E-02	8,64E-01	NA	No
ENSG00000222924	RNU6-1148P	snRNA	NA	NA	NA	NA	6,68E-02	9,56E-01	NA	No
ENSG00000200756	RNU6-236P	snRNA	NA	NA	NA	NA	8,64E-02	8,64E-01	NA	No
ENSG00000251946	RNU6-1023P	snRNA	NA	NA	NA	NA	6,17E-02	9,56E-01	NA	No
ENSG00000207309	RNU4-70P	snRNA	NA	NA	NA	NA	6,54E-02	9,56E-01	NA	No

ENSG00000199932	RNU1-18P	snRNA	NA	NA	NA	NA	2,60E-01	2,90E-01	NA	No
ENSG00000201119	RNU1-33P	snRNA	NA	NA	NA	NA	6,17E-02	9,56E-01	NA	No
ENSG00000201796	RNU6-392P	snRNA	NA	NA	NA	NA	6,68E-02	9,56E-01	NA	No
ENSG00000251712	RNU7-20P	snRNA	NA	NA	NA	NA	8,55E-02	8,64E-01	NA	No
ENSG00000238709	RNU7-56P	snRNA	NA	NA	NA	NA	6,67E-02	9,56E-01	NA	No
ENSG00000199885	RNU6-330P	snRNA	NA	NA	NA	NA	6,23E-02	9,56E-01	NA	No
ENSG00000207220	RNU1-57P	snRNA	NA	NA	NA	NA	6,67E-02	9,56E-01	NA	No
ENSG00000207041	RNU6-3P	snRNA	NA	NA	NA	NA	1,22E-01	5,33E-01	NA	No
ENSG00000200713	RNU6-682P	snRNA	NA	NA	NA	NA	8,58E-02	8,64E-01	NA	No
ENSG00000201761	RNU6-336P	snRNA	NA	NA	NA	NA	8,51E-02	8,64E-01	NA	No
ENSG00000207201	RNU1-148P	snRNA	NA	NA	NA	NA	6,19E-02	9,56E-01	NA	No
ENSG00000222862	RNU6-1086P	snRNA	NA	NA	NA	NA	1,11E-01	6,71E-01	NA	No
ENSG00000207361	RNU6-178P	snRNA	NA	NA	NA	NA	9,18E-02	8,64E-01	NA	No
ENSG00000223215	RNU6-607P	snRNA	NA	NA	NA	NA	8,64E-02	8,64E-01	NA	No
ENSG00000252753	RNU6-1202P	snRNA	NA	NA	NA	NA	9,81E-02	7,75E-01	NA	No
ENSG00000222231	RNU2-54P	snRNA	NA	NA	NA	NA	8,58E-02	8,64E-01	NA	No
ENSG00000238884	RNU7-85P	snRNA	NA	NA	NA	NA	6,23E-02	9,56E-01	NA	No
ENSG00000200887	RNU6-598P	snRNA	NA	NA	NA	NA	1,42E-01	5,71E-01	NA	No
ENSG00000252893	RNU7-58P	snRNA	NA	NA	NA	NA	9,20E-02	8,64E-01	NA	No
ENSG00000252033	RNU6-311P	snRNA	NA	NA	NA	NA	8,77E-02	8,64E-01	NA	No
ENSG00000207113	RNU6-16P	snRNA	NA	NA	NA	NA	9,85E-02	7,75E-01	NA	No
ENSG00000252351	RNU6ATAC12P	snRNA	NA	NA	NA	NA	8,77E-02	8,64E-01	NA	No
ENSG00000201909	RNU6-590P	snRNA	NA	NA	NA	NA	8,61E-02	8,64E-01	NA	No
ENSG00000207339	RNU1-69P	snRNA	NA	NA	NA	NA	6,68E-02	9,56E-01	NA	No
ENSG00000200296	RNU1-83P	snRNA	NA	NA	NA	NA	6,54E-02	9,56E-01	NA	No
ENSG00000238842	RNU7-106P	snRNA	NA	NA	NA	NA	6,68E-02	9,56E-01	NA	No
ENSG00000199506	RNU6-247P	snRNA	NA	NA	NA	NA	1,04E-01	7,75E-01	NA	No
ENSG00000206899	RNU6-36P	snRNA	NA	NA	NA	NA	6,23E-02	9,56E-01	NA	No
ENSG00000252863	RNU6-1183P	snRNA	NA	NA	NA	NA	8,77E-02	8,64E-01	NA	No
ENSG00000200483	RNU6-1017P	snRNA	NA	NA	NA	NA	1,01E-01	7,75E-01	NA	No
ENSG00000207203	RNU6-71P	snRNA	NA	NA	NA	NA	9,18E-02	8,64E-01	NA	No
ENSG00000202398	RNU6-61P	snRNA	NA	NA	NA	NA	8,51E-02	8,64E-01	NA	No
ENSG00000252508	RNU4ATAC3P	snRNA	NA	NA	NA	NA	1,63E-01	4,77E-01	NA	No
ENSG00000252746	RNU6-273P	snRNA	NA	NA	NA	NA	8,58E-02	8,64E-01	NA	No
ENSG00000221114	RNU6ATAC30P	snRNA	NA	NA	NA	NA	6,67E-02	9,56E-01	NA	No
ENSG00000251929	RNU6-189P	snRNA	NA	NA	NA	NA	8,87E-02	8,64E-01	NA	No
ENSG00000200653	RNU4-92P	snRNA	NA	NA	NA	NA	6,54E-02	9,56E-01	NA	No
ENSG00000222761	RNU6-684P	snRNA	NA	NA	NA	NA	1,37E-01	5,97E-01	NA	No
ENSG00000207514	RNU6-385P	snRNA	NA	NA	NA	NA	8,77E-02	8,64E-01	NA	No
ENSG00000207242	RNU6-1065P	snRNA	NA	NA	NA	NA	8,77E-02	8,64E-01	NA	No
ENSG00000206730	RNU6-468P	snRNA	NA	NA	NA	NA	6,19E-02	9,56E-01	NA	No
ENSG00000222363	RNU4-34P	snRNA	NA	NA	NA	NA	6,17E-02	9,56E-01	NA	No
ENSG00000210709	RNU6ATAC3P	snRNA	NA	NA	NA	NA	8,77E-02	8,64E-01	NA	No
ENSG00000199426	RNU1-108P	snRNA	NA	NA	NA	NA	8,98E-02	8,64E-01	NA	No
ENSG00000212572	RNU6-903P	snRNA	NA	NA	NA	NA	8,55E-02	8,64E-01	NA	No
ENSG00000199803	RNU6-1159P	snRNA	NA	NA	NA	NA	1,10E-01	6,87E-01	NA	No
ENSG00000238468	RNU7-14P	snRNA	NA	NA	NA	NA	6,19E-02	9,56E-01	NA	No
ENSG00000252089	RNU4ATAC7P	snRNA	NA	NA	NA	NA	6,17E-02	9,56E-01	NA	No
ENSG00000252661	RNU6-782P	snRNA	NA	NA	NA	NA	6,17E-02	9,56E-01	NA	No
ENSG00000252273	RNU6-426P	snRNA	NA	NA	NA	NA	8,55E-02	8,64E-01	NA	No
ENSG00000238850	U2	snRNA	NA	NA	NA	NA	1,33E-01	4,74E-01	NA	No
ENSG00000238471	U2	snRNA	NA	NA	NA	NA	8,64E-02	8,64E-01	NA	No
