# Supplementary material for: Epigenetic regulation of transcription factor binding motifs promotes Th1 response in Chagas disease cardiomyopathy
Source: Front Immunol. 2022 Aug 22;13:958200. doi: 10.3389/fimmu.2022.958200 (PMC9441916; doi:10.3389/fimmu.2022.958200)
Supplement: Supplementary Table 1 — Biological samples included in this study. [file DataSheet_1.zip › Supplementary Material/Supplementary Table 8.pdf]

Supplementary table 8. List of the Differentially Methylated CpGs on heart tissues samples (controls versus CC

ID	Chromosome	Position	Gene	Feature	deltaBeta	pvalue	Corrected pva
cg18978163	9	131448476	SET	Body	-0,467	5,73E-16	4,14E-10
cg16795263	2	11915747	LPIN1	Body	-0,409	1,58E-14	2,99E-09
cg13974632	11	27740813	BDNF	Body	0,34	1,07E-14	2,99E-09
cg14885748	12	127211262		IGR	0,229	2,49E-14	2,99E-09
cg05651496	3	186463943		IGR	0,305	4,51E-14	3,72E-09
cg26333652	5	2750758	IRX2	Body	0,335	4,20E-14	3,72E-09
cg09017434	5	16179660	MARCH11	1stExon	0,379	4,64E-14	3,72E-09
cg05937737	3	170303540	SLC7A14	5'UTR	0,277	6,72E-14	3,99E-09
cg11482794	5	135364827	TGFBI	1stExon	0,321	7,18E-14	3,99E-09
cg18422586	16	22825856	HS3ST2	TSS200	0,231	6,35E-14	3,99E-09
cg27391267	19	58545333	ZSCAN1	TSS200	0,247	5,70E-14	3,99E-09
cg02987928	1	16553456		IGR	0,255	1,83E-13	4,38E-09
cg16023545	2	237076726	GBX2	TSS200	0,266	1,35E-13	4,38E-09
cg14532827	2	237076820	GBX2	TSS200	0,363	1,47E-13	4,38E-09
cg18863595	3	6902845	GRM7	1stExon	0,36	1,72E-13	4,38E-09
cg00688962	4	21950567	KCNIP4	TSS200	0,333	1,48E-13	4,38E-09
cg02297541	6	33180841		IGR	0,257	1,21E-13	4,38E-09
cg17536595	6	43612908	RSPH9	1stExon	0,292	1,22E-13	4,38E-09
cg17555373	13	53775108		IGR	0,34	1,45E-13	4,38E-09
cg26444528	13	58207859	PCDH17	1stExon	0,271	1,91E-13	4,38E-09
cg09469566	13	112717244		IGR	0,293	1,87E-13	4,38E-09
cg10919522	14	74227441	C14orf43	5'UTR	-0,227	1,26E-13	4,38E-09
cg22556683	15	91427738	FES	TSS1500	0,304	1,94E-13	4,38E-09
cg23883696	18	70534298	NETO1	TSS1500	0,318	1,40E-13	4,38E-09
cg23331484	19	33718148	SLC7A10	TSS1500	0,327	1,76E-13	4,38E-09
cg17780246	19	54481467	CACNG8	Body	0,275	1,34E-13	4,38E-09
cg10659886	19	58629719	ZSCAN18	Body	0,32	1,21E-13	4,38E-09
cg11784767	22	17718022		IGR	-0,378	1,86E-13	4,38E-09
cg09141965	7	1269385		IGR	0,265	2,07E-13	4,52E-09
cg04679040	2	74726700	LBX2	TSS200	0,341	2,14E-13	4,55E-09
cg05903444	5	2751041	IRX2	Body	0,324	2,45E-13	4,68E-09
cg02926165	5	3595963	IRX1	TSS1500	0,218	2,41E-13	4,68E-09
cg08446900	17	38501519	RARA	Body	0,277	2,39E-13	4,68E-09
cg20340302	20	55841149	BMP7	1stExon	0,258	2,46E-13	4,68E-09
cg21816330	17	27044629	RAB34	Body	0,344	2,53E-13	4,69E-09
cg10702770	1	39283780		IGR	0,33	2,92E-13	5,10E-09
cg19858756	5	155829432	SGCD	Body	-0,325	2,97E-13	5,10E-09
cg12751948	10	133794454	BNIP3	Body	0,233	3,35E-13	5,38E-09
cg13319542	2	71580473	ZNF638	Body	-0,703	3,82E-13	5,85E-09
cg16768018	3	147108843	ZIC4	Body	0,352	3,77E-13	5,85E-09
cg05602531	6	84418222	SNAP91	5'UTR	0,28	3,89E-13	5,85E-09
cg15050390	11	111847984	DIXDC1	TSS200	0,307	4,28E-13	6,05E-09
cg18301583	19	53635967	ZNF415	5'UTR	0,248	4,23E-13	6,05E-09
cg14309283	6	33282890	ZBTB22	Body	0,216	4,43E-13	6,15E-09
cg10263370	3	44754102	ZNF502	TSS200	0,272	5,24E-13	7,08E-09
cg18954541	20	45279985	SLC13A3	1stExon	0,237	5,30E-13	7,08E-09
cg06547966	3	6903067	GRM7	5'UTR	0,221	5,94E-13	7,37E-09

cg26133769	4	30723855	PCDH7	1stExon	0,319	6,44E-13	7,37E-09
cg16272981	5	1489889	LPCAT1	Body	-0,315	6,47E-13	7,37E-09
cg19248557	6	32407838	HLA-DRA	Body	-0,349	6,54E-13	7,37E-09
cg08641579	7	79083447	MAGI2	TSS1500	0,261	6,52E-13	7,37E-09
cg15427886	8	55379663		IGR	0,388	6,09E-13	7,37E-09
cg04449479	9	137967397	OLFM1	5'UTR	0,332	6,52E-13	7,37E-09
cg22633280	10	110671907		IGR	0,251	5,94E-13	7,37E-09
cg09757109	11	111848638	DIXDC1	Body	0,324	5,87E-13	7,37E-09
cg10747242	21	45033804	HSF2BP	Body	-0,479	6,37E-13	7,37E-09
cg03451670	2	236579469	AGAP1	Body	0,349	6,75E-13	7,50E-09
cg18331412	5	135364986	TGFBI	Body	0,339	7,27E-13	7,83E-09
cg08688023	10	60936925	PHYHIPL	TSS1500	0,207	7,20E-13	7,83E-09
cg22331862	3	10857717	SLC6A11	TSS200	0,242	7,56E-13	8,02E-09
cg04236622	14	61995566	PRKCH	Body	-0,204	7,68E-13	8,04E-09
cg06638795	2	42719933	KCNG3	Body	0,326	8,10E-13	8,23E-09
cg09607446	16	2286703	DNASE1L2	5'UTR	0,237	8,21E-13	8,23E-09
cg11574975	1	112058304	ADORA3	Body	0,293	8,70E-13	8,27E-09
cg05191076	4	66536186	EPHA5	TSS1500	0,207	8,55E-13	8,27E-09
cg09907509	13	37248244	C13orf36	5'UTR	0,434	8,68E-13	8,27E-09
cg02155398	2	45160490		IGR	0,233	8,93E-13	8,37E-09
cg00386408	5	135364970	TGFBI	Body	0,228	9,14E-13	8,46E-09
cg06861426	5	179244317	SQSTM1	5'UTR	0,323	9,73E-13	8,78E-09
cg09167044	17	38501412	RARA	Body	0,291	9,67E-13	8,78E-09
cg03172796	20	46115993		IGR	-0,285	9,85E-13	8,78E-09
cg26824216	3	187086147	RTP4	TSS200	-0,275	1,03E-12	8,94E-09
cg09767822	11	20178040	DBX1	Body	0,304	1,02E-12	8,94E-09
cg20605134	6	15400462	JARID2	Body	0,269	1,04E-12	8,94E-09
cg06012215	10	111216867		IGR	0,254	1,06E-12	9,03E-09
cg22165175	1	111148915	KCNA2	TSS1500	0,259	1,19E-12	9,04E-09
cg05378938	1	179560983	TDRD5	TSS200	0,322	1,23E-12	9,04E-09
cg10148764	2	101618394	RPL31	TSS1500	0,212	1,12E-12	9,04E-09
cg18115040	2	176981328	HOXD10	TSS200	0,237	1,14E-12	9,04E-09
cg26855918	3	64431038		IGR	0,223	1,20E-12	9,04E-09
cg00735667	3	74663796		IGR	0,283	1,14E-12	9,04E-09
cg09829265	8	145925704		IGR	0,233	1,26E-12	9,04E-09
cg18301891	14	102027172	DIO3	TSS1500	0,209	1,13E-12	9,04E-09
cg11254700	19	53561386		IGR	0,368	1,11E-12	9,04E-09
cg10794804	20	44035675	DBNDD2	5'UTR	0,285	1,27E-12	9,04E-09
cg19343611	22	28010528		IGR	0,202	1,14E-12	9,04E-09
cg07171956	22	37558687		IGR	-0,38	1,17E-12	9,04E-09
cg05704952	4	113040636		IGR	-0,688	1,33E-12	9,25E-09
cg03964940	6	144477779	STX11	5'UTR	-0,518	1,33E-12	9,25E-09
cg03209642	15	91427605	FES	TSS200	0,287	1,33E-12	9,25E-09
cg11006267	5	139017424		IGR	0,444	1,38E-12	9,49E-09
cg18273840	5	45695643	HCN1	Body	0,279	1,50E-12	9,79E-09
cg22305167	6	29521420		IGR	0,264	1,51E-12	9,79E-09
cg09310718	9	19441929	ACER2	Body	-0,311	1,47E-12	9,79E-09
cg21494776	19	10397780	ICAM4	1stExon	0,241	1,47E-12	9,79E-09
cg11354682	19	10978833	C19orf38	Body	-0,651	1,47E-12	9,79E-09
cg13300273	1	200842890	GPR25	1stExon	0,401	1,53E-12	9,84E-09

cg27207470	11	111848326	DIXDC1	1stExon	0,275	1,62E-12	1,03E-08
cg21762610	2	210655030	UNC80	Body	-0,379	1,69E-12	1,05E-08
cg05184377	10	50976646		IGR	0,223	1,68E-12	1,05E-08
cg11756029	12	112568357	TRAFD1	Body	-0,23	1,72E-12	1,05E-08
cg02918910	1	151032962	MLLT11	5'UTR	0,22	1,80E-12	1,08E-08
cg03599078	10	8078314		IGR	0,231	1,81E-12	1,08E-08
cg25008217	19	41882654	TMEM91	TSS1500	0,332	1,82E-12	1,08E-08
cg00449763	12	31559410	DENND5B	Body	-0,595	1,89E-12	1,10E-08
cg23823076	15	91432732	FES	Body	-0,243	2,22E-12	1,27E-08
cg05134945	3	188665466		IGR	0,278	2,31E-12	1,31E-08
cg25874782	8	59508474	NSMAF	Body	-0,256	2,32E-12	1,31E-08
cg02656891	1	34642519	C1orf94	5'UTR	0,268	2,35E-12	1,31E-08
cg04993082	1	226178964	C1orf55	Body	-0,335	2,41E-12	1,33E-08
cg11625005	5	1295737	TERT	TSS1500	0,366	2,40E-12	1,33E-08
cg06424065	4	6247640		IGR	0,316	2,45E-12	1,33E-08
cg03129384	10	128994644	FAM196A	TSS1500	0,212	2,45E-12	1,33E-08
cg25174438	4	85403915		IGR	0,232	2,56E-12	1,35E-08
cg21694941	8	57358590	PENK	5'UTR	0,259	2,53E-12	1,35E-08
cg00672359	9	137967411	OLFM1	5'UTR	0,31	2,51E-12	1,35E-08
cg21123160	3	10857719	SLC6A11	TSS200	0,29	2,67E-12	1,35E-08
cg20596329	11	67895842		IGR	0,271	2,68E-12	1,35E-08
cg12423667	16	2287078	DNASE1L2	Body	0,308	2,71E-12	1,35E-08
cg09115713	16	88832476	FAM38A	Body	-0,262	2,61E-12	1,35E-08
cg12639019	17	38734157		IGR	-0,506	2,71E-12	1,35E-08
cg09540961	20	4229561	ADRA1D	1stExon	0,208	2,64E-12	1,35E-08
cg24713204	19	57019373	ZNF471	5'UTR	0,258	2,75E-12	1,36E-08
cg26886462	5	155108449		IGR	0,27	2,77E-12	1,36E-08
cg18871648	14	74227431	ELMSAN1	TSS1500	-0,217	2,79E-12	1,36E-08
cg15439862	18	28622593	DSC3	1stExon	0,209	2,82E-12	1,37E-08
cg24496475	4	1400189		IGR	0,337	2,96E-12	1,42E-08
cg07466705	3	11034749	SLC6A1	5'UTR	0,208	3,11E-12	1,42E-08
cg21512644	4	156130016	NPY2R	5'UTR	0,235	3,20E-12	1,42E-08
cg18912728	6	45631106		IGR	0,237	3,03E-12	1,42E-08
cg07015393	7	150946356	SMARCD3	TSS1500	0,253	3,20E-12	1,42E-08
cg09638473	8	60176739		IGR	-0,378	3,19E-12	1,42E-08
cg03730428	11	32009004		IGR	0,316	3,05E-12	1,42E-08
cg24237328	14	70007511		IGR	-0,226	3,11E-12	1,42E-08
cg05747555	17	60778881	MARCH10	3'UTR	-0,282	3,10E-12	1,42E-08
cg10190416	19	40168876	LOC400696	TSS1500	-0,377	3,21E-12	1,42E-08
cg23244095	1	1476269	C1orf70	TSS1500	0,239	3,32E-12	1,45E-08
cg24392878	14	100256100		IGR	-0,303	3,36E-12	1,45E-08
cg23668184	2	5836713	SOX11	3'UTR	0,282	3,47E-12	1,46E-08
cg14585186	12	104974102	CHST11	Body	-0,505	3,49E-12	1,46E-08
cg12161228	11	89224506	NOX4	1stExon	0,237	3,56E-12	1,47E-08
cg05095318	4	17783610	FAM184B	TSS1500	0,252	3,62E-12	1,48E-08
cg26106778	6	33175147	RING1	TSS1500	0,215	3,70E-12	1,48E-08
cg25497250	10	95326974	GPR120	1stExon	0,268	3,70E-12	1,48E-08
cg10836392	22	44258132	SULT4A1	1stExon	0,295	3,67E-12	1,48E-08
cg24442740	1	27902069	AHDC1	5'UTR	0,294	3,92E-12	1,49E-08
cg08854628	6	30785152	LINC00243	Body	-0,54	3,97E-12	1,49E-08

cg19356389	6	33175140	RING1	TSS1500	0,254	3,87E-12	1,49E-08
cg11705975	10	120354248	PRLHR	Body	0,297	3,94E-12	1,49E-08
cg08490115	11	30038675	KCNA4	TSS200	0,223	3,77E-12	1,49E-08
cg15916004	11	65816646	GAL3ST3	5'UTR	0,269	3,96E-12	1,49E-08
cg23368787	19	36049342	ATP4A	Body	0,257	3,87E-12	1,49E-08
cg12682684	4	66535403	EPHA5	1stExon	0,22	4,00E-12	1,50E-08
cg18722847	3	44754129	ZNF502	TSS200	0,211	4,28E-12	1,50E-08
cg07374210	3	177096170		IGR	-0,449	4,13E-12	1,50E-08
cg00084669	3	188665440	TPRG1-AS1	TSS200	0,336	4,17E-12	1,50E-08
cg13904970	5	123987667	ZNF608	Body	0,289	4,26E-12	1,50E-08
cg25019722	6	37503610		IGR	0,282	4,29E-12	1,50E-08
cg17486097	8	35093411	UNC5D	Body	0,209	4,18E-12	1,50E-08
cg02919422	8	55370544	SOX17	5'UTR	0,222	4,34E-12	1,50E-08
cg20213416	11	30037943	KCNA4	5'UTR	0,257	4,18E-12	1,50E-08
cg06675821	16	70622659	IL34	5'UTR	-0,249	4,26E-12	1,50E-08
cg11282657	17	79615101	TSPAN10	Body	0,269	4,32E-12	1,50E-08
cg04656042	19	39755901		IGR	0,276	4,23E-12	1,50E-08
cg15909981	22	37465178	TMPRSS6	Body	0,224	4,14E-12	1,50E-08
cg25843003	6	31431312	HCP5	3'UTR	-0,284	4,55E-12	1,52E-08
cg17176573	11	120110887	POU2F3	TSS200	0,251	4,48E-12	1,52E-08
cg12647054	12	4228689		IGR	-0,294	4,55E-12	1,52E-08
cg12894649	20	11871396	BTBD3	TSS200	0,223	4,52E-12	1,52E-08
cg15989068	2	5837057	SOX11	3'UTR	0,228	4,70E-12	1,55E-08
cg19802138	13	112722719	SOX1	1stExon	0,203	4,73E-12	1,55E-08
cg04611801	17	56402029	BZRAP1	Body	0,226	4,81E-12	1,57E-08
cg00492348	4	681130	MFSD7	Body	0,235	4,87E-12	1,58E-08
cg05733135	11	27740876	BDNF	Body	0,224	4,91E-12	1,58E-08
cg03045471	11	32962661	QSER1	Body	-0,603	4,90E-12	1,58E-08
cg10359823	17	1509752	SLC43A2	TSS1500	-0,32	4,93E-12	1,58E-08
cg23326607	11	330945		IGR	-0,301	5,08E-12	1,62E-08
cg08185241	1	209848864	G0S2	1stExon	0,239	5,18E-12	1,63E-08
cg05678749	15	27216819	GABRG3	Body	0,277	5,36E-12	1,67E-08
cg06383163	8	139509073	FAM135B	TSS200	0,281	5,40E-12	1,68E-08
cg05802994	5	135364812	TGFBI	1stExon	0,215	5,48E-12	1,70E-08
cg07954091	12	124864913	NCOR2	Body	0,262	5,65E-12	1,72E-08
cg15296767	11	64122743	CCDC88B	Body	0,354	5,72E-12	1,73E-08
cg14730445	12	101603581	SLC5A8	1stExon	0,2	5,79E-12	1,73E-08
cg00221794	2	20776620		IGR	-0,353	5,88E-12	1,74E-08
cg13790603	3	24536478	THRB	TSS200	0,287	5,92E-12	1,74E-08
cg03171770	10	43393728		IGR	0,269	5,91E-12	1,74E-08
cg01011118	12	54813590	ITGA5	TSS1500	0,208	6,07E-12	1,76E-08
cg18686527	17	27044685	RAB34	Body	0,237	6,09E-12	1,76E-08
cg23828876	3	196001706	PCYT1A	5'UTR	-0,428	6,17E-12	1,77E-08
cg02724824	10	64374721	ZNF365	Body	-0,303	6,22E-12	1,78E-08
cg20505704	16	6069411	RBFOX1	TSS1500	0,281	6,36E-12	1,81E-08
cg24235581	12	58290452		IGR	0,254	6,49E-12	1,84E-08
cg04992056	16	4620301	C16orf96	Body	-0,209	6,66E-12	1,88E-08
cg24869601	6	10414350	TFAP2A	Body	0,231	6,77E-12	1,89E-08
cg19918700	6	24743575		IGR	-0,392	6,73E-12	1,89E-08
cg18235050	12	126676048		IGR	0,293	6,77E-12	1,89E-08

cg17370163	5	63461654	RNF180	TSS200	0,32	6,80E-12	1,89E-08
cg13057875	1	8343004		IGR	-0,284	6,90E-12	1,91E-08
cg02397768	4	113430989		IGR	0,206	6,95E-12	1,91E-08
cg04822822	9	116356457	RGS3	1stExon	0,204	6,94E-12	1,91E-08
cg10679301	6	50818268		IGR	0,201	7,18E-12	1,94E-08
cg08305436	19	22806184		IGR	0,264	7,11E-12	1,94E-08
cg09684160	1	17054957		IGR	-0,221	7,25E-12	1,95E-08
cg20586431	7	116963479	WNT2	TSS200	0,254	7,27E-12	1,95E-08
cg12763919	9	125137580	PTGS1	5'UTR	0,264	7,29E-12	1,95E-08
cg24428877	6	21664652		IGR	0,298	7,38E-12	1,96E-08
cg03599009	17	38501491	RARA	Body	0,307	7,39E-12	1,96E-08
cg08512167	19	45901567	PPP1R13L	Body	0,303	7,40E-12	1,96E-08
cg01956976	1	34642502	C1orf94	TSS200	0,343	7,45E-12	1,96E-08
cg17241310	1	91182856	BARHL2	TSS200	0,206	7,48E-12	1,96E-08
cg17807479	17	11145042	SHISA6	1stExon	0,262	7,53E-12	1,97E-08
cg08122691	12	46378401	SCAF11	5'UTR	-0,473	7,60E-12	1,98E-08
cg10132208	19	58545542	ZSCAN1	5'UTR	0,288	7,66E-12	1,98E-08
cg18525486	3	62357176	FEZF2	Body	0,21	7,80E-12	2,00E-08
cg22894896	17	29886890	MIR193A	TSS200	0,332	7,79E-12	2,00E-08
cg08952337	10	52397056		IGR	-0,241	7,84E-12	2,00E-08
cg15638709	10	110226387		IGR	0,321	8,09E-12	2,06E-08
cg10182317	6	123317487	CLVS2	TSS200	0,272	8,16E-12	2,06E-08
cg23809124	3	37717578	ITGA9	Body	-0,389	8,32E-12	2,09E-08
cg24772819	11	122940919		IGR	-0,325	8,37E-12	2,09E-08
cg26336059	1	153958977	RAB13	TSS200	0,266	8,53E-12	2,10E-08
cg14473643	6	33282885	ZBTB22	Body	0,267	8,44E-12	2,10E-08
cg07355841	16	67427339	TPPP3	5'UTR	0,216	8,52E-12	2,10E-08
cg06290690	9	100850337	TRIM14	Body	0,285	8,56E-12	2,10E-08
cg02699218	5	132150128	ANKRD43	1stExon	0,24	9,00E-12	2,17E-08
cg26722381	17	9903866	GAS7	Body	-0,215	9,02E-12	2,17E-08
cg08136809	19	41882642	TMEM91	1stExon	0,305	8,99E-12	2,17E-08
cg04360793	1	79472361	ELTD1	5'UTR	0,312	9,09E-12	2,17E-08
cg05790197	12	130646752	FZD10-AS1	TSS1500	0,258	9,08E-12	2,17E-08
cg11888651	15	75940778	SNX33	TSS1500	0,204	9,18E-12	2,18E-08
cg10191314	16	20955404	DNAH3	Body	-0,472	9,17E-12	2,18E-08
cg23933241	17	33823690	SLFN12L	Body	0,297	9,24E-12	2,19E-08
cg11964823	6	31466726	MICB	Body	0,22	9,41E-12	2,22E-08
cg20587213	17	39679000		IGR	-0,267	9,50E-12	2,23E-08
cg17066494	9	116327218	RGS3	TSS200	0,276	9,55E-12	2,24E-08
cg10298052	10	57391055		IGR	0,249	9,82E-12	2,28E-08
cg26875805	1	166890429	ILDR2	Body	0,205	9,94E-12	2,29E-08
cg18932726	10	128594141	DOCK1	Body	0,293	9,96E-12	2,29E-08
cg15299835	11	66034835	KLC2	3'UTR	0,26	9,91E-12	2,29E-08
cg13321967	4	53617679		IGR	0,246	1,04E-11	2,36E-08
cg19505546	5	139017263		IGR	0,279	1,04E-11	2,36E-08
cg22556008	6	116711983	DSE	5'UTR	-0,625	1,04E-11	2,36E-08
cg13576552	9	100149720		IGR	0,208	1,08E-11	2,43E-08
cg11095383	5	140174887	PCDHA1	Body	0,281	1,10E-11	2,44E-08
cg05628049	3	42113624		IGR	0,232	1,11E-11	2,45E-08
cg09232937	5	3595970	IRX1	TSS200	0,222	1,14E-11	2,49E-08

cg04402007	8	35093901	UNC5D	Body	0,209	1,15E-11	2,50E-08
cg11671688	6	110301075	GPR6	1stExon	0,273	1,19E-11	2,52E-08
cg25399158	1	156828821	INSRR	TSS200	0,205	1,21E-11	2,53E-08
cg19908812	4	164253006	NPY1R	5'UTR	0,281	1,19E-11	2,53E-08
cg24103927	14	67955485	TMEM229B	5'UTR	-0,294	1,20E-11	2,53E-08
cg10909080	17	79881468	MAFG	5'UTR	0,239	1,21E-11	2,53E-08
cg26008365	1	153958797	RAB13	1stExon	0,365	1,23E-11	2,54E-08
cg11372636	6	110299485	GPR6	1stExon	0,416	1,23E-11	2,54E-08
cg14761417	7	130636860	FLJ43663	Body	-0,373	1,22E-11	2,54E-08
cg01718742	16	62070184	CDH8	TSS200	0,224	1,23E-11	2,54E-08
cg13203135	21	45565328	C21orf33	3'UTR	-0,318	1,23E-11	2,54E-08
cg08184652	2	162270993		IGR	0,263	1,28E-11	2,62E-08
cg03355526	5	178368415	ZNF454	5'UTR	0,209	1,28E-11	2,62E-08
cg10928112	2	16500445		IGR	-0,222	1,28E-11	2,62E-08
cg17772171	6	144608178		IGR	0,301	1,30E-11	2,64E-08
cg12426092	15	31689570		IGR	0,219	1,30E-11	2,65E-08
cg19391456	16	55690418	SLC6A2	5'UTR	0,265	1,31E-11	2,65E-08
cg04355675	3	36953952	TRANK1	Body	-0,258	1,35E-11	2,66E-08
cg21884231	3	170303721	SLC7A14	5'UTR	0,22	1,34E-11	2,66E-08
cg20840157	16	81369438	GAN	Body	-0,331	1,35E-11	2,66E-08
cg21123446	22	43356001	PACSIN2	TSS200	0,278	1,35E-11	2,66E-08
cg12595013	3	147128123	ZIC1	1stExon	0,211	1,37E-11	2,68E-08
cg13985485	1	239550283		IGR	0,267	1,40E-11	2,71E-08
cg21277729	8	23305122	ENTPD4	Body	-0,373	1,41E-11	2,71E-08
cg07489381	8	29326884		IGR	-0,319	1,41E-11	2,71E-08
cg16065021	12	127940654		IGR	0,257	1,41E-11	2,71E-08
cg25923214	17	74864205	MGAT5B	TSS1500	0,223	1,40E-11	2,71E-08
cg11863813	19	42627663	POU2F2	Body	-0,216	1,42E-11	2,71E-08
cg06188545	1	156863643	PEAR1	5'UTR	0,254	1,48E-11	2,72E-08
cg15736127	2	157292127	GPD2	1stExon	0,323	1,43E-11	2,72E-08
cg22668995	3	168558640		IGR	-0,278	1,47E-11	2,72E-08
cg00064733	7	153584038	DPP6	TSS200	0,349	1,43E-11	2,72E-08
cg08792700	9	86911279	SLC28A3	Body	-0,303	1,46E-11	2,72E-08
cg23062535	9	115653295	SLC46A2	TSS200	0,205	1,47E-11	2,72E-08
cg12204732	10	128994605	DOCK1	Body	0,273	1,44E-11	2,72E-08
cg16477774	11	65325249	LTBP3	1stExon	0,278	1,47E-11	2,72E-08
cg19490307	11	69863374		IGR	-0,252	1,45E-11	2,72E-08
cg23944804	20	11871384	BTBD3	TSS200	0,271	1,46E-11	2,72E-08
cg16473762	20	57797319	ZNF831	Body	0,228	1,47E-11	2,72E-08
cg20846213	10	125425707	GPR26	TSS200	0,252	1,51E-11	2,75E-08
cg03964958	2	176964720	HOXD12	1stExon	0,221	1,53E-11	2,75E-08
cg01468420	6	33393112	SYNGAP1	Body	0,236	1,51E-11	2,75E-08
cg26198737	11	117150905	RNF214	ExonBnd	-0,223	1,54E-11	2,75E-08
cg03000848	16	396164	AXIN1	Body	-0,285	1,52E-11	2,75E-08
cg05829104	12	51789042	SLC4A8	5'UTR	-0,296	1,55E-11	2,77E-08
cg13327545	10	22623548		IGR	0,289	1,56E-11	2,77E-08
cg07536910	2	176948105	EVX2	1stExon	0,222	1,59E-11	2,78E-08
cg18337222	3	99595254	C3orf26	Body	0,215	1,58E-11	2,78E-08
cg02143559	3	147138968	LOC440982	TSS200	0,264	1,58E-11	2,78E-08
cg19346645	4	141490153	UCP1	TSS200	0,217	1,61E-11	2,80E-08

cg20816889	11	111848087	DIXDC1	1stExon	0,229	1,62E-11	2,80E-08
cg12473912	3	136751656		IGR	0,267	1,63E-11	2,80E-08
cg12349858	3	160822545	B3GALNT1	5'UTR	0,218	1,64E-11	2,80E-08
cg18127680	3	187871034	LPP	TSS1500	0,264	1,65E-11	2,80E-08
cg07055616	10	134600600	NKX6-2	TSS1500	0,277	1,65E-11	2,80E-08
cg20644402	11	74424521	CHRDL2	5'UTR	-0,276	1,64E-11	2,80E-08
cg18490614	15	27213362		IGR	0,249	1,63E-11	2,80E-08
cg10137837	17	6926742	BCL6B	5'UTR	0,208	1,64E-11	2,80E-08
cg01355374	1	89531025	GBP1	5'UTR	-0,303	1,77E-11	2,88E-08
cg01142635	4	6224117		IGR	0,217	1,75E-11	2,88E-08
cg13971504	5	74344416		IGR	-0,369	1,76E-11	2,88E-08
cg09284708	6	39901897	MOCS1	Body	0,24	1,78E-11	2,88E-08
cg03574723	10	125425994	GPR26	1stExon	0,224	1,79E-11	2,88E-08
cg02423817	11	67203661	PTPRCAP	Body	-0,33	1,74E-11	2,88E-08
cg17007640	14	29243504	C14orf23	Body	0,227	1,77E-11	2,88E-08
cg10501704	14	30396444	PRKD1	Body	0,246	1,76E-11	2,88E-08
cg20900050	15	31689509		IGR	0,259	1,73E-11	2,88E-08
cg19332452	19	12936719	RTBDN	Body	0,224	1,78E-11	2,88E-08
cg26923490	19	49575475	KCNA7	1stExon	0,236	1,78E-11	2,88E-08
cg26115228	2	85116113		IGR	-0,276	1,80E-11	2,89E-08
cg05337387	7	116681233	ST7	Body	-0,259	1,83E-11	2,93E-08
cg14156751	10	50976571		IGR	0,317	1,83E-11	2,93E-08
cg13595655	15	41238325		IGR	-0,335	1,84E-11	2,93E-08
cg11377136	22	46658960	PKDREJ	1stExon	0,261	1,84E-11	2,93E-08
cg11929284	19	18731797	TMEM59L	3'UTR	-0,287	1,86E-11	2,93E-08
cg17349389	2	80530770	CTNNA2	Body	0,21	1,89E-11	2,94E-08
cg12301981	2	100379392	AFF3	Body	-0,48	1,88E-11	2,94E-08
cg12523932	4	39448653	KLB	Body	0,356	1,88E-11	2,94E-08
cg07362060	2	11915711	LPIN1	Body	-0,412	1,89E-11	2,94E-08
cg13620240	9	126298781	DENND1A	Body	-0,244	1,90E-11	2,94E-08
cg09596674	3	46607350	LRRC2	5'UTR	0,293	1,93E-11	2,95E-08
cg18878242	3	186848672	RPL39L	5'UTR	-0,301	1,94E-11	2,95E-08
cg01047555	6	127835990	C6orf174	Body	0,239	1,94E-11	2,95E-08
cg19202014	11	417578	SIGIRR	TSS200	0,209	1,93E-11	2,95E-08
cg13598335	15	73367847	NEO1	Body	-0,383	1,93E-11	2,95E-08
cg19517476	19	14591301	GIPC1	Body	-0,277	1,92E-11	2,95E-08
cg12936304	1	59943123	FGGY	Body	-0,32	1,95E-11	2,96E-08
cg05732750	7	6704045		IGR	0,293	1,97E-11	2,98E-08
cg21359303	10	106068573		IGR	-0,31	2,02E-11	3,02E-08
cg19181162	14	38091824		IGR	0,206	2,02E-11	3,03E-08
cg27583010	16	30198505	CORO1A	Body	-0,367	2,03E-11	3,03E-08
cg22694818	1	75595970	LHX8	5'UTR	0,264	2,04E-11	3,03E-08
cg24673765	19	36247869	HSPB6	1stExon	0,237	2,05E-11	3,04E-08
cg09322259	6	106434131		IGR	0,264	2,08E-11	3,07E-08
cg15685693	7	70597351	WBSCR17	TSS200	0,226	2,11E-11	3,12E-08
cg05863502	9	140771990	CACNA1B	TSS1500	0,207	2,13E-11	3,14E-08
cg07699978	12	63194001	PPM1H	Body	-0,253	2,19E-11	3,20E-08
cg10454572	1	48222012		IGR	-0,268	2,25E-11	3,22E-08
cg04567445	1	65730409	DNAJC6	TSS200	0,223	2,23E-11	3,22E-08
cg20780180	5	122251808	SNX24	Body	-0,458	2,22E-11	3,22E-08

cg13246235	6	12749978	PHACTR1	Body	0,324	2,24E-11	3,22E-08
cg15854154	10	73592958	PSAP	Body	-0,378	2,24E-11	3,22E-08
cg12387754	11	35991209	LDLRAD3	5'UTR	-0,271	2,22E-11	3,22E-08
cg22853588	20	35169881	MYL9	TSS200	0,257	2,25E-11	3,22E-08
cg17478979	6	149772150	ZC3H12D	Body	0,359	2,27E-11	3,22E-08
cg08400159	20	45947786	ZMYND8	Body	0,24	2,26E-11	3,22E-08
cg20779964	20	54580070	CBLN4	TSS200	0,32	2,28E-11	3,23E-08
cg15726707	18	44775558	SKOR2	TSS200	0,246	2,29E-11	3,24E-08
cg21399064	15	93156705		IGR	-0,208	2,31E-11	3,26E-08
cg10161614	3	17193484		IGR	-0,33	2,32E-11	3,26E-08
cg11439596	5	1931376		IGR	0,221	2,32E-11	3,26E-08
cg11666365	5	131824796	IRF1	Body	-0,246	2,31E-11	3,26E-08
cg16582419	6	30038998	RNF39	Body	0,238	2,35E-11	3,28E-08
cg04340435	13	114145973	TMCO3	5'UTR	0,213	2,36E-11	3,28E-08
cg00052472	20	46116225		IGR	-0,323	2,35E-11	3,28E-08
cg10679103	2	56463190	CCDC85A	Body	-0,215	2,36E-11	3,28E-08
cg23128634	5	170288788	RANBP17	TSS200	0,247	2,37E-11	3,28E-08
cg10234998	6	29601491	GABBR1	TSS1500	0,278	2,40E-11	3,30E-08
cg20286236	17	29886672	MIR193A	TSS1500	0,239	2,40E-11	3,30E-08
cg19761848	2	237076815	GBX2	TSS200	0,323	2,45E-11	3,34E-08
cg21856067	20	13975853	MACROD2	TSS1500	0,205	2,45E-11	3,34E-08
cg13867963	8	35092878	UNC5D	TSS200	0,29	2,49E-11	3,37E-08
cg09399937	3	6902843	GRM7	5'UTR	0,226	2,51E-11	3,38E-08
cg09344033	11	123047766	CLMP	Body	-0,266	2,52E-11	3,38E-08
cg15278374	12	131576088	GPR133	Body	-0,264	2,51E-11	3,38E-08
cg20473937	15	36338126		IGR	-0,239	2,54E-11	3,39E-08
cg11879465	22	43041118	CYB5R3	TSS1500	0,254	2,54E-11	3,39E-08
cg25570278	14	23306509	MMP14	Body	0,303	2,59E-11	3,42E-08
cg16387176	18	44048991		IGR	-0,292	2,59E-11	3,42E-08
cg24239148	10	63422841	C10orf107	1stExon	0,203	2,60E-11	3,42E-08
cg16651768	1	222340714		IGR	-0,324	2,63E-11	3,43E-08
cg20127733	10	33635645		IGR	-0,25	2,62E-11	3,43E-08
cg08358907	19	17877508	FCHO1	Body	0,248	2,63E-11	3,43E-08
cg20267559	3	71772939	EIF4E3	Body	-0,362	2,64E-11	3,44E-08
cg22163059	12	51610927	POU6F1	Body	0,207	2,64E-11	3,44E-08
cg15283373	12	57998317	DTX3	TSS1500	0,211	2,71E-11	3,49E-08
cg22533356	4	82415477		IGR	-0,38	2,75E-11	3,53E-08
cg22726155	21	45575559		IGR	-0,369	2,76E-11	3,53E-08
cg05259958	7	106408701		IGR	-0,25	2,79E-11	3,56E-08
cg05768427	3	127634188		IGR	0,267	2,80E-11	3,56E-08
cg15605858	15	29395932	APBA2	Body	0,281	2,80E-11	3,56E-08
cg08182193	14	105942243	CRIP2	Body	0,27	2,83E-11	3,58E-08
cg11563680	6	100917397		IGR	0,256	2,85E-11	3,59E-08
cg07437373	3	134125814		IGR	0,241	2,86E-11	3,59E-08
cg17885226	6	105388731		IGR	0,23	2,87E-11	3,59E-08
cg19098522	12	6059526	VWF	Body	-0,231	2,88E-11	3,60E-08
cg02965878	16	50719042		IGR	-0,335	2,88E-11	3,60E-08
cg19275261	18	5630072		IGR	0,246	2,89E-11	3,60E-08
cg18044383	18	59001158		IGR	0,209	2,90E-11	3,61E-08
cg26584653	15	96875354	NR2F2	1stExon	0,206	2,96E-11	3,65E-08

cg21113740	1	79472343	ELTD1	5'UTR	0,223	2,99E-11	3,66E-08
cg16248329	4	187644739	FAT1	5'UTR	0,288	3,00E-11	3,66E-08
cg17187521	12	125003379	NCOR2	5'UTR	0,231	2,99E-11	3,66E-08
cg04454086	5	140345966	PCDHAC2	1stExon	0,214	3,06E-11	3,69E-08
cg11404915	12	127610471		IGR	-0,295	3,05E-11	3,69E-08
cg22752533	20	44657948	SLC12A5	1stExon	0,243	3,06E-11	3,69E-08
cg03132134	8	131577301		IGR	-0,247	3,07E-11	3,69E-08
cg16697438	10	72043538		IGR	0,243	3,09E-11	3,72E-08
cg11011640	6	33175177	RING1	TSS1500	0,213	3,10E-11	3,72E-08
cg27537252	15	45006400	B2M	Body	-0,457	3,10E-11	3,72E-08
cg16454432	2	235748808		IGR	-0,226	3,13E-11	3,73E-08
cg18026197	3	126422536	CHCHD6	TSS1500	0,216	3,14E-11	3,73E-08
cg06128198	10	128593922	DOCK1	TSS200	0,2	3,13E-11	3,73E-08
cg08418079	11	11448117	GALNT18	Body	-0,24	3,17E-11	3,74E-08
cg22731637	11	45231545	PRDM11	Body	0,271	3,18E-11	3,74E-08
cg07533239	12	6422131	PLEKHG6	1stExon	-0,333	3,15E-11	3,74E-08
cg08837627	19	22715300		IGR	0,222	3,17E-11	3,74E-08
cg04624110	20	13976093	MACROD2	TSS200	0,313	3,20E-11	3,74E-08
cg07796897	10	6018225	IL15RA	Body	-0,22	3,21E-11	3,75E-08
cg26200580	6	170615273	FAM120B	TSS1500	0,216	3,23E-11	3,77E-08
cg07203423	2	74743009	TLX2	Body	0,241	3,25E-11	3,79E-08
cg26931862	12	54349169	HOXC12	1stExon	0,242	3,27E-11	3,79E-08
cg15961744	5	140228182	PCDHA6	Body	0,204	3,30E-11	3,81E-08
cg01373667	11	11451344	GALNT18	Body	-0,217	3,33E-11	3,81E-08
cg16159688	16	56651523	MT1L	Body	0,211	3,33E-11	3,81E-08
cg16863375	6	55443990	HMGCLL1	1stExon	0,251	3,43E-11	3,90E-08
cg13141458	15	61349870	RORA	Body	-0,345	3,43E-11	3,90E-08
cg00453232	1	93343575	FAM69A	TSS1500	-0,264	3,44E-11	3,90E-08
cg08362273	17	46719577		IGR	0,22	3,45E-11	3,90E-08
cg00929635	20	44035918	DBNDD2	5'UTR	0,375	3,47E-11	3,91E-08
cg07843760	2	68547531	CNRIP1	TSS1500	0,209	3,47E-11	3,91E-08
cg17199870	9	92505134		IGR	-0,204	3,48E-11	3,91E-08
cg02084848	16	69380204	TMED6	Body	-0,276	3,53E-11	3,94E-08
cg22049881	4	82428030		IGR	-0,219	3,54E-11	3,94E-08
cg10941566	22	30117148	CABP7	Body	0,276	3,54E-11	3,94E-08
cg14222229	1	149137747		IGR	0,307	3,57E-11	3,96E-08
cg24679890	19	17246356	MYO9B	Body	0,324	3,57E-11	3,96E-08
cg16776065	11	6340486	PRKCDBP	Body	0,243	3,59E-11	3,98E-08
cg02290590	6	72037946		IGR	-0,277	3,61E-11	3,99E-08
cg15452970	1	91190620		IGR	0,336	3,62E-11	3,99E-08
cg11636504	2	99439883	C2orf55	Body	0,273	3,64E-11	3,99E-08
cg20985755	3	24536474	THRB	TSS200	0,294	3,65E-11	3,99E-08
cg05543049	3	173115741	NLGN1	TSS1500	0,216	3,63E-11	3,99E-08
cg15903956	15	74676231		IGR	-0,297	3,65E-11	3,99E-08
cg23527945	5	62079718		IGR	-0,399	3,66E-11	3,99E-08
cg01360627	6	31544931	TNF	Body	-0,292	3,67E-11	3,99E-08
cg13718729	9	140056619	GRIN1	Body	0,278	3,71E-11	4,00E-08
cg05983814	17	72276388	DNAI2	5'UTR	-0,252	3,71E-11	4,00E-08
cg22827250	5	134363823	PITX1	3'UTR	0,259	3,73E-11	4,01E-08
cg09221888	1	172715611		IGR	-0,31	3,85E-11	4,03E-08

cg26574680	1	235811860	GNG4	5'UTR	0,256	3,83E-11	4,03E-08
cg02630214	3	147098585		IGR	0,211	3,78E-11	4,03E-08
cg04675542	5	150284416	ZNF300	TSS200	0,298	3,85E-11	4,03E-08
cg13528873	6	1770669	GMDS	Body	-0,299	3,80E-11	4,03E-08
cg16533901	8	57901234	IMPAD1	Body	-0,227	3,87E-11	4,03E-08
cg14742234	11	12914823	TEAD1	Body	-0,239	3,81E-11	4,03E-08
cg18625627	14	81426015	TSHR	Body	-0,379	3,87E-11	4,03E-08
cg06360427	18	74962672	GALR1	1stExon	0,258	3,86E-11	4,03E-08
cg03192598	19	12305854		IGR	0,285	3,78E-11	4,03E-08
cg15873320	20	4230374	ADRA1D	TSS1500	0,264	3,81E-11	4,03E-08
cg23169957	20	13976106	MACROD2	TSS200	0,294	3,87E-11	4,03E-08
cg02294411	17	7461678	TNFSF13	1stExon	0,228	3,90E-11	4,06E-08
cg05215127	2	115918775	DPP10	TSS1500	0,226	3,92E-11	4,07E-08
cg11063729	6	10415653	TFAP2A	Body	0,229	3,93E-11	4,08E-08
cg15662465	13	70682004	KLHL1	1stExon	0,303	3,95E-11	4,09E-08
cg26331172	1	151118299	SEMA6C	5'UTR	0,218	4,02E-11	4,14E-08
cg12690978	12	8801526	MFAP5	Body	-0,32	4,10E-11	4,20E-08
cg24707200	1	156833163	NTRK1	Body	-0,267	4,15E-11	4,23E-08
cg22213391	1	200860537	C1orf106	TSS200	0,389	4,14E-11	4,23E-08
cg10530883	5	3596207	IRX1	1stExon	0,335	4,13E-11	4,23E-08
cg11189004	20	55750671	BMP7	Body	-0,355	4,17E-11	4,23E-08
cg25357163	1	39323275	RRAGC	Body	-0,418	4,20E-11	4,25E-08
cg21169203	11	111847968	DIXDC1	TSS200	0,23	4,21E-11	4,25E-08
cg04224064	14	36992233		IGR	0,316	4,27E-11	4,27E-08
cg06629130	18	44787585		IGR	0,236	4,25E-11	4,27E-08
cg10453719	4	165305050	MARCH1	TSS1500	0,266	4,30E-11	4,29E-08
cg08708961	1	227070630	PSEN2	Body	-0,278	4,41E-11	4,29E-08
cg00355656	1	229692519	ABCB10	Body	-0,293	4,38E-11	4,29E-08
cg06702607	3	32470351	CMTM7	Body	-0,286	4,34E-11	4,29E-08
cg04597433	4	9783206	DRD5	TSS200	0,259	4,40E-11	4,29E-08
cg12363722	4	187476703	MTNR1A	TSS200	0,257	4,39E-11	4,29E-08
cg04268312	11	102043542	YAP1	Body	-0,3	4,39E-11	4,29E-08
cg18161890	12	52995250	KRT72	1stExon	0,259	4,37E-11	4,29E-08
cg17306339	12	124198578	ATP6V0A2	Body	-0,322	4,41E-11	4,29E-08
cg00765705	12	124865130	NCOR2	Body	0,391	4,37E-11	4,29E-08
cg15447787	15	29967531		IGR	0,224	4,39E-11	4,29E-08
cg04079760	16	68269694	ESRP2	Body	0,218	4,37E-11	4,29E-08
cg01237056	22	43739992	SCUBE1	TSS1500	0,228	4,33E-11	4,29E-08
cg21497056	7	70597354	WBSCR17	TSS200	0,241	4,42E-11	4,30E-08
cg02167856	2	122936339		IGR	-0,311	4,44E-11	4,30E-08
cg24104815	4	147053856		IGR	-0,37	4,46E-11	4,31E-08
cg19335413	1	153599812	S100A13	TSS200	0,29	4,48E-11	4,32E-08
cg17101296	8	145925708		IGR	0,236	4,50E-11	4,33E-08
cg04560304	3	119225648	TIMMDC1	Body	-0,512	4,52E-11	4,33E-08
cg01552272	20	13976096	MACROD2	TSS200	0,26	4,52E-11	4,33E-08
cg05181290	1	203733914	LAX1	TSS1500	-0,288	4,54E-11	4,33E-08
cg08441806	10	134599149	NKX6-2	1stExon	0,238	4,54E-11	4,33E-08
cg10237903	8	139509075	FAM135B	TSS200	0,331	4,56E-11	4,33E-08
cg22139665	10	103733410	C10orf76	Body	-0,316	4,58E-11	4,34E-08
cg19303748	11	844085	TSPAN4	5'UTR	0,218	4,58E-11	4,34E-08

cg25984344	10	128994608	DOCK1	Body	0,258	4,61E-11	4,35E-08
cg17619311	3	42947565	ZNF662	TSS200	0,23	4,65E-11	4,37E-08
cg02741521	3	9745610	CPNE9	1stExon	0,216	4,68E-11	4,38E-08
cg07143083	7	70597921	WBSCR17	1stExon	0,34	4,67E-11	4,38E-08
cg11656175	1	203040823	PPFIA4	Body	-0,3	4,74E-11	4,40E-08
cg02697127	2	64628677		IGR	-0,347	4,75E-11	4,40E-08
cg08259261	4	89978400	FAM13A	TSS200	0,202	4,72E-11	4,40E-08
cg15989608	6	31545321	TNF	3'UTR	-0,321	4,75E-11	4,40E-08
cg15310492	11	30038677	KCNA4	TSS200	0,219	4,73E-11	4,40E-08
cg08518667	12	58290447		IGR	0,26	4,76E-11	4,40E-08
cg06062984	3	22413960		IGR	0,229	4,78E-11	4,40E-08
cg18543758	17	33868252		IGR	-0,31	4,84E-11	4,43E-08
cg17220933	1	206729613	RASSF5	Body	-0,207	4,88E-11	4,45E-08
cg13723420	2	208527045		IGR	-0,263	4,91E-11	4,45E-08
cg00884040	16	6069401	RBFOX1	TSS1500	0,316	4,97E-11	4,49E-08
cg07746943	3	238615	CHL1	TSS200	0,261	4,99E-11	4,49E-08
cg08500510	10	6216350	PFKFB3	Body	0,247	4,99E-11	4,49E-08
cg02623400	1	50513749	ELAVL4	1stExon	0,251	5,03E-11	4,51E-08
cg10084644	7	99775521	STAG3	TSS200	0,233	5,03E-11	4,51E-08
cg20271029	1	46859845	FAAH	TSS200	0,228	5,05E-11	4,52E-08
cg12582959	19	56159199	CCDC106	5'UTR	0,227	5,04E-11	4,52E-08
cg26844246	5	170736277	TLX3	TSS200	0,236	5,08E-11	4,52E-08
cg25810938	2	241497060	ANKMY1	5'UTR	0,297	5,09E-11	4,53E-08
cg16387380	19	44860856	ZNF112	TSS200	0,252	5,09E-11	4,53E-08
cg10906284	12	63544430	AVPR1A	1stExon	0,267	5,13E-11	4,55E-08
cg01154283	2	36603543	CRIM1	Body	0,263	5,24E-11	4,64E-08
cg03578193	2	63671845	C2orf86	Body	-0,466	5,28E-11	4,66E-08
cg08907868	4	185342501	IRF2	Body	-0,425	5,27E-11	4,66E-08
cg02501779	20	54579355	CBLN4	5'UTR	0,251	5,31E-11	4,68E-08
cg27020216	3	153840347	SGEF	Body	0,203	5,45E-11	4,75E-08
cg09660365	3	10858395	SLC6A11	Body	0,23	5,47E-11	4,75E-08
cg06577205	5	15500714	FBXL7	5'UTR	0,276	5,46E-11	4,75E-08
cg21609640	1	166890380	ILDR2	Body	0,204	5,48E-11	4,76E-08
cg04823169	9	100850334	TRIM14	Body	0,264	5,49E-11	4,76E-08
cg16823083	1	3567412	WDR8	TSS1500	0,343	5,53E-11	4,79E-08
cg04631347	1	206588542	SRGAP2	Body	-0,223	5,54E-11	4,79E-08
cg14603345	20	11871375	BTBD3	TSS200	0,264	5,57E-11	4,80E-08
cg11323506	5	80529135	RNU5E	Body	0,25	5,61E-11	4,82E-08
cg25233914	12	27484462	ARNTL2	TSS1500	-0,28	5,63E-11	4,83E-08
cg10990006	17	65243288		IGR	-0,304	5,72E-11	4,88E-08
cg10205045	15	69087809	ANP32A	Body	0,244	5,73E-11	4,88E-08
cg03859162	13	99404887	SLC15A1	1stExon	0,208	5,81E-11	4,92E-08
cg05059480	15	40633202	C15orf52	TSS200	0,238	5,80E-11	4,92E-08
cg05881290	6	38322959	BTBD9	Body	-0,326	5,82E-11	4,92E-08
cg02198144	16	85462102		IGR	-0,236	5,82E-11	4,92E-08
cg07480608	3	15352493	SH3BP5	Body	-0,232	5,86E-11	4,94E-08
cg22195627	19	12305869		IGR	0,338	5,88E-11	4,95E-08
cg27273788	1	15416720	KAZN	Body	-0,21	5,90E-11	4,95E-08
cg07535790	3	114528931	ZBTB20	Body	-0,47	5,94E-11	4,95E-08
cg01286685	6	30039025	RNF39	Body	0,347	5,94E-11	4,95E-08

cg15287611	18	55500861		IGR	-0,342	5,90E-11	4,95E-08
cg10552126	7	154002064	DPP6	TSS1500	0,229	6,01E-11	4,98E-08
cg11857363	1	201604628		IGR	-0,207	6,03E-11	4,99E-08
cg20305576	13	28968481	FLT1	Body	-0,278	6,07E-11	5,00E-08
cg26287345	20	35169886	MYL9	TSS200	0,205	6,11E-11	5,01E-08
cg10207600	9	130640172	AK1	TSS200	0,283	6,16E-11	5,04E-08
cg06749872	16	30198509	CORO1A	Body	-0,234	6,20E-11	5,04E-08
cg08634938	5	122218767	SNX24	Body	-0,431	6,33E-11	5,13E-08
cg04388093	7	50358218	IKZF1	5'UTR	-0,218	6,33E-11	5,13E-08
cg22620221	7	153584416	DPP6	TSS200	0,286	6,32E-11	5,13E-08
cg09693588	3	66633255		IGR	-0,25	6,37E-11	5,15E-08
cg23248887	14	38679643	SSTR1	Body	0,293	6,46E-11	5,18E-08
cg15548099	1	218884690	MIR548F3	Body	-0,351	6,61E-11	5,23E-08
cg08777941	5	56515425	GPBP1	Body	-0,345	6,62E-11	5,23E-08
cg09873933	5	135364580	TGFBI	TSS200	0,251	6,62E-11	5,23E-08
cg13860849	11	66084469	CD248	1stExon	0,234	6,60E-11	5,23E-08
cg09611279	11	86792640	TMEM135	Body	-0,344	6,58E-11	5,23E-08
cg13525276	14	81426012	TSHR	Body	-0,441	6,57E-11	5,23E-08
cg14792774	16	11833132	TXNDC11	Body	-0,304	6,55E-11	5,23E-08
cg24057642	19	17357641	NR2F6	TSS1500	0,223	6,62E-11	5,23E-08
cg12595335	6	164159736		IGR	-0,267	6,63E-11	5,23E-08
cg01723826	11	125819942		IGR	-0,235	6,69E-11	5,27E-08
cg01599709	5	139725677	HBEGF	Body	0,215	6,75E-11	5,30E-08
cg14696038	2	24308243	TP53I3	TSS1500	0,247	6,77E-11	5,31E-08
cg13680423	4	122502303		IGR	-0,259	6,77E-11	5,31E-08
cg24229304	2	233020971	DIS3L2	Body	-0,389	6,82E-11	5,31E-08
cg18599514	7	156544275	LMBR1	Body	-0,319	6,83E-11	5,31E-08
cg18655807	8	104418702	SLC25A32	Body	-0,48	6,91E-11	5,31E-08
cg08451967	10	99549843		IGR	-0,304	6,90E-11	5,31E-08
cg17282904	10	102826005		IGR	0,205	6,91E-11	5,31E-08
cg03254336	10	114635839		IGR	-0,257	6,90E-11	5,31E-08
cg15156941	15	76578733	ETFA	Body	-0,419	6,90E-11	5,31E-08
cg06532379	15	85360319	ALPK3	1stExon	0,226	6,86E-11	5,31E-08
cg10377921	19	15391946	BRD4	TSS1500	0,248	6,87E-11	5,31E-08
cg10743390	19	30020728	VSTM2B	Body	0,227	6,87E-11	5,31E-08
cg08039560	21	45575832		IGR	-0,273	6,85E-11	5,31E-08
cg08838158	2	118594167		IGR	0,311	6,94E-11	5,32E-08
cg08790440	2	124782831	CNTNAP5	TSS200	0,201	6,95E-11	5,32E-08
cg05684430	6	15400474	JARID2	Body	0,282	6,95E-11	5,32E-08
cg15843567	10	26223206	MYO3A	1stExon	0,261	7,01E-11	5,34E-08
cg02994956	22	29876534	NEFH	1stExon	0,249	7,02E-11	5,34E-08
cg09095033	2	157292214	GPD2	TSS1500	0,202	7,04E-11	5,35E-08
cg22491141	18	56932314		IGR	0,234	7,04E-11	5,35E-08
cg03595140	9	132772857	FNBP1	Body	-0,29	7,08E-11	5,36E-08
cg01664864	14	102027677	DIO3	TSS200	0,261	7,09E-11	5,36E-08
cg11379081	1	209405050		IGR	0,317	7,12E-11	5,37E-08
cg10603275	3	239665	CHL1	5'UTR	0,282	7,12E-11	5,37E-08
cg15341124	14	102027734	DIO3	5'UTR	0,211	7,13E-11	5,37E-08
cg18470101	4	26032635		IGR	-0,249	7,15E-11	5,37E-08
cg16249416	5	148783952		IGR	-0,314	7,17E-11	5,37E-08

cg08136772	13	53420933	PCDH8	1stExon	0,209	7,16E-11	5,37E-08
cg12452298	15	67134587		IGR	0,213	7,16E-11	5,37E-08
cg20317748	15	40633124	C15orf52	1stExon	0,284	7,18E-11	5,37E-08
cg00046073	6	15401039	JARID2	Body	0,304	7,30E-11	5,44E-08
cg05702092	2	74726710	LBX2	TSS200	0,288	7,37E-11	5,48E-08
cg14799482	1	90135931	LRRC8C	5'UTR	-0,289	7,40E-11	5,50E-08
cg03783907	6	26446387	BTN3A3	Body	-0,305	7,42E-11	5,50E-08
cg19274890	7	153584042	DPP6	TSS200	0,376	7,40E-11	5,50E-08
cg26734440	19	35481930		IGR	-0,269	7,42E-11	5,50E-08
cg26388182	1	44331632	ST3GAL3	Body	-0,329	7,51E-11	5,52E-08
cg26903130	12	124864543	NCOR2	Body	0,204	7,50E-11	5,52E-08
cg20065463	2	220283156	DES	1stExon	0,238	7,55E-11	5,54E-08
cg10384676	5	35130385	PRLR	5'UTR	-0,279	7,60E-11	5,54E-08
cg01758512	6	96463902	FUT9	5'UTR	0,212	7,58E-11	5,54E-08
cg25645553	6	17470599	CAP2	Body	-0,323	7,65E-11	5,56E-08
cg17241776	13	51417469	DLEU7	1stExon	0,241	7,68E-11	5,57E-08
cg21055045	2	47266465	TTC7A	Body	-0,276	7,70E-11	5,57E-08
cg14366490	19	17571825	NXNL1	TSS200	-0,202	7,69E-11	5,57E-08
cg01939453	6	166077483		IGR	0,224	7,71E-11	5,58E-08
cg10225090	8	95175640	CDH17	Body	-0,306	7,74E-11	5,58E-08
cg08575537	7	100318626	EPO	Body	0,219	7,76E-11	5,58E-08
cg11675876	3	188665436	TPRG1-AS1	TSS200	0,269	7,85E-11	5,63E-08
cg14431602	19	36247942	PROSER3	TSS1500	0,278	7,85E-11	5,63E-08
cg14555811	5	847052	ZDHHC11	Body	-0,226	7,88E-11	5,63E-08
cg13400720	6	16413855	ATXN1	5'UTR	-0,421	7,95E-11	5,64E-08
cg05200037	6	149772346	ZC3H12D	Body	0,231	7,97E-11	5,65E-08
cg27617640	2	206849310		IGR	-0,381	8,11E-11	5,68E-08
cg21753092	2	238071771		IGR	-0,234	8,08E-11	5,68E-08
cg23244910	6	106434169		IGR	0,264	8,04E-11	5,68E-08
cg01210432	10	23652593		IGR	-0,348	8,10E-11	5,68E-08
cg23944251	10	25464719	LOC10012881	Body	0,237	8,10E-11	5,68E-08
cg03793270	11	89224684	NOX4	5'UTR	0,219	8,06E-11	5,68E-08
cg26305896	16	2286937	DNASE1L2	Body	0,212	8,09E-11	5,68E-08
cg10815291	19	22611500		IGR	0,213	8,09E-11	5,68E-08
cg26415566	16	2287095	DNASE1L2	Body	0,242	8,13E-11	5,69E-08
cg04021697	1	3567303	WDR8	TSS1500	0,249	8,19E-11	5,71E-08
cg14410016	2	176986659	HOXD9	TSS1500	0,247	8,18E-11	5,71E-08
cg23314364	8	29230998		IGR	0,294	8,33E-11	5,78E-08
cg22476458	8	102996241	LOC1040541	TSS1500	-0,208	8,32E-11	5,78E-08
cg10597734	15	65682326	IGDCC4	Body	-0,312	8,33E-11	5,78E-08
cg13083127	2	131128151	PTPN18	Body	-0,241	8,34E-11	5,79E-08
cg06728274	2	175863238	CHN1	Body	0,228	8,40E-11	5,82E-08
cg22010052	5	115298704	LVRN	1stExon	0,236	8,40E-11	5,82E-08
cg15636887	17	38023480	ZPBP2	TSS1500	-0,268	8,39E-11	5,82E-08
cg07382454	19	19383976	TM6SF2	1stExon	0,321	8,42E-11	5,82E-08
cg14481502	18	51787171		IGR	-0,266	8,46E-11	5,84E-08
cg24013620	8	144798688	MAPK15	Body	0,202	8,58E-11	5,90E-08
cg25537993	19	58545182	ZSCAN1	TSS1500	0,282	8,58E-11	5,90E-08
cg14273450	2	26624934	C2orf39	1stExon	0,281	8,61E-11	5,90E-08
cg10575395	4	154837585		IGR	-0,242	8,60E-11	5,90E-08

cg19590483	19	36623725		IGR	-0,292	8,61E-11	5,90E-08
cg08915603	4	187476569	MTNR1A	TSS200	0,223	8,63E-11	5,90E-08
cg18362281	7	157204364	DNAJB6	Body	-0,225	8,66E-11	5,90E-08
cg19293468	17	1973400	SMG6	Body	-0,245	8,64E-11	5,90E-08
cg13763181	10	71087979	HK1	Body	0,254	8,68E-11	5,91E-08
cg21115558	6	39816319	DAAM2	5'UTR	-0,255	8,69E-11	5,91E-08
cg10269548	4	53728923	RASL11B	Body	0,214	8,71E-11	5,92E-08
cg02402436	6	31540051	LTA	TSS200	-0,215	8,72E-11	5,92E-08
cg00234200	15	58723769	LIPC	TSS1500	-0,298	8,75E-11	5,93E-08
cg25500080	5	140346199	PCDHAC2	1stExon	0,235	8,81E-11	5,96E-08
cg16015423	19	15217974	SYDE1	TSS1500	0,257	8,81E-11	5,96E-08
cg22438763	1	47900256	FOXD2	TSS1500	0,266	8,90E-11	5,98E-08
cg18768136	10	52096068	SGMS1	Body	-0,495	8,90E-11	5,98E-08
cg11382055	1	233749410	KCNK1	TSS1500	0,208	8,95E-11	6,00E-08
cg13637654	10	103538719		IGR	0,252	8,98E-11	6,01E-08
cg04478147	16	786393	NARFL	Body	-0,316	9,02E-11	6,02E-08
cg08509172	19	19383838	TM6SF2	Body	0,242	9,02E-11	6,02E-08
cg09614415	10	129535509	FOXI2	TSS200	0,249	9,03E-11	6,02E-08
cg08044694	19	15391927	BRD4	TSS1500	0,231	9,06E-11	6,03E-08
cg27606499	3	147111120	ZIC4	Body	0,208	9,08E-11	6,04E-08
cg15410978	16	17473034	XYLT1	Body	0,207	9,12E-11	6,04E-08
cg20208009	22	19974048	ARVCF	Body	0,227	9,13E-11	6,04E-08
cg23378736	1	23953785	MDS2	TSS200	-0,217	9,16E-11	6,05E-08
cg13627479	7	5741213	RNF216	Body	-0,26	9,19E-11	6,06E-08
cg24346629	3	74663689		IGR	0,229	9,30E-11	6,10E-08
cg15124215	4	172734426	GALNTL6	TSS200	0,318	9,35E-11	6,10E-08
cg27596513	5	66391438	MAST4	ExonBnd	0,285	9,34E-11	6,10E-08
cg00257101	9	19031179	SAXO1	Body	-0,244	9,29E-11	6,10E-08
cg14286363	10	64493241		IGR	-0,424	9,34E-11	6,10E-08
cg11136145	16	72988399	ZFHX3	Body	-0,272	9,36E-11	6,10E-08
cg06444390	14	105421098	AHNAK2	Body	-0,367	9,51E-11	6,15E-08
cg00916884	16	56666334	MT1M	TSS200	0,257	9,49E-11	6,15E-08
cg14509403	22	30476281	HORMAD2	TSS200	0,263	9,52E-11	6,15E-08
cg20917552	21	26945251	MIR155	TSS1500	-0,502	9,54E-11	6,16E-08
cg27216880	19	19384162	TM6SF2	TSS200	0,26	9,61E-11	6,20E-08
cg14373410	14	81426234	TSHR	Body	-0,297	9,67E-11	6,21E-08
cg16437908	2	85640810		IGR	0,231	9,74E-11	6,22E-08
cg19429861	4	122710195		IGR	-0,22	9,73E-11	6,22E-08
cg26558485	1	47489282	CYP4X1	1stExon	0,308	9,76E-11	6,22E-08
cg10978355	5	80529340	RNU5E	Body	0,225	9,81E-11	6,23E-08
cg11201447	8	128808063	MIR1204	TSS200	-0,496	9,81E-11	6,23E-08
cg08362102	15	69087774	ANP32A	Body	0,241	9,86E-11	6,26E-08
cg04938381	17	66374702	ARSG	Body	0,206	9,94E-11	6,31E-08
cg08110170	11	72474129	STARD10	Body	-0,244	9,96E-11	6,31E-08
cg18763165	19	1316091		IGR	0,212	9,96E-11	6,31E-08
cg18065557	3	58596027	FAM107A	Body	-0,264	9,98E-11	6,31E-08
cg18505752	6	32808752	PSMB8	Body	-0,339	1,00E-10	6,32E-08
cg03744748	15	73595458	NEO1	3'UTR	-0,291	1,00E-10	6,33E-08
cg00991504	12	109011364		IGR	-0,339	1,01E-10	6,35E-08
cg02891293	10	134188295	LRRC27	Body	-0,214	1,01E-10	6,37E-08

cg04895445	3	11342661	ATG7	Body	-0,354	1,01E-10	6,37E-08
cg16664405	10	111216962		IGR	0,229	1,02E-10	6,38E-08
cg11748354	6	53200697	ELOVL5	5'UTR	-0,343	1,02E-10	6,39E-08
cg19282042	5	73872818		IGR	-0,223	1,02E-10	6,40E-08
cg09287629	2	203036208		IGR	0,204	1,02E-10	6,40E-08
cg03323696	5	59189120	PDE4D	Body	0,235	1,03E-10	6,41E-08
cg06804992	8	128695231		IGR	-0,236	1,03E-10	6,41E-08
cg22534145	20	23015936	SSTR4	TSS200	0,291	1,03E-10	6,41E-08
cg27642691	1	156828819	INSRR	TSS200	0,263	1,03E-10	6,42E-08
cg23469673	1	181057362	IER5	TSS1500	0,267	1,04E-10	6,42E-08
cg00969154	6	15401024	JARID2	Body	0,433	1,04E-10	6,42E-08
cg26641076	2	74208602		IGR	0,293	1,04E-10	6,42E-08
cg21399832	4	117847410		IGR	0,275	1,04E-10	6,43E-08
cg10034096	6	72892300	RIMS1	Body	0,263	1,05E-10	6,43E-08
cg19153494	9	129376716	LMX1B	TSS200	0,262	1,04E-10	6,43E-08
cg14287557	15	40633232	C15orf52	TSS200	0,206	1,05E-10	6,44E-08
cg27394911	16	23304561		IGR	-0,245	1,05E-10	6,46E-08
cg04585112	2	24308215	TP53I3	TSS1500	0,247	1,07E-10	6,53E-08
cg13443627	1	59043070	TACSTD2	5'UTR	0,269	1,07E-10	6,53E-08
cg24186458	4	185338596	IRF2	Body	-0,219	1,08E-10	6,57E-08
cg11727282	9	23826368	ELAVL2	TSS1500	0,255	1,08E-10	6,57E-08
cg22648996	10	63946213		IGR	-0,243	1,08E-10	6,57E-08
cg11651237	15	27216529	GABRG3	5'UTR	0,214	1,08E-10	6,57E-08
cg00080081	17	33701776	SLFN11	TSS1500	-0,241	1,09E-10	6,61E-08
cg08891071	5	150399909	GPX3	TSS200	0,278	1,10E-10	6,62E-08
cg04739593	12	106640902	CKAP4	Body	0,228	1,10E-10	6,62E-08
cg15696906	13	38444134	TRPC4	TSS200	0,27	1,11E-10	6,64E-08
cg22585927	3	9958613	IL17RC	TSS200	0,224	1,11E-10	6,66E-08
cg25804542	11	63619042	MARK2	Body	-0,335	1,12E-10	6,66E-08
cg15661311	11	504551	RNH1	5'UTR	0,202	1,12E-10	6,66E-08
cg23494847	2	198104386	ANKRD44	Body	-0,461	1,12E-10	6,67E-08
cg11460314	20	56195574	ZBP1	1stExon	-0,313	1,13E-10	6,68E-08
cg08697251	5	1489875	LPCAT1	Body	-0,347	1,13E-10	6,68E-08
cg27553048	1	111772613	CHI3L2	5'UTR	-0,314	1,13E-10	6,68E-08
cg21930227	1	109504047	CLCC1	5'UTR	-0,27	1,14E-10	6,71E-08
cg04813787	6	167814397		IGR	-0,317	1,14E-10	6,71E-08
cg07058058	12	29256578		IGR	-0,401	1,14E-10	6,71E-08
cg11183072	17	37894397	GRB7	TSS200	0,275	1,14E-10	6,71E-08
cg07851567	18	48695911		IGR	-0,293	1,14E-10	6,74E-08
cg15107575	8	134151332		IGR	-0,242	1,15E-10	6,74E-08
cg14676407	10	125651370	CPXM2	1stExon	0,201	1,16E-10	6,77E-08
cg07102546	9	133689990	ABL1	Body	-0,255	1,18E-10	6,83E-08
cg26898567	17	10630621	TMEM220	Body	-0,289	1,18E-10	6,83E-08
cg26519249	8	123682301		IGR	-0,304	1,18E-10	6,86E-08
cg18840589	6	100441867	MCHR2-AS1	TSS200	0,267	1,19E-10	6,88E-08
cg27294813	2	109791599	SH3RF3	Body	-0,255	1,20E-10	6,89E-08
cg01942962	4	158143439	GRIA2	Body	0,241	1,20E-10	6,89E-08
cg16026943	15	75641093	NEIL1	5'UTR	0,22	1,21E-10	6,91E-08
cg10135483	6	87647527	HTR1E	1stExon	0,235	1,22E-10	6,95E-08
cg12827530	5	80529121	RNU5E	Body	0,243	1,22E-10	6,95E-08

cg10257049	5	154230308	C5orf4	TSS200	0,269	1,22E-10	6,96E-08
cg22245446	2	218316451	DIRC3	Body	-0,233	1,23E-10	6,96E-08
cg15044957	11	30038672	KCNA4	TSS200	0,244	1,23E-10	6,96E-08
cg20170271	22	38034681	SH3BP1	TSS1500	-0,212	1,23E-10	6,96E-08
cg23189410	3	147125712	ZIC4	TSS1500	0,28	1,23E-10	6,97E-08
cg10804656	10	22623460		IGR	0,25	1,24E-10	7,00E-08
cg06078093	12	125404808		IGR	-0,432	1,24E-10	7,00E-08
cg03767970	1	33721234	ZNF362	TSS1500	0,217	1,25E-10	7,02E-08
cg11723801	1	156646485	NES	1stExon	0,256	1,25E-10	7,02E-08
cg01032200	1	155290641	RUSC1-AS1	Body	0,232	1,26E-10	7,03E-08
cg17756730	1	172608644		IGR	-0,277	1,27E-10	7,06E-08
cg08314989	3	160822444	B3GALNT1	5'UTR	0,205	1,28E-10	7,09E-08
cg07407886	6	116711971	DSE	5'UTR	-0,541	1,28E-10	7,09E-08
cg08936483	22	48475730		IGR	-0,22	1,28E-10	7,09E-08
cg26284844	13	24829503	SPATA13-AS1	TSS1500	-0,287	1,28E-10	7,10E-08
cg11443656	19	42928408	LIPE	Body	0,203	1,28E-10	7,10E-08
cg17688211	4	139657467		IGR	-0,202	1,29E-10	7,11E-08
cg07900372	5	78857250		IGR	-0,393	1,29E-10	7,11E-08
cg11293190	19	1523856	PLK5P	TSS1500	0,225	1,29E-10	7,11E-08
cg12024104	12	6662581	IFFO1	Body	0,253	1,30E-10	7,12E-08
cg17813891	14	100532036	EVL	Body	-0,292	1,31E-10	7,17E-08
cg00264136	19	13326697	CACNA1A	Body	-0,329	1,31E-10	7,17E-08
cg08653292	11	58920029	FAM111A	Body	-0,266	1,32E-10	7,21E-08
cg14326196	9	116860650	KIF12	5'UTR	0,322	1,32E-10	7,23E-08
cg26635451	12	89749142		IGR	0,217	1,32E-10	7,23E-08
cg06922393	11	72064370	CLPB	Body	-0,268	1,33E-10	7,24E-08
cg05005082	2	24799727		IGR	-0,282	1,33E-10	7,26E-08
cg02142972	9	116327210	RGS3	TSS200	0,314	1,33E-10	7,26E-08
cg04864807	2	121412139		IGR	0,306	1,34E-10	7,27E-08
cg18385447	10	106027915	GSTO2	TSS1500	-0,331	1,34E-10	7,29E-08
cg22374940	1	26551782		IGR	0,216	1,35E-10	7,33E-08
cg24764939	1	48484851		IGR	-0,227	1,35E-10	7,33E-08
cg00056066	10	25010738	ARHGAP21	Body	0,233	1,35E-10	7,34E-08
cg01458054	20	62200603	PRIC285	Body	-0,24	1,36E-10	7,34E-08
cg04519775	2	231849693		IGR	-0,343	1,36E-10	7,34E-08
cg12258042	8	4852036	CSMD1	5'UTR	0,222	1,36E-10	7,34E-08
cg09048251	5	77253974		IGR	0,204	1,37E-10	7,39E-08
cg02927346	17	34059260	RASL10B	5'UTR	0,218	1,37E-10	7,42E-08
cg14120129	5	135364575	TGFBI	TSS200	0,293	1,38E-10	7,45E-08
cg24453664	11	33758413	CD59	TSS1500	0,226	1,39E-10	7,48E-08
cg09526806	9	131003043	DNM1	Body	-0,206	1,39E-10	7,50E-08
cg04259907	19	43969884	LYPD3	TSS200	0,229	1,39E-10	7,50E-08
cg14920979	11	12540983	PARVA	Body	-0,22	1,40E-10	7,50E-08
cg22478317	2	110271930		IGR	-0,251	1,40E-10	7,51E-08
cg00764612	1	150255213	C1orf51	TSS200	0,23	1,40E-10	7,52E-08
cg10882476	6	47669434	ADGRF4	5'UTR	-0,226	1,40E-10	7,52E-08
cg06271970	15	96889169		IGR	0,208	1,40E-10	7,52E-08
cg00548552	19	57183205	ZNF835	TSS200	0,219	1,41E-10	7,55E-08
cg22570970	6	15401067	JARID2	Body	0,296	1,42E-10	7,56E-08
cg01228271	3	142663294		IGR	-0,431	1,42E-10	7,56E-08

cg22294181	15	85431307	SLC28A1	Body	-0,24	1,43E-10	7,62E-08
cg08051678	6	154570975	IPCEF1	Body	-0,31	1,45E-10	7,69E-08
cg00248297	10	128943833	FAM196A	Body	-0,31	1,45E-10	7,69E-08
cg05678584	20	13975883	MACROD2	TSS1500	0,282	1,46E-10	7,69E-08
cg25968569	13	77461335	KCTD12	TSS1500	0,249	1,46E-10	7,70E-08
cg22015555	15	78358486	TBC1D2B	Body	-0,281	1,46E-10	7,70E-08
cg24257776	3	47051546	LOC1001293!	TSS1500	0,272	1,47E-10	7,72E-08
cg22119447	6	14060075		IGR	-0,204	1,47E-10	7,72E-08
cg12800105	12	121972412	KDM2B	Body	-0,237	1,47E-10	7,72E-08
cg11245369	18	21580850	TTC39C-AS1	Body	-0,301	1,48E-10	7,74E-08
cg23280305	1	17834982		IGR	-0,291	1,49E-10	7,77E-08
cg06501536	7	2298920	SNX8	Body	-0,227	1,49E-10	7,77E-08
cg20535085	1	160616604	SLAMF1	Body	-0,291	1,50E-10	7,78E-08
cg26348243	6	31540461	LTA	5'UTR	-0,368	1,50E-10	7,78E-08
cg02512902	17	25799447	KSR1	5'UTR	-0,202	1,50E-10	7,80E-08
cg17100943	19	6459300	SLC25A23	Body	0,266	1,51E-10	7,81E-08
cg05473666	5	149664993	CAMK2A	Body	-0,266	1,51E-10	7,82E-08
cg10975897	6	15504844	JARID2	Body	0,248	1,51E-10	7,84E-08
cg26442186	18	46307622	CTIF	Body	0,261	1,52E-10	7,87E-08
cg03083290	12	90257533		IGR	-0,307	1,53E-10	7,88E-08
cg15689410	2	74727000	LBX2	Body	0,346	1,53E-10	7,88E-08
cg27461178	10	72359588	PRF1	Body	-0,215	1,53E-10	7,88E-08
cg05912212	11	119622531		IGR	-0,207	1,54E-10	7,89E-08
cg19197158	12	116498620	MED13L	Body	-0,465	1,54E-10	7,89E-08
cg02781614	1	28843701	RCC1	TSS1500	-0,249	1,55E-10	7,91E-08
cg12950645	15	71410605		IGR	-0,278	1,55E-10	7,91E-08
cg03831847	16	88832485	FAM38A	Body	-0,245	1,55E-10	7,91E-08
cg10671478	20	52790299	CYP24A1	1stExon	0,247	1,56E-10	7,92E-08
cg21027742	5	130730032	CDC42SE2	3'UTR	-0,427	1,56E-10	7,93E-08
cg05207354	1	42107405	HIVEP3	5'UTR	-0,205	1,57E-10	7,93E-08
cg25465322	19	44860658	ZFP112	5'UTR	0,207	1,57E-10	7,93E-08
cg14273269	15	57927978	GCOM1	Body	0,411	1,57E-10	7,95E-08
cg16391517	3	172241008	TNFSF10	Body	-0,227	1,58E-10	7,97E-08
cg02086195	1	12656232	DHRS3	Body	0,275	1,59E-10	7,97E-08
cg16313807	18	77723226	HSBP1L1	TSS1500	-0,352	1,59E-10	7,97E-08
cg03004374	3	114879405		IGR	-0,438	1,59E-10	7,98E-08
cg05256420	10	133980556	JAKMIP3	3'UTR	-0,38	1,59E-10	7,98E-08
cg21767299	5	41172854	C6	Body	-0,245	1,60E-10	8,00E-08
cg09005378	5	139560714	CYSTM1	5'UTR	-0,457	1,61E-10	8,06E-08
cg16729415	15	35047203	GJD2	TSS1500	0,221	1,62E-10	8,07E-08
cg21754246	5	78712589	HOMER1	Body	-0,451	1,62E-10	8,08E-08
cg05293861	12	4262708		IGR	-0,307	1,63E-10	8,10E-08
cg10455196	22	44851731		IGR	-0,333	1,63E-10	8,11E-08
cg09755547	9	116327183	RGS3	TSS200	0,204	1,63E-10	8,11E-08
cg26832294	4	122078348	TNIP3	Body	0,26	1,64E-10	8,12E-08
cg00606255	1	32807686		IGR	-0,321	1,64E-10	8,12E-08
cg00446123	20	62367888	LIME1	TSS200	-0,371	1,64E-10	8,12E-08
cg00738635	1	158966231		IGR	-0,286	1,64E-10	8,13E-08
cg05099387	2	177053292	HOXD1	TSS200	0,203	1,64E-10	8,13E-08
cg27302806	12	3338502	TSPAN9	Body	-0,266	1,64E-10	8,13E-08

cg27156259	12	120688673	PXN	Body	-0,222	1,65E-10	8,16E-08
cg25136495	12	6729718	LPAR5	Body	0,261	1,66E-10	8,20E-08
cg04897299	6	21402890		IGR	-0,295	1,67E-10	8,21E-08
cg07811198	7	153585368	DPP6	Body	0,225	1,68E-10	8,24E-08
cg18274480	5	145713863		IGR	0,203	1,68E-10	8,26E-08
cg20405017	17	50235393	CA10	1stExon	0,227	1,68E-10	8,26E-08
cg14641506	11	27431603	LGR4	Body	-0,304	1,69E-10	8,28E-08
cg07996532	1	21620812	ECE1	Body	-0,322	1,70E-10	8,29E-08
cg17186168	2	11810403	NTSR2	TSS200	0,264	1,70E-10	8,29E-08
cg11266624	13	43571711		IGR	-0,288	1,70E-10	8,29E-08
cg17169196	6	31540026	LTA	TSS200	-0,232	1,70E-10	8,30E-08
cg07786657	1	167487633	CD247	Body	-0,234	1,71E-10	8,31E-08
cg15084543	1	79472408	ELTD1	5'UTR	0,285	1,72E-10	8,33E-08
cg22731432	10	80300328		IGR	-0,257	1,72E-10	8,33E-08
cg03900143	3	147111660	ZIC4	Body	0,203	1,73E-10	8,35E-08
cg25283465	1	111323341		IGR	0,224	1,73E-10	8,35E-08
cg15293582	10	72362866	PRF1	TSS1500	-0,276	1,73E-10	8,35E-08
cg22828602	7	122338579	CADPS2	Body	-0,226	1,73E-10	8,36E-08
cg08603642	1	64501127	ROR1	Body	-0,24	1,75E-10	8,40E-08
cg06138905	3	171825439	FNDC3B	5'UTR	-0,314	1,75E-10	8,40E-08
cg20903160	6	139117274		IGR	0,266	1,75E-10	8,40E-08
cg00884731	9	85678133	RASEF	TSS200	0,3	1,75E-10	8,40E-08
cg26357587	11	120110882	POU2F3	TSS200	0,215	1,75E-10	8,40E-08
cg09839635	5	63461803	RNF180	5'UTR	0,272	1,76E-10	8,41E-08
cg03749201	12	8115842		IGR	0,207	1,75E-10	8,41E-08
cg02988397	15	45007647	B2M	Body	-0,4	1,76E-10	8,41E-08
cg19811761	19	57019022	ZNF471	TSS200	0,261	1,76E-10	8,41E-08
cg10192893	4	41747895	PHOX2B	Body	0,219	1,77E-10	8,43E-08
cg17394978	5	131824502	IRF1	Body	-0,375	1,78E-10	8,44E-08
cg12019893	3	42950090	ZNF662	Body	-0,251	1,78E-10	8,45E-08
cg15701237	3	187086154	RTP4	TSS200	-0,283	1,79E-10	8,46E-08
cg03544320	4	5894691	CRMP1	1stExon	0,375	1,79E-10	8,47E-08
cg26999345	22	37584441	C1QTNF6	TSS200	0,209	1,79E-10	8,47E-08
cg18197392	20	23015908	SSTR4	TSS200	0,226	1,80E-10	8,48E-08
cg16113883	6	138190021	TNFAIP3	5'UTR	-0,485	1,80E-10	8,48E-08
cg26595643	10	118899291	VAX1	TSS1500	0,211	1,80E-10	8,48E-08
cg18076732	13	49832342	CDADC1	Body	-0,537	1,80E-10	8,48E-08
cg01822807	5	53615682	LINC01033	TSS1500	-0,221	1,82E-10	8,55E-08
cg03308652	3	62354991		IGR	0,202	1,83E-10	8,56E-08
cg01437142	15	60942549	RORA	Body	-0,372	1,83E-10	8,56E-08
cg22367191	6	10425648		IGR	0,206	1,85E-10	8,60E-08
cg26336395	7	91687107	AKAP9	Body	0,305	1,85E-10	8,60E-08
cg20207763	10	13392088		IGR	0,222	1,85E-10	8,60E-08
cg11076280	17	64733766	PRKCA	Body	0,249	1,86E-10	8,62E-08
cg01145975	6	106562613		IGR	-0,359	1,87E-10	8,64E-08
cg02355112	1	24603424		IGR	0,36	1,88E-10	8,68E-08
cg03449297	14	76101532	FLVCR2	Body	-0,214	1,89E-10	8,72E-08
cg16465027	6	12595019		IGR	-0,311	1,89E-10	8,73E-08
cg26059468	8	55370579	SOX17	5'UTR	0,232	1,91E-10	8,76E-08
cg15699226	13	78492678	EDNRB	1stExon	0,227	1,91E-10	8,76E-08

cg03425812	15	45005363	B2M	Body	-0,445	1,91E-10	8,78E-08
cg15278204	12	111126594	HVCN1	5'UTR	0,208	1,92E-10	8,79E-08
cg18384778	19	57618136		IGR	0,274	1,95E-10	8,87E-08
cg07243762	12	125030744		IGR	-0,268	1,95E-10	8,88E-08
cg08875995	17	25887224	KSR1	5'UTR	0,2	1,95E-10	8,89E-08
cg23400222	5	124672706		IGR	-0,251	1,96E-10	8,91E-08
cg22300839	1	32169770	COL16A1	TSS200	0,207	1,97E-10	8,92E-08
cg24843380	5	178367827	ZNF454	TSS1500	0,24	1,97E-10	8,92E-08
cg18446736	20	37626622	DHX35	Body	-0,257	1,97E-10	8,92E-08
cg19563510	17	79881483	MAFG	5'UTR	0,326	1,98E-10	8,93E-08
cg19687311	3	5063546		IGR	-0,243	1,99E-10	8,94E-08
cg08815968	3	143550727	SLC9A9	Body	-0,305	1,99E-10	8,94E-08
cg02878244	11	20181911	DBX1	TSS200	0,227	1,99E-10	8,94E-08
cg24895173	17	33825374	SLFN12L	Body	0,249	1,99E-10	8,94E-08
cg00066511	17	42346877	SLC4A1	TSS1500	-0,207	1,99E-10	8,94E-08
cg04270489	11	85827505		IGR	-0,372	2,00E-10	8,98E-08
cg26188692	15	67173012		IGR	-0,286	2,00E-10	8,98E-08
cg15331633	4	25095343		IGR	-0,237	2,01E-10	8,98E-08
cg05560983	10	106402168	SORCS3	Body	0,208	2,01E-10	9,00E-08
cg01140711	12	58290450		IGR	0,214	2,02E-10	9,00E-08
cg11145160	3	170136278	CLDN11	TSS1500	0,219	2,02E-10	9,00E-08
cg08965235	11	65325158	LTBP3	1stExon	0,221	2,03E-10	9,04E-08
cg16270990	11	130542897		IGR	-0,254	2,04E-10	9,04E-08
cg05213896	19	50393653	IL4I1	Body	0,26	2,05E-10	9,08E-08
cg14153689	4	10732403		IGR	-0,238	2,05E-10	9,09E-08
cg06571552	13	100195258	TM9SF2	Body	-0,315	2,05E-10	9,09E-08
cg19383430	3	187086187	RTP4	5'UTR	-0,263	2,06E-10	9,10E-08
cg19867107	8	142255537		IGR	-0,213	2,06E-10	9,10E-08
cg20454946	5	159658949	FABP6	Body	-0,243	2,06E-10	9,11E-08
cg03738025	6	105388694		IGR	0,288	2,07E-10	9,13E-08
cg20465027	1	19372076		IGR	-0,243	2,07E-10	9,14E-08
cg11236515	2	74213762		IGR	-0,252	2,09E-10	9,18E-08
cg07605285	10	26680729		IGR	0,206	2,09E-10	9,19E-08
cg26743587	10	114993079		IGR	-0,328	2,09E-10	9,19E-08
cg13858847	10	129172498	DOCK1	Body	-0,272	2,09E-10	9,19E-08
cg10372921	15	74218733	LOXL1	TSS200	0,249	2,09E-10	9,19E-08
cg16247328	1	12656375	DHRS3	Body	0,224	2,10E-10	9,22E-08
cg04573706	1	22216432	HSPG2	Body	-0,256	2,12E-10	9,25E-08
cg16846645	2	14773973	FAM84A	5'UTR	0,223	2,12E-10	9,25E-08
cg25168494	2	63286355		IGR	0,254	2,12E-10	9,25E-08
cg00957466	20	43984001		IGR	-0,405	2,12E-10	9,25E-08
cg21330949	12	19557320		IGR	-0,245	2,13E-10	9,27E-08
cg00109264	9	116282531	RGS3	Body	-0,26	2,14E-10	9,27E-08
cg11148130	3	73674068	PDZRN3	5'UTR	0,267	2,15E-10	9,30E-08
cg25089643	12	116773963		IGR	-0,244	2,15E-10	9,31E-08
cg12215340	20	4229610	ADRA1D	1stExon	0,243	2,16E-10	9,32E-08
cg10183989	14	69289150		IGR	-0,315	2,17E-10	9,33E-08
cg15490177	4	151540382	LRBA	Body	-0,331	2,17E-10	9,33E-08
cg23588204	1	212003001	LPGAT1	5'UTR	0,245	2,17E-10	9,34E-08
cg09473510	4	187476573	MTNR1A	TSS200	0,229	2,18E-10	9,35E-08

cg22512011	13	77402717		IGR	-0,279	2,18E-10	9,35E-08
cg11403708	20	62605086		IGR	0,279	2,18E-10	9,35E-08
cg00015664	9	140655655	EHMT1	Body	-0,41	2,19E-10	9,37E-08
cg12648537	8	101118463	RGS22	TSS1500	0,203	2,20E-10	9,39E-08
cg21726551	12	131590460	GPR133	Body	-0,204	2,20E-10	9,39E-08
cg14736819	2	107157603		IGR	-0,238	2,21E-10	9,44E-08
cg09941405	7	18901296	HDAC9	Body	-0,336	2,22E-10	9,44E-08
cg04793333	16	57712673	ADGRG3	Body	-0,276	2,23E-10	9,47E-08
cg25541209	20	45947892	ZMYND8	Body	0,212	2,23E-10	9,47E-08
cg04252061	22	37921738		IGR	-0,254	2,23E-10	9,47E-08
cg07005444	17	3820796	P2RX1	TSS1500	0,211	2,24E-10	9,47E-08
cg25806603	17	30851285	MYO1D	Body	-0,266	2,24E-10	9,47E-08
cg16952287	9	117762730		IGR	-0,285	2,24E-10	9,47E-08
cg15094819	17	11501999	DNAH9	1stExon	0,223	2,26E-10	9,51E-08
cg08776660	16	1670473	CRAMP1L	Body	-0,305	2,27E-10	9,54E-08
cg11727383	10	102988389	LBX1	1stExon	0,239	2,28E-10	9,58E-08
cg17387577	12	124864657	NCOR2	Body	0,214	2,28E-10	9,58E-08
cg22449811	15	96382748		IGR	-0,202	2,28E-10	9,58E-08
cg05954453	14	52064259	FRMD6-AS2	Body	-0,202	2,29E-10	9,60E-08
cg00405112	3	137728329	CLDN18	TSS1500	-0,238	2,30E-10	9,63E-08
cg18430478	1	153958792	RAB13	Body	0,308	2,31E-10	9,65E-08
cg09032544	1	167487295	CD247	Body	-0,268	2,31E-10	9,65E-08
cg01414654	6	15776970		IGR	-0,299	2,31E-10	9,65E-08
cg10502231	1	224363449		IGR	0,274	2,33E-10	9,67E-08
cg02082843	14	92339595	FBLN5	Body	-0,219	2,33E-10	9,67E-08
cg04046669	22	30476206	HORMAD2	TSS1500	0,27	2,34E-10	9,71E-08
cg09340693	9	112890072	AKAP2	5'UTR	0,217	2,35E-10	9,73E-08
cg15571640	3	32463663	CMTM7	Body	0,201	2,36E-10	9,74E-08
cg01235533	6	18211899	KDM1B	Body	-0,223	2,37E-10	9,78E-08
cg17368297	16	11552396		IGR	-0,365	2,37E-10	9,78E-08
cg07492680	1	156915620	ARHGEF11	Body	-0,267	2,38E-10	9,79E-08
cg14473924	3	73674170	PDZRN3	TSS200	0,235	2,38E-10	9,81E-08
cg13320138	12	6419575	PLEKHG6	TSS1500	0,222	2,39E-10	9,82E-08
cg16661098	2	191185669	HIBCH	TSS1500	-0,27	2,40E-10	9,87E-08
cg06912990	4	159131518	TMEM144	1stExon	0,226	2,40E-10	9,87E-08
cg07377422	19	46318633	RSPH6A	TSS200	0,295	2,40E-10	9,87E-08
cg04919489	11	120283690	ARHGEF12	Body	-0,282	2,41E-10	9,90E-08
cg05083067	11	4661335	OR51D1	1stExon	-0,281	2,42E-10	9,92E-08
cg00812438	12	127940086		IGR	0,213	2,43E-10	9,93E-08
cg27434596	11	85894417		IGR	-0,252	2,43E-10	9,93E-08
cg01295539	15	63100998	TLN2	Body	-0,251	2,44E-10	9,94E-08
cg19987210	12	63544752	AVPR1A	1stExon	0,215	2,46E-10	9,98E-08
cg03171478	22	37572916		IGR	0,229	2,46E-10	1,00E-07
cg07755390	3	124931736	SLC12A8	TSS200	0,22	2,47E-10	1,00E-07
cg21536671	1	55951958		IGR	-0,219	2,49E-10	1,01E-07
cg04153193	2	38264963	RMDN2-AS1	TSS1500	-0,327	2,50E-10	1,01E-07
cg23704150	1	223853352	CAPN8	1stExon	-0,269	2,52E-10	1,02E-07
cg25066857	2	85921438	GNLY	5'UTR	-0,282	2,52E-10	1,02E-07
cg21402921	15	27112305	GABRA5	1stExon	0,301	2,53E-10	1,02E-07
cg21161394	1	167408709	CD247	Body	0,282	2,54E-10	1,02E-07

cg14752356	6	166247032		IGR	-0,347	2,56E-10	1,03E-07
cg08835847	17	72450692		IGR	0,233	2,56E-10	1,03E-07
cg20513976	20	62367893	LIME1	TSS200	-0,343	2,56E-10	1,03E-07
cg21145524	3	239207	CHL1	5'UTR	0,228	2,58E-10	1,03E-07
cg21305777	6	71361112		IGR	-0,279	2,57E-10	1,03E-07
cg02488878	12	92950912		IGR	-0,261	2,58E-10	1,03E-07
cg02294230	19	13326629	CACNA1A	Body	-0,278	2,58E-10	1,03E-07
cg02550268	22	21926094	UBE2L3	Body	-0,312	2,57E-10	1,03E-07
cg13315222	1	111174338	KCNA2	TSS1500	-0,297	2,58E-10	1,03E-07
cg17137424	1	231299352	TRIM67	1stExon	0,266	2,58E-10	1,03E-07
cg25759064	13	111805337	ARHGEF7	TSS1500	0,28	2,58E-10	1,03E-07
cg04299757	9	133028341	HMCN2	1stExon	0,249	2,59E-10	1,03E-07
cg01927645	15	75423359		IGR	-0,6	2,60E-10	1,03E-07
cg02374745	17	6679564	FBXO39	1stExon	0,238	2,62E-10	1,04E-07
cg17245726	1	180541206		IGR	-0,316	2,62E-10	1,04E-07
cg06598836	3	2140699		IGR	0,255	2,62E-10	1,04E-07
cg05279172	17	7113697	DLG4	Body	-0,238	2,63E-10	1,04E-07
cg10222552	7	69781854	AUTS2	Body	-0,262	2,64E-10	1,04E-07
cg05413285	2	151537993		IGR	-0,286	2,65E-10	1,04E-07
cg24119006	2	240226243	MIR4269	TSS1500	-0,275	2,65E-10	1,04E-07
cg00759807	16	89390789	ANKRD11	5'UTR	-0,266	2,71E-10	1,06E-07
cg14669379	1	112058559	ADORA3	Body	0,282	2,72E-10	1,06E-07
cg22552055	5	144808911		IGR	-0,212	2,72E-10	1,06E-07
cg18342026	6	29521046		IGR	0,21	2,73E-10	1,06E-07
cg14079719	6	154551098	OPRM1	Body	-0,291	2,72E-10	1,06E-07
cg07814763	7	73770020	CLIP2	Body	-0,379	2,73E-10	1,06E-07
cg06583921	3	193495351		IGR	-0,214	2,74E-10	1,06E-07
cg01875986	2	10678797		IGR	-0,237	2,77E-10	1,07E-07
cg18143940	2	72111267		IGR	-0,256	2,78E-10	1,07E-07
cg12619509	16	2286601	DNASE1L2	5'UTR	0,221	2,79E-10	1,08E-07
cg12641739	1	235062535		IGR	-0,308	2,81E-10	1,08E-07
cg14291919	11	93583693	C11orf90	TSS200	0,209	2,81E-10	1,08E-07
cg12030632	12	18318647		IGR	-0,275	2,81E-10	1,08E-07
cg23619365	13	112712009		IGR	0,247	2,81E-10	1,08E-07
cg08243981	6	42325547	TRERF1	5'UTR	-0,273	2,83E-10	1,09E-07
cg00081270	20	13975880	MACROD2	TSS1500	0,226	2,84E-10	1,09E-07
cg01867395	11	31839628	PAX6	TSS200	0,24	2,86E-10	1,09E-07
cg14239348	6	164094293		IGR	-0,218	2,87E-10	1,09E-07
cg21525141	15	70592930		IGR	-0,356	2,87E-10	1,09E-07
cg25258740	12	75603230	KCNC2	5'UTR	0,379	2,89E-10	1,10E-07
cg14303464	9	73135040		IGR	-0,216	2,89E-10	1,10E-07
cg04347477	12	125002007	NCOR2	5'UTR	0,315	2,89E-10	1,10E-07
cg06097537	6	126226312	NCOA7	Body	-0,266	2,90E-10	1,10E-07
cg09887059	12	113916664		IGR	0,319	2,91E-10	1,10E-07
cg02807882	6	125534847	TPD52L1	5'UTR	-0,282	2,92E-10	1,10E-07
cg18952945	7	120689323	C7orf58	Body	-0,362	2,92E-10	1,10E-07
cg21987716	8	129577000	LINC00824	TSS200	-0,214	2,92E-10	1,10E-07
cg26332187	17	47826445	FAM117A	Body	-0,379	2,94E-10	1,11E-07
cg00002080	21	30390417	RWDD2B	Body	-0,297	2,94E-10	1,11E-07
cg17680611	19	16191217	TPM4	Body	-0,326	2,95E-10	1,11E-07

cg06067885	3	122084677	CCDC58	Body	-0,321	2,98E-10	1,12E-07
cg21218758	19	11591970	ELAVL3	TSS200	0,214	2,98E-10	1,12E-07
cg06793436	2	232173844	ARMC9	Body	-0,243	2,99E-10	1,12E-07
cg27452612	5	142418568	ARHGAP26	Body	-0,225	3,00E-10	1,12E-07
cg21070688	2	72028629		IGR	-0,207	3,01E-10	1,12E-07
cg17012964	3	61400295		IGR	-0,261	3,02E-10	1,12E-07
cg03357721	13	111956695	ARHGEF7	3'UTR	-0,244	3,02E-10	1,12E-07
cg11150303	1	198333105		IGR	-0,287	3,03E-10	1,12E-07
cg00207352	3	48470181	PLXNB1	5'UTR	0,204	3,05E-10	1,13E-07
cg24524285	11	64405919	NRXN2	Body	0,205	3,05E-10	1,13E-07
cg06831576	16	62070086	CDH8	TSS200	0,204	3,08E-10	1,13E-07
cg09503219	12	49295318		IGR	-0,214	3,08E-10	1,13E-07
cg16055410	6	31146124	PSORS1C3	TSS1500	-0,227	3,09E-10	1,14E-07
cg18810664	10	31074367		IGR	0,313	3,11E-10	1,14E-07
cg17525495	17	56401734	BZRAP1	Body	0,378	3,11E-10	1,14E-07
cg06143806	11	110244072		IGR	-0,362	3,12E-10	1,14E-07
cg04234104	10	6051242		IGR	-0,262	3,13E-10	1,15E-07
cg08399904	15	45413336	DUOXA1	Body	-0,255	3,13E-10	1,15E-07
cg10829592	5	170912457		IGR	-0,247	3,14E-10	1,15E-07
cg13985784	9	25678015	TUSC1	1stExon	0,273	3,14E-10	1,15E-07
cg18090634	11	47530711	CELF1	5'UTR	-0,352	3,14E-10	1,15E-07
cg12123019	19	18197853	IL12RB1	TSS200	-0,227	3,14E-10	1,15E-07
cg03546977	5	78985489	CMYA5	TSS200	0,254	3,16E-10	1,15E-07
cg15075241	2	10587052	SNORA80B	TSS200	-0,248	3,16E-10	1,15E-07
cg09147985	11	40314618	LRRC4C	5'UTR	0,249	3,17E-10	1,15E-07
cg08315613	19	11074303	SMARCA4	5'UTR	-0,337	3,17E-10	1,15E-07
cg26708144	11	119211650	C1QTNF5	5'UTR	0,263	3,18E-10	1,15E-07
cg02343823	5	150284419	ZNF300	TSS200	0,267	3,18E-10	1,15E-07
cg07773434	11	64015289	PPP1R14B	TSS1500	0,255	3,20E-10	1,16E-07
cg26337070	2	85999873	ATOH8	Body	0,339	3,21E-10	1,16E-07
cg00829809	17	48709989		IGR	-0,294	3,23E-10	1,16E-07
cg18091385	14	99502466		IGR	0,2	3,24E-10	1,16E-07
cg12404344	14	100117143	HHIPL1	Body	-0,289	3,25E-10	1,16E-07
cg16234027	17	15862624	ADORA2B	Body	-0,269	3,25E-10	1,16E-07
cg11701471	8	54164051	OPRK1	5'UTR	0,26	3,27E-10	1,17E-07
cg05159292	10	89870787		IGR	-0,32	3,28E-10	1,17E-07
cg26698347	12	48214620	HDAC7	TSS1500	0,226	3,28E-10	1,17E-07
cg26458072	10	102279694	SEC31B	TSS200	0,273	3,29E-10	1,17E-07
cg02473540	19	58570454	ZNF135	TSS200	0,279	3,30E-10	1,18E-07
cg24028809	6	153414101	RGS17	5'UTR	-0,273	3,32E-10	1,18E-07
cg16856285	3	25502270	RARB	5'UTR	-0,218	3,32E-10	1,18E-07
cg10868706	11	62789179		IGR	-0,216	3,33E-10	1,18E-07
cg14699728	11	66188522	NPAS4	5'UTR	0,275	3,33E-10	1,18E-07
cg16408081	11	67205869	CORO1B	Body	-0,267	3,33E-10	1,18E-07
cg18322569	1	91182777	BARHL2	5'UTR	0,254	3,35E-10	1,18E-07
cg24247923	7	116416924	MET	Body	-0,28	3,35E-10	1,18E-07
cg13561879	8	35092687	UNC5D	TSS1500	0,213	3,35E-10	1,18E-07
cg17330838	11	57267101	SLC43A1	Body	0,285	3,34E-10	1,18E-07
cg24196592	17	79678105	SLC25A10	TSS1500	0,218	3,35E-10	1,18E-07
cg08760589	1	26002451	MAN1C1	Body	-0,259	3,37E-10	1,19E-07

cg12190917	2	58250834	VRK2	5'UTR	-0,402	3,42E-10	1,20E-07
cg19011590	16	89831437	FANCA	Body	-0,538	3,44E-10	1,21E-07
cg27459630	17	28019440	SSH2	Body	-0,287	3,45E-10	1,21E-07
cg06584478	7	16974789		IGR	-0,259	3,49E-10	1,22E-07
cg01966091	16	69141478	HAS3	5'UTR	0,205	3,49E-10	1,22E-07
cg09895920	1	153941186	CREB3L4	Body	0,289	3,49E-10	1,22E-07
cg05361559	8	22549103	EGR3	Body	0,213	3,50E-10	1,22E-07
cg21108628	8	28022272	ELP3	Body	-0,272	3,50E-10	1,22E-07
cg08478619	10	48372128	ZNF488	3'UTR	-0,269	3,51E-10	1,22E-07
cg06906472	2	107502801	ST6GAL2	TSS200	0,297	3,52E-10	1,23E-07
cg17861230	19	18343901	PDE4C	Body	0,238	3,52E-10	1,23E-07
cg00578917	21	27945542	CYYR1	1stExon	0,22	3,53E-10	1,23E-07
cg18477674	10	133793527	BNIP3	Body	0,252	3,53E-10	1,23E-07
cg22411799	4	55432937		IGR	-0,237	3,54E-10	1,23E-07
cg08315202	7	98246006	NPTX2	TSS1500	0,232	3,57E-10	1,24E-07
cg09270922	10	11020464		IGR	-0,23	3,57E-10	1,24E-07
cg03984560	7	135396495	SLC13A4	Body	-0,291	3,58E-10	1,24E-07
cg13473196	11	46848312	CKAP5	5'UTR	0,228	3,58E-10	1,24E-07
cg01829241	4	8582249	GPR78	TSS200	0,232	3,59E-10	1,24E-07
cg09425481	12	53338607		IGR	-0,266	3,59E-10	1,24E-07
cg02738988	2	173178612		IGR	-0,215	3,61E-10	1,24E-07
cg08311647	10	11596182	USP6NL	Body	-0,49	3,61E-10	1,24E-07
cg11668923	10	128076936	ADAM12	1stExon	0,237	3,61E-10	1,24E-07
cg10155599	18	10423983		IGR	-0,261	3,61E-10	1,24E-07
cg16037947	8	22305714	PPP3CC	Body	-0,331	3,61E-10	1,24E-07
cg11591032	4	182940206		IGR	-0,238	3,63E-10	1,25E-07
cg05196858	15	57833021	CGNL1	Body	-0,327	3,64E-10	1,25E-07
cg11177980	1	41982115	HIVEP3	Body	0,253	3,66E-10	1,25E-07
cg22999502	2	87052595	CD8B	Body	-0,226	3,65E-10	1,25E-07
cg25247520	8	128808017	MIR1204	TSS200	-0,399	3,65E-10	1,25E-07
cg18651870	18	70535005	NETO1	TSS200	0,217	3,65E-10	1,25E-07
cg03190219	7	25898688		IGR	0,317	3,67E-10	1,25E-07
cg15791171	1	218829778	MIR548F3	Body	-0,296	3,68E-10	1,25E-07
cg01804934	6	33041229	HLA-DPA1	Body	-0,245	3,70E-10	1,26E-07
cg20518430	19	52848430	ZNF610	5'UTR	-0,205	3,71E-10	1,26E-07
cg27149109	4	3087964	HTT	Body	-0,238	3,72E-10	1,26E-07
cg13698224	9	87309394	NTRK2	Body	-0,475	3,73E-10	1,26E-07
cg26441055	1	42995495		IGR	-0,313	3,75E-10	1,26E-07
cg06665075	14	100895393	WDR25	Body	-0,218	3,74E-10	1,26E-07
cg04266908	4	873633	GAK	Body	-0,414	3,76E-10	1,27E-07
cg01428589	19	33167718	RGS9BP	1stExon	0,22	3,76E-10	1,27E-07
cg10250426	6	158182413		IGR	-0,348	3,76E-10	1,27E-07
cg27451576	1	117827168		IGR	-0,237	3,78E-10	1,27E-07
cg02159419	22	40797675	SGSM3	Body	-0,339	3,79E-10	1,27E-07
cg13784559	2	131128163	PTPN18	Body	-0,289	3,81E-10	1,28E-07
cg26719940	6	2976764		IGR	-0,327	3,81E-10	1,28E-07
cg07609862	11	92702912	MTNR1B	1stExon	0,301	3,80E-10	1,28E-07
cg07728874	11	118213272	CD3D	1stExon	-0,258	3,80E-10	1,28E-07
cg02782152	15	59882466		IGR	-0,297	3,80E-10	1,28E-07
cg16406669	8	133930827	TG	Body	-0,362	3,82E-10	1,28E-07

cg04974290	17	5001047		IGR	0,24	3,83E-10	1,28E-07
cg22667851	16	11405878		IGR	-0,246	3,84E-10	1,28E-07
cg23725321	10	102279690	SEC31B	TSS200	0,288	3,85E-10	1,28E-07
cg07274618	17	74070698	GALR2	TSS200	0,223	3,85E-10	1,28E-07
cg09521703	19	55944864	SHISA7	Body	0,221	3,85E-10	1,28E-07
cg20443778	2	142887886	LRP1B	Body	0,233	3,86E-10	1,28E-07
cg18182399	2	220283175	DES	1stExon	0,206	3,86E-10	1,28E-07
cg11442606	5	130610319	CDC42SE2	5'UTR	-0,329	3,87E-10	1,29E-07
cg19254434	1	61198697	LOC1019269(	Body	-0,207	3,88E-10	1,29E-07
cg26242531	14	104190678	ZFYVE21	Body	0,229	3,89E-10	1,29E-07
cg09635667	17	2300514	MNT	Body	0,212	3,89E-10	1,29E-07
cg10550302	5	31995926	PDZD2	Body	-0,22	3,90E-10	1,29E-07
cg03622700	4	6918740	TBC1D14	5'UTR	-0,246	3,92E-10	1,29E-07
cg00146437	1	209529869		IGR	-0,202	3,93E-10	1,30E-07
cg23954153	1	44402353	ARTN	Body	0,279	3,97E-10	1,30E-07
cg14100191	2	178034634		IGR	-0,301	3,98E-10	1,30E-07
cg25922279	8	141577061	EIF2C2	Body	0,24	3,97E-10	1,30E-07
cg14488317	11	3181446	OSBPL5	5'UTR	0,253	3,95E-10	1,30E-07
cg24520862	11	75943228		IGR	-0,305	3,97E-10	1,30E-07
cg12063490	19	12759131	MAN2B1	Body	0,27	3,97E-10	1,30E-07
cg00674365	19	57019069	ZNF471	TSS200	0,303	3,97E-10	1,30E-07
cg12094065	2	181802439		IGR	-0,353	3,98E-10	1,30E-07
cg25529477	2	127845211	BIN1	Body	0,223	3,99E-10	1,30E-07
cg06351682	2	106974831		IGR	-0,226	4,01E-10	1,30E-07
cg20813589	11	504930	RNH1	5'UTR	0,278	4,01E-10	1,30E-07
cg23474890	12	122467179	BCL7A	Body	-0,239	4,01E-10	1,30E-07
cg01282639	18	60820159	BCL2	Body	-0,242	4,03E-10	1,31E-07
cg09361368	22	38037250	SH3BP1	Body	-0,262	4,03E-10	1,31E-07
cg01434649	1	3567870	TP73	TSS1500	0,243	4,06E-10	1,31E-07
cg17965945	2	217954903		IGR	-0,234	4,06E-10	1,31E-07
cg26766875	13	99202291	STK24	Body	-0,263	4,06E-10	1,31E-07
cg05499559	20	48789176	LINC01273	Body	0,217	4,05E-10	1,31E-07
cg02910018	12	29903855	TMTC1	Body	-0,362	4,07E-10	1,31E-07
cg26404881	10	126405899	FAM53B	5'UTR	-0,219	4,08E-10	1,31E-07
cg23947417	1	208718201		IGR	-0,253	4,08E-10	1,31E-07
cg09934892	16	3559551	CLUAP1	Body	-0,302	4,08E-10	1,31E-07
cg13283952	2	121412005		IGR	0,244	4,10E-10	1,32E-07
cg15615312	16	28997939	LAT	Body	-0,27	4,10E-10	1,32E-07
cg03172657	16	89163625	ACSF3	Body	-0,294	4,10E-10	1,32E-07
cg16548787	22	27503825		IGR	-0,285	4,09E-10	1,32E-07
cg10937802	4	26177098		IGR	-0,228	4,12E-10	1,32E-07
cg27565277	16	17228474	XYLT1	Body	-0,26	4,13E-10	1,32E-07
cg05424884	2	63882416		IGR	-0,269	4,13E-10	1,32E-07
cg01975156	4	15783402	CD38	Body	-0,357	4,13E-10	1,32E-07
cg22850158	5	123920010		IGR	-0,251	4,14E-10	1,32E-07
cg10798745	1	145715636	CD160	TSS200	-0,318	4,15E-10	1,32E-07
cg12106899	16	6069069	RBFOX1	TSS1500	0,264	4,16E-10	1,32E-07
cg24943066	21	27009142		IGR	-0,284	4,18E-10	1,33E-07
cg00563824	1	209405064		IGR	0,23	4,21E-10	1,33E-07
cg07183362	5	1536762		IGR	-0,335	4,21E-10	1,33E-07

cg09500443	5	180018465	SCGB3A1	5'UTR	0,201	4,20E-10	1,33E-07
cg17268758	13	100780551	PCCA	Body	-0,264	4,20E-10	1,33E-07
cg25255847	20	62360072	ZGPAT	Body	-0,285	4,21E-10	1,33E-07
cg26333902	8	17354701	SLC7A2	1stExon	0,418	4,23E-10	1,34E-07
cg03602288	17	46641863	HOXB3	5'UTR	0,259	4,27E-10	1,34E-07
cg27072996	19	822635	LPPR3	TSS1500	0,214	4,29E-10	1,35E-07
cg10391228	8	59222640		IGR	-0,222	4,29E-10	1,35E-07
cg12166610	8	31498256	NRG1	Body	0,203	4,30E-10	1,35E-07
cg06836238	8	95031979		IGR	-0,283	4,30E-10	1,35E-07
cg20134880	7	5622551		IGR	-0,316	4,31E-10	1,35E-07
cg01161042	4	2322052	ZFYVE28	Body	-0,214	4,32E-10	1,35E-07
cg08733789	3	37724048	ITGA9	Body	-0,289	4,33E-10	1,36E-07
cg13995073	19	54481594	CACNG8	Body	0,202	4,34E-10	1,36E-07
cg23425533	5	80529187	RNU5E	Body	0,246	4,36E-10	1,36E-07
cg13460643	1	209848845	G0S2	1stExon	0,255	4,36E-10	1,36E-07
cg16170385	8	103902386	AZIN1-AS1	Body	-0,275	4,38E-10	1,36E-07
cg03546360	1	12675968	DHRS3	Body	0,265	4,39E-10	1,36E-07
cg08692937	1	200860499	C1orf106	TSS200	0,328	4,39E-10	1,36E-07
cg17705767	2	71777794	DYSF	Body	-0,246	4,39E-10	1,36E-07
cg07625529	2	176981283	HOXD10	TSS1500	0,248	4,39E-10	1,37E-07
cg01818220	9	23821773	ELAVL2	1stExon	0,21	4,40E-10	1,37E-07
cg26444727	11	11629695	GALNT18	Body	-0,257	4,40E-10	1,37E-07
cg07211259	9	5510497	PDCD1LG2	TSS200	-0,258	4,41E-10	1,37E-07
cg25256924	11	67205739	CORO1B	3'UTR	-0,262	4,43E-10	1,37E-07
cg03532926	7	153584839	DPP6	Body	0,204	4,44E-10	1,37E-07
cg03062768	7	47900669	PKD1L1	Body	-0,234	4,45E-10	1,37E-07
cg10499126	2	47137142	MCFD2	Body	-0,245	4,45E-10	1,37E-07
cg12924224	3	39374587	CCR8	Body	-0,205	4,51E-10	1,39E-07
cg05963759	2	238336866		IGR	-0,372	4,55E-10	1,39E-07
cg27201914	8	21533486		IGR	-0,216	4,55E-10	1,39E-07
cg15244352	12	49327916		IGR	-0,312	4,56E-10	1,39E-07
cg14217091	14	103285756	TRAF3	5'UTR	-0,32	4,57E-10	1,39E-07
cg02935338	18	25755847	CDH2	Body	0,205	4,57E-10	1,40E-07
cg00973848	15	99793877	LRRC28	5'UTR	-0,259	4,58E-10	1,40E-07
cg16077279	1	11714269	FBXO2	Body	0,243	4,58E-10	1,40E-07
cg25291396	9	129376721	LMX1B	5'UTR	0,255	4,59E-10	1,40E-07
cg11777886	5	171493808	STK10	Body	-0,224	4,62E-10	1,41E-07
cg16519100	15	83869266	HDGFRP3	Body	-0,41	4,65E-10	1,41E-07
cg00873050	1	32828016	TSSK3	1stExon	0,233	4,68E-10	1,41E-07
cg19554338	9	89407232		IGR	-0,233	4,68E-10	1,41E-07
cg06525651	10	128994297	FAM196A	1stExon	0,249	4,68E-10	1,41E-07
cg26023019	21	31311859	GRIK1	1stExon	0,214	4,67E-10	1,41E-07
cg02054003	1	40652215	RLF	Body	-0,345	4,69E-10	1,41E-07
cg03593550	19	54485404	CACNG8	Body	0,288	4,71E-10	1,42E-07
cg18566452	3	139305596	NMNAT3	Body	-0,433	4,73E-10	1,42E-07
cg00892518	15	53485623		IGR	0,244	4,73E-10	1,42E-07
cg12312386	12	129291774	SLC15A4	Body	-0,425	4,73E-10	1,42E-07
cg09923648	10	722301	DIP2C	Body	-0,245	4,74E-10	1,42E-07
cg01877352	11	60775233	CD6	Body	0,229	4,74E-10	1,42E-07
cg08465346	12	54441458	HOXC4	5'UTR	0,273	4,75E-10	1,42E-07

cg02792829	15	40762807	CHST14	TSS1500	0,27	4,74E-10	1,42E-07
cg25547580	18	55103734	ONECUT2	1stExon	0,229	4,74E-10	1,42E-07
cg12377473	11	128494797		IGR	-0,364	4,77E-10	1,43E-07
cg19044062	3	152364975		IGR	-0,244	4,78E-10	1,43E-07
cg14160170	10	73239376	CDH23	Body	-0,286	4,78E-10	1,43E-07
cg12737198	11	110220620		IGR	-0,284	4,78E-10	1,43E-07
cg24430140	19	57276642		IGR	0,223	4,78E-10	1,43E-07
cg17526573	2	50574708	NRXN1	Body	0,215	4,79E-10	1,43E-07
cg17539824	7	45196400	RAMP3	TSS1500	-0,276	4,80E-10	1,43E-07
cg08324705	7	92264450	CDK6	Body	-0,225	4,79E-10	1,43E-07
cg05407714	11	45722308		IGR	-0,246	4,80E-10	1,43E-07
cg12348202	7	158381023	PTPRN2	TSS1500	0,245	4,82E-10	1,43E-07
cg12043818	10	47008202		IGR	0,206	4,82E-10	1,43E-07
cg12696761	18	60848167	BCL2	Body	-0,351	4,82E-10	1,43E-07
cg26492368	10	22634733	SPAG6	Body	0,273	4,82E-10	1,43E-07
cg08606497	8	28244769	ZNF395	TSS1500	0,209	4,84E-10	1,43E-07
cg01528542	12	81468232		IGR	-0,239	4,84E-10	1,44E-07
cg06449396	22	46382247		IGR	-0,247	4,85E-10	1,44E-07
cg02523270	3	193987651		IGR	0,208	4,86E-10	1,44E-07
cg14634531	15	52975970		IGR	-0,294	4,86E-10	1,44E-07
cg25734842	1	228657381		IGR	0,268	4,86E-10	1,44E-07
cg02463418	4	66535575	EPHA5	1stExon	0,214	4,87E-10	1,44E-07
cg21684411	6	31431573	HCP5	3'UTR	-0,306	4,87E-10	1,44E-07
cg18207164	14	29254973	LINC01551	Body	0,223	4,87E-10	1,44E-07
cg03219362	1	212865360	BATF3	Body	-0,305	4,90E-10	1,44E-07
cg06179011	11	843897	TSPAN4	TSS1500	0,28	4,91E-10	1,44E-07
cg20500085	8	119122667	EXT1	1stExon	0,208	4,92E-10	1,45E-07
cg01630660	2	217854369		IGR	-0,245	4,94E-10	1,45E-07
cg02513778	16	21658718	IGSF6	Body	-0,287	4,95E-10	1,45E-07
cg26426867	18	60121710		IGR	-0,254	4,96E-10	1,45E-07
cg04210130	7	2891373		IGR	-0,329	4,97E-10	1,45E-07
cg06810647	16	1665094	CRAMP1L	Body	0,206	4,98E-10	1,46E-07
cg22095604	18	31803241	NOL4	1stExon	0,216	5,00E-10	1,46E-07
cg01595951	5	54834655		IGR	-0,364	5,02E-10	1,47E-07
cg09874992	1	31314608		IGR	-0,286	5,03E-10	1,47E-07
cg02696607	7	151492863	PRKAG2	5'UTR	-0,367	5,03E-10	1,47E-07
cg19897071	5	35991382	UGT3A1	5'UTR	0,201	5,03E-10	1,47E-07
cg17875555	8	23564490	NKX2-6	TSS1500	0,202	5,04E-10	1,47E-07
cg02130906	3	187520901		IGR	-0,304	5,05E-10	1,47E-07
cg10016435	10	3784156	LOC10537636	Body	-0,385	5,06E-10	1,47E-07
cg08714341	9	126973204		IGR	-0,243	5,07E-10	1,47E-07
cg16769152	11	77734206	KCTD14	1stExon	0,279	5,07E-10	1,47E-07
cg02458065	19	52598653	ZNF841	5'UTR	0,202	5,07E-10	1,47E-07
cg11703784	1	55723736		IGR	-0,259	5,07E-10	1,47E-07
cg06430509	21	44853470		IGR	-0,3	5,08E-10	1,47E-07
cg01284698	2	206260068	PARD3B	Body	-0,249	5,09E-10	1,47E-07
cg24135088	12	27361176		IGR	-0,277	5,10E-10	1,47E-07
cg09641390	14	91711520	GPR68	TSS1500	-0,265	5,10E-10	1,47E-07
cg19904805	13	70681978	KLHL1	1stExon	0,228	5,10E-10	1,47E-07
cg07351894	7	94960164		IGR	-0,23	5,12E-10	1,48E-07

cg25124241	7	121944931	FEZF1	TSS1500	0,202	5,15E-10	1,48E-07
cg00840332	7	127881269	LEP	TSS200	0,223	5,15E-10	1,48E-07
cg26903873	10	75635186	CAMK2G	TSS1500	-0,312	5,15E-10	1,48E-07
cg26692804	2	238699193		IGR	-0,325	5,16E-10	1,49E-07
cg01896761	3	13590444	FBLN2	TSS200	0,214	5,20E-10	1,49E-07
cg15030584	13	27927676		IGR	-0,26	5,20E-10	1,49E-07
cg05522449	14	100220613		IGR	-0,218	5,21E-10	1,50E-07
cg03696781	7	45742854	ADCY1	Body	-0,271	5,22E-10	1,50E-07
cg19835045	13	50801738	DLEU1	Body	-0,25	5,23E-10	1,50E-07
cg23708361	7	145813432	CNTNAP2	TSS200	0,243	5,25E-10	1,50E-07
cg10504392	12	110044639		IGR	0,255	5,24E-10	1,50E-07
cg01629240	17	46642011	HOXB3	5'UTR	0,227	5,24E-10	1,50E-07
cg13554246	20	1317746	FKBP1A-SDCE	Body	0,246	5,25E-10	1,50E-07
cg08405630	9	36383478	RNF38	Body	-0,411	5,26E-10	1,50E-07
cg02115412	4	57127406	KIAA1211	5'UTR	-0,212	5,27E-10	1,50E-07
cg13570972	11	31839632	PAX6	TSS200	0,224	5,27E-10	1,50E-07
cg09459403	3	4864036	ITPR1	Body	-0,327	5,31E-10	1,51E-07
cg08003353	13	31407120		IGR	0,208	5,32E-10	1,51E-07
cg00593536	2	139538222	NXPH2	TSS1500	0,207	5,33E-10	1,51E-07
cg19568380	4	2012845		IGR	-0,213	5,35E-10	1,51E-07
cg20055861	15	68055293	MAP2K5	Body	-0,323	5,35E-10	1,51E-07
cg17651247	16	1578024	TMEM204	TSS1500	0,207	5,35E-10	1,51E-07
cg17001328	19	2495886		IGR	0,242	5,35E-10	1,51E-07
cg12188986	11	93063886	CCDC67	1stExon	0,217	5,36E-10	1,51E-07
cg13790545	20	44035808	DBNDD2	5'UTR	0,217	5,36E-10	1,51E-07
cg05565022	2	71837721	DYSF	Body	-0,216	5,39E-10	1,52E-07
cg23495279	8	4849827	CSMD1	Body	0,244	5,38E-10	1,52E-07
cg05662003	3	46720909	ALS2CL	Body	-0,228	5,40E-10	1,52E-07
cg21905972	15	90845254		IGR	-0,221	5,40E-10	1,52E-07
cg01763935	17	40493229	STAT3	Body	-0,332	5,40E-10	1,52E-07
cg05637536	1	154475068	TDRD10	5'UTR	0,214	5,41E-10	1,52E-07
cg05783585	1	201118532	TMEM9	Body	-0,226	5,42E-10	1,52E-07
cg18110428	6	168157526		IGR	-0,242	5,42E-10	1,52E-07
cg11639950	12	125003407	NCOR2	5'UTR	0,276	5,42E-10	1,52E-07
cg06844988	14	32351434		IGR	-0,33	5,42E-10	1,52E-07
cg27596890	16	49621173	ZNF423	Body	-0,294	5,41E-10	1,52E-07
cg16105687	19	22805801		IGR	0,204	5,41E-10	1,52E-07
cg14315130	6	11786679		IGR	-0,308	5,42E-10	1,52E-07
cg08645488	14	65263560	SPTB	Body	-0,278	5,42E-10	1,52E-07
cg09996019	2	70296389	PCBP1-AS1	Body	-0,364	5,43E-10	1,52E-07
cg26422458	1	79472452	ELTD1	5'UTR	0,227	5,43E-10	1,52E-07
cg10194759	4	53655574	LOC152578	TSS1500	-0,236	5,45E-10	1,52E-07
cg17775765	13	95354021		IGR	0,205	5,45E-10	1,52E-07
cg24755459	14	101908865		IGR	-0,252	5,45E-10	1,52E-07
cg07866765	15	72073756	THSD4	3'UTR	-0,308	5,46E-10	1,52E-07
cg13189271	6	15365693	JARID2	Body	0,277	5,50E-10	1,53E-07
cg23331421	19	30866202	ZNF536	5'UTR	0,212	5,50E-10	1,53E-07
cg02406032	18	77195886	NFATC1	5'UTR	-0,464	5,53E-10	1,54E-07
cg16836311	1	25944712	MAN1C1	1stExon	0,305	5,58E-10	1,55E-07
cg25429672	11	73692155	UCP2	5'UTR	-0,27	5,58E-10	1,55E-07

cg24368848	19	58545160	ZSCAN1	TSS1500	0,235	5,60E-10	1,55E-07
cg02071516	3	64497342		IGR	-0,216	5,61E-10	1,55E-07
cg18697636	5	524503	SLC9A3	5'UTR	0,264	5,62E-10	1,55E-07
cg05085838	17	8643262	CCDC42	Body	-0,268	5,64E-10	1,56E-07
cg07382920	1	3567646	TP73	TSS1500	0,254	5,65E-10	1,56E-07
cg17974477	2	9490525	ASAP2	Body	-0,29	5,66E-10	1,56E-07
cg21672829	2	236619825	AGAP1	Body	-0,202	5,65E-10	1,56E-07
cg19078576	5	17217877	BASP1	5'UTR	0,305	5,65E-10	1,56E-07
cg18349527	12	48214579	HDAC7	TSS1500	0,224	5,66E-10	1,56E-07
cg04100611	14	73102243	DPF3	Body	-0,282	5,66E-10	1,56E-07
cg01783070	20	21686293	PAX1	TSS200	0,21	5,67E-10	1,56E-07
cg14912355	11	86454834		IGR	-0,227	5,68E-10	1,56E-07
cg17547295	5	157282503	CLINT1	Body	0,225	5,68E-10	1,56E-07
cg14929173	10	29111009		IGR	-0,275	5,69E-10	1,56E-07
cg08798862	16	88832532	FAM38A	Body	-0,3	5,70E-10	1,56E-07
cg09985344	16	84648441	COTL1	Body	-0,372	5,72E-10	1,57E-07
cg07511014	10	135191624	PAOX	TSS1500	0,251	5,75E-10	1,57E-07
cg09727935	6	105388471	LINC00577	TSS200	0,263	5,75E-10	1,57E-07
cg02861298	9	112889844	AKAP2	5'UTR	0,25	5,76E-10	1,58E-07
cg03035489	7	30362155	ZNRF2	Body	-0,314	5,79E-10	1,58E-07
cg09417728	11	116087186		IGR	-0,241	5,79E-10	1,58E-07
cg01560045	17	41953079	MPP2	3'UTR	-0,258	5,79E-10	1,58E-07
cg02450248	9	16145918		IGR	-0,265	5,80E-10	1,58E-07
cg24033471	12	2735579	CACNA1C	Body	-0,232	5,84E-10	1,59E-07
cg03070297	10	128994603	DOCK1	Body	0,242	5,85E-10	1,59E-07
cg02250548	2	134879167		IGR	0,211	5,86E-10	1,59E-07
cg20449685	19	58545728	ZSCAN1	5'UTR	0,236	5,87E-10	1,59E-07
cg18758626	20	57589049		IGR	-0,224	5,88E-10	1,60E-07
cg21173447	21	42218964	DSCAM	1stExon	0,229	5,89E-10	1,60E-07
cg15599826	5	42550096	GHR	TSS200	-0,221	5,91E-10	1,60E-07
cg09666051	4	41611109	LIMCH1	Body	-0,275	5,93E-10	1,61E-07
cg08111847	9	86088428	FRMD3	Body	-0,245	5,94E-10	1,61E-07
cg17945231	19	34841182	KIAA0355	Body	-0,35	5,95E-10	1,61E-07
cg23887194	4	140514190		IGR	-0,243	5,97E-10	1,61E-07
cg20388916	3	133682290	SLCO2A1	Body	-0,276	5,98E-10	1,61E-07
cg17155010	7	130602744	LOC10050686	Body	-0,346	5,99E-10	1,61E-07
cg03794801	17	33817159	SLFN12L	Body	0,212	5,99E-10	1,61E-07
cg00123104	1	9884732	CLSTN1	TSS200	0,243	6,00E-10	1,62E-07
cg16440309	1	89776303		IGR	-0,234	6,01E-10	1,62E-07
cg06883126	5	140457585		IGR	0,201	6,01E-10	1,62E-07
cg12371372	11	62569485	NXF1	Body	-0,269	6,01E-10	1,62E-07
cg20078270	3	30290161		IGR	-0,557	6,02E-10	1,62E-07
cg25690723	5	133228759		IGR	-0,238	6,02E-10	1,62E-07
cg00939495	4	9783398	DRD5	1stExon	0,257	6,04E-10	1,62E-07
cg14231297	19	58629901	ZSCAN18	TSS200	0,213	6,05E-10	1,62E-07
cg20671059	20	57875735	EDN3	5'UTR	0,249	6,06E-10	1,62E-07
cg14965398	13	27705648	USP12-AS1	Body	-0,28	6,07E-10	1,63E-07
cg23478547	11	69259265		IGR	0,238	6,08E-10	1,63E-07
cg06302284	4	76945319	CXCL10	TSS1500	-0,27	6,09E-10	1,63E-07
cg10379517	9	132639412	USP20	Body	-0,29	6,11E-10	1,63E-07

cg09088496	13	24825973	SPATA13	Body	-0,315	6,13E-10	1,64E-07
cg07141441	2	36582270	CRIM1	TSS1500	0,206	6,16E-10	1,64E-07
cg14663940	3	183894397	AP2M1	5'UTR	0,231	6,16E-10	1,64E-07
cg19074747	4	4639746	STX18-AS1	Body	-0,276	6,16E-10	1,64E-07
cg02624791	10	33314085		IGR	-0,263	6,16E-10	1,64E-07
cg05044185	15	27112197	GABRA5	TSS200	0,201	6,15E-10	1,64E-07
cg09304470	18	76916901	ATP9B	Body	-0,377	6,16E-10	1,64E-07
cg09702161	1	36843511	STK40	5'UTR	-0,277	6,17E-10	1,64E-07
cg14908576	19	45880430		IGR	-0,234	6,17E-10	1,64E-07
cg08914638	3	121373739	HCLS1	Body	-0,222	6,19E-10	1,64E-07
cg18836852	15	71952255	THSD4	Body	-0,287	6,19E-10	1,64E-07
cg12036619	4	80929171	ANTXR2	Body	-0,29	6,21E-10	1,65E-07
cg03909935	13	113609067		IGR	-0,235	6,21E-10	1,65E-07
cg19599666	3	187871024	LPP	TSS1500	0,247	6,23E-10	1,65E-07
cg04472685	6	31545473	TNF	3'UTR	-0,304	6,26E-10	1,65E-07
cg06538141	20	30229335	COX4I2	Body	-0,278	6,27E-10	1,66E-07
cg07965255	15	38342973	LOC10192822[illegible]	Body	-0,219	6,28E-10	1,66E-07
cg21585306	3	133476947	TF	Body	-0,25	6,32E-10	1,67E-07
cg08284263	10	92958627		IGR	-0,278	6,32E-10	1,67E-07
cg00705130	21	35649196		IGR	-0,203	6,33E-10	1,67E-07
cg11638347	21	38352880	HLCS	5'UTR	0,279	6,33E-10	1,67E-07
cg01201432	6	106235379		IGR	-0,267	6,35E-10	1,67E-07
cg10858945	10	116528318		IGR	0,206	6,36E-10	1,67E-07
cg14822490	10	124909889	HMX2	3'UTR	0,224	6,41E-10	1,68E-07
cg09439920	3	99979117	TBC1D23	TSS1500	0,246	6,42E-10	1,68E-07
cg06078830	2	26986550	C2orf18	TSS1500	0,232	6,43E-10	1,68E-07
cg23906738	14	36987301	NKX2-1	Body	0,283	6,44E-10	1,68E-07
cg08468008	14	51203813	NIN	Body	-0,252	6,44E-10	1,68E-07
cg00123055	8	55370951	SOX17	1stExon	0,222	6,49E-10	1,69E-07
cg04652097	7	79083445	MAGI2	TSS1500	0,213	6,51E-10	1,70E-07
cg26137915	14	69247337		IGR	-0,258	6,52E-10	1,70E-07
cg06028605	16	24865363	SLC5A11	5'UTR	0,21	6,52E-10	1,70E-07
cg04643822	4	82965250		IGR	0,227	6,52E-10	1,70E-07
cg10536276	2	113956344	PSD4	Body	0,246	6,53E-10	1,70E-07
cg27560783	20	46654667		IGR	-0,334	6,56E-10	1,70E-07
cg06640822	1	25291472	RUNX3	1stExon	-0,239	6,58E-10	1,70E-07
cg26389280	17	16191219	PIGL	Body	-0,258	6,60E-10	1,71E-07
cg01486321	2	238064341		IGR	-0,244	6,62E-10	1,71E-07
cg23630145	9	36144545	GLIPR2	Body	-0,231	6,62E-10	1,71E-07
cg15653143	11	60822690		IGR	-0,259	6,62E-10	1,71E-07
cg02349866	15	90391877	AP3S2	Body	-0,238	6,62E-10	1,71E-07
cg12560987	7	1287065		IGR	0,232	6,63E-10	1,71E-07
cg25824571	15	60874596	RORA-AS1	Body	-0,309	6,64E-10	1,71E-07
cg21573200	5	2739602		IGR	0,235	6,65E-10	1,71E-07
cg08965387	17	48140739	ITGA3	Body	-0,242	6,67E-10	1,71E-07
cg03499808	11	73020459	ARHGEF17	1stExon	0,259	6,68E-10	1,72E-07
cg21863296	20	45989595		IGR	0,205	6,69E-10	1,72E-07
cg10235051	3	133676992	SLCO2A1	Body	-0,266	6,70E-10	1,72E-07
cg15598762	6	148598455		IGR	-0,269	6,77E-10	1,74E-07
cg19324139	3	34290115		IGR	-0,254	6,79E-10	1,74E-07

cg02372723	11	48103048	PTPRJ	Body	0,349	6,81E-10	1,74E-07
cg23247281	17	60937887	MIR548W	Body	-0,248	6,81E-10	1,74E-07
cg19920923	5	150463164	TNIP1	5'UTR	-0,343	6,84E-10	1,75E-07
cg14853776	8	144301894		IGR	-0,213	6,84E-10	1,75E-07
cg23029655	12	53591756	ITGB7	Body	0,216	6,84E-10	1,75E-07
cg07967627	7	46028360		IGR	-0,304	6,87E-10	1,75E-07
cg11893955	8	28918821		IGR	-0,231	6,89E-10	1,75E-07
cg03634806	18	54777662		IGR	-0,222	6,91E-10	1,76E-07
cg15572575	17	75438133	sept-09	Body	-0,304	6,93E-10	1,76E-07
cg26252281	6	146864885	RAB32	1stExon	0,207	6,94E-10	1,76E-07
cg11497957	8	125985846	ZNF572	5'UTR	0,267	6,94E-10	1,76E-07
cg19383445	3	107661189		IGR	-0,235	6,98E-10	1,77E-07
cg17895697	3	129255805		IGR	-0,235	6,98E-10	1,77E-07
cg26078977	5	59189467	PDE4D	Body	0,228	6,98E-10	1,77E-07
cg09354050	21	43824262	UBASH3A	Body	-0,278	6,99E-10	1,77E-07
cg04759756	20	35273933	SLA2	5'UTR	-0,294	7,01E-10	1,77E-07
cg24332305	4	140819621	MAML3	Body	-0,347	7,04E-10	1,78E-07
cg18417954	19	55672513	C19orf51	Body	0,248	7,08E-10	1,79E-07
cg07044770	6	5206209	LOC1001294(	Body	0,208	7,12E-10	1,79E-07
cg19439123	17	17687621	RAI1	5'UTR	0,203	7,12E-10	1,79E-07
cg21947597	10	72491899	ADAMTS14	Body	-0,219	7,13E-10	1,79E-07
cg16661800	6	149805292	ZC3H12D	5'UTR	-0,267	7,14E-10	1,79E-07
cg15862137	13	42980762		IGR	-0,251	7,14E-10	1,79E-07
cg10387551	11	30038615	KCNA4	TSS200	0,219	7,14E-10	1,79E-07
cg17072965	8	105759976		IGR	-0,23	7,18E-10	1,80E-07
cg13870516	11	16145130	SOX6	Body	-0,25	7,18E-10	1,80E-07
cg04211806	11	95433839		IGR	-0,415	7,18E-10	1,80E-07
cg17833746	17	40489785	STAT3	Body	-0,381	7,19E-10	1,80E-07
cg25724461	2	235384049		IGR	-0,299	7,19E-10	1,80E-07
cg25104397	10	104535920	C10orf26	5'UTR	0,213	7,21E-10	1,80E-07
cg20557454	16	29019437		IGR	-0,212	7,24E-10	1,80E-07
cg02223905	3	46720206	ALS2CL	TSS1500	-0,293	7,27E-10	1,81E-07
cg05926722	14	26674202		IGR	0,229	7,27E-10	1,81E-07
cg08445602	3	72683664		IGR	-0,292	7,31E-10	1,81E-07
cg26212321	6	685077	EXOC2	Body	-0,276	7,33E-10	1,82E-07
cg17384806	10	105808267	MIR936	TSS1500	-0,289	7,34E-10	1,82E-07
cg02769781	1	1713676		IGR	0,215	7,39E-10	1,83E-07
cg11581653	5	176085973	TSPAN17	3'UTR	-0,281	7,39E-10	1,83E-07
cg24010658	8	144544041	ZC3H3	Body	-0,291	7,39E-10	1,83E-07
cg15810184	12	105324638		IGR	-0,356	7,39E-10	1,83E-07
cg11824827	16	31075547	ZNF668	Body	-0,321	7,41E-10	1,83E-07
cg21426003	2	237076811	GBX2	TSS200	0,244	7,42E-10	1,83E-07
cg20991988	7	28459911	CREB5	5'UTR	-0,288	7,52E-10	1,85E-07
cg24251448	8	141577100	EIF2C2	Body	0,217	7,52E-10	1,85E-07
cg14305476	14	95969736		IGR	-0,212	7,54E-10	1,85E-07
cg12220236	20	3364800	C20orf194	Body	-0,26	7,56E-10	1,85E-07
cg17037282	5	50678928	ISL1	TSS200	0,216	7,57E-10	1,85E-07
cg04773818	8	31496966	NRG1	TSS1500	0,273	7,58E-10	1,85E-07
cg12044599	11	67206308	CORO1B	Body	-0,217	7,61E-10	1,86E-07
cg19915620	4	110509477	CCDC109B	Body	-0,279	7,65E-10	1,87E-07

cg14892936	6	15400500	JARID2	Body	0,212	7,65E-10	1,87E-07
cg04537282	11	331179		IGR	-0,283	7,66E-10	1,87E-07
cg16660091	16	56995856	CETP	5'UTR	0,208	7,66E-10	1,87E-07
cg15010616	5	149093472		IGR	-0,249	7,66E-10	1,87E-07
cg18320160	12	53591607	ITGB7	Body	0,269	7,68E-10	1,87E-07
cg27304668	5	124562285	LOC10192742	Body	-0,222	7,69E-10	1,87E-07
cg07991686	7	2111950	MAD1L1	Body	-0,244	7,69E-10	1,87E-07
cg10047041	12	3446781		IGR	-0,311	7,69E-10	1,87E-07
cg12339752	3	189559609	TP63	Body	-0,279	7,70E-10	1,87E-07
cg25622154	1	228657536		IGR	0,222	7,72E-10	1,87E-07
cg25468274	1	31280338		IGR	0,203	7,74E-10	1,88E-07
cg09907936	19	58570466	ZNF135	TSS200	0,247	7,74E-10	1,88E-07
cg03724964	19	39465969	FBXO17	5'UTR	0,263	7,75E-10	1,88E-07
cg12748266	3	111395538	PLCXD2-AS1	Body	-0,283	7,78E-10	1,88E-07
cg07924282	9	85678135	RASEF	TSS200	0,246	7,77E-10	1,88E-07
cg25818611	2	235872830	SH3BP4	5'UTR	-0,268	7,79E-10	1,88E-07
cg16326504	4	24987424		IGR	-0,335	7,81E-10	1,89E-07
cg14094409	12	122712093	DIABLO	TSS200	-0,261	7,81E-10	1,89E-07
cg03841028	1	200860378	C1orf106	TSS1500	0,225	7,82E-10	1,89E-07
cg18395269	18	60827075	BCL2	Body	-0,305	7,83E-10	1,89E-07
cg10183670	6	168521614		IGR	-0,217	7,84E-10	1,89E-07
cg27454412	7	1067447	C7orf50	Body	0,255	7,92E-10	1,90E-07
cg18728921	2	8295355	LINC00299	Body	-0,226	7,96E-10	1,91E-07
cg01645892	17	45378625	ITGB3	Body	-0,218	8,00E-10	1,91E-07
cg16097315	11	9093160	SCUBE2	Body	-0,244	8,02E-10	1,92E-07
cg09518226	9	129174148	NRON	TSS1500	-0,442	8,08E-10	1,93E-07
cg18864691	1	23933764		IGR	-0,2	8,09E-10	1,93E-07
cg22690561	7	22441955		IGR	-0,203	8,09E-10	1,93E-07
cg23690893	19	14671371	TECR	Body	0,218	8,12E-10	1,93E-07
cg13697891	17	73312128		IGR	-0,304	8,14E-10	1,93E-07
cg24980995	2	218219798	DIRC3	Body	-0,261	8,16E-10	1,94E-07
cg13407975	19	18705946	CRLF1	Body	0,267	8,17E-10	1,94E-07
cg25693279	10	77888507	C10orf11	Body	-0,26	8,20E-10	1,94E-07
cg04833533	3	38080323	DLEC1	TSS1500	-0,273	8,23E-10	1,95E-07
cg23548201	3	195623792	TNK2	TSS1500	0,306	8,24E-10	1,95E-07
cg06594404	4	380396		IGR	0,226	8,24E-10	1,95E-07
cg27273242	6	3802297		IGR	-0,218	8,26E-10	1,95E-07
cg17388248	11	34645707	EHF	5'UTR	-0,233	8,27E-10	1,96E-07
cg06083887	11	105962468	AASDHPPT	Body	-0,336	8,29E-10	1,96E-07
cg22037030	1	56139081		IGR	-0,255	8,30E-10	1,96E-07
cg26498475	20	35575417	SAMHD1	Body	-0,207	8,30E-10	1,96E-07
cg13164395	1	180878044		IGR	-0,248	8,32E-10	1,96E-07
cg02836135	6	108052093	SCML4	Body	-0,259	8,32E-10	1,96E-07
cg11144351	1	209759842	CAMK1G	5'UTR	-0,3	8,34E-10	1,97E-07
cg14473102	2	176994448	HOXD8	TSS200	0,246	8,34E-10	1,97E-07
cg12777794	3	60072801	FHIT	Body	-0,231	8,34E-10	1,97E-07
cg19713330	15	71033447	UACA	Body	0,202	8,34E-10	1,97E-07
cg17407893	16	16119200	ABCC1	Body	-0,264	8,34E-10	1,97E-07
cg27074995	19	17953473	JAK3	Body	0,296	8,39E-10	1,98E-07
cg02573472	5	76940356		IGR	0,241	8,40E-10	1,98E-07

cg25931721	12	120449805	CCDC64	Body	-0,362	8,40E-10	1,98E-07
cg19230730	15	51171384		IGR	-0,425	8,39E-10	1,98E-07
cg03766763	11	125080240	PKNOX2	5'UTR	-0,24	8,42E-10	1,98E-07
cg22803223	12	64587031	C12orf66	3'UTR	-0,321	8,42E-10	1,98E-07
cg14811700	17	3947417	ZZEF1	Body	-0,246	8,43E-10	1,98E-07
cg10093938	1	19248952	IFFO2	Body	0,259	8,45E-10	1,98E-07
cg13084154	11	117444338	DSCAML1	Body	-0,24	8,45E-10	1,98E-07
cg12135269	2	3642586	COLEC11	1stExon	0,345	8,47E-10	1,98E-07
cg24332995	6	154482894	OPRM1	Body	-0,254	8,47E-10	1,98E-07
cg02056062	3	149374914	WWTR1	Body	0,222	8,54E-10	1,99E-07
cg10063403	6	167364065	RNASET2	Body	-0,336	8,54E-10	1,99E-07
cg23450038	19	2462961		IGR	0,217	8,54E-10	1,99E-07
cg24087723	8	125274521		IGR	-0,219	8,55E-10	2,00E-07
cg07565505	5	1887300		IGR	0,258	8,57E-10	2,00E-07
cg17765669	3	17193366		IGR	-0,28	8,61E-10	2,00E-07
cg23165348	11	36172658	LDLRAD3	5'UTR	-0,315	8,62E-10	2,00E-07
cg27128939	7	40351128	SUGCT	Body	-0,308	8,64E-10	2,01E-07
cg03732999	2	33433548	LTBP1	Body	-0,333	8,65E-10	2,01E-07
cg15653173	13	112723034	SOX1	1stExon	0,205	8,67E-10	2,01E-07
cg08964513	19	4566759		IGR	-0,276	8,69E-10	2,01E-07
cg22341683	15	61342360	RORA	Body	-0,272	8,71E-10	2,02E-07
cg01692482	20	52198378	ZNF217	1stExon	-0,407	8,72E-10	2,02E-07
cg14312386	1	9344435		IGR	-0,26	8,72E-10	2,02E-07
cg12260811	16	79115817	WWOX	Body	-0,229	8,74E-10	2,02E-07
cg20132590	3	73673893	PDZRN3	1stExon	0,202	8,76E-10	2,02E-07
cg25684999	10	23480803	PTF1A	TSS1500	0,223	8,79E-10	2,03E-07
cg20465980	3	125029710	ZNF148	Body	-0,27	8,79E-10	2,03E-07
cg09716921	3	66519998	LRIG1	Body	-0,272	8,81E-10	2,03E-07
cg21789023	22	40915746	MKL1	5'UTR	-0,434	8,85E-10	2,04E-07
cg17660890	1	3392473	ARHGEF16	Body	-0,256	8,86E-10	2,04E-07
cg00738608	6	113608887		IGR	-0,475	8,87E-10	2,04E-07
cg10432797	13	113607851		IGR	-0,233	8,87E-10	2,04E-07
cg00432884	9	36148527	GLIPR2	ExonBnd	-0,309	8,90E-10	2,04E-07
cg06392637	15	39487809		IGR	-0,296	8,90E-10	2,04E-07
cg20454249	2	217815182		IGR	-0,242	8,91E-10	2,04E-07
cg01248932	18	44121822	LOXHD1	Body	-0,219	8,97E-10	2,05E-07
cg26902263	12	120640065	PXN-AS1	Body	-0,277	8,98E-10	2,05E-07
cg05210804	6	33282896	ZBTB22	Body	0,208	8,99E-10	2,05E-07
cg03753191	13	43566902	EPSTI1	TSS1500	-0,262	8,99E-10	2,05E-07
cg11587466	1	245855322	KIF26B	Body	-0,209	9,00E-10	2,05E-07
cg14981961	16	89414269	ANKRD11	Body	-0,282	9,02E-10	2,06E-07
cg25147376	1	166890293	ILDR2	Body	0,214	9,05E-10	2,06E-07
cg19112952	5	150466688	TNIP1	1stExon	-0,231	9,05E-10	2,06E-07
cg26354128	14	93897195	KIAA1409	5'UTR	0,22	9,05E-10	2,06E-07
cg08076717	17	9880790	GAS7	Body	-0,24	9,09E-10	2,07E-07
cg26993745	5	169820433	CTD-2270F17	Body	-0,405	9,09E-10	2,07E-07
cg18689454	21	45705694	AIRE	TSS200	0,262	9,10E-10	2,07E-07
cg18743485	3	195839069		IGR	-0,201	9,12E-10	2,07E-07
cg06058170	5	78808668	HOMER1	1stExon	0,208	9,12E-10	2,07E-07
cg21163717	8	21769903	DOK2	Body	-0,266	9,12E-10	2,07E-07

cg17585031	17	25798942	KSR1	TSS200	-0,221	9,13E-10	2,07E-07
cg09486548	1	200407213		IGR	-0,434	9,19E-10	2,08E-07
cg02787852	16	27414536	IL21R	1stExon	-0,254	9,20E-10	2,08E-07
cg08965143	2	24308246	TP53I3	TSS1500	0,23	9,23E-10	2,09E-07
cg15586429	2	150187225	LYPD6	1stExon	0,222	9,22E-10	2,09E-07
cg23420260	2	177053285	HOXD1	TSS200	0,213	9,25E-10	2,09E-07
cg26296455	1	95198014	LINC01057	Body	-0,259	9,26E-10	2,09E-07
cg07176264	2	120281999	SCTR	1stExon	0,251	9,25E-10	2,09E-07
cg18915452	10	133999471	DPYSL4	TSS1500	0,252	9,25E-10	2,09E-07
cg08856161	2	232070095	ARMC9	5'UTR	-0,269	9,28E-10	2,09E-07
cg07676859	20	23015932	SSTR4	TSS200	0,331	9,28E-10	2,09E-07
cg13228395	20	47005243		IGR	-0,227	9,28E-10	2,09E-07
cg20299399	1	15237260	KIAA1026	Body	-0,227	9,30E-10	2,09E-07
cg21187226	17	38718229	CCR7	TSS1500	-0,315	9,31E-10	2,09E-07
cg17915021	21	34757377		IGR	-0,275	9,36E-10	2,10E-07
cg02858512	17	1992954	SMG6	Body	-0,211	9,40E-10	2,11E-07
cg02320481	13	110438578	IRS2	5'UTR	0,215	9,45E-10	2,11E-07
cg03078269	12	54354974		IGR	0,251	9,49E-10	2,12E-07
cg20031327	7	151033415		IGR	-0,357	9,61E-10	2,14E-07
cg05786622	6	16652128	ATXN1	5'UTR	-0,236	9,64E-10	2,14E-07
cg25362652	6	106429769		IGR	0,219	9,64E-10	2,14E-07
cg22561587	9	139298132	SDCCAG3	Body	-0,374	9,64E-10	2,14E-07
cg01962143	13	24825649	SPATA13	Body	-0,353	9,64E-10	2,14E-07
cg04086935	6	5314959	FARS2	5'UTR	-0,227	9,65E-10	2,14E-07
cg24688803	12	105478590	ALDH1L2	TSS1500	0,237	9,66E-10	2,14E-07
cg03092631	10	33406660		IGR	-0,29	9,67E-10	2,14E-07
cg02268417	1	225951743		IGR	0,208	9,71E-10	2,15E-07
cg06653796	20	62367805	LIME1	TSS200	-0,244	9,75E-10	2,15E-07
cg10661378	1	217655643	GPATCH2	Body	-0,24	9,76E-10	2,16E-07
cg23426156	8	126557901		IGR	-0,314	9,83E-10	2,16E-07
cg07506530	10	35022125	PARD3	Body	-0,336	9,83E-10	2,16E-07
cg08435137	16	89696327	DPEP1	5'UTR	-0,293	9,83E-10	2,16E-07
cg08550353	6	134497627	SGK1	TSS1500	0,225	9,86E-10	2,17E-07
cg18031307	2	74726649	LBX2	1stExon	0,258	9,87E-10	2,17E-07
cg14609929	1	219562002		IGR	-0,247	9,90E-10	2,17E-07
cg24815273	4	23780009		IGR	-0,204	9,90E-10	2,17E-07
cg26316423	10	6104137	IL2RA	1stExon	-0,286	9,90E-10	2,17E-07
cg16981614	12	107216023	RIC8B	Body	-0,249	9,91E-10	2,17E-07
cg14538944	2	218340518	DIRC3	Body	-0,293	9,93E-10	2,17E-07
cg00422699	19	12759093	MAN2B1	Body	0,205	9,93E-10	2,17E-07
cg00639010	7	553693	PDGFA	Body	-0,251	9,95E-10	2,18E-07
cg02226192	16	89461734	ANKRD11	5'UTR	0,213	9,95E-10	2,18E-07
cg19187756	3	23753801		IGR	-0,231	1,00E-09	2,19E-07
cg01898698	2	105472759	POU3F3	1stExon	0,207	1,00E-09	2,19E-07
cg06912936	5	169935197	KCNIP1	Body	-0,234	1,01E-09	2,19E-07
cg13080254	9	134388788	POMT1	Body	-0,204	1,01E-09	2,19E-07
cg17638055	18	2960691	LPIN2	Body	-0,38	1,01E-09	2,20E-07
cg07145284	11	66084631	CD248	TSS200	0,247	1,01E-09	2,20E-07
cg23621365	18	8412379		IGR	-0,266	1,01E-09	2,20E-07
cg16307144	19	38704933	DPF1	Body	-0,256	1,01E-09	2,20E-07

cg07270947	6	42007707	CCND3	5'UTR	-0,23	1,02E-09	2,20E-07
cg00797703	4	81093567		IGR	-0,239	1,02E-09	2,20E-07
cg11324999	2	36582398	CRIM1	TSS1500	0,201	1,02E-09	2,22E-07
cg13428477	3	122748086		IGR	-0,265	1,03E-09	2,22E-07
cg26930588	7	45683471	ADCY1	Body	-0,411	1,03E-09	2,22E-07
cg23649619	2	98461039	TMEM131	Body	0,212	1,03E-09	2,22E-07
cg14921966	8	103786546		IGR	-0,27	1,03E-09	2,23E-07
cg03854427	9	116924818	COL27A1	Body	-0,241	1,03E-09	2,23E-07
cg21223520	6	149354166	UST	Body	-0,326	1,03E-09	2,23E-07
cg24255149	5	163767110		IGR	-0,202	1,04E-09	2,24E-07
cg20106459	19	55866087	COX6B2	5'UTR	0,213	1,04E-09	2,24E-07
cg26391408	1	230954520		IGR	-0,234	1,04E-09	2,24E-07
cg01798595	6	21664778		IGR	0,207	1,04E-09	2,24E-07
cg03995649	10	11425457		IGR	-0,248	1,05E-09	2,24E-07
cg15392364	1	206976689	IL19	Body	-0,31	1,05E-09	2,24E-07
cg24437859	12	7066614	PTPN6	Body	-0,337	1,05E-09	2,25E-07
cg07983472	12	111214586		IGR	-0,268	1,05E-09	2,25E-07
cg24055703	20	46417680		IGR	-0,204	1,05E-09	2,25E-07
cg23471279	4	76608281		IGR	-0,292	1,05E-09	2,25E-07
cg17389538	17	4528269		IGR	-0,201	1,05E-09	2,25E-07
cg07416733	11	66834385	RHOD	Body	-0,264	1,06E-09	2,25E-07
cg04212842	14	50549167	LINC01599	Body	-0,238	1,06E-09	2,25E-07
cg17993419	6	125364093	RNF217	Body	-0,21	1,06E-09	2,26E-07
cg05746708	15	91221258	CRTC3-AS1	Body	-0,24	1,06E-09	2,26E-07
cg18049167	6	32121261	PPT2	TSS200	0,205	1,06E-09	2,26E-07
cg15961775	2	174806008	SP3	Body	-0,211	1,06E-09	2,26E-07
cg13429876	11	19530093	NAV2-AS5	TSS1500	-0,226	1,06E-09	2,26E-07
cg25206992	3	185555472		IGR	0,272	1,07E-09	2,26E-07
cg14239460	12	111260933		IGR	-0,214	1,07E-09	2,27E-07
cg16528039	11	67329816		IGR	-0,504	1,07E-09	2,27E-07
cg21511941	15	72489609	GRAMD2	Body	0,259	1,07E-09	2,27E-07
cg01046945	22	38034539	SH3BP1	TSS1500	-0,321	1,08E-09	2,28E-07
cg05979583	3	45984851	CXCR6	TSS200	-0,248	1,08E-09	2,28E-07
cg04214488	14	35860760		IGR	-0,255	1,08E-09	2,28E-07
cg17661686	8	73986912	C8orf84	Body	-0,249	1,08E-09	2,29E-07
cg12630082	2	42275740	PKDCC	1stExon	0,261	1,08E-09	2,29E-07
cg05068866	3	66633374		IGR	-0,33	1,08E-09	2,29E-07
cg01907911	3	4931065		IGR	-0,433	1,09E-09	2,29E-07
cg05429448	3	101659630	LOC152225	TSS200	0,233	1,09E-09	2,29E-07
cg14129729	11	125083077	PKNOX2	5'UTR	-0,225	1,09E-09	2,29E-07
cg01659176	1	214154936		IGR	-0,236	1,09E-09	2,29E-07
cg02088943	8	11719079	CTSB	5'UTR	-0,221	1,09E-09	2,29E-07
cg03986829	3	62358234	FEZF2	Body	0,231	1,09E-09	2,29E-07
cg18603228	3	13590439	FBLN2	TSS200	0,207	1,10E-09	2,30E-07
cg21156998	8	38574072		IGR	-0,251	1,09E-09	2,30E-07
cg03776194	16	88770966	RNF166	Body	-0,289	1,10E-09	2,30E-07
cg08077942	2	61596228	USP34	Body	-0,285	1,10E-09	2,30E-07
cg17383853	15	63166381		IGR	-0,318	1,10E-09	2,30E-07
cg11012463	1	33804514	PHC2	5'UTR	0,204	1,10E-09	2,30E-07
cg14947846	2	129227604		IGR	-0,201	1,10E-09	2,31E-07

cg19985495	16	83073842	CDH13	5'UTR	-0,287	1,11E-09	2,31E-07
cg10741422	19	21769315		IGR	0,207	1,11E-09	2,31E-07
cg24405951	1	19337477		IGR	-0,215	1,12E-09	2,33E-07
cg01586074	2	101586915	NPAS2	Body	-0,28	1,12E-09	2,33E-07
cg12671632	6	422935		IGR	-0,275	1,12E-09	2,33E-07
cg20464719	19	17889512	FCHO1	Body	0,246	1,13E-09	2,34E-07
cg05746985	18	45703599		IGR	-0,244	1,13E-09	2,34E-07
cg20139305	20	34497362	PHF20	Body	-0,295	1,13E-09	2,34E-07
cg10853746	14	50864245	CDKL1	TSS200	0,222	1,14E-09	2,35E-07
cg18508824	4	76440715	RCHY1	TSS1500	-0,4	1,14E-09	2,35E-07
cg20499290	10	38015579		IGR	0,242	1,14E-09	2,35E-07
cg07408835	5	140306377	PCDHAC1	1stExon	0,207	1,14E-09	2,35E-07
cg25595762	5	35130547	PRLR	5'UTR	-0,28	1,14E-09	2,35E-07
cg26096887	7	110723678	IMMP2L	Body	-0,269	1,14E-09	2,35E-07
cg26328951	1	28906514	SNHG12	Body	-0,315	1,15E-09	2,37E-07
cg17694130	4	14858290		IGR	-0,303	1,15E-09	2,37E-07
cg05806864	19	12758681	MAN2B1	Body	0,274	1,15E-09	2,37E-07
cg06958535	1	203734478	LAX1	1stExon	-0,352	1,16E-09	2,37E-07
cg00012522	15	98503952	ARRDC4	1stExon	0,215	1,16E-09	2,37E-07
cg06848034	9	109266213		IGR	-0,226	1,16E-09	2,37E-07
cg25405138	10	34488427	PARD3	Body	-0,284	1,16E-09	2,38E-07
cg25843174	11	12811716	TEAD1	Body	-0,273	1,16E-09	2,38E-07
cg01844201	13	40963299	LINC00598	Body	-0,314	1,17E-09	2,39E-07
cg16410618	7	65542775	ASL	Body	-0,242	1,17E-09	2,39E-07
cg26249510	10	112624792		IGR	-0,288	1,17E-09	2,39E-07
cg11719608	14	51910138		IGR	-0,285	1,17E-09	2,39E-07
cg15815235	1	20247384	PLA2G2E	Body	-0,236	1,17E-09	2,40E-07
cg23880374	10	124092636	BTBD16	Body	-0,256	1,18E-09	2,40E-07
cg21991633	19	9515211		IGR	-0,235	1,18E-09	2,40E-07
cg03692651	19	22444593		IGR	0,29	1,18E-09	2,41E-07
cg00854429	7	65520870		IGR	-0,271	1,19E-09	2,41E-07
cg14865219	19	18954235	UPF1	Body	-0,295	1,19E-09	2,42E-07
cg18832142	3	101659642	LOC152225	TSS200	0,251	1,19E-09	2,42E-07
cg23379818	15	89892276		IGR	-0,254	1,19E-09	2,42E-07
cg10961604	1	27709771	CD164L2	1stExon	0,244	1,19E-09	2,42E-07
cg23510357	22	31934920	SFI1	Body	-0,309	1,19E-09	2,42E-07
cg25273707	11	76037066		IGR	-0,291	1,20E-09	2,43E-07
cg16598790	15	71389060		IGR	0,208	1,20E-09	2,44E-07
cg13116138	12	51582638	POU6F1	3'UTR	-0,341	1,20E-09	2,44E-07
cg06125825	9	123657230	PHF19	TSS200	0,202	1,20E-09	2,44E-07
cg18436544	13	24825781	SPATA13	Body	-0,382	1,20E-09	2,44E-07
cg13327797	1	218695426	MIR548F3	Body	-0,234	1,20E-09	2,44E-07
cg19459342	12	2513226	CACNA1C	Body	-0,27	1,21E-09	2,44E-07
cg22845912	8	134059874	SLA	Body	-0,299	1,22E-09	2,45E-07
cg07921759	14	55203969	SAMD4A	Body	-0,259	1,22E-09	2,45E-07
cg22914993	5	31747854		IGR	-0,283	1,22E-09	2,46E-07
cg06975684	2	159248551	CCDC148	Body	-0,245	1,22E-09	2,46E-07
cg20563910	5	174152033	MSX2	1stExon	0,204	1,22E-09	2,46E-07
cg12377607	21	35565044		IGR	-0,201	1,22E-09	2,46E-07
cg03957124	6	37016869		IGR	-0,272	1,23E-09	2,47E-07

cg26890181	19	44285940	KCNN4	TSS1500	-0,221	1,23E-09	2,47E-07
cg10300362	6	23421100		IGR	-0,231	1,23E-09	2,47E-07
cg15218868	6	42357731	TRERF1	5'UTR	-0,276	1,23E-09	2,47E-07
cg04698728	8	136685858		IGR	-0,219	1,23E-09	2,47E-07
cg11360729	8	105385495		IGR	-0,354	1,24E-09	2,48E-07
cg14520148	5	118664824	TNFAIP8	5'UTR	-0,229	1,24E-09	2,48E-07
cg08428266	7	55238412	EGFR	3'UTR	-0,222	1,24E-09	2,48E-07
cg11358199	2	8453529		IGR	-0,315	1,24E-09	2,48E-07
cg09029902	20	52199594	ZNF217	5'UTR	-0,253	1,24E-09	2,48E-07
cg26114043	4	128544375		IGR	0,208	1,24E-09	2,48E-07
cg20510033	1	8960134		IGR	-0,296	1,24E-09	2,48E-07
cg08815939	7	55635255	VOPP1	Body	-0,282	1,25E-09	2,49E-07
cg13101226	1	183260600	NMNAT2	Body	-0,248	1,25E-09	2,50E-07
cg15036326	17	27048708	RPL23A	Body	-0,269	1,26E-09	2,50E-07
cg00533206	9	100130170	LOC1004994ε	Body	-0,33	1,26E-09	2,50E-07
cg07878065	18	2641871		IGR	-0,241	1,26E-09	2,50E-07
cg08676970	2	8453146	LINC00299	Body	-0,284	1,26E-09	2,50E-07
cg16528895	2	108994311	SULT1C4	TSS200	0,213	1,26E-09	2,50E-07
cg16694198	2	127069963		IGR	-0,255	1,26E-09	2,50E-07
cg23627550	2	227354641		IGR	-0,361	1,26E-09	2,50E-07
cg24491078	9	129246144	MVB12B	Body	-0,294	1,26E-09	2,50E-07
cg13997759	19	39230902	CAPN12	Body	-0,236	1,26E-09	2,50E-07
cg23025873	7	77487860	PHTF2	Body	-0,22	1,27E-09	2,50E-07
cg16928046	17	45812243	TBX21	Body	-0,266	1,27E-09	2,50E-07
cg11846112	1	227729906		IGR	0,236	1,27E-09	2,50E-07
cg23200336	1	182273206	LINC01344	Body	-0,328	1,27E-09	2,51E-07
cg10817441	6	32823728	PSMB9	Body	-0,43	1,27E-09	2,51E-07
cg15463550	13	21904723	LINC00539	ExonBnd	-0,308	1,27E-09	2,51E-07
cg12419669	6	160820176	SLC22A3	Body	-0,335	1,27E-09	2,51E-07
cg11866391	3	10479269	ATP2B2	Body	-0,248	1,28E-09	2,51E-07
cg08679183	12	31161954		IGR	-0,207	1,28E-09	2,51E-07
cg11970327	7	40529988	SUGCT	Body	-0,209	1,28E-09	2,52E-07
cg01249544	10	69372655	CTNNA3	Body	-0,218	1,28E-09	2,52E-07
cg02388865	1	26737318	LIN28	1stExon	0,23	1,28E-09	2,52E-07
cg17994196	1	15617106	FHAD1	Body	-0,261	1,29E-09	2,53E-07
cg08479533	7	90680162	CDK14	Body	-0,275	1,29E-09	2,53E-07
cg06808751	8	54163693	OPRK1	5'UTR	0,203	1,29E-09	2,53E-07
cg23687466	11	504937	RNH1	5'UTR	0,291	1,29E-09	2,53E-07
cg25006194	12	94288553		IGR	0,213	1,29E-09	2,53E-07
cg18058689	13	93879670	GPC6	1stExon	0,238	1,29E-09	2,53E-07
cg03165391	12	120266281	CIT	Body	-0,285	1,30E-09	2,54E-07
cg07260070	19	45519636	RELB	Body	-0,334	1,30E-09	2,54E-07
cg26872137	8	35092870	UNC5D	TSS200	0,24	1,30E-09	2,54E-07
cg00329656	1	17054246		IGR	-0,23	1,30E-09	2,54E-07
cg24391385	2	65526509		IGR	-0,28	1,30E-09	2,55E-07
cg05914697	9	116327278	RGS3	1stExon	0,285	1,30E-09	2,55E-07
cg01772743	21	45576085		IGR	-0,324	1,30E-09	2,55E-07
cg02795981	10	81045119	ZMIZ1	Body	-0,278	1,31E-09	2,55E-07
cg21580797	13	96004438		IGR	-0,216	1,31E-09	2,55E-07
cg02910979	15	78339457	TBC1D2B	Body	-0,317	1,31E-09	2,55E-07

cg02315315	2	47055138	LOC10013425	Body	-0,267	1,31E-09	2,55E-07
cg00995999	3	72314869		IGR	-0,23	1,31E-09	2,55E-07
cg27213416	5	131595932	PDLIM4	Body	-0,202	1,31E-09	2,55E-07
cg26582375	4	4639639	STX18-AS1	Body	-0,246	1,31E-09	2,56E-07
cg04599341	16	2029855	NOXO1	Body	0,205	1,32E-09	2,56E-07
cg05246522	17	25798973	KSR1	TSS200	-0,289	1,32E-09	2,56E-07
cg17682392	14	23013010		IGR	-0,255	1,32E-09	2,56E-07
cg10001244	18	12840815	PTPN2	1stExon	-0,23	1,32E-09	2,56E-07
cg12199261	8	145871380	ARHGAP39	5'UTR	-0,215	1,32E-09	2,56E-07
cg26609631	13	28366814	GSX1	5'UTR	0,262	1,33E-09	2,57E-07
cg19371105	13	29159914		IGR	-0,372	1,33E-09	2,57E-07
cg12542255	19	45976195	FOSB	Body	0,25	1,33E-09	2,57E-07
cg26071135	11	843943	TSPAN4	TSS1500	0,204	1,33E-09	2,58E-07
cg13698548	8	128808194	MIR1204	TSS200	-0,348	1,34E-09	2,58E-07
cg14952249	12	116777931		IGR	-0,305	1,34E-09	2,58E-07
cg10102102	14	62129058		IGR	-0,237	1,34E-09	2,58E-07
cg14265755	2	236473728	AGAP1	Body	-0,232	1,34E-09	2,58E-07
cg08899626	4	16900199	LDB2	1stExon	0,208	1,34E-09	2,58E-07
cg01295399	10	50820278	SLC18A3	1stExon	0,265	1,35E-09	2,59E-07
cg25826082	10	13503388	BEND7	Body	-0,214	1,35E-09	2,59E-07
cg21519781	2	88849576		IGR	0,257	1,35E-09	2,60E-07
cg16111461	22	51154009	SHANK3	Body	-0,289	1,35E-09	2,60E-07
cg05775517	15	93158989		IGR	-0,317	1,36E-09	2,60E-07
cg13321077	2	220196755	RESP18	Body	0,204	1,36E-09	2,61E-07
cg17569958	10	31036797		IGR	-0,28	1,36E-09	2,61E-07
cg01452308	3	193682169	LOC647323	Body	-0,309	1,37E-09	2,61E-07
cg05026039	5	52067603		IGR	-0,39	1,37E-09	2,61E-07
cg14095677	12	116014459		IGR	-0,24	1,37E-09	2,61E-07
cg03700780	14	76005972	BATF	Body	-0,277	1,37E-09	2,61E-07
cg07047068	11	844686	TSPAN4	5'UTR	0,235	1,37E-09	2,61E-07
cg00055876	2	106497031	NCK2	Body	-0,297	1,37E-09	2,61E-07
cg17539402	1	232320035		IGR	-0,264	1,37E-09	2,62E-07
cg19656282	2	74742786	TLX2	Body	0,245	1,37E-09	2,62E-07
cg24436906	2	242498081	BOK	TSS200	0,215	1,37E-09	2,62E-07
cg26647600	3	39369720	CCR8	TSS1500	-0,245	1,37E-09	2,62E-07
cg07490756	18	45698336		IGR	-0,232	1,37E-09	2,62E-07
cg03230364	18	55681687		IGR	-0,252	1,38E-09	2,62E-07
cg10198479	16	85268435		IGR	0,271	1,38E-09	2,62E-07
cg14772925	11	100998943	PGR	1stExon	0,228	1,38E-09	2,63E-07
cg07394965	16	27787070	KIAA0556	Body	-0,252	1,38E-09	2,63E-07
cg15820574	1	156406711		IGR	0,229	1,39E-09	2,63E-07
cg23040259	18	21390641	LAMA3	Body	-0,284	1,38E-09	2,63E-07
cg07156249	6	32822911	PSMB9	Body	-0,23	1,39E-09	2,64E-07
cg18934050	11	34645726	EHF	5'UTR	-0,216	1,39E-09	2,64E-07
cg03680790	12	131281661	STX2	Body	-0,284	1,39E-09	2,64E-07
cg23266258	14	23000539		IGR	-0,281	1,39E-09	2,64E-07
cg09532889	15	63738508		IGR	-0,283	1,39E-09	2,64E-07
cg12417457	16	67018923		IGR	-0,27	1,40E-09	2,65E-07
cg21461564	8	63999030	TTPA	TSS1500	0,215	1,40E-09	2,65E-07
cg03602280	18	11148914	FAM38B	TSS200	0,208	1,41E-09	2,65E-07

cg26317934	16	85775380	MIR1910	TSS200	-0,242	1,41E-09	2,66E-07
cg12390720	6	42342438	TRERF1	5'UTR	-0,233	1,41E-09	2,66E-07
cg01316152	10	116168351		IGR	-0,229	1,41E-09	2,66E-07
cg03644845	4	111437428	ENPEP	Body	-0,326	1,42E-09	2,67E-07
cg00637477	20	62525797	DNAJC5	TSS1500	0,212	1,42E-09	2,67E-07
cg13494769	1	243509235	MIR4677	TSS1500	-0,295	1,42E-09	2,68E-07
cg13980719	2	217725560	TNP1	TSS1500	-0,219	1,42E-09	2,68E-07
cg14579651	12	27429400	STK38L	5'UTR	0,276	1,43E-09	2,68E-07
cg17804959	15	63961165	HERC1	Body	-0,334	1,43E-09	2,68E-07
cg25140783	1	24861872	RCAN3	3'UTR	-0,221	1,43E-09	2,69E-07
cg01710179	20	30150916	HM13	Body	0,201	1,43E-09	2,69E-07
cg12831208	14	77219295		IGR	-0,241	1,44E-09	2,69E-07
cg20683482	16	29002173		IGR	-0,237	1,44E-09	2,69E-07
cg20073327	19	34842652	KIAA0355	Body	-0,222	1,44E-09	2,69E-07
cg10132627	22	43355971	PACSIN2	TSS200	0,216	1,44E-09	2,69E-07
cg07575207	16	75046592	ZNRF1	Body	-0,256	1,44E-09	2,69E-07
cg04901718	2	16380415		IGR	-0,206	1,44E-09	2,70E-07
cg06501366	5	78365687	BHMT2	Body	0,256	1,45E-09	2,70E-07
cg16407511	11	2763633	KCNQ1	Body	-0,225	1,45E-09	2,70E-07
cg02498441	2	191992936	STAT4	Body	-0,252	1,45E-09	2,70E-07
cg00813378	1	1475209	C1orf70	Body	0,228	1,45E-09	2,71E-07
cg13347761	10	4075946		IGR	-0,317	1,46E-09	2,71E-07
cg21109025	17	32580869	CCL2	TSS1500	-0,233	1,46E-09	2,71E-07
cg17496659	1	3568245	TP73	TSS1500	0,269	1,47E-09	2,72E-07
cg24561297	1	155016363	DCST1	Body	-0,231	1,47E-09	2,72E-07
cg12234697	4	80117230	LINC01088	Body	-0,202	1,47E-09	2,72E-07
cg05288075	7	77022728	GSAP	Body	-0,27	1,47E-09	2,72E-07
cg13127598	16	23893106	PRKCB	Body	-0,231	1,47E-09	2,72E-07
cg21567022	11	93067324	CCDC67	Body	-0,211	1,47E-09	2,72E-07
cg05072547	16	19883549	GPRC5B	Body	-0,297	1,47E-09	2,72E-07
cg26919283	20	34795295	EPB41L1	Body	-0,205	1,48E-09	2,72E-07
cg27554953	6	108062292	SCML4	Body	-0,322	1,48E-09	2,73E-07
cg05161791	9	125987802	STRBP	Body	-0,298	1,48E-09	2,73E-07
cg11823511	1	91183697	BARHL2	TSS1500	0,212	1,48E-09	2,73E-07
cg15197065	5	78985562	CMYA5	TSS200	0,312	1,48E-09	2,73E-07
cg16354502	2	71697410	DYSF	Body	-0,206	1,48E-09	2,73E-07
cg19655993	2	206798844		IGR	-0,303	1,49E-09	2,74E-07
cg07761351	2	43199639		IGR	-0,39	1,49E-09	2,75E-07
cg20477259	6	31544960	TNF	Body	-0,277	1,49E-09	2,75E-07
cg03453870	15	39563291		IGR	-0,226	1,50E-09	2,75E-07
cg15592555	2	161797388		IGR	-0,245	1,50E-09	2,75E-07
cg03782453	4	7043935	CCDC96	1stExon	0,24	1,50E-09	2,75E-07
cg21018698	5	148986280	ARHGEF37	Body	-0,295	1,50E-09	2,75E-07
cg24212696	2	111812810	ACOXL	Body	-0,247	1,50E-09	2,75E-07
cg10808914	7	6660940	ZNF853	Body	-0,283	1,50E-09	2,75E-07
cg08771567	9	116327270	RGS3	1stExon	0,23	1,50E-09	2,75E-07
cg05784862	17	25799018	KSR1	TSS200	-0,314	1,50E-09	2,75E-07
cg26816268	1	206763683		IGR	-0,292	1,50E-09	2,75E-07
cg23692114	2	75154873	LINC01291	Body	-0,219	1,51E-09	2,76E-07
cg10723736	11	19431887	NAV2	Body	-0,224	1,51E-09	2,76E-07

cg26165277	18	33153698		IGR	-0,32	1,51E-09	2,76E-07
cg03739340	2	1720509	PXDN	Body	-0,239	1,51E-09	2,76E-07
cg10599444	14	23305941	MMP14	5'UTR	0,265	1,51E-09	2,77E-07
cg15995296	11	67210812	CORO1B	5'UTR	0,235	1,52E-09	2,77E-07
cg05146480	1	172630463	FASLG	Body	-0,264	1,52E-09	2,77E-07
cg21577598	17	80084751	CCDC57	Body	-0,335	1,52E-09	2,77E-07
cg05835105	4	111543401	PITX2	Body	0,271	1,52E-09	2,77E-07
cg23927970	11	504933	RNH1	5'UTR	0,233	1,52E-09	2,77E-07
cg12811871	4	2322078	ZFYVE28	Body	-0,242	1,53E-09	2,78E-07
cg08592912	20	56403926		IGR	-0,322	1,53E-09	2,78E-07
cg25606842	1	86052061		IGR	-0,268	1,53E-09	2,78E-07
cg13879776	3	170136263	CLDN11	TSS1500	0,201	1,53E-09	2,78E-07
cg14595291	11	35993855	LDLRAD3	5'UTR	-0,315	1,53E-09	2,78E-07
cg22794405	14	50864238	CDKL1	TSS200	0,202	1,53E-09	2,78E-07
cg01151584	11	64627528	EHD1	Body	-0,238	1,54E-09	2,79E-07
cg06478249	3	186743256	ST6GAL1	5'UTR	-0,233	1,54E-09	2,79E-07
cg04609265	10	81045085	ZMIZ1	Body	-0,299	1,54E-09	2,79E-07
cg04376808	3	184267741	EIF2B5-AS1	Body	0,208	1,54E-09	2,79E-07
cg24124443	12	107711648	BTBD11	TSS1500	0,25	1,55E-09	2,79E-07
cg00622905	9	137283224	RXRA	5'UTR	-0,283	1,55E-09	2,80E-07
cg07902730	16	10360788		IGR	-0,202	1,55E-09	2,80E-07
cg02726722	20	48510804		IGR	-0,362	1,55E-09	2,80E-07
cg02157078	1	114888769		IGR	0,269	1,55E-09	2,80E-07
cg26573334	12	113669935	TPCN1	Body	0,239	1,55E-09	2,80E-07
cg02263051	18	3811216	DLGAP1	Body	-0,248	1,55E-09	2,80E-07
cg12422228	3	107804925	CD47	Body	-0,294	1,56E-09	2,80E-07
cg13156207	2	28873060		IGR	-0,288	1,56E-09	2,80E-07
cg17900199	10	1156548	WDR37	Body	-0,215	1,56E-09	2,81E-07
cg07112491	17	71733231	LOC1001343‹	TSS1500	-0,219	1,56E-09	2,81E-07
cg19312305	18	56117016	MIR122	TSS1500	-0,253	1,56E-09	2,81E-07
cg01361348	14	67907924		IGR	-0,203	1,57E-09	2,82E-07
cg05171197	2	240230850	HDAC4	Body	0,23	1,57E-09	2,82E-07
cg26429850	16	4464427	CORO7-PAM1	Body	-0,349	1,57E-09	2,82E-07
cg18414488	17	27198308		IGR	-0,239	1,57E-09	2,82E-07
cg21800196	3	48673931	CELSR3	3'UTR	0,212	1,59E-09	2,84E-07
cg23679992	12	6421513	PLEKHG6	TSS1500	-0,267	1,59E-09	2,84E-07
cg17283407	5	146889238	DPYSL3	1stExon	0,219	1,59E-09	2,85E-07
cg07169399	2	239276221	TRAF3IP1	Body	-0,21	1,60E-09	2,85E-07
cg15393399	16	50715700	SNX20	TSS1500	-0,273	1,60E-09	2,86E-07
cg27584318	1	48319220	TRABD2B	Body	-0,238	1,60E-09	2,86E-07
cg08366458	5	41172659	C6	Body	-0,239	1,60E-09	2,86E-07
cg19426625	2	9518324	ASAP2	Body	-0,211	1,61E-09	2,86E-07
cg09111477	5	10635405	ANKRD33B	Body	-0,24	1,61E-09	2,86E-07
cg05392803	17	79443222		IGR	-0,262	1,61E-09	2,86E-07
cg23505180	3	194614099		IGR	-0,236	1,61E-09	2,87E-07
cg08495827	10	81206525	ZCCHC24	TSS1500	0,208	1,62E-09	2,87E-07
cg19867917	2	3642629	COLEC11	TSS200	0,381	1,62E-09	2,87E-07
cg09563940	7	130873558	MKLN1	Body	-0,26	1,62E-09	2,87E-07
cg14289511	16	24682446		IGR	0,201	1,62E-09	2,88E-07
cg26551200	7	2681369	TTYH3	Body	0,285	1,63E-09	2,88E-07

cg21812313	1	94158618		IGR	-0,295	1,63E-09	2,88E-07
cg22021794	5	54177306		IGR	-0,253	1,63E-09	2,89E-07
cg27518279	20	55205792	TFAP2C	Body	0,214	1,63E-09	2,89E-07
cg21689664	18	7525014		IGR	-0,259	1,63E-09	2,89E-07
cg04096150	15	40098188	GPR176	Body	-0,21	1,63E-09	2,89E-07
cg11232362	2	65047387		IGR	-0,213	1,64E-09	2,89E-07
cg05683165	22	18920088	PRODH	Body	-0,299	1,64E-09	2,90E-07
cg02739094	4	16122488		IGR	-0,246	1,65E-09	2,90E-07
cg20772512	1	38022359	DNALI1	TSS200	0,234	1,65E-09	2,90E-07
cg06088445	14	23837451		IGR	-0,25	1,65E-09	2,90E-07
cg13439189	9	93926553		IGR	-0,263	1,65E-09	2,91E-07
cg19103712	14	21498725	TPPP2	5'UTR	-0,243	1,65E-09	2,91E-07
cg03072600	14	53657673		IGR	-0,203	1,65E-09	2,91E-07
cg23467561	17	73841592	UNC13D	TSS1500	0,234	1,65E-09	2,91E-07
cg11811625	22	45079203	PRR5	5'UTR	-0,255	1,65E-09	2,91E-07
cg22011254	11	2739975	KCNQ1	Body	-0,297	1,66E-09	2,91E-07
cg03602064	2	133204592	GPR39	Body	-0,228	1,66E-09	2,91E-07
cg12937391	7	29900403	WIPF3	Body	-0,245	1,66E-09	2,91E-07
cg19014705	1	44026770	PTPRF	Body	-0,295	1,67E-09	2,91E-07
cg12609247	7	130611417		IGR	-0,229	1,66E-09	2,91E-07
cg16014491	9	123267471	CDK5RAP2	Body	-0,228	1,66E-09	2,91E-07
cg17531025	16	29002219		IGR	-0,241	1,67E-09	2,91E-07
cg22328786	20	35491355	C20orf117	1stExon	0,21	1,67E-09	2,91E-07
cg08476925	11	82690000	RAB30	3'UTR	-0,34	1,67E-09	2,92E-07
cg10073338	10	89521994	ATAD1	Body	-0,269	1,67E-09	2,92E-07
cg19420599	1	17966166	ARHGEF10L	Body	-0,264	1,68E-09	2,93E-07
cg24066052	7	128358822	FAM71F1	Body	-0,229	1,68E-09	2,93E-07
cg23074260	18	77723195	HSBP1L1	TSS1500	-0,368	1,68E-09	2,93E-07
cg15747192	20	5824829	C20orf196	Body	-0,237	1,69E-09	2,93E-07
cg04560229	20	50098437	NFATC2	Body	-0,298	1,68E-09	2,93E-07
cg03771015	2	15831147	LOC10192696	Body	-0,244	1,69E-09	2,93E-07
cg13912311	9	127265348	NR5A1	Body	0,25	1,69E-09	2,93E-07
cg06040872	17	34394215	CCL18	Body	-0,22	1,69E-09	2,93E-07
cg27183400	18	47315112	ACAA2	Body	-0,301	1,69E-09	2,93E-07
cg25631863	3	71922382		IGR	-0,242	1,69E-09	2,94E-07
cg19308985	16	85950321	IRF8	Body	0,218	1,69E-09	2,94E-07
cg08555226	11	60746131	CD6	Body	-0,311	1,70E-09	2,95E-07
cg08446539	12	25541565		IGR	-0,25	1,71E-09	2,95E-07
cg14201249	14	25288746	STXBP6	Body	-0,236	1,70E-09	2,95E-07
cg23963682	21	40508807		IGR	-0,209	1,71E-09	2,95E-07
cg13247871	7	88632493	ZNF804B	Body	-0,334	1,71E-09	2,96E-07
cg25330665	7	69922036	AUTS2	Body	-0,289	1,71E-09	2,96E-07
cg18886274	7	84789871		IGR	-0,308	1,71E-09	2,96E-07
cg08033142	2	46260367	PRKCE	Body	-0,319	1,72E-09	2,97E-07
cg26491791	10	128593800	DOCK1	TSS200	0,245	1,72E-09	2,97E-07
cg15631007	10	46967589	SYT15	Body	-0,235	1,72E-09	2,97E-07
cg02374486	10	72362809	PRF1	TSS1500	-0,322	1,73E-09	2,97E-07
cg05388840	1	41981911	HIVEP3	Body	0,271	1,73E-09	2,97E-07
cg09097594	1	22183322	HSPG2	Body	-0,211	1,73E-09	2,98E-07
cg18770350	1	236849966	ACTN2	1stExon	0,21	1,73E-09	2,98E-07

cg23305420	11	109948734		IGR	-0,312	1,74E-09	2,98E-07
cg23452498	5	144889607		IGR	0,291	1,74E-09	2,98E-07
cg20331795	2	139537843	NXPH2	TSS200	0,25	1,74E-09	2,98E-07
cg07015803	14	99655593	BCL11B	Body	-0,237	1,74E-09	2,98E-07
cg20063214	2	46132923	PRKCE	Body	-0,208	1,75E-09	2,99E-07
cg06871578	1	112143839	LINC01160	Body	-0,216	1,75E-09	2,99E-07
cg04131610	3	46411447	CCR5	TSS200	-0,258	1,75E-09	2,99E-07
cg21887530	5	123919649		IGR	-0,31	1,75E-09	2,99E-07
cg20219381	8	101118083	RGS22	Body	0,213	1,75E-09	2,99E-07
cg24507266	8	145027948	PLEC1	1stExon	0,293	1,75E-09	2,99E-07
cg02438816	17	73368189	GRB2	Body	-0,387	1,75E-09	2,99E-07
cg21238882	6	83796488	DOPEY1	5'UTR	-0,368	1,76E-09	3,00E-07
cg15496807	7	47119580		IGR	-0,303	1,76E-09	3,00E-07
cg20814813	1	247506185		IGR	-0,222	1,76E-09	3,00E-07
cg12700402	10	104470719	ARL3	Body	-0,221	1,76E-09	3,00E-07
cg20175587	16	88771044	RNF166	TSS1500	-0,32	1,76E-09	3,00E-07
cg09239439	4	129584769		IGR	-0,242	1,77E-09	3,01E-07
cg08910705	8	79711103	IL7	Body	0,284	1,77E-09	3,01E-07
cg07057579	15	95870112		IGR	0,245	1,77E-09	3,01E-07
cg15584219	8	124050114	DERL1	Body	-0,403	1,77E-09	3,01E-07
cg23024967	3	40150853	MYRIP	Body	-0,22	1,78E-09	3,02E-07
cg03377916	16	10758658	TEKT5	Body	-0,237	1,78E-09	3,02E-07
cg24155515	6	15387512	JARID2	Body	-0,222	1,79E-09	3,03E-07
cg17929770	19	46318514	RSPH6A	1stExon	0,222	1,79E-09	3,03E-07
cg07474825	19	10677826	KRI1	TSS1500	-0,208	1,79E-09	3,03E-07
cg04024218	10	35003357	PARD3	Body	-0,273	1,80E-09	3,04E-07
cg23786205	10	77057425		IGR	-0,283	1,80E-09	3,04E-07
cg24545166	6	154674363	IPCEF1	5'UTR	-0,267	1,80E-09	3,04E-07
cg14518276	9	123687270	TRAF1	Body	-0,335	1,80E-09	3,04E-07
cg01917852	11	2772759	KCNQ1	Body	-0,23	1,80E-09	3,04E-07
cg07713291	6	16577265	ATXN1	5'UTR	-0,301	1,80E-09	3,04E-07
cg16064512	2	135472142	TMEM163	Body	-0,271	1,81E-09	3,05E-07
cg11856917	5	159632639	FABP6	Body	-0,243	1,81E-09	3,05E-07
cg11210357	8	144543951	ZC3H3	Body	-0,22	1,81E-09	3,05E-07
cg16501323	21	45705618	AIRE	TSS200	0,232	1,81E-09	3,05E-07
cg15043350	11	60214279	MS4A5	Body	-0,28	1,81E-09	3,05E-07
cg10542606	6	163748837		IGR	-0,234	1,82E-09	3,05E-07
cg16811856	3	124829683	SLC12A8	Body	-0,301	1,82E-09	3,05E-07
cg20483374	11	119211646	C1QTNF5	5'UTR	0,27	1,82E-09	3,05E-07
cg19104979	2	23847714	KLHL29	Body	-0,28	1,82E-09	3,05E-07
cg17210801	3	140808151	SPSB4	Body	-0,213	1,82E-09	3,05E-07
cg25044876	22	43041146	CYB5R3	5'UTR	0,21	1,82E-09	3,05E-07
cg01941619	18	29770081	MEP1B	1stExon	-0,214	1,83E-09	3,05E-07
cg22900088	3	100784989		IGR	-0,276	1,83E-09	3,05E-07
cg26766164	6	502440	EXOC2	Body	-0,219	1,83E-09	3,05E-07
cg06488536	6	147225846		IGR	-0,212	1,83E-09	3,05E-07
cg13598010	7	72838776		IGR	0,251	1,83E-09	3,05E-07
cg00500913	11	331288		IGR	-0,274	1,83E-09	3,05E-07
cg18941817	14	50864369	CDKL1	TSS1500	0,209	1,83E-09	3,05E-07
cg22627753	1	988623	AGRN	Body	-0,34	1,84E-09	3,06E-07

cg17604908	4	165931056		IGR	-0,211	1,84E-09	3,06E-07
cg10536898	5	50673227		IGR	0,201	1,85E-09	3,07E-07
cg04552852	11	844390	TSPAN4	TSS200	0,2	1,85E-09	3,07E-07
cg24576535	3	113417997		IGR	0,207	1,86E-09	3,08E-07
cg03752765	14	50864315	CDKL1	TSS200	0,205	1,86E-09	3,08E-07
cg12737452	13	52548992	ATP7B	Body	-0,228	1,86E-09	3,09E-07
cg08496742	19	56159485	CCDC106	5'UTR	0,215	1,86E-09	3,09E-07
cg05404091	12	133182886		IGR	-0,225	1,87E-09	3,09E-07
cg16053890	2	231091055	SP110	TSS1500	-0,29	1,87E-09	3,09E-07
cg05515866	2	242298062	FARP2	5'UTR	-0,483	1,87E-09	3,09E-07
cg01804715	7	35762825		IGR	-0,286	1,87E-09	3,09E-07
cg00747372	2	102398839	MAP4K4	Body	-0,245	1,87E-09	3,09E-07
cg19120513	11	102189303	BIRC3	5'UTR	-0,33	1,87E-09	3,09E-07
cg20436086	10	71615835	COL13A1	Body	-0,257	1,87E-09	3,09E-07
cg25705792	21	35320017	LINC00649	TSS1500	-0,247	1,88E-09	3,10E-07
cg04215897	10	73843982	SPOCK2	Body	-0,308	1,88E-09	3,10E-07
cg07465171	3	4818475	ITPR1	Body	-0,23	1,89E-09	3,11E-07
cg21861324	5	81661638		IGR	-0,214	1,90E-09	3,12E-07
cg08403891	20	31957417	CDK5RAP1	Body	-0,317	1,90E-09	3,12E-07
cg21927325	3	127556254		IGR	-0,257	1,90E-09	3,13E-07
cg08817540	3	108020727	HHLA2	TSS1500	-0,213	1,90E-09	3,13E-07
cg25237113	2	80551812	CTNNA2	Body	-0,202	1,90E-09	3,13E-07
cg03912518	8	19221205	SH2D4A	Body	-0,315	1,91E-09	3,13E-07
cg06063903	1	40783063	COL9A2	TSS200	0,228	1,91E-09	3,13E-07
cg17147638	2	236509769	AGAP1	Body	-0,227	1,91E-09	3,13E-07
cg19269473	17	55939590		IGR	0,246	1,91E-09	3,13E-07
cg15167367	2	113933123	PSD4	5'UTR	-0,289	1,91E-09	3,13E-07
cg15852130	18	12102699	ANKRD62	Body	-0,259	1,91E-09	3,13E-07
cg14134551	1	204598444	LRRN2	5'UTR	-0,255	1,91E-09	3,13E-07
cg27334376	7	47335209	TNS3	Body	-0,248	1,91E-09	3,13E-07
cg20457195	18	7904834	PTPRM	Body	-0,32	1,91E-09	3,13E-07
cg15134801	5	60138838	ELOVL7	5'UTR	0,215	1,91E-09	3,13E-07
cg18335129	6	133194303		IGR	-0,207	1,91E-09	3,13E-07
cg23163724	17	48404379		IGR	-0,245	1,92E-09	3,14E-07
cg10479082	7	28996639	TRIL	1stExon	0,201	1,92E-09	3,14E-07
cg16890093	6	32813084	PSMB8	TSS1500	-0,279	1,92E-09	3,14E-07
cg18287053	3	133780356		IGR	-0,357	1,92E-09	3,14E-07
cg12336777	2	145210222	ZEB2	Body	0,236	1,93E-09	3,15E-07
cg07000480	12	124724894	ZNF664-FAM:	5'UTR	-0,258	1,93E-09	3,15E-07
cg22384423	8	128808262	MIR1204	Body	-0,289	1,95E-09	3,18E-07
cg21545720	1	32405083	PTP4A2	TSS1500	-0,276	1,95E-09	3,18E-07
cg14660461	4	147379854	SLC10A7	Body	-0,273	1,95E-09	3,19E-07
cg20916656	4	36350465		IGR	-0,209	1,96E-09	3,19E-07
cg18109874	19	14115611	RFX1	5'UTR	0,206	1,96E-09	3,19E-07
cg04498270	20	3646703		IGR	-0,208	1,96E-09	3,19E-07
cg23839599	1	161708999		IGR	-0,267	1,96E-09	3,19E-07
cg02431562	2	96809970	DUSP2	Body	-0,277	1,97E-09	3,20E-07
cg21321611	4	39530047	UGDH	TSS1500	0,254	1,97E-09	3,20E-07
cg09442613	12	76054089		IGR	-0,291	1,97E-09	3,20E-07
cg04706229	14	50550589	C14orf183	Body	-0,249	1,97E-09	3,20E-07

cg14441276	6	31539735	LTA	TSS1500	-0,265	1,97E-09	3,20E-07
cg11784870	22	33188718	SYN3	Body	-0,247	1,97E-09	3,20E-07
cg07766263	3	170303045	SLC7A14	5'UTR	0,265	1,98E-09	3,20E-07
cg01897428	12	57457953	NEMP1	ExonBnd	-0,471	1,98E-09	3,20E-07
cg09122679	2	46897833		IGR	-0,24	1,98E-09	3,21E-07
cg04553173	5	138783103		IGR	-0,295	1,98E-09	3,21E-07
cg16408805	14	50864343	CDKL1	TSS1500	0,233	1,98E-09	3,21E-07
cg14332225	7	141402926	KIAA1147	TSS1500	0,298	1,99E-09	3,21E-07
cg04255230	2	74727010	LBX2	Body	0,301	1,99E-09	3,22E-07
cg11753018	10	45469396	RASSF4	Body	-0,255	2,00E-09	3,22E-07
cg09895458	20	49596978		IGR	-0,282	2,00E-09	3,22E-07
cg09880551	21	42218932	DSCAM	1stExon	0,214	2,00E-09	3,22E-07
cg18953506	20	48639596		IGR	-0,228	2,00E-09	3,23E-07
cg19003958	15	101926453	PCSK6	Body	-0,217	2,01E-09	3,24E-07
cg20670361	1	25258679	RUNX3	Body	0,203	2,01E-09	3,24E-07
cg07157830	1	236228741	NID1	TSS1500	0,235	2,01E-09	3,24E-07
cg06354543	10	130376572		IGR	-0,265	2,01E-09	3,24E-07
cg06256438	2	121260279		IGR	-0,236	2,02E-09	3,24E-07
cg23834593	20	42983920	HNF4A	TSS1500	-0,217	2,02E-09	3,24E-07
cg20120216	1	41293379	KCNQ4	Body	-0,219	2,02E-09	3,24E-07
cg22786472	4	26198975		IGR	-0,29	2,02E-09	3,24E-07
cg26343116	5	172045960		IGR	-0,306	2,02E-09	3,24E-07
cg22318901	13	34253578		IGR	-0,383	2,02E-09	3,24E-07
cg16672562	19	46801672	HIF3A	5'UTR	0,261	2,02E-09	3,24E-07
cg02102872	4	79338837	FRAS1	Body	-0,238	2,02E-09	3,24E-07
cg23799091	11	75365642	MAP6	Body	-0,296	2,02E-09	3,24E-07
cg15173780	3	111043756		IGR	-0,293	2,02E-09	3,24E-07
cg24304186	18	20846733		IGR	-0,282	2,03E-09	3,24E-07
cg20417024	5	76028950	F2R	Body	-0,319	2,03E-09	3,25E-07
cg06619939	11	15732102		IGR	-0,226	2,03E-09	3,25E-07
cg01280849	8	141678964	PTK2	Body	0,221	2,04E-09	3,26E-07
cg17869789	12	76373265		IGR	-0,306	2,04E-09	3,26E-07
cg07495637	2	26143450		IGR	-0,219	2,05E-09	3,26E-07
cg25892035	6	86395559		IGR	-0,242	2,05E-09	3,26E-07
cg02981639	7	42155502	GLI3	Body	-0,245	2,05E-09	3,26E-07
cg17730271	21	34578205		IGR	-0,217	2,05E-09	3,26E-07
cg13104938	11	843956	TSPAN4	TSS1500	0,214	2,05E-09	3,27E-07
cg16419354	1	179713349	FAM163A	5'UTR	0,219	2,06E-09	3,27E-07
cg04214995	2	27503849	DNAJC5G	Body	-0,266	2,06E-09	3,27E-07
cg00527254	11	82707269	RAB30	Body	-0,257	2,06E-09	3,27E-07
cg08575699	14	100546923	EVL	Body	-0,235	2,06E-09	3,27E-07
cg03407747	17	6899364	ALOX12	TSS200	0,26	2,06E-09	3,27E-07
cg10140148	1	160678957	CD48	Body	-0,246	2,07E-09	3,27E-07
cg00312486	1	209877438	HSD11B1	TSS1500	-0,386	2,07E-09	3,27E-07
cg06930314	2	37851357		IGR	-0,314	2,07E-09	3,27E-07
cg09831549	2	68947188		IGR	-0,245	2,07E-09	3,27E-07
cg17329494	5	65018961	SGTB	TSS1500	0,22	2,07E-09	3,27E-07
cg16941430	6	45678413		IGR	-0,276	2,07E-09	3,27E-07
cg26248201	7	73875457	GTF2IRD1	5'UTR	-0,202	2,06E-09	3,27E-07
cg17002670	7	155453394	RBM33	Body	-0,255	2,07E-09	3,27E-07

cg13555716	8	1747170		IGR	-0,229	2,07E-09	3,27E-07
cg16443910	10	34813513	PARD3	Body	-0,269	2,07E-09	3,27E-07
cg12620322	10	77969251	C10orf11	Body	-0,227	2,07E-09	3,27E-07
cg11959746	12	50321909		IGR	-0,242	2,07E-09	3,27E-07
cg07879213	14	63749933	RHOJ	ExonBnd	-0,27	2,06E-09	3,27E-07
cg26517663	15	60295294	FOXB1	TSS1500	0,217	2,06E-09	3,27E-07
cg10777887	15	63484315	RAB8B	Body	-0,203	2,07E-09	3,27E-07
cg14512563	15	96897190		IGR	0,237	2,06E-09	3,27E-07
cg00549574	17	74350942	PRPSAP1	TSS1500	0,21	2,07E-09	3,27E-07
cg21904337	3	46131862		IGR	-0,208	2,09E-09	3,29E-07
cg25107254	4	187476455	MTNR1A	1stExon	0,214	2,09E-09	3,29E-07
cg19769182	16	29823868	PRRT2	5'UTR	0,216	2,09E-09	3,29E-07
cg10258381	10	73486251	C10orf105	5'UTR	-0,308	2,10E-09	3,29E-07
cg26444411	3	47469671	SCAP	Body	-0,324	2,11E-09	3,30E-07
cg13989603	3	151974942		IGR	-0,277	2,11E-09	3,30E-07
cg23991388	15	100654760	ADAMTS17	Body	0,202	2,11E-09	3,30E-07
cg13032272	17	63096456	LOC1005070(	TSS1500	-0,314	2,11E-09	3,30E-07
cg26145176	3	193820496		IGR	-0,235	2,11E-09	3,30E-07
cg06628133	20	1811563		IGR	-0,238	2,11E-09	3,30E-07
cg19771694	22	30038921	NF2	Body	-0,227	2,11E-09	3,30E-07
cg00025278	11	17429271	ABCC8	Body	-0,278	2,12E-09	3,31E-07
cg26490299	5	173146735	LINC01484	Body	-0,29	2,12E-09	3,31E-07
cg17328407	5	157288521		IGR	-0,21	2,13E-09	3,32E-07
cg21201401	20	62367884	LIME1	TSS200	-0,361	2,13E-09	3,32E-07
cg08189464	4	123505847		IGR	-0,217	2,13E-09	3,32E-07
cg04333495	17	18763778	PRPSAP2	5'UTR	-0,361	2,13E-09	3,32E-07
cg21718253	12	10471124		IGR	-0,209	2,13E-09	3,32E-07
cg02606496	9	130640137	AK1	TSS200	0,238	2,13E-09	3,32E-07
cg05334066	15	36715027		IGR	-0,207	2,14E-09	3,33E-07
cg26408118	2	158599659	ACVR1	Body	-0,274	2,15E-09	3,34E-07
cg25284252	18	19433356	MIB1	Body	-0,285	2,15E-09	3,34E-07
cg05358504	9	137682814	COL5A1	Body	-0,303	2,16E-09	3,35E-07
cg27512316	8	27516631	SCARA3	Body	-0,218	2,16E-09	3,35E-07
cg03817667	7	54609953	VSTM2A	TSS200	0,265	2,16E-09	3,36E-07
cg11198334	14	75040680	LTBP2	Body	-0,241	2,17E-09	3,36E-07
cg08393788	2	53357721		IGR	-0,212	2,17E-09	3,36E-07
cg18660064	1	23504632		IGR	0,216	2,17E-09	3,36E-07
cg09116870	5	176724043	NSD1	3'UTR	0,238	2,17E-09	3,36E-07
cg14062244	3	170352694		IGR	-0,266	2,18E-09	3,36E-07
cg00234176	5	156642893	ITK	Body	-0,241	2,18E-09	3,37E-07
cg05899688	5	55448527	ANKRD55	Body	-0,209	2,18E-09	3,37E-07
cg26644170	5	121495831	LOC1005058[illegible]	TSS200	-0,231	2,18E-09	3,37E-07
cg00808196	1	183344122	NMNAT2	Body	-0,227	2,19E-09	3,37E-07
cg12669088	12	25541364		IGR	-0,228	2,19E-09	3,37E-07
cg13268590	3	65940167	MAGI1	Body	0,246	2,19E-09	3,38E-07
cg02434259	3	27391622	NEK10	Body	-0,212	2,19E-09	3,38E-07
cg10377582	12	51612794	POU6F1	TSS1500	0,301	2,19E-09	3,38E-07
cg20732539	16	27416077	IL21R	5'UTR	-0,308	2,19E-09	3,38E-07
cg03988568	2	65683094		IGR	-0,24	2,20E-09	3,38E-07
cg22340508	19	22891978		IGR	0,256	2,20E-09	3,39E-07

cg03363318	2	196467488		IGR	0,206	2,21E-09	3,40E-07
cg05948940	16	68481342	SMPD3	5'UTR	0,224	2,21E-09	3,40E-07
cg24363299	19	15391923	BRD4	TSS1500	0,239	2,21E-09	3,40E-07
cg25302142	1	54604196		IGR	-0,251	2,22E-09	3,40E-07
cg10806146	8	42356871	SLC20A2	5'UTR	0,225	2,22E-09	3,40E-07
cg07512258	10	75619420	CAMK2G	Body	-0,222	2,22E-09	3,40E-07
cg13404054	19	15311666	NOTCH3	1stExon	0,21	2,22E-09	3,40E-07
cg20248758	8	446004	TDRP	Body	-0,201	2,23E-09	3,41E-07
cg17445097	17	27263598	PHF12	Body	-0,308	2,23E-09	3,41E-07
cg27363829	3	164914614	SLITRK3	TSS200	0,234	2,23E-09	3,42E-07
cg26502666	2	29034052	SPDYA	5'UTR	0,212	2,23E-09	3,42E-07
cg03256424	12	13099884	GPRC5D	Body	-0,237	2,24E-09	3,42E-07
cg16814884	20	49347058	PARD6B	TSS1500	0,245	2,24E-09	3,42E-07
cg22926424	12	70721074	CNOT2	Body	-0,208	2,24E-09	3,42E-07
cg06267139	1	23704878		IGR	-0,385	2,24E-09	3,42E-07
cg10695868	13	33954699	STARD13	Body	-0,26	2,24E-09	3,42E-07
cg07426802	8	60885377		IGR	-0,296	2,25E-09	3,42E-07
cg15264991	12	7066563	PTPN6	Body	-0,223	2,25E-09	3,43E-07
cg12630461	15	91500009	RCCD1	Body	0,204	2,25E-09	3,43E-07
cg13670411	7	6422688	RAC1	Body	-0,279	2,26E-09	3,43E-07
cg05634376	11	66659993	PC	5'UTR	-0,213	2,26E-09	3,44E-07
cg26165117	10	17553514		IGR	-0,371	2,26E-09	3,44E-07
cg04972065	12	53591766	ITGB7	Body	0,207	2,27E-09	3,44E-07
cg00806547	5	125184891		IGR	-0,243	2,27E-09	3,44E-07
cg08116693	4	77675652	SHROOM3	Body	-0,22	2,27E-09	3,45E-07
cg06631881	2	3675626	COLEC11	Body	-0,294	2,28E-09	3,46E-07
cg13146184	19	16696698	MED26	Body	-0,364	2,29E-09	3,46E-07
cg23205610	3	124567375	ITGB5	Body	-0,296	2,29E-09	3,47E-07
cg07312943	15	40069872	FSIP1	5'UTR	-0,245	2,29E-09	3,47E-07
cg05557110	7	46218895		IGR	-0,214	2,30E-09	3,47E-07
cg01774645	1	161040044	ARHGAP30	TSS1500	-0,263	2,31E-09	3,48E-07
cg27198652	1	159928680		IGR	-0,255	2,31E-09	3,49E-07
cg08525429	7	1553444		IGR	0,202	2,31E-09	3,49E-07
cg00160027	16	49583602	ZNF423	Body	-0,201	2,32E-09	3,49E-07
cg17685004	2	217419293		IGR	-0,252	2,32E-09	3,50E-07
cg13272029	22	20088401	DGCR8	Body	-0,225	2,33E-09	3,50E-07
cg06555842	2	108979649		IGR	-0,214	2,33E-09	3,50E-07
cg12240358	15	83619523	HOMER2	Body	-0,304	2,33E-09	3,50E-07
cg13460223	6	158221921		IGR	-0,236	2,33E-09	3,51E-07
cg09489567	2	43364053		IGR	-0,216	2,34E-09	3,51E-07
cg16845382	2	236478646	AGAP1	Body	-0,274	2,35E-09	3,52E-07
cg09914304	10	72362292	PRF1	5'UTR	-0,308	2,35E-09	3,53E-07
cg27520924	3	151845056		IGR	-0,217	2,35E-09	3,53E-07
cg00432262	7	46400029		IGR	-0,204	2,36E-09	3,53E-07
cg07494299	10	5064396		IGR	-0,237	2,37E-09	3,55E-07
cg18096987	3	11623873	VGLL4	Body	0,223	2,38E-09	3,55E-07
cg19959061	13	51320134	DLEU7	Body	-0,234	2,38E-09	3,55E-07
cg06994248	12	110168451	FAM222A	5'UTR	-0,226	2,40E-09	3,57E-07
cg18811907	22	38037190	SH3BP1	ExonBnd	-0,249	2,41E-09	3,58E-07
cg16853770	12	123355562	VPS37B	Body	0,204	2,41E-09	3,58E-07

cg01936718	10	4144300		IGR	-0,202	2,42E-09	3,59E-07
cg15805300	11	14117382	SPON1	Body	-0,216	2,42E-09	3,59E-07
cg00532474	18	43652594	PSTPIP2	TSS1500	0,322	2,42E-09	3,59E-07
cg25557432	20	13976117	MACROD2	TSS200	0,215	2,42E-09	3,60E-07
cg00859858	1	37944257	ZC3H12A	Body	-0,376	2,43E-09	3,60E-07
cg02484781	11	102459860	MMP20	Body	-0,278	2,43E-09	3,60E-07
cg08421685	3	238611	CHL1	TSS200	0,209	2,43E-09	3,60E-07
cg00573079	10	114292138	VTI1A	Body	-0,326	2,43E-09	3,60E-07
cg23834123	17	73291891		IGR	-0,459	2,43E-09	3,60E-07
cg13928868	3	17064669	PLCL2	Body	-0,253	2,44E-09	3,60E-07
cg00875989	18	44774054		IGR	0,211	2,44E-09	3,60E-07
cg14103848	12	337962	SLC6A13	Body	-0,208	2,44E-09	3,61E-07
cg10488012	5	3306368		IGR	-0,261	2,44E-09	3,61E-07
cg25632577	8	128048403		IGR	-0,23	2,44E-09	3,61E-07
cg05019211	22	33343603	SYN3	Body	-0,242	2,44E-09	3,61E-07
cg01089498	4	11428985	HS3ST1	5'UTR	0,226	2,45E-09	3,61E-07
cg08096750	8	101348741	RNF19A	TSS1500	0,227	2,45E-09	3,62E-07
cg04393958	10	123964964	TACC2	5'UTR	-0,279	2,46E-09	3,62E-07
cg15063561	10	71643542	COL13A1	Body	-0,265	2,47E-09	3,63E-07
cg16986485	2	99074891	INPP4A	5'UTR	-0,29	2,47E-09	3,63E-07
cg20666917	2	99439848	C2orf55	Body	0,238	2,47E-09	3,63E-07
cg23436304	22	27889107		IGR	-0,216	2,48E-09	3,64E-07
cg25616829	1	112535356		IGR	0,29	2,49E-09	3,64E-07
cg18426664	16	66952840	CDH16	5'UTR	-0,225	2,49E-09	3,64E-07
cg07710335	17	15151869	PMP22	Body	-0,232	2,49E-09	3,64E-07
cg13504858	11	117874181		IGR	-0,275	2,50E-09	3,66E-07
cg12446199	1	167408841	CD247	Body	0,239	2,52E-09	3,68E-07
cg13676800	9	89722432		IGR	-0,253	2,52E-09	3,68E-07
cg24414363	22	42336273	CENPM	TSS200	-0,372	2,52E-09	3,68E-07
cg18343957	14	48144197	MDGA2	TSS200	0,299	2,52E-09	3,68E-07
cg08072391	6	41711422	PGC	Body	-0,219	2,52E-09	3,68E-07
cg05295536	6	30749628		IGR	0,231	2,52E-09	3,68E-07
cg07899411	12	123604434		IGR	-0,228	2,53E-09	3,68E-07
cg23556358	1	203738697	LAX1	TSS200	0,21	2,53E-09	3,69E-07
cg11846577	20	49744880		IGR	-0,218	2,54E-09	3,70E-07
cg06824858	1	62773825	KANK4	5'UTR	-0,221	2,54E-09	3,70E-07
cg24533227	1	42145514	HIVEP3	5'UTR	-0,316	2,54E-09	3,70E-07
cg02251850	17	78851503	RPTOR	Body	-0,321	2,54E-09	3,70E-07
cg13152690	14	91695017		IGR	-0,3	2,55E-09	3,71E-07
cg16920250	3	99979119	TBC1D23	TSS1500	0,221	2,55E-09	3,71E-07
cg16615154	6	40567553		IGR	0,201	2,55E-09	3,71E-07
cg01725130	14	92994158	RIN3	Body	-0,21	2,55E-09	3,71E-07
cg07033395	1	28906538	SNHG12	Body	-0,218	2,56E-09	3,71E-07
cg24405417	6	138059484	LOC1005074(	Body	-0,272	2,56E-09	3,72E-07
cg13947871	1	174126546	LOC1027246(	Body	-0,274	2,56E-09	3,72E-07
cg08445550	5	16784821	MYO10	Body	-0,24	2,57E-09	3,73E-07
cg23670794	3	111313065	ZBED2	5'UTR	-0,257	2,58E-09	3,73E-07
cg19750864	11	64846935	CDCA5	Body	-0,208	2,58E-09	3,73E-07
cg05096672	3	195959987	SLC51A	Body	-0,247	2,59E-09	3,74E-07
cg00580291	13	21606284	LATS2	Body	-0,217	2,59E-09	3,74E-07

cg02715491	7	111761042	DOCK4	Body	-0,224	2,60E-09	3,75E-07
cg03495913	5	149652881	CAMK2A	Body	-0,253	2,60E-09	3,75E-07
cg04438162	18	72707209	ZNF407	Body	-0,292	2,60E-09	3,75E-07
cg07997797	6	162081846	PARK2	Body	-0,267	2,60E-09	3,75E-07
cg13867052	2	208332688		IGR	-0,213	2,61E-09	3,76E-07
cg09655666	17	34820190		IGR	0,249	2,61E-09	3,76E-07
cg16602508	2	155352542		IGR	-0,238	2,61E-09	3,76E-07
cg23652093	11	75347560	MAP6	Body	-0,209	2,61E-09	3,76E-07
cg12340037	12	102193720	GNPTAB	Body	-0,412	2,62E-09	3,76E-07
cg15935860	1	201282499	PKP1	Body	-0,207	2,62E-09	3,77E-07
cg06463964	9	116741586	ZNF618	Body	-0,258	2,63E-09	3,77E-07
cg08816569	7	114568475	MDFIC	Body	-0,316	2,63E-09	3,78E-07
cg24749015	11	122049594	MIR100HG	Body	-0,243	2,64E-09	3,78E-07
cg14964374	1	201637064	NAV1	Body	-0,255	2,64E-09	3,78E-07
cg13894134	10	3799642		IGR	-0,337	2,64E-09	3,78E-07
cg27300125	2	64713000		IGR	-0,279	2,64E-09	3,79E-07
cg05790451	15	29396200	APBA2	Body	0,218	2,64E-09	3,79E-07
cg27081230	16	50715529	SNX20	TSS1500	-0,346	2,65E-09	3,79E-07
cg23161218	17	7461638	TNFSF13	5'UTR	0,229	2,66E-09	3,81E-07
cg25547902	3	40271748	MYRIP	Body	-0,211	2,67E-09	3,81E-07
cg09123760	12	122712212	DIABLO	TSS200	-0,277	2,67E-09	3,81E-07
cg24737324	20	19867143		IGR	0,206	2,68E-09	3,83E-07
cg15547513	4	141889815	RNF150	Body	-0,25	2,69E-09	3,83E-07
cg00872726	14	97499908		IGR	0,202	2,70E-09	3,85E-07
cg20783780	11	112191546	LOC283140	Body	0,202	2,70E-09	3,85E-07
cg15123984	5	174151634	MSX2	5'UTR	0,211	2,71E-09	3,85E-07
cg01919669	11	62782872	SLC22A8	5'UTR	-0,249	2,71E-09	3,85E-07
cg21632158	6	29521356		IGR	0,232	2,71E-09	3,85E-07
cg08337633	7	55602109	VOPP1	Body	-0,247	2,72E-09	3,86E-07
cg06660550	18	67563048	CD226	Body	-0,216	2,72E-09	3,86E-07
cg16292250	18	55802511	NEDD4L	Body	-0,264	2,72E-09	3,86E-07
cg19420817	11	35992808	LDLRAD3	5'UTR	-0,252	2,72E-09	3,86E-07
cg11658419	12	58290850		IGR	0,224	2,72E-09	3,86E-07
cg12421755	15	53080933	ONECUT1	Body	0,206	2,73E-09	3,87E-07
cg15187982	17	42675980		IGR	-0,208	2,73E-09	3,87E-07
cg06897797	9	21070497		IGR	0,207	2,73E-09	3,87E-07
cg11052143	2	202484020	ALS2CR11	TSS200	0,202	2,74E-09	3,87E-07
cg11965327	6	32997101		IGR	-0,213	2,74E-09	3,87E-07
cg04127242	9	139990710	MAN1B1	Body	-0,356	2,74E-09	3,87E-07
cg11224579	10	34794060	PARD3	Body	-0,274	2,74E-09	3,87E-07
cg24321269	2	129260370		IGR	-0,225	2,74E-09	3,87E-07
cg00364704	7	2750071	AMZ1	Body	-0,298	2,74E-09	3,87E-07
cg01425348	13	30949446	LINC00426	TSS1500	-0,271	2,74E-09	3,87E-07
cg16923681	22	32361109		IGR	-0,225	2,74E-09	3,87E-07
cg11625986	8	19372890	CSGALNACT1	Body	-0,251	2,75E-09	3,87E-07
cg26856257	1	32805350		IGR	-0,328	2,76E-09	3,89E-07
cg22977420	3	11279984	HRH1	5'UTR	-0,246	2,76E-09	3,89E-07
cg04288619	7	97913809	BRI3	Body	0,227	2,76E-09	3,89E-07
cg04573564	5	2012303		IGR	0,208	2,77E-09	3,89E-07
cg06630731	17	71344483	SDK2	Body	-0,216	2,77E-09	3,89E-07

cg08587313	8	130215373		IGR	-0,211	2,78E-09	3,90E-07
cg20879057	11	36396962	PRR5L	TSS1500	-0,209	2,78E-09	3,90E-07
cg02982740	12	122429384	WDR66	Body	-0,283	2,78E-09	3,90E-07
cg15569292	11	3022005		IGR	0,228	2,78E-09	3,91E-07
cg24613080	17	32484035	ACCN1	TSS1500	0,227	2,78E-09	3,91E-07
cg21333884	15	85859614		IGR	-0,315	2,79E-09	3,91E-07
cg05223210	11	117630397	DSCAML1	Body	-0,224	2,79E-09	3,91E-07
cg21570108	1	178542596		IGR	-0,235	2,80E-09	3,92E-07
cg06560379	6	44231305	NFKBIE	Body	-0,205	2,80E-09	3,92E-07
cg12172089	6	135555373		IGR	-0,205	2,81E-09	3,93E-07
cg05916539	20	34763531	EPB41L1	Body	-0,213	2,81E-09	3,93E-07
cg10226149	12	76036209		IGR	-0,283	2,82E-09	3,94E-07
cg19741032	13	115078824	CHAMP1	TSS1500	-0,266	2,82E-09	3,94E-07
cg18922843	9	132359485		IGR	0,225	2,82E-09	3,94E-07
cg22865550	2	174235085		IGR	-0,258	2,83E-09	3,95E-07
cg23488266	3	156237918	KCNAB1	Body	-0,238	2,84E-09	3,95E-07
cg02954987	3	49170599	LAMB2	TSS200	0,213	2,84E-09	3,96E-07
cg02768790	11	69152387		IGR	-0,341	2,85E-09	3,96E-07
cg12111137	17	80880759	TBCD	Body	-0,203	2,85E-09	3,96E-07
cg07222852	2	47137221	MCFD2	Body	-0,238	2,87E-09	3,98E-07
cg02039404	2	70368784		IGR	-0,232	2,87E-09	3,98E-07
cg12520042	11	12092637		IGR	-0,247	2,87E-09	3,98E-07
cg24680171	18	44015531	RNF165	5'UTR	-0,27	2,87E-09	3,98E-07
cg19947104	11	17757342	KCNC1	TSS200	0,221	2,87E-09	3,98E-07
cg04499701	11	111312453		IGR	-0,301	2,87E-09	3,98E-07
cg02576468	19	41074006	SPTBN4	Body	0,216	2,87E-09	3,99E-07
cg20188980	2	135471997	TMEM163	Body	-0,254	2,88E-09	3,99E-07
cg19230755	7	65878503		IGR	0,241	2,88E-09	3,99E-07
cg10096208	2	231067424	SP110	ExonBnd	-0,244	2,88E-09	3,99E-07
cg02576975	19	5334189	PTPRS	5'UTR	-0,282	2,88E-09	3,99E-07
cg14555967	2	198470913	RFTN2	Body	-0,237	2,89E-09	4,00E-07
cg15497761	1	155147397	TRIM46	Body	0,299	2,89E-09	4,00E-07
cg09924653	5	139189331	PSD2	Body	-0,223	2,89E-09	4,00E-07
cg01637169	3	13459281	NUP210	Body	-0,21	2,90E-09	4,01E-07
cg14037991	3	167409467	PDCD10	Body	-0,325	2,90E-09	4,01E-07
cg03625177	5	135364556	TGFBI	TSS200	0,217	2,91E-09	4,02E-07
cg12812233	4	40752838	NSUN7	Body	0,256	2,91E-09	4,02E-07
cg01682400	4	109029545	LEF1	Body	-0,211	2,91E-09	4,02E-07
cg01856865	8	58492007		IGR	-0,33	2,91E-09	4,02E-07
cg17900288	19	50227645		IGR	-0,262	2,92E-09	4,02E-07
cg22222858	9	36092680	RECK	Body	-0,469	2,92E-09	4,03E-07
cg10169852	11	125147091	PKNOX2	5'UTR	-0,246	2,92E-09	4,03E-07
cg13580827	9	72081155	APBA1	Body	-0,234	2,93E-09	4,03E-07
cg16616467	16	89642914	CPNE7	Body	0,346	2,93E-09	4,04E-07
cg07093667	10	23652849		IGR	-0,27	2,94E-09	4,04E-07
cg17718904	14	93570132	ITPK1	Body	-0,217	2,94E-09	4,04E-07
cg08781015	10	30095273		IGR	-0,233	2,94E-09	4,05E-07
cg15151492	6	48039722		IGR	-0,238	2,94E-09	4,05E-07
cg13836216	1	89594773		IGR	-0,291	2,95E-09	4,05E-07
cg14677380	3	32470449	CMTM7	Body	-0,255	2,95E-09	4,05E-07

cg08536989	9	116355621	RGS3	TSS1500	-0,285	2,95E-09	4,05E-07
cg20701556	1	95698924	RWDD3	TSS1500	0,296	2,96E-09	4,06E-07
cg16919130	15	89179302	ISG20	5'UTR	-0,218	2,96E-09	4,06E-07
cg09196959	6	30104440	TRIM40	TSS200	-0,255	2,96E-09	4,06E-07
cg24529771	3	178978849	KCNMB3	TSS1500	0,202	2,96E-09	4,06E-07
cg23378546	17	76352717		IGR	-0,328	2,97E-09	4,06E-07
cg20837066	10	72742406		IGR	-0,297	2,97E-09	4,06E-07
cg24375409	1	38200920	EPHA10	Body	0,317	2,97E-09	4,06E-07
cg11812438	4	48067995		IGR	-0,206	2,97E-09	4,06E-07
cg05762671	16	67345737	KCTD19	Body	-0,277	2,97E-09	4,06E-07
cg15611912	10	121300857	RGS10	Body	-0,207	2,97E-09	4,06E-07
cg15209808	22	30476254	HORMAD2	TSS200	0,246	2,98E-09	4,07E-07
cg26981586	6	135858988	LINC00271	Body	-0,303	2,98E-09	4,07E-07
cg08226857	7	101264582	MYL10	Body	-0,217	2,98E-09	4,07E-07
cg00027083	18	5543801	EPB41L3	5'UTR	0,28	2,98E-09	4,07E-07
cg14525062	22	22121889	MAPK1	3'UTR	-0,248	2,98E-09	4,07E-07
cg26002686	22	26019477	ADRBK2	Body	-0,355	2,99E-09	4,07E-07
cg18857552	22	28023266		IGR	-0,292	2,99E-09	4,07E-07
cg11107120	12	122350289	PSMD9	Body	-0,297	2,99E-09	4,07E-07
cg00849491	18	6510785	LINC01387	TSS1500	-0,268	2,99E-09	4,08E-07
cg19853612	5	122059384	LOC10192737	Body	-0,265	2,99E-09	4,08E-07
cg22595230	20	3766560	CENPB	1stExon	0,229	2,99E-09	4,08E-07
cg20246452	14	103871434	MARK3	Body	0,319	3,00E-09	4,08E-07
cg11635903	12	53602918		IGR	-0,237	3,01E-09	4,09E-07
cg10531986	9	132652466	FNBP1	3'UTR	-0,253	3,01E-09	4,09E-07
cg05408896	3	59372081		IGR	-0,287	3,01E-09	4,10E-07
cg05260789	1	43772827	TIE1	ExonBnd	-0,226	3,02E-09	4,11E-07
cg16789764	14	89066213	ZC3H14	Body	-0,293	3,03E-09	4,11E-07
cg24693741	18	46886163	DYM	Body	-0,203	3,03E-09	4,11E-07
cg06082897	1	7767716	CAMTA1	Body	-0,237	3,03E-09	4,12E-07
cg10830544	1	180554833		IGR	-0,213	3,04E-09	4,12E-07
cg07900312	22	36362528	RBFOX2	Body	-0,256	3,04E-09	4,12E-07
cg02986287	11	2798509	KCNQ1	Body	-0,216	3,04E-09	4,12E-07
cg23589151	15	72468522	GRAMD2	Body	-0,208	3,04E-09	4,12E-07
cg14165142	20	3778655	CDC25B	Body	-0,296	3,04E-09	4,12E-07
cg02017926	12	123754328	CDK2AP1	Body	0,291	3,05E-09	4,12E-07
cg27230697	11	58673577		IGR	0,227	3,05E-09	4,13E-07
cg01724702	20	1613146	SIRPG-AS1	TSS1500	-0,28	3,06E-09	4,13E-07
cg01570297	2	238569373	LRRFIP1	Body	-0,296	3,06E-09	4,13E-07
cg04369211	5	138783504		IGR	-0,201	3,06E-09	4,13E-07
cg00831710	8	144240945	LY6H	Body	0,214	3,06E-09	4,13E-07
cg27073830	16	70622736	IL34	5'UTR	-0,296	3,06E-09	4,13E-07
cg01172604	20	39212526		IGR	-0,202	3,06E-09	4,13E-07
cg02879290	2	217525287	IGFBP2	ExonBnd	-0,222	3,06E-09	4,13E-07
cg25708990	1	203443667	PRELP	TSS1500	-0,252	3,08E-09	4,14E-07
cg06475911	2	219446965	RQCD1	Body	-0,219	3,07E-09	4,14E-07
cg03614034	1	87783910		IGR	-0,268	3,08E-09	4,14E-07
cg13613174	2	242702881	D2HGDH	Body	-0,37	3,08E-09	4,14E-07
cg04763867	9	124452177	DAB2IP	Body	-0,217	3,09E-09	4,15E-07
cg12834086	14	22988556		IGR	-0,214	3,10E-09	4,16E-07

cg16671138	14	50864320	CDKL1	TSS200	0,223	3,10E-09	4,16E-07
cg09637963	6	14798944		IGR	-0,254	3,10E-09	4,16E-07
cg08277216	2	242808803		IGR	0,201	3,10E-09	4,16E-07
cg07317449	2	127964374	CYP27C1	TSS1500	-0,237	3,11E-09	4,17E-07
cg20330447	6	31539539	LTA	TSS1500	-0,242	3,11E-09	4,17E-07
cg21493516	12	54446033	HOXC4	5'UTR	0,234	3,12E-09	4,17E-07
cg17280106	17	77767084		IGR	0,25	3,12E-09	4,17E-07
cg19356022	1	154943932	SHC1	5'UTR	0,215	3,13E-09	4,18E-07
cg14384416	2	193393		IGR	-0,238	3,13E-09	4,18E-07
cg19193964	11	85836030		IGR	-0,266	3,14E-09	4,19E-07
cg01068944	20	9049273		IGR	0,225	3,14E-09	4,19E-07
cg16437202	16	12630008	SNX29	Body	-0,289	3,15E-09	4,20E-07
cg05701438	15	74523417		IGR	-0,214	3,15E-09	4,20E-07
cg01095328	17	78238779	RNF213	Body	-0,26	3,15E-09	4,20E-07
cg00479390	9	100130105	LOC1004994٤	Body	-0,271	3,15E-09	4,20E-07
cg14799457	18	19927125		IGR	0,202	3,15E-09	4,20E-07
cg16239783	11	125058338	PKNOX2	5'UTR	-0,306	3,16E-09	4,21E-07
cg18926797	14	69523229	DCAF5	Body	0,241	3,16E-09	4,21E-07
cg19583359	10	71686735	COL13A1	Body	-0,273	3,17E-09	4,21E-07
cg10530851	14	37051417	NKX2-8	Body	0,236	3,17E-09	4,21E-07
cg12066624	4	25032060	LGI2	Body	0,216	3,17E-09	4,21E-07
cg18652900	19	15580445	PGLYRP2	Body	0,208	3,17E-09	4,22E-07
cg03343571	6	30039175	RNF39	Body	0,372	3,18E-09	4,22E-07
cg10754002	2	121276805		IGR	-0,221	3,18E-09	4,22E-07
cg03864696	17	66180795		IGR	-0,263	3,18E-09	4,22E-07
cg19762764	15	83833947	HDGFRP3	Body	-0,311	3,19E-09	4,23E-07
cg01419727	5	131438977		IGR	-0,225	3,20E-09	4,23E-07
cg04779083	9	137617035	COL5A1	Body	-0,244	3,20E-09	4,23E-07
cg07668739	16	89852101	FANCA	Body	-0,216	3,20E-09	4,23E-07
cg04935965	21	40822476	SH3BGR	5'UTR	0,244	3,21E-09	4,24E-07
cg16426366	8	1919236		IGR	-0,248	3,21E-09	4,25E-07
cg13369611	9	79634544	FOXB2	TSS200	0,271	3,22E-09	4,25E-07
cg24126437	9	114678662	UGCG	Body	-0,461	3,22E-09	4,25E-07
cg16901197	14	85883852	LINC00911	Body	-0,29	3,22E-09	4,26E-07
cg10282890	5	73611882		IGR	-0,322	3,22E-09	4,26E-07
cg12367009	18	55673626		IGR	-0,298	3,23E-09	4,26E-07
cg19011089	20	33307672	NCOA6	Body	-0,236	3,23E-09	4,26E-07
cg27138697	8	18589116	PSD3	Body	-0,268	3,24E-09	4,27E-07
cg02034951	2	47086248		IGR	-0,221	3,24E-09	4,27E-07
cg06311215	5	149593619		IGR	-0,314	3,25E-09	4,27E-07
cg15149219	14	23024952		IGR	-0,335	3,25E-09	4,27E-07
cg19872095	1	37941263	ZC3H12A	Body	-0,263	3,25E-09	4,28E-07
cg10721834	19	22990066		IGR	0,234	3,26E-09	4,28E-07
cg17448192	3	186718821	ST6GAL1	5'UTR	-0,256	3,26E-09	4,28E-07
cg06335867	7	8482325	NXPH1	Body	0,253	3,26E-09	4,28E-07
cg12564453	16	56995840	CETP	5'UTR	0,233	3,27E-09	4,28E-07
cg07540520	1	14925170	KAZN	TSS200	0,282	3,27E-09	4,29E-07
cg03221073	1	186157555	HMCN1	Body	-0,245	3,27E-09	4,29E-07
cg27592079	6	18016000		IGR	-0,244	3,28E-09	4,30E-07
cg26415370	10	65226379	JMJD1C	TSS1500	0,232	3,28E-09	4,30E-07

cg17791651	1	38513489	POU3F1	TSS1500	0,223	3,29E-09	4,30E-07
cg25010149	5	38456022	EGFLAM	Body	-0,252	3,29E-09	4,30E-07
cg01372811	16	85970529		IGR	-0,262	3,29E-09	4,30E-07
cg19151402	20	51825316	TSHZ2	Body	-0,212	3,30E-09	4,31E-07
cg25922180	4	693321		IGR	-0,309	3,30E-09	4,31E-07
cg18869624	19	2260400		IGR	-0,289	3,30E-09	4,31E-07
cg05988583	10	7356258	SFMBT2	Body	-0,2	3,31E-09	4,32E-07
cg08541935	22	34202573	LARGE	5'UTR	-0,201	3,32E-09	4,32E-07
cg06273318	2	64553381		IGR	-0,327	3,33E-09	4,33E-07
cg09875213	5	134786596	TIFAB	5'UTR	-0,225	3,33E-09	4,33E-07
cg03771840	6	30140145	TRIM15	3'UTR	0,21	3,33E-09	4,33E-07
cg17523488	6	47469225	CD2AP	Body	0,307	3,34E-09	4,34E-07
cg13895867	2	237076164	GBX2	1stExon	0,233	3,34E-09	4,34E-07
cg03105083	21	46628758	ADARB1	Body	-0,272	3,34E-09	4,34E-07
cg23279355	5	78985592	CMYA5	TSS200	0,322	3,34E-09	4,34E-07
cg25325918	2	120413260	CFAP221	Body	-0,253	3,34E-09	4,34E-07
cg13093457	16	83826869	CDH13	Body	-0,208	3,34E-09	4,34E-07
cg26279070	2	130971343		IGR	0,208	3,35E-09	4,34E-07
cg12563898	22	32978938	SYN3	Body	-0,232	3,35E-09	4,35E-07
cg19009471	14	101908998		IGR	-0,28	3,35E-09	4,35E-07
cg13213594	5	115471173	COMMD10	Body	-0,327	3,35E-09	4,35E-07
cg05007923	13	49721418	FNDC3A	Body	-0,22	3,36E-09	4,36E-07
cg07623696	1	167487060	CD247	Body	-0,268	3,37E-09	4,37E-07
cg13423838	10	4818881		IGR	-0,221	3,37E-09	4,37E-07
cg16443148	16	776667	CCDC78	TSS200	0,206	3,37E-09	4,37E-07
cg18443253	14	38678420	SSTR1	5'UTR	0,201	3,37E-09	4,37E-07
cg00444151	17	33823619	SLFN12L	Body	0,401	3,37E-09	4,37E-07
cg22750001	11	58981095	MPEG1	TSS1500	-0,322	3,38E-09	4,37E-07
cg17178170	1	184129500		IGR	-0,268	3,38E-09	4,37E-07
cg09655403	5	78985495	CMYA5	TSS200	0,319	3,38E-09	4,37E-07
cg02330816	5	130601240	CDC42SE2	5'UTR	-0,231	3,38E-09	4,37E-07
cg15852732	1	235115059		IGR	-0,286	3,38E-09	4,37E-07
cg00358727	3	183156168		IGR	-0,212	3,39E-09	4,37E-07
cg11802175	12	96190765		IGR	-0,245	3,40E-09	4,39E-07
cg00864458	8	56985462	RPS20	Body	-0,208	3,41E-09	4,39E-07
cg11575507	2	45795183	SRBD1	Body	-0,21	3,42E-09	4,40E-07
cg12959966	3	56759314		IGR	-0,263	3,41E-09	4,40E-07
cg17951713	8	130698161		IGR	0,215	3,41E-09	4,40E-07
cg13771402	15	82172817		IGR	-0,289	3,42E-09	4,40E-07
cg17098965	20	52199520	ZNF217	5'UTR	-0,318	3,41E-09	4,40E-07
cg09221598	5	177983059	COL23A1	Body	0,276	3,42E-09	4,40E-07
cg05557874	4	89449097		IGR	-0,232	3,42E-09	4,41E-07
cg08869700	1	151318680	RFX5	Body	-0,28	3,43E-09	4,41E-07
cg02331910	1	153599831	S100A13	5'UTR	0,271	3,44E-09	4,42E-07
cg14339466	13	27927490		IGR	-0,268	3,44E-09	4,42E-07
cg20473072	15	48106646	LINC01491	Body	-0,291	3,44E-09	4,42E-07
cg23939846	6	3585854		IGR	-0,29	3,44E-09	4,42E-07
cg23449696	3	147125942	ZIC1	TSS1500	0,218	3,44E-09	4,42E-07
cg25476513	14	69453815	ACTN1-AS1	Body	-0,315	3,44E-09	4,42E-07
cg21431650	1	236621205	EDARADD	Body	-0,257	3,45E-09	4,43E-07

cg10381153	1	65062855	CACHD1	Body	-0,226	3,45E-09	4,43E-07
cg19051666	3	24045729		IGR	-0,272	3,46E-09	4,43E-07
cg18104919	5	7851396	C5orf49	TSS200	0,229	3,46E-09	4,43E-07
cg17334468	7	55606341	VOPP1	TSS1500	-0,366	3,46E-09	4,44E-07
cg07760161	17	7588378	TP53	5'UTR	-0,219	3,46E-09	4,44E-07
cg08936230	7	2079167	MAD1L1	Body	-0,235	3,47E-09	4,44E-07
cg17579667	11	40315404	LRRC4C	5'UTR	0,203	3,47E-09	4,44E-07
cg02632441	2	218711232	TNS1	Body	-0,272	3,48E-09	4,45E-07
cg17644949	7	135477741		IGR	-0,261	3,48E-09	4,45E-07
cg03661577	12	92864525		IGR	-0,282	3,48E-09	4,45E-07
cg26706380	14	48144186	MDGA2	TSS200	0,22	3,48E-09	4,45E-07
cg03413902	6	47155543		IGR	-0,209	3,48E-09	4,45E-07
cg09321817	6	33041343	HLA-DPA1	1stExon	-0,252	3,51E-09	4,48E-07
cg13626005	11	16177409	SOX6	Body	-0,26	3,51E-09	4,48E-07
cg09458566	11	116857794	SIK3	Body	-0,417	3,51E-09	4,48E-07
cg08665903	7	137822735		IGR	-0,225	3,52E-09	4,49E-07
cg24306397	7	93718680		IGR	-0,231	3,53E-09	4,49E-07
cg19401538	12	2757744	CACNA1C	Body	-0,278	3,55E-09	4,52E-07
cg10410946	5	171610343	STK10	Body	-0,309	3,56E-09	4,52E-07
cg06268694	22	46932642	CELSR1	1stExon	0,219	3,55E-09	4,52E-07
cg07111834	1	114402309	PTPN22	Body	-0,364	3,56E-09	4,52E-07
cg12262378	17	6899522	ALOX12	1stExon	0,225	3,57E-09	4,53E-07
cg14245804	11	68081226	LRP5	Body	0,224	3,57E-09	4,53E-07
cg07060551	19	51198381	SHANK1	Body	0,251	3,57E-09	4,53E-07
cg22693055	6	107897986	SOBP	Body	-0,237	3,57E-09	4,53E-07
cg01719405	14	62401258		IGR	-0,275	3,57E-09	4,53E-07
cg20275646	10	10911683		IGR	-0,323	3,58E-09	4,53E-07
cg16265859	1	19401069	UBR4	3'UTR	-0,28	3,58E-09	4,53E-07
cg10792302	12	54376019		IGR	0,242	3,58E-09	4,53E-07
cg17540296	14	20835697	TEP1	3'UTR	-0,229	3,59E-09	4,54E-07
cg05418680	2	10883188	ATP6V1C2	Body	-0,213	3,59E-09	4,54E-07
cg03301085	3	42680224	NKTR	Body	-0,447	3,60E-09	4,55E-07
cg19669790	1	57154147	PRKAA2	Body	-0,205	3,60E-09	4,55E-07
cg16610605	18	46146308	CTIF	Body	-0,222	3,60E-09	4,55E-07
cg01001246	16	85398020		IGR	-0,239	3,61E-09	4,56E-07
cg13017929	7	116909621		IGR	-0,28	3,61E-09	4,56E-07
cg03750165	5	144915975		IGR	0,21	3,62E-09	4,56E-07
cg17357548	15	50429829		IGR	-0,201	3,62E-09	4,56E-07
cg05886275	17	1444869	PITPNA	ExonBnd	-0,256	3,62E-09	4,56E-07
cg15048611	12	32487523	BICD1	Body	-0,296	3,62E-09	4,57E-07
cg15295132	7	116273734		IGR	-0,258	3,63E-09	4,57E-07
cg20162694	20	47384125	PREX1	Body	-0,205	3,63E-09	4,57E-07
cg03743508	8	6312162	MCPH1	Body	-0,235	3,63E-09	4,57E-07
cg27005749	8	141608646	EIF2C2	Body	0,227	3,63E-09	4,57E-07
cg05578480	1	231174269	FAM89A	Body	0,312	3,64E-09	4,58E-07
cg07464423	11	2869473	KCNQ1	3'UTR	-0,215	3,64E-09	4,58E-07
cg09497880	9	95918949		IGR	-0,258	3,65E-09	4,58E-07
cg18176633	19	50508844	VRK3	Body	-0,227	3,65E-09	4,58E-07
cg17558906	2	120220754	SCTR	Body	-0,223	3,66E-09	4,59E-07
cg13452646	6	36747145	CPNE5	Body	-0,289	3,66E-09	4,59E-07

cg15838320	20	49461129	BCAS4	Body	-0,284	3,67E-09	4,60E-07
cg14030719	20	32266794	E2F1	Body	-0,221	3,67E-09	4,61E-07
cg13301442	2	120990322		IGR	-0,294	3,68E-09	4,61E-07
cg09092528	11	126175760	DCPS	Body	-0,216	3,68E-09	4,61E-07
cg10257870	5	78985484	CMYA5	TSS200	0,253	3,68E-09	4,61E-07
cg18399451	6	16421273	ATXN1	5'UTR	-0,304	3,68E-09	4,61E-07
cg04813880	16	21171067	TMEM159	5'UTR	0,294	3,68E-09	4,61E-07
cg24379812	2	74213392	TET3	TSS200	-0,328	3,68E-09	4,61E-07
cg23115387	1	47882936	FOXE3	1stExon	0,222	3,69E-09	4,61E-07
cg11354101	17	25798880	KSR1	TSS200	-0,336	3,69E-09	4,61E-07
cg19646491	1	15575718	FHAD1	5'UTR	-0,298	3,70E-09	4,62E-07
cg26922780	16	88769443	RNF166	Body	-0,217	3,70E-09	4,62E-07
cg05041430	18	13279300	LDLRAD4	5'UTR	-0,241	3,70E-09	4,62E-07
cg01649611	2	43521066	THADA	Body	-0,287	3,70E-09	4,62E-07
cg15972984	2	99756731	C2orf15	TSS1500	0,342	3,70E-09	4,62E-07
cg01797899	14	22978195		IGR	-0,213	3,71E-09	4,63E-07
cg06399164	3	42695035	ZBTB47	TSS200	0,218	3,71E-09	4,63E-07
cg10197057	1	42612518		IGR	0,241	3,71E-09	4,63E-07
cg14779951	3	129147541	C3orf25	TSS200	0,255	3,72E-09	4,63E-07
cg05830425	13	21654356		IGR	0,211	3,72E-09	4,63E-07
cg04892766	3	72570318		IGR	-0,222	3,72E-09	4,63E-07
cg27571604	10	80722277	ZMIZ1-AS1	Body	-0,313	3,72E-09	4,63E-07
cg00448292	3	4863959	ITPR1	Body	-0,216	3,73E-09	4,64E-07
cg16693245	11	116390360		IGR	-0,254	3,74E-09	4,65E-07
cg14599440	18	11909634	MPPE1	TSS1500	-0,212	3,75E-09	4,65E-07
cg06484274	3	142164840	XRN1	Body	-0,339	3,75E-09	4,65E-07
cg11936289	10	33407061		IGR	-0,277	3,75E-09	4,66E-07
cg06011000	20	35722944	RBL1	Body	-0,259	3,75E-09	4,66E-07
cg00824681	7	41279912		IGR	-0,215	3,75E-09	4,66E-07
cg11051132	20	11852574	LINC00687	TSS1500	-0,253	3,75E-09	4,66E-07
cg09221159	10	90031426		IGR	-0,281	3,76E-09	4,66E-07
cg02492280	11	12217666	MICAL2	Body	-0,252	3,76E-09	4,66E-07
cg16483188	1	117306469	CD2	Body	-0,234	3,76E-09	4,66E-07
cg09914323	2	127900763		IGR	-0,249	3,77E-09	4,66E-07
cg15777575	5	92679522		IGR	-0,208	3,77E-09	4,66E-07
cg01204452	2	136814763		IGR	-0,227	3,77E-09	4,66E-07
cg14255337	12	121972602	KDM2B	Body	-0,341	3,77E-09	4,66E-07
cg20097985	3	124003076	KALRN	Body	-0,286	3,78E-09	4,67E-07
cg19529732	12	122712101	DIABLO	TSS200	-0,291	3,78E-09	4,67E-07
cg24659054	10	134600656	NKX6-2	TSS1500	0,219	3,78E-09	4,67E-07
cg01840860	19	1102868	GPX4	TSS1500	-0,346	3,78E-09	4,67E-07
cg22466595	8	135632168	ZFAT	Body	-0,251	3,78E-09	4,67E-07
cg01018492	4	184746611		IGR	-0,212	3,80E-09	4,68E-07
cg00554616	16	79747651		IGR	-0,34	3,80E-09	4,68E-07
cg03055671	3	172231528	TNFSF10	Body	-0,265	3,80E-09	4,69E-07
cg10766007	16	75077059	ZNRF1	Body	0,201	3,82E-09	4,70E-07
cg01309418	6	111278585	GTF3C6	TSS1500	0,24	3,83E-09	4,71E-07
cg19721221	1	181081214		IGR	-0,232	3,83E-09	4,71E-07
cg19984355	5	1794232		IGR	-0,233	3,85E-09	4,73E-07
cg27649239	15	68120393	LBXCOR1	Body	0,232	3,85E-09	4,73E-07

cg26239233	16	10970801	CIITA	TSS1500	-0,217	3,85E-09	4,73E-07
cg24790158	19	51893935	C19orf84	TSS200	0,211	3,86E-09	4,74E-07
cg27434114	12	66007516	LOC1005070(	Body	-0,23	3,87E-09	4,74E-07
cg12775201	3	112724732	C3orf17	Body	-0,215	3,87E-09	4,74E-07
cg07866399	8	39771219	IDO1	TSS200	-0,365	3,87E-09	4,74E-07
cg14379968	21	33968392		IGR	-0,254	3,87E-09	4,75E-07
cg21058458	2	232783546		IGR	-0,335	3,88E-09	4,75E-07
cg21898884	6	22568278	HDGFL1	TSS1500	-0,249	3,88E-09	4,75E-07
cg27398117	5	56612736		IGR	-0,251	3,88E-09	4,75E-07
cg01128504	7	45954981	IGFBP3	Body	-0,216	3,89E-09	4,76E-07
cg17511819	6	56755762	DST	Body	-0,313	3,89E-09	4,76E-07
cg00700609	21	44993442	HSF2BP	Body	-0,277	3,90E-09	4,77E-07
cg14459193	5	37112232	C5orf42	Body	-0,298	3,90E-09	4,77E-07
cg02976351	4	57623782		IGR	-0,321	3,91E-09	4,78E-07
cg10276354	8	134070272	SLA	Body	-0,279	3,92E-09	4,78E-07
cg18502743	3	46335416		IGR	-0,319	3,92E-09	4,78E-07
cg24035245	1	119535928		IGR	0,244	3,92E-09	4,78E-07
cg06010416	2	121211251		IGR	-0,228	3,93E-09	4,79E-07
cg11481431	15	78833962	PSMA4	5'UTR	-0,208	3,94E-09	4,80E-07
cg17893439	16	786418	NARFL	Body	-0,26	3,94E-09	4,80E-07
cg13339155	18	12461096	SPIRE1	Body	0,247	3,95E-09	4,81E-07
cg09761442	4	140925793	MAML3	Body	0,333	3,95E-09	4,81E-07
cg14256841	2	237571681		IGR	-0,301	3,95E-09	4,81E-07
cg26841013	1	228248013	WNT3A	3'UTR	0,209	3,96E-09	4,81E-07
cg19436429	3	178978985	KCNMB3	TSS1500	0,2	3,96E-09	4,81E-07
cg13515233	7	158448712	NCAPG2	Body	-0,233	3,97E-09	4,82E-07
cg21587469	12	56323816	DGKA	TSS1500	-0,284	3,97E-09	4,82E-07
cg21884962	2	218225246	DIRC3	Body	-0,282	3,98E-09	4,82E-07
cg21371487	11	113712188	USP28	Body	-0,365	3,98E-09	4,83E-07
cg07345937	19	1175444	SBNO2	TSS1500	-0,286	3,99E-09	4,83E-07
cg24457403	17	39770337	KRT16	TSS1500	-0,277	3,99E-09	4,83E-07
cg24096902	1	27754893	WASF2	Body	0,224	3,99E-09	4,84E-07
cg05090186	1	244215443	ZBTB18	5'UTR	0,282	4,00E-09	4,84E-07
cg12810670	11	13134412		IGR	-0,257	4,01E-09	4,84E-07
cg25028832	9	34652038	IL11RA	TSS200	0,283	4,01E-09	4,85E-07
cg22495957	6	138191135	TNFAIP3	5'UTR	-0,264	4,02E-09	4,85E-07
cg04650982	6	159430221		IGR	-0,246	4,03E-09	4,86E-07
cg04376028	15	62414449		IGR	-0,263	4,03E-09	4,86E-07
cg19915738	19	44952730	ZNF229	TSS200	0,218	4,03E-09	4,86E-07
cg02449608	19	58486600	C19orf18	TSS1500	-0,218	4,03E-09	4,86E-07
cg06670979	9	124303014		IGR	-0,275	4,05E-09	4,87E-07
cg04611437	10	102279697	SEC31B	TSS200	0,223	4,05E-09	4,87E-07
cg13862848	13	44272416	ENOX1	5'UTR	-0,286	4,05E-09	4,87E-07
cg05790136	18	33550437		IGR	-0,327	4,06E-09	4,87E-07
cg08763984	10	44872504	CXCL12	Body	-0,278	4,06E-09	4,87E-07
cg15949239	15	62988566	TLN2	Body	-0,29	4,06E-09	4,87E-07
cg26365358	8	40202292		IGR	-0,253	4,08E-09	4,90E-07
cg11616457	4	38525723	LINC01258	TSS1500	-0,333	4,08E-09	4,90E-07
cg06917617	1	207818818	CR1L	Body	0,209	4,09E-09	4,90E-07
cg19897964	20	56416164		IGR	-0,265	4,09E-09	4,90E-07

cg26596278	1	206227162	AVPR1B	Body	-0,279	4,09E-09	4,90E-07
cg13449306	3	115775303	LSAMP	Body	-0,515	4,10E-09	4,91E-07
cg22562942	8	24772309	NEFM	TSS200	0,238	4,10E-09	4,91E-07
cg24236025	6	874435	LOC10192769	Body	-0,242	4,11E-09	4,91E-07
cg06218310	12	3367472	TSPAN9	Body	-0,302	4,11E-09	4,91E-07
cg18215692	7	30890848	FAM188B	Body	-0,26	4,12E-09	4,92E-07
cg09007236	17	78228820		IGR	-0,245	4,12E-09	4,92E-07
cg07637006	18	46147848	CTIF	Body	-0,231	4,12E-09	4,92E-07
cg18170385	10	330411	DIP2C	Body	-0,229	4,13E-09	4,93E-07
cg00440194	11	95436310		IGR	-0,256	4,13E-09	4,93E-07
cg20769177	17	44928516	WNT9B	TSS1500	0,26	4,14E-09	4,93E-07
cg13954457	5	167956819	FBLL1	Body	0,241	4,14E-09	4,93E-07
cg07454492	6	31665801	ABHD16A	Body	-0,287	4,14E-09	4,93E-07
cg07132966	1	68742237		IGR	-0,264	4,14E-09	4,93E-07
cg15407257	20	23621136		IGR	-0,208	4,14E-09	4,93E-07
cg14132982	1	157931698		IGR	-0,219	4,15E-09	4,93E-07
cg07175536	16	15991691		IGR	-0,263	4,15E-09	4,94E-07
cg17193974	5	118664518	TNFAIP8	5'UTR	-0,273	4,16E-09	4,94E-07
cg13040992	21	40032457	ERG	1stExon	0,211	4,16E-09	4,94E-07
cg07583615	12	80572738		IGR	-0,253	4,18E-09	4,96E-07
cg21465150	17	3433428	TRPV3	Body	0,203	4,18E-09	4,96E-07
cg11523366	7	2798106	GNA12	Body	-0,208	4,18E-09	4,96E-07
cg19728051	1	31328091		IGR	-0,203	4,19E-09	4,96E-07
cg11344566	2	124782885	CNTNAP5	5'UTR	0,206	4,19E-09	4,96E-07
cg05673506	14	97176849		IGR	-0,234	4,19E-09	4,96E-07
cg27382666	4	120056287	MYOZ2	TSS1500	-0,211	4,20E-09	4,97E-07
cg11979298	3	101707603	LOC152225	Body	-0,359	4,21E-09	4,97E-07
cg22423974	3	156379194		IGR	-0,328	4,21E-09	4,97E-07
cg03801404	21	19166327	C21orf91	Body	-0,319	4,22E-09	4,98E-07
cg21727635	2	20415243	SDC1	Body	-0,263	4,23E-09	4,99E-07
cg15920942	2	70743881	TGFA	Body	-0,215	4,23E-09	4,99E-07
cg11300244	9	137193619		IGR	-0,226	4,24E-09	5,00E-07
cg24307601	19	12371781		IGR	-0,224	4,25E-09	5,00E-07
cg14488043	11	8788397	ST5	5'UTR	-0,259	4,25E-09	5,00E-07
cg09503954	10	50565220		IGR	-0,294	4,27E-09	5,01E-07
cg01621606	2	189261761	GULP1	5'UTR	-0,208	4,28E-09	5,03E-07
cg12418544	1	208190130		IGR	-0,255	4,29E-09	5,03E-07
cg09433254	6	143888314	LOC285740	Body	-0,296	4,29E-09	5,03E-07
cg06257110	16	21658497	METTL9	Body	-0,267	4,29E-09	5,03E-07
cg14016177	9	131965610		IGR	0,203	4,30E-09	5,04E-07
cg00661018	8	145769086	KIAA1688	Body	-0,244	4,31E-09	5,04E-07
cg18281107	16	67443389	ZDHHC1	5'UTR	-0,212	4,31E-09	5,05E-07
cg08477714	17	41671170		IGR	-0,239	4,32E-09	5,05E-07
cg11749141	15	91432980	FES	Body	-0,249	4,32E-09	5,06E-07
cg17239057	1	224363575		IGR	0,317	4,33E-09	5,06E-07
cg20217345	3	41075813		IGR	-0,254	4,33E-09	5,06E-07
cg07225017	17	74913153	MGAT5B	Body	-0,293	4,33E-09	5,06E-07
cg20043176	12	4255525		IGR	-0,269	4,33E-09	5,06E-07
cg04911510	3	124556321	ITGB5	Body	0,301	4,33E-09	5,06E-07
cg07790489	1	232756306		IGR	0,235	4,35E-09	5,08E-07

cg03666441	17	7254671	KCTD11	TSS1500	0,29	4,35E-09	5,08E-07
cg05475649	15	45007015	B2M	Body	-0,295	4,36E-09	5,09E-07
cg01820962	6	116511817	NT5DC1	Body	0,244	4,37E-09	5,10E-07
cg09501025	10	73312955	CDH23	Body	-0,265	4,37E-09	5,10E-07
cg06127067	12	111984723	ATXN2	Body	-0,402	4,38E-09	5,11E-07
cg12433559	10	72362730	PRF1	TSS200	-0,284	4,39E-09	5,11E-07
cg23746497	6	105388668		IGR	0,278	4,39E-09	5,11E-07
cg12043187	1	226974433		IGR	-0,267	4,40E-09	5,12E-07
cg20948431	4	2941316	C4orf10	Body	-0,225	4,39E-09	5,12E-07
cg04552811	3	149708132		IGR	-0,217	4,40E-09	5,12E-07
cg16244648	5	141555043		IGR	-0,228	4,40E-09	5,12E-07
cg20352718	10	80899340	ZMIZ1	5'UTR	0,219	4,41E-09	5,12E-07
cg01710361	1	51769687	TTC39A	Body	-0,203	4,41E-09	5,13E-07
cg05144700	6	41301695		IGR	-0,204	4,41E-09	5,13E-07
cg14797899	7	69882555	AUTS2	Body	-0,219	4,42E-09	5,13E-07
cg26398797	20	52302538		IGR	-0,256	4,42E-09	5,13E-07
cg22724228	6	154826964	CNKSR3	Body	-0,246	4,43E-09	5,14E-07
cg21863949	21	43198431		IGR	0,211	4,43E-09	5,14E-07
cg12149114	20	20315300	CFAP61	Body	-0,232	4,43E-09	5,14E-07
cg25744767	7	79764178	GNAI1	1stExon	0,235	4,43E-09	5,14E-07
cg27526665	3	24537050	THRB	TSS1500	0,212	4,45E-09	5,15E-07
cg22071944	3	134032379		IGR	0,209	4,45E-09	5,15E-07
cg23509922	8	119323702	SAMD12	Body	-0,206	4,46E-09	5,16E-07
cg20676788	13	49379791		IGR	-0,285	4,48E-09	5,18E-07
cg20918393	20	19867136		IGR	0,292	4,48E-09	5,18E-07
cg12775233	5	95933440	LOC10192971	Body	-0,217	4,48E-09	5,18E-07
cg08708949	9	137972023	OLFM1	Body	-0,219	4,48E-09	5,18E-07
cg01208726	5	6829916		IGR	-0,229	4,49E-09	5,18E-07
cg23847712	4	9783196	DRD5	TSS200	0,268	4,49E-09	5,19E-07
cg18376017	15	91407407		IGR	-0,216	4,50E-09	5,19E-07
cg02654940	6	34206400	HMGA1	5'UTR	-0,318	4,50E-09	5,19E-07
cg16213217	13	50074189	PHF11	Body	-0,376	4,50E-09	5,19E-07
cg09609957	14	91711676	GPR68	TSS1500	-0,234	4,52E-09	5,21E-07
cg13558754	19	36247867	HSPB6	1stExon	0,298	4,53E-09	5,22E-07
cg24976875	17	47792648	FAM117A	Body	-0,268	4,54E-09	5,23E-07
cg26282731	4	148939288	ARHGAP10	Body	0,209	4,54E-09	5,23E-07
cg23384708	6	31544934	TNF	Body	-0,298	4,56E-09	5,24E-07
cg13796381	9	139872395	PTGDS	Body	0,215	4,57E-09	5,25E-07
cg18986767	15	38963215		IGR	-0,224	4,58E-09	5,25E-07
cg05635114	16	88832572	FAM38A	Body	-0,226	4,58E-09	5,25E-07
cg06410591	19	6741181	TRIP10	Body	0,217	4,58E-09	5,26E-07
cg16406674	7	41140977	LINC01449	TSS1500	-0,208	4,59E-09	5,26E-07
cg18835815	8	54757048	ATP6V1H	TSS1500	-0,227	4,59E-09	5,26E-07
cg00470050	15	72206554	MYO9A	Body	-0,206	4,59E-09	5,26E-07
cg24985438	2	1644626	PXDN	Body	-0,227	4,60E-09	5,27E-07
cg25345520	7	116210196		IGR	-0,213	4,60E-09	5,27E-07
cg02323129	1	17965346	ARHGEF10L	Body	-0,293	4,61E-09	5,27E-07
cg20449670	1	153498959		IGR	0,228	4,61E-09	5,28E-07
cg05232977	10	24745236	KIAA1217	5'UTR	-0,295	4,62E-09	5,28E-07
cg02043127	10	72299368	PALD1	Body	-0,257	4,62E-09	5,28E-07

cg16063783	1	25291720	RUNX3	TSS1500	-0,291	4,63E-09	5,29E-07
cg09203111	4	169770398	PALLD	5'UTR	0,23	4,63E-09	5,29E-07
cg19928195	3	123813191	KALRN	TSS1500	-0,277	4,63E-09	5,29E-07
cg13815445	11	88028475	CTSC	Body	-0,246	4,64E-09	5,29E-07
cg26194172	5	96221731	ERAP2	Body	-0,225	4,64E-09	5,29E-07
cg15719903	12	6570167	TAPBPL	Body	-0,331	4,66E-09	5,31E-07
cg27381549	22	24824483	ADORA2A	5'UTR	-0,223	4,67E-09	5,32E-07
cg02193640	22	25456676	KIAA1671	Body	-0,262	4,67E-09	5,32E-07
cg17055704	6	33282879	ZBTB22	Body	0,251	4,67E-09	5,32E-07
cg00501919	6	31540750	LTA	Body	-0,276	4,68E-09	5,33E-07
cg04659754	1	208716074		IGR	-0,222	4,69E-09	5,33E-07
cg27617244	3	123813090	KALRN	TSS1500	-0,252	4,69E-09	5,34E-07
cg08222626	7	5732732	RNF216	Body	0,227	4,69E-09	5,34E-07
cg20157782	1	206751776	RASSF5	Body	-0,303	4,70E-09	5,34E-07
cg15209200	4	38511227	LINC01258	Body	-0,296	4,70E-09	5,34E-07
cg08516641	10	129789772	PTPRE	5'UTR	-0,238	4,70E-09	5,34E-07
cg08744125	16	71692816	PHLPP2	Body	-0,337	4,70E-09	5,34E-07
cg20728464	12	46610134	SLC38A1	Body	-0,231	4,70E-09	5,34E-07
cg15113672	15	61224544	RORA	Body	-0,258	4,71E-09	5,35E-07
cg13693278	16	86976371		IGR	-0,235	4,72E-09	5,35E-07
cg04753467	12	53569794	CSAD	5'UTR	-0,261	4,72E-09	5,35E-07
cg22317385	14	91544974		IGR	-0,269	4,72E-09	5,35E-07
cg00691123	3	11632974	VGLL4	Body	0,226	4,72E-09	5,35E-07
cg17213402	2	5813650		IGR	0,274	4,73E-09	5,35E-07
cg12216397	5	170020912	KCNIP1	5'UTR	-0,242	4,72E-09	5,35E-07
cg01268058	15	75738663	SIN3A	5'UTR	-0,224	4,73E-09	5,35E-07
cg24617344	16	30483713	ITGAL	TSS1500	-0,269	4,73E-09	5,35E-07
cg01519464	1	24861818	RCAN3	3'UTR	-0,267	4,74E-09	5,36E-07
cg12221087	17	78237432	RNF213	5'UTR	-0,212	4,74E-09	5,37E-07
cg10491774	18	46394982		IGR	-0,27	4,75E-09	5,37E-07
cg11725018	4	2414055	ZFYVE28	Body	-0,272	4,75E-09	5,37E-07
cg10175829	21	15907213	SAMSN1	5'UTR	-0,285	4,76E-09	5,37E-07
cg06865026	18	12015910	IMPA2	Body	0,202	4,77E-09	5,38E-07
cg08005460	17	38473382	RARA	TSS1500	0,205	4,77E-09	5,38E-07
cg17295699	6	15474332	JARID2	Body	0,212	4,77E-09	5,38E-07
cg06406209	8	103596294		IGR	-0,233	4,77E-09	5,38E-07
cg18379532	20	17973048		IGR	-0,26	4,78E-09	5,39E-07
cg13352520	5	65418807		IGR	-0,217	4,79E-09	5,39E-07
cg14076124	19	35924960		IGR	-0,229	4,79E-09	5,40E-07
cg14200350	9	35690051	TPM2	1stExon	0,253	4,80E-09	5,40E-07
cg10195901	22	30476278	HORMAD2	TSS200	0,244	4,80E-09	5,40E-07
cg19594666	7	127881280	LEP	TSS200	0,273	4,82E-09	5,41E-07
cg05963085	3	112359648	CCDC80	5'UTR	0,226	4,82E-09	5,42E-07
cg07441944	11	44623459	CD82	Body	-0,247	4,83E-09	5,42E-07
cg17279458	3	44753948	ZNF502	TSS200	0,22	4,84E-09	5,42E-07
cg05746574	11	3111966	OSBPL5	Body	-0,28	4,83E-09	5,42E-07
cg25769127	3	61543023		IGR	-0,24	4,84E-09	5,42E-07
cg25635522	2	217663710		IGR	-0,266	4,85E-09	5,42E-07
cg12416878	3	23431400	UBE2E2	Body	-0,286	4,84E-09	5,42E-07
cg13479204	17	46641708	HOXB3	5'UTR	0,258	4,84E-09	5,42E-07

cg23080355	1	95119714		IGR	0,359	4,85E-09	5,43E-07
cg00945209	17	76801579	USP36	Body	-0,244	4,85E-09	5,43E-07
cg00127167	5	5140029	ADAMTS16	TSS1500	0,217	4,85E-09	5,43E-07
cg04009575	1	160714382	SLAMF7	5'UTR	-0,292	4,86E-09	5,43E-07
cg09757644	6	447497		IGR	-0,272	4,86E-09	5,43E-07
cg05775049	1	177905610	SEC16B	Body	-0,222	4,86E-09	5,43E-07
cg11045943	7	127881293	LEP	TSS200	0,289	4,87E-09	5,44E-07
cg27291904	17	25802477	KSR1	5'UTR	-0,243	4,87E-09	5,44E-07
cg24263958	20	2270450		IGR	0,267	4,87E-09	5,44E-07
cg14622909	2	191709127		IGR	-0,335	4,87E-09	5,44E-07
cg15188217	6	106589718		IGR	-0,259	4,89E-09	5,45E-07
cg09337141	2	197624239		IGR	0,248	4,89E-09	5,45E-07
cg03921587	11	95444955		IGR	-0,239	4,90E-09	5,46E-07
cg07584721	1	167246402	POU2F1	Body	-0,208	4,91E-09	5,46E-07
cg10787865	1	226632601		IGR	-0,243	4,92E-09	5,47E-07
cg21910196	14	72402619	RGS6	5'UTR	-0,239	4,92E-09	5,47E-07
cg26381313	10	102279455	SEC31B	5'UTR	0,253	4,93E-09	5,48E-07
cg04813697	10	22920025	PIP4K2A	Body	-0,281	4,94E-09	5,48E-07
cg07126444	14	75608010	TMED10	Body	-0,254	4,95E-09	5,49E-07
cg00368259	18	67602690	CD226	Body	-0,211	4,95E-09	5,49E-07
cg00155799	2	172904495	METAP1D	Body	-0,207	4,97E-09	5,50E-07
cg03234557	4	99404186	TSPAN5	Body	-0,293	4,97E-09	5,51E-07
cg00679763	17	46993161	UBE2Z	Body	-0,397	4,98E-09	5,51E-07
cg18018129	21	35929748	RCAN1	Body	-0,244	4,99E-09	5,52E-07
cg01722932	11	92702653	MTNR1B	TSS200	0,23	4,99E-09	5,52E-07
cg00656990	16	78812407	WWOX	Body	-0,291	4,99E-09	5,52E-07
cg08638320	1	47900265	FOXD2	TSS1500	0,252	5,00E-09	5,52E-07
cg12524168	5	76028910	F2R	Body	-0,286	5,00E-09	5,52E-07
cg05084776	10	97115496	SORBS1	Body	-0,283	5,00E-09	5,52E-07
cg05280806	17	79366205		IGR	0,255	5,00E-09	5,52E-07
cg25346025	18	77743882	TXNL4A	Body	-0,203	5,00E-09	5,52E-07
cg14422392	7	129629691		IGR	-0,284	5,01E-09	5,53E-07
cg27403618	1	209907350	HSD11B1	Body	-0,271	5,01E-09	5,53E-07
cg05133205	6	32121249	PPT2	TSS200	0,254	5,03E-09	5,54E-07
cg11182499	9	132754630	FNBP1	Body	-0,232	5,03E-09	5,54E-07
cg06675248	17	5019611	ZNF232	5'UTR	0,237	5,03E-09	5,54E-07
cg27243312	3	65364943	MAGI1	Body	-0,224	5,03E-09	5,55E-07
cg02193859	18	77960683	PARD6G	Body	-0,266	5,04E-09	5,55E-07
cg19072817	5	176057992	EIF4E1B	5'UTR	0,241	5,04E-09	5,55E-07
cg07363202	9	131185138	CERCAM	5'UTR	-0,208	5,04E-09	5,55E-07
cg01615704	2	110873396	MALL	5'UTR	0,209	5,05E-09	5,55E-07
cg07091607	4	153919440		IGR	-0,235	5,05E-09	5,55E-07
cg25724842	6	163574564	PACRG	Body	-0,259	5,05E-09	5,55E-07
cg07168526	7	116797907	ST7	Body	0,238	5,04E-09	5,55E-07
cg01175610	12	12224246	BCL2L14	5'UTR	-0,376	5,05E-09	5,55E-07
cg05404236	13	110437093	IRS2	1stExon	0,253	5,05E-09	5,55E-07
cg17614731	22	25464836	KIAA1671	Body	-0,331	5,05E-09	5,55E-07
cg02381488	10	80679729		IGR	-0,308	5,07E-09	5,56E-07
cg02344871	8	131054654		IGR	-0,202	5,07E-09	5,56E-07
cg12010784	22	23487588	RAB36	1stExon	0,236	5,07E-09	5,56E-07

cg13344413	4	39034272	TMEM156	TSS1500	-0,207	5,08E-09	5,57E-07
cg07993586	7	157204960	DNAJB6	Body	-0,275	5,08E-09	5,57E-07
cg04115680	7	75889229	SRRM3	Body	0,264	5,08E-09	5,57E-07
cg07094833	2	98330493	ZAP70	5'UTR	-0,262	5,09E-09	5,57E-07
cg12552994	10	64374314	ZNF365	Body	-0,25	5,09E-09	5,57E-07
cg14349231	9	103340474	MURC	1stExon	0,332	5,09E-09	5,57E-07
cg17709873	6	31540456	LTA	5'UTR	-0,273	5,09E-09	5,58E-07
cg22747092	9	138987818	NACC2	TSS1500	0,204	5,10E-09	5,58E-07
cg12114602	16	11145869	CLEC16A	Body	-0,299	5,10E-09	5,58E-07
cg01926616	11	115796067		IGR	-0,325	5,11E-09	5,59E-07
cg26004771	1	178456093		IGR	0,272	5,12E-09	5,59E-07
cg26239078	11	115420727		IGR	-0,257	5,13E-09	5,60E-07
cg08232500	8	8143585		IGR	-0,288	5,14E-09	5,61E-07
cg26570844	7	101579003	CUX1	Body	0,352	5,14E-09	5,61E-07
cg17826679	19	10736038	SLC44A2	TSS200	0,247	5,15E-09	5,62E-07
cg01786128	5	176117988		IGR	-0,203	5,16E-09	5,63E-07
cg09433910	17	1626509	WDR81	TSS1500	-0,214	5,16E-09	5,63E-07
cg01931614	2	120007053	STEAP3-AS1	TSS1500	-0,266	5,17E-09	5,63E-07
cg17336354	3	34298905		IGR	-0,226	5,17E-09	5,63E-07
cg21277502	2	25546091	DNMT3A	5'UTR	-0,269	5,18E-09	5,63E-07
cg22615078	18	71931546	CYB5A	Body	-0,228	5,18E-09	5,64E-07
cg20412902	16	85398906		IGR	-0,249	5,20E-09	5,65E-07
cg05682482	10	79956845		IGR	-0,232	5,21E-09	5,65E-07
cg09586927	18	43454862	EPG5	Body	-0,261	5,21E-09	5,66E-07
cg03733470	3	196613114	SENP5	Body	-0,228	5,22E-09	5,67E-07
cg24258592	10	119806883	CASC2	Body	0,219	5,22E-09	5,67E-07
cg05213174	14	54810056		IGR	-0,247	5,23E-09	5,67E-07
cg09099868	10	22622793		IGR	0,219	5,23E-09	5,67E-07
cg17672740	11	67255424	AIP	Body	-0,284	5,24E-09	5,67E-07
cg09079666	5	138956692	UBE2D2	5'UTR	-0,253	5,24E-09	5,67E-07
cg23600475	1	155084807		IGR	-0,252	5,25E-09	5,68E-07
cg08859278	2	98329691	ZAP70	TSS1500	-0,267	5,25E-09	5,68E-07
cg18306593	14	50488142	LINC01599	Body	-0,328	5,25E-09	5,68E-07
cg03014019	7	70113224	AUTS2	Body	-0,228	5,26E-09	5,68E-07
cg07370751	12	52364935	ACVR1B	Body	-0,335	5,26E-09	5,68E-07
cg09811263	20	20477152	RALGAPA2	Body	-0,208	5,26E-09	5,68E-07
cg09852346	6	111688745	REV3L	Body	-0,251	5,26E-09	5,69E-07
cg16744314	20	30446221		IGR	-0,211	5,26E-09	5,69E-07
cg11694222	9	110853383		IGR	-0,21	5,27E-09	5,69E-07
cg01371776	20	57623249		IGR	-0,203	5,27E-09	5,69E-07
cg18094203	1	112302896	DDX20	Body	-0,235	5,27E-09	5,69E-07
cg07349899	11	11443325	GALNTL4	Body	-0,223	5,28E-09	5,70E-07
cg02196386	6	109793534	ZBTB24	Body	-0,261	5,30E-09	5,71E-07
cg16158513	6	164124190		IGR	-0,259	5,30E-09	5,72E-07
cg11160054	8	72460431		IGR	0,23	5,31E-09	5,72E-07
cg10566012	13	40121146	LHFP	Body	-0,254	5,31E-09	5,72E-07
cg08279186	14	72053158	SIPA1L1	TSS1500	0,231	5,32E-09	5,72E-07
cg05662436	9	13351377		IGR	-0,283	5,33E-09	5,73E-07
cg18343437	8	142528415		IGR	0,242	5,34E-09	5,74E-07
cg19927816	19	46319153	SYMPK	Body	0,242	5,36E-09	5,76E-07

cg17184593	2	74213565	TET3	1stExon	-0,286	5,37E-09	5,77E-07
cg14888243	1	210858559	KCNH1	Body	-0,306	5,38E-09	5,77E-07
cg11067966	2	15317374	NBAS	Body	-0,257	5,39E-09	5,78E-07
cg07214120	12	49647060	TUBA1C	5'UTR	-0,245	5,39E-09	5,78E-07
cg10674398	5	169913452	KCNIP1	Body	-0,207	5,40E-09	5,79E-07
cg23607975	6	11386826		IGR	-0,274	5,40E-09	5,79E-07
cg09408629	6	138192297	TNFAIP3	5'UTR	-0,339	5,41E-09	5,80E-07
cg24642169	1	212731669		IGR	0,203	5,42E-09	5,80E-07
cg17019053	2	74875263	C2orf65	TSS200	0,225	5,43E-09	5,81E-07
cg16569650	7	2773072	GNA12	Body	0,243	5,43E-09	5,81E-07
cg15001714	1	48459813		IGR	-0,312	5,44E-09	5,81E-07
cg19214657	3	192151711	FGF12	Body	-0,224	5,44E-09	5,82E-07
cg11993583	20	56195541	ZBP1	1stExon	-0,299	5,44E-09	5,82E-07
cg14438812	10	574670	DIP2C	Body	-0,245	5,45E-09	5,82E-07
cg08558360	1	94544894	ABCA4	ExonBnd	-0,254	5,46E-09	5,83E-07
cg15814717	6	29521228		IGR	0,222	5,47E-09	5,84E-07
cg21920303	1	160617356	SLAMF1	TSS1500	-0,258	5,47E-09	5,84E-07
cg11380570	6	28271503		IGR	-0,294	5,48E-09	5,84E-07
cg03017297	11	15886708		IGR	-0,249	5,49E-09	5,84E-07
cg17295389	6	110717270	DDO	Body	-0,214	5,49E-09	5,85E-07
cg03515479	13	77206729		IGR	-0,207	5,50E-09	5,85E-07
cg14034882	3	37637413	ITGA9	Body	-0,235	5,51E-09	5,86E-07
cg13840239	9	89218392		IGR	-0,227	5,52E-09	5,87E-07
cg00020590	2	173891947	RAPGEF4	Body	0,284	5,53E-09	5,87E-07
cg08882528	3	135689675	PPP2R3A	5'UTR	-0,238	5,53E-09	5,87E-07
cg23550589	4	24894154	CCDC149	Body	-0,233	5,53E-09	5,87E-07
cg13236889	5	170186645		IGR	-0,236	5,53E-09	5,87E-07
cg17313218	11	1875716	LSP1	Body	-0,329	5,54E-09	5,88E-07
cg10171724	7	138605500	KIAA1549	Body	-0,242	5,54E-09	5,88E-07
cg07962143	19	57149436		IGR	0,207	5,55E-09	5,88E-07
cg14143723	3	27638612		IGR	-0,274	5,55E-09	5,88E-07
cg07241660	11	69259336		IGR	0,23	5,55E-09	5,88E-07
cg27559997	1	42105137	HIVEP3	5'UTR	-0,223	5,59E-09	5,90E-07
cg12767180	21	47162336		IGR	-0,238	5,59E-09	5,90E-07
cg18372930	5	137939469		IGR	-0,218	5,60E-09	5,91E-07
cg10374424	5	156896196	NIPAL4	Body	-0,228	5,60E-09	5,91E-07
cg07248223	17	38717275	CCR7	Body	-0,277	5,60E-09	5,91E-07
cg06905401	1	210306176	SYT14	Body	0,26	5,61E-09	5,92E-07
cg14626325	10	33432425		IGR	-0,256	5,62E-09	5,93E-07
cg07136210	1	89765537		IGR	-0,377	5,63E-09	5,93E-07
cg26945145	5	107339091	FBXL17	Body	-0,215	5,64E-09	5,94E-07
cg26848395	19	4915401	UHRF1	Body	-0,272	5,64E-09	5,94E-07
cg07248315	15	59676920		IGR	-0,304	5,66E-09	5,95E-07
cg17700352	17	62168737	ERN1	Body	-0,207	5,66E-09	5,95E-07
cg25914821	8	128899514		IGR	-0,397	5,67E-09	5,95E-07
cg03151645	2	102517122		IGR	-0,292	5,68E-09	5,96E-07
cg27058497	1	25291546	RUNX3	TSS200	-0,311	5,70E-09	5,97E-07
cg15152331	2	160958810	ITGB6	Body	-0,318	5,70E-09	5,97E-07
cg16674025	13	47167669	LRCH1	Body	-0,312	5,70E-09	5,97E-07
cg24631526	20	62367961	LIME1	TSS200	-0,203	5,70E-09	5,97E-07

cg23155853	2	10930911	PDIA6	Body	-0,424	5,70E-09	5,97E-07
cg11362926	16	87041023		IGR	-0,247	5,71E-09	5,97E-07
cg15236453	6	17043034		IGR	0,246	5,72E-09	5,98E-07
cg26116556	6	150954381	PLEKHG1	5'UTR	-0,358	5,72E-09	5,98E-07
cg24999823	15	40353812	SRP14-AS1	Body	-0,267	5,72E-09	5,98E-07
cg22325734	20	23236654		IGR	-0,232	5,72E-09	5,98E-07
cg20849025	11	58981043	MPEG1	TSS1500	-0,28	5,74E-09	5,99E-07
cg26071872	16	86858339		IGR	-0,258	5,74E-09	5,99E-07
cg23475710	9	137188193		IGR	-0,257	5,75E-09	5,99E-07
cg04622024	1	38201001	EPHA10	Body	0,208	5,75E-09	6,00E-07
cg19138376	2	149360870		IGR	-0,234	5,75E-09	6,00E-07
cg07338787	19	47690096	SAE1	Body	-0,268	5,77E-09	6,01E-07
cg08586855	1	9375862	SPSB1	5'UTR	-0,274	5,80E-09	6,04E-07
cg02744525	1	67397410	MIER1	5'UTR	-0,24	5,81E-09	6,05E-07
cg02089409	4	185324303	IRF2	Body	-0,223	5,81E-09	6,05E-07
cg10636442	10	73306741	CDH23	Body	-0,239	5,83E-09	6,06E-07
cg26418434	11	57267120	SLC43A1	Body	0,274	5,83E-09	6,07E-07
cg03516026	1	16564651	C1orf89	TSS1500	-0,226	5,84E-09	6,07E-07
cg07592258	16	57910413		IGR	-0,221	5,84E-09	6,07E-07
cg21296632	5	90355362	ADGRV1	Body	-0,365	5,86E-09	6,07E-07
cg06195354	6	14733316		IGR	-0,283	5,86E-09	6,07E-07
cg14505896	4	154188400	TRIM2	Body	0,247	5,86E-09	6,08E-07
cg17645898	8	27527745	SCARA3	Body	-0,204	5,87E-09	6,08E-07
cg03950000	1	160617327	SLAMF1	TSS1500	-0,277	5,87E-09	6,09E-07
cg07942554	11	17523279	USH1C	Body	-0,21	5,88E-09	6,09E-07
cg03438097	16	11009795	CIITA	Body	-0,267	5,89E-09	6,10E-07
cg26946015	16	66178419		IGR	-0,25	5,89E-09	6,10E-07
cg04042333	17	1104665		IGR	-0,283	5,89E-09	6,10E-07
cg15901239	16	4192436		IGR	-0,206	5,89E-09	6,10E-07
cg07751331	2	26205865	KIF3C	TSS1500	0,207	5,91E-09	6,11E-07
cg04816013	3	123987591	KALRN	Body	-0,219	5,91E-09	6,11E-07
cg14491827	18	36124953		IGR	-0,203	5,91E-09	6,11E-07
cg06211203	12	5607385		IGR	-0,269	5,91E-09	6,11E-07
cg16588056	5	122614190		IGR	-0,23	5,92E-09	6,12E-07
cg07448060	7	79083753	MAGI2	TSS1500	0,207	5,92E-09	6,12E-07
cg25459558	6	33161290	COL11A2	TSS1500	0,224	5,93E-09	6,13E-07
cg03614193	18	55021542	ST8SIA3	Body	0,234	5,96E-09	6,15E-07
cg10575547	6	42283846	TRERF1	5'UTR	-0,266	5,97E-09	6,16E-07
cg11577089	2	38264725	RMDN2-AS1	TSS1500	-0,265	5,97E-09	6,16E-07
cg22394272	2	208181989		IGR	-0,211	5,98E-09	6,17E-07
cg07852628	8	27755022	SCARA5	Body	-0,282	5,98E-09	6,17E-07
cg13944175	19	52222698	HAS1	Body	0,275	5,98E-09	6,17E-07
cg09530267	10	105014933		IGR	-0,251	5,99E-09	6,17E-07
cg16149401	8	101348747	RNF19A	TSS1500	0,24	5,99E-09	6,18E-07
cg14780632	11	65816521	GAL3ST3	5'UTR	0,221	6,00E-09	6,18E-07
cg11080540	5	54897272		IGR	0,218	6,01E-09	6,18E-07
cg17761226	11	75900775	WNT11	Body	-0,256	6,02E-09	6,19E-07
cg19016296	5	67570687	PIK3R1	Body	-0,24	6,02E-09	6,19E-07
cg13823169	9	139776893		IGR	-0,201	6,02E-09	6,20E-07
cg08070771	3	147125758	ZIC4	TSS1500	0,232	6,03E-09	6,20E-07

cg07410412	1	48287762	TRABD2B	Body	-0,222	6,03E-09	6,20E-07
cg17708685	20	62666933	LINC00176	Body	-0,247	6,04E-09	6,20E-07
cg26459819	11	111169457	C11orf93	TSS1500	0,204	6,04E-09	6,21E-07
cg18128887	1	24861708	RCAN3	Body	-0,258	6,06E-09	6,22E-07
cg13150854	1	34005181	CSMD2	Body	-0,284	6,07E-09	6,23E-07
cg03111921	1	174756146	RABGAP1L	Body	-0,239	6,09E-09	6,24E-07
cg20547015	12	111165713	PPP1CC	Body	-0,336	6,09E-09	6,24E-07
cg13787354	7	140076239	SLC37A3	Body	-0,247	6,09E-09	6,24E-07
cg06699216	8	19333253	CSGALNACT1	Body	-0,306	6,10E-09	6,24E-07
cg13007324	15	38855619	RASGRP1	Body	-0,215	6,10E-09	6,24E-07
cg02225847	16	67563418	FAM65A	5'UTR	0,24	6,11E-09	6,24E-07
cg16000361	2	120182330		IGR	-0,206	6,11E-09	6,25E-07
cg26300517	4	38732661		IGR	-0,255	6,11E-09	6,25E-07
cg04825215	6	31466722	MICB	Body	0,208	6,11E-09	6,25E-07
cg27102941	15	99679368	TTC23	Body	-0,203	6,12E-09	6,25E-07
cg25154236	10	44871372	CXCL12	3'UTR	-0,229	6,13E-09	6,26E-07
cg14602393	12	133343405		IGR	0,206	6,13E-09	6,26E-07
cg14488265	7	107284118		IGR	-0,225	6,14E-09	6,26E-07
cg20626249	11	12708608	TEAD1	5'UTR	-0,231	6,14E-09	6,26E-07
cg16436686	1	186157568	HMCN1	Body	-0,245	6,14E-09	6,27E-07
cg26234900	6	32820214	TAP1	Body	-0,357	6,15E-09	6,27E-07
cg13990107	15	63663946	CA12	Body	-0,324	6,15E-09	6,27E-07
cg20095507	10	3027087		IGR	-0,264	6,16E-09	6,27E-07
cg09379489	12	12224360	BCL2L14	5'UTR	-0,25	6,16E-09	6,27E-07
cg11851174	17	17712609	RAI1	Body	-0,231	6,16E-09	6,27E-07
cg00474561	6	134557109	SGK1	Body	-0,323	6,17E-09	6,28E-07
cg12819762	16	83221020	CDH13	5'UTR	-0,204	6,20E-09	6,31E-07
cg13725885	20	24679131		IGR	-0,302	6,20E-09	6,31E-07
cg27253301	2	68949089		IGR	-0,286	6,21E-09	6,31E-07
cg24066284	3	193836817		IGR	-0,252	6,22E-09	6,32E-07
cg05555283	14	105109967		IGR	-0,202	6,24E-09	6,33E-07
cg02426376	1	63793987		IGR	0,225	6,25E-09	6,34E-07
cg21833554	4	147255130	SLC10A7	Body	-0,288	6,25E-09	6,34E-07
cg16108059	14	81421989	TSHR	5'UTR	0,251	6,25E-09	6,34E-07
cg09563119	14	100223977		IGR	-0,313	6,25E-09	6,34E-07
cg01306003	2	24802612		IGR	-0,219	6,26E-09	6,34E-07
cg17284326	13	98749760		IGR	-0,246	6,26E-09	6,34E-07
cg21628404	6	44035746	LOC1019297(	Body	-0,283	6,27E-09	6,35E-07
cg01407107	1	12107431		IGR	-0,21	6,27E-09	6,35E-07
cg24194775	9	35791475	NPR2	TSS1500	0,304	6,28E-09	6,35E-07
cg12027254	17	76055290	TNRC6C	Body	0,212	6,28E-09	6,35E-07
cg07788493	15	86236291	AKAP13	Body	-0,22	6,28E-09	6,35E-07
cg14767360	18	46695469	DYM	Body	-0,366	6,28E-09	6,35E-07
cg14688272	17	80673958	FN3KRP	TSS1500	0,222	6,30E-09	6,36E-07
cg09878888	11	57529614	CTNND1	5'UTR	0,207	6,30E-09	6,36E-07
cg27494086	20	3365025	C20orf194	Body	-0,28	6,30E-09	6,36E-07
cg11466837	11	120009682	TRIM29	TSS1500	-0,226	6,30E-09	6,37E-07
cg25030049	1	56169508		IGR	-0,236	6,31E-09	6,37E-07
cg16047279	17	38717242	CCR7	Body	-0,261	6,32E-09	6,38E-07
cg21704050	3	151063814	P2RY12	5'UTR	-0,235	6,32E-09	6,38E-07

cg05290065	12	123509672	PITPNM2	Body	-0,255	6,32E-09	6,38E-07
cg11722578	16	11419616		IGR	-0,44	6,32E-09	6,38E-07
cg05747633	18	39527524		IGR	-0,302	6,33E-09	6,38E-07
cg09292377	14	91010398	TTC7B	Body	-0,201	6,33E-09	6,38E-07
cg15295209	20	43596460	STK4-AS1	TSS1500	-0,343	6,34E-09	6,38E-07
cg00809772	11	6768084		IGR	-0,228	6,34E-09	6,39E-07
cg00444906	1	184712389	EDEM3	Body	-0,234	6,34E-09	6,39E-07
cg07322402	15	66393410	MEGF11	Body	-0,207	6,34E-09	6,39E-07
cg18924324	6	100057143	PRDM13	Body	0,2	6,35E-09	6,39E-07
cg11665416	21	45485710	TRAPPC10	Body	-0,273	6,36E-09	6,40E-07
cg05500904	2	10266949	RRM2	Body	0,247	6,36E-09	6,40E-07
cg09623279	10	45395734		IGR	-0,236	6,36E-09	6,40E-07
cg21223625	14	59332349		IGR	0,215	6,37E-09	6,41E-07
cg16012597	11	3021854		IGR	0,235	6,39E-09	6,42E-07
cg16193547	17	53536548		IGR	-0,289	6,40E-09	6,43E-07
cg07880098	21	32090978	KRTAP21-3	1stExon	-0,201	6,40E-09	6,43E-07
cg00545580	17	78571214	RPTOR	Body	-0,268	6,41E-09	6,44E-07
cg21207429	5	38827787	OSMR-AS1	Body	-0,258	6,43E-09	6,45E-07
cg22489329	11	36653370	C11orf74	Body	-0,286	6,43E-09	6,45E-07
cg23384668	14	96556170	C14orf132	3'UTR	-0,233	6,42E-09	6,45E-07
cg14318467	10	98781674	SLIT1	Body	-0,316	6,43E-09	6,45E-07
cg21761307	1	21475984	EIF4G3	5'UTR	0,252	6,44E-09	6,46E-07
cg07300934	9	111240896		IGR	-0,291	6,44E-09	6,46E-07
cg08119046	6	14543106		IGR	-0,266	6,46E-09	6,47E-07
cg23250489	5	172300158	ERGIC1	Body	0,292	6,47E-09	6,47E-07
cg09251429	11	124735128	ROBO3	TSS200	0,217	6,48E-09	6,48E-07
cg12828896	15	45005365	B2M	Body	-0,411	6,48E-09	6,49E-07
cg16099404	2	202336282	STRADB	Body	-0,205	6,49E-09	6,49E-07
cg24786434	16	84231249		IGR	-0,211	6,49E-09	6,49E-07
cg20318166	15	85360664	ALPK3	1stExon	0,263	6,50E-09	6,49E-07
cg02058741	1	94724340		IGR	-0,225	6,52E-09	6,51E-07
cg18031557	11	120706637	GRIK4	Body	-0,244	6,53E-09	6,51E-07
cg23893629	1	203733971	LAX1	TSS1500	-0,237	6,54E-09	6,51E-07
cg03110996	2	191883483		IGR	-0,237	6,55E-09	6,52E-07
cg09274385	12	115346510		IGR	-0,217	6,54E-09	6,52E-07
cg00810173	14	100195612		IGR	-0,29	6,55E-09	6,52E-07
cg20456448	8	25232904	DOCK5	Body	-0,231	6,56E-09	6,52E-07
cg03460623	11	119579144	PVRL1	Body	-0,222	6,58E-09	6,54E-07
cg13819611	16	83431995	CDH13	Body	-0,267	6,60E-09	6,55E-07
cg07121219	10	21644842		IGR	-0,25	6,60E-09	6,55E-07
cg11721913	10	102825997		IGR	0,244	6,63E-09	6,57E-07
cg14306663	12	66291943	HMGA2	Body	-0,201	6,64E-09	6,58E-07
cg16886414	17	78851149	RPTOR	Body	-0,266	6,64E-09	6,58E-07
cg15188623	15	90608429	ZNF710	5'UTR	0,255	6,65E-09	6,58E-07
cg14529767	18	53664618		IGR	-0,215	6,65E-09	6,58E-07
cg23615741	10	101297642		IGR	0,319	6,66E-09	6,58E-07
cg01631024	5	158563352		IGR	-0,201	6,67E-09	6,59E-07
cg18111733	1	110306809	EPS8L3	TSS200	-0,242	6,67E-09	6,60E-07
cg19624029	2	10161972		IGR	-0,218	6,68E-09	6,60E-07
cg16113371	5	66040195	MAST4	Body	-0,2	6,69E-09	6,61E-07

cg11428758	22	28397015	TTC28-AS1	Body	-0,254	6,70E-09	6,61E-07
cg04920032	12	50262986	FAIM2	3'UTR	0,208	6,71E-09	6,62E-07
cg17975791	4	156561415		IGR	-0,224	6,72E-09	6,63E-07
cg10194843	15	67168298		IGR	-0,204	6,74E-09	6,64E-07
cg07323399	4	24800823	SOD3	5'UTR	-0,233	6,76E-09	6,65E-07
cg01457185	6	47252055	TNFRSF21	Body	-0,202	6,76E-09	6,66E-07
cg18036501	14	21500078	TPPP2	Body	-0,226	6,76E-09	6,66E-07
cg23959474	17	40195832		IGR	-0,216	6,76E-09	6,66E-07
cg25215608	10	44820772		IGR	-0,317	6,77E-09	6,66E-07
cg09220123	1	117099363	CD58	Body	-0,235	6,77E-09	6,66E-07
cg12176682	20	23018803		IGR	-0,285	6,78E-09	6,66E-07
cg07872280	7	35765161		IGR	-0,388	6,78E-09	6,67E-07
cg15571933	22	19186646	CLTCL1	Body	-0,232	6,78E-09	6,67E-07
cg24289952	3	113942043		IGR	-0,226	6,79E-09	6,67E-07
cg10170847	19	41834029		IGR	-0,202	6,79E-09	6,67E-07
cg11486744	5	118679509	TNFAIP8	Body	-0,273	6,80E-09	6,67E-07
cg11042276	3	172521595	ECT2	Body	-0,21	6,81E-09	6,67E-07
cg25757700	6	53692338	LRRC1	Body	-0,208	6,81E-09	6,67E-07
cg10111084	6	37001154		IGR	-0,248	6,81E-09	6,67E-07
cg12646452	2	144560348		IGR	-0,206	6,81E-09	6,67E-07
cg18717288	20	327377	NRSN2	1stExon	0,239	6,82E-09	6,68E-07
cg13741422	1	41229900	NFYC	Body	-0,209	6,83E-09	6,69E-07
cg04719202	2	10646702		IGR	-0,237	6,83E-09	6,69E-07
cg16160417	8	128579407		IGR	-0,327	6,83E-09	6,69E-07
cg04350675	6	111873161		IGR	0,206	6,83E-09	6,69E-07
cg08031790	17	28032758	SSH2	Body	-0,291	6,83E-09	6,69E-07
cg03444838	10	1156482	WDR37	Body	-0,281	6,84E-09	6,69E-07
cg04513764	10	129144998	DOCK1	Body	-0,255	6,84E-09	6,69E-07
cg23149098	7	133169151	EXOC4	Body	-0,258	6,84E-09	6,69E-07
cg09977449	17	50236098	CA10	1stExon	0,203	6,87E-09	6,71E-07
cg14445459	5	175280246	CPLX2	5'UTR	-0,249	6,87E-09	6,72E-07
cg10802680	12	122712075	DIABLO	TSS200	-0,239	6,89E-09	6,73E-07
cg23234811	1	204230657	PLEKHA6	Body	-0,212	6,90E-09	6,73E-07
cg09111892	5	14415656	TRIO	Body	-0,303	6,90E-09	6,73E-07
cg19837174	10	6389707		IGR	-0,301	6,90E-09	6,73E-07
cg02286091	5	78407678	BHMT	5'UTR	0,212	6,92E-09	6,74E-07
cg23799246	2	99085310	INPP4A	5'UTR	-0,343	6,92E-09	6,74E-07
cg22033476	2	43532275	THADA	Body	0,234	6,93E-09	6,75E-07
cg19148362	12	3146948	TEAD4	Body	-0,223	6,94E-09	6,75E-07
cg03577433	2	174731517		IGR	-0,243	6,95E-09	6,76E-07
cg01424078	1	22486183		IGR	-0,244	6,95E-09	6,77E-07
cg09027879	1	25914071		IGR	-0,263	6,95E-09	6,77E-07
cg13634319	12	56323727	DGKA	TSS1500	-0,234	6,97E-09	6,77E-07
cg13298616	9	131169508	MIR1268A	Body	-0,213	6,97E-09	6,77E-07
cg08154048	7	105447488	ATXN7L1	Body	-0,25	6,98E-09	6,78E-07
cg06241170	10	124249994	HTRA1	Body	-0,281	7,00E-09	6,79E-07
cg12050175	10	44754714		IGR	-0,286	7,00E-09	6,79E-07
cg23939182	4	54461431		IGR	-0,24	7,01E-09	6,80E-07
cg14991769	7	150497157	TMEM176B	TSS1500	0,206	7,02E-09	6,81E-07
cg12651029	14	91502375	RPS6KA5	Body	-0,281	7,02E-09	6,81E-07

cg12108397	18	35032140	CELF4	Body	-0,207	7,02E-09	6,81E-07
cg00440472	6	150623355		IGR	-0,255	7,03E-09	6,81E-07
cg10831212	11	57529419	CTNND1	5'UTR	0,225	7,04E-09	6,82E-07
cg03714754	15	68993073	CORO2B	Body	0,201	7,06E-09	6,83E-07
cg25096622	7	70144961	AUTS2	Body	-0,351	7,06E-09	6,83E-07
cg05335423	5	40436872		IGR	-0,249	7,06E-09	6,83E-07
cg06981132	8	145857950	ARHGAP39	5'UTR	-0,209	7,08E-09	6,85E-07
cg05573378	15	42876795	STARD9	Body	0,207	7,09E-09	6,85E-07
cg13267551	6	30915371	DPCR1	Body	-0,279	7,10E-09	6,86E-07
cg16322513	9	92096295		IGR	-0,24	7,10E-09	6,86E-07
cg06136645	7	148907120	ZNF282	Body	-0,235	7,10E-09	6,86E-07
cg19156729	9	124452422	DAB2IP	Body	-0,205	7,11E-09	6,86E-07
cg06752040	10	14647373	FAM107B	Body	-0,238	7,12E-09	6,87E-07
cg01456695	11	48108785	PTPRJ	Body	0,227	7,12E-09	6,87E-07
cg16653724	8	18286687		IGR	-0,231	7,13E-09	6,87E-07
cg10740902	4	55991683	KDR	1stExon	0,209	7,13E-09	6,88E-07
cg02931208	15	77866341		IGR	-0,261	7,13E-09	6,88E-07
cg12464134	7	5578193		IGR	-0,296	7,14E-09	6,88E-07
cg06664357	19	39993390	DLL3	Body	0,229	7,14E-09	6,88E-07
cg22108311	1	46766456	LRRC41	Body	0,276	7,14E-09	6,88E-07
cg09805254	6	164520673		IGR	0,245	7,15E-09	6,89E-07
cg16442638	2	120989893		IGR	-0,22	7,16E-09	6,89E-07
cg09737314	17	6899359	ALOX12	TSS200	0,243	7,16E-09	6,90E-07
cg15653090	18	32440614	DTNA	Body	-0,343	7,16E-09	6,90E-07
cg24587057	1	181601850	CACNA1E	Body	-0,211	7,17E-09	6,90E-07
cg10533409	3	11766469		IGR	-0,246	7,17E-09	6,90E-07
cg27032232	7	153584609	DPP6	1stExon	0,243	7,20E-09	6,92E-07
cg12413156	20	62368256	LIME1	5'UTR	-0,328	7,20E-09	6,92E-07
cg05243517	11	95610702	MTMR2	Body	-0,27	7,21E-09	6,92E-07
cg07361830	2	217513101	IGFBP2	Body	-0,277	7,21E-09	6,93E-07
cg02118562	3	124082907	KALRN	Body	-0,268	7,22E-09	6,94E-07
cg18148314	1	6425319	ACOT7	Body	-0,254	7,22E-09	6,94E-07
cg13514641	2	86740268	CHMP3	Body	0,274	7,23E-09	6,94E-07
cg18645642	1	8631756	RERE	Body	0,33	7,23E-09	6,94E-07
cg26856867	11	11562311	GALNT18	Body	-0,294	7,25E-09	6,95E-07
cg25701417	3	101109159	SENP7	Body	-0,343	7,26E-09	6,96E-07
cg07456387	22	33819282	LARGE	Body	-0,209	7,26E-09	6,96E-07
cg04750316	1	42935595	PPCS	Body	-0,261	7,27E-09	6,96E-07
cg23134239	3	150370943		IGR	-0,251	7,27E-09	6,96E-07
cg03404292	10	126437642		IGR	-0,232	7,27E-09	6,96E-07
cg18594663	14	105533022	GPR132	TSS1500	-0,279	7,28E-09	6,97E-07
cg04494298	7	31851648	PDE1C	Body	-0,3	7,29E-09	6,97E-07
cg16293569	11	2870208	KCNQ1	3'UTR	-0,203	7,29E-09	6,97E-07
cg13888763	11	69023300		IGR	-0,219	7,29E-09	6,97E-07
cg13055252	16	85186068		IGR	-0,271	7,29E-09	6,97E-07
cg19646897	5	156536379	HAVCR2	TSS1500	-0,225	7,30E-09	6,98E-07
cg02231590	2	231737958	ITM2C	Body	-0,229	7,31E-09	6,98E-07
cg01090020	5	14415593	TRIO	Body	-0,266	7,31E-09	6,99E-07
cg11819201	1	171105925	FMO6P	TSS1500	-0,279	7,32E-09	6,99E-07
cg07197948	12	96131706	NTN4	Body	-0,237	7,35E-09	7,02E-07

cg24885369	6	167364028	RNASET2	Body	-0,324	7,36E-09	7,02E-07
cg19167338	8	62581684	ASPH	Body	-0,271	7,36E-09	7,02E-07
cg17980364	11	86748241	TMEM135	TSS1500	0,209	7,36E-09	7,02E-07
cg08310756	16	79019230	WWOX	Body	0,203	7,36E-09	7,02E-07
cg06948120	8	17743177	FGL1	5'UTR	0,22	7,38E-09	7,03E-07
cg10606725	21	42551825	PLAC4	5'UTR	-0,243	7,38E-09	7,03E-07
cg15258711	3	187087086	RTP4	Body	-0,305	7,38E-09	7,03E-07
cg12534371	16	83382775	CDH13	Body	-0,2	7,39E-09	7,04E-07
cg03048210	17	35903966	SYNRG	Body	-0,216	7,40E-09	7,04E-07
cg09430819	10	5607078		IGR	-0,247	7,42E-09	7,05E-07
cg21697426	6	112311269		IGR	-0,282	7,42E-09	7,05E-07
cg27528326	11	14396120		IGR	0,201	7,43E-09	7,06E-07
cg08450017	3	45984838	CXCR6	TSS200	-0,248	7,44E-09	7,07E-07
cg02025583	17	10632861	TMEM220	Body	0,215	7,46E-09	7,08E-07
cg12648510	5	78365677	DMGDH	TSS200	0,272	7,46E-09	7,08E-07
cg11666630	21	35322924	LINC00649	Body	-0,235	7,48E-09	7,09E-07
cg25396287	7	17301295		IGR	-0,258	7,48E-09	7,10E-07
cg24292761	6	117584665		IGR	0,231	7,49E-09	7,10E-07
cg23280720	6	139483193	HECA	Body	-0,292	7,51E-09	7,11E-07
cg04729140	9	100926300	CORO2A	5'UTR	-0,223	7,52E-09	7,12E-07
cg12961607	2	38976250	SFRS7	Body	-0,228	7,54E-09	7,13E-07
cg09917274	2	152144828	NMI	5'UTR	-0,326	7,56E-09	7,15E-07
cg08529982	14	100485220		IGR	-0,229	7,56E-09	7,15E-07
cg24392635	4	100865450	DNAJB14	Body	-0,203	7,56E-09	7,15E-07
cg26704331	9	137790811		IGR	-0,254	7,56E-09	7,15E-07
cg22388897	14	77508811	LOC283575	Body	0,225	7,57E-09	7,15E-07
cg16472834	2	231533021		IGR	-0,294	7,59E-09	7,17E-07
cg04203138	13	100087080		IGR	-0,229	7,59E-09	7,17E-07
cg21026460	3	13539059	HDAC11	Body	-0,234	7,60E-09	7,17E-07
cg10567463	5	40490647		IGR	-0,356	7,61E-09	7,18E-07
cg23847017	1	28764854	PHACTR4	1stExon	0,2	7,62E-09	7,18E-07
cg21809516	14	50988575	MAP4K5	Body	-0,366	7,62E-09	7,18E-07
cg11112979	16	66260403		IGR	-0,256	7,62E-09	7,18E-07
cg05758692	6	4790999	CDYL	Body	-0,21	7,63E-09	7,19E-07
cg17542742	1	226788200	C1orf95	Body	-0,233	7,64E-09	7,19E-07
cg14644686	9	88126383		IGR	-0,231	7,64E-09	7,19E-07
cg06988064	7	33771735		IGR	-0,227	7,65E-09	7,20E-07
cg04590386	8	128538307		IGR	-0,223	7,66E-09	7,20E-07
cg08428292	16	85981373		IGR	-0,237	7,66E-09	7,20E-07
cg12706260	4	89146418	ABCG2	5'UTR	0,243	7,67E-09	7,21E-07
cg11370748	6	153296563	FBXO5	Body	-0,477	7,67E-09	7,21E-07
cg24809973	8	72468820		IGR	0,214	7,70E-09	7,23E-07
cg12670295	11	129150579		IGR	-0,222	7,70E-09	7,23E-07
cg02593918	1	22164880	HSPG2	Body	-0,285	7,71E-09	7,23E-07
cg18019234	2	12959467		IGR	0,206	7,71E-09	7,23E-07
cg06492235	2	205823322	PARD3B	Body	-0,294	7,71E-09	7,23E-07
cg19119071	6	83904067	PGM3	TSS1500	0,21	7,71E-09	7,23E-07
cg15799450	7	94210097		IGR	-0,224	7,71E-09	7,23E-07
cg22341908	1	197747884		IGR	-0,258	7,71E-09	7,23E-07
cg27297315	2	23704185	KLHL29	5'UTR	-0,222	7,72E-09	7,23E-07

cg02746044	2	106521576		IGR	-0,33	7,72E-09	7,23E-07
cg11438011	5	148033882	HTR4	TSS200	0,248	7,72E-09	7,23E-07
cg02772121	6	30130881	TRIM15	TSS200	-0,236	7,72E-09	7,23E-07
cg13873813	16	22825440	HS3ST2	TSS1500	0,206	7,72E-09	7,23E-07
cg16394871	4	26163878		IGR	-0,212	7,73E-09	7,23E-07
cg14298140	13	76452681	LMO7DN-IT1	Body	-0,204	7,77E-09	7,27E-07
cg20076442	8	72745197		IGR	0,224	7,78E-09	7,27E-07
cg18608055	19	1130866	SBNO2	Body	-0,346	7,78E-09	7,27E-07
cg13018715	5	108105182	FER	5'UTR	-0,266	7,79E-09	7,28E-07
cg01631657	11	14259494	SPON1	Body	-0,225	7,80E-09	7,28E-07
cg21553436	3	33701149	CLASP2	TSS1500	0,203	7,80E-09	7,28E-07
cg03046424	2	220420867	OBSL1	Body	-0,211	7,82E-09	7,30E-07
cg10773266	10	43895225	HNRNPF	5'UTR	-0,331	7,82E-09	7,30E-07
cg03394824	7	50246097		IGR	-0,219	7,82E-09	7,30E-07
cg06610978	2	114437300		IGR	-0,233	7,83E-09	7,30E-07
cg12654620	8	90741449	LOC1019297(	Body	-0,219	7,84E-09	7,31E-07
cg04118514	4	873212	GAK	Body	-0,265	7,84E-09	7,31E-07
cg16279575	6	12275455		IGR	-0,215	7,85E-09	7,32E-07
cg22997113	7	27170241	HOXA4	1stExon	0,202	7,85E-09	7,32E-07
cg23130711	9	78526426	PCSK5	Body	-0,237	7,86E-09	7,32E-07
cg25002426	2	26150583	KIF3C	3'UTR	-0,25	7,87E-09	7,33E-07
cg26631003	5	149948662		IGR	-0,215	7,87E-09	7,33E-07
cg06432655	19	36523405	CLIP3	5'UTR	0,205	7,87E-09	7,33E-07
cg22983119	10	35710333	CCNY	5'UTR	0,269	7,88E-09	7,34E-07
cg06658591	14	91709304	GPR68	5'UTR	-0,264	7,91E-09	7,36E-07
cg07106625	6	11870215		IGR	0,308	7,92E-09	7,36E-07
cg25581222	15	64539239	CSNK1G1	Body	-0,281	7,92E-09	7,36E-07
cg15480653	9	132751589	FNBP1	Body	-0,24	7,93E-09	7,36E-07
cg09199338	4	178674883	LINC01098	Body	-0,448	7,93E-09	7,37E-07
cg25713524	1	203970557		IGR	-0,217	7,94E-09	7,37E-07
cg02869808	4	40841006	APBB2	5'UTR	-0,229	7,96E-09	7,39E-07
cg13343960	3	10735291		IGR	0,226	7,97E-09	7,39E-07
cg14384158	9	140348828	NELF	Body	0,216	7,98E-09	7,40E-07
cg13657077	8	61906032		IGR	-0,246	8,01E-09	7,42E-07
cg01937809	1	37941854	ZC3H12A	Body	-0,201	8,01E-09	7,42E-07
cg03111079	11	12900975	TEAD1	Body	-0,223	8,01E-09	7,42E-07
cg11910675	2	27587064		IGR	-0,209	8,01E-09	7,42E-07
cg17722508	1	31228006	LAPTM5	Body	-0,28	8,03E-09	7,43E-07
cg16878549	11	19840651	NAV2	Body	-0,208	8,03E-09	7,44E-07
cg07698102	2	205746734	PARD3B	Body	-0,275	8,03E-09	7,44E-07
cg08568472	6	151476184		IGR	-0,235	8,04E-09	7,44E-07
cg18103825	11	116653916	ZPR1	Body	-0,202	8,04E-09	7,44E-07
cg10909101	11	12927423	TEAD1	Body	-0,272	8,05E-09	7,45E-07
cg04352083	2	46619888		IGR	-0,278	8,05E-09	7,45E-07
cg23518039	5	148579190	ABLIM3	Body	-0,213	8,06E-09	7,45E-07
cg21445207	3	187518146		IGR	-0,241	8,07E-09	7,46E-07
cg16606773	20	19955806	RIN2	Body	0,337	8,09E-09	7,47E-07
cg27472156	3	4793415	EGOT	TSS200	0,225	8,11E-09	7,48E-07
cg24103965	15	93365153		IGR	-0,209	8,11E-09	7,48E-07
cg19907913	17	455636	VPS53	Body	-0,225	8,11E-09	7,48E-07

cg08731961	3	50988282	DOCK3	Body	-0,305	8,14E-09	7,50E-07
cg17973115	1	25333445		IGR	0,207	8,15E-09	7,51E-07
cg21517495	15	93114995	LINC00930	Body	-0,225	8,15E-09	7,51E-07
cg06728793	7	644702	PRKAR1B	Body	-0,286	8,15E-09	7,51E-07
cg09375033	3	137833634	DZIP1L	5'UTR	0,233	8,17E-09	7,52E-07
cg21999022	9	137735674	COL5A1	3'UTR	-0,24	8,19E-09	7,53E-07
cg02694800	6	42237575	TRERF1	ExonBnd	-0,228	8,19E-09	7,53E-07
cg08504214	19	1133528	SBNO2	TSS1500	-0,312	8,20E-09	7,54E-07
cg07751349	3	114884353		IGR	-0,26	8,21E-09	7,55E-07
cg11723850	14	24020053		IGR	0,206	8,21E-09	7,55E-07
cg02986632	8	136680012		IGR	-0,281	8,23E-09	7,56E-07
cg17799614	21	36607318		IGR	-0,379	8,24E-09	7,56E-07
cg21554704	2	225282008		IGR	0,222	8,24E-09	7,57E-07
cg15264393	10	98763300	SLIT1	Body	-0,293	8,25E-09	7,57E-07
cg24603102	8	119011173	EXT1	Body	-0,311	8,25E-09	7,57E-07
cg11943988	16	1056222		IGR	-0,258	8,25E-09	7,57E-07
cg02830178	2	25576057		IGR	-0,242	8,27E-09	7,58E-07
cg09818863	1	28624451		IGR	-0,235	8,27E-09	7,58E-07
cg24073452	2	236987322	AGAP1	Body	-0,216	8,27E-09	7,58E-07
cg23518805	4	139443334		IGR	-0,223	8,29E-09	7,59E-07
cg22292738	9	93926074		IGR	-0,217	8,29E-09	7,59E-07
cg14489474	18	905611	ADCYAP1	Body	0,213	8,29E-09	7,59E-07
cg24605886	14	72065164	SIPA1L1	Body	-0,201	8,29E-09	7,60E-07
cg27417606	14	21503144	RNASE13	TSS1500	-0,298	8,32E-09	7,61E-07
cg18789887	3	16216100	GALNTL2	TSS200	0,206	8,32E-09	7,62E-07
cg13331196	10	88295591		IGR	0,246	8,32E-09	7,62E-07
cg02885907	12	275754	IQSEC3	Body	-0,321	8,33E-09	7,62E-07
cg05746406	21	43889627		IGR	-0,208	8,34E-09	7,62E-07
cg01373166	22	24823389	ADORA2A	TSS200	-0,221	8,35E-09	7,63E-07
cg18732047	8	102275500		IGR	-0,207	8,36E-09	7,63E-07
cg18081515	14	104630064	KIF26A	Body	-0,215	8,36E-09	7,63E-07
cg19927885	20	39969058	LPIN3	TSS1500	0,202	8,36E-09	7,63E-07
cg23774950	8	102996800	LOC1040541	Body	-0,301	8,37E-09	7,64E-07
cg16999147	5	138404196	SIL1	Body	-0,274	8,38E-09	7,64E-07
cg20563072	11	48030718	PTPRJ	Body	-0,285	8,38E-09	7,64E-07
cg02498886	5	95066236	RHOBTB3	TSS1500	0,208	8,39E-09	7,64E-07
cg08525461	8	40958085		IGR	-0,265	8,40E-09	7,65E-07
cg17234273	8	71529977	LACTB2-AS1	Body	-0,203	8,40E-09	7,65E-07
cg17379405	2	200716738		IGR	0,261	8,41E-09	7,65E-07
cg02913734	1	85988934	DDAH1	5'UTR	-0,316	8,42E-09	7,66E-07
cg14802587	2	113579048		IGR	-0,323	8,42E-09	7,66E-07
cg05057720	14	38724675	CLEC14A	1stExon	0,234	8,42E-09	7,66E-07
cg11692194	17	53639752		IGR	-0,226	8,43E-09	7,66E-07
cg20845670	4	16080944	PROM1	5'UTR	-0,311	8,43E-09	7,66E-07
cg16822992	5	41213503	C6	1stExon	-0,24	8,43E-09	7,66E-07
cg21834207	1	50489827	AGBL4	TSS1500	0,253	8,43E-09	7,66E-07
cg09840155	11	10716592	MRVI1	TSS1500	-0,261	8,48E-09	7,70E-07
cg03076972	17	6436043	PITPNM3	Body	-0,22	8,48E-09	7,70E-07
cg18404374	3	26663551	LRRC3B	TSS1500	0,205	8,48E-09	7,70E-07
cg01951972	9	109685580	ZNF462	5'UTR	-0,235	8,49E-09	7,70E-07

cg12094604	17	41891026	MPP3	Body	-0,233	8,48E-09	7,70E-07
cg12621285	11	129724659	TMEM45B	Body	-0,206	8,49E-09	7,70E-07
cg27212386	1	38411997	INPP5B	5'UTR	0,203	8,49E-09	7,70E-07
cg21281167	1	62799248		IGR	-0,245	8,50E-09	7,71E-07
cg03683583	11	118314223	KMT2A	Body	-0,258	8,51E-09	7,71E-07
cg08560458	19	42627608	POU2F2	Body	-0,221	8,52E-09	7,72E-07
cg09256360	19	12111146		IGR	-0,217	8,54E-09	7,73E-07
cg16686158	22	30476098	HORMAD2	TSS1500	0,213	8,54E-09	7,73E-07
cg15564650	1	43520973		IGR	-0,236	8,54E-09	7,73E-07
cg01803729	1	52000373		IGR	-0,203	8,54E-09	7,73E-07
cg01337349	12	131724157		IGR	-0,218	8,54E-09	7,73E-07
cg15996739	14	90890298		IGR	-0,21	8,54E-09	7,73E-07
cg25072336	17	43306802	FMNL1	Body	-0,21	8,55E-09	7,73E-07
cg11373428	2	28496822	BRE	Body	-0,299	8,57E-09	7,75E-07
cg21062226	20	61554004	DIDO1	5'UTR	-0,22	8,58E-09	7,75E-07
cg01890120	8	133930777	TG	Body	-0,317	8,58E-09	7,75E-07
cg07380906	5	102201102	PAM	TSS1500	0,219	8,59E-09	7,75E-07
cg16075393	14	35829997		IGR	-0,267	8,61E-09	7,77E-07
cg22608179	19	39290548		IGR	-0,223	8,61E-09	7,77E-07
cg05329720	9	137718814	COL5A1	Body	-0,249	8,62E-09	7,77E-07
cg25998967	6	111727497	REV3L	Body	-0,295	8,62E-09	7,77E-07
cg08738340	16	82685721	CDH13	5'UTR	-0,225	8,63E-09	7,77E-07
cg26301662	6	57070622	RAB23	Body	-0,246	8,63E-09	7,78E-07
cg13825098	11	15934588		IGR	-0,246	8,65E-09	7,79E-07
cg14438134	21	40170587		IGR	-0,201	8,65E-09	7,79E-07
cg05991902	2	9385082	ASAP2	Body	-0,246	8,66E-09	7,80E-07
cg17062279	17	3658505	ITGAE	Body	0,203	8,67E-09	7,80E-07
cg26453818	6	14755099		IGR	-0,211	8,68E-09	7,81E-07
cg10271993	8	110593242	SYBU	Body	-0,248	8,70E-09	7,82E-07
cg04914198	1	203019107	PPFIA4	TSS1500	-0,234	8,70E-09	7,82E-07
cg19635805	10	72115214	LRRC20	5'UTR	-0,312	8,71E-09	7,82E-07
cg21327112	7	642639	LOC1019269(	TSS1500	-0,26	8,71E-09	7,83E-07
cg26930374	14	23013826		IGR	-0,269	8,72E-09	7,83E-07
cg25030470	3	194850472	XXYLT1	Body	-0,239	8,73E-09	7,84E-07
cg17783174	2	235950763	SH3BP4	Body	-0,242	8,74E-09	7,84E-07
cg02873163	1	153599829	S100A13	TSS200	0,286	8,74E-09	7,84E-07
cg12506165	12	116997072	MAP1LC3B2	TSS200	-0,245	8,74E-09	7,84E-07
cg04997275	2	208423225	CREB1	Body	0,219	8,75E-09	7,85E-07
cg09451832	9	116246461	RGS3	Body	0,218	8,75E-09	7,85E-07
cg00755688	10	128994434	FAM196A	TSS200	0,242	8,76E-09	7,85E-07
cg26986871	12	10545083		IGR	-0,329	8,76E-09	7,85E-07
cg11518764	15	31751448		IGR	-0,284	8,77E-09	7,86E-07
cg11531491	22	19984541	ARVCF	5'UTR	-0,263	8,77E-09	7,86E-07
cg13429095	1	206913187		IGR	-0,297	8,79E-09	7,87E-07
cg12197357	1	228930267		IGR	-0,224	8,80E-09	7,87E-07
cg08767286	2	237077265	GBX2	TSS1500	0,278	8,80E-09	7,88E-07
cg26764799	10	94044301	CPEB3	5'UTR	-0,273	8,80E-09	7,88E-07
cg11596404	1	153599772	S100A13	TSS200	0,302	8,82E-09	7,89E-07
cg08548560	9	4100149	GLIS3	Body	-0,267	8,82E-09	7,89E-07
cg06246469	10	134336223		IGR	-0,317	8,82E-09	7,89E-07

cg16134988	3	40961681		IGR	-0,225	8,83E-09	7,89E-07
cg10242565	7	47420961	TNS3	Body	-0,312	8,85E-09	7,91E-07
cg23422835	22	33919338	LARGE	Body	-0,245	8,88E-09	7,93E-07
cg13680038	2	15930883		IGR	-0,203	8,90E-09	7,94E-07
cg16847141	8	98988226	MATN2	Body	-0,24	8,91E-09	7,95E-07
cg25022233	11	68545369	CPT1A	Body	0,21	8,91E-09	7,95E-07
cg26119215	15	42436660	PLA2G4F	ExonBnd	-0,247	8,91E-09	7,95E-07
cg04180419	3	39336933		IGR	-0,227	8,92E-09	7,95E-07
cg10545142	19	42070902	CEACAM21	5'UTR	0,207	8,92E-09	7,95E-07
cg08535361	12	96177524	NTN4	Body	-0,26	8,94E-09	7,96E-07
cg17583667	1	36039356	TFAP2E	Body	0,207	8,94E-09	7,96E-07
cg05327844	11	330872		IGR	-0,215	8,95E-09	7,97E-07
cg12858998	2	33212211	LTBP1	Body	-0,21	8,98E-09	7,98E-07
cg23969417	2	5313194		IGR	-0,213	9,00E-09	8,00E-07
cg00255275	8	131668478		IGR	-0,204	9,00E-09	8,00E-07
cg03861089	4	3036239	GRK4	Body	-0,21	9,02E-09	8,01E-07
cg26806588	1	65446986	LINC01359	Body	-0,32	9,04E-09	8,02E-07
cg15370388	10	72497230	ADAMTS14	Body	-0,256	9,04E-09	8,02E-07
cg26919551	10	75681949	C10orf55	5'UTR	-0,265	9,04E-09	8,02E-07
cg00232772	20	1612808	SIRPG-AS1	TSS1500	-0,248	9,04E-09	8,02E-07
cg26849382	5	140901022	DIAPH1	Body	0,231	9,05E-09	8,02E-07
cg13618712	12	122228527	RHOF	Body	-0,238	9,06E-09	8,03E-07
cg11103049	17	3433632	TRPV3	Body	0,273	9,06E-09	8,03E-07
cg12059732	8	37818407		IGR	-0,226	9,06E-09	8,03E-07
cg24851946	11	15781897		IGR	-0,2	9,06E-09	8,03E-07
cg23688350	2	110317956	sept-10	Body	-0,241	9,07E-09	8,03E-07
cg04275692	7	45610627		IGR	-0,268	9,07E-09	8,04E-07
cg06487943	16	11319398		IGR	-0,27	9,08E-09	8,04E-07
cg27378899	1	62696669		IGR	-0,253	9,11E-09	8,06E-07
cg26755038	22	18264535		IGR	-0,284	9,11E-09	8,06E-07
cg08657492	7	27170832	HOXA4	TSS1500	0,201	9,13E-09	8,08E-07
cg06715204	3	171594651	TMEM212-AS	Body	-0,244	9,14E-09	8,08E-07
cg12023979	12	106467048	NUAK1	Body	-0,206	9,14E-09	8,08E-07
cg19783560	15	65655740	IGDCC3	Body	-0,244	9,14E-09	8,08E-07
cg21870038	17	33390736	RFFL	1stExon	-0,212	9,15E-09	8,09E-07
cg25729445	6	29595347	GABBR1	Body	0,21	9,18E-09	8,10E-07
cg20447966	8	41009724		IGR	-0,233	9,19E-09	8,10E-07
cg19646503	9	133227712	HMCN2	Body	-0,325	9,19E-09	8,10E-07
cg05739254	22	24916084	UPB1	Body	-0,224	9,19E-09	8,11E-07
cg19018034	3	188057596	LPP	5'UTR	-0,214	9,20E-09	8,11E-07
cg11730023	10	35636096	CCNY	Body	0,255	9,21E-09	8,12E-07
cg00928122	6	149057979		IGR	-0,277	9,22E-09	8,12E-07
cg10071009	9	19229618	DENND4C	TSS1500	0,244	9,22E-09	8,12E-07
cg14946295	18	74701280	MBP	Body	-0,325	9,22E-09	8,12E-07
cg16328549	20	50041045	NFATC2	Body	-0,293	9,22E-09	8,12E-07
cg23105877	9	136542466	SARDH	Body	-0,272	9,23E-09	8,13E-07
cg05312882	6	43114278	PTK7	Body	-0,255	9,23E-09	8,13E-07
cg25211066	9	136726942	VAV2	Body	-0,267	9,23E-09	8,13E-07
cg05933218	17	48151298	ITGA3	ExonBnd	-0,274	9,24E-09	8,13E-07
cg10903525	5	156967339	ADAM19	Body	-0,231	9,25E-09	8,14E-07

cg04747693	16	88111362		IGR	0,287	9,25E-09	8,14E-07
cg08314949	17	78851213	RPTOR	Body	-0,36	9,26E-09	8,14E-07
cg00894216	9	123688885	TRAF1	5'UTR	-0,34	9,27E-09	8,14E-07
cg06974428	10	22972299	PIP4K2A	Body	-0,327	9,28E-09	8,15E-07
cg22065614	4	157997360	GLRB	1stExon	0,206	9,30E-09	8,15E-07
cg13028554	19	36233893	U2AF1L4	Body	-0,248	9,34E-09	8,19E-07
cg11315621	17	57829517	VMP1	Body	-0,234	9,34E-09	8,19E-07
cg16935597	17	76129099	TMC8	Body	-0,23	9,34E-09	8,19E-07
cg22347637	2	40467425	SLC8A1-AS1	Body	0,237	9,35E-09	8,19E-07
cg07351322	7	104738380	MLL5	Body	0,282	9,36E-09	8,19E-07
cg23418857	15	42309323	PLA2G4E	Body	-0,209	9,35E-09	8,19E-07
cg14316278	7	2089966	MAD1L1	Body	-0,228	9,36E-09	8,19E-07
cg26388816	12	570155	B4GALNT3	Body	0,224	9,36E-09	8,19E-07
cg22443330	15	31196599	MTMR15	5'UTR	0,2	9,37E-09	8,20E-07
cg12078154	17	78851262	RPTOR	Body	-0,327	9,37E-09	8,20E-07
cg02325250	5	131409289	CSF2	TSS200	-0,252	9,40E-09	8,21E-07
cg12035123	3	111261303	CD96	Body	-0,253	9,43E-09	8,23E-07
cg00049674	3	123058535	ADCY5	Body	-0,212	9,48E-09	8,27E-07
cg07506970	21	36522256		IGR	-0,206	9,49E-09	8,28E-07
cg15893925	6	134758804	LINC01010	TSS200	-0,358	9,50E-09	8,28E-07
cg23149770	5	171393566	FBXW11	Body	0,213	9,50E-09	8,28E-07
cg25914433	22	37986622		IGR	-0,229	9,51E-09	8,29E-07
cg10734415	5	132634425	FSTL4	Body	-0,206	9,53E-09	8,30E-07
cg14206684	5	32004817	PDZD2	Body	-0,256	9,57E-09	8,32E-07
cg12743068	2	71779926	DYSF	Body	-0,219	9,57E-09	8,32E-07
cg06410824	18	74701492	MBP	Body	-0,212	9,58E-09	8,32E-07
cg12134633	15	32934004	SCG5	5'UTR	0,28	9,60E-09	8,34E-07
cg18422094	9	93926097		IGR	-0,26	9,61E-09	8,35E-07
cg14620572	9	127215669	GPR144	Body	0,225	9,61E-09	8,35E-07
cg19230867	3	42103367		IGR	0,223	9,62E-09	8,35E-07
cg13325133	17	56616586	sept-04	ExonBnd	0,233	9,62E-09	8,35E-07
cg04463187	22	23518694		IGR	-0,213	9,62E-09	8,35E-07
cg18247179	22	19436867	C22orf39	TSS1500	-0,249	9,65E-09	8,37E-07
cg05472466	15	80870768	ARNT2	Body	-0,222	9,66E-09	8,38E-07
cg13925011	1	111216387	KCNA3	1stExon	-0,253	9,68E-09	8,39E-07
cg03436397	7	102067162		IGR	-0,389	9,68E-09	8,39E-07
cg10583769	15	71481461	THSD4-AS1	TSS200	-0,218	9,68E-09	8,39E-07
cg16469046	2	99062999	INPP4A	5'UTR	-0,233	9,69E-09	8,40E-07
cg19182267	3	57132190	IL17RD	Body	-0,237	9,70E-09	8,40E-07
cg12419864	5	31800978	PDZD2	Body	-0,296	9,70E-09	8,40E-07
cg15933120	14	102102737		IGR	-0,269	9,70E-09	8,40E-07
cg23629809	11	11411778	GALNT18	Body	-0,222	9,71E-09	8,40E-07
cg00957585	15	86189830	AKAP13	Body	-0,316	9,72E-09	8,41E-07
cg14091103	3	187870929	LPP	TSS1500	0,234	9,74E-09	8,43E-07
cg15391057	3	38017188	CTDSPL	ExonBnd	-0,25	9,76E-09	8,44E-07
cg13182163	1	219616112		IGR	-0,288	9,77E-09	8,44E-07
cg13257436	8	145726034	PPP1R16A	Body	0,21	9,77E-09	8,44E-07
cg20310170	10	72698709		IGR	-0,242	9,78E-09	8,45E-07
cg06667434	2	74191125	DGUOK-AS1	Body	-0,29	9,79E-09	8,45E-07
cg27512858	10	11222525	CELF2	Body	-0,358	9,80E-09	8,46E-07

cg06008378	5	176925570	PDLIM7	TSS1500	0,203	9,80E-09	8,46E-07
cg09695403	22	18920114	PRODH	Body	-0,25	9,81E-09	8,46E-07
cg19871597	13	59592287		IGR	-0,263	9,82E-09	8,46E-07
cg05490616	19	12111658		IGR	-0,226	9,81E-09	8,46E-07
cg05777037	9	130448586	STXBP1	3'UTR	-0,228	9,84E-09	8,48E-07
cg25045228	10	14146672	FRMD4A	Body	-0,205	9,84E-09	8,48E-07
cg01557411	1	28587248	SESN2	Body	0,212	9,85E-09	8,49E-07
cg06784574	16	46745827	MYLK3	Body	-0,215	9,86E-09	8,49E-07
cg26247345	3	114175762	LOC10192975	Body	-0,27	9,87E-09	8,49E-07
cg18640536	5	126211060	MARCH3	Body	0,215	9,87E-09	8,50E-07
cg20754980	1	227154520	ADCK3	Body	0,216	9,89E-09	8,51E-07
cg00078456	1	1564422	MIB2	Body	-0,281	9,90E-09	8,51E-07
cg06476693	11	130283454	ADAMTS8	Body	-0,242	9,92E-09	8,53E-07
cg12103951	10	88162314		IGR	0,264	9,92E-09	8,53E-07
cg08052751	18	42366943	SETBP1	Body	-0,332	9,94E-09	8,54E-07
cg15370320	14	61802813	PRKCH	Body	-0,265	9,95E-09	8,55E-07
cg19377673	5	40480645		IGR	-0,254	9,97E-09	8,56E-07
cg09009517	15	69580779		IGR	-0,256	9,98E-09	8,56E-07
cg13151664	3	123813232	KALRN	TSS1500	-0,283	1,00E-08	8,57E-07
cg11472725	6	138938931		IGR	-0,231	1,00E-08	8,58E-07
cg10975391	1	201632478	NAV1	Body	-0,229	1,00E-08	8,58E-07
cg25523477	17	79410702	BAHCC1	Body	-0,239	1,00E-08	8,59E-07
cg23196421	9	130541452	SH2D3C	TSS1500	-0,201	1,00E-08	8,60E-07
cg09042262	8	126711187		IGR	-0,293	1,00E-08	8,60E-07
cg19054358	5	78219426	ARSB	Body	-0,222	1,01E-08	8,60E-07
cg11755558	4	122103800	TNIP3	Body	-0,234	1,01E-08	8,61E-07
cg26227465	12	68553577	IFNG	TSS200	-0,219	1,01E-08	8,61E-07
cg15889621	2	85521810	TCF7L1	Body	-0,32	1,01E-08	8,62E-07
cg04891708	6	25027496	FAM65B	Body	-0,211	1,01E-08	8,62E-07
cg21684179	1	110576933	FAM40A	TSS1500	-0,249	1,01E-08	8,63E-07
cg02593099	1	27011393		IGR	-0,303	1,01E-08	8,63E-07
cg07353116	8	29028701	KIF13B	Body	-0,385	1,01E-08	8,64E-07
cg07480639	1	88330500		IGR	-0,24	1,01E-08	8,64E-07
cg04885759	3	49170498	LAMB2	1stExon	0,237	1,01E-08	8,64E-07
cg26213453	15	89455860	MFGE8	Body	0,219	1,01E-08	8,65E-07
cg16892729	22	29372982	ZNRF3	Body	-0,248	1,02E-08	8,66E-07
cg14094689	1	55892562		IGR	-0,267	1,02E-08	8,66E-07
cg02920848	5	149773839	TCOF1	Body	-0,245	1,02E-08	8,67E-07
cg20090731	2	177001667	HOXD-AS2	Body	0,236	1,02E-08	8,69E-07
cg01100915	18	60848285	BCL2	Body	-0,268	1,02E-08	8,69E-07
cg21048162	14	75988356	BATF	TSS1500	-0,208	1,02E-08	8,70E-07
cg22624568	20	36271857		IGR	-0,265	1,03E-08	8,71E-07
cg12864235	5	27038782	CDH9	TSS200	0,221	1,03E-08	8,72E-07
cg07839457	16	57023022	NLRC5	TSS1500	-0,299	1,03E-08	8,72E-07
cg17311122	5	156167820	SGCD	Body	-0,215	1,03E-08	8,73E-07
cg18279094	1	63790044	FOXD3	1stExon	0,268	1,03E-08	8,75E-07
cg04479264	19	41831759		IGR	0,207	1,03E-08	8,76E-07
cg16134718	2	204973770		IGR	-0,22	1,03E-08	8,76E-07
cg06824978	4	14692240	LINC00504	Body	-0,225	1,03E-08	8,76E-07
cg18577231	7	127743667		IGR	0,264	1,03E-08	8,76E-07

cg23569968	11	62185488	SCGB1A1	TSS1500	-0,219	1,03E-08	8,76E-07
cg13456688	9	124965049	LHX6	3'UTR	-0,216	1,04E-08	8,78E-07
cg01329245	16	79303284		IGR	-0,277	1,04E-08	8,78E-07
cg21506638	1	8263912		IGR	-0,281	1,04E-08	8,79E-07
cg14608581	2	70122240	SNRNP27	Body	0,288	1,04E-08	8,79E-07
cg26868829	2	40511921	SLC8A1	Body	0,246	1,04E-08	8,80E-07
cg13066930	18	77369529		IGR	-0,268	1,04E-08	8,80E-07
cg00947782	6	30039142	RNF39	Body	0,321	1,05E-08	8,82E-07
cg07995927	2	161995135	TANK	5'UTR	-0,341	1,05E-08	8,82E-07
cg13178361	1	162532502	UAP1	5'UTR	0,207	1,05E-08	8,83E-07
cg19896563	3	21371992		IGR	-0,224	1,05E-08	8,83E-07
cg10801719	3	183245249	KLHL6	Body	-0,375	1,05E-08	8,83E-07
cg18654674	7	92794982		IGR	-0,262	1,05E-08	8,83E-07
cg23561220	8	82019431	PAG1	5'UTR	-0,212	1,05E-08	8,84E-07
cg02399746	20	35899171		IGR	-0,233	1,05E-08	8,85E-07
cg15284257	8	12802721	KIAA1456	TSS1500	-0,212	1,05E-08	8,86E-07
cg00103543	2	45225295		IGR	-0,238	1,05E-08	8,86E-07
cg02109167	4	41187789	APBB2	5'UTR	-0,239	1,05E-08	8,86E-07
cg00299719	3	40964878		IGR	-0,252	1,06E-08	8,87E-07
cg02615376	5	141755319		IGR	-0,212	1,05E-08	8,87E-07
cg06631916	18	60826611	BCL2	Body	-0,37	1,06E-08	8,87E-07
cg05302350	4	141013724	MAML3	Body	0,225	1,06E-08	8,88E-07
cg17298516	1	94131533	BCAR3	Body	-0,286	1,06E-08	8,88E-07
cg07698025	2	130055329		IGR	-0,215	1,06E-08	8,88E-07
cg16496477	2	114606877	LOC1010600	Body	-0,241	1,06E-08	8,89E-07
cg16759787	14	85667733		IGR	-0,252	1,06E-08	8,89E-07
cg08437914	4	140651985	MGST2	3'UTR	-0,257	1,06E-08	8,90E-07
cg23190561	4	41621533	LIMCH1	Body	-0,212	1,06E-08	8,90E-07
cg19190762	13	44806055		IGR	-0,259	1,06E-08	8,90E-07
cg13322557	11	74173518	KCNE3	5'UTR	-0,263	1,06E-08	8,90E-07
cg10874168	1	44298113	ST3GAL3	Body	-0,273	1,07E-08	8,92E-07
cg16435343	13	106741468		IGR	-0,287	1,07E-08	8,92E-07
cg20994118	1	209780966	CAMK1G	Body	-0,282	1,07E-08	8,92E-07
cg10996413	18	45325666		IGR	-0,286	1,07E-08	8,93E-07
cg23780193	2	64926154		IGR	-0,383	1,07E-08	8,93E-07
cg04437881	17	2697740		IGR	-0,223	1,07E-08	8,93E-07
cg14970802	16	56848917	NUP93	Body	0,243	1,07E-08	8,94E-07
cg23130596	19	57178386	ZNF835	5'UTR	-0,213	1,07E-08	8,95E-07
cg04327143	5	135226781		IGR	-0,325	1,07E-08	8,95E-07
cg13184736	1	68299409	GNG12	TSS1500	0,235	1,07E-08	8,95E-07
cg06791592	6	32823292	PSMB9	Body	-0,336	1,07E-08	8,95E-07
cg00870161	12	12584171	LOH12CR1	Body	-0,217	1,07E-08	8,95E-07
cg20549165	9	79634539	FOXB2	TSS200	0,213	1,07E-08	8,95E-07
cg04100284	22	39486356		IGR	-0,232	1,07E-08	8,95E-07
cg23572512	3	185435540	IGF2BP2-AS1	Body	-0,216	1,07E-08	8,96E-07
cg00187889	6	159463103	TAGAP	5'UTR	-0,284	1,07E-08	8,96E-07
cg03531140	13	74644456	KLF12	5'UTR	-0,259	1,07E-08	8,96E-07
cg12626166	20	50332000	ATP9A	Body	-0,244	1,07E-08	8,96E-07
cg00217420	16	88771527	CTU2	TSS1500	-0,292	1,08E-08	8,96E-07
cg04171808	11	35188437	CD44	Body	-0,345	1,08E-08	8,97E-07

cg09137165	20	56456874		IGR	-0,223	1,08E-08	8,98E-07
cg10859442	11	73716367	UCP3	Body	-0,249	1,08E-08	8,98E-07
cg14440203	1	28836475	SNHG3	Body	-0,231	1,08E-08	9,00E-07
cg17163387	7	132342320	FLJ40288	Body	-0,238	1,08E-08	9,00E-07
cg16324694	8	101372117		IGR	-0,265	1,08E-08	9,00E-07
cg14792357	1	147041822	BCL9	5'UTR	-0,238	1,08E-08	9,01E-07
cg14148016	9	138874126		IGR	-0,233	1,08E-08	9,01E-07
cg01470330	3	153970918	ARHGEF26	ExonBnd	0,258	1,08E-08	9,01E-07
cg08452292	10	126710009	CTBP2	Body	-0,234	1,08E-08	9,01E-07
cg08680218	14	89743161	FOXN3	Body	-0,243	1,08E-08	9,02E-07
cg11272244	9	111616366		IGR	-0,217	1,09E-08	9,02E-07
cg01165817	13	101086489	PCCA	Body	-0,237	1,09E-08	9,02E-07
cg08530036	1	19496406	UBR4	Body	-0,308	1,09E-08	9,03E-07
cg13178170	1	92270513	TGFBR3	Body	-0,267	1,09E-08	9,04E-07
cg23712018	6	30039132	RNF39	Body	0,228	1,09E-08	9,05E-07
cg23475018	3	124277638	KALRN	Body	-0,225	1,09E-08	9,05E-07
cg13825926	2	197099526	HECW2	Body	-0,302	1,09E-08	9,06E-07
cg21444116	11	126020475		IGR	-0,3	1,09E-08	9,06E-07
cg23672659	6	30648020	KIAA1949	Body	-0,203	1,09E-08	9,06E-07
cg25737087	8	131373613	ASAP1	Body	-0,271	1,09E-08	9,06E-07
cg02493798	17	6899577	ALOX12	Body	0,234	1,10E-08	9,07E-07
cg18168435	22	36552934	APOL3	Body	-0,205	1,10E-08	9,07E-07
cg05894882	3	32613083	DYNC1LI1	TSS1500	0,229	1,10E-08	9,09E-07
cg18195006	22	37317797	CSF2RB	5'UTR	-0,231	1,10E-08	9,09E-07
cg12362977	1	23928846		IGR	-0,281	1,10E-08	9,09E-07
cg26838015	4	78072602		IGR	-0,243	1,10E-08	9,10E-07
cg01553231	5	141072866		IGR	-0,257	1,10E-08	9,10E-07
cg01762827	16	69141442	HAS3	5'UTR	0,241	1,11E-08	9,11E-07
cg06202778	4	57514827	HOPX	3'UTR	-0,207	1,11E-08	9,12E-07
cg22039960	2	218416812	DIRC3	Body	-0,237	1,11E-08	9,13E-07
cg24424889	22	39494175	APOBEC3H	5'UTR	-0,286	1,11E-08	9,13E-07
cg20991819	1	218617769	TGFB2	3'UTR	-0,227	1,11E-08	9,16E-07
cg06757498	5	137193140		IGR	0,234	1,11E-08	9,16E-07
cg12172057	7	47621687		IGR	0,278	1,11E-08	9,16E-07
cg06022561	2	242702553	D2HGDH	Body	-0,285	1,11E-08	9,16E-07
cg13531460	2	231790813	GPR55	TSS1500	-0,266	1,11E-08	9,16E-07
cg25191235	12	125772262		IGR	-0,332	1,12E-08	9,17E-07
cg19248264	17	1774149	RPA1	Body	-0,213	1,12E-08	9,18E-07
cg26745060	13	50741803	DLEU1	Body	-0,244	1,12E-08	9,18E-07
cg20368231	17	75269862		IGR	-0,201	1,12E-08	9,18E-07
cg12585232	10	44918789		IGR	-0,262	1,12E-08	9,18E-07
cg07476426	19	46825002	HIF3A	Body	-0,257	1,12E-08	9,19E-07
cg22051760	7	50358075	IKZF1	5'UTR	-0,232	1,12E-08	9,20E-07
cg17972058	1	159796231	SLAMF8	TSS1500	-0,236	1,12E-08	9,22E-07
cg05853441	8	8998915	PPP1R3B	Body	0,307	1,13E-08	9,23E-07
cg07633072	2	218879105		IGR	-0,253	1,13E-08	9,23E-07
cg07728206	14	50809390	CDKL1	Body	0,201	1,13E-08	9,25E-07
cg12023331	2	20675440		IGR	-0,241	1,13E-08	9,26E-07
cg26170623	4	38511215	LINC01258	Body	-0,298	1,13E-08	9,26E-07
cg14957089	11	331092		IGR	-0,34	1,13E-08	9,27E-07

cg26720913	5	142814934	NR3C1	1stExon	-0,39	1,13E-08	9,27E-07
cg16232058	11	47965571		IGR	0,233	1,14E-08	9,30E-07
cg25383425	21	40259710	LOC400867	Body	-0,213	1,14E-08	9,30E-07
cg24837370	2	5833774	SOX11	1stExon	0,208	1,14E-08	9,30E-07
cg00190294	3	52873158	TMEM110-M	Body	-0,263	1,14E-08	9,30E-07
cg06763464	5	54164486		IGR	-0,284	1,14E-08	9,31E-07
cg03388043	17	80084554	CCDC57	Body	-0,261	1,14E-08	9,31E-07
cg03826704	12	12162168		IGR	-0,328	1,15E-08	9,34E-07
cg07418159	19	13056052	RAD23A	TSS1500	-0,266	1,15E-08	9,35E-07
cg06987246	7	6659785	ZNF853	Body	-0,316	1,15E-08	9,35E-07
cg17953764	4	48492845	ZAR1	1stExon	0,292	1,15E-08	9,35E-07
cg10850293	15	99780127	TTC23	5'UTR	-0,395	1,15E-08	9,36E-07
cg25172521	18	20818349	CABLES1	Body	0,218	1,15E-08	9,37E-07
cg27608964	13	80237459		IGR	0,209	1,15E-08	9,38E-07
cg12274324	9	125583959	PDCL	Body	0,212	1,15E-08	9,38E-07
cg03187166	3	107807805	CD47	Body	-0,223	1,16E-08	9,39E-07
cg03012235	8	129009289	PVT1	Body	-0,294	1,16E-08	9,39E-07
cg01254505	19	17516470	BST2	TSS200	-0,213	1,16E-08	9,39E-07
cg18728493	3	48264021	CAMP	TSS1500	-0,231	1,16E-08	9,41E-07
cg00521221	8	128319661	CASC21	Body	-0,245	1,16E-08	9,41E-07
cg20262857	8	81718227	ZNF704	Body	-0,262	1,16E-08	9,41E-07
cg14875171	2	50574196	NRXN1	Body	0,231	1,16E-08	9,44E-07
cg16952026	6	106968674	AIM1	Body	-0,325	1,16E-08	9,44E-07
cg20073039	16	8774580	ABAT	5'UTR	-0,241	1,17E-08	9,44E-07
cg08154998	1	53540497	PODN	Body	-0,2	1,17E-08	9,44E-07
cg15892872	20	56412647		IGR	-0,271	1,17E-08	9,44E-07
cg00678892	2	53543334		IGR	-0,209	1,17E-08	9,46E-07
cg13938098	6	112575966	LAMA4	TSS200	0,226	1,17E-08	9,46E-07
cg03764226	20	30942880		IGR	-0,227	1,17E-08	9,46E-07
cg16017696	4	186461002		IGR	-0,243	1,17E-08	9,47E-07
cg26547750	2	152495247	NEB	Body	-0,208	1,17E-08	9,48E-07
cg01499518	7	75890022	SRRM3	Body	0,219	1,17E-08	9,48E-07
cg20275771	1	201981574	ELF3	ExonBnd	-0,264	1,18E-08	9,49E-07
cg03690469	9	137544870	COL5A1	Body	-0,204	1,18E-08	9,49E-07
cg05931439	14	23305957	MMP14	5'UTR	0,222	1,18E-08	9,49E-07
cg23171055	6	134786314	LOC10192823	Body	-0,2	1,18E-08	9,52E-07
cg00410951	12	123604538		IGR	-0,272	1,18E-08	9,52E-07
cg03425163	13	24671500	SPATA13	Body	-0,259	1,18E-08	9,52E-07
cg03600588	1	32127319	COL16A1	ExonBnd	-0,204	1,18E-08	9,52E-07
cg07510596	4	38511195	LINC01258	Body	-0,216	1,19E-08	9,56E-07
cg21534006	19	30261677		IGR	-0,242	1,19E-08	9,57E-07
cg12693548	7	101136665	COL26A1	Body	-0,203	1,19E-08	9,57E-07
cg10626982	14	32410938		IGR	-0,236	1,19E-08	9,57E-07
cg12097222	8	16885000	EFHA2	1stExon	0,218	1,19E-08	9,59E-07
cg24875463	10	34864942	PARD3	Body	-0,2	1,20E-08	9,61E-07
cg15551881	9	123688715	TRAF1	5'UTR	-0,327	1,20E-08	9,61E-07
cg12372610	1	211694647		IGR	-0,227	1,20E-08	9,61E-07
cg26471580	11	7556080	PPFIBP2	5'UTR	-0,201	1,20E-08	9,62E-07
cg08652441	17	40489513	STAT3	Body	-0,324	1,20E-08	9,62E-07
cg09584785	1	162333354	NOS1AP	Body	-0,281	1,20E-08	9,62E-07

cg17007693	18	12041356		IGR	-0,222	1,20E-08	9,63E-07
cg24645812	3	18153374	LOC339862	Body	-0,254	1,20E-08	9,64E-07
cg25143652	20	62168670	PTK6	1stExon	-0,242	1,20E-08	9,64E-07
cg00038446	1	172635668	FASLG	3'UTR	-0,228	1,20E-08	9,64E-07
cg13140220	9	110514313		IGR	-0,359	1,20E-08	9,64E-07
cg04191678	1	171153458	FMO2	TSS1500	-0,229	1,21E-08	9,65E-07
cg16864385	11	8108831	TUB	Body	-0,217	1,21E-08	9,66E-07
cg05299718	20	49528511	ADNP	5'UTR	-0,26	1,21E-08	9,66E-07
cg09476130	1	159870086	CCDC19	TSS200	0,242	1,21E-08	9,66E-07
cg04484798	14	105419905	AHNAK2	Body	-0,2	1,21E-08	9,66E-07
cg18122958	9	98583167	LINC00476	Body	-0,212	1,21E-08	9,66E-07
cg12999267	12	94376970		IGR	-0,273	1,21E-08	9,66E-07
cg22432250	1	218661554	MIR548F3	Body	-0,22	1,21E-08	9,66E-07
cg27353963	5	156452438		IGR	-0,306	1,21E-08	9,66E-07
cg20869070	10	74489461	MCU	Body	-0,221	1,21E-08	9,66E-07
cg06100161	15	60987894	RORA	Body	-0,335	1,21E-08	9,66E-07
cg04597711	1	200022291	NR5A2	Body	-0,201	1,21E-08	9,67E-07
cg21551745	18	46548378		IGR	-0,266	1,21E-08	9,68E-07
cg15626350	6	152130207	ESR1	Body	0,243	1,22E-08	9,71E-07
cg22292345	3	47051533	LOC10012935	TSS1500	0,231	1,22E-08	9,71E-07
cg08441133	1	201982957	ELF3	ExonBnd	-0,203	1,22E-08	9,73E-07
cg06605984	5	138210906	LRRTM2	1stExon	0,219	1,22E-08	9,73E-07
cg03029723	16	18799331	RPS15A	Body	-0,242	1,23E-08	9,76E-07
cg07717331	20	49663138		IGR	-0,203	1,23E-08	9,77E-07
cg06633978	16	75353806	CFDP1	Body	-0,355	1,23E-08	9,77E-07
cg13917925	3	35701161	ARPP21	5'UTR	-0,239	1,23E-08	9,78E-07
cg11226198	6	161920107	PARK2	Body	-0,226	1,23E-08	9,79E-07
cg10174466	8	11739341		IGR	-0,246	1,23E-08	9,79E-07
cg04682135	3	157260764	C3orf55	TSS1500	0,215	1,23E-08	9,79E-07
cg05215884	15	58670807		IGR	-0,223	1,23E-08	9,79E-07
cg17503977	2	160473461	LOC643072	Body	0,275	1,24E-08	9,81E-07
cg14356799	6	33044345	HLA-DPB1	Body	-0,21	1,24E-08	9,81E-07
cg25130912	1	201982886	ELF3	Body	-0,224	1,24E-08	9,81E-07
cg10250335	8	80745965	LOC10192704	Body	-0,234	1,24E-08	9,82E-07
cg00761225	13	25851201	MTMR6	Body	-0,231	1,24E-08	9,82E-07
cg03616532	10	79583991	DLG5	Body	-0,242	1,24E-08	9,82E-07
cg02298788	3	69337696	FRMD4B	Body	-0,262	1,24E-08	9,82E-07
cg03252361	14	21493853	NDRG2	5'UTR	0,201	1,24E-08	9,82E-07
cg10742523	4	55650393		IGR	0,242	1,24E-08	9,83E-07
cg24781296	9	112320102		IGR	-0,219	1,24E-08	9,83E-07
cg20440638	10	33415933		IGR	-0,24	1,24E-08	9,83E-07
cg07465344	17	4849412	PFN1	Body	-0,256	1,24E-08	9,83E-07
cg22721334	19	58609618	ZSCAN18	1stExon	0,254	1,24E-08	9,83E-07
cg12909619	2	136806951		IGR	-0,226	1,24E-08	9,84E-07
cg02723558	11	27536073	BDNFOS	Body	0,277	1,25E-08	9,85E-07
cg14630001	3	66633408		IGR	-0,227	1,25E-08	9,86E-07
cg09596131	2	19986567		IGR	-0,292	1,25E-08	9,87E-07
cg12399197	15	71667720	THSD4	Body	-0,25	1,25E-08	9,87E-07
cg08282589	1	217794428	GPATCH2	Body	-0,23	1,25E-08	9,88E-07
cg06560547	10	32707038		IGR	0,229	1,25E-08	9,88E-07

cg26422488	5	16935950	MYO10	1stExon	0,236	1,25E-08	9,89E-07
cg10075819	2	109229337	LIMS1	5'UTR	-0,267	1,25E-08	9,89E-07
cg07904045	3	8782064	CAV3	Body	-0,288	1,25E-08	9,90E-07
cg16038030	6	106589908		IGR	-0,235	1,26E-08	9,90E-07
cg07474842	6	136915088	MAP3K5	Body	-0,252	1,26E-08	9,90E-07
cg07388313	18	72654549	ZNF407	Body	-0,268	1,26E-08	9,93E-07
cg20910519	9	129854630	ANGPTL2	Body	-0,221	1,26E-08	9,93E-07
cg23365999	1	198460992		IGR	0,299	1,26E-08	9,93E-07
cg01309328	6	32811253	PSMB8	Body	-0,244	1,26E-08	9,94E-07
cg06726019	10	63642670		IGR	-0,245	1,26E-08	9,94E-07
cg02456062	17	37912327		IGR	-0,256	1,26E-08	9,94E-07
cg17557530	9	90193634	DAPK1	Body	-0,29	1,26E-08	9,94E-07
cg06450563	15	80475159	FAH	Body	0,236	1,26E-08	9,95E-07
cg01293725	11	76816834	CAPN5	Body	-0,245	1,27E-08	9,95E-07
cg25608041	4	37891834	TBC1D1	TSS1500	0,228	1,27E-08	9,96E-07
cg00012362	1	153599487	S100A13	5'UTR	0,235	1,27E-08	9,96E-07
cg01382864	2	220174251	PTPRN	TSS200	0,218	1,27E-08	9,96E-07
cg10167235	14	100807646	WARS	Body	-0,256	1,27E-08	9,97E-07
cg16730908	18	43652592	PSTPIP2	TSS1500	0,212	1,27E-08	9,97E-07
cg17341568	2	216486163	LINC00607	Body	-0,317	1,27E-08	9,99E-07
cg04505972	2	173508040		IGR	-0,228	1,27E-08	9,99E-07
cg21184074	15	76473995	LOC1019294[illegible]	TSS200	-0,228	1,28E-08	1,00E-06
cg00898920	2	241504756		IGR	-0,318	1,28E-08	1,00E-06
cg01704474	11	504918	RNH1	5'UTR	0,253	1,28E-08	1,00E-06
cg12188699	8	126342991	NSMCE2	Body	-0,23	1,28E-08	1,00E-06
cg04598281	5	172057647		IGR	-0,201	1,28E-08	1,00E-06
cg18477949	15	69760994		IGR	-0,238	1,28E-08	1,00E-06
cg18339303	7	73922315	GTF2IRD1	5'UTR	-0,211	1,28E-08	1,00E-06
cg01413354	9	136017755	RALGDS	Body	-0,217	1,28E-08	1,00E-06
cg11612354	5	157023724		IGR	-0,324	1,28E-08	1,00E-06
cg09758805	7	54870727	LOC1009966[illegible]	Body	-0,31	1,29E-08	1,01E-06
cg02663352	11	35167041	CD44	Body	-0,245	1,29E-08	1,01E-06
cg23851932	11	64627633	EHD1	Body	-0,219	1,29E-08	1,01E-06
cg18850465	16	50718807		IGR	-0,357	1,29E-08	1,01E-06
cg20327324	4	24590014		IGR	0,269	1,29E-08	1,01E-06
cg12253732	6	41030303	APOBEC2	3'UTR	0,217	1,29E-08	1,01E-06
cg26079312	4	139269254	LINC00499	Body	-0,215	1,29E-08	1,01E-06
cg14784010	5	169083252	DOCK2	Body	-0,231	1,30E-08	1,01E-06
cg10529845	16	49522337		IGR	-0,23	1,29E-08	1,01E-06
cg13109155	2	204735897	CTLA4	Body	-0,311	1,30E-08	1,01E-06
cg16888658	2	25142117	ADCY3	TSS200	-0,257	1,30E-08	1,01E-06
cg09753321	16	79312619		IGR	-0,382	1,30E-08	1,01E-06
cg25231123	1	32120171	COL16A1	Body	-0,22	1,30E-08	1,01E-06
cg00838934	18	8720328	MTCL1	ExonBnd	-0,201	1,30E-08	1,01E-06
cg08304353	15	39921659	FSIP1	Body	-0,247	1,30E-08	1,02E-06
cg27192248	15	65285669		IGR	-0,313	1,30E-08	1,02E-06
cg10295325	11	66814384	SYT12	Body	-0,2	1,31E-08	1,02E-06
cg11150581	2	189735264		IGR	-0,279	1,31E-08	1,02E-06
cg05970811	12	64237202	SRGAP1	TSS1500	0,236	1,31E-08	1,02E-06
cg08159978	5	951967		IGR	-0,219	1,31E-08	1,02E-06

cg05368740	19	36246882	HSPB6	Body	0,21	1,31E-08	1,02E-06
cg04524735	17	77108934	RBFOX3	Body	-0,25	1,31E-08	1,02E-06
cg19804889	3	29691496	RBMS3	Body	-0,342	1,32E-08	1,02E-06
cg04815890	16	84628855	COTL1	Body	-0,327	1,32E-08	1,02E-06
cg11293868	21	33535444		IGR	-0,248	1,32E-08	1,02E-06
cg13647725	11	69241093		IGR	-0,201	1,32E-08	1,02E-06
cg11606195	12	124145110	GTF2H3	3'UTR	-0,314	1,32E-08	1,02E-06
cg18011672	2	190522601		IGR	-0,216	1,32E-08	1,02E-06
cg15929078	6	33267996	TAPBP	3'UTR	0,203	1,32E-08	1,02E-06
cg12318500	2	135428158	TMEM163	Body	-0,214	1,32E-08	1,02E-06
cg06953756	22	42427436		IGR	-0,323	1,32E-08	1,02E-06
cg18723737	8	81489559		IGR	0,262	1,32E-08	1,03E-06
cg05284050	10	80665368		IGR	-0,208	1,32E-08	1,03E-06
cg26632446	11	18302340	HPS5	Body	-0,307	1,32E-08	1,03E-06
cg15167811	9	115094058	MIR3134	Body	-0,294	1,33E-08	1,03E-06
cg09839120	7	150365999		IGR	-0,223	1,33E-08	1,03E-06
cg13190942	10	73203851	CDH23	Body	-0,21	1,33E-08	1,03E-06
cg19465320	17	40426890	STAT5B	5'UTR	-0,264	1,33E-08	1,03E-06
cg14621698	1	109935710	SORT1	1stExon	0,269	1,34E-08	1,03E-06
cg03833948	12	21678044	C12orf39	TSS1500	-0,224	1,34E-08	1,03E-06
cg13837026	5	148784173		IGR	-0,221	1,34E-08	1,03E-06
cg17148976	2	131594925		IGR	0,222	1,34E-08	1,04E-06
cg09687907	12	57915636	MBD6	TSS1500	0,211	1,35E-08	1,04E-06
cg05161803	6	12393258	RNU6-48P	TSS1500	-0,262	1,35E-08	1,04E-06
cg15352013	16	49497733		IGR	-0,242	1,35E-08	1,04E-06
cg13553829	4	122200097		IGR	-0,223	1,35E-08	1,04E-06
cg20306087	4	5044013		IGR	-0,23	1,35E-08	1,04E-06
cg25280810	19	16480643	EPS15L1	Body	-0,245	1,35E-08	1,04E-06
cg03364444	13	99176134	STK24	Body	-0,268	1,35E-08	1,04E-06
cg27054084	5	122851781	CSNK1G3	5'UTR	-0,21	1,35E-08	1,04E-06
cg13161865	13	24825312	SPATA13	Body	-0,284	1,35E-08	1,04E-06
cg17312754	13	106804496		IGR	-0,237	1,35E-08	1,04E-06
cg17592363	7	104930547	SRPK2	5'UTR	-0,218	1,35E-08	1,04E-06
cg11293097	6	14277467		IGR	-0,237	1,35E-08	1,04E-06
cg16464007	3	188002729	LPP	5'UTR	-0,259	1,36E-08	1,04E-06
cg04666122	10	127876309	ADAM12	Body	-0,219	1,36E-08	1,04E-06
cg26758857	22	36649135	APOL1	5'UTR	-0,247	1,36E-08	1,04E-06
cg27190410	8	101348639	RNF19A	TSS200	0,214	1,36E-08	1,04E-06
cg13795321	15	71398102		IGR	-0,26	1,36E-08	1,04E-06
cg19776366	17	79032662	BAIAP2	Body	-0,241	1,36E-08	1,04E-06
cg03903527	17	16956218	MPRIP	Body	0,202	1,36E-08	1,04E-06
cg11923631	21	45705721	AIRE	TSS200	0,239	1,36E-08	1,04E-06
cg19344545	21	45575573		IGR	-0,282	1,37E-08	1,05E-06
cg22762215	6	29521272		IGR	0,203	1,37E-08	1,05E-06
cg01479491	3	188059305	LPP	5'UTR	-0,204	1,37E-08	1,05E-06
cg13381984	7	127881344	LEP	1stExon	0,253	1,37E-08	1,05E-06
cg08063428	2	217664322		IGR	-0,208	1,37E-08	1,05E-06
cg20872930	21	40259397	LOC400867	Body	-0,283	1,37E-08	1,05E-06
cg01297984	8	22992397		IGR	-0,25	1,38E-08	1,05E-06
cg07137411	11	117395264	DSCAML1	Body	-0,203	1,38E-08	1,05E-06

cg26102687	11	130635926		IGR	-0,233	1,38E-08	1,05E-06
cg21510305	7	28522887	CREB5	5'UTR	-0,227	1,38E-08	1,05E-06
cg14874110	2	134778245		IGR	-0,252	1,38E-08	1,05E-06
cg17792666	10	84836338		IGR	-0,254	1,38E-08	1,05E-06
cg09791475	12	14993624	ART4	Body	-0,217	1,38E-08	1,05E-06
cg16671586	22	25464299	KIAA1671	Body	-0,293	1,38E-08	1,05E-06
cg09487449	19	47983663	KPTN	Body	0,217	1,39E-08	1,06E-06
cg22909322	3	71775267	EIF4E3	TSS1500	-0,28	1,39E-08	1,06E-06
cg12307197	2	72190769		IGR	-0,241	1,39E-08	1,06E-06
cg16373141	7	151492698	PRKAG2	5'UTR	-0,334	1,39E-08	1,06E-06
cg04907151	3	135684161	PPP2R3A	TSS1500	0,205	1,39E-08	1,06E-06
cg06516865	12	11883921	ETV6	Body	-0,248	1,40E-08	1,06E-06
cg27444225	15	74594705	CCDC33	Body	-0,243	1,40E-08	1,06E-06
cg22846767	5	156543653		IGR	-0,217	1,40E-08	1,06E-06
cg21151701	1	36738670	THRAP3	5'UTR	0,2	1,40E-08	1,06E-06
cg22814474	3	12512848		IGR	-0,238	1,41E-08	1,06E-06
cg10857558	4	90035421	TIGD2	1stExon	-0,272	1,41E-08	1,07E-06
cg13714691	4	18573144		IGR	-0,206	1,41E-08	1,07E-06
cg24136205	13	100624293	ZIC5	TSS200	0,22	1,41E-08	1,07E-06
cg25117875	12	14413581		IGR	0,211	1,41E-08	1,07E-06
cg17329115	20	62690171	TCEA2	5'UTR	-0,243	1,41E-08	1,07E-06
cg00624378	1	94158163	BCAR3	5'UTR	-0,24	1,41E-08	1,07E-06
cg12208064	12	52539620		IGR	-0,239	1,41E-08	1,07E-06
cg05685272	11	132511149	OPCML	Body	-0,288	1,42E-08	1,07E-06
cg11348478	18	67834126	RTTN	Body	-0,379	1,42E-08	1,07E-06
cg04137004	11	13140079		IGR	-0,252	1,42E-08	1,07E-06
cg21805118	12	122712153	DIABLO	TSS200	-0,285	1,42E-08	1,07E-06
cg14970311	18	77839582		IGR	-0,218	1,42E-08	1,07E-06
cg22831508	8	95556145	KIAA1429	Body	0,231	1,42E-08	1,07E-06
cg23051349	14	104094779	KLC1	TSS1500	0,214	1,42E-08	1,07E-06
cg26231768	8	75912188	CRISPLD1	TSS200	-0,323	1,42E-08	1,07E-06
cg04477628	6	137999653		IGR	-0,309	1,43E-08	1,07E-06
cg23460823	3	170444635		IGR	-0,276	1,43E-08	1,07E-06
cg12233654	8	145853541	ARHGAP39	5'UTR	-0,229	1,43E-08	1,07E-06
cg25223823	13	33593349	KL	Body	-0,256	1,43E-08	1,07E-06
cg23031196	12	46664096	SLC38A1	TSS1500	-0,3	1,43E-08	1,07E-06
cg17605007	2	20776435		IGR	-0,356	1,43E-08	1,07E-06
cg22012156	1	155916405		IGR	-0,2	1,43E-08	1,07E-06
cg20179674	21	35828181	KCNE1	TSS200	0,232	1,43E-08	1,08E-06
cg10462961	11	130493253		IGR	-0,246	1,43E-08	1,08E-06
cg17816219	6	90788825	BACH2	5'UTR	-0,21	1,43E-08	1,08E-06
cg06713314	1	201233340		IGR	-0,246	1,43E-08	1,08E-06
cg24732049	13	20994266	CRYL1	Body	-0,253	1,44E-08	1,08E-06
cg05093455	19	58907454	RNF225	TSS200	0,245	1,44E-08	1,08E-06
cg17357062	9	137810348	FCN1	TSS1500	-0,209	1,44E-08	1,08E-06
cg23925513	17	6923526		IGR	0,245	1,44E-08	1,08E-06
cg05324121	14	50988384	MAP4K5	Body	-0,305	1,44E-08	1,08E-06
cg19431670	12	51481817		IGR	-0,25	1,44E-08	1,08E-06
cg24553037	3	41137721		IGR	-0,234	1,44E-08	1,08E-06
cg10203740	1	204107231	ETNK2	Body	-0,223	1,45E-08	1,08E-06

cg20270835	2	61204501	PUS10	Body	-0,233	1,45E-08	1,09E-06
cg08613636	14	91271482	TTC7B	Body	-0,225	1,45E-08	1,09E-06
cg18029321	5	140230326	PCDHA6	Body	0,391	1,45E-08	1,09E-06
cg06937409	14	91281948	TTC7B	Body	0,229	1,45E-08	1,09E-06
cg21185662	12	45662797	ANO6	Body	0,211	1,45E-08	1,09E-06
cg00735574	9	137282509	RXRA	5'UTR	-0,301	1,45E-08	1,09E-06
cg03874089	1	14925172	KAZN	TSS200	0,252	1,46E-08	1,09E-06
cg18751141	7	27138173		IGR	0,253	1,46E-08	1,09E-06
cg15930380	1	67399983	MIER1	Body	-0,352	1,46E-08	1,09E-06
cg01132893	17	46712086		IGR	0,224	1,46E-08	1,09E-06
cg09659400	20	43744673	WFDC5	TSS1500	-0,246	1,46E-08	1,09E-06
cg25645302	16	67642367	CTCF	5'UTR	-0,238	1,46E-08	1,09E-06
cg11562727	1	236323243	GPR137B	Body	-0,235	1,46E-08	1,09E-06
cg17855595	1	249148077	ZNF692	Body	-0,288	1,46E-08	1,09E-06
cg11651615	2	85667381		IGR	-0,243	1,47E-08	1,09E-06
cg09649473	3	149071364		IGR	-0,243	1,47E-08	1,09E-06
cg22257386	6	139603902	TXLNB	Body	-0,231	1,46E-08	1,09E-06
cg14502847	9	18490009	ADAMTSL1	Body	-0,214	1,47E-08	1,09E-06
cg00521255	5	139726689	HBEGF	TSS1500	0,204	1,47E-08	1,09E-06
cg00017709	6	99638132		IGR	-0,214	1,47E-08	1,10E-06
cg16608498	5	158612132	RNF145	Body	-0,286	1,47E-08	1,10E-06
cg12433043	6	100619893		IGR	-0,238	1,47E-08	1,10E-06
cg14668271	8	72743243		IGR	-0,239	1,47E-08	1,10E-06
cg25497767	12	92528765	C12orf79	Body	-0,308	1,47E-08	1,10E-06
cg01305093	20	14937482	MACROD2	Body	-0,219	1,47E-08	1,10E-06
cg09390241	4	8174148		IGR	-0,271	1,47E-08	1,10E-06
cg23954655	13	99223562	STK24	Body	-0,201	1,48E-08	1,10E-06
cg25438961	14	54307520		IGR	-0,262	1,48E-08	1,10E-06
cg11593225	20	17742003		IGR	-0,23	1,48E-08	1,10E-06
cg18510160	2	30488615		IGR	-0,224	1,48E-08	1,10E-06
cg01272053	12	62650638		IGR	0,279	1,48E-08	1,10E-06
cg16126286	1	243646235	SDCCAG8	Body	0,221	1,48E-08	1,10E-06
cg11367948	9	90254311	DAPK1	Body	-0,224	1,48E-08	1,10E-06
cg17847474	16	78802628	WWOX	Body	-0,289	1,48E-08	1,10E-06
cg11786338	18	56809717	SEC11C	Body	-0,235	1,48E-08	1,10E-06
cg05100294	11	69200673		IGR	-0,206	1,49E-08	1,10E-06
cg04298242	5	133706139	UBE2B	TSS1500	-0,256	1,49E-08	1,10E-06
cg11745672	10	43900830	HNRNPF	5'UTR	-0,228	1,49E-08	1,10E-06
cg00927104	9	116764618	ZNF618	Body	-0,276	1,49E-08	1,10E-06
cg27280172	10	114741791	TCF7L2	Body	-0,253	1,49E-08	1,10E-06
cg13327664	15	77898183		IGR	-0,228	1,49E-08	1,10E-06
cg25478797	8	141064908	TRAPPC9	Body	-0,27	1,49E-08	1,10E-06
cg17837191	17	62318673	TEX2	5'UTR	0,208	1,49E-08	1,10E-06
cg13307058	6	134497542	SGK1	TSS1500	0,202	1,49E-08	1,10E-06
cg13107347	2	238494558	RAB17	Body	-0,243	1,50E-08	1,11E-06
cg26165146	12	27484656	ARNTL2	TSS1500	-0,29	1,50E-08	1,11E-06
cg17716500	22	40600549	TNRC6B	Body	0,275	1,50E-08	1,11E-06
cg08388413	2	225751034	DOCK10	Body	-0,288	1,50E-08	1,11E-06
cg13660174	9	136238392	SURF4	Body	-0,265	1,50E-08	1,11E-06
cg11438825	3	10390474	ATP2B2	Body	-0,211	1,50E-08	1,11E-06

cg27570351	6	53759098	LRRC1	Body	-0,262	1,50E-08	1,11E-06
cg24213240	7	98446784	TMEM130	Body	-0,212	1,50E-08	1,11E-06
cg07487555	1	9778058	PIK3CD	Body	-0,228	1,51E-08	1,11E-06
cg10808534	16	69502772		IGR	-0,202	1,51E-08	1,11E-06
cg21565575	20	35274281	SLA2	5'UTR	-0,211	1,51E-08	1,11E-06
cg24261666	1	181412476		IGR	-0,2	1,51E-08	1,11E-06
cg09481252	13	22260911	FGF9	Body	-0,202	1,51E-08	1,11E-06
cg09647993	21	35321467	LINC00649	5'UTR	-0,214	1,51E-08	1,11E-06
cg03903701	5	110563123	CAMK4	Body	-0,239	1,52E-08	1,11E-06
cg07638723	3	134060450		IGR	-0,285	1,52E-08	1,12E-06
cg05142982	8	495736	C8orf42	TSS1500	0,214	1,52E-08	1,12E-06
cg09138207	11	95962058	MAML2	Body	0,266	1,52E-08	1,12E-06
cg15811515	16	31580989	CSDAP1	TSS200	0,254	1,52E-08	1,12E-06
cg01402099	12	122651715	LRRC43	TSS1500	-0,281	1,53E-08	1,12E-06
cg00430036	9	99145401	SLC35D2	Body	0,204	1,53E-08	1,12E-06
cg22016077	11	75470515	LOC283214	Body	-0,235	1,53E-08	1,12E-06
cg05314930	9	96077634	WNK2	Body	-0,233	1,53E-08	1,12E-06
cg10314082	20	39291107		IGR	-0,24	1,53E-08	1,12E-06
cg13309012	21	26946167	MIR155HG	Body	-0,375	1,54E-08	1,13E-06
cg13557947	8	1900613	ARHGEF10	Body	-0,277	1,54E-08	1,13E-06
cg17008160	1	232315535		IGR	-0,253	1,54E-08	1,13E-06
cg01280674	1	236439326	ERO1B	Body	-0,231	1,55E-08	1,13E-06
cg04119029	16	22369415	CDR2	Body	-0,216	1,55E-08	1,13E-06
cg05382022	1	95698917	RWDD3	TSS1500	0,24	1,55E-08	1,13E-06
cg19325365	10	26850419	APBB1IP	Body	0,294	1,55E-08	1,13E-06
cg26716823	7	33709329		IGR	-0,296	1,55E-08	1,14E-06
cg02763536	17	40932515	WNK4	TSS200	0,214	1,56E-08	1,14E-06
cg08449348	5	38673959		IGR	-0,201	1,56E-08	1,14E-06
cg22342710	15	44838476	EIF3J	Body	-0,265	1,56E-08	1,14E-06
cg01359822	21	40176597	ETS2	TSS1500	-0,217	1,56E-08	1,14E-06
cg20387068	1	94008257	FNBP1L	Body	0,201	1,56E-08	1,14E-06
cg01556706	3	59804435	FHIT	Body	0,206	1,56E-08	1,14E-06
cg25344862	11	1876365	LSP1	Body	-0,28	1,56E-08	1,14E-06
cg12935023	2	231839161		IGR	-0,226	1,57E-08	1,14E-06
cg16352612	7	76054035	ZP3	TSS1500	-0,217	1,57E-08	1,14E-06
cg20097000	15	71400306		IGR	-0,269	1,57E-08	1,14E-06
cg09287933	6	33384473	CUTA	Body	-0,249	1,57E-08	1,14E-06
cg21651444	1	116800256		IGR	-0,222	1,57E-08	1,14E-06
cg00457494	1	44034998	PTPRF	Body	-0,209	1,58E-08	1,15E-06
cg04309234	6	106441468		IGR	0,223	1,58E-08	1,15E-06
cg20752373	11	34645224	EHF	5'UTR	-0,244	1,58E-08	1,15E-06
cg24841244	11	118213330	CD3D	5'UTR	-0,214	1,58E-08	1,15E-06
cg00045592	1	160714299	SLAMF7	5'UTR	-0,241	1,58E-08	1,15E-06
cg26262500	18	46064965	CTIF	TSS1500	0,258	1,58E-08	1,15E-06
cg10610528	6	34206540	HMGA1	5'UTR	-0,326	1,58E-08	1,15E-06
cg17089889	18	55505440		IGR	-0,213	1,58E-08	1,15E-06
cg12839556	17	8243756	ODF4	1stExon	-0,224	1,58E-08	1,15E-06
cg03046247	7	55106050	EGFR	Body	-0,216	1,59E-08	1,15E-06
cg04262640	7	122636149	TAS2R16	TSS1500	-0,203	1,59E-08	1,15E-06
cg06325565	11	63889842	MACROD1	Body	-0,231	1,59E-08	1,15E-06

cg05483692	12	216093	IQSEC3	5'UTR	-0,254	1,59E-08	1,15E-06
cg17708276	16	78812460	WWOX	Body	-0,236	1,59E-08	1,15E-06
cg01938725	2	102600505	LINC01127	Body	0,229	1,59E-08	1,15E-06
cg27569829	18	60984485	BCL2	Body	-0,213	1,59E-08	1,15E-06
cg17672432	2	240425476		IGR	-0,204	1,59E-08	1,15E-06
cg01681396	1	27676122	SYTL1	ExonBnd	0,268	1,60E-08	1,15E-06
cg13680196	1	117099318	CD58	Body	-0,248	1,60E-08	1,15E-06
cg04516672	10	126411663	FAM53B	5'UTR	-0,264	1,60E-08	1,16E-06
cg13901712	12	28696068	CCDC91	Body	-0,26	1,60E-08	1,16E-06
cg02238624	11	119533790	PVRL1	3'UTR	-0,225	1,60E-08	1,16E-06
cg00933182	17	43298345	FMNL1	TSS1500	-0,225	1,61E-08	1,16E-06
cg13299824	1	92414737	BRDT	TSS200	-0,271	1,61E-08	1,16E-06
cg18371789	19	45116368	IGSF23	TSS1500	-0,234	1,61E-08	1,16E-06
cg18530324	18	46064992	KIAA0427	TSS1500	0,201	1,61E-08	1,16E-06
cg14754555	2	157292018	GPD2	1stExon	0,284	1,62E-08	1,16E-06
cg14989316	10	80757927	LOC283050	Body	-0,243	1,62E-08	1,17E-06
cg14263174	18	28962209	DSG1-AS1	Body	-0,253	1,62E-08	1,17E-06
cg03421440	1	92414520	BRDT	TSS1500	-0,232	1,62E-08	1,17E-06
cg12756880	6	106612416		IGR	-0,236	1,62E-08	1,17E-06
cg17879774	12	95496740	FGD6	Body	-0,206	1,62E-08	1,17E-06
cg25760426	10	73189015	CDH23	5'UTR	-0,21	1,63E-08	1,17E-06
cg13394762	14	103871452	MARK3	Body	0,243	1,63E-08	1,17E-06
cg16185421	17	5404345	LOC728392	TSS200	-0,237	1,63E-08	1,17E-06
cg05596876	15	29969720		IGR	-0,258	1,63E-08	1,17E-06
cg20005350	5	111478628		IGR	0,218	1,63E-08	1,17E-06
cg26927413	3	33002183		IGR	-0,26	1,63E-08	1,17E-06
cg13106133	17	38607840	IGFBP4	Body	0,383	1,64E-08	1,17E-06
cg22161666	16	49709752	ZNF423	Body	-0,323	1,64E-08	1,17E-06
cg10281249	1	160546979	CD84	Body	-0,238	1,64E-08	1,18E-06
cg26893022	4	38511077	LINC01258	Body	-0,271	1,64E-08	1,18E-06
cg18048341	15	48043013	SEMA6D	5'UTR	-0,234	1,64E-08	1,18E-06
cg00308680	18	2963259	LPIN2	5'UTR	-0,261	1,64E-08	1,18E-06
cg02117102	1	22210229	HSPG2	Body	-0,266	1,64E-08	1,18E-06
cg16781287	11	118267111	UBE4A	Body	-0,227	1,65E-08	1,18E-06
cg15732576	1	48281875	TRABD2B	Body	-0,299	1,65E-08	1,18E-06
cg18156685	6	53097064		IGR	-0,23	1,65E-08	1,18E-06
cg25087340	11	11590279	GALNT18	Body	-0,224	1,65E-08	1,18E-06
cg17633736	8	27237985	PTK2B	5'UTR	-0,31	1,66E-08	1,18E-06
cg11071523	17	16891339		IGR	-0,251	1,66E-08	1,18E-06
cg12188528	8	49334186		IGR	-0,231	1,66E-08	1,18E-06
cg16550264	12	115813214		IGR	-0,227	1,66E-08	1,19E-06
cg05487134	17	40489569	STAT3	Body	-0,392	1,66E-08	1,19E-06
cg03287930	9	35933869		IGR	-0,216	1,66E-08	1,19E-06
cg08079760	7	130148702	COPG2	Body	-0,211	1,66E-08	1,19E-06
cg10689504	8	41863031	KAT6A	Body	0,21	1,67E-08	1,19E-06
cg06244482	3	188044365	LPP	5'UTR	0,291	1,67E-08	1,19E-06
cg13700250	4	77702233	SHROOM3	3'UTR	-0,237	1,67E-08	1,19E-06
cg18983694	20	42121012		IGR	-0,243	1,67E-08	1,19E-06
cg07845483	19	10990043	CARM1	Body	-0,234	1,67E-08	1,19E-06
cg21931628	11	76353533		IGR	-0,212	1,67E-08	1,19E-06

cg07263283	10	73458450	CDH23	Body	-0,26	1,68E-08	1,19E-06
cg02115302	16	27237719	NSMCE1	Body	-0,292	1,68E-08	1,19E-06
cg26556101	19	44285954	KCNN4	TSS1500	-0,224	1,68E-08	1,20E-06
cg23821020	13	99962835	UBAC2	Body	-0,277	1,68E-08	1,20E-06
cg12296772	8	17271067	MTMR7	TSS200	0,255	1,68E-08	1,20E-06
cg12033075	1	9788767	PIK3CD	3'UTR	-0,248	1,69E-08	1,20E-06
cg00045515	20	35723332	RBL1	Body	-0,229	1,69E-08	1,20E-06
cg14584292	11	122063558	MIR100HG	Body	-0,293	1,69E-08	1,20E-06
cg22934200	2	61407270	AHSA2	5'UTR	0,242	1,69E-08	1,20E-06
cg02473334	1	89828988	GBP6	TSS1500	-0,247	1,70E-08	1,20E-06
cg05728281	15	60840435	RORA-AS1	Body	-0,258	1,70E-08	1,20E-06
cg09279736	6	30039403	RNF39	Body	0,404	1,70E-08	1,20E-06
cg02745605	13	40794828	LINC00548	TSS200	-0,215	1,70E-08	1,21E-06
cg00041030	14	91694672		IGR	-0,24	1,71E-08	1,21E-06
cg18675097	6	28227127	NKAPL	5'UTR	0,228	1,71E-08	1,21E-06
cg09813306	13	98168041		IGR	-0,232	1,71E-08	1,21E-06
cg19821751	2	121138001		IGR	-0,228	1,71E-08	1,21E-06
cg23941537	13	73676019		IGR	-0,206	1,71E-08	1,21E-06
cg04760526	15	41859436	TYRO3	Body	-0,217	1,71E-08	1,21E-06
cg08732993	22	24797222	SPECC1L-ADC	Body	-0,257	1,71E-08	1,21E-06
cg05756492	1	206729685	RASSF5	Body	-0,278	1,72E-08	1,21E-06
cg03035162	14	22993190		IGR	-0,264	1,72E-08	1,21E-06
cg16051589	9	5438508	PLGRKT	TSS1500	-0,342	1,72E-08	1,21E-06
cg12966876	2	111873850	ACOXL	Body	-0,268	1,72E-08	1,21E-06
cg04588680	1	178431014	RASAL2	Body	-0,203	1,72E-08	1,21E-06
cg05280527	14	80328450	NRXN3	3'UTR	-0,253	1,72E-08	1,21E-06
cg12820310	2	218531045	DIRC3	Body	-0,263	1,72E-08	1,22E-06
cg20273931	2	106490359	NCK2	Body	-0,232	1,72E-08	1,22E-06
cg26576047	7	131874478	PLXNA4	Body	-0,242	1,73E-08	1,22E-06
cg14965131	9	82246594	TLE4	Body	-0,223	1,73E-08	1,22E-06
cg26369282	1	229991967		IGR	-0,248	1,73E-08	1,22E-06
cg14438279	5	142806343	NR3C1	5'UTR	-0,265	1,74E-08	1,22E-06
cg26283496	3	146269126		IGR	-0,257	1,74E-08	1,22E-06
cg03035881	15	34627596	SLC12A6	Body	-0,218	1,74E-08	1,22E-06
cg07948951	6	45649741		IGR	-0,209	1,74E-08	1,22E-06
cg24495488	6	151308884	MTHFD1L	Body	-0,287	1,74E-08	1,22E-06
cg17221251	14	98164265		IGR	-0,204	1,74E-08	1,22E-06
cg13946767	1	153599671	S100A13	5'UTR	0,215	1,74E-08	1,22E-06
cg11257888	1	24828096	RCAN3	TSS1500	-0,274	1,74E-08	1,22E-06
cg00416386	2	75149526		IGR	-0,226	1,74E-08	1,22E-06
cg19761245	4	41366989	LIMCH1	Body	-0,248	1,74E-08	1,22E-06
cg14088196	11	70211408	PPFIA1	Body	-0,374	1,74E-08	1,22E-06
cg07630274	10	71583235	COL13A1	Body	-0,201	1,75E-08	1,23E-06
cg10634403	12	19585266		IGR	-0,237	1,75E-08	1,23E-06
cg08053658	11	12229145	MICAL2	Body	-0,234	1,75E-08	1,23E-06
cg07214406	1	16312238		IGR	-0,21	1,75E-08	1,23E-06
cg09473049	3	31294342		IGR	-0,267	1,75E-08	1,23E-06
cg01946790	13	30951587		IGR	-0,21	1,76E-08	1,23E-06
cg25695391	7	28077169	JAZF1	Body	-0,269	1,76E-08	1,23E-06
cg13868356	9	102573997		IGR	-0,213	1,76E-08	1,23E-06

cg00703481	20	52198279	ZNF217	1stExon	-0,246	1,76E-08	1,23E-06
cg02209488	4	185882527		IGR	-0,277	1,76E-08	1,23E-06
cg27298253	22	24806281	SPECC1L-ADC	Body	-0,245	1,76E-08	1,23E-06
cg04945608	20	43118723	TTPAL	3'UTR	-0,254	1,77E-08	1,24E-06
cg14419893	4	141049164	MAML3	Body	-0,269	1,77E-08	1,24E-06
cg08403064	11	44068709	ACCSL	TSS1500	-0,23	1,77E-08	1,24E-06
cg26611815	14	52329157	GNG2	5'UTR	-0,209	1,78E-08	1,24E-06
cg18397877	14	72906773	RGS6	Body	-0,215	1,78E-08	1,24E-06
cg01163193	3	169012590	MECOM	5'UTR	-0,211	1,78E-08	1,24E-06
cg09825327	1	211503219	TRAF5	5'UTR	-0,225	1,78E-08	1,24E-06
cg01019262	1	155095195		IGR	-0,215	1,78E-08	1,24E-06
cg18587063	11	122940457		IGR	-0,275	1,78E-08	1,24E-06
cg22492274	20	10657313		IGR	-0,202	1,79E-08	1,24E-06
cg08982961	2	227476235		IGR	-0,226	1,79E-08	1,24E-06
cg07285237	6	139447669		IGR	-0,307	1,79E-08	1,24E-06
cg17754500	3	99224966		IGR	-0,307	1,79E-08	1,24E-06
cg01245155	2	205919517	PARD3B	Body	-0,212	1,79E-08	1,24E-06
cg11750300	15	68503423	CLN6	Body	0,203	1,79E-08	1,24E-06
cg20090783	10	50347920	FAM170B-AS	Body	-0,227	1,79E-08	1,24E-06
cg20596790	2	42256787		IGR	-0,244	1,79E-08	1,24E-06
cg23059461	10	72362694	PRF1	TSS200	-0,27	1,79E-08	1,25E-06
cg00730561	10	102279703	SEC31B	TSS200	0,207	1,79E-08	1,25E-06
cg18363129	9	117130097	AKNA	Body	-0,335	1,79E-08	1,25E-06
cg01217984	7	27143235	HOXA2	TSS1500	0,216	1,80E-08	1,25E-06
cg16964394	5	170292807	RANBP17	Body	-0,207	1,80E-08	1,25E-06
cg09780035	19	50508881	VRK3	Body	-0,233	1,80E-08	1,25E-06
cg26757279	16	85238227		IGR	-0,229	1,80E-08	1,25E-06
cg04085571	11	1872753	LSP1	TSS1500	-0,273	1,80E-08	1,25E-06
cg08677535	9	505721	KANK1	5'UTR	0,207	1,81E-08	1,25E-06
cg18005079	15	76109893		IGR	-0,445	1,81E-08	1,25E-06
cg11822211	17	1737837	RPA1	Body	-0,273	1,81E-08	1,25E-06
cg02381279	19	16394366		IGR	0,254	1,81E-08	1,26E-06
cg13004663	18	7754953	PTPRM	Body	0,248	1,82E-08	1,26E-06
cg08838106	4	111791019		IGR	-0,286	1,82E-08	1,26E-06
cg06297614	4	108664968		IGR	-0,223	1,82E-08	1,26E-06
cg22925274	14	71288210		IGR	0,205	1,82E-08	1,26E-06
cg13730497	14	21494253	NDRG2	TSS1500	0,263	1,83E-08	1,26E-06
cg01628181	3	99385215	COL8A1	5'UTR	-0,222	1,83E-08	1,26E-06
cg06337041	11	118502632	PHLDB1	ExonBnd	-0,24	1,83E-08	1,26E-06
cg03902135	3	13672669	FBLN2	Body	-0,258	1,83E-08	1,26E-06
cg09517873	1	12656315	DHRS3	Body	0,22	1,83E-08	1,26E-06
cg05664833	13	40107878	LHFP	Body	-0,219	1,83E-08	1,27E-06
cg02182110	12	6417135		IGR	-0,239	1,84E-08	1,27E-06
cg04024781	17	49107365	SPAG9	Body	-0,228	1,84E-08	1,27E-06
cg21144928	1	88928678		IGR	0,243	1,84E-08	1,27E-06
cg09647108	17	41934923	CD300LG	Body	-0,234	1,84E-08	1,27E-06
cg17118478	2	191543902	NAB1	Body	-0,257	1,84E-08	1,27E-06
cg03961389	13	33696479	STARD13	Body	-0,225	1,84E-08	1,27E-06
cg17785683	18	21322159	LAMA3	Body	-0,266	1,84E-08	1,27E-06
cg16362815	7	42111762	GLI3	Body	-0,203	1,85E-08	1,27E-06

cg07045857	9	13323113		IGR	-0,265	1,85E-08	1,27E-06
cg15604182	2	62687964		IGR	-0,213	1,85E-08	1,27E-06
cg02289653	20	34738710	EPB41L1	5'UTR	-0,233	1,85E-08	1,27E-06
cg13588517	6	170562720		IGR	-0,245	1,85E-08	1,27E-06
cg03123541	1	95699097	RWDD3	TSS1500	0,226	1,86E-08	1,27E-06
cg15244786	12	54446279	HOXC4	5'UTR	0,263	1,86E-08	1,28E-06
cg19712159	13	100087002		IGR	-0,246	1,86E-08	1,28E-06
cg22401808	5	108654232		IGR	-0,222	1,86E-08	1,28E-06
cg18258397	5	149853625		IGR	-0,256	1,86E-08	1,28E-06
cg03352496	15	58064595		IGR	-0,262	1,86E-08	1,28E-06
cg23067317	3	16216094	GALNTL2	TSS200	0,219	1,86E-08	1,28E-06
cg21536074	7	42105481	GLI3	Body	-0,204	1,86E-08	1,28E-06
cg02329183	10	96996226		IGR	-0,304	1,86E-08	1,28E-06
cg10388536	1	244173761	LOC339529	Body	-0,208	1,87E-08	1,28E-06
cg02722539	2	68654573		IGR	-0,335	1,87E-08	1,28E-06
cg15969263	21	45287137	AGPAT3	5'UTR	-0,255	1,87E-08	1,28E-06
cg11481063	20	6036814		IGR	-0,209	1,88E-08	1,29E-06
cg05566691	19	42882932		IGR	-0,215	1,88E-08	1,29E-06
cg19091176	20	11212622		IGR	-0,256	1,88E-08	1,29E-06
cg13096923	11	62785058		IGR	-0,207	1,88E-08	1,29E-06
cg07267782	6	54711227	FAM83B	TSS1500	0,23	1,88E-08	1,29E-06
cg17286920	16	49666052	ZNF423	Body	-0,221	1,89E-08	1,29E-06
cg22366787	12	123569700	PITPNM2	5'UTR	-0,25	1,89E-08	1,29E-06
cg07237196	2	159884408	TANC1	5'UTR	-0,236	1,89E-08	1,29E-06
cg20310100	11	68081207	LRP5	Body	0,238	1,89E-08	1,29E-06
cg01882126	11	108604316	DDX10	Body	-0,259	1,90E-08	1,29E-06
cg01036611	17	2169272	SMG6	1stExon	-0,263	1,90E-08	1,29E-06
cg00356897	1	24866792	LOC1005069ɛ	Body	-0,244	1,90E-08	1,30E-06
cg07901571	6	47665223	GPR111	3'UTR	-0,248	1,91E-08	1,30E-06
cg17327423	3	62821363	CADPS	Body	-0,27	1,91E-08	1,30E-06
cg17872886	2	3642732	COLEC11	1stExon	0,278	1,91E-08	1,30E-06
cg23205936	6	170560006		IGR	-0,222	1,91E-08	1,30E-06
cg03225882	11	121987054	LOC399959	Body	-0,243	1,91E-08	1,30E-06
cg26021815	13	23895705	SGCG	Body	-0,258	1,91E-08	1,30E-06
cg00182170	9	21852933	MTAP	Body	-0,211	1,91E-08	1,30E-06
cg16688467	17	57902930	VMP1	Body	-0,202	1,91E-08	1,30E-06
cg05962022	20	2198719		IGR	-0,297	1,91E-08	1,30E-06
cg10836540	10	124405496		IGR	-0,208	1,92E-08	1,30E-06
cg03677056	17	33817217	SLFN12L	Body	0,218	1,92E-08	1,30E-06
cg24234212	6	45678425		IGR	-0,285	1,92E-08	1,31E-06
cg26430230	7	69228260	AUTS2	Body	-0,208	1,92E-08	1,31E-06
cg11218048	6	489238	EXOC2	Body	-0,221	1,93E-08	1,31E-06
cg03739402	19	36204551	ZBTB32	5'UTR	-0,229	1,93E-08	1,31E-06
cg12699756	6	33173482	HSD17B8	Body	-0,29	1,93E-08	1,31E-06
cg13732582	4	39448975	KLB	Body	0,278	1,93E-08	1,31E-06
cg10683668	6	138191600	TNFAIP3	5'UTR	-0,381	1,93E-08	1,31E-06
cg15472659	17	75663362		IGR	-0,298	1,93E-08	1,31E-06
cg26399994	1	198649046	PTPRC	Body	-0,365	1,93E-08	1,31E-06
cg10461784	10	50245385	VSTM4	Body	-0,26	1,93E-08	1,31E-06
cg10904547	15	58262083	ALDH1A2	Body	-0,245	1,93E-08	1,31E-06

cg03518098	9	138172812		IGR	-0,213	1,94E-08	1,31E-06
cg22125902	1	26002535	MAN1C1	Body	-0,253	1,94E-08	1,31E-06
cg00565688	1	3568212	TP73	TSS1500	0,265	1,94E-08	1,31E-06
cg08419476	11	12739670	TEAD1	5'UTR	-0,252	1,94E-08	1,31E-06
cg06016120	3	5041728		IGR	-0,217	1,94E-08	1,31E-06
cg04988171	7	86847564	TMEM243	Body	-0,241	1,94E-08	1,31E-06
cg23884217	4	41147318	APBB2	5'UTR	-0,264	1,95E-08	1,31E-06
cg18736474	1	46770512	LRRC41	TSS1500	0,203	1,95E-08	1,31E-06
cg22114934	1	26645093	CD52	Body	-0,319	1,95E-08	1,32E-06
cg15382025	17	55957555	CUEDC1	Body	-0,208	1,95E-08	1,32E-06
cg14158201	16	85231695		IGR	-0,223	1,96E-08	1,32E-06
cg25109721	17	48125064		IGR	-0,2	1,96E-08	1,32E-06
cg15267998	11	34373473	ABTB2	Body	-0,202	1,96E-08	1,32E-06
cg22974245	8	133930507	TG	Body	-0,264	1,96E-08	1,32E-06
cg06442859	7	131854833	PLXNA4	Body	-0,219	1,96E-08	1,32E-06
cg11527862	10	131701292	EBF3	Body	-0,225	1,96E-08	1,32E-06
cg09744501	16	14050769		IGR	-0,237	1,97E-08	1,32E-06
cg27027055	16	79306292		IGR	-0,222	1,97E-08	1,32E-06
cg13408416	1	152549813		IGR	-0,237	1,97E-08	1,32E-06
cg07470597	17	1974029	SMG6	Body	-0,276	1,97E-08	1,32E-06
cg12972164	18	21695725	TTC39C	Body	-0,202	1,97E-08	1,32E-06
cg26748726	3	112934444	BOC	5'UTR	-0,249	1,97E-08	1,32E-06
cg16248376	2	175472517	WIPF1	5'UTR	-0,201	1,98E-08	1,33E-06
cg26738034	1	8964112		IGR	0,229	1,98E-08	1,33E-06
cg06815976	6	32164816	NOTCH4	Body	0,206	1,98E-08	1,33E-06
cg00088620	16	73224998		IGR	-0,226	1,98E-08	1,33E-06
cg08640904	19	47288915	SLC1A5	5'UTR	-0,301	1,98E-08	1,33E-06
cg27253035	2	106761185	UXS1	Body	-0,217	1,98E-08	1,33E-06
cg05649009	2	175629850	CHRNA1	TSS1500	-0,201	1,98E-08	1,33E-06
cg25433552	5	159560548		IGR	0,205	1,98E-08	1,33E-06
cg15857470	7	104945248	SRPK2	Body	-0,233	1,98E-08	1,33E-06
cg04891836	19	6670390	TNFSF14	1stExon	-0,239	1,99E-08	1,33E-06
cg01837846	2	23755343	KLHL29	5'UTR	-0,242	1,99E-08	1,33E-06
cg18901644	4	8582227	GPR78	TSS200	0,245	1,99E-08	1,33E-06
cg20687848	16	87203817		IGR	-0,217	1,99E-08	1,33E-06
cg03628117	12	68553980	IFNG	TSS1500	-0,203	1,99E-08	1,34E-06
cg13271643	13	21036583	CRYL1	Body	0,235	1,99E-08	1,34E-06
cg03641729	19	46636173		IGR	-0,206	1,99E-08	1,34E-06
cg10848373	21	39047855	KCNJ6	Body	0,21	2,00E-08	1,34E-06
cg15106851	15	74000381	CD276	Body	-0,236	2,00E-08	1,34E-06
cg15243488	13	40677131		IGR	-0,206	2,00E-08	1,34E-06
cg12475507	1	3567852	TP73	TSS1500	0,209	2,00E-08	1,34E-06
cg14793844	2	100516880	AFF3	Body	-0,257	2,00E-08	1,34E-06
cg08634560	5	58296040	PDE4D	TSS1500	-0,206	2,00E-08	1,34E-06
cg02315870	7	26236721	HNRNPA2B1	Body	-0,225	2,00E-08	1,34E-06
cg16600822	11	12898443	TEAD1	Body	-0,235	2,00E-08	1,34E-06
cg12223358	10	115782893		IGR	0,223	2,00E-08	1,34E-06
cg24759521	3	142851615		IGR	-0,25	2,00E-08	1,34E-06
cg05099035	17	60815033	MARCH10	Body	-0,202	2,01E-08	1,34E-06
cg20315739	15	42303032	PLA2G4E	TSS1500	-0,211	2,01E-08	1,34E-06

cg10569147	9	85816185		IGR	-0,232	2,01E-08	1,34E-06
cg26444911	3	49142698	QARS	TSS200	0,248	2,01E-08	1,34E-06
cg13488761	5	168200244	SLIT3	Body	-0,21	2,01E-08	1,34E-06
cg27442125	14	93043537	RIN3	Body	-0,249	2,01E-08	1,34E-06
cg10787842	11	75212660	GDPD5	5'UTR	-0,215	2,01E-08	1,34E-06
cg14743346	8	124050185	DERL1	Body	-0,295	2,01E-08	1,34E-06
cg25571136	6	127612751	ECHDC1	3'UTR	-0,358	2,01E-08	1,34E-06
cg01980817	4	113208944		IGR	-0,258	2,02E-08	1,34E-06
cg24857943	6	37791201	ZFAND3	Body	0,203	2,02E-08	1,34E-06
cg25477843	8	145061318	PARP10	TSS1500	-0,249	2,02E-08	1,34E-06
cg25674102	10	24684432	KIAA1217	Body	-0,281	2,02E-08	1,35E-06
cg11005229	1	67114525	SGIP1	Body	-0,265	2,02E-08	1,35E-06
cg06883317	7	12297351		IGR	-0,373	2,03E-08	1,35E-06
cg21187770	2	26205876	KIF3C	TSS1500	0,23	2,03E-08	1,35E-06
cg10424751	16	32289757		IGR	0,258	2,03E-08	1,35E-06
cg07882213	8	21541227		IGR	-0,253	2,03E-08	1,35E-06
cg09919947	2	8190454	LINC00299	Body	-0,273	2,03E-08	1,35E-06
cg18509272	3	3143430	IL5RA	Body	-0,214	2,03E-08	1,35E-06
cg16755500	17	27918338		IGR	0,238	2,04E-08	1,35E-06
cg20352108	6	161676037	AGPAT4	5'UTR	0,268	2,04E-08	1,36E-06
cg26712743	10	100164594	PYROXD2	Body	-0,269	2,05E-08	1,36E-06
cg05908144	2	54268386		IGR	-0,216	2,05E-08	1,36E-06
cg09510531	21	45705742	AIRE	TSS200	0,2	2,05E-08	1,36E-06
cg12890080	12	91401688		IGR	-0,221	2,05E-08	1,36E-06
cg03882250	2	8453321	LINC00299	Body	-0,273	2,06E-08	1,36E-06
cg05090695	11	2907670	CDKN1C	TSS1500	0,204	2,06E-08	1,36E-06
cg15991632	16	47678468	PHKB	Body	0,287	2,06E-08	1,36E-06
cg15080295	22	27619629		IGR	-0,223	2,07E-08	1,36E-06
cg14241454	17	30851049	MYO1D	Body	-0,266	2,07E-08	1,37E-06
cg18436957	12	131477312	LACAT8	Body	-0,217	2,07E-08	1,37E-06
cg00645755	7	1514031	INTS1	Body	-0,202	2,07E-08	1,37E-06
cg12810837	12	9822287	CLEC2D	TSS200	-0,242	2,07E-08	1,37E-06
cg00907288	3	9178249	SRGAP3	Body	0,322	2,07E-08	1,37E-06
cg17633917	1	111764738		IGR	-0,227	2,07E-08	1,37E-06
cg18344468	9	91182899	NXNL2	Body	-0,304	2,07E-08	1,37E-06
cg00989596	2	110037917	SH3RF3	Body	-0,225	2,08E-08	1,37E-06
cg07238065	22	20920729	MED15	Body	-0,255	2,08E-08	1,37E-06
cg03275459	15	95889928		IGR	-0,209	2,08E-08	1,37E-06
cg13077099	9	6429665	UHRF2	Body	-0,209	2,08E-08	1,37E-06
cg15375424	5	131823451	IRF1	Body	-0,336	2,08E-08	1,37E-06
cg08582164	9	84442861		IGR	-0,229	2,08E-08	1,37E-06
cg24043628	1	225960108		IGR	0,203	2,09E-08	1,37E-06
cg13777844	19	1754195	ONECUT3	1stExon	0,221	2,09E-08	1,37E-06
cg22904711	19	44278628	KCNN4	Body	0,215	2,09E-08	1,37E-06
cg26457678	11	124953628	SLC37A2	Body	-0,223	2,09E-08	1,38E-06
cg09572366	7	130692936	LINC-PINT	Body	-0,22	2,09E-08	1,38E-06
cg21302796	2	71913841	DYSF	3'UTR	-0,2	2,10E-08	1,38E-06
cg19631651	3	55005522	CACNA2D3	Body	-0,225	2,10E-08	1,38E-06
cg05157433	6	29527885	UBD	TSS200	-0,243	2,10E-08	1,38E-06
cg02366772	7	105662809	FLJ23834	Body	-0,265	2,11E-08	1,38E-06

cg23987891	8	142068624		IGR	-0,206	2,11E-08	1,38E-06
cg13391244	7	120629638	C7orf58	5'UTR	0,225	2,11E-08	1,38E-06
cg03736473	10	112610085		IGR	-0,203	2,11E-08	1,38E-06
cg10043535	13	43728589	LINC00400	Body	0,243	2,11E-08	1,38E-06
cg13515047	16	75298429	BCAR1	5'UTR	0,211	2,11E-08	1,38E-06
cg18628732	6	30095295		IGR	0,225	2,12E-08	1,39E-06
cg27509293	6	14919597		IGR	-0,222	2,12E-08	1,39E-06
cg25061755	2	48808114	STON1-GTF2A	Body	-0,231	2,12E-08	1,39E-06
cg06363968	16	84629008	COTL1	Body	-0,271	2,12E-08	1,39E-06
cg18700043	15	74516719		IGR	-0,245	2,12E-08	1,39E-06
cg02219601	14	35835511		IGR	-0,266	2,12E-08	1,39E-06
cg11605100	1	221098640		IGR	-0,239	2,12E-08	1,39E-06
cg12413881	6	170547477		IGR	-0,255	2,13E-08	1,39E-06
cg14615152	1	34093099	CSMD2	Body	-0,246	2,13E-08	1,39E-06
cg26550874	7	4183528	SDK1	Body	-0,203	2,13E-08	1,39E-06
cg27199820	3	6903019	GRM7	1stExon	0,205	2,13E-08	1,39E-06
cg24151755	1	230238754	GALNT2	Body	-0,235	2,14E-08	1,39E-06
cg20212364	4	101745102		IGR	-0,202	2,13E-08	1,39E-06
cg10590815	12	43359134		IGR	-0,2	2,14E-08	1,39E-06
cg12500811	4	177648950	VEGFC	Body	-0,201	2,14E-08	1,39E-06
cg00128509	5	56869195	LINCR-0003	Body	-0,213	2,14E-08	1,39E-06
cg10444228	3	8701782		IGR	-0,277	2,14E-08	1,40E-06
cg23079808	12	6493003	LTBR	TSS1500	0,249	2,14E-08	1,40E-06
cg23460472	1	203023236	PPFIA4	Body	-0,202	2,14E-08	1,40E-06
cg11774909	10	24527412	KIAA1217	Body	-0,211	2,14E-08	1,40E-06
cg15045153	3	189693045	LEPREL1	Body	-0,204	2,14E-08	1,40E-06
cg26216323	8	37722193	RAB11FIP1	Body	0,205	2,14E-08	1,40E-06
cg10125465	1	10169029	UBE4B	Body	0,21	2,15E-08	1,40E-06
cg16906497	18	6836103	ARHGAP28	5'UTR	-0,258	2,15E-08	1,40E-06
cg22292326	2	106060764		IGR	0,223	2,15E-08	1,40E-06
cg08574044	5	142361829	ARHGAP26	Body	0,205	2,16E-08	1,40E-06
cg05603440	16	48482984	MIR5095	Body	-0,235	2,16E-08	1,40E-06
cg01060950	20	49178355	PTPN1	5'UTR	-0,226	2,16E-08	1,40E-06
cg07610498	10	6312774		IGR	-0,325	2,17E-08	1,41E-06
cg20380768	7	130125511	MEST	TSS1500	-0,237	2,17E-08	1,41E-06
cg13963980	1	204670417		IGR	-0,226	2,17E-08	1,41E-06
cg02196805	5	131409637	CSF2	1stExon	-0,248	2,17E-08	1,41E-06
cg09760963	4	154680808	RNF175	Body	0,218	2,17E-08	1,41E-06
cg23679344	17	37560925	MED1	3'UTR	-0,323	2,17E-08	1,41E-06
cg04470054	17	78830472	RPTOR	Body	-0,423	2,17E-08	1,41E-06
cg18735920	9	90919260		IGR	-0,219	2,18E-08	1,41E-06
cg01092582	8	129000122	PVT1	Body	-0,233	2,18E-08	1,41E-06
cg12816876	14	32363503		IGR	-0,272	2,18E-08	1,41E-06
cg19426454	1	26872016	RPS6KA1	TSS1500	-0,242	2,19E-08	1,41E-06
cg07713288	3	194417388		IGR	-0,299	2,19E-08	1,42E-06
cg15794184	1	174933932	RABGAP1L	5'UTR	-0,242	2,20E-08	1,42E-06
cg26244013	12	6470176	SCNN1A	Body	-0,255	2,20E-08	1,42E-06
cg25635022	3	170147199	CLDN11	Body	-0,237	2,20E-08	1,42E-06
cg08946713	2	191844998	STAT1	Body	0,255	2,20E-08	1,42E-06
cg21830683	16	84634696	COTL1	Body	-0,343	2,20E-08	1,42E-06

cg20444209	22	42418735	WBP2NL	Body	-0,229	2,20E-08	1,42E-06
cg11940839	19	16230553	RAB8A	Body	-0,279	2,21E-08	1,43E-06
cg11804414	12	122712137	DIABLO	TSS200	-0,282	2,21E-08	1,43E-06
cg24570371	4	36258130	LOC439933	TSS200	-0,221	2,21E-08	1,43E-06
cg00158122	10	101290029		IGR	0,249	2,21E-08	1,43E-06
cg21322043	10	11905941	PROSER2	Body	-0,289	2,22E-08	1,43E-06
cg09162514	7	40740462	SUGCT	Body	-0,233	2,22E-08	1,43E-06
cg18329700	6	170258257		IGR	-0,22	2,22E-08	1,43E-06
cg03849185	17	61472444	TANC2	Body	-0,231	2,22E-08	1,43E-06
cg15253892	6	151308594	MTHFD1L	Body	-0,313	2,23E-08	1,43E-06
cg07153995	5	171875680	SH3PXD2B	Body	-0,271	2,23E-08	1,43E-06
cg08006125	14	35755071	PSMA6	Body	-0,254	2,23E-08	1,43E-06
cg22471376	15	89179370	ISG20	5'UTR	-0,253	2,23E-08	1,43E-06
cg16747463	21	41632420	DSCAM	Body	-0,215	2,23E-08	1,43E-06
cg27277179	8	21032107		IGR	-0,204	2,23E-08	1,43E-06
cg13585930	10	72027357	NPFFR1	TSS1500	-0,25	2,23E-08	1,43E-06
cg14727981	6	37074912		IGR	0,286	2,24E-08	1,44E-06
cg05352689	10	45407522	TMEM72	Body	-0,25	2,24E-08	1,44E-06
cg21111613	14	32473981		IGR	-0,229	2,24E-08	1,44E-06
cg13299325	6	447777		IGR	-0,21	2,24E-08	1,44E-06
cg25231776	8	10480438	RP1L1	Body	-0,217	2,25E-08	1,44E-06
cg15401399	1	4751172	AJAP1	Body	-0,211	2,25E-08	1,44E-06
cg14994032	1	192500435		IGR	-0,227	2,25E-08	1,44E-06
cg20168230	1	37499649	GRIK3	1stExon	0,23	2,26E-08	1,44E-06
cg09199614	5	56946761	LOC10192850	Body	-0,257	2,26E-08	1,44E-06
cg25571189	2	133427612	LYPD1	1stExon	0,214	2,26E-08	1,44E-06
cg09648454	16	48157560	ABCC12	Body	-0,209	2,26E-08	1,44E-06
cg00448868	16	66775493	DYNC1LI2	Body	0,202	2,26E-08	1,44E-06
cg02198653	19	36204918	ZBTB32	5'UTR	-0,211	2,27E-08	1,45E-06
cg00428134	5	168229751	SLIT3	Body	-0,208	2,27E-08	1,45E-06
cg23017728	17	40110371	TTC25	Body	-0,209	2,27E-08	1,45E-06
cg00366917	18	74845154	MBP	TSS1500	0,228	2,27E-08	1,45E-06
cg26724841	7	5816628	RNF216	5'UTR	-0,378	2,28E-08	1,45E-06
cg24098086	2	232585925		IGR	-0,238	2,28E-08	1,46E-06
cg12213910	6	150211211	RAET1E	Body	-0,21	2,28E-08	1,46E-06
cg13952106	15	68614106	ITGA11	Body	-0,206	2,29E-08	1,46E-06
cg11869499	15	89872984	POLG	Body	-0,258	2,29E-08	1,46E-06
cg03407562	8	1682921		IGR	-0,266	2,29E-08	1,46E-06
cg07362492	1	232814481		IGR	-0,237	2,29E-08	1,46E-06
cg17011173	5	123981378	ZNF608	Body	-0,215	2,29E-08	1,46E-06
cg19236287	20	23215597		IGR	-0,23	2,29E-08	1,46E-06
cg00228553	12	96031625		IGR	-0,222	2,29E-08	1,46E-06
cg07633702	18	45612462		IGR	-0,235	2,29E-08	1,46E-06
cg03771282	2	198062750	ANKRD44	5'UTR	-0,297	2,29E-08	1,46E-06
cg17141263	17	80851473	TBCD	Body	-0,204	2,29E-08	1,46E-06
cg14082886	11	35164485	CD44	Body	-0,432	2,30E-08	1,46E-06
cg04542940	14	100358511	EML1	Body	-0,21	2,30E-08	1,46E-06
cg09736286	12	122712925	DIABLO	TSS1500	-0,231	2,30E-08	1,46E-06
cg24965116	11	102754061		IGR	-0,212	2,30E-08	1,46E-06
cg02879171	1	45955199	TESK2	5'UTR	-0,251	2,30E-08	1,46E-06

cg09966085	18	42588939	SETBP1	Body	-0,275	2,31E-08	1,47E-06
cg20460771	1	28521540	PTAFR	TSS1500	-0,245	2,31E-08	1,47E-06
cg24134018	18	10413897		IGR	-0,237	2,31E-08	1,47E-06
cg08841318	6	125283740	RNF217	1stExon	0,212	2,31E-08	1,47E-06
cg01334831	13	114832833	RASA3	Body	-0,348	2,31E-08	1,47E-06
cg22507154	1	91185233		IGR	0,229	2,32E-08	1,47E-06
cg14527645	9	116977924	COL27A1	Body	-0,207	2,32E-08	1,47E-06
cg08035822	12	121471970	OASL	Body	-0,335	2,33E-08	1,47E-06
cg00626263	7	5741049	RNF216	Body	-0,299	2,33E-08	1,47E-06
cg07658637	2	199236982	LOC10192761	Body	-0,303	2,33E-08	1,47E-06
cg07661704	4	139144433	SLC7A11	Body	0,261	2,33E-08	1,48E-06
cg23635935	8	98596240		IGR	-0,212	2,33E-08	1,48E-06
cg22905543	15	70977270	UACA	Body	-0,229	2,33E-08	1,48E-06
cg17172946	10	112440727	RBM20	Body	0,205	2,34E-08	1,48E-06
cg02417408	3	52280688	PPM1M	5'UTR	-0,223	2,35E-08	1,49E-06
cg11474081	8	129234520		IGR	-0,346	2,35E-08	1,49E-06
cg03156626	22	37579635	C1QTNF6	Body	-0,266	2,36E-08	1,49E-06
cg02479744	6	16303089	ATXN1	3'UTR	0,229	2,36E-08	1,49E-06
cg04295144	19	10407184	ICAM5	Body	0,267	2,36E-08	1,49E-06
cg11619961	5	149792840	CD74	TSS1500	-0,228	2,36E-08	1,49E-06
cg24315450	8	134494619	ST3GAL1	5'UTR	-0,285	2,36E-08	1,49E-06
cg16367786	1	17940280	ARHGEF10L	Body	-0,282	2,37E-08	1,49E-06
cg08574423	19	12724912	ZNF791	Body	-0,202	2,37E-08	1,49E-06
cg06635975	6	74232158	EEF1A1	TSS1500	-0,325	2,37E-08	1,49E-06
cg19574388	4	10075174		IGR	-0,263	2,37E-08	1,49E-06
cg15205884	6	35286844	DEF6	Body	0,253	2,38E-08	1,49E-06
cg25718284	5	40437913		IGR	-0,281	2,38E-08	1,50E-06
cg21694210	8	48565455	SPIDR	Body	-0,243	2,38E-08	1,50E-06
cg05299836	16	31119067	BCKDK	TSS1500	-0,259	2,39E-08	1,50E-06
cg05969983	14	65146958		IGR	-0,239	2,39E-08	1,50E-06
cg09947542	1	158085438		IGR	-0,236	2,39E-08	1,50E-06
cg00739155	17	36832423	C17orf96	TSS1500	-0,239	2,39E-08	1,50E-06
cg02731237	1	48387877	TRABD2B	Body	-0,263	2,39E-08	1,50E-06
cg26841655	1	113525976		IGR	0,217	2,39E-08	1,50E-06
cg11473417	8	145946869	ZNF251	3'UTR	-0,251	2,39E-08	1,50E-06
cg19485202	19	18902081	COMP	1stExon	0,202	2,39E-08	1,50E-06
cg09417176	10	123993026	TACC2	Body	-0,22	2,40E-08	1,50E-06
cg06296880	7	11445870	THSD7A	Body	-0,23	2,40E-08	1,50E-06
cg03642261	22	43160131		IGR	-0,216	2,40E-08	1,50E-06
cg14430754	18	46360298	CTIF	Body	-0,228	2,40E-08	1,50E-06
cg04494602	17	62982045		IGR	-0,241	2,41E-08	1,51E-06
cg20858481	15	52369365		IGR	-0,251	2,41E-08	1,51E-06
cg09803930	1	204660745		IGR	-0,212	2,41E-08	1,51E-06
cg01510609	2	3751134	DCDC2C	TSS200	0,241	2,41E-08	1,51E-06
cg15128003	7	96243777		IGR	-0,219	2,42E-08	1,51E-06
cg21736686	15	101503596	LRRK1	Body	0,243	2,42E-08	1,51E-06
cg22213050	5	132028185		IGR	-0,231	2,43E-08	1,52E-06
cg11343938	3	72978007	GXYLT2	Body	0,256	2,43E-08	1,52E-06
cg15033085	3	172334072		IGR	-0,377	2,44E-08	1,52E-06
cg01135588	1	212440983	LOC1019295	Body	-0,255	2,44E-08	1,52E-06

cg02981636	3	46311184		IGR	-0,233	2,44E-08	1,52E-06
cg04154027	5	78985588	CMYA5	TSS200	0,309	2,44E-08	1,52E-06
cg24960057	3	194293992		IGR	-0,278	2,44E-08	1,52E-06
cg22318959	1	159770368	FCRL6	5'UTR	-0,23	2,44E-08	1,52E-06
cg17905009	7	83690400	SEMA3A	Body	-0,238	2,45E-08	1,53E-06
cg14543402	15	61503408	RORA	Body	-0,204	2,45E-08	1,53E-06
cg02882775	5	168377734	SLIT3	Body	-0,241	2,45E-08	1,53E-06
cg16456533	1	208280512	PLXNA2	Body	-0,206	2,45E-08	1,53E-06
cg14016406	3	32474566	CMTM7	Body	-0,352	2,45E-08	1,53E-06
cg25762006	16	73398539		IGR	-0,252	2,45E-08	1,53E-06
cg11796827	7	6435978	RAC1	Body	0,212	2,45E-08	1,53E-06
cg08766184	2	204734884	CTLA4	Body	-0,249	2,46E-08	1,53E-06
cg14002739	18	59680370		IGR	-0,21	2,46E-08	1,53E-06
cg05998899	20	32150043	CBFA2T2	TSS200	0,288	2,46E-08	1,53E-06
cg08144943	3	52280702	PPM1M	5'UTR	-0,22	2,47E-08	1,53E-06
cg23169499	10	120939330	PRDX3	TSS1500	-0,248	2,47E-08	1,53E-06
cg12051592	18	73033528		IGR	-0,224	2,47E-08	1,54E-06
cg21686287	2	73021348	EXOC6B	Body	0,202	2,48E-08	1,54E-06
cg17227300	11	122617067	UBASH3B	Body	-0,256	2,48E-08	1,54E-06
cg07829075	11	67174623	TBC1D10C	Body	-0,299	2,48E-08	1,54E-06
cg21319157	12	110405554	GIT2	Body	-0,205	2,48E-08	1,54E-06
cg21663431	19	10736355	SLC44A2	Body	0,259	2,48E-08	1,54E-06
cg25600410	1	205550669	MFSD4	Body	-0,241	2,48E-08	1,54E-06
cg26371422	5	39256706	FYB	Body	-0,412	2,48E-08	1,54E-06
cg02680909	6	39899321	MOCS1	Body	-0,243	2,49E-08	1,54E-06
cg00331226	1	100110954	PALMD	TSS1500	0,294	2,49E-08	1,54E-06
cg15292513	11	12257627	MICAL2	Body	-0,227	2,49E-08	1,54E-06
cg22798822	12	29860019	TMTC1	Body	-0,218	2,49E-08	1,54E-06
cg26324159	5	71490901	MAP1B	Body	-0,208	2,49E-08	1,54E-06
cg13519829	14	104551502	ASPG	TSS1500	0,214	2,49E-08	1,54E-06
cg08552853	2	102875372		IGR	-0,29	2,49E-08	1,54E-06
cg06488371	1	24392896	MYOM3	Body	-0,214	2,50E-08	1,54E-06
cg20937594	6	15876596		IGR	-0,213	2,50E-08	1,54E-06
cg19238161	18	44944311		IGR	-0,228	2,50E-08	1,55E-06
cg01936702	2	208393204	CREB1	TSS1500	-0,204	2,51E-08	1,55E-06
cg18016288	13	95834131	ABCC4	Body	-0,281	2,51E-08	1,55E-06
cg11083595	4	58044400	IGFBP7-AS1	Body	-0,228	2,51E-08	1,55E-06
cg05624387	7	36068970		IGR	-0,264	2,52E-08	1,55E-06
cg18679976	7	3927032	SDK1	Body	-0,224	2,52E-08	1,55E-06
cg13879937	15	42265413	EHD4	TSS1500	-0,249	2,52E-08	1,55E-06
cg17338146	2	147142856		IGR	-0,284	2,52E-08	1,55E-06
cg01476567	9	14585991		IGR	-0,248	2,52E-08	1,56E-06
cg02717454	16	3928799	CREBBP	Body	0,201	2,52E-08	1,56E-06
cg26508142	19	44814671		IGR	-0,202	2,53E-08	1,56E-06
cg01685665	15	68905043	CORO2B	Body	-0,253	2,53E-08	1,56E-06
cg12685166	2	175455876	WIPF1	5'UTR	-0,287	2,53E-08	1,56E-06
cg12564406	12	109027086	SELPLG	TSS1500	-0,224	2,53E-08	1,56E-06
cg06120527	7	148204377		IGR	-0,21	2,54E-08	1,56E-06
cg26800786	11	105922059	KBTBD3	3'UTR	0,249	2,54E-08	1,56E-06
cg24904958	15	58834543	LIPC	Body	-0,271	2,54E-08	1,56E-06

cg27215752	7	65613549	CRCP	Body	-0,251	2,54E-08	1,56E-06
cg02553170	1	84556762	PRKACB	Body	-0,306	2,54E-08	1,56E-06
cg18787224	7	130645353	LINC-PINT	Body	-0,426	2,54E-08	1,56E-06
cg12155541	17	48124267		IGR	-0,317	2,54E-08	1,56E-06
cg24185736	1	224020061	TP53BP2	Body	-0,291	2,54E-08	1,56E-06
cg11958949	6	151701920	ZBTB2	5'UTR	-0,303	2,54E-08	1,56E-06
cg18622049	1	174756086	RABGAP1L	Body	-0,213	2,54E-08	1,56E-06
cg08363345	7	4752063	FOXK1	Body	-0,248	2,54E-08	1,56E-06
cg23192604	12	46151535	ARID2	Body	0,25	2,55E-08	1,56E-06
cg12288551	7	50801220	GRB10	5'UTR	-0,229	2,55E-08	1,56E-06
cg24475773	5	10441239	ROPN1L	TSS1500	0,212	2,55E-08	1,56E-06
cg19272720	1	204289444	PLEKHA6	5'UTR	-0,225	2,55E-08	1,56E-06
cg04945379	16	10970200	CIITA	TSS1500	-0,255	2,55E-08	1,57E-06
cg03556972	13	24249366	TNFRSF19	3'UTR	-0,229	2,55E-08	1,57E-06
cg12052401	16	56951035		IGR	-0,33	2,56E-08	1,57E-06
cg01649307	17	3699034	ITGAE	Body	-0,222	2,56E-08	1,57E-06
cg00704592	7	92534346	LOC10192749	Body	-0,244	2,56E-08	1,57E-06
cg27609154	13	107027105		IGR	-0,247	2,56E-08	1,57E-06
cg05529890	16	28997459	LAT	ExonBnd	-0,27	2,56E-08	1,57E-06
cg10459935	16	89518843	LOC10192781	Body	-0,224	2,56E-08	1,57E-06
cg16086620	14	93389557	CHGA	5'UTR	0,258	2,56E-08	1,57E-06
cg01235820	20	642782	SCRT2	3'UTR	0,229	2,57E-08	1,57E-06
cg21281086	5	55067187	DDX4	Body	-0,263	2,57E-08	1,57E-06
cg04089246	7	47579217	TNS3	TSS200	-0,22	2,57E-08	1,57E-06
cg14487805	17	3433970	TRPV3	Body	0,227	2,57E-08	1,57E-06
cg20833278	5	90522401		IGR	-0,238	2,57E-08	1,57E-06
cg22121892	13	100195189	TM9SF2	Body	-0,269	2,58E-08	1,58E-06
cg05106936	12	104962891	CHST11	Body	-0,302	2,58E-08	1,58E-06
cg13565707	16	85316434	LINC00311	TSS200	-0,292	2,58E-08	1,58E-06
cg14658067	7	1273394	UNCX	Body	0,201	2,58E-08	1,58E-06
cg20964248	11	111564787	SIK2	Body	0,235	2,59E-08	1,58E-06
cg24683185	15	31214501	MTMR15	Body	0,244	2,59E-08	1,58E-06
cg23218897	17	2169522	SMG6	TSS200	-0,232	2,59E-08	1,58E-06
cg05588074	20	17486490	BFSP1	Body	-0,264	2,59E-08	1,58E-06
cg06397732	13	22141970	MICU2	Body	-0,292	2,59E-08	1,58E-06
cg08084319	8	127466696		IGR	-0,209	2,60E-08	1,58E-06
cg17692821	9	117769024		IGR	-0,202	2,60E-08	1,58E-06
cg08682036	10	43898033	HNRNPF	5'UTR	-0,283	2,60E-08	1,58E-06
cg13493207	16	57662079	ADGRG1	TSS200	0,202	2,60E-08	1,58E-06
cg21756223	18	10317090		IGR	-0,227	2,60E-08	1,58E-06
cg10888155	1	225896735		IGR	-0,283	2,60E-08	1,58E-06
cg09704544	15	85616433	PDE8A	Body	-0,255	2,60E-08	1,58E-06
cg05793752	9	129939432	RALGPS1	Body	-0,219	2,61E-08	1,59E-06
cg26937637	5	130749792		IGR	-0,244	2,61E-08	1,59E-06
cg13375187	15	39886363	THBS1	Body	-0,211	2,61E-08	1,59E-06
cg03220210	11	101732946		IGR	-0,202	2,61E-08	1,59E-06
cg00277591	17	4079652	ANKFY1	Body	-0,245	2,62E-08	1,59E-06
cg17161250	2	106682209	C2orf40	1stExon	0,207	2,62E-08	1,59E-06
cg21862081	14	77769687	POMT2	Body	-0,239	2,62E-08	1,59E-06
cg06970884	17	2300100	MNT	Body	0,239	2,62E-08	1,59E-06

cg26054764	1	214152805		IGR	-0,266	2,62E-08	1,59E-06
cg09594160	8	18918361		IGR	-0,211	2,63E-08	1,59E-06
cg11993578	7	18509097	HDAC9	Body	-0,278	2,63E-08	1,59E-06
cg06221509	20	46612085	LINC01522	Body	-0,252	2,63E-08	1,59E-06
cg20126910	8	102644374	GRHL2	Body	-0,2	2,63E-08	1,60E-06
cg14114133	6	10839408		IGR	-0,304	2,64E-08	1,60E-06
cg10061361	4	122078167	TNIP3	Body	0,256	2,64E-08	1,60E-06
cg08697689	13	84457340	SLITRK1	TSS1500	0,218	2,64E-08	1,60E-06
cg27140633	11	63685905		IGR	0,276	2,64E-08	1,60E-06
cg07489959	4	57746442		IGR	-0,284	2,64E-08	1,60E-06
cg11405200	6	14212128		IGR	0,225	2,65E-08	1,60E-06
cg15083288	2	111551024	ACOXL	Body	-0,219	2,65E-08	1,60E-06
cg00778996	8	6275498	MCPH1	Body	-0,214	2,65E-08	1,60E-06
cg09981814	6	141647780		IGR	-0,325	2,65E-08	1,60E-06
cg16596294	10	94607768	EXOC6	TSS1500	0,207	2,65E-08	1,60E-06
cg02491871	12	11911896	ETV6	Body	0,202	2,65E-08	1,60E-06
cg03385871	18	46311648	CTIF	Body	-0,352	2,65E-08	1,60E-06
cg02578087	3	8671361	C3orf32	Body	-0,266	2,66E-08	1,61E-06
cg21470699	3	69492121		IGR	-0,242	2,67E-08	1,61E-06
cg11213983	17	63652954	CCDC46	Body	-0,274	2,67E-08	1,61E-06
cg02045981	21	45882643		IGR	-0,203	2,67E-08	1,61E-06
cg03785755	6	26196794		IGR	-0,26	2,67E-08	1,61E-06
cg27089703	17	33373905	RFFL	Body	-0,295	2,68E-08	1,61E-06
cg11327513	1	199694221		IGR	-0,277	2,68E-08	1,61E-06
cg24743237	2	242702932	D2HGDH	Body	-0,23	2,68E-08	1,61E-06
cg27367170	10	5488628	NET1	Body	0,209	2,68E-08	1,61E-06
cg11403831	5	108082959	FER	TSS1500	0,244	2,68E-08	1,61E-06
cg18704597	4	154138519	TRIM2	5'UTR	-0,223	2,69E-08	1,62E-06
cg04533691	2	29353221	CLIP4	Body	-0,211	2,69E-08	1,62E-06
cg11892200	20	44572943	PCIF1	Body	-0,306	2,69E-08	1,62E-06
cg13958456	20	23014939	SSTR4	TSS1500	-0,244	2,69E-08	1,62E-06
cg04451259	11	35358924	SLC1A2	Body	-0,328	2,70E-08	1,62E-06
cg03054432	1	65721125		IGR	0,209	2,70E-08	1,62E-06
cg23057567	19	47017629		IGR	-0,214	2,70E-08	1,62E-06
cg13940215	14	68974689	RAD51B	Body	-0,224	2,70E-08	1,62E-06
cg18006637	4	89713820	FAM13A	Body	-0,294	2,71E-08	1,62E-06
cg09468228	2	231815170		IGR	-0,24	2,71E-08	1,62E-06
cg24939283	3	185785561	ETV5	Body	-0,46	2,72E-08	1,63E-06
cg11846559	19	55788089	HSPBP1	Body	-0,262	2,73E-08	1,64E-06
cg03173502	6	15505345	JARID2	Body	0,271	2,73E-08	1,64E-06
cg27461687	9	98313948		IGR	-0,219	2,74E-08	1,64E-06
cg06001734	2	54467214	ACYP2	Body	-0,232	2,74E-08	1,64E-06
cg00676801	2	191876673	STAT1	5'UTR	-0,432	2,74E-08	1,64E-06
cg11899448	13	45816538	GTF2F2	Body	0,238	2,74E-08	1,64E-06
cg24473180	8	22048846	BMP1	Body	-0,229	2,74E-08	1,64E-06
cg15092343	4	4860061	MSX1	TSS1500	0,276	2,74E-08	1,64E-06
cg09271318	14	53209092	STYX	Body	-0,258	2,74E-08	1,64E-06
cg04576377	7	73769375	CLIP2	Body	-0,215	2,75E-08	1,64E-06
cg04813726	3	58097766	FLNB	Body	-0,241	2,75E-08	1,64E-06
cg16562296	2	61046710		IGR	-0,217	2,75E-08	1,64E-06

cg20127419	1	112289991	FAM212B-AS	Body	-0,277	2,75E-08	1,64E-06
cg02542684	4	185209748		IGR	-0,212	2,75E-08	1,64E-06
cg16452866	14	99655676	BCL11B	Body	-0,204	2,75E-08	1,64E-06
cg09330125	18	60884450	BCL2	Body	-0,3	2,76E-08	1,64E-06
cg06118256	9	4310629		IGR	-0,21	2,76E-08	1,65E-06
cg09010484	10	78083603	C10orf11	Body	-0,252	2,76E-08	1,65E-06
cg23510258	17	80840821	TBCD	Body	-0,272	2,76E-08	1,65E-06
cg16553721	19	6668742	TNFSF14	Body	-0,235	2,77E-08	1,65E-06
cg15507486	19	46627896	IGFL3	1stExon	-0,288	2,77E-08	1,65E-06
cg02289020	16	83037335	CDH13	Body	-0,229	2,77E-08	1,65E-06
cg17692230	4	103360578		IGR	0,258	2,77E-08	1,65E-06
cg16592182	10	91127497		IGR	-0,219	2,78E-08	1,65E-06
cg25784220	19	58609602	ZSCAN18	1stExon	0,211	2,78E-08	1,66E-06
cg19339726	2	217524431	IGFBP2	Body	-0,257	2,79E-08	1,66E-06
cg16648880	8	18304637		IGR	-0,205	2,79E-08	1,66E-06
cg06884055	14	74163654	DNAL1	3'UTR	-0,205	2,79E-08	1,66E-06
cg02618151	14	105838415	PACS2	Body	-0,209	2,79E-08	1,66E-06
cg10680210	11	64107158	CCDC88B	TSS1500	-0,264	2,79E-08	1,66E-06
cg18122743	2	41921124		IGR	-0,245	2,79E-08	1,66E-06
cg05525455	11	128835715	ARHGAP32	3'UTR	-0,281	2,80E-08	1,66E-06
cg13928272	20	33992946	UQCC1	Body	-0,272	2,80E-08	1,66E-06
cg27251412	21	45705740	AIRE	TSS200	0,212	2,80E-08	1,66E-06
cg20778731	2	37595280	QPCT	Body	-0,246	2,80E-08	1,66E-06
cg04472592	12	52585786	KRT80	TSS200	-0,274	2,81E-08	1,67E-06
cg17404083	3	39449544	SNORA6	TSS1500	-0,219	2,81E-08	1,67E-06
cg19249814	9	90257654	DAPK1	Body	-0,246	2,81E-08	1,67E-06
cg07830362	3	153685391		IGR	-0,253	2,82E-08	1,67E-06
cg07180988	12	59325286		IGR	-0,261	2,82E-08	1,67E-06
cg16759041	17	53521675		IGR	-0,206	2,83E-08	1,67E-06
cg23348081	12	14413690		IGR	0,239	2,83E-08	1,67E-06
cg04666591	7	197206	FAM20C	Body	-0,249	2,83E-08	1,68E-06
cg18172358	1	76734569	ST6GALNAC3	Body	-0,284	2,84E-08	1,68E-06
cg12736877	6	30039010	RNF39	Body	0,312	2,84E-08	1,68E-06
cg18279004	3	111802305		IGR	0,216	2,84E-08	1,68E-06
cg21308365	6	119031399		IGR	-0,224	2,84E-08	1,68E-06
cg17010349	12	6884128	LAG3	Body	-0,239	2,84E-08	1,68E-06
cg01173814	1	8762310	RERE	5'UTR	0,203	2,84E-08	1,68E-06
cg11563064	2	198169819	ANKRD44	Body	-0,247	2,85E-08	1,68E-06
cg21745456	2	102332995	MAP4K4	Body	-0,251	2,85E-08	1,68E-06
cg20189274	15	69339228	NOX5	Body	-0,252	2,85E-08	1,68E-06
cg21194040	3	11862088	TAMM41	Body	-0,249	2,86E-08	1,69E-06
cg21754296	8	9118076		IGR	-0,241	2,86E-08	1,69E-06
cg12684621	19	48589790	PLA2G4C	Body	-0,209	2,86E-08	1,69E-06
cg27370393	3	178104333		IGR	-0,258	2,87E-08	1,69E-06
cg03498271	5	38427885	EGFLAM	Body	-0,25	2,87E-08	1,69E-06
cg11079441	8	131432739	ASAP1	5'UTR	-0,249	2,88E-08	1,69E-06
cg12483843	10	61667087	CCDC6	TSS1500	-0,219	2,88E-08	1,70E-06
cg25289604	5	33850228	ADAMTS12	Body	-0,354	2,89E-08	1,70E-06
cg11824750	20	3888037	PANK2	5'UTR	-0,2	2,89E-08	1,70E-06
cg10002193	6	3526492		IGR	-0,215	2,89E-08	1,70E-06

cg27636058	11	57443308	ZDHHC5	Body	-0,225	2,90E-08	1,70E-06
cg17456265	2	9492809	ASAP2	Body	-0,207	2,90E-08	1,70E-06
cg19374755	2	160471238	LOC643072	TSS1500	0,233	2,90E-08	1,70E-06
cg21334513	6	30095248		IGR	0,209	2,90E-08	1,70E-06
cg13614617	15	65656635	IGDCC3	Body	-0,297	2,90E-08	1,70E-06
cg14651189	14	25361589	STXBP6	Body	-0,206	2,90E-08	1,70E-06
cg11055000	18	45843994		IGR	-0,253	2,90E-08	1,70E-06
cg19540824	17	1687348	SMYD4	Body	0,216	2,91E-08	1,70E-06
cg20824939	1	205255082		IGR	-0,364	2,91E-08	1,71E-06
cg15668843	2	75833084		IGR	-0,221	2,91E-08	1,71E-06
cg04276058	1	19254031	IFFO2	Body	-0,203	2,91E-08	1,71E-06
cg14867900	12	69067768		IGR	-0,241	2,92E-08	1,71E-06
cg03390370	2	190478061		IGR	0,221	2,92E-08	1,71E-06
cg07163839	2	113601124		IGR	-0,205	2,92E-08	1,71E-06
cg18236571	4	135122410	PABPC4L	5'UTR	0,205	2,92E-08	1,71E-06
cg18774048	3	50611151	HEMK1	Body	-0,258	2,92E-08	1,71E-06
cg10582357	2	33090774	LINC00486	Body	-0,302	2,92E-08	1,71E-06
cg24776142	6	16513829	ATXN1	5'UTR	0,231	2,92E-08	1,71E-06
cg03574832	4	184746764		IGR	-0,235	2,93E-08	1,71E-06
cg27255534	10	6170886		IGR	0,228	2,93E-08	1,71E-06
cg20162679	5	140186706	PCDHA4	5'UTR	0,224	2,93E-08	1,71E-06
cg16478616	14	103272089	TRAF3	5'UTR	-0,226	2,93E-08	1,72E-06
cg02155073	15	39915812	FSIP1	Body	-0,278	2,93E-08	1,72E-06
cg23937608	12	49317231	FKBP11	Body	-0,268	2,94E-08	1,72E-06
cg14506192	12	122711988	DIABLO	Body	-0,27	2,94E-08	1,72E-06
cg20580788	17	189410	RPH3AL	5'UTR	-0,227	2,94E-08	1,72E-06
cg13593263	7	46300412		IGR	-0,23	2,95E-08	1,72E-06
cg02056404	5	88263995	MEF2C-AS1	Body	-0,234	2,95E-08	1,72E-06
cg18980148	8	134552558	ST3GAL1	5'UTR	0,206	2,95E-08	1,72E-06
cg13520090	17	8986764	NTN1	Body	-0,209	2,96E-08	1,72E-06
cg27302675	6	90385873	MDN1	Body	0,259	2,96E-08	1,72E-06
cg24874802	5	149618974	CAMK2A	Body	-0,241	2,96E-08	1,73E-06
cg19092837	1	10271724	KIF1B	5'UTR	0,213	2,96E-08	1,73E-06
cg27578247	3	50510521	CACNA2D2	Body	-0,248	2,96E-08	1,73E-06
cg07706695	1	111179008		IGR	-0,256	2,97E-08	1,73E-06
cg13199569	10	44409382	LINC00841	Body	-0,216	2,97E-08	1,73E-06
cg05105226	5	138238954	CTNNA1	Body	0,215	2,98E-08	1,74E-06
cg16730327	1	223054067	DISP1	5'UTR	-0,275	2,98E-08	1,74E-06
cg16646463	2	74610527	DCTN1	Body	0,228	2,98E-08	1,74E-06
cg00508023	14	74296957		IGR	-0,341	2,99E-08	1,74E-06
cg25582867	4	56860292	CEP135	Body	-0,314	2,99E-08	1,74E-06
cg05254221	10	61050062	FAM13C	Body	-0,273	3,00E-08	1,74E-06
cg08250738	5	73772012		IGR	-0,236	3,01E-08	1,75E-06
cg08038110	18	47400743	MYO5B	Body	-0,206	3,01E-08	1,75E-06
cg27434809	17	48880966		IGR	-0,251	3,01E-08	1,75E-06
cg12049875	20	19955868	RIN2	Body	0,211	3,01E-08	1,75E-06
cg19270965	17	40856988	EZH1	Body	-0,22	3,02E-08	1,75E-06
cg13205848	12	5675505	ANO2	Body	-0,25	3,02E-08	1,75E-06
cg05113537	8	103169068		IGR	-0,246	3,02E-08	1,75E-06
cg08031024	2	121617597	GLI2	Body	-0,225	3,02E-08	1,75E-06

cg10139191	2	216847583	MREG	Body	-0,22	3,02E-08	1,75E-06
cg24539090	1	158978504	IFI16	TSS1500	-0,221	3,02E-08	1,75E-06
cg18633711	6	37123271		IGR	-0,23	3,03E-08	1,75E-06
cg18125510	14	100841768	WARS	1stExon	-0,235	3,03E-08	1,75E-06
cg24119251	3	193627593		IGR	-0,203	3,03E-08	1,76E-06
cg26570257	1	223404230	SUSD4	Body	-0,223	3,04E-08	1,76E-06
cg24172173	3	124775523	HEG1	TSS1500	0,216	3,04E-08	1,76E-06
cg21136104	3	142666320		IGR	0,287	3,04E-08	1,76E-06
cg18366639	11	14096911	SPON1	Body	-0,225	3,04E-08	1,76E-06
cg19707677	1	207271346	C4BPB	Body	-0,221	3,04E-08	1,76E-06
cg01132696	6	33048558	HLA-DPB1	Body	0,223	3,04E-08	1,76E-06
cg08746853	12	14924953	HIST4H4	TSS1500	-0,209	3,04E-08	1,76E-06
cg19865770	1	221510889	LOC400804	TSS1500	-0,216	3,05E-08	1,76E-06
cg05860978	5	148758992	IL17B	TSS200	-0,236	3,05E-08	1,76E-06
cg16178097	7	56045724	GBAS	Body	0,228	3,05E-08	1,76E-06
cg01891782	3	178036764		IGR	-0,203	3,05E-08	1,76E-06
cg17516825	1	111213827		IGR	-0,209	3,05E-08	1,76E-06
cg27188243	11	12851817	TEAD1	Body	-0,255	3,06E-08	1,76E-06
cg08131884	7	135414087	SLC13A4	TSS1500	-0,27	3,06E-08	1,76E-06
cg00882967	21	44484325	CBS	Body	-0,218	3,06E-08	1,76E-06
cg10718267	2	9783004		IGR	-0,238	3,06E-08	1,76E-06
cg08153812	20	50021369	NFATC2	Body	-0,289	3,07E-08	1,77E-06
cg23665568	1	85745053	BCL10	TSS1500	-0,216	3,07E-08	1,77E-06
cg21057323	14	103412980	CDC42BPB	Body	-0,382	3,07E-08	1,77E-06
cg00678701	1	245382355	KIF26B	Body	-0,223	3,07E-08	1,77E-06
cg03383651	19	18954880	UPF1	Body	-0,241	3,07E-08	1,77E-06
cg25727293	20	2846624	VPS16	Body	-0,203	3,07E-08	1,77E-06
cg24141382	1	41446726	CTPS	5'UTR	-0,278	3,07E-08	1,77E-06
cg20750319	7	625089	PRKAR1B	Body	-0,279	3,07E-08	1,77E-06
cg15617059	13	99175893	STK24	Body	-0,221	3,07E-08	1,77E-06
cg00611549	2	206504322		IGR	-0,235	3,08E-08	1,77E-06
cg22212250	5	132232586	AFF4	Body	-0,314	3,08E-08	1,77E-06
cg25895948	15	85360655	ALPK3	1stExon	0,218	3,08E-08	1,77E-06
cg02193425	6	3848280	FAM50B	TSS1500	-0,207	3,08E-08	1,77E-06
cg20529074	4	20286834	SLIT2	Body	-0,209	3,08E-08	1,77E-06
cg22713649	2	178034599		IGR	-0,242	3,09E-08	1,77E-06
cg06682450	2	218231928	DIRC3	Body	-0,223	3,09E-08	1,77E-06
cg19916943	5	95992957		IGR	-0,344	3,09E-08	1,77E-06
cg23077820	2	223154176	PAX3	Body	0,262	3,09E-08	1,78E-06
cg02523640	10	129534613	FOXI2	TSS1500	0,24	3,09E-08	1,78E-06
cg19250932	20	1800881		IGR	-0,29	3,10E-08	1,78E-06
cg14611086	7	93689119		IGR	-0,252	3,10E-08	1,78E-06
cg19811144	18	46286151	KIAA0427	Body	-0,265	3,10E-08	1,78E-06
cg10201299	7	11100805	PHF14	Body	0,311	3,10E-08	1,78E-06
cg02803819	7	27141067	HOXA2	Body	0,22	3,10E-08	1,78E-06
cg13574913	10	81045166	ZMIZ1	Body	-0,28	3,11E-08	1,78E-06
cg21208028	10	113831817		IGR	-0,207	3,11E-08	1,78E-06
cg04864378	7	151037217		IGR	-0,247	3,11E-08	1,78E-06
cg06332620	5	80903690	SSBP2	Body	-0,205	3,11E-08	1,78E-06
cg09948419	7	45019965	MYO1G	TSS1500	-0,292	3,11E-08	1,78E-06

cg16274385	22	39684821		IGR	-0,202	3,11E-08	1,78E-06
cg00565412	21	26933554	MIR155HG	TSS1500	-0,322	3,12E-08	1,78E-06
cg11601400	6	52859336	GSTA4	5'UTR	0,246	3,12E-08	1,79E-06
cg25695232	2	202397506	ALS2CR11	Body	-0,202	3,12E-08	1,79E-06
cg10876297	19	16706125	MED26	Body	-0,231	3,12E-08	1,79E-06
cg15855021	5	141468137		IGR	-0,247	3,13E-08	1,79E-06
cg05408649	12	54446019	HOXC4	5'UTR	0,241	3,13E-08	1,79E-06
cg05620847	6	90695223	BACH2	Body	0,22	3,13E-08	1,79E-06
cg00568949	10	34985257	PARD3	ExonBnd	-0,213	3,13E-08	1,79E-06
cg05952367	14	75605586	TMED10	Body	-0,23	3,13E-08	1,79E-06
cg14916983	15	40674883	C15orf23	TSS200	0,206	3,14E-08	1,79E-06
cg19419575	12	53808961	SP1	3'UTR	0,214	3,14E-08	1,79E-06
cg01065078	1	209941646	TRAF3IP3	TSS200	-0,204	3,14E-08	1,79E-06
cg13126984	10	33398884		IGR	-0,321	3,14E-08	1,79E-06
cg11508674	14	89651548	FOXN3	Body	0,28	3,14E-08	1,79E-06
cg24480540	2	39356161		IGR	-0,378	3,14E-08	1,79E-06
cg07236647	2	106472964	NCK2	Body	-0,328	3,15E-08	1,79E-06
cg13700089	3	65940340	MAGI1	Body	0,232	3,15E-08	1,79E-06
cg00307819	17	47817938	FAM117A	Body	-0,314	3,15E-08	1,79E-06
cg12032027	20	52198225	ZNF217	1stExon	-0,323	3,15E-08	1,79E-06
cg01140515	20	50419033	SALL4	1stExon	0,355	3,15E-08	1,80E-06
cg23264234	5	73875179		IGR	-0,291	3,16E-08	1,80E-06
cg02517189	19	42553354	GRIK5	Body	-0,351	3,16E-08	1,80E-06
cg25601025	13	33906662	STARD13	Body	-0,266	3,18E-08	1,80E-06
cg25343560	19	13155130	NFIX	Body	-0,217	3,18E-08	1,80E-06
cg01316476	7	25899194		IGR	0,221	3,18E-08	1,80E-06
cg12832565	1	145715672	CD160	TSS200	-0,214	3,18E-08	1,81E-06
cg13465381	1	47282666	CYP4B1	Body	-0,227	3,18E-08	1,81E-06
cg27424995	1	27902555	AHDC1	5'UTR	0,271	3,18E-08	1,81E-06
cg19526450	5	142254301	ARHGAP26	Body	0,229	3,18E-08	1,81E-06
cg21105443	7	7576792	COL28A1	TSS1500	0,228	3,18E-08	1,81E-06
cg04432875	13	113411561	ATP11A	Body	0,204	3,18E-08	1,81E-06
cg22593668	18	13284606	LDLRAD4	5'UTR	-0,217	3,18E-08	1,81E-06
cg26847571	15	99664049	SYNM	Body	0,202	3,19E-08	1,81E-06
cg21735522	10	87501662	GRID1	Body	-0,206	3,19E-08	1,81E-06
cg03309738	6	13764242		IGR	-0,281	3,20E-08	1,81E-06
cg08486371	1	226768661	C1orf95	Body	-0,255	3,20E-08	1,81E-06
cg09987521	9	36603035	MELK	5'UTR	-0,213	3,20E-08	1,81E-06
cg06347851	5	124347050		IGR	-0,285	3,20E-08	1,81E-06
cg04207180	21	37682600		IGR	-0,266	3,21E-08	1,82E-06
cg05933789	2	97190408		IGR	-0,219	3,21E-08	1,82E-06
cg05683586	1	46010892		IGR	-0,265	3,22E-08	1,82E-06
cg18268547	17	79615552	TSPAN10	3'UTR	0,217	3,22E-08	1,82E-06
cg01772658	3	45610544		IGR	-0,273	3,23E-08	1,82E-06
cg14761754	1	94798711		IGR	-0,244	3,23E-08	1,82E-06
cg08491437	1	78442533	FUBP1	Body	-0,276	3,23E-08	1,83E-06
cg26277556	14	54507223		IGR	-0,203	3,23E-08	1,83E-06
cg01611783	7	140016443		IGR	-0,292	3,23E-08	1,83E-06
cg07491975	3	4448600	SUMF1	Body	-0,224	3,23E-08	1,83E-06
cg11063110	7	129985205	CPA5	5'UTR	0,21	3,24E-08	1,83E-06

cg05250640	11	74907144	SLCO2B1	Body	-0,235	3,24E-08	1,83E-06
cg16286874	4	77923247	sept-11	Body	0,237	3,25E-08	1,83E-06
cg20001810	12	52829316	KRT75	TSS1500	-0,271	3,26E-08	1,84E-06
cg17552471	7	4678661		IGR	-0,234	3,26E-08	1,84E-06
cg16424396	10	71022363	HKDC1	Body	-0,255	3,27E-08	1,84E-06
cg17702736	6	35941709	SLC26A8	Body	-0,204	3,27E-08	1,84E-06
cg18448581	18	70534767	NETO1	1stExon	0,24	3,27E-08	1,84E-06
cg03102603	2	201239016	SPATS2L	5'UTR	-0,441	3,27E-08	1,84E-06
cg06650563	2	165777079	SLC38A11	Body	-0,302	3,27E-08	1,84E-06
cg02892925	8	60032926	TOX	TSS1500	0,232	3,27E-08	1,84E-06
cg18975250	2	146515156		IGR	-0,243	3,27E-08	1,84E-06
cg09362796	4	3204792	HTT	Body	-0,2	3,28E-08	1,84E-06
cg19011176	2	121186502		IGR	-0,224	3,28E-08	1,84E-06
cg23079252	6	30095199		IGR	0,226	3,28E-08	1,84E-06
cg24657691	15	64286419	DAPK2	Body	0,205	3,29E-08	1,85E-06
cg09373727	1	32405360	PTP4A2	TSS1500	-0,261	3,29E-08	1,85E-06
cg09568130	19	35480440		IGR	-0,263	3,29E-08	1,85E-06
cg25262361	3	178978993	KCNMB3	TSS1500	0,215	3,30E-08	1,85E-06
cg16529592	1	25292215	RUNX3	TSS1500	-0,239	3,30E-08	1,85E-06
cg10028097	5	125296986		IGR	-0,267	3,31E-08	1,85E-06
cg03926767	11	122268864		IGR	-0,252	3,31E-08	1,85E-06
cg11577310	16	11405816		IGR	-0,226	3,31E-08	1,85E-06
cg08450079	15	81633740	TMC3	ExonBnd	-0,239	3,31E-08	1,86E-06
cg10130628	6	30958445		IGR	-0,212	3,32E-08	1,86E-06
cg21044995	11	75310137	MAP6	Body	-0,225	3,32E-08	1,86E-06
cg10081941	2	189269489	GULP1	5'UTR	-0,239	3,32E-08	1,86E-06
cg08065408	6	29520774		IGR	0,217	3,32E-08	1,86E-06
cg05440269	1	204172023	GOLT1A	ExonBnd	-0,207	3,33E-08	1,86E-06
cg24299174	6	112375383	WISP3	5'UTR	-0,232	3,33E-08	1,86E-06
cg04837025	1	236228744	NID1	TSS1500	0,215	3,33E-08	1,86E-06
cg20831708	10	102279373	SEC31B	5'UTR	0,246	3,33E-08	1,86E-06
cg15991124	19	34841438	KIAA0355	Body	-0,306	3,33E-08	1,86E-06
cg18451373	3	189755966	P3H2	Body	0,246	3,34E-08	1,86E-06
cg10488454	10	50146756	WDFY4	Body	-0,207	3,34E-08	1,86E-06
cg11061136	5	175969423	CDHR2	TSS200	-0,227	3,35E-08	1,87E-06
cg04438074	10	63422539	C10orf107	TSS200	0,271	3,35E-08	1,87E-06
cg01044476	12	7865756	DPPA3	Body	-0,224	3,35E-08	1,87E-06
cg16268759	5	16657611		IGR	-0,214	3,35E-08	1,87E-06
cg04580625	2	7966002		IGR	-0,266	3,35E-08	1,87E-06
cg23441763	5	55906354		IGR	-0,246	3,36E-08	1,87E-06
cg18434246	5	123912954		IGR	-0,237	3,36E-08	1,87E-06
cg24488891	10	25464132	LOC10012881	Body	0,211	3,36E-08	1,87E-06
cg26757673	22	37545423	IL2RB	5'UTR	-0,227	3,37E-08	1,87E-06
cg00193581	1	77879926	AK5	Body	-0,23	3,37E-08	1,88E-06
cg18720818	16	67412708	LRRC36	Body	-0,235	3,37E-08	1,88E-06
cg20713968	8	1737564		IGR	0,217	3,37E-08	1,88E-06
cg10022184	5	158338266	EBF1	Body	-0,247	3,38E-08	1,88E-06
cg12645561	1	51339561	FAF1	Body	-0,243	3,38E-08	1,88E-06
cg26356061	19	58446960	ZNF418	TSS1500	0,234	3,38E-08	1,88E-06
cg10863207	17	33412186	RFFL	5'UTR	-0,263	3,38E-08	1,88E-06

cg20460006	1	192955285		IGR	-0,214	3,40E-08	1,89E-06
cg23819330	4	186459047		IGR	-0,221	3,40E-08	1,89E-06
cg05058138	5	135049034		IGR	-0,307	3,40E-08	1,89E-06
cg24127061	11	65839402	PACS1	Body	-0,243	3,40E-08	1,89E-06
cg22886659	6	498807	EXOC2	Body	-0,242	3,40E-08	1,89E-06
cg16898345	5	149645151	CAMK2A	Body	-0,253	3,41E-08	1,89E-06
cg09827588	1	31353769	SDC3	Body	-0,238	3,41E-08	1,89E-06
cg06056332	1	160765923	LY9	5'UTR	-0,231	3,41E-08	1,89E-06
cg01446492	15	85859864		IGR	-0,288	3,42E-08	1,89E-06
cg10111335	6	105935915		IGR	-0,291	3,42E-08	1,89E-06
cg22920603	20	49509062	ADNP	Body	-0,251	3,42E-08	1,89E-06
cg18404652	8	42009622	AP3M2	TSS1500	-0,214	3,43E-08	1,90E-06
cg23471274	19	43955029		IGR	-0,303	3,44E-08	1,90E-06
cg03423665	20	46788649		IGR	-0,212	3,44E-08	1,90E-06
cg11870407	4	47533850	ATP10D	Body	-0,208	3,44E-08	1,90E-06
cg21919820	7	28081543	JAZF1	Body	-0,255	3,45E-08	1,90E-06
cg06012220	18	13291163	LDLRAD4	5'UTR	-0,251	3,45E-08	1,91E-06
cg17474560	2	238364539		IGR	-0,22	3,45E-08	1,91E-06
cg11951897	15	77286957	PSTPIP1	TSS1500	-0,244	3,45E-08	1,91E-06
cg12464638	11	844400	TSPAN4	TSS200	0,246	3,46E-08	1,91E-06
cg22146612	6	17715219		IGR	-0,262	3,46E-08	1,91E-06
cg04194449	10	73633607		IGR	-0,25	3,46E-08	1,91E-06
cg10139216	8	101441196		IGR	-0,273	3,46E-08	1,91E-06
cg14038959	1	42136440	HIVEP3	5'UTR	-0,241	3,47E-08	1,91E-06
cg10811275	11	130332787	ADAMTS15	Body	-0,227	3,47E-08	1,91E-06
cg14977069	20	62367698	LIME1	TSS1500	-0,27	3,48E-08	1,92E-06
cg09827400	3	194676597		IGR	-0,308	3,48E-08	1,92E-06
cg06045844	22	39487493		IGR	-0,248	3,48E-08	1,92E-06
cg16086570	5	141924566		IGR	-0,244	3,49E-08	1,92E-06
cg06043840	1	94305094	BCAR3	5'UTR	-0,273	3,49E-08	1,92E-06
cg05817709	11	63304098	RARRES3	TSS200	-0,296	3,49E-08	1,92E-06
cg06766034	22	47082260	CERK	3'UTR	0,232	3,49E-08	1,92E-06
cg02501827	11	11862879	USP47	TSS200	0,225	3,50E-08	1,92E-06
cg27648405	17	6922960	MIR497HG	Body	0,249	3,50E-08	1,92E-06
cg01592226	22	36740752	MYH9	Body	-0,262	3,50E-08	1,92E-06
cg21608953	21	16273878		IGR	-0,212	3,50E-08	1,93E-06
cg19750908	7	51086038	COBL	Body	-0,221	3,51E-08	1,93E-06
cg26198959	14	65673250		IGR	-0,323	3,51E-08	1,93E-06
cg01482418	1	225642131		IGR	-0,23	3,51E-08	1,93E-06
cg06147895	10	133791401	BNIP3	Body	0,237	3,51E-08	1,93E-06
cg25348374	6	42488845		IGR	-0,263	3,52E-08	1,93E-06
cg06618740	1	1100126		IGR	0,219	3,52E-08	1,93E-06
cg06660497	3	107704588		IGR	-0,205	3,52E-08	1,93E-06
cg09525260	15	84523205	ADAMTSL3	Body	-0,254	3,52E-08	1,93E-06
cg26031327	4	7975403	ABLIM2	Body	-0,232	3,52E-08	1,93E-06
cg06795963	17	40932359	WNK4	TSS1500	0,231	3,53E-08	1,93E-06
cg27617011	20	57070142	APCDD1L	Body	-0,207	3,53E-08	1,93E-06
cg14768545	4	139416093		IGR	-0,213	3,53E-08	1,94E-06
cg20604274	2	217523078	IGFBP2	Body	-0,266	3,53E-08	1,94E-06
cg14512523	11	17230914		IGR	0,201	3,53E-08	1,94E-06

cg19004775	13	106063150		IGR	0,228	3,54E-08	1,94E-06
cg07846220	18	7117680	LAMA1	1stExon	0,22	3,54E-08	1,94E-06
cg16035777	1	205550148	MFSD4	Body	-0,243	3,54E-08	1,94E-06
cg23427549	6	168307882	MLLT4	Body	0,312	3,54E-08	1,94E-06
cg15823097	4	5883699	CRMP1	Body	-0,265	3,55E-08	1,94E-06
cg05640676	12	68513016		IGR	-0,246	3,55E-08	1,94E-06
cg15510965	1	46505350		IGR	0,217	3,56E-08	1,94E-06
cg26144027	3	69047209	EOGT	Body	-0,207	3,56E-08	1,95E-06
cg11867253	10	99642327	CRTAC1	Body	-0,215	3,56E-08	1,95E-06
cg13900216	21	35881375	KCNE1	5'UTR	-0,209	3,57E-08	1,95E-06
cg16383863	5	79097013		IGR	-0,262	3,57E-08	1,95E-06
cg10501835	18	77723201	HSBP1L1	TSS1500	-0,31	3,57E-08	1,95E-06
cg15162818	2	23858940	KLHL29	Body	-0,251	3,57E-08	1,95E-06
cg03954045	12	43247617		IGR	-0,205	3,58E-08	1,95E-06
cg23318908	6	112113492	FYN	5'UTR	-0,249	3,58E-08	1,96E-06
cg01904296	11	45713046		IGR	-0,247	3,58E-08	1,96E-06
cg18843496	3	129261646	H1FOO	TSS1500	-0,277	3,59E-08	1,96E-06
cg18287455	6	121936444		IGR	-0,236	3,59E-08	1,96E-06
cg11993277	11	67172526	TBC1D10C	Body	-0,259	3,59E-08	1,96E-06
cg03579392	14	101002170		IGR	-0,229	3,59E-08	1,96E-06
cg01989456	15	75469169		IGR	-0,26	3,59E-08	1,96E-06
cg26680608	6	136915556	MAP3K5	Body	-0,27	3,59E-08	1,96E-06
cg16972619	12	28078288		IGR	-0,227	3,59E-08	1,96E-06
cg10772230	18	59415294	LINC01544	TSS200	0,251	3,60E-08	1,96E-06
cg09152451	5	31742848		IGR	-0,23	3,60E-08	1,96E-06
cg15885703	11	118094830	AMICA1	5'UTR	-0,302	3,61E-08	1,96E-06
cg18582260	13	25085301	PARP4	5'UTR	-0,312	3,61E-08	1,96E-06
cg05318546	2	221864241		IGR	-0,234	3,61E-08	1,96E-06
cg23138978	17	27876689	TAOK1	3'UTR	0,215	3,61E-08	1,96E-06
cg04233620	21	46629219	ADARB1	Body	-0,235	3,61E-08	1,97E-06
cg06501716	22	19436948	C22orf39	TSS1500	-0,242	3,61E-08	1,97E-06
cg21324642	18	36521390		IGR	-0,211	3,62E-08	1,97E-06
cg07569918	1	212002970	LPGAT1	5'UTR	0,305	3,62E-08	1,97E-06
cg08283465	18	45547783		IGR	-0,218	3,62E-08	1,97E-06
cg12492653	11	11862867	USP47	TSS200	0,262	3,62E-08	1,97E-06
cg23095383	1	198649189	PTPRC	Body	-0,34	3,63E-08	1,97E-06
cg13194738	1	169332649	NME7	5'UTR	-0,231	3,63E-08	1,97E-06
cg19968742	2	33311713	LTBP1	Body	0,228	3,64E-08	1,98E-06
cg00918181	18	11947875		IGR	0,235	3,64E-08	1,98E-06
cg02394664	10	6572395	PRKCQ	5'UTR	-0,228	3,64E-08	1,98E-06
cg00754989	15	72530044		IGR	-0,315	3,64E-08	1,98E-06
cg18725599	5	149590502	SLC6A7	3'UTR	-0,208	3,65E-08	1,98E-06
cg00050618	16	27414418	IL21R	TSS200	-0,238	3,66E-08	1,98E-06
cg01613414	10	126693304	CTBP2	Body	-0,234	3,66E-08	1,98E-06
cg13112004	12	12509612	LOH12CR2	Body	-0,21	3,67E-08	1,99E-06
cg19403364	5	67611940		IGR	-0,248	3,67E-08	1,99E-06
cg17605629	1	7625636	CAMTA1	Body	-0,239	3,67E-08	1,99E-06
cg26736952	11	34645679	EHF	5'UTR	-0,207	3,67E-08	1,99E-06
cg01226193	11	125909557	CDON	5'UTR	-0,287	3,67E-08	1,99E-06
cg06835941	19	16449199		IGR	-0,271	3,67E-08	1,99E-06

cg15449419	4	184202832	WWC2	Body	0,273	3,68E-08	1,99E-06
cg03718013	14	103871417	MARK3	ExonBnd	0,261	3,68E-08	1,99E-06
cg17468185	3	129147397	C3orf25	5'UTR	0,263	3,69E-08	1,99E-06
cg16463956	14	100530076		IGR	-0,262	3,69E-08	1,99E-06
cg27616929	20	47377425	PREX1	Body	-0,241	3,69E-08	1,99E-06
cg09189074	3	134068113		IGR	-0,212	3,69E-08	1,99E-06
cg07288713	12	64324997	SRGAP1	Body	-0,225	3,69E-08	1,99E-06
cg26011170	15	101667529		IGR	-0,337	3,70E-08	2,00E-06
cg19456061	18	74076380	ZNF516	Body	-0,239	3,70E-08	2,00E-06
cg15664323	6	4427110		IGR	-0,224	3,71E-08	2,00E-06
cg06203637	10	31933573		IGR	-0,239	3,71E-08	2,00E-06
cg25223532	2	206606411	NRP2	Body	-0,285	3,71E-08	2,00E-06
cg18559618	17	1848171	RTN4RL1	Body	-0,221	3,72E-08	2,01E-06
cg10092779	1	57294085		IGR	-0,227	3,72E-08	2,01E-06
cg11223361	6	32023102	TNXB	Body	-0,227	3,72E-08	2,01E-06
cg01020567	17	75469976	sept-09	TSS1500	-0,237	3,73E-08	2,01E-06
cg23146957	12	95015658	TMCC3	Body	-0,368	3,73E-08	2,01E-06
cg22981158	19	47156401	DACT3	Body	-0,259	3,73E-08	2,01E-06
cg05274664	18	12840211	PTPN2	Body	-0,32	3,73E-08	2,01E-06
cg20419869	3	145898583		IGR	-0,272	3,73E-08	2,01E-06
cg17371189	2	61555931	USP34	Body	-0,281	3,73E-08	2,01E-06
cg01591160	10	129901854	MKI67	Body	-0,216	3,74E-08	2,01E-06
cg02936931	6	134856977		IGR	-0,243	3,75E-08	2,02E-06
cg06648277	10	134600489	NKX6-2	TSS1500	0,21	3,75E-08	2,02E-06
cg04894218	5	112614911	MCC	Body	-0,296	3,75E-08	2,02E-06
cg12303488	11	67176300	TBC1D10C	Body	-0,21	3,75E-08	2,02E-06
cg25724800	4	71602849	RUFY3	5'UTR	-0,356	3,76E-08	2,02E-06
cg12278018	1	35266042		IGR	-0,365	3,76E-08	2,02E-06
cg09219302	20	47435660	PREX1	Body	-0,225	3,76E-08	2,02E-06
cg08644046	2	241929774		IGR	-0,254	3,77E-08	2,02E-06
cg04768479	6	30095292		IGR	0,233	3,77E-08	2,02E-06
cg16132337	7	63498261		IGR	0,208	3,77E-08	2,02E-06
cg23590603	7	47010866		IGR	-0,219	3,77E-08	2,02E-06
cg26354493	19	4305072	FSD1	Body	0,29	3,77E-08	2,02E-06
cg13988440	11	69240805		IGR	-0,278	3,77E-08	2,02E-06
cg04388124	16	10870426	TVP23A	Body	-0,27	3,78E-08	2,03E-06
cg05422344	11	114033783	ZBTB16	Body	0,23	3,78E-08	2,03E-06
cg20786697	11	67205650	PTPRCAP	TSS1500	-0,225	3,78E-08	2,03E-06
cg24371856	5	31882166	PDZD2	Body	-0,231	3,79E-08	2,03E-06
cg03730529	14	89892521	FOXN3	5'UTR	-0,235	3,79E-08	2,03E-06
cg26719625	17	16322482	TRPV2	Body	-0,289	3,79E-08	2,03E-06
cg26585452	13	50702914		IGR	-0,26	3,79E-08	2,03E-06
cg14070534	6	166163813		IGR	-0,206	3,80E-08	2,03E-06
cg25368412	15	91790678	SV2B	Body	-0,213	3,80E-08	2,03E-06
cg05496913	5	130618957	CDC42SE2	5'UTR	-0,253	3,80E-08	2,03E-06
cg02965092	12	72347052	TPH2	Body	-0,235	3,80E-08	2,03E-06
cg25286579	1	56069436		IGR	-0,24	3,80E-08	2,03E-06
cg24559044	5	142435797	ARHGAP26	Body	-0,232	3,80E-08	2,03E-06
cg11335171	6	33173333	HSD17B8	Body	-0,248	3,80E-08	2,04E-06
cg10334053	1	2078117	PRKCZ	Body	-0,241	3,81E-08	2,04E-06

cg11258257	9	140514683	ARRDC1-AS1	TSS1500	-0,31	3,81E-08	2,04E-06
cg06121748	10	34511567	PARD3	Body	-0,221	3,81E-08	2,04E-06
cg04748516	11	62230794	AHNAK	Body	-0,204	3,82E-08	2,04E-06
cg25230907	13	20624124	ZMYM2	Body	-0,313	3,82E-08	2,04E-06
cg27382744	17	3289663		IGR	0,242	3,82E-08	2,04E-06
cg20588566	17	19209758	EPN2-AS1	TSS200	-0,255	3,82E-08	2,04E-06
cg14001486	14	61801201	PRKCH	Body	-0,246	3,83E-08	2,04E-06
cg10389741	10	77794758	C10orf11	Body	-0,225	3,83E-08	2,04E-06
cg04945753	2	201320027	SPATS2L	Body	-0,235	3,83E-08	2,04E-06
cg15214071	20	57740069		IGR	-0,209	3,84E-08	2,05E-06
cg08658634	1	184319916		IGR	-0,215	3,85E-08	2,05E-06
cg08815220	15	81789941		IGR	-0,252	3,85E-08	2,05E-06
cg11043993	10	50536966		IGR	-0,203	3,87E-08	2,06E-06
cg03379664	2	197877514	ANKRD44	Body	0,239	3,87E-08	2,06E-06
cg13880193	2	120209611	SCTR	Body	-0,206	3,87E-08	2,06E-06
cg02188024	8	14718513	SGCZ	Body	0,21	3,87E-08	2,06E-06
cg25210796	1	65089808	CACHD1	Body	-0,247	3,87E-08	2,06E-06
cg05901971	6	152630996	SYNE1	Body	-0,302	3,87E-08	2,06E-06
cg03512007	8	90739332	LOC1019297(	Body	-0,229	3,87E-08	2,06E-06
cg08561684	12	92474176	C12orf79	Body	-0,243	3,87E-08	2,06E-06
cg07383370	7	40766405	SUGCT	Body	-0,319	3,88E-08	2,06E-06
cg12155188	20	47255254	PREX1	Body	-0,276	3,88E-08	2,06E-06
cg01115116	10	30323383	KIAA1462	Body	-0,229	3,88E-08	2,06E-06
cg18303615	10	74085233		IGR	0,21	3,89E-08	2,06E-06
cg08822136	3	127345113		IGR	-0,203	3,89E-08	2,07E-06
cg01796929	12	57352514	RDH16	TSS1500	-0,201	3,89E-08	2,07E-06
cg02883147	12	9217769	LOC144571	TSS200	0,223	3,90E-08	2,07E-06
cg22210682	5	81354741	ATG10	Body	-0,338	3,90E-08	2,07E-06
cg19820287	5	71240614		IGR	-0,212	3,90E-08	2,07E-06
cg02824202	18	6729810		IGR	0,227	3,91E-08	2,07E-06
cg06050350	20	12225088		IGR	-0,206	3,91E-08	2,07E-06
cg18728029	2	197041315		IGR	-0,256	3,91E-08	2,08E-06
cg06850464	22	18525602		IGR	0,212	3,92E-08	2,08E-06
cg14067840	3	133825419		IGR	-0,205	3,92E-08	2,08E-06
cg01369027	10	11133829	CELF2	Body	-0,266	3,92E-08	2,08E-06
cg07371504	17	3433451	TRPV3	Body	0,276	3,94E-08	2,08E-06
cg07056241	4	139821219		IGR	-0,238	3,94E-08	2,08E-06
cg17847345	21	17980718	MIR99AHG	Body	0,216	3,94E-08	2,09E-06
cg27317179	9	100870993	TRIM14	Body	-0,255	3,94E-08	2,09E-06
cg08474396	16	27237656	NSMCE1	Body	-0,222	3,94E-08	2,09E-06
cg25711701	18	8420876		IGR	-0,21	3,94E-08	2,09E-06
cg10867870	1	116138161		IGR	-0,264	3,95E-08	2,09E-06
cg02562604	2	145417369		IGR	-0,216	3,95E-08	2,09E-06
cg10461264	12	125131065		IGR	-0,244	3,95E-08	2,09E-06
cg01628128	16	15647369	C16orf45	Body	-0,206	3,95E-08	2,09E-06
cg21173790	6	491184	EXOC2	ExonBnd	-0,219	3,96E-08	2,09E-06
cg20229798	5	131802741	C5orf56	Body	-0,3	3,96E-08	2,09E-06
cg06388647	2	121467863		IGR	0,258	3,97E-08	2,09E-06
cg22027075	4	184746662		IGR	-0,257	3,97E-08	2,10E-06
cg05096734	22	20186356	LOC284865	Body	-0,208	3,98E-08	2,10E-06

cg05593775	10	102778743	PDZD7	Body	0,204	3,98E-08	2,10E-06
cg21723993	22	33665299		IGR	-0,368	3,98E-08	2,10E-06
cg12685837	15	57625838		IGR	-0,209	3,99E-08	2,10E-06
cg09448009	7	23163400	KLHL7	ExonBnd	0,301	3,99E-08	2,10E-06
cg26014782	3	124840721	SLC12A8	Body	-0,233	3,99E-08	2,10E-06
cg07539983	5	151067341	SPARC	TSS1500	-0,213	3,99E-08	2,10E-06
cg10921592	6	33039414	HLA-DPA1	Body	-0,31	3,99E-08	2,10E-06
cg12703269	15	77286232	PSTPIP1	TSS1500	-0,214	3,99E-08	2,10E-06
cg07568841	7	30362781	ZNRF2	Body	-0,323	4,00E-08	2,10E-06
cg24943575	2	15933717		IGR	-0,236	4,00E-08	2,10E-06
cg12120261	5	125898727	ALDH7A1	Body	-0,373	4,00E-08	2,10E-06
cg24499333	5	171876294	SH3PXD2B	Body	-0,216	4,00E-08	2,10E-06
cg11108023	11	86004529		IGR	-0,206	4,00E-08	2,10E-06
cg15046489	17	48857353		IGR	-0,206	4,00E-08	2,10E-06
cg16953297	3	183993906	ECE2	1stExon	0,208	4,01E-08	2,11E-06
cg22550233	12	2782103	CACNA1C-AS:	TSS1500	-0,213	4,01E-08	2,11E-06
cg15671775	8	39957750		IGR	-0,253	4,01E-08	2,11E-06
cg05719140	1	63782426		IGR	0,214	4,01E-08	2,11E-06
cg00303422	2	217571639		IGR	-0,287	4,02E-08	2,11E-06
cg21591192	1	221612851		IGR	-0,33	4,02E-08	2,11E-06
cg26168873	20	56547387		IGR	-0,205	4,02E-08	2,11E-06
cg09248826	9	130860839	SLC25A25	5'UTR	0,221	4,02E-08	2,11E-06
cg00091524	11	17516772	USH1C	Body	-0,214	4,02E-08	2,11E-06
cg12469665	4	101940221		IGR	-0,279	4,02E-08	2,11E-06
cg13983640	6	41010223	TSPO2	TSS1500	0,21	4,03E-08	2,11E-06
cg07906046	16	4131584	ADCY9	Body	-0,254	4,03E-08	2,11E-06
cg15794638	16	49722796	ZNF423	Body	-0,216	4,04E-08	2,12E-06
cg04291149	6	34880563	ANKS1A	Body	-0,309	4,04E-08	2,12E-06
cg23918296	14	103397019	AMN	3'UTR	0,207	4,05E-08	2,12E-06
cg03484346	1	203433415		IGR	-0,237	4,05E-08	2,12E-06
cg07848660	6	3796243		IGR	0,202	4,05E-08	2,12E-06
cg17431952	5	113806337	KCNN2	Body	-0,214	4,05E-08	2,12E-06
cg00560747	1	31279227		IGR	-0,27	4,06E-08	2,12E-06
cg05184456	8	145557639	SCRT1	Body	0,216	4,06E-08	2,12E-06
cg07823639	11	9048816	SCUBE2	Body	-0,243	4,06E-08	2,12E-06
cg07033577	17	55540785	MSI2	Body	-0,261	4,06E-08	2,13E-06
cg12684820	1	23917192		IGR	-0,242	4,06E-08	2,13E-06
cg09159198	8	23713035	STC1	TSS1500	-0,231	4,06E-08	2,13E-06
cg21247923	13	103532435	LOC121952	TSS200	0,209	4,06E-08	2,13E-06
cg03259445	8	134065658	SLA	Body	-0,203	4,07E-08	2,13E-06
cg07920734	12	14405823		IGR	-0,265	4,07E-08	2,13E-06
cg25727825	3	195542580		IGR	-0,263	4,07E-08	2,13E-06
cg00629513	2	205125813		IGR	-0,231	4,07E-08	2,13E-06
cg14607496	8	81662179	ZNF704	Body	-0,245	4,08E-08	2,13E-06
cg18802773	2	3360115	TSSC1	Body	0,213	4,08E-08	2,13E-06
cg10002668	7	107665708	LAMB4	Body	-0,224	4,08E-08	2,13E-06
cg02487233	3	107810687	CD47	TSS1500	0,241	4,09E-08	2,13E-06
cg18330617	13	111776208	ARHGEF7	Body	0,23	4,09E-08	2,13E-06
cg03626187	16	8834851	ABAT	Body	0,293	4,09E-08	2,14E-06
cg03117444	10	34368563		IGR	-0,212	4,10E-08	2,14E-06

cg05509789	4	74966407	CXCL2	TSS1500	-0,201	4,11E-08	2,14E-06
cg02660277	7	157438616	PTPRN2	Body	-0,221	4,11E-08	2,14E-06
cg13811092	5	66010095	MAST4	Body	-0,245	4,12E-08	2,14E-06
cg01284438	5	175250977	CPLX2	5'UTR	0,249	4,12E-08	2,15E-06
cg24601030	11	111847747	DIXDC1	TSS1500	0,246	4,12E-08	2,15E-06
cg08419235	16	68702501	CDH3	Body	-0,263	4,12E-08	2,15E-06
cg09291478	13	106095894		IGR	-0,214	4,13E-08	2,15E-06
cg18501783	15	100509864		IGR	-0,205	4,13E-08	2,15E-06
cg16829755	15	55562786	RAB27A	TSS200	-0,211	4,13E-08	2,15E-06
cg09510085	16	84518355	KIAA1609	Body	0,335	4,13E-08	2,15E-06
cg26436927	3	98484917	ST3GAL6	5'UTR	0,221	4,14E-08	2,15E-06
cg03559915	11	67201998	RPS6KB2	Body	-0,231	4,14E-08	2,15E-06
cg03988297	1	219060547		IGR	-0,304	4,15E-08	2,15E-06
cg16519911	4	135122914	PABPC4L	TSS200	0,214	4,15E-08	2,15E-06
cg01527805	13	24850703	SPATA13	5'UTR	-0,218	4,15E-08	2,15E-06
cg14528486	20	62666862	LINC00176	Body	-0,239	4,16E-08	2,16E-06
cg16860435	5	159179752		IGR	0,312	4,17E-08	2,16E-06
cg20140488	22	25463866	KIAA1671	Body	-0,266	4,17E-08	2,16E-06
cg07955409	18	46144678	CTIF	5'UTR	-0,213	4,17E-08	2,16E-06
cg26893685	6	2523615		IGR	-0,2	4,18E-08	2,17E-06
cg07774680	7	29127983	CPVL	Body	-0,285	4,18E-08	2,17E-06
cg18630030	17	60900969	MIR548W	Body	-0,211	4,19E-08	2,17E-06
cg16406315	22	39270139		IGR	-0,289	4,19E-08	2,17E-06
cg27109588	3	128930686		IGR	0,234	4,19E-08	2,17E-06
cg05147453	8	71043691	NCOA2	Body	-0,21	4,19E-08	2,17E-06
cg09805466	2	223566483	MOGAT1	Body	-0,343	4,20E-08	2,17E-06
cg19617373	11	61548003	C11orf9	Body	-0,209	4,20E-08	2,17E-06
cg18892169	1	150737762	CTSS	5'UTR	-0,22	4,21E-08	2,18E-06
cg02070740	1	146763914	CHD1L	Body	-0,297	4,21E-08	2,18E-06
cg03516301	1	7362873	CAMTA1	Body	-0,249	4,23E-08	2,19E-06
cg10902504	9	116263582	RGS3	TSS200	0,263	4,23E-08	2,19E-06
cg07266431	7	92460113	CDK6	Body	-0,209	4,23E-08	2,19E-06
cg02012279	17	37974860	IKZF3	Body	-0,242	4,24E-08	2,19E-06
cg04780380	20	57412945	GNASAS	Body	-0,249	4,24E-08	2,19E-06
cg00183786	2	71817620	DYSF	Body	-0,205	4,24E-08	2,19E-06
cg05408442	1	24691920	STPG1	Body	-0,305	4,24E-08	2,19E-06
cg22328208	8	98289745	TSPYL5	1stExon	0,215	4,24E-08	2,19E-06
cg07589149	2	38162540	RMDN2	Body	-0,245	4,25E-08	2,19E-06
cg03789415	17	76813470	USP36	Body	-0,267	4,25E-08	2,19E-06
cg07941916	5	131762292	C5orf56	Body	-0,214	4,25E-08	2,19E-06
cg04693169	1	49187460	AGBL4	Body	-0,216	4,25E-08	2,19E-06
cg12193327	2	31454631		IGR	0,261	4,25E-08	2,19E-06
cg16531578	6	42370098	TRERF1	5'UTR	-0,326	4,26E-08	2,19E-06
cg03117976	18	11149435	FAM38B	TSS1500	0,21	4,26E-08	2,19E-06
cg08792630	6	108883909	FOXO3	Body	0,205	4,26E-08	2,20E-06
cg14483137	12	12837495	GPR19	5'UTR	0,232	4,27E-08	2,20E-06
cg22714134	15	101922258	PCSK6	Body	-0,217	4,27E-08	2,20E-06
cg13603599	11	67251939	AIP	Body	-0,252	4,27E-08	2,20E-06
cg00290758	10	121171859	GRK5	Body	0,218	4,28E-08	2,20E-06
cg20994481	6	167742801	TTLL2	Body	-0,231	4,28E-08	2,20E-06

cg03517226	16	89408322	ANKRD11	5'UTR	-0,215	4,29E-08	2,21E-06
cg21826978	6	32823588	PSMB9	Body	-0,277	4,29E-08	2,21E-06
cg17761826	21	18913093	CXADR	Body	0,268	4,30E-08	2,21E-06
cg15762892	2	169489435	CERS6	Body	0,232	4,30E-08	2,21E-06
cg13287515	1	170209726		IGR	-0,285	4,30E-08	2,21E-06
cg20325131	16	55689851	SLC6A2	1stExon	0,207	4,30E-08	2,21E-06
cg18141714	18	48697784		IGR	-0,259	4,30E-08	2,21E-06
cg07277549	7	30509141	NOD1	5'UTR	0,262	4,30E-08	2,21E-06
cg05602862	2	14775046	FAM84A	3'UTR	0,219	4,31E-08	2,21E-06
cg22144875	19	46032431	OPA3	Body	0,203	4,31E-08	2,21E-06
cg04878498	10	1219979		IGR	-0,201	4,31E-08	2,21E-06
cg22486834	6	52859107	GSTA4	5'UTR	0,257	4,31E-08	2,21E-06
cg10236239	2	108994514	SULT1C4	5'UTR	0,22	4,31E-08	2,21E-06
cg02852959	12	2944436	NRIP2	TSS1500	0,222	4,32E-08	2,21E-06
cg11683242	1	32716557	LCK	TSS1500	-0,241	4,33E-08	2,22E-06
cg17055489	6	143888190	LOC285740	Body	-0,274	4,33E-08	2,22E-06
cg08139247	14	38725312	CLEC14A	1stExon	0,291	4,34E-08	2,22E-06
cg22609984	2	161126801		IGR	0,201	4,34E-08	2,22E-06
cg18297960	7	1142765	C7orf50	Body	-0,239	4,35E-08	2,23E-06
cg26829071	12	131590596	GPR133	Body	-0,213	4,35E-08	2,23E-06
cg21150108	13	34750105		IGR	-0,204	4,36E-08	2,23E-06
cg18992943	3	123445008	MYLK	Body	-0,212	4,36E-08	2,23E-06
cg04465154	8	9045558		IGR	-0,246	4,37E-08	2,23E-06
cg12469306	1	230850110	AGT	5'UTR	0,205	4,37E-08	2,24E-06
cg26791242	1	87596895	LOC339524	Body	-0,222	4,38E-08	2,24E-06
cg08329501	9	79056558	GCNT1	TSS200	-0,297	4,38E-08	2,24E-06
cg14814749	5	176080659	TSPAN17	Body	-0,205	4,38E-08	2,24E-06
cg08193130	12	106114929	CASC18	Body	-0,246	4,38E-08	2,24E-06
cg12702760	18	47216848		IGR	-0,274	4,38E-08	2,24E-06
cg16078649	6	30039466	RNF39	Body	0,304	4,39E-08	2,24E-06
cg03353654	10	80677715		IGR	-0,209	4,39E-08	2,24E-06
cg02890820	10	105750013	SLK	Body	-0,237	4,40E-08	2,25E-06
cg18362003	11	122051630	LOC399959	Body	-0,303	4,41E-08	2,25E-06
cg02085507	19	6739192	TRIP10	TSS1500	0,227	4,41E-08	2,25E-06
cg10110288	5	55274030	IL6ST	Body	-0,29	4,41E-08	2,25E-06
cg27618173	8	134069863	SLA	Body	-0,325	4,41E-08	2,25E-06
cg24091315	10	97080852	SORBS1	Body	-0,236	4,42E-08	2,25E-06
cg11235869	1	245857639	KIF26B	Body	-0,214	4,42E-08	2,25E-06
cg18603250	11	94501052	AMOTL1	TSS1500	0,204	4,42E-08	2,25E-06
cg05477778	19	16480028	EPS15L1	Body	-0,285	4,43E-08	2,25E-06
cg02970507	3	101707435	LOC152225	Body	-0,238	4,43E-08	2,25E-06
cg13315147	10	135341528	CYP2E1	Body	0,206	4,44E-08	2,26E-06
cg04374617	4	139262791	LINC00499	Body	-0,256	4,45E-08	2,26E-06
cg14321810	4	36292218	DTHD1	Body	0,224	4,46E-08	2,26E-06
cg01097384	11	1358675		IGR	0,272	4,47E-08	2,27E-06
cg07972624	7	41525		IGR	-0,249	4,47E-08	2,27E-06
cg26575450	2	64834280		IGR	-0,244	4,47E-08	2,27E-06
cg03478487	5	35822800		IGR	-0,212	4,47E-08	2,27E-06
cg23535171	1	171226354	FMO1	5'UTR	0,231	4,48E-08	2,27E-06
cg06585566	19	57276794		IGR	0,274	4,48E-08	2,27E-06

cg24545125	2	217724866	TNP1	TSS200	-0,22	4,48E-08	2,27E-06
cg02916283	14	90087792		IGR	-0,287	4,49E-08	2,27E-06
cg16723255	1	225928050		IGR	-0,226	4,49E-08	2,27E-06
cg17818435	12	53497138	SOAT2	TSS200	-0,259	4,49E-08	2,28E-06
cg06484854	1	3516471	MEGF6	Body	-0,262	4,50E-08	2,28E-06
cg08452038	22	28426107	TTC28	Body	-0,265	4,50E-08	2,28E-06
cg08418111	11	18433745	LDHC	TSS200	0,25	4,50E-08	2,28E-06
cg08797194	13	96705123	UGGT2	Body	0,223	4,51E-08	2,28E-06
cg17813524	5	14415171	TRIO	Body	-0,203	4,51E-08	2,28E-06
cg14637146	1	192508256		IGR	-0,253	4,51E-08	2,28E-06
cg11688093	13	111178359	RAB20	Body	-0,201	4,53E-08	2,29E-06
cg21583467	2	237591649		IGR	-0,232	4,53E-08	2,29E-06
cg20706070	6	112035786	FYN	Body	-0,246	4,53E-08	2,29E-06
cg22596511	1	42164849	HIVEP3	5'UTR	-0,238	4,54E-08	2,29E-06
cg02459648	12	104613282	TXNRD1	Body	0,217	4,55E-08	2,29E-06
cg26510452	20	39991461	EMILIN3	Body	-0,226	4,54E-08	2,29E-06
cg10377643	15	96164040		IGR	-0,255	4,55E-08	2,29E-06
cg18916494	2	134263033	NCKAP5	Body	-0,233	4,55E-08	2,29E-06
cg11943574	10	90170867	RNLS	Body	0,289	4,55E-08	2,29E-06
cg12240237	7	73946402	GTF2IRD1	Body	-0,265	4,55E-08	2,29E-06
cg08150816	1	169281716	NME7	Body	-0,303	4,56E-08	2,29E-06
cg03838641	8	95254549		IGR	-0,293	4,56E-08	2,30E-06
cg09791316	11	67404589	TBX10	Body	-0,263	4,56E-08	2,30E-06
cg25391492	5	138078269		IGR	-0,229	4,57E-08	2,30E-06
cg02008113	7	48127251	UPP1	TSS1500	-0,201	4,57E-08	2,30E-06
cg00357551	5	169407472	FAM196B	5'UTR	0,243	4,57E-08	2,30E-06
cg02184632	8	123983086	ZHX2	3'UTR	-0,302	4,59E-08	2,31E-06
cg05777268	11	76898500	MYO7A	Body	-0,218	4,59E-08	2,31E-06
cg21641482	6	41539244	FOXP4	Body	-0,212	4,60E-08	2,31E-06
cg19385474	2	96764052		IGR	-0,204	4,60E-08	2,31E-06
cg00295572	1	3568236	TP73	TSS1500	0,22	4,60E-08	2,31E-06
cg10606698	6	76203642	FILIP1	TSS200	0,317	4,61E-08	2,31E-06
cg05922456	15	77860052		IGR	-0,23	4,61E-08	2,31E-06
cg04647828	2	72255204		IGR	-0,215	4,62E-08	2,32E-06
cg14967987	4	3204843	HTT	Body	-0,336	4,62E-08	2,32E-06
cg16822208	6	149434103		IGR	-0,226	4,62E-08	2,32E-06
cg11065747	9	131349902	SPTAN1	ExonBnd	-0,213	4,63E-08	2,32E-06
cg10728351	4	122628020		IGR	-0,371	4,64E-08	2,32E-06
cg18339718	19	12759034	MAN2B1	Body	0,247	4,64E-08	2,32E-06
cg17007711	16	86948010		IGR	-0,206	4,65E-08	2,32E-06
cg04579254	2	231090745	SP140	Body	-0,293	4,65E-08	2,32E-06
cg09088602	16	67563608	LOC1005059	TSS1500	0,208	4,65E-08	2,32E-06
cg09683413	2	16400723		IGR	-0,215	4,65E-08	2,32E-06
cg16020346	1	11761078	C1orf187	5'UTR	0,246	4,65E-08	2,32E-06
cg08059719	12	122444450		IGR	-0,295	4,65E-08	2,33E-06
cg23489236	5	1536491		IGR	-0,203	4,66E-08	2,33E-06
cg03392965	7	30931622	FAM188B	3'UTR	-0,205	4,66E-08	2,33E-06
cg19433225	1	78512997	GIPC2	Body	0,237	4,67E-08	2,33E-06
cg10016364	1	159770136		IGR	-0,273	4,67E-08	2,33E-06
cg11303839	7	75405967	CCL26	5'UTR	-0,208	4,67E-08	2,33E-06

cg21947565	20	37650634	DHX35	Body	-0,217	4,67E-08	2,33E-06
cg12679070	1	19786286	CAPZB	Body	-0,3	4,67E-08	2,33E-06
cg11955117	10	52588271	A1CF	Body	-0,215	4,67E-08	2,33E-06
cg05239504	7	35755671		IGR	0,258	4,68E-08	2,33E-06
cg05155018	19	46078660	OPA3	Body	-0,25	4,70E-08	2,34E-06
cg17900249	19	51879206		IGR	-0,254	4,70E-08	2,34E-06
cg05647013	11	124807726	HEPACAM	TSS1500	-0,23	4,71E-08	2,34E-06
cg06754496	10	52268375	SGMS1	5'UTR	-0,234	4,72E-08	2,34E-06
cg08024910	6	156830177		IGR	-0,234	4,74E-08	2,35E-06
cg04352272	17	3289798		IGR	0,218	4,74E-08	2,35E-06
cg11993828	4	38525620	LINC01258	TSS1500	-0,31	4,74E-08	2,35E-06
cg23846704	20	46115856		IGR	-0,222	4,74E-08	2,35E-06
cg05115314	21	45301421	AGPAT3	5'UTR	-0,252	4,74E-08	2,35E-06
cg01416838	18	56333177		IGR	-0,227	4,75E-08	2,35E-06
cg18870424	16	73238968		IGR	-0,246	4,76E-08	2,36E-06
cg09446656	22	33356753	SYN3	Body	-0,207	4,76E-08	2,36E-06
cg09318763	11	128798565		IGR	-0,202	4,76E-08	2,36E-06
cg26665670	5	137958778		IGR	-0,262	4,76E-08	2,36E-06
cg01456831	8	126557912		IGR	-0,326	4,76E-08	2,36E-06
cg13655082	9	125109046		IGR	0,28	4,77E-08	2,36E-06
cg18682028	3	46035439	FYCO1	5'UTR	-0,211	4,77E-08	2,36E-06
cg25623524	19	58862063	NCRNA00181	TSS1500	0,204	4,77E-08	2,36E-06
cg09668936	1	49196418	BEND5	Body	-0,208	4,77E-08	2,36E-06
cg25135018	1	154435948	IL6R	Body	-0,255	4,77E-08	2,36E-06
cg18766016	16	10970773	CIITA	TSS1500	-0,277	4,77E-08	2,36E-06
cg13788117	19	35324528		IGR	-0,207	4,78E-08	2,36E-06
cg01563309	18	21552432		IGR	-0,211	4,78E-08	2,37E-06
cg08946644	6	111855700	TRAF3IP2-AS1	Body	0,207	4,78E-08	2,37E-06
cg06092694	5	66503017		IGR	-0,279	4,80E-08	2,37E-06
cg03222176	1	96948323		IGR	0,238	4,81E-08	2,37E-06
cg06612432	8	134073797	SLA	TSS1500	-0,266	4,81E-08	2,37E-06
cg14475478	10	105303766	NEURL	Body	-0,248	4,81E-08	2,38E-06
cg06760356	19	17546188		IGR	-0,245	4,82E-08	2,38E-06
cg19024002	2	69397161	ANTXR1	Body	-0,246	4,82E-08	2,38E-06
cg07937170	1	219498589		IGR	-0,257	4,83E-08	2,38E-06
cg11404464	3	129267034	H1FOO	TSS200	-0,203	4,83E-08	2,38E-06
cg27057509	6	30883762	VARS2	Body	0,243	4,84E-08	2,38E-06
cg16725262	13	36444273	MIR548F5	Body	-0,209	4,84E-08	2,38E-06
cg25153505	2	134322791	NCKAP5	5'UTR	-0,23	4,84E-08	2,38E-06
cg10160946	7	105662697	CDHR3	Body	-0,254	4,84E-08	2,38E-06
cg19318393	1	223936508	CAPN2	Body	0,29	4,85E-08	2,38E-06
cg16524839	10	80717418	ZMIZ1-AS1	Body	-0,227	4,85E-08	2,39E-06
cg06961290	17	28535040	SLC6A4	Body	0,285	4,85E-08	2,39E-06
cg16992839	11	76353433		IGR	-0,226	4,85E-08	2,39E-06
cg08109681	6	166825084	RPS6KA2	3'UTR	-0,201	4,86E-08	2,39E-06
cg02160272	10	112185666		IGR	-0,3	4,86E-08	2,39E-06
cg18946280	19	53580403	ZNF160	Body	0,339	4,86E-08	2,39E-06
cg01737554	17	33866071	SLFN12L	TSS1500	-0,239	4,87E-08	2,39E-06
cg08343600	10	43894095	HNRNPF	5'UTR	-0,209	4,87E-08	2,39E-06
cg04415176	2	176957842	HOXD13	1stExon	0,286	4,89E-08	2,40E-06

cg23831998	10	112628942	PDCD4-AS1	Body	-0,249	4,89E-08	2,40E-06
cg04128583	9	34690407	CCL19	Body	-0,22	4,89E-08	2,40E-06
cg05229457	12	7985739	SLC2A14	Body	-0,209	4,90E-08	2,40E-06
cg05658107	4	40198392	RHOH	TSS200	-0,261	4,90E-08	2,40E-06
cg15117901	4	186682695	SORBS2	5'UTR	0,225	4,90E-08	2,40E-06
cg04587858	2	236484874	AGAP1	Body	-0,244	4,90E-08	2,40E-06
cg04483596	8	75065106		IGR	-0,217	4,90E-08	2,40E-06
cg08025585	15	92611535	SLCO3A1	Body	-0,303	4,90E-08	2,40E-06
cg07647771	16	84786436	USP10	Body	-0,201	4,90E-08	2,40E-06
cg24141280	14	76035419		IGR	-0,254	4,91E-08	2,41E-06
cg27501190	2	160473496	LOC643072	Body	0,283	4,91E-08	2,41E-06
cg05638011	11	73691625	UCP2	5'UTR	-0,295	4,91E-08	2,41E-06
cg14828411	1	149817540		IGR	-0,219	4,92E-08	2,41E-06
cg06452665	13	43148436	TNFSF11	1stExon	0,23	4,92E-08	2,41E-06
cg11832040	5	157566574		IGR	-0,248	4,92E-08	2,41E-06
cg22878091	11	125046070	PKNOX2	5'UTR	-0,256	4,93E-08	2,41E-06
cg24457304	9	98524869		IGR	-0,224	4,93E-08	2,41E-06
cg00523973	10	17069055	CUBN	Body	-0,277	4,93E-08	2,41E-06
cg09447640	17	42854853	ADAM11	ExonBnd	-0,207	4,93E-08	2,41E-06
cg25252197	10	112580109	RBM20	Body	0,232	4,93E-08	2,41E-06
cg24818200	5	140501365	PCDHB4	TSS1500	0,245	4,93E-08	2,41E-06
cg04864826	15	79086705	ADAMTS7	Body	-0,22	4,93E-08	2,41E-06
cg03233185	12	26123616	RASSF8	5'UTR	-0,216	4,95E-08	2,41E-06
cg25514244	13	114122903	DCUN1D2	Body	0,236	4,95E-08	2,42E-06
cg08711342	9	123682913	TRAF1	Body	-0,262	4,95E-08	2,42E-06
cg05412410	7	3033173	CARD11	5'UTR	0,238	4,96E-08	2,42E-06
cg22027471	22	32651776	SLC5A4	TSS1500	-0,252	4,97E-08	2,42E-06
cg05588396	2	73007743	EXOC6B	Body	0,237	4,97E-08	2,42E-06
cg14158710	6	24937034	FAM65B	TSS1500	-0,346	4,97E-08	2,42E-06
cg05460647	10	121464085		IGR	-0,267	4,98E-08	2,43E-06
cg04236980	14	55800924	FBXO34	5'UTR	-0,226	4,98E-08	2,43E-06
cg06826760	1	193076307	GLRX2	TSS1500	-0,206	4,99E-08	2,43E-06
cg02283238	5	118691126	TNFAIP8	Body	-0,263	4,99E-08	2,43E-06
cg14226680	5	142023939	FGF1	TSS200	-0,252	4,99E-08	2,43E-06
cg02383228	6	160687392		IGR	-0,212	4,99E-08	2,43E-06
cg15159247	19	2082122	MOBKL2A	5'UTR	-0,369	5,00E-08	2,43E-06
cg14242696	14	73361964	DPF3	TSS1500	0,238	5,00E-08	2,43E-06
cg02023986	1	15668855	FHAD1	Body	-0,219	5,00E-08	2,43E-06
cg19329986	8	124751904		IGR	0,209	5,02E-08	2,44E-06
cg02324835	8	28919075		IGR	-0,206	5,04E-08	2,45E-06
cg20391784	1	47653579	PDZK1IP1	Body	-0,24	5,04E-08	2,45E-06
cg04447362	2	237654635		IGR	-0,221	5,04E-08	2,45E-06
cg13081720	5	150399890	GPX3	TSS200	0,201	5,05E-08	2,45E-06
cg00298230	3	66352837	SLC25A26	Body	-0,222	5,05E-08	2,45E-06
cg09325036	17	32307434	ASIC2	Body	-0,201	5,06E-08	2,45E-06
cg03567055	9	84108400		IGR	-0,265	5,06E-08	2,45E-06
cg11291869	2	174150971		IGR	-0,213	5,06E-08	2,45E-06
cg21344159	12	131609316	GPR133	Body	-0,209	5,06E-08	2,45E-06
cg11465795	5	65015646	SGTB	Body	-0,238	5,07E-08	2,46E-06
cg22856222	19	13233013	NACC1	5'UTR	-0,218	5,08E-08	2,46E-06

cg02297226	9	91428853		IGR	-0,204	5,08E-08	2,46E-06
cg16322736	5	6496603	UBE2QL1	3'UTR	-0,201	5,10E-08	2,46E-06
cg19848778	11	12217329	MICAL2	Body	-0,28	5,10E-08	2,46E-06
cg11351908	17	75402473	sept-09	Body	-0,217	5,10E-08	2,46E-06
cg22557167	9	98778703	ERCC6L2	3'UTR	-0,288	5,11E-08	2,47E-06
cg27440592	11	125971784		IGR	-0,207	5,11E-08	2,47E-06
cg08197493	11	82838289		IGR	-0,237	5,12E-08	2,47E-06
cg13860688	20	37650491	DHX35	ExonBnd	-0,246	5,12E-08	2,47E-06
cg03040292	1	200847096		IGR	-0,223	5,12E-08	2,47E-06
cg06719359	2	110092518	SH3RF3	Body	-0,222	5,12E-08	2,47E-06
cg21516670	15	31215156	FAN1	Body	0,253	5,12E-08	2,47E-06
cg26974013	19	7402704		IGR	-0,279	5,13E-08	2,47E-06
cg22070401	5	79351924	THBS4	Body	-0,212	5,13E-08	2,47E-06
cg12931148	22	20232415	RTN4R	Body	-0,223	5,13E-08	2,47E-06
cg23156711	15	85376403	ALPK3	Body	-0,218	5,13E-08	2,47E-06
cg15693299	12	7916371	LOC360030	TSS1500	-0,223	5,13E-08	2,47E-06
cg08571810	9	102276165		IGR	-0,247	5,14E-08	2,48E-06
cg11745838	1	224007693	TP53BP2	Body	0,23	5,14E-08	2,48E-06
cg26336232	2	38838729		IGR	-0,255	5,15E-08	2,48E-06
cg25356759	1	48342535	TRABD2B	Body	-0,246	5,16E-08	2,48E-06
cg10107890	2	44314289		IGR	0,24	5,16E-08	2,48E-06
cg26099918	10	44409234	LINC00841	Body	-0,243	5,16E-08	2,48E-06
cg24609140	5	78249305	ARSB	Body	-0,202	5,17E-08	2,48E-06
cg21091841	13	34251239		IGR	0,274	5,17E-08	2,48E-06
cg22553423	2	68961955	ARHGAP25	5'UTR	-0,206	5,17E-08	2,48E-06
cg22747843	1	109936194	SORT1	Body	0,2	5,17E-08	2,48E-06
cg15424377	2	37971270		IGR	0,444	5,18E-08	2,48E-06
cg07646765	11	125046910	PKNOX2	5'UTR	-0,224	5,18E-08	2,48E-06
cg24988684	17	76128556	TMC8	Body	-0,216	5,18E-08	2,48E-06
cg00532502	1	246953273	LOC149134	Body	-0,206	5,18E-08	2,49E-06
cg20361407	2	242497928	BOK-AS1	Body	0,247	5,18E-08	2,49E-06
cg08060386	3	44637866		IGR	-0,24	5,18E-08	2,49E-06
cg21477302	20	46701128		IGR	-0,226	5,18E-08	2,49E-06
cg24337158	12	1099342	ERC1	TSS1500	0,204	5,18E-08	2,49E-06
cg05949889	11	106336898		IGR	-0,218	5,19E-08	2,49E-06
cg02825243	2	197041490		IGR	-0,26	5,19E-08	2,49E-06
cg26002422	19	19488846		IGR	0,216	5,19E-08	2,49E-06
cg17810176	19	36035831	GAPDHS	Body	0,208	5,20E-08	2,49E-06
cg15027446	4	56484062	NMU	Body	-0,228	5,20E-08	2,49E-06
cg04622720	7	158657028	WDR60	Body	0,219	5,20E-08	2,49E-06
cg27473165	1	15943123	DDI2	TSS1500	-0,282	5,21E-08	2,49E-06
cg06071593	18	29819065		IGR	-0,233	5,21E-08	2,50E-06
cg13180703	4	36161034	ARAP2	Body	0,23	5,24E-08	2,51E-06
cg14553042	5	173369264	CPEB4	Body	0,276	5,25E-08	2,51E-06
cg00834585	6	99396115	FBXL4	TSS1500	0,202	5,25E-08	2,51E-06
cg03570900	10	7682790	ITIH5	Body	-0,256	5,25E-08	2,51E-06
cg04419723	9	74398117		IGR	-0,264	5,25E-08	2,51E-06
cg08999906	12	91331583	LINC00615	Body	-0,242	5,26E-08	2,51E-06
cg04324158	3	167932012		IGR	-0,228	5,26E-08	2,51E-06
cg14830274	4	139813683		IGR	-0,254	5,26E-08	2,51E-06

cg07290413	2	16383611		IGR	-0,224	5,26E-08	2,51E-06
cg04594619	1	27095543	ARID1A	Body	0,211	5,27E-08	2,51E-06
cg12831026	1	160752312		IGR	-0,32	5,27E-08	2,51E-06
cg21818126	1	223422132	SUSD4	Body	-0,258	5,27E-08	2,51E-06
cg05082111	9	112888903	AKAP2	5'UTR	0,204	5,27E-08	2,51E-06
cg05266989	14	74296969		IGR	-0,363	5,28E-08	2,52E-06
cg22157099	2	15830172		IGR	-0,271	5,29E-08	2,52E-06
cg26886572	11	3009206	NAP1L4	5'UTR	-0,245	5,29E-08	2,52E-06
cg22895728	12	93531972	LOC643339	Body	-0,291	5,29E-08	2,52E-06
cg19418009	1	16564689	RSG1	TSS1500	-0,247	5,29E-08	2,52E-06
cg19340031	14	73956407	HEATR4	Body	0,226	5,30E-08	2,52E-06
cg14204222	14	32474740		IGR	-0,208	5,30E-08	2,52E-06
cg16266809	20	30943371		IGR	-0,235	5,30E-08	2,52E-06
cg19513654	4	139314416	LINC00499	Body	-0,236	5,31E-08	2,52E-06
cg12473781	5	149558804	CDX1	Body	-0,277	5,31E-08	2,52E-06
cg04376645	15	67040057	SMAD6	Body	-0,223	5,31E-08	2,53E-06
cg01201959	2	8748898		IGR	-0,277	5,32E-08	2,53E-06
cg23394633	2	208087996	LOC10192786	Body	-0,235	5,32E-08	2,53E-06
cg12451887	3	183155777		IGR	-0,205	5,33E-08	2,53E-06
cg15925478	1	94081080	BCAR3	Body	-0,256	5,33E-08	2,53E-06
cg08859309	13	30948103	LOC1001889	TSS200	-0,235	5,33E-08	2,53E-06
cg23700156	3	131272446	CPNE4	Body	-0,253	5,33E-08	2,53E-06
cg24366261	3	111327532	CD96	Body	-0,223	5,34E-08	2,53E-06
cg24339671	17	75780450		IGR	-0,274	5,35E-08	2,54E-06
cg21150026	2	207904284		IGR	-0,24	5,35E-08	2,54E-06
cg09397653	15	68643403	ITGA11	Body	-0,203	5,36E-08	2,54E-06
cg00151680	10	26720032		IGR	-0,204	5,37E-08	2,54E-06
cg04318494	11	64640770	EHD1	Body	-0,228	5,37E-08	2,54E-06
cg01741606	22	34199319	LARGE	5'UTR	-0,21	5,38E-08	2,55E-06
cg15454110	6	33384380	CUTA	3'UTR	-0,241	5,38E-08	2,55E-06
cg09772661	19	7794952	CLEC4G	Body	0,209	5,38E-08	2,55E-06
cg15796641	1	205913709	SLC26A9	TSS1500	-0,258	5,38E-08	2,55E-06
cg08425810	12	58132558	AGAP2	TSS1500	0,202	5,39E-08	2,55E-06
cg13994550	5	73617380	LINC01333	TSS1500	-0,211	5,39E-08	2,55E-06
cg21495850	1	222165992		IGR	-0,268	5,40E-08	2,55E-06
cg06854637	7	22702493	LOC401312	Body	-0,212	5,40E-08	2,55E-06
cg13447080	8	131054408		IGR	-0,268	5,40E-08	2,55E-06
cg08741022	11	76455305		IGR	-0,223	5,40E-08	2,55E-06
cg14614038	2	28858407	PLB1	Body	0,235	5,40E-08	2,55E-06
cg08972831	12	68091432		IGR	-0,214	5,40E-08	2,55E-06
cg24391912	15	100259320		IGR	-0,297	5,41E-08	2,55E-06
cg00352505	15	96777737	NR2F2-AS1	Body	-0,266	5,41E-08	2,56E-06
cg17360199	7	45263685		IGR	-0,243	5,41E-08	2,56E-06
cg09061423	1	67802018	IL12RB2	Body	-0,235	5,42E-08	2,56E-06
cg08549335	7	30387954	ZNRF2	Body	-0,338	5,42E-08	2,56E-06
cg14568422	18	53069269	TCF4	Body	0,308	5,42E-08	2,56E-06
cg13266327	2	11485561	ROCK2	TSS1500	0,205	5,42E-08	2,56E-06
cg18941665	3	150300618	EIF2A	Body	-0,256	5,43E-08	2,56E-06
cg24182205	20	5471597		IGR	-0,293	5,43E-08	2,56E-06
cg27208362	3	133575205	RAB6B	Body	-0,207	5,43E-08	2,56E-06

cg02561376	4	39035483	TMEM156	TSS1500	-0,214	5,43E-08	2,56E-06
cg02079584	15	74528719	CCDC33	1stExon	-0,279	5,44E-08	2,56E-06
cg20387993	10	4277124	LINC00702	Body	-0,24	5,44E-08	2,56E-06
cg06586886	11	130671465		IGR	-0,255	5,44E-08	2,56E-06
cg18623314	4	55711209		IGR	-0,263	5,45E-08	2,57E-06
cg06929583	10	88172435		IGR	-0,237	5,45E-08	2,57E-06
cg06798483	3	98504973	ST3GAL6	Body	-0,26	5,46E-08	2,57E-06
cg10163211	6	43672630		IGR	-0,251	5,46E-08	2,57E-06
cg10841025	11	127924442		IGR	-0,365	5,46E-08	2,57E-06
cg12888360	5	142732502	NR3C1	Body	-0,263	5,47E-08	2,57E-06
cg23750514	11	2790418	KCNQ1	Body	-0,231	5,47E-08	2,57E-06
cg25363387	17	63823864	CCDC46	TSS1500	-0,202	5,48E-08	2,58E-06
cg01218206	11	116933977	SIK3	Body	-0,328	5,48E-08	2,58E-06
cg04728310	19	45947905		IGR	0,227	5,48E-08	2,58E-06
cg04572071	12	109278022	DAO	5'UTR	-0,212	5,48E-08	2,58E-06
cg24828864	8	38279600	FGFR1	Body	-0,242	5,48E-08	2,58E-06
cg00460983	10	91133941		IGR	0,234	5,49E-08	2,58E-06
cg19842239	11	12204022	MICAL2	Body	0,314	5,49E-08	2,58E-06
cg21814290	8	10466918	RP1L1	Body	-0,206	5,49E-08	2,58E-06
cg08542725	8	75010420		IGR	-0,223	5,51E-08	2,59E-06
cg19094050	5	71256127		IGR	-0,209	5,51E-08	2,59E-06
cg18030943	3	182876556	LAMP3	Body	-0,241	5,52E-08	2,59E-06
cg10939719	1	117311712	CD2	3'UTR	-0,285	5,52E-08	2,59E-06
cg05350944	3	133794122		IGR	-0,265	5,52E-08	2,59E-06
cg08239610	18	7958788	PTPRM	Body	-0,283	5,52E-08	2,59E-06
cg02457866	1	84900707		IGR	-0,286	5,53E-08	2,59E-06
cg02212491	10	100103851		IGR	-0,207	5,53E-08	2,59E-06
cg06222397	16	86953019		IGR	-0,221	5,53E-08	2,60E-06
cg15892950	8	23168643	LOXL2	Body	-0,233	5,54E-08	2,60E-06
cg25485235	16	78776702	WWOX	Body	-0,247	5,54E-08	2,60E-06
cg12072789	1	110735112	SLC6A17	Body	-0,222	5,55E-08	2,60E-06
cg12871593	6	108038173	SCML4	Body	-0,209	5,55E-08	2,60E-06
cg14445012	9	115926922		IGR	0,267	5,56E-08	2,60E-06
cg16765678	10	35047821	PARD3	Body	-0,216	5,56E-08	2,60E-06
cg09248869	5	131808798	C5orf56	Body	-0,228	5,56E-08	2,60E-06
cg22014112	6	138192658	TNFAIP3	Body	-0,274	5,56E-08	2,60E-06
cg09761846	11	57117162	P2RX3	Body	0,262	5,56E-08	2,60E-06
cg04223044	22	45608428	C22orf9	Body	0,222	5,56E-08	2,60E-06
cg09395562	3	123501051	MYLK	Body	-0,22	5,56E-08	2,60E-06
cg23322640	6	111675295	REV3L	Body	0,212	5,57E-08	2,61E-06
cg24855402	2	201979988	CFLAR	TSS1500	-0,291	5,57E-08	2,61E-06
cg18835865	14	52020726	FRMD6	5'UTR	-0,216	5,57E-08	2,61E-06
cg22857194	4	142419075		IGR	-0,208	5,57E-08	2,61E-06
cg14349078	9	78547359	PCSK5	Body	-0,206	5,57E-08	2,61E-06
cg01070751	6	12372429		IGR	-0,213	5,58E-08	2,61E-06
cg15961092	14	45681130	C14orf106	Body	0,204	5,58E-08	2,61E-06
cg08863939	2	172067553	TLK1	5'UTR	-0,235	5,59E-08	2,61E-06
cg14830003	1	38022657	DNALI1	1stExon	0,257	5,59E-08	2,61E-06
cg11906021	17	47467221		IGR	-0,232	5,60E-08	2,61E-06
cg12894524	12	116997069	MAP1LC3B2	TSS200	-0,203	5,60E-08	2,61E-06

cg02793158	2	128417186	LIMS2	Body	0,226	5,61E-08	2,62E-06
cg23733945	6	43056688	PTK7	Body	-0,213	5,62E-08	2,62E-06
cg06707620	1	226714963		IGR	-0,26	5,63E-08	2,62E-06
cg19536401	19	6504797		IGR	-0,239	5,63E-08	2,62E-06
cg19449565	2	240230892	HDAC4	Body	0,236	5,63E-08	2,62E-06
cg01335986	3	46320769		IGR	-0,247	5,64E-08	2,63E-06
cg02453236	16	67438518	ZDHHC1	Body	-0,217	5,64E-08	2,63E-06
cg16621816	2	47798256	KCNK12	TSS1500	0,224	5,64E-08	2,63E-06
cg11580135	12	109725038	FOXN4	Body	-0,212	5,64E-08	2,63E-06
cg01243685	13	52923325		IGR	-0,246	5,64E-08	2,63E-06
cg15635633	2	161850423		IGR	0,22	5,64E-08	2,63E-06
cg08667164	2	192797930		IGR	-0,227	5,65E-08	2,63E-06
cg06412241	2	201333656	SPATS2L	Body	-0,202	5,65E-08	2,63E-06
cg26763394	3	45957664		IGR	-0,224	5,65E-08	2,63E-06
cg17848521	5	127685050	FBN2	ExonBnd	0,211	5,66E-08	2,63E-06
cg14908307	12	106069481		IGR	-0,236	5,66E-08	2,63E-06
cg06282952	6	148859147	SASH1	Body	-0,222	5,67E-08	2,63E-06
cg07171609	8	91640288	TMEM64	Body	-0,221	5,67E-08	2,63E-06
cg14500160	12	19705654		IGR	-0,238	5,67E-08	2,64E-06
cg26872780	2	149478494	EPC2	Body	0,225	5,68E-08	2,64E-06
cg01235448	14	51940896	FRMD6-AS2	Body	-0,21	5,68E-08	2,64E-06
cg07753480	10	72363272	PRF1	TSS1500	-0,236	5,69E-08	2,64E-06
cg20001791	6	16239799	GMPR	Body	0,217	5,70E-08	2,65E-06
cg18282243	2	204667641		IGR	-0,229	5,71E-08	2,65E-06
cg24610349	7	98696198	SMURF1	Body	-0,201	5,71E-08	2,65E-06
cg11143193	12	123713553		IGR	-0,228	5,72E-08	2,65E-06
cg07546433	8	102063914		IGR	0,312	5,72E-08	2,65E-06
cg11935547	8	80929565	MRPS28	Body	-0,228	5,73E-08	2,65E-06
cg12853247	13	97927719	MBNL2	5'UTR	0,292	5,77E-08	2,66E-06
cg12860679	1	198265258	NEK7	Body	0,205	5,78E-08	2,67E-06
cg01338762	2	48125586	FBXO11	Body	-0,2	5,78E-08	2,67E-06
cg01122516	20	48484368	SLC9A8	Body	-0,202	5,79E-08	2,67E-06
cg25496760	16	11051651	CLEC16A	ExonBnd	-0,246	5,80E-08	2,67E-06
cg04790487	16	3031471	PKMYT1	TSS1500	0,232	5,80E-08	2,68E-06
cg07802484	3	33103528	GLB1	Body	-0,256	5,80E-08	2,68E-06
cg24783211	4	55093038		IGR	0,205	5,81E-08	2,68E-06
cg24183514	14	51863868		IGR	-0,251	5,81E-08	2,68E-06
cg26055899	4	154462759	KIAA0922	Body	-0,272	5,81E-08	2,68E-06
cg17022548	5	139294892	NRG2	Body	-0,229	5,82E-08	2,68E-06
cg26350431	4	146451571	SMAD1	Body	-0,208	5,82E-08	2,68E-06
cg21280719	6	42927975	GNMT	TSS1500	0,226	5,83E-08	2,68E-06
cg17316874	12	123478048	PITPNM2	Body	-0,258	5,84E-08	2,69E-06
cg02476270	1	156481647		IGR	-0,224	5,84E-08	2,69E-06
cg09178666	8	12894534		IGR	-0,212	5,84E-08	2,69E-06
cg04455641	11	2055361		IGR	-0,227	5,84E-08	2,69E-06
cg09344348	6	170581085		IGR	-0,278	5,84E-08	2,69E-06
cg17376712	11	44802564	TSPAN18	5'UTR	0,202	5,84E-08	2,69E-06
cg20825110	1	33854504		IGR	-0,236	5,85E-08	2,69E-06
cg13245380	14	91544928		IGR	-0,247	5,85E-08	2,69E-06
cg13521267	18	66401186	CCDC102B	5'UTR	0,23	5,86E-08	2,69E-06

cg13336515	11	78619091	TENM4	Body	-0,226	5,86E-08	2,69E-06
cg13391235	16	85316611		IGR	-0,282	5,86E-08	2,69E-06
cg18517540	18	32446543	DTNA	3'UTR	-0,276	5,86E-08	2,69E-06
cg11343553	6	42346034	TRERF1	5'UTR	-0,237	5,87E-08	2,70E-06
cg20509001	9	95911768		IGR	-0,324	5,87E-08	2,70E-06
cg04371413	2	207395846	ADAM23	Body	-0,231	5,87E-08	2,70E-06
cg27424261	2	15317393	NBAS	Body	-0,267	5,87E-08	2,70E-06
cg10114358	11	35993896	LDLRAD3	5'UTR	-0,225	5,87E-08	2,70E-06
cg01268711	3	137799724	DZIP1L	Body	-0,203	5,87E-08	2,70E-06
cg10641655	2	238441378	MLPH	Body	-0,217	5,88E-08	2,70E-06
cg07775431	13	107160426	EFNB2	Body	-0,204	5,88E-08	2,70E-06
cg22474767	8	96133173		IGR	-0,203	5,89E-08	2,70E-06
cg10145196	6	30647649	KIAA1949	Body	-0,204	5,89E-08	2,70E-06
cg26161921	20	56475055		IGR	-0,2	5,90E-08	2,71E-06
cg07506247	4	54365684	LNX1-AS1	TSS1500	-0,214	5,90E-08	2,71E-06
cg12102398	1	205792512		IGR	-0,222	5,91E-08	2,71E-06
cg08900864	17	27696160		IGR	0,22	5,91E-08	2,71E-06
cg22338715	11	68450267		IGR	-0,208	5,91E-08	2,71E-06
cg19571211	2	71828198	DYSF	Body	-0,266	5,91E-08	2,71E-06
cg16696044	7	130636436	LINC-PINT	Body	-0,254	5,91E-08	2,71E-06
cg03038548	9	124049158	GSN-AS1	TSS1500	0,202	5,92E-08	2,71E-06
cg07195319	5	167908725		IGR	-0,211	5,92E-08	2,71E-06
cg18661872	5	131436611		IGR	-0,307	5,93E-08	2,72E-06
cg26441038	5	169739831		IGR	-0,255	5,93E-08	2,72E-06
cg04075796	1	178343677	RASAL2	Body	-0,21	5,94E-08	2,72E-06
cg09914444	1	46972183	DMBX1	TSS1500	-0,211	5,94E-08	2,72E-06
cg07009717	1	203680904	ATP2B4	Body	-0,256	5,95E-08	2,72E-06
cg01249670	3	71707453		IGR	0,215	5,95E-08	2,72E-06
cg10661163	7	116592959	ST7OT4	TSS1500	0,206	5,96E-08	2,72E-06
cg00160143	7	17442316	KCCAT333	Body	-0,316	5,96E-08	2,73E-06
cg15058727	12	6454662		IGR	-0,223	5,96E-08	2,73E-06
cg25824411	20	44650062	SLC12A5	TSS1500	0,212	5,96E-08	2,73E-06
cg10577087	6	125552200	TPD52L1	Body	-0,25	5,97E-08	2,73E-06
cg25222052	20	49247600	FAM65C	5'UTR	-0,279	5,97E-08	2,73E-06
cg00868206	6	149641149	TAB2	Body	-0,263	5,97E-08	2,73E-06
cg23138413	8	49320596		IGR	-0,33	5,97E-08	2,73E-06
cg19884937	6	33872665		IGR	-0,272	5,99E-08	2,73E-06
cg11521318	22	40572844	TNRC6B	TSS1500	-0,23	5,99E-08	2,73E-06
cg06557644	7	30510463	NOD1	5'UTR	0,209	6,00E-08	2,74E-06
cg21814178	12	51720755		IGR	-0,205	6,00E-08	2,74E-06
cg11055449	10	91136405	IFIT1L	TSS1500	-0,221	6,00E-08	2,74E-06
cg11820913	14	101021865	BEGAIN	Body	-0,22	6,01E-08	2,74E-06
cg18735973	1	207929700	CD46	Body	-0,248	6,01E-08	2,74E-06
cg24174557	17	57903544	TMEM49	Body	-0,336	6,01E-08	2,74E-06
cg03242819	10	128994432	DOCK1	Body	0,215	6,01E-08	2,74E-06
cg02863807	13	113327910	ATP11AUN	5'UTR	-0,229	6,02E-08	2,74E-06
cg11369830	8	59291008		IGR	-0,217	6,02E-08	2,74E-06
cg00512333	8	40018174		IGR	-0,234	6,03E-08	2,75E-06
cg02023548	11	68748461	MRGPRD	TSS200	-0,221	6,04E-08	2,75E-06
cg05890855	2	216783091		IGR	-0,306	6,04E-08	2,75E-06

cg15273517	5	154236651	CNOT8	TSS1500	0,202	6,04E-08	2,75E-06
cg11564369	4	105888053		IGR	-0,226	6,04E-08	2,75E-06
cg11425902	12	52534939		IGR	-0,228	6,04E-08	2,75E-06
cg01374580	15	62521056		IGR	-0,243	6,05E-08	2,75E-06
cg06128194	5	168035109		IGR	-0,21	6,05E-08	2,75E-06
cg23409713	3	38215772	OXSR1	Body	-0,273	6,06E-08	2,75E-06
cg05659486	6	53792689		IGR	-0,244	6,06E-08	2,75E-06
cg10139653	15	90608388	ZNF710	5'UTR	0,202	6,06E-08	2,76E-06
cg12466037	10	126331909	FAM53B	Body	0,254	6,06E-08	2,76E-06
cg24041556	19	10736059	SLC44A2	TSS200	0,252	6,06E-08	2,76E-06
cg11897902	7	140076235	SLC37A3	Body	-0,238	6,06E-08	2,76E-06
cg17754737	7	30362179	ZNRF2	Body	-0,31	6,07E-08	2,76E-06
cg08592104	11	8761869	ST5	Body	-0,233	6,08E-08	2,76E-06
cg18530645	19	12759156	MAN2B1	Body	0,261	6,08E-08	2,76E-06
cg13487614	6	42192461		IGR	-0,371	6,08E-08	2,76E-06
cg22680823	18	56459606		IGR	-0,314	6,08E-08	2,76E-06
cg23867498	16	86959212		IGR	-0,202	6,10E-08	2,77E-06
cg14540739	9	115248156	KIAA1958	TSS1500	0,218	6,10E-08	2,77E-06
cg16174204	6	88689693		IGR	-0,264	6,11E-08	2,77E-06
cg19223119	10	574732	DIP2C	Body	-0,258	6,11E-08	2,77E-06
cg26993334	2	69281647	ANTXR1	Body	-0,235	6,11E-08	2,77E-06
cg09547502	11	2590685	KCNQ1	Body	-0,222	6,11E-08	2,77E-06
cg12521010	9	96907980		IGR	-0,221	6,11E-08	2,77E-06
cg07194829	1	17053974		IGR	-0,28	6,13E-08	2,78E-06
cg07162700	3	8499933		IGR	-0,222	6,13E-08	2,78E-06
cg00841849	2	8683604		IGR	-0,265	6,13E-08	2,78E-06
cg12123300	7	139910412		IGR	-0,255	6,13E-08	2,78E-06
cg14564439	7	102067066	PRKRIP1	3'UTR	-0,296	6,14E-08	2,78E-06
cg15273860	9	130789692		IGR	-0,201	6,16E-08	2,79E-06
cg13800769	12	4916913	KCNA6	TSS1500	-0,225	6,16E-08	2,79E-06
cg12604331	1	156906485	ARHGEF11	Body	-0,211	6,17E-08	2,79E-06
cg11333222	4	7795124	AFAP1	Body	-0,214	6,17E-08	2,79E-06
cg21774136	1	8593495	RERE	Body	-0,213	6,19E-08	2,79E-06
cg26216618	22	23480087	RTDR1	Body	-0,254	6,19E-08	2,79E-06
cg04908668	6	32823941	PSMB9	Body	-0,258	6,19E-08	2,79E-06
cg24898914	6	32810706	PSMB8	Body	-0,304	6,19E-08	2,80E-06
cg00213900	18	45867023		IGR	-0,237	6,20E-08	2,80E-06
cg04626879	15	91432866	FES	Body	-0,221	6,22E-08	2,80E-06
cg23259038	1	235656143	B3GALNT2	Body	-0,23	6,23E-08	2,81E-06
cg01579531	10	28897137	WAC	Body	0,225	6,24E-08	2,81E-06
cg21526106	15	32392647	CHRNA7	Body	-0,219	6,24E-08	2,81E-06
cg27331241	7	751830	PRKAR1B	5'UTR	0,214	6,25E-08	2,82E-06
cg21399203	4	57623913		IGR	-0,303	6,26E-08	2,82E-06
cg07135931	11	69232813		IGR	-0,226	6,26E-08	2,82E-06
cg16487097	14	75800941		IGR	-0,273	6,26E-08	2,82E-06
cg00695286	6	21354634		IGR	-0,205	6,28E-08	2,82E-06
cg10166825	5	125052595		IGR	-0,241	6,28E-08	2,82E-06
cg04978197	3	16164375		IGR	-0,257	6,28E-08	2,82E-06
cg09866553	2	46467131		IGR	-0,275	6,29E-08	2,83E-06
cg03199887	19	49973476	ALDH16A1	Body	-0,231	6,29E-08	2,83E-06

cg24787400	17	44097757	MAPT	Body	-0,227	6,29E-08	2,83E-06
cg25570767	9	85214282		IGR	-0,259	6,29E-08	2,83E-06
cg23752752	7	4778908	FOXK1	Body	0,213	6,30E-08	2,83E-06
cg18209791	8	125274169		IGR	-0,218	6,31E-08	2,83E-06
cg24206084	13	44687020	SMIM2-AS1	Body	-0,235	6,31E-08	2,83E-06
cg17799287	13	92001764	MIR19A	TSS1500	-0,277	6,32E-08	2,83E-06
cg04554131	1	25291540	RUNX3	TSS200	-0,275	6,32E-08	2,83E-06
cg09646737	22	36206677	RBFOX2	Body	-0,201	6,32E-08	2,84E-06
cg11869500	2	158755155		IGR	-0,291	6,32E-08	2,84E-06
cg26433674	4	15230035	LOC1019290	Body	-0,254	6,32E-08	2,84E-06
cg11371646	10	29634996		IGR	-0,206	6,33E-08	2,84E-06
cg24984777	17	40636525	ATP6V0A1	Body	-0,216	6,33E-08	2,84E-06
cg01292394	12	321723	SLC6A12	5'UTR	-0,269	6,34E-08	2,84E-06
cg04086871	4	113570169	MIR367	TSS1500	0,217	6,34E-08	2,84E-06
cg06717747	8	23626859		IGR	-0,206	6,34E-08	2,84E-06
cg12339259	4	109029347	LEF1	Body	-0,21	6,34E-08	2,84E-06
cg08817105	14	100905738	WDR25	Body	-0,247	6,35E-08	2,84E-06
cg12801030	1	203297311		IGR	-0,23	6,35E-08	2,84E-06
cg19131272	13	106457767		IGR	-0,226	6,35E-08	2,84E-06
cg03895810	5	171612595	STK10	Body	-0,368	6,37E-08	2,85E-06
cg20822693	2	230934399	SLC16A14	TSS1500	-0,25	6,37E-08	2,85E-06
cg15188947	1	15383673	KAZN	Body	-0,28	6,37E-08	2,85E-06
cg08950751	11	67255492	AIP	Body	-0,226	6,38E-08	2,85E-06
cg08267612	3	69484913		IGR	-0,31	6,39E-08	2,86E-06
cg24290487	11	119763422		IGR	0,208	6,40E-08	2,86E-06
cg09421136	2	202125175	CASP8	TSS200	-0,329	6,40E-08	2,86E-06
cg09232378	10	89898420		IGR	-0,219	6,42E-08	2,87E-06
cg17187001	19	6229011	MLLT1	Body	0,242	6,42E-08	2,87E-06
cg13020868	14	25522307		IGR	-0,213	6,42E-08	2,87E-06
cg05900080	22	48494607		IGR	-0,265	6,43E-08	2,87E-06
cg14640279	22	28119044		IGR	-0,217	6,43E-08	2,87E-06
cg11986433	9	111330500		IGR	-0,233	6,43E-08	2,87E-06
cg16949299	10	98807514	SLIT1	Body	-0,244	6,44E-08	2,88E-06
cg16770601	22	27067721	MIATNB	TSS1500	-0,363	6,45E-08	2,88E-06
cg08267286	1	60598894		IGR	-0,239	6,45E-08	2,88E-06
cg00100538	4	184067792	WWC2	Body	0,242	6,46E-08	2,88E-06
cg08710520	1	219090191	MIR548F3	Body	-0,224	6,48E-08	2,88E-06
cg19180905	9	111232084		IGR	-0,369	6,49E-08	2,89E-06
cg02878739	2	236840858	AGAP1	Body	-0,22	6,49E-08	2,89E-06
cg21341645	7	155091523	INSIG1	Body	-0,235	6,50E-08	2,89E-06
cg00109764	3	43811308		IGR	0,25	6,50E-08	2,89E-06
cg24612198	11	118175631	CD3E	5'UTR	-0,223	6,50E-08	2,89E-06
cg07642463	19	57703860	ZNF264	Body	0,201	6,50E-08	2,89E-06
cg26857141	3	127005632		IGR	-0,216	6,51E-08	2,89E-06
cg25701472	1	78392479	NEXN	Body	-0,315	6,52E-08	2,89E-06
cg12170314	11	15136505	INSC	Body	0,27	6,53E-08	2,90E-06
cg04651570	14	64117156		IGR	-0,228	6,53E-08	2,90E-06
cg16717549	21	45705699	AIRE	TSS200	0,261	6,54E-08	2,90E-06
cg15087376	3	149113539		IGR	0,222	6,55E-08	2,90E-06
cg25920381	22	38539629	PLA2G6	Body	-0,241	6,55E-08	2,91E-06

cg02205957	1	89737118	GBP5	5'UTR	-0,277	6,55E-08	2,91E-06
cg05475524	11	66326798	ACTN3	Body	0,214	6,55E-08	2,91E-06
cg17210999	1	224954584		IGR	-0,202	6,56E-08	2,91E-06
cg02505225	5	31933966	PDZD2	Body	-0,212	6,58E-08	2,91E-06
cg00530015	6	14755359		IGR	-0,202	6,58E-08	2,92E-06
cg21113478	3	36782467	DCLK3	TSS1500	-0,264	6,59E-08	2,92E-06
cg09047090	20	49109706		IGR	-0,275	6,59E-08	2,92E-06
cg25099516	1	48288813		IGR	-0,256	6,61E-08	2,92E-06
cg25701076	15	88484067	NTRK3	Body	-0,201	6,61E-08	2,92E-06
cg23100365	8	41500461		IGR	-0,234	6,62E-08	2,93E-06
cg09870892	14	73859699	NUMB	5'UTR	-0,276	6,62E-08	2,93E-06
cg26691898	6	25833199	SLC17A1	TSS1500	0,219	6,62E-08	2,93E-06
cg15757838	5	143569897	KCTD16	5'UTR	-0,23	6,64E-08	2,93E-06
cg09228833	20	52199778	ZNF217	TSS200	-0,277	6,64E-08	2,93E-06
cg17485530	20	36974799	LBP	TSS200	-0,225	6,64E-08	2,93E-06
cg25392995	22	50625926	TRABD	5'UTR	-0,25	6,65E-08	2,93E-06
cg27501686	12	2184874	CACNA1C	Body	0,222	6,66E-08	2,94E-06
cg02002324	8	64107602	YTHDF3	Body	0,26	6,66E-08	2,94E-06
cg10738683	2	205357452		IGR	-0,203	6,67E-08	2,94E-06
cg17520215	12	100967016	GAS2L3	TSS1500	0,219	6,67E-08	2,94E-06
cg20317872	1	111743202	DENND2D	1stExon	-0,293	6,68E-08	2,95E-06
cg16651537	19	51226536	CLEC11A	TSS200	0,245	6,69E-08	2,95E-06
cg08356262	5	75838704	IQGAP2	Body	-0,206	6,69E-08	2,95E-06
cg24386456	13	27536371		IGR	-0,216	6,69E-08	2,95E-06
cg24348488	6	13681972	RANBP9	Body	-0,268	6,69E-08	2,95E-06
cg23246095	2	162931638	DPP4	TSS1500	0,25	6,70E-08	2,95E-06
cg00703830	8	61471845	RAB2A	Body	-0,328	6,70E-08	2,95E-06
cg18972304	14	22999023		IGR	-0,215	6,70E-08	2,95E-06
cg03360944	7	92324872	CDK6	Body	0,393	6,72E-08	2,96E-06
cg14563251	3	113345875	SIDT1	Body	0,201	6,72E-08	2,96E-06
cg15821498	14	32498045		IGR	-0,271	6,72E-08	2,96E-06
cg06813482	20	20499757	RALGAPA2	Body	-0,211	6,72E-08	2,96E-06
cg11066140	11	3114741	OSBPL5	ExonBnd	-0,202	6,73E-08	2,96E-06
cg12802876	8	72751354		IGR	-0,26	6,73E-08	2,96E-06
cg18151041	18	52917041	TCF4	Body	-0,291	6,73E-08	2,96E-06
cg26296928	6	125292227	RNF217	Body	-0,28	6,74E-08	2,96E-06
cg14217987	3	71471505	FOXP1	5'UTR	-0,279	6,74E-08	2,96E-06
cg15259233	6	170712708	FAM120B	Body	0,213	6,74E-08	2,96E-06
cg15585341	1	145525080	ITGA10	1stExon	0,305	6,74E-08	2,96E-06
cg04098585	2	204571215	CD28	5'UTR	-0,32	6,74E-08	2,96E-06
cg05377824	16	89431687	ANKRD11	Body	-0,222	6,75E-08	2,96E-06
cg23694723	12	115096427		IGR	-0,208	6,76E-08	2,97E-06
cg26012482	2	8423155		IGR	-0,229	6,77E-08	2,97E-06
cg22756138	1	245819890	KIF26B	Body	-0,224	6,77E-08	2,97E-06
cg03086003	11	120192715		IGR	-0,224	6,77E-08	2,97E-06
cg13174306	1	115822971		IGR	-0,298	6,78E-08	2,97E-06
cg19234495	1	43748663	C1orf210	Body	-0,222	6,78E-08	2,97E-06
cg16513984	1	12261884	TNFRSF1B	Body	-0,259	6,78E-08	2,97E-06
cg06632549	6	31529993		IGR	-0,282	6,79E-08	2,97E-06
cg17367708	13	38060376	LINC01048	TSS1500	-0,203	6,79E-08	2,97E-06

cg08173216	15	41524250	CHP	Body	0,233	6,79E-08	2,98E-06
cg09154880	6	32813715	TAP1	Body	-0,362	6,79E-08	2,98E-06
cg07547279	7	151433873	PRKAG2	TSS1500	0,211	6,81E-08	2,98E-06
cg16197161	1	111307245		IGR	0,213	6,81E-08	2,98E-06
cg07894331	22	22121119	MAPK1	3'UTR	-0,232	6,81E-08	2,98E-06
cg13588403	14	73209128	DPF3	Body	0,21	6,82E-08	2,98E-06
cg03555201	1	153327578		IGR	-0,239	6,83E-08	2,99E-06
cg05714960	1	25291695	RUNX3	TSS200	-0,261	6,83E-08	2,99E-06
cg08542560	5	14671841	OTULIN	Body	-0,233	6,83E-08	2,99E-06
cg13074682	15	40113167	GPR176	Body	-0,202	6,86E-08	2,99E-06
cg02186222	2	39914417	TMEM178A	5'UTR	-0,257	6,86E-08	3,00E-06
cg16138454	1	201267940	PKP1	Body	-0,2	6,87E-08	3,00E-06
cg13038250	5	142388655	ARHGAP26	Body	-0,361	6,87E-08	3,00E-06
cg24062313	4	41610944	LIMCH1	Body	-0,273	6,87E-08	3,00E-06
cg12717745	14	61852241	PRKCH	Body	-0,279	6,88E-08	3,00E-06
cg27483478	5	175969167	CDHR2	TSS1500	-0,268	6,88E-08	3,00E-06
cg23195488	10	80605565		IGR	-0,217	6,88E-08	3,00E-06
cg22606224	19	12266489	ZNF625	5'UTR	0,216	6,88E-08	3,00E-06
cg18302273	22	40306116	GRAP2	5'UTR	-0,231	6,88E-08	3,00E-06
cg06516608	2	8661038		IGR	-0,276	6,89E-08	3,00E-06
cg02016809	19	48246427		IGR	0,222	6,89E-08	3,00E-06
cg06837088	4	173917918	GALNTL6	Body	-0,309	6,89E-08	3,00E-06
cg14634110	2	71908344	DYSF	Body	-0,225	6,89E-08	3,00E-06
cg00322212	22	28072555		IGR	-0,213	6,91E-08	3,01E-06
cg25960366	9	71764492	TJP2	5'UTR	0,203	6,91E-08	3,01E-06
cg10748220	1	87827958	LINC01364	Body	-0,203	6,92E-08	3,01E-06
cg04390191	2	231090628	SP110	TSS200	-0,304	6,94E-08	3,02E-06
cg05083839	12	6270003		IGR	-0,216	6,94E-08	3,02E-06
cg15747045	10	14373423	FRMD4A	TSS1500	-0,229	6,94E-08	3,02E-06
cg12580582	12	13759540	GRIN2B	Body	-0,213	6,95E-08	3,02E-06
cg10249243	16	83150088	CDH13	5'UTR	-0,272	6,96E-08	3,02E-06
cg12938127	13	114908785		IGR	-0,279	6,96E-08	3,03E-06
cg05499690	2	187469863	ITGAV	Body	0,249	6,96E-08	3,03E-06
cg18296770	16	10906666	TVP23A	Body	-0,263	6,97E-08	3,03E-06
cg19580930	5	114750644		IGR	-0,201	6,98E-08	3,03E-06
cg15682985	8	56904810	LYN	Body	-0,243	6,98E-08	3,03E-06
cg20366862	3	47098794	SETD2	Body	-0,288	6,98E-08	3,03E-06
cg05284937	2	217943593		IGR	-0,233	6,99E-08	3,03E-06
cg19527959	6	168394191		IGR	0,211	7,00E-08	3,03E-06
cg03357456	7	102067072	PRKRIP1	3'UTR	-0,232	7,00E-08	3,03E-06
cg15512958	14	91711930	GPR68	TSS1500	-0,237	7,00E-08	3,04E-06
cg25018731	14	73181273	DPF3	Body	-0,252	7,00E-08	3,04E-06
cg22387369	15	85360691	ALPK3	1stExon	0,216	7,01E-08	3,04E-06
cg21618273	5	14414947	TRIO	Body	-0,224	7,01E-08	3,04E-06
cg23051059	8	89072565	MMP16	Body	-0,213	7,01E-08	3,04E-06
cg16596317	1	85755348		IGR	-0,262	7,03E-08	3,04E-06
cg14541541	11	36382508	PRR5L	5'UTR	-0,23	7,03E-08	3,04E-06
cg11754911	5	27450806		IGR	0,276	7,03E-08	3,04E-06
cg13900989	14	90730393	PSMC1	ExonBnd	-0,246	7,03E-08	3,04E-06
cg15253026	1	38596819		IGR	-0,2	7,04E-08	3,05E-06

cg20515679	21	39056255	KCNJ6	Body	-0,219	7,04E-08	3,05E-06
cg10497391	18	71839713		IGR	-0,242	7,05E-08	3,05E-06
cg03322922	20	62609249	SAMD10	Body	-0,272	7,05E-08	3,05E-06
cg24234899	10	118922493		IGR	0,22	7,05E-08	3,05E-06
cg06545937	19	39107006	MAP4K1	Body	-0,281	7,06E-08	3,05E-06
cg12183347	8	71587009	XKR9	5'UTR	-0,304	7,06E-08	3,05E-06
cg16524818	1	236162295	NID1	Body	-0,215	7,06E-08	3,05E-06
cg06290379	5	133377824		IGR	-0,212	7,06E-08	3,05E-06
cg19636625	7	158575868	ESYT2	Body	0,228	7,06E-08	3,05E-06
cg23033755	1	65062243	CACHD1	Body	-0,301	7,07E-08	3,06E-06
cg23353945	11	33718369		IGR	-0,211	7,07E-08	3,06E-06
cg24262206	18	9140610	ANKRD12	5'UTR	-0,339	7,07E-08	3,06E-06
cg13523559	2	10375851		IGR	-0,239	7,08E-08	3,06E-06
cg25382214	1	3105252	PRDM16	Body	0,202	7,09E-08	3,06E-06
cg19970121	11	93822184	HEPHL1	Body	0,205	7,09E-08	3,06E-06
cg26871703	15	62929846	MGC15885	Body	-0,217	7,09E-08	3,06E-06
cg04130728	12	104888396	CHST11	Body	-0,238	7,10E-08	3,06E-06
cg00931644	13	77461368	KCTD12	TSS1500	0,268	7,10E-08	3,06E-06
cg19337219	5	169407459	FAM196B	1stExon	0,276	7,10E-08	3,07E-06
cg22090271	3	112992570	BOC	Body	-0,215	7,11E-08	3,07E-06
cg22740441	4	102249652	PPP3CA	Body	-0,272	7,12E-08	3,07E-06
cg15282616	3	24045994		IGR	-0,239	7,12E-08	3,07E-06
cg26689077	12	53599806	ITGB7	5'UTR	-0,247	7,13E-08	3,07E-06
cg17254386	5	70897396	MCCC2	Body	-0,234	7,14E-08	3,08E-06
cg25112068	14	51940935	FRMD6-AS2	Body	-0,203	7,14E-08	3,08E-06
cg18329187	14	103989711	CKB	TSS1500	0,28	7,14E-08	3,08E-06
cg09900268	17	41607971	ETV4	TSS200	0,206	7,14E-08	3,08E-06
cg00735586	1	1711716	NADK	TSS1500	-0,241	7,15E-08	3,08E-06
cg21371950	6	2578733		IGR	-0,21	7,15E-08	3,08E-06
cg02294561	18	55732481	NEDD4L	Body	-0,205	7,16E-08	3,08E-06
cg19682762	6	37470483		IGR	-0,255	7,16E-08	3,08E-06
cg10505658	17	80084571	CCDC57	Body	-0,252	7,17E-08	3,08E-06
cg16889557	12	50426531		IGR	0,217	7,17E-08	3,09E-06
cg01165781	1	27676117	SYTL1	Body	0,285	7,18E-08	3,09E-06
cg03821216	2	71775047	DYSF	Body	-0,223	7,19E-08	3,09E-06
cg07519280	15	34186541	AVEN	Body	-0,351	7,19E-08	3,09E-06
cg01360261	20	49218101	FAM65C	Body	-0,228	7,20E-08	3,09E-06
cg25398315	8	135938022		IGR	-0,221	7,20E-08	3,09E-06
cg20058848	2	96852116	STARD7	3'UTR	0,201	7,21E-08	3,09E-06
cg03268699	1	162331801	NOS1AP	TSS1500	-0,225	7,21E-08	3,09E-06
cg21775463	8	25538546		IGR	-0,207	7,21E-08	3,09E-06
cg09847541	22	25404660		IGR	-0,217	7,21E-08	3,09E-06
cg04968249	9	88967853	ZCCHC6	Body	-0,202	7,21E-08	3,09E-06
cg14178991	4	76440699	RCHY1	TSS1500	-0,4	7,22E-08	3,10E-06
cg05099985	1	16245931	SPEN	Body	-0,329	7,23E-08	3,10E-06
cg14446827	1	160825797	CD244	Body	-0,223	7,23E-08	3,10E-06
cg09315367	19	10736006	SLC44A2	TSS200	0,238	7,23E-08	3,10E-06
cg06103377	15	39771589		IGR	-0,22	7,24E-08	3,10E-06
cg02677386	2	114653298	ACTR3	5'UTR	-0,203	7,24E-08	3,10E-06
cg04608356	1	27668433	SYTL1	TSS200	-0,208	7,24E-08	3,10E-06

cg07027444	6	39893930	MOCS1	Body	-0,234	7,25E-08	3,10E-06
cg25660884	18	57324466	CCBE1	Body	-0,233	7,25E-08	3,11E-06
cg20863321	2	242250819	HDLBP	5'UTR	-0,211	7,26E-08	3,11E-06
cg15727694	2	151183013		IGR	-0,248	7,27E-08	3,11E-06
cg08855630	3	68978377	FAM19A4	5'UTR	-0,207	7,27E-08	3,11E-06
cg21615593	11	60850715		IGR	-0,21	7,29E-08	3,12E-06
cg00248242	2	240867059		IGR	-0,24	7,29E-08	3,12E-06
cg13353002	17	27582351		IGR	0,21	7,29E-08	3,12E-06
cg24360137	6	90598826		IGR	-0,254	7,30E-08	3,12E-06
cg26852739	1	207144061	FCAMR	TSS200	-0,203	7,31E-08	3,12E-06
cg16348380	11	70336520	SHANK2	Body	-0,212	7,31E-08	3,12E-06
cg04790887	15	58515544		IGR	-0,205	7,34E-08	3,13E-06
cg19115548	3	39147888	TTC21A	TSS1500	0,215	7,36E-08	3,14E-06
cg17543324	12	125030232	NCOR2	5'UTR	-0,207	7,36E-08	3,14E-06
cg23713934	1	167073025	DUSP27	Body	-0,232	7,36E-08	3,14E-06
cg21194730	20	48616106		IGR	-0,236	7,36E-08	3,14E-06
cg08591538	16	533228	RAB11FIP3	Body	0,215	7,36E-08	3,14E-06
cg27058093	20	1798766		IGR	-0,213	7,37E-08	3,14E-06
cg00278655	5	173741058		IGR	-0,202	7,37E-08	3,14E-06
cg10546150	15	100537605	ADAMTS17	ExonBnd	-0,234	7,37E-08	3,14E-06
cg07103517	7	50348485	IKZF1	5'UTR	-0,269	7,38E-08	3,14E-06
cg08969925	3	33015641		IGR	-0,21	7,38E-08	3,14E-06
cg06588915	13	31619253		IGR	-0,265	7,39E-08	3,15E-06
cg17434901	11	133913868		IGR	-0,215	7,41E-08	3,15E-06
cg23004019	1	48570799	SKINTL	Body	-0,28	7,41E-08	3,15E-06
cg23640925	1	110599679		IGR	-0,227	7,41E-08	3,15E-06
cg06700060	12	65859647	MSRB3	3'UTR	-0,223	7,41E-08	3,16E-06
cg00026412	10	62059894	ANK3	Body	0,262	7,42E-08	3,16E-06
cg25761074	15	74251604		IGR	-0,273	7,42E-08	3,16E-06
cg07958316	17	67853145	LINC01483	Body	-0,229	7,42E-08	3,16E-06
cg04011469	2	82864753		IGR	-0,203	7,42E-08	3,16E-06
cg06084610	3	195623768	TNK2	TSS1500	0,245	7,42E-08	3,16E-06
cg01116137	20	25034263	ACSS1	Body	0,236	7,43E-08	3,16E-06
cg09665975	2	172739219	SLC25A12	Body	-0,356	7,43E-08	3,16E-06
cg16900775	10	34202112		IGR	-0,252	7,43E-08	3,16E-06
cg12729623	4	101816814		IGR	-0,268	7,46E-08	3,17E-06
cg01019843	11	119024496	ABCG4	Body	-0,262	7,46E-08	3,17E-06
cg20427318	6	134757763		IGR	-0,315	7,47E-08	3,17E-06
cg00496462	7	93577610		IGR	-0,249	7,46E-08	3,17E-06
cg24944290	8	72246997	EYA1	5'UTR	-0,226	7,46E-08	3,17E-06
cg23546097	16	15740943	NDE1	5'UTR	-0,265	7,46E-08	3,17E-06
cg04375504	4	185955237		IGR	-0,212	7,47E-08	3,17E-06
cg01706991	8	104185501	BAALC	Body	-0,265	7,47E-08	3,17E-06
cg14174367	1	202128508	PTPN7	1stExon	-0,239	7,48E-08	3,17E-06
cg23414759	7	133245852	EXOC4	Body	-0,201	7,48E-08	3,17E-06
cg16580362	1	227961708	SNAP47	Body	-0,226	7,48E-08	3,17E-06
cg08590732	4	77624601	SHROOM3	Body	-0,211	7,48E-08	3,17E-06
cg07582789	6	53171642	ELOVL5	5'UTR	-0,234	7,48E-08	3,17E-06
cg19554257	12	116996931	MAP1LC3B2	TSS1500	-0,302	7,48E-08	3,17E-06
cg19004771	22	51173065		IGR	-0,205	7,49E-08	3,17E-06

cg12980774	3	119630889	GSK3B	Body	-0,298	7,50E-08	3,18E-06
cg01171339	6	27831894	HIST1H2AL	TSS1500	0,25	7,51E-08	3,18E-06
cg21880733	11	77342278	CLNS1A	Body	-0,335	7,51E-08	3,18E-06
cg03149532	2	217523702	IGFBP2	Body	-0,264	7,51E-08	3,18E-06
cg02343254	2	3583963		IGR	0,226	7,52E-08	3,18E-06
cg07298772	2	176306492		IGR	0,273	7,54E-08	3,19E-06
cg11722533	18	60119699		IGR	-0,351	7,54E-08	3,19E-06
cg03805839	17	71876436		IGR	-0,212	7,56E-08	3,19E-06
cg08857221	1	37941361	ZC3H12A	Body	-0,265	7,56E-08	3,20E-06
cg21777700	12	116763201		IGR	-0,315	7,57E-08	3,20E-06
cg07135032	1	165322099	LMX1A	Body	0,225	7,57E-08	3,20E-06
cg01811153	13	98852934	FARP1	5'UTR	-0,247	7,57E-08	3,20E-06
cg13228642	1	181057360	IER5	TSS1500	0,22	7,59E-08	3,20E-06
cg05305753	6	43222155	TTBK1	Body	-0,226	7,59E-08	3,20E-06
cg21695167	3	141271397	RASA2	Body	-0,261	7,60E-08	3,21E-06
cg17665505	5	10685203	DAP	Body	-0,21	7,60E-08	3,21E-06
cg05631224	3	4560725	ITPR1	Body	-0,202	7,61E-08	3,21E-06
cg07945582	7	26206579	NFE2L3	Body	-0,292	7,61E-08	3,21E-06
cg17458850	14	33165403	AKAP6	Body	-0,233	7,62E-08	3,21E-06
cg09127129	11	67205642	PTPRCAP	TSS1500	-0,208	7,62E-08	3,21E-06
cg17974515	1	2252516		IGR	-0,212	7,62E-08	3,21E-06
cg07053784	3	178735033	ZMAT3	3'UTR	-0,282	7,62E-08	3,21E-06
cg26565676	20	57538303		IGR	0,24	7,63E-08	3,21E-06
cg24032668	1	48092677		IGR	-0,24	7,63E-08	3,21E-06
cg10950125	6	106047526		IGR	-0,211	7,63E-08	3,21E-06
cg20758789	15	82206091		IGR	-0,222	7,63E-08	3,21E-06
cg12047375	8	54757132	ATP6V1H	TSS1500	-0,281	7,64E-08	3,22E-06
cg02347043	16	11277202		IGR	-0,255	7,64E-08	3,22E-06
cg19010214	2	106488957	NCK2	Body	-0,234	7,65E-08	3,22E-06
cg21689782	2	158301252	CYTIP	TSS1500	-0,218	7,66E-08	3,22E-06
cg26891574	1	56288471		IGR	-0,267	7,69E-08	3,23E-06
cg18691720	18	59619669		IGR	-0,256	7,69E-08	3,23E-06
cg09147822	14	76273218	TTLL5	Body	0,23	7,70E-08	3,23E-06
cg19682870	14	99715826	BCL11B	Body	-0,212	7,70E-08	3,23E-06
cg22281120	19	7186372	INSR	Body	-0,267	7,72E-08	3,24E-06
cg25992016	13	77387018		IGR	-0,208	7,72E-08	3,24E-06
cg03224992	1	51750976		IGR	-0,258	7,74E-08	3,24E-06
cg04661335	3	156060002	KCNAB1	Body	-0,236	7,75E-08	3,25E-06
cg15162876	15	74495276	STRA6	5'UTR	-0,243	7,75E-08	3,25E-06
cg14620941	9	139776929		IGR	-0,202	7,75E-08	3,25E-06
cg21535253	19	12758416	MAN2B1	Body	0,231	7,76E-08	3,25E-06
cg19235283	1	89725209	GBP5	3'UTR	-0,389	7,76E-08	3,25E-06
cg09006572	10	3293547		IGR	-0,256	7,76E-08	3,25E-06
cg24408488	20	31123411	NOL4L	Body	-0,208	7,76E-08	3,25E-06
cg01168166	1	235062594		IGR	-0,32	7,77E-08	3,25E-06
cg03404566	17	6899310	ALOX12	TSS200	0,216	7,77E-08	3,25E-06
cg27108059	1	154412397	IL6R	Body	-0,227	7,77E-08	3,26E-06
cg02857074	7	81746161	CACNA2D1	Body	-0,218	7,78E-08	3,26E-06
cg23295921	18	21292539	LAMA3	Body	-0,219	7,80E-08	3,26E-06
cg13569051	9	124051703	GSN	5'UTR	0,337	7,81E-08	3,27E-06

cg17353890	1	149908753	MTMR11	TSS1500	0,215	7,81E-08	3,27E-06
cg14485407	2	45063342		IGR	-0,314	7,82E-08	3,27E-06
cg25504607	13	30728534		IGR	0,283	7,82E-08	3,27E-06
cg02964085	5	131808970	C5orf56	Body	-0,221	7,82E-08	3,27E-06
cg21787323	11	85338252	TMEM126B	TSS1500	0,242	7,83E-08	3,27E-06
cg15541798	11	124763926	ROBO4	ExonBnd	-0,217	7,83E-08	3,27E-06
cg26561615	3	19973206	EFHB	Body	-0,256	7,83E-08	3,27E-06
cg12484022	10	31253602	ZNF438	Body	0,228	7,83E-08	3,27E-06
cg12693179	12	71863439	LGR5	Body	0,211	7,83E-08	3,27E-06
cg21929481	3	33089501	GLB1	Body	-0,228	7,83E-08	3,27E-06
cg17501459	2	177506985	LINC01117	Body	-0,231	7,85E-08	3,28E-06
cg05940538	8	102063934	FLJ42969	TSS1500	0,212	7,85E-08	3,28E-06
cg04070007	10	11218007	CELF2	Body	-0,364	7,85E-08	3,28E-06
cg00747043	11	11366051	GALNT18	Body	-0,256	7,87E-08	3,28E-06
cg06005544	19	4423831	CHAF1A	Body	-0,201	7,87E-08	3,28E-06
cg04646374	2	208672498		IGR	-0,262	7,87E-08	3,28E-06
cg18573891	5	141559610		IGR	-0,211	7,87E-08	3,28E-06
cg13166115	3	113940596		IGR	-0,285	7,91E-08	3,29E-06
cg09911401	10	29827566	SVIL	Body	-0,235	7,91E-08	3,29E-06
cg10825315	14	81425912	TSHR	Body	-0,233	7,92E-08	3,30E-06
cg26358483	22	42674091		IGR	-0,227	7,92E-08	3,30E-06
cg12356793	17	13299004		IGR	-0,215	7,94E-08	3,30E-06
cg11848788	1	39041399		IGR	-0,237	7,94E-08	3,30E-06
cg23824801	12	54653403	CBX5	TSS200	0,246	7,94E-08	3,30E-06
cg22161562	2	25198596		IGR	0,201	7,94E-08	3,30E-06
cg08729573	18	74476651		IGR	-0,216	7,94E-08	3,30E-06
cg06314450	2	121044892	RALB	Body	-0,321	7,95E-08	3,30E-06
cg09551155	1	209559561		IGR	-0,21	7,95E-08	3,31E-06
cg15908654	17	66484111	PRKAR1A	5'UTR	-0,213	7,95E-08	3,31E-06
cg17505469	10	8373450		IGR	-0,262	7,95E-08	3,31E-06
cg07862768	8	105379411		IGR	0,21	7,96E-08	3,31E-06
cg16224163	3	187870621	LPP	TSS1500	0,214	7,96E-08	3,31E-06
cg05377120	12	10456885	KLRD1	TSS200	-0,279	7,97E-08	3,31E-06
cg25711246	12	4917426	KCNA6	TSS1500	-0,209	7,97E-08	3,31E-06
cg13508482	13	44147687	ENOX1	5'UTR	0,226	7,98E-08	3,31E-06
cg24636814	2	191980241	STAT4	Body	-0,225	7,99E-08	3,32E-06
cg23482724	14	101322548	MEG3	Body	-0,209	7,99E-08	3,32E-06
cg19633645	15	76428015	C15orf27	Body	-0,269	7,99E-08	3,32E-06
cg05313644	20	47400500	PREX1	Body	-0,268	7,99E-08	3,32E-06
cg05897122	4	53112707		IGR	-0,261	8,00E-08	3,32E-06
cg21541534	4	86684656	ARHGAP24	Body	-0,202	8,00E-08	3,32E-06
cg05157266	2	183901614	NCKAP1	Body	0,201	8,00E-08	3,32E-06
cg22537347	22	43053535		IGR	-0,209	8,01E-08	3,32E-06
cg24526441	2	231528350		IGR	-0,258	8,01E-08	3,32E-06
cg18947120	3	13015508	IQSEC1	Body	-0,308	8,01E-08	3,32E-06
cg15535471	7	139332087	HIPK2	Body	-0,251	8,03E-08	3,33E-06
cg00100361	12	110752302	ATP2A2	Body	-0,243	8,03E-08	3,33E-06
cg05994850	6	30095341		IGR	0,253	8,04E-08	3,33E-06
cg07972114	2	157520453		IGR	-0,278	8,05E-08	3,33E-06
cg14367527	20	62858816	MYT1	ExonBnd	-0,239	8,05E-08	3,34E-06

cg13391490	20	38907508		IGR	-0,203	8,06E-08	3,34E-06
cg19331423	9	2818246	KIAA0020	Body	0,207	8,06E-08	3,34E-06
cg19843271	11	116644707	BUD13	TSS1500	-0,207	8,07E-08	3,34E-06
cg05209917	3	1134730	CNTN6	5'UTR	0,233	8,08E-08	3,34E-06
cg19208848	9	80177513	GNA14	Body	-0,222	8,08E-08	3,34E-06
cg02182856	5	133474100	TCF7	Body	-0,22	8,09E-08	3,34E-06
cg05828605	8	134401000		IGR	-0,29	8,09E-08	3,34E-06
cg11339384	2	113405125	SLC20A1	Body	-0,226	8,10E-08	3,35E-06
cg13602358	3	14885560	FGD5	Body	-0,203	8,10E-08	3,35E-06
cg25291653	20	19867145		IGR	0,211	8,11E-08	3,35E-06
cg10252315	1	156879551	PEAR1	ExonBnd	-0,212	8,11E-08	3,35E-06
cg13353542	9	80826125		IGR	-0,206	8,11E-08	3,35E-06
cg15938090	1	10570921	PEX14	Body	-0,304	8,12E-08	3,35E-06
cg22702857	12	4269985		IGR	-0,222	8,12E-08	3,35E-06
cg25984584	16	50532151		IGR	-0,21	8,12E-08	3,35E-06
cg03126946	12	2944310	NRIP2	TSS200	0,29	8,12E-08	3,35E-06
cg19309256	2	9795717		IGR	-0,255	8,13E-08	3,36E-06
cg16912386	17	9336502	STX8	Body	-0,297	8,14E-08	3,36E-06
cg08670831	12	116610481	MED13L	Body	-0,258	8,15E-08	3,36E-06
cg13618787	1	205500511	CDK18	3'UTR	-0,261	8,16E-08	3,37E-06
cg06596563	9	97628146	C9orf3	Body	0,202	8,16E-08	3,37E-06
cg24820936	8	101270745	RNF19A	3'UTR	-0,209	8,18E-08	3,37E-06
cg19754515	2	224749380	WDFY1	ExonBnd	-0,279	8,18E-08	3,37E-06
cg13661983	10	50286560	VSTM4	Body	-0,21	8,20E-08	3,38E-06
cg25641635	15	39704033		IGR	-0,245	8,21E-08	3,38E-06
cg19633390	13	110381307		IGR	0,301	8,21E-08	3,38E-06
cg23621149	10	6824325		IGR	0,24	8,22E-08	3,39E-06
cg01069505	17	44971405		IGR	-0,21	8,23E-08	3,39E-06
cg09047179	10	11846999		IGR	-0,209	8,25E-08	3,39E-06
cg10473466	11	68951380		IGR	-0,241	8,26E-08	3,39E-06
cg10334489	17	25798878	KSR1	TSS200	-0,227	8,26E-08	3,40E-06
cg20053001	12	44766066	TMEM117	Body	-0,202	8,27E-08	3,40E-06
cg06101607	1	48283575	TRABD2B	Body	-0,242	8,29E-08	3,40E-06
cg16704560	2	15777810		IGR	0,204	8,30E-08	3,41E-06
cg05235104	8	62038992		IGR	-0,276	8,30E-08	3,41E-06
cg21111416	3	148804720	HLTF	TSS1500	0,23	8,31E-08	3,41E-06
cg10198294	10	28616726		IGR	0,25	8,32E-08	3,41E-06
cg01752257	15	34531718	SLC12A6	Body	-0,377	8,32E-08	3,41E-06
cg26653677	17	33863922		IGR	-0,388	8,32E-08	3,41E-06
cg01006334	3	170957383	TNIK	Body	0,219	8,33E-08	3,41E-06
cg15439725	4	299311		IGR	0,221	8,33E-08	3,41E-06
cg16310145	11	12890339	TEAD1	Body	-0,248	8,33E-08	3,41E-06
cg08936231	10	112296001		IGR	-0,218	8,33E-08	3,41E-06
cg20804799	11	122007253	MIR100HG	Body	-0,204	8,34E-08	3,42E-06
cg22777062	21	38353114	HLCS	5'UTR	0,211	8,34E-08	3,42E-06
cg04733856	3	24961439		IGR	-0,209	8,34E-08	3,42E-06
cg26931934	17	47419801	ZNF652	5'UTR	-0,274	8,35E-08	3,42E-06
cg10527707	2	10054932	TAF1B	Body	-0,234	8,35E-08	3,42E-06
cg22416963	5	159632402	FABP6	Body	-0,216	8,35E-08	3,42E-06
cg14599380	4	17310680		IGR	-0,223	8,35E-08	3,42E-06

cg00592046	18	69848574		IGR	-0,207	8,36E-08	3,42E-06
cg25423364	14	73480891	ZFYVE1	Body	-0,211	8,37E-08	3,42E-06
cg26736341	6	31545342	TNF	3'UTR	-0,225	8,37E-08	3,43E-06
cg00582194	6	169930709	WDR27	Body	-0,248	8,37E-08	3,43E-06
cg03732846	18	11096230	PIEZO2	Body	-0,28	8,38E-08	3,43E-06
cg07068406	3	134647778	EPHB1	Body	-0,237	8,39E-08	3,43E-06
cg05297088	14	88339576		IGR	-0,216	8,41E-08	3,44E-06
cg12260336	5	23446830		IGR	0,215	8,43E-08	3,44E-06
cg27001818	22	33724826	LARGE	Body	-0,278	8,43E-08	3,44E-06
cg12099018	11	47921550		IGR	-0,265	8,44E-08	3,44E-06
cg14886500	6	23447204		IGR	-0,298	8,44E-08	3,44E-06
cg19361800	2	208014177	KLF7	Body	0,211	8,45E-08	3,45E-06
cg05322916	6	36880416	C6orf89	Body	-0,367	8,46E-08	3,45E-06
cg10529022	11	60694470	TMEM132A	Body	-0,222	8,47E-08	3,45E-06
cg13465598	8	38401333		IGR	-0,215	8,47E-08	3,45E-06
cg14378167	12	93113838		IGR	-0,229	8,49E-08	3,46E-06
cg27381396	15	101966898	PCSK6	Body	-0,21	8,49E-08	3,46E-06
cg04304059	11	12048600		IGR	-0,256	8,49E-08	3,46E-06
cg24063411	6	135449953		IGR	-0,26	8,49E-08	3,46E-06
cg13901957	17	29907534		IGR	-0,282	8,49E-08	3,46E-06
cg02751745	6	52532536	LOC730101	Body	-0,207	8,50E-08	3,46E-06
cg02492678	1	49226411	BEND5	Body	-0,203	8,50E-08	3,46E-06
cg22417613	7	138560796	KIAA1549	Body	-0,28	8,50E-08	3,46E-06
cg20979153	20	52199748	ZNF217	TSS200	-0,237	8,50E-08	3,46E-06
cg23676423	8	81869656		IGR	-0,319	8,51E-08	3,46E-06
cg25142327	13	113497950	ATP11A	Body	0,206	8,51E-08	3,46E-06
cg09728869	15	85951572	AKAP13	5'UTR	-0,291	8,53E-08	3,47E-06
cg10418410	12	2751699	CACNA1C	Body	-0,231	8,53E-08	3,47E-06
cg01656396	9	27157924	TEK	Body	-0,214	8,53E-08	3,47E-06
cg10662390	1	24046776		IGR	-0,229	8,55E-08	3,47E-06
cg16568840	14	32436704		IGR	-0,269	8,56E-08	3,47E-06
cg25246741	5	148438389	SH3TC2	Body	-0,243	8,56E-08	3,48E-06
cg03009213	14	86400582	LOC10192876	TSS1500	-0,34	8,57E-08	3,48E-06
cg23837109	10	75670435	PLAU	TSS1500	0,214	8,57E-08	3,48E-06
cg26320771	11	19472257	NAV2	Body	-0,225	8,57E-08	3,48E-06
cg13881547	17	40545151		IGR	-0,29	8,58E-08	3,48E-06
cg17603988	7	56160766	PHKG1	TSS200	0,214	8,58E-08	3,48E-06
cg20041729	13	80934176		IGR	0,215	8,60E-08	3,49E-06
cg08494738	16	4369512		IGR	-0,308	8,60E-08	3,49E-06
cg15365795	11	69318319		IGR	-0,292	8,61E-08	3,49E-06
cg03809021	16	89831123	FANCA	Body	-0,275	8,61E-08	3,49E-06
cg11728953	10	72488950	ADAMTS14	Body	-0,222	8,62E-08	3,49E-06
cg06001894	4	122148579	TNIP3	1stExon	-0,246	8,63E-08	3,49E-06
cg17610535	1	209897614	HSD11B1	Body	-0,256	8,64E-08	3,50E-06
cg10423607	8	68418480	CPA6	Body	-0,378	8,64E-08	3,50E-06
cg13226737	16	24682412	LINC01567	TSS200	0,281	8,65E-08	3,50E-06
cg20600379	6	32909282	HLA-DMB	TSS1500	-0,211	8,65E-08	3,50E-06
cg13393479	15	68591493		IGR	-0,214	8,66E-08	3,50E-06
cg15643078	12	22562854		IGR	-0,23	8,67E-08	3,50E-06
cg26029453	7	47818048	PKD1L1	Body	-0,236	8,67E-08	3,51E-06

cg07085167	14	55740496	FBXO34	5'UTR	0,201	8,68E-08	3,51E-06
cg26581287	9	104349265	GRIN3A	Body	-0,22	8,68E-08	3,51E-06
cg25773259	12	94019073		IGR	-0,308	8,68E-08	3,51E-06
cg21817764	12	15154690	LINC01489	TSS200	0,214	8,70E-08	3,51E-06
cg26140366	2	1482385	TPO	Body	-0,206	8,70E-08	3,51E-06
cg09993145	1	25291905	RUNX3	TSS1500	-0,308	8,71E-08	3,51E-06
cg10420609	6	7538349		IGR	0,214	8,71E-08	3,52E-06
cg09387559	1	161695606	FCRLB	ExonBnd	0,202	8,72E-08	3,52E-06
cg21650640	2	237783849		IGR	-0,216	8,72E-08	3,52E-06
cg20120438	19	4305239	FSD1	Body	0,209	8,72E-08	3,52E-06
cg16478871	16	28199217	XPO6	5'UTR	-0,252	8,73E-08	3,52E-06
cg04654829	10	134776718	LINC01167	TSS1500	-0,247	8,74E-08	3,52E-06
cg23597094	2	33335706	LTBP1	Body	-0,249	8,76E-08	3,53E-06
cg05247773	14	103018580		IGR	-0,219	8,76E-08	3,53E-06
cg04929544	1	7450634	CAMTA1	Body	-0,222	8,77E-08	3,53E-06
cg08898866	17	13941263		IGR	0,201	8,77E-08	3,53E-06
cg05080154	18	76739409	SALL3	TSS1500	0,229	8,77E-08	3,53E-06
cg24251058	3	50626119		IGR	-0,206	8,79E-08	3,54E-06
cg26705250	13	100512311	CLYBL	Body	-0,287	8,79E-08	3,54E-06
cg01466232	12	121998505	KDM2B	Body	-0,25	8,80E-08	3,54E-06
cg21990700	12	7260776	LOC283314	TSS200	-0,243	8,80E-08	3,54E-06
cg00147172	22	46423604		IGR	0,231	8,80E-08	3,54E-06
cg18780396	12	3165771		IGR	-0,22	8,80E-08	3,54E-06
cg23895467	6	119373649	FAM184A	Body	-0,203	8,81E-08	3,54E-06
cg14312538	18	25757710	CDH2	TSS1500	0,203	8,82E-08	3,55E-06
cg13736004	12	24974836	BCAT1	Body	-0,24	8,83E-08	3,55E-06
cg00649318	9	124409433	DAB2IP	Body	-0,218	8,83E-08	3,55E-06
cg05145922	4	36287903	DTHD1	Body	-0,254	8,83E-08	3,55E-06
cg01294868	12	15103680	ARHGDIB	5'UTR	-0,249	8,84E-08	3,55E-06
cg07565956	17	7381288	ZBTB4	5'UTR	0,278	8,85E-08	3,56E-06
cg01059421	6	88447049		IGR	-0,207	8,85E-08	3,56E-06
cg14010720	20	62168878	PTK6	TSS200	-0,227	8,87E-08	3,56E-06
cg12914151	17	62309147	TEX2	5'UTR	0,222	8,87E-08	3,56E-06
cg03605454	4	150806283		IGR	-0,209	8,87E-08	3,56E-06
cg00336700	2	176943704		IGR	0,214	8,88E-08	3,56E-06
cg07065343	3	48166681		IGR	-0,214	8,89E-08	3,57E-06
cg13160637	1	160737558		IGR	-0,201	8,90E-08	3,57E-06
cg14822564	3	156420921	TIPARP	Body	0,213	8,90E-08	3,57E-06
cg22056425	9	96344590	PHF2	Body	0,215	8,90E-08	3,57E-06
cg13284849	3	133167599	BFSP2	Body	-0,23	8,91E-08	3,57E-06
cg22058537	16	87067771		IGR	0,245	8,91E-08	3,57E-06
cg25902889	19	4305090	FSD1	Body	0,31	8,92E-08	3,57E-06
cg25978218	1	15738732	EFHD2	Body	-0,262	8,93E-08	3,57E-06
cg05933681	10	126053180		IGR	-0,238	8,94E-08	3,58E-06
cg06152215	12	124422259	CCDC92	Body	-0,303	8,94E-08	3,58E-06
cg15026743	1	95003968	F3	Body	0,223	8,95E-08	3,58E-06
cg20757133	3	21370390		IGR	0,229	8,95E-08	3,58E-06
cg07333191	4	13526769		IGR	0,231	8,96E-08	3,58E-06
cg23545544	6	150962421	PLEKHG1	5'UTR	-0,224	8,96E-08	3,58E-06
cg12788694	17	39873380		IGR	-0,228	8,96E-08	3,58E-06

cg08810073	20	33214972	PIGU	Body	0,312	8,96E-08	3,58E-06
cg10083098	14	75305100		IGR	-0,215	8,97E-08	3,58E-06
cg17560073	8	124850602		IGR	-0,287	8,97E-08	3,58E-06
cg26676376	1	7530055	CAMTA1	Body	-0,201	8,98E-08	3,59E-06
cg00424315	16	17120799		IGR	-0,209	8,98E-08	3,59E-06
cg01755037	1	170492043	LOC10192865	Body	-0,25	8,99E-08	3,59E-06
cg16596691	19	41260294	SNRPA	Body	-0,2	8,99E-08	3,59E-06
cg03721175	5	173390427		IGR	0,226	8,99E-08	3,59E-06
cg02747254	16	29938183	KCTD13	TSS1500	0,227	9,00E-08	3,59E-06
cg00712311	3	42039734		IGR	0,246	9,01E-08	3,60E-06
cg13233355	11	72472164	STARD10	Body	0,213	9,03E-08	3,60E-06
cg04254052	5	170029226	KCNIP1	5'UTR	-0,243	9,06E-08	3,61E-06
cg24699005	19	1192342		IGR	-0,23	9,06E-08	3,61E-06
cg12819431	3	193990908		IGR	0,258	9,06E-08	3,61E-06
cg12986110	19	48551504	PLA2G4C	3'UTR	-0,211	9,07E-08	3,61E-06
cg16898391	12	12867916		IGR	-0,229	9,08E-08	3,61E-06
cg24312520	17	40489584	STAT3	Body	-0,364	9,09E-08	3,62E-06
cg26532208	17	46506244	SKAP1	Body	-0,255	9,09E-08	3,62E-06
cg08267433	1	205554300	MFSD4	Body	-0,238	9,10E-08	3,62E-06
cg15674181	10	3158688	PFKP	Body	0,21	9,10E-08	3,62E-06
cg18812257	20	48212528		IGR	-0,216	9,10E-08	3,62E-06
cg05726764	3	179399579	USP13	Body	0,232	9,12E-08	3,62E-06
cg16122778	16	86671056		IGR	-0,215	9,12E-08	3,62E-06
cg00826632	19	46969832	PNMAL1	3'UTR	-0,233	9,12E-08	3,62E-06
cg24457337	4	10094737	WDR1	Body	0,25	9,13E-08	3,63E-06
cg17217296	1	159046937	AIM2	TSS1500	-0,257	9,14E-08	3,63E-06
cg02735988	20	11125380		IGR	-0,232	9,15E-08	3,63E-06
cg27578275	5	169696692	LCP2	Body	-0,305	9,16E-08	3,63E-06
cg00156230	7	45073692	CCM2	Body	0,244	9,16E-08	3,63E-06
cg13385114	1	9656493	TMEM201	Body	0,201	9,17E-08	3,64E-06
cg26645700	1	149908766	MTMR11	TSS1500	0,207	9,17E-08	3,64E-06
cg03708142	2	72100639		IGR	-0,206	9,17E-08	3,64E-06
cg13547213	2	223684678		IGR	-0,275	9,17E-08	3,64E-06
cg04376403	22	25411874		IGR	0,242	9,17E-08	3,64E-06
cg25957677	4	55147657	PDGFRA	Body	0,209	9,17E-08	3,64E-06
cg09775648	2	190309186	WDR75	Body	-0,239	9,18E-08	3,64E-06
cg11113436	17	63071046		IGR	0,256	9,20E-08	3,65E-06
cg11462667	3	160698705	PPM1L	Body	-0,25	9,22E-08	3,65E-06
cg27660627	16	89461803	ANKRD11	5'UTR	0,22	9,23E-08	3,65E-06
cg17609780	2	86693233	KDM3A	Body	0,263	9,24E-08	3,66E-06
cg20126656	17	76801712	USP36	Body	-0,237	9,24E-08	3,66E-06
cg02462252	3	23988942	NR1D2	5'UTR	0,22	9,25E-08	3,66E-06
cg00287009	2	201273195	SPATS2L	5'UTR	-0,222	9,25E-08	3,66E-06
cg03326064	1	234542953	TARBP1	Body	0,259	9,26E-08	3,66E-06
cg11112948	2	231306379	SP100	Body	-0,239	9,26E-08	3,66E-06
cg21512660	19	15427469		IGR	-0,286	9,27E-08	3,66E-06
cg18322510	1	236686618	LGALS8	TSS200	0,229	9,28E-08	3,67E-06
cg14062002	6	139117468		IGR	0,217	9,29E-08	3,67E-06
cg19904126	22	20919808	MED15	Body	-0,232	9,29E-08	3,67E-06
cg00755378	6	28953126		IGR	0,234	9,31E-08	3,67E-06

cg20416762	14	95766570	CLMN	Body	-0,258	9,31E-08	3,67E-06
cg06873351	17	60770405	MRC2	3'UTR	-0,266	9,31E-08	3,67E-06
cg13343931	5	124318074		IGR	-0,231	9,32E-08	3,68E-06
cg15655369	20	49135042	PTPN1	5'UTR	-0,359	9,33E-08	3,68E-06
cg16767183	21	47531343	COL6A2	ExonBnd	-0,222	9,33E-08	3,68E-06
cg20810954	14	56552265		IGR	-0,224	9,34E-08	3,68E-06
cg11783901	17	47102000	IGF2BP1	Body	-0,214	9,36E-08	3,69E-06
cg24000206	1	218550762	TGFB2	Body	-0,413	9,38E-08	3,70E-06
cg24141156	12	54758422	GPR84	TSS200	-0,219	9,38E-08	3,70E-06
cg11343894	1	153599704	S100A13	5'UTR	0,207	9,39E-08	3,70E-06
cg21890667	22	30476089	HORMAD2	TSS1500	0,201	9,39E-08	3,70E-06
cg17926236	3	151975490		IGR	-0,243	9,40E-08	3,70E-06
cg07084358	4	157997086	GLRB	TSS1500	0,235	9,40E-08	3,70E-06
cg13689667	7	26007848		IGR	-0,231	9,43E-08	3,71E-06
cg06869641	2	71947929		IGR	-0,203	9,44E-08	3,71E-06
cg04990420	22	24824362	ADORA2A	5'UTR	-0,203	9,46E-08	3,72E-06
cg14189583	19	42703852	DEDD2	Body	-0,284	9,47E-08	3,72E-06
cg26712087	8	18704653	PSD3	Body	-0,263	9,48E-08	3,72E-06
cg03214420	19	44257589	C19orf61	5'UTR	-0,287	9,49E-08	3,72E-06
cg09748936	3	127356529	PODXL2	Body	-0,22	9,50E-08	3,73E-06
cg02639359	19	17862017	FCHO1	5'UTR	-0,338	9,51E-08	3,73E-06
cg11886884	3	138062860		IGR	-0,206	9,51E-08	3,73E-06
cg23991209	14	68973600	RAD51B	Body	-0,289	9,51E-08	3,73E-06
cg26176717	17	45364717	ITGB3	Body	-0,2	9,52E-08	3,73E-06
cg05866504	2	12644930	LOC10050645	Body	-0,272	9,52E-08	3,73E-06
cg10311217	6	47171647		IGR	-0,211	9,52E-08	3,73E-06
cg23299807	17	19313486	RNF112	TSS1500	-0,235	9,53E-08	3,73E-06
cg20380214	12	9217688	LOC144571	TSS200	0,229	9,53E-08	3,74E-06
cg21021089	13	78292473	SLAIN1	Body	0,244	9,54E-08	3,74E-06
cg21048159	11	13030929	RASSF10	TSS200	0,239	9,55E-08	3,74E-06
cg17360256	2	64099621	UGP2	Body	0,285	9,56E-08	3,74E-06
cg03179867	11	34645372	EHF	5'UTR	-0,244	9,56E-08	3,74E-06
cg16839726	8	42359064	SLC20A2	TSS200	0,363	9,56E-08	3,74E-06
cg27227156	11	114430285	FAM55A	5'UTR	0,24	9,56E-08	3,74E-06
cg04133658	3	129161504	IFT122	Body	0,219	9,56E-08	3,74E-06
cg08008959	7	143007688		IGR	-0,292	9,57E-08	3,75E-06
cg25181651	2	1748132	PXDN	1stExon	0,21	9,58E-08	3,75E-06
cg07382347	6	30039408	RNF39	Body	0,345	9,58E-08	3,75E-06
cg04632367	10	80199052		IGR	-0,208	9,58E-08	3,75E-06
cg14278300	1	167486978	CD247	Body	-0,219	9,59E-08	3,75E-06
cg13390284	1	65531864		IGR	0,223	9,60E-08	3,75E-06
cg11260483	11	12031826	DKK3	TSS1500	-0,204	9,60E-08	3,75E-06
cg03797115	4	110625010	CASP6	TSS1500	0,202	9,60E-08	3,75E-06
cg07467492	17	55173766	AKAP1	5'UTR	0,203	9,62E-08	3,76E-06
cg14205511	8	48572137	SPIDR	Body	0,229	9,62E-08	3,76E-06
cg15886317	12	4228378		IGR	-0,225	9,62E-08	3,76E-06
cg10004150	22	36335438	RBFOX2	Body	-0,208	9,62E-08	3,76E-06
cg12964261	6	36332842	ETV7	Body	-0,262	9,63E-08	3,76E-06
cg00749672	7	26437192		IGR	0,208	9,63E-08	3,76E-06
cg21281441	2	20565253		IGR	-0,201	9,64E-08	3,76E-06

cg13590876	14	105154689	INF2	TSS1500	0,228	9,64E-08	3,76E-06
cg23902073	11	10709788	MRVI1	5'UTR	-0,204	9,65E-08	3,76E-06
cg26842280	20	44692501	NCOA5	Body	-0,229	9,65E-08	3,76E-06
cg15709243	18	67957767	SOCS6	5'UTR	0,285	9,65E-08	3,77E-06
cg14123900	9	92444737		IGR	-0,211	9,67E-08	3,77E-06
cg26695909	8	81639869	ZNF704	Body	0,229	9,67E-08	3,77E-06
cg05035315	11	132691484	OPCML	Body	-0,203	9,67E-08	3,77E-06
cg15806306	11	2893385		IGR	-0,212	9,68E-08	3,77E-06
cg10754646	11	41453445	LRRC4C	5'UTR	0,24	9,67E-08	3,77E-06
cg05674377	20	43275684	ADA	Body	-0,272	9,69E-08	3,77E-06
cg03536657	15	69099834	C15orf28	TSS1500	0,214	9,69E-08	3,77E-06
cg07210763	2	223654386		IGR	-0,227	9,70E-08	3,77E-06
cg03068437	1	170037915	KIFAP3	1stExon	-0,232	9,72E-08	3,78E-06
cg22187952	5	16509635	FAM134B	TSS1500	0,247	9,71E-08	3,78E-06
cg22875875	4	72978321	NPFFR2	5'UTR	-0,259	9,72E-08	3,78E-06
cg24934137	8	98869237		IGR	0,22	9,73E-08	3,79E-06
cg09511741	19	49668729	TRPM4	Body	0,22	9,74E-08	3,79E-06
cg12996520	17	45819275	TBX21	Body	-0,221	9,75E-08	3,79E-06
cg05890804	2	20716912		IGR	-0,225	9,75E-08	3,79E-06
cg09434667	15	67078890		IGR	-0,222	9,75E-08	3,79E-06
cg17104429	20	572111		IGR	-0,228	9,76E-08	3,79E-06
cg11707219	8	21905756	FGF17	Body	0,218	9,77E-08	3,80E-06
cg01864026	15	101639459		IGR	-0,276	9,77E-08	3,80E-06
cg13962111	17	25574608		IGR	0,208	9,79E-08	3,80E-06
cg16196595	1	156192737	PMF1	Body	-0,215	9,79E-08	3,80E-06
cg00152117	1	156928845	ARHGEF11	Body	-0,2	9,79E-08	3,80E-06
cg07617283	7	127913660		IGR	0,257	9,79E-08	3,80E-06
cg03499256	8	72912611	MSC-AS1	Body	-0,205	9,81E-08	3,81E-06
cg13763649	12	109554277		IGR	0,21	9,81E-08	3,81E-06
cg07475177	11	64178090		IGR	-0,222	9,82E-08	3,81E-06
cg08057255	14	69036779	RAD51B	Body	0,236	9,83E-08	3,81E-06
cg15177986	12	50411007	RACGAP1	5'UTR	0,216	9,84E-08	3,82E-06
cg21223380	11	11193606		IGR	-0,218	9,88E-08	3,83E-06
cg24722198	13	113567732		IGR	-0,217	9,89E-08	3,83E-06
cg22296190	3	31138258		IGR	-0,207	9,90E-08	3,83E-06
cg08117738	6	147851430	SAMD5	Body	-0,243	9,90E-08	3,83E-06
cg04428305	6	25085161	CMAHP	Body	0,222	9,93E-08	3,84E-06
cg06214339	5	171416481	FBXW11	Body	-0,204	9,95E-08	3,85E-06
cg26213458	12	5918191	ANO2	Body	-0,211	9,95E-08	3,85E-06
cg01845559	8	93647215	LOC10272471	Body	-0,253	9,96E-08	3,85E-06
cg23457705	8	107071197	ZFPM2-AS1	Body	-0,255	9,96E-08	3,85E-06
cg19139322	2	217669382		IGR	-0,312	9,96E-08	3,85E-06
cg23523057	5	79150013		IGR	-0,216	9,96E-08	3,85E-06
cg23262619	15	41721497	RTF1	Body	-0,232	9,97E-08	3,85E-06
cg12094903	6	32808689	PSMB8	3'UTR	-0,271	9,97E-08	3,85E-06
cg13928306	16	3162397	ZNF205	TSS200	0,207	9,99E-08	3,86E-06
cg14011070	1	235811811	GNG4	5'UTR	0,241	1,00E-07	3,86E-06
cg04255947	6	34833130	UHRF1BP1	Body	0,243	1,00E-07	3,86E-06
cg07645590	2	155314687	LOC10014459	TSS1500	-0,262	1,00E-07	3,87E-06
cg20535450	5	75903464	IQGAP2	TSS1500	0,202	1,00E-07	3,87E-06

cg27293130	7	106245083		IGR	-0,201	1,00E-07	3,87E-06
cg19621475	10	11242921	CELF2	Body	-0,259	1,00E-07	3,87E-06
cg02634805	1	27936307		IGR	-0,282	1,00E-07	3,87E-06
cg15718287	13	36702658	DCLK1	5'UTR	-0,261	1,00E-07	3,87E-06
cg19235180	3	155945853	KCNAB1-AS2	TSS200	-0,205	1,01E-07	3,88E-06
cg16060214	4	86936666	MAPK10	3'UTR	-0,252	1,01E-07	3,89E-06
cg14806636	11	19761062	NAV2	Body	-0,228	1,01E-07	3,89E-06
cg07907474	7	80502612	SEMA3C	Body	-0,214	1,01E-07	3,90E-06
cg12754085	15	36893949	C15orf41	5'UTR	0,237	1,01E-07	3,90E-06
cg19926385	15	62764577		IGR	-0,2	1,01E-07	3,90E-06
cg09721047	2	68962012	ARHGAP25	5'UTR	-0,24	1,01E-07	3,90E-06
cg06980941	10	44397116		IGR	-0,207	1,01E-07	3,90E-06
cg01002264	10	121137808	GRK5	Body	0,21	1,01E-07	3,90E-06
cg11223933	17	43319137	FMNL1	Body	0,208	1,02E-07	3,90E-06
cg06200967	1	55456822	TMEM61	Body	-0,217	1,02E-07	3,91E-06
cg24916768	20	62413537	ZBTB46	Body	-0,24	1,02E-07	3,91E-06
cg18154954	19	39055500	RYR1	Body	0,211	1,02E-07	3,91E-06
cg14306709	6	31547704		IGR	-0,249	1,02E-07	3,91E-06
cg04744626	11	15931625		IGR	-0,224	1,02E-07	3,91E-06
cg10184771	17	62102410		IGR	-0,215	1,02E-07	3,91E-06
cg00698646	9	136417855	ADAMTSL2	Body	-0,202	1,02E-07	3,91E-06
cg06728488	9	136691294	VAV2	Body	-0,256	1,02E-07	3,92E-06
cg10244390	16	575700		IGR	-0,232	1,02E-07	3,92E-06
cg02771117	8	11279352	FAM167A	3'UTR	-0,218	1,02E-07	3,92E-06
cg07734975	11	9780844		IGR	0,225	1,02E-07	3,92E-06
cg22931622	3	65129155		IGR	-0,215	1,02E-07	3,92E-06
cg20248790	4	150999029	DCLK2	TSS1500	0,244	1,02E-07	3,92E-06
cg05745631	6	30095178		IGR	0,291	1,02E-07	3,92E-06
cg10534455	21	34775001	IFNGR2	TSS200	0,21	1,02E-07	3,93E-06
cg26322315	7	7758995	RPA3	TSS1500	-0,361	1,02E-07	3,93E-06
cg11033835	3	77087860	ROBO2	TSS1500	0,211	1,02E-07	3,93E-06
cg05360682	14	105536542		IGR	-0,304	1,03E-07	3,93E-06
cg10349208	7	37590645		IGR	-0,226	1,03E-07	3,94E-06
cg09692653	3	171122711	TNIK	Body	0,258	1,03E-07	3,94E-06
cg00779348	14	91708201	GPR68	5'UTR	-0,303	1,03E-07	3,94E-06
cg11722595	6	116766585		IGR	-0,24	1,03E-07	3,94E-06
cg20311002	1	244763541	C1orf101	Body	-0,271	1,03E-07	3,95E-06
cg03116755	3	15598133		IGR	-0,274	1,03E-07	3,95E-06
cg26975460	20	24761330		IGR	-0,228	1,03E-07	3,95E-06
cg22069272	13	67205408	PCDH9	Body	-0,23	1,04E-07	3,96E-06
cg03738384	16	84628969	COTL1	Body	-0,209	1,04E-07	3,96E-06
cg19000815	15	60802542	RORA-AS1	Body	0,335	1,04E-07	3,97E-06
cg06754309	10	3977396		IGR	-0,209	1,04E-07	3,97E-06
cg09777386	19	9648349	ZNF426	5'UTR	0,217	1,04E-07	3,97E-06
cg10281604	22	40732359		IGR	-0,307	1,04E-07	3,97E-06
cg10037005	19	49838478	CD37	TSS200	-0,235	1,04E-07	3,97E-06
cg25350511	2	175766041	CHN1	Body	-0,215	1,04E-07	3,97E-06
cg04515533	1	231295786		IGR	-0,212	1,04E-07	3,97E-06
cg19441317	3	123512314	MYLK	Body	-0,252	1,04E-07	3,98E-06
cg11752130	11	46321127	CREB3L1	Body	-0,203	1,04E-07	3,98E-06

cg14208041	15	40080369		IGR	-0,233	1,04E-07	3,98E-06
cg22823725	2	192900649	TMEFF2	Body	-0,214	1,04E-07	3,99E-06
cg00889260	8	25867125	EBF2	Body	0,217	1,05E-07	3,99E-06
cg21769514	8	2743697		IGR	0,309	1,05E-07	3,99E-06
cg22566500	11	15640579		IGR	-0,222	1,05E-07	3,99E-06
cg27638284	20	39127000		IGR	-0,31	1,05E-07	3,99E-06
cg13826452	6	26758395		IGR	0,213	1,05E-07	3,99E-06
cg12512875	9	71784067	TJP2	5'UTR	-0,34	1,05E-07	3,99E-06
cg16768840	18	67625835	CD226	TSS1500	-0,238	1,05E-07	3,99E-06
cg22514722	3	127473755	MGLL	Body	-0,303	1,05E-07	4,00E-06
cg07616471	3	46413711	CCR5	5'UTR	-0,203	1,05E-07	4,00E-06
cg14484251	2	70122167	SNRNP27	Body	0,259	1,05E-07	4,00E-06
cg07192409	10	99173040		IGR	-0,256	1,05E-07	4,01E-06
cg12655260	5	142562569	ARHGAP26	Body	-0,241	1,05E-07	4,01E-06
cg20169450	12	108728433	CMKLR1	5'UTR	-0,22	1,06E-07	4,01E-06
cg00353243	3	47652338	SMARCC1	Body	0,201	1,06E-07	4,02E-06
cg27545630	16	2013058	SNORA64	Body	-0,299	1,06E-07	4,02E-06
cg06784563	18	77284509	NFATC1	Body	-0,297	1,06E-07	4,02E-06
cg21681030	2	46777652	RHOQ	Body	0,219	1,06E-07	4,02E-06
cg08752726	21	36254259	RUNX1	Body	-0,312	1,06E-07	4,03E-06
cg19528789	1	12232415	TNFRSF1B	Body	-0,208	1,06E-07	4,03E-06
cg20542798	4	170930060	MFAP3L	5'UTR	-0,22	1,06E-07	4,03E-06
cg19269033	5	131419179		IGR	-0,229	1,06E-07	4,03E-06
cg25614758	22	28110926		IGR	-0,2	1,06E-07	4,03E-06
cg11733245	10	6104312	IL2RA	1stExon	-0,286	1,06E-07	4,04E-06
cg11001890	5	133184202		IGR	-0,266	1,07E-07	4,04E-06
cg08058560	1	117544206	CD101	TSS200	-0,312	1,07E-07	4,05E-06
cg07537705	2	121082687		IGR	-0,217	1,07E-07	4,05E-06
cg18183774	15	75200015	C15orf17	TSS1500	0,228	1,07E-07	4,05E-06
cg00717988	11	125105030	PKNOX2	ExonBnd	-0,207	1,07E-07	4,05E-06
cg00602743	13	114890696	RASA3	Body	-0,218	1,07E-07	4,05E-06
cg02551980	5	112628419	MCC	Body	0,33	1,07E-07	4,05E-06
cg21327055	2	203182277		IGR	0,264	1,07E-07	4,06E-06
cg24708182	3	133709280	SLCO2A1	Body	-0,207	1,07E-07	4,06E-06
cg08177070	15	96935641		IGR	-0,209	1,07E-07	4,06E-06
cg13145193	7	105926817	NAMPT	TSS1500	-0,233	1,07E-07	4,06E-06
cg06754513	7	90899699		IGR	0,209	1,07E-07	4,06E-06
cg07536737	2	98630091		IGR	-0,346	1,07E-07	4,07E-06
cg13204320	7	137194074	DGKI	Body	-0,203	1,07E-07	4,07E-06
cg03960066	1	150255503	C1orf51	1stExon	0,21	1,07E-07	4,07E-06
cg10808934	6	109059268		IGR	-0,236	1,07E-07	4,07E-06
cg20677901	1	3568210	TP73	TSS1500	0,232	1,07E-07	4,07E-06
cg21026639	12	54656343	CBX5	5'UTR	0,2	1,07E-07	4,07E-06
cg25468450	16	69167903	CHTF8	TSS1500	-0,21	1,07E-07	4,07E-06
cg23389172	15	56760907		IGR	-0,315	1,07E-07	4,07E-06
cg27092594	2	227517346		IGR	-0,2	1,08E-07	4,07E-06
cg27158659	2	46077243	PRKCE	Body	-0,274	1,08E-07	4,07E-06
cg15646529	3	15367892	SH3BP5	Body	0,205	1,08E-07	4,07E-06
cg17198132	6	152489472	MIR3163	Body	0,209	1,08E-07	4,08E-06
cg27475774	5	131879688	IL5	TSS1500	-0,248	1,08E-07	4,08E-06

cg17477578	8	129552914		IGR	-0,251	1,08E-07	4,08E-06
cg09355011	3	45926450	LZTFL1	5'UTR	-0,258	1,08E-07	4,08E-06
cg16204830	16	66223850		IGR	-0,317	1,08E-07	4,08E-06
cg27453761	16	66624346		IGR	-0,347	1,08E-07	4,08E-06
cg24996659	14	23024312		IGR	-0,269	1,08E-07	4,08E-06
cg16014076	2	225811640	DOCK10	1stExon	-0,256	1,08E-07	4,09E-06
cg03930313	17	36621405	ARHGAP23	Body	0,235	1,08E-07	4,09E-06
cg15981982	2	8685837		IGR	-0,214	1,08E-07	4,09E-06
cg17572948	6	4408181		IGR	-0,311	1,08E-07	4,09E-06
cg17967260	6	166242715		IGR	-0,216	1,09E-07	4,09E-06
cg15312943	11	17843855	SERGEF	Body	0,237	1,09E-07	4,09E-06
cg21885872	2	205099023		IGR	-0,231	1,09E-07	4,10E-06
cg21036280	19	35089191		IGR	-0,201	1,09E-07	4,10E-06
cg03477042	5	126424350		IGR	-0,233	1,09E-07	4,10E-06
cg27310092	1	151945663		IGR	-0,276	1,09E-07	4,10E-06
cg24718015	17	40489721	STAT3	Body	-0,274	1,09E-07	4,11E-06
cg15922680	1	167325517	POU2F1	Body	0,238	1,09E-07	4,11E-06
cg15978357	4	40201884	RHOH	5'UTR	-0,22	1,09E-07	4,11E-06
cg14419424	10	65388604		IGR	0,23	1,09E-07	4,11E-06
cg04687131	20	50236499	ATP9A	Body	-0,247	1,09E-07	4,11E-06
cg17280346	3	147126703	ZIC1	TSS1500	0,216	1,09E-07	4,11E-06
cg15723468	1	230387268	GALNT2	Body	-0,226	1,09E-07	4,11E-06
cg14983463	3	177545386	KCCAT211	Body	-0,217	1,09E-07	4,12E-06
cg14664530	1	16076951		IGR	-0,203	1,10E-07	4,12E-06
cg20953324	13	34021438	STARD13	Body	-0,229	1,10E-07	4,12E-06
cg10753610	17	45335202	ITGB3	Body	-0,244	1,10E-07	4,12E-06
cg00565242	3	167582594		IGR	0,383	1,10E-07	4,13E-06
cg08573566	6	159727573		IGR	-0,204	1,10E-07	4,13E-06
cg15179400	1	15670452	FHAD1	Body	0,284	1,10E-07	4,13E-06
cg27025738	14	72006683	SIPA1L1	5'UTR	0,201	1,10E-07	4,13E-06
cg24524702	15	100109440	MEF2A	5'UTR	0,251	1,10E-07	4,13E-06
cg26628907	17	38195238	MED24	Body	0,245	1,10E-07	4,14E-06
cg03066525	7	138199097	TRIM24	Body	0,215	1,10E-07	4,14E-06
cg03845654	2	153492884	FMNL2	Body	-0,212	1,10E-07	4,14E-06
cg01954305	8	135817117		IGR	0,304	1,10E-07	4,14E-06
cg10620151	3	12512896		IGR	-0,224	1,10E-07	4,14E-06
cg26679261	5	94891733	TTC37	TSS1500	0,201	1,10E-07	4,14E-06
cg05382301	16	22046638	C16orf52	Body	-0,249	1,10E-07	4,14E-06
cg25321854	18	6916835		IGR	-0,203	1,10E-07	4,14E-06
cg02139965	5	169739749		IGR	-0,23	1,11E-07	4,14E-06
cg15145453	4	99120553		IGR	0,23	1,11E-07	4,14E-06
cg02652185	2	99387621	LOC10192707	Body	-0,238	1,11E-07	4,15E-06
cg10449711	16	65668310		IGR	-0,233	1,11E-07	4,15E-06
cg20728901	18	67553469	CD226	Body	-0,244	1,11E-07	4,15E-06
cg14979828	8	75912393	CRISPLD1	1stExon	-0,232	1,11E-07	4,15E-06
cg12337011	2	28002424	MRPL33	3'UTR	0,219	1,11E-07	4,15E-06
cg15175457	20	50097296	NFATC2	Body	-0,332	1,11E-07	4,15E-06
cg00293660	6	55103999	HCRTR2	Body	0,281	1,11E-07	4,15E-06
cg20354777	3	140814133	SPSB4	Body	0,22	1,11E-07	4,16E-06
cg16213139	5	38609835	LIFR-AS1	Body	-0,207	1,11E-07	4,16E-06

cg11970797	13	20994066	CRYL1	Body	-0,326	1,11E-07	4,16E-06
cg06092869	1	225642746		IGR	-0,206	1,11E-07	4,16E-06
cg02488934	1	156422580		IGR	-0,228	1,11E-07	4,17E-06
cg16389078	22	17569018	IL17RA	Body	-0,3	1,11E-07	4,17E-06
cg17067696	6	17930180	KIF13A	Body	0,231	1,11E-07	4,17E-06
cg00360175	7	156405820		IGR	-0,281	1,12E-07	4,17E-06
cg10501349	14	75961925		IGR	-0,216	1,12E-07	4,17E-06
cg10142997	13	67240245	PCDH9	Body	-0,261	1,12E-07	4,18E-06
cg05564251	2	231090640	SP140	Body	-0,322	1,12E-07	4,18E-06
cg00443121	8	21770233	DOK2	Body	-0,233	1,12E-07	4,18E-06
cg12995384	6	151706546	ZBTB2	5'UTR	-0,243	1,12E-07	4,18E-06
cg08226276	5	73918270		IGR	-0,264	1,12E-07	4,18E-06
cg12250498	5	156616356	ITK	Body	-0,255	1,12E-07	4,18E-06
cg09251291	15	63483946	RAB8B	Body	-0,229	1,12E-07	4,18E-06
cg10683392	3	154901913		IGR	-0,242	1,12E-07	4,18E-06
cg03514139	7	50732341	GRB10	Body	-0,28	1,12E-07	4,18E-06
cg13521018	10	134429846	INPP5A	Body	-0,245	1,12E-07	4,18E-06
cg22046455	10	99052786	ARHGAP19	TSS1500	-0,23	1,12E-07	4,19E-06
cg04752731	17	16891388		IGR	-0,261	1,12E-07	4,19E-06
cg10929246	3	50612972	HEMK1	Body	-0,22	1,13E-07	4,20E-06
cg00484421	7	8166010	ICA1	Body	-0,227	1,13E-07	4,20E-06
cg02867196	18	44051322		IGR	-0,209	1,13E-07	4,20E-06
cg11368679	4	20194824		IGR	-0,23	1,13E-07	4,20E-06
cg16095032	14	103693100		IGR	-0,202	1,13E-07	4,21E-06
cg11141696	1	161700167		IGR	-0,28	1,13E-07	4,21E-06
cg03004286	7	68014		IGR	-0,247	1,13E-07	4,21E-06
cg18397314	14	100047630	CCDC85C	Body	-0,225	1,13E-07	4,21E-06
cg17279138	21	43657930	ABCG1	Body	-0,212	1,13E-07	4,21E-06
cg18580274	6	30070074		IGR	0,201	1,13E-07	4,22E-06
cg25636481	6	33241410	RPS18	Body	-0,238	1,13E-07	4,22E-06
cg10754659	7	38110151		IGR	0,211	1,13E-07	4,22E-06
cg04168676	8	69902612	LINC01592	Body	-0,223	1,13E-07	4,22E-06
cg10866548	1	41511511	SCMH1	Body	0,234	1,13E-07	4,22E-06
cg22483213	15	95019591	MCTP2	Body	0,228	1,13E-07	4,22E-06
cg08711521	15	70861740		IGR	0,21	1,13E-07	4,22E-06
cg12899747	3	25391527		IGR	-0,288	1,14E-07	4,22E-06
cg12514207	7	73384346		IGR	-0,202	1,14E-07	4,23E-06
cg20275841	13	94392671	GPC6	Body	0,266	1,14E-07	4,24E-06
cg04300151	10	65226360	JMJD1C	TSS1500	0,232	1,14E-07	4,24E-06
cg20156237	8	144543750	ZC3H3	Body	-0,202	1,14E-07	4,24E-06
cg12847371	19	13056058	RAD23A	TSS1500	-0,205	1,14E-07	4,24E-06
cg19455189	2	231742734	ITM2C	Body	-0,232	1,14E-07	4,24E-06
cg10766876	2	98897033	VWA3B	Body	0,275	1,14E-07	4,24E-06
cg08082321	8	130587834	CCDC26	TSS1500	-0,21	1,15E-07	4,25E-06
cg03900565	1	203031815	PPFIA4	Body	-0,204	1,15E-07	4,25E-06
cg18132479	20	48869583		IGR	-0,201	1,15E-07	4,26E-06
cg07405796	6	30104552	TRIM40	1stExon	-0,231	1,15E-07	4,26E-06
cg05239225	15	86022419	AKAP13	5'UTR	0,225	1,15E-07	4,26E-06
cg13175329	6	25035073		IGR	-0,237	1,15E-07	4,26E-06
cg02288610	8	125898153		IGR	-0,205	1,15E-07	4,26E-06

cg00687117	15	76129654		IGR	-0,278	1,15E-07	4,26E-06
cg05313151	11	35104878		IGR	-0,334	1,15E-07	4,26E-06
cg18733230	7	4763182	FOXK1	Body	0,299	1,15E-07	4,27E-06
cg07975212	1	26057394	MAN1C1	Body	-0,308	1,15E-07	4,27E-06
cg15989810	12	131617481	GPR133	Body	-0,216	1,15E-07	4,27E-06
cg17356268	8	105360676	DCSTAMP	5'UTR	-0,231	1,16E-07	4,28E-06
cg02020801	9	93893852		IGR	-0,204	1,16E-07	4,28E-06
cg20509903	15	69609912	PAQR5	5'UTR	-0,232	1,16E-07	4,28E-06
cg21890646	14	91816120	CCDC88C	Body	-0,28	1,16E-07	4,28E-06
cg00274965	21	34405681		IGR	0,228	1,16E-07	4,28E-06
cg07346751	2	174223564	CDCA7	ExonBnd	-0,212	1,16E-07	4,29E-06
cg08290207	22	22117567	MAPK1	3'UTR	-0,237	1,16E-07	4,29E-06
cg23701226	3	122516277	DIRC2	Body	-0,295	1,16E-07	4,29E-06
cg14064762	9	123688745	TRAF1	5'UTR	-0,271	1,16E-07	4,30E-06
cg01673485	6	42391208	TRERF1	5'UTR	-0,234	1,17E-07	4,31E-06
cg08538037	13	41015415	LINC00598	Body	0,27	1,17E-07	4,31E-06
cg06599814	9	130452752	STXBP1	3'UTR	-0,208	1,17E-07	4,31E-06
cg00149121	12	70388822		IGR	0,242	1,17E-07	4,32E-06
cg07762764	3	24565992		IGR	-0,203	1,17E-07	4,32E-06
cg02217975	7	92325242	CDK6	Body	0,218	1,17E-07	4,32E-06
cg03202009	14	60031299	CCDC175	Body	0,288	1,18E-07	4,33E-06
cg19450830	13	40795891	LINC00548	TSS1500	-0,205	1,18E-07	4,33E-06
cg17833958	5	118689317	TNFAIP8	TSS1500	-0,204	1,18E-07	4,33E-06
cg01026233	2	206713941		IGR	-0,212	1,18E-07	4,33E-06
cg13703804	11	35187061	CD44	Body	-0,214	1,18E-07	4,33E-06
cg06249604	6	30039206	RNF39	Body	0,478	1,18E-07	4,33E-06
cg24330456	6	30038955	RNF39	Body	0,298	1,18E-07	4,34E-06
cg24652786	1	160615240	SLAMF1	Body	-0,239	1,18E-07	4,34E-06
cg08946205	18	10169877		IGR	-0,201	1,18E-07	4,34E-06
cg17965690	19	10736049	SLC44A2	TSS200	0,212	1,18E-07	4,34E-06
cg19061068	12	43703894		IGR	-0,206	1,18E-07	4,34E-06
cg12526997	2	242974096		IGR	-0,235	1,18E-07	4,34E-06
cg15032909	5	170726353	RANBP17	3'UTR	-0,239	1,18E-07	4,34E-06
cg10276968	15	60547697		IGR	-0,238	1,18E-07	4,34E-06
cg02234529	8	134783646		IGR	-0,209	1,18E-07	4,34E-06
cg10547050	17	27253274	PHF12	Body	-0,233	1,18E-07	4,34E-06
cg01219426	11	95856658	MAML2	Body	-0,228	1,18E-07	4,35E-06
cg14947993	6	106562851		IGR	-0,306	1,18E-07	4,35E-06
cg10077978	11	35062246		IGR	-0,259	1,19E-07	4,36E-06
cg09938784	9	35689902	TPM2	1stExon	0,272	1,19E-07	4,36E-06
cg04462329	14	91697876		IGR	-0,201	1,19E-07	4,36E-06
cg15868622	6	138149370	LOC10013047	Body	-0,235	1,19E-07	4,37E-06
cg14390141	1	109734442	KIAA1324	Body	-0,235	1,19E-07	4,37E-06
cg08563032	14	30973098		IGR	0,303	1,19E-07	4,37E-06
cg12793826	8	141538019		IGR	0,312	1,19E-07	4,37E-06
cg20250397	14	92807379	SLC24A4	Body	-0,234	1,19E-07	4,37E-06
cg21448937	5	122885347	CSNK1G3	5'UTR	-0,215	1,19E-07	4,37E-06
cg15593298	3	142681996	PAQR9	1stExon	0,203	1,19E-07	4,37E-06
cg00886225	9	87374383	NTRK2	Body	-0,231	1,19E-07	4,37E-06
cg18989133	11	17518482	USH1C	Body	-0,208	1,19E-07	4,37E-06

cg20230870	2	121469286		IGR	-0,238	1,20E-07	4,38E-06
cg06562283	11	78781557	ODZ4	5'UTR	-0,222	1,20E-07	4,38E-06
cg19510565	1	31217240	LAPTM5	Body	-0,212	1,20E-07	4,38E-06
cg04880086	22	33019617	SYN3	Body	-0,219	1,20E-07	4,38E-06
cg05249074	17	48648690	CACNA1G	Body	-0,233	1,20E-07	4,39E-06
cg06023838	10	71273679		IGR	-0,236	1,20E-07	4,39E-06
cg19288944	16	1926486		IGR	-0,242	1,20E-07	4,39E-06
cg04152643	4	17494161	QDPR	Body	0,224	1,20E-07	4,39E-06
cg03038146	6	24936374	FAM65B	TSS200	-0,297	1,20E-07	4,39E-06
cg12691330	6	159070656	SYTL3	TSS1500	-0,235	1,20E-07	4,39E-06
cg00284386	8	1786207	ARHGEF10	5'UTR	-0,213	1,20E-07	4,39E-06
cg18914226	9	129744805	RALGPS1	Body	-0,244	1,20E-07	4,39E-06
cg01751584	9	100159780	LOC286359	TSS1500	-0,286	1,20E-07	4,40E-06
cg04589151	6	148130124		IGR	-0,208	1,21E-07	4,40E-06
cg06777568	7	135528072		IGR	-0,336	1,21E-07	4,40E-06
cg25634041	3	185348303	SENP2	3'UTR	0,254	1,21E-07	4,40E-06
cg20536289	4	148499207		IGR	-0,212	1,21E-07	4,41E-06
cg15602215	8	102772842	NCALD	5'UTR	0,208	1,21E-07	4,41E-06
cg26829395	15	40217855		IGR	-0,243	1,21E-07	4,41E-06
cg04999352	11	63304614	RARRES3	Body	-0,239	1,21E-07	4,41E-06
cg16379283	14	75801023		IGR	-0,216	1,21E-07	4,41E-06
cg15563070	3	193450115		IGR	-0,223	1,21E-07	4,42E-06
cg00887881	2	242067251	PASK	Body	-0,23	1,21E-07	4,42E-06
cg08828552	1	201231001		IGR	-0,217	1,21E-07	4,42E-06
cg12595444	20	47356166	PREX1	Body	-0,242	1,21E-07	4,42E-06
cg26191960	9	87359419	NTRK2	Body	-0,24	1,21E-07	4,42E-06
cg23173894	11	9200508	DENND5A	Body	0,269	1,21E-07	4,42E-06
cg15057323	1	245496060	KIF26B	Body	-0,206	1,22E-07	4,42E-06
cg07931189	2	53463158		IGR	-0,297	1,22E-07	4,42E-06
cg09427944	16	30579519		IGR	-0,226	1,22E-07	4,43E-06
cg03368318	4	86744925	ARHGAP24	Body	0,233	1,22E-07	4,43E-06
cg11414693	5	158547920		IGR	-0,229	1,22E-07	4,43E-06
cg03620939	10	2653325		IGR	0,255	1,22E-07	4,43E-06
cg20063106	14	23938147	NGDN	TSS1500	0,205	1,22E-07	4,43E-06
cg07106169	2	95740362		IGR	-0,223	1,22E-07	4,43E-06
cg21261924	12	124680327	ZNF664-FAM:	5'UTR	-0,231	1,22E-07	4,43E-06
cg24039541	13	97943199	MBNL2	Body	-0,246	1,22E-07	4,43E-06
cg01824618	16	85315212		IGR	-0,232	1,22E-07	4,43E-06
cg17913946	12	53443877	TNS2	5'UTR	0,217	1,22E-07	4,43E-06
cg13088089	20	34079093	CEP250	Body	-0,254	1,22E-07	4,43E-06
cg21060317	5	74241536		IGR	-0,326	1,22E-07	4,43E-06
cg04059696	2	27211953	MAPRE3	5'UTR	-0,211	1,22E-07	4,43E-06
cg12419482	11	72933282	P2RY2	5'UTR	-0,214	1,22E-07	4,43E-06
cg19253379	1	56206919		IGR	-0,214	1,22E-07	4,44E-06
cg13209475	1	234979009		IGR	-0,277	1,22E-07	4,44E-06
cg07375256	7	99222196	ZNF498	Body	-0,278	1,22E-07	4,44E-06
cg03485608	2	139535785	NXPH2	Body	0,255	1,22E-07	4,44E-06
cg06962639	18	61939606	LOC284294	Body	-0,215	1,23E-07	4,45E-06
cg01480218	8	23341715		IGR	-0,217	1,23E-07	4,45E-06
cg02423856	10	33795998		IGR	-0,283	1,23E-07	4,45E-06

cg01712647	2	159746267		IGR	-0,227	1,23E-07	4,46E-06
cg00209786	20	56752779		IGR	-0,2	1,23E-07	4,46E-06
cg14913647	1	183760973	RGL1	Body	-0,239	1,23E-07	4,46E-06
cg02019495	20	43599168	STK4	Body	-0,257	1,23E-07	4,46E-06
cg10340048	8	74258684		IGR	-0,226	1,23E-07	4,46E-06
cg20299572	11	1750763	HCCA2	Body	-0,227	1,23E-07	4,46E-06
cg25337674	21	37852979	CLDN14	TSS1500	-0,216	1,23E-07	4,46E-06
cg00753748	3	134053141		IGR	-0,202	1,23E-07	4,46E-06
cg09972723	1	66733475	PDE4B	Body	0,222	1,23E-07	4,46E-06
cg12927730	2	97260774	KIAA1310	3'UTR	-0,284	1,23E-07	4,46E-06
cg20861261	2	18944917		IGR	-0,22	1,23E-07	4,47E-06
cg03302578	21	38598289	DSCR3	Body	-0,241	1,24E-07	4,47E-06
cg22856976	1	181047581		IGR	-0,251	1,24E-07	4,48E-06
cg21057107	19	10908762	DNM2	Body	-0,202	1,24E-07	4,48E-06
cg16072296	22	32042760		IGR	-0,322	1,24E-07	4,48E-06
cg12004641	2	218750749	TNS1	Body	-0,206	1,24E-07	4,49E-06
cg06680852	10	22722951		IGR	-0,229	1,24E-07	4,49E-06
cg09151882	19	7459355	ARHGEF18	TSS1500	0,215	1,24E-07	4,49E-06
cg22484898	9	132772665	FNBP1	Body	-0,218	1,24E-07	4,49E-06
cg26567697	16	28567218	CCDC101	5'UTR	-0,24	1,24E-07	4,49E-06
cg26052635	2	29339307	CLIP4	5'UTR	-0,28	1,24E-07	4,50E-06
cg23229770	2	129491004		IGR	-0,21	1,25E-07	4,50E-06
cg13549174	9	133910264	LAMC3	Body	-0,256	1,25E-07	4,50E-06
cg01627770	22	40781416	SGSM3	5'UTR	-0,202	1,25E-07	4,50E-06
cg25196374	2	152231104	LOC10192931	Body	-0,224	1,25E-07	4,50E-06
cg00789792	2	179245704	MIR548N	TSS1500	-0,214	1,25E-07	4,50E-06
cg25372085	10	80528720		IGR	-0,227	1,25E-07	4,50E-06
cg11983398	4	153954958		IGR	-0,202	1,25E-07	4,50E-06
cg09901765	3	154897269	MME	Body	-0,224	1,25E-07	4,51E-06
cg16916782	13	113261142		IGR	0,243	1,25E-07	4,51E-06
cg08092463	5	176526586		IGR	-0,226	1,25E-07	4,51E-06
cg00326690	19	34283376		IGR	-0,221	1,25E-07	4,51E-06
cg22495968	2	5622155		IGR	-0,201	1,25E-07	4,51E-06
cg24908617	2	48755777	STON1-GTF2/	TSS1500	0,264	1,25E-07	4,51E-06
cg24426255	12	94703450	CEP83	Body	-0,275	1,25E-07	4,52E-06
cg24723883	19	2608495	GNG7	5'UTR	-0,207	1,25E-07	4,52E-06
cg06144762	3	4560682	ITPR1	Body	-0,313	1,25E-07	4,52E-06
cg13531667	5	112825132	MCC	TSS1500	-0,25	1,26E-07	4,52E-06
cg14648920	4	169770406	PALLD	5'UTR	0,209	1,26E-07	4,53E-06
cg24924050	8	13123122	DLC1	Body	-0,208	1,26E-07	4,53E-06
cg11476571	18	60880519	BCL2	Body	-0,268	1,26E-07	4,53E-06
cg20066118	2	3751440	DCDC2C	1stExon	0,218	1,26E-07	4,53E-06
cg18328154	18	44479410	PIAS2	Body	-0,29	1,26E-07	4,53E-06
cg23739048	1	165393661	RXRG	5'UTR	-0,248	1,26E-07	4,54E-06
cg04442723	10	29506476		IGR	0,238	1,26E-07	4,54E-06
cg04217016	1	201305661		IGR	-0,22	1,26E-07	4,54E-06
cg22232707	10	10274047		IGR	-0,222	1,26E-07	4,54E-06
cg21424270	3	33700962	CLASP2	TSS200	0,23	1,26E-07	4,54E-06
cg05168410	4	75261557		IGR	0,248	1,26E-07	4,54E-06
cg27571329	1	59859555	FGGY	Body	0,252	1,27E-07	4,55E-06

cg12963323	21	46361528	FAM207A	Body	0,237	1,27E-07	4,55E-06
cg24097496	1	26615344	UBXN11	Body	-0,314	1,27E-07	4,55E-06
cg15406709	11	847042	TSPAN4	5'UTR	-0,217	1,27E-07	4,55E-06
cg03731202	1	36574795		IGR	-0,211	1,27E-07	4,55E-06
cg12081304	2	38346853		IGR	-0,251	1,27E-07	4,55E-06
cg10131808	8	82824929		IGR	0,239	1,27E-07	4,55E-06
cg02500392	6	138539372	PBOV1	1stExon	-0,246	1,27E-07	4,56E-06
cg13093012	7	32246897	PDE1C	Body	0,325	1,27E-07	4,56E-06
cg08818984	5	142814827	NR3C1	1stExon	-0,297	1,27E-07	4,56E-06
cg03174319	2	28582155		IGR	-0,213	1,28E-07	4,57E-06
cg09283991	17	72726802	RAB37	Body	-0,218	1,28E-07	4,57E-06
cg16139075	2	121679057	GLI2	Body	-0,203	1,28E-07	4,57E-06
cg00338113	14	100208367		IGR	-0,243	1,28E-07	4,57E-06
cg12818557	8	89340139	MMP16	TSS1500	0,23	1,28E-07	4,58E-06
cg21011434	8	39971253		IGR	-0,234	1,28E-07	4,58E-06
cg06919472	2	174104433	ZAK	Body	-0,261	1,28E-07	4,58E-06
cg15511785	10	126340361	FAM53B	Body	-0,219	1,28E-07	4,58E-06
cg01898254	5	81048083	SSBP2	TSS1500	0,226	1,28E-07	4,58E-06
cg25721832	3	81913557		IGR	0,206	1,28E-07	4,58E-06
cg22358291	4	10101553	WDR1	Body	-0,241	1,28E-07	4,58E-06
cg16370620	20	1338659	FKBP1A-SDCE	Body	-0,207	1,28E-07	4,58E-06
cg21273878	7	116594917	ST7-OT4	Body	0,247	1,28E-07	4,58E-06
cg15661950	15	82172767		IGR	-0,219	1,28E-07	4,59E-06
cg11309785	2	72922245	EXOC6B	Body	0,31	1,28E-07	4,59E-06
cg09471471	12	56323020	WIBG	TSS1500	-0,264	1,28E-07	4,59E-06
cg22693272	22	45724948	FAM118A	Body	0,212	1,28E-07	4,59E-06
cg00059459	1	8343575		IGR	-0,21	1,28E-07	4,59E-06
cg24177868	11	118510867	PHLDB1	Body	0,208	1,29E-07	4,59E-06
cg14236062	18	60119693		IGR	-0,343	1,29E-07	4,59E-06
cg13772414	2	222383060	EPHA4	Body	-0,275	1,29E-07	4,59E-06
cg22563732	5	172331733	ERGIC1	Body	0,229	1,29E-07	4,59E-06
cg17673013	2	105688706	MRPS9	Body	-0,271	1,29E-07	4,59E-06
cg19546693	20	35397574	DSN1	Body	0,249	1,29E-07	4,60E-06
cg12345785	4	71587440	RUFY3	TSS200	0,224	1,29E-07	4,60E-06
cg03508824	6	105931438		IGR	-0,223	1,29E-07	4,60E-06
cg02690881	12	68767945		IGR	-0,255	1,29E-07	4,61E-06
cg23583448	18	56648561	ZNF532	Body	-0,206	1,29E-07	4,61E-06
cg20874718	15	29862801	FAM189A1	1stExon	0,277	1,29E-07	4,61E-06
cg06648791	12	69511510		IGR	-0,202	1,30E-07	4,62E-06
cg12177513	6	111360314		IGR	-0,261	1,30E-07	4,62E-06
cg14731400	2	42300755		IGR	-0,224	1,30E-07	4,62E-06
cg01679017	20	51825417	TSHZ2	Body	-0,244	1,30E-07	4,62E-06
cg15762106	1	15558548		IGR	-0,251	1,30E-07	4,62E-06
cg09273059	10	71077221	HK1	Body	-0,204	1,30E-07	4,62E-06
cg08750473	19	45301587	CBLC	Body	-0,209	1,30E-07	4,62E-06
cg02099010	8	99466796		IGR	0,244	1,30E-07	4,62E-06
cg00490406	1	159046773	AIM2	TSS200	-0,341	1,30E-07	4,63E-06
cg06729532	19	14376770		IGR	0,215	1,30E-07	4,63E-06
cg05920383	11	43291188		IGR	0,24	1,30E-07	4,64E-06
cg17442683	1	8664311	RERE	Body	-0,229	1,31E-07	4,64E-06

cg01178899	3	45985168	CXCR6	5'UTR	-0,252	1,31E-07	4,64E-06
cg27321175	10	15689009	ITGA8	Body	-0,23	1,31E-07	4,64E-06
cg09661228	1	161932212	ATF6	3'UTR	-0,28	1,31E-07	4,64E-06
cg19954877	8	82275341		IGR	0,249	1,31E-07	4,64E-06
cg09146350	11	8795619	LOC1027247ٰ8	Body	-0,207	1,31E-07	4,65E-06
cg25426203	10	88600793	BMPR1A	5'UTR	0,296	1,31E-07	4,65E-06
cg02209936	9	131983681		IGR	-0,288	1,31E-07	4,65E-06
cg19423402	3	138763993	PRR23C	TSS1500	0,342	1,31E-07	4,65E-06
cg02802073	19	13243586	NACC1	5'UTR	-0,26	1,31E-07	4,66E-06
cg12084965	7	107342265	SLC26A4	ExonBnd	-0,205	1,31E-07	4,66E-06
cg00146027	10	33299147		IGR	-0,254	1,31E-07	4,66E-06
cg16363413	15	68311899		IGR	-0,203	1,31E-07	4,66E-06
cg23325384	2	223724754	ACSL3	TSS1500	0,288	1,32E-07	4,66E-06
cg19621176	18	43735827		IGR	-0,237	1,32E-07	4,66E-06
cg16804814	1	23771037	ASAP3	Body	-0,212	1,32E-07	4,67E-06
cg14446709	11	72315744	PDE2A	Body	-0,23	1,32E-07	4,67E-06
cg03165356	2	216882347		IGR	-0,271	1,32E-07	4,67E-06
cg08715935	8	26429697	DPYSL2	Body	-0,241	1,32E-07	4,67E-06
cg18855351	13	30920638	LINC00426	Body	-0,256	1,32E-07	4,67E-06
cg00470636	8	26440349	DPYSL2	Body	0,279	1,32E-07	4,67E-06
cg16547038	8	135505342	ZFAT	Body	-0,209	1,32E-07	4,68E-06
cg03615933	5	142586466	ARHGAP26	Body	-0,208	1,32E-07	4,68E-06
cg18729664	6	76203675	FILIP1	TSS200	0,276	1,32E-07	4,68E-06
cg09974897	2	232261864	B3GNT7	Body	-0,217	1,32E-07	4,68E-06
cg13091627	1	153518476	S100A4	TSS200	-0,239	1,32E-07	4,68E-06
cg12650265	12	77766400		IGR	-0,21	1,32E-07	4,68E-06
cg13038178	16	56233377	GNAO1	Body	-0,269	1,32E-07	4,68E-06
cg23302570	2	139538601	NXPH2	TSS1500	0,32	1,32E-07	4,68E-06
cg03037039	4	16787920	LDB2	Body	0,232	1,32E-07	4,68E-06
cg04293724	3	150478499	SIAH2	Body	-0,229	1,33E-07	4,69E-06
cg09157586	18	56486573		IGR	-0,227	1,33E-07	4,69E-06
cg00824087	17	48058055		IGR	-0,229	1,33E-07	4,69E-06
cg02088086	20	50226012	ATP9A	Body	-0,263	1,33E-07	4,70E-06
cg19313408	21	46827585	COL18A1	Body	-0,23	1,33E-07	4,70E-06
cg12001491	1	26645487	CD52	Body	-0,304	1,33E-07	4,71E-06
cg05803533	12	52966447	KRT74	Body	-0,206	1,34E-07	4,71E-06
cg07768107	4	40201943	RHOH	5'UTR	-0,259	1,34E-07	4,72E-06
cg24800754	19	6234327	MLLT1	Body	0,202	1,34E-07	4,72E-06
cg16253681	17	38696964		IGR	-0,216	1,34E-07	4,72E-06
cg21765597	21	29660975		IGR	-0,207	1,34E-07	4,72E-06
cg10001987	17	7381769	ZBTB4	5'UTR	0,243	1,34E-07	4,72E-06
cg12688234	5	169407439	FAM196B	5'UTR	0,279	1,34E-07	4,72E-06
cg12428546	9	117281041		IGR	-0,263	1,34E-07	4,72E-06
cg13235059	3	149192304	TM4SF4	TSS200	-0,219	1,34E-07	4,73E-06
cg22243685	17	41052543	G6PC	TSS1500	-0,214	1,34E-07	4,73E-06
cg01937806	7	68737874		IGR	-0,22	1,34E-07	4,73E-06
cg02328601	11	129908164		IGR	-0,242	1,34E-07	4,73E-06
cg19048031	11	68976362		IGR	-0,217	1,34E-07	4,73E-06
cg26537335	12	105136339	CHST11	Body	0,201	1,34E-07	4,73E-06
cg25020550	8	134028958	TG	Body	-0,264	1,35E-07	4,74E-06

cg02840645	1	118221263		IGR	0,205	1,35E-07	4,74E-06
cg25691038	7	104597785		IGR	-0,245	1,35E-07	4,74E-06
cg05267394	20	32856898	ASIP	Body	0,204	1,35E-07	4,74E-06
cg07459714	6	116786636		IGR	-0,278	1,35E-07	4,74E-06
cg06705767	3	25483002	RARB	5'UTR	-0,214	1,35E-07	4,74E-06
cg02864956	10	22935362	PIP4K2A	Body	-0,251	1,35E-07	4,75E-06
cg06748006	5	175969350	CDHR2	TSS200	-0,311	1,35E-07	4,75E-06
cg20481604	7	93807606		IGR	-0,226	1,35E-07	4,75E-06
cg09326780	3	192561283	MB21D2	Body	-0,219	1,35E-07	4,75E-06
cg04570316	6	24776902	GMNN	5'UTR	-0,246	1,35E-07	4,75E-06
cg04334826	9	137561990	COL5A1	Body	0,225	1,35E-07	4,75E-06
cg25738688	12	89138236		IGR	0,211	1,35E-07	4,75E-06
cg05392949	13	77091333		IGR	-0,203	1,35E-07	4,75E-06
cg19984681	3	64479957		IGR	-0,209	1,35E-07	4,75E-06
cg09720734	10	129046280	DOCK1	ExonBnd	-0,272	1,35E-07	4,76E-06
cg00092623	19	28462158		IGR	0,268	1,36E-07	4,76E-06
cg01676127	11	15541144		IGR	-0,233	1,36E-07	4,76E-06
cg00774089	5	118676727	TNFAIP8	Body	-0,291	1,36E-07	4,77E-06
cg00378510	19	2291020	LINGO3	Body	0,256	1,36E-07	4,77E-06
cg10639435	8	146104221	ZNF250	3'UTR	-0,221	1,36E-07	4,77E-06
cg07996482	20	45718331	EYA2	Body	-0,212	1,36E-07	4,77E-06
cg18300998	15	52323904	MAPK6	5'UTR	-0,204	1,36E-07	4,77E-06
cg23186333	11	35161900	CD44	Body	-0,342	1,36E-07	4,78E-06
cg10575026	11	94886618	LOC10192929	Body	-0,215	1,36E-07	4,78E-06
cg01425718	1	81974159		IGR	-0,217	1,37E-07	4,78E-06
cg02206980	6	13574034	SIRT5	TSS1500	0,208	1,37E-07	4,78E-06
cg13289177	2	109204340	LIMS1	TSS1500	0,207	1,37E-07	4,78E-06
cg03801881	9	138833750	UBAC1	Body	0,225	1,37E-07	4,79E-06
cg16867466	8	117316880	LINC00536	Body	0,244	1,37E-07	4,80E-06
cg16118839	11	88069169	CTSC	Body	-0,206	1,37E-07	4,80E-06
cg19966212	5	149792783	CD74	TSS1500	-0,201	1,37E-07	4,80E-06
cg03954917	2	137063332		IGR	-0,245	1,37E-07	4,80E-06
cg17210535	3	182984429	MCF2L2	Body	-0,231	1,37E-07	4,80E-06
cg26501007	8	126614261		IGR	-0,28	1,37E-07	4,80E-06
cg16146253	6	25597934	LRRC16A	Body	-0,228	1,38E-07	4,81E-06
cg05930266	12	122887949	CLIP1	5'UTR	-0,253	1,38E-07	4,81E-06
cg21178208	22	18189131	BCL2L13	TSS1500	-0,269	1,38E-07	4,81E-06
cg03952672	21	45126620		IGR	-0,277	1,38E-07	4,81E-06
cg09904216	15	44968015	PATL2	Body	-0,203	1,38E-07	4,81E-06
cg04257163	12	89620072		IGR	-0,21	1,38E-07	4,81E-06
cg18908419	10	75800004	VCL	Body	0,245	1,38E-07	4,81E-06
cg08539991	19	36203832	ZBTB32	5'UTR	-0,29	1,38E-07	4,82E-06
cg05740247	2	43070600		IGR	-0,219	1,38E-07	4,82E-06
cg16908501	1	23212017	EPHB2	Body	-0,208	1,38E-07	4,82E-06
cg25937003	3	72309147		IGR	-0,233	1,38E-07	4,82E-06
cg05770389	6	88722796		IGR	-0,228	1,38E-07	4,82E-06
cg04435740	10	11220040	CELF2	Body	-0,258	1,38E-07	4,82E-06
cg16150105	19	41882368	TMEM91	TSS1500	0,21	1,38E-07	4,82E-06
cg01997665	11	10765070		IGR	-0,268	1,39E-07	4,83E-06
cg13502568	6	134757461	LOC10192823	Body	-0,261	1,39E-07	4,83E-06

cg13345129	5	54358229		IGR	-0,271	1,39E-07	4,83E-06
cg18994497	5	111333693	NREP-AS1	Body	-0,261	1,39E-07	4,83E-06
cg02494958	12	46760490	SLC38A2	Body	0,202	1,39E-07	4,84E-06
cg16258657	3	29338542	RBMS3	Body	-0,274	1,39E-07	4,84E-06
cg00692173	1	53217358	ZYG11B	Body	0,209	1,39E-07	4,84E-06
cg12967050	19	36233297	TMEM149	1stExon	-0,247	1,39E-07	4,84E-06
cg15314855	6	116879209	FAM26D	Body	0,302	1,40E-07	4,85E-06
cg01229865	13	113528751	ATP11A	Body	0,207	1,40E-07	4,86E-06
cg23671708	17	7758291	TMEM88	TSS200	0,207	1,40E-07	4,86E-06
cg26101485	8	142264769		IGR	0,233	1,40E-07	4,86E-06
cg12568669	8	11666485	FDFT1	Body	0,233	1,40E-07	4,86E-06
cg06893785	9	16705153	BNC2	Body	0,203	1,40E-07	4,86E-06
cg21341012	10	126378832	FAM53B	Body	-0,236	1,40E-07	4,86E-06
cg23481102	7	45442673		IGR	-0,217	1,40E-07	4,86E-06
cg15130132	1	21619582	LOC1005068(	TSS200	-0,207	1,40E-07	4,87E-06
cg17875595	2	69085258		IGR	-0,295	1,40E-07	4,87E-06
cg27380537	1	162114131	NOS1AP	Body	-0,232	1,41E-07	4,87E-06
cg14116294	9	124218386	GGTA1P	Body	-0,285	1,41E-07	4,88E-06
cg19280785	3	134340945	KY	Body	-0,26	1,41E-07	4,88E-06
cg23494498	11	117572655	DSCAML1	Body	-0,205	1,41E-07	4,88E-06
cg21187312	14	93048746	RIN3	Body	-0,268	1,41E-07	4,89E-06
cg27230705	10	124380473	DMBT1	Body	-0,216	1,41E-07	4,89E-06
cg23704412	2	136893882		IGR	-0,294	1,41E-07	4,89E-06
cg07868088	11	47531942	CELF1	5'UTR	-0,201	1,41E-07	4,89E-06
cg04586376	16	77624154		IGR	-0,261	1,42E-07	4,90E-06
cg23361764	11	78542948	ODZ4	Body	-0,213	1,42E-07	4,90E-06
cg07620562	3	124933890		IGR	-0,213	1,42E-07	4,90E-06
cg23652065	5	111092139	NREP	5'UTR	0,213	1,42E-07	4,91E-06
cg16567216	17	59385695	BCAS3	Body	-0,215	1,42E-07	4,91E-06
cg02181920	6	32820029	TAP1	Body	-0,286	1,42E-07	4,91E-06
cg26861995	3	127831673	RUVBL1	Body	-0,258	1,42E-07	4,91E-06
cg13495373	7	28474813	CREB5	TSS1500	-0,248	1,42E-07	4,91E-06
cg05833095	12	111015746	PPTC7	Body	-0,259	1,42E-07	4,92E-06
cg04348441	10	115955087	TDRD1	Body	-0,27	1,42E-07	4,92E-06
cg26114124	12	9217669	LOC144571	TSS200	0,403	1,42E-07	4,92E-06
cg18896452	17	42981993		IGR	-0,21	1,43E-07	4,93E-06
cg12161349	13	100089630		IGR	-0,206	1,43E-07	4,93E-06
cg23451443	8	101349044	RNF19A	TSS1500	0,229	1,43E-07	4,93E-06
cg06968859	2	80724209	CTNNA2	Body	-0,264	1,43E-07	4,93E-06
cg16236276	8	102436880		IGR	-0,213	1,43E-07	4,93E-06
cg07890553	3	119182858	TMEM39A	TSS1500	-0,267	1,43E-07	4,94E-06
cg07811261	16	73116575		IGR	-0,203	1,43E-07	4,94E-06
cg06393558	11	69982916	ANO1	Body	-0,236	1,44E-07	4,95E-06
cg00762397	1	203454010	PRELP	Body	-0,203	1,44E-07	4,96E-06
cg27287833	7	93690314		IGR	-0,3	1,44E-07	4,97E-06
cg08655071	1	209928895	TRAF3IP3	TSS1500	-0,266	1,44E-07	4,97E-06
cg17228456	2	237724426		IGR	-0,241	1,44E-07	4,97E-06
cg18871892	5	68035750		IGR	-0,212	1,44E-07	4,97E-06
cg26876834	16	2013573	SNHG9	TSS1500	-0,273	1,44E-07	4,97E-06
cg15233992	2	178031033		IGR	-0,279	1,45E-07	4,97E-06

cg16422113	18	31332083		IGR	0,222	1,45E-07	4,97E-06
cg23144598	1	30309219		IGR	-0,258	1,45E-07	4,98E-06
cg24647253	7	94997769	PON3	Body	-0,213	1,45E-07	4,98E-06
cg15361231	1	193075191	GLRX2	1stExon	0,224	1,45E-07	4,98E-06
cg05221629	5	55593516		IGR	-0,233	1,45E-07	4,98E-06
cg03233624	17	55740464	MSI2	Body	0,245	1,45E-07	4,98E-06
cg20445630	9	98256776	PTCH1	Body	-0,226	1,45E-07	4,98E-06
cg22595391	17	80407031	C17orf62	Body	-0,252	1,45E-07	4,98E-06
cg09863368	12	2862419		IGR	0,226	1,45E-07	4,99E-06
cg16473103	5	131434800		IGR	-0,231	1,46E-07	4,99E-06
cg22399236	18	20651637		IGR	-0,27	1,46E-07	4,99E-06
cg09116068	2	74775635	DOK1	TSS1500	0,23	1,46E-07	4,99E-06
cg03889381	10	31484004		IGR	-0,233	1,46E-07	4,99E-06
cg11183587	12	13213698	KIAA1467	Body	-0,267	1,46E-07	4,99E-06
cg04738237	2	121622953	GLI2	Body	-0,205	1,46E-07	5,00E-06
cg21957311	13	45623331		IGR	-0,205	1,46E-07	5,00E-06
cg11156560	17	42816286	DBF4B	Body	-0,247	1,46E-07	5,00E-06
cg15601452	1	28906382	SNHG12	Body	-0,2	1,46E-07	5,00E-06
cg07903098	10	3136907	PFKP	Body	0,21	1,46E-07	5,00E-06
cg05582310	4	5760808	EVC	Body	-0,218	1,46E-07	5,00E-06
cg08413352	2	214053906		IGR	0,263	1,46E-07	5,00E-06
cg24419094	2	10266986	RRM2	Body	0,256	1,46E-07	5,01E-06
cg11389889	20	2634874	NOP56	Body	-0,204	1,46E-07	5,01E-06
cg02970086	20	35272793	SLA2	5'UTR	-0,229	1,46E-07	5,01E-06
cg09078103	6	158314257	SNX9	Body	-0,203	1,46E-07	5,01E-06
cg16014085	16	30406122	ZNF48	TSS1500	0,213	1,46E-07	5,01E-06
cg04375897	4	159603427	ETFDH	1stExon	0,227	1,46E-07	5,01E-06
cg20251591	6	74232674		IGR	-0,206	1,47E-07	5,01E-06
cg13739348	1	218880529	MIR548F3	Body	-0,248	1,47E-07	5,02E-06
cg07252010	4	89146395	ABCG2	5'UTR	0,329	1,47E-07	5,02E-06
cg24185913	12	68575158		IGR	-0,213	1,47E-07	5,02E-06
cg04523558	12	12736011		IGR	-0,214	1,47E-07	5,02E-06
cg07874964	1	91406655	ZNF644	Body	0,254	1,47E-07	5,03E-06
cg01616440	15	84319908		IGR	-0,202	1,47E-07	5,03E-06
cg22040527	15	65053596	RBPMS2	Body	0,204	1,47E-07	5,03E-06
cg13738686	3	185599118		IGR	-0,245	1,47E-07	5,03E-06
cg11953335	4	141037812	MAML3	Body	-0,311	1,47E-07	5,03E-06
cg01806880	2	72426232	EXOC6B	Body	-0,275	1,47E-07	5,03E-06
cg06754698	3	52828393	ITIH3	TSS1500	-0,279	1,47E-07	5,04E-06
cg10402417	11	67171476	TBC1D10C	5'UTR	-0,2	1,47E-07	5,04E-06
cg12805374	17	43306622	FMNL1	Body	-0,201	1,48E-07	5,04E-06
cg03398785	9	127633544	ARPC5L	Body	-0,255	1,48E-07	5,04E-06
cg01297872	6	45674277		IGR	-0,226	1,48E-07	5,04E-06
cg10072237	6	33872861		IGR	-0,239	1,48E-07	5,05E-06
cg16332831	15	101483070	LRRK1	Body	-0,263	1,48E-07	5,05E-06
cg17353823	3	186656591	ST6GAL1	5'UTR	0,21	1,48E-07	5,05E-06
cg19683655	10	85440299		IGR	-0,244	1,48E-07	5,05E-06
cg15052335	18	3011810	LPIN2	5'UTR	-0,211	1,48E-07	5,05E-06
cg00613788	2	101175596		IGR	0,239	1,48E-07	5,06E-06
cg05714732	1	32409096		IGR	-0,206	1,49E-07	5,06E-06

cg23064082	11	30428652	MPPED2	Body	-0,231	1,49E-07	5,06E-06
cg00041829	2	7870747		IGR	-0,238	1,49E-07	5,06E-06
cg03269019	13	62895082		IGR	0,362	1,49E-07	5,06E-06
cg02252421	5	127914655		IGR	-0,217	1,49E-07	5,07E-06
cg08370430	17	12927578		IGR	0,206	1,49E-07	5,07E-06
cg21484910	2	40097661		IGR	-0,224	1,49E-07	5,07E-06
cg02671445	5	73614960		IGR	-0,241	1,49E-07	5,07E-06
cg24392515	14	24578415		IGR	-0,254	1,49E-07	5,07E-06
cg21052932	14	51342320	ABHD12B	5'UTR	-0,226	1,49E-07	5,07E-06
cg00632533	3	131045564	NEK11	Body	-0,257	1,49E-07	5,07E-06
cg14789818	1	227748712		IGR	0,259	1,49E-07	5,08E-06
cg23362937	17	2169852	SMG6	TSS1500	-0,31	1,49E-07	5,08E-06
cg13100117	1	230516116	PGBD5	Body	-0,225	1,49E-07	5,08E-06
cg07781301	1	227953053	SNAP47	Body	-0,237	1,50E-07	5,09E-06
cg16490596	11	35205279	CD44	Body	-0,231	1,50E-07	5,09E-06
cg02606936	22	39337729		IGR	-0,343	1,50E-07	5,09E-06
cg01984743	5	172751331	STC2	Body	0,212	1,50E-07	5,10E-06
cg25002649	6	43022044	CUL7	TSS1500	0,231	1,50E-07	5,10E-06
cg18357911	12	12889955	APOLD1	Body	-0,27	1,50E-07	5,10E-06
cg16147511	14	99655820	BCL11B	Body	-0,221	1,50E-07	5,11E-06
cg19118751	17	26577636		IGR	-0,311	1,51E-07	5,11E-06
cg11904429	19	6592554	CD70	TSS1500	-0,239	1,51E-07	5,11E-06
cg09245312	11	72316926	PDE2A	Body	-0,211	1,51E-07	5,12E-06
cg12702368	1	86035283	DDAH1	5'UTR	-0,224	1,51E-07	5,12E-06
cg21161568	9	117047688	COL27A1	Body	-0,208	1,51E-07	5,12E-06
cg17499833	7	93690381		IGR	-0,275	1,51E-07	5,12E-06
cg18856478	1	43814358	MPL	Body	0,243	1,51E-07	5,13E-06
cg14109813	3	69963596	MITF	Body	-0,212	1,52E-07	5,14E-06
cg13549474	5	95066422	RHOBTB3	TSS1500	0,202	1,52E-07	5,14E-06
cg04066552	21	43837842	UBASH3A	Body	-0,209	1,52E-07	5,14E-06
cg14454186	10	3800461		IGR	-0,319	1,52E-07	5,14E-06
cg11206506	18	28509936		IGR	-0,251	1,52E-07	5,14E-06
cg04415023	5	67435702		IGR	-0,256	1,52E-07	5,15E-06
cg13136287	6	3868172		IGR	-0,255	1,52E-07	5,15E-06
cg19520512	3	23839056		IGR	-0,235	1,53E-07	5,15E-06
cg03510041	6	41559210	FOXP4	Body	-0,216	1,53E-07	5,16E-06
cg07413155	2	64242795	VPS54	5'UTR	0,232	1,53E-07	5,16E-06
cg13996386	2	174150974		IGR	-0,221	1,53E-07	5,16E-06
cg18543142	3	23137484		IGR	-0,221	1,53E-07	5,16E-06
cg06557390	1	177905133	SEC16B	Body	-0,257	1,53E-07	5,17E-06
cg24782378	15	100672379	ADAMTS17	Body	0,205	1,53E-07	5,17E-06
cg20664269	18	56021135	NEDD4L	Body	-0,201	1,53E-07	5,18E-06
cg07920076	20	35224478	TGIF2-C20orf	Body	-0,311	1,53E-07	5,18E-06
cg25454161	13	34987107		IGR	-0,218	1,54E-07	5,18E-06
cg01251360	16	68772225	CDH1	Body	0,231	1,54E-07	5,18E-06
cg07999090	13	28882299	FLT1	Body	-0,201	1,54E-07	5,18E-06
cg19952385	3	143829074		IGR	-0,207	1,54E-07	5,18E-06
cg25070442	8	881586		IGR	0,236	1,54E-07	5,18E-06
cg25144893	1	63792210		IGR	0,223	1,54E-07	5,18E-06
cg01240412	3	134142342		IGR	-0,223	1,54E-07	5,18E-06

cg00058290	6	400090	IRF4	Body	-0,233	1,54E-07	5,19E-06
cg20427865	16	57406353	CX3CL1	TSS200	0,295	1,54E-07	5,20E-06
cg07616791	15	101661720		IGR	0,206	1,54E-07	5,20E-06
cg07410210	2	69662765	NFU1	Body	0,26	1,54E-07	5,20E-06
cg02949759	22	39025137	FAM227A	Body	-0,208	1,54E-07	5,20E-06
cg18499340	8	119496067	SAMD12	Body	-0,203	1,54E-07	5,20E-06
cg17311132	12	88693546		IGR	-0,322	1,54E-07	5,20E-06
cg06545367	7	110731527	LRRN3	5'UTR	0,25	1,55E-07	5,21E-06
cg00025216	5	118781384		IGR	0,227	1,55E-07	5,21E-06
cg19508464	1	62744697	KANK4	Body	0,212	1,55E-07	5,22E-06
cg24685296	18	11308716		IGR	-0,231	1,56E-07	5,23E-06
cg25844708	13	53270248		IGR	-0,229	1,56E-07	5,23E-06
cg20832054	10	35759633	CCNY	5'UTR	0,242	1,56E-07	5,23E-06
cg08311343	7	92324861	CDK6	Body	0,268	1,56E-07	5,24E-06
cg09207745	2	8715640	LOC10192956	Body	-0,2	1,56E-07	5,24E-06
cg26217424	3	111577657	PHLDB2	TSS1500	0,223	1,56E-07	5,24E-06
cg03100639	16	31483137	TGFB1I1	TSS1500	0,228	1,56E-07	5,24E-06
cg20911456	1	15403913	KAZN	Body	-0,227	1,56E-07	5,24E-06
cg15253080	14	31560390	AP4S1	Body	0,277	1,56E-07	5,25E-06
cg09306458	15	70994682	UACA	TSS200	0,274	1,57E-07	5,25E-06
cg26357596	5	54397592	GZMA	TSS1500	-0,274	1,57E-07	5,25E-06
cg15819345	7	28579607	CREB5	Body	-0,229	1,57E-07	5,25E-06
cg02330172	2	37603618		IGR	-0,257	1,57E-07	5,25E-06
cg08666143	20	49952074		IGR	-0,219	1,57E-07	5,25E-06
cg25305972	2	198070713	ANKRD44	Body	-0,222	1,57E-07	5,26E-06
cg21578351	15	68864164		IGR	-0,202	1,57E-07	5,26E-06
cg18937561	10	14879252	HSPA14	TSS1500	0,212	1,57E-07	5,26E-06
cg19557480	17	30470448	RHOT1	5'UTR	0,229	1,57E-07	5,26E-06
cg13411784	9	140473680	WDR85	TSS1500	0,201	1,57E-07	5,26E-06
cg20463995	15	96817359		IGR	0,208	1,57E-07	5,26E-06
cg25576706	4	142693136		IGR	-0,203	1,57E-07	5,26E-06
cg23130369	2	129245177		IGR	-0,202	1,57E-07	5,27E-06
cg23235468	7	11413714	THSD7A	3'UTR	-0,204	1,57E-07	5,27E-06
cg12580783	12	49362475	WNT10B	Body	-0,267	1,57E-07	5,27E-06
cg06893362	20	47897124	C20orf199	Body	-0,23	1,57E-07	5,27E-06
cg01602822	3	56951917	ARHGEF3	TSS1500	-0,343	1,57E-07	5,27E-06
cg08462127	8	2017365	MYOM2	Body	0,207	1,57E-07	5,27E-06
cg24410453	6	53919041	C6orf142	Body	0,288	1,57E-07	5,27E-06
cg26180867	4	114598560	CAMK2D	Body	-0,277	1,58E-07	5,28E-06
cg01547915	2	38947762	GALM	Body	0,202	1,58E-07	5,28E-06
cg26916162	5	142986108		IGR	-0,244	1,58E-07	5,28E-06
cg05115279	4	129442982		IGR	0,27	1,58E-07	5,28E-06
cg22122098	2	170934512	UBR3	Body	-0,355	1,58E-07	5,29E-06
cg11752788	7	123438230		IGR	0,235	1,58E-07	5,29E-06
cg11114141	2	206633633	NRP2	Body	-0,25	1,58E-07	5,29E-06
cg08023852	3	65864381	MAGI1	Body	-0,223	1,59E-07	5,30E-06
cg16261489	9	91969068	SECISBP2	Body	0,203	1,59E-07	5,30E-06
cg17158941	7	1073255	C7orf50	Body	-0,225	1,59E-07	5,30E-06
cg11713660	17	33545616		IGR	-0,247	1,59E-07	5,30E-06
cg19281794	3	112218761	BTLA	TSS1500	-0,203	1,59E-07	5,30E-06

cg18344365	2	30585859		IGR	0,229	1,59E-07	5,30E-06
cg21380077	6	143164338	HIVEP2	5'UTR	-0,219	1,59E-07	5,31E-06
cg08559711	6	20978738	CDKAL1	Body	-0,218	1,60E-07	5,32E-06
cg16994669	8	101325645	RNF19A	5'UTR	-0,209	1,60E-07	5,32E-06
cg05792935	4	79560054		IGR	-0,263	1,60E-07	5,32E-06
cg18880384	6	32365113	BTNL2	Body	-0,21	1,60E-07	5,32E-06
cg20960017	11	111195850		IGR	-0,244	1,60E-07	5,32E-06
cg19899972	1	61624321	NFIA	Body	-0,247	1,60E-07	5,32E-06
cg24529549	10	77716422	C10orf11	Body	-0,222	1,60E-07	5,32E-06
cg02508438	15	64730989	TRIP4	Body	-0,27	1,60E-07	5,33E-06
cg06417385	10	11847111		IGR	-0,216	1,60E-07	5,33E-06
cg10179694	3	188164137	LPP	Body	-0,276	1,60E-07	5,33E-06
cg14274656	10	61900584	ANK3	1stExon	0,347	1,60E-07	5,33E-06
cg19643431	2	110259569	SH3RF3	3'UTR	-0,22	1,60E-07	5,34E-06
cg23810282	5	43037519		IGR	0,242	1,60E-07	5,34E-06
cg07268431	3	47436106	PTPN23	Body	0,22	1,60E-07	5,34E-06
cg15844008	17	55957633	CUEDC1	Body	-0,246	1,60E-07	5,34E-06
cg16245868	1	55547090	USP24	Body	0,256	1,61E-07	5,34E-06
cg23933261	1	94127802	BCAR3	Body	-0,298	1,61E-07	5,34E-06
cg19585100	14	73703023	PAPLN	TSS1500	-0,201	1,61E-07	5,34E-06
cg01677804	1	212469953	PPP2R5A	Body	0,26	1,61E-07	5,34E-06
cg20697575	6	38571941	BTBD9	5'UTR	0,33	1,61E-07	5,34E-06
cg05573542	20	52724619		IGR	0,226	1,61E-07	5,34E-06
cg05774900	12	33579112	SYT10	Body	-0,203	1,61E-07	5,35E-06
cg07692435	16	29743682		IGR	-0,21	1,61E-07	5,35E-06
cg22897419	3	147449682		IGR	-0,201	1,61E-07	5,36E-06
cg17355820	14	72516174	RGS6	5'UTR	-0,239	1,61E-07	5,36E-06
cg19123356	8	130898833	FAM49B	5'UTR	-0,242	1,61E-07	5,36E-06
cg05125353	3	169477189		IGR	-0,338	1,62E-07	5,36E-06
cg21660452	11	64397807	NRXN2	Body	0,263	1,62E-07	5,36E-06
cg27549730	11	111867678	DIXDC1	Body	0,214	1,62E-07	5,37E-06
cg02855936	19	42449790		IGR	-0,303	1,62E-07	5,37E-06
cg14535274	3	121151186	POLQ	Body	-0,202	1,62E-07	5,37E-06
cg27231850	2	207956157	KLF7	Body	0,201	1,62E-07	5,37E-06
cg21710377	15	70006832		IGR	-0,208	1,62E-07	5,38E-06
cg13009608	8	81034420	TPD52	Body	-0,275	1,62E-07	5,38E-06
cg18097707	10	32702584		IGR	-0,222	1,62E-07	5,38E-06
cg14987298	16	27475382	GTF3C1	Body	-0,224	1,62E-07	5,38E-06
cg17846091	3	156821310	LINC00880	Body	-0,23	1,63E-07	5,39E-06
cg01399402	19	47221403	PRKD2	TSS1500	-0,296	1,63E-07	5,39E-06
cg02321197	2	158755934		IGR	-0,291	1,63E-07	5,39E-06
cg14863272	3	188632580		IGR	-0,254	1,63E-07	5,39E-06
cg21245642	4	178244875	NEIL3	Body	0,216	1,63E-07	5,40E-06
cg22199864	10	22233858	DNAJC1	Body	0,228	1,63E-07	5,40E-06
cg07301061	12	80663711		IGR	0,255	1,63E-07	5,40E-06
cg10335612	21	43507256	UMODL1	Body	-0,21	1,64E-07	5,41E-06
cg12137682	2	173694210	RAPGEF4	Body	0,216	1,64E-07	5,41E-06
cg13060531	1	84466098	TTLL7	TSS1500	0,247	1,64E-07	5,41E-06
cg06211062	18	8176696	PTPRM	Body	0,268	1,64E-07	5,42E-06
cg24053394	13	46747837	LCP1	5'UTR	-0,301	1,64E-07	5,42E-06

cg14856755	13	49379799		IGR	-0,231	1,64E-07	5,42E-06
cg00305624	19	2237672	PLEKHJ1	TSS1500	-0,252	1,64E-07	5,42E-06
cg13356808	19	6613813		IGR	-0,354	1,64E-07	5,43E-06
cg08987713	9	25716571		IGR	0,232	1,65E-07	5,43E-06
cg25168581	10	13276816	UCMA	TSS1500	-0,209	1,65E-07	5,43E-06
cg27488176	15	60866344	RORA-AS1	Body	-0,209	1,65E-07	5,44E-06
cg27276993	3	64367255		IGR	-0,27	1,65E-07	5,44E-06
cg14562076	21	44869785	C21orf125	TSS200	-0,228	1,65E-07	5,44E-06
cg02913946	11	46926130	LRP4	Body	-0,234	1,65E-07	5,44E-06
cg05390091	4	48298462		IGR	-0,218	1,65E-07	5,45E-06
cg03384701	2	12643011	LOC10050645	Body	-0,301	1,66E-07	5,45E-06
cg15411952	2	234777881	MSL3P1	TSS1500	0,327	1,66E-07	5,45E-06
cg20395295	3	5239540	EDEM1	Body	-0,306	1,66E-07	5,46E-06
cg12553238	14	20960017		IGR	-0,237	1,66E-07	5,46E-06
cg04526449	1	46471562	MAST2	Body	0,285	1,66E-07	5,47E-06
cg03950924	21	42551539	PLAC4	1stExon	-0,233	1,66E-07	5,47E-06
cg25206113	1	151788262	RORC	Body	-0,219	1,66E-07	5,47E-06
cg11855693	12	96632551	ELK3	Body	0,305	1,66E-07	5,47E-06
cg14772639	2	128711564	SAP130	Body	0,21	1,66E-07	5,47E-06
cg05561193	4	150999042	DCLK2	TSS1500	0,211	1,67E-07	5,48E-06
cg25102614	12	40602692		IGR	-0,227	1,67E-07	5,49E-06
cg23200218	19	39050485	RYR1	Body	-0,227	1,67E-07	5,49E-06
cg05876737	5	58653705	PDE4D	TSS1500	0,208	1,67E-07	5,49E-06
cg17432748	2	235959556	SH3BP4	Body	0,218	1,67E-07	5,49E-06
cg19426522	3	27766009		IGR	0,201	1,67E-07	5,49E-06
cg14533567	3	52705872	PBRM1	Body	-0,223	1,67E-07	5,49E-06
cg26363363	12	7060386	PTPN6	TSS200	-0,302	1,67E-07	5,49E-06
cg01982088	1	4714986	AJAP1	TSS200	0,224	1,68E-07	5,50E-06
cg23044178	11	12136405	MICAL2	5'UTR	0,283	1,68E-07	5,50E-06
cg01566695	10	496874	DIP2C	Body	-0,203	1,68E-07	5,50E-06
cg21659576	17	47467409		IGR	-0,311	1,68E-07	5,50E-06
cg04960440	10	14606714	FAM107B	5'UTR	-0,206	1,68E-07	5,50E-06
cg07462448	10	115441840	CASP7	5'UTR	-0,317	1,68E-07	5,51E-06
cg00329307	5	131808892	C5orf56	Body	-0,295	1,68E-07	5,51E-06
cg01199321	5	169694363	LCP2	Body	-0,212	1,68E-07	5,52E-06
cg14054883	9	93619467	SYK	Body	-0,232	1,69E-07	5,52E-06
cg11977716	18	77284742	NFATC1	Body	-0,201	1,69E-07	5,52E-06
cg26691477	2	119605281	EN1	1stExon	0,226	1,69E-07	5,52E-06
cg08903069	2	85500887	TCF7L1	Body	-0,206	1,69E-07	5,52E-06
cg21857724	2	231523609		IGR	-0,219	1,69E-07	5,53E-06
cg24026273	2	3255919	TSSC1	Body	0,203	1,69E-07	5,53E-06
cg26462892	15	86039647	AKAP13	Body	0,317	1,69E-07	5,53E-06
cg20057060	6	90544812	CASP8AP2	5'UTR	-0,248	1,69E-07	5,53E-06
cg06827792	7	36723794	AOAH	Body	-0,233	1,69E-07	5,53E-06
cg11704582	10	104814269	CNNM2	Body	-0,242	1,69E-07	5,53E-06
cg13009839	1	3088978	PRDM16	Body	-0,204	1,69E-07	5,54E-06
cg17618942	19	2668273	GNG7	5'UTR	-0,241	1,69E-07	5,54E-06
cg00014825	17	65602810	PITPNC1	Body	0,235	1,69E-07	5,54E-06
cg08270236	4	160027432		IGR	0,236	1,70E-07	5,55E-06
cg02567269	6	90326298	ANKRD6	Body	-0,208	1,70E-07	5,55E-06

cg10298479	7	156796929		IGR	0,212	1,70E-07	5,55E-06
cg06255986	7	55254148	EGFR-AS1	Body	-0,251	1,70E-07	5,56E-06
cg07856430	18	42339206	SETBP1	Body	-0,289	1,70E-07	5,56E-06
cg13355129	12	58637434		IGR	-0,245	1,70E-07	5,56E-06
cg00402941	10	36783791		IGR	-0,221	1,71E-07	5,57E-06
cg03260458	3	152100288	MBNL1	Body	-0,207	1,71E-07	5,57E-06
cg11845417	11	111789613	C11orf52	1stExon	0,201	1,71E-07	5,57E-06
cg27650654	11	122567595	UBASH3B	Body	-0,305	1,71E-07	5,57E-06
cg06703222	16	69598327	NFAT5	TSS1500	-0,209	1,71E-07	5,57E-06
cg27209741	7	97663876		IGR	0,217	1,71E-07	5,57E-06
cg03204155	22	40915817	MKL1	5'UTR	-0,248	1,71E-07	5,57E-06
cg19283890	3	193896367		IGR	-0,285	1,71E-07	5,58E-06
cg05810221	15	101602954	LRRK1	Body	-0,202	1,71E-07	5,58E-06
cg07088064	1	177905658	SEC16B	Body	-0,236	1,71E-07	5,58E-06
cg08801849	5	170224936	GABRP	Body	-0,244	1,71E-07	5,58E-06
cg08684834	2	68994403	ARHGAP25	Body	-0,268	1,71E-07	5,59E-06
cg08354007	2	170618192		IGR	-0,209	1,71E-07	5,59E-06
cg16248584	16	83413155	CDH13	Body	-0,26	1,71E-07	5,59E-06
cg04681088	18	52918079	TCF4	Body	-0,253	1,71E-07	5,59E-06
cg25191711	11	8269255	LMO1	Body	-0,201	1,72E-07	5,60E-06
cg08241745	2	15908508		IGR	-0,237	1,72E-07	5,60E-06
cg20734664	2	47979699		IGR	-0,252	1,72E-07	5,60E-06
cg08415762	20	50224747	ATP9A	Body	-0,251	1,72E-07	5,60E-06
cg16126275	21	38010568		IGR	-0,24	1,72E-07	5,60E-06
cg20832643	6	111873828	TRAF3IP2-AS1	Body	0,216	1,72E-07	5,60E-06
cg25515258	3	46581056	LRRC2	Body	-0,232	1,72E-07	5,61E-06
cg01720337	3	1134711	CNTN6	5'UTR	0,225	1,72E-07	5,61E-06
cg01314930	11	119544518	PVRL1	Body	-0,297	1,72E-07	5,61E-06
cg12633154	6	30039435	RNF39	Body	0,428	1,72E-07	5,61E-06
cg07425204	17	66511183	PRKAR1A	5'UTR	0,207	1,72E-07	5,61E-06
cg03252313	7	148117075	CNTNAP2	3'UTR	-0,281	1,73E-07	5,62E-06
cg21564045	13	39726023		IGR	-0,239	1,73E-07	5,62E-06
cg07234571	20	35362404	NDRG3	Body	-0,226	1,73E-07	5,63E-06
cg13170967	5	38695390	OSMR-AS1	Body	-0,202	1,73E-07	5,63E-06
cg16264581	18	3061278		IGR	-0,304	1,74E-07	5,63E-06
cg09350801	1	15391073	KAZN	Body	-0,2	1,74E-07	5,64E-06
cg26286901	8	22595307	PEBP4	Body	0,211	1,74E-07	5,64E-06
cg20078006	11	128751307		IGR	-0,212	1,74E-07	5,64E-06
cg26345770	15	69972079	PCAT29	Body	-0,203	1,74E-07	5,64E-06
cg27525033	4	10180984		IGR	-0,2	1,74E-07	5,65E-06
cg09603514	1	175052941	TNN	Body	-0,208	1,74E-07	5,65E-06
cg24104567	17	40932508	WNK4	TSS200	0,204	1,74E-07	5,65E-06
cg07069868	10	75416206	SYNPO2L	TSS1500	0,21	1,74E-07	5,65E-06
cg01395330	13	24825727	SPATA13	Body	-0,279	1,75E-07	5,66E-06
cg12426470	17	1104627		IGR	-0,248	1,75E-07	5,66E-06
cg08903181	12	89018232		IGR	-0,208	1,75E-07	5,66E-06
cg18204964	5	38487146	LIFR	Body	-0,227	1,76E-07	5,68E-06
cg06520521	11	62323216		IGR	-0,223	1,76E-07	5,68E-06
cg19918599	3	112366281		IGR	0,2	1,76E-07	5,69E-06
cg25522867	11	34236648	ABTB2	Body	-0,24	1,76E-07	5,69E-06

cg24859236	1	9750213	PIK3CD	5'UTR	0,276	1,76E-07	5,69E-06
cg09214779	3	61367509		IGR	-0,241	1,76E-07	5,70E-06
cg22991506	14	50468241	C14orf182	Body	-0,252	1,77E-07	5,70E-06
cg00574412	17	27892866	ABHD15	Body	-0,201	1,77E-07	5,70E-06
cg04669218	4	166966753	TLL1	Body	-0,221	1,77E-07	5,71E-06
cg17357341	2	58603854		IGR	-0,315	1,77E-07	5,71E-06
cg11601093	10	80339031		IGR	-0,229	1,78E-07	5,73E-06
cg08811117	5	79050913	CMYA5	Body	-0,244	1,78E-07	5,73E-06
cg06303168	12	56322959	WIBG	TSS1500	-0,276	1,78E-07	5,73E-06
cg00184457	8	8946301		IGR	-0,287	1,78E-07	5,74E-06
cg07626553	22	44494494	PARVB	Body	0,332	1,78E-07	5,74E-06
cg13945265	5	17802512		IGR	0,208	1,78E-07	5,75E-06
cg02124853	14	69410155	ACTN1	Body	-0,235	1,79E-07	5,75E-06
cg04201335	7	144524837	TPK1	Body	0,247	1,79E-07	5,75E-06
cg09873533	2	114452193		IGR	0,214	1,79E-07	5,75E-06
cg20330256	16	57648576		IGR	-0,206	1,79E-07	5,75E-06
cg23061725	2	202126379	CASP8	5'UTR	-0,201	1,79E-07	5,75E-06
cg21552104	8	9189178	LOC157273	Body	-0,315	1,79E-07	5,75E-06
cg11803027	12	66150884		IGR	-0,232	1,79E-07	5,76E-06
cg23579062	9	34457500	C9orf25	Body	0,216	1,80E-07	5,77E-06
cg17352276	15	66795208	SNORD16	Body	-0,222	1,80E-07	5,77E-06
cg23409348	5	142179573	ARHGAP26	Body	-0,235	1,80E-07	5,77E-06
cg23403004	7	27162780	HOXA3	5'UTR	0,222	1,80E-07	5,77E-06
cg05798059	11	30444984	MPPED2	Body	-0,202	1,80E-07	5,78E-06
cg05198507	5	138735248	SPATA24	Body	-0,232	1,80E-07	5,78E-06
cg25755851	1	9335794		IGR	-0,239	1,81E-07	5,79E-06
cg18358988	15	58600237		IGR	-0,232	1,81E-07	5,79E-06
cg07245447	1	156184331	PMF1	Body	-0,205	1,81E-07	5,80E-06
cg04854505	3	13634590	FBLN2	Body	-0,209	1,81E-07	5,80E-06
cg10343206	4	71872103	DCK	Body	-0,333	1,81E-07	5,80E-06
cg07641497	22	33195343	SYN3	Body	-0,288	1,81E-07	5,81E-06
cg15150336	8	145773927	KIAA1688	Body	-0,209	1,81E-07	5,81E-06
cg15130548	17	40566710	PTRF	Body	0,23	1,82E-07	5,81E-06
cg16581692	2	162019868	TANK	5'UTR	-0,211	1,82E-07	5,82E-06
cg04222358	3	2140256		IGR	0,213	1,82E-07	5,82E-06
cg04083145	17	64028625	CEP112	Body	-0,264	1,82E-07	5,82E-06
cg00498162	6	125551273	TPD52L1	Body	-0,211	1,82E-07	5,82E-06
cg15986314	6	24256161	DCDC2	Body	-0,261	1,82E-07	5,82E-06
cg25090204	7	148169569		IGR	-0,262	1,82E-07	5,82E-06
cg16504108	15	71510536	THSD4	Body	-0,214	1,82E-07	5,82E-06
cg22032706	7	120498729	TSPAN12	TSS1500	0,237	1,82E-07	5,83E-06
cg24699146	1	24152579	HMGCL	TSS1500	-0,218	1,82E-07	5,83E-06
cg12287291	11	122319811		IGR	-0,274	1,82E-07	5,83E-06
cg02654003	20	32810251		IGR	-0,307	1,83E-07	5,83E-06
cg27048989	6	150397765		IGR	-0,337	1,83E-07	5,83E-06
cg09066377	18	19922996		IGR	-0,253	1,83E-07	5,83E-06
cg21847454	1	194032120		IGR	0,207	1,83E-07	5,83E-06
cg01861257	2	237520134		IGR	-0,276	1,83E-07	5,84E-06
cg05616584	5	133447785		IGR	-0,215	1,83E-07	5,84E-06
cg10622566	18	20651839		IGR	-0,226	1,83E-07	5,85E-06

cg04651018	14	68715849	RAD51B	Body	0,211	1,84E-07	5,85E-06
cg04022595	2	217860011		IGR	-0,214	1,84E-07	5,86E-06
cg19426434	3	183272159	KLHL6	Body	-0,278	1,84E-07	5,86E-06
cg26897744	3	158450246	RARRES1	1stExon	0,202	1,84E-07	5,87E-06
cg18935506	17	30230000	UTP6	TSS1500	-0,238	1,85E-07	5,88E-06
cg12509933	2	42398315	LOC10272382	TSS1500	-0,227	1,85E-07	5,88E-06
cg19961945	19	7192407	INSR	Body	-0,242	1,85E-07	5,89E-06
cg24843790	21	44727494		IGR	-0,24	1,85E-07	5,89E-06
cg04287259	1	9730401	PIK3CD	5'UTR	-0,234	1,85E-07	5,90E-06
cg07158040	2	191185285	HIBCH	TSS1500	-0,218	1,85E-07	5,90E-06
cg00533182	8	145726136	PPP1R16A	Body	0,217	1,85E-07	5,90E-06
cg21308322	4	8374697	ACOX3	Body	0,224	1,86E-07	5,91E-06
cg04124151	7	27401424		IGR	0,232	1,86E-07	5,91E-06
cg22946147	7	88425148	ZNF804B	Body	0,212	1,86E-07	5,92E-06
cg15781610	6	36992554	FGD2	Body	-0,2	1,86E-07	5,92E-06
cg24613135	8	129253074		IGR	-0,249	1,87E-07	5,92E-06
cg15358723	1	245750662	KIF26B	Body	-0,254	1,87E-07	5,93E-06
cg07929642	16	89390685	ANKRD11	5'UTR	-0,344	1,87E-07	5,93E-06
cg00514575	5	180231155	MGAT1	TSS1500	-0,26	1,87E-07	5,93E-06
cg06518838	6	170403264		IGR	-0,287	1,87E-07	5,93E-06
cg12073436	1	206958014		IGR	-0,242	1,87E-07	5,93E-06
cg06431655	15	58605733		IGR	-0,215	1,87E-07	5,93E-06
cg25097480	6	45511238	RUNX2	Body	0,27	1,87E-07	5,94E-06
cg21033632	5	64486421	ADAMTS6	Body	-0,218	1,87E-07	5,94E-06
cg03961112	11	57559256	CTNND1	Body	0,231	1,87E-07	5,94E-06
cg18716014	1	47125994	ATPAF1	5'UTR	-0,242	1,88E-07	5,94E-06
cg25582220	6	30130914	TRIM15	TSS200	-0,237	1,88E-07	5,94E-06
cg17066452	6	33173501	HSD17B8	Body	-0,219	1,87E-07	5,94E-06
cg06105555	6	22017093	FLJ22536	Body	-0,204	1,88E-07	5,94E-06
cg17803840	20	11946703		IGR	-0,236	1,88E-07	5,94E-06
cg15352367	22	36236732	RBM9	Body	0,21	1,88E-07	5,94E-06
cg09004167	1	17053983		IGR	-0,321	1,88E-07	5,94E-06
cg20002901	2	238777656	RAMP1	Body	-0,237	1,88E-07	5,95E-06
cg11499323	11	45114907	PRDM11	TSS1500	-0,264	1,88E-07	5,96E-06
cg16363276	1	55617192	USP24	Body	-0,259	1,89E-07	5,96E-06
cg06494960	12	47613263	PCED1B	5'UTR	-0,217	1,89E-07	5,96E-06
cg03210827	10	104574906	C10orf26	3'UTR	0,201	1,89E-07	5,97E-06
cg25817655	1	8487870	LOC10272455	Body	-0,308	1,89E-07	5,97E-06
cg23882945	4	113300580	ALPK1	Body	0,205	1,89E-07	5,97E-06
cg19303305	4	8277560	HTRA3	Body	-0,21	1,89E-07	5,98E-06
cg22337620	18	3689357	DLGAP1	Body	-0,207	1,89E-07	5,98E-06
cg03157210	5	168569592	SLIT3	Body	0,208	1,89E-07	5,98E-06
cg20821276	17	65243119		IGR	-0,22	1,89E-07	5,98E-06
cg04322298	2	190635899	ORMDL1	3'UTR	0,301	1,90E-07	5,99E-06
cg25342423	6	158088897	ZDHHC14	Body	-0,203	1,90E-07	5,99E-06
cg06736189	18	60173617		IGR	-0,209	1,90E-07	5,99E-06
cg16151262	8	12954058	DLC1	Body	0,251	1,90E-07	5,99E-06
cg05649684	5	169701216	LCP2	Body	-0,274	1,90E-07	5,99E-06
cg05287437	3	16215248	GALNTL2	TSS1500	-0,22	1,90E-07	6,00E-06
cg13654573	14	53576363	DDHD1	Body	-0,209	1,90E-07	6,00E-06

cg07698196	7	150485638	LOC10012854	Body	0,224	1,90E-07	6,00E-06
cg23251761	1	9750061	PIK3CD	5'UTR	0,313	1,90E-07	6,00E-06
cg14280382	12	96094182	NTN4	Body	-0,247	1,90E-07	6,00E-06
cg01320510	17	8772194	PIK3R6	TSS1500	-0,225	1,90E-07	6,00E-06
cg06134364	3	124919714	SLC12A8	Body	-0,229	1,91E-07	6,01E-06
cg27342720	8	120033982		IGR	-0,229	1,91E-07	6,01E-06
cg07127888	8	41400789	GINS4	3'UTR	0,235	1,91E-07	6,01E-06
cg08872577	1	42385480	HIVEP3	TSS1500	-0,25	1,91E-07	6,02E-06
cg02696428	13	46743535	LCP1	5'UTR	-0,205	1,91E-07	6,02E-06
cg10290803	19	2535750	GNG7	5'UTR	0,201	1,91E-07	6,02E-06
cg06223926	10	33626522		IGR	0,205	1,91E-07	6,02E-06
cg26579713	15	65701865	IGDCC4	Body	-0,364	1,91E-07	6,02E-06
cg06784420	2	236299218		IGR	-0,291	1,91E-07	6,02E-06
cg05117638	6	113952661		IGR	-0,352	1,91E-07	6,02E-06
cg18688142	6	144657165	UTRN	Body	-0,265	1,91E-07	6,02E-06
cg16622061	16	86888736		IGR	-0,212	1,92E-07	6,03E-06
cg05193179	22	17679799	CECR1	Body	0,249	1,92E-07	6,03E-06
cg01082299	6	31431969	HCP5	3'UTR	-0,243	1,92E-07	6,03E-06
cg26465532	10	79446426		IGR	-0,204	1,92E-07	6,03E-06
cg03274046	10	127813782	ADAM12	Body	-0,201	1,92E-07	6,03E-06
cg03494648	11	2322642	C11orf21	Body	-0,217	1,92E-07	6,03E-06
cg05957847	16	10706567		IGR	-0,217	1,92E-07	6,04E-06
cg07094521	11	132227828		IGR	-0,223	1,92E-07	6,04E-06
cg02887458	19	19495540	GATAD2A	TSS1500	0,272	1,92E-07	6,04E-06
cg08120210	15	66682733	MAP2K1	Body	-0,228	1,92E-07	6,04E-06
cg09238199	13	111097777	COL4A2	Body	-0,263	1,93E-07	6,05E-06
cg00181497	14	71822206		IGR	0,25	1,93E-07	6,05E-06
cg05787705	6	166830864	RPS6KA2	Body	-0,341	1,93E-07	6,05E-06
cg03807411	19	46020926	VASP	ExonBnd	0,209	1,93E-07	6,06E-06
cg09327855	1	236156587	NID1	Body	-0,201	1,93E-07	6,06E-06
cg01186595	11	89954679	CHORDC1	Body	-0,256	1,93E-07	6,06E-06
cg05106502	17	46506947	SKAP1	Body	-0,238	1,93E-07	6,06E-06
cg04341591	5	54357769		IGR	-0,231	1,93E-07	6,06E-06
cg26499547	14	69425439	ACTN1	Body	0,205	1,93E-07	6,06E-06
cg10293217	17	42678919		IGR	-0,214	1,94E-07	6,07E-06
cg05685023	3	187870940	LPP	TSS1500	0,24	1,94E-07	6,07E-06
cg17587997	6	112085781	FYN	5'UTR	-0,233	1,94E-07	6,07E-06
cg14860676	20	48393157		IGR	-0,238	1,94E-07	6,07E-06
cg12950181	5	93447648	FAM172A	TSS1500	0,206	1,94E-07	6,07E-06
cg20322837	11	57005876	APLNR	TSS1500	-0,209	1,94E-07	6,08E-06
cg02867709	14	60689765		IGR	0,224	1,94E-07	6,08E-06
cg12700710	22	25472360	KIAA1671	Body	-0,27	1,94E-07	6,08E-06
cg16979165	9	97786891	C9orf3	Body	0,251	1,95E-07	6,09E-06
cg08841257	8	10191002	MSRA	Body	-0,229	1,95E-07	6,09E-06
cg12904262	16	10937069		IGR	-0,204	1,95E-07	6,09E-06
cg04764187	2	148703364	ORC4	Body	0,293	1,96E-07	6,11E-06
cg06948187	9	34403522	FAM219A	Body	-0,27	1,96E-07	6,11E-06
cg04792365	12	5126150		IGR	-0,203	1,96E-07	6,11E-06
cg10929685	7	2446794	CHST12	5'UTR	-0,224	1,96E-07	6,11E-06
cg21733226	10	135214398	MTG1	Body	-0,381	1,96E-07	6,12E-06

cg02264229	14	95240560		IGR	0,219	1,96E-07	6,13E-06
cg00613369	1	28521427	PTAFR	TSS1500	-0,213	1,96E-07	6,13E-06
cg03416917	15	71404087	CT62	Body	-0,211	1,97E-07	6,14E-06
cg20708282	5	127234683		IGR	-0,239	1,97E-07	6,14E-06
cg18211315	8	98437059	LOC1019270(	Body	-0,215	1,97E-07	6,14E-06
cg05372679	15	48064730	SEMA6D	3'UTR	-0,207	1,97E-07	6,14E-06
cg11177552	3	25347174		IGR	-0,213	1,97E-07	6,15E-06
cg21531916	7	75608553	POR	Body	0,248	1,97E-07	6,15E-06
cg13804090	6	143105744	HIVEP2	5'UTR	-0,271	1,97E-07	6,15E-06
cg10243030	20	16156233		IGR	-0,207	1,98E-07	6,15E-06
cg02554272	12	3835876	CRACR2A	5'UTR	0,266	1,98E-07	6,16E-06
cg08663999	3	150089701		IGR	0,256	1,98E-07	6,16E-06
cg01967102	3	20145874	KAT2B	Body	-0,281	1,98E-07	6,17E-06
cg02350755	4	55898322		IGR	-0,271	1,98E-07	6,17E-06
cg14104356	6	15715552		IGR	-0,208	1,98E-07	6,17E-06
cg00065944	13	77015067		IGR	-0,223	1,98E-07	6,17E-06
cg02820318	9	135331299	C9orf171	Body	-0,202	1,98E-07	6,17E-06
cg12721429	10	45220304		IGR	-0,211	1,98E-07	6,17E-06
cg07599737	9	35929835		IGR	-0,259	1,99E-07	6,18E-06
cg00348836	7	128367251	FAM71F1	Body	-0,207	1,99E-07	6,18E-06
cg24367957	1	161931993		IGR	-0,248	1,99E-07	6,18E-06
cg16874554	7	35756275	LOC1005067:	Body	0,207	1,99E-07	6,18E-06
cg13896387	11	122357689		IGR	-0,233	1,99E-07	6,18E-06
cg13133338	11	33485781		IGR	-0,208	1,99E-07	6,18E-06
cg19214097	2	202814249		IGR	0,308	1,99E-07	6,18E-06
cg10823818	18	9025668		IGR	-0,201	1,99E-07	6,19E-06
cg10952495	19	31792367	TSHZ3	Body	-0,246	1,99E-07	6,19E-06
cg18716255	1	38184739	EPHA10	Body	-0,215	1,99E-07	6,19E-06
cg21471707	11	18433748	LDHC	TSS200	0,246	1,99E-07	6,20E-06
cg17618902	14	51133534	SAV1	Body	0,287	2,00E-07	6,20E-06
cg19159978	14	91029235	TTC7B	Body	-0,213	2,00E-07	6,20E-06
cg11191814	1	41311012		IGR	-0,313	2,00E-07	6,20E-06
cg17779063	5	142922486		IGR	0,212	2,00E-07	6,20E-06
cg22759823	19	57610870		IGR	0,204	2,00E-07	6,20E-06
cg07818846	4	88964580	PKD2	Body	0,229	2,00E-07	6,20E-06
cg19903071	2	160473251	BAZ2B	TSS200	0,236	2,00E-07	6,20E-06
cg27252325	12	49745406	DNAJC22	3'UTR	-0,211	2,00E-07	6,21E-06
cg14288848	6	33282873	ZBTB22	Body	0,207	2,00E-07	6,21E-06
cg14094841	20	3692262		IGR	-0,251	2,00E-07	6,21E-06
cg07696516	4	41614958	LIMCH1	1stExon	0,264	2,00E-07	6,21E-06
cg21468885	6	88758044	SPACA1	Body	0,22	2,00E-07	6,21E-06
cg06847624	5	176827671	PFN3	TSS200	0,23	2,00E-07	6,22E-06
cg10291238	13	21423447	XPO4	Body	0,242	2,01E-07	6,22E-06
cg07884764	11	64107517	CCDC88B	TSS200	-0,233	2,01E-07	6,23E-06
cg15779457	1	244481810		IGR	-0,22	2,01E-07	6,23E-06
cg18377857	5	81144352		IGR	0,228	2,01E-07	6,23E-06
cg00969893	15	63133069	TLN2	3'UTR	-0,256	2,01E-07	6,23E-06
cg00045114	1	172674159		IGR	-0,215	2,02E-07	6,24E-06
cg18071712	7	117417798	CTTNBP2	Body	-0,213	2,02E-07	6,24E-06
cg14063916	6	43351415		IGR	-0,317	2,02E-07	6,24E-06

cg03689403	12	89749377		IGR	0,203	2,02E-07	6,24E-06
cg15989436	5	150465875		IGR	-0,217	2,02E-07	6,24E-06
cg16790524	22	23161353		IGR	-0,215	2,02E-07	6,24E-06
cg05414516	1	42268969	HIVEP3	5'UTR	-0,308	2,02E-07	6,25E-06
cg17607024	10	126630765	ZRANB1	1stExon	0,244	2,02E-07	6,25E-06
cg14442455	10	15726980	ITGA8	Body	-0,378	2,02E-07	6,26E-06
cg27080019	22	28103394		IGR	-0,211	2,03E-07	6,26E-06
cg12596733	6	2124644	GMDS	Body	0,203	2,03E-07	6,26E-06
cg27303578	6	110813794		IGR	-0,211	2,03E-07	6,26E-06
cg12024811	3	123979446	KALRN	Body	-0,226	2,03E-07	6,26E-06
cg17189671	8	61846477		IGR	-0,207	2,03E-07	6,26E-06
cg22123915	1	86047807	CYR61	Body	0,237	2,03E-07	6,28E-06
cg26416745	1	34093069	CSMD2	Body	-0,216	2,04E-07	6,28E-06
cg04069273	20	31340348		IGR	0,222	2,04E-07	6,28E-06
cg09453312	5	156608118	ITK	1stExon	-0,204	2,04E-07	6,28E-06
cg06636678	11	104576302		IGR	-0,275	2,04E-07	6,29E-06
cg11970806	17	56066485	VEZF1	TSS1500	0,218	2,04E-07	6,29E-06
cg01055594	2	20190349	WDR35	TSS1500	0,269	2,04E-07	6,29E-06
cg11988321	5	138725622	MZB1	TSS200	-0,264	2,04E-07	6,29E-06
cg07725303	5	82730775		IGR	-0,26	2,04E-07	6,29E-06
cg08854008	1	9714397	C1orf200	Body	-0,229	2,04E-07	6,29E-06
cg25372631	12	115446961		IGR	-0,209	2,04E-07	6,30E-06
cg06611805	3	56722340		IGR	-0,232	2,05E-07	6,30E-06
cg00317852	10	126728201	CTBP2	5'UTR	0,221	2,05E-07	6,30E-06
cg00958197	7	28474389	CREB5	TSS1500	-0,226	2,05E-07	6,30E-06
cg12251779	22	45608440	C22orf9	Body	0,21	2,05E-07	6,31E-06
cg23521163	14	77287324		IGR	-0,221	2,05E-07	6,31E-06
cg16936094	10	15164788	NMT2	Body	-0,227	2,05E-07	6,32E-06
cg11321893	22	32931606	SYN3	Body	-0,232	2,06E-07	6,32E-06
cg08569460	5	149722259		IGR	-0,285	2,06E-07	6,32E-06
cg25230111	19	56159817	CCDC106	5'UTR	0,213	2,06E-07	6,32E-06
cg06567920	4	129582864		IGR	-0,234	2,06E-07	6,33E-06
cg05097447	16	17399733	XYLT1	Body	-0,237	2,06E-07	6,33E-06
cg14362105	1	91000048		IGR	-0,211	2,06E-07	6,33E-06
cg16524733	11	117070046	TAGLN	5'UTR	0,353	2,06E-07	6,33E-06
cg24813323	12	63242527	PPM1H	Body	-0,256	2,06E-07	6,33E-06
cg14928893	9	129836981	RALGPS1	Body	0,208	2,06E-07	6,34E-06
cg11919577	8	119291748	SAMD12	Body	-0,287	2,06E-07	6,34E-06
cg00942756	5	43703187	NNT	Body	0,261	2,07E-07	6,35E-06
cg00607050	4	89733526	FAM13A	Body	-0,211	2,07E-07	6,35E-06
cg10985150	6	150989765	PLEKHG1	5'UTR	0,31	2,07E-07	6,35E-06
cg10688755	9	130788900		IGR	-0,35	2,07E-07	6,35E-06
cg15174246	22	30663901	OSM	TSS1500	-0,282	2,07E-07	6,35E-06
cg23076537	10	93643678		IGR	0,202	2,07E-07	6,36E-06
cg17250929	1	153513810	S100A5	Body	-0,237	2,07E-07	6,36E-06
cg07931368	14	73927768		IGR	0,245	2,08E-07	6,36E-06
cg13748799	10	11056954	CUGBP2	Body	0,221	2,08E-07	6,36E-06
cg17676618	10	80563421		IGR	-0,212	2,08E-07	6,36E-06
cg09847717	11	126318677	KIRREL3	Body	-0,211	2,08E-07	6,36E-06
cg09366265	17	40858947	EZH1	Body	-0,206	2,08E-07	6,37E-06

cg26491653	12	1280948	ERC1	Body	0,244	2,08E-07	6,37E-06
cg26842802	2	202125212	CASP8	5'UTR	-0,346	2,09E-07	6,38E-06
cg27607995	1	17886632	ARHGEF10L	5'UTR	-0,212	2,09E-07	6,38E-06
cg10725892	2	175460353	WIPF1	5'UTR	-0,311	2,09E-07	6,38E-06
cg15113334	16	55600544	CAPNS2	TSS200	-0,25	2,09E-07	6,38E-06
cg20033737	22	28059945		IGR	-0,227	2,09E-07	6,39E-06
cg23610213	6	4802019	CDYL	Body	-0,213	2,09E-07	6,39E-06
cg08928261	4	40240766	RHOH	5'UTR	-0,264	2,09E-07	6,39E-06
cg26158194	11	1874320	LSP1	5'UTR	-0,284	2,09E-07	6,40E-06
cg13002740	20	62367632	LIME1	5'UTR	-0,289	2,09E-07	6,40E-06
cg23012133	11	72320716	PDE2A	Body	-0,201	2,10E-07	6,40E-06
cg26732155	15	37236977	MEIS2	Body	0,212	2,10E-07	6,40E-06
cg14047387	9	4080919	GLIS3	Body	-0,265	2,10E-07	6,41E-06
cg21692534	3	142663498		IGR	-0,302	2,10E-07	6,41E-06
cg01673307	6	32819911	TAP1	Body	-0,297	2,10E-07	6,41E-06
cg03948182	17	62348168		IGR	-0,222	2,10E-07	6,41E-06
cg11860896	6	37476192	LOC1005055[illegible]	Body	-0,2	2,10E-07	6,42E-06
cg12231009	14	69152471		IGR	-0,279	2,11E-07	6,42E-06
cg17521868	10	24755299	KIAA1217	TSS200	-0,286	2,11E-07	6,43E-06
cg19214331	20	35169260	MYL9	TSS1500	0,208	2,11E-07	6,43E-06
cg26175448	17	39072167		IGR	-0,202	2,11E-07	6,43E-06
cg04936213	9	124009589		IGR	0,226	2,11E-07	6,43E-06
cg02691035	16	89163800	ACSF3	Body	-0,251	2,11E-07	6,43E-06
cg18615133	12	116997095	MAP1LC3B2	TSS200	-0,33	2,11E-07	6,43E-06
cg00792015	2	218466469	DIRC3	Body	-0,215	2,11E-07	6,43E-06
cg00167836	11	112131713	C11orf34	TSS200	-0,216	2,11E-07	6,43E-06
cg14063530	22	17702092	CECR1	5'UTR	-0,214	2,11E-07	6,44E-06
cg23197939	17	78039368	CCDC40	Body	0,232	2,12E-07	6,46E-06
cg01456691	6	11382556	NEDD9	1stExon	-0,232	2,13E-07	6,46E-06
cg04994397	15	89673485	ABHD2	Body	-0,231	2,13E-07	6,46E-06
cg08426081	1	43313732	ZNF691	5'UTR	-0,211	2,13E-07	6,47E-06
cg02789354	1	25357289		IGR	-0,203	2,13E-07	6,47E-06
cg14408997	9	34050096	UBAP2	TSS1500	0,234	2,13E-07	6,47E-06
cg04244937	6	127663631	ECHDC1	TSS200	0,209	2,13E-07	6,47E-06
cg13397939	9	91273538		IGR	0,207	2,13E-07	6,47E-06
cg04197436	3	15847439	ANKRD28	Body	-0,253	2,13E-07	6,48E-06
cg06893296	6	30095136		IGR	0,272	2,13E-07	6,48E-06
cg00771752	14	25142840		IGR	-0,264	2,13E-07	6,48E-06
cg17934470	5	49959703		IGR	0,224	2,14E-07	6,49E-06
cg10405645	1	160407908		IGR	-0,274	2,14E-07	6,49E-06
cg23461448	2	62541312		IGR	-0,297	2,14E-07	6,49E-06
cg00321478	1	197238398	CRB1	Body	0,224	2,14E-07	6,50E-06
cg11512009	17	38220694	THRA	5'UTR	0,221	2,14E-07	6,50E-06
cg21400303	2	197048025		IGR	-0,233	2,15E-07	6,51E-06
cg18001457	1	229828242		IGR	-0,299	2,15E-07	6,51E-06
cg01965984	4	7983493	ABLIM2	Body	-0,245	2,15E-07	6,51E-06
cg03078767	11	111154742	C11orf53	Body	0,235	2,15E-07	6,52E-06
cg20538211	4	58027287		IGR	-0,257	2,15E-07	6,52E-06
cg04685228	5	172462626		IGR	-0,237	2,15E-07	6,52E-06
cg12744966	15	96715626	NR2F2-AS1	Body	-0,211	2,15E-07	6,52E-06

cg16879857	2	56274495	MIR217HG	TSS200	0,222	2,15E-07	6,52E-06
cg09456758	14	61329252	MNAT1	Body	0,218	2,15E-07	6,52E-06
cg02510381	2	25815015	DTNB	Body	-0,294	2,15E-07	6,52E-06
cg06483899	9	130667623	ST6GALNAC6	1stExon	-0,246	2,15E-07	6,52E-06
cg03253449	9	14213559	NFIB	Body	-0,288	2,15E-07	6,52E-06
cg06189126	14	25211322		IGR	-0,289	2,15E-07	6,52E-06
cg07795766	22	45608516	C22orf9	Body	0,304	2,15E-07	6,52E-06
cg27009285	5	66563232		IGR	-0,205	2,16E-07	6,52E-06
cg16861615	10	90031307		IGR	-0,325	2,16E-07	6,52E-06
cg14717263	2	101505759	NPAS2	5'UTR	-0,203	2,16E-07	6,53E-06
cg16564576	6	109771173	MICAL1	ExonBnd	-0,234	2,16E-07	6,54E-06
cg18483459	2	203294144	BMPR2	Body	0,277	2,17E-07	6,54E-06
cg06769820	4	54554571		IGR	-0,219	2,17E-07	6,55E-06
cg08422803	21	46341067	ITGB2	TSS200	-0,22	2,17E-07	6,55E-06
cg12413381	1	231719135	TSNAX-DISC1	Body	0,209	2,17E-07	6,56E-06
cg04244970	1	160708987	SLAMF7	TSS200	-0,224	2,17E-07	6,56E-06
cg24476449	17	25799212	KSR1	5'UTR	-0,247	2,17E-07	6,56E-06
cg18884865	3	151911580		IGR	-0,335	2,18E-07	6,58E-06
cg02573566	1	153599479	S100A13	5'UTR	0,213	2,18E-07	6,58E-06
cg06898048	10	33614690	NRP1	Body	0,26	2,18E-07	6,58E-06
cg01362959	6	110632518	METTL24	Body	-0,258	2,18E-07	6,58E-06
cg03699176	1	183860765	RGL1	Body	-0,252	2,18E-07	6,58E-06
cg03459185	10	15250411		IGR	-0,205	2,18E-07	6,58E-06
cg25241888	20	36615579	TTI1	Body	0,301	2,18E-07	6,59E-06
cg08595555	11	44942110	TSPAN18	Body	-0,206	2,19E-07	6,59E-06
cg21536828	12	853169		IGR	-0,242	2,19E-07	6,59E-06
cg03413950	6	128581074	PTPRK	Body	-0,295	2,19E-07	6,59E-06
cg01394709	1	221243086		IGR	-0,367	2,19E-07	6,59E-06
cg06559878	5	156642345	ITK	Body	-0,201	2,19E-07	6,60E-06
cg10732871	19	1102675	GPX4	TSS1500	-0,202	2,19E-07	6,60E-06
cg20855972	2	11057244		IGR	-0,212	2,19E-07	6,61E-06
cg06926377	15	40443853		IGR	0,311	2,20E-07	6,61E-06
cg19641804	12	54653065	CBX5	1stExon	0,221	2,20E-07	6,61E-06
cg13647033	2	38645840		IGR	-0,226	2,20E-07	6,62E-06
cg10847870	11	13359223	ARNTL	5'UTR	-0,202	2,20E-07	6,62E-06
cg10205038	14	93052501	RIN3	Body	-0,255	2,20E-07	6,62E-06
cg05544413	6	127663627	ECHDC1	5'UTR	0,211	2,21E-07	6,63E-06
cg04603068	18	42290070	SETBP1	Body	-0,274	2,21E-07	6,63E-06
cg24188163	8	39782018	IDO1	Body	0,236	2,21E-07	6,64E-06
cg07369569	6	55444821	HMGCLL1	TSS1500	-0,227	2,21E-07	6,64E-06
cg07595606	15	49372235		IGR	-0,217	2,21E-07	6,64E-06
cg14643892	12	64784070	C12orf56	Body	0,206	2,22E-07	6,65E-06
cg04617967	6	53687400	LRRC1	Body	0,258	2,22E-07	6,65E-06
cg08004377	2	200231190	SATB2	Body	-0,292	2,22E-07	6,65E-06
cg03402144	4	114495910	CAMK2D	Body	0,212	2,22E-07	6,65E-06
cg10571421	9	91405027		IGR	-0,206	2,22E-07	6,66E-06
cg24221738	4	166125552		IGR	-0,25	2,22E-07	6,66E-06
cg15100426	2	219187432	PNKD	TSS1500	0,28	2,22E-07	6,66E-06
cg07697234	10	33405714		IGR	-0,212	2,22E-07	6,67E-06
cg06422189	6	31322766	HLA-B	Body	-0,331	2,23E-07	6,67E-06

cg10813010	7	92360796	CDK6	Body	-0,251	2,23E-07	6,68E-06
cg18776616	10	72235645		IGR	-0,234	2,23E-07	6,68E-06
cg09373825	18	33198928		IGR	0,306	2,23E-07	6,68E-06
cg18575710	22	28569035	TTC28	Body	-0,223	2,23E-07	6,68E-06
cg22910748	1	21910078		IGR	-0,216	2,23E-07	6,68E-06
cg17430666	6	140330573	LOC10050747	Body	0,228	2,23E-07	6,68E-06
cg19451463	3	37137955	LRRFIP2	Body	0,26	2,23E-07	6,69E-06
cg00031346	6	168478519	FRMD1	Body	-0,206	2,24E-07	6,69E-06
cg02091185	5	170288766	RANBP17	TSS1500	0,293	2,24E-07	6,69E-06
cg10613215	6	143234755	HIVEP2	5'UTR	-0,3	2,24E-07	6,71E-06
cg10170944	12	57599717	LRP1	Body	-0,238	2,24E-07	6,71E-06
cg05635798	18	573825		IGR	-0,211	2,24E-07	6,71E-06
cg26219843	5	72247648		IGR	-0,23	2,25E-07	6,72E-06
cg04157658	11	110243994		IGR	-0,251	2,25E-07	6,72E-06
cg17794604	11	111789615	C11orf52	5'UTR	0,253	2,25E-07	6,72E-06
cg20433386	17	48702158	CACNA1G	Body	-0,221	2,25E-07	6,72E-06
cg19316123	2	190043537	COL5A2	Body	0,268	2,25E-07	6,72E-06
cg07331725	18	61157478	SERPINB5	Body	0,208	2,25E-07	6,72E-06
cg08423443	20	56621504		IGR	-0,284	2,25E-07	6,73E-06
cg26036406	10	123754994	TACC2	5'UTR	0,232	2,26E-07	6,73E-06
cg22685215	1	178232856	RASAL2	Body	-0,272	2,26E-07	6,73E-06
cg11274856	3	185301563		IGR	0,222	2,26E-07	6,73E-06
cg17168836	1	68256161	GNG12	5'UTR	-0,21	2,26E-07	6,73E-06
cg17325206	15	91396109		IGR	-0,294	2,26E-07	6,73E-06
cg19446492	11	44278707		IGR	-0,217	2,26E-07	6,74E-06
cg17031697	12	94602306	PLXNC1	Body	-0,224	2,26E-07	6,75E-06
cg03764544	1	33835013	PHC2	Body	-0,201	2,26E-07	6,75E-06
cg23352530	10	98136448	TLL2	ExonBnd	-0,204	2,26E-07	6,75E-06
cg04593523	12	11912203	ETV6	Body	0,207	2,26E-07	6,75E-06
cg16705489	7	128500263		IGR	0,222	2,26E-07	6,75E-06
cg07732735	15	99790022	TTC23	TSS200	0,279	2,26E-07	6,75E-06
cg11401986	9	91604088		IGR	-0,278	2,26E-07	6,75E-06
cg13479077	20	30150918	HM13	Body	0,204	2,27E-07	6,76E-06
cg21929472	8	27527865	SCARA3	Body	-0,204	2,27E-07	6,76E-06
cg06246510	8	101504720		IGR	-0,289	2,27E-07	6,76E-06
cg00843973	12	10367690	GABARAPL1	Body	0,222	2,27E-07	6,76E-06
cg27590090	7	21963488	CDCA7L	Body	0,238	2,27E-07	6,77E-06
cg12905085	1	32054510		IGR	0,207	2,27E-07	6,77E-06
cg06363465	2	125044021	CNTNAP5	Body	-0,209	2,27E-07	6,77E-06
cg12741639	22	37678728	CYTH4	Body	-0,218	2,28E-07	6,77E-06
cg24976037	6	54101786	MLIP	Body	-0,222	2,28E-07	6,78E-06
cg18806561	3	23803797		IGR	-0,241	2,28E-07	6,78E-06
cg21459985	8	995913	ERICH1-AS1	Body	0,203	2,28E-07	6,78E-06
cg13721515	8	74335868	STAU2-AS1	Body	0,239	2,28E-07	6,78E-06
cg24486037	1	151345181	SELENBP1	TSS200	0,309	2,28E-07	6,79E-06
cg24302752	6	80853625	BCKDHB	Body	0,234	2,28E-07	6,79E-06
cg06743153	12	104194370	NT5DC3	Body	0,253	2,28E-07	6,79E-06
cg12741572	18	57565356		IGR	0,225	2,28E-07	6,79E-06
cg14145667	19	909533	C19orf22	Body	-0,278	2,29E-07	6,80E-06
cg14591730	11	65902164	PACS1	Body	-0,248	2,29E-07	6,80E-06

cg16103542	10	112127915		IGR	-0,2	2,30E-07	6,82E-06
cg15579084	10	129294626		IGR	0,22	2,30E-07	6,83E-06
cg01353265	2	95716533	MAL	Body	0,218	2,30E-07	6,83E-06
cg03249723	9	98880057	LOC158434	TSS1500	-0,247	2,30E-07	6,83E-06
cg26483574	1	205543936	MFSD4	Body	-0,254	2,30E-07	6,83E-06
cg20187011	21	28214928	ADAMTS1	Body	0,258	2,31E-07	6,83E-06
cg03746749	3	185471179	IGF2BP2	Body	-0,201	2,31E-07	6,84E-06
cg02452586	1	233497903	KIAA1804	Body	0,236	2,31E-07	6,84E-06
cg17364089	21	40139776	LINC00114	Body	-0,264	2,31E-07	6,84E-06
cg11578213	6	144636859	UTRN	Body	0,244	2,31E-07	6,84E-06
cg21259553	11	63818078	MACROD1	Body	-0,206	2,31E-07	6,85E-06
cg25818097	2	134975778		IGR	-0,324	2,31E-07	6,85E-06
cg06738030	2	217487494		IGR	-0,228	2,32E-07	6,86E-06
cg23984989	21	40383685		IGR	-0,209	2,32E-07	6,87E-06
cg22842189	3	24401786	THRB	5'UTR	-0,215	2,32E-07	6,87E-06
cg14937293	1	177928080	SEC16B	Body	-0,207	2,32E-07	6,87E-06
cg13646880	2	102685846	IL1R1	TSS1500	0,246	2,32E-07	6,88E-06
cg27646412	10	77501817	C10orf11	Body	-0,222	2,33E-07	6,88E-06
cg27372467	10	124201479		IGR	-0,2	2,33E-07	6,88E-06
cg07225555	8	142264800	SLC45A4	TSS200	0,234	2,33E-07	6,89E-06
cg02197634	6	33048875	HLA-DPB1	Body	0,321	2,33E-07	6,90E-06
cg15526535	1	12238546	TNFRSF1B	Body	-0,223	2,34E-07	6,90E-06
cg04907595	1	25258264	RUNX3	TSS1500	0,205	2,34E-07	6,91E-06
cg00765992	1	210462861		IGR	-0,201	2,34E-07	6,91E-06
cg03043243	2	172172701		IGR	0,218	2,34E-07	6,91E-06
cg20699586	1	227748719		IGR	0,253	2,34E-07	6,91E-06
cg27367066	15	34662635		IGR	-0,215	2,34E-07	6,91E-06
cg11080513	2	135426596	TMEM163	Body	-0,244	2,34E-07	6,92E-06
cg10699171	6	24936965		IGR	-0,321	2,34E-07	6,92E-06
cg26098117	6	125803002		IGR	-0,256	2,34E-07	6,92E-06
cg11549713	2	55001373	EML6	Body	-0,224	2,34E-07	6,92E-06
cg12286415	13	30948122	LOC1001889	TSS200	-0,235	2,34E-07	6,92E-06
cg10380221	16	31075618	ZNF668	Body	-0,219	2,35E-07	6,92E-06
cg08471952	1	30569592		IGR	-0,202	2,35E-07	6,93E-06
cg04202047	17	28671936		IGR	0,213	2,35E-07	6,93E-06
cg20766052	6	14744370		IGR	-0,346	2,35E-07	6,93E-06
cg09144495	3	64585981	ADAMTS9	Body	-0,203	2,35E-07	6,93E-06
cg22178121	4	25022049	LGI2	Body	-0,233	2,36E-07	6,94E-06
cg18625270	12	47777775		IGR	0,287	2,36E-07	6,94E-06
cg18791617	3	52409637	DNAH1	Body	-0,203	2,36E-07	6,94E-06
cg27526774	1	235099089		IGR	-0,203	2,36E-07	6,94E-06
cg01441041	16	19448742	TMC5	5'UTR	-0,208	2,36E-07	6,94E-06
cg09516664	21	46514212	ADARB1	5'UTR	0,253	2,36E-07	6,95E-06
cg25715498	1	160492991	SLAMF6	1stExon	-0,239	2,37E-07	6,96E-06
cg15137734	3	11703398	VGLL4	Body	0,217	2,37E-07	6,96E-06
cg17603813	5	151007330		IGR	0,221	2,37E-07	6,96E-06
cg26857432	16	68306547	SLC7A6	5'UTR	0,213	2,37E-07	6,96E-06
cg15814766	17	2140942	SMG6	TSS1500	0,212	2,37E-07	6,96E-06
cg22109433	18	13472332	LDLRAD4	Body	0,207	2,37E-07	6,97E-06
cg07732037	12	123707827	MPHOSPH9	TSS1500	-0,211	2,37E-07	6,97E-06

cg18537923	3	114171375	LOC10192975	TSS1500	0,218	2,37E-07	6,97E-06
cg04149015	16	31159854	PRSS36	Body	0,214	2,38E-07	6,98E-06
cg18113803	1	220977858	MOSC1	Body	-0,243	2,38E-07	6,99E-06
cg11828888	19	9977094	OLFM2	Body	-0,224	2,38E-07	7,00E-06
cg13588483	12	24549226	SOX5	5'UTR	0,212	2,39E-07	7,00E-06
cg11380577	10	81082872		IGR	-0,321	2,39E-07	7,01E-06
cg23200931	14	45367690	C14orf28	5'UTR	0,246	2,39E-07	7,01E-06
cg07036235	22	44705216	KIAA1644	5'UTR	-0,22	2,39E-07	7,01E-06
cg13881376	1	183259923	NMNAT2	Body	-0,209	2,39E-07	7,01E-06
cg05149104	18	23795837		IGR	-0,237	2,39E-07	7,01E-06
cg24363732	22	43333586	PACSIN2	5'UTR	-0,225	2,39E-07	7,01E-06
cg08949181	22	46352496	WNT7B	Body	0,276	2,39E-07	7,02E-06
cg03575584	14	69248973		IGR	-0,267	2,40E-07	7,02E-06
cg02499214	12	122230490	RHOF	Body	-0,279	2,40E-07	7,02E-06
cg10025166	8	41400782	GINS4	3'UTR	0,215	2,40E-07	7,02E-06
cg00811567	2	240250595	HDAC4	Body	0,201	2,40E-07	7,02E-06
cg24258347	11	13983851	SPON1	TSS200	0,231	2,40E-07	7,02E-06
cg02811647	5	179757414	GFPT2	Body	-0,211	2,40E-07	7,03E-06
cg16036025	12	111028784		IGR	-0,373	2,40E-07	7,03E-06
cg00619263	5	134471974		IGR	-0,201	2,40E-07	7,03E-06
cg21949194	2	39355435		IGR	-0,218	2,41E-07	7,04E-06
cg11819637	3	184095505	THPO	5'UTR	-0,203	2,41E-07	7,05E-06
cg12569246	6	76203666	FILIP1	TSS200	0,277	2,41E-07	7,05E-06
cg06976598	10	53639124	PRKG1	Body	-0,202	2,41E-07	7,06E-06
cg12386151	2	121628408	GLI2	Body	-0,238	2,42E-07	7,06E-06
cg25550323	11	117694062	FXYD2	Body	-0,246	2,42E-07	7,07E-06
cg17941730	4	119744738	SEC24D	Body	0,248	2,42E-07	7,07E-06
cg18034978	1	179479373	AXDND1	Body	-0,213	2,42E-07	7,08E-06
cg05202888	8	124666629	KLHL38	TSS1500	0,204	2,42E-07	7,08E-06
cg16018427	5	180110371		IGR	0,207	2,43E-07	7,08E-06
cg02243303	6	52147973	MCM3	Body	-0,218	2,43E-07	7,08E-06
cg01785099	8	22756144	PEBP4	Body	-0,25	2,43E-07	7,09E-06
cg18414019	13	99156719	STK24	Body	-0,227	2,43E-07	7,09E-06
cg13667234	17	44256396	KANSL1	5'UTR	-0,243	2,43E-07	7,09E-06
cg01116484	11	116943299	SIK3	Body	-0,222	2,43E-07	7,10E-06
cg12169306	4	40230325	RHOH	5'UTR	-0,248	2,44E-07	7,10E-06
cg22332066	1	210501621	HHAT	TSS1500	0,203	2,44E-07	7,11E-06
cg12300750	3	98497349	ST3GAL6	5'UTR	-0,275	2,44E-07	7,11E-06
cg25867318	17	40494745	STAT3	Body	-0,296	2,44E-07	7,11E-06
cg25816160	1	93053203	EVI5	Body	-0,226	2,44E-07	7,11E-06
cg25666403	22	30662994	OSM	TSS200	-0,211	2,44E-07	7,12E-06
cg15979123	6	155632375	TFB1M	Body	0,281	2,44E-07	7,12E-06
cg05647041	12	24897401		IGR	0,261	2,45E-07	7,12E-06
cg15479997	22	37441214		IGR	-0,257	2,45E-07	7,12E-06
cg03185009	17	3974329	ZZEF1	Body	0,245	2,45E-07	7,12E-06
cg04134539	9	97623475	C9orf3	Body	-0,328	2,45E-07	7,13E-06
cg02768585	6	109557073	LOC10099663	TSS1500	-0,258	2,45E-07	7,13E-06
cg03664302	6	135407598		IGR	-0,206	2,45E-07	7,13E-06
cg18983337	17	19406038		IGR	-0,227	2,45E-07	7,13E-06
cg14710896	10	24671718	KIAA1217	Body	-0,259	2,45E-07	7,13E-06

cg18046677	19	49220142	MAMSTR	1stExon	0,288	2,45E-07	7,13E-06
cg08752141	3	58149979	FLNB	Body	0,24	2,45E-07	7,13E-06
cg17706411	6	109780058		IGR	-0,264	2,45E-07	7,13E-06
cg14979034	9	132342382		IGR	-0,273	2,45E-07	7,13E-06
cg01903374	1	151129844	TNFAIP8L2	5'UTR	-0,252	2,46E-07	7,14E-06
cg07623567	6	32909523	HLA-DMB	TSS1500	-0,267	2,46E-07	7,14E-06
cg16720578	14	54410717		IGR	-0,228	2,46E-07	7,14E-06
cg13112407	2	97204931	ARID5A	Body	-0,245	2,46E-07	7,15E-06
cg07777362	12	88785541		IGR	-0,292	2,46E-07	7,15E-06
cg23790641	8	25110025	DOCK5	Body	0,235	2,46E-07	7,15E-06
cg17306848	14	61793064	PRKCH	Body	-0,228	2,46E-07	7,15E-06
cg02902546	16	50136493	HEATR3	Body	-0,27	2,46E-07	7,15E-06
cg19040173	17	61356602	TANC2	Body	-0,221	2,46E-07	7,16E-06
cg07110142	5	142435493	ARHGAP26	Body	-0,26	2,46E-07	7,16E-06
cg15836151	8	142264807	SLC45A4	TSS200	0,257	2,46E-07	7,16E-06
cg03387716	4	36244174	ARAP2	5'UTR	-0,242	2,47E-07	7,16E-06
cg18367727	6	167810961		IGR	-0,203	2,47E-07	7,16E-06
cg11403608	7	96125487	C7orf76	Body	-0,284	2,47E-07	7,17E-06
cg16338736	16	3117771	IL32	Body	-0,219	2,47E-07	7,17E-06
cg21321626	15	68325193		IGR	-0,231	2,47E-07	7,17E-06
cg14764476	5	143138515		IGR	-0,26	2,47E-07	7,17E-06
cg03812676	11	67254381	AIP	Body	-0,226	2,47E-07	7,17E-06
cg04864107	21	46046393	KRTAP10-9	TSS1500	-0,253	2,47E-07	7,17E-06
cg09018746	1	117312085		IGR	-0,257	2,47E-07	7,17E-06
cg20042412	2	178100858	NFE2L2	Body	0,218	2,47E-07	7,18E-06
cg00260810	3	183894469	AP2M1	5'UTR	0,206	2,48E-07	7,18E-06
cg14087199	10	103850639		IGR	0,256	2,48E-07	7,18E-06
cg12696328	1	227729680		IGR	0,206	2,48E-07	7,18E-06
cg10364923	11	124952899	SLC37A2	Body	-0,224	2,48E-07	7,19E-06
cg13974615	1	60209701	FGGY	Body	0,251	2,48E-07	7,19E-06
cg12774649	2	216468225		IGR	-0,2	2,49E-07	7,20E-06
cg02973778	6	17634009	NUP153	Body	-0,323	2,49E-07	7,20E-06
cg09966895	11	78738326	ODZ4	Body	-0,299	2,49E-07	7,21E-06
cg02392278	3	171279890		IGR	-0,208	2,49E-07	7,21E-06
cg01703004	12	51718155	BIN2	TSS200	-0,302	2,49E-07	7,21E-06
cg23683779	13	52755638	MRPS31P5	Body	0,214	2,49E-07	7,21E-06
cg03181524	19	39048014	RYR1	Body	-0,223	2,50E-07	7,22E-06
cg05851594	8	23883186		IGR	-0,24	2,50E-07	7,22E-06
cg04548204	12	9162872	KLRG1	3'UTR	-0,223	2,50E-07	7,23E-06
cg14579734	8	142065135		IGR	-0,22	2,50E-07	7,23E-06
cg02684057	18	53778561	LINC01539	Body	-0,27	2,50E-07	7,23E-06
cg16697344	18	3319784		IGR	-0,239	2,51E-07	7,24E-06
cg00659629	9	37300859	ZCCHC7	Body	-0,207	2,51E-07	7,24E-06
cg01884891	12	116975346		IGR	-0,314	2,51E-07	7,25E-06
cg24639679	6	32847590	PPP1R2P1	Body	0,209	2,51E-07	7,25E-06
cg00362812	14	69201112		IGR	-0,227	2,51E-07	7,25E-06
cg20486668	12	127610222		IGR	-0,216	2,51E-07	7,26E-06
cg03771986	13	51804094	FAM124A	Body	0,227	2,52E-07	7,27E-06
cg09234582	6	33048286	HLA-DPB1	Body	0,244	2,52E-07	7,27E-06
cg13984913	16	72013790	PKD1L3	Body	0,3	2,53E-07	7,28E-06

cg16426858	15	61487998	RORA	Body	-0,256	2,53E-07	7,28E-06
cg03515844	1	16277545	ZBTB17	5'UTR	-0,22	2,53E-07	7,29E-06
cg15369466	22	24124070	MMP11	Body	-0,24	2,53E-07	7,29E-06
cg16627587	5	45268733	HCN1	Body	-0,209	2,53E-07	7,29E-06
cg22677379	3	195106412	ACAP2	Body	0,216	2,54E-07	7,30E-06
cg07800907	12	124950669	NCOR2	Body	-0,217	2,54E-07	7,30E-06
cg12628550	14	91817627	CCDC88C	Body	-0,368	2,54E-07	7,30E-06
cg15236136	2	70336118		IGR	-0,224	2,54E-07	7,30E-06
cg00942219	16	85203635		IGR	0,222	2,54E-07	7,30E-06
cg19954602	6	76203532	FILIP1	TSS200	0,209	2,54E-07	7,31E-06
cg07941983	4	36245138	ARAP2	5'UTR	-0,2	2,54E-07	7,31E-06
cg10608681	5	37723176	WDR70	Body	-0,317	2,55E-07	7,31E-06
cg17173881	15	90388953	AP3S2	Body	-0,225	2,55E-07	7,33E-06
cg16797514	2	202860995		IGR	0,205	2,55E-07	7,33E-06
cg00264110	15	70772760		IGR	-0,342	2,55E-07	7,34E-06
cg27452231	5	126255379	MARCH3	5'UTR	0,204	2,56E-07	7,34E-06
cg16853860	6	32823116	PSMB9	Body	-0,222	2,56E-07	7,34E-06
cg09658958	8	56904259	LYN	Body	-0,278	2,56E-07	7,34E-06
cg22304799	2	28877884		IGR	0,26	2,56E-07	7,34E-06
cg04859027	2	175629635	CHRNA1	TSS1500	-0,3	2,56E-07	7,34E-06
cg03602119	20	39190789		IGR	-0,213	2,56E-07	7,35E-06
cg13926208	4	15239597	LOC10192909	Body	-0,207	2,56E-07	7,35E-06
cg02987618	10	30054988		IGR	-0,205	2,56E-07	7,35E-06
cg22443212	17	78253912	RNF213	Body	0,333	2,56E-07	7,35E-06
cg17293973	2	39948248		IGR	-0,212	2,57E-07	7,35E-06
cg02058870	14	72053146	SIPA1L1	5'UTR	0,27	2,57E-07	7,36E-06
cg11089592	6	155371793		IGR	-0,27	2,57E-07	7,36E-06
cg18315468	2	234330835	DGKD	Body	-0,22	2,57E-07	7,36E-06
cg20320455	1	82014804	LOC10192743	Body	-0,235	2,57E-07	7,36E-06
cg15809195	19	14037542	CC2D1A	ExonBnd	0,322	2,57E-07	7,36E-06
cg25498969	3	136062740	STAG1	Body	0,233	2,57E-07	7,36E-06
cg14281039	19	55669189	TNNI3	TSS200	0,212	2,57E-07	7,36E-06
cg09012633	16	90114173	LOC10013001	TSS200	0,206	2,57E-07	7,36E-06
cg05318454	19	46117805	EML2	Body	-0,216	2,57E-07	7,37E-06
cg23538701	2	60795719		IGR	0,23	2,58E-07	7,37E-06
cg13401893	6	30039432	RNF39	Body	0,424	2,58E-07	7,38E-06
cg26912602	1	206644843	IKBKE	5'UTR	-0,219	2,58E-07	7,38E-06
cg03016445	4	110915704	EGF	Body	-0,219	2,58E-07	7,38E-06
cg27643697	8	123997804		IGR	-0,245	2,59E-07	7,39E-06
cg24704344	21	44154904	PDE9A	5'UTR	-0,271	2,59E-07	7,39E-06
cg00965617	1	162328124	NOS1AP	Body	-0,201	2,59E-07	7,39E-06
cg06695406	12	106589841		IGR	-0,23	2,59E-07	7,40E-06
cg21513728	4	23932841		IGR	0,219	2,59E-07	7,40E-06
cg09949775	19	18902107	COMP	1stExon	0,21	2,59E-07	7,40E-06
cg06286328	20	52325081		IGR	-0,276	2,59E-07	7,40E-06
cg21380883	8	143824491	SLURP1	TSS1500	-0,222	2,59E-07	7,41E-06
cg17048700	15	95985725	LOC145820	Body	-0,205	2,59E-07	7,41E-06
cg00902374	3	25461111		IGR	-0,266	2,60E-07	7,41E-06
cg15838148	10	28700847		IGR	-0,245	2,60E-07	7,41E-06
cg06949933	2	61406491	AHSA2	5'UTR	-0,2	2,60E-07	7,41E-06

cg16066235	6	164169838		IGR	-0,234	2,60E-07	7,41E-06
cg25265519	13	111927942	ARHGEF7	Body	0,236	2,60E-07	7,42E-06
cg04902429	17	43301721	FMNL1	Body	-0,237	2,60E-07	7,42E-06
cg21655992	2	61465487	USP34	Body	-0,266	2,60E-07	7,42E-06
cg26725862	10	112404774	RBM20	Body	0,219	2,60E-07	7,43E-06
cg01630046	2	42332600		IGR	-0,31	2,61E-07	7,43E-06
cg17376000	3	14730295	C3orf20	Body	-0,211	2,61E-07	7,43E-06
cg04066596	2	230812360	FBXO36	Body	-0,266	2,61E-07	7,43E-06
cg25308111	2	242383075	FARP2	Body	-0,279	2,61E-07	7,44E-06
cg13767515	3	172633879	SPATA16	Body	-0,22	2,61E-07	7,45E-06
cg22952142	15	68549178		IGR	0,239	2,62E-07	7,45E-06
cg00874480	12	16512895	MGST1	Body	0,227	2,62E-07	7,45E-06
cg17117310	19	3133726		IGR	-0,218	2,62E-07	7,46E-06
cg06751920	1	12704007	AADACL4	TSS1500	-0,217	2,62E-07	7,46E-06
cg03190868	1	178208094	RASAL2	Body	-0,249	2,62E-07	7,46E-06
cg13460464	2	23728776	KLHL29	5'UTR	-0,239	2,62E-07	7,46E-06
cg15574405	2	71776472	DYSF	ExonBnd	-0,214	2,62E-07	7,46E-06
cg26646061	1	93441156		IGR	-0,218	2,62E-07	7,46E-06
cg09500975	15	30202604	TJP1	Body	0,257	2,62E-07	7,46E-06
cg24347498	1	160456459	SLAMF6	3'UTR	0,267	2,63E-07	7,47E-06
cg23397013	10	10571132		IGR	-0,213	2,63E-07	7,47E-06
cg23642827	10	98751284		IGR	-0,251	2,63E-07	7,47E-06
cg08871244	16	88848307	FAM38A	Body	-0,241	2,63E-07	7,47E-06
cg14314352	10	90760909	FAS	Body	-0,233	2,63E-07	7,47E-06
cg12204149	17	43124246	DCAKD	5'UTR	-0,216	2,63E-07	7,48E-06
cg26800264	1	185530998	LINC01350	Body	-0,275	2,63E-07	7,48E-06
cg25769767	22	40859466	MKL1	TSS200	-0,338	2,63E-07	7,48E-06
cg16068833	1	26644515	UBXN11	5'UTR	-0,262	2,63E-07	7,49E-06
cg26841311	1	168118258		IGR	-0,294	2,63E-07	7,49E-06
cg10141611	6	25580716	LRRC16A	Body	-0,235	2,64E-07	7,49E-06
cg23683984	4	105836811		IGR	0,233	2,64E-07	7,50E-06
cg09238502	8	41901211	KAT6A	Body	0,234	2,65E-07	7,51E-06
cg25498638	6	7996863	BLOC1S5-TXN	Body	0,221	2,65E-07	7,51E-06
cg10500147	12	6881601	LAG3	TSS200	-0,225	2,65E-07	7,51E-06
cg02343652	21	30476730	MAP3K7CL	5'UTR	-0,211	2,65E-07	7,52E-06
cg10528361	10	101780405		IGR	-0,206	2,65E-07	7,52E-06
cg01998671	5	127207218		IGR	0,208	2,65E-07	7,52E-06
cg08464190	16	49315302	CBLN1	1stExon	0,205	2,65E-07	7,52E-06
cg21283720	17	6922957	MIR497HG	Body	0,225	2,65E-07	7,52E-06
cg14404727	4	170146550	SH3RF1	Body	-0,237	2,66E-07	7,52E-06
cg11818811	1	151608392	SNX27	Body	-0,215	2,66E-07	7,52E-06
cg11262246	13	96406302	DNAJC3	Body	0,259	2,66E-07	7,53E-06
cg27320439	17	60745135	MRC2	Body	-0,201	2,66E-07	7,54E-06
cg09803092	15	91381597		IGR	-0,283	2,67E-07	7,54E-06
cg12054653	18	9575664	PPP4R1	Body	0,225	2,67E-07	7,54E-06
cg00240178	6	74232108	EEF1A1	TSS1500	-0,2	2,67E-07	7,55E-06
cg24603464	2	238395852	MLPH	TSS200	0,202	2,67E-07	7,55E-06
cg24815934	21	46332181	ITGB2	5'UTR	-0,259	2,67E-07	7,55E-06
cg07161588	3	12779524	TMEM40	Body	-0,242	2,67E-07	7,55E-06
cg17594256	13	46014654		IGR	0,276	2,68E-07	7,57E-06

cg10861599	1	173176523	TNFSF4	TSS200	-0,203	2,68E-07	7,57E-06
cg03365569	17	74507970		IGR	-0,231	2,68E-07	7,57E-06
cg00610748	13	111935077	ARHGEF7	Body	0,244	2,69E-07	7,59E-06
cg11244005	22	26943564	TPST2	5'UTR	-0,227	2,69E-07	7,59E-06
cg20834178	4	6944071	TBC1D14	Body	-0,234	2,69E-07	7,59E-06
cg04744409	6	105388191		IGR	0,238	2,69E-07	7,59E-06
cg16922323	2	169673305	NOSTRIN	5'UTR	-0,233	2,69E-07	7,59E-06
cg03815480	16	48492439	MIR5095	Body	-0,252	2,69E-07	7,60E-06
cg06624369	9	114800463	MIR3134	Body	-0,233	2,69E-07	7,60E-06
cg07570498	1	33813321	PHC2	5'UTR	0,216	2,69E-07	7,60E-06
cg09196534	7	77975971	RPL13AP17	TSS1500	-0,218	2,70E-07	7,61E-06
cg01367992	1	160766535	LY9	Body	-0,251	2,70E-07	7,61E-06
cg08767044	16	11678785	LITAF	5'UTR	-0,316	2,70E-07	7,61E-06
cg18213495	6	17985408	KIF13A	Body	0,234	2,70E-07	7,61E-06
cg08528629	6	50061065		IGR	0,223	2,70E-07	7,62E-06
cg25364041	17	33815814	SLFN12L	TSS1500	0,217	2,70E-07	7,62E-06
cg26916625	21	46385436	FAM207A	Body	-0,264	2,70E-07	7,62E-06
cg11065972	14	92998059	RIN3	Body	-0,289	2,71E-07	7,62E-06
cg12029697	8	66863584		IGR	-0,219	2,71E-07	7,62E-06
cg22549540	2	145388197		IGR	-0,202	2,71E-07	7,62E-06
cg10209670	15	93128681		IGR	-0,203	2,71E-07	7,63E-06
cg22877504	2	216844602	MREG	Body	-0,251	2,71E-07	7,63E-06
cg04450994	6	3318592	SLC22A23	Body	-0,215	2,71E-07	7,63E-06
cg11935738	6	29520752		IGR	0,236	2,71E-07	7,63E-06
cg17298751	11	22363370	SLC17A6	Body	0,252	2,71E-07	7,64E-06
cg21416692	1	33803805	PHC2	5'UTR	0,213	2,72E-07	7,64E-06
cg10709220	9	96337635	PHF2	TSS1500	0,203	2,72E-07	7,65E-06
cg13070581	12	92530939	C12orf79	Body	-0,247	2,72E-07	7,65E-06
cg23170410	6	147964028		IGR	-0,227	2,72E-07	7,65E-06
cg24605511	5	175969064	CDHR2	TSS1500	-0,272	2,72E-07	7,66E-06
cg14889891	16	30482593	ITGAL	TSS1500	-0,21	2,72E-07	7,66E-06
cg09781900	5	177983205	COL23A1	Body	0,248	2,73E-07	7,67E-06
cg10128806	4	78977322	FRAS1	TSS1500	0,217	2,73E-07	7,67E-06
cg12323574	3	23097631		IGR	-0,315	2,73E-07	7,68E-06
cg06496648	1	47488961	CYP4X1	TSS1500	0,234	2,73E-07	7,68E-06
cg01748255	18	56349813	MALT1	Body	0,218	2,73E-07	7,68E-06
cg15408889	5	118623661	TNFAIP8	Body	-0,219	2,73E-07	7,68E-06
cg05210646	11	32843775		IGR	-0,241	2,74E-07	7,69E-06
cg10600295	20	1789945		IGR	-0,245	2,74E-07	7,69E-06
cg12768975	19	6721965	C3	TSS1500	-0,208	2,74E-07	7,69E-06
cg07201319	6	139909641		IGR	0,239	2,74E-07	7,70E-06
cg18733612	1	17964771	ARHGEF10L	Body	-0,234	2,74E-07	7,70E-06
cg12973514	19	16442807		IGR	0,207	2,75E-07	7,71E-06
cg17072045	2	174224060	CDCA7	Body	-0,221	2,75E-07	7,71E-06
cg13203474	5	127873873	FBN2	TSS200	0,238	2,75E-07	7,71E-06
cg25920590	8	29265970		IGR	-0,306	2,75E-07	7,71E-06
cg08611416	3	13284191		IGR	-0,209	2,75E-07	7,71E-06
cg11586570	7	63498133		IGR	0,202	2,75E-07	7,71E-06
cg07230209	1	89164037	PKN2	Body	0,233	2,75E-07	7,72E-06
cg18514720	16	2286395	DNASE1L2	TSS200	0,205	2,76E-07	7,73E-06

cg04270401	10	102806871		IGR	0,214	2,76E-07	7,74E-06
cg16732780	5	175969439	CDHR2	TSS200	-0,245	2,76E-07	7,74E-06
cg11592082	1	27683345	MAP3K6	Body	-0,227	2,77E-07	7,75E-06
cg15892682	1	219836686		IGR	-0,259	2,77E-07	7,75E-06
cg24567724	9	72435964	C9orf135-AS1	TSS1500	0,293	2,77E-07	7,75E-06
cg21786295	19	34352841		IGR	-0,213	2,77E-07	7,75E-06
cg18721145	18	19344185	MIB1	Body	0,257	2,77E-07	7,75E-06
cg12424298	21	44176857	PDE9A	Body	-0,208	2,77E-07	7,75E-06
cg08774089	8	123703052	LINC01151	Body	-0,207	2,77E-07	7,76E-06
cg05673431	17	25871504	KSR1	5'UTR	-0,284	2,77E-07	7,76E-06
cg23512079	2	31349757	GALNT14	5'UTR	-0,237	2,78E-07	7,76E-06
cg14170627	16	87929406	CA5A	Body	-0,229	2,78E-07	7,77E-06
cg25643644	11	118210017	CD3D	Body	-0,208	2,78E-07	7,78E-06
cg22861969	5	139631508	PFDN1	Body	-0,267	2,79E-07	7,78E-06
cg05473258	2	153150111		IGR	-0,202	2,79E-07	7,79E-06
cg03605208	8	133876029		IGR	-0,209	2,79E-07	7,79E-06
cg09424006	16	5657230		IGR	0,256	2,79E-07	7,79E-06
cg12772987	19	4841297	PLIN3	Body	-0,332	2,79E-07	7,79E-06
cg12270180	3	11806486		IGR	-0,267	2,79E-07	7,80E-06
cg17673017	8	49336317		IGR	-0,21	2,79E-07	7,80E-06
cg03191032	3	186130038		IGR	-0,207	2,79E-07	7,80E-06
cg06901890	9	132803508	FNBP1	Body	-0,303	2,79E-07	7,80E-06
cg03510732	10	114871931	TCF7L2	Body	-0,223	2,79E-07	7,80E-06
cg13521620	17	57410687	YPEL2	5'UTR	0,237	2,79E-07	7,80E-06
cg19514721	5	141813298		IGR	0,203	2,79E-07	7,80E-06
cg02416536	12	98577192		IGR	-0,247	2,80E-07	7,81E-06
cg06934426	12	10373481	GABARAPL1	Body	0,254	2,80E-07	7,82E-06
cg21158528	1	211501629	TRAF5	5'UTR	-0,269	2,80E-07	7,82E-06
cg02956301	20	52489563		IGR	-0,245	2,80E-07	7,82E-06
cg23314870	8	19755888		IGR	-0,216	2,80E-07	7,82E-06
cg01926175	8	128671375		IGR	-0,2	2,81E-07	7,82E-06
cg02450981	1	32825803		IGR	-0,207	2,81E-07	7,83E-06
cg07306826	22	37938106		IGR	-0,286	2,81E-07	7,83E-06
cg19139358	5	111400717		IGR	-0,219	2,81E-07	7,83E-06
cg17004737	6	166832975	RPS6KA2	Body	-0,367	2,81E-07	7,84E-06
cg22298430	17	5404330	LOC728392	1stExon	-0,205	2,82E-07	7,85E-06
cg14234394	1	212787866	ATF3	5'UTR	-0,254	2,82E-07	7,85E-06
cg13148524	1	223363874		IGR	-0,318	2,82E-07	7,85E-06
cg24525238	8	130305094		IGR	-0,203	2,82E-07	7,86E-06
cg01675134	5	139155182		IGR	-0,204	2,82E-07	7,86E-06
cg17552829	14	23653110	SLC7A8	TSS1500	0,2	2,82E-07	7,86E-06
cg09905178	1	171226352	FMO1	5'UTR	0,238	2,82E-07	7,86E-06
cg00628788	18	33529997		IGR	0,211	2,82E-07	7,86E-06
cg00394261	5	42483732	GHR	5'UTR	-0,332	2,83E-07	7,86E-06
cg02760152	1	57886427	DAB1	5'UTR	-0,203	2,83E-07	7,87E-06
cg01454698	4	140793545	MAML3	Body	-0,261	2,83E-07	7,87E-06
cg26273013	11	14468109		IGR	-0,305	2,83E-07	7,87E-06
cg05976481	12	92524968		IGR	-0,208	2,83E-07	7,87E-06
cg27596096	16	83025351	LOC10192841	TSS1500	-0,204	2,83E-07	7,88E-06
cg19784198	11	67038917	ADRBK1	Body	-0,267	2,83E-07	7,88E-06

cg02053407	6	119047242		IGR	-0,208	2,84E-07	7,88E-06
cg16388513	5	90713140	ARRDC3-AS1	Body	-0,203	2,84E-07	7,89E-06
cg08786644	9	107594238	ABCA1	Body	-0,211	2,84E-07	7,89E-06
cg02012622	5	55936747		IGR	-0,265	2,84E-07	7,89E-06
cg22949256	1	67135552	SGIP1	Body	-0,245	2,84E-07	7,89E-06
cg12183789	7	2026368	MAD1L1	Body	-0,215	2,84E-07	7,90E-06
cg06797389	15	44969481	PATL2	TSS1500	-0,254	2,85E-07	7,90E-06
cg23967739	12	8997529	A2ML1	TSS200	0,229	2,85E-07	7,90E-06
cg18720720	5	173305796		IGR	0,241	2,85E-07	7,91E-06
cg17203617	10	98313180	TM9SF3	Body	0,214	2,85E-07	7,91E-06
cg17620027	1	55754205		IGR	-0,205	2,85E-07	7,91E-06
cg27470208	10	8373416		IGR	-0,201	2,85E-07	7,92E-06
cg12226265	7	9229589		IGR	0,21	2,85E-07	7,92E-06
cg08986803	7	86847333	TMEM243	Body	-0,216	2,86E-07	7,92E-06
cg08461692	17	8481454	MYH10	Body	-0,208	2,86E-07	7,92E-06
cg21939215	11	60738995	CD6	TSS200	-0,337	2,86E-07	7,92E-06
cg23084416	10	80936005	ZMIZ1	5'UTR	0,217	2,86E-07	7,92E-06
cg21597480	17	66511166	PRKAR1A	5'UTR	0,209	2,86E-07	7,92E-06
cg15747595	8	98289880	TSPYL5	1stExon	0,206	2,86E-07	7,93E-06
cg05138702	10	126827000	CTBP2	5'UTR	0,223	2,86E-07	7,93E-06
cg15438184	3	124358997	KALRN	Body	-0,217	2,86E-07	7,93E-06
cg09711214	11	12924825	TEAD1	Body	-0,23	2,87E-07	7,94E-06
cg19836199	21	36421941	RUNX1	TSS1500	-0,275	2,87E-07	7,95E-06
cg02113446	6	167508537		IGR	-0,27	2,87E-07	7,95E-06
cg10919111	10	74075783		IGR	0,257	2,87E-07	7,96E-06
cg14154784	9	84208253	TLE1	Body	0,203	2,87E-07	7,96E-06
cg26802494	11	93263353	SMCO4	5'UTR	-0,232	2,88E-07	7,96E-06
cg14583300	2	238358457		IGR	0,213	2,88E-07	7,96E-06
cg16020672	19	18586017	ELL	Body	-0,289	2,88E-07	7,96E-06
cg26049648	17	41607973	ETV4	TSS200	0,257	2,88E-07	7,97E-06
cg21999735	15	38963943		IGR	-0,312	2,89E-07	7,98E-06
cg00664406	3	51740875	GRM2	TSS1500	0,228	2,89E-07	7,98E-06
cg19275754	10	16688827	RSU1	Body	-0,302	2,89E-07	7,98E-06
cg19464252	16	30675233	FBRS	TSS1500	-0,204	2,89E-07	7,99E-06
cg06817454	1	48360454		IGR	-0,236	2,90E-07	7,99E-06
cg22799934	13	42067563		IGR	-0,216	2,90E-07	7,99E-06
cg08177522	18	59431787		IGR	-0,256	2,90E-07	7,99E-06
cg08021237	1	42149162	HIVEP3	5'UTR	-0,238	2,90E-07	8,00E-06
cg01450133	11	12136467	MICAL2	5'UTR	0,252	2,90E-07	8,00E-06
cg00201393	12	93568010	LOC10272493	TSS200	0,223	2,90E-07	8,00E-06
cg07859288	8	132003249	ADCY8	Body	0,249	2,90E-07	8,01E-06
cg12937276	7	47925161	PKD1L1	Body	-0,236	2,91E-07	8,02E-06
cg14647944	9	73095740		IGR	0,259	2,91E-07	8,02E-06
cg22199044	1	83346298		IGR	-0,204	2,91E-07	8,02E-06
cg01647693	8	143513441		IGR	-0,226	2,91E-07	8,03E-06
cg03483713	7	27162766	HOXA3	5'UTR	0,27	2,91E-07	8,03E-06
cg26675764	7	130081470	CEP41	TSS1500	0,22	2,92E-07	8,03E-06
cg09758039	2	237240826	IQCA1	Body	-0,215	2,92E-07	8,03E-06
cg21972557	6	161940503	PARK2	Body	-0,258	2,92E-07	8,04E-06
cg21768844	5	130719929	CDC42SE2	Body	-0,267	2,93E-07	8,05E-06

cg14326161	20	8229198	PLCB1	Body	-0,216	2,93E-07	8,05E-06
cg00115458	6	33141305	COL11A2	Body	-0,208	2,93E-07	8,05E-06
cg24990674	20	8048453		IGR	-0,237	2,93E-07	8,05E-06
cg18701870	2	102063454	RFX8	Body	-0,235	2,93E-07	8,06E-06
cg22640092	2	11530707		IGR	-0,209	2,93E-07	8,06E-06
cg01656547	21	47970842	DIP2A	Body	-0,288	2,93E-07	8,06E-06
cg25369553	1	61522373		IGR	0,262	2,94E-07	8,07E-06
cg17115419	10	5593926		IGR	-0,234	2,94E-07	8,07E-06
cg11392231	4	55181809		IGR	-0,23	2,94E-07	8,07E-06
cg21319458	2	160472940	BAZ2B	1stExon	0,207	2,94E-07	8,08E-06
cg01435613	13	75952601	TBC1D4	Body	0,213	2,95E-07	8,09E-06
cg01380376	4	40765329	NSUN7	Body	-0,223	2,95E-07	8,10E-06
cg00515282	12	42978494	PRICKLE1	5'UTR	0,229	2,95E-07	8,10E-06
cg25785200	17	56066543	VEZF1	TSS1500	0,207	2,96E-07	8,11E-06
cg15263802	1	39318607	RRAGC	Body	-0,22	2,96E-07	8,11E-06
cg24234141	11	47376519	SPI1	3'UTR	0,234	2,96E-07	8,11E-06
cg07091301	4	25028641	LGI2	Body	-0,218	2,96E-07	8,12E-06
cg08265950	16	69220400	SNTB2	TSS1500	-0,331	2,96E-07	8,12E-06
cg06640123	10	21638844		IGR	0,271	2,96E-07	8,12E-06
cg24925303	1	112141421		IGR	-0,251	2,96E-07	8,12E-06
cg16589830	1	212284214		IGR	0,229	2,97E-07	8,13E-06
cg24674703	11	60869960	CD5	5'UTR	-0,232	2,97E-07	8,14E-06
cg05698763	7	91685		IGR	-0,203	2,98E-07	8,15E-06
cg05845376	5	140683632	SLC25A2	TSS200	0,283	2,98E-07	8,16E-06
cg02585938	14	59800395	DAAM1	Body	0,221	2,98E-07	8,16E-06
cg05802075	10	63721212	ARID5B	Body	-0,241	2,98E-07	8,16E-06
cg03818715	6	7591348	SNRNP48	Body	0,293	2,98E-07	8,16E-06
cg19852147	16	78795483	WWOX	Body	-0,251	2,99E-07	8,17E-06
cg14160518	20	50418952	SALL4	1stExon	0,249	2,99E-07	8,17E-06
cg03540148	17	56419621	BZRAP1-AS1	Body	-0,255	2,99E-07	8,17E-06
cg22730004	1	158656718	SPTA1	TSS1500	-0,224	2,99E-07	8,17E-06
cg23078228	3	64711384	MIR548A2	Body	-0,2	2,99E-07	8,17E-06
cg06962909	3	8518704	LMCD1-AS1	Body	-0,21	2,99E-07	8,17E-06
cg12048225	6	32808669	PSMB8	3'UTR	-0,242	2,99E-07	8,18E-06
cg05690805	9	574139	KANK1	5'UTR	0,264	3,00E-07	8,19E-06
cg06798429	5	130560473		IGR	-0,273	3,00E-07	8,19E-06
cg10761553	22	32970518	SYN3	Body	-0,302	3,00E-07	8,19E-06
cg03102442	8	125738432	MTSS1	Body	0,22	3,00E-07	8,19E-06
cg02074078	13	23754150	SGCG	TSS1500	0,22	3,00E-07	8,19E-06
cg03893872	1	210501634	HHAT	TSS1500	0,213	3,00E-07	8,19E-06
cg16387372	3	71775503	EIF4E3	TSS1500	-0,313	3,00E-07	8,19E-06
cg11962479	17	76126032	TMC6	5'UTR	-0,306	3,00E-07	8,20E-06
cg14562930	11	61595050	FADS2	TSS1500	0,208	3,00E-07	8,20E-06
cg05646531	12	68515202		IGR	-0,248	3,00E-07	8,20E-06
cg23989012	1	147791203		IGR	-0,261	3,01E-07	8,20E-06
cg00524271	2	198668185	PLCL1	TSS1500	0,201	3,01E-07	8,20E-06
cg20428356	9	134857358	MED27	Body	0,215	3,01E-07	8,21E-06
cg19018844	10	617607	DIP2C	Body	-0,218	3,01E-07	8,21E-06
cg01738164	14	90147503		IGR	-0,257	3,01E-07	8,21E-06
cg06084526	2	47445633	LOC1019270	Body	-0,25	3,01E-07	8,21E-06

cg26965801	1	200122591	NR5A2	Body	-0,262	3,01E-07	8,21E-06
cg04807696	12	120574721	GCN1	Body	0,224	3,01E-07	8,21E-06
cg18262580	17	8215160	ARHGEF15	5'UTR	0,214	3,01E-07	8,22E-06
cg23381998	1	88867081		IGR	-0,222	3,02E-07	8,22E-06
cg03058727	11	121011127	TECTA	Body	-0,201	3,02E-07	8,22E-06
cg26562691	16	23850404	PRKCB	Body	-0,242	3,02E-07	8,23E-06
cg08163193	9	34692051	CCL19	TSS1500	-0,237	3,02E-07	8,23E-06
cg01775612	16	31484199	TGFB1I1	Body	0,21	3,02E-07	8,23E-06
cg24627508	21	44182156	PDE9A	Body	-0,221	3,02E-07	8,24E-06
cg00900628	5	176784352	RGS14	TSS1500	-0,226	3,03E-07	8,25E-06
cg05341260	17	37730450		IGR	0,232	3,03E-07	8,25E-06
cg06026160	21	34766787		IGR	-0,272	3,03E-07	8,25E-06
cg25114597	2	48698251	PPP1R21	Body	0,295	3,03E-07	8,25E-06
cg16063553	12	47058897		IGR	-0,235	3,04E-07	8,26E-06
cg12485744	1	15258456	KIAA1026	Body	-0,228	3,04E-07	8,26E-06
cg18307618	12	25242587	LRMP	Body	-0,266	3,04E-07	8,26E-06
cg16200257	15	57180471	LOC145783	Body	0,216	3,04E-07	8,26E-06
cg18647259	17	36610306		IGR	0,201	3,04E-07	8,27E-06
cg25123566	12	47610734	FAM113B	5'UTR	-0,372	3,04E-07	8,27E-06
cg27576485	17	40558063	PTRF	Body	0,243	3,05E-07	8,29E-06
cg09153462	8	62602643	ASPH	Body	0,255	3,05E-07	8,29E-06
cg01526396	1	24516287		IGR	-0,237	3,05E-07	8,29E-06
cg14362370	9	133589654	ABL1	5'UTR	0,219	3,05E-07	8,29E-06
cg13199963	1	27126172		IGR	0,201	3,06E-07	8,30E-06
cg01399475	4	26755598	TBC1D19	Body	0,262	3,06E-07	8,30E-06
cg26137190	2	69453290	ANTXR1	Body	-0,225	3,06E-07	8,31E-06
cg13283817	13	24133614		IGR	0,258	3,06E-07	8,31E-06
cg04816803	10	44426803		IGR	-0,318	3,06E-07	8,31E-06
cg17059829	15	39933961	FSIP1	Body	-0,21	3,06E-07	8,31E-06
cg18752327	3	169404841		IGR	-0,21	3,06E-07	8,31E-06
cg09473725	1	167485873	CD247	Body	-0,251	3,07E-07	8,32E-06
cg25092052	10	74014582		IGR	0,26	3,07E-07	8,32E-06
cg02044895	11	58343318	LPXN	Body	-0,318	3,07E-07	8,32E-06
cg07569984	14	24759069		IGR	-0,3	3,07E-07	8,32E-06
cg20211533	18	64969433		IGR	-0,237	3,07E-07	8,32E-06
cg19269426	1	235500468	GGPS1	Body	-0,272	3,08E-07	8,34E-06
cg04011173	3	10326540	GHRLOS	TSS1500	-0,296	3,08E-07	8,34E-06
cg25122752	5	114713211		IGR	0,217	3,08E-07	8,34E-06
cg27524944	6	140295369		IGR	0,221	3,08E-07	8,34E-06
cg02203224	12	123464087	ARL6IP4	TSS1500	0,209	3,08E-07	8,34E-06
cg00953665	10	4003468		IGR	-0,239	3,08E-07	8,34E-06
cg18880737	1	44219108	ST3GAL3	Body	-0,242	3,08E-07	8,35E-06
cg08289130	16	3210357		IGR	0,283	3,08E-07	8,35E-06
cg10743544	12	50067424	FMNL3	Body	0,221	3,08E-07	8,35E-06
cg11188451	12	6163963	VWF	Body	-0,203	3,08E-07	8,35E-06
cg26645082	11	70563264	SHANK2	Body	-0,262	3,10E-07	8,38E-06
cg02854972	4	8327406		IGR	-0,222	3,10E-07	8,38E-06
cg02775989	17	47465656		IGR	-0,286	3,10E-07	8,38E-06
cg09590468	22	39601450		IGR	-0,235	3,10E-07	8,38E-06
cg02007288	8	30513708	GTF2E2	5'UTR	-0,265	3,10E-07	8,38E-06

cg25130728	7	73483053	ELN	3'UTR	-0,201	3,10E-07	8,38E-06
cg23731826	15	90371692		IGR	0,223	3,10E-07	8,38E-06
cg22178178	14	102699183	MOK	Body	-0,243	3,10E-07	8,39E-06
cg08999291	4	48263525	TEC	5'UTR	-0,207	3,11E-07	8,40E-06
cg14297410	2	63351486	WDPCP	Body	-0,265	3,11E-07	8,41E-06
cg08216686	12	116761955		IGR	-0,228	3,12E-07	8,41E-06
cg05447290	5	158275960	EBF1	Body	-0,271	3,12E-07	8,41E-06
cg01070399	12	53454300	TNS2	Body	-0,202	3,12E-07	8,42E-06
cg17958242	4	40197954	RHOH	TSS1500	-0,238	3,12E-07	8,42E-06
cg03301579	19	3783755	MATK	Body	0,223	3,12E-07	8,43E-06
cg01738152	17	43301209	FMNL1	Body	-0,266	3,13E-07	8,44E-06
cg05608541	11	2321770	C11orf21	Body	-0,232	3,13E-07	8,44E-06
cg03739132	5	171601028	STK10	Body	-0,235	3,13E-07	8,44E-06
cg14953379	7	141285961	AGK	Body	-0,216	3,13E-07	8,45E-06
cg05770909	12	131493506	ADGRD1	Body	-0,248	3,14E-07	8,45E-06
cg05128437	1	245209111	EFCAB2	Body	0,201	3,14E-07	8,45E-06
cg26572896	2	151386104		IGR	-0,236	3,14E-07	8,46E-06
cg25770393	11	78459659	TENM4	Body	-0,212	3,14E-07	8,46E-06
cg16522719	5	86636497	RASA1	Body	-0,271	3,14E-07	8,46E-06
cg04628881	15	49477971	GALK2	5'UTR	-0,288	3,14E-07	8,46E-06
cg01394116	5	150081499	RBM22	TSS1500	-0,202	3,14E-07	8,46E-06
cg06756927	2	16499491		IGR	-0,267	3,14E-07	8,47E-06
cg01944137	14	64974907	ZBTB1	5'UTR	-0,202	3,14E-07	8,47E-06
cg22874664	20	57752991		IGR	-0,277	3,14E-07	8,47E-06
cg19551813	15	101251061		IGR	-0,24	3,15E-07	8,47E-06
cg03447908	3	45076599	CLEC3B	Body	-0,274	3,15E-07	8,47E-06
cg03974637	3	141118465	ZBTB38	5'UTR	-0,237	3,15E-07	8,48E-06
cg11228724	3	192543891	MB21D2	Body	-0,221	3,15E-07	8,48E-06
cg06246967	15	38962858		IGR	-0,225	3,15E-07	8,48E-06
cg01965560	17	7758222	TMEM88	TSS200	0,264	3,15E-07	8,48E-06
cg11147886	16	69598170	NFAT5	TSS1500	-0,295	3,16E-07	8,48E-06
cg07027528	18	30590300	CCDC178	Body	0,218	3,16E-07	8,48E-06
cg12647920	12	109144744		IGR	-0,21	3,16E-07	8,49E-06
cg05444524	16	88119777		IGR	-0,205	3,16E-07	8,49E-06
cg03278514	15	70779346		IGR	-0,203	3,16E-07	8,49E-06
cg10605084	12	9217763	LOC144571	TSS200	0,218	3,16E-07	8,49E-06
cg12027161	11	45692152		IGR	-0,228	3,17E-07	8,50E-06
cg15304322	6	42313795	TRERF1	5'UTR	-0,207	3,17E-07	8,51E-06
cg21237687	17	6899380	ALOX12	TSS200	0,216	3,17E-07	8,51E-06
cg09715958	10	97240553	SORBS1	5'UTR	0,203	3,17E-07	8,51E-06
cg14567804	3	123757344		IGR	-0,235	3,17E-07	8,51E-06
cg23313665	9	130517848	SH2D3C	Body	0,275	3,17E-07	8,51E-06
cg08530445	1	55944299		IGR	-0,237	3,17E-07	8,51E-06
cg03822198	12	116997535	MAP1LC3B2	5'UTR	-0,286	3,18E-07	8,52E-06
cg23089564	22	30847650		IGR	0,275	3,18E-07	8,52E-06
cg04351205	2	143886326	ARHGAP15	TSS1500	-0,288	3,18E-07	8,53E-06
cg22262670	2	68994821	ARHGAP25	Body	-0,345	3,18E-07	8,53E-06
cg08481075	6	33161328	COL11A2	TSS1500	0,23	3,18E-07	8,53E-06
cg06288727	4	172735549	GALNTL6	5'UTR	0,211	3,19E-07	8,54E-06
cg19622623	12	86230825	RASSF9	TSS1500	-0,215	3,19E-07	8,54E-06

cg08035694	1	192921988		IGR	-0,209	3,19E-07	8,55E-06
cg24462980	1	19762766	CAPZB	Body	-0,226	3,19E-07	8,55E-06
cg07929956	16	70437897	ST3GAL2	5'UTR	-0,21	3,19E-07	8,55E-06
cg16674280	19	37063457	ZNF529-AS1	TSS1500	0,217	3,19E-07	8,56E-06
cg10797624	20	62382338	ZBTB46	Body	-0,2	3,20E-07	8,56E-06
cg21066685	1	207924376	CD46	TSS1500	0,243	3,20E-07	8,56E-06
cg08646535	2	46131730	PRKCE	Body	-0,219	3,20E-07	8,56E-06
cg00673404	2	231523953		IGR	-0,32	3,20E-07	8,57E-06
cg15913312	4	111009988	ELOVL6	Body	-0,209	3,20E-07	8,57E-06
cg17412989	3	177310640	LINC00578	Body	-0,21	3,21E-07	8,58E-06
cg02306995	3	122635049	SEMA5B	Body	-0,221	3,21E-07	8,59E-06
cg00339726	13	36293991	MIR548F5	Body	-0,243	3,21E-07	8,59E-06
cg13618145	10	3917475		IGR	-0,207	3,21E-07	8,60E-06
cg27256074	12	46780872	LOC1002887	Body	0,267	3,21E-07	8,60E-06
cg07348418	1	44903330	RNF220	Body	-0,238	3,21E-07	8,60E-06
cg17751510	15	72408898	SENP8	TSS1500	0,216	3,22E-07	8,61E-06
cg00903584	1	202128682	PTPN7	5'UTR	-0,328	3,22E-07	8,61E-06
cg02673849	12	11856257	ETV6	Body	-0,292	3,22E-07	8,61E-06
cg15241611	6	22221564		IGR	-0,3	3,22E-07	8,61E-06
cg07856138	16	29673465	SPN	TSS1500	-0,22	3,23E-07	8,62E-06
cg24775298	5	130718942	CDC42SE2	Body	-0,238	3,23E-07	8,62E-06
cg04360967	6	14636504		IGR	-0,41	3,23E-07	8,62E-06
cg00031042	6	158254767	SNX9	Body	-0,304	3,23E-07	8,63E-06
cg19273826	20	37491253	PPP1R16B	Body	-0,236	3,24E-07	8,63E-06
cg15475323	19	49220102	MAMSTR	1stExon	0,232	3,24E-07	8,65E-06
cg13493426	14	34111564	NPAS3	Body	0,237	3,25E-07	8,65E-06
cg00528079	16	10060873	GRIN2A	Body	-0,202	3,25E-07	8,65E-06
cg10037068	2	175500072	WIPF1	5'UTR	-0,231	3,25E-07	8,66E-06
cg01805547	21	42626439	BACE2	Body	-0,221	3,26E-07	8,68E-06
cg16488065	1	204656750		IGR	-0,202	3,26E-07	8,68E-06
cg22196750	5	96309629	LNPEP	5'UTR	-0,215	3,26E-07	8,68E-06
cg21882416	10	112117365		IGR	-0,245	3,26E-07	8,68E-06
cg05200998	2	242497924	BOK-AS1	Body	0,25	3,27E-07	8,69E-06
cg10888461	4	113300821	ALPK1	Body	0,226	3,27E-07	8,70E-06
cg06940267	8	19188674	SH2D4A	Body	-0,21	3,27E-07	8,70E-06
cg24989900	2	18952548		IGR	-0,214	3,28E-07	8,71E-06
cg04502145	3	77285505	ROBO2	5'UTR	0,262	3,28E-07	8,72E-06
cg03854993	7	38784604	VPS41	Body	-0,241	3,28E-07	8,72E-06
cg14928510	19	55449439	NLRP7	Body	-0,206	3,29E-07	8,73E-06
cg14828066	1	151250056		IGR	-0,2	3,29E-07	8,73E-06
cg06806711	11	60223291	MS4A1	1stExon	-0,205	3,29E-07	8,73E-06
cg07584516	13	42269266	VWA8	Body	0,241	3,29E-07	8,73E-06
cg19114721	2	103126336	SLC9A4	Body	-0,252	3,29E-07	8,73E-06
cg15021109	15	60881681	RORA-AS1	Body	-0,297	3,29E-07	8,73E-06
cg04385511	4	54561824	LOC1005064	TSS1500	-0,213	3,30E-07	8,74E-06
cg22790915	9	91450296		IGR	-0,238	3,30E-07	8,75E-06
cg06526997	16	19868612	IQCK	3'UTR	-0,214	3,30E-07	8,75E-06
cg01051524	2	106415145	NCK2	5'UTR	-0,265	3,30E-07	8,75E-06
cg09372007	4	77881312	sept-11	5'UTR	-0,254	3,31E-07	8,76E-06
cg00242423	2	65782280		IGR	-0,204	3,31E-07	8,76E-06

cg21831210	2	15454056	NBAS	Body	-0,208	3,31E-07	8,77E-06
cg09994678	5	157030768		IGR	-0,23	3,31E-07	8,77E-06
cg07345374	17	76772464	CYTH1	Body	-0,224	3,31E-07	8,77E-06
cg15484406	14	94461913		IGR	0,201	3,31E-07	8,77E-06
cg13686952	17	37926322	IKZF3	Body	-0,219	3,31E-07	8,77E-06
cg15962969	6	138866887	NHSL1	Body	0,232	3,31E-07	8,77E-06
cg00803692	3	46411474	CCR5	TSS200	-0,208	3,32E-07	8,78E-06
cg15243610	18	3037629		IGR	-0,237	3,32E-07	8,78E-06
cg01378335	11	74870090	SLCO2B1	TSS1500	-0,251	3,32E-07	8,78E-06
cg15239796	3	38547167	EXOG	Body	0,245	3,32E-07	8,78E-06
cg06725641	2	197347708	HECW2	5'UTR	-0,264	3,32E-07	8,78E-06
cg18227169	14	60723315	PPM1A	Body	0,22	3,32E-07	8,78E-06
cg17210803	10	129653554		IGR	-0,248	3,32E-07	8,79E-06
cg21221767	14	65673646		IGR	-0,304	3,34E-07	8,81E-06
cg25546991	2	96811841	DUSP2	TSS1500	-0,21	3,34E-07	8,81E-06
cg06976795	2	125567771	CNTNAP5	Body	0,245	3,34E-07	8,82E-06
cg21031775	16	69224803	SNTB2	Body	-0,272	3,34E-07	8,82E-06
cg06810231	14	51906822		IGR	0,249	3,34E-07	8,82E-06
cg10464262	4	40435823	RBM47	Body	-0,229	3,34E-07	8,82E-06
cg22372285	11	125951005		IGR	-0,201	3,34E-07	8,82E-06
cg09430344	16	27237355	NSMCE1	Body	-0,211	3,34E-07	8,83E-06
cg16501452	8	9534629	TNKS	Body	0,208	3,35E-07	8,83E-06
cg14665891	10	77536561	C10orf11	Body	-0,226	3,36E-07	8,85E-06
cg23797100	16	28996053	LAT	TSS200	-0,24	3,36E-07	8,86E-06
cg21984889	19	5330654	PTPRS	5'UTR	-0,215	3,36E-07	8,86E-06
cg12196720	1	162307228	NOS1AP	Body	-0,306	3,36E-07	8,86E-06
cg25108994	2	114456848		IGR	-0,205	3,36E-07	8,86E-06
cg17841267	10	112117449		IGR	-0,201	3,36E-07	8,86E-06
cg14839649	8	8775310		IGR	-0,21	3,36E-07	8,87E-06
cg13028856	6	114147161		IGR	0,258	3,37E-07	8,87E-06
cg25576362	2	202125088	CASP8	TSS200	-0,295	3,37E-07	8,88E-06
cg26094719	3	179139405	GNB4	Body	0,287	3,37E-07	8,88E-06
cg25173785	6	52022616		IGR	-0,221	3,37E-07	8,88E-06
cg00520410	16	81558154	CMIP	Body	-0,244	3,37E-07	8,88E-06
cg09353563	15	70994435	UACA	5'UTR	0,266	3,37E-07	8,88E-06
cg26657550	4	150998949	DCLK2	TSS1500	0,226	3,38E-07	8,89E-06
cg26079664	3	51740956	GRM2	TSS200	0,242	3,38E-07	8,89E-06
cg07839742	6	84761960	MRAP2	5'UTR	0,271	3,38E-07	8,89E-06
cg26979423	1	246038364	SMYD3	Body	0,274	3,38E-07	8,89E-06
cg07709932	6	52859109	GSTA4	5'UTR	0,253	3,38E-07	8,90E-06
cg22406037	2	203590985	FAM117B	ExonBnd	0,208	3,38E-07	8,90E-06
cg21565508	8	103816801		IGR	-0,208	3,39E-07	8,91E-06
cg16725974	14	64341178	SYNE2	5'UTR	0,278	3,39E-07	8,92E-06
cg18010302	11	94579436	AMOTL1	Body	-0,219	3,39E-07	8,92E-06
cg23533154	4	160022566		IGR	-0,203	3,39E-07	8,92E-06
cg11850468	5	180231185	MGAT1	TSS1500	-0,298	3,39E-07	8,92E-06
cg23933684	9	120494719		IGR	-0,218	3,40E-07	8,93E-06
cg08091949	5	169740799		IGR	-0,277	3,40E-07	8,93E-06
cg09610578	3	129407542	TMCC1	TSS200	0,232	3,40E-07	8,93E-06
cg05092777	7	131368648		IGR	-0,251	3,40E-07	8,93E-06

cg04957729	3	4312607		IGR	-0,269	3,40E-07	8,93E-06
cg15307353	2	237781809		IGR	-0,203	3,40E-07	8,94E-06
cg03562367	21	17106209	USP25	Body	-0,243	3,41E-07	8,94E-06
cg03651043	14	55542565		IGR	-0,247	3,41E-07	8,95E-06
cg10540573	2	218869684		IGR	-0,218	3,41E-07	8,95E-06
cg05663417	2	238165425		IGR	-0,202	3,42E-07	8,97E-06
cg21854324	3	197198944		IGR	-0,209	3,42E-07	8,97E-06
cg23089272	15	39985431	FSIP1	Body	-0,242	3,43E-07	8,98E-06
cg08546725	10	67705174	CTNNA3	Body	-0,264	3,43E-07	8,98E-06
cg14050761	4	139586881		IGR	-0,221	3,43E-07	8,99E-06
cg04473618	8	49661550		IGR	-0,203	3,43E-07	8,99E-06
cg22827694	1	1356442	ANKRD65	5'UTR	-0,223	3,43E-07	8,99E-06
cg16133681	12	25801621	IFLTD1	TSS200	0,226	3,43E-07	9,00E-06
cg17576140	11	11172009		IGR	-0,258	3,44E-07	9,00E-06
cg26371056	1	221348170		IGR	0,227	3,44E-07	9,00E-06
cg14830367	1	178459176		IGR	-0,224	3,44E-07	9,02E-06
cg18703515	10	44830587		IGR	-0,203	3,45E-07	9,02E-06
cg08926477	3	171918983	FNDC3B	Body	0,218	3,45E-07	9,02E-06
cg25644284	2	235903440	SH3BP4	5'UTR	-0,219	3,45E-07	9,03E-06
cg07377755	9	114568286		IGR	-0,277	3,46E-07	9,03E-06
cg11680590	15	92701885	SLCO3A1	Body	-0,202	3,46E-07	9,03E-06
cg04462378	11	71749760	NUMA1	5'UTR	0,206	3,46E-07	9,04E-06
cg26510778	12	124416341	DNAH10	Body	-0,227	3,46E-07	9,04E-06
cg08474748	5	74350214		IGR	0,238	3,46E-07	9,04E-06
cg19846353	11	119585587	PVRL1	Body	-0,22	3,46E-07	9,04E-06
cg08680693	17	21077675	DHRS7B	Body	0,218	3,46E-07	9,04E-06
cg07492937	10	35443057	CREM	5'UTR	0,228	3,46E-07	9,04E-06
cg06660109	12	28218448		IGR	-0,203	3,46E-07	9,04E-06
cg05951474	1	54155470	GLIS1	5'UTR	-0,209	3,46E-07	9,05E-06
cg25385940	15	99789637	TTC23	1stExon	0,266	3,46E-07	9,05E-06
cg18838698	5	10415611	MARCH6	ExonBnd	0,219	3,47E-07	9,05E-06
cg11320244	18	21795073	OSBPL1A	Body	0,257	3,47E-07	9,05E-06
cg25636629	16	82805427	LOC1019284	TSS1500	-0,248	3,47E-07	9,06E-06
cg03219235	14	88336254		IGR	-0,256	3,47E-07	9,06E-06
cg11640769	4	185386045	IRF2	5'UTR	0,215	3,47E-07	9,06E-06
cg04562757	1	17882998	ARHGEF10L	5'UTR	-0,216	3,47E-07	9,06E-06
cg22594214	8	135842299		IGR	0,295	3,48E-07	9,07E-06
cg08994526	9	112887640	AKAP2	TSS200	0,269	3,48E-07	9,07E-06
cg10884539	6	2752360	MYLK4	TSS1500	-0,242	3,48E-07	9,07E-06
cg17691837	6	151203458	MTHFD1L	Body	0,223	3,48E-07	9,07E-06
cg24687276	15	89163928	AEN	TSS1500	0,219	3,48E-07	9,07E-06
cg24576172	19	52408518	ZNF649	TSS1500	0,214	3,48E-07	9,08E-06
cg26472518	12	27332626		IGR	-0,27	3,48E-07	9,08E-06
cg10734164	8	145726283	PPP1R16A	Body	0,231	3,49E-07	9,09E-06
cg15311766	10	16908364	CUBN	Body	-0,316	3,49E-07	9,09E-06
cg23261319	2	182713409		IGR	-0,249	3,49E-07	9,10E-06
cg01144823	3	66004339	MAGI1	Body	-0,203	3,49E-07	9,10E-06
cg00045273	3	197119863		IGR	0,204	3,49E-07	9,10E-06
cg00380176	3	128505627	RAB7A	5'UTR	0,239	3,50E-07	9,11E-06
cg00454770	10	116286974	ABLIM1	Body	-0,219	3,50E-07	9,11E-06

cg09444023	7	131294967		IGR	-0,228	3,50E-07	9,11E-06
cg22187085	20	6716982		IGR	0,216	3,50E-07	9,11E-06
cg19153228	2	173902435	RAPGEF4	Body	0,264	3,50E-07	9,11E-06
cg16799191	3	112395154		IGR	-0,213	3,50E-07	9,11E-06
cg17680767	5	73623208		IGR	-0,279	3,50E-07	9,12E-06
cg13772431	1	161039601	ARHGAP30	1stExon	-0,2	3,51E-07	9,12E-06
cg14386061	8	21771446	DOK2	TSS1500	-0,317	3,51E-07	9,12E-06
cg12483545	1	2198879	SKI	Body	0,228	3,51E-07	9,12E-06
cg05654164	1	85725892	C1orf52	TSS1500	0,209	3,51E-07	9,13E-06
cg13581859	6	33048706	HLA-DPB1	Body	0,26	3,51E-07	9,13E-06
cg16580762	19	8634137	MYO1F	Body	-0,266	3,51E-07	9,13E-06
cg14978830	2	21645829		IGR	-0,21	3,51E-07	9,13E-06
cg15958074	1	219875907		IGR	-0,212	3,51E-07	9,14E-06
cg15784618	12	69264028	CPM	Body	-0,207	3,52E-07	9,14E-06
cg07370683	19	423000	SHC2	Body	-0,241	3,52E-07	9,15E-06
cg02153286	6	436928		IGR	0,259	3,52E-07	9,15E-06
cg21758962	11	30929224		IGR	-0,201	3,52E-07	9,15E-06
cg02556924	15	101661693		IGR	0,208	3,53E-07	9,16E-06
cg06055086	8	106539890	ZFPM2	Body	-0,207	3,53E-07	9,16E-06
cg19759093	21	40410304		IGR	-0,211	3,53E-07	9,16E-06
cg03300664	13	48795010		IGR	-0,246	3,53E-07	9,17E-06
cg10131026	8	122823862		IGR	-0,279	3,53E-07	9,17E-06
cg12116027	12	58130410	AGAP2	Body	0,223	3,53E-07	9,17E-06
cg22756896	20	36847423	KIAA1755	Body	-0,236	3,54E-07	9,18E-06
cg10736902	1	184836216	FAM129A	Body	-0,275	3,55E-07	9,20E-06
cg24359358	1	78358425	NEXN	5'UTR	0,248	3,55E-07	9,20E-06
cg11741818	8	10367873		IGR	-0,22	3,55E-07	9,20E-06
cg24366563	7	642485	LOC1019269(	TSS1500	-0,261	3,55E-07	9,21E-06
cg26226661	1	223212906		IGR	-0,22	3,55E-07	9,21E-06
cg22021226	8	43015521	HGSNAT	Body	-0,257	3,56E-07	9,21E-06
cg13782346	1	228901387		IGR	-0,213	3,56E-07	9,22E-06
cg05792501	9	112621743	PALM2	Body	-0,221	3,57E-07	9,23E-06
cg04671742	12	6881997	LAG3	1stExon	-0,23	3,57E-07	9,23E-06
cg22889646	8	29107750	KIF13B	Body	-0,2	3,57E-07	9,25E-06
cg19742687	11	88025975		IGR	0,235	3,58E-07	9,25E-06
cg26692749	1	24861919	RCAN3	3'UTR	-0,246	3,58E-07	9,26E-06
cg08997191	8	133788834	PHF20L1	5'UTR	0,22	3,58E-07	9,26E-06
cg02051599	17	55804630		IGR	-0,219	3,59E-07	9,26E-06
cg12745332	6	14639068		IGR	0,214	3,59E-07	9,27E-06
cg12920843	3	152541641		IGR	-0,209	3,59E-07	9,28E-06
cg03257930	9	110399293		IGR	-0,255	3,59E-07	9,28E-06
cg24411719	6	12356579		IGR	-0,218	3,59E-07	9,28E-06
cg25835351	10	13388524	SEPHS1	5'UTR	0,259	3,60E-07	9,28E-06
cg27234747	2	120525972	PTPN4	5'UTR	0,213	3,60E-07	9,28E-06
cg14925240	11	73067487	ARHGEF17	Body	-0,217	3,60E-07	9,29E-06
cg22952189	11	9091264	SCUBE2	Body	-0,227	3,60E-07	9,29E-06
cg08751348	10	120850686		IGR	-0,213	3,61E-07	9,30E-06
cg20228731	7	130646051	FLJ43663	Body	-0,336	3,61E-07	9,31E-06
cg00446763	3	124625655	MUC13	3'UTR	-0,223	3,61E-07	9,31E-06
cg10165864	2	173419899	PDK1	TSS1500	0,207	3,62E-07	9,33E-06

cg18080303	6	112576342	LAMA4	TSS1500	0,278	3,62E-07	9,33E-06
cg12102016	14	89259748	EML5	TSS1500	0,208	3,63E-07	9,33E-06
cg17446488	6	6772111		IGR	-0,332	3,63E-07	9,34E-06
cg18719106	15	57845204		IGR	-0,256	3,63E-07	9,34E-06
cg27549720	1	19992167	HTR6	5'UTR	0,204	3,63E-07	9,34E-06
cg17454920	14	74185993	C14orf43	3'UTR	0,204	3,63E-07	9,34E-06
cg18710458	10	30927198		IGR	-0,274	3,64E-07	9,36E-06
cg14582550	9	97786879	C9orf3	Body	0,252	3,65E-07	9,37E-06
cg16450086	7	50753269	GRB10	5'UTR	0,222	3,65E-07	9,38E-06
cg18903093	10	63422511	C10orf107	TSS1500	0,228	3,65E-07	9,38E-06
cg24969098	8	21768710	DOK2	Body	-0,209	3,65E-07	9,38E-06
cg15970145	22	33834204	LARGE	Body	-0,203	3,65E-07	9,38E-06
cg24381458	6	12203576		IGR	0,203	3,66E-07	9,39E-06
cg23463494	16	29832769	PAGR1	3'UTR	-0,237	3,66E-07	9,39E-06
cg22363520	6	35558488	FKBP5	Body	0,247	3,66E-07	9,39E-06
cg09249793	1	76257000	RABGGTB	Body	-0,235	3,66E-07	9,40E-06
cg17117459	13	25085405	PARP4	5'UTR	-0,319	3,66E-07	9,40E-06
cg00491064	11	19681786	NAV2	Body	-0,221	3,67E-07	9,41E-06
cg06522118	14	23024876		IGR	-0,255	3,67E-07	9,41E-06
cg26013712	10	22972662	PIP4K2A	Body	-0,243	3,67E-07	9,41E-06
cg21142789	1	203331138		IGR	-0,28	3,67E-07	9,42E-06
cg02159157	11	32365507		IGR	-0,203	3,68E-07	9,43E-06
cg05537706	2	46453098		IGR	0,202	3,68E-07	9,43E-06
cg23392269	14	75963787		IGR	-0,312	3,68E-07	9,44E-06
cg00098345	15	69605418	PAQR5	TSS1500	-0,267	3,68E-07	9,44E-06
cg00783433	1	200122721	NR5A2	Body	-0,239	3,69E-07	9,44E-06
cg16375118	3	13024075	IQSEC1	Body	-0,226	3,69E-07	9,44E-06
cg15964468	6	28351351	ZSCAN12	Body	0,2	3,69E-07	9,45E-06
cg18924562	12	10242785	CLEC1A	Body	-0,283	3,69E-07	9,45E-06
cg14951497	2	191875807	STAT1	5'UTR	-0,406	3,69E-07	9,45E-06
cg17016275	3	124148564	KALRN	Body	-0,203	3,69E-07	9,45E-06
cg15208882	1	120178965	ZNF697	5'UTR	0,207	3,70E-07	9,46E-06
cg25578535	7	130098336		IGR	-0,253	3,70E-07	9,46E-06
cg14069239	3	13581003		IGR	-0,201	3,70E-07	9,47E-06
cg12191163	18	12838280	PTPN2	Body	-0,232	3,71E-07	9,48E-06
cg18216249	12	48100805	RPAP3	TSS1500	-0,216	3,71E-07	9,48E-06
cg09395195	17	29645782	EVI2A	Body	-0,286	3,71E-07	9,48E-06
cg05946080	2	160777247		IGR	-0,219	3,71E-07	9,49E-06
cg19592637	20	25063052	VSX1	TSS1500	0,203	3,71E-07	9,49E-06
cg24303598	5	35884755		IGR	-0,335	3,71E-07	9,49E-06
cg15102540	11	120497515	GRIK4	5'UTR	-0,205	3,71E-07	9,49E-06
cg26064794	1	204435434	PIK3C2B	Body	0,222	3,72E-07	9,50E-06
cg25066845	5	115949876		IGR	-0,226	3,72E-07	9,50E-06
cg26884773	1	235256863		IGR	0,229	3,72E-07	9,50E-06
cg13501527	9	123640474	PHF19	TSS1500	-0,213	3,72E-07	9,50E-06
cg24096763	3	170061985		IGR	0,251	3,72E-07	9,51E-06
cg13872789	5	150845740	SLC36A1	Body	0,252	3,72E-07	9,51E-06
cg24527695	6	4937087	CDYL	Body	0,223	3,73E-07	9,51E-06
cg26842815	12	7060263	PTPN6	TSS200	-0,263	3,73E-07	9,51E-06
cg27409514	19	1169138	SBNO2	5'UTR	-0,233	3,73E-07	9,51E-06

cg09272951	5	139926789	EIF4EBP3	TSS1500	0,207	3,73E-07	9,51E-06
cg23961198	12	6298436		IGR	-0,256	3,73E-07	9,52E-06
cg01104230	3	65940327	MAGI1	Body	0,209	3,73E-07	9,52E-06
cg18209560	17	74060086	SRP68	Body	-0,204	3,74E-07	9,53E-06
cg27176715	1	60595457		IGR	-0,203	3,74E-07	9,53E-06
cg22978730	3	194733121		IGR	-0,243	3,74E-07	9,53E-06
cg01003666	7	139929429		IGR	-0,268	3,75E-07	9,54E-06
cg22300541	3	65160432		IGR	-0,23	3,75E-07	9,55E-06
cg04457177	3	159572833	SCHIP1	Body	0,226	3,75E-07	9,55E-06
cg11239720	4	152967415		IGR	0,229	3,75E-07	9,55E-06
cg02803228	18	42777673		IGR	-0,214	3,75E-07	9,55E-06
cg15245598	8	94709667		IGR	0,215	3,76E-07	9,57E-06
cg12860635	6	30095140		IGR	0,31	3,76E-07	9,57E-06
cg26824071	8	103110169	NCALD	5'UTR	-0,201	3,76E-07	9,57E-06
cg04333785	10	31446813		IGR	0,209	3,77E-07	9,60E-06
cg24351577	3	134068270		IGR	-0,222	3,78E-07	9,61E-06
cg03949284	6	33161333	COL11A2	TSS1500	0,207	3,78E-07	9,61E-06
cg18680181	14	35629311	KIAA0391	Body	-0,234	3,79E-07	9,62E-06
cg03649649	17	56408197		IGR	-0,229	3,79E-07	9,63E-06
cg06192079	11	33942988		IGR	-0,231	3,79E-07	9,63E-06
cg19669199	19	7584241	ZNF358	Body	-0,229	3,79E-07	9,63E-06
cg23039425	4	99812050	EIF4E	Body	0,273	3,80E-07	9,63E-06
cg21387773	4	57964342	IGFBP7	Body	0,245	3,80E-07	9,64E-06
cg05703230	4	14112376		IGR	-0,212	3,80E-07	9,64E-06
cg03240518	11	111849360	DIXDC1	Body	0,22	3,80E-07	9,65E-06
cg25828480	2	171366488	MYO3B	Body	-0,235	3,81E-07	9,65E-06
cg17607231	2	231090329	SP140	TSS200	-0,244	3,81E-07	9,65E-06
cg15551816	21	36418766	RUNX1	Body	-0,299	3,81E-07	9,65E-06
cg07235958	1	155974953		IGR	-0,245	3,81E-07	9,65E-06
cg00641207	6	93499856		IGR	0,22	3,81E-07	9,65E-06
cg05860426	16	1014765	LMF1	Body	-0,209	3,81E-07	9,66E-06
cg06858379	3	16244848	GALNT15	Body	-0,206	3,82E-07	9,67E-06
cg21143956	8	125769926		IGR	-0,295	3,82E-07	9,67E-06
cg17177006	3	5232410	EDEM1	Body	-0,255	3,82E-07	9,67E-06
cg16104837	15	77320672	PSTPIP1	Body	0,222	3,82E-07	9,67E-06
cg26890815	8	141828343	PTK2	Body	0,284	3,82E-07	9,67E-06
cg16094186	4	147044796		IGR	-0,238	3,82E-07	9,68E-06
cg08143698	1	165471527	LOC400794	Body	-0,21	3,83E-07	9,69E-06
cg00748566	5	108747559		IGR	-0,223	3,83E-07	9,69E-06
cg13840445	6	53530628	KLHL31	TSS200	0,238	3,83E-07	9,69E-06
cg21690978	11	12133333	MICAL2	5'UTR	0,203	3,83E-07	9,70E-06
cg05192497	1	147052131	BCL9	5'UTR	-0,327	3,83E-07	9,70E-06
cg20314480	15	81591736	IL16	5'UTR	-0,273	3,84E-07	9,71E-06
cg02400938	11	66141076	SLC29A2	TSS1500	0,221	3,84E-07	9,71E-06
cg14038575	1	214432990		IGR	-0,279	3,84E-07	9,72E-06
cg24627036	15	99644088	SYNM	TSS1500	-0,21	3,84E-07	9,72E-06
cg23218559	19	2250956	AMH	Body	0,216	3,84E-07	9,72E-06
cg16560595	15	89648789	ABHD2	5'UTR	0,207	3,85E-07	9,73E-06
cg16967578	1	218575437	TGFB2	Body	-0,238	3,86E-07	9,75E-06
cg08070200	4	187572960	FAT1	Body	0,284	3,87E-07	9,76E-06

cg20176142	8	81408357	ZBTB10	Body	0,225	3,87E-07	9,76E-06
cg16505233	3	5239311	EDEM1	Body	-0,268	3,87E-07	9,77E-06
cg00679477	11	78023985	GAB2	5'UTR	-0,216	3,87E-07	9,77E-06
cg06568744	9	128047310	GAPVD1	5'UTR	0,219	3,87E-07	9,77E-06
cg21089993	12	109022454	SELPLG	Body	-0,203	3,87E-07	9,77E-06
cg20100801	12	95987654		IGR	0,226	3,88E-07	9,77E-06
cg02652344	9	123553595	FBXW2	5'UTR	0,277	3,88E-07	9,78E-06
cg09838062	3	185407926	IGF2BP2	Body	-0,211	3,88E-07	9,78E-06
cg03413960	22	31136755	OSBP2	Body	-0,207	3,88E-07	9,78E-06
cg03593731	2	201606256	AOX2P	Body	-0,231	3,88E-07	9,78E-06
cg27642002	11	122958609	CLMP	Body	-0,323	3,88E-07	9,78E-06
cg24958342	10	22375787		IGR	0,26	3,88E-07	9,79E-06
cg18480318	2	133667120	MIR7853	Body	0,253	3,89E-07	9,80E-06
cg12403514	19	13124343	NFIX	Body	-0,217	3,89E-07	9,80E-06
cg14209247	3	14307176		IGR	-0,202	3,89E-07	9,80E-06
cg27519828	16	85394177		IGR	-0,229	3,90E-07	9,81E-06
cg10497447	13	77427321		IGR	-0,222	3,90E-07	9,81E-06
cg06427571	4	189863116		IGR	-0,217	3,91E-07	9,83E-06
cg03404493	20	5153652	CDS2	Body	0,212	3,91E-07	9,84E-06
cg16509569	12	54891634	NCKAP1L	1stExon	-0,267	3,92E-07	9,84E-06
cg09445682	8	142013703		IGR	-0,236	3,92E-07	9,85E-06
cg04762199	1	181048756		IGR	-0,229	3,92E-07	9,85E-06
cg10809828	1	32722278	LCK	5'UTR	-0,234	3,93E-07	9,86E-06
cg00856041	6	27798455		IGR	-0,222	3,93E-07	9,87E-06
cg11954763	3	132928979	TMEM108	5'UTR	-0,302	3,93E-07	9,87E-06
cg01695954	2	10262019	RRM2	TSS1500	-0,21	3,93E-07	9,88E-06
cg10920240	5	139986986		IGR	0,306	3,94E-07	9,88E-06
cg22775310	3	137833648	DZIP1L	5'UTR	0,234	3,94E-07	9,89E-06
cg05260411	11	67331241		IGR	0,21	3,94E-07	9,89E-06
cg25060738	6	108108559	SCML4	5'UTR	-0,233	3,95E-07	9,90E-06
cg13376219	16	67422624		IGR	-0,209	3,95E-07	9,90E-06
cg04751078	2	165730100		IGR	-0,221	3,95E-07	9,90E-06
cg01343543	2	235887633	SH3BP4	5'UTR	0,205	3,95E-07	9,90E-06
cg05546847	2	85975173		IGR	-0,308	3,95E-07	9,91E-06
cg19748086	12	70300410	MYRFL	Body	-0,283	3,95E-07	9,91E-06
cg18970534	8	38862157	ADAM9	Body	0,208	3,96E-07	9,92E-06
cg24790126	4	83057688		IGR	-0,241	3,96E-07	9,92E-06
cg19895047	8	19460197	CSGALNACT1	TSS200	0,227	3,96E-07	9,92E-06
cg20555589	20	39343310		IGR	-0,205	3,96E-07	9,92E-06
cg26209441	1	12501684	VPS13D	Body	0,228	3,96E-07	9,92E-06
cg14229655	9	98052350	FANCC	5'UTR	-0,226	3,96E-07	9,93E-06
cg07703370	17	52938144		IGR	0,23	3,97E-07	9,93E-06
cg10267515	1	151117829	SEMA6C	5'UTR	0,227	3,97E-07	9,93E-06
cg25011396	8	103395444	UBR5	Body	0,244	3,97E-07	9,93E-06
cg04931655	16	27414210	IL21R	5'UTR	-0,205	3,97E-07	9,93E-06
cg08044927	16	33948849		IGR	0,205	3,97E-07	9,93E-06
cg11851656	12	10396552		IGR	-0,361	3,97E-07	9,94E-06
cg01000850	1	209941843	TRAF3IP3	1stExon	-0,35	3,97E-07	9,94E-06
cg16808149	3	124782311		IGR	0,296	3,97E-07	9,94E-06
cg01247693	2	101468450	NPAS2	5'UTR	-0,22	3,98E-07	9,94E-06

cg24497361	11	3858493	RHOG	5'UTR	-0,2	3,98E-07	9,94E-06
cg04679295	12	12686360	DUSP16	5'UTR	0,324	3,97E-07	9,94E-06
cg18417313	7	41910163		IGR	-0,309	3,98E-07	9,94E-06
cg26282283	7	2704368	TTYH3	3'UTR	-0,202	3,98E-07	9,94E-06
cg22124241	4	152022611	RPS3A	Body	-0,227	3,99E-07	9,96E-06
cg00871487	5	170288779	RANBP17	TSS200	0,286	3,99E-07	9,96E-06
cg23128495	12	106254483		IGR	-0,204	3,99E-07	9,97E-06
cg08666741	9	116861440	KIF12	TSS200	0,201	3,99E-07	9,97E-06
cg15782344	2	69016925	ARHGAP25	Body	-0,208	4,00E-07	9,98E-06
cg01270709	11	57519364	BTBD18	TSS200	0,219	4,00E-07	9,98E-06
cg16137493	7	37382349	ELMO1	ExonBnd	-0,362	4,00E-07	9,98E-06
cg23346933	6	46012788	CLIC5	Body	0,265	4,00E-07	9,99E-06
cg11554605	7	95115354	ASB4	1stExon	0,215	4,00E-07	9,99E-06
cg13854271	9	82208129	TLE4	5'UTR	-0,273	4,00E-07	9,99E-06
cg09443153	10	100156205	PYROXD2	Body	-0,203	4,00E-07	9,99E-06
cg25678587	11	6129408	OR56B4	1stExon	-0,218	4,00E-07	9,99E-06
cg09946623	16	29673933	SPN	TSS1500	-0,239	4,00E-07	9,99E-06
cg05631194	6	31146417	PSORS1C3	TSS1500	-0,253	4,00E-07	9,99E-06
cg15754627	6	70589534	COL19A1	ExonBnd	0,285	4,01E-07	1,00E-05
cg18210414	1	46113135	RPS15AP10	TSS1500	0,221	4,02E-07	1,00E-05
cg18680612	20	52456134		IGR	-0,224	4,02E-07	1,00E-05
cg26104464	9	112983796		IGR	-0,204	4,02E-07	1,00E-05
cg10489463	2	33546572	LTBP1	Body	-0,256	4,02E-07	1,00E-05
cg14385738	1	114414340	PTPN22	5'UTR	-0,223	4,03E-07	1,00E-05
cg22955778	6	27798556		IGR	-0,206	4,03E-07	1,00E-05
cg26945376	12	122897502	CLIP1	5'UTR	-0,242	4,03E-07	1,00E-05
cg03634452	15	71626380	THSD4	Body	-0,248	4,04E-07	1,01E-05
cg24970080	13	75549875		IGR	-0,235	4,04E-07	1,01E-05
cg19247448	3	187653484		IGR	-0,225	4,05E-07	1,01E-05
cg17155068	5	114567574	PGGT1B	Body	-0,211	4,05E-07	1,01E-05
cg08626131	5	42812509	SEPP1	TSS1500	0,281	4,05E-07	1,01E-05
cg01509792	21	43608929		IGR	-0,218	4,05E-07	1,01E-05
cg16225947	15	30011038	TJP1	Body	-0,207	4,05E-07	1,01E-05
cg27079371	21	19165713	C21orf91	3'UTR	-0,232	4,05E-07	1,01E-05
cg10523399	10	12606492	CAMK1D	Body	-0,213	4,05E-07	1,01E-05
cg23778422	14	72053177	SIPA1L1	TSS1500	0,289	4,05E-07	1,01E-05
cg16565154	1	151129062	TNFAIP8L2	TSS200	-0,294	4,06E-07	1,01E-05
cg04737115	1	65356919	JAK1	5'UTR	0,208	4,06E-07	1,01E-05
cg06031797	1	228889801		IGR	0,22	4,06E-07	1,01E-05
cg05927789	2	47171414	TTC7A	Body	-0,245	4,06E-07	1,01E-05
cg22625504	20	36615509	TTI1	Body	0,248	4,06E-07	1,01E-05
cg21980864	20	62666983	LINC00176	Body	-0,207	4,06E-07	1,01E-05
cg09393083	11	13462227	BTBD10	TSS1500	0,236	4,07E-07	1,01E-05
cg23923934	6	31322914	HLA-B	Body	-0,245	4,08E-07	1,01E-05
cg08211591	8	48513516	SPIDR	Body	-0,303	4,08E-07	1,01E-05
cg19983391	13	29115045		IGR	-0,215	4,08E-07	1,01E-05
cg06943751	6	154566747	OPRM1	Body	-0,236	4,08E-07	1,01E-05
cg21351795	2	173422338	PDK1	Body	0,211	4,08E-07	1,01E-05
cg08059631	8	6274876	MCPH1	Body	-0,233	4,09E-07	1,01E-05
cg18828137	12	52968145	KRT74	TSS1500	-0,204	4,09E-07	1,01E-05

cg11927451	4	77511497	SHROOM3	Body	0,243	4,09E-07	1,01E-05
cg21117978	2	64957132		IGR	-0,204	4,09E-07	1,01E-05
cg19090437	4	38080799	TBC1D1	Body	-0,245	4,09E-07	1,01E-05
cg03601752	19	2621851	GNG7	5'UTR	-0,342	4,09E-07	1,01E-05
cg09130377	19	10215638	PPAN-P2RY11	TSS1500	-0,211	4,10E-07	1,01E-05
cg00875832	1	169646803		IGR	-0,289	4,10E-07	1,02E-05
cg15174220	2	202125310	CASP8	1stExon	-0,276	4,10E-07	1,02E-05
cg08194377	6	34984836	ANKS1A	Body	0,292	4,10E-07	1,02E-05
cg16128973	15	42749836	ZNF106	TSS200	0,241	4,11E-07	1,02E-05
cg03694035	11	59342627	OSBP	3'UTR	0,207	4,11E-07	1,02E-05
cg12273426	9	5438510	PLGRKT	TSS1500	-0,285	4,12E-07	1,02E-05
cg02039225	5	180231650	MGAT1	TSS1500	-0,209	4,12E-07	1,02E-05
cg03611552	4	36131126	ARAP2	Body	0,272	4,12E-07	1,02E-05
cg07109453	1	234669825		IGR	0,224	4,12E-07	1,02E-05
cg02643971	17	68071130	KCNJ16	TSS1500	-0,259	4,12E-07	1,02E-05
cg10284884	11	121000571	TECTA	Body	0,256	4,12E-07	1,02E-05
cg01707219	15	68605424	ITGA11	Body	-0,245	4,12E-07	1,02E-05
cg14753757	3	127682324	KBTBD12	Body	-0,214	4,13E-07	1,02E-05
cg15073784	2	105406666		IGR	0,237	4,13E-07	1,02E-05
cg16836719	22	47146104		IGR	-0,263	4,13E-07	1,02E-05
cg00004996	8	17271535	MTMR7	TSS1500	0,201	4,13E-07	1,02E-05
cg18860449	2	30488769		IGR	-0,349	4,13E-07	1,02E-05
cg26508164	2	182650931		IGR	-0,218	4,13E-07	1,02E-05
cg15493737	13	29113107		IGR	-0,254	4,14E-07	1,02E-05
cg14169134	6	14749088		IGR	-0,245	4,14E-07	1,02E-05
cg05265707	5	157986062		IGR	-0,204	4,14E-07	1,02E-05
cg03555751	6	4299961		IGR	-0,208	4,14E-07	1,02E-05
cg01996116	3	42846907	HIGD1A	TSS1500	0,226	4,14E-07	1,02E-05
cg05692712	20	37502192	PPP1R16B	Body	-0,278	4,14E-07	1,02E-05
cg04487296	7	149389669		IGR	0,266	4,15E-07	1,02E-05
cg13572776	12	76118945		IGR	0,257	4,15E-07	1,02E-05
cg06382922	11	11524269	GALNT18	Body	0,213	4,15E-07	1,02E-05
cg25986527	5	175969383	CDHR2	TSS200	-0,263	4,15E-07	1,02E-05
cg08155032	20	17828838		IGR	-0,232	4,16E-07	1,03E-05
cg27506442	13	30948716	LOC1001889	TSS1500	-0,262	4,16E-07	1,03E-05
cg00240828	14	54692574		IGR	0,242	4,16E-07	1,03E-05
cg05360477	2	12959313		IGR	0,209	4,16E-07	1,03E-05
cg05651417	7	26333515	SNX10	5'UTR	-0,265	4,17E-07	1,03E-05
cg14824005	3	105895856		IGR	-0,24	4,18E-07	1,03E-05
cg09953116	21	27787702		IGR	-0,218	4,18E-07	1,03E-05
cg04266019	16	71963109	IST1	3'UTR	0,264	4,18E-07	1,03E-05
cg11947295	17	75471214	sept-09	TSS200	-0,307	4,18E-07	1,03E-05
cg00736744	1	66748057	PDE4B	Body	-0,268	4,19E-07	1,03E-05
cg07987463	6	143833179	FUCA2	TSS200	0,212	4,19E-07	1,03E-05
cg09785483	14	72985239	RGS6	ExonBnd	-0,239	4,19E-07	1,03E-05
cg27225309	2	213969340	IKZF2	Body	0,218	4,19E-07	1,03E-05
cg08577426	6	31876831	C2	5'UTR	-0,228	4,20E-07	1,03E-05
cg25286679	1	245879613		IGR	-0,204	4,20E-07	1,03E-05
cg14883961	8	127846077		IGR	0,204	4,20E-07	1,03E-05
cg22368454	16	19528985	GDE1	Body	-0,238	4,21E-07	1,03E-05

cg09979692	7	771007	DNAAF5	Body	0,213	4,21E-07	1,03E-05
cg17524466	3	46411369	CCR5	TSS1500	-0,235	4,21E-07	1,04E-05
cg12011727	15	96831838	NR2F2-AS1	Body	-0,232	4,21E-07	1,04E-05
cg20604028	8	29413587		IGR	0,201	4,21E-07	1,04E-05
cg16897937	3	53136880	RFT1	Body	-0,276	4,22E-07	1,04E-05
cg02234653	2	224625080	AP1S3	Body	-0,212	4,22E-07	1,04E-05
cg12185402	4	143337884	INPP4B	Body	-0,231	4,22E-07	1,04E-05
cg19409962	11	67309816		IGR	-0,218	4,23E-07	1,04E-05
cg09092102	10	43870071	FXYD4	Body	-0,209	4,23E-07	1,04E-05
cg13654873	14	31974893		IGR	0,278	4,23E-07	1,04E-05
cg22196855	18	389881	COLEC12	Body	-0,201	4,23E-07	1,04E-05
cg00886738	15	62798237		IGR	0,254	4,24E-07	1,04E-05
cg04635482	8	121045979	DEPTOR	Body	-0,209	4,24E-07	1,04E-05
cg22158366	2	121437519		IGR	-0,211	4,24E-07	1,04E-05
cg23166376	20	57127543	APCDD1L-AS1	Body	-0,229	4,25E-07	1,04E-05
cg10271794	4	163033864	FSTL5	5'UTR	-0,213	4,25E-07	1,04E-05
cg08338683	3	170048267		IGR	-0,242	4,25E-07	1,04E-05
cg17722435	7	22604019		IGR	-0,215	4,25E-07	1,04E-05
cg20484417	12	81763492	PPFIA2	Body	-0,202	4,26E-07	1,04E-05
cg10251354	18	45682442		IGR	-0,24	4,26E-07	1,04E-05
cg13440692	1	1186357		IGR	-0,255	4,26E-07	1,04E-05
cg17834343	3	112013162	SLC9C1	TSS200	0,273	4,26E-07	1,04E-05
cg03090734	10	44525757		IGR	-0,262	4,26E-07	1,04E-05
cg23825512	15	50476073	SLC27A2	Body	-0,204	4,26E-07	1,04E-05
cg26357116	5	76675528	PDE8B	Body	-0,314	4,27E-07	1,04E-05
cg06484182	6	2861746	SERPINB9P1	Body	-0,239	4,27E-07	1,04E-05
cg24808901	1	95698954	RWDD3	TSS1500	0,223	4,27E-07	1,04E-05
cg17281840	8	134106388	TG	Body	-0,283	4,27E-07	1,04E-05
cg16596069	2	16059540	MYCNUT	TSS1500	-0,23	4,27E-07	1,05E-05
cg18046575	13	27628354		IGR	0,207	4,27E-07	1,05E-05
cg19073708	20	23075550		IGR	-0,205	4,28E-07	1,05E-05
cg20551080	22	28012700		IGR	-0,245	4,28E-07	1,05E-05
cg03352173	3	112013130	SLC9A10	TSS200	0,278	4,28E-07	1,05E-05
cg02363526	7	150674012	KCNH2	Body	0,214	4,29E-07	1,05E-05
cg04224025	1	10992454		IGR	-0,202	4,29E-07	1,05E-05
cg05249479	14	100542584	EVL	Body	-0,245	4,30E-07	1,05E-05
cg27415283	17	75437699	sept-09	Body	-0,27	4,30E-07	1,05E-05
cg09992746	15	60285821		IGR	0,258	4,30E-07	1,05E-05
cg05345154	19	2250901	AMH	Body	0,213	4,30E-07	1,05E-05
cg20485084	1	27945005	FGR	Body	-0,283	4,31E-07	1,05E-05
cg15810415	4	111397134	ENPEP	TSS200	0,246	4,31E-07	1,05E-05
cg26457248	18	74840216	MBP	5'UTR	-0,215	4,31E-07	1,05E-05
cg09638208	16	29757318	C16orf54	1stExon	-0,29	4,32E-07	1,05E-05
cg14375300	8	131146876	ASAP1	Body	-0,217	4,32E-07	1,05E-05
cg27531236	4	47854334	NFXL1	Body	0,26	4,32E-07	1,05E-05
cg06754803	1	154407300	IL6R	Body	0,206	4,32E-07	1,05E-05
cg24373619	10	96059372	PLCE1	Body	-0,291	4,32E-07	1,05E-05
cg14495538	1	245118072		IGR	0,246	4,33E-07	1,05E-05
cg01296173	15	34535560	SLC12A6	Body	0,201	4,33E-07	1,05E-05
cg23634134	14	21082393		IGR	0,248	4,33E-07	1,05E-05

cg04184094	2	219187299	PNKD	TSS1500	0,205	4,33E-07	1,06E-05
cg03337057	19	50249776	TSKS	Body	0,215	4,34E-07	1,06E-05
cg08028179	1	23786288	ASAP3	Body	-0,236	4,34E-07	1,06E-05
cg13156132	2	235399596		IGR	-0,256	4,34E-07	1,06E-05
cg20002207	4	57957811	IGFBP7	Body	0,272	4,34E-07	1,06E-05
cg14010405	1	89357911	GTF2B	TSS1500	0,225	4,35E-07	1,06E-05
cg22466032	22	24681310	SPECC1L	5'UTR	-0,209	4,36E-07	1,06E-05
cg16530086	10	11064196	CUGBP2	Body	0,287	4,36E-07	1,06E-05
cg15928106	7	130646078	FLJ43663	Body	-0,347	4,36E-07	1,06E-05
cg27474900	3	71775763	EIF4E3	TSS1500	-0,203	4,36E-07	1,06E-05
cg23485591	7	25601773		IGR	-0,201	4,37E-07	1,06E-05
cg12084990	10	91276070	SLC16A12	5'UTR	0,31	4,37E-07	1,06E-05
cg18742182	4	14918788	CPEB2-AS1	Body	0,208	4,37E-07	1,06E-05
cg21121843	4	3203982	HTT	Body	-0,319	4,38E-07	1,06E-05
cg12515939	7	130641999	LINC-PINT	Body	-0,26	4,38E-07	1,06E-05
cg17537683	2	55239067	RTN4	Body	0,259	4,38E-07	1,06E-05
cg25780496	15	101137253	LINS	5'UTR	-0,33	4,39E-07	1,06E-05
cg13520278	15	70994404	UACA	1stExon	0,235	4,39E-07	1,07E-05
cg20472746	13	42039334	C13orf15	Body	-0,206	4,40E-07	1,07E-05
cg02669047	1	206931233		IGR	-0,274	4,40E-07	1,07E-05
cg08625564	17	1553453	RILP	TSS200	0,203	4,40E-07	1,07E-05
cg09569569	2	74645769	C2orf81	TSS1500	0,205	4,40E-07	1,07E-05
cg08540215	7	150548765	ABP1	TSS1500	-0,25	4,40E-07	1,07E-05
cg14407819	1	110477054		IGR	-0,208	4,40E-07	1,07E-05
cg09997693	5	138263581	CTNNA1	Body	0,25	4,40E-07	1,07E-05
cg20973735	11	67251677	AIP	Body	-0,251	4,41E-07	1,07E-05
cg23787348	4	144110710	USP38	5'UTR	-0,244	4,41E-07	1,07E-05
cg05596756	12	47610220	FAM113B	5'UTR	-0,3	4,41E-07	1,07E-05
cg22687798	4	147111879		IGR	0,216	4,41E-07	1,07E-05
cg01798341	17	80842262	TBCD	Body	-0,212	4,41E-07	1,07E-05
cg11555385	9	124738213	TTLL11	Body	-0,224	4,41E-07	1,07E-05
cg17080168	12	120690045	PXN	Body	-0,291	4,41E-07	1,07E-05
cg24586978	13	99612723	DOCK9	Body	0,247	4,42E-07	1,07E-05
cg08460026	2	204732474	CTLA4	TSS200	-0,257	4,43E-07	1,07E-05
cg11864477	1	184130680		IGR	-0,208	4,43E-07	1,07E-05
cg26950531	19	38704515	DPF1	Body	-0,287	4,43E-07	1,07E-05
cg07217653	15	101591436	LRRK1	Body	-0,201	4,44E-07	1,07E-05
cg02288628	18	60829008	BCL2	Body	-0,339	4,44E-07	1,07E-05
cg26528744	10	75240045	PPP3CB	5'UTR	-0,244	4,44E-07	1,07E-05
cg20792833	11	67205195	PTPRCAP	TSS200	-0,239	4,44E-07	1,07E-05
cg26033526	6	32819858	TAP1	Body	-0,346	4,45E-07	1,07E-05
cg03065503	4	4442273	STX18	Body	-0,241	4,45E-07	1,07E-05
cg04043486	6	127663644	ECHDC1	TSS200	0,201	4,45E-07	1,07E-05
cg25123362	10	133793734	BNIP3	Body	0,235	4,45E-07	1,07E-05
cg16576288	11	33201323	CSTF3-AS1	Body	-0,225	4,45E-07	1,07E-05
cg22359581	19	48229109	EHD2	Body	0,231	4,45E-07	1,07E-05
cg05595142	1	228004967	PRSS38	Body	-0,231	4,45E-07	1,07E-05
cg24744014	2	11113304		IGR	-0,251	4,45E-07	1,07E-05
cg16777413	5	138211013	LRRTM2	1stExon	0,201	4,45E-07	1,07E-05
cg01110839	11	47437783	SLC39A13	3'UTR	0,236	4,46E-07	1,08E-05

cg21210903	18	55335379	ATP8B1	Body	0,215	4,46E-07	1,08E-05
cg04589048	2	223724730	ACSL3	TSS1500	0,267	4,46E-07	1,08E-05
cg18168364	18	9083394		IGR	-0,247	4,47E-07	1,08E-05
cg22032364	13	26112093	ATP8A2	Body	-0,24	4,47E-07	1,08E-05
cg22922632	2	40165857	SLC8A1-AS1	Body	-0,205	4,47E-07	1,08E-05
cg19438746	4	174191964	GALNT7	Body	0,233	4,47E-07	1,08E-05
cg18478319	13	99405122	SLC15A1	TSS200	0,205	4,47E-07	1,08E-05
cg07685795	14	54418880	BMP4	Body	0,208	4,48E-07	1,08E-05
cg03635766	17	38607865	IGFBP4	Body	0,278	4,48E-07	1,08E-05
cg19911385	13	99856297	UBAC2	Body	-0,224	4,48E-07	1,08E-05
cg16044109	3	124762597	HEG1	Body	0,226	4,49E-07	1,08E-05
cg17788468	10	112628934	PDCD4-AS1	Body	-0,267	4,49E-07	1,08E-05
cg17607238	6	154569104	IPCEF1	Body	-0,208	4,49E-07	1,08E-05
cg10029065	10	22895256	PIP4K2A	Body	-0,24	4,49E-07	1,08E-05
cg00996231	6	108910471	FOXO3	Body	0,236	4,50E-07	1,08E-05
cg04600297	19	42750575		IGR	0,207	4,50E-07	1,08E-05
cg16171281	18	73167422		IGR	0,211	4,50E-07	1,08E-05
cg07303091	12	42863385	PRICKLE1	Body	-0,305	4,51E-07	1,08E-05
cg01902081	16	79019007	WWOX	Body	0,227	4,51E-07	1,08E-05
cg14967268	5	37198289	C5orf42	Body	-0,267	4,51E-07	1,08E-05
cg19343518	6	157508679	ARID1B	Body	0,212	4,51E-07	1,09E-05
cg00186224	10	21369318	NEBL	Body	-0,224	4,52E-07	1,09E-05
cg08518551	1	110161942	AMPD2	TSS1500	-0,215	4,52E-07	1,09E-05
cg09247619	1	198648849	PTPRC	Body	-0,27	4,53E-07	1,09E-05
cg02120866	6	6804127		IGR	-0,247	4,53E-07	1,09E-05
cg17366276	14	93052524	RIN3	Body	-0,24	4,54E-07	1,09E-05
cg25524245	6	105674581		IGR	0,237	4,54E-07	1,09E-05
cg21952207	2	231091920	SP110	TSS1500	-0,257	4,54E-07	1,09E-05
cg20442926	22	27431824		IGR	-0,226	4,55E-07	1,09E-05
cg26106365	19	38530902	SIPA1L3	5'UTR	-0,21	4,55E-07	1,09E-05
cg21464565	7	27141139	HOXA2	Body	0,247	4,55E-07	1,09E-05
cg24032265	8	128807841	MIR1204	TSS1500	-0,349	4,55E-07	1,09E-05
cg21414092	15	81248254	MESDC2	Body	-0,21	4,55E-07	1,09E-05
cg00475490	11	86517110	PRSS23	5'UTR	-0,201	4,56E-07	1,09E-05
cg02441149	13	107159723	EFNB2	Body	-0,207	4,56E-07	1,09E-05
cg04705737	1	48417583	TRABD2B	Body	-0,208	4,56E-07	1,09E-05
cg25763709	9	34191833	UBAP1	5'UTR	0,208	4,57E-07	1,09E-05
cg08651417	8	141172195	TRAPPC9	Body	-0,213	4,57E-07	1,09E-05
cg23404351	5	21751337	CDH12	3'UTR	0,281	4,57E-07	1,09E-05
cg12253357	2	237574509		IGR	-0,236	4,57E-07	1,09E-05
cg09481212	12	32756971	FGD4	5'UTR	-0,245	4,59E-07	1,10E-05
cg27567319	4	153001160		IGR	-0,213	4,59E-07	1,10E-05
cg10303013	1	219378340	LYPLAL1	Body	0,208	4,59E-07	1,10E-05
cg04877689	11	35166136	CD44	Body	-0,258	4,59E-07	1,10E-05
cg07230600	17	36207457	YWHAEP7	Body	-0,202	4,60E-07	1,10E-05
cg00845602	10	31270080	ZNF438	Body	0,208	4,61E-07	1,10E-05
cg21104276	7	90896572	FZD1	1stExon	0,243	4,61E-07	1,10E-05
cg22784513	17	29895360		IGR	-0,244	4,61E-07	1,10E-05
cg20402826	2	198077081		IGR	-0,205	4,61E-07	1,10E-05
cg07803375	7	811206	HEATR2	Body	0,241	4,61E-07	1,10E-05

cg03655775	18	13558635	LDLRAD4	Body	0,235	4,62E-07	1,10E-05
cg26868659	3	150019285	LINC01214	Body	-0,251	4,62E-07	1,10E-05
cg11564884	9	71040282	PGM5	Body	-0,225	4,62E-07	1,10E-05
cg12031524	8	82396390	FABP4	TSS1500	-0,218	4,63E-07	1,10E-05
cg13524257	10	80698358		IGR	-0,21	4,63E-07	1,10E-05
cg24275848	7	80231487	CD36	TSS200	0,201	4,63E-07	1,10E-05
cg03896542	16	56378687	GNAO1	Body	-0,208	4,64E-07	1,11E-05
cg11499236	4	103520170	NFKB1	Body	-0,268	4,64E-07	1,11E-05
cg26014699	19	47282122	SLC1A5	Body	-0,275	4,64E-07	1,11E-05
cg01167556	12	56323639	DGKA	TSS1500	-0,314	4,64E-07	1,11E-05
cg18555277	6	170581248		IGR	-0,358	4,64E-07	1,11E-05
cg09311683	20	2801995		IGR	0,272	4,65E-07	1,11E-05
cg21902394	17	19883326		IGR	0,204	4,65E-07	1,11E-05
cg03483626	1	111218276	KCNA3	TSS1500	0,201	4,65E-07	1,11E-05
cg00640479	7	218726	FAM20C	Body	-0,204	4,66E-07	1,11E-05
cg03994381	2	160471518	LOC643072	TSS1500	0,216	4,66E-07	1,11E-05
cg23608915	2	102333795	MAP4K4	Body	-0,219	4,66E-07	1,11E-05
cg00170295	6	46720370	LOC1002877[illegible]	Body	-0,214	4,66E-07	1,11E-05
cg10672754	18	56486327		IGR	-0,253	4,66E-07	1,11E-05
cg19690404	7	129933646	CPA4	Body	-0,225	4,66E-07	1,11E-05
cg26539571	6	16731770	ATXN1	5'UTR	-0,225	4,66E-07	1,11E-05
cg11860632	1	9486173	LOC1005060[illegible]	Body	-0,232	4,67E-07	1,11E-05
cg14763798	2	133431884	NCKAP5	Body	-0,214	4,67E-07	1,11E-05
cg16162843	11	12983482		IGR	-0,23	4,68E-07	1,11E-05
cg20459035	5	128433455	ISOC1	Body	0,213	4,68E-07	1,11E-05
cg09015797	6	27791239	HIST1H4J	TSS1500	0,254	4,68E-07	1,11E-05
cg03043417	1	111415781	CD53	1stExon	-0,209	4,69E-07	1,11E-05
cg01562556	10	49677004	ARHGAP22	Body	-0,236	4,69E-07	1,11E-05
cg03809883	10	3920005		IGR	-0,225	4,69E-07	1,12E-05
cg25939505	15	74693354		IGR	-0,208	4,69E-07	1,12E-05
cg08182501	2	177003406		IGR	0,215	4,69E-07	1,12E-05
cg26686403	14	91710747	GPR68	5'UTR	-0,218	4,69E-07	1,12E-05
cg26096333	8	42358481	SLC20A2	5'UTR	0,335	4,70E-07	1,12E-05
cg14684434	5	53829957	SNX18	Body	0,218	4,70E-07	1,12E-05
cg27548323	11	35294221	SLC1A2	Body	-0,322	4,70E-07	1,12E-05
cg06573787	8	143070187		IGR	0,205	4,70E-07	1,12E-05
cg09262230	6	159052937	TMEM181	3'UTR	0,203	4,70E-07	1,12E-05
cg24640583	22	41122692		IGR	-0,292	4,70E-07	1,12E-05
cg14557185	3	149374763	WWTR1	Body	0,246	4,71E-07	1,12E-05
cg11744047	9	92195050		IGR	-0,208	4,71E-07	1,12E-05
cg12053199	8	94029904		IGR	0,267	4,72E-07	1,12E-05
cg01916610	15	75342802	PPCDC	3'UTR	0,219	4,72E-07	1,12E-05
cg22144990	21	45509539	TRAPPC10	Body	-0,23	4,72E-07	1,12E-05
cg11559014	18	61576332		IGR	-0,213	4,72E-07	1,12E-05
cg10797702	5	1313931		IGR	-0,218	4,73E-07	1,12E-05
cg21553980	17	109444	RPH3AL	Body	0,205	4,73E-07	1,12E-05
cg27563420	15	31758204		IGR	-0,221	4,74E-07	1,12E-05
cg06881898	1	95006539	F3	Body	0,209	4,74E-07	1,12E-05
cg08821232	8	134074665	TG	Body	-0,251	4,74E-07	1,12E-05
cg26932455	2	136577732	LOC1005076[illegible]	TSS200	-0,292	4,74E-07	1,12E-05

cg06601098	16	11319090		IGR	-0,441	4,74E-07	1,12E-05
cg19809645	2	31031251	CAPN13	TSS1500	-0,2	4,75E-07	1,12E-05
cg26227523	15	77287243	PSTPIP1	TSS1500	-0,321	4,75E-07	1,12E-05
cg12122057	11	12133015	MICAL2	5'UTR	0,321	4,76E-07	1,12E-05
cg19576099	6	106531491		IGR	-0,23	4,76E-07	1,13E-05
cg12144393	11	117480983	DSCAML1	Body	-0,207	4,76E-07	1,13E-05
cg22392451	6	10839494	MAK	TSS1500	-0,214	4,76E-07	1,13E-05
cg22562550	1	236039927	LYST	Body	-0,212	4,77E-07	1,13E-05
cg03444495	10	6342269		IGR	-0,227	4,77E-07	1,13E-05
cg19272171	13	97960392	MBNL2	Body	0,256	4,77E-07	1,13E-05
cg20527746	4	160149260		IGR	0,303	4,78E-07	1,13E-05
cg02368064	17	66342776	ARSG	Body	-0,304	4,79E-07	1,13E-05
cg01970322	6	33173489	HSD17B8	Body	-0,203	4,80E-07	1,13E-05
cg07016145	5	154034166		IGR	0,274	4,80E-07	1,13E-05
cg05405150	4	100591876		IGR	0,261	4,80E-07	1,13E-05
cg05370351	5	14203488	TRIO	Body	0,211	4,81E-07	1,13E-05
cg15668538	17	15931082	TTC19	3'UTR	-0,209	4,81E-07	1,13E-05
cg05982345	11	48191068	PTPRJ	3'UTR	0,244	4,81E-07	1,13E-05
cg04147906	17	26373775	NLK	Body	-0,256	4,82E-07	1,14E-05
cg09220563	14	54261853		IGR	-0,23	4,84E-07	1,14E-05
cg03476473	8	81851811		IGR	-0,249	4,84E-07	1,14E-05
cg01049995	3	188353178	LPP	Body	0,219	4,84E-07	1,14E-05
cg23462514	4	1107585	RNF212	TSS200	0,202	4,84E-07	1,14E-05
cg01620410	16	46791076		IGR	0,214	4,84E-07	1,14E-05
cg06180822	8	48812543	PRKDC	Body	-0,206	4,84E-07	1,14E-05
cg18259253	5	60745986	ZSWIM6	Body	-0,207	4,85E-07	1,14E-05
cg17426273	10	21462441	NEBL	Body	0,212	4,85E-07	1,14E-05
cg12068600	1	118353410		IGR	-0,204	4,86E-07	1,14E-05
cg07499032	7	102066852	PRKRIP1	3'UTR	-0,334	4,87E-07	1,15E-05
cg07242032	10	130424244		IGR	-0,25	4,87E-07	1,15E-05
cg22916645	1	209931334	TRAF3IP3	5'UTR	-0,261	4,88E-07	1,15E-05
cg17924421	3	57875594	SLMAP	TSS200	0,249	4,88E-07	1,15E-05
cg01233795	3	143533434	SLC9A9	Body	-0,309	4,88E-07	1,15E-05
cg03505813	15	48744110	FBN1	Body	0,205	4,88E-07	1,15E-05
cg20622669	6	128293285	PTPRK	Body	-0,236	4,88E-07	1,15E-05
cg27345524	15	67393714	SMAD3	Body	-0,282	4,89E-07	1,15E-05
cg15041443	2	28959354		IGR	0,204	4,89E-07	1,15E-05
cg02352716	17	1104805		IGR	-0,262	4,89E-07	1,15E-05
cg10602219	8	142040925		IGR	-0,209	4,89E-07	1,15E-05
cg10094825	10	8642275		IGR	-0,248	4,89E-07	1,15E-05
cg05214708	2	207961006	KLF7	Body	0,241	4,90E-07	1,15E-05
cg14161940	9	14027419		IGR	0,207	4,90E-07	1,15E-05
cg10559416	2	158300485	CYTIP	1stExon	-0,328	4,90E-07	1,15E-05
cg20683207	7	138583193	KIAA1549	Body	-0,292	4,90E-07	1,15E-05
cg22371961	1	169132356	NME7	Body	-0,226	4,90E-07	1,15E-05
cg22559497	16	79662727		IGR	-0,24	4,90E-07	1,15E-05
cg19996939	6	135466480		IGR	-0,272	4,91E-07	1,15E-05
cg26319038	2	218723627	TNS1	Body	0,242	4,91E-07	1,15E-05
cg16365807	21	42559652	BACE2	Body	-0,208	4,91E-07	1,15E-05
cg00221400	18	34235154	FHOD3	Body	0,241	4,92E-07	1,15E-05

cg12081643	13	110802645	COL4A1	3'UTR	-0,223	4,92E-07	1,15E-05
cg11311865	13	79768628		IGR	-0,255	4,92E-07	1,15E-05
cg13900763	9	137160473		IGR	-0,242	4,93E-07	1,15E-05
cg10888620	15	64992351	OAZ2	Body	0,274	4,93E-07	1,15E-05
cg25370025	2	85231178	KCMF1	Body	-0,274	4,94E-07	1,16E-05
cg02695343	12	6881595	LAG3	TSS200	-0,236	4,94E-07	1,16E-05
cg14756780	16	15888406	MYH11	Body	0,222	4,94E-07	1,16E-05
cg00636363	7	112474547	C7orf60	Body	0,211	4,94E-07	1,16E-05
cg05396392	20	57817959	ZNF831	Body	-0,203	4,94E-07	1,16E-05
cg12364786	15	64056664	HERC1	Body	0,241	4,94E-07	1,16E-05
cg14110548	19	36393696	HCST	Body	-0,257	4,95E-07	1,16E-05
cg06982089	8	42359099	SLC20A2	TSS200	0,278	4,95E-07	1,16E-05
cg13811417	9	25677628	TUSC1	1stExon	0,247	4,95E-07	1,16E-05
cg11586258	16	89865681	FANCA	Body	0,306	4,95E-07	1,16E-05
cg17345620	13	97640663	OXGR1	5'UTR	-0,219	4,96E-07	1,16E-05
cg26531286	13	98799358	FARP1	5'UTR	-0,254	4,96E-07	1,16E-05
cg00308563	2	106473189	NCK2	Body	-0,244	4,97E-07	1,16E-05
cg12630243	3	150067033		IGR	-0,213	4,97E-07	1,16E-05
cg15546356	2	10795765	NOL10	Body	-0,242	4,98E-07	1,16E-05
cg26382029	11	70034700	ANO1	3'UTR	-0,232	4,98E-07	1,16E-05
cg01818579	20	55982315	RBM38	Body	0,209	4,98E-07	1,16E-05
cg23111342	11	116857480	SIK3	Body	-0,272	4,98E-07	1,16E-05
cg18953183	5	156607793	ITK	TSS200	-0,284	4,99E-07	1,16E-05
cg01136169	2	55339820		IGR	0,253	4,99E-07	1,16E-05
cg04736120	4	100325677		IGR	-0,223	5,00E-07	1,16E-05
cg27286790	9	85914710	FRMD3	Body	-0,246	5,00E-07	1,17E-05
cg23595884	4	82430098		IGR	-0,216	5,00E-07	1,17E-05
cg20458968	10	121463688		IGR	-0,214	5,00E-07	1,17E-05
cg06580770	6	32054790	TNXB	Body	-0,303	5,00E-07	1,17E-05
cg18824549	17	47807591	FAM117A	Body	0,22	5,00E-07	1,17E-05
cg09340617	2	47212928	TTC7A	Body	-0,218	5,00E-07	1,17E-05
cg19746337	7	112094063	IFRD1	5'UTR	0,266	5,00E-07	1,17E-05
cg11984636	18	74845706	MBP	TSS1500	0,241	5,00E-07	1,17E-05
cg14049706	22	36080711		IGR	0,234	5,00E-07	1,17E-05
cg19040077	2	242702749	D2HGDH	Body	-0,313	5,01E-07	1,17E-05
cg14349855	13	92419985	GPC5	Body	0,201	5,01E-07	1,17E-05
cg20701897	2	179586761	TTN	Body	0,215	5,01E-07	1,17E-05
cg02534418	1	33383395		IGR	-0,295	5,02E-07	1,17E-05
cg24030804	11	11232879		IGR	0,265	5,02E-07	1,17E-05
cg03226737	11	7273049	SYT9	TSS200	0,21	5,03E-07	1,17E-05
cg04202120	12	31883115	AMN1	TSS1500	0,251	5,03E-07	1,17E-05
cg10027161	6	162959683	PARK2	Body	0,23	5,03E-07	1,17E-05
cg02215242	11	62166156		IGR	0,258	5,03E-07	1,17E-05
cg18503195	3	151911602		IGR	-0,333	5,04E-07	1,17E-05
cg18083402	8	100675936	VPS13B	Body	-0,253	5,04E-07	1,17E-05
cg08554554	20	62330618	ARFRP1	3'UTR	-0,204	5,05E-07	1,17E-05
cg20023079	20	34078800	CEP250	Body	-0,262	5,05E-07	1,17E-05
cg18081338	6	17212963		IGR	0,256	5,06E-07	1,17E-05
cg08818207	6	32820355	TAP1	Body	-0,214	5,06E-07	1,17E-05
cg09154959	6	109198550	ARMC2	Body	0,299	5,06E-07	1,18E-05

cg18233931	1	36670099		IGR	0,251	5,06E-07	1,18E-05
cg14741236	5	171985354		IGR	-0,229	5,06E-07	1,18E-05
cg15412371	19	7881574		IGR	-0,205	5,07E-07	1,18E-05
cg09196251	14	95437786		IGR	-0,233	5,07E-07	1,18E-05
cg14607332	1	8658322	RERE	Body	0,202	5,07E-07	1,18E-05
cg15069235	6	114289969	HDAC2	Body	0,218	5,08E-07	1,18E-05
cg16411101	1	54831653	SSBP3	Body	0,24	5,08E-07	1,18E-05
cg08964563	7	116319695	MET	5'UTR	-0,224	5,09E-07	1,18E-05
cg26684689	11	10690857	MRVI1	5'UTR	-0,297	5,10E-07	1,18E-05
cg12459798	12	122230734	RHOF	Body	-0,26	5,11E-07	1,18E-05
cg27664085	15	89157815		IGR	0,282	5,11E-07	1,18E-05
cg10443091	21	26859614		IGR	-0,277	5,12E-07	1,19E-05
cg06425373	9	111725888	CTNNAL1	Body	0,233	5,13E-07	1,19E-05
cg27400216	18	56702913	LOC390858	TSS200	-0,204	5,14E-07	1,19E-05
cg01400004	9	16461676	BNC2	Body	-0,2	5,14E-07	1,19E-05
cg11174024	11	9060910	SCUBE2	Body	-0,21	5,15E-07	1,19E-05
cg26581481	10	115720695		IGR	-0,209	5,15E-07	1,19E-05
cg08398441	10	97096451	SORBS1	Body	-0,241	5,15E-07	1,19E-05
cg24692636	4	88939348	PKD2	Body	0,213	5,15E-07	1,19E-05
cg04517263	9	123689193	TRAF1	TSS200	-0,279	5,15E-07	1,19E-05
cg09365105	9	71838646	TJP2	Body	0,218	5,15E-07	1,19E-05
cg14555284	4	184244765	CLDN24	TSS1500	-0,225	5,15E-07	1,19E-05
cg06156640	2	3704363	ALLC	TSS1500	0,25	5,15E-07	1,19E-05
cg25060269	18	77283883	NFATC1	Body	-0,25	5,16E-07	1,19E-05
cg11252862	7	108167515	PNPLA8	5'UTR	0,207	5,16E-07	1,19E-05
cg05931177	17	73333529	GRB2	Body	-0,226	5,17E-07	1,19E-05
cg24069884	13	111116765	COL4A2	Body	-0,302	5,18E-07	1,20E-05
cg06763443	5	137675015	FAM53C	5'UTR	0,206	5,18E-07	1,20E-05
cg23360308	1	198648220	PTPRC	Body	-0,253	5,19E-07	1,20E-05
cg12120701	5	139926783	EIF4EBP3	TSS1500	0,218	5,19E-07	1,20E-05
cg24226193	7	28191663	JAZF1	Body	-0,305	5,20E-07	1,20E-05
cg04011474	2	28904455		IGR	-0,208	5,21E-07	1,20E-05
cg15788572	13	41059835		IGR	-0,22	5,21E-07	1,20E-05
cg06467945	5	12691677	LINC01194	Body	0,233	5,21E-07	1,20E-05
cg25077558	5	156991510	ADAM19	Body	0,222	5,21E-07	1,20E-05
cg01612110	9	138967027	NACC2	5'UTR	-0,221	5,21E-07	1,20E-05
cg05830760	20	20528394	RALGAPA2	Body	-0,272	5,21E-07	1,20E-05
cg04976933	9	101463365	GABBR2	Body	-0,226	5,23E-07	1,20E-05
cg03421046	9	137856067		IGR	-0,231	5,23E-07	1,20E-05
cg17683940	15	65763347	DPP8	Body	-0,22	5,23E-07	1,20E-05
cg00728566	8	70556437	SULF1	Body	-0,225	5,23E-07	1,20E-05
cg07822203	3	177719585		IGR	-0,203	5,25E-07	1,21E-05
cg24861859	1	175474533	TNR	5'UTR	0,207	5,26E-07	1,21E-05
cg07092748	7	151771858	GALNT11	5'UTR	0,273	5,26E-07	1,21E-05
cg14127705	20	4172792	LINC01433	TSS1500	-0,219	5,26E-07	1,21E-05
cg22803549	14	93215468	LGMN	TSS1500	0,208	5,26E-07	1,21E-05
cg11784053	3	107517460	BBX	Body	0,29	5,27E-07	1,21E-05
cg15695155	12	121973871	KDM2B	Body	0,215	5,27E-07	1,21E-05
cg21035962	13	49142310		IGR	0,207	5,27E-07	1,21E-05
cg03526142	17	33864734	SLFN12L	5'UTR	-0,239	5,28E-07	1,21E-05

cg02532700	22	37257404	NCF4	Body	-0,308	5,28E-07	1,21E-05
cg10228162	9	133589493	ABL1	5'UTR	0,229	5,28E-07	1,21E-05
cg26770787	4	101986946	PPP3CA	Body	0,217	5,29E-07	1,21E-05
cg14780427	17	78754372	RPTOR	Body	-0,268	5,29E-07	1,21E-05
cg13696898	2	37593113	QPCT	Body	-0,265	5,29E-07	1,21E-05
cg21187386	12	12016730	ETV6	Body	0,23	5,30E-07	1,22E-05
cg00769161	12	58130183	AGAP2	Body	0,307	5,30E-07	1,22E-05
cg02471987	5	148170888		IGR	-0,243	5,30E-07	1,22E-05
cg00916635	1	114414312	PTPN22	5'UTR	-0,285	5,31E-07	1,22E-05
cg23750151	6	32808504	PSMB8	3'UTR	-0,237	5,31E-07	1,22E-05
cg18361892	18	56383450	MALT1	Body	-0,253	5,32E-07	1,22E-05
cg17290000	2	12085854		IGR	-0,278	5,32E-07	1,22E-05
cg11462121	12	3201961	TSPAN9	5'UTR	-0,202	5,32E-07	1,22E-05
cg01647795	15	44969244	PATL2	TSS200	-0,212	5,32E-07	1,22E-05
cg18277315	16	88771002	RNF166	TSS1500	-0,316	5,33E-07	1,22E-05
cg02032559	18	33078287	INO80C	TSS1500	0,208	5,33E-07	1,22E-05
cg16927606	19	36233324	TMEM149	1stExon	-0,252	5,33E-07	1,22E-05
cg21453668	10	44839517		IGR	-0,218	5,34E-07	1,22E-05
cg27246361	22	23161339		IGR	-0,213	5,34E-07	1,22E-05
cg00045607	1	161039690	ARHGAP30	1stExon	-0,219	5,35E-07	1,22E-05
cg15035421	3	182631849	ATP11B	Body	-0,227	5,35E-07	1,22E-05
cg25511667	6	33048732	HLA-DPB1	Body	0,231	5,35E-07	1,22E-05
cg23253179	11	68801241		IGR	-0,232	5,35E-07	1,22E-05
cg06495055	4	151505192	MAB21L2	1stExon	0,254	5,35E-07	1,22E-05
cg15046675	19	49838777	CD37	1stExon	-0,31	5,36E-07	1,22E-05
cg11360546	7	1094263	C7orf50	Body	0,217	5,36E-07	1,22E-05
cg24544716	14	88339690		IGR	-0,212	5,37E-07	1,23E-05
cg16599983	6	32168969	NOTCH4	Body	-0,202	5,37E-07	1,23E-05
cg07328962	1	236680069	LGALS8	TSS1500	-0,235	5,37E-07	1,23E-05
cg01170124	12	108962900	ISCU	3'UTR	-0,227	5,37E-07	1,23E-05
cg15832085	6	7861530	BMP6	Body	-0,201	5,37E-07	1,23E-05
cg08660971	3	153778467		IGR	0,211	5,38E-07	1,23E-05
cg09236158	12	68122344	LOC1019279(	Body	0,206	5,38E-07	1,23E-05
cg14643264	9	27408242	MOBKL2B	Body	-0,227	5,38E-07	1,23E-05
cg26534847	20	36932479	BPI	TSS200	-0,214	5,38E-07	1,23E-05
cg07725235	21	18188123		IGR	-0,203	5,40E-07	1,23E-05
cg27264684	18	56517674		IGR	-0,258	5,40E-07	1,23E-05
cg17974015	11	120076520		IGR	-0,259	5,40E-07	1,23E-05
cg05859264	6	36097692	MAPK13	TSS1500	-0,209	5,40E-07	1,23E-05
cg23144046	15	77294905	PSTPIP1	Body	-0,24	5,40E-07	1,23E-05
cg17750024	1	175538621	TNR	5'UTR	-0,227	5,40E-07	1,23E-05
cg04175911	10	99172893		IGR	-0,262	5,40E-07	1,23E-05
cg01138448	15	45028595	TRIM69	5'UTR	-0,203	5,41E-07	1,23E-05
cg11889721	3	188668845		IGR	-0,206	5,41E-07	1,23E-05
cg17216165	3	11475784	ATG7	Body	0,216	5,41E-07	1,23E-05
cg23811850	6	24936246	FAM65B	1stExon	-0,239	5,41E-07	1,23E-05
cg13536866	10	116375533	ABLIM1	Body	-0,226	5,41E-07	1,23E-05
cg21697378	21	43947935	SLC37A1	Body	-0,233	5,42E-07	1,23E-05
cg02881595	4	57958129	IGFBP7	Body	0,236	5,42E-07	1,23E-05
cg25320587	6	1684567	GMDS	Body	0,298	5,42E-07	1,23E-05

cg10713881	12	125390804		IGR	-0,236	5,43E-07	1,24E-05
cg22608108	17	74625283	ST6GALNAC1	Body	-0,201	5,43E-07	1,24E-05
cg19946091	19	54869073	LAIR1	Body	0,241	5,43E-07	1,24E-05
cg06518107	15	49715460	FGF7	1stExon	0,27	5,44E-07	1,24E-05
cg12934686	12	53443962	TNS2	5'UTR	0,204	5,45E-07	1,24E-05
cg13862214	20	51559933		IGR	-0,28	5,45E-07	1,24E-05
cg19281135	7	92271366	CDK6	Body	-0,248	5,45E-07	1,24E-05
cg10438589	4	14531493		IGR	0,203	5,45E-07	1,24E-05
cg07854436	2	177356448		IGR	-0,214	5,46E-07	1,24E-05
cg15910205	1	59859015	FGGY	Body	0,238	5,47E-07	1,24E-05
cg22378449	10	94331624	IDE	Body	0,208	5,47E-07	1,24E-05
cg17996830	11	78616440	ODZ4	Body	-0,234	5,47E-07	1,24E-05
cg05352838	6	33384391	CUTA	3'UTR	-0,224	5,47E-07	1,24E-05
cg10407469	7	144550836		IGR	-0,214	5,47E-07	1,24E-05
cg04629404	4	133046482		IGR	0,278	5,47E-07	1,24E-05
cg16993620	20	46969252		IGR	-0,223	5,47E-07	1,24E-05
cg19997662	15	101784653	CHSY1	Body	-0,341	5,48E-07	1,24E-05
cg07592095	6	149805596	ZC3H12D	5'UTR	-0,24	5,50E-07	1,25E-05
cg00338095	10	36964853		IGR	-0,224	5,50E-07	1,25E-05
cg16651792	18	715830		IGR	-0,285	5,51E-07	1,25E-05
cg09063663	1	16484811		IGR	-0,223	5,51E-07	1,25E-05
cg25876794	19	15054695		IGR	-0,212	5,51E-07	1,25E-05
cg23052776	14	71609158		IGR	-0,283	5,51E-07	1,25E-05
cg09424828	8	26101536		IGR	-0,221	5,52E-07	1,25E-05
cg02841912	6	152955983	SYNE1	5'UTR	-0,288	5,52E-07	1,25E-05
cg03195881	17	75437840	sept-09	Body	-0,309	5,52E-07	1,25E-05
cg13719901	3	46608139	LRRC2	5'UTR	0,223	5,53E-07	1,25E-05
cg05968833	2	86266051	POLR1A	Body	-0,285	5,53E-07	1,25E-05
cg05804702	14	75696910		IGR	-0,292	5,53E-07	1,25E-05
cg00409842	15	86076110	AKAP13	Body	0,23	5,53E-07	1,25E-05
cg01403030	9	19026479	FAM154A	Body	-0,311	5,53E-07	1,25E-05
cg14172108	21	34405553		IGR	0,21	5,54E-07	1,25E-05
cg21150330	3	185060664	MAP3K13	5'UTR	-0,274	5,54E-07	1,25E-05
cg10206601	8	10183609	MSRA	Body	-0,211	5,54E-07	1,25E-05
cg06774142	14	71122191	TTC9	Body	-0,223	5,55E-07	1,26E-05
cg17619803	19	41196047	NUMBL	5'UTR	0,217	5,56E-07	1,26E-05
cg04397137	17	76886912	TIMP2	Body	-0,306	5,57E-07	1,26E-05
cg06391776	8	87417251	WWP1	Body	0,212	5,57E-07	1,26E-05
cg02003199	8	145579422	TMEM249	TSS1500	-0,276	5,57E-07	1,26E-05
cg07143720	17	12453166		IGR	0,294	5,58E-07	1,26E-05
cg18623836	2	10261907	RRM2	TSS1500	-0,225	5,58E-07	1,26E-05
cg00835279	2	3642710	COLEC11	1stExon	0,212	5,58E-07	1,26E-05
cg22902505	4	81119473	PRDM8	5'UTR	0,223	5,59E-07	1,26E-05
cg19233405	1	154988721	ZBTB7B	Body	-0,236	5,59E-07	1,26E-05
cg08806156	15	77913143	LINGO1	Body	-0,262	5,60E-07	1,26E-05
cg03466198	7	50727932	GRB10	Body	-0,23	5,60E-07	1,26E-05
cg13810114	12	26267890		IGR	0,287	5,60E-07	1,26E-05
cg12743248	8	49636840	EFCAB1	3'UTR	-0,21	5,61E-07	1,26E-05
cg11489945	11	122974340	CLMP	Body	0,24	5,61E-07	1,26E-05
cg00260664	7	37105350	ELMO1	Body	-0,211	5,61E-07	1,26E-05

cg14157768	2	239414104		IGR	-0,325	5,61E-07	1,26E-05
cg02778243	2	239563383		IGR	-0,229	5,62E-07	1,27E-05
cg21226201	7	94539112	PPP1R9A	5'UTR	-0,21	5,62E-07	1,27E-05
cg24359397	9	136390696	TMEM8C	TSS1500	-0,233	5,62E-07	1,27E-05
cg25350105	10	3846495		IGR	-0,231	5,62E-07	1,27E-05
cg12521091	1	204642599	LRRN2	5'UTR	-0,21	5,63E-07	1,27E-05
cg03013486	8	17449854	PDGFRL	Body	-0,2	5,63E-07	1,27E-05
cg01063146	20	49134119	PTPN1	5'UTR	-0,218	5,63E-07	1,27E-05
cg13004792	8	1080555		IGR	0,264	5,64E-07	1,27E-05
cg01925738	5	140480770	PCDHB3	1stExon	0,222	5,64E-07	1,27E-05
cg25491066	15	58328952	ALDH1A2	Body	-0,236	5,65E-07	1,27E-05
cg09739536	5	178339445	ZFP2	5'UTR	-0,262	5,65E-07	1,27E-05
cg09499316	20	37987086		IGR	-0,223	5,65E-07	1,27E-05
cg00412514	17	80158796	CCDC57	Body	-0,224	5,66E-07	1,27E-05
cg12749467	2	28551104	BRE	3'UTR	-0,208	5,66E-07	1,27E-05
cg13902777	4	82646474		IGR	-0,205	5,66E-07	1,27E-05
cg04997749	22	20885323	MED15	5'UTR	0,232	5,66E-07	1,27E-05
cg14335536	15	93347244		IGR	-0,273	5,66E-07	1,27E-05
cg27230784	17	5404337	LOC728392	1stExon	-0,208	5,66E-07	1,27E-05
cg04180046	7	45002736	MYO1G	Body	0,239	5,66E-07	1,27E-05
cg20070323	15	72520350	PKM	Body	-0,217	5,67E-07	1,27E-05
cg19297788	21	45602432		IGR	-0,223	5,67E-07	1,27E-05
cg03898321	16	24190035	PRKCB	Body	0,256	5,67E-07	1,27E-05
cg19880425	17	28650886	TMIGD1	Body	-0,258	5,68E-07	1,27E-05
cg10917910	12	95541112	FGD6	Body	-0,231	5,68E-07	1,27E-05
cg09045073	12	78392948	NAV3	Body	-0,234	5,68E-07	1,28E-05
cg11591813	17	59458263	BCAS3	Body	-0,215	5,68E-07	1,28E-05
cg03318985	1	8215809		IGR	0,211	5,69E-07	1,28E-05
cg23848062	20	20018854	CRNKL1	Body	0,209	5,69E-07	1,28E-05
cg20857280	15	51632905	GLDN	TSS1500	-0,222	5,70E-07	1,28E-05
cg05708367	8	66868175		IGR	-0,254	5,70E-07	1,28E-05
cg07143733	21	26938557	MIR155HG	Body	-0,24	5,70E-07	1,28E-05
cg14913407	1	227953546	SNAP47	Body	-0,209	5,71E-07	1,28E-05
cg26205979	11	46443014	AMBRA1	Body	0,2	5,71E-07	1,28E-05
cg11952809	20	52459046		IGR	-0,216	5,71E-07	1,28E-05
cg26418147	1	205743515	RAB7L1	Body	-0,206	5,71E-07	1,28E-05
cg06639053	12	111837470		IGR	-0,227	5,71E-07	1,28E-05
cg10743102	3	177055590		IGR	-0,253	5,71E-07	1,28E-05
cg12148262	2	121415957		IGR	-0,244	5,72E-07	1,28E-05
cg04484639	3	81773021	GBE1	Body	0,207	5,74E-07	1,28E-05
cg23060965	8	42206026	POLB	Body	0,253	5,74E-07	1,28E-05
cg17083132	14	78668470	NRXN3	Body	-0,236	5,74E-07	1,28E-05
cg00021659	2	161996385	TANK	5'UTR	-0,274	5,75E-07	1,29E-05
cg15113110	12	5724577	ANO2	Body	-0,238	5,75E-07	1,29E-05
cg05968188	6	16483997	ATXN1	5'UTR	-0,217	5,75E-07	1,29E-05
cg08231525	21	32505284	TIAM1	Body	-0,2	5,75E-07	1,29E-05
cg06939970	4	26235129		IGR	-0,202	5,76E-07	1,29E-05
cg00729654	20	31123895		IGR	-0,236	5,76E-07	1,29E-05
cg20692845	3	181416497	SOX2-OT	Body	0,226	5,77E-07	1,29E-05
cg26059418	12	24991545	BCAT1	Body	-0,214	5,77E-07	1,29E-05

cg03202693	13	113637903	MCF2L	Body	0,201	5,77E-07	1,29E-05
cg16330369	8	71357702		IGR	0,297	5,77E-07	1,29E-05
cg15911349	11	70189805	PPFIA1	Body	0,208	5,77E-07	1,29E-05
cg14283887	8	35040351		IGR	0,213	5,77E-07	1,29E-05
cg02358647	6	25698362	SCGN	Body	-0,203	5,78E-07	1,29E-05
cg10613387	8	37738443	RAB11FIP1	Body	-0,23	5,78E-07	1,29E-05
cg20788584	10	116004773	VWA2	5'UTR	-0,211	5,79E-07	1,29E-05
cg17749649	12	32635437		IGR	0,235	5,81E-07	1,30E-05
cg27300870	2	99438264	C2orf55	Body	0,21	5,82E-07	1,30E-05
cg03473096	10	76250491	ADK	Body	0,247	5,82E-07	1,30E-05
cg16345972	6	161829484	PARK2	Body	-0,203	5,82E-07	1,30E-05
cg09000502	3	37944377	CTDSPL	Body	-0,299	5,82E-07	1,30E-05
cg08717672	14	100346149	EML1	Body	-0,205	5,83E-07	1,30E-05
cg20066792	20	4664695		IGR	-0,221	5,83E-07	1,30E-05
cg15165122	2	71206291	ANKRD53	Body	0,205	5,84E-07	1,30E-05
cg11424970	2	71969999		IGR	-0,222	5,85E-07	1,30E-05
cg15139596	19	36233380	TMEM149	TSS200	-0,249	5,86E-07	1,30E-05
cg23318800	1	150656569	GOLPH3L	Body	-0,234	5,86E-07	1,30E-05
cg13858974	9	86149293	FRMD3	Body	-0,266	5,86E-07	1,30E-05
cg00461077	8	93149010		IGR	-0,237	5,88E-07	1,31E-05
cg00598772	8	119909801		IGR	-0,221	5,89E-07	1,31E-05
cg06138935	16	49586623	ZNF423	Body	-0,202	5,89E-07	1,31E-05
cg07791987	2	189193728	GULP1	5'UTR	-0,224	5,91E-07	1,31E-05
cg10485410	2	26586435	EPT1	Body	-0,221	5,92E-07	1,31E-05
cg04993815	12	123978747	RILPL1	Body	-0,208	5,92E-07	1,31E-05
cg09367901	14	54418851	BMP4	Body	0,225	5,92E-07	1,31E-05
cg04660147	20	62716443	OPRL1	5'UTR	0,206	5,92E-07	1,31E-05
cg26370312	3	171052399	TNIK	Body	0,214	5,92E-07	1,31E-05
cg16886936	2	240355846		IGR	-0,237	5,92E-07	1,31E-05
cg25788857	16	69872614	WWP2	TSS200	0,204	5,94E-07	1,32E-05
cg17388996	6	108145374	SCML4	1stExon	-0,296	5,94E-07	1,32E-05
cg11324978	6	14110577		IGR	-0,201	5,94E-07	1,32E-05
cg26197008	3	12392024	PPARG	TSS1500	0,207	5,94E-07	1,32E-05
cg08787832	6	34452932	PACSIN1	5'UTR	-0,21	5,94E-07	1,32E-05
cg01516645	1	28471495		IGR	-0,286	5,95E-07	1,32E-05
cg27606137	4	37891816	TBC1D1	TSS1500	0,258	5,95E-07	1,32E-05
cg24442506	1	89737833	GBP5	5'UTR	-0,322	5,95E-07	1,32E-05
cg06348275	1	227507059	CDC42BPA	TSS1500	0,214	5,95E-07	1,32E-05
cg20171892	7	27169584	HOXA4	Body	0,34	5,95E-07	1,32E-05
cg03420443	3	132439678	NPHP3	Body	0,219	5,95E-07	1,32E-05
cg05494436	6	155753714	NOX3	Body	-0,221	5,96E-07	1,32E-05
cg00353676	9	108675455		IGR	-0,206	5,96E-07	1,32E-05
cg01543570	3	15835938	ANKRD28	5'UTR	-0,218	5,96E-07	1,32E-05
cg25386499	3	58335732	PXK	5'UTR	-0,219	5,96E-07	1,32E-05
cg01986630	16	53946263	FTO	Body	-0,267	5,96E-07	1,32E-05
cg02684341	13	21644106		IGR	-0,266	5,96E-07	1,32E-05
cg10692140	6	30496072		IGR	-0,257	5,96E-07	1,32E-05
cg08027708	1	185410958		IGR	0,282	5,97E-07	1,32E-05
cg17926756	3	55217960		IGR	-0,241	5,98E-07	1,32E-05
cg22574343	6	152489702	MIR3163	Body	0,215	5,98E-07	1,32E-05

cg22580512	12	125002474	NCOR2	5'UTR	0,216	5,99E-07	1,32E-05
cg13810195	4	106227800	TET2-AS1	Body	-0,354	5,99E-07	1,32E-05
cg26511276	4	170263013		IGR	-0,219	5,99E-07	1,32E-05
cg05478082	7	134233797	AKR1B15	TSS200	-0,24	6,00E-07	1,32E-05
cg03699843	16	50701064	SNX20	3'UTR	-0,288	6,00E-07	1,32E-05
cg11910667	20	61812321		IGR	-0,21	6,00E-07	1,33E-05
cg26296836	11	11564925	GALNT18	Body	-0,25	6,00E-07	1,33E-05
cg00482389	3	45867935	LZTFL1	Body	-0,261	6,01E-07	1,33E-05
cg14571479	1	217668391	GPATCH2	Body	-0,205	6,01E-07	1,33E-05
cg19851816	22	50657907	TUBGCP6	Body	0,24	6,01E-07	1,33E-05
cg00442513	11	120157719	POU2F3	Body	-0,227	6,01E-07	1,33E-05
cg21303980	2	106356028		IGR	-0,276	6,02E-07	1,33E-05
cg20157127	6	166658103	LOC10192929	Body	-0,291	6,02E-07	1,33E-05
cg00287773	5	139594619	C5orf32	Body	-0,296	6,02E-07	1,33E-05
cg00919781	7	122279066	CADPS2	Body	-0,204	6,02E-07	1,33E-05
cg23212761	2	9505705	ASAP2	Body	-0,213	6,02E-07	1,33E-05
cg04243682	6	86395706		IGR	-0,221	6,03E-07	1,33E-05
cg03048919	2	8656829		IGR	-0,211	6,03E-07	1,33E-05
cg05047361	8	142303030		IGR	0,224	6,04E-07	1,33E-05
cg21011403	2	43302362		IGR	0,264	6,04E-07	1,33E-05
cg00720598	7	3632934	SDK1	Body	-0,209	6,04E-07	1,33E-05
cg11483911	3	58860839	C3orf67	Body	-0,213	6,04E-07	1,33E-05
cg21234471	9	92023797	SEMA4D	5'UTR	-0,302	6,05E-07	1,33E-05
cg22593894	20	1781910		IGR	-0,239	6,05E-07	1,33E-05
cg15103566	2	12443780	LOC10050645	Body	-0,261	6,05E-07	1,33E-05
cg18699940	6	151203416	MTHFD1L	Body	0,246	6,05E-07	1,33E-05
cg19090382	9	89891828		IGR	-0,222	6,06E-07	1,33E-05
cg24326325	5	123920955		IGR	-0,232	6,06E-07	1,33E-05
cg25312229	11	65246873		IGR	0,212	6,07E-07	1,34E-05
cg08147389	14	73756251	NUMB	Body	-0,251	6,07E-07	1,34E-05
cg14351952	9	5515324	PDCD1LG2	5'UTR	0,202	6,07E-07	1,34E-05
cg21449597	4	154036828		IGR	-0,232	6,08E-07	1,34E-05
cg11065036	13	39828976		IGR	0,207	6,08E-07	1,34E-05
cg12154454	6	150137069		IGR	-0,236	6,09E-07	1,34E-05
cg03290748	15	68370885	PIAS1	Body	0,214	6,09E-07	1,34E-05
cg09737078	16	4366839		IGR	0,21	6,10E-07	1,34E-05
cg07551060	10	121075316	GRK5	Body	-0,281	6,10E-07	1,34E-05
cg27467282	13	52116184		IGR	0,227	6,10E-07	1,34E-05
cg00208274	7	4779342	FOXK1	Body	0,223	6,10E-07	1,34E-05
cg21573329	15	56635031		IGR	0,215	6,10E-07	1,34E-05
cg05678715	11	65724470	TSGA10IP	Body	-0,209	6,11E-07	1,34E-05
cg16106768	14	71709281		IGR	0,215	6,11E-07	1,34E-05
cg06559421	4	186697078	SORBS2	TSS200	0,288	6,12E-07	1,34E-05
cg22462726	3	184209261		IGR	0,204	6,12E-07	1,34E-05
cg27506338	14	21567554	ZNF219	5'UTR	0,253	6,13E-07	1,35E-05
cg13661519	7	27141088	HOXA2	Body	0,208	6,14E-07	1,35E-05
cg20944852	12	81172316		IGR	-0,214	6,15E-07	1,35E-05
cg20408104	6	157399325	ARID1B	Body	0,241	6,15E-07	1,35E-05
cg10312186	6	170403583		IGR	-0,29	6,15E-07	1,35E-05
cg21812292	8	141518396		IGR	0,205	6,15E-07	1,35E-05

cg02703606	3	38150443	DLEC1	Body	-0,224	6,15E-07	1,35E-05
cg26486972	10	27390386	ANKRD26	TSS1500	-0,255	6,15E-07	1,35E-05
cg21733531	7	63560711		IGR	0,218	6,16E-07	1,35E-05
cg00612672	15	59620380	MYO1E	Body	-0,201	6,16E-07	1,35E-05
cg14190344	10	133729189		IGR	0,208	6,17E-07	1,35E-05
cg13237947	6	152481678	MIR3163	Body	-0,205	6,17E-07	1,35E-05
cg00348168	19	30196030	C19orf12	Body	-0,302	6,17E-07	1,35E-05
cg23808165	22	25421713		IGR	0,215	6,18E-07	1,35E-05
cg17666418	6	106612002		IGR	-0,247	6,18E-07	1,35E-05
cg06561106	9	37034166	PAX5	1stExon	0,273	6,18E-07	1,35E-05
cg25522143	13	95071712		IGR	-0,202	6,18E-07	1,35E-05
cg08293002	11	65246590		IGR	0,205	6,18E-07	1,35E-05
cg08150312	16	46847274	C16orf87	Body	-0,224	6,18E-07	1,35E-05
cg01225948	1	22366897		IGR	-0,225	6,19E-07	1,35E-05
cg23010084	6	69428797	ADGRB3	Body	0,237	6,19E-07	1,36E-05
cg15787037	16	73834612		IGR	-0,223	6,20E-07	1,36E-05
cg09397397	12	29671478	TMTC1	Body	-0,223	6,21E-07	1,36E-05
cg02745459	15	96207497		IGR	-0,235	6,24E-07	1,36E-05
cg11057205	8	134588243		IGR	-0,255	6,24E-07	1,36E-05
cg25370657	2	44383264		IGR	0,231	6,24E-07	1,36E-05
cg17593384	4	88000269	AFF1	Body	0,247	6,25E-07	1,36E-05
cg02831474	10	1783879		IGR	-0,218	6,25E-07	1,36E-05
cg19002902	9	27053909	IFT74	Body	0,234	6,25E-07	1,36E-05
cg14132895	11	95433641		IGR	-0,256	6,25E-07	1,36E-05
cg14580870	9	2656530		IGR	0,261	6,25E-07	1,36E-05
cg00999338	11	116724606	APOA1-AS	Body	-0,276	6,25E-07	1,36E-05
cg14312063	3	85010606	CADM2	Body	0,21	6,26E-07	1,36E-05
cg20008885	1	193670163		IGR	-0,216	6,26E-07	1,37E-05
cg14260632	1	84139163	LOC10192758	Body	-0,214	6,26E-07	1,37E-05
cg23093496	16	29757323	C16orf54	1stExon	-0,25	6,26E-07	1,37E-05
cg00816652	8	131199076	ASAP1	Body	-0,259	6,28E-07	1,37E-05
cg02947862	10	129979375		IGR	-0,209	6,28E-07	1,37E-05
cg11603350	8	87534931	CPNE3	5'UTR	0,279	6,28E-07	1,37E-05
cg03316149	2	198062696	ANKRD44	Body	-0,203	6,29E-07	1,37E-05
cg02558751	2	218027522		IGR	-0,291	6,29E-07	1,37E-05
cg11883141	8	134081000	TG	Body	-0,23	6,29E-07	1,37E-05
cg06027964	15	65504597	CILP	TSS1500	-0,251	6,29E-07	1,37E-05
cg19604703	4	16261642		IGR	-0,223	6,29E-07	1,37E-05
cg23649326	5	80604119	ZCCHC9	Body	0,202	6,29E-07	1,37E-05
cg11768715	12	3152477		IGR	-0,223	6,29E-07	1,37E-05
cg05737535	10	130316398		IGR	0,228	6,30E-07	1,37E-05
cg04489548	1	110616863		IGR	-0,206	6,31E-07	1,37E-05
cg03876150	2	102369368	MAP4K4	Body	0,227	6,32E-07	1,37E-05
cg25383510	9	92229357		IGR	-0,221	6,32E-07	1,37E-05
cg11185765	11	48009578	PTPRJ	Body	0,217	6,32E-07	1,38E-05
cg21258705	2	97628429	FAM178B	Body	-0,211	6,33E-07	1,38E-05
cg16811988	3	42103138		IGR	0,249	6,33E-07	1,38E-05
cg15665949	16	30196549	CORO1A	Body	-0,21	6,33E-07	1,38E-05
cg15129183	5	169760269	LOC257358	Body	-0,334	6,34E-07	1,38E-05
cg14209244	9	72877684	SMC5	Body	-0,21	6,34E-07	1,38E-05

cg21156263	9	19926448		IGR	0,22	6,34E-07	1,38E-05
cg08838842	3	111577634	PHLDB2	TSS1500	0,247	6,35E-07	1,38E-05
cg10509626	11	70333993	SHANK2	Body	-0,201	6,35E-07	1,38E-05
cg24835939	15	81460792		IGR	-0,215	6,36E-07	1,38E-05
cg17242596	11	132527296	OPCML	Body	-0,217	6,36E-07	1,38E-05
cg26485452	1	193559038		IGR	0,204	6,36E-07	1,38E-05
cg05989693	3	50376155	RASSF1	TSS1500	-0,261	6,37E-07	1,38E-05
cg15758525	7	139952594		IGR	-0,272	6,38E-07	1,38E-05
cg07917644	15	101766122	CHSY1	Body	0,259	6,38E-07	1,39E-05
cg17635080	12	109030115		IGR	-0,241	6,38E-07	1,39E-05
cg22650197	11	44227997	EXT2	Body	0,261	6,40E-07	1,39E-05
cg17379666	17	71304784	CDC42EP4	5'UTR	0,227	6,41E-07	1,39E-05
cg16849000	11	68548426	CPT1A	Body	0,216	6,41E-07	1,39E-05
cg21049302	17	53702388		IGR	-0,229	6,41E-07	1,39E-05
cg01776094	9	3385979	RFX3	Body	-0,237	6,42E-07	1,39E-05
cg06640997	12	32081573		IGR	-0,209	6,42E-07	1,39E-05
cg03482710	1	226976946		IGR	-0,213	6,43E-07	1,39E-05
cg17502127	5	142024012	FGF1	TSS200	-0,25	6,43E-07	1,39E-05
cg24956715	2	32157955	MEMO1	5'UTR	-0,22	6,43E-07	1,39E-05
cg15924566	4	57969541	IGFBP7	Body	0,203	6,43E-07	1,39E-05
cg06427233	7	134133904	AKR1B1	Body	0,204	6,44E-07	1,39E-05
cg20506659	6	33132943	COL11A2	Body	-0,216	6,44E-07	1,39E-05
cg02326386	19	2085286	MOBKL2A	5'UTR	-0,267	6,44E-07	1,39E-05
cg08460812	4	109087419	LOC641518	TSS1500	-0,204	6,44E-07	1,39E-05
cg17243737	7	107110488	GPR22	TSS200	0,252	6,44E-07	1,39E-05
cg08717751	13	37881008		IGR	-0,3	6,44E-07	1,39E-05
cg14367715	17	50868219		IGR	0,205	6,44E-07	1,39E-05
cg03804474	5	67760417		IGR	-0,297	6,44E-07	1,39E-05
cg01231543	16	68741748		IGR	0,226	6,44E-07	1,39E-05
cg23244545	18	25621544	CDH2	Body	0,333	6,44E-07	1,39E-05
cg16280624	3	39476251		IGR	-0,202	6,45E-07	1,39E-05
cg14014990	3	29321523	RBMS3	TSS1500	0,214	6,45E-07	1,39E-05
cg02976355	9	14312435	NFIB	Body	0,252	6,45E-07	1,40E-05
cg25508633	19	10218184	SNORD105	TSS200	-0,213	6,46E-07	1,40E-05
cg05540100	7	137367050	DGKI	Body	-0,26	6,46E-07	1,40E-05
cg00572630	12	12716638	DUSP16	TSS1500	0,244	6,46E-07	1,40E-05
cg17552686	3	114818612	ZBTB20-AS4	TSS1500	0,201	6,46E-07	1,40E-05
cg03534770	9	34653294	IL11RA	5'UTR	0,314	6,46E-07	1,40E-05
cg12685640	1	208714976		IGR	-0,208	6,46E-07	1,40E-05
cg26406857	20	23148111		IGR	-0,213	6,48E-07	1,40E-05
cg25766424	2	162357826	AHCTF1P1	Body	-0,215	6,48E-07	1,40E-05
cg18462342	7	117448542	CTTNBP2	Body	0,267	6,49E-07	1,40E-05
cg19458020	17	38473119	RARA	TSS1500	0,242	6,49E-07	1,40E-05
cg25564008	16	65613872		IGR	-0,205	6,49E-07	1,40E-05
cg02790744	17	45246276	CDC27	Body	0,235	6,49E-07	1,40E-05
cg07493097	2	158957632	UPP2	TSS1500	-0,238	6,50E-07	1,40E-05
cg13687834	10	3514783		IGR	-0,24	6,50E-07	1,40E-05
cg12934374	20	48815077		IGR	0,21	6,50E-07	1,40E-05
cg17045432	19	7554359	PEX11G	TSS1500	0,205	6,50E-07	1,40E-05
cg17988640	17	7358474	CHRNB1	Body	-0,215	6,51E-07	1,40E-05

cg01687102	8	61380148		IGR	-0,204	6,51E-07	1,40E-05
cg15851462	4	144538111	FREM3	Body	-0,278	6,52E-07	1,41E-05
cg04394036	10	130408883		IGR	-0,206	6,52E-07	1,41E-05
cg20598124	17	57456381	YPEL2	Body	0,222	6,52E-07	1,41E-05
cg16635972	15	70994663	UACA	TSS200	0,298	6,52E-07	1,41E-05
cg04742369	11	6224927		IGR	-0,3	6,52E-07	1,41E-05
cg13550493	8	29948482	MIR548O2	Body	-0,285	6,52E-07	1,41E-05
cg04290171	1	207924482	CD46	TSS1500	0,228	6,52E-07	1,41E-05
cg06287731	14	75988251	BATF	TSS1500	-0,235	6,52E-07	1,41E-05
cg01144086	16	50715260	SNX20	1stExon	-0,216	6,52E-07	1,41E-05
cg22637006	2	706036		IGR	-0,218	6,53E-07	1,41E-05
cg20427362	1	206955971		IGR	-0,273	6,53E-07	1,41E-05
cg09219038	9	34651962	IL11RA	TSS1500	0,221	6,53E-07	1,41E-05
cg04911139	17	25871790	KSR1	5'UTR	-0,315	6,53E-07	1,41E-05
cg00625303	1	44949534	RNF220	Body	-0,226	6,54E-07	1,41E-05
cg01298194	4	122612779	ANXA5	Body	0,218	6,54E-07	1,41E-05
cg06229614	8	29381775		IGR	-0,218	6,54E-07	1,41E-05
cg02947427	1	209897658	HSD11B1	Body	-0,208	6,54E-07	1,41E-05
cg11644424	6	157258856	ARID1B	Body	0,219	6,55E-07	1,41E-05
cg16517419	5	3980447		IGR	-0,214	6,55E-07	1,41E-05
cg11550950	13	32297002		IGR	0,227	6,55E-07	1,41E-05
cg20073153	10	30238815		IGR	-0,25	6,55E-07	1,41E-05
cg04365878	1	92311629	TGFBR3	Body	0,233	6,56E-07	1,41E-05
cg03663509	2	12647691	LOC10050645	Body	-0,296	6,57E-07	1,41E-05
cg03203179	1	92275181	TGFBR3	Body	-0,288	6,57E-07	1,41E-05
cg04559928	7	102056278	LOC10063092	Body	-0,256	6,57E-07	1,41E-05
cg02755130	4	184186563	WWC2	Body	-0,216	6,57E-07	1,41E-05
cg07737503	19	1132895	SBNO2	Body	-0,219	6,58E-07	1,41E-05
cg24441810	2	120436039	TMEM177	TSS1500	-0,213	6,58E-07	1,42E-05
cg17608171	14	50562852	LINC01599	Body	-0,223	6,58E-07	1,42E-05
cg13527169	4	88452108	SPARCL1	TSS1500	0,231	6,60E-07	1,42E-05
cg01556275	1	59245597		IGR	0,252	6,61E-07	1,42E-05
cg13457462	8	49801972		IGR	-0,229	6,62E-07	1,42E-05
cg00864618	8	141518479		IGR	0,248	6,62E-07	1,42E-05
cg13107596	2	191886156		IGR	-0,257	6,62E-07	1,42E-05
cg16527405	2	15830137	LOC10192696	TSS1500	-0,211	6,64E-07	1,42E-05
cg24785074	7	12611108	SCIN	Body	0,221	6,64E-07	1,42E-05
cg24771111	5	73893040		IGR	-0,239	6,64E-07	1,42E-05
cg07220750	7	35769215		IGR	-0,207	6,65E-07	1,43E-05
cg04400628	10	76737777	KAT6B	Body	-0,3	6,65E-07	1,43E-05
cg25503999	18	74774466	MBP	Body	-0,305	6,65E-07	1,43E-05
cg12989088	22	28403796	TTC28	Body	-0,225	6,65E-07	1,43E-05
cg03651509	1	179268330	SOAT1	5'UTR	-0,213	6,65E-07	1,43E-05
cg09895103	9	137713454	COL5A1	Body	-0,212	6,66E-07	1,43E-05
cg12457361	2	8716988	LOC10192956	Body	-0,255	6,66E-07	1,43E-05
cg26361535	8	144576604	ZC3H3	Body	0,217	6,67E-07	1,43E-05
cg18560551	11	34654233	EHF	5'UTR	0,21	6,68E-07	1,43E-05
cg00442389	6	32160074	GPSM3	Body	-0,295	6,68E-07	1,43E-05
cg12274898	1	153586108	S100A16	TSS1500	-0,21	6,69E-07	1,43E-05
cg02185843	21	45348632	AGPAT3	5'UTR	0,254	6,69E-07	1,43E-05

cg02700164	8	42046567	PLAT	Body	-0,239	6,69E-07	1,43E-05
cg08683307	3	33701580	CLASP2	TSS1500	0,23	6,70E-07	1,43E-05
cg23671920	11	27338979		IGR	-0,213	6,70E-07	1,43E-05
cg23092956	2	54846562	SPTBN1	Body	0,263	6,70E-07	1,43E-05
cg07642569	3	151910095		IGR	-0,227	6,70E-07	1,43E-05
cg27133780	3	32474793	CMTM7	Body	-0,263	6,70E-07	1,43E-05
cg14732540	1	92414722	BRDT	TSS1500	-0,287	6,71E-07	1,43E-05
cg09488594	5	60602794		IGR	-0,2	6,71E-07	1,44E-05
cg06838703	9	22765724	LINC01239	Body	0,227	6,72E-07	1,44E-05
cg03677952	22	50524541	MLC1	TSS1500	-0,204	6,72E-07	1,44E-05
cg07910110	10	61062665	FAM13C	Body	-0,247	6,73E-07	1,44E-05
cg10592926	6	157876961	ZDHHC14	Body	0,223	6,74E-07	1,44E-05
cg17733649	7	151387983	PRKAG2	Body	0,227	6,74E-07	1,44E-05
cg04836028	6	31554263	LST1	Body	-0,203	6,74E-07	1,44E-05
cg10543419	3	37489393		IGR	-0,23	6,74E-07	1,44E-05
cg03652456	6	1670293	GMDS	Body	-0,241	6,75E-07	1,44E-05
cg04497870	1	42277284	HIVEP3	5'UTR	-0,225	6,76E-07	1,44E-05
cg10407744	9	97715349	C9orf3	Body	0,251	6,76E-07	1,44E-05
cg26929272	10	88295210		IGR	0,263	6,76E-07	1,44E-05
cg27280037	3	124782069		IGR	0,205	6,76E-07	1,44E-05
cg03802185	7	101927846	SH2B2	TSS1500	-0,243	6,76E-07	1,44E-05
cg08829107	10	3520065	LOC10537636	Body	-0,203	6,77E-07	1,44E-05
cg20441135	10	93999132	CPEB3	Body	0,235	6,77E-07	1,44E-05
cg22705268	11	85430159	SYTL2	1stExon	0,215	6,77E-07	1,44E-05
cg10417386	17	8772067	PIK3R6	TSS1500	-0,256	6,77E-07	1,44E-05
cg11942332	13	32859382	FRY	Body	0,333	6,77E-07	1,45E-05
cg04803153	19	37329859	ZNF790	TSS1500	0,206	6,78E-07	1,45E-05
cg18768872	22	26922334	TPST2	3'UTR	0,205	6,79E-07	1,45E-05
cg10677943	2	224403756		IGR	0,237	6,79E-07	1,45E-05
cg00190116	14	22986483		IGR	-0,25	6,80E-07	1,45E-05
cg08584947	8	93468317		IGR	-0,26	6,80E-07	1,45E-05
cg03591690	3	166884766		IGR	0,211	6,81E-07	1,45E-05
cg00936935	10	52271899	SGMS1	5'UTR	-0,202	6,81E-07	1,45E-05
cg07177413	19	35225819	ZNF181	5'UTR	0,253	6,81E-07	1,45E-05
cg19359072	15	51598433	CYP19A1	5'UTR	-0,203	6,81E-07	1,45E-05
cg19941833	4	83785731	SEC31A	ExonBnd	-0,235	6,81E-07	1,45E-05
cg03297901	17	76117767	TMC6	Body	-0,285	6,82E-07	1,45E-05
cg20175766	6	112513208	LAMA4	Body	-0,259	6,82E-07	1,45E-05
cg27144368	14	100286635	EML1	Body	-0,245	6,82E-07	1,45E-05
cg22624902	2	28475342	BRE	Body	-0,266	6,83E-07	1,45E-05
cg02470808	5	17118758		IGR	-0,255	6,83E-07	1,45E-05
cg05932538	11	13190514		IGR	-0,211	6,83E-07	1,45E-05
cg23079727	3	11598978	VGLL4	3'UTR	0,227	6,83E-07	1,45E-05
cg16432182	4	89152511	ABCG2	TSS200	0,243	6,83E-07	1,45E-05
cg06504753	20	56743843		IGR	-0,24	6,84E-07	1,46E-05
cg22994830	7	623846	PRKAR1B	Body	-0,309	6,84E-07	1,46E-05
cg11543399	5	176837851	F12	TSS1500	-0,207	6,85E-07	1,46E-05
cg23055617	2	109223691	LIMS1	5'UTR	0,252	6,85E-07	1,46E-05
cg21188037	7	45018658	MYO1G	5'UTR	-0,323	6,86E-07	1,46E-05
cg22833382	14	65395368	CHURC1	Body	-0,261	6,86E-07	1,46E-05

cg16489118	18	53158947	TCF4	Body	0,261	6,86E-07	1,46E-05
cg06034609	14	50532020	LINC01599	Body	-0,26	6,86E-07	1,46E-05
cg13819877	14	93029893	RIN3	Body	-0,302	6,86E-07	1,46E-05
cg19793537	4	148750768	ARHGAP10	Body	0,206	6,87E-07	1,46E-05
cg19565738	2	174083367	ZAK	Body	0,263	6,87E-07	1,46E-05
cg19155346	1	112054157	TMIGD3	Body	-0,21	6,88E-07	1,46E-05
cg10631752	13	52985702		IGR	-0,209	6,88E-07	1,46E-05
cg15104839	19	46932612		IGR	-0,227	6,88E-07	1,46E-05
cg11510403	15	79101981	ADAMTS7	Body	0,234	6,89E-07	1,46E-05
cg09961946	12	9820221		IGR	-0,272	6,90E-07	1,46E-05
cg16144533	1	87677564	LOC1019278	TSS1500	-0,21	6,90E-07	1,46E-05
cg17484090	18	323534	COLEC12	Body	-0,295	6,90E-07	1,46E-05
cg19268695	1	111743411	DENND2D	TSS200	-0,251	6,91E-07	1,47E-05
cg09185582	9	273074	DOCK8	1stExon	-0,25	6,91E-07	1,47E-05
cg20614854	17	71331689	SDK2	3'UTR	-0,208	6,91E-07	1,47E-05
cg16472369	17	4618322	ARRB2	Body	-0,299	6,91E-07	1,47E-05
cg25780687	2	160563271	BAZ2B	Body	0,274	6,91E-07	1,47E-05
cg24538975	15	64992573	OAZ2	Body	0,218	6,91E-07	1,47E-05
cg00971309	6	32809960	PSMB8	Body	-0,251	6,93E-07	1,47E-05
cg10917109	7	75637742	STYXL1	Body	-0,272	6,93E-07	1,47E-05
cg09469870	6	22785550		IGR	0,239	6,94E-07	1,47E-05
cg08060673	11	117922264	TMPRSS4-AS	Body	-0,221	6,94E-07	1,47E-05
cg05598246	6	29624414	MOG	TSS1500	0,248	6,94E-07	1,47E-05
cg16000803	7	32619660	AVL9	Body	0,255	6,94E-07	1,47E-05
cg19908615	11	61270498		IGR	-0,216	6,95E-07	1,47E-05
cg03016153	1	117296954	CD2	TSS200	-0,205	6,95E-07	1,47E-05
cg07285490	1	32071681		IGR	-0,25	6,96E-07	1,47E-05
cg15655027	8	41575780	ANK1	Body	-0,218	6,96E-07	1,47E-05
cg11102369	10	114674303		IGR	-0,214	6,97E-07	1,48E-05
cg13888748	9	107525704	NIPSNAP3B	TSS1500	0,298	6,97E-07	1,48E-05
cg04334651	16	4366968		IGR	0,207	6,97E-07	1,48E-05
cg19569526	5	155754265	SGCD	5'UTR	0,237	6,98E-07	1,48E-05
cg06566239	10	131361472	MGMT	Body	-0,216	6,99E-07	1,48E-05
cg17605084	12	54891491	NCKAP1L	TSS200	-0,234	6,99E-07	1,48E-05
cg00314029	2	161348665	RBMS1	Body	0,241	7,00E-07	1,48E-05
cg19913557	16	34726852		IGR	0,262	7,00E-07	1,48E-05
cg21740139	17	60753158	MRC2	ExonBnd	-0,225	7,01E-07	1,48E-05
cg17885415	7	43661792	STK17A	Body	-0,202	7,01E-07	1,48E-05
cg03494429	12	4417093		IGR	0,253	7,01E-07	1,48E-05
cg23963252	3	40919573		IGR	0,202	7,01E-07	1,48E-05
cg09152136	1	178464723		IGR	-0,23	7,02E-07	1,48E-05
cg16911583	19	36233447	TMEM149	TSS200	-0,263	7,02E-07	1,48E-05
cg09093656	17	16323481	TRPV2	Body	-0,252	7,02E-07	1,48E-05
cg15367056	7	92271711	CDK6	Body	-0,289	7,02E-07	1,48E-05
cg01167502	6	106441506		IGR	0,202	7,02E-07	1,48E-05
cg25843543	16	83977610		IGR	-0,212	7,03E-07	1,48E-05
cg03210579	1	24311230		IGR	0,201	7,04E-07	1,49E-05
cg23956960	7	79758274		IGR	-0,22	7,04E-07	1,49E-05
cg01095006	3	179674585	PEX5L	5'UTR	-0,251	7,05E-07	1,49E-05
cg13208687	2	177373823		IGR	-0,226	7,05E-07	1,49E-05

cg13151103	10	38147465	ZNF248	TSS1500	0,257	7,05E-07	1,49E-05
cg27325579	12	29318120	FAR2	5'UTR	-0,212	7,06E-07	1,49E-05
cg05110803	15	99385323	IGF1R	Body	-0,307	7,07E-07	1,49E-05
cg04899990	16	84026531	NECAB2	Body	0,214	7,08E-07	1,49E-05
cg13501042	6	75308261	LOC10192851	Body	-0,262	7,08E-07	1,49E-05
cg08250921	16	88111009		IGR	0,275	7,09E-07	1,49E-05
cg10411272	4	186559707	SORBS2	Body	-0,24	7,09E-07	1,49E-05
cg25273767	4	151953574		IGR	-0,287	7,10E-07	1,49E-05
cg07277017	11	1874093	LSP1	TSS200	-0,215	7,10E-07	1,49E-05
cg09046664	1	21501349	EIF4G3	TSS200	0,3	7,11E-07	1,50E-05
cg00404280	8	144631887		IGR	-0,204	7,11E-07	1,50E-05
cg08606110	1	147245812	GJA5	TSS1500	0,248	7,11E-07	1,50E-05
cg25574939	2	112456709		IGR	-0,221	7,11E-07	1,50E-05
cg13212814	13	52195288	WDFY2	Body	0,303	7,11E-07	1,50E-05
cg00147801	17	3699818	ITGAE	Body	-0,238	7,11E-07	1,50E-05
cg23911696	12	62652900	USP15	TSS1500	-0,286	7,11E-07	1,50E-05
cg15101392	11	117748132	FXYD6	TSS1500	0,254	7,11E-07	1,50E-05
cg13252206	8	16970768	EFHA2	Body	-0,204	7,12E-07	1,50E-05
cg13442606	11	3071269	CARS	Body	-0,212	7,13E-07	1,50E-05
cg26963367	15	89157841		IGR	0,274	7,13E-07	1,50E-05
cg18869840	2	85436586	TCF7L1	Body	-0,203	7,13E-07	1,50E-05
cg10375559	13	60026399		IGR	-0,251	7,14E-07	1,50E-05
cg19631251	16	86098382		IGR	-0,208	7,14E-07	1,50E-05
cg00192535	11	34609696		IGR	0,24	7,14E-07	1,50E-05
cg26260558	21	39661796	KCNJ15	5'UTR	-0,207	7,15E-07	1,50E-05
cg09824150	16	59239308		IGR	-0,227	7,15E-07	1,50E-05
cg07135454	2	236479620	AGAP1	Body	-0,214	7,16E-07	1,50E-05
cg05020854	19	2710761		IGR	-0,251	7,16E-07	1,50E-05
cg25598890	12	117501447	TESC	Body	-0,259	7,17E-07	1,50E-05
cg05193461	1	101707876		IGR	0,283	7,18E-07	1,51E-05
cg26340050	14	105771879		IGR	-0,241	7,18E-07	1,51E-05
cg20591728	17	46683606	LOC404266	Body	0,212	7,18E-07	1,51E-05
cg15450369	6	116467227	NT5DC1	Body	0,238	7,19E-07	1,51E-05
cg05575639	8	61593696	CHD7	5'UTR	0,218	7,19E-07	1,51E-05
cg06630915	9	92097137		IGR	-0,239	7,19E-07	1,51E-05
cg16877498	3	65469183	MAGI1	Body	0,241	7,19E-07	1,51E-05
cg02704103	3	72846991	SHQ1	Body	0,257	7,19E-07	1,51E-05
cg00593365	8	68403258	CPA6	Body	0,231	7,20E-07	1,51E-05
cg14609862	5	80121879	MSH3	Body	0,217	7,21E-07	1,51E-05
cg08953836	20	56704172		IGR	-0,247	7,21E-07	1,51E-05
cg13860281	7	55638724	VOPP1	Body	-0,279	7,21E-07	1,51E-05
cg26165081	11	14562955	PSMA1	Body	0,21	7,22E-07	1,51E-05
cg04026789	7	101487021	CUX1	Body	0,249	7,22E-07	1,51E-05
cg08491889	2	30490303		IGR	-0,211	7,22E-07	1,51E-05
cg04664516	10	89638807	PTEN	5'UTR	0,284	7,22E-07	1,51E-05
cg03089651	1	161039695	ARHGAP30	1stExon	-0,225	7,23E-07	1,51E-05
cg16385758	20	4795919	RASSF2	5'UTR	-0,286	7,23E-07	1,51E-05
cg03715731	16	80266039	LOC10272408	Body	-0,204	7,23E-07	1,51E-05
cg00843917	1	33415247	RNF19B	Body	0,213	7,23E-07	1,51E-05
cg24376039	5	126404739	C5orf63	5'UTR	0,213	7,23E-07	1,51E-05

cg12541223	22	41628523	CHADL	Body	0,208	7,24E-07	1,51E-05
cg12332383	5	89908984	ADGRV1	Body	-0,223	7,24E-07	1,51E-05
cg04153061	2	70174164		IGR	-0,267	7,24E-07	1,52E-05
cg14984221	3	133776543		IGR	-0,226	7,25E-07	1,52E-05
cg11729823	12	109178017	SSH1	3'UTR	0,223	7,25E-07	1,52E-05
cg14972155	5	54281198	ESM1	1stExon	0,219	7,26E-07	1,52E-05
cg25792999	15	98065822		IGR	0,209	7,26E-07	1,52E-05
cg16890490	6	108053402	SCML4	1stExon	-0,209	7,27E-07	1,52E-05
cg13521286	9	71939182	FAM189A2	TSS1500	0,228	7,27E-07	1,52E-05
cg20020452	1	231108544	TTC13	Body	-0,256	7,27E-07	1,52E-05
cg07398791	5	118676053	TNFAIP8	Body	-0,259	7,27E-07	1,52E-05
cg00055603	1	48508839		IGR	-0,201	7,27E-07	1,52E-05
cg20716653	5	65124049		IGR	0,205	7,28E-07	1,52E-05
cg26390509	5	515549	SLC9A3	Body	0,25	7,28E-07	1,52E-05
cg27069628	2	218570889	DIRC3	Body	-0,211	7,29E-07	1,52E-05
cg20567385	15	77829818		IGR	-0,268	7,29E-07	1,52E-05
cg00766002	19	33651237	WDR88	Body	-0,234	7,29E-07	1,52E-05
cg00851518	1	27676652	SYTL1	Body	0,204	7,29E-07	1,52E-05
cg08094466	17	72635946		IGR	-0,205	7,29E-07	1,52E-05
cg07171504	8	103662317	KLF10	3'UTR	-0,2	7,30E-07	1,52E-05
cg25370658	6	7673306		IGR	0,237	7,31E-07	1,53E-05
cg13821008	2	175499113	WIPF1	5'UTR	-0,263	7,32E-07	1,53E-05
cg14751669	20	44330126	WFDC13	TSS1500	0,239	7,32E-07	1,53E-05
cg12034641	14	55799643	FBXO34	5'UTR	-0,203	7,33E-07	1,53E-05
cg25531488	3	133465869	TF	Body	-0,201	7,33E-07	1,53E-05
cg11982214	7	37007063	ELMO1	Body	0,256	7,34E-07	1,53E-05
cg08605199	7	870865	SUN1	TSS1500	0,251	7,35E-07	1,53E-05
cg08626800	7	42979643		IGR	-0,208	7,35E-07	1,53E-05
cg03886135	10	112557340	RBM20	Body	0,211	7,38E-07	1,54E-05
cg06101959	2	8615754		IGR	-0,319	7,38E-07	1,54E-05
cg21241410	2	43267816		IGR	-0,289	7,38E-07	1,54E-05
cg13663303	5	35046992	AGXT2	Body	-0,208	7,39E-07	1,54E-05
cg22793601	18	43953569	RNF165	5'UTR	-0,206	7,39E-07	1,54E-05
cg06970699	7	2117046	MAD1L1	Body	-0,228	7,40E-07	1,54E-05
cg11382203	11	76470635		IGR	-0,243	7,40E-07	1,54E-05
cg23082347	11	117150989	RNF214	ExonBnd	-0,235	7,40E-07	1,54E-05
cg05236728	12	47609513	PCED1B-AS1	Body	-0,345	7,40E-07	1,54E-05
cg26802813	3	47014128	CCDC12	Body	-0,204	7,40E-07	1,54E-05
cg26398044	1	58862917		IGR	-0,238	7,41E-07	1,54E-05
cg00001801	8	107299451	OXR1	5'UTR	-0,253	7,41E-07	1,54E-05
cg06239064	12	66697621	HELB	Body	-0,257	7,41E-07	1,54E-05
cg06342406	10	7306599	SFMBT2	Body	0,282	7,42E-07	1,54E-05
cg17583432	8	129005567	PVT1	Body	-0,274	7,43E-07	1,54E-05
cg17163404	1	19414438	UBR4	Body	-0,22	7,43E-07	1,54E-05
cg10895511	2	158277352	CYTIP	Body	-0,21	7,43E-07	1,54E-05
cg05290572	18	55923122	NEDD4L	5'UTR	0,248	7,44E-07	1,54E-05
cg22400059	1	10571508	PEX14	Body	-0,262	7,44E-07	1,55E-05
cg13888154	5	95236305	ELL2	Body	0,223	7,45E-07	1,55E-05
cg23468728	7	29325157	CHN2	Body	-0,334	7,45E-07	1,55E-05
cg11728747	7	29037910	CPVL	Body	0,221	7,45E-07	1,55E-05

cg20074750	1	89511130		IGR	0,227	7,46E-07	1,55E-05
cg09936080	2	43653785	THADA	Body	-0,29	7,46E-07	1,55E-05
cg25346136	8	55051838	MRPL15	Body	0,205	7,47E-07	1,55E-05
cg10016185	9	109725150	MIR548Q	Body	0,221	7,47E-07	1,55E-05
cg25508830	15	100893944		IGR	-0,23	7,47E-07	1,55E-05
cg09828381	12	63177404	PPM1H	Body	-0,201	7,47E-07	1,55E-05
cg12705392	6	12586532		IGR	-0,207	7,49E-07	1,55E-05
cg00484899	8	110309784	NUDCD1	Body	0,202	7,49E-07	1,55E-05
cg06869619	21	36208575	RUNX1	Body	-0,223	7,50E-07	1,55E-05
cg13584568	6	91182585		IGR	-0,248	7,50E-07	1,55E-05
cg14656259	7	658218	PRKAR1B	Body	-0,243	7,51E-07	1,56E-05
cg08340378	8	86176183	CA13	Body	-0,228	7,51E-07	1,56E-05
cg08847476	3	14467397	SLC6A6	5'UTR	-0,239	7,52E-07	1,56E-05
cg19894966	6	108670013	LACE1	Body	-0,218	7,52E-07	1,56E-05
cg04579403	4	111775789		IGR	0,249	7,52E-07	1,56E-05
cg16434326	15	74463370		IGR	-0,206	7,53E-07	1,56E-05
cg08954948	10	6100768	IL2RA	Body	-0,286	7,54E-07	1,56E-05
cg04690464	14	104711952		IGR	-0,231	7,54E-07	1,56E-05
cg20740005	8	88217551	CNBD1	Body	0,236	7,54E-07	1,56E-05
cg20792919	8	66655233	PDE7A	Body	0,202	7,55E-07	1,56E-05
cg07830269	1	240363446	FMN2	Body	0,202	7,56E-07	1,56E-05
cg15690495	11	92762522		IGR	-0,247	7,57E-07	1,56E-05
cg17675492	11	62679111	CHRM1	5'UTR	-0,29	7,57E-07	1,56E-05
cg09834652	8	8998765	PPP1R3B	Body	0,215	7,59E-07	1,57E-05
cg10026930	10	105277936	NEURL1-AS1	TSS1500	-0,21	7,59E-07	1,57E-05
cg04053295	10	80716301	ZMIZ1-AS1	Body	-0,22	7,60E-07	1,57E-05
cg19013682	15	101503560	LRRK1	Body	0,216	7,60E-07	1,57E-05
cg24065354	20	11105791		IGR	-0,206	7,60E-07	1,57E-05
cg09126123	14	59730061	DAAM1	TSS200	0,217	7,60E-07	1,57E-05
cg23252259	6	31148612		IGR	0,217	7,61E-07	1,57E-05
cg13553455	10	105846002	COL17A1	TSS1500	-0,221	7,61E-07	1,57E-05
cg02621376	1	85461303	MCOLN2	Body	-0,227	7,61E-07	1,57E-05
cg13414010	22	43118522		IGR	-0,219	7,62E-07	1,57E-05
cg14180330	5	40224461		IGR	-0,275	7,62E-07	1,57E-05
cg11161150	5	98119892	RGMB	Body	-0,209	7,63E-07	1,57E-05
cg27486585	6	30459595	HLA-E	Body	-0,235	7,63E-07	1,57E-05
cg06013042	1	114414532	PTPN22	TSS200	-0,295	7,63E-07	1,57E-05
cg14241836	7	48124921		IGR	-0,209	7,64E-07	1,58E-05
cg09015246	16	10971105	CIITA	1stExon	-0,267	7,64E-07	1,58E-05
cg23611326	5	126211278	MARCH3	Body	0,207	7,64E-07	1,58E-05
cg02304377	5	42812512	SEPP1	TSS1500	0,242	7,64E-07	1,58E-05
cg11476866	17	41920415		IGR	-0,212	7,65E-07	1,58E-05
cg24104237	3	45649408	LIMD1	Body	0,209	7,67E-07	1,58E-05
cg18849004	15	33094797	FMN1	Body	-0,202	7,68E-07	1,58E-05
cg08755525	3	110344467		IGR	0,28	7,68E-07	1,58E-05
cg13885437	2	216922738	PECR	Body	-0,318	7,68E-07	1,58E-05
cg15102466	19	38540035	SIPA1L3	5'UTR	-0,269	7,68E-07	1,58E-05
cg09012411	1	95167783		IGR	-0,233	7,68E-07	1,58E-05
cg05444304	2	31442675		IGR	-0,21	7,68E-07	1,58E-05
cg13027206	14	91866325	CCDC88C	Body	-0,26	7,69E-07	1,58E-05

cg21355828	7	149389814		IGR	0,203	7,70E-07	1,58E-05
cg00294562	22	39487550		IGR	-0,222	7,70E-07	1,58E-05
cg20152501	13	78602831	LINC00446	Body	-0,253	7,70E-07	1,58E-05
cg10173633	12	107273512	RIC8B	Body	0,282	7,70E-07	1,58E-05
cg17897728	3	171850081	FNDC3B	Body	-0,236	7,71E-07	1,58E-05
cg07324083	1	198590276		IGR	-0,237	7,71E-07	1,59E-05
cg06442437	2	102679203		IGR	-0,238	7,72E-07	1,59E-05
cg02733803	20	43275344	ADA	Body	-0,31	7,73E-07	1,59E-05
cg11963638	18	61157839	SERPINB5	Body	0,219	7,74E-07	1,59E-05
cg15003132	3	2553182	CNTN4	5'UTR	0,254	7,74E-07	1,59E-05
cg11117717	14	31560529	AP4S1	Body	0,253	7,74E-07	1,59E-05
cg08114094	14	73061477		IGR	-0,231	7,75E-07	1,59E-05
cg11679871	10	13388567	SEPHS1	5'UTR	0,282	7,77E-07	1,59E-05
cg08133268	17	78061119	CCDC40	Body	-0,229	7,77E-07	1,59E-05
cg18313416	3	13420961	NUP210	Body	0,211	7,77E-07	1,59E-05
cg19334977	10	483555	DIP2C	Body	-0,204	7,77E-07	1,59E-05
cg11262216	17	41723227	MEOX1	Body	0,261	7,77E-07	1,59E-05
cg07572451	3	160168238	TRIM59	TSS1500	0,223	7,79E-07	1,60E-05
cg05455249	10	11054243	CELF2	Body	-0,255	7,79E-07	1,60E-05
cg04232282	16	14107433		IGR	0,207	7,79E-07	1,60E-05
cg07230471	20	42792883	JPH2	Body	-0,232	7,79E-07	1,60E-05
cg23545734	1	21619571	LOC1005068(	TSS1500	-0,227	7,80E-07	1,60E-05
cg04462774	12	50368110	AQP6	Body	0,202	7,80E-07	1,60E-05
cg22372173	20	24941563		IGR	-0,205	7,80E-07	1,60E-05
cg09249114	19	6828446	VAV1	ExonBnd	0,21	7,80E-07	1,60E-05
cg19730379	10	104542002	C10orf26	Body	0,207	7,81E-07	1,60E-05
cg01450441	1	94136079	BCAR3	Body	0,266	7,81E-07	1,60E-05
cg05712639	14	52819386		IGR	-0,292	7,81E-07	1,60E-05
cg16470816	12	58566565		IGR	-0,216	7,82E-07	1,60E-05
cg20121904	9	102969487	INVS	Body	0,257	7,82E-07	1,60E-05
cg09815885	12	93478385	LOC643339	Body	-0,203	7,84E-07	1,60E-05
cg05623986	6	36459598		IGR	0,228	7,84E-07	1,60E-05
cg20063728	7	553423	PDGFA	Body	-0,2	7,84E-07	1,60E-05
cg00053851	1	226270442		IGR	-0,209	7,84E-07	1,60E-05
cg06998765	14	75389618	RPS6KL1	TSS1500	0,219	7,84E-07	1,60E-05
cg06383241	12	116997022	MAP1LC3B2	TSS200	-0,285	7,85E-07	1,61E-05
cg09563025	20	22783191		IGR	-0,235	7,85E-07	1,61E-05
cg19616624	20	1638811	SIRPG	TSS1500	-0,203	7,86E-07	1,61E-05
cg19566482	11	33404678		IGR	-0,231	7,87E-07	1,61E-05
cg03385667	12	3979037	PARP11	5'UTR	-0,267	7,87E-07	1,61E-05
cg14854525	1	31263794		IGR	-0,202	7,87E-07	1,61E-05
cg21126403	3	114343227	ZBTB20	TSS200	0,448	7,87E-07	1,61E-05
cg09803321	10	104913480	NT5C2	Body	0,214	7,88E-07	1,61E-05
cg26931601	1	67030196	SGIP1	Body	-0,295	7,88E-07	1,61E-05
cg13504059	17	38721942	CCR7	TSS1500	-0,227	7,88E-07	1,61E-05
cg14328004	6	105778350	PREP	Body	0,213	7,89E-07	1,61E-05
cg20945738	13	108922032	TNFSF13B	5'UTR	-0,254	7,90E-07	1,61E-05
cg25471572	10	37091904		IGR	0,214	7,90E-07	1,61E-05
cg08250187	2	17482455		IGR	-0,236	7,90E-07	1,61E-05
cg12289092	2	97009820	NCAPH	Body	-0,245	7,91E-07	1,61E-05

cg19418618	8	119658437	SAMD12-AS1	Body	-0,213	7,91E-07	1,62E-05
cg24360606	17	8837741	PIK3R5	5'UTR	-0,211	7,92E-07	1,62E-05
cg06679537	9	112210147	PTPN3	Body	-0,266	7,92E-07	1,62E-05
cg10921624	14	64549863	SYNE2	Body	0,217	7,94E-07	1,62E-05
cg14251798	19	19545333	MIR640	TSS1500	0,246	7,94E-07	1,62E-05
cg16115024	13	49136607		IGR	-0,264	7,94E-07	1,62E-05
cg20167074	1	151967023	S100A10	TSS1500	-0,207	7,95E-07	1,62E-05
cg12193485	5	106954123	EFNA5	Body	0,215	7,96E-07	1,62E-05
cg08828627	1	230341642	GALNT2	Body	0,221	7,96E-07	1,62E-05
cg25484252	3	72184399		IGR	-0,274	7,96E-07	1,62E-05
cg13386331	1	25788495	TMEM57	Body	0,206	7,97E-07	1,62E-05
cg21554616	14	55226755	SAMD4A	5'UTR	-0,207	7,97E-07	1,62E-05
cg19989663	7	115981565		IGR	-0,232	7,98E-07	1,62E-05
cg23074881	15	60872858	RORA	Body	-0,215	7,98E-07	1,62E-05
cg05308976	6	164365285		IGR	0,214	7,98E-07	1,62E-05
cg03913374	4	32418872		IGR	0,31	7,99E-07	1,63E-05
cg25719023	17	18084816		IGR	-0,225	7,99E-07	1,63E-05
cg08413096	12	118025471	KSR2	Body	0,2	7,99E-07	1,63E-05
cg24387487	14	74094162		IGR	-0,239	8,01E-07	1,63E-05
cg00832661	2	231530291		IGR	-0,212	8,01E-07	1,63E-05
cg14106719	9	80524371	GNAQ	Body	-0,324	8,02E-07	1,63E-05
cg09283818	6	155492543	TIAM2	Body	-0,289	8,02E-07	1,63E-05
cg01190276	3	24284272	THRB	5'UTR	-0,201	8,03E-07	1,63E-05
cg06524165	13	30948114	LOC1001889	TSS200	-0,249	8,03E-07	1,63E-05
cg12092651	19	4109096	MAP2K2	Body	-0,382	8,03E-07	1,63E-05
cg08701621	19	58570419	ZNF135	TSS200	0,209	8,03E-07	1,63E-05
cg03909295	5	61086467		IGR	-0,209	8,03E-07	1,63E-05
cg12226475	2	191426880		IGR	0,251	8,04E-07	1,63E-05
cg14784833	1	147323659		IGR	-0,226	8,04E-07	1,63E-05
cg11631143	12	88954901	KITLG	Body	-0,202	8,04E-07	1,63E-05
cg20548013	6	12887210	PHACTR1	Body	0,216	8,05E-07	1,63E-05
cg11526752	2	175462540	WIPF1	5'UTR	-0,254	8,05E-07	1,64E-05
cg12308675	2	239169537	PER2	Body	0,204	8,06E-07	1,64E-05
cg01751057	8	134261928	NDRG1	Body	-0,201	8,06E-07	1,64E-05
cg26767081	3	194293260		IGR	0,246	8,08E-07	1,64E-05
cg15669090	12	6359376		IGR	-0,206	8,08E-07	1,64E-05
cg11507517	1	246743403	CNST	5'UTR	0,214	8,08E-07	1,64E-05
cg04790574	1	6285067	ICMT	3'UTR	0,214	8,09E-07	1,64E-05
cg04880464	8	67447920		IGR	-0,272	8,09E-07	1,64E-05
cg11983038	13	34251128		IGR	0,201	8,09E-07	1,64E-05
cg10556337	3	194482220	LOC1005073	Body	-0,223	8,10E-07	1,64E-05
cg20446176	17	6922932	MIR497HG	Body	0,293	8,10E-07	1,64E-05
cg11453837	6	32810551	PSMB8	Body	-0,243	8,11E-07	1,64E-05
cg17990510	1	209941813	TRAF3IP3	TSS200	-0,275	8,13E-07	1,65E-05
cg02632314	13	41593416	ELF1	5'UTR	-0,221	8,13E-07	1,65E-05
cg27032905	13	51878551		IGR	-0,21	8,14E-07	1,65E-05
cg09430000	3	16556065	RFTN1	TSS1500	-0,205	8,14E-07	1,65E-05
cg26192328	4	76956571	CXCL11	Body	-0,373	8,15E-07	1,65E-05
cg12598114	14	100550249	EVL	Body	-0,258	8,15E-07	1,65E-05
cg11811217	5	178650853	ADAMTS2	Body	-0,228	8,16E-07	1,65E-05

cg06905216	2	128300072	MYO7B	5'UTR	-0,202	8,18E-07	1,65E-05
cg19135414	11	1875098	LSP1	Body	-0,203	8,18E-07	1,65E-05
cg10140197	1	95186105	LINC01057	Body	-0,333	8,19E-07	1,65E-05
cg22014557	7	123410655		IGR	-0,263	8,19E-07	1,65E-05
cg16080308	21	45087640	RRP1B	Body	-0,223	8,19E-07	1,65E-05
cg21580043	17	17929070	ATPAF2	Body	-0,236	8,19E-07	1,65E-05
cg09374763	12	92863806		IGR	0,23	8,20E-07	1,66E-05
cg19100779	7	95115232	ASB4	TSS200	0,221	8,20E-07	1,66E-05
cg12815829	10	14614200	FAM107B	TSS200	-0,23	8,20E-07	1,66E-05
cg04822893	10	16612764		IGR	-0,202	8,20E-07	1,66E-05
cg07157398	17	81006821	B3GNTL1	Body	-0,338	8,20E-07	1,66E-05
cg10960268	2	156002497		IGR	-0,231	8,21E-07	1,66E-05
cg03280235	6	32158953	PBX2	TSS1500	-0,228	8,21E-07	1,66E-05
cg08502128	14	93049592	RIN3	Body	-0,273	8,22E-07	1,66E-05
cg13879455	12	58133008	AGAP2	TSS1500	0,269	8,22E-07	1,66E-05
cg22193385	12	52638005	KRT7	Body	-0,212	8,22E-07	1,66E-05
cg12642431	15	70994672	UACA	Body	0,273	8,22E-07	1,66E-05
cg03708153	4	110510998	CCDC109B	Body	0,227	8,23E-07	1,66E-05
cg15005526	2	73438142	NOTO	3'UTR	-0,236	8,24E-07	1,66E-05
cg11197258	12	124845001	NCOR2	Body	0,254	8,24E-07	1,66E-05
cg16415863	19	17516339	BST2	1stExon	-0,201	8,24E-07	1,66E-05
cg12884780	1	3078148	PRDM16	Body	0,22	8,25E-07	1,66E-05
cg26886885	16	85603371		IGR	-0,416	8,25E-07	1,66E-05
cg15404001	22	45996826	FBLN1	3'UTR	-0,206	8,26E-07	1,66E-05
cg05574076	3	142694271	PAQR9-AS1	Body	-0,247	8,26E-07	1,66E-05
cg25090121	17	43407096		IGR	-0,272	8,26E-07	1,66E-05
cg03328754	22	43093450	A4GALT	5'UTR	0,251	8,27E-07	1,67E-05
cg10776914	6	16583467	ATXN1	5'UTR	-0,268	8,27E-07	1,67E-05
cg16823927	12	105900263		IGR	-0,255	8,28E-07	1,67E-05
cg07362977	15	75941087	SNX33	TSS1500	0,256	8,28E-07	1,67E-05
cg07604117	6	152702365	SYNE1	Body	0,222	8,28E-07	1,67E-05
cg19560927	19	46995312	PNMAL2	1stExon	-0,228	8,29E-07	1,67E-05
cg13045294	19	41113556	LTBP4	Body	0,205	8,29E-07	1,67E-05
cg22590674	5	145356185	SH3RF2	Body	-0,217	8,30E-07	1,67E-05
cg21225548	9	100864335	TRIM14	Body	-0,203	8,30E-07	1,67E-05
cg06682371	3	32291082	CMTM8	Body	-0,258	8,30E-07	1,67E-05
cg16381609	11	70002745	ANO1	Body	-0,209	8,30E-07	1,67E-05
cg16857771	7	56160409	PHKG1	5'UTR	0,207	8,31E-07	1,67E-05
cg13780718	20	44513768	ZSWIM1	3'UTR	-0,209	8,32E-07	1,67E-05
cg03221819	10	75647445		IGR	0,255	8,32E-07	1,67E-05
cg23851638	19	47803550		IGR	-0,224	8,32E-07	1,67E-05
cg11043002	5	177876863	COL23A1	Body	0,206	8,33E-07	1,67E-05
cg03208825	12	6178792	VWF	Body	-0,271	8,34E-07	1,68E-05
cg06984255	10	104991193		IGR	0,353	8,34E-07	1,68E-05
cg18379332	16	79124177	WWOX	Body	-0,23	8,35E-07	1,68E-05
cg12368503	2	196428888		IGR	-0,233	8,35E-07	1,68E-05
cg16202812	6	149396105	UST	3'UTR	0,213	8,35E-07	1,68E-05
cg02743518	4	81019101		IGR	0,263	8,35E-07	1,68E-05
cg10740660	6	31740849	C6orf27	Body	0,216	8,36E-07	1,68E-05
cg08714510	2	106755481	UXS1	TSS200	0,241	8,36E-07	1,68E-05

cg14073946	9	73103756		IGR	0,216	8,36E-07	1,68E-05
cg14424070	14	75988820	BATF	5'UTR	-0,234	8,37E-07	1,68E-05
cg20133559	2	64566124		IGR	-0,204	8,37E-07	1,68E-05
cg13089904	1	8343271		IGR	-0,21	8,39E-07	1,68E-05
cg12134349	8	142264816		IGR	0,242	8,39E-07	1,68E-05
cg15712869	15	39703790		IGR	-0,297	8,40E-07	1,68E-05
cg22568270	3	69811867	MITF	TSS1500	-0,291	8,40E-07	1,68E-05
cg18974450	16	86421336		IGR	-0,216	8,41E-07	1,69E-05
cg07163687	9	129767752	RALGPS1	Body	-0,266	8,41E-07	1,69E-05
cg24205387	17	1464972	PITPNA	Body	0,249	8,42E-07	1,69E-05
cg18554868	20	62117537		IGR	-0,259	8,42E-07	1,69E-05
cg12064913	5	158208089	EBF1	Body	-0,227	8,42E-07	1,69E-05
cg17321346	2	68954175		IGR	-0,245	8,42E-07	1,69E-05
cg10832714	12	70901437		IGR	0,229	8,43E-07	1,69E-05
cg21187882	1	205756683		IGR	0,211	8,43E-07	1,69E-05
cg27583677	5	80528430	CKMT2	TSS1500	0,212	8,44E-07	1,69E-05
cg26508775	20	55050494	RTFDC1	Body	0,237	8,44E-07	1,69E-05
cg05622862	4	2292692	ZFYVE28	Body	-0,285	8,44E-07	1,69E-05
cg03364760	1	199138268		IGR	-0,224	8,44E-07	1,69E-05
cg02702424	3	30654871	TGFBR2	Body	-0,235	8,45E-07	1,69E-05
cg12658634	13	44599207	LINC00284	Body	-0,212	8,45E-07	1,69E-05
cg14543169	10	72546833		IGR	-0,219	8,45E-07	1,69E-05
cg19474015	5	126129089	LMNB1	5'UTR	-0,237	8,45E-07	1,69E-05
cg23827476	20	39863272	ZHX3	5'UTR	0,248	8,46E-07	1,69E-05
cg12177677	2	158300475	CYTIP	1stExon	-0,286	8,46E-07	1,69E-05
cg25466169	1	31745458	SNRNP40	Body	0,28	8,47E-07	1,69E-05
cg06920237	9	110819868		IGR	-0,207	8,47E-07	1,69E-05
cg08188060	1	246561637	SMYD3	5'UTR	0,217	8,47E-07	1,69E-05
cg21411014	12	4616445	C12orf4	Body	-0,2	8,48E-07	1,70E-05
cg00482489	17	34892036	PIGW	5'UTR	-0,201	8,49E-07	1,70E-05
cg04011423	3	141107482	ZBTB38	5'UTR	-0,261	8,50E-07	1,70E-05
cg17108303	2	133165082		IGR	-0,232	8,51E-07	1,70E-05
cg11357008	19	55449446	NLRP7	Body	-0,23	8,51E-07	1,70E-05
cg26515213	12	94171127	CRADD	Body	0,216	8,51E-07	1,70E-05
cg03228624	20	32979070	ITCH	5'UTR	0,223	8,53E-07	1,70E-05
cg19797536	7	91909243	ANKIB1	5'UTR	0,218	8,54E-07	1,70E-05
cg07744695	19	44229909		IGR	0,208	8,54E-07	1,70E-05
cg15167754	1	234764021	LINC00184	TSS1500	-0,203	8,55E-07	1,71E-05
cg11974430	2	241561007	GPR35	Body	-0,289	8,55E-07	1,71E-05
cg14661631	2	42368489		IGR	0,228	8,56E-07	1,71E-05
cg24930541	5	118664652	TNFAIP8	5'UTR	-0,239	8,57E-07	1,71E-05
cg20933483	22	23856505		IGR	-0,336	8,58E-07	1,71E-05
cg09538777	4	39362341	MIR1273H	Body	0,228	8,58E-07	1,71E-05
cg07581811	3	184491461		IGR	0,206	8,62E-07	1,72E-05
cg17451255	3	13455702	NUP210	Body	-0,252	8,62E-07	1,72E-05
cg14531564	1	1154853	SDF4	Body	-0,238	8,62E-07	1,72E-05
cg25611940	5	39191068	FYB	Body	-0,264	8,63E-07	1,72E-05
cg24675236	19	36009358		IGR	0,219	8,63E-07	1,72E-05
cg07826424	3	50628064		IGR	-0,241	8,64E-07	1,72E-05
cg17526952	6	36643854		IGR	-0,218	8,64E-07	1,72E-05

cg06650819	3	179283451	ACTL6A	5'UTR	-0,201	8,64E-07	1,72E-05
cg20963443	2	45939688	PRKCE	Body	-0,238	8,65E-07	1,72E-05
cg16940259	1	236323313	GPR137B	Body	-0,248	8,66E-07	1,72E-05
cg16072937	5	64344256		IGR	-0,243	8,66E-07	1,72E-05
cg25224001	15	71228855	LRRC49	Body	0,229	8,67E-07	1,72E-05
cg18825221	14	68749962	RAD51L1	Body	-0,33	8,67E-07	1,72E-05
cg17341404	11	269327		IGR	-0,254	8,67E-07	1,72E-05
cg16450309	3	170626822	EIF5A2	TSS1500	0,229	8,67E-07	1,72E-05
cg07302884	14	72064925	SIPA1L1	Body	-0,303	8,67E-07	1,72E-05
cg07432009	1	1815199	GNB1	5'UTR	0,209	8,68E-07	1,72E-05
cg26337602	17	792516	NXN	Body	-0,261	8,68E-07	1,72E-05
cg15718528	1	244327251		IGR	0,259	8,69E-07	1,73E-05
cg10815470	6	142508602	VTA1	Body	0,239	8,69E-07	1,73E-05
cg01514075	1	7832357	VAMP3	Body	0,236	8,70E-07	1,73E-05
cg00541303	1	208962374		IGR	-0,254	8,70E-07	1,73E-05
cg06362176	9	127048628	NEK6	Body	-0,273	8,71E-07	1,73E-05
cg02920954	7	106496406		IGR	-0,225	8,71E-07	1,73E-05
cg05658748	9	107818550		IGR	0,228	8,71E-07	1,73E-05
cg12883629	6	25042497		IGR	-0,379	8,71E-07	1,73E-05
cg06164122	13	99909260	GPR18	5'UTR	-0,239	8,72E-07	1,73E-05
cg19683021	12	2775658	CACNA1C	Body	-0,201	8,72E-07	1,73E-05
cg03990003	1	19275238	IFFO2	Body	-0,231	8,73E-07	1,73E-05
cg01692956	6	88435515		IGR	-0,229	8,73E-07	1,73E-05
cg27337902	2	175581955		IGR	-0,204	8,75E-07	1,73E-05
cg15114787	5	31745603		IGR	-0,212	8,75E-07	1,73E-05
cg06693983	19	55889216	TMEM190	Body	0,325	8,75E-07	1,74E-05
cg17344353	10	32702863		IGR	-0,225	8,76E-07	1,74E-05
cg13054007	13	81018323		IGR	-0,215	8,77E-07	1,74E-05
cg11067146	3	172229988	TNFSF10	Body	-0,293	8,78E-07	1,74E-05
cg14545963	8	21868607		IGR	0,203	8,79E-07	1,74E-05
cg08428677	3	177167200	LINC00578	Body	-0,324	8,79E-07	1,74E-05
cg07905034	9	92041302	SEMA4D	5'UTR	-0,325	8,80E-07	1,74E-05
cg00724003	15	77790658		IGR	0,251	8,80E-07	1,74E-05
cg10942056	1	223101848	DISP1	1stExon	0,27	8,80E-07	1,74E-05
cg07274406	21	46334192	ITGB2	5'UTR	-0,28	8,81E-07	1,74E-05
cg19101624	1	63871257	ALG6	Body	0,258	8,82E-07	1,74E-05
cg01089538	3	186745642	ST6GAL1	5'UTR	-0,232	8,82E-07	1,74E-05
cg25001923	1	76760322	ST6GALNAC3	Body	-0,211	8,82E-07	1,75E-05
cg05244428	6	101008489	ASCC3	Body	0,209	8,82E-07	1,75E-05
cg02352281	3	46139305		IGR	-0,258	8,83E-07	1,75E-05
cg17375396	11	67202808	RPS6KB2	3'UTR	-0,239	8,83E-07	1,75E-05
cg08261478	10	71922832	SAR1A	5'UTR	-0,249	8,83E-07	1,75E-05
cg21542303	4	103474041	NFKB1	Body	-0,254	8,84E-07	1,75E-05
cg06399398	1	235299630	RBM34	Body	0,246	8,84E-07	1,75E-05
cg06209576	1	198734078		IGR	-0,259	8,84E-07	1,75E-05
cg00647658	16	54227814		IGR	0,201	8,84E-07	1,75E-05
cg15139163	3	11408424	ATG7	Body	0,239	8,85E-07	1,75E-05
cg10310792	8	17708956		IGR	0,229	8,85E-07	1,75E-05
cg15978890	2	231615652	CAB39	5'UTR	0,232	8,85E-07	1,75E-05
cg06794612	11	65695774		IGR	-0,384	8,87E-07	1,75E-05

cg20701676	2	64782942	AFTPH	Body	-0,255	8,88E-07	1,75E-05
cg09113284	7	92327995	CDK6	Body	0,307	8,88E-07	1,75E-05
cg04417677	7	150039105	RARRES2	TSS1500	0,201	8,88E-07	1,75E-05
cg05025391	6	16583476	ATXN1	5'UTR	-0,267	8,88E-07	1,75E-05
cg13172549	7	27153636	HOXA3	5'UTR	0,277	8,88E-07	1,75E-05
cg25638946	6	85823949		IGR	0,26	8,88E-07	1,75E-05
cg00822433	12	129286685	SLC15A4	Body	0,232	8,89E-07	1,75E-05
cg11778638	14	104829666		IGR	-0,218	8,90E-07	1,76E-05
cg06065769	2	99065000	INPP4A	5'UTR	-0,213	8,90E-07	1,76E-05
cg08182911	3	187520832		IGR	-0,237	8,90E-07	1,76E-05
cg24556660	7	106842632	COG5	3'UTR	0,254	8,91E-07	1,76E-05
cg18397073	19	42600278	POU2F2	Body	0,245	8,91E-07	1,76E-05
cg09087669	18	658937	TYMSOS	TSS1500	-0,209	8,92E-07	1,76E-05
cg21165870	10	112627861		IGR	-0,218	8,92E-07	1,76E-05
cg08721974	11	47575259	CELF1	TSS1500	0,276	8,92E-07	1,76E-05
cg18054375	10	81194201	ZCCHC24	Body	-0,302	8,92E-07	1,76E-05
cg08264265	2	231091012	SP110	TSS1500	-0,218	8,93E-07	1,76E-05
cg02319097	12	10373231	GABARAPL1	Body	0,227	8,93E-07	1,76E-05
cg24538352	2	207687959		IGR	0,208	8,93E-07	1,76E-05
cg22365834	1	186646027	PTGS2	Body	0,228	8,93E-07	1,76E-05
cg15289708	6	46143406		IGR	-0,237	8,93E-07	1,76E-05
cg18582253	22	36034719		IGR	0,202	8,94E-07	1,76E-05
cg18996876	21	43483090	UMODL1	1stExon	-0,203	8,94E-07	1,76E-05
cg08806779	16	86965605		IGR	0,222	8,94E-07	1,76E-05
cg04588052	5	111268485	NREP-AS1	Body	0,21	8,94E-07	1,76E-05
cg02052157	8	26211126	PPP2R2A	Body	-0,206	8,95E-07	1,76E-05
cg07205203	2	28940400		IGR	0,255	8,95E-07	1,76E-05
cg22093077	2	231249869	SP140L	Body	0,231	8,95E-07	1,76E-05
cg01378515	3	124773106	HEG1	Body	0,22	8,96E-07	1,76E-05
cg06391839	8	134510900	ST3GAL1	5'UTR	-0,258	8,96E-07	1,76E-05
cg22435586	14	35834860		IGR	-0,234	8,97E-07	1,77E-05
cg04567124	11	3970505	STIM1	Body	-0,269	8,97E-07	1,77E-05
cg25116200	5	156698854	CYFIP2	5'UTR	-0,263	8,97E-07	1,77E-05
cg14018072	1	8157705		IGR	-0,243	8,98E-07	1,77E-05
cg23680242	11	8833885	ST5	TSS1500	-0,223	8,98E-07	1,77E-05
cg24793264	5	39461619		IGR	-0,25	8,98E-07	1,77E-05
cg10390354	4	88040748	AFF1	Body	0,212	8,99E-07	1,77E-05
cg12134752	19	5249431	PTPRS	Body	-0,201	8,99E-07	1,77E-05
cg02025944	4	80998921		IGR	-0,28	8,99E-07	1,77E-05
cg02077256	17	15133981	PMP22	3'UTR	-0,221	8,99E-07	1,77E-05
cg03252196	12	113646285	IQCD	5'UTR	-0,275	9,00E-07	1,77E-05
cg22257479	15	93684746		IGR	-0,204	9,01E-07	1,77E-05
cg27077827	15	60943536	RORA	Body	0,211	9,03E-07	1,77E-05
cg12967579	2	65173818		IGR	-0,269	9,03E-07	1,77E-05
cg21800127	22	40503479	TNRC6B	5'UTR	-0,226	9,03E-07	1,77E-05
cg07787301	17	76325712		IGR	-0,265	9,04E-07	1,77E-05
cg15384589	5	54873393		IGR	0,271	9,04E-07	1,77E-05
cg16145913	5	35858063	IL7R	Body	-0,251	9,04E-07	1,78E-05
cg11258813	14	53593556	DDHD1	Body	-0,264	9,05E-07	1,78E-05
cg21379388	3	195854275		IGR	-0,207	9,05E-07	1,78E-05

cg10225865	8	54605566		IGR	0,202	9,05E-07	1,78E-05
cg00834923	16	85393998		IGR	-0,229	9,06E-07	1,78E-05
cg04151727	4	71645025	RUFY3	Body	0,23	9,06E-07	1,78E-05
cg07389058	6	140858452		IGR	0,208	9,07E-07	1,78E-05
cg02831295	8	94030034		IGR	0,24	9,07E-07	1,78E-05
cg15899972	17	40425728	STAT5B	5'UTR	-0,246	9,07E-07	1,78E-05
cg26516510	11	2170779	IGF2	1stExon	-0,222	9,08E-07	1,78E-05
cg10446995	11	17265834		IGR	-0,231	9,08E-07	1,78E-05
cg27095222	11	88090861		IGR	-0,272	9,08E-07	1,78E-05
cg06181674	5	147646592		IGR	0,241	9,08E-07	1,78E-05
cg27119456	10	42863173	LOC441666	Body	0,213	9,09E-07	1,78E-05
cg02041561	11	76234319	C11orf30	ExonBnd	0,289	9,09E-07	1,78E-05
cg16794139	1	111174123	KCNA2	TSS200	-0,247	9,09E-07	1,78E-05
cg13399698	12	53443059	TNS2	TSS1500	0,304	9,09E-07	1,78E-05
cg01413054	2	231658979	CAB39	Body	-0,294	9,11E-07	1,78E-05
cg01025818	4	119679656	SEC24D	Body	0,248	9,11E-07	1,78E-05
cg02375313	6	658301	EXOC2	5'UTR	0,21	9,12E-07	1,78E-05
cg14225372	16	84200627	DNAAF1	Body	-0,242	9,12E-07	1,79E-05
cg23513337	10	24813690	KIAA1217	ExonBnd	-0,266	9,13E-07	1,79E-05
cg06256735	12	8815894	MFAP5	TSS1500	-0,216	9,14E-07	1,79E-05
cg14573876	19	4105153	MAP2K2	Body	-0,301	9,14E-07	1,79E-05
cg01110291	22	20402637		IGR	0,28	9,15E-07	1,79E-05
cg19050450	12	104871585	CHST11	Body	-0,221	9,15E-07	1,79E-05
cg14053644	10	90923971		IGR	-0,228	9,16E-07	1,79E-05
cg22226904	1	27819060		IGR	0,249	9,17E-07	1,79E-05
cg12476455	1	46052453	NASP	Body	-0,223	9,17E-07	1,79E-05
cg20827174	11	62195389		IGR	-0,217	9,17E-07	1,79E-05
cg19744045	4	22457802	ADGRA3	Body	-0,224	9,18E-07	1,79E-05
cg16471283	6	149816288		IGR	-0,239	9,18E-07	1,79E-05
cg16212207	3	187685215		IGR	0,215	9,21E-07	1,80E-05
cg00386405	19	30863275	ZNF536	TSS200	0,249	9,21E-07	1,80E-05
cg05258935	19	39086923	MAP4K1	Body	0,216	9,21E-07	1,80E-05
cg18840355	4	164033061		IGR	0,204	9,21E-07	1,80E-05
cg17027262	5	169704214	LCP2	Body	-0,201	9,23E-07	1,80E-05
cg01895805	8	141266703	TRAPPC9	Body	-0,282	9,23E-07	1,80E-05
cg18222768	18	77284207	NFATC1	Body	-0,244	9,24E-07	1,80E-05
cg19429281	19	53496738	ZNF702P	Body	0,255	9,25E-07	1,80E-05
cg03482769	19	10445593	RAVER1	TSS1500	0,209	9,25E-07	1,80E-05
cg24387544	11	122614858	UBASH3B	Body	-0,222	9,25E-07	1,80E-05
cg16312628	3	128393800		IGR	-0,308	9,27E-07	1,81E-05
cg23757168	12	116202497		IGR	-0,208	9,28E-07	1,81E-05
cg03519577	20	4795886	RASSF2	5'UTR	-0,278	9,29E-07	1,81E-05
cg09060360	14	81406605	CEP128	TSS1500	-0,219	9,29E-07	1,81E-05
cg11100581	6	75910011	COL12A1	Body	-0,201	9,29E-07	1,81E-05
cg04348305	20	25230959	PYGB	Body	0,223	9,30E-07	1,81E-05
cg04817532	1	56432132		IGR	-0,283	9,30E-07	1,81E-05
cg26461710	10	91470135	KIF20B	Body	0,249	9,30E-07	1,81E-05
cg02923856	3	23989124	NR1D2	5'UTR	0,212	9,31E-07	1,81E-05
cg01732224	11	4659876	OR51D1	TSS1500	-0,203	9,31E-07	1,81E-05
cg05870166	2	144707254	GTDC1	3'UTR	0,217	9,32E-07	1,81E-05

cg18666492	5	22242842	CDH12	5'UTR	0,201	9,32E-07	1,81E-05
cg12995376	13	51102160	DLEU1-AS1	TSS1500	-0,206	9,32E-07	1,81E-05
cg05587373	4	88189110		IGR	-0,233	9,33E-07	1,81E-05
cg19398425	11	108799455	DDX10	Body	-0,245	9,33E-07	1,82E-05
cg18084609	14	31345041	COCH	Body	0,24	9,34E-07	1,82E-05
cg07024786	7	29883787	WIPF3	Body	-0,204	9,35E-07	1,82E-05
cg04767756	16	10652706	EMP2	5'UTR	0,218	9,35E-07	1,82E-05
cg15120743	2	9211406		IGR	-0,21	9,35E-07	1,82E-05
cg07581623	8	26498830	DPYSL2	Body	0,214	9,35E-07	1,82E-05
cg20951694	3	120245478		IGR	0,215	9,36E-07	1,82E-05
cg23334660	4	187573046	FAT1	Body	0,288	9,36E-07	1,82E-05
cg09283701	9	71440805	PIP5K1B	Body	0,236	9,36E-07	1,82E-05
cg00806644	10	126889815		IGR	-0,341	9,37E-07	1,82E-05
cg09251078	15	59461599	MYO1E	Body	-0,217	9,37E-07	1,82E-05
cg04146883	11	46062035	PHF21A	Body	0,221	9,37E-07	1,82E-05
cg09337852	7	134464288	CALD1	5'UTR	0,234	9,37E-07	1,82E-05
cg14847662	6	131350835	EPB41L2	5'UTR	0,263	9,38E-07	1,82E-05
cg00702417	1	6054867		IGR	-0,291	9,38E-07	1,82E-05
cg14887620	18	53501191		IGR	-0,203	9,38E-07	1,82E-05
cg25646701	2	121443256		IGR	-0,202	9,39E-07	1,82E-05
cg12190768	4	100737691	DAPP1	TSS1500	-0,263	9,39E-07	1,82E-05
cg19628064	12	89550231		IGR	-0,236	9,39E-07	1,82E-05
cg25320889	17	66342532	ARSG	Body	-0,322	9,39E-07	1,82E-05
cg12819393	9	127240449		IGR	0,204	9,40E-07	1,82E-05
cg07027613	12	7260608	C1RL	Body	-0,273	9,41E-07	1,83E-05
cg02628177	8	134079254	TG	Body	-0,217	9,41E-07	1,83E-05
cg07663893	4	122121190	TNIP3	Body	-0,252	9,44E-07	1,83E-05
cg02401078	10	49660642	ARHGAP22	Body	-0,217	9,45E-07	1,83E-05
cg21643095	11	105009375	CARD18	3'UTR	-0,229	9,45E-07	1,83E-05
cg16558214	13	110981828	COL4A2	Body	-0,208	9,46E-07	1,83E-05
cg14339182	1	39190061		IGR	-0,208	9,46E-07	1,83E-05
cg15097913	1	36021962	KIAA0319L	5'UTR	0,207	9,47E-07	1,83E-05
cg02152417	1	7326345	CAMTA1	Body	0,257	9,47E-07	1,83E-05
cg11705496	1	209941848	TRAF3IP3	1stExon	-0,337	9,48E-07	1,83E-05
cg18653372	6	112647973		IGR	-0,231	9,48E-07	1,83E-05
cg19753209	16	4211240		IGR	-0,238	9,48E-07	1,84E-05
cg08274633	15	38988533	C15orf53	TSS1500	-0,299	9,49E-07	1,84E-05
cg18096764	20	52226460		IGR	-0,267	9,49E-07	1,84E-05
cg07267367	5	32099275	PDZD2	Body	0,262	9,50E-07	1,84E-05
cg05201209	22	22152374	MAPK1	Body	0,262	9,50E-07	1,84E-05
cg25165932	12	109025846	SELPLG	5'UTR	-0,216	9,51E-07	1,84E-05
cg07871859	7	120504674		IGR	-0,213	9,52E-07	1,84E-05
cg07128770	2	38593166	ATL2	Body	0,267	9,52E-07	1,84E-05
cg03858102	1	33515278		IGR	-0,291	9,52E-07	1,84E-05
cg02524864	8	9321300		IGR	-0,225	9,53E-07	1,84E-05
cg13864304	5	159782556	C1QTNF2	Body	-0,218	9,55E-07	1,84E-05
cg17204289	7	151433358	PRKAG2	1stExon	0,229	9,56E-07	1,85E-05
cg06059025	5	104369506		IGR	0,329	9,56E-07	1,85E-05
cg03700670	17	2169502	SMG6	TSS200	-0,241	9,56E-07	1,85E-05
cg24332577	20	50419248	SALL4	TSS1500	0,224	9,57E-07	1,85E-05

cg06064964	20	62199181	PRIC285	Body	-0,311	9,57E-07	1,85E-05
cg20816330	8	22697267	PEBP4	Body	-0,245	9,58E-07	1,85E-05
cg27112045	3	111521399	PLCXD2	Body	0,209	9,59E-07	1,85E-05
cg12709196	1	26616481	UBXN11	Body	-0,318	9,59E-07	1,85E-05
cg04367503	19	13121571	NFIX	Body	-0,203	9,59E-07	1,85E-05
cg13973086	2	469105		IGR	0,21	9,60E-07	1,85E-05
cg06465011	16	84860871	CRISPLD2	5'UTR	0,204	9,61E-07	1,85E-05
cg12612465	6	37533112		IGR	0,273	9,61E-07	1,85E-05
cg01775245	8	6452110	MCPH1	Body	0,307	9,62E-07	1,85E-05
cg15314957	8	135332875		IGR	-0,224	9,63E-07	1,86E-05
cg22273285	17	46683120	HOXB6	TSS1500	0,257	9,63E-07	1,86E-05
cg23722588	1	100996682		IGR	-0,223	9,63E-07	1,86E-05
cg00734838	10	14614164	FAM107B	1stExon	-0,223	9,64E-07	1,86E-05
cg14714222	20	47440761	PREX1	Body	-0,261	9,65E-07	1,86E-05
cg21250978	7	106684541	PRKAR2B	TSS1500	0,244	9,65E-07	1,86E-05
cg09560488	8	107295414	OXR1	5'UTR	-0,232	9,66E-07	1,86E-05
cg15903937	11	88092892		IGR	-0,282	9,66E-07	1,86E-05
cg00403484	9	36030087		IGR	-0,206	9,67E-07	1,86E-05
cg07278971	8	22717823	PEBP4	Body	-0,203	9,68E-07	1,86E-05
cg11603365	14	61970209	PRKCH	Body	-0,221	9,68E-07	1,86E-05
cg19458022	7	158215688	PTPRN2	Body	0,323	9,68E-07	1,86E-05
cg25404570	10	74472750	MCU	Body	0,257	9,68E-07	1,86E-05
cg11776045	5	15937472	FBXL7	3'UTR	-0,239	9,69E-07	1,86E-05
cg07575466	1	40769708	COL9A2	Body	0,23	9,69E-07	1,86E-05
cg15731221	2	106005609	FHL2	5'UTR	0,265	9,69E-07	1,86E-05
cg04990314	2	9889094		IGR	-0,215	9,70E-07	1,86E-05
cg12903499	11	57549042	TMX2-CTNND	Body	0,26	9,70E-07	1,87E-05
cg24377495	19	6660075		IGR	0,25	9,71E-07	1,87E-05
cg05052463	4	153274105	FBXW7	Body	0,216	9,72E-07	1,87E-05
cg13294644	8	10183929	MSRA	Body	-0,25	9,72E-07	1,87E-05
cg10295572	7	27173518		IGR	0,287	9,73E-07	1,87E-05
cg12528835	19	50001058	RPS11	Body	-0,222	9,73E-07	1,87E-05
cg08687753	11	89234928	NOX4	5'UTR	-0,223	9,74E-07	1,87E-05
cg21528163	7	155075730		IGR	-0,205	9,74E-07	1,87E-05
cg09721612	8	109321336		IGR	-0,21	9,75E-07	1,87E-05
cg00024437	4	146828472	ZNF827	Body	0,231	9,75E-07	1,87E-05
cg06088244	1	38171423	CDCA8	Body	-0,208	9,77E-07	1,87E-05
cg24054898	3	148721868	GYG1	Body	-0,23	9,77E-07	1,87E-05
cg02707799	8	41814854	MYST3	Body	-0,259	9,77E-07	1,88E-05
cg20602359	3	52120287	POC1A	Body	-0,203	9,77E-07	1,88E-05
cg03372243	12	124718467	ZNF664-FAM:	5'UTR	0,253	9,77E-07	1,88E-05
cg15971442	10	76660199	KAT6B	Body	-0,277	9,78E-07	1,88E-05
cg01256665	2	69951589		IGR	0,349	9,79E-07	1,88E-05
cg08473139	20	56579268		IGR	-0,211	9,79E-07	1,88E-05
cg00582562	17	46388465	SKAP1	Body	0,263	9,79E-07	1,88E-05
cg18598146	20	47896448	SNORD12B	TSS1500	-0,249	9,79E-07	1,88E-05
cg11589567	1	175570022	TNR	5'UTR	-0,209	9,80E-07	1,88E-05
cg25222848	14	52792015	PTGER2	Body	-0,252	9,81E-07	1,88E-05
cg23121975	7	139618125	TBXAS1	Body	0,225	9,81E-07	1,88E-05
cg08873865	11	15924912		IGR	-0,21	9,82E-07	1,88E-05

cg11620863	18	55435948	ATP8B1	5'UTR	0,245	9,82E-07	1,88E-05
cg03884924	2	106355341		IGR	-0,243	9,82E-07	1,88E-05
cg02637352	5	148961536	FLJ41603	5'UTR	0,21	9,83E-07	1,88E-05
cg08001909	2	160473325	LOC643072	Body	0,219	9,84E-07	1,88E-05
cg18254356	5	43044573	LOC153684	Body	0,246	9,85E-07	1,88E-05
cg00303269	14	85706085		IGR	-0,212	9,85E-07	1,89E-05
cg01320698	1	9788517	PIK3CD	3'UTR	-0,243	9,88E-07	1,89E-05
cg12434312	10	129845509	PTPRE	Body	-0,213	9,88E-07	1,89E-05
cg14760590	14	70870160	SYNJ2BP-COX	Body	0,207	9,88E-07	1,89E-05
cg25875565	3	139078139	COPB2	Body	0,21	9,89E-07	1,89E-05
cg04330884	10	126339578	FAM53B	Body	-0,213	9,89E-07	1,89E-05
cg23782426	5	134692343	H2AFY	Body	0,223	9,89E-07	1,89E-05
cg26665444	15	63791209		IGR	-0,347	9,90E-07	1,89E-05
cg11101698	17	762846	NXN	Body	0,202	9,91E-07	1,89E-05
cg07476513	15	40324712	EIF2AK4	Body	0,237	9,92E-07	1,90E-05
cg17627654	11	70508410	SHANK2	Body	0,202	9,92E-07	1,90E-05
cg04479071	5	41794623	OXCT1	Body	0,201	9,93E-07	1,90E-05
cg16286285	5	124064299	ZNF608	Body	-0,251	9,93E-07	1,90E-05
cg05697191	9	21432880		IGR	-0,284	9,94E-07	1,90E-05
cg15751602	3	49142815	QARS	TSS1500	0,205	9,94E-07	1,90E-05
cg01856529	12	54653091	CBX5	1stExon	0,229	9,94E-07	1,90E-05
cg26450106	20	24929607	CST7	TSS1500	-0,233	9,94E-07	1,90E-05
cg08915171	2	105273376		IGR	0,246	9,94E-07	1,90E-05
cg02697979	11	34265361	ABTB2	Body	-0,22	9,94E-07	1,90E-05
cg10200202	10	121277385	RGS10	Body	-0,219	9,95E-07	1,90E-05
cg25423428	22	44254463	SULT4A1	Body	-0,222	9,95E-07	1,90E-05
cg21350324	6	158452623	SYNJ2	5'UTR	-0,202	9,96E-07	1,90E-05
cg19249516	4	156667493		IGR	-0,221	9,96E-07	1,90E-05
cg26441627	11	2044066		IGR	-0,207	9,96E-07	1,90E-05
cg00382999	3	136649333	NCK1	Body	0,213	9,97E-07	1,90E-05
cg14178436	1	212362064		IGR	-0,23	9,98E-07	1,90E-05
cg07235981	17	30470872	RHOT1	5'UTR	0,219	9,98E-07	1,90E-05
cg25892587	10	3666169		IGR	0,319	9,99E-07	1,90E-05
cg17942388	11	113649065	CLDN25	TSS1500	0,231	1,00E-06	1,91E-05
cg23497683	2	100759159	AFF3	TSS200	-0,23	1,00E-06	1,91E-05
cg13376973	6	170437946		IGR	0,235	1,00E-06	1,91E-05
cg27326113	8	91953125	NECAB1	Body	0,231	1,00E-06	1,91E-05
cg05726450	17	8771947	PIK3R6	TSS1500	-0,215	1,00E-06	1,91E-05
cg18177548	2	226329535	NYAP2	Body	-0,221	1,00E-06	1,91E-05
cg17277833	2	43558544	THADA	Body	0,222	1,00E-06	1,91E-05
cg26512925	16	85519061		IGR	-0,209	1,00E-06	1,91E-05
cg26909276	16	11448916		IGR	-0,224	1,00E-06	1,91E-05
cg03050508	14	62011349	PRKCH	Body	0,222	1,00E-06	1,91E-05
cg21302651	17	19976569		IGR	-0,254	1,00E-06	1,91E-05
cg23182003	6	117003752	KPNA5	Body	-0,229	1,01E-06	1,91E-05
cg15423654	1	158217942		IGR	-0,289	1,01E-06	1,91E-05
cg22103474	11	18611215	UEVLD	TSS1500	0,219	1,01E-06	1,91E-05
cg00892294	13	49323918		IGR	-0,209	1,01E-06	1,91E-05
cg18123418	11	118146284		IGR	0,219	1,01E-06	1,91E-05
cg05700689	16	15834098	MYH11	Body	-0,212	1,01E-06	1,91E-05

cg09902130	11	60739178	CD6	5'UTR	-0,236	1,01E-06	1,92E-05
cg06202958	10	6312422		IGR	-0,236	1,01E-06	1,92E-05
cg26669984	9	99249066	HABP4	Body	0,256	1,01E-06	1,92E-05
cg22863637	7	42287188		IGR	0,206	1,01E-06	1,92E-05
cg18068443	11	69812114		IGR	-0,206	1,01E-06	1,92E-05
cg20861716	8	29357029		IGR	-0,278	1,01E-06	1,92E-05
cg09367522	5	154172825	LARP1	Body	0,205	1,01E-06	1,92E-05
cg23250405	4	77380623	SHROOM3	Body	0,254	1,01E-06	1,92E-05
cg16996281	11	119217884	MFRP	TSS1500	-0,205	1,01E-06	1,92E-05
cg04487654	9	88353585	AGTPBP1	Body	0,265	1,01E-06	1,92E-05
cg06419398	11	59952352	MS4A6A	TSS1500	-0,217	1,01E-06	1,92E-05
cg25394605	20	16374118	KIF16B	Body	0,236	1,01E-06	1,92E-05
cg25651608	3	42073868		IGR	0,243	1,01E-06	1,92E-05
cg18754118	19	2588376	GNG7	5'UTR	0,2	1,01E-06	1,92E-05
cg10515989	17	32528422		IGR	-0,224	1,01E-06	1,92E-05
cg20984949	20	52226582		IGR	-0,251	1,02E-06	1,92E-05
cg21336878	4	4286680	LYAR	5'UTR	0,221	1,02E-06	1,93E-05
cg01759870	14	105936409	MTA1	Body	0,286	1,02E-06	1,93E-05
cg03081173	6	31166502	HCG27	Body	-0,273	1,02E-06	1,93E-05
cg01735788	22	27520354		IGR	-0,216	1,02E-06	1,93E-05
cg13488556	15	43532047	TGM5	Body	-0,205	1,02E-06	1,93E-05
cg17244719	8	144661001	NAPRT	TSS1500	0,245	1,02E-06	1,93E-05
cg05400854	8	38523411		IGR	-0,221	1,02E-06	1,93E-05
cg24723731	11	15643912		IGR	-0,21	1,02E-06	1,93E-05
cg13333736	18	293336		IGR	-0,207	1,02E-06	1,93E-05
cg16213164	12	25823509		IGR	0,207	1,02E-06	1,93E-05
cg24530225	2	102701960	IL1R1	5'UTR	-0,21	1,02E-06	1,93E-05
cg08683522	2	7960281		IGR	0,265	1,02E-06	1,93E-05
cg04543014	11	121039874	TECTA	Body	-0,214	1,02E-06	1,93E-05
cg17677968	12	121100923	CABP1	Body	-0,313	1,02E-06	1,93E-05
cg03235140	20	4215839	ADRA1D	Body	-0,208	1,02E-06	1,94E-05
cg07054292	2	127963330	CYP27C1	1stExon	0,24	1,02E-06	1,94E-05
cg17839611	17	47286802	ABI3	TSS1500	-0,21	1,03E-06	1,94E-05
cg01530803	22	30877800	SDC4P	TSS200	-0,208	1,03E-06	1,94E-05
cg22256354	8	131554148		IGR	-0,214	1,03E-06	1,94E-05
cg06004060	19	18434815	LSM4	TSS1500	0,225	1,03E-06	1,94E-05
cg00757404	1	45050279	RNF220	Body	-0,223	1,03E-06	1,95E-05
cg24321566	5	171844324	SH3PXD2B	Body	-0,203	1,03E-06	1,95E-05
cg06324869	21	22474516	NCAM2	Body	0,238	1,03E-06	1,95E-05
cg17449107	12	105014903	CHST11	Body	0,207	1,03E-06	1,95E-05
cg13359998	1	230241764	GALNT2	Body	-0,21	1,03E-06	1,95E-05
cg07085900	19	9488547	ZNF177	5'UTR	-0,204	1,03E-06	1,95E-05
cg23953831	1	117544416	CD101	5'UTR	-0,28	1,03E-06	1,95E-05
cg04051649	7	105088822		IGR	-0,248	1,04E-06	1,95E-05
cg08939481	14	21494269	NDRG2	TSS1500	0,241	1,04E-06	1,95E-05
cg08098128	2	220112465	STK16	Body	-0,263	1,04E-06	1,96E-05
cg09460688	12	43085391		IGR	-0,222	1,04E-06	1,96E-05
cg14093103	1	61546214	NFIA	Body	0,239	1,04E-06	1,96E-05
cg22449863	3	49968246	MON1A	TSS1500	0,228	1,04E-06	1,96E-05
cg18477166	2	19131738		IGR	0,257	1,04E-06	1,96E-05

cg14884828	10	14603607	FAM107B	Body	-0,234	1,04E-06	1,96E-05
cg12712122	6	88435312		IGR	-0,226	1,04E-06	1,96E-05
cg25587107	17	68078868	KCNJ16	5'UTR	-0,254	1,04E-06	1,96E-05
cg22439390	19	14480797		IGR	-0,241	1,04E-06	1,96E-05
cg18163955	17	40833833	CNTNAP1	TSS1500	-0,263	1,04E-06	1,96E-05
cg02973057	22	31494860	SMTN	Body	0,201	1,04E-06	1,97E-05
cg05184826	3	149051368	TM4SF18	5'UTR	0,241	1,05E-06	1,97E-05
cg26188685	15	50540269	HDC	Body	-0,252	1,05E-06	1,97E-05
cg23600719	3	53204418	PRKCD	5'UTR	-0,272	1,05E-06	1,97E-05
cg06459984	17	79381472	BAHCC1	Body	-0,215	1,05E-06	1,97E-05
cg27215666	8	30600962	UBXN8	TSS1500	0,203	1,05E-06	1,97E-05
cg16738327	16	28204624	XPO6	5'UTR	-0,269	1,05E-06	1,97E-05
cg22480109	7	158575681	ESYT2	Body	0,252	1,05E-06	1,97E-05
cg22354983	21	33344786	HUNK	Body	-0,203	1,05E-06	1,97E-05
cg24252262	5	23014515		IGR	0,21	1,05E-06	1,97E-05
cg20140023	10	22278414	DNAJC1	Body	0,216	1,05E-06	1,98E-05
cg12552289	11	132813577	OPCML	TSS1500	0,206	1,05E-06	1,98E-05
cg14974591	12	6081629	VWF	Body	-0,217	1,05E-06	1,98E-05
cg13225810	12	68730832		IGR	0,239	1,05E-06	1,98E-05
cg19425289	5	34917257	RAD1	TSS1500	-0,238	1,05E-06	1,98E-05
cg24137448	17	46189113	SNX11	5'UTR	-0,211	1,05E-06	1,98E-05
cg24480379	1	151345123	SELENBP1	5'UTR	0,236	1,05E-06	1,98E-05
cg18316328	15	70994416	UACA	1stExon	0,285	1,05E-06	1,98E-05
cg12126243	14	59742285	DAAM1	Body	-0,225	1,06E-06	1,98E-05
cg05213092	9	112887565	AKAP2	TSS1500	0,217	1,06E-06	1,98E-05
cg20571751	2	109187875	LIMS1	Body	0,201	1,06E-06	1,98E-05
cg22568423	19	8590567	MYO1F	Body	0,209	1,06E-06	1,98E-05
cg10814308	10	97087287	SORBS1	Body	0,21	1,06E-06	1,98E-05
cg18151410	15	92367692		IGR	0,215	1,06E-06	1,98E-05
cg22987568	19	2621982	GNG7	5'UTR	-0,225	1,06E-06	1,98E-05
cg03683087	10	114746071	TCF7L2	Body	-0,24	1,06E-06	1,98E-05
cg02058807	5	77852006	LHFPL2	5'UTR	-0,204	1,06E-06	1,98E-05
cg03951267	14	50552617	LINC01599	Body	-0,227	1,06E-06	1,99E-05
cg00816396	5	58839530	PDE4D	Body	0,248	1,06E-06	1,99E-05
cg09998038	10	75654563		IGR	-0,272	1,06E-06	1,99E-05
cg15885891	3	182592736	ATP11B	Body	0,265	1,06E-06	1,99E-05
cg22064834	4	141024933	MAML3	Body	-0,252	1,06E-06	1,99E-05
cg18022258	7	105449844	ATXN7L1	Body	-0,256	1,06E-06	1,99E-05
cg24759562	3	48777860		IGR	0,243	1,06E-06	1,99E-05
cg27480497	13	48784568		IGR	-0,302	1,06E-06	1,99E-05
cg08578765	7	131919970	PLXNA4	Body	-0,208	1,06E-06	1,99E-05
cg19981301	2	63820005	MDH1	Body	0,228	1,07E-06	2,00E-05
cg02948555	15	42749848	ZNF106	TSS200	0,205	1,07E-06	2,00E-05
cg26036831	10	13723418	FRMD4A	Body	-0,227	1,07E-06	2,00E-05
cg26084032	1	235017899		IGR	-0,298	1,07E-06	2,00E-05
cg25815177	6	164506829		IGR	0,208	1,07E-06	2,00E-05
cg08748036	18	13577725	LDLRAD4	Body	-0,257	1,07E-06	2,00E-05
cg12968732	2	67788868		IGR	0,209	1,07E-06	2,00E-05
cg19089201	7	45002287	MYO1G	3'UTR	0,209	1,07E-06	2,00E-05
cg16429748	14	72170517	SIPA1L1	Body	0,236	1,07E-06	2,00E-05

cg19070952	15	66080290	DENND4A	5'UTR	-0,21	1,07E-06	2,00E-05
cg08279097	1	114455336	DCLRE1B	3'UTR	0,332	1,07E-06	2,00E-05
cg07086380	5	118691033	TNFAIP8	Body	-0,232	1,07E-06	2,00E-05
cg24770256	17	80486771	FOXK2	Body	0,222	1,07E-06	2,00E-05
cg21555762	2	109834751	SH3RF3	Body	-0,257	1,07E-06	2,00E-05
cg22033823	15	52640656	MYO5A	Body	0,227	1,07E-06	2,00E-05
cg22356268	19	58693616	ZNF274	TSS1500	0,205	1,07E-06	2,01E-05
cg18907362	4	72352852	SLC4A4	Body	0,283	1,08E-06	2,01E-05
cg18081487	1	197411408	CRB1	ExonBnd	0,28	1,08E-06	2,01E-05
cg07910043	1	90167408	LRRC8C	Body	0,261	1,08E-06	2,01E-05
cg05755408	8	27157515	TRIM35	Body	-0,245	1,08E-06	2,01E-05
cg07717559	17	2699021	RAP1GAP2	TSS1500	-0,23	1,08E-06	2,01E-05
cg10263494	12	45995746		IGR	0,211	1,08E-06	2,01E-05
cg00363337	3	111329355	CD96	Body	-0,215	1,08E-06	2,02E-05
cg23642047	5	111265808	NREP-AS1	Body	-0,247	1,08E-06	2,02E-05
cg05335944	10	13749010	FRMD4A	Body	0,272	1,08E-06	2,02E-05
cg19306332	9	13282594		IGR	-0,239	1,08E-06	2,02E-05
cg07945618	15	99926398	LRRC28	3'UTR	0,238	1,08E-06	2,02E-05
cg20586169	2	85855952	USP39	Body	-0,201	1,08E-06	2,02E-05
cg23786580	7	43204506	HECW1	5'UTR	-0,228	1,08E-06	2,02E-05
cg18413706	21	39759139	ERG	Body	-0,238	1,08E-06	2,02E-05
cg09793303	1	212774506	ATF3	5'UTR	-0,233	1,09E-06	2,02E-05
cg12591668	1	192520335		IGR	-0,31	1,09E-06	2,02E-05
cg02784513	2	68430960	PPP3R1	Body	-0,274	1,09E-06	2,02E-05
cg11137074	14	95652357	CLMN	3'UTR	-0,287	1,09E-06	2,02E-05
cg09289622	15	76041384		IGR	-0,318	1,09E-06	2,02E-05
cg07899244	17	4219377	UBE2G1	Body	0,223	1,09E-06	2,02E-05
cg23895418	20	34078780	CEP250	Body	-0,302	1,09E-06	2,02E-05
cg09795903	2	33444985	LTBP1	Body	-0,268	1,09E-06	2,02E-05
cg00626573	1	166895404	ILDR2	Body	0,216	1,09E-06	2,03E-05
cg12875711	2	40326460	SLC8A1-AS1	Body	0,26	1,09E-06	2,03E-05
cg12419334	4	184121800	WWC2	Body	0,208	1,09E-06	2,03E-05
cg26161816	19	7414043		IGR	-0,25	1,09E-06	2,03E-05
cg24692969	5	55555411		IGR	-0,22	1,09E-06	2,03E-05
cg22953759	5	10632397	ANKRD33B	Body	-0,283	1,09E-06	2,03E-05
cg21267919	1	118665061	SPAG17	Body	0,255	1,09E-06	2,03E-05
cg08497939	4	114605002	CAMK2D	Body	-0,214	1,09E-06	2,03E-05
cg23688956	1	230167197		IGR	-0,202	1,09E-06	2,03E-05
cg14347002	6	41066169	NFYA	3'UTR	0,228	1,09E-06	2,03E-05
cg12298429	1	239883464	CHRM3-AS2	TSS1500	-0,22	1,10E-06	2,04E-05
cg12432168	10	4284253	LINC00702	Body	0,292	1,10E-06	2,04E-05
cg19100091	21	44358654		IGR	0,228	1,10E-06	2,04E-05
cg02408464	11	103746841		IGR	-0,256	1,10E-06	2,04E-05
cg20979800	2	238136548		IGR	-0,323	1,10E-06	2,04E-05
cg16961769	10	123274709	FGFR2	Body	0,216	1,10E-06	2,04E-05
cg12734107	11	57519426	BTBD18	TSS200	0,204	1,10E-06	2,04E-05
cg18836974	1	52133824	OSBPL9	Body	0,229	1,10E-06	2,04E-05
cg13952483	1	223101782	DISP1	1stExon	0,259	1,10E-06	2,04E-05
cg06165742	7	114297860	FOXP2	Body	0,216	1,10E-06	2,04E-05
cg24497819	12	109027683	SELPLG	TSS200	-0,237	1,10E-06	2,04E-05

cg00304793	2	106003911	FHL2	5'UTR	0,203	1,10E-06	2,04E-05
cg14327393	9	75141633	TMC1	5'UTR	0,209	1,10E-06	2,04E-05
cg17502037	17	3289666		IGR	0,296	1,10E-06	2,04E-05
cg00407926	1	27982013		IGR	0,211	1,10E-06	2,04E-05
cg04167430	15	99847223	LRRC28	Body	0,251	1,11E-06	2,04E-05
cg06401459	9	134128240		IGR	-0,301	1,11E-06	2,04E-05
cg02713706	1	247681749	GCSAML	TSS200	0,21	1,11E-06	2,05E-05
cg16968681	19	452803	SHC2	Body	-0,207	1,11E-06	2,05E-05
cg11853918	6	17159487		IGR	-0,214	1,11E-06	2,05E-05
cg03531211	6	32920102	HLA-DMA	Body	-0,232	1,11E-06	2,05E-05
cg14160521	7	18336912	HDAC9	Body	0,219	1,11E-06	2,05E-05
cg12241243	1	164740849	LOC10050579	Body	0,202	1,11E-06	2,05E-05
cg12583320	14	74296935		IGR	-0,256	1,11E-06	2,05E-05
cg12833765	2	69470878	ANTXR1	Body	-0,259	1,11E-06	2,05E-05
cg03495173	9	14251737	NFIB	Body	0,261	1,11E-06	2,05E-05
cg17345994	11	57567448	CTNND1	Body	0,255	1,11E-06	2,05E-05
cg07237979	11	1874017	LSP1	TSS200	-0,297	1,11E-06	2,05E-05
cg24468939	18	25738353	CDH2	Body	0,212	1,11E-06	2,05E-05
cg25132782	15	84047082		IGR	-0,252	1,11E-06	2,05E-05
cg03616958	3	154579208		IGR	-0,202	1,11E-06	2,06E-05
cg04027736	7	27143403	HOXA2	TSS1500	0,233	1,11E-06	2,06E-05
cg23489671	10	14614245	FAM107B	TSS200	-0,256	1,11E-06	2,06E-05
cg12853789	20	57738826		IGR	-0,213	1,11E-06	2,06E-05
cg11796219	3	194854342	C3orf21	Body	-0,222	1,12E-06	2,06E-05
cg22809683	1	182998916	LAMC1	Body	-0,229	1,12E-06	2,06E-05
cg24646487	14	100214704		IGR	-0,207	1,12E-06	2,06E-05
cg25542319	5	112540429	MCC	Body	0,21	1,12E-06	2,06E-05
cg05050910	1	231108572	TTC13	Body	-0,228	1,12E-06	2,06E-05
cg25242968	16	79317678		IGR	-0,249	1,12E-06	2,06E-05
cg23912266	6	33139581	COL11A2	Body	-0,207	1,12E-06	2,06E-05
cg03803789	11	111854003	DIXDC1	Body	0,205	1,12E-06	2,06E-05
cg23263641	19	44128109	CADM4	Body	0,221	1,12E-06	2,06E-05
cg16491284	11	33723462	C11orf91	TSS1500	0,217	1,12E-06	2,06E-05
cg13710341	21	33829932	EVA1C	Body	0,248	1,12E-06	2,06E-05
cg14302428	9	4139392	GLIS3	Body	-0,263	1,12E-06	2,07E-05
cg13509731	2	204676448		IGR	-0,224	1,12E-06	2,07E-05
cg13520893	9	136006118	RALGDS	Body	-0,225	1,12E-06	2,07E-05
cg10464462	17	33417059	RFFL	TSS1500	-0,228	1,12E-06	2,07E-05
cg24538401	7	106684555	PRKAR2B	TSS1500	0,276	1,12E-06	2,07E-05
cg15518883	9	35650561	SIT1	Body	-0,203	1,12E-06	2,07E-05
cg20578731	2	179188138	OSBPL6	Body	-0,204	1,12E-06	2,07E-05
cg14629475	20	36432615	CTNNBL1	Body	-0,219	1,12E-06	2,07E-05
cg16611208	18	3058547		IGR	-0,227	1,12E-06	2,07E-05
cg16067079	9	133708845	ABL1	Body	0,209	1,13E-06	2,07E-05
cg14414682	2	175700729	CHN1	Body	-0,212	1,13E-06	2,07E-05
cg23429794	6	119131733		IGR	-0,208	1,13E-06	2,07E-05
cg26186509	2	136577654	LOC10050760	TSS200	-0,282	1,13E-06	2,08E-05
cg27175112	2	11515031		IGR	0,22	1,13E-06	2,08E-05
cg04496782	3	31741190	OSBPL10	Body	-0,256	1,13E-06	2,08E-05
cg04071264	7	130881686	MKLN1	Body	0,244	1,13E-06	2,08E-05

cg21658656	2	120413205	CFAP221	Body	-0,26	1,13E-06	2,08E-05
cg11389866	11	69240780		IGR	-0,24	1,13E-06	2,08E-05
cg05748342	3	51721635	TEX264	Body	0,245	1,13E-06	2,08E-05
cg23304605	14	91866373	CCDC88C	Body	-0,212	1,13E-06	2,08E-05
cg07852774	7	24575660		IGR	0,256	1,13E-06	2,08E-05
cg00991994	3	99639553	C3orf26	Body	0,313	1,13E-06	2,08E-05
cg09953778	20	56097887	CTCFL	Body	-0,212	1,13E-06	2,09E-05
cg24036116	14	91281636	TTC7B	Body	0,265	1,14E-06	2,09E-05
cg27204197	7	50731484	GRB10	Body	-0,205	1,14E-06	2,09E-05
cg20691469	4	2691863	FAM193A	Body	-0,24	1,14E-06	2,09E-05
cg02066322	5	150478162		IGR	-0,287	1,14E-06	2,09E-05
cg06648780	3	105133817	ALCAM	Body	0,249	1,14E-06	2,09E-05
cg24759279	10	7128104		IGR	0,209	1,14E-06	2,09E-05
cg19409133	1	28843744	SNHG3-RCC1	Body	-0,216	1,14E-06	2,09E-05
cg06490868	10	79725233		IGR	-0,208	1,14E-06	2,09E-05
cg08240068	9	134460967	RAPGEF1	Body	-0,206	1,14E-06	2,09E-05
cg16053158	12	47610006	PCED1B-AS1	Body	-0,239	1,14E-06	2,09E-05
cg04161859	10	73404657	CDH23	Body	-0,214	1,14E-06	2,09E-05
cg18624930	14	61970191	PRKCH	Body	-0,271	1,14E-06	2,09E-05
cg27154651	6	30796209		IGR	0,208	1,14E-06	2,09E-05
cg02456998	3	124293098	KALRN	Body	-0,232	1,14E-06	2,09E-05
cg19662219	15	34260719	AVEN	Body	0,224	1,14E-06	2,09E-05
cg26976693	17	8802846	PIK3R5	Body	-0,214	1,14E-06	2,09E-05
cg25638056	21	40234113		IGR	-0,204	1,14E-06	2,09E-05
cg08376583	6	32222492		IGR	0,215	1,14E-06	2,10E-05
cg20622308	15	75039031		IGR	0,226	1,14E-06	2,10E-05
cg23243176	18	45420696	SMAD2	Body	0,227	1,14E-06	2,10E-05
cg25625968	5	38464319	EGFLAM	3'UTR	0,23	1,14E-06	2,10E-05
cg21171858	5	131792825	C5orf56	Body	-0,31	1,14E-06	2,10E-05
cg16081687	8	845388	ERICH1-AS1	Body	0,253	1,14E-06	2,10E-05
cg26992245	8	29848579		IGR	-0,211	1,14E-06	2,10E-05
cg09328744	20	50031354	NFATC2	Body	-0,228	1,15E-06	2,10E-05
cg11303634	2	191980337	STAT4	Body	-0,209	1,15E-06	2,10E-05
cg17602444	7	644819	PRKAR1B	Body	-0,203	1,15E-06	2,10E-05
cg08844365	6	112576393	LAMA4	TSS1500	0,215	1,15E-06	2,11E-05
cg01629993	12	127545014		IGR	0,219	1,15E-06	2,11E-05
cg07207692	4	88452129	SPARCL1	TSS1500	0,224	1,15E-06	2,11E-05
cg07573937	3	14465629	SLC6A6	5'UTR	-0,255	1,15E-06	2,11E-05
cg24875405	12	54653411	CBX5	TSS200	0,214	1,15E-06	2,11E-05
cg06975179	1	181119042		IGR	-0,277	1,15E-06	2,11E-05
cg05247914	19	35629701	FXYD1	TSS200	0,221	1,16E-06	2,11E-05
cg16544318	6	76322352	SENP6	Body	-0,255	1,16E-06	2,12E-05
cg24523415	19	16404594		IGR	0,227	1,16E-06	2,12E-05
cg03272225	19	17957106	JAK3	5'UTR	-0,283	1,16E-06	2,12E-05
cg18233424	12	48384159	COL2A1	Body	-0,267	1,16E-06	2,12E-05
cg08796342	14	92334029	TC2N	TSS200	-0,231	1,16E-06	2,12E-05
cg21554180	1	116272679	CASQ2	Body	-0,25	1,16E-06	2,12E-05
cg12305431	7	27157855	HOXA3	5'UTR	0,246	1,16E-06	2,12E-05
cg03917666	10	3977608		IGR	-0,207	1,16E-06	2,12E-05
cg02795540	20	34078980	CEP250	Body	-0,271	1,16E-06	2,12E-05

cg26814835	20	30195969		IGR	0,211	1,16E-06	2,12E-05
cg01054110	12	125002332	NCOR2	5'UTR	0,225	1,16E-06	2,12E-05
cg24418581	5	124703928		IGR	-0,213	1,16E-06	2,12E-05
cg06966110	11	469881	PTDSS2	Body	0,24	1,16E-06	2,12E-05
cg15560745	14	21624796	OR5AU1	TSS1500	-0,213	1,16E-06	2,12E-05
cg06600936	15	85361858	ALPK3	Body	0,209	1,16E-06	2,13E-05
cg15627721	2	232045255		IGR	0,202	1,16E-06	2,13E-05
cg08759860	14	100134437	HHIPL1	Body	-0,209	1,16E-06	2,13E-05
cg06484638	17	79402455	BAHCC1	ExonBnd	-0,225	1,17E-06	2,13E-05
cg03722295	19	10519375		IGR	-0,23	1,17E-06	2,13E-05
cg25691442	15	63345512	TPM1	Body	0,207	1,17E-06	2,13E-05
cg03655142	1	153950843	JTB	TSS1500	-0,208	1,17E-06	2,13E-05
cg11600161	11	67171585	TBC1D10C	5'UTR	-0,228	1,17E-06	2,13E-05
cg01111414	7	33711078		IGR	-0,212	1,17E-06	2,13E-05
cg12901592	11	44527562		IGR	-0,206	1,17E-06	2,13E-05
cg18493761	11	125386885		IGR	0,209	1,17E-06	2,13E-05
cg09315305	6	82916096	IBTK	Body	0,22	1,17E-06	2,13E-05
cg10666916	6	33872206		IGR	-0,228	1,17E-06	2,14E-05
cg16471179	10	104914352	NT5C2	Body	0,272	1,17E-06	2,14E-05
cg23041929	16	86922147		IGR	-0,242	1,17E-06	2,14E-05
cg14025893	3	30794667	GADL1	Body	-0,208	1,17E-06	2,14E-05
cg00427618	2	218280252	DIRC3	Body	-0,295	1,17E-06	2,14E-05
cg22687244	15	59815173	FAM81A	3'UTR	-0,229	1,17E-06	2,14E-05
cg08247321	13	33479697	LINC00423	Body	-0,228	1,17E-06	2,14E-05
cg00686838	6	107730183	PDSS2	Body	0,216	1,18E-06	2,14E-05
cg04429417	6	163850011	QKI	Body	0,246	1,18E-06	2,14E-05
cg00007809	2	242275777	sept-02	Body	-0,206	1,18E-06	2,14E-05
cg12172916	17	32705657		IGR	-0,278	1,18E-06	2,14E-05
cg20790742	10	11651797	USP6NL	Body	-0,215	1,18E-06	2,14E-05
cg21670717	13	30733388		IGR	-0,24	1,18E-06	2,14E-05
cg15615645	10	104543040	C10orf26	Body	0,201	1,18E-06	2,14E-05
cg04234016	12	7062109	PTPN6	Body	-0,27	1,18E-06	2,14E-05
cg09659000	1	13820748	LRRC38	Body	-0,225	1,18E-06	2,15E-05
cg09328921	1	221341100		IGR	-0,258	1,18E-06	2,15E-05
cg01147106	17	8269762		IGR	0,24	1,18E-06	2,15E-05
cg00015530	8	145052950	PARP10	Body	-0,222	1,18E-06	2,15E-05
cg10296382	6	4518960		IGR	-0,208	1,18E-06	2,15E-05
cg20959701	10	97239456	SORBS1	5'UTR	0,221	1,18E-06	2,15E-05
cg10850532	2	158325604		IGR	-0,225	1,18E-06	2,15E-05
cg07737180	16	87339877	C16orf95	Body	0,203	1,19E-06	2,15E-05
cg26694386	1	244868765	PPPDE1	Body	0,208	1,19E-06	2,15E-05
cg26168154	4	86739979	ARHGAP24	Body	-0,207	1,19E-06	2,16E-05
cg14719129	4	113569835	MIR302D	TSS1500	0,245	1,19E-06	2,16E-05
cg19473377	2	38891148		IGR	-0,204	1,19E-06	2,16E-05
cg07765820	6	30973372	MUC22	TSS1500	0,219	1,19E-06	2,16E-05
cg21533262	7	107110551	COG5	Body	0,253	1,19E-06	2,16E-05
cg16458589	2	103303534	SLC9A2	Body	-0,23	1,19E-06	2,16E-05
cg15026474	3	170891516	TNIK	Body	0,227	1,19E-06	2,16E-05
cg04785083	1	9031262	CA6	Body	-0,252	1,19E-06	2,16E-05
cg06915667	3	128272723		IGR	-0,202	1,19E-06	2,16E-05

cg10895198	1	204436206	PIK3C2B	Body	0,241	1,19E-06	2,16E-05
cg11299543	9	5510595	PDCD1LG2	1stExon	-0,244	1,20E-06	2,17E-05
cg17453840	15	83317526	CPEB1	TSS1500	0,2	1,20E-06	2,17E-05
cg21897315	3	81811448	GBE1	TSS1500	0,245	1,20E-06	2,17E-05
cg04985566	15	96517205		IGR	-0,218	1,20E-06	2,17E-05
cg11085762	19	17942317	JAK3	Body	0,254	1,20E-06	2,17E-05
cg07050692	8	61664695	CHD7	Body	0,277	1,20E-06	2,17E-05
cg12326699	1	240400726	FMN2	Body	0,23	1,20E-06	2,17E-05
cg23855093	3	100328713	GPR128	1stExon	-0,227	1,20E-06	2,17E-05
cg24692944	17	41608258	ETV4	TSS1500	0,252	1,20E-06	2,17E-05
cg10784511	3	148426295	AGTR1	5'UTR	-0,241	1,20E-06	2,17E-05
cg21235950	19	14496535	ADGRE5	Body	-0,296	1,20E-06	2,17E-05
cg07921503	7	2445843	CHST12	5'UTR	-0,226	1,20E-06	2,17E-05
cg12448285	1	245534821	KIF26B	Body	0,263	1,20E-06	2,18E-05
cg06051411	17	73044991	KCTD2	Body	0,206	1,20E-06	2,18E-05
cg02187937	1	21005254	KIF17	Body	-0,259	1,20E-06	2,18E-05
cg23602489	7	101508949	CUX1	Body	0,205	1,20E-06	2,18E-05
cg19695053	21	25113130		IGR	0,251	1,20E-06	2,18E-05
cg02635625	10	69838818		IGR	0,256	1,21E-06	2,18E-05
cg27156771	6	34326474	NUDT3	Body	0,235	1,21E-06	2,18E-05
cg22707239	13	27488892		IGR	-0,242	1,21E-06	2,18E-05
cg15928808	3	64188875	PRICKLE2	5'UTR	-0,214	1,21E-06	2,18E-05
cg25978327	1	8065039		IGR	0,216	1,21E-06	2,18E-05
cg13668346	22	35871548		IGR	-0,209	1,21E-06	2,18E-05
cg27658518	3	183723888	ABCC5-AS1	TSS1500	0,221	1,21E-06	2,19E-05
cg22954906	13	103531058	LOC121952	TSS1500	0,295	1,21E-06	2,19E-05
cg05331789	1	229461833	CCSAP	Body	-0,21	1,21E-06	2,19E-05
cg26908868	6	139961862		IGR	-0,213	1,21E-06	2,19E-05
cg06585511	6	2434392		IGR	-0,221	1,22E-06	2,19E-05
cg08692046	1	21501347	EIF4G3	TSS200	0,202	1,22E-06	2,19E-05
cg12822165	12	50997854	DIP2B	Body	0,271	1,22E-06	2,19E-05
cg06471596	7	101579936	CUX1	Body	0,247	1,22E-06	2,19E-05
cg01510278	16	11456238		IGR	-0,279	1,22E-06	2,19E-05
cg22633096	10	64451393		IGR	-0,202	1,22E-06	2,19E-05
cg16477262	3	37949403	CTDSPL	Body	-0,27	1,22E-06	2,19E-05
cg07119830	10	104412306	TRIM8	Body	-0,26	1,22E-06	2,19E-05
cg21301387	11	128457475	ETS1	TSS200	0,228	1,22E-06	2,20E-05
cg11596947	11	15966774		IGR	-0,248	1,22E-06	2,20E-05
cg16078269	18	45568004	ZBTB7C	TSS1500	-0,267	1,22E-06	2,20E-05
cg07727170	15	70458214		IGR	0,272	1,22E-06	2,20E-05
cg14394126	4	27005430	STIM2	Body	-0,232	1,22E-06	2,20E-05
cg00041401	1	114414408	PTPN22	TSS200	-0,261	1,22E-06	2,20E-05
cg11861562	11	117069780	TAGLN	TSS1500	0,29	1,22E-06	2,20E-05
cg08077981	12	109026956	SELPLG	TSS1500	-0,243	1,23E-06	2,20E-05
cg22633111	5	56989399		IGR	0,22	1,23E-06	2,20E-05
cg05194426	10	135343193	CYP2E1	Body	0,259	1,23E-06	2,20E-05
cg11194182	11	60838995		IGR	-0,203	1,23E-06	2,21E-05
cg04229482	8	58983086	FAM110B	5'UTR	0,215	1,23E-06	2,21E-05
cg20975835	11	64107374	CCDC88B	TSS1500	-0,23	1,23E-06	2,21E-05
cg14202820	21	28853537	MIR5009	Body	-0,253	1,23E-06	2,21E-05

cg03726628	1	6088407	KCNAB2	5'UTR	-0,306	1,23E-06	2,21E-05
cg12722782	16	72509753	LINC01572	Body	0,231	1,23E-06	2,21E-05
cg00311883	1	235099120		IGR	-0,233	1,23E-06	2,21E-05
cg02164561	7	8215977	ICA1	Body	-0,23	1,23E-06	2,21E-05
cg22249529	22	50985797	KLHDC7B	TSS1500	0,221	1,23E-06	2,21E-05
cg10034963	12	46616664	SLC38A1	Body	-0,256	1,23E-06	2,21E-05
cg17596493	13	24143068	TNFRSF19	TSS1500	-0,216	1,23E-06	2,21E-05
cg14044785	2	43560368	THADA	Body	0,248	1,23E-06	2,21E-05
cg02286857	2	47297177	TTC7A	Body	0,228	1,23E-06	2,21E-05
cg04244624	1	85991560	DDAH1	5'UTR	0,239	1,23E-06	2,21E-05
cg10453443	1	224022797	TP53BP2	Body	-0,296	1,23E-06	2,21E-05
cg08206357	7	131803380		IGR	-0,201	1,23E-06	2,21E-05
cg08155109	4	173731209	GALNTL6	Body	-0,233	1,24E-06	2,22E-05
cg11536788	9	37800138	DCAF10	TSS1500	0,203	1,24E-06	2,22E-05
cg07661899	12	53443057	TENC1	TSS1500	0,268	1,24E-06	2,22E-05
cg19063065	1	95635395	TMEM56	Body	0,224	1,24E-06	2,22E-05
cg14891553	19	39144278	ACTN4	Body	0,237	1,24E-06	2,22E-05
cg04136344	2	47452737	LOC1019270[illegible]	Body	0,287	1,24E-06	2,22E-05
cg06920833	18	3666099	DLGAP1	Body	0,211	1,24E-06	2,22E-05
cg17122157	21	44819445		IGR	-0,249	1,24E-06	2,22E-05
cg10406499	15	67478816	SMAD3	Body	0,232	1,24E-06	2,22E-05
cg11302913	11	44989471		IGR	-0,215	1,24E-06	2,22E-05
cg26489737	10	3930603		IGR	-0,232	1,24E-06	2,23E-05
cg04361487	8	128425921	CASC8	Body	-0,234	1,24E-06	2,23E-05
cg24111025	6	32819921	TAP1	Body	-0,246	1,24E-06	2,23E-05
cg08856326	9	124382072	DAB2IP	Body	0,263	1,25E-06	2,23E-05
cg18516609	10	31747241	ZEB1	Body	-0,46	1,25E-06	2,23E-05
cg03493768	4	76996690	ART3	5'UTR	0,22	1,25E-06	2,23E-05
cg05992786	12	54446308	HOXC4	5'UTR	0,205	1,25E-06	2,23E-05
cg18588298	8	141562576	AGO2	Body	0,209	1,25E-06	2,23E-05
cg05256490	2	74643745	C2orf81	Body	-0,2	1,25E-06	2,23E-05
cg06872436	5	61040674		IGR	0,2	1,25E-06	2,23E-05
cg02471028	4	1203653	LOC1001308[illegible]	TSS1500	0,238	1,25E-06	2,23E-05
cg04933317	4	39314460	MIR1273H	Body	0,21	1,25E-06	2,23E-05
cg02686689	21	30195182		IGR	0,29	1,25E-06	2,23E-05
cg00415613	1	204597392	LRRN2	5'UTR	-0,236	1,25E-06	2,23E-05
cg16133703	5	95206387		IGR	-0,2	1,25E-06	2,23E-05
cg22974301	8	54566797		IGR	-0,204	1,25E-06	2,23E-05
cg09493223	2	68962172	ARHGAP25	5'UTR	-0,202	1,25E-06	2,24E-05
cg06460227	10	21951196	MLLT10	Body	0,234	1,25E-06	2,24E-05
cg20654632	2	188412311	TFPI	5'UTR	-0,22	1,25E-06	2,24E-05
cg06294561	3	141161580	ZBTB38	Body	0,258	1,25E-06	2,24E-05
cg16116639	15	89683968	ABHD2	Body	-0,258	1,25E-06	2,24E-05
cg14241289	15	55612304	PIGBOS1	TSS1500	-0,309	1,25E-06	2,24E-05
cg22772527	3	71181342	FOXP1	TSS1500	0,21	1,25E-06	2,24E-05
cg11958233	1	40736075	ZMPSTE24	Body	-0,22	1,26E-06	2,24E-05
cg17995626	2	38405469	CYP1B1-AS1	Body	-0,26	1,26E-06	2,24E-05
cg06536724	17	64544418	PRKCA	Body	-0,225	1,26E-06	2,24E-05
cg27339426	3	134013359		IGR	-0,214	1,26E-06	2,24E-05
cg13320146	1	150954826	ANXA9	1stExon	-0,297	1,26E-06	2,24E-05

cg05641698	2	201195632	SPATS2L	5'UTR	0,244	1,26E-06	2,24E-05
cg08400424	5	142357042	ARHGAP26	Body	-0,264	1,26E-06	2,25E-05
cg01363734	12	117482953	TESC	Body	0,201	1,26E-06	2,25E-05
cg27580567	18	61108394		IGR	-0,212	1,26E-06	2,25E-05
cg02320084	16	85769350	C16orf74	5'UTR	-0,309	1,26E-06	2,25E-05
cg14873515	5	149887461	NDST1	TSS1500	-0,238	1,26E-06	2,25E-05
cg21809161	1	59617037		IGR	0,213	1,26E-06	2,25E-05
cg24848259	1	223827339	CAPN8	Body	-0,286	1,26E-06	2,25E-05
cg14463790	13	26337396	ATP8A2	Body	-0,23	1,26E-06	2,25E-05
cg26870745	19	3133503		IGR	-0,203	1,26E-06	2,25E-05
cg22390040	8	17220621	MTMR7	Body	0,204	1,26E-06	2,25E-05
cg15601264	17	54864697		IGR	-0,272	1,26E-06	2,25E-05
cg11175473	6	165933115	PDE10A	Body	-0,204	1,26E-06	2,25E-05
cg12447100	19	52734532		IGR	0,221	1,27E-06	2,25E-05
cg15060367	19	51775821		IGR	-0,214	1,27E-06	2,25E-05
cg02180006	1	35681117		IGR	0,222	1,27E-06	2,26E-05
cg15325154	7	88425122	ZNF804B	Body	0,221	1,27E-06	2,26E-05
cg16394709	8	74493032	STAU2	Body	0,219	1,27E-06	2,26E-05
cg26381514	12	26963489	ITPR2	Body	0,305	1,27E-06	2,26E-05
cg15313507	17	76393108	PGS1	Body	0,218	1,27E-06	2,26E-05
cg14375890	6	13355672		IGR	-0,235	1,27E-06	2,27E-05
cg00474549	3	187707374		IGR	0,268	1,27E-06	2,27E-05
cg10570745	2	201640187	AOX2P	Body	0,212	1,28E-06	2,27E-05
cg00520689	8	131469811		IGR	-0,271	1,28E-06	2,27E-05
cg08258740	18	58560613		IGR	-0,208	1,28E-06	2,27E-05
cg01661564	20	52509854		IGR	-0,295	1,28E-06	2,27E-05
cg12244644	18	60655398		IGR	-0,201	1,28E-06	2,27E-05
cg02052531	5	81692895		IGR	0,237	1,28E-06	2,27E-05
cg27474897	2	199480893		IGR	-0,207	1,28E-06	2,27E-05
cg13311440	1	160681404	CD48	Body	-0,229	1,28E-06	2,27E-05
cg03080336	4	186033378		IGR	0,216	1,28E-06	2,28E-05
cg26700919	18	13375474	C18orf1	5'UTR	-0,255	1,28E-06	2,28E-05
cg24533117	2	54622005		IGR	-0,217	1,28E-06	2,28E-05
cg03663556	7	50900556		IGR	-0,278	1,28E-06	2,28E-05
cg15061330	10	115580593		IGR	0,209	1,28E-06	2,28E-05
cg17329393	13	32820875	FRY	Body	0,223	1,28E-06	2,28E-05
cg03449922	7	132953368	EXOC4	Body	-0,262	1,29E-06	2,28E-05
cg22522688	12	109221232	SSH1	1stExon	0,232	1,29E-06	2,28E-05
cg14854112	5	34732408	RAI14	Body	-0,214	1,29E-06	2,28E-05
cg21043695	13	99890787	UBAC2	Body	-0,24	1,29E-06	2,28E-05
cg12073656	7	116475018		IGR	0,256	1,29E-06	2,28E-05
cg00168191	19	3097565	GNA11	Body	0,234	1,29E-06	2,28E-05
cg00618275	5	142488469	ARHGAP26	Body	0,254	1,29E-06	2,29E-05
cg26187339	6	43602925	MAD2L1BP	TSS1500	0,223	1,29E-06	2,29E-05
cg06596013	17	70927385	SLC39A11	Body	-0,207	1,29E-06	2,29E-05
cg26389811	4	127697722		IGR	0,243	1,29E-06	2,29E-05
cg14392772	9	123666481	TRAF1	3'UTR	-0,275	1,29E-06	2,29E-05
cg14158968	11	11590474	GALNTL4	Body	-0,206	1,29E-06	2,29E-05
cg16870048	6	4600735		IGR	-0,238	1,29E-06	2,29E-05
cg15662224	20	62266902		IGR	-0,285	1,30E-06	2,29E-05

cg00179272	1	112050099	TMIGD3	Body	-0,242	1,30E-06	2,29E-05
cg23377551	12	58130154	AGAP2	Body	0,234	1,30E-06	2,29E-05
cg27127608	5	158488659	EBF1	Body	0,211	1,30E-06	2,29E-05
cg08389995	3	131645295	CPNE4	Body	-0,201	1,30E-06	2,30E-05
cg14592839	3	49506547	DAG1	TSS1500	0,239	1,30E-06	2,30E-05
cg00411203	5	57753407	PLK2	ExonBnd	0,256	1,30E-06	2,30E-05
cg11874091	2	109228889	LIMS1	5'UTR	-0,265	1,30E-06	2,30E-05
cg01564661	14	105942636	CRIP2	Body	0,216	1,30E-06	2,30E-05
cg14481528	1	110933996	SLC16A4	TSS1500	0,216	1,30E-06	2,30E-05
cg08136020	6	14735175		IGR	-0,25	1,30E-06	2,30E-05
cg23166590	8	142247880		IGR	0,219	1,30E-06	2,30E-05
cg17274057	1	164599431	PBX1	Body	-0,225	1,30E-06	2,30E-05
cg01034094	1	117101030	CD58	Body	-0,218	1,30E-06	2,30E-05
cg12468774	3	49236860	CCDC36	TSS200	0,32	1,30E-06	2,31E-05
cg18674464	8	74395934	STAU2	Body	0,213	1,31E-06	2,31E-05
cg06294319	8	127387971		IGR	0,227	1,30E-06	2,31E-05
cg23696593	10	28200623	ARMC4	Body	0,233	1,30E-06	2,31E-05
cg12042598	2	74097426		IGR	0,204	1,31E-06	2,31E-05
cg07799572	5	70309843	NAIP	Body	-0,278	1,31E-06	2,31E-05
cg24511258	3	195634428	TNK2	5'UTR	-0,278	1,31E-06	2,31E-05
cg04931656	16	3118215	IL32	Body	-0,23	1,31E-06	2,31E-05
cg13620369	2	109182706	LIMS1	Body	-0,224	1,31E-06	2,31E-05
cg10556868	6	16413526	ATXN1	5'UTR	-0,226	1,31E-06	2,31E-05
cg16740910	20	34788104	EPB41L1	Body	-0,205	1,31E-06	2,31E-05
cg23501836	14	89629974	FOXN3	Body	0,205	1,31E-06	2,32E-05
cg06722212	3	66119580	SLC25A26	1stExon	-0,222	1,31E-06	2,32E-05
cg06145110	3	108013109		IGR	-0,27	1,31E-06	2,32E-05
cg12584858	11	110326628	FDX1	Body	-0,225	1,31E-06	2,32E-05
cg19954357	18	13496835	LDLRAD4	Body	-0,267	1,31E-06	2,32E-05
cg09749064	11	85464041	SYTL2	5'UTR	-0,238	1,32E-06	2,32E-05
cg14201617	2	47382452	C2orf61	1stExon	0,246	1,32E-06	2,32E-05
cg07758286	15	64989000	OAZ2	Body	0,221	1,32E-06	2,32E-05
cg19229759	14	77051233		IGR	-0,238	1,32E-06	2,32E-05
cg11251253	5	130693810	CDC42SE2	5'UTR	-0,295	1,32E-06	2,32E-05
cg12269161	6	143178039	HIVEP2	5'UTR	-0,209	1,32E-06	2,32E-05
cg04889100	17	66342801	ARSG	Body	-0,278	1,32E-06	2,32E-05
cg23564931	17	66511075	PRKAR1A	5'UTR	0,23	1,32E-06	2,32E-05
cg14727405	9	19999663		IGR	-0,213	1,32E-06	2,32E-05
cg20611731	1	15389011	KAZN	Body	-0,204	1,32E-06	2,32E-05
cg08260676	5	31962746	PDZD2	Body	0,226	1,32E-06	2,32E-05
cg03772020	6	148879122		IGR	-0,282	1,32E-06	2,32E-05
cg19873536	10	116475283		IGR	0,212	1,32E-06	2,33E-05
cg01951274	17	56409518	MIR142	TSS1500	-0,226	1,32E-06	2,33E-05
cg07913430	4	115609343		IGR	0,209	1,32E-06	2,33E-05
cg02998883	4	170477098	NEK1	Body	0,234	1,33E-06	2,33E-05
cg23495022	12	129315279		IGR	-0,241	1,33E-06	2,33E-05
cg16861537	16	55542330	LPCAT2	TSS1500	-0,231	1,33E-06	2,33E-05
cg07721663	5	139675220	PFDN1	Body	0,261	1,33E-06	2,33E-05
cg19925780	1	101509557		IGR	0,207	1,33E-06	2,34E-05
cg25735583	5	95681194	LOC10192971	Body	-0,249	1,33E-06	2,34E-05

cg11234839	4	185189248		IGR	-0,232	1,33E-06	2,34E-05
cg15878300	17	73008137	ICT1	TSS1500	0,201	1,33E-06	2,34E-05
cg20185994	11	125823933		IGR	-0,26	1,33E-06	2,34E-05
cg03024301	6	52440589	TRAM2	Body	0,208	1,33E-06	2,34E-05
cg20061654	22	45608492	C22orf9	Body	0,21	1,33E-06	2,34E-05
cg00718594	12	56968168	RBMS2	Body	-0,229	1,33E-06	2,34E-05
cg09306332	15	72470685	GRAMD2	Body	-0,232	1,33E-06	2,34E-05
cg03035427	2	201787406	ORC2	Body	-0,24	1,33E-06	2,34E-05
cg19758142	3	172365942	NCEH1	5'UTR	0,219	1,33E-06	2,34E-05
cg09451188	13	45989144	SLC25A30	5'UTR	0,222	1,33E-06	2,34E-05
cg16324747	2	225811610	DOCK10	1stExon	-0,27	1,33E-06	2,34E-05
cg18051527	6	119864698		IGR	-0,242	1,33E-06	2,34E-05
cg00223569	12	25064560	BCAT1	Body	0,201	1,34E-06	2,35E-05
cg06052829	22	33158075	SYN3	Body	-0,221	1,34E-06	2,35E-05
cg00515954	12	99548504	ANKS1B	TSS200	0,2	1,34E-06	2,35E-05
cg12149795	21	47882121	DIP2A	Body	0,207	1,34E-06	2,35E-05
cg14169496	4	36288123	DTHD1	Body	-0,241	1,34E-06	2,35E-05
cg02292475	15	58921725	ADAM10	Body	0,213	1,34E-06	2,35E-05
cg07564690	14	32597733	ARHGAP5	Body	0,225	1,34E-06	2,35E-05
cg20449614	8	22438122	PDLIM2	Body	-0,29	1,34E-06	2,35E-05
cg16134349	10	61900552	ANK3	1stExon	0,225	1,34E-06	2,35E-05
cg22987448	19	8591364	MYO1F	Body	0,231	1,34E-06	2,35E-05
cg13709923	9	95726377	FGD3	1stExon	-0,209	1,34E-06	2,35E-05
cg10746278	11	116061735		IGR	-0,227	1,34E-06	2,35E-05
cg25406657	15	63342033	TPM1	Body	0,218	1,34E-06	2,36E-05
cg10091996	16	31548640		IGR	0,209	1,35E-06	2,36E-05
cg18345549	12	80737811	OTOGL	Body	0,241	1,35E-06	2,36E-05
cg02760167	16	69457337	CYB5B	TSS1500	0,213	1,35E-06	2,36E-05
cg26032419	1	81578539		IGR	-0,203	1,35E-06	2,36E-05
cg09977847	2	217070953	XRCC5	3'UTR	-0,241	1,35E-06	2,36E-05
cg07324869	12	9268730	A2M	TSS200	0,201	1,35E-06	2,36E-05
cg21397177	4	143017332	INPP4B	Body	0,231	1,35E-06	2,36E-05
cg00227790	15	93347048		IGR	-0,281	1,35E-06	2,36E-05
cg22913231	5	72456190		IGR	-0,256	1,35E-06	2,37E-05
cg07946977	1	221886639	DUSP10	5'UTR	-0,286	1,35E-06	2,37E-05
cg01691592	12	25801556	LMNTD1	TSS200	0,241	1,35E-06	2,37E-05
cg06535918	18	72778537		IGR	0,201	1,35E-06	2,37E-05
cg19215003	1	111422722	CD53	5'UTR	-0,231	1,35E-06	2,37E-05
cg13067553	1	147782452		IGR	0,231	1,35E-06	2,37E-05
cg20293874	22	29070191	TTC28	Body	-0,222	1,35E-06	2,37E-05
cg27478635	20	39632437		IGR	-0,269	1,36E-06	2,37E-05
cg18990068	11	92062752		IGR	-0,208	1,36E-06	2,37E-05
cg10455038	3	100372896	ADGRG7	Body	0,234	1,36E-06	2,37E-05
cg07621169	3	53277395	TKT	Body	0,23	1,36E-06	2,37E-05
cg12703852	8	98209210	LOC1019270(	Body	0,257	1,36E-06	2,38E-05
cg01048287	8	29762857		IGR	0,252	1,36E-06	2,38E-05
cg18313661	7	63560590		IGR	0,246	1,36E-06	2,38E-05
cg19606569	4	39029049	TMEM156	Body	-0,268	1,36E-06	2,38E-05
cg13401703	15	99789777	TTC23	1stExon	0,225	1,36E-06	2,38E-05
cg23198707	3	112023284		IGR	0,221	1,36E-06	2,38E-05

cg12767903	3	114027886	TIGIT	3'UTR	-0,222	1,36E-06	2,38E-05
cg07194102	21	36386792	RUNX1	Body	0,226	1,36E-06	2,38E-05
cg07645228	7	73867009	GTF2IRD1	TSS1500	-0,229	1,37E-06	2,38E-05
cg03908400	10	118450818	HSPA12A	Body	0,229	1,37E-06	2,38E-05
cg21359538	4	76440740	RCHY1	TSS1500	-0,228	1,37E-06	2,38E-05
cg24106052	13	31228831	USPL1	Body	-0,204	1,37E-06	2,38E-05
cg09853238	6	149532290		IGR	-0,227	1,37E-06	2,38E-05
cg05914150	5	67522332	PIK3R1	TSS200	0,22	1,37E-06	2,39E-05
cg24644188	5	39178563	FYB	Body	-0,212	1,37E-06	2,39E-05
cg23208066	6	138037318		IGR	-0,203	1,37E-06	2,39E-05
cg08799570	7	103125102	LOC10192787	Body	-0,216	1,37E-06	2,39E-05
cg19384289	2	176994365	HOXD8	TSS200	0,251	1,37E-06	2,39E-05
cg20959189	3	141122632	ZBTB38	5'UTR	-0,302	1,37E-06	2,39E-05
cg19913422	17	72232430	TTYH2	Body	0,215	1,37E-06	2,39E-05
cg03412953	6	69433703	ADGRB3	Body	0,208	1,37E-06	2,39E-05
cg02898721	17	38473222	RARA	TSS1500	0,211	1,37E-06	2,39E-05
cg14972210	10	106340238		IGR	0,213	1,37E-06	2,39E-05
cg02285791	6	27550747		IGR	0,216	1,38E-06	2,39E-05
cg23698062	12	66221667	RPSAP52	TSS1500	-0,28	1,38E-06	2,39E-05
cg09623974	8	141607092	AGO2	Body	-0,208	1,38E-06	2,39E-05
cg27039868	8	29357644		IGR	-0,215	1,38E-06	2,40E-05
cg14472551	11	33563060	C11orf41	TSS1500	-0,252	1,38E-06	2,40E-05
cg20872940	5	76663728	PDE8B	Body	-0,244	1,38E-06	2,40E-05
cg07812887	5	175793492	ARL10	Body	0,246	1,38E-06	2,40E-05
cg10532358	2	161080729		IGR	-0,361	1,38E-06	2,40E-05
cg21239428	15	35085212	ACTC1	Body	0,214	1,38E-06	2,40E-05
cg17220055	1	42248998	HIVEP3	5'UTR	-0,234	1,38E-06	2,40E-05
cg20288617	4	22239907		IGR	0,247	1,38E-06	2,40E-05
cg11551901	10	102270793	SEC31B	Body	-0,267	1,39E-06	2,41E-05
cg16932827	3	193988639		IGR	0,228	1,39E-06	2,41E-05
cg00164997	6	25042548		IGR	-0,272	1,39E-06	2,41E-05
cg13681992	10	123823129	TACC2	Body	0,255	1,39E-06	2,41E-05
cg13081009	1	8430745	RERE	5'UTR	0,254	1,39E-06	2,41E-05
cg05484710	9	90094857		IGR	-0,228	1,39E-06	2,41E-05
cg03548415	11	111991342		IGR	-0,214	1,39E-06	2,41E-05
cg12926596	12	56111944	BLOC1S1	Body	0,212	1,39E-06	2,41E-05
cg05964212	3	48936979	SLC25A20	TSS1500	-0,278	1,39E-06	2,41E-05
cg00183886	4	53524296	USP46	Body	0,213	1,39E-06	2,41E-05
cg18252328	2	8549078		IGR	-0,241	1,40E-06	2,42E-05
cg17372426	5	57786271	GAPT	TSS1500	-0,207	1,40E-06	2,42E-05
cg11995037	6	17923647	KIF13A	Body	0,314	1,40E-06	2,42E-05
cg01448132	16	28996533	LAT	5'UTR	-0,218	1,40E-06	2,42E-05
cg24738346	1	27479312	SLC9A1	Body	0,21	1,40E-06	2,42E-05
cg15253698	7	75523398		IGR	-0,208	1,40E-06	2,42E-05
cg12513025	17	66511045	PRKAR1A	5'UTR	0,274	1,40E-06	2,42E-05
cg14231974	4	183247161	ODZ3	Body	0,224	1,40E-06	2,42E-05
cg09609116	4	124410274		IGR	-0,208	1,40E-06	2,42E-05
cg14056226	3	64277063		IGR	-0,202	1,40E-06	2,42E-05
cg25968721	16	10674992	EMP2	TSS1500	0,226	1,40E-06	2,42E-05
cg22473770	17	29647977	EVI2A	5'UTR	-0,282	1,40E-06	2,43E-05

cg23370051	1	180784581	XPR1	Body	0,258	1,40E-06	2,43E-05
cg03684062	19	3179364	S1PR4	1stExon	-0,222	1,40E-06	2,43E-05
cg14849597	4	56225057	SRD5A3	Body	-0,217	1,40E-06	2,43E-05
cg10682957	11	117756485		IGR	0,203	1,40E-06	2,43E-05
cg26495442	21	44210836		IGR	-0,214	1,41E-06	2,43E-05
cg14845962	12	58120237	AGAP2	3'UTR	0,23	1,41E-06	2,43E-05
cg21907708	13	50095594	PHF11	Body	0,229	1,41E-06	2,44E-05
cg21772500	21	38417551		IGR	-0,271	1,41E-06	2,44E-05
cg23706819	4	89152212		IGR	0,26	1,41E-06	2,44E-05
cg24802860	17	73514515	TSEN54	Body	-0,296	1,41E-06	2,44E-05
cg24348980	1	232073393	DISC1-IT1	Body	0,216	1,41E-06	2,44E-05
cg00534502	17	80822180	TBCD	Body	-0,217	1,41E-06	2,44E-05
cg18071894	14	105260530	AKT1	5'UTR	0,2	1,42E-06	2,45E-05
cg17614506	7	47803072		IGR	-0,255	1,42E-06	2,45E-05
cg05090939	6	143228657	HIVEP2	5'UTR	-0,227	1,42E-06	2,45E-05
cg02873419	15	52545136	MYO5C	Body	-0,259	1,42E-06	2,45E-05
cg05603896	6	108145574	SCML4	TSS200	-0,303	1,42E-06	2,45E-05
cg24670552	13	110436780	IRS2	1stExon	0,261	1,42E-06	2,45E-05
cg25436826	1	188751462		IGR	0,218	1,42E-06	2,45E-05
cg09176246	10	125503602		IGR	0,201	1,42E-06	2,45E-05
cg20489425	5	40386735		IGR	-0,207	1,42E-06	2,45E-05
cg23415052	17	33778427		IGR	0,23	1,42E-06	2,45E-05
cg21159844	10	5314490		IGR	-0,2	1,42E-06	2,45E-05
cg01143097	20	32149650	CBFA2T2	TSS1500	0,207	1,42E-06	2,45E-05
cg15027328	8	35452489	UNC5D	Body	-0,221	1,43E-06	2,45E-05
cg16733505	2	183756458		IGR	-0,23	1,43E-06	2,45E-05
cg02606840	2	99280930	MGAT4A	TSS1500	-0,255	1,43E-06	2,46E-05
cg12891637	6	36560264		IGR	-0,234	1,43E-06	2,46E-05
cg26590199	12	67138296		IGR	0,207	1,43E-06	2,46E-05
cg24593488	8	49533682	LOC10192921	Body	0,221	1,43E-06	2,46E-05
cg05781767	3	127347362	PODXL2	TSS1500	0,21	1,43E-06	2,46E-05
cg21402860	15	57374444	TCF12	Body	0,241	1,43E-06	2,46E-05
cg00050692	2	25524877	DNMT3A	Body	-0,303	1,43E-06	2,46E-05
cg16672582	4	99760695		IGR	0,235	1,43E-06	2,46E-05
cg13590475	6	133137827	RPS12	Body	-0,201	1,43E-06	2,46E-05
cg24323726	3	111314186	ZBED2	TSS200	-0,225	1,43E-06	2,46E-05
cg25809784	5	131349251	ACSL6	TSS1500	0,203	1,43E-06	2,46E-05
cg07085156	10	64959524	JMJD1C	Body	0,233	1,43E-06	2,46E-05
cg15959113	5	167655079	TENM2	Body	-0,236	1,43E-06	2,46E-05
cg03330872	4	46477671		IGR	0,214	1,43E-06	2,46E-05
cg13523134	8	131305669	ASAP1	Body	-0,243	1,43E-06	2,46E-05
cg11466449	8	135842613		IGR	0,215	1,44E-06	2,47E-05
cg03489660	9	36091578	RECK	Body	0,27	1,44E-06	2,47E-05
cg17364031	13	46279429	SPERT	Body	0,208	1,44E-06	2,47E-05
cg22126171	1	203047830	PPFIA4	3'UTR	-0,208	1,44E-06	2,48E-05
cg22351602	21	40310774	LOC400867	TSS1500	-0,228	1,44E-06	2,48E-05
cg12656272	3	100328186	GPR128	TSS1500	-0,247	1,44E-06	2,48E-05
cg26327040	12	131561080	GPR133	Body	-0,205	1,44E-06	2,48E-05
cg02060460	17	80164137	CCDC57	5'UTR	-0,204	1,44E-06	2,48E-05
cg26828835	20	35476822	SOGA1	Body	0,276	1,44E-06	2,48E-05

cg20266220	22	50524032	MLC1	5'UTR	-0,209	1,45E-06	2,48E-05
cg02522196	12	44226393		IGR	-0,24	1,45E-06	2,48E-05
cg14323364	7	114556701		IGR	-0,204	1,45E-06	2,48E-05
cg10806462	2	240072244	HDAC4	Body	-0,219	1,45E-06	2,48E-05
cg00749878	2	38880756		IGR	-0,248	1,45E-06	2,49E-05
cg11012749	4	24479782		IGR	0,205	1,45E-06	2,49E-05
cg23704675	2	219347615	USP37	Body	-0,213	1,45E-06	2,49E-05
cg19712663	6	3371264	SLC22A23	Body	-0,22	1,45E-06	2,49E-05
cg03062284	2	122994061		IGR	-0,214	1,45E-06	2,49E-05
cg27543981	7	132427039		IGR	0,283	1,45E-06	2,49E-05
cg15901949	17	17654304	RAI1	5'UTR	-0,213	1,46E-06	2,49E-05
cg04288108	11	68717764		IGR	-0,206	1,46E-06	2,49E-05
cg17362900	6	33047944	HLA-DPB1	Body	0,209	1,46E-06	2,49E-05
cg11047500	14	89629859	FOXN3	Body	0,255	1,46E-06	2,49E-05
cg10442549	1	230818260	COG2	Body	0,215	1,46E-06	2,49E-05
cg16965605	12	66312027	HMGA2	Body	-0,274	1,46E-06	2,50E-05
cg01607625	6	32847830	PPP1R2P1	Body	0,202	1,46E-06	2,50E-05
cg01206378	1	95698827	RWDD3	TSS1500	0,234	1,46E-06	2,50E-05
cg06759423	18	72114620	FAM69C	Body	-0,218	1,46E-06	2,50E-05
cg03750478	16	29757375	C16orf54	TSS200	-0,237	1,46E-06	2,50E-05
cg10126903	16	29675214	SPN	Body	-0,203	1,46E-06	2,50E-05
cg15286094	7	116660213	ST7	Body	0,336	1,46E-06	2,50E-05
cg05689766	7	26220561	NFE2L3	Body	0,326	1,47E-06	2,50E-05
cg26996371	3	73129911		IGR	-0,211	1,47E-06	2,51E-05
cg24265771	5	176856550	GRK6	Body	-0,284	1,47E-06	2,51E-05
cg05390356	1	10277983	KIF1B	5'UTR	0,215	1,47E-06	2,51E-05
cg14701867	10	64193068	ZNF365	Body	-0,22	1,47E-06	2,51E-05
cg24347070	6	26382817	BTN2A2	TSS1500	0,225	1,47E-06	2,51E-05
cg12389653	4	162982402	FSTL5	Body	0,234	1,47E-06	2,51E-05
cg20465954	12	53496921	SOAT2	TSS1500	-0,218	1,47E-06	2,51E-05
cg17198361	21	44101541	PDE9A	5'UTR	-0,246	1,47E-06	2,51E-05
cg12368690	7	19958878	LOC10192766	Body	0,203	1,47E-06	2,51E-05
cg16357662	14	66104562	FUT8	Body	-0,227	1,47E-06	2,51E-05
cg23112946	5	10762094	DAP	TSS1500	0,203	1,47E-06	2,52E-05
cg16782524	8	141584720	EIF2C2	Body	-0,306	1,48E-06	2,52E-05
cg17081013	4	52969325		IGR	-0,24	1,48E-06	2,52E-05
cg24595844	10	80590256		IGR	-0,203	1,48E-06	2,52E-05
cg00093129	2	216574866	LINC00607	Body	-0,228	1,48E-06	2,52E-05
cg21954684	2	64341404	PELI1	5'UTR	-0,271	1,48E-06	2,52E-05
cg16454316	6	32847845	PPP1R2P1	Body	0,222	1,48E-06	2,52E-05
cg10585941	1	214461479	SMYD2	Body	-0,219	1,48E-06	2,52E-05
cg13969674	7	129918047	CPA2	Body	0,221	1,48E-06	2,52E-05
cg12135543	7	26428126		IGR	-0,228	1,48E-06	2,52E-05
cg20400196	21	19156681	C21orf91-OT1	Body	-0,294	1,48E-06	2,52E-05
cg24462021	7	102514737	FBXL13	Body	-0,243	1,48E-06	2,53E-05
cg05384127	22	27067406	MIATNB	TSS1500	-0,218	1,48E-06	2,53E-05
cg25557396	10	62296386	ANK3	Body	-0,206	1,49E-06	2,53E-05
cg15134506	10	75336540	USP54	TSS1500	-0,217	1,49E-06	2,53E-05
cg07921501	9	101820391	COL15A1	Body	-0,312	1,49E-06	2,53E-05
cg10269350	5	55376713		IGR	-0,258	1,49E-06	2,53E-05

cg09511513	8	20060014	ATP6V1B2	Body	-0,303	1,49E-06	2,53E-05
cg17947181	3	150967900	P2RY14	TSS1500	0,222	1,49E-06	2,53E-05
cg10894085	14	91817232	CCDC88C	Body	-0,323	1,49E-06	2,53E-05
cg08411558	3	43254978		IGR	-0,22	1,49E-06	2,53E-05
cg15859496	12	118490276	WSB2	Body	0,261	1,49E-06	2,53E-05
cg03545404	5	149556350	CDX1	Body	-0,207	1,49E-06	2,53E-05
cg20150640	1	23697656	C1orf213	3'UTR	0,266	1,49E-06	2,53E-05
cg24407833	13	24040091		IGR	0,232	1,49E-06	2,53E-05
cg11696200	13	33768013	STARD13	5'UTR	0,255	1,49E-06	2,53E-05
cg24067652	5	139626136	PFDN1	Body	0,242	1,49E-06	2,54E-05
cg03141675	5	150875990		IGR	0,224	1,49E-06	2,54E-05
cg26840970	16	71523432	ZNF19	TSS200	0,21	1,49E-06	2,54E-05
cg14213105	9	126980322		IGR	-0,259	1,50E-06	2,54E-05
cg20373544	2	202814226		IGR	0,204	1,50E-06	2,54E-05
cg25273160	11	56955011	LRRC55	3'UTR	-0,229	1,50E-06	2,54E-05
cg07349815	3	123751269		IGR	0,26	1,50E-06	2,54E-05
cg04973912	4	58044450	IGFBP7-AS1	Body	-0,203	1,50E-06	2,54E-05
cg15417249	6	114131273		IGR	-0,278	1,50E-06	2,55E-05
cg02952945	21	27276157	APP	Body	-0,204	1,50E-06	2,55E-05
cg19513181	1	209923132		IGR	-0,211	1,50E-06	2,55E-05
cg15362386	17	53134147	STXBP4	Body	0,255	1,50E-06	2,55E-05
cg20399011	5	180231194	MGAT1	TSS1500	-0,315	1,50E-06	2,55E-05
cg03672117	13	35353939		IGR	-0,232	1,50E-06	2,55E-05
cg20893956	6	152126736	ESR1	5'UTR	0,201	1,50E-06	2,55E-05
cg21171746	18	10950846	PIEZO2	Body	-0,203	1,50E-06	2,55E-05
cg15834833	7	41751411	INHBA-AS1	Body	0,249	1,50E-06	2,55E-05
cg24010993	18	43620112	PSTPIP2	Body	0,235	1,51E-06	2,55E-05
cg22100553	7	46098450		IGR	-0,223	1,51E-06	2,55E-05
cg26896911	3	45986498	CXCR6	5'UTR	-0,203	1,51E-06	2,55E-05
cg17842918	13	113540400	ATP11A	3'UTR	0,241	1,51E-06	2,56E-05
cg25492345	20	42733927		IGR	0,2	1,51E-06	2,56E-05
cg20723815	8	17061850	ZDHHC2	Body	-0,237	1,51E-06	2,56E-05
cg06804334	1	192706599		IGR	0,214	1,51E-06	2,56E-05
cg11935287	9	4138322	GLIS3	5'UTR	-0,205	1,51E-06	2,56E-05
cg03120983	15	80215045	ST20-MTHFS	5'UTR	0,211	1,51E-06	2,56E-05
cg22962123	7	27153605	HOXA3	5'UTR	0,228	1,51E-06	2,56E-05
cg01167274	21	46334214	ITGB2	5'UTR	-0,224	1,51E-06	2,56E-05
cg25502312	15	90389012	AP3S2	Body	-0,232	1,51E-06	2,56E-05
cg12388888	19	55128473	LILRB1	1stExon	-0,205	1,51E-06	2,56E-05
cg25738176	17	3848506	ATP2A3	Body	0,25	1,51E-06	2,56E-05
cg05612839	16	85768907	C16orf74	5'UTR	-0,223	1,51E-06	2,56E-05
cg20086047	12	9268711	A2M	TSS200	0,212	1,51E-06	2,56E-05
cg21473142	3	27762095	EOMES	Body	0,244	1,52E-06	2,56E-05
cg11979414	1	57443964		IGR	-0,274	1,52E-06	2,56E-05
cg06002516	12	57666521	R3HDM2	Body	0,243	1,52E-06	2,57E-05
cg19758893	11	15932004		IGR	-0,26	1,52E-06	2,57E-05
cg01570137	20	62199539	HELZ2	TSS1500	-0,23	1,52E-06	2,57E-05
cg19515360	3	156086146	KCNAB1	Body	-0,225	1,52E-06	2,57E-05
cg24452349	2	232778721		IGR	-0,218	1,52E-06	2,57E-05
cg12828741	11	18600741	UEVLD	5'UTR	-0,225	1,52E-06	2,57E-05

cg13472050	16	10707708		IGR	-0,229	1,52E-06	2,57E-05
cg10766282	6	106630507		IGR	-0,201	1,52E-06	2,58E-05
cg13733684	15	42749819	ZNF106	TSS200	0,229	1,52E-06	2,58E-05
cg16339042	5	118689638	TNFAIP8	TSS1500	-0,247	1,53E-06	2,58E-05
cg01581024	8	142009794	PTK2	5'UTR	0,276	1,53E-06	2,58E-05
cg22693837	15	81591058	IL16	5'UTR	-0,281	1,53E-06	2,58E-05
cg01733904	13	110493045		IGR	-0,209	1,53E-06	2,58E-05
cg02293044	22	29703543	GAS2L1	5'UTR	0,208	1,53E-06	2,58E-05
cg21482942	20	44033595	DBNDD2	TSS1500	0,22	1,53E-06	2,58E-05
cg15172734	3	57743377	SLMAP	5'UTR	0,231	1,53E-06	2,58E-05
cg16594913	12	5607455		IGR	-0,22	1,53E-06	2,58E-05
cg03332250	3	16342086	OXNAD1	Body	-0,219	1,53E-06	2,58E-05
cg21540802	7	7984512		IGR	-0,284	1,53E-06	2,59E-05
cg24801892	8	131194705	ASAP1	Body	-0,239	1,53E-06	2,59E-05
cg15979178	17	13416546	HS3ST3A1	Body	0,274	1,53E-06	2,59E-05
cg05869563	4	6233652	LOC285484	Body	0,224	1,54E-06	2,59E-05
cg25386676	5	172175721		IGR	0,24	1,54E-06	2,59E-05
cg15183772	9	84436693		IGR	-0,21	1,54E-06	2,59E-05
cg14488715	9	139407288	NOTCH1	Body	0,219	1,54E-06	2,59E-05
cg05333648	11	117886486	TMPRSS4-AS1	Body	-0,243	1,54E-06	2,59E-05
cg18352935	11	4843021	OR51F2	1stExon	-0,207	1,54E-06	2,59E-05
cg05081225	10	114803007	TCF7L2	Body	-0,225	1,54E-06	2,59E-05
cg13030790	21	36421503	RUNX1	5'UTR	-0,209	1,54E-06	2,60E-05
cg10017626	2	65085243		IGR	0,233	1,54E-06	2,60E-05
cg18734877	8	27297419	PTK2B	Body	-0,219	1,54E-06	2,60E-05
cg14622879	6	12234001		IGR	-0,26	1,54E-06	2,60E-05
cg10918624	4	89627891	HERC3	Body	0,21	1,54E-06	2,60E-05
cg23999572	7	130716402	LINC-PINT	Body	-0,248	1,54E-06	2,60E-05
cg13305373	17	63180156	RGS9	Body	0,276	1,54E-06	2,60E-05
cg02988816	8	19333274	CSGALNACT1	Body	-0,216	1,55E-06	2,60E-05
cg07519262	9	131932860		IGR	-0,215	1,55E-06	2,60E-05
cg13443797	7	50133024	ZPBP	TSS200	0,229	1,55E-06	2,60E-05
cg10044458	1	93400465	FAM69A	Body	0,32	1,55E-06	2,60E-05
cg27531206	1	165820531	UCK2	Body	-0,281	1,55E-06	2,60E-05
cg06763161	1	33813745	PHC2	5'UTR	0,212	1,55E-06	2,60E-05
cg06853339	17	76117687	TMC6	Body	-0,322	1,55E-06	2,61E-05
cg00513941	19	42437245		IGR	0,289	1,55E-06	2,61E-05
cg00443788	10	1072690	C10orf110	Body	0,21	1,55E-06	2,61E-05
cg13638593	13	67240484	PCDH9	Body	-0,295	1,55E-06	2,61E-05
cg01153946	20	37062621	LOC388796	Body	-0,212	1,55E-06	2,61E-05
cg26289965	18	73880304		IGR	0,202	1,55E-06	2,61E-05
cg10721066	12	109028610	SELPLG	TSS1500	-0,282	1,55E-06	2,61E-05
cg26413855	6	30796199		IGR	0,251	1,55E-06	2,61E-05
cg02457308	20	45403872		IGR	-0,214	1,55E-06	2,61E-05
cg05742096	5	134959834		IGR	-0,292	1,56E-06	2,61E-05
cg06751794	22	28693706	TTC28	Body	-0,208	1,56E-06	2,61E-05
cg01697163	22	38035301	SH3BP1	TSS1500	-0,246	1,56E-06	2,61E-05
cg13199720	4	154475566	KIAA0922	Body	0,238	1,56E-06	2,61E-05
cg12915460	2	109760376	SH3RF3	Body	-0,241	1,56E-06	2,62E-05
cg03405785	5	148869379		IGR	0,321	1,56E-06	2,62E-05

cg18648037	3	106917496	LOC1003026	Body	0,211	1,56E-06	2,62E-05
cg24833079	16	3996400		IGR	0,224	1,56E-06	2,62E-05
cg19766900	7	137999287		IGR	-0,268	1,56E-06	2,62E-05
cg22485289	20	4795999	RASSF2	5'UTR	-0,238	1,56E-06	2,62E-05
cg06794543	15	42749748	ZFP106	TSS200	0,249	1,56E-06	2,62E-05
cg17518427	12	65076857	RASSF3	Body	0,249	1,57E-06	2,63E-05
cg17711402	22	17784963		IGR	-0,203	1,57E-06	2,63E-05
cg00818853	8	30660495	PPP2CB	Body	0,237	1,57E-06	2,63E-05
cg03880642	10	105560568	SH3PXD2A	Body	-0,288	1,57E-06	2,63E-05
cg08762229	17	12453280	LINC00670	TSS200	0,209	1,57E-06	2,63E-05
cg13850921	7	13869826		IGR	0,228	1,57E-06	2,63E-05
cg02268965	2	40739515	SLC8A1	5'UTR	0,224	1,57E-06	2,63E-05
cg15926420	1	160159672	CASQ1	TSS1500	0,223	1,57E-06	2,63E-05
cg23045972	3	118230228		IGR	-0,21	1,57E-06	2,63E-05
cg17996413	18	44287235	ST8SIA5	Body	-0,235	1,57E-06	2,63E-05
cg11605450	3	49332009	USP4	Body	0,208	1,57E-06	2,63E-05
cg16882376	16	2046352		IGR	-0,221	1,57E-06	2,64E-05
cg21652108	12	12944951		IGR	0,212	1,57E-06	2,64E-05
cg11535366	2	128422284	LIMS2	Body	0,209	1,58E-06	2,64E-05
cg18363918	19	51829984	IGLON5	Body	0,271	1,58E-06	2,64E-05
cg04890026	18	74842101	MBP	5'UTR	-0,261	1,58E-06	2,64E-05
cg09952332	7	69088087	AUTS2	Body	-0,248	1,58E-06	2,64E-05
cg14423620	1	236118257		IGR	-0,296	1,58E-06	2,64E-05
cg22921619	6	35025887	ANKS1A	Body	0,211	1,58E-06	2,64E-05
cg10982443	14	20903611	KLHL33	5'UTR	0,244	1,58E-06	2,64E-05
cg23666951	5	32099080	PDZD2	Body	0,222	1,58E-06	2,64E-05
cg26139949	2	234296280	DGKD	Body	0,267	1,58E-06	2,64E-05
cg27160726	2	128587544		IGR	-0,214	1,58E-06	2,64E-05
cg20902666	6	96605114	FUT9	5'UTR	-0,201	1,58E-06	2,64E-05
cg06063479	2	145184893	ZEB2	Body	0,258	1,58E-06	2,64E-05
cg21348406	17	76361166		IGR	-0,273	1,58E-06	2,64E-05
cg09391916	16	54273455		IGR	0,208	1,58E-06	2,64E-05
cg21801637	15	64185283		IGR	-0,337	1,58E-06	2,64E-05
cg16336556	2	33295138	LTBP1	Body	-0,211	1,58E-06	2,64E-05
cg11656483	15	101779889	CHSY1	Body	-0,29	1,58E-06	2,65E-05
cg01171954	14	68713369	RAD51B	Body	0,261	1,59E-06	2,65E-05
cg20315577	10	74075151		IGR	0,207	1,59E-06	2,65E-05
cg13399952	9	132652889	FNBP1	Body	-0,284	1,59E-06	2,65E-05
cg12712755	5	79402130		IGR	0,311	1,59E-06	2,65E-05
cg20423781	2	31031734	CAPN13	TSS1500	0,221	1,59E-06	2,65E-05
cg13453168	10	129845660	PTPRE	TSS200	-0,259	1,59E-06	2,66E-05
cg13435326	3	178907655	PIK3CA	5'UTR	0,27	1,59E-06	2,66E-05
cg15078271	17	73472441	KIAA0195	Body	0,205	1,59E-06	2,66E-05
cg09169117	2	153302353	FMNL2	Body	-0,221	1,59E-06	2,66E-05
cg03862987	4	76596784	G3BP2	5'UTR	-0,209	1,59E-06	2,66E-05
cg19681056	15	66121734		IGR	-0,237	1,59E-06	2,66E-05
cg20184499	17	8888813		IGR	-0,236	1,59E-06	2,66E-05
cg01131890	2	17931375	SMC6	5'UTR	-0,208	1,60E-06	2,66E-05
cg05632420	7	130793732	MKLN1	TSS1500	-0,221	1,60E-06	2,66E-05
cg24440441	18	67522377		IGR	0,224	1,60E-06	2,66E-05

cg16141702	15	25651598	UBE3A	TSS1500	-0,357	1,60E-06	2,66E-05
cg03297297	3	45957266	LZTFL1	TSS200	-0,213	1,60E-06	2,66E-05
cg04061696	2	136577747	LOC1005076(	TSS200	-0,259	1,60E-06	2,66E-05
cg05507002	5	68728606	MARVELD2	Body	-0,272	1,60E-06	2,66E-05
cg03951662	17	28709931	CPD	Body	0,246	1,60E-06	2,66E-05
cg13868361	1	89357908	GTF2B	TSS1500	0,203	1,60E-06	2,67E-05
cg14131256	2	174812038	SP3	Body	0,252	1,60E-06	2,67E-05
cg26720543	2	233947027	INPP5D	Body	-0,255	1,60E-06	2,67E-05
cg09765299	2	38947733	GALM	Body	0,227	1,61E-06	2,68E-05
cg18237178	6	106574273		IGR	-0,205	1,61E-06	2,68E-05
cg08674206	18	8910781		IGR	-0,246	1,61E-06	2,68E-05
cg26214630	16	27264942	NSMCE1	Body	-0,413	1,61E-06	2,68E-05
cg27585914	7	134475235	CALD1	5'UTR	0,263	1,61E-06	2,68E-05
cg11707896	3	152154849	MBNL1	Body	0,227	1,61E-06	2,68E-05
cg20783697	17	56407147	BZRAP1	TSS1500	-0,279	1,61E-06	2,68E-05
cg18680977	7	27155039	HOXA3	5'UTR	0,225	1,61E-06	2,68E-05
cg00913360	7	151594392		IGR	0,203	1,61E-06	2,68E-05
cg20778786	6	29483989		IGR	-0,246	1,61E-06	2,68E-05
cg22977481	2	134523563		IGR	0,214	1,62E-06	2,69E-05
cg03224396	1	221017300		IGR	0,239	1,62E-06	2,69E-05
cg24148368	1	2145653	FAAP20	TSS1500	0,227	1,62E-06	2,69E-05
cg09828507	3	148921851	CP	Body	-0,275	1,62E-06	2,69E-05
cg14928932	6	33401520	SYNGAP1	Body	0,223	1,62E-06	2,69E-05
cg02682566	9	71985689	FAM189A2	Body	0,31	1,62E-06	2,69E-05
cg18937354	2	68943968		IGR	-0,217	1,62E-06	2,69E-05
cg23695037	1	245778964	KIF26B	Body	-0,228	1,62E-06	2,69E-05
cg17866022	17	19209889	EPN2-AS1	TSS1500	-0,301	1,62E-06	2,69E-05
cg13058623	1	233040477		IGR	-0,235	1,62E-06	2,70E-05
cg10371155	17	37561101	MED1	3'UTR	-0,246	1,62E-06	2,70E-05
cg11414268	4	17076406		IGR	-0,21	1,63E-06	2,70E-05
cg24670537	2	228383135	AGFG1	Body	0,206	1,63E-06	2,70E-05
cg10916401	5	3286167		IGR	0,207	1,63E-06	2,70E-05
cg04570984	5	58223316		IGR	-0,246	1,63E-06	2,70E-05
cg03824139	1	67235477	TCTEX1D1	Body	-0,23	1,63E-06	2,70E-05
cg17245251	8	103603330		IGR	0,213	1,63E-06	2,70E-05
cg17048233	8	28560467	EXTL3	5'UTR	0,286	1,63E-06	2,70E-05
cg16059434	6	135450366		IGR	-0,261	1,63E-06	2,70E-05
cg26219523	5	154214787	FAXDC2	Body	0,22	1,63E-06	2,70E-05
cg09409342	12	6525854		IGR	-0,313	1,63E-06	2,70E-05
cg19377661	3	142605811	PCOLCE2	Body	-0,227	1,63E-06	2,70E-05
cg05306637	1	26345353		IGR	0,211	1,63E-06	2,70E-05
cg00008629	9	115093661	ROD1	Body	-0,213	1,63E-06	2,70E-05
cg08546130	12	96618698	ELK3	Body	0,302	1,63E-06	2,70E-05
cg16405455	17	37934378	IKZF3	5'UTR	-0,209	1,63E-06	2,70E-05
cg16147002	12	113697418	TPCN1	Body	0,206	1,63E-06	2,71E-05
cg00730887	4	160149604		IGR	0,278	1,63E-06	2,71E-05
cg20515823	12	47610276	PCED1B-AS1	TSS200	-0,275	1,64E-06	2,71E-05
cg18011382	18	53361589		IGR	-0,248	1,64E-06	2,71E-05
cg00426382	3	170973243	TNIK	Body	-0,271	1,64E-06	2,71E-05
cg07938266	4	6182894	JAKMIP1	5'UTR	-0,255	1,64E-06	2,71E-05

cg07864377	9	102825692	ERP44	Body	0,272	1,64E-06	2,71E-05
cg06766732	11	35099490		IGR	-0,223	1,64E-06	2,71E-05
cg00678862	1	6113057	KCNAB2	Body	0,258	1,64E-06	2,71E-05
cg04358957	6	2542813		IGR	-0,206	1,64E-06	2,71E-05
cg21917510	11	85887699		IGR	-0,267	1,64E-06	2,72E-05
cg14879248	2	235963473	SH3BP4	3'UTR	-0,208	1,64E-06	2,72E-05
cg04379703	3	13053208	IQSEC1	Body	-0,279	1,64E-06	2,72E-05
cg18605938	2	242679736	D2HGDH	5'UTR	-0,268	1,64E-06	2,72E-05
cg11721464	19	42703763	DEDD2	Body	-0,303	1,64E-06	2,72E-05
cg07682037	11	63974153	FERMT3	1stExon	-0,25	1,64E-06	2,72E-05
cg15853475	16	29757565	C16orf54	TSS1500	-0,202	1,65E-06	2,72E-05
cg23584087	13	33626629	KL	Body	-0,21	1,65E-06	2,72E-05
cg19508726	17	46094964		IGR	-0,234	1,65E-06	2,73E-05
cg06224161	10	75751945		IGR	-0,277	1,65E-06	2,73E-05
cg03100752	19	12847402	ASNA1	TSS1500	0,214	1,65E-06	2,73E-05
cg12142326	4	74089113	ANKRD17	TSS1500	0,209	1,65E-06	2,73E-05
cg26445440	10	104754422	CNNM2	Body	-0,273	1,65E-06	2,73E-05
cg11837076	1	1711588	NADK	TSS1500	-0,207	1,66E-06	2,73E-05
cg26091808	16	89660453	CPNE7	Body	-0,21	1,66E-06	2,73E-05
cg14751721	1	232791337		IGR	-0,286	1,66E-06	2,74E-05
cg00755296	9	128668590	PBX3	Body	0,202	1,66E-06	2,74E-05
cg11778321	18	61157826	SERPINB5	Body	0,244	1,66E-06	2,74E-05
cg06822249	1	24345274		IGR	0,246	1,66E-06	2,74E-05
cg14082893	15	75400931		IGR	-0,228	1,66E-06	2,74E-05
cg15032861	2	2791584		IGR	0,227	1,66E-06	2,74E-05
cg27659622	6	108145539	SCML4	TSS200	-0,235	1,66E-06	2,74E-05
cg14725810	2	2791596		IGR	0,217	1,66E-06	2,74E-05
cg15820868	2	133105045		IGR	0,213	1,66E-06	2,74E-05
cg17198320	10	695877	DIP2C	Body	0,218	1,66E-06	2,74E-05
cg24674368	14	97879637		IGR	0,204	1,67E-06	2,75E-05
cg14515506	9	14203578	NFIB	Body	0,294	1,67E-06	2,75E-05
cg26132097	1	2145655	FAAP20	TSS1500	0,224	1,67E-06	2,75E-05
cg16284456	1	164679394	PBX1	Body	-0,213	1,67E-06	2,75E-05
cg13192602	3	151976532		IGR	-0,279	1,67E-06	2,75E-05
cg26045457	17	59316817	BCAS3	Body	-0,249	1,67E-06	2,75E-05
cg14374463	15	81590933	IL16	5'UTR	-0,288	1,67E-06	2,75E-05
cg03240696	15	99495967	IGF1R	Body	-0,277	1,67E-06	2,75E-05
cg20254265	15	91306178	BLM	ExonBnd	0,2	1,67E-06	2,75E-05
cg17689490	10	128952444	DOCK1	Body	-0,244	1,67E-06	2,75E-05
cg00609731	17	66342646	ARSG	Body	-0,258	1,67E-06	2,76E-05
cg03920403	7	23114829		IGR	0,251	1,67E-06	2,76E-05
cg23592919	1	28213868		IGR	-0,291	1,68E-06	2,76E-05
cg09473282	6	35115955		IGR	0,272	1,68E-06	2,76E-05
cg14429919	6	3054820		IGR	0,256	1,68E-06	2,76E-05
cg08894837	10	5119369	AKR1C3	Body	-0,253	1,68E-06	2,76E-05
cg22436753	1	209929546	TRAF3IP3	1stExon	-0,282	1,68E-06	2,76E-05
cg00282706	12	6055430	ANO2	TSS200	-0,261	1,68E-06	2,76E-05
cg05457684	11	2919808	SLC22A18AS	Body	-0,201	1,68E-06	2,77E-05
cg04630141	3	14607952		IGR	-0,257	1,68E-06	2,77E-05
cg22410826	13	110396942		IGR	0,239	1,68E-06	2,77E-05

cg11478105	4	77130254	SCARB2	Body	0,222	1,68E-06	2,77E-05
cg11604377	3	106983926	LINC00883	Body	0,223	1,69E-06	2,77E-05
cg23538418	8	39957279		IGR	-0,226	1,69E-06	2,77E-05
cg17736308	15	52774434	MYO5A	Body	-0,264	1,69E-06	2,77E-05
cg19818218	7	76752140	CCDC146	5'UTR	0,23	1,69E-06	2,77E-05
cg17967673	19	38040009		IGR	0,206	1,69E-06	2,77E-05
cg21033653	2	111808598	ACOXL	Body	-0,213	1,69E-06	2,77E-05
cg00230725	18	7453101		IGR	-0,231	1,69E-06	2,77E-05
cg26650383	13	29290660	SLC46A3	Body	0,28	1,69E-06	2,77E-05
cg15512736	12	75885093	GLIPR1	Body	0,22	1,69E-06	2,77E-05
cg00819233	1	162601805	DDR2	TSS1500	0,242	1,69E-06	2,77E-05
cg03810451	5	90086869	ADGRV1	Body	-0,201	1,69E-06	2,77E-05
cg17619566	16	50715411	SNX20	TSS200	-0,226	1,69E-06	2,77E-05
cg06912965	21	36422112	RUNX1	TSS1500	-0,202	1,69E-06	2,77E-05
cg24973993	12	120242513	CIT	Body	0,211	1,69E-06	2,77E-05
cg07290269	12	31479853	FAM60A	TSS1500	0,247	1,69E-06	2,78E-05
cg12869058	1	94587872	ABCA4	TSS1500	-0,208	1,69E-06	2,78E-05
cg08313893	8	105022801	RIMS2	Body	0,234	1,70E-06	2,78E-05
cg25917966	21	19157142	C21orf91-OT1	Body	-0,314	1,70E-06	2,78E-05
cg13182010	14	23235291	OXA1L	TSS1500	0,206	1,70E-06	2,78E-05
cg05189667	5	54742635	PPAP2A	Body	0,202	1,70E-06	2,78E-05
cg16318442	2	231526084		IGR	-0,274	1,70E-06	2,79E-05
cg22598496	3	190980644		IGR	-0,224	1,70E-06	2,79E-05
cg00760950	13	38066983		IGR	-0,204	1,70E-06	2,79E-05
cg10297997	18	72446092	ZNF407	Body	0,222	1,70E-06	2,79E-05
cg18143317	11	2037124		IGR	-0,214	1,70E-06	2,79E-05
cg11584042	1	229977122		IGR	-0,207	1,70E-06	2,79E-05
cg13308713	7	70065077	AUTS2	Body	-0,201	1,71E-06	2,79E-05
cg11839596	20	62833186	MYT1	Body	-0,376	1,71E-06	2,79E-05
cg08639424	6	31542556	TNF	TSS1500	-0,223	1,71E-06	2,79E-05
cg13853450	18	64271503	CDH19	TSS1500	0,258	1,71E-06	2,79E-05
cg17137562	9	36143401	GLIPR2	Body	-0,215	1,71E-06	2,80E-05
cg14462534	17	66311247	ARSG	Body	-0,229	1,71E-06	2,80E-05
cg10817237	5	73698657	LINC01331	Body	-0,236	1,71E-06	2,80E-05
cg22552736	9	132258136		IGR	0,204	1,71E-06	2,80E-05
cg01038597	16	31120649	BCKDK	Body	0,21	1,71E-06	2,80E-05
cg22601415	10	61900940	ANK3	Body	0,204	1,71E-06	2,80E-05
cg08414647	3	119963930	GPR156	TSS1500	-0,211	1,71E-06	2,80E-05
cg10636246	1	159046973	AIM2	TSS1500	-0,288	1,71E-06	2,80E-05
cg09645007	5	160282282		IGR	0,208	1,72E-06	2,80E-05
cg15482893	1	32837667	BSDC1	Body	-0,21	1,72E-06	2,81E-05
cg13029400	3	141087190	ZBTB38	5'UTR	0,233	1,72E-06	2,81E-05
cg21050356	10	116415614	ABLIM1	Body	0,207	1,72E-06	2,81E-05
cg13761321	9	97768771	C9orf3	Body	0,208	1,72E-06	2,81E-05
cg10850215	6	33048469	HLA-DPB1	Body	0,226	1,72E-06	2,81E-05
cg26904555	2	10587170	ODC1	5'UTR	-0,222	1,72E-06	2,81E-05
cg11955514	16	74958045	WDR59	Body	0,208	1,72E-06	2,81E-05
cg06381455	17	73472178	KIAA0195	Body	0,204	1,72E-06	2,81E-05
cg27568751	14	59730071	DAAM1	TSS200	0,21	1,72E-06	2,81E-05
cg04292941	11	6518485	DNHD1	TSS200	0,214	1,72E-06	2,81E-05

cg11242602	1	40099015	HEYL	Body	0,226	1,72E-06	2,81E-05
cg20389635	12	28120054	PTHLH	Body	-0,21	1,73E-06	2,81E-05
cg14265474	3	170948986	TNIK	Body	0,221	1,73E-06	2,82E-05
cg04717644	7	130916200	MKLN1	Body	-0,23	1,73E-06	2,82E-05
cg14480116	2	65594890	SPRED2	TSS1500	0,207	1,73E-06	2,82E-05
cg03557731	5	39063337	RICTOR	Body	0,238	1,73E-06	2,82E-05
cg01042564	4	147145675		IGR	0,214	1,73E-06	2,82E-05
cg13935577	12	107974897	BTBD11	Body	0,231	1,73E-06	2,82E-05
cg25644447	1	36408500	AGO3	5'UTR	-0,21	1,73E-06	2,82E-05
cg27123208	12	46940886	LOC1002887!	Body	-0,293	1,73E-06	2,82E-05
cg27262778	21	43557248	UMODL1	Body	-0,2	1,74E-06	2,83E-05
cg21236845	5	98115329	RGMB	Body	0,239	1,74E-06	2,83E-05
cg11751848	16	58328846	PRSS54	5'UTR	-0,214	1,74E-06	2,83E-05
cg12877532	7	116180662	CAV1	Body	0,208	1,74E-06	2,83E-05
cg04913766	6	25042678	FAM65B	TSS1500	-0,34	1,74E-06	2,83E-05
cg22401240	18	45902784		IGR	-0,207	1,74E-06	2,83E-05
cg15410675	4	154144800	TRIM2	Body	0,208	1,74E-06	2,83E-05
cg07128466	9	132033069		IGR	-0,221	1,74E-06	2,83E-05
cg26345969	3	48506717	TREX1	TSS1500	0,202	1,74E-06	2,83E-05
cg22111043	7	45019005	MYO1G	TSS1500	-0,238	1,74E-06	2,83E-05
cg01979223	3	138998997		IGR	-0,207	1,74E-06	2,83E-05
cg22070062	10	61901370	ANK3	TSS1500	0,268	1,75E-06	2,84E-05
cg07336045	14	77587986		IGR	-0,208	1,75E-06	2,84E-05
cg04643892	7	75247307	HIP1	Body	-0,203	1,75E-06	2,84E-05
cg16209351	11	85850230		IGR	-0,251	1,75E-06	2,84E-05
cg21935981	22	45608465	C22orf9	Body	0,238	1,75E-06	2,84E-05
cg25532340	14	78047326	SPTLC2	Body	-0,209	1,75E-06	2,84E-05
cg19431282	12	53985580	ATF7	Body	0,287	1,75E-06	2,85E-05
cg09452445	20	4792685	RASSF2	5'UTR	-0,289	1,76E-06	2,85E-05
cg08513547	5	150323558	LOC134466	Body	-0,252	1,76E-06	2,85E-05
cg23691182	22	29305360	ZNRF3	Body	-0,219	1,76E-06	2,85E-05
cg20611129	14	102345424	PPP2R5C	Body	-0,301	1,76E-06	2,85E-05
cg04371671	13	99830888		IGR	-0,222	1,76E-06	2,86E-05
cg07600533	22	50986031	KLHDC7B	TSS1500	0,291	1,76E-06	2,86E-05
cg02082929	2	109068933	GCC2	Body	-0,299	1,76E-06	2,86E-05
cg25545260	18	9653626		IGR	0,204	1,76E-06	2,86E-05
cg21951648	17	53784724		IGR	0,201	1,76E-06	2,86E-05
cg17593958	20	62199034	PRIC285	5'UTR	-0,232	1,77E-06	2,86E-05
cg08303079	9	132345286		IGR	-0,262	1,77E-06	2,86E-05
cg14253655	19	10842463	DNM2	Body	-0,293	1,77E-06	2,86E-05
cg04903930	4	78352562		IGR	0,281	1,77E-06	2,86E-05
cg17037271	7	75638542	STYXL1	Body	-0,212	1,77E-06	2,86E-05
cg08025954	22	24823455	ADORA2A	TSS200	-0,228	1,77E-06	2,86E-05
cg23910073	9	87324564	NTRK2	Body	-0,214	1,77E-06	2,87E-05
cg15481638	19	45150251	PVR	Body	0,235	1,77E-06	2,87E-05
cg20029923	1	91868079	HFM1	5'UTR	-0,207	1,77E-06	2,87E-05
cg22797773	14	65239943	SPTB	Body	-0,2	1,77E-06	2,87E-05
cg22443345	22	33941052	LARGE	Body	-0,273	1,77E-06	2,87E-05
cg10910512	6	112576009	LAMA4	TSS200	0,225	1,77E-06	2,87E-05
cg27482619	10	30818479		IGR	-0,284	1,78E-06	2,87E-05

cg11185549	12	116996871	MAP1LC3B2	TSS1500	-0,205	1,78E-06	2,87E-05
cg16084822	18	9969105		IGR	-0,237	1,78E-06	2,87E-05
cg22274074	7	27220839	HOXA10-HOX	TSS1500	0,237	1,78E-06	2,88E-05
cg25574765	11	70211531	PPFIA1	Body	-0,224	1,78E-06	2,88E-05
cg07689597	16	87337090		IGR	0,205	1,78E-06	2,88E-05
cg00852548	10	98898335	SLIT1	Body	0,206	1,78E-06	2,88E-05
cg22213076	2	174834787		IGR	0,233	1,78E-06	2,88E-05
cg19354017	2	101220624		IGR	0,263	1,78E-06	2,88E-05
cg03188253	2	240211637	HDAC4	Body	-0,272	1,79E-06	2,88E-05
cg14970222	2	230024870	PID1	Body	-0,232	1,79E-06	2,89E-05
cg15534755	11	117069859	TAGLN	TSS200	0,234	1,79E-06	2,89E-05
cg25739715	22	30663881	OSM	TSS1500	-0,235	1,79E-06	2,89E-05
cg18473388	15	81488867	IL16	TSS1500	-0,216	1,79E-06	2,89E-05
cg04449166	19	52110485		IGR	-0,236	1,79E-06	2,89E-05
cg21199093	1	35836009	ZMYM4	Body	0,201	1,79E-06	2,89E-05
cg02502727	20	11780146		IGR	-0,228	1,79E-06	2,89E-05
cg11714327	16	80603608	LINC01227	Body	-0,239	1,80E-06	2,90E-05
cg08529295	1	245132782	EFCAB2	TSS1500	0,232	1,80E-06	2,90E-05
cg15339596	10	26805756	APBB1IP	Body	0,244	1,80E-06	2,90E-05
cg18019929	2	242680597	D2HGDH	5'UTR	-0,283	1,80E-06	2,90E-05
cg15324331	10	30085133		IGR	-0,231	1,80E-06	2,90E-05
cg00753399	2	74404556	MOBKL1B	Body	-0,22	1,80E-06	2,90E-05
cg09058170	2	54394228	ACYP2	Body	0,22	1,80E-06	2,90E-05
cg09973676	8	82006417	PAG1	5'UTR	-0,256	1,80E-06	2,91E-05
cg01740633	4	87770576	SLC10A6	TSS200	0,265	1,80E-06	2,91E-05
cg21114453	1	235998720	LYST	Body	0,218	1,80E-06	2,91E-05
cg10919760	8	100816724	VPS13B	Body	0,227	1,81E-06	2,91E-05
cg21150327	12	96889770	CFAP54	Body	-0,23	1,81E-06	2,91E-05
cg19198470	1	169860336	SCYL3	5'UTR	-0,251	1,81E-06	2,92E-05
cg02299931	17	4631697		IGR	-0,282	1,81E-06	2,92E-05
cg07207982	6	34984930	ANKS1A	Body	0,278	1,81E-06	2,92E-05
cg02696960	8	19554461		IGR	-0,213	1,81E-06	2,92E-05
cg04443978	17	57762474	CLTC	Body	0,224	1,81E-06	2,92E-05
cg14067068	2	40357649	SLC8A1-AS1	Body	0,219	1,81E-06	2,92E-05
cg03195100	1	209941780	TRAF3IP3	TSS200	-0,237	1,82E-06	2,92E-05
cg22646760	10	43636142	CSGALNACT2	5'UTR	-0,283	1,82E-06	2,92E-05
cg21657758	11	11535011	GALNT18	Body	-0,233	1,82E-06	2,92E-05
cg11938738	6	134999799		IGR	0,269	1,82E-06	2,92E-05
cg05469229	2	39743856	LOC728730	Body	0,213	1,82E-06	2,92E-05
cg12906713	1	40889886		IGR	0,239	1,82E-06	2,92E-05
cg04344331	1	97109279		IGR	-0,207	1,82E-06	2,92E-05
cg21861651	5	10479220		IGR	-0,25	1,82E-06	2,92E-05
cg11587118	5	35659072	SPEF2	Body	0,271	1,82E-06	2,92E-05
cg22024390	19	38704760	DPF1	Body	-0,286	1,82E-06	2,93E-05
cg12091331	8	42065314	PLAT	TSS200	0,216	1,82E-06	2,93E-05
cg00651016	16	552754	RAB11FIP3	Body	0,223	1,82E-06	2,93E-05
cg08981537	2	54858444	SPTBN1	Body	0,253	1,82E-06	2,93E-05
cg00876175	3	179615032	LOC10192879	TSS1500	0,275	1,82E-06	2,93E-05
cg00952960	7	55477450	LANCL2	Body	0,22	1,82E-06	2,93E-05
cg20214404	1	66741905	PDE4B	Body	-0,309	1,82E-06	2,93E-05

cg22502776	14	24540516	CPNE6	TSS200	-0,225	1,83E-06	2,93E-05
cg01010073	7	45065573	CCM2	Body	-0,231	1,83E-06	2,93E-05
cg24994863	9	95970064	WNK2	Body	0,205	1,83E-06	2,93E-05
cg10334750	8	101348456		IGR	0,244	1,83E-06	2,93E-05
cg13841043	11	73980757	P4HA3	ExonBnd	-0,235	1,83E-06	2,93E-05
cg03760483	17	6899297	ALOX12	TSS200	0,2	1,83E-06	2,93E-05
cg16249322	20	62199475	HELZ2	TSS1500	-0,262	1,83E-06	2,94E-05
cg27269002	1	223927665	CAPN2	Body	0,218	1,83E-06	2,94E-05
cg15956680	2	29195923		IGR	-0,272	1,83E-06	2,94E-05
cg17853039	2	69084431		IGR	-0,251	1,84E-06	2,94E-05
cg17474786	13	40981077	LINC00598	Body	0,212	1,84E-06	2,95E-05
cg06871497	19	2087708	MOB3A	5'UTR	-0,288	1,84E-06	2,95E-05
cg04484243	15	85938337	AKAP13	5'UTR	0,242	1,84E-06	2,95E-05
cg19268652	6	12119552	HIVEP1	Body	-0,256	1,84E-06	2,95E-05
cg03791164	11	35293872	SLC1A2	Body	-0,237	1,84E-06	2,95E-05
cg27436261	16	4587672	CDIP1	5'UTR	0,239	1,84E-06	2,95E-05
cg26164910	4	183897293		IGR	-0,248	1,84E-06	2,95E-05
cg09624120	17	58156912	HEATR6	TSS1500	0,231	1,85E-06	2,95E-05
cg12560020	15	93823645		IGR	0,211	1,85E-06	2,95E-05
cg04543901	17	63519783		IGR	-0,244	1,85E-06	2,96E-05
cg06173543	2	43848195		IGR	0,209	1,85E-06	2,96E-05
cg12030017	4	40893064	APBB2	Body	-0,275	1,85E-06	2,96E-05
cg12317144	3	14419512		IGR	-0,213	1,85E-06	2,96E-05
cg16566353	3	37860954	ITGA9	3'UTR	0,271	1,85E-06	2,96E-05
cg09655719	1	12513560	VPS13D	Body	0,222	1,85E-06	2,96E-05
cg08965435	11	129034338	ARHGAP32	ExonBnd	-0,204	1,85E-06	2,96E-05
cg01089914	2	218843229		IGR	0,262	1,85E-06	2,96E-05
cg15367150	2	239046275	KLHL30	TSS1500	-0,204	1,85E-06	2,96E-05
cg26832165	8	107748800	OXR1	Body	-0,287	1,85E-06	2,96E-05
cg09779687	9	128307665	MAPKAP1	Body	0,201	1,85E-06	2,96E-05
cg21944402	4	77184893	FAM47E	Body	0,229	1,86E-06	2,97E-05
cg10238145	2	114644207		IGR	-0,201	1,86E-06	2,97E-05
cg03051116	10	30720703		IGR	0,233	1,86E-06	2,97E-05
cg02033582	17	29394814		IGR	-0,218	1,86E-06	2,97E-05
cg00028135	3	114343673	ZBTB20	5'UTR	0,215	1,86E-06	2,97E-05
cg01202651	15	41533639	CHP1	Body	0,232	1,86E-06	2,97E-05
cg27215601	8	126275042	NSMCE2	Body	-0,237	1,86E-06	2,97E-05
cg09760098	10	11251111	CELF2	Body	-0,206	1,86E-06	2,97E-05
cg23318600	19	50867153	NAPSA	Body	-0,233	1,86E-06	2,97E-05
cg05303901	7	28338625	CREB5	TSS1500	0,204	1,86E-06	2,98E-05
cg23666829	17	80842382	TBCD	Body	-0,21	1,87E-06	2,98E-05
cg09995544	13	114015757	GRTP1-AS1	Body	-0,249	1,87E-06	2,98E-05
cg22573746	2	111629330	ACOXL	Body	-0,236	1,87E-06	2,98E-05
cg04630800	15	52850728	ARPP19	Body	-0,262	1,88E-06	2,99E-05
cg24145118	10	2777041		IGR	0,248	1,88E-06	2,99E-05
cg16051561	5	11529629	CTNND2	Body	0,224	1,88E-06	2,99E-05
cg16297799	1	225934486		IGR	0,208	1,88E-06	3,00E-05
cg05937787	16	72594726	LINC01572	Body	0,222	1,88E-06	3,00E-05
cg25614924	11	117821717		IGR	-0,21	1,88E-06	3,00E-05
cg27069285	12	110427408	GIT2	Body	0,215	1,88E-06	3,00E-05

cg04811411	1	173834585	SNORD80	TSS1500	-0,211	1,89E-06	3,00E-05
cg12223243	2	60643473		IGR	0,303	1,89E-06	3,01E-05
cg02286554	6	72129214	LINC00472	Body	0,214	1,89E-06	3,01E-05
cg26286826	18	65085608		IGR	-0,203	1,89E-06	3,01E-05
cg02451691	6	105721586		IGR	0,218	1,89E-06	3,01E-05
cg07389305	2	231809311		IGR	-0,286	1,89E-06	3,01E-05
cg27518324	13	99630505	DOCK9	Body	0,227	1,89E-06	3,01E-05
cg27297010	1	101649098		IGR	0,237	1,89E-06	3,01E-05
cg13211657	12	9768149		IGR	-0,218	1,89E-06	3,01E-05
cg00894467	20	23339931	LINC01431	TSS1500	0,23	1,89E-06	3,01E-05
cg25598376	3	15084348	NR2C2	Body	0,244	1,89E-06	3,01E-05
cg24724232	18	34044170	FHOD3	Body	0,239	1,89E-06	3,01E-05
cg17408891	11	121211073		IGR	-0,211	1,89E-06	3,01E-05
cg02507181	12	10251536	CLEC1A	5'UTR	0,21	1,89E-06	3,01E-05
cg16949914	10	6104515	IL2RA	TSS200	-0,243	1,90E-06	3,01E-05
cg09122208	20	46377882	SULF2	Body	-0,277	1,90E-06	3,01E-05
cg00700039	18	2659555	SMCHD1	Body	-0,202	1,90E-06	3,01E-05
cg23194363	6	138087898		IGR	0,207	1,90E-06	3,02E-05
cg12492087	15	42749885	ZFP106	TSS200	0,228	1,90E-06	3,02E-05
cg12630354	16	10970709	CIITA	TSS1500	-0,209	1,90E-06	3,02E-05
cg15043384	3	149374761	WWTR1	Body	0,207	1,90E-06	3,02E-05
cg16673522	18	33485594	MIR187	TSS1500	0,289	1,90E-06	3,02E-05
cg11438039	3	62145697	PTPRG	Body	0,262	1,90E-06	3,02E-05
cg20734448	6	27568835		IGR	-0,244	1,90E-06	3,02E-05
cg08665528	6	11215243	NEDD9	Body	0,278	1,91E-06	3,03E-05
cg06764229	6	14876085		IGR	-0,239	1,91E-06	3,03E-05
cg08652536	2	32490065	NLRC4	TSS200	-0,219	1,91E-06	3,03E-05
cg04002181	22	51067515	ARSA	TSS1500	0,213	1,91E-06	3,03E-05
cg06535804	15	99990114		IGR	0,226	1,91E-06	3,03E-05
cg20787639	3	111040847		IGR	0,201	1,91E-06	3,03E-05
cg19615017	3	30673459	TGFBR2	Body	-0,206	1,91E-06	3,03E-05
cg10143205	7	36985363	ELMO1	Body	0,223	1,91E-06	3,04E-05
cg11916753	7	69161398	AUTS2	Body	-0,219	1,92E-06	3,04E-05
cg26498365	6	151088907	PLEKHG1	Body	0,3	1,92E-06	3,04E-05
cg10495393	2	208402967	CREB1	5'UTR	0,219	1,92E-06	3,04E-05
cg13360848	5	106822319	EFNA5	Body	-0,227	1,92E-06	3,04E-05
cg10813029	18	3715502	DLGAP1	Body	-0,241	1,92E-06	3,04E-05
cg17566737	15	96456154		IGR	-0,206	1,92E-06	3,04E-05
cg05101643	5	61163044		IGR	-0,221	1,92E-06	3,04E-05
cg18958437	11	129820917	PRDM10	Body	0,219	1,92E-06	3,04E-05
cg23711394	6	2450948		IGR	0,269	1,92E-06	3,05E-05
cg03762572	1	7692325	CAMTA1	Body	-0,225	1,92E-06	3,05E-05
cg25237333	6	17993827		IGR	-0,266	1,92E-06	3,05E-05
cg08173263	19	14276911	LPHN1	Body	-0,214	1,92E-06	3,05E-05
cg08880790	4	153059761		IGR	-0,262	1,92E-06	3,05E-05
cg25840838	11	47488535	CELF1	3'UTR	0,224	1,93E-06	3,05E-05
cg23627149	1	193155273	B3GALT2	5'UTR	0,288	1,93E-06	3,05E-05
cg06369187	14	50506033	LINC01599	Body	-0,237	1,93E-06	3,05E-05
cg10737415	15	34190524	AVEN	Body	-0,209	1,93E-06	3,05E-05
cg00902153	3	188506715	LPP	Body	-0,245	1,93E-06	3,06E-05

cg12335214	4	39661877		IGR	-0,234	1,93E-06	3,06E-05
cg13641532	1	29502946	SRSF4	Body	0,215	1,93E-06	3,06E-05
cg05403469	11	2322781	C11orf21	Body	-0,222	1,93E-06	3,06E-05
cg17897629	17	9292737	STX8	Body	-0,27	1,93E-06	3,06E-05
cg13454493	22	40859630	MKL1	TSS200	-0,281	1,94E-06	3,06E-05
cg01611115	18	2978833	LPIN2	5'UTR	-0,209	1,94E-06	3,06E-05
cg25218322	10	8446838		IGR	-0,275	1,94E-06	3,06E-05
cg09409898	17	2169829	SMG6	TSS1500	-0,201	1,94E-06	3,06E-05
cg16061590	17	36179637		IGR	0,202	1,94E-06	3,06E-05
cg07656865	17	27363765		IGR	-0,217	1,94E-06	3,06E-05
cg12722640	10	13276561	UCMA	TSS1500	0,217	1,94E-06	3,07E-05
cg03214468	5	128432489	MIR4633	TSS1500	0,226	1,94E-06	3,07E-05
cg14678223	2	158300811	CYTIP	TSS1500	-0,257	1,94E-06	3,07E-05
cg10297007	6	16699991	ATXN1	5'UTR	-0,222	1,94E-06	3,07E-05
cg10248037	5	131792820	C5orf56	Body	-0,332	1,94E-06	3,07E-05
cg02750582	6	34314143	NUDT3	Body	0,235	1,95E-06	3,07E-05
cg12644561	16	75098546	ZNRF1	Body	-0,25	1,95E-06	3,07E-05
cg21690653	5	36369252		IGR	-0,259	1,95E-06	3,07E-05
cg05794556	7	47528807	TNS3	5'UTR	0,272	1,95E-06	3,08E-05
cg03333443	10	44730855		IGR	-0,252	1,95E-06	3,08E-05
cg26164488	2	64440295		IGR	-0,208	1,96E-06	3,08E-05
cg12806049	14	51208379	NIN	Body	-0,27	1,96E-06	3,09E-05
cg22514294	20	62199502	HELZ2	TSS1500	-0,224	1,96E-06	3,09E-05
cg26709782	22	17682403	CECR1	Body	0,277	1,96E-06	3,09E-05
cg24129356	6	32920735	HLA-DMA	1stExon	-0,228	1,96E-06	3,09E-05
cg10612251	6	137960552		IGR	-0,221	1,96E-06	3,09E-05
cg19442101	5	173001933		IGR	-0,254	1,96E-06	3,09E-05
cg02456934	16	75108509	ZNRF1	Body	-0,248	1,96E-06	3,09E-05
cg03770370	5	42718755	GHR	3'UTR	-0,221	1,96E-06	3,09E-05
cg17115619	2	86898569	RNF103-CHM	5'UTR	0,206	1,97E-06	3,09E-05
cg00688819	14	55604543	LGALS3	Body	0,228	1,97E-06	3,10E-05
cg11265882	4	18013937	LCORL	Body	0,251	1,97E-06	3,10E-05
cg04847265	3	46482949	LTF	Body	-0,223	1,97E-06	3,10E-05
cg14120750	8	104197384	BAALC	Body	-0,263	1,97E-06	3,10E-05
cg02721693	1	182922705	C1orf14	TSS200	-0,207	1,97E-06	3,10E-05
cg14989243	6	76203530	FILIP1	TSS200	0,206	1,97E-06	3,10E-05
cg22624907	1	80447732		IGR	0,234	1,98E-06	3,11E-05
cg26435773	6	131520782	AKAP7	Body	-0,215	1,98E-06	3,11E-05
cg04195062	6	145199929		IGR	0,227	1,98E-06	3,11E-05
cg24931844	12	47642790		IGR	0,262	1,98E-06	3,11E-05
cg08963067	3	115502443		IGR	0,213	1,98E-06	3,11E-05
cg02181808	12	27577672	ARNTL2-AS1	Body	0,267	1,98E-06	3,11E-05
cg20032280	3	113160640	CFAP44	TSS1500	0,207	1,98E-06	3,11E-05
cg20846192	7	43012776		IGR	0,221	1,98E-06	3,11E-05
cg13254269	12	110717818	ATP2A2	TSS1500	0,205	1,98E-06	3,11E-05
cg17759095	6	56755566	DST	Body	-0,266	1,98E-06	3,11E-05
cg25307097	12	118745017	TAOK3	5'UTR	-0,229	1,98E-06	3,11E-05
cg16476235	8	21771668	DOK2	TSS1500	-0,26	1,98E-06	3,11E-05
cg23503775	6	108145601	SCML4	TSS200	-0,264	1,98E-06	3,11E-05
cg17657502	3	196358703		IGR	-0,205	1,98E-06	3,11E-05

cg03754825	7	104626086	LINC01004	Body	0,208	1,98E-06	3,11E-05
cg16624069	13	99630210	DOCK9	Body	0,201	1,98E-06	3,12E-05
cg17293845	1	42186714	HIVEP3	5'UTR	-0,21	1,98E-06	3,12E-05
cg23235142	7	45066841	CCM2	Body	-0,318	1,98E-06	3,12E-05
cg00943384	15	98064827		IGR	0,278	1,98E-06	3,12E-05
cg13572782	18	74799495	MBP	Body	0,217	1,99E-06	3,12E-05
cg20761636	1	234129649	SLC35F3	Body	-0,239	1,99E-06	3,12E-05
cg04388548	13	114905640		IGR	-0,222	1,99E-06	3,12E-05
cg08618451	5	96117388	ERAP1	Body	0,248	1,99E-06	3,12E-05
cg19736179	14	50364197		IGR	-0,215	1,99E-06	3,12E-05
cg24112628	6	150174215	LRP11	Body	-0,204	1,99E-06	3,13E-05
cg00216055	2	136889249		IGR	-0,29	2,00E-06	3,13E-05
cg09787640	4	169646378	PALLD	Body	-0,235	2,00E-06	3,13E-05
cg16288834	17	33400277	RFFL	Body	-0,227	2,00E-06	3,13E-05
cg20219287	3	193374120	OPA1	Body	0,206	2,00E-06	3,13E-05
cg20705287	8	118894213	EXT1	Body	-0,21	2,00E-06	3,14E-05
cg00625436	11	34706961		IGR	-0,256	2,00E-06	3,14E-05
cg06649716	12	31985870		IGR	-0,216	2,00E-06	3,14E-05
cg10949072	4	152488554	FAM160A1	Body	-0,241	2,00E-06	3,14E-05
cg23026554	10	42862978	LOC441666	Body	0,219	2,01E-06	3,14E-05
cg23332989	7	134854388	C7orf49	5'UTR	-0,27	2,01E-06	3,14E-05
cg25126728	16	27242311	NSMCE1	Body	-0,237	2,01E-06	3,14E-05
cg14640661	1	29299015	EPB41	5'UTR	-0,207	2,01E-06	3,14E-05
cg17219660	1	202091880	GPR37L1	TSS200	0,225	2,01E-06	3,14E-05
cg01274809	10	34526008	PARD3	Body	-0,215	2,01E-06	3,14E-05
cg03423979	11	12204246	MICAL2	Body	0,23	2,01E-06	3,15E-05
cg17578639	3	176862949	TBL1XR1	5'UTR	-0,217	2,01E-06	3,15E-05
cg10697032	4	3438850	RGS12	Body	-0,22	2,01E-06	3,15E-05
cg14479884	7	80267943	CD36	5'UTR	0,207	2,01E-06	3,15E-05
cg00255726	8	21769114	DOK2	Body	-0,257	2,01E-06	3,15E-05
cg18065686	8	23411171	SLC25A37	Body	0,203	2,01E-06	3,15E-05
cg23149418	8	134706823		IGR	-0,267	2,02E-06	3,15E-05
cg15348877	2	17854374	SMC6	Body	-0,203	2,02E-06	3,15E-05
cg05427189	2	169964245		IGR	-0,203	2,02E-06	3,15E-05
cg12428326	20	39807896	ZHX3	3'UTR	0,205	2,02E-06	3,15E-05
cg02392359	15	55572079	RAB27A	5'UTR	-0,256	2,02E-06	3,15E-05
cg19867914	2	144234430	ARHGAP15	Body	-0,202	2,02E-06	3,15E-05
cg13796884	1	154417817	IL6R	Body	-0,256	2,02E-06	3,16E-05
cg04276755	3	72504716		IGR	-0,233	2,02E-06	3,16E-05
cg26076905	5	67522298	PIK3R1	TSS200	0,236	2,02E-06	3,16E-05
cg07166703	1	46471211	MAST2	Body	0,245	2,02E-06	3,16E-05
cg07076751	6	30647539	KIAA1949	Body	-0,204	2,02E-06	3,16E-05
cg00811865	13	77304603		IGR	0,262	2,03E-06	3,16E-05
cg13510849	6	164572718		IGR	0,303	2,03E-06	3,16E-05
cg26354908	12	49610040		IGR	-0,212	2,03E-06	3,16E-05
cg23439593	5	54734076	PPAP2A	Body	0,205	2,03E-06	3,17E-05
cg05341689	1	25120473	CLIC4	Body	0,218	2,03E-06	3,17E-05
cg07259711	4	87221920	MAPK10	5'UTR	0,212	2,03E-06	3,17E-05
cg06095802	11	102843294		IGR	-0,211	2,03E-06	3,17E-05
cg11146691	12	47219737	SLC38A4	1stExon	0,2	2,03E-06	3,17E-05

cg11091478	1	173421266	LOC10050602	Body	0,204	2,03E-06	3,17E-05
cg04165577	2	54281229		IGR	-0,251	2,04E-06	3,17E-05
cg06335364	12	50221272	BCDIN3D-AS1	TSS1500	0,21	2,04E-06	3,17E-05
cg17006260	13	111075313	COL4A2	Body	-0,241	2,04E-06	3,17E-05
cg00975238	17	54437076	ANKFN1	Body	-0,242	2,04E-06	3,18E-05
cg05732487	13	94709668	GPC6	Body	-0,203	2,04E-06	3,18E-05
cg13422047	16	22037961	C16orf52	Body	-0,213	2,04E-06	3,18E-05
cg14944538	18	3220532	MYOM1	TSS1500	0,251	2,04E-06	3,18E-05
cg26469215	4	105888253		IGR	-0,228	2,04E-06	3,18E-05
cg06068373	3	42594510	SEC22C	3'UTR	0,21	2,04E-06	3,18E-05
cg26915629	4	156536695		IGR	-0,212	2,04E-06	3,18E-05
cg12581967	12	127632023		IGR	-0,214	2,04E-06	3,18E-05
cg14969976	19	909019	R3HDM4	Body	-0,24	2,04E-06	3,18E-05
cg18062196	17	57642675	DHX40	TSS1500	0,229	2,04E-06	3,18E-05
cg07701307	5	13986034		IGR	0,259	2,04E-06	3,18E-05
cg16478718	1	205255989		IGR	-0,306	2,05E-06	3,18E-05
cg27319662	4	41415813	LIMCH1	5'UTR	-0,254	2,05E-06	3,18E-05
cg26854588	17	38440015		IGR	-0,229	2,05E-06	3,19E-05
cg08673728	17	2140831	SMG6	TSS1500	0,205	2,05E-06	3,19E-05
cg05601847	4	83826691	THAP9	Body	-0,291	2,05E-06	3,19E-05
cg18908499	1	247712237	C1orf150	TSS1500	-0,29	2,05E-06	3,19E-05
cg19799762	2	102755019	IL1R1	5'UTR	-0,205	2,05E-06	3,19E-05
cg02461460	11	77962471	GAB2	Body	0,305	2,05E-06	3,19E-05
cg16494471	3	193748385		IGR	-0,216	2,05E-06	3,19E-05
cg11073846	11	94391770		IGR	0,203	2,05E-06	3,19E-05
cg12774311	2	238617736	LRRFIP1	Body	0,235	2,05E-06	3,19E-05
cg03140624	1	235931377	LYST	Body	-0,204	2,06E-06	3,19E-05
cg21294861	14	91125291	TTC7B	Body	-0,213	2,06E-06	3,20E-05
cg08845659	2	55727415		IGR	-0,208	2,06E-06	3,20E-05
cg24544047	7	101927904	SH2B2	TSS1500	-0,203	2,06E-06	3,20E-05
cg07846061	16	74732434	MLKL	5'UTR	-0,221	2,06E-06	3,20E-05
cg01437204	1	202130344	PTPN7	TSS1500	-0,236	2,06E-06	3,20E-05
cg00316508	4	146828698	ZNF827	Body	0,264	2,06E-06	3,20E-05
cg25973537	2	111562588	ACOXL	Body	-0,295	2,07E-06	3,21E-05
cg05496549	1	175045286	TNN	5'UTR	-0,2	2,07E-06	3,21E-05
cg11779113	20	62199156	PRIC285	Body	-0,291	2,07E-06	3,21E-05
cg22350027	3	17036922	PLCL2	Body	0,209	2,07E-06	3,21E-05
cg14885266	22	45297226	PHF21B	Body	-0,303	2,07E-06	3,21E-05
cg07843390	19	2541015	GNG7	5'UTR	0,202	2,07E-06	3,21E-05
cg14112978	10	74804603	P4HA1	Body	0,217	2,07E-06	3,21E-05
cg07378424	7	70143763	AUTS2	Body	-0,344	2,07E-06	3,21E-05
cg04899656	15	62718755		IGR	-0,214	2,07E-06	3,21E-05
cg11011736	17	33934776	AP2B1	Body	0,217	2,08E-06	3,22E-05
cg11839415	1	43814764	MPL	Body	0,236	2,08E-06	3,22E-05
cg13025388	11	118098752	MPZL3	3'UTR	-0,231	2,08E-06	3,22E-05
cg09938845	13	44848747		IGR	-0,236	2,08E-06	3,22E-05
cg03429643	2	99280963	MGAT4A	TSS1500	-0,24	2,08E-06	3,22E-05
cg19280572	11	1873884	LSP1	TSS1500	-0,301	2,08E-06	3,22E-05
cg01987353	17	32569640		IGR	-0,235	2,08E-06	3,22E-05
cg08500472	21	19901838		IGR	0,246	2,08E-06	3,22E-05

cg01242422	1	244215387	ZBTB18	5'UTR	0,214	2,08E-06	3,22E-05
cg27000059	15	95579265		IGR	-0,208	2,08E-06	3,22E-05
cg11042452	5	171560840	STK10	Body	-0,294	2,08E-06	3,22E-05
cg06625914	4	119747132	SEC24D	Body	-0,21	2,08E-06	3,22E-05
cg12420081	8	114384888	CSMD3	Body	0,266	2,08E-06	3,23E-05
cg01529769	12	70049884	BEST3	Body	0,242	2,09E-06	3,23E-05
cg16185386	13	29077350		IGR	-0,242	2,09E-06	3,23E-05
cg06895754	3	151917359		IGR	-0,275	2,09E-06	3,23E-05
cg07519259	2	31520167		IGR	0,246	2,09E-06	3,23E-05
cg02968445	2	68972158	ARHGAP25	Body	-0,225	2,09E-06	3,23E-05
cg12273284	10	12490844	CAMK1D	Body	-0,217	2,09E-06	3,23E-05
cg26157361	3	105434386	CBLB	Body	-0,206	2,09E-06	3,23E-05
cg06453717	9	33446442	AQP3	Body	-0,22	2,09E-06	3,23E-05
cg13327258	15	67141531		IGR	-0,215	2,09E-06	3,24E-05
cg16118028	19	6066473	RFX2	5'UTR	0,214	2,10E-06	3,24E-05
cg05716270	16	1138344	C1QTNF8	3'UTR	0,228	2,10E-06	3,24E-05
cg03398090	1	6525501	TNFRSF25	Body	-0,202	2,10E-06	3,24E-05
cg27522733	1	193448591		IGR	-0,299	2,10E-06	3,24E-05
cg01152055	20	62266767		IGR	-0,208	2,10E-06	3,24E-05
cg15612193	17	46206041		IGR	0,251	2,10E-06	3,24E-05
cg24577892	12	44919734	NELL2	Body	0,202	2,10E-06	3,25E-05
cg01719718	13	36458809	MIR548F5	Body	-0,219	2,10E-06	3,25E-05
cg16243571	15	25982360	ATP10A	Body	0,263	2,10E-06	3,25E-05
cg22190361	2	174886880		IGR	-0,229	2,10E-06	3,25E-05
cg24215459	4	122110906	TNIP3	5'UTR	-0,219	2,10E-06	3,25E-05
cg02060515	6	24936756	FAM65B	TSS1500	-0,206	2,11E-06	3,25E-05
cg15919165	2	11530611		IGR	-0,217	2,11E-06	3,25E-05
cg22208022	12	14553107	ATF7IP	5'UTR	-0,216	2,11E-06	3,25E-05
cg15512344	8	130502881	CCDC26	Body	-0,21	2,11E-06	3,25E-05
cg09005612	1	186047680	HMCN1	Body	-0,239	2,11E-06	3,25E-05
cg17080882	1	92191625	TGFBR3	Body	0,263	2,11E-06	3,25E-05
cg21004490	1	26872337	RPS6KA1	Body	-0,205	2,11E-06	3,25E-05
cg05875421	14	91709951	GPR68	5'UTR	-0,321	2,11E-06	3,26E-05
cg15795081	21	19220782	CHODL-AS1	Body	-0,239	2,11E-06	3,26E-05
cg02099418	2	204571146	CD28	TSS200	-0,202	2,11E-06	3,26E-05
cg23387569	12	58120011	AGAP2	3'UTR	0,2	2,11E-06	3,26E-05
cg09351082	10	97319133	SORBS1	5'UTR	0,216	2,11E-06	3,26E-05
cg14540650	9	6683274		IGR	-0,225	2,12E-06	3,26E-05
cg20578258	10	97732488		IGR	-0,258	2,12E-06	3,26E-05
cg11781335	10	30818581		IGR	-0,245	2,12E-06	3,26E-05
cg03217204	6	131893980	ARG1	TSS1500	0,202	2,12E-06	3,27E-05
cg15002347	1	186590321		IGR	-0,218	2,12E-06	3,27E-05
cg25338636	19	36233695	U2AF1L4	Body	-0,23	2,12E-06	3,27E-05
cg23392845	7	2660962		IGR	0,216	2,12E-06	3,27E-05
cg03731740	1	29062689	YTHDF2	TSS1500	0,247	2,12E-06	3,27E-05
cg00645687	10	115704988		IGR	0,219	2,13E-06	3,28E-05
cg16851046	13	21291173	IL17D	Body	0,206	2,13E-06	3,28E-05
cg00164282	17	30830732	MYO1D	Body	-0,259	2,14E-06	3,28E-05
cg24681304	2	150992998		IGR	-0,246	2,14E-06	3,28E-05
cg02842010	11	63350252	PLA2G16	Body	-0,265	2,14E-06	3,28E-05

cg02584094	17	6461275	PITPNM3	TSS1500	-0,207	2,14E-06	3,28E-05
cg21533897	11	64377229	NRXN2	Body	-0,242	2,14E-06	3,29E-05
cg03634145	6	484044		IGR	-0,203	2,14E-06	3,29E-05
cg12859146	9	124644444	TTLL11	Body	-0,252	2,14E-06	3,29E-05
cg06160539	1	244573354	ADSS	Body	-0,213	2,15E-06	3,29E-05
cg17775994	20	12338067		IGR	-0,217	2,15E-06	3,30E-05
cg07615351	16	75087014	ZNRF1	Body	0,232	2,15E-06	3,30E-05
cg06242807	13	24828692	SPATA13-AS1	TSS200	-0,301	2,15E-06	3,30E-05
cg14995816	6	47571870	CD2AP	Body	0,247	2,15E-06	3,30E-05
cg24386065	22	27897650		IGR	-0,228	2,15E-06	3,30E-05
cg03109660	4	37684505	RELL1	Body	-0,227	2,15E-06	3,30E-05
cg12517471	1	162147093	NOS1AP	Body	-0,236	2,15E-06	3,30E-05
cg06225328	15	77307757	PSTPIP1	Body	-0,235	2,15E-06	3,30E-05
cg19698870	12	110253132	TRPV4	TSS1500	-0,207	2,15E-06	3,31E-05
cg08279280	18	2967603	LPIN2	5'UTR	-0,394	2,15E-06	3,31E-05
cg24601480	6	138814716	NHSL1	Body	0,213	2,16E-06	3,31E-05
cg02969929	18	57588692		IGR	-0,256	2,16E-06	3,31E-05
cg25805115	1	81964207		IGR	0,205	2,16E-06	3,31E-05
cg25391231	8	130428001	CCDC26	Body	-0,218	2,16E-06	3,31E-05
cg09045128	12	106132053	CASC18	Body	0,23	2,16E-06	3,31E-05
cg03257308	13	30722898		IGR	-0,288	2,16E-06	3,31E-05
cg18758238	17	53626325		IGR	-0,215	2,17E-06	3,32E-05
cg16728653	19	909521	R3HDM4	Body	-0,307	2,17E-06	3,32E-05
cg14452932	17	49785422	CA10	Body	-0,203	2,17E-06	3,32E-05
cg05940452	11	118842484	FOXR1	5'UTR	0,207	2,17E-06	3,32E-05
cg14443399	3	122334476	PARP15	5'UTR	0,232	2,17E-06	3,32E-05
cg12293949	2	218469425	DIRC3	Body	0,206	2,17E-06	3,33E-05
cg08801804	1	244277190		IGR	-0,204	2,17E-06	3,33E-05
cg27576325	10	92731756	XLOC_00855	Body	0,255	2,17E-06	3,33E-05
cg05816365	1	111758969		IGR	-0,205	2,18E-06	3,33E-05
cg06680511	11	15672258		IGR	-0,215	2,18E-06	3,33E-05
cg19812944	7	767893	PRKAR1B	TSS1500	0,326	2,18E-06	3,33E-05
cg25671438	17	7240223	ACAP1	Body	-0,236	2,18E-06	3,33E-05
cg02563407	11	269468		IGR	-0,266	2,18E-06	3,33E-05
cg25607267	12	122362708	WDR66	Body	0,247	2,18E-06	3,33E-05
cg22851959	16	84641514	COTL1	Body	-0,223	2,18E-06	3,33E-05
cg03729251	4	151501035	LRBA	Body	0,211	2,18E-06	3,33E-05
cg03396047	3	188118309	LPP	5'UTR	-0,215	2,18E-06	3,33E-05
cg22543173	12	39681866		IGR	-0,21	2,18E-06	3,34E-05
cg24106132	11	33366388	HIPK3	Body	0,2	2,18E-06	3,34E-05
cg26961240	6	90813309	BACH2	5'UTR	-0,243	2,19E-06	3,34E-05
cg10551193	15	63790246		IGR	-0,308	2,19E-06	3,34E-05
cg14491284	4	74846046		IGR	-0,206	2,19E-06	3,35E-05
cg09021952	1	172852813		IGR	-0,224	2,19E-06	3,35E-05
cg00911180	2	175357981		IGR	-0,349	2,19E-06	3,35E-05
cg24329798	22	27592445		IGR	-0,266	2,20E-06	3,35E-05
cg23513363	10	43817997		IGR	0,283	2,20E-06	3,35E-05
cg25116398	7	129017343	AHCYL2	Body	-0,237	2,20E-06	3,35E-05
cg07389319	3	196368700	NRROS	5'UTR	-0,22	2,20E-06	3,35E-05
cg17500986	13	110836388	COL4A1	Body	-0,231	2,20E-06	3,35E-05

cg15035143	17	56408688	MIR142	TSS200	-0,211	2,20E-06	3,35E-05
cg23162647	11	11994913	DKK3	Body	0,244	2,20E-06	3,35E-05
cg09764435	1	230348493	GALNT2	Body	-0,273	2,20E-06	3,36E-05
cg20448594	7	73940352	GTF2IRD1	Body	-0,207	2,20E-06	3,36E-05
cg23774857	3	120118430	FSTL1	Body	-0,237	2,20E-06	3,36E-05
cg11511175	12	58119979	AGAP2	3'UTR	0,255	2,20E-06	3,36E-05
cg10685628	1	46251517		IGR	-0,2	2,21E-06	3,36E-05
cg00567542	6	53826994		IGR	0,271	2,21E-06	3,36E-05
cg13582966	3	183724015	ABCC5-AS1	TSS200	0,327	2,21E-06	3,36E-05
cg03437769	2	28937060		IGR	-0,204	2,21E-06	3,36E-05
cg04640972	10	8373522		IGR	-0,206	2,21E-06	3,36E-05
cg24559147	1	36020018	KIAA0319L	Body	0,245	2,21E-06	3,36E-05
cg19740353	6	53394492	GCLC	Body	0,251	2,21E-06	3,36E-05
cg17660183	12	105065386	CHST11	Body	-0,255	2,21E-06	3,37E-05
cg23966363	19	937630	ARID3A	Body	-0,266	2,22E-06	3,37E-05
cg15292593	2	36374912		IGR	-0,28	2,22E-06	3,37E-05
cg23053977	6	46434547		IGR	0,251	2,22E-06	3,37E-05
cg26877720	10	14647530	FAM107B	Body	-0,358	2,22E-06	3,37E-05
cg13391259	10	73600040	PSAP	Body	0,213	2,22E-06	3,37E-05
cg06986667	15	97037828		IGR	-0,235	2,22E-06	3,37E-05
cg07906724	8	42623946	CHRNA6	TSS1500	-0,209	2,22E-06	3,38E-05
cg12527995	1	53103294	FAM159A	Body	-0,221	2,22E-06	3,38E-05
cg20764603	16	30672510	FBRS	Body	-0,2	2,22E-06	3,38E-05
cg09066349	20	22817536		IGR	-0,209	2,22E-06	3,38E-05
cg20630926	1	46601365	LOC10192962	Body	0,238	2,22E-06	3,38E-05
cg03006376	7	28102470	JAZF1	Body	-0,23	2,23E-06	3,38E-05
cg02210967	7	5572794		IGR	-0,246	2,23E-06	3,38E-05
cg11072637	7	65773051	TPST1	Body	-0,237	2,23E-06	3,38E-05
cg15215348	19	18344520	PDE4C	5'UTR	0,267	2,23E-06	3,39E-05
cg07186392	1	33088944	ZBTB8OS	Body	-0,265	2,23E-06	3,39E-05
cg11961656	5	41789350	OXCT1	Body	0,274	2,23E-06	3,39E-05
cg09317554	4	151505084	LRBA	Body	0,223	2,23E-06	3,39E-05
cg19104050	12	108701064	CMKLR1	5'UTR	-0,244	2,24E-06	3,39E-05
cg17615706	10	126858335		IGR	-0,21	2,24E-06	3,40E-05
cg24821680	2	198542050	RFTN2	TSS1500	0,274	2,24E-06	3,40E-05
cg12849108	5	55452517	ANKRD55	Body	-0,21	2,24E-06	3,40E-05
cg03466765	7	3458230	SDK1	Body	-0,218	2,25E-06	3,40E-05
cg07345108	10	49893463	WDFY4	TSS200	-0,24	2,25E-06	3,40E-05
cg23841699	9	2054119	SMARCA2	Body	-0,25	2,25E-06	3,41E-05
cg11990488	12	2159166		IGR	-0,257	2,25E-06	3,41E-05
cg20957179	5	38806773	OSMR-AS1	Body	-0,208	2,25E-06	3,41E-05
cg04499015	6	33141696	COL11A2	Body	-0,206	2,25E-06	3,41E-05
cg12421481	12	112191077	ACAD10	Body	0,211	2,25E-06	3,41E-05
cg17964016	8	121062275	DEPDC6	3'UTR	0,201	2,26E-06	3,41E-05
cg00557430	14	90848490		IGR	0,206	2,26E-06	3,41E-05
cg06009645	6	112036099	FYN	Body	-0,267	2,26E-06	3,42E-05
cg15942368	7	139118969		IGR	0,205	2,26E-06	3,42E-05
cg14180787	5	32099270	PDZD2	Body	0,249	2,26E-06	3,42E-05
cg23467627	12	7260403	C1RL-AS1	TSS1500	-0,26	2,26E-06	3,42E-05
cg08540835	15	77775185	HMG20A	3'UTR	0,23	2,27E-06	3,42E-05

cg09381022	4	120465302	PDE5A	Body	0,235	2,27E-06	3,43E-05
cg22644372	17	15183987		IGR	-0,203	2,27E-06	3,43E-05
cg24499891	6	25007637	FAM65B	Body	-0,236	2,27E-06	3,43E-05
cg25832175	10	14614385	FAM107B	TSS200	-0,263	2,27E-06	3,43E-05
cg18854474	5	174948722	SFXN1	Body	-0,263	2,27E-06	3,43E-05
cg11823443	16	57406340	CX3CL1	TSS200	0,283	2,27E-06	3,43E-05
cg22968137	7	91654971	AKAP9	Body	0,217	2,27E-06	3,43E-05
cg08788250	5	13986021		IGR	0,262	2,28E-06	3,44E-05
cg01915345	2	237445829		IGR	-0,233	2,28E-06	3,44E-05
cg08841199	11	86438265		IGR	-0,22	2,28E-06	3,44E-05
cg00250196	2	16836324	FAM49A	5'UTR	-0,274	2,28E-06	3,44E-05
cg12067423	15	85141633		IGR	-0,215	2,28E-06	3,44E-05
cg09931909	6	74160206	C6orf150	Body	-0,281	2,28E-06	3,44E-05
cg14764819	2	202125092	CASP8	TSS200	-0,242	2,28E-06	3,44E-05
cg24180910	4	26841435		IGR	0,231	2,28E-06	3,44E-05
cg04616027	10	7837642	ATP5C1	Body	0,236	2,28E-06	3,44E-05
cg05473387	3	47485244	SCAP	5'UTR	0,225	2,28E-06	3,44E-05
cg18527651	3	10284280	IRAK2	3'UTR	-0,25	2,28E-06	3,44E-05
cg11586448	12	123383399		IGR	-0,212	2,28E-06	3,44E-05
cg18748970	20	33935061	UQCC1	ExonBnd	0,245	2,28E-06	3,44E-05
cg17981620	17	6449778	PITPNM3	Body	-0,281	2,30E-06	3,46E-05
cg04947350	6	42336360	TRERF1	5'UTR	-0,234	2,30E-06	3,46E-05
cg26159440	2	33217280	LTBP1	Body	-0,213	2,30E-06	3,46E-05
cg15999216	11	118214295	CD3G	TSS1500	-0,302	2,30E-06	3,46E-05
cg27177808	1	162527478		IGR	0,201	2,30E-06	3,46E-05
cg05067125	5	76146148	S100Z	5'UTR	-0,222	2,30E-06	3,46E-05
cg23176316	2	20784406		IGR	0,211	2,30E-06	3,46E-05
cg25045942	6	33048291	HLA-DPB1	Body	0,264	2,31E-06	3,47E-05
cg02022386	3	188789524		IGR	-0,22	2,31E-06	3,48E-05
cg13581101	11	72985796	P2RY6	5'UTR	-0,27	2,31E-06	3,48E-05
cg25920850	2	6111617	LINC01105	Body	-0,219	2,31E-06	3,48E-05
cg21171115	9	112887903	AKAP2	Body	0,242	2,31E-06	3,48E-05
cg00404703	2	64504695		IGR	-0,22	2,31E-06	3,48E-05
cg10214492	17	80377647	HEXDC	5'UTR	-0,204	2,32E-06	3,48E-05
cg07380416	11	60739172	CD6	5'UTR	-0,244	2,32E-06	3,48E-05
cg12375586	7	43698429	C7orf44	5'UTR	-0,204	2,32E-06	3,48E-05
cg05898307	10	60082329		IGR	-0,237	2,32E-06	3,48E-05
cg19046858	20	52061151	TSHZ2	3'UTR	0,212	2,32E-06	3,48E-05
cg23023643	12	97527928		IGR	-0,221	2,32E-06	3,48E-05
cg08046836	3	177548306	KCCAT211	Body	0,223	2,32E-06	3,48E-05
cg07244095	13	114833435	RASA3	Body	-0,246	2,32E-06	3,49E-05
cg07920321	16	30481009		IGR	-0,231	2,33E-06	3,49E-05
cg18359575	1	32721190	LCK	5'UTR	-0,267	2,33E-06	3,49E-05
cg00426976	22	51140977	SHANK3	Body	-0,21	2,33E-06	3,49E-05
cg12904135	2	171669275		IGR	0,249	2,33E-06	3,49E-05
cg15404463	9	90124734	DAPK1	Body	0,205	2,33E-06	3,49E-05
cg06499262	16	69598417	NFAT5	TSS1500	-0,278	2,33E-06	3,49E-05
cg23031939	11	134216076	GLB1L2	Body	0,234	2,33E-06	3,49E-05
cg18719938	11	43457668	TTC17	Body	0,227	2,33E-06	3,50E-05
cg05130485	2	202098257	CASP8	5'UTR	-0,232	2,33E-06	3,50E-05

cg04075144	6	76078031	FILIP1	Body	0,251	2,33E-06	3,50E-05
cg16599596	9	137828883		IGR	-0,29	2,34E-06	3,50E-05
cg23739746	13	114905867		IGR	-0,318	2,34E-06	3,50E-05
cg11971771	20	691098		IGR	0,204	2,34E-06	3,50E-05
cg16312900	17	48628397	SPATA20	Body	0,209	2,34E-06	3,51E-05
cg02662525	14	51288521	NIN	Body	-0,274	2,34E-06	3,51E-05
cg20140933	8	103915888	AZIN1-AS1	Body	-0,207	2,34E-06	3,51E-05
cg27537772	6	108143682	SCML4	5'UTR	-0,201	2,34E-06	3,51E-05
cg02795249	3	37986365	CTDSPL	Body	0,287	2,35E-06	3,51E-05
cg09062708	1	61649907	NFIA	Body	0,217	2,35E-06	3,51E-05
cg18922987	8	101303492	RNF19A	5'UTR	0,22	2,35E-06	3,51E-05
cg19474047	19	10207448	ANGPTL6	5'UTR	0,212	2,35E-06	3,52E-05
cg20790161	2	204553666		IGR	0,211	2,35E-06	3,52E-05
cg23945334	1	15738831	EFHD2	Body	-0,214	2,35E-06	3,52E-05
cg20775850	12	105076986	CHST11	Body	-0,307	2,35E-06	3,52E-05
cg15108521	1	160680856	CD48	Body	-0,228	2,35E-06	3,52E-05
cg20216069	11	86651685	PRSS23	Body	0,21	2,35E-06	3,52E-05
cg23458989	10	433254	DIP2C	Body	-0,214	2,35E-06	3,52E-05
cg06728252	6	26598149	ABT1	Body	0,293	2,35E-06	3,52E-05
cg25159519	8	18413278	PSD3	Body	0,215	2,36E-06	3,53E-05
cg00138547	3	47002303	CCDC12	Body	0,205	2,36E-06	3,53E-05
cg25405962	1	9852785	CLSTN1	Body	0,211	2,36E-06	3,53E-05
cg17449964	7	127307454	SND1	Body	-0,24	2,36E-06	3,53E-05
cg22292910	1	12493605	VPS13D	Body	0,309	2,36E-06	3,53E-05
cg11756387	1	32401244	PTP4A2	5'UTR	-0,212	2,37E-06	3,54E-05
cg12879425	4	68424495	STAP1	5'UTR	-0,271	2,37E-06	3,54E-05
cg23408990	2	222864086		IGR	-0,212	2,37E-06	3,54E-05
cg10338697	10	3542788	LOC10537636	Body	0,212	2,37E-06	3,54E-05
cg13051263	3	16883899		IGR	-0,269	2,37E-06	3,54E-05
cg21078144	14	81790203	STON2	Body	0,244	2,37E-06	3,54E-05
cg03341276	17	65255104		IGR	-0,334	2,37E-06	3,54E-05
cg12201616	2	111920341	BCL2L11	Body	-0,218	2,37E-06	3,54E-05
cg27405647	5	42547112	GHR	TSS1500	-0,217	2,37E-06	3,54E-05
cg18698225	1	171366338		IGR	0,255	2,38E-06	3,55E-05
cg26131315	4	140787316	MAML3	Body	-0,202	2,38E-06	3,55E-05
cg15847015	13	52288933	WDFY2	Body	-0,24	2,38E-06	3,55E-05
cg12513352	10	11204958	CUGBP2	Body	-0,263	2,38E-06	3,55E-05
cg23474135	2	46072444	PRKCE	Body	-0,255	2,38E-06	3,56E-05
cg11936410	15	63345124	TPM1	Body	0,259	2,39E-06	3,56E-05
cg19948167	13	55819841		IGR	0,231	2,39E-06	3,56E-05
cg13461622	1	25291385	RUNX3	1stExon	-0,222	2,39E-06	3,56E-05
cg15793613	2	29538328	ALK	Body	-0,246	2,40E-06	3,57E-05
cg08015164	5	172330073	ERGIC1	Body	0,21	2,40E-06	3,57E-05
cg15954484	1	100608293	TRMT13	Body	0,235	2,40E-06	3,58E-05
cg04939882	5	36880750	NIPBL	5'UTR	0,215	2,40E-06	3,58E-05
cg26537635	12	22671186	C2CD5	Body	0,209	2,40E-06	3,58E-05
cg07860918	16	90092755	GAS8	Body	-0,275	2,40E-06	3,58E-05
cg17479843	2	231818776		IGR	-0,201	2,41E-06	3,59E-05
cg09844573	20	62199190	PRIC285	Body	-0,234	2,41E-06	3,59E-05
cg03216144	5	175969569	CDHR2	1stExon	-0,227	2,41E-06	3,59E-05

cg17496788	1	162601807	DDR2	TSS1500	0,267	2,42E-06	3,59E-05
cg12532604	3	15823696	ANKRD28	5'UTR	0,238	2,42E-06	3,59E-05
cg11130987	15	42226096	EHD4	Body	0,225	2,42E-06	3,59E-05
cg10127822	9	112887568	AKAP2	TSS1500	0,246	2,42E-06	3,59E-05
cg01539436	21	40176506	ETS2	TSS1500	-0,218	2,42E-06	3,60E-05
cg23434216	1	9291085		IGR	-0,222	2,42E-06	3,60E-05
cg14087045	14	66963158	LINC00238	Body	0,223	2,42E-06	3,60E-05
cg12422539	6	2515337		IGR	0,228	2,42E-06	3,60E-05
cg20536048	11	110237244		IGR	0,273	2,42E-06	3,60E-05
cg00994207	1	167774542		IGR	-0,235	2,43E-06	3,60E-05
cg16010672	2	238199234		IGR	0,312	2,43E-06	3,60E-05
cg12356730	7	151433246	PRKAG2	1stExon	0,207	2,43E-06	3,60E-05
cg23093954	2	170370646	KLHL41	Body	0,223	2,43E-06	3,61E-05
cg25541213	8	116612735	TRPS1	Body	0,209	2,43E-06	3,61E-05
cg13761818	10	116473420		IGR	0,221	2,43E-06	3,61E-05
cg05579124	16	23522995	GGA2	TSS1500	0,253	2,43E-06	3,61E-05
cg04915566	21	36421472	RUNX1	5'UTR	-0,276	2,43E-06	3,61E-05
cg09614754	6	26367571	BTN3A2	5'UTR	-0,262	2,43E-06	3,61E-05
cg20526668	3	56785436	ARHGEF3	ExonBnd	0,25	2,43E-06	3,61E-05
cg07782794	14	34155739	NPAS3	Body	-0,236	2,44E-06	3,61E-05
cg15054595	3	49910483		IGR	-0,231	2,44E-06	3,61E-05
cg18379793	13	111868162	ARHGEF7	5'UTR	0,27	2,44E-06	3,61E-05
cg15908125	1	27668098	SYTL1	TSS1500	-0,239	2,44E-06	3,61E-05
cg08991104	7	44500003	NUDCD3	Body	-0,255	2,44E-06	3,61E-05
cg21966500	12	95656624	VEZT	Body	0,222	2,44E-06	3,61E-05
cg18020869	13	24828544	SPATA13-AS1	Body	-0,321	2,44E-06	3,62E-05
cg19473656	14	23623663	SLC7A8	Body	0,267	2,44E-06	3,62E-05
cg21978532	4	26133589		IGR	-0,215	2,44E-06	3,62E-05
cg21144383	18	3046721		IGR	-0,207	2,44E-06	3,62E-05
cg13104335	3	188342353	LPP	Body	0,22	2,44E-06	3,62E-05
cg01794245	8	145839459	KIAA1688	TSS1500	-0,257	2,44E-06	3,62E-05
cg19647783	10	24496967	KIAA1217	TSS1500	0,21	2,44E-06	3,62E-05
cg12325700	7	48338551	ABCA13	Body	-0,217	2,44E-06	3,62E-05
cg04212021	5	180231084	MGAT1	5'UTR	-0,296	2,44E-06	3,62E-05
cg13854135	2	68997641	ARHGAP25	Body	-0,265	2,45E-06	3,62E-05
cg10417140	5	68258787		IGR	0,231	2,45E-06	3,63E-05
cg25564990	10	19923218	MALRD1	Body	-0,209	2,45E-06	3,63E-05
cg03077671	19	13211225	LYL1	Body	-0,271	2,45E-06	3,63E-05
cg03601372	19	37267026		IGR	0,203	2,45E-06	3,63E-05
cg25240363	17	27990001	SSH2	Body	0,202	2,45E-06	3,63E-05
cg27526617	12	68876570		IGR	-0,214	2,45E-06	3,63E-05
cg16700163	16	85169956		IGR	-0,244	2,45E-06	3,63E-05
cg01862052	5	77836817	LHFPL2	5'UTR	-0,268	2,45E-06	3,63E-05
cg02726274	1	57527647	DAB1	Body	-0,206	2,46E-06	3,63E-05
cg25693349	6	32808596	PSMB8	3'UTR	-0,229	2,46E-06	3,64E-05
cg22471316	22	42940998		IGR	0,251	2,46E-06	3,64E-05
cg19677267	1	26645161	CD52	Body	-0,259	2,46E-06	3,64E-05
cg25587431	3	177542099		IGR	0,222	2,46E-06	3,64E-05
cg20770857	3	195633813	TNK2	5'UTR	-0,22	2,46E-06	3,64E-05
cg03489712	7	143076881	ZYX	TSS1500	-0,213	2,46E-06	3,64E-05

cg11784753	1	206853217		IGR	-0,272	2,46E-06	3,64E-05
cg24685134	1	55267152	TTC22	TSS1500	-0,217	2,46E-06	3,64E-05
cg04919516	1	201708419	NAV1	TSS1500	0,284	2,46E-06	3,64E-05
cg25990402	6	41302256	NCR2	TSS1500	-0,218	2,47E-06	3,64E-05
cg05688588	4	25237268	PI4K2B	Body	-0,202	2,47E-06	3,64E-05
cg10548185	5	64242958	CWC27	Body	0,24	2,47E-06	3,65E-05
cg27326208	2	164917513		IGR	-0,202	2,47E-06	3,65E-05
cg05642789	1	1564482	MIB2	Body	-0,226	2,47E-06	3,65E-05
cg21780171	10	30889252		IGR	-0,204	2,47E-06	3,65E-05
cg25567805	6	112572766	LAMA4	Body	0,25	2,47E-06	3,65E-05
cg18008345	19	1096084	POLR2E	TSS1500	-0,277	2,47E-06	3,65E-05
cg26477169	17	75196864	SEC14L1	Body	0,217	2,48E-06	3,65E-05
cg21261235	11	35028557		IGR	-0,279	2,48E-06	3,65E-05
cg13747967	16	57392618	CCL22	TSS200	-0,229	2,48E-06	3,65E-05
cg09365107	2	99055444		IGR	-0,324	2,48E-06	3,65E-05
cg10786043	8	38643568	TACC1	TSS1500	0,213	2,48E-06	3,66E-05
cg07858796	2	201826595	ORC2	5'UTR	0,207	2,48E-06	3,66E-05
cg08519799	7	28279693	JAZF1-AS1	Body	-0,283	2,48E-06	3,66E-05
cg13110775	5	32028055	PDZD2	Body	0,208	2,48E-06	3,66E-05
cg06492558	16	80625384		IGR	-0,201	2,48E-06	3,66E-05
cg24095374	5	176799366	RGS14	3'UTR	-0,246	2,48E-06	3,66E-05
cg21648072	6	144683853	UTRN	Body	0,203	2,49E-06	3,66E-05
cg10741463	1	18020474	ARHGEF10L	Body	-0,2	2,49E-06	3,67E-05
cg01012572	5	16785051	MYO10	Body	-0,257	2,49E-06	3,67E-05
cg05851542	3	9989156	PRRT3	Body	0,227	2,49E-06	3,67E-05
cg26607686	12	56514081	ZC3H10	5'UTR	-0,248	2,49E-06	3,67E-05
cg23700377	2	167997585	XIRP2-AS1	TSS200	0,279	2,49E-06	3,67E-05
cg10192933	9	127194043		IGR	0,207	2,49E-06	3,67E-05
cg01140143	6	33039396	HLA-DPA1	Body	-0,231	2,49E-06	3,67E-05
cg05687083	7	5371160	TNRC18	Body	-0,22	2,50E-06	3,67E-05
cg06990571	8	132986509	EFR3A	Body	0,204	2,50E-06	3,68E-05
cg00191181	13	47150059	LRCH1	Body	0,2	2,50E-06	3,68E-05
cg15833565	1	44741190	ERI3	Body	0,213	2,50E-06	3,68E-05
cg00970057	1	42272694	HIVEP3	5'UTR	-0,208	2,50E-06	3,68E-05
cg08658551	22	24097660	VPREB3	TSS1500	0,21	2,51E-06	3,68E-05
cg11683471	22	42057447	XRCC6	ExonBnd	0,211	2,51E-06	3,69E-05
cg11197533	3	129205338	IFT122	Body	-0,248	2,51E-06	3,69E-05
cg07781212	8	87535689	CPNE3	5'UTR	-0,303	2,51E-06	3,69E-05
cg20081771	2	10058418	TAF1B	Body	0,205	2,51E-06	3,69E-05
cg02636670	3	84774189	LINC00971	Body	0,315	2,51E-06	3,69E-05
cg02258751	2	144487774	ARHGAP15	Body	-0,21	2,51E-06	3,69E-05
cg04355169	14	24700477	GMPR2	TSS1500	0,242	2,51E-06	3,69E-05
cg01439230	11	2696198	KCNQ1OT1	Body	0,217	2,51E-06	3,69E-05
cg05323251	19	10449942	ICAM3	Body	-0,25	2,51E-06	3,69E-05
cg18581405	19	3180035	S1PR4	3'UTR	-0,266	2,51E-06	3,69E-05
cg07892915	4	99478858	TSPAN5	Body	0,236	2,51E-06	3,69E-05
cg04737434	16	80640392	CDYL2	Body	-0,207	2,52E-06	3,70E-05
cg12575672	10	13968042	FRMD4A	Body	-0,265	2,52E-06	3,70E-05
cg08061755	11	9781826		IGR	0,207	2,52E-06	3,70E-05
cg24255859	7	90227416	CDK14	Body	0,225	2,52E-06	3,70E-05

cg01247454	2	190028935	COL5A2	Body	0,253	2,52E-06	3,70E-05
cg25431366	2	238887209	UBE2F	Body	0,21	2,52E-06	3,70E-05
cg16959688	12	77350297		IGR	-0,24	2,53E-06	3,71E-05
cg03261565	10	29312799		IGR	-0,229	2,53E-06	3,71E-05
cg23409168	8	117101690		IGR	0,235	2,53E-06	3,71E-05
cg08472738	21	32965774		IGR	0,268	2,53E-06	3,72E-05
cg26749761	19	13372030	CACNA1A	Body	-0,288	2,54E-06	3,72E-05
cg14316320	5	124293017		IGR	-0,224	2,54E-06	3,72E-05
cg17093539	4	144303012	GAB1	Body	0,201	2,54E-06	3,72E-05
cg17818763	22	32998791	SYN3	Body	0,321	2,54E-06	3,72E-05
cg02665650	6	34984838	ANKS1A	Body	0,254	2,54E-06	3,72E-05
cg15412519	20	51416086		IGR	-0,233	2,54E-06	3,72E-05
cg25643361	6	26361389		IGR	-0,205	2,54E-06	3,73E-05
cg24673985	18	22252839		IGR	-0,217	2,54E-06	3,73E-05
cg07328351	21	48026298	S100B	TSS1500	-0,23	2,54E-06	3,73E-05
cg16238620	6	21434110		IGR	0,211	2,54E-06	3,73E-05
cg11304291	15	85504412		IGR	-0,214	2,54E-06	3,73E-05
cg24897996	2	202937892	KIAA2012	TSS200	0,213	2,55E-06	3,73E-05
cg07281385	7	46799529		IGR	-0,205	2,55E-06	3,73E-05
cg09792879	2	202126492	CASP8	Body	-0,237	2,55E-06	3,73E-05
cg20820367	2	69116791		IGR	0,21	2,55E-06	3,73E-05
cg26078301	17	40426127	STAT5B	5'UTR	-0,208	2,55E-06	3,73E-05
cg10945573	7	99809205	STAG3	Body	-0,244	2,55E-06	3,73E-05
cg10123669	2	168970900	STK39	Body	0,251	2,56E-06	3,74E-05
cg11761483	17	70723386	SLC39A11	Body	-0,208	2,56E-06	3,74E-05
cg08288130	8	21771540	DOK2	TSS1500	-0,271	2,56E-06	3,74E-05
cg22410499	9	33749216	PRSS3	TSS1500	-0,235	2,56E-06	3,74E-05
cg12855906	21	44092508	PDE9A	5'UTR	-0,296	2,56E-06	3,74E-05
cg09235719	1	208374810	PLXNA2	Body	0,252	2,56E-06	3,75E-05
cg26271170	18	72166402	CNDP2	5'UTR	-0,266	2,56E-06	3,75E-05
cg15164173	11	13461837	BTBD10	TSS200	0,201	2,56E-06	3,75E-05
cg20103054	2	218620242	DIRC3	Body	-0,253	2,57E-06	3,75E-05
cg05800416	8	19460097	CSGALNACT1	TSS200	0,205	2,57E-06	3,75E-05
cg20543950	15	80929325		IGR	0,203	2,57E-06	3,75E-05
cg25409990	2	198162738	ANKRD44-IT1	Body	-0,271	2,57E-06	3,75E-05
cg01997629	1	209929622	TRAF3IP3	1stExon	-0,297	2,57E-06	3,75E-05
cg10066186	14	23083160		IGR	0,224	2,57E-06	3,76E-05
cg16734744	12	105081903	CHST11	Body	-0,347	2,57E-06	3,76E-05
cg03110104	2	17240457		IGR	0,225	2,57E-06	3,76E-05
cg23929682	11	2321842	C11orf21	Body	-0,214	2,57E-06	3,76E-05
cg17299812	9	128652652	PBX3	Body	0,253	2,57E-06	3,76E-05
cg07797709	15	74258320		IGR	-0,256	2,58E-06	3,76E-05
cg12228683	7	5673716	RNF216	Body	0,249	2,58E-06	3,76E-05
cg12434132	2	25268065	EFR3B	Body	-0,243	2,58E-06	3,76E-05
cg06922269	20	25013918	ACSS1	TSS1500	0,207	2,58E-06	3,76E-05
cg08045462	2	3491169		IGR	0,209	2,58E-06	3,77E-05
cg08559364	3	11634536	VGLL4	Body	0,277	2,58E-06	3,77E-05
cg11642880	2	242020988	SNED1	Body	0,27	2,58E-06	3,77E-05
cg14256263	20	52273084		IGR	-0,203	2,59E-06	3,77E-05
cg03061778	10	10337051		IGR	0,207	2,59E-06	3,77E-05

cg06627717	17	80240045		IGR	-0,203	2,59E-06	3,77E-05
cg14432130	2	441802		IGR	-0,202	2,59E-06	3,77E-05
cg10289789	17	55517638	MSI2	Body	0,22	2,59E-06	3,78E-05
cg14503399	8	97866363	CPQ	Body	-0,276	2,59E-06	3,78E-05
cg24025961	1	59448553		IGR	0,225	2,59E-06	3,78E-05
cg22366321	8	38628418	TACC1	5'UTR	0,214	2,59E-06	3,78E-05
cg25900515	1	32701825	MTMR9LP	Body	-0,211	2,59E-06	3,78E-05
cg25467224	22	50218031	BRD1	5'UTR	-0,249	2,59E-06	3,78E-05
cg15626913	10	24755359	KIAA1217	TSS200	-0,239	2,60E-06	3,78E-05
cg11148876	14	91436403	RPS6KA5	Body	-0,229	2,60E-06	3,78E-05
cg08804124	15	64634343	CSNK1G1	5'UTR	0,232	2,60E-06	3,78E-05
cg10130564	11	117069849	TAGLN	TSS200	0,283	2,60E-06	3,78E-05
cg12738529	14	56039435		IGR	0,212	2,60E-06	3,79E-05
cg14144175	11	44126666	EXT2	5'UTR	-0,231	2,60E-06	3,79E-05
cg27270412	10	33622332	NRP1	Body	0,223	2,60E-06	3,79E-05
cg02156070	1	25985801	MAN1C1	Body	0,224	2,60E-06	3,79E-05
cg24320779	8	59949670	TOX	Body	0,265	2,61E-06	3,80E-05
cg21664636	3	70432664		IGR	-0,22	2,61E-06	3,80E-05
cg10764013	8	42358746	SLC20A2	1stExon	0,291	2,62E-06	3,80E-05
cg23942160	16	10719960		IGR	-0,204	2,62E-06	3,80E-05
cg25602756	1	247611881	NLRP3	3'UTR	0,229	2,62E-06	3,80E-05
cg05430997	13	110397189		IGR	0,224	2,62E-06	3,80E-05
cg19811337	11	65374663	MAP3K11	Body	0,225	2,62E-06	3,81E-05
cg08423851	3	11658043	VGLL4	Body	0,248	2,62E-06	3,81E-05
cg05371331	14	105586785		IGR	-0,208	2,62E-06	3,81E-05
cg01630060	8	101629886	SNX31	Body	-0,225	2,62E-06	3,81E-05
cg05517976	7	27220049	HOXA10	TSS200	0,269	2,62E-06	3,81E-05
cg06952940	6	83765357	UBE3D	Body	0,236	2,63E-06	3,82E-05
cg24035107	17	2394399	METT10D	Body	0,212	2,63E-06	3,82E-05
cg18826637	2	145116633		IGR	-0,219	2,63E-06	3,82E-05
cg23752260	11	27602844	BDNF-AS	Body	-0,219	2,63E-06	3,82E-05
cg03630576	2	227526737		IGR	0,215	2,63E-06	3,82E-05
cg05493812	21	47157612		IGR	-0,207	2,63E-06	3,82E-05
cg05851858	6	36302712	C6orf222	5'UTR	0,25	2,63E-06	3,82E-05
cg21006866	17	3666925	ITGAE	Body	-0,205	2,63E-06	3,82E-05
cg06975262	5	171805000	SH3PXD2B	Body	-0,202	2,64E-06	3,82E-05
cg08126344	8	42359079	SLC20A2	TSS200	0,364	2,64E-06	3,82E-05
cg10099795	21	16681618		IGR	-0,237	2,64E-06	3,82E-05
cg07423489	1	46915458	LINC01398	TSS200	-0,205	2,64E-06	3,83E-05
cg20909234	2	83065254		IGR	-0,228	2,64E-06	3,83E-05
cg19078081	5	163616087		IGR	0,256	2,64E-06	3,83E-05
cg23175406	6	34992755	ANKS1A	Body	0,314	2,65E-06	3,83E-05
cg10936796	2	20650757		IGR	0,256	2,65E-06	3,83E-05
cg12181407	5	54052513		IGR	-0,215	2,65E-06	3,83E-05
cg10065115	8	19554113		IGR	-0,29	2,65E-06	3,84E-05
cg00156265	15	77265459		IGR	0,249	2,65E-06	3,84E-05
cg13716852	22	27375547		IGR	0,205	2,65E-06	3,84E-05
cg07288447	2	27371818	TCF23	TSS200	0,226	2,65E-06	3,84E-05
cg02083271	16	20826285	LOC81691	Body	-0,247	2,65E-06	3,84E-05
cg04052730	17	80841933	TBCD	Body	-0,206	2,65E-06	3,84E-05

cg03131729	11	129183282		IGR	0,213	2,65E-06	3,84E-05
cg26763486	10	124013672	TACC2	3'UTR	-0,202	2,66E-06	3,84E-05
cg11431144	9	123657192	PHF19	TSS200	0,233	2,66E-06	3,84E-05
cg01998606	2	145161543	ZEB2	Body	0,22	2,66E-06	3,85E-05
cg18696027	15	45002597	B2M	TSS1500	-0,218	2,66E-06	3,85E-05
cg10598456	5	88589895		IGR	-0,317	2,66E-06	3,85E-05
cg24443032	14	78555510		IGR	0,201	2,66E-06	3,85E-05
cg25040418	3	77861475		IGR	-0,21	2,66E-06	3,85E-05
cg21332420	3	58191826	DNASE1L3	Body	-0,201	2,66E-06	3,85E-05
cg02188582	15	48254179		IGR	-0,271	2,66E-06	3,85E-05
cg07364520	6	37789512	ZFAND3	Body	0,24	2,66E-06	3,85E-05
cg24999588	8	61839426		IGR	-0,265	2,66E-06	3,85E-05
cg05449373	11	118095405	AMICA1	5'UTR	-0,238	2,66E-06	3,85E-05
cg12846715	10	7487926		IGR	-0,2	2,66E-06	3,85E-05
cg15175599	20	11833806	LINC00687	Body	-0,209	2,66E-06	3,85E-05
cg18666944	6	16737421	ATXN1	5'UTR	0,201	2,67E-06	3,85E-05
cg02914427	16	29674184	SPN	TSS200	-0,223	2,67E-06	3,86E-05
cg20264214	20	13781473	NDUFAF5	Body	0,232	2,67E-06	3,86E-05
cg07537738	3	40279993	MYRIP	Body	0,207	2,67E-06	3,86E-05
cg14265043	12	96643154	ELK3	Body	0,267	2,67E-06	3,86E-05
cg10026093	12	27143468	TM7SF3	Body	0,244	2,67E-06	3,86E-05
cg08417104	4	123542548	IL21	TSS1500	-0,228	2,68E-06	3,87E-05
cg18914863	12	96133983	NTN4	Body	0,254	2,68E-06	3,87E-05
cg14461613	21	38417634		IGR	-0,245	2,68E-06	3,87E-05
cg20306863	1	3593812	TP73	5'UTR	-0,23	2,68E-06	3,87E-05
cg06183267	2	100759134	AFF3	TSS200	-0,25	2,68E-06	3,87E-05
cg18874265	2	12411599	LOC10050645	Body	-0,258	2,69E-06	3,88E-05
cg27356296	7	134565928	CALD1	Body	-0,212	2,69E-06	3,88E-05
cg00214140	9	97786649	C9orf3	Body	0,232	2,69E-06	3,89E-05
cg09169366	9	74894067		IGR	-0,213	2,70E-06	3,89E-05
cg01740922	9	130473596	C9orf117	Body	-0,274	2,70E-06	3,89E-05
cg27347391	12	49855209	SPATS2	Body	0,212	2,70E-06	3,89E-05
cg06413718	1	184861111	FAM129A	Body	-0,258	2,70E-06	3,89E-05
cg06595211	16	3999188		IGR	0,217	2,70E-06	3,89E-05
cg26630411	16	47891132	LOC10192713	TSS1500	-0,242	2,70E-06	3,89E-05
cg24923566	2	97019472	NCAPH	Body	0,306	2,70E-06	3,89E-05
cg07965566	14	73934847		IGR	-0,222	2,70E-06	3,89E-05
cg23446377	4	102127231	PPP3CA	Body	-0,21	2,70E-06	3,89E-05
cg21162395	4	129376224	LOC10050748	Body	0,203	2,71E-06	3,90E-05
cg08517938	20	39590677		IGR	-0,268	2,71E-06	3,90E-05
cg09853467	2	23824418	KLHL29	Body	-0,296	2,71E-06	3,90E-05
cg01288904	8	80947440	TPD52	3'UTR	0,231	2,71E-06	3,90E-05
cg21816128	1	222826779	MIA3	Body	-0,203	2,71E-06	3,90E-05
cg11807829	4	54458109	LNX1	TSS1500	-0,219	2,71E-06	3,90E-05
cg06502892	7	103073259	SLC26A5	5'UTR	-0,236	2,71E-06	3,90E-05
cg20345556	6	30175305	TRIM26	5'UTR	0,283	2,71E-06	3,90E-05
cg08782420	5	141508150	NDFIP1	Body	0,244	2,71E-06	3,90E-05
cg07350732	17	2169571	SMG6	TSS200	-0,202	2,71E-06	3,90E-05
cg18185285	1	144008886		IGR	0,207	2,71E-06	3,91E-05
cg08403885	2	149373142		IGR	0,205	2,71E-06	3,91E-05

cg01855540	12	12716653	DUSP16	TSS1500	0,205	2,72E-06	3,91E-05
cg11774233	2	127117494		IGR	-0,248	2,72E-06	3,91E-05
cg17771150	13	46756209	LCP1	5'UTR	-0,28	2,73E-06	3,92E-05
cg00757207	17	7456223	TNFSF12	Body	-0,275	2,73E-06	3,92E-05
cg08478621	3	183723836	ABCC5-AS1	TSS1500	0,266	2,73E-06	3,92E-05
cg00404923	4	184882211	STOX2	Body	0,255	2,73E-06	3,92E-05
cg11060940	9	110022767		IGR	-0,218	2,73E-06	3,93E-05
cg04062576	12	53496842	SOAT2	TSS1500	-0,252	2,74E-06	3,93E-05
cg10025586	15	83501699	WHAMM	Body	-0,255	2,74E-06	3,93E-05
cg05603736	1	40860592	SMAP2	Body	-0,214	2,74E-06	3,94E-05
cg03538499	1	27941694	FGR	Body	0,234	2,75E-06	3,94E-05
cg16628838	14	65938016	FUT8	5'UTR	-0,214	2,75E-06	3,94E-05
cg14419496	4	2283688	ZFYVE28	Body	-0,225	2,75E-06	3,94E-05
cg05130482	8	98271949		IGR	-0,232	2,75E-06	3,94E-05
cg03591954	17	80964433	B3GNTL1	Body	0,232	2,75E-06	3,94E-05
cg23445294	3	81533970		IGR	0,264	2,75E-06	3,94E-05
cg24705426	3	11550659	ATG7	Body	0,29	2,75E-06	3,94E-05
cg26038366	20	32380839	ZNF341-AS1	Body	0,204	2,75E-06	3,94E-05
cg13810673	14	88473038	GPR65	5'UTR	-0,211	2,75E-06	3,95E-05
cg13440206	9	133736192	ABL1	Body	0,233	2,75E-06	3,95E-05
cg07450210	13	99880947	UBAC2	Body	-0,283	2,75E-06	3,95E-05
cg26259131	18	30111370		IGR	-0,244	2,76E-06	3,95E-05
cg00087804	22	37761817	ELFN2	Body	-0,212	2,76E-06	3,95E-05
cg25721906	3	66545802	LRIG1	Body	0,223	2,76E-06	3,95E-05
cg18692507	13	36726031		IGR	-0,23	2,76E-06	3,96E-05
cg05053752	7	140059315	SLC37A3	Body	-0,329	2,76E-06	3,96E-05
cg01828742	1	230305461	GALNT2	Body	-0,284	2,76E-06	3,96E-05
cg08477106	4	75895596	PARM1	Body	0,25	2,76E-06	3,96E-05
cg16398747	17	75471105	sept-09	TSS1500	-0,214	2,76E-06	3,96E-05
cg26572435	22	44589353	PARVG	Body	-0,221	2,77E-06	3,96E-05
cg21843317	13	40683714		IGR	-0,237	2,77E-06	3,96E-05
cg19567035	10	112220496		IGR	-0,267	2,77E-06	3,97E-05
cg26475740	21	26890817		IGR	0,228	2,77E-06	3,97E-05
cg03419151	17	6658728	XAF1	TSS1500	0,247	2,77E-06	3,97E-05
cg11365440	3	46608179	LRRC2	5'UTR	0,204	2,78E-06	3,97E-05
cg23416131	21	36392191	RUNX1	Body	0,237	2,78E-06	3,97E-05
cg00947809	10	73124633		IGR	-0,229	2,78E-06	3,97E-05
cg07504154	4	177159387	ASB5	Body	0,264	2,78E-06	3,98E-05
cg17152462	14	50692300	SOS2	Body	0,268	2,79E-06	3,98E-05
cg11755039	12	27032146		IGR	0,24	2,79E-06	3,98E-05
cg08482167	6	144066847	PHACTR2	Body	0,273	2,79E-06	3,98E-05
cg07755896	2	460967		IGR	-0,266	2,79E-06	3,98E-05
cg08454936	19	39149389	ACTN4	Body	0,203	2,79E-06	3,98E-05
cg14097517	6	25042369	FAM65B	1stExon	-0,329	2,79E-06	3,99E-05
cg11991607	6	155337438		IGR	0,239	2,79E-06	3,99E-05
cg18740968	6	21438909		IGR	-0,263	2,80E-06	3,99E-05
cg22867816	4	16081205	PROM1	5'UTR	-0,229	2,80E-06	3,99E-05
cg11711543	12	68981281		IGR	-0,254	2,80E-06	4,00E-05
cg02205015	19	7413482		IGR	-0,21	2,81E-06	4,00E-05
cg08242636	16	67062389	CBFB	TSS1500	-0,217	2,81E-06	4,01E-05

cg14585331	1	109455881	GPSM2	Body	0,219	2,81E-06	4,01E-05
cg05037348	5	134452611	C5orf66	5'UTR	0,23	2,81E-06	4,01E-05
cg24545533	9	133721366	ABL1	Body	0,273	2,81E-06	4,01E-05
cg19284549	4	185425261		IGR	-0,202	2,81E-06	4,01E-05
cg12573049	14	61795149	PRKCH	Body	-0,209	2,81E-06	4,01E-05
cg12600843	6	30179881	TRIM26	5'UTR	-0,229	2,82E-06	4,01E-05
cg01299462	15	55575244	RAB27A	5'UTR	-0,236	2,82E-06	4,01E-05
cg02722633	5	142180618	ARHGAP26	Body	-0,257	2,82E-06	4,01E-05
cg05152479	12	109246508	SSH1	Body	0,219	2,82E-06	4,01E-05
cg13815212	17	59018561	BCAS3	Body	0,25	2,82E-06	4,02E-05
cg06800222	17	10195469		IGR	0,21	2,82E-06	4,02E-05
cg05417498	13	88445300		IGR	0,207	2,82E-06	4,02E-05
cg25219359	2	3497553		IGR	0,239	2,82E-06	4,02E-05
cg22513455	3	118955844	B4GALT4	5'UTR	-0,271	2,82E-06	4,02E-05
cg05739933	7	30025184	SCRN1	Body	-0,22	2,82E-06	4,02E-05
cg04215287	3	149310862	WWTR1	Body	0,235	2,83E-06	4,02E-05
cg19240869	21	40140783	LINC00114	Body	-0,282	2,83E-06	4,02E-05
cg13575601	2	68962431	ARHGAP25	Body	-0,267	2,83E-06	4,03E-05
cg09364744	17	55373884	MSI2	Body	0,212	2,83E-06	4,03E-05
cg26003222	17	75347770	sept-09	5'UTR	0,227	2,83E-06	4,03E-05
cg20273531	5	16509481	FAM134B	TSS1500	0,275	2,84E-06	4,03E-05
cg03449946	1	61586195	NFIA	Body	0,257	2,84E-06	4,03E-05
cg04773587	1	206736644	RASSF5	Body	-0,332	2,84E-06	4,03E-05
cg14040735	5	149857155	LOC10254629	Body	-0,253	2,84E-06	4,03E-05
cg06625638	7	69336997	AUTS2	Body	0,207	2,84E-06	4,03E-05
cg26065909	1	151345173	SELENBP1	TSS200	0,261	2,84E-06	4,04E-05
cg02343446	12	11699363		IGR	-0,209	2,84E-06	4,04E-05
cg03705817	9	140600122	EHMT1	Body	-0,242	2,85E-06	4,04E-05
cg15737719	10	22743835		IGR	0,205	2,85E-06	4,04E-05
cg07387444	16	49727717	ZNF423	Body	-0,213	2,85E-06	4,05E-05
cg25346893	1	154407366	IL6R	Body	0,218	2,85E-06	4,05E-05
cg08433504	15	39872071	THBS1	TSS1500	0,208	2,85E-06	4,05E-05
cg15077193	19	802989	PTBP1	Body	0,227	2,85E-06	4,05E-05
cg14153437	5	118689974	TNFAIP8	TSS1500	-0,208	2,86E-06	4,05E-05
cg14259717	11	18433732	LDHC	TSS200	0,204	2,86E-06	4,05E-05
cg19918549	14	100531800	EVL	1stExon	-0,322	2,86E-06	4,05E-05
cg26110991	21	45578724		IGR	-0,246	2,86E-06	4,05E-05
cg02775243	10	102831287		IGR	-0,224	2,86E-06	4,06E-05
cg09837977	7	110731201	LRRN3	5'UTR	0,304	2,86E-06	4,06E-05
cg15031685	7	128494060	FLNC	Body	-0,212	2,86E-06	4,06E-05
cg10767216	7	130130187	MESTIT1	Body	0,222	2,86E-06	4,06E-05
cg05945608	6	42739639		IGR	-0,245	2,87E-06	4,06E-05
cg25417842	7	150822987	AGAP3	Body	0,211	2,87E-06	4,06E-05
cg12295485	2	236325529		IGR	0,204	2,87E-06	4,06E-05
cg15737177	12	8717467		IGR	0,209	2,87E-06	4,07E-05
cg13663116	9	116372034		IGR	0,209	2,87E-06	4,07E-05
cg12577427	5	88507857		IGR	-0,211	2,87E-06	4,07E-05
cg12924105	3	45957730	LZTFL1	TSS1500	-0,239	2,87E-06	4,07E-05
cg17102851	21	35828017	KCNE1	5'UTR	0,216	2,87E-06	4,07E-05
cg26439401	12	103849421	C12orf42	Body	-0,265	2,87E-06	4,07E-05

cg13464662	5	59909226	DEPDC1B	Body	0,241	2,88E-06	4,07E-05
cg12749653	2	225713461	DOCK10	Body	0,259	2,88E-06	4,07E-05
cg20780851	7	112094008	IFRD1	5'UTR	0,27	2,88E-06	4,07E-05
cg10265502	4	72095404	SLC4A4	5'UTR	0,234	2,88E-06	4,08E-05
cg03835698	10	4284219	LINC00702	Body	0,232	2,88E-06	4,08E-05
cg19133531	10	7514073		IGR	-0,212	2,88E-06	4,08E-05
cg07656025	1	231174350	FAM89A	Body	0,243	2,88E-06	4,08E-05
cg15676292	7	141069418		IGR	0,216	2,89E-06	4,09E-05
cg07403350	13	108867111	LIG4	5'UTR	0,228	2,89E-06	4,09E-05
cg06060758	21	33927488		IGR	0,235	2,89E-06	4,09E-05
cg01157612	10	105604205	SH3PXD2A	Body	0,211	2,89E-06	4,09E-05
cg07384439	17	69334706		IGR	0,221	2,89E-06	4,09E-05
cg04957377	14	21507259		IGR	0,212	2,89E-06	4,09E-05
cg21546022	12	47052349		IGR	0,211	2,89E-06	4,09E-05
cg05865954	10	63224906	TMEM26-AS1	Body	-0,22	2,90E-06	4,09E-05
cg24765162	7	23224700	NUPL2	ExonBnd	0,251	2,90E-06	4,09E-05
cg13980799	10	119000215	SLC18A2	TSS1500	0,212	2,90E-06	4,10E-05
cg03058083	2	12185086	LOC10050645	Body	-0,215	2,90E-06	4,10E-05
cg10468933	5	38781281	OSMR-AS1	Body	0,255	2,90E-06	4,10E-05
cg00387133	6	71021740		IGR	-0,209	2,90E-06	4,10E-05
cg21960512	6	138002351		IGR	-0,232	2,90E-06	4,10E-05
cg03760457	15	70326230		IGR	0,208	2,90E-06	4,10E-05
cg17184651	1	212885934		IGR	-0,213	2,91E-06	4,10E-05
cg23087931	1	19467440	UBR4	Body	0,228	2,91E-06	4,11E-05
cg27377644	8	59442595		IGR	0,287	2,92E-06	4,12E-05
cg21205089	2	19674070		IGR	-0,253	2,92E-06	4,12E-05
cg13387113	17	74696737	MXRA7	Body	0,227	2,92E-06	4,12E-05
cg14672407	16	68163196	NFATC3	Body	0,216	2,93E-06	4,12E-05
cg18141115	14	56076483	KTN1	5'UTR	0,277	2,93E-06	4,12E-05
cg21008998	22	32134789	PRR14L	Body	0,227	2,93E-06	4,13E-05
cg05246100	7	55246275	EGFR	Body	-0,221	2,93E-06	4,13E-05
cg12560076	11	10481217	AMPD3	5'UTR	-0,262	2,93E-06	4,13E-05
cg21556281	7	27154720	HOXA3	5'UTR	0,207	2,93E-06	4,13E-05
cg02250877	6	139909393		IGR	0,233	2,94E-06	4,13E-05
cg16233524	4	48250320	TEC	5'UTR	0,22	2,94E-06	4,14E-05
cg20148334	1	209815452	LAMB3	Body	0,207	2,94E-06	4,14E-05
cg12568198	6	12061273	HIVEP1	Body	-0,215	2,94E-06	4,14E-05
cg02868516	17	1737955	RPA1	Body	-0,24	2,94E-06	4,14E-05
cg13558774	4	1196025	LOC10013087	Body	-0,23	2,94E-06	4,14E-05
cg01051098	14	55604454	LGALS3	Body	0,219	2,94E-06	4,14E-05
cg20192752	14	20903683	KLHL33	5'UTR	0,227	2,95E-06	4,14E-05
cg14698646	17	46684750	HOXB7	3'UTR	0,334	2,95E-06	4,15E-05
cg00558031	4	8466291	C4orf23	Body	0,28	2,95E-06	4,15E-05
cg11795809	9	92271335	UNQ6494	Body	-0,21	2,95E-06	4,15E-05
cg14289461	6	112575929	LAMA4	TSS200	0,227	2,95E-06	4,15E-05
cg05745299	12	3790647	CRACR2A	Body	0,22	2,96E-06	4,15E-05
cg14847622	22	28106439		IGR	-0,263	2,96E-06	4,16E-05
cg12683410	20	2108416	STK35	3'UTR	0,214	2,96E-06	4,16E-05
cg11558867	6	47757408	OPN5	Body	-0,217	2,96E-06	4,16E-05
cg00631856	10	101673866	DNMBP	Body	0,252	2,96E-06	4,16E-05

cg01301319	7	27153580	HOXA3	5'UTR	0,247	2,96E-06	4,16E-05
cg24851112	6	7074199		IGR	-0,311	2,96E-06	4,16E-05
cg11131599	1	204224211	PLEKHA6	Body	-0,2	2,97E-06	4,16E-05
cg22783664	6	144479349	STX11	5'UTR	0,294	2,97E-06	4,17E-05
cg24953428	5	121815152	MGC32805	TSS1500	-0,214	2,97E-06	4,17E-05
cg13461821	8	97280827	PTDSS1	Body	0,205	2,97E-06	4,17E-05
cg20433641	15	52945342	FAM214A	TSS1500	0,262	2,97E-06	4,17E-05
cg27531927	13	98598462		IGR	-0,201	2,98E-06	4,18E-05
cg02572427	8	17611495	MTUS1	Body	0,208	2,98E-06	4,18E-05
cg15120701	6	4008556		IGR	0,233	2,98E-06	4,18E-05
cg15645309	14	75988747	BATF	TSS200	-0,253	2,98E-06	4,18E-05
cg20691539	16	51051363	LOC1019273	TSS1500	-0,2	2,98E-06	4,18E-05
cg00938816	17	8058149		IGR	0,227	2,98E-06	4,18E-05
cg26316384	2	216972625	XRCC5	TSS1500	0,2	2,98E-06	4,18E-05
cg03926552	12	92987767		IGR	-0,207	2,98E-06	4,18E-05
cg02394186	1	6088550	KCNAB2	5'UTR	-0,219	2,98E-06	4,18E-05
cg27582546	1	1062237		IGR	-0,206	2,98E-06	4,18E-05
cg20066040	3	132975933	TMEM108-AS	Body	-0,237	2,98E-06	4,18E-05
cg08038178	6	39905662		IGR	-0,253	2,99E-06	4,19E-05
cg09799676	7	27169586	HOXA4	Body	0,218	2,99E-06	4,19E-05
cg14083015	6	25042090		IGR	-0,252	2,99E-06	4,19E-05
cg23807718	20	42810587	JPH2	Body	0,215	2,99E-06	4,19E-05
cg05651762	3	176844015	TBL1XR1	5'UTR	0,282	2,99E-06	4,19E-05
cg16477554	3	24198189	LOC1019278	Body	-0,251	2,99E-06	4,19E-05
cg13362736	19	6249479	MLLT1	Body	0,26	2,99E-06	4,19E-05
cg26758524	18	45902012		IGR	-0,211	2,99E-06	4,19E-05
cg25656283	10	50733196	ERCC6	Body	0,238	2,99E-06	4,19E-05
cg18854004	2	8444004		IGR	-0,273	3,00E-06	4,20E-05
cg08962305	3	46622647	LRRC2	TSS1500	-0,231	3,00E-06	4,20E-05
cg07473959	8	134088519	TG	Body	-0,223	3,01E-06	4,20E-05
cg08293824	3	172313318		IGR	-0,231	3,01E-06	4,21E-05
cg07451222	13	95602734		IGR	0,251	3,01E-06	4,21E-05
cg14276268	3	194457354	LOC1005073	Body	-0,268	3,01E-06	4,21E-05
cg17620798	18	21444805	LAMA3	Body	-0,236	3,01E-06	4,21E-05
cg04850453	2	62189480	COMMD1	Body	0,264	3,01E-06	4,21E-05
cg14106027	12	10396521		IGR	-0,283	3,02E-06	4,21E-05
cg13322350	13	114914275		IGR	-0,283	3,02E-06	4,22E-05
cg18908811	22	36600770	APOL4	5'UTR	-0,283	3,02E-06	4,22E-05
cg26236143	3	29377160	RBMS3	Body	0,218	3,02E-06	4,22E-05
cg03981793	15	49716270	C15orf33	Body	0,209	3,03E-06	4,23E-05
cg21725986	1	159770253	FCRL6	TSS200	-0,224	3,03E-06	4,23E-05
cg12900404	2	225811669	DOCK10	1stExon	-0,228	3,04E-06	4,24E-05
cg08418472	8	127889346		IGR	0,213	3,04E-06	4,24E-05
cg07965110	1	24863166		IGR	-0,259	3,04E-06	4,24E-05
cg20304938	8	103050519	NCALD	5'UTR	0,207	3,04E-06	4,24E-05
cg07698039	10	4540959		IGR	0,304	3,04E-06	4,24E-05
cg06974672	2	129397288		IGR	-0,211	3,04E-06	4,24E-05
cg09083359	5	3854537		IGR	0,216	3,04E-06	4,24E-05
cg08661112	10	91405677	PANK1	TSS1500	0,21	3,04E-06	4,24E-05
cg02744249	20	57582918	CTSZ	TSS1500	-0,328	3,04E-06	4,24E-05

cg21193729	11	65374665	MAP3K11	Body	0,251	3,04E-06	4,24E-05
cg01188191	6	32047349	TNXB	Body	-0,203	3,05E-06	4,25E-05
cg07641530	1	175796062		IGR	-0,207	3,05E-06	4,25E-05
cg24784042	3	33034475		IGR	-0,275	3,05E-06	4,25E-05
cg11877812	10	19918310		IGR	0,205	3,05E-06	4,25E-05
cg21686703	16	89479349	ANKRD11	Body	0,234	3,05E-06	4,25E-05
cg22464884	1	101644652		IGR	-0,239	3,05E-06	4,25E-05
cg14253027	8	21602343	GFRA2	Body	0,292	3,06E-06	4,25E-05
cg25794831	18	29018594		IGR	-0,236	3,06E-06	4,26E-05
cg15263821	6	25042651	FAM65B	TSS1500	-0,343	3,06E-06	4,26E-05
cg15229994	17	75471203	sept-09	Body	-0,246	3,06E-06	4,26E-05
cg03173528	13	40175221	LHFP	Body	0,229	3,07E-06	4,26E-05
cg17020792	10	5082700		IGR	-0,214	3,07E-06	4,27E-05
cg05772903	18	65296539	LOC643542	Body	-0,234	3,07E-06	4,27E-05
cg10599156	3	141145231	ZBTB38	5'UTR	0,264	3,07E-06	4,27E-05
cg02317313	12	122235206	LOC338799	Body	0,219	3,07E-06	4,27E-05
cg14172897	1	37965113	MEAF6	Body	0,258	3,07E-06	4,27E-05
cg24058402	22	39488814		IGR	-0,201	3,07E-06	4,27E-05
cg14761799	3	197759096	LMLN	Body	0,245	3,08E-06	4,27E-05
cg25602225	11	45355222		IGR	0,266	3,08E-06	4,27E-05
cg16735692	4	173734719	GALNTL6	Body	-0,223	3,08E-06	4,27E-05
cg14588855	7	3067293	CARD11	5'UTR	0,211	3,08E-06	4,27E-05
cg11422237	14	84503142		IGR	-0,235	3,08E-06	4,28E-05
cg08586669	7	50727761	GRB10	Body	-0,262	3,08E-06	4,28E-05
cg02710534	5	141225619		IGR	0,2	3,08E-06	4,28E-05
cg08771678	12	106495651	NUAK1	Body	0,202	3,09E-06	4,28E-05
cg02322889	4	25101274		IGR	0,204	3,09E-06	4,29E-05
cg26344732	4	41527343	LIMCH1	5'UTR	0,253	3,09E-06	4,29E-05
cg04322572	10	43447618		IGR	0,227	3,09E-06	4,29E-05
cg24507760	1	207992403	LOC148696	Body	0,208	3,09E-06	4,29E-05
cg21917656	8	119988101		IGR	-0,222	3,09E-06	4,29E-05
cg25555753	19	3133435		IGR	-0,264	3,09E-06	4,29E-05
cg18710751	15	56189462	NEDD4	Body	0,212	3,09E-06	4,29E-05
cg19817070	10	20064377		IGR	-0,235	3,09E-06	4,29E-05
cg22120714	17	40332420	KCNH4	Body	0,2	3,10E-06	4,29E-05
cg06541222	8	74019754		IGR	-0,233	3,10E-06	4,29E-05
cg16694837	14	99945655	SETD3	5'UTR	0,205	3,10E-06	4,30E-05
cg03137972	1	173380251	LOC10050602	Body	-0,248	3,10E-06	4,30E-05
cg04939902	19	47660875	SAE1	Body	0,203	3,10E-06	4,30E-05
cg27575100	11	27410771	LGR4	Body	-0,217	3,10E-06	4,30E-05
cg08456334	1	151298699	PI4KB	1stExon	0,231	3,11E-06	4,31E-05
cg11640793	7	17862007	SNX13	Body	0,212	3,11E-06	4,31E-05
cg08732984	21	15938259	SAMSN1	Body	0,263	3,11E-06	4,31E-05
cg05845393	3	171172141	TNIK	Body	0,267	3,12E-06	4,31E-05
cg17109198	14	24891647		IGR	-0,257	3,12E-06	4,32E-05
cg06795340	9	107546652	ABCA1	Body	-0,24	3,12E-06	4,32E-05
cg08174191	8	61967144		IGR	0,2	3,12E-06	4,32E-05
cg22669352	5	151025079		IGR	-0,217	3,13E-06	4,32E-05
cg13344118	16	56295857	GNAO1	Body	-0,203	3,13E-06	4,33E-05
cg15557489	17	59228220	BCAS3	Body	-0,207	3,13E-06	4,33E-05

cg16324121	3	9954273	IL17RE	Body	-0,259	3,13E-06	4,33E-05
cg25751205	6	168703559	DACT2	Body	0,228	3,13E-06	4,33E-05
cg09367815	21	30463648	MAP3K7CL	TSS1500	-0,244	3,13E-06	4,33E-05
cg12360935	5	169757754	LOC257358	TSS1500	-0,26	3,13E-06	4,33E-05
cg24600366	16	69482837	CYB5B	Body	0,275	3,13E-06	4,33E-05
cg20025658	2	219031640	CXCR1	1stExon	-0,267	3,14E-06	4,33E-05
cg19801553	11	45070822		IGR	0,203	3,14E-06	4,34E-05
cg25012274	17	9130163	NTN1	Body	-0,224	3,14E-06	4,34E-05
cg08206267	19	17862104	FCHO1	5'UTR	-0,254	3,14E-06	4,34E-05
cg12364136	7	158331217	PTPRN2	Body	-0,207	3,14E-06	4,34E-05
cg05438336	12	19699832		IGR	0,227	3,14E-06	4,34E-05
cg21188977	6	25718316		IGR	0,281	3,15E-06	4,35E-05
cg00807698	1	168678926	DPT	Body	0,203	3,15E-06	4,35E-05
cg06437840	6	33048529	HLA-DPB1	Body	0,206	3,15E-06	4,35E-05
cg03892989	9	126979656		IGR	-0,209	3,15E-06	4,35E-05
cg20175324	13	50858812	DLEU1	Body	-0,263	3,15E-06	4,35E-05
cg14134256	11	94502879	AMOTL1	Body	0,205	3,15E-06	4,35E-05
cg18126557	1	247611842	NLRP3	3'UTR	0,257	3,16E-06	4,36E-05
cg24497954	5	39210223	FYB	5'UTR	-0,273	3,17E-06	4,37E-05
cg11268227	1	67833522	IL12RB2	ExonBnd	0,307	3,17E-06	4,37E-05
cg18354041	10	2834411		IGR	-0,219	3,17E-06	4,37E-05
cg05982301	5	121751763	SNCAIP	Body	-0,218	3,17E-06	4,37E-05
cg12634306	1	40098811	HEYL	Body	0,202	3,17E-06	4,37E-05
cg19824502	2	175615819	CHRNA1	Body	-0,261	3,18E-06	4,38E-05
cg15069496	14	100530153		IGR	-0,209	3,18E-06	4,38E-05
cg04368942	7	134571320	CALD1	Body	0,218	3,18E-06	4,38E-05
cg07669182	5	39203755	FYB	5'UTR	-0,203	3,18E-06	4,38E-05
cg24850585	12	50350354	AQP2	3'UTR	-0,211	3,18E-06	4,38E-05
cg23339720	3	140814055	SPSB4	Body	0,225	3,18E-06	4,38E-05
cg23361265	17	15917660	TTC19	Body	0,221	3,18E-06	4,38E-05
cg10191799	22	39436141	APOBEC3F	TSS1500	0,236	3,18E-06	4,38E-05
cg16193045	2	163823957		IGR	0,227	3,19E-06	4,39E-05
cg07893159	22	43091206	A4GALT	5'UTR	0,218	3,19E-06	4,39E-05
cg19311890	15	90721869		IGR	-0,228	3,19E-06	4,39E-05
cg16131257	15	67212075		IGR	0,23	3,19E-06	4,39E-05
cg12605568	2	178463258		IGR	0,219	3,19E-06	4,39E-05
cg12609052	12	3335597	TSPAN9	Body	-0,203	3,19E-06	4,39E-05
cg03055976	16	3996416		IGR	0,215	3,19E-06	4,40E-05
cg20502797	9	101881628	TGFBR1	Body	-0,271	3,20E-06	4,40E-05
cg18164357	11	77534497	C11orf67	5'UTR	0,213	3,20E-06	4,40E-05
cg12970937	7	93188125	CALCR	5'UTR	0,241	3,20E-06	4,40E-05
cg19998294	10	18629497	CACNB2	Body	0,251	3,20E-06	4,40E-05
cg07913403	5	34735977	RAI14	Body	-0,258	3,20E-06	4,40E-05
cg06879746	6	30883768	VARS2	Body	0,207	3,21E-06	4,41E-05
cg09548638	5	148443744	SH3TC2	TSS1500	0,22	3,21E-06	4,41E-05
cg11065271	6	159070607	SYTL3	TSS1500	-0,226	3,21E-06	4,41E-05
cg05189835	1	220187223	RNU5F-1	Body	-0,311	3,21E-06	4,41E-05
cg03877953	17	56757566	TEX14	5'UTR	0,215	3,21E-06	4,41E-05
cg07364631	1	228917168		IGR	0,214	3,21E-06	4,42E-05
cg16953763	3	52281290	PPM1M	Body	-0,21	3,22E-06	4,42E-05

cg25900902	17	7253340	ACAP1	Body	0,296	3,22E-06	4,42E-05
cg01904580	16	16113527	ABCC1	Body	-0,216	3,22E-06	4,42E-05
cg20385959	18	45986063		IGR	-0,202	3,22E-06	4,42E-05
cg24338091	19	46270244	SIX5	Body	0,203	3,22E-06	4,43E-05
cg07838943	1	12493840	VPS13D	Body	0,201	3,22E-06	4,43E-05
cg18973416	10	35843536	CCNY	Body	0,201	3,23E-06	4,43E-05
cg24632427	3	15333998	SH3BP5	Body	-0,241	3,23E-06	4,43E-05
cg01949952	15	59034841	ADAM10	Body	0,3	3,23E-06	4,43E-05
cg21757169	7	33966501	BMPER	Body	-0,226	3,23E-06	4,43E-05
cg01017212	7	43652291	STK17A	Body	-0,202	3,23E-06	4,43E-05
cg09428934	14	71468614	PCNX	Body	0,278	3,23E-06	4,43E-05
cg14464829	11	36029266	LDLRAD3	5'UTR	-0,213	3,23E-06	4,43E-05
cg22760668	14	69152424		IGR	-0,231	3,23E-06	4,43E-05
cg05394244	6	64281439	PTP4A1	TSS1500	0,229	3,23E-06	4,43E-05
cg20947760	10	3392776	LOC1053763(	Body	-0,208	3,24E-06	4,44E-05
cg24774517	6	42742564		IGR	-0,299	3,24E-06	4,44E-05
cg01795160	9	95727505	FGD3	5'UTR	-0,278	3,24E-06	4,44E-05
cg19909613	8	109487153	TTC35	Body	0,299	3,24E-06	4,44E-05
cg05080285	2	204400815	RAPH1	TSS1500	0,277	3,24E-06	4,44E-05
cg00601491	3	16296686		IGR	-0,21	3,24E-06	4,44E-05
cg25085195	11	66927799	KDM2A	Body	0,211	3,24E-06	4,44E-05
cg24530147	3	138763894	PRR23C	TSS200	0,337	3,24E-06	4,44E-05
cg05826162	18	72166704	CNDP2	5'UTR	-0,273	3,25E-06	4,44E-05
cg01235116	16	50705762	SNX20	Body	0,202	3,25E-06	4,44E-05
cg18726748	1	221951837		IGR	-0,301	3,25E-06	4,45E-05
cg16727006	16	87470545	ZCCHC14	Body	-0,214	3,25E-06	4,45E-05
cg21208154	14	23013104		IGR	-0,207	3,25E-06	4,45E-05
cg23753807	17	1090291	ABR	1stExon	0,257	3,25E-06	4,45E-05
cg02254461	3	39195904	CSRNP1	TSS1500	0,229	3,25E-06	4,45E-05
cg19908827	1	204591505	LRRN2	5'UTR	-0,254	3,25E-06	4,45E-05
cg24769398	3	188666022		IGR	0,215	3,26E-06	4,46E-05
cg23345618	4	139812537		IGR	0,211	3,26E-06	4,46E-05
cg02800033	18	46481907		IGR	0,203	3,26E-06	4,46E-05
cg02597698	22	30662987	OSM	TSS200	-0,24	3,26E-06	4,46E-05
cg15949108	16	84109949	MBTPS1	Body	0,257	3,27E-06	4,47E-05
cg08006519	17	41822590		IGR	-0,243	3,27E-06	4,47E-05
cg22536405	2	198145382	ANKRD44-IT1	Body	0,241	3,27E-06	4,47E-05
cg03214649	10	112389401		IGR	0,23	3,27E-06	4,47E-05
cg02101707	1	201819402	IPO9	Body	0,222	3,27E-06	4,47E-05
cg20818222	6	167493614		IGR	-0,267	3,27E-06	4,47E-05
cg23695504	1	247275344	C1orf229	1stExon	0,207	3,27E-06	4,47E-05
cg16017420	12	1642879		IGR	-0,247	3,28E-06	4,48E-05
cg26126178	3	42138999	TRAK1	Body	0,211	3,28E-06	4,48E-05
cg26924109	5	122915359	CSNK1G3	Body	0,255	3,28E-06	4,48E-05
cg03778207	11	86230664	ME3	Body	0,217	3,28E-06	4,48E-05
cg04176141	12	121366732		IGR	0,204	3,28E-06	4,48E-05
cg23178195	19	35629703	FXYD1	TSS200	0,222	3,28E-06	4,48E-05
cg11000969	17	75337274	sept-09	Body	-0,203	3,28E-06	4,48E-05
cg27119612	15	57491001	TCF12	Body	0,218	3,29E-06	4,48E-05
cg07107726	11	91526980		IGR	-0,255	3,29E-06	4,48E-05

cg16004722	15	95674587		IGR	0,281	3,29E-06	4,49E-05
cg12139878	11	62324558		IGR	-0,205	3,29E-06	4,49E-05
cg17395885	12	116602340	MED13L	Body	0,249	3,29E-06	4,49E-05
cg15233025	14	59100662	DACT1	TSS200	0,238	3,30E-06	4,50E-05
cg13254958	16	84548506		IGR	-0,321	3,30E-06	4,50E-05
cg24339395	14	35844969		IGR	-0,246	3,30E-06	4,50E-05
cg17418085	1	31229122	LAPTM5	Body	-0,307	3,30E-06	4,50E-05
cg08260413	17	54037217		IGR	-0,24	3,30E-06	4,50E-05
cg26836479	19	42706353	DEDD2	Body	-0,232	3,31E-06	4,51E-05
cg01522006	3	129333962		IGR	0,2	3,31E-06	4,51E-05
cg03879120	10	26768835	APBB1IP	5'UTR	-0,292	3,31E-06	4,51E-05
cg14412134	14	64912417	MTHFD1	Body	-0,219	3,31E-06	4,51E-05
cg03477732	12	125403600		IGR	-0,203	3,31E-06	4,51E-05
cg15272153	4	38525916	LINC01258	TSS1500	-0,247	3,32E-06	4,51E-05
cg01519261	21	36421467	RUNX1	5'UTR	-0,205	3,32E-06	4,52E-05
cg09121624	1	1918541	CFAP74	Body	-0,215	3,33E-06	4,53E-05
cg14864167	8	66751182	PDE7A	Body	-0,273	3,33E-06	4,53E-05
cg23878024	2	204488580		IGR	-0,236	3,33E-06	4,53E-05
cg24736886	13	72195560	DACH1	Body	0,343	3,33E-06	4,53E-05
cg00240653	12	55378411	KIAA0748	1stExon	-0,323	3,33E-06	4,53E-05
cg11214115	6	34286233	NUDT3	Body	0,206	3,33E-06	4,53E-05
cg18792365	8	13132967	DLC1	Body	0,274	3,33E-06	4,53E-05
cg10277204	9	456556	DOCK8	Body	0,216	3,34E-06	4,53E-05
cg17053687	3	155742871		IGR	0,299	3,34E-06	4,54E-05
cg12178835	14	69186571		IGR	-0,225	3,34E-06	4,54E-05
cg08944029	5	112553445	MCC	Body	0,247	3,34E-06	4,54E-05
cg27538957	18	13615792	LDLRAD4	5'UTR	-0,221	3,34E-06	4,54E-05
cg10680328	16	83881192		IGR	-0,227	3,34E-06	4,54E-05
cg09406387	5	58592636	PDE4D	Body	0,24	3,34E-06	4,54E-05
cg10129391	4	54582825		IGR	-0,218	3,35E-06	4,55E-05
cg21151381	15	40690968		IGR	-0,316	3,35E-06	4,55E-05
cg09722826	16	50780248	CYLD	5'UTR	-0,229	3,35E-06	4,55E-05
cg12406839	8	992025	ERICH1-AS1	Body	0,203	3,35E-06	4,55E-05
cg23514621	11	76338130		IGR	-0,243	3,36E-06	4,55E-05
cg16090701	13	24844242	SPATA13	TSS1500	-0,202	3,36E-06	4,56E-05
cg09304968	7	76977748	PION	Body	0,247	3,36E-06	4,56E-05
cg15725542	1	7990871	TNFRSF9	Body	-0,222	3,36E-06	4,56E-05
cg14478011	11	72567752	FCHSD2	Body	0,213	3,36E-06	4,56E-05
cg00426127	6	167461262		IGR	-0,252	3,36E-06	4,56E-05
cg20827484	20	23097651		IGR	-0,214	3,37E-06	4,57E-05
cg13702000	17	38966101		IGR	0,233	3,37E-06	4,57E-05
cg22577540	5	171540398	STK10	Body	-0,332	3,37E-06	4,57E-05
cg08379738	19	6477033	DENND1C	Body	0,242	3,37E-06	4,57E-05
cg13513652	1	48671325		IGR	-0,2	3,37E-06	4,57E-05
cg04340918	8	19577028		IGR	-0,225	3,37E-06	4,57E-05
cg09333487	16	25155451	LCMT1-AS2	Body	0,251	3,38E-06	4,58E-05
cg07953344	2	206614079	NRP2	Body	-0,255	3,38E-06	4,58E-05
cg02586023	6	76198240	FILIP1	5'UTR	0,271	3,38E-06	4,58E-05
cg22419425	3	101441433		IGR	-0,216	3,38E-06	4,58E-05
cg02004661	13	43652125	DNAJC15	Body	0,227	3,38E-06	4,58E-05

cg14551458	5	123149103		IGR	-0,219	3,38E-06	4,58E-05
cg00921266	7	27153663	HOXA3	5'UTR	0,282	3,39E-06	4,59E-05
cg06197201	2	111528479	ACOXL	Body	0,209	3,39E-06	4,59E-05
cg15606135	12	55378169	TESPA1	5'UTR	-0,344	3,39E-06	4,59E-05
cg10376838	4	26165511		IGR	0,234	3,39E-06	4,59E-05
cg04539111	16	67997858	SLC12A4	Body	0,213	3,39E-06	4,59E-05
cg12090026	6	119752021		IGR	0,247	3,39E-06	4,59E-05
cg13784195	21	27104564	ATP5J	5'UTR	0,282	3,39E-06	4,59E-05
cg22232164	1	32610876	KPNA6	Body	-0,254	3,39E-06	4,59E-05
cg24118856	19	10223710	PPAN-P2RY11	Body	-0,206	3,40E-06	4,59E-05
cg11405216	5	178654006	ADAMTS2	Body	0,2	3,40E-06	4,60E-05
cg08891559	10	119480849		IGR	0,205	3,40E-06	4,60E-05
cg26909924	6	112516524	LAMA4	Body	-0,212	3,41E-06	4,60E-05
cg21011913	17	7392218	POLR2A	Body	0,296	3,41E-06	4,61E-05
cg25733272	7	36764082	AOAH	1stExon	-0,212	3,41E-06	4,61E-05
cg00965578	22	36560801	APOL3	5'UTR	-0,285	3,41E-06	4,61E-05
cg12681119	1	232593528	SIPA1L2	Body	-0,223	3,41E-06	4,61E-05
cg11572080	6	111925158	TRAF3IP2	Body	0,242	3,41E-06	4,61E-05
cg04064050	6	127663586	ECHDC1	5'UTR	0,219	3,42E-06	4,62E-05
cg24076537	4	139858980		IGR	-0,2	3,42E-06	4,62E-05
cg15930219	1	71510804	PTGER3	Body	0,211	3,42E-06	4,62E-05
cg07099553	15	89178856		IGR	-0,241	3,42E-06	4,62E-05
cg02936292	11	16826170	PLEKHA7	Body	0,222	3,43E-06	4,62E-05
cg14921479	17	66342543	ARSG	Body	-0,334	3,43E-06	4,62E-05
cg05305434	11	1874049	LSP1	TSS200	-0,244	3,43E-06	4,62E-05
cg01134662	17	39165631	KRTAP3-1	TSS1500	0,236	3,43E-06	4,62E-05
cg09126320	8	22778500	PEBP4	Body	-0,218	3,43E-06	4,63E-05
cg03478739	2	175712381	CHN1	Body	0,222	3,43E-06	4,63E-05
cg01020308	8	102679791	GRHL2	3'UTR	-0,218	3,43E-06	4,63E-05
cg10922453	9	21444258		IGR	-0,232	3,43E-06	4,63E-05
cg02350144	1	160545376	CD84	Body	-0,245	3,44E-06	4,63E-05
cg18722402	5	138477779	SIL1	5'UTR	0,237	3,44E-06	4,63E-05
cg22464785	15	58809601	LIPC	Body	-0,203	3,44E-06	4,63E-05
cg26382679	19	30215768		IGR	0,235	3,44E-06	4,64E-05
cg04945634	17	74434961	UBE2O	Body	0,203	3,44E-06	4,64E-05
cg18240967	14	98011083	LOC10192924	Body	-0,217	3,44E-06	4,64E-05
cg03261473	1	208529522		IGR	-0,265	3,45E-06	4,64E-05
cg19652271	6	122722069	HSF2	Body	0,218	3,45E-06	4,64E-05
cg24094541	15	52789119	MYO5A	Body	-0,204	3,45E-06	4,65E-05
cg16930438	3	72846958	SHQ1	Body	0,208	3,45E-06	4,65E-05
cg06608119	3	28368305	AZI2	ExonBnd	-0,202	3,45E-06	4,65E-05
cg19506623	2	161265259	RBMS1	Body	0,261	3,45E-06	4,65E-05
cg26731143	1	42357199	HIVEP3	5'UTR	-0,201	3,46E-06	4,65E-05
cg04731861	2	219085781	ARPC2	Body	-0,222	3,46E-06	4,65E-05
cg26601310	11	36397123	PRR5L	5'UTR	-0,202	3,46E-06	4,65E-05
cg14998516	16	22041228	C16orf52	Body	0,239	3,46E-06	4,65E-05
cg02036430	5	80992249	SSBP2	Body	0,203	3,46E-06	4,66E-05
cg02517337	20	51590841	TSHZ2	Body	-0,235	3,46E-06	4,66E-05
cg03441770	4	81128845		IGR	0,202	3,46E-06	4,66E-05
cg11425848	2	193510695		IGR	0,258	3,46E-06	4,66E-05

cg07118556	8	135816995		IGR	0,269	3,46E-06	4,66E-05
cg06350853	1	168890140		IGR	0,252	3,46E-06	4,66E-05
cg16615161	20	57649682		IGR	-0,265	3,46E-06	4,66E-05
cg03240883	17	4173233	UBE2G1	3'UTR	0,207	3,47E-06	4,66E-05
cg15006682	4	102875309	BANK1	Body	0,267	3,47E-06	4,66E-05
cg02779913	17	40088680	TTC25	Body	-0,261	3,47E-06	4,66E-05
cg25983622	19	2700849	GNG7	5'UTR	0,234	3,47E-06	4,66E-05
cg20031843	8	17656574	MTUS1	5'UTR	0,258	3,47E-06	4,66E-05
cg05988980	10	73582215	PSAP	Body	-0,235	3,47E-06	4,66E-05
cg18551822	6	2110875	GMDS	Body	-0,285	3,47E-06	4,66E-05
cg20397384	12	46862172	LOC1002887	Body	0,207	3,47E-06	4,66E-05
cg22439500	13	30720170		IGR	-0,239	3,47E-06	4,67E-05
cg09596958	12	58132105	AGAP2	TSS200	0,223	3,47E-06	4,67E-05
cg05564182	1	221219852		IGR	-0,247	3,48E-06	4,67E-05
cg26314722	1	234867300		IGR	-0,223	3,48E-06	4,68E-05
cg09138272	13	95826930	ABCC4	Body	0,225	3,48E-06	4,68E-05
cg07654200	6	28952955		IGR	0,206	3,48E-06	4,68E-05
cg01817364	5	43037411		IGR	0,258	3,49E-06	4,68E-05
cg15397593	12	2273119	CACNA1C	Body	0,214	3,49E-06	4,68E-05
cg11877875	10	90031655		IGR	-0,225	3,49E-06	4,69E-05
cg04426621	20	52444699		IGR	0,262	3,49E-06	4,69E-05
cg15387403	17	61553170	ACE	TSS1500	0,209	3,49E-06	4,69E-05
cg27060793	4	88775480		IGR	-0,202	3,49E-06	4,69E-05
cg17266581	18	74799572	MBP	Body	0,232	3,49E-06	4,69E-05
cg03581822	5	126377032		IGR	0,203	3,50E-06	4,70E-05
cg11584380	10	6094836	IL2RA	Body	-0,289	3,51E-06	4,70E-05
cg02690559	14	53499160		IGR	0,318	3,51E-06	4,70E-05
cg09417586	2	38587860	ATL2	Body	0,23	3,51E-06	4,70E-05
cg05516344	5	78204870	ARSB	Body	0,246	3,51E-06	4,70E-05
cg00672802	1	226296871		IGR	0,21	3,51E-06	4,71E-05
cg11598403	18	74799500	MBP	Body	0,221	3,52E-06	4,71E-05
cg22816091	1	42611670		IGR	0,211	3,52E-06	4,71E-05
cg27111050	2	139260866	SPOPL	5'UTR	-0,235	3,52E-06	4,71E-05
cg26507839	6	148525282		IGR	-0,234	3,52E-06	4,72E-05
cg23482132	2	153415027	FMNL2	Body	-0,219	3,53E-06	4,72E-05
cg11312495	5	115697370		IGR	0,2	3,53E-06	4,72E-05
cg25610697	9	676996	KANK1	ExonBnd	0,224	3,54E-06	4,73E-05
cg23543318	4	1195845	LOC1001308	Body	-0,263	3,54E-06	4,73E-05
cg15569829	10	69551089		IGR	-0,209	3,54E-06	4,74E-05
cg02077648	1	8456619	RERE	5'UTR	0,2	3,55E-06	4,74E-05
cg27353361	6	108145420	SCML4	1stExon	-0,235	3,55E-06	4,74E-05
cg18613324	22	36236760	RBM9	Body	0,259	3,55E-06	4,74E-05
cg20194367	14	61929688	PRKCH	Body	0,254	3,55E-06	4,74E-05
cg20372886	3	42094749		IGR	0,204	3,55E-06	4,74E-05
cg00260499	19	3405140	NFIC	Body	0,288	3,55E-06	4,75E-05
cg00952352	13	46752424	LCP1	5'UTR	-0,277	3,56E-06	4,76E-05
cg15932059	1	218946128	MIR548F3	Body	-0,208	3,57E-06	4,76E-05
cg26873042	5	67363338		IGR	-0,217	3,58E-06	4,77E-05
cg07586285	1	200994978		IGR	-0,215	3,58E-06	4,78E-05
cg08783050	16	80201665	LOC1027240	Body	-0,201	3,58E-06	4,78E-05

cg14359153	15	33493358		IGR	0,271	3,59E-06	4,78E-05
cg21274723	11	34842625		IGR	-0,235	3,59E-06	4,78E-05
cg08132573	1	235159410		IGR	-0,233	3,60E-06	4,79E-05
cg12418071	19	15919836	OR10H1	TSS1500	0,22	3,60E-06	4,79E-05
cg18729493	2	161244580	RBMS1	Body	0,201	3,60E-06	4,79E-05
cg25960393	8	9106559		IGR	-0,266	3,60E-06	4,79E-05
cg08578641	9	34457440	C9orf25	Body	0,227	3,61E-06	4,80E-05
cg19848140	2	168822706	STK39	Body	-0,2	3,61E-06	4,80E-05
cg08995327	16	66070379		IGR	0,274	3,61E-06	4,80E-05
cg06977852	3	52532867	STAB1	Body	-0,279	3,61E-06	4,81E-05
cg01588379	7	151433326	PRKAG2	Body	0,202	3,61E-06	4,81E-05
cg06289802	17	72748189	SLC9A3R1	Body	-0,249	3,62E-06	4,81E-05
cg01065213	7	14876447	DGKB	Body	0,254	3,63E-06	4,83E-05
cg20092942	3	177538695	KCCAT211	Body	-0,237	3,64E-06	4,83E-05
cg19218929	6	18386873	RNF144B	TSS1500	0,228	3,64E-06	4,84E-05
cg12049174	17	37055904	LASP1	Body	-0,257	3,64E-06	4,84E-05
cg11683706	3	150811929	MED12L	Body	-0,273	3,65E-06	4,84E-05
cg00533183	6	32810742	PSMB8	Body	-0,271	3,65E-06	4,84E-05
cg16116735	5	54623336	SKIV2L2	Body	-0,233	3,65E-06	4,85E-05
cg06757178	12	96068576	NTN4	Body	0,219	3,66E-06	4,85E-05
cg13346442	7	112124984	LSMEM1	Body	0,311	3,66E-06	4,85E-05
cg17333269	3	71137113	FOXP1	Body	-0,207	3,66E-06	4,86E-05
cg06880420	1	200119987	NR5A2	Body	-0,232	3,66E-06	4,86E-05
cg02148426	7	32801268		IGR	-0,223	3,66E-06	4,86E-05
cg16608652	1	193155390	B3GALT2	5'UTR	0,201	3,66E-06	4,86E-05
cg16335583	11	122714824	CRTAM	Body	-0,223	3,66E-06	4,86E-05
cg19933220	5	142024805	FGF1	TSS1500	0,202	3,66E-06	4,86E-05
cg22309167	1	235017787		IGR	-0,214	3,67E-06	4,86E-05
cg10692728	4	140872429	MAML3	Body	-0,287	3,67E-06	4,86E-05
cg26515755	16	15878612	MYH11	Body	-0,23	3,67E-06	4,86E-05
cg02152068	18	60606857	PHLPP1	Body	0,261	3,67E-06	4,86E-05
cg12426640	3	38415918	XYLB	Body	-0,264	3,67E-06	4,87E-05
cg14442312	12	3838009	CRACR2A	5'UTR	-0,263	3,68E-06	4,87E-05
cg16533830	3	9988662	PRRT3-AS1	TSS1500	0,21	3,68E-06	4,88E-05
cg20479688	12	53046513	KRT2	TSS1500	-0,264	3,68E-06	4,88E-05
cg13415831	9	92137791		IGR	-0,312	3,69E-06	4,88E-05
cg11257009	2	8615454		IGR	-0,292	3,69E-06	4,89E-05
cg10322779	17	38966084		IGR	0,259	3,70E-06	4,89E-05
cg14315054	1	221068730		IGR	0,201	3,70E-06	4,89E-05
cg17266406	2	60743021	BCL11A	Body	-0,239	3,70E-06	4,89E-05
cg19824242	2	105273432		IGR	0,248	3,70E-06	4,89E-05
cg16163382	2	37938640		IGR	-0,289	3,70E-06	4,89E-05
cg16576033	1	235268621		IGR	-0,273	3,71E-06	4,90E-05
cg26282299	8	142189729	DENND3	Body	0,209	3,71E-06	4,90E-05
cg23904955	10	101282759		IGR	0,206	3,71E-06	4,90E-05
cg10653245	20	56283721	PMEPA1	5'UTR	0,21	3,71E-06	4,90E-05
cg18205668	7	45066895	CCM2	Body	-0,282	3,71E-06	4,90E-05
cg16031515	1	205743344	RAB7L1	Body	-0,244	3,71E-06	4,91E-05
cg05036991	2	155089854	GALNT13	Body	-0,219	3,71E-06	4,91E-05
cg08484337	6	31590563	SNORA38	TSS1500	0,208	3,71E-06	4,91E-05

cg05185749	9	117692759	TNFSF8	1stExon	-0,214	3,72E-06	4,91E-05
cg24314564	3	188668940		IGR	-0,288	3,72E-06	4,91E-05
cg05639068	2	27938112		IGR	0,206	3,72E-06	4,91E-05
cg06206987	6	130897017		IGR	-0,235	3,73E-06	4,92E-05
cg24881262	3	81792918	GBE1	Body	0,22	3,73E-06	4,92E-05
cg08371406	9	6598558	GLDC	Body	-0,21	3,73E-06	4,92E-05
cg03887098	3	32002750	OSBPL10	Body	-0,247	3,73E-06	4,92E-05
cg12177851	15	89178776		IGR	-0,3	3,73E-06	4,92E-05
cg10470108	2	86625453		IGR	0,212	3,73E-06	4,92E-05
cg00598693	2	10261684	RRM2	TSS1500	-0,205	3,74E-06	4,93E-05
cg07282778	10	80391121		IGR	-0,238	3,74E-06	4,93E-05
cg26286479	12	69924501	FRS2	5'UTR	0,229	3,74E-06	4,93E-05
cg26112797	17	56409011	MIR142	TSS1500	-0,259	3,74E-06	4,93E-05
cg00083188	10	79314844	KCNMA1	Body	-0,224	3,75E-06	4,94E-05
cg07369274	3	58200599		IGR	-0,251	3,75E-06	4,94E-05
cg08614769	6	1679393	GMDS	Body	0,238	3,75E-06	4,94E-05
cg06663149	4	41457558	LIMCH1	Body	0,203	3,76E-06	4,95E-05
cg26379553	5	171542053	STK10	Body	-0,202	3,76E-06	4,95E-05
cg10097215	6	132834914	STX7	TSS1500	0,207	3,76E-06	4,95E-05
cg23330281	11	19372234	NAV2	TSS200	-0,222	3,76E-06	4,95E-05
cg23228346	9	102193601		IGR	0,229	3,77E-06	4,96E-05
cg06551854	6	164405318		IGR	-0,235	3,77E-06	4,96E-05
cg01559302	19	48246266	EHD2	3'UTR	0,219	3,77E-06	4,96E-05
cg13537590	12	26451968		IGR	-0,32	3,79E-06	4,98E-05
cg05264639	2	216980171	XRCC5	Body	0,215	3,79E-06	4,98E-05
cg02489169	6	81233024		IGR	0,208	3,80E-06	4,99E-05
cg01209614	1	39851370	MACF1	Body	0,211	3,80E-06	4,99E-05
cg03365455	15	62852118		IGR	-0,2	3,81E-06	5,00E-05
cg09108686	1	203582462		IGR	-0,233	3,81E-06	5,00E-05
cg17248139	6	114241874	FLJ34503	Body	0,24	3,81E-06	5,00E-05
cg12790664	18	9083065		IGR	-0,238	3,81E-06	5,00E-05
cg00413596	14	53982283		IGR	-0,238	3,82E-06	5,01E-05
cg26353738	6	46698987	PLA2G7	5'UTR	-0,207	3,82E-06	5,01E-05
cg22728262	11	45922657	MAPK8IP1	Body	0,217	3,82E-06	5,01E-05
cg09982773	7	128550860	KCP	TSS200	0,204	3,82E-06	5,01E-05
cg09991519	8	97571853	SDC2	Body	-0,237	3,82E-06	5,01E-05
cg00270878	11	64334217	SLC22A11	Body	0,237	3,82E-06	5,01E-05
cg17522423	12	111218502		IGR	-0,227	3,83E-06	5,02E-05
cg19886272	16	69872272	WWP2	TSS1500	0,222	3,83E-06	5,02E-05
cg15956720	12	132550715	EP400	Body	-0,231	3,83E-06	5,02E-05
cg16638607	15	97579756		IGR	0,248	3,83E-06	5,02E-05
cg27129153	10	21839422	MLLT10	Body	0,203	3,83E-06	5,02E-05
cg01411101	11	86651671	PRSS23	Body	0,275	3,83E-06	5,02E-05
cg12733396	1	40783234	COL9A2	TSS1500	0,209	3,84E-06	5,02E-05
cg13508369	9	129884026	ANGPTL2	5'UTR	0,298	3,84E-06	5,03E-05
cg18109011	6	111920611	TRAF3IP2-AS:	Body	0,243	3,85E-06	5,04E-05
cg12058490	12	9268379	A2M	1stExon	0,268	3,85E-06	5,04E-05
cg21174110	3	149956337	LINC01213	TSS200	-0,236	3,85E-06	5,04E-05
cg01117339	11	73737124		IGR	-0,282	3,85E-06	5,04E-05
cg05469118	11	122215103		IGR	0,25	3,85E-06	5,04E-05

cg09030622	2	10529903	HPCAL1	5'UTR	0,24	3,85E-06	5,04E-05
cg07429146	7	150822655	AGAP3	Body	0,208	3,85E-06	5,04E-05
cg06641416	1	244489020		IGR	-0,218	3,86E-06	5,04E-05
cg00397739	22	47010373		IGR	-0,313	3,86E-06	5,05E-05
cg22701940	2	152773518	CACNB4	Body	0,261	3,86E-06	5,05E-05
cg13399593	17	26674140	POLDIP2	3'UTR	0,218	3,86E-06	5,05E-05
cg12769118	1	247712377	C1orf150	TSS200	-0,219	3,86E-06	5,05E-05
cg23829949	1	244214679	ZNF238	1stExon	0,233	3,87E-06	5,05E-05
cg17349352	7	45066738	CCM2	Body	-0,282	3,87E-06	5,06E-05
cg11991267	10	36823765		IGR	-0,205	3,87E-06	5,06E-05
cg26972389	5	169407753	FAM196B	TSS200	0,246	3,87E-06	5,06E-05
cg08706746	15	52975231		IGR	-0,274	3,88E-06	5,06E-05
cg03573445	14	105154858	INF2	TSS1500	0,2	3,88E-06	5,07E-05
cg12804755	11	15796715		IGR	-0,208	3,88E-06	5,07E-05
cg04791291	22	45525789		IGR	-0,2	3,88E-06	5,07E-05
cg22994849	7	149578441		IGR	0,248	3,88E-06	5,07E-05
cg13855435	17	57449015	YPEL2	Body	0,213	3,88E-06	5,07E-05
cg17415380	3	71447574	FOXP1	5'UTR	-0,21	3,88E-06	5,07E-05
cg01764953	11	70814657	SHANK2	Body	0,211	3,89E-06	5,07E-05
cg11891887	1	200323547	LINC00862	Body	-0,242	3,89E-06	5,08E-05
cg19741107	4	140872103	MAML3	Body	-0,282	3,89E-06	5,08E-05
cg18282005	1	221881576	DUSP10	Body	-0,207	3,89E-06	5,08E-05
cg13472369	19	57678865	DUXA	TSS200	0,202	3,89E-06	5,08E-05
cg11248646	12	50645711	LIMA1	5'UTR	-0,201	3,89E-06	5,08E-05
cg15989167	10	133639339		IGR	0,207	3,90E-06	5,08E-05
cg05984244	16	31488238	TGFB1I1	Body	-0,237	3,90E-06	5,08E-05
cg10908334	9	93913894		IGR	-0,278	3,90E-06	5,08E-05
cg21072457	5	151192481		IGR	0,212	3,90E-06	5,09E-05
cg06727578	11	98438938		IGR	0,225	3,91E-06	5,09E-05
cg20663432	12	5696809	ANO2	Body	-0,213	3,91E-06	5,09E-05
cg14145194	19	10450022	ICAM3	Body	-0,285	3,91E-06	5,10E-05
cg18621299	14	100531591	EVL	TSS200	-0,219	3,91E-06	5,10E-05
cg02807047	5	65850214		IGR	-0,279	3,91E-06	5,10E-05
cg01110618	9	130853728	SLC25A25	TSS200	0,235	3,92E-06	5,10E-05
cg14122836	2	71815722	DYSF	Body	-0,256	3,92E-06	5,10E-05
cg03536993	9	80524274	GNAQ	Body	-0,213	3,92E-06	5,10E-05
cg09549949	2	240240119	HDAC4	Body	0,265	3,92E-06	5,11E-05
cg10628699	13	103531040	LOC121952	TSS1500	0,228	3,92E-06	5,11E-05
cg10419493	10	33561323	NRP1	Body	0,222	3,93E-06	5,11E-05
cg25374097	2	208939842		IGR	0,216	3,93E-06	5,12E-05
cg09739024	7	86758549		IGR	0,207	3,93E-06	5,12E-05
cg27309649	11	107583988	SLN	TSS1500	0,239	3,94E-06	5,12E-05
cg01312898	5	126097217		IGR	-0,281	3,94E-06	5,12E-05
cg08370082	3	196616876	SENP5	Body	0,23	3,94E-06	5,13E-05
cg03085275	2	172182919	METTL8	Body	-0,301	3,94E-06	5,13E-05
cg10248116	7	38272733		IGR	-0,296	3,94E-06	5,13E-05
cg01382502	16	22252504	EEF2K	Body	-0,307	3,95E-06	5,13E-05
cg14589358	2	42291824		IGR	0,212	3,95E-06	5,13E-05
cg08202226	1	153896590	GATAD2B	TSS1500	-0,267	3,95E-06	5,13E-05
cg22437335	13	49136803		IGR	-0,266	3,95E-06	5,13E-05

cg12675180	11	132730007	OPCML	Body	-0,207	3,95E-06	5,13E-05
cg22803353	1	118198949		IGR	-0,26	3,96E-06	5,14E-05
cg20026393	14	62070628	FLJ22447	Body	-0,254	3,96E-06	5,14E-05
cg07149121	4	151660960	LRBA	Body	0,256	3,96E-06	5,14E-05
cg02584306	9	112877683	PALM2-AKAP	Body	0,206	3,96E-06	5,14E-05
cg18227843	12	47610257	PCED1B-AS1	TSS200	-0,219	3,97E-06	5,15E-05
cg26427498	7	105987258		IGR	-0,237	3,97E-06	5,15E-05
cg03767003	1	230259588	GALNT2	Body	-0,231	3,97E-06	5,15E-05
cg01693697	15	74212896	LOXL1-AS1	Body	-0,255	3,97E-06	5,15E-05
cg00080816	6	160411080	IGF2R	Body	-0,247	3,97E-06	5,15E-05
cg14760640	6	10965215	SYCP2L	3'UTR	-0,224	3,97E-06	5,15E-05
cg15565987	1	89357945	GTF2B	TSS1500	0,224	3,97E-06	5,15E-05
cg14066163	17	17311866		IGR	-0,206	3,98E-06	5,16E-05
cg22903543	1	203059772		IGR	-0,212	3,98E-06	5,16E-05
cg00839850	4	87872419	AFF1	Body	0,206	3,99E-06	5,17E-05
cg12125614	17	76886835	TIMP2	Body	-0,244	3,99E-06	5,17E-05
cg00390215	9	129233076	MVB12B	Body	-0,221	3,99E-06	5,17E-05
cg01587424	8	142173149	DENND3	Body	-0,23	4,00E-06	5,18E-05
cg21206147	2	23749087	KLHL29	5'UTR	-0,248	4,00E-06	5,18E-05
cg07162704	6	91113687		IGR	0,242	4,00E-06	5,18E-05
cg09150130	20	3083101		IGR	-0,232	4,00E-06	5,18E-05
cg20322685	7	97978097	BAIAP2L1	Body	-0,245	4,00E-06	5,18E-05
cg19948701	17	75471193	sept-09	Body	-0,236	4,00E-06	5,18E-05
cg19485227	1	68216572	GNG12	5'UTR	-0,227	4,00E-06	5,18E-05
cg08407014	19	2541104	GNG7	5'UTR	0,212	4,00E-06	5,18E-05
cg03629872	7	122849941		IGR	0,237	4,00E-06	5,18E-05
cg10181698	12	58187866	TSFM	Body	-0,243	4,00E-06	5,18E-05
cg16422731	5	43711766		IGR	0,203	4,00E-06	5,18E-05
cg13549673	6	138975950		IGR	-0,253	4,01E-06	5,19E-05
cg08886988	2	46974797	SOCS5	5'UTR	-0,221	4,01E-06	5,19E-05
cg08092318	16	75625571		IGR	0,206	4,01E-06	5,19E-05
cg16536892	6	129993667	ARHGAP18	Body	-0,275	4,01E-06	5,19E-05
cg17747924	14	75988651	BATF	TSS200	-0,326	4,01E-06	5,19E-05
cg26919014	16	58061992	MMP15	Body	0,205	4,01E-06	5,19E-05
cg14776033	19	49240808	RASIP1	Body	0,249	4,02E-06	5,20E-05
cg16406967	7	27155036	HOXA3	5'UTR	0,221	4,02E-06	5,20E-05
cg14204499	8	29891282		IGR	0,215	4,03E-06	5,21E-05
cg24818060	9	101595573	GALNT12	Body	0,283	4,03E-06	5,21E-05
cg03835671	6	155475560	TIAM2	Body	-0,204	4,03E-06	5,21E-05
cg14122188	8	130282087		IGR	-0,238	4,03E-06	5,21E-05
cg24135211	14	64342282	SYNE2	5'UTR	0,267	4,03E-06	5,21E-05
cg22363909	15	75081871	MIR4513	TSS1500	-0,27	4,04E-06	5,22E-05
cg27232352	1	168332300		IGR	-0,208	4,04E-06	5,22E-05
cg25451702	2	225440982	CUL3	Body	-0,222	4,04E-06	5,22E-05
cg26698460	19	58716004	ZNF274	Body	0,21	4,04E-06	5,22E-05
cg15843975	11	59963564		IGR	-0,213	4,04E-06	5,22E-05
cg08101036	7	27153655	HOXA3	5'UTR	0,303	4,05E-06	5,23E-05
cg15823733	9	116225992	RGS3	5'UTR	0,201	4,05E-06	5,23E-05
cg14350325	16	58916375		IGR	-0,243	4,05E-06	5,23E-05
cg24127748	22	17680531	CECR1	Body	0,222	4,05E-06	5,23E-05

cg13376507	2	47432782	LOC1019270(	Body	0,246	4,05E-06	5,23E-05
cg03573679	7	2445727	CHST12	5'UTR	-0,247	4,05E-06	5,23E-05
cg00562553	7	27169740	HOXA4	1stExon	0,241	4,05E-06	5,23E-05
cg26039479	18	19137760	ESCO1	Body	0,217	4,06E-06	5,24E-05
cg23403122	16	50718139		IGR	-0,243	4,06E-06	5,24E-05
cg19961151	5	68207879		IGR	0,261	4,06E-06	5,24E-05
cg12386721	11	68265892	SAPS3	5'UTR	0,258	4,06E-06	5,24E-05
cg12941480	8	101708858		IGR	0,235	4,07E-06	5,24E-05
cg21494379	5	88275243		IGR	-0,253	4,07E-06	5,25E-05
cg05528187	9	112887616	AKAP2	TSS200	0,22	4,07E-06	5,25E-05
cg02707071	7	27143806	HOXA2	TSS1500	0,247	4,08E-06	5,25E-05
cg09947611	11	1542360	HCCA2	Body	0,256	4,08E-06	5,26E-05
cg17570354	12	27374821		IGR	-0,203	4,08E-06	5,26E-05
cg04754652	3	150921051	MED12L	Body	-0,229	4,08E-06	5,26E-05
cg06800235	1	7692367	CAMTA1	Body	-0,235	4,09E-06	5,27E-05
cg19498400	2	24308302	TP53I3	TSS1500	0,214	4,09E-06	5,27E-05
cg09089020	14	66472735		IGR	0,212	4,09E-06	5,27E-05
cg05548425	17	1553415	RILP	TSS200	0,224	4,10E-06	5,28E-05
cg10294555	11	18713331		IGR	0,28	4,11E-06	5,28E-05
cg26398636	3	42162632	TRAK1	Body	0,2	4,11E-06	5,29E-05
cg05998030	5	95965947	LOC10192971	Body	0,218	4,11E-06	5,29E-05
cg17116738	3	113334964	SIDT1	ExonBnd	0,215	4,11E-06	5,29E-05
cg08396875	2	225847045	DOCK10	Body	0,223	4,11E-06	5,29E-05
cg06246351	18	72067761		IGR	-0,262	4,11E-06	5,29E-05
cg22300949	3	35710038	ARPP21	5'UTR	-0,225	4,12E-06	5,29E-05
cg26466773	16	84211947	TAF1C	3'UTR	0,215	4,12E-06	5,29E-05
cg02928663	6	25042023	FAM65B	Body	-0,305	4,12E-06	5,29E-05
cg00220248	15	38164845		IGR	-0,209	4,12E-06	5,29E-05
cg07321742	20	8696066	PLCB1	Body	-0,217	4,12E-06	5,29E-05
cg20771596	1	247681766	GCSAML	1stExon	0,273	4,12E-06	5,29E-05
cg00098053	17	16025423	NCOR1	Body	0,207	4,12E-06	5,30E-05
cg14216068	7	27146445	HOXA3	3'UTR	0,235	4,12E-06	5,30E-05
cg05270710	9	74600025		IGR	0,206	4,12E-06	5,30E-05
cg13374172	8	56861055	LYN	Body	-0,205	4,12E-06	5,30E-05
cg10530767	17	56409028	MIR142	TSS1500	-0,242	4,13E-06	5,30E-05
cg15389995	7	24861196	OSBPL3	Body	-0,28	4,13E-06	5,30E-05
cg15514521	11	85854937		IGR	-0,204	4,13E-06	5,31E-05
cg07235562	10	14613892	FAM107B	1stExon	-0,223	4,14E-06	5,31E-05
cg08504062	5	95384662	LOC10192971	Body	0,219	4,14E-06	5,32E-05
cg27213872	2	206667827		IGR	-0,24	4,15E-06	5,32E-05
cg23359289	7	122830311	SLC13A1	Body	-0,24	4,15E-06	5,32E-05
cg08761535	16	75079000	ZNRF1	Body	0,235	4,15E-06	5,32E-05
cg07453857	3	170806983	TNIK	Body	0,203	4,15E-06	5,32E-05
cg10907866	3	42190778	TRAK1	Body	0,213	4,15E-06	5,33E-05
cg03919766	9	134608778	RAPGEF1	Body	-0,23	4,15E-06	5,33E-05
cg11985825	17	72074845		IGR	0,216	4,15E-06	5,33E-05
cg13784312	9	134609065	RAPGEF1	Body	-0,225	4,16E-06	5,33E-05
cg06051311	6	30131001	TRIM15	5'UTR	-0,2	4,16E-06	5,33E-05
cg04776469	21	26945612	MIR155	TSS1500	-0,246	4,16E-06	5,33E-05
cg23648444	3	57330391	DNAH12	Body	0,215	4,16E-06	5,33E-05

cg20781880	19	3179741	S1PR4	1stExon	-0,218	4,16E-06	5,34E-05
cg01059398	3	172235808	TNFSF10	Body	-0,23	4,17E-06	5,34E-05
cg01291088	16	67687648	RLTPR	Body	0,223	4,18E-06	5,35E-05
cg01450522	3	13465878		IGR	0,237	4,18E-06	5,35E-05
cg05343159	10	129347529	NPS	TSS200	-0,206	4,18E-06	5,35E-05
cg18928312	19	8580456	ZNF414	TSS1500	-0,221	4,18E-06	5,35E-05
cg18993359	15	39846753		IGR	0,205	4,19E-06	5,36E-05
cg24246165	2	54435033	ACYP2	Body	0,224	4,19E-06	5,36E-05
cg15049495	10	28029852	MKX	Body	0,203	4,19E-06	5,36E-05
cg23714123	10	60228239		IGR	0,22	4,19E-06	5,36E-05
cg16603374	1	6113068	KCNAB2	Body	0,216	4,19E-06	5,36E-05
cg16934684	17	49317989	MBTD1	5'UTR	0,208	4,20E-06	5,37E-05
cg17125837	11	358595		IGR	0,226	4,20E-06	5,37E-05
cg03861514	6	53883755	MLIP	5'UTR	0,222	4,20E-06	5,37E-05
cg00903577	6	28831109		IGR	-0,239	4,20E-06	5,37E-05
cg01037756	1	33565228	AZIN2	Body	-0,255	4,20E-06	5,37E-05
cg08780527	17	62232316	TEX2	ExonBnd	0,207	4,20E-06	5,37E-05
cg11909250	9	100700917	HEMGN	5'UTR	-0,271	4,20E-06	5,37E-05
cg04526498	7	3067279	CARD11	5'UTR	0,234	4,20E-06	5,37E-05
cg16340422	17	17110120	PLD6	TSS1500	-0,247	4,21E-06	5,38E-05
cg25727671	7	27193351	HOXA7	3'UTR	0,221	4,21E-06	5,38E-05
cg03662545	16	85254209		IGR	0,236	4,21E-06	5,38E-05
cg16627949	14	21526351	RNASE8	1stExon	-0,201	4,21E-06	5,38E-05
cg18722852	1	55621237	USP24	Body	0,243	4,21E-06	5,38E-05
cg13686184	21	37258354		IGR	-0,299	4,21E-06	5,38E-05
cg14591511	9	114375915		IGR	0,206	4,21E-06	5,38E-05
cg13288195	15	63889711	FBXL22	1stExon	0,245	4,22E-06	5,38E-05
cg04011795	7	37157001	ELMO1	Body	0,223	4,22E-06	5,39E-05
cg02376745	16	12184181	SNX29	Body	0,202	4,22E-06	5,39E-05
cg19184885	17	45699322	NPEPPS	3'UTR	0,231	4,23E-06	5,40E-05
cg23490275	12	23088168		IGR	0,223	4,23E-06	5,40E-05
cg08663890	1	111746277		IGR	-0,282	4,23E-06	5,40E-05
cg05224770	12	68553819	IFNG	TSS1500	-0,233	4,23E-06	5,40E-05
cg12024826	10	49893336	WDFY4	TSS200	-0,234	4,23E-06	5,40E-05
cg02167190	17	35872723	DUSP14	Body	0,225	4,24E-06	5,40E-05
cg01081456	6	123072613		IGR	-0,21	4,24E-06	5,41E-05
cg24666970	7	45447395		IGR	0,211	4,24E-06	5,41E-05
cg16325432	7	27176250		IGR	0,22	4,25E-06	5,41E-05
cg19460817	11	47575278	CUGBP1	TSS1500	0,234	4,25E-06	5,41E-05
cg11107598	13	44833178		IGR	-0,24	4,25E-06	5,42E-05
cg19195279	4	54457798	LNX1-AS2	TSS1500	-0,22	4,25E-06	5,42E-05
cg05054998	21	46378625	C21orf70	Body	0,225	4,26E-06	5,42E-05
cg20002204	19	695582	PRSS57	TSS200	-0,216	4,26E-06	5,42E-05
cg06352730	7	47621692		IGR	0,203	4,26E-06	5,42E-05
cg13453436	17	19209859	EPN2-AS1	TSS1500	-0,261	4,26E-06	5,43E-05
cg12697259	18	29674098	RNF138	Body	-0,236	4,26E-06	5,43E-05
cg25601481	6	149522472		IGR	0,243	4,26E-06	5,43E-05
cg10767970	13	45431256		IGR	-0,207	4,27E-06	5,43E-05
cg13781678	1	3103106	PRDM16	Body	0,221	4,27E-06	5,43E-05
cg08548486	1	24866714	LOC1005069[illegible]	Body	-0,222	4,27E-06	5,43E-05

cg11570752	21	44166975	PDE9A	Body	-0,219	4,27E-06	5,44E-05
cg21814905	10	13812297	FRMD4A	Body	-0,241	4,28E-06	5,44E-05
cg18830699	15	47970510	SEMA6D	5'UTR	-0,273	4,28E-06	5,45E-05
cg00286773	11	12207449	MICAL2	Body	0,264	4,28E-06	5,45E-05
cg17808440	17	602596	VPS53	Body	0,202	4,28E-06	5,45E-05
cg09863266	14	51125203	SAV1	Body	0,209	4,28E-06	5,45E-05
cg27228611	13	31144200		IGR	-0,221	4,28E-06	5,45E-05
cg04031279	15	86169244	AKAP13	Body	-0,201	4,29E-06	5,45E-05
cg02755759	9	133567787	EXOSC2	TSS1500	0,203	4,29E-06	5,45E-05
cg15718838	18	5867839		IGR	0,204	4,29E-06	5,46E-05
cg19212779	4	48777471	FRYL	5'UTR	0,208	4,30E-06	5,46E-05
cg24419550	10	71243993	TSPAN15	Body	-0,2	4,30E-06	5,47E-05
cg26859158	2	202127542	CASP8	Body	-0,228	4,30E-06	5,47E-05
cg09020840	17	73087391	SLC16A5	5'UTR	0,245	4,31E-06	5,47E-05
cg12253175	12	58132093	AGAP2	TSS200	0,241	4,31E-06	5,47E-05
cg26883434	5	111091560	C5orf13	5'UTR	0,281	4,31E-06	5,47E-05
cg03050022	15	55559759	RAB27A	5'UTR	-0,275	4,31E-06	5,47E-05
cg22480404	3	11333113	ATG7	5'UTR	-0,222	4,31E-06	5,47E-05
cg09703114	12	132515752	EP400	Body	0,267	4,31E-06	5,47E-05
cg02905876	6	161778032	PARK2	Body	-0,216	4,31E-06	5,47E-05
cg07158402	7	50413851	IKZF1	Body	-0,212	4,31E-06	5,47E-05
cg01133103	3	4765015	ITPR1	Body	-0,2	4,32E-06	5,48E-05
cg04802986	1	202255958	LGR6	Body	0,262	4,32E-06	5,48E-05
cg04787138	20	36945238	BPI	Body	-0,206	4,32E-06	5,48E-05
cg11664931	3	58335621	PXK	5'UTR	-0,28	4,32E-06	5,48E-05
cg25693497	2	1566094		IGR	0,202	4,32E-06	5,48E-05
cg19573457	22	25893657		IGR	0,214	4,33E-06	5,49E-05
cg15809837	1	186419148	PDC	TSS1500	0,239	4,33E-06	5,49E-05
cg27444700	10	24783256	KIAA1217	Body	-0,251	4,33E-06	5,49E-05
cg06507043	6	29945728	HCG9	Body	0,209	4,33E-06	5,50E-05
cg12849710	5	58952314	PDE4D	Body	-0,251	4,34E-06	5,50E-05
cg16929712	3	73024037	GXYLT2	Body	-0,203	4,34E-06	5,50E-05
cg00577167	9	104197675	ALDOB	5'UTR	0,202	4,34E-06	5,50E-05
cg23720384	21	23747055		IGR	0,246	4,35E-06	5,51E-05
cg23521905	12	129298690	SLC15A4	Body	-0,292	4,35E-06	5,51E-05
cg03331514	7	1577016	MAFK	5'UTR	0,203	4,35E-06	5,51E-05
cg23453322	4	14235703		IGR	-0,212	4,36E-06	5,52E-05
cg11484413	11	127645018		IGR	-0,239	4,36E-06	5,52E-05
cg22607706	11	13351671	ARNTL	5'UTR	0,202	4,36E-06	5,52E-05
cg09619064	2	171692781	GAD1	Body	0,217	4,37E-06	5,53E-05
cg25643123	19	49493928	GYS1	Body	0,23	4,37E-06	5,53E-05
cg24496264	15	91006854	IQGAP1	Body	0,233	4,37E-06	5,53E-05
cg04987438	20	42380335		IGR	-0,283	4,37E-06	5,53E-05
cg06832339	7	23842119	STK31	Body	0,249	4,37E-06	5,53E-05
cg13100449	1	184836205	FAM129A	Body	-0,308	4,37E-06	5,53E-05
cg21439672	12	7260546	C1RL	Body	-0,205	4,38E-06	5,54E-05
cg02643580	12	95671146	VEZT	Body	0,266	4,38E-06	5,54E-05
cg07349094	2	100759014	AFF3	1stExon	-0,26	4,38E-06	5,54E-05
cg09767076	12	124199914	ATP6V0A2	Body	-0,21	4,38E-06	5,54E-05
cg22517705	1	26645313	CD52	Body	-0,228	4,38E-06	5,54E-05

cg22834580	10	13546267	BEND7	TSS1500	-0,207	4,38E-06	5,54E-05
cg22064155	1	218716543	MIR548F3	Body	-0,211	4,40E-06	5,56E-05
cg09489900	2	64083493	UGP2	Body	-0,245	4,40E-06	5,56E-05
cg16490336	21	30451812	MAP3K7CL	5'UTR	-0,23	4,40E-06	5,56E-05
cg02767825	4	90022810		IGR	-0,209	4,41E-06	5,57E-05
cg16636885	1	159252119		IGR	0,215	4,41E-06	5,57E-05
cg22853813	6	157743451	C6orf35	Body	0,233	4,41E-06	5,57E-05
cg03498995	20	30656899	HCK	5'UTR	-0,212	4,41E-06	5,57E-05
cg19115997	4	41575976	LIMCH1	5'UTR	0,264	4,42E-06	5,57E-05
cg18500174	11	78164660	NARS2	Body	-0,26	4,42E-06	5,57E-05
cg04880990	12	52887566	KRT6A	TSS1500	-0,246	4,42E-06	5,57E-05
cg13430338	1	246997341		IGR	-0,216	4,42E-06	5,57E-05
cg04452432	16	57406511	CX3CL1	1stExon	0,216	4,42E-06	5,58E-05
cg04765589	18	61550142		IGR	0,271	4,42E-06	5,58E-05
cg08787647	3	129213480	IFT122	Body	0,222	4,42E-06	5,58E-05
cg06009834	13	44833543		IGR	-0,272	4,43E-06	5,58E-05
cg00854448	1	205288877	NUAK2	Body	-0,205	4,43E-06	5,59E-05
cg18047221	3	177167606	LINC00578	Body	-0,264	4,43E-06	5,59E-05
cg05719612	10	18629545	CACNB2	Body	0,224	4,44E-06	5,59E-05
cg07031251	8	8945555		IGR	-0,215	4,44E-06	5,60E-05
cg04424002	1	235099406	LOC10192785	Body	-0,209	4,45E-06	5,60E-05
cg18211729	18	55832105	NEDD4L	Body	-0,211	4,46E-06	5,61E-05
cg11704879	16	56932897	SLC12A3	Body	-0,205	4,46E-06	5,61E-05
cg10666909	6	32820249	TAP1	Body	-0,201	4,46E-06	5,62E-05
cg04723343	2	238647913	LRRFIP1	Body	0,22	4,46E-06	5,62E-05
cg04893733	19	31831060	TSHZ3	Body	-0,311	4,47E-06	5,62E-05
cg07881400	19	47803608		IGR	-0,227	4,47E-06	5,63E-05
cg00971789	17	66342787	ARSG	Body	-0,336	4,47E-06	5,63E-05
cg06033320	8	66750110	PDE7A	Body	-0,24	4,48E-06	5,63E-05
cg01774304	1	235133721		IGR	0,213	4,48E-06	5,63E-05
cg08088593	1	183814672	RGL1	Body	-0,287	4,49E-06	5,64E-05
cg14354844	10	108326914		IGR	0,23	4,49E-06	5,64E-05
cg26244646	17	59391924	BCAS3	Body	0,214	4,49E-06	5,65E-05
cg26069093	12	122048642		IGR	0,227	4,49E-06	5,65E-05
cg26540441	2	236120905		IGR	-0,222	4,49E-06	5,65E-05
cg07849997	1	100110875	PALMD	TSS1500	0,205	4,50E-06	5,65E-05
cg14282004	19	46270700	SIX5	Body	0,218	4,50E-06	5,65E-05
cg25064712	10	90611646	ANKRD22	1stExon	-0,206	4,50E-06	5,65E-05
cg18354714	12	54891655	NCKAP1L	TSS1500	-0,231	4,50E-06	5,65E-05
cg14610236	5	143581812	KCTD16	5'UTR	0,23	4,50E-06	5,65E-05
cg16559259	10	92810359		IGR	0,22	4,50E-06	5,65E-05
cg21486148	19	1070688	HMHA1	TSS1500	0,242	4,50E-06	5,65E-05
cg18858583	1	231954065	DISC2	Body	0,248	4,50E-06	5,66E-05
cg15696506	4	57947735	IGFBP7	Body	0,21	4,50E-06	5,66E-05
cg02901006	19	8117024	CCL25	TSS1500	-0,223	4,51E-06	5,66E-05
cg15298831	11	60754076	CD6	Body	-0,251	4,51E-06	5,66E-05
cg12654941	13	47106176		IGR	-0,207	4,51E-06	5,66E-05
cg22265430	3	30396206		IGR	0,204	4,51E-06	5,67E-05
cg18866825	5	150477744		IGR	-0,23	4,52E-06	5,67E-05
cg05641432	3	38542463	EXOG	Body	0,21	4,52E-06	5,67E-05

cg01313764	1	15400100	KAZN	Body	-0,21	4,52E-06	5,67E-05
cg27582384	1	24345233		IGR	0,26	4,52E-06	5,68E-05
cg16900185	6	119131888		IGR	-0,281	4,52E-06	5,68E-05
cg15691199	14	23589419	CEBPE	TSS1500	-0,283	4,52E-06	5,68E-05
cg03337218	10	25189866	PRTFDC1	Body	-0,235	4,53E-06	5,68E-05
cg24569082	6	45888588	CLIC5	Body	-0,26	4,53E-06	5,68E-05
cg03604842	6	53177151	ELOVL5	5'UTR	-0,227	4,53E-06	5,68E-05
cg08619842	17	66292963	ARSG	5'UTR	-0,269	4,53E-06	5,68E-05
cg25381747	10	115425899		IGR	0,225	4,53E-06	5,69E-05
cg02645135	16	69516238		IGR	0,215	4,54E-06	5,69E-05
cg00017639	15	85438982	SLC28A1	Body	-0,242	4,54E-06	5,69E-05
cg21094122	2	202298223	TRAK2	5'UTR	0,245	4,54E-06	5,69E-05
cg14688167	21	43321496	C2CD2	Body	-0,222	4,54E-06	5,69E-05
cg07655126	1	236019440	LYST	Body	-0,214	4,54E-06	5,69E-05
cg01951337	20	10762181		IGR	-0,244	4,54E-06	5,69E-05
cg07507951	1	247096025	AHCTF1	TSS1500	0,208	4,54E-06	5,69E-05
cg02895591	1	204513560	MDM4	Body	-0,226	4,55E-06	5,70E-05
cg07917052	13	51110982		IGR	-0,212	4,55E-06	5,70E-05
cg04874286	12	95477853	FGD6	Body	0,279	4,55E-06	5,70E-05
cg20546648	1	9764863	PIK3CD	5'UTR	-0,256	4,55E-06	5,70E-05
cg13739938	8	30401046	RBPMS	Body	0,217	4,55E-06	5,70E-05
cg08744986	1	183274583	NMNAT2	TSS1500	0,204	4,55E-06	5,70E-05
cg00747734	20	25014764	ACSS1	TSS1500	0,223	4,55E-06	5,70E-05
cg15575375	1	61649973	NFIA	Body	0,27	4,56E-06	5,70E-05
cg06473288	6	32820102	TAP1	Body	-0,234	4,56E-06	5,71E-05
cg23261280	2	197106919	HECW2	ExonBnd	-0,249	4,56E-06	5,71E-05
cg23821328	10	81083393		IGR	-0,294	4,56E-06	5,71E-05
cg20567570	8	98992580	MATN2	Body	-0,219	4,56E-06	5,71E-05
cg22801799	8	134072526	SLA	5'UTR	-0,279	4,57E-06	5,71E-05
cg08949143	13	30947713	LOC1001889	Body	-0,276	4,57E-06	5,71E-05
cg26787217	15	44267249	FRMD5	Body	0,225	4,57E-06	5,71E-05
cg25337907	15	68712931	ITGA11	Body	-0,202	4,57E-06	5,71E-05
cg23222446	6	63991020	LGSN	Body	0,272	4,57E-06	5,71E-05
cg08018572	16	1014592	LMF1	Body	-0,223	4,57E-06	5,72E-05
cg19470226	3	57882055	SLMAP	5'UTR	0,212	4,57E-06	5,72E-05
cg05306897	13	112282409		IGR	0,216	4,57E-06	5,72E-05
cg18661463	13	103360028		IGR	0,202	4,57E-06	5,72E-05
cg19314470	8	30401028	RBPMS	Body	0,221	4,58E-06	5,73E-05
cg05550603	17	71287339	CDC42EP4	5'UTR	-0,225	4,59E-06	5,73E-05
cg07813031	1	61298421		IGR	-0,232	4,60E-06	5,74E-05
cg00167820	8	131663355		IGR	0,284	4,60E-06	5,74E-05
cg10368128	19	57702442	ZNF264	TSS1500	0,227	4,60E-06	5,75E-05
cg00246969	13	99159656	STK24	Body	0,209	4,61E-06	5,75E-05
cg22334140	3	143550518	SLC9A9	Body	-0,223	4,61E-06	5,75E-05
cg13027299	1	86146992	ZNHIT6	Body	0,254	4,62E-06	5,76E-05
cg02895724	18	48719384	MEX3C	Body	-0,21	4,62E-06	5,76E-05
cg06134770	3	36527654	STAC	Body	-0,203	4,62E-06	5,76E-05
cg15714546	4	148031965		IGR	-0,265	4,62E-06	5,77E-05
cg06999856	10	135260170		IGR	0,235	4,63E-06	5,77E-05
cg16100244	15	93121689		IGR	-0,236	4,63E-06	5,77E-05

cg20182454	10	7662882	ITIH5	TSS1500	-0,22	4,64E-06	5,78E-05
cg10166664	5	60624229		IGR	-0,262	4,64E-06	5,78E-05
cg20806182	10	11056987	CELF2	Body	0,228	4,64E-06	5,78E-05
cg24795351	8	68863603	PREX2	TSS1500	0,258	4,65E-06	5,79E-05
cg15800420	17	47528166		IGR	-0,2	4,65E-06	5,79E-05
cg04777348	1	92952897	GFI1	TSS1500	-0,239	4,65E-06	5,79E-05
cg01820007	5	52858131	NDUFS4	Body	0,26	4,65E-06	5,79E-05
cg17148876	12	95730175		IGR	0,296	4,66E-06	5,80E-05
cg06333164	14	68752237	RAD51B	Body	-0,238	4,66E-06	5,80E-05
cg09707649	6	2340991	GMDS-AS1	Body	0,215	4,66E-06	5,80E-05
cg27100370	19	1082612	HMHA1	Body	0,218	4,66E-06	5,80E-05
cg20024393	3	33131138	GLB1	Body	-0,226	4,67E-06	5,81E-05
cg13377771	17	78398120	ENDOV	Body	-0,205	4,68E-06	5,81E-05
cg09889997	1	7692321	CAMTA1	Body	-0,231	4,68E-06	5,81E-05
cg03883520	11	65042801	POLA2	Body	0,224	4,68E-06	5,81E-05
cg19058202	5	41794804	OXCT1	Body	0,222	4,68E-06	5,81E-05
cg27304798	11	118095739	AMICA1	5'UTR	-0,21	4,68E-06	5,82E-05
cg08384379	14	73664343	PSEN1	Body	0,253	4,69E-06	5,82E-05
cg05659657	13	42972801		IGR	0,224	4,69E-06	5,82E-05
cg00292675	5	96045083	CAST	5'UTR	0,239	4,69E-06	5,83E-05
cg26433975	7	45066858	CCM2	Body	-0,241	4,69E-06	5,83E-05
cg08871701	7	38453574	AMPH	Body	-0,227	4,69E-06	5,83E-05
cg20694619	1	209929496	TRAF3IP3	TSS200	-0,203	4,69E-06	5,83E-05
cg22222999	1	234894712		IGR	-0,255	4,70E-06	5,83E-05
cg12393201	15	52428848	GNB5	Body	-0,204	4,70E-06	5,84E-05
cg00452882	16	67687119	RLTPR	Body	0,204	4,70E-06	5,84E-05
cg11466109	6	34482482	PACSIN1	TSS200	0,209	4,71E-06	5,84E-05
cg19448065	3	11703612	VGLL4	Body	0,233	4,71E-06	5,84E-05
cg16404243	17	78958777		IGR	0,224	4,71E-06	5,84E-05
cg08669954	16	1582105	IFT140	Body	0,202	4,71E-06	5,85E-05
cg05783384	2	218843735		IGR	0,253	4,71E-06	5,85E-05
cg16190128	20	14693044	MACROD2	Body	0,263	4,72E-06	5,85E-05
cg09123897	11	9774828		IGR	-0,261	4,72E-06	5,85E-05
cg21205855	8	61563866		IGR	0,23	4,72E-06	5,85E-05
cg11420667	11	1750353	HCCA2	Body	-0,244	4,73E-06	5,86E-05
cg05981711	13	53495627		IGR	-0,21	4,73E-06	5,86E-05
cg00901741	4	70999690	CSN1S2B	Body	0,203	4,73E-06	5,86E-05
cg00184907	4	124426192		IGR	0,223	4,73E-06	5,86E-05
cg02372905	18	67901983		IGR	0,217	4,73E-06	5,86E-05
cg22328644	12	109028385	SELPLG	TSS1500	-0,219	4,74E-06	5,87E-05
cg23415995	4	128704651	HSPA4L	Body	0,21	4,74E-06	5,87E-05
cg20257761	19	8521635	HNRNPM	5'UTR	0,219	4,74E-06	5,87E-05
cg19929449	2	85932339		IGR	-0,263	4,74E-06	5,87E-05
cg03062689	4	154477094	KIAA0922	Body	0,283	4,74E-06	5,87E-05
cg05692055	11	118743365		IGR	-0,216	4,75E-06	5,87E-05
cg20880354	2	135339607	TMEM163	Body	-0,212	4,75E-06	5,88E-05
cg18714582	12	32143047	KIAA1551	Body	0,204	4,75E-06	5,88E-05
cg23791011	10	124219564	HTRA1	TSS1500	-0,281	4,75E-06	5,88E-05
cg25588348	14	76334455	TTLL5	Body	0,267	4,75E-06	5,88E-05
cg16258854	2	20648194	RHOB	1stExon	0,265	4,76E-06	5,88E-05

cg01905489	1	26616534	UBXN11	Body	-0,244	4,76E-06	5,88E-05
cg12351651	10	105525816	SH3PXD2A	Body	-0,215	4,76E-06	5,88E-05
cg17697611	15	57170022		IGR	-0,278	4,76E-06	5,89E-05
cg06389019	17	72755647	SLC9A3R1	Body	-0,255	4,76E-06	5,89E-05
cg17692798	20	52489234		IGR	-0,29	4,76E-06	5,89E-05
cg07915896	15	65129816		IGR	-0,251	4,77E-06	5,89E-05
cg08344080	14	74607384	LIN52	Body	0,209	4,77E-06	5,90E-05
cg07322667	8	104349038		IGR	-0,247	4,77E-06	5,90E-05
cg09428582	12	7039396	ATN1	5'UTR	0,21	4,77E-06	5,90E-05
cg03366425	6	112572936	LAMA4	Body	0,203	4,78E-06	5,90E-05
cg05648964	12	8833355		IGR	0,201	4,78E-06	5,90E-05
cg27078890	11	128457459	ETS1	TSS200	0,281	4,78E-06	5,91E-05
cg00625298	5	64468549	ADAMTS6	Body	-0,276	4,78E-06	5,91E-05
cg19411952	8	61936514		IGR	0,24	4,79E-06	5,91E-05
cg20570458	3	124069933	KALRN	Body	-0,212	4,80E-06	5,93E-05
cg04472856	12	59197803	LOC1005068(	Body	-0,231	4,80E-06	5,93E-05
cg19321684	6	32159933	GPSM3	Body	-0,227	4,81E-06	5,93E-05
cg10661781	4	103600345	MANBA	Body	0,214	4,81E-06	5,94E-05
cg06590856	14	104042936	APOPT1	Body	0,212	4,82E-06	5,94E-05
cg25865120	15	48625058	DUT	Body	-0,203	4,82E-06	5,94E-05
cg22588023	1	24469840	IL22RA1	TSS200	0,247	4,82E-06	5,94E-05
cg01640319	13	106467378		IGR	-0,216	4,82E-06	5,94E-05
cg02366100	16	85295997		IGR	-0,23	4,82E-06	5,95E-05
cg03729676	5	71855774	LOC1025034:	Body	-0,233	4,82E-06	5,95E-05
cg09052537	1	166921447	ILDR2	Body	0,212	4,83E-06	5,95E-05
cg12684105	13	29114924		IGR	-0,206	4,84E-06	5,96E-05
cg12281219	3	193924501	LOC1005059:	TSS1500	-0,205	4,84E-06	5,96E-05
cg09754663	8	28918446		IGR	-0,268	4,84E-06	5,97E-05
cg15787438	2	161992907	TANK	TSS1500	-0,231	4,84E-06	5,97E-05
cg13076095	14	77542600	LOC1027241(	TSS200	0,215	4,85E-06	5,97E-05
cg17202840	13	43294719		IGR	-0,235	4,85E-06	5,97E-05
cg24101670	5	140748861	PCDHGB3	TSS1500	-0,235	4,85E-06	5,97E-05
cg11404831	1	147766912	NBPF8	Body	0,225	4,86E-06	5,98E-05
cg08278167	1	50641617	ELAVL4	Body	0,202	4,86E-06	5,98E-05
cg19182683	4	183730519		IGR	-0,294	4,86E-06	5,98E-05
cg08492145	7	157648386	PTPRN2	Body	0,217	4,87E-06	5,99E-05
cg25304680	5	14229352	TRIO	Body	0,207	4,87E-06	5,99E-05
cg19017553	10	34817409	PARD3	Body	-0,201	4,87E-06	5,99E-05
cg01870058	1	27668112	SYTL1	TSS1500	-0,256	4,88E-06	5,99E-05
cg24966555	5	37910554		IGR	-0,207	4,88E-06	6,00E-05
cg07897530	6	97098016		IGR	-0,201	4,88E-06	6,00E-05
cg05904364	2	20650792		IGR	0,229	4,88E-06	6,00E-05
cg20778071	6	43729725		IGR	-0,212	4,88E-06	6,00E-05
cg17754130	18	47937500		IGR	0,228	4,88E-06	6,00E-05
cg10392613	19	55836777	TMEM150B	TSS200	-0,21	4,89E-06	6,01E-05
cg17125018	12	80319514	PPP1R12A	Body	0,217	4,89E-06	6,01E-05
cg13554864	5	118666304	TNFAIP8	5'UTR	-0,232	4,89E-06	6,01E-05
cg14670793	2	102686810	IL1R1	TSS200	0,242	4,90E-06	6,02E-05
cg14112569	3	124604450	ITGB5	Body	0,215	4,91E-06	6,03E-05
cg01931766	12	109062040	CORO1C	Body	0,223	4,91E-06	6,03E-05

cg23007755	14	32979292	AKAP6	Body	0,294	4,92E-06	6,03E-05
cg25788001	4	38688565	KLF3	Body	0,241	4,92E-06	6,04E-05
cg25341032	10	111766049	ADD3	5'UTR	0,239	4,92E-06	6,04E-05
cg25440680	4	77226417	STBD1	TSS1500	-0,228	4,93E-06	6,04E-05
cg23375598	14	104662377		IGR	0,213	4,93E-06	6,04E-05
cg01871498	16	4855523	GLYR1	Body	0,205	4,93E-06	6,05E-05
cg00554631	4	155340841	DCHS2	Body	-0,205	4,93E-06	6,05E-05
cg09677663	6	130573714	SAMD3	5'UTR	0,272	4,94E-06	6,05E-05
cg26913563	5	172517927	C5orf41	Body	-0,201	4,94E-06	6,06E-05
cg07769299	10	111766297	ADD3	TSS1500	0,202	4,94E-06	6,06E-05
cg08091601	22	38248464	EIF3L	Body	0,262	4,95E-06	6,06E-05
cg11623339	5	138725482	MGC29506	1stExon	-0,213	4,95E-06	6,06E-05
cg21250756	17	36077300	HNF1B	Body	0,238	4,95E-06	6,07E-05
cg27079614	12	110442647	ANKRD13A	Body	-0,219	4,96E-06	6,07E-05
cg26632177	12	110555921		IGR	0,238	4,97E-06	6,08E-05
cg11217193	1	12538341	VPS13D	Body	0,206	4,97E-06	6,08E-05
cg06678269	12	12889835	APOLD1	Body	-0,223	4,97E-06	6,08E-05
cg27656728	18	70710710		IGR	0,207	4,98E-06	6,09E-05
cg19742736	20	439960	TBC1D20	Body	0,211	4,98E-06	6,09E-05
cg04252075	9	137716306	COL5A1	Body	-0,23	4,98E-06	6,09E-05
cg07581070	6	40291048		IGR	-0,217	4,99E-06	6,09E-05
cg09228095	2	234143023		IGR	-0,244	4,99E-06	6,10E-05
cg21592232	22	23430449	GNAZ	5'UTR	-0,264	4,99E-06	6,10E-05
cg11500952	9	135362985	C9orf171	Body	-0,246	5,00E-06	6,10E-05
cg15435155	2	3149996		IGR	0,23	5,00E-06	6,11E-05
cg24189218	15	102230227	TARSL2	Body	-0,207	5,00E-06	6,11E-05
cg26578156	16	49875061		IGR	-0,203	5,00E-06	6,11E-05
cg00763961	9	108519972	TMEM38B	Body	-0,251	5,01E-06	6,11E-05
cg16947583	2	189352810	GULP1	Body	-0,227	5,01E-06	6,11E-05
cg20684197	5	142066938	FGF1	5'UTR	-0,204	5,01E-06	6,11E-05
cg02219026	3	48282209	ZNF589	TSS1500	0,21	5,01E-06	6,11E-05
cg25622325	16	75282317	BCAR1	TSS200	0,221	5,02E-06	6,12E-05
cg25588283	6	3076008	RIPK1	TSS1500	-0,241	5,02E-06	6,12E-05
cg19390810	10	71001680	HKDC1	Body	-0,203	5,03E-06	6,13E-05
cg27188968	6	89674197	RNGTT	TSS1500	0,213	5,03E-06	6,13E-05
cg23997074	21	35897782	RCAN1	TSS200	0,234	5,03E-06	6,13E-05
cg17972013	5	9547468	SNORD123	TSS1500	0,243	5,03E-06	6,14E-05
cg05506818	16	50717778		IGR	-0,201	5,04E-06	6,14E-05
cg09071007	9	26954308	IFT74	5'UTR	-0,201	5,04E-06	6,14E-05
cg09185165	4	55898268		IGR	-0,234	5,05E-06	6,15E-05
cg13054029	3	32087017		IGR	0,225	5,06E-06	6,16E-05
cg07195224	1	159047034	AIM2	TSS1500	-0,254	5,06E-06	6,16E-05
cg13859468	5	11322414	CTNND2	Body	-0,233	5,06E-06	6,16E-05
cg00166343	17	29150100	CRLF3	Body	-0,249	5,07E-06	6,17E-05
cg13976338	5	165423657		IGR	0,251	5,07E-06	6,17E-05
cg26866325	4	74920559	PPBPL2	Body	-0,219	5,07E-06	6,17E-05
cg21911021	19	58095011	ZIK1	TSS1500	0,21	5,08E-06	6,18E-05
cg16823042	12	58119992	AGAP2	3'UTR	0,215	5,08E-06	6,18E-05
cg11530289	8	67350852	ADHFE1	Body	0,202	5,09E-06	6,18E-05
cg23954423	6	46291216	RCAN2	Body	-0,263	5,09E-06	6,18E-05

cg19324996	16	50403566	BRD7	TSS1500	0,231	5,09E-06	6,19E-05
cg14062243	17	269351		IGR	-0,24	5,09E-06	6,19E-05
cg04018779	14	38759725		IGR	0,213	5,09E-06	6,19E-05
cg04799410	3	187717711		IGR	-0,215	5,10E-06	6,20E-05
cg23821329	10	17269643	VIM	TSS1500	-0,209	5,10E-06	6,20E-05
cg20311481	20	4206624	ADRA1D	Body	0,224	5,11E-06	6,20E-05
cg14078714	9	97711748	C9orf3	Body	0,257	5,11E-06	6,21E-05
cg05967163	5	17157976	LOC285696	Body	-0,208	5,12E-06	6,21E-05
cg23352695	17	29648811	NF1	Body	-0,25	5,12E-06	6,21E-05
cg23109996	5	142023934	FGF1	TSS200	-0,209	5,12E-06	6,22E-05
cg17311022	3	119379792	POPDC2	TSS1500	0,219	5,12E-06	6,22E-05
cg02848389	4	10626874	CLNK	Body	0,217	5,12E-06	6,22E-05
cg00590230	17	71707450		IGR	-0,231	5,13E-06	6,22E-05
cg00961679	19	47458233	ARHGAP35	Body	-0,251	5,13E-06	6,22E-05
cg12156344	4	141156039		IGR	-0,222	5,14E-06	6,23E-05
cg27604873	10	111832333	ADD3	5'UTR	-0,233	5,14E-06	6,23E-05
cg13393830	19	52391605	ZNF577	TSS1500	0,241	5,14E-06	6,23E-05
cg24890964	20	36153642	BLCAP	5'UTR	-0,211	5,14E-06	6,24E-05
cg05522718	2	19740639		IGR	-0,211	5,15E-06	6,24E-05
cg03550349	3	150012001	LINC01214	Body	-0,239	5,15E-06	6,24E-05
cg09801339	14	72985417	RGS6	Body	-0,231	5,15E-06	6,24E-05
cg11633177	11	119499044		IGR	-0,204	5,16E-06	6,24E-05
cg10727316	17	65431313	PITPNC1	Body	-0,286	5,16E-06	6,24E-05
cg10969521	21	36355537	RUNX1	Body	-0,202	5,17E-06	6,26E-05
cg21620078	5	55307817		IGR	-0,283	5,17E-06	6,26E-05
cg03889877	22	47550024	TBC1D22A	Body	0,235	5,18E-06	6,26E-05
cg14346046	11	27494792	LGR4	TSS1500	0,227	5,18E-06	6,26E-05
cg23221375	15	65053605	RBPMS2	Body	0,225	5,18E-06	6,26E-05
cg00055073	2	43398118		IGR	-0,227	5,18E-06	6,27E-05
cg02010763	15	61044829	RORA	Body	0,21	5,18E-06	6,27E-05
cg03437106	1	225696324	ENAH	Body	0,23	5,19E-06	6,27E-05
cg22865470	1	113286757		IGR	0,205	5,19E-06	6,27E-05
cg00329429	3	38193513		IGR	-0,218	5,19E-06	6,27E-05
cg21655771	3	183721438	ABCC5	Body	0,216	5,20E-06	6,28E-05
cg18042427	14	86053716	FLRT2	5'UTR	-0,203	5,20E-06	6,28E-05
cg01895008	2	170370674	KLHL41	Body	0,223	5,20E-06	6,28E-05
cg14080071	8	82009570	PAG1	5'UTR	-0,219	5,21E-06	6,29E-05
cg14536372	7	25992501		IGR	0,208	5,21E-06	6,29E-05
cg08423585	20	17557271	DSTN	5'UTR	0,252	5,21E-06	6,30E-05
cg01623879	13	107559229		IGR	-0,254	5,22E-06	6,30E-05
cg13441927	10	93220988	HECTD2	Body	0,202	5,22E-06	6,30E-05
cg07093324	2	114652143	ACTR3	Body	-0,315	5,22E-06	6,30E-05
cg20036097	11	18432571	LDHC	TSS1500	-0,254	5,22E-06	6,30E-05
cg13798585	7	1980479	MAD1L1	Body	-0,244	5,23E-06	6,30E-05
cg12309938	13	98968161	FARP1	Body	0,23	5,24E-06	6,32E-05
cg22712983	2	219187374	PNKD	TSS1500	0,287	5,24E-06	6,32E-05
cg00981413	3	132296421	ACAD11	Body	0,239	5,24E-06	6,32E-05
cg12192582	7	37392880	ELMO1	5'UTR	-0,201	5,25E-06	6,33E-05
cg14737484	6	32054561	TNXB	Body	-0,217	5,26E-06	6,33E-05
cg26811313	7	1959989	MAD1L1	Body	-0,264	5,26E-06	6,33E-05

cg13207860	2	224890850	SERPINE2	Body	-0,263	5,26E-06	6,34E-05
cg18984825	20	55563146		IGR	0,273	5,27E-06	6,34E-05
cg06073139	16	58718950	SLC38A7	TSS1500	0,204	5,27E-06	6,34E-05
cg23977489	14	23083219		IGR	0,226	5,27E-06	6,34E-05
cg02841199	6	80247907	LCA5	TSS1500	0,209	5,27E-06	6,35E-05
cg21051791	6	137355264	IL20RA	Body	-0,205	5,28E-06	6,35E-05
cg05275595	19	43857874	CD177	1stExon	0,227	5,28E-06	6,36E-05
cg15408080	12	90057674		IGR	-0,221	5,28E-06	6,36E-05
cg21448352	2	23824291	KLHL29	Body	-0,26	5,29E-06	6,36E-05
cg17226349	5	157342933		IGR	0,28	5,29E-06	6,36E-05
cg01893100	3	167799377	GOLIM4	Body	0,224	5,29E-06	6,36E-05
cg10012517	5	66255015	MAST4	5'UTR	0,251	5,29E-06	6,36E-05
cg04497535	17	38459739		IGR	0,219	5,29E-06	6,36E-05
cg03795219	13	47732605		IGR	0,274	5,30E-06	6,37E-05
cg26906383	2	33260139	LTBP1	Body	0,232	5,30E-06	6,37E-05
cg18838367	15	41896680		IGR	0,22	5,30E-06	6,38E-05
cg17466952	8	22844396	RHOBTB2	TSS1500	0,203	5,30E-06	6,38E-05
cg17283266	11	111717611	ALG9	Body	0,201	5,31E-06	6,38E-05
cg05784996	18	10787643	PIEZO2	Body	-0,241	5,31E-06	6,38E-05
cg02496392	1	27916318	AHDC1	5'UTR	0,291	5,31E-06	6,38E-05
cg13288164	5	140501380	PCDHB4	TSS200	0,201	5,32E-06	6,39E-05
cg03849927	1	67396905	MIER1	5'UTR	-0,202	5,32E-06	6,39E-05
cg15761265	11	109948791		IGR	-0,231	5,32E-06	6,39E-05
cg22270593	5	169757829	LINC01366	TSS1500	-0,214	5,33E-06	6,40E-05
cg11295574	20	11830345	LINC00687	Body	0,219	5,33E-06	6,40E-05
cg19931491	11	125328008	FEZ1	Body	0,247	5,33E-06	6,40E-05
cg04898289	5	38596712	LIFR	TSS1500	-0,2	5,33E-06	6,40E-05
cg21008693	21	38290190	HLCS	Body	0,22	5,34E-06	6,41E-05
cg07790104	6	129989275	ARHGAP18	Body	0,217	5,34E-06	6,41E-05
cg13858127	13	113498128	ATP11A	Body	0,247	5,34E-06	6,41E-05
cg04072771	15	55574912	RAB27A	5'UTR	-0,265	5,35E-06	6,41E-05
cg12643710	10	18448187	CACNB2	Body	0,301	5,35E-06	6,42E-05
cg00791764	4	53727839	RASL11B	TSS1500	0,212	5,36E-06	6,42E-05
cg10632899	7	21711693	DNAH11	Body	-0,247	5,36E-06	6,42E-05
cg01088900	8	134532543	ST3GAL1	5'UTR	-0,226	5,38E-06	6,44E-05
cg15596749	1	205410562	BLACAT1	Body	-0,244	5,38E-06	6,44E-05
cg15964472	5	19038955		IGR	0,254	5,38E-06	6,44E-05
cg00048458	19	39034307	RYR1	ExonBnd	0,22	5,38E-06	6,45E-05
cg12656085	2	85173360		IGR	-0,243	5,38E-06	6,45E-05
cg01936220	13	114906016		IGR	-0,201	5,39E-06	6,45E-05
cg04609499	6	139919028		IGR	0,229	5,39E-06	6,45E-05
cg10068516	9	109678810	ZNF462	5'UTR	0,221	5,39E-06	6,45E-05
cg12492840	21	30967249	GRIK1-AS2	TSS1500	-0,302	5,39E-06	6,45E-05
cg00457087	1	162602295	DDR2	1stExon	0,21	5,39E-06	6,46E-05
cg03411865	6	166829166	RPS6KA2	Body	-0,225	5,39E-06	6,46E-05
cg14768892	15	39915931	FSIP1	Body	-0,248	5,40E-06	6,46E-05
cg11417323	1	41160271	NFYC	5'UTR	-0,217	5,40E-06	6,46E-05
cg01928794	1	151427769	POGZ	5'UTR	0,254	5,40E-06	6,47E-05
cg19276668	18	9632080		IGR	0,253	5,41E-06	6,47E-05
cg11221687	17	5083868	ZNF594	3'UTR	0,212	5,41E-06	6,47E-05

cg24023487	20	12988727	SPTLC3	TSS1500	-0,202	5,41E-06	6,47E-05
cg08976381	13	111854649	ARHGEF7	5'UTR	0,227	5,42E-06	6,48E-05
cg02063173	3	107853573	LINC01215	Body	-0,224	5,43E-06	6,49E-05
cg25298490	14	57724183	EXOC5	Body	0,216	5,43E-06	6,49E-05
cg05335806	1	55088543	FAM151A	Body	-0,214	5,44E-06	6,50E-05
cg22378524	15	61994558		IGR	0,215	5,44E-06	6,50E-05
cg18306198	11	116819384	SIK3	Body	-0,253	5,45E-06	6,51E-05
cg04783959	7	48430756	ABCA13	Body	-0,2	5,45E-06	6,51E-05
cg13086593	14	35885253		IGR	-0,226	5,46E-06	6,52E-05
cg02308359	5	128446115	ISOC1	Body	0,207	5,46E-06	6,52E-05
cg19761466	8	134072706	SLA	TSS200	-0,269	5,46E-06	6,52E-05
cg24320816	6	13372573	GFOD1	Body	0,211	5,47E-06	6,53E-05
cg23055518	8	30656033	PPP2CB	Body	0,205	5,47E-06	6,53E-05
cg19153095	8	106811143	ZFPM2	Body	-0,2	5,48E-06	6,53E-05
cg18204684	4	99859711		IGR	-0,204	5,48E-06	6,54E-05
cg23398487	13	107408915		IGR	0,207	5,48E-06	6,54E-05
cg09594622	5	118689955	TNFAIP8	TSS1500	-0,219	5,49E-06	6,54E-05
cg27584013	1	23012439		IGR	0,212	5,49E-06	6,54E-05
cg02330548	20	22598097		IGR	0,214	5,49E-06	6,54E-05
cg12737327	5	114633004	CCDC112	TSS1500	0,227	5,49E-06	6,55E-05
cg26577523	2	70270826	PCBP1-AS1	Body	-0,257	5,49E-06	6,55E-05
cg01883287	8	58744154		IGR	-0,212	5,50E-06	6,56E-05
cg23243690	12	109026944	SELPLG	TSS1500	-0,252	5,50E-06	6,56E-05
cg06949673	1	56463319		IGR	-0,206	5,51E-06	6,56E-05
cg18666104	12	109062076	CORO1C	Body	0,312	5,51E-06	6,56E-05
cg21128206	9	71409945	PIP5K1B	5'UTR	0,205	5,51E-06	6,56E-05
cg10217863	10	99188343	PGAM1	Body	-0,219	5,51E-06	6,56E-05
cg15393941	1	59408670		IGR	-0,236	5,52E-06	6,57E-05
cg04804748	15	52028433	LYSMD2	Body	-0,225	5,52E-06	6,57E-05
cg12949547	12	58718950		IGR	-0,226	5,52E-06	6,57E-05
cg19730248	1	197873880	C1orf53	Body	0,211	5,52E-06	6,57E-05
cg07388969	15	38509709		IGR	-0,218	5,54E-06	6,58E-05
cg23770814	1	90175638	LRRC8C	Body	0,224	5,55E-06	6,59E-05
cg20337696	20	44465613	SNX21	Body	0,229	5,55E-06	6,59E-05
cg11809342	2	160007430	TANC1	Body	0,204	5,55E-06	6,60E-05
cg22708897	15	39577128		IGR	-0,267	5,55E-06	6,60E-05
cg01107874	10	77165618	C10orf41	Body	0,255	5,56E-06	6,60E-05
cg25060018	15	87475309	AGBL1	Body	0,206	5,56E-06	6,60E-05
cg23071186	19	6669849	TNFSF14	Body	-0,238	5,56E-06	6,61E-05
cg11515089	17	18553533		IGR	0,21	5,56E-06	6,61E-05
cg23006040	4	188528319		IGR	-0,22	5,57E-06	6,61E-05
cg02300750	10	108575704	SORCS1	Body	-0,22	5,57E-06	6,61E-05
cg23822407	8	123981935	ZHX2	3'UTR	0,217	5,57E-06	6,62E-05
cg16148959	17	29648466	EVI2A	5'UTR	-0,23	5,57E-06	6,62E-05
cg00033440	12	47601952	PCED1B	5'UTR	-0,223	5,58E-06	6,62E-05
cg19645616	19	10444917	RAVER1	TSS1500	0,215	5,58E-06	6,63E-05
cg05290397	3	17020229	PLCL2	Body	0,207	5,58E-06	6,63E-05
cg17806020	22	40859498	MKL1	TSS200	-0,242	5,59E-06	6,63E-05
cg15681465	12	26857320	ITPR2	Body	0,227	5,59E-06	6,63E-05
cg18679236	4	178612295		IGR	-0,207	5,60E-06	6,64E-05

cg25113391	5	33825223	ADAMTS12	Body	0,235	5,60E-06	6,64E-05
cg17522588	6	53556672		IGR	0,228	5,60E-06	6,64E-05
cg17359975	6	132273157	CTGF	TSS1500	0,23	5,60E-06	6,64E-05
cg06858852	5	169706415	LCP2	Body	-0,282	5,60E-06	6,65E-05
cg25481160	3	71111489	FOXP1	Body	0,226	5,62E-06	6,66E-05
cg00002776	8	145264938	MROH1	Body	-0,238	5,63E-06	6,67E-05
cg02839658	16	10836231	NUBP1	TSS1500	0,202	5,63E-06	6,67E-05
cg20388038	18	11751315	GNAL	TSS200	0,219	5,63E-06	6,67E-05
cg20822521	14	33383070		IGR	0,218	5,63E-06	6,67E-05
cg24922884	6	100250342		IGR	0,22	5,64E-06	6,68E-05
cg15142335	1	43938212		IGR	0,229	5,64E-06	6,68E-05
cg20934731	8	4439787	CSMD1	Body	0,286	5,64E-06	6,68E-05
cg06975248	3	37125416	LRRFIP2	Body	0,209	5,65E-06	6,68E-05
cg15308565	16	84785543	USP10	Body	-0,223	5,65E-06	6,68E-05
cg20890243	7	123241798		IGR	0,225	5,65E-06	6,68E-05
cg02294690	4	17581984	LAP3	Body	-0,29	5,65E-06	6,68E-05
cg27001914	1	97215359	PTBP2	5'UTR	0,286	5,65E-06	6,69E-05
cg00347818	8	130692530	CCDC26	TSS200	-0,211	5,67E-06	6,70E-05
cg19038462	10	31715394	ZEB1	Body	0,26	5,67E-06	6,70E-05
cg00813837	13	50588620	TRIM13	3'UTR	0,265	5,67E-06	6,70E-05
cg20776362	5	75647612	SV2C	Body	-0,206	5,67E-06	6,71E-05
cg18809074	12	29394828	FAR2	5'UTR	0,213	5,69E-06	6,72E-05
cg01841762	5	156698925	CYFIP2	5'UTR	-0,204	5,69E-06	6,72E-05
cg06282956	12	133613433	ZNF84	TSS1500	0,207	5,69E-06	6,72E-05
cg09101004	18	59028139		IGR	-0,237	5,69E-06	6,72E-05
cg23494813	5	118605346	TNFAIP8	5'UTR	-0,209	5,69E-06	6,72E-05
cg12466556	22	30443509	HORMAD2-AS	Body	-0,205	5,70E-06	6,73E-05
cg13574337	16	4016720	ADCY9	Body	-0,236	5,70E-06	6,73E-05
cg17789933	4	11288456		IGR	-0,204	5,70E-06	6,73E-05
cg12251895	2	207980866	KLF7	Body	0,219	5,70E-06	6,73E-05
cg04589034	5	114717195		IGR	0,244	5,70E-06	6,73E-05
cg10753065	7	10062707		IGR	0,228	5,70E-06	6,73E-05
cg19778015	3	11625154	VGLL4	Body	0,265	5,71E-06	6,74E-05
cg19650300	4	149357960	NR3C2	Body	0,231	5,71E-06	6,74E-05
cg22432956	2	43364319		IGR	-0,284	5,71E-06	6,74E-05
cg17233422	3	66467994	LRIG1	Body	0,226	5,72E-06	6,74E-05
cg10224345	2	82461714		IGR	-0,228	5,72E-06	6,74E-05
cg02521361	2	136766281		IGR	-0,235	5,72E-06	6,75E-05
cg24180759	5	167551234	ODZ2	Body	-0,214	5,73E-06	6,76E-05
cg25373429	13	33851629	STARD13-AS	TSS200	0,241	5,74E-06	6,76E-05
cg10130476	20	36945546	BPI	Body	-0,21	5,74E-06	6,76E-05
cg18693985	5	173351052	CPEB4	Body	0,244	5,74E-06	6,76E-05
cg01495918	2	204605898		IGR	-0,203	5,76E-06	6,78E-05
cg21987515	4	153274118	FBXW7	Body	0,208	5,76E-06	6,78E-05
cg18522464	1	9253044		IGR	-0,22	5,76E-06	6,78E-05
cg14526664	8	67033824		IGR	-0,214	5,76E-06	6,78E-05
cg13434525	13	98627196	IPO5	Body	0,241	5,77E-06	6,78E-05
cg18316974	1	92947035	GFI1	Body	0,204	5,77E-06	6,79E-05
cg21026358	17	62595484	SMURF2	Body	-0,226	5,77E-06	6,79E-05
cg09543819	6	49881441		IGR	0,253	5,78E-06	6,79E-05

cg21944234	3	177234095		IGR	0,221	5,78E-06	6,79E-05
cg04991327	9	117720937		IGR	-0,26	5,78E-06	6,80E-05
cg25639280	16	31188734		IGR	-0,297	5,78E-06	6,80E-05
cg25206536	4	11369007	MIR572	TSS1500	0,207	5,78E-06	6,80E-05
cg07212067	18	25583526	CDH2	Body	0,217	5,78E-06	6,80E-05
cg19257297	2	208840029	PLEKHM3	Body	-0,202	5,80E-06	6,81E-05
cg18429317	6	150488893	PPP1R14C	Body	0,221	5,80E-06	6,81E-05
cg09753632	5	95413861	MIR583	TSS1500	0,248	5,80E-06	6,81E-05
cg00832811	5	154346390	MRPL22	Body	0,204	5,80E-06	6,81E-05
cg20363271	8	55181528		IGR	-0,205	5,80E-06	6,82E-05
cg07977170	18	54456700	WDR7	Body	0,235	5,81E-06	6,82E-05
cg08961985	11	103886604	PDGFD	Body	-0,208	5,81E-06	6,82E-05
cg04803214	19	42278668		IGR	-0,207	5,81E-06	6,82E-05
cg11412129	1	88928839		IGR	0,228	5,82E-06	6,83E-05
cg07028929	13	60197945		IGR	-0,215	5,82E-06	6,83E-05
cg12359995	11	33193082	CSTF3-AS1	Body	0,258	5,82E-06	6,83E-05
cg21543918	6	138893159	NHSL1	1stExon	0,221	5,82E-06	6,83E-05
cg07150062	14	104552032	ASPG	TSS200	0,207	5,82E-06	6,83E-05
cg00923927	18	60960289	BCL2	Body	-0,202	5,83E-06	6,83E-05
cg19814032	10	50415394		IGR	-0,295	5,83E-06	6,83E-05
cg07696673	7	122184594	CADPS2	Body	-0,212	5,83E-06	6,84E-05
cg06034014	16	82671807	CDH13	5'UTR	0,263	5,83E-06	6,84E-05
cg20207327	3	30512797		IGR	0,24	5,83E-06	6,84E-05
cg06793565	1	184790782	FAM129A	Body	0,295	5,83E-06	6,84E-05
cg26362417	11	69317541		IGR	0,203	5,84E-06	6,84E-05
cg02284278	1	110777870		IGR	0,223	5,84E-06	6,85E-05
cg04787166	8	68738438		IGR	-0,268	5,84E-06	6,85E-05
cg17974185	20	36323340	CTNNBL1	Body	0,265	5,84E-06	6,85E-05
cg06611720	1	156569655	GPATCH4	5'UTR	-0,23	5,86E-06	6,86E-05
cg10372188	11	117748169	FXYD6	TSS1500	0,213	5,86E-06	6,86E-05
cg01894614	18	67565361	CD226	Body	-0,204	5,86E-06	6,87E-05
cg05747963	19	7492066	ARHGEF18	5'UTR	-0,211	5,86E-06	6,87E-05
cg18628646	4	184908739	STOX2	Body	0,296	5,86E-06	6,87E-05
cg14796658	22	18429965	MICAL3	5'UTR	0,225	5,87E-06	6,88E-05
cg02139485	14	93215447	LGMN	TSS1500	0,228	5,88E-06	6,89E-05
cg23669081	17	46685353	HOXB7	Body	0,223	5,88E-06	6,89E-05
cg10437842	15	42213760	EHD4	Body	0,202	5,88E-06	6,89E-05
cg27315111	3	186157146		IGR	0,217	5,89E-06	6,89E-05
cg10686502	7	43556694	LOC10050689	Body	-0,223	5,89E-06	6,89E-05
cg22887597	9	97788247	C9orf3	Body	0,205	5,89E-06	6,90E-05
cg08687322	2	144019668	ARHGAP15	Body	-0,249	5,90E-06	6,90E-05
cg18809126	3	11623526	VGLL4	Body	0,202	5,90E-06	6,90E-05
cg05109569	7	27146430	HOXA3	3'UTR	0,242	5,90E-06	6,91E-05
cg10006314	2	134777581		IGR	0,212	5,90E-06	6,91E-05
cg08944961	12	111537195	CUX2	Body	-0,243	5,91E-06	6,91E-05
cg06169746	3	119419493		IGR	-0,309	5,91E-06	6,91E-05
cg11193799	2	74304989	TET3	Body	0,22	5,91E-06	6,91E-05
cg10104480	16	72955250	ZFHX3	Body	-0,215	5,92E-06	6,92E-05
cg01860774	14	64969374	ZBTB25	5'UTR	-0,211	5,92E-06	6,92E-05
cg25430205	6	72129198	LINC00472	Body	0,221	5,92E-06	6,92E-05

cg00577905	12	12659359	DUSP16	Body	0,221	5,93E-06	6,93E-05
cg15112076	7	20856285		IGR	0,222	5,93E-06	6,93E-05
cg21715189	2	38304802	CYP1B1	TSS1500	0,208	5,94E-06	6,94E-05
cg04657000	6	112115430	FYN	5'UTR	-0,207	5,94E-06	6,94E-05
cg03124507	2	43361267		IGR	-0,234	5,95E-06	6,94E-05
cg24528851	6	109158951		IGR	-0,276	5,95E-06	6,95E-05
cg09802688	1	209942066	TRAF3IP3	Body	-0,29	5,96E-06	6,95E-05
cg13675621	17	75673452		IGR	0,209	5,97E-06	6,96E-05
cg24452392	2	204732461	CTLA4	TSS200	-0,223	5,97E-06	6,96E-05
cg01679611	8	121744480	SNTB1	Body	-0,234	5,97E-06	6,97E-05
cg06742794	12	14576011	ATF7IP	5'UTR	0,203	5,98E-06	6,97E-05
cg06155414	13	97893311	MBNL2	5'UTR	0,209	5,98E-06	6,97E-05
cg14451791	9	16040625		IGR	-0,212	5,98E-06	6,97E-05
cg04314986	13	107569408		IGR	0,252	5,98E-06	6,97E-05
cg10337088	16	83946845	MLYCD	Body	0,232	5,98E-06	6,98E-05
cg05008070	14	59106662	DACT1	Body	0,283	5,99E-06	6,98E-05
cg03850957	20	16713359	SNRPB2	Body	-0,202	5,99E-06	6,98E-05
cg12524318	17	269342		IGR	-0,252	5,99E-06	6,98E-05
cg15331609	13	26422660	ATP8A2	Body	-0,227	6,00E-06	6,99E-05
cg09530779	3	112218257	BTLA	1stExon	-0,249	6,01E-06	6,99E-05
cg19590895	15	40632399	C15orf52	Body	0,202	6,01E-06	6,99E-05
cg01052291	6	133036573	VNN1	TSS1500	0,249	6,01E-06	7,00E-05
cg10576392	16	65759562		IGR	0,227	6,01E-06	7,00E-05
cg02844810	1	208345500	PLXNA2	Body	-0,256	6,03E-06	7,01E-05
cg12200215	3	57881959	SLMAP	5'UTR	0,225	6,03E-06	7,02E-05
cg08599446	12	96571208		IGR	-0,261	6,03E-06	7,02E-05
cg20023762	6	11224219	NEDD9	Body	0,217	6,03E-06	7,02E-05
cg10441250	4	53708510		IGR	0,217	6,03E-06	7,02E-05
cg17970299	12	54772804	ZNF385A	Body	0,32	6,03E-06	7,02E-05
cg04596499	8	42213370	POLB	Body	-0,245	6,03E-06	7,02E-05
cg21159568	7	2445331	CHST12	5'UTR	-0,211	6,04E-06	7,02E-05
cg13575798	7	127641221	SND1	Body	-0,231	6,04E-06	7,03E-05
cg09201221	5	112358411	MCC	3'UTR	-0,236	6,05E-06	7,03E-05
cg12851638	3	71707392		IGR	0,229	6,05E-06	7,04E-05
cg06607764	17	76719635	CYTH1	Body	0,213	6,06E-06	7,04E-05
cg26691434	16	29675160	SPN	Body	-0,227	6,06E-06	7,04E-05
cg26724018	11	5716255	TRIM22	5'UTR	-0,232	6,07E-06	7,05E-05
cg02562540	13	49684114	FNDC3A	TSS1500	0,224	6,07E-06	7,05E-05
cg00452034	12	65108408	GNS	3'UTR	-0,23	6,09E-06	7,07E-05
cg22520587	6	129829269	LAMA2	Body	0,214	6,09E-06	7,07E-05
cg19543867	7	112124979	C7orf53	Body	0,357	6,09E-06	7,07E-05
cg03922814	15	90395025	MIR5094	TSS1500	-0,202	6,09E-06	7,07E-05
cg20101398	6	114384045	HS3ST5	TSS200	0,213	6,09E-06	7,07E-05
cg02670123	2	173330395	ITGA6	Body	0,201	6,10E-06	7,07E-05
cg08145377	20	22327527		IGR	-0,219	6,11E-06	7,09E-05
cg11820377	2	187460089	ITGAV	Body	0,244	6,12E-06	7,09E-05
cg13848363	1	206749188	RASSF5	Body	-0,239	6,13E-06	7,10E-05
cg05545915	20	52226492		IGR	-0,211	6,14E-06	7,11E-05
cg23092565	8	73998859	SBSPON	Body	-0,202	6,14E-06	7,11E-05
cg11231949	8	63161616	NKAIN3	1stExon	0,273	6,16E-06	7,13E-05

cg16853390	9	21832180	MTAP	Body	-0,267	6,16E-06	7,13E-05
cg12888725	14	85983384		IGR	0,21	6,17E-06	7,13E-05
cg20827128	6	13274284	PHACTR1	Body	-0,23	6,17E-06	7,14E-05
cg02616418	4	154125681	TRIM2	5'UTR	0,202	6,17E-06	7,14E-05
cg06889370	4	102181304	PPP3CA	Body	-0,201	6,18E-06	7,14E-05
cg20214179	5	49998639	PARP8	Body	-0,249	6,18E-06	7,14E-05
cg02786073	11	68625231		IGR	0,232	6,18E-06	7,14E-05
cg12654340	14	91247576	TTC7B	Body	-0,226	6,19E-06	7,15E-05
cg27021709	15	81433929	C15orf26	Body	-0,232	6,20E-06	7,16E-05
cg03977303	20	30628112		IGR	-0,239	6,20E-06	7,16E-05
cg03131724	11	58731433		IGR	0,214	6,20E-06	7,16E-05
cg05352016	2	202873789		IGR	0,206	6,21E-06	7,17E-05
cg00142933	2	128420784	LIMS2	Body	0,217	6,21E-06	7,17E-05
cg22579471	5	55199404	IL31RA	Body	-0,211	6,22E-06	7,18E-05
cg07955762	13	111074260	COL4A2	Body	-0,265	6,23E-06	7,18E-05
cg26407316	14	50801745	CDKL1	Body	0,224	6,23E-06	7,18E-05
cg22757535	2	237418897		IGR	-0,269	6,24E-06	7,19E-05
cg20613889	2	49381495	FSHR	1stExon	0,212	6,24E-06	7,20E-05
cg20945560	9	20362156	MLLT3	Body	-0,204	6,24E-06	7,20E-05
cg24870415	11	6121200		IGR	-0,245	6,25E-06	7,20E-05
cg26195495	8	1906338	ARHGEF10	3'UTR	0,209	6,25E-06	7,20E-05
cg17344223	3	129295781	PLXND1	Body	-0,206	6,25E-06	7,20E-05
cg26288692	1	120230026		IGR	-0,202	6,26E-06	7,21E-05
cg20631820	13	95086158		IGR	0,248	6,26E-06	7,21E-05
cg07933297	19	45879792		IGR	0,205	6,27E-06	7,22E-05
cg14963724	18	72166303	CNDP2	TSS1500	-0,244	6,28E-06	7,23E-05
cg00607627	16	28995994	LAT	TSS200	-0,243	6,28E-06	7,23E-05
cg21815742	8	29035023	KIF13B	Body	0,209	6,28E-06	7,23E-05
cg09536315	2	179729091	CCDC141	Body	0,231	6,28E-06	7,23E-05
cg23238315	7	156259194		IGR	0,225	6,29E-06	7,23E-05
cg17148532	18	10483580	APCDD1	Body	-0,273	6,30E-06	7,24E-05
cg03879808	2	69140146		IGR	-0,231	6,30E-06	7,25E-05
cg25569396	6	11382769	NEDD9	TSS200	-0,246	6,31E-06	7,25E-05
cg14093293	6	151910755	CCDC170	Body	0,248	6,31E-06	7,25E-05
cg04828580	10	93639778		IGR	0,235	6,32E-06	7,26E-05
cg23004599	4	126656451		IGR	0,223	6,32E-06	7,26E-05
cg25817165	18	72167213	CNDP2	1stExon	-0,348	6,33E-06	7,27E-05
cg07670068	8	142457310	MROH5	Body	-0,527	6,34E-06	7,27E-05
cg26751588	17	14109671	COX10	Body	-0,222	6,35E-06	7,28E-05
cg21581865	1	32782332	HDAC1	Body	0,298	6,36E-06	7,29E-05
cg18860868	12	117588951	FBXO21	Body	0,222	6,37E-06	7,30E-05
cg20997662	9	6412392	UHRF2	TSS1500	0,204	6,38E-06	7,30E-05
cg10081621	15	94808566		IGR	0,229	6,38E-06	7,31E-05
cg20790207	4	160169851		IGR	0,251	6,38E-06	7,31E-05
cg27154217	5	50023127	PARP8	Body	-0,234	6,38E-06	7,31E-05
cg00070635	1	46358168	MAST2	Body	0,351	6,39E-06	7,31E-05
cg20245379	18	20887297	TMEM241	Body	-0,297	6,40E-06	7,32E-05
cg01470839	1	198625128	PTPRC	Body	-0,216	6,40E-06	7,32E-05
cg22073530	8	39771450	IDO1	1stExon	-0,256	6,40E-06	7,32E-05
cg01561780	9	101253517	GABBR2	Body	-0,228	6,40E-06	7,32E-05

cg12536660	11	12246400	MICAL2	Body	-0,236	6,40E-06	7,32E-05
cg22691687	8	134073176	SLA	TSS1500	-0,237	6,40E-06	7,33E-05
cg06001396	14	55603957	LGALS3	5'UTR	0,214	6,40E-06	7,33E-05
cg00437441	9	92024239	SEMA4D	5'UTR	-0,208	6,41E-06	7,33E-05
cg13340947	12	40229749	SLC2A13	Body	-0,213	6,41E-06	7,33E-05
cg03788242	11	45070809		IGR	0,225	6,41E-06	7,34E-05
cg08517455	2	99439997	C2orf55	Body	0,27	6,42E-06	7,34E-05
cg18573082	6	136425179	PDE7B	Body	0,222	6,42E-06	7,34E-05
cg25041035	9	140008039	DPP7	Body	-0,202	6,42E-06	7,34E-05
cg06806788	16	86665450		IGR	-0,208	6,43E-06	7,35E-05
cg19941257	9	18824985	ADAMTSL1	Body	0,217	6,44E-06	7,36E-05
cg06485332	2	152945945	CACNB4	Body	-0,211	6,44E-06	7,36E-05
cg02684208	8	130814219		IGR	0,215	6,45E-06	7,37E-05
cg11021321	20	57471660	GNAS	Body	0,202	6,46E-06	7,37E-05
cg01039128	11	12436394	PARVA	Body	-0,216	6,46E-06	7,37E-05
cg18393717	10	17552431		IGR	-0,201	6,46E-06	7,37E-05
cg04942605	22	37699966	CYTH4	Body	-0,242	6,46E-06	7,37E-05
cg12710531	11	88032774	CTSC	Body	0,21	6,46E-06	7,38E-05
cg04071964	3	100328745	GPR128	1stExon	-0,221	6,47E-06	7,38E-05
cg10861135	9	14345348		IGR	-0,216	6,47E-06	7,38E-05
cg00870689	11	109855902		IGR	-0,216	6,49E-06	7,40E-05
cg01318963	10	98339870	TM9SF3	Body	0,214	6,50E-06	7,40E-05
cg01420103	5	94237541	MCTP1	Body	-0,201	6,51E-06	7,41E-05
cg11408613	2	28959117		IGR	0,208	6,51E-06	7,41E-05
cg21655900	11	33731253	CD59	3'UTR	0,201	6,51E-06	7,41E-05
cg03213322	5	160039911	ATP10B	Body	0,229	6,51E-06	7,41E-05
cg26605006	16	30197364	CORO1A	Body	-0,266	6,51E-06	7,42E-05
cg10939445	2	238334022		IGR	0,213	6,52E-06	7,42E-05
cg05089197	6	11194815	NEDD9	Body	0,222	6,52E-06	7,43E-05
cg09285543	14	101296134	MEG3	Body	-0,215	6,52E-06	7,43E-05
cg19373778	1	9354516	SPSB1	5'UTR	-0,244	6,53E-06	7,43E-05
cg27341789	4	37667175	RELL1	Body	0,22	6,53E-06	7,43E-05
cg08103988	17	6558365		IGR	0,254	6,54E-06	7,44E-05
cg04139396	15	82420926		IGR	-0,284	6,54E-06	7,45E-05
cg15348550	10	12163898	DHTKD1	3'UTR	-0,207	6,55E-06	7,45E-05
cg12306427	6	13428245	GFOD1	Body	-0,214	6,55E-06	7,45E-05
cg00027619	2	46948734	SOCS5	5'UTR	0,217	6,55E-06	7,45E-05
cg24111443	15	40583709	PLCB2	Body	0,231	6,56E-06	7,46E-05
cg20407708	3	171816827	FNDC3B	5'UTR	0,266	6,56E-06	7,46E-05
cg06485521	6	53530600	KLHL31	TSS200	0,256	6,57E-06	7,46E-05
cg16509173	11	8781014	ST5	5'UTR	-0,225	6,57E-06	7,47E-05
cg03883098	1	26398992		IGR	0,229	6,57E-06	7,47E-05
cg06918449	12	23405060		IGR	-0,216	6,57E-06	7,47E-05
cg12294118	2	202879585		IGR	0,219	6,58E-06	7,47E-05
cg15303062	2	236328380		IGR	-0,203	6,58E-06	7,48E-05
cg17498346	12	10349040		IGR	-0,226	6,59E-06	7,48E-05
cg26504529	5	149999033	SYNPO	Body	0,205	6,59E-06	7,48E-05
cg14816594	3	141623203	ATP1B3	Body	-0,2	6,59E-06	7,49E-05
cg08135617	1	92317088	TGFBR3	Body	0,205	6,60E-06	7,49E-05
cg10163536	4	174845485		IGR	-0,2	6,60E-06	7,49E-05

cg23641658	14	103864655	MARK3	Body	0,217	6,61E-06	7,50E-05
cg11345703	11	61338462	SYT7	Body	-0,206	6,61E-06	7,50E-05
cg05914005	1	10992446		IGR	-0,245	6,61E-06	7,50E-05
cg19827883	9	100755480	ANP32B	Body	-0,247	6,61E-06	7,50E-05
cg14928672	16	74291175		IGR	-0,222	6,62E-06	7,51E-05
cg20768919	8	96248248		IGR	-0,207	6,62E-06	7,51E-05
cg10397389	4	100738011	DAPP1	1stExon	-0,233	6,62E-06	7,51E-05
cg15433588	4	100763828	DAPP1	Body	0,23	6,62E-06	7,51E-05
cg16542345	11	19213578	CSRP3	Body	0,211	6,64E-06	7,53E-05
cg07939845	10	3182623	PITRM1-AS1	TSS1500	0,26	6,64E-06	7,53E-05
cg13114095	17	46652532	HOXB3	TSS1500	0,252	6,65E-06	7,53E-05
cg18263988	3	111582491	PHLDB2	5'UTR	0,206	6,65E-06	7,53E-05
cg07626206	7	55200657	EGFR	Body	-0,278	6,65E-06	7,53E-05
cg12232222	13	98825268	FARP1	5'UTR	-0,205	6,66E-06	7,54E-05
cg06373086	20	48658164	TRERNA1	Body	-0,205	6,68E-06	7,56E-05
cg09300932	12	2081583	DCP1B	Body	0,222	6,68E-06	7,56E-05
cg03874542	10	8338503		IGR	0,221	6,69E-06	7,56E-05
cg21487856	2	54828502	SPTBN1	Body	0,221	6,69E-06	7,57E-05
cg01512626	17	29982200		IGR	-0,215	6,69E-06	7,57E-05
cg04998805	12	15115389	ARHGDIB	TSS1500	-0,219	6,71E-06	7,58E-05
cg00043234	15	80324492		IGR	-0,21	6,71E-06	7,58E-05
cg26187205	3	159659697		IGR	-0,227	6,71E-06	7,58E-05
cg02269207	12	120433323	CCDC64	Body	0,216	6,71E-06	7,59E-05
cg04778178	7	27162294	HOXA3	5'UTR	0,237	6,71E-06	7,59E-05
cg01872544	4	57985260	IGFBP7-AS1	Body	0,21	6,72E-06	7,59E-05
cg01211137	11	13993807	SPON1	Body	0,25	6,73E-06	7,60E-05
cg14747453	1	40063592		IGR	-0,272	6,73E-06	7,60E-05
cg07832674	3	9943884	IL17RE	TSS1500	-0,204	6,73E-06	7,60E-05
cg14166756	15	90894880	ZNF774	TSS1500	0,207	6,74E-06	7,61E-05
cg17624315	2	202289200	TRAK2	5'UTR	0,226	6,74E-06	7,61E-05
cg07584861	3	11551341	ATG7	Body	0,218	6,74E-06	7,61E-05
cg10657837	3	136604874	NCK1	5'UTR	-0,234	6,75E-06	7,62E-05
cg25491704	6	33048879	HLA-DPB1	Body	0,21	6,75E-06	7,62E-05
cg01046382	2	169658950	NOSTRIN	TSS200	0,224	6,76E-06	7,62E-05
cg24835232	6	24165957		IGR	-0,21	6,76E-06	7,63E-05
cg05913211	8	66864146		IGR	-0,268	6,76E-06	7,63E-05
cg06820782	11	83800924	DLG2	Body	0,208	6,77E-06	7,63E-05
cg02676457	12	93381925		IGR	-0,247	6,77E-06	7,63E-05
cg11483865	8	38643342	TACC1	TSS1500	0,236	6,77E-06	7,63E-05
cg06135810	22	43042002	CYB5R3	TSS1500	0,201	6,77E-06	7,64E-05
cg25496764	17	78735398	RPTOR	Body	-0,255	6,78E-06	7,64E-05
cg27019749	7	112635781	HRAT17	TSS200	0,226	6,79E-06	7,65E-05
cg10051213	9	456725	DOCK8	Body	0,236	6,79E-06	7,65E-05
cg12264517	5	66255020	MAST4	5'UTR	0,248	6,79E-06	7,65E-05
cg13442319	10	58356579		IGR	0,203	6,79E-06	7,65E-05
cg13635516	20	31123396		IGR	-0,26	6,80E-06	7,66E-05
cg03693012	10	95715050		IGR	-0,208	6,81E-06	7,67E-05
cg16417028	1	207070825	IL24	1stExon	-0,201	6,82E-06	7,67E-05
cg22388683	5	169763426		IGR	-0,282	6,82E-06	7,67E-05
cg00037681	12	47610371	FAM113B	5'UTR	-0,256	6,82E-06	7,67E-05

cg10881225	2	9984929	TAF1B	Body	-0,234	6,83E-06	7,69E-05
cg11166453	1	247681781		IGR	0,242	6,84E-06	7,69E-05
cg04255037	12	95034515	TMCC3	Body	-0,277	6,84E-06	7,69E-05
cg02846651	1	61546179	NFIA	TSS1500	0,209	6,86E-06	7,71E-05
cg24448003	4	151153475	DCLK2	ExonBnd	0,239	6,87E-06	7,72E-05
cg05492387	4	99298930	RAP1GDS1	Body	-0,255	6,88E-06	7,72E-05
cg27002794	2	160474347	LOC643072	Body	0,253	6,88E-06	7,72E-05
cg17575906	5	111090585	C5orf13	Body	0,212	6,88E-06	7,72E-05
cg01072003	6	135828046	LINC00271	Body	-0,26	6,88E-06	7,72E-05
cg14484844	9	139590683		IGR	-0,213	6,88E-06	7,73E-05
cg00253248	17	48948878		IGR	-0,233	6,89E-06	7,73E-05
cg06341731	14	99641017	BCL11B	Body	0,207	6,89E-06	7,73E-05
cg21804327	12	53496729	SOAT2	TSS1500	-0,25	6,89E-06	7,73E-05
cg09114848	13	99930686	UBAC2	Body	-0,234	6,89E-06	7,73E-05
cg10551374	12	95818059		IGR	0,244	6,89E-06	7,74E-05
cg20018022	17	35851415	DUSP14	5'UTR	0,21	6,90E-06	7,74E-05
cg24239418	3	52278436	PPM1M	TSS1500	-0,228	6,90E-06	7,74E-05
cg16243532	3	138617437		IGR	0,26	6,91E-06	7,74E-05
cg21455600	7	66096806	KCTD7	Body	-0,253	6,92E-06	7,76E-05
cg07591518	18	60119756		IGR	-0,259	6,93E-06	7,77E-05
cg24584488	6	88618959	LOC10192891	Body	0,217	6,94E-06	7,77E-05
cg02292367	6	157112830	ARID1B	Body	0,208	6,94E-06	7,77E-05
cg24358781	1	178067721	RASAL2	Body	0,217	6,95E-06	7,78E-05
cg11949335	1	55247408	TTC22	Body	0,288	6,96E-06	7,79E-05
cg01565209	9	122696951		IGR	0,234	6,97E-06	7,80E-05
cg03178823	4	3033304	GRK4	Body	0,225	6,98E-06	7,80E-05
cg26083537	5	38541503	LIFR	5'UTR	-0,242	6,98E-06	7,81E-05
cg12524553	20	32952045	ITCH	5'UTR	0,203	6,98E-06	7,81E-05
cg14227558	2	213983851	IKZF2	Body	-0,23	7,00E-06	7,82E-05
cg06401532	16	24220008	PRKCB	Body	0,23	7,00E-06	7,82E-05
cg01631215	3	67706047	SUCLG2	TSS1500	-0,374	7,00E-06	7,82E-05
cg02171814	17	1645925	SERPINF2	TSS1500	-0,245	7,01E-06	7,83E-05
cg10518811	2	183008922	PDE1A	3'UTR	0,291	7,01E-06	7,83E-05
cg12302880	12	57671076	R3HDM2	Body	0,223	7,01E-06	7,83E-05
cg05810129	10	14597004	FAM107B	Body	-0,218	7,02E-06	7,84E-05
cg18792031	8	94574750		IGR	0,201	7,02E-06	7,84E-05
cg00849854	17	17780937	TOM1L2	Body	0,232	7,03E-06	7,85E-05
cg26395478	1	209929437	TRAF3IP3	1stExon	-0,262	7,04E-06	7,86E-05
cg02390801	15	74528565	CCDC33	TSS200	-0,204	7,05E-06	7,86E-05
cg03484312	1	49216204	BEND5	Body	-0,228	7,06E-06	7,87E-05
cg12410597	1	199132660		IGR	0,231	7,06E-06	7,87E-05
cg05046784	19	14570558	PKN1	Body	-0,213	7,06E-06	7,87E-05
cg08777963	4	83934806	LIN54	TSS1500	0,209	7,06E-06	7,87E-05
cg17708080	10	112186035		IGR	-0,218	7,07E-06	7,88E-05
cg18335117	3	7588182	GRM7	Body	0,204	7,08E-06	7,88E-05
cg06847417	7	144515907	TPK1	Body	-0,24	7,10E-06	7,91E-05
cg19224759	6	144619004	UTRN	Body	0,261	7,11E-06	7,91E-05
cg04099876	2	9232531		IGR	-0,206	7,12E-06	7,92E-05
cg11855643	1	184473341	C1orf21	Body	0,203	7,13E-06	7,93E-05
cg06437892	15	43387093	UBR1	Body	0,206	7,13E-06	7,93E-05

cg02976723	6	3825927		IGR	0,223	7,14E-06	7,94E-05
cg08924131	22	43438584	LOC10050667	Body	-0,36	7,14E-06	7,94E-05
cg21471231	10	80010338	LINC00856	Body	-0,251	7,14E-06	7,94E-05
cg02717112	4	160149377		IGR	0,251	7,14E-06	7,94E-05
cg23873580	12	96133946	NTN4	Body	0,214	7,15E-06	7,94E-05
cg06717633	14	30194536	PRKD1	Body	-0,24	7,15E-06	7,95E-05
cg02987264	21	43482721	UMODL1	TSS1500	-0,212	7,15E-06	7,95E-05
cg12284093	21	32535242	TIAM1	Body	0,239	7,16E-06	7,95E-05
cg21085744	12	27020210		IGR	-0,202	7,16E-06	7,95E-05
cg15440524	3	177534482	KCCAT211	TSS200	0,231	7,17E-06	7,97E-05
cg12823025	16	53490806	RBL2	Body	-0,284	7,17E-06	7,97E-05
cg07010192	6	114056449		IGR	-0,273	7,18E-06	7,97E-05
cg19307003	11	60775715	CD6	Body	0,278	7,18E-06	7,97E-05
cg13930375	10	6558770	PRKCQ	5'UTR	0,2	7,18E-06	7,97E-05
cg09341491	16	50707795	SNX20	Body	0,222	7,19E-06	7,98E-05
cg03457445	20	47374685	PREX1	Body	-0,24	7,19E-06	7,98E-05
cg16852369	8	1843398	ARHGEF10	Body	-0,233	7,19E-06	7,98E-05
cg23209432	21	33105773	SCAF4	TSS1500	-0,205	7,19E-06	7,98E-05
cg15934389	6	35362046	PPARD	5'UTR	-0,207	7,19E-06	7,98E-05
cg22368847	10	63843925	ARID5B	Body	0,26	7,20E-06	7,99E-05
cg07547492	4	89512588	HERC3	TSS1500	0,211	7,20E-06	7,99E-05
cg06438216	11	10746826		IGR	-0,204	7,21E-06	8,00E-05
cg13669152	6	130923610		IGR	-0,221	7,22E-06	8,01E-05
cg06566100	18	25184831		IGR	0,231	7,22E-06	8,01E-05
cg25260906	5	15073606		IGR	-0,203	7,23E-06	8,01E-05
cg05710777	2	65089821	LOC10192743	Body	-0,23	7,23E-06	8,02E-05
cg12497360	12	58135235	AGAP2	Body	-0,236	7,23E-06	8,02E-05
cg12337227	2	225876576	MIR4439	TSS1500	0,218	7,24E-06	8,02E-05
cg22275629	1	212475117	PPP2R5A	TSS200	0,245	7,24E-06	8,02E-05
cg05218090	4	182230345		IGR	0,222	7,24E-06	8,02E-05
cg04415269	22	18269958		IGR	0,206	7,24E-06	8,02E-05
cg08840164	10	17551207		IGR	-0,228	7,25E-06	8,02E-05
cg15384400	4	36257279		IGR	-0,209	7,25E-06	8,03E-05
cg19045353	9	37954074	SHB	Body	-0,21	7,25E-06	8,03E-05
cg19128982	4	90431081		IGR	-0,224	7,25E-06	8,03E-05
cg00372249	4	185745711	ACSL1	5'UTR	-0,207	7,26E-06	8,03E-05
cg01076874	19	39797966	LRFN1	3'UTR	0,278	7,26E-06	8,04E-05
cg23729101	5	55059745	DDX4	Body	-0,2	7,26E-06	8,04E-05
cg17791799	4	16036123	PROM1	Body	0,204	7,27E-06	8,04E-05
cg05974249	17	64229272		IGR	0,219	7,27E-06	8,05E-05
cg19498595	4	159957085	C4orf45	TSS1500	-0,227	7,27E-06	8,05E-05
cg05807666	8	121769788	SNTB1	Body	-0,229	7,27E-06	8,05E-05
cg22518417	16	67192409	FBXL8	TSS1500	-0,243	7,28E-06	8,05E-05
cg21224972	4	8308063	HTRA3	3'UTR	0,217	7,28E-06	8,05E-05
cg05309683	18	34107658	FHOD3	Body	-0,209	7,28E-06	8,06E-05
cg26581146	3	99654279	FILIP1L	5'UTR	0,203	7,29E-06	8,06E-05
cg09719807	1	33565386	AZIN2	Body	-0,271	7,30E-06	8,07E-05
cg02304303	4	77483299	SHROOM3	Body	0,218	7,31E-06	8,08E-05
cg15994792	12	14730525	PLBD1-AS1	Body	-0,256	7,31E-06	8,08E-05
cg16097858	2	46765700		IGR	-0,229	7,32E-06	8,08E-05

cg05792588	5	113015126		IGR	0,216	7,32E-06	8,09E-05
cg06725421	13	52367810	DHRS12	Body	-0,218	7,33E-06	8,09E-05
cg05800561	1	39680296	MACF1	Body	0,2	7,33E-06	8,09E-05
cg01336162	1	92350133	TGFBR3	5'UTR	0,227	7,34E-06	8,10E-05
cg16952945	10	80758294	ZMIZ1-AS1	Body	-0,212	7,34E-06	8,10E-05
cg03172765	2	232034629	PSMD1	Body	0,208	7,35E-06	8,11E-05
cg03170036	2	202814433		IGR	0,216	7,36E-06	8,12E-05
cg18066206	22	46459890		IGR	0,213	7,36E-06	8,12E-05
cg25892281	1	220266860	IARS2	TSS1500	0,242	7,37E-06	8,13E-05
cg19002297	3	100475484	ABI3BP	Body	0,229	7,37E-06	8,13E-05
cg03771939	10	96161916	TBC1D12	TSS1500	0,205	7,38E-06	8,14E-05
cg12058677	8	58905974	FAM110B	TSS1500	0,222	7,38E-06	8,14E-05
cg13381976	12	85269223	SLC6A15	Body	0,206	7,38E-06	8,14E-05
cg18316621	6	144283348	PLAGL1	5'UTR	0,226	7,38E-06	8,14E-05
cg03402235	15	42749336	ZFP106	1stExon	0,253	7,39E-06	8,14E-05
cg03158822	20	46658750		IGR	-0,224	7,39E-06	8,15E-05
cg26531195	11	64097053		IGR	0,245	7,40E-06	8,15E-05
cg03328727	9	92034102	SEMA4D	5'UTR	-0,22	7,41E-06	8,16E-05
cg22067466	1	246873298		IGR	-0,217	7,41E-06	8,16E-05
cg10130636	10	92179206	LOC1019269	Body	0,28	7,41E-06	8,16E-05
cg25067702	17	70723227	SLC39A11	Body	-0,218	7,42E-06	8,17E-05
cg05677900	8	59969254	TOX	Body	0,238	7,42E-06	8,17E-05
cg17560895	9	100069891	CCDC180	TSS200	0,213	7,43E-06	8,18E-05
cg16301617	17	76128906	TMC8	Body	-0,204	7,44E-06	8,19E-05
cg03526671	9	80422800	GNAQ	Body	0,261	7,45E-06	8,19E-05
cg27391445	1	231954090	DISC2	Body	0,208	7,46E-06	8,20E-05
cg05217312	10	24417827	KIAA1217	5'UTR	-0,235	7,48E-06	8,22E-05
cg20071413	5	157034949		IGR	-0,242	7,48E-06	8,22E-05
cg05995448	6	52849140	GSTA4	Body	-0,211	7,49E-06	8,23E-05
cg16842634	12	46780880	LOC1002887	Body	0,24	7,49E-06	8,23E-05
cg08578703	6	25042495		IGR	-0,232	7,49E-06	8,23E-05
cg16765394	6	149545554	TAB2	Body	0,262	7,50E-06	8,23E-05
cg17520018	8	72680291		IGR	0,215	7,51E-06	8,24E-05
cg11176159	1	28213800		IGR	-0,208	7,52E-06	8,25E-05
cg19716228	3	171276438		IGR	0,218	7,53E-06	8,26E-05
cg23109910	4	88026471	AFF1	Body	0,215	7,53E-06	8,26E-05
cg21666996	1	99999627		IGR	0,262	7,53E-06	8,26E-05
cg24292689	19	4104584	MAP2K2	Body	-0,203	7,54E-06	8,27E-05
cg00649425	11	116761535	SIK3	Body	0,202	7,54E-06	8,27E-05
cg24699685	1	95268325		IGR	-0,261	7,54E-06	8,27E-05
cg02196651	4	54457842	LNX1	TSS200	-0,247	7,54E-06	8,27E-05
cg26192769	15	77286576	PSTPIP1	TSS1500	-0,245	7,54E-06	8,27E-05
cg15872760	22	33119230	SYN3	Body	-0,236	7,55E-06	8,28E-05
cg13436655	21	27762440		IGR	0,219	7,57E-06	8,29E-05
cg11496404	6	88428902		IGR	-0,273	7,57E-06	8,29E-05
cg15794584	2	191332000	MFSD6	Body	0,213	7,57E-06	8,29E-05
cg17852507	4	154125720	TRIM2	Body	0,28	7,57E-06	8,29E-05
cg09903698	2	218798029	TNS1	5'UTR	0,256	7,58E-06	8,30E-05
cg27273515	16	80690594	CDYL2	Body	-0,202	7,59E-06	8,31E-05
cg26827450	1	235299614	RBM34	Body	0,224	7,59E-06	8,31E-05

cg22141921	5	175866105		IGR	0,203	7,59E-06	8,31E-05
cg01409343	17	57915740	TMEM49	Body	-0,222	7,60E-06	8,32E-05
cg14081262	9	92034950	SEMA4D	5'UTR	-0,219	7,60E-06	8,32E-05
cg15198736	5	32709396		IGR	-0,226	7,60E-06	8,32E-05
cg27027024	18	53756914	LINC01539	Body	-0,231	7,61E-06	8,32E-05
cg15978558	17	48945892	TOB1	TSS1500	0,22	7,62E-06	8,33E-05
cg27209641	17	74994501		IGR	-0,257	7,62E-06	8,34E-05
cg05106778	8	97598420	SDC2	Body	-0,209	7,63E-06	8,34E-05
cg03291037	15	94642624	LINC01581	Body	0,249	7,64E-06	8,34E-05
cg03084920	5	148127220		IGR	0,238	7,64E-06	8,35E-05
cg20633370	17	847577	NXN	Body	-0,218	7,66E-06	8,37E-05
cg00047501	2	201680860	BZW1	Body	0,231	7,67E-06	8,37E-05
cg09596624	3	146235970	PLSCR1	Body	0,301	7,67E-06	8,37E-05
cg03398002	1	15272082	KIAA1026	Body	0,212	7,67E-06	8,38E-05
cg01078772	20	30920486	KIF3B	3'UTR	-0,235	7,67E-06	8,38E-05
cg24949723	4	92758965		IGR	0,222	7,68E-06	8,38E-05
cg00242096	1	236074111		IGR	-0,244	7,68E-06	8,38E-05
cg07775813	15	69223368	MIR548H4	Body	0,223	7,68E-06	8,39E-05
cg04724326	2	42291847		IGR	0,215	7,69E-06	8,39E-05
cg08210637	16	58160654	C16orf80	Body	0,216	7,69E-06	8,39E-05
cg03972071	18	72917163	ZADH2	Body	0,217	7,72E-06	8,42E-05
cg06513139	19	4455923	UBXN6	Body	0,225	7,73E-06	8,42E-05
cg18904605	12	93758071	LOC643339	Body	-0,231	7,74E-06	8,43E-05
cg27541570	8	98619765		IGR	0,257	7,74E-06	8,44E-05
cg10061129	1	83445408		IGR	-0,241	7,75E-06	8,44E-05
cg20617256	5	57753051	PLK2	Body	0,232	7,76E-06	8,44E-05
cg21834604	3	11610595	VGLL4	Body	0,207	7,76E-06	8,45E-05
cg07053851	17	61352831	TANC2	Body	-0,255	7,77E-06	8,45E-05
cg05823823	12	26493991	ITPR2	Body	-0,21	7,77E-06	8,46E-05
cg04203587	4	75895612	PARM1	Body	0,25	7,77E-06	8,46E-05
cg02401789	15	68577185	FEM1B	Body	0,211	7,77E-06	8,46E-05
cg05567143	22	50980910		IGR	-0,273	7,77E-06	8,46E-05
cg15705512	2	53248645		IGR	0,215	7,77E-06	8,46E-05
cg00315837	11	59244789	OR4D10	TSS200	0,225	7,78E-06	8,46E-05
cg18115132	1	107819805	NTNG1	Body	0,215	7,78E-06	8,46E-05
cg15775060	3	17380911	TBC1D5	Body	0,223	7,79E-06	8,47E-05
cg02728634	20	326465	NRSN2	TSS1500	0,208	7,79E-06	8,47E-05
cg21414363	4	123107257	KIAA1109	Body	0,243	7,81E-06	8,49E-05
cg00495428	9	23765199	ELAVL2	5'UTR	-0,209	7,81E-06	8,49E-05
cg11596009	1	36916744	OSCP1	TSS1500	0,221	7,81E-06	8,49E-05
cg03124318	12	109491989	USP30	Body	-0,236	7,81E-06	8,49E-05
cg24496563	1	67842041	IL12RB2	Body	0,253	7,82E-06	8,50E-05
cg03856504	7	1478120	MICALL2	Body	-0,22	7,83E-06	8,50E-05
cg04395431	14	45619710	FANCM	Body	0,218	7,84E-06	8,51E-05
cg23471351	12	48552752	ASB8	TSS1500	0,264	7,88E-06	8,54E-05
cg09264140	17	43302776	FMNL1	Body	-0,257	7,88E-06	8,55E-05
cg02983911	20	36932397	BPI	TSS200	-0,341	7,88E-06	8,55E-05
cg00444050	2	28809988	PLB1	Body	-0,271	7,88E-06	8,55E-05
cg07083043	17	17397972	RASD1	3'UTR	0,286	7,89E-06	8,55E-05
cg10376312	12	124296473	DNAH10	Body	-0,273	7,89E-06	8,55E-05

cg05629195	3	183903129	ABCF3	TSS1500	0,217	7,90E-06	8,56E-05
cg18347209	2	208669062		IGR	-0,215	7,91E-06	8,57E-05
cg03603946	5	115412334		IGR	-0,276	7,91E-06	8,57E-05
cg23592288	2	224761669	WDFY1	Body	-0,27	7,91E-06	8,57E-05
cg06671623	15	99262351	IGF1R	Body	0,263	7,91E-06	8,57E-05
cg20809153	1	225769034	ENAH	Body	0,209	7,92E-06	8,58E-05
cg03868783	5	142159393	ARHGAP26	Body	0,257	7,92E-06	8,58E-05
cg07906170	8	126204062	NSMCE2	Body	-0,228	7,92E-06	8,58E-05
cg20270052	10	63703062	ARID5B	Body	-0,216	7,92E-06	8,58E-05
cg11154733	5	127148500		IGR	-0,248	7,93E-06	8,59E-05
cg12288256	12	92805752		IGR	0,205	7,94E-06	8,59E-05
cg05784193	1	192847589		IGR	0,208	7,94E-06	8,59E-05
cg13643794	7	123241154	NDUFA5	5'UTR	0,229	7,95E-06	8,60E-05
cg07187576	22	50730852	PLXNB2	5'UTR	-0,206	7,95E-06	8,60E-05
cg12058875	2	145770038		IGR	0,227	7,95E-06	8,60E-05
cg01073602	5	118645177	TNFAIP8	5'UTR	-0,207	7,97E-06	8,62E-05
cg23395553	5	137290112	FAM13B	Body	0,22	7,98E-06	8,63E-05
cg03680338	6	22043967	FLJ22536	Body	0,209	7,98E-06	8,63E-05
cg02653010	16	57636203		IGR	-0,255	7,98E-06	8,63E-05
cg15645660	1	55247356	TTC22	Body	0,226	7,99E-06	8,63E-05
cg24193524	12	89560643		IGR	-0,201	7,99E-06	8,63E-05
cg02529737	15	47714991	SEMA6D	5'UTR	-0,207	7,99E-06	8,64E-05
cg08421051	12	122235560	LOC338799	Body	0,229	7,99E-06	8,64E-05
cg11559446	8	141728568	PTK2	Body	0,219	7,99E-06	8,64E-05
cg11233741	9	139826759		IGR	-0,243	8,00E-06	8,64E-05
cg15786558	18	65052975		IGR	0,252	8,01E-06	8,65E-05
cg19889321	12	95639222	VEZT	Body	0,233	8,02E-06	8,66E-05
cg01058368	5	24645487	CDH10	TSS1500	0,228	8,03E-06	8,67E-05
cg18266783	1	156466729	MEF2D	5'UTR	0,232	8,03E-06	8,67E-05
cg17342469	22	46473074		IGR	0,288	8,03E-06	8,67E-05
cg02756373	5	167997991	PANK3	Body	0,219	8,04E-06	8,67E-05
cg23495647	4	170284641		IGR	-0,23	8,05E-06	8,69E-05
cg24145401	1	159047177	AIM2	TSS1500	-0,201	8,06E-06	8,69E-05
cg17438167	2	160601669	MARCH7	Body	0,21	8,07E-06	8,70E-05
cg19902005	11	57545678	TMX2-CTNND	Body	0,231	8,07E-06	8,70E-05
cg12908797	2	234240596	SAG	Body	-0,222	8,08E-06	8,71E-05
cg02328020	6	138168326	LOC10013047	Body	-0,221	8,08E-06	8,71E-05
cg12772086	6	107783986		IGR	0,203	8,09E-06	8,72E-05
cg23012875	20	50527332		IGR	0,201	8,09E-06	8,72E-05
cg18095302	16	84133451	MBTPS1	Body	-0,249	8,10E-06	8,72E-05
cg17508434	7	97831974	LMTK2	Body	-0,245	8,11E-06	8,74E-05
cg09796146	16	33937240		IGR	0,207	8,12E-06	8,74E-05
cg16492341	10	26856105	APBB1IP	Body	0,227	8,12E-06	8,74E-05
cg17979881	18	7754943	PTPRM	Body	0,23	8,13E-06	8,75E-05
cg12374911	2	111897426	BCL2L11	Body	0,239	8,13E-06	8,75E-05
cg03551062	16	88041289	BANP	Body	0,201	8,13E-06	8,75E-05
cg01486979	5	126147721	LMNB1	Body	-0,248	8,14E-06	8,76E-05
cg00969405	7	27184441	HOXA5	TSS1500	0,211	8,14E-06	8,76E-05
cg24025012	3	171024707	TNIK	Body	0,252	8,15E-06	8,76E-05
cg25293821	11	12185892	MICAL2	Body	0,2	8,15E-06	8,76E-05

cg24587080	6	160016373		IGR	-0,23	8,15E-06	8,76E-05
cg05691747	12	92578794		IGR	-0,243	8,15E-06	8,76E-05
cg06961771	16	80729230	CDYL2	Body	-0,203	8,15E-06	8,76E-05
cg00972690	3	43252363		IGR	0,21	8,19E-06	8,79E-05
cg00587301	6	156717406		IGR	-0,215	8,19E-06	8,80E-05
cg04849506	12	44383073	TMEM117	5'UTR	-0,232	8,20E-06	8,80E-05
cg12614949	2	159458777	PKP4	Body	0,293	8,20E-06	8,80E-05
cg17424516	14	64458783	SYNE2	Body	-0,223	8,20E-06	8,81E-05
cg01463779	9	3472120	RFX3	5'UTR	0,29	8,20E-06	8,81E-05
cg02031314	5	81180943		IGR	0,218	8,21E-06	8,81E-05
cg23093664	2	197831702		IGR	0,239	8,21E-06	8,81E-05
cg27497578	14	23915878		IGR	0,236	8,22E-06	8,82E-05
cg00211883	3	176378514		IGR	-0,214	8,22E-06	8,82E-05
cg25036341	16	46708292	VPS35	Body	0,208	8,23E-06	8,83E-05
cg10023565	6	157405733	ARID1B	Body	0,233	8,23E-06	8,83E-05
cg12803053	8	126142264	NSMCE2	Body	0,284	8,24E-06	8,84E-05
cg15029037	4	146729164	ZNF827	Body	-0,242	8,24E-06	8,84E-05
cg00661777	7	106511741	PIK3CG	Body	-0,257	8,25E-06	8,84E-05
cg06031268	6	2770510	WRNIP1	Body	0,211	8,25E-06	8,84E-05
cg26120787	2	178516770	PDE11A	Body	0,234	8,29E-06	8,88E-05
cg08442934	4	1773246		IGR	-0,237	8,30E-06	8,89E-05
cg09744840	10	74469027	CCDC109A	Body	0,245	8,30E-06	8,89E-05
cg03199285	13	29598098	MTUS2	TSS1500	-0,2	8,31E-06	8,89E-05
cg15946524	2	47714346		IGR	-0,23	8,31E-06	8,89E-05
cg01339630	14	101376887	MIR370	TSS1500	-0,217	8,31E-06	8,89E-05
cg04421582	9	20242730		IGR	-0,212	8,31E-06	8,89E-05
cg16394702	12	104194358	NT5DC3	Body	0,225	8,32E-06	8,90E-05
cg16647921	4	41867533		IGR	0,203	8,32E-06	8,90E-05
cg26480890	1	185615787		IGR	0,252	8,34E-06	8,92E-05
cg15049460	3	24720314		IGR	-0,241	8,37E-06	8,94E-05
cg19055098	12	52165097	SCN8A	Body	0,204	8,38E-06	8,94E-05
cg23002982	11	70323008	SHANK2	Body	-0,216	8,38E-06	8,95E-05
cg13258313	4	160266429	RAPGEF2	Body	0,228	8,38E-06	8,95E-05
cg21394729	15	31316860	TRPM1	Body	0,218	8,38E-06	8,95E-05
cg13995811	16	927533	LMF1	Body	-0,215	8,39E-06	8,95E-05
cg25035008	4	100011046	ADH5	TSS1500	0,235	8,39E-06	8,95E-05
cg02991901	3	31269031		IGR	-0,229	8,40E-06	8,96E-05
cg20553277	4	96336870	UNC5C	Body	-0,207	8,42E-06	8,97E-05
cg04441857	10	14701815	FAM107B	Body	-0,267	8,42E-06	8,98E-05
cg14451276	7	36764019	AOAH	1stExon	-0,317	8,42E-06	8,98E-05
cg05511872	11	70824260	SHANK2	Body	0,221	8,42E-06	8,98E-05
cg24715776	4	77437377	SHROOM3	Body	-0,21	8,43E-06	8,99E-05
cg13843993	5	10474634		IGR	-0,235	8,44E-06	8,99E-05
cg02910483	2	148055636		IGR	-0,23	8,44E-06	8,99E-05
cg11241750	5	125694354	GRAMD3	TSS1500	-0,208	8,44E-06	9,00E-05
cg26024214	15	58215163		IGR	0,212	8,44E-06	9,00E-05
cg16739092	20	17519424	BFSP1	Body	-0,234	8,45E-06	9,00E-05
cg22014955	15	92401373	SLCO3A1	Body	-0,301	8,45E-06	9,00E-05
cg06353847	2	174928222		IGR	0,2	8,45E-06	9,00E-05
cg17351801	11	18640326	SPTY2D1	Body	0,231	8,46E-06	9,01E-05

cg07050611	12	117468529	FBXW8	3'UTR	0,22	8,48E-06	9,02E-05
cg20611253	1	150743628		IGR	-0,212	8,48E-06	9,02E-05
cg05160909	2	217381364		IGR	0,219	8,48E-06	9,03E-05
cg01532051	17	71224078	FAM104A	TSS1500	-0,23	8,48E-06	9,03E-05
cg09120724	17	56494602	RNF43	5'UTR	0,261	8,49E-06	9,03E-05
cg13015243	6	131318735	EPB41L2	5'UTR	0,225	8,49E-06	9,03E-05
cg02772995	2	161348781	RBMS1	Body	0,223	8,49E-06	9,03E-05
cg21833157	7	107237359	BCAP29	Body	0,21	8,50E-06	9,04E-05
cg21886807	8	17463091	PDGFRL	Body	0,24	8,50E-06	9,04E-05
cg20303075	8	80804323		IGR	-0,227	8,51E-06	9,05E-05
cg21099332	5	39270715		IGR	-0,227	8,51E-06	9,05E-05
cg17206555	7	90227371		IGR	0,218	8,52E-06	9,05E-05
cg18259291	15	52945358	FAM214A	TSS1500	0,231	8,52E-06	9,06E-05
cg12453687	11	82745712	RAB30	5'UTR	0,211	8,52E-06	9,06E-05
cg13935919	1	52837342		IGR	0,227	8,52E-06	9,06E-05
cg03880362	11	108874967		IGR	-0,201	8,53E-06	9,06E-05
cg25783189	1	27953220	FGR	TSS1500	-0,213	8,54E-06	9,07E-05
cg10477778	2	12271436	LOC10050645	Body	-0,202	8,55E-06	9,08E-05
cg26537719	11	122874364		IGR	0,256	8,55E-06	9,08E-05
cg21187669	17	17929033	ATPAF2	Body	-0,227	8,56E-06	9,08E-05
cg24040694	22	24882671	ADORA2A-AS	Body	-0,237	8,56E-06	9,09E-05
cg12402265	3	9954187	IL17RE	Body	-0,257	8,56E-06	9,09E-05
cg11174855	10	134598352	NKX6-2	3'UTR	0,218	8,57E-06	9,09E-05
cg02612766	3	49156707	USP19	5'UTR	0,251	8,57E-06	9,09E-05
cg20616856	1	221951356		IGR	-0,286	8,58E-06	9,10E-05
cg00347981	18	3604036	DLGAP1-AS2	Body	0,218	8,58E-06	9,11E-05
cg03558688	10	106059925		IGR	0,279	8,59E-06	9,11E-05
cg01105418	1	244214593	ZNF238	1stExon	0,269	8,60E-06	9,12E-05
cg05970238	8	131321814	ASAP1	Body	-0,251	8,61E-06	9,12E-05
cg11938624	1	244443743		IGR	-0,21	8,61E-06	9,13E-05
cg07885792	11	74303296	POLD3	TSS1500	0,22	8,61E-06	9,13E-05
cg12897600	7	126339236	GRM8	Body	0,253	8,62E-06	9,13E-05
cg19980369	5	121506564		IGR	0,264	8,62E-06	9,13E-05
cg11487379	2	9428037	ASAP2	Body	-0,212	8,63E-06	9,14E-05
cg14096828	7	110731116	LRRN3	1stExon	0,247	8,63E-06	9,14E-05
cg16860863	5	112610818	MCC	Body	0,226	8,64E-06	9,15E-05
cg12433575	6	30881464	VARS2	TSS1500	0,202	8,64E-06	9,15E-05
cg09821400	10	33654419		IGR	-0,204	8,64E-06	9,15E-05
cg17692879	1	32314458		IGR	-0,268	8,65E-06	9,15E-05
cg02853497	1	46113000	RPS15AP10	TSS1500	0,246	8,66E-06	9,16E-05
cg23463787	12	124155297	TCTN2	TSS1500	0,296	8,66E-06	9,16E-05
cg04420916	4	160149048		IGR	0,215	8,67E-06	9,17E-05
cg06522188	1	117509410	PTGFRN	Body	-0,234	8,67E-06	9,17E-05
cg12681370	2	218794733	TNS1	5'UTR	0,26	8,67E-06	9,17E-05
cg06640991	11	104576519		IGR	-0,263	8,67E-06	9,17E-05
cg06000934	10	31875842		IGR	-0,201	8,69E-06	9,18E-05
cg11886187	17	56033756		IGR	-0,208	8,70E-06	9,19E-05
cg26368594	10	62491439	ANK3	Body	-0,203	8,70E-06	9,20E-05
cg23985447	5	118691066	TNFAIP8	Body	-0,211	8,72E-06	9,21E-05
cg02906061	2	178623883	PDE11A	Body	0,214	8,73E-06	9,22E-05

cg11248246	18	7562058		IGR	0,212	8,73E-06	9,22E-05
cg23591302	12	42876090	PRICKLE1	5'UTR	0,204	8,74E-06	9,22E-05
cg15475485	21	43924037	SLC37A1	5'UTR	-0,233	8,75E-06	9,24E-05
cg23902821	21	31012599	GRIK1	Body	-0,231	8,76E-06	9,24E-05
cg03290188	2	3674266	COLEC11	Body	0,265	8,77E-06	9,25E-05
cg09730457	3	107363673	BBX	5'UTR	-0,23	8,78E-06	9,25E-05
cg04405165	6	70684187	COL19A1	Body	0,329	8,80E-06	9,27E-05
cg03485672	2	130237627		IGR	0,249	8,80E-06	9,27E-05
cg20908789	8	11551964		IGR	-0,238	8,80E-06	9,27E-05
cg08621315	7	3039048	CARD11	5'UTR	0,206	8,81E-06	9,28E-05
cg05304815	8	65669140	CYP7B1	Body	-0,221	8,82E-06	9,29E-05
cg18470427	17	33842301	SLFN12L	Body	0,209	8,82E-06	9,29E-05
cg20254830	6	116333646	FRK	Body	0,284	8,84E-06	9,31E-05
cg25042675	21	36666547		IGR	0,213	8,86E-06	9,32E-05
cg16662477	17	33401430	RFFL	5'UTR	-0,209	8,87E-06	9,33E-05
cg10302857	17	29133390	CRLF3	Body	-0,209	8,87E-06	9,33E-05
cg27311880	16	70575432	SF3B3	Body	0,267	8,87E-06	9,33E-05
cg10433819	11	76289700		IGR	0,203	8,88E-06	9,34E-05
cg22556505	7	148004033	CNTNAP2	Body	-0,209	8,89E-06	9,34E-05
cg02258759	12	89528126		IGR	0,284	8,89E-06	9,35E-05
cg05682942	7	142942530		IGR	-0,203	8,90E-06	9,36E-05
cg13623749	6	15329886	JARID2	Body	0,237	8,91E-06	9,37E-05
cg24048497	6	14866284		IGR	-0,223	8,91E-06	9,37E-05
cg14393813	5	16679416	MYO10	Body	-0,21	8,92E-06	9,37E-05
cg02928476	7	98311382		IGR	-0,204	8,93E-06	9,38E-05
cg05613080	21	46378013	FAM207A	Body	0,21	8,93E-06	9,38E-05
cg08195271	4	174530370		IGR	-0,207	8,93E-06	9,38E-05
cg13711385	2	218843411		IGR	0,213	8,94E-06	9,39E-05
cg06795995	5	56204613	SETD9	TSS1500	0,291	8,95E-06	9,39E-05
cg01822145	17	17621774	RAI1	5'UTR	-0,229	8,95E-06	9,39E-05
cg16497714	11	4597246	C11orf40	Body	-0,231	8,95E-06	9,39E-05
cg08065344	6	163839882	QKI	Body	0,207	8,97E-06	9,41E-05
cg21964662	13	79234715	RNF219	TSS1500	0,272	8,97E-06	9,41E-05
cg13071107	2	187778658		IGR	-0,24	8,97E-06	9,41E-05
cg17594004	4	183247132	ODZ3	Body	0,201	8,97E-06	9,41E-05
cg15271220	1	94108287	BCAR3	Body	-0,203	8,97E-06	9,41E-05
cg11669284	5	37721782	WDR70	Body	-0,253	8,97E-06	9,41E-05
cg11546385	11	269375		IGR	-0,263	8,98E-06	9,42E-05
cg06635478	5	124347068		IGR	-0,254	8,98E-06	9,42E-05
cg26666676	4	122035021		IGR	-0,254	8,99E-06	9,42E-05
cg00117514	8	38776417	PLEKHA2	Body	0,214	8,99E-06	9,42E-05
cg20736887	15	75400438		IGR	-0,283	8,99E-06	9,43E-05
cg15599614	4	160148591		IGR	0,278	8,99E-06	9,43E-05
cg21146761	3	16884339		IGR	-0,208	9,00E-06	9,43E-05
cg01595702	5	52365620	ITGA2	Body	-0,209	9,00E-06	9,43E-05
cg21671094	6	112349238		IGR	-0,208	9,00E-06	9,43E-05
cg05087623	6	29527870	UBD	TSS200	-0,278	9,00E-06	9,43E-05
cg08451044	11	102670426	MMP1	TSS1500	0,23	9,00E-06	9,43E-05
cg26537640	1	160642715		IGR	-0,235	9,01E-06	9,44E-05
cg20473155	2	10688228		IGR	-0,228	9,01E-06	9,44E-05

cg21775245	3	129407548	TMCC1	TSS200	0,252	9,01E-06	9,44E-05
cg09307985	19	36643070	COX7A1	Body	0,225	9,02E-06	9,44E-05
cg07077953	14	59623180		IGR	-0,218	9,04E-06	9,47E-05
cg12392667	1	234534701	TARBP1	Body	-0,255	9,04E-06	9,47E-05
cg17691545	4	87770586	SLC10A6	TSS200	0,237	9,04E-06	9,47E-05
cg07310406	22	50524374	MLC1	TSS1500	-0,252	9,05E-06	9,47E-05
cg00791074	6	151186169	MTHFD1L	TSS1500	-0,208	9,06E-06	9,48E-05
cg18758923	12	63126696	PPM1H	Body	-0,219	9,07E-06	9,49E-05
cg20940250	21	16595275		IGR	-0,295	9,07E-06	9,49E-05
cg15318881	5	167614840	TENM2	Body	0,216	9,07E-06	9,49E-05
cg19535999	16	49677798	ZNF423	Body	-0,244	9,08E-06	9,49E-05
cg02809083	3	123123167	ADCY5	Body	0,217	9,08E-06	9,49E-05
cg25757164	1	201707021	NAV1	Body	0,217	9,09E-06	9,50E-05
cg14216322	2	218225443	DIRC3	Body	-0,2	9,11E-06	9,52E-05
cg26524547	1	201820022	IPO9	Body	0,2	9,11E-06	9,52E-05
cg11065462	9	14323694	NFIB	Body	0,274	9,12E-06	9,53E-05
cg06882849	6	75856164	COL12A1	Body	-0,232	9,14E-06	9,54E-05
cg12426896	18	64271465	CDH19	TSS200	0,323	9,14E-06	9,54E-05
cg27075679	1	63186797		IGR	0,243	9,14E-06	9,54E-05
cg11855699	10	118721376	SHTN1	Body	0,264	9,14E-06	9,54E-05
cg24522085	2	169556419	LASS6	Body	0,205	9,15E-06	9,54E-05
cg17497790	5	34494483		IGR	-0,206	9,15E-06	9,55E-05
cg11597973	6	34150573		IGR	-0,258	9,17E-06	9,56E-05
cg26206347	9	90452455		IGR	0,239	9,17E-06	9,56E-05
cg24464915	5	66254431	MAST4	TSS1500	0,32	9,17E-06	9,56E-05
cg06729408	8	110380981	PKHD1L1	Body	0,208	9,18E-06	9,58E-05
cg23517743	2	238623585	LRRFIP1	Body	0,247	9,21E-06	9,59E-05
cg12062175	6	56734221	DST	Body	-0,227	9,21E-06	9,59E-05
cg15717858	12	109956918	UBE3B	Body	0,257	9,21E-06	9,59E-05
cg17841550	2	170683621	UBR3	TSS1500	0,221	9,22E-06	9,60E-05
cg00903453	3	179045853	ZNF639	5'UTR	0,223	9,23E-06	9,61E-05
cg09310774	4	170273207		IGR	0,225	9,23E-06	9,61E-05
cg26336265	6	25042955		IGR	-0,247	9,24E-06	9,61E-05
cg09369619	3	187529583		IGR	-0,215	9,26E-06	9,63E-05
cg01291947	21	34305156		IGR	-0,207	9,26E-06	9,63E-05
cg00478942	2	75854312		IGR	-0,216	9,26E-06	9,63E-05
cg16207184	6	116796960		IGR	-0,26	9,26E-06	9,64E-05
cg10465805	8	134911885	LOC10192782	Body	-0,275	9,28E-06	9,65E-05
cg06349264	1	173106049		IGR	0,213	9,29E-06	9,65E-05
cg02702215	13	91578815	LINC00410	Body	0,212	9,29E-06	9,66E-05
cg14497054	12	34261116		IGR	0,244	9,29E-06	9,66E-05
cg12982531	6	71938489		IGR	-0,27	9,30E-06	9,66E-05
cg14791193	1	16702476	C1orf144	Body	0,278	9,30E-06	9,67E-05
cg23530834	1	114220113	MAGI3	Body	0,204	9,31E-06	9,67E-05
cg08657146	9	134872430	MED27	Body	0,219	9,32E-06	9,68E-05
cg13618623	9	126693384	DENND1A	TSS1500	0,201	9,32E-06	9,68E-05
cg04607003	10	54515301		IGR	-0,222	9,33E-06	9,68E-05
cg21502751	20	50633639		IGR	-0,2	9,34E-06	9,70E-05
cg18489994	4	15235419		IGR	-0,211	9,34E-06	9,70E-05
cg02403419	9	130853593	SLC25A25	TSS200	0,213	9,35E-06	9,70E-05

cg09385371	6	142959676	LOC153910	TSS1500	-0,226	9,35E-06	9,70E-05
cg11883423	4	153273933	FBXW7	1stExon	0,25	9,35E-06	9,70E-05
cg06547898	2	120524715	PTPN4	5'UTR	0,228	9,35E-06	9,71E-05
cg04124281	15	80190137	MTHFS	TSS1500	-0,238	9,35E-06	9,71E-05
cg19541916	2	19674105		IGR	-0,264	9,36E-06	9,71E-05
cg22602379	2	8369887	LINC00299	Body	-0,211	9,37E-06	9,72E-05
cg09342985	6	47497590	CD2AP	Body	0,275	9,37E-06	9,72E-05
cg25779355	11	86235351	ME3	Body	-0,202	9,37E-06	9,72E-05
cg23214480	11	6424058	APBB1	Body	-0,277	9,38E-06	9,72E-05
cg18384097	1	202129566	PTPN7	TSS1500	-0,216	9,39E-06	9,74E-05
cg06363937	10	75336152	USP54	TSS1500	0,253	9,40E-06	9,74E-05
cg10655885	20	5824213	C20orf196	Body	-0,213	9,40E-06	9,74E-05
cg00191458	5	169757859	LOC257358	TSS1500	-0,238	9,42E-06	9,76E-05
cg00231528	1	9714280	C1orf200	Body	-0,226	9,42E-06	9,76E-05
cg19238141	20	9441312	PLCB4	Body	0,204	9,42E-06	9,76E-05
cg17137186	2	101928209		IGR	-0,201	9,43E-06	9,77E-05
cg04342202	7	1961968	MAD1L1	Body	-0,258	9,44E-06	9,78E-05
cg20427486	2	162849137	DPP4	3'UTR	-0,203	9,46E-06	9,79E-05
cg12015737	7	27184030	HOXA5	TSS1500	0,272	9,46E-06	9,79E-05
cg18807940	3	124358702	KALRN	Body	-0,263	9,47E-06	9,80E-05
cg17076222	4	2979112	GRK4	Body	0,211	9,47E-06	9,80E-05
cg01930890	7	28332374		IGR	-0,2	9,48E-06	9,81E-05
cg18109417	5	90540438		IGR	0,2	9,49E-06	9,81E-05
cg10888348	7	4763137	FOXK1	Body	0,221	9,49E-06	9,81E-05
cg12853970	11	103522978		IGR	-0,214	9,50E-06	9,82E-05
cg04863884	7	37059138	ELMO1	Body	0,262	9,51E-06	9,83E-05
cg19756830	2	216769058		IGR	-0,247	9,52E-06	9,84E-05
cg25774094	7	41771743	INHBA-AS1	Body	-0,223	9,53E-06	9,84E-05
cg02464822	6	13733592		IGR	0,223	9,55E-06	9,86E-05
cg13890447	15	101897346	PCSK6	Body	-0,222	9,55E-06	9,86E-05
cg04979427	6	16109573		IGR	0,205	9,56E-06	9,87E-05
cg26754179	7	136836288	LOC349160	Body	0,245	9,56E-06	9,87E-05
cg07138399	1	200272215		IGR	0,214	9,56E-06	9,87E-05
cg20973561	7	133218612	EXOC4	Body	0,213	9,57E-06	9,88E-05
cg05590294	12	9913395	CD69	1stExon	-0,247	9,57E-06	9,88E-05
cg26794606	10	89942354		IGR	-0,226	9,58E-06	9,88E-05
cg12017702	11	126278603	ST3GAL4	Body	0,325	9,59E-06	9,89E-05
cg13989888	5	94120872	MCTP1	Body	0,213	9,59E-06	9,89E-05
cg15804669	8	17718512		IGR	0,272	9,59E-06	9,89E-05
cg20268292	13	46065593	COG3	Body	0,229	9,60E-06	9,90E-05
cg15594977	2	109830595	SH3RF3	Body	-0,28	9,61E-06	9,91E-05
cg00553355	6	43019994	CUL7	Body	0,216	9,62E-06	9,91E-05
cg05897307	5	111016518	STARD4-AS1	Body	0,213	9,62E-06	9,91E-05
cg23916522	12	26283729		IGR	-0,201	9,62E-06	9,92E-05
cg20703928	2	106364229	NCK2	5'UTR	-0,223	9,63E-06	9,92E-05
cg21051815	11	1542394	HCCA2	Body	0,235	9,63E-06	9,93E-05
cg14534144	13	114150007	TMCO3	Body	0,225	9,63E-06	9,93E-05
cg21751556	21	32451135		IGR	-0,23	9,64E-06	9,93E-05
cg11462099	13	113540512	ATP11A	3'UTR	0,202	9,65E-06	9,94E-05
cg12754527	9	134460562	RAPGEF1	Body	-0,257	9,65E-06	9,94E-05

cg10227363	20	49133173	PTPN1	5'UTR	-0,222	9,66E-06	9,95E-05
cg21358336	17	6558440		IGR	0,204	9,66E-06	9,95E-05
cg24703167	4	48027025	NIPAL1	Body	0,226	9,66E-06	9,95E-05
cg07599828	17	30053217		IGR	-0,234	9,66E-06	9,95E-05
cg03795157	12	133531927	ZNF605	5'UTR	0,22	9,66E-06	9,95E-05
cg19841228	6	112098309	FYN	5'UTR	-0,214	9,67E-06	9,96E-05
cg23328295	5	118670788	TNFAIP8	Body	0,293	9,67E-06	9,96E-05
cg20739713	13	82576962		IGR	0,202	9,68E-06	9,96E-05
cg02619087	12	103838906	C12orf42	Body	0,249	9,68E-06	9,96E-05
cg24798010	19	6464885	CRB3	Body	0,228	9,70E-06	9,98E-05
cg02427468	7	158575934	ESYT2	Body	0,215	9,71E-06	9,98E-05
cg27155939	15	40347163		IGR	-0,203	9,71E-06	9,99E-05
cg07118895	17	54398673	ANKFN1	Body	0,23	9,72E-06	1,00E-04
cg19750223	1	181175885		IGR	0,203	9,73E-06	1,00E-04
cg10211193	10	44782092		IGR	-0,214	9,73E-06	1,00E-04
cg18669861	3	16555789	RFTN1	TSS1500	-0,212	9,74E-06	1,00E-04
cg01287173	2	36923444	VIT	TSS1500	-0,237	9,74E-06	1,00E-04
cg20504007	10	49892954	WDFY4	TSS1500	-0,232	9,75E-06	1,00E-04
cg17237907	6	109780119	MICAL1	Body	-0,234	9,77E-06	1,00E-04
cg26498958	1	169498218	F5	Body	-0,23	9,77E-06	1,00E-04
cg25147804	14	60810476		IGR	-0,214	9,77E-06	1,00E-04
cg23656872	1	167633584	RCSD1	Body	0,231	9,78E-06	1,00E-04
cg03099144	6	125323425	RNF217	5'UTR	0,225	9,78E-06	1,00E-04
cg06564875	5	157353780		IGR	0,21	9,78E-06	1,00E-04
cg05494668	11	100433017		IGR	0,224	9,81E-06	1,01E-04
cg03817685	1	78567411	GIPC2	Body	0,276	9,82E-06	1,01E-04
cg10794973	6	43692678		IGR	-0,243	9,82E-06	1,01E-04
cg04131952	9	71994536	FAM189A2	Body	0,238	9,82E-06	1,01E-04
cg05152903	12	10872218	CSDA	Body	0,275	9,83E-06	1,01E-04
cg04188070	13	95086170		IGR	0,314	9,83E-06	1,01E-04
cg08271577	9	6683284		IGR	-0,215	9,83E-06	1,01E-04
cg08014182	6	33241594	RPS18	Body	-0,222	9,85E-06	1,01E-04
cg15613349	11	62304055	AHNAK	ExonBnd	0,21	9,85E-06	1,01E-04
cg15495906	6	105232862	HACE1	ExonBnd	0,219	9,86E-06	1,01E-04
cg13699428	3	48154442		IGR	-0,213	9,86E-06	1,01E-04
cg11224418	21	35897663	RCAN1	1stExon	0,248	9,87E-06	1,01E-04
cg16662370	5	14830500	ANKH	Body	0,207	9,89E-06	1,01E-04
cg03633969	11	6222973		IGR	-0,219	9,89E-06	1,01E-04
cg17166288	11	67979344	SUV420H1	5'UTR	0,212	9,89E-06	1,01E-04
cg17299042	21	32958653		IGR	0,2	9,90E-06	1,01E-04
cg04398539	15	63140217		IGR	0,201	9,90E-06	1,01E-04
cg20340034	15	70284710		IGR	0,208	9,90E-06	1,01E-04
cg14776616	10	24745320	KIAA1217	5'UTR	-0,21	9,91E-06	1,01E-04
cg14260002	8	29971104	LEPROTL1	Body	0,235	9,92E-06	1,01E-04
cg04488894	10	5067466		IGR	-0,239	9,92E-06	1,01E-04
cg09153080	11	60739183	CD6	5'UTR	-0,233	9,92E-06	1,01E-04
cg13816042	9	137615839	COL5A1	Body	-0,225	9,93E-06	1,02E-04
cg12592387	12	131701888		IGR	0,207	9,93E-06	1,02E-04
cg14939821	10	128947230	DOCK1	Body	-0,22	9,95E-06	1,02E-04
cg20017590	2	54852195	SPTBN1	Body	0,218	9,95E-06	1,02E-04

cg18974949	5	90515112		IGR	-0,203	9,95E-06	1,02E-04
cg15359122	11	113958057	ZBTB16	Body	-0,32	9,95E-06	1,02E-04
cg17798857	15	70994632	UACA	Body	0,222	9,96E-06	1,02E-04
cg10545616	9	108645859		IGR	-0,257	9,96E-06	1,02E-04
cg13831575	15	48834416	FBN1	Body	-0,217	9,98E-06	1,02E-04
cg27171569	16	83987465	OSGIN1	5'UTR	0,22	9,98E-06	1,02E-04
cg21620736	11	48037919	PTPRJ	Body	-0,252	9,99E-06	1,02E-04
cg22268403	4	151703778	LRBA	Body	0,205	1,00E-05	1,02E-04
cg02770724	7	73132837	STX1A	Body	-0,209	1,00E-05	1,02E-04
cg14491582	13	34250241	STARD13	5'UTR	0,252	1,00E-05	1,02E-04
cg00819895	13	34361495		IGR	-0,222	1,00E-05	1,02E-04
cg11389191	12	16017042		IGR	-0,223	1,00E-05	1,02E-04
cg01030479	11	78066119	GAB2	Body	-0,204	1,00E-05	1,02E-04
cg17971017	12	108168122	ASCL4	TSS200	-0,234	1,00E-05	1,02E-04
cg21836475	13	76535057		IGR	-0,219	1,00E-05	1,02E-04
cg24128292	8	30419935	RBPMS	3'UTR	0,209	1,00E-05	1,02E-04
cg15000222	12	117486164	TESC	Body	0,213	1,00E-05	1,02E-04
cg22526076	2	99072069	INPP4A	5'UTR	-0,243	1,01E-05	1,03E-04
cg21807628	1	226856091	ITPKB-IT1	Body	0,204	1,01E-05	1,03E-04
cg15294391	10	95069090	MYOF	Body	0,201	1,01E-05	1,03E-04
cg03638567	1	206290355		IGR	-0,26	1,01E-05	1,03E-04
cg17638418	3	30560307		IGR	0,239	1,01E-05	1,03E-04
cg09762808	4	144152055		IGR	-0,236	1,01E-05	1,03E-04
cg26688775	6	167704942	UNC93A	5'UTR	-0,214	1,01E-05	1,03E-04
cg06090404	17	8644134	CCDC42	Body	-0,223	1,01E-05	1,03E-04
cg09860956	14	51210430	NIN	Body	0,241	1,01E-05	1,03E-04
cg17944300	7	68899546		IGR	-0,331	1,02E-05	1,03E-04
cg07677157	12	66050928		IGR	-0,26	1,02E-05	1,03E-04
cg14286208	14	20903445	KLHL33	5'UTR	0,207	1,02E-05	1,03E-04
cg00829747	13	86594661		IGR	0,231	1,02E-05	1,03E-04
cg13994842	7	151433414	PRKAG2	TSS200	0,208	1,02E-05	1,03E-04
cg11311283	1	51970518	EPS15	Body	-0,211	1,02E-05	1,03E-04
cg19776337	11	86636890	PRSS23	Body	-0,208	1,02E-05	1,03E-04
cg14627404	3	111327958	CD96	Body	-0,203	1,02E-05	1,04E-04
cg08459862	5	148174584		IGR	-0,231	1,02E-05	1,04E-04
cg02257635	3	121745511		IGR	0,221	1,02E-05	1,04E-04
cg11530213	8	42037966	PLAT	Body	-0,256	1,02E-05	1,04E-04
cg01010753	6	116355605	FRK	Body	0,261	1,02E-05	1,04E-04
cg14374614	8	38261764	LETM2	Body	0,217	1,02E-05	1,04E-04
cg08284097	12	32178932		IGR	-0,229	1,02E-05	1,04E-04
cg03234777	11	118095544	AMICA1	5'UTR	-0,229	1,03E-05	1,04E-04
cg18935477	20	15653971	MACROD2	Body	0,219	1,03E-05	1,04E-04
cg17665601	8	131162292	ASAP1	Body	0,213	1,03E-05	1,04E-04
cg03646131	20	49161847	PTPN1	5'UTR	-0,229	1,03E-05	1,04E-04
cg23017826	13	22027670	ZDHHC20	Body	-0,234	1,03E-05	1,04E-04
cg08455089	6	37292135	TBC1D22B	Body	-0,206	1,03E-05	1,04E-04
cg01425762	16	81666633	CMIP	Body	-0,228	1,03E-05	1,04E-04
cg22961212	5	66564534		IGR	0,221	1,03E-05	1,05E-04
cg17236752	4	40303738		IGR	-0,251	1,03E-05	1,05E-04
cg26100071	11	17493151	ABCC8	Body	-0,205	1,03E-05	1,05E-04

cg27458033	14	72021237	SIPA1L1	5'UTR	-0,226	1,03E-05	1,05E-04
cg14716923	18	35061080	CELF4	Body	0,208	1,04E-05	1,05E-04
cg09901201	1	53515142	SCP2	3'UTR	0,254	1,04E-05	1,05E-04
cg07031797	22	50483123	TTLL8	Body	-0,281	1,04E-05	1,05E-04
cg02515217	17	57918600	MIR21	TSS200	-0,242	1,04E-05	1,05E-04
cg14161159	2	132481826	C2orf27A	5'UTR	0,256	1,04E-05	1,05E-04
cg11351522	6	27791241	HIST1H4J	TSS1500	0,21	1,04E-05	1,05E-04
cg01262952	10	92680914	ANKRD1	1stExon	0,202	1,04E-05	1,05E-04
cg15934958	3	37212084	LRRFIP2	5'UTR	0,234	1,04E-05	1,05E-04
cg15143370	9	132625508	USP20	Body	-0,225	1,04E-05	1,05E-04
cg02586348	4	95410089	PDLIM5	5'UTR	0,217	1,04E-05	1,05E-04
cg09685096	1	86157111	ZNHIT6	Body	0,201	1,04E-05	1,05E-04
cg20225766	3	111414672	PLCXD2	Body	-0,224	1,04E-05	1,05E-04
cg27010141	22	43259569		IGR	-0,231	1,04E-05	1,06E-04
cg03001119	8	62692844		IGR	-0,218	1,05E-05	1,06E-04
cg14557202	12	54764371	ZNF385A	Body	0,228	1,05E-05	1,06E-04
cg16780862	19	2346131	SPPL2B	Body	0,212	1,05E-05	1,06E-04
cg00220415	4	177752334		IGR	0,222	1,05E-05	1,06E-04
cg17557284	3	93865479		IGR	0,235	1,05E-05	1,06E-04
cg17036418	17	33417113	RFFL	TSS1500	-0,248	1,05E-05	1,06E-04
cg21568193	1	173496518	SLC9C2	Body	0,261	1,05E-05	1,06E-04
cg04875514	12	53497147	SOAT2	TSS200	-0,219	1,05E-05	1,06E-04
cg14368661	5	111313948	NREP	TSS1500	-0,282	1,05E-05	1,06E-04
cg10212247	1	170406421		IGR	-0,2	1,05E-05	1,06E-04
cg00681462	14	75800724		IGR	-0,206	1,05E-05	1,06E-04
cg19703946	2	120127622	DBI	Body	0,203	1,05E-05	1,06E-04
cg16683060	13	99974673	UBAC2	Body	-0,256	1,05E-05	1,06E-04
cg16719404	1	117296242	CD2	TSS1500	-0,22	1,05E-05	1,06E-04
cg05541910	7	42120541	GLI3	Body	-0,217	1,05E-05	1,06E-04
cg17894064	5	112657195	MCC	Body	0,256	1,05E-05	1,06E-04
cg12229151	11	6340283	PRKCDBP	3'UTR	0,207	1,05E-05	1,06E-04
cg11084532	6	132268051		IGR	-0,237	1,05E-05	1,06E-04
cg10803722	21	46713166	LOC642852	Body	-0,204	1,05E-05	1,06E-04
cg21949305	22	24828655	C22orf45	Body	0,229	1,05E-05	1,06E-04
cg06499993	14	23905057	MYH7	TSS200	0,232	1,05E-05	1,06E-04
cg09858955	2	58135951	VRK2	5'UTR	-0,207	1,06E-05	1,06E-04
cg11757130	10	112434043	RBM20	Body	0,234	1,06E-05	1,06E-04
cg07828121	21	43607978		IGR	-0,209	1,06E-05	1,06E-04
cg20044923	8	117698655	EIF3H	Body	0,204	1,06E-05	1,07E-04
cg22519160	13	59939001		IGR	0,256	1,06E-05	1,07E-04
cg02782634	17	57916643	TMEM49	Body	-0,291	1,06E-05	1,07E-04
cg20022223	17	80260410		IGR	-0,221	1,06E-05	1,07E-04
cg18057688	4	65188897	TECRL	Body	0,213	1,06E-05	1,07E-04
cg02925872	6	30010005	NCRNA00171	Body	0,241	1,06E-05	1,07E-04
cg26224381	2	202118126	CASP8	5'UTR	-0,279	1,06E-05	1,07E-04
cg20844427	13	106470099		IGR	-0,202	1,06E-05	1,07E-04
cg23612220	1	151129298	TNFAIP8L2	5'UTR	-0,261	1,06E-05	1,07E-04
cg16331689	14	105915268	MTA1	Body	0,2	1,06E-05	1,07E-04
cg09204638	16	4536324	HMOX2	5'UTR	0,208	1,06E-05	1,07E-04
cg26343990	7	31069579		IGR	-0,244	1,06E-05	1,07E-04

cg14871101	17	37437492	FBXL20	Body	0,233	1,06E-05	1,07E-04
cg15602272	7	55219876	EGFR	Body	-0,24	1,07E-05	1,07E-04
cg06096208	3	186464151		IGR	0,228	1,07E-05	1,07E-04
cg20798124	3	111682537	PHLDB2	Body	-0,203	1,07E-05	1,07E-04
cg17407629	5	102200701	PAM	TSS1500	0,22	1,07E-05	1,07E-04
cg23619100	16	73289073		IGR	0,227	1,07E-05	1,07E-04
cg19706707	19	16599761	CALR3	Body	0,225	1,07E-05	1,07E-04
cg24889694	8	101522535		IGR	0,266	1,07E-05	1,07E-04
cg11664375	1	203713227		IGR	-0,207	1,07E-05	1,08E-04
cg03595091	7	130081452	CEP41	TSS1500	0,22	1,07E-05	1,08E-04
cg07044749	10	97310310	SORBS1	5'UTR	0,215	1,07E-05	1,08E-04
cg03424206	8	62279200	CLVS1	Body	-0,229	1,07E-05	1,08E-04
cg15791438	22	31893238	SFI1	5'UTR	-0,238	1,07E-05	1,08E-04
cg09729147	15	60802526	RORA-AS1	Body	0,285	1,07E-05	1,08E-04
cg12945054	7	12841881		IGR	0,222	1,07E-05	1,08E-04
cg17171539	1	59398690		IGR	-0,202	1,07E-05	1,08E-04
cg03362667	15	85928046	AKAP13	5'UTR	0,217	1,07E-05	1,08E-04
cg16173847	18	77565614		IGR	-0,217	1,08E-05	1,08E-04
cg11012616	14	35835542		IGR	-0,203	1,08E-05	1,08E-04
cg07046870	3	122803153	PDIA5	Body	0,211	1,08E-05	1,08E-04
cg25809059	15	90581649	ZNF710	5'UTR	-0,229	1,08E-05	1,08E-04
cg01205058	6	167508191		IGR	0,217	1,08E-05	1,08E-04
cg24686983	14	33832387	NPAS3	Body	-0,212	1,08E-05	1,08E-04
cg26692531	6	152702370	SYNE1-AS1	Body	0,218	1,08E-05	1,08E-04
cg03980424	10	3526723		IGR	0,299	1,08E-05	1,08E-04
cg17218026	1	154582156	ADAR	5'UTR	-0,256	1,08E-05	1,08E-04
cg10653297	2	96905832	LOC285033	TSS200	0,226	1,08E-05	1,08E-04
cg05432558	17	62632136	SMURF2	Body	-0,267	1,08E-05	1,08E-04
cg23843597	7	16473518		IGR	-0,217	1,08E-05	1,08E-04
cg09481121	5	78985425	CMYA5	TSS1500	0,299	1,08E-05	1,08E-04
cg24839608	3	158414519		IGR	0,23	1,09E-05	1,09E-04
cg20873066	17	72101242		IGR	-0,204	1,09E-05	1,09E-04
cg23724027	6	139284923	REPS1	Body	0,222	1,09E-05	1,09E-04
cg19653890	15	55547488	RAB27A	5'UTR	-0,256	1,09E-05	1,09E-04
cg17515347	1	159047163	AIM2	TSS1500	-0,207	1,09E-05	1,09E-04
cg24441922	2	235401950	ARL4C	3'UTR	-0,223	1,09E-05	1,09E-04
cg04214961	5	156812167	CYFIP2	Body	0,209	1,09E-05	1,09E-04
cg11473433	10	6013477	IL15RA	Body	-0,264	1,09E-05	1,09E-04
cg10331073	10	26856128	APBB1IP	Body	0,202	1,09E-05	1,09E-04
cg03675258	19	13124691	NFIX	Body	-0,232	1,09E-05	1,09E-04
cg10750730	14	90067476	FOXN3	5'UTR	0,213	1,09E-05	1,09E-04
cg12235695	10	131969555	GLRX3	Body	-0,245	1,09E-05	1,09E-04
cg03490965	5	167614859	TENM2	Body	0,223	1,10E-05	1,09E-04
cg05291674	15	68480210	PIAS1	3'UTR	0,23	1,10E-05	1,09E-04
cg16850027	13	50932050	DLEU1	Body	0,218	1,10E-05	1,09E-04
cg14989202	1	9714843	C1orf200	TSS200	-0,27	1,10E-05	1,09E-04
cg22411162	14	98601450		IGR	0,202	1,10E-05	1,10E-04
cg19146916	9	101998297		IGR	-0,227	1,10E-05	1,10E-04
cg14646451	8	121792636	SNTB1	Body	-0,217	1,10E-05	1,10E-04
cg12991634	4	45319563		IGR	0,211	1,10E-05	1,10E-04

cg15284082	10	77142527		IGR	-0,213	1,10E-05	1,10E-04
cg24961480	11	13893152		IGR	-0,203	1,10E-05	1,10E-04
cg02695390	3	148708091	GYG1	TSS1500	-0,222	1,10E-05	1,10E-04
cg05485060	9	111776779	CTNNAL1	TSS1500	0,219	1,10E-05	1,10E-04
cg04574896	1	109247738		IGR	0,226	1,10E-05	1,10E-04
cg14332439	6	90819008	BACH2	5'UTR	-0,262	1,10E-05	1,10E-04
cg09651651	1	92208626	TGFBR3	Body	0,218	1,10E-05	1,10E-04
cg05812666	1	231954814	TSNAX-DISC1	Body	0,244	1,10E-05	1,10E-04
cg14836103	11	17290680		IGR	-0,204	1,11E-05	1,10E-04
cg07790232	2	148386625		IGR	-0,27	1,11E-05	1,10E-04
cg23564869	2	227566082		IGR	-0,256	1,11E-05	1,10E-04
cg05508074	14	52720463		IGR	0,218	1,11E-05	1,10E-04
cg03081563	7	142903058		IGR	-0,243	1,11E-05	1,10E-04
cg26705494	1	15473623	TMEM51-AS1	Body	-0,224	1,11E-05	1,10E-04
cg10880006	4	81122687	PRDM8	Body	0,217	1,11E-05	1,10E-04
cg24835948	5	14331816	TRIO	Body	-0,249	1,11E-05	1,10E-04
cg03607573	11	93471889	TAF1D	Body	-0,21	1,11E-05	1,10E-04
cg08635395	16	15747286	NDE1	5'UTR	0,223	1,11E-05	1,10E-04
cg19755435	14	88473001	GPR65	5'UTR	-0,216	1,11E-05	1,11E-04
cg25652454	17	414740	VPS53	3'UTR	0,215	1,11E-05	1,11E-04
cg18700166	13	103279421	TPP2	Body	0,206	1,11E-05	1,11E-04
cg25978276	11	15914689		IGR	-0,246	1,11E-05	1,11E-04
cg24612000	20	20065873	CFAP61	Body	0,221	1,11E-05	1,11E-04
cg24505803	6	80248448	LCA5	TSS1500	0,242	1,11E-05	1,11E-04
cg16039157	15	45007336	B2M	Body	-0,219	1,12E-05	1,11E-04
cg15729315	4	40992667	APBB2	Body	-0,234	1,12E-05	1,11E-04
cg23462985	5	142178218	ARHGAP26	Body	-0,265	1,12E-05	1,11E-04
cg23393593	2	238644583	LRRFIP1	Body	0,241	1,12E-05	1,11E-04
cg21616755	12	3838094	CRACR2A	5'UTR	-0,206	1,12E-05	1,11E-04
cg09580278	13	42222676	VWA8	Body	0,201	1,12E-05	1,11E-04
cg05651277	9	133360349	ASS1	Body	-0,244	1,12E-05	1,11E-04
cg12423387	7	130871960	MKLN1	Body	0,256	1,13E-05	1,12E-04
cg26799474	2	202098951	CASP8	5'UTR	-0,23	1,13E-05	1,12E-04
cg16988998	3	52755038	NEK4	Body	-0,208	1,13E-05	1,12E-04
cg26708596	8	49577641	LOC1019292(	Body	-0,23	1,13E-05	1,12E-04
cg15781838	2	128422717	LIMS2	Body	0,218	1,13E-05	1,12E-04
cg07471614	8	125855152		IGR	0,209	1,13E-05	1,12E-04
cg09385667	3	73543236	PDZRN3	Body	0,232	1,13E-05	1,12E-04
cg18924738	1	111743038	DENND2D	1stExon	-0,214	1,13E-05	1,12E-04
cg07566505	5	171023156		IGR	-0,241	1,13E-05	1,12E-04
cg23467838	2	67788837		IGR	0,252	1,14E-05	1,12E-04
cg10169653	21	36599791		IGR	-0,2	1,14E-05	1,12E-04
cg03639782	2	216532743	LINC00607	Body	0,239	1,14E-05	1,12E-04
cg04947157	17	76128481	TMC8	Body	-0,233	1,14E-05	1,13E-04
cg20318045	8	127601070		IGR	0,225	1,14E-05	1,13E-04
cg05697873	10	10337312		IGR	0,203	1,14E-05	1,13E-04
cg04523597	17	44235235	KANSL1	Body	0,26	1,14E-05	1,13E-04
cg20069757	2	182177430	LOC10192715	Body	-0,216	1,14E-05	1,13E-04
cg02952121	2	230330508	DNER	Body	-0,212	1,14E-05	1,13E-04
cg25280513	1	209931457	TRAF3IP3	5'UTR	-0,21	1,14E-05	1,13E-04

cg25450819	5	37717768	WDR70	Body	0,294	1,15E-05	1,13E-04
cg26437697	14	55143755	SAMD4A	Body	0,205	1,15E-05	1,13E-04
cg21497499	1	101708126		IGR	0,205	1,15E-05	1,13E-04
cg25715827	22	32111456	PRR14L	Body	0,228	1,15E-05	1,14E-04
cg11345909	1	22270751		IGR	0,265	1,15E-05	1,14E-04
cg23517999	2	202814523		IGR	0,207	1,15E-05	1,14E-04
cg09584188	16	58364996		IGR	-0,21	1,16E-05	1,14E-04
cg06112977	14	68869769	RAD51B	Body	0,219	1,16E-05	1,14E-04
cg25096745	11	35195513	CD44	Body	-0,205	1,16E-05	1,14E-04
cg15178610	1	185033187	RNF2	5'UTR	0,227	1,16E-05	1,14E-04
cg21376090	6	106201547		IGR	0,218	1,16E-05	1,14E-04
cg17949057	2	158297335	CYTIP	Body	-0,206	1,16E-05	1,14E-04
cg22925712	1	171221979	FMO1	5'UTR	-0,204	1,16E-05	1,14E-04
cg11793796	12	133706128	ZNF891	5'UTR	0,232	1,17E-05	1,15E-04
cg01098479	22	24699599	SPECC1L-ADC	Body	0,207	1,17E-05	1,15E-04
cg25716814	19	13124960	NFIX	Body	-0,218	1,17E-05	1,15E-04
cg03302605	7	34264887		IGR	-0,21	1,17E-05	1,15E-04
cg23144799	16	66556133	TK2	Body	-0,31	1,17E-05	1,15E-04
cg25602603	19	22320744		IGR	0,246	1,17E-05	1,15E-04
cg19633100	13	50028789	SETDB2	Body	-0,211	1,17E-05	1,15E-04
cg04094225	6	24930971		IGR	0,232	1,17E-05	1,15E-04
cg25588844	2	10037561	TAF1B	Body	-0,207	1,17E-05	1,15E-04
cg01372366	11	48041783	PTPRJ	Body	-0,244	1,17E-05	1,15E-04
cg19324188	11	69390826		IGR	-0,202	1,17E-05	1,15E-04
cg20495738	12	2338399	CACNA1C	Body	-0,244	1,17E-05	1,15E-04
cg06862227	19	47448286	ARHGAP35	Body	0,219	1,17E-05	1,15E-04
cg10033958	12	45729326	ANO6	Body	0,256	1,17E-05	1,15E-04
cg06962918	7	18127112		IGR	0,227	1,17E-05	1,15E-04
cg24020353	13	110991132	COL4A2	Body	0,251	1,18E-05	1,15E-04
cg20921268	9	438110	DOCK8	Body	0,229	1,18E-05	1,15E-04
cg06350844	4	141071263	MAML3	Body	0,234	1,18E-05	1,15E-04
cg07681806	15	70920794		IGR	-0,243	1,18E-05	1,16E-04
cg23449993	1	46563930	PIK3R3	Body	-0,204	1,18E-05	1,16E-04
cg07572714	11	9588384		IGR	0,203	1,18E-05	1,16E-04
cg08853277	12	75874140	GLIPR1	TSS1500	-0,205	1,18E-05	1,16E-04
cg10185277	3	57486443	DNAH12	Body	0,216	1,18E-05	1,16E-04
cg25217269	6	46619688	SLC25A27	TSS1500	0,213	1,18E-05	1,16E-04
cg03466858	3	69107805	UBA3	Body	-0,241	1,18E-05	1,16E-04
cg09574009	13	55211764		IGR	0,212	1,18E-05	1,16E-04
cg09047486	10	104082860	GBF1	Body	-0,216	1,19E-05	1,16E-04
cg20991420	2	173061715		IGR	-0,229	1,19E-05	1,16E-04
cg27084310	20	46370782	SULF2	Body	0,202	1,19E-05	1,16E-04
cg10519437	14	35184556	CFL2	TSS1500	0,204	1,19E-05	1,16E-04
cg05977696	2	154669330		IGR	0,224	1,19E-05	1,16E-04
cg22675660	17	2827599	RAP1GAP2	Body	0,205	1,19E-05	1,17E-04
cg22098672	4	25442127		IGR	0,215	1,19E-05	1,17E-04
cg11713770	12	92637878		IGR	0,234	1,19E-05	1,17E-04
cg01937212	6	32295097	C6orf10	Body	-0,222	1,20E-05	1,17E-04
cg02330500	14	95731916	CLMN	Body	-0,201	1,20E-05	1,17E-04
cg15213052	11	57549269	TMX2-CTNNL	Body	0,226	1,20E-05	1,17E-04

cg26005196	12	67197984		IGR	0,213	1,20E-05	1,17E-04
cg26305486	8	99033221	MATN2	Body	0,216	1,20E-05	1,17E-04
cg17313986	3	134945028	EPHB1	Body	-0,233	1,20E-05	1,17E-04
cg07254413	11	59940485	MS4A6A	Body	0,233	1,20E-05	1,17E-04
cg01083804	2	45405216	LINC01121	Body	-0,263	1,20E-05	1,17E-04
cg09226986	6	160852328	SLC22A3	Body	0,233	1,20E-05	1,18E-04
cg14999168	11	70211301	PPFIA1	Body	-0,255	1,21E-05	1,18E-04
cg03073269	16	54008588	FTO	Body	0,206	1,21E-05	1,18E-04
cg17783368	7	75899435	SRRM3	Body	0,21	1,21E-05	1,18E-04
cg09022900	8	853170	ERICH1-AS1	Body	0,21	1,21E-05	1,18E-04
cg08874886	1	48054045		IGR	0,241	1,21E-05	1,18E-04
cg08534512	7	32079181	PDE1C	Body	0,219	1,21E-05	1,18E-04
cg17468459	1	92208474	TGFBR3	Body	0,224	1,22E-05	1,18E-04
cg17025221	20	1450873		IGR	0,214	1,22E-05	1,18E-04
cg18735312	8	6594783	AGPAT5	Body	0,231	1,22E-05	1,19E-04
cg08671460	12	51912686		IGR	-0,237	1,22E-05	1,19E-04
cg01834979	12	58119918	AGAP2	3'UTR	0,222	1,22E-05	1,19E-04
cg07538187	13	45285150		IGR	-0,233	1,22E-05	1,19E-04
cg03755420	9	106517808		IGR	0,205	1,22E-05	1,19E-04
cg00105102	1	156217178	PAQR6	5'UTR	0,202	1,22E-05	1,19E-04
cg07290761	10	5859460		IGR	-0,2	1,22E-05	1,19E-04
cg27178401	16	10652711	EMP2	5'UTR	0,228	1,22E-05	1,19E-04
cg11967372	14	72064773	SIPA1L1	Body	-0,201	1,22E-05	1,19E-04
cg02696430	1	54237367	NDC1	Body	0,232	1,22E-05	1,19E-04
cg09935667	12	117164627	C12orf49	Body	0,245	1,23E-05	1,19E-04
cg24757533	17	74494286	RHBDF2	5'UTR	0,204	1,23E-05	1,19E-04
cg25207870	10	34491448	PARD3	Body	-0,21	1,23E-05	1,19E-04
cg12417775	1	212463238	PPP2R5A	Body	0,201	1,23E-05	1,19E-04
cg11676189	2	69875470		IGR	-0,235	1,23E-05	1,19E-04
cg16545019	9	14277484	NFIB	Body	0,238	1,23E-05	1,19E-04
cg22847647	11	75056970	ARRB1	Body	-0,201	1,23E-05	1,19E-04
cg07555832	13	31456553	LINC00545	TSS200	-0,203	1,23E-05	1,19E-04
cg06567829	16	89440878	ANKRD11	5'UTR	0,206	1,23E-05	1,19E-04
cg04382450	1	61800816	NFIA	Body	0,232	1,23E-05	1,19E-04
cg15391590	6	52913427	ICK	5'UTR	-0,273	1,23E-05	1,20E-04
cg18783806	9	115293024	KIAA1958	5'UTR	0,208	1,23E-05	1,20E-04
cg12939519	6	35130226		IGR	0,219	1,23E-05	1,20E-04
cg16976870	8	144631524		IGR	-0,218	1,23E-05	1,20E-04
cg25682171	13	110885015	COL4A1	Body	0,2	1,23E-05	1,20E-04
cg21701585	17	37303498	PLXDC1	Body	0,206	1,23E-05	1,20E-04
cg04629955	4	185203736		IGR	-0,226	1,23E-05	1,20E-04
cg09395612	16	86018936		IGR	-0,22	1,24E-05	1,20E-04
cg04002524	1	32037272		IGR	-0,228	1,24E-05	1,20E-04
cg11907323	8	122685859		IGR	-0,235	1,24E-05	1,20E-04
cg01136460	1	28383024	EYA3	Body	-0,235	1,24E-05	1,20E-04
cg12811582	16	21620253	METTL9	Body	-0,209	1,24E-05	1,20E-04
cg18409436	7	41245355		IGR	-0,251	1,24E-05	1,20E-04
cg12206359	13	36430134	MIR548F5	Body	-0,229	1,24E-05	1,20E-04
cg19893874	19	21205975	ZNF430	Body	-0,235	1,24E-05	1,20E-04
cg16331599	3	121796627	CD86	TSS200	-0,233	1,24E-05	1,20E-04

cg17849569	6	28058911	ZSCAN12L1	TSS200	0,207	1,24E-05	1,20E-04
cg04974594	16	75098576	ZNRF1	Body	-0,23	1,24E-05	1,20E-04
cg01947523	1	235098925	LOC10192785	Body	-0,233	1,24E-05	1,20E-04
cg23392273	1	108211585	VAV3	Body	0,209	1,25E-05	1,20E-04
cg02275985	10	74877855	NUDT13	5'UTR	0,21	1,25E-05	1,21E-04
cg13857915	1	66822848	PDE4B	Body	-0,265	1,25E-05	1,21E-04
cg07310671	16	85253980		IGR	0,206	1,25E-05	1,21E-04
cg15022880	2	135035221	MGAT5	Body	0,216	1,25E-05	1,21E-04
cg04797958	12	26880038	ITPR2	Body	0,218	1,25E-05	1,21E-04
cg25556008	19	2525384	GNG7	5'UTR	0,215	1,25E-05	1,21E-04
cg26584309	14	91864737	CCDC88C	Body	-0,219	1,25E-05	1,21E-04
cg09302031	6	24313867	DCDC2	Body	0,259	1,25E-05	1,21E-04
cg07493260	19	6630909		IGR	-0,236	1,25E-05	1,21E-04
cg01162866	22	42241482	SREBF2	Body	0,217	1,26E-05	1,21E-04
cg19264599	9	126504364	DENND1A	Body	0,213	1,26E-05	1,21E-04
cg17415730	9	81880073		IGR	-0,213	1,26E-05	1,22E-04
cg17330403	14	73919241	NUMB	5'UTR	-0,224	1,26E-05	1,22E-04
cg20348196	6	32408284	HLA-DRA	Body	-0,207	1,26E-05	1,22E-04
cg19885871	14	68853810	RAD51B	Body	0,226	1,26E-05	1,22E-04
cg11149194	14	27245119		IGR	0,23	1,26E-05	1,22E-04
cg25426743	7	27166103	HOXA3	5'UTR	0,325	1,26E-05	1,22E-04
cg09888670	20	49032344		IGR	0,257	1,27E-05	1,22E-04
cg10946742	12	94208017	CRADD	Body	0,287	1,27E-05	1,22E-04
cg03795776	6	90687632	BACH2	Body	0,262	1,27E-05	1,22E-04
cg12032795	11	10886441	ZBED5-AS1	Body	0,26	1,27E-05	1,22E-04
cg15502231	7	107235907	BCAP29	Body	0,242	1,27E-05	1,22E-04
cg15078203	2	20650041		IGR	0,217	1,27E-05	1,22E-04
cg26874367	2	129381792		IGR	-0,25	1,27E-05	1,22E-04
cg16301825	2	2791276		IGR	0,221	1,27E-05	1,22E-04
cg17489312	1	9376039	SPSB1	5'UTR	-0,22	1,27E-05	1,23E-04
cg18089847	12	111598683	CUX2	Body	0,201	1,28E-05	1,23E-04
cg05499031	10	126755512	CTBP2	5'UTR	0,21	1,28E-05	1,23E-04
cg01129641	2	241561237		IGR	-0,271	1,28E-05	1,23E-04
cg08637269	11	122524689		IGR	0,209	1,28E-05	1,23E-04
cg06911238	1	46859671	FAAH	TSS1500	0,24	1,28E-05	1,23E-04
cg20265748	14	89017615	PTPN21	5'UTR	0,21	1,28E-05	1,23E-04
cg18977617	18	32291165	DTNA	5'UTR	0,235	1,28E-05	1,23E-04
cg08331345	16	28995882	LAT	TSS1500	-0,207	1,28E-05	1,23E-04
cg08368849	14	103663129		IGR	-0,208	1,28E-05	1,23E-04
cg26556684	2	102764618	IL1R1	5'UTR	-0,24	1,28E-05	1,23E-04
cg11016270	6	52974621		IGR	-0,202	1,28E-05	1,23E-04
cg11967111	4	24105842		IGR	0,256	1,28E-05	1,23E-04
cg21064698	9	14308890	NFIB	Body	0,22	1,28E-05	1,23E-04
cg06469326	17	73507843	CASKIN2	Body	0,222	1,28E-05	1,23E-04
cg02640147	5	170024515	KCNIP1	Body	-0,295	1,28E-05	1,23E-04
cg12538222	2	7576494	LOC10050627	Body	-0,201	1,28E-05	1,23E-04
cg26345663	5	36652280	SLC1A3	Body	0,201	1,28E-05	1,23E-04
cg06796314	10	7088654		IGR	-0,216	1,28E-05	1,23E-04
cg25437822	7	50357133	IKZF1	5'UTR	-0,238	1,29E-05	1,23E-04
cg21871746	7	99616820	ZKSCAN1	5'UTR	0,207	1,29E-05	1,23E-04

cg27484857	7	30370507	ZNRF2	Body	-0,266	1,29E-05	1,23E-04
cg10198174	2	24331829	FAM228B	5'UTR	0,226	1,29E-05	1,24E-04
cg24366693	7	7608860	MIOS	5'UTR	-0,321	1,29E-05	1,24E-04
cg14223695	6	106968434	AIM1	Body	-0,206	1,29E-05	1,24E-04
cg23398454	16	50608327	NKD1	Body	0,212	1,29E-05	1,24E-04
cg26338428	4	148767763	ARHGAP10	Body	-0,201	1,29E-05	1,24E-04
cg08828261	6	3913611		IGR	0,251	1,29E-05	1,24E-04
cg14387854	2	182321282	ITGA4	TSS1500	-0,238	1,29E-05	1,24E-04
cg08310863	6	111390240		IGR	0,209	1,30E-05	1,24E-04
cg09608949	11	117748189	FXYD6	TSS1500	0,203	1,30E-05	1,24E-04
cg06238667	8	29086832	KIF13B	Body	-0,246	1,30E-05	1,24E-04
cg25350169	2	44347690		IGR	-0,205	1,30E-05	1,24E-04
cg20983265	2	21647170		IGR	-0,2	1,30E-05	1,24E-04
cg17856019	14	53389611	FERMT2	Body	0,212	1,30E-05	1,24E-04
cg04318619	17	1090300	ABR	1stExon	0,23	1,30E-05	1,24E-04
cg04799018	4	183167241		IGR	0,22	1,30E-05	1,24E-04
cg16082108	2	33906275		IGR	0,204	1,30E-05	1,25E-04
cg21648978	1	114201416	MAGI3	Body	0,227	1,30E-05	1,25E-04
cg18932278	5	169407941	FAM196B	TSS200	0,271	1,30E-05	1,25E-04
cg10082647	12	107348855	C12orf23	TSS1500	0,204	1,30E-05	1,25E-04
cg00081417	6	140400137		IGR	0,259	1,31E-05	1,25E-04
cg24143645	6	149347812	UST	Body	0,227	1,31E-05	1,25E-04
cg06017039	12	72423683	TPH2	Body	-0,218	1,31E-05	1,25E-04
cg25252839	1	235285886	TOMM20	Body	0,214	1,31E-05	1,25E-04
cg10350284	1	178908349		IGR	0,205	1,31E-05	1,25E-04
cg23654401	7	55606442	VOPP1	Body	-0,239	1,31E-05	1,25E-04
cg08991108	5	172752277	STC2	Body	0,216	1,31E-05	1,25E-04
cg04195774	5	118677172	TNFAIP8	Body	-0,227	1,31E-05	1,25E-04
cg20929180	4	20700668	PACRGL	TSS1500	0,293	1,31E-05	1,25E-04
cg17898279	1	201959647	RNPEP	Body	0,237	1,31E-05	1,25E-04
cg08867031	15	25937582	ATP10A	Body	0,221	1,31E-05	1,25E-04
cg17501566	4	54457916	LNX1-AS2	TSS1500	-0,228	1,31E-05	1,25E-04
cg27348620	1	48169751		IGR	-0,205	1,32E-05	1,26E-04
cg04501558	6	1665686	GMDS	Body	-0,216	1,32E-05	1,26E-04
cg25000900	5	73197088	RGNEF	Body	0,232	1,32E-05	1,26E-04
cg14273411	8	61656679	CHD7	Body	0,259	1,32E-05	1,26E-04
cg04780401	18	2959542	LPIN2	Body	-0,21	1,32E-05	1,26E-04
cg04801961	2	46017286	PRKCE	Body	0,243	1,32E-05	1,26E-04
cg15295089	6	109381878	SESN1	Body	0,262	1,32E-05	1,26E-04
cg26826793	6	36172020	BRPF3	Body	-0,214	1,32E-05	1,26E-04
cg19093687	3	145874048	PLOD2	Body	0,243	1,32E-05	1,26E-04
cg17987203	13	44048036	ENOX1	5'UTR	0,231	1,32E-05	1,26E-04
cg18136633	12	99050719	APAF1	Body	-0,241	1,32E-05	1,26E-04
cg08248287	3	171024841	TNIK	Body	0,274	1,32E-05	1,26E-04
cg07847812	6	21856464	CASC15	Body	-0,262	1,32E-05	1,26E-04
cg11701069	12	96133818	NTN4	Body	0,218	1,33E-05	1,26E-04
cg13291688	4	139753296		IGR	-0,203	1,33E-05	1,27E-04
cg11732322	5	172193903		IGR	0,208	1,33E-05	1,27E-04
cg15195938	5	137387730		IGR	0,294	1,33E-05	1,27E-04
cg00245615	7	141674668	TAS2R38	TSS1500	0,211	1,33E-05	1,27E-04

cg24951224	4	103730655	UBE2D3	Body	0,262	1,33E-05	1,27E-04
cg15730828	8	124838057		IGR	0,211	1,33E-05	1,27E-04
cg08016578	2	47042548	LINC01118	TSS1500	0,205	1,34E-05	1,27E-04
cg12741256	18	29232383	B4GALT6	Body	-0,224	1,34E-05	1,27E-04
cg22368524	11	94283990		IGR	-0,239	1,34E-05	1,27E-04
cg19137417	17	79362935		IGR	0,214	1,34E-05	1,27E-04
cg18290725	1	101571581		IGR	0,236	1,34E-05	1,27E-04
cg19432886	5	124394386		IGR	0,218	1,34E-05	1,27E-04
cg04593197	2	172811676	HAT1	Body	-0,228	1,34E-05	1,27E-04
cg08334000	22	28792294	TTC28	Body	-0,269	1,34E-05	1,27E-04
cg08166631	1	40599011		IGR	0,209	1,34E-05	1,27E-04
cg17696898	18	11160476		IGR	0,23	1,34E-05	1,27E-04
cg05335855	3	94647969		IGR	0,247	1,34E-05	1,27E-04
cg05479962	16	31488985	TGFB1I1	3'UTR	-0,267	1,34E-05	1,27E-04
cg12589798	20	44839734	CDH22	Body	0,213	1,34E-05	1,28E-04
cg12553873	5	142159386	ARHGAP26	Body	0,288	1,34E-05	1,28E-04
cg08004685	5	117897966	HRAT56	TSS200	0,253	1,35E-05	1,28E-04
cg08545995	6	41990244	CCND3	5'UTR	-0,295	1,35E-05	1,28E-04
cg12010857	4	102112901	PPP3CA	Body	0,252	1,35E-05	1,28E-04
cg16231595	1	31129056		IGR	0,204	1,35E-05	1,28E-04
cg06929425	7	84315906		IGR	-0,204	1,35E-05	1,28E-04
cg08435386	20	11791080	LINC00687	Body	-0,213	1,35E-05	1,28E-04
cg03957495	3	42084438		IGR	0,257	1,35E-05	1,28E-04
cg03737199	2	3105346	LINC01250	Body	0,258	1,36E-05	1,28E-04
cg19561274	2	96905601	LOC285033	TSS1500	0,224	1,36E-05	1,28E-04
cg12127758	8	1707330		IGR	0,243	1,36E-05	1,28E-04
cg13252498	21	42588294	BACE2	Body	-0,24	1,36E-05	1,28E-04
cg26728382	6	163848720	QKI	Body	0,208	1,36E-05	1,29E-04
cg12431891	3	114958699		IGR	0,234	1,36E-05	1,29E-04
cg15915594	8	93063888	RUNX1T1	Body	0,272	1,36E-05	1,29E-04
cg19709608	3	16558965		IGR	0,229	1,36E-05	1,29E-04
cg19903896	6	154579470	IPCEF1	Body	-0,248	1,36E-05	1,29E-04
cg24237035	11	9023437	NRIP3	Body	-0,229	1,36E-05	1,29E-04
cg01879420	6	111194645	AMD1	TSS1500	-0,215	1,37E-05	1,29E-04
cg20340429	10	64967610	JMJD1C	Body	0,21	1,37E-05	1,29E-04
cg12105042	4	160150415		IGR	0,261	1,37E-05	1,29E-04
cg07301793	3	167631492	LINC01330	Body	-0,223	1,37E-05	1,29E-04
cg26606574	18	63711791		IGR	0,206	1,37E-05	1,29E-04
cg25390683	3	42391487		IGR	0,221	1,37E-05	1,30E-04
cg00543474	10	134260981	C10orf91	Body	-0,22	1,37E-05	1,30E-04
cg08112356	12	8187244	FOXJ2	5'UTR	0,203	1,37E-05	1,30E-04
cg15319576	11	127909557		IGR	-0,214	1,38E-05	1,30E-04
cg02539809	7	120725046	C7orf58	Body	0,203	1,38E-05	1,30E-04
cg19733073	9	73007962	KLF9	Body	0,211	1,38E-05	1,30E-04
cg18081760	1	114483597	HIPK1	Body	-0,266	1,38E-05	1,30E-04
cg17370665	14	69162065		IGR	-0,208	1,38E-05	1,30E-04
cg00563031	5	119782696		IGR	0,213	1,38E-05	1,30E-04
cg00188579	7	115806045		IGR	-0,224	1,38E-05	1,30E-04
cg23963437	1	68101162		IGR	-0,206	1,38E-05	1,30E-04
cg12378055	1	74929525	FPGT-TNNI3K	Body	-0,224	1,38E-05	1,30E-04

cg14892011	1	61582339	NFIA	Body	0,212	1,38E-05	1,30E-04
cg12779727	5	135423888		IGR	0,225	1,38E-05	1,30E-04
cg22894824	5	102143635		IGR	0,207	1,38E-05	1,30E-04
cg14286144	2	53285758		IGR	0,223	1,38E-05	1,30E-04
cg27552340	2	233647291	GIGYF2	Body	0,217	1,39E-05	1,31E-04
cg10572465	1	114414042	PTPN22	Body	-0,242	1,39E-05	1,31E-04
cg06374595	2	66723348	MEIS1	Body	0,201	1,39E-05	1,31E-04
cg11508123	6	129796679	LAMA2	Body	0,214	1,39E-05	1,31E-04
cg26118759	1	117058474	CD58	Body	-0,287	1,39E-05	1,31E-04
cg04471498	6	36818903		IGR	0,204	1,39E-05	1,31E-04
cg18790427	2	204547598		IGR	0,216	1,39E-05	1,31E-04
cg17971841	2	192140872	MYO1B	5'UTR	-0,228	1,39E-05	1,31E-04
cg09290894	9	19665584	SLC24A2	Body	-0,252	1,39E-05	1,31E-04
cg04984847	8	29894504		IGR	0,233	1,40E-05	1,31E-04
cg01810967	2	86693259	KDM3A	Body	0,222	1,40E-05	1,31E-04
cg04973863	12	18866655	PLCZ1	Body	-0,203	1,40E-05	1,31E-04
cg21359528	3	69762275		IGR	0,203	1,40E-05	1,31E-04
cg04838382	2	235400052		IGR	-0,208	1,40E-05	1,32E-04
cg17603082	11	95650701	MTMR2	Body	0,247	1,40E-05	1,32E-04
cg02438950	3	176816524	TBL1XR1	5'UTR	0,234	1,41E-05	1,32E-04
cg06530081	12	50127515		IGR	0,207	1,41E-05	1,32E-04
cg07297115	15	42800679	SNAP23	5'UTR	0,232	1,41E-05	1,32E-04
cg00902185	22	20232832	RTN4R	Body	-0,283	1,41E-05	1,32E-04
cg04903269	15	72598763	CELF6	TSS200	-0,216	1,41E-05	1,32E-04
cg16495782	17	64260237		IGR	-0,27	1,41E-05	1,32E-04
cg18278790	19	56187851	EPN1	5'UTR	0,216	1,41E-05	1,33E-04
cg22782019	2	189856425	COL3A1	Body	0,202	1,41E-05	1,33E-04
cg24543939	5	53952507		IGR	0,278	1,42E-05	1,33E-04
cg05209630	12	72205041		IGR	-0,221	1,42E-05	1,33E-04
cg04965094	13	45854777	GTF2F2	Body	0,223	1,42E-05	1,33E-04
cg16776350	1	160549158	CD84	Body	-0,211	1,42E-05	1,33E-04
cg23199203	22	50976939		IGR	-0,236	1,42E-05	1,33E-04
cg01058888	14	70478540	SMOC1	Body	0,213	1,42E-05	1,33E-04
cg03537657	5	73527843		IGR	-0,245	1,42E-05	1,33E-04
cg02661473	6	6798768		IGR	-0,207	1,42E-05	1,33E-04
cg13996193	8	19533749	CSGALNACT1	Body	0,218	1,42E-05	1,33E-04
cg03534504	5	171594998	STK10	Body	0,214	1,42E-05	1,33E-04
cg17283031	4	40185812		IGR	-0,21	1,42E-05	1,33E-04
cg13932370	12	70992723	PTPRB	Body	0,275	1,43E-05	1,33E-04
cg00821201	20	17557257	DSTN	5'UTR	0,22	1,43E-05	1,33E-04
cg22588412	6	36082889		IGR	-0,279	1,43E-05	1,33E-04
cg18599303	6	159108144	SYTL3	Body	-0,21	1,43E-05	1,33E-04
cg20079922	12	47610418	PCED1B-AS1	TSS200	-0,27	1,43E-05	1,33E-04
cg03179020	8	77914291	PEX2	TSS1500	0,227	1,43E-05	1,34E-04
cg07526040	8	94744456	RBM12B	3'UTR	0,222	1,43E-05	1,34E-04
cg15234668	20	35925236	MANBAL	5'UTR	0,219	1,43E-05	1,34E-04
cg18023339	11	130541481	C11orf44	TSS1500	-0,212	1,43E-05	1,34E-04
cg12825867	11	19416398	NAV2	Body	-0,272	1,43E-05	1,34E-04
cg04951594	6	144587505		IGR	0,229	1,43E-05	1,34E-04
cg05046814	15	77320670	PSTPIP1	Body	0,204	1,43E-05	1,34E-04

cg17007339	11	85430629	SYTL2	Body	0,215	1,44E-05	1,34E-04
cg20371437	13	36296872	MIR548F5	Body	-0,203	1,44E-05	1,34E-04
cg15834355	12	54442075	HOXC4	5'UTR	0,261	1,44E-05	1,34E-04
cg01460604	7	102584030	LRRC17	3'UTR	-0,263	1,44E-05	1,34E-04
cg13829717	10	26741185	APBB1IP	5'UTR	0,213	1,44E-05	1,34E-04
cg00940054	9	73216478	TRPM3	Body	0,203	1,44E-05	1,34E-04
cg15976080	15	47809041	SEMA6D	5'UTR	-0,219	1,44E-05	1,34E-04
cg15176005	1	228122465	WNT9A	Body	-0,247	1,44E-05	1,35E-04
cg20942931	11	10806733		IGR	0,23	1,44E-05	1,35E-04
cg11880074	1	236917178	ACTN2	Body	0,216	1,45E-05	1,35E-04
cg14202467	12	71426855		IGR	0,208	1,45E-05	1,35E-04
cg01552711	13	21291353	IL17D	Body	0,203	1,45E-05	1,35E-04
cg02628581	6	149641011	TAB2	Body	-0,206	1,45E-05	1,35E-04
cg03578022	17	67057578	ABCA9	TSS1500	0,202	1,45E-05	1,35E-04
cg23057326	1	42207150	HIVEP3	5'UTR	-0,207	1,45E-05	1,35E-04
cg16342538	2	31607367	XDH	Body	-0,218	1,45E-05	1,35E-04
cg08424981	11	5586092		IGR	-0,251	1,45E-05	1,35E-04
cg07965441	14	86523568	LOC1019287(	Body	0,215	1,45E-05	1,35E-04
cg14924131	13	74207962		IGR	-0,202	1,46E-05	1,36E-04
cg19695507	10	13526193	BEND7	Body	-0,201	1,46E-05	1,36E-04
cg11781963	8	22210276	PIWIL2	Body	-0,234	1,46E-05	1,36E-04
cg12221689	2	98827983	VWA3B	Body	-0,206	1,46E-05	1,36E-04
cg22417827	11	108471742		IGR	0,281	1,46E-05	1,36E-04
cg01410923	2	192198841	MYO1B	Body	-0,227	1,47E-05	1,36E-04
cg12934382	3	51741135	GRM2	1stExon	0,268	1,47E-05	1,36E-04
cg24676384	1	154929455	PBXIP1	TSS1500	0,22	1,47E-05	1,36E-04
cg11460161	13	109933938		IGR	0,206	1,47E-05	1,36E-04
cg03116124	1	231293208		IGR	-0,245	1,47E-05	1,36E-04
cg19810767	13	31146447		IGR	0,236	1,47E-05	1,36E-04
cg20342432	2	240667900		IGR	0,203	1,47E-05	1,37E-04
cg12160575	9	117129367	AKNA	Body	-0,202	1,47E-05	1,37E-04
cg13376953	11	1750381	HCCA2	Body	-0,204	1,47E-05	1,37E-04
cg11240057	6	136444593	PDE7B	Body	0,204	1,47E-05	1,37E-04
cg11049075	17	42199063	HDAC5	5'UTR	0,211	1,48E-05	1,37E-04
cg16919019	7	45066650	CCM2	TSS1500	-0,237	1,48E-05	1,37E-04
cg09808606	16	21620545	METTL9	Body	-0,254	1,48E-05	1,37E-04
cg08669718	4	121568	ZNF718	Body	-0,217	1,48E-05	1,37E-04
cg07508429	7	44448689	NUDCD3	Body	0,201	1,48E-05	1,37E-04
cg18016034	10	61900573	ANK3	1stExon	0,217	1,48E-05	1,37E-04
cg13794868	17	9998770	GAS7	Body	0,204	1,48E-05	1,37E-04
cg18588194	11	30304009		IGR	-0,221	1,48E-05	1,37E-04
cg22234941	7	114571546	MDFIC	Body	-0,3	1,48E-05	1,37E-04
cg12718972	17	1737862	RPA1	Body	-0,206	1,48E-05	1,37E-04
cg10594473	4	106112932	TET2	5'UTR	0,216	1,48E-05	1,37E-04
cg05471051	1	185623946		IGR	0,235	1,49E-05	1,38E-04
cg02750262	18	72916776	ZADH2	Body	0,217	1,49E-05	1,38E-04
cg08010783	8	143324994	TSNARE1	Body	0,206	1,50E-05	1,38E-04
cg11030720	9	3937059	GLIS3	Body	0,276	1,50E-05	1,38E-04
cg02468320	12	2404134	CACNA1C	Body	-0,267	1,50E-05	1,38E-04
cg04464475	3	13054327	IQSEC1	Body	-0,209	1,50E-05	1,38E-04

cg03328892	9	93913460		IGR	-0,217	1,50E-05	1,39E-04
cg16763954	13	44379437		IGR	-0,211	1,50E-05	1,39E-04
cg16198905	1	153609060	CHTOP	5'UTR	0,2	1,50E-05	1,39E-04
cg07172334	21	31655261	KRTAP24-1	1stExon	-0,203	1,50E-05	1,39E-04
cg14178003	5	70778203	BDP1	Body	0,244	1,50E-05	1,39E-04
cg06810986	16	85394259		IGR	-0,243	1,50E-05	1,39E-04
cg25349276	4	41614591	LIMCH1	Body	0,221	1,50E-05	1,39E-04
cg15125963	3	176868150	TBL1XR1	5'UTR	0,209	1,50E-05	1,39E-04
cg21097817	1	178840498	ANGPTL1	TSS1500	0,249	1,51E-05	1,39E-04
cg10669368	12	76414305		IGR	-0,217	1,51E-05	1,39E-04
cg14171343	9	114219932	KIAA0368	Body	0,215	1,51E-05	1,40E-04
cg04066265	8	19460243	CSGALNACT1	TSS200	0,235	1,52E-05	1,40E-04
cg16065769	4	52903163	SGCB	Body	0,212	1,52E-05	1,40E-04
cg23668476	7	65715904	TPST1	Body	-0,21	1,52E-05	1,40E-04
cg04210301	17	47710727	SPOP	5'UTR	0,213	1,52E-05	1,40E-04
cg23111696	1	41626466	SCMH1	5'UTR	0,209	1,53E-05	1,40E-04
cg24140860	4	182986612		IGR	0,201	1,53E-05	1,40E-04
cg17511997	12	69555776		IGR	-0,22	1,53E-05	1,41E-04
cg08705351	1	21384744	EIF4G3	5'UTR	0,246	1,53E-05	1,41E-04
cg27549963	12	53991101	ATF7	Body	0,219	1,53E-05	1,41E-04
cg15129265	2	183292922	PDE1A	Body	0,216	1,53E-05	1,41E-04
cg01946656	10	71086072	HK1	Body	0,208	1,54E-05	1,41E-04
cg11504063	9	132682473	FNBP1	Body	-0,207	1,54E-05	1,41E-04
cg27130197	8	29028955	KIF13B	Body	-0,243	1,54E-05	1,41E-04
cg18957794	3	194210793		IGR	-0,219	1,54E-05	1,41E-04
cg06528787	1	110500956		IGR	0,267	1,54E-05	1,42E-04
cg27300637	8	25110030	DOCK5	Body	0,206	1,54E-05	1,42E-04
cg09592900	12	18582318	PIK3C2G	Body	0,209	1,54E-05	1,42E-04
cg01001115	9	95244844	ASPN	TSS200	0,266	1,54E-05	1,42E-04
cg21047695	8	63614552	NKAIN3	Body	0,22	1,55E-05	1,42E-04
cg10138338	8	142289648		IGR	0,237	1,55E-05	1,42E-04
cg13697804	1	171153187	FMO2	TSS1500	-0,215	1,55E-05	1,42E-04
cg14357164	10	20002879	LOC10192883	Body	0,211	1,55E-05	1,42E-04
cg08587314	10	90106505	RNLS	Body	0,279	1,55E-05	1,42E-04
cg01482958	1	85357136	LPAR3	5'UTR	0,203	1,55E-05	1,42E-04
cg26932379	16	26609755		IGR	-0,209	1,55E-05	1,42E-04
cg14818794	2	47074519	LINC01119	Body	0,233	1,55E-05	1,42E-04
cg26786571	3	182832789		IGR	0,211	1,55E-05	1,42E-04
cg16421726	10	126629326	ZRANB1	TSS1500	0,228	1,55E-05	1,42E-04
cg11802899	14	52867870		IGR	-0,219	1,56E-05	1,43E-04
cg22328249	9	92080039	SEMA4D	5'UTR	-0,222	1,56E-05	1,43E-04
cg23533275	5	36575671		IGR	0,213	1,56E-05	1,43E-04
cg05185926	5	142602110	ARHGAP26	3'UTR	-0,283	1,56E-05	1,43E-04
cg02141472	21	35897406	RCAN1	1stExon	0,204	1,56E-05	1,43E-04
cg05898591	5	174805715		IGR	-0,215	1,56E-05	1,43E-04
cg10098353	2	204571344	CD28	5'UTR	-0,214	1,56E-05	1,43E-04
cg00213192	2	159363889	PKP4	5'UTR	-0,212	1,56E-05	1,43E-04
cg11003133	1	159046391	AIM2	5'UTR	-0,221	1,56E-05	1,43E-04
cg03011271	6	108927579	FOXO3	Body	0,205	1,57E-05	1,43E-04
cg24031653	6	73349745	KCNQ5-IT1	Body	0,218	1,57E-05	1,43E-04

cg17376609	13	88328813	SLITRK5	Body	0,204	1,57E-05	1,43E-04
cg16964728	15	61340524	RORA	Body	-0,213	1,57E-05	1,43E-04
cg01184901	1	6113023	KCNAB2	Body	0,247	1,57E-05	1,44E-04
cg14264204	12	131510557	ADGRD1	Body	-0,222	1,57E-05	1,44E-04
cg22668906	11	128180077		IGR	0,212	1,58E-05	1,44E-04
cg18669406	6	138145161		IGR	-0,237	1,58E-05	1,44E-04
cg02151918	11	70211268	PPFIA1	Body	-0,258	1,58E-05	1,44E-04
cg02283964	6	108563766	SNX3	Body	0,209	1,58E-05	1,44E-04
cg12670405	4	109145949		IGR	0,206	1,58E-05	1,44E-04
cg08439787	11	28779514		IGR	0,22	1,58E-05	1,44E-04
cg08549566	18	77861279		IGR	0,213	1,58E-05	1,44E-04
cg24498417	2	46964839	SOCS5	5'UTR	0,277	1,58E-05	1,44E-04
cg15652863	10	61697741		IGR	0,23	1,58E-05	1,44E-04
cg02410766	10	6969426		IGR	-0,258	1,58E-05	1,44E-04
cg26453900	1	66868107		IGR	0,22	1,59E-05	1,45E-04
cg25737397	2	170725297	UBR3	Body	0,211	1,59E-05	1,45E-04
cg07628084	6	112576119	LAMA4	TSS1500	0,216	1,59E-05	1,45E-04
cg06038367	16	30198370	CORO1A	Body	-0,203	1,59E-05	1,45E-04
cg18324583	5	142975083		IGR	-0,205	1,59E-05	1,45E-04
cg10674392	1	67813535	IL12RB2	Body	0,214	1,60E-05	1,45E-04
cg22902340	21	46332291	ITGB2	5'UTR	-0,218	1,60E-05	1,45E-04
cg15148691	6	29404733		IGR	0,25	1,60E-05	1,45E-04
cg06811300	6	16504474	ATXN1	5'UTR	-0,226	1,60E-05	1,46E-04
cg03613557	8	11666810	FDFT1	1stExon	0,203	1,60E-05	1,46E-04
cg23108778	1	7616070	CAMTA1	Body	-0,228	1,61E-05	1,46E-04
cg12971325	12	109027932	SELPLG	TSS1500	-0,232	1,61E-05	1,46E-04
cg02330501	1	2997409	PRDM16	Body	0,204	1,61E-05	1,46E-04
cg10174146	10	6540795	PRKCQ	Body	-0,256	1,61E-05	1,46E-04
cg27446233	12	48516484	PFKM	5'UTR	0,209	1,61E-05	1,46E-04
cg00632940	2	189651242	DIRC1	5'UTR	0,202	1,61E-05	1,46E-04
cg06397398	19	54023732	ZNF331	TSS1500	0,256	1,61E-05	1,46E-04
cg20146767	12	102065524	MYBPC1	Body	-0,202	1,61E-05	1,47E-04
cg00533132	6	80853181	BCKDHB	Body	0,219	1,62E-05	1,47E-04
cg16935031	15	98539090		IGR	-0,213	1,62E-05	1,47E-04
cg11532216	1	183063517	LAMC1	Body	-0,216	1,62E-05	1,47E-04
cg20423756	19	47805662		IGR	-0,217	1,62E-05	1,47E-04
cg16536855	10	71038317	HK1	5'UTR	0,204	1,62E-05	1,47E-04
cg04546262	21	40365716		IGR	0,244	1,63E-05	1,47E-04
cg12751277	1	231954287	TSNAX-DISC1	Body	0,244	1,63E-05	1,48E-04
cg15037137	7	27144302		IGR	0,215	1,63E-05	1,48E-04
cg20342073	2	33013352	TTC27	Body	0,26	1,63E-05	1,48E-04
cg05674928	12	121410996	C12orf27	TSS1500	0,212	1,63E-05	1,48E-04
cg00979661	1	28144006	STX12	Body	0,226	1,64E-05	1,48E-04
cg24697911	5	153454971		IGR	-0,238	1,64E-05	1,48E-04
cg13410614	9	136341915	SLC2A6	Body	-0,231	1,64E-05	1,48E-04
cg13425294	8	125934433		IGR	0,292	1,64E-05	1,48E-04
cg00653455	3	53914075	ACTR8	Body	0,201	1,64E-05	1,48E-04
cg25315420	16	87984024	BANP	TSS1500	0,202	1,64E-05	1,48E-04
cg17758611	3	112130582		IGR	0,255	1,64E-05	1,48E-04
cg15416948	17	53658125		IGR	-0,208	1,64E-05	1,48E-04

cg02923966	10	73483782	CDH23	ExonBnd	-0,219	1,64E-05	1,49E-04
cg17454283	4	96549942		IGR	-0,217	1,65E-05	1,49E-04
cg12011343	6	20320423		IGR	-0,212	1,65E-05	1,49E-04
cg17518550	1	247712383	C1orf150	TSS200	-0,203	1,65E-05	1,49E-04
cg15408855	12	106736581	TCP11L2	Body	0,21	1,65E-05	1,49E-04
cg22480773	6	49466589	GLYATL3	TSS1500	0,209	1,65E-05	1,49E-04
cg24501493	8	12951315	DLC1	Body	0,247	1,66E-05	1,49E-04
cg12767645	18	62295302		IGR	-0,212	1,66E-05	1,49E-04
cg22057263	2	106825600		IGR	0,219	1,66E-05	1,49E-04
cg01663277	1	109246717		IGR	-0,209	1,66E-05	1,50E-04
cg15838333	14	60554440		IGR	0,421	1,66E-05	1,50E-04
cg05225461	5	102596755	C5orf30	5'UTR	-0,205	1,66E-05	1,50E-04
cg16657434	8	135817588	MIR30D	TSS1500	0,216	1,66E-05	1,50E-04
cg00345372	3	112404450		IGR	0,204	1,66E-05	1,50E-04
cg21066950	2	241564473	GPR35	TSS200	-0,204	1,66E-05	1,50E-04
cg23279152	4	75856823	PARM1	TSS1500	0,237	1,66E-05	1,50E-04
cg22382309	9	95726447	FGD3	1stExon	-0,235	1,67E-05	1,50E-04
cg17643230	17	16950619	MPRIP	Body	0,211	1,67E-05	1,50E-04
cg10768932	1	198590738		IGR	-0,214	1,67E-05	1,50E-04
cg09161245	10	73604666	PSAP	Body	-0,229	1,67E-05	1,50E-04
cg26083380	11	87036674	TMEM135	3'UTR	0,228	1,67E-05	1,51E-04
cg27616666	2	168193427		IGR	-0,211	1,67E-05	1,51E-04
cg23156245	2	212300337	ERBB4	Body	0,235	1,68E-05	1,51E-04
cg18781031	5	127598507	FBN2	Body	-0,229	1,68E-05	1,51E-04
cg24791478	8	129214157		IGR	0,209	1,68E-05	1,51E-04
cg20432732	1	19451037	UBR4	Body	-0,219	1,69E-05	1,51E-04
cg26406238	2	19172689		IGR	0,282	1,69E-05	1,52E-04
cg24636368	17	46388447	SKAP1	Body	0,21	1,69E-05	1,52E-04
cg06702484	2	8443774	LINC00299	Body	-0,203	1,69E-05	1,52E-04
cg22365339	14	70193468		IGR	-0,215	1,70E-05	1,52E-04
cg01062564	2	235670719		IGR	0,236	1,70E-05	1,52E-04
cg07192082	14	75981936		IGR	-0,226	1,70E-05	1,52E-04
cg02767093	13	99130655	STK24	Body	-0,212	1,70E-05	1,53E-04
cg15360037	3	41002920		IGR	-0,209	1,71E-05	1,53E-04
cg24622589	3	58613446		IGR	0,215	1,71E-05	1,53E-04
cg13334937	2	149839048	KIF5C	Body	0,239	1,71E-05	1,53E-04
cg07151737	13	41137364	FOXO1	Body	-0,242	1,72E-05	1,54E-04
cg18156583	2	103035227	IL18RAP	TSS200	-0,224	1,72E-05	1,54E-04
cg08072911	15	95119831		IGR	-0,224	1,72E-05	1,54E-04
cg21376733	12	54891636	NCKAP1L	1stExon	-0,265	1,72E-05	1,54E-04
cg01024668	15	69099680	C15orf28	TSS1500	0,237	1,72E-05	1,54E-04
cg21460765	6	157117157	ARID1B	Body	0,213	1,72E-05	1,54E-04
cg13640737	4	146729642	ZNF827	Body	-0,207	1,72E-05	1,54E-04
cg22740684	17	66343093	ARSG	Body	-0,281	1,72E-05	1,54E-04
cg27118576	13	22688794		IGR	-0,237	1,73E-05	1,54E-04
cg26198483	13	103560417		IGR	-0,207	1,73E-05	1,54E-04
cg11352292	2	39732246	LOC728730	Body	0,208	1,73E-05	1,54E-04
cg01643513	12	57674078	R3HDM2	Body	-0,206	1,73E-05	1,54E-04
cg06765837	19	16705189	MED26	Body	-0,211	1,73E-05	1,55E-04
cg27284288	11	60739005	CD6	TSS200	-0,255	1,73E-05	1,55E-04

cg12244961	5	63984998	FAM159B	TSS1500	0,261	1,74E-05	1,55E-04
cg03928246	1	89742686		IGR	0,299	1,74E-05	1,55E-04
cg07653960	3	4876145	ITPR1	Body	-0,237	1,74E-05	1,55E-04
cg24637724	21	18811330	LINC01549	Body	0,209	1,74E-05	1,56E-04
cg18257485	4	2956627	NOP14	Body	-0,211	1,75E-05	1,56E-04
cg16272728	15	55569254	RAB27A	5'UTR	-0,247	1,75E-05	1,56E-04
cg04960147	1	32423584		IGR	-0,284	1,75E-05	1,56E-04
cg06123179	13	81447974		IGR	-0,233	1,75E-05	1,56E-04
cg14642392	6	32026740	TNXB	Body	-0,214	1,75E-05	1,56E-04
cg06055935	1	220324931	RAB3GAP2	ExonBnd	0,248	1,75E-05	1,56E-04
cg17810003	10	21589610		IGR	-0,245	1,75E-05	1,56E-04
cg08093733	18	74754635	MBP	Body	0,259	1,75E-05	1,56E-04
cg24160437	6	34309455	NUDT3	Body	0,261	1,76E-05	1,56E-04
cg21899520	18	71957896	CYB5A	Body	0,226	1,76E-05	1,57E-04
cg08802890	1	52199980	OSBPL9	5'UTR	0,22	1,76E-05	1,57E-04
cg22714290	1	207992548	LOC148696	Body	0,204	1,76E-05	1,57E-04
cg24392204	4	26940348	STIM2	Body	0,219	1,76E-05	1,57E-04
cg03029255	8	144120681	C8orf31	1stExon	0,215	1,76E-05	1,57E-04
cg19333433	6	159463387	TAGAP	Body	-0,201	1,77E-05	1,57E-04
cg24464500	1	215196452	KCNK2	Body	-0,228	1,77E-05	1,57E-04
cg23632791	8	90794647	RIPK2	Body	-0,258	1,77E-05	1,57E-04
cg24045348	7	111797383	DOCK4	Body	-0,228	1,77E-05	1,57E-04
cg09797465	8	103449351		IGR	0,21	1,77E-05	1,57E-04
cg15872107	3	71771244	EIF4E3	Body	-0,213	1,77E-05	1,57E-04
cg25682700	13	42493215	VWA8	Body	0,212	1,77E-05	1,57E-04
cg21826552	6	159465218	TAGAP	ExonBnd	-0,22	1,78E-05	1,58E-04
cg07660029	1	87169387	SH3GLB1	TSS1500	-0,202	1,78E-05	1,58E-04
cg14105271	5	66254655	MAST4	TSS200	0,222	1,78E-05	1,58E-04
cg17944368	1	28495978	PTAFR	5'UTR	-0,213	1,78E-05	1,58E-04
cg07645237	2	99119127	INPP4A	5'UTR	0,263	1,78E-05	1,58E-04
cg13689742	6	76491683	MYO6	5'UTR	-0,225	1,78E-05	1,58E-04
cg11927190	5	1125351		IGR	0,257	1,78E-05	1,58E-04
cg12453154	13	39925024	LHFP	Body	0,25	1,79E-05	1,58E-04
cg10421515	8	119098754	EXT1	Body	0,253	1,79E-05	1,59E-04
cg15064963	6	82854937		IGR	0,256	1,79E-05	1,59E-04
cg24474200	8	87334734		IGR	-0,204	1,79E-05	1,59E-04
cg08635276	2	12997124		IGR	-0,223	1,79E-05	1,59E-04
cg24219822	1	160808976	CD244	Body	-0,204	1,80E-05	1,59E-04
cg20654468	11	58342997	LPXN	Body	-0,226	1,80E-05	1,59E-04
cg22800543	14	91616033	C14orf159	5'UTR	0,211	1,80E-05	1,59E-04
cg23920151	12	121474312	OASL	Body	-0,233	1,80E-05	1,59E-04
cg24710395	15	77774208	HMG20A	3'UTR	0,252	1,80E-05	1,60E-04
cg24784245	2	135019597	MGAT5	Body	0,206	1,80E-05	1,60E-04
cg25422938	7	7735220	RPA3	5'UTR	0,262	1,81E-05	1,60E-04
cg08634510	21	46382810	FAM207A	Body	0,239	1,81E-05	1,60E-04
cg19189864	10	105372137	SH3PXD2A	Body	-0,223	1,81E-05	1,60E-04
cg03542374	15	93448465	CHD2	Body	0,211	1,81E-05	1,60E-04
cg04055345	10	116264862	ABLIM1	Body	0,225	1,81E-05	1,60E-04
cg22502153	17	46687312	HOXB7	Body	0,21	1,81E-05	1,60E-04
cg05917713	6	42264793	TRERF1	5'UTR	0,222	1,81E-05	1,60E-04

cg20155584	14	77617489		IGR	0,211	1,82E-05	1,60E-04
cg16355233	6	166648800		IGR	-0,208	1,82E-05	1,60E-04
cg14596588	18	59759046	PIGN	Body	0,244	1,82E-05	1,61E-04
cg14088532	9	97991725	FANCC	Body	-0,216	1,82E-05	1,61E-04
cg22180580	6	138867046	NHSL1	Body	0,226	1,82E-05	1,61E-04
cg18650307	4	17809468	DCAF16	5'UTR	-0,207	1,82E-05	1,61E-04
cg13056926	13	30916564	LINC00426	Body	0,213	1,82E-05	1,61E-04
cg25032089	17	46643351	HOXB3	5'UTR	0,229	1,82E-05	1,61E-04
cg10204058	14	64358561	SYNE2	5'UTR	0,229	1,82E-05	1,61E-04
cg03257014	11	111391945	C11orf88	Body	-0,218	1,83E-05	1,61E-04
cg07013148	8	146233339		IGR	0,213	1,83E-05	1,61E-04
cg22021972	11	108430798	EXPH5	Body	0,306	1,83E-05	1,61E-04
cg25895236	8	6547857	MCPH1-AS1	Body	-0,22	1,83E-05	1,61E-04
cg17692028	12	77175743	ZDHHC17	Body	0,207	1,83E-05	1,61E-04
cg16313554	7	111709228	DOCK4	Body	0,212	1,83E-05	1,62E-04
cg21559140	10	64288630	ZNF365	Body	-0,214	1,84E-05	1,62E-04
cg06827192	6	154730156	CNKSR3	Body	0,222	1,84E-05	1,62E-04
cg25163837	6	138225759		IGR	-0,234	1,84E-05	1,62E-04
cg24237074	7	51370544	COBL	Body	0,244	1,84E-05	1,62E-04
cg20043447	20	13665232		IGR	-0,224	1,84E-05	1,62E-04
cg11532575	14	78429641		IGR	0,22	1,85E-05	1,62E-04
cg14649073	13	113437118	ATP11A	Body	0,265	1,85E-05	1,63E-04
cg02217269	19	18146040		IGR	-0,237	1,85E-05	1,63E-04
cg27523193	21	44167104	PDE9A	Body	-0,217	1,85E-05	1,63E-04
cg08975914	7	115994254		IGR	0,291	1,85E-05	1,63E-04
cg14526028	8	139840496	COL22A1	Body	0,208	1,85E-05	1,63E-04
cg26942031	6	163912396	QKI	Body	0,273	1,85E-05	1,63E-04
cg05332446	17	28004714	SSH2	Body	0,234	1,85E-05	1,63E-04
cg12982075	1	81771553		IGR	0,207	1,86E-05	1,63E-04
cg00575665	20	39315211	MAFB	3'UTR	-0,213	1,86E-05	1,63E-04
cg01517680	16	49499006		IGR	0,203	1,86E-05	1,63E-04
cg17175521	1	79627622		IGR	-0,223	1,86E-05	1,63E-04
cg09933108	8	96002841		IGR	0,212	1,86E-05	1,64E-04
cg12193833	17	30244370		IGR	0,217	1,87E-05	1,64E-04
cg22753548	19	55668066	TNNI3	Body	0,219	1,87E-05	1,64E-04
cg22504849	5	171385088	FBXW11	Body	-0,228	1,87E-05	1,64E-04
cg07554357	13	36051749	MIR548F5	Body	-0,21	1,87E-05	1,64E-04
cg16557204	11	27264214		IGR	-0,225	1,87E-05	1,64E-04
cg24030964	4	174926748		IGR	0,236	1,87E-05	1,64E-04
cg10457968	4	176892125	GPM6A	Body	0,205	1,87E-05	1,64E-04
cg17127379	17	65678548	PITPNC1	Body	0,251	1,88E-05	1,64E-04
cg14902763	14	67137966	GPHN	Body	0,218	1,88E-05	1,65E-04
cg01512223	12	121829954		IGR	-0,21	1,88E-05	1,65E-04
cg16271200	20	6074933	FERMT1	Body	-0,213	1,88E-05	1,65E-04
cg02806715	6	32920567	HLA-DMA	Body	-0,222	1,88E-05	1,65E-04
cg03284061	5	127194342		IGR	0,217	1,88E-05	1,65E-04
cg17290591	6	164817337		IGR	0,273	1,88E-05	1,65E-04
cg06202829	16	47426181	ITFG1	Body	-0,238	1,89E-05	1,65E-04
cg08699608	13	115029228	CDC16	Body	-0,217	1,89E-05	1,65E-04
cg18290639	5	106741019	EFNA5	Body	0,245	1,89E-05	1,65E-04

cg04603413	3	52056618		IGR	0,211	1,90E-05	1,66E-04
cg06003834	5	66354967	MAST4	Body	0,24	1,90E-05	1,66E-04
cg20891326	3	179389971	USP13	Body	0,213	1,90E-05	1,66E-04
cg16987437	2	231282895	SP100	Body	-0,213	1,90E-05	1,66E-04
cg26486044	19	50353548	PTOV1	TSS1500	0,215	1,90E-05	1,66E-04
cg27459529	10	49892943	WDFY4	TSS1500	-0,206	1,90E-05	1,66E-04
cg27476331	10	51543368	TIMM23B	Body	0,218	1,90E-05	1,66E-04
cg14317770	14	38016000	MIPOL1	Body	0,231	1,91E-05	1,66E-04
cg13883576	12	13152467	HEBP1	Body	0,207	1,91E-05	1,67E-04
cg15895854	9	97765296	C9orf3	Body	0,201	1,91E-05	1,67E-04
cg17235791	9	127230821		IGR	-0,241	1,91E-05	1,67E-04
cg03639825	18	5455802	EPB41L3	Body	-0,21	1,92E-05	1,67E-04
cg02909952	5	169404456	DOCK2	Body	0,211	1,92E-05	1,67E-04
cg09657114	15	62853598		IGR	0,253	1,92E-05	1,67E-04
cg21557108	15	50410962	ATP8B4	5'UTR	-0,242	1,92E-05	1,68E-04
cg22914291	12	58288081		IGR	0,204	1,93E-05	1,68E-04
cg21199465	7	156556796	LMBR1	Body	0,245	1,93E-05	1,68E-04
cg12583076	12	65082713	RASSF3	Body	0,304	1,94E-05	1,68E-04
cg14222615	2	216262472	FN1	Body	0,202	1,94E-05	1,68E-04
cg07723932	17	75712761		IGR	0,242	1,94E-05	1,68E-04
cg18405631	3	171155686	TNIK	Body	0,206	1,94E-05	1,68E-04
cg12717995	18	10499521		IGR	-0,228	1,94E-05	1,69E-04
cg05432627	4	89680450	FAM13A	Body	0,217	1,94E-05	1,69E-04
cg01935992	17	76408791	PGS1	Body	-0,206	1,94E-05	1,69E-04
cg27374269	20	35893721		IGR	-0,214	1,94E-05	1,69E-04
cg05466804	13	62895148		IGR	0,244	1,95E-05	1,69E-04
cg13707945	3	4714992	ITPR1	Body	0,213	1,95E-05	1,69E-04
cg25421409	13	107026684		IGR	-0,211	1,95E-05	1,69E-04
cg27049722	6	46621856	CYP39A1	TSS1500	0,201	1,96E-05	1,70E-04
cg26159063	12	28376427		IGR	-0,226	1,96E-05	1,70E-04
cg16094166	20	32007380	SNTA1	Body	0,213	1,96E-05	1,70E-04
cg14467794	3	32086860		IGR	0,234	1,96E-05	1,70E-04
cg17519715	9	116225947	RGS3	TSS200	0,205	1,96E-05	1,70E-04
cg13884147	3	48111329	MAP4	5'UTR	0,224	1,96E-05	1,70E-04
cg02972607	17	926146	MIR3183	TSS1500	0,214	1,97E-05	1,70E-04
cg26529329	3	43252505		IGR	0,216	1,97E-05	1,70E-04
cg10476288	17	49101055	SPAG9	Body	0,221	1,97E-05	1,70E-04
cg15744650	8	133976701	TG	Body	0,204	1,97E-05	1,70E-04
cg14193001	8	8710208	MFHAS1	Body	0,205	1,97E-05	1,71E-04
cg09037968	2	37828140		IGR	-0,269	1,98E-05	1,71E-04
cg02883503	16	75423900	CFDP1	Body	0,208	1,98E-05	1,71E-04
cg21894525	1	184839487	FAM129A	Body	-0,203	1,98E-05	1,71E-04
cg14942863	19	37894762		IGR	0,251	1,98E-05	1,71E-04
cg22249300	9	135326868	C9orf171	Body	-0,234	1,98E-05	1,71E-04
cg14393693	6	166045279	PDE10A	Body	-0,283	1,98E-05	1,71E-04
cg10258107	7	95722949	DYNC1I1	Body	-0,217	1,98E-05	1,71E-04
cg08061524	22	46472568		IGR	0,27	1,99E-05	1,72E-04
cg02266086	6	33161336	COL11A2	TSS1500	0,262	1,99E-05	1,72E-04
cg05229723	12	104526470	NFYB	Body	0,224	1,99E-05	1,72E-04
cg14007128	17	72864722	FDXR	1stExon	0,232	1,99E-05	1,72E-04

cg14101959	9	128229793	MAPKAP1	Body	0,242	1,99E-05	1,72E-04
cg06412349	12	64190065	TMEM5	5'UTR	0,207	1,99E-05	1,72E-04
cg25252965	12	8695028	CLEC4E	TSS1500	-0,254	2,00E-05	1,72E-04
cg23364517	15	86623057		IGR	0,202	2,00E-05	1,72E-04
cg18447488	1	150295033	PRPF3	5'UTR	-0,218	2,00E-05	1,72E-04
cg07852840	3	140813919	SPSB4	Body	0,237	2,00E-05	1,73E-04
cg14473030	9	119336987	ASTN2	Body	0,242	2,00E-05	1,73E-04
cg20200594	3	111697388	ABHD10	TSS1500	0,231	2,00E-05	1,73E-04
cg11889800	15	86253551	AKAP13	Body	-0,202	2,00E-05	1,73E-04
cg07922290	16	11146606	CLEC16A	Body	-0,258	2,01E-05	1,73E-04
cg08064683	4	187572844	FAT1	Body	0,259	2,01E-05	1,73E-04
cg11996423	5	5341921		IGR	-0,202	2,01E-05	1,73E-04
cg22261694	6	106967720	AIM1	Body	-0,333	2,01E-05	1,73E-04
cg14128332	8	134706976		IGR	-0,298	2,01E-05	1,73E-04
cg05713666	11	30030873		IGR	0,212	2,01E-05	1,73E-04
cg24501676	6	89949442		IGR	-0,202	2,02E-05	1,74E-04
cg26251599	13	67722165	PCDH9	Body	0,229	2,02E-05	1,74E-04
cg11289747	1	42328785	HIVEP3	5'UTR	-0,203	2,02E-05	1,74E-04
cg18216046	9	73484191	TRPM3	TSS1500	0,277	2,02E-05	1,74E-04
cg13927454	9	90798982		IGR	0,213	2,02E-05	1,74E-04
cg17132446	7	27169342	HOXA4	Body	0,202	2,03E-05	1,74E-04
cg12853765	6	137224053	PEX7	Body	0,202	2,03E-05	1,74E-04
cg14641757	21	36410464	C21orf96	Body	-0,315	2,04E-05	1,75E-04
cg22613769	1	214463441	SMYD2	Body	0,245	2,04E-05	1,75E-04
cg23215587	1	2842673		IGR	0,211	2,04E-05	1,75E-04
cg08425678	1	14029478	PRDM2	5'UTR	-0,2	2,04E-05	1,75E-04
cg07903472	2	168846657	STK39	Body	0,226	2,04E-05	1,75E-04
cg16582814	8	27228210	PTK2B	5'UTR	-0,206	2,04E-05	1,75E-04
cg08592160	13	108003584	FAM155A	Body	0,294	2,04E-05	1,75E-04
cg16703660	6	41207506		IGR	0,207	2,04E-05	1,75E-04
cg08671543	6	138421227	PERP	Body	0,219	2,04E-05	1,75E-04
cg11663570	10	97033671	PDLIM1	Body	0,248	2,04E-05	1,75E-04
cg24723615	7	12751962		IGR	0,207	2,05E-05	1,76E-04
cg15663705	4	14527347	LINC00504	Body	0,282	2,05E-05	1,76E-04
cg06501991	17	79023201	BAIAP2	Body	-0,255	2,05E-05	1,76E-04
cg02473484	20	52371088		IGR	-0,247	2,05E-05	1,76E-04
cg10165209	17	46715533		IGR	0,226	2,05E-05	1,76E-04
cg19269039	1	111743200	DENND2D	1stExon	-0,229	2,05E-05	1,76E-04
cg01948217	20	36932385	BPI	TSS200	-0,217	2,05E-05	1,76E-04
cg17359787	9	96299230	FAM120A	Body	0,233	2,06E-05	1,76E-04
cg18641655	8	125025636	FER1L6	Body	-0,216	2,06E-05	1,76E-04
cg12374834	1	31227605	LAPTM5	Body	-0,242	2,06E-05	1,77E-04
cg01648751	5	38931978	OSMR	ExonBnd	-0,218	2,06E-05	1,77E-04
cg21871091	21	38349937	HLCS	5'UTR	-0,267	2,07E-05	1,77E-04
cg17613629	2	43355158		IGR	-0,206	2,07E-05	1,77E-04
cg05545634	2	161992157	TANK	TSS1500	-0,227	2,07E-05	1,77E-04
cg13152204	3	196007766	PCYT1A	5'UTR	-0,207	2,07E-05	1,77E-04
cg04031093	1	233027124		IGR	-0,242	2,07E-05	1,77E-04
cg08805940	1	171535791	PRRC2C	Body	0,227	2,07E-05	1,77E-04
cg20432211	4	77342104		IGR	0,209	2,08E-05	1,77E-04

cg24578343	9	27460981	MOB3B	5'UTR	-0,236	2,08E-05	1,78E-04
cg23279380	2	191333798	MFSD6	Body	0,213	2,08E-05	1,78E-04
cg12827601	5	73196969	RGNEF	Body	0,203	2,08E-05	1,78E-04
cg07063908	7	37714403		IGR	-0,207	2,08E-05	1,78E-04
cg06856555	4	184908648	STOX2	Body	0,241	2,08E-05	1,78E-04
cg13033202	1	214601486	PTPN14	Body	-0,278	2,09E-05	1,78E-04
cg10290679	11	128457400	ETS1	1stExon	0,209	2,09E-05	1,78E-04
cg02188185	6	30039524	RNF39	Body	0,2	2,09E-05	1,78E-04
cg08033285	16	48638251	N4BP1	Body	0,205	2,09E-05	1,79E-04
cg00891184	1	10272185	KIF1B	5'UTR	0,232	2,09E-05	1,79E-04
cg24885425	13	103836611		IGR	-0,201	2,10E-05	1,79E-04
cg06905129	9	31825001		IGR	0,222	2,10E-05	1,79E-04
cg05763378	8	101349699	RNF19A	TSS1500	0,299	2,10E-05	1,79E-04
cg20779404	9	80719050		IGR	-0,209	2,10E-05	1,79E-04
cg02529312	6	39850612	DAAM2	Body	0,216	2,10E-05	1,79E-04
cg09414280	12	120130253	CIT	Body	0,224	2,10E-05	1,79E-04
cg13273540	3	176850227	TBL1XR1	5'UTR	-0,203	2,10E-05	1,79E-04
cg01765152	8	129005526	PVT1	Body	-0,248	2,11E-05	1,79E-04
cg15619756	16	17436933	XYLT1	Body	-0,243	2,11E-05	1,80E-04
cg08556541	2	54861563	SPTBN1	Body	0,206	2,11E-05	1,80E-04
cg10491637	2	102421970	MAP4K4	Body	0,228	2,11E-05	1,80E-04
cg19365810	7	152064079	MLL3	Body	0,245	2,11E-05	1,80E-04
cg16296417	8	126285443	NSMCE2	Body	-0,214	2,11E-05	1,80E-04
cg10504573	12	66876894	GRIP1	Body	0,243	2,11E-05	1,80E-04
cg23559544	14	95961108		IGR	-0,227	2,12E-05	1,80E-04
cg18649319	8	144631768		IGR	-0,231	2,12E-05	1,80E-04
cg19100992	12	105244251	SLC41A2	Body	0,215	2,12E-05	1,80E-04
cg09574499	7	27168962	HOXA4	Body	0,281	2,12E-05	1,81E-04
cg09088279	17	25609077		IGR	-0,232	2,13E-05	1,81E-04
cg15134787	7	57471759		IGR	0,225	2,13E-05	1,81E-04
cg19268623	18	32783409		IGR	0,251	2,13E-05	1,81E-04
cg24671886	1	19756839	CAPZB	Body	0,241	2,14E-05	1,81E-04
cg20287910	3	11625160	VGLL4	TSS1500	0,242	2,14E-05	1,81E-04
cg18946602	1	61549982	NFIA	Body	0,271	2,14E-05	1,82E-04
cg05935800	1	226023590	EPHX1	Body	0,206	2,14E-05	1,82E-04
cg26990625	7	16969027		IGR	-0,225	2,14E-05	1,82E-04
cg20815560	18	60718358		IGR	-0,238	2,14E-05	1,82E-04
cg12931839	13	95675859	ABCC4	Body	0,228	2,15E-05	1,82E-04
cg19961411	14	85881982	LINC00911	Body	0,217	2,15E-05	1,82E-04
cg25509343	5	171986770		IGR	-0,203	2,15E-05	1,82E-04
cg24078375	3	170070814		IGR	-0,219	2,15E-05	1,82E-04
cg25631874	11	85430657	SYTL2	Body	0,215	2,15E-05	1,82E-04
cg07453440	18	11948154		IGR	0,211	2,15E-05	1,82E-04
cg26693584	16	79127896	WWOX	Body	-0,237	2,15E-05	1,82E-04
cg22259344	13	34068089	STARD13	Body	0,23	2,15E-05	1,82E-04
cg00860726	2	129508082		IGR	-0,276	2,15E-05	1,82E-04
cg17792843	10	26764523	APBB1IP	5'UTR	-0,214	2,16E-05	1,83E-04
cg04890478	15	48253784		IGR	-0,231	2,16E-05	1,83E-04
cg01369817	3	47435787	PTPN23	5'UTR	0,232	2,16E-05	1,83E-04
cg04944524	17	54954220		IGR	0,203	2,16E-05	1,83E-04

cg09302575	9	73484010	TRPM3	TSS200	0,263	2,16E-05	1,83E-04
cg01352267	8	67064467	TRIM55	Body	0,23	2,16E-05	1,83E-04
cg19085165	11	42107926		IGR	-0,432	2,17E-05	1,83E-04
cg15609257	2	192775447		IGR	-0,202	2,17E-05	1,83E-04
cg19659833	1	180821196	XPR1	Body	-0,209	2,17E-05	1,83E-04
cg26890646	12	109062025	CORO1C	Body	0,209	2,17E-05	1,84E-04
cg07581883	3	125676865		IGR	0,217	2,17E-05	1,84E-04
cg26457901	4	86935060		IGR	0,244	2,18E-05	1,84E-04
cg16926051	10	82420606		IGR	-0,228	2,18E-05	1,84E-04
cg14592487	13	95086046		IGR	0,271	2,18E-05	1,84E-04
cg26922585	7	30960956	AQP1	Body	0,233	2,18E-05	1,84E-04
cg15258980	2	68961597	ARHGAP25	TSS1500	-0,205	2,18E-05	1,84E-04
cg05491166	11	19885901	NAV2	Body	0,207	2,18E-05	1,84E-04
cg07684775	12	54772688	ZNF385A	Body	0,25	2,18E-05	1,84E-04
cg24937675	6	134639482	SGK1	TSS1500	0,23	2,18E-05	1,84E-04
cg05550145	10	73631213		IGR	-0,241	2,18E-05	1,84E-04
cg17122758	2	65804208		IGR	-0,224	2,18E-05	1,84E-04
cg12471814	1	199911355		IGR	-0,229	2,19E-05	1,85E-04
cg16217445	5	18466528		IGR	0,272	2,19E-05	1,85E-04
cg12227401	4	185189393		IGR	-0,239	2,19E-05	1,85E-04
cg15454737	12	94657408	PLXNC1	Body	-0,211	2,19E-05	1,85E-04
cg12166603	2	136897419		IGR	-0,218	2,19E-05	1,85E-04
cg19720186	7	17015614		IGR	-0,213	2,20E-05	1,85E-04
cg19066171	4	72061917	SLC4A4	5'UTR	0,217	2,20E-05	1,85E-04
cg01235375	2	66836203	LOC10050707	Body	-0,21	2,20E-05	1,85E-04
cg15158754	13	42138012		IGR	0,222	2,20E-05	1,86E-04
cg20974102	8	127080252		IGR	0,247	2,21E-05	1,86E-04
cg14694366	4	129762160	JADE1	Body	0,203	2,21E-05	1,86E-04
cg22142865	10	7294334	SFMBT2	Body	0,238	2,22E-05	1,86E-04
cg02044846	3	143162505	SLC9A9	Body	0,204	2,22E-05	1,87E-04
cg00406211	10	121077022	GRK5	Body	0,209	2,22E-05	1,87E-04
cg19304422	6	24436179	GPLD1	Body	-0,202	2,23E-05	1,87E-04
cg11366178	4	54457737	LNX1	TSS200	-0,221	2,23E-05	1,87E-04
cg25684105	12	104613662	TXNRD1	Body	0,213	2,23E-05	1,87E-04
cg16692435	1	65361326	JAK1	5'UTR	-0,233	2,23E-05	1,88E-04
cg21593432	18	74919823		IGR	0,215	2,24E-05	1,88E-04
cg18263166	7	92533866		IGR	-0,2	2,24E-05	1,88E-04
cg23269221	8	133994740	TG	Body	-0,239	2,24E-05	1,88E-04
cg08957564	16	12071814	RUNDC2A	Body	0,224	2,24E-05	1,88E-04
cg08168669	6	26367580	BTN3A2	5'UTR	-0,236	2,24E-05	1,88E-04
cg13685563	2	23969041		IGR	0,211	2,25E-05	1,88E-04
cg25028792	19	998978	GRIN3B	TSS1500	0,228	2,25E-05	1,88E-04
cg14407840	12	106736599	TCP11L2	Body	0,219	2,25E-05	1,88E-04
cg04229258	8	67664535	C8orf44-SGK3	5'UTR	-0,21	2,25E-05	1,88E-04
cg16597045	1	9714845	C1orf200	TSS1500	-0,238	2,25E-05	1,89E-04
cg26349266	19	57352074	ZIM2	1stExon	0,275	2,25E-05	1,89E-04
cg08270005	8	96179452		IGR	0,21	2,26E-05	1,89E-04
cg05197228	1	194337692		IGR	0,219	2,26E-05	1,89E-04
cg14462124	2	197031748	STK17B	5'UTR	-0,207	2,26E-05	1,89E-04
cg07734259	8	17995219		IGR	0,207	2,26E-05	1,89E-04

cg13149574	4	170601084	CLCN3	Body	0,209	2,26E-05	1,89E-04
cg19701577	7	27181418	HOXA5	3'UTR	0,289	2,27E-05	1,90E-04
cg04202945	5	127675452	FBN2	Body	-0,276	2,27E-05	1,90E-04
cg26956141	1	235099122	LOC10192785	Body	-0,21	2,27E-05	1,90E-04
cg17434852	13	31151963		IGR	-0,25	2,27E-05	1,90E-04
cg08853735	8	129139526		IGR	-0,219	2,29E-05	1,91E-04
cg16339225	20	3058312		IGR	-0,209	2,29E-05	1,91E-04
cg07677794	2	219574787	TTLL4	TSS1500	0,207	2,29E-05	1,91E-04
cg12343591	2	16031275		IGR	-0,211	2,29E-05	1,91E-04
cg21824243	8	60513879		IGR	0,314	2,30E-05	1,92E-04
cg10148702	6	144076558	PHACTR2	Body	0,224	2,31E-05	1,92E-04
cg12908597	2	204799035		IGR	-0,228	2,31E-05	1,92E-04
cg17906152	11	105846433	GRIA4	3'UTR	0,2	2,31E-05	1,93E-04
cg18045149	6	75278756	LOC10192851	Body	0,214	2,31E-05	1,93E-04
cg06508263	3	173916037	NLGN1	Body	0,263	2,32E-05	1,93E-04
cg25017985	5	40406395		IGR	-0,2	2,32E-05	1,93E-04
cg09383151	13	81028585		IGR	-0,247	2,33E-05	1,94E-04
cg10757709	6	491347	EXOC2	Body	-0,241	2,33E-05	1,94E-04
cg14567957	6	76204172	FILIP1	TSS1500	0,228	2,33E-05	1,94E-04
cg21614925	6	114318487	LOC10192776	Body	0,23	2,34E-05	1,94E-04
cg07136652	15	39627235		IGR	-0,246	2,34E-05	1,94E-04
cg01110837	13	50383231		IGR	-0,203	2,34E-05	1,94E-04
cg13925809	9	84646696		IGR	0,205	2,34E-05	1,94E-04
cg04776040	1	210607796	HHAT	Body	0,203	2,34E-05	1,94E-04
cg09937388	7	28279278	JAZF1-AS1	Body	-0,202	2,34E-05	1,95E-04
cg22819738	3	98705169		IGR	0,246	2,34E-05	1,95E-04
cg03681807	18	8468436		IGR	-0,221	2,35E-05	1,95E-04
cg06727965	5	67437515		IGR	-0,2	2,35E-05	1,95E-04
cg03680517	10	43632967	CSGALNACT2	TSS1500	0,206	2,35E-05	1,95E-04
cg26400954	13	76291075	LMO7	Body	-0,216	2,35E-05	1,95E-04
cg23025750	12	93489359	LOC643339	Body	-0,207	2,36E-05	1,96E-04
cg15686608	9	124461008	DAB2IP	Body	-0,21	2,36E-05	1,96E-04
cg06466407	9	128312369	MAPKAP1	Body	0,204	2,37E-05	1,96E-04
cg23520084	1	244525494	C1orf100	5'UTR	0,209	2,37E-05	1,96E-04
cg07296841	12	13307263		IGR	-0,219	2,37E-05	1,96E-04
cg12067736	1	8412849	RERE	3'UTR	0,225	2,37E-05	1,96E-04
cg02719634	11	2924899	SLC22A18AS	1stExon	0,201	2,37E-05	1,96E-04
cg18512628	6	124811541	NKAIN2	Body	0,219	2,37E-05	1,96E-04
cg18950099	8	37459344		IGR	-0,25	2,37E-05	1,96E-04
cg24159575	1	39662235	MACF1	Body	-0,206	2,37E-05	1,96E-04
cg09211160	6	7148832	RREB1	5'UTR	-0,238	2,38E-05	1,97E-04
cg24423435	20	2687363	EBF4	Body	-0,238	2,38E-05	1,97E-04
cg21497549	9	135991297	RALGDS	Body	-0,22	2,38E-05	1,97E-04
cg27067922	6	147643548	STXBP5	Body	-0,22	2,38E-05	1,97E-04
cg13211181	12	25801455	IFLTD1	1stExon	0,21	2,39E-05	1,97E-04
cg22066911	17	72588425	C17orf77	Body	-0,21	2,39E-05	1,97E-04
cg27531197	17	16171299	PIGL	Body	-0,235	2,39E-05	1,97E-04
cg16722078	8	121432283	MRPL13	Body	0,21	2,39E-05	1,98E-04
cg14541801	5	40437301		IGR	-0,243	2,39E-05	1,98E-04
cg25554911	3	115060104		IGR	-0,202	2,39E-05	1,98E-04

cg21675285	17	53392259	HLF	Body	-0,225	2,40E-05	1,98E-04
cg06614754	13	107569395		IGR	0,223	2,40E-05	1,98E-04
cg03741874	9	130073953	GARNL3	Body	0,254	2,40E-05	1,98E-04
cg17010254	5	73445334		IGR	-0,214	2,41E-05	1,98E-04
cg17316760	1	198625883	PTPRC	Body	-0,225	2,41E-05	1,99E-04
cg24499199	20	2665626		IGR	-0,241	2,41E-05	1,99E-04
cg13766434	6	17981180	KIF13A	Body	-0,223	2,41E-05	1,99E-04
cg03499328	21	22603302	NCAM2	Body	0,235	2,42E-05	1,99E-04
cg19853902	1	181042900		IGR	0,213	2,42E-05	1,99E-04
cg23706319	5	137708073	KDM3B	Body	-0,202	2,42E-05	2,00E-04
cg01050106	5	60254699	NDUFAF2	Body	0,213	2,42E-05	2,00E-04
cg27631256	9	117692745	TNFSF8	1stExon	-0,248	2,43E-05	2,00E-04
cg13248581	22	17680545	CECR1	TSS200	0,212	2,43E-05	2,00E-04
cg15167169	6	142727580	ADGRG6	Body	0,203	2,43E-05	2,00E-04
cg14205164	5	37717742	WDR70	Body	0,248	2,44E-05	2,00E-04
cg02948125	17	7560317	ATP1B2	3'UTR	0,218	2,44E-05	2,01E-04
cg19189688	10	64532056		IGR	0,238	2,44E-05	2,01E-04
cg02153041	12	131502934	GPR133	Body	-0,208	2,45E-05	2,01E-04
cg02306139	5	148638033	ABLIM3	3'UTR	-0,214	2,45E-05	2,01E-04
cg01579024	5	170288757	RANBP17	TSS1500	0,22	2,45E-05	2,01E-04
cg12821663	22	38459051	PICK1	Body	0,251	2,45E-05	2,01E-04
cg03040477	21	38753088	DYRK1A	5'UTR	0,204	2,45E-05	2,02E-04
cg22272994	10	48481389		IGR	-0,246	2,46E-05	2,02E-04
cg27031099	8	126620534		IGR	-0,21	2,47E-05	2,02E-04
cg17032757	15	73886305	NPTN	Body	0,292	2,47E-05	2,02E-04
cg20423899	3	194461795	LOC10050739	Body	0,214	2,47E-05	2,02E-04
cg21880903	4	39408665	KLB	1stExon	0,251	2,47E-05	2,02E-04
cg23087531	1	66716368	PDE4B	Body	-0,203	2,47E-05	2,03E-04
cg13215473	2	198010355	ANKRD44	Body	-0,203	2,47E-05	2,03E-04
cg01448062	15	73598635		IGR	0,207	2,47E-05	2,03E-04
cg24842659	22	48534775		IGR	-0,216	2,47E-05	2,03E-04
cg15711720	12	77156933	ZDHHC17	TSS1500	0,207	2,47E-05	2,03E-04
cg17808195	3	150690585	CLRN1	1stExon	0,201	2,48E-05	2,03E-04
cg15628377	6	151937653	CCDC170	Body	0,234	2,48E-05	2,03E-04
cg02891455	3	170901125	TNIK	Body	0,238	2,50E-05	2,04E-04
cg02000623	18	55513061		IGR	0,22	2,50E-05	2,04E-04
cg02676654	19	45704185		IGR	0,251	2,50E-05	2,04E-04
cg00528779	12	124926062	NCOR2	Body	0,219	2,50E-05	2,04E-04
cg12763511	1	170701445	PRRX1	3'UTR	0,225	2,50E-05	2,05E-04
cg23813732	2	102392841	MAP4K4	Body	0,253	2,50E-05	2,05E-04
cg07804470	13	99201496	STK24	Body	-0,206	2,51E-05	2,05E-04
cg07811915	7	144509662	TPK1	Body	-0,237	2,52E-05	2,05E-04
cg26821000	2	773393		IGR	-0,201	2,52E-05	2,05E-04
cg12920851	17	79630153		IGR	-0,288	2,52E-05	2,06E-04
cg21209212	15	60802563	RORA-AS1	Body	0,273	2,53E-05	2,06E-04
cg23229016	1	26872525	RPS6KA1	1stExon	-0,204	2,53E-05	2,06E-04
cg24529650	9	14031418		IGR	0,247	2,53E-05	2,06E-04
cg21289613	19	34774239	KIAA0355	5'UTR	0,209	2,53E-05	2,06E-04
cg23321751	18	34429726	KIAA1328	Body	0,208	2,53E-05	2,06E-04
cg09965593	11	33776130	FBXO3	Body	0,242	2,53E-05	2,06E-04

cg13226072	3	168809227	MECOM	Body	-0,213	2,53E-05	2,07E-04
cg06606563	13	29157266		IGR	-0,209	2,53E-05	2,07E-04
cg25655189	14	78609989		IGR	-0,225	2,54E-05	2,07E-04
cg23258717	3	195615577	TNK2	Body	0,29	2,54E-05	2,07E-04
cg14003693	9	14308247	NFIB	Body	0,263	2,54E-05	2,07E-04
cg15722631	1	214835694	CENPF	Body	0,214	2,54E-05	2,07E-04
cg10807450	6	35110744		IGR	-0,203	2,54E-05	2,07E-04
cg05772390	2	137140148		IGR	-0,222	2,54E-05	2,07E-04
cg08718293	1	10399783	KIF1B	Body	0,241	2,54E-05	2,07E-04
cg22141203	22	24912340	UPB1	Body	-0,233	2,55E-05	2,07E-04
cg19776306	5	118781406		IGR	0,205	2,56E-05	2,08E-04
cg03849254	11	13741341	FAR1	Body	0,262	2,56E-05	2,08E-04
cg19573931	3	121450086	GOLGB1	5'UTR	0,201	2,56E-05	2,08E-04
cg18358650	16	50307938	ADCY7	5'UTR	-0,238	2,56E-05	2,08E-04
cg09897604	10	49893549	WDFY4	5'UTR	-0,222	2,56E-05	2,08E-04
cg08922436	9	100265926	TMOD1	5'UTR	0,2	2,57E-05	2,09E-04
cg17838334	20	10732416	LOC1019293!	TSS1500	-0,239	2,57E-05	2,09E-04
cg24024260	2	182174340	LOC1019271!	Body	-0,225	2,57E-05	2,09E-04
cg20471600	22	39808500	TAB1	Body	-0,24	2,57E-05	2,09E-04
cg21234506	15	80263132	BCL2A1	1stExon	-0,206	2,58E-05	2,09E-04
cg05918327	16	72955295	ZFHX3	Body	-0,203	2,58E-05	2,09E-04
cg03557441	6	42391254	TRERF1	5'UTR	-0,23	2,58E-05	2,09E-04
cg05600412	17	66251877	AMZ2	Body	-0,219	2,58E-05	2,09E-04
cg13022679	5	140207498	PCDHA2	Body	0,204	2,58E-05	2,09E-04
cg20384542	6	42533883	UBR2	Body	-0,228	2,58E-05	2,10E-04
cg06432055	2	171364407	MYO3B	Body	-0,26	2,58E-05	2,10E-04
cg23485147	14	23445909	AJUBA	1stExon	0,201	2,59E-05	2,10E-04
cg06031622	20	35923593	MANBAL	5'UTR	-0,201	2,59E-05	2,10E-04
cg09941995	5	28404014		IGR	0,262	2,59E-05	2,10E-04
cg01433914	10	111792543	ADD3	5'UTR	0,222	2,59E-05	2,10E-04
cg05006285	12	6501933		IGR	0,211	2,60E-05	2,10E-04
cg21707106	1	42804929		IGR	0,202	2,60E-05	2,10E-04
cg24205793	10	52267147	SGMS1	5'UTR	0,235	2,60E-05	2,10E-04
cg20532937	10	13748985	FRMD4A	Body	0,217	2,60E-05	2,11E-04
cg12906712	5	111033085	STARD4-AS1	Body	0,201	2,60E-05	2,11E-04
cg07820966	4	185859052		IGR	0,216	2,61E-05	2,11E-04
cg18959453	6	143168347	HIVEP2	5'UTR	0,222	2,61E-05	2,11E-04
cg01316378	10	63629580		IGR	0,204	2,61E-05	2,11E-04
cg27255456	20	33479235	ACSS2	Body	-0,224	2,61E-05	2,11E-04
cg07926147	14	72005807	SIPA1L1	5'UTR	0,206	2,61E-05	2,12E-04
cg18687624	12	28466975	CCDC91	Body	0,269	2,62E-05	2,12E-04
cg15963499	1	178368509	RASAL2	Body	-0,205	2,62E-05	2,12E-04
cg13650024	2	149433604	EPC2	Body	0,243	2,62E-05	2,12E-04
cg25155064	11	118100782	MPZL3	Body	-0,201	2,62E-05	2,12E-04
cg19482216	14	100900810	WDR25	Body	-0,247	2,63E-05	2,13E-04
cg08192798	15	83501835	WHAMM	Body	-0,214	2,63E-05	2,13E-04
cg10142008	13	46698747		IGR	-0,226	2,64E-05	2,13E-04
cg02686793	6	112340333		IGR	0,211	2,64E-05	2,13E-04
cg21097354	7	27179161		IGR	0,21	2,64E-05	2,13E-04
cg17083429	10	89627854	PTEN	Body	0,229	2,65E-05	2,14E-04

cg09333299	6	144462191		IGR	-0,225	2,65E-05	2,14E-04
cg06398242	2	46049176	PRKCE	Body	0,241	2,66E-05	2,14E-04
cg13537416	9	124506555	DAB2IP	Body	0,204	2,66E-05	2,14E-04
cg01547466	18	22813556	ZNF521	5'UTR	0,218	2,66E-05	2,15E-04
cg14731761	3	12553969	TSEN2	Body	0,22	2,66E-05	2,15E-04
cg00297832	1	1565541	MIB2	Body	-0,27	2,66E-05	2,15E-04
cg05825053	16	11697522		IGR	0,248	2,66E-05	2,15E-04
cg12468255	1	201617056	NAV1	TSS1500	0,298	2,67E-05	2,15E-04
cg09569224	9	108026834	SLC44A1	Body	-0,261	2,68E-05	2,15E-04
cg21479422	7	46584088		IGR	-0,285	2,68E-05	2,15E-04
cg27183791	16	89381904	ANKRD11	Body	-0,206	2,68E-05	2,15E-04
cg10095795	6	144095294	PHACTR2	Body	0,215	2,68E-05	2,16E-04
cg26340968	19	3798367	MATK	5'UTR	-0,211	2,69E-05	2,16E-04
cg13315362	3	60744009	FHIT	5'UTR	-0,21	2,69E-05	2,17E-04
cg19839601	4	134459203		IGR	0,212	2,70E-05	2,17E-04
cg03091413	8	42356068	SLC20A2	5'UTR	0,218	2,70E-05	2,17E-04
cg17915436	10	8179599		IGR	-0,226	2,70E-05	2,17E-04
cg26528644	4	93155635		IGR	0,245	2,70E-05	2,17E-04
cg11643041	3	169067194	MECOM	5'UTR	-0,203	2,70E-05	2,17E-04
cg27582527	1	15734285		IGR	-0,21	2,70E-05	2,17E-04
cg15573664	11	12833710	TEAD1	Body	-0,259	2,71E-05	2,17E-04
cg16973808	18	29238851	B4GALT6	Body	-0,226	2,71E-05	2,18E-04
cg24533189	1	209925718		IGR	-0,23	2,71E-05	2,18E-04
cg08042254	1	21399544	EIF4G3	5'UTR	0,213	2,73E-05	2,19E-04
cg01609889	4	185308004		IGR	0,215	2,73E-05	2,19E-04
cg13760479	2	241564507	GPR35	TSS200	-0,221	2,73E-05	2,19E-04
cg09975110	15	81588897	IL16	TSS1500	-0,218	2,73E-05	2,19E-04
cg24808001	2	19673800		IGR	-0,221	2,74E-05	2,19E-04
cg16831889	15	50558051	HDC	TSS200	0,219	2,74E-05	2,19E-04
cg19244722	12	2048973		IGR	0,226	2,74E-05	2,20E-04
cg09487819	15	42197367	EHD4	Body	0,222	2,75E-05	2,20E-04
cg26939107	16	82171045		IGR	0,212	2,75E-05	2,20E-04
cg01766850	6	170580966		IGR	-0,205	2,75E-05	2,20E-04
cg05750926	1	159886341		IGR	0,244	2,75E-05	2,20E-04
cg14462215	15	72598823	CELF6	TSS200	-0,208	2,75E-05	2,20E-04
cg07048527	17	46700449	HOXB9	Body	0,235	2,75E-05	2,20E-04
cg24764848	14	92814814	SLC24A4	Body	0,206	2,76E-05	2,20E-04
cg25193114	8	119001969	EXT1	Body	-0,266	2,76E-05	2,20E-04
cg17053902	10	127790337	ADAM12	Body	-0,207	2,76E-05	2,21E-04
cg20132609	13	99698423	DOCK9	Body	-0,202	2,76E-05	2,21E-04
cg26240235	1	197607830	DENND1B	Body	0,233	2,76E-05	2,21E-04
cg07649832	12	18082030		IGR	0,204	2,77E-05	2,21E-04
cg22074858	1	89488430	GBP3	1stExon	-0,234	2,77E-05	2,21E-04
cg20617328	6	33040610	HLA-DPA1	Body	-0,206	2,77E-05	2,21E-04
cg16975335	13	113928287		IGR	-0,228	2,77E-05	2,21E-04
cg15080351	6	3851517	FAM50B	3'UTR	-0,201	2,78E-05	2,22E-04
cg03351487	2	169003820	STK39	Body	0,209	2,78E-05	2,22E-04
cg20003058	10	26726503	APBB1IP	TSS1500	-0,235	2,79E-05	2,22E-04
cg08238472	14	27289660	LOC1027248	Body	0,213	2,79E-05	2,22E-04
cg18203005	2	165755156	SLC38A11	Body	0,236	2,80E-05	2,23E-04

cg14919044	1	167652688	RCSD1	Body	0,245	2,81E-05	2,23E-04
cg18060909	5	150534782	ANXA6	5'UTR	-0,23	2,81E-05	2,24E-04
cg22768101	11	35208949	CD44	Body	-0,213	2,81E-05	2,24E-04
cg07639701	7	92268886	CDK6	Body	-0,248	2,81E-05	2,24E-04
cg08242070	17	29247612	ADAP2	TSS1500	0,21	2,81E-05	2,24E-04
cg15129144	2	46527958	EPAS1	Body	0,209	2,82E-05	2,24E-04
cg10202638	2	173754319	RAPGEF4	Body	0,207	2,82E-05	2,24E-04
cg16774479	18	33529461		IGR	0,202	2,83E-05	2,25E-04
cg18877006	3	167583761		IGR	0,24	2,83E-05	2,25E-04
cg10331119	1	7209666	CAMTA1	Body	0,235	2,83E-05	2,25E-04
cg23882282	10	4448187	LINC00703	TSS1500	0,201	2,83E-05	2,25E-04
cg01007821	2	106019543	FHL2	5'UTR	0,216	2,84E-05	2,25E-04
cg25982946	7	141437322	SSBP1	TSS1500	-0,229	2,84E-05	2,26E-04
cg02709407	2	106016462	FHL2	TSS1500	0,229	2,85E-05	2,26E-04
cg04315865	13	47103926		IGR	-0,213	2,85E-05	2,26E-04
cg06284172	2	102422343	MAP4K4	Body	0,22	2,85E-05	2,26E-04
cg26534238	16	3628485	NLRC3	TSS1500	-0,208	2,85E-05	2,26E-04
cg00164641	16	79030890	WWOX	Body	0,231	2,85E-05	2,26E-04
cg09122558	18	67513540	DOK6	3'UTR	0,254	2,85E-05	2,26E-04
cg02126353	12	111078234	TCTN1	Body	0,205	2,85E-05	2,26E-04
cg07231911	1	94141047	BCAR3	5'UTR	0,22	2,85E-05	2,26E-04
cg10930308	6	30039476	RNF39	Body	0,245	2,86E-05	2,27E-04
cg07269574	2	227343345		IGR	0,222	2,86E-05	2,27E-04
cg05843672	10	26774178	APBB1IP	5'UTR	0,238	2,86E-05	2,27E-04
cg24067911	6	16729610	ATXN1	5'UTR	-0,222	2,86E-05	2,27E-04
cg10926412	13	22685842		IGR	0,204	2,86E-05	2,27E-04
cg11257041	4	6918589	TBC1D14	5'UTR	-0,237	2,86E-05	2,27E-04
cg23462338	12	132588323	EP400NL	Body	0,201	2,86E-05	2,27E-04
cg16616070	4	102339255		IGR	-0,213	2,87E-05	2,27E-04
cg15077070	2	102803290	IL1RL2	TSS200	0,222	2,88E-05	2,28E-04
cg24833674	1	166914191	ILDR2	Body	-0,212	2,89E-05	2,28E-04
cg10207061	18	65686571		IGR	0,213	2,89E-05	2,28E-04
cg04478678	17	46642878	HOXB3	5'UTR	0,285	2,89E-05	2,29E-04
cg02743284	2	72833370	EXOC6B	Body	0,224	2,89E-05	2,29E-04
cg24045194	2	140122875		IGR	0,246	2,90E-05	2,29E-04
cg17274605	5	53550834	ARL15	Body	0,22	2,90E-05	2,29E-04
cg04451448	4	110130	ZNF718	Body	0,205	2,90E-05	2,29E-04
cg05711445	7	139528855	TBXAS1	TSS200	-0,206	2,91E-05	2,29E-04
cg22206517	6	12232846		IGR	-0,224	2,92E-05	2,30E-04
cg08715862	16	67275028	FHOD1	Body	0,246	2,94E-05	2,31E-04
cg04686545	12	107348824	C12orf23	TSS1500	0,319	2,94E-05	2,32E-04
cg17509989	5	176798049	RGS14	Body	0,246	2,94E-05	2,32E-04
cg00525782	2	105943452	TGFBRAP1	5'UTR	-0,206	2,94E-05	2,32E-04
cg17596409	4	153186305		IGR	0,205	2,95E-05	2,32E-04
cg04989278	1	66839134	PDE4B	3'UTR	-0,228	2,96E-05	2,33E-04
cg25233900	10	133793646	BNIP3	Body	0,203	2,96E-05	2,33E-04
cg13456408	5	147286084	C5orf46	1stExon	0,223	2,96E-05	2,33E-04
cg14784664	11	123302579		IGR	-0,209	2,97E-05	2,33E-04
cg18422587	7	3134670		IGR	-0,242	2,97E-05	2,34E-04
cg14135616	13	67944216		IGR	0,2	2,97E-05	2,34E-04

cg11330155	13	47179523	LRCH1	Body	0,243	2,97E-05	2,34E-04
cg21901577	4	118736876		IGR	0,373	2,98E-05	2,34E-04
cg01683757	5	169720600	LCP2	Body	-0,248	2,98E-05	2,34E-04
cg01081584	15	40268610	EIF2AK4	Body	0,244	2,99E-05	2,34E-04
cg17346647	2	154842584	GALNT13	Body	0,216	2,99E-05	2,35E-04
cg16002660	17	2310278	LOC284009	Body	-0,218	2,99E-05	2,35E-04
cg25758863	6	163760593		IGR	0,211	2,99E-05	2,35E-04
cg01068375	4	13415898	RAB28	Body	0,228	3,00E-05	2,35E-04
cg22320183	6	33161577	COL11A2	TSS1500	0,23	3,00E-05	2,35E-04
cg02682092	11	124647347	MSANTD2	Body	-0,212	3,00E-05	2,35E-04
cg25769469	5	71643841	PTCD2	Body	-0,218	3,00E-05	2,35E-04
cg06438531	9	101760846	COL15A1	Body	0,201	3,01E-05	2,36E-04
cg14320625	19	54384186	PRKCG	TSS1500	-0,206	3,01E-05	2,36E-04
cg01255193	16	88848972	PIEZO1	Body	-0,236	3,01E-05	2,36E-04
cg04794720	15	60719572	ICE2	Body	-0,203	3,02E-05	2,36E-04
cg22956084	8	80597489		IGR	0,219	3,02E-05	2,36E-04
cg15881795	12	106361273		IGR	0,21	3,03E-05	2,37E-04
cg01700683	15	63510942	RAB8B	Body	0,207	3,04E-05	2,38E-04
cg07104583	2	65459346	ACTR2	Body	-0,218	3,04E-05	2,38E-04
cg18037808	12	109059679	CORO1C	Body	0,213	3,05E-05	2,38E-04
cg22571393	13	113498308	ATP11A	Body	0,223	3,05E-05	2,38E-04
cg00459354	8	8708997	MFHAS1	Body	0,207	3,05E-05	2,38E-04
cg09646593	4	125631168	ANKRD50	5'UTR	0,216	3,06E-05	2,39E-04
cg25099892	13	113313905	C13orf35	5'UTR	-0,219	3,06E-05	2,39E-04
cg19938436	1	170278698		IGR	0,243	3,06E-05	2,39E-04
cg08803531	1	1090812		IGR	0,218	3,07E-05	2,39E-04
cg23933723	17	9572135	USP43	Body	-0,253	3,07E-05	2,40E-04
cg26943987	6	139446639		IGR	-0,213	3,08E-05	2,40E-04
cg06153757	1	85968010	DDAH1	5'UTR	0,247	3,08E-05	2,40E-04
cg27497781	1	111833467	CHIA	TSS200	0,225	3,08E-05	2,40E-04
cg00253649	1	239184853		IGR	0,208	3,09E-05	2,41E-04
cg00524773	16	121668	RHBDF1	5'UTR	0,213	3,09E-05	2,41E-04
cg26150263	2	161924456		IGR	0,21	3,10E-05	2,41E-04
cg03031330	1	72964136		IGR	0,243	3,10E-05	2,41E-04
cg05796838	19	17953157	JAK3	Body	0,202	3,11E-05	2,42E-04
cg05067022	10	30707911		IGR	-0,204	3,11E-05	2,42E-04
cg01485838	14	100532680	EVL	Body	-0,236	3,11E-05	2,42E-04
cg23549972	3	58389654	PXK	Body	-0,2	3,12E-05	2,42E-04
cg24039042	6	170580947		IGR	-0,214	3,12E-05	2,42E-04
cg10720644	14	60768693		IGR	0,214	3,12E-05	2,43E-04
cg12503949	9	120445501		IGR	-0,201	3,13E-05	2,43E-04
cg23357708	19	8580509	ZNF414	TSS1500	-0,229	3,13E-05	2,43E-04
cg27491799	10	29003413		IGR	0,232	3,13E-05	2,43E-04
cg25030097	8	24062213		IGR	0,215	3,17E-05	2,46E-04
cg02698900	8	1094770		IGR	-0,258	3,18E-05	2,46E-04
cg15305836	7	151423562	PRKAG2	Body	0,205	3,18E-05	2,46E-04
cg18273457	13	76445152	LMO7DN	TSS200	0,226	3,19E-05	2,46E-04
cg03132276	4	15732097	BST1	Body	0,221	3,19E-05	2,46E-04
cg09237454	7	152063907	MLL3	Body	0,218	3,19E-05	2,47E-04
cg04606421	7	84177901	LOC1019273[illegible]	Body	0,205	3,20E-05	2,47E-04

cg09644404	11	118174785	CD3E	TSS1500	-0,25	3,20E-05	2,47E-04
cg18739675	2	43041125		IGR	-0,21	3,20E-05	2,47E-04
cg01421140	1	57470853	DAB1	3'UTR	-0,208	3,20E-05	2,48E-04
cg22707210	2	10029993	TAF1B	Body	0,295	3,20E-05	2,48E-04
cg18647382	3	183298319		IGR	0,229	3,21E-05	2,48E-04
cg10452799	1	66911669		IGR	-0,202	3,21E-05	2,48E-04
cg13029130	2	68593246	PLEK	Body	-0,243	3,21E-05	2,48E-04
cg26896861	11	122568671	UBASH3B	Body	-0,236	3,21E-05	2,48E-04
cg09212074	6	130909550		IGR	0,203	3,21E-05	2,48E-04
cg17684765	2	3833816		IGR	0,22	3,21E-05	2,48E-04
cg23507945	6	137477184	IL22RA2	Body	-0,244	3,22E-05	2,48E-04
cg03921837	14	53419337		IGR	0,213	3,22E-05	2,48E-04
cg12455681	12	15059241		IGR	0,219	3,23E-05	2,49E-04
cg14298774	2	174060199	ZAK	Body	0,217	3,24E-05	2,50E-04
cg08274405	5	66254395	MAST4	TSS1500	0,262	3,24E-05	2,50E-04
cg23418968	2	9816010		IGR	0,219	3,24E-05	2,50E-04
cg08714975	16	73332746		IGR	-0,216	3,24E-05	2,50E-04
cg25616055	12	89638890		IGR	-0,222	3,25E-05	2,50E-04
cg08970648	3	50644192	CISH	3'UTR	-0,204	3,26E-05	2,51E-04
cg21428990	7	155790277		IGR	-0,212	3,26E-05	2,51E-04
cg23923308	7	37006604	ELMO1	Body	0,221	3,26E-05	2,51E-04
cg21021168	4	140975098	MAML3	Body	-0,205	3,26E-05	2,51E-04
cg08266180	11	33027070		IGR	-0,202	3,27E-05	2,51E-04
cg19398349	7	69642889	AUTS2	Body	-0,204	3,27E-05	2,51E-04
cg17371020	4	154125535	TRIM2	TSS200	0,206	3,27E-05	2,52E-04
cg24577159	11	111748532	FDXACB1	Body	0,203	3,27E-05	2,52E-04
cg10906729	17	46682390	LOC404266	Body	0,218	3,28E-05	2,52E-04
cg26672376	1	38481965	UTP11L	Body	0,234	3,29E-05	2,53E-04
cg05008948	7	99160714	ZNF655	Body	-0,246	3,29E-05	2,53E-04
cg22301128	4	77011716	ART3	Body	0,22	3,30E-05	2,53E-04
cg02313909	21	48081257	PRMT2	Body	0,225	3,30E-05	2,53E-04
cg17923947	14	50584005	C14orf138	TSS1500	0,221	3,30E-05	2,53E-04
cg01284193	9	27109111	TEK	TSS200	0,229	3,30E-05	2,54E-04
cg01884851	3	45906498	LZTFL1	5'UTR	-0,229	3,32E-05	2,54E-04
cg00411072	17	46660940		IGR	0,286	3,33E-05	2,55E-04
cg08799655	6	35183390	SCUBE3	Body	0,23	3,33E-05	2,55E-04
cg03748202	1	35811540	ZMYM4	Body	-0,237	3,33E-05	2,55E-04
cg19344315	6	16669649	ATXN1	5'UTR	-0,202	3,34E-05	2,56E-04
cg05036422	10	76684214	KAT6B	Body	0,207	3,35E-05	2,56E-04
cg04420294	7	130641745	LINC-PINT	Body	-0,203	3,35E-05	2,56E-04
cg04425263	14	69446981	ACTN1	TSS1500	0,209	3,35E-05	2,56E-04
cg26492631	4	148721238	ARHGAP10	Body	-0,247	3,35E-05	2,56E-04
cg06907356	21	45664138		IGR	-0,283	3,36E-05	2,57E-04
cg20442128	10	1817005		IGR	0,25	3,36E-05	2,57E-04
cg07113325	20	30104660	HM13	Body	-0,266	3,37E-05	2,57E-04
cg15859867	12	57735981		IGR	0,227	3,37E-05	2,57E-04
cg03463994	2	189046152		IGR	-0,255	3,38E-05	2,58E-04
cg06839158	8	41844932	KAT6A	Body	-0,222	3,38E-05	2,58E-04
cg21767615	10	14478569	MIR1265	TSS200	-0,207	3,38E-05	2,58E-04
cg03521246	4	89680660	FAM13A	Body	0,259	3,38E-05	2,58E-04

cg00160260	8	142039278		IGR	-0,217	3,39E-05	2,59E-04
cg05498195	2	438323		IGR	-0,233	3,39E-05	2,59E-04
cg18853981	3	188302105	LPP	Body	-0,251	3,40E-05	2,59E-04
cg04448881	5	65814772		IGR	0,24	3,41E-05	2,60E-04
cg13969271	3	150451639		IGR	0,213	3,41E-05	2,60E-04
cg17027335	9	126504286	DENND1A	Body	0,227	3,42E-05	2,60E-04
cg12819548	11	120256570	ARHGEF12	Body	0,239	3,42E-05	2,60E-04
cg14441312	1	198879201	MIR181A1HG	Body	0,204	3,43E-05	2,61E-04
cg13354414	7	88425139	ZNF804B	Body	0,227	3,43E-05	2,61E-04
cg08500929	17	80788219	ZNF750	Body	0,236	3,44E-05	2,61E-04
cg04708975	19	40459334		IGR	-0,218	3,45E-05	2,62E-04
cg15827559	3	99816799	FILIP1L	5'UTR	0,205	3,45E-05	2,62E-04
cg26799368	2	52449445		IGR	0,271	3,46E-05	2,63E-04
cg15196806	7	27171213	HOXA4	TSS1500	0,247	3,46E-05	2,63E-04
cg01779906	4	160195650	RAPGEF2	Body	0,218	3,46E-05	2,63E-04
cg13110623	14	62334031		IGR	-0,214	3,46E-05	2,63E-04
cg17156867	15	95019541	MCTP2	Body	0,233	3,47E-05	2,63E-04
cg06511149	19	30469222	URI1	Body	-0,223	3,47E-05	2,63E-04
cg08161364	3	101726208		IGR	0,232	3,47E-05	2,63E-04
cg26438679	6	13762892		IGR	-0,239	3,47E-05	2,64E-04
cg05218470	5	56326114		IGR	-0,225	3,48E-05	2,64E-04
cg02347984	6	26170601	HIST1H2BD	3'UTR	-0,212	3,48E-05	2,64E-04
cg22035088	17	66352599	ARSG	Body	-0,241	3,48E-05	2,64E-04
cg04873792	12	15082096	ERP27	TSS200	0,201	3,49E-05	2,64E-04
cg08362592	15	69841259		IGR	0,203	3,49E-05	2,65E-04
cg11375919	2	5918059		IGR	-0,211	3,50E-05	2,65E-04
cg03434907	11	128323520		IGR	0,256	3,50E-05	2,65E-04
cg00338685	8	144883443	SCRIB	Body	0,212	3,50E-05	2,65E-04
cg02301648	14	70962479		IGR	0,205	3,51E-05	2,66E-04
cg17285448	7	27173480		IGR	0,235	3,51E-05	2,66E-04
cg04617267	2	65460005	ACTR2	Body	-0,224	3,51E-05	2,66E-04
cg20390513	13	59757719		IGR	-0,236	3,52E-05	2,66E-04
cg19569053	3	119289119		IGR	-0,214	3,52E-05	2,67E-04
cg08471801	5	149640795	CAMK2A	Body	-0,265	3,53E-05	2,67E-04
cg05864401	8	135850231	NCRNA00250	TSS200	0,231	3,54E-05	2,67E-04
cg26075732	3	49331846	USP4	ExonBnd	0,219	3,55E-05	2,68E-04
cg25439420	2	158970512	UPP2	Body	-0,214	3,56E-05	2,69E-04
cg18587674	3	23919329	UBE2E1	Body	0,207	3,57E-05	2,69E-04
cg16262572	2	122479703	NIFK-AS1	Body	-0,225	3,57E-05	2,69E-04
cg00238353	10	129785537	PTPRE	5'UTR	0,205	3,57E-05	2,69E-04
cg14036584	4	48702085	FRYL	5'UTR	-0,22	3,57E-05	2,69E-04
cg10812980	12	96754479	CDK17	5'UTR	0,213	3,58E-05	2,69E-04
cg23865211	17	16275350		IGR	0,203	3,58E-05	2,70E-04
cg06489993	19	47082996	PPP5D1	Body	-0,303	3,58E-05	2,70E-04
cg10745669	1	214470760	SMYD2	Body	0,203	3,58E-05	2,70E-04
cg03731458	10	11246763	CUGBP2	Body	0,205	3,59E-05	2,70E-04
cg10901608	13	31314334	ALOX5AP	Body	-0,213	3,60E-05	2,71E-04
cg16345789	20	30000680	DEFB121	TSS200	0,232	3,60E-05	2,71E-04
cg16754788	8	97803770	PGCP	Body	0,234	3,62E-05	2,72E-04
cg24907176	2	54747944	SPTBN1	5'UTR	-0,213	3,62E-05	2,72E-04

cg26385891	10	121276899	RGS10	Body	-0,209	3,62E-05	2,72E-04
cg10809434	16	27243530	NSMCE1	Body	-0,208	3,62E-05	2,72E-04
cg17500416	7	92484049	LOC10192749	Body	0,2	3,64E-05	2,73E-04
cg10197846	5	123144349		IGR	0,289	3,64E-05	2,73E-04
cg21065964	2	86161043		IGR	0,222	3,64E-05	2,73E-04
cg08744519	8	2308391		IGR	0,231	3,64E-05	2,73E-04
cg26905258	13	77898407	MYCBP2	Body	-0,211	3,65E-05	2,74E-04
cg06496300	16	88686428	ZC3H18	Body	-0,201	3,65E-05	2,74E-04
cg07530359	3	4554673	ITPR1	5'UTR	0,203	3,66E-05	2,74E-04
cg18929251	7	107744567	LAMB4	Body	-0,211	3,67E-05	2,75E-04
cg15172885	12	57037506	SNORD59B	Body	0,207	3,67E-05	2,75E-04
cg04058669	3	70410237		IGR	-0,24	3,68E-05	2,75E-04
cg19173056	2	137068818		IGR	-0,203	3,68E-05	2,76E-04
cg10512448	14	77479632		IGR	-0,205	3,72E-05	2,78E-04
cg19793962	1	9714738	PIK3CD-AS1	TSS200	-0,215	3,72E-05	2,78E-04
cg24160371	5	107333404	FBXL17	Body	0,204	3,72E-05	2,78E-04
cg04772968	19	12404112	ZNF44	Body	0,201	3,73E-05	2,78E-04
cg01297020	13	106117425	DAOA	TSS1500	-0,244	3,73E-05	2,78E-04
cg01582648	14	73926538	NUMB	TSS1500	0,218	3,73E-05	2,78E-04
cg15530035	2	221547499		IGR	0,252	3,74E-05	2,79E-04
cg11296933	2	143886567	ARHGAP15	TSS1500	-0,207	3,75E-05	2,79E-04
cg24193140	5	143581779	KCTD16	5'UTR	0,201	3,75E-05	2,79E-04
cg00377691	2	74413238		IGR	-0,202	3,75E-05	2,80E-04
cg04706063	8	102920721	NCALD	5'UTR	-0,225	3,76E-05	2,80E-04
cg02600199	8	135793179		IGR	-0,236	3,77E-05	2,80E-04
cg02806733	2	231731972	ITM2C	Body	-0,206	3,77E-05	2,81E-04
cg05858280	1	217251410	ESRRG	TSS1500	0,271	3,78E-05	2,81E-04
cg05297352	10	14585169	FAM107B	5'UTR	-0,203	3,78E-05	2,81E-04
cg06831584	2	242249417	HDLBP	5'UTR	-0,214	3,78E-05	2,81E-04
cg26909981	19	49377294	PPP1R15A	Body	0,219	3,80E-05	2,82E-04
cg25338707	11	63973846	FERMT3	TSS1500	-0,235	3,80E-05	2,82E-04
cg01385870	5	124164760		IGR	-0,23	3,80E-05	2,82E-04
cg11914189	1	86022243	DDAH1	5'UTR	0,202	3,80E-05	2,82E-04
cg12127605	19	43910505	TEX101	5'UTR	0,218	3,80E-05	2,83E-04
cg20842915	7	39665132	RALA	5'UTR	0,206	3,81E-05	2,83E-04
cg13049997	6	166744404	SFT2D1	Body	0,236	3,81E-05	2,83E-04
cg05177308	12	106674076		IGR	0,253	3,82E-05	2,83E-04
cg13413719	6	3592887		IGR	-0,231	3,82E-05	2,84E-04
cg05608389	1	66796739	PDE4B	TSS1500	-0,227	3,83E-05	2,84E-04
cg04371749	10	15251036		IGR	-0,301	3,83E-05	2,84E-04
cg10334642	6	141998825		IGR	0,227	3,83E-05	2,84E-04
cg24107088	14	35399820		IGR	-0,226	3,83E-05	2,84E-04
cg25874123	18	3220124	MYOM1	TSS200	0,206	3,84E-05	2,84E-04
cg04858322	13	114831550	RASA3	Body	-0,219	3,84E-05	2,85E-04
cg04871807	5	95186505	C5orf27	TSS1500	-0,217	3,84E-05	2,85E-04
cg10466421	21	27010696	JAM2	TSS1500	0,209	3,84E-05	2,85E-04
cg08076955	17	46608462	HOXB1	TSS200	0,322	3,84E-05	2,85E-04
cg18392999	6	139043760		IGR	-0,202	3,85E-05	2,85E-04
cg05174290	2	24114887	ATAD2B	Body	-0,251	3,85E-05	2,85E-04
cg27211295	19	55485068	NLRP2	Body	-0,201	3,86E-05	2,86E-04

cg03773136	1	202252791	LGR6	Body	-0,246	3,86E-05	2,86E-04
cg10237142	14	25479210	STXBP6	5'UTR	0,232	3,87E-05	2,86E-04
cg07354583	12	9911289	CD69	Body	-0,326	3,87E-05	2,87E-04
cg27330193	3	114137930	ZBTB20	5'UTR	0,244	3,87E-05	2,87E-04
cg06621763	12	77214912	ZDHHC17	Body	0,247	3,87E-05	2,87E-04
cg10973622	2	86423274	IMMT	TSS1500	0,253	3,88E-05	2,87E-04
cg20802358	6	135547984		IGR	-0,202	3,88E-05	2,87E-04
cg01062942	19	15568935	RASAL3	Body	0,214	3,89E-05	2,88E-04
cg18788642	5	38634990	LIFR-AS1	Body	-0,242	3,90E-05	2,88E-04
cg19227210	13	51466481	RNASEH2B-A!	Body	-0,234	3,90E-05	2,88E-04
cg23606396	6	29717917	LOC285830	TSS1500	-0,212	3,90E-05	2,88E-04
cg16270169	11	94350032	PIWIL4	Body	-0,201	3,91E-05	2,88E-04
cg26218487	6	52086161		IGR	-0,243	3,91E-05	2,89E-04
cg04868886	15	38963972		IGR	-0,202	3,92E-05	2,89E-04
cg09356555	8	58489984		IGR	0,224	3,92E-05	2,89E-04
cg24295032	14	89976955	FOXN3	5'UTR	-0,23	3,94E-05	2,90E-04
cg22742747	6	159064007	DYNLT1	Body	-0,211	3,94E-05	2,90E-04
cg13700680	7	23748334	STK31	TSS1500	0,228	3,95E-05	2,91E-04
cg07851964	5	83535548	EDIL3	Body	0,233	3,95E-05	2,91E-04
cg23821889	14	75736234		IGR	-0,202	3,95E-05	2,91E-04
cg24601404	2	197124825	LOC10192748	Body	-0,248	3,96E-05	2,91E-04
cg14968926	5	64267561	CWC27	Body	0,246	3,96E-05	2,92E-04
cg04885749	12	64855513	TBK1	Body	-0,228	3,97E-05	2,92E-04
cg13316720	17	25840984	KSR1	5'UTR	0,208	3,97E-05	2,92E-04
cg10738358	4	57968182	IGFBP7	Body	0,219	3,98E-05	2,93E-04
cg22742992	2	99077511	INPP4A	5'UTR	-0,24	3,99E-05	2,93E-04
cg02138684	11	72864108		IGR	-0,227	3,99E-05	2,93E-04
cg09295050	12	56105981	ITGA7	Body	0,202	4,00E-05	2,94E-04
cg20879720	5	77882295	LHFPL2	5'UTR	-0,249	4,01E-05	2,94E-04
cg25637972	6	17116181		IGR	0,226	4,02E-05	2,95E-04
cg23015343	13	50484082		IGR	0,25	4,03E-05	2,95E-04
cg20037328	11	78003389	GAB2	5'UTR	-0,211	4,03E-05	2,96E-04
cg24770230	5	115194332	AP3S1	Body	-0,296	4,04E-05	2,96E-04
cg16120275	14	92042040	C14orf184	TSS1500	0,228	4,04E-05	2,96E-04
cg14299115	9	85020050		IGR	0,213	4,04E-05	2,96E-04
cg23861944	1	80243103		IGR	0,205	4,05E-05	2,97E-04
cg23497569	7	19417585		IGR	0,252	4,05E-05	2,97E-04
cg20845627	10	18762622	CACNB2	Body	0,234	4,06E-05	2,97E-04
cg21336122	1	62417788	INADL	Body	0,201	4,06E-05	2,97E-04
cg08640619	1	157989520	KIRREL	Body	0,206	4,07E-05	2,98E-04
cg26888760	2	161351126	RBMS1	TSS1500	0,223	4,08E-05	2,98E-04
cg23270724	5	23466515		IGR	0,275	4,08E-05	2,98E-04
cg02088292	1	235099151		IGR	-0,249	4,09E-05	2,99E-04
cg09376558	12	96407225	LTA4H	Body	0,242	4,10E-05	2,99E-04
cg07964754	16	84650202	COTL1	Body	-0,23	4,12E-05	3,00E-04
cg08281426	4	67953781		IGR	-0,213	4,12E-05	3,00E-04
cg18877567	1	169903761	KIFAP3	Body	0,21	4,13E-05	3,01E-04
cg16067528	2	202655138		IGR	0,223	4,14E-05	3,02E-04
cg08764927	1	7844895	PER3	5'UTR	0,208	4,15E-05	3,02E-04
cg13016619	2	527786		IGR	0,222	4,15E-05	3,02E-04

cg12066530	6	90504299	MDN1	ExonBnd	-0,218	4,15E-05	3,02E-04
cg23561791	1	27952665	FGR	1stExon	-0,259	4,16E-05	3,03E-04
cg22979265	8	114435951	CSMD3	Body	0,209	4,16E-05	3,03E-04
cg16538953	22	17089603	TPTEP1	Body	0,228	4,16E-05	3,03E-04
cg26110562	2	236877461	AGAP1	Body	0,218	4,16E-05	3,03E-04
cg08818829	5	13810279	DNAH5	Body	0,239	4,16E-05	3,03E-04
cg26673784	10	80787497	LOC283050	Body	-0,214	4,16E-05	3,03E-04
cg07152607	2	168793702	LOC1056169(	Body	0,203	4,17E-05	3,03E-04
cg05773762	2	169579076	CERS6	Body	0,205	4,18E-05	3,04E-04
cg00291006	15	26056773	ATP10A	Body	-0,233	4,18E-05	3,04E-04
cg07696277	4	146097432	OTUD4	5'UTR	0,212	4,19E-05	3,04E-04
cg18112235	2	196451226		IGR	0,202	4,19E-05	3,05E-04
cg26349474	17	46705637		IGR	0,219	4,20E-05	3,05E-04
cg25550003	15	39719270		IGR	-0,21	4,21E-05	3,06E-04
cg06150841	1	61606693	NFIA	Body	-0,236	4,23E-05	3,06E-04
cg21388639	4	159740817	FNIP2	Body	0,227	4,24E-05	3,07E-04
cg01022869	5	117897813	HRAT56	TSS200	0,226	4,25E-05	3,08E-04
cg27468302	13	73458477	PIBF1	Body	0,228	4,26E-05	3,08E-04
cg19290238	2	233764049	NGEF	Body	0,219	4,26E-05	3,08E-04
cg14533030	5	119771163		IGR	0,212	4,27E-05	3,09E-04
cg10400707	1	160369840	VANGL2	TSS1500	0,23	4,27E-05	3,09E-04
cg09952002	17	46640774	HOXB3	5'UTR	0,317	4,29E-05	3,10E-04
cg24362952	1	109189641		IGR	-0,285	4,29E-05	3,10E-04
cg15812586	7	139953117		IGR	-0,254	4,29E-05	3,10E-04
cg17643692	2	145198903	ZEB2	Body	0,224	4,30E-05	3,10E-04
cg01411934	5	150643908	GM2A	Body	-0,231	4,30E-05	3,10E-04
cg23665662	3	152166698	MBNL1	Body	0,22	4,30E-05	3,11E-04
cg11070989	6	113768503		IGR	-0,272	4,31E-05	3,11E-04
cg00796611	1	206603444	SRGAP2	Body	0,284	4,32E-05	3,12E-04
cg10664406	6	166878682	RPS6KA2-IT1	Body	-0,253	4,32E-05	3,12E-04
cg07613153	14	104436947	TDRD9	Body	-0,204	4,32E-05	3,12E-04
cg25182066	10	30743637	MAP3K8	Body	0,212	4,33E-05	3,12E-04
cg11102682	2	62266795	COMMD1	Body	-0,24	4,33E-05	3,12E-04
cg27358165	15	86215313	AKAP13	Body	0,208	4,33E-05	3,12E-04
cg12075011	2	44411547	PPM1B	5'UTR	-0,215	4,33E-05	3,12E-04
cg23070085	15	70550213		IGR	-0,272	4,34E-05	3,13E-04
cg21002957	19	15568894	RASAL3	Body	0,21	4,34E-05	3,13E-04
cg12192529	18	10020398		IGR	-0,207	4,34E-05	3,13E-04
cg04439720	3	33467812	UBP1	Body	0,212	4,35E-05	3,13E-04
cg12582728	17	36162433		IGR	-0,215	4,35E-05	3,14E-04
cg24685239	3	105159482	ALCAM	Body	0,215	4,36E-05	3,14E-04
cg06040990	5	66254876	MAST4	5'UTR	0,238	4,37E-05	3,14E-04
cg24180975	6	53883752	C6orf142	5'UTR	0,207	4,37E-05	3,15E-04
cg24076069	3	138609914		IGR	-0,209	4,39E-05	3,15E-04
cg15942601	5	172000372		IGR	-0,25	4,39E-05	3,16E-04
cg18061711	5	71683955		IGR	0,238	4,41E-05	3,17E-04
cg22182936	13	36070148	NBEA	5'UTR	-0,226	4,42E-05	3,17E-04
cg24515352	6	52257884	PAQR8	5'UTR	-0,206	4,42E-05	3,18E-04
cg26921036	4	48214533	TEC	Body	-0,215	4,43E-05	3,18E-04
cg21868496	2	218700130	TNS1	Body	-0,206	4,43E-05	3,18E-04

cg06456935	1	185537796	LINC01350	Body	-0,23	4,43E-05	3,18E-04
cg21972431	7	17812356		IGR	0,202	4,43E-05	3,18E-04
cg09054339	3	188265293	LPP	Body	0,204	4,44E-05	3,18E-04
cg13195276	5	127203128		IGR	-0,229	4,45E-05	3,19E-04
cg08600456	8	81963035	PAG1	5'UTR	0,227	4,46E-05	3,20E-04
cg23916896	5	368804	AHRR	Body	-0,233	4,47E-05	3,20E-04
cg16515952	6	113787865		IGR	-0,23	4,47E-05	3,20E-04
cg19551848	7	146478972	CNTNAP2	Body	0,202	4,48E-05	3,20E-04
cg20306694	12	51718251	BIN2	TSS1500	-0,204	4,49E-05	3,21E-04
cg03731316	6	149102334	UST	Body	-0,202	4,50E-05	3,22E-04
cg00359365	1	26880547	MIR1976	TSS1500	-0,249	4,50E-05	3,22E-04
cg24202390	1	52017771		IGR	-0,214	4,51E-05	3,22E-04
cg00299070	1	237946534	RYR2	Body	0,234	4,53E-05	3,24E-04
cg07968094	14	64901479	MIR548AZ	Body	0,242	4,54E-05	3,24E-04
cg04948285	10	64973486	JMJD1C	Body	0,211	4,54E-05	3,24E-04
cg26221442	7	7869872	UMAD1	Body	0,23	4,54E-05	3,24E-04
cg24752836	10	63733787	ARID5B	Body	0,258	4,56E-05	3,25E-04
cg15889594	17	36605927		IGR	0,232	4,56E-05	3,25E-04
cg25115455	17	11944836	MAP2K4	Body	-0,236	4,57E-05	3,25E-04
cg14849608	11	109897980		IGR	-0,208	4,58E-05	3,26E-04
cg20775007	6	12052395	HIVEP1	Body	-0,237	4,58E-05	3,26E-04
cg02220419	2	36712809	CRIM1	Body	0,206	4,59E-05	3,27E-04
cg10438023	1	231519776	EGLN1	Body	0,235	4,61E-05	3,28E-04
cg00421693	5	118689293	TNFAIP8	Body	-0,212	4,61E-05	3,28E-04
cg08529212	11	110001556	ZC3H12C	Body	0,21	4,61E-05	3,28E-04
cg06684410	20	55017557	CASS4	Body	-0,211	4,61E-05	3,28E-04
cg06324719	15	70118762		IGR	0,204	4,61E-05	3,28E-04
cg23091585	4	48855768	OCIAD1	Body	0,205	4,62E-05	3,28E-04
cg14288925	7	107223076	BCAP29	Body	0,23	4,62E-05	3,28E-04
cg20997448	5	76814429		IGR	0,247	4,62E-05	3,28E-04
cg00459613	1	176050414	RFWD2	ExonBnd	0,228	4,63E-05	3,29E-04
cg16292136	13	49448643		IGR	0,212	4,64E-05	3,29E-04
cg17651403	8	28090642		IGR	0,208	4,65E-05	3,30E-04
cg17191650	18	13471280	LDLRAD4	Body	0,212	4,66E-05	3,30E-04
cg08617160	19	345312	MIER2	TSS1500	0,216	4,66E-05	3,31E-04
cg26891135	1	89521065	GBP1	Body	0,205	4,68E-05	3,32E-04
cg14066916	16	28193292	XPO6	5'UTR	-0,211	4,68E-05	3,32E-04
cg22356061	1	227954102	SNAP47	Body	-0,212	4,68E-05	3,32E-04
cg08210019	7	107121395	COG5	Body	0,229	4,69E-05	3,33E-04
cg17749967	19	18160301		IGR	0,232	4,70E-05	3,33E-04
cg09922561	7	116193371	CAV1	Body	0,229	4,71E-05	3,33E-04
cg21112241	5	54203516		IGR	0,231	4,71E-05	3,33E-04
cg07988091	6	116567403		IGR	0,228	4,72E-05	3,34E-04
cg07689590	15	48138213	LINC01491	Body	-0,261	4,72E-05	3,34E-04
cg26351453	7	91685854	AKAP9	Body	0,205	4,73E-05	3,34E-04
cg14458615	3	118864952	C3orf30	TSS200	0,241	4,73E-05	3,35E-04
cg06742884	15	60411061		IGR	0,22	4,73E-05	3,35E-04
cg07387286	8	39172120	ADAM5P	TSS200	0,202	4,74E-05	3,35E-04
cg18784912	3	189176507		IGR	0,258	4,75E-05	3,35E-04
cg00567588	3	12596831		IGR	0,227	4,76E-05	3,36E-04

cg25076039	5	169128937	DOCK2	Body	0,215	4,77E-05	3,37E-04
cg06873024	1	215130954		IGR	-0,294	4,78E-05	3,37E-04
cg11575782	3	142573154	PCOLCE2	Body	0,202	4,78E-05	3,37E-04
cg02524112	16	73206624		IGR	0,2	4,79E-05	3,38E-04
cg19831563	5	44886106		IGR	-0,283	4,80E-05	3,38E-04
cg22781205	4	150855245		IGR	0,229	4,80E-05	3,38E-04
cg13660320	1	76430157		IGR	0,292	4,80E-05	3,38E-04
cg16552271	1	15392907	KIAA1026	Body	-0,232	4,81E-05	3,39E-04
cg22583953	16	69872200	WWP2	TSS1500	0,218	4,81E-05	3,39E-04
cg03181382	2	131839563	FAM168B	Body	0,201	4,84E-05	3,40E-04
cg14559139	2	88367362	SMYD1	TSS200	0,248	4,84E-05	3,40E-04
cg16708623	4	154125208	TRIM2	TSS1500	0,227	4,84E-05	3,40E-04
cg14443849	22	44505040	PARVB	Body	0,2	4,84E-05	3,40E-04
cg14071298	2	42333048		IGR	-0,206	4,84E-05	3,41E-04
cg18988395	9	4152606	GLIS3	TSS1500	-0,226	4,85E-05	3,41E-04
cg18647039	5	138753161	DNAJC18	Body	0,214	4,85E-05	3,41E-04
cg03915704	1	40851486	SMAP2	Body	-0,218	4,85E-05	3,41E-04
cg25766274	8	128352772	CASC21	Body	-0,229	4,85E-05	3,41E-04
cg11207540	12	52399731	GRASP	TSS1500	-0,201	4,86E-05	3,41E-04
cg17880661	1	214169870	PROX1	5'UTR	0,208	4,86E-05	3,42E-04
cg12661316	1	7029235	CAMTA1	Body	-0,233	4,86E-05	3,42E-04
cg09293182	2	231192275	SP140L	Body	-0,201	4,87E-05	3,42E-04
cg25927838	2	183807007	NCKAP1	Body	0,237	4,89E-05	3,43E-04
cg09456576	5	3285988		IGR	0,202	4,90E-05	3,44E-04
cg19511748	5	94220272	MCTP1	Body	0,258	4,91E-05	3,44E-04
cg24622817	22	40859826	MKL1	TSS1500	-0,237	4,92E-05	3,44E-04
cg24605932	11	110145293	RDX	Body	0,216	4,95E-05	3,46E-04
cg07997634	7	95818282	SLC25A13	Body	0,228	4,96E-05	3,47E-04
cg23263857	12	95999854		IGR	0,206	4,96E-05	3,47E-04
cg17926918	5	88964896		IGR	0,209	4,97E-05	3,47E-04
cg26965687	2	88367360	SMYD1	5'UTR	0,211	4,98E-05	3,48E-04
cg14792180	5	10632056	ANKRD33B	Body	-0,204	4,99E-05	3,48E-04
cg06530044	9	128239551	MAPKAP1	Body	0,212	5,00E-05	3,49E-04
cg01349489	21	32720030	TIAM1	5'UTR	0,228	5,03E-05	3,50E-04
cg18810919	3	119361179	POPDC2	3'UTR	0,211	5,03E-05	3,50E-04
cg17656945	3	189428406	TP63	Body	0,268	5,04E-05	3,51E-04
cg12813441	2	55239331	RTN4	Body	0,25	5,04E-05	3,51E-04
cg13243685	9	4145286	GLIS3	5'UTR	-0,202	5,05E-05	3,52E-04
cg18408335	18	3305544		IGR	-0,203	5,05E-05	3,52E-04
cg00533994	6	34274189	NUDT3	Body	0,223	5,07E-05	3,53E-04
cg23446277	1	226876751	ITPKB	Body	0,206	5,07E-05	3,53E-04
cg02095290	11	17008895	PLEKHA7	Body	0,246	5,08E-05	3,54E-04
cg25562417	12	21679891	SPX	Body	0,201	5,09E-05	3,54E-04
cg00661485	5	169533372	FOXI1	1stExon	-0,215	5,09E-05	3,54E-04
cg04615639	17	77997950	TBC1D16	5'UTR	0,213	5,09E-05	3,54E-04
cg07088118	14	29712761		IGR	0,226	5,09E-05	3,54E-04
cg27302255	12	20336617		IGR	0,237	5,10E-05	3,55E-04
cg08091497	3	16545999	RFTN1	5'UTR	-0,234	5,11E-05	3,55E-04
cg20467260	13	46751170	LCP1	5'UTR	-0,238	5,11E-05	3,55E-04
cg06047184	7	2347654	SNX8	Body	-0,219	5,12E-05	3,55E-04

cg25201372	13	75941163	TBC1D4	Body	0,244	5,12E-05	3,56E-04
cg17510121	6	35182910	SCUBE3	Body	0,21	5,13E-05	3,56E-04
cg23610447	6	113650073		IGR	0,203	5,13E-05	3,56E-04
cg04633764	4	26194902		IGR	-0,217	5,15E-05	3,57E-04
cg12933553	8	24052851		IGR	-0,217	5,15E-05	3,57E-04
cg10726346	9	14308118	NFIB	Body	0,211	5,15E-05	3,57E-04
cg18229341	18	64271643	CDH19	TSS1500	0,206	5,15E-05	3,57E-04
cg07368827	2	225787372	DOCK10	Body	-0,214	5,16E-05	3,58E-04
cg02584678	19	15310520	NOTCH3	Body	0,223	5,17E-05	3,58E-04
cg09839862	3	65990185	MAGI1	Body	0,218	5,17E-05	3,58E-04
cg01920735	2	197237764	HECW2	Body	0,229	5,18E-05	3,59E-04
cg20663448	6	76204308	FILIP1	TSS1500	0,24	5,18E-05	3,59E-04
cg01668330	14	20955311		IGR	-0,222	5,18E-05	3,59E-04
cg25565925	12	7586119	CD163L1	Body	0,202	5,20E-05	3,60E-04
cg08757868	6	90832027	BACH2	5'UTR	-0,221	5,20E-05	3,60E-04
cg04693040	1	99999596		IGR	0,22	5,21E-05	3,61E-04
cg04749316	10	49893346	WDFY4	TSS200	-0,209	5,21E-05	3,61E-04
cg27165960	5	39177381	FYB	Body	-0,209	5,22E-05	3,61E-04
cg23105515	2	143903655	ARHGAP15	5'UTR	0,205	5,22E-05	3,61E-04
cg13212315	3	194300269		IGR	0,206	5,24E-05	3,62E-04
cg11409139	6	30292481	HCG18	Body	0,235	5,24E-05	3,62E-04
cg10774331	3	156529497	PA2G4P4	Body	0,244	5,25E-05	3,62E-04
cg04995521	1	8468871	RERE	5'UTR	0,254	5,25E-05	3,62E-04
cg14787959	21	17906739	MIR99AHG	Body	0,219	5,25E-05	3,63E-04
cg22088248	18	72917387	ZADH2	Body	0,233	5,26E-05	3,63E-04
cg03555881	6	31275551		IGR	0,2	5,26E-05	3,63E-04
cg07102509	17	46131897	NFE2L1	Body	0,21	5,26E-05	3,63E-04
cg27288886	18	20898196	TMEM241	Body	-0,264	5,27E-05	3,64E-04
cg13157426	1	198087386		IGR	0,22	5,28E-05	3,64E-04
cg09223871	18	55869140	NEDD4L	Body	0,245	5,30E-05	3,65E-04
cg02384491	1	162307064	NOS1AP	Body	-0,272	5,30E-05	3,65E-04
cg06854882	2	61646507	USP34	Body	-0,213	5,31E-05	3,65E-04
cg14078306	14	65548222	MAX	Body	-0,245	5,31E-05	3,66E-04
cg18463607	4	56718320	EXOC1	TSS1500	-0,242	5,31E-05	3,66E-04
cg13037572	8	128310106	CASC21	Body	0,213	5,32E-05	3,66E-04
cg12784701	5	66093004	MAST4	Body	0,204	5,33E-05	3,67E-04
cg25987285	20	25014771	ACSS1	TSS1500	0,257	5,34E-05	3,67E-04
cg07616499	10	30324789	KIAA1462	Body	0,233	5,34E-05	3,67E-04
cg18826274	17	76335184		IGR	-0,221	5,35E-05	3,68E-04
cg07104639	20	15125595	MACROD2	Body	0,27	5,37E-05	3,69E-04
cg15589135	5	96294128	LNPEP	TSS200	-0,214	5,37E-05	3,69E-04
cg18320356	12	10103812		IGR	-0,241	5,37E-05	3,69E-04
cg26849111	3	124438600	KALRN	3'UTR	-0,259	5,38E-05	3,69E-04
cg13377896	5	98274424		IGR	0,216	5,39E-05	3,70E-04
cg04828105	6	113681338		IGR	0,247	5,40E-05	3,70E-04
cg05762432	4	169663078	PALLD	Body	-0,223	5,42E-05	3,72E-04
cg05932885	3	193769720		IGR	-0,221	5,43E-05	3,72E-04
cg13377232	3	192747867		IGR	-0,214	5,43E-05	3,72E-04
cg14873380	6	47647971	ADGRF2	Body	0,211	5,43E-05	3,72E-04
cg07241416	5	39177998	FYB	Body	-0,21	5,45E-05	3,73E-04

cg11235594	2	110907413	NPHP1	Body	-0,273	5,46E-05	3,74E-04
cg26162691	3	124170647	KALRN	Body	-0,248	5,49E-05	3,75E-04
cg14036627	6	31148657		IGR	0,207	5,49E-05	3,75E-04
cg01638185	17	64530027	PRKCA	Body	0,206	5,50E-05	3,76E-04
cg26379120	3	49229490	C3orf84	TSS200	0,209	5,51E-05	3,76E-04
cg26904005	6	29676792		IGR	-0,204	5,52E-05	3,77E-04
cg03450336	11	84208983	DLG2	Body	-0,221	5,53E-05	3,77E-04
cg20576322	4	85886384	WDFY3	5'UTR	0,203	5,53E-05	3,77E-04
cg24969417	6	107140115		IGR	0,207	5,53E-05	3,77E-04
cg25979526	15	52778344	MYO5A	Body	0,217	5,53E-05	3,77E-04
cg04578004	17	46637400	HOXB3	5'UTR	0,273	5,53E-05	3,77E-04
cg17015260	1	8035582	PARK7	Body	0,201	5,55E-05	3,78E-04
cg19487738	5	87785433		IGR	0,242	5,57E-05	3,79E-04
cg26872543	14	61910902	PRKCH	Body	-0,229	5,57E-05	3,79E-04
cg08633209	5	140071946	HARS2	5'UTR	0,216	5,58E-05	3,79E-04
cg15531995	12	94602562	PLXNC1	Body	-0,301	5,58E-05	3,80E-04
cg05119709	5	39385456	DAB2	Body	-0,215	5,61E-05	3,81E-04
cg19510688	11	126101694	FAM118B	5'UTR	0,22	5,61E-05	3,81E-04
cg16915477	20	36339624	CTNNBL1	5'UTR	0,219	5,61E-05	3,81E-04
cg16915733	3	62571255	CADPS	Body	-0,216	5,62E-05	3,82E-04
cg07665923	7	1174784	C7orf50	5'UTR	0,218	5,62E-05	3,82E-04
cg07003097	3	111458198	PLCXD2	Body	0,208	5,63E-05	3,82E-04
cg14756006	6	94489271		IGR	0,206	5,66E-05	3,84E-04
cg17870688	2	182753233		IGR	-0,201	5,66E-05	3,84E-04
cg03787837	6	32605385	HLA-DQA1	Body	-0,255	5,68E-05	3,85E-04
cg14618239	5	86417299	LOC10192938	Body	-0,21	5,70E-05	3,86E-04
cg17610333	6	168107313		IGR	-0,21	5,70E-05	3,86E-04
cg23047137	3	24089778		IGR	-0,216	5,70E-05	3,86E-04
cg02877743	4	153271092	FBXW7	Body	0,28	5,71E-05	3,87E-04
cg11637898	11	90815210		IGR	0,207	5,72E-05	3,87E-04
cg11453820	15	57719605	CGNL1	5'UTR	0,21	5,72E-05	3,87E-04
cg10373607	20	51592177	TSHZ2	Body	-0,204	5,73E-05	3,88E-04
cg20462500	10	102513301	PAX2	Body	0,252	5,74E-05	3,88E-04
cg20163038	19	7145750	INSR	Body	0,214	5,74E-05	3,88E-04
cg15658676	6	53530538	KLHL31	TSS200	0,205	5,75E-05	3,88E-04
cg19935951	4	151258915	LRBA	Body	0,237	5,75E-05	3,88E-04
cg26709988	16	84860918	CRISPLD2	5'UTR	0,208	5,76E-05	3,89E-04
cg03687650	11	3144685	OSBPL5	Body	0,216	5,77E-05	3,90E-04
cg13440073	16	73577682		IGR	-0,219	5,77E-05	3,90E-04
cg20711585	1	46330040	MAST2	Body	0,213	5,79E-05	3,91E-04
cg25968247	3	57581113	ARF4	Body	0,223	5,79E-05	3,91E-04
cg18461866	3	59996864	FHIT	Body	-0,219	5,80E-05	3,91E-04
cg05258531	16	53673620	RPGRIP1L	Body	0,23	5,80E-05	3,91E-04
cg13802433	2	68403007	PNO1	3'UTR	0,203	5,80E-05	3,92E-04
cg24006939	16	82958126	CDH13	5'UTR	-0,257	5,83E-05	3,93E-04
cg22686132	6	28725957		IGR	0,206	5,84E-05	3,93E-04
cg16981938	8	125622651	MTSS1	Body	0,242	5,86E-05	3,95E-04
cg27011118	8	30179144		IGR	-0,215	5,87E-05	3,95E-04
cg09666717	4	106071543	TET2	5'UTR	0,284	5,87E-05	3,95E-04
cg24197781	1	188355450		IGR	0,234	5,88E-05	3,96E-04

cg16947138	11	100610569	FLJ32810	Body	0,231	5,88E-05	3,96E-04
cg26255314	11	60869910	CD5	TSS200	-0,246	5,89E-05	3,96E-04
cg14136698	16	85342729		IGR	-0,202	5,89E-05	3,96E-04
cg13792714	3	114103345	ZBTB20	5'UTR	0,224	5,90E-05	3,97E-04
cg25461106	6	1079945	LOC285768	Body	0,216	5,91E-05	3,97E-04
cg26629369	15	40040665	FSIP1	Body	0,207	5,91E-05	3,97E-04
cg08479514	7	23043767	FAM126A	5'UTR	0,216	5,93E-05	3,98E-04
cg17943647	4	154125374	TRIM2	TSS1500	0,202	5,93E-05	3,98E-04
cg09496585	3	178038488		IGR	0,241	5,95E-05	4,00E-04
cg10208777	8	23775498		IGR	-0,226	5,96E-05	4,00E-04
cg20516845	21	43823604	UBASH3A	TSS1500	-0,21	5,96E-05	4,00E-04
cg15549827	15	80104246		IGR	0,219	5,97E-05	4,00E-04
cg05124953	5	90524312		IGR	-0,209	5,97E-05	4,00E-04
cg05932560	16	87279321		IGR	0,218	5,97E-05	4,00E-04
cg27486935	3	118865135	C3orf30	1stExon	0,216	5,99E-05	4,01E-04
cg17449339	3	15823705	ANKRD28	5'UTR	0,239	6,00E-05	4,02E-04
cg02879143	17	68618551		IGR	0,241	6,03E-05	4,03E-04
cg20476329	6	75804472	COL12A1	Body	0,205	6,08E-05	4,06E-04
cg16705969	9	71409959	PIP5K1B	5'UTR	0,32	6,10E-05	4,07E-04
cg15786238	6	170441907		IGR	0,209	6,10E-05	4,07E-04
cg08735211	6	32920657	HLA-DMA	Body	-0,22	6,11E-05	4,08E-04
cg10302019	9	14180824	NFIB	TSS200	0,235	6,12E-05	4,08E-04
cg16618104	12	104853100	CHST11	Body	-0,256	6,15E-05	4,10E-04
cg11373114	20	25096068		IGR	0,253	6,15E-05	4,10E-04
cg18116670	1	21649784	ECE1	Body	0,225	6,16E-05	4,10E-04
cg12469355	2	238633681	LRRFIP1	Body	0,206	6,17E-05	4,11E-04
cg02390954	5	15620858	FBXL7	Body	-0,201	6,18E-05	4,11E-04
cg20758219	3	138738597	PRR23B	1stExon	0,204	6,18E-05	4,11E-04
cg14792595	22	50980925		IGR	-0,223	6,19E-05	4,12E-04
cg01863495	3	190581232	GMNC	TSS1500	0,209	6,19E-05	4,12E-04
cg04410632	5	70897464	MCCC2	Body	-0,202	6,19E-05	4,12E-04
cg27023597	17	57918262	MIR21	TSS1500	-0,227	6,20E-05	4,12E-04
cg02627455	7	27157818	HOXA3	5'UTR	0,257	6,20E-05	4,12E-04
cg05848378	17	39341988	KRTAP4-1	TSS1500	0,237	6,21E-05	4,13E-04
cg07270545	9	35195884	UNC13B	Body	-0,215	6,21E-05	4,13E-04
cg15139930	2	223788811	ACSL3	Body	0,221	6,21E-05	4,13E-04
cg17371064	10	3464176	LOC1053763(	Body	-0,219	6,22E-05	4,13E-04
cg18822905	5	77882454	LHFPL2	5'UTR	-0,206	6,22E-05	4,13E-04
cg04121996	4	41052430	APBB2	5'UTR	0,221	6,22E-05	4,13E-04
cg25778564	15	81425747	C15orf26	TSS1500	-0,209	6,23E-05	4,14E-04
cg26373662	5	169740386		IGR	-0,204	6,23E-05	4,14E-04
cg07084675	18	56666239		IGR	0,243	6,24E-05	4,15E-04
cg18637843	4	38566968		IGR	0,219	6,25E-05	4,15E-04
cg25785177	4	114445662	CAMK2D	Body	0,219	6,26E-05	4,16E-04
cg18933133	8	38705090	TACC1	Body	0,21	6,28E-05	4,17E-04
cg10043414	13	109754127	MYO16	Body	-0,201	6,28E-05	4,17E-04
cg22139615	4	141048272	MAML3	Body	-0,203	6,29E-05	4,17E-04
cg24954186	4	172560790		IGR	-0,27	6,30E-05	4,17E-04
cg19557019	8	56481188		IGR	-0,209	6,31E-05	4,18E-04
cg11060532	17	46652399	HOXB3	TSS1500	0,227	6,31E-05	4,18E-04

cg03002264	2	147788732		IGR	0,269	6,32E-05	4,18E-04
cg01093476	5	32020512	PDZD2	Body	-0,203	6,33E-05	4,19E-04
cg18053725	12	47788146		IGR	-0,203	6,35E-05	4,20E-04
cg17415486	19	42057419	CEACAM21	5'UTR	-0,248	6,37E-05	4,21E-04
cg25649910	10	116526504	LOC10192769	Body	0,26	6,37E-05	4,21E-04
cg08473006	12	110981484	PPTC7	Body	0,215	6,40E-05	4,23E-04
cg21748464	7	20204222	MACC1	5'UTR	-0,241	6,42E-05	4,24E-04
cg16029876	2	216541896	LINC00607	Body	-0,206	6,43E-05	4,24E-04
cg16830225	5	83074876		IGR	-0,202	6,44E-05	4,25E-04
cg17623923	9	117003237	COL27A1	Body	-0,217	6,45E-05	4,25E-04
cg13148429	18	46688124	DYM	Body	-0,246	6,45E-05	4,25E-04
cg25963419	2	102618414	IL1R2	5'UTR	-0,213	6,45E-05	4,25E-04
cg10882756	5	167282267	ODZ2	Body	0,21	6,46E-05	4,25E-04
cg08443563	2	112456811		IGR	-0,205	6,46E-05	4,26E-04
cg10351869	9	18057067		IGR	-0,201	6,47E-05	4,26E-04
cg07536036	12	78169496		IGR	-0,202	6,49E-05	4,27E-04
cg02370734	8	66702198	PDE7A	TSS1500	0,203	6,51E-05	4,28E-04
cg16288421	4	54213525	SCFD2	Body	0,212	6,51E-05	4,28E-04
cg14069619	17	8275273	KRBA2	TSS1500	0,21	6,51E-05	4,28E-04
cg12303318	6	130031784	ARHGAP18	TSS1500	0,207	6,52E-05	4,29E-04
cg26261540	17	57171138	TRIM37	Body	0,249	6,54E-05	4,30E-04
cg04259892	8	130281734		IGR	-0,231	6,56E-05	4,31E-04
cg14234063	10	75289990	USP54	Body	-0,204	6,58E-05	4,32E-04
cg06615316	18	67482658	DOK6	Body	0,207	6,60E-05	4,33E-04
cg25612399	13	60252260	DIAPH3	Body	0,235	6,61E-05	4,33E-04
cg11290762	8	79717969	IL7	TSS1500	-0,207	6,62E-05	4,34E-04
cg10843076	6	55956609	COL21A1	Body	0,238	6,62E-05	4,34E-04
cg18261588	10	74513912	MCU	Body	-0,208	6,63E-05	4,34E-04
cg11234339	11	10907448		IGR	0,236	6,63E-05	4,34E-04
cg07131313	7	17221322		IGR	0,209	6,64E-05	4,35E-04
cg05101019	12	109029473	MIR4496	TSS200	-0,242	6,65E-05	4,35E-04
cg14929130	6	159166120	SYTL3	Body	0,218	6,66E-05	4,36E-04
cg09928045	10	104913440	NT5C2	Body	0,23	6,67E-05	4,36E-04
cg09414187	17	16258107	CENPV	TSS1500	0,261	6,70E-05	4,38E-04
cg00539581	5	66477808		IGR	-0,225	6,71E-05	4,39E-04
cg04015097	20	17519317	BFSP1	Body	-0,2	6,72E-05	4,39E-04
cg14634386	5	82582024	XRCC4	Body	-0,21	6,73E-05	4,40E-04
cg01109694	15	33176640	FMN1	Body	0,204	6,75E-05	4,40E-04
cg13532787	3	30389169		IGR	-0,223	6,75E-05	4,40E-04
cg04904531	1	118405119		IGR	0,202	6,75E-05	4,41E-04
cg08734066	10	80338775		IGR	-0,208	6,81E-05	4,44E-04
cg12617598	1	193222046	CDC73	3'UTR	0,219	6,83E-05	4,45E-04
cg02773737	11	61657498	FADS3	Body	0,231	6,84E-05	4,45E-04
cg06779847	14	77687015	TMEM63C	Body	0,2	6,84E-05	4,45E-04
cg11636765	7	11747365	THSD7A	Body	-0,207	6,85E-05	4,46E-04
cg10903440	13	113588478		IGR	-0,257	6,85E-05	4,46E-04
cg06637961	2	207327408	ADAM23	Body	0,206	6,87E-05	4,47E-04
cg17211409	2	202297914	TRAK2	5'UTR	0,262	6,89E-05	4,48E-04
cg07672071	2	191223603	INPP1	5'UTR	-0,213	6,90E-05	4,48E-04
cg15431103	5	55457410	ANKRD55	Body	-0,22	6,91E-05	4,49E-04

cg19523166	6	14117344	CD83	TSS1500	0,224	6,92E-05	4,50E-04
cg20786422	6	159070491	SYTL3	TSS1500	-0,204	6,96E-05	4,51E-04
cg21872327	2	15451590	NBAS	Body	-0,222	6,98E-05	4,52E-04
cg02871154	11	72915874		IGR	-0,214	6,98E-05	4,53E-04
cg16136238	4	57929310	IGFBP7	Body	0,231	6,99E-05	4,53E-04
cg14105327	18	42175181		IGR	0,309	7,00E-05	4,53E-04
cg19256291	10	133156155		IGR	0,202	7,01E-05	4,54E-04
cg24543552	6	73935270	KHDC1L	TSS200	-0,243	7,01E-05	4,54E-04
cg22642498	19	54312855	NLRP12	Body	0,205	7,01E-05	4,54E-04
cg02488702	2	142889392	LRP1B	TSS200	0,201	7,02E-05	4,54E-04
cg03627980	1	193305239	LINC01031	Body	0,214	7,02E-05	4,55E-04
cg01888131	6	91247068	MAP3K7	Body	0,214	7,02E-05	4,55E-04
cg22706647	5	76465803		IGR	-0,22	7,04E-05	4,55E-04
cg00854503	5	178749073	ADAMTS2	Body	-0,21	7,04E-05	4,56E-04
cg08864725	15	42212924	EHD4-AS1	TSS1500	0,261	7,05E-05	4,56E-04
cg20326248	13	24217863	TNFRSF19	Body	0,205	7,07E-05	4,57E-04
cg24289237	13	60240868	DIAPH3	Body	0,258	7,09E-05	4,58E-04
cg26049187	1	196976319	CFHR5	Body	-0,206	7,09E-05	4,58E-04
cg23519637	7	1121738	C7orf50	Body	0,241	7,10E-05	4,58E-04
cg11811828	6	31148666		IGR	0,223	7,10E-05	4,59E-04
cg01471034	20	40208239	CHD6	5'UTR	0,253	7,11E-05	4,59E-04
cg12832929	5	130615081	CDC42SE2	5'UTR	-0,21	7,12E-05	4,59E-04
cg23683079	10	44353285		IGR	-0,207	7,17E-05	4,62E-04
cg10180415	1	117057968	CD58	Body	-0,263	7,19E-05	4,63E-04
cg27011674	6	15399125	JARID2	Body	-0,201	7,20E-05	4,63E-04
cg02119091	8	11553749		IGR	-0,206	7,20E-05	4,64E-04
cg03169021	5	140242486	PCDHA1	Body	0,208	7,21E-05	4,64E-04
cg21444496	18	55454141	ATP8B1	5'UTR	0,204	7,24E-05	4,65E-04
cg24540730	6	106972012	AIM1	Body	-0,211	7,24E-05	4,66E-04
cg01341014	5	71435001	MAP1B	Body	-0,206	7,24E-05	4,66E-04
cg08677579	2	200468559		IGR	0,202	7,24E-05	4,66E-04
cg22713460	9	72289582		IGR	-0,202	7,28E-05	4,68E-04
cg00770754	16	89440949	ANKRD11	5'UTR	0,207	7,30E-05	4,69E-04
cg26019160	4	185616485	CENPU	Body	0,235	7,31E-05	4,69E-04
cg11924141	12	41041648		IGR	0,224	7,31E-05	4,69E-04
cg05138695	5	14687325	OTULIN	Body	0,201	7,32E-05	4,70E-04
cg01092809	7	29373307	CHN2	Body	0,22	7,32E-05	4,70E-04
cg21243394	4	79482294	ANXA3	Body	0,203	7,33E-05	4,70E-04
cg21247889	16	79485198		IGR	-0,208	7,37E-05	4,72E-04
cg20541527	13	50811383	DLEU1	Body	-0,238	7,38E-05	4,73E-04
cg16346169	13	99880353	UBAC2	Body	-0,22	7,40E-05	4,74E-04
cg07537187	9	88669060	GOLM1	Body	0,242	7,42E-05	4,74E-04
cg08787791	13	43355737	C13orf30	1stExon	0,228	7,42E-05	4,75E-04
cg04960065	4	185189300		IGR	-0,221	7,49E-05	4,78E-04
cg03813151	6	149778058	ZC3H12D	Body	0,23	7,49E-05	4,78E-04
cg08576856	1	96700043		IGR	0,214	7,51E-05	4,79E-04
cg22758653	7	116066796		IGR	0,216	7,55E-05	4,81E-04
cg04235280	2	103097094	SLC9A4	Body	-0,203	7,55E-05	4,81E-04
cg24705158	5	177876830	COL23A1	Body	0,202	7,56E-05	4,82E-04
cg18225955	3	72021276		IGR	0,292	7,56E-05	4,82E-04

cg01059738	3	162003213		IGR	0,209	7,57E-05	4,82E-04
cg06394677	16	57636195		IGR	-0,262	7,58E-05	4,83E-04
cg11986282	8	80943724	MRPS28	TSS1500	0,21	7,58E-05	4,83E-04
cg09022477	9	14097058	NFIB	Body	0,25	7,60E-05	4,84E-04
cg22399458	11	112038039	TEX12	TSS200	0,203	7,61E-05	4,84E-04
cg07226481	2	202298388	TRAK2	5'UTR	0,211	7,61E-05	4,84E-04
cg14503204	8	14017320	SGCZ	Body	0,237	7,61E-05	4,84E-04
cg08918879	19	46977118		IGR	-0,202	7,61E-05	4,84E-04
cg05432003	11	312518	IFITM1	TSS1500	-0,237	7,63E-05	4,85E-04
cg11236008	2	7133766	RNF144A	5'UTR	0,212	7,65E-05	4,86E-04
cg12526797	16	83466984	CDH13	Body	-0,257	7,65E-05	4,86E-04
cg05247838	6	52167742		IGR	0,208	7,67E-05	4,87E-04
cg13333800	5	125694418	GRAMD3	TSS1500	-0,229	7,69E-05	4,88E-04
cg12029639	13	36050993	MIR548F5	Body	-0,237	7,69E-05	4,88E-04
cg19503826	19	56187810	EPN1	TSS200	0,201	7,70E-05	4,89E-04
cg13978337	6	11825273		IGR	-0,316	7,74E-05	4,90E-04
cg07872794	5	117897957	HRAT56	TSS200	0,217	7,74E-05	4,91E-04
cg18602819	4	105828377		IGR	0,229	7,75E-05	4,91E-04
cg02523647	15	47690188	SEMA6D	5'UTR	0,206	7,76E-05	4,92E-04
cg21195480	1	170699473	PRRX1	ExonBnd	0,251	7,76E-05	4,92E-04
cg06124667	3	178110731		IGR	-0,226	7,76E-05	4,92E-04
cg07823492	17	46608099	HOXB1	1stExon	0,248	7,77E-05	4,92E-04
cg24236334	6	149534584		IGR	-0,252	7,78E-05	4,92E-04
cg08626823	6	47246556	TNFRSF21	Body	-0,225	7,78E-05	4,93E-04
cg23297125	4	111714932		IGR	0,268	7,78E-05	4,93E-04
cg15852150	12	42875816	PRICKLE1	5'UTR	0,229	7,80E-05	4,94E-04
cg22711741	19	57742444	AURKC	5'UTR	0,221	7,81E-05	4,94E-04
cg26707200	1	27953026	FGR	TSS1500	-0,2	7,82E-05	4,95E-04
cg22884714	4	85505574	CDS1	Body	0,229	7,84E-05	4,96E-04
cg08651611	7	76832226	CCDC146	Body	-0,24	7,86E-05	4,97E-04
cg26979216	5	142210381	ARHGAP26	Body	0,237	7,87E-05	4,97E-04
cg22286382	6	25875233	SLC17A3	TSS1500	-0,302	7,88E-05	4,98E-04
cg14658235	17	62198338	ERN1	Body	-0,202	7,90E-05	4,99E-04
cg00338530	8	76664974		IGR	0,211	7,92E-05	4,99E-04
cg18667222	20	47654878		IGR	-0,206	7,92E-05	5,00E-04
cg14383952	1	162205449	NOS1AP	Body	-0,207	7,93E-05	5,00E-04
cg08380850	14	100605006	EVL	Body	-0,201	7,97E-05	5,02E-04
cg19326543	2	128422715	LIMS2	Body	0,21	7,98E-05	5,03E-04
cg15705813	2	70297499		IGR	-0,201	7,99E-05	5,03E-04
cg21017065	13	61001645	TDRD3	5'UTR	-0,23	8,01E-05	5,04E-04
cg07450920	22	44701799	KIAA1644	5'UTR	-0,207	8,01E-05	5,04E-04
cg22612792	2	227632034	IRS1	3'UTR	0,242	8,02E-05	5,04E-04
cg04050978	4	95818478	BMPR1B	5'UTR	0,227	8,04E-05	5,05E-04
cg26498349	12	95034650	TMCC3	Body	-0,213	8,04E-05	5,06E-04
cg12370929	9	34701219		IGR	0,252	8,07E-05	5,07E-04
cg05630725	12	24104115	SOX5	5'UTR	0,254	8,08E-05	5,07E-04
cg07522913	7	27150403	HOXA3	5'UTR	0,203	8,08E-05	5,07E-04
cg25046649	12	60014351	SLC16A7	5'UTR	0,208	8,10E-05	5,08E-04
cg23596764	8	121116685		IGR	-0,224	8,10E-05	5,08E-04
cg13444270	3	177412572	LINC00578	Body	0,24	8,10E-05	5,08E-04

cg10599273	9	95156166	CENPP	Body	0,211	8,11E-05	5,09E-04
cg03343128	1	247712140	C1orf150	TSS1500	-0,211	8,11E-05	5,09E-04
cg10628634	6	17713043		IGR	0,227	8,13E-05	5,10E-04
cg08784911	1	204346839		IGR	0,218	8,14E-05	5,10E-04
cg26935681	14	59275736		IGR	0,218	8,14E-05	5,10E-04
cg16562126	1	95788900		IGR	0,217	8,15E-05	5,11E-04
cg05458118	2	154990557	GALNT13	Body	0,215	8,16E-05	5,11E-04
cg04912297	8	17943293	ASAH1	TSS1500	0,318	8,17E-05	5,12E-04
cg11209631	4	72204651	SLC4A4	Body	0,244	8,18E-05	5,12E-04
cg14010086	17	47235170	B4GALNT2	Body	0,219	8,20E-05	5,13E-04
cg17576688	10	35643454	CCNY	5'UTR	0,225	8,23E-05	5,15E-04
cg10260093	22	50586318	MOV10L1	Body	-0,287	8,24E-05	5,15E-04
cg10660353	1	235403054	ARID4B	Body	0,207	8,32E-05	5,19E-04
cg11630392	3	150920964	GPR171	1stExon	-0,227	8,33E-05	5,20E-04
cg14564722	3	71251601	FOXP1	5'UTR	0,213	8,34E-05	5,20E-04
cg01301885	10	128258570		IGR	0,213	8,35E-05	5,21E-04
cg00932624	14	67975805	TMEM229B	5'UTR	-0,231	8,36E-05	5,21E-04
cg10818657	2	74967690		IGR	0,223	8,37E-05	5,21E-04
cg19244380	11	46612744	AMBRA1	5'UTR	0,207	8,39E-05	5,22E-04
cg09469649	7	122225817	CADPS2	Body	0,241	8,40E-05	5,23E-04
cg16666675	10	31663016	ZEB1	Body	0,224	8,43E-05	5,24E-04
cg04089637	13	105987461		IGR	0,26	8,44E-05	5,25E-04
cg06003231	8	67867717	TCF24	Body	0,284	8,44E-05	5,25E-04
cg00490859	12	6662254	IFFO1	Body	0,213	8,49E-05	5,27E-04
cg17707057	7	43630711	STK17A	Body	-0,245	8,51E-05	5,28E-04
cg16267913	8	27138829		IGR	-0,204	8,52E-05	5,29E-04
cg08164294	7	27154387	HOXA3	5'UTR	0,208	8,55E-05	5,30E-04
cg15968539	10	3846317		IGR	-0,211	8,55E-05	5,30E-04
cg14515801	6	80411369	SH3BGRL2	3'UTR	-0,232	8,57E-05	5,31E-04
cg05088463	12	50411104	RACGAP1	5'UTR	0,209	8,58E-05	5,32E-04
cg06910290	3	58372856	PXK	Body	0,202	8,60E-05	5,32E-04
cg07971716	1	199773294		IGR	-0,22	8,60E-05	5,33E-04
cg17263819	12	119697735		IGR	-0,226	8,61E-05	5,33E-04
cg27029927	4	10613575	CLNK	Body	0,242	8,61E-05	5,33E-04
cg20256286	14	100535030	EVL	Body	-0,235	8,62E-05	5,34E-04
cg07413747	10	20221082	PLXDC2	Body	0,205	8,63E-05	5,34E-04
cg25106676	21	17452671	MIR99AHG	Body	-0,204	8,66E-05	5,35E-04
cg07913379	1	200325145	LINC00862	Body	-0,213	8,66E-05	5,35E-04
cg10958362	5	2754016	C5orf38	Body	-0,204	8,69E-05	5,37E-04
cg18392242	11	32338931		IGR	-0,238	8,69E-05	5,37E-04
cg05793390	3	194659868		IGR	0,222	8,69E-05	5,37E-04
cg01515515	1	92414295	BRDT	TSS1500	-0,204	8,70E-05	5,37E-04
cg22812151	7	50796075	GRB10	Body	0,239	8,70E-05	5,37E-04
cg00476022	9	114811681	MIR3134	Body	0,205	8,71E-05	5,38E-04
cg13313385	18	55876097	NEDD4L	Body	0,231	8,72E-05	5,38E-04
cg22398619	3	196540928	PAK2	Body	0,203	8,72E-05	5,38E-04
cg19041076	18	72917390	ZADH2	Body	0,232	8,73E-05	5,39E-04
cg00332393	10	54277038		IGR	0,206	8,74E-05	5,40E-04
cg25077243	5	41809304	OXCT1	Body	-0,268	8,76E-05	5,40E-04
cg05193970	3	58523649	ACOX2	TSS1500	0,215	8,76E-05	5,40E-04

cg18581972	21	17680819	MIR99AHG	Body	-0,22	8,77E-05	5,41E-04
cg11284299	10	115784904		IGR	-0,208	8,81E-05	5,43E-04
cg11110934	6	161856377	PARK2	Body	0,219	8,85E-05	5,45E-04
cg05277425	2	191065479	C2orf88	3'UTR	0,221	8,86E-05	5,45E-04
cg14573817	12	107348689	C12orf23	TSS1500	0,212	8,87E-05	5,46E-04
cg10589332	12	90458958		IGR	0,208	8,89E-05	5,47E-04
cg25988256	12	92754465		IGR	-0,213	8,90E-05	5,47E-04
cg12618270	8	8667411	MFHAS1	Body	0,208	8,92E-05	5,48E-04
cg00169303	10	90227592	RNLS	Body	0,209	8,92E-05	5,48E-04
cg00881257	9	137828892		IGR	-0,206	8,92E-05	5,48E-04
cg13254359	16	48029413		IGR	-0,22	8,98E-05	5,51E-04
cg17359931	3	20080218	KAT2B	TSS1500	0,21	8,99E-05	5,51E-04
cg01719070	13	99652024	DOCK9	Body	0,201	9,00E-05	5,52E-04
cg26723237	15	65643176	IGDCC3	Body	-0,207	9,01E-05	5,53E-04
cg03288515	5	149588739	SLC6A7	Body	-0,209	9,01E-05	5,53E-04
cg00943287	1	245474722	KIF26B	Body	-0,217	9,02E-05	5,53E-04
cg18466213	17	65117644	HELZ	Body	0,208	9,03E-05	5,53E-04
cg03245423	2	112989626	ZC3H8	Body	0,2	9,04E-05	5,54E-04
cg06817669	15	77518882	C15orf5	TSS1500	0,223	9,05E-05	5,55E-04
cg04946387	11	134253884	B3GAT1	Body	0,219	9,10E-05	5,57E-04
cg18470397	2	75076967	HK2	Body	-0,225	9,15E-05	5,59E-04
cg16112050	18	713200	ENOSF1	TSS1500	0,208	9,18E-05	5,61E-04
cg25714927	1	59909200	FGGY	Body	0,21	9,21E-05	5,62E-04
cg16829246	8	17532941	MTUS1	Body	0,229	9,22E-05	5,63E-04
cg07195118	8	145162905	KIAA1875	Body	0,208	9,23E-05	5,63E-04
cg23098512	2	66017710		IGR	0,233	9,24E-05	5,63E-04
cg10551856	2	10774704	NOL10	Body	0,229	9,24E-05	5,64E-04
cg27078862	3	12360753	PPARG	5'UTR	0,255	9,25E-05	5,64E-04
cg07974500	8	121723792	SNTB1	Body	0,207	9,28E-05	5,65E-04
cg02147978	14	33172537	AKAP6	Body	0,21	9,29E-05	5,66E-04
cg08344351	16	81507328	CMIP	Body	0,2	9,30E-05	5,67E-04
cg16618413	3	30478953		IGR	0,221	9,37E-05	5,70E-04
cg03577157	5	80716263	RNU5E	Body	-0,21	9,39E-05	5,71E-04
cg25736125	17	33236198		IGR	-0,202	9,41E-05	5,72E-04
cg20038219	7	73241729		IGR	-0,224	9,42E-05	5,72E-04
cg17980432	17	28591078	BLMH	Body	0,22	9,47E-05	5,74E-04
cg16267060	2	135684318	CCNT2	Body	0,214	9,47E-05	5,75E-04
cg18685013	7	139463355	HIPK2	Body	0,2	9,48E-05	5,75E-04
cg23387623	11	14532758	PSMA1	Body	0,287	9,50E-05	5,76E-04
cg07677634	9	80435507	GNAQ	Body	-0,218	9,50E-05	5,76E-04
cg22061445	12	41220247	CNTN1	TSS1500	0,208	9,51E-05	5,77E-04
cg20064462	13	109582080	MYO16	Body	0,234	9,52E-05	5,77E-04
cg16153943	8	93112104	RUNX1T1	TSS200	0,224	9,53E-05	5,78E-04
cg13293524	17	46651822	HOXB3	TSS200	0,23	9,56E-05	5,79E-04
cg02695541	19	37837190	HKR1	Body	0,203	9,58E-05	5,80E-04
cg11560792	14	84442097		IGR	0,2	9,62E-05	5,82E-04
cg09486474	13	100953771	PCCA	Body	0,222	9,63E-05	5,82E-04
cg08283187	2	223223673		IGR	-0,22	9,63E-05	5,82E-04
cg26753518	21	17909993	MIR99A	TSS1500	0,248	9,64E-05	5,83E-04
cg02319016	3	196469777	PAK2	5'UTR	0,21	9,64E-05	5,83E-04

cg11259289	7	65606104	CRCP	Body	0,231	9,65E-05	5,83E-04
cg05411594	10	86056416		IGR	0,206	9,68E-05	5,85E-04
cg05128156	2	42135223		IGR	0,268	9,69E-05	5,85E-04
cg01206418	12	92698650		IGR	0,207	9,70E-05	5,86E-04
cg03059188	12	93643383	LOC643339	Body	-0,244	9,74E-05	5,88E-04
cg13970118	4	184659523		IGR	0,218	9,77E-05	5,89E-04
cg15079483	4	37887467		IGR	0,245	9,78E-05	5,89E-04
cg13116350	2	28686966		IGR	-0,207	9,80E-05	5,90E-04
cg09580822	10	130834003		IGR	-0,222	9,80E-05	5,90E-04
cg06544951	8	59555116	NSMAF	Body	-0,207	9,85E-05	5,93E-04
cg02703145	9	93922525		IGR	0,232	9,87E-05	5,94E-04
cg24191225	12	9885824	CLECL1	1stExon	-0,209	9,87E-05	5,94E-04
cg14768836	13	44643116		IGR	0,205	9,93E-05	5,96E-04
cg05606089	5	77306302	AP3B1	Body	0,234	1,01E-04	6,02E-04
cg00798281	6	33041697	HLA-DPA1	TSS1500	-0,324	1,01E-04	6,03E-04
cg15846907	17	64501368	PRKCA	Body	0,233	1,01E-04	6,04E-04
cg18309897	12	116695047	MED13L	Body	0,212	1,03E-04	6,12E-04
cg23965132	17	27969965	SSH2	Body	0,217	1,03E-04	6,13E-04
cg04606644	10	96256027	TBC1D12	Body	-0,202	1,03E-04	6,14E-04
cg22128724	2	85402928	TCF7L1	Body	0,208	1,03E-04	6,15E-04
cg21110034	5	130752683		IGR	-0,237	1,03E-04	6,15E-04
cg15571518	2	212273301	ERBB4	Body	0,234	1,04E-04	6,16E-04
cg02379695	6	35064541		IGR	0,235	1,04E-04	6,17E-04
cg16387467	18	72166016	CNDP2	TSS1500	-0,211	1,04E-04	6,18E-04
cg07703515	7	69163584	AUTS2	Body	-0,238	1,04E-04	6,18E-04
cg21118780	2	102379567	MAP4K4	Body	0,246	1,04E-04	6,19E-04
cg17648080	17	2141841	SMG6	Body	0,211	1,05E-04	6,21E-04
cg02550398	18	55366264	ATP8B1	Body	0,239	1,05E-04	6,22E-04
cg00118342	2	106777038	UXS1	Body	-0,21	1,05E-04	6,24E-04
cg05210365	8	123486109	LOC10537573	Body	-0,206	1,05E-04	6,25E-04
cg09673812	19	30469225	URI1	Body	-0,228	1,06E-04	6,27E-04
cg07124052	6	142145643		IGR	0,218	1,06E-04	6,30E-04
cg15624376	7	27171391	HOXA4	TSS1500	0,283	1,07E-04	6,32E-04
cg24118526	7	90397517	CDK14	Body	-0,226	1,07E-04	6,33E-04
cg19187110	8	94372725		IGR	0,211	1,08E-04	6,36E-04
cg01925594	8	1320786		IGR	0,228	1,08E-04	6,37E-04
cg12744859	17	46669492	LOC404266	TSS200	0,232	1,08E-04	6,37E-04
cg24544278	12	9822238	CLEC2D	TSS200	-0,235	1,08E-04	6,39E-04
cg25511621	6	53883681	C6orf142	TSS200	0,208	1,08E-04	6,40E-04
cg14527108	7	27144854		IGR	0,277	1,09E-04	6,41E-04
cg12723019	5	14298377	TRIO	Body	-0,208	1,09E-04	6,42E-04
cg22431381	6	64219743		IGR	0,271	1,09E-04	6,43E-04
cg10297617	14	91610405	C14orf159	5'UTR	0,215	1,09E-04	6,44E-04
cg15118537	10	52828851	LOC10272471	TSS1500	0,2	1,10E-04	6,45E-04
cg11120154	8	101455661		IGR	-0,204	1,10E-04	6,45E-04
cg10502957	12	123757070	CDK2AP1	TSS1500	0,206	1,10E-04	6,45E-04
cg18390596	9	117691764	TNFSF8	Body	-0,236	1,10E-04	6,45E-04
cg16672270	8	105549699	LRP12	Body	0,239	1,10E-04	6,45E-04
cg23839180	2	16805111	FAM49A	5'UTR	-0,212	1,10E-04	6,46E-04
cg27109284	17	45681383	NPEPPS	Body	0,221	1,10E-04	6,46E-04

cg11019891	1	42950191		IGR	0,218	1,10E-04	6,47E-04
cg16516295	3	129147846	C3orf25	TSS1500	0,238	1,10E-04	6,47E-04
cg22164224	10	29095760	LINC01517	Body	0,264	1,10E-04	6,48E-04
cg22493759	16	3839567	CREBBP	Body	-0,21	1,10E-04	6,49E-04
cg09693239	2	65632739	SPRED2	Body	-0,221	1,11E-04	6,51E-04
cg07715663	5	142721796	NR3C1	Body	0,256	1,12E-04	6,54E-04
cg24889708	17	46678719	LOC404266	Body	0,205	1,12E-04	6,55E-04
cg02696675	2	161265366	RBMS1	Body	0,216	1,12E-04	6,55E-04
cg05655397	5	151573780	CTB-12O2.1	Body	0,223	1,12E-04	6,57E-04
cg24527252	15	85951725	AKAP13	5'UTR	-0,284	1,13E-04	6,61E-04
cg08832695	17	46676375	LOC404266	Body	0,235	1,13E-04	6,61E-04
cg20966920	18	40593068	RIT2	Body	-0,221	1,13E-04	6,61E-04
cg15339033	4	151882901	LRBA	Body	0,222	1,14E-04	6,62E-04
cg06292784	20	41595708	PTPRT	Body	0,209	1,14E-04	6,65E-04
cg11160654	15	60369068		IGR	0,237	1,14E-04	6,65E-04
cg07214466	12	104892360	CHST11	Body	-0,219	1,14E-04	6,66E-04
cg22682201	6	41020847	APOBEC2	TSS200	0,259	1,15E-04	6,67E-04
cg19363337	12	95609720	FGD6	Body	0,201	1,15E-04	6,69E-04
cg03290741	1	94792064		IGR	0,221	1,15E-04	6,69E-04
cg20059080	3	130791858	NEK11	Body	0,204	1,15E-04	6,70E-04
cg24767968	17	46651945	HOXB3	TSS200	0,222	1,15E-04	6,70E-04
cg18787963	17	76409193	PGS1	Body	-0,21	1,15E-04	6,71E-04
cg05552343	1	42893241		IGR	0,208	1,15E-04	6,71E-04
cg24597512	15	26874788	GABRB3	Body	0,233	1,16E-04	6,72E-04
cg15733810	14	67819734	ATP6V1D	Body	0,206	1,16E-04	6,74E-04
cg13620184	21	28647755		IGR	-0,25	1,17E-04	6,79E-04
cg00752223	18	379845	COLEC12	Body	-0,225	1,18E-04	6,81E-04
cg21222554	10	104941444	NT5C2	5'UTR	0,21	1,18E-04	6,81E-04
cg01331261	1	119688618	LOC10192914	Body	0,236	1,18E-04	6,82E-04
cg23151403	19	52070868		IGR	0,202	1,18E-04	6,85E-04
cg01323723	6	114126547		IGR	-0,229	1,18E-04	6,85E-04
cg18609120	6	29945409	HCG9	Body	0,218	1,19E-04	6,87E-04
cg14498722	6	27034685		IGR	0,215	1,19E-04	6,87E-04
cg25641330	6	54670167		IGR	0,216	1,19E-04	6,88E-04
cg08442291	8	73056397		IGR	0,253	1,19E-04	6,88E-04
cg14242904	5	149151262	PPARGC1B	TSS1500	0,2	1,19E-04	6,89E-04
cg26352652	12	133013600		IGR	0,204	1,19E-04	6,89E-04
cg01741924	16	31453406	ZNF843	5'UTR	0,227	1,20E-04	6,90E-04
cg27482662	4	89541350	HERC3	Body	0,202	1,21E-04	6,94E-04
cg07741184	6	167504864		IGR	-0,253	1,21E-04	6,96E-04
cg24220948	1	168527152		IGR	-0,222	1,21E-04	6,98E-04
cg05954551	9	336313	DOCK8	Body	0,203	1,21E-04	6,99E-04
cg03768569	3	112851921		IGR	0,26	1,21E-04	6,99E-04
cg25661571	17	63543769	AXIN2	Body	0,217	1,22E-04	6,99E-04
cg04265964	11	121806961		IGR	-0,218	1,22E-04	7,00E-04
cg04874580	8	30389210	RBPMS	Body	0,211	1,22E-04	7,00E-04
cg21897425	17	21744126		IGR	0,21	1,22E-04	7,03E-04
cg15500907	6	112438951	LAMA4	Body	-0,221	1,22E-04	7,03E-04
cg00769600	3	9867308	ARPC4-TTLL3	Body	0,216	1,23E-04	7,03E-04
cg02631791	21	31077585	GRIK1	Body	0,228	1,23E-04	7,05E-04

cg01012713	8	1960217		IGR	0,225	1,23E-04	7,06E-04
cg06229048	10	94457696		IGR	-0,249	1,23E-04	7,06E-04
cg08657654	7	27138974		IGR	0,227	1,23E-04	7,07E-04
cg01613294	22	36557607	APOL3	5'UTR	-0,216	1,23E-04	7,08E-04
cg09733528	2	169832726	ABCB11	Body	0,231	1,24E-04	7,08E-04
cg17360849	1	201688419	MIR5191	TSS1500	-0,209	1,24E-04	7,08E-04
cg08390770	12	2127025		IGR	0,202	1,24E-04	7,09E-04
cg25837126	5	37901381		IGR	0,204	1,24E-04	7,10E-04
cg10039523	1	12517834	VPS13D	Body	0,235	1,24E-04	7,10E-04
cg00568177	19	37177434	LINC01534	Body	0,204	1,25E-04	7,16E-04
cg14123840	12	80906091	PTPRQ	Body	0,22	1,25E-04	7,16E-04
cg14015502	10	104535020	C10orf26	TSS1500	0,223	1,26E-04	7,18E-04
cg24167667	3	30482466		IGR	0,225	1,26E-04	7,19E-04
cg25103731	11	35704400	TRIM44	Body	0,211	1,26E-04	7,19E-04
cg03737629	1	78343253	FAM73A	3'UTR	0,259	1,26E-04	7,20E-04
cg27356001	2	144475341	ARHGAP15	Body	0,23	1,26E-04	7,20E-04
cg24243620	1	154420919	IL6R	Body	0,225	1,26E-04	7,21E-04
cg00986598	3	129369724	TMCC1	3'UTR	-0,217	1,26E-04	7,21E-04
cg14455887	5	66254474	MAST4	TSS1500	0,211	1,27E-04	7,21E-04
cg12471378	19	31705093		IGR	-0,205	1,27E-04	7,22E-04
cg00077302	11	83353224	DLG2	5'UTR	0,203	1,27E-04	7,22E-04
cg07618453	11	2502181	KCNQ1	Body	-0,215	1,27E-04	7,22E-04
cg18982550	12	1756191	WNT5B	3'UTR	-0,209	1,27E-04	7,24E-04
cg27512077	4	113209777		IGR	-0,21	1,27E-04	7,25E-04
cg10874403	3	87325835	POU1F1	TSS200	0,204	1,28E-04	7,29E-04
cg09154166	10	95608979		IGR	-0,225	1,28E-04	7,29E-04
cg01070987	3	149687002	PFN2	Body	0,248	1,28E-04	7,29E-04
cg20540235	8	91683243		IGR	0,223	1,28E-04	7,29E-04
cg07500432	18	77918588	PARD6G	Body	0,201	1,29E-04	7,30E-04
cg02056098	2	167233372	SCN9A	TSS1500	0,205	1,29E-04	7,31E-04
cg26582754	6	144127643	PHACTR2	Body	-0,22	1,29E-04	7,32E-04
cg13090364	21	38491741	TTC3	Body	0,225	1,30E-04	7,35E-04
cg03915932	17	46591291		IGR	0,268	1,30E-04	7,37E-04
cg11535210	7	143072448		IGR	-0,231	1,30E-04	7,38E-04
cg06188746	7	27179800	HOXA-AS3	TSS200	0,263	1,30E-04	7,38E-04
cg26881511	5	173513516	HMP19	Body	-0,413	1,31E-04	7,39E-04
cg03532030	2	242824433		IGR	-0,221	1,31E-04	7,42E-04
cg25683810	2	204547817		IGR	0,262	1,31E-04	7,43E-04
cg08475514	11	9520327	ZNF143	Body	0,21	1,32E-04	7,46E-04
cg11387576	9	18260848		IGR	-0,234	1,32E-04	7,46E-04
cg18206764	6	152283837	ESR1	Body	-0,203	1,32E-04	7,47E-04
cg15977317	2	228648394		IGR	-0,201	1,32E-04	7,48E-04
cg26648735	21	40351741		IGR	-0,215	1,33E-04	7,50E-04
cg06395298	17	46651225	HOXB3	5'UTR	0,312	1,33E-04	7,51E-04
cg26910169	20	30000665	DEFB121	TSS200	0,264	1,33E-04	7,51E-04
cg01477835	4	87989696	AFF1	Body	0,238	1,33E-04	7,51E-04
cg14236118	4	70697558		IGR	-0,247	1,33E-04	7,51E-04
cg11257573	9	27185147	TEK	Body	0,221	1,33E-04	7,51E-04
cg12561945	8	64615707		IGR	0,203	1,33E-04	7,51E-04
cg05109049	17	29641333	NF1	Body	-0,244	1,33E-04	7,52E-04

cg20139176	4	126245214	FAT4	Body	0,212	1,33E-04	7,52E-04
cg06493985	5	139672838	PFDN1	Body	0,206	1,34E-04	7,53E-04
cg23082221	6	146276439	SHPRH	Body	0,212	1,34E-04	7,53E-04
cg09454417	6	152085247	ESR1	5'UTR	0,201	1,34E-04	7,55E-04
cg06272272	10	112684117	SHOC2	5'UTR	0,221	1,35E-04	7,58E-04
cg01153368	2	52840342		IGR	0,215	1,36E-04	7,62E-04
cg14971718	6	30175327	TRIM26	5'UTR	0,209	1,36E-04	7,64E-04
cg16536561	14	59100737	DACT1	TSS200	0,239	1,36E-04	7,65E-04
cg21018784	3	98696537		IGR	0,236	1,37E-04	7,66E-04
cg01853974	6	108913164	FOXO3	Body	0,213	1,37E-04	7,67E-04
cg09336446	2	103793135		IGR	0,208	1,37E-04	7,68E-04
cg25102832	18	66401194	CCDC102B	5'UTR	0,236	1,38E-04	7,70E-04
cg08251704	2	234296312	DGKD	Body	0,224	1,38E-04	7,70E-04
cg25195136	3	187664174		IGR	0,212	1,38E-04	7,72E-04
cg02948188	10	33272417		IGR	-0,243	1,39E-04	7,76E-04
cg13237945	13	43355739	C13orf30	1stExon	0,207	1,39E-04	7,77E-04
cg02977388	10	90253615	RNLS	Body	0,226	1,39E-04	7,78E-04
cg24272697	7	27159883	HOXA3	5'UTR	0,287	1,40E-04	7,79E-04
cg05369141	6	42052839		IGR	0,22	1,40E-04	7,80E-04
cg16403049	13	97888209	MBNL2	5'UTR	0,201	1,40E-04	7,80E-04
cg11203827	10	100175560	PYROXD2	TSS1500	0,239	1,41E-04	7,85E-04
cg07568313	1	120508705	NOTCH2	Body	0,207	1,42E-04	7,90E-04
cg10008950	1	153358924		IGR	-0,225	1,42E-04	7,91E-04
cg20488226	20	48295805	B4GALT5	Body	-0,207	1,42E-04	7,91E-04
cg07700028	18	34423826	KIAA1328	Body	-0,21	1,42E-04	7,91E-04
cg11362211	8	93080591	RUNX1T1	5'UTR	0,223	1,43E-04	7,93E-04
cg24123120	1	51954650	EPS15	Body	0,256	1,43E-04	7,94E-04
cg18184219	1	243388524	CEP170	Body	0,207	1,43E-04	7,94E-04
cg22397254	17	13679687		IGR	0,201	1,43E-04	7,95E-04
cg18693345	5	2754148	C5orf38	Body	-0,266	1,43E-04	7,95E-04
cg23382043	9	121092760		IGR	0,212	1,44E-04	7,98E-04
cg10063407	8	124425615		IGR	0,211	1,44E-04	7,99E-04
cg21251785	9	73484407	TRPM3	TSS1500	0,241	1,44E-04	7,99E-04
cg26888807	3	79634890	ROBO1	Body	0,234	1,44E-04	8,01E-04
cg06244022	5	65652549		IGR	0,201	1,45E-04	8,05E-04
cg20426532	6	64185918		IGR	0,205	1,46E-04	8,06E-04
cg24948406	17	46608268	HOXB1	5'UTR	0,253	1,46E-04	8,07E-04
cg15639652	6	99428885		IGR	0,218	1,46E-04	8,08E-04
cg09178064	6	141702457		IGR	-0,251	1,46E-04	8,09E-04
cg14312557	2	197107109	HECW2	Body	-0,205	1,47E-04	8,10E-04
cg09380798	2	172195925	METTL8	Body	0,263	1,47E-04	8,10E-04
cg00814542	11	9228272	DENND5A	Body	0,2	1,47E-04	8,11E-04
cg10750493	18	64899739		IGR	0,215	1,47E-04	8,11E-04
cg26847046	9	3490307	RFX3	TSS1500	0,242	1,47E-04	8,12E-04
cg01583875	4	80979859	ANTXR2	Body	-0,21	1,48E-04	8,16E-04
cg24158931	14	92116289	CATSPERB	Body	0,204	1,49E-04	8,19E-04
cg14559537	13	77430364		IGR	-0,228	1,50E-04	8,23E-04
cg18684142	17	46682394	LOC404266	Body	0,273	1,50E-04	8,24E-04
cg19494811	6	34356253	NUDT3	Body	0,239	1,50E-04	8,25E-04
cg12518799	17	74188153	RNF157	Body	-0,223	1,51E-04	8,28E-04

cg15735030	12	20152586		IGR	0,202	1,51E-04	8,29E-04
cg12857372	20	61091405		IGR	0,278	1,51E-04	8,30E-04
cg15619439	11	102700944		IGR	-0,228	1,52E-04	8,32E-04
cg02285620	16	57162866	CPNE2	Body	-0,204	1,52E-04	8,32E-04
cg22500906	13	48700296		IGR	0,339	1,53E-04	8,39E-04
cg17561932	2	230989623		IGR	-0,208	1,54E-04	8,41E-04
cg09710521	4	103605622	MANBA	Body	0,207	1,54E-04	8,42E-04
cg20909645	8	99985049		IGR	0,223	1,54E-04	8,44E-04
cg27513568	12	70359551		IGR	0,202	1,55E-04	8,45E-04
cg14381255	13	113437214	ATP11A	Body	0,209	1,55E-04	8,45E-04
cg05832312	9	18767398	ADAMTSL1	Body	-0,213	1,55E-04	8,46E-04
cg26944755	19	52267708	FPR2	5'UTR	-0,224	1,55E-04	8,48E-04
cg20087386	1	7168403	CAMTA1	Body	-0,208	1,56E-04	8,49E-04
cg12121162	6	144904561	UTRN	Body	-0,203	1,56E-04	8,50E-04
cg20956520	12	88242651		IGR	0,23	1,56E-04	8,51E-04
cg21029666	14	106968325		IGR	0,209	1,57E-04	8,54E-04
cg13977827	9	14307195	NFIB	Body	0,251	1,59E-04	8,62E-04
cg22709100	7	91322751		IGR	0,32	1,59E-04	8,63E-04
cg07492031	3	177196087	LINC00578	Body	-0,22	1,59E-04	8,64E-04
cg07891716	1	65744567	DNAJC6	5'UTR	0,261	1,59E-04	8,64E-04
cg09237583	14	70272490		IGR	0,219	1,60E-04	8,67E-04
cg23639072	10	17045334	CUBN	Body	-0,277	1,61E-04	8,71E-04
cg11932091	12	8717391		IGR	0,217	1,61E-04	8,74E-04
cg15753546	2	12626487		IGR	0,233	1,63E-04	8,81E-04
cg04048472	7	90227413	CDK14	Body	0,234	1,64E-04	8,85E-04
cg24556252	11	31149217		IGR	0,205	1,64E-04	8,86E-04
cg21853871	17	46628717	HOXB3	Body	0,263	1,65E-04	8,89E-04
cg15919924	7	129780475		IGR	0,208	1,65E-04	8,90E-04
cg26717763	6	28832567		IGR	0,211	1,65E-04	8,91E-04
cg02344527	11	102702414		IGR	0,21	1,65E-04	8,91E-04
cg01572694	17	46657555	MIR10A	TSS1500	0,202	1,66E-04	8,93E-04
cg22107525	17	66951611	ABCA8	TSS200	0,244	1,66E-04	8,93E-04
cg07516591	2	122338632	CLASP1	Body	0,216	1,67E-04	8,99E-04
cg23807580	4	90419210		IGR	0,226	1,69E-04	9,06E-04
cg10658072	19	45260756	MIR8085	TSS1500	0,205	1,69E-04	9,07E-04
cg18027004	5	9631798	TAS2R1	TSS1500	-0,211	1,71E-04	9,14E-04
cg20750932	4	159963035		IGR	0,2	1,71E-04	9,14E-04
cg24540763	12	122377170	WDR66	Body	-0,206	1,71E-04	9,14E-04
cg23551720	17	46633726	HOXB3	5'UTR	0,242	1,71E-04	9,15E-04
cg20849818	12	10283548	CLEC7A	TSS1500	-0,203	1,71E-04	9,17E-04
cg22223182	17	56494544	RNF43	5'UTR	0,223	1,72E-04	9,18E-04
cg23173042	6	140160925	LOC1001327[illegible]	Body	-0,204	1,72E-04	9,20E-04
cg11222700	2	225792829	DOCK10	Body	-0,211	1,73E-04	9,22E-04
cg07245037	19	49863818	TEAD2	5'UTR	-0,215	1,73E-04	9,24E-04
cg27396830	1	11760709	C1orf187	5'UTR	0,216	1,73E-04	9,24E-04
cg17385867	11	69196986		IGR	0,223	1,73E-04	9,26E-04
cg07043325	6	87909742	ZNF292	Body	0,237	1,74E-04	9,29E-04
cg22053945	17	46651360	HOXB3	5'UTR	0,299	1,75E-04	9,31E-04
cg21009648	13	74251359		IGR	0,213	1,75E-04	9,34E-04
cg06508738	3	138763852	PRR23C	TSS200	0,256	1,76E-04	9,35E-04

cg05691311	8	93895754		IGR	0,219	1,76E-04	9,36E-04
cg08268757	19	57742132	AURKC	TSS1500	0,268	1,76E-04	9,38E-04
cg12457427	1	237176227		IGR	0,25	1,77E-04	9,39E-04
cg07554637	8	41363217	GOLGA7	Body	0,201	1,77E-04	9,42E-04
cg00249374	1	168678879	DPT	Body	0,266	1,78E-04	9,44E-04
cg12519676	4	174912210		IGR	0,206	1,78E-04	9,45E-04
cg20796635	11	82826902		IGR	0,226	1,78E-04	9,46E-04
cg17135225	3	171814909	FNDC3B	5'UTR	0,21	1,79E-04	9,48E-04
cg17761654	4	172560793		IGR	-0,279	1,79E-04	9,48E-04
cg01247535	6	15418354	JARID2	Body	-0,205	1,79E-04	9,48E-04
cg11078242	12	69878548	FRS2	5'UTR	0,203	1,79E-04	9,50E-04
cg24777290	4	48625648	FRYL	Body	0,212	1,79E-04	9,50E-04
cg22504837	10	75809922	VCL	Body	0,253	1,79E-04	9,51E-04
cg00644322	10	97099497	SORBS1	Body	-0,205	1,81E-04	9,56E-04
cg11781506	17	13209886		IGR	0,281	1,83E-04	9,64E-04
cg10800095	7	30362971	ZNRF2	Body	-0,241	1,85E-04	9,72E-04
cg02005162	4	141852934	RNF150	Body	-0,202	1,86E-04	9,79E-04
cg13470673	3	114790477	ZBTB20	5'UTR	0,207	1,86E-04	9,80E-04
cg22838050	19	872690	MED16	Body	-0,221	1,86E-04	9,80E-04
cg15230057	6	158444863	SYNJ2	5'UTR	0,209	1,87E-04	9,81E-04
cg22061832	2	173293627	ITGA6	Body	0,206	1,87E-04	9,83E-04
cg22756426	4	15757965		IGR	-0,223	1,87E-04	9,84E-04
cg05425114	19	45620752	PPP1R37	Body	-0,204	1,88E-04	9,85E-04
cg16456816	1	99337082		IGR	-0,249	1,88E-04	9,87E-04
cg15031550	2	57661686		IGR	-0,255	1,89E-04	9,89E-04
cg14106310	20	57659637		IGR	0,207	1,90E-04	9,93E-04
cg18008653	11	33185376		IGR	0,217	1,90E-04	9,93E-04
cg13975855	17	46652550	HOXB3	TSS1500	0,225	1,90E-04	9,93E-04
cg14974749	7	27181480	HOXA5	Body	0,265	1,91E-04	1,00E-03
cg25707745	3	34884326		IGR	0,207	1,91E-04	1,00E-03
cg09509462	1	53207187	ZYG11B	Body	-0,2	1,92E-04	1,00E-03
cg20251528	6	89674094	RNGTT	TSS1500	0,204	1,92E-04	1,00E-03
cg03281572	10	114502318	VTI1A	Body	0,223	1,93E-04	1,01E-03
cg12910797	17	46651722	HOXB3	5'UTR	0,294	1,94E-04	1,01E-03
cg11977413	7	148642571		IGR	0,232	1,94E-04	1,01E-03
cg19614169	11	110216762		IGR	-0,22	1,94E-04	1,01E-03
cg17616537	17	46628663	HOXB3	Body	0,271	1,95E-04	1,01E-03
cg11937577	17	46661340		IGR	0,234	1,95E-04	1,01E-03
cg20168658	13	89645919		IGR	0,244	1,95E-04	1,01E-03
cg25238274	10	127914098	ADAM12	Body	0,206	1,96E-04	1,02E-03
cg25205993	8	60513808		IGR	0,257	1,96E-04	1,02E-03
cg18649943	2	12626802	LOC10050645	Body	-0,232	1,97E-04	1,02E-03
cg17514855	15	48872722	FBN1	Body	0,212	1,98E-04	1,03E-03
cg23745469	8	120166885		IGR	0,288	1,98E-04	1,03E-03
cg23794358	6	137690400		IGR	0,203	1,98E-04	1,03E-03
cg20860265	5	88082175	MEF2C	Body	0,215	1,99E-04	1,03E-03
cg11938672	6	170055155	WDR27	Body	0,229	1,99E-04	1,03E-03
cg08201041	1	210513958	HHAT	5'UTR	-0,22	2,00E-04	1,03E-03
cg23427909	6	131018394		IGR	0,208	2,00E-04	1,03E-03
cg02561820	3	195910638		IGR	-0,225	2,00E-04	1,04E-03

cg10558129	17	46608538	HOXB1	TSS1500	0,272	2,01E-04	1,04E-03
cg04274128	17	72631810		IGR	0,225	2,02E-04	1,04E-03
cg06829120	6	114741679		IGR	0,214	2,03E-04	1,05E-03
cg14134003	9	88952877	ZCCHC6	Body	-0,255	2,03E-04	1,05E-03
cg17820085	2	163100992	FAP	TSS1500	-0,218	2,04E-04	1,05E-03
cg22792232	3	55398548		IGR	-0,208	2,05E-04	1,06E-03
cg11821245	11	18433683	LDHC	TSS200	0,202	2,05E-04	1,06E-03
cg04988216	1	64471626	ROR1	Body	0,209	2,05E-04	1,06E-03
cg13630204	18	32288159	DTNA	5'UTR	0,223	2,06E-04	1,06E-03
cg17135459	6	158464398	SYNJ2	Body	0,236	2,06E-04	1,06E-03
cg11104088	10	7859676	TAF3	TSS1500	0,206	2,06E-04	1,06E-03
cg02517528	12	132068115		IGR	0,205	2,07E-04	1,07E-03
cg03944495	5	147300781		IGR	-0,211	2,07E-04	1,07E-03
cg13794460	4	175181359	FBXO8	Body	0,204	2,07E-04	1,07E-03
cg13131370	4	70804758	CSN1S1	Body	0,204	2,08E-04	1,07E-03
cg15140721	2	131010115		IGR	-0,219	2,09E-04	1,07E-03
cg23882683	14	90975893		IGR	-0,204	2,09E-04	1,07E-03
cg03686111	8	26061959		IGR	0,218	2,10E-04	1,08E-03
cg12379611	10	17045387	CUBN	Body	-0,207	2,11E-04	1,08E-03
cg06915310	1	183865731	RGL1	Body	-0,22	2,11E-04	1,08E-03
cg23658987	1	175047784	TNN	Body	-0,262	2,11E-04	1,08E-03
cg18087248	7	34492988	NPSR1-AS1	Body	0,222	2,12E-04	1,08E-03
cg11591809	14	78059873	SPTLC2	Body	-0,218	2,12E-04	1,09E-03
cg17066349	12	120130646	CIT	Body	0,202	2,12E-04	1,09E-03
cg11344148	14	70193476		IGR	-0,202	2,14E-04	1,09E-03
cg02275861	18	33214547		IGR	0,231	2,17E-04	1,10E-03
cg03331474	7	27163331	HOXA3	5'UTR	0,221	2,17E-04	1,11E-03
cg26634219	17	46608277	HOXB1	TSS200	0,24	2,17E-04	1,11E-03
cg05342469	4	183601649	ODZ3	Body	0,239	2,19E-04	1,11E-03
cg19522262	4	77512495	SHROOM3	Body	0,202	2,19E-04	1,12E-03
cg06287548	21	34773372		IGR	0,217	2,19E-04	1,12E-03
cg24360871	7	27163929	HOXA3	5'UTR	0,237	2,20E-04	1,12E-03
cg06019613	2	18717524		IGR	-0,212	2,22E-04	1,13E-03
cg07107116	8	43097631		IGR	0,216	2,23E-04	1,13E-03
cg06770076	1	197782247		IGR	0,242	2,24E-04	1,13E-03
cg10734259	9	22103886	CDKN2B-AS1	Body	0,216	2,26E-04	1,14E-03
cg06723619	6	30346055		IGR	-0,263	2,27E-04	1,14E-03
cg21827912	4	86763803	ARHGAP24	Body	-0,202	2,27E-04	1,15E-03
cg05413866	12	30054758		IGR	0,225	2,27E-04	1,15E-03
cg15542608	6	12827379	PHACTR1	Body	0,259	2,28E-04	1,15E-03
cg18270328	11	88546376	GRM5	Body	0,217	2,29E-04	1,16E-03
cg11040181	8	2074935	MYOM2	Body	0,212	2,29E-04	1,16E-03
cg01952553	5	75500607	SV2C	Body	0,223	2,31E-04	1,16E-03
cg25597117	1	161349116		IGR	0,212	2,32E-04	1,16E-03
cg13837962	17	63561164		IGR	0,21	2,32E-04	1,16E-03
cg24266330	2	149447611	EPC2	Body	0,244	2,33E-04	1,17E-03
cg20076516	22	46481816	LOC400931	TSS200	0,23	2,34E-04	1,17E-03
cg23014425	17	46648525	HOXB3	5'UTR	0,219	2,34E-04	1,17E-03
cg24853868	1	146555624		IGR	0,248	2,36E-04	1,18E-03
cg24381100	16	74514272	GLG1	ExonBnd	0,211	2,36E-04	1,18E-03

cg24241823	17	46635529	HOXB3	5'UTR	0,217	2,37E-04	1,18E-03
cg13575625	3	172575508		IGR	0,216	2,37E-04	1,19E-03
cg02997851	3	97542295		IGR	0,218	2,38E-04	1,19E-03
cg00825764	11	77833983	ALG8	Body	0,201	2,38E-04	1,19E-03
cg26916621	17	46657346	MIR10A	TSS200	0,205	2,39E-04	1,19E-03
cg10771315	3	3505881		IGR	0,255	2,39E-04	1,19E-03
cg22008020	3	175140651	MIR548AY	Body	-0,201	2,41E-04	1,20E-03
cg24186108	2	173724798	RAPGEF4	1stExon	0,208	2,44E-04	1,21E-03
cg09871315	7	27142682	HOXA2	TSS1500	0,206	2,44E-04	1,21E-03
cg17817521	8	3086479	CSMD1	Body	0,245	2,44E-04	1,21E-03
cg27534367	1	77532151		IGR	0,209	2,44E-04	1,21E-03
cg04275792	3	188841676		IGR	0,202	2,47E-04	1,22E-03
cg09062922	5	131436890		IGR	-0,241	2,47E-04	1,22E-03
cg05719146	3	179389712	USP13	Body	0,208	2,48E-04	1,23E-03
cg16148696	6	111784159	REV3L	Body	0,205	2,50E-04	1,24E-03
cg09621868	13	58210058	PCDH17	Body	0,231	2,51E-04	1,24E-03
cg01206211	2	36825736	FEZ2	TSS1500	0,332	2,54E-04	1,25E-03
cg23336143	2	36635519	CRIM1	Body	0,227	2,55E-04	1,26E-03
cg26010395	3	122977688	SEC22A	Body	0,21	2,56E-04	1,26E-03
cg02852397	8	56660763	TMEM68	Body	0,214	2,57E-04	1,26E-03
cg17464436	1	184922883	FAM129A	Body	-0,212	2,58E-04	1,27E-03
cg12139369	17	71284979	CDC42EP4	5'UTR	0,207	2,58E-04	1,27E-03
cg13525280	12	123357517	VPS37B	Body	0,231	2,59E-04	1,27E-03
cg11367172	7	55605450	VOPP1	TSS200	-0,258	2,60E-04	1,28E-03
cg07850899	20	6889112		IGR	0,214	2,61E-04	1,28E-03
cg08125218	6	8000706	BLOC1S5-TXN	Body	0,213	2,62E-04	1,29E-03
cg10508439	3	152226825		IGR	-0,206	2,65E-04	1,30E-03
cg12667940	9	15420634		IGR	0,203	2,66E-04	1,30E-03
cg26668911	12	92168481		IGR	0,202	2,68E-04	1,31E-03
cg04709373	16	69832614	WWP2	Body	0,201	2,68E-04	1,31E-03
cg20671506	9	100505352		IGR	-0,203	2,69E-04	1,31E-03
cg18969175	2	9838106		IGR	-0,201	2,69E-04	1,31E-03
cg06565641	8	141359674	TRAPPC9	Body	0,271	2,69E-04	1,31E-03
cg18392798	2	102407455	MAP4K4	Body	0,215	2,70E-04	1,32E-03
cg21576187	15	29963223		IGR	-0,205	2,70E-04	1,32E-03
cg14361252	13	111465457		IGR	0,211	2,74E-04	1,33E-03
cg18905886	8	18656149	PSD3	Body	-0,206	2,76E-04	1,34E-03
cg23962148	5	132121270		IGR	0,201	2,78E-04	1,35E-03
cg23776302	3	37362953	GOLGA4	Body	-0,21	2,79E-04	1,35E-03
cg25620989	5	162861766		IGR	0,212	2,79E-04	1,35E-03
cg26332114	19	57742260	AURKC	TSS200	0,242	2,80E-04	1,35E-03
cg04117801	17	46651867	HOXB3	TSS200	0,263	2,82E-04	1,36E-03
cg21641008	6	15319176	JARID2	Body	0,219	2,84E-04	1,37E-03
cg14844468	12	12540867	LOH12CR1	Body	0,271	2,86E-04	1,38E-03
cg16387151	16	85369142		IGR	-0,22	2,86E-04	1,38E-03
cg26267333	16	83614791	CDH13	Body	-0,205	2,86E-04	1,38E-03
cg01972843	22	17680706	CECR1	TSS200	0,22	2,87E-04	1,38E-03
cg05684375	6	37956634	ZFAND3	Body	0,225	2,87E-04	1,38E-03
cg20630344	16	80604078	LINC01227	Body	-0,223	2,89E-04	1,39E-03
cg08259796	6	49431865	CENPQ	5'UTR	0,244	2,89E-04	1,39E-03

cg11931284	22	41492370	EP300	Body	0,219	2,89E-04	1,39E-03
cg18156625	3	67040025		IGR	0,203	2,90E-04	1,39E-03
cg18934605	4	74207894		IGR	0,201	2,90E-04	1,39E-03
cg21864868	17	46673002	LOC404266	Body	0,237	2,91E-04	1,40E-03
cg07433407	1	154370818		IGR	-0,241	2,92E-04	1,40E-03
cg24888214	11	129012999	ARHGAP32	Body	-0,224	2,92E-04	1,40E-03
cg10443496	1	160881549		IGR	0,372	2,92E-04	1,40E-03
cg10004550	14	88482145		IGR	0,206	2,94E-04	1,41E-03
cg25521406	12	20523517	PDE3A	Body	0,206	2,95E-04	1,41E-03
cg22813133	17	46643727	HOXB3	5'UTR	0,234	2,95E-04	1,41E-03
cg08290373	9	8633541	PTPRD	Body	-0,223	2,96E-04	1,41E-03
cg09465394	4	140764922	MAML3	Body	-0,207	2,97E-04	1,42E-03
cg06647068	12	104853274	CHST11	Body	-0,24	3,01E-04	1,43E-03
cg26176362	1	227379480	CDC42BPA	Body	-0,206	3,02E-04	1,44E-03
cg09591524	7	27150031	HOXA3	5'UTR	0,275	3,03E-04	1,44E-03
cg02788834	7	150517855		IGR	0,21	3,04E-04	1,45E-03
cg12092726	18	41002542		IGR	0,213	3,05E-04	1,45E-03
cg20485607	1	120217696		IGR	0,353	3,06E-04	1,45E-03
cg07752095	4	128630266	INTU	Body	0,206	3,08E-04	1,46E-03
cg00035526	3	2396810	CNTN4	5'UTR	0,215	3,08E-04	1,46E-03
cg12701606	11	131905819	NTM	Body	0,202	3,12E-04	1,48E-03
cg19384241	2	55393977		IGR	-0,515	3,14E-04	1,49E-03
cg15343157	6	15936946		IGR	-0,255	3,16E-04	1,49E-03
cg06676354	10	127950914	ADAM12	Body	0,211	3,17E-04	1,50E-03
cg22772747	7	27164285	HOXA3	5'UTR	0,217	3,17E-04	1,50E-03
cg23320368	11	28557421		IGR	-0,253	3,19E-04	1,50E-03
cg23029597	12	123009494	RSRC2	5'UTR	-0,212	3,19E-04	1,50E-03
cg06362482	8	16021835	MSR1	Body	-0,225	3,21E-04	1,51E-03
cg23106378	8	144631921		IGR	-0,209	3,26E-04	1,53E-03
cg20928596	1	70742678	ANKRD13C	Body	0,216	3,27E-04	1,53E-03
cg26585701	6	30418565		IGR	-0,23	3,27E-04	1,53E-03
cg10636490	3	149687315	PFN2	Body	0,23	3,27E-04	1,53E-03
cg22641704	2	131648444		IGR	0,209	3,28E-04	1,54E-03
cg24040595	7	27185512	HOXA6	Body	0,209	3,30E-04	1,54E-03
cg27144903	2	238382860		IGR	-0,237	3,30E-04	1,54E-03
cg00641437	3	138763875	PRR23C	TSS200	0,235	3,30E-04	1,54E-03
cg08057919	20	6645952		IGR	-0,213	3,30E-04	1,54E-03
cg25434864	8	83353753		IGR	0,288	3,30E-04	1,54E-03
cg17230535	7	32626962		IGR	0,203	3,31E-04	1,55E-03
cg21183455	22	50052396	C22orf34	TSS1500	0,214	3,31E-04	1,55E-03
cg09579760	1	163132916	RGS5	Body	0,238	3,33E-04	1,55E-03
cg06638556	11	81681870	LOC1019289ٍ8	Body	0,249	3,37E-04	1,57E-03
cg22807166	18	60545325	PHLPP1	Body	0,209	3,42E-04	1,59E-03
cg12393620	7	111645567	DOCK4	Body	0,201	3,42E-04	1,59E-03
cg20605886	10	77472147		IGR	0,227	3,42E-04	1,59E-03
cg03538821	16	22248009	EEF2K	Body	0,202	3,44E-04	1,60E-03
cg19889856	7	134856654	C7orf49	TSS1500	0,235	3,46E-04	1,60E-03
cg14874905	5	39165082	FYB	Body	-0,225	3,46E-04	1,60E-03
cg22012473	11	104700958		IGR	0,222	3,49E-04	1,61E-03
cg02219607	4	70504750	UGT2A2	1stExon	0,262	3,53E-04	1,63E-03

cg04599946	16	85936480	IRF8	5'UTR	-0,226	3,54E-04	1,63E-03
cg15078822	7	52341469		IGR	0,201	3,54E-04	1,63E-03
cg17475117	4	26880696	STIM2	Body	-0,224	3,55E-04	1,64E-03
cg19047868	17	46669485	LOC404266	TSS200	0,214	3,55E-04	1,64E-03
cg02986791	19	1062178	ABCA7	Body	0,211	3,55E-04	1,64E-03
cg10996039	7	27178861		IGR	0,206	3,55E-04	1,64E-03
cg08941355	7	27133106	HOXA1	3'UTR	0,243	3,55E-04	1,64E-03
cg15848945	7	15601842	AGMO	TSS1500	0,243	3,56E-04	1,64E-03
cg09937190	20	15177509	MACROD2	5'UTR	0,201	3,56E-04	1,64E-03
cg14227773	9	2419272		IGR	-0,238	3,60E-04	1,66E-03
cg15254822	7	564137		IGR	-0,31	3,67E-04	1,68E-03
cg03237474	6	149778775	ZC3H12D	Body	0,204	3,68E-04	1,69E-03
cg13874969	15	26874480	GABRB3	TSS200	0,207	3,69E-04	1,69E-03
cg24835861	1	174130261	RABGAP1L	5'UTR	-0,277	3,72E-04	1,70E-03
cg17713488	19	10077935	COL5A3	Body	-0,254	3,72E-04	1,70E-03
cg25792462	3	121786916	CD86	5'UTR	-0,212	3,73E-04	1,70E-03
cg06613763	10	125061286		IGR	0,2	3,74E-04	1,70E-03
cg22793564	13	80489687	LINC00382	Body	0,207	3,78E-04	1,72E-03
cg14094245	12	108878085		IGR	-0,206	3,78E-04	1,72E-03
cg15294781	3	93747482	STX19	TSS200	0,255	3,83E-04	1,74E-03
cg10730786	6	47632998	ADGRF2	5'UTR	-0,203	3,85E-04	1,75E-03
cg12771637	11	56371643		IGR	0,234	3,87E-04	1,75E-03
cg20541870	11	46671034	KIAA0652	Body	0,209	3,87E-04	1,75E-03
cg27335855	5	60787422	ZSWIM6	Body	0,22	3,90E-04	1,76E-03
cg18517818	4	10956577		IGR	-0,204	3,91E-04	1,77E-03
cg03924551	15	25930203	ATP10A	Body	0,223	3,97E-04	1,79E-03
cg00632736	12	68798875		IGR	0,207	3,99E-04	1,80E-03
cg08843330	12	69126191	NUP107	Body	0,201	4,02E-04	1,81E-03
cg17347063	22	36043021	APOL6	TSS1500	0,262	4,03E-04	1,81E-03
cg03726490	5	57752122	PLK2	Body	0,21	4,03E-04	1,81E-03
cg26899284	1	9714553	PIK3CD-AS1	Body	-0,269	4,03E-04	1,81E-03
cg02912291	20	17944845	SNORD17	TSS1500	-0,208	4,06E-04	1,82E-03
cg01495494	18	53179320	TCF4	TSS1500	0,205	4,14E-04	1,85E-03
cg23944400	15	36249835		IGR	-0,2	4,16E-04	1,86E-03
cg02778759	6	91156138		IGR	0,212	4,17E-04	1,86E-03
cg10214340	11	31663468	ELP4	Body	0,216	4,17E-04	1,86E-03
cg23217463	17	46676973	LOC404266	Body	0,224	4,20E-04	1,87E-03
cg12317346	22	17680704	CECR1	TSS200	0,271	4,20E-04	1,87E-03
cg25747783	7	105603622	FLJ23834	TSS200	0,208	4,25E-04	1,89E-03
cg26767593	8	30015647	DCTN6	Body	0,207	4,25E-04	1,89E-03
cg16366056	7	93365934		IGR	-0,233	4,26E-04	1,89E-03
cg04412444	3	149686860	PFN2	Body	0,246	4,26E-04	1,89E-03
cg13723078	14	54734241		IGR	-0,201	4,28E-04	1,90E-03
cg16869647	8	120137099		IGR	-0,245	4,28E-04	1,90E-03
cg03650232	4	181538451		IGR	0,202	4,30E-04	1,91E-03
cg04118610	4	62707027	LPHN3	Body	-0,303	4,31E-04	1,91E-03
cg03453744	17	46627484	HOXB3	3'UTR	0,205	4,32E-04	1,92E-03
cg13285698	8	141455681	TRAPPC9	Body	0,224	4,33E-04	1,92E-03
cg21144218	12	60082988	SLC16A7	TSS200	0,232	4,34E-04	1,92E-03
cg19751670	8	23088262	LOC389641	Body	-0,202	4,35E-04	1,93E-03

cg07472199	16	31453477	ZNF843	5'UTR	0,232	4,35E-04	1,93E-03
cg01703736	2	20649480		IGR	0,225	4,37E-04	1,93E-03
cg14176755	2	176803677	KIAA1715	Body	0,22	4,44E-04	1,96E-03
cg20016599	11	104508637		IGR	0,231	4,45E-04	1,96E-03
cg22206740	20	37208947	ADIG	TSS1500	0,206	4,47E-04	1,97E-03
cg24430528	4	76599500	G3BP2	TSS1500	0,204	4,50E-04	1,98E-03
cg20132775	3	142444202	TRPC1	Body	0,217	4,51E-04	1,98E-03
cg15628518	8	145025059	PLEC1	Body	0,206	4,53E-04	1,99E-03
cg17415216	20	38413520		IGR	-0,216	4,56E-04	2,00E-03
cg00127176	10	88545884	BMPR1A	5'UTR	0,211	4,59E-04	2,01E-03
cg14952037	15	95023640	MCTP2	3'UTR	0,208	4,59E-04	2,01E-03
cg14111048	8	114390137	CSMD3	TSS1500	0,217	4,60E-04	2,01E-03
cg02369029	3	20310722		IGR	0,219	4,61E-04	2,02E-03
cg21636610	5	55655747		IGR	-0,206	4,64E-04	2,03E-03
cg13413777	8	30015678	MIR548O2	Body	0,223	4,66E-04	2,03E-03
cg24669389	4	169330464	DDX60L	Body	0,215	4,69E-04	2,05E-03
cg07740669	13	111856202	ARHGEF7	5'UTR	0,209	4,69E-04	2,05E-03
cg23078267	12	59310932	LRIG3	Body	0,229	4,70E-04	2,05E-03
cg11755407	11	62370490	EML3	Body	0,221	4,72E-04	2,06E-03
cg10926360	10	112902090		IGR	0,206	4,79E-04	2,08E-03
cg23467487	1	174784282	RABGAP1L	Body	-0,206	4,81E-04	2,09E-03
cg25432232	19	57742423	AURKC	5'UTR	0,201	4,82E-04	2,09E-03
cg07409153	2	76839078		IGR	-0,212	4,86E-04	2,10E-03
cg06683487	16	936480	LMF1	Body	0,238	4,86E-04	2,10E-03
cg14232218	9	93922330		IGR	0,206	4,93E-04	2,13E-03
cg16984089	10	90591639	ANKRD22	Body	-0,202	4,96E-04	2,14E-03
cg13548543	9	34460044	DNAI1	Body	-0,25	5,05E-04	2,17E-03
cg24461183	9	85107437		IGR	-0,223	5,13E-04	2,20E-03
cg11447849	15	25842111		IGR	0,219	5,17E-04	2,21E-03
cg24974158	8	129001476	PVT1	Body	0,214	5,17E-04	2,21E-03
cg19585676	6	29913343	HLA-A	3'UTR	-0,294	5,19E-04	2,22E-03
cg00693915	1	24290099		IGR	0,2	5,20E-04	2,22E-03
cg08938925	15	64110513	HERC1	5'UTR	0,208	5,22E-04	2,23E-03
cg07622712	14	88461259	GALC	TSS1500	-0,22	5,22E-04	2,23E-03
cg20308819	14	52889825		IGR	0,216	5,24E-04	2,24E-03
cg14951064	6	164526907		IGR	0,202	5,27E-04	2,24E-03
cg01320533	1	183049085	LAMC1	Body	-0,218	5,31E-04	2,26E-03
cg13342844	10	77166420	ZNF503-AS2	Body	0,236	5,34E-04	2,27E-03
cg17823769	3	152006158	MBNL1	5'UTR	0,212	5,35E-04	2,27E-03
cg01182076	4	183601697	ODZ3	Body	0,223	5,40E-04	2,29E-03
cg02836478	17	46652501	HOXB3	TSS1500	0,243	5,42E-04	2,30E-03
cg09254210	14	61873622	PRKCH	Body	0,206	5,43E-04	2,30E-03
cg00567448	3	121271467		IGR	0,25	5,49E-04	2,32E-03
cg08203284	7	27171528	HOXA4	TSS1500	0,23	5,51E-04	2,33E-03
cg04904318	17	46607828	HOXB1	1stExon	0,207	5,67E-04	2,38E-03
cg11143671	1	180904018	KIAA1614	Body	-0,201	5,67E-04	2,38E-03
cg20013151	11	3009702	NAP1L4	5'UTR	-0,232	5,71E-04	2,39E-03
cg01148098	2	102383972	MAP4K4	Body	0,244	5,75E-04	2,41E-03
cg18075505	2	183292822	PDE1A	TSS1500	0,215	5,80E-04	2,43E-03
cg11815473	7	135771715		IGR	0,217	5,82E-04	2,43E-03

cg23367392	7	42267337	GLI3	5'UTR	-0,2	5,83E-04	2,44E-03
cg19055270	6	137301377		IGR	0,207	5,86E-04	2,44E-03
cg08923669	16	420230	MRPL28	5'UTR	0,271	5,86E-04	2,45E-03
cg11348760	11	118955271	HMBS	TSS1500	0,23	5,94E-04	2,47E-03
cg02693607	7	27150598	HOXA3	5'UTR	0,215	6,00E-04	2,49E-03
cg17375248	2	66811157		IGR	-0,224	6,07E-04	2,52E-03
cg23295647	14	34269637	NPAS3	Body	0,217	6,20E-04	2,56E-03
cg26884463	7	29996206	SCRN1	Body	-0,203	6,25E-04	2,58E-03
cg17869231	2	198938221	PLCL1	Body	0,208	6,33E-04	2,60E-03
cg06000162	5	73146672	ARHGEF28	Body	0,206	6,39E-04	2,62E-03
cg27640794	4	169555699	PALLD	5'UTR	-0,201	6,52E-04	2,66E-03
cg23681866	6	29895175		IGR	-0,213	6,57E-04	2,68E-03
cg04345852	15	88782143	NTRK3	Body	0,207	6,58E-04	2,68E-03
cg04329291	6	74484508	CD109	Body	0,217	6,59E-04	2,69E-03
cg20897616	15	26874515	GABRB3	Body	0,223	6,69E-04	2,72E-03
cg19189010	11	5840774	OR52N2	TSS1500	0,213	6,70E-04	2,72E-03
cg03645730	3	149673434	RNF13	Body	0,216	6,79E-04	2,75E-03
cg04579734	12	10869507	YBX3	Body	0,207	6,79E-04	2,75E-03
cg10157512	15	29989603		IGR	-0,203	6,86E-04	2,78E-03
cg17143945	12	117354631	FBXW8	Body	-0,232	6,91E-04	2,79E-03
cg09803351	12	113661644	TPCN1	5'UTR	0,219	6,91E-04	2,79E-03
cg04974402	17	54199232		IGR	-0,201	6,94E-04	2,80E-03
cg23204512	1	42748289	FOXJ3	Body	0,205	6,98E-04	2,82E-03
cg16787431	17	46629350	HOXB3	Body	0,221	7,01E-04	2,82E-03
cg06186155	17	46648582	HOXB3	5'UTR	0,234	7,06E-04	2,84E-03
cg01251603	15	26874098	GABRB3	Body	0,228	7,08E-04	2,85E-03
cg18091117	7	27179832	HOXA-AS3	TSS200	0,26	7,10E-04	2,85E-03
cg03986471	13	60345900	DIAPH3	Body	0,211	7,21E-04	2,89E-03
cg12082025	19	1064218	ABCA7	Body	0,248	7,27E-04	2,91E-03
cg21225740	13	44362256	ENOX1	TSS1500	0,217	7,30E-04	2,92E-03
cg11173014	16	419916	MRPL28	Body	0,259	7,57E-04	3,01E-03
cg05107618	6	167470163		IGR	-0,203	7,59E-04	3,01E-03
cg16676373	14	65175332	PLEKHG3	5'UTR	-0,224	7,60E-04	3,02E-03
cg20675391	3	118864895	C3orf30	TSS200	0,223	7,63E-04	3,02E-03
cg10409366	9	94586898	ROR2	Body	0,239	7,63E-04	3,02E-03
cg17422692	16	420245	MRPL28	5'UTR	0,331	7,67E-04	3,04E-03
cg05199232	12	43548942		IGR	-0,216	7,68E-04	3,04E-03
cg20391993	14	62452805		IGR	0,204	7,70E-04	3,05E-03
cg14702570	2	74259524		IGR	-0,211	7,81E-04	3,08E-03
cg08732636	3	16949571	PLCL2	Body	0,207	7,83E-04	3,09E-03
cg11921111	13	62884315		IGR	0,241	7,85E-04	3,09E-03
cg20837474	1	28765433	PHACTR4	Body	0,2	7,90E-04	3,11E-03
cg19814209	20	18411869	DZANK1	Body	0,208	7,90E-04	3,11E-03
cg01911613	3	196325238		IGR	-0,235	7,90E-04	3,11E-03
cg16305787	10	19705290		IGR	0,211	7,95E-04	3,13E-03
cg26949055	15	34144832	RYR3	Body	0,213	8,01E-04	3,14E-03
cg02771997	7	102475694	FBXL13	Body	-0,235	8,30E-04	3,24E-03
cg03026888	6	128388899	LOC1019281	Body	0,215	8,44E-04	3,28E-03
cg16431720	6	32607509	HLA-DQA1	Body	-0,28	8,46E-04	3,29E-03
cg00925947	4	134932828		IGR	0,214	8,56E-04	3,32E-03

cg10185505	1	150335496	RPRD2	TSS1500	0,269	8,88E-04	3,42E-03
cg14404008	5	54995287	SLC38A9	5'UTR	-0,233	8,98E-04	3,45E-03
cg15841167	6	29633622	MOG	3'UTR	0,201	9,04E-04	3,47E-03
cg19711314	2	182853053	PPP1R1C	Body	0,205	9,07E-04	3,48E-03
cg03909863	11	638404	DRD4	Body	0,202	9,14E-04	3,50E-03
cg24420706	10	4574886		IGR	-0,22	9,14E-04	3,50E-03
cg23987077	16	73012207	ZFHX3	5'UTR	-0,214	9,22E-04	3,52E-03
cg05699681	21	15645969	ABCC13	TSS200	-0,247	9,30E-04	3,55E-03
cg21507367	12	27446544	STK38L	5'UTR	0,303	9,30E-04	3,55E-03
cg02447476	1	241308012	RGS7	Body	0,207	9,31E-04	3,55E-03
cg22295857	5	6497496		IGR	0,201	9,35E-04	3,56E-03
cg08532525	2	228375421	AGFG1	Body	0,204	9,39E-04	3,57E-03
cg14772660	2	108605769	SLC5A7	Body	0,202	9,39E-04	3,58E-03
cg03999491	20	12788857		IGR	0,217	9,58E-04	3,63E-03
cg00215019	10	50311040	C10orf72	Body	-0,213	9,87E-04	3,72E-03
cg14552882	8	93091082	RUNX1T1	5'UTR	0,21	9,92E-04	3,74E-03
cg16759545	6	6316435	F13A1	Body	-0,341	1,01E-03	3,79E-03
cg26805839	9	4575020	SLC1A1	Body	-0,206	1,01E-03	3,80E-03
cg06092290	3	71180818	FOXP1	TSS1500	0,212	1,01E-03	3,80E-03
cg20415092	2	4183644		IGR	0,221	1,02E-03	3,81E-03
cg01975684	8	125283894		IGR	0,222	1,05E-03	3,92E-03
cg08296601	16	420255	MRPL28	5'UTR	0,306	1,06E-03	3,96E-03
cg16989719	2	238392110		IGR	-0,229	1,07E-03	3,97E-03
cg01946824	3	118864870	C3orf30	TSS200	0,215	1,07E-03	3,98E-03
cg16805884	8	131469585		IGR	-0,22	1,08E-03	4,00E-03
cg08124399	6	74104868	DDX43	1stExon	0,204	1,09E-03	4,02E-03
cg21720451	12	104193772	NT5DC3	Body	0,214	1,09E-03	4,04E-03
cg17733104	6	136081144		IGR	0,254	1,09E-03	4,05E-03
cg00253526	14	93421782	ITPK1	Body	0,214	1,10E-03	4,08E-03
cg24241305	13	114826075	RASA3	Body	0,214	1,13E-03	4,14E-03
cg01214346	17	406501		IGR	0,217	1,14E-03	4,18E-03
cg13037951	8	53125923	ST18	Body	0,22	1,14E-03	4,19E-03
cg06643849	19	57742421	AURKC	5'UTR	0,202	1,16E-03	4,23E-03
cg09507411	5	82395650	XRCC4	5'UTR	0,235	1,16E-03	4,24E-03
cg19408827	17	46651407	HOXB3	1stExon	0,269	1,17E-03	4,27E-03
cg26557993	4	122723875	EXOSC9	Body	0,209	1,17E-03	4,29E-03
cg18102738	17	4487109	SMTNL2	TSS200	0,206	1,18E-03	4,29E-03
cg25550461	3	119488815		IGR	0,211	1,18E-03	4,31E-03
cg18333653	11	75794702	UVRAG	Body	0,214	1,20E-03	4,37E-03
cg06382895	4	174094774	GALNT7	Body	-0,2	1,23E-03	4,44E-03
cg23371413	19	57742394	AURKC	5'UTR	0,228	1,23E-03	4,44E-03
cg02573091	5	74908125		IGR	0,203	1,24E-03	4,47E-03
cg23682246	8	136661041		IGR	0,209	1,24E-03	4,49E-03
cg25267487	19	29217858		IGR	-0,244	1,25E-03	4,49E-03
cg19043516	6	53500338		IGR	0,211	1,25E-03	4,51E-03
cg20618651	4	56718365	EXOC1	TSS1500	-0,209	1,27E-03	4,57E-03
cg00510795	5	98099238		IGR	0,205	1,27E-03	4,57E-03
cg13863938	15	68821540		IGR	-0,226	1,30E-03	4,66E-03
cg03961133	3	57256135		IGR	0,249	1,31E-03	4,70E-03
cg15871215	5	81402204	ATG10	Body	0,221	1,32E-03	4,70E-03

cg18089380	3	55232663		IGR	0,218	1,33E-03	4,75E-03
cg21477591	5	54049046		IGR	-0,246	1,34E-03	4,76E-03
cg22100175	6	167419092	FGFR1OP	Body	0,204	1,38E-03	4,89E-03
cg21428318	13	23727146		IGR	0,207	1,41E-03	4,98E-03
cg05066391	15	85951812	AKAP13	5'UTR	-0,209	1,42E-03	5,00E-03
cg18213661	11	93681423		IGR	-0,462	1,51E-03	5,24E-03
cg09864843	1	111890799	C1orf88	Body	0,209	1,52E-03	5,28E-03
cg26364809	7	27145159		IGR	0,233	1,53E-03	5,31E-03
cg07298985	8	22133076	PIWIL2	5'UTR	0,231	1,53E-03	5,32E-03
cg07356135	8	49965470	C8orf22	TSS1500	0,223	1,56E-03	5,39E-03
cg21750589	6	28321301	ZNF323	5'UTR	0,207	1,58E-03	5,45E-03
cg18212298	17	579835	VPS53	Body	0,269	1,58E-03	5,47E-03
cg16210690	1	23537939		IGR	-0,202	1,61E-03	5,54E-03
cg08197588	6	22477409		IGR	0,226	1,64E-03	5,63E-03
cg22519482	6	21387005		IGR	0,232	1,65E-03	5,64E-03
cg26048849	14	59679170	DAAM1	5'UTR	0,251	1,66E-03	5,68E-03
cg07113653	6	13428657	GFOD1	Body	-0,206	1,71E-03	5,82E-03
cg14375496	17	440392	VPS53	Body	0,207	1,74E-03	5,91E-03
cg12562660	2	121689039	GLI2	Body	-0,254	1,74E-03	5,91E-03
cg08166072	2	46213920	PRKCE	Body	0,248	1,75E-03	5,92E-03
cg10460168	5	112198022	SRP19	Body	0,2	1,75E-03	5,93E-03
cg14093255	9	14081508		IGR	-0,272	1,76E-03	5,94E-03
cg07748963	1	82627662		IGR	-0,206	1,76E-03	5,95E-03
cg07631144	17	46657393	MIR10A	TSS200	0,228	1,88E-03	6,28E-03
cg23248424	5	179741104	GFPT2	Body	0,247	1,95E-03	6,46E-03
cg14301048	22	36043070	APOL6	TSS1500	0,223	1,97E-03	6,51E-03
cg19039140	16	84627664	COTL1	Body	-0,211	1,98E-03	6,55E-03
cg05555194	14	95600370	DICER1	5'UTR	0,202	2,00E-03	6,60E-03
cg26681889	12	113680495	TPCN1	Body	-0,224	2,00E-03	6,60E-03
cg18091269	17	63822838	CCDC46	TSS200	-0,219	2,02E-03	6,66E-03
cg09929763	11	358332		IGR	0,216	2,02E-03	6,66E-03
cg12368969	20	50021968	NFATC2	Body	-0,225	2,02E-03	6,67E-03
cg05927610	9	115821053		IGR	0,238	2,03E-03	6,68E-03
cg14370847	19	57742331	AURKC	TSS200	0,213	2,03E-03	6,68E-03
cg08776296	7	134856544	C7orf49	TSS1500	0,373	2,03E-03	6,69E-03
cg07247255	2	166489360	CSRNP3	Body	-0,203	2,05E-03	6,75E-03
cg06451467	1	98369409	DPYD	Body	0,211	2,08E-03	6,83E-03
cg03864958	4	10174168		IGR	0,22	2,09E-03	6,84E-03
cg10313988	11	83352844	DLG2	5'UTR	0,207	2,11E-03	6,91E-03
cg24135491	17	4487099	SMTNL2	TSS200	0,247	2,13E-03	6,95E-03
cg22904437	8	81064304	TPD52	Body	0,216	2,19E-03	7,12E-03
cg00742851	3	3843342	LRRN1	5'UTR	0,201	2,20E-03	7,15E-03
cg13289490	6	134965825		IGR	-0,21	2,24E-03	7,25E-03
cg01513157	16	82031434	SDR42E1	3'UTR	0,213	2,24E-03	7,25E-03
cg00470469	7	6071095	ANKRD61	1stExon	0,204	2,25E-03	7,28E-03
cg04546413	19	29218101		IGR	-0,231	2,26E-03	7,31E-03
cg25134647	12	25454990		IGR	0,532	2,28E-03	7,35E-03
cg01974803	2	100853843	LINC01104	Body	0,205	2,30E-03	7,39E-03
cg12126656	10	134405295	INPP5A	Body	0,234	2,32E-03	7,45E-03
cg26446133	18	72167187	CNDP2	5'UTR	-0,228	2,35E-03	7,52E-03

cg18322025	6	170055332	WDR27	Body	0,211	2,36E-03	7,57E-03
cg27009374	10	47655296		IGR	0,267	2,42E-03	7,72E-03
cg09255157	4	106553472	ARHGEF38	Body	-0,236	2,48E-03	7,87E-03
cg22620746	19	22234992	ZNF257	TSS1500	0,241	2,56E-03	8,07E-03
cg02891314	5	179741120	GFPT2	Body	0,273	2,58E-03	8,12E-03
cg25092186	14	46916528		IGR	0,211	2,66E-03	8,34E-03
cg26749518	19	51506165	KLK9	3'UTR	0,216	2,69E-03	8,42E-03
cg13678441	4	36387674		IGR	-0,212	2,69E-03	8,43E-03
cg20095237	7	615919	PRKAR1B	Body	-0,241	2,75E-03	8,56E-03
cg02439266	7	27150042	HOXA3	5'UTR	0,241	2,88E-03	8,89E-03
cg12188187	20	8583715	PLCB1	Body	-0,213	2,98E-03	9,15E-03
cg14781242	1	32738251	LCK	5'UTR	-0,308	2,99E-03	9,17E-03
cg01368132	7	35698654	HERPUD2	Body	-0,214	3,11E-03	9,47E-03
cg25403368	5	130554320		IGR	-0,439	3,14E-03	9,54E-03
cg13375589	17	4487125	SMTNL2	TSS200	0,265	3,14E-03	9,54E-03
cg09935308	19	341861	MIER2	Body	0,201	3,17E-03	9,62E-03
cg22061769	8	17942896	ASAH1	TSS1500	0,222	3,19E-03	9,68E-03
cg07199764	1	5347468		IGR	0,271	3,27E-03	9,87E-03
cg02230774	5	51108149		IGR	0,2	3,35E-03	1,01E-02
cg10233674	3	112493304		IGR	-0,258	3,41E-03	1,02E-02
cg02872767	19	1525453	PLK5P	Body	0,31	3,41E-03	1,02E-02
cg19603903	19	57742345	AURKC	TSS200	0,207	3,52E-03	1,05E-02
cg14321373	8	140627494	KCNK9	3'UTR	-0,366	3,54E-03	1,05E-02
cg03771436	7	81977646	CACNA2D1	Body	0,203	3,58E-03	1,06E-02
cg10632894	6	32552453	HLA-DRB1	Body	0,256	3,60E-03	1,07E-02
cg13786083	1	19110734		IGR	0,225	3,65E-03	1,08E-02
cg00635994	18	48564941	SMAD4	5'UTR	0,233	3,65E-03	1,08E-02
cg07414487	6	31107186	PSORS1C1	Body	0,217	3,67E-03	1,09E-02
cg05287581	7	15601822	AGMO	TSS200	0,204	3,79E-03	1,11E-02
cg27236896	7	80128051	GNAT3	Body	0,22	3,79E-03	1,11E-02
cg02164656	5	108089396	FER	5'UTR	0,212	3,82E-03	1,12E-02
cg16345059	1	210536172	HHAT	Body	0,218	4,45E-03	1,27E-02
cg13405775	22	32599648	RFPL2	TSS200	-0,206	4,55E-03	1,29E-02
cg01124132	22	32599511	RFPL2	TSS200	-0,236	4,62E-03	1,31E-02
cg18537979	5	180402690		IGR	-0,225	4,86E-03	1,36E-02
cg08060588	7	70527670		IGR	0,201	4,97E-03	1,39E-02
cg05859533	16	57727230	CCDC135	TSS1500	-0,245	5,32E-03	1,47E-02
cg13609544	17	46664647		IGR	0,226	5,50E-03	1,51E-02
cg11265221	4	175241684	CEP44	Body	0,218	5,65E-03	1,54E-02
cg17119047	13	106840208		IGR	-0,222	5,67E-03	1,55E-02
cg16288713	13	79234144	RNF219	TSS1500	0,234	5,83E-03	1,58E-02
cg03288922	2	118616237		IGR	-0,204	5,83E-03	1,58E-02
cg09992818	17	46591533		IGR	0,207	5,84E-03	1,59E-02
cg09686326	17	3879761		IGR	0,227	6,03E-03	1,63E-02
cg14285533	7	63386328		IGR	0,235	6,11E-03	1,65E-02
cg12794147	18	12255567	CIDEA	Body	0,209	6,31E-03	1,69E-02
cg26913058	16	419975	MRPL28	Body	0,202	6,32E-03	1,69E-02
cg18929511	8	17942993	ASAH1	TSS1500	0,218	6,44E-03	1,72E-02
cg08517984	12	16431562	SLC15A5	TSS1500	-0,267	6,45E-03	1,72E-02
cg02447542	3	125710226		IGR	0,239	6,68E-03	1,77E-02

cg14378539	1	218698011	MIR548F3	Body	0,29	7,16E-03	1,87E-02
cg21847720	8	2075777	MYOM2	Body	-0,281	7,17E-03	1,88E-02
cg24713959	12	71709550		IGR	-0,26	7,37E-03	1,92E-02
cg10075506	2	1817351	MYT1L	Body	0,2	7,87E-03	2,03E-02
cg19683494	5	74908142		IGR	0,217	7,87E-03	2,03E-02
cg19707454	7	6616677	ZDHHC4	TSS1500	-0,24	8,08E-03	2,07E-02
cg20694160	3	17761543	TBC1D5	5'UTR	0,21	9,18E-03	2,30E-02
cg12690462	21	43822540	UBASH3A	TSS1500	0,236	9,39E-03	2,34E-02
cg24160354	4	23867593	PPARGC1A	Body	-0,289	9,88E-03	2,44E-02
cg07510230	7	30361323	ZNRF2	Body	-0,224	1,02E-02	2,51E-02
cg01200585	1	228362443	C1orf69	Body	0,204	1,06E-02	2,58E-02
cg09781987	6	4828434	CDYL	Body	-0,378	1,07E-02	2,60E-02
cg22676075	6	135203613		IGR	0,207	1,07E-02	2,60E-02
cg21248347	15	78568978	DNAJA4	Body	0,201	1,14E-02	2,74E-02
cg17939448	4	77200832	FAM47E	Body	-0,205	1,20E-02	2,86E-02
cg23442650	14	70690296		IGR	0,235	1,24E-02	2,94E-02
cg20124547	2	175062978	OLA1	5'UTR	0,206	1,29E-02	3,04E-02
cg08564027	20	61660810		IGR	0,29	1,29E-02	3,04E-02
cg10110780	1	28573139		IGR	-0,214	1,34E-02	3,13E-02
cg08716255	11	95433344		IGR	-0,227	1,34E-02	3,13E-02
cg27425262	2	113953981	PSD4	Body	0,226	1,36E-02	3,18E-02
cg24951781	5	132114055	sept-08	TSS1500	-0,233	1,37E-02	3,20E-02
cg26035071	1	209982407		IGR	-0,362	1,37E-02	3,20E-02
cg05064286	5	157850416		IGR	0,2	1,57E-02	3,58E-02
cg05199761	10	6105145	IL2RA	TSS1500	0,206	1,67E-02	3,76E-02
cg17611936	7	151411526	PRKAG2	Body	0,245	1,75E-02	3,90E-02
cg16814680	8	91681699		IGR	-0,272	1,79E-02	3,97E-02
cg14683065	10	134149184	LRRC27	Body	-0,329	1,84E-02	4,07E-02
cg23085500	12	14952637	WBP11	Body	0,214	1,85E-02	4,10E-02
cg12844592	13	37610025	SUPT20H	Body	0,239	1,86E-02	4,12E-02
cg00101154	16	420108	MRPL28	Body	0,247	1,93E-02	4,24E-02
cg09467607	2	36825704	FEZ2	TSS1500	0,264	2,01E-02	4,39E-02
cg01820934	2	102614569	IL1R2	5'UTR	-0,277	2,03E-02	4,41E-02
cg02042823	16	6714429	A2BP1	5'UTR	0,201	2,24E-02	4,79E-02
cg20088245	8	1321375		IGR	0,231	2,27E-02	4,84E-02
cg09152353	2	10134152	GRHL1	Body	-0,205	2,36E-02	4,99E-02
