# Supplementary material for: Epigenetic regulation of transcription factor binding motifs promotes Th1 response in Chagas disease cardiomyopathy
Source: Front Immunol. 2022 Aug 22;13:958200. doi: 10.3389/fimmu.2022.958200 (PMC9441916; doi:10.3389/fimmu.2022.958200)
Supplement: Supplementary Table 1 — Biological samples included in this study. [file DataSheet_1.zip › Supplementary Material/Supplementary Table 9.pdf]

Supplementary table 9. List of the regulatory DMRs associated to the 89 DEGs.

DMR id	chromosome	start	end	Region covered by this DMR	Gene
DMR82	chr1	9714843	9714845	TSS	C1orf200
DMR2	chr1	25291385	25292215	1stExon,TSS	RUNX3
DMR41	chr1	27668098	27668433	TSS	SYTL1
DMR51	chr1	27952665	27953220	TSS,1stExon	FGR
DMR52	chr1	28521427	28521540	TSS	PTAFR
DMR57	chr1	40783063	40783234	TSS	COL9A2
DMR92	chr1	111415781	111416181	1stExon,5'UTR	CD53
DMR17	chr1	111743038	111743411	1stExon,TSS	DENND2D
DMR20	chr1	114414312	114414532	5'UTR,TSS	PTPN22
DMR72	chr1	151129062	151129298	TSS,5'UTR	TNFAIP8L2
DMR3	chr1	159046391	159047177	TSS,5'UTR	AIM2
DMR74	chr1	159770253	159770368	5'UTR,TSS	FCRL6
DMR50	chr1	160714299	160714382	5'UTR	SLAMF7
DMR16	chr1	161039601	161040044	TSS,1stExon	ARHGAP30
DMR55	chr1	202128508	202128682	1stExon,5'UTR	PTPN7
DMR28	chr1	203733914	203733971	TSS	LAX1
DMR23	chr1	209929437	209929622	1stExon,TSS	TRAF3IP3
DMR56	chr1	209931334	209931457	5'UTR	TRAF3IP3
DMR9	chr1	209941646	209941848	TSS,1stExon	TRAF3IP3
DMR8	chr10	49892943	49893549	TSS,5'UTR	WDFY4
DMR7	chr10	72362694	72362866	TSS	PRF1
DMR34	chr10	125651034	125651370	1stExon	CPXM2
DMR33	chr11	58981043	58981095	TSS	MPEG1
DMR5	chr11	60738971	60739183	TSS,5'UTR	CD6
DMR83	chr11	60869910	60869960	5'UTR,TSS	CD5

DMR29	chr11	64107158	64107517	TSS	CCDC88B
DMR81	chr11	67171476	67171585	5'UTR	TBC1D10C
DMR63	chr11	67205642	67205650	TSS	PTPRCAP
DMR38	chr11	118095405	118095739	5'UTR	AMICA1
DMR54	chr11	118213272	118213330	1stExon,5'UTR	CD3D
DMR30	chr12	6881595	6881997	1stExon,TSS	LAG3
DMR61	chr12	7060263	7060386	TSS	PTPN6
DMR49	chr12	12224246	12224360	5'UTR	BCL2L14
DMR43	chr12	47610257	47610418	TSS	PCED1B-AS1
DMR71	chr12	51718155	51718251	TSS	BIN2
DMR15	chr12	53496729	53497147	TSS	SOAT2
DMR22	chr12	54891491	54891655	1stExon,TSS	NCKAP1L
DMR26	chr12	68553577	68553980	TSS	IFNG
DMR32	chr12	109026944	109027086	TSS	SELPLG
DMR87	chr12	109027683	109027932	TSS	SELPLG
DMR76	chr12	109028385	109028610	TSS	SELPLG
DMR14	chr14	75988251	75988820	TSS,5'UTR	BATF
DMR89	chr15	38988533	38988755	TSS	C15orf53
DMR62	chr15	44969244	44969481	TSS	PATL2
DMR10	chr15	77286232	77287243	TSS	PSTPIP1
DMR69	chr15	81590933	81591058	5'UTR	IL16
DMR18	chr16	27414210	27414536	1stExon,TSS,5'UTR	IL21R
DMR80	chr16	29673933	29674184	TSS	SPN
DMR21	chr16	29757318	29757565	1stExon,TSS	C16orf54
DMR11	chr16	50715260	50715700	TSS,1stExon	SNX20
DMR94	chr17	4487099	4487125	TSS	SMTNL2
DMR37	chr17	56408688	56409028	TSS	MIR142
DMR40	chr18	43652592	43652594	TSS	PSTPIP2
DMR45	chr19	3179364	3179741	1stExon	S1PR4

DMR60	chr19	17862017	17862104	5'UTR	FCHO1
DMR46	chr19	36204551	36204918	5'UTR	ZBTB32
DMR48	chr19	44285940	44285954	TSS	KCNN4
DMR64	chr19	49838478	49838777	TSS,1stExon	CD37
DMR25	chr2	10261684	10262019	TSS	RRM2
DMR79	chr2	143886326	143886567	TSS	ARHGAP15
DMR27	chr2	158300475	158300811	1stExon,TSS	CYTIP
DMR6	chr2	202125088	202125310	TSS,5'UTR,1stExon	CASP8
DMR66	chr2	204732461	204732474	TSS	CTLA4
DMR35	chr2	225811610	225811669	1stExon	DOCK10
DMR39	chr20	35273933	35274281	5'UTR	SLA2
DMR42	chr20	56195541	56195574	1stExon	ZBP1
DMR68	chr21	46332181	46332291	5'UTR	ITGB2
DMR77	chr21	46334192	46334214	5'UTR	ITGB2
DMR44	chr22	50524032	50524541	TSS,5'UTR	MLC1
DMR84	chr22	50985797	50986031	TSS	KLHDC7B
DMR19	chr3	45984838	45985168	TSS,5'UTR	CXCR6
DMR24	chr3	46411369	46411474	TSS	CCR5
DMR67	chr4	40201884	40201943	5'UTR	RHOH
DMR85	chr4	100737691	100738011	TSS,1stExon	DAPP1
DMR65	chr5	149792783	149792840	TSS	CD74
DMR58	chr5	156607793	156608118	1stExon,TSS	ITK
DMR4	chr5	169407439	169407941	1stExon,5'UTR,TSS	FAM196B
DMR88	chr6	26367571	26367580	5'UTR	BTN3A2
DMR53	chr6	29527870	29527885	TSS	UBD
DMR1	chr6	31539539	31540461	TSS,5'UTR	LTA
DMR70	chr6	32909282	32909523	TSS	HLA-DMB
DMR78	chr6	33041343	33041697	1stExon,TSS	HLA-DPA1
DMR59	chr6	42391208	42391254	5'UTR	TRERF1

DMR13	chr6	108145374	108145601	1stExon,TSS	SCML4
DMR47	chr6	149805292	149805596	5'UTR	ZC3H12D
DMR91	chr7	3067279	3067293	5'UTR	CARD11
DMR86	chr7	36764019	36764082	1stExon	AOAH
DMR73	chr7	45018658	45019005	5'UTR,TSS	MYO1G
DMR36	chr7	50358075	50358218	5'UTR	IKZF1
DMR31	chr8	21771446	21771668	TSS	DOK2
DMR75	chr8	134072526	134072706	5'UTR,TSS	SLA
DMR90	chr9	95726377	95726447	1stExon	FGD3
DMR93	chr9	117692745	117692759	1stExon	TNFSF8
DMR12	chr9	123688715	123689193	5'UTR,TSS	TRAF1
